# Extracellular Vesicles Bearing Vimentin Drive Epithelial–Mesenchymal Transition

**DOI:** 10.1016/j.mcpro.2025.101028

**Published:** 2025-07-04

**Authors:** Sepideh Parvanian, Leila S. Coelho-Rato, Michael Santos Silva, Giulia Sultana, Arun P. Venu, Pallavi Vilas Devre, Mayank Kumar Modi, John E. Eriksson

**Affiliations:** 1 Center for Systems Biology, Massachusetts General Hospital Research Institute and Harvard Medical School, Boston, Massachusetts, USA; 2 Turku Bioscience Centre, University of Turku and Åbo Akademi University, Turku, Finland; 3 Faculty of Science and Engineering, Cell Biology, Åbo Akademi University, Turku, Finland; 4 Euro-Bioimaging ERIC, Turku, Finland

**Keywords:** epithelial–mesenchymal transition, extracellular vesicles, fibroblast, extracellular vimentin

## Abstract

Epithelial–mesenchymal transition (EMT) is a key biological process in physiological and pathological conditions, spanning development, wound healing, and cancer. Vimentin, a key cytoskeletal intermediate filament (IF) protein, is an established intracellular determinant of EMT. Recently, extracellular vimentin has also emerged with important functions, and we demonstrated that vimentin from fibroblast-derived extracellular vesicles (EVs) promotes wound healing. Building on these findings, we explored whether extracellular vimentin regulates EMT. We employed fibroblast-derived EVs to assess their EMT-driving capacity. Using coculture models and EV treatments from WT and vimentin-KO fibroblasts, we observed that fibroblasts induce an EMT phenotype in epithelial cells, marked by elevated mesenchymal markers and reduced epithelial markers. EVs from vimentin-deficient fibroblasts showed a decreased EMT-inducing capacity and failed to stimulate cell cover closure, underscoring vimentin’s critical role in orchestrating these processes. Coculturing epithelial cells with WT fibroblasts mirrored these outcomes, while vimentin-deficient fibroblasts produced similarly poor EMT induction. Proteomic profiling revealed that WT EVs contained an enriched set of EMT-associated proteins, including those involved in cytoskeletal organization, cell adhesion, and EMT-regulating signaling pathways. Notably, these proteins, such as fibronectin and N-cadherin, were significantly diminished in vimentin-deficient EVs. Moreover, we identified over 600 additional proteins uniquely present in WT-derived EVs, with enrichment in key biological processes like wound healing and cell migration. These findings demonstrate that vimentin-positive EVs drive EMT by transmitting a specific protein cargo that supports EMT-related cellular changes. The vimentin-positive EV proteome will help understand EMT mechanisms and develop targeted therapies for pathological conditions related to abnormal EMT.

## Highlights

• Vimentin-carrying fibroblast EVs trigger EMT by raising mesenchymal markers.
• Vimentin-deficient fibroblast EVs do not promote EMT, migration, or cell invasion.
• Over 600 proteins unique to vimentin-positive EVs are linked to EMT and cell remodeling.
• Vimentin shapes EV cargo, impacting recipient epithelial cell signaling and EMT.
• Key EMT proteins like fibronectin and N-cadherin are depleted in vimentin-deficient EVs.

## Graphical Abstract

**Figure:**
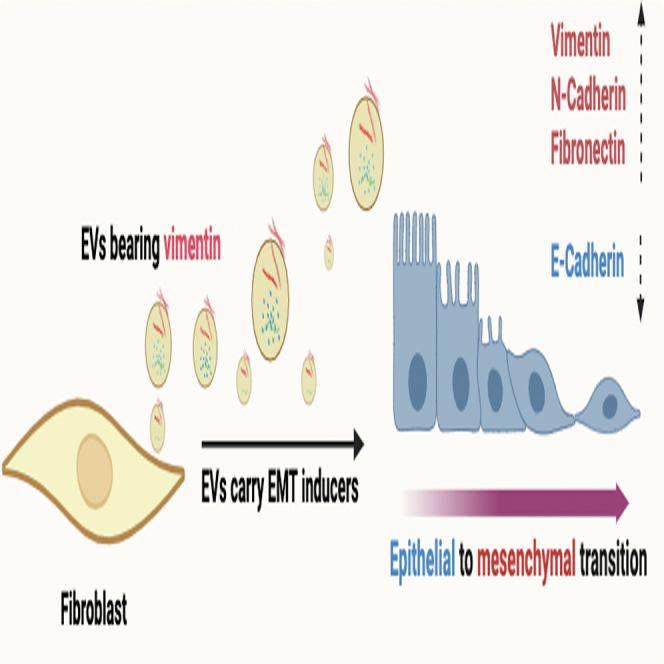

## In Brief

Fibroblast-derived extracellular vesicles containing vimentin drive epithelial–mesenchymal transition (EMT) by delivering a protein cargo enriched in cytoskeletal and adhesion regulators. These findings reveal a critical extracellular role for vimentin in modulating epithelial cell plasticity and suggest new therapeutic targets for EMT-associated pathologies.

Epithelial–mesenchymal transition (EMT) is a crucial biological process through which epithelial cells lose their characteristic cell-cell adhesions and polarity while acquiring migratory and invasive properties typical of mesenchymal cells (1, 2). This transition plays crucial roles in diverse physiological and pathological contexts, including embryogenesis, tissue regeneration, fibrosis, and cancer progression (3). EMT is driven by complex regulatory networks that integrate epigenetic modifications, cytoskeletal rearrangements, and signaling pathways such as transforming growth factor beta (TGF-β), alongside key transcription factors including SNAIL, SLUG, and TWIST, as well as various environmental cues (4, 5, 6, 7). Vimentin, a type III intermediate filament protein, is a hallmark of the mesenchymal phenotype and has been shown to be a key intracellular determinant of EMT (8, 9, 10, 11, 12). Vimentin, beyond its role as an EMT marker, actively promotes EMT and tumor progression in vivo. Studies show that increased vimentin expression correlates with EMT induction and metastasis in models of colorectal carcinoma and lung cancer (13, 14). Vimentin also interacts with key EMT signaling pathways, such as TGF-β, to drive tumor invasion (15). Beyond cancer, vimentin plays essential roles in multiple biological systems. Vimentin-null mice display alterations in EMT-dependent developmental processes, highlighting its broader role in tissue remodeling and cellular plasticity. In mammary gland development, vimentin is crucial for proper ductal outgrowth, and its loss results in dilated ducts and a reduced basal-to-luminal mammary epithelial cell ratio, indicating its role in regulating mammary stem cell function and epithelial cell dynamics (16). In renal fibrosis, vimentin expression is essential for EMT-driven fibrotic tissue formation, and its absence impairs the progression of fibrosis in the kidneys (17). Similarly, in lens development and integrity, vimentin is required for normal lens epithelial cell function and transparency, as mutations or loss of vimentin have been linked to cataract formation and disrupted lens homeostasis (18, 19). This accumulating evidence supports the notion that vimentin is not just a biomarker of EMT but a functional driver that regulates tumor invasion, fibrosis, organ development, and cellular plasticity across multiple biological contexts.

Importantly, we obtained evidence highlighting that vimentin also functions extracellularly (20, 21, 22, 23, 24, 25, 26, 27), suggesting that vimentin is likely to have a potential role in mediating cell-cell communication during EMT.

Extracellular vesicles (EVs) are lipid-bound particles secreted by most cell types that play crucial roles in intercellular communication by transporting a variety of bioactive molecules, including proteins, lipids, and nucleic acids (28, 29). Recent studies suggest that fibroblast-derived EVs can transmit EMT-inducing signals to epithelial cells, influencing key cellular processes such as cytoskeletal reorganization, adhesion dynamics, and the activation of signaling pathways critical for EMT regulation (30, 31, 32, 33, 34, 35, 36, 37). Given the established role of intracellular vimentin in EMT (10, 38, 39, 40, 41, 42), we hypothesized that fibroblast-derived EVs containing vimentin could drive EMT in epithelial cells by carrying a specific proteomic cargo enriched in EMT-associated factors.

In our study, we performed in-depth proteomic profiling of EVs derived from wild-type (WT) and vimentin-KO (vim −/−) fibroblasts to characterize the composition and functional implications of the EV protein cargo. Our analysis identified over 600 proteins uniquely present in WT-derived EVs, with significant enrichment in biological processes related to cytoskeletal organization, cell adhesion, and extracellular matrix (ECM) remodeling, all key elements in EMT regulation. Notably, these EVs were enriched in EMT markers such as fibronectin and N-cadherin, along with proteins involved in critical signaling pathways like the epidermal growth factor receptor (EGFR) tyrosine kinase inhibitor resistance pathway and the PD-L1/PD-1 checkpoint pathway, suggesting a potential role in creating an immunosuppressive microenvironment conducive to metastasis. In contrast, vim −/− EVs displayed a diminished presence of these key EMT-promoting proteins, underscoring the essential role of extracellular vimentin in determining the EV cargo composition and function.

These findings indicate that WT-derived, vimentin-positive EVs transmit a unique set of EMT-associated proteins, supporting mesenchymal reprogramming and promoting cellular behaviors associated with EMT. The enrichment of proteins involved in cytoskeletal rearrangements, cell adhesion, and immune modulation highlights the multifaceted impact of vimentin-containing EVs on epithelial plasticity and the tumor microenvironment. By extending the role of vimentin beyond its intracellular functions, this study provides new insights into how extracellular vimentin can facilitate EMT through its presence within EVs, shaping both the local tissue environment and distant metastatic sites.

Understanding the proteomic landscape of vimentin-positive EVs opens avenues for targeted therapeutic strategies aimed at modulating EMT in pathological conditions such as wound healing, fibrosis, and cancer metastasis. Specifically, targeting extracellular vimentin or its EV-associated cargo could offer novel interventions to disrupt EMT-driven processes, thereby impacting disease progression.

## Experimental Procedures

### Cell Lines and Cell Culture

HDFs, MCF7, and MDA MB 231 were purchased from The American Type Culture Collection (ATCC). All these cell lines were cultured in Dulbecco’s modified Eagle medium (DMEM) (Lonza) supplemented with 1% l-glutamine (Lonza), 0.5% penicillin/streptomycin (Gibco Life Technologies Ltd), and 10% fetal bovine serum (Thermo Fisher Scientific). MCF10a cells were purchased from The American Type Culture Collection and cultured in DMEM/F12 media (Invitrogen) supplemented with 5% horse serum (Thermo Fisher Scientific), 20 ng mL−1 EGF (Thermo Fisher Scientific), 0.5 mg mL−1 hydrocortisone (Sigma-Aldrich), 10 μg mL−1 insulin (Sigma-Aldrich), and 100 ng mL−1 cholera toxin (Sigma-Aldrich). All cells were maintained in a humidified incubator at 37 °C with 5% CO2. All cell lines have been tested for *mycoplasma* contamination.

### Coculture Using a Transwell System

In parallel, coculture experiments were performed by transwell system using cell inserts (Sarstedt). Briefly, in separate experiments, epithelial cells, including MCF7 and MCF10a cells, were seeded onto the top chambers of the transwell plates, while WT and vim −/− HDFs were seeded onto the lower chambers. The transwell inserts allowed for the exchange of soluble factors between the two chambers while physically separating the 2 cell populations. This coculture setup was designed to study the influence of HDFs and their EVs on MCF7 and MCF10a cells in a controlled manner.

For each epithelial cell line, six different culture conditions were employed, including treatment with media supplemented with 1X StemXVivo EMT Inducing Media Supplement (R&D Systems), EVs from WT and vim −/− HDFs (100 μg/ml), and cocultures with WT and vim −/− HDFs. Untreated MCF7, MCF10a, and MDA MB231 have been used as negative and positive controls, respectively. Two types of cell inserts, including 6-well and 24-well plates, were utilized based on the specific objectives of the experiments.

### Generation of vim −/− HDFs Using CRISPR-Cas9

Primary HDFs were immortalized and successful immortalization was validated via western blotting using the SV40 T large antigen. HDFs lacking vimentin (vim −/−) were generated using CRISPR genome editing by deleting a specific 488 bp region within the vimentin-encoding gene with a single guide RNA (GeneScript, 5′- ATTGCTGACGTACGTCACGC-3′). Single-cell sorting was carried out using the SH800 Cell Sorter (Sony). The absence of vimentin in the 2 cell lines was further confirmed through western blotting, reverse transcription quantitative polymerase chain reaction, and imaging.

### Extracellular Vesicles Isolation and Characterization

EVs were isolated from both WT and vim −/− HDFs. At 70% confluency, cells were washed with PBS, and growth media were replaced with media supplemented with 0.5% exosome-depleted fetal bovine serum (Thermo Fisher Scientific). After 24 h, culture supernatant was collected, and EVs were isolated using a differential centrifugation protocol. Collected conditioned media were centrifuged at 300*g* for 10 min to remove cellular debris. The supernatant was then transferred to a new 50 ml conical tube and centrifuged at 2000*g* for 20 min to isolate apoptotic bodies. Supernatants were transferred to a sterile Ultra-Clear tube (Beckman Coulter) and centrifugation in a Beckman Coulter Optima L-80XP Ultracentrifuge to isolate microvesicles by 40 min centrifugation at 100,00*g* and then again at 1,000,00*g* for 90 min to pellet EVs. A 1% Protease Inhibitor Cocktail (Thermo Fisher Scientific) was added to the conditioned media and each wash step. The resulting EV pellet was resuspended in 1×PBS and stored at −80 °C for future use. All procedures were performed at 4 °C.

To ensure consistency and reproducibility, we optimized the EV concentration based on our previous studies, which established an effective range for biological effects. For each isolation, we typically used five 15-cm culture plates at 70 to 80% confluency, yielding approximately 30 million cells in total. This protocol consistently resulted in 1 to 3 × 10^9^ particles/ml (as measured by nanoparticle tracking analysis [NTA]) and 0.1 to 1 mg protein/ml (as measured by bicinchoninic acid assay [BCA]). For recipient epithelial cell treatments, we used 100 μg/ml EVs, a dose optimized in our previous studies to induce measurable biological effects.

### Transmission Electron Microscopy

The morphology of EVs was examined by the EV Core at the University of Helsinki using transmission electron microscopy (TEM). In this process, EVs were loaded onto carbon-coated and glow-discharged 200 mesh copper grids with pioloform support membrane. Subsequently, the samples were fixed using 2.0% paraformaldehyde (PFA) in NaPO4 buffer and stained with 2% neutral uranyl acetate before being embedded in a mixture of uranyl acetate and methylcellulose (1.8/0.4%). For immunostaining, the samples were initially blocked with 0.5% bovine serum albumin (BSA) in 0.1 M NaPO4 buffer (pH 7.0). They were then incubated with anti-vimentin antibodies (1:200 dilution, Novus Biologicals) in 0.1% BSA/NaPO4 buffer. Subsequent steps involved incubation with 15 nm goat anti-rabbit IgG (1:80 dilution, BBI Solutions) in 0.1% BSA/NaPO4 buffer, followed by washing with NaPO4 buffer, deionized water, and negative staining. The EVs were visualized using transmission electron microscopy with a Jeol JEM-1400 microscope (Jeol Ltd) operating at 80 kV. Images were captured with a Gatan Orius SC 1000B CCD camera (Gatan Inc) with a 4008 × 2672 px image size and no binning.

### Nanoparticle Tracking Analysis

Particle number and size distribution of the EVs were performed by the EV Core at the University of Helsinki. EVs were diluted with PBS and measured by NTA instrument LM14C (NanoSight LTD) equipped with a blue (404 nm, 70 mW) laser and sCMOS camera. Data were analyzed with Nanosight software v3.0, using a threshold five and a gain 10.

### Western Blot

EVs and cell lysate from both WT and vim −/− HDFs were lysed in radio-immunoprecipitation assay buffer buffer (Thermo Scientific Pierce) containing protease/phosphatase inhibitor cocktail (Cell Signaling), heated to 95 °C for 5 to 10 min and subsequently cooled on ice. Total protein concentration in the samples was measured by Pierce BCA protein assay kit (Thermo Scientific Pierce) and a spectrophotometer at 562 nm (Hidex Plate Reader). Samples (30 μg of protein per well) were separated on one-dimensional SDS-PAGE 12% gel. Proteins were transferred to nitrocellulose membrane (Bio-Rad Laboratories), blocked in 5% nonfat powdered milk in TBS-T (0.5% Tween-20), and probed with EVs markers including CD9 (1:500), CD63 (1:1000), CD81 (1:1000), GAPDH (1:1000) (all from System Biosciences) antibodies, and EMT markers including vimentin (1:1000, Biolegend), E-cadherin (1:1000, Genetex), N-cadherin (1:1000, Genetex) and fibronectin (1:1000, Novus Biologicals) antibodies overnight. Membranes were washed three times, 5 min in TBS-T, and probed with their respective secondary antibodies at a 1:10,000 dilution. The signals were visualized by the ECL Prime Western Blotting Detection Reagent (Advansta) and iBright CL1500 western blot imaging system (Thermo Fisher Scientific).

### EVs Labeling and Uptake Assays

Purified EVs were fluorescently labeled with PKH67 (Sigma-Aldrich), and HDFs were labeled with 1,10-Dioctadecyl3,3,30,30-tetramethylindocarbocyanine perchlorate (Dil) dye (Thermo Fisher Scientific), following the manufacturer's protocol. For EV labeling, briefly, a 2x dye solution was prepared by adding the PKH dye solution to the diluent C. An EVs/dye mixture with a 1:1 dilution containing at least 100 μg of EVs was incubated at room temperature for 10 min, followed by centrifugation at 14,000*g* for 10 min at 4 °C. The resulting EV pellet was washed with PBS through repeated centrifugation steps. For DiI staining, 1 × 10^4^ cells/ml were seeded on top of sterile coverslips (Greiner Bio-One GmbH) exposed to a working concentration of 5 to 10 μg/ml of dye and incubated at 37 °C for 15 to 30 min. Subsequently, the cells were washed with PBS to remove any excess, unincorporated dye. Cells were incubated with labeled EVs for 0, 6, 12, and 24 h, fixed with 4% PFA for 15 min, and mounted onto glass slides using Mowiol (Sigma-Aldrich). Nuclei were counterstained with 4',6-diamidino-2-phenylindole (DAPI). Cells were imaged using a 3i spinning disk confocal microscope (20 × objective, numerical Aperture: 0.8) equipped with an Orca Flash 4 v2 C11440-22CU Scientific CMOS camera (Hamamatsu). For quantification, the percentage of EV-positive cells was calculated by dividing the number of PKH67-positive cells (green signal in the cytoplasm) by the total number of DAPI-stained nuclei. Mean fluorescence intensity per image was quantified using ImageJ to assess relative EV uptake per cell.

### Immunofluorescence Staining

For both normal cell culture and transwells, a cell density of 1 × 10^4^ cells/ml was seeded in the respective wells. For normal cell culture, cells were seeded on top of sterile coverslips. After 4 days of culture, both coverslips and the cell inserts were fixed with 4% PFA for 15 min. Following fixation, cells were permeabilized with 0.1% Triton X-100 in 1% PBS and blocked with PBS containing 3% BSA. Cells were then stained for nuclei using 0.3 μg/ml DAPI (Thermo Fisher Scientific) and EMT markers including N-cadherin (1: 1000), E-cadherin (1: 1000), and vimentin (1: 500) antibodies. For transwell cultures, inserts were carefully removed from the well, and membranes were cut using a scalpel. The membrane was then mounted onto glass slides using Mowiol (Sigma-Aldrich). Cells were imaged using a 3i spinning disk confocal microscope (20 × objective, numerical Aperture: 0.8) equipped with an Orca Flash 4 v2 C11440-22CU Scientific CMOS camera. Fluorescent intensity was measured by ImageJ software. Three samples of each condition were measured for the mean intensity (n = 10). Subsequently, the values from individual immunofluorescence images with maximum intensity projection were used for the statistical analysis by using a paired *t* test; *p* < 0.05 was considered significant.

### Western Blot

To measure the expression of EMT markers, cells were seeded at a density of 1 x 10^4^ cells/ml in 6-well plates, either in normal adherent culture or on transwell inserts. The cells were treated as previously described. Following a 4-day incubation period, the cells were lysed using radio-immunoprecipitation assay buffer buffer. Subsequently, Western blot analysis was conducted using specific antibodies against N-cadherin (1:1000), E-cadherin (1:1000), vimentin (1:500), and β-actin (1:1000, Cell Signaling Technology) as a loading control. The blots were probed with corresponding secondary antibodies, and the signals were quantified using ImageJ software.

### Wound Scratch Assay

To study cell migration, MCF7 and MCF10a cells were seeded in 96-well ImageLock plates (Essen BioScience) at 2.4 × 10^4^ cells/well and allowed to reach 100% confluence over 48 h. Subsequently, a wound was introduced in the confluent monolayer using a WoundMaker device (Essen Bioscience). Cells were washed with PBS, and culture medium was immediately replaced with serum-free media. Six different culture conditions were employed, including treatment with media supplemented with 1X StemXVivo EMT Inducing Media Supplement, EVs from WT and vim −/− HDFs (100 μg/ml), and conditioned media from WT or vim −/− HDFs. Cells without any treatments served as negative controls, while MDA MB231 cells were utilized as positive controls. Three positions along the scratch were imaged continuously for 24 h using the IncuCyte ZOOM (Essen Bioscience). The acquired images were subsequently subjected to analysis for relative wound density using the IncuCyte ZOOM software. All scratch assays were performed in triplicate to ensure robustness and consistency of results.

### Invasion Assay

For the invasion assay, Transwell inserts were coated with 50 to 100 μl of Matrigel (Corning) dilution mixed in a 1:5 ratio with serum-free medium. After incubating at 37 °C for 30 min, the Matrigel-coated insert solidified. A cell suspension containing 5 × 10^4^ cells in serum-free medium was added to the upper chamber on the Matrigel-coated insert, while the lower chamber contained media supplemented with serum. Six distinct culture conditions were implemented, including the use of media supplemented with 1X StemXVivo EMT Inducing Media Supplement, EVs from WT and vim −/− HDFs (100 μg/ml), and conditioned media from WT or vim −/− HDFs. Cells were allowed to invade through the Matrigel for 48 h. The cells that had invaded through the Matrigel were fixed with 4% PFA for 15 min and stained with Crystal violet (Sigma-Aldrich). The quantification of the invaded cells was performed under a light microscope.

### Proliferation Assay

Cell proliferation was evaluated using an MTT (3-[4.5-Dimethylthiazol-2-yl]-2.5-diphenyltetrazolium bromide; Life Technologies) assay. MCF7 and MCF10a cells were seeded in 96-well plates at an initial density of 5 × 10^3^ cells per well and subjected to various treatments, including media supplemented with 1X StemXVivo EMT Inducing Media Supplement, EVs from WT and vim −/− HDFs (100 μg/ml), and conditioned media from WT or vim −/− HDFs for 72 h. The MTT assay was conducted after 24, 48, and 72 h of treatment. Cultures were exposed to 0.5 mg/ml of MTT for 3 to 5 h. Subsequently, 100 μl of dimethyl sulfoxide (Sigma-Aldrich) was introduced into each well, and the absorbance was measured at 540 nm using a Hidex Plate Reader. Throughout the experimental period, the culture medium was replaced with fresh medium containing the same supplements, ensuring consistent conditions for accurate assessments of cell proliferation.

### Mass Spectrometry

For our mass spectrometry pipeline, we used four biological replicates for each condition. Samples were lysed in a buffer containing 20 mM Hepes pH 7.4, 0.5% NP40, 2 mM EDTA, 150 mM NaCl, and one tablet of protease and phosphatase inhibitors (Thermo Fisher Scientific) for 30 min on ice, and then cleared by centrifugation. Protein was precipitated using 4 to 5 volumes of cold acetone overnight at −20 °C, followed by 15-min centrifugation using 16,000g at 4 °C. The pellet was dissolved in 100 μl of 6 M urea in 25 mM ammonium bicarbonate, and the protein concentration was accessed using Pierce 660 nm. The in-solution digestion was started with 60 μg of protein for whole cell lysates and 30 μg for EV samples. The samples were reduced with 10 mM DTT for 1 hour at 37 °C and alkylated with 40 mM iodoacetamide for 1 hour in the dark. The alkylation was quenched with 40 mM DTT, and the urea concentration was diluted by adding 900 μl of ammonium bicarbonate. Sequencing grade trypsin was added to each tube in a 1:30 ratio to total protein, and then samples were incubated overnight at 37 °C. The digestion was quenched with trifluoroacetic acid to a final concentration of 0.5%. Samples were then desalted using Sep Pak C18 columns (100 mg, Waters) according to the instructions of the manufacturer. Finally, they were dried on a speed-vac centrifuge and kept at −80 °C. Before analysis, all samples were dissolved in 0.1% formic acid. Approximately, 400 ng of each sample was loaded for liquid chromatography-tandem mass spectrometry analysis. The analysis was conducted using an Easy-nLC 1000 liquid chromatograph (Thermo Fisher Scientific) coupled to an Orbitrap Fusion Lumos Mass Spectrometer (Thermo Fisher Scientific) equipped with a FAIMS Pro interface (Thermo Fisher Scientific). Compensation voltages of −50 and −70 were used. The peptides were loaded on a precolumn (0.1 × 20 mm), followed by separation in an analytical column (75 μm × 150 mm), both packed with ReproSil-Pur 3 μm 120 Å C18-AQ (Dr Maisch HPLC GmbH). The peptides were separated using a 60-min gradient (5–21% B in 28 min, 21–36% B in 22 min, 36–100% B in 5 min, and 100% B for 5 min) in which solvent B was acetonitrile/water (80:20), at a flow rate of 300 nl/min. MS/MS data were acquired in positive ionization mode using data-dependent acquisition and a 1.5 s cycle time. The mass spectrometry survey scans were acquired with a resolution of 120,000 across the range of 300 to 1750 m/z, an automatic gain control target of 700,000, and a maximum injection time of 50 ms. Ions with charge >+2 were selected for higher-energy collisional dissociation fragmentation using an isolation window of 1.6  m/z, a dynamic exclusion window of 30 s, and 10 ppm mass tolerance.

Raw liquid chromatography-tandem mass spectrometry were processed using Fragpipe software (version 20.0) with MSFragger (version 3.8), IonQuant (version 1.9.8), and Philosopher (version 5.0.0). The data were searched against the UniProt human protein database with added common contaminants, downloaded on September 5, 2023. The database contained a total of 40,912 entries, with 50% designated as decoys. Trypsin digestion with a maximum of two missed cleavages, cysteine carbamidomethylation as a fixed modification, and methionine oxidation as variable modifications were selected as the parameters of these searches. The LFQ-MBR workflow was used with default settings, using a mass tolerance of 20 ppm for both precursor and fragment ions, along with a 1% false discovery rate at both the peptide and protein levels. The database search results were analyzed using Perseus (v1.6.15) (Tyanova *et al*., 2016). Data were transformed and filtered for at least three values (four for the MCF10A dataset) in at least one group. At least three replicates were used for analysis. Venn diagrams were made with the median average of replicates. Data were imputed based on a normal distribution in Perseus and filtered for ANOVA- or student *t*-test-significant (*p*-value <0.05). Hierarchical clustering was performed on z-scored data using Euclidean distance. Venn diagrams were generated with InteractiVenn (Heberle *et al*., 2015). Gene enrichment analysis (top 15) was carried out with Shiny 0.80 (Ge *et al*., 2020), using a human background with a minimum pathway size of two and an false discovery rate cutoff of 0.05. Vesiclepedia (Pathan *et al*., 2019) was used in Venn diagrams to compare EV cargo. Data can be found in Supplemental Data 1.

The mass spectrometry proteomics data have been deposited in the ProteomeXchange Consortium via the PRIDE (43) partner repository with the dataset identifier PXD060764.

### Statistics

Statistical analyses were conducted using data obtained from a minimum of three independent biological replicates. Differences between groups were assessed using either Student's *t* test or one-way ANOVA, followed by post hoc multiple comparisons. A significance level of *p* < 0.05 was considered indicative of statistically significant differences between the groups.

## Results

### Characterization of Fibroblast-Derived EVs

**Fig. 1:**
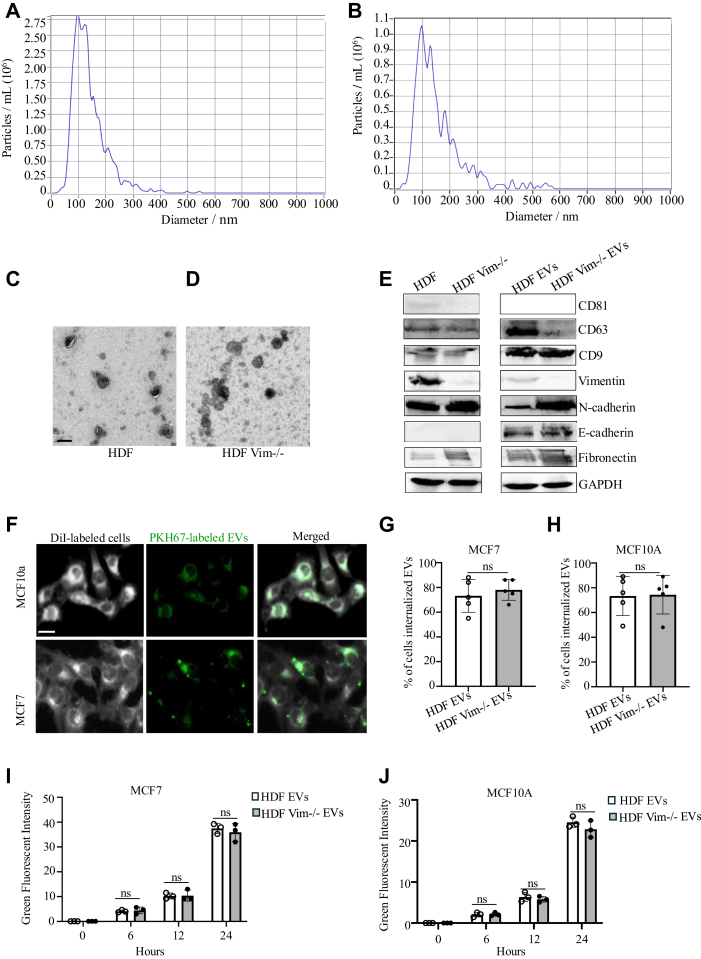
**Characterization and cellular uptake of HDF and HDF vim −/− derived EVs.** Nanoparticle tracking analysis (NTA) of EVs derived from HDFs (*A*) and vim −/− HDFs (*B*) confirmed the size of a small EV subpopulation within the size range of 30 to 150 nm. Representative transmission electron microscopy (TEM) images of EVs isolated from HDFs (*C*) and vim −/− HDFs (*D*). The scale bar represents 100 nm. *E*, western blotting analysis showing detection of EV markers including CD81, CD63, and CD9, vimentin, N-cadherin, and fibronectin as mesenchymal markers, and E-cadherin as epithelial marker in HDF, vim −/− HDF, and their respective EVs. GAPDH is used as the loading control. Fluorescence microscopy images (*F*) of MCF7 and MCF10A cells incubated with PKH67-labeled EVs for 24 h, showing intracellular localization of EVs. Quantification of EV uptake is shown as percentage of EV-positive cells (*G* and *H*) and mean green fluorescence intensity (*I* and *J*) in MCF7 and MCF10A cells, respectively. The *scale bar* represents 100 μm. EV, extracellular vesicle.

After isolating EVs from WT and vim −/− HDFs, NTA was employed to confirm the isolation of a small EV subpopulation within the size range of 30 to 150 nm (Fig. 1, *A* and *B*). We visualized the morphology of EVs using TEM (Fig. 1, *C* and *D*). We then tested whether WT and vim −/− EVs could be internalized by MCF7 and MCF10A cells (Fig. 1*E*). We incubated PKH67-labeled EVs with DiI-labeled cells and observed successful uptake of EVs after 24 h by imaging (Fig. 1, *F* and *G*). In addition, we conducted western blotting to check the presence of EV markers, including tetraspanins CD81, CD9, and CD63. Vim −/− HDFs and their EVs showcased an absence of vimentin expression (Fig. 1*H*).

### Vimentin-Positive HDFs and Their EVs Act as Strong Inducers of EMT in Epithelial Cells

To investigate the impact of fibroblasts on epithelial cells, we established a coculture model employing transwell cell inserts. In this configuration, epithelial cells, including MCF7 and MCF10a, were grown on the upper surface of the inserts, while WT and vim −/− fibroblasts were cultivated on the lower side. This arrangement facilitated the transmission of any potentially secreted material from the HDFs to the upper compartment through the insert. In parallel experiments, in a normal cell culture, we treated epithelial cells with EVs isolated from WT and vim −/− HDFs. As a positive control when evaluating the EMT induction potential, we employed cells that had been stimulated to undergo EMT by using 1X StemXVivo EMT induction media and MDA MB 231. Cells with no treatment were considered as negative controls. N-cadherin and vimentin were used as markers of mesenchymal state, and E-cadherin as a marker of epithelial state as measured in both MCF7 and MCF10a cells by both fluorescence microscopy and western blot analysis.

**Fig. 2:**
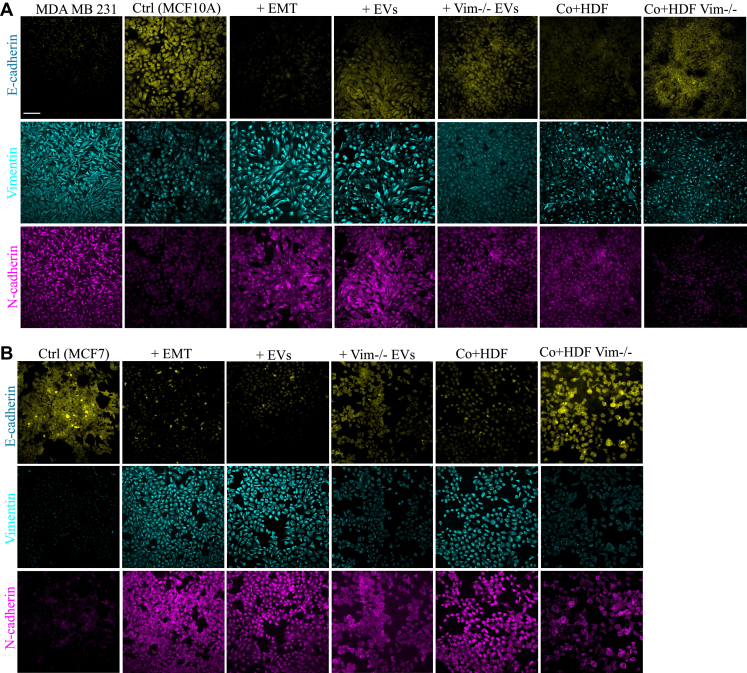

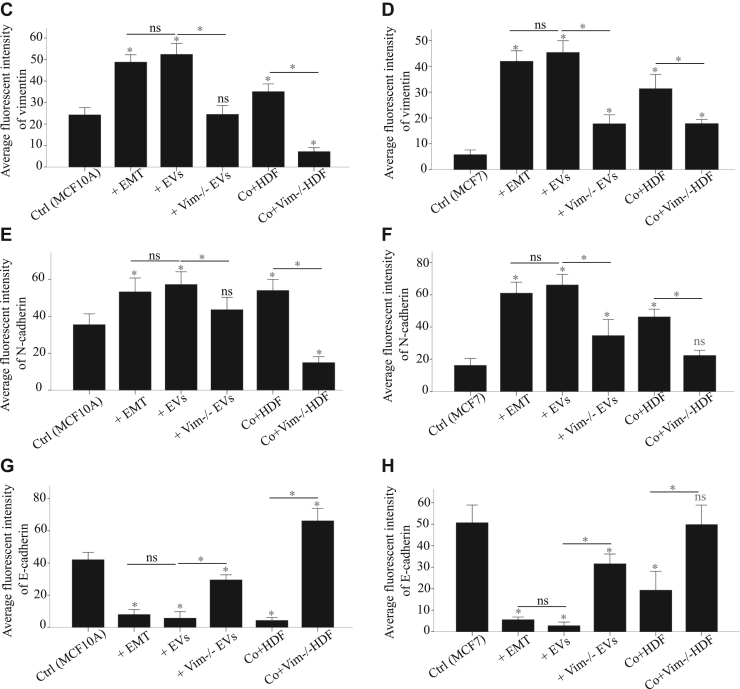
**The expression of EMT-associated markers is increased in epithelial cells treated with vimentin-positive HDFs and their EVs**. Expression of vimentin, N-cadherin, and fibronectin as mesenchymal markers and E-cadherin as epithelial markers in MCF10A (*A*) and MCF7 cells (*B*) treated with StemXVivo EMT inducing media, EVs from HDF and vim −/− HDFs (100 μg/ml), and cocultures with HDF and vim −/− HDFs. Cells without treatments and MDA MB231 have been used as negative and positive controls, respectively. Average fluorescent intensity quantification of EMT-associated protein markers expression including vimentin (*C* and *D*), N-cadherin (*E* and *F*), and E-cadherin (*G* and *H*) by MCF10A and MCF7 cells. Vimentin and N-cadherin levels were significantly elevated in cells treated with WT-EVs or cocultured with WT HDFs, similar to those treated with StemXVivo EMT induction media. Cells treated with or cocultured with vim −/− HDFs showed no EMT induction, matching negative controls. E-cadherin expression decreased in cells treated with StemXVivo, WT-EVs, and WT HDF coculture, while coculture with vim −/− HDFs increased E-cadherin. Data were normalized to surface area and represented as mean ± standard error of the mean (n = 10). Each treatment was compared first to untreated control and then to its related treatment group. The *scale bar* represents 100 μm. Statistical significance: ∗*p* < 0.05, ∗∗*p* < 0.01, ∗∗∗*p* < 0.001. EMT, epithelial–mesenchymal transition; EV, extracellular vesicle.

From the microscopy results, we observed a significant elevation of vimentin (Fig. 2*C*) and N-cadherin (Fig. 2*E*) levels in MCF10a cells treated with WT-EVs and when cocultured with both WT HDFs to similar levels as when the MCF10a cells were stimulated with the StemXVivo EMT induction media. In contrast, MCF10a cells treated with vim −/− HDFs, por cocultured with vim −/− HDFs, showed no EMT induction, corresponding to the signals of the negative controls. Moreover, E-cadherin expression was significantly reduced to corresponding levels in StemXVivo-treated, EV-treated, and HDF cocultured with MCF10a cells, whereas coculture with vim −/− HDFs increased E-cadherin expression. (Fig. 2*G*).

We obtained similar results with MCF7 cells, although MCF7 cells characteristically displayed lower vimentin (Fig. 2*D*) and N-cadherin (Fig. 2*F*) levels. Additionally, MCF10a cells showed more pronounced alterations after treatments. Both cell lines exhibited a significant difference between treatments involving WT and vim −/− conditions, whether through coculturing or exposure to EVs (Fig. 2, *A* and *B*).

**Fig. 3:**
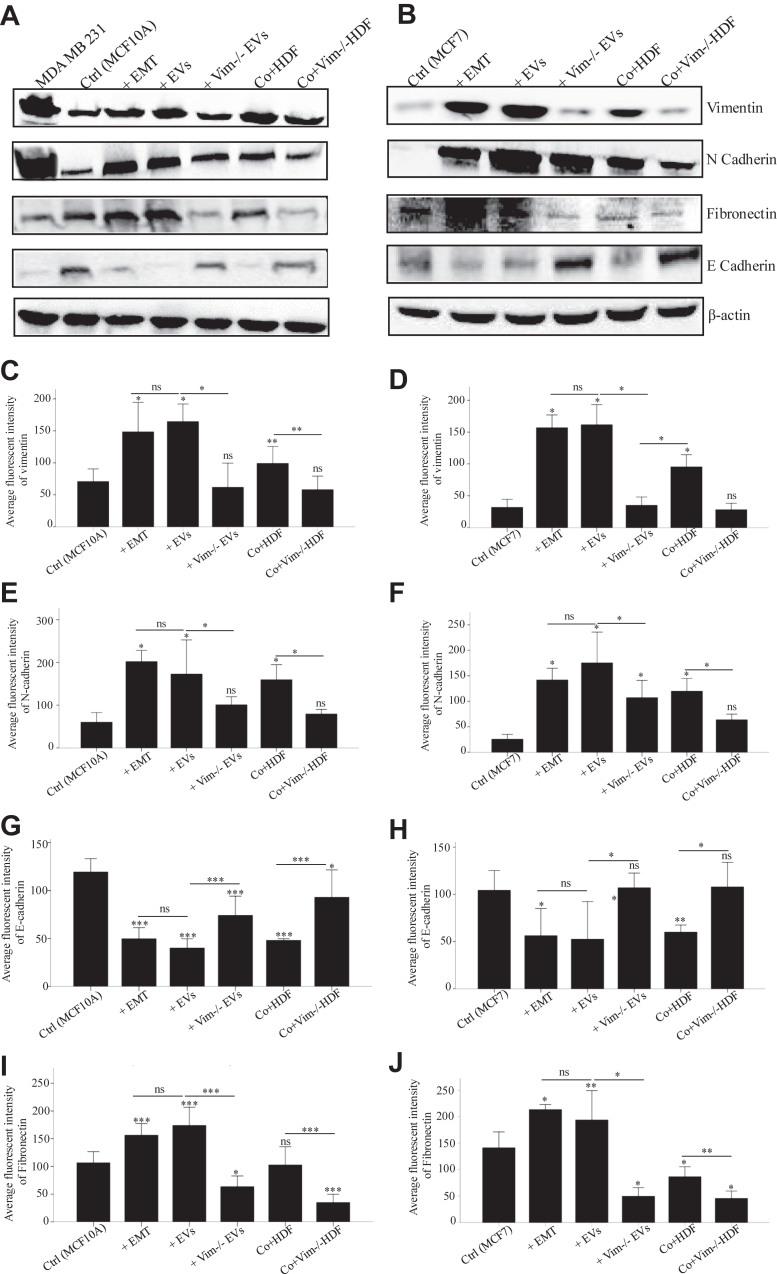
**EMT-associated markers are elevated in epithelial cells treated with vimentin-positive HDFs and their EVs**. Expression of vimentin, N-cadherin, and fibronectin as mesenchymal markers and E-cadherin as epithelial markers in MCF10A (*A*) and MCF7 cells (*B*) treated with StemXVivo EMT Inducing media, EVs from HDF and vim −/− HDFs (100 μg/ml), and cocultures with HDF and vim −/− HDFs. Cells without treatments and MDA MB231 cells were used as negative and positive controls, respectively. β-Actin was used as a normalization control for the total lysate. Pixel density quantification of vimentin (*C* and *D*), N-cadherin (*E* and *F*), E-cadherin (*G* and *H*), and fibronectin (*I* and *J*) proteins from three independent experiments. The western blot results confirmed the microscopy findings, with minor variations attributed to methodological differences. Vimentin and N-cadherin levels were significantly elevated in cells treated with WT-EVs or cocultured with WT HDFs, similar to those treated with StemXVivo EMT induction media. Cells treated with or cocultured with vim −/− HDFs showed no EMT induction, matching negative controls. E-cadherin expression decreased in cells treated with StemXVivo, WT-EVs, and WT HDF coculture, while coculture with vim −/− HDFs increased E-cadherin. Each treatment was compared first to untreated control and then to its related treatment group. The *scale bar* represents 100 μm. Statistical significance: ∗*p* < 0.05, ∗∗*p* < 0.01, ∗∗∗*p* < 0.001. EMT, epithelial–mesenchymal transition; EV, extracellular vesicle.

The western blot results confirmed the findings from microscopy, with slight variations in marker expressions, variations attributed to the inherent differences in the methodologies employed (Fig. 3). Overall, these changes in marker expression are consistent with EMT-related transformations, further validating a key role for vimentin in the HDF secretion and EV-mediated EMT.

### EV-Associated Vimentin Promotes Cell Migration

**Fig. 4:**
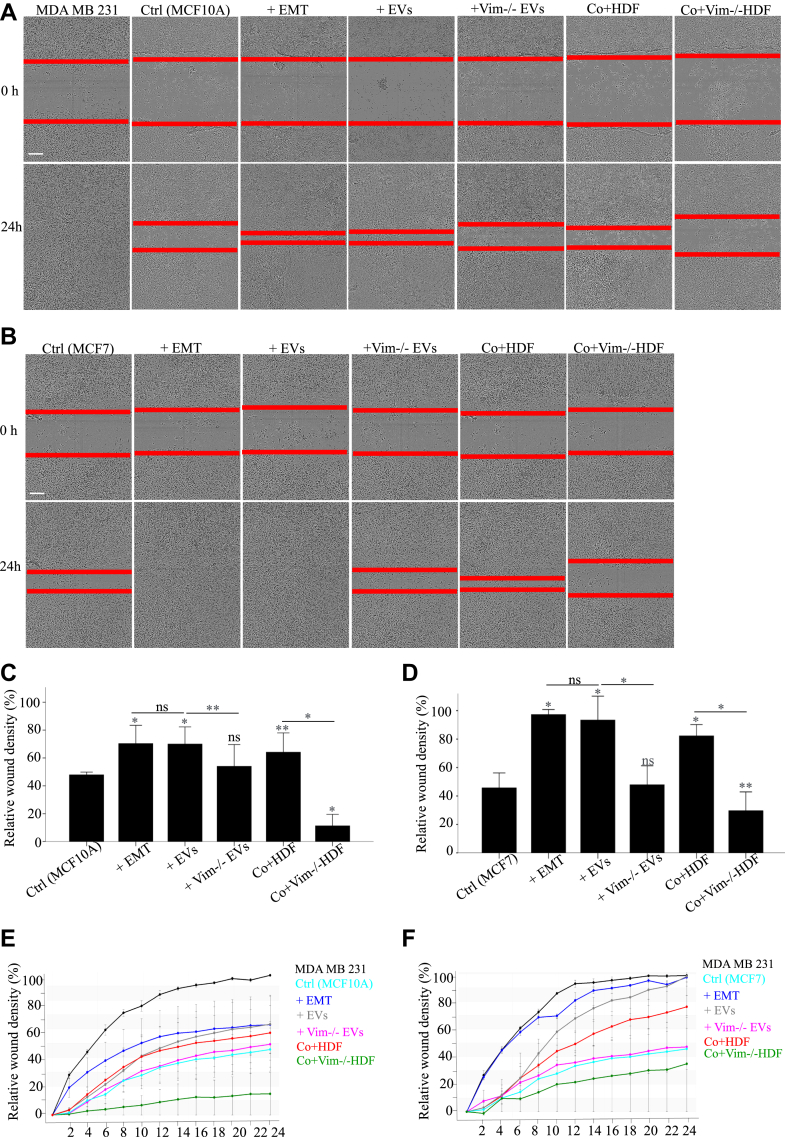
**EV-associated vimentin promotes cell migration**. Representative images of MCF10A (*A*) and MCF7 (*B*) cell migration following treatment with StemXVivo EMT Inducing media, EVs from HDF and vim −/− HDFs (100 μg/ml) and HDF and vim −/− HDFs conditioned media after 0 and 24 hours of scratch wounding. Relative wound density percentage of MCF10A (*C* and *E*) and MCF7 (*D* and *F*) cells. Cells without treatments and MDA MB231 have been used as negative and positive controls, respectively. The migration rate is represented as relative wound density in percentage. Each bar represents the mean ± SEM of three independent experiments. Treatment with WT-EVs and coculture with WT HDFs increased wound density and enhanced epithelial cell migration, equaling the efficiency of StemXVivo-mediated induction. Conversely, treatment with conditioned media from vim −/− HDFs reduced wound density, indicating inhibited migration, similar to results with vim −/− EVs. Both cell lines showed similar trends, with MCF10a cells generally migrating more slowly than MCF7 cells. Each treatment was compared first to untreated control and then to its related treatment group. The scale bar represents 500 μm. ∗*p* < 0.05, ∗∗*p* < 0.01, ∗∗∗*p* < 0.001. EMT, epithelial–mesenchymal transition; EV, extracellular vesicle.

As vim-positive HDFs and their EVs potently induced EMT epithelial cells, we wanted to assess the impact of HDF-derived conditioned media and their EVs on cell migration. Due to challenges encountered during the wound scratch assay using the transwell model, we adopted an alternative approach. We utilized conditioned media obtained from both HDFs and vim −/− HDFs to treat epithelial cells, thus approximating a coculture model. Treatment with WT-EVs and coculture with WT-HDFs resulted in increased wound density and enhanced migration of epithelial cells (Fig. 4). Notably, these treatments were equally efficient inducing EMT as the StemXVivo-mediated induction, highlighting the potential efficacy of WT-EVs in enhancing cell migration and proliferation. In contrast, we observed a reduction in wound density after treatment with conditioned media from vim −/− HDFs implying an inhibition of migratory potential. Similar results were obtained when cells were treated with vim −/− EVs. The outcome of the wound scratch assay conducted on both cell lines revealed similar trends with a generally slower rate of cell migration for MCF10a cells (Fig. 4, *A*, *C* and *E*) in comparison to MCF7 (Fig. 4, *B*, *D* and *F*).

### EV-Associated Vimentin Drives Cell Invasion

**Fig. 5:**
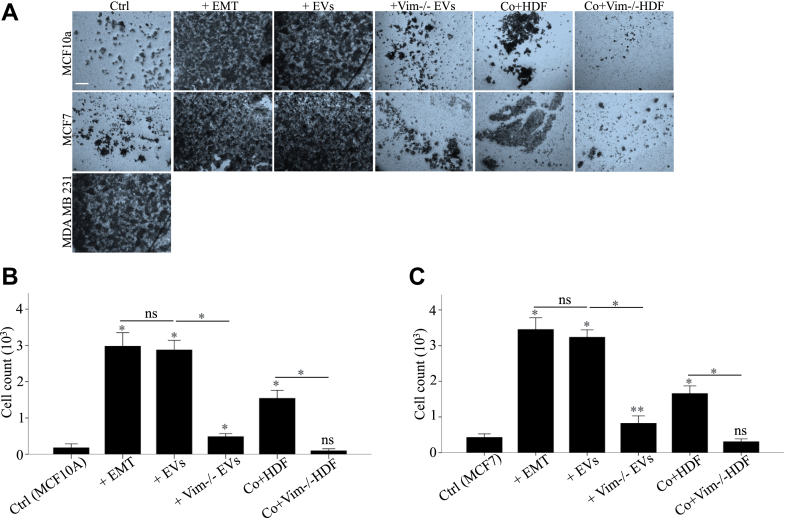
**EV-associated vimentin promotes cell invasion**. Representative images of MCF10A and MCF7 (*A*) cells following treatment with StemXVivo EMT Inducing media, EVs from HDF and vim −/− HDFs (100 μg/ml) and HDF and vim −/− HDFs conditioned media after 48 h of treatment. Cells without treatments and MDA MB231 have been used as negative and positive controls respectively. MTT colorimetric assay of MCF10A (*B*) and MCF7 (*C*) cells at 24, 48, and 72 h post treatment. WT EVs had a significant impact on invasion, similar to the effect of StemXVivo treatment. Coculture with WT HDFs resulted in a modest increase in invasion. In contrast, stimulation with vim −/− EVs and coculture with vim −/− HDFs did not promote invasion. Each treatment was compared first to untreated control and then to its related treatment group. The *scale bar* represents 100 μm. ∗*p* < 0.05, ∗∗*p* < 0.01, ∗∗∗*p* < 0.001. EMT, epithelial–mesenchymal transition; EV, extracellular vesicle; MTT, 3-[4.5-Dimethylthiazol-2-yl]-2.5-diphenyltetrazolium bromide.

To investigate cell invasion, we employed a transwell model with cell inserts. After 48 h of treatments, we quantified the number of cells that had invaded through the Matrigel. Both epithelial cell lines responded similarly to the treatments. WT EVs had a remarkable effect on the invasion corresponding to that of the StemXVivo treatment. Also, the coculture with WT HDFs gave a modest increase in invasion, whereas stimulation with vim −/− EVs and coculture with vim −/− HDFs did not stimulate invasion (Fig. 5).

### EV-Associated Vimentin Promotes MCF7 and MCF10a Proliferation

**Fig. 6:**
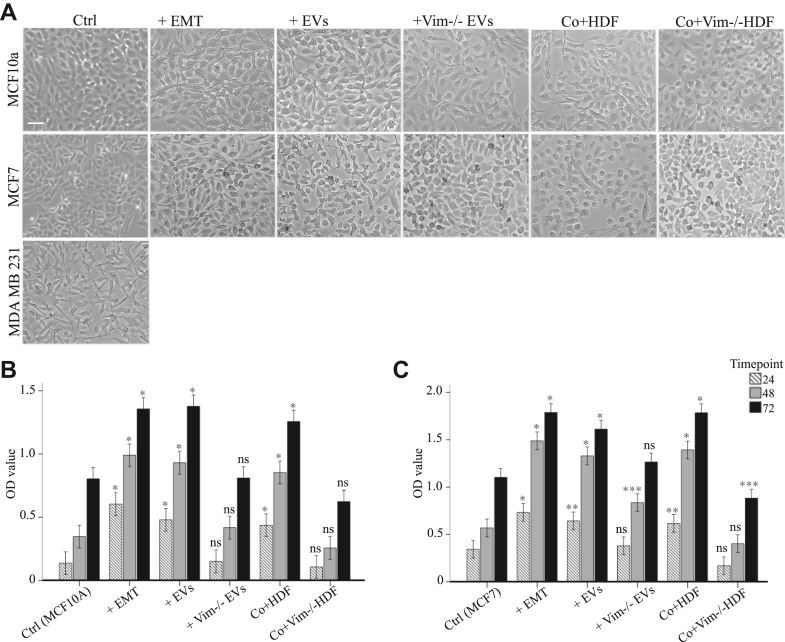
**EV-associated vimentin promotes MCF7 and MCF10a proliferation**. Representative images of MCF10A and MCF7 (*A*) cells following treatment with StemXVivo EMT Inducing media, EVs from HDF and vim −/− HDFs (100 μg/ml) and HDF and vim −/− HDFs conditioned media after 5 days of treatment. Cells without treatments and MDA MB231 have been used as negative and positive controls, respectively. MTT colorimetric assay of MCF10A (*B*) and MCF7 (*C*) cells at 24, 48, and 72 h post treatment. Cells exposed to WT-EVs and conditioned media from HDFs exhibited migration morphology and heightened proliferation rates similar to StemXVivo-treated cells. In contrast, cells treated with vim −/− EVs and conditioned media from vim −/− HDFs showed no significant differences compared to nontreated cells. The scale bar represents 100 μm. ∗*p* < 0.05, ∗∗*p* < 0.01, ∗∗∗*p* < 0.001. EMT, epithelial–mesenchymal transition; EV, extracellular vesicle; MTT, 3-[4.5-Dimethylthiazol-2-yl]-2.5-diphenyltetrazolium bromide.

To investigate the influence of fibroblast secretion, particularly extracellular vimentin, on epithelial cell proliferation, we monitored cell numbers using MTT assay at 24, 48, and 72-h intervals post treatment. The findings unveiled those cells exposed to WT-EVs and conditioned media from HDFs displayed migration morphology similar to that of StemXVivo-treated cells, concomitant with heightened proliferation rates (Fig. 6*A*). Intriguingly, MCF10A cells treated with vim −/− EVs and conditioned media from vim −/− HDFs, as well as MCF7 cells under similar treatments at certain time points, demonstrated no significant variance compared to nontreated cells (Fig. 6, *B* and *C*). This was particularly evident for all three time intervals in MCF10A cells and during the 24 and 72-h durations in MCF7 cells treated with vim −/− EVs, along with the 24 and 48-h time points in MCF7 cells treated with vim −/− HDFs' conditioned media.

### Fibroblast-Derived EVs Carry EMT-Associated Markers Including Vimentin

**Fig. 7:**
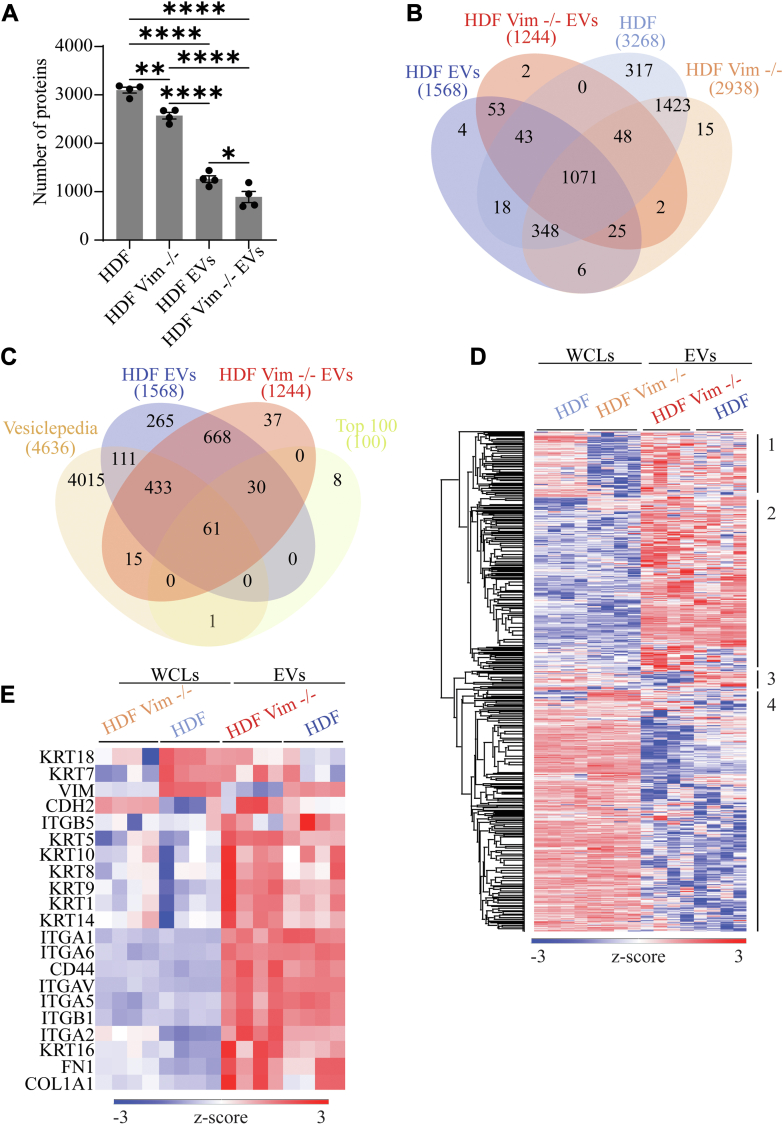
**Mass spectrometry-based profiling of HDF and HDF vim −/− and their EVs**. *A*, bar plot showing the total number of identified proteins, filtered for three values in at list one group in HDF, HDF vim −/−, and EVs. *B*, Venn diagram of averaged biological replicates illustrating differences in protein content in HDF, HDF vim −/−, and EVs. *C*, EV samples are compared with proteins annotated in vesiclepedia. *D*, hierarchical clustering of ANOVA significant proteins, calculated on imputed data, based on Euclidean distance, normalized by z-score and divided into four clusters. Clusters one and two contain proteins that are enriched in EVs, cluster three shows proteins enriched in WT samples only and cluster four comprises proteins enriched in whole cell lysate samples. *E*, filtered heatmap illustrating epithelial–mesenchymal transition (EMT) protein markers. Gene ontology analysis of each cluster can be found in Supplemental Figures S2 and S3. EV, extracellular vesicle.

Considering the striking effect observed in our coculture and EV treatments to cause behavior changes in the epithelial cells, we set out to investigate the protein cargo of WT and vim −/− HDFs and their derived EVs. First, we assessed the isolated EVs for the expression of markers associated with EMT, including vimentin, fibronectin, E-cadherin, and N-cadherin proteins using western blot. Both WT and HDF vim −/− EVs expressed fibronectin, E-cadherin, and N-cadherin proteins (Fig. 1*E*). Delving deeper, we assessed the isolated EVs for the expression of markers associated with EMT using a shotgun proteomics approach. As expected, the number of proteins identified in cell lysates exceeded that of their respective EVs (approximately double the amount), and we observed a good overlap of the sample groups (Fig. 7, *A* and *B*). Moreover, WT-derived EVs contained a higher number of proteins compared to vim −/− EVs, which was in line with the respective whole cell lysate samples (Fig. 7*A*). When we compared the EV cargos with vesiclepedia, publicly available collection of proteins present in EVs, we found that our samples contained nearly all of the top 100 proteins found in EVs (Fig. 7*C*). Notably, our EV datasets contain over 600 proteins not present in the database. Further inspection with gene ontology over-enrichment analysis revealed that these are part of cellular components such as ribosomes, cell-substrate junction, focal adhesion, extracellular space, and vesicles (Supplemental Fig. S1*A*) corresponding to biosynthesis-related processes (Supplemental Fig. S1*B*). Although some of these proteins might stem from cross-contamination during the protocol of EV isolation, much of this novel cargo warrants further inspection and should perhaps be included in current databases are EV-related proteins. To further understand the effects of EV protein cargo in inducing EMT, we conducted an ANOVA test on imputed data upon inspection of replicate grouping (Supplemental Fig. S1*C*) and performed hierarchical clustering. The clustering results show four distinct clusters: clusters one and two contain proteins that are highly enriched in EVs, while cluster three shows proteins enriched in WT samples only and cluster four comprises proteins enriched in whole cell lysate samples (Fig. 7*D*). Gene ontology analysis of each of these clusters showed that while the EV-rich clusters contain proteins involved wound healing, cell migration, and adhesion, they also contain proteins involved in the cell cycle (Supplemental Fig. S2, *A* and *B*). Moreover, we confirmed that these proteins are part of the extracellular matrix, EVs, and focal adhesions, while also containing nuclear proteins (Supplemental Fig. S3, *A* and *B*). Importantly, cluster three contains proteins involved with cytoskeletal organization (Supplemental Fig. S2*C*), belonging to cell components including lamellipodium, focal adhesion, and cell leading edge (Supplemental Fig. S3*C*). Finally, cluster four, the WCL-rich, contains proteins involved in translation, RNA processing, and macromolecule biosynthesis (Supplemental Figs. S2*D*, S3*D*).

Although we verified that EMT-associated markers such as keratin, integrin, and collagen were enriched in both EV types and saw the expected difference in vimentin expression (Fig. 7*E*), our previous results show that WT-derived EVs are better equipped to induce EMT. This may be due to the differences in cargo we observed, especially in clusters one and three on the hierarchical clustering (Fig. 7*D*), i.e. mitosis and cytoskeletal-related processes, respectively.

**Fig. 8:**
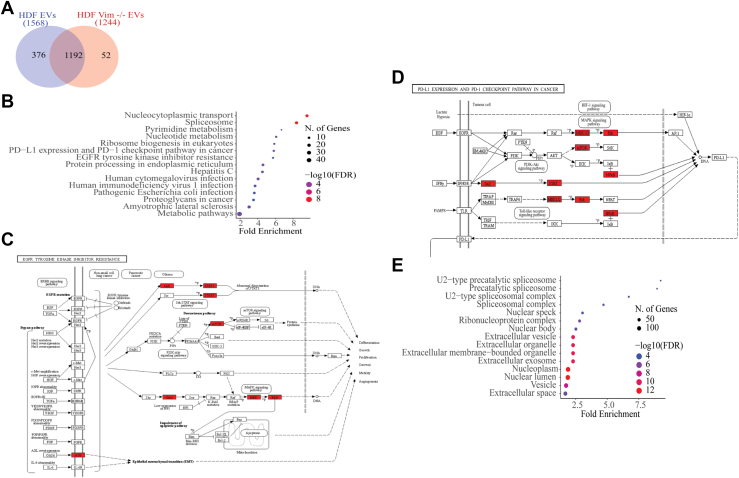
**EVs from HDF WT contain cargo that supports cancer-related activities**. *A*, Venn diagram of average biological replicates illustrating differences in protein content between HDF and HDF vim −/− EVs. *B–E*, functional enrichment analysis of proteins found only in WT EVs, conducted with ShinyGO 0.80. *B*, KEGG pathway enrichment analysis, showing significantly enriched pathways. *C*, visual representation of the KEGG pathway “EGFR tyrosine kinase inhibitor resistance” and (*D*) “PD-L1 expression and PD-1 checkpoint pathway in cancer,” with proteins found in the dataset highlighted in *red*. (*E*) GO enrichment analysis, representing enriched cellular components. KEGG, Kyoto Encyclopedia of Genes and Genomes; EV, extracellular vesicle; GO, gene ontology; EGFR, epidermal growth factor receptor.

Next, we wanted to understand what in the EV cargos could be playing additional roles to support EMT. We compared WT versus vim −/− derived EV cargo (Fig. 8*A*) and saw that 376 proteins appeared only in WT-derived EVs. Through gene ontology analysis, specifically Kyoto Encyclopedia of Genes and Genomes pathways, we found these proteins show enrichment for the pathways EGFR tyrosine kinase inhibitor resistance and PD-L1 expression and PD-1 checkpoint pathway in cancer, among others (Fig. 8, *B*–*D*) and contain proteins that are part of EVs and extracellular space (Fig. 8*D*). As tumor cells with higher expression levels of PD-L1 exhibit higher immunosuppressive activity, these results suggest that vimentin-containing vesicles might contribute to this phenomenon.

**Fig. 9:**
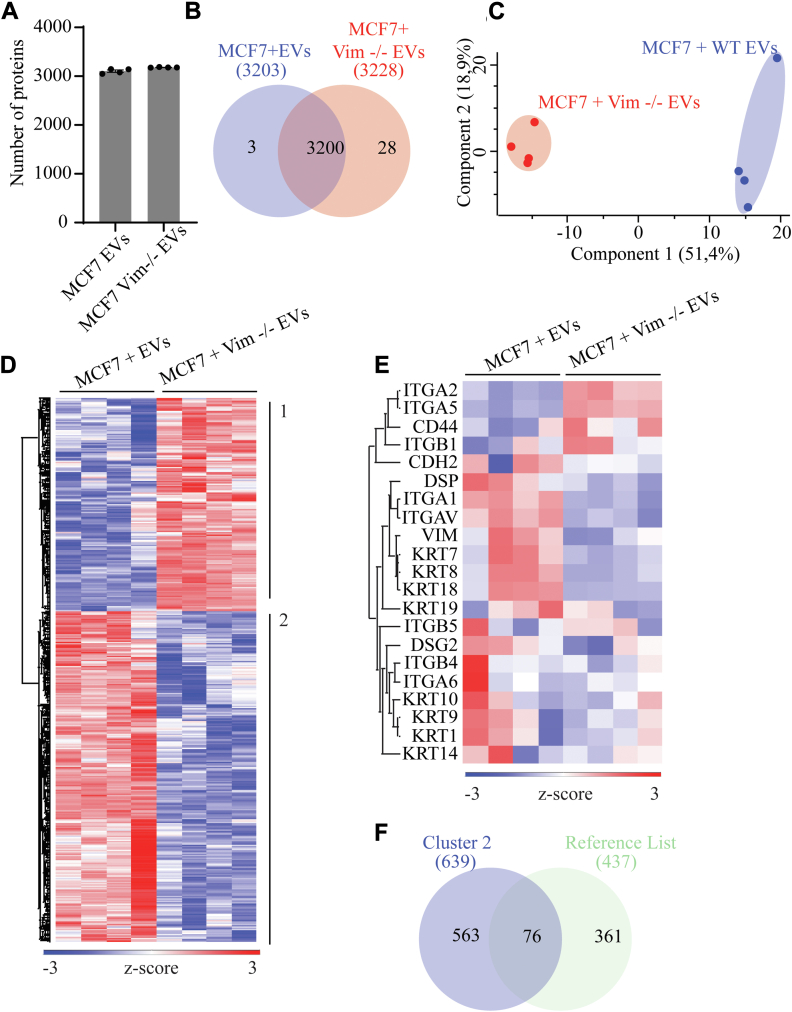
**Mass spectrometry-based profiling of MCF7 proteomes after treatment with EVs from HDF and vim −/− HDFs**. *A*, bar plot of the total number of identified proteins in each sample group. Data were filtered for three values in at least one group. *B*, Venn diagram depicting differences between sample groups. *C*, principal component analysis of imputed data used for statistical testing, showing the two distinct sample groups. *D*, hierarchical clustering of Student *t* test significant proteins, calculated from imputed data, based on Euclidean distance and divided into two clusters (EVs high and vim −/− EVs high). Data were normalized by z-score. *E*, filtered heatmap illustrating the epithelial–mesenchymal transition (EMT) protein markers in the dataset before statistical analysis. Gene ontology analysis of each cluster can be found in Supplemental Fig. S4. *F*, Venn diagram showing the overlap between proteins in cluster two (EVs high) of *D*. and a reference list of proteins comprised of proteins in only WT EVs (Fig. 8*A*) and proteins from cluster three (enriched in WT samples) of Fig. 7*D*. Gene ontology analysis of the 76 overlapping proteins can be found in Supplemental Fig. S5. EV, extracellular vesicle.

**Fig. 10:**
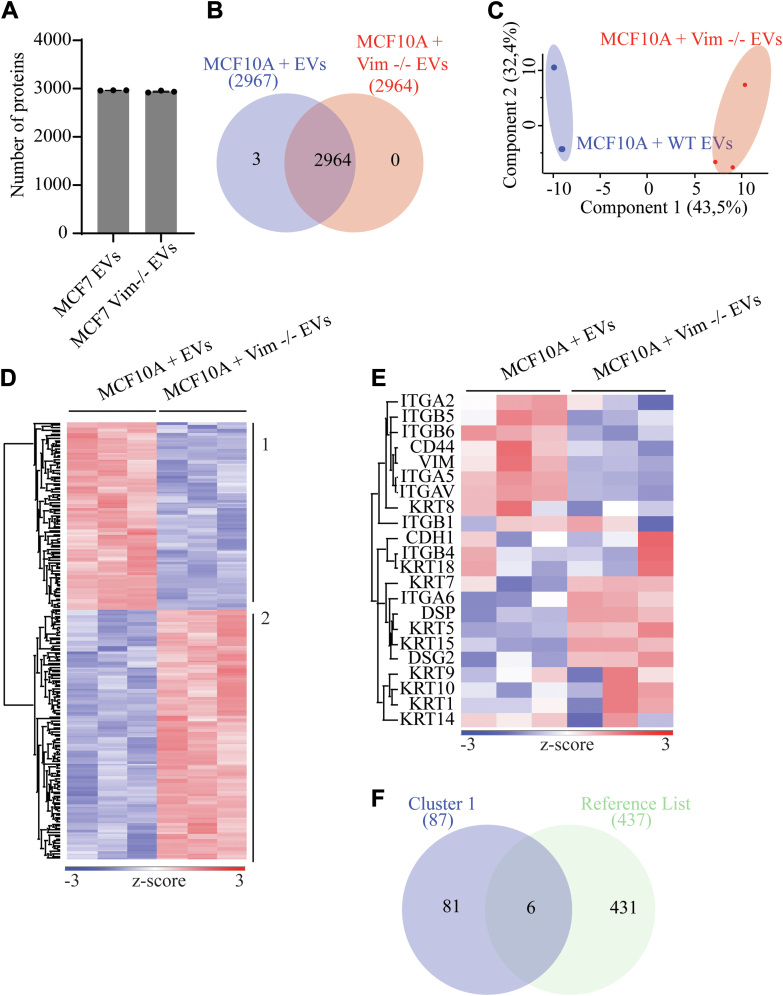
**Mass spectrometry-based profiling of MCF10A proteomes after treatment with EVs from HDF and vim −/− HDFs**. *A*, bar plot of the total number of identified proteins in each sample group. Data were filtered for three values in at least one group. *B*, Venn diagram depicting differences between sample groups. *C*, principal component analysis of imputed data used for statistical testing, showing the two distinct sample groups. *D*, hierarchical clustering of Student *t* test significant proteins, calculated from imputed data, based on Euclidean distance and divided into two clusters (EVs high and vim −/− EVs high). Data were normalized by z-score analysis. Gene ontology analysis of each cluster can be found in Supplemental Fig. S6. *E*, filtered heatmap illustrating the epithelial–mesenchymal transition (EMT) protein markers in the dataset before statistical analysis. *F*, Venn diagram showing the overlap between proteins in cluster one (EVs high) of (*D*) and a reference list of proteins comprised of proteins in only EVs from WT cells (Fig. 8*A*) and proteins from cluster three (enriched in WT samples) of Fig. 7*D*. EV, extracellular vesicle.

As we previously showed, after 24 h, cells treated with these EVs have taken up their cargo, which could explain, in part, the behavior changes observed upon treatment. To test this, we analyzed the differences in proteomes of MCF7 (Fig. 9) and MCF10A (Fig. 10) treated with WT or vim −/− EVs. Expectedly, the number of proteins is nearly the same, and the overlap between samples is seamless for both the MCF7 (Fig. 9, *A* and *B*) and MCF10A (Fig. 10, *A* and *B*) datasets. Similarly to the HDF dataset, we conducted statistical analysis on imputed data upon inspection of replicate grouping (Figs. 8*C* and 9*C*) and performed hierarchical clustering. The clustering results show two distinct clusters in both the cases, one EVs-high and one vim −/− EVs-high (Figs. 8*E* and 9*D*).

In MCF7 cells, while the vim −/− EVs-high cluster, i.e., cluster one, contains proteins involved in metabolic processes (Supplemental Fig. S4*A*) with EV- and extracellular proteins (Supplemental Fig. S4*B*), the EV-high cluster (cluster two) also contains proteins involved in translation and biosynthesis-related processes (Supplemental Fig. S4*C*). Moreover, besides EV- and extracellular-related proteins, the EV-high cluster also contains proteins from cellular components such as ribosomes, focal adhesions, and cell-substrate junctions (Supplemental Fig. S4*D*). These terms were also enriched in the EV cargo we analyzed above (Supplemental Fig. S1*A*) which suggests that EVs are uptaken by those cells and that EV cargo supports EMT-related functions. Although both sample groups possess EMT markers (Fig. 9*E*), albeit with different abundances, we see upregulation of vimentin, several integrins, and n-cadherin in cells treated with WT-derived EVs, not in the ones treated with vim −/− EVs. Also, upon the comparison of the EV-high cluster with a reference list of proteins comprised of proteins found in WT-derived EVs alone (Fig. 8*A*) together with proteins significantly more abundant in WT-derived EVs (cluster three of Fig. 7*D*), we saw a 76-protein overlap (Fig. 9). Notably, gene ontology analysis of these proteins (Supplemental Fig. S5, *A*–*C*) confirms the presence of proteins involved in the regulation of the actin cytoskeleton and proteins from PD-L1 expression and PD-1 checkpoint pathway in cancer, further highlighting the idea that WT-derived EVs have the potential to modulate EMT-related functions.

Looking at the MCF10A dataset, we see that the EV-high cluster (cluster one) contains proteins involved in the organization of actin cytoskeleton as well as cell adhesion and migration (Supplemental Fig. S6*A*), i.e., proteins belonging to focal adhesions, cell-substrate junctions, and actin cytoskeleton (Supplemental Fig. S6*B*), which are absent in the vim −/− high cluster (Fig. S6, *C* and *D*). Once again, both groups possess varied abundances of EMT markers, and cells treated with WT-EVs show upregulation of integrins and vimentin (Fig. 10*E*). However, when compared to the reference list of WT-derived EVs mentioned above, the MCF10A cells treated with WT-derived EVs show an overlap of just six proteins (CLTB, AP1M1, TWF2, TXNDC17, ARPC4, and ARL6IP5; Fig. 10*F*) involved in the regulation of the actin cytoskeleton and endocytosis. This could be attributed to the fact that the MCF10A dataset is much smaller than the MCF7 dataset due to harsher filtering strategies to compensate for lower sample quality. It is also important to consider the underlying differences between these two distinct cell lines, as MCF10A cells are normal epithelial cells, and MCF7 cells are ER- and PR- PR-positive epithelial breast cancer cells. Thus, the differences in MCF7 cells are likely more pronounced than the ones in MCF10A cells, which is in line with our previous findings (Fig. 2, Fig. 3, Fig. 4, Fig. 5, Fig. 6). Nevertheless, both MCF7 and MCF10A cells treated with WT-derived EVs show enrichment of processes regulating EMT activities.

Our analysis of EV protein cargo from WT and vim −/− HDFs revealed significant differences in their potential to induce EMT. The proteomic profiling of these EVs confirmed the presence of EMT-associated markers such as N-cadherin and fibronectin. Moreover, the presence of additional proteins unique to the WT-derived EVs, especially those involved in processes like cytoskeletal organization and cell adhesion, likely contributes to their enhanced capacity to drive EMT. Interestingly, our study identifies the presence of S100A4 protein in the WT EVs, which is known to regulate key EMT transcription factors, including ZEB1, ZEB2, SNAI1, SNAI2, TWIST1, and TWIST2. S100A4 plays a crucial role in activating these transcription factors, thereby promoting EMT and enhancing cellular plasticity (44, 45, 46, 47). As a known upstream regulator of EMT transcriptional programs, S100A4 may act as an effector molecule within EVs (Supplemental Table S1). In addition, the enrichment of protein involved in PD-L1 signaling in these EVs suggests that vimentin-positive EVs from WT fibroblasts are not only more effective at transmitting mesenchymal properties compared to their vim −/− counterparts but are also better equipped to support metastasis-related functions.

This finding underscores the direct influence of vimentin controlling EV cargo that can modulate recipient cell behavior and suggests an alternative regulatory layer influencing EMT, involving vimentin-mediated extracellular delivery of transcriptional regulators through EVs.

## Discussion

Our study reveals a novel role for extracellular vimentin in fibroblast-derived EVs as a driver of EMT. Although vimentin is a well-known mesenchymal marker and EMT regulator, its presence in EVs adds an extracellular dimension to its function. By showing that EV-associated vimentin induces EMT in epithelial cells, we highlight its role in cell-cell communication, particularly in the tumor microenvironment and wound healing.

Through comprehensive proteomic profiling of EVs, we revealed that vimentin-positive EVs derived from WT fibroblasts contain a distinct set of proteins enriched in EMT-related processes, such as cytoskeletal organization, cell adhesion, and ECM remodeling. Vim −/− EVs showed a marked depletion in these proteins, which strongly correlates with their reduced capacity to induce EMT in recipient cells. Notably, we identified over 600 proteins uniquely present in WT-derived EVs, many of which are involved in critical signaling pathways, such as the EGFR tyrosine kinase inhibitor resistance pathway and the PD-L1/PD-1 checkpoint pathway. This enrichment suggests that these vesicles are equipped not only to drive cellular changes typical of EMT but also to potentially contribute to immune modulation within the microenvironment.

Our proteomic analysis also confirmed that key EMT markers, such as N-cadherin and fibronectin, were more abundant in WT-derived EVs, reflecting the mesenchymal programming these vesicles can induce. The finding that these proteins are diminished in vim −/− EVs highlights the critical role of vimentin in determining EV cargo composition. Moreover, the identification of EMT-associated signaling molecules in the proteome of vimentin-positive EVs suggests that these vesicles may be able to propagate and reinforce EMT signaling networks across epithelial cell populations. This could have significant implications in pathological contexts such as cancer metastasis, where the creation of a supportive microenvironment is crucial for disease progression.

The functional assays in this study reinforce the importance of vimentin-positive EVs in promoting EMT. We observed that epithelial cells treated with WT EVs exhibited elevated levels of mesenchymal markers like N-cadherin and decreased levels of epithelial markers such as E-cadherin. These changes were consistent across multiple methods, including fluorescence microscopy and western blot analysis, indicating robust phenotypic shifts toward a mesenchymal state. In addition, treatments with WT EVs significantly enhanced cellular migration and invasion capabilities, while vim −/− EVs had a minimal impact. This suggests that extracellular vimentin may be a key regulator in EV-mediated EMT, possibly by influencing the loading or release of EMT-inducing proteins within EVs.

The findings from this study highlight several therapeutic opportunities centered around the modulation of extracellular vimentin and vimentin-positive EVs. Given their demonstrated role in driving epithelial–mesenchymal transition EMT, targeting these vesicles or their cargo could present a strategic pathway to influence EMT-related processes in various pathological conditions.

One promising therapeutic opportunity involves wound healing. Our results indicate that vimentin-positive EVs enhance the migratory and invasive properties of epithelial cells, key components of the wound healing process. By using vimentin-positive EVs or by modulating the levels of extracellular vimentin in EVs and thereby affecting their cargo, it may be possible to accelerate tissue regeneration and improve healing outcomes. Such approaches could potentially benefit patients suffering from chronic wounds, where delayed healing is a significant clinical challenge.

Beyond wound healing, vimentin-positive EVs also open avenues for targeting EMT in cancer progression and metastasis. The presence of proteins linked to immune regulation pathways like PD-L1/PD-1 in WT EVs is particularly noteworthy, as it hints at a potential mechanism by which these EVs may contribute to immune evasion and metastasis in cancer. Tumors that express higher levels of PD-L1 often exhibit increased immunosuppressive activity, allowing cancer cells to evade immune detection. The enrichment of these pathways in WT EVs implies that extracellular vimentin might play a multifaceted role in modulating both cellular plasticity and the immune landscape within the tumor microenvironment.

Furthermore, our study detected S100A4 in WT-EVs, which is a key regulator of EMT transcription factors that drive mesenchymal transitions. S100A4 activates EMT-associated genes like COL1A1 and α-SMA via the Wnt/β-catenin/TCF-4 pathway while suppressing epithelial markers such as E-cadherin (48). This mirrors observations in chronic inflammatory and fibrotic conditions, where S100A4-enriched EVs promote collagen deposition and ECM remodeling through matrix metalloproteinases activity (49). The presence of S100A4 and vimentin in WT-EVs suggests their combined role in shaping tumor plasticity and immune evasion (50).

Understanding the proteomic landscape of vimentin-positive EVs provides valuable insights into their role in EMT and provides mechanistic approaches to explore avenues for targeted therapeutic interventions. For instance, disrupting the formation or uptake of vimentin-containing EVs could be a promising strategy to inhibit EMT-driven cancer metastasis or fibrosis. By targeting these extracellular components, it might be possible to impair the signaling networks that drive pathological EMT, potentially improving treatment outcomes for cancer and other EMT-related conditions.

The observed differences in EV protein content between WT and vim −/− cells likely stem from vimentin multifaceted role in cellular processes. Beyond its cytoskeletal function, vimentin influences mTOR-dependent protein synthesis and cell size, and participates in EV biogenesis. Vimentin deficiency may disrupt intracellular trafficking pathways, unconventional protein secretion mechanisms, and Rab7a-mediated late endosomal maturation, ultimately altering both EV cargo composition and secretion efficiency.

The differential abundance of EMT-associated proteins between WT and vim −/− EVs raises interesting questions about how vimentin regulates the sorting and packaging of proteins into EVs. As vimentin has been shown to act as both a scaffold and modulator of many signaling proteins (10, 21, 22, 51), one possible explanation is that vimentin acts as a scaffold within fibroblasts, facilitating the selective loading of mesenchymal proteins into EVs.

Interestingly, in vim −/− EVs, we observed a reduction in CD63, a common exosomal marker, compared to WT EVs. This decrease is likely attributable to the structural and trafficking roles vimentin plays within the cell, potentially disrupting endosomal trafficking, exosome biogenesis, and membrane dynamics, leading to a reduction in CD63+ EVs. However, our prior EV labeling and uptake assays demonstrated successful uptake of both WT and vim −/− EVs in recipient cells, suggesting that alternative mechanisms may compensate for the reduced CD63 in facilitating EV uptake.

Another intriguing observation from our study was the expression of keratins in vim −/− fibroblasts and their derived EVs. Although fibroblasts are not typically known for expressing high levels of keratins, the absence of vimentin could lead to compensatory upregulation of other structural proteins. This may indicate a broader role for vimentin in maintaining cytoskeletal integrity and determining the composition of EVs. However, the potential for contamination from skin keratins cannot be entirely ruled out and warrants further investigation.

Although vimentin presence in EVs adds an extracellular dimension to its function, its specific role in mediating EMT through EVs is likely dependent on its dynamic interactions during EV biogenesis rather than its mere presence. Future studies exploring controlled models of vimentin incorporation in EVs could provide additional mechanistic insights into its role in EMT.

## Conclusions

The proteomic results from this study reveal significant insights into the role of vimentin-positive EVs in regulating critical cellular processes such as EMT and immune modulation. The identification of a specific enrichment of EMT-associated proteins and immunomodulatory signals, such as PD-L1, within these EVs suggests that extracellular vimentin may be a key player in driving both cellular migration and immune evasion in the tumor microenvironment.

These findings have several important ramifications. First, they highlight extracellular vimentin as a potential biomarker for aggressive cancer phenotypes characterized by enhanced migratory capabilities and resistance to immune surveillance. The presence of PD-L1 in vimentin-positive EVs also suggests a link between these vesicles and the suppression of antitumor immune responses, which could contribute to immune escape and facilitate metastatic spread. This reinforces the need to explore extracellular vimentin and its associated EVs as targets for immunotherapeutic interventions, potentially improving the efficacy of current immune checkpoint inhibitors.

Moreover, the role of vimentin-positive EVs in wound healing adds a new dimension to our understanding of tissue regeneration. By enhancing the migratory and invasive properties of epithelial cells, these vesicles may play a crucial role in orchestrating the wound repair process. This opens up therapeutic avenues to modulate EV-mediated signaling to accelerate healing, especially in chronic or nonhealing wounds where impaired cell migration is a known barrier.

In summary, the proteomic characterization of vimentin-positive EVs not only broadens our knowledge of the molecular drivers of EMT and immune modulation but also provides a promising target for therapeutic interventions in both cancer and regenerative medicine. Future studies should aim to develop targeted strategies to modulate these vesicles, thereby mitigating metastatic progression and enhancing wound healing, potentially leading to novel treatments for cancer patients and those with chronic wounds.

## Data availability

The mass spectrometry proteomics data generated in this study have been deposited in the ProteomeXchange Consortium via the PRIDE partner repository with the dataset identifier PXD060764. All other relevant data supporting the findings of this study are available from the corresponding authors upon reasonable request. Supplementary datasets and additional materials are included with the online version of the manuscript.

## Supplemental Data

This article contains supplemental data.

## Conflict of Interest

The authors declare no competing interests.
