# Supplementary material for: Extracellular Vesicles Bearing Vimentin Drive Epithelial–Mesenchymal Transition
Source: Mol Cell Proteomics. 2025 Jul 4;24(12):101028. doi: 10.1016/j.mcpro.2025.101028 (PMC12719745; doi:10.1016/j.mcpro.2025.101028)
Supplement: Supplemental Figures [file mmc1.docx]

**Supplementary figures**


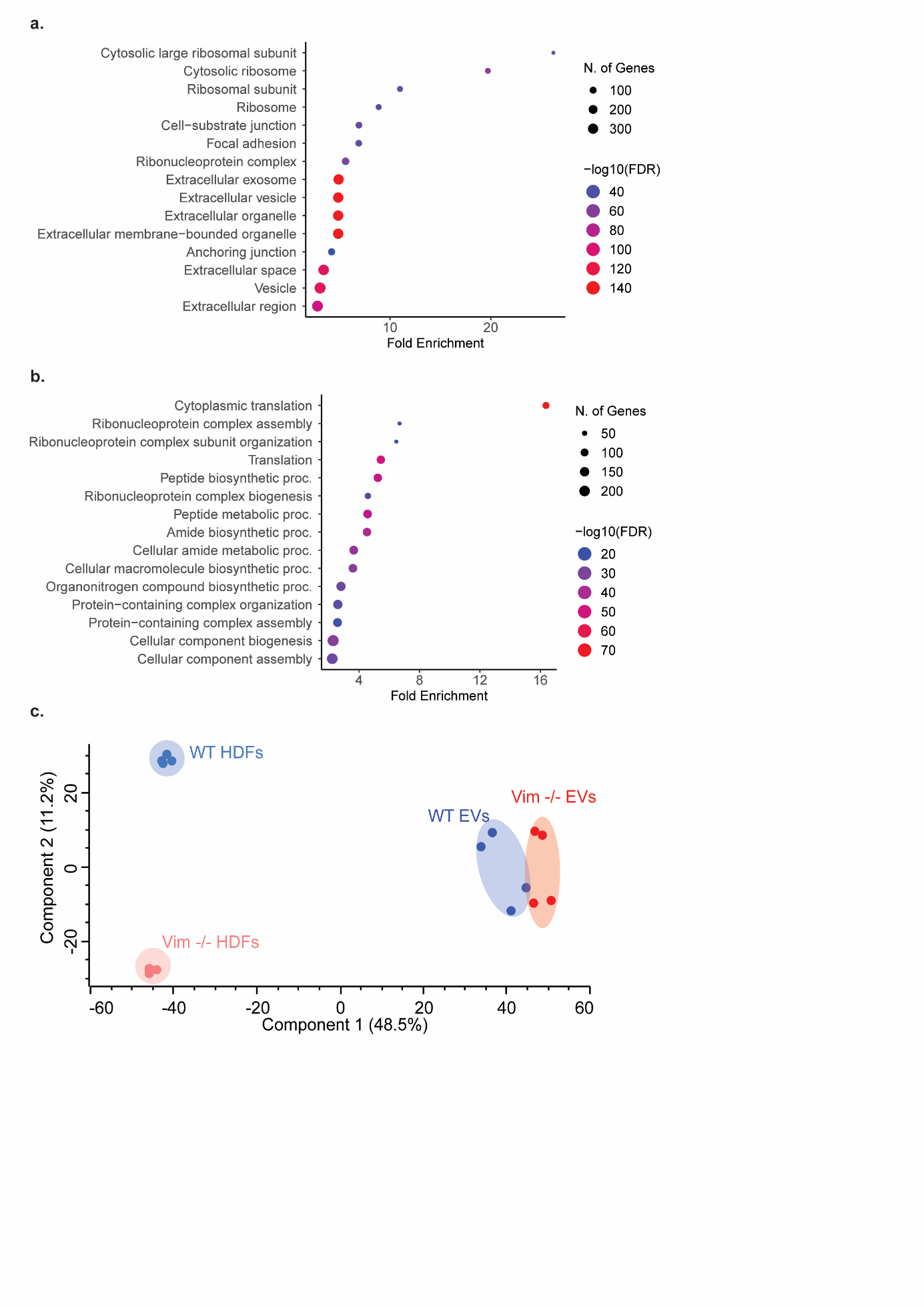


Figure S1. Gene ontology analysis for (a) cellular component and (b) biological processing of the 668 proteins present in EVs and absent in the Vesiclepedia database from Fig.1c. (c) Principal component analysis of imputed data used for ANOVA testing.

**
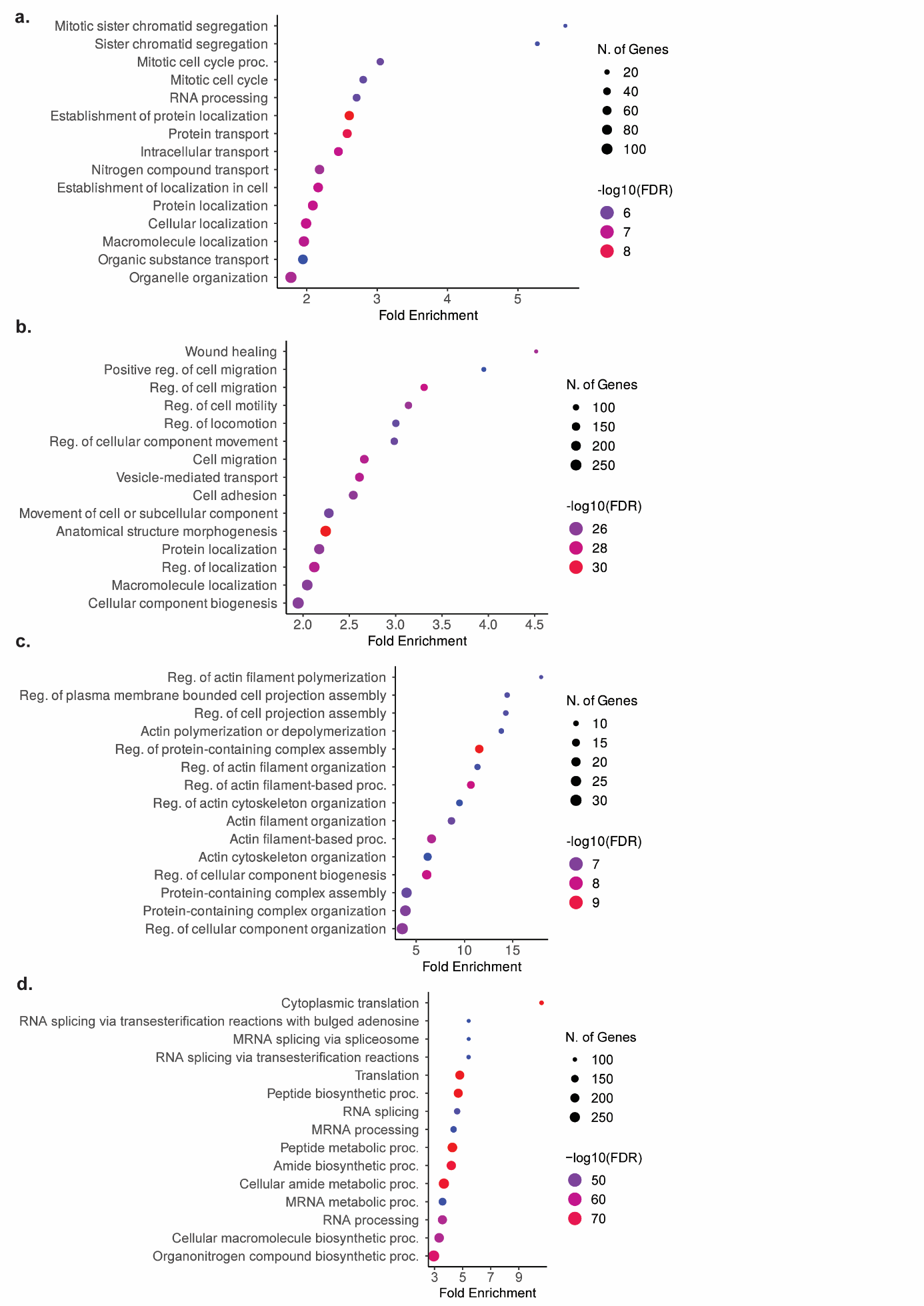
**

Figure S2. Gene ontology enrichment analysis of proteins in the clusters (a) 1, (b) 2, (c) 3, and (d) 4 from Fig.4d. Analysis was conducted with ShinyGO 0.80 and represents biological processes.


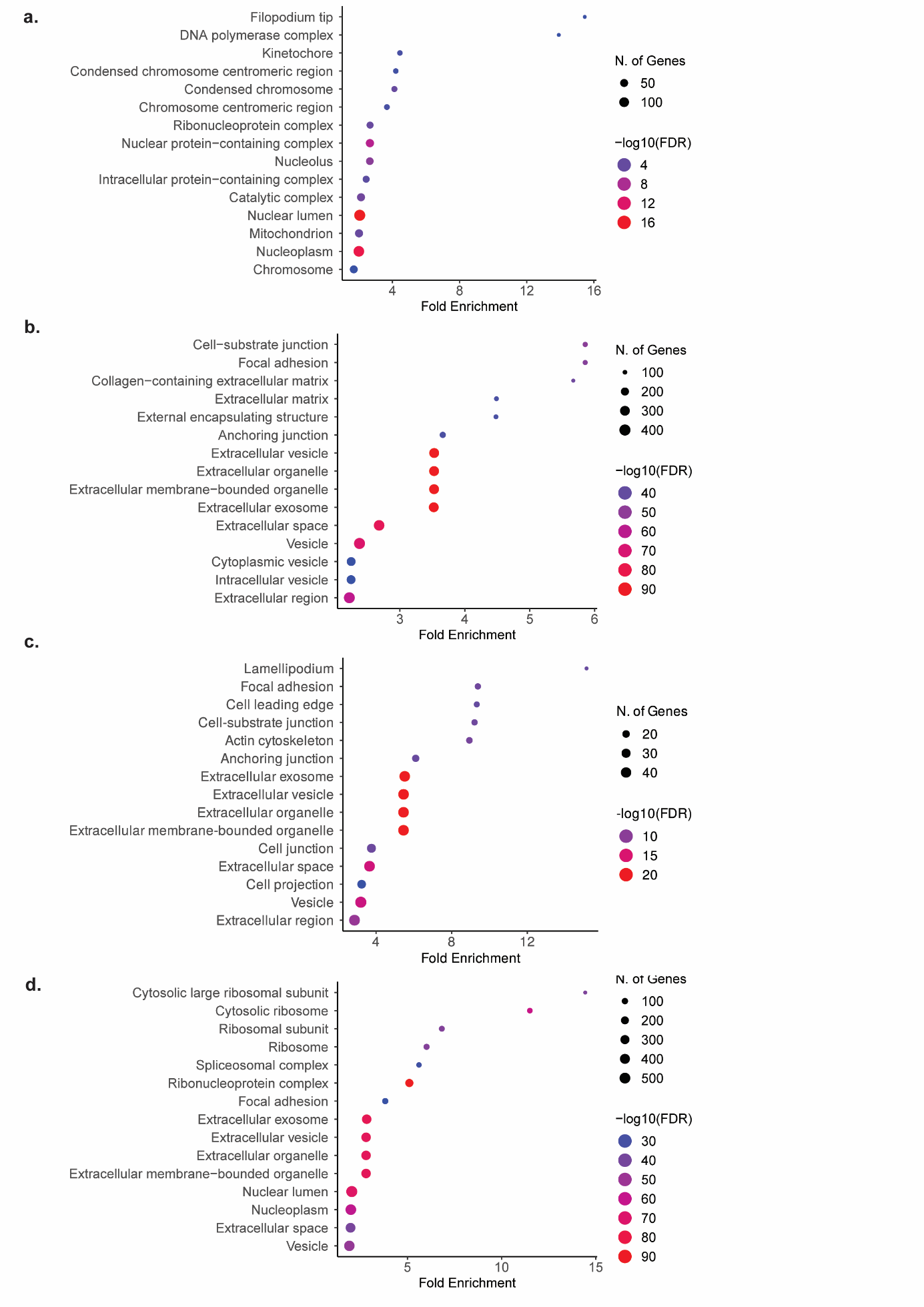


Figure S3. Gene ontology enrichment analysis of proteins in the clusters (a) 1, (b) 2, (c) 3, and (d) 4 from Fig. 4d. Analysis was conducted with ShinyGO 0.80 and represents cellular components.

**
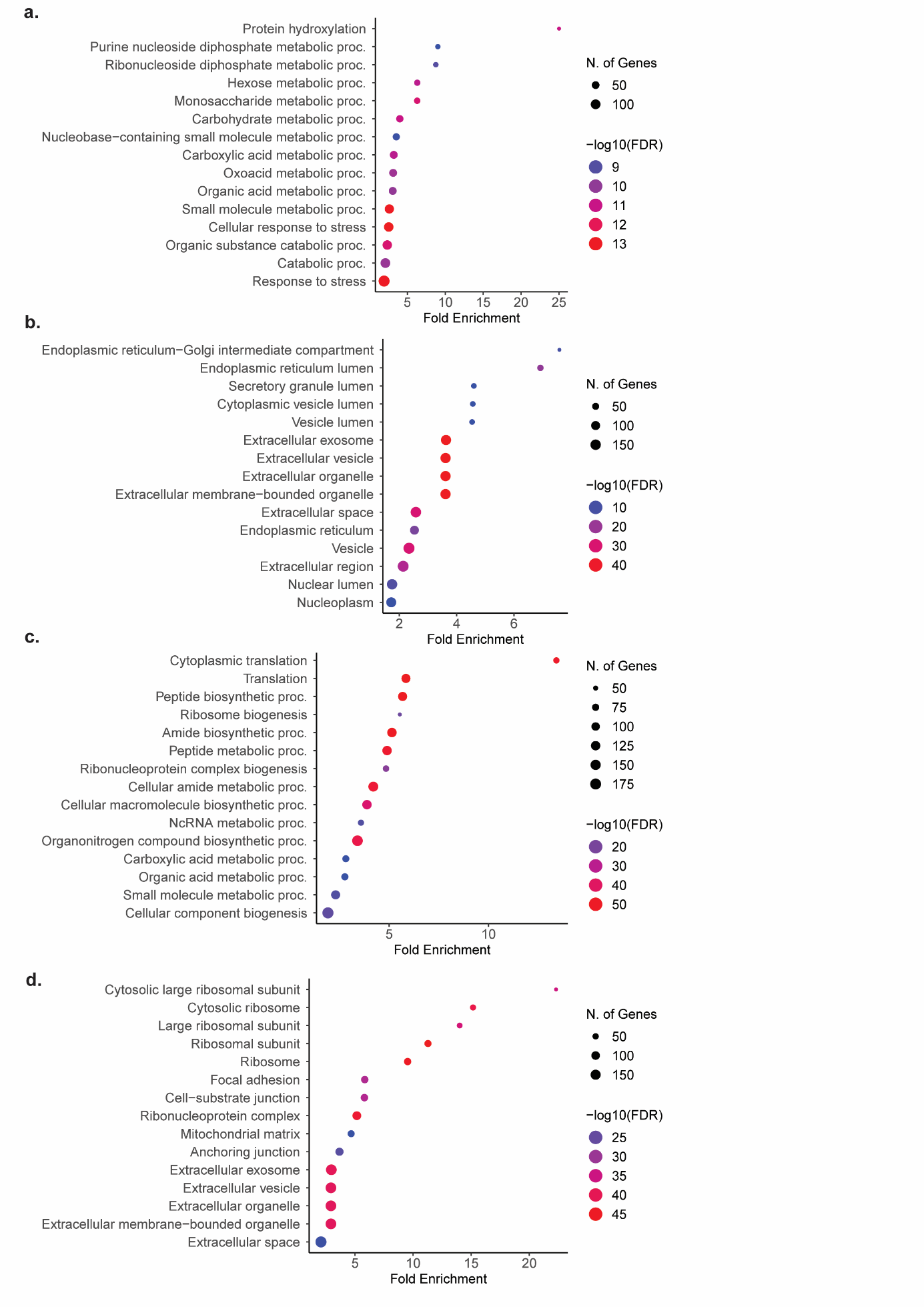
**

Figure S4. Gene ontology enrichment analysis of proteins in clusters (a-b) 1 and (c-d) 2 from Fig.9 (MCF7). Analysis was conducted with ShinyGO 0.80 and represents (a,c) biological processes and (b,d) cellular components.


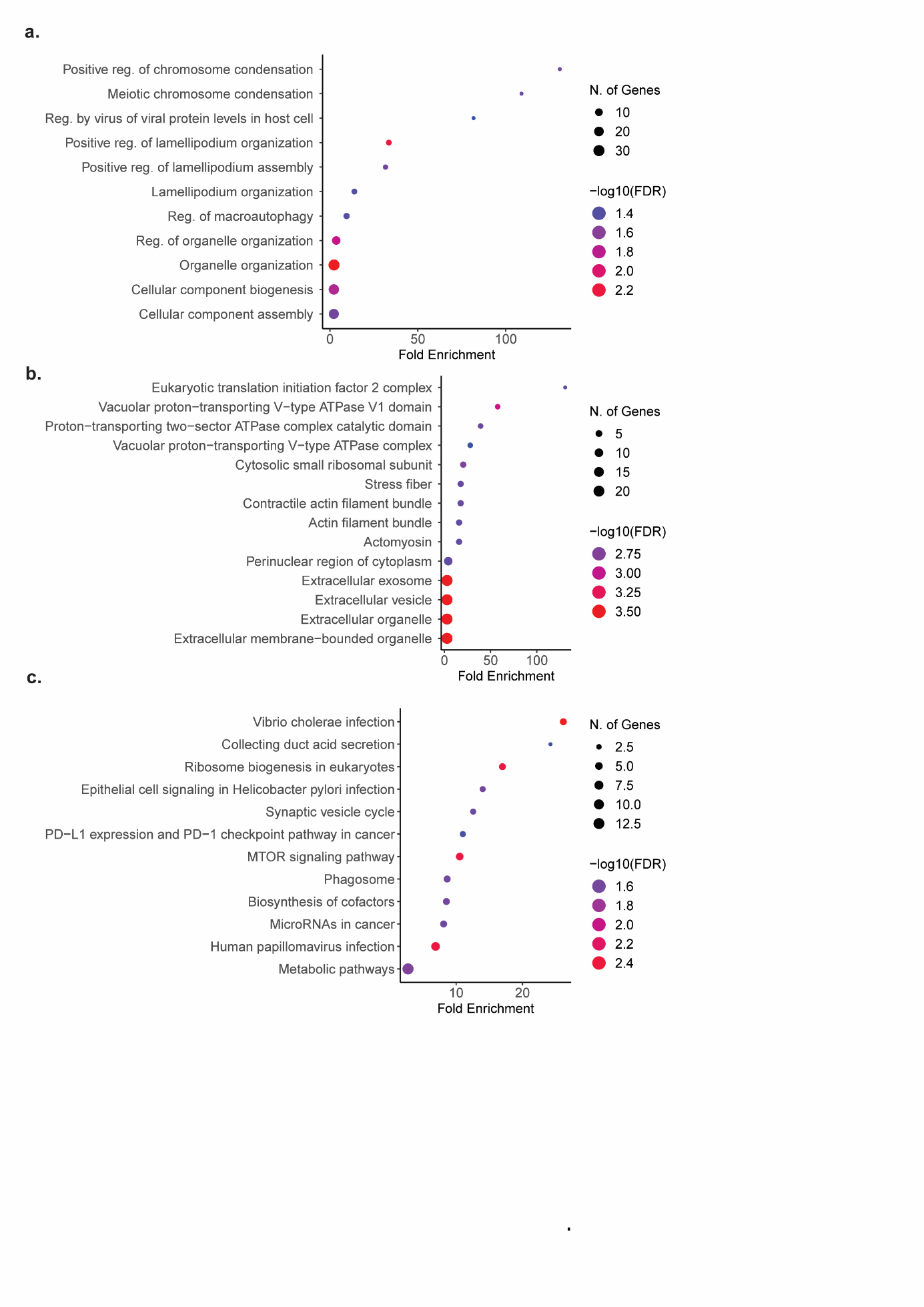


Figure S5. Gene ontology analysis for (a) cellular component and (b) biological processing (c) KEEG pathway of the 76 proteins from the Venn diagram in Fig. 9f.


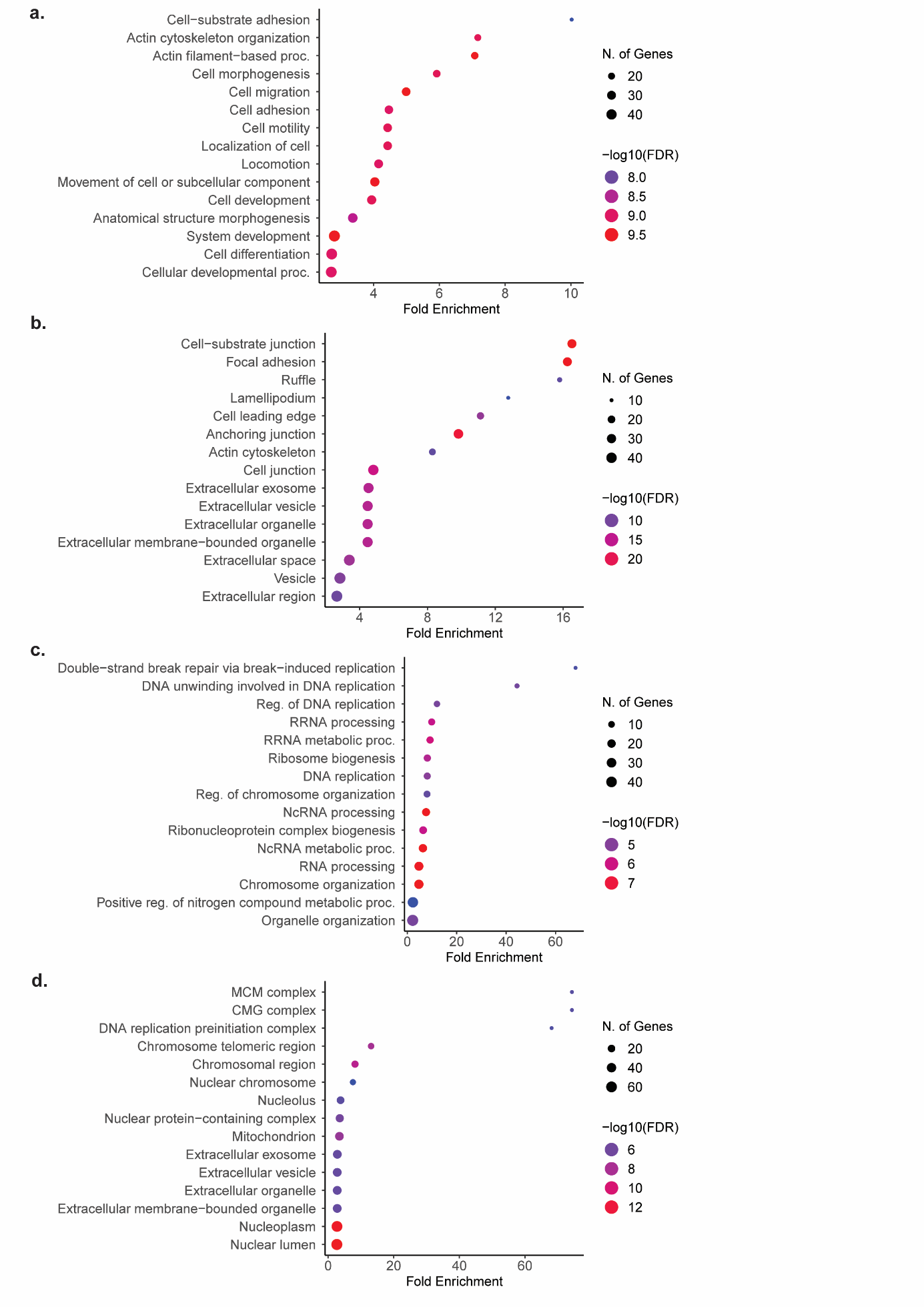


Figure S6. Gene ontology enrichment analysis of proteins in clusters (a-b) 1 and (c-d) 2 from Fig.10 (MCF10A). Analysis was conducted with ShinyGO 0.80 and represents (a,c) biological processes and (b,d) cellular components.
