# Supplementary material for: Extracellular Vesicles Bearing Vimentin Drive Epithelial–Mesenchymal Transition
Source: Mol Cell Proteomics. 2025 Jul 4;24(12):101028. doi: 10.1016/j.mcpro.2025.101028 (PMC12719745; doi:10.1016/j.mcpro.2025.101028)
Supplement: Supplemental Data 1 [file mmc4.pdf]

Sheet 1

GENE SYMBOL	NUMBER OF TIMES IDENTIFIED
CD63	1119
CD9	972
PDCD6IP	884
TSG101	834
CD81	704
GAPDH	593
FLOT1	549
ACTB	538
ANXA2	514
SDCBP	481
HSP90AA1	457
HSPA8	453
ANXA5	447
ENO1	441
PKM	436
HSP90AB1	422
YWHAZ	420
PGK 1.00	408
YWHAE	404
FLOT2	404
ANXA1	400
VCP	396
PPIA	395
EEF1A1	394
ALB	391
ALDOA	386
ITGB1	383
MYH9	377
CLTC	376

TPI1	374
CFL1	371
EEF2	370
MSN	369
GNB1	367
PRDX1	366
ANXA6	363
SLC3A2	363
EZR	357
LDHA	356
LDHB	355
Bsg	354
Cdc42	354
PFN1	352
ATP1A1	351
ACTN4	351
HSPA1A	350
FLNA	348
YWHAB	348
GNAI2	347
YWHAQ	346
FASN	344
CLIC1	340
PRDX2	336
GSN	333
CCT2	333
RAB5C	333
HIST1H4A	333
RAP1B	333
GNB2	332
LGALS3BP	332
YWHAG	331

RAB10	330
HLA-A	329
ACTN1	328
ANXA7	328
FN1	327
TFRC	323
ran	322
GDI2	322
CCT3	320
AHCY	318
HSPA5	317
CCT4	316
ACLY	314
C3	312
Uba1	311
ANXA11	310
TUBB4B	309
KPNB1	309
CAP1	309
Rac1	308
MFGE8	307
TCP1	306
RHOA	304
TLN1	303
CCT6A	302
GNAS	302
Cct5	301
Rala	300
EHD1	300
CCT8	298
PGAM1	298
IQGAP1	297

VCL	297
GPI	294
EIF4A1	294
RAB7A	293
EEF1G	293
ADAM10	292
A2M	290
