# Supplementary material for: Extracellular Vesicles Bearing Vimentin Drive Epithelial–Mesenchymal Transition
Source: Mol Cell Proteomics. 2025 Jul 4;24(12):101028. doi: 10.1016/j.mcpro.2025.101028 (PMC12719745; doi:10.1016/j.mcpro.2025.101028)
Supplement: Supplemental Data 3 [file mmc6.pdf]

sheet 3

HDF_EV_1 MaxLFQ Intensity	HDF_EV_2 MaxLFQ Intensity	HDF_EV_3 MaxLFQ Intensity	HDF_EV_4 MaxLFQ Intensity	HDF_EV_KO_1 MaxLFQ Intensity	HDF_EV_KO_2 MaxLFQ Intensity	HDF_EV_KO_3 MaxLFQ Intensity	HDF_EV_KO_4 MaxLFQ Intensity	HDF_KO_1 MaxLFQ Intensity	HDF_KO_2 MaxLFQ Intensity	HDF_KO_3 MaxLFQ Intensity	HDF_KO_4 MaxLFQ Intensity
29.0717	29.0055	30.0029	29.819	#NUM!	28.6302	#NUM!	#NUM!	30.0626	29.9319	29.7319	29.6637
#NUM!	#NUM!	#NUM!	#NUM!	#NUM!	#NUM!	#NUM!	#NUM!	#NUM!	#NUM!	#NUM!	#NUM!
#NUM!	#NUM!	#NUM!	#NUM!	#NUM!	#NUM!	#NUM!	#NUM!	#NUM!	#NUM!	#NUM!	26.8267
#NUM!	#NUM!	#NUM!	#NUM!	#NUM!	#NUM!	#NUM!	#NUM!	#NUM!	27.5349	#NUM!	27.4944
#NUM!	#NUM!	#NUM!	#NUM!	#NUM!	#NUM!	#NUM!	#NUM!	#NUM!	#NUM!	26.869	26.7474
28.6221	28.717	28.1434	#NUM!	#NUM!	27.6406	#NUM!	#NUM!	#NUM!	#NUM!	#NUM!	#NUM!
#NUM!	#NUM!	#NUM!	#NUM!	#NUM!	#NUM!	#NUM!	#NUM!	#NUM!	#NUM!	27.7321	#NUM!
31.4805	31.523	28.6471	28.6934	30.0487	31.0446	29.4684	31.7495	28.8749	29.2068	28.7002	28.952
29.334	28.9714	#NUM!	27.7393	#NUM!	29.0546	27.9526	29.6309	#NUM!	28.715	28.6954	28.3621
31.6316	31.7265	29.8632	29.7106	31.4038	30.8642	31.3731	31.4971	#NUM!	#NUM!	#NUM!	#NUM!
31.9374	31.991	32.9524	33.8527	29.7596	#NUM!	29.4699	#NUM!	#NUM!	#NUM!	#NUM!	#NUM!
#NUM!	#NUM!	#NUM!	#NUM!	#NUM!	#NUM!	#NUM!	#NUM!	#NUM!	#NUM!	#NUM!	26.3577
#NUM!	#NUM!	#NUM!	#NUM!	#NUM!	#NUM!	#NUM!	#NUM!	#NUM!	#NUM!	#NUM!	#NUM!
#NUM!	#NUM!	#NUM!	#NUM!	#NUM!	#NUM!	#NUM!	#NUM!	#NUM!	#NUM!	#NUM!	#NUM!
27.1809	27.1762	#NUM!	#NUM!	#NUM!	#NUM!	#NUM!	#NUM!	26.8552	26.8546	27.2514	27.4052
#NUM!	#NUM!	#NUM!	#NUM!	#NUM!	#NUM!	#NUM!	#NUM!	25.5736	25.0975	25.5579	25.4215
#NUM!	#NUM!	#NUM!	#NUM!	#NUM!	#NUM!	#NUM!	#NUM!	#NUM!	#NUM!	#NUM!	#NUM!
28.9636	29.2487	28.3329	28.3982	#NUM!	#NUM!	#NUM!	27.7842	#NUM!	#NUM!	#NUM!	#NUM!
#NUM!	#NUM!	#NUM!	#NUM!	#NUM!	#NUM!	#NUM!	#NUM!	#NUM!	25.1901	25.289	25.3415
#NUM!	#NUM!	#NUM!	#NUM!	#NUM!	#NUM!	#NUM!	#NUM!	#NUM!	#NUM!	26.2952	26.9806
#NUM!	#NUM!	#NUM!	#NUM!	#NUM!	#NUM!	#NUM!	#NUM!	#NUM!	#NUM!	#NUM!	#NUM!
#NUM!	#NUM!	#NUM!	#NUM!	#NUM!	#NUM!	#NUM!	#NUM!	#NUM!	#NUM!	#NUM!	#NUM!
29.6026	29.7446	29.1825	29.1066	29.1078	28.2486	29.0007	28.1616	#NUM!	#NUM!	#NUM!	#NUM!
#NUM!	#NUM!	#NUM!	#NUM!	#NUM!	#NUM!	#NUM!	#NUM!	#NUM!	#NUM!	#NUM!	#NUM!
#NUM!	#NUM!	#NUM!	#NUM!	#NUM!	#NUM!	#NUM!	#NUM!	25.7888	#NUM!	#NUM!	25.3718
28.5008	28.7401	26.7628	#NUM!	28.2084	29.0742	#NUM!	29.9463	#NUM!	#NUM!	#NUM!	#NUM!
#NUM!	#NUM!	#NUM!	#NUM!	#NUM!	#NUM!	#NUM!	#NUM!	#NUM!	#NUM!	#NUM!	26.301
27.8705	27.6189	25.905	25.9434	27.8152	28.2756	#NUM!	27.2946	31.5538	30.7771	30.5119	30.8791
#NUM!	#NUM!	#NUM!	#NUM!	#NUM!	#NUM!	#NUM!	#NUM!	25.4184	25.3532	25.0473	24.9095
27.4064	27.6202	26.5389	#NUM!	#NUM!	#NUM!	#NUM!	#NUM!	#NUM!	#NUM!	#NUM!	#NUM!
29.4518	29.4427	28.9646	29.3312	28.7168	29.6886	#NUM!	28.9588	#NUM!	#NUM!	#NUM!	#NUM!
28.7227	28.2288	27.544	27.4489	#NUM!	28.7011	#NUM!	28.0044	#NUM!	#NUM!	#NUM!	#NUM!
29.3779	29.6736	29.5526	28.9073	29.1082	#NUM!	28.9444	28.416	#NUM!	#NUM!	#NUM!	#NUM!
#NUM!	#NUM!	#NUM!	#NUM!	#NUM!	#NUM!	#NUM!	#NUM!	26.4933	25.7748	#NUM!	#NUM!
#NUM!	#NUM!	#NUM!	#NUM!	#NUM!	#NUM!	#NUM!	#NUM!	#NUM!	#NUM!	#NUM!	#NUM!
#NUM!	#NUM!	#NUM!	#NUM!	#NUM!	#NUM!	#NUM!	#NUM!	25.7204	26.3088	25.5654	25.6687
29.2926	29.7279	29.2229	28.9228	29.8822	29.7107	29.8853	29.077	#NUM!	#NUM!	#NUM!	#NUM!
#NUM!	#NUM!	#NUM!	#NUM!	#NUM!	#NUM!	#NUM!	#NUM!	#NUM!	#NUM!	#NUM!	#NUM!
#NUM!	#NUM!	#NUM!	#NUM!	#NUM!	#NUM!	#NUM!	#NUM!	26.4194	#NUM!	26.5437	26.9233
33.2335	33.4789	29.1955	#NUM!	29.7628	33.5544	#NUM!	#NUM!	#NUM!	29.5628	30.7153	30.314
#NUM!	#NUM!	#NUM!	#NUM!	#NUM!	#NUM!	#NUM!	#NUM!	26.3749	26.2696	25.3786	#NUM!
29.0328	29.199	29.1714	29.0457	30.57	#NUM!	30.4733	29.4134	#NUM!	#NUM!	#NUM!	#NUM!
#NUM!	#NUM!	#NUM!	#NUM!	#NUM!	#NUM!	#NUM!	#NUM!	#NUM!	#NUM!	#NUM!	#NUM!
#NUM!	#NUM!	#NUM!	#NUM!	#NUM!	#NUM!	#NUM!	#NUM!	#NUM!	#NUM!	#NUM!	25.9297
29.0469	29.0638	#NUM!	#NUM!	#NUM!	#NUM!	#NUM!	#NUM!	26.0877	26.3617	26.794	26.8387
#NUM!	#NUM!	#NUM!	#NUM!	#NUM!	#NUM!	#NUM!	#NUM!	#NUM!	#NUM!	#NUM!	#NUM!
#NUM!	#NUM!	#NUM!	#NUM!	#NUM!	#NUM!	#NUM!	#NUM!	#NUM!	#NUM!	#NUM!	#NUM!
#NUM!	#NUM!	#NUM!	#NUM!	28.7152	28.3969	28.7794	28.5324	#NUM!	#NUM!	#NUM!	#NUM!
#NUM!	27.9304	#NUM!	#NUM!	#NUM!	#NUM!	#NUM!	#NUM!	27.1081	27.5711	27.6587	27.5859
#NUM!	#NUM!	#NUM!	#NUM!	#NUM!	#NUM!	#NUM!	#NUM!	#NUM!	25.572	25.7544	25.8748
#NUM!	#NUM!	#NUM!	#NUM!	#NUM!	#NUM!	#NUM!	#NUM!	#NUM!	26.1992	#NUM!	26.4536
#NUM!	#NUM!	#NUM!	#NUM!	#NUM!	#NUM!	#NUM!	#NUM!	#NUM!	#NUM!	#NUM!	#NUM!
29.8609	29.8999	28.565	28.4969	#NUM!	30.2499	#NUM!	#NUM!	#NUM!	#NUM!	#NUM!	#NUM!
#NUM!	#NUM!	#NUM!	27.8063	#NUM!	#NUM!	#NUM!	#NUM!	25.639	25.5819	#NUM!	#NUM!
#NUM!	#NUM!	#NUM!	#NUM!	#NUM!	#NUM!	#NUM!	#NUM!	#NUM!	#NUM!	#NUM!	#NUM!
#NUM!	#NUM!	#NUM!	#NUM!	#NUM!	#NUM!	#NUM!	#NUM!	#NUM!	#NUM!	24.8489	#NUM!
#NUM!	#NUM!	#NUM!	#NUM!	#NUM!	#NUM!	#NUM!	#NUM!	25.1993	25.0563	24.4074	24.8948
29.3337	29.4571	27.6337	27.3222	#NUM!	29.6243	#NUM!	#NUM!	#NUM!	#NUM!	#NUM!	#NUM!
#NUM!	#NUM!	#NUM!	#NUM!	#NUM!	#NUM!	#NUM!	#NUM!	#NUM!	#NUM!	#NUM!	#NUM!
#NUM!	#NUM!	#NUM!	#NUM!	#NUM!	28.3132	#NUM!	28.3017	25.9657	26.5599	26.7329	26.946
#NUM!	#NUM!	#NUM!	#NUM!	#NUM!	#NUM!	#NUM!	#NUM!	#NUM!	#NUM!	26.9197	#NUM!
27.5138	#NUM!	#NUM!	28.8122	31.0511	#NUM!	30.249	27.974	27.2506	#NUM!	26.8252	26.5835
31.1976	31.0523	28.4857	28.2007	29.4634	31.1189	29.327	31.8285	#NUM!	#NUM!	#NUM!	#NUM!
#NUM!	#NUM!	#NUM!	#NUM!	#NUM!	#NUM!	#NUM!	#NUM!	28.7223	29.367	30.6944	31.0392
#NUM!	#NUM!	#NUM!	#NUM!	#NUM!	#NUM!	#NUM!	#NUM!	28.5115	28.1115	#NUM!	28.2623
#NUM!	#NUM!	#NUM!	#NUM!	#NUM!	#NUM!	#NUM!	#NUM!	27.6858	26.972	27.0921	26.9962
#NUM!	#NUM!	#NUM!	#NUM!	#NUM!	#NUM!	#NUM!	#NUM!	#NUM!	#NUM!	#NUM!	#NUM!
#NUM!	#NUM!	#NUM!	#NUM!	#NUM!	#NUM!	#NUM!	#NUM!	26.6345	#NUM!	25.8409	26.0245
#NUM!	26.8901	#NUM!	27.6915	#NUM!	#NUM!	#NUM!	#NUM!	#NUM!	#NUM!	#NUM!	#NUM!
#NUM!	#NUM!	#NUM!	#NUM!	#NUM!	#NUM!	#NUM!	#NUM!	#NUM!	#NUM!	#NUM!	#NUM!
#NUM!	#NUM!	#NUM!	#NUM!	#NUM!	#NUM!	#NUM!	#NUM!	#NUM!	#NUM!	#NUM!	#NUM!
#NUM!	#NUM!	#NUM!	#NUM!	#NUM!	#NUM!	#NUM!	#NUM!	#NUM!	#NUM!	#NUM!	#NUM!
#NUM!	#NUM!	#NUM!	#NUM!	#NUM!	#NUM!	#NUM!	#NUM!	#NUM!	#NUM!	#NUM!	#NUM!
26.7108	#NUM!	#NUM!	#NUM!	#NUM!	#NUM!	#NUM!	#NUM!	28.034	28.2232	27.6765	27.5833
#NUM!	29.668	29.688	29.4116	29.606	#NUM!	29.5456	#NUM!	27.5153	#NUM!	27.2761	26.9169
#NUM!	#NUM!	#NUM!	#NUM!	#NUM!	#NUM!	#NUM!	#NUM!	26.7324	26.9115	25.9303	26.1587
#NUM!	#NUM!	#NUM!	#NUM!	#NUM!	#NUM!	#NUM!	#NUM!	#NUM!	#NUM!	28.1726	27.6526
#NUM!	#NUM!	#NUM!	#NUM!	#NUM!	#NUM!	#NUM!	#NUM!	#NUM!	#NUM!	#NUM!	25.858
#NUM!	#NUM!	#NUM!	#NUM!	#NUM!	#NUM!	#NUM!	#NUM!	28.2821	28.1732	28.6982	28.167
#NUM!	#NUM!	#NUM!	#NUM!	#NUM!	#NUM!	#NUM!	#NUM!	#NUM!	23.8987	#NUM!	#NUM!
#NUM!	#NUM!	#NUM!	#NUM!	#NUM!	#NUM!	#NUM!	#NUM!	#NUM!	#NUM!	25.1844	25.4804
#NUM!	#NUM!	#NUM!	#NUM!	#NUM!	#NUM!	#NUM!	#NUM!	#NUM!	28.7597	#NUM!	#NUM!
#NUM!	#NUM!	#NUM!	#NUM!	#NUM!	#NUM!	#NUM!	#NUM!	#NUM!	25.1691	25.8992	#NUM!
#NUM!	#NUM!	#NUM!	#NUM!	#NUM!	#NUM!	#NUM!	#NUM!	#NUM!	#NUM!	25.0969	25.1811
#NUM!	#NUM!	#NUM!	#NUM!	#NUM!	#NUM!	#NUM!	#NUM!	#NUM!	#NUM!	#NUM!	#NUM!
28.5392	28.6069	28.2856	28.3492	28.4283	27.8924	28.2806	28.0898	#NUM!	#NUM!	25.8807	#NUM!
#NUM!	#NUM!	#NUM!	#NUM!	#NUM!	#NUM!	#NUM!	#NUM!	#NUM!	25.3771	#NUM!	25.2462
#NUM!	#NUM!	#NUM!	#NUM!	#NUM!	#NUM!	#NUM!	#NUM!	#NUM!	24.9903	#NUM!	#NUM!
#NUM!	#NUM!	#NUM!	#NUM!	#NUM!	#NUM!	#NUM!	#NUM!	#NUM!	#NUM!	#NUM!	#NUM!
#NUM!	#NUM!	#NUM!	#NUM!	#NUM!	#NUM!	#NUM!	#NUM!	26.1452	26.2375	26.0908	25.9613
#NUM!	#NUM!	#NUM!	#NUM!	#NUM!	#NUM!	#NUM!	#NUM!	#NUM!	#NUM!	25.8551	25.9449
#NUM!	#NUM!	#NUM!	#NUM!	#NUM!	#NUM!	#NUM!	#NUM!	#NUM!	#NUM!	#NUM!	#NUM!
#NUM!	#NUM!	#NUM!	#NUM!	#NUM!	#NUM!	#NUM!	#NUM!	#NUM!	#NUM!	#NUM!	25.4948
#NUM!	#NUM!	#NUM!	#NUM!	#NUM!	#NUM!	#NUM!	#NUM!	#NUM!	#NUM!	#NUM!	#NUM!
#NUM!	#NUM!	#NUM!	#NUM!	#NUM!	#NUM!	#NUM!	#NUM!	#NUM!	#NUM!	24.5841	#NUM!
#NUM!	#NUM!	#NUM!	#NUM!	#NUM!	#NUM!	#NUM!	#NUM!	#NUM!	#NUM!	#NUM!	#NUM!
#NUM!	#NUM!	#NUM!	#NUM!	#NUM!	#NUM!	#NUM!	#NUM!	26.0935	26.2074	25.8367	26.0399
#NUM!	#NUM!	#NUM!	#NUM!	#NUM!	#NUM!	#NUM!	#NUM!	26.8151	26.8561	25.7726	26.0774
#NUM!	#NUM!	#NUM!	#NUM!	#NUM!	#NUM!	#NUM!	#NUM!	25.8344	25.8895	26.5056	26.113
#NUM!	#NUM!	#NUM!	#NUM!	#NUM!	#NUM!	#NUM!	#NUM!	#NUM!	#NUM!	#NUM!	#NUM!
#NUM!	26.9788	#NUM!	27.1215	#NUM!	#NUM!	#NUM!	#NUM!	#NUM!	#NUM!	#NUM!	#NUM!
#NUM!	27.9874	#NUM!	#NUM!	29.9091	28.4111	29.9495	#NUM!	30.6911	30.3983	31.6041	31.4333
#NUM!	#NUM!	#NUM!	#NUM!	#NUM!	#NUM!	#NUM!	#NUM!	26.8943	26.7591	26.6654	26.5797
#NUM!	#NUM!	#NUM!	#NUM!	#NUM!	#NUM!	#NUM!	#NUM!	#NUM!	#NUM!	#NUM!	#NUM!
#NUM!	#NUM!	#NUM!	#NUM!	#NUM!	#NUM!	#NUM!	#NUM!	23.8884	24.107	#NUM!	#NUM!
#NUM!	#NUM!	#NUM!	#NUM!	#NUM!	#NUM!	#NUM!	#NUM!	26.916	#NUM!	#NUM!	#NUM!
#NUM!	#NUM!	#NUM!	#NUM!	#NUM!	#NUM!	#NUM!	#NUM!	#NUM!	#NUM!	#NUM!	#NUM!
#NUM!	#NUM!	#NUM!	#NUM!	#NUM!	#NUM!	#NUM!	#NUM!	26.0591	25.8551	24.8843	24.9966
#NUM!	#NUM!	#NUM!	#NUM!	#NUM!	#NUM!	#NUM!	#NUM!	25.1033	#NUM!	#NUM!	#NUM!
#NUM!	#NUM!	#NUM!	#NUM!	#NUM!	#NUM!	#NUM!	#NUM!	#NUM!	#NUM!	27.7526	28.2283
#NUM!	#NUM!	#NUM!	#NUM!	#NUM!	#NUM!	#NUM!	#NUM!	#NUM!	#NUM!	#NUM!	#NUM!
#NUM!	#NUM!	#NUM!	#NUM!	#NUM!	#NUM!	#NUM!	#NUM!	#NUM!	#NUM!	#NUM!	#NUM!
#NUM!	#NUM!	#NUM!	#NUM!	#NUM!	#NUM!	#NUM!	#NUM!	#NUM!	#NUM!	#NUM!	#NUM!
#NUM!	#NUM!	#NUM!	#NUM!	#NUM!	#NUM!	#NUM!	#NUM!	#NUM!	#NUM!	26.7944	26.9614
#NUM!	#NUM!	#NUM!	#NUM!	#NUM!	#NUM!	#NUM!	#NUM!	#NUM!	#NUM!	25.8893	25.3913
#NUM!	#NUM!	#NUM!	#NUM!	#NUM!	#NUM!	#NUM!	#NUM!	#NUM!	#NUM!	#NUM!	#NUM!
#NUM!	#NUM!	#NUM!	#NUM!	#NUM!	#NUM!	#NUM!	#NUM!	#NUM!	#NUM!	#NUM!	#NUM!
#NUM!	#NUM!	#NUM!	#NUM!	#NUM!	#NUM!	#NUM!	#NUM!	25.9422	25.974	25.458	25.4061
#NUM!	#NUM!	#NUM!	#NUM!	#NUM!	#NUM!	#NUM!	#NUM!	#NUM!	23.7325	24.339	24.4187
29.9062	30.0651	29.0532	28.87	29.4256	29.8734	29.1715	28.6637	#NUM!	#NUM!	#NUM!	#NUM!
#NUM!	26.5711	#NUM!	#NUM!	#NUM!	#NUM!	#NUM!	#NUM!	#NUM!	#NUM!	#NUM!	26.3457
#NUM!	#NUM!	#NUM!	#NUM!	#NUM!	#NUM!	#NUM!	#NUM!	26.5856	26.5603	26.5426	26.0246
#NUM!	#NUM!	#NUM!	#NUM!	#NUM!	#NUM!	#NUM!	#NUM!	#NUM!	#NUM!	#NUM!	#NUM!
#NUM!	#NUM!	#NUM!	#NUM!	#NUM!	#NUM!	#NUM!	#NUM!	#NUM!	#NUM!	#NUM!	#NUM!
#NUM!	#NUM!	#NUM!	#NUM!	#NUM!	#NUM!	#NUM!	#NUM!	#NUM!	#NUM!	#NUM!	#NUM!
#NUM!	27.3192	27.7809	27.4135	27.8483	28.2157	#NUM!	29.1663	#NUM!	#NUM!	#NUM!	#NUM!

#NUM!	#NUM!	#NUM!	#NUM!	#NUM!	#NUM!	#NUM!	#NUM!	#NUM!	26.3593	25.5487	25.0544
#NUM!	#NUM!	#NUM!	#NUM!	#NUM!	#NUM!	#NUM!	#NUM!	#NUM!	25.5202	#NUM!	25.6293
#NUM!	#NUM!	#NUM!	#NUM!	#NUM!	#NUM!	#NUM!	#NUM!	#NUM!	25.3594	24.6889	#NUM!
#NUM!	#NUM!	#NUM!	#NUM!	#NUM!	#NUM!	#NUM!	#NUM!	27.1588	#NUM!	#NUM!	#NUM!
#NUM!	#NUM!	#NUM!	#NUM!	#NUM!	#NUM!	#NUM!	#NUM!	#NUM!	#NUM!	#NUM!	#NUM!
#NUM!	#NUM!	#NUM!	#NUM!	#NUM!	#NUM!	#NUM!	#NUM!	25.8442	#NUM!	25.8309	25.6745
29.5494	29.3977	30.0303	30.0901	#NUM!	30.0922	30.8899	29.7571	#NUM!	26.0414	#NUM!	25.8211
#NUM!	#NUM!	#NUM!	#NUM!	#NUM!	#NUM!	#NUM!	#NUM!	25.9812	#NUM!	#NUM!	#NUM!
#NUM!	#NUM!	#NUM!	#NUM!	#NUM!	#NUM!	#NUM!	#NUM!	#NUM!	#NUM!	#NUM!	#NUM!
#NUM!	#NUM!	#NUM!	#NUM!	#NUM!	#NUM!	#NUM!	#NUM!	24.4318	#NUM!	#NUM!	#NUM!
#NUM!	#NUM!	#NUM!	#NUM!	#NUM!	#NUM!	#NUM!	#NUM!	#NUM!	#NUM!	#NUM!	#NUM!
#NUM!	#NUM!	#NUM!	#NUM!	#NUM!	#NUM!	#NUM!	#NUM!	#NUM!	#NUM!	#NUM!	#NUM!
#NUM!	#NUM!	#NUM!	#NUM!	#NUM!	#NUM!	#NUM!	#NUM!	24.9295	#NUM!	#NUM!	#NUM!
#NUM!	#NUM!	#NUM!	#NUM!	#NUM!	#NUM!	#NUM!	#NUM!	#NUM!	#NUM!	#NUM!	#NUM!
#NUM!	#NUM!	#NUM!	#NUM!	#NUM!	#NUM!	#NUM!	#NUM!	#NUM!	23.7171	#NUM!	24.1525
#NUM!	#NUM!	#NUM!	#NUM!	#NUM!	#NUM!	#NUM!	#NUM!	#NUM!	#NUM!	#NUM!	#NUM!
#NUM!	#NUM!	#NUM!	#NUM!	#NUM!	#NUM!	#NUM!	#NUM!	#NUM!	#NUM!	27.0686	27.1517
#NUM!	#NUM!	#NUM!	#NUM!	#NUM!	#NUM!	#NUM!	#NUM!	#NUM!	#NUM!	#NUM!	#NUM!
#NUM!	#NUM!	#NUM!	#NUM!	#NUM!	#NUM!	#NUM!	#NUM!	26.7347	#NUM!	#NUM!	#NUM!
#NUM!	#NUM!	#NUM!	#NUM!	#NUM!	#NUM!	#NUM!	#NUM!	#NUM!	#NUM!	#NUM!	26.1959
#NUM!	#NUM!	#NUM!	#NUM!	#NUM!	#NUM!	#NUM!	#NUM!	#NUM!	#NUM!	#NUM!	#NUM!
#NUM!	#NUM!	#NUM!	#NUM!	#NUM!	#NUM!	#NUM!	#NUM!	#NUM!	#NUM!	#NUM!	#NUM!
#NUM!	#NUM!	#NUM!	#NUM!	#NUM!	#NUM!	#NUM!	#NUM!	#NUM!	24.5427	#NUM!	#NUM!
#NUM!	#NUM!	#NUM!	#NUM!	#NUM!	#NUM!	#NUM!	#NUM!	25.7567	25.8582	26.1448	26.1458
#NUM!	#NUM!	#NUM!	#NUM!	#NUM!	#NUM!	#NUM!	#NUM!	#NUM!	#NUM!	#NUM!	#NUM!
#NUM!	#NUM!	#NUM!	#NUM!	#NUM!	#NUM!	#NUM!	#NUM!	27.2919	27.5071	27.654	#NUM!
#NUM!	#NUM!	#NUM!	#NUM!	#NUM!	#NUM!	#NUM!	#NUM!	#NUM!	25.5097	26.5851	#NUM!
#NUM!	#NUM!	#NUM!	#NUM!	#NUM!	#NUM!	#NUM!	#NUM!	#NUM!	#NUM!	#NUM!	#NUM!
#NUM!	#NUM!	#NUM!	#NUM!	#NUM!	#NUM!	#NUM!	#NUM!	#NUM!	25.8324	25.5388	25.8601
31.1974	31.3607	30.1007	29.9934	29.9396	31.4355	30.6201	30.6528	27.5333	27.9847	27.0779	27.696
#NUM!	#NUM!	#NUM!	#NUM!	#NUM!	#NUM!	#NUM!	#NUM!	28.3972	28.651	28.2331	28.3825
#NUM!	#NUM!	#NUM!	26.0953	#NUM!	#NUM!	#NUM!	#NUM!	26.3451	26.62	26.6647	26.6937
#NUM!	#NUM!	#NUM!	#NUM!	#NUM!	#NUM!	#NUM!	#NUM!	#NUM!	#NUM!	#NUM!	#NUM!
#NUM!	#NUM!	#NUM!	#NUM!	#NUM!	#NUM!	#NUM!	#NUM!	#NUM!	24.7717	#NUM!	#NUM!
#NUM!	27.0371	#NUM!	#NUM!	#NUM!	#NUM!	#NUM!	#NUM!	29.4339	29.5778	29.609	29.6309
#NUM!	#NUM!	#NUM!	#NUM!	#NUM!	#NUM!	#NUM!	#NUM!	26.8214	27.0407	27.4155	27.1183
36.3244	36.3428	35.4974	35.5324	37.2688	36.3314	37.0717	37.1692	38.5449	38.2374	38.6438	38.5435
30.4479	30.2451	29.1047	29.2349	29.0185	29.7424	28.4175	30.5344	32.675	32.2458	31.797	31.9823
#NUM!	28.3788	#NUM!	#NUM!	#NUM!	28.3995	#NUM!	29.1687	28.5952	28.5856	28.397	28.5403
29.7171	29.2601	28.68	29.5456	27.7143	27.5464	27.6204	27.73	26.2483	26.4047	#NUM!	#NUM!
27.8024	27.4369	#NUM!	27.5124	#NUM!	28.2193	#NUM!	28.6572	29.8158	29.7262	29.6702	29.6654
#NUM!	#NUM!	#NUM!	#NUM!	#NUM!	#NUM!	#NUM!	#NUM!	#NUM!	#NUM!	#NUM!	#NUM!
30.0681	30.1752	29.8876	29.8559	30.8009	30.2711	30.6371	29.6173	29.2158	29.0051	29.1633	29.0347
28.7678	28.828	28.2609	#NUM!	#NUM!	29.6121	#NUM!	28.1872	#NUM!	#NUM!	#NUM!	#NUM!
#NUM!	#NUM!	#NUM!	#NUM!	#NUM!	#NUM!	#NUM!	#NUM!	#NUM!	25.8828	#NUM!	25.9696
#NUM!	#NUM!	#NUM!	#NUM!	#NUM!	#NUM!	#NUM!	#NUM!	#NUM!	#NUM!	#NUM!	#NUM!
28.7416	28.7067	#NUM!	#NUM!	26.9946	28.7976	#NUM!	29.6546	#NUM!	#NUM!	#NUM!	#NUM!
#NUM!	#NUM!	#NUM!	#NUM!	28.1471	28.5699	28.1017	28.7329	#NUM!	#NUM!	#NUM!	#NUM!
#NUM!	#NUM!	#NUM!	#NUM!	#NUM!	#NUM!	#NUM!	#NUM!	27.3091	27.2313	27.2118	27.8896
27.714	27.4784	26.581	26.6558	28.5081	28.8627	28.49	29.2082	#NUM!	#NUM!	#NUM!	#NUM!
#NUM!	#NUM!	#NUM!	#NUM!	#NUM!	#NUM!	#NUM!	#NUM!	#NUM!	#NUM!	#NUM!	#NUM!
#NUM!	#NUM!	#NUM!	#NUM!	#NUM!	#NUM!	#NUM!	#NUM!	28.5769	28.3838	28.633	28.6415
#NUM!	#NUM!	#NUM!	#NUM!	#NUM!	29.0989	#NUM!	#NUM!	31.1463	30.9426	31.2335	31.1768
#NUM!	#NUM!	#NUM!	#NUM!	#NUM!	#NUM!	#NUM!	#NUM!	#NUM!	#NUM!	26.8987	#NUM!
29.6928	29.3511	#NUM!	26.8799	29.2499	30.6793	29.029	29.6022	#NUM!	#NUM!	#NUM!	#NUM!
#NUM!	#NUM!	#NUM!	#NUM!	#NUM!	#NUM!	#NUM!	#NUM!	28.9109	28.7968	28.6445	28.4103
#NUM!	25.919	#NUM!	#NUM!	#NUM!	#NUM!	#NUM!	#NUM!	#NUM!	#NUM!	#NUM!	26.6803
28.3625	28.3922	28.4124	28.3301	#NUM!	28.0474	#NUM!	#NUM!	#NUM!	#NUM!	#NUM!	#NUM!
29.1217	28.9511	28.066	28.6443	28.0584	#NUM!	28.0064	#NUM!	#NUM!	#NUM!	#NUM!	#NUM!
#NUM!	#NUM!	#NUM!	#NUM!	#NUM!	#NUM!	#NUM!	#NUM!	#NUM!	#NUM!	#NUM!	#NUM!
#NUM!	#NUM!	#NUM!	#NUM!	#NUM!	#NUM!	#NUM!	#NUM!	#NUM!	27.6186	27.5467	27.6869
#NUM!	#NUM!	#NUM!	#NUM!	#NUM!	#NUM!	#NUM!	#NUM!	#NUM!	#NUM!	#NUM!	#NUM!
#NUM!	#NUM!	#NUM!	#NUM!	#NUM!	#NUM!	#NUM!	#NUM!	27.9563	27.6467	27.5364	#NUM!
27.351	27.4899	27.9153	27.4713	#NUM!	#NUM!	#NUM!	#NUM!	#NUM!	#NUM!	#NUM!	#NUM!
#NUM!	#NUM!	#NUM!	#NUM!	#NUM!	#NUM!	#NUM!	#NUM!	27.2715	27.76	27.2889	26.6572
#NUM!	#NUM!	#NUM!	#NUM!	#NUM!	#NUM!	#NUM!	#NUM!	#NUM!	#NUM!	#NUM!	#NUM!
29.9091	29.8478	29.8107	30.0147	29.5746	29.1823	29.6597	29.1638	26.4855	26.056	26.4007	27.1958
#NUM!	#NUM!	#NUM!	#NUM!	#NUM!	#NUM!	#NUM!	#NUM!	24.9677	25.3431	25.1044	25.2035
#NUM!	#NUM!	#NUM!	#NUM!	#NUM!	#NUM!	#NUM!	#NUM!	#NUM!	#NUM!	#NUM!	#NUM!
29.7484	29.782	27.7791	27.7435	28.9453	29.6916	28.6814	30.0884	27.5541	27.9594	27.3594	27.2292
28.0155	28.2589	29.0453	28.8751	28.0704	28.038	28.009	#NUM!	#NUM!	#NUM!	#NUM!	#NUM!
27.6117	27.5734	#NUM!	#NUM!	#NUM!	27.8184	#NUM!	28.4218	26.5997	26.4423	26.3273	26.8019
28.6346	28.748	#NUM!	27.1177	29.0856	29.8767	29.3675	30.7796	26.6203	26.5532	26.2529	26.2113
#NUM!	#NUM!	#NUM!	#NUM!	#NUM!	#NUM!	#NUM!	#NUM!	#NUM!	26.1113	26.3323	#NUM!
#NUM!	#NUM!	#NUM!	#NUM!	#NUM!	#NUM!	#NUM!	#NUM!	#NUM!	#NUM!	#NUM!	#NUM!
#NUM!	#NUM!	#NUM!	#NUM!	#NUM!	#NUM!	#NUM!	26.9875	25.6	25.7718	25.955	25.9706
#NUM!	#NUM!	#NUM!	#NUM!	#NUM!	#NUM!	#NUM!	#NUM!	#NUM!	#NUM!	#NUM!	#NUM!
#NUM!	#NUM!	#NUM!	#NUM!	#NUM!	#NUM!	#NUM!	#NUM!	#NUM!	#NUM!	#NUM!	#NUM!
#NUM!	#NUM!	#NUM!	#NUM!	#NUM!	#NUM!	#NUM!	#NUM!	#NUM!	#NUM!	#NUM!	#NUM!
#NUM!	#NUM!	#NUM!	#NUM!	#NUM!	#NUM!	#NUM!	#NUM!	#NUM!	#NUM!	#NUM!	#NUM!
#NUM!	#NUM!	#NUM!	#NUM!	#NUM!	#NUM!	#NUM!	#NUM!	#NUM!	#NUM!	#NUM!	#NUM!
#NUM!	#NUM!	#NUM!	#NUM!	#NUM!	#NUM!	#NUM!	#NUM!	27.411	#NUM!	#NUM!	#NUM!
#NUM!	#NUM!	#NUM!	#NUM!	#NUM!	#NUM!	#NUM!	#NUM!	#NUM!	#NUM!	#NUM!	#NUM!
#NUM!	#NUM!	#NUM!	#NUM!	#NUM!	#NUM!	#NUM!	#NUM!	#NUM!	#NUM!	#NUM!	#NUM!
#NUM!	#NUM!	#NUM!	#NUM!	#NUM!	#NUM!	#NUM!	#NUM!	#NUM!	#NUM!	#NUM!	#NUM!
#NUM!	#NUM!	#NUM!	#NUM!	#NUM!	#NUM!	#NUM!	#NUM!	#NUM!	#NUM!	#NUM!	#NUM!
28.2407	28.4935	28.4342	28.1975	#NUM!	#NUM!	#NUM!	28.3758	#NUM!	#NUM!	#NUM!	#NUM!
#NUM!	#NUM!	#NUM!	#NUM!	#NUM!	#NUM!	#NUM!	#NUM!	#NUM!	#NUM!	#NUM!	#NUM!
#NUM!	#NUM!	#NUM!	#NUM!	#NUM!	#NUM!	#NUM!	#NUM!	#NUM!	#NUM!	26.1232	26.049
27.6441	27.8297	27.6861	27.6067	#NUM!	#NUM!	#NUM!	#NUM!	#NUM!	27.1465	#NUM!	#NUM!
#NUM!	#NUM!	#NUM!	#NUM!	#NUM!	#NUM!	#NUM!	#NUM!	#NUM!	#NUM!	#NUM!	25.158
#NUM!	#NUM!	#NUM!	#NUM!	#NUM!	#NUM!	#NUM!	#NUM!	#NUM!	24.6081	24.3538	24.1697
#NUM!	#NUM!	#NUM!	#NUM!	#NUM!	#NUM!	#NUM!	#NUM!	#NUM!	#NUM!	#NUM!	#NUM!
#NUM!	#NUM!	#NUM!	#NUM!	#NUM!	#NUM!	#NUM!	#NUM!	#NUM!	#NUM!	#NUM!	#NUM!
#NUM!	#NUM!	#NUM!	#NUM!	#NUM!	#NUM!	#NUM!	#NUM!	#NUM!	#NUM!	#NUM!	#NUM!
#NUM!	#NUM!	#NUM!	#NUM!	#NUM!	#NUM!	#NUM!	#NUM!	#NUM!	#NUM!	#NUM!	#NUM!
#NUM!	26.9	26.9334	26.7082	28.3078	28.4862	#NUM!	28.5846	#NUM!	#NUM!	#NUM!	#NUM!
29.6973	29.9831	29.9519	29.4866	#NUM!	29.4712	#NUM!	29.6143	#NUM!	#NUM!	#NUM!	#NUM!
#NUM!	#NUM!	#NUM!	#NUM!	#NUM!	#NUM!	#NUM!	#NUM!	#NUM!	#NUM!	#NUM!	#NUM!
#NUM!	25.9497	#NUM!	26.5658	#NUM!	#NUM!	#NUM!	#NUM!	26.3655	26.1479	25.7143	#NUM!
#NUM!	#NUM!	#NUM!	#NUM!	#NUM!	#NUM!	#NUM!	#NUM!	#NUM!	#NUM!	27.027	27.0164
#NUM!	#NUM!	#NUM!	#NUM!	#NUM!	#NUM!	#NUM!	#NUM!	26.4942	26.4219	26.6159	26.5224
26.3187	26.0185	#NUM!	#NUM!	#NUM!	26.3284	#NUM!	#NUM!	26.9286	26.7787	27.9571	27.5841
#NUM!	#NUM!	#NUM!	#NUM!	#NUM!	#NUM!	#NUM!	#NUM!	27.2517	27.1705	26.9211	26.7537
#NUM!	#NUM!	#NUM!	#NUM!	#NUM!	#NUM!	#NUM!	#NUM!	#NUM!	26.8209	27.1816	27.1701
#NUM!	#NUM!	#NUM!	#NUM!	#NUM!	#NUM!	#NUM!	#NUM!	#NUM!	#NUM!	#NUM!	#NUM!
#NUM!	#NUM!	#NUM!	#NUM!	#NUM!	#NUM!	#NUM!	#NUM!	26.2941	26.2545	26.0364	#NUM!
29.6325	29.4085	28.7519	28.9307	29.5791	29.3898	29.5093	#NUM!	#NUM!	#NUM!	#NUM!	27.1061
#NUM!	#NUM!	#NUM!	#NUM!	#NUM!	#NUM!	#NUM!	#NUM!	26.1442	#NUM!	#NUM!	26.1864
#NUM!	#NUM!	#NUM!	#NUM!	#NUM!	#NUM!	#NUM!	#NUM!	#NUM!	#NUM!	#NUM!	#NUM!
#NUM!	#NUM!	#NUM!	#NUM!	#NUM!	#NUM!	#NUM!	#NUM!	#NUM!	#NUM!	26.39	25.7227
#NUM!	#NUM!	#NUM!	#NUM!	#NUM!	#NUM!	#NUM!	#NUM!	26.6079	26.7912	26.4847	26.5579
#NUM!	#NUM!	#NUM!	#NUM!	#NUM!	#NUM!	#NUM!	#NUM!	#NUM!	#NUM!	#NUM!	#NUM!
#NUM!	#NUM!	#NUM!	#NUM!	#NUM!	#NUM!	#NUM!	#NUM!	#NUM!	#NUM!	#NUM!	#NUM!
#NUM!	#NUM!	#NUM!	#NUM!	#NUM!	#NUM!	#NUM!	#NUM!	#NUM!	#NUM!	#NUM!	#NUM!
#NUM!	#NUM!	#NUM!	#NUM!	#NUM!	#NUM!	#NUM!	#NUM!	#NUM!	#NUM!	#NUM!	#NUM!
#NUM!	#NUM!	#NUM!	#NUM!	#NUM!	#NUM!	#NUM!	#NUM!	27.215	26.9041	26.4876	26.7738
#NUM!	#NUM!	#NUM!	#NUM!	#NUM!	#NUM!	#NUM!	#NUM!	#NUM!	#NUM!	#NUM!	#NUM!
#NUM!	#NUM!	#NUM!	#NUM!	#NUM!	#NUM!	#NUM!	#NUM!	#NUM!	#NUM!	#NUM!	#NUM!
#NUM!	#NUM!	#NUM!	#NUM!	#NUM!	#NUM!	#NUM!	#NUM!	#NUM!	25.2897	25.1103	24.6633
#NUM!	#NUM!	#NUM!	#NUM!	#NUM!	#NUM!	#NUM!	#NUM!	#NUM!	#NUM!	#NUM!	#NUM!
#NUM!	#NUM!	#NUM!	#NUM!	#NUM!	#NUM!	#NUM!	#NUM!	#NUM!	#NUM!	#NUM!	#NUM!
#NUM!	#NUM!	#NUM!	#NUM!	#NUM!	#NUM!	#NUM!	#NUM!	#NUM!	#NUM!	#NUM!	#NUM!
#NUM!	#NUM!	#NUM!	#NUM!	#NUM!	#NUM!	#NUM!	#NUM!	#NUM!	24.8874	25.2526	25.1764
#NUM!	#NUM!	#NUM!	#NUM!	#NUM!	#NUM!	#NUM!	#NUM!	#NUM!	#NUM!	28.7979	28.4588
#NUM!	#NUM!	#NUM!	#NUM!	#NUM!	#NUM!	#NUM!	#NUM!	#NUM!	26.3403	26.1414	25.9269
#NUM!	#NUM!	#NUM!	#NUM!	#NUM!	#NUM!	#NUM!	#NUM!	#NUM!	#NUM!	25.0018	#NUM!
#NUM!	#NUM!	#NUM!	#NUM!	#NUM!	#NUM!	#NUM!	#NUM!	26.5704	26.2969	25.8629	25.9481
#NUM!	#NUM!	#NUM!	#NUM!	#NUM!	#NUM!	#NUM!	#NUM!	#NUM!	#NUM!	#NUM!	#NUM!

#NUM!	28.39	#NUM!	#NUM!	#NUM!	28.4227	#NUM!	#NUM!	26.8918	27.2932	27.0322	27.2154
#NUM!	#NUM!	#NUM!	#NUM!	#NUM!	#NUM!	#NUM!	#NUM!	23.6781	#NUM!	24.9241	24.3213
#NUM!	#NUM!	#NUM!	#NUM!	#NUM!	#NUM!	#NUM!	#NUM!	#NUM!	#NUM!	#NUM!	#NUM!
#NUM!	#NUM!	#NUM!	#NUM!	#NUM!	#NUM!	#NUM!	#NUM!	27.3554	#NUM!	27.8878	27.3648
#NUM!	#NUM!	#NUM!	#NUM!	#NUM!	#NUM!	#NUM!	#NUM!	24.9153	25.2019	#NUM!	#NUM!
#NUM!	#NUM!	#NUM!	#NUM!	#NUM!	#NUM!	#NUM!	#NUM!	32.0465	#NUM!	#NUM!	#NUM!
#NUM!	#NUM!	#NUM!	#NUM!	#NUM!	#NUM!	#NUM!	#NUM!	24.9394	25.5446	25.8906	#NUM!
#NUM!	#NUM!	#NUM!	#NUM!	#NUM!	#NUM!	#NUM!	#NUM!	25.6605	25.916	25.622	25.5648
#NUM!	#NUM!	#NUM!	#NUM!	#NUM!	#NUM!	#NUM!	#NUM!	25.86	25.511	#NUM!	#NUM!
#NUM!	#NUM!	#NUM!	#NUM!	#NUM!	#NUM!	#NUM!	#NUM!	#NUM!	27.1587	27.3924	27.4678
#NUM!	#NUM!	#NUM!	#NUM!	#NUM!	#NUM!	#NUM!	#NUM!	#NUM!	#NUM!	#NUM!	#NUM!
#NUM!	26.7808	#NUM!	26.1164	#NUM!	#NUM!	#NUM!	#NUM!	#NUM!	#NUM!	#NUM!	#NUM!
27.586	27.6055	27.434	27.3708	#NUM!	27.4443	27.4056	27.2687	#NUM!	#NUM!	#NUM!	#NUM!
#NUM!	#NUM!	#NUM!	#NUM!	#NUM!	#NUM!	#NUM!	#NUM!	#NUM!	#NUM!	25.0834	#NUM!
#NUM!	#NUM!	#NUM!	#NUM!	#NUM!	#NUM!	#NUM!	#NUM!	#NUM!	#NUM!	25.3917	25.2119
#NUM!	#NUM!	#NUM!	#NUM!	#NUM!	#NUM!	#NUM!	#NUM!	27.7386	27.4041	27.3029	26.788
#NUM!	#NUM!	#NUM!	#NUM!	#NUM!	#NUM!	#NUM!	#NUM!	27.2983	27.2196	27.2673	27.3069
#NUM!	#NUM!	#NUM!	#NUM!	#NUM!	#NUM!	#NUM!	#NUM!	26.4875	#NUM!	#NUM!	#NUM!
#NUM!	#NUM!	#NUM!	#NUM!	#NUM!	#NUM!	#NUM!	#NUM!	25.7629	25.9891	25.6459	25.6078
#NUM!	#NUM!	#NUM!	#NUM!	#NUM!	#NUM!	#NUM!	#NUM!	#NUM!	#NUM!	#NUM!	26.463
#NUM!	#NUM!	#NUM!	#NUM!	#NUM!	#NUM!	#NUM!	#NUM!	26.6658	#NUM!	27.1489	27.1796
#NUM!	#NUM!	#NUM!	#NUM!	#NUM!	#NUM!	#NUM!	#NUM!	#NUM!	#NUM!	#NUM!	#NUM!
#NUM!	#NUM!	#NUM!	#NUM!	#NUM!	#NUM!	#NUM!	#NUM!	26.924	26.6679	26.0647	26.2
#NUM!	#NUM!	#NUM!	#NUM!	#NUM!	#NUM!	#NUM!	#NUM!	29.8877	29.6911	29.3701	29.8653
#NUM!	#NUM!	#NUM!	#NUM!	#NUM!	#NUM!	#NUM!	#NUM!	#NUM!	#NUM!	26.1523	25.8501
#NUM!	#NUM!	#NUM!	#NUM!	#NUM!	#NUM!	#NUM!	#NUM!	#NUM!	#NUM!	#NUM!	#NUM!
28.0755	29.8275	#NUM!	#NUM!	#NUM!	27.5804	#NUM!	27.3202	29.2885	29.2569	29.1162	28.4951
#NUM!	#NUM!	#NUM!	#NUM!	#NUM!	#NUM!	#NUM!	#NUM!	27.7549	27.1229	27.3584	26.7327
#NUM!	#NUM!	#NUM!	#NUM!	#NUM!	#NUM!	#NUM!	#NUM!	26.018	25.9452	26.2591	#NUM!
30.1808	30.0395	30.3952	30.7635	28.4037	28.6427	28.7494	28.2941	#NUM!	#NUM!	#NUM!	#NUM!
#NUM!	#NUM!	#NUM!	#NUM!	#NUM!	#NUM!	#NUM!	#NUM!	#NUM!	#NUM!	#NUM!	#NUM!
#NUM!	#NUM!	#NUM!	#NUM!	#NUM!	#NUM!	#NUM!	#NUM!	27.8818	27.5726	27.6182	27.4269
27.8904	27.9249	27.9432	#NUM!	#NUM!	28.7551	#NUM!	#NUM!	27.3505	27.2844	27.0743	26.7834
#NUM!	#NUM!	#NUM!	#NUM!	#NUM!	#NUM!	#NUM!	#NUM!	#NUM!	#NUM!	#NUM!	#NUM!
#NUM!	#NUM!	#NUM!	#NUM!	#NUM!	#NUM!	#NUM!	#NUM!	#NUM!	27.3743	#NUM!	#NUM!
#NUM!	#NUM!	#NUM!	#NUM!	#NUM!	#NUM!	#NUM!	#NUM!	#NUM!	27.6555	26.7718	26.7929
#NUM!	#NUM!	#NUM!	#NUM!	#NUM!	#NUM!	#NUM!	#NUM!	26.9209	#NUM!	#NUM!	#NUM!
#NUM!	#NUM!	#NUM!	#NUM!	#NUM!	#NUM!	#NUM!	#NUM!	26.0912	26.0068	26.1384	26.7579
#NUM!	#NUM!	#NUM!	#NUM!	#NUM!	#NUM!	#NUM!	#NUM!	#NUM!	#NUM!	#NUM!	#NUM!
29.2624	29.5986	28.576	28.3064	#NUM!	29.6567	#NUM!	28.711	#NUM!	#NUM!	#NUM!	#NUM!
#NUM!	#NUM!	#NUM!	#NUM!	#NUM!	#NUM!	#NUM!	#NUM!	#NUM!	#NUM!	#NUM!	#NUM!
#NUM!	#NUM!	#NUM!	#NUM!	#NUM!	#NUM!	#NUM!	#NUM!	#NUM!	#NUM!	#NUM!	#NUM!
29.6212	29.6151	29.8595	29.484	30.0462	29.2426	#NUM!	28.8083	#NUM!	#NUM!	#NUM!	#NUM!
#NUM!	#NUM!	#NUM!	#NUM!	#NUM!	#NUM!	#NUM!	#NUM!	#NUM!	#NUM!	#NUM!	#NUM!
#NUM!	#NUM!	#NUM!	#NUM!	#NUM!	#NUM!	#NUM!	#NUM!	#NUM!	26.169	26.2504	26.0155
#NUM!	#NUM!	#NUM!	#NUM!	#NUM!	#NUM!	#NUM!	#NUM!	#NUM!	24.6183	24.1072	23.9181
28.3116	28.7344	27.0283	#NUM!	#NUM!	29.9196	#NUM!	30.02	#NUM!	#NUM!	#NUM!	#NUM!
31.2691	31.5743	29.0672	28.9748	30.421	32.0321	30.5657	32.3085	#NUM!	#NUM!	#NUM!	#NUM!
#NUM!	#NUM!	#NUM!	#NUM!	#NUM!	#NUM!	#NUM!	#NUM!	#NUM!	#NUM!	#NUM!	#NUM!
30.9181	31.267	29.9478	30.1507	#NUM!	30.6236	#NUM!	32.0537	32.6825	32.756	32.4974	32.5298
28.8032	28.8939	29.6456	29.6217	#NUM!	27.1377	#NUM!	26.6638	27.3771	27.2042	27.6035	27.9291
31.2106	31.0317	29.1167	28.9144	30.5909	30.9316	30.2776	31.6343	29.9427	30.0578	29.9822	30.1174
#NUM!	#NUM!	#NUM!	#NUM!	#NUM!	#NUM!	#NUM!	#NUM!	#NUM!	#NUM!	#NUM!	#NUM!
#NUM!	#NUM!	#NUM!	#NUM!	#NUM!	#NUM!	#NUM!	#NUM!	#NUM!	#NUM!	#NUM!	#NUM!
#NUM!	#NUM!	#NUM!	#NUM!	#NUM!	#NUM!	#NUM!	#NUM!	27.7582	27.8402	28.1892	28.1191
27.7023	27.6537	25.7607	25.1344	27.7622	29.7223	27.6171	28.5798	#NUM!	#NUM!	#NUM!	#NUM!
30.156	30.3756	29.2439	29.9162	31.265	30.694	30.9582	30.3721	#NUM!	#NUM!	28.9566	28.6922
27.7434	27.0638	#NUM!	26.6069	#NUM!	#NUM!	#NUM!	29.7569	#NUM!	26.5564	27.2366	26.8623
28.6577	28.6565	28.1925	28.2154	29.1607	28.9224	#NUM!	28.2034	26.7111	26.523	26.2357	#NUM!
#NUM!	#NUM!	#NUM!	#NUM!	#NUM!	#NUM!	#NUM!	#NUM!	25.9436	#NUM!	26.4082	26.6406
#NUM!	#NUM!	#NUM!	#NUM!	#NUM!	#NUM!	#NUM!	28.6981	26.9071	26.7963	27.2134	26.9823
#NUM!	27.1703	#NUM!	27.4532	#NUM!	#NUM!	#NUM!	#NUM!	#NUM!	#NUM!	#NUM!	#NUM!
#NUM!	#NUM!	#NUM!	#NUM!	#NUM!	#NUM!	#NUM!	#NUM!	26.8973	#NUM!	27.4722	27.2339
#NUM!	#NUM!	#NUM!	#NUM!	#NUM!	#NUM!	#NUM!	#NUM!	#NUM!	#NUM!	29.7259	#NUM!
#NUM!	#NUM!	#NUM!	#NUM!	#NUM!	#NUM!	#NUM!	#NUM!	#NUM!	#NUM!	27.2565	26.6437
#NUM!	26.8111	#NUM!	#NUM!	#NUM!	#NUM!	#NUM!	#NUM!	26.7325	26.938	#NUM!	26.9152
25.716	#NUM!	#NUM!	#NUM!	#NUM!	#NUM!	#NUM!	#NUM!	#NUM!	26.8156	26.7963	26.8321
#NUM!	#NUM!	#NUM!	#NUM!	#NUM!	#NUM!	#NUM!	#NUM!	#NUM!	#NUM!	25.784	#NUM!
#NUM!	#NUM!	#NUM!	#NUM!	#NUM!	#NUM!	#NUM!	#NUM!	#NUM!	27.2471	26.7573	27.2407
#NUM!	#NUM!	#NUM!	#NUM!	#NUM!	#NUM!	#NUM!	#NUM!	#NUM!	24.8364	25.8051	25.6063
#NUM!	#NUM!	#NUM!	#NUM!	#NUM!	#NUM!	#NUM!	#NUM!	#NUM!	#NUM!	25.0874	#NUM!
28.1214	28.2302	#NUM!	#NUM!	31.4224	29.2969	31.0851	29.5488	27.2988	27.0817	26.9077	26.8643
#NUM!	#NUM!	#NUM!	#NUM!	#NUM!	#NUM!	#NUM!	#NUM!	25.1441	#NUM!	#NUM!	#NUM!
29.2971	29.4669	29.3159	29.1117	30.3404	30.1344	30.2906	29.3347	26.7985	27.1396	26.608	26.8645
#NUM!	#NUM!	#NUM!	#NUM!	#NUM!	#NUM!	#NUM!	#NUM!	26.9609	#NUM!	24.9897	#NUM!
#NUM!	#NUM!	#NUM!	#NUM!	#NUM!	#NUM!	#NUM!	#NUM!	#NUM!	25.2398	25.7194	25.836
#NUM!	#NUM!	#NUM!	#NUM!	#NUM!	#NUM!	#NUM!	#NUM!	25.3021	25.67	25.6142	25.7976
29.0545	29.1161	#NUM!	28.4107	#NUM!	28.6946	#NUM!	28.8933	29.4436	29.6591	29.6855	29.665
28.8477	28.8291	#NUM!	#NUM!	#NUM!	#NUM!	#NUM!	#NUM!	#NUM!	#NUM!	#NUM!	#NUM!
#NUM!	#NUM!	#NUM!	#NUM!	#NUM!	#NUM!	#NUM!	#NUM!	#NUM!	26.0676	25.482	26.1098
#NUM!	#NUM!	#NUM!	#NUM!	#NUM!	#NUM!	#NUM!	#NUM!	#NUM!	#NUM!	#NUM!	#NUM!
#NUM!	#NUM!	#NUM!	#NUM!	#NUM!	#NUM!	#NUM!	#NUM!	#NUM!	#NUM!	26.836	#NUM!
#NUM!	#NUM!	#NUM!	28.3463	#NUM!	#NUM!	#NUM!	#NUM!	#NUM!	#NUM!	#NUM!	#NUM!
29.2729	29.1733	#NUM!	#NUM!	#NUM!	29.4234	#NUM!	29.2745	28.1862	28.4641	28.825	28.9659
29.0772	28.931	28.6012	28.4776	27.8783	28.1555	27.923	27.8031	30.1169	30.3415	29.6309	29.6636
#NUM!	#NUM!	#NUM!	26.9709	#NUM!	#NUM!	#NUM!	#NUM!	#NUM!	27.3109	27.7309	#NUM!
#NUM!	25.8021	#NUM!	#NUM!	#NUM!	#NUM!	#NUM!	#NUM!	#NUM!	26.466	26.2502	#NUM!
#NUM!	#NUM!	#NUM!	#NUM!	#NUM!	#NUM!	#NUM!	#NUM!	#NUM!	#NUM!	#NUM!	#NUM!
28.7503	27.0325	27.5908	28.9765	28.4381	29.6735	27.7026	27.3237	#NUM!	#NUM!	#NUM!	#NUM!
#NUM!	#NUM!	#NUM!	#NUM!	#NUM!	#NUM!	#NUM!	#NUM!	32.3511	31.5886	27.0029	26.9458
#NUM!	#NUM!	#NUM!	#NUM!	#NUM!	#NUM!	#NUM!	#NUM!	#NUM!	30.0624	#NUM!	#NUM!
#NUM!	#NUM!	#NUM!	#NUM!	#NUM!	#NUM!	#NUM!	#NUM!	26.337	26.1018	25.8868	26.0838
#NUM!	#NUM!	#NUM!	#NUM!	#NUM!	#NUM!	#NUM!	#NUM!	#NUM!	#NUM!	26.2664	26.2841
27.0038	#NUM!	28.5183	28.4674	#NUM!	#NUM!	#NUM!	#NUM!	#NUM!	#NUM!	#NUM!	#NUM!
#NUM!	#NUM!	#NUM!	#NUM!	#NUM!	#NUM!	#NUM!	#NUM!	27.3317	27.9938	27.7561	27.5579
#NUM!	#NUM!	#NUM!	#NUM!	#NUM!	#NUM!	#NUM!	#NUM!	#NUM!	#NUM!	#NUM!	25.6788
26.9181	27.1191	#NUM!	#NUM!	27.6303	29.0076	#NUM!	28.5632	#NUM!	#NUM!	#NUM!	#NUM!
#NUM!	#NUM!	#NUM!	#NUM!	#NUM!	#NUM!	#NUM!	#NUM!	#NUM!	#NUM!	#NUM!	#NUM!
#NUM!	#NUM!	#NUM!	#NUM!	#NUM!	#NUM!	#NUM!	#NUM!	#NUM!	#NUM!	#NUM!	#NUM!
#NUM!	#NUM!	#NUM!	#NUM!	#NUM!	#NUM!	#NUM!	#NUM!	#NUM!	#NUM!	#NUM!	#NUM!
#NUM!	#NUM!	#NUM!	#NUM!	#NUM!	29.6079	29.7384	#NUM!	30.811	30.7551	30.8233	31.0246
#NUM!	#NUM!	#NUM!	#NUM!	#NUM!	#NUM!	#NUM!	#NUM!	#NUM!	#NUM!	#NUM!	24.039
32.1899	32.442	29.5003	29.374	30.8428	31.704	30.4176	32.723	31.1412	30.9905	30.9208	31.5052
#NUM!	#NUM!	#NUM!	#NUM!	#NUM!	#NUM!	#NUM!	#NUM!	#NUM!	#NUM!	#NUM!	#NUM!
#NUM!	#NUM!	#NUM!	#NUM!	#NUM!	#NUM!	#NUM!	#NUM!	25.8729	25.7844	25.9738	26.0576
#NUM!	#NUM!	#NUM!	#NUM!	#NUM!	#NUM!	#NUM!	#NUM!	#NUM!	#NUM!	#NUM!	#NUM!
28.7849	28.4928	27.9201	27.9381	29.0971	29.3173	29.0014	30.1027	26.4031	26.437	26.4551	26.5241
28.1773	28.2239	28.8003	28.661	29.1407	28.2816	29.0911	#NUM!	25.3404	#NUM!	#NUM!	#NUM!
#NUM!	#NUM!	#NUM!	#NUM!	#NUM!	#NUM!	#NUM!	#NUM!	26.2433	25.7924	26.3267	25.8803
#NUM!	#NUM!	#NUM!	#NUM!	#NUM!	#NUM!	#NUM!	#NUM!	26.0655	25.9572	#NUM!	25.413
#NUM!	#NUM!	#NUM!	#NUM!	#NUM!	#NUM!	#NUM!	#NUM!	#NUM!	#NUM!	#NUM!	#NUM!
#NUM!	#NUM!	#NUM!	#NUM!	#NUM!	#NUM!	#NUM!	#NUM!	#NUM!	#NUM!	26.2923	26.6641
#NUM!	#NUM!	#NUM!	#NUM!	#NUM!	#NUM!	#NUM!	#NUM!	26.4087	26.3778	26.1189	26.2399
27.6311	#NUM!	#NUM!	#NUM!	29.691	28.9283	28.8222	29.0672	#NUM!	#NUM!	#NUM!	#NUM!
#NUM!	#NUM!	#NUM!	#NUM!	#NUM!	#NUM!	#NUM!	#NUM!	24.3281	24.6338	24.5219	24.8362
#NUM!	26.732	#NUM!	26.0936	#NUM!	#NUM!	#NUM!	#NUM!	#NUM!	#NUM!	#NUM!	#NUM!
#NUM!	#NUM!	#NUM!	#NUM!	#NUM!	#NUM!	#NUM!	#NUM!	#NUM!	#NUM!	25.4154	#NUM!
#NUM!	#NUM!	#NUM!	#NUM!	#NUM!	#NUM!	#NUM!	#NUM!	#NUM!	24.5131	#NUM!	25.3154
#NUM!	#NUM!	#NUM!	#NUM!	#NUM!	#NUM!	#NUM!	#NUM!	#NUM!	26.878	27.3355	27.7898
28.7022	28.7733	27.8918	27.8535	28.5337	28.4915	28.283	28.1611	#NUM!	#NUM!	#NUM!	#NUM!
26.9912	26.9771	#NUM!	#NUM!	27.9703	27.9019	27.7834	27.8694	#NUM!	#NUM!	#NUM!	#NUM!
#NUM!	#NUM!	#NUM!	#NUM!	#NUM!	#NUM!	#NUM!	#NUM!	#NUM!	#NUM!	24.9875	#NUM!
#NUM!	27.6138	#NUM!	#NUM!	#NUM!	#NUM!	#NUM!	#NUM!	#NUM!	25.2058	23.627	#NUM!
#NUM!	#NUM!	#NUM!	#NUM!	#NUM!	#NUM!	#NUM!	#NUM!	#NUM!	25.8293	26.0157	25.885
#NUM!	#NUM!	#NUM!	#NUM!	#NUM!	#NUM!	#NUM!	#NUM!	#NUM!	#NUM!	#NUM!	#NUM!
#NUM!	#NUM!	#NUM!	#NUM!	#NUM!	#NUM!	#NUM!	#NUM!	#NUM!	#NUM!	#NUM!	#NUM!
#NUM!	#NUM!	#NUM!	#NUM!	#NUM!	#NUM!	#NUM!	#NUM!	26.7406	26.5249	26.4898	26.2795
#NUM!	#NUM!	#NUM!	#NUM!	#NUM!	#NUM!	#NUM!	#NUM!	25.0223	#NUM!	#NUM!	#NUM!
#NUM!	#NUM!	#NUM!	#NUM!	#NUM!	#NUM!	#NUM!	#NUM!	#NUM!	#NUM!	#NUM!	#NUM!

#NUM!	#NUM!	#NUM!	#NUM!	#NUM!	#NUM!	#NUM!	#NUM!	25.6014	25.5218	25.837	#NUM!
#NUM!	#NUM!	#NUM!	#NUM!	#NUM!	#NUM!	#NUM!	#NUM!	24.4997	25.2679	24.6324	24.5473
#NUM!	#NUM!	#NUM!	#NUM!	#NUM!	#NUM!	#NUM!	#NUM!	#NUM!	26.888	26.7531	26.5618
#NUM!	#NUM!	#NUM!	#NUM!	#NUM!	#NUM!	#NUM!	#NUM!	#NUM!	26.8213	27.1149	27.4012
#NUM!	#NUM!	#NUM!	#NUM!	#NUM!	#NUM!	#NUM!	#NUM!	#NUM!	#NUM!	25.8134	#NUM!
#NUM!	#NUM!	#NUM!	#NUM!	#NUM!	#NUM!	#NUM!	#NUM!	#NUM!	#NUM!	#NUM!	26.9093
27.3153	27.5739	#NUM!	26.2759	27.9029	28.3314	27.5989	27.7728	#NUM!	#NUM!	#NUM!	#NUM!
#NUM!	#NUM!	#NUM!	#NUM!	#NUM!	#NUM!	#NUM!	#NUM!	26.5421	26.3283	#NUM!	26.317
#NUM!	#NUM!	#NUM!	#NUM!	#NUM!	#NUM!	#NUM!	#NUM!	26.3392	26.6556	26.545	#NUM!
#NUM!	#NUM!	#NUM!	#NUM!	#NUM!	#NUM!	#NUM!	#NUM!	#NUM!	#NUM!	25.2682	25.891
#NUM!	#NUM!	#NUM!	#NUM!	#NUM!	#NUM!	#NUM!	#NUM!	26.4586	#NUM!	26.3263	26.877
#NUM!	#NUM!	#NUM!	#NUM!	#NUM!	#NUM!	#NUM!	#NUM!	#NUM!	#NUM!	#NUM!	#NUM!
#NUM!	#NUM!	#NUM!	#NUM!	#NUM!	#NUM!	#NUM!	#NUM!	#NUM!	#NUM!	#NUM!	#NUM!
#NUM!	#NUM!	#NUM!	#NUM!	#NUM!	#NUM!	#NUM!	#NUM!	26.2496	#NUM!	#NUM!	26.3927
#NUM!	#NUM!	#NUM!	#NUM!	#NUM!	#NUM!	#NUM!	#NUM!	26.943	26.8199	26.1186	25.7678
#NUM!	#NUM!	#NUM!	#NUM!	#NUM!	#NUM!	#NUM!	#NUM!	25.9697	26.24	26.5273	#NUM!
#NUM!	#NUM!	#NUM!	#NUM!	#NUM!	#NUM!	#NUM!	#NUM!	#NUM!	#NUM!	#NUM!	#NUM!
#NUM!	#NUM!	#NUM!	#NUM!	#NUM!	#NUM!	#NUM!	#NUM!	#NUM!	25.3381	25.2077	25.1449
#NUM!	#NUM!	#NUM!	#NUM!	#NUM!	#NUM!	#NUM!	#NUM!	25.5839	#NUM!	25.2349	#NUM!
#NUM!	#NUM!	#NUM!	#NUM!	#NUM!	#NUM!	#NUM!	#NUM!	27.1812	26.286	26.3414	25.8855
#NUM!	#NUM!	#NUM!	#NUM!	#NUM!	#NUM!	#NUM!	#NUM!	#NUM!	#NUM!	#NUM!	27.6524
#NUM!	#NUM!	#NUM!	#NUM!	#NUM!	#NUM!	#NUM!	#NUM!	34.6064	34.7814	35.3812	35.4528
#NUM!	#NUM!	#NUM!	#NUM!	#NUM!	#NUM!	#NUM!	#NUM!	#NUM!	#NUM!	25.5656	#NUM!
#NUM!	#NUM!	27.7346	#NUM!	#NUM!	27.0421	#NUM!	#NUM!	27.4422	27.4199	27.4498	27.2904
#NUM!	#NUM!	#NUM!	#NUM!	#NUM!	#NUM!	#NUM!	#NUM!	#NUM!	#NUM!	#NUM!	#NUM!
28.6736	28.6113	27.4757	27.5832	27.9858	29.1676	28.1761	28.3834	#NUM!	#NUM!	#NUM!	#NUM!
#NUM!	#NUM!	#NUM!	#NUM!	#NUM!	#NUM!	#NUM!	#NUM!	25.9868	26.0138	25.738	25.6086
#NUM!	#NUM!	#NUM!	#NUM!	#NUM!	#NUM!	#NUM!	#NUM!	25.8582	25.8841	25.0887	#NUM!
#NUM!	#NUM!	#NUM!	#NUM!	#NUM!	#NUM!	#NUM!	#NUM!	#NUM!	#NUM!	#NUM!	#NUM!
#NUM!	27.9195	#NUM!	27.7723	#NUM!	#NUM!	#NUM!	#NUM!	28.6016	28.9201	28.555	28.3768
#NUM!	#NUM!	#NUM!	#NUM!	#NUM!	#NUM!	#NUM!	#NUM!	27.6025	#NUM!	28.2619	28.0648
#NUM!	27.9489	#NUM!	#NUM!	#NUM!	#NUM!	#NUM!	#NUM!	27.0575	27.205	26.5723	26.3504
#NUM!	#NUM!	#NUM!	#NUM!	#NUM!	#NUM!	#NUM!	#NUM!	#NUM!	#NUM!	#NUM!	#NUM!
#NUM!	#NUM!	#NUM!	#NUM!	#NUM!	#NUM!	#NUM!	#NUM!	#NUM!	#NUM!	26.6416	#NUM!
#NUM!	#NUM!	#NUM!	#NUM!	#NUM!	#NUM!	#NUM!	#NUM!	#NUM!	25.8312	26.1434	#NUM!
#NUM!	#NUM!	#NUM!	#NUM!	#NUM!	#NUM!	#NUM!	#NUM!	#NUM!	#NUM!	#NUM!	#NUM!
#NUM!	#NUM!	#NUM!	#NUM!	#NUM!	#NUM!	#NUM!	#NUM!	#NUM!	#NUM!	#NUM!	#NUM!
#NUM!	#NUM!	#NUM!	#NUM!	#NUM!	#NUM!	#NUM!	#NUM!	#NUM!	24.5459	24.1768	24.4123
#NUM!	#NUM!	#NUM!	#NUM!	#NUM!	#NUM!	#NUM!	#NUM!	#NUM!	#NUM!	25.8016	25.5255
#NUM!	#NUM!	#NUM!	#NUM!	#NUM!	#NUM!	#NUM!	#NUM!	#NUM!	#NUM!	#NUM!	#NUM!
#NUM!	#NUM!	#NUM!	#NUM!	#NUM!	#NUM!	#NUM!	#NUM!	25.3664	25.6095	25.3574	25.7061
#NUM!	#NUM!	#NUM!	#NUM!	#NUM!	#NUM!	#NUM!	#NUM!	27.062	#NUM!	27.5259	27.7837
#NUM!	#NUM!	#NUM!	#NUM!	#NUM!	#NUM!	#NUM!	#NUM!	#NUM!	#NUM!	#NUM!	#NUM!
#NUM!	#NUM!	#NUM!	#NUM!	#NUM!	#NUM!	#NUM!	#NUM!	25.1886	25.0644	24.9332	#NUM!
#NUM!	#NUM!	#NUM!	#NUM!	#NUM!	#NUM!	#NUM!	#NUM!	#NUM!	#NUM!	#NUM!	#NUM!
32.042	31.911	30.3228	30.4024	30.9222	31.2156	31.0791	30.3099	29.4273	#NUM!	#NUM!	30.2755
#NUM!	#NUM!	#NUM!	#NUM!	#NUM!	#NUM!	#NUM!	#NUM!	#NUM!	#NUM!	#NUM!	#NUM!
29.051	28.9717	28.5255	28.2126	#NUM!	27.7108	#NUM!	#NUM!	25.9086	25.5559	#NUM!	#NUM!
#NUM!	#NUM!	#NUM!	#NUM!	#NUM!	#NUM!	#NUM!	#NUM!	26.2696	26.5561	26.6498	26.4771
#NUM!	#NUM!	#NUM!	#NUM!	#NUM!	#NUM!	#NUM!	#NUM!	#NUM!	#NUM!	#NUM!	#NUM!
#NUM!	#NUM!	#NUM!	#NUM!	#NUM!	#NUM!	#NUM!	#NUM!	27.8219	27.6936	27.4991	27.57
#NUM!	#NUM!	#NUM!	#NUM!	#NUM!	#NUM!	#NUM!	#NUM!	26.6522	27.3832	27.311	27.4675
#NUM!	#NUM!	#NUM!	#NUM!	#NUM!	#NUM!	#NUM!	#NUM!	#NUM!	#NUM!	#NUM!	25.43
#NUM!	#NUM!	#NUM!	#NUM!	#NUM!	#NUM!	#NUM!	#NUM!	#NUM!	25.2317	25.2925	25.1384
28.6579	28.9843	27.786	28.0536	28.3764	27.6047	28.2434	27.5515	#NUM!	#NUM!	#NUM!	#NUM!
#NUM!	#NUM!	#NUM!	#NUM!	#NUM!	#NUM!	#NUM!	#NUM!	#NUM!	25.2626	25.6569	26.1177
#NUM!	#NUM!	#NUM!	#NUM!	#NUM!	#NUM!	#NUM!	#NUM!	27.1204	26.9445	27.1908	27.1381
#NUM!	#NUM!	#NUM!	#NUM!	#NUM!	#NUM!	#NUM!	#NUM!	26.442	26.4176	26.4303	26.0706
#NUM!	#NUM!	#NUM!	#NUM!	#NUM!	#NUM!	#NUM!	#NUM!	27.3188	26.8836	27.0439	27.1223
#NUM!	#NUM!	#NUM!	#NUM!	#NUM!	#NUM!	#NUM!	#NUM!	#NUM!	#NUM!	26.8554	#NUM!
#NUM!	#NUM!	#NUM!	#NUM!	#NUM!	#NUM!	#NUM!	#NUM!	25.4588	28.8771	31.1904	30.8223
#NUM!	#NUM!	#NUM!	#NUM!	#NUM!	#NUM!	#NUM!	#NUM!	26.7012	26.3808	#NUM!	#NUM!
#NUM!	#NUM!	#NUM!	#NUM!	#NUM!	#NUM!	#NUM!	#NUM!	#NUM!	#NUM!	#NUM!	#NUM!
#NUM!	#NUM!	#NUM!	#NUM!	#NUM!	#NUM!	#NUM!	#NUM!	#NUM!	#NUM!	26.4063	26.0219
#NUM!	#NUM!	#NUM!	#NUM!	#NUM!	#NUM!	#NUM!	#NUM!	27.6968	27.3	27.5262	27.5689
#NUM!	#NUM!	#NUM!	#NUM!	#NUM!	#NUM!	#NUM!	#NUM!	#NUM!	#NUM!	#NUM!	#NUM!
28.1048	28.3627	28.9221	28.6062	28.4487	28.4903	28.4042	#NUM!	27.1788	26.8881	#NUM!	26.9305
28.2084	28.3015	27.7003	27.712	27.96	27.8722	28.1278	27.669	#NUM!	27.1877	#NUM!	27.4351
#NUM!	#NUM!	#NUM!	#NUM!	#NUM!	#NUM!	#NUM!	#NUM!	28.2423	28.4534	27.589	27.9926
#NUM!	#NUM!	#NUM!	#NUM!	#NUM!	#NUM!	#NUM!	#NUM!	#NUM!	25.3832	25.0931	#NUM!
#NUM!	#NUM!	#NUM!	#NUM!	#NUM!	#NUM!	#NUM!	#NUM!	#NUM!	#NUM!	#NUM!	#NUM!
#NUM!	#NUM!	#NUM!	#NUM!	#NUM!	#NUM!	#NUM!	#NUM!	#NUM!	#NUM!	#NUM!	#NUM!
27.3366	27.0887	#NUM!	27.1931	#NUM!	27.1517	#NUM!	27.0582	27.2609	27.0112	27.459	27.3443
#NUM!	#NUM!	#NUM!	#NUM!	#NUM!	#NUM!	#NUM!	#NUM!	#NUM!	#NUM!	26.7786	26.7378
#NUM!	#NUM!	#NUM!	#NUM!	#NUM!	#NUM!	#NUM!	#NUM!	27.4795	27.0729	27.3286	27.2817
#NUM!	#NUM!	#NUM!	#NUM!	#NUM!	#NUM!	#NUM!	#NUM!	28.5111	28.116	27.9181	27.417
#NUM!	#NUM!	#NUM!	#NUM!	#NUM!	#NUM!	#NUM!	#NUM!	#NUM!	#NUM!	#NUM!	#NUM!
#NUM!	#NUM!	#NUM!	#NUM!	#NUM!	#NUM!	#NUM!	#NUM!	25.9829	#NUM!	#NUM!	#NUM!
#NUM!	#NUM!	#NUM!	#NUM!	#NUM!	#NUM!	#NUM!	#NUM!	28.4145	#NUM!	28.178	29.0908
#NUM!	#NUM!	#NUM!	#NUM!	#NUM!	#NUM!	#NUM!	#NUM!	#NUM!	#NUM!	23.3029	#NUM!
#NUM!	#NUM!	#NUM!	#NUM!	#NUM!	#NUM!	#NUM!	#NUM!	27.4598	27.5586	27.3275	27.1527
#NUM!	#NUM!	#NUM!	#NUM!	#NUM!	#NUM!	#NUM!	#NUM!	#NUM!	#NUM!	24.5044	25.1481
#NUM!	#NUM!	#NUM!	#NUM!	#NUM!	#NUM!	#NUM!	#NUM!	#NUM!	#NUM!	27.2571	25.9483
#NUM!	#NUM!	#NUM!	#NUM!	#NUM!	#NUM!	#NUM!	#NUM!	#NUM!	27.1116	#NUM!	#NUM!
#NUM!	#NUM!	#NUM!	#NUM!	#NUM!	#NUM!	#NUM!	#NUM!	25.441	24.9932	24.3178	#NUM!
#NUM!	#NUM!	#NUM!	#NUM!	#NUM!	#NUM!	#NUM!	#NUM!	27.835	27.6689	#NUM!	27.466
26.9856	27.0169	#NUM!	27.4034	#NUM!	26.6939	#NUM!	#NUM!	26.447	26.7572	26.6852	26.7813
28.6425	28.2475	28.7168	28.5218	29.8716	29.1444	29.4234	#NUM!	31.7654	31.6603	31.1251	30.8533
#NUM!	#NUM!	#NUM!	#NUM!	#NUM!	#NUM!	#NUM!	#NUM!	27.8382	#NUM!	27.5677	27.7865
#NUM!	#NUM!	#NUM!	#NUM!	#NUM!	#NUM!	#NUM!	#NUM!	#NUM!	#NUM!	#NUM!	#NUM!
#NUM!	#NUM!	#NUM!	#NUM!	#NUM!	#NUM!	#NUM!	#NUM!	27.3354	26.9019	27.1676	27.4296
28.604	28.6774	#NUM!	27.5086	#NUM!	27.6168	28.4226	28.034	#NUM!	#NUM!	#NUM!	#NUM!
31.8368	32.0224	30.3492	30.0636	29.7343	31.149	29.5192	29.6951	29.0457	28.6093	28.8945	28.7288
27.4706	27.2728	#NUM!	#NUM!	#NUM!	#NUM!	#NUM!	#NUM!	26.4639	26.3831	26.5492	26.4367
#NUM!	#NUM!	#NUM!	#NUM!	#NUM!	#NUM!	#NUM!	#NUM!	#NUM!	#NUM!	26.212	26.2039
#NUM!	#NUM!	#NUM!	#NUM!	#NUM!	#NUM!	#NUM!	#NUM!	26.9214	26.4216	25.8901	26.155
#NUM!	#NUM!	#NUM!	#NUM!	#NUM!	#NUM!	#NUM!	#NUM!	24.8237	24.4301	24.7679	24.793
29.5793	29.4914	27.6963	28.3861	#NUM!	28.9382	#NUM!	29.259	#NUM!	#NUM!	#NUM!	#NUM!
#NUM!	#NUM!	#NUM!	#NUM!	#NUM!	#NUM!	#NUM!	#NUM!	29.0799	27.4415	#NUM!	28.2054
#NUM!	#NUM!	#NUM!	#NUM!	#NUM!	#NUM!	#NUM!	#NUM!	27.3753	27.212	28.2881	27.4227
#NUM!	28.1859	28.4426	28.323	#NUM!	28.5402	#NUM!	#NUM!	27.9252	27.8367	28.3021	27.7719
31.169	31.2994	28.1934	28.3838	30.0216	31.7244	29.844	31.5252	28.9863	29.148	29.319	29.3617
#NUM!	#NUM!	#NUM!	#NUM!	#NUM!	#NUM!	#NUM!	#NUM!	#NUM!	26.4307	26.4663	26.5539
#NUM!	#NUM!	#NUM!	#NUM!	#NUM!	#NUM!	#NUM!	#NUM!	26.748	#NUM!	27.0706	27.2852
#NUM!	#NUM!	#NUM!	#NUM!	#NUM!	#NUM!	#NUM!	#NUM!	#NUM!	25.5402	25.6863	25.3657
#NUM!	#NUM!	#NUM!	#NUM!	#NUM!	#NUM!	#NUM!	#NUM!	#NUM!	#NUM!	#NUM!	25.6477
#NUM!	#NUM!	#NUM!	#NUM!	#NUM!	#NUM!	#NUM!	#NUM!	27.8006	27.9645	24.951	24.9232
#NUM!	#NUM!	#NUM!	#NUM!	#NUM!	#NUM!	#NUM!	#NUM!	#NUM!	#NUM!	#NUM!	27.0016
27.3928	#NUM!	#NUM!	27.0198	#NUM!	#NUM!	#NUM!	#NUM!	27.2852	27.4012	27.0948	27.2614
#NUM!	#NUM!	#NUM!	#NUM!	#NUM!	#NUM!	#NUM!	#NUM!	27.7414	27.3469	27.3274	27.8335
#NUM!	#NUM!	#NUM!	#NUM!	#NUM!	#NUM!	#NUM!	#NUM!	#NUM!	#NUM!	#NUM!	#NUM!
#NUM!	#NUM!	#NUM!	#NUM!	#NUM!	#NUM!	#NUM!	#NUM!	28.4088	28.4086	27.8391	27.8485
#NUM!	#NUM!	#NUM!	#NUM!	#NUM!	#NUM!	#NUM!	#NUM!	30.0727	29.0673	27.7087	27.5451
#NUM!	#NUM!	#NUM!	#NUM!	#NUM!	#NUM!	#NUM!	#NUM!	#NUM!	#NUM!	26.8761	27.2033
28.0245	27.784	27.7577	27.5454	27.5931	27.2425	27.4014	27.2588	#NUM!	25.3794	25.5005	25.4591
#NUM!	#NUM!	#NUM!	#NUM!	#NUM!	#NUM!	#NUM!	#NUM!	27.9716	28.3849	27.988	27.9397
#NUM!	#NUM!	#NUM!	#NUM!	#NUM!	#NUM!	#NUM!	#NUM!	#NUM!	#NUM!	#NUM!	#NUM!
28.2027	28.0958	27.801	27.447	#NUM!	#NUM!	#NUM!	#NUM!	27.9277	#NUM!	29.0064	28.9269
32.928	32.6718	31.0378	31.2576	32.6357	33.1382	32.5038	32.554	#NUM!	#NUM!	#NUM!	#NUM!
#NUM!	#NUM!	#NUM!	#NUM!	#NUM!	#NUM!	#NUM!	#NUM!	28.0433	27.639	28.1334	28.2532
29.1914	#NUM!	29.0711	28.7326	28.5166	28.9026	28.2916	28.3923	29.2043	29.1765	29.0805	28.6676
#NUM!	#NUM!	#NUM!	#NUM!	#NUM!	#NUM!	#NUM!	#NUM!	27.3674	27.0419	28.0781	26.8542
#NUM!	26.0797	#NUM!	#NUM!	#NUM!	#NUM!	#NUM!	#NUM!	27.3039	27.1201	27.6414	27.5382
28.3502	28.6807	#NUM!	#NUM!	#NUM!	28.519	#NUM!	#NUM!	29.9017	29.82	29.8382	30.1266
#NUM!	#NUM!	#NUM!	#NUM!	#NUM!	#NUM!	#NUM!	#NUM!	27.9099	#NUM!	28.0357	28.1418
#NUM!	#NUM!	#NUM!	#NUM!	#NUM!	#NUM!	#NUM!	#NUM!	#NUM!	#NUM!	#NUM!	#NUM!
#NUM!	#NUM!	#NUM!	#NUM!	#NUM!	#NUM!	#NUM!	#NUM!	25.6937	#NUM!	#NUM!	#NUM!
#NUM!	#NUM!	#NUM!	#NUM!	#NUM!	#NUM!	#NUM!	#NUM!	26.2819	26.338	26.0439	26.0515
#NUM!	#NUM!	#NUM!	#NUM!	#NUM!	#NUM!	#NUM!	#NUM!	26.974	26.9382	26.7261	26.7924

#NUM!	#NUM!	#NUM!	#NUM!	#NUM!	#NUM!	#NUM!	#NUM!	#NUM!	#NUM!	#NUM!	#NUM!
#NUM!	#NUM!	#NUM!	#NUM!	#NUM!	#NUM!	#NUM!	#NUM!	#NUM!	#NUM!	27.2439	#NUM!
#NUM!	#NUM!	#NUM!	#NUM!	#NUM!	#NUM!	#NUM!	#NUM!	#NUM!	25.1363	#NUM!	#NUM!
#NUM!	#NUM!	#NUM!	#NUM!	#NUM!	#NUM!	#NUM!	#NUM!	26.5381	26.5516	26.5419	26.6409
#NUM!	28.6356	#NUM!	#NUM!	#NUM!	#NUM!	#NUM!	#NUM!	27.0283	27.107	#NUM!	26.6633
#NUM!	#NUM!	#NUM!	#NUM!	#NUM!	#NUM!	#NUM!	#NUM!	#NUM!	#NUM!	#NUM!	#NUM!
#NUM!	#NUM!	#NUM!	#NUM!	#NUM!	#NUM!	#NUM!	#NUM!	#NUM!	#NUM!	#NUM!	24.4802
#NUM!	#NUM!	#NUM!	#NUM!	#NUM!	#NUM!	#NUM!	#NUM!	#NUM!	26.7153	#NUM!	#NUM!
28.6018	28.7502	28.6269	28.4288	29.411	29.285	29.4075	28.6226	26.3156	26.6972	26.7573	26.7602
#NUM!	#NUM!	#NUM!	#NUM!	#NUM!	#NUM!	#NUM!	#NUM!	#NUM!	#NUM!	#NUM!	#NUM!
#NUM!	#NUM!	#NUM!	#NUM!	#NUM!	#NUM!	#NUM!	#NUM!	26.2485	26.4439	26.3495	26.5173
#NUM!	#NUM!	#NUM!	#NUM!	#NUM!	#NUM!	#NUM!	#NUM!	25.8669	#NUM!	#NUM!	#NUM!
#NUM!	#NUM!	#NUM!	#NUM!	#NUM!	#NUM!	#NUM!	#NUM!	#NUM!	25.777	24.9985	#NUM!
#NUM!	#NUM!	#NUM!	#NUM!	#NUM!	#NUM!	#NUM!	#NUM!	#NUM!	26.1291	#NUM!	25.7108
#NUM!	#NUM!	#NUM!	#NUM!	#NUM!	#NUM!	#NUM!	#NUM!	#NUM!	#NUM!	#NUM!	#NUM!
#NUM!	#NUM!	#NUM!	#NUM!	#NUM!	#NUM!	#NUM!	#NUM!	#NUM!	#NUM!	#NUM!	#NUM!
#NUM!	#NUM!	#NUM!	#NUM!	#NUM!	#NUM!	#NUM!	#NUM!	#NUM!	#NUM!	#NUM!	#NUM!
#NUM!	#NUM!	#NUM!	#NUM!	#NUM!	#NUM!	#NUM!	#NUM!	25.7504	25.8357	25.5996	25.4101
#NUM!	#NUM!	#NUM!	#NUM!	#NUM!	#NUM!	#NUM!	#NUM!	27.0046	26.9159	26.9124	26.9432
#NUM!	#NUM!	#NUM!	#NUM!	#NUM!	#NUM!	#NUM!	#NUM!	#NUM!	#NUM!	24.3517	24.2892
#NUM!	#NUM!	#NUM!	#NUM!	#NUM!	#NUM!	#NUM!	#NUM!	#NUM!	#NUM!	#NUM!	#NUM!
#NUM!	#NUM!	#NUM!	#NUM!	#NUM!	#NUM!	#NUM!	#NUM!	30.7243	30.532	#NUM!	30.4178
#NUM!	#NUM!	#NUM!	28.5863	#NUM!	#NUM!	#NUM!	#NUM!	28.4727	28.8687	28.4572	28.5388
#NUM!	#NUM!	#NUM!	#NUM!	#NUM!	#NUM!	#NUM!	#NUM!	#NUM!	#NUM!	#NUM!	#NUM!
#NUM!	#NUM!	#NUM!	#NUM!	#NUM!	#NUM!	#NUM!	#NUM!	#NUM!	#NUM!	26.9889	27.0737
#NUM!	#NUM!	#NUM!	#NUM!	#NUM!	#NUM!	#NUM!	#NUM!	#NUM!	#NUM!	#NUM!	25.7471
#NUM!	#NUM!	#NUM!	#NUM!	#NUM!	#NUM!	#NUM!	#NUM!	#NUM!	#NUM!	26.9929	#NUM!
#NUM!	#NUM!	#NUM!	#NUM!	#NUM!	#NUM!	#NUM!	#NUM!	#NUM!	#NUM!	#NUM!	#NUM!
#NUM!	#NUM!	#NUM!	#NUM!	#NUM!	#NUM!	#NUM!	#NUM!	#NUM!	#NUM!	#NUM!	#NUM!
#NUM!	#NUM!	#NUM!	#NUM!	#NUM!	#NUM!	#NUM!	#NUM!	25.2171	25.4711	#NUM!	#NUM!
#NUM!	#NUM!	#NUM!	#NUM!	#NUM!	#NUM!	#NUM!	#NUM!	#NUM!	#NUM!	25.4689	#NUM!
#NUM!	#NUM!	#NUM!	#NUM!	#NUM!	#NUM!	#NUM!	#NUM!	#NUM!	26.5794	#NUM!	#NUM!
#NUM!	#NUM!	#NUM!	#NUM!	#NUM!	#NUM!	#NUM!	#NUM!	#NUM!	24.9862	24.9575	25.5526
#NUM!	#NUM!	#NUM!	#NUM!	#NUM!	#NUM!	#NUM!	#NUM!	#NUM!	#NUM!	#NUM!	#NUM!
#NUM!	#NUM!	#NUM!	#NUM!	#NUM!	#NUM!	#NUM!	#NUM!	26.4385	26.8452	26.6036	26.4416
#NUM!	#NUM!	#NUM!	#NUM!	#NUM!	#NUM!	#NUM!	#NUM!	26.8784	27.185	26.9964	26.9291
#NUM!	#NUM!	#NUM!	#NUM!	#NUM!	#NUM!	#NUM!	#NUM!	#NUM!	#NUM!	25.2582	#NUM!
#NUM!	#NUM!	#NUM!	#NUM!	#NUM!	#NUM!	#NUM!	#NUM!	#NUM!	27.7841	26.7322	27.2233
#NUM!	#NUM!	#NUM!	#NUM!	#NUM!	#NUM!	#NUM!	#NUM!	#NUM!	24.1894	24.8858	25.961
#NUM!	#NUM!	#NUM!	#NUM!	#NUM!	#NUM!	#NUM!	#NUM!	#NUM!	#NUM!	#NUM!	#NUM!
#NUM!	#NUM!	#NUM!	#NUM!	#NUM!	#NUM!	#NUM!	#NUM!	25.7618	#NUM!	#NUM!	#NUM!
28.4724	28.3637	29.2792	29.0928	28.7588	28.403	28.6228	#NUM!	27.3621	#NUM!	26.1764	#NUM!
#NUM!	#NUM!	#NUM!	#NUM!	#NUM!	#NUM!	#NUM!	#NUM!	25.4079	25.555	25.0493	24.831
27.7549	#NUM!	#NUM!	#NUM!	#NUM!	#NUM!	#NUM!	#NUM!	25.4818	25.401	25.9285	25.8614
28.7541	28.7163	28.9197	28.745	28.2121	28.488	28.0013	27.7262	#NUM!	#NUM!	25.2978	#NUM!
#NUM!	#NUM!	#NUM!	#NUM!	#NUM!	#NUM!	#NUM!	#NUM!	25.3862	24.8064	#NUM!	#NUM!
28.406	28.6367	#NUM!	26.274	#NUM!	#NUM!	#NUM!	#NUM!	#NUM!	#NUM!	#NUM!	#NUM!
#NUM!	#NUM!	#NUM!	#NUM!	#NUM!	#NUM!	#NUM!	#NUM!	#NUM!	#NUM!	#NUM!	#NUM!
#NUM!	#NUM!	#NUM!	#NUM!	#NUM!	#NUM!	#NUM!	#NUM!	23.8729	#NUM!	24.5637	24.9364
#NUM!	#NUM!	#NUM!	#NUM!	#NUM!	#NUM!	#NUM!	#NUM!	#NUM!	24.8839	25.3513	25.1886
#NUM!	#NUM!	#NUM!	#NUM!	#NUM!	#NUM!	#NUM!	#NUM!	#NUM!	#NUM!	#NUM!	#NUM!
#NUM!	#NUM!	#NUM!	#NUM!	#NUM!	#NUM!	#NUM!	#NUM!	28.4555	28.4368	28.2108	28.5062
#NUM!	#NUM!	#NUM!	#NUM!	#NUM!	#NUM!	#NUM!	#NUM!	#NUM!	#NUM!	25.9606	25.3585
#NUM!	#NUM!	#NUM!	#NUM!	#NUM!	#NUM!	#NUM!	#NUM!	25.8293	25.9801	26.2099	26.3873
#NUM!	#NUM!	#NUM!	#NUM!	#NUM!	#NUM!	#NUM!	#NUM!	26.9591	27.1024	26.6178	26.5013
#NUM!	#NUM!	#NUM!	#NUM!	#NUM!	#NUM!	#NUM!	#NUM!	25.9579	26.1228	26.0259	26.2749
#NUM!	#NUM!	#NUM!	#NUM!	#NUM!	#NUM!	#NUM!	#NUM!	27.789	28.0926	27.9441	28.0245
#NUM!	#NUM!	#NUM!	#NUM!	#NUM!	#NUM!	#NUM!	#NUM!	25.3194	#NUM!	#NUM!	#NUM!
27.9432	28.1673	27.115	26.7699	#NUM!	28.2999	27.4289	27.9362	#NUM!	#NUM!	#NUM!	#NUM!
#NUM!	#NUM!	#NUM!	#NUM!	#NUM!	#NUM!	#NUM!	#NUM!	#NUM!	#NUM!	27.1395	#NUM!
#NUM!	#NUM!	#NUM!	#NUM!	#NUM!	#NUM!	#NUM!	#NUM!	#NUM!	#NUM!	#NUM!	#NUM!
#NUM!	#NUM!	#NUM!	#NUM!	#NUM!	#NUM!	#NUM!	#NUM!	#NUM!	26.9711	26.6756	26.4891
#NUM!	#NUM!	#NUM!	#NUM!	#NUM!	#NUM!	#NUM!	#NUM!	26.5248	#NUM!	#NUM!	26.288
#NUM!	#NUM!	#NUM!	26.6947	#NUM!	#NUM!	#NUM!	#NUM!	#NUM!	26.057	26.0332	26.6994
#NUM!	#NUM!	#NUM!	#NUM!	#NUM!	#NUM!	#NUM!	#NUM!	26.8242	26.2125	26.7715	25.9792
#NUM!	#NUM!	#NUM!	#NUM!	#NUM!	#NUM!	#NUM!	#NUM!	25.9368	#NUM!	25.9579	25.6734
#NUM!	#NUM!	#NUM!	#NUM!	#NUM!	#NUM!	#NUM!	#NUM!	#NUM!	#NUM!	#NUM!	#NUM!
#NUM!	27.7589	#NUM!	#NUM!	#NUM!	#NUM!	#NUM!	#NUM!	25.8725	25.9281	26.4179	26.371
#NUM!	#NUM!	#NUM!	#NUM!	#NUM!	#NUM!	#NUM!	#NUM!	#NUM!	#NUM!	#NUM!	#NUM!
#NUM!	#NUM!	27.055	#NUM!	#NUM!	#NUM!	#NUM!	#NUM!	25.9965	26.1283	26.2148	26.4966
#NUM!	#NUM!	#NUM!	#NUM!	#NUM!	#NUM!	#NUM!	#NUM!	#NUM!	#NUM!	#NUM!	#NUM!
#NUM!	#NUM!	#NUM!	#NUM!	#NUM!	#NUM!	#NUM!	#NUM!	#NUM!	#NUM!	#NUM!	#NUM!
#NUM!	#NUM!	#NUM!	#NUM!	#NUM!	#NUM!	#NUM!	#NUM!	#NUM!	#NUM!	#NUM!	#NUM!
#NUM!	#NUM!	#NUM!	#NUM!	#NUM!	#NUM!	#NUM!	#NUM!	29.7325	#NUM!	29.3379	29.481
#NUM!	#NUM!	#NUM!	#NUM!	#NUM!	#NUM!	#NUM!	#NUM!	#NUM!	25.6632	#NUM!	#NUM!
#NUM!	#NUM!	#NUM!	#NUM!	#NUM!	#NUM!	#NUM!	#NUM!	#NUM!	#NUM!	#NUM!	24.0381
28.022	28.3668	27.6667	27.9181	#NUM!	28.2826	#NUM!	#NUM!	#NUM!	27.2315	27.7656	27.1509
#NUM!	26.3232	#NUM!	#NUM!	#NUM!	#NUM!	#NUM!	#NUM!	#NUM!	#NUM!	#NUM!	#NUM!
28.776	28.1447	#NUM!	28.7491	#NUM!	#NUM!	#NUM!	#NUM!	29.1031	28.6219	29.1046	28.8409
#NUM!	#NUM!	#NUM!	#NUM!	#NUM!	#NUM!	#NUM!	#NUM!	28.9097	28.5068	27.8154	28.0378
#NUM!	#NUM!	#NUM!	#NUM!	#NUM!	#NUM!	#NUM!	#NUM!	26.0318	25.9843	26.1917	#NUM!
#NUM!	#NUM!	#NUM!	#NUM!	#NUM!	#NUM!	#NUM!	#NUM!	#NUM!	26.6263	26.7831	#NUM!
#NUM!	#NUM!	#NUM!	#NUM!	#NUM!	#NUM!	#NUM!	#NUM!	#NUM!	#NUM!	25.9912	26.2851
#NUM!	#NUM!	#NUM!	#NUM!	#NUM!	#NUM!	#NUM!	#NUM!	#NUM!	#NUM!	#NUM!	#NUM!
#NUM!	#NUM!	#NUM!	#NUM!	#NUM!	#NUM!	#NUM!	#NUM!	25.7388	26.1623	#NUM!	26.0918
#NUM!	#NUM!	#NUM!	#NUM!	#NUM!	#NUM!	#NUM!	#NUM!	#NUM!	26.928	26.5668	26.6295
#NUM!	#NUM!	#NUM!	#NUM!	#NUM!	#NUM!	#NUM!	#NUM!	26.2622	#NUM!	26.2654	26.0563
#NUM!	#NUM!	#NUM!	#NUM!	#NUM!	#NUM!	#NUM!	#NUM!	#NUM!	#NUM!	26.4873	26.5522
#NUM!	#NUM!	#NUM!	#NUM!	#NUM!	#NUM!	#NUM!	#NUM!	26.5473	26.9433	26.1548	26.1585
#NUM!	#NUM!	#NUM!	#NUM!	#NUM!	#NUM!	#NUM!	#NUM!	#NUM!	24.7576	24.38	24.3777
#NUM!	#NUM!	#NUM!	#NUM!	#NUM!	#NUM!	#NUM!	#NUM!	#NUM!	#NUM!	#NUM!	#NUM!
#NUM!	#NUM!	#NUM!	#NUM!	#NUM!	#NUM!	#NUM!	#NUM!	#NUM!	#NUM!	#NUM!	27.1195
#NUM!	26.9715	27.7528	#NUM!	#NUM!	27.7122	#NUM!	#NUM!	25.5427	25.8611	26.1489	25.9787
#NUM!	#NUM!	#NUM!	#NUM!	#NUM!	#NUM!	#NUM!	#NUM!	#NUM!	26.5602	25.614	25.4172
#NUM!	#NUM!	#NUM!	#NUM!	#NUM!	#NUM!	#NUM!	#NUM!	#NUM!	#NUM!	#NUM!	26.3997
#NUM!	#NUM!	#NUM!	#NUM!	#NUM!	#NUM!	#NUM!	#NUM!	28.8459	28.4086	27.9183	28.4703
#NUM!	#NUM!	#NUM!	#NUM!	#NUM!	#NUM!	#NUM!	#NUM!	26.7322	26.9688	27.093	26.8671
#NUM!	#NUM!	#NUM!	#NUM!	#NUM!	#NUM!	#NUM!	#NUM!	#NUM!	26.3982	26.5262	26.1371
#NUM!	#NUM!	#NUM!	#NUM!	#NUM!	#NUM!	#NUM!	#NUM!	#NUM!	#NUM!	#NUM!	#NUM!
#NUM!	#NUM!	#NUM!	#NUM!	#NUM!	#NUM!	#NUM!	#NUM!	#NUM!	#NUM!	27.2251	#NUM!
#NUM!	#NUM!	#NUM!	#NUM!	#NUM!	#NUM!	#NUM!	#NUM!	#NUM!	#NUM!	25.65	#NUM!
#NUM!	#NUM!	#NUM!	#NUM!	#NUM!	#NUM!	#NUM!	#NUM!	#NUM!	#NUM!	26.7032	26.7108
#NUM!	#NUM!	#NUM!	#NUM!	#NUM!	#NUM!	#NUM!	#NUM!	24.6764	#NUM!	25.7247	25.5851
#NUM!	#NUM!	#NUM!	#NUM!	#NUM!	#NUM!	#NUM!	#NUM!	#NUM!	24.831	24.9352	#NUM!
#NUM!	#NUM!	#NUM!	#NUM!	#NUM!	#NUM!	#NUM!	#NUM!	#NUM!	#NUM!	#NUM!	#NUM!
#NUM!	#NUM!	#NUM!	#NUM!	#NUM!	#NUM!	#NUM!	#NUM!	26.2582	26.9294	#NUM!	27.311
#NUM!	#NUM!	#NUM!	#NUM!	#NUM!	#NUM!	#NUM!	#NUM!	#NUM!	#NUM!	#NUM!	#NUM!
#NUM!	#NUM!	#NUM!	#NUM!	#NUM!	#NUM!	#NUM!	#NUM!	#NUM!	#NUM!	#NUM!	#NUM!
#NUM!	#NUM!	#NUM!	#NUM!	#NUM!	#NUM!	#NUM!	#NUM!	#NUM!	#NUM!	#NUM!	#NUM!
#NUM!	#NUM!	#NUM!	#NUM!	#NUM!	#NUM!	#NUM!	#NUM!	26.3757	#NUM!	26.1239	25.8673
#NUM!	#NUM!	#NUM!	#NUM!	#NUM!	#NUM!	#NUM!	#NUM!	#NUM!	26.1249	26.4059	26.497
#NUM!	#NUM!	#NUM!	#NUM!	#NUM!	#NUM!	#NUM!	#NUM!	29.5181	29.1736	#NUM!	27.9524
#NUM!	#NUM!	#NUM!	#NUM!	#NUM!	#NUM!	#NUM!	#NUM!	#NUM!	27.4326	#NUM!	#NUM!
#NUM!	#NUM!	#NUM!	#NUM!	#NUM!	#NUM!	#NUM!	#NUM!	#NUM!	#NUM!	#NUM!	#NUM!
#NUM!	#NUM!	#NUM!	#NUM!	#NUM!	#NUM!	#NUM!	#NUM!	#NUM!	#NUM!	#NUM!	#NUM!
#NUM!	#NUM!	#NUM!	#NUM!	#NUM!	#NUM!	#NUM!	#NUM!	27.4166	27.2514	26.8761	26.6579
#NUM!	#NUM!	#NUM!	#NUM!	#NUM!	#NUM!	#NUM!	#NUM!	26.8876	26.8434	26.9311	26.8192
#NUM!	#NUM!	#NUM!	#NUM!	#NUM!	#NUM!	#NUM!	#NUM!	#NUM!	#NUM!	25.2707	25.0814
#NUM!	#NUM!	#NUM!	#NUM!	#NUM!	#NUM!	#NUM!	#NUM!	27.2665	27.3309	#NUM!	27.3835
#NUM!	#NUM!	#NUM!	#NUM!	#NUM!	#NUM!	#NUM!	#NUM!	28.3419	27.7418	27.8722	27.5134
#NUM!	#NUM!	#NUM!	#NUM!	#NUM!	#NUM!	#NUM!	#NUM!	26.1289	24.5938	25.8572	#NUM!
#NUM!	#NUM!	#NUM!	#NUM!	#NUM!	#NUM!	#NUM!	#NUM!	#NUM!	#NUM!	#NUM!	#NUM!
#NUM!	#NUM!	#NUM!	#NUM!	#NUM!	#NUM!	#NUM!	#NUM!	26.442	26.3859	26.8756	26.7562
#NUM!	#NUM!	#NUM!	#NUM!	#NUM!	#NUM!	#NUM!	#NUM!	#NUM!	#NUM!	25.6658	25.7484
#NUM!	#NUM!	#NUM!	#NUM!	#NUM!	#NUM!	#NUM!	#NUM!	26.1332	26.7983	#NUM!	25.6695
#NUM!	#NUM!	#NUM!	#NUM!	#NUM!	#NUM!	#NUM!	#NUM!	27.4272	#NUM!	27.658	27.7785
#NUM!	#NUM!	#NUM!	#NUM!	#NUM!	#NUM!	#NUM!	#NUM!	25.8135	25.8606	25.8218	25.694
#NUM!	#NUM!	#NUM!	#NUM!	#NUM!	#NUM!	#NUM!	#NUM!	#NUM!	28.0865	28.1554	27.6983
#NUM!	#NUM!	#NUM!	#NUM!	#NUM!	#NUM!	#NUM!	#NUM!	#NUM!	#NUM!	#NUM!	#NUM!

#NUM!	#NUM!	#NUM!	#NUM!	#NUM!	#NUM!	#NUM!	#NUM!	26.5794	26.92	27.1315	27.1348
#NUM!	#NUM!	#NUM!	#NUM!	#NUM!	#NUM!	#NUM!	#NUM!	#NUM!	#NUM!	#NUM!	#NUM!
#NUM!	#NUM!	#NUM!	#NUM!	#NUM!	#NUM!	#NUM!	#NUM!	#NUM!	#NUM!	#NUM!	#NUM!
#NUM!	#NUM!	#NUM!	#NUM!	#NUM!	#NUM!	#NUM!	#NUM!	#NUM!	#NUM!	#NUM!	#NUM!
#NUM!	#NUM!	#NUM!	#NUM!	#NUM!	#NUM!	#NUM!	#NUM!	26.3184	26.4515	26.356	26.3525
#NUM!	26.3487	#NUM!	#NUM!	#NUM!	#NUM!	#NUM!	#NUM!	28.7518	28.6065	28.6997	28.5908
28.6948	28.7555	26.7805	27.096	#NUM!	27.7977	27.5198	27.1192	#NUM!	#NUM!	#NUM!	#NUM!
28.7587	28.8205	27.935	27.5258	28.0162	27.5772	27.7955	27.269	#NUM!	#NUM!	#NUM!	#NUM!
31.8193	32.0006	29.106	29.2493	30.9692	31.7579	30.9388	33.1734	30.7834	30.6567	30.9678	30.9132
32.2035	32.5207	28.3793	28.838	29.4197	32.6808	29.7191	33.1247	28.7063	28.7707	28.6601	28.6751
#NUM!	#NUM!	#NUM!	#NUM!	#NUM!	#NUM!	#NUM!	#NUM!	#NUM!	#NUM!	26.1471	26.4153
#NUM!	#NUM!	#NUM!	#NUM!	#NUM!	#NUM!	#NUM!	#NUM!	27.3359	27.5214	28.1179	27.878
28.3037	28.1611	28.2361	28.3044	#NUM!	27.1998	27.2039	26.6273	28.1417	28.2763	27.7192	27.9343
#NUM!	#NUM!	#NUM!	#NUM!	#NUM!	#NUM!	#NUM!	#NUM!	27.0343	26.981	26.8752	26.8427
#NUM!	27.3099	#NUM!	#NUM!	#NUM!	#NUM!	#NUM!	#NUM!	31.3313	30.8742	30.5348	30.3628
#NUM!	#NUM!	#NUM!	#NUM!	#NUM!	#NUM!	#NUM!	#NUM!	25.5694	25.5967	#NUM!	25.295
#NUM!	#NUM!	#NUM!	#NUM!	#NUM!	#NUM!	#NUM!	#NUM!	#NUM!	28.4774	27.8497	#NUM!
27.5387	26.7473	27.6357	28.2681	#NUM!	27.4607	#NUM!	28.0284	27.5544	27.2838	27.8232	27.8305
30.1326	30.213	28.2427	28.1259	29.0163	30.4967	29.443	30.8209	27.3489	27.1892	28.0269	#NUM!
#NUM!	#NUM!	#NUM!	#NUM!	#NUM!	#NUM!	#NUM!	#NUM!	29.9366	29.8943	29.031	29.6115
31.4291	31.7313	28.6248	28.7305	29.9627	31.2326	29.8087	32.1404	29.1622	28.9124	29.2085	28.9282
29.259	28.8914	26.9522	27.8926	29.263	30.9493	29.1668	31.0482	#NUM!	#NUM!	#NUM!	#NUM!
#NUM!	#NUM!	#NUM!	#NUM!	#NUM!	#NUM!	#NUM!	#NUM!	#NUM!	#NUM!	#NUM!	#NUM!
29.7351	29.7821	28.5438	29.005	29.2897	30.0747	29.0309	29.9336	#NUM!	26.6827	27.2351	#NUM!
26.7821	26.6181	#NUM!	#NUM!	#NUM!	#NUM!	#NUM!	#NUM!	26.5406	26.6531	27.1712	#NUM!
28.8433	28.8996	28.3563	28.6728	#NUM!	#NUM!	#NUM!	#NUM!	27.9472	28.0712	28.251	27.9589
32.9311	33.1074	32.5562	32.9403	32.5007	31.9638	32.7092	30.8285	28.9154	28.8799	28.4863	29.0896
29.9393	29.9736	30.1629	30.2378	30.3535	29.1375	29.9727	28.9445	26.4915	26.6195	26.6237	26.553
#NUM!	#NUM!	#NUM!	#NUM!	#NUM!	#NUM!	#NUM!	#NUM!	26.3688	26.5129	26.7616	27.0647
#NUM!	#NUM!	#NUM!	#NUM!	#NUM!	#NUM!	#NUM!	#NUM!	28.137	27.812	28.572	28.6631
#NUM!	#NUM!	#NUM!	#NUM!	#NUM!	#NUM!	#NUM!	#NUM!	26.3873	26.3921	26.3302	26.5237
32.9729	33.5015	32.4697	32.358	33.2504	32.9871	33.3183	33.5433	#NUM!	#NUM!	28.9153	28.8103
#NUM!	#NUM!	#NUM!	#NUM!	#NUM!	#NUM!	#NUM!	#NUM!	26.2791	26.5058	#NUM!	26.3459
#NUM!	#NUM!	#NUM!	#NUM!	#NUM!	#NUM!	#NUM!	#NUM!	#NUM!	25.2763	24.7351	24.9718
#NUM!	#NUM!	#NUM!	#NUM!	#NUM!	#NUM!	#NUM!	#NUM!	#NUM!	#NUM!	#NUM!	#NUM!
#NUM!	#NUM!	#NUM!	#NUM!	#NUM!	#NUM!	#NUM!	#NUM!	#NUM!	#NUM!	28.0556	28.1602
#NUM!	28.3009	#NUM!	27.0787	#NUM!	#NUM!	#NUM!	#NUM!	#NUM!	#NUM!	#NUM!	#NUM!
#NUM!	#NUM!	#NUM!	27.5846	#NUM!	#NUM!	#NUM!	#NUM!	#NUM!	24.7964	25.304	24.8429
29.6882	29.8116	28.9569	28.654	29.3225	29.3914	29.0334	29.3648	26.5701	27.2781	27.4239	27.5817
#NUM!	#NUM!	#NUM!	#NUM!	#NUM!	#NUM!	#NUM!	#NUM!	23.419	24.0464	#NUM!	24.2283
29.9617	30.0288	29.989	30.0251	28.8091	#NUM!	28.762	28.0619	29.6787	29.338	29.2279	29.8275
#NUM!	#NUM!	#NUM!	#NUM!	#NUM!	#NUM!	#NUM!	#NUM!	26.7475	26.7593	26.5705	26.827
#NUM!	#NUM!	#NUM!	#NUM!	#NUM!	#NUM!	#NUM!	#NUM!	#NUM!	#NUM!	#NUM!	26.0475
#NUM!	#NUM!	27.6655	#NUM!	#NUM!	#NUM!	#NUM!	#NUM!	27.4901	26.9787	26.3084	27.0917
#NUM!	#NUM!	#NUM!	#NUM!	#NUM!	#NUM!	#NUM!	#NUM!	#NUM!	#NUM!	25.4613	25.2859
#NUM!	#NUM!	#NUM!	#NUM!	#NUM!	#NUM!	#NUM!	#NUM!	#NUM!	25.3886	#NUM!	25.5705
29.8564	30.3397	27.6339	#NUM!	28.2001	29.8828	28.336	30.6208	28.7965	28.8389	28.9532	29.1935
#NUM!	#NUM!	#NUM!	#NUM!	#NUM!	#NUM!	#NUM!	#NUM!	#NUM!	#NUM!	#NUM!	#NUM!
27.7125	27.4783	26.3556	26.4682	28.027	28.347	27.5142	28.6052	#NUM!	25.5796	26.3859	#NUM!
#NUM!	#NUM!	#NUM!	#NUM!	#NUM!	#NUM!	#NUM!	#NUM!	#NUM!	#NUM!	#NUM!	#NUM!
33.3299	33.3399	30.7954	31.0486	32.3259	32.9856	32.0535	33.8069	31.291	30.8564	31.0321	31.1068
#NUM!	#NUM!	#NUM!	#NUM!	#NUM!	#NUM!	#NUM!	#NUM!	25.4618	25.7555	25.6372	25.6764
#NUM!	#NUM!	#NUM!	#NUM!	#NUM!	#NUM!	#NUM!	#NUM!	#NUM!	#NUM!	#NUM!	25.8685
#NUM!	#NUM!	#NUM!	#NUM!	#NUM!	#NUM!	#NUM!	#NUM!	25.9325	#NUM!	#NUM!	#NUM!
28.33	28.6681	27.9475	27.8129	28.3697	29.3198	28.0992	28.619	#NUM!	#NUM!	#NUM!	#NUM!
#NUM!	29.0809	#NUM!	29.7953	28.9972	#NUM!	#NUM!	28.643	#NUM!	27.9277	28.2999	28.3136
#NUM!	#NUM!	#NUM!	#NUM!	#NUM!	#NUM!	#NUM!	#NUM!	27.6885	27.8488	27.3619	27.7952
#NUM!	#NUM!	#NUM!	#NUM!	#NUM!	#NUM!	#NUM!	#NUM!	30.1922	29.6017	28.9876	28.6989
28.477	#NUM!	#NUM!	28.1308	#NUM!	#NUM!	#NUM!	#NUM!	29.4175	29.4285	29.7935	29.9321
27.1882	#NUM!	#NUM!	#NUM!	#NUM!	#NUM!	#NUM!	#NUM!	27.4943	26.9925	27.5878	27.4935
27.6672	#NUM!	27.9499	27.5417	#NUM!	#NUM!	#NUM!	#NUM!	29.9846	30.0012	30.0532	30.2546
#NUM!	#NUM!	#NUM!	#NUM!	#NUM!	#NUM!	#NUM!	#NUM!	#NUM!	#NUM!	27.3452	27.6354
29.4064	29.4292	28.5543	28.2625	#NUM!	#NUM!	#NUM!	#NUM!	30.1652	30.0425	30.0582	#NUM!
#NUM!	#NUM!	#NUM!	#NUM!	#NUM!	#NUM!	#NUM!	#NUM!	26.5021	26.2398	26.3015	26.2863
#NUM!	#NUM!	#NUM!	#NUM!	#NUM!	#NUM!	#NUM!	#NUM!	26.8173	27.2027	27.7931	27.5598
#NUM!	#NUM!	#NUM!	#NUM!	#NUM!	#NUM!	#NUM!	#NUM!	27.1523	27.3524	27.2302	27.3035
30.767	30.7625	30.1862	29.9865	31.0011	31.4937	31.094	30.844	28.5666	28.185	28.208	28.3751
#NUM!	#NUM!	#NUM!	#NUM!	#NUM!	#NUM!	#NUM!	#NUM!	#NUM!	24.0843	24.5805	24.5363
#NUM!	#NUM!	#NUM!	#NUM!	#NUM!	#NUM!	#NUM!	#NUM!	#NUM!	#NUM!	#NUM!	#NUM!
#NUM!	#NUM!	#NUM!	#NUM!	#NUM!	#NUM!	#NUM!	#NUM!	26.8103	26.6883	26.9212	27.1865
#NUM!	#NUM!	#NUM!	#NUM!	#NUM!	#NUM!	#NUM!	#NUM!	27.0915	27.2253	27.4683	27.4833
#NUM!	#NUM!	#NUM!	#NUM!	#NUM!	#NUM!	#NUM!	#NUM!	#NUM!	#NUM!	27.3929	27.0929
28.8676	28.9567	28.5261	28.497	29.4271	28.8023	29.4793	28.4422	#NUM!	#NUM!	#NUM!	#NUM!
#NUM!	#NUM!	#NUM!	#NUM!	#NUM!	#NUM!	#NUM!	#NUM!	26.3576	26.2783	26.3478	26.0086
#NUM!	#NUM!	#NUM!	#NUM!	#NUM!	#NUM!	#NUM!	#NUM!	29.1626	#NUM!	28.7235	28.6424
#NUM!	#NUM!	#NUM!	#NUM!	#NUM!	#NUM!	#NUM!	#NUM!	#NUM!	#NUM!	#NUM!	#NUM!
#NUM!	#NUM!	#NUM!	#NUM!	#NUM!	#NUM!	#NUM!	#NUM!	28.1963	28.1755	27.751	27.4996
#NUM!	#NUM!	#NUM!	#NUM!	#NUM!	#NUM!	#NUM!	#NUM!	26.7966	27.2106	27.4108	27.5884
#NUM!	27.629	#NUM!	#NUM!	#NUM!	27.1706	#NUM!	#NUM!	29.378	29.3495	29.4916	29.1707
#NUM!	#NUM!	#NUM!	#NUM!	#NUM!	#NUM!	#NUM!	#NUM!	28.4036	29.1348	28.6542	28.639
#NUM!	#NUM!	#NUM!	#NUM!	#NUM!	#NUM!	#NUM!	#NUM!	#NUM!	#NUM!	#NUM!	#NUM!
28.127	27.8608	27.8025	28.0877	#NUM!	#NUM!	#NUM!	#NUM!	28.5539	28.1862	28.2738	28.246
#NUM!	#NUM!	#NUM!	#NUM!	#NUM!	#NUM!	#NUM!	#NUM!	25.9872	26.0497	26.367	26.3801
#NUM!	#NUM!	#NUM!	#NUM!	#NUM!	#NUM!	#NUM!	#NUM!	#NUM!	#NUM!	#NUM!	#NUM!
#NUM!	#NUM!	#NUM!	#NUM!	#NUM!	#NUM!	#NUM!	#NUM!	26.1067	26.2936	26.1796	26.4374
#NUM!	#NUM!	#NUM!	#NUM!	#NUM!	#NUM!	#NUM!	#NUM!	#NUM!	#NUM!	#NUM!	#NUM!
#NUM!	#NUM!	#NUM!	#NUM!	#NUM!	#NUM!	#NUM!	#NUM!	#NUM!	#NUM!	#NUM!	#NUM!
#NUM!	#NUM!	#NUM!	#NUM!	#NUM!	#NUM!	#NUM!	#NUM!	#NUM!	#NUM!	#NUM!	#NUM!
#NUM!	#NUM!	#NUM!	#NUM!	#NUM!	#NUM!	#NUM!	#NUM!	#NUM!	#NUM!	25.3563	25.1449
#NUM!	#NUM!	#NUM!	#NUM!	#NUM!	#NUM!	#NUM!	#NUM!	#NUM!	#NUM!	#NUM!	#NUM!
#NUM!	#NUM!	#NUM!	#NUM!	#NUM!	#NUM!	#NUM!	#NUM!	#NUM!	#NUM!	#NUM!	25.5827
#NUM!	#NUM!	#NUM!	#NUM!	#NUM!	#NUM!	#NUM!	#NUM!	26.4424	#NUM!	25.5034	#NUM!
#NUM!	#NUM!	#NUM!	#NUM!	#NUM!	#NUM!	#NUM!	#NUM!	#NUM!	#NUM!	#NUM!	#NUM!
#NUM!	#NUM!	#NUM!	#NUM!	#NUM!	#NUM!	#NUM!	#NUM!	25.8258	25.9861	25.5291	25.4362
#NUM!	#NUM!	#NUM!	#NUM!	#NUM!	#NUM!	#NUM!	#NUM!	25.2157	#NUM!	25.8852	25.3121
#NUM!	27.3163	#NUM!	#NUM!	#NUM!	#NUM!	#NUM!	#NUM!	27.9982	27.6945	28.2894	28.1314
29.6868	29.791	28.1292	#NUM!	#NUM!	30.3431	28.8328	30.8272	#NUM!	#NUM!	#NUM!	#NUM!
#NUM!	26.0601	#NUM!	#NUM!	#NUM!	#NUM!	#NUM!	#NUM!	26.2834	26.4615	26.8637	26.5427
#NUM!	#NUM!	#NUM!	#NUM!	#NUM!	#NUM!	#NUM!	#NUM!	#NUM!	27.1572	26.0135	#NUM!
27.8242	27.8629	#NUM!	#NUM!	29.0269	29.1073	28.8437	29.0804	#NUM!	#NUM!	#NUM!	#NUM!
#NUM!	#NUM!	#NUM!	#NUM!	#NUM!	#NUM!	#NUM!	#NUM!	#NUM!	#NUM!	#NUM!	26.9841
#NUM!	#NUM!	#NUM!	#NUM!	#NUM!	#NUM!	#NUM!	#NUM!	28.3919	28.4957	28.0759	27.8853
#NUM!	#NUM!	#NUM!	#NUM!	#NUM!	#NUM!	#NUM!	#NUM!	#NUM!	#NUM!	25.6569	#NUM!
#NUM!	#NUM!	#NUM!	#NUM!	#NUM!	#NUM!	#NUM!	#NUM!	#NUM!	25.7777	#NUM!	#NUM!
#NUM!	#NUM!	#NUM!	#NUM!	#NUM!	#NUM!	#NUM!	#NUM!	26.687	26.9446	26.9069	26.9125
#NUM!	#NUM!	#NUM!	#NUM!	#NUM!	#NUM!	#NUM!	#NUM!	#NUM!	#NUM!	#NUM!	#NUM!
#NUM!	#NUM!	#NUM!	#NUM!	#NUM!	#NUM!	#NUM!	#NUM!	27.61	27.6805	27.1522	27.6681
#NUM!	27.1677	#NUM!	#NUM!	#NUM!	28.0866	#NUM!	#NUM!	26.6243	26.6063	26.5527	26.6138
#NUM!	#NUM!	#NUM!	#NUM!	#NUM!	#NUM!	#NUM!	#NUM!	26.5528	26.5712	27.4869	27.3472
29.5819	29.55	29.4293	29.4823	29.7754	29.2989	29.8097	29.4184	#NUM!	#NUM!	#NUM!	#NUM!
28.3599	28.1889	26.2527	#NUM!	27.8797	29.0107	27.9313	28.9909	#NUM!	#NUM!	#NUM!	#NUM!
#NUM!	#NUM!	#NUM!	#NUM!	#NUM!	#NUM!	#NUM!	#NUM!	#NUM!	#NUM!	#NUM!	#NUM!
#NUM!	#NUM!	#NUM!	#NUM!	#NUM!	#NUM!	#NUM!	#NUM!	26.2383	26.8326	26.2149	26.4155
#NUM!	#NUM!	#NUM!	#NUM!	#NUM!	#NUM!	#NUM!	#NUM!	26.4818	#NUM!	#NUM!	27.8722
#NUM!	#NUM!	#NUM!	#NUM!	#NUM!	#NUM!	#NUM!	#NUM!	#NUM!	#NUM!	#NUM!	#NUM!
#NUM!	#NUM!	#NUM!	#NUM!	#NUM!	#NUM!	#NUM!	#NUM!	#NUM!	#NUM!	#NUM!	#NUM!
#NUM!	#NUM!	#NUM!	#NUM!	#NUM!	#NUM!	#NUM!	#NUM!	#NUM!	#NUM!	25.6594	26.1754
#NUM!	#NUM!	#NUM!	#NUM!	#NUM!	#NUM!	#NUM!	#NUM!	25.3255	25.6779	25.5865	25.763
#NUM!	#NUM!	#NUM!	#NUM!	#NUM!	#NUM!	#NUM!	#NUM!	#NUM!	#NUM!	#NUM!	#NUM!
#NUM!	#NUM!	#NUM!	#NUM!	#NUM!	#NUM!	#NUM!	#NUM!	26.3333	26.0648	26.4731	#NUM!
#NUM!	27.0973	#NUM!	27.3446	#NUM!	27.9182	#NUM!	#NUM!	27.8282	27.7193	28.1457	28.0769
#NUM!	#NUM!	#NUM!	#NUM!	#NUM!	#NUM!	#NUM!	#NUM!	#NUM!	#NUM!	#NUM!	#NUM!
29.7494	29.7128	27.5311	27.5897	29.2744	29.8327	29.3196	30.1594	#NUM!	#NUM!	#NUM!	#NUM!
#NUM!	#NUM!	#NUM!	#NUM!	#NUM!	#NUM!	#NUM!	#NUM!	#NUM!	#NUM!	27.0132	26.8093
#NUM!	#NUM!	#NUM!	#NUM!	#NUM!	#NUM!	#NUM!	#NUM!	#NUM!	#NUM!	#NUM!	#NUM!
#NUM!	#NUM!	#NUM!	#NUM!	#NUM!	#NUM!	#NUM!	#NUM!	#NUM!	#NUM!	27.4386	27.5521
#NUM!	#NUM!	#NUM!	#NUM!	#NUM!	#NUM!	#NUM!	#NUM!	25.8001	25.7165	25.6988	25.6761
#NUM!	#NUM!	#NUM!	#NUM!	#NUM!	#NUM!	#NUM!	#NUM!	27.3839	27.642	27.5229	27.6504
#NUM!	#NUM!	#NUM!	#NUM!	#NUM!	#NUM!	#NUM!	#NUM!	26.8812	27.0791	27.3664	27.2238

#NUM!	#NUM!	#NUM!	#NUM!	#NUM!	#NUM!	#NUM!	#NUM!	#NUM!	#NUM!	27.3119	#NUM!
#NUM!	#NUM!	#NUM!	#NUM!	#NUM!	#NUM!	#NUM!	#NUM!	#NUM!	24.8827	25.7044	25.6049
#NUM!	#NUM!	#NUM!	#NUM!	#NUM!	#NUM!	#NUM!	#NUM!	24.5245	25.0102	24.7792	24.4296
#NUM!	#NUM!	#NUM!	#NUM!	#NUM!	#NUM!	#NUM!	#NUM!	#NUM!	#NUM!	25.7293	25.8919
#NUM!	#NUM!	#NUM!	#NUM!	#NUM!	#NUM!	#NUM!	#NUM!	#NUM!	#NUM!	28.0347	27.8504
#NUM!	#NUM!	#NUM!	#NUM!	#NUM!	#NUM!	#NUM!	#NUM!	26.8389	27.8151	27.9189	27.879
#NUM!	#NUM!	#NUM!	#NUM!	#NUM!	#NUM!	#NUM!	#NUM!	26.9506	#NUM!	26.5523	26.8714
#NUM!	#NUM!	#NUM!	#NUM!	#NUM!	#NUM!	#NUM!	#NUM!	27.002	26.4194	26.5904	26.3852
#NUM!	#NUM!	#NUM!	#NUM!	#NUM!	#NUM!	#NUM!	#NUM!	#NUM!	#NUM!	#NUM!	26.0159
#NUM!	#NUM!	#NUM!	#NUM!	#NUM!	#NUM!	#NUM!	#NUM!	#NUM!	#NUM!	#NUM!	#NUM!
#NUM!	#NUM!	#NUM!	#NUM!	#NUM!	#NUM!	#NUM!	#NUM!	#NUM!	25.0336	#NUM!	#NUM!
#NUM!	#NUM!	#NUM!	#NUM!	#NUM!	#NUM!	#NUM!	#NUM!	#NUM!	#NUM!	#NUM!	#NUM!
#NUM!	#NUM!	#NUM!	#NUM!	#NUM!	#NUM!	#NUM!	#NUM!	#NUM!	25.1657	25.4998	25.4688
#NUM!	#NUM!	#NUM!	#NUM!	#NUM!	#NUM!	#NUM!	#NUM!	28.6047	28.4028	27.6785	27.5753
#NUM!	#NUM!	#NUM!	#NUM!	#NUM!	28.3203	#NUM!	#NUM!	28.6377	28.368	28.3029	28.4666
#NUM!	#NUM!	#NUM!	#NUM!	#NUM!	#NUM!	#NUM!	#NUM!	#NUM!	#NUM!	#NUM!	#NUM!
#NUM!	#NUM!	#NUM!	#NUM!	#NUM!	#NUM!	#NUM!	#NUM!	26.7198	#NUM!	26.0251	25.8316
#NUM!	#NUM!	#NUM!	#NUM!	#NUM!	#NUM!	#NUM!	#NUM!	#NUM!	#NUM!	#NUM!	25.8622
#NUM!	#NUM!	#NUM!	#NUM!	#NUM!	#NUM!	#NUM!	#NUM!	#NUM!	#NUM!	26.977	27.2105
#NUM!	#NUM!	#NUM!	#NUM!	#NUM!	#NUM!	#NUM!	#NUM!	26.4244	#NUM!	25.799	26.3147
#NUM!	#NUM!	#NUM!	#NUM!	#NUM!	#NUM!	#NUM!	#NUM!	#NUM!	26.0757	25.9503	26.0118
#NUM!	#NUM!	#NUM!	#NUM!	#NUM!	#NUM!	#NUM!	#NUM!	25.6128	25.3574	25.7279	24.9672
#NUM!	#NUM!	#NUM!	#NUM!	#NUM!	#NUM!	#NUM!	#NUM!	26.1005	26.2573	26.3948	26.6141
#NUM!	#NUM!	#NUM!	#NUM!	#NUM!	#NUM!	#NUM!	#NUM!	#NUM!	#NUM!	#NUM!	#NUM!
#NUM!	#NUM!	#NUM!	#NUM!	#NUM!	#NUM!	#NUM!	#NUM!	26.4507	26.5368	26.3688	26.6213
#NUM!	#NUM!	#NUM!	#NUM!	#NUM!	#NUM!	#NUM!	#NUM!	#NUM!	25.1032	25.3783	#NUM!
27.9742	27.7523	#NUM!	#NUM!	#NUM!	27.9909	#NUM!	#NUM!	27.3973	27.007	28.0525	27.8917
#NUM!	#NUM!	#NUM!	#NUM!	#NUM!	#NUM!	#NUM!	#NUM!	25.0064	#NUM!	24.7514	#NUM!
#NUM!	#NUM!	#NUM!	#NUM!	#NUM!	#NUM!	#NUM!	#NUM!	25.8107	25.73	25.128	24.9862
#NUM!	#NUM!	#NUM!	#NUM!	#NUM!	#NUM!	#NUM!	#NUM!	#NUM!	#NUM!	#NUM!	#NUM!
#NUM!	#NUM!	#NUM!	#NUM!	#NUM!	#NUM!	#NUM!	#NUM!	26.5854	26.4627	26.6609	26.4207
29.5723	29.5857	29.0908	29.0059	29.7074	29.5	29.5623	28.6909	27.5636	27.8727	27.7304	27.6828
#NUM!	26.967	#NUM!	#NUM!	#NUM!	#NUM!	#NUM!	#NUM!	27.8037	27.6039	27.6605	27.6374
28.9085	29.3293	#NUM!	28.5936	29.1484	29.5561	28.9947	28.7155	28.0132	#NUM!	28.3706	28.2636
28.044	28.3101	28.2427	28.3686	28.8328	28.9971	28.8785	28.6845	28.2985	27.7005	27.8945	27.9133
#NUM!	#NUM!	#NUM!	#NUM!	#NUM!	#NUM!	#NUM!	#NUM!	25.6246	25.609	26.1324	#NUM!
#NUM!	#NUM!	#NUM!	#NUM!	#NUM!	#NUM!	#NUM!	#NUM!	#NUM!	#NUM!	#NUM!	#NUM!
#NUM!	#NUM!	#NUM!	#NUM!	#NUM!	#NUM!	#NUM!	#NUM!	26.575	26.4863	26.6892	26.8444
#NUM!	#NUM!	#NUM!	#NUM!	#NUM!	#NUM!	#NUM!	#NUM!	#NUM!	#NUM!	26.0532	25.8195
#NUM!	#NUM!	#NUM!	27.717	#NUM!	#NUM!	#NUM!	#NUM!	28.8161	28.8375	29.1969	29.1331
28.8063	28.4247	29.0085	#NUM!	#NUM!	#NUM!	#NUM!	#NUM!	26.7771	26.5853	26.5538	26.4799
#NUM!	#NUM!	#NUM!	#NUM!	#NUM!	#NUM!	#NUM!	#NUM!	#NUM!	#NUM!	#NUM!	#NUM!
#NUM!	#NUM!	#NUM!	#NUM!	#NUM!	#NUM!	#NUM!	#NUM!	#NUM!	#NUM!	27.4398	27.4733
#NUM!	#NUM!	#NUM!	#NUM!	#NUM!	#NUM!	#NUM!	#NUM!	26.308	26.079	25.8084	25.7592
#NUM!	#NUM!	#NUM!	#NUM!	#NUM!	#NUM!	#NUM!	#NUM!	26.2898	27.0641	26.4607	26.785
26.9847	27.2088	27.5901	27.5686	#NUM!	#NUM!	#NUM!	#NUM!	28.1485	27.6728	27.385	27.6442
#NUM!	#NUM!	#NUM!	#NUM!	#NUM!	#NUM!	#NUM!	#NUM!	#NUM!	#NUM!	24.7379	#NUM!
#NUM!	#NUM!	#NUM!	#NUM!	#NUM!	#NUM!	#NUM!	#NUM!	27.0785	27.3423	27.4495	27.4674
28.1981	28.3816	28.3077	28.0455	#NUM!	27.2595	#NUM!	27.8693	27.2052	26.9584	27.3167	27.6758
#NUM!	#NUM!	#NUM!	#NUM!	#NUM!	#NUM!	#NUM!	#NUM!	26.5936	26.751	26.6947	27.03
#NUM!	#NUM!	#NUM!	#NUM!	#NUM!	#NUM!	#NUM!	#NUM!	28.4854	27.7025	27.3155	27.0519
#NUM!	#NUM!	#NUM!	#NUM!	#NUM!	#NUM!	#NUM!	#NUM!	#NUM!	#NUM!	#NUM!	25.4057
#NUM!	#NUM!	#NUM!	#NUM!	#NUM!	#NUM!	#NUM!	#NUM!	24.9791	25.752	#NUM!	25.0435
28.4559	28.3908	28.0073	27.8212	28.3799	28.5285	28.3442	28.6726	#NUM!	#NUM!	#NUM!	#NUM!
#NUM!	#NUM!	#NUM!	#NUM!	#NUM!	#NUM!	#NUM!	#NUM!	27.2697	27.5588	#NUM!	#NUM!
#NUM!	#NUM!	#NUM!	#NUM!	#NUM!	#NUM!	#NUM!	#NUM!	#NUM!	25.2784	26.3695	26.2726
#NUM!	26.5532	#NUM!	#NUM!	#NUM!	#NUM!	#NUM!	#NUM!	28.9364	#NUM!	28.1158	27.9704
#NUM!	26.7924	#NUM!	26.7341	#NUM!	#NUM!	#NUM!	#NUM!	27.4383	26.5327	27.634	27.3926
27.1997	26.8266	#NUM!	26.4167	#NUM!	#NUM!	#NUM!	#NUM!	27.6049	27.6669	27.4644	27.4571
#NUM!	#NUM!	#NUM!	#NUM!	#NUM!	#NUM!	#NUM!	#NUM!	#NUM!	#NUM!	26.0921	25.9603
#NUM!	#NUM!	#NUM!	#NUM!	#NUM!	#NUM!	#NUM!	#NUM!	#NUM!	#NUM!	#NUM!	#NUM!
#NUM!	#NUM!	#NUM!	#NUM!	#NUM!	#NUM!	#NUM!	#NUM!	#NUM!	#NUM!	#NUM!	#NUM!
#NUM!	25.8529	#NUM!	#NUM!	#NUM!	27.115	#NUM!	#NUM!	#NUM!	26.6447	26.9531	26.9963
#NUM!	#NUM!	#NUM!	#NUM!	#NUM!	#NUM!	#NUM!	#NUM!	25.9046	26.1241	27.3391	27.0073
28.9724	28.9074	29.571	29.0725	31.8403	30.9517	31.7854	31.4034	#NUM!	#NUM!	#NUM!	#NUM!
#NUM!	#NUM!	#NUM!	#NUM!	#NUM!	#NUM!	#NUM!	#NUM!	#NUM!	#NUM!	#NUM!	#NUM!
#NUM!	#NUM!	#NUM!	#NUM!	#NUM!	#NUM!	#NUM!	#NUM!	#NUM!	#NUM!	#NUM!	#NUM!
30.1805	30.2747	28.3228	28.2863	29.5131	31.3196	29.1939	31.8343	28.1014	28.0725	28.3017	28.8069
31.8952	32.0778	32.604	32.5638	#NUM!	31.9425	#NUM!	#NUM!	30.1757	30.0691	29.8066	30.0183
#NUM!	28.1201	#NUM!	#NUM!	#NUM!	#NUM!	#NUM!	27.8197	27.9525	27.5752	27.7575	#NUM!
32.5427	32.3924	28.7563	29.898	30.0613	35.1765	30.3322	34.3696	30.6099	31.0252	31.35	30.9559
#NUM!	#NUM!	#NUM!	#NUM!	#NUM!	#NUM!	#NUM!	#NUM!	25.6626	25.6618	25.8132	25.6003
27.7619	27.4933	#NUM!	#NUM!	#NUM!	#NUM!	#NUM!	28.3761	27.2529	27.6265	27.3076	27.2141
#NUM!	#NUM!	#NUM!	#NUM!	#NUM!	#NUM!	#NUM!	#NUM!	27.0534	#NUM!	26.8064	27.024
#NUM!	#NUM!	#NUM!	#NUM!	#NUM!	#NUM!	#NUM!	#NUM!	25.9992	26.6965	#NUM!	#NUM!
#NUM!	#NUM!	#NUM!	#NUM!	#NUM!	#NUM!	#NUM!	#NUM!	28.6086	29.132	29.3688	29.0152
#NUM!	26.813	#NUM!	28.4815	#NUM!	#NUM!	#NUM!	#NUM!	#NUM!	24.0351	#NUM!	#NUM!
28.6512	29.087	28.368	28.2459	28.1185	28.0494	28.078	27.3487	#NUM!	#NUM!	#NUM!	#NUM!
#NUM!	#NUM!	#NUM!	#NUM!	#NUM!	#NUM!	#NUM!	#NUM!	25.2647	24.9843	25.0457	25.3178
#NUM!	#NUM!	#NUM!	#NUM!	#NUM!	#NUM!	#NUM!	#NUM!	#NUM!	26.9914	26.3027	#NUM!
#NUM!	#NUM!	#NUM!	#NUM!	#NUM!	#NUM!	#NUM!	#NUM!	#NUM!	#NUM!	#NUM!	#NUM!
#NUM!	#NUM!	#NUM!	#NUM!	#NUM!	#NUM!	#NUM!	#NUM!	26.8487	#NUM!	26.5893	26.5971
28.9684	29.0262	29.4986	29.3076	#NUM!	29.5518	28.4136	#NUM!	30.7565	30.3038	30.4172	30.2873
#NUM!	#NUM!	#NUM!	#NUM!	#NUM!	#NUM!	#NUM!	#NUM!	#NUM!	25.3536	25.5019	25.6531
#NUM!	#NUM!	#NUM!	#NUM!	#NUM!	#NUM!	#NUM!	#NUM!	#NUM!	27.3937	#NUM!	27.0089
28.799	28.6156	27.6633	27.6245	#NUM!	27.7362	#NUM!	28.3643	28.3736	28.8217	28.9022	28.7342
29.0745	29.3303	28.8609	28.4251	28.5624	29.1033	28.7837	28.507	29.3938	29.6045	29.822	30.0702
#NUM!	#NUM!	#NUM!	#NUM!	#NUM!	#NUM!	#NUM!	#NUM!	27.645	27.5549	#NUM!	#NUM!
#NUM!	#NUM!	#NUM!	#NUM!	#NUM!	#NUM!	#NUM!	#NUM!	#NUM!	#NUM!	26.7188	26.4703
31.3953	31.7109	29.0453	28.9955	29.7106	31.3778	29.4832	31.8613	29.4821	29.5239	29.3415	29.3725
#NUM!	#NUM!	#NUM!	#NUM!	#NUM!	#NUM!	#NUM!	#NUM!	#NUM!	#NUM!	#NUM!	#NUM!
#NUM!	#NUM!	#NUM!	#NUM!	#NUM!	#NUM!	#NUM!	#NUM!	#NUM!	#NUM!	#NUM!	#NUM!
27.8031	27.8872	26.1643	#NUM!	#NUM!	27.279	#NUM!	27.4313	27.7733	27.7295	27.7345	27.7286
#NUM!	#NUM!	#NUM!	#NUM!	#NUM!	#NUM!	#NUM!	#NUM!	#NUM!	25.9061	26.2918	26.3639
#NUM!	#NUM!	#NUM!	#NUM!	#NUM!	#NUM!	#NUM!	#NUM!	#NUM!	#NUM!	25.3408	#NUM!
#NUM!	#NUM!	#NUM!	#NUM!	#NUM!	#NUM!	#NUM!	#NUM!	27.5945	27.6178	27.7137	27.9212
28.8856	28.8059	#NUM!	#NUM!	#NUM!	28.9515	#NUM!	28.6513	#NUM!	#NUM!	#NUM!	#NUM!
#NUM!	#NUM!	#NUM!	#NUM!	#NUM!	#NUM!	#NUM!	#NUM!	#NUM!	#NUM!	#NUM!	#NUM!
32.4835	32.4875	33.9584	33.6028	33.6872	32.9069	33.5388	31.437	30.841	30.4479	30.3021	30.5889
#NUM!	#NUM!	#NUM!	#NUM!	#NUM!	#NUM!	#NUM!	#NUM!	29.4744	29.2979	30.0648	29.703
28.4744	28.2171	28.7034	28.4546	#NUM!	#NUM!	#NUM!	#NUM!	28.493	28.4783	27.5598	27.7067
36.9841	37.07	33.5492	33.7015	35.1384	37.5294	34.8385	37.6305	34.1606	34.1208	34.3577	34.2615
#NUM!	#NUM!	#NUM!	#NUM!	#NUM!	#NUM!	#NUM!	#NUM!	27.894	27.6603	28.5247	28.5255
#NUM!	#NUM!	#NUM!	#NUM!	#NUM!	#NUM!	#NUM!	#NUM!	26.2998	27.2458	26.7196	26.7296
#NUM!	#NUM!	#NUM!	#NUM!	#NUM!	#NUM!	#NUM!	#NUM!	#NUM!	#NUM!	#NUM!	#NUM!
#NUM!	#NUM!	#NUM!	#NUM!	#NUM!	#NUM!	#NUM!	#NUM!	#NUM!	#NUM!	#NUM!	#NUM!
#NUM!	#NUM!	#NUM!	#NUM!	#NUM!	#NUM!	#NUM!	#NUM!	26.941	27.4379	27.2537	27.6419
#NUM!	#NUM!	#NUM!	#NUM!	#NUM!	#NUM!	#NUM!	#NUM!	#NUM!	#NUM!	26.1085	26.2519
#NUM!	#NUM!	#NUM!	#NUM!	#NUM!	#NUM!	#NUM!	#NUM!	#NUM!	25.6246	27.4845	#NUM!
#NUM!	#NUM!	#NUM!	#NUM!	#NUM!	#NUM!	#NUM!	#NUM!	#NUM!	#NUM!	26.5504	26.7355
#NUM!	#NUM!	#NUM!	#NUM!	#NUM!	#NUM!	#NUM!	#NUM!	#NUM!	#NUM!	#NUM!	#NUM!
#NUM!	#NUM!	#NUM!	#NUM!	#NUM!	#NUM!	#NUM!	#NUM!	25.7907	25.8985	25.3848	25.6307
29.8222	30.0465	29.2101	29.3337	28.4568	28.745	28.5793	28.4054	28.6183	29.0345	28.9493	29.0746
30.5733	30.5608	28.2246	28.3115	29.6612	30.6768	29.6252	30.7706	27.6688	27.0575	27.0678	26.8373
27.9176	28.2119	#NUM!	28.3324	#NUM!	28.4541	#NUM!	#NUM!	27.869	27.9179	28.1997	28.0042
29.9082	29.8185	29.3911	29.5732	#NUM!	29.8015	#NUM!	#NUM!	29.0645	#NUM!	29.713	28.4804
#NUM!	#NUM!	#NUM!	#NUM!	#NUM!	#NUM!	#NUM!	#NUM!	24.8387	24.8602	#NUM!	#NUM!
#NUM!	#NUM!	#NUM!	#NUM!	#NUM!	#NUM!	#NUM!	#NUM!	#NUM!	#NUM!	#NUM!	#NUM!
#NUM!	#NUM!	#NUM!	#NUM!	#NUM!	#NUM!	#NUM!	#NUM!	#NUM!	#NUM!	#NUM!	25.3668
26.9097	27.0538	27.3015	27.1858	#NUM!	28.8202	#NUM!	27.1396	#NUM!	#NUM!	#NUM!	#NUM!
#NUM!	#NUM!	#NUM!	#NUM!	#NUM!	#NUM!	#NUM!	#NUM!	#NUM!	#NUM!	#NUM!	26.0228
#NUM!	#NUM!	#NUM!	#NUM!	#NUM!	#NUM!	#NUM!	#NUM!	#NUM!	#NUM!	#NUM!	25.507
#NUM!	#NUM!	#NUM!	#NUM!	#NUM!	#NUM!	#NUM!	#NUM!	#NUM!	#NUM!	24.9214	25.0695
#NUM!	#NUM!	#NUM!	#NUM!	#NUM!	#NUM!	#NUM!	#NUM!	25.027	24.1662	25.3684	24.2713
#NUM!	#NUM!	#NUM!	#NUM!	#NUM!	#NUM!	#NUM!	#NUM!	27.0137	26.9725	26.6811	26.6826
#NUM!	#NUM!	#NUM!	#NUM!	#NUM!	#NUM!	#NUM!	#NUM!	#NUM!	25.2716	#NUM!	#NUM!
#NUM!	#NUM!	#NUM!	#NUM!	#NUM!	#NUM!	#NUM!	#NUM!	#NUM!	#NUM!	#NUM!	#NUM!
#NUM!	#NUM!	#NUM!	26.3588	27.7618	28.8513	#NUM!	27.5459	#NUM!	#NUM!	#NUM!	#NUM!
#NUM!	#NUM!	#NUM!	#NUM!	#NUM!	#NUM!	#NUM!	#NUM!	#NUM!	#NUM!	#NUM!	#NUM!

#NUM!	#NUM!	#NUM!	#NUM!	#NUM!	#NUM!	#NUM!	#NUM!	#NUM!	#NUM!	#NUM!	#NUM!
#NUM!	#NUM!	#NUM!	#NUM!	#NUM!	#NUM!	#NUM!	#NUM!	#NUM!	#NUM!	#NUM!	#NUM!
#NUM!	#NUM!	#NUM!	#NUM!	#NUM!	#NUM!	#NUM!	#NUM!	#NUM!	26.0811	25.6779	25.9081
27.4976	27.7527	#NUM!	26.5408	#NUM!	28.0254	#NUM!	#NUM!	27.3229	27.1651	27.3078	27.2652
#NUM!	#NUM!	#NUM!	#NUM!	#NUM!	#NUM!	#NUM!	#NUM!	#NUM!	26.2752	25.8391	#NUM!
#NUM!	#NUM!	#NUM!	#NUM!	#NUM!	#NUM!	#NUM!	#NUM!	26.3246	26.3823	27.2564	26.6863
#NUM!	#NUM!	#NUM!	#NUM!	#NUM!	#NUM!	#NUM!	#NUM!	25.5697	25.4393	25.7027	25.9216
#NUM!	#NUM!	#NUM!	#NUM!	#NUM!	#NUM!	#NUM!	#NUM!	#NUM!	25.8186	26.253	25.8312
#NUM!	#NUM!	#NUM!	#NUM!	#NUM!	#NUM!	#NUM!	#NUM!	26.9216	27.6806	27.1367	26.9198
29.9416	30.1653	29.1211	29.3119	29.5516	29.9855	29.3641	29.4458	#NUM!	#NUM!	#NUM!	#NUM!
#NUM!	#NUM!	#NUM!	#NUM!	#NUM!	#NUM!	#NUM!	#NUM!	#NUM!	26.2344	#NUM!	#NUM!
#NUM!	#NUM!	#NUM!	#NUM!	#NUM!	#NUM!	#NUM!	#NUM!	#NUM!	#NUM!	25.8481	25.4073
#NUM!	#NUM!	#NUM!	#NUM!	#NUM!	#NUM!	#NUM!	#NUM!	25.8622	#NUM!	25.7783	25.9504
#NUM!	#NUM!	#NUM!	#NUM!	#NUM!	#NUM!	#NUM!	#NUM!	25.7571	25.9869	26.6118	26.0451
#NUM!	26.6103	#NUM!	27.173	#NUM!	#NUM!	#NUM!	#NUM!	27.5787	26.612	26.9233	26.7279
29.0165	28.8296	28.0121	#NUM!	27.8695	29.0202	#NUM!	29.7178	28.8574	28.8096	#NUM!	#NUM!
#NUM!	#NUM!	#NUM!	#NUM!	#NUM!	#NUM!	#NUM!	#NUM!	#NUM!	#NUM!	#NUM!	#NUM!
#NUM!	#NUM!	#NUM!	#NUM!	#NUM!	#NUM!	#NUM!	#NUM!	26.6077	27.3252	27.3277	27.3359
#NUM!	#NUM!	#NUM!	#NUM!	#NUM!	#NUM!	#NUM!	#NUM!	26.4112	26.4692	27.1074	27.0979
#NUM!	#NUM!	#NUM!	#NUM!	#NUM!	#NUM!	#NUM!	#NUM!	#NUM!	26.3063	26.0641	25.8468
#NUM!	#NUM!	#NUM!	#NUM!	#NUM!	#NUM!	#NUM!	#NUM!	#NUM!	#NUM!	#NUM!	#NUM!
#NUM!	#NUM!	#NUM!	#NUM!	#NUM!	#NUM!	#NUM!	#NUM!	26.9688	26.6711	#NUM!	26.0624
#NUM!	#NUM!	#NUM!	#NUM!	#NUM!	#NUM!	#NUM!	#NUM!	#NUM!	25.8161	26.042	26.47
#NUM!	25.3207	#NUM!	#NUM!	#NUM!	#NUM!	#NUM!	#NUM!	#NUM!	#NUM!	#NUM!	#NUM!
#NUM!	#NUM!	#NUM!	#NUM!	#NUM!	#NUM!	#NUM!	#NUM!	28.6366	27.7659	#NUM!	28.1697
#NUM!	#NUM!	#NUM!	#NUM!	#NUM!	#NUM!	#NUM!	#NUM!	27.8058	#NUM!	#NUM!	#NUM!
#NUM!	#NUM!	#NUM!	#NUM!	#NUM!	#NUM!	#NUM!	#NUM!	#NUM!	#NUM!	25.1489	#NUM!
#NUM!	#NUM!	#NUM!	#NUM!	#NUM!	#NUM!	#NUM!	#NUM!	#NUM!	23.9149	#NUM!	24.3244
#NUM!	#NUM!	#NUM!	#NUM!	#NUM!	#NUM!	#NUM!	#NUM!	25.4467	26.0451	26.1957	26.0059
#NUM!	#NUM!	#NUM!	#NUM!	#NUM!	#NUM!	#NUM!	#NUM!	28.245	28.1833	28.3981	28.3369
#NUM!	#NUM!	#NUM!	#NUM!	#NUM!	#NUM!	#NUM!	#NUM!	#NUM!	#NUM!	#NUM!	#NUM!
#NUM!	#NUM!	#NUM!	#NUM!	#NUM!	#NUM!	#NUM!	#NUM!	#NUM!	#NUM!	#NUM!	#NUM!
#NUM!	#NUM!	#NUM!	#NUM!	#NUM!	#NUM!	#NUM!	#NUM!	27.5486	27.9069	28.0629	27.8405
#NUM!	#NUM!	#NUM!	#NUM!	#NUM!	#NUM!	#NUM!	#NUM!	#NUM!	#NUM!	25.7139	25.7055
#NUM!	#NUM!	#NUM!	#NUM!	#NUM!	#NUM!	#NUM!	#NUM!	#NUM!	#NUM!	#NUM!	#NUM!
#NUM!	#NUM!	#NUM!	#NUM!	#NUM!	#NUM!	#NUM!	#NUM!	#NUM!	#NUM!	#NUM!	#NUM!
#NUM!	#NUM!	#NUM!	#NUM!	#NUM!	#NUM!	#NUM!	#NUM!	28.4562	28.216	28.3587	27.6963
#NUM!	#NUM!	#NUM!	#NUM!	#NUM!	#NUM!	#NUM!	#NUM!	#NUM!	#NUM!	25.7693	25.5707
28.4698	28.2661	#NUM!	27.9041	#NUM!	#NUM!	#NUM!	27.9664	26.6447	#NUM!	26.8378	26.9964
#NUM!	#NUM!	#NUM!	#NUM!	#NUM!	#NUM!	#NUM!	#NUM!	#NUM!	#NUM!	#NUM!	#NUM!
#NUM!	#NUM!	#NUM!	#NUM!	#NUM!	#NUM!	#NUM!	#NUM!	#NUM!	#NUM!	#NUM!	#NUM!
#NUM!	#NUM!	#NUM!	#NUM!	#NUM!	#NUM!	#NUM!	#NUM!	#NUM!	#NUM!	#NUM!	25.1119
#NUM!	#NUM!	#NUM!	#NUM!	#NUM!	#NUM!	#NUM!	#NUM!	24.9226	25.3001	25.2044	25.157
#NUM!	#NUM!	#NUM!	#NUM!	#NUM!	#NUM!	#NUM!	#NUM!	25.4377	25.4973	25.2581	#NUM!
#NUM!	26.7293	#NUM!	#NUM!	#NUM!	#NUM!	#NUM!	#NUM!	25.2801	#NUM!	25.3848	25.6263
#NUM!	#NUM!	#NUM!	#NUM!	#NUM!	#NUM!	#NUM!	#NUM!	#NUM!	26.7004	26.7032	27.0479
#NUM!	#NUM!	#NUM!	#NUM!	#NUM!	#NUM!	#NUM!	#NUM!	#NUM!	27.585	#NUM!	27.597
28.0386	28.2406	#NUM!	28.0274	#NUM!	#NUM!	#NUM!	27.0257	29.0478	28.9989	28.388	28.3803
#NUM!	#NUM!	#NUM!	#NUM!	#NUM!	#NUM!	#NUM!	#NUM!	#NUM!	28.5239	27.4871	28.9319
#NUM!	#NUM!	#NUM!	#NUM!	#NUM!	#NUM!	#NUM!	#NUM!	25.0299	#NUM!	#NUM!	#NUM!
#NUM!	#NUM!	#NUM!	#NUM!	#NUM!	#NUM!	#NUM!	#NUM!	#NUM!	27.641	#NUM!	#NUM!
#NUM!	#NUM!	#NUM!	#NUM!	#NUM!	#NUM!	#NUM!	#NUM!	#NUM!	#NUM!	26.5578	#NUM!
#NUM!	#NUM!	#NUM!	#NUM!	#NUM!	#NUM!	#NUM!	#NUM!	#NUM!	#NUM!	#NUM!	#NUM!
#NUM!	#NUM!	#NUM!	#NUM!	#NUM!	#NUM!	#NUM!	#NUM!	26.9146	26.9981	27.3326	26.8793
#NUM!	#NUM!	#NUM!	#NUM!	#NUM!	#NUM!	#NUM!	#NUM!	#NUM!	#NUM!	24.5942	25.12
#NUM!	#NUM!	#NUM!	#NUM!	#NUM!	#NUM!	#NUM!	#NUM!	#NUM!	#NUM!	#NUM!	#NUM!
#NUM!	#NUM!	#NUM!	#NUM!	#NUM!	#NUM!	#NUM!	#NUM!	26.414	26.6793	26.7471	26.8954
#NUM!	#NUM!	#NUM!	#NUM!	#NUM!	#NUM!	#NUM!	#NUM!	27.5557	26.9171	26.7616	26.7207
#NUM!	#NUM!	#NUM!	#NUM!	#NUM!	#NUM!	#NUM!	#NUM!	#NUM!	27.3405	#NUM!	#NUM!
28.4041	28.5683	28.4378	28.2002	#NUM!	#NUM!	#NUM!	27.3615	27.3188	27.5674	27.362	27.5325
#NUM!	#NUM!	#NUM!	#NUM!	#NUM!	#NUM!	#NUM!	#NUM!	#NUM!	#NUM!	#NUM!	#NUM!
27.994	28.4589	28.09	27.8484	27.8548	27.7078	#NUM!	27.0639	27.5135	27.0145	27.5601	26.8603
#NUM!	#NUM!	#NUM!	#NUM!	#NUM!	#NUM!	#NUM!	#NUM!	28.0696	28.2884	28.2707	28.1724
#NUM!	#NUM!	#NUM!	#NUM!	#NUM!	#NUM!	#NUM!	#NUM!	26.3356	25.9806	25.9103	26.045
#NUM!	#NUM!	#NUM!	#NUM!	#NUM!	#NUM!	#NUM!	#NUM!	#NUM!	25.9759	26.5579	26.429
#NUM!	28.5926	#NUM!	28.3111	#NUM!	#NUM!	#NUM!	#NUM!	27.5605	#NUM!	#NUM!	#NUM!
#NUM!	#NUM!	#NUM!	#NUM!	#NUM!	#NUM!	#NUM!	#NUM!	#NUM!	26.3115	26.4891	26.3873
27.1505	27.2868	27.4394	#NUM!	#NUM!	#NUM!	#NUM!	#NUM!	26.7584	26.6591	26.9334	26.8172
#NUM!	#NUM!	#NUM!	#NUM!	#NUM!	#NUM!	#NUM!	#NUM!	24.7242	24.6418	25.6244	#NUM!
#NUM!	#NUM!	#NUM!	#NUM!	#NUM!	#NUM!	#NUM!	#NUM!	26.1946	26.1123	26.5633	26.4384
#NUM!	#NUM!	#NUM!	#NUM!	#NUM!	#NUM!	#NUM!	#NUM!	26.0321	26.7568	25.7728	25.9457
27.3891	27.3347	26.9818	26.9724	#NUM!	27.1645	#NUM!	27.7895	27.1771	#NUM!	27.3307	27.3706
27.9451	28.422	#NUM!	29.047	#NUM!	#NUM!	#NUM!	#NUM!	27.8232	27.7988	27.6304	27.3485
#NUM!	#NUM!	#NUM!	#NUM!	#NUM!	#NUM!	#NUM!	#NUM!	25.3459	25.2981	25.3331	25.3953
#NUM!	#NUM!	#NUM!	#NUM!	#NUM!	#NUM!	#NUM!	#NUM!	#NUM!	#NUM!	#NUM!	#NUM!
27.2337	#NUM!	#NUM!	#NUM!	#NUM!	#NUM!	#NUM!	#NUM!	27.4318	27.0407	27.4489	27.4524
#NUM!	27.7551	#NUM!	#NUM!	#NUM!	27.4301	#NUM!	#NUM!	28.2967	#NUM!	27.9972	27.8004
28.4605	28.456	27.5524	27.7029	#NUM!	28.1268	#NUM!	#NUM!	28.67	28.8198	29.0861	29.2683
#NUM!	#NUM!	#NUM!	#NUM!	#NUM!	#NUM!	#NUM!	#NUM!	27.2339	27.081	27.0005	26.6444
#NUM!	#NUM!	#NUM!	#NUM!	#NUM!	#NUM!	#NUM!	#NUM!	26.1653	26.1781	26.1635	#NUM!
#NUM!	#NUM!	#NUM!	#NUM!	#NUM!	#NUM!	#NUM!	#NUM!	#NUM!	#NUM!	#NUM!	#NUM!
#NUM!	27.7488	27.5105	#NUM!	#NUM!	27.784	#NUM!	27.4144	27.4811	27.1159	27.3564	26.643
29.0538	29.0448	#NUM!	#NUM!	#NUM!	#NUM!	#NUM!	#NUM!	29.7251	30.3993	29.0954	29.7696
29.3613	29.5717	29.2438	29.4357	30.0346	29.5139	29.8681	29.2518	27.7726	27.6858	27.7615	27.3785
#NUM!	#NUM!	#NUM!	#NUM!	#NUM!	#NUM!	#NUM!	#NUM!	#NUM!	#NUM!	#NUM!	#NUM!
#NUM!	#NUM!	#NUM!	#NUM!	#NUM!	#NUM!	#NUM!	#NUM!	27.4457	27.497	27.4207	27.6385
27.5988	27.569	#NUM!	27.4131	#NUM!	#NUM!	#NUM!	#NUM!	26.6321	26.8595	#NUM!	27.4088
#NUM!	#NUM!	#NUM!	#NUM!	#NUM!	#NUM!	#NUM!	#NUM!	#NUM!	#NUM!	#NUM!	#NUM!
27.0774	27.2836	#NUM!	#NUM!	#NUM!	26.4874	#NUM!	#NUM!	27.1086	27.369	27.3841	27.558
#NUM!	27.937	#NUM!	#NUM!	#NUM!	#NUM!	#NUM!	27.3857	28.0211	28.1301	28.2144	27.7563
#NUM!	#NUM!	#NUM!	#NUM!	#NUM!	#NUM!	#NUM!	#NUM!	27.3442	27.6525	28.4101	27.5844
#NUM!	#NUM!	#NUM!	#NUM!	#NUM!	#NUM!	#NUM!	#NUM!	#NUM!	#NUM!	#NUM!	#NUM!
#NUM!	#NUM!	#NUM!	#NUM!	#NUM!	#NUM!	#NUM!	#NUM!	#NUM!	24.8836	24.6013	#NUM!
#NUM!	#NUM!	#NUM!	#NUM!	#NUM!	#NUM!	#NUM!	#NUM!	25.7123	26.2859	26.1932	26.0377
#NUM!	#NUM!	#NUM!	#NUM!	#NUM!	#NUM!	#NUM!	#NUM!	#NUM!	#NUM!	25.6951	26.0794
#NUM!	#NUM!	#NUM!	#NUM!	#NUM!	#NUM!	#NUM!	#NUM!	26.1849	25.9186	26.2575	26.059
29.5711	29.585	28.8283	28.6073	#NUM!	28.3763	#NUM!	27.179	#NUM!	27.2884	#NUM!	27.6996
#NUM!	#NUM!	#NUM!	#NUM!	#NUM!	#NUM!	#NUM!	#NUM!	27.0628	26.7446	27.0506	26.7645
#NUM!	#NUM!	#NUM!	25.9634	#NUM!	#NUM!	#NUM!	#NUM!	#NUM!	27.0079	#NUM!	26.5853
#NUM!	#NUM!	#NUM!	#NUM!	#NUM!	#NUM!	#NUM!	#NUM!	27.9336	28.1132	28.5074	28.6052
#NUM!	#NUM!	#NUM!	#NUM!	#NUM!	#NUM!	#NUM!	#NUM!	#NUM!	26.1682	25.9634	26.5049
33.6847	33.7934	32.655	32.7835	33.4821	33.732	33.2383	34.3091	35.3959	34.5915	35.0012	35.3434
#NUM!	27.705	#NUM!	#NUM!	#NUM!	#NUM!	#NUM!	28.4762	26.0742	26.5685	25.8301	26.0827
#NUM!	28.3518	#NUM!	#NUM!	#NUM!	#NUM!	#NUM!	#NUM!	28.6112	28.4112	28.0371	27.7878
27.8738	27.7746	26.9833	27.0563	#NUM!	28.0632	#NUM!	28.1249	27.3918	26.9847	27.3223	27.1961
#NUM!	#NUM!	#NUM!	#NUM!	#NUM!	#NUM!	#NUM!	#NUM!	#NUM!	26.8921	27.5739	27.2267
#NUM!	#NUM!	#NUM!	#NUM!	#NUM!	#NUM!	#NUM!	#NUM!	27.3917	27.3242	26.8283	27.1302
#NUM!	#NUM!	#NUM!	#NUM!	#NUM!	#NUM!	#NUM!	#NUM!	26.8016	27.1881	27.0425	27.0927
32.7018	32.4487	31.6739	32.6175	31.8417	31.7443	31.5622	#NUM!	32.1705	32.1146	31.0318	30.8641
#NUM!	#NUM!	#NUM!	#NUM!	#NUM!	#NUM!	#NUM!	#NUM!	26.5174	26.3273	26.3268	25.5883
#NUM!	#NUM!	#NUM!	27.0394	#NUM!	#NUM!	#NUM!	#NUM!	29.003	28.557	28.3566	28.1715
#NUM!	28.4433	26.6457	26.2153	26.6949	28.6304	#NUM!	#NUM!	28.6085	28.5595	28.7917	28.5776
#NUM!	#NUM!	#NUM!	#NUM!	#NUM!	#NUM!	#NUM!	#NUM!	27.5654	26.9945	27.39	26.8316
#NUM!	#NUM!	#NUM!	#NUM!	#NUM!	#NUM!	#NUM!	#NUM!	29.5986	29.5516	29.7829	29.6487
31.8517	31.4667	28.8829	#NUM!	#NUM!	#NUM!	#NUM!	32.0789	#NUM!	#NUM!	#NUM!	#NUM!
#NUM!	#NUM!	#NUM!	#NUM!	#NUM!	#NUM!	#NUM!	#NUM!	25.1617	25.451	25.572	25.4189
#NUM!	#NUM!	#NUM!	#NUM!	#NUM!	#NUM!	#NUM!	#NUM!	27.5473	26.7608	27.6742	26.773
30.9145	31.0564	28.6654	28.8952	30.058	30.3857	29.6513	31.351	31.2071	30.9112	30.4665	30.3968
#NUM!	#NUM!	#NUM!	#NUM!	#NUM!	#NUM!	#NUM!	#NUM!	27.5326	27.3379	26.9547	26.934
#NUM!	#NUM!	#NUM!	#NUM!	#NUM!	#NUM!	#NUM!	#NUM!	#NUM!	#NUM!	25.1027	24.5922
#NUM!	#NUM!	#NUM!	#NUM!	#NUM!	#NUM!	#NUM!	#NUM!	#NUM!	#NUM!	#NUM!	#NUM!
#NUM!	#NUM!	#NUM!	#NUM!	#NUM!	#NUM!	#NUM!	#NUM!	#NUM!	27.4387	26.2913	26.3158
#NUM!	#NUM!	#NUM!	#NUM!	#NUM!	#NUM!	#NUM!	#NUM!	27.1005	26.8371	26.9351	26.7897
28.9762	28.8421	28.9575	28.3114	#NUM!	#NUM!	#NUM!	#NUM!	#NUM!	27.8759	26.9617	27.2526
#NUM!	#NUM!	#NUM!	#NUM!	#NUM!	#NUM!	#NUM!	#NUM!	#NUM!	#NUM!	26.5359	26.4507
#NUM!	#NUM!	#NUM!	#NUM!	#NUM!	#NUM!	#NUM!	#NUM!	#NUM!	#NUM!	27.7797	27.9663
#NUM!	#NUM!	#NUM!	#NUM!	#NUM!	#NUM!	#NUM!	#NUM!	26.7522	26.5658	27.289	26.586
29.3701	29.3656	27.6921	28.6212	30.3158	29.6896	30.0013	30.0645	27.9783	27.4901	26.8313	27.0632
29.6088	29.7072	29.7771	29.2493	29.7778	30.3344	29.6602	29.488	28.0129	#NUM!	27.4711	27.4107

#NUM!	#NUM!	#NUM!	#NUM!	#NUM!	#NUM!	#NUM!	#NUM!	#NUM!	#NUM!	#NUM!	#NUM!
#NUM!	#NUM!	#NUM!	#NUM!	#NUM!	#NUM!	#NUM!	#NUM!	#NUM!	#NUM!	#NUM!	#NUM!
#NUM!	#NUM!	#NUM!	#NUM!	#NUM!	#NUM!	#NUM!	#NUM!	#NUM!	#NUM!	#NUM!	#NUM!
#NUM!	#NUM!	#NUM!	#NUM!	#NUM!	#NUM!	#NUM!	#NUM!	26.2657	26.2816	26.5906	26.332
#NUM!	#NUM!	#NUM!	#NUM!	#NUM!	#NUM!	#NUM!	#NUM!	26.2315	25.9591	26.0721	26.6578
#NUM!	#NUM!	#NUM!	#NUM!	#NUM!	#NUM!	#NUM!	#NUM!	#NUM!	#NUM!	#NUM!	#NUM!
#NUM!	#NUM!	#NUM!	#NUM!	#NUM!	#NUM!	#NUM!	#NUM!	27.1641	27.0112	27.4304	27.3424
#NUM!	#NUM!	#NUM!	#NUM!	#NUM!	#NUM!	#NUM!	#NUM!	28.0936	27.5254	#NUM!	#NUM!
26.3368	#NUM!	#NUM!	#NUM!	#NUM!	#NUM!	#NUM!	#NUM!	27.6307	27.9405	27.8085	28.1627
#NUM!	27.6517	#NUM!	27.9054	#NUM!	#NUM!	#NUM!	#NUM!	27.3157	26.8141	26.8901	26.6524
31.3422	30.9589	29.6622	30.6213	29.3753	30.3884	29.3495	30.9514	30.4149	30.4594	31.1063	31.0281
27.7844	27.8905	#NUM!	#NUM!	#NUM!	#NUM!	#NUM!	#NUM!	29.1779	28.9209	28.9764	28.6626
33.1416	32.7231	32.8193	33.0574	34.5112	33.4708	33.4899	32.6474	34.5239	34.1311	33.7163	33.8835
#NUM!	28.7539	28.0625	28.5799	#NUM!	28.4022	#NUM!	#NUM!	30.4223	30.2924	30.6449	30.2389
29.5988	30.1108	29.5901	29.7515	28.9563	28.9975	28.639	29.1785	27.7378	27.7351	27.2442	27.415
#NUM!	#NUM!	#NUM!	#NUM!	#NUM!	#NUM!	#NUM!	#NUM!	28.2356	28.4573	27.7908	27.528
#NUM!	#NUM!	#NUM!	#NUM!	#NUM!	#NUM!	#NUM!	#NUM!	26.4536	26.3276	26.2942	26.3284
27.9157	28.0993	#NUM!	28.1174	#NUM!	28.6244	#NUM!	29.3179	28.3825	27.8669	27.8757	27.888
#NUM!	#NUM!	#NUM!	#NUM!	#NUM!	#NUM!	#NUM!	#NUM!	27.2918	27.4298	27.8681	#NUM!
#NUM!	#NUM!	#NUM!	#NUM!	#NUM!	#NUM!	#NUM!	#NUM!	#NUM!	#NUM!	#NUM!	#NUM!
#NUM!	#NUM!	#NUM!	#NUM!	#NUM!	#NUM!	#NUM!	#NUM!	26.3155	26.5998	26.4292	25.9681
#NUM!	#NUM!	#NUM!	#NUM!	#NUM!	#NUM!	#NUM!	#NUM!	28.2723	#NUM!	28.5942	28.596
#NUM!	#NUM!	#NUM!	#NUM!	#NUM!	#NUM!	#NUM!	#NUM!	#NUM!	25.8504	#NUM!	#NUM!
#NUM!	26.2195	#NUM!	26.4897	#NUM!	#NUM!	#NUM!	#NUM!	26.9403	26.3559	26.6244	26.7417
#NUM!	#NUM!	#NUM!	#NUM!	#NUM!	#NUM!	#NUM!	#NUM!	27.9488	27.4541	27.5388	27.5538
#NUM!	#NUM!	#NUM!	#NUM!	#NUM!	#NUM!	#NUM!	#NUM!	26.0843	25.8589	26.4054	26.4037
#NUM!	#NUM!	#NUM!	#NUM!	#NUM!	#NUM!	#NUM!	#NUM!	#NUM!	#NUM!	#NUM!	#NUM!
#NUM!	#NUM!	#NUM!	#NUM!	#NUM!	#NUM!	#NUM!	#NUM!	#NUM!	#NUM!	24.9815	25.4489
#NUM!	26.471	#NUM!	#NUM!	#NUM!	#NUM!	#NUM!	#NUM!	25.805	26.0234	25.5531	25.8461
#NUM!	#NUM!	#NUM!	#NUM!	#NUM!	#NUM!	#NUM!	#NUM!	28.5167	28.6429	28.2863	28.4692
#NUM!	#NUM!	#NUM!	#NUM!	#NUM!	#NUM!	#NUM!	#NUM!	#NUM!	25.5867	25.4324	25.4477
#NUM!	#NUM!	#NUM!	#NUM!	#NUM!	#NUM!	#NUM!	#NUM!	28.5411	27.1885	27.9932	27.4647
#NUM!	#NUM!	#NUM!	#NUM!	#NUM!	#NUM!	#NUM!	#NUM!	25.9562	26.5285	27.1214	26.6498
#NUM!	#NUM!	#NUM!	#NUM!	#NUM!	#NUM!	#NUM!	#NUM!	26.1678	#NUM!	#NUM!	#NUM!
#NUM!	#NUM!	#NUM!	#NUM!	#NUM!	#NUM!	#NUM!	#NUM!	26.8888	26.4077	26.9126	26.8493
#NUM!	#NUM!	#NUM!	#NUM!	#NUM!	#NUM!	#NUM!	#NUM!	28.2767	27.7749	27.5227	27.5822
#NUM!	#NUM!	#NUM!	#NUM!	#NUM!	#NUM!	#NUM!	#NUM!	#NUM!	26.5698	26.9015	26.8359
#NUM!	#NUM!	#NUM!	#NUM!	#NUM!	#NUM!	#NUM!	#NUM!	27.3355	26.96	26.5876	#NUM!
#NUM!	#NUM!	#NUM!	#NUM!	#NUM!	#NUM!	#NUM!	#NUM!	27.7048	27.6406	27.5484	27.5023
#NUM!	26.6428	#NUM!	#NUM!	#NUM!	#NUM!	#NUM!	#NUM!	27.0844	26.8041	26.9533	26.5029
#NUM!	#NUM!	#NUM!	#NUM!	#NUM!	#NUM!	#NUM!	#NUM!	#NUM!	#NUM!	#NUM!	#NUM!
#NUM!	#NUM!	#NUM!	#NUM!	#NUM!	#NUM!	#NUM!	#NUM!	#NUM!	#NUM!	26.3389	26.6768
#NUM!	#NUM!	#NUM!	#NUM!	#NUM!	#NUM!	#NUM!	#NUM!	26.4279	26.1409	25.9251	25.576
#NUM!	#NUM!	#NUM!	#NUM!	#NUM!	#NUM!	#NUM!	#NUM!	26.289	#NUM!	26.7474	26.408
#NUM!	#NUM!	#NUM!	#NUM!	#NUM!	#NUM!	#NUM!	#NUM!	25.3962	26.2315	26.1348	26.4965
#NUM!	#NUM!	#NUM!	#NUM!	#NUM!	#NUM!	#NUM!	#NUM!	#NUM!	26.7539	26.7606	26.9231
#NUM!	#NUM!	#NUM!	#NUM!	#NUM!	#NUM!	#NUM!	#NUM!	27.1119	26.9439	27.2342	27.1607
27.4387	27.0435	#NUM!	26.5663	#NUM!	28.2807	#NUM!	28.5919	27.2875	26.3004	26.2703	26.309
#NUM!	#NUM!	#NUM!	#NUM!	#NUM!	#NUM!	#NUM!	#NUM!	#NUM!	#NUM!	#NUM!	#NUM!
#NUM!	#NUM!	#NUM!	#NUM!	#NUM!	#NUM!	#NUM!	#NUM!	27.8913	28.215	27.1318	26.9694
#NUM!	#NUM!	#NUM!	#NUM!	#NUM!	#NUM!	#NUM!	#NUM!	#NUM!	#NUM!	#NUM!	#NUM!
#NUM!	#NUM!	#NUM!	#NUM!	#NUM!	#NUM!	#NUM!	#NUM!	27.4528	26.6656	26.6568	26.5119
28.2433	28.2923	26.3161	#NUM!	#NUM!	29.0218	#NUM!	28.4738	#NUM!	#NUM!	#NUM!	#NUM!
#NUM!	#NUM!	#NUM!	#NUM!	#NUM!	#NUM!	#NUM!	#NUM!	#NUM!	#NUM!	#NUM!	#NUM!
#NUM!	#NUM!	#NUM!	#NUM!	#NUM!	#NUM!	#NUM!	#NUM!	#NUM!	26.7117	26.7075	27.2449
#NUM!	#NUM!	#NUM!	#NUM!	#NUM!	#NUM!	#NUM!	#NUM!	#NUM!	#NUM!	#NUM!	#NUM!
#NUM!	#NUM!	#NUM!	#NUM!	#NUM!	#NUM!	#NUM!	#NUM!	26.8851	26.8627	26.4924	#NUM!
#NUM!	#NUM!	#NUM!	#NUM!	#NUM!	#NUM!	#NUM!	#NUM!	26.3212	26.3548	26.7894	26.7884
#NUM!	#NUM!	#NUM!	#NUM!	#NUM!	#NUM!	#NUM!	#NUM!	#NUM!	26.8819	26.3547	26.094
#NUM!	#NUM!	#NUM!	#NUM!	#NUM!	#NUM!	#NUM!	#NUM!	#NUM!	25.3063	24.8309	25.0486
#NUM!	#NUM!	#NUM!	#NUM!	#NUM!	#NUM!	#NUM!	#NUM!	25.6531	26.2858	26.3148	26.6474
#NUM!	#NUM!	#NUM!	#NUM!	#NUM!	#NUM!	#NUM!	#NUM!	27.0022	#NUM!	26.7685	27.0138
#NUM!	27.2052	26.7385	#NUM!	#NUM!	#NUM!	#NUM!	#NUM!	29.5935	29.3392	29.5895	29.6597
#NUM!	#NUM!	#NUM!	#NUM!	#NUM!	#NUM!	#NUM!	#NUM!	#NUM!	#NUM!	#NUM!	25.2724
#NUM!	#NUM!	#NUM!	#NUM!	#NUM!	#NUM!	#NUM!	#NUM!	25.9335	25.5523	25.9203	#NUM!
#NUM!	#NUM!	#NUM!	#NUM!	#NUM!	#NUM!	#NUM!	#NUM!	26.8186	26.6103	27.0444	26.8982
#NUM!	#NUM!	#NUM!	#NUM!	#NUM!	#NUM!	#NUM!	#NUM!	#NUM!	#NUM!	#NUM!	#NUM!
#NUM!	#NUM!	#NUM!	#NUM!	#NUM!	#NUM!	#NUM!	#NUM!	26.8642	27.0984	27.5479	27.2308
#NUM!	#NUM!	#NUM!	#NUM!	#NUM!	#NUM!	#NUM!	#NUM!	#NUM!	#NUM!	#NUM!	#NUM!
#NUM!	#NUM!	#NUM!	#NUM!	#NUM!	#NUM!	#NUM!	#NUM!	26.8772	27.4752	26.7114	26.3189
#NUM!	#NUM!	#NUM!	#NUM!	#NUM!	#NUM!	#NUM!	#NUM!	#NUM!	#NUM!	#NUM!	25.1759
#NUM!	#NUM!	#NUM!	#NUM!	#NUM!	#NUM!	#NUM!	#NUM!	25.2907	25.2347	25.2114	25.4623
29.3582	29.2095	28.4808	28.5145	#NUM!	28.9569	#NUM!	28.5698	29.3993	29.2915	29.7183	29.6118
#NUM!	#NUM!	#NUM!	#NUM!	#NUM!	#NUM!	#NUM!	#NUM!	#NUM!	25.6881	25.03	25.7149
#NUM!	#NUM!	#NUM!	#NUM!	#NUM!	#NUM!	#NUM!	#NUM!	28.1562	27.8452	28.572	28.5918
#NUM!	#NUM!	#NUM!	#NUM!	#NUM!	28.5021	#NUM!	#NUM!	26.7677	26.6862	26.4217	26.79
#NUM!	#NUM!	#NUM!	#NUM!	#NUM!	#NUM!	#NUM!	#NUM!	#NUM!	25.2542	#NUM!	24.8182
#NUM!	#NUM!	#NUM!	#NUM!	#NUM!	#NUM!	#NUM!	#NUM!	#NUM!	#NUM!	26.3846	26.4148
#NUM!	#NUM!	#NUM!	#NUM!	#NUM!	#NUM!	#NUM!	#NUM!	26.5562	26.9407	26.6982	27.0363
#NUM!	#NUM!	#NUM!	#NUM!	#NUM!	#NUM!	#NUM!	#NUM!	26.1135	26.4303	26.4242	26.5753
#NUM!	#NUM!	#NUM!	#NUM!	#NUM!	#NUM!	#NUM!	#NUM!	25.3236	25.0123	25.1626	24.7098
#NUM!	#NUM!	#NUM!	#NUM!	#NUM!	#NUM!	#NUM!	#NUM!	#NUM!	#NUM!	#NUM!	#NUM!
#NUM!	#NUM!	#NUM!	#NUM!	#NUM!	#NUM!	#NUM!	#NUM!	26.3612	26.2631	26.1463	26.1805
#NUM!	#NUM!	#NUM!	#NUM!	#NUM!	#NUM!	#NUM!	#NUM!	26.0272	26.3208	26.4535	26.5061
#NUM!	#NUM!	#NUM!	#NUM!	#NUM!	#NUM!	#NUM!	#NUM!	#NUM!	#NUM!	26.9322	#NUM!
#NUM!	#NUM!	#NUM!	#NUM!	#NUM!	#NUM!	#NUM!	#NUM!	#NUM!	#NUM!	#NUM!	#NUM!
#NUM!	#NUM!	#NUM!	#NUM!	#NUM!	#NUM!	#NUM!	#NUM!	#NUM!	27.4041	27.0039	26.6764
#NUM!	#NUM!	#NUM!	#NUM!	#NUM!	#NUM!	#NUM!	#NUM!	#NUM!	#NUM!	#NUM!	#NUM!
#NUM!	#NUM!	#NUM!	#NUM!	#NUM!	#NUM!	#NUM!	#NUM!	#NUM!	#NUM!	#NUM!	#NUM!
#NUM!	#NUM!	#NUM!	#NUM!	#NUM!	#NUM!	#NUM!	#NUM!	#NUM!	#NUM!	#NUM!	#NUM!
28.3314	28.2686	#NUM!	28.0961	#NUM!	#NUM!	#NUM!	27.167	26.9801	27.2446	26.8834	27.3141
#NUM!	#NUM!	#NUM!	#NUM!	#NUM!	#NUM!	#NUM!	#NUM!	#NUM!	28.0263	27.3975	29.5633
28.124	28.652	#NUM!	#NUM!	#NUM!	#NUM!	#NUM!	27.5305	28.3646	28.7427	28.8796	28.8206
#NUM!	#NUM!	#NUM!	#NUM!	#NUM!	#NUM!	#NUM!	#NUM!	#NUM!	25.0202	26.0069	25.9314
#NUM!	27.3446	#NUM!	27.6197	#NUM!	#NUM!	#NUM!	#NUM!	26.2931	26.1768	26.3202	26.0002
#NUM!	#NUM!	#NUM!	#NUM!	#NUM!	#NUM!	#NUM!	#NUM!	27.3212	27.4014	26.3836	26.6519
26.9852	27.2349	27.4688	#NUM!	#NUM!	#NUM!	#NUM!	#NUM!	28.1937	27.9841	28.1173	27.8585
#NUM!	#NUM!	#NUM!	#NUM!	#NUM!	#NUM!	#NUM!	#NUM!	#NUM!	#NUM!	25.311	25.1459
#NUM!	26.2638	#NUM!	#NUM!	#NUM!	#NUM!	#NUM!	#NUM!	#NUM!	#NUM!	#NUM!	#NUM!
#NUM!	#NUM!	#NUM!	#NUM!	#NUM!	#NUM!	#NUM!	#NUM!	28.0742	28.2203	28.1942	29.2199
#NUM!	#NUM!	#NUM!	#NUM!	#NUM!	#NUM!	#NUM!	#NUM!	28.4609	#NUM!	27.9853	27.3834
#NUM!	#NUM!	#NUM!	#NUM!	#NUM!	#NUM!	#NUM!	#NUM!	#NUM!	26.8482	25.31	#NUM!
#NUM!	#NUM!	#NUM!	#NUM!	#NUM!	#NUM!	#NUM!	#NUM!	26.6583	26.3306	26.6275	26.3542
#NUM!	#NUM!	#NUM!	#NUM!	#NUM!	#NUM!	#NUM!	#NUM!	25.8793	25.8648	25.739	26.3326
31.6821	31.5477	31.1199	31.4088	31.3504	31.2394	31.5125	30.9428	28.175	28.1568	28.116	28.0074
#NUM!	#NUM!	#NUM!	#NUM!	#NUM!	#NUM!	#NUM!	#NUM!	#NUM!	26.5275	#NUM!	#NUM!
#NUM!	#NUM!	#NUM!	#NUM!	#NUM!	#NUM!	#NUM!	#NUM!	#NUM!	#NUM!	#NUM!	#NUM!
#NUM!	#NUM!	#NUM!	#NUM!	#NUM!	#NUM!	#NUM!	#NUM!	24.1461	#NUM!	#NUM!	#NUM!
#NUM!	#NUM!	#NUM!	#NUM!	#NUM!	#NUM!	#NUM!	#NUM!	#NUM!	26.2258	26.5981	26.8677
#NUM!	#NUM!	#NUM!	#NUM!	#NUM!	#NUM!	#NUM!	#NUM!	28.1674	28.0938	27.7609	27.7072
#NUM!	#NUM!	#NUM!	#NUM!	#NUM!	#NUM!	#NUM!	#NUM!	#NUM!	#NUM!	#NUM!	#NUM!
#NUM!	#NUM!	#NUM!	#NUM!	#NUM!	#NUM!	#NUM!	#NUM!	#NUM!	#NUM!	#NUM!	#NUM!
#NUM!	#NUM!	#NUM!	#NUM!	#NUM!	#NUM!	#NUM!	#NUM!	26.6171	26.5449	26.3439	26.6873
#NUM!	#NUM!	#NUM!	#NUM!	#NUM!	#NUM!	#NUM!	#NUM!	27.749	27.7707	27.8754	27.6613
#NUM!	#NUM!	#NUM!	#NUM!	#NUM!	#NUM!	#NUM!	#NUM!	26.4944	26.4947	#NUM!	26.1587
#NUM!	#NUM!	#NUM!	#NUM!	#NUM!	#NUM!	#NUM!	#NUM!	#NUM!	26.2536	26.2617	26.7084
28.959	29.7145	29.6471	29.2172	29.0604	29.2981	28.8401	28.6022	27.9493	27.9884	#NUM!	#NUM!
27.473	27.4423	#NUM!	26.9973	#NUM!	27.3859	#NUM!	#NUM!	26.1255	26.1986	25.8864	26.0012
#NUM!	#NUM!	#NUM!	#NUM!	#NUM!	#NUM!	#NUM!	#NUM!	26.7811	26.8848	26.6684	26.6822
#NUM!	#NUM!	#NUM!	#NUM!	#NUM!	#NUM!	#NUM!	#NUM!	26.0972	26.5308	26.3135	26.3381
#NUM!	#NUM!	#NUM!	#NUM!	#NUM!	#NUM!	#NUM!	#NUM!	26.6676	27.1265	27.3962	26.6391
27.3819	#NUM!	27.8061	#NUM!	#NUM!	27.546	#NUM!	#NUM!	28.2474	27.9747	28.0108	27.9726
27.8643	#NUM!	#NUM!	#NUM!	#NUM!	27.763	#NUM!	#NUM!	27.9094	27.6725	27.276	27.2596
#NUM!	#NUM!	#NUM!	#NUM!	#NUM!	#NUM!	#NUM!	#NUM!	#NUM!	25.9068	26.5065	#NUM!
#NUM!	#NUM!	#NUM!	#NUM!	#NUM!	#NUM!	#NUM!	#NUM!	#NUM!	#NUM!	25.6689	25.2828
#NUM!	26.4593	#NUM!	#NUM!	#NUM!	#NUM!	#NUM!	#NUM!	26.6834	26.8093	27.2108	26.7524
#NUM!	#NUM!	#NUM!	#NUM!	#NUM!	#NUM!	#NUM!	#NUM!	26.8531	26.8291	26.9068	26.46
#NUM!	#NUM!	#NUM!	#NUM!	#NUM!	#NUM!	#NUM!	#NUM!	26.1613	26.2014	26.1647	26.3267
#NUM!	#NUM!	#NUM!	#NUM!	#NUM!	#NUM!	#NUM!	#NUM!	27.3736	27.2003	27.4124	27.2311

#NUM!	#NUM!	#NUM!	#NUM!	#NUM!	#NUM!	#NUM!	#NUM!	27.7961	27.384	#NUM!	27.1821
30.0559	29.7546	28.0376	28.6222	28.7767	29.6702	29.0407	29.3866	27.0597	#NUM!	28.1193	28.7491
#NUM!	#NUM!	#NUM!	#NUM!	#NUM!	#NUM!	#NUM!	#NUM!	26.9982	26.7379	26.5642	26.6122
#NUM!	#NUM!	#NUM!	#NUM!	#NUM!	#NUM!	#NUM!	#NUM!	#NUM!	#NUM!	25.8531	25.6431
26.7586	#NUM!	26.5752	26.9006	#NUM!	#NUM!	#NUM!	#NUM!	26.2885	#NUM!	27.1813	27.1321
30.2508	30.2948	30.8109	30.5612	#NUM!	#NUM!	29.245	#NUM!	#NUM!	27.2978	26.8648	27.0318
#NUM!	#NUM!	#NUM!	#NUM!	#NUM!	#NUM!	#NUM!	#NUM!	#NUM!	#NUM!	#NUM!	#NUM!
#NUM!	#NUM!	#NUM!	#NUM!	#NUM!	#NUM!	#NUM!	#NUM!	25.9081	25.2583	25.1271	25.0368
#NUM!	#NUM!	#NUM!	#NUM!	#NUM!	#NUM!	#NUM!	#NUM!	24.7389	24.9217	25.6545	25.4863
#NUM!	#NUM!	#NUM!	#NUM!	#NUM!	#NUM!	#NUM!	#NUM!	#NUM!	#NUM!	#NUM!	#NUM!
#NUM!	#NUM!	#NUM!	#NUM!	#NUM!	#NUM!	#NUM!	#NUM!	26.3331	26.5571	26.3442	25.8674
#NUM!	#NUM!	#NUM!	28.0137	#NUM!	#NUM!	#NUM!	#NUM!	#NUM!	#NUM!	#NUM!	26.71
#NUM!	27.7645	#NUM!	#NUM!	#NUM!	#NUM!	#NUM!	#NUM!	27.181	#NUM!	#NUM!	#NUM!
#NUM!	#NUM!	#NUM!	#NUM!	#NUM!	#NUM!	#NUM!	#NUM!	27.6711	27.8615	27.13	#NUM!
#NUM!	#NUM!	#NUM!	#NUM!	#NUM!	#NUM!	#NUM!	#NUM!	27.9609	27.6705	27.4567	27.5757
32.5417	32.6955	29.2895	28.8191	31.0816	32.4768	30.9024	32.7448	28.6492	29.2095	29.1411	29.0542
31.8283	32.1278	31.1816	30.9608	32.5418	31.3197	32.4029	32.3994	33.5742	33.4826	31.0998	33.4021
#NUM!	#NUM!	#NUM!	#NUM!	#NUM!	#NUM!	#NUM!	#NUM!	27.5249	27.6507	28.4436	#NUM!
#NUM!	#NUM!	#NUM!	#NUM!	#NUM!	#NUM!	#NUM!	#NUM!	#NUM!	#NUM!	25.5552	25.2809
29.0617	29.2518	29.1716	29.0947	29.4914	29.7841	29.7187	29.1404	#NUM!	#NUM!	#NUM!	#NUM!
#NUM!	#NUM!	#NUM!	#NUM!	#NUM!	#NUM!	#NUM!	#NUM!	26.079	26.0538	26.641	26.4237
#NUM!	#NUM!	#NUM!	#NUM!	#NUM!	27.4773	#NUM!	#NUM!	27.5176	#NUM!	#NUM!	28.0066
#NUM!	#NUM!	#NUM!	#NUM!	#NUM!	#NUM!	#NUM!	#NUM!	29.16	28.7923	28.3103	28.184
29.0946	29.5872	29.3085	29.6465	29.8021	28.9858	30.3743	29.0176	#NUM!	26.6261	#NUM!	#NUM!
#NUM!	#NUM!	#NUM!	#NUM!	#NUM!	27.5947	#NUM!	#NUM!	27.5312	27.6305	27.0208	27.3446
27.6128	27.5249	27.7134	27.7052	#NUM!	26.6177	#NUM!	#NUM!	27.1738	26.4595	27.0816	27.0933
27.8463	27.7983	27.3042	27.1061	28.6003	29.7093	28.5205	28.9233	#NUM!	#NUM!	#NUM!	#NUM!
#NUM!	#NUM!	#NUM!	#NUM!	#NUM!	#NUM!	#NUM!	#NUM!	#NUM!	#NUM!	27.0874	#NUM!
30.0715	30.1695	29.7626	29.4931	29.1869	29.6096	29.0165	29.2045	30.2487	30.0844	30.1588	30.5839
#NUM!	#NUM!	#NUM!	#NUM!	#NUM!	#NUM!	#NUM!	#NUM!	#NUM!	24.76	24.443	24.4771
30.2379	30.5312	28.6734	28.2646	29.5569	29.7106	29.4601	30.7291	29.9308	29.9433	29.2497	29.7115
27.2336	27.0186	27.789	27.793	31.4144	29.3202	31.1589	29.9341	25.6462	25.7438	#NUM!	#NUM!
27.7827	27.751	27.7781	#NUM!	#NUM!	27.8648	#NUM!	#NUM!	29.9979	29.8928	30.0687	29.7978
27.6425	27.5311	27.0354	28.0148	#NUM!	26.9939	#NUM!	#NUM!	29.0118	29.1764	29.3036	29.5003
#NUM!	#NUM!	#NUM!	#NUM!	#NUM!	#NUM!	#NUM!	#NUM!	#NUM!	#NUM!	#NUM!	#NUM!
28.1549	27.9315	27.6562	27.2762	29.5396	31.4642	29.583	30.6877	#NUM!	#NUM!	#NUM!	#NUM!
27.7464	#NUM!	#NUM!	28.2049	#NUM!	27.9152	#NUM!	#NUM!	29.2022	28.5572	30.2081	29.3425
28.177	28.0087	#NUM!	27.5526	#NUM!	28.0816	#NUM!	#NUM!	28.2185	28.226	28.1567	28.3193
31.2817	31.2123	31.221	31.2246	30.0326	30.4513	29.9788	29.8311	28.8753	28.7063	28.6004	28.7043
28.8854	29.0556	29.0303	28.4404	28.8822	29.161	28.8233	28.7421	25.8288	26.0982	26.0121	26.5591
#NUM!	27.158	27.4154	26.8526	#NUM!	#NUM!	#NUM!	#NUM!	27.1022	27.3854	27.0625	27.24
#NUM!	27.8592	#NUM!	27.7974	#NUM!	#NUM!	#NUM!	#NUM!	27.5709	27.6945	27.4379	28.0874
#NUM!	#NUM!	#NUM!	#NUM!	#NUM!	#NUM!	#NUM!	#NUM!	27.9763	28.0012	27.8207	28.1454
#NUM!	#NUM!	#NUM!	26.4239	#NUM!	#NUM!	#NUM!	#NUM!	26.2558	26.3936	26.6612	25.7517
28.1171	28.3554	27.8619	27.8517	29.4355	30.8337	29.5727	30.6009	#NUM!	#NUM!	#NUM!	#NUM!
27.7764	27.3915	#NUM!	#NUM!	#NUM!	#NUM!	#NUM!	#NUM!	26.8759	26.4875	26.5526	26.7449
#NUM!	30.3456	#NUM!	#NUM!	#NUM!	#NUM!	#NUM!	#NUM!	31.5318	31.128	30.6083	30.8281
#NUM!	#NUM!	#NUM!	#NUM!	#NUM!	#NUM!	#NUM!	#NUM!	26.166	#NUM!	#NUM!	25.5627
#NUM!	#NUM!	#NUM!	#NUM!	#NUM!	#NUM!	#NUM!	27.1011	27.4731	27.1402	26.8508	27.2133
28.0291	28.259	27.5991	27.5102	#NUM!	28.0522	#NUM!	#NUM!	28.2496	28.3665	28.5971	28.3265
#NUM!	#NUM!	#NUM!	#NUM!	#NUM!	#NUM!	#NUM!	#NUM!	#NUM!	#NUM!	#NUM!	#NUM!
#NUM!	#NUM!	#NUM!	#NUM!	#NUM!	#NUM!	#NUM!	#NUM!	25.4247	25.6358	#NUM!	25.4814
#NUM!	#NUM!	#NUM!	#NUM!	#NUM!	#NUM!	#NUM!	#NUM!	#NUM!	#NUM!	#NUM!	#NUM!
#NUM!	#NUM!	#NUM!	#NUM!	#NUM!	#NUM!	#NUM!	#NUM!	28.9448	28.9312	28.7562	28.9868
#NUM!	#NUM!	27.4489	#NUM!	#NUM!	#NUM!	#NUM!	#NUM!	28.8479	29.1098	29.4617	29.0698
#NUM!	#NUM!	#NUM!	#NUM!	#NUM!	#NUM!	#NUM!	#NUM!	#NUM!	#NUM!	26.9471	26.992
#NUM!	#NUM!	#NUM!	#NUM!	#NUM!	#NUM!	#NUM!	#NUM!	26.3573	26.5308	26.0008	26.1995
#NUM!	#NUM!	#NUM!	#NUM!	#NUM!	#NUM!	#NUM!	#NUM!	#NUM!	#NUM!	25.0542	#NUM!
29.1948	28.7319	28.2004	28.3024	#NUM!	27.6385	#NUM!	27.1139	28.4186	28.2166	28.211	28.1857
28.665	28.4084	29.3142	28.8527	28.6048	29.9296	28.5374	29.0156	#NUM!	#NUM!	28.5363	28.3964
33.0804	32.4611	33.2471	33.4522	33.5904	32.8081	33.4286	32.0579	34.9548	34.1891	34.4221	34.2748
#NUM!	#NUM!	#NUM!	#NUM!	#NUM!	#NUM!	#NUM!	#NUM!	#NUM!	27.1315	27.1574	#NUM!
29.1859	29.1031	28.4856	28.5912	#NUM!	28.3173	#NUM!	#NUM!	31.2521	31.0629	30.9147	30.814
#NUM!	#NUM!	#NUM!	#NUM!	#NUM!	#NUM!	#NUM!	#NUM!	#NUM!	#NUM!	#NUM!	#NUM!
#NUM!	28.9109	#NUM!	#NUM!	#NUM!	#NUM!	#NUM!	#NUM!	27.145	26.204	26.2715	26.4654
#NUM!	#NUM!	#NUM!	#NUM!	#NUM!	#NUM!	#NUM!	#NUM!	#NUM!	#NUM!	26.4972	26.3778
29.5416	29.5322	29.8769	29.6565	29.8794	29.7744	29.8926	29.453	#NUM!	#NUM!	#NUM!	#NUM!
#NUM!	#NUM!	#NUM!	#NUM!	#NUM!	27.2663	#NUM!	#NUM!	26.4445	26.6536	26.72	26.7198
#NUM!	#NUM!	#NUM!	#NUM!	#NUM!	#NUM!	#NUM!	#NUM!	#NUM!	26.3629	26.33	#NUM!
#NUM!	#NUM!	#NUM!	#NUM!	#NUM!	#NUM!	#NUM!	#NUM!	27.8192	27.8146	27.8465	27.6661
#NUM!	27.7921	#NUM!	27.5055	#NUM!	#NUM!	#NUM!	#NUM!	27.5259	27.5103	27.9511	27.9936
#NUM!	#NUM!	#NUM!	#NUM!	#NUM!	#NUM!	#NUM!	#NUM!	26.074	26.2955	26.2228	25.8692
28.0726	28.2636	28.4419	28.5683	28.0891	28.1456	28.035	27.7515	29.1292	29.2281	29.6208	29.6507
27.7624	27.5538	27.5614	27.7573	#NUM!	#NUM!	#NUM!	27.4652	28.2446	28.3301	28.2172	28.0901
#NUM!	27.0232	#NUM!	#NUM!	#NUM!	#NUM!	#NUM!	#NUM!	#NUM!	#NUM!	27.0415	#NUM!
31.8822	32.1616	31.6572	31.5352	31.5509	31.2429	31.295	30.3086	28.2581	28.148	27.8355	27.7651
27.9144	27.9013	26.546	26.2385	27.679	29.0603	27.5637	29.1745	#NUM!	#NUM!	#NUM!	#NUM!
#NUM!	#NUM!	#NUM!	#NUM!	#NUM!	#NUM!	#NUM!	#NUM!	26.6712	26.8704	27.7258	27.5644
27.7373	27.8779	28.3154	27.5192	28.2299	28.2708	28.2168	27.3364	25.1994	25.4788	24.8932	24.8795
#NUM!	#NUM!	#NUM!	#NUM!	#NUM!	#NUM!	#NUM!	#NUM!	26.9942	26.7315	27.147	27.5184
#NUM!	#NUM!	#NUM!	#NUM!	#NUM!	#NUM!	#NUM!	#NUM!	#NUM!	#NUM!	25.2783	25.414
#NUM!	#NUM!	#NUM!	#NUM!	#NUM!	#NUM!	#NUM!	#NUM!	26.4139	25.6527	26.0566	25.9928
28.1307	28.3043	28.6735	28.1628	29.0389	28.3853	28.9733	28.2808	27.2591	27.0614	#NUM!	26.8092
#NUM!	#NUM!	#NUM!	#NUM!	#NUM!	#NUM!	#NUM!	#NUM!	26.3131	26.2548	26.8561	26.5111
26.0536	26.5283	#NUM!	#NUM!	#NUM!	27.0069	#NUM!	#NUM!	25.9552	25.8327	26.0828	26.1075
#NUM!	#NUM!	#NUM!	#NUM!	#NUM!	#NUM!	#NUM!	#NUM!	#NUM!	#NUM!	#NUM!	#NUM!
#NUM!	26.6806	#NUM!	#NUM!	#NUM!	#NUM!	#NUM!	#NUM!	26.5298	26.7815	27.0634	26.8815
#NUM!	#NUM!	#NUM!	#NUM!	#NUM!	#NUM!	#NUM!	#NUM!	26.5839	26.7288	26.8164	26.8906
#NUM!	#NUM!	#NUM!	#NUM!	#NUM!	#NUM!	#NUM!	#NUM!	#NUM!	#NUM!	24.146	24.5849
#NUM!	#NUM!	#NUM!	#NUM!	#NUM!	#NUM!	#NUM!	#NUM!	#NUM!	25.0707	24.922	#NUM!
29.0062	28.6799	29.2165	28.9066	29.0964	29.7775	29.4227	29.3186	#NUM!	#NUM!	#NUM!	#NUM!
#NUM!	#NUM!	#NUM!	#NUM!	#NUM!	#NUM!	#NUM!	#NUM!	26.5134	26.4293	26.7247	26.9623
#NUM!	#NUM!	#NUM!	#NUM!	#NUM!	#NUM!	#NUM!	#NUM!	#NUM!	#NUM!	#NUM!	#NUM!
#NUM!	#NUM!	#NUM!	#NUM!	#NUM!	#NUM!	#NUM!	#NUM!	26.633	#NUM!	#NUM!	#NUM!
#NUM!	#NUM!	#NUM!	#NUM!	#NUM!	#NUM!	#NUM!	#NUM!	#NUM!	26.7256	26.4848	25.514
#NUM!	#NUM!	#NUM!	#NUM!	#NUM!	#NUM!	#NUM!	#NUM!	#NUM!	26.5708	#NUM!	#NUM!
#NUM!	#NUM!	#NUM!	#NUM!	#NUM!	#NUM!	#NUM!	#NUM!	25.6826	25.9871	25.7141	25.9291
#NUM!	#NUM!	#NUM!	#NUM!	#NUM!	#NUM!	#NUM!	#NUM!	#NUM!	#NUM!	26.5653	26.1488
27.5538	27.9753	#NUM!	#NUM!	#NUM!	#NUM!	#NUM!	#NUM!	28.4176	28.8288	28.0992	27.9972
#NUM!	#NUM!	#NUM!	#NUM!	#NUM!	#NUM!	#NUM!	#NUM!	27.8465	27.7118	27.5878	27.4918
#NUM!	#NUM!	#NUM!	#NUM!	#NUM!	#NUM!	#NUM!	#NUM!	25.6344	25.5628	#NUM!	25.7071
#NUM!	#NUM!	#NUM!	#NUM!	#NUM!	#NUM!	#NUM!	#NUM!	#NUM!	#NUM!	#NUM!	25.6502
#NUM!	#NUM!	#NUM!	#NUM!	#NUM!	#NUM!	#NUM!	#NUM!	#NUM!	25.1378	25.2032	24.867
#NUM!	#NUM!	#NUM!	#NUM!	#NUM!	#NUM!	#NUM!	#NUM!	#NUM!	25.1028	24.8674	#NUM!
#NUM!	#NUM!	#NUM!	#NUM!	#NUM!	#NUM!	#NUM!	#NUM!	#NUM!	#NUM!	#NUM!	#NUM!
#NUM!	#NUM!	#NUM!	#NUM!	#NUM!	#NUM!	#NUM!	#NUM!	26.9893	26.713	26.932	#NUM!
#NUM!	#NUM!	#NUM!	#NUM!	#NUM!	#NUM!	#NUM!	#NUM!	#NUM!	26.0566	26.4331	26.3002
#NUM!	27.8161	#NUM!	#NUM!	#NUM!	#NUM!	#NUM!	#NUM!	28.6196	28.8911	28.1163	27.9809
#NUM!	#NUM!	#NUM!	#NUM!	#NUM!	#NUM!	#NUM!	#NUM!	27.4961	27.4793	#NUM!	27.0078
#NUM!	#NUM!	#NUM!	#NUM!	#NUM!	#NUM!	#NUM!	#NUM!	#NUM!	#NUM!	26.2213	26.419
32.1523	32.2482	29.9835	30.2667	31.1821	31.8314	31.0797	32.0891	29.2481	28.634	29.5465	29.0422
#NUM!	#NUM!	#NUM!	#NUM!	#NUM!	#NUM!	#NUM!	#NUM!	25.1212	25.3555	#NUM!	#NUM!
#NUM!	#NUM!	#NUM!	#NUM!	#NUM!	#NUM!	#NUM!	#NUM!	#NUM!	27.0674	26.5399	26.9491
#NUM!	#NUM!	#NUM!	#NUM!	#NUM!	#NUM!	#NUM!	#NUM!	27.0745	26.7917	27.1109	27.6
26.3317	26.406	#NUM!	#NUM!	#NUM!	25.5026	#NUM!	#NUM!	27.6305	27.6684	26.908	26.5918
#NUM!	#NUM!	#NUM!	#NUM!	#NUM!	#NUM!	#NUM!	#NUM!	#NUM!	27.0253	26.7745	#NUM!
#NUM!	#NUM!	#NUM!	#NUM!	#NUM!	#NUM!	#NUM!	#NUM!	24.908	24.7983	24.5747	25.087
#NUM!	#NUM!	#NUM!	#NUM!	#NUM!	#NUM!	#NUM!	#NUM!	#NUM!	#NUM!	#NUM!	#NUM!
#NUM!	#NUM!	#NUM!	#NUM!	#NUM!	#NUM!	#NUM!	#NUM!	#NUM!	#NUM!	#NUM!	#NUM!
#NUM!	#NUM!	#NUM!	#NUM!	#NUM!	#NUM!	#NUM!	#NUM!	25.883	26.0773	26.1997	25.6838
#NUM!	#NUM!	#NUM!	#NUM!	#NUM!	#NUM!	#NUM!	#NUM!	26.299	26.4795	25.9523	25.8388
#NUM!	28.8521	28.2591	28.6559	#NUM!	#NUM!	#NUM!	#NUM!	28.8276	28.2659	28.2355	28.5996
#NUM!	26.5629	#NUM!	#NUM!	#NUM!	#NUM!	#NUM!	#NUM!	26.6856	26.8824	27.0224	27.121
#NUM!	#NUM!	#NUM!	#NUM!	#NUM!	#NUM!	#NUM!	#NUM!	25.303	25.3118	25.3359	25.5433
#NUM!	#NUM!	#NUM!	#NUM!	#NUM!	#NUM!	#NUM!	#NUM!	25.7283	24.846	25.3815	25.2115
#NUM!	#NUM!	#NUM!	#NUM!	#NUM!	#NUM!	#NUM!	#NUM!	#NUM!	#NUM!	#NUM!	26.7011
#NUM!	#NUM!	#NUM!	#NUM!	#NUM!	#NUM!	#NUM!	#NUM!	26.1921	26.3141	#NUM!	25.986
#NUM!	#NUM!	#NUM!	#NUM!	#NUM!	#NUM!	#NUM!	#NUM!	26.9117	26.8367	26.8806	26.9195
#NUM!	#NUM!	#NUM!	#NUM!	#NUM!	#NUM!	#NUM!	#NUM!	25.2076	24.9142	25.2999	25.0461

#NUM!	#NUM!	#NUM!	#NUM!	#NUM!	#NUM!	#NUM!	#NUM!	#NUM!	#NUM!	#NUM!	#NUM!
#NUM!	#NUM!	#NUM!	#NUM!	#NUM!	#NUM!	#NUM!	#NUM!	#NUM!	#NUM!	26.7155	26.7748
#NUM!	#NUM!	#NUM!	#NUM!	#NUM!	#NUM!	#NUM!	#NUM!	#NUM!	26.0499	25.6296	#NUM!
#NUM!	#NUM!	#NUM!	#NUM!	#NUM!	#NUM!	#NUM!	#NUM!	#NUM!	27.1501	27.4887	27.1427
#NUM!	#NUM!	#NUM!	#NUM!	#NUM!	#NUM!	#NUM!	#NUM!	#NUM!	#NUM!	25.2757	25.1604
#NUM!	#NUM!	#NUM!	#NUM!	#NUM!	#NUM!	#NUM!	#NUM!	#NUM!	25.5623	#NUM!	#NUM!
#NUM!	#NUM!	#NUM!	#NUM!	#NUM!	#NUM!	#NUM!	#NUM!	#NUM!	#NUM!	25.2762	25.1697
#NUM!	#NUM!	#NUM!	#NUM!	#NUM!	#NUM!	#NUM!	#NUM!	#NUM!	#NUM!	#NUM!	24.7175
#NUM!	#NUM!	#NUM!	#NUM!	#NUM!	#NUM!	#NUM!	#NUM!	#NUM!	#NUM!	#NUM!	#NUM!
#NUM!	#NUM!	#NUM!	#NUM!	#NUM!	#NUM!	#NUM!	#NUM!	#NUM!	#NUM!	#NUM!	#NUM!
#NUM!	#NUM!	#NUM!	#NUM!	#NUM!	#NUM!	#NUM!	#NUM!	27.3969	27.046	26.6491	#NUM!
30.6521	30.5908	#NUM!	#NUM!	#NUM!	30.2878	#NUM!	#NUM!	33.3498	32.0339	30.6775	32.4819
#NUM!	#NUM!	#NUM!	#NUM!	#NUM!	#NUM!	#NUM!	#NUM!	#NUM!	#NUM!	#NUM!	#NUM!
#NUM!	#NUM!	#NUM!	#NUM!	#NUM!	#NUM!	#NUM!	#NUM!	27.1673	27.5668	26.7051	26.9089
28.1509	28.1341	#NUM!	#NUM!	#NUM!	#NUM!	#NUM!	#NUM!	#NUM!	#NUM!	#NUM!	#NUM!
27.8797	#NUM!	#NUM!	27.9485	27.9077	#NUM!	27.9403	#NUM!	29.0172	28.768	29.0611	29.1346
#NUM!	#NUM!	#NUM!	#NUM!	#NUM!	#NUM!	#NUM!	#NUM!	26.7645	26.7355	26.8057	27.1676
#NUM!	#NUM!	#NUM!	#NUM!	#NUM!	#NUM!	#NUM!	#NUM!	25.5254	25.3185	25.5378	25.5784
#NUM!	#NUM!	#NUM!	#NUM!	#NUM!	#NUM!	#NUM!	#NUM!	#NUM!	25.6006	26.1948	#NUM!
#NUM!	#NUM!	#NUM!	#NUM!	#NUM!	#NUM!	#NUM!	#NUM!	26.8507	27.0361	26.8203	26.5878
#NUM!	#NUM!	#NUM!	#NUM!	#NUM!	#NUM!	#NUM!	#NUM!	27.6569	#NUM!	#NUM!	#NUM!
#NUM!	#NUM!	#NUM!	#NUM!	#NUM!	#NUM!	#NUM!	#NUM!	#NUM!	25.6181	25.5443	25.0297
28.7832	28.7717	29.7941	29.3798	#NUM!	28.5869	#NUM!	27.2935	28.697	28.4465	28.5744	28.2812
#NUM!	#NUM!	#NUM!	#NUM!	#NUM!	#NUM!	#NUM!	#NUM!	25.9059	26.0231	25.6327	25.4846
#NUM!	#NUM!	#NUM!	#NUM!	#NUM!	#NUM!	#NUM!	#NUM!	#NUM!	#NUM!	#NUM!	#NUM!
26.8366	26.8037	#NUM!	26.9998	#NUM!	#NUM!	#NUM!	#NUM!	27.3425	27.1704	26.8786	26.6179
#NUM!	#NUM!	#NUM!	#NUM!	#NUM!	#NUM!	#NUM!	#NUM!	26.1543	26.1298	26.4995	26.7991
#NUM!	#NUM!	#NUM!	#NUM!	#NUM!	#NUM!	#NUM!	#NUM!	#NUM!	#NUM!	27.8873	27.1361
#NUM!	#NUM!	#NUM!	#NUM!	#NUM!	#NUM!	#NUM!	#NUM!	26.9817	27.1971	26.9073	26.7285
#NUM!	29.9198	#NUM!	#NUM!	#NUM!	#NUM!	#NUM!	#NUM!	29.7103	29.6704	30.1032	29.735
#NUM!	#NUM!	#NUM!	#NUM!	#NUM!	#NUM!	#NUM!	#NUM!	29.6207	29.3816	28.9891	28.9998
#NUM!	26.584	#NUM!	#NUM!	#NUM!	#NUM!	#NUM!	#NUM!	27.581	27.5241	27.2914	27.3273
#NUM!	#NUM!	#NUM!	#NUM!	#NUM!	#NUM!	#NUM!	#NUM!	#NUM!	#NUM!	25.7499	25.4121
28.4765	28.5026	28.814	28.5434	#NUM!	#NUM!	#NUM!	#NUM!	#NUM!	#NUM!	#NUM!	#NUM!
#NUM!	#NUM!	#NUM!	#NUM!	#NUM!	#NUM!	#NUM!	#NUM!	28.5004	28.5919	28.5105	28.6275
27.1568	27.3441	28.6249	28.6023	#NUM!	#NUM!	#NUM!	#NUM!	#NUM!	25.4586	25.5527	25.8941
#NUM!	#NUM!	#NUM!	#NUM!	#NUM!	#NUM!	#NUM!	#NUM!	27.8016	27.8864	27.6416	27.6021
#NUM!	#NUM!	#NUM!	#NUM!	#NUM!	#NUM!	#NUM!	#NUM!	25.9583	26.0972	26.7938	26.5455
27.3573	27.5385	27.8648	27.7324	#NUM!	27.1801	#NUM!	#NUM!	27.8987	27.7691	27.7764	27.804
#NUM!	#NUM!	#NUM!	#NUM!	#NUM!	#NUM!	#NUM!	#NUM!	25.0568	26.1621	25.877	26.1555
#NUM!	#NUM!	#NUM!	#NUM!	#NUM!	#NUM!	#NUM!	#NUM!	#NUM!	27.5524	#NUM!	#NUM!
#NUM!	#NUM!	#NUM!	#NUM!	#NUM!	#NUM!	#NUM!	#NUM!	#NUM!	#NUM!	#NUM!	#NUM!
28.6987	28.7157	28.425	28.2227	#NUM!	#NUM!	28.0471	#NUM!	27.8558	27.4746	27.9275	27.1921
#NUM!	#NUM!	#NUM!	#NUM!	#NUM!	#NUM!	#NUM!	#NUM!	#NUM!	#NUM!	27.0499	26.6016
#NUM!	#NUM!	#NUM!	#NUM!	#NUM!	#NUM!	#NUM!	#NUM!	25.2546	25.0715	#NUM!	#NUM!
27.0581	26.7898	27.978	27.5494	#NUM!	26.915	#NUM!	#NUM!	27.2703	#NUM!	27.0259	26.7516
#NUM!	#NUM!	#NUM!	#NUM!	#NUM!	#NUM!	#NUM!	#NUM!	27.7306	27.5506	27.959	27.8771
#NUM!	#NUM!	#NUM!	#NUM!	#NUM!	#NUM!	#NUM!	#NUM!	26.8906	26.8971	26.9439	27.1762
#NUM!	#NUM!	#NUM!	#NUM!	#NUM!	#NUM!	#NUM!	#NUM!	30.4658	29.1981	28.5825	28.6173
#NUM!	#NUM!	#NUM!	#NUM!	#NUM!	#NUM!	#NUM!	#NUM!	#NUM!	25.5461	25.764	25.7901
27.7363	27.9968	27.8534	28.0744	#NUM!	27.4239	#NUM!	27.1039	28.3045	28.1504	28.5093	28.6132
#NUM!	#NUM!	#NUM!	#NUM!	#NUM!	#NUM!	#NUM!	#NUM!	#NUM!	#NUM!	#NUM!	#NUM!
#NUM!	#NUM!	#NUM!	#NUM!	#NUM!	#NUM!	#NUM!	#NUM!	27.0587	26.739	26.9025	27.1596
#NUM!	#NUM!	#NUM!	#NUM!	#NUM!	#NUM!	#NUM!	#NUM!	#NUM!	#NUM!	#NUM!	#NUM!
32.6356	32.9616	30.1893	30.5223	32.1206	32.1534	31.8909	33.4556	31.5785	31.0223	31.1228	31.1258
28.8803	29.1536	27.2787	26.8299	29.2313	28.8128	#NUM!	29.6903	31.3184	31.1907	31.0927	31.3673
29.8485	29.8153	30.0843	29.7637	29.9946	29.8224	30.1449	29.7797	27.4371	27.1432	27.3706	27.7242
29.8462	29.2167	#NUM!	#NUM!	#NUM!	#NUM!	#NUM!	#NUM!	30.676	30.2068	28.4987	28.2244
29.014	28.6445	28.0661	28.986	28.6395	27.7522	30.5119	28.3866	29.7185	29.4189	29.962	29.8364
27.6633	27.7136	#NUM!	#NUM!	#NUM!	#NUM!	#NUM!	#NUM!	25.2475	#NUM!	26.3905	#NUM!
27.624	#NUM!	#NUM!	27.2102	#NUM!	27.9852	#NUM!	27.5027	#NUM!	#NUM!	#NUM!	28.0941
#NUM!	#NUM!	#NUM!	#NUM!	#NUM!	#NUM!	#NUM!	#NUM!	27.6515	27.8895	27.9189	27.847
33.1082	32.5791	33.3105	34.2379	31.999	31.8809	32.2963	31.7836	30.1985	29.7112	28.8138	28.4838
30.9469	31.0691	30.306	30.8344	31.9473	31.9856	32.1012	31.4325	29.1796	29.0713	29.0583	29.123
30.9399	31.1393	30.3348	30.8265	30.6301	30.7376	30.8269	30.5023	28.4032	28.452	28.8019	28.3701
31.2154	31.3767	30.7486	30.4545	30.0964	30.3033	29.9984	29.539	29.6633	29.7063	29.9916	29.8595
#NUM!	27.3536	#NUM!	#NUM!	#NUM!	#NUM!	#NUM!	#NUM!	#NUM!	26.4581	25.5033	26.4232
#NUM!	#NUM!	#NUM!	#NUM!	#NUM!	#NUM!	#NUM!	#NUM!	#NUM!	28.5669	27.8568	26.554
#NUM!	#NUM!	#NUM!	#NUM!	#NUM!	#NUM!	#NUM!	#NUM!	28.2149	27.9397	29.0542	28.8691
#NUM!	#NUM!	#NUM!	#NUM!	#NUM!	#NUM!	#NUM!	#NUM!	27.1215	26.5631	#NUM!	#NUM!
#NUM!	#NUM!	#NUM!	#NUM!	#NUM!	#NUM!	#NUM!	#NUM!	26.2222	26.2162	26.3946	26.301
27.8035	27.9284	28.2642	28.0267	28.9098	27.8707	28.7036	#NUM!	26.0166	26.153	25.7426	26.0431
#NUM!	28.7145	28.4683	28.1387	#NUM!	28.0931	#NUM!	#NUM!	28.3122	28.4644	28.6615	28.5214
#NUM!	28.1382	#NUM!	28.7694	#NUM!	#NUM!	#NUM!	#NUM!	28.1098	28.5712	28.0037	27.9156
#NUM!	27.7103	#NUM!	27.1059	#NUM!	#NUM!	#NUM!	#NUM!	29.5895	28.7951	28.6521	28.6299
30.0593	30.48	27.6355	#NUM!	#NUM!	31.1739	#NUM!	31.5536	#NUM!	#NUM!	#NUM!	#NUM!
#NUM!	#NUM!	#NUM!	#NUM!	#NUM!	#NUM!	#NUM!	#NUM!	26.7296	26.945	27.0948	27.1749
27.6592	27.4937	27.8956	28.0795	#NUM!	27.7464	28.3431	27.7209	26.7738	26.7154	26.8607	26.9749
#NUM!	#NUM!	#NUM!	#NUM!	#NUM!	#NUM!	#NUM!	#NUM!	26.5167	26.7889	26.9832	27.2361
#NUM!	#NUM!	#NUM!	#NUM!	#NUM!	#NUM!	#NUM!	#NUM!	#NUM!	25.7339	25.6931	#NUM!
29.1057	28.0224	27.4625	27.5873	#NUM!	32.0331	#NUM!	32.0812	27.6222	27.8275	27.2156	27.5739
#NUM!	#NUM!	#NUM!	#NUM!	#NUM!	#NUM!	#NUM!	#NUM!	27.6704	27.3359	27.3322	27.1231
28.7985	28.9747	29.1558	28.7869	28.9495	28.8234	29.4813	#NUM!	27.405	27.8256	27.6801	27.2494
27.7973	27.3048	#NUM!	#NUM!	#NUM!	#NUM!	#NUM!	#NUM!	28.3841	28.521	27.8515	28.0607
#NUM!	#NUM!	#NUM!	#NUM!	#NUM!	#NUM!	#NUM!	#NUM!	26.5456	26.4557	25.6063	#NUM!
#NUM!	#NUM!	#NUM!	#NUM!	#NUM!	#NUM!	#NUM!	#NUM!	#NUM!	26.2393	25.862	26.1644
27.7304	27.9655	27.7553	27.9858	#NUM!	#NUM!	#NUM!	#NUM!	27.1407	27.1343	28.1344	27.7945
32.1353	32.5687	31.5149	31.4793	31.7281	32.1105	31.7173	32.2798	29.7853	29.2775	29.6067	29.5468
28.3128	28.217	#NUM!	28.2085	#NUM!	28.1805	#NUM!	#NUM!	29.2054	29.2683	29.353	28.9869
28.4942	#NUM!	#NUM!	28.3582	#NUM!	#NUM!	#NUM!	#NUM!	28.1417	27.9657	28.6727	28.322
27.7988	27.9016	27.6279	#NUM!	#NUM!	28.1734	#NUM!	#NUM!	28.4304	28.295	28.5897	28.3393
#NUM!	26.7171	#NUM!	27.404	#NUM!	#NUM!	#NUM!	#NUM!	27.0789	#NUM!	#NUM!	#NUM!
#NUM!	#NUM!	#NUM!	26.5727	#NUM!	#NUM!	#NUM!	#NUM!	29.3973	28.9794	29.0774	28.9041
#NUM!	#NUM!	#NUM!	#NUM!	#NUM!	#NUM!	#NUM!	#NUM!	27.6652	27.7791	27.0407	27.4641
#NUM!	#NUM!	#NUM!	#NUM!	#NUM!	#NUM!	#NUM!	#NUM!	25.5418	#NUM!	#NUM!	#NUM!
#NUM!	#NUM!	#NUM!	#NUM!	#NUM!	#NUM!	#NUM!	#NUM!	#NUM!	25.5947	#NUM!	#NUM!
#NUM!	#NUM!	#NUM!	#NUM!	#NUM!	#NUM!	#NUM!	#NUM!	#NUM!	#NUM!	26.1671	#NUM!
28.0241	27.736	28.1572	28.5075	27.9588	27.7693	#NUM!	27.4601	28.1065	28.1881	28.0709	27.842
#NUM!	#NUM!	#NUM!	#NUM!	#NUM!	#NUM!	#NUM!	#NUM!	#NUM!	#NUM!	27.0928	26.8401
#NUM!	#NUM!	#NUM!	#NUM!	#NUM!	#NUM!	#NUM!	#NUM!	24.9488	24.9796	25.4433	25.3724
#NUM!	#NUM!	#NUM!	#NUM!	#NUM!	#NUM!	#NUM!	#NUM!	#NUM!	#NUM!	25.4106	24.9555
#NUM!	#NUM!	#NUM!	#NUM!	#NUM!	#NUM!	#NUM!	#NUM!	26.1519	#NUM!	27.0826	26.5964
26.6326	26.8092	#NUM!	#NUM!	27.8675	29.4464	28.1718	28.9466	#NUM!	#NUM!	#NUM!	#NUM!
#NUM!	#NUM!	#NUM!	#NUM!	#NUM!	#NUM!	#NUM!	#NUM!	25.3856	#NUM!	25.5661	26.0376
27.2374	27.3445	28.0675	28.0388	28.9883	28.2526	28.6865	#NUM!	26.2372	26.2453	26.0243	27.0259
#NUM!	#NUM!	#NUM!	#NUM!	#NUM!	#NUM!	#NUM!	#NUM!	25.6578	25.9598	26.3332	26.1006
29.0903	28.9547	#NUM!	27.9095	#NUM!	28.9639	#NUM!	29.5564	26.6158	26.9061	26.7499	26.6311
27.266	27.2315	28.7179	28.7291	#NUM!	27.5347	28.8741	27.9861	27.4028	27.1028	26.8086	26.6643
#NUM!	#NUM!	#NUM!	#NUM!	#NUM!	#NUM!	#NUM!	#NUM!	#NUM!	#NUM!	25.7174	25.693
#NUM!	#NUM!	#NUM!	#NUM!	#NUM!	#NUM!	#NUM!	#NUM!	#NUM!	#NUM!	#NUM!	#NUM!
#NUM!	27.1725	#NUM!	27.0472	#NUM!	#NUM!	#NUM!	#NUM!	26.442	26.2738	26.3362	26.3796
#NUM!	#NUM!	#NUM!	#NUM!	#NUM!	#NUM!	#NUM!	#NUM!	#NUM!	26.7293	#NUM!	#NUM!
#NUM!	#NUM!	#NUM!	#NUM!	#NUM!	#NUM!	#NUM!	#NUM!	26.0196	25.7121	26.429	26.0396
#NUM!	#NUM!	#NUM!	#NUM!	#NUM!	#NUM!	#NUM!	#NUM!	28.7149	#NUM!	28.4851	28.1679
#NUM!	#NUM!	#NUM!	#NUM!	#NUM!	#NUM!	#NUM!	#NUM!	26.7405	26.8795	26.881	26.8955
#NUM!	#NUM!	#NUM!	29.694	#NUM!	#NUM!	#NUM!	#NUM!	27.5831	#NUM!	27.8574	27.9747
#NUM!	#NUM!	#NUM!	26.758	#NUM!	#NUM!	#NUM!	#NUM!	26.9402	26.6826	26.5804	26.1968
#NUM!	#NUM!	#NUM!	#NUM!	#NUM!	#NUM!	#NUM!	#NUM!	#NUM!	#NUM!	#NUM!	24.37
#NUM!	#NUM!	#NUM!	#NUM!	#NUM!	#NUM!	#NUM!	#NUM!	26.1933	26.3374	26.4146	26.318
#NUM!	#NUM!	#NUM!	#NUM!	#NUM!	#NUM!	#NUM!	#NUM!	#NUM!	#NUM!	#NUM!	#NUM!
#NUM!	#NUM!	#NUM!	#NUM!	#NUM!	#NUM!	#NUM!	#NUM!	25.6285	26.1352	26.3271	26.1606
#NUM!	#NUM!	#NUM!	#NUM!	#NUM!	#NUM!	#NUM!	#NUM!	25.1618	25.2257	25.0466	25.2043
#NUM!	#NUM!	#NUM!	#NUM!	#NUM!	#NUM!	#NUM!	#NUM!	#NUM!	25.5323	26.0668	25.547
#NUM!	#NUM!	27.4408	27.5173	#NUM!	#NUM!	#NUM!	#NUM!	26.0592	25.9061	25.8955	25.8524
#NUM!	#NUM!	#NUM!	#NUM!	#NUM!	#NUM!	#NUM!	#NUM!	25.5079	25.5307	25.7507	25.5939
#NUM!	#NUM!	#NUM!	#NUM!	#NUM!	#NUM!	#NUM!	#NUM!	#NUM!	25.2921	#NUM!	#NUM!
#NUM!	#NUM!	#NUM!	#NUM!	#NUM!	#NUM!	#NUM!	#NUM!	24.7932	#NUM!	#NUM!	#NUM!
#NUM!	28.0159	#NUM!	#NUM!	#NUM!	#NUM!	#NUM!	#NUM!	27.4649	#NUM!	#NUM!	#NUM!
27.0625	26.866	#NUM!	#NUM!	#NUM!	#NUM!	#NUM!	#NUM!	26.6926	26.8094	26.6164	26.6886

#NUM!	#NUM!	#NUM!	#NUM!	#NUM!	#NUM!	#NUM!	#NUM!	26.8806	26.5914	27.6332	27.7118
#NUM!	#NUM!	#NUM!	#NUM!	#NUM!	#NUM!	#NUM!	#NUM!	26.4727	26.3463	26.9049	26.5672
#NUM!	#NUM!	#NUM!	#NUM!	#NUM!	#NUM!	#NUM!	#NUM!	#NUM!	#NUM!	#NUM!	#NUM!
#NUM!	#NUM!	#NUM!	#NUM!	#NUM!	#NUM!	#NUM!	#NUM!	25.8933	25.8821	25.7598	25.7603
#NUM!	25.5162	#NUM!	25.8972	#NUM!	#NUM!	#NUM!	#NUM!	25.883	26.1621	26.2277	26.04
27.3174	27.2084	27.7876	27.2339	#NUM!	26.0796	#NUM!	#NUM!	27.5413	27.042	27.3034	27.5714
#NUM!	#NUM!	#NUM!	26.1566	#NUM!	#NUM!	#NUM!	#NUM!	27.8766	27.9487	28.1261	28.1723
27.7156	27.6708	#NUM!	27.8783	#NUM!	#NUM!	#NUM!	27.394	26.0447	26.4118	26.2381	#NUM!
#NUM!	27.5134	#NUM!	#NUM!	#NUM!	#NUM!	#NUM!	#NUM!	#NUM!	26.9481	27.5281	27.5081
#NUM!	#NUM!	#NUM!	#NUM!	#NUM!	#NUM!	#NUM!	#NUM!	25.7928	#NUM!	25.5726	25.9314
#NUM!	#NUM!	#NUM!	#NUM!	#NUM!	#NUM!	#NUM!	#NUM!	#NUM!	27.3985	27.4221	27.6107
#NUM!	#NUM!	#NUM!	#NUM!	#NUM!	#NUM!	#NUM!	#NUM!	25.7433	25.7492	26.3083	26.047
#NUM!	#NUM!	#NUM!	#NUM!	#NUM!	#NUM!	#NUM!	#NUM!	24.9517	#NUM!	#NUM!	#NUM!
#NUM!	27.161	#NUM!	28.2245	#NUM!	28.3908	#NUM!	28.6535	26.8404	26.5912	27.1894	27.0682
#NUM!	#NUM!	#NUM!	#NUM!	#NUM!	#NUM!	#NUM!	#NUM!	27.1602	26.4529	27.4519	27.198
#NUM!	#NUM!	#NUM!	#NUM!	#NUM!	#NUM!	#NUM!	#NUM!	26.6915	26.3679	#NUM!	27.3657
#NUM!	#NUM!	#NUM!	#NUM!	#NUM!	#NUM!	#NUM!	#NUM!	27.3258	27.426	26.9842	26.8174
#NUM!	#NUM!	#NUM!	#NUM!	#NUM!	#NUM!	#NUM!	#NUM!	26.9229	26.8855	27.0839	27.2639
#NUM!	#NUM!	#NUM!	#NUM!	#NUM!	#NUM!	#NUM!	#NUM!	#NUM!	24.3363	#NUM!	24.6276
27.9309	27.9686	26.5642	25.668	#NUM!	28.8216	#NUM!	28.5153	#NUM!	#NUM!	#NUM!	#NUM!
30.7853	30.7592	31.7749	31.3718	30.0475	30.6442	30.0032	29.2985	31.7322	31.6469	31.6099	31.2882
#NUM!	#NUM!	#NUM!	#NUM!	#NUM!	#NUM!	#NUM!	#NUM!	26.0482	26.0751	26.0568	26.2871
26.1909	26.5908	26.8121	26.9645	#NUM!	#NUM!	#NUM!	#NUM!	27.4044	27.6969	27.3062	27.2923
27.422	27.6613	#NUM!	#NUM!	#NUM!	27.2633	#NUM!	#NUM!	28.7054	28.5779	28.7229	28.9053
28.7601	28.7064	28.5952	28.653	28.5913	29.1052	#NUM!	28.5963	27.1478	28.4086	#NUM!	27.8003
#NUM!	#NUM!	#NUM!	#NUM!	#NUM!	#NUM!	#NUM!	#NUM!	#NUM!	#NUM!	#NUM!	#NUM!
#NUM!	#NUM!	#NUM!	#NUM!	#NUM!	#NUM!	#NUM!	#NUM!	25.5609	25.7748	25.0239	25.0838
#NUM!	#NUM!	#NUM!	#NUM!	#NUM!	#NUM!	#NUM!	#NUM!	#NUM!	#NUM!	#NUM!	25.2833
27.3476	27.2296	28.0742	28.3489	#NUM!	27.5929	#NUM!	#NUM!	28.6476	28.3245	28.7881	28.9605
#NUM!	#NUM!	#NUM!	#NUM!	#NUM!	#NUM!	#NUM!	#NUM!	25.7071	26.2548	25.9079	25.9624
#NUM!	#NUM!	#NUM!	#NUM!	#NUM!	#NUM!	#NUM!	#NUM!	#NUM!	25.653	25.3404	25.2735
#NUM!	#NUM!	#NUM!	#NUM!	#NUM!	#NUM!	#NUM!	#NUM!	#NUM!	#NUM!	24.7272	#NUM!
#NUM!	#NUM!	#NUM!	#NUM!	#NUM!	#NUM!	#NUM!	#NUM!	26.7744	26.7267	26.397	26.3953
#NUM!	#NUM!	#NUM!	#NUM!	#NUM!	#NUM!	#NUM!	#NUM!	27.9068	27.2824	27.5308	27.4902
31.5813	32.1132	28.2754	28.8134	30.5871	30.6202	31.111	#NUM!	#NUM!	#NUM!	#NUM!	#NUM!
#NUM!	#NUM!	#NUM!	#NUM!	#NUM!	#NUM!	#NUM!	#NUM!	#NUM!	#NUM!	#NUM!	#NUM!
#NUM!	#NUM!	#NUM!	#NUM!	#NUM!	#NUM!	#NUM!	#NUM!	#NUM!	26.6383	26.561	26.4622
#NUM!	#NUM!	#NUM!	#NUM!	#NUM!	#NUM!	#NUM!	#NUM!	#NUM!	#NUM!	#NUM!	26.6367
#NUM!	#NUM!	27.853	#NUM!	#NUM!	#NUM!	#NUM!	#NUM!	29.242	29.0647	28.9787	28.7015
#NUM!	#NUM!	#NUM!	#NUM!	#NUM!	#NUM!	#NUM!	#NUM!	#NUM!	26.1056	26.5325	#NUM!
#NUM!	#NUM!	#NUM!	#NUM!	#NUM!	#NUM!	#NUM!	#NUM!	27.7507	27.8923	27.5874	27.5869
#NUM!	#NUM!	#NUM!	26.1367	#NUM!	#NUM!	#NUM!	#NUM!	25.5412	25.4557	25.5761	25.567
#NUM!	#NUM!	#NUM!	#NUM!	#NUM!	#NUM!	#NUM!	#NUM!	28.075	28.096	28.016	29.4001
#NUM!	#NUM!	#NUM!	#NUM!	#NUM!	#NUM!	#NUM!	#NUM!	#NUM!	#NUM!	#NUM!	#NUM!
#NUM!	#NUM!	#NUM!	#NUM!	#NUM!	#NUM!	#NUM!	#NUM!	#NUM!	#NUM!	#NUM!	#NUM!
#NUM!	#NUM!	#NUM!	#NUM!	#NUM!	#NUM!	#NUM!	#NUM!	26.2041	26.558	25.698	25.3472
#NUM!	#NUM!	#NUM!	#NUM!	#NUM!	#NUM!	#NUM!	#NUM!	#NUM!	#NUM!	#NUM!	#NUM!
#NUM!	#NUM!	#NUM!	#NUM!	#NUM!	#NUM!	#NUM!	#NUM!	#NUM!	26.9335	25.8828	26.0757
#NUM!	#NUM!	#NUM!	#NUM!	#NUM!	#NUM!	#NUM!	#NUM!	24.9836	25.3293	25.3052	25.1031
#NUM!	#NUM!	#NUM!	#NUM!	#NUM!	#NUM!	#NUM!	#NUM!	#NUM!	#NUM!	#NUM!	25.4672
#NUM!	#NUM!	#NUM!	#NUM!	#NUM!	#NUM!	#NUM!	#NUM!	#NUM!	#NUM!	#NUM!	#NUM!
#NUM!	#NUM!	#NUM!	#NUM!	#NUM!	#NUM!	#NUM!	#NUM!	27.0027	#NUM!	#NUM!	#NUM!
29.2655	29.7086	29.2931	28.6006	#NUM!	28.6901	#NUM!	#NUM!	27.4931	27.7019	27.9417	28.1851
#NUM!	#NUM!	#NUM!	#NUM!	#NUM!	#NUM!	#NUM!	#NUM!	#NUM!	#NUM!	24.9346	#NUM!
#NUM!	#NUM!	#NUM!	#NUM!	#NUM!	#NUM!	#NUM!	#NUM!	#NUM!	25.7679	25.4576	25.4839
#NUM!	#NUM!	#NUM!	#NUM!	#NUM!	#NUM!	#NUM!	#NUM!	27.3472	27.6104	27.3851	27.1962
#NUM!	#NUM!	#NUM!	#NUM!	#NUM!	#NUM!	#NUM!	#NUM!	27.5405	27.5687	27.02	26.8571
27.5144	27.5795	27.3431	27.3791	#NUM!	27.3346	#NUM!	27.5482	27.5587	27.7763	28.4546	28.3223
#NUM!	#NUM!	#NUM!	#NUM!	#NUM!	#NUM!	#NUM!	#NUM!	26.047	26.3743	25.8037	25.6982
#NUM!	#NUM!	#NUM!	#NUM!	#NUM!	#NUM!	#NUM!	#NUM!	#NUM!	#NUM!	25.478	#NUM!
#NUM!	#NUM!	#NUM!	#NUM!	#NUM!	#NUM!	#NUM!	#NUM!	#NUM!	#NUM!	#NUM!	#NUM!
#NUM!	#NUM!	#NUM!	#NUM!	#NUM!	#NUM!	#NUM!	#NUM!	#NUM!	26.2853	26.4877	26.5517
31.8978	31.7235	31.2601	31.6222	34.6377	33.1749	34.4664	34.1056	28.6509	27.9737	28.2698	28.7143
#NUM!	#NUM!	#NUM!	#NUM!	#NUM!	#NUM!	#NUM!	#NUM!	#NUM!	#NUM!	24.3775	#NUM!
26.2455	#NUM!	#NUM!	#NUM!	#NUM!	#NUM!	#NUM!	#NUM!	27.4428	27.7274	28.2759	27.9613
#NUM!	#NUM!	#NUM!	#NUM!	#NUM!	#NUM!	#NUM!	#NUM!	27.3185	27.5756	27.5344	27.6051
28.028	#NUM!	#NUM!	28.7742	#NUM!	#NUM!	#NUM!	#NUM!	28.3891	28.3241	28.5801	28.9317
#NUM!	#NUM!	#NUM!	#NUM!	#NUM!	#NUM!	#NUM!	#NUM!	26.819	26.7801	26.6537	26.7435
26.7073	26.831	26.5712	26.5637	27.806	28.1009	#NUM!	27.9486	#NUM!	#NUM!	#NUM!	#NUM!
#NUM!	#NUM!	#NUM!	#NUM!	#NUM!	#NUM!	#NUM!	#NUM!	25.0644	25.5761	25.7034	25.9657
28.9581	#NUM!	27.4385	27.0135	#NUM!	29.3526	#NUM!	29.9345	#NUM!	#NUM!	25.9239	26.346
28.5882	28.2236	27.8377	28.2756	27.624	27.2292	27.7697	27.339	28.3124	28.2205	28.5554	28.3747
29.2748	29.1828	28.8132	28.8271	28.7874	29.008	28.7961	28.8174	#NUM!	#NUM!	#NUM!	#NUM!
32.8339	33.1957	33.2471	32.9058	32.8799	32.3067	32.9591	31.8809	30.0911	30.434	30.7771	30.9602
#NUM!	#NUM!	#NUM!	#NUM!	#NUM!	26.564	#NUM!	#NUM!	27.5059	27.3681	27.2396	27.1453
#NUM!	#NUM!	#NUM!	#NUM!	#NUM!	#NUM!	#NUM!	#NUM!	28.504	28.2021	28.5621	28.3289
28.9846	28.6886	#NUM!	28.2893	#NUM!	29.095	#NUM!	29.5644	29.0301	28.9595	28.9511	29.1643
29.0462	28.9243	28.1627	28.0623	28.0857	29.8286	28.1462	29.2048	29.416	29.1998	29.1485	29.4984
#NUM!	#NUM!	#NUM!	#NUM!	#NUM!	#NUM!	#NUM!	#NUM!	26.904	26.6884	26.5971	26.5194
#NUM!	#NUM!	#NUM!	#NUM!	#NUM!	#NUM!	#NUM!	#NUM!	27.561	27.4659	27.5626	27.2797
#NUM!	#NUM!	#NUM!	#NUM!	#NUM!	#NUM!	#NUM!	#NUM!	#NUM!	26.8944	28.2517	28.0983
28.0241	27.9921	27.31	27.2837	#NUM!	27.6495	#NUM!	27.6125	#NUM!	#NUM!	#NUM!	#NUM!
#NUM!	27.0159	#NUM!	#NUM!	#NUM!	28.0374	#NUM!	#NUM!	27.5016	27.3619	27.8053	27.8358
#NUM!	#NUM!	#NUM!	#NUM!	#NUM!	#NUM!	#NUM!	#NUM!	24.789	24.7046	24.7537	#NUM!
30.3786	30.2883	29.4958	29.6855	29.4546	29.6869	29.4336	29.9872	29.7953	29.7114	30.2945	29.8166
28.3142	28.1531	28.7598	28.6734	#NUM!	#NUM!	#NUM!	#NUM!	28.1846	28.2059	28.1075	28.0657
#NUM!	#NUM!	#NUM!	#NUM!	#NUM!	#NUM!	#NUM!	#NUM!	27.0628	27.0331	27.1519	26.8866
30.2405	30.1988	28.8042	28.832	29.1414	29.3054	29.2251	29.4688	26.9569	27.4601	27.6565	27.6598
#NUM!	#NUM!	#NUM!	#NUM!	#NUM!	#NUM!	#NUM!	#NUM!	#NUM!	26.8311	27.1061	26.9512
25.82	26.0885	#NUM!	#NUM!	#NUM!	#NUM!	#NUM!	#NUM!	25.5939	25.4208	26.0221	25.9564
#NUM!	#NUM!	#NUM!	28.2661	#NUM!	27.1175	#NUM!	#NUM!	27.9308	27.8635	28.2913	27.9725
#NUM!	#NUM!	#NUM!	#NUM!	#NUM!	#NUM!	#NUM!	#NUM!	29.5956	29.3571	29.5744	29.6278
27.3547	27.501	27.4741	#NUM!	#NUM!	27.7809	#NUM!	#NUM!	26.7555	26.8665	27.0473	27.0849
30.9337	31.0013	30.1619	30.3996	30.1143	30.5469	30.1311	29.9863	29.7453	29.8893	29.9315	29.8728
#NUM!	27.0288	#NUM!	#NUM!	#NUM!	#NUM!	#NUM!	#NUM!	26.1502	25.9756	26.5832	26.6625
#NUM!	#NUM!	#NUM!	#NUM!	#NUM!	#NUM!	#NUM!	#NUM!	27.5612	27.2386	27.6301	27.5099
#NUM!	#NUM!	#NUM!	#NUM!	#NUM!	#NUM!	#NUM!	#NUM!	27.6709	27.1707	27.4713	27.3622
28.4312	28.3643	27.8675	27.8682	#NUM!	28.1618	27.0639	28.004	26.8388	26.5876	27.1163	26.9598
#NUM!	#NUM!	#NUM!	#NUM!	#NUM!	#NUM!	#NUM!	#NUM!	#NUM!	26.5797	26.2254	#NUM!
#NUM!	#NUM!	#NUM!	#NUM!	#NUM!	#NUM!	#NUM!	#NUM!	26.2073	26.1336	25.6662	25.695
#NUM!	#NUM!	#NUM!	#NUM!	#NUM!	#NUM!	#NUM!	#NUM!	27.7307	27.6787	27.8479	27.6013
28.9978	29.0906	28.7443	28.7586	#NUM!	28.1295	#NUM!	27.906	28.4952	28.1883	28.6485	28.2856
#NUM!	#NUM!	#NUM!	#NUM!	#NUM!	#NUM!	#NUM!	#NUM!	27.0182	#NUM!	26.4659	#NUM!
29.6854	29.289	#NUM!	29.1948	29.0195	#NUM!	28.8172	#NUM!	30.0945	29.9053	29.9564	30.1476
#NUM!	#NUM!	#NUM!	26.4993	#NUM!	#NUM!	#NUM!	#NUM!	27.0706	27.008	26.9411	26.9367
28.1857	27.9366	#NUM!	#NUM!	#NUM!	28.055	#NUM!	#NUM!	27.9153	27.5505	28.1873	27.8073
27.8542	28.0068	26.6888	26.8547	28.9517	29.9593	28.9653	29.7944	#NUM!	#NUM!	#NUM!	#NUM!
#NUM!	#NUM!	#NUM!	#NUM!	#NUM!	29.1384	#NUM!	#NUM!	29.5736	#NUM!	26.5392	26.0801
28.1503	27.676	29.2299	28.7984	#NUM!	29.5706	#NUM!	#NUM!	31.3083	31.0673	31.2015	30.9631
#NUM!	#NUM!	#NUM!	#NUM!	#NUM!	27.9461	#NUM!	#NUM!	27.6445	27.7284	27.5046	27.4524
#NUM!	#NUM!	#NUM!	#NUM!	#NUM!	#NUM!	#NUM!	#NUM!	27.4447	27.4898	27.5615	27.6665
#NUM!	#NUM!	#NUM!	#NUM!	#NUM!	#NUM!	#NUM!	#NUM!	#NUM!	#NUM!	#NUM!	#NUM!
30.4418	30.3497	29.3355	28.9339	#NUM!	28.9226	28.5263	28.4897	28.4765	28.7491	28.3431	28.722
29.4685	29.5186	29.9484	30.0975	29.6668	28.3666	29.8517	28.5167	#NUM!	#NUM!	#NUM!	#NUM!
#NUM!	#NUM!	#NUM!	#NUM!	#NUM!	#NUM!	#NUM!	#NUM!	27.9372	27.5244	27.9044	27.6763
#NUM!	#NUM!	#NUM!	#NUM!	#NUM!	#NUM!	#NUM!	#NUM!	#NUM!	26.2783	25.4178	25.6499
27.5363	27.5787	27.3248	27.9776	#NUM!	27.7476	27.5357	27.2584	#NUM!	#NUM!	26.0819	26.2514
#NUM!	#NUM!	#NUM!	#NUM!	#NUM!	#NUM!	#NUM!	#NUM!	#NUM!	#NUM!	27.5148	27.1555
27.9989	28.075	28.4013	28.3482	28.7856	28.6104	28.775	28.1238	26.3241	26.0713	26.0063	26.2976
#NUM!	#NUM!	#NUM!	#NUM!	#NUM!	#NUM!	#NUM!	#NUM!	#NUM!	#NUM!	#NUM!	#NUM!
29.1211	29.1567	27.2541	27.3635	#NUM!	29.6388	27.5777	29.7181	#NUM!	#NUM!	#NUM!	26.8741
27.5737	27.456	27.3657	27.1453	#NUM!	26.9392	#NUM!	#NUM!	27.8267	27.579	27.9349	27.9554
#NUM!	#NUM!	#NUM!	#NUM!	#NUM!	#NUM!	#NUM!	#NUM!	26.9896	27.4135	27.8232	27.7945
27.6212	27.8102	27.3398	27.4917	#NUM!	27.9586	27.4738	27.9712	27.7657	27.574	28.4163	28.0882
#NUM!	#NUM!	#NUM!	#NUM!	#NUM!	#NUM!	#NUM!	#NUM!	27.0119	26.7575	27.0097	27.1077
#NUM!	#NUM!	#NUM!	#NUM!	#NUM!	#NUM!	#NUM!	#NUM!	#NUM!	#NUM!	24.8434	24.6591
#NUM!	#NUM!	#NUM!	#NUM!	#NUM!	#NUM!	#NUM!	#NUM!	#NUM!	#NUM!	#NUM!	#NUM!
31.0896	31.3303	30.8715	30.5107	32.2953	33.7134	32.1638	32.224	32.0908	31.9711	31.6522	31.6735
#NUM!	#NUM!	#NUM!	#NUM!	#NUM!	#NUM!	#NUM!	#NUM!	#NUM!	#NUM!	#NUM!	#NUM!

#NUM!	#NUM!	#NUM!	#NUM!	#NUM!	#NUM!	#NUM!	#NUM!	#NUM!	#NUM!	25.4887	25.5329
#NUM!	#NUM!	#NUM!	#NUM!	#NUM!	#NUM!	#NUM!	#NUM!	#NUM!	#NUM!	25.6132	25.4511
#NUM!	#NUM!	#NUM!	#NUM!	#NUM!	#NUM!	#NUM!	#NUM!	25.94	#NUM!	26.6399	26.7638
#NUM!	#NUM!	#NUM!	#NUM!	#NUM!	#NUM!	#NUM!	#NUM!	27.1665	27.2341	27.2899	27.0812
#NUM!	#NUM!	#NUM!	#NUM!	#NUM!	#NUM!	#NUM!	#NUM!	25.191	25.4544	25.4548	25.4064
#NUM!	#NUM!	#NUM!	#NUM!	#NUM!	#NUM!	#NUM!	#NUM!	#NUM!	#NUM!	#NUM!	#NUM!
#NUM!	#NUM!	#NUM!	#NUM!	#NUM!	#NUM!	#NUM!	#NUM!	28.287	28.0139	28.2621	28.5111
#NUM!	#NUM!	#NUM!	#NUM!	#NUM!	#NUM!	#NUM!	#NUM!	#NUM!	24.9298	24.8787	24.8777
28.1431	27.7082	28.8302	#NUM!	30.1441	30.571	#NUM!	#NUM!	26.4257	24.7365	25.8928	25.8912
#NUM!	#NUM!	#NUM!	26.1994	#NUM!	#NUM!	#NUM!	#NUM!	26.5465	26.6167	26.7483	26.5482
#NUM!	#NUM!	#NUM!	#NUM!	#NUM!	#NUM!	#NUM!	#NUM!	#NUM!	25.2999	25.649	25.1916
#NUM!	#NUM!	#NUM!	#NUM!	#NUM!	#NUM!	#NUM!	#NUM!	26.2555	26.2218	25.9904	26.2868
#NUM!	#NUM!	#NUM!	#NUM!	#NUM!	#NUM!	#NUM!	#NUM!	#NUM!	25.8169	#NUM!	26.3319
#NUM!	#NUM!	#NUM!	#NUM!	#NUM!	#NUM!	#NUM!	#NUM!	#NUM!	26.7253	26.5148	26.8493
#NUM!	#NUM!	#NUM!	#NUM!	#NUM!	#NUM!	#NUM!	#NUM!	#NUM!	#NUM!	#NUM!	#NUM!
#NUM!	#NUM!	#NUM!	#NUM!	#NUM!	#NUM!	#NUM!	#NUM!	#NUM!	#NUM!	#NUM!	#NUM!
#NUM!	#NUM!	#NUM!	#NUM!	#NUM!	#NUM!	#NUM!	#NUM!	26.0875	25.9933	26.0041	25.8583
#NUM!	#NUM!	#NUM!	#NUM!	#NUM!	#NUM!	#NUM!	#NUM!	26.5229	26.4061	26.243	26.4786
27.9203	27.9083	27.4475	27.5933	28.602	28.3419	28.4833	28.5984	#NUM!	25.5762	24.5326	24.8101
#NUM!	#NUM!	#NUM!	#NUM!	#NUM!	#NUM!	#NUM!	#NUM!	26.2051	#NUM!	25.5294	25.3212
#NUM!	#NUM!	#NUM!	#NUM!	#NUM!	#NUM!	#NUM!	#NUM!	25.6175	25.7781	26.8389	25.7933
#NUM!	#NUM!	#NUM!	#NUM!	#NUM!	#NUM!	#NUM!	#NUM!	#NUM!	#NUM!	26.0157	26.4403
#NUM!	#NUM!	#NUM!	#NUM!	#NUM!	#NUM!	#NUM!	#NUM!	28.0901	28.025	27.5643	27.9411
#NUM!	#NUM!	#NUM!	#NUM!	#NUM!	#NUM!	#NUM!	#NUM!	27.7461	27.4588	27.3122	27.365
#NUM!	#NUM!	#NUM!	#NUM!	#NUM!	#NUM!	#NUM!	#NUM!	24.8224	25.7653	25.2845	24.9112
#NUM!	#NUM!	#NUM!	#NUM!	#NUM!	#NUM!	#NUM!	#NUM!	26.0378	#NUM!	#NUM!	25.9177
#NUM!	#NUM!	#NUM!	#NUM!	#NUM!	#NUM!	#NUM!	#NUM!	#NUM!	#NUM!	25.6476	25.5959
#NUM!	#NUM!	#NUM!	#NUM!	#NUM!	#NUM!	#NUM!	#NUM!	#NUM!	#NUM!	#NUM!	#NUM!
#NUM!	#NUM!	#NUM!	#NUM!	#NUM!	#NUM!	#NUM!	#NUM!	#NUM!	#NUM!	26.5977	26.5483
#NUM!	#NUM!	#NUM!	#NUM!	#NUM!	#NUM!	#NUM!	#NUM!	27.1477	27.3379	27.1292	26.9359
#NUM!	#NUM!	#NUM!	#NUM!	#NUM!	#NUM!	#NUM!	#NUM!	26.9824	26.5683	26.3076	26.6655
#NUM!	#NUM!	#NUM!	#NUM!	#NUM!	#NUM!	#NUM!	#NUM!	26.8262	27.0025	26.7347	26.8139
#NUM!	#NUM!	#NUM!	#NUM!	#NUM!	#NUM!	#NUM!	#NUM!	25.278	25.3445	#NUM!	25.6706
#NUM!	#NUM!	#NUM!	#NUM!	#NUM!	#NUM!	#NUM!	#NUM!	#NUM!	#NUM!	#NUM!	#NUM!
#NUM!	#NUM!	#NUM!	26.8173	#NUM!	#NUM!	#NUM!	#NUM!	28.3448	28.4818	27.9011	27.9398
#NUM!	#NUM!	#NUM!	#NUM!	#NUM!	#NUM!	#NUM!	#NUM!	#NUM!	#NUM!	#NUM!	#NUM!
#NUM!	#NUM!	#NUM!	#NUM!	#NUM!	#NUM!	#NUM!	#NUM!	#NUM!	25.3405	#NUM!	#NUM!
26.9662	26.8835	27.728	27.2211	#NUM!	29.3289	28.2755	27.5715	26.1966	25.8195	25.2749	25.85
#NUM!	#NUM!	#NUM!	#NUM!	#NUM!	#NUM!	#NUM!	#NUM!	26.6224	26.221	26.5165	26.1138
#NUM!	#NUM!	#NUM!	#NUM!	#NUM!	#NUM!	#NUM!	#NUM!	24.8352	25.2933	25.6139	24.9882
#NUM!	#NUM!	#NUM!	#NUM!	#NUM!	#NUM!	#NUM!	#NUM!	#NUM!	#NUM!	27.1974	28.2106
#NUM!	#NUM!	#NUM!	#NUM!	#NUM!	#NUM!	#NUM!	#NUM!	#NUM!	27.6423	27.9723	28.1049
31.6136	32	30.188	30.4504	29.414	29.8647	29.3998	29.9064	29.8693	30.2498	30.4428	30.5753
#NUM!	#NUM!	#NUM!	#NUM!	#NUM!	#NUM!	#NUM!	#NUM!	#NUM!	#NUM!	#NUM!	23.9409
#NUM!	#NUM!	28.5624	#NUM!	#NUM!	#NUM!	#NUM!	#NUM!	28.281	28.0134	28.2123	28.5099
29.0269	29.1613	28.3131	28.7241	29.7081	30.5833	29.788	30.8145	#NUM!	#NUM!	#NUM!	#NUM!
#NUM!	#NUM!	#NUM!	#NUM!	#NUM!	#NUM!	#NUM!	#NUM!	#NUM!	25.2769	25.3475	25.4714
#NUM!	#NUM!	#NUM!	#NUM!	#NUM!	#NUM!	#NUM!	#NUM!	26.3016	26.1588	#NUM!	26.7493
#NUM!	#NUM!	#NUM!	#NUM!	#NUM!	#NUM!	#NUM!	#NUM!	28.8295	28.5709	28.5214	28.5869
#NUM!	#NUM!	#NUM!	#NUM!	#NUM!	#NUM!	#NUM!	#NUM!	#NUM!	25.1386	#NUM!	24.6618
28.8673	28.5299	#NUM!	27.2291	#NUM!	28.6151	#NUM!	#NUM!	27.0132	26.5016	27.1484	26.9738
#NUM!	#NUM!	#NUM!	#NUM!	#NUM!	#NUM!	#NUM!	#NUM!	#NUM!	25.3082	25.6863	25.8035
#NUM!	#NUM!	#NUM!	#NUM!	#NUM!	#NUM!	#NUM!	#NUM!	26.418	26.3932	26.7935	26.7278
#NUM!	#NUM!	#NUM!	#NUM!	#NUM!	#NUM!	#NUM!	#NUM!	26.1936	27.2137	26.5952	26.3278
27.3046	26.6854	27.5478	27.6504	#NUM!	#NUM!	#NUM!	#NUM!	26.9464	27.0026	27.209	27.368
28.1804	27.9194	#NUM!	28.7155	#NUM!	#NUM!	#NUM!	#NUM!	28.6124	28.0261	27.9784	27.8137
#NUM!	27.4066	27.0062	#NUM!	#NUM!	27.2942	#NUM!	#NUM!	27.4514	27.2943	27.4555	27.5655
#NUM!	#NUM!	#NUM!	#NUM!	#NUM!	#NUM!	#NUM!	#NUM!	24.9745	#NUM!	25.4496	#NUM!
#NUM!	#NUM!	#NUM!	#NUM!	#NUM!	#NUM!	#NUM!	#NUM!	#NUM!	26.6933	25.9256	26.4762
#NUM!	#NUM!	#NUM!	28.0923	#NUM!	#NUM!	#NUM!	#NUM!	28.088	27.9991	28.2849	27.9639
#NUM!	#NUM!	#NUM!	27.1904	#NUM!	#NUM!	#NUM!	#NUM!	27.3704	27.0054	27.7157	27.6197
#NUM!	#NUM!	#NUM!	#NUM!	#NUM!	#NUM!	#NUM!	#NUM!	27.9181	28.9891	28.0527	27.7609
#NUM!	#NUM!	#NUM!	#NUM!	#NUM!	#NUM!	#NUM!	#NUM!	27.1261	27.3799	27.6704	27.5125
28.409	28.3521	27.3234	27.918	#NUM!	28.5108	#NUM!	28.9609	28.5265	28.5636	29.1207	28.8835
#NUM!	#NUM!	#NUM!	#NUM!	#NUM!	#NUM!	#NUM!	#NUM!	#NUM!	#NUM!	25.4464	26.1333
#NUM!	#NUM!	#NUM!	#NUM!	#NUM!	#NUM!	#NUM!	#NUM!	26.7416	26.5079	26.6546	26.853
#NUM!	#NUM!	#NUM!	#NUM!	#NUM!	#NUM!	#NUM!	#NUM!	25.0372	#NUM!	25.5253	25.696
#NUM!	#NUM!	#NUM!	28.0081	#NUM!	#NUM!	#NUM!	#NUM!	29.6986	28.8221	28.3847	28.74
#NUM!	#NUM!	#NUM!	#NUM!	#NUM!	#NUM!	#NUM!	#NUM!	27.2762	27.8982	#NUM!	#NUM!
28.0033	27.7342	27.9488	27.8163	#NUM!	#NUM!	#NUM!	#NUM!	28.4618	28.4632	28.3826	28.3232
29.6559	29.2738	29.2304	29.6049	#NUM!	29.2669	#NUM!	29.048	30.9137	30.895	30.4657	30.0032
#NUM!	26.7059	#NUM!	27.0164	#NUM!	#NUM!	#NUM!	#NUM!	27.7109	27.5456	28.0029	27.9739
#NUM!	26.8254	#NUM!	#NUM!	#NUM!	#NUM!	#NUM!	#NUM!	26.4427	26.8163	26.7799	26.8158
30.9477	30.8986	30.0841	30.1567	30.5155	31.2596	30.3714	31.2903	28.4114	28.625	28.641	28.553
#NUM!	#NUM!	#NUM!	#NUM!	#NUM!	#NUM!	#NUM!	#NUM!	#NUM!	#NUM!	24.8992	#NUM!
28.9631	29.0671	29.2834	28.9943	#NUM!	28.8231	#NUM!	28.1141	29.6133	29.5366	29.7922	29.7935
27.726	27.984	27.9737	27.9482	27.6918	27.8148	#NUM!	28.1013	27.7664	27.7822	28.3306	28.2635
#NUM!	#NUM!	#NUM!	#NUM!	#NUM!	#NUM!	#NUM!	#NUM!	28.2947	27.6695	27.8957	27.6655
#NUM!	#NUM!	#NUM!	#NUM!	#NUM!	#NUM!	#NUM!	#NUM!	26.1036	26.1132	26.448	26.3009
#NUM!	28.7126	29.0625	28.9604	#NUM!	#NUM!	#NUM!	#NUM!	28.9314	28.6591	28.6176	28.6204
#NUM!	#NUM!	#NUM!	28.6311	#NUM!	#NUM!	#NUM!	#NUM!	28.7035	29.0008	29.2477	29.2929
#NUM!	#NUM!	#NUM!	27.2306	#NUM!	#NUM!	#NUM!	#NUM!	27.3989	27.7882	#NUM!	27.4669
28.4742	28.6131	28.6135	28.5179	#NUM!	28.5711	29.0334	27.8417	25.7749	25.9949	26.1729	25.9268
#NUM!	#NUM!	#NUM!	#NUM!	#NUM!	#NUM!	#NUM!	#NUM!	#NUM!	24.6832	25.0664	25.1324
#NUM!	#NUM!	#NUM!	#NUM!	#NUM!	#NUM!	#NUM!	#NUM!	27.5007	27.3145	27.2166	26.9478
#NUM!	#NUM!	#NUM!	#NUM!	#NUM!	#NUM!	#NUM!	#NUM!	27.9638	28.1225	28.0551	28.1384
#NUM!	#NUM!	#NUM!	#NUM!	#NUM!	#NUM!	#NUM!	#NUM!	28.2281	27.9901	27.0049	28.1294
#NUM!	#NUM!	#NUM!	#NUM!	#NUM!	#NUM!	#NUM!	#NUM!	26.9901	27.0165	26.6131	26.9878
#NUM!	#NUM!	#NUM!	#NUM!	#NUM!	#NUM!	#NUM!	#NUM!	26.6883	26.2253	26.8986	26.7522
32.0953	31.6424	30.5922	31.153	31.7653	31.9275	31.1212	32.8633	34.4706	34.4805	34.6857	34.5823
28.4992	28.3917	28.1006	28.0755	#NUM!	28.2459	#NUM!	28.5065	28.2769	28.4214	28.6077	28.3766
#NUM!	#NUM!	#NUM!	#NUM!	#NUM!	#NUM!	#NUM!	#NUM!	#NUM!	#NUM!	#NUM!	26.3426
30.9053	30.8966	30.5379	30.7883	30.0766	30.0442	29.883	29.4571	31.9151	31.8318	32.5787	32.2977
#NUM!	#NUM!	#NUM!	#NUM!	#NUM!	#NUM!	#NUM!	#NUM!	28.7855	28.3915	28.6746	28.4181
#NUM!	#NUM!	#NUM!	26.3396	#NUM!	#NUM!	#NUM!	#NUM!	28.2123	28.3243	28.5723	28.5671
#NUM!	#NUM!	#NUM!	#NUM!	#NUM!	#NUM!	#NUM!	#NUM!	26.3944	26.7339	26.6689	26.6521
#NUM!	27.4721	28.7141	28.0005	#NUM!	28.5496	#NUM!	#NUM!	27.7198	27.8237	27.3317	27.0231
28.3742	28.1413	28.661	28.5761	28.3799	28.0441	28.2697	27.3219	28.3253	28.4983	28.4076	28.4416
28.4918	28.5126	27.9786	28.0956	28.5105	27.7459	28.3261	27.3711	#NUM!	#NUM!	26.119	#NUM!
#NUM!	#NUM!	#NUM!	#NUM!	#NUM!	#NUM!	#NUM!	#NUM!	25.885	25.9504	26.6353	26.4786
28.033	28.5602	28.8839	28.6206	#NUM!	28.0276	28.1822	#NUM!	28.7058	28.5876	28.7257	28.385
28.5788	29.0774	#NUM!	28.1938	#NUM!	29.3783	#NUM!	28.8767	29.8716	29.7959	29.9715	29.8806
#NUM!	#NUM!	#NUM!	#NUM!	#NUM!	27.5136	#NUM!	#NUM!	27.281	27.4674	27.0587	#NUM!
29.4325	29.5516	28.4002	28.695	#NUM!	29.7126	27.7848	28.9157	29.9586	29.7421	29.8176	29.8288
29.527	29.6674	29.3803	29.5392	28.8389	28.5257	28.7634	27.7992	25.8161	25.6032	25.9868	25.8708
#NUM!	#NUM!	#NUM!	#NUM!	#NUM!	#NUM!	#NUM!	#NUM!	27.7836	27.5604	27.5227	27.7992
#NUM!	#NUM!	#NUM!	#NUM!	#NUM!	#NUM!	#NUM!	#NUM!	#NUM!	#NUM!	#NUM!	#NUM!
32.2191	32.499	31.9112	31.6105	32.0919	32.3198	31.9956	31.7386	29.2197	28.9312	28.8365	28.8212
#NUM!	#NUM!	#NUM!	#NUM!	27.4546	27.0346	#NUM!	#NUM!	27.327	27.0216	26.5011	27.3467
#NUM!	#NUM!	#NUM!	#NUM!	#NUM!	#NUM!	#NUM!	#NUM!	26.4276	26.2258	26.5652	26.4258
#NUM!	#NUM!	#NUM!	#NUM!	#NUM!	#NUM!	#NUM!	#NUM!	26.7043	27.0177	27.4163	27.3531
27.9368	27.8756	#NUM!	28.3375	#NUM!	28.628	28.7744	#NUM!	27.9909	27.9246	28.3922	28.3593
#NUM!	#NUM!	#NUM!	#NUM!	#NUM!	#NUM!	#NUM!	#NUM!	27.3238	27.0351	26.9284	27.0968
#NUM!	#NUM!	#NUM!	#NUM!	#NUM!	#NUM!	#NUM!	#NUM!	#NUM!	26.0985	26.7024	26.6802
31.3172	31.1451	30.4326	30.8089	29.4734	29.8999	29.3439	29	27.1558	27.4271	27.4815	27.2398
28.8645	28.8757	27.6731	28.0621	27.8087	28.386	27.9467	28.6155	27.774	27.8161	28.5447	28.4812
32.6678	32.9134	31.701	32.1558	32.3299	32.0016	32.2857	32.85	29.0957	29.3237	28.7071	28.7894
28.3294	27.9574	#NUM!	28.0858	#NUM!	#NUM!	#NUM!	#NUM!	28.997	29.2169	29.3176	29.2139
#NUM!	#NUM!	#NUM!	#NUM!	#NUM!	#NUM!	#NUM!	#NUM!	28.657	28.7818	28.7285	29.0023
29.3911	30.0106	29.3844	28.1168	28.3246	30.0857	28.2026	29.3018	29.7905	29.4443	30.0694	29.958
31.6125	31.8269	31.4119	31.5871	31.7008	31.7432	31.7322	31.2971	29.8287	29.9205	29.7991	30.1373
#NUM!	#NUM!	#NUM!	#NUM!	#NUM!	#NUM!	#NUM!	#NUM!	28.0253	27.8558	28.3679	28.2254
#NUM!	#NUM!	#NUM!	#NUM!	#NUM!	#NUM!	#NUM!	#NUM!	28.0622	28.0303	27.6209	27.6698
#NUM!	#NUM!	#NUM!	#NUM!	#NUM!	#NUM!	#NUM!	#NUM!	25.939	#NUM!	25.6799	25.1168
27.8179	27.6434	28.5023	28.2445	#NUM!	27.9604	#NUM!	27.4926	28.7346	28.5599	28.5138	28.5472
#NUM!	#NUM!	#NUM!	#NUM!	#NUM!	#NUM!	#NUM!	#NUM!	26.1105	25.9114	26.3992	26.8366
#NUM!	#NUM!	#NUM!	#NUM!	#NUM!	26.8396	#NUM!	#NUM!	27.0821	27.138	27.2079	27.5787
27.5112	27.7162	27.6158	27.1028	#NUM!	27.7928	#NUM!	27.1531	28.1051	28.2821	28.1144	28.1239
#NUM!	#NUM!	#NUM!	#NUM!	#NUM!	#NUM!	#NUM!	#NUM!	27.3895	27.7641	27.927	28.0057

#NUM!	#NUM!	#NUM!	#NUM!	#NUM!	#NUM!	#NUM!	#NUM!	#NUM!	26.9604	26.8529	27.1369
29.1266	29.0916	28.8908	28.4585	28.6917	28.3272	28.5375	28.0434	26.6362	26.4887	26.5756	26.7386
28.2791	28.2959	28.3368	28.3336	28.0087	28.666	28.4597	28.1364	30.2576	30.21	30.2013	29.9333
#NUM!	#NUM!	#NUM!	26.5351	#NUM!	#NUM!	#NUM!	#NUM!	26.9174	27.4456	26.894	26.898
#NUM!	#NUM!	#NUM!	#NUM!	#NUM!	#NUM!	#NUM!	#NUM!	26.3169	26.0057	#NUM!	26.1323
28.9679	29.2178	28.6123	28.0677	28.6278	27.8502	28.8076	29.4002	26.3514	#NUM!	26.5933	26.1958
26.0908	#NUM!	#NUM!	#NUM!	#NUM!	#NUM!	#NUM!	#NUM!	27.3104	27.4672	27.7587	27.5552
#NUM!	#NUM!	#NUM!	#NUM!	#NUM!	#NUM!	#NUM!	#NUM!	26.1534	26.3276	26.4875	26.5708
#NUM!	#NUM!	#NUM!	#NUM!	#NUM!	#NUM!	#NUM!	#NUM!	25.3428	25.5013	25.7445	25.698
#NUM!	#NUM!	#NUM!	#NUM!	#NUM!	27.2331	#NUM!	#NUM!	28.0425	28.0477	27.8345	27.7832
29.5109	29.5805	29.6358	29.4265	28.9523	29.0306	28.6918	28.8287	29.9459	29.9131	30.2577	30.1793
#NUM!	#NUM!	#NUM!	#NUM!	#NUM!	#NUM!	#NUM!	29.5047	#NUM!	#NUM!	28.6351	28.0365
27.8525	27.4129	28.4925	28.2392	#NUM!	28.2639	#NUM!	#NUM!	27.9406	27.9457	27.9932	28.3326
#NUM!	#NUM!	27.0022	#NUM!	#NUM!	#NUM!	#NUM!	#NUM!	27.4162	27.1186	27.3792	27.3604
#NUM!	#NUM!	#NUM!	#NUM!	#NUM!	#NUM!	#NUM!	#NUM!	#NUM!	24.8549	25.1819	#NUM!
#NUM!	#NUM!	#NUM!	#NUM!	#NUM!	#NUM!	#NUM!	#NUM!	#NUM!	26.2379	25.8552	26.1907
#NUM!	#NUM!	#NUM!	#NUM!	#NUM!	#NUM!	#NUM!	#NUM!	27.444	27.4696	27.5957	27.6687
#NUM!	#NUM!	#NUM!	#NUM!	#NUM!	#NUM!	#NUM!	#NUM!	#NUM!	#NUM!	#NUM!	#NUM!
#NUM!	#NUM!	#NUM!	#NUM!	#NUM!	#NUM!	#NUM!	#NUM!	#NUM!	#NUM!	25.1558	#NUM!
#NUM!	#NUM!	#NUM!	#NUM!	#NUM!	#NUM!	#NUM!	#NUM!	25.2839	25.1832	25.728	25.6572
#NUM!	#NUM!	#NUM!	#NUM!	#NUM!	#NUM!	#NUM!	#NUM!	#NUM!	#NUM!	#NUM!	#NUM!
#NUM!	#NUM!	#NUM!	#NUM!	#NUM!	#NUM!	#NUM!	#NUM!	#NUM!	#NUM!	25.2661	25.8536
#NUM!	#NUM!	#NUM!	#NUM!	#NUM!	#NUM!	#NUM!	#NUM!	25.9428	26.3766	26.2719	26.2751
#NUM!	#NUM!	#NUM!	#NUM!	#NUM!	#NUM!	#NUM!	#NUM!	#NUM!	#NUM!	#NUM!	26.3619
#NUM!	27.1243	#NUM!	27.0298	#NUM!	#NUM!	#NUM!	#NUM!	#NUM!	27.7579	27.9677	27.8535
#NUM!	#NUM!	#NUM!	#NUM!	#NUM!	#NUM!	#NUM!	#NUM!	25.0817	24.7182	25.0672	25.1063
#NUM!	#NUM!	#NUM!	#NUM!	#NUM!	#NUM!	#NUM!	#NUM!	#NUM!	25.7032	26.0559	#NUM!
#NUM!	#NUM!	#NUM!	#NUM!	#NUM!	#NUM!	#NUM!	26.919	26.2032	26.3474	26.4135	26.4891
27.4945	27.6288	27.709	27.3661	#NUM!	28.3044	#NUM!	28.4047	27.6841	27.7986	27.49	27.3227
#NUM!	#NUM!	#NUM!	#NUM!	#NUM!	26.9506	#NUM!	#NUM!	26.0432	26.6233	26.5429	26.6828
28.4956	28.5252	28.5986	28.4835	27.9201	27.4545	#NUM!	26.8141	28.3294	28.2551	28.5468	28.3527
#NUM!	28.4661	#NUM!	#NUM!	#NUM!	#NUM!	#NUM!	#NUM!	29.8974	29.7029	29.5767	29.7701
#NUM!	#NUM!	#NUM!	#NUM!	#NUM!	#NUM!	#NUM!	#NUM!	26.7968	26.8018	26.6301	26.674
#NUM!	#NUM!	#NUM!	#NUM!	#NUM!	#NUM!	#NUM!	#NUM!	#NUM!	25.5319	27.4186	26.353
#NUM!	#NUM!	#NUM!	#NUM!	#NUM!	#NUM!	#NUM!	#NUM!	#NUM!	25.3844	25.6544	25.7851
25.9813	#NUM!	#NUM!	#NUM!	#NUM!	#NUM!	#NUM!	#NUM!	25.666	25.891	25.1808	25.0388
#NUM!	#NUM!	#NUM!	#NUM!	#NUM!	#NUM!	#NUM!	#NUM!	27.5345	27.3146	27.0192	27.0371
#NUM!	#NUM!	#NUM!	#NUM!	#NUM!	#NUM!	#NUM!	#NUM!	#NUM!	24.6703	#NUM!	#NUM!
#NUM!	#NUM!	27.6715	#NUM!	#NUM!	#NUM!	#NUM!	#NUM!	27.5671	27.7129	27.8169	27.6223
#NUM!	#NUM!	#NUM!	#NUM!	#NUM!	#NUM!	#NUM!	#NUM!	#NUM!	23.6269	23.9971	#NUM!
#NUM!	#NUM!	#NUM!	#NUM!	#NUM!	#NUM!	#NUM!	#NUM!	27.8813	#NUM!	27.9286	27.9531
#NUM!	#NUM!	#NUM!	#NUM!	#NUM!	#NUM!	#NUM!	#NUM!	#NUM!	25.6577	26.8901	27.2321
#NUM!	26.7249	#NUM!	27.4457	#NUM!	#NUM!	#NUM!	#NUM!	27.7687	27.8258	28.2131	27.8215
27.6625	28.0041	27.9741	27.849	#NUM!	28.681	27.9781	27.5392	27.549	27.4886	27.6499	27.9301
25.7133	25.7463	25.633	25.3702	#NUM!	26.1828	#NUM!	25.7462	#NUM!	#NUM!	#NUM!	#NUM!
26.7487	26.7369	#NUM!	#NUM!	#NUM!	#NUM!	#NUM!	#NUM!	27.0456	26.6289	27.4942	27.6392
28.3358	28.5279	28.1646	28.1234	28.3467	28.4741	28.0687	28.8367	28.8421	28.7416	28.7569	28.8835
29.6054	29.8339	#NUM!	27.5546	#NUM!	#NUM!	#NUM!	30.1922	29.3002	29.2204	28.5957	29.1049
#NUM!	#NUM!	#NUM!	#NUM!	#NUM!	#NUM!	#NUM!	#NUM!	25.9466	26.2941	25.9048	26.0811
#NUM!	#NUM!	#NUM!	#NUM!	#NUM!	#NUM!	#NUM!	#NUM!	#NUM!	26.7216	26.9214	#NUM!
#NUM!	25.1319	#NUM!	#NUM!	#NUM!	#NUM!	#NUM!	#NUM!	26.0149	26.299	25.936	26.33
#NUM!	#NUM!	#NUM!	#NUM!	#NUM!	#NUM!	#NUM!	#NUM!	26.3165	26.2962	26.2705	26.0635
26.9714	#NUM!	#NUM!	#NUM!	#NUM!	#NUM!	#NUM!	#NUM!	25.8748	26.5651	26.5619	26.4493
#NUM!	26.4015	#NUM!	#NUM!	#NUM!	#NUM!	#NUM!	#NUM!	26.4402	26.2072	26.2621	26.4915
#NUM!	#NUM!	#NUM!	#NUM!	#NUM!	#NUM!	#NUM!	#NUM!	28.8746	28.5857	28.604	28.5961
#NUM!	#NUM!	#NUM!	#NUM!	#NUM!	#NUM!	#NUM!	#NUM!	27.2516	26.9759	26.5638	26.6461
#NUM!	#NUM!	#NUM!	#NUM!	#NUM!	#NUM!	#NUM!	#NUM!	#NUM!	#NUM!	#NUM!	#NUM!
#NUM!	#NUM!	#NUM!	#NUM!	#NUM!	#NUM!	#NUM!	#NUM!	#NUM!	26.3176	25.844	25.9156
28.8923	29.1767	29.431	29.2462	29.0841	29.1571	29.4539	28.7242	26.8377	26.4286	26.648	26.3862
#NUM!	27.5332	#NUM!	#NUM!	#NUM!	#NUM!	#NUM!	#NUM!	27.8858	27.8382	28.1266	28.4979
#NUM!	#NUM!	#NUM!	#NUM!	#NUM!	#NUM!	#NUM!	#NUM!	26.3693	26.6729	26.6456	26.7036
27.3461	#NUM!	#NUM!	27.3275	#NUM!	#NUM!	#NUM!	#NUM!	27.7894	27.4162	#NUM!	27.3638
29.3711	29.3042	29.3682	29.3101	28.5429	28.7596	28.3944	28.6787	28.7698	29.1908	29.0763	28.9515
28.6112	28.5181	28.0538	28.0121	28.7158	29.0311	28.1629	28.4516	#NUM!	27.2882	26.6505	26.5604
27.0197	27.1569	27.5159	27.1731	#NUM!	#NUM!	#NUM!	#NUM!	28.3345	28.3762	28.5235	28.5305
#NUM!	#NUM!	#NUM!	#NUM!	#NUM!	#NUM!	#NUM!	#NUM!	28.5405	28.4607	28.5361	28.496
#NUM!	#NUM!	#NUM!	#NUM!	#NUM!	#NUM!	#NUM!	#NUM!	#NUM!	27.5141	#NUM!	27.3625
#NUM!	#NUM!	#NUM!	#NUM!	#NUM!	#NUM!	#NUM!	#NUM!	26.4718	26.1471	#NUM!	#NUM!
28.3743	28.3015	28.8787	28.6029	28.5727	27.5749	28.2024	28.0862	26.4432	27.0119	26.5287	26.6559
33.7348	33.1379	32.7618	32.6216	33.7723	33.0001	33.131	32.8987	34.4562	33.7563	33.5889	33.1928
30.6203	30.6913	27.9025	27.5181	28.8807	30.7287	28.89	30.2802	#NUM!	#NUM!	#NUM!	#NUM!
#NUM!	#NUM!	#NUM!	#NUM!	#NUM!	#NUM!	#NUM!	#NUM!	29.0979	29.0803	29.1912	29.0803
29.6053	29.826	29.3553	29.4417	29.0373	29.0472	29.0459	28.6025	26.2601	26.6638	26.6807	26.6283
30.7173	31.0557	30.9136	32.0884	32.0088	30.9619	32.1418	30.5835	29.2446	29.1976	29.8138	29.6404
#NUM!	#NUM!	#NUM!	#NUM!	#NUM!	#NUM!	#NUM!	#NUM!	#NUM!	#NUM!	#NUM!	#NUM!
#NUM!	27.2708	27.3899	#NUM!	#NUM!	#NUM!	#NUM!	#NUM!	27.7337	27.6835	27.963	27.9352
#NUM!	#NUM!	#NUM!	#NUM!	#NUM!	#NUM!	#NUM!	#NUM!	27.3856	27.8385	27.5817	27.7035
28.3254	28.1744	28.1613	27.9271	#NUM!	28.4877	27.7209	28.7562	27.1548	27.3071	27.3659	27.7398
29.1927	28.6823	29.0779	28.5882	#NUM!	29.8763	#NUM!	27.4205	28.4831	28.736	29.0043	28.9824
#NUM!	#NUM!	#NUM!	#NUM!	#NUM!	#NUM!	#NUM!	#NUM!	28.8106	28.5111	28.8935	28.4951
29.1885	29.1625	27.9906	28.5139	28.5195	29.1289	28.4991	28.776	28.4412	27.825	27.4416	27.4851
#NUM!	#NUM!	#NUM!	#NUM!	#NUM!	#NUM!	#NUM!	#NUM!	#NUM!	#NUM!	#NUM!	#NUM!
#NUM!	#NUM!	#NUM!	#NUM!	#NUM!	#NUM!	#NUM!	#NUM!	#NUM!	25.8292	25.9583	25.92
28.9894	29.0111	28.6762	28.6482	28.1442	28.4123	28.081	28.2027	28.2323	28.0274	28.2659	28.0581
28.9015	28.775	28.4496	28.6872	#NUM!	28.5011	#NUM!	28.0936	29.2236	29.1477	29.441	29.4322
#NUM!	#NUM!	#NUM!	#NUM!	#NUM!	#NUM!	#NUM!	#NUM!	#NUM!	26.2048	25.0189	25.4593
#NUM!	#NUM!	#NUM!	#NUM!	#NUM!	#NUM!	#NUM!	#NUM!	27.0226	26.7452	27.0663	27.0237
30.2842	30.4073	30.2882	29.9586	30.3237	30.0171	30.1623	29.9704	30.4617	30.2905	29.9591	29.8115
#NUM!	#NUM!	#NUM!	#NUM!	#NUM!	28.6417	#NUM!	#NUM!	27.0687	27.1902	27.3502	27.6396
#NUM!	#NUM!	#NUM!	#NUM!	#NUM!	#NUM!	#NUM!	#NUM!	27.5235	27.659	27.3147	27.5906
#NUM!	26.2252	#NUM!	25.5312	#NUM!	#NUM!	#NUM!	#NUM!	27.6029	27.7639	27.4046	27.5217
28.4206	28.372	28.0824	27.9852	28.66	28.7603	28.742	28.5199	#NUM!	#NUM!	26.2002	25.7573
29.3958	29.3484	28.2588	28.2019	29.027	30.067	29.201	29.8442	26.477	26.5041	26.127	25.9203
27.3277	27.3386	#NUM!	27.3725	#NUM!	27.7202	#NUM!	#NUM!	27.6063	27.6453	27.975	27.7756
29.3978	28.7941	28.2828	28.4788	#NUM!	27.4681	27.8367	#NUM!	#NUM!	#NUM!	26.0527	25.9117
#NUM!	#NUM!	#NUM!	#NUM!	#NUM!	#NUM!	#NUM!	#NUM!	27.1746	#NUM!	27.8193	28.2448
#NUM!	27.8232	#NUM!	#NUM!	#NUM!	#NUM!	#NUM!	#NUM!	27.4339	26.9879	28.2354	27.709
#NUM!	#NUM!	26.7522	#NUM!	#NUM!	#NUM!	#NUM!	#NUM!	27.5803	27.6691	27.1515	27.3732
#NUM!	#NUM!	#NUM!	#NUM!	#NUM!	#NUM!	#NUM!	#NUM!	28.4453	28.1948	27.9244	27.7913
#NUM!	#NUM!	#NUM!	#NUM!	#NUM!	#NUM!	#NUM!	#NUM!	26.6864	27.0325	26.6438	27.0769
29.1817	29.2851	28.9955	29.3245	28.5545	28.4037	28.6283	28.0993	#NUM!	#NUM!	26.6224	26.5031
28.3856	27.9664	29.1054	28.5146	#NUM!	27.8265	#NUM!	#NUM!	28.7462	28.5943	28.7852	28.6435
#NUM!	#NUM!	#NUM!	#NUM!	#NUM!	#NUM!	#NUM!	#NUM!	27.5617	28.4737	28.4588	28.4928
#NUM!	#NUM!	#NUM!	#NUM!	#NUM!	#NUM!	#NUM!	#NUM!	#NUM!	#NUM!	25.7452	25.9017
#NUM!	#NUM!	#NUM!	#NUM!	#NUM!	#NUM!	#NUM!	#NUM!	27.2	27.1159	26.7936	27.1552
31.0728	31.101	30.2997	30.6763	30.8179	30.1254	30.8345	29.999	28.2679	28.3999	28.3368	28.0334
28.3342	27.6569	27.7751	#NUM!	#NUM!	27.7296	#NUM!	#NUM!	28.8777	29.088	29.34	29.1231
#NUM!	#NUM!	28.499	28.6301	#NUM!	#NUM!	#NUM!	#NUM!	29.0519	28.5234	29.3329	29.2467
29.5523	29.6469	29.486	28.5057	#NUM!	29.5177	#NUM!	#NUM!	30.6423	30.7861	30.6924	30.4213
29.8106	29.7588	29.4681	29.408	29.3479	29.6717	29.4114	29.177	26.6	#NUM!	26.7621	26.7651
29.9163	29.8837	29.5584	29.5392	29.0377	29.5409	29.1401	29.1304	30.6366	30.6297	30.4172	30.5594
29.8596	29.9422	29.2313	28.8291	#NUM!	29.3231	#NUM!	#NUM!	29.8598	29.7002	29.2232	29.248
27.9945	28.3792	28.101	28.3301	#NUM!	29.2666	#NUM!	29.2523	29.4601	29.3608	29.4727	29.4713
#NUM!	#NUM!	#NUM!	#NUM!	#NUM!	27.9257	#NUM!	#NUM!	29.0029	29.0929	27.6742	27.4528
#NUM!	#NUM!	#NUM!	#NUM!	#NUM!	#NUM!	#NUM!	#NUM!	#NUM!	#NUM!	26.2275	#NUM!
30.0135	#NUM!	#NUM!	#NUM!	#NUM!	29.548	#NUM!	#NUM!	30.9662	30.6828	30.666	30.5662
#NUM!	#NUM!	#NUM!	#NUM!	#NUM!	#NUM!	#NUM!	#NUM!	#NUM!	#NUM!	#NUM!	#NUM!
#NUM!	#NUM!	#NUM!	#NUM!	#NUM!	#NUM!	#NUM!	#NUM!	#NUM!	#NUM!	26.5529	26.4449
#NUM!	#NUM!	#NUM!	#NUM!	#NUM!	#NUM!	#NUM!	#NUM!	26.3014	26.4153	26.3096	26.4466
#NUM!	#NUM!	#NUM!	#NUM!	#NUM!	#NUM!	#NUM!	#NUM!	#NUM!	#NUM!	#NUM!	#NUM!
31.993	31.9748	29.4894	29.2865	29.8397	31.6552	30.1402	32.5451	31.3111	31.0595	30.9288	31.3428
#NUM!	#NUM!	#NUM!	#NUM!	#NUM!	#NUM!	#NUM!	#NUM!	#NUM!	26.5903	25.9782	25.8699
29.1345	28.7711	27.9853	28.3354	#NUM!	28.5525	#NUM!	28.4376	26.912	26.7358	27.5479	27.7233
#NUM!	26.9824	#NUM!	27.4189	#NUM!	#NUM!	#NUM!	#NUM!	#NUM!	26.4433	26.5422	26.606
#NUM!	26.6179	#NUM!	#NUM!	#NUM!	#NUM!	#NUM!	#NUM!	26.9624	26.7458	27.5654	27.2926
29.1006	29.4062	28.5471	28.3894	28.2469	28.3031	28.141	28.1052	27.7163	27.6718	28.4203	28.505
#NUM!	#NUM!	#NUM!	#NUM!	#NUM!	#NUM!	#NUM!	#NUM!	#NUM!	#NUM!	#NUM!	#NUM!
#NUM!	#NUM!	#NUM!	#NUM!	#NUM!	#NUM!	#NUM!	#NUM!	#NUM!	25.9919	26.1273	26.3655
#NUM!	#NUM!	#NUM!	#NUM!	#NUM!	#NUM!	#NUM!	#NUM!	#NUM!	#NUM!	#NUM!	#NUM!

#NUM!	#NUM!	#NUM!	#NUM!	#NUM!	#NUM!	#NUM!	#NUM!	26.2034	26.6903	26.3764	26.6351
26.1673	#NUM!	27.6734	27.4146	#NUM!	#NUM!	#NUM!	#NUM!	26.1631	26.5269	26.1506	26.415
#NUM!	#NUM!	#NUM!	#NUM!	#NUM!	#NUM!	#NUM!	#NUM!	#NUM!	25.9184	25.8799	26.0452
#NUM!	#NUM!	#NUM!	#NUM!	#NUM!	#NUM!	#NUM!	#NUM!	25.5654	#NUM!	25.7869	25.8526
#NUM!	#NUM!	#NUM!	#NUM!	#NUM!	#NUM!	#NUM!	#NUM!	27.3709	27.7674	27.6923	27.2123
#NUM!	#NUM!	#NUM!	#NUM!	#NUM!	#NUM!	#NUM!	#NUM!	#NUM!	#NUM!	25.3581	#NUM!
#NUM!	#NUM!	#NUM!	#NUM!	#NUM!	#NUM!	#NUM!	#NUM!	25.7274	#NUM!	25.9578	26.4544
#NUM!	#NUM!	#NUM!	#NUM!	#NUM!	#NUM!	#NUM!	#NUM!	27.0017	27.497	27.3289	27.1551
#NUM!	#NUM!	#NUM!	#NUM!	#NUM!	#NUM!	#NUM!	#NUM!	26.0324	26.1816	25.7463	25.9208
#NUM!	#NUM!	#NUM!	#NUM!	#NUM!	#NUM!	#NUM!	#NUM!	26.1631	26.2088	26.346	26.1461
#NUM!	#NUM!	#NUM!	#NUM!	#NUM!	#NUM!	#NUM!	#NUM!	#NUM!	#NUM!	26.0518	#NUM!
#NUM!	#NUM!	#NUM!	#NUM!	#NUM!	#NUM!	#NUM!	#NUM!	28.1371	28.3626	27.8938	27.9397
#NUM!	#NUM!	#NUM!	#NUM!	#NUM!	#NUM!	#NUM!	#NUM!	25.2988	25.6981	25.4495	25.8728
27.9746	27.3525	#NUM!	27.7165	#NUM!	#NUM!	#NUM!	#NUM!	#NUM!	#NUM!	#NUM!	#NUM!
27.8567	27.567	#NUM!	27.465	#NUM!	27.3337	#NUM!	27.5601	27.0684	27.0075	27.0947	26.5211
27.8464	27.3564	27.5735	28.371	28.6869	27.3688	#NUM!	27.4647	25.975	26.0702	25.7394	25.6999
#NUM!	#NUM!	#NUM!	#NUM!	#NUM!	#NUM!	#NUM!	#NUM!	27.4084	27.4762	27.3658	27.3776
26.145	26.2752	#NUM!	#NUM!	#NUM!	27.3312	#NUM!	#NUM!	26.4581	26.911	26.4614	26.4436
27.1359	#NUM!	#NUM!	26.8121	#NUM!	27.1309	#NUM!	26.9619	27.2831	27.2473	26.9455	27.2247
#NUM!	#NUM!	#NUM!	#NUM!	#NUM!	#NUM!	#NUM!	#NUM!	26.5589	26.9471	26.6137	26.2623
#NUM!	#NUM!	#NUM!	#NUM!	#NUM!	#NUM!	#NUM!	#NUM!	26.2912	26.3124	26.3572	26.595
#NUM!	#NUM!	#NUM!	#NUM!	#NUM!	#NUM!	#NUM!	#NUM!	27.0505	26.7241	26.7911	26.9669
#NUM!	#NUM!	#NUM!	#NUM!	#NUM!	#NUM!	#NUM!	#NUM!	#NUM!	#NUM!	25.6319	#NUM!
29.5602	29.4556	29.3101	29.347	29.5762	30.6613	29.6473	29.3237	28.4939	28.2593	28.3568	28.5151
#NUM!	#NUM!	#NUM!	#NUM!	#NUM!	#NUM!	#NUM!	#NUM!	27.0561	27.2035	27.658	27.4555
#NUM!	#NUM!	#NUM!	#NUM!	#NUM!	#NUM!	#NUM!	#NUM!	25.3789	#NUM!	25.547	25.5951
#NUM!	#NUM!	#NUM!	#NUM!	#NUM!	#NUM!	#NUM!	#NUM!	#NUM!	#NUM!	#NUM!	#NUM!
#NUM!	#NUM!	#NUM!	26.7519	#NUM!	#NUM!	#NUM!	#NUM!	26.3287	26.4858	26.932	26.7113
#NUM!	#NUM!	#NUM!	#NUM!	#NUM!	#NUM!	#NUM!	#NUM!	#NUM!	27.1379	26.7535	26.2483
#NUM!	#NUM!	#NUM!	#NUM!	#NUM!	#NUM!	#NUM!	#NUM!	27.1729	27.2034	27.2285	27.3604
#NUM!	#NUM!	#NUM!	#NUM!	#NUM!	#NUM!	#NUM!	#NUM!	#NUM!	25.7502	26.0676	#NUM!
#NUM!	28.4949	#NUM!	#NUM!	#NUM!	28.9841	#NUM!	#NUM!	28.2411	27.9211	28.3792	28.665
#NUM!	#NUM!	#NUM!	#NUM!	#NUM!	#NUM!	#NUM!	#NUM!	26.1541	26.2672	26.7894	26.7165
#NUM!	#NUM!	#NUM!	#NUM!	#NUM!	#NUM!	#NUM!	#NUM!	#NUM!	#NUM!	#NUM!	#NUM!
#NUM!	#NUM!	#NUM!	#NUM!	#NUM!	#NUM!	#NUM!	#NUM!	#NUM!	#NUM!	#NUM!	26.7945
27.4633	#NUM!	#NUM!	#NUM!	#NUM!	#NUM!	#NUM!	#NUM!	25.9074	26.2704	25.5889	25.4185
29.2024	28.6592	27.8619	28.875	28.7331	28.5803	28.5636	28.6604	26.3667	#NUM!	25.9793	26.0909
29.5228	29.3872	28.6066	28.5882	29.0805	28.8871	29.0466	28.8525	#NUM!	27.9366	26.9767	27.4938
27.955	28.2687	#NUM!	#NUM!	28.167	27.314	#NUM!	#NUM!	27.5171	27.7876	28.2419	27.9895
29.2762	29.4165	29.9025	29.2754	#NUM!	28.2497	28.671	#NUM!	29.2907	29.6033	29.3082	29.4085
#NUM!	#NUM!	#NUM!	#NUM!	#NUM!	#NUM!	#NUM!	#NUM!	#NUM!	26.1793	26.6706	26.7678
#NUM!	#NUM!	#NUM!	#NUM!	#NUM!	#NUM!	#NUM!	#NUM!	#NUM!	#NUM!	26.5882	25.9974
#NUM!	#NUM!	#NUM!	#NUM!	#NUM!	#NUM!	#NUM!	#NUM!	#NUM!	#NUM!	#NUM!	#NUM!
#NUM!	#NUM!	#NUM!	#NUM!	#NUM!	#NUM!	#NUM!	#NUM!	26.1355	27.1486	27.6184	27.4621
#NUM!	28.0775	#NUM!	#NUM!	#NUM!	#NUM!	#NUM!	#NUM!	28.4721	28.0723	28.2494	27.6877
#NUM!	#NUM!	#NUM!	#NUM!	#NUM!	#NUM!	#NUM!	#NUM!	27.621	27.7391	27.8574	27.4129
#NUM!	#NUM!	#NUM!	#NUM!	#NUM!	28.4438	#NUM!	#NUM!	26.4037	26.4567	26.4538	26.1432
28.4183	28.1882	27.6993	28.1146	#NUM!	27.5529	#NUM!	28.9456	28.8538	29.2234	29.5526	29.8438
28.4588	27.6633	#NUM!	#NUM!	#NUM!	#NUM!	#NUM!	#NUM!	29.145	29.7299	29.4488	29.4137
31.9305	31.7739	31.7918	31.8307	31.3506	31.5567	31.4762	31.1646	30.9802	30.3646	30.4789	30.4069
#NUM!	#NUM!	#NUM!	#NUM!	#NUM!	#NUM!	#NUM!	#NUM!	#NUM!	#NUM!	#NUM!	#NUM!
30.1462	30.3473	28.7905	28.8757	30.0178	31.0957	29.9824	30.9948	29.0745	29.0405	29.6771	29.6767
31.8445	32.4532	31.1055	31.0901	33.5082	34.3195	33.5332	34.5769	31.9997	32.1488	32.3291	32.0954
30.8871	31.0171	28.7175	28.766	30.203	32.1041	29.8173	31.9165	#NUM!	#NUM!	#NUM!	#NUM!
#NUM!	#NUM!	#NUM!	#NUM!	#NUM!	#NUM!	#NUM!	#NUM!	29.2514	29.2236	29.6479	29.5717
#NUM!	#NUM!	#NUM!	#NUM!	#NUM!	#NUM!	#NUM!	#NUM!	28.0733	26.8729	#NUM!	26.3875
#NUM!	#NUM!	#NUM!	#NUM!	#NUM!	#NUM!	#NUM!	#NUM!	#NUM!	#NUM!	#NUM!	#NUM!
29.8068	29.39	28.0315	29.0591	#NUM!	28.7829	#NUM!	28.2844	29.0888	28.8894	29.7556	29.5767
#NUM!	#NUM!	#NUM!	#NUM!	#NUM!	#NUM!	#NUM!	#NUM!	#NUM!	25.7387	#NUM!	26.385
#NUM!	#NUM!	#NUM!	#NUM!	#NUM!	#NUM!	#NUM!	#NUM!	27.1604	27.1392	27.2208	27.2208
28.1028	28.0076	28.4946	28.7601	#NUM!	27.4593	#NUM!	27.3913	27.0103	26.9878	26.6058	26.6617
#NUM!	26.4715	#NUM!	#NUM!	#NUM!	#NUM!	#NUM!	#NUM!	27.6819	27.3153	26.6992	26.6995
#NUM!	#NUM!	#NUM!	#NUM!	#NUM!	#NUM!	#NUM!	#NUM!	#NUM!	26.304	26.3092	#NUM!
#NUM!	#NUM!	#NUM!	#NUM!	#NUM!	26.1459	#NUM!	#NUM!	28.8588	28.889	29.3602	29.2661
#NUM!	#NUM!	#NUM!	26.9246	#NUM!	27.5418	#NUM!	#NUM!	30.1573	30.1305	30.1436	29.909
#NUM!	#NUM!	#NUM!	#NUM!	#NUM!	#NUM!	#NUM!	#NUM!	26.2712	#NUM!	26.1531	25.574
#NUM!	#NUM!	#NUM!	#NUM!	#NUM!	26.7558	#NUM!	#NUM!	27.8201	27.651	27.4851	27.3584
32.4717	32.4024	31.4866	31.5678	32.1978	32.0294	32.1007	31.9046	29.7117	30.0588	29.806	30.0774
#NUM!	27.0036	#NUM!	#NUM!	#NUM!	28.2061	#NUM!	#NUM!	27.8372	27.9458	27.5955	27.8413
26.343	26.4769	#NUM!	26.4667	#NUM!	26.9345	#NUM!	#NUM!	28.2938	28.5415	28.3816	28.4292
30.2794	30.3342	28.7062	29.0733	27.4074	29.9067	28.8088	29.0305	30.8768	30.6348	30.0836	30.0117
#NUM!	27.8205	27.8362	#NUM!	#NUM!	28.0184	#NUM!	#NUM!	28.0033	27.7705	27.8548	27.9996
#NUM!	#NUM!	#NUM!	#NUM!	#NUM!	#NUM!	#NUM!	#NUM!	25.4282	25.4561	25.7755	25.8032
#NUM!	26.8343	#NUM!	26.7559	#NUM!	#NUM!	#NUM!	#NUM!	27.0532	27.0608	27.4478	27.6355
#NUM!	#NUM!	#NUM!	#NUM!	#NUM!	#NUM!	#NUM!	#NUM!	27.5334	27.3094	27.5324	27.5039
#NUM!	#NUM!	#NUM!	#NUM!	#NUM!	#NUM!	#NUM!	#NUM!	27.255	27.3844	27.3866	27.4688
#NUM!	#NUM!	#NUM!	#NUM!	#NUM!	30.2218	#NUM!	#NUM!	27.5889	27.3202	28.2807	28.3005
28.4613	28.5209	27.9317	28.0668	28.5211	27.6258	28.5878	29.0218	28.1703	27.8823	28.289	28.1792
28.5269	28.1301	#NUM!	#NUM!	#NUM!	26.9928	#NUM!	27.0431	25.4754	25.3536	26.2186	26.0781
31.6787	31.9992	31.6107	31.4853	31.5519	31.2897	31.5066	31.0207	30.3814	30.5189	30.5322	30.5526
29.2258	29.0953	28.6705	28.6304	#NUM!	29.4261	#NUM!	29.6366	29.4725	29.1357	29.5724	29.5381
#NUM!	#NUM!	#NUM!	#NUM!	#NUM!	#NUM!	#NUM!	#NUM!	27.6596	27.3874	27.8986	27.7728
#NUM!	#NUM!	#NUM!	#NUM!	#NUM!	#NUM!	#NUM!	#NUM!	#NUM!	#NUM!	26.1453	#NUM!
29.4167	29.5904	28.5296	28.4558	29.2753	30.23	#NUM!	29.6936	30.162	30.2996	30.6081	30.5499
29.5839	29.0841	28.8348	28.9407	#NUM!	30.3059	27.9395	29.8849	29.4146	29.4725	29.7938	29.6826
#NUM!	27.2033	#NUM!	26.9895	#NUM!	28.2585	27.8175	#NUM!	26.3103	25.316	25.4096	#NUM!
#NUM!	#NUM!	26.9591	#NUM!	#NUM!	#NUM!	#NUM!	#NUM!	27.9639	27.7783	27.4395	27.4341
29.163	29.2242	29.3827	29.1376	28.7653	29.1618	28.7623	28.3229	29.4501	29.4137	29.6522	29.8524
#NUM!	#NUM!	#NUM!	#NUM!	#NUM!	#NUM!	#NUM!	#NUM!	26.2996	26.6552	26.257	26.2195
28.0032	27.9471	28.5389	#NUM!	#NUM!	26.9768	#NUM!	#NUM!	29.312	28.8814	29.3319	29.3915
#NUM!	27.1614	#NUM!	#NUM!	#NUM!	#NUM!	#NUM!	#NUM!	26.42	26.3399	26.555	26.801
#NUM!	#NUM!	#NUM!	27.6841	#NUM!	#NUM!	#NUM!	#NUM!	26.7162	27.0135	27.2908	27.1618
30.0526	30.1981	30.0484	29.976	29.6633	29.4556	29.6822	28.8172	27.7975	27.6956	27.6597	27.5785
28.6735	28.754	26.0328	26.9577	28.3704	29.175	28.0266	28.8899	#NUM!	#NUM!	#NUM!	#NUM!
#NUM!	#NUM!	#NUM!	#NUM!	#NUM!	#NUM!	#NUM!	#NUM!	#NUM!	26.2293	25.1299	25.2741
#NUM!	#NUM!	#NUM!	#NUM!	#NUM!	#NUM!	#NUM!	#NUM!	25.7765	25.9514	25.5551	25.5726
#NUM!	#NUM!	#NUM!	#NUM!	#NUM!	#NUM!	#NUM!	#NUM!	27.2701	27.2304	27.2369	27.5002
27.7766	27.9559	#NUM!	27.8215	#NUM!	27.2664	#NUM!	#NUM!	29.026	29.0226	28.8183	28.6071
27.9143	27.9227	28.0127	27.7529	#NUM!	27.9376	#NUM!	27.4719	27.2153	26.5623	26.6931	27.2183
#NUM!	26.819	#NUM!	26.8366	#NUM!	#NUM!	#NUM!	#NUM!	29.1428	29.006	29.1293	29.0644
#NUM!	#NUM!	#NUM!	#NUM!	#NUM!	#NUM!	#NUM!	#NUM!	27.4014	27.3513	27.2014	27.1603
#NUM!	26.3595	#NUM!	#NUM!	#NUM!	#NUM!	#NUM!	#NUM!	27.3895	27.5495	27.6323	27.7029
#NUM!	#NUM!	#NUM!	#NUM!	#NUM!	#NUM!	#NUM!	#NUM!	26.1567	26.3651	27.2747	27.194
#NUM!	#NUM!	#NUM!	#NUM!	#NUM!	#NUM!	#NUM!	#NUM!	26.6656	26.2396	#NUM!	#NUM!
#NUM!	#NUM!	#NUM!	#NUM!	#NUM!	#NUM!	#NUM!	#NUM!	#NUM!	#NUM!	25.6567	25.5637
#NUM!	#NUM!	#NUM!	#NUM!	#NUM!	#NUM!	#NUM!	#NUM!	28.0937	#NUM!	28.2256	27.7905
#NUM!	#NUM!	#NUM!	#NUM!	#NUM!	#NUM!	#NUM!	#NUM!	25.1463	25.0243	25.4911	25.3884
#NUM!	#NUM!	#NUM!	#NUM!	#NUM!	#NUM!	#NUM!	#NUM!	26.4227	25.9562	#NUM!	25.859
#NUM!	#NUM!	#NUM!	#NUM!	#NUM!	#NUM!	#NUM!	#NUM!	#NUM!	#NUM!	#NUM!	25.5157
#NUM!	#NUM!	#NUM!	#NUM!	#NUM!	#NUM!	#NUM!	#NUM!	28.6316	#NUM!	28.4638	29.2331
#NUM!	27.4824	#NUM!	#NUM!	#NUM!	#NUM!	#NUM!	27.6836	26.9929	27.3324	27.5669	27.7098
28.6874	28.6995	28.4544	28.2347	29.0638	29.6389	29.0295	28.6351	27.6629	28.0471	27.5393	27.7981
#NUM!	#NUM!	#NUM!	#NUM!	#NUM!	#NUM!	#NUM!	#NUM!	26.8235	#NUM!	26.8853	#NUM!
#NUM!	#NUM!	#NUM!	#NUM!	#NUM!	#NUM!	#NUM!	#NUM!	26.2287	26.2521	26.1956	26.1188
#NUM!	#NUM!	#NUM!	#NUM!	#NUM!	#NUM!	#NUM!	#NUM!	26.5505	26.4469	27.2929	27.1095
#NUM!	#NUM!	#NUM!	#NUM!	#NUM!	#NUM!	#NUM!	#NUM!	28.3431	#NUM!	#NUM!	27.4219
#NUM!	28.6577	#NUM!	28.8842	28.0963	27.0372	#NUM!	#NUM!	25.9778	25.9302	25.5943	25.4824
#NUM!	#NUM!	#NUM!	#NUM!	#NUM!	#NUM!	#NUM!	#NUM!	26.6076	26.4381	26.7309	26.8836
#NUM!	#NUM!	#NUM!	#NUM!	#NUM!	#NUM!	#NUM!	#NUM!	#NUM!	26.3582	26.6592	26.6167
#NUM!	#NUM!	#NUM!	#NUM!	#NUM!	#NUM!	#NUM!	#NUM!	#NUM!	#NUM!	24.9094	24.6519
#NUM!	28.4483	28.9944	#NUM!	#NUM!	#NUM!	#NUM!	#NUM!	26.9068	27.1847	27.0749	27.1907
#NUM!	28.9486	#NUM!	28.8044	#NUM!	#NUM!	#NUM!	#NUM!	29.1955	29.3915	29.2093	29.1204
#NUM!	#NUM!	#NUM!	#NUM!	#NUM!	#NUM!	#NUM!	#NUM!	27.1203	26.742	26.7972	26.9592
#NUM!	#NUM!	#NUM!	#NUM!	#NUM!	#NUM!	#NUM!	#NUM!	26.3299	26.0082	26.452	26.0755
#NUM!	#NUM!	#NUM!	#NUM!	#NUM!	#NUM!	#NUM!	#NUM!	27.1509	27.4384	27.4192	27.5158
#NUM!	#NUM!	#NUM!	#NUM!	#NUM!	#NUM!	#NUM!	#NUM!	29.2936	29.0745	29.3637	29.2959
#NUM!	#NUM!	#NUM!	#NUM!	#NUM!	#NUM!	#NUM!	#NUM!	25.8824	25.7424	25.4811	25.7778
#NUM!	#NUM!	#NUM!	#NUM!	#NUM!	#NUM!	#NUM!	#NUM!	27.8814	27.671	27.7355	27.7357
#NUM!	#NUM!	#NUM!	#NUM!	#NUM!	#NUM!	#NUM!	#NUM!	26.3144	26.5586	26.8526	26.6591

#NUM!	#NUM!	#NUM!	#NUM!	#NUM!	#NUM!	#NUM!	#NUM!	26.5329	26.5585	26.603	26.6537
#NUM!	#NUM!	#NUM!	#NUM!	#NUM!	#NUM!	#NUM!	#NUM!	27.0493	27.1974	27.6593	27.8906
#NUM!	#NUM!	#NUM!	#NUM!	#NUM!	#NUM!	#NUM!	#NUM!	28.4239	27.6973	27.358	27.473
#NUM!	#NUM!	#NUM!	#NUM!	#NUM!	#NUM!	#NUM!	#NUM!	27.8275	27.9595	26.9472	#NUM!
#NUM!	#NUM!	#NUM!	#NUM!	#NUM!	#NUM!	#NUM!	#NUM!	28.0011	28.1328	28.5971	28.6082
27.5673	27.8499	#NUM!	27.854	#NUM!	#NUM!	#NUM!	#NUM!	27.072	27.156	27.4726	27.2291
#NUM!	#NUM!	#NUM!	#NUM!	#NUM!	#NUM!	#NUM!	#NUM!	25.0772	#NUM!	25.5692	#NUM!
31.3305	31.5265	29.1037	29.5008	30.3703	32.1818	29.8696	32.7109	#NUM!	#NUM!	26.8507	28.1761
28.7263	28.795	28.6151	28.4637	28.5893	29.1154	28.5452	28.3413	27.6712	27.4916	26.994	27.3838
#NUM!	#NUM!	#NUM!	#NUM!	#NUM!	#NUM!	#NUM!	#NUM!	#NUM!	26.9066	#NUM!	#NUM!
#NUM!	29.5458	#NUM!	29.1565	#NUM!	#NUM!	#NUM!	#NUM!	#NUM!	26.2921	26.8698	26.4121
#NUM!	27.8863	#NUM!	#NUM!	#NUM!	28.858	#NUM!	28.8512	28.0184	27.9362	28.1327	28.1163
#NUM!	#NUM!	#NUM!	#NUM!	#NUM!	#NUM!	#NUM!	#NUM!	26.8008	26.7713	27.0059	27.4278
#NUM!	#NUM!	#NUM!	#NUM!	#NUM!	#NUM!	#NUM!	#NUM!	26.9487	27.44	27.6034	26.9256
#NUM!	#NUM!	#NUM!	#NUM!	#NUM!	#NUM!	#NUM!	#NUM!	27.88	27.7384	27.5876	27.7207
#NUM!	28.6131	28.0702	27.9884	#NUM!	28.7564	#NUM!	#NUM!	26.9932	27.227	27.0578	27.2371
#NUM!	26.8794	#NUM!	#NUM!	#NUM!	#NUM!	#NUM!	#NUM!	27.4543	#NUM!	27.7681	27.8468
#NUM!	#NUM!	#NUM!	#NUM!	#NUM!	#NUM!	#NUM!	#NUM!	#NUM!	24.9808	26.0194	25.5055
#NUM!	#NUM!	#NUM!	#NUM!	#NUM!	#NUM!	#NUM!	#NUM!	26.2039	26.4511	26.6974	26.2935
#NUM!	#NUM!	#NUM!	#NUM!	#NUM!	#NUM!	#NUM!	#NUM!	26.0565	#NUM!	26.4291	26.2454
28.4245	28.4358	#NUM!	#NUM!	29.1336	31.0933	#NUM!	29.9779	#NUM!	#NUM!	#NUM!	#NUM!
#NUM!	#NUM!	#NUM!	#NUM!	#NUM!	#NUM!	#NUM!	#NUM!	#NUM!	25.9998	#NUM!	#NUM!
#NUM!	#NUM!	#NUM!	#NUM!	#NUM!	#NUM!	#NUM!	#NUM!	28.2429	28.2518	28.4621	28.4195
27.7853	27.543	27.5503	27.3393	#NUM!	28.4838	#NUM!	27.3984	27.2006	26.9416	26.8526	27.5857
#NUM!	#NUM!	#NUM!	#NUM!	#NUM!	#NUM!	#NUM!	#NUM!	#NUM!	#NUM!	26.0626	26.1222
#NUM!	#NUM!	#NUM!	#NUM!	#NUM!	#NUM!	#NUM!	#NUM!	26.0286	26.0893	#NUM!	26.0824
27.9915	28.2474	28.0421	27.9387	#NUM!	27.1431	#NUM!	26.8034	29.172	28.9135	29.2815	29.0445
#NUM!	#NUM!	#NUM!	#NUM!	#NUM!	#NUM!	#NUM!	#NUM!	27.2058	27.2355	27.3237	26.8016
#NUM!	28.074	#NUM!	28.1203	#NUM!	#NUM!	#NUM!	#NUM!	28.2093	28.3563	28.8466	28.7423
#NUM!	#NUM!	#NUM!	#NUM!	#NUM!	#NUM!	#NUM!	#NUM!	#NUM!	#NUM!	26.9341	27.3639
27.1191	26.9262	27.3516	27.1451	#NUM!	#NUM!	#NUM!	#NUM!	27.168	27.7629	27.5892	27.4189
30.4607	30.2671	30.9658	30.9726	30.2672	29.9539	30.2104	28.3747	31.2357	30.4954	31.3301	31.1424
#NUM!	#NUM!	#NUM!	#NUM!	#NUM!	#NUM!	#NUM!	#NUM!	#NUM!	#NUM!	26.6374	25.4309
29.9268	29.7857	29.2306	29.7545	29.2008	29.8225	29.1815	29.8393	28.9701	29.0901	28.7367	28.5715
30.5345	30.9796	31.1189	31.3162	29.934	29.3342	29.3293	28.782	30.4331	30.4165	31.0474	30.978
30.4744	30.155	29.4204	29.5486	29.3562	29.4219	29.4627	29.1489	29.9744	29.7041	29.9842	29.8849
#NUM!	#NUM!	#NUM!	#NUM!	#NUM!	#NUM!	#NUM!	#NUM!	26.9578	#NUM!	26.5029	26.445
#NUM!	#NUM!	#NUM!	#NUM!	#NUM!	#NUM!	#NUM!	#NUM!	27.1157	26.9747	26.6398	26.9492
29.736	29.6918	29.2994	28.9954	29.4975	29.9942	29.4479	29.3162	#NUM!	#NUM!	#NUM!	#NUM!
28.5007	28.5247	28.3739	28.1806	27.6088	27.7178	27.6957	#NUM!	28.1627	28.325	27.7675	27.5267
33.9507	34.1915	32.6017	32.7239	34.8238	33.5234	34.4964	34.9952	33.384	33.2427	33.3241	33.3287
27.5233	27.4038	28.8812	29.0569	#NUM!	#NUM!	#NUM!	#NUM!	#NUM!	#NUM!	#NUM!	#NUM!
29.8617	29.7304	29.723	29.2447	28.2781	29.0781	#NUM!	#NUM!	30.5056	30.4857	30.4597	30.2849
32.7809	32.8149	30.5259	30.7858	29.5029	30.4658	29.4408	29.2213	#NUM!	#NUM!	#NUM!	24.7485
28.4531	28.8085	#NUM!	#NUM!	#NUM!	#NUM!	#NUM!	#NUM!	29.0575	29.0818	28.889	28.7417
28.9693	28.8139	29.1202	28.9966	28.4395	28.7182	28.4459	28.7343	29.1269	28.9538	29.315	29.0071
#NUM!	#NUM!	#NUM!	#NUM!	#NUM!	#NUM!	#NUM!	#NUM!	#NUM!	27.1051	#NUM!	#NUM!
#NUM!	26.8281	#NUM!	27.2283	#NUM!	#NUM!	#NUM!	#NUM!	27.0259	27.1708	27.4916	27.3709
32.9713	33.0552	32.4461	32.2191	30.9351	30.6702	30.9	30.0726	30.8934	30.8189	31.1974	31.3639
#NUM!	#NUM!	#NUM!	#NUM!	#NUM!	#NUM!	#NUM!	#NUM!	26.9901	27.1224	27.1631	27.2179
#NUM!	#NUM!	#NUM!	#NUM!	#NUM!	#NUM!	#NUM!	#NUM!	25.0649	24.2578	25.1214	25.1146
#NUM!	#NUM!	#NUM!	#NUM!	#NUM!	#NUM!	#NUM!	#NUM!	#NUM!	26.5269	26.7444	26.5669
29.6518	29.7727	29.7815	29.6259	29.8251	29.6548	29.8199	29.1058	31.0944	30.9182	31.1525	31.1663
#NUM!	25.9103	#NUM!	#NUM!	#NUM!	#NUM!	#NUM!	#NUM!	27.4832	27.6136	27.585	27.5902
28.1872	28.2216	28.9145	27.3892	#NUM!	#NUM!	#NUM!	#NUM!	29.0081	29.1379	29.1575	28.9489
#NUM!	#NUM!	#NUM!	#NUM!	#NUM!	#NUM!	#NUM!	#NUM!	27.0133	26.7419	26.4131	26.6139
#NUM!	#NUM!	#NUM!	27.2858	#NUM!	#NUM!	#NUM!	#NUM!	26.8327	26.7084	26.4779	26.837
27.4852	27.5077	#NUM!	#NUM!	#NUM!	#NUM!	#NUM!	#NUM!	27.6237	27.6229	28.082	27.9793
#NUM!	29.9172	28.5935	28.5328	#NUM!	29.2013	#NUM!	#NUM!	29.7369	29.9148	29.6328	29.6083
#NUM!	#NUM!	#NUM!	#NUM!	#NUM!	#NUM!	#NUM!	#NUM!	27.9493	27.1529	26.9188	27.2474
#NUM!	27.9341	#NUM!	#NUM!	#NUM!	27.9626	#NUM!	#NUM!	27.7621	27.7822	27.9613	28.1773
#NUM!	#NUM!	#NUM!	#NUM!	#NUM!	#NUM!	#NUM!	#NUM!	28.5779	28.6281	28.7027	28.8119
#NUM!	#NUM!	#NUM!	#NUM!	#NUM!	#NUM!	#NUM!	#NUM!	#NUM!	26.0781	26.0769	26.4205
#NUM!	#NUM!	#NUM!	#NUM!	#NUM!	#NUM!	#NUM!	#NUM!	25.982	#NUM!	26.1704	#NUM!
#NUM!	#NUM!	#NUM!	#NUM!	#NUM!	#NUM!	#NUM!	#NUM!	#NUM!	#NUM!	24.8102	#NUM!
#NUM!	#NUM!	#NUM!	#NUM!	#NUM!	#NUM!	#NUM!	#NUM!	27.6247	27.536	27.6405	27.9965
28.1447	28.565	27.4573	27.0333	30.0462	30.2341	30.1899	28.7126	30.6701	30.3622	30.1066	30.7989
30.4284	30.6617	30.4651	30.7202	30.951	30.9552	31.377	30.2276	28.0291	28.3804	27.9816	28.3605
#NUM!	#NUM!	#NUM!	#NUM!	#NUM!	#NUM!	#NUM!	#NUM!	26.6582	26.3434	26.2219	26.2466
#NUM!	#NUM!	#NUM!	#NUM!	#NUM!	#NUM!	#NUM!	#NUM!	24.0273	24.7959	25.3919	24.1656
#NUM!	#NUM!	#NUM!	#NUM!	#NUM!	#NUM!	#NUM!	#NUM!	#NUM!	27.3473	27.0446	26.9763
#NUM!	#NUM!	#NUM!	#NUM!	#NUM!	#NUM!	#NUM!	#NUM!	28.0287	27.7835	28.4981	28.9123
#NUM!	#NUM!	#NUM!	#NUM!	#NUM!	#NUM!	#NUM!	#NUM!	26.5284	26.8946	26.6353	26.5091
#NUM!	#NUM!	#NUM!	#NUM!	#NUM!	26.5423	#NUM!	#NUM!	26.9662	26.3924	26.9465	26.8102
#NUM!	#NUM!	#NUM!	#NUM!	#NUM!	#NUM!	#NUM!	#NUM!	26.8508	26.9733	27.1936	26.9599
#NUM!	#NUM!	#NUM!	#NUM!	#NUM!	#NUM!	#NUM!	#NUM!	#NUM!	#NUM!	25.9451	26.0174
#NUM!	#NUM!	#NUM!	#NUM!	#NUM!	#NUM!	#NUM!	#NUM!	27.18	27.125	26.8836	26.9443
#NUM!	#NUM!	#NUM!	#NUM!	#NUM!	#NUM!	#NUM!	#NUM!	26.6169	26.5424	26.7719	26.7444
#NUM!	#NUM!	#NUM!	#NUM!	#NUM!	#NUM!	#NUM!	#NUM!	#NUM!	#NUM!	25.7939	25.4855
#NUM!	#NUM!	#NUM!	#NUM!	#NUM!	#NUM!	#NUM!	#NUM!	26.2889	26.2811	26.4274	25.9192
29.4758	29.6325	29.639	29.3342	#NUM!	29.4395	29.2574	28.8358	26.4938	26.364	26.596	26.7957
#NUM!	#NUM!	#NUM!	#NUM!	#NUM!	#NUM!	#NUM!	#NUM!	26.4402	27.4095	26.0201	26.184
26.7562	26.7795	#NUM!	26.4254	#NUM!	26.1108	#NUM!	#NUM!	27.8584	28.01	27.8853	28.0211
#NUM!	#NUM!	#NUM!	#NUM!	#NUM!	#NUM!	#NUM!	#NUM!	#NUM!	#NUM!	#NUM!	#NUM!
#NUM!	#NUM!	#NUM!	#NUM!	#NUM!	#NUM!	#NUM!	#NUM!	26.5759	#NUM!	25.0699	#NUM!
#NUM!	#NUM!	#NUM!	#NUM!	#NUM!	#NUM!	#NUM!	#NUM!	26.6283	26.2296	26.0942	25.9297
#NUM!	#NUM!	#NUM!	#NUM!	#NUM!	#NUM!	#NUM!	#NUM!	#NUM!	#NUM!	#NUM!	#NUM!
#NUM!	#NUM!	#NUM!	#NUM!	#NUM!	#NUM!	#NUM!	#NUM!	27.4746	27.4305	27.2206	26.9736
#NUM!	#NUM!	#NUM!	#NUM!	#NUM!	#NUM!	#NUM!	#NUM!	#NUM!	#NUM!	#NUM!	#NUM!
#NUM!	#NUM!	#NUM!	#NUM!	#NUM!	#NUM!	#NUM!	#NUM!	26.7326	26.9003	26.6504	26.4998
#NUM!	#NUM!	#NUM!	#NUM!	#NUM!	#NUM!	#NUM!	#NUM!	26.0998	26.223	#NUM!	26.167
#NUM!	#NUM!	#NUM!	#NUM!	#NUM!	#NUM!	#NUM!	#NUM!	25.6301	26.4456	26.8655	26.5945
28.521	28.9769	27.2091	27.292	#NUM!	#NUM!	#NUM!	#NUM!	28.9726	28.6937	28.5745	28.7052
#NUM!	#NUM!	#NUM!	#NUM!	#NUM!	#NUM!	#NUM!	#NUM!	26.4832	26.2303	#NUM!	#NUM!
27.8913	27.7396	#NUM!	27.7109	#NUM!	27.5275	#NUM!	#NUM!	28.2588	28.1939	28.0606	28.2495
#NUM!	#NUM!	#NUM!	#NUM!	#NUM!	#NUM!	#NUM!	#NUM!	27.4266	27.3826	27.8935	27.9929
#NUM!	#NUM!	#NUM!	#NUM!	#NUM!	#NUM!	#NUM!	#NUM!	26.2334	27.3506	26.5908	27.3655
#NUM!	#NUM!	#NUM!	#NUM!	#NUM!	#NUM!	#NUM!	#NUM!	26.297	26.8138	26.5348	26.2919
#NUM!	#NUM!	#NUM!	#NUM!	#NUM!	#NUM!	#NUM!	#NUM!	26.0217	26.536	#NUM!	25.1847
#NUM!	#NUM!	#NUM!	#NUM!	#NUM!	#NUM!	#NUM!	#NUM!	25.1023	25.2577	25.6801	25.6497
#NUM!	#NUM!	#NUM!	#NUM!	#NUM!	#NUM!	#NUM!	#NUM!	27.5289	27.647	26.8937	27.6894
#NUM!	#NUM!	#NUM!	#NUM!	#NUM!	#NUM!	#NUM!	#NUM!	27.8479	27.8809	28.0218	27.8944
28.0978	27.8974	28.1192	27.9688	28.346	28.6131	28.2319	28.3088	27.4453	27.407	27.1067	27.0691
29.4324	29.3135	29.3805	29.1902	#NUM!	29.2714	28.5869	27.9975	28.763	28.8842	29.48	29.4663
#NUM!	#NUM!	#NUM!	#NUM!	#NUM!	#NUM!	#NUM!	#NUM!	25.8235	25.801	25.4104	25.3285
#NUM!	#NUM!	#NUM!	#NUM!	#NUM!	#NUM!	#NUM!	#NUM!	#NUM!	#NUM!	#NUM!	23.9544
27.5061	27.3765	#NUM!	#NUM!	#NUM!	27.3782	#NUM!	#NUM!	26.5588	26.5755	26.8723	26.7267
#NUM!	#NUM!	#NUM!	#NUM!	#NUM!	#NUM!	#NUM!	#NUM!	25.3187	#NUM!	26.0432	#NUM!
#NUM!	#NUM!	#NUM!	#NUM!	#NUM!	#NUM!	#NUM!	#NUM!	25.5612	25.6799	25.9915	25.6308
#NUM!	26.9747	#NUM!	#NUM!	#NUM!	#NUM!	#NUM!	#NUM!	27.9062	27.884	28.0821	28.1009
#NUM!	#NUM!	#NUM!	#NUM!	#NUM!	#NUM!	#NUM!	#NUM!	26.9225	26.7987	26.8308	26.8641
#NUM!	#NUM!	#NUM!	26.2153	#NUM!	#NUM!	#NUM!	#NUM!	26.5579	26.0321	25.6884	25.7939
28.5758	28.4762	28.1567	27.9505	29.218	29.1332	28.9653	28.5745	27.4196	27.3544	27.4588	27.2333
28.4609	27.7643	27.9543	28.0094	29.0254	28.3893	28.8389	#NUM!	29.8903	29.9169	29.6381	29.6534
28.1845	28.3749	27.9893	27.9222	27.8167	28.1824	27.9269	#NUM!	28.5918	28.5629	28.5534	28.2729
#NUM!	27.4568	#NUM!	#NUM!	#NUM!	28.1599	#NUM!	#NUM!	27.2337	27.2886	27.3624	27.3464
#NUM!	#NUM!	#NUM!	#NUM!	#NUM!	#NUM!	#NUM!	#NUM!	26.9206	27.1019	26.69	27.1676
#NUM!	#NUM!	#NUM!	#NUM!	#NUM!	#NUM!	#NUM!	#NUM!	25.8233	26.6148	27.2186	26.027
#NUM!	#NUM!	#NUM!	#NUM!	#NUM!	#NUM!	#NUM!	#NUM!	24.9971	25.3842	#NUM!	25.5171
#NUM!	#NUM!	#NUM!	#NUM!	#NUM!	#NUM!	#NUM!	#NUM!	#NUM!	#NUM!	#NUM!	#NUM!
28.8717	28.6846	28.2022	28.0306	#NUM!	27.1039	27.4832	#NUM!	28.1472	28.1982	28.4357	28.1622
#NUM!	#NUM!	#NUM!	#NUM!	#NUM!	#NUM!	#NUM!	#NUM!	26.8748	26.7956	27.0388	26.9927
29.7318	29.6037	29.5554	29.5781	29.0075	30.0325	28.8502	29.99	29.3256	29.0954	29.2102	29.0689
#NUM!	#NUM!	#NUM!	#NUM!	#NUM!	#NUM!	#NUM!	#NUM!	29.2994	29.1956	29.0608	29.2686
30.2514	30.1286	30.0601	29.969	29.475	29.5137	29.3873	29.524	31.213	31.0722	31.37	31.194
#NUM!	#NUM!	#NUM!	#NUM!	#NUM!	#NUM!	#NUM!	#NUM!	#NUM!	#NUM!	#NUM!	#NUM!
27.6178	27.3262	#NUM!	26.8851	#NUM!	#NUM!	#NUM!	#NUM!	27.0285	26.9603	27.039	27.0591
27.6463	27.7634	27.0597	#NUM!	#NUM!	28.6346	#NUM!	28.523	27.8196	27.6127	27.4059	27.1154
#NUM!	27.803	26.6268	27.5962	#NUM!	28.1882	#NUM!	28.5056	27.8606	27.8797	28.2323	28.255

28.5722	27.7699	28.6487	28.5046	#NUM!	28.1091	29.2355	27.6134	29.3518	29.1826	28.9741	28.8635
#NUM!	#NUM!	#NUM!	#NUM!	#NUM!	#NUM!	#NUM!	#NUM!	28.5547	28.6444	28.8876	28.6717
27.8644	27.8486	28.5198	28.5305	#NUM!	26.9735	#NUM!	26.8598	27.5998	27.5798	27.6457	27.5873
#NUM!	#NUM!	#NUM!	#NUM!	#NUM!	#NUM!	#NUM!	#NUM!	#NUM!	#NUM!	26.2767	#NUM!
#NUM!	#NUM!	#NUM!	27.0739	#NUM!	#NUM!	#NUM!	#NUM!	27.1174	27.4465	27.0483	26.8674
29.1612	28.8689	28.9956	29.4752	#NUM!	28.7422	#NUM!	28.5081	29.5408	29.5146	30.0176	29.8244
#NUM!	#NUM!	#NUM!	#NUM!	#NUM!	27.3004	#NUM!	27.2681	29.1933	29.2835	29.5842	29.5811
29.4299	30.0915	30.784	29.8408	30.7121	28.6387	30.6116	28.7174	30.6347	30.6157	30.3656	30.2819
29.0447	29.07	28.6588	28.6253	#NUM!	28.6893	28.7098	28.1879	28.9787	29.0069	29.2659	29.2261
#NUM!	#NUM!	#NUM!	#NUM!	#NUM!	#NUM!	#NUM!	#NUM!	25.6088	25.7963	25.5814	25.5165
27.4437	27.2169	26.9727	27.2941	#NUM!	27.3892	#NUM!	#NUM!	28.0613	28.0719	28.4725	28.2328
#NUM!	#NUM!	#NUM!	#NUM!	#NUM!	#NUM!	#NUM!	#NUM!	29.7549	29.0903	28.1175	28.2803
#NUM!	26.7714	26.6562	#NUM!	#NUM!	#NUM!	#NUM!	#NUM!	27.5086	27.903	27.3341	27.4516
29.4561	29.3217	28.7742	28.9947	28.2615	29.4214	28.7338	29.4026	29.2882	29.1254	29.3167	29.2334
26.7647	27.2823	#NUM!	#NUM!	#NUM!	#NUM!	#NUM!	#NUM!	27.7753	27.7811	27.9228	27.9395
32.1912	32.3649	31.5532	32.1061	31.6953	32.0782	32.0313	31.6725	31.2268	31.3022	31.2033	31.2253
#NUM!	#NUM!	#NUM!	#NUM!	#NUM!	#NUM!	#NUM!	#NUM!	29.4241	29.3105	28.212	28.0746
29.2289	29.2362	28.8024	29.0876	28.4142	29.0091	28.5218	28.8086	29.4326	29.0794	29.5266	29.286
28.3307	28.2004	28.3673	28.3991	27.8175	28.6204	28.2921	28.221	28.6782	28.8034	28.8638	28.8573
29.2712	29.5009	28.3526	28.2881	28.1885	29.1661	#NUM!	28.862	30.5246	30.2569	30.8062	30.8787
27.9341	28.1257	27.4698	27.8963	#NUM!	28.0077	#NUM!	28.0768	28.3163	28.4467	28.8887	28.4704
29.21	29.443	29.319	29.2709	#NUM!	28.1963	29.7075	28.4902	30.4925	30.2971	30.4149	30.4967
29.5397	29.718	29.7651	29.9266	#NUM!	28.4583	28.7783	27.3071	31.007	30.9431	30.711	30.7017
28.7464	29.1373	28.6894	28.2764	29.0707	28.8738	28.7907	27.9083	26.577	26.604	26.1142	26.2806
#NUM!	#NUM!	#NUM!	#NUM!	#NUM!	#NUM!	#NUM!	#NUM!	#NUM!	#NUM!	#NUM!	#NUM!
28.9259	29.0478	28.6522	28.7487	#NUM!	27.9169	#NUM!	28.2757	28.7258	28.7999	29.325	29.1871
#NUM!	#NUM!	#NUM!	#NUM!	#NUM!	#NUM!	#NUM!	#NUM!	26.2705	26.2853	26.4519	26.5578
25.8775	26.7546	#NUM!	#NUM!	#NUM!	27.4587	#NUM!	#NUM!	27.5195	27.7111	27.0603	27.1806
#NUM!	#NUM!	#NUM!	#NUM!	#NUM!	#NUM!	#NUM!	#NUM!	#NUM!	#NUM!	25.3903	25.5545
#NUM!	#NUM!	#NUM!	#NUM!	#NUM!	#NUM!	#NUM!	#NUM!	25.7873	25.9002	25.8461	25.7174
#NUM!	#NUM!	#NUM!	#NUM!	#NUM!	#NUM!	#NUM!	#NUM!	25.4776	25.3659	25.8272	26.2638
#NUM!	#NUM!	#NUM!	#NUM!	#NUM!	#NUM!	#NUM!	#NUM!	#NUM!	#NUM!	24.7838	24.7772
#NUM!	#NUM!	#NUM!	#NUM!	#NUM!	#NUM!	#NUM!	#NUM!	#NUM!	27.0954	27.2363	27.1312
29.0893	29.1174	27.8705	28.2198	28.945	29.5294	28.7873	30.2866	30.1508	29.8637	30.1327	30.0641
27.6921	27.3058	27.1168	27.6555	#NUM!	#NUM!	#NUM!	#NUM!	28.1601	28.032	28.3632	27.9204
#NUM!	#NUM!	#NUM!	#NUM!	#NUM!	#NUM!	#NUM!	#NUM!	#NUM!	25.1616	25.8169	25.1833
#NUM!	#NUM!	#NUM!	#NUM!	#NUM!	#NUM!	#NUM!	#NUM!	26.0849	25.9467	#NUM!	#NUM!
27.8093	27.0188	#NUM!	27.617	#NUM!	29.7272	#NUM!	30.3743	26.6625	26.7576	26.5763	26.5566
#NUM!	27.5574	28.0436	27.2691	#NUM!	28.578	#NUM!	#NUM!	28.5455	28.2412	28.23	28.2274
#NUM!	#NUM!	#NUM!	#NUM!	#NUM!	#NUM!	#NUM!	#NUM!	25.3286	25.088	25.51	#NUM!
#NUM!	#NUM!	#NUM!	#NUM!	#NUM!	#NUM!	#NUM!	#NUM!	27.0289	26.7049	27.0622	27.0277
#NUM!	#NUM!	#NUM!	#NUM!	#NUM!	#NUM!	#NUM!	#NUM!	25.5832	25.9215	26.1703	26.2673
#NUM!	26.3869	#NUM!	27.0669	#NUM!	#NUM!	#NUM!	#NUM!	27.9139	28.6405	28.1722	28.2258
29.4044	29.1773	27.7223	28.0094	28.2463	29.1756	28.6425	29.0165	27.8172	27.8623	28.4893	28.3328
30.0545	30.078	30.0483	30.1514	30.3701	29.5517	29.622	29.8515	31.1035	30.6933	31.1639	31.0543
29.0585	28.7845	29.1455	28.5378	#NUM!	#NUM!	#NUM!	#NUM!	30.0636	29.7671	29.8389	30.1161
27.1666	26.8644	27.2296	27.0213	#NUM!	#NUM!	#NUM!	#NUM!	28.5705	28.4004	28.377	28.5013
#NUM!	#NUM!	#NUM!	#NUM!	#NUM!	#NUM!	#NUM!	#NUM!	#NUM!	25.3245	25.6332	25.1952
#NUM!	#NUM!	#NUM!	#NUM!	#NUM!	#NUM!	#NUM!	#NUM!	25.4707	25.7247	#NUM!	#NUM!
27.4472	#NUM!	#NUM!	28.4071	#NUM!	#NUM!	#NUM!	#NUM!	28.3344	28.0537	28.5177	28.1576
28.189	28.4258	#NUM!	#NUM!	#NUM!	29.1922	#NUM!	28.8795	29.0333	29.0039	29.4864	29.2802
26.9263	26.9818	#NUM!	#NUM!	#NUM!	#NUM!	#NUM!	#NUM!	27.563	27.6193	27.8293	27.9232
#NUM!	#NUM!	#NUM!	#NUM!	#NUM!	#NUM!	#NUM!	#NUM!	#NUM!	#NUM!	#NUM!	#NUM!
#NUM!	#NUM!	#NUM!	#NUM!	#NUM!	#NUM!	#NUM!	#NUM!	26.2971	26.6928	25.9278	25.765
#NUM!	#NUM!	#NUM!	#NUM!	#NUM!	#NUM!	#NUM!	#NUM!	#NUM!	#NUM!	26.1352	#NUM!
#NUM!	#NUM!	#NUM!	#NUM!	#NUM!	#NUM!	#NUM!	#NUM!	27.7482	27.6772	28.2025	27.7091
#NUM!	#NUM!	#NUM!	#NUM!	#NUM!	#NUM!	#NUM!	#NUM!	#NUM!	26.1976	26.1683	25.7584
#NUM!	#NUM!	#NUM!	#NUM!	#NUM!	#NUM!	#NUM!	#NUM!	25.9377	25.8606	26.7358	26.3613
#NUM!	#NUM!	#NUM!	#NUM!	#NUM!	#NUM!	#NUM!	#NUM!	27.5004	27.6234	27.4168	27.6155
#NUM!	#NUM!	#NUM!	#NUM!	#NUM!	#NUM!	#NUM!	#NUM!	#NUM!	#NUM!	#NUM!	#NUM!
#NUM!	#NUM!	#NUM!	#NUM!	#NUM!	#NUM!	#NUM!	#NUM!	#NUM!	#NUM!	#NUM!	#NUM!
#NUM!	#NUM!	#NUM!	#NUM!	#NUM!	#NUM!	#NUM!	#NUM!	#NUM!	25.8557	26.3323	26.385
#NUM!	#NUM!	#NUM!	#NUM!	#NUM!	#NUM!	#NUM!	#NUM!	26.8735	26.7974	26.8612	26.9448
#NUM!	#NUM!	#NUM!	#NUM!	#NUM!	#NUM!	#NUM!	#NUM!	27.5557	27.4652	27.5396	26.9426
#NUM!	#NUM!	28.6896	27.9023	#NUM!	#NUM!	#NUM!	#NUM!	27.8979	27.7072	27.6386	27.4066
29.557	29.8364	28.6611	28.4104	29.3761	30.4337	29.4605	29.2088	#NUM!	#NUM!	#NUM!	#NUM!
#NUM!	#NUM!	#NUM!	#NUM!	#NUM!	#NUM!	#NUM!	#NUM!	25.7532	26.0496	#NUM!	25.6381
#NUM!	#NUM!	#NUM!	#NUM!	#NUM!	#NUM!	#NUM!	#NUM!	27.1029	26.9587	26.7591	27.0179
#NUM!	#NUM!	#NUM!	#NUM!	#NUM!	#NUM!	#NUM!	#NUM!	#NUM!	26.7316	26.8202	26.7058
#NUM!	#NUM!	#NUM!	#NUM!	#NUM!	#NUM!	#NUM!	#NUM!	27.1325	26.4481	26.0718	26.8494
28.6166	28.392	28.2561	28.9948	#NUM!	#NUM!	#NUM!	#NUM!	27.8794	28.0587	28.0944	28.2648
30.3649	30.582	30.5608	30.465	30.5488	29.5203	30.4192	29.6895	29.6424	29.544	29.6479	29.802
30.1428	30.1784	29.7198	29.6636	29.5959	29.3868	29.4127	29.4935	28.5491	28.6495	28.8747	28.6635
#NUM!	#NUM!	#NUM!	#NUM!	#NUM!	#NUM!	#NUM!	#NUM!	#NUM!	27.2095	27.2985	26.6246
#NUM!	#NUM!	#NUM!	#NUM!	#NUM!	#NUM!	#NUM!	#NUM!	26.3898	26.4103	26.6853	27.0372
#NUM!	#NUM!	#NUM!	#NUM!	#NUM!	#NUM!	#NUM!	#NUM!	#NUM!	26.1034	25.9457	26.3836
#NUM!	#NUM!	#NUM!	#NUM!	#NUM!	#NUM!	#NUM!	#NUM!	25.2803	25.4756	25.7166	26.1292
#NUM!	#NUM!	#NUM!	#NUM!	#NUM!	#NUM!	#NUM!	#NUM!	26.979	26.5898	26.9234	26.7806
#NUM!	#NUM!	#NUM!	#NUM!	#NUM!	#NUM!	#NUM!	#NUM!	#NUM!	26.0245	#NUM!	25.6293
#NUM!	#NUM!	#NUM!	#NUM!	#NUM!	#NUM!	#NUM!	#NUM!	#NUM!	#NUM!	#NUM!	26.3822
#NUM!	#NUM!	#NUM!	#NUM!	#NUM!	#NUM!	#NUM!	#NUM!	#NUM!	25.17	25.497	25.5642
#NUM!	#NUM!	#NUM!	#NUM!	#NUM!	#NUM!	#NUM!	#NUM!	26.4451	26.3243	26.235	26.2509
28.0051	28.0532	28.5601	29.0683	#NUM!	#NUM!	#NUM!	#NUM!	28.7248	28.7086	28.6674	28.7895
#NUM!	#NUM!	#NUM!	#NUM!	#NUM!	#NUM!	#NUM!	#NUM!	27.976	28.2224	28.255	28.531
#NUM!	#NUM!	#NUM!	#NUM!	#NUM!	#NUM!	#NUM!	#NUM!	#NUM!	#NUM!	26.3015	26.4729
#NUM!	#NUM!	#NUM!	#NUM!	#NUM!	#NUM!	#NUM!	#NUM!	27.0586	27.0054	27.5961	27.2715
#NUM!	#NUM!	#NUM!	#NUM!	#NUM!	#NUM!	#NUM!	#NUM!	25.9641	26.5737	26.6453	26.6198
#NUM!	#NUM!	#NUM!	27.9937	#NUM!	#NUM!	#NUM!	#NUM!	26.5341	27.7189	27.731	27.5561
#NUM!	#NUM!	#NUM!	#NUM!	#NUM!	#NUM!	#NUM!	#NUM!	27.8966	27.9066	27.857	28.0793
#NUM!	#NUM!	#NUM!	#NUM!	#NUM!	#NUM!	#NUM!	#NUM!	26.5624	27.0765	27.1242	27.3935
27.9526	27.9235	27.5885	27.7361	27.2403	27.1644	#NUM!	27.1337	28.4586	28.4117	28.7686	28.7228
#NUM!	#NUM!	#NUM!	#NUM!	#NUM!	#NUM!	#NUM!	#NUM!	#NUM!	26.2591	26.1793	26.0547
#NUM!	#NUM!	#NUM!	#NUM!	#NUM!	#NUM!	#NUM!	#NUM!	26.4476	26.3629	26.625	26.4573
25.3162	#NUM!	#NUM!	#NUM!	#NUM!	#NUM!	#NUM!	#NUM!	26.4062	26.615	26.6359	26.9473
#NUM!	27.9319	#NUM!	28.127	#NUM!	28.0826	#NUM!	#NUM!	28.4689	28.4198	28.5251	28.3207
#NUM!	#NUM!	#NUM!	#NUM!	#NUM!	#NUM!	#NUM!	#NUM!	26.3387	26.6779	26.4036	26.4209
#NUM!	#NUM!	#NUM!	#NUM!	#NUM!	#NUM!	#NUM!	#NUM!	#NUM!	26.6969	26.7393	26.8737
#NUM!	27.6655	27.0886	#NUM!	#NUM!	#NUM!	#NUM!	#NUM!	26.7195	26.6555	26.611	26.7109
27.8352	#NUM!	28.1642	27.7482	#NUM!	28.3237	#NUM!	#NUM!	27.9219	27.7506	27.3308	27.5823
#NUM!	#NUM!	#NUM!	#NUM!	#NUM!	#NUM!	#NUM!	#NUM!	26.0781	26.1716	25.5575	25.6318
27.9375	27.3944	#NUM!	27.661	#NUM!	28.9124	#NUM!	#NUM!	26.8658	26.7061	26.9697	26.7846
#NUM!	#NUM!	#NUM!	#NUM!	#NUM!	#NUM!	#NUM!	#NUM!	27.0043	#NUM!	27.5623	27.4495
#NUM!	28.1922	#NUM!	#NUM!	#NUM!	28.7227	#NUM!	#NUM!	29.3619	29.321	29.2211	29.1084
28.1358	28.0776	#NUM!	28.0228	27.6098	27.8568	#NUM!	#NUM!	28.414	28.4154	28.6898	28.6047
27.7435	27.0055	#NUM!	27.4199	#NUM!	27.1557	#NUM!	#NUM!	28.1467	28.0321	28.3142	28.2765
#NUM!	#NUM!	#NUM!	#NUM!	#NUM!	#NUM!	#NUM!	#NUM!	27.3094	27.0479	26.8293	27.0165
#NUM!	#NUM!	#NUM!	#NUM!	#NUM!	#NUM!	#NUM!	#NUM!	#NUM!	#NUM!	#NUM!	#NUM!
#NUM!	#NUM!	#NUM!	#NUM!	#NUM!	#NUM!	#NUM!	#NUM!	26.4539	26.636	26.6071	26.6109
28.2269	28.3056	27.9388	27.6313	31.4758	30.8902	31.3488	31.4609	27.5221	27.2121	26.7525	26.6907
#NUM!	#NUM!	#NUM!	#NUM!	#NUM!	#NUM!	#NUM!	#NUM!	28.7682	28.7237	28.9567	28.814
29.8997	30.0575	#NUM!	24.748	#NUM!	29.4435	#NUM!	28.3735	#NUM!	#NUM!	#NUM!	#NUM!
29.397	29.643	28.9212	29.038	29.2622	29.955	29.5276	29.1783	26.6815	#NUM!	#NUM!	25.2352
29.757	29.8372	29.7265	29.8464	28.6992	28.579	28.7154	27.9108	29.6472	29.6852	30.0387	30.088
30.0726	30.255	27.955	28.0752	29.2936	29.0603	28.9767	28.6527	#NUM!	#NUM!	#NUM!	#NUM!
#NUM!	#NUM!	#NUM!	27.8781	#NUM!	#NUM!	#NUM!	#NUM!	27.8059	28.304	#NUM!	27.4163
33.2458	33.4571	33.0575	33.2402	34.0055	33.4191	33.9586	33.2017	31.4659	31.4032	31.3692	31.4177
32.7389	32.7711	32.2899	32.2292	31.959	31.778	31.3961	31.45	33.0627	33.0545	33.0346	33.0811
#NUM!	#NUM!	#NUM!	#NUM!	#NUM!	#NUM!	#NUM!	#NUM!	25.6866	#NUM!	26.1855	26.2012
#NUM!	25.9379	27.139	27.0012	#NUM!	#NUM!	#NUM!	#NUM!	26.8438	26.8385	26.9116	26.7658
31.9689	32.2549	32.4343	32.0219	32.5295	31.7241	32.265	31.5616	27.7647	27.5794	27.772	27.8069
#NUM!	26.7607	#NUM!	#NUM!	#NUM!	#NUM!	#NUM!	#NUM!	27.1509	27.0208	#NUM!	#NUM!
#NUM!	#NUM!	#NUM!	#NUM!	#NUM!	#NUM!	#NUM!	#NUM!	28.8064	28.4838	29.4786	29.0975
29.4347	29.4399	28.1467	28.8569	29.2447	29.3408	29.0521	29.3595	30.1114	30.088	30.0726	29.9993
#NUM!	26.3022	#NUM!	#NUM!	#NUM!	#NUM!	#NUM!	#NUM!	27.4337	27.2473	26.9931	26.8903
#NUM!	#NUM!	#NUM!	#NUM!	#NUM!	#NUM!	#NUM!	#NUM!	26.8211	26.5084	26.9049	26.5628
#NUM!	#NUM!	#NUM!	#NUM!	#NUM!	#NUM!	#NUM!	#NUM!	29.406	29.0434	29.6816	29.8572
27.8203	28.4466	28.3315	#NUM!	#NUM!	29.2711	#NUM!	27.635	29.1684	29.2225	28.5288	28.3363
31.1355	31.0895	30.4507	30.4511	30.07	30.1764	29.7993	30.204	29.3697	29.5181	29.534	29.6008
#NUM!	#NUM!	#NUM!	#NUM!	#NUM!	#NUM!	#NUM!	#NUM!	#NUM!	#NUM!	#NUM!	#NUM!

#NUM!	#NUM!	#NUM!	#NUM!	#NUM!	#NUM!	#NUM!	#NUM!	#NUM!	27.0644	26.6574	26.678
#NUM!	26.2048	#NUM!	#NUM!	#NUM!	#NUM!	#NUM!	#NUM!	27.2306	27.5262	27.2432	27.2712
#NUM!	#NUM!	#NUM!	#NUM!	#NUM!	27.5457	#NUM!	#NUM!	28.135	28.1042	28.2413	28.1878
#NUM!	#NUM!	#NUM!	#NUM!	#NUM!	#NUM!	#NUM!	#NUM!	27.2394	27.4547	27.2703	27.2775
29.9778	29.9198	29.0442	29.7685	28.8866	29.8826	28.868	29.627	30.15	30.2488	30.7678	30.5348
31.2801	31.0481	31.5053	31.6887	30.7131	31.3233	30.6032	30.9404	32.1555	31.8727	32.2352	32.2737
#NUM!	#NUM!	27.4644	#NUM!	#NUM!	#NUM!	#NUM!	#NUM!	28.2883	28.3386	27.8912	27.6286
#NUM!	#NUM!	#NUM!	#NUM!	#NUM!	26.8313	#NUM!	#NUM!	27.425	27.1834	27.5622	27.5536
#NUM!	27.2261	#NUM!	#NUM!	#NUM!	28.5255	#NUM!	#NUM!	27.0642	27.2872	#NUM!	27.7015
#NUM!	#NUM!	#NUM!	#NUM!	#NUM!	#NUM!	#NUM!	#NUM!	#NUM!	#NUM!	#NUM!	#NUM!
#NUM!	#NUM!	#NUM!	#NUM!	#NUM!	#NUM!	#NUM!	#NUM!	#NUM!	25.3114	25.116	25.744
28.1892	28.1056	28.424	28.2035	#NUM!	28.3395	#NUM!	28.0139	28.4446	28.2102	28.2529	28.3419
27.5308	27.5755	28.1795	27.9912	#NUM!	26.9459	#NUM!	27.3012	28.9554	28.8502	28.9569	28.801
#NUM!	#NUM!	#NUM!	#NUM!	#NUM!	#NUM!	#NUM!	#NUM!	28.0311	27.9273	28.0045	28.0837
#NUM!	#NUM!	#NUM!	#NUM!	#NUM!	27.0056	#NUM!	#NUM!	27.6479	27.2888	28.2632	28.2011
#NUM!	#NUM!	#NUM!	26.7258	#NUM!	#NUM!	#NUM!	#NUM!	28.2963	28.2734	28.3394	28.1448
#NUM!	27.5409	27.4205	26.9735	#NUM!	27.7106	#NUM!	#NUM!	28.7326	28.9716	28.6961	28.7751
31.0744	30.9522	30.5776	30.4562	30.4695	30.579	30.3452	31.1042	31.2863	31.3581	31.1535	31.2202
#NUM!	#NUM!	#NUM!	#NUM!	#NUM!	#NUM!	#NUM!	#NUM!	27.6253	27.6752	27.9267	27.758
#NUM!	#NUM!	#NUM!	#NUM!	#NUM!	#NUM!	#NUM!	#NUM!	26.6938	26.8667	26.5526	26.4907
#NUM!	#NUM!	#NUM!	27.2896	#NUM!	#NUM!	#NUM!	#NUM!	25.943	26.8828	26.5685	26.4513
#NUM!	#NUM!	#NUM!	#NUM!	#NUM!	#NUM!	#NUM!	#NUM!	24.8465	25.161	25.3877	25.3684
28.291	28.4333	28.5461	27.7704	28.4081	28.4015	28.4858	28.2671	#NUM!	#NUM!	#NUM!	#NUM!
#NUM!	#NUM!	#NUM!	#NUM!	#NUM!	#NUM!	#NUM!	#NUM!	#NUM!	25.3766	25.8491	25.9801
#NUM!	#NUM!	#NUM!	#NUM!	#NUM!	#NUM!	#NUM!	#NUM!	25.4025	25.9565	25.0517	25.3172
#NUM!	#NUM!	#NUM!	#NUM!	#NUM!	#NUM!	#NUM!	#NUM!	27.1103	26.9104	26.5028	26.9725
#NUM!	27.1592	#NUM!	28.113	#NUM!	#NUM!	#NUM!	#NUM!	27.492	27.4915	26.9859	26.9841
#NUM!	#NUM!	#NUM!	#NUM!	#NUM!	#NUM!	#NUM!	#NUM!	24.5361	24.3905	24.581	#NUM!
#NUM!	#NUM!	#NUM!	#NUM!	#NUM!	#NUM!	#NUM!	#NUM!	27.544	27.7478	27.6743	27.5454
27.6695	27.7084	26.8587	27.0832	#NUM!	#NUM!	#NUM!	#NUM!	28.9082	28.8243	29.262	29.2601
#NUM!	#NUM!	27.3646	#NUM!	#NUM!	#NUM!	#NUM!	#NUM!	29.7557	29.56	29.7588	29.7558
27.9728	27.8089	#NUM!	27.8331	#NUM!	28.0021	#NUM!	#NUM!	27.2103	27.2991	27.1092	27.4313
#NUM!	#NUM!	#NUM!	#NUM!	#NUM!	#NUM!	#NUM!	#NUM!	#NUM!	25.6803	26.0518	26.2032
#NUM!	#NUM!	#NUM!	#NUM!	#NUM!	#NUM!	#NUM!	#NUM!	#NUM!	#NUM!	#NUM!	27.3643
28.5699	28.4744	27.0441	27.4836	#NUM!	27.6278	#NUM!	#NUM!	28.0554	27.9053	28.256	28.4903
#NUM!	28.0194	28.6896	28.5315	28.7334	28.667	28.6945	#NUM!	28.589	28.6062	28.0889	28.4661
#NUM!	#NUM!	#NUM!	#NUM!	#NUM!	#NUM!	#NUM!	#NUM!	#NUM!	26.5513	#NUM!	26.9094
27.7344	27.7533	#NUM!	27.1828	#NUM!	27.4244	#NUM!	#NUM!	27.3695	27.3987	27.5985	28.1218
#NUM!	#NUM!	#NUM!	#NUM!	#NUM!	#NUM!	#NUM!	#NUM!	#NUM!	#NUM!	24.8821	25.0396
28.9593	28.4889	28.5932	28.4289	#NUM!	29.5506	#NUM!	29.6952	26.9848	27.3281	27.46	27.8091
#NUM!	#NUM!	#NUM!	#NUM!	#NUM!	#NUM!	#NUM!	#NUM!	#NUM!	25.9857	26.0184	26.0085
#NUM!	#NUM!	#NUM!	#NUM!	#NUM!	#NUM!	#NUM!	#NUM!	27.0499	27.1534	27.321	27.2544
#NUM!	#NUM!	#NUM!	#NUM!	#NUM!	#NUM!	#NUM!	#NUM!	26.8197	26.8965	26.5526	26.8565
28.5992	28.4517	28.9433	#NUM!	#NUM!	28.5063	#NUM!	28.1219	28.1099	27.7833	27.854	27.8258
#NUM!	#NUM!	#NUM!	#NUM!	#NUM!	#NUM!	#NUM!	#NUM!	28.2834	27.8382	28.0159	27.679
#NUM!	#NUM!	#NUM!	#NUM!	#NUM!	#NUM!	#NUM!	#NUM!	25.9787	26.0589	26.9006	26.7458
#NUM!	#NUM!	#NUM!	#NUM!	#NUM!	#NUM!	#NUM!	#NUM!	25.6983	26.0292	26.4029	26.2182
#NUM!	#NUM!	#NUM!	#NUM!	#NUM!	#NUM!	#NUM!	#NUM!	#NUM!	#NUM!	25.6216	25.4163
#NUM!	#NUM!	#NUM!	#NUM!	#NUM!	#NUM!	#NUM!	#NUM!	26.1855	26.7844	#NUM!	#NUM!
#NUM!	#NUM!	#NUM!	#NUM!	#NUM!	#NUM!	#NUM!	#NUM!	28.7046	28.4921	28.1321	28.2285
27.8317	27.9329	27.9826	28.0744	#NUM!	#NUM!	27.8748	#NUM!	27.5683	27.1212	27.0322	26.7652
#NUM!	#NUM!	#NUM!	#NUM!	#NUM!	#NUM!	#NUM!	#NUM!	27.6578	27.7054	28.2415	28.1049
#NUM!	#NUM!	#NUM!	#NUM!	#NUM!	#NUM!	#NUM!	#NUM!	29.9525	29.7946	29.7737	29.5762
29.5342	29.6582	28.5202	28.8377	28.2765	28.9949	28.4906	28.3987	30.0363	29.9927	29.7382	30.0142
27.1465	27.146	#NUM!	27.7024	#NUM!	#NUM!	#NUM!	#NUM!	26.3053	26.6218	27.1296	26.7707
28.3694	28.2428	#NUM!	#NUM!	#NUM!	28.9817	#NUM!	28.5358	27.371	27.3663	27.8166	27.7777
#NUM!	#NUM!	#NUM!	27.4515	#NUM!	#NUM!	#NUM!	#NUM!	26.5846	26.6334	26.4467	26.2562
#NUM!	#NUM!	#NUM!	#NUM!	#NUM!	#NUM!	#NUM!	#NUM!	26.7007	27.1082	26.7128	26.6872
#NUM!	#NUM!	#NUM!	#NUM!	#NUM!	#NUM!	#NUM!	#NUM!	26.1132	26.5099	26.3734	26.1813
#NUM!	27.6924	#NUM!	#NUM!	#NUM!	#NUM!	#NUM!	#NUM!	26.7281	26.5241	27.1844	27.4993
#NUM!	#NUM!	#NUM!	#NUM!	#NUM!	#NUM!	#NUM!	#NUM!	27.1565	27.295	27.0918	27.1262
#NUM!	#NUM!	#NUM!	#NUM!	#NUM!	#NUM!	#NUM!	#NUM!	#NUM!	#NUM!	25.5401	25.815
#NUM!	27.7527	#NUM!	27.8337	#NUM!	#NUM!	#NUM!	#NUM!	28.1816	27.8322	#NUM!	27.7065
#NUM!	#NUM!	#NUM!	#NUM!	#NUM!	#NUM!	#NUM!	#NUM!	27.0666	26.3175	26.8411	27.4885
#NUM!	26.1582	#NUM!	#NUM!	#NUM!	#NUM!	#NUM!	26.7604	28.7008	28.6296	28.9717	28.944
28.0364	28.2958	#NUM!	27.159	#NUM!	28.4249	#NUM!	#NUM!	#NUM!	#NUM!	26.3915	26.5029
27.7379	27.7552	27.3794	26.7042	#NUM!	27.5077	#NUM!	#NUM!	27.4772	27.7245	27.3602	27.4064
33.6821	33.5471	33.6324	34.0182	33.2586	32.5261	33.1263	32.2734	34.1328	34.0892	34.4266	34.255
29.9978	29.9094	30.162	30.3104	30.4149	29.8564	30.3781	29.4177	26.9776	26.7924	27.3653	26.9751
#NUM!	#NUM!	#NUM!	#NUM!	#NUM!	#NUM!	#NUM!	#NUM!	25.8363	26.5665	26.6696	26.7761
30.5436	30.5892	30.1521	30.4932	30.3195	30.0647	29.9003	29.3434	31.5303	31.4164	31.5409	31.5063
#NUM!	#NUM!	#NUM!	#NUM!	#NUM!	#NUM!	#NUM!	#NUM!	27.5006	27.557	27.4525	28.1085
#NUM!	#NUM!	#NUM!	#NUM!	#NUM!	#NUM!	#NUM!	#NUM!	#NUM!	#NUM!	24.6919	24.8163
28.9313	29.2302	28.7413	28.9663	28.1921	28.4177	27.5997	28.1671	30.0594	29.8509	30.4634	30.6213
28.6148	28.1746	28.3696	28.0654	27.6765	28.0014	#NUM!	#NUM!	28.2501	28.2863	28.6942	28.6555
#NUM!	#NUM!	#NUM!	#NUM!	#NUM!	#NUM!	#NUM!	#NUM!	28.4288	28.3071	28.3271	28.3174
27.2498	27.3249	#NUM!	27.6475	#NUM!	27.5129	#NUM!	27.7273	28.6293	28.4298	28.2742	28.4866
27.0592	26.5738	27.6597	27.6183	#NUM!	27.9575	27.9884	27.6033	30.1098	30.1597	30.2261	30.2251
#NUM!	#NUM!	#NUM!	#NUM!	#NUM!	27.9098	#NUM!	27.6484	28.3474	28.4782	28.7272	28.6199
#NUM!	#NUM!	#NUM!	#NUM!	#NUM!	#NUM!	#NUM!	#NUM!	25.9813	#NUM!	27.5652	27.131
28.8064	28.8829	28.9472	29.026	29.8403	29.3932	29.7182	28.7699	27.5383	27.4878	27.4756	27.4892
29.8884	29.6619	29.4248	29.8115	29.1999	29.6625	29.1964	29.0978	30.0678	29.8609	30.2088	30.0404
28.7258	28.76	28.3835	28.5138	27.7415	28.4679	27.5365	28.1782	27.8334	27.973	28.4036	27.8652
30.252	30.3305	29.9683	29.7553	29.8185	30.3047	29.9285	30.1813	27.7194	28.0847	28.2767	28.2381
25.4699	26.7799	#NUM!	#NUM!	#NUM!	27.2992	#NUM!	#NUM!	27.435	27.7109	27.7164	27.5275
29.9535	30.127	29.8032	29.8676	30.0479	30.1293	30.0154	30.0709	28.8115	28.9052	29.1872	29.4203
30.1209	29.9901	29.2135	29.2281	29.5865	30.1135	29.7115	29.7447	29.2317	28.7857	29.1819	29.2746
28.1316	28.016	#NUM!	#NUM!	#NUM!	28.3741	#NUM!	#NUM!	26.9512	27.3449	26.9605	27.5184
28.6225	28.9195	29.5011	28.9382	29.1891	28.7134	29.6275	27.6496	29.8503	29.7221	29.6829	29.8038
28.1914	28.2613	27.7096	27.998	#NUM!	28.6386	#NUM!	#NUM!	29.5666	29.3238	29.4385	29.4226
28.4772	28.4648	27.9254	27.7864	#NUM!	28.4935	#NUM!	27.9263	28.8002	28.716	29.03	28.6787
30.1426	30.1652	29.1177	28.8675	29.1373	30.2191	28.8893	29.8564	30.7901	30.6454	30.6507	30.3348
#NUM!	#NUM!	#NUM!	#NUM!	#NUM!	#NUM!	#NUM!	#NUM!	27.2403	27.4484	27.7521	27.2426
28.2935	28.7411	#NUM!	28.6833	#NUM!	28.2801	#NUM!	#NUM!	29.1869	29.0148	29.2514	28.9809
#NUM!	#NUM!	#NUM!	#NUM!	#NUM!	#NUM!	#NUM!	#NUM!	28.0903	27.6741	28.1734	28.1654
#NUM!	#NUM!	#NUM!	#NUM!	#NUM!	#NUM!	#NUM!	#NUM!	26.819	26.7606	26.6088	26.649
#NUM!	#NUM!	#NUM!	#NUM!	#NUM!	#NUM!	#NUM!	#NUM!	#NUM!	#NUM!	26.0488	26.1554
27.2903	27.426	#NUM!	#NUM!	#NUM!	#NUM!	#NUM!	#NUM!	26.6409	26.9063	26.9112	27.1117
#NUM!	#NUM!	#NUM!	#NUM!	#NUM!	27.1318	#NUM!	#NUM!	26.9091	26.9468	27.158	26.6361
#NUM!	#NUM!	#NUM!	#NUM!	#NUM!	#NUM!	#NUM!	#NUM!	#NUM!	26.3462	26.2233	25.8897
#NUM!	#NUM!	#NUM!	#NUM!	#NUM!	#NUM!	#NUM!	#NUM!	27.4944	27.6204	27.7039	27.4971
#NUM!	#NUM!	#NUM!	#NUM!	#NUM!	#NUM!	#NUM!	#NUM!	#NUM!	27.2948	27.5681	27.5752
28.7872	28.8323	28.93	29.1549	28.8952	28.6487	28.8794	28.2528	29.1638	29.0003	29.1238	28.9207
#NUM!	#NUM!	#NUM!	#NUM!	#NUM!	#NUM!	#NUM!	#NUM!	#NUM!	#NUM!	#NUM!	27.4211
#NUM!	#NUM!	#NUM!	#NUM!	#NUM!	#NUM!	#NUM!	#NUM!	26.2276	#NUM!	25.9867	26.2646
27.7192	27.5959	28.6746	28.5929	#NUM!	28.8667	27.9639	#NUM!	30.2973	30.3739	29.9279	29.8923
#NUM!	#NUM!	#NUM!	#NUM!	#NUM!	#NUM!	#NUM!	#NUM!	27.1933	27.3475	27.603	27.8795
#NUM!	28.2414	28.7068	28.9224	#NUM!	#NUM!	#NUM!	#NUM!	27.8875	27.8563	27.7178	27.7146
#NUM!	#NUM!	#NUM!	#NUM!	#NUM!	#NUM!	#NUM!	#NUM!	27.0727	27.1201	27.0929	27.217
#NUM!	#NUM!	#NUM!	#NUM!	#NUM!	#NUM!	#NUM!	#NUM!	#NUM!	26.2668	26.4323	#NUM!
#NUM!	#NUM!	#NUM!	#NUM!	#NUM!	#NUM!	#NUM!	#NUM!	25.6747	25.592	25.5061	25.1971
#NUM!	#NUM!	#NUM!	#NUM!	#NUM!	#NUM!	#NUM!	#NUM!	26.2271	26.1178	26.3404	26.4412
27.5772	27.6462	27.6557	27.6031	27.985	27.7105	27.9097	28.2424	26.3232	26.2386	#NUM!	26.6384
#NUM!	#NUM!	#NUM!	#NUM!	#NUM!	#NUM!	#NUM!	#NUM!	26.8122	#NUM!	26.8655	26.4515
#NUM!	#NUM!	#NUM!	#NUM!	#NUM!	#NUM!	#NUM!	#NUM!	#NUM!	28.4395	28.0724	28.5108
#NUM!	#NUM!	#NUM!	#NUM!	#NUM!	30.1558	#NUM!	#NUM!	28.2066	27.9048	28.4584	28.1267
#NUM!	#NUM!	#NUM!	#NUM!	#NUM!	#NUM!	#NUM!	#NUM!	27.3958	27.2598	26.931	26.8265
#NUM!	#NUM!	#NUM!	30.5368	#NUM!	#NUM!	#NUM!	#NUM!	27.9242	27.4012	27.2153	27.61
#NUM!	#NUM!	#NUM!	#NUM!	#NUM!	#NUM!	#NUM!	#NUM!	#NUM!	#NUM!	25.974	26.637
#NUM!	#NUM!	#NUM!	#NUM!	#NUM!	#NUM!	#NUM!	#NUM!	#NUM!	#NUM!	25.7644	25.8558
#NUM!	#NUM!	#NUM!	#NUM!	#NUM!	#NUM!	#NUM!	#NUM!	28.0747	28.0233	28.1019	28.1749
#NUM!	#NUM!	#NUM!	#NUM!	#NUM!	#NUM!	#NUM!	#NUM!	26.5691	26.1906	#NUM!	#NUM!
#NUM!	#NUM!	#NUM!	#NUM!	#NUM!	#NUM!	#NUM!	#NUM!	25.4779	25.9961	#NUM!	#NUM!
28.5683	28.6198	27.8258	27.7846	27.3469	27.2192	27.5132	27.2724	#NUM!	#NUM!	#NUM!	#NUM!
#NUM!	#NUM!	27.2578	27.5789	#NUM!	#NUM!	#NUM!	#NUM!	29.0203	28.6112	28.2879	28.3966
#NUM!	#NUM!	#NUM!	#NUM!	#NUM!	#NUM!	#NUM!	#NUM!	#NUM!	#NUM!	26.3523	26.7014
27.6032	29.1528	#NUM!	28.0638	#NUM!	29.1431	#NUM!	#NUM!	28.6897	28.7108	28.9702	29.0482
#NUM!	#NUM!	#NUM!	#NUM!	#NUM!	27.2212	#NUM!	#NUM!	27.9787	27.9899	27.9492	27.8175
#NUM!	#NUM!	#NUM!	#NUM!	#NUM!	#NUM!	#NUM!	#NUM!	26.9419	26.8219	27.1673	26.7721

30.6359	30.7148	30.6961	30.9297	29.8529	29.6096	29.5728	29.7122	31.0527	30.82	31.0786	31.2079
#NUM!	#NUM!	27.9289	27.4098	#NUM!	27.9301	#NUM!	#NUM!	27.9063	27.8326	28.0974	28.0125
#NUM!	27.3899	27.248	27.5797	#NUM!	#NUM!	#NUM!	#NUM!	28.3929	28.0213	27.7111	27.8639
#NUM!	#NUM!	#NUM!	#NUM!	#NUM!	#NUM!	#NUM!	#NUM!	26.4823	26.6892	27.402	26.9999
#NUM!	#NUM!	#NUM!	#NUM!	#NUM!	#NUM!	#NUM!	#NUM!	27.0559	27.3885	27.4425	27.4117
28.411	28.0202	#NUM!	28.0719	#NUM!	#NUM!	#NUM!	#NUM!	27.7774	27.8106	27.7002	27.6327
#NUM!	#NUM!	#NUM!	#NUM!	#NUM!	#NUM!	#NUM!	#NUM!	25.9673	24.8965	25.629	25.5661
#NUM!	#NUM!	#NUM!	#NUM!	#NUM!	#NUM!	#NUM!	#NUM!	25.6666	25.6295	25.8104	25.5055
29.2319	29.1554	29.0317	29.1091	28.2884	28.5434	28.5228	28.2479	29.8164	29.6799	30.0278	29.9893
#NUM!	#NUM!	#NUM!	#NUM!	#NUM!	#NUM!	#NUM!	#NUM!	#NUM!	#NUM!	26.066	25.6472
27.2472	27.4165	#NUM!	#NUM!	#NUM!	27.1526	#NUM!	#NUM!	29.1621	29.2543	29.3336	29.6049
#NUM!	#NUM!	#NUM!	#NUM!	#NUM!	#NUM!	#NUM!	#NUM!	27.1148	27.515	27.3723	26.7436
#NUM!	#NUM!	#NUM!	#NUM!	#NUM!	#NUM!	#NUM!	#NUM!	#NUM!	#NUM!	26.0833	26.828
30.3084	30.4529	29.5785	29.6382	31.2746	26.239	31.4635	28.8568	#NUM!	#NUM!	26.3538	#NUM!
28.3532	28.4237	28.1047	28.5786	#NUM!	28.7436	#NUM!	28.8609	28.4538	28.2882	28.4299	28.4305
31.7935	32.1936	32.7602	32.6614	32.2722	32.6688	32.0973	31.3231	33.2123	33.023	33.1487	33.1869
27.415	27.2071	27.3125	27.0812	#NUM!	#NUM!	#NUM!	#NUM!	29.0841	29.0227	28.9641	28.7934
28.5408	27.8506	27.8724	28.1254	#NUM!	27.6467	#NUM!	#NUM!	27.6856	27.6566	28.2211	27.9567
29.9669	30.161	29.7171	29.6891	29.5031	29.8815	29.5108	29.7829	31.2913	31.3033	31.642	31.4544
27.351	27.4456	27.5561	27.7701	29.7309	27.9553	29.1338	27.7988	26.8169	26.8087	26.7051	26.8782
#NUM!	#NUM!	#NUM!	#NUM!	#NUM!	#NUM!	#NUM!	#NUM!	27.5703	27.759	27.4454	27.5459
#NUM!	#NUM!	#NUM!	#NUM!	#NUM!	#NUM!	#NUM!	#NUM!	27.4398	27.5567	27.1634	27.2481
28.4452	28.0112	#NUM!	27.0758	28.7178	28.5009	#NUM!	29.1692	27.7328	27.4376	27.4352	27.4193
#NUM!	#NUM!	#NUM!	#NUM!	#NUM!	#NUM!	#NUM!	#NUM!	28.3521	28.3805	28.5742	28.5235
29.8337	29.7197	28.6931	28.8382	28.873	29.3641	29.0331	29.6853	29.8733	29.6995	29.9894	29.838
28.3906	28.2314	28.0358	28.058	#NUM!	#NUM!	#NUM!	27.2125	28.9439	29.0568	29.3933	29.3255
#NUM!	#NUM!	#NUM!	#NUM!	#NUM!	#NUM!	#NUM!	#NUM!	#NUM!	25.1799	#NUM!	26.5839
27.8896	28.1002	28.4927	28.3502	#NUM!	#NUM!	#NUM!	#NUM!	#NUM!	26.7427	26.8933	26.3901
#NUM!	#NUM!	#NUM!	#NUM!	#NUM!	#NUM!	#NUM!	#NUM!	26.9866	27.0519	27.3558	27.4237
#NUM!	27.6042	#NUM!	#NUM!	#NUM!	#NUM!	#NUM!	#NUM!	27.3901	27.134	27.2634	27.365
27.6614	27.9096	28.0001	27.814	#NUM!	#NUM!	#NUM!	#NUM!	28.5514	27.9668	27.8754	28.0985
#NUM!	#NUM!	#NUM!	#NUM!	#NUM!	#NUM!	#NUM!	#NUM!	27.5307	27.6914	27.4645	27.4996
#NUM!	#NUM!	#NUM!	#NUM!	#NUM!	#NUM!	#NUM!	#NUM!	#NUM!	25.1465	26.0121	26.0864
#NUM!	#NUM!	#NUM!	#NUM!	#NUM!	#NUM!	#NUM!	#NUM!	27.9113	27.8603	27.6234	28.0234
28.1723	28.1309	27.6684	27.2161	#NUM!	28.939	27.9269	29.1586	26.9085	26.9276	26.4955	26.5977
#NUM!	#NUM!	#NUM!	#NUM!	#NUM!	#NUM!	#NUM!	#NUM!	26.7143	27.3992	27.2763	27.2817
31.3128	31.4714	30.8004	30.9761	30.4662	30.6663	30.5736	29.9832	32.1733	32.0867	32.3355	32.4281
30.6965	30.6332	29.6263	30.0032	29.2352	31.1582	29.3899	30.749	30.7357	30.4198	30.5507	30.6233
#NUM!	#NUM!	#NUM!	#NUM!	#NUM!	#NUM!	#NUM!	#NUM!	28.3817	28.5602	28.5283	28.3772
#NUM!	#NUM!	#NUM!	#NUM!	#NUM!	#NUM!	#NUM!	#NUM!	27.5068	27.592	27.9157	27.8861
31.527	31.788	30.8504	31.076	30.9386	30.6341	30.9508	30.4057	#NUM!	#NUM!	25.9097	25.6542
#NUM!	#NUM!	#NUM!	#NUM!	#NUM!	#NUM!	#NUM!	#NUM!	27.0244	27.2328	27.1895	27.1771
#NUM!	#NUM!	#NUM!	#NUM!	#NUM!	#NUM!	#NUM!	#NUM!	#NUM!	#NUM!	#NUM!	#NUM!
#NUM!	28.9573	#NUM!	#NUM!	#NUM!	#NUM!	#NUM!	#NUM!	29.6688	29.6852	30.0884	29.9656
28.4502	28.4409	#NUM!	#NUM!	#NUM!	29.6002	#NUM!	28.7372	29.9131	30.0139	29.6473	29.6516
#NUM!	#NUM!	#NUM!	#NUM!	#NUM!	#NUM!	#NUM!	#NUM!	25.3481	25.513	26.1701	26.2424
#NUM!	#NUM!	#NUM!	#NUM!	#NUM!	#NUM!	#NUM!	#NUM!	27.8623	27.5498	27.1794	27.4648
#NUM!	#NUM!	#NUM!	#NUM!	#NUM!	#NUM!	#NUM!	#NUM!	27.0492	27.3727	27.714	27.8663
#NUM!	#NUM!	#NUM!	#NUM!	#NUM!	#NUM!	#NUM!	#NUM!	26.0572	25.3576	24.9482	25.1157
28.6207	28.5656	28.5656	28.561	27.9077	28.5214	#NUM!	29.1157	29.2773	29.4241	29.7921	29.6429
#NUM!	#NUM!	#NUM!	#NUM!	#NUM!	#NUM!	#NUM!	#NUM!	26.5563	26.2599	26.2583	26.3749
#NUM!	27.7411	#NUM!	#NUM!	#NUM!	#NUM!	#NUM!	#NUM!	27.033	27.3761	27.9462	28.1303
#NUM!	#NUM!	#NUM!	#NUM!	#NUM!	#NUM!	#NUM!	#NUM!	27.9726	27.7762	27.9871	27.943
29.5604	29.7127	28.6092	29.0898	31.4264	31.525	31.1776	31.2444	29.4173	29.1159	29.4465	29.6269
#NUM!	#NUM!	#NUM!	#NUM!	#NUM!	#NUM!	#NUM!	#NUM!	26.0038	26.1407	25.6593	25.6485
#NUM!	#NUM!	#NUM!	27.8887	#NUM!	#NUM!	#NUM!	#NUM!	29.7602	29.4942	29.4585	29.3008
26.8162	27.1684	27.3237	27.3556	#NUM!	#NUM!	#NUM!	#NUM!	27.9965	27.9792	28.4309	28.1569
#NUM!	#NUM!	#NUM!	#NUM!	#NUM!	#NUM!	#NUM!	#NUM!	#NUM!	#NUM!	25.3849	25.2949
#NUM!	#NUM!	#NUM!	#NUM!	#NUM!	#NUM!	#NUM!	#NUM!	27.153	27.4787	27.2792	26.988
#NUM!	#NUM!	#NUM!	#NUM!	#NUM!	#NUM!	#NUM!	#NUM!	#NUM!	#NUM!	28.0639	#NUM!
#NUM!	#NUM!	26.6035	26.4255	#NUM!	#NUM!	#NUM!	#NUM!	28.3741	27.9558	27.812	27.6329
#NUM!	#NUM!	#NUM!	#NUM!	#NUM!	#NUM!	#NUM!	#NUM!	27.446	26.9491	26.8367	27.1154
27.8744	28.0344	27.7855	27.6227	#NUM!	27.6548	#NUM!	#NUM!	28.277	28.348	28.5649	28.5213
#NUM!	27.0701	#NUM!	#NUM!	#NUM!	#NUM!	#NUM!	#NUM!	28.0606	27.9735	28.2445	28.2279
28.9551	29.2186	29.5097	29.5177	28.2015	28.6286	28.2974	28.2262	#NUM!	#NUM!	#NUM!	26.2114
27.1401	27.3194	26.6842	27.6085	#NUM!	#NUM!	#NUM!	#NUM!	#NUM!	#NUM!	#NUM!	#NUM!
#NUM!	#NUM!	#NUM!	#NUM!	#NUM!	#NUM!	#NUM!	#NUM!	26.3084	26.2177	26.9294	26.9315
27.4548	27.3779	27.3689	26.9051	#NUM!	27.2513	#NUM!	26.5769	26.3763	26.6554	26.5365	26.5272
#NUM!	#NUM!	#NUM!	#NUM!	#NUM!	#NUM!	#NUM!	#NUM!	26.6402	26.6662	26.629	26.7275
#NUM!	#NUM!	#NUM!	#NUM!	#NUM!	#NUM!	#NUM!	#NUM!	#NUM!	25.2279	#NUM!	#NUM!
#NUM!	#NUM!	#NUM!	#NUM!	#NUM!	#NUM!	#NUM!	#NUM!	28.0965	27.933	28.7033	28.5591
#NUM!	27.8817	#NUM!	#NUM!	#NUM!	27.8017	#NUM!	#NUM!	27.052	26.8	27.3314	26.877
#NUM!	27.8631	#NUM!	#NUM!	#NUM!	#NUM!	#NUM!	#NUM!	27.815	27.9565	27.7756	27.7357
29.9699	30.0676	30.2503	30.7062	29.7177	29.5511	29.6321	29.1835	29.9101	30.1711	29.6022	29.818
26.9092	#NUM!	#NUM!	#NUM!	#NUM!	27.1348	#NUM!	#NUM!	27.1212	26.9388	27.1044	26.968
#NUM!	#NUM!	#NUM!	#NUM!	#NUM!	#NUM!	#NUM!	#NUM!	26.8016	27.1482	26.4388	#NUM!
#NUM!	#NUM!	#NUM!	#NUM!	#NUM!	#NUM!	#NUM!	#NUM!	26.2744	25.9963	26.7232	26.7993
28.9534	28.9967	28.9951	28.4794	28.6731	29.1922	28.9323	28.6531	26.7666	26.2632	26.1358	26.1156
#NUM!	#NUM!	#NUM!	#NUM!	#NUM!	#NUM!	#NUM!	#NUM!	27.1108	27.4877	27.8734	28.0012
36.9736	37.3332	34.3876	34.2857	35.7328	36.0178	35.6369	37.1499	34.4065	34.3566	34.4118	34.3254
30.5088	30.1087	29.1765	29.106	30.8548	31.1236	30.7981	31.3619	#NUM!	#NUM!	26.1784	26.5342
31.4577	31.4841	31.4594	31.344	30.0333	29.8074	29.8361	29.055	26.4672	26.6593	26.9879	27.1553
29.7345	29.804	28.8532	28.5942	29.1834	29.1066	29.1452	28.3258	25.6355	24.7756	25.5208	25.5863
29.207	28.9039	28.0873	28.5868	27.1165	27.2896	#NUM!	#NUM!	27.8915	27.7384	27.5879	27.5697
#NUM!	#NUM!	#NUM!	#NUM!	#NUM!	#NUM!	#NUM!	#NUM!	27.7597	27.4255	27.4977	27.705
#NUM!	#NUM!	#NUM!	#NUM!	#NUM!	27.3309	#NUM!	#NUM!	28.0112	27.7704	27.9363	27.8463
28.725	28.4872	28.459	28.0959	#NUM!	28.557	#NUM!	#NUM!	28.6433	28.3877	28.4987	28.5789
#NUM!	#NUM!	#NUM!	#NUM!	#NUM!	#NUM!	#NUM!	#NUM!	27.7777	27.4732	27.5849	27.5908
29.5547	29.4524	29.3267	29.558	29.766	30.1558	29.8592	29.0828	29.2579	29.3567	29.3159	29.264
#NUM!	29.024	29.9259	29.3289	#NUM!	30.6022	#NUM!	28.3363	29.9919	29.8334	29.857	29.7985
#NUM!	28.2378	#NUM!	#NUM!	#NUM!	#NUM!	#NUM!	#NUM!	29.2604	29.2308	28.6892	29.0329
27.8431	28.021	28.1496	27.7529	28.1164	28.3925	28.0585	28.4133	28.483	28.3705	28.5083	28.6555
#NUM!	#NUM!	#NUM!	#NUM!	#NUM!	#NUM!	#NUM!	#NUM!	26.0431	25.9186	26.0381	26.3543
#NUM!	28.1116	28.0632	#NUM!	#NUM!	28.387	#NUM!	#NUM!	29.1325	29.1816	28.9485	29.029
#NUM!	#NUM!	#NUM!	#NUM!	#NUM!	#NUM!	#NUM!	#NUM!	29.3483	29.3854	29.5254	29.4033
29.4992	29.5785	28.8235	28.7844	28.2253	29.2523	28.4188	29.1012	#NUM!	#NUM!	#NUM!	#NUM!
29.4141	29.4937	29.4777	30.4342	32.1156	31.7167	30.8366	31.0538	29.0599	28.9911	28.8968	28.7768
31.7235	31.8899	31.3984	31.3902	30.9874	31.0819	30.9754	31.0028	29.54	29.2196	28.8622	28.9343
28.2159	28.4335	27.9947	27.8555	#NUM!	28.6622	#NUM!	#NUM!	29.7724	29.6728	29.5776	29.7788
27.8525	28.1179	#NUM!	27.7311	#NUM!	#NUM!	#NUM!	#NUM!	28.028	27.8522	27.9926	27.6849
#NUM!	#NUM!	#NUM!	#NUM!	#NUM!	#NUM!	#NUM!	#NUM!	#NUM!	#NUM!	26.3011	#NUM!
28.8016	28.357	28.4954	28.3035	#NUM!	#NUM!	27.8322	#NUM!	28.8441	28.7539	28.8605	28.9027
#NUM!	#NUM!	#NUM!	#NUM!	#NUM!	#NUM!	#NUM!	#NUM!	26.3954	26.9141	26.7403	26.9354
#NUM!	#NUM!	#NUM!	#NUM!	#NUM!	#NUM!	#NUM!	#NUM!	#NUM!	#NUM!	#NUM!	29.7491
#NUM!	#NUM!	#NUM!	#NUM!	#NUM!	#NUM!	#NUM!	#NUM!	28.1368	28.0763	27.9573	28.0911
#NUM!	#NUM!	27.4412	26.9025	#NUM!	#NUM!	#NUM!	#NUM!	27.0106	27.2126	27.2146	27.1962
#NUM!	#NUM!	#NUM!	#NUM!	#NUM!	#NUM!	#NUM!	#NUM!	26.2155	26.2285	26.3257	26.7053
28.1766	28.1743	#NUM!	#NUM!	#NUM!	#NUM!	#NUM!	#NUM!	26.7189	26.9779	27.0028	27.1659
#NUM!	#NUM!	27.6299	27.5825	#NUM!	#NUM!	#NUM!	#NUM!	27.3793	27.4494	27.1441	27.0876
#NUM!	#NUM!	#NUM!	#NUM!	#NUM!	#NUM!	#NUM!	#NUM!	26.4342	26.39	26.2778	26.3848
#NUM!	#NUM!	#NUM!	#NUM!	#NUM!	#NUM!	#NUM!	#NUM!	26.7384	26.732	26.5891	26.6309
#NUM!	#NUM!	#NUM!	#NUM!	#NUM!	#NUM!	#NUM!	#NUM!	25.1811	25.3991	25.4518	25.6801
#NUM!	#NUM!	#NUM!	#NUM!	#NUM!	#NUM!	#NUM!	#NUM!	26.2142	26.4134	26.834	26.9602
#NUM!	26.895	#NUM!	27.4073	#NUM!	#NUM!	#NUM!	#NUM!	27.5131	27.3064	27.3156	27.4061
#NUM!	#NUM!	28.0099	28.1237	#NUM!	28.2993	#NUM!	#NUM!	28.803	28.7285	28.5635	28.7305
#NUM!	#NUM!	#NUM!	#NUM!	#NUM!	#NUM!	#NUM!	#NUM!	26.2651	26.311	26.5642	26.7558
#NUM!	28.1908	27.6119	27.0508	#NUM!	28.2481	#NUM!	#NUM!	28.5857	28.6089	28.6712	28.5008
#NUM!	#NUM!	#NUM!	#NUM!	#NUM!	#NUM!	#NUM!	#NUM!	26.599	26.4081	26.7283	26.5741
29.1298	29.2456	28.9471	29.1378	28.8435	28.5446	28.8007	28.3833	27.061	27.0168	27.2346	27.3103
#NUM!	#NUM!	#NUM!	#NUM!	#NUM!	#NUM!	#NUM!	#NUM!	#NUM!	#NUM!	27.0365	#NUM!
#NUM!	#NUM!	#NUM!	#NUM!	#NUM!	#NUM!	#NUM!	#NUM!	25.8139	#NUM!	26.0969	25.741
#NUM!	#NUM!	#NUM!	#NUM!	#NUM!	#NUM!	#NUM!	#NUM!	29.0836	29.0042	28.9303	29.0153
#NUM!	#NUM!	#NUM!	#NUM!	#NUM!	#NUM!	#NUM!	#NUM!	26.1064	26.0006	25.7607	25.8839
29.9045	29.9574	27.6202	28.0518	29.6392	30.8454	29.4175	30.6633	28.1784	27.91	27.9794	27.7831
#NUM!	#NUM!	27.4454	27.7427	#NUM!	#NUM!	27.7822	#NUM!	27.5564	27.3104	27.2523	27.4678
#NUM!	#NUM!	#NUM!	#NUM!	#NUM!	#NUM!	#NUM!	#NUM!	27.5268	27.5451	27.6985	27.5207
#NUM!	#NUM!	#NUM!	#NUM!	#NUM!	#NUM!	#NUM!	#NUM!	27.3208	27.4592	27.6674	27.4131
27.24	27.115	#NUM!	#NUM!	#NUM!	#NUM!	#NUM!	#NUM!	28.1427	28.1679	28.1405	28.0491
#NUM!	26.6083	#NUM!	26.9528	#NUM!	#NUM!	#NUM!	#NUM!	27.2386	27.2741	27.057	27.0618

#NUM!	#NUM!	#NUM!	#NUM!	#NUM!	#NUM!	#NUM!	#NUM!	27.4911	27.3365	27.1627	27.6179
#NUM!	28.682	#NUM!	#NUM!	#NUM!	28.7859	#NUM!	#NUM!	28.8323	28.87	29.4124	29.2579
27.1889	27.622	28.5238	28.4257	#NUM!	#NUM!	#NUM!	#NUM!	#NUM!	#NUM!	#NUM!	#NUM!
#NUM!	#NUM!	#NUM!	#NUM!	#NUM!	#NUM!	#NUM!	#NUM!	26.7549	26.5305	26.9955	26.9487
32.515	32.5354	32.3032	32.5944	32.7263	32.309	32.8019	31.9998	30.5672	30.6045	30.7422	30.5832
29.2873	29.4266	29.1968	29.2895	29.4492	29.7375	29.2503	29.3664	29.6054	29.612	29.9348	30.0196
#NUM!	#NUM!	#NUM!	#NUM!	#NUM!	#NUM!	#NUM!	#NUM!	29.346	28.1317	28.7073	28.2235
27.4204	27.1903	#NUM!	#NUM!	#NUM!	#NUM!	#NUM!	#NUM!	26.698	26.2843	27.164	26.5193
#NUM!	27.4483	#NUM!	27.4603	#NUM!	#NUM!	#NUM!	#NUM!	27.3665	27.1531	27.3561	27.4396
29.1055	28.8825	#NUM!	28.8199	#NUM!	29.2796	#NUM!	29.0225	28.544	28.7949	29.0923	29.2981
29.1721	29.0905	28.5947	28.4799	#NUM!	29.3873	#NUM!	#NUM!	30.5704	30.4548	30.234	30.158
31.8212	32.12	29.7423	29.3115	#NUM!	31.4804	30.481	32.2068	26.2736	26.6261	26.6767	26.82
27.6077	27.5569	27.1538	27.2632	27.9154	27.6718	27.8174	27.4354	29.0054	29.1424	29.1863	29.0299
#NUM!	#NUM!	#NUM!	#NUM!	#NUM!	#NUM!	#NUM!	#NUM!	28.4118	28.1739	28.3472	28.3052
#NUM!	26.8716	#NUM!	#NUM!	#NUM!	27.4471	#NUM!	#NUM!	27.2431	27.1429	27.3957	27.1731
#NUM!	#NUM!	#NUM!	29.3866	#NUM!	#NUM!	#NUM!	#NUM!	28.6967	28.5357	28.7689	28.628
30.7195	30.6389	29.7884	29.7736	30.4502	30.8196	29.9888	29.8893	31.2671	31.2904	30.9802	31.1269
31.5913	31.5848	30.5762	30.5934	32.0789	33.4989	31.9601	32.8245	30.3067	30.2015	30.5951	30.5981
29.23	29.2074	28.4872	28.8159	28.3459	27.9253	28.278	28.125	29.4549	29.4958	29.8509	29.77
29.6768	29.6212	28.8376	28.9362	28.9752	29.5063	29.4787	29.0934	30.5502	30.438	30.0928	29.9386
#NUM!	#NUM!	#NUM!	#NUM!	#NUM!	#NUM!	#NUM!	#NUM!	25.7205	25.7627	25.7748	26.1478
27.3498	#NUM!	#NUM!	#NUM!	#NUM!	#NUM!	#NUM!	#NUM!	25.9962	25.7916	25.8548	26.2877
30.009	30.0569	28.7408	28.6516	28.5924	30.1997	29.0368	29.7759	30.1842	30.2525	30.0652	29.8982
#NUM!	#NUM!	#NUM!	#NUM!	#NUM!	#NUM!	#NUM!	#NUM!	28.9841	29.287	29.191	29.0058
#NUM!	#NUM!	#NUM!	#NUM!	#NUM!	#NUM!	#NUM!	#NUM!	27.7253	28.2808	27.9849	28.1635
#NUM!	#NUM!	#NUM!	#NUM!	#NUM!	#NUM!	#NUM!	#NUM!	26.3533	26.2119	#NUM!	#NUM!
#NUM!	#NUM!	#NUM!	#NUM!	#NUM!	#NUM!	#NUM!	#NUM!	28.2794	28.2601	28.0984	28.3322
29.3104	28.9473	29.0299	29.1168	29.0005	29.0393	29.1987	28.3924	30.2104	30.2525	30.2021	30.3886
30.8784	30.9467	30.72	30.569	30.3723	30.6765	30.283	30.4273	29.4157	29.808	29.6504	29.568
#NUM!	#NUM!	#NUM!	#NUM!	#NUM!	#NUM!	#NUM!	#NUM!	26.8318	27.0378	27.043	27.1598
#NUM!	#NUM!	#NUM!	#NUM!	#NUM!	#NUM!	#NUM!	#NUM!	27.555	27.6567	27.3362	27.3319
#NUM!	#NUM!	#NUM!	#NUM!	#NUM!	#NUM!	#NUM!	#NUM!	26.7962	26.7986	27.1159	27.1633
#NUM!	#NUM!	#NUM!	#NUM!	#NUM!	#NUM!	#NUM!	#NUM!	27.7183	27.7863	27.8678	27.7918
#NUM!	27.2789	26.6453	#NUM!	#NUM!	#NUM!	#NUM!	#NUM!	27.1705	27.2336	27.6718	27.7503
28.0908	28.2345	#NUM!	27.5299	#NUM!	27.9543	#NUM!	27.9498	28.6098	28.5507	28.9405	28.9109
#NUM!	#NUM!	#NUM!	#NUM!	#NUM!	#NUM!	#NUM!	#NUM!	27.7988	27.8515	27.6105	27.782
#NUM!	#NUM!	#NUM!	#NUM!	#NUM!	#NUM!	#NUM!	#NUM!	26.2979	26.5539	26.2405	26.2505
#NUM!	#NUM!	#NUM!	#NUM!	#NUM!	#NUM!	#NUM!	#NUM!	26.3887	25.7314	26.3527	26.2526
28.0665	28.2359	#NUM!	28.2414	#NUM!	#NUM!	#NUM!	#NUM!	29.1229	29.234	29.1911	29.4062
28.3095	27.8694	28.5029	28.5914	#NUM!	28.0098	#NUM!	27.9582	28.4952	28.294	28.3123	28.2485
#NUM!	#NUM!	29.5678	29.9751	#NUM!	#NUM!	#NUM!	#NUM!	28.6705	28.5947	28.1925	28.0405
#NUM!	#NUM!	#NUM!	#NUM!	#NUM!	#NUM!	#NUM!	#NUM!	#NUM!	#NUM!	25.8759	#NUM!
29.7805	29.7848	30.5565	30.6298	29.4828	29.4043	29.3898	29.2902	27.664	27.3799	27.8762	27.8172
#NUM!	#NUM!	#NUM!	#NUM!	#NUM!	#NUM!	#NUM!	#NUM!	28.2429	28.0748	28.2517	28.2623
#NUM!	#NUM!	#NUM!	#NUM!	#NUM!	#NUM!	#NUM!	#NUM!	26.3771	27.627	26.4259	27.1572
28.5499	29.0235	28.6345	28.2716	#NUM!	29.1394	#NUM!	28.3602	28.9172	29.0342	29.0624	29.0624
28.7278	28.7082	28.6118	28.4699	#NUM!	29.4737	#NUM!	#NUM!	28.4477	28.7905	28.7221	28.7043
#NUM!	#NUM!	#NUM!	#NUM!	#NUM!	#NUM!	#NUM!	#NUM!	#NUM!	#NUM!	#NUM!	#NUM!
#NUM!	#NUM!	#NUM!	#NUM!	#NUM!	#NUM!	#NUM!	#NUM!	26.0703	26.3746	26.1752	26.2177
#NUM!	27.2665	#NUM!	#NUM!	#NUM!	#NUM!	#NUM!	#NUM!	26.3112	26.3344	27.0646	26.7857
#NUM!	28.4685	28.9696	28.8702	#NUM!	28.3214	#NUM!	30.212	29.0386	29.1212	29.0499	28.7338
#NUM!	26.9572	#NUM!	27.4669	#NUM!	27.9272	#NUM!	#NUM!	28.417	28.3361	28.6572	28.795
27.5437	28.0613	27.3695	27.5346	#NUM!	28.2341	#NUM!	27.9909	27.9705	27.8904	28.301	28.3276
#NUM!	#NUM!	#NUM!	#NUM!	#NUM!	#NUM!	#NUM!	#NUM!	26.869	27.0962	26.7216	27.0045
#NUM!	#NUM!	#NUM!	#NUM!	#NUM!	#NUM!	#NUM!	#NUM!	27.4139	27.2177	27.6075	27.3229
28.3996	28.1209	28.6346	28.1178	28.4876	28.3449	#NUM!	#NUM!	28.1794	28.0137	27.996	27.9741
#NUM!	#NUM!	#NUM!	#NUM!	#NUM!	#NUM!	#NUM!	#NUM!	27.223	28.0335	27.3266	27.6411
31.3594	31.5673	30.2576	30.1329	31.9676	32.4075	31.9497	30.927	27.3615	27.2596	27.512	27.3489
27.8141	27.6506	27.9913	28.1439	#NUM!	#NUM!	#NUM!	#NUM!	27.9893	27.4347	28.1828	27.9809
28.8712	28.568	29.1441	29.0282	#NUM!	28.2152	#NUM!	#NUM!	30.2992	30.0839	30.3141	30.1853
29.0538	28.837	28.907	28.8845	28.8877	29.835	#NUM!	29.793	29.2884	29.5051	29.6485	29.6024
#NUM!	27.4976	#NUM!	28.2194	#NUM!	#NUM!	#NUM!	27.0926	31.4635	31.2519	31.3927	31.4782
#NUM!	#NUM!	#NUM!	#NUM!	#NUM!	#NUM!	#NUM!	#NUM!	#NUM!	#NUM!	#NUM!	25.7871
#NUM!	28.0586	#NUM!	#NUM!	#NUM!	#NUM!	#NUM!	#NUM!	27.8054	27.9297	27.666	27.7506
29.4521	29.4451	28.3973	28.5982	#NUM!	28.7912	28.513	28.5483	30.3604	30.3003	30.7204	30.7089
#NUM!	#NUM!	#NUM!	#NUM!	#NUM!	#NUM!	#NUM!	#NUM!	27.384	27.5717	27.7794	27.5456
27.9777	28.4652	#NUM!	#NUM!	#NUM!	#NUM!	#NUM!	#NUM!	30.567	30.6234	30.7998	30.6913
#NUM!	#NUM!	#NUM!	#NUM!	#NUM!	#NUM!	#NUM!	#NUM!	26.7027	26.9105	26.9613	27.3343
29.505	29.5534	29.0036	28.8974	27.8959	29.7013	#NUM!	#NUM!	30.4797	30.6964	30.3465	30.2265
30.8494	30.6981	29.9749	29.9353	29.2289	30.7211	28.9417	30.0533	31.7794	31.8311	31.962	31.7474
29.2383	29.4515	29.1892	29.1177	28.8972	29.1008	28.9706	28.754	29.7719	29.6848	29.423	29.5588
#NUM!	#NUM!	#NUM!	#NUM!	#NUM!	#NUM!	#NUM!	#NUM!	27.0801	26.9677	26.9058	26.8014
29.0593	30.3153	28.3809	28.8097	#NUM!	#NUM!	#NUM!	#NUM!	27.4624	27.4092	27.0213	26.9836
#NUM!	#NUM!	#NUM!	#NUM!	#NUM!	#NUM!	#NUM!	#NUM!	#NUM!	#NUM!	#NUM!	25.8658
#NUM!	27.3023	#NUM!	28.49	#NUM!	#NUM!	#NUM!	#NUM!	#NUM!	26.4045	26.1329	26.227
27.3002	27.3581	#NUM!	27.2248	#NUM!	#NUM!	#NUM!	#NUM!	28.0372	28.3996	28.4013	28.4843
28.2002	28.1203	28.1566	27.869	28.0112	27.8123	#NUM!	#NUM!	28.2749	28.6892	28.8748	28.8216
27.6076	27.607	27.0449	26.9036	#NUM!	#NUM!	#NUM!	#NUM!	26.7116	26.8851	26.9035	27.1377
29.4874	29.5784	28.6077	28.8529	28.7896	29.0461	28.9935	28.7665	29.4968	29.5347	29.6781	29.6465
#NUM!	27.1507	#NUM!	#NUM!	#NUM!	#NUM!	#NUM!	#NUM!	27.906	28.1748	28.2623	28.0697
29.381	29.2779	29.3252	29.4033	29.1928	29.0589	29.3184	28.5399	29.4266	29.6541	29.549	29.4292
29.302	29.1763	27.8526	27.5705	28.4257	28.99	28.2208	28.8851	29.1747	29.073	29.5727	29.3447
#NUM!	27.2932	#NUM!	27.15	#NUM!	#NUM!	#NUM!	#NUM!	27.4929	27.4621	28.278	27.9284
#NUM!	#NUM!	#NUM!	#NUM!	#NUM!	#NUM!	#NUM!	#NUM!	25.433	25.9602	26.1533	25.9894
#NUM!	#NUM!	#NUM!	#NUM!	#NUM!	#NUM!	#NUM!	#NUM!	26.8958	26.8655	26.4776	26.5019
#NUM!	28.1999	#NUM!	#NUM!	#NUM!	#NUM!	#NUM!	#NUM!	27.9339	28.0795	28.2952	28.4496
29.5036	29.3584	28.5853	28.4432	#NUM!	#NUM!	#NUM!	#NUM!	27.781	27.4848	27.6204	27.9845
#NUM!	#NUM!	#NUM!	#NUM!	#NUM!	#NUM!	#NUM!	#NUM!	#NUM!	26.2658	26.626	26.4706
29.7894	29.8984	29.2153	28.7707	28.4587	29.864	28.4511	29.2749	30.8243	30.8755	30.9351	30.7175
30.8121	#NUM!	#NUM!	29.4077	#NUM!	31.412	#NUM!	#NUM!	29.389	29.5585	29.816	29.9181
30.6656	30.8324	30.2474	29.96	28.8299	30.3669	29.0692	30.198	31.3099	31.5321	31.3503	31.505
30.1917	30.4126	30.8356	30.6065	31.337	30.4468	31.2038	29.8568	27.8066	27.6701	27.2077	27.239
29.1665	29.1115	28.9172	28.6975	29.0294	#NUM!	29.2107	#NUM!	#NUM!	#NUM!	#NUM!	26.7446
#NUM!	#NUM!	#NUM!	#NUM!	#NUM!	#NUM!	#NUM!	#NUM!	26.9631	26.916	26.7091	26.574
28.8398	28.4999	27.7749	28.3461	28.3273	28.2668	#NUM!	27.8498	28.0948	28.1762	28.1372	28.3159
#NUM!	#NUM!	#NUM!	#NUM!	#NUM!	#NUM!	#NUM!	#NUM!	27.2338	27.1075	27.1325	26.988
#NUM!	28.3539	28.9492	#NUM!	#NUM!	#NUM!	#NUM!	#NUM!	28.6043	27.2127	27.1091	26.8436
#NUM!	#NUM!	#NUM!	#NUM!	#NUM!	#NUM!	#NUM!	#NUM!	31.7754	31.3554	31.1736	31.3175
27.597	27.6975	27.0431	27.0155	#NUM!	27.8762	#NUM!	#NUM!	27.5195	27.6509	27.9301	27.8747
27.6975	27.3666	#NUM!	27.2353	#NUM!	28.1641	#NUM!	28.1831	27.3676	27.3625	27.7699	27.8381
27.0019	27.6276	#NUM!	#NUM!	#NUM!	#NUM!	#NUM!	#NUM!	27.2309	27.0845	27.2609	27.2789
#NUM!	#NUM!	#NUM!	#NUM!	#NUM!	#NUM!	#NUM!	#NUM!	#NUM!	#NUM!	#NUM!	#NUM!
29.4885	29.9287	29.5936	30.0374	29.7802	30.0192	29.8819	29.5909	27.5724	27.8774	28.3131	28.5401
25.9676	#NUM!	#NUM!	26.9665	#NUM!	#NUM!	#NUM!	#NUM!	27.6854	27.6035	27.782	27.7569
30.1019	28.5506	27.3851	27.4155	28.462	29.1935	28.172	28.9249	25.5666	25.5467	25.2169	24.9841
#NUM!	27.5552	#NUM!	28.056	#NUM!	#NUM!	#NUM!	#NUM!	27.9054	28.0496	27.9518	27.9752
#NUM!	28.3016	#NUM!	#NUM!	#NUM!	#NUM!	#NUM!	#NUM!	27.5451	27.5155	27.4727	27.1767
#NUM!	28.3605	27.3722	27.1568	#NUM!	28.4748	#NUM!	#NUM!	27.2266	27.6017	27.1907	27.1982
#NUM!	28.5674	28.7016	28.4718	#NUM!	28.2294	#NUM!	#NUM!	27.9182	27.6631	27.7396	27.8101
#NUM!	#NUM!	#NUM!	#NUM!	#NUM!	#NUM!	#NUM!	#NUM!	27.2507	27.6405	27.6263	27.6204
#NUM!	#NUM!	#NUM!	#NUM!	#NUM!	#NUM!	#NUM!	26.6214	26.5667	26.5277	26.8808	27.042
#NUM!	#NUM!	#NUM!	#NUM!	#NUM!	#NUM!	#NUM!	#NUM!	25.9644	26.1695	26.1702	26.0346
#NUM!	#NUM!	#NUM!	#NUM!	#NUM!	#NUM!	#NUM!	#NUM!	27.2614	27.3711	27.6083	27.4809
#NUM!	#NUM!	#NUM!	#NUM!	#NUM!	#NUM!	#NUM!	#NUM!	26.4799	26.7243	26.4868	26.455
#NUM!	26.9589	#NUM!	#NUM!	#NUM!	28.6754	#NUM!	28.2799	26.5508	26.7338	26.742	26.8797
#NUM!	#NUM!	#NUM!	#NUM!	#NUM!	#NUM!	#NUM!	#NUM!	28.2433	28.2361	28.4071	28.3385
#NUM!	27.7448	#NUM!	27.7506	#NUM!	#NUM!	#NUM!	#NUM!	29.9874	29.6853	29.8561	29.9244
26.8876	#NUM!	24.9661	26.6196	#NUM!	#NUM!	28.286	27.0033	29.4344	29.4252	29.6047	29.5909
32.8663	32.8211	32.471	32.8919	32.329	32.5481	32.0465	31.9584	32.9961	33.0179	32.9906	32.9498
30.5712	30.4809	28.488	28.6254	29.8375	31.3588	29.7452	30.8913	27.9555	27.8446	27.9871	28.2802
31.6235	31.9224	31.8802	32.065	32.0083	31.558	32.0595	31.0208	28.7701	28.9078	29.1656	29.2394
28.7603	28.7947	28.0726	28.086	28.3424	28.0978	#NUM!	28.6436	28.2811	28.2882	28.594	28.4847
#NUM!	#NUM!	#NUM!	#NUM!	#NUM!	#NUM!	#NUM!	#NUM!	27.0527	26.7745	#NUM!	26.7953
29.4547	29.8398	28.6905	28.8765	28.7799	29.3328	28.6337	29.588	30.2098	30.1368	29.4922	29.6512
#NUM!	#NUM!	#NUM!	#NUM!	#NUM!	#NUM!	#NUM!	#NUM!	28.5906	28.9575	28.9017	28.912
27.4816	27.9325	27.4056	27.4117	#NUM!	27.3259	#NUM!	27.1492	29.2077	29.2134	29.2988	29.2281
29.0557	29.1372	29.28	29.2482	29.0276	29.0469	29.1624	28.9366	29.1109	29.179	29.3214	29.4057
28.3277	28.3037	28.6172	#NUM!	#NUM!	28.674	#NUM!	#NUM!	28.122	28.434	28.1137	28.0783
#NUM!	#NUM!	#NUM!	#NUM!	#NUM!	#NUM!	#NUM!	#NUM!	26.545	26.7647	26.6297	26.5658

28.5065	28.5153	28.8407	28.4852	31.7779	29.9079	31.6522	30.7564	#NUM!	#NUM!	#NUM!	#NUM!
#NUM!	#NUM!	#NUM!	#NUM!	#NUM!	#NUM!	#NUM!	#NUM!	26.6207	27.2073	27.3611	27.1327
#NUM!	25.8854	#NUM!	#NUM!	#NUM!	#NUM!	#NUM!	#NUM!	28.7508	28.7805	29.0435	29.0628
#NUM!	#NUM!	#NUM!	#NUM!	#NUM!	#NUM!	#NUM!	#NUM!	26.5243	26.4301	26.8591	26.9504
28.1597	28.1203	28.02	28.5159	#NUM!	#NUM!	#NUM!	#NUM!	29.0739	28.8696	28.7045	28.6963
29.1292	29.1334	29.8591	29.6828	28.5345	28.9039	28.7833	28.4462	29.6162	29.7203	29.4276	29.3614
29.5525	29.702	28.2655	28.5646	27.8242	29.6322	27.8615	28.5306	30.4098	30.4863	30.5482	30.5653
28.9321	29.0776	28.7354	28.5406	28.019	30.0426	27.9984	29.0895	30.0947	30.1158	30.156	30.187
29.6783	29.7787	29.6007	28.6283	28.9547	29.5874	#NUM!	29.1595	30.0836	30.0974	29.8813	30.0104
29.4216	29.4743	#NUM!	#NUM!	#NUM!	29.2746	#NUM!	26.9949	29.1894	29.3962	29.3459	29.4745
#NUM!	#NUM!	#NUM!	#NUM!	#NUM!	#NUM!	#NUM!	#NUM!	30.0513	30.2013	30.3972	30.6217
29.4824	29.1241	29.4836	29.2129	28.5077	29.5329	#NUM!	28.9918	30.0197	29.899	30.0399	29.9184
27.407	27.4658	#NUM!	27.88	#NUM!	27.4632	#NUM!	#NUM!	27.3719	27.4411	27.489	27.4525
28.8603	28.6244	28.6077	28.9517	27.9094	28.8551	#NUM!	28.3107	28.9834	28.9722	28.966	28.9639
#NUM!	#NUM!	#NUM!	#NUM!	#NUM!	#NUM!	#NUM!	#NUM!	26.3559	#NUM!	26.1415	26.0254
30.2545	30.2336	30.4465	30.4777	29.7679	29.8304	29.9564	29.5201	31.1228	31.0343	31.295	31.3114
27.5305	27.4051	27.2418	#NUM!	#NUM!	#NUM!	#NUM!	#NUM!	27.9307	28.5437	28.376	28.4973
#NUM!	#NUM!	#NUM!	#NUM!	#NUM!	#NUM!	#NUM!	#NUM!	26.4768	26.6776	26.6413	26.5585
#NUM!	#NUM!	#NUM!	#NUM!	#NUM!	#NUM!	#NUM!	#NUM!	27.6003	27.7587	27.7045	27.6285
#NUM!	#NUM!	#NUM!	#NUM!	#NUM!	#NUM!	#NUM!	#NUM!	25.8441	26.4633	26.3179	26.4156
#NUM!	#NUM!	#NUM!	#NUM!	#NUM!	#NUM!	#NUM!	#NUM!	26.8571	26.1889	26.5373	26.2051
#NUM!	#NUM!	#NUM!	#NUM!	#NUM!	#NUM!	#NUM!	#NUM!	26.0374	26.0572	26.4488	26.4219
#NUM!	#NUM!	#NUM!	#NUM!	#NUM!	#NUM!	#NUM!	#NUM!	#NUM!	#NUM!	#NUM!	#NUM!
#NUM!	#NUM!	#NUM!	#NUM!	#NUM!	#NUM!	#NUM!	#NUM!	#NUM!	#NUM!	26.6118	26.7372
29.4459	29.1414	28.9158	28.9954	#NUM!	28.9617	#NUM!	29.1261	29.7535	29.6742	29.8152	29.8679
27.2119	27.9805	27.6654	27.6123	#NUM!	28.1783	#NUM!	#NUM!	29.2951	29.2919	29.2955	29.068
#NUM!	#NUM!	#NUM!	#NUM!	#NUM!	#NUM!	#NUM!	#NUM!	26.1664	26.7726	26.3615	26.5757
#NUM!	#NUM!	#NUM!	#NUM!	#NUM!	#NUM!	#NUM!	#NUM!	26.5652	#NUM!	27.2607	27.4614
#NUM!	27.6935	27.4423	28.085	#NUM!	26.6449	#NUM!	#NUM!	28.6169	28.8247	28.5593	28.7261
#NUM!	#NUM!	#NUM!	#NUM!	#NUM!	#NUM!	#NUM!	#NUM!	27.3292	27.2734	27.3407	27.4972
#NUM!	#NUM!	#NUM!	#NUM!	#NUM!	#NUM!	#NUM!	#NUM!	26.355	26.5031	27.0798	27.1641
29.0979	28.5581	29.0641	29.1528	28.1539	28.5505	28.2835	29.1629	28.6151	28.3446	28.8566	28.8524
#NUM!	#NUM!	#NUM!	#NUM!	#NUM!	#NUM!	#NUM!	#NUM!	#NUM!	#NUM!	#NUM!	26.7066
28.9262	28.8554	28.6397	28.8658	#NUM!	28.4589	27.8403	28.2485	28.5855	28.3911	28.7097	28.747
#NUM!	#NUM!	#NUM!	#NUM!	#NUM!	#NUM!	#NUM!	#NUM!	26.9193	27.464	27.5756	27.7017
29.6488	29.712	29.6252	29.7164	29.7132	29.6173	29.8684	29.1412	26.2322	26.2211	26.8172	26.5541
30.5852	30.5295	30.2074	30.0848	29.3185	29.9602	29.4992	29.2434	30.8776	30.6931	30.3226	30.2198
30.7187	30.892	30.7435	30.5939	31.4473	31.2943	31.4697	30.7371	27.936	27.988	27.7921	27.8602
#NUM!	#NUM!	#NUM!	#NUM!	#NUM!	#NUM!	#NUM!	#NUM!	27.061	27.01	26.829	26.7853
30.0836	30.0204	29.766	30.2607	32.2573	31.2316	32.1596	32.1881	31.2611	30.8377	28.6705	28.3941
#NUM!	#NUM!	#NUM!	#NUM!	#NUM!	#NUM!	#NUM!	#NUM!	27.7356	27.911	28.0587	28.0994
32.467	32.1199	31.9681	32.0866	31.5706	32.2548	31.0382	32.4009	32.7738	32.9229	33.0707	32.7249
28.4658	28.5745	28.278	27.8698	27.8491	28.0349	27.8496	28.1311	27.9168	27.9252	28.052	27.9602
#NUM!	29.1066	#NUM!	#NUM!	#NUM!	#NUM!	#NUM!	#NUM!	27.1025	27.1555	27.5152	27.7469
#NUM!	#NUM!	#NUM!	#NUM!	#NUM!	#NUM!	#NUM!	#NUM!	27.3437	27.4831	26.9245	27.1383
28.0518	27.9333	27.7419	27.9049	#NUM!	27.7432	30.6209	#NUM!	28.4403	28.5286	28.7466	28.7781
27.8188	27.5834	27.897	27.1081	#NUM!	27.5061	#NUM!	#NUM!	27.775	27.8478	27.7744	27.8826
27.4651	26.8995	27.2144	27.2034	#NUM!	27.3912	#NUM!	27.1827	28.5077	28.3179	28.5703	28.6302
27.1801	27.4293	27.8755	27.4607	#NUM!	#NUM!	#NUM!	#NUM!	28.688	28.9091	29.2022	29.2434
30.8575	31.17	31.3797	31.1979	30.4428	30.7876	30.4562	29.9506	30.9871	31.2626	30.8261	30.8627
#NUM!	29.2038	#NUM!	27.5526	#NUM!	#NUM!	#NUM!	#NUM!	26.9887	26.954	27.309	27.2933
28.25	28.2613	28.0212	28.0495	#NUM!	28.212	#NUM!	27.4353	28.4228	28.4727	28.6831	28.682
31.9339	31.9288	31.1072	31.2428	33.4299	34.1598	33.3712	34.2137	32.5014	32.5414	31.8083	31.7982
28.2927	28.49	28.6197	#NUM!	#NUM!	28.31	#NUM!	29.5459	28.7304	28.9328	29.2723	29.0888
30.5305	30.487	29.9672	30.2323	29.4672	29.8996	29.6767	29.9193	29.2332	29.4006	29.3845	29.0844
32.531	32.5755	32.6777	32.9785	33.1887	32.416	33.1935	32.3229	29.6164	29.8375	30.0096	29.9672
#NUM!	#NUM!	#NUM!	#NUM!	#NUM!	#NUM!	#NUM!	#NUM!	27.3039	27.7059	27.479	27.4952
29.5294	28.7211	28.5147	28.7484	#NUM!	30.9943	29.6091	#NUM!	30.5825	30.324	30.5204	30.4069
#NUM!	#NUM!	#NUM!	#NUM!	#NUM!	#NUM!	#NUM!	#NUM!	#NUM!	26.7369	#NUM!	#NUM!
#NUM!	26.8382	#NUM!	#NUM!	#NUM!	#NUM!	#NUM!	#NUM!	28.7841	28.5055	28.3456	28.1974
33.5439	33.438	32.6849	32.6006	33.3448	33.9052	32.9284	32.5371	#NUM!	#NUM!	#NUM!	#NUM!
#NUM!	#NUM!	#NUM!	#NUM!	#NUM!	#NUM!	#NUM!	#NUM!	26.8	26.8171	26.8082	26.8072
#NUM!	#NUM!	28.2754	#NUM!	#NUM!	#NUM!	#NUM!	#NUM!	29.2188	29.1501	29.0319	28.6931
28.2376	28.1782	#NUM!	28.5149	#NUM!	#NUM!	#NUM!	28.4243	28.2238	28.299	28.3463	28.4121
28.5154	28.5486	28.467	28.4503	28.2345	28.2351	27.9915	27.8107	29.3752	29.4431	29.5318	29.5674
28.8376	29.1131	28.8843	28.9516	#NUM!	28.4946	28.3149	27.4522	29.5622	29.2394	29.7592	29.3397
#NUM!	#NUM!	#NUM!	#NUM!	#NUM!	#NUM!	#NUM!	#NUM!	25.2833	25.715	25.9766	26.3917
29.2755	29.3873	29.7989	29.5095	29.3969	29.815	29.3475	29.2405	30.8116	30.6068	30.7892	30.8188
#NUM!	#NUM!	#NUM!	#NUM!	#NUM!	#NUM!	#NUM!	#NUM!	27.7379	27.7834	28.2289	28.2342
#NUM!	#NUM!	#NUM!	#NUM!	#NUM!	26.6923	#NUM!	#NUM!	28.1223	28.0263	27.9082	27.8977
#NUM!	#NUM!	#NUM!	#NUM!	#NUM!	#NUM!	#NUM!	#NUM!	#NUM!	#NUM!	25.7429	25.8492
#NUM!	#NUM!	#NUM!	#NUM!	#NUM!	#NUM!	#NUM!	#NUM!	26.6226	26.6034	26.7321	26.6721
27.8839	27.5397	27.4141	28.0085	28.0523	28.393	28.3654	27.5703	27.7439	27.485	27.3947	27.4388
#NUM!	#NUM!	#NUM!	#NUM!	#NUM!	#NUM!	#NUM!	#NUM!	25.0567	25.7313	25.4738	25.207
26.9706	27.6183	#NUM!	#NUM!	#NUM!	#NUM!	#NUM!	#NUM!	27.6187	27.6257	27.8782	27.9074
29.7719	29.6654	29.7881	29.8674	29.3297	29.0955	29.2751	28.7457	30.4569	30.5865	30.4086	30.4371
27.3721	27.5131	27.265	26.807	29.5267	31.4946	29.455	31.0882	#NUM!	24.8174	25.3816	25.1586
28.1241	28.0709	27.9843	28.3837	#NUM!	28.6902	#NUM!	28.8791	26.9713	27.2552	26.4928	26.9211
#NUM!	27.0215	#NUM!	27.5027	#NUM!	#NUM!	#NUM!	#NUM!	27.1296	27.2686	27.4979	27.8462
27.9686	27.4821	#NUM!	27.092	#NUM!	27.8566	#NUM!	28.4606	26.7715	26.7278	26.961	27.1317
28.9774	28.8479	29.2693	29.6516	#NUM!	28.9617	30.2743	28.3934	28.5883	28.5847	28.9811	28.9766
#NUM!	28.0345	#NUM!	28.072	#NUM!	#NUM!	#NUM!	#NUM!	27.7291	27.3529	28.1282	27.7144
27.0628	27.1854	#NUM!	26.6813	#NUM!	#NUM!	#NUM!	#NUM!	27.7494	27.9006	28.0102	28.0116
28.531	28.3181	28.6845	28.7271	28.5635	28.7297	28.5945	27.8547	27.7335	28.0131	28.1823	28.2352
#NUM!	#NUM!	27.9853	#NUM!	#NUM!	#NUM!	#NUM!	#NUM!	27.5141	27.8603	27.7076	28.3098
28.3164	28.5784	27.4787	27.9961	#NUM!	28.2583	27.6117	28.4573	28.4853	28.3193	28.8907	28.7669
#NUM!	#NUM!	#NUM!	#NUM!	#NUM!	#NUM!	#NUM!	#NUM!	26.9788	27.1134	27.0061	27.5635
31.1732	31.2113	31.2673	31.1759	30.2769	30.8068	30.6333	30.3964	30.8798	31.062	31.2218	31.1082
28.9037	28.949	28.7166	28.3805	28.0796	28.2218	28.3472	28.5139	29.0966	29.0733	29.4081	29.2449
#NUM!	#NUM!	#NUM!	#NUM!	#NUM!	#NUM!	#NUM!	#NUM!	25.3566	25.0886	#NUM!	25.364
29.4804	29.5434	28.4532	28.7503	28.8232	28.6918	28.783	28.5337	26.8771	26.6308	26.7699	27.1154
#NUM!	#NUM!	#NUM!	#NUM!	#NUM!	#NUM!	#NUM!	#NUM!	#NUM!	25.9804	26.5762	26.3762
#NUM!	#NUM!	#NUM!	#NUM!	#NUM!	27.1484	#NUM!	#NUM!	26.0769	26.1194	26.4721	26.2403
#NUM!	25.4222	#NUM!	#NUM!	#NUM!	#NUM!	#NUM!	#NUM!	27.443	27.261	27.6306	27.6337
28.4815	28.4403	28.2202	28.3738	#NUM!	28.1356	#NUM!	27.9006	27.9829	28.2418	27.9292	28.0256
33.0432	33.176	28.7841	28.9023	29.3408	33.3616	29.4557	33.9989	30.8692	30.5611	30.6738	30.8082
29.7107	29.7077	29.2492	29.5382	29.8388	29.3582	29.7436	29.4806	31.0894	30.9714	31.3663	31.3155
29.4549	29.1159	28.7771	29.1498	28.7001	29.0237	28.9189	29.4551	29.4481	29.4646	29.5586	29.4352
27.5771	#NUM!	26.1646	26.5385	#NUM!	#NUM!	#NUM!	#NUM!	27.7044	27.8046	28.0273	28.012
#NUM!	#NUM!	#NUM!	#NUM!	#NUM!	#NUM!	#NUM!	#NUM!	28.6623	28.1409	28.5727	28.8588
#NUM!	#NUM!	#NUM!	#NUM!	#NUM!	#NUM!	#NUM!	#NUM!	29.0858	29.1457	29.2243	29.2367
27.8298	27.4063	#NUM!	26.0756	26.9868	28.5944	#NUM!	28.8451	28.7351	28.9316	29.2501	29.318
#NUM!	#NUM!	27.7662	#NUM!	#NUM!	29.3029	#NUM!	#NUM!	28.506	28.557	28.7211	28.7224
28.8682	29.2648	29.3305	28.1426	29.2034	29.5371	28.7488	29.9762	27.4991	27.2712	27.4656	27.198
28.8549	28.8657	28.2578	28.3166	28.3852	29.2942	27.9225	29.5132	28.0006	28.2867	28.1464	28.04
28.4835	28.6673	28.1453	28.0097	#NUM!	28.8879	27.5959	29.2608	28.909	28.8859	29.2203	29.2524
29.4127	29.5452	28.0802	28.3309	27.2746	29.3449	27.8982	28.6577	29.2771	29.3627	29.8295	29.5569
32.61	32.8402	32.7867	32.6355	32.6909	32.5501	32.653	31.9727	29.5609	29.8743	30.226	29.8187
#NUM!	#NUM!	#NUM!	#NUM!	#NUM!	#NUM!	#NUM!	#NUM!	27.4938	27.3657	27.4437	27.6586
29.2139	28.6413	#NUM!	#NUM!	#NUM!	29.0709	#NUM!	29.8018	30.408	30.3265	30.6642	30.6241
26.8403	26.7278	26.752	26.9816	#NUM!	#NUM!	#NUM!	#NUM!	27.0659	27.3529	27.1484	27.2147
#NUM!	#NUM!	#NUM!	#NUM!	#NUM!	#NUM!	#NUM!	#NUM!	25.8687	25.9327	25.9841	25.8808
#NUM!	#NUM!	#NUM!	#NUM!	#NUM!	#NUM!	#NUM!	#NUM!	27.1744	27.2945	27.6877	27.4668
31.088	31.1479	30.1926	30.6212	30.4551	30.607	30.2559	30.7394	29.6123	29.8577	29.8768	29.9399
#NUM!	26.2967	#NUM!	#NUM!	#NUM!	#NUM!	#NUM!	#NUM!	26.6786	26.4211	26.9339	26.8195
#NUM!	27.2689	#NUM!	27.5867	#NUM!	#NUM!	#NUM!	#NUM!	28.5622	28.3623	27.6473	28.0318
26.295	26.5988	#NUM!	26.5549	#NUM!	27.6446	#NUM!	26.688	28.7615	28.9371	29.0769	28.7439
28.6818	28.7736	27.927	27.9341	28.2687	28.1082	28.2099	#NUM!	28.6764	28.482	28.8874	29.0471
#NUM!	#NUM!	27.0783	#NUM!	#NUM!	#NUM!	#NUM!	#NUM!	28.0037	27.8624	28.1311	28.2309
#NUM!	#NUM!	#NUM!	#NUM!	#NUM!	#NUM!	#NUM!	#NUM!	#NUM!	#NUM!	26.3248	27.1815
#NUM!	#NUM!	#NUM!	#NUM!	#NUM!	#NUM!	#NUM!	#NUM!	26.6654	26.3376	26.5947	26.7429
29.0227	28.5799	28.2369	28.7218	27.753	#NUM!	#NUM!	28.334	28.2399	27.91	28.1273	28.1452
#NUM!	#NUM!	#NUM!	#NUM!	#NUM!	#NUM!	#NUM!	#NUM!	28.2315	28.2848	28.896	28.7977
#NUM!	#NUM!	#NUM!	#NUM!	#NUM!	#NUM!	#NUM!	#NUM!	26.3297	26.2424	26.9541	26.6488
28.9308	28.7253	#NUM!	#NUM!	#NUM!	30.5415	#NUM!	29.4071	32.89	32.8142	32.5524	32.4759
28.0058	28.1364	28.5421	28.1885	27.9658	28.252	28.0438	27.3497	29.0137	28.6969	28.7989	28.9852
#NUM!	26.7049	#NUM!	26.7482	#NUM!	#NUM!	#NUM!	#NUM!	28.0159	28.0963	28.0133	27.9543
32.0323	32.2715	32.8354	32.4457	30.9053	31.4061	30.8695	30.6124	31.7971	31.4057	31.8079	31.6669
#NUM!	#NUM!	#NUM!	#NUM!	#NUM!	#NUM!	#NUM!	#NUM!	32.1778	32.534	29.1872	29.2274

28.4085	28.1602	27.9529	28.2617	#NUM!	28.157	#NUM!	27.7361	28.311	28.5944	28.3668	28.3181
#NUM!	#NUM!	#NUM!	27.0964	#NUM!	#NUM!	#NUM!	#NUM!	27.1769	27.0461	27.232	27.2644
#NUM!	#NUM!	#NUM!	#NUM!	#NUM!	28.1074	#NUM!	#NUM!	26.8599	26.427	26.3658	26.5049
30.2569	30.3126	29.8373	29.5663	29.736	30.2774	29.4702	29.7442	30.8389	30.9919	30.7202	30.6112
#NUM!	#NUM!	#NUM!	#NUM!	#NUM!	#NUM!	#NUM!	#NUM!	26.4375	26.885	26.3715	26.3948
#NUM!	#NUM!	#NUM!	#NUM!	#NUM!	#NUM!	#NUM!	#NUM!	26.9644	27.1928	27.0981	26.9864
28.892	28.9562	29.677	29.5705	29.2353	28.8272	29.2318	28.4	26.3511	26.4937	26.1629	26.195
31.1367	31.1385	30.0759	30.1634	29.7094	30.5427	29.5319	30.0191	31.4076	31.4068	31.3852	31.2858
#NUM!	#NUM!	#NUM!	#NUM!	#NUM!	#NUM!	#NUM!	#NUM!	26.5771	26.2974	26.2431	26.0893
#NUM!	#NUM!	#NUM!	#NUM!	#NUM!	#NUM!	#NUM!	#NUM!	28.4614	28.0132	28.8212	28.5323
27.9728	27.4568	#NUM!	#NUM!	#NUM!	#NUM!	#NUM!	#NUM!	28.1646	28.0613	28.4525	28.4341
29.4797	27.4937	29.3769	28.1847	#NUM!	29.6069	#NUM!	#NUM!	28.5558	28.4005	28.7253	28.8831
#NUM!	28.0263	#NUM!	#NUM!	#NUM!	#NUM!	#NUM!	#NUM!	27.6215	27.3664	27.5769	27.5454
#NUM!	#NUM!	#NUM!	#NUM!	#NUM!	#NUM!	#NUM!	#NUM!	28.5739	28.9592	28.4819	28.3624
29.0108	28.8647	28.6125	28.5801	#NUM!	28.5996	#NUM!	28.7525	28.997	29.1983	29.3497	29.2697
26.6042	26.5925	#NUM!	#NUM!	#NUM!	#NUM!	#NUM!	#NUM!	26.5988	26.656	27.2923	26.972
28.4779	28.4779	28.5334	28.9633	28.3461	28.446	28.447	28.1281	27.6438	27.5894	27.9602	27.9086
#NUM!	#NUM!	#NUM!	26.7147	#NUM!	#NUM!	#NUM!	#NUM!	28.649	28.6666	28.5345	28.6223
28.5077	27.983	28.7705	27.924	#NUM!	28.6992	#NUM!	27.7827	29.3199	29.2438	29.1835	29.2383
27.9136	27.824	27.659	27.4813	#NUM!	27.4939	#NUM!	28.0792	28.0887	28.0247	28.19	28.086
#NUM!	26.5966	27.1542	26.8569	#NUM!	27.4831	#NUM!	#NUM!	27.2564	27.4022	27.2974	27.3016
27.5172	27.3521	#NUM!	27.4524	#NUM!	27.074	#NUM!	#NUM!	27.2552	27.3528	27.4936	27.2896
27.1237	28.5073	#NUM!	#NUM!	#NUM!	#NUM!	#NUM!	#NUM!	28.8449	29.0756	29.2056	29.1042
28.7809	28.684	28.6304	29.1738	#NUM!	28.8173	28.9113	28.932	28.9651	29.3565	29.1469	29.0332
27.9065	28.0208	27.6508	27.5754	#NUM!	28.3094	28.0755	27.8572	28.1481	28.1388	28.4276	28.4306
29.6982	29.8642	30.9394	30.6149	31.6871	33.2923	31.6039	33.4287	27.0087	27.2118	26.4714	25.6744
#NUM!	26.9664	27.6768	#NUM!	#NUM!	27.4095	#NUM!	#NUM!	28.7557	28.6783	28.4744	28.4016
28.7117	28.84	28.9914	29.1933	29.6451	29.2056	29.66	28.5909	26.0331	26.6408	26.1399	26.2855
28.9091	29.0848	28.8072	28.9748	28.3461	29.0445	28.4085	28.6483	28.5713	28.5948	28.8339	29.0171
#NUM!	#NUM!	#NUM!	#NUM!	#NUM!	#NUM!	#NUM!	#NUM!	28.302	27.899	28.617	28.1714
26.9426	#NUM!	#NUM!	#NUM!	#NUM!	26.9663	#NUM!	#NUM!	27.8923	28.5286	28.9343	28.7948
#NUM!	27.4467	27.6171	27.5165	#NUM!	#NUM!	#NUM!	#NUM!	26.7035	26.3547	26.7596	26.7455
27.2914	28.088	27.541	28.2108	#NUM!	#NUM!	#NUM!	#NUM!	30.5018	30.501	30.2811	30.2405
30.0655	30.0718	28.8178	29.0196	29.0027	29.7174	29.1864	29.484	27.8645	27.779	27.9713	28.0515
28.9521	28.5114	28.1833	28.7281	#NUM!	28.5065	28.3724	28.2287	31.3662	31.2274	31.497	31.5101
#NUM!	#NUM!	#NUM!	#NUM!	#NUM!	#NUM!	#NUM!	#NUM!	29.5279	29.6585	29.5381	29.5176
#NUM!	#NUM!	#NUM!	#NUM!	#NUM!	#NUM!	#NUM!	#NUM!	#NUM!	25.7063	26.4539	26.525
30.0316	30.1412	29.9367	30.3716	29.6474	29.2146	29.922	28.8242	30.1113	30.311	29.9604	29.947
#NUM!	28.6287	#NUM!	28.7736	#NUM!	#NUM!	#NUM!	#NUM!	28.429	28.5749	28.6427	28.8054
29.6123	29.5952	29.0378	29.3931	29.5897	29.7732	29.4508	29.7048	29.7708	29.6788	29.557	29.6712
#NUM!	#NUM!	#NUM!	#NUM!	#NUM!	#NUM!	#NUM!	#NUM!	27.0952	27.3474	26.9705	27.2002
#NUM!	27.4909	#NUM!	27.2371	#NUM!	#NUM!	#NUM!	#NUM!	29.1553	28.578	28.968	29.0125
29.1726	29.1521	28.6444	28.6596	28.4487	28.5592	28.4639	28.6843	29.1673	29.3166	29.2866	29.078
27.686	27.4856	26.7581	26.677	#NUM!	27.2459	#NUM!	#NUM!	30.3975	30.0298	30.4438	30.5304
30.8432	31.0454	29.8678	29.704	29.2521	30.8916	29.2979	30.2483	30.8188	31.0513	31.1619	31.1964
30.4823	30.5589	29.1508	29.2887	29.057	30.1672	29.0797	29.9655	30.5992	30.7972	31.0971	30.9788
29.5171	29.4913	29.2228	29.0618	#NUM!	29.5179	28.9997	29.0785	29.1403	29.3301	29.1741	29.15
#NUM!	27.3837	#NUM!	26.8038	#NUM!	#NUM!	#NUM!	#NUM!	27.2341	27.0664	27.5239	27.3764
28.0522	27.5863	28.1873	27.9846	27.6756	28.0789	#NUM!	27.5731	29.2191	29.3269	29.7388	29.5389
#NUM!	28.1221	#NUM!	#NUM!	#NUM!	#NUM!	#NUM!	#NUM!	#NUM!	#NUM!	#NUM!	#NUM!
#NUM!	#NUM!	#NUM!	#NUM!	#NUM!	#NUM!	#NUM!	#NUM!	26.7986	#NUM!	26.1855	#NUM!
28.1236	28.3515	27.4847	27.6078	#NUM!	27.5958	#NUM!	#NUM!	27.3808	27.2743	27.7373	27.7094
29.6152	29.5466	#NUM!	#NUM!	#NUM!	29.4581	#NUM!	28.9438	28.3522	27.8647	27.01	27.1231
#NUM!	#NUM!	#NUM!	#NUM!	#NUM!	#NUM!	#NUM!	#NUM!	29.8749	29.7979	29.8569	29.9876
#NUM!	#NUM!	#NUM!	#NUM!	#NUM!	#NUM!	#NUM!	#NUM!	25.7986	25.9044	25.9969	25.9369
26.2512	26.1628	#NUM!	26.8668	#NUM!	#NUM!	#NUM!	#NUM!	26.4554	26.1649	25.975	25.9006
#NUM!	#NUM!	#NUM!	#NUM!	#NUM!	#NUM!	#NUM!	#NUM!	26.4375	26.6702	26.9897	26.5823
29.2172	29.2713	29.5242	29.3418	28.724	28.9929	28.604	28.9925	28.354	28.3168	28.6374	28.5012
#NUM!	#NUM!	#NUM!	#NUM!	#NUM!	#NUM!	#NUM!	#NUM!	27.0917	26.9515	27.125	26.9735
#NUM!	#NUM!	#NUM!	26.9945	#NUM!	#NUM!	#NUM!	#NUM!	26.3741	26.678	26.407	26.1827
28.5544	28.4359	28.402	28.4451	#NUM!	27.5626	#NUM!	27.4161	29.2134	29.1687	29.1982	29.2065
27.7877	27.6121	#NUM!	27.5299	#NUM!	28.6435	#NUM!	28.1727	27.2171	27.2239	27.3414	27.3853
27.7182	27.7783	#NUM!	#NUM!	#NUM!	28.2764	#NUM!	26.8644	28.019	28.0232	28.1448	28.195
#NUM!	#NUM!	#NUM!	#NUM!	#NUM!	#NUM!	#NUM!	#NUM!	25.011	25.3742	#NUM!	25.0074
#NUM!	#NUM!	#NUM!	#NUM!	#NUM!	#NUM!	#NUM!	#NUM!	26.971	27.0576	27.2832	27.135
#NUM!	27.5541	#NUM!	27.6397	#NUM!	#NUM!	#NUM!	#NUM!	27.0526	27.0613	27.2797	27.216
#NUM!	#NUM!	#NUM!	#NUM!	#NUM!	#NUM!	#NUM!	#NUM!	#NUM!	25.7148	25.5573	26.1082
#NUM!	25.9953	#NUM!	#NUM!	#NUM!	#NUM!	#NUM!	#NUM!	26.6984	26.5053	26.8268	27.0425
28.4026	28.2189	28.0796	28.3712	27.909	28.6578	28.0822	28.7359	28.6519	28.6175	29.1774	28.9047
#NUM!	#NUM!	#NUM!	#NUM!	#NUM!	#NUM!	#NUM!	#NUM!	26.1225	25.4642	26.0964	26.029
31.4307	31.4927	31.3553	31.6125	31.7271	31.3926	31.8797	30.9931	28.4167	28.5422	28.8836	28.7642
29.6496	29.9282	29.846	30.1408	29.799	29.5195	29.8025	29.1986	27.7231	27.6168	27.5939	27.6073
29.4234	28.7221	29.0071	28.9024	#NUM!	29.6785	#NUM!	30.296	29.1161	29.0457	28.9373	28.9467
27.9829	27.2192	#NUM!	28.1332	#NUM!	#NUM!	#NUM!	28.8784	28.1978	28.202	28.3891	28.2707
#NUM!	#NUM!	#NUM!	#NUM!	#NUM!	#NUM!	#NUM!	#NUM!	#NUM!	#NUM!	#NUM!	#NUM!
#NUM!	#NUM!	#NUM!	#NUM!	#NUM!	#NUM!	#NUM!	#NUM!	26.41	25.7811	26.2108	26.1598
#NUM!	#NUM!	#NUM!	#NUM!	#NUM!	#NUM!	#NUM!	#NUM!	27.1344	26.9337	26.2093	26.636
28.7684	28.5652	28.7951	28.5473	28.7457	28.7632	29.199	27.7975	29.0573	28.9337	29.4482	29.1984
29.882	29.9689	28.8571	28.5396	28.4923	30.161	28.757	28.7897	27.9255	26.7277	26.5736	27.9396
28.3885	28.1877	28.94	29.1834	#NUM!	27.2995	#NUM!	27.0207	27.2865	27.406	27.6112	27.5474
27.7369	28.0384	27.8945	27.797	27.6661	28.2457	27.6677	28.3068	27.8208	27.817	28.0474	27.9426
29.09	29.3305	27.9771	27.982	28.7886	28.759	28.3953	28.0989	30.2895	30.1634	29.919	29.858
30.2164	30.3414	30.2651	30.4379	29.4669	29.8955	29.6219	29.4255	29.8786	29.8622	29.9426	30.1957
29.9615	29.2803	28.9535	29.0295	29.1137	30.9821	29.0092	30.1646	28.1506	28.1893	28.2062	28.1575
#NUM!	#NUM!	#NUM!	#NUM!	#NUM!	#NUM!	#NUM!	#NUM!	26.4286	#NUM!	26.8424	26.3156
29.0039	29.1126	28.951	28.7351	28.6388	29.5146	28.6406	29.1415	29.146	29.1913	29.2524	29.2487
32.4954	32.6508	31.4108	31.7253	31.0353	32.6953	31.0046	32.8937	31.941	31.9664	32.0508	32.0574
30.3655	30.1244	29.4798	29.6416	29.1665	30.175	28.8348	30.13	30.8669	30.9504	30.6136	30.8545
33.5445	33.4281	33.0895	33.5175	33.0488	33.1764	32.99	32.9233	33.9319	33.7776	33.7487	33.5586
27.8214	28.0633	#NUM!	28.2914	#NUM!	29.1539	#NUM!	#NUM!	27.831	27.923	27.8197	27.851
27.7755	27.942	27.8999	27.166	#NUM!	27.8469	#NUM!	28.2129	28.138	28.0254	27.822	27.707
27.8343	27.8777	26.8901	27.658	#NUM!	28.0213	#NUM!	27.6153	#NUM!	#NUM!	26.1258	26.0498
#NUM!	#NUM!	#NUM!	#NUM!	#NUM!	#NUM!	#NUM!	28.0778	27.5186	27.5606	28.0051	27.7428
#NUM!	#NUM!	#NUM!	#NUM!	#NUM!	#NUM!	#NUM!	#NUM!	#NUM!	25.1442	25.5561	25.6613
#NUM!	#NUM!	#NUM!	#NUM!	#NUM!	#NUM!	#NUM!	#NUM!	26.9901	28.0868	26.2227	26.2594
27.6307	27.3608	27.819	27.2851	#NUM!	27.1053	#NUM!	#NUM!	30.0852	30.08	30.176	30.0245
#NUM!	#NUM!	#NUM!	#NUM!	#NUM!	#NUM!	#NUM!	#NUM!	27.2937	27.5658	27.805	27.4514
#NUM!	#NUM!	#NUM!	#NUM!	#NUM!	#NUM!	#NUM!	#NUM!	27.8761	27.7405	28.0941	28.0876
#NUM!	#NUM!	#NUM!	#NUM!	#NUM!	#NUM!	#NUM!	#NUM!	25.5078	25.758	#NUM!	#NUM!
#NUM!	27.9905	#NUM!	#NUM!	#NUM!	#NUM!	#NUM!	#NUM!	27.5192	28.3317	28.2966	27.9526
28.0129	27.9311	28.4058	28.1466	30.778	31.0287	30.8808	29.8892	#NUM!	#NUM!	25.3184	25.6725
27.939	28.0862	28.1458	27.876	#NUM!	27.7339	#NUM!	#NUM!	28.5495	28.598	28.5872	28.4809
#NUM!	#NUM!	#NUM!	#NUM!	#NUM!	#NUM!	#NUM!	#NUM!	27.1518	27.5385	27.4191	27.7052
#NUM!	27.0541	#NUM!	27.1378	#NUM!	27.6243	#NUM!	28.0225	25.6296	26.1342	26.2966	26.6795
#NUM!	#NUM!	#NUM!	#NUM!	#NUM!	#NUM!	#NUM!	#NUM!	27.8247	27.6895	27.3316	27.6064
28.799	28.7797	28.835	29.051	28.6786	28.9083	29.7218	#NUM!	29.7064	29.627	29.168	29.4829
30.9281	30.8294	30.0571	29.5812	29.56	30.8475	29.6912	30.5362	30.7257	30.9197	31.0945	31.1201
#NUM!	28.9876	#NUM!	#NUM!	#NUM!	31.2721	#NUM!	#NUM!	28.0538	27.9797	27.6962	27.7339
27.9626	27.3039	#NUM!	#NUM!	#NUM!	28.6009	#NUM!	28.8976	29.5448	29.3455	29.7843	29.721
28.7001	28.8925	27.645	27.5454	28.1957	28.0694	27.9553	27.5526	26.711	26.8535	27.0796	27.5442
28.5762	28.2862	29.5504	29.4337	29.2662	29.2372	29.3082	28.7779	27.5096	27.5898	27.4849	27.5235
#NUM!	#NUM!	#NUM!	#NUM!	#NUM!	#NUM!	#NUM!	#NUM!	29.0723	28.973	29.4258	29.3367
#NUM!	#NUM!	#NUM!	#NUM!	#NUM!	#NUM!	#NUM!	#NUM!	26.9941	27.1045	27.138	27.3639
#NUM!	#NUM!	#NUM!	#NUM!	#NUM!	27.1796	#NUM!	#NUM!	27.094	27.2785	27.3696	27.3301
30.2488	30.3328	29.6781	29.5736	29.4724	29.6815	29.4704	29.6334	#NUM!	25.2876	26.1256	25.5129
#NUM!	#NUM!	#NUM!	#NUM!	#NUM!	#NUM!	#NUM!	#NUM!	25.791	26.0846	25.9873	26.273
27.5155	#NUM!	#NUM!	#NUM!	#NUM!	#NUM!	#NUM!	#NUM!	28.0552	28.0515	28.0332	27.9699
28.3442	28.1789	28.9156	28.5052	29.1496	28.2244	28.9724	28.4456	28.2845	28.3903	28.8165	28.9096
#NUM!	#NUM!	#NUM!	#NUM!	#NUM!	#NUM!	#NUM!	#NUM!	27.8821	27.6905	28.0032	27.9217
#NUM!	27.7888	#NUM!	26.1796	#NUM!	28.1742	#NUM!	#NUM!	28.1536	27.8687	27.6463	27.7847
27.886	27.674	#NUM!	#NUM!	#NUM!	27.7539	#NUM!	#NUM!	28.4501	28.5634	28.4468	28.3747
28.0375	27.7028	28.0289	27.4858	#NUM!	28.7902	#NUM!	28.8434	29.9582	29.7861	30.0547	29.9045
27.8721	27.9922	28.1596	27.8216	28.8379	27.4859	28.9138	28.0496	28.2873	28.4328	28.6054	28.3135
#NUM!	#NUM!	#NUM!	#NUM!	#NUM!	#NUM!	#NUM!	#NUM!	27.4422	27.3584	26.9521	26.9079
#NUM!	#NUM!	#NUM!	#NUM!	#NUM!	#NUM!	#NUM!	#NUM!	26.758	26.554	26.8064	26.8999
26.3875	26.6153	26.0588	26.2697	#NUM!	27.7358	#NUM!	27.751	27.707	27.6937	27.2931	27.4194
28.521	28.6068	28.3309	28.3571	#NUM!	29.3547	#NUM!	28.4234	28.4162	28.4507	28.7079	28.5893
#NUM!	26.947	#NUM!	#NUM!	#NUM!	#NUM!	#NUM!	#NUM!	28.345	28.1216	28.0437	28.1882
28.5506	28.5223	#NUM!	#NUM!	#NUM!	27.4277	#NUM!	#NUM!	28.2203	28.4044	28.1393	28.0144

31.2457	31.3294	30.9202	31.1873	30.2211	30.6044	30.3888	31.0026	31.4526	31.6053	31.5379	31.7659
30.5329	30.7331	29.9335	29.9813	30.8809	31.2065	30.9548	30.7824	28.3313	28.3455	28.864	28.5851
27.4201	26.866	#NUM!	26.6586	28.5712	29.6198	#NUM!	29.0744	#NUM!	#NUM!	#NUM!	#NUM!
26.9807	27.2373	#NUM!	#NUM!	#NUM!	28.6075	#NUM!	28.2377	27.4914	27.973	28.1541	28.1036
27.5514	27.9691	27.9615	27.3515	28.6458	28.3992	28.2906	28.709	26.4609	25.8978	25.2435	25.9705
28.7905	28.7837	28.9199	29.6139	28.8891	28.2522	28.5196	28.6604	29.3165	29.4367	29.6583	29.5572
30.8895	31.3029	31.0662	31.0491	31.35	30.4734	31.0674	30.1369	28.6999	28.703	28.5391	28.3397
#NUM!	#NUM!	#NUM!	#NUM!	#NUM!	#NUM!	#NUM!	#NUM!	27.2217	27.2757	27.1798	27.3301
30.0969	30.4947	30.4233	29.8858	28.7199	29.2708	28.6912	28.6274	31.0617	30.7916	31.256	31.0159
29.353	29.3942	29.0643	29.1029	28.7761	29.0727	28.9385	28.5905	30.3983	30.4908	30.4666	30.5226
28.0918	27.8035	#NUM!	27.324	#NUM!	28.4596	#NUM!	28.2474	28.7297	28.8254	29.0207	29.0066
28.3492	28.4676	28.0506	28.2613	#NUM!	27.6826	#NUM!	27.2171	28.7206	29.0023	29.0024	29.1203
28.2988	28.0016	#NUM!	27.7238	#NUM!	#NUM!	#NUM!	26.5917	27.9478	27.8279	28.2926	28.0077
#NUM!	#NUM!	#NUM!	#NUM!	#NUM!	#NUM!	#NUM!	#NUM!	24.8003	24.6317	25.0046	25.0682
#NUM!	28.8622	#NUM!	28.2173	#NUM!	#NUM!	#NUM!	#NUM!	27.835	28.0348	28.1242	28.0248
30.2001	30.2933	29.2601	29.6767	#NUM!	30.8392	#NUM!	30.1446	30.804	30.7736	30.5693	30.5899
30.1608	30.264	29.1737	29.0282	28.7576	29.6865	28.6645	29.393	30.9725	31.0598	30.8076	30.9546
28.2673	28.2361	27.6986	28.1636	#NUM!	28.6504	28.2913	28.4747	28.086	28.2007	28.6851	28.5333
28.373	28.517	27.2074	27.4671	27.653	29.5859	27.9051	29.5412	27.6799	27.5904	27.9943	27.8918
#NUM!	#NUM!	#NUM!	#NUM!	#NUM!	#NUM!	#NUM!	#NUM!	27.3804	26.8555	27.0661	27.0309
28.2873	28.1122	28.399	28.5705	#NUM!	#NUM!	27.8812	27.4612	28.4116	28.5392	28.8114	28.8233
#NUM!	26.8327	#NUM!	#NUM!	#NUM!	#NUM!	#NUM!	#NUM!	26.9446	26.917	26.6335	26.7138
#NUM!	#NUM!	#NUM!	#NUM!	#NUM!	#NUM!	#NUM!	#NUM!	26.822	26.8348	26.3954	26.8215
27.4399	27.0589	27.3988	27.4635	#NUM!	27.1365	#NUM!	27.2725	28.4665	28.2881	28.5789	28.9452
30.0761	30.1559	30.2804	30.503	29.665	30.0535	29.8413	29.3934	27.8137	28.0361	27.9223	28.1223
28.0331	#NUM!	28.7814	#NUM!	#NUM!	28.9418	#NUM!	29.1082	27.5086	27.8628	27.8712	27.8861
28.7822	28.3127	28.1337	28.1931	#NUM!	28.3196	#NUM!	27.7999	29.6128	29.6231	29.7082	29.9913
28.4003	28.5922	28.6602	28.9393	#NUM!	28.3279	#NUM!	28.3313	27.0707	27.059	27.1971	27.0856
#NUM!	#NUM!	#NUM!	#NUM!	#NUM!	#NUM!	#NUM!	#NUM!	#NUM!	#NUM!	26.8682	26.3908
29.0113	28.7639	29.5141	29.1145	29.4936	29.3136	29.4726	29.0989	27.2576	27.1281	27.0257	27.4121
27.9133	27.9884	27.7243	27.9426	27.6871	28.6693	27.5629	28.8034	27.3225	27.7917	27.5032	27.4105
27.5924	28.3046	30.0439	28.3328	#NUM!	27.8401	28.2252	#NUM!	25.5583	25.2068	25.6994	25.6958
29.5699	29.5375	29.8631	29.6961	30.5155	29.6884	30.1919	29.4633	27.3376	27.5401	27.3954	27.3944
28.1283	27.7041	#NUM!	#NUM!	#NUM!	28.4617	#NUM!	27.9905	28.5837	28.5177	28.7865	28.6557
#NUM!	#NUM!	#NUM!	#NUM!	#NUM!	#NUM!	#NUM!	#NUM!	28.169	28.1523	28.1803	28.0732
26.5366	26.8192	26.8039	#NUM!	#NUM!	28.7135	#NUM!	27.7827	32.3261	32.2315	31.657	31.889
27.8345	27.7816	27.6351	27.6387	27.9031	28.0517	27.9375	28.236	28.2328	28.3986	28.8975	28.7104
31.1944	31.3919	30.6973	31.0583	30.6341	30.2385	30.8162	30.6756	27.0387	27.7683	27.4805	27.15
#NUM!	26.9275	#NUM!	#NUM!	#NUM!	27.6132	#NUM!	27.2622	27.8211	28.0364	28.2259	28.1866
29.3021	29.6313	28.8382	28.5798	28.5966	29.6335	28.1699	29.0277	30.1649	30.1752	29.8408	29.9593
#NUM!	#NUM!	#NUM!	#NUM!	#NUM!	#NUM!	#NUM!	#NUM!	26.2992	26.8165	26.9156	27.0399
28.1559	28.0318	28.4564	27.8074	#NUM!	#NUM!	27.8515	#NUM!	29.0132	29.0772	28.9922	29.1381
#NUM!	#NUM!	#NUM!	#NUM!	#NUM!	#NUM!	#NUM!	#NUM!	27.4364	26.3937	26.7733	26.6915
31.177	31.1563	31.0162	30.7821	30.409	31.6522	30.6286	31.5044	30.1348	30.2584	30.6824	30.6372
29.9137	29.9025	28.5076	28.8408	28.5976	29.6704	28.2169	29.2238	30.3347	30.4148	30.5352	30.4932
29.9662	29.8153	29.43	29.3788	29.1166	29.2461	29.0937	29.8297	29.4744	29.3757	29.582	29.4542
#NUM!	#NUM!	#NUM!	#NUM!	#NUM!	#NUM!	#NUM!	#NUM!	26.6862	26.917	26.9201	26.7446
#NUM!	#NUM!	#NUM!	#NUM!	#NUM!	#NUM!	#NUM!	#NUM!	27.5581	27.2658	27.6763	27.8817
28.2666	28.4043	28.4956	28.4636	#NUM!	28.5373	#NUM!	#NUM!	28.2749	28.5686	28.4338	28.4971
30.4952	30.6341	28.9472	29.3221	29.0873	30.5469	28.9451	29.8433	30.1414	30.4376	30.879	30.779
29.2308	29.2838	28.4818	28.6159	28.3412	28.8844	28.4726	28.7071	29.6864	29.6722	29.6219	29.6156
28.6354	28.6729	28.2626	28.4466	27.409	28.2603	27.6144	28.6973	28.5916	28.8003	28.638	28.7228
27.0426	26.9289	28.228	27.5731	#NUM!	28.0923	#NUM!	27.9589	27.0707	27.2237	27.3027	27.2276
#NUM!	#NUM!	#NUM!	#NUM!	#NUM!	26.0617	#NUM!	#NUM!	27.4361	27.3372	27.1803	27.24
27.8125	27.6981	27.3567	27.2705	#NUM!	27.7998	#NUM!	#NUM!	28.6971	28.3478	28.7019	28.7345
#NUM!	27.6456	28.3875	28.666	#NUM!	#NUM!	#NUM!	#NUM!	30.4245	30.5234	30.608	30.5234
28.0645	27.9703	27.2747	27.7472	#NUM!	27.26	#NUM!	#NUM!	29.2312	29.1048	29.28	29.0213
#NUM!	#NUM!	#NUM!	#NUM!	#NUM!	#NUM!	#NUM!	#NUM!	#NUM!	#NUM!	#NUM!	24.4785
#NUM!	#NUM!	#NUM!	#NUM!	#NUM!	#NUM!	#NUM!	#NUM!	27.0164	27.117	27.183	27.134
#NUM!	#NUM!	#NUM!	#NUM!	#NUM!	#NUM!	#NUM!	#NUM!	#NUM!	26.1102	26.053	26.4387
27.7734	27.8064	27.2692	#NUM!	#NUM!	#NUM!	#NUM!	#NUM!	28.1686	28.0926	27.9963	27.8889
29.8423	29.9867	27.5921	27.6843	28.6797	29.5779	28.052	28.7248	#NUM!	25.0276	#NUM!	#NUM!
#NUM!	#NUM!	#NUM!	#NUM!	#NUM!	26.9517	#NUM!	#NUM!	28.259	28.5045	28.3486	28.4106
28.3324	28.5174	28.6452	28.534	28.4874	28.832	28.5694	#NUM!	27.2272	27.042	27.4698	27.4261
27.9746	27.9354	27.9462	27.9411	#NUM!	28.0789	#NUM!	28.0269	27.8822	27.6704	27.7449	27.7507
#NUM!	#NUM!	#NUM!	#NUM!	#NUM!	#NUM!	#NUM!	#NUM!	27.3486	27.4826	27.4386	27.6002
#NUM!	#NUM!	#NUM!	#NUM!	#NUM!	#NUM!	#NUM!	#NUM!	30.7918	#NUM!	30.4198	30.5961
29.1321	29.4732	28.7696	28.4433	28.1004	28.7892	27.961	28.2316	29.9479	30.0312	29.7262	29.5105
#NUM!	#NUM!	#NUM!	#NUM!	#NUM!	#NUM!	#NUM!	#NUM!	#NUM!	#NUM!	25.3232	25.3311
29.7462	29.738	29.2847	28.997	29.5516	29.4001	29.3863	29.4076	29.772	29.7696	29.4682	29.5659
29.216	29.3885	29.1352	29.276	29.087	28.7331	29.6488	29.6263	32.3633	32.3087	32.5126	32.7689
31.0029	30.9168	30.2672	30.4304	29.3924	29.5874	29.4505	29.9432	30.493	30.4811	30.8843	30.8849
29.5748	29.7954	29.4172	29.4285	28.8608	29.4428	28.88	29.3477	29.8111	29.7113	29.8187	29.7111
28.607	28.6947	28.4115	28.5	28.3172	28.9004	28.456	28.6918	28.9217	29.0429	28.8144	29.0674
29.415	29.4054	28.2572	27.8395	28.206	29.8649	28.1926	28.6338	29.5306	29.4886	29.6307	30.1222
#NUM!	#NUM!	#NUM!	#NUM!	#NUM!	#NUM!	#NUM!	#NUM!	28.1196	27.9572	28.01	28.1421
28.3829	28.1485	#NUM!	28.3228	#NUM!	#NUM!	#NUM!	#NUM!	28.4165	28.5524	28.8874	28.773
31.845	31.8562	32.0071	31.9658	31.7272	32.0684	31.8007	31.1715	32.7457	32.6247	32.7832	32.6036
28.832	28.892	29.2862	29.1413	#NUM!	28.4411	28.1521	28.3425	28.5691	28.7178	29.01	29.0082
30.0472	30.0993	29.0187	29.0096	28.6568	30.0052	28.8255	29.1158	30.2915	30.0855	30.1301	30.1453
30.636	30.6695	29.7605	29.8851	29.0749	30.2144	29.1659	29.2844	31.279	31.349	31.1755	31.215
29.5441	29.6475	29.1252	28.9436	28.8954	29.8045	28.8453	29.3446	30.6586	30.6512	30.8599	30.8097
29.3926	29.4886	29.5885	29.2011	28.962	28.8734	28.9684	29.5051	28.5732	28.737	28.4847	28.8254
31.0554	31.0883	30.9422	31.1058	30.8831	30.7248	30.872	30.6274	31.0297	31.1265	31.1036	31.1104
31.9735	31.9837	31.7138	32.0731	31.3076	31.453	31.2451	31.0031	32.4477	32.4554	32.8787	32.5921
28.6345	28.5377	#NUM!	27.3695	#NUM!	28.9381	#NUM!	28.6923	29.4679	29.4961	29.5269	29.6033
#NUM!	27.8756	#NUM!	#NUM!	#NUM!	#NUM!	#NUM!	#NUM!	27.5399	27.5727	27.6277	27.7868
30.1498	30.1735	28.9621	29.2888	29.4827	30.8626	29.2644	31.0943	27.8889	28.1208	27.9775	28.3072
#NUM!	#NUM!	#NUM!	#NUM!	#NUM!	#NUM!	#NUM!	#NUM!	#NUM!	#NUM!	25.7848	25.8371
#NUM!	#NUM!	#NUM!	#NUM!	#NUM!	#NUM!	#NUM!	#NUM!	26.0947	26.2547	25.9965	26.0942
28.9583	28.9981	28.8774	29.3478	28.6772	28.8191	28.671	28.1324	30.2269	30.1787	30.2662	30.2153
#NUM!	26.0353	#NUM!	#NUM!	#NUM!	#NUM!	#NUM!	#NUM!	27.2412	27.3743	27.2685	27.0475
29.2382	29.1893	29.1546	29.2679	#NUM!	28.6808	#NUM!	27.6774	29.2761	29.3107	29.5058	29.6195
29.3916	29.3369	29.3235	29.7484	29.0198	29.3603	28.8519	29.2321	29.4842	29.4066	29.3223	29.3611
#NUM!	26.5991	#NUM!	#NUM!	#NUM!	#NUM!	#NUM!	#NUM!	28.1659	28.2639	27.983	28.1566
28.2454	28.3564	28.122	27.9035	#NUM!	28.2637	27.5442	27.9657	28.4529	28.4925	28.6427	28.5318
28.3782	28.4919	28.1961	28.5781	#NUM!	28.385	#NUM!	27.5641	29.4027	29.2326	29.6039	29.6307
28.1443	28.175	27.7784	27.4477	#NUM!	27.934	#NUM!	#NUM!	27.8391	27.8952	28.1493	28.3783
#NUM!	#NUM!	#NUM!	#NUM!	#NUM!	#NUM!	#NUM!	#NUM!	26.782	26.9158	27.1783	27.11
#NUM!	26.6195	27.3566	27.3833	#NUM!	#NUM!	#NUM!	#NUM!	27.066	27.2227	27.2996	27.0606
29.5807	29.463	29.508	29.7703	28.3502	28.3982	29.0649	27.7356	30.7965	30.2174	30.2746	30.061
30.4105	28.8959	28.0908	28.4615	29.9792	29.3027	#NUM!	29.1165	29.7829	29.7939	29.8485	29.8822
28.0076	28.3845	27.7344	27.0326	#NUM!	28.8049	#NUM!	28.1403	28.4163	28.5184	28.2708	28.2401
30.2156	30.3638	29.5593	29.619	29.2091	29.6548	29.5286	29.4422	25.7275	25.747	25.3245	24.9861
#NUM!	#NUM!	27.5439	27.7	#NUM!	27.2027	#NUM!	#NUM!	29.9765	30.0945	30.185	30.078
30.2308	30.3633	29.1564	29.1449	29.0406	30.6989	29.8837	30.2945	30.5248	30.662	30.9133	30.9781
30.335	30.3759	28.9775	28.7963	29.4963	30.9343	29.2065	31.2024	28.9674	29.202	29.1602	29.317
29.4764	29.407	28.8915	29.0739	28.7923	29.8009	28.9206	29.2719	30.6206	30.7325	30.6512	30.6941
#NUM!	#NUM!	#NUM!	#NUM!	#NUM!	#NUM!	#NUM!	#NUM!	27.1185	27.308	26.8872	27.2422
29.927	29.8433	30.1146	29.9441	30.1484	30.4616	30.1952	30.1559	27.8705	27.7864	28.3126	28.0011
#NUM!	#NUM!	#NUM!	#NUM!	#NUM!	#NUM!	#NUM!	#NUM!	28.8245	28.5561	28.2741	28.6272
27.8946	27.8125	27.6268	27.9695	28.2538	28.5309	28.779	28.2635	28.4238	27.9811	27.9428	27.8231
29.3301	29.2874	29.3885	28.8607	#NUM!	29.6188	#NUM!	29.4619	30.2352	30.1947	30.2312	30.2023
28.8516	28.8102	28.007	28.2126	28.0568	28.696	28.6907	28.4201	29.1944	29.2813	29.3908	29.4389
28.8217	28.9355	28.3236	28.5207	#NUM!	29.12	27.6813	29.0276	28.5108	28.5803	28.9545	28.9061
#NUM!	#NUM!	#NUM!	#NUM!	#NUM!	#NUM!	#NUM!	#NUM!	27.4785	26.9072	27.2959	27.3581
28.972	28.9659	28.8521	28.394	28.3477	28.9167	28.5937	28.3109	30.0117	29.9853	30.4049	30.3083
29.5645	29.4649	28.5141	29.3358	28.8576	29.9393	29.0412	29.7448	30.2048	30.2862	30.5877	30.404
27.1587	27.1061	#NUM!	#NUM!	#NUM!	#NUM!	#NUM!	#NUM!	28.3989	28.1878	28.5501	28.566
29.1587	29.0912	28.6964	28.6651	27.8951	28.6644	28.2644	28.8953	28.2455	28.3963	28.4069	28.5664
28.7326	28.6701	28.9175	28.9059	28.5106	28.2725	29.0231	28.2216	30.1181	30.1805	30.3301	30.2788
29.2948	29.2499	29.4563	29.6033	28.7762	28.9625	28.5588	28.3798	30.1426	29.8103	29.6348	29.4825
#NUM!	#NUM!	#NUM!	#NUM!	#NUM!	28.0912	#NUM!	28.4989	27.2545	27.5456	27.7525	27.826
26.409	#NUM!	#NUM!	#NUM!	#NUM!	26.1874	#NUM!	#NUM!	26.6103	26.8711	27.0382	27.2076
29.4382	28.9469	28.6412	28.5926	#NUM!	29.1823	#NUM!	28.9063	27.9139	27.688	27.5289	27.2533
#NUM!	27.2324	27.4284	#NUM!	#NUM!	#NUM!	#NUM!	#NUM!	27.1264	27.3106	27.2797	27.4005
#NUM!	27.7198	#NUM!	28.2926	#NUM!	27.9532	#NUM!	#NUM!	27.6836	27.8373	27.7693	27.8415
30.3966	30.5399	30.5523	30.3995	29.5097	30.1734	29.1479	30.2234	30.2034	30.2425	30.0831	30.0488
#NUM!	#NUM!	#NUM!	#NUM!	#NUM!	#NUM!	#NUM!	#NUM!	27.0815	26.7116	27.0774	27.1121

26.6186	26.6768	#NUM!	#NUM!	#NUM!	#NUM!	#NUM!	#NUM!	26.2907	26.5518	26.4487	26.3035
#NUM!	26.8731	#NUM!	27.394	#NUM!	#NUM!	#NUM!	#NUM!	26.1155	26.2059	26.5655	26.886
26.1799	26.2397	#NUM!	#NUM!	#NUM!	#NUM!	#NUM!	#NUM!	26.7661	26.7961	26.6459	26.862
#NUM!	27.0654	#NUM!	#NUM!	#NUM!	27.9085	#NUM!	26.6152	27.3119	27.204	27.0672	26.9944
29.762	29.7633	29.7023	29.7537	30.1281	29.7954	30.1539	29.4175	27.3305	27.0157	27.0303	27.1198
30.0421	29.9607	29.9611	29.7928	29.2923	28.8177	29.2668	28.6106	29.7124	29.636	30	30.0009
27.6136	27.7864	27.7735	27.2426	#NUM!	27.6383	#NUM!	27.7632	26.6166	27.1554	26.6779	26.9572
28.504	28.4151	28.4494	28.4536	#NUM!	27.5529	#NUM!	#NUM!	29.5665	29.7166	29.5882	29.5573
29.4085	29.3098	28.9421	29.2125	28.2716	28.2429	28.7834	28.4003	29.1078	29.0762	29.1206	28.9069
#NUM!	#NUM!	#NUM!	#NUM!	#NUM!	#NUM!	#NUM!	#NUM!	27.254	27.0627	27.4733	27.5332
#NUM!	#NUM!	#NUM!	27.0349	#NUM!	#NUM!	#NUM!	#NUM!	#NUM!	26.4712	25.4862	#NUM!
26.9469	27.2844	#NUM!	27.3435	#NUM!	27.4377	#NUM!	#NUM!	29.8736	29.8744	29.7309	29.9663
#NUM!	#NUM!	#NUM!	#NUM!	#NUM!	#NUM!	#NUM!	#NUM!	#NUM!	26.0345	25.5358	26.056
31.1153	31.0379	30.6181	30.9464	30.7454	30.8357	30.7584	30.5806	30.9604	30.9078	30.8846	31.1273
29.4164	29.3726	28.6745	28.857	#NUM!	29.3051	28.735	28.8397	29.6061	29.7907	29.3902	29.3742
26.6559	26.5231	#NUM!	#NUM!	#NUM!	#NUM!	#NUM!	#NUM!	27.3886	27.6024	27.7152	27.7515
#NUM!	#NUM!	#NUM!	#NUM!	#NUM!	#NUM!	#NUM!	#NUM!	31.9238	27.3552	27.405	31.3064
#NUM!	#NUM!	#NUM!	#NUM!	#NUM!	#NUM!	#NUM!	#NUM!	26.3339	26.5497	26.5201	26.5626
#NUM!	#NUM!	#NUM!	#NUM!	#NUM!	#NUM!	#NUM!	#NUM!	26.4408	26.6664	26.7495	26.8583
#NUM!	26.6629	#NUM!	26.0178	#NUM!	27.4242	#NUM!	#NUM!	28.0086	28.1209	28.1048	28.2419
#NUM!	28.6344	28.2638	28.3192	#NUM!	#NUM!	#NUM!	#NUM!	26.7325	26.6551	26.4083	26.2073
#NUM!	26.8237	#NUM!	#NUM!	#NUM!	28.0751	#NUM!	28.352	27.3779	27.5733	27.7975	27.7229
#NUM!	#NUM!	#NUM!	#NUM!	#NUM!	#NUM!	#NUM!	#NUM!	26.8632	27.0114	26.938	26.9607
29.9502	29.8259	29.5846	30.2652	29.3299	29.4377	29.3927	28.9117	30.3215	30.3626	30.4838	30.3354
#NUM!	#NUM!	#NUM!	#NUM!	#NUM!	#NUM!	#NUM!	#NUM!	26.2601	26.0912	26.7658	26.874
30.6337	30.8368	29.1797	29.1311	30.5727	31.0809	30.3345	30.2969	26.3601	26.2047	25.8412	25.9169
28.1966	28.2075	27.3255	27.8168	#NUM!	27.9379	27.524	28.0049	28.1657	28.3604	28.6645	28.7716
28.3907	28.2481	27.7079	27.8295	27.9831	28.5509	#NUM!	28.5169	28.1108	28.1729	28.2444	28.2668
28.847	28.7639	28.9389	28.7847	28.7267	29.0946	28.4497	29.4263	28.852	28.7703	28.9812	28.9589
28.5748	28.523	#NUM!	27.4851	#NUM!	#NUM!	#NUM!	28.8487	28.2735	28.4474	28.5307	28.8052
#NUM!	28.0464	#NUM!	27.0852	#NUM!	#NUM!	#NUM!	#NUM!	27.9839	27.9883	28.3125	28.284
27.4291	27.3932	#NUM!	#NUM!	#NUM!	#NUM!	#NUM!	#NUM!	25.9614	26.0856	26.4519	26.6265
32.3241	32.4688	31.8248	31.8865	34.2918	32.4929	34.5441	33.3038	29.3429	29.5862	29.6705	29.6399
30.0957	30.1095	30.6987	30.6565	29.9257	30.3386	29.9628	29.106	31.4251	31.0083	31.4298	31.3737
29.052	28.9314	28.7869	29.0399	28.9978	29.4306	28.7676	29.1023	29.5135	29.3251	29.4429	29.3714
31.9127	31.8776	31.7747	31.8861	31.7103	31.2924	31.6735	31.3097	31.8819	31.9116	31.6981	31.9414
31.1596	31.3577	30.0002	29.962	29.3166	30.5403	30.1036	29.1669	31.7766	31.5602	31.4631	31.4238
27.8559	27.7768	27.4212	27.7119	#NUM!	27.7565	28.353	29.2473	28.2117	28.3015	27.9114	28.1161
27.7876	28.4379	28.0622	27.9674	#NUM!	28.3933	28.1528	28.635	28.2663	28.2262	28.5764	28.4524
28.1918	28.096	27.6155	27.7492	#NUM!	27.8745	#NUM!	27.9769	28.481	28.5037	28.6973	28.4524
29.1546	28.9065	28.9143	29.2279	30.2847	28.5616	#NUM!	29.621	30.2774	30.3497	30.4751	30.4539
28.327	27.9486	28.5911	28.794	#NUM!	28.9692	#NUM!	28.6923	30.1515	30.0897	30.1543	30.2419
#NUM!	#NUM!	#NUM!	#NUM!	#NUM!	#NUM!	#NUM!	#NUM!	28.0121	27.9893	27.8682	28.1205
29.4766	29.477	29.6836	29.551	28.8875	28.9897	29.0119	28.9472	28.9753	29.0475	29.1347	29.1861
#NUM!	26.9883	#NUM!	27.8365	#NUM!	#NUM!	#NUM!	#NUM!	27.8021	27.5795	27.2346	27.5212
28.9647	28.9717	29.172	29.2285	28.092	29.1727	28.0083	28.5962	28.3079	28.3493	28.5125	28.4963
#NUM!	#NUM!	#NUM!	#NUM!	#NUM!	#NUM!	#NUM!	#NUM!	26.5374	26.8539	27.0012	27.2062
#NUM!	#NUM!	#NUM!	#NUM!	#NUM!	#NUM!	#NUM!	#NUM!	26.8574	26.8514	#NUM!	#NUM!
28.3639	28.4113	28.8701	28.389	#NUM!	28.1512	#NUM!	28.7393	28.6759	28.6503	28.3645	28.1616
28.5233	28.5066	28.1528	28.2771	27.9963	28.2667	28.1859	28.1838	28.0058	28.1915	28.4349	28.4667
29.2204	29.0085	28.8535	28.5816	28.173	28.0601	27.8698	27.5147	28.2302	28.1276	28.3559	28.418
#NUM!	#NUM!	#NUM!	#NUM!	#NUM!	26.9468	#NUM!	#NUM!	28.1438	28.0279	27.648	27.8232
#NUM!	#NUM!	#NUM!	#NUM!	#NUM!	#NUM!	#NUM!	#NUM!	#NUM!	#NUM!	#NUM!	28.4177
28.4162	28.2877	27.8	28.0796	27.5845	28.8712	27.9011	29.0584	30.5884	30.6046	30.9201	31.0113
#NUM!	#NUM!	#NUM!	#NUM!	#NUM!	#NUM!	#NUM!	#NUM!	28.7495	28.4475	28.8947	28.8944
32.252	30.5495	29.9619	31.8335	31.4185	33.2616	30.8568	31.6956	30.1394	29.2595	29.1799	30.9533
29.3812	29.8809	29.5937	29.7771	30.5468	29.3074	30.2864	29.0659	27.9613	27.9401	27.9965	28.1736
29.1531	29.2969	29.0163	28.9108	28.9582	29.2761	28.9147	28.7696	#NUM!	26.0909	26.2274	25.6451
30.0764	30.3817	29.2796	29.1507	28.8538	30.0495	28.8154	29.5015	31.2429	30.8953	30.9279	30.8353
#NUM!	#NUM!	#NUM!	#NUM!	#NUM!	#NUM!	#NUM!	#NUM!	26.4475	26.6879	26.8069	26.476
30.3425	30.5695	30.5057	30.6532	30.5674	30.2715	30.0778	30.0721	30.601	30.3981	30.514	30.3901
#NUM!	#NUM!	#NUM!	#NUM!	#NUM!	#NUM!	#NUM!	#NUM!	28.0213	28.1247	28.179	28.4299
#NUM!	#NUM!	#NUM!	#NUM!	#NUM!	#NUM!	#NUM!	#NUM!	28.9831	29.1019	29.0893	28.9776
#NUM!	26.923	#NUM!	26.8505	#NUM!	#NUM!	#NUM!	#NUM!	27.223	27.2498	27.6486	27.615
#NUM!	28.1961	27.9738	#NUM!	#NUM!	27.8345	#NUM!	28.1214	28.5881	28.5846	28.2557	28.7368
#NUM!	#NUM!	#NUM!	#NUM!	#NUM!	#NUM!	#NUM!	#NUM!	28.962	28.6344	28.7925	28.7036
29.9208	29.9106	29.7462	30.3325	28.6586	29.7169	28.7494	29.9223	27.4765	27.0995	27.8411	27.736
#NUM!	27.3346	27.4967	27.8866	#NUM!	28.1093	#NUM!	28.6655	27.4305	27.4841	27.5272	27.6013
28.2246	28.5108	28.4859	28.6073	#NUM!	27.9959	27.714	28.1021	28.5306	28.5565	28.505	28.79
28.5417	28.1299	#NUM!	27.9807	#NUM!	28.3778	#NUM!	28.6787	28.168	28.0731	28.4715	28.5634
32.2577	32.1759	31.5767	32.274	31.5903	31.6141	31.7064	31.1302	32.3859	32.25	32.2902	32.1898
28.4036	28.5125	28.3663	28.8674	28.5958	27.6042	28.2803	27.4324	31.0875	31.0088	31.3114	31.1808
31.5018	30.4774	30.9836	30.5657	32.5448	33.8475	31.7558	31.8914	28.7638	27.5832	28.9615	29.5537
28.1862	28.1774	27.7137	27.8724	28.3104	28.1896	27.938	27.4228	30.8234	30.5952	30.8652	30.7906
28.0085	28.4036	28.2921	28.0931	#NUM!	28.3433	#NUM!	#NUM!	28.493	28.7153	28.6018	28.6353
28.6338	28.988	28.6436	28.7354	28.3611	28.6859	28.6012	28.1312	29.9426	29.8854	30.2104	30.0772
31.3466	31.4861	30.3561	30.2824	29.6489	31.0368	29.4869	30.6393	31.8134	31.8086	31.5236	31.6456
27.8266	27.8168	28.0436	28.0601	#NUM!	27.9436	27.7919	27.0997	28.1441	28.2381	28.4705	28.5951
#NUM!	28.4003	#NUM!	#NUM!	#NUM!	27.4221	#NUM!	#NUM!	28.622	28.6143	28.5254	28.3515
#NUM!	26.1399	#NUM!	26.3148	#NUM!	#NUM!	#NUM!	#NUM!	27.557	27.5238	27.4383	27.6735
#NUM!	#NUM!	#NUM!	#NUM!	#NUM!	#NUM!	#NUM!	#NUM!	#NUM!	#NUM!	25.8251	25.2553
#NUM!	#NUM!	#NUM!	#NUM!	#NUM!	#NUM!	#NUM!	#NUM!	26.6961	27.3064	27.1434	26.8016
27.6701	27.8062	28.4877	28.1933	28.8247	27.8132	#NUM!	28.117	27.2137	27.2659	26.924	26.9421
#NUM!	25.7057	27.2014	27.0817	#NUM!	#NUM!	#NUM!	#NUM!	27.0711	27.1603	27.3032	27.3351
#NUM!	27.9223	28.5696	28.2425	#NUM!	28.7416	#NUM!	28.9158	28.3834	28.4284	28.6341	28.4298
28.0632	28.2242	28.0752	27.8987	#NUM!	27.5895	#NUM!	#NUM!	27.7681	28.4123	28.2313	28.0193
28.9034	28.3792	29.0595	28.6224	#NUM!	29.5594	#NUM!	29.4087	28.7564	28.8623	28.504	28.7277
#NUM!	#NUM!	#NUM!	#NUM!	#NUM!	#NUM!	#NUM!	#NUM!	27.4053	27.3889	26.8746	27.1974
29.2278	29.3117	29.1555	29.1935	28.6474	29.0579	28.5274	29.1314	28.0324	28.0485	28.3762	28.3764
29.0948	28.8303	29.0871	28.8239	28.3916	28.4689	28.3777	28.0972	28.9908	29.2462	29.4124	29.3005
#NUM!	#NUM!	#NUM!	#NUM!	#NUM!	#NUM!	#NUM!	#NUM!	28.0625	27.952	27.8373	27.8728
27.0996	27.4244	#NUM!	25.6994	#NUM!	28.6172	28.0675	29.0423	29.6526	29.5752	29.5052	29.5172
28.1262	27.9471	27.9875	27.9092	#NUM!	28.2736	28.2621	28.3402	28.4083	28.8113	28.6742	28.6716
24.9436	#NUM!	#NUM!	#NUM!	#NUM!	#NUM!	#NUM!	#NUM!	24.2106	24.8737	25.2239	24.9032
30.2541	30.5332	29.4993	29.4219	29.0717	30.3458	28.9642	29.8281	30.6402	30.7497	30.9921	30.8866
33.549	33.5014	33.4711	33.4914	33.5024	33.3663	33.5798	33.0768	33.5591	33.4295	33.5783	33.5292
#NUM!	27.1205	#NUM!	#NUM!	#NUM!	27.513	#NUM!	#NUM!	28.0885	28.151	28.2206	28.3038
#NUM!	#NUM!	#NUM!	#NUM!	#NUM!	#NUM!	#NUM!	#NUM!	27.9077	27.9423	27.8218	27.607
#NUM!	27.5605	26.1352	27.6219	#NUM!	27.0827	#NUM!	#NUM!	28.3878	28.4649	28.5282	28.6401
#NUM!	27.4814	#NUM!	27.4932	#NUM!	27.0569	#NUM!	#NUM!	27.9334	27.9722	28.131	28.1775
#NUM!	#NUM!	#NUM!	#NUM!	#NUM!	#NUM!	#NUM!	#NUM!	29.8505	29.7306	29.7709	29.6672
#NUM!	#NUM!	24.5284	#NUM!	#NUM!	#NUM!	#NUM!	#NUM!	27.5963	27.9124	27.6514	27.804
29.2292	29.2273	29.0272	28.9909	#NUM!	29.2283	#NUM!	29.0476	30.0997	30.1121	30.2309	30.1378
28.9049	27.336	#NUM!	#NUM!	#NUM!	27.3025	#NUM!	28.8182	28.4853	28.3886	28.3947	28.4254
29.9701	29.8679	29.5734	29.7251	30.0195	29.5887	30.0195	29.6283	30.2356	30.2139	30.0585	30.1257
#NUM!	27.054	#NUM!	#NUM!	#NUM!	27.7837	#NUM!	#NUM!	29.4515	29.4001	29.4039	29.354
#NUM!	#NUM!	#NUM!	#NUM!	#NUM!	#NUM!	#NUM!	#NUM!	26.8085	26.7092	26.5215	26.8523
26.9857	27.3211	27.7784	27.5308	#NUM!	26.5904	#NUM!	#NUM!	27.6633	27.8895	27.7629	27.8773
30.3204	30.0157	29.6734	29.5101	29.1415	30.2812	29.2816	29.5645	33.1329	33.0884	33.1805	33.1542
28.3251	28.7419	28.159	28.776	#NUM!	27.9578	27.744	28.0775	27.5795	27.7117	28.0318	27.7844
30.87	30.8854	30.7134	30.9745	32.9782	30.9438	33.0003	31.6684	27.5115	27.4903	27.9981	28.0331
28.2655	28.1313	28.3331	28.4511	27.9623	28.7544	#NUM!	28.7058	29.282	29.169	29.4404	29.3116
28.6696	28.7212	27.3606	27.4979	28.3108	29.5806	28.2698	29.7598	28.3759	28.4796	28.751	28.7597
28.651	28.5918	28.6	28.5891	#NUM!	28.739	#NUM!	28.6368	28.7543	28.6557	28.8438	28.9267
30.2733	30.2238	29.4237	29.3861	28.8836	29.7508	29.2037	29.6408	30.0105	30.1567	30.4075	30.4075
28.4297	28.4694	28.9318	28.9521	#NUM!	29.3798	#NUM!	28.3325	29.8697	29.6782	29.8244	29.9854
28.1474	28.3437	#NUM!	#NUM!	#NUM!	28.7363	#NUM!	29.6983	27.551	27.8998	27.9345	28.1377
28.1313	27.8982	27.2593	27.4442	#NUM!	27.8007	#NUM!	28.1428	27.9997	27.9036	28.3001	28.2957
29.9061	30.0153	29.2731	29.0314	28.7017	29.4624	28.2996	29.0315	30.4285	30.4708	30.2271	30.2766
#NUM!	27.7974	26.898	27.9961	#NUM!	#NUM!	#NUM!	#NUM!	28.4267	28.4532	28.3354	28.2193
29.0814	28.6903	29.1399	29.5366	#NUM!	28.4	28.6419	28.2024	29.3834	29.7358	29.4542	28.9886
#NUM!	#NUM!	#NUM!	#NUM!	#NUM!	#NUM!	#NUM!	#NUM!	27.1336	27.1456	26.9639	27.1361
27.1467	27.6355	27.2832	27.3355	#NUM!	28.8777	#NUM!	28.56	27.5299	27.5242	27.4386	27.5983
#NUM!	#NUM!	#NUM!	#NUM!	#NUM!	#NUM!	#NUM!	#NUM!	26.5751	25.8223	25.8386	25.8877
#NUM!	28.6805	27.6924	#NUM!	#NUM!	29.7174	#NUM!	29.9012	31.6163	31.6804	31.6028	31.5705
29.0794	28.967	28.2977	28.4956	27.9482	30.2352	#NUM!	29.2954	31.2424	31.1795	31.4848	31.3176
29.2534	28.9564	29.958	30.0643	28.8474	29.03	29.6	#NUM!	31.5568	31.0221	31.3158	31.3124
27.4356	27.6162	27.8935	27.3314	#NUM!	#NUM!	#NUM!	#NUM!	27.2645	27.1654	27.7461	27.5578
28.5555	28.5147	28.5707	28.5942	#NUM!	28.5647	#NUM!	28.3149	29.7808	29.853	30.2226	29.9371

29.0807	29.1883	29.2151	29.2154	30.5468	29.2717	30.7658	29.9324	28.2293	28.0111	28.4253	28.278
30.9679	29.578	30.1783	30.2546	31.7215	32.5599	31.1934	31.7672	29.233	28.2237	28.1898	30.034
29.6249	29.7048	29.4699	29.5657	29.3394	29.4967	29.2613	29.1868	30.238	30.4057	30.4056	30.4166
30.4032	30.7207	29.5019	29.1839	28.8979	30.4741	29.1366	30.1574	31.0453	30.8393	30.9655	30.8825
29.441	29.612	29.5439	29.478	28.9649	30.1747	28.9055	29.1483	30.9428	30.8548	31.1473	31.1738
32.1623	32.3171	32.5575	32.2565	32.319	32.3474	32.3235	31.5405	32.4584	32.3138	32.3443	32.4034
33.4643	34.1212	33.2862	33.4987	32.8119	31.8989	32.3863	32.9808	33.5268	33.7783	34.2769	34.2021
27.281	27.1642	27.2837	27.5274	#NUM!	27.2661	#NUM!	#NUM!	27.5106	27.5378	27.4684	27.6644
27.5122	27.5917	#NUM!	#NUM!	#NUM!	27.9723	#NUM!	#NUM!	27.3061	26.9959	27.8047	27.4721
27.5475	28.2074	28.7182	27.8984	#NUM!	28.7731	#NUM!	#NUM!	28.5225	28.6327	28.5682	28.5543
#NUM!	27.9989	27.7694	#NUM!	#NUM!	#NUM!	#NUM!	#NUM!	28.6435	28.6863	28.9237	28.97
27.5231	27.315	27.3231	27.0213	#NUM!	28.5064	#NUM!	#NUM!	27.9599	28.0146	27.92	27.9731
27.5343	27.5385	26.4901	26.7285	#NUM!	28.1568	#NUM!	28.5497	26.7963	26.9621	27.2812	27.0388
27.4924	27.5473	28.039	28.192	#NUM!	27.8973	#NUM!	#NUM!	27.871	27.8674	27.7516	27.8281
28.5964	#NUM!	#NUM!	#NUM!	#NUM!	#NUM!	#NUM!	#NUM!	28.5601	28.7343	28.5128	28.5563
27.7662	28.0358	27.967	27.7795	#NUM!	27.702	#NUM!	28.3453	28.5514	28.4776	28.5894	28.5194
30.5092	30.478	29.3233	29.2489	29.1814	30.571	29.042	29.9999	30.924	30.9176	30.9352	30.9926
#NUM!	#NUM!	#NUM!	#NUM!	#NUM!	#NUM!	#NUM!	#NUM!	27.7701	27.7872	28.1744	27.8958
27.8736	27.0374	#NUM!	#NUM!	#NUM!	28.5247	#NUM!	#NUM!	26.8551	27.037	27.2027	27.2592
30.5162	26.9075	#NUM!	#NUM!	#NUM!	26.4372	#NUM!	#NUM!	28.0312	27.8724	28.0558	28.0234
29.2105	29.1251	28.658	29.0022	28.5607	29.4689	28.7138	29.3976	30.2066	30.2084	30.2865	30.2214
28.1457	28.4871	28.4452	28.4703	#NUM!	27.8143	#NUM!	#NUM!	28.2156	28.304	28.5592	28.4859
32.5886	32.5602	29.9374	30.0962	31.6532	33.6834	31.5863	33.1301	29.8006	29.6636	29.9924	30.1935
29.5571	#NUM!	27.7349	29.3403	28.1014	28.1197	28.3202	27.5517	31.6318	31.8165	31.8494	31.7358
33.158	33.3388	33.0836	33.2235	33.8598	33.2495	33.8556	32.8829	30.2853	30.2098	30.6877	30.7814
29.4133	29.2994	29.0675	29.2033	29.0715	29.1061	29.0919	29.1636	29.1006	28.9498	29.2353	29.2692
28.7195	28.7247	28.4578	28.4268	#NUM!	28.159	#NUM!	#NUM!	26.9246	27.1838	26.8945	27.038
27.8036	27.9331	27.1407	27.487	28.3576	28.3909	28.4125	28.7305	29.2491	29.2477	29.2647	29.4293
#NUM!	27.1031	#NUM!	#NUM!	#NUM!	#NUM!	#NUM!	#NUM!	28.0986	28.1352	28.2822	28.2832
31.4747	31.258	29.5366	29.6451	32.2645	34.1537	32.3313	32.9569	27.8584	27.9881	28.3302	28.271
29.3468	29.4284	29.5906	29.3752	29.0577	29.603	29.113	29.2994	27.737	27.5853	27.7306	27.845
#NUM!	#NUM!	#NUM!	#NUM!	#NUM!	#NUM!	#NUM!	#NUM!	27.313	27.2628	27.0529	27.1682
29.397	29.6128	29.2153	29.1302	#NUM!	28.6723	28.2476	28.2044	29.2479	29.2015	29.5954	29.5874
#NUM!	#NUM!	#NUM!	#NUM!	#NUM!	#NUM!	#NUM!	#NUM!	26.5063	26.5291	25.7755	#NUM!
30.8312	30.9453	28.4999	28.3117	29.4816	30.3237	29.3254	30.0638	28.0369	28.1031	28.2809	28.2137
#NUM!	#NUM!	#NUM!	#NUM!	#NUM!	#NUM!	#NUM!	#NUM!	28.8604	28.5887	29.101	28.989
28.2973	28.4891	28.1992	28.0022	#NUM!	28.4239	28.1107	28.0705	28.4881	28.3869	28.689	28.6948
27.9779	27.1821	28.4193	28.067	#NUM!	27.6543	#NUM!	26.9918	26.8282	26.5638	26.5585	26.7716
#NUM!	27.6837	#NUM!	#NUM!	#NUM!	26.6628	#NUM!	#NUM!	28.4168	28.1898	28.2462	28.1954
#NUM!	27.349	27.5525	27.8375	#NUM!	27.3864	#NUM!	27.3831	26.7815	26.725	26.7106	26.9025
#NUM!	#NUM!	#NUM!	#NUM!	#NUM!	#NUM!	#NUM!	#NUM!	29.1403	29.0989	28.8251	28.8434
27.897	27.8731	27.5554	27.4458	#NUM!	27.972	#NUM!	27.4799	27.8988	27.8551	27.8944	28.0958
#NUM!	#NUM!	#NUM!	#NUM!	#NUM!	#NUM!	#NUM!	#NUM!	27.368	27.3768	27.3248	27.2266
32.6465	32.7187	30.5873	30.4645	31.1276	32.8267	31.0846	33.8718	30.8913	30.72	30.6919	30.8499
29.4424	29.4035	30.3388	30.1396	29.162	29.453	29.0796	28.2985	31.8496	31.647	31.5718	32.0048
28.9334	28.7999	29.5462	28.7359	30.8355	31.3542	30.5095	30.6862	27.7206	#NUM!	#NUM!	28.1404
29.6611	29.7238	29.988	30.3091	29.9664	29.6005	30.061	29.5727	28.7543	28.6857	28.8609	28.813
29.3345	29.3126	28.3398	28.3122	30.4484	31.1075	30.3621	28.987	26.6764	26.7907	26.5266	26.4664
28.2216	28.127	#NUM!	27.5992	#NUM!	#NUM!	#NUM!	#NUM!	28.6435	28.6951	28.7038	28.7135
#NUM!	#NUM!	#NUM!	#NUM!	#NUM!	#NUM!	#NUM!	#NUM!	27.0357	27.0387	27.367	27.4731
30.7625	30.814	30.4844	30.4981	30.384	30.3973	30.3977	30.2402	31.1692	31.0802	31.2567	31.4244
#NUM!	#NUM!	#NUM!	#NUM!	#NUM!	#NUM!	#NUM!	27.719	27.129	27.3733	27.267	27.3771
28.1504	28.0297	28.3178	27.9991	#NUM!	27.5801	#NUM!	#NUM!	29.1269	28.9694	29.0515	29.1228
#NUM!	#NUM!	#NUM!	#NUM!	#NUM!	#NUM!	#NUM!	#NUM!	26.9759	26.7495	26.8193	27.0295
26.656	26.2278	#NUM!	27.2479	#NUM!	27.0804	#NUM!	#NUM!	27.3214	27.6097	27.8633	27.8828
31.0388	30.2278	30.2792	30.574	31.8287	33.2569	31.1002	32.1872	30.0326	29.2342	29.4714	31.026
29.0978	29.1129	29.199	29.2494	#NUM!	28.8011	28.9307	28.4763	29.9276	29.5459	29.6463	29.7266
#NUM!	#NUM!	#NUM!	#NUM!	#NUM!	#NUM!	#NUM!	#NUM!	27.9474	28.0547	28.2781	28.2434
28.6438	28.6164	28.1348	28.6205	27.7782	29.0315	27.9689	29.2947	29.4389	29.4377	29.9371	29.9103
26.9733	27.053	#NUM!	26.3073	#NUM!	#NUM!	#NUM!	#NUM!	26.7561	26.7154	26.911	26.835
#NUM!	#NUM!	#NUM!	28.0254	#NUM!	#NUM!	#NUM!	#NUM!	28.4017	28.2493	28.4761	28.3778
29.6689	29.6219	30.0008	30.2406	29.121	29.6546	29.7168	29.0776	28.5534	28.585	28.9589	28.8555
30.115	30.0559	30.1175	30.1871	29.9536	30.2672	30.6855	29.4749	31.6521	31.4832	31.8166	31.6482
28.9848	28.967	28.8279	28.884	28.5185	29.6492	28.2421	29.935	29.2658	29.1991	29.7242	29.5715
30.1052	30.1366	30.0273	29.9076	29.4943	29.4579	29.5426	29.6888	29.9322	30.055	30.2617	30.4583
27.9636	27.6736	28.6722	28.7533	#NUM!	28.8366	#NUM!	27.1363	30.1318	29.9778	30.0348	30.0762
#NUM!	#NUM!	#NUM!	#NUM!	#NUM!	#NUM!	#NUM!	#NUM!	#NUM!	#NUM!	24.8085	24.7607
26.8133	27.7824	27.0828	#NUM!	#NUM!	28.1862	#NUM!	#NUM!	27.9991	28.2818	28.3867	28.4434
#NUM!	#NUM!	#NUM!	27.184	#NUM!	#NUM!	#NUM!	#NUM!	27.0514	26.7895	26.6376	26.7087
#NUM!	#NUM!	#NUM!	#NUM!	#NUM!	#NUM!	#NUM!	#NUM!	28.364	28.2918	28.3065	28.3855
27.4958	27.9557	27.3602	27.1468	#NUM!	27.9728	#NUM!	27.9585	27.9274	28.268	28.1923	28.1688
#NUM!	#NUM!	#NUM!	#NUM!	#NUM!	#NUM!	#NUM!	#NUM!	25.4045	25.5984	25.6718	25.4033
#NUM!	#NUM!	#NUM!	#NUM!	#NUM!	#NUM!	#NUM!	#NUM!	28.9617	28.91	29.2143	29.2425
29.7699	29.9051	29.0272	28.3137	29.7049	29.1852	29.3312	28.46	27.8706	27.9769	28.2409	28.2095
#NUM!	#NUM!	#NUM!	#NUM!	#NUM!	#NUM!	#NUM!	#NUM!	#NUM!	#NUM!	25.3416	#NUM!
30.3094	30.5184	29.1289	29.1142	29.2812	30.1809	29.0846	29.8083	30.7069	30.8967	30.7882	30.9686
27.2287	27.3948	27.7159	#NUM!	#NUM!	28.7164	#NUM!	#NUM!	29.0624	29.1007	29.1305	29.0579
30.0893	30.0489	29.1	29.5223	29.1685	30.3995	29.6626	30.2815	30.6481	30.551	30.5944	30.491
29.0599	29.074	28.8389	28.7656	28.2475	28.5484	28.1211	28.454	29.3747	29.6156	29.6594	29.7753
#NUM!	#NUM!	#NUM!	#NUM!	#NUM!	#NUM!	#NUM!	#NUM!	27.2939	27.4321	27.5642	27.6922
31.2394	31.3581	30.93	31.1127	30.4245	31.3529	30.6236	30.9748	32.1731	32.2656	32.4797	32.4803
32.49	32.3853	32.5826	32.9377	31.5106	31.7226	31.4423	30.7185	33.5611	33.2787	33.417	33.4774
#NUM!	#NUM!	#NUM!	#NUM!	#NUM!	#NUM!	#NUM!	#NUM!	27.2482	27.3193	27.4267	27.2114
#NUM!	#NUM!	#NUM!	#NUM!	#NUM!	#NUM!	#NUM!	#NUM!	25.4465	27.0893	25.5666	25.7177
#NUM!	#NUM!	#NUM!	#NUM!	#NUM!	#NUM!	#NUM!	#NUM!	27.1426	27.3334	27.9094	27.9026
29.513	29.6094	26.7702	27.0505	28.5271	28.8524	28.446	29.0322	26.9132	27.0014	27.3141	27.0276
#NUM!	#NUM!	#NUM!	#NUM!	#NUM!	#NUM!	#NUM!	#NUM!	27.4457	27.4853	27.5355	27.5263
29.4551	29.4644	29.6877	29.7559	29.6271	29.5141	29.7821	29.5556	30.4673	30.494	30.7027	30.6218
#NUM!	#NUM!	#NUM!	#NUM!	#NUM!	#NUM!	#NUM!	#NUM!	#NUM!	#NUM!	#NUM!	#NUM!
31.9651	32.0785	31.3853	31.467	32.146	31.493	32.0813	31.2899	28.0644	28.245	28.2869	28.3699
28.7229	28.9114	28.3414	28.4071	27.9864	28.3123	27.916	27.8188	28.4896	28.7846	28.6569	28.6639
30.4714	30.3111	29.815	30.2888	29.8797	30.3231	30.0417	30.5764	30.5144	30.6503	30.5626	30.5481
28.916	28.8305	26.943	27.2944	#NUM!	29.6942	#NUM!	30.1289	26.8806	27.1249	27.1157	27.0346
29.7116	29.7545	29.3756	29.3331	29.2894	29.7391	29.3533	29.4308	29.9176	29.9931	30.2944	30.34
#NUM!	27.6232	#NUM!	#NUM!	#NUM!	#NUM!	#NUM!	#NUM!	30.0466	30.0379	30.0283	30.236
34.2378	34.0303	32.8249	32.9674	33.2719	35.1271	33.0657	35.4442	32.4672	32.4199	32.9297	32.7105
31.4251	31.5517	31.411	31.6877	30.3506	31.1646	30.7606	31.2138	31.9536	31.9891	32.5692	32.1792
#NUM!	26.8989	#NUM!	27.2844	#NUM!	#NUM!	#NUM!	#NUM!	26.7105	26.6171	26.4673	26.9267
29.3237	29.1436	29.0144	29.5772	#NUM!	28.1448	29.1199	29.2563	29.4112	29.2903	29.4015	29.2369
32.715	31.2633	31.273	31.8014	32.8036	34.3563	32.1708	32.7717	30.0913	29.1807	29.7737	31.0382
34.0452	34.1935	33.7175	33.9012	33.3725	34.1562	33.4035	34.237	34.6976	34.718	34.8478	34.7961
28.7747	28.7503	28.3491	#NUM!	#NUM!	29.3399	#NUM!	29.4257	30.8469	30.7382	30.9618	31.0039
27.811	27.8151	26.8744	27.4763	#NUM!	27.59	#NUM!	28.3582	29.4254	29.6134	29.8651	29.9759
29.0096	29.1546	29.4536	29.6839	28.2499	29.0396	28.3186	28.3376	31.3736	31.1731	31.4601	31.1563
26.8978	#NUM!	#NUM!	#NUM!	#NUM!	26.0812	#NUM!	#NUM!	27.5049	27.5824	27.5447	27.4101
29.7682	29.7546	29.3058	29.4081	29.318	28.6092	29.2172	28.4408	29.6317	29.5077	30.0605	29.9861
#NUM!	27.3765	#NUM!	26.4731	#NUM!	27.3183	#NUM!	27.595	27.9566	28.133	28.181	28.1824
29.5863	29.8296	29.5288	29.362	29.1743	29.4691	29.2565	29.6063	30.3371	30.2559	30.2626	30.2309
29.4826	29.3763	29.5198	29.667	29.3832	30.086	29.4092	30.0977	29.1793	29.0506	29.307	29.2514
26.869	27.0339	#NUM!	#NUM!	#NUM!	28.1083	#NUM!	#NUM!	27.5041	27.4695	27.5522	27.7191
27.1015	27.3546	27.3256	27.0754	#NUM!	26.8414	#NUM!	26.3768	27.4993	27.8142	28.0127	28.0207
27.4079	27.3317	26.9061	29.3443	#NUM!	27.9141	#NUM!	28.1542	27.8361	27.8231	28.0456	28.0482
#NUM!	#NUM!	#NUM!	#NUM!	#NUM!	#NUM!	#NUM!	#NUM!	27.3932	27.5318	27.375	27.3827
28.4513	28.5135	29.2169	29.321	29.1276	28.9816	29.1059	28.7093	27.3238	27.2874	27.2215	27.442
27.8375	27.7887	28.4476	27.9607	#NUM!	28.2168	#NUM!	#NUM!	29.8179	29.6441	29.4877	29.4539
28.4317	28.6549	28.6275	28.5477	#NUM!	28.8949	#NUM!	#NUM!	29.0972	29.0694	29.2586	29.2365
28.3096	28.3975	28.1371	28.0993	#NUM!	27.8605	#NUM!	28.0089	27.8659	28.0204	28.019	28.1996
29.7366	29.7834	28.9966	28.9847	30.3121	29.7487	30.423	30.1762	28.7682	28.8049	28.7441	28.9493
#NUM!	26.9511	#NUM!	#NUM!	#NUM!	26.9149	#NUM!	#NUM!	27.4374	27.9485	27.8551	27.7397
#NUM!	#NUM!	#NUM!	#NUM!	#NUM!	#NUM!	#NUM!	#NUM!	26.275	26.4286	26.4866	26.4211
34.8956	34.8172	34.3965	34.6407	34.1815	35.0054	34.2092	35.3947	34.9326	34.9455	35.3663	35.1262
31.3675	30.5251	30.9435	31.2807	31.558	33.0503	30.9499	32.0787	29.2191	28.9417	28.9002	29.8719
32.5088	32.7659	31.5166	31.5592	33.9794	32.691	33.7648	33.7495	30.2038	30.306	30.1829	30.1634
#NUM!	#NUM!	#NUM!	#NUM!	#NUM!	#NUM!	#NUM!	#NUM!	#NUM!	#NUM!	#NUM!	#NUM!
30.7797	30.7992	30.4007	30.6031	29.6587	30.1986	29.6675	30.1746	29.5936	29.3539	29.6272	29.508
27.0869	27.0884	26.4318	26.6822	#NUM!	27.084	#NUM!	#NUM!	28.2705	28.204	28.53	28.5341
28.8775	28.8537	28.3948	28.4012	#NUM!	27.7856	#NUM!	27.9218	28.4932	28.8167	28.6065	28.7828
29.5217	29.6694	29.4712	29.8005	29.1424	29.1392	29.2374	29.3285	29.9017	29.7969	30.1387	30.2425
27.4674	27.5983	#NUM!	#NUM!	#NUM!	27.1739	#NUM!	27.3282	28.1831	28.2176	28.3741	28.2794

27.5019	27.2747	27.6483	27.5176	#NUM!	27.7509	#NUM!	#NUM!	29.2461	29.3256	29.5193	29.5334
33.7547	33.5878	32.6556	32.9848	32.6535	32.4613	32.9507	31.9359	31.1961	31.2871	31.5737	31.5925
27.6712	28.1155	27.6053	27.7533	28.2357	28.9093	28.3961	28.5651	28.8229	28.9076	29.1901	29.0207
28.5219	28.6125	28.7983	28.4347	#NUM!	28.6336	28.941	28.5863	29.2031	29.2898	29.3777	29.4592
31.6984	30.5838	30.449	30.6149	31.4275	33.4495	30.2297	31.9489	29.248	28.1303	28.15	29.6358
29.9667	29.8791	30.063	30.0702	28.7689	29.1934	29.0489	29.0725	29.5541	29.4955	29.5274	29.4785
28.8985	28.8918	28.6411	28.8417	28.2893	27.6697	27.9095	#NUM!	29.8205	29.8078	29.9715	29.8992
31.7712	31.8367	29.6986	29.694	30.9527	30.4612	30.8611	31.0078	27.7954	27.6141	27.7604	28.1165
#NUM!	#NUM!	#NUM!	#NUM!	#NUM!	#NUM!	#NUM!	#NUM!	28.0514	28.1142	28.0775	28.0314
#NUM!	#NUM!	#NUM!	#NUM!	#NUM!	#NUM!	#NUM!	#NUM!	27.9324	27.9493	28.086	27.7995
27.4321	27.6156	#NUM!	#NUM!	#NUM!	27.4072	#NUM!	#NUM!	28.7148	28.6927	28.8256	28.7275
27.9028	27.674	28.1454	27.9995	27.6806	28.7659	28.1249	28.7076	28.6433	28.8407	29.1438	29.0354
32.5164	32.6354	31.923	32.123	31.6402	31.8504	31.541	31.103	32.4614	32.4942	32.7146	32.7015
28.9582	28.5205	27.4936	27.0173	#NUM!	29.0346	#NUM!	29.2814	27.138	26.9559	26.6307	27.0666
27.7698	28.0032	27.9485	27.8752	#NUM!	27.1977	27.5685	#NUM!	29.4128	29.6941	29.8647	29.7571
30.0714	30.2364	29.6354	29.82	29.3195	29.8885	29.5267	29.9797	30.0398	30.3004	30.4855	30.3727
28.2742	27.9712	28.0457	28.3186	#NUM!	27.6638	#NUM!	#NUM!	29.3178	29.2996	29.5507	29.6907
30.7177	30.8032	29.6116	29.825	30.5334	31.0313	30.7467	30.5575	28.476	28.4869	28.4848	28.7131
28.0036	28.0167	28.0845	28.106	27.9387	28.7625	28.231	28.7965	28.3218	28.4643	28.3448	28.4224
27.9387	27.8919	#NUM!	27.4828	#NUM!	28.5455	#NUM!	#NUM!	27.4962	27.4923	27.9235	28.0002
32.85	32.9521	32.8929	33.0879	32.7623	32.4686	32.6788	32.237	33.1376	33.1194	33.2829	33.2168
29.0677	29.2861	29.9146	29.7095	#NUM!	29.002	#NUM!	29.1837	27.7167	27.513	27.5985	27.6822
28.532	28.6182	28.4849	28.337	#NUM!	28.2763	#NUM!	28.1938	28.6321	28.865	28.8915	28.9295
29.9296	29.7947	29.437	29.3667	29.2817	29.5651	29.243	29.7899	30.2533	30.2678	30.4182	30.5086
28.3188	28.4027	27.8857	27.8449	#NUM!	28.8964	#NUM!	#NUM!	30.0455	29.7992	29.9392	29.8337
#NUM!	#NUM!	#NUM!	#NUM!	#NUM!	#NUM!	#NUM!	#NUM!	27.2473	27.1092	27.4045	27.465
#NUM!	#NUM!	#NUM!	#NUM!	#NUM!	#NUM!	#NUM!	#NUM!	27.9816	27.9228	28.1691	28.1163
36.6093	36.9217	37.0289	36.78	36.4219	36.3973	36.2458	35.8455	36.6231	36.5445	36.6945	36.6605
27.1529	27.1951	28.2945	28.4737	#NUM!	27.0595	#NUM!	#NUM!	29.3182	29.0736	29.1639	29.139
28.6538	28.6378	28.7802	28.8626	28.4786	28.8649	28.4728	28.4176	29.6403	29.5945	29.9479	29.9856
#NUM!	#NUM!	28.0529	28.4943	#NUM!	#NUM!	#NUM!	#NUM!	27.276	27.418	27.4314	27.3678
#NUM!	#NUM!	#NUM!	#NUM!	#NUM!	#NUM!	#NUM!	27.4109	28.4557	27.999	28.1299	28.2538
#NUM!	27.009	#NUM!	28.4033	#NUM!	27.998	#NUM!	#NUM!	28.1732	28.1498	28.3523	28.3903
27.5341	27.413	27.2897	27.6172	27.1374	27.6351	#NUM!	27.7556	27.6838	27.6185	27.7755	27.58
28.9157	29.2127	29.2501	29.1429	28.7116	29.2929	28.4618	29.3844	28.7467	28.71	28.9037	28.9331
28.3086	27.2641	#NUM!	#NUM!	#NUM!	#NUM!	#NUM!	#NUM!	28.6765	28.6605	28.786	28.846
27.288	26.9917	26.7438	27.3544	#NUM!	#NUM!	#NUM!	27.1994	28.6598	28.7394	28.7168	28.7781
#NUM!	#NUM!	#NUM!	#NUM!	#NUM!	#NUM!	#NUM!	#NUM!	28.4376	#NUM!	#NUM!	29.0073
30.9114	30.9971	28.6162	28.3139	28.9763	30.5669	29.0904	29.8368	27.5525	27.8225	27.6592	27.6772
30.8171	30.8249	30.8881	30.9687	30.3158	30.4629	30.2033	30.7864	30.566	30.6752	30.8951	30.8181
33.6316	33.5719	32.0886	32.5312	31.5394	31.7815	31.6601	31.4014	29.8863	29.8697	30.3979	30.4203
35.0241	34.922	34.274	34.3442	34.3238	35.1976	34.081	35.1622	34.7829	34.5793	34.9829	34.795
25.6036	26.7379	27.5444	26.9739	#NUM!	26.7448	#NUM!	#NUM!	30.6381	30.5891	30.7176	30.7289
#NUM!	27.5156	27.1708	26.945	#NUM!	#NUM!	#NUM!	#NUM!	28.7912	28.7297	28.8073	28.85
#NUM!	#NUM!	#NUM!	#NUM!	#NUM!	#NUM!	#NUM!	#NUM!	27.4306	27.6056	27.4509	27.5024
#NUM!	#NUM!	#NUM!	#NUM!	#NUM!	#NUM!	#NUM!	#NUM!	27.2395	27.2547	27.319	27.4803
29.8915	29.9266	29.6051	29.6191	29.0693	29.5546	29.1448	29.3858	30.365	30.4768	30.3742	30.4307
29.3518	29.4568	29.0106	28.9826	28.9912	29.2428	28.7503	29.6291	29.4901	29.4798	29.6097	29.6237
28.2581	28.6616	28.4813	28.4501	#NUM!	28.5092	#NUM!	28.4793	28.3049	28.5411	28.7253	28.6402
30.157	30.1413	29.4125	29.4513	28.9682	30.1438	28.747	29.6375	30.8111	30.7499	30.9813	30.9071
31.8447	29.7021	29.6099	30.0292	31.2876	32.6949	30.7422	31.2348	29.8049	29.3045	28.3222	29.8288
30.0371	28.4485	28.7608	28.3583	#NUM!	28.121	#NUM!	#NUM!	29.5874	29.5804	29.5719	29.4116
33.9179	33.9851	33.5504	33.6022	33.4214	34.0757	33.3861	34.0149	34.5162	34.4625	34.694	34.7692
#NUM!	#NUM!	#NUM!	#NUM!	#NUM!	#NUM!	#NUM!	#NUM!	27.4069	27.5124	27.9388	27.9886
28.4996	28.3654	27.8164	28.3859	#NUM!	29.0831	27.8616	28.8903	28.8597	29.0623	29.0889	29.0413
29.1771	29.3139	29.0234	29.0582	28.9242	29.4493	28.9767	29.6312	29.1943	29.2757	29.5195	29.4736
27.2544	27.3237	26.9752	25.9299	#NUM!	27.4592	#NUM!	28.3479	27.031	27.0789	27.1808	27.0209
29.5139	29.3757	29.3951	29.6367	29.0115	28.5595	28.6806	28.4659	26.0849	26.7213	26.8927	26.8146
31.7325	32.0497	31.8728	31.6288	32.3928	31.5625	32.3888	31.1392	29.0786	29.2951	29.0191	29.1706
29.3804	29.7151	29.4447	29.4113	29.2067	29.8202	29.3517	29.775	29.7414	29.6799	29.6488	29.684
27.9198	28.1764	28.1131	28.3016	27.9557	28.0568	28.1451	28.4199	27.9566	28.0276	28.3144	28.3462
27.2175	27.4813	27.7491	27.8433	#NUM!	27.6768	27.7839	26.6662	30.5606	30.4744	30.8644	30.8452
29.2337	29.196	29.2759	29.0342	28.4194	29.0268	28.2895	28.4779	29.875	29.7948	30.1584	30.2227
31.3625	31.4088	30.565	30.7444	31.0929	31.7692	31.1788	31.8066	30.8288	30.8159	31.181	31.0892
29.3206	29.1713	32.4452	32.3137	#NUM!	29.4317	33.139	33.46	31.3418	31.24	31.5295	31.5534
30.3233	30.398	28.9971	29.1395	28.8055	29.7035	28.6343	29.5565	30.8498	30.8125	30.658	30.5715
28.1647	28.3991	27.875	28.207	28.3385	28.4056	28.5758	28.1547	29.6913	29.722	29.9376	29.9518
28.2729	27.9173	28.0603	27.5195	#NUM!	28.2009	#NUM!	27.5033	29.5347	29.5182	30.0568	29.9873
29.1255	28.9015	28.5231	28.5611	30.0493	29.985	28.5211	29.8124	29.5122	29.4568	29.3712	29.4514
#NUM!	26.9445	#NUM!	27.2832	#NUM!	28.109	#NUM!	#NUM!	27.6366	27.6318	27.6446	27.6413
26.4955	#NUM!	#NUM!	#NUM!	#NUM!	#NUM!	#NUM!	#NUM!	28.4424	28.438	28.3104	28.4394
29.8032	30.0671	29.5216	29.7693	29.1008	29.4362	29.4603	29.0481	30.927	30.8253	30.9084	31.0505
29.1542	29.3432	29.4539	29.5486	29.0913	29.3917	28.864	29.3274	31.849	31.8839	31.9609	31.8892
30.1942	30.3308	30.65	30.5936	30.4832	29.9958	30.405	29.8667	28.3459	28.4903	28.3775	28.4249
29.9545	29.9201	28.4524	28.3658	29.419	30.4511	29.4716	30.3546	25.8951	26.375	25.8598	25.9826
31.8808	31.731	31.0731	31.4655	31.2409	31.3564	31.4335	31.5606	31.8483	31.8768	32.0072	32.1165
27.32	27.5307	27.862	27.1239	#NUM!	28.6644	#NUM!	28.8838	28.1527	28.1433	28.3229	28.2017
#NUM!	27.7433	27.9235	#NUM!	#NUM!	#NUM!	#NUM!	28.8837	27.8946	28.11	27.9381	28.0245
28.5368	28.5591	28.4869	28.4695	#NUM!	28.589	#NUM!	#NUM!	28.7845	28.7358	29.1991	29.0257
27.9646	27.7639	27.0841	#NUM!	27.1477	27.8819	27.5338	28.2287	27.9524	28.0647	28.2487	28.376
28.6116	28.5442	28.3272	28.2381	28.5373	28.4376	28.1713	27.8907	31.1164	31.1446	31.3082	31.3678
30.5988	30.622	30.8186	30.9609	30.1009	30.4987	30.2213	29.8372	29.8533	29.9197	29.8956	29.8507
31.8945	31.7442	28.7687	29.0968	30.5443	32.3787	30.4515	31.661	26.7752	26.8561	26.9937	26.7553
#NUM!	27.3117	26.9098	27.307	#NUM!	28.0258	#NUM!	28.6356	28.2217	28.2874	28.3382	28.2291
29.6629	29.8902	29.5339	29.6822	28.7602	28.6746	28.7547	28.0453	29.1699	29.1982	29.5319	29.4781
28.9349	28.7393	28.5257	28.8376	#NUM!	28.4122	#NUM!	#NUM!	29.9033	29.8673	29.9375	30.0114
27.5895	28.1369	27.5145	27.5805	28.4395	28.3189	#NUM!	#NUM!	30.7442	30.5508	30.4424	30.3906
29.033	28.8676	28.2646	28.1351	28.8231	29.6112	28.1536	29.1039	30.8262	30.7603	30.9331	30.9082
28.9913	28.8868	28.3607	28.5738	#NUM!	30.0272	27.7787	29.8176	30.38	30.2831	30.4277	30.5244
28.7679	28.8345	27.751	27.8838	#NUM!	28.5369	28.6482	29.01	28.2906	28.3849	28.4004	28.463
30.2314	30.3462	29.9079	30.1462	30.0835	29.9483	29.8319	30.0967	30.7251	30.7391	30.8466	30.856
29.0925	29.1015	28.7485	28.9445	#NUM!	28.7102	28.3161	29.2742	29.8633	29.842	29.8746	29.9509
#NUM!	#NUM!	#NUM!	#NUM!	#NUM!	#NUM!	#NUM!	#NUM!	#NUM!	25.7937	26.1645	26.6769
35.0563	34.9607	33.8207	34.3313	33.7815	33.7153	33.8427	32.9508	30.0627	30.1855	30.4337	30.4803
32.8888	33.0397	32.3554	32.4956	32.2073	32.8727	32.1685	32.3642	32.96	32.8469	32.7254	32.7107
30.3533	30.2478	29.4671	29.2764	28.9398	30.6912	29.6316	30.8775	31.6227	31.4742	31.6611	31.5694
#NUM!	#NUM!	#NUM!	#NUM!	#NUM!	#NUM!	#NUM!	#NUM!	26.5278	26.3979	25.8443	26.7165
28.9317	28.7504	28.9508	28.8113	28.5216	28.9811	28.8017	28.9113	32.4182	32.3517	32.3872	32.3699
28.6341	28.4737	27.3749	27.6426	28.1264	29.3335	27.9494	29.3294	28.1508	28.0819	28.5066	28.4284
29.5083	29.3903	29.4982	29.2996	28.8794	28.6418	28.8249	28.4745	29.604	29.435	29.4274	29.5491
29.1859	29.1153	28.7781	28.8635	29.3259	30.3465	29.0475	30.1349	29.8979	29.8678	29.8359	30.0005
30.2164	30.5481	29.4722	29.193	29.0826	30.2431	29.2111	29.4863	30.9009	30.932	30.7996	30.9281
#NUM!	#NUM!	#NUM!	#NUM!	#NUM!	27.9437	#NUM!	#NUM!	28.3128	28.3507	28.5716	28.6739
#NUM!	#NUM!	#NUM!	#NUM!	#NUM!	#NUM!	#NUM!	#NUM!	#NUM!	26.8771	28.0679	#NUM!
33.4995	33.5538	33.3076	33.3347	33.0751	33.1227	33.0904	32.8979	33.8304	33.5701	33.4102	33.3495
30.4383	30.4795	30.2004	31.0458	29.5768	29.7275	29.6313	29.85	31.6257	31.6303	32.0846	31.9382
27.6867	27.7683	28.4396	28.0158	#NUM!	#NUM!	#NUM!	#NUM!	27.5812	27.5333	27.5863	27.8185
29.187	29.1693	29.2455	29.2122	28.7826	29.2055	28.7474	29.3378	30.0531	30.0198	30.0289	30.0475
30.0716	30.217	29.1692	29.4527	32.0403	33.068	32.0256	32.9889	27.1986	27.2834	27.3307	27.3158
26.5804	26.4696	27.112	26.7172	#NUM!	27.5301	27.5738	26.7349	31.5242	31.4393	31.7159	31.722
30.9101	31.1216	29.7802	29.8551	29.8093	30.3499	29.6907	30.7432	28.346	28.5158	28.6834	28.5834
33.0921	33.1457	31.5416	31.7862	30.7928	30.7118	30.8854	30.4441	27.0011	26.84	27.2855	27.0225
29.5476	29.5208	29.4991	29.5622	29.5083	29.5554	29.4345	29.4992	29.8563	29.9643	30.0749	30.1312
28.8231	29.0063	29.0214	28.7908	28.349	28.8696	28.1218	28.9184	29.0561	29.0974	29.1468	29.094
26.8865	26.9535	#NUM!	#NUM!	#NUM!	27.3398	#NUM!	27.6978	27.9184	27.8444	27.8711	27.8882
27.8	27.7798	27.6502	27.8618	#NUM!	27.8982	#NUM!	27.7987	28.5699	28.602	28.8285	28.8178
27.615	27.7331	#NUM!	28.9722	#NUM!	#NUM!	#NUM!	#NUM!	28.3209	28.399	28.4904	28.5164
29.0938	29.3138	29.1983	29.2332	29.2421	29.5968	29.1703	29.4037	32.5323	32.6564	32.8433	32.8373
#NUM!	#NUM!	28.0824	27.5269	#NUM!	27.0162	#NUM!	#NUM!	28.9607	28.9622	29.138	29.0986
32.7893	32.7655	32.6854	32.8802	32.0154	32.5509	31.9602	31.9921	31.6331	31.7545	32.1577	32.1728
28.7929	28.9519	28.9341	28.7615	28.581	28.6233	28.5867	28.4787	29.1332	29.122	29.3138	29.2511
31.2896	31.4007	31.0945	31.2629	30.5548	30.6116	30.5017	30.585	31.7652	31.861	31.9459	31.9034
32.76	32.7154	29.8281	29.8141	31.162	33.1936	31.2499	33.4078	28.6067	28.2662	28.2328	28.3821
34.0993	34.0382	33.8275	33.9445	33.9897	34.2646	33.7151	34.5937	34.0087	34.0898	34.1707	33.9914
28.5771	28.5848	29.4818	29.195	28.2471	28.7666	28.8005	27.6647	31.0489	30.9642	30.762	30.8162
32.1399	32.1645	31.5689	31.6897	31.3459	31.9873	31.4792	31.7907	32.7414	32.8207	33.0334	32.9606
#NUM!	#NUM!	28.1077	30.0594	#NUM!	#NUM!	#NUM!	#NUM!	27.579	27.6591	27.7339	27.6361
34.5785	34.2065	34.0099	34.5587	#NUM!	29.066	#NUM!	#NUM!	29.5522	29.2548	29.6115	29.4528
30.3711	30.3374	30.6813	30.7385	29.8188	30.4324	29.7538	30.473	29.3341	29.325	29.4417	29.4671

30.4784	30.5223	30.2812	30.8954	29.6115	29.8163	29.5176	29.6033	30.813	30.6893	30.8926	30.8403
#NUM!	#NUM!	#NUM!	#NUM!	#NUM!	#NUM!	#NUM!	#NUM!	26.7476	26.5143	26.5898	26.7452
27.8479	27.9512	28.5102	28.5834	27.3109	27.5908	#NUM!	#NUM!	27.5644	27.5886	27.7971	27.7728
31.3087	30.9568	31.1251	30.8965	30.5383	30.4196	30.0027	29.0392	32.0668	32.0351	32.1482	32.1234
32.4283	32.5379	31.8508	32.003	31.5484	31.8545	31.5469	31.6814	33.0352	33.0296	33.1486	33.0807
29.3551	29.3423	29.1951	29.151	29.1797	30.0768	29.2082	30.1494	30.0766	30.1132	30.1209	30.103
28.9979	27.0091	#NUM!	26.8622	#NUM!	#NUM!	#NUM!	#NUM!	27.7639	27.6239	27.8516	27.8252
27.6975	27.1423	#NUM!	#NUM!	#NUM!	#NUM!	#NUM!	28.3008	27.2046	27.5831	27.4201	27.5375
30.652	30.8151	30.5861	30.5524	30.0759	29.7949	30.222	29.4317	28.6115	28.7605	28.9104	28.9492
28.7745	28.738	28.5503	28.6148	#NUM!	28.8562	#NUM!	28.3883	29.0856	29.0251	29.1873	29.2005
#NUM!	#NUM!	26.8009	27.7719	#NUM!	#NUM!	#NUM!	#NUM!	29.1725	29.1888	29.3851	29.3582
27.9275	28.4679	28.0261	28.3448	30.0343	28.772	28.5037	29.5638	28.1531	28.1853	28.6065	28.6664
#NUM!	26.6313	#NUM!	#NUM!	#NUM!	#NUM!	#NUM!	#NUM!	27.5214	27.4182	27.559	27.5934
26.7734	27.2722	#NUM!	26.0834	#NUM!	#NUM!	#NUM!	#NUM!	28.5557	28.5733	28.8316	28.8881
32.1693	32.2968	30.5942	30.6679	30.5152	31.3512	30.5903	30.9199	30.5212	30.5509	30.7596	30.6973
31.6239	31.7945	31.5425	31.5603	33.9329	31.6346	33.9237	32.3837	28.2606	28.4557	28.5759	28.6114
#NUM!	#NUM!	#NUM!	#NUM!	#NUM!	#NUM!	#NUM!	#NUM!	#NUM!	29.4186	#NUM!	#NUM!
31.6429	31.6022	30.9688	31.2288	30.6201	31.706	30.6226	31.8395	31.825	31.9493	32.1225	32.1049
28.4996	28.457	28.4629	28.7061	28.8261	28.0956	28.125	28.3343	28.9292	29.036	29.2783	29.327
29.2969	29.3701	29.7325	29.6959	29.492	29.467	29.3546	28.9689	29.0208	29.0541	29.1596	29.1549
31.3581	31.4895	30.2776	30.2542	30.369	31.6851	30.2779	32.0345	30.4311	30.5286	30.5606	30.5217
29.9753	30.0928	29.5508	29.6023	29.4632	30.4806	29.4261	30.6358	29.6242	29.7048	29.9095	29.7938
27.7412	27.8412	#NUM!	27.5771	#NUM!	28.6974	#NUM!	28.1184	29.6529	29.7201	29.9671	30.0138
31.113	31.2494	31.268	31.3437	31.0805	31.9405	31.0633	31.6626	32.3178	32.306	32.6835	32.646
31.22	31.0009	31.3015	31.2684	30.9343	30.3036	30.6305	29.8798	29.3217	29.2303	29.483	29.4301
29.2451	29.1689	29.6379	29.6046	28.7502	29.2478	29.1367	28.5244	30.2254	30.2613	30.4326	30.4223

HDF_WT_1 MaxLFQ Intensity	HDF_WT_2 MaxLFQ Intensity	HDF_WT_3 MaxLFQ Intensity	HDF_WT_4 MaxLFQ Intensity	C: Protein Existence	C: GOBP name
29.6676	29.9415	30.1536	30.4561	1:Experimental evidence at protein level	establishment of localization;establishment of protein localization;protein transport;transport
26.6761	#NUM!	27.0683	26.7011	2:Experimental evidence at transcript level	biological regulation;calcium ion homeostasis;cation homeostasis;cellular calcium ion homeostasis;cellular cation homeostasis;cellular chemical homeostasis;cellular divalent inorganic cation homeostasis;cellular homeostasi
24.941	25.4176	25.6122	#NUM!	3:Protein inferred from homology	biological regulation;cell surface receptor linked signaling pathway;cellular process;cellular response to stimulus;detection of chemical stimulus;detection of chemical stimulus involved in sensory perception;detection of chem
27.771	27.8558	27.5065	27.3537	1:Experimental evidence at protein level	biological regulation;biosynthetic process;cellular biosynthetic process;cellular macromolecule biosynthetic process;cellular macromolecule metabolic process;cellular metabolic process;cellular process;cellular protein metab
26.1802	26.4495	26.6989	26.4822	1:Experimental evidence at protein level	catabolic process;cellular catabolic process;cellular macromolecule catabolic process;cellular macromolecule metabolic process;cellular metabolic process;cellular nitrogen compound metabolic process;cellular process;dea
#NUM!	#NUM!	#NUM!	#NUM!	1:Experimental evidence at protein level	
27.8587	#NUM!	27.7486	27.0598	1:Experimental evidence at protein level	ATP biosynthetic process;ATP metabolic process;ATP synthesis coupled proton transport;biosynthetic process;cation transport;cellular biosynthetic process;cellular metabolic process;cellular nitrogen compound biosynthetic
#NUM!	#NUM!	#NUM!	#NUM!	1:Experimental evidence at protein level	biological regulation;blood coagulation;coagulation;hemostasis;multicellular organismal process;regulation of biological quality;regulation of body fluid levels
#NUM!	#NUM!	#NUM!	#NUM!	1:Experimental evidence at protein level	aging;amine transport;amino acid transport;biological regulation;carboxylic acid transport;developmental process;establishment of localization;hormone transport;modified amino acid transport;negative regulation of catalytic
#NUM!	#NUM!	#NUM!	#NUM!	1:Experimental evidence at protein level	acute inflammatory response;acute-phase response;anatomical structure morphogenesis;biological regulation;blood coagulation;blood vessel morphogenesis;cell activation;cellular component organization;cellular componer
#NUM!	29.0542	#NUM!	#NUM!	1:Experimental evidence at protein level	biological regulation;cell surface receptor linked signaling pathway;cellular process;cellular response to chemical stimulus;cellular response to cytokine stimulus;cellular response to organic substance;cellular response to stim
26.6485	27.023	27.3794	27.4169	1:Experimental evidence at protein level	biological regulation;cell cycle phase;cell cycle process;cell division;cell proliferation;cellular process;meiosis I;regulation of biological process;regulation of catalytic activity;regulation of cell cycle;regulation of cellular metabo
28.4826	28.4283	28.0849	28.8551	1:Experimental evidence at protein level	biological regulation;biosynthetic process;cellular biosynthetic process;cellular macromolecule biosynthetic process;cellular macromolecule metabolic process;cellular metabolic process;cellular process;cellular protein metab
#NUM!	26.8948	26.8115	26.84	1:Experimental evidence at protein level	activation of immune response;activation of innate immune response;biological regulation;cell surface receptor linked signaling pathway;cellular macromolecule metabolic process;cellular metabolic process;cellular process;c
#NUM!	#NUM!	#NUM!	#NUM!	1:Experimental evidence at protein level	biological regulation;biosynthetic process;cell motility;cellular biosynthetic process;cellular component movement;cellular component organization;cellular component organization or biogenesis;cellular metabolic process;cel
#NUM!	#NUM!	#NUM!	#NUM!	1:Experimental evidence at protein level	apoptosis;apoptosis in response to endoplasmic reticulum stress;biological regulation;calcium ion homeostasis;cation homeostasis;cell death;cellular calcium ion homeostasis;cellular cation homeostasis;cellular chemical ho
26.6935	26.9444	26.9111	26.6367	1:Experimental evidence at protein level	
#NUM!	#NUM!	#NUM!	#NUM!	1:Experimental evidence at protein level	biological regulation;establishment of localization;establishment of protein localization;establishment of protein localization in membrane;establishment of protein localization in plasma membrane;macromolecule metabolic pr
#NUM!	#NUM!	#NUM!	#NUM!	1:Experimental evidence at protein level	biological regulation;cellular macromolecule metabolic process;cellular metabolic process;cellular process;cellular protein metabolic process;cellular response to stimulus;intracellular signal transduction;macromolecule metal
#NUM!	27.0564	26.5956	26.5599	1:Experimental evidence at protein level	anatomical structure development;brain development;cation transport;cellular metabolic process;cellular process;copper ion transport;developmental process;establishment of localization;generation of precursor metabolites
27.7287	27.3886	27.6081	27.694	1:Experimental evidence at protein level	biological regulation;biosynthetic process;cellular biosynthetic process;cellular component organization;cellular component organization at cellular level;cellular component organization or biogenesis;cellular component orgar
23.9452	23.7397	23.9899	#NUM!	1:Experimental evidence at protein level	antigen processing and presentation;antigen processing and presentation of peptide antigen;antigen processing and presentation of peptide antigen via MHC class I;biological regulation;biosynthetic process;BMP signaling p
#NUM!	#NUM!	#NUM!	#NUM!	1:Experimental evidence at protein level	biological regulation;cation homeostasis;cellular cation homeostasis;cellular chemical homeostasis;cellular homeostasis;cellular ion homeostasis;cellular iron ion homeostasis;cellular metal ion homeostasis;cellular process;ch
26.4071	27.1364	26.8316	26.9335	1:Experimental evidence at protein level	
25.6852	25.4026	26.0599	26.1211	1:Experimental evidence at protein level	autophagic vacuole assembly;autophagy;catabolic process;cell communication;cellular catabolic process;cellular component assembly;cellular component assembly at cellular level;cellular component organization;cellular co
#NUM!	#NUM!	#NUM!	#NUM!	1:Experimental evidence at protein level	biological adhesion;biological regulation;carbohydrate metabolic process;catabolic process;cell activation;cell adhesion;cell surface receptor linked signaling pathway;cell-matrix adhesion;cell-substrate adhesion;cellular carb
26.5242	25.9452	26.2004	#NUM!	1:Experimental evidence at protein level	apoptosis;cell death;cellular process;death;programmed cell death
29.0402	29.5188	29.0937	29.5861	2:Experimental evidence at transcript level	response to chemical stimulus;response to organic substance;response to pheromone;response to stimulus
23.7699	#NUM!	24.8257	24.6391	1:Experimental evidence at protein level	
#NUM!	#NUM!	#NUM!	#NUM!	1:Experimental evidence at protein level	
26.9639	#NUM!	#NUM!	27.0058	1:Experimental evidence at protein level	
#NUM!	#NUM!	#NUM!	#NUM!	1:Experimental evidence at protein level	biological regulation;cell cycle cytokinesis;cell cycle process;cellular process;cytokinesis;cytokinesis after mitosis;cytokinetic cell separation;cytokinetic process;establishment of localization;negative regulation of binding;neg
#NUM!	#NUM!	#NUM!	#NUM!	1:Experimental evidence at protein level	activation of MAPK activity;activation of phospholipase C activity;axon ensheathment;biological regulation;bleb assembly;calcium ion homeostasis;cation homeostasis;cell projection assembly;cell projection organization;cell
25.4182	#NUM!	25.4153	25.8952	1:Experimental evidence at protein level	cellular macromolecule metabolic process;cellular metabolic process;cellular process;cellular protein metabolic process;establishment of localization;establishment of localization in cell;establishment of protein localization;es
#NUM!	25.1358	25.1352	24.6042	1:Experimental evidence at protein level	biological regulation;cellular macromolecule metabolic process;cellular metabolic process;cellular process;cellular protein metabolic process;macromolecule metabolic process;macromolecule modification;metabolic process
25.9861	#NUM!	25.7507	26.2901	1:Experimental evidence at protein level	biological regulation;biosynthetic process;cellular biosynthetic process;cellular macromolecule metabolic process;cellular metabolic process;cellular nitrogen compound metabolic process;cellular process;developmental proc
#NUM!	26.4303	25.8988	#NUM!	1:Experimental evidence at protein level	aging;apoptosis;biological regulation;biosynthetic process;cell death;cellular biosynthetic process;cellular macromolecule biosynthetic process;cellular macromolecule metabolic process;cellular metabolic process;cellular nitr
25.0674	#NUM!	25.0132	25.3434	2:Experimental evidence at transcript level	anatomical structure development;anatomical structure morphogenesis;appendage morphogenesis;artery morphogenesis;biological regulation;blood vessel morphogenesis;cartilage development;developmental process;emb
#NUM!	#NUM!	26.5033	26.5266	1:Experimental evidence at protein level	anatomical structure development;anatomical structure formation involved in morphogenesis;anatomical structure morphogenesis;biological regulation;BMP signaling pathway;camera-type eye development;cell differentiatio
#NUM!	#NUM!	#NUM!	27.1449	1:Experimental evidence at protein level	biological adhesion;biological regulation;calcium ion homeostasis;calcium ion transport;calcium-dependent cell-cell adhesion;cation homeostasis;cation transport;cell adhesion;cell-cell adhesion;cellular calcium ion homeosta
#NUM!	#NUM!	26.1881	#NUM!	1:Experimental evidence at protein level	biosynthetic process;cellular biosynthetic process;cellular macromolecule metabolic process;cellular metabolic process;cellular process;cellular protein metabolic process;macromolecule metabolic process;macromolecule m
#NUM!	#NUM!	#NUM!	#NUM!	1:Experimental evidence at protein level	biological regulation;positive regulation of biological process;positive regulation of cell communication;positive regulation of cell development;positive regulation of cell differentiation;positive regulation of cell migration;positiv
25.8776	26.1002	26.2308	26.1135	1:Experimental evidence at protein level	cellular component assembly;cellular component organization;cellular component organization or biogenesis;macromolecular complex assembly;macromolecular complex subunit organization;metabolic process;protein com
26.5898	#NUM!	26.2138	26.4154	1:Experimental evidence at protein level	adherens junction organization;anatomical structure morphogenesis;biological adhesion;cell adhesion;cell junction assembly;cell junction organization;cell-cell adhesion;cell-cell junction organization;cellular component asser
#NUM!	#NUM!	27.017	25.1516	1:Experimental evidence at protein level	amine transport;amino acid transport;biological regulation;biosynthetic process;carboxylic acid transport;cellular biosynthetic process;cellular lipid metabolic process;cellular metabolic process;cellular process;establishment
26.5772	26.3506	26.431	26.529	1:Experimental evidence at protein level	biological regulation;biosynthetic process;cellular biosynthetic process;cellular macromolecule biosynthetic process;cellular macromolecule metabolic process;cellular metabolic process;cellular nitrogen compound metabolic
25.5174	26.1243	26.3081	26.3909	1:Experimental evidence at protein level	cellular macromolecule metabolic process;cellular metabolic process;cellular process;cellular protein metabolic process;macromolecule metabolic process;macromolecule modification;metabolic process;primary metabolic p
#NUM!	#NUM!	#NUM!	#NUM!	1:Experimental evidence at protein level	anatomical structure development;anatomical structure formation involved in morphogenesis;biological regulation;cell surface receptor linked signaling pathway;cellular process;cellular response to stimulus;developmental pr
#NUM!	#NUM!	#NUM!	#NUM!	1:Experimental evidence at protein level	amine transport;amino acid transport;anatomical structure development;biological adhesion;biological regulation;blood coagulation;brain development;carboxylic acid transport;cell adhesion;cell differentiation;cell migration;c
25.9302	25.9057	25.9205	#NUM!	1:Experimental evidence at protein level	
26.4975	26.783	27.0219	26.4864	1:Experimental evidence at protein level	acylglycerol metabolic process;biosynthetic process;CDP-diacylglycerol metabolic process;cellular biosynthetic process;cellular lipid metabolic process;cellular metabolic process;cellular process;diacylglycerol metabolic pro
25.4486	25.9214	#NUM!	25.7278	1:Experimental evidence at protein level	anatomical structure development;biological regulation;biosynthetic process;cellular biosynthetic process;cellular macromolecule biosynthetic process;cellular macromolecule metabolic process;cellular metabolic process;ce
#NUM!	#NUM!	#NUM!	#NUM!	1:Experimental evidence at protein level	cellular component assembly;cellular component organization;cellular component organization or biogenesis;macromolecular complex assembly;macromolecular complex subunit organization;protein complex assembly;prot
27.1218	27.2504	26.9505	27.0746	1:Experimental evidence at protein level	biological regulation;biosynthetic process;catabolic process;cell surface receptor linked signaling pathway;cellular biosynthetic process;cellular catabolic process;cellular macromolecule biosynthetic process;cellular macrom
25.5177	25.9326	#NUM!	26.5898	1:Experimental evidence at protein level	biological regulation;biosynthetic process;cell cycle;cell cycle checkpoint;cell cycle phase;cell cycle process;cell division;cellular biosynthetic process;cellular component assembly;cellular component assembly at cellular lev
#NUM!	25.6782	25.4922	25.6679	1:Experimental evidence at protein level	
23.6927	#NUM!	#NUM!	#NUM!	1:Experimental evidence at protein level	activation of immune response;activation of innate immune response;activation of MAPK activity;adaptive immune response;antigen receptor-mediated signaling pathway;apoptosis;biological regulation;cell activation;cell dea
25.5767	#NUM!	#NUM!	25.4034	1:Experimental evidence at protein level	biological regulation;cellular process;cellular response to stimulus;positive regulation of biological process;positive regulation of cell communication;positive regulation of cellular process;positive regulation of I-kappaB kinase
25.4533	25.0165	25.2718	25.3788	1:Experimental evidence at protein level	anatomical structure morphogenesis;angiotensin maturation;anterior/posterior pattern specification;biological regulation;cellular macromolecule metabolic process;cellular metabolic process;cellular process;cellular protein m
#NUM!	#NUM!	#NUM!	#NUM!	1:Experimental evidence at protein level	anatomical structure development;biological regulation;calcium ion homeostasis;cation homeostasis;cellular calcium ion homeostasis;cellular cation homeostasis;cellular chemical homeostasis;cellular divalent inorganic catio
27.1565	27.6909	27.3188	27.1585	1:Experimental evidence at protein level	cellular component organization;cellular component organization at cellular level;cellular component organization or biogenesis;cellular component organization or biogenesis at cellular level;cellular process;endocytic recyclin
#NUM!	#NUM!	#NUM!	#NUM!	1:Experimental evidence at protein level	aging;biological regulation;blood coagulation;cell activation;cellular component disassembly;cellular component disassembly at cellular level;cellular component organization;cellular component organization at cellular level;c
#NUM!	#NUM!	#NUM!	#NUM!	1:Experimental evidence at protein level	activation of immune response;biological regulation;cell killing;complement activation;complement activation, alternative pathway;complement activation, classical pathway;cytolysis by symbiont of host cells;cytolysis in othe
#NUM!	26.0812	#NUM!	26.7947	1:Experimental evidence at protein level	
28.2639	#NUM!	#NUM!	#NUM!	1:Experimental evidence at protein level	anatomical structure morphogenesis;biological regulation;cellular component organization;cellular component organization at cellular level;cellular component organization or biogenesis;cellular component organization or bic
#NUM!	#NUM!	#NUM!	#NUM!	1:Experimental evidence at protein level	axon guidance;biological adhesion;biological regulation;cell adhesion;cell surface receptor linked signaling pathway;cellular component organization;cellular component organization at cellular level;cellular component organiz
25.3358	#NUM!	24.5145	25.0205	1:Experimental evidence at protein level	biological regulation;biosynthetic process;cell death;cellular biosynthetic process;cellular macromolecule biosynthetic process;cellular macromolecule metabolic process;cellular metabolic process;cellular nitrogen compounc
#NUM!	#NUM!	#NUM!	#NUM!	1:Experimental evidence at protein level	activation of immune response;activation of innate immune response;adaptive immune response;antigen processing and presentation;antigen processing and presentation of exogenous antigen;antigen processing and prese
#NUM!	26.5594	26.9433	26.8197	1:Experimental evidence at protein level	biological regulation;cellular component organization;cellular component organization or biogenesis;cellular process;cellular response to stimulus;establishment of localization;establishment of protein localization;intracellular
25.119	25.4812	#NUM!	25.3886	1:Experimental evidence at protein level	biological regulation;cellular process;cellular response to stimulus;endosome transport;establishment of localization;establishment of localization in cell;establishment of protein localization;intracellular protein transport;intrac
26.5821	26.255	26.8667	26.4585	1:Experimental evidence at protein level	anion transport;biological regulation;cell volume homeostasis;cellular component assembly;cellular component assembly at cellular level;cellular component organization;cellular component organization at cellular level;cellula
28.5527	28.6298	28.7304	28.5455	1:Experimental evidence at protein level	ATP biosynthetic process;ATP metabolic process;ATP synthesis coupled proton transport;biosynthetic process;cation transport;cellular biosynthetic process;cellular metabolic process;cellular nitrogen compound biosynthetic
27.6205	27.9786	27.7353	27.5133	1:Experimental evidence at protein level	anatomical structure development;apoptosis;biological regulation;blastocyst development;carbohydrate metabolic process;cell death;cellular carbohydrate metabolic process;cellular macromolecule metabolic process;cellula
#NUM!	#NUM!	#NUM!	#NUM!	1:Experimental evidence at protein level	macromolecule metabolic process;metabolic process;primary metabolic process;protein metabolic process;proteolysis
26.54	#NUM!	26.8643	26.7407	1:Experimental evidence at protein level	biological adhesion;biological regulation;blood coagulation;cell adhesion;cell migration;cell motility;cell surface receptor linked signaling pathway;cellular component movement;cellular component organization;cellular compo
27.0955	#NUM!	26.5281	27.0426	1:Experimental evidence at protein level	activation of anaphase-promoting complex activity;anaphase-promoting complex-dependent proteasomal ubiquitin-dependent protein catabolic process;biological regulation;catabolic process;cell cycle;cell cycle checkpoint
26.7711	#NUM!	27.1126	26.675	1:Experimental evidence at protein level	amine biosynthetic process;amine metabolic process;anatomical structure morphogenesis;biological regulation;biosynthetic process;carboxylic acid biosynthetic process;carboxylic acid metabolic process;cellular amine met
26.4914	26.2188	25.8968	25.8084	1:Experimental evidence at protein level	biological regulation;cation homeostasis;cation transport;cellular cation homeostasis;cellular chemical homeostasis;cellular divalent inorganic cation homeostasis;cellular homeostasis;cellular ion homeostasis;cellular process
28.5022	#NUM!	#NUM!	28.596	1:Experimental evidence at protein level	alcohol metabolic process;anatomical structure development;biological regulation;biosynthetic process;cellular process;cellular response to stimulus;cholesterol biosynthetic process;cholesterol metabolic process;developme
25.3902	#NUM!	25.0604	24.8449	1:Experimental evidence at protein level	anatomical structure development;biological regulation;biosynthetic process;cell cycle arrest;cell cycle process;cellular biosynthetic process;cellular component organization;cellular component organization at cellular level;ce
25.662	#NUM!	25.6068	25.5137	1:Experimental evidence at protein level	biological regulation;cellular process;endosome transport;establishment of localization;establishment of localization in cell;intracellular transport;positive regulation of catalytic activity;positive regulation of GTPase activity;pos
29.1147	#NUM!	28.6357	28.6418	1:Experimental evidence at protein level	biological regulation;cellular process;cellular response to stimulus;intracellular signal transduction;positive regulation of catalytic activity;positive regulation of GTPase activity;positive regulation of hydrolase activity;positive re
25.869	26.1569	25.9639	#NUM!	1:Experimental evidence at protein level	anatomical structure formation involved in morphogenesis;anterior/posterior axis specification;axis specification;biological regulation;cell surface receptor linked signaling pathway;cellular process;cellular response to stimulu
26.0865	26.0003	25.9602	25.8668	1:Experimental evidence at protein level	biological regulation;cell proliferation;muscle cell proliferation;negative regulation of apoptosis;negative regulation of biological process;negative regulation of cell death;negative regulation of cellular process;negative regulatic
24.5574	#NUM!	24.4544	24.5066	1:Experimental evidence at protein level	cellular macromolecule metabolic process;cellular metabolic process;cellular process;cellular protein metabolic process;macromolecule metabolic process;macromolecule modification;metabolic process;primary metabolic p
26.1568	25.8395	25.7802	25.8266	1:Experimental evidence at protein level	biological regulation;cell activation;cell proliferation;cellular process;immune response;immune system process;leukocyte activation;lymphocyte activation;negative regulation of biological process;negative regulation of biosy
26.0646	24.7007	24.7714	25.3617	1:Experimental evidence at protein level	anatomical structure development;biological regulation;cellular homeostasis;cellular process;cellular response to stimulus;collagen metabolic process;developmental process;growth;homeostatic process;intracellular signal tr
25.3227	25.8799	25.5061	26.0194	1:Experimental evidence at protein level	cellular macromolecule metabolic process;cellular metabolic process;cellular nitrogen compound metabolic process;cellular process;establishment of localization;establishment of localization in cell;establishment of RNA loca
25.3963	25.3049	25.7544	25.3322	1:Experimental evidence at protein level	anatomical structure formation involved in morphogenesis;anatomical structure morphogenesis;angiogenesis;biological regulation;blood vessel morphogenesis;developmental process;lymphangiogenesis;positive regulation c
26.4584	26.3518	26.3583	26.3186	2:Experimental evidence at transcript level	
#NUM!	25.5594	25.1984	25.4265	1:Experimental evidence at protein level	activation of immune response;activation of innate immune response;biological regulation;cellular process;cellular response to chemical stimulus;cellular response to dsRNA;cellular response to exogenous dsRNA;cellular res
26.224	#NUM!	26.9006	27.1021	1:Experimental evidence at protein level	biosynthetic process;cellular biosynthetic process;cellular macromolecule metabolic process;cellular metabolic process;cellular nitrogen compound metabolic process;cellular process;gene expression;macromolecule metabo
25.3027	25.1354	25.3897	#NUM!	1:Experimental evidence at protein level	cellular process;establishment of localization;transport;vesicle-mediated transport
25.1275	25.2025	25.3937	#NUM!	1:Experimental evidence at protein level	cellular macromolecule metabolic process;cellular metabolic process;cellular nitrogen compound metabolic process;cellular process;gene expression;macromolecule metabolic process;metabolic process;mRNA metabolic p
25.021	25.1091	24.8633	#NUM!	1:Experimental evidence at protein level	
24.9612	24.9494	24.5992	24.8796	1:Experimental evidence at protein level	
#NUM!	#NUM!	#NUM!	#NUM!	1:Experimental evidence at protein level	biological regulation;cell surface receptor linked signaling pathway;cellular component organization;cellular component organization at cellular level;cellular component organization or biogenesis;cellular component organizat
#NUM!	#NUM!	#NUM!	#NUM!	1:Experimental evidence at protein level	cellular localization;cellular macromolecule localization;cellular process;cellular protein localization;localization;macromolecule localization;protein localization;protein localization in endoplasmic reticulum;protein localization t
25.4296	25.6326	25.4528	25.3532	1:Experimental evidence at protein level	biological regulation;cation transport;cellular macromolecule metabolic process;cellular metabolic process;cellular process;cellular protein metabolic process;divalent inorganic cation transport;divalent metal ion transport;est
24.3621	24.4803	24.9546	24.6808	1:Experimental evidence at protein level	catabolic process;cellular catabolic process;cellular component assembly;cellular component assembly at cellular level;cellular component organization;cellular component organization at cellular level;cellular component org
25.6731	26.0994	25.8409	25.7661	1:Experimental evidence at protein level	biological regulation;cellular localization;cellular macromolecule localization;cellular process;cellular protein complex localization;cellular protein localization;establishment of localization;establishment of localization in cell;exc
30.4849	30.0793	30.0097	30.1609	1:Experimental evidence at protein level	biological regulation;cell differentiation;cellular component organization;cellular component organization or biogenesis;cellular developmental process;cellular membrane organization;cellular process;developmental process;e
26.9626	#NUM!	#NUM!	27.3086	1:Experimental evidence at protein level	cation transport;cellular process;divalent inorganic cation transport;divalent metal ion transport;establishment of localization;ion transport;metal ion transport;transition metal ion transport;transmembrane transport;transport;z
25.393	#NUM!	25.1846	25.8618	1:Experimental evidence at protein level	anatomical structure formation involved in morphogenesis;biological regulation;cell differentiation;cellular developmental process;cellular process;developmental process;muscle cell differentiation;negative regulation of biolog
24.6896	24.6486	25.4388	25.075	1:Experimental evidence at protein level	biological regulation;biosynthetic process;cellular biosynthetic process;cellular macromolecule biosynthetic process;cellular macromolecule metabolic process;cellular metabolic process;cellular nitrogen compound metabolic
25.3858	26.051	25.9736	25.9123	1:Experimental evidence at protein level	biological regulation;cellular process;cellular response to stimulus;intracellular signal transduction;regulation of biological process;regulation of cell migration;regulation of cell motility;regulation of cellular component moveme
25.4954	#NUM!	25.3489	25.5525	1:Experimental evidence at protein level	biosynthetic process;cellular biosynthetic process;cellular component disassembly;cellular component disassembly at cellular level;cellular component organization;cellular component organization at cellular level;cellular cor
25.7832	#NUM!	#NUM!	25.8575	1:Experimental evidence at protein level	autophagy;biological adhesion;biological regulation;catabolic process;cell adhesion;cell junction assembly;cell junction organization;cell-cell adhesion;cellular catabolic process;cellular component assembly;cellular compone
25.6565	25.3201	25.3709	25.2842	1:Experimental evidence at protein level	biological adhesion;biological regulation;cell adhesion;cell-cell adhesion;cellular component organization;cellular component organization or biogenesis;cellular process;establishment of localization;excretion;multicellular orga
#NUM!	26.0779	26.1477	26.1532	1:Experimental evidence at protein level	
24.2887	25.8075	#NUM!	26.0072	1:Experimental evidence at protein level	
25.0953	25.6503	#NUM!	25.4489	1:Experimental evidence at protein level	
25.5711	25.2781	25.5649	25.4903	1:Experimental evidence at protein level	anatomical structure development;biological regulation;developmental process;immune response;immune system process;muscle organ development;muscle structure development;organ development;positive regulation of
27.1322	27.1625	26.9332	26.8442	1:Experimental evidence at protein level	activation of NF-kappaB-inducing kinase activity;activation of protein kinase activity;biological regulation;cellular macromolecule metabolic process;cellular metabolic process;cellular process;cellular protein metabolic proces
25.2519	25.3863	25.5923	25.3801	1:Experimental evidence at protein level	biosynthetic process;carbohydrate biosynthetic process;carbohydrate metabolic process;cellular biosynthetic process;cellular carbohydrate biosynthetic process;cellular carbohydrate metabolic process;cellular lipid metabol
24.5264	24.6015	24.3115	24.5401	1:Experimental evidence at protein level	cell cycle phase;cell cycle process;cell division;cellular component organization;cellular component organization at cellular level;cellular component organization or biogenesis;cellular component organization or biogenesis at
26.3406	26.4449	25.9986	26.6313	1:Experimental evidence at protein level	biosynthetic process;cellular biosynthetic process;cellular component disassembly;cellular component disassembly at cellular level;cellular component organization;cellular component organization at cellular level;cellular cor
24.8503	25.0484	24.9833	25.0843	1:Experimental evidence at protein level	anatomical structure formation involved in morphogenesis;cell cycle;cell cycle process;cell projection assembly;cell projection organization;cellular component assembly;cellular component assembly at cellular level;cellular c
24.3466	23.83	24.0078	#NUM!	1:Experimental evidence at protein level	
26.9301	#NUM!	27.1001	#NUM!	1:Experimental evidence at protein level	anion transport;cellular process;chloride transport;establishment of localization;inorganic anion transport;ion transmembrane transport;ion transport;transmembrane transport;transport
26.1622	26.6275	26.5781	26.4651	1:Experimental evidence at protein level	biological regulation;cell differentiation;cellular developmental process;cellular process;developmental process;neuron differentiation;positive regulation of biological process;positive regulation of biosynthetic process;positive
26.0752	#NUM!	26.3251	26.1908	1:Experimental evidence at protein level	
25.9112	25.7427	25.4782	#NUM!	1:Experimental evidence at protein level	cellular component organization;cellular component organization at cellular level;cellular component organization or biogenesis;cellular component organization or biogenesis at cellular level;cellular process;chromatin organiz
25.4827	#NUM!	25.7149	25.641	1:Experimental evidence at protein level	
24.8128	24.2674	24.6521	24.3854	1:Experimental evidence at protein level	activation of protein kinase activity;anatomical structure morphogenesis;biological regulation;cell junction assembly;cell junction organization;cell morphogenesis;cell surface receptor linked signaling pathway;cell-cell junctior
24.9535	#NUM!	24.5411	24.5124	1:Experimental evidence at protein level	axon guidance;biological regulation;cell communication;cell maturation;cellular component organization;cellular component organization at cellular level;cellular component organization or biogenesis;cellular component orga

25.9006	26.2247	#NUM!	#NUM!	1:Experimental evidence at protein level	
25.7437	#NUM!	25.9265	25.4818	1:Experimental evidence at protein level	cellular macromolecule metabolic process;cellular metabolic process;cellular nitrogen compound metabolic process;cellular process;macromolecule metabolic process;macromolecule modification;metabolic process;ncRNA
24.7426	24.8566	24.7269	24.6766	1:Experimental evidence at protein level	actin cytoskeleton organization;actin filament organization;actin filament-based process;anatomical structure development;anatomical structure morphogenesis;barrier septum formation;biological regulation;cell development
26.9424	26.507	27.1613	26.9909	1:Experimental evidence at protein level	biosynthetic process;cellular biosynthetic process;cellular component organization;cellular component organization at cellular level;cellular component organization or biogenesis;cellular component organization or biogenesis
26.663	27.4061	26.8153	27.2762	1:Experimental evidence at protein level	biosynthetic process;cellular biosynthetic process;cellular component organization;cellular component organization at cellular level;cellular component organization or biogenesis;cellular component organization or biogenesis
24.4243	#NUM!	#NUM!	#NUM!	1:Experimental evidence at protein level	
26.5382	#NUM!	#NUM!	26.6448	1:Experimental evidence at protein level	
25.6278	#NUM!	25.5391	25.8374	1:Experimental evidence at protein level	biological regulation;biosynthetic process;cellular biosynthetic process;cellular macromolecule biosynthetic process;cellular macromolecule metabolic process;cellular metabolic process;cellular nitrogen compound metabolic
#NUM!	26.4203	26.8914	26.536	1:Experimental evidence at protein level	cellular macromolecule metabolic process;cellular metabolic process;cellular process;cellular protein metabolic process;chaperone-mediated protein folding;macromolecule metabolic process;macromolecule modification;me
24.8833	25.7975	25.5642	25.637	1:Experimental evidence at protein level	biological regulation;biosynthetic process;cation transport;cellular biosynthetic process;cellular macromolecule biosynthetic process;cellular macromolecule metabolic process;cellular metabolic process;cellular nitrogen com
25.1602	24.9486	24.8996	25.0169	1:Experimental evidence at protein level	anatomical structure homeostasis;biological regulation;bone resorption;cell projection organization;cellular component organization;cellular component organization at cellular level;cellular component organization or biogene
25.0772	#NUM!	24.892	24.7696	1:Experimental evidence at protein level	cellular component organization;cellular component organization or biogenesis;cellular process;establishment of localization;establishment of localization in cell;establishment of protein localization;Golgi vesicle transport;intra
25.0219	#NUM!	25.3192	25.0422	1:Experimental evidence at protein level	anatomical structure morphogenesis;biological regulation;biosynthetic process;cell differentiation;cellular biosynthetic process;cellular developmental process;cellular macromolecule biosynthetic process;cellular macromolec
25.4337	25.2061	25.522	25.2208	1:Experimental evidence at protein level	cellular metabolic process;cellular nitrogen compound metabolic process;cellular process;metabolic process;nitrogen compound metabolic process;nucleobase-containing compound metabolic process;nucleobase-containi
25.0011	24.5438	24.9919	24.6621	1:Experimental evidence at protein level	biological regulation;biosynthetic process;cellular biosynthetic process;cellular macromolecule biosynthetic process;cellular macromolecule metabolic process;cellular metabolic process;cellular nitrogen compound metabolic
25.9366	#NUM!	25.0828	25.9547	1:Experimental evidence at protein level	cell cycle;cellular process;meiotic cell cycle
27.4877	27.5421	28.1334	27.8298	1:Experimental evidence at protein level	
#NUM!	26.0642	26.3043	26.4922	1:Experimental evidence at protein level	cellular macromolecule metabolic process;cellular metabolic process;cellular process;cellular protein metabolic process;chaperone-mediated protein folding;macromolecule metabolic process;macromolecule modification;me
26.5594	26.7512	26.516	26.5617	1:Experimental evidence at protein level	autophagic vacuole assembly;autophagy;catabolic process;cell communication;cellular catabolic process;cellular component assembly;cellular component assembly at cellular level;cellular component organization;cellular co
26.9532	27.3753	27.1419	27.3517	1:Experimental evidence at protein level	biological regulation;regulation of biological process;regulation of multicellular organismal process;regulation of muscle contraction;regulation of muscle system process;regulation of system process
26.6322	26.2556	26.7772	26.5206	1:Experimental evidence at protein level	biosynthetic process;carboxylic acid biosynthetic process;carboxylic acid metabolic process;cellular biosynthetic process;cellular ketone metabolic process;cellular lipid metabolic process;cellular metabolic process;cellular p
26.175	26.0816	25.9291	26.3536	1:Experimental evidence at protein level	biosynthetic process;cellular biosynthetic process;cellular component disassembly;cellular component disassembly at cellular level;cellular component organization;cellular component organization at cellular level;cellular cor
24.8329	#NUM!	25.3007	25.038	1:Experimental evidence at protein level	biological regulation;biosynthetic process;catabolic process;cellular biosynthetic process;cellular catabolic process;cellular component assembly;cellular component assembly at cellular level;cellular component organization;
25.7814	#NUM!	#NUM!	25.3542	1:Experimental evidence at protein level	
26.9269	25.9536	26.437	26.4292	1:Experimental evidence at protein level	biological regulation;cellular localization;cellular macromolecule localization;cellular macromolecule metabolic process;cellular metabolic process;cellular nitrogen compound metabolic process;cellular process;cellular protein
26.6343	27.6635	27.5215	27.8386	1:Experimental evidence at protein level	cell cycle phase;cell cycle process;cell division;cellular component organization;cellular component organization at cellular level;cellular component organization or biogenesis;cellular component organization or biogenesis at
26.5152	26.8811	26.8508	26.6304	1:Experimental evidence at protein level	biological regulation;cell surface receptor linked signaling pathway;cellular process;cellular response to chemical stimulus;cellular response to cytokine stimulus;cellular response to organic substance;cellular response to stim
25.159	#NUM!	25.4433	24.9952	1:Experimental evidence at protein level	amine metabolic process;aminoglycan catabolic process;aminoglycan metabolic process;carbohydrate catabolic process;carbohydrate metabolic process;catabolic process;glycosaminoglycan catabolic process;glycosamin
25.5342	#NUM!	#NUM!	25.705	1:Experimental evidence at protein level	biological regulation;cellular component assembly;cellular component organization;cellular component organization at cellular level;cellular component organization or biogenesis;cellular component organization or biogenesis
28.1196	#NUM!	27.6359	27.681	1:Experimental evidence at protein level	axon ensheathment;biological adhesion;calcium-independent cell-cell adhesion;cell adhesion;cell-cell adhesion;cellular process;ensheathment of neurons;gamete generation;male gamete generation;multicellular organismal
28.3334	28.4807	28.3458	28.3587	1:Experimental evidence at protein level	biological regulation;cation homeostasis;cation transport;cellular cation homeostasis;cellular chemical homeostasis;cellular component assembly;cellular component assembly at cellular level;cellular component organization;
26.2868	25.8415	26.2286	26.3903	1:Experimental evidence at protein level	biological regulation;regulation of biological process;regulation of cell proliferation;regulation of cellular process
25.4407	#NUM!	25.5274	25.0159	1:Experimental evidence at protein level	biological regulation;cellular process;cellular response to chemical stimulus;cellular response to endogenous stimulus;cellular response to growth factor stimulus;cellular response to hormone stimulus;cellular response to org
25.4682	25.5626	25.3121	25.4519	1:Experimental evidence at protein level	biological regulation;cellular macromolecule metabolic process;cellular metabolic process;cellular process;cellular protein metabolic process;macromolecule metabolic process;macromolecule modification;metabolic process
#NUM!	#NUM!	#NUM!	#NUM!	1:Experimental evidence at protein level	acute inflammatory response;amine metabolic process;biological adhesion;biological regulation;carboxylic acid metabolic process;cell adhesion;cell-cell adhesion;cellular amine metabolic process;cellular amino acid metabo
26.479	26.7168	27.1112	27.4636	1:Experimental evidence at protein level	cellular macromolecule metabolic process;cellular metabolic process;cellular nitrogen compound metabolic process;cellular process;macromolecule metabolic process;metabolic process;mRNA metabolic process;mRNA pro
33.92	34.561	34.0701	34.9252	1:Experimental evidence at protein level	acute inflammatory response;acute-phase response;anatomical structure development;biological regulation;cellular component organization;cellular component organization or biogenesis;cellular membrane organization;cellu
27.6379	28.237	28.1556	28.5028	1:Experimental evidence at protein level	anatomical structure homeostasis;biological regulation;blood coagulation;cation homeostasis;cation transport;cell activation;cellular cation homeostasis;cellular chemical homeostasis;cellular homeostasis;cellular ion homeos
#NUM!	#NUM!	#NUM!	#NUM!	1:Experimental evidence at protein level	biological regulation;blood coagulation;blood coagulation, intrinsic pathway;coagulation;hemostasis;macromolecule metabolic process;metabolic process;multicellular organismal process;negative regulation of biological pro
28.1255	27.9712	27.7081	27.8693	1:Experimental evidence at protein level	activation of immune response;anatomical structure development;anatomical structure formation involved in morphogenesis;angiogenesis;antigen receptor-mediated signaling pathway;biological adhesion;biological regulatio
#NUM!	25.9087	#NUM!	26.6713	1:Experimental evidence at protein level	anatomical structure development;biological regulation;biomineral tissue development;bone mineralization;cellular process;cellular response to chemical stimulus;cellular response to endogenous stimulus;cellular response to
25.4437	#NUM!	25.3425	26.04	1:Experimental evidence at protein level	cellular localization;cellular macromolecule localization;cellular macromolecule metabolic process;cellular metabolic process;cellular process;cellular protein localization;cellular protein metabolic process;establishment of loca
29.0386	29.5079	29.2743	29.5426	1:Experimental evidence at protein level	biological adhesion;cell adhesion;cellular process
26.3081	#NUM!	#NUM!	25.7624	1:Experimental evidence at protein level	adenine nucleotide transport;adult heart development;anatomical structure development;anatomical structure morphogenesis;apoptosis;ATP transport;atrial ventricular junction remodeling;biological regulation;blood vessel m
26.2318	#NUM!	25.8603	25.7457	1:Experimental evidence at protein level	cellular metabolic process;cellular process;electron transport chain;generation of precursor metabolites and energy;metabolic process;mitochondrial electron transport, NADH to ubiquinone;oxidation-reduction process;respi
26.6244	26.37	26.8609	26.7398	1:Experimental evidence at protein level	biological regulation;biosynthetic process;cellular biosynthetic process;cellular macromolecule biosynthetic process;cellular macromolecule metabolic process;cellular metabolic process;cellular nitrogen compound metabolic
#NUM!	#NUM!	#NUM!	#NUM!	1:Experimental evidence at protein level	amine metabolic process;aminoglycan biosynthetic process;aminoglycan catabolic process;aminoglycan metabolic process;biological regulation;biosynthetic process;carbohydrate biosynthetic process;carbohydrate catabol
#NUM!	#NUM!	#NUM!	#NUM!	1:Experimental evidence at protein level	aging;anatomical structure development;anatomical structure formation involved in morphogenesis;anatomical structure homeostasis;angiogenesis;biological adhesion;biological regulation;biosynthetic process;calcium ion tr
27.4282	26.3559	26.4364	26.93	1:Experimental evidence at protein level	adrenal gland development;aging;anatomical structure development;anatomical structure morphogenesis;anion transport;apoptosis;axon regeneration;axonogenesis;behavior;behavioral response to pain;biological regulation
#NUM!	#NUM!	#NUM!	#NUM!	1:Experimental evidence at protein level	anatomical structure formation involved in morphogenesis;anatomical structure morphogenesis;angiogenesis;biological adhesion;biological regulation;cell adhesion;cell morphogenesis;cell morphogenesis involved in differen
26.1027	26.3243	26.6104	26.6722	1:Experimental evidence at protein level	biological regulation;carbohydrate metabolic process;cellular carbohydrate metabolic process;cellular glucan metabolic process;cellular macromolecule metabolic process;cellular metabolic process;cellular polysaccharide m
28.5294	27.823	28.5337	28.5786	1:Experimental evidence at protein level	biological regulation;dosage compensation;dosage compensation, by inactivation of X chromosome;posttranscriptional regulation of gene expression;regulation of biological process;regulation of biosynthetic process;regulat
#NUM!	#NUM!	#NUM!	#NUM!	1:Experimental evidence at protein level	establishment of localization;transport;vitamin transport
25.9591	#NUM!	26.1152	25.685	1:Experimental evidence at protein level	alcohol metabolic process;carbohydrate catabolic process;carbohydrate metabolic process;catabolic process;cellular carbohydrate catabolic process;cellular carbohydrate metabolic process;cellular glucan metabolic proces
#NUM!	#NUM!	#NUM!	#NUM!	1:Experimental evidence at protein level	adult behavior;adult locomotory behavior;ameboidal cell migration;anatomical structure development;anatomical structure morphogenesis;axon guidance;behavior;biological adhesion;biological regulation;blood circulation;br
27.8791	#NUM!	27.6391	27.7405	1:Experimental evidence at protein level	aging;anatomical structure morphogenesis;base-excision repair;biological regulation;cell aging;cell communication;cell cycle checkpoint;cell cycle phase;cell cycle process;cell differentiation;cell division;cell morphogenesis;c
26.6119	26.7661	26.3681	26.762	1:Experimental evidence at protein level	cellular macromolecule metabolic process;cellular metabolic process;cellular process;cellular protein metabolic process;macromolecule metabolic process;macromolecule modification;metabolic process;multicellular organis
28.2891	27.7849	27.8778	27.6516	1:Experimental evidence at protein level	antigen processing and presentation;antigen processing and presentation of exogenous antigen;antigen processing and presentation of exogenous peptide antigen;antigen processing and presentation of exogenous peptide
#NUM!	27.6132	27.1841	27.2459	1:Experimental evidence at protein level	developmental process;establishment of localization;establishment of localization in cell;establishment of protein localization;intracellular protein transport;intracellular transport;multicellular organismal development;multicellu
25.8358	26.348	25.0945	25.9964	1:Experimental evidence at protein level	cellular metabolic process;cellular process;electron transport chain;generation of precursor metabolites and energy;metabolic process;mitochondrial electron transport, NADH to ubiquinone;oxidation-reduction process;respi
27.7498	#NUM!	27.4526	27.3927	1:Experimental evidence at protein level	aging;biological regulation;cell aging;cellular process;cellular response to chemical stimulus;cellular response to oxidative stress;cellular response to reactive oxygen species;cellular response to stimulus;cellular response to s
#NUM!	26.5397	26.6461	26.5944	1:Experimental evidence at protein level	carbohydrate metabolic process;catabolic process;cellular carbohydrate metabolic process;cellular catabolic process;cellular macromolecule catabolic process;cellular macromolecule metabolic process;cellular metabolic pr
27.2516	27.709	27.698	27.9587	1:Experimental evidence at protein level	biological regulation;cell cycle;cell cycle process;cell division;cell proliferation;cellular process;G1/S transition of mitotic cell cycle;mitotic cell cycle;regulation of biological process;regulation of catalytic activity;regulation of c
#NUM!	25.5925	#NUM!	25.4923	1:Experimental evidence at protein level	actin cytoskeleton organization;actin filament-based process;biological adhesion;biological regulation;cell adhesion;cellular component organization;cellular component organization at cellular level;cellular component organiz
27.7352	27.7665	27.7899	27.1774	1:Experimental evidence at protein level	biosynthetic process;cellular biosynthetic process;cellular component assembly;cellular component assembly at cellular level;cellular component organization;cellular component organization at cellular level;cellular compone
28.242	28.9328	28.7967	28.4804	1:Experimental evidence at protein level	antibacterial humoral response;antimicrobial humoral response;biosynthetic process;catabolic process;cellular biosynthetic process;cellular catabolic process;cellular component disassembly;cellular component disassembly
27.2989	27.1623	27.1184	26.9556	1:Experimental evidence at protein level	biological regulation;cell communication;cell surface receptor linked signaling pathway;cell-cell signaling;cellular metabolic process;cellular process;cellular response to chemical stimulus;cellular response to endogenous stin
25.2064	#NUM!	#NUM!	24.8912	1:Experimental evidence at protein level	biosynthetic process;cellular biosynthetic process;cellular component disassembly;cellular component disassembly at cellular level;cellular component organization;cellular component organization at cellular level;cellular cor
#NUM!	25.4143	25.2471	24.9114	1:Experimental evidence at protein level	amine catabolic process;amine metabolic process;branched chain family amino acid catabolic process;branched chain family amino acid metabolic process;brown fat cell differentiation;carboxylic acid catabolic process;carb
#NUM!	#NUM!	#NUM!	#NUM!	1:Experimental evidence at protein level	amine metabolic process;aminoglycan biosynthetic process;aminoglycan catabolic process;aminoglycan metabolic process;biosynthetic process;carbohydrate biosynthetic process;carbohydrate catabolic process;carbohyd
#NUM!	#NUM!	#NUM!	#NUM!	1:Experimental evidence at protein level	biological regulation;developmental process;humoral immune response;immune response;immune system process;metabolic process;multicellular organismal development;multicellular organismal process;positive regulation
#NUM!	#NUM!	#NUM!	#NUM!	1:Experimental evidence at protein level	anatomical structure development;axon guidance;biological adhesion;biological regulation;cell adhesion;cell surface receptor linked signaling pathway;cellular process;cellular response to stimulus;cerebellum development;c
#NUM!	#NUM!	#NUM!	#NUM!	1:Experimental evidence at protein level	biological adhesion;cell adhesion;cellular process
27.1975	26.9253	26.969	26.7938	1:Experimental evidence at protein level	activation of immune response;activation of innate immune response;apoptosis;biological regulation;biosynthetic process;cell death;cell surface receptor linked signaling pathway;cellular biosynthetic process;cellular macrom
25.8772	26.6854	26.4412	26.6294	1:Experimental evidence at protein level	activation of anaphase-promoting complex activity;anaphase-promoting complex-dependent proteasomal ubiquitin-dependent protein catabolic process;biological regulation;catabolic process;cell cycle process;cell division;
26.0169	25.7041	25.7255	25.7244	1:Experimental evidence at protein level	biological regulation;cellular component organization;cellular component organization or biogenesis;cellular macromolecule metabolic process;cellular membrane organization;cellular metabolic process;cellular process;cellula
24.3014	#NUM!	24.0698	24.9166	1:Experimental evidence at protein level	autophagic vacuole assembly;autophagy;catabolic process;cell communication;cellular catabolic process;cellular component assembly;cellular component assembly at cellular level;cellular component organization;cellular co
26.8497	#NUM!	27.6626	27.5142	1:Experimental evidence at protein level	biological regulation;biosynthetic process;cellular biosynthetic process;cellular macromolecule biosynthetic process;cellular macromolecule metabolic process;cellular metabolic process;cellular nitrogen compound metabolic
25.9779	25.8821	26.3125	#NUM!	1:Experimental evidence at protein level	activation of immune response;activation of innate immune response;biological regulation;carbohydrate mediated signaling;cell recognition;cellular component assembly;cellular component organization;cellular component o
29.1948	#NUM!	29.7715	29.4277	1:Experimental evidence at protein level	cellular component assembly;cellular component organization;cellular component organization or biogenesis;macromolecular complex assembly;macromolecular complex subunit organization;protein complex assembly;prot
25.4749	#NUM!	26.0295	26.3173	1:Experimental evidence at protein level	apoptosis;biological regulation;cell death;cellular process;cellular response to chemical stimulus;cellular response to cytokine stimulus;cellular response to organic substance;cellular response to stimulus;cellular response to
27.625	27.7873	#NUM!	27.4158	1:Experimental evidence at protein level	
#NUM!	24.3269	25.2897	25.6046	1:Experimental evidence at protein level	cellular macromolecule metabolic process;cellular metabolic process;cellular nitrogen compound metabolic process;cellular process;cellular protein metabolic process;macromolecule metabolic process;macromolecule modi
25.6402	#NUM!	25.7779	26.0702	1:Experimental evidence at protein level	apoptosis;biological regulation;cell communication;cell death;cellular localization;cellular macromolecule localization;cellular process;cellular protein localization;cellular response to acid;cellular response to amine stimulus;ce
24.5802	#NUM!	24.613	24.1821	1:Experimental evidence at protein level	anatomical structure development;biological adhesion;biological regulation;cell adhesion;cell cycle;cell development;cell division;cell maturation;cellular component organization;cellular component organization at cellular leve
26.6377	#NUM!	26.9487	26.4592	1:Experimental evidence at protein level	
25.2906	#NUM!	#NUM!	#NUM!	1:Experimental evidence at protein level	biological regulation;cellular macromolecule metabolic process;cellular metabolic process;cellular process;cellular protein metabolic process;establishment of localization;establishment of localization in cell;establishment of p
#NUM!	26.5239	26.4495	26.5716	1:Experimental evidence at protein level	
25.4239	25.7389	26.772	25.9044	1:Experimental evidence at protein level	anatomical structure development;biological regulation;cell redox homeostasis;cellular homeostasis;cellular process;developmental process;hemopoiesis;hemopoietic or lymphoid organ development;homeostatic process;or
#NUM!	26.1737	25.8651	26.2577	1:Experimental evidence at protein level	
25.5172	25.5173	25.5218	25.0415	1:Experimental evidence at protein level	anatomical structure development;biological regulation;cell differentiation;cellular developmental process;cellular process;developmental process;nervous system development;regulation of biological process;regulation of ce
25.3435	25.0255	25.101	25.0158	1:Experimental evidence at protein level	
#NUM!	24.5666	24.6138	24.6211	1:Experimental evidence at protein level	anatomical structure morphogenesis;biological regulation;cellular process;cellular response to stimulus;developmental process;intracellular signal transduction;morphogenesis of an epithelial sheet;morphogenesis of an epith
25.547	25.5799	25.4338	25.5219	1:Experimental evidence at protein level	biological regulation;calcium ion homeostasis;calcium ion transport;cation homeostasis;cation transport;cellular calcium ion homeostasis;cellular cation homeostasis;cellular chemical homeostasis;cellular divalent inorganic c
25.3826	25.6527	25.5135	#NUM!	1:Experimental evidence at protein level	biological regulation;cellular metabolic process;cellular process;electron transport chain;generation of precursor metabolites and energy;metabolic process;negative regulation of biological process;negative regulation of cellu
27.5293	27.4648	27.6442	26.972	1:Experimental evidence at protein level	cation transport;cellular process;divalent inorganic cation transport;divalent metal ion transport;establishment of localization;ion transport;magnesium ion transport;metal ion transport;transmembrane transport;transport
#NUM!	#NUM!	#NUM!	#NUM!	1:Experimental evidence at protein level	amine metabolic process;aminoglycan biosynthetic process;aminoglycan metabolic process;biosynthetic process;carbohydrate biosynthetic process;carbohydrate metabolic process;cellular biosynthetic process;cellular mac
#NUM!	#NUM!	#NUM!	#NUM!	1:Experimental evidence at protein level	biological regulation;cellular process;cellular response to stimulus;regulation of biological process;regulation of cellular process;response to stimulus;signal transduction
26.747	#NUM!	26.8161	25.8379	1:Experimental evidence at protein level	cellular macromolecule metabolic process;cellular metabolic process;cellular nitrogen compound metabolic process;cellular process;macromolecule metabolic process;metabolic process;nitrogen compound metabolic proce
25.3465	26.0192	25.6336	25.9548	1:Experimental evidence at protein level	
27.0327	#NUM!	26.6579	26.8467	1:Experimental evidence at protein level	3'-phosphoadenosine 5'-phosphosulfate biosynthetic process;3'-phosphoadenosine 5'-phosphosulfate metabolic process;3'-phosphoadenosine 5'-phosphosulfate transport;amine metabolic process;aminoglycan metabolic
26.0258	26.5545	26.8234	26.7847	1:Experimental evidence at protein level	biological regulation;biosynthetic process;cellular biosynthetic process;cellular component organization;cellular component organization at cellular level;cellular component organization or biogenesis;cellular component orga
27.3024	27.1136	27.7101	27.2242	1:Experimental evidence at protein level	amine metabolic process;carboxylic acid metabolic process;cellular amine metabolic process;cellular amino acid metabolic process;cellular ketone metabolic process;cellular metabolic process;cellular nitrogen compound m
26.9868	27.6368	27.5656	27.7809	1:Experimental evidence at protein level	biological regulation;posttranscriptional regulation of gene expression;protein destabilization;regulation of biological process;regulation of biological quality;regulation of gene expression;regulation of macromolecule metaboli
27.0268	26.9063	27.0767	26.9557	1:Experimental evidence at protein level	cellular macromolecule metabolic process;cellular metabolic process;cellular nitrogen compound metabolic process;cellular process;macromolecule metabolic process;macromolecule methylation;macromolecule modificatio
26.4325	26.6562	26.581	26.6275	1:Experimental evidence at protein level	
25.9229	26.8092	26.8816	27.0419	1:Experimental evidence at protein level	
26.9357	#NUM!	#NUM!	#NUM!	1:Experimental evidence at protein level	amine transport;amino acid transmembrane transport;amino acid transport;biological regulation;carboxylic acid transport;cation transport;cell communication;cell-cell signaling;cellular process;establishment of localization;es
27.3578	27.2864	27.3488	27.266	1:Experimental evidence at protein level	activation of immune response;activation of innate immune response;antigen processing and presentation;antigen processing and presentation of exogenous antigen;antigen processing and presentation of exogenous peptic
26.1303	#NUM!	26.4326	25.8014	1:Experimental evidence at protein level	cellular macromolecule metabolic process;cellular metabolic process;cellular nitrogen compound metabolic process;cellular process;macromolecule metabolic process;metabolic process;mRNA metabolic process;mRNA pro
26.614	26.8471	26.7524	27.362	1:Experimental evidence at protein level	acylglycerol catabolic process;acylglycerol metabolic process;arachidonic acid metabolic process;biological regulation;biosynthetic process;blood coagulation;carboxylic acid biosynthetic process;carboxylic acid metabolic p
26.4258	26.7528	26.3333	26.5771	1:Experimental evidence at protein level	anatomical structure development;biological regulation;biosynthetic process;cellular biosynthetic process;cellular macromolecule biosynthetic process;cellular macromolecule metabolic process;cellular metabolic process;ce
26.7508	26.3877	#NUM!	26.5804	1:Experimental evidence at protein level	biological regulation;biosynthetic process;cellular biosynthetic process;cellular component organization;cellular component organization or biogenesis;cellular macromolecule biosynthetic process;cellular macromolecule met
25.744	25.3155	#NUM!	25.6627	1:Experimental evidence at protein level	viral reproduction
#NUM!	25.9806	25.9588	25.8816	1:Experimental evidence at protein level	apoptotic mitochondrial changes;biological regulation;cellular component organization;cellular component organization at cellular level;cellular component organization or biogenesis;cellular component organization or bioger
24.9051	26.0425	25.7734	26.2948	1:Experimental evidence at protein level	biosynthetic process;cellular biosynthetic process;cellular macromolecule biosynthetic process;cellular macromolecule metabolic process;cellular metabolic process;cellular nitrogen compound metabolic process;cellular pro
25.7684	#NUM!	26.5446	26.2847	1:Experimental evidence at protein level	alcohol metabolic process;cellular component assembly;cellular component organization;cellular component organization or biogenesis;cellular ketone metabolic process;cellular metabolic process;cellular process;lipid meta
26.7775	#NUM!	27.184	27.2727	1:Experimental evidence at protein level	biological regulation;catabolic process;cellular catabolic process;cellular macromolecule catabolic process;cellular macromolecule metabolic process;cellular metabolic process;cellular process;cellular protein metabolic proc
25.9548	26.0302	25.8057	25.5637	1:Experimental evidence at protein level	
#NUM!	#NUM!	#NUM!	#NUM!	1:Experimental evidence at protein level	biological regulation;cell surface receptor linked signaling pathway;cellular component movement;cellular component organization;cellular component organization at cellular level;cellular component organization or biogenes
25.3295	25.1292	25.3234	#NUM!	1:Experimental evidence at protein level	amine metabolic process;aminoglycan catabolic process;aminoglycan metabolic process;carbohydrate catabolic process;carbohydrate metabolic process;catabolic process;chitin catabolic process;chitin metabolic process;
26.311	#NUM!	26.2332	26.5075	1:Experimental evidence at protein level	biological regulation;developmental process;multicellular organismal development;multicellular organismal process;posttranscriptional regulation of gene expression;regulation of biological process;regulation of biosynthetic p
25.9958	25.9713	26.1555	26.4324	1:Experimental evidence at protein level	biological regulation;cellular macromolecule metabolic process;cellular metabolic process;cellular nitrogen compound metabolic process;cellular process;developmental process;establishment of localization;establishment of
24.7356	24.4233	24.4306	24.2486	1:Experimental evidence at protein level	anatomical structure development;apoptosis;autophagic vacuole assembly;autophagy;biological regulation;blood vessel remodeling;cardiac cell development;cardiac muscle cell development;catabolic process;cell communi
28.479	#NUM!	29.3123	28.8216	1:Experimental evidence at protein level	apoptosis;biological regulation;biosynthetic process;cell communication;cell death;cell growth;cellular biosynthetic process;cellular localization;cellular macromolecule biosynthetic process;cellular macromolecule localization
25.9064	26.2413	#NUM!	#NUM!	1:Experimental evidence at protein level	biological regulation;biosynthetic process;carbohydrate metabolic process;cellular biosynthetic process;cellular carbohydrate metabolic process;cellular component organization;cellular component organization at cellular lev
26.0013	26.3491	26.6017	26.9797	1:Experimental evidence at protein level	biological regulation;cellular macromolecule metabolic process;cellular metabolic process;cellular nitrogen compound metabolic process;cellular process;cellular protein metabolic process;dephosphorylation;dephosphorylati
26.4407	#NUM!	26.4501	26.7083	1:Experimental evidence at protein level	
26.1842	#NUM!	26.6607	25.9869	1:Experimental evidence at protein level	biological regulation;catabolic process;cellular catabolic process;cellular macromolecule metabolic process;cellular metabolic process;cellular nitrogen compound catabolic process;cellular nitrogen compound metabolic proc

27.3962	27.0823	26.8198	27.1583	1:Experimental evidence at protein level	biological regulation;cellular component organization;cellular component organization or biogenesis;cellular membrane organization;cellular process;drug transmembrane transport;drug transport;establishment of localization;
24.8799	25.0726	24.8365	24.8777	1:Experimental evidence at protein level	biological regulation;biosynthetic process;cell differentiation;cellular biosynthetic process;cellular component assembly;cellular component assembly at cellular level;cellular component organization;cellular component organi
25.5418	25.4473	25.9092	25.565	1:Experimental evidence at protein level	box C/D snoRNP assembly;cell differentiation;cellular component assembly;cellular component assembly at cellular level;cellular component organization;cellular component organization at cellular level;cellular component o
27.3536	27.6632	28.0347	27.9113	1:Experimental evidence at protein level	
25.3266	#NUM!	23.7214	24.3109	1:Experimental evidence at protein level	
29.2151	27.7846	27.9635	28.4006	1:Experimental evidence at protein level	actin cytoskeleton organization;actin filament capping;actin filament organization;actin filament-based process;actomyosin structure organization;anatomical structure development;anatomical structure formation involved in
#NUM!	#NUM!	#NUM!	#NUM!	1:Experimental evidence at protein level	biological regulation;cell surface receptor linked signaling pathway;cellular component assembly;cellular component organization;cellular component organization or biogenesis;cellular process;cellular response to stimulus;c
25.8879	#NUM!	#NUM!	25.8188	1:Experimental evidence at protein level	cellular component organization;cellular component organization or biogenesis;cellular membrane organization;cellular process;endoplasmic reticulum membrane organization;establishment of localization;establishment of lo
25.6548	25.7822	26.0774	26.2718	1:Experimental evidence at protein level	catabolic process;cellular catabolic process;cellular macromolecule catabolic process;cellular macromolecule metabolic process;cellular metabolic process;cellular process;cellular protein metabolic process;macromolecule
26.4485	#NUM!	25.7147	#NUM!	1:Experimental evidence at protein level	macromolecule metabolic process;metabolic process;primary metabolic process;protein metabolic process;proteolysis;response to calcium ion;response to chemical stimulus;response to inorganic substance;response to me
24.9402	24.2815	24.423	24.5413	1:Experimental evidence at protein level	cellular metabolic process;cellular process;electron transport chain;generation of precursor metabolites and energy;metabolic process;multicellular organismal process;oxidation-reduction process;respiratory electron transpo
26.6732	26.7234	26.2637	26.6629	1:Experimental evidence at protein level	
26.3621	#NUM!	27.2644	#NUM!	1:Experimental evidence at protein level	cellular metabolic process;cellular process;metabolic process;peptide metabolic process
26.5072	25.6437	#NUM!	25.8581	1:Experimental evidence at protein level	
25.0691	#NUM!	25.8151	25.552	1:Experimental evidence at protein level	biological regulation;cell surface receptor linked signaling pathway;cellular process;cellular response to stimulus;hippo signaling cascade;intracellular signal transduction;regulation of biological process;regulation of cellular p
27.4118	27.6638	27.3468	27.625	1:Experimental evidence at protein level	biological regulation;cellular component assembly;cellular component assembly at cellular level;cellular component biogenesis;cellular component organization;cellular component organization at cellular level;cellular compor
#NUM!	#NUM!	#NUM!	#NUM!	1:Experimental evidence at protein level	adenosine metabolic process;cellular component organization;cellular component organization at cellular level;cellular component organization or biogenesis;cellular component organization or biogenesis at cellular level;cell
26.7099	27.2501	27.1234	26.9513	1:Experimental evidence at protein level	biological regulation;cell redox homeostasis;cellular homeostasis;cellular process;homeostatic process;regulation of biological process;regulation of biological quality;regulation of cellular process
25.8249	#NUM!	#NUM!	25.793	1:Experimental evidence at protein level	apoptosis;biological regulation;cell death;cell surface receptor linked signaling pathway;cellular process;cellular response to stimulus;circadian regulation of gene expression;circadian rhythm;death;enzyme linked receptor pr
26.6691	27.4561	#NUM!	26.6067	1:Experimental evidence at protein level	biosynthetic process;cellular biosynthetic process;cellular metabolic process;cellular process;cofactor biosynthetic process;cofactor metabolic process;iron-sulfur cluster assembly;metabolic process;metallo-sulfur cluster as
26.1867	26.5439	26.5517	25.856	1:Experimental evidence at protein level	
25.931	#NUM!	26.2404	25.944	3:Protein inferred from homology	
26.0504	26.3903	25.8521	26.1041	1:Experimental evidence at protein level	establishment of localization;establishment of protein localization;establishment of RNA localization;mRNA transport;nucleic acid transport;nucleobase-containing compound transport;protein transport;RNA transport;transpo
29.4833	#NUM!	29.6365	29.8322	5:Protein uncertain	cellular macromolecule metabolic process;cellular metabolic process;cellular nitrogen compound metabolic process;cellular process;macromolecule metabolic process;metabolic process;mRNA metabolic process;mRNA pro
26.7565	26.6623	27.1426	27.0541	4:Protein predicted	
24.9407	#NUM!	24.8916	24.4676	3:Protein inferred from homology	
28.581	28.78	28.801	29.0217	1:Experimental evidence at protein level	anatomical structure development;anatomical structure homeostasis;biological regulation;bone resorption;catabolic process;cell differentiation;cellular catabolic process;cellular component organization;cellular component or
26.8658	26.4947	26.7592	27.2305	1:Experimental evidence at protein level	biological regulation;cellular metabolic process;cellular process;cellular response to stimulus;lipid metabolic process;metabolic process;oxidation-reduction process;primary metabolic process;regulation of biological process
25.8515	25.8008	#NUM!	25.7291	1:Experimental evidence at protein level	axon ensheathment;biological regulation;cell cycle process;cellular component assembly;cellular component assembly at cellular level;cellular component organization;cellular component organization at cellular level;cellular
#NUM!	#NUM!	#NUM!	#NUM!	1:Experimental evidence at protein level	acute inflammatory response;acute-phase response;apoptosis;biological regulation;biosynthetic process;cell activation;cell death;cellular biosynthetic process;cellular component organization;cellular component organization
26.8612	26.0588	26.6959	26.9226	1:Experimental evidence at protein level	biological regulation;biosynthetic process;cell surface receptor linked signaling pathway;cellular biosynthetic process;cellular macromolecule biosynthetic process;cellular macromolecule metabolic process;cellular metabolic
26.8785	27.0705	27.0349	27.107	1:Experimental evidence at protein level	amide transport;antigen processing and presentation;antigen processing and presentation of endogenous antigen;antigen processing and presentation of endogenous peptide antigen;antigen processing and presentation of
26.3723	#NUM!	#NUM!	27.1115	1:Experimental evidence at protein level	anatomical structure morphogenesis;androgen receptor signaling pathway;biological adhesion;biological regulation;biosynthetic process;catabolic process;cell adhesion;cell differentiation;cell fate commitment;cell surface re
26.3114	26.8418	27.0131	27.0157	1:Experimental evidence at protein level	cellular metabolic process;cellular process;electron transport chain;generation of precursor metabolites and energy;metabolic process;mitochondrial electron transport, NADH to ubiquinone;oxidation-reduction process;respi
28.1676	28.2807	28.4406	28.535	1:Experimental evidence at protein level	biological adhesion;biological regulation;biosynthetic process;cell adhesion;cellular biosynthetic process;cellular component assembly;cellular component organization;cellular component organization or biogenesis;cellular m
27.1273	27.3319	27.4902	26.9698	1:Experimental evidence at protein level	acidic amino acid transport;amine transport;amino acid transport;anion transport;carboxylic acid transport;establishment of localization;establishment of protein localization;ion transport;L-glutamate transport;nitrogen comp
27.9167	27.828	27.9758	27.8576	1:Experimental evidence at protein level	apoptosis;cell death;cellular component assembly;cellular component assembly at cellular level;cellular component organization;cellular component organization at cellular level;cellular component organization or biogenesis;
26.5641	#NUM!	26.807	26.609	1:Experimental evidence at protein level	
25.4513	25.1997	25.3144	25.1486	1:Experimental evidence at protein level	anatomical structure development;biological regulation;cell cycle;cell cycle phase;cell cycle process;cell division;cellular component disassembly;cellular component disassembly at cellular level;cellular component organizati
#NUM!	#NUM!	#NUM!	#NUM!	1:Experimental evidence at protein level	
27.1764	27.8419	#NUM!	27.0278	1:Experimental evidence at protein level	cellular metabolic process;cellular process;electron transport chain;generation of precursor metabolites and energy;metabolic process;mitochondrial electron transport, NADH to ubiquinone;oxidation-reduction process;respi
26.4965	#NUM!	25.9693	26.3389	1:Experimental evidence at protein level	cellular component organization;cellular component organization or biogenesis;cellular process;endosome transport;ER to Golgi vesicle-mediated transport;establishment of localization;establishment of localization in cell;est
#NUM!	#NUM!	#NUM!	#NUM!	1:Experimental evidence at protein level	biological regulation;cell communication;cell surface receptor linked signaling pathway;cell-cell signaling;cellular process;cellular response to stimulus;enzyme linked receptor protein signaling pathway;regulation of biological
26.639	26.2933	26.8041	26.3126	1:Experimental evidence at protein level	assembly of spliceosomal tri-snRNP;biological regulation;cellular component assembly;cellular component assembly at cellular level;cellular component organization;cellular component organization at cellular level;cellular co
26.7354	26.5834	26.976	26.6485	1:Experimental evidence at protein level	
24.4694	#NUM!	#NUM!	24.4864	1:Experimental evidence at protein level	bis(5'-nucleosidyl) oligophosphate catabolic process;bis(5'-nucleosidyl) oligophosphate metabolic process;catabolic process;cell communication;cell-cell signaling;cellular catabolic process;cellular metabolic process;cellular
#NUM!	#NUM!	#NUM!	#NUM!	1:Experimental evidence at protein level	biological regulation;blood coagulation;cell activation;cellular macromolecule metabolic process;cellular metabolic process;cellular process;cellular protein metabolic process;coagulation;establishment of localization;establis
#NUM!	#NUM!	#NUM!	#NUM!	1:Experimental evidence at protein level	biological regulation;blood coagulation;blood coagulation, extrinsic pathway;blood coagulation, intrinsic pathway;cellular macromolecule metabolic process;cellular metabolic process;cellular process;cellular protein metaboli
25.3017	25.4409	25.3338	25.2645	1:Experimental evidence at protein level	biological regulation;blood coagulation;cell communication;cell migration;cell motility;cell surface receptor linked signaling pathway;cell-cell signaling;cellular component movement;cellular macromolecule metabolic process;
#NUM!	#NUM!	27.9538	28.1832	1:Experimental evidence at protein level	acylglycerol metabolic process;acylglycerol transport;biological regulation;blood coagulation, intrinsic pathway;cellular lipid metabolic process;cellular metabolic process;cellular process;establishment of localization;glycerol
28.2394	28.1746	28.0105	28.1065	1:Experimental evidence at protein level	biological regulation;biosynthetic process;catabolic process;cell surface receptor linked signaling pathway;cellular biosynthetic process;cellular catabolic process;cellular macromolecule biosynthetic process;cellular macrom
#NUM!	#NUM!	26.5067	#NUM!	1:Experimental evidence at protein level	biological regulation;blood coagulation;chemotaxis;coagulation;hemostasis;locomotion;multicellular organismal process;negative regulation of catalytic activity;negative regulation of endopeptidase activity;negative regulation
25.6954	#NUM!	25.8472	25.9845	1:Experimental evidence at protein level	activation of MAPK activity;amine metabolic process;aminoglycan catabolic process;aminoglycan metabolic process;anatomical structure development;anatomical structure morphogenesis;biological regulation;biosynthetic
24.8846	25.0868	24.7784	24.6798	1:Experimental evidence at protein level	apoptotic mitochondrial changes;biological regulation;cell surface receptor linked signaling pathway;cellular component organization;cellular component organization at cellular level;cellular component organization or biogen
27.8504	27.5818	27.7808	27.7453	1:Experimental evidence at protein level	biological regulation;biosynthetic process;carbohydrate biosynthetic process;carbohydrate metabolic process;cellular carbohydrate metabolic process;cellular lipid metabolic process;cellular macromolecule metabolic proces
#NUM!	#NUM!	#NUM!	#NUM!	1:Experimental evidence at protein level	activation of immune response;anatomical structure morphogenesis;apoptotic mitochondrial changes;biological regulation;blood coagulation;cell activation;cell morphogenesis;cellular component assembly;cellular compone
#NUM!	28.9975	29.2696	#NUM!	1:Experimental evidence at protein level	activation of blood coagulation via clotting cascade;activation of caspase activity;activation of plasma proteins involved in acute inflammatory response;biological regulation;blood coagulation;blood coagulation, extrinsic pat
26.4965	25.8177	26.0035	25.7373	1:Experimental evidence at protein level	acute inflammatory response;alcohol metabolic process;amine metabolic process;aminoglycan biosynthetic process;aminoglycan metabolic process;anatomical structure development;anatomical structure formation involve
25.762	#NUM!	26.0635	26.3934	1:Experimental evidence at protein level	aging;anatomical structure development;biological regulation;cellular component disassembly;cellular component disassembly at cellular level;cellular component organization;cellular component organization at cellular level;
26.6311	26.1962	26.7276	26.6357	1:Experimental evidence at protein level	activation of immune response;activation of innate immune response;biological regulation;cell surface receptor linked signaling pathway;cellular component assembly;cellular component assembly at cellular level;cellular com
#NUM!	26.5361	#NUM!	#NUM!	1:Experimental evidence at protein level	adherens junction organization;anatomical structure morphogenesis;biological adhesion;biological regulation;blood vessel morphogenesis;calcium-dependent cell-cell adhesion;cell adhesion;cell adhesion mediated by integri
#NUM!	26.1375	25.6223	25.6866	1:Experimental evidence at protein level	biological regulation;cell differentiation;cell migration;cell motility;cell surface receptor linked signaling pathway;cellular component movement;cellular developmental process;cellular macromolecule metabolic process;cellula
#NUM!	#NUM!	26.941	27.0101	1:Experimental evidence at protein level	activation of immune response;activation of innate immune response;anaphase-promoting complex-dependent proteasomal ubiquitin-dependent protein catabolic process;antigen processing and presentation;antigen proces
28.6541	28.9305	29.4405	29.1837	1:Experimental evidence at protein level	ATP biosynthetic process;ATP metabolic process;ATP synthesis coupled proton transport;biosynthetic process;cation transport;cellular biosynthetic process;cellular metabolic process;cellular nitrogen compound biosyntheti
26.5217	26.2294	26.1361	#NUM!	1:Experimental evidence at protein level	activation of phospholipase D activity;biological regulation;cellular component organization;cellular component organization at cellular level;cellular component organization or biogenesis;cellular component organization or bi
26.9684	26.8218	27.0881	27.0802	1:Experimental evidence at protein level	alcohol metabolic process;biosynthetic process;carbohydrate biosynthetic process;carbohydrate catabolic process;carbohydrate metabolic process;catabolic process;cellular biosynthetic process;cellular carbohydrate biosy
26.8112	25.994	25.8367	#NUM!	1:Experimental evidence at protein level	
25.4653	25.9083	26.0293	25.9813	1:Experimental evidence at protein level	amine biosynthetic process;amine catabolic process;amine metabolic process;biological regulation;biosynthetic process;carbohydrate homeostasis;carboxylic acid biosynthetic process;carboxylic acid metabolic process;cat
27.3439	27.2511	27.2873	27.4869	1:Experimental evidence at protein level	biological regulation;cellular component organization;cellular component organization at cellular level;cellular component organization or biogenesis;cellular component organization or biogenesis at cellular level;cellular macr
24.2671	24.4966	#NUM!	24.5392	1:Experimental evidence at protein level	apoptosis;cell death;cellular metabolic process;cellular nitrogen compound metabolic process;cellular process;death;metabolic process;nitrogen compound metabolic process;nucleobase-containing compound metabolic pr
25.9381	26.0759	26.0273	26.1862	1:Experimental evidence at protein level	adult heart development;anatomical structure development;biological regulation;biosynthetic process;catabolic process;cell cycle;cell cycle process;cell proliferation;cellular biosynthetic process;cellular catabolic process;cel
25.0698	#NUM!	#NUM!	25.5764	1:Experimental evidence at protein level	anatomical structure development;anatomical structure morphogenesis;axis specification;biological regulation;bone development;calcium ion homeostasis;cation homeostasis;cell morphogenesis;cell morphogenesis involve
26.1817	25.7281	25.8705	25.769	1:Experimental evidence at protein level	activation of anaphase-promoting complex activity;activation of mitotic anaphase-promoting complex activity;anaphase-promoting complex-dependent proteasomal ubiquitin-dependent protein catabolic process;biological r
#NUM!	#NUM!	#NUM!	#NUM!	1:Experimental evidence at protein level	adherens junction organization;anatomical structure formation involved in morphogenesis;angiogenesis;biological adhesion;biological regulation;calcium-dependent cell-cell adhesion;cell adhesion;cell junction assembly;cell
#NUM!	26.5732	26.1611	26.7329	1:Experimental evidence at protein level	biological regulation;cell cycle;cell cycle phase;cell cycle process;cell division;cell surface receptor linked signaling pathway;cellular component organization;cellular component organization at cellular level;cellular componen
24.5387	24.7771	24.6565	24.8412	1:Experimental evidence at protein level	actin cytoskeleton organization;actin filament organization;actin filament-based process;activation of immune response;antigen receptor-mediated signaling pathway;B cell receptor signaling pathway;biological adhesion;biol
25.5766	25.6992	25.4345	25.3718	1:Experimental evidence at protein level	
29.8992	29.8766	29.6091	29.8564	1:Experimental evidence at protein level	activation of immune response;activation of innate immune response;apoptosis;biological regulation;biosynthetic process;BMP signaling pathway;catabolic process;cell death;cell surface receptor linked signaling pathway;ce
27.1369	27.079	27.3891	26.8735	1:Experimental evidence at protein level	anatomical structure development;anatomical structure formation involved in morphogenesis;ATP hydrolysis coupled proton transport;biological regulation;brain development;cation homeostasis;cation transport;cell projectio
26.0369	26.4218	26.356	26.332	1:Experimental evidence at protein level	cellular macromolecule metabolic process;cellular metabolic process;cellular process;cellular protein metabolic process;developmental process;macromolecule metabolic process;macromolecule modification;metabolic proc
#NUM!	26.6548	26.9397	26.2687	1:Experimental evidence at protein level	anatomical structure development;biological regulation;biosynthetic process;cellular biosynthetic process;cellular component organization;cellular component organization at cellular level;cellular component organization or b
25.706	25.6874	26.4336	26.3504	1:Experimental evidence at protein level	cellular component organization;cellular component organization at cellular level;cellular component organization or biogenesis;cellular component organization or biogenesis at cellular level;cellular macromolecule metabolic
29.2735	28.842	28.9844	28.9328	1:Experimental evidence at protein level	biosynthetic process;cellular biosynthetic process;cellular component assembly;cellular component assembly at cellular level;cellular component organization;cellular component organization at cellular level;cellular compone
29.2314	28.8664	28.67	29.0041	1:Experimental evidence at protein level	biological regulation;cellular component assembly;cellular component assembly at cellular level;cellular component organization;cellular component organization at cellular level;cellular component organization or biogenesis;
30.4195	30.1668	30.2559	30.4698	1:Experimental evidence at protein level	antibacterial humoral response;antimicrobial humoral response;biosynthetic process;catabolic process;cellular biosynthetic process;cellular catabolic process;cellular component disassembly;cellular component disassembly
26.9635	27.5983	27.4725	27.4324	1:Experimental evidence at protein level	biological regulation;biosynthetic process;catabolic process;cell surface receptor linked signaling pathway;cellular biosynthetic process;cellular catabolic process;cellular macromolecule biosynthetic process;cellular macrom
25.931	26.2144	26.1675	26.0381	1:Experimental evidence at protein level	biological regulation;biosynthetic process;cellular biosynthetic process;cellular component organization;cellular component organization at cellular level;cellular component organization or biogenesis;cellular component orga
24.6497	#NUM!	24.669	24.4675	1:Experimental evidence at protein level	cellular macromolecule metabolic process;cellular metabolic process;cellular nitrogen compound metabolic process;cellular process;macromolecule metabolic process;metabolic process;nitrogen compound metabolic proce
#NUM!	#NUM!	#NUM!	#NUM!	1:Experimental evidence at protein level	cell killing;defense response;defense response to bacterium;defense response to fungus;killing of cells of other organism;multi-organism process;response to bacterium;response to biotic stimulus;response to fungus;respons
27.726	28.7324	#NUM!	29.1267	1:Experimental evidence at protein level	acrosome assembly;anatomical structure formation involved in morphogenesis;biological regulation;biosynthetic process;cell differentiation;cell motility;cellular biosynthetic process;cellular component assembly;cellular comp
29.3032	29.6562	#NUM!	29.8325	1:Experimental evidence at protein level	antigen processing and presentation;antigen processing and presentation of exogenous antigen;antigen processing and presentation of exogenous peptide antigen;antigen processing and presentation of exogenous peptide
26.0461	#NUM!	26.043	26.2886	1:Experimental evidence at protein level	anatomical structure development;anatomical structure morphogenesis;B cell activation;B cell differentiation;biological regulation;biosynthetic process;cell activation;cell differentiation;cellular biosynthetic process;cellular de
25.6818	#NUM!	26.0575	25.4397	1:Experimental evidence at protein level	anatomical structure development;androgen receptor signaling pathway;biological regulation;brain development;catabolic process;cellular catabolic process;cellular component organization;cellular component organization o
#NUM!	#NUM!	#NUM!	#NUM!	1:Experimental evidence at protein level	B cell activation;biological regulation;cell activation;cell communication;cell proliferation;cell surface receptor linked signaling pathway;cell-cell signaling;cellular process;cellular response to chemical stimulus;cellular respons
#NUM!	#NUM!	#NUM!	#NUM!	1:Experimental evidence at protein level	biological regulation;cell surface receptor linked signaling pathway;cellular component organization;cellular component organization at cellular level;cellular component organization or biogenesis;cellular component organizat
25.3462	26.0198	25.7044	26.0115	1:Experimental evidence at protein level	cellular macromolecule metabolic process;cellular metabolic process;cellular process;cellular protein metabolic process;dephosphorylation;macromolecule metabolic process;macromolecule modification;metabolic process;
#NUM!	#NUM!	#NUM!	#NUM!	1:Experimental evidence at protein level	axon guidance;cell communication;cell-cell signaling;cellular process;chemotaxis;locomotion;response to chemical stimulus;response to external stimulus;response to stimulus;signaling;taxis
#NUM!	26.134	26.6267	26.4982	1:Experimental evidence at protein level	biological regulation;catabolic process;cellular catabolic process;cellular macromolecule catabolic process;cellular macromolecule metabolic process;cellular metabolic process;cellular process;cellular protein metabolic proc
#NUM!	27.2829	27.3918	27.7395	1:Experimental evidence at protein level	anatomical structure development;biological regulation;biosynthetic process;cap-independent translational initiation;cell cycle process;cell surface receptor linked signaling pathway;cellular biosynthetic process;cellular macr
25.2169	25.2957	25.0189	25.0781	1:Experimental evidence at protein level	catabolic process;cellular catabolic process;cellular macromolecule catabolic process;cellular macromolecule metabolic process;cellular metabolic process;cellular process;cellular protein metabolic process;macromolecule
29.9868	29.7426	29.9732	30.0629	1:Experimental evidence at protein level	biological regulation;biosynthetic process;cellular biosynthetic process;cellular macromolecule biosynthetic process;cellular macromolecule metabolic process;cellular metabolic process;cellular nitrogen compound metaboli
24.7685	#NUM!	25.3832	25.1386	1:Experimental evidence at protein level	anatomical structure development;cellular macromolecule metabolic process;cellular metabolic process;cellular nitrogen compound metabolic process;cellular process;developmental process;macromolecule metabolic proce
#NUM!	#NUM!	#NUM!	#NUM!	1:Experimental evidence at protein level	acute inflammatory response;acute-phase response;amine metabolic process;aminoglycan metabolic process;biological regulation;carbohydrate metabolic process;defense response;glycosaminoglycan metabolic process;h
26.0936	25.8749	25.875	25.901	1:Experimental evidence at protein level	biological regulation;biosynthetic process;cellular biosynthetic process;cellular component organization;cellular component organization at cellular level;cellular component organization or biogenesis;cellular component orga
25.406	25.6951	25.6863	25.8296	1:Experimental evidence at protein level	apoptosis;cell death;cellular component organization;cellular component organization or biogenesis;cellular membrane organization;cellular process;death;endocytosis;establishment of localization;establishment of protein lo
25.1099	#NUM!	25.5355	24.8552	1:Experimental evidence at protein level	
26.7001	26.928	27.2082	27.2839	1:Experimental evidence at protein level	anatomical structure development;anatomical structure formation involved in morphogenesis;angiogenesis;biological regulation;biomineral tissue development;cellular process;cellular response to stimulus;defense response;
26.6983	#NUM!	26.3213	26.8715	1:Experimental evidence at protein level	cell differentiation;cellular developmental process;cellular process;developmental process;osteoblast differentiation
25.3424	26.063	26.1797	26.2418	1:Experimental evidence at protein level	
25.0153	26.2009	25.3222	25.3643	1:Experimental evidence at protein level	biological regulation;cellular process;cellular response to stimulus;establishment of localization;establishment of localization in cell;establishment of protein localization;intracellular protein transport;intracellular signal transdu
26.1663	#NUM!	26.0525	25.9367	1:Experimental evidence at protein level	cellular macromolecule metabolic process;cellular metabolic process;cellular nitrogen compound metabolic process;cellular process;cleavage in ITS2 between 5.8S rRNA and LSU-rRNA of tricistronic rRNA transcript (SSU-r
26.0936	26.2675	25.874	26.2001	1:Experimental evidence at protein level	actin filament-based movement;actin filament-based process;anatomical structure formation involved in morphogenesis;autophagy;biological regulation;catabolic process;cell projection assembly;cell projection organization;
25.6532	#NUM!	25.4678	25.8398	1:Experimental evidence at protein level	
#NUM!	#NUM!	#NUM!	#NUM!	1:Experimental evidence at protein level	
24.5733	25.5326	24.6559	25.1435	1:Experimental evidence at protein level	actin cytoskeleton organization;actin filament-based process;biological regulation;cellular component organization;cellular component organization at cellular level;cellular component organization or biogenesis;cellular comp
26.5985	26.5664	26.7981	26.4398	1:Experimental evidence at protein level	alcohol metabolic process;biosynthetic process;lipid biosynthetic process;lipid metabolic process;metabolic process;oxidation-reduction process;primary metabolic process;small molecule metabolic process;steroid biosynth
26.0546	26.2048	26.4323	26.2533	1:Experimental evidence at protein level	biological regulation;biosynthetic process;cellular biosynthetic process;cellular macromolecule biosynthetic process;cellular macromolecule metabolic process;cellular metabolic process;cellular nitrogen compound metaboli
25.7714	#NUM!	24.9026	24.5561	1:Experimental evidence at protein level	
26.6468	26.4587	26.3482	26.8985	1:Experimental evidence at protein level	anatomical structure development;biological regulation;cardiovascular system development;cell differentiation;cell surface receptor linked signaling pathway;cellular developmental process;cellular process;cellular response t
#NUM!	#NUM!	#NUM!	25.8219	1:Experimental evidence at protein level	
#NUM!	#NUM!	#NUM!	#NUM!	1:Experimental evidence at protein level	apoptosis;biological regulation;carbohydrate metabolic process;cell death;cellular carbohydrate metabolic process;cellular component organization;cellular component organization at cellular level;cellular component organiza
25.852	#NUM!	25.8483	25.8318	1:Experimental evidence at protein level	
#NUM!	25.4798	25.5746	25.0496	1:Experimental evidence at protein level	lipid metabolic process;metabolic process;primary metabolic process
25.9724	25.9604	25.9389	25.3808	1:Experimental evidence at protein level	biological regulation;defense response;defense response to virus;immune effector process;immune system process;multi-organism process;negative regulation of biological process;negative regulation of cell cycle process;n
26.0554	#NUM!	26.0877	26.0396	1:Experimental evidence at protein level	
25.6826	#NUM!	25.8391	25.6836	1:Experimental evidence at protein level	activation of immune response;activation of innate immune response;apoptosis;biological regulation;cell death;cellular process;cellular response to chemical stimulus;cellular response to cytokine stimulus;cellular response t
26.5217	#NUM!	26.6113	26.2232	1:Experimental evidence at protein level	
24.8806	#NUM!	24.4327	24.7678	1:Experimental evidence at protein level	cellular macromolecule metabolic process;cellular metabolic process;cellular nitrogen compound metabolic process;cellular process;macromolecule metabolic process;metabolic process;mRNA metabolic process;mRNA pro
25.7687	#NUM!	25.2542	25.5315	1:Experimental evidence at protein level	

26.483	26.9316	26.6122	26.7736	1:Experimental evidence at protein level	
25.4944	25.5587	25.2509	25.2523	1:Experimental evidence at protein level	anatomical structure development;biological regulation;carbohydrate metabolic process;cardiovascular system development;cell surface receptor linked signaling pathway;cellular carbohydrate metabolic process;cellular ma
27.3034	27.3124	27.6088	27.4166	1:Experimental evidence at protein level	biological regulation;cell cycle;cell cycle phase;cell cycle process;cell division;cellular component organization;cellular component organization at cellular level;cellular component organization or biogenesis;cellular componen
26.6957	26.7761	26.7999	26.4703	1:Experimental evidence at protein level	
26.4985	26.1703	26.2576	26.1697	1:Experimental evidence at protein level	biological regulation;cell surface receptor linked signaling pathway;cellular process;cellular response to chemical stimulus;cellular response to fibroblast growth factor stimulus;cellular response to growth factor stimulus;cellul
26.1776	27.3348	28.0368	27.0334	1:Experimental evidence at protein level	biological regulation;biosynthetic process;carbohydrate metabolic process;carbohydrate transport;cell cycle;cell cycle phase;cell cycle process;cell division;cell surface receptor linked signaling pathway;cellular biosynthetic p
#NUM!	#NUM!	#NUM!	#NUM!	1:Experimental evidence at protein level	adhesion to host;adhesion to other organism involved in symbiotic interaction;alcohol metabolic process;androgen biosynthetic process;androgen metabolic process;biological adhesion;biological regulation;biosynthetic proc
26.6526	26.3883	26.1509	26.2343	1:Experimental evidence at protein level	biological regulation;biosynthetic process;catabolic process;cell surface receptor linked signaling pathway;cellular biosynthetic process;cellular catabolic process;cellular macromolecule biosynthetic process;cellular macrom
26.5198	#NUM!	26.4934	26.6102	1:Experimental evidence at protein level	catabolic process;cellular catabolic process;cellular macromolecule catabolic process;cellular macromolecule metabolic process;cellular metabolic process;cellular nitrogen compound metabolic process;cellular process;exo
25.5398	26.1613	25.7529	25.7817	1:Experimental evidence at protein level	alcohol metabolic process;carbohydrate metabolic process;cellular carbohydrate metabolic process;cellular metabolic process;cellular process;galactose metabolic process;glucose metabolic process;hexose metabolic proc
27.4531	26.927	26.9959	27.1348	1:Experimental evidence at protein level	biological regulation;catabolic process;cellular catabolic process;cellular macromolecule catabolic process;cellular macromolecule metabolic process;cellular metabolic process;cellular nitrogen compound metabolic process
26.1912	25.7126	26.1031	25.6281	1:Experimental evidence at protein level	biological regulation;cell cycle;cell cycle checkpoint;cell cycle process;cell division;cellular component organization;cellular component organization at cellular level;cellular component organization or biogenesis;cellular comp
25.5845	25.9285	25.7988	#NUM!	1:Experimental evidence at protein level	biological regulation;biosynthetic process;cellular biosynthetic process;cellular component organization;cellular component organization at cellular level;cellular component organization or biogenesis;cellular component orga
26.4975	26.1374	25.8241	25.8371	1:Experimental evidence at protein level	biosynthetic process;cellular biosynthetic process;cellular component disassembly;cellular component disassembly at cellular level;cellular component organization;cellular component organization at cellular level;cellular cor
26.1818	#NUM!	#NUM!	26.4512	1:Experimental evidence at protein level	biosynthetic process;cellular biosynthetic process;cellular component disassembly;cellular component disassembly at cellular level;cellular component organization;cellular component organization at cellular level;cellular cor
27.0217	27.024	26.8742	26.7438	1:Experimental evidence at protein level	abscission;aging;anaphase-promoting complex-dependent proteasomal ubiquitin-dependent protein catabolic process;attachment of spindle microtubules to chromosome;attachment of spindle microtubules to kinetochore;
#NUM!	25.4986	25.1151	25.2824	1:Experimental evidence at protein level	biological regulation;cellular macromolecule metabolic process;cellular metabolic process;cellular nitrogen compound metabolic process;cellular process;cellular response to stimulus;cellular response to stress;cytoplasmic s
25.4889	#NUM!	25.6777	25.6287	1:Experimental evidence at protein level	abscission;biological regulation;cell cycle;cell division;cellular process;developmental process;negative regulation of biological process;negative regulation of cell division;negative regulation of cellular process;negative regula
25.7454	25.6392	25.8906	25.836	1:Experimental evidence at protein level	
26.3358	26.2949	26.5707	26.6619	1:Experimental evidence at protein level	
27.8231	27.8374	27.7061	27.9602	1:Experimental evidence at protein level	biological regulation;cell cycle cytokinesis;cell cycle phase;cell cycle process;cellular component organization;cellular component organization at cellular level;cellular component organization or biogenesis;cellular componen
28.955	#NUM!	#NUM!	#NUM!	1:Experimental evidence at protein level	multicellular organismal process;neurological system process;response to stimulus;sensory perception;sensory perception of light stimulus;system process;visual perception
25.7907	26.0113	25.8923	25.7251	1:Experimental evidence at protein level	axon guidance;biological regulation;cellular component assembly;cellular component organization;cellular component organization at cellular level;cellular component organization or biogenesis;cellular component organizatio
27.1167	27.0954	27.5753	27.5521	1:Experimental evidence at protein level	biological regulation;biosynthetic process;cellular biosynthetic process;cellular macromolecule biosynthetic process;cellular macromolecule metabolic process;cellular metabolic process;cellular nitrogen compound metabolic
25.4676	25.3451	25.9155	25.9178	1:Experimental evidence at protein level	cellular macromolecule metabolic process;cellular metabolic process;cellular nitrogen compound metabolic process;cellular process;macromolecule metabolic process;maturation of 5.8S rRNA;metabolic process;ncRNA me
26.2458	26.0958	26.0528	25.8313	1:Experimental evidence at protein level	behavior;biological regulation;body fluid secretion;carbohydrate homeostasis;cellular chemical homeostasis;cellular glucose homeostasis;cellular homeostasis;cellular ion homeostasis;cellular metabolic process;cellular nitrog
24.9953	24.6278	#NUM!	25.3055	1:Experimental evidence at protein level	biological regulation;cellular component assembly;cellular component assembly at cellular level;cellular component biogenesis;cellular component organization;cellular component organization at cellular level;cellular compor
26.1245	#NUM!	#NUM!	26.2305	1:Experimental evidence at protein level	cell cycle phase;cell cycle process;cell division;cellular component assembly;cellular component assembly at cellular level;cellular component organization;cellular component organization at cellular level;cellular component
26.1438	26.2457	26.1263	26.3047	1:Experimental evidence at protein level	establishment of localization;lipid transport;organic substance transport;transport
29.4463	29.6249	29.6676	29.4377	1:Experimental evidence at protein level	biological regulation;cellular process;cellular response to abiotic stimulus;cellular response to gamma radiation;cellular response to ionizing radiation;cellular response to radiation;cellular response to stimulus;cellular respons
28.4071	27.7947	28.3241	28.6175	1:Experimental evidence at protein level	cellular macromolecule metabolic process;cellular metabolic process;cellular nitrogen compound metabolic process;cellular process;gene expression;macromolecule metabolic process;metabolic process;mRNA metabolic p
#NUM!	#NUM!	#NUM!	#NUM!	1:Experimental evidence at protein level	biological regulation;cell differentiation;cellular chemical homeostasis;cellular component assembly;cellular component organization;cellular component organization or biogenesis;cellular developmental process;cellular home
26.3711	26.4652	26.3739	26.374	1:Experimental evidence at protein level	cell differentiation;cellular developmental process;cellular process;developmental process
26.2528	#NUM!	26.1817	26.3681	1:Experimental evidence at protein level	anatomical structure morphogenesis;apoptotic mitochondrial changes;biological regulation;cell part morphogenesis;cellular component assembly;cellular component morphogenesis;cellular component organization;cellular c
25.757	26.0848	25.738	#NUM!	1:Experimental evidence at protein level	biosynthetic process;cellular biosynthetic process;cellular component disassembly;cellular component disassembly at cellular level;cellular component organization;cellular component organization at cellular level;cellular cor
24.5002	#NUM!	24.082	24.0669	1:Experimental evidence at protein level	
25.07	25.479	24.8819	25.4422	1:Experimental evidence at protein level	activation of MAPKK activity;activation of protein kinase activity;anatomical structure development;biological regulation;cell cycle checkpoint;cell cycle process;cellular macromolecule metabolic process;cellular metabolic pro
24.266	#NUM!	#NUM!	#NUM!	1:Experimental evidence at protein level	biological regulation;cell cycle;cell cycle phase;cell cycle process;cell division;cellular component organization;cellular component organization at cellular level;cellular component organization or biogenesis;cellular componen
26.5509	26.5113	26.7168	26.4562	1:Experimental evidence at protein level	
26.4088	#NUM!	26.1308	26.2279	1:Experimental evidence at protein level	cellular macromolecule metabolic process;cellular metabolic process;cellular process;cellular protein metabolic process;cullin deneddylation;macromolecule metabolic process;macromolecule modification;metabolic process
25.4712	#NUM!	#NUM!	25.6486	1:Experimental evidence at protein level	cell differentiation;cellular developmental process;cellular process;developmental process;osteoblast differentiation
27.2735	27.557	27.4713	27.2985	1:Experimental evidence at protein level	biological regulation;calcium ion homeostasis;calcium ion transport;carbohydrate metabolic process;cation homeostasis;cation transport;cellular calcium ion homeostasis;cellular carbohydrate metabolic process;cellular catio
24.4651	#NUM!	24.6995	24.5007	1:Experimental evidence at protein level	biological regulation;biosynthetic process;cellular biosynthetic process;cellular component organization;cellular component organization at cellular level;cellular component organization or biogenesis;cellular component orga
25.4709	25.1462	25.382	25.0922	1:Experimental evidence at protein level	biological regulation;cell cycle arrest;cell cycle process;cellular process;negative regulation of autophagy;negative regulation of biological process;negative regulation of catabolic process;negative regulation of cell cycle;nega
25.1235	25.2937	25.3144	25.129	1:Experimental evidence at protein level	glomerular filtration;macromolecule metabolic process;metabolic process;multicellular organismal process;primary metabolic process;protein maturation;protein metabolic process;protein processing;renal system process;sy
27.8255	27.7665	27.9942	27.9626	1:Experimental evidence at protein level	apoptosis;biological regulation;carbohydrate homeostasis;cell death;cellular component organization;cellular component organization or biogenesis;cellular membrane organization;cellular process;cellular response to biotic s
#NUM!	26.178	25.5857	25.844	1:Experimental evidence at protein level	anatomical structure morphogenesis;asymmetric protein localization;biological regulation;cell communication;cell-cell signaling;cellular process;developmental process;establishment of localization;establishment of localizatio
26.4453	#NUM!	#NUM!	26.339	1:Experimental evidence at protein level	anatomical structure development;biological regulation;cell surface receptor linked signaling pathway;cellular process;cellular response to stimulus;developmental process;enzyme linked receptor protein signaling pathway;in
26.3563	26.0977	26.5958	26.308	1:Experimental evidence at protein level	
25.249	25.3617	#NUM!	25.0845	1:Experimental evidence at protein level	
27.6139	27.9143	27.7587	27.7921	1:Experimental evidence at protein level	biological regulation;biosynthetic process;cellular biosynthetic process;cellular component assembly;cellular component assembly at cellular level;cellular component organization;cellular component organization at cellular le
26.2789	26.2059	26.1793	26.3785	1:Experimental evidence at protein level	cellular metabolic process;cellular process;electron transport chain;generation of precursor metabolites and energy;metabolic process;mitochondrial electron transport, ubiquinol to cytochrome c;oxidation-reduction process;
25.2608	#NUM!	25.1164	25.039	1:Experimental evidence at protein level	autophagy;catabolic process;cellular catabolic process;cellular metabolic process;cellular process;endosome transport;establishment of localization;establishment of localization in cell;establishment of protein localization;int
24.675	#NUM!	25.2953	24.8836	1:Experimental evidence at protein level	amine metabolic process;aspartate family amino acid metabolic process;carboxylic acid metabolic process;cellular amine metabolic process;cellular amino acid metabolic process;cellular ketone metabolic process;cellular m
#NUM!	#NUM!	#NUM!	#NUM!	1:Experimental evidence at protein level	anatomical structure development;anatomical structure morphogenesis;biological regulation;blood vessel morphogenesis;cardiac atrium morphogenesis;cardiac chamber morphogenesis;cardiac muscle tissue growth;cardiac
25.7242	#NUM!	25.2986	25.1523	1:Experimental evidence at protein level	cellular macromolecule metabolic process;cellular metabolic process;cellular nitrogen compound metabolic process;cellular process;macromolecule metabolic process;metabolic process;mRNA metabolic process;mRNA pro
27.3113	27.4007	27.9655	27.6783	1:Experimental evidence at protein level	
25.4134	26.2446	#NUM!	26.5882	1:Experimental evidence at protein level	cellular macromolecule metabolic process;cellular metabolic process;cellular process;cellular protein metabolic process;chaperone-mediated protein transport;establishment of localization;establishment of localization in cell;
25.6465	26.3832	26.5898	26.6901	1:Experimental evidence at protein level	cellular macromolecule metabolic process;cellular metabolic process;cellular process;cellular protein metabolic process;chaperone-mediated protein transport;establishment of localization;establishment of localization in cell;
26.8836	26.6935	26.426	26.6316	1:Experimental evidence at protein level	alcohol biosynthetic process;alcohol metabolic process;biosynthetic process;carbohydrate biosynthetic process;carbohydrate metabolic process;cellular biosynthetic process;cellular carbohydrate biosynthetic process;cellul
27.4569	27.2386	27.1004	27.2985	1:Experimental evidence at protein level	cellular component organization;cellular component organization at cellular level;cellular component organization or biogenesis;cellular component organization or biogenesis at cellular level;cellular membrane organization;ce
26.0818	#NUM!	25.7502	26.21	1:Experimental evidence at protein level	biological regulation;biosynthetic process;cell cycle;cell surface receptor linked signaling pathway;cellular biosynthetic process;cellular component organization;cellular component organization at cellular level;cellular compor
26.2432	26.039	25.7924	26.3451	1:Experimental evidence at protein level	anatomical structure development;biological regulation;cell surface receptor linked signaling pathway;cellular component organization;cellular component organization or biogenesis;cellular membrane organization;cellular pro
26.853	27.1869	27.5009	27.5802	1:Experimental evidence at protein level	catabolic process;cell differentiation;cellular catabolic process;cellular developmental process;cellular macromolecule catabolic process;cellular macromolecule metabolic process;cellular metabolic process;cellular nitrogen c
27.8815	28.5442	28.311	28.4306	1:Experimental evidence at protein level	activation of anaphase-promoting complex activity;anaphase-promoting complex-dependent proteasomal ubiquitin-dependent protein catabolic process;biological regulation;catabolic process;cell cycle;cell cycle checkpoint
24.5429	24.7283	#NUM!	24.5832	1:Experimental evidence at protein level	cellular component assembly;cellular component organization;cellular component organization at cellular level;cellular component organization or biogenesis;cellular component organization or biogenesis at cellular level;cellu
27.7217	27.9612	27.8388	28.1766	1:Experimental evidence at protein level	biological regulation;cell communication;cell migration;cell motility;cell surface receptor linked signaling pathway;cell-cell signaling;cellular component movement;cellular process;cellular response to stimulus;endothelial cell r
27.2716	#NUM!	#NUM!	27.2364	1:Experimental evidence at protein level	cellular process;establishment of localization;establishment of localization in cell;establishment of protein localization;Golgi vesicle transport;intracellular transport;post-Golgi vesicle-mediated transport;protein transport;trans
28.2149	28.1755	28.1002	28.3063	1:Experimental evidence at protein level	biological regulation;cellular component organization;cellular component organization or biogenesis;cellular localization;cellular macromolecule localization;cellular membrane organization;cellular process;cellular protein local
24.8394	25.1212	#NUM!	24.8812	1:Experimental evidence at protein level	
28.1523	28.1681	27.8968	28.0691	1:Experimental evidence at protein level	metabolic process;oxidation-reduction process
26.0534	26.6669	26.4615	#NUM!	1:Experimental evidence at protein level	biological adhesion;biological regulation;cell adhesion;cell surface receptor linked signaling pathway;cell-cell adhesion;cellular process;cellular response to chemical stimulus;cellular response to fibroblast growth factor stimu
27.5156	27.9316	27.8168	27.7984	1:Experimental evidence at protein level	cellular process;establishment of localization;transport;vesicle-mediated transport
26.7522	26.5686	26.7191	26.5819	1:Experimental evidence at protein level	cellular metabolic process;cellular process;electron transport chain;generation of precursor metabolites and energy;metabolic process;mitochondrial electron transport, NADH to ubiquinone;oxidation-reduction process;respi
25.4865	26.6714	26.8876	27.0052	1:Experimental evidence at protein level	anatomical structure development;cellular macromolecule metabolic process;cellular metabolic process;cellular process;cellular protein metabolic process;chaperone-mediated protein transport;developmental process;estab
28.0631	27.9939	28.3384	28.3093	1:Experimental evidence at protein level	alternative nuclear mRNA splicing, via spliceosome;biological regulation;biosynthetic process;cell projection organization;cellular biosynthetic process;cellular component organization;cellular component organization at cellul
24.8571	#NUM!	24.5464	24.5792	1:Experimental evidence at protein level	biological regulation;cell cycle arrest;cell cycle process;cell division;cellular process;negative regulation of biological process;negative regulation of cell cycle;negative regulation of cellular process;positive regulation of biolog
25.9945	26.6821	26.4944	26.0493	1:Experimental evidence at protein level	cellular macromolecule metabolic process;cellular metabolic process;cellular nitrogen compound metabolic process;cellular process;macromolecule metabolic process;metabolic process;ncRNA metabolic process;ncRNA pr
28.7747	28.9417	28.8527	28.6008	1:Experimental evidence at protein level	ATP biosynthetic process;ATP metabolic process;ATP synthesis coupled proton transport;biosynthetic process;cation transport;cellular biosynthetic process;cellular metabolic process;cellular nitrogen compound biosyntheti
24.8077	#NUM!	25.3042	25.0538	1:Experimental evidence at protein level	cellular component assembly;cellular component assembly at cellular level;cellular component organization;cellular component organization at cellular level;cellular component organization or biogenesis;cellular component c
27.5106	27.4165	27.2056	27.1983	1:Experimental evidence at protein level	cellular metabolic process;cellular process;electron transport chain;generation of precursor metabolites and energy;metabolic process;mitochondrial electron transport, NADH to ubiquinone;oxidation-reduction process;respi
25.9993	25.4542	25.4441	25.609	1:Experimental evidence at protein level	cellular macromolecule metabolic process;cellular metabolic process;cellular nitrogen compound metabolic process;cellular process;macromolecule metabolic process;metabolic process;ncRNA metabolic process;ncRNA pr
26.2794	26.3648	26.3452	26.5444	1:Experimental evidence at protein level	biological regulation;cell cycle cytokinesis;cell cycle process;cellular macromolecule metabolic process;cellular metabolic process;cellular process;cellular protein metabolic process;cellular response to stimulus;cytokinesis;c
27.4578	27.1937	26.97	26.9219	1:Experimental evidence at protein level	
26.1218	#NUM!	25.844	26.2107	1:Experimental evidence at protein level	anatomical structure development;ATP-dependent chromatin remodeling;biological regulation;biosynthetic process;cellular biosynthetic process;cellular component organization;cellular component organization at cellular leve
28.0503	28.1333	28.0185	28.2003	1:Experimental evidence at protein level	carboxylic acid metabolic process;cellular ketone metabolic process;cellular metabolic process;cellular process;L-ascorbic acid metabolic process;metabolic process;organic acid metabolic process;oxoacid metabolic proce
27.025	27.2797	27.0811	27.4241	1:Experimental evidence at protein level	amine biosynthetic process;amine metabolic process;aromatic compound biosynthetic process;biological regulation;biosynthetic process;carboxylic acid biosynthetic process;carboxylic acid metabolic process;cell cycle;cell
25.9973	27.671	27.299	27.6708	1:Experimental evidence at protein level	alcohol biosynthetic process;alcohol metabolic process;anatomical structure development;anatomical structure formation involved in morphogenesis;anatomical structure homeostasis;anatomical structure morphogenesis;ap
27.4085	28.2694	28.165	28.4714	1:Experimental evidence at protein level	adult behavior;adult locomotory behavior;behavior;biological regulation;locomotory behavior;multicellular organismal process;negative regulation of biological process;negative regulation of catalytic activity;negative regulatio
#NUM!	25.8977	25.2624	25.2251	1:Experimental evidence at protein level	biological regulation;carbohydrate catabolic process;carbohydrate metabolic process;catabolic process;cellular carbohydrate catabolic process;cellular carbohydrate metabolic process;cellular catabolic process;cellular lipid
26.6154	28.0561	28.0285	27.7967	1:Experimental evidence at protein level	activation of immune response;activation of innate immune response;adaptive immune response;antigen processing and presentation;antigen processing and presentation of exogenous antigen;antigen processing and prese
#NUM!	#NUM!	#NUM!	#NUM!	1:Experimental evidence at protein level	anatomical structure formation involved in morphogenesis;anatomical structure maturation;anatomical structure morphogenesis;angiogenesis;axon guidance;biological regulation;blood vessel maturation;bone trabecula form
28.1647	28.1629	27.5797	27.7752	1:Experimental evidence at protein level	biological adhesion;biological regulation;blood coagulation;cell activation;cell adhesion;cell migration;cell motility;cell-matrix adhesion;cell-substrate adhesion;cellular component movement;cellular localization;cellular macror
26.1369	26.5516	26.7391	26.7352	1:Experimental evidence at protein level	amine catabolic process;amine metabolic process;anatomical structure development;aromatic amino acid family catabolic process;aromatic amino acid family metabolic process;aromatic compound biosynthetic process;aro
26.5309	25.903	#NUM!	26.1373	1:Experimental evidence at protein level	biosynthetic process;cellular biosynthetic process;cellular macromolecule metabolic process;cellular metabolic process;cellular nitrogen compound metabolic process;cellular process;electron transport chain;gene expressio
27.2341	#NUM!	26.6311	#NUM!	1:Experimental evidence at protein level	biological regulation;biosynthetic process;cellular biosynthetic process;cellular macromolecule biosynthetic process;cellular macromolecule metabolic process;cellular metabolic process;cellular nitrogen compound metabolic
25.4597	#NUM!	#NUM!	#NUM!	1:Experimental evidence at protein level	apoptosis;biological regulation;biosynthetic process;catabolic process;cell death;cell differentiation;cellular biosynthetic process;cellular catabolic process;cellular developmental process;cellular macromolecule biosynthetic p
#NUM!	#NUM!	#NUM!	#NUM!	1:Experimental evidence at protein level	
28.1021	28.4976	28.2004	28.4368	1:Experimental evidence at protein level	cellular macromolecule metabolic process;cellular metabolic process;cellular process;cellular protein metabolic process;macromolecule metabolic process;macromolecule modification;metabolic process;peptidyl-amino acid
27.5139	27.4137	28.0936	27.7023	1:Experimental evidence at protein level	anatomical structure homeostasis;base-excision repair;biological regulation;biosynthetic process;catabolic process;cell cycle;cell cycle process;cellular biosynthetic process;cellular catabolic process;cellular component asse
27.6469	27.9757	28.4829	28.2872	1:Experimental evidence at protein level	anatomical structure development;anatomical structure morphogenesis;biological regulation;cell development;cell part morphogenesis;cell projection morphogenesis;cell projection organization;cell surface receptor linked sig
#NUM!	#NUM!	#NUM!	#NUM!	1:Experimental evidence at protein level	aging;anatomical structure development;behavior;biological regulation;carbohydrate homeostasis;cell communication;cell proliferation;cellular chemical homeostasis;cellular glucose homeostasis;cellular homeostasis;cellular
25.6857	25.735	25.5488	#NUM!	1:Experimental evidence at protein level	aging;amine metabolic process;arachidonic acid metabolic process;biological regulation;carboxylic acid metabolic process;catabolic process;cellular amine metabolic process;cellular amino acid metabolic process;cellular ca
27.3549	27.244	27.4108	27.5334	1:Experimental evidence at protein level	aging;biological regulation;biosynthetic process;carbohydrate metabolic process;carbohydrate transport;cell aging;cell cycle;cell cycle process;cell death;cell surface receptor linked signaling pathway;cellular biosynthetic pro
26.7092	26.7243	26.7939	26.5275	1:Experimental evidence at protein level	acute inflammatory response;anatomical structure formation involved in morphogenesis;angiogenesis;angiogenesis involved in wound healing;biological regulation;blood coagulation;blood coagulation, intrinsic pathway;carb
#NUM!	25.315	25.4724	25.8869	1:Experimental evidence at protein level	cellular macromolecule metabolic process;cellular metabolic process;cellular process;cellular protein metabolic process;macromolecule metabolic process;macromolecule modification;metabolic process;peptidyl-amino acid
27.3749	#NUM!	#NUM!	28.609	1:Experimental evidence at protein level	activation of immune response;activation of innate immune response;anatomical structure development;apoptotic mitochondrial changes;biological regulation;cell projection organization;cellular component organization;cellu
#NUM!	25.7881	26.0424	25.9706	1:Experimental evidence at protein level	biological regulation;cell cycle checkpoint;cellular component organization;cellular component organization at cellular level;cellular component organization or biogenesis;cellular component organization or biogenesis at cellu
26.8634	#NUM!	26.7013	26.7634	1:Experimental evidence at protein level	amine biosynthetic process;amine metabolic process;apoptotic mitochondrial changes;biological regulation;biosynthetic process;carboxylic acid biosynthetic process;carboxylic acid metabolic process;cellular amine metabo
27.3268	28.5436	28.4815	29.0676	1:Experimental evidence at protein level	anatomical structure development;anatomical structure morphogenesis;biological regulation;cell proliferation;cellular process;cellular response to abiotic stimulus;cellular response to antibiotic;cellular response to chemical st
25.785	#NUM!	26.056	26.2885	1:Experimental evidence at protein level	androgen receptor signaling pathway;biological regulation;biosynthetic process;catabolic process;cell cycle;cell cycle arrest;cell cycle process;cell division;cell proliferation;cellular biosynthetic process;cellular catabolic proce
27.9742	28.3345	28.4027	28.3121	1:Experimental evidence at protein level	adult behavior;adult locomotory behavior;anatomical structure development;associative learning;behavior;biological regulation;brain development;catabolic process;cation homeostasis;cell development;cellular catabolic pro
26.8455	#NUM!	#NUM!	28.045	1:Experimental evidence at protein level	activation of caspase activity;apoptosis;autophagy;biological regulation;catabolic process;cell communication;cell death;cellular catabolic process;cellular metabolic process;cellular process;cellular response to amino acid st
25.7535	26.268	26.0346	25.8859	1:Experimental evidence at protein level	acetyl-CoA catabolic process;acetyl-CoA metabolic process;carbohydrate metabolic process;carboxylic acid metabolic process;catabolic process;cellular catabolic process;cellular ketone metabolic process;cellular metabol
#NUM!	#NUM!	#NUM!	#NUM!	1:Experimental evidence at protein level	biological regulation;calcium ion transmembrane transport;calcium ion transport;cation transport;cellular process;divalent inorganic cation transport;divalent metal ion transport;establishment of localization;ion transmembran
28.0314	27.682	27.7112	27.7742	1:Experimental evidence at protein level	ATP biosynthetic process;ATP metabolic process;ATP synthesis coupled proton transport;biosynthetic process;cation transport;cellular biosynthetic process;cellular metabolic process;cellular nitrogen compound biosyntheti
28.5306	27.669	28.0486	28.1166	1:Experimental evidence at protein level	cellular component assembly;cellular component assembly at cellular level;cellular component organization;cellular component organization at cellular level;cellular component organization or biogenesis;cellular component c
28.2171	28.5368	29.0665	28.9803	1:Experimental evidence at protein level	alcohol metabolic process;amine metabolic process;anatomical structure development;anatomical structure morphogenesis;benzene-containing compound metabolic process;biological regulation;catecholamine metabolic p
#NUM!	#NUM!	#NUM!	#NUM!	1:Experimental evidence at protein level	activation of MAPK activity;biological regulation;biosynthetic process;cell proliferation;cellular biosynthetic process;cellular lipid metabolic process;cellular macromolecule metabolic process;cellular metabolic process;cellular
28.5057	28.2107	28.1762	27.9508	1:Experimental evidence at protein level	cellular component organization;cellular component organization at cellular level;cellular component organization or biogenesis;cellular component organization or biogenesis at cellular level;cellular process;COPI coating of G
27.7952	29.3703	29.1148	28.9015	1:Experimental evidence at protein level	establishment of localization;establishment of localization in cell;establishment of protein localization;intracellular protein transport;intracellular transport;nuclear export;nuclear transport;nucleocytoplasmic transport;protein ex
27.3005	27.5153	27.5534	27.7325	1:Experimental evidence at protein level	catabolic process;cellular catabolic process;cellular macromolecule catabolic process;cellular macromolecule metabolic process;cellular metabolic process;cellular nitrogen compound metabolic process;cellular process;exo
27.4936	27.2768	27.6192	27.5014	1:Experimental evidence at protein level	catabolic process;cellular catabolic process;cellular macromolecule catabolic process;cellular macromolecule metabolic process;cellular metabolic process;cellular nitrogen compound metabolic process;cellular process;exo
30.7864	29.9914	30.621	30.5077	1:Experimental evidence at protein level	90S preribosome assembly;anatomical structure development;anatomical structure morphogenesis;axial mesoderm development;biological regulation;biosynthetic process;catabolic process;cellular biosynthetic process;cell
28.1458	27.7544	27.9412	27.4878	1:Experimental evidence at protein level	adiponectin-mediated signaling pathway;anatomical structure morphogenesis;axon guidance;biological regulation;cell cycle;cell surface receptor linked signaling pathway;cellular component assembly;cellular component ass
25.5521	25.5857	25.2333	25.2102	1:Experimental evidence at protein level	anatomical structure formation involved in morphogenesis;anatomical structure morphogenesis;axon cargo transport;axon guidance;biological adhesion;biological regulation;cell adhesion;cell junction assembly;cell junction c
25.7425	#NUM!	25.4697	25.5745	1:Experimental evidence at protein level	biosynthetic process;cellular biosynthetic process;cellular component disassembly;cellular component disassembly at cellular level;cellular component organization;cellular component organization at cellular level;cellular cor
25.7531	25.3031	25.596	25.9369	1:Experimental evidence at protein level	
26.0069	26.1486	26.4354	26.5246	1:Experimental evidence at protein level	biological regulation;cellular component organization;cellular component organization or biogenesis;cellular process;cellular response to stimulus;intracellular signal transduction;positive regulation of actin filament polymeriza

#NUM!	24.515	25.0549	24.2434	1:Experimental evidence at protein level	biological regulation;biosynthetic process;body fluid secretion;cellular aromatic compound metabolic process;cellular biosynthetic process;cellular metabolic process;cellular nitrogen compound biosynthetic process;cellular
26.9034	27.3011	26.9567	26.7733	1:Experimental evidence at protein level	antigen processing and presentation;antigen processing and presentation of endogenous antigen;antigen processing and presentation of endogenous peptide antigen;antigen processing and presentation of endogenous pep
25.252	25.2863	24.9557	24.8903	1:Experimental evidence at protein level	amine metabolic process;anatomical structure development;anatomical structure morphogenesis;brain development;carboxylic acid metabolic process;cellular amine metabolic process;cellular amino acid metabolic process
26.3752	25.4066	26.0777	26.0714	1:Experimental evidence at protein level	anatomical structure development;biological regulation;cardiac muscle fiber development;cell development;cellular developmental process;cellular macromolecule metabolic process;cellular metabolic process;cellular proces
26.9434	27.0122	27.229	26.9973	1:Experimental evidence at protein level	biological regulation;blood coagulation;cell activation;cellular component assembly;cellular component assembly at cellular level;cellular component organization;cellular component organization at cellular level;cellular comp
26.0254	26.0177	26.1326	26.0128	1:Experimental evidence at protein level	actin crosslink formation;actin cytoskeleton organization;actin cytoskeleton reorganization;actin filament bundle assembly;actin filament capping;actin filament organization;actin filament-based process;actin polymerization-c
26.3595	#NUM!	26.3095	26.097	1:Experimental evidence at protein level	defense response;defense response to virus;immune effector process;immune response;immune system process;innate immune response;multi-organism process;response to biotic stimulus;response to other organism;resp
27.0543	#NUM!	26.7292	26.6714	1:Experimental evidence at protein level	anatomical structure development;biological regulation;biosynthetic process;cell development;cellular biosynthetic process;cellular developmental process;cellular macromolecule biosynthetic process;cellular macromolecule
26.8474	27.0323	26.8301	26.8571	1:Experimental evidence at protein level	anatomical structure development;anatomical structure morphogenesis;biological regulation;canonical Wnt receptor signaling pathway;cell communication;cell development;cell differentiation;cell surface receptor linked signa
24.7051	24.7735	25.0885	#NUM!	1:Experimental evidence at protein level	biological regulation;developmental process;multicellular organismal development;multicellular organismal process;negative regulation of biological process;negative regulation of gene expression;negative regulation of macro
26.7045	26.664	26.604	26.6232	1:Experimental evidence at protein level	biological regulation;cell cycle;cellular macromolecule metabolic process;cellular metabolic process;cellular nitrogen compound metabolic process;cellular process;macromolecule metabolic process;macromolecule methylat
25.9404	#NUM!	25.527	25.563	1:Experimental evidence at protein level	biological regulation;biosynthetic process;cellular biosynthetic process;cellular component organization;cellular component organization at cellular level;cellular component organization or biogenesis;cellular component orga
25.6204	#NUM!	25.295	25.3109	1:Experimental evidence at protein level	biological regulation;cellular process;cellular response to stimulus;chemotaxis;establishment of localization;intracellular signal transduction;locomotion;metabolic process;positive regulation of catalytic activity;positive regulat
27.0968	#NUM!	26.6992	26.9804	1:Experimental evidence at protein level	cellular macromolecule metabolic process;cellular metabolic process;cellular nitrogen compound metabolic process;cellular process;macromolecule metabolic process;metabolic process;nitrogen compound metabolic proce
25.2097	24.4423	#NUM!	24.5668	1:Experimental evidence at protein level	biological regulation;cell surface receptor linked signaling pathway;cellular process;cellular response to chemical stimulus;cellular response to cytokine stimulus;cellular response to interferon-gamma;cellular response to orga
26.3718	#NUM!	25.9204	26.3203	1:Experimental evidence at protein level	activin receptor signaling pathway;anatomical structure development;anatomical structure formation involved in morphogenesis;anatomical structure morphogenesis;anterior/posterior pattern specification;axis specification;b
25.6193	#NUM!	25.4989	25.9516	1:Experimental evidence at protein level	
25.9755	25.5862	26.1996	25.9761	1:Experimental evidence at protein level	
26.6179	27.0412	26.4461	26.2439	1:Experimental evidence at protein level	acyl-CoA biosynthetic process;acyl-CoA metabolic process;acylglycerol biosynthetic process;acylglycerol metabolic process;biological regulation;biosynthetic process;carboxylic acid biosynthetic process;carboxylic acid me
25.0164	#NUM!	24.7717	24.7255	1:Experimental evidence at protein level	
24.6711	#NUM!	24.3535	24.3305	1:Experimental evidence at protein level	
28.6608	#NUM!	28.2786	#NUM!	1:Experimental evidence at protein level	cellular macromolecule metabolic process;cellular metabolic process;cellular nitrogen compound metabolic process;cellular process;macromolecule metabolic process;macromolecule methylation;macromolecule modificatio
28.534	28.3549	28.4266	28.6684	1:Experimental evidence at protein level	biological regulation;cellular process;cellular response to chemical stimulus;cellular response to hypoxia;cellular response to oxygen levels;cellular response to redox state;cellular response to stimulus;cellular response to stre
#NUM!	26.1225	25.838	25.9908	1:Experimental evidence at protein level	cell cycle phase;cell cycle process;cell division;cellular component organization;cellular component organization at cellular level;cellular component organization or biogenesis;cellular component organization or biogenesis at
26.4947	26.4585	26.63	26.7621	1:Experimental evidence at protein level	
25.2331	24.7021	24.6829	24.9204	1:Experimental evidence at protein level	
27.2987	27.1574	27.0317	27.0629	1:Experimental evidence at protein level	2-oxoglutarate metabolic process;amine catabolic process;amine metabolic process;aromatic amino acid family catabolic process;aromatic amino acid family metabolic process;aromatic compound catabolic process;biosyn
25.656	25.8044	25.7736	#NUM!	1:Experimental evidence at protein level	alcohol metabolic process;amine metabolic process;biosynthetic process;cellular amine metabolic process;cellular biogenic amine metabolic process;cellular biosynthetic process;cellular component organization;cellular com
26.3828	#NUM!	26.0731	25.7249	1:Experimental evidence at protein level	actin cytoskeleton organization;actin filament-based process;biological regulation;cellular component organization;cellular component organization at cellular level;cellular component organization or biogenesis;cellular comp
26.633	26.6347	26.5687	26.5632	1:Experimental evidence at protein level	
25.2729	25.1114	24.9073	#NUM!	1:Experimental evidence at protein level	
25.8974	26.4917	25.7396	26.3342	1:Experimental evidence at protein level	cell cycle;cell cycle phase;cell cycle process;cell division;cell proliferation;cellular component organization;cellular component organization at cellular level;cellular component organization or biogenesis;cellular component org
25.2409	#NUM!	25.3587	25.6523	1:Experimental evidence at protein level	biological regulation;catabolic process;cellular catabolic process;cellular macromolecule catabolic process;cellular macromolecule metabolic process;cellular metabolic process;cellular nitrogen compound metabolic process
24.6574	#NUM!	24.0768	24.5444	1:Experimental evidence at protein level	biological regulation;biosynthetic process;cellular biosynthetic process;cellular macromolecule biosynthetic process;cellular macromolecule metabolic process;cellular metabolic process;cellular nitrogen compound metabolic
26.9169	#NUM!	#NUM!	26.5089	1:Experimental evidence at protein level	
27.0072	27.3181	26.8371	27.0715	1:Experimental evidence at protein level	
24.9875	#NUM!	24.9863	25.0777	1:Experimental evidence at protein level	cellular macromolecule metabolic process;cellular metabolic process;cellular nitrogen compound metabolic process;cellular process;macromolecule metabolic process;macromolecule modification;metabolic process;nitroge
26.4248	#NUM!	26.6297	#NUM!	1:Experimental evidence at protein level	biological regulation;biosynthetic process;cell communication;cell cycle arrest;cell cycle process;cell growth;cell surface receptor linked signaling pathway;cellular biosynthetic process;cellular component organization;cellular
26.2555	#NUM!	25.6934	25.1441	1:Experimental evidence at protein level	biological regulation;regulation of biological process;regulation of cellular component organization;regulation of cellular process;regulation of cytoskeleton organization;regulation of organelle organization;regulation of respons
26.366	#NUM!	26.2248	25.0613	1:Experimental evidence at protein level	cell cycle;cell cycle phase;cell cycle process;cell division;cellular component organization;cellular component organization at cellular level;cellular component organization or biogenesis;cellular component organization or bio
25.3006	25.7984	26.0346	25.9216	1:Experimental evidence at protein level	cellular macromolecule metabolic process;cellular metabolic process;cellular process;cellular protein metabolic process;macromolecule metabolic process;macromolecule modification;metabolic process;peptidyl-amino acid
26.5846	27.4794	27.1635	28.0109	1:Experimental evidence at protein level	actin cytoskeleton organization;actin filament-based process;biological regulation;cell motility;cell surface receptor linked signaling pathway;cellular component assembly;cellular component movement;cellular component or
25.9763	#NUM!	#NUM!	26.0053	1:Experimental evidence at protein level	apoptosis;biological regulation;cell cycle cytokinesis;cell cycle process;cell death;cellular process;cytokinesis;cytokinesis after mitosis;death;negative regulation of biological process;negative regulation of cellular component
25.2794	#NUM!	25.8277	25.5883	1:Experimental evidence at protein level	amyloid precursor protein catabolic process;amyloid precursor protein metabolic process;axon guidance;beta-amyloid metabolic process;biological regulation;catabolic process;cell activation;cell proliferation;cell surface rec
26.8748	27.137	26.7602	26.9136	1:Experimental evidence at protein level	acrosome assembly;adherens junction organization;adhesion to host;adhesion to other organism involved in symbiotic interaction;anatomical structure development;anatomical structure formation involved in morphogenesis;
25.7305	25.893	26.0436	26.1573	1:Experimental evidence at protein level	apoptosis;biological regulation;biosynthetic process;cell death;cellular biosynthetic process;cellular macromolecule biosynthetic process;cellular macromolecule metabolic process;cellular metabolic process;cellular nitrogen
#NUM!	#NUM!	#NUM!	#NUM!	1:Experimental evidence at protein level	amine metabolic process;aminoglycan biosynthetic process;aminoglycan metabolic process;anatomical structure development;anatomical structure morphogenesis;biological regulation;biosynthetic process;bone morphoge
26.3938	26.0701	26.5442	26.415	1:Experimental evidence at protein level	biological regulation;developmental process;dorsal/ventral pattern formation;negative regulation of biological process;negative regulation of catalytic activity;negative regulation of cell communication;negative regulation of ce
24.9814	#NUM!	25.0582	25.2126	1:Experimental evidence at protein level	acylglycerol metabolic process;aging;anatomical structure development;anatomical structure formation involved in morphogenesis;angiogenesis;behavioral response to starvation;biological regulation;biosynthetic process;ca
#NUM!	#NUM!	#NUM!	#NUM!	1:Experimental evidence at protein level	
26.2481	25.5902	25.9576	25.5828	1:Experimental evidence at protein level	activation of caspase activity;activation-induced cell death of T cells;aging;apoptosis;biological regulation;biosynthetic process;cell activation;cell aging;cell death;cell differentiation;cellular biosynthetic process;cellular comp
28.2571	28.5058	28.6927	28.7763	1:Experimental evidence at protein level	
25.8966	25.2537	25.4752	25.3032	1:Experimental evidence at protein level	cellular component organization;cellular component organization at cellular level;cellular component organization or biogenesis;cellular component organization or biogenesis at cellular level;cellular macromolecule metabolic
25.9651	25.2009	26.2529	25.7541	1:Experimental evidence at protein level	
27.4384	27.4069	27.3953	27.3722	1:Experimental evidence at protein level	biological regulation;cellular macromolecule metabolic process;cellular metabolic process;cellular process;cellular protein metabolic process;cellular response to chemical stimulus;cellular response to organic substance;cellu
26.4388	26.3701	26.6195	26.8935	1:Experimental evidence at protein level	anatomical structure morphogenesis;anterior/posterior axis specification;axis specification;biological regulation;biosynthetic process;cell cycle;cellular biosynthetic process;cellular component organization;cellular componen
25.1604	25.6689	25.5819	25.7596	1:Experimental evidence at protein level	carboxylic acid transport;cellular lipid metabolic process;cellular metabolic process;cellular process;establishment of localization;fatty acid transport;lipid localization;lipid metabolic process;lipid storage;lipid transport;localiza
25.7036	26.4027	25.4301	26.2909	1:Experimental evidence at protein level	biological regulation;biosynthetic process;cellular biosynthetic process;cellular macromolecule metabolic process;cellular metabolic process;cellular nitrogen compound metabolic process;cellular process;cellular response to
#NUM!	26.7061	#NUM!	#NUM!	1:Experimental evidence at protein level	biological regulation;catabolic process;cell cycle arrest;cell cycle process;cell differentiation;cell division;cellular catabolic process;cellular component assembly;cellular component assembly at cellular level;cellular componen
25.908	#NUM!	25.7868	25.6902	1:Experimental evidence at protein level	acylglycerol biosynthetic process;acylglycerol metabolic process;biological regulation;biosynthetic process;carboxylic acid biosynthetic process;carboxylic acid metabolic process;CDP-diacylglycerol biosynthetic process;CD
25.2704	24.7681	24.8061	24.6311	1:Experimental evidence at protein level	
26.3561	26.6371	26.712	26.8336	1:Experimental evidence at protein level	activation of immune response;activation of innate immune response;biological regulation;cellular process;cellular response to stimulus;defense response;immune response;immune response-activating signal transduction;im
25.6609	#NUM!	25.8495	26.4151	1:Experimental evidence at protein level	
26.6257	26.2938	25.8901	26.0555	1:Experimental evidence at protein level	biological regulation;biosynthetic process;carboxylic acid catabolic process;carboxylic acid metabolic process;catabolic process;cation homeostasis;cell differentiation;cellular biosynthetic process;cellular catabolic process;
26.5999	26.9623	26.9766	26.8178	1:Experimental evidence at protein level	cellular component organization;cellular component organization at cellular level;cellular component organization or biogenesis;cellular component organization or biogenesis at cellular level;cellular macromolecule metabolic
25.7972	25.2101	25.2495	25.3815	1:Experimental evidence at protein level	cellular macromolecule metabolic process;cellular metabolic process;cellular nitrogen compound metabolic process;cellular process;macromolecule metabolic process;maturation of SSU-rRNA;maturation of SSU-rRNA from
26.1446	25.5575	#NUM!	25.3224	1:Experimental evidence at protein level	biosynthetic process;cellular biosynthetic process;cellular component disassembly;cellular component disassembly at cellular level;cellular component organization;cellular component organization at cellular level;cellular cor
25.625	25.8138	25.3365	25.6731	1:Experimental evidence at protein level	antigen processing and presentation;biological regulation;cellular component organization;cellular component organization at cellular level;cellular component organization or biogenesis;cellular component organization or bio
25.9188	25.4596	#NUM!	25.5927	1:Experimental evidence at protein level	biological regulation;biosynthetic process;canonical Wnt receptor signaling pathway;catabolic process;cell surface receptor linked signaling pathway;cellular biosynthetic process;cellular catabolic process;cellular component
26.3437	26.7067	26.0934	26.439	1:Experimental evidence at protein level	cellular macromolecule metabolic process;cellular metabolic process;cellular nitrogen compound metabolic process;cellular process;cellular protein metabolic process;macromolecule metabolic process;macromolecule modi
25.7623	#NUM!	25.876	25.9121	1:Experimental evidence at protein level	activation of immune response;anatomical structure formation involved in morphogenesis;anatomical structure morphogenesis;angiogenesis;antigen receptor-mediated signaling pathway;biological adhesion;biological regula
#NUM!	25.4041	25.0984	25.2711	1:Experimental evidence at protein level	
26.4276	26.2855	26.8248	26.892	1:Experimental evidence at protein level	anatomical structure development;cranial nerve development;developmental process;nerve development;optic nerve development
28.4738	29.242	28.6844	28.8031	1:Experimental evidence at protein level	biological regulation;cell redox homeostasis;cellular homeostasis;cellular macromolecule metabolic process;cellular metabolic process;cellular process;cellular protein metabolic process;cellular response to stimulus;cellular r
25.4169	25.3817	25.0979	25.4094	1:Experimental evidence at protein level	biosynthetic process;carbohydrate biosynthetic process;carbohydrate metabolic process;cellular carbohydrate metabolic process;cellular macromolecule metabolic process;cellular metabolic process;cellular process;cellular
25.0015	#NUM!	24.7959	25.0122	1:Experimental evidence at protein level	biosynthetic process;cellular biosynthetic process;cellular macromolecule biosynthetic process;cellular macromolecule metabolic process;cellular metabolic process;cellular nitrogen compound metabolic process;cellular pro
27.4038	27.592	27.5499	27.5508	1:Experimental evidence at protein level	biological regulation;cellular process;cellular response to stimulus;hippo signaling cascade;intracellular signal transduction;regulation of biological process;regulation of cellular process;response to stimulus;signal transductio
26.215	26.7764	26.5203	26.651	1:Experimental evidence at protein level	anatomical structure development;anatomical structure regression;biological regulation;cell migration;cell motility;cellular component movement;cellular process;developmental process;hemopoietic or lymphoid organ develo
28.1808	28.8098	29.0065	28.8667	1:Experimental evidence at protein level	behavior;cellular component movement;cellular component organization;cellular component organization at cellular level;cellular component organization or biogenesis;cellular component organization or biogenesis at cellula
28.0507	27.7805	27.7353	27.6607	1:Experimental evidence at protein level	
25.8179	25.6416	25.8325	25.9956	1:Experimental evidence at protein level	biological regulation;catabolic process;cellular catabolic process;cellular macromolecule catabolic process;cellular macromolecule metabolic process;cellular metabolic process;cellular nitrogen compound metabolic process
26.689	#NUM!	26.4466	26.5143	1:Experimental evidence at protein level	catabolic process;cellular catabolic process;cellular macromolecule catabolic process;cellular macromolecule metabolic process;cellular metabolic process;cellular nitrogen compound metabolic process;cellular process;defe
25.9685	25.9938	26.0949	26.1787	1:Experimental evidence at protein level	activation of caspase activity;activation of caspase activity by cytochrome c;apoptosis;biological regulation;cell death;cellular process;death;induction of apoptosis;induction of apoptosis by extracellular signals;induction of a
28.026	#NUM!	27.8471	28.1663	1:Experimental evidence at protein level	amine metabolic process;biosynthetic process;carboxylic acid metabolic process;cellular amine metabolic process;cellular amino acid metabolic process;cellular biosynthetic process;cellular ketone metabolic process;cellula
26.6404	26.1399	26.3271	26.1614	1:Experimental evidence at protein level	behavior;biological regulation;biosynthetic process;carbohydrate metabolic process;carbohydrate transport;cell cycle;cell cycle process;cell surface receptor linked signaling pathway;cellular biosynthetic process;cellular com
27.1568	26.6121	26.8184	26.2446	1:Experimental evidence at protein level	cation transport;establishment of localization;ion transport;metal ion transport;transport
26.8309	26.8538	26.8381	26.5055	1:Experimental evidence at protein level	cellular macromolecule metabolic process;cellular metabolic process;cellular nitrogen compound metabolic process;cellular process;macromolecule metabolic process;macromolecule modification;metabolic process;ncRNA
26.4004	25.5966	26.1862	26.2457	1:Experimental evidence at protein level	cellular process;cellular response to chemical stimulus;cellular response to oxidative stress;cellular response to oxygen radical;cellular response to reactive oxygen species;cellular response to stimulus;cellular response to st
25.9807	#NUM!	25.3166	25.9287	1:Experimental evidence at protein level	alcohol biosynthetic process;alcohol metabolic process;amine metabolic process;aminoglycan metabolic process;anatomical structure development;anatomical structure morphogenesis;biosynthetic process;carbohydrate bi
#NUM!	#NUM!	#NUM!	#NUM!	1:Experimental evidence at protein level	biological regulation;negative regulation of biological process;negative regulation of catalytic activity;negative regulation of cell death;negative regulation of cellular process;negative regulation of kinase activity;negative regula
26.5039	26.3439	26.1557	26.3662	1:Experimental evidence at protein level	cation transport;cellular macromolecule metabolic process;cellular metabolic process;cellular nitrogen compound metabolic process;cellular process;establishment of localization;ion transport;macromolecule metabolic proce
26.4361	#NUM!	26.9531	26.538	1:Experimental evidence at protein level	anatomical structure homeostasis;biological regulation;biosynthetic process;cellular biosynthetic process;cellular component organization;cellular component organization at cellular level;cellular component organization or bi
26.256	#NUM!	#NUM!	25.7474	1:Experimental evidence at protein level	biological regulation;cellular component organization;cellular component organization or biogenesis;cellular membrane organization;cellular process;cellular response to acid;cellular response to amine stimulus;cellular respor
25.9766	#NUM!	#NUM!	27.0946	1:Experimental evidence at protein level	biosynthetic process;cell differentiation;cellular biosynthetic process;cellular developmental process;cellular macromolecule biosynthetic process;cellular macromolecule metabolic process;cellular metabolic process;cellular
26.1712	#NUM!	26.4902	26.0926	1:Experimental evidence at protein level	biological regulation;cellular localization;cellular macromolecule localization;cellular process;cellular protein localization;localization;macromolecule localization;positive regulation of biological process;positive regulation of bio
27.931	27.8934	28.3849	28.1028	1:Experimental evidence at protein level	biological regulation;regulation of autophagic vacuole assembly;regulation of autophagy;regulation of biological process;regulation of catabolic process;regulation of cell communication;regulation of cellular catabolic process
27.3226	26.998	27.2436	26.7486	1:Experimental evidence at protein level	biosynthetic process;cellular biosynthetic process;cellular macromolecule metabolic process;cellular metabolic process;cellular nitrogen compound metabolic process;cellular process;ER to Golgi vesicle-mediated transport;
26.1249	27.1186	26.2913	26.2341	1:Experimental evidence at protein level	amine biosynthetic process;amine metabolic process;amino acid salvage;aspartate family amino acid biosynthetic process;aspartate family amino acid metabolic process;biosynthetic process;carboxylic acid biosynthetic pro
25.826	25.9259	26.2627	25.7908	1:Experimental evidence at protein level	establishment of localization;establishment of localization in cell;establishment of protein localization;intracellular protein transport;intracellular transport;nuclear import;nuclear transport;nucleocytoplasmic transport;protein in
26.9181	#NUM!	27.4239	26.9553	1:Experimental evidence at protein level	cellular macromolecule metabolic process;cellular metabolic process;cellular process;cellular protein metabolic process;macromolecule metabolic process;macromolecule methylation;macromolecule modification;metabolic
25.4386	24.7961	25.0511	25.4146	1:Experimental evidence at protein level	biological regulation;cellular component organization;cellular component organization at cellular level;cellular component organization or biogenesis;cellular component organization or biogenesis at cellular level;cellular meml
26.629	#NUM!	26.6962	26.6287	1:Experimental evidence at protein level	biological regulation;biosynthetic process;blood coagulation;cell differentiation;cellular biosynthetic process;cellular developmental process;cellular macromolecule biosynthetic process;cellular macromolecule metabolic proc
24.8359	25.0978	25.6734	#NUM!	1:Experimental evidence at protein level	antigen processing and presentation;biological regulation;blood coagulation;cell migration;cell motility;cellular component movement;cellular process;coagulation;hemostasis;immune response;immune system process;leuko
24.9491	25.9745	25.6055	#NUM!	1:Experimental evidence at protein level	biological regulation;biosynthetic process;cell surface receptor linked signaling pathway;cellular biosynthetic process;cellular macromolecule biosynthetic process;cellular macromolecule metabolic process;cellular metabolic
25.7108	25.6992	25.5513	25.0523	1:Experimental evidence at protein level	biosynthetic process;cation transport;cellular biosynthetic process;cellular lipid metabolic process;cellular macromolecule metabolic process;cellular metabolic process;cellular potassium ion transport;cellular process;cellular
26.7961	26.3687	26.6373	26.286	1:Experimental evidence at protein level	cellular process;establishment of localization;transport;vesicle-mediated transport
26.9261	26.9792	27.1208	26.8946	1:Experimental evidence at protein level	catabolic process;cellular catabolic process;cellular macromolecule catabolic process;cellular macromolecule metabolic process;cellular metabolic process;cellular nitrogen compound metabolic process;cellular process;exo
26.1221	26.0674	25.9708	26.1199	1:Experimental evidence at protein level	catabolic process;cellular catabolic process;cellular macromolecule catabolic process;cellular macromolecule metabolic process;cellular metabolic process;cellular nitrogen compound metabolic process;cellular process;exo
26.8579	26.1311	26.2862	#NUM!	1:Experimental evidence at protein level	apoptosis;biological regulation;cell cycle;cell death;cellular component assembly;cellular component assembly at cellular level;cellular component organization;cellular component organization at cellular level;cellular compon
26.2377	#NUM!	#NUM!	26.1711	1:Experimental evidence at protein level	biological regulation;cellular macromolecule metabolic process;cellular metabolic process;cellular nitrogen compound metabolic process;cellular process;humoral immune response;immune response;immune system process
26.2068	26.2191	25.9927	25.7288	1:Experimental evidence at protein level	
27.6187	28.2723	28.1276	28.712	1:Experimental evidence at protein level	biological regulation;biosynthetic process;cellular biosynthetic process;cellular macromolecule biosynthetic process;cellular macromolecule metabolic process;cellular metabolic process;cellular nitrogen compound metabolic
27.3054	27.2174	27.0315	#NUM!	1:Experimental evidence at protein level	cellular macromolecule metabolic process;cellular metabolic process;cellular nitrogen compound metabolic process;cellular process;macromolecule metabolic process;metabolic process;nitrogen compound metabolic proce
24.4986	#NUM!	24.3963	23.7647	1:Experimental evidence at protein level	biological regulation;cellular macromolecule metabolic process;cellular metabolic process;cellular nitrogen compound metabolic process;cellular process;macromolecule metabolic process;maturation of SSU-rRNA;metabolic
26.7848	26.9352	27.2822	26.3251	1:Experimental evidence at protein level	attachment of spindle microtubules to chromosome;attachment of spindle microtubules to kinetochore;attachment of spindle microtubules to kinetochore involved in mitotic sister chromatid segregation;biological regulation;
#NUM!	#NUM!	25.7336	#NUM!	1:Experimental evidence at protein level	binding of sperm to zona pellucida;cell recognition;cell-cell recognition;cellular component assembly;cellular component organization;cellular component organization or biogenesis;cellular macromolecule metabolic process;
26.5635	26.0883	26.8275	26.4971	1:Experimental evidence at protein level	biological regulation;cellular component assembly;cellular component assembly at cellular level;cellular component organization;cellular component organization at cellular level;cellular component organization or biogenesis;
25.2356	25.2344	25.5192	25.273	1:Experimental evidence at protein level	activation of immune response;activation of innate immune response;activation of MAPK activity;activation of MAPKK activity;activation of NF-kappaB-inducing kinase activity;activation of protein kinase activity;anatomical s
27.1123	27.1016	27.1108	27.4494	1:Experimental evidence at protein level	
26.7891	28.1021	28.2913	28.4602	1:Experimental evidence at protein level	biological regulation;cell cycle;cell cycle phase;cell cycle process;cell division;cellular component organization;cellular component organization at cellular level;cellular component organization or biogenesis;cellular componer
25.151	#NUM!	25.1115	24.8544	1:Experimental evidence at protein level	cellular component assembly;cellular component assembly at cellular level;cellular component biogenesis;cellular component organization;cellular component organization at cellular level;cellular component organization or b
25.249	25.6971	25.4581	25.7341	1:Experimental evidence at protein level	establishment of localization;establishment of protein localization;protein transport;transport
26.8322	26.605	26.6156	26.4925	1:Experimental evidence at protein level	alcohol metabolic process;biosynthetic process;carbohydrate biosynthetic process;carbohydrate metabolic process;cellular biosynthetic process;cellular carbohydrate metabolic process;cellular lipid metabolic process;cellul
25.965	26.4359	26.2868	25.9343	1:Experimental evidence at protein level	biological regulation;cation homeostasis;cellular cation homeostasis;cellular chemical homeostasis;cellular copper ion homeostasis;cellular homeostasis;cellular ion homeostasis;cellular process;chemical homeostasis;coppe
26.7074	26.7295	26.4025	26.6082	1:Experimental evidence at protein level	anatomical structure morphogenesis;biological regulation;cellular macromolecule metabolic process;cellular metabolic process;cellular nitrogen compound metabolic process;cellular process;cellular protein metabolic proces
26.5601	27.3102	27.224	27.5835	1:Experimental evidence at protein level	biological regulation;carboxylic acid metabolic process;catabolic process;cellular catabolic process;cellular ketone metabolic process;cellular lipid metabolic process;cellular macromolecule catabolic process;cellular macrom
25.534	26.148	26.039	26.071	1:Experimental evidence at protein level	biological regulation;cell surface receptor linked signaling pathway;cellular macromolecule metabolic process;cellular metabolic process;cellular process;cellular protein metabolic process;cellular response to chemical stimul
26.6733	27.0698	27.3528	27.0186	1:Experimental evidence at protein level	activation of immune response;activation of innate immune response;anaphase-promoting complex-dependent proteasomal ubiquitin-dependent protein catabolic process;antigen processing and presentation;antigen proces
26.0421	#NUM!	26.4543	26.8517	1:Experimental evidence at protein level	

26.4411	26.1961	26.2872	26.1812	1:Experimental evidence at protein level	biological regulation;cellular process;establishment of localization;regulation of biological process;regulation of biosynthetic process;regulation of cellular biosynthetic process;regulation of cellular macromolecule biosynthetic
27.4766	26.8482	27.157	27.0579	1:Experimental evidence at protein level	cellular metabolic process;cellular process;electron transport chain;generation of precursor metabolites and energy;metabolic process;mitochondrial electron transport, NADH to ubiquinone;oxidation-reduction process;respi
25.72	25.7411	26.2925	26.7128	1:Experimental evidence at protein level	cell cycle;cell cycle cytokinesis;cell cycle process;cellular component movement;cellular component organization;cellular component organization at cellular level;cellular component organization or biogenesis;cellular compo
26.4901	26.5933	26.2374	26.4986	1:Experimental evidence at protein level	cell growth;cell proliferation;cellular macromolecule metabolic process;cellular metabolic process;cellular process;cellular protein metabolic process;developmental cell growth;developmental growth;developmental process;g
26.387	#NUM!	26.5148	26.916	1:Experimental evidence at protein level	cell proliferation;cell proliferation in external granule layer;cell proliferation in hindbrain;cellular component assembly;cellular component assembly at cellular level;cellular component organization;cellular component organizat
28.2771	29.0804	28.9957	29.1698	1:Experimental evidence at protein level	biological regulation;cell redox homeostasis;cellular homeostasis;cellular process;homeostatic process;regulation of biological process;regulation of biological quality;regulation of cellular process
#NUM!	#NUM!	#NUM!	#NUM!	1:Experimental evidence at protein level	cellular component organization;cellular component organization at cellular level;cellular component organization or biogenesis;cellular component organization or biogenesis at cellular level;cellular process;extracellular matr
#NUM!	#NUM!	#NUM!	#NUM!	1:Experimental evidence at protein level	activation of immune response;biological regulation;complement activation;complement activation, classical pathway;defense response;humoral immune response;immune effector process;immune response;immune system
#NUM!	#NUM!	#NUM!	#NUM!	1:Experimental evidence at protein level	biological regulation;blood coagulation;cell activation;cellular component disassembly;cellular component disassembly at cellular level;cellular component organization;cellular component organization at cellular level;cellular
#NUM!	#NUM!	#NUM!	#NUM!	1:Experimental evidence at protein level	antibacterial humoral response;antimicrobial humoral response;biological adhesion;biological regulation;blood coagulation;cell activation;cell adhesion;cell-cell adhesion;cell-matrix adhesion;cell-substrate adhesion;cellular co
26.3943	26.5057	26.3791	25.9222	1:Experimental evidence at protein level	activation of adenylate cyclase activity;activation of immune response;activation of innate immune response;activation of MAPKK activity;activation of protein kinase activity;anatomical structure development;apoptosis;axon
27.1906	27.3025	26.9888	26.9508	1:Experimental evidence at protein level	alcohol biosynthetic process;alcohol metabolic process;amine metabolic process;anatomical structure morphogenesis;biological regulation;biosynthetic process;carbohydrate metabolic process;catabolic process;cellular bio
28.722	29.255	28.9518	29.078	1:Experimental evidence at protein level	anatomical structure development;biosynthetic process;cell proliferation;cellular aromatic compound metabolic process;cellular biosynthetic process;cellular component assembly;cellular component organization;cellular com
27.2466	27.4807	27.5614	27.3607	1:Experimental evidence at protein level	2'-deoxyribonucleotide biosynthetic process;2'-deoxyribonucleotide metabolic process;aging;anatomical structure development;anatomical structure morphogenesis;biological regulation;biosynthetic process;cartilage develo
30.2049	30.2942	30.4812	31.0398	1:Experimental evidence at protein level	biosynthetic process;cellular biosynthetic process;cellular macromolecule biosynthetic process;cellular macromolecule metabolic process;cellular metabolic process;cellular nitrogen compound metabolic process;cellular pro
#NUM!	25.5705	25.9369	25.8817	1:Experimental evidence at protein level	biosynthetic process;cellular aromatic compound metabolic process;cellular biosynthetic process;cellular macromolecule metabolic process;cellular metabolic process;cellular nitrogen compound biosynthetic process;cellula
27.7989	28.2934	27.8837	28.5527	1:Experimental evidence at protein level	cellular component assembly;cellular component assembly at cellular level;cellular component organization;cellular component organization at cellular level;cellular component organization or biogenesis;cellular component c
27.0583	27.2681	27.6701	27.3004	1:Experimental evidence at protein level	anatomical structure development;biological regulation;blood coagulation;bone development;cell activation;cellular component organization;cellular component organization at cellular level;cellular component organization or
#NUM!	#NUM!	#NUM!	#NUM!	1:Experimental evidence at protein level	
28.5558	29.2499	29.342	29.4929	1:Experimental evidence at protein level	biosynthetic process;cellular biosynthetic process;cellular macromolecule metabolic process;cellular metabolic process;cellular nitrogen compound metabolic process;cellular process;electron transport chain;gene expressio
#NUM!	#NUM!	#NUM!	#NUM!	1:Experimental evidence at protein level	activation of immune response;biological regulation;cation homeostasis;cell death;cellular cation homeostasis;cellular chemical homeostasis;cellular homeostasis;cellular ion homeostasis;cellular metal ion homeostasis;cellula
#NUM!	#NUM!	#NUM!	#NUM!	1:Experimental evidence at protein level	biological regulation;blood coagulation;blood coagulation, extrinsic pathway;coagulation;hemostasis;macromolecule metabolic process;metabolic process;multicellular organismal process;negative regulation of catalytic activ
26.4542	26.1556	#NUM!	26.4517	1:Experimental evidence at protein level	anatomical structure development;cellular component organization;cellular component organization at cellular level;cellular component organization or biogenesis;cellular component organization or biogenesis at cellular level
27.5312	27.4959	27.5831	27.237	1:Experimental evidence at protein level	carbohydrate metabolic process;carbohydrate transmembrane transport;carbohydrate transport;carboxylic acid metabolic process;cation transport;cellular ketone metabolic process;cellular metabolic process;cellular proces
#NUM!	#NUM!	#NUM!	#NUM!	1:Experimental evidence at protein level	anatomical structure development;anatomical structure morphogenesis;biological adhesion;cardiac muscle tissue morphogenesis;cartilage condensation;cell adhesion;cell development;cell differentiation;cell-cell adhesion;ce
26.7987	27.0933	27.0407	27.2636	1:Experimental evidence at protein level	biological regulation;blood coagulation;cell activation;cellular process;coagulation;establishment of localization;establishment of localization in cell;exocytosis;hemostasis;multicellular organismal process;platelet activation;pla
30.2362	30.2313	29.9801	30.2265	1:Experimental evidence at protein level	biological regulation;blood coagulation;cell activation;cell surface receptor linked signaling pathway;cellular process;cellular response to stimulus;coagulation;defense response;hemostasis;immune response;immune system
27.3193	27.8033	27.3863	27.5863	1:Experimental evidence at protein level	aging;anatomical structure development;anatomical structure morphogenesis;artery morphogenesis;biological adhesion;biological regulation;blood vessel morphogenesis;BMP signaling pathway;bone development;branching
26.7428	26.676	27.2621	26.981	1:Experimental evidence at protein level	biological regulation;biosynthetic process;cellular biosynthetic process;cellular macromolecule biosynthetic process;cellular macromolecule metabolic process;cellular metabolic process;cellular nitrogen compound metabolic
26.7567	27.8597	28.0817	28.3164	1:Experimental evidence at protein level	anatomical structure development;ATP biosynthetic process;ATP metabolic process;ATP synthesis coupled proton transport;biosynthetic process;cation transport;cellular biosynthetic process;cellular metabolic process;cellu
26.7824	26.5837	26.8599	26.6253	1:Experimental evidence at protein level	activation of MAPKK activity;activation of protein kinase activity;avoidance of defenses of other organism involved in symbiotic interaction;avoidance of host defenses;biological regulation;biosynthetic process;cellular biosyn
29.4364	#NUM!	#NUM!	29.1776	1:Experimental evidence at protein level	anatomical structure development;anatomical structure formation involved in morphogenesis;biological adhesion;biological regulation;blood coagulation;brain development;cell activation;cell adhesion;cell development;cell ju
27.001	27.215	26.8887	27.0548	1:Experimental evidence at protein level	arachidonic acid metabolic process;biological regulation;biosynthetic process;carboxylic acid biosynthetic process;carboxylic acid metabolic process;cellular biosynthetic process;cellular ketone metabolic process;cellular lip
25.0817	#NUM!	24.67	#NUM!	1:Experimental evidence at protein level	biological regulation;biosynthetic process;cellular biosynthetic process;cellular component assembly;cellular component assembly at cellular level;cellular component organization;cellular component organization at cellular le
26.3823	26.665	27.1823	26.7815	1:Experimental evidence at protein level	biosynthetic process;cellular aromatic compound metabolic process;cellular biosynthetic process;cellular metabolic compound salvage;cellular metabolic process;cellular nitrogen compound biosynthetic process;cellular nitr
26.9411	#NUM!	27.3528	27.1418	1:Experimental evidence at protein level	activation of immune response;activation of innate immune response;anaphase-promoting complex-dependent proteasomal ubiquitin-dependent protein catabolic process;antigen processing and presentation;antigen proces
26.8874	27.6377	27.0859	27.1207	1:Experimental evidence at protein level	anatomical structure development;blood vessel development;cellular component assembly;cellular component assembly at cellular level;cellular component organization;cellular component organization at cellular level;cellula
26.621	26.5161	26.5749	26.4238	1:Experimental evidence at protein level	anatomical structure development;anatomical structure morphogenesis;apoptotic cell clearance;biological adhesion;biological regulation;blood vessel remodeling;cell activation;cell adhesion;cell communication;cell differenti
27.4914	27.0484	27.0125	27.2599	1:Experimental evidence at protein level	amine transport;amino acid transport;carboxylic acid transport;cellular process;establishment of localization;ion transport;nitrogen compound transport;organic acid transport;organic substance transport;transmembrane tran
25.1626	24.3512	24.8481	24.21	1:Experimental evidence at protein level	biological regulation;cell cycle checkpoint;cell cycle process;cellular component organization;cellular component organization at cellular level;cellular component organization or biogenesis;cellular component organization or
29.0852	29.948	29.6966	30.0168	1:Experimental evidence at protein level	actin cytoskeleton organization;actin filament-based process;biological regulation;cellular component organization;cellular component organization at cellular level;cellular component organization or biogenesis;cellular compo
25.3306	25.8507	25.8514	26.0048	1:Experimental evidence at protein level	acyl-CoA metabolic process;amine catabolic process;amine metabolic process;anatomical structure development;biosynthetic process;branched chain family amino acid catabolic process;branched chain family amino acid
25.1233	26.2457	#NUM!	25.9553	1:Experimental evidence at protein level	activation of immune response;activation of innate immune response;anaphase-promoting complex-dependent proteasomal ubiquitin-dependent protein catabolic process;anatomical structure morphogenesis;antigen proces
28.053	27.8999	27.8798	27.6635	1:Experimental evidence at protein level	activation of caspase activity;activation of caspase activity by cytochrome c;anatomical structure development;anatomical structure formation involved in morphogenesis;apoptosis;apoptotic mitochondrial changes;B cell ho
25.5402	25.6035	25.1438	25.1073	1:Experimental evidence at protein level	anatomical structure homeostasis;biological regulation;biosynthetic process;cell cycle;cell cycle process;cellular biosynthetic process;cellular component organization;cellular component organization at cellular level;cellular c
26.2122	25.5598	25.3693	25.5987	1:Experimental evidence at protein level	apoptosis;apoptosis in response to endoplasmic reticulum stress;biological regulation;cell death;cellular process;death;macromolecule metabolic process;metabolic process;primary metabolic process;programmed cell death
#NUM!	#NUM!	#NUM!	#NUM!	1:Experimental evidence at protein level	anatomical structure development;anatomical structure morphogenesis;apoptosis;appendage development;biological adhesion;biological regulation;cartilage development;cartilage development involved in endochondral bo
24.5451	24.761	24.9646	#NUM!	1:Experimental evidence at protein level	biosynthetic process;cellular aromatic compound metabolic process;cellular biosynthetic process;cellular metabolic process;cellular nitrogen compound biosynthetic process;cellular nitrogen compound metabolic process;ce
25.6273	25.6775	25.1917	25.2424	1:Experimental evidence at protein level	antigen processing and presentation;biological regulation;blood coagulation;cell activation;cell activation involved in immune response;cell differentiation;cellular developmental process;cellular localization;cellular macromole
25.8058	25.8771	25.9328	25.8897	1:Experimental evidence at protein level	cellular metabolic process;cellular nitrogen compound metabolic process;cellular process;metabolic process;methylation;nitrogen compound metabolic process;nucleobase-containing compound metabolic process;one-carb
#NUM!	#NUM!	#NUM!	#NUM!	1:Experimental evidence at protein level	amine metabolic process;aminoglycan biosynthetic process;aminoglycan catabolic process;aminoglycan metabolic process;anatomical structure development;biological regulation;biosynthetic process;carbohydrate biosynth
25.666	25.7541	25.5873	25.7265	1:Experimental evidence at protein level	biological regulation;biosynthetic process;catabolic process;cell cycle;cell cycle process;cellular biosynthetic process;cellular catabolic process;cellular macromolecule biosynthetic process;cellular macromolecule catabolic p
26.6975	26.8056	26.7979	26.6419	1:Experimental evidence at protein level	biological regulation;biosynthetic process;cell surface receptor linked signaling pathway;cellular biosynthetic process;cellular macromolecule biosynthetic process;cellular macromolecule metabolic process;cellular metabolic
25.1793	25.7815	25.5979	25.4169	1:Experimental evidence at protein level	actin cytoskeleton organization;actin filament-based process;biological regulation;cell projection assembly;cell projection organization;cellular component assembly;cellular component assembly at cellular level;cellular comp
26.8254	26.6116	26.5759	26.6275	1:Experimental evidence at protein level	abscission;biological regulation;cell division;cellular process;cytokinesis;developmental process;establishment of localization;establishment of protein localization;localization;macromolecule localization;positive regulation of
28.075	28.1072	28.0095	28.0762	1:Experimental evidence at protein level	adherens junction organization;anatomical structure development;anatomical structure morphogenesis;axonogenesis;biological adhesion;cell adhesion;cell junction assembly;cell junction organization;cell part morphogenesis
27.2582	27.1966	27.4017	27.4539	1:Experimental evidence at protein level	activation of caspase activity;anatomical structure development;apoptosis;apoptotic mitochondrial changes;biological regulation;brain development;cell death;cellular component assembly;cellular component organization;ce
28.3689	29.3449	28.5589	28.8564	1:Experimental evidence at protein level	ATP biosynthetic process;ATP metabolic process;ATP synthesis coupled proton transport;biosynthetic process;cation transport;cellular biosynthetic process;cellular metabolic process;cellular nitrogen compound biosyntheti
29.5268	29.258	29.5908	29.5491	1:Experimental evidence at protein level	cellular component assembly;cellular component assembly at cellular level;cellular component biogenesis;cellular component biogenesis at cellular level;cellular component organization;cellular component organization at ce
27.2805	27.2218	27.0825	27.1536	1:Experimental evidence at protein level	cellular component assembly;cellular component assembly at cellular level;cellular component organization;cellular component organization at cellular level;cellular component organization or biogenesis;cellular component c
29.354	30.4692	29.1896	29.4543	1:Experimental evidence at protein level	antigen processing and presentation;antigen processing and presentation of exogenous antigen;antigen processing and presentation of exogenous peptide antigen;antigen processing and presentation of exogenous peptide
27.1611	27.0846	27.6523	27.6385	1:Experimental evidence at protein level	antigen processing and presentation;biological regulation;cellular component organization;cellular component organization at cellular level;cellular component organization or biogenesis;cellular component organization or bio
29.0982	#NUM!	#NUM!	30.2555	1:Experimental evidence at protein level	biological regulation;biosynthetic process;catabolic process;cellular biosynthetic process;cellular catabolic process;cellular component disassembly;cellular component disassembly at cellular level;cellular component organiz
26.3359	26.2213	26.6426	26.4985	1:Experimental evidence at protein level	B cell activation;biological regulation;cell activation;cellular process;cellular response to stimulus;immune system process;leukocyte activation;lymphocyte activation;negative regulation of biological process;negative regulatio
27.2238	27.1715	27.657	27.2685	1:Experimental evidence at protein level	biological regulation;negative regulation of biological process;negative regulation of biosynthetic process;negative regulation of cellular biosynthetic process;negative regulation of cellular macromolecule biosynthetic process
27.4677	27.433	27.1719	27.5656	1:Experimental evidence at protein level	anatomical structure development;apoptosis;biological regulation;cell death;cell development;cell division;cellular developmental process;cellular process;cellular process involved in reproduction;death;defense response;dev
27.1527	27.428	27.4643	27.138	1:Experimental evidence at protein level	amine metabolic process;amine transport;amino acid transmembrane transport;amino acid transport;anatomical structure development;aromatic amino acid transport;biological regulation;blood coagulation;branched-chain a
24.7075	#NUM!	#NUM!	24.605	1:Experimental evidence at protein level	cellular macromolecule metabolic process;cellular metabolic process;cellular process;cellular protein metabolic process;macromolecule metabolic process;macromolecule modification;metabolic process;primary metabolic p
25.5285	25.4162	26.0235	#NUM!	1:Experimental evidence at protein level	anatomical structure morphogenesis;biological regulation;biosynthetic process;cell surface receptor linked signaling pathway;cellular biosynthetic process;cellular macromolecule biosynthetic process;cellular macromolecule
27.0217	26.7506	26.9219	26.7365	1:Experimental evidence at protein level	catabolic process;cellular catabolic process;cellular component assembly;cellular component organization;cellular component organization or biogenesis;cellular metabolic process;cellular process;macromolecular complex a
27.0821	26.9869	26.8095	27.0379	1:Experimental evidence at protein level	biological regulation;biosynthetic process;cellular biosynthetic process;cellular lipid metabolic process;cellular metabolic process;cellular process;cellular response to stimulus;glycerolipid biosynthetic process;glycerolipid me
26.2984	26.1434	26.1339	26.3883	1:Experimental evidence at protein level	biological regulation;cellular component disassembly;cellular component disassembly at cellular level;cellular component organization;cellular component organization at cellular level;cellular component organization or bioge
27.1286	26.6006	26.7075	26.5579	1:Experimental evidence at protein level	biological adhesion;cell adhesion;cellular process
26.5789	26.7623	26.7995	26.6496	1:Experimental evidence at protein level	anatomical structure formation involved in morphogenesis;angiogenesis;biological regulation;cell differentiation;cell migration;cell motility;cellular component movement;cellular developmental process;cellular process;develo
27.6609	27.9401	27.9644	28.0035	1:Experimental evidence at protein level	anatomical structure development;biological regulation;biosynthetic process;cell activation;cell differentiation;cell maturation;cellular biosynthetic process;cellular developmental process;cellular macromolecule biosynthetic p
25.7113	#NUM!	25.4116	26.0046	1:Experimental evidence at protein level	biosynthetic process;cellular biosynthetic process;cellular component disassembly;cellular component disassembly at cellular level;cellular component organization;cellular component organization at cellular level;cellular cor
27.4754	27.8325	27.9739	28.0263	1:Experimental evidence at protein level	
27.3377	27.0757	27.46	27.5146	1:Experimental evidence at protein level	anatomical structure development;biological regulation;cell surface receptor linked signaling pathway;cellular localization;cellular macromolecule localization;cellular macromolecule metabolic process;cellular metabolic proce
29.5866	29.0897	28.9998	29.0523	1:Experimental evidence at protein level	biological regulation;biosynthetic process;catabolic process;cellular biosynthetic process;cellular catabolic process;cellular macromolecule biosynthetic process;cellular macromolecule catabolic process;cellular macromolec
28.2374	27.9828	27.9608	27.8221	1:Experimental evidence at protein level	anatomical structure development;anatomical structure morphogenesis;biological regulation;cargo loading into vesicle;cellular component assembly;cellular component assembly at cellular level;cellular component organizati
26.6814	26.6035	26.3425	26.4061	1:Experimental evidence at protein level	anatomical structure morphogenesis;biological regulation;biosynthetic process;cellular biosynthetic process;cellular macromolecule biosynthetic process;cellular macromolecule metabolic process;cellular metabolic process;
28.2276	28.4308	28.4352	28.5475	1:Experimental evidence at protein level	anatomical structure morphogenesis;biological regulation;catabolic process;cell surface receptor linked signaling pathway;cellular catabolic process;cellular macromolecule catabolic process;cellular macromolecule metaboli
26.2629	26.0564	26.4396	26.2976	1:Experimental evidence at protein level	cellular metabolic process;cellular process;electron transport chain;generation of precursor metabolites and energy;metabolic process;mitochondrial electron transport, NADH to ubiquinone;oxidation-reduction process;respi
#NUM!	25.5692	25.825	25.4953	1:Experimental evidence at protein level	2'-deoxyribonucleotide biosynthetic process;2'-deoxyribonucleotide metabolic process;alcohol metabolic process;ATP metabolic process;biosynthetic process;carbohydrate metabolic process;cellular biosynthetic process;c
26.4659	26.1996	26.494	26.4054	1:Experimental evidence at protein level	
27.5521	28.9086	27.6516	28.4317	5:Protein uncertain	cellular macromolecule metabolic process;cellular metabolic process;cellular process;cellular protein metabolic process;macromolecule metabolic process;metabolic process;primary metabolic process;protein folding;protein
26.4084	26.9302	26.3686	26.4521	1:Experimental evidence at protein level	anatomical structure development;biological regulation;cellular macromolecule metabolic process;cellular metabolic process;cellular nitrogen compound metabolic process;cellular process;developmental process;hemopoiet
26.6865	26.1596	26.5426	26.1858	1:Experimental evidence at protein level	biological regulation;catabolic process;cellular catabolic process;cellular component organization;cellular component organization at cellular level;cellular component organization or biogenesis;cellular component organizatio
25.6488	25.6034	25.5322	25.4486	1:Experimental evidence at protein level	
26.2636	26.4951	26.0024	26.3469	1:Experimental evidence at protein level	
#NUM!	24.6709	24.3078	24.6258	1:Experimental evidence at protein level	
26.357	#NUM!	26.5806	26.7195	1:Experimental evidence at protein level	biological regulation;cell differentiation;cellular developmental process;cellular macromolecule metabolic process;cellular metabolic process;cellular nitrogen compound metabolic process;cellular process;demethylation;deve
25.9501	#NUM!	25.8167	25.7011	1:Experimental evidence at protein level	alcohol metabolic process;carbohydrate metabolic process;cellular carbohydrate metabolic process;cellular metabolic process;cellular process;inositol metabolic process;inositol phosphate metabolic process;metabolic proc
26.5449	#NUM!	#NUM!	25.9211	1:Experimental evidence at protein level	
25.58	25.3772	25.8342	25.9346	1:Experimental evidence at protein level	anatomical structure development;axis elongation;biological regulation;branch elongation of an epithelium;bud outgrowth involved in lung branching;cell development;cellular developmental process;cellular process;developm
27.7142	27.5936	28.2425	27.9865	1:Experimental evidence at protein level	biological regulation;biosynthetic process;cellular biosynthetic process;cellular macromolecule biosynthetic process;cellular macromolecule metabolic process;cellular metabolic process;cellular nitrogen compound metabolic
#NUM!	#NUM!	#NUM!	#NUM!	1:Experimental evidence at protein level	anatomical structure morphogenesis;biological adhesion;biological regulation;cell adhesion;cell morphogenesis;cell morphogenesis involved in differentiation;cell surface receptor linked signaling pathway;cell-cell adhesion;c
26.5284	26.4933	26.4254	26.3039	1:Experimental evidence at protein level	biological regulation;cellular component organization;cellular component organization at cellular level;cellular component organization or biogenesis;cellular component organization or biogenesis at cellular level;cellular locali
26.6805	26.7674	27.1251	26.5498	1:Experimental evidence at protein level	biological regulation;cellular component assembly;cellular component assembly at cellular level;cellular component organization;cellular component organization at cellular level;cellular component organization or biogenesis;
#NUM!	#NUM!	#NUM!	#NUM!	1:Experimental evidence at protein level	anatomical structure development;anatomical structure formation involved in morphogenesis;anatomical structure morphogenesis;axis specification;biological regulation;bone trabecula formation;branching involved in prosta
27.0843	26.9924	27.011	27.2743	1:Experimental evidence at protein level	
27.8152	28.2676	27.2658	27.7627	1:Experimental evidence at protein level	cell differentiation;cellular developmental process;cellular process;developmental process;gamete generation;male gamete generation;multicellular organismal development;multicellular organismal process;multicellular organ
25.5101	25.6055	26.1058	25.6298	1:Experimental evidence at protein level	developmental process;keratinization
25.5662	25.4464	25.8397	25.7269	1:Experimental evidence at protein level	biological regulation;biosynthetic process;carbohydrate metabolic process;carbohydrate transport;cell cycle;cell cycle process;cell surface receptor linked signaling pathway;cellular biosynthetic process;cellular component c
26.5226	26.789	26.1844	26.2244	1:Experimental evidence at protein level	cell cycle;cell cycle phase;cell cycle process;cell division;cellular component disassembly;cellular component disassembly at cellular level;cellular component organization;cellular component organization at cellular level;cellu
25.8746	25.9638	25.3125	25.6558	1:Experimental evidence at protein level	cellular component organization;cellular component organization at cellular level;cellular component organization or biogenesis;cellular component organization or biogenesis at cellular level;cellular macromolecule metabolic
27.6539	28.0372	27.7034	27.8466	1:Experimental evidence at protein level	response to chemical stimulus;response to inorganic substance;response to oxidative stress;response to reactive oxygen species;response to stimulus;response to stress
25.9109	#NUM!	25.7863	25.811	1:Experimental evidence at protein level	apoptosis;biological regulation;biosynthetic process;cell death;cell differentiation;cellular biosynthetic process;cellular developmental process;cellular macromolecule biosynthetic process;cellular macromolecule metabolic pr
26.8285	26.882	27.0685	26.5681	1:Experimental evidence at protein level	
#NUM!	#NUM!	26.0536	25.8055	1:Experimental evidence at protein level	alcohol metabolic process;amine metabolic process;amine transport;biosynthetic process;cation transport;cellular amine metabolic process;cellular biogenic amine metabolic process;cellular biosynthetic process;cellular lipi
#NUM!	#NUM!	#NUM!	#NUM!	1:Experimental evidence at protein level	biological regulation;macromolecule metabolic process;metabolic process;negative regulation of biological process;negative regulation of BMP signaling pathway;negative regulation of cell communication;negative regulation
25.8372	25.643	#NUM!	25.8248	1:Experimental evidence at protein level	activation of immune response;activation of innate immune response;anatomical structure morphogenesis;biological regulation;biosynthetic process;cell surface receptor linked signaling pathway;cellular biosynthetic process
26.4581	26.931	26.6029	26.6504	1:Experimental evidence at protein level	anatomical structure development;biosynthetic process;catabolic process;cellular biosynthetic process;cellular catabolic process;cellular macromolecule biosynthetic process;cellular macromolecule catabolic process;cellula
25.2064	#NUM!	26.708	26.2984	1:Experimental evidence at protein level	biosynthetic process;cellular biosynthetic process;cellular component disassembly;cellular component disassembly at cellular level;cellular component organization;cellular component organization at cellular level;cellular cor
25.8608	27.3926	26.6879	26.3266	1:Experimental evidence at protein level	biological regulation;cell cycle checkpoint;cellular component assembly;cellular component assembly at cellular level;cellular component organization;cellular component organization at cellular level;cellular component orga
28.4471	29.192	29.5497	29.5595	1:Experimental evidence at protein level	biological regulation;defense response;immune response;immune system process;innate immune response;regulation of biological process;regulation of cellular metabolic process;regulation of cellular process;regulation of m
25.6671	25.3036	25.5101	25.6177	1:Experimental evidence at protein level	apoptosis;biological regulation;biosynthetic process;cell death;cellular biosynthetic process;cellular macromolecule biosynthetic process;cellular macromolecule metabolic process;cellular metabolic process;cellular nitrogen
26.4135	#NUM!	25.7337	25.7817	1:Experimental evidence at protein level	
26.0414	25.9988	26.0257	26.1679	1:Experimental evidence at protein level	
25.6837	#NUM!	#NUM!	#NUM!	1:Experimental evidence at protein level	biological regulation;cellular process;cellular response to stimulus;cellular response to stress;positive regulation of molecular function;positive regulation of NF-kappaB transcription factor activity;positive regulation of sequen
27.2029	27.0434	27.2861	27.204	1:Experimental evidence at protein level	biological regulation;metabolic process;positive regulation of biological process;positive regulation of biosynthetic process;positive regulation of cellular biosynthetic process;positive regulation of cellular metabolic process;p
25.9352	26.0308	25.7622	25.6379	1:Experimental evidence at protein level	biological regulation;cell cycle process;cell division;cellular component organization;cellular component organization at cellular level;cellular component organization or biogenesis;cellular component organization or biogenes
#NUM!	#NUM!	#NUM!	#NUM!	1:Experimental evidence at protein level	anatomical structure development;biological regulation;cell communication;cell development;cell surface receptor linked signaling pathway;cell-cell signaling;cellular developmental process;cellular process;cellular response
26.9667	27.1383	27.4661	27.3002	1:Experimental evidence at protein level	
25.3839	25.8356	25.6298	#NUM!	1:Experimental evidence at protein level	cellular macromolecule metabolic process;cellular metabolic process;cellular nitrogen compound metabolic process;cellular process;cellular protein metabolic process;cellular response to stimulus;cellular response to stress;
26.4966	26.4933	26.705	26.5209	1:Experimental evidence at protein level	biological regulation;cell differentiation;cellular component assembly;cellular component assembly at cellular level;cellular component organization;cellular component organization at cellular level;cellular component organiza
25.6789	25.5882	26.0382	26.0394	1:Experimental evidence at protein level	
27.7472	27.4216	27.5234	27.2399	1:Experimental evidence at protein level	
#NUM!	26.4343	25.7673	26.2841	1:Experimental evidence at protein level	apoptosis;biosynthetic process;cell death;cellular process;death;lipid biosynthetic process;lipid metabolic process;metabolic process;primary metabolic process;programmed cell death;steroid biosynthetic process;steroid m

26.7231	#NUM!	26.4908	26.6726	1:Experimental evidence at protein level	catabolic process;cellular catabolic process;cellular component organization;cellular component organization at cellular level;cellular component organization or biogenesis;cellular component organization or biogenesis at ce
26.0909	25.5245	25.9676	25.1828	1:Experimental evidence at protein level	biosynthetic process;cellular biosynthetic process;cellular component disassembly;cellular component disassembly at cellular level;cellular component organization;cellular component organization at cellular level;cellular cor
25.5623	25.0919	25.2365	25.1373	1:Experimental evidence at protein level	biological regulation;cellular component assembly;cellular component assembly at cellular level;cellular component organization;cellular component organization at cellular level;cellular component organization or biogenesis;
26.3254	26.3176	26.3859	25.9375	1:Experimental evidence at protein level	behavior;biosynthetic process;cellular aromatic compound metabolic process;cellular biosynthetic process;cellular metabolic compound salvage;cellular metabolic process;cellular nitrogen compound biosynthetic process;ce
26.8724	#NUM!	27.4009	27.9516	1:Experimental evidence at protein level	biological regulation;biosynthetic process;cellular biosynthetic process;cellular component organization;cellular component organization at cellular level;cellular component organization or biogenesis;cellular component orga
27.9607	27.6333	27.5607	27.4489	1:Experimental evidence at protein level	carbohydrate metabolic process;cation transport;cellular carbohydrate metabolic process;cellular macromolecule metabolic process;cellular metabolic process;cellular process;cellular protein metabolic process;cognition;div
26.2628	#NUM!	#NUM!	26.4388	1:Experimental evidence at protein level	biological regulation;biosynthetic process;cation homeostasis;cellular biosynthetic process;cellular cation homeostasis;cellular chemical homeostasis;cellular homeostasis;cellular ion homeostasis;cellular iron ion homeostasis
26.0891	26.2708	26.2649	26.1431	1:Experimental evidence at protein level	cellular component organization;cellular component organization at cellular level;cellular component organization or biogenesis;cellular component organization or biogenesis at cellular level;cellular localization;cellular proces
26.12	25.7987	25.7731	25.9465	1:Experimental evidence at protein level	cellular process;establishment of localization;establishment of localization in cell;establishment of protein localization;intracellular protein transport;intracellular transport;protein transport;transport;vesicle-mediated transport
27.8902	27.7067	28.4961	28.6744	1:Experimental evidence at protein level	cellular macromolecule metabolic process;cellular metabolic process;cellular nitrogen compound metabolic process;cellular process;cellular protein metabolic process;macromolecule metabolic process;macromolecule modi
24.1058	#NUM!	24.6507	24.1424	1:Experimental evidence at protein level	
27.1834	27.1578	27.0473	27.4617	1:Experimental evidence at protein level	establishment of localization;establishment of localization in cell;establishment of protein localization;intracellular transport;protein transport;transport
#NUM!	#NUM!	#NUM!	#NUM!	1:Experimental evidence at protein level	cellular component assembly;cellular component assembly at cellular level;cellular component organization;cellular component organization at cellular level;cellular component organization or biogenesis;cellular component c
27.4521	27.667	27.6047	27.6623	1:Experimental evidence at protein level	biological regulation;catabolic process;cellular catabolic process;cellular macromolecule catabolic process;cellular macromolecule metabolic process;cellular metabolic process;cellular process;cellular response to chemical s
28.2056	28.2257	28.1711	28.0355	1:Experimental evidence at protein level	
25.9451	26.323	25.0464	25.2925	1:Experimental evidence at protein level	apoptosis;biosynthetic process;cell death;cellular biosynthetic process;cellular component disassembly;cellular component disassembly at cellular level;cellular component organization;cellular component organization at cel
26.0304	#NUM!	#NUM!	#NUM!	1:Experimental evidence at protein level	
25.6984	25.7372	25.736	25.4102	1:Experimental evidence at protein level	biological regulation;biosynthetic process;cell activation;cell cycle phase;cell cycle process;cell division;cell proliferation;cellular biosynthetic process;cellular component assembly;cellular component assembly at cellular leve
26.5999	26.3758	26.1429	26.5034	1:Experimental evidence at protein level	
25.456	25.4359	25.2426	25.5045	1:Experimental evidence at protein level	biosynthetic process;carboxylic acid biosynthetic process;carboxylic acid metabolic process;cellular biosynthetic process;cellular ketone metabolic process;cellular lipid metabolic process;cellular metabolic process;cellular p
25.7452	25.7995	26.2198	26.0118	1:Experimental evidence at protein level	carbohydrate catabolic process;carbohydrate metabolic process;catabolic process;cellular carbohydrate catabolic process;cellular carbohydrate metabolic process;cellular metabolic process;cellular process;glucuronoside c
26.3195	26.3596	26.2018	26.0964	1:Experimental evidence at protein level	biological regulation;positive regulation of biological process;positive regulation of cytokine production;positive regulation of interleukin-6 production;positive regulation of multicellular organismal process;regulation of biologi
26.9935	27.0918	26.9659	26.9518	1:Experimental evidence at protein level	cellular component organization;cellular component organization or biogenesis;cellular membrane organization;cellular process;endocytosis;establishment of localization;establishment of protein localization;membrane invagi
25.6184	#NUM!	24.919	25.277	1:Experimental evidence at protein level	biological regulation;cellular process;cellular response to stimulus;cellular response to stress;negative regulation of biological process;negative regulation of cell growth;negative regulation of cellular process;negative regulatio
25.7051	26.3967	26.1578	26.0626	1:Experimental evidence at protein level	biological regulation;cellular component organization;cellular component organization at cellular level;cellular component organization or biogenesis;cellular component organization or biogenesis at cellular level;cellular proce
26.1716	25.4674	26.0907	25.8716	1:Experimental evidence at protein level	biological regulation;cellular macromolecule metabolic process;cellular metabolic process;cellular process;cellular protein metabolic process;dephosphorylation;macromolecule metabolic process;macromolecule modification
26.1267	27.3778	27.3417	27.5637	1:Experimental evidence at protein level	cellular process;endosome transport;establishment of localization;establishment of localization in cell;establishment of protein localization;intracellular protein transport;intracellular transport;protein transport;retrograde trans
25.3142	25.8359	25.7603	25.869	1:Experimental evidence at protein level	anatomical structure development;biological regulation;biosynthetic process;cell development;cell migration;cell motility;cellular biosynthetic process;cellular component assembly;cellular component assembly at cellular leve
24.9129	25.4645	#NUM!	25.5406	1:Experimental evidence at protein level	viral reproduction
25.0125	#NUM!	24.2981	24.4004	1:Experimental evidence at protein level	biological regulation;cellular component organization;cellular component organization or biogenesis;cellular membrane organization;cellular process;endocytosis;establishment of localization;establishment of localization in ce
26.6418	26.6134	26.4885	26.5588	1:Experimental evidence at protein level	cellular macromolecule metabolic process;cellular metabolic process;cellular nitrogen compound metabolic process;cellular process;macromolecule metabolic process;macromolecule methylation;macromolecule modificatio
28.0326	27.7592	27.9683	27.6898	1:Experimental evidence at protein level	biological regulation;catabolic process;cellular catabolic process;cellular component assembly;cellular component assembly at cellular level;cellular component organization;cellular component organization at cellular level;ce
27.4877	26.9113	27.6948	27.6183	1:Experimental evidence at protein level	catabolic process;cellular catabolic process;cellular macromolecule catabolic process;cellular macromolecule metabolic process;cellular metabolic process;cellular nitrogen compound metabolic process;cellular process;exo
27.5957	27.975	27.7395	27.8079	1:Experimental evidence at protein level	biological regulation;cellular process;cellular response to stimulus;establishment of localization;establishment of localization in cell;establishment of protein localization;intracellular protein transport;intracellular signal transdu
27.6061	26.9891	26.8395	26.4148	1:Experimental evidence at protein level	anatomical structure formation involved in morphogenesis;anatomical structure morphogenesis;autophagic vacuole assembly;biological regulation;cell projection assembly;cell projection organization;cellular component asse
25.6155	25.9675	#NUM!	25.5316	1:Experimental evidence at protein level	biosynthetic process;cellular biosynthetic process;cellular macromolecule biosynthetic process;cellular macromolecule metabolic process;cellular metabolic process;cellular process;cellular protein metabolic process;cotrans
26.8137	#NUM!	26.7843	27.2407	1:Experimental evidence at protein level	
26.8397	26.8718	26.7038	26.5949	1:Experimental evidence at protein level	biological regulation;regulation of biological process;regulation of catalytic activity;regulation of cellular metabolic process;regulation of cellular process;regulation of dephosphorylation;regulation of hydrolase activity;regulatio
25.541	25.891	25.6437	25.5689	1:Experimental evidence at protein level	biological regulation;cell surface receptor linked signaling pathway;cellular process;cellular response to chemical stimulus;cellular response to fibroblast growth factor stimulus;cellular response to growth factor stimulus;cellul
28.6379	28.6439	28.721	28.2357	1:Experimental evidence at protein level	biological regulation;biosynthetic process;cellular biosynthetic process;cellular macromolecule biosynthetic process;cellular macromolecule metabolic process;cellular metabolic process;cellular nitrogen compound metabolic
27.0791	28.1954	28.4015	28.4187	1:Experimental evidence at protein level	cellular metabolic process;cellular nitrogen compound metabolic process;cellular process;metabolic process;nitrogen compound metabolic process;nucleic acid metabolic process;nucleic acid phosphodiester bond hydrolys
26.0913	25.8581	#NUM!	25.9265	1:Experimental evidence at protein level	biosynthetic process;cellular biosynthetic process;cellular component disassembly;cellular component disassembly at cellular level;cellular component organization;cellular component organization at cellular level;cellular cor
27.6081	27.6099	#NUM!	27.1416	1:Experimental evidence at protein level	actin filament-based movement;actin filament-based process;anatomical structure morphogenesis;axon ensheathment;biological regulation;biosynthetic process;carboxylic acid biosynthetic process;carboxylic acid metaboli
25.8444	#NUM!	#NUM!	25.7297	1:Experimental evidence at protein level	biological regulation;regulation of biological process;regulation of cellular process;regulation of response to stimulus;regulation of signal transduction;regulation of signaling;regulation of TOR signaling cascade
26.534	26.3565	26.6034	26.3365	1:Experimental evidence at protein level	biosynthetic process;cellular biosynthetic process;cellular component disassembly;cellular component disassembly at cellular level;cellular component organization;cellular component organization at cellular level;cellular cor
26.906	28.1565	28.1394	28.1222	1:Experimental evidence at protein level	biological regulation;cation homeostasis;cation transport;cellular cation homeostasis;cellular chemical homeostasis;cellular copper ion homeostasis;cellular homeostasis;cellular ion homeostasis;cellular process;chemical hor
24.9911	24.7068	24.9468	24.4357	1:Experimental evidence at protein level	adaptive immune response;biological regulation;biosynthetic process;cell activation;cell chemotaxis;cell migration;cell motility;cell surface receptor linked signaling pathway;cellular biosynthetic process;cellular component as
27.8352	27.8185	27.7381	27.3672	1:Experimental evidence at protein level	cellular metabolic process;cellular process;electron transport chain;generation of precursor metabolites and energy;metabolic process;mitochondrial electron transport, NADH to ubiquinone;oxidation-reduction process;respi
26.1332	#NUM!	#NUM!	26.0143	1:Experimental evidence at protein level	anatomical structure development;anatomical structure formation involved in morphogenesis;biological adhesion;biological regulation;cell adhesion;cell development;cell migration;cell motility;cellular component movement;
26.5538	27.12	27.2437	27.2817	1:Experimental evidence at protein level	biological regulation;cation transport;cellular metabolic process;cellular process;cellular response to chemical stimulus;cellular response to oxidative stress;cellular response to oxygen radical;cellular response to reactive oxy
26.5243	#NUM!	26.0783	26.5072	1:Experimental evidence at protein level	acute inflammatory response;arachidonic acid metabolic process;biological regulation;biosynthetic process;carboxylic acid biosynthetic process;carboxylic acid metabolic process;cellular biosynthetic process;cellular ketone
24.82	24.6781	24.7729	24.4815	1:Experimental evidence at protein level	activation of caspase activity;activation of NF-kappaB-inducing kinase activity;activation of protein kinase activity;apoptosis;apoptosis in response to endoplasmic reticulum stress;biological regulation;cell death;cell surface r
25.8134	#NUM!	25.6362	25.5869	1:Experimental evidence at protein level	anaphase-promoting complex-dependent proteasomal ubiquitin-dependent protein catabolic process;anterior/posterior axis specification;axis specification;biological regulation;catabolic process;cell cycle;cell cycle phase;c
#NUM!	#NUM!	#NUM!	#NUM!	1:Experimental evidence at protein level	biological regulation;cellular process;cellular response to stimulus;macromolecule metabolic process;metabolic process;primary metabolic process;protein metabolic process;proteolysis;regulation of biological process;regula
27.4102	27.684	27.2758	27.0659	1:Experimental evidence at protein level	apoptosis;biological regulation;biosynthetic process;cell death;cell differentiation;cellular biosynthetic process;cellular component organization;cellular component organization at cellular level;cellular component organization
26.6771	26.7029	27.2647	26.9039	1:Experimental evidence at protein level	biological regulation;negative regulation of biological process;negative regulation of cell communication;negative regulation of cellular process;negative regulation of G-protein coupled receptor protein signaling pathway;nega
27.6891	27.8421	28.0072	28.0813	1:Experimental evidence at protein level	attachment of spindle microtubules to chromosome;attachment of spindle microtubules to kinetochore;biological regulation;cell cycle checkpoint;cell cycle process;cell division;cellular component organization;cellular compo
27.0507	27.6166	27.7478	27.7648	1:Experimental evidence at protein level	viral reproduction
27.5353	27.8406	27.7036	27.8104	1:Experimental evidence at protein level	biosynthetic process;cellular biosynthetic process;cellular component assembly;cellular component organization;cellular component organization or biogenesis;cellular macromolecule biosynthetic process;cellular macromole
25.9961	25.749	25.9771	26.0423	1:Experimental evidence at protein level	anatomical structure development;biological regulation;biosynthetic process;blood coagulation;cell development;cell differentiation;cell proliferation;cellular biosynthetic process;cellular component organization;cellular comp
25.9546	25.6876	25.4479	#NUM!	1:Experimental evidence at protein level	biosynthetic process;cellular biosynthetic process;cellular macromolecule biosynthetic process;cellular macromolecule metabolic process;cellular metabolic process;cellular nitrogen compound metabolic process;cellular pro
26.5468	25.7661	26.1382	#NUM!	1:Experimental evidence at protein level	actin cytoskeleton organization;actin filament organization;actin filament polymerization;actin filament-based process;actin polymerization or depolymerization;biological regulation;cellular component assembly;cellular compo
26.9555	26.5902	26.7204	26.7295	1:Experimental evidence at protein level	biological regulation;cellular component organization;cellular component organization at cellular level;cellular component organization or biogenesis;cellular component organization or biogenesis at cellular level;cellular macr
26.1843	#NUM!	#NUM!	26.715	1:Experimental evidence at protein level	biological regulation;biosynthetic process;cellular biosynthetic process;cellular metabolic process;cellular process;chromosome segregation;cofactor biosynthetic process;cofactor metabolic process;iron-sulfur cluster assem
#NUM!	25.3729	#NUM!	#NUM!	1:Experimental evidence at protein level	anatomical structure development;anatomical structure formation involved in morphogenesis;anatomical structure morphogenesis;appendage morphogenesis;biological regulation;cell morphogenesis;cell morphogenesis invo
27.1337	27.4732	27.3653	27.842	1:Experimental evidence at protein level	carboxylic acid metabolic process;cellular ketone metabolic process;cellular lipid metabolic process;cellular metabolic process;cellular process;fatty acid metabolic process;lipid metabolic process;metabolic process;monoca
25.6258	25.8473	25.8537	26.1588	1:Experimental evidence at protein level	activation of immune response;activation of innate immune response;adaptive immune response;adaptive immune response based on somatic recombination of immune receptors built from immunoglobulin superfamily dom
#NUM!	#NUM!	#NUM!	#NUM!	1:Experimental evidence at protein level	activation of immune response;activation of MAPK activity;biological regulation;calcium ion homeostasis;carbohydrate homeostasis;cation homeostasis;cell chemotaxis;cell death;cell migration;cell motility;cell surface recept
29.3678	#NUM!	#NUM!	29.5424	5:Protein uncertain	antigen processing and presentation;antigen processing and presentation of peptide antigen;antigen processing and presentation of peptide antigen via MHC class I;biological regulation;immune response;immune system pr
27.2358	26.7769	27.1007	26.9774	1:Experimental evidence at protein level	aging;anatomical structure development;anatomical structure morphogenesis;aorta smooth muscle tissue morphogenesis;axon guidance;biological adhesion;biological regulation;cell activation;cell adhesion;cell surface recep
26.9124	#NUM!	#NUM!	27.121	1:Experimental evidence at protein level	biological adhesion;biological regulation;cell adhesion;cell adhesion mediated by integrin;cell differentiation;cell-matrix adhesion;cell-substrate adhesion;cellular component organization;cellular component organization at cel
24.5567	24.2989	24.4812	#NUM!	1:Experimental evidence at protein level	alcohol metabolic process;amine metabolic process;aminoglycan catabolic process;aminoglycan metabolic process;carbohydrate catabolic process;carbohydrate metabolic process;catabolic process;cellular carbohydrate c
29.4021	#NUM!	#NUM!	#NUM!	1:Experimental evidence at protein level	acylglycerol catabolic process;acylglycerol metabolic process;alcohol metabolic process;anatomical structure development;anatomical structure morphogenesis;artery morphogenesis;biological regulation;biosynthetic proces
26.6365	26.7829	26.6661	26.7877	1:Experimental evidence at protein level	bile acid metabolic process;carboxylic acid catabolic process;carboxylic acid metabolic process;catabolic process;cellular catabolic process;cellular ketone metabolic process;cellular lipid catabolic process;cellular lipid meta
26.5267	26.6695	26.5272	26.5683	1:Experimental evidence at protein level	biosynthetic process;cellular biosynthetic process;cellular macromolecule biosynthetic process;cellular macromolecule metabolic process;cellular metabolic process;cellular process;cellular protein metabolic process;cotrans
28.9897	28.7651	28.6433	28.5501	1:Experimental evidence at protein level	biosynthetic process;cellular biosynthetic process;cellular macromolecule metabolic process;cellular metabolic process;cellular nitrogen compound metabolic process;cellular process;electron transport chain;gene expressio
25.8191	26.3748	26.0667	26.1107	1:Experimental evidence at protein level	biological regulation;cell surface receptor linked signaling pathway;cellular process;cellular response to chemical stimulus;cellular response to cytokine stimulus;cellular response to dsRNA;cellular response to exogenous dsR
26.9754	26.9234	27.0734	26.9254	1:Experimental evidence at protein level	adherens junction organization;biological adhesion;biological regulation;cell adhesion;cell junction assembly;cell junction organization;cell-cell junction organization;cellular component assembly;cellular component assembly
25.8063	#NUM!	#NUM!	#NUM!	1:Experimental evidence at protein level	anatomical structure development;biological regulation;biosynthetic process;blood vessel development;cell differentiation;cell proliferation;cell surface receptor linked signaling pathway;cellular biosynthetic process;cellular de
25.3468	#NUM!	26.2715	26.4505	1:Experimental evidence at protein level	behavior;biological regulation;carbohydrate catabolic process;carbohydrate metabolic process;catabolic process;cellular catabolic process;cellular lipid catabolic process;cellular lipid metabolic process;cellular metabolic pro
25.9443	26.5445	26.2007	26.2949	1:Experimental evidence at protein level	anatomical structure homeostasis;anatomical structure morphogenesis;base-excision repair;biological regulation;biosynthetic process;cell cycle;cell division;cellular biosynthetic process;cellular component organization;cellu
26.9722	27.7678	27.4762	27.0476	1:Experimental evidence at protein level	biological regulation;blood coagulation;cellular component organization;cellular component organization or biogenesis;cellular macromolecule metabolic process;cellular membrane organization;cellular metabolic process;cel
29.9501	30.1323	30.7729	30.427	1:Experimental evidence at protein level	biosynthetic process;cellular biosynthetic process;cellular macromolecule biosynthetic process;cellular macromolecule metabolic process;cellular metabolic process;cellular nitrogen compound metabolic process;cellular pro
26.0113	25.8354	25.969	25.8721	1:Experimental evidence at protein level	apoptosis;biological regulation;cell cycle phase;cell cycle process;cell death;cell proliferation;cellular component organization;cellular component organization at cellular level;cellular component organization or biogenesis;cel
26.1601	26.937	26.7237	27.0917	1:Experimental evidence at protein level	
28.0412	28.2384	28.423	28.5901	1:Experimental evidence at protein level	activation of immune response;activation of innate immune response;anaphase-promoting complex-dependent proteasomal ubiquitin-dependent protein catabolic process;antigen processing and presentation;antigen proces
29.1694	29.2218	29.6727	29.3672	1:Experimental evidence at protein level	activation of immune response;activation of innate immune response;anaphase-promoting complex-dependent proteasomal ubiquitin-dependent protein catabolic process;antigen processing and presentation;antigen proces
27.8468	28.1209	28.044	28.2333	1:Experimental evidence at protein level	2'-deoxyribonucleotide metabolic process;base-excision repair;base-excision repair, AP site formation;catabolic process;cellular catabolic process;cellular macromolecule catabolic process;cellular macromolecule metabolic
25.6716	26.0946	25.8692	26.0181	1:Experimental evidence at protein level	alcohol catabolic process;alcohol metabolic process;amine catabolic process;amine metabolic process;branched chain family amino acid catabolic process;branched chain family amino acid metabolic process;carbohydrate
#NUM!	#NUM!	#NUM!	#NUM!	1:Experimental evidence at protein level	behavior;behavioral response to pain;biological adhesion;biological regulation;cell adhesion;cell migration;cell motility;cell-cell adhesion;cellular component movement;cellular process;cellular response to stimulus;cellular res
25.0543	24.5964	24.783	24.6152	1:Experimental evidence at protein level	cap-independent translational initiation;cellular component assembly;cellular component assembly at cellular level;cellular component disassembly;cellular component disassembly at cellular level;cellular component organiza
26.0375	26.3449	#NUM!	25.5255	1:Experimental evidence at protein level	actin cytoskeleton organization;actin filament organization;actin filament-based process;anatomical structure development;biological regulation;cell development;cell junction assembly;cell junction organization;cell migration
27.6303	27.4583	27.9503	27.8728	1:Experimental evidence at protein level	activation of immune response;activation of innate immune response;anaphase-promoting complex-dependent proteasomal ubiquitin-dependent protein catabolic process;antigen processing and presentation;antigen proces
26.8727	26.9595	26.9416	26.8657	1:Experimental evidence at protein level	anatomical structure development;anatomical structure morphogenesis;biological regulation;carbohydrate metabolic process;cardiac chamber morphogenesis;cardiac left ventricle morphogenesis;cardiac ventricle morphoge
26.1359	#NUM!	25.9878	25.7842	1:Experimental evidence at protein level	biosynthetic process;cellular biosynthetic process;cellular macromolecule biosynthetic process;cellular macromolecule metabolic process;cellular metabolic process;cellular nitrogen compound metabolic process;cellular pro
28.045	#NUM!	28.3609	28.219	1:Experimental evidence at protein level	acrosome assembly;anatomical structure formation involved in morphogenesis;cellular component assembly;cellular component assembly at cellular level;cellular component assembly involved in morphogenesis;cellular com
26.4207	26.4193	26.5557	26.67	1:Experimental evidence at protein level	anion transport;behavioral response to nutrient;biological regulation;blood coagulation;carbohydrate homeostasis;carboxylic acid metabolic process;carboxylic acid transport;cell migration;cell motility;cellular component mo
25.259	25.682	25.7724	25.8334	1:Experimental evidence at protein level	activation of caspase activity;activation of caspase activity by cytochrome c;apoptosis;biological regulation;cell death;cellular component disassembly;cellular component disassembly at cellular level;cellular component disa
29.5623	29.7252	30.5993	30.1869	1:Experimental evidence at protein level	anatomical structure homeostasis;antigen processing and presentation;antigen processing and presentation of exogenous antigen;antigen processing and presentation of exogenous peptide antigen;antigen processing and p
29.9473	29.4327	30.2424	29.6751	1:Experimental evidence at protein level	biosynthetic process;catabolic process;cell differentiation;cellular biosynthetic process;cellular catabolic process;cellular component assembly;cellular component assembly at cellular level;cellular component biogenesis;cell
28.5716	28.6376	28.8679	29.2372	1:Experimental evidence at protein level	anatomical structure development;biological regulation;cell surface receptor linked signaling pathway;cellular component organization;cellular component organization at cellular level;cellular component organization or bioge
29.7931	30.3555	30.1529	30.2459	1:Experimental evidence at protein level	anion transport;bicarbonate transport;biological regulation;catabolic process;cellular catabolic process;cellular component assembly;cellular component organization;cellular component organization or biogenesis;cellular me
27.3264	#NUM!	28.0903	27.357	1:Experimental evidence at protein level	biological regulation;cellular process;cellular response to stimulus;establishment of localization;establishment of protein localization;intracellular signal transduction;metabolic process;protein transport;regulation of biological
26.6046	26.3472	26.0481	26.1317	1:Experimental evidence at protein level	antigen processing and presentation;antigen processing and presentation of endogenous antigen;antigen processing and presentation of endogenous peptide antigen;antigen processing and presentation of endogenous pep
26.1746	26.2996	26.5082	26.5189	1:Experimental evidence at protein level	biological regulation;biosynthetic process;catabolic process;cell proliferation;cellular biosynthetic process;cellular catabolic process;cellular macromolecule biosynthetic process;cellular macromolecule catabolic process;cellu
26.0651	26.4896	25.9861	#NUM!	1:Experimental evidence at protein level	activation of MAPKK activity;activation of protein kinase activity;anatomical structure development;anatomical structure formation involved in morphogenesis;anatomical structure morphogenesis;apoptosis;biological regulatio
27.6164	27.6904	27.2898	27.7015	1:Experimental evidence at protein level	anaphase-promoting complex-dependent proteasomal ubiquitin-dependent protein catabolic process;biological regulation;catabolic process;cell cycle;cell cycle checkpoint;cell cycle process;cell division;cellular catabolic pr
26.6605	26.4294	26.2761	26.3961	1:Experimental evidence at protein level	cellular macromolecule metabolic process;cellular metabolic process;cellular process;cellular protein metabolic process;establishment of localization;establishment of localization in cell;establishment of protein localization;es
26.9	26.9065	26.9498	26.8812	1:Experimental evidence at protein level	antigen processing and presentation;biological regulation;cellular component organization;cellular component organization at cellular level;cellular component organization or biogenesis;cellular component organization or bio
27.3189	27.4485	27.1747	27.134	1:Experimental evidence at protein level	anatomical structure development;apoptosis;biological regulation;camera-type eye development;cell death;cell fate specification;cell surface receptor linked signaling pathway;cellular developmental process;cellular macrom
25.139	25.5855	25.5302	25.2944	1:Experimental evidence at protein level	activation of caspase activity;activation of immune response;activation of innate immune response;anatomical structure development;anatomical structure formation involved in morphogenesis;angiogenesis;apoptosis;B cell a
25.5206	#NUM!	25.2948	25.2034	1:Experimental evidence at protein level	cellular process;cellular response to cold;cellular response to freezing;cellular response to stimulus;cellular response to stress;response to abiotic stimulus;response to cold;response to freezing;response to stimulus;response
28.5375	28.6096	28.3194	28.2517	1:Experimental evidence at protein level	cotranslational protein targeting to membrane;establishment of localization;establishment of localization in cell;establishment of protein localization;intracellular protein transport;intracellular transport;protein targeting;protein
27.3331	27.5611	27.192	27.2297	1:Experimental evidence at protein level	biological regulation;developmental process;macromolecule metabolic process;metabolic process;multicellular organismal development;multicellular organismal process;positive regulation of catalytic activity;positive regulati
28.0975	28.1679	28.0899	28.0405	1:Experimental evidence at protein level	biological regulation;biosynthetic process;cell cycle arrest;cell cycle process;cell surface receptor linked signaling pathway;cellular biosynthetic process;cellular macromolecule metabolic process;cellular metabolic process;c
29.0474	29.4	29.0635	29.0968	1:Experimental evidence at protein level	anatomical structure morphogenesis;biological adhesion;biological regulation;calcium ion-dependent exocytosis;cell adhesion;cell morphogenesis;cell morphogenesis involved in differentiation;cell-substrate adhesion;cellula
26.2534	26.1808	26.1246	26.1574	1:Experimental evidence at protein level	biological regulation;cell cycle;cell cycle phase;cell cycle process;cell division;cellular component organization;cellular component organization at cellular level;cellular component organization or biogenesis;cellular componen
25.6821	25.357	25.5797	25.3031	1:Experimental evidence at protein level	activation of protein kinase activity;activation of protein kinase B activity;biological regulation;biosynthetic process;cell migration;cell motility;cell projection assembly;cell projection organization;cellular biosynthetic process;c
25.6267	25.7717	25.2692	25.3377	1:Experimental evidence at protein level	alcohol catabolic process;alcohol metabolic process;carbohydrate catabolic process;carbohydrate metabolic process;catabolic process;cellular carbohydrate catabolic process;cellular carbohydrate metabolic process;cellula
26.1602	25.8071	26.258	26.127	1:Experimental evidence at protein level	cell cycle cytokinesis;cell cycle phase;cell cycle process;cellular component organization;cellular component organization at cellular level;cellular component organization or biogenesis;cellular component organization or biog
25.7762	25.9024	26.0144	26.0501	1:Experimental evidence at protein level	
25.7395	25.5188	25.1429	25.4706	1:Experimental evidence at protein level	cellular process;establishment of localization;establishment of localization in cell;establishment of protein localization;Golgi to plasma membrane transport;Golgi vesicle transport;intracellular transport;post-Golgi vesicle-med
25.3525	24.9328	25.1724	#NUM!	1:Experimental evidence at protein level	
26.2308	#NUM!	26.0655	26.0662	1:Experimental evidence at protein level	anatomical structure development;anatomical structure morphogenesis;apoptosis;autophagy;biological regulation;brain development;catabolic process;cell death;cell part morphogenesis;cell projection morphogenesis;cell p
27.3977	#NUM!	27.2168	27.2809	1:Experimental evidence at protein level	apoptosis;cell death;cellular process;death;programmed cell death
26.0693	25.5493	25.7026	25.5772	1:Experimental evidence at protein level	cell differentiation;cellular developmental process;cellular process;developmental process;hemopoietic progenitor cell differentiation
25.8682	26.0162	25.8669	26.0775	1:Experimental evidence at protein level	biological regulation;cell redox homeostasis;cellular homeostasis;cellular macromolecule metabolic process;cellular metabolic process;cellular process;cellular protein metabolic process;homeostatic process;macromolecule
#NUM!	#NUM!	26.4449	#NUM!	1:Experimental evidence at protein level	anatomical structure morphogenesis;developmental process;hair cycle process;hair follicle morphogenesis;molting cycle;molting cycle process;multicellular organismal process
25.6061	#NUM!	25.8905	25.7221	1:Experimental evidence at protein level	biological regulation;directional locomotion;locomotion;negative regulation of actin nucleation;negative regulation of biological process;negative regulation of cell migration;negative regulation of cell motility;negative regulatio

25.5152	25.2578	25.6255	26.0005	1:Experimental evidence at protein level	catabolic process;cellular catabolic process;cellular component assembly;cellular component assembly at cellular level;cellular component organization;cellular component organization at cellular level;cellular component org
26.1759	25.6997	25.6596	25.8524	1:Experimental evidence at protein level	biological regulation;catabolic process;cellular catabolic process;cellular macromolecule catabolic process;cellular macromolecule metabolic process;cellular metabolic process;cellular process;cellular protein metabolic proc
26.4793	26.3145	26.3238	26.4686	1:Experimental evidence at protein level	adipose tissue development;anatomical structure development;biological regulation;carbohydrate homeostasis;carboxylic acid metabolic process;cell differentiation;cellular chemical homeostasis;cellular developmental proc
26.7119	26.7862	26.8767	26.9293	1:Experimental evidence at protein level	biological regulation;cellular component organization;cellular component organization at cellular level;cellular component organization or biogenesis;cellular component organization or biogenesis at cellular level;cellular meml
26.2804	26.4804	26.4805	26.6325	1:Experimental evidence at protein level	biological regulation;cellular component organization;cellular component organization at cellular level;cellular component organization or biogenesis;cellular component organization or biogenesis at cellular level;cellular proce
26.451	26.1492	26.2473	26.2437	1:Experimental evidence at protein level	acylglycerol catabolic process;acylglycerol metabolic process;adult behavior;adult locomotory behavior;adult walking behavior;behavior;catabolic process;cellular catabolic process;cellular lipid catabolic process;cellular lipid
26.5457	26.0039	26.2875	25.8991	1:Experimental evidence at protein level	biological regulation;cellular process;cellular response to stimulus;intracellular signal transduction;regulation of biological process;regulation of cellular process;regulation of response to stimulus;regulation of signal transducti
25.6842	25.8053	25.5051	25.2747	1:Experimental evidence at protein level	biological regulation;cell proliferation;cellular component assembly;cellular component assembly at cellular level;cellular component organization;cellular component organization at cellular level;cellular component organizatio
26.7898	27.408	26.0407	26.8414	1:Experimental evidence at protein level	aging;autophagy;biological regulation;catabolic process;cellular catabolic process;cellular metabolic process;cellular process;developmental process;metabolic process;mitochondrion degradation;multicellular organismal ag
27.0991	26.513	26.7645	26.6036	1:Experimental evidence at protein level	cellular process;establishment of localization;transmembrane transport;transport
25.9804	26.9215	26.7349	26.8226	1:Experimental evidence at protein level	cellular macromolecule metabolic process;cellular metabolic process;cellular process;cellular protein metabolic process;macromolecule metabolic process;macromolecule modification;metabolic process;post-translational pr
25.457	25.4083	25.4567	25.0602	1:Experimental evidence at protein level	biological regulation;calcium ion homeostasis;calcium ion transport;calcium-mediated signaling;carbohydrate homeostasis;cation homeostasis;cation transport;cellular calcium ion homeostasis;cellular cation homeostasis;ce
25.7189	25.8654	25.5086	25.5627	1:Experimental evidence at protein level	
25.3643	#NUM!	26.0726	25.5526	1:Experimental evidence at protein level	biological regulation;positive regulation of catalytic activity;positive regulation of GTPase activity;positive regulation of hydrolase activity;positive regulation of molecular function;regulation of biological process;regulation of ca
28.1251	27.9879	28.0514	27.8532	1:Experimental evidence at protein level	biological regulation;cellular component assembly;cellular component assembly at cellular level;cellular component organization;cellular component organization at cellular level;cellular component organization or biogenesis;
29.174	29.0907	28.6013	29.1132	1:Experimental evidence at protein level	actin cytoskeleton organization;actin filament depolymerization;actin filament organization;actin filament-based process;actin polymerization or depolymerization;biological regulation;cellular component disassembly;cellular c
25.8262	26.0899	26.4737	#NUM!	1:Experimental evidence at protein level	
27.3411	27.7179	27.2209	27.3725	1:Experimental evidence at protein level	activation of immune response;activation of innate immune response;anaphase-promoting complex-dependent proteasomal ubiquitin-dependent protein catabolic process;antigen processing and presentation;antigen proces
26.5963	26.4377	26.5437	26.3937	1:Experimental evidence at protein level	
25.6013	#NUM!	#NUM!	25.3956	1:Experimental evidence at protein level	autophagy;biological regulation;catabolic process;cellular catabolic process;cellular metabolic process;cellular process;endosome transport;establishment of localization;establishment of localization in cell;establishment of p
27.048	28.4034	26.999	28.102	1:Experimental evidence at protein level	biological regulation;cellular process;cellular response to stimulus;multicellular organismal process;ossification;regulation of biological process;regulation of cellular process;response to stimulus;signal transduction
26.013	27.0363	26.7053	26.9651	1:Experimental evidence at protein level	alcohol catabolic process;alcohol metabolic process;carbohydrate catabolic process;carbohydrate metabolic process;catabolic process;cellular carbohydrate catabolic process;cellular carbohydrate metabolic process;cellula
26.2649	26.4237	26.2407	26.2575	1:Experimental evidence at protein level	anatomical structure formation involved in morphogenesis;biological regulation;cell differentiation;cellular developmental process;cellular localization;cellular process;developmental process;establishment of localization;estab
26.2743	26.3007	26.1249	26.0943	1:Experimental evidence at protein level	cellular localization;cellular process;establishment of localization;establishment of localization in cell;establishment of organelle localization;establishment of protein localization;establishment of ribosome localization;intracellu
27.8522	28.0665	27.7963	27.9427	1:Experimental evidence at protein level	biosynthetic process;cellular biosynthetic process;cellular lipid metabolic process;cellular metabolic process;cellular process;ceramide biosynthetic process;ceramide metabolic process;lipid biosynthetic process;lipid metabo
26.5079	26.8997	27.0706	27.1036	1:Experimental evidence at protein level	actin cytoskeleton organization;actin filament bundle assembly;actin filament organization;actin filament-based process;apoptosis;biological regulation;biosynthetic process;cell death;cellular biosynthetic process;cellular con
24.9492	24.7126	24.7087	24.7527	1:Experimental evidence at protein level	apoptosis;biological regulation;cell chemotaxis;cell death;cell migration;cell motility;cell surface receptor linked signaling pathway;cellular component movement;cellular process;cellular response to chemical stimulus;cellular
24.6594	24.6087	27.6174	24.2304	1:Experimental evidence at protein level	biological regulation;cellular component organization;cellular component organization at cellular level;cellular component organization or biogenesis;cellular component organization or biogenesis at cellular level;cellular macro
26.3006	25.9	26.1907	25.74	1:Experimental evidence at protein level	biological regulation;cellular process;cellular response to stimulus;endosome to lysosome transport;endosome transport;establishment of cell polarity;establishment of localization;establishment of localization in cell;establish
27.5205	27.6392	28.0303	27.8323	1:Experimental evidence at protein level	
28.2581	28.3831	27.9668	28.0404	1:Experimental evidence at protein level	anatomical structure development;anatomical structure formation involved in morphogenesis;biosynthetic process;cellular biosynthetic process;cellular lipid metabolic process;cellular metabolic process;cellular process;deve
25.0825	24.8717	24.5998	24.9739	1:Experimental evidence at protein level	cellular macromolecule metabolic process;cellular metabolic process;cellular nitrogen compound metabolic process;cellular process;macromolecule metabolic process;metabolic process;ncRNA metabolic process;ncRNA pr
27.8896	28.0184	28.3717	28.1542	1:Experimental evidence at protein level	3'-UTR-mediated mRNA destabilization;anatomical structure development;axon ensheathment;biological regulation;biosynthetic process;carboxylic acid biosynthetic process;carboxylic acid metabolic process;cell developm
26.2411	25.8156	25.8835	26.304	1:Experimental evidence at protein level	establishment of localization;establishment of localization in cell;establishment of protein localization;intracellular protein transport;intracellular transport;nuclear export;nuclear transport;nucleocytoplasmic transport;protein e
26.2839	26.16	26.2911	25.9326	1:Experimental evidence at protein level	biological regulation;biosynthetic process;cellular biosynthetic process;cellular macromolecule biosynthetic process;cellular macromolecule metabolic process;cellular metabolic process;cellular nitrogen compound metabolic
25.0557	26.4553	26.5225	26.6205	1:Experimental evidence at protein level	carbohydrate metabolic process;cellular carbohydrate metabolic process;cellular macromolecule metabolic process;cellular metabolic process;cellular process;cellular protein metabolic process;glycosylation;macromolecule
28.221	28.3873	28.7021	28.7824	1:Experimental evidence at protein level	
25.8215	25.8927	#NUM!	25.3833	1:Experimental evidence at protein level	cell cycle;cell cycle process;cellular component assembly;cellular component organization;cellular component organization at cellular level;cellular component organization or biogenesis;cellular component organization or bio
26.8496	26.3938	26.38	26.2724	1:Experimental evidence at protein level	biosynthetic process;cellular biosynthetic process;cellular lipid metabolic process;cellular metabolic process;cellular process;glycerolipid biosynthetic process;glycerolipid metabolic process;glycerophospholipid biosynthetic
#NUM!	24.4283	24.822	24.5052	1:Experimental evidence at protein level	anatomical structure formation involved in morphogenesis;angiogenesis;apoptosis;biological regulation;cell death;cell differentiation;cellular developmental process;cellular process;death;developmental process;establishmer
25.9435	26.8457	26.6436	26.8673	1:Experimental evidence at protein level	amine biosynthetic process;amine metabolic process;amino acid salvage;aspartate family amino acid biosynthetic process;aspartate family amino acid metabolic process;biosynthetic process;carboxylic acid biosynthetic pro
25.582	25.0078	25.0875	#NUM!	1:Experimental evidence at protein level	biosynthetic process;carboxylic acid biosynthetic process;carboxylic acid metabolic process;cellular biosynthetic process;cellular ketone metabolic process;cellular lipid metabolic process;cellular metabolic process;cellular p
25.0856	24.6656	25.3474	24.2018	1:Experimental evidence at protein level	establishment of localization;lipid transport;organic substance transport;transport
24.9998	#NUM!	24.6527	25.0916	1:Experimental evidence at protein level	biosynthetic process;cellular biosynthetic process;cellular component disassembly;cellular component disassembly at cellular level;cellular component organization;cellular component organization at cellular level;cellular cor
26.5332	26.2153	26.0522	26.109	1:Experimental evidence at protein level	biological regulation;cell cycle;cell cycle phase;cell cycle process;cell division;cellular component organization;cellular component organization at cellular level;cellular component organization or biogenesis;cellular componer
26.3904	26.5015	26.6818	26.7972	1:Experimental evidence at protein level	biological regulation;biosynthetic process;catabolic process;cell surface receptor linked signaling pathway;cellular biosynthetic process;cellular catabolic process;cellular component organization;cellular component organizat
27.5473	28.0328	28.0733	28.1842	1:Experimental evidence at protein level	anatomical structure development;anatomical structure morphogenesis;biological regulation;biosynthetic process;cell development;cell motility;cell part morphogenesis;cellular biosynthetic process;cellular component morph
28.928	29.131	28.729	28.9829	1:Experimental evidence at protein level	cell cycle process;cellular component organization;cellular component organization at cellular level;cellular component organization or biogenesis;cellular component organization or biogenesis at cellular level;cellular macrom
26.9887	26.7907	26.9608	26.4423	1:Experimental evidence at protein level	biological regulation;cellular macromolecule metabolic process;cellular metabolic process;cellular nitrogen compound metabolic process;cellular process;induction of apoptosis;induction of apoptosis by extracellular signals;i
24.8192	#NUM!	25.606	25.5521	1:Experimental evidence at protein level	metabolic process
27.1984	27.7597	28.0037	28.0896	1:Experimental evidence at protein level	catabolic process;cellular catabolic process;cellular component assembly;cellular component organization;cellular component organization or biogenesis;cellular metabolic process;cellular nitrogen compound catabolic proce
26.6061	26.7691	27.2017	27.3205	1:Experimental evidence at protein level	cellular component assembly;cellular component assembly at cellular level;cellular component organization;cellular component organization at cellular level;cellular component organization or biogenesis;cellular component c
#NUM!	24.1342	24.0911	24.6009	1:Experimental evidence at protein level	
26.8636	26.7816	27.2186	26.9656	1:Experimental evidence at protein level	cell cycle;cellular component organization;cellular component organization at cellular level;cellular component organization or biogenesis;cellular component organization or biogenesis at cellular level;cellular process;mitotic c
25.3087	#NUM!	24.7046	24.382	1:Experimental evidence at protein level	biological regulation;cellular macromolecule metabolic process;cellular metabolic process;cellular nitrogen compound metabolic process;cellular process;macromolecule metabolic process;metabolic process;mRNA metabol
25.0661	25.8046	25.2982	25.2282	1:Experimental evidence at protein level	anatomical structure development;ATP-dependent chromatin remodeling;biological regulation;biosynthetic process;brain development;canonical Wnt receptor signaling pathway;cell surface receptor linked signaling pathway
26.6731	26.3534	26.3402	26.2392	1:Experimental evidence at protein level	biosynthetic process;cellular biosynthetic process;cellular component disassembly;cellular component disassembly at cellular level;cellular component organization;cellular component organization at cellular level;cellular cor
26.633	27.2284	26.9105	26.609	1:Experimental evidence at protein level	cellular component organization;cellular component organization at cellular level;cellular component organization or biogenesis;cellular component organization or biogenesis at cellular level;cellular process;establishment of l
26.8407	27.1728	27.4527	27.3592	1:Experimental evidence at protein level	biological regulation;regulation of biological process;regulation of catabolic process;regulation of cellular catabolic process;regulation of cellular metabolic process;regulation of cellular process;regulation of cellular protein me
26.9618	27.5577	27.7615	27.4653	1:Experimental evidence at protein level	circadian rhythm;rhythmic process
#NUM!	24.6418	24.8631	24.5079	1:Experimental evidence at protein level	anatomical structure development;anatomical structure formation involved in morphogenesis;axon guidance;biological regulation;biosynthetic process;camera-type eye development;cellular biosynthetic process;cellular mac
#NUM!	26.358	26.266	26.1987	1:Experimental evidence at protein level	biological regulation;cell cycle;cell division;cellular process;cellular response to stimulus;chromosome segregation;intracellular signal transduction;metabolic process;regulation of biological process;regulation of cellular proce
28.0591	27.7176	28.3602	28.2879	1:Experimental evidence at protein level	biological regulation;cell cycle checkpoint;cellular component organization;cellular component organization at cellular level;cellular component organization or biogenesis;cellular component organization or biogenesis at cellu
26.4066	26.7315	26.5906	26.2085	1:Experimental evidence at protein level	
26.8636	26.5417	26.8886	26.7134	1:Experimental evidence at protein level	anatomical structure formation involved in morphogenesis;angiogenesis;biological regulation;biosynthetic process;cellular biosynthetic process;cellular macromolecule biosynthetic process;cellular macromolecule metabolic p
26.7988	27.3818	27.1277	27.6946	1:Experimental evidence at protein level	biological regulation;cell cycle process;cellular component organization;cellular component organization at cellular level;cellular component organization or biogenesis;cellular component organization or biogenesis at cellular
26.7649	26.5642	26.5959	26.535	1:Experimental evidence at protein level	biological regulation;cell communication;cellular component organization;cellular component organization at cellular level;cellular component organization or biogenesis;cellular component organization or biogenesis at cellula
26.6765	26.613	26.5908	26.5419	1:Experimental evidence at protein level	biosynthetic process;cellular aromatic compound metabolic process;cellular biosynthetic process;cellular metabolic compound salvage;cellular metabolic process;cellular nitrogen compound biosynthetic process;cellular nitro
25.1141	#NUM!	26.3452	25.1982	1:Experimental evidence at protein level	biological regulation;biosynthetic process;cell proliferation;cellular biosynthetic process;cellular component organization;cellular component organization at cellular level;cellular component organization or biogenesis;cellular c
26.4802	26.6676	27.1559	26.7936	1:Experimental evidence at protein level	biological regulation;catabolic process;cell surface receptor linked signaling pathway;cellular catabolic process;cellular macromolecule catabolic process;cellular macromolecule metabolic process;cellular metabolic process;c
26.1901	26.2144	26.0643	26.3172	1:Experimental evidence at protein level	activation of immune response;activation of innate immune response;biological regulation;cellular process;cellular response to stimulus;cytokine production;defense response;defense response to bacterium;defense respons
27.4039	27.0103	27.1126	27.388	1:Experimental evidence at protein level	antigen processing and presentation;antigen processing and presentation of exogenous antigen;antigen processing and presentation of exogenous peptide antigen;antigen processing and presentation of exogenous peptide
27.9032	28.6566	28.9493	29.0483	1:Experimental evidence at protein level	alcohol catabolic process;alcohol metabolic process;carbohydrate catabolic process;carbohydrate metabolic process;catabolic process;cellular carbohydrate catabolic process;cellular carbohydrate metabolic process;cellula
25.4633	25.6269	25.6446	25.3763	1:Experimental evidence at protein level	anatomical structure morphogenesis;cell part morphogenesis;cell projection morphogenesis;cell projection organization;cellular component morphogenesis;cellular component organization;cellular component organization at
27.4843	27.0138	27.3046	27.0637	1:Experimental evidence at protein level	apoptosis;biological regulation;catabolic process;cell death;cellular catabolic process;cellular macromolecule catabolic process;cellular macromolecule metabolic process;cellular metabolic process;cellular process;cytoplasm
27.4484	27.7884	27.7439	27.4776	1:Experimental evidence at protein level	cellular macromolecule metabolic process;cellular metabolic process;cellular nitrogen compound metabolic process;cellular process;macromolecule metabolic process;metabolic process;ncRNA metabolic process;ncRNA pr
28.4534	28.4495	28.1701	28.2747	1:Experimental evidence at protein level	cellular macromolecule metabolic process;cellular metabolic process;cellular nitrogen compound metabolic process;cellular process;gene expression;macromolecule metabolic process;metabolic process;mRNA metabolic p
28.5672	28.4276	28.2513	28.2556	1:Experimental evidence at protein level	cellular macromolecule metabolic process;cellular metabolic process;cellular nitrogen compound metabolic process;cellular process;cellular protein metabolic process;macromolecule metabolic process;macromolecule modi
26.5573	26.7542	26.818	26.4535	1:Experimental evidence at protein level	apoptosis;biological regulation;cell death;cellular process;death;metabolic process;negative regulation of anoikis;negative regulation of apoptosis;negative regulation of biological process;negative regulation of cell death;neg
26.3774	26.4486	26.7447	26.3328	1:Experimental evidence at protein level	biological regulation;BMP signaling pathway;catabolic process;cell surface receptor linked signaling pathway;cellular catabolic process;cellular component organization;cellular component organization at cellular level;cellular
26.7007	27.1098	26.8008	27.0208	1:Experimental evidence at protein level	activation of JUN kinase activity;activation of MAPK activity;activation of MAPKKK activity;activation of protein kinase activity;biological regulation;cellular macromolecule metabolic process;cellular metabolic process;cellular
27.2781	27.5417	27.5853	27.6077	1:Experimental evidence at protein level	biological regulation;catabolic process;cellular catabolic process;cellular macromolecule catabolic process;cellular macromolecule metabolic process;cellular metabolic process;cellular nitrogen compound metabolic process
29.4611	29.0935	29.8471	29.5494	1:Experimental evidence at protein level	biological regulation;biosynthetic process;catabolic process;cellular biosynthetic process;cellular catabolic process;cellular macromolecule biosynthetic process;cellular macromolecule catabolic process;cellular macromolecu
27.7825	27.5242	27.3525	27.7126	1:Experimental evidence at protein level	biological adhesion;biological regulation;calcium ion homeostasis;cation homeostasis;cell adhesion;cell surface receptor linked signaling pathway;cell-cell adhesion;cellular calcium ion homeostasis;cellular cation homeostasi
26.321	25.9334	26.1358	26.1824	1:Experimental evidence at protein level	activation of protein kinase activity;apoptosis;biological regulation;cell death;cellular component disassembly;cellular component disassembly at cellular level;cellular component disassembly involved in apoptosis;cellular cor
27.1208	#NUM!	26.9926	26.98	1:Experimental evidence at protein level	biological regulation;biosynthetic process;cellular biosynthetic process;cellular component organization;cellular component organization at cellular level;cellular component organization or biogenesis;cellular component orga
27.0854	27.5369	27.7919	27.7282	1:Experimental evidence at protein level	cell cycle process;cellular macromolecule metabolic process;cellular metabolic process;cellular process;cellular protein metabolic process;dephosphorylation;G1/S transition of mitotic cell cycle;macromolecule metabolic pro
25.8943	26.0598	25.9066	25.9981	1:Experimental evidence at protein level	cellular component organization;cellular component organization or biogenesis;cellular membrane organization;cellular process;endocytosis;establishment of localization;establishment of localization in cell;establishment of p
27.6754	27.1989	27.3803	27.1429	1:Experimental evidence at protein level	biological regulation;cellular process;cellular response to chemical stimulus;cellular response to endogenous stimulus;cellular response to hormone stimulus;cellular response to organic substance;cellular response to steroid
27.7749	27.8676	28.0549	27.7247	1:Experimental evidence at protein level	amine metabolic process;amino acid activation;biological regulation;carboxylic acid metabolic process;cellular amine metabolic process;cellular amino acid metabolic process;cellular ketone metabolic process;cellular macro
26.0736	26.557	26.6212	26.8905	1:Experimental evidence at protein level	biological regulation;catabolic process;cell differentiation;cell proliferation;cellular catabolic process;cellular developmental process;cellular metabolic process;cellular nitrogen compound catabolic process;cellular nitrogen co
24.9402	#NUM!	24.8935	24.972	1:Experimental evidence at protein level	anatomical structure homeostasis;B cell activation;B cell activation involved in immune response;biological regulation;blastocyst growth;cell activation;cell activation involved in immune response;cell cycle;cell cycle arrest;ce
24.9719	24.3806	#NUM!	24.2358	1:Experimental evidence at protein level	biological regulation;cation homeostasis;cellular cation homeostasis;cellular chemical homeostasis;cellular homeostasis;cellular ion homeostasis;cellular iron ion homeostasis;cellular metal ion homeostasis;cellular process;ch
25.7237	26.0463	25.9079	25.7053	1:Experimental evidence at protein level	ATP biosynthetic process;ATP metabolic process;biological regulation;biosynthetic process;cellular biosynthetic process;cellular metabolic process;cellular nitrogen compound biosynthetic process;cellular nitrogen compoun
26.0622	25.9298	26.1229	25.9799	1:Experimental evidence at protein level	biological regulation;biosynthetic process;cellular biosynthetic process;cellular component assembly;cellular component assembly at cellular level;cellular component organization;cellular component organization at cellular le
26.2314	25.9927	25.9302	26.0789	1:Experimental evidence at protein level	
28.3151	28.0009	27.6748	27.9055	1:Experimental evidence at protein level	apoptosis;cell death;cellular component organization;cellular component organization at cellular level;cellular component organization or biogenesis;cellular component organization or biogenesis at cellular level;cellular proce
26.7243	26.3663	26.5933	26.7344	1:Experimental evidence at protein level	cellular metabolic process;cellular process;electron transport chain;generation of precursor metabolites and energy;metabolic process;mitochondrial electron transport, NADH to ubiquinone;oxidation-reduction process;respi
27.3619	27.9978	27.6319	27.8085	1:Experimental evidence at protein level	biological regulation;cell differentiation;cellular developmental process;cellular process;developmental process;multicellular organismal development;multicellular organismal process;regulation of biological process;regulation
28.4735	28.2357	28.3917	28.2172	1:Experimental evidence at protein level	biosynthetic process;cellular biosynthetic process;cellular macromolecule metabolic process;cellular metabolic process;cellular nitrogen compound metabolic process;cellular process;electron transport chain;gene expressio
26.1431	25.8824	26.0946	25.9461	1:Experimental evidence at protein level	2'-deoxyribonucleotide metabolic process;adenosine catabolic process;adenosine metabolic process;aging;amine transport;anatomical structure development;B cell activation;B cell activation involved in immune response;B
30.131	31.6412	31.931	31.5764	1:Experimental evidence at protein level	anatomical structure homeostasis;anatomical structure morphogenesis;antibacterial humoral response;antifungal humoral response;antimicrobial humoral response;biological regulation;biosynthetic process;bone morphogen
26.1761	26.3015	25.939	26.3226	1:Experimental evidence at protein level	amide biosynthetic process;amine biosynthetic process;amine catabolic process;amine metabolic process;arginine biosynthetic process;arginine biosynthetic process via ornithine;arginine catabolic process;arginine metabol
27.3564	#NUM!	#NUM!	27.6079	1:Experimental evidence at protein level	
27.4627	27.6573	28.0038	28.1221	1:Experimental evidence at protein level	biosynthetic process;cellular aromatic compound metabolic process;cellular biosynthetic process;cellular metabolic process;cellular nitrogen compound biosynthetic process;cellular nitrogen compound metabolic process;ce
25.5071	26.0281	26.1118	25.6737	1:Experimental evidence at protein level	acylglycerol metabolic process;aging;amine metabolic process;anatomical structure development;anatomical structure formation involved in morphogenesis;anatomical structure morphogenesis;angiogenesis;angiogenesis in
27.1431	26.8237	27.0045	27.2028	1:Experimental evidence at protein level	biological regulation;biosynthetic process;cellular biosynthetic process;cellular macromolecule biosynthetic process;cellular macromolecule metabolic process;cellular metabolic process;cellular nitrogen compound metabolic
27.0447	27.1716	27.2779	27.1267	1:Experimental evidence at protein level	aromatic compound biosynthetic process;biosynthetic process;cellular aromatic compound metabolic process;cellular biosynthetic process;cellular metabolic process;cellular nitrogen compound biosynthetic process;cellular
28.5826	31.8067	30.8509	31.7138	1:Experimental evidence at protein level	aging;biological regulation;biosynthetic process;carboxylic acid biosynthetic process;carboxylic acid metabolic process;cell aging;cell proliferation;cell surface receptor linked signaling pathway;cellular biosynthetic process;c
27.2198	27.0646	27.1869	26.7235	1:Experimental evidence at protein level	anaphase-promoting complex-dependent proteasomal ubiquitin-dependent protein catabolic process;anatomical structure development;anatomical structure morphogenesis;biological regulation;cardiac cell development;ca
26.3172	27.9719	27.6186	28.7509	1:Experimental evidence at protein level	anatomical structure development;biosynthetic process;cellular biosynthetic process;cellular macromolecule metabolic process;cellular metabolic process;cellular nitrogen compound metabolic process;cellular process;deve
28.5109	28.7871	29.1351	29.0043	1:Experimental evidence at protein level	amine metabolic process;aminoglycan catabolic process;aminoglycan metabolic process;carbohydrate catabolic process;carbohydrate metabolic process;catabolic process;cellular catabolic process;cellular metabolic proce
27.0325	26.8008	26.6472	26.6586	1:Experimental evidence at protein level	activation of immune response;activation of MAPKK activity;activation of phospholipase C activity;activation of protein kinase activity;antigen receptor-mediated signaling pathway;axon guidance;biological regulation;blood c
28.3601	29.1798	29.4292	29.228	1:Experimental evidence at protein level	biosynthetic process;cellular biosynthetic process;cellular macromolecule metabolic process;cellular metabolic process;cellular nitrogen compound metabolic process;cellular process;electron transport chain;gene expressio
#NUM!	#NUM!	#NUM!	#NUM!	1:Experimental evidence at protein level	biological regulation;female pregnancy;multi-organism process;negative regulation of catalytic activity;negative regulation of endopeptidase activity;negative regulation of hydrolase activity;negative regulation of molecular fur
25.7582	25.3775	25.7128	25.2391	1:Experimental evidence at protein level	amine metabolic process;carboxylic acid catabolic process;carboxylic acid metabolic process;catabolic process;cellular amine metabolic process;cellular amino acid metabolic process;cellular catabolic process;cellular keto
26.7636	26.8701	26.8104	26.888	1:Experimental evidence at protein level	alcohol metabolic process;biological regulation;biosynthetic process;cation homeostasis;cell differentiation;cellular aromatic compound metabolic process;cellular biosynthetic process;cellular component assembly;cellular co
27.6066	28.2645	27.8439	28.2856	1:Experimental evidence at protein level	cellular component organization;cellular component organization at cellular level;cellular component organization or biogenesis;cellular component organization or biogenesis at cellular level;cellular process;embryo implantat
26.4847	27.2208	27.7923	27.7032	1:Experimental evidence at protein level	cellular macromolecule metabolic process;cellular metabolic process;cellular process;cellular protein metabolic process;chaperone-mediated protein folding;macromolecule metabolic process;macromolecule modification;me
24.9105	#NUM!	24.1761	24.2266	1:Experimental evidence at protein level	anatomical structure development;biological regulation;biosynthetic process;cardiac cell differentiation;cardiac muscle cell differentiation;cardiac muscle cell proliferation;cell communication;cell differentiation;cell proliferation
25.2045	#NUM!	25.0108	24.447	1:Experimental evidence at protein level	alcohol metabolic process;apoptosis;biological regulation;biosynthetic process;carbohydrate biosynthetic process;carbohydrate metabolic process;carbohydrate transport;cell death;cell differentiation;cell surface receptor lin
26.3861	26.6393	26.4028	26.3541	1:Experimental evidence at protein level	alcohol biosynthetic process;alcohol metabolic process;biosynthetic process;carbohydrate biosynthetic process;carbohydrate metabolic process;cellular biosynthetic process;cellular carbohydrate biosynthetic process;cellul
26.8159	26.3193	26.3094	26.556	1:Experimental evidence at protein level	amine metabolic process;aminoglycan biosynthetic process;aminoglycan catabolic process;aminoglycan metabolic process;anatomical structure formation involved in morphogenesis;axon guidance;biological regulation;bios
27.289	28.86	28.29	29.3059	1:Experimental evidence at protein level	biological regulation;cell redox homeostasis;cellular chemical homeostasis;cellular homeostasis;cellular ion homeostasis;cellular macromolecule metabolic process;cellular metabolic process;cellular nitrogen compound meta
25.8704	25.9029	25.8242	26.1081	1:Experimental evidence at protein level	biological regulation;biosynthetic process;cell cycle arrest;cell cycle process;cell surface receptor linked signaling pathway;cellular biosynthetic process;cellular macromolecule biosynthetic process;cellular macromolecule me
27.5992	27.4935	27.4718	27.2798	1:Experimental evidence at protein level	activation of adenylate cyclase activity;axon guidance;biological regulation;cellular component organization;cellular component organization at cellular level;cellular component organization or biogenesis;cellular component c
26.9477	27.1082	26.9516	26.9542	1:Experimental evidence at protein level	carboxylic acid catabolic process;carboxylic acid metabolic process;catabolic process;cellular catabolic process;cellular ketone metabolic process;cellular lipid catabolic process;cellular lipid metabolic process;cellular metab
26.127	25.9979	26.143	26.5219	1:Experimental evidence at protein level	biological regulation;blood coagulation;coagulation;hemostasis;multicellular organismal process;regulation of biological quality;regulation of body fluid levels
26.9103	27.2615	27.4233	27.7324	1:Experimental evidence at protein level	biological regulation;positive regulation of biological process;positive regulation of cell proliferation;positive regulation of cellular process;regulation of biological process;regulation of cell proliferation;regulation of cellular proc

26.181	26.1027	25.6162	25.9684	1:Experimental evidence at protein level	3'-UTR-mediated mRNA stabilization;activation of immune response;activation of innate immune response;activation of MAPK activity;anatomical structure development;arachidonic acid metabolic process;biological regulati
26.3005	25.4562	25.8639	25.4513	1:Experimental evidence at protein level	anatomical structure development;biological regulation;biosynthetic process;cell cycle;cell cycle checkpoint;cell cycle phase;cell cycle process;cell differentiation;cell division;cell proliferation;cellular biosynthetic process;cellu
26.0524	25.9732	25.6883	25.699	1:Experimental evidence at protein level	anatomical structure homeostasis;biological regulation;biosynthetic process;cell cycle;cell cycle process;cellular biosynthetic process;cellular component organization;cellular component organization at cellular level;cellular c
26.6715	26.9848	26.9481	26.9464	1:Experimental evidence at protein level	cellular macromolecule metabolic process;cellular metabolic process;cellular process;cellular protein metabolic process;macromolecule metabolic process;macromolecule modification;metabolic process;primary metabolic p
25.9924	25.8332	26.1639	26.0379	1:Experimental evidence at protein level	biological regulation;biosynthetic process;catabolic process;cellular biosynthetic process;cellular catabolic process;cellular component assembly;cellular component assembly at cellular level;cellular component organization;
26.6334	26.174	26.0722	26.5001	1:Experimental evidence at protein level	biological regulation;establishment of localization;establishment of localization in cell;establishment of protein localization;intracellular protein transport;intracellular transport;protein transport;regulation of actin cytoskeleton c
26.6775	26.9525	26.8767	26.9995	1:Experimental evidence at protein level	biological regulation;biosynthetic process;cation homeostasis;cell growth;cellular biosynthetic process;cellular cation homeostasis;cellular chemical homeostasis;cellular homeostasis;cellular ion homeostasis;cellular iron ion
26.6331	26.7382	27.2358	27.2151	1:Experimental evidence at protein level	cellular component biogenesis;cellular component biogenesis at cellular level;cellular component organization or biogenesis;cellular component organization or biogenesis at cellular level;cellular macromolecule metabolic pro
28.1952	28.117	28.1667	28.0962	1:Experimental evidence at protein level	biological regulation;biosynthetic process;cellular biosynthetic process;cellular macromolecule biosynthetic process;cellular macromolecule metabolic process;cellular metabolic process;cellular process;cellular protein metab
27.7721	27.5804	27.567	27.7435	1:Experimental evidence at protein level	apoptosis;apoptosis in response to endoplasmic reticulum stress;biological regulation;catabolic process;cell death;cellular catabolic process;cellular macromolecule catabolic process;cellular macromolecule metabolic proce
30.6866	30.5476	30.6459	30.3571	1:Experimental evidence at protein level	biological regulation;biosynthetic process;cellular biosynthetic process;cellular lipid metabolic process;cellular metabolic process;cellular process;cellular response to stimulus;establishment of localization;establishment of pr
28.2203	29.7959	27.7323	28.809	1:Experimental evidence at protein level	biological regulation;cellular macromolecule metabolic process;cellular metabolic process;cellular process;cellular protein metabolic process;macromolecule metabolic process;macromolecule modification;metabolic process
32.0968	34.3164	33.6284	34.8092	1:Experimental evidence at protein level	actin cytoskeleton organization;actin filament organization;actin filament-based process;biological regulation;blood coagulation;cell activation;cellular component organization;cellular component organization at cellular level;c
30.637	30.1799	30.3764	30.5581	1:Experimental evidence at protein level	actin cytoskeleton organization;actin filament-based process;anatomical structure development;anatomical structure morphogenesis;antigen processing and presentation;antigen processing and presentation of exogenous a
27.646	28.6198	28.1117	28.301	1:Experimental evidence at protein level	actin cytoskeleton organization;actin filament-based process;axon guidance;biological regulation;blood coagulation;cell activation;cell chemotaxis;cell migration;cell motility;cellular component movement;cellular component
28.3338	#NUM!	28.246	28.7662	1:Experimental evidence at protein level	
26.4692	26.2786	26.566	26.3094	1:Experimental evidence at protein level	anatomical structure development;anatomical structure morphogenesis;biological regulation;cell differentiation;cellular developmental process;cellular process;developmental process;muscle organ development;muscle struc
27.9876	28.1501	28.2114	28.3729	1:Experimental evidence at protein level	biological regulation;biosynthetic process;cellular biosynthetic process;cellular macromolecule biosynthetic process;cellular macromolecule metabolic process;cellular metabolic process;cellular nitrogen compound metabolic
28.0419	27.959	27.8519	27.5718	1:Experimental evidence at protein level	biosynthetic process;cellular biosynthetic process;cellular macromolecule biosynthetic process;cellular macromolecule metabolic process;cellular metabolic process;cellular nitrogen compound metabolic process;cellular pro
25.3869	25.4434	25.4233	25.4705	1:Experimental evidence at protein level	activation of immune response;activation of innate immune response;activation of phospholipase C activity;adaptive immune response;adaptive immune response based on somatic recombination of immune receptors built f
26.4176	26.4387	26.4708	26.6156	1:Experimental evidence at protein level	biological regulation;catabolic process;cellular catabolic process;cellular macromolecule catabolic process;cellular macromolecule metabolic process;cellular metabolic process;cellular nitrogen compound metabolic process
27.0595	27.4107	27.0481	26.9247	1:Experimental evidence at protein level	activation of caspase activity;activation of NF-kappaB-inducing kinase activity;activation of protein kinase activity;apoptosis;apoptosis in response to endoplasmic reticulum stress;biological regulation;catabolic process;cell c
25.9387	26.1632	25.3376	26.5412	1:Experimental evidence at protein level	cellular macromolecule metabolic process;cellular metabolic process;cellular nitrogen compound metabolic process;cellular process;macromolecule metabolic process;maturation of SSU-rRNA;maturation of SSU-rRNA from
26.1738	26.3485	26.7137	26.7902	1:Experimental evidence at protein level	actin cytoskeleton organization;actin filament-based process;anatomical structure development;cellular component organization;cellular component organization at cellular level;cellular component organization or biogenesis;
27.0228	27.2109	27.3806	27.4054	1:Experimental evidence at protein level	antigen processing and presentation;antigen processing and presentation of peptide antigen;antigen processing and presentation of peptide antigen via MHC class I;cellular macromolecule metabolic process;cellular metabo
26.1905	#NUM!	26.03	25.9801	1:Experimental evidence at protein level	axon guidance;biological regulation;cell differentiation;cell junction assembly;cell junction organization;cell projection assembly;cell projection organization;cell surface receptor linked signaling pathway;cell-substrate junction
26.9083	27.1022	26.8511	26.9264	1:Experimental evidence at protein level	behavior;behavioral response to pain;biological regulation;multicellular organismal process;multicellular organismal response to stress;regulation of biological process;regulation of cellular process;regulation of G-protein coup
24.6812	24.739	#NUM!	24.055	1:Experimental evidence at protein level	biosynthetic process;carbohydrate biosynthetic process;carbohydrate metabolic process;cellular carbohydrate metabolic process;cellular component organization;cellular component organization at cellular level;cellular com
26.5379	26.1336	26.2407	25.9669	1:Experimental evidence at protein level	activation of immune response;activation of innate immune response;anaphase-promoting complex-dependent proteasomal ubiquitin-dependent protein catabolic process;antigen processing and presentation;antigen proces
28.0707	28.2863	27.9971	28.2453	1:Experimental evidence at protein level	
26.3727	26.2499	25.9282	26.0878	1:Experimental evidence at protein level	anatomical structure morphogenesis;cell morphogenesis;cellular component morphogenesis;cellular component organization;cellular component organization or biogenesis;cellular developmental process;cellular macromole
27.0207	27.3196	27.692	27.3734	1:Experimental evidence at protein level	biological regulation;biosynthetic process;cellular biosynthetic process;cellular component assembly;cellular component assembly at cellular level;cellular component organization;cellular component organization at cellular le
26.3281	26.6543	26.4241	26.2845	1:Experimental evidence at protein level	alcohol metabolic process;anatomical structure development;anatomical structure morphogenesis;biological regulation;carbohydrate metabolic process;cellular carbohydrate metabolic process;cellular component organizati
26.3332	26.2307	25.9665	25.8459	1:Experimental evidence at protein level	amine catabolic process;amine metabolic process;carboxylic acid catabolic process;carboxylic acid metabolic process;catabolic process;cell differentiation;cellular amine metabolic process;cellular amino acid catabolic proc
26.2187	#NUM!	26.7957	26.7867	1:Experimental evidence at protein level	amine biosynthetic process;amine metabolic process;biosynthetic process;carboxylic acid biosynthetic process;carboxylic acid metabolic process;cellular amine metabolic process;cellular amino acid biosynthetic process;ce
27.1447	27.5546	27.2495	27.5599	1:Experimental evidence at protein level	biosynthetic process;catabolic process;cell communication;cellular aromatic compound metabolic process;cellular biosynthetic process;cellular catabolic process;cellular metabolic compound salvage;cellular metabolic proc
26.1788	#NUM!	26.4473	26.6958	1:Experimental evidence at protein level	
27.6508	27.7067	27.6185	27.703	1:Experimental evidence at protein level	biological regulation;regulation of biological process;regulation of catalytic activity;regulation of cellular metabolic process;regulation of cellular process;regulation of dephosphorylation;regulation of hydrolase activity;regulatio
27.4069	27.2193	27.1875	27.5215	1:Experimental evidence at protein level	B cell activation;B cell activation involved in immune response;catabolic process;cell activation;cell activation involved in immune response;cellular catabolic process;cellular macromolecule catabolic process;cellular macrom
26.8398	27.1808	26.7774	27.0264	1:Experimental evidence at protein level	
25.3357	#NUM!	25.5527	25.4643	1:Experimental evidence at protein level	cellular metabolic process;cellular process;dephosphorylation;metabolic process;phosphate-containing compound metabolic process;phosphorus metabolic process
26.4542	26.5226	26.2577	26.304	1:Experimental evidence at protein level	cellular macromolecule metabolic process;cellular metabolic process;cellular nitrogen compound metabolic process;cellular process;macromolecule metabolic process;metabolic process;mRNA metabolic process;mRNA pro
25.9281	26.5659	26.0285	26.211	1:Experimental evidence at protein level	biological regulation;cellular process;cellular response to stimulus;intracellular signal transduction;regulation of biological process;regulation of cellular process;regulation of response to stimulus;regulation of signal transducti
26.1913	26.1867	26.5335	26.4811	1:Experimental evidence at protein level	cellular macromolecule metabolic process;cellular metabolic process;cellular nitrogen compound metabolic process;cellular process;macromolecule metabolic process;metabolic process;mRNA metabolic process;mRNA pro
26.6835	#NUM!	#NUM!	#NUM!	1:Experimental evidence at protein level	biosynthetic process;cellular biosynthetic process;cellular macromolecule biosynthetic process;cellular macromolecule metabolic process;cellular metabolic process;cellular process;cellular protein metabolic process;macron
27.6676	28.0508	27.625	27.7	1:Experimental evidence at protein level	catabolic process;lipid catabolic process;lipid metabolic process;metabolic process;primary metabolic process
26.5918	26.9278	26.6275	26.8028	1:Experimental evidence at protein level	biological regulation;cellular component organization;cellular component organization at cellular level;cellular component organization or biogenesis;cellular component organization or biogenesis at cellular level;cellular proce
25.4979	25.951	25.8426	#NUM!	1:Experimental evidence at protein level	alcohol metabolic process;carbohydrate metabolic process;cellular carbohydrate metabolic process;cellular component assembly;cellular component organization;cellular component organization or biogenesis;cellular metab
26.1827	27.1937	26.0627	25.9459	1:Experimental evidence at protein level	biological regulation;cellular component assembly;cellular component assembly at cellular level;cellular component organization;cellular component organization at cellular level;cellular component organization or biogenesis;
26.5191	27.2949	27.0355	26.228	1:Experimental evidence at protein level	biological regulation;biosynthetic process;cellular biosynthetic process;cellular macromolecule biosynthetic process;cellular macromolecule metabolic process;cellular metabolic process;cellular nitrogen compound metabolic
26.6027	26.8859	27.2031	26.9346	1:Experimental evidence at protein level	biosynthetic process;catabolic process;cellular biosynthetic process;cellular lipid metabolic process;cellular metabolic process;cellular process;glycerolipid biosynthetic process;glycerolipid metabolic process;glycerophosph
26.8595	26.9709	#NUM!	27.2006	1:Experimental evidence at protein level	cellular component assembly;cellular component assembly at cellular level;cellular component organization;cellular component organization at cellular level;cellular component organization or biogenesis;cellular component c
#NUM!	#NUM!	#NUM!	#NUM!	1:Experimental evidence at protein level	carbohydrate metabolic process;cellular carbohydrate metabolic process;cellular macromolecule metabolic process;cellular metabolic process;cellular process;cellular protein metabolic process;glycosylation;macromolecule
25.3978	#NUM!	25.2099	24.9555	1:Experimental evidence at protein level	
26.2549	26.6261	26.5644	26.3716	1:Experimental evidence at protein level	
#NUM!	24.9844	24.647	24.8309	1:Experimental evidence at protein level	androgen catabolic process;androgen metabolic process;biological regulation;biosynthetic process;catabolic process;cellular hormone metabolic process;cellular metabolic process;cellular process;hormone catabolic proces
25.7955	25.6166	25.6358	25.6606	1:Experimental evidence at protein level	behavior;biological regulation;cellular component organization;cellular component organization at cellular level;cellular component organization or biogenesis;cellular component organization or biogenesis at cellular level;cellu
26.3648	25.7741	25.8794	25.9421	1:Experimental evidence at protein level	biological regulation;biosynthetic process;carbohydrate metabolic process;carbohydrate transport;cell cycle;cell cycle phase;cell cycle process;cell division;cell surface receptor linked signaling pathway;cellular biosynthetic p
26.0246	#NUM!	26.279	26.5799	1:Experimental evidence at protein level	biological regulation;biosynthetic process;cell differentiation;cell junction assembly;cell junction organization;cell surface receptor linked signaling pathway;cell-cell junction assembly;cell-cell junction organization;cellular bios
25.743	26.4784	26.162	26.1557	1:Experimental evidence at protein level	
26.5921	26.2185	26.4302	26.021	1:Experimental evidence at protein level	biological regulation;biosynthetic process;cell fate commitment;cell fate commitment involved in formation of primary germ layers;cell surface receptor linked signaling pathway;cellular biosynthetic process;cellular componer
27.0582	27.0274	26.868	26.7928	1:Experimental evidence at protein level	biological regulation;biosynthetic process;cellular biosynthetic process;cellular macromolecule biosynthetic process;cellular macromolecule metabolic process;cellular metabolic process;cellular nitrogen compound metabolic
28.754	29.3084	29.6451	29.6381	1:Experimental evidence at protein level	anatomical structure formation involved in morphogenesis;angiogenesis;apoptosis;biological regulation;cell death;cellular macromolecule metabolic process;cellular metabolic process;cellular process;cellular protein metabol
25.5525	26.086	26.0391	26.0735	1:Experimental evidence at protein level	activation of caspase activity;apoptosis;biological regulation;cell death;cellular process;cellular response to chemical stimulus;cellular response to hypoxia;cellular response to oxygen levels;cellular response to stimulus;cellu
25.3664	#NUM!	26.3637	26.3309	1:Experimental evidence at protein level	
26.3559	26.7841	26.9354	27.0682	1:Experimental evidence at protein level	
#NUM!	26.4144	26.5448	26.6315	1:Experimental evidence at protein level	carboxylic acid metabolic process;cellular ketone metabolic process;cellular lipid metabolic process;cellular metabolic process;cellular process;fatty acid metabolic process;lipid metabolic process;metabolic process;monoca
27.4609	27.3337	27.236	27.231	1:Experimental evidence at protein level	anatomical structure development;cell differentiation;cellular developmental process;cellular process;developmental process;establishment of localization;establishment of localization in cell;intracellular transport;mitochondri
26.0537	26.3477	26.1672	25.9946	1:Experimental evidence at protein level	catabolic process;cellular catabolic process;cellular macromolecule catabolic process;cellular macromolecule metabolic process;cellular metabolic process;cellular nitrogen compound metabolic process;cellular process;exo
25.2409	25.303	#NUM!	25.1369	1:Experimental evidence at protein level	amine catabolic process;amine metabolic process;aspartate family amino acid catabolic process;aspartate family amino acid metabolic process;carboxylic acid catabolic process;carboxylic acid metabolic process;catabolic
25.595	#NUM!	25.8226	25.3331	1:Experimental evidence at protein level	attachment of spindle microtubules to chromosome;attachment of spindle microtubules to kinetochore;attachment of spindle microtubules to kinetochore involved in mitotic sister chromatid segregation;biological regulation;
25.7379	25.8148	26.0938	25.9406	1:Experimental evidence at protein level	activation of NF-kappaB-inducing kinase activity;activation of protein kinase activity;biological regulation;cellular macromolecule metabolic process;cellular metabolic process;cellular process;cellular protein metabolic proces
29.3534	29.4356	29.6719	29.5781	1:Experimental evidence at protein level	activation of immune response;activation of innate immune response;anaphase-promoting complex-dependent proteasomal ubiquitin-dependent protein catabolic process;antigen processing and presentation;antigen proces
25.2803	#NUM!	25.7183	25.9326	1:Experimental evidence at protein level	alcohol metabolic process;amine biosynthetic process;amine metabolic process;biosynthetic process;cellular amine metabolic process;cellular biogenic amine biosynthetic process;cellular biogenic amine metabolic process;
27.5142	27.5269	27.959	27.9454	1:Experimental evidence at protein level	anatomical structure development;biological regulation;cellular macromolecule metabolic process;cellular metabolic process;cellular process;cellular protein metabolic process;'de novo' posttranslational protein folding;'de n
26.7391	26.3261	26.5028	26.0994	1:Experimental evidence at protein level	biological regulation;cell cycle;cell cycle phase;cell cycle process;cell division;cell surface receptor linked signaling pathway;cellular component disassembly;cellular component disassembly at cellular level;cellular componer
25.357	25.009	24.8976	24.7707	1:Experimental evidence at protein level	biological regulation;calcium ion homeostasis;calcium ion import;calcium ion transmembrane transport;calcium ion transport;cation homeostasis;cation transport;cellular calcium ion homeostasis;cellular cation homeostasis;
26.0925	26.1786	26.2722	25.986	1:Experimental evidence at protein level	biosynthetic process;cellular biosynthetic process;cellular component disassembly;cellular component disassembly at cellular level;cellular component organization;cellular component organization at cellular level;cellular cor
26.8271	26.6883	26.8372	26.4678	1:Experimental evidence at protein level	developmental process;multicellular organismal development;multicellular organismal process
27.0227	26.9011	26.9348	26.8999	1:Experimental evidence at protein level	
25.8573	26.1595	26.3522	26.0744	1:Experimental evidence at protein level	biological regulation;cell cycle cytokinesis;cell cycle process;cellular component organization;cellular component organization at cellular level;cellular component organization or biogenesis;cellular component organization or
26.1001	26.143	25.8939	26.1734	1:Experimental evidence at protein level	
25.7332	#NUM!	25.6347	26.1111	1:Experimental evidence at protein level	adipose tissue development;anatomical structure development;biological regulation;cellular macromolecule metabolic process;cellular metabolic process;cellular nitrogen compound metabolic process;cellular process;deme
26.3293	26.0776	26.56	26.0677	1:Experimental evidence at protein level	
26.1417	#NUM!	26.1588	26.5251	1:Experimental evidence at protein level	biological regulation;catabolic process;cellular catabolic process;cellular macromolecule catabolic process;cellular macromolecule metabolic process;cellular metabolic process;cellular nitrogen compound metabolic process
26.1404	25.7595	26.1671	26.1211	1:Experimental evidence at protein level	viral reproduction
26.5755	26.3291	26.4827	26.2593	1:Experimental evidence at protein level	
26.983	#NUM!	26.4387	26.5304	1:Experimental evidence at protein level	
26.104	26.1386	#NUM!	26.0434	1:Experimental evidence at protein level	
25.2688	25.0197	25.105	24.9264	1:Experimental evidence at protein level	anatomical structure development;biological regulation;biosynthetic process;cell migration;cell motility;cellular biosynthetic process;cellular component movement;cellular component organization;cellular component organiza
27.6353	27.6406	27.4914	27.8186	1:Experimental evidence at protein level	anatomical structure development;biological regulation;brain development;cellular component organization;cellular component organization at cellular level;cellular component organization or biogenesis;cellular component o
27.2981	27.2864	27.8799	27.7843	1:Experimental evidence at protein level	anatomical structure development;biological regulation;biosynthetic process;cell development;cellular biosynthetic process;cellular developmental process;cellular macromolecule metabolic process;cellular metabolic proces
28.9618	29.2073	29.058	29.2594	1:Experimental evidence at protein level	cellular process;establishment of localization;establishment of localization in cell;establishment of protein localization;intracellular protein transport;intracellular transport;protein transport;transport;vesicle-mediated transport
25.9742	25.5769	25.6482	25.4617	1:Experimental evidence at protein level	
26.3446	27.0515	26.7537	26.8358	1:Experimental evidence at protein level	apoptotic mitochondrial changes;autophagic vacuole assembly;autophagy;catabolic process;cellular catabolic process;cellular component assembly;cellular component assembly at cellular level;cellular component organiza
26.1572	26.2721	26.3015	26.1201	1:Experimental evidence at protein level	
27.274	27.6663	27.7765	27.8598	1:Experimental evidence at protein level	
25.7477	24.7881	25.1322	25.0072	1:Experimental evidence at protein level	biosynthetic process;catabolic process;cellular biosynthetic process;cellular catabolic process;cellular lipid catabolic process;cellular lipid metabolic process;cellular metabolic process;cellular process;cellular response to ch
26.1014	25.9166	26.1858	26.1224	1:Experimental evidence at protein level	actin cytoskeleton organization;actin filament organization;actin filament-based process;actin polymerization or depolymerization;biological regulation;cell migration;cell motility;cellular component movement;cellular compon
27.322	27.9348	27.1841	27.0528	1:Experimental evidence at protein level	biological regulation;regulation of biological process;regulation of cellular metabolic process;regulation of cellular process;regulation of cellular respiration;regulation of generation of precursor metabolites and energy;regulatio
27.743	27.5992	27.5892	27.6821	1:Experimental evidence at protein level	activation of caspase activity;biological regulation;cellular component organization;cellular component organization or biogenesis;cellular membrane organization;cellular process;establishment of localization;membrane orga
25.9083	26.6286	26.3326	26.5103	1:Experimental evidence at protein level	biosynthetic process;cellular biosynthetic process;cellular component disassembly;cellular component disassembly at cellular level;cellular component organization;cellular component organization at cellular level;cellular cor
26.5443	26.5775	26.5789	26.5895	1:Experimental evidence at protein level	biosynthetic process;cellular biosynthetic process;cellular macromolecule biosynthetic process;cellular macromolecule metabolic process;cellular metabolic process;cellular nitrogen compound metabolic process;cellular pro
26.5724	26.6539	26.3454	26.7104	1:Experimental evidence at protein level	biological regulation;cell surface receptor linked signaling pathway;cellular component organization;cellular component organization or biogenesis;cellular membrane organization;cellular process;cellular response to stimulus
29.2743	29.0178	29.06	28.9131	1:Experimental evidence at protein level	anatomical structure development;biological regulation;cell communication;cell surface receptor linked signaling pathway;cell-cell signaling;cellular metabolic process;cellular process;cellular response to chemical stimulus;c
26.8155	26.9853	26.9669	26.6235	1:Experimental evidence at protein level	cellular process;establishment of localization;transmembrane transport;transport
26.6816	#NUM!	26.0818	26.1269	1:Experimental evidence at protein level	apoptosis;cell death;cell differentiation;cellular developmental process;cellular localization;cellular macromolecule localization;cellular process;cellular protein localization;death;developmental process;ER to Golgi vesicle-med
24.47	24.7027	24.6973	24.4859	1:Experimental evidence at protein level	carboxylic acid catabolic process;carboxylic acid metabolic process;catabolic process;cellular catabolic process;cellular component assembly;cellular component organization;cellular component organization or biogenesis;
26.4447	#NUM!	25.9111	#NUM!	1:Experimental evidence at protein level	anaphase-promoting complex-dependent proteasomal ubiquitin-dependent protein catabolic process;biological regulation;catabolic process;cell cycle;cell cycle checkpoint;cell cycle phase;cell cycle process;cell division;cel
27.5379	27.6221	27.3742	27.516	1:Experimental evidence at protein level	
26.6683	27.1987	#NUM!	26.6827	1:Experimental evidence at protein level	biological regulation;cell cycle;cell cycle process;cell differentiation;cellular component movement;cellular component organization;cellular component organization at cellular level;cellular component organization or biogenes
26.6875	26.3927	26.7599	26.4511	1:Experimental evidence at protein level	anterograde axon cargo transport;anterograde synaptic vesicle transport;axon cargo transport;cellular component movement;cellular process;cytoskeleton-dependent intracellular transport;establishment of localization;estab
27.3432	26.9404	27.1585	27.2791	1:Experimental evidence at protein level	biological regulation;metabolic process;negative regulation of catalytic activity;negative regulation of molecular function;negative regulation of monooxygenase activity;negative regulation of nitric-oxide synthase activity;nega
27.4025	26.7476	#NUM!	26.8001	1:Experimental evidence at protein level	establishment of localization;establishment of protein localization;protein transport;transport
26.4472	#NUM!	26.5806	26.6841	1:Experimental evidence at protein level	biosynthetic process;cellular biosynthetic process;cellular component disassembly;cellular component disassembly at cellular level;cellular component organization;cellular component organization at cellular level;cellular cor
26.6947	26.7307	26.2538	26.8796	1:Experimental evidence at protein level	2'-deoxyribonucleotide metabolic process;biological regulation;catabolic process;cell surface receptor linked signaling pathway;cellular catabolic process;cellular component assembly;cellular component organization;cellula
27.0018	26.9907	#NUM!	26.7975	1:Experimental evidence at protein level	anatomical structure morphogenesis;biological regulation;cell part morphogenesis;cell projection morphogenesis;cell projection organization;cellular component assembly;cellular component assembly at cellular level;cellular
26.8783	25.9498	26.4025	26.4253	1:Experimental evidence at protein level	anatomical structure formation involved in morphogenesis;biological regulation;cation homeostasis;cation transport;cell projection assembly;cell projection organization;cell surface receptor linked signaling pathway;cellular c
26.456	26.4758	26.4475	26.1698	1:Experimental evidence at protein level	biosynthetic process;carbohydrate biosynthetic process;carbohydrate metabolic process;cellular carbohydrate metabolic process;cellular macromolecule metabolic process;cellular metabolic process;cellular process;cellular
26.3457	26.3082	26.5664	26.4435	1:Experimental evidence at protein level	activation of immune response;activation of innate immune response;activation of MAPK activity;activation of NF-kappaB-inducing kinase activity;activation of protein kinase activity;antigen receptor-mediated signaling pathv
26.8933	26.17	26.6868	26.518	1:Experimental evidence at protein level	cellular component assembly;cellular component assembly at cellular level;cellular component biogenesis;cellular component organization;cellular component organization at cellular level;cellular component organization or b
27.912	28.1521	28.4944	28.4216	1:Experimental evidence at protein level	activation of immune response;activation of innate immune response;anaphase-promoting complex-dependent proteasomal ubiquitin-dependent protein catabolic process;antigen processing and presentation;antigen proces
26.9593	27.3414	27.2689	27.4233	1:Experimental evidence at protein level	alcohol metabolic process;carbohydrate metabolic process;cellular carbohydrate metabolic process;cellular macromolecule metabolic process;cellular metabolic process;cellular process;cellular protein metabolic process;he
26.8678	26.614	26.4454	26.3844	1:Experimental evidence at protein level	apoptosis;biological regulation;carbohydrate metabolic process;cell communication;cell death;cellular carbohydrate metabolic process;cellular component organization;cellular component organization at cellular level;cellular
26.8268	27.4676	27.0688	27.3478	1:Experimental evidence at protein level	amine biosynthetic process;amine catabolic process;amine metabolic process;biological regulation;biosynthetic process;branched chain family amino acid biosynthetic process;branched chain family amino acid catabolic pro
27.0839	26.748	26.9066	26.6491	1:Experimental evidence at protein level	cellular macromolecule metabolic process;cellular metabolic process;cellular nitrogen compound metabolic process;cellular process;gene expression;macromolecule metabolic process;metabolic process;mRNA metabolic p
26.1883	#NUM!	26.7884	27.1317	1:Experimental evidence at protein level	biological regulation;cellular component assembly;cellular component organization;cellular component organization or biogenesis;cellular macromolecule metabolic process;cellular metabolic process;cellular nitrogen compo
26.646	26.6615	26.3681	26.3879	1:Experimental evidence at protein level	biological regulation;cell cycle process;cellular process;cellular response to stimulus;cytokinesis;intracellular signal transduction;microtubule-based process;mitotic spindle elongation;regulation of biological process;regulatio
26.5584	26.7724	26.8706	26.9732	1:Experimental evidence at protein level	cellular component organization;cellular component organization or biogenesis;cellular membrane organization;cellular process;endocytosis;endosome transport;establishment of localization;establishment of localization in c

26.7878	#NUM!	26.3195	26.4446	1:Experimental evidence at protein level	biological regulation;biosynthetic process;catabolic process;cell differentiation;cellular biosynthetic process;cellular catabolic process;cellular developmental process;cellular macromolecule biosynthetic process;cellular macr
26.8526	27.0914	27.0896	27.0337	1:Experimental evidence at protein level	activation of caspase activity;anatomical structure formation involved in morphogenesis;angiogenesis;biological regulation;cellular process;cellular response to heat;cellular response to stimulus;cellular response to stress;dev
25.9108	25.933	26.0574	26.2086	1:Experimental evidence at protein level	anatomical structure development;biological regulation;cell cycle;cell differentiation;cell division;cell proliferation;cellular component organization;cellular component organization at cellular level;cellular component organizatio
26.3584	#NUM!	25.9789	25.9897	1:Experimental evidence at protein level	cellular macromolecule metabolic process;cellular metabolic process;cellular process;cellular protein metabolic process;establishment of localization;establishment of localization in cell;establishment of protein localization;es
27.4001	27.4671	27.3123	26.9952	1:Experimental evidence at protein level	acidic amino acid transport;amine transport;amino acid transport;anion transport;biological regulation;carboxylic acid transport;cellular chemical homeostasis;cellular homeostasis;cellular ion homeostasis;cellular process;che
28.6112	28.9053	28.8081	29.1941	1:Experimental evidence at protein level	amine catabolic process;amine metabolic process;arginine catabolic process;arginine metabolic process;biological regulation;carboxylic acid catabolic process;carboxylic acid metabolic process;catabolic process;cellular am
29.3103	29.2844	29.6852	28.7756	1:Experimental evidence at protein level	alcohol biosynthetic process;alcohol metabolic process;amine metabolic process;amino sugar biosynthetic process;amino sugar metabolic process;biosynthetic process;carbohydrate biosynthetic process;carbohydrate meta
25.4073	25.8736	25.6176	25.7804	1:Experimental evidence at protein level	cellular macromolecule metabolic process;cellular metabolic process;cellular process;cellular protein metabolic process;macromolecule metabolic process;macromolecule modification;metabolic process;primary metabolic p
25.3551	24.9793	25.3575	25.0273	1:Experimental evidence at protein level	anatomical structure formation involved in morphogenesis;anatomical structure morphogenesis;apoptosis;autophagy;biological regulation;blastocyst formation;blood coagulation;camera-type eye morphogenesis;catabolic pr
25.7376	25.9323	26.0605	26.1037	1:Experimental evidence at protein level	biological regulation;catabolic process;cellular catabolic process;cellular macromolecule catabolic process;cellular macromolecule metabolic process;cellular metabolic process;cellular process;cellular protein metabolic proc
25.7465	#NUM!	25.8504	25.9784	1:Experimental evidence at protein level	adult behavior;adult locomotory behavior;adult walking behavior;amine metabolic process;anatomical structure development;anatomical structure morphogenesis;autophagy;axonogenesis;behavior;biological regulation;carbo
27.7101	27.6557	27.7671	27.7555	1:Experimental evidence at protein level	
27.8651	27.8693	28.1331	27.9813	1:Experimental evidence at protein level	biological regulation;cellular macromolecule metabolic process;cellular metabolic process;cellular process;cellular protein metabolic process;cellular response to stimulus;establishment of localization;establishment of localiza
27.2979	27.4233	27.022	27.1202	1:Experimental evidence at protein level	activation of protein kinase activity;biological regulation;cellular hypotonic response;cellular macromolecule metabolic process;cellular metabolic process;cellular process;cellular protein metabolic process;cellular response to
28.334	27.7276	28.1044	27.9434	1:Experimental evidence at protein level	cellular macromolecule metabolic process;cellular metabolic process;cellular process;cellular protein metabolic process;establishment of localization;establishment of localization in cell;establishment of protein localization;es
#NUM!	#NUM!	#NUM!	#NUM!	1:Experimental evidence at protein level	acylglycerol catabolic process;acylglycerol metabolic process;aging;alcohol metabolic process;alpha-amino-3-hydroxy-5-methyl-4-isoxazole propionate receptor clustering;anatomical structure morphogenesis;artery morpho
28.9996	29.3852	29.6117	29.6756	1:Experimental evidence at protein level	biological regulation;C21-steroid hormone metabolic process;cellular hormone metabolic process;cellular metabolic process;cellular process;cellular response to stimulus;establishment of localization;hormone metabolic proc
27.4526	28.0498	28.2755	28.1767	1:Experimental evidence at protein level	age-dependent response to oxidative stress;age-dependent response to reactive oxygen species;amine metabolic process;anatomical structure development;apoptotic mitochondrial changes;behavior;biological regulation;b
25.4629	25.7774	25.5464	25.7328	1:Experimental evidence at protein level	adult behavior;adult locomotory behavior;alcohol metabolic process;anatomical structure development;anatomical structure morphogenesis;apoptosis;associative learning;axon cargo transport;axon choice point recognition;
26.8972	26.606	27.3951	#NUM!	1:Experimental evidence at protein level	axon guidance;biological regulation;blood coagulation;carbohydrate transport;cell differentiation;cell migration;cell motility;cell surface receptor linked signaling pathway;cellular component movement;cellular developmental p
26.5933	26.2712	26.296	26.1692	1:Experimental evidence at protein level	biosynthetic process;cellular biosynthetic process;cellular component disassembly;cellular component disassembly at cellular level;cellular component organization;cellular component organization at cellular level;cellular cor
29.6357	#NUM!	28.067	28.1831	1:Experimental evidence at protein level	biological regulation;cellular process;cellular response to chemical stimulus;cellular response to endogenous stimulus;cellular response to estradiol stimulus;cellular response to estrogen stimulus;cellular response to hormone
27.5084	27.1991	27.3513	27.2985	1:Experimental evidence at protein level	amine metabolic process;carboxylic acid metabolic process;cell differentiation;cellular amine metabolic process;cellular amino acid metabolic process;cellular component assembly;cellular component organization;cellular co
27.6247	26.7797	26.8574	27.1738	1:Experimental evidence at protein level	apoptosis;biological regulation;cell communication;cell death;cell surface receptor linked signaling pathway;cellular process;cellular response to chemical stimulus;cellular response to dsRNA;cellular response to exogenous d
27.601	27.8611	27.8514	27.8669	1:Experimental evidence at protein level	anatomical structure development;anatomical structure morphogenesis;biological regulation;cell cycle;cell cycle process;cell division;cellular component organization;cellular component organization at cellular level;cellular co
26.5712	26.9087	27.0439	27.1687	1:Experimental evidence at protein level	amine metabolic process;carboxylic acid metabolic process;cellular amine metabolic process;cellular amino acid metabolic process;cellular ketone metabolic process;cellular metabolic process;cellular nitrogen compound m
#NUM!	#NUM!	#NUM!	#NUM!	1:Experimental evidence at protein level	anatomical structure development;biological adhesion;biological regulation;cartilage condensation;cell adhesion;cell differentiation;cell-cell adhesion;cellular component disassembly;cellular component disassembly at cellula
27.8086	26.1807	26.6626	26.4534	1:Experimental evidence at protein level	actin cytoskeleton organization;actin filament organization;actin filament-based process;activation of immune response;antigen receptor-mediated signaling pathway;axon guidance;biological regulation;cell activation;cell pro
30.2084	29.9723	29.9774	30.143	1:Experimental evidence at protein level	activation of phospholipase D activity;anatomical structure development;biological regulation;brain development;cell migration;cell motility;cell surface receptor linked signaling pathway;cellular component movement;cellular
25.9006	26.1148	25.6721	25.5721	1:Experimental evidence at protein level	anatomical structure development;base-excision repair;cellular macromolecule metabolic process;cellular metabolic process;cellular nitrogen compound metabolic process;cellular process;cellular response to stimulus;cellula
#NUM!	26.0471	25.6295	25.8082	1:Experimental evidence at protein level	amine metabolic process;aminoglycan metabolic process;carbohydrate metabolic process;glycosaminoglycan metabolic process;hyaluronan metabolic process;macromolecule metabolic process;metabolic process;nitrogen
#NUM!	#NUM!	#NUM!	25.1815	1:Experimental evidence at protein level	anatomical structure development;anatomical structure morphogenesis;biological regulation;BMP signaling pathway;cell differentiation;cell proliferation;cell surface receptor linked signaling pathway;cellular developmental pr
30.0161	30.1001	30.5244	30.2304	1:Experimental evidence at protein level	biological regulation;biosynthetic process;cellular biosynthetic process;cellular macromolecule biosynthetic process;cellular macromolecule metabolic process;cellular metabolic process;cellular nitrogen compound metabolic
28.7442	28.9999	28.8547	28.726	1:Experimental evidence at protein level	alcohol catabolic process;alcohol metabolic process;amine catabolic process;amine metabolic process;behavior;benzene-containing compound metabolic process;biological regulation;biosynthetic process;catabolic process
26.0845	26.1061	26.1396	25.8826	1:Experimental evidence at protein level	biological regulation;cell surface receptor linked signaling pathway;cellular macromolecule metabolic process;cellular metabolic process;cellular process;cellular protein metabolic process;cellular response to chemical stimul
#NUM!	#NUM!	#NUM!	#NUM!	1:Experimental evidence at protein level	alcohol metabolic process;anatomical structure morphogenesis;biological regulation;carbohydrate homeostasis;carbohydrate metabolic process;cell differentiation;cell proliferation;cellular carbohydrate metabolic process;ce
28.3144	28.5342	29.1345	28.5616	1:Experimental evidence at protein level	axon guidance;biological adhesion;biological regulation;cell adhesion;cell surface receptor linked signaling pathway;cell-cell adhesion;cellular process;cellular response to stimulus;chemotaxis;enzyme linked receptor protein
27.8332	27.8361	28.4056	28.1475	1:Experimental evidence at protein level	activation of immune response;activation of innate immune response;anaphase-promoting complex-dependent proteasomal ubiquitin-dependent protein catabolic process;antigen processing and presentation;antigen proces
28.7187	29.5362	29.1443	29.5497	1:Experimental evidence at protein level	biological regulation;cellular metabolic process;cellular process;energy derivation by oxidation of organic compounds;energy reserve metabolic process;generation of precursor metabolites and energy;metabolic process;oxid
26.6403	26.3999	26.1527	26.2307	1:Experimental evidence at protein level	activation of phospholipase C activity;activation of phospholipase C activity by dopamine receptor signaling pathway;activation of phospholipase C activity by G-protein coupled receptor protein signaling pathway coupled to
26.8126	27.176	26.9348	27.2886	1:Experimental evidence at protein level	biosynthetic process;cellular aromatic compound metabolic process;cellular biosynthetic process;cellular metabolic process;cellular nitrogen compound biosynthetic process;cellular nitrogen compound metabolic process;ce
27.2939	27.3022	27.4558	27.2757	1:Experimental evidence at protein level	amine biosynthetic process;amine catabolic process;amine metabolic process;betaine biosynthetic process;betaine metabolic process;biosynthetic process;carboxylic acid biosynthetic process;carboxylic acid catabolic proc
27.4496	#NUM!	#NUM!	#NUM!	1:Experimental evidence at protein level	alkaloid metabolic process;anatomical structure development;anatomical structure formation involved in morphogenesis;angiogenesis;arachidonic acid metabolic process;behavior;biological regulation;biomineral tissue deve
27.205	26.9364	27.0105	27.0885	1:Experimental evidence at protein level	actin cytoskeleton organization;actin filament bundle assembly;actin filament capping;actin filament organization;actin filament-based process;anatomical structure homeostasis;anatomical structure morphogenesis;apoptosi
#NUM!	#NUM!	#NUM!	#NUM!	1:Experimental evidence at protein level	biological regulation;cellular process;cellular response to chemical stimulus;cellular response to organic substance;cellular response to stimulus;multicellular organismal process;negative regulation of biological process;negat
26.3269	26.058	26.2127	26.1167	1:Experimental evidence at protein level	ATP hydrolysis coupled proton transport;biological regulation;cation homeostasis;cation transport;cell surface receptor linked signaling pathway;cellular cation homeostasis;cellular chemical homeostasis;cellular component c
31.3077	31.0464	30.9412	31.3628	1:Experimental evidence at protein level	biological regulation;biosynthetic process;catabolic process;cell cycle;cell proliferation;cellular biosynthetic process;cellular catabolic process;cellular component disassembly;cellular component disassembly at cellular level;
25.8099	#NUM!	25.783	25.6659	1:Experimental evidence at protein level	acidic amino acid transport;aging;amide biosynthetic process;amine metabolic process;amine transport;amino acid import;amino acid transport;anatomical structure development;anatomical structure formation involved in m
27.0162	27.0129	26.9624	26.8074	1:Experimental evidence at protein level	activation of immune response;activation of innate immune response;activation of MAPK activity;biological regulation;cardiac muscle contraction;cellular macromolecule metabolic process;cellular metabolic process;cellular p
28.3311	28.2664	28.307	28.0641	1:Experimental evidence at protein level	activation of immune response;activation of innate immune response;anaphase-promoting complex-dependent proteasomal ubiquitin-dependent protein catabolic process;antigen processing and presentation;antigen proces
26.3797	25.8961	26.1918	26.1276	1:Experimental evidence at protein level	anatomical structure homeostasis;base-excision repair;biological regulation;biosynthetic process;cell cycle;cellular biosynthetic process;cellular component organization;cellular component organization at cellular level;cellula
25.6708	25.6727	25.8967	25.86	1:Experimental evidence at protein level	biological regulation;cellular component assembly;cellular component assembly at cellular level;cellular component organization;cellular component organization at cellular level;cellular component organization or biogenesis;
25.9443	25.6233	25.5777	25.9802	1:Experimental evidence at protein level	adherens junction organization;anatomical structure development;axon guidance;biological regulation;cell differentiation;cell division;cell junction organization;cell surface receptor linked signaling pathway;cell-cell junction or
29.0951	28.9572	28.8738	28.8403	1:Experimental evidence at protein level	anatomical structure development;biological regulation;cell cycle;cell cycle process;cell differentiation;cellular component disassembly;cellular component disassembly at cellular level;cellular component organization;cellular
28.6629	28.9311	28.8956	28.8562	1:Experimental evidence at protein level	biosynthetic process;cellular biosynthetic process;cellular macromolecule biosynthetic process;cellular macromolecule metabolic process;cellular metabolic process;cellular process;cellular protein metabolic process;cotrans
26.0077	#NUM!	26.7893	26.4198	1:Experimental evidence at protein level	cellular metabolic process;cellular process;electron transport chain;generation of precursor metabolites and energy;metabolic process;mitochondrial electron transport, NADH to ubiquinone;oxidation-reduction process;respi
26.0765	26.3803	26.0354	26.2096	1:Experimental evidence at protein level	anatomical structure morphogenesis;developmental process;metabolic process;organ morphogenesis;oxidation-reduction process
24.9698	24.8767	25.0695	24.8296	1:Experimental evidence at protein level	actin cytoskeleton organization;actin filament organization;actin filament polymerization;actin filament-based process;actin polymerization or depolymerization;cellular component assembly;cellular component assembly at ce
28.7291	29.0092	28.6622	28.7129	1:Experimental evidence at protein level	activation of immune response;activation of innate immune response;antigen receptor-mediated signaling pathway;biological regulation;catabolic process;cell surface receptor linked signaling pathway;cellular catabolic proce
27.8102	27.5045	27.5742	27.5826	1:Experimental evidence at protein level	actin cytoskeleton organization;actin filament-based process;anatomical structure development;apoptosis;biological adhesion;biological regulation;cell adhesion;cell cycle;cell death;cell division;cell projection organization;ce
32.3449	34.838	34.2779	35.0306	1:Experimental evidence at protein level	actin cytoskeleton organization;actin filament organization;actin filament-based process;biological regulation;cellular component organization;cellular component organization at cellular level;cellular component organization o
27.0176	26.7578	#NUM!	27.0384	1:Experimental evidence at protein level	cellular macromolecule metabolic process;cellular metabolic process;cellular nitrogen compound metabolic process;cellular process;macromolecule metabolic process;metabolic process;ncRNA metabolic process;ncRNA pr
31.0308	30.9648	30.9378	31.1955	1:Experimental evidence at protein level	biological regulation;biosynthetic process;cell cycle;cell differentiation;cellular biosynthetic process;cellular developmental process;cellular metabolic process;cellular nitrogen compound biosynthetic process;cellular nitrogen
25.1573	25.3234	25.3903	25.1393	1:Experimental evidence at protein level	alcohol metabolic process;anatomical structure development;behavior;behavioral interaction between organisms;biological regulation;cation homeostasis;cell surface receptor linked signaling pathway;cellular cation homeost
26.7317	27.0371	27.1123	27.3028	1:Experimental evidence at protein level	biosynthetic process;cellular biosynthetic process;cellular macromolecule biosynthetic process;cellular macromolecule metabolic process;cellular metabolic process;cellular nitrogen compound metabolic process;cellular pro
#NUM!	27.2989	26.8323	27.5046	1:Experimental evidence at protein level	alcohol biosynthetic process;alcohol metabolic process;biosynthetic process;cellular biosynthetic process;cellular lipid metabolic process;cellular metabolic process;cellular process;cholesterol biosynthetic process;cholester
27.2396	28.0459	27.6932	27.3857	1:Experimental evidence at protein level	activation of adenylate cyclase activity;activation of adenylate cyclase activity by G-protein signaling pathway;activation of phospholipase D activity;anatomical structure morphogenesis;biological regulation;blood coagulatio
26.517	26.7409	26.9385	26.6239	1:Experimental evidence at protein level	biological regulation;biosynthetic process;cellular biosynthetic process;cellular metabolic process;cellular nitrogen compound biosynthetic process;cellular nitrogen compound metabolic process;cellular process;metabolic pr
26.5642	26.1763	26.9177	26.9554	1:Experimental evidence at protein level	catabolic process;cellular catabolic process;cellular macromolecule catabolic process;cellular macromolecule metabolic process;cellular metabolic process;cellular nitrogen compound metabolic process;cellular process;exo
27.6863	28.0229	28.4092	28.3481	1:Experimental evidence at protein level	apoptosis;biological regulation;carbohydrate transport;cell cycle checkpoint;cell death;cellular process;cellular response to stimulus;cellular response to stress;death;DNA damage checkpoint;DNA integrity checkpoint;establi
27.4649	28.0819	27.6901	27.5156	1:Experimental evidence at protein level	biological regulation;cell cycle process;cellular component organization;cellular component organization at cellular level;cellular component organization or biogenesis;cellular component organization or biogenesis at cellular
25.3085	#NUM!	#NUM!	25.4501	1:Experimental evidence at protein level	biological adhesion;biological regulation;cell adhesion;cell junction assembly;cell junction organization;cell migration;cell motility;cell surface receptor linked signaling pathway;cell-substrate junction assembly;cellular compor
28.9816	29.2126	29.2418	29.0243	1:Experimental evidence at protein level	biological regulation;biosynthetic process;cellular biosynthetic process;cellular macromolecule biosynthetic process;cellular macromolecule metabolic process;cellular metabolic process;cellular nitrogen compound metabolic
27.948	28.0116	27.9566	28.1052	1:Experimental evidence at protein level	biological regulation;biosynthetic process;cellular biosynthetic process;cellular component assembly;cellular component organization;cellular component organization or biogenesis;cellular macromolecule biosynthetic proces
26.7242	26.7689	26.9248	27.0524	1:Experimental evidence at protein level	biological regulation;cellular process;chromosome segregation;positive regulation of biological process;positive regulation of cellular metabolic process;positive regulation of cellular process;positive regulation of cellular prote
29.1096	29.4093	29.115	29.0079	1:Experimental evidence at protein level	amine transport;amino acid transport;carboxylic acid transport;cellular process;establishment of localization;extracellular amino acid transport;extracellular transport;glutamine transport;ion transport;neutral amino acid transp
#NUM!	#NUM!	#NUM!	#NUM!	1:Experimental evidence at protein level	amine metabolic process;aminoglycan biosynthetic process;aminoglycan metabolic process;anatomical structure development;anatomical structure morphogenesis;axon guidance;biological regulation;biosynthetic process;c
26.7734	27.1243	27.0667	27.0157	1:Experimental evidence at protein level	
25.3443	25.5789	25.5231	25.323	1:Experimental evidence at protein level	activation of blood coagulation via clotting cascade;anatomical structure development;anion transport;biological regulation;biomineral tissue development;blood coagulation;bone mineralization;bone mineralization involved i
27.1326	26.8701	27.1463	27.0643	1:Experimental evidence at protein level	
25.3876	25.1273	25.2188	#NUM!	1:Experimental evidence at protein level	cellular macromolecule metabolic process;cellular metabolic process;cellular nitrogen compound metabolic process;cellular process;developmental process;macromolecule metabolic process;maturation of SSU-rRNA;matur
26.3308	26.272	26.0037	26.3539	1:Experimental evidence at protein level	
26.0448	26.4101	25.988	26.4865	1:Experimental evidence at protein level	
26.6136	#NUM!	26.9698	27.0426	1:Experimental evidence at protein level	cellular macromolecule metabolic process;cellular metabolic process;cellular nitrogen compound metabolic process;cellular process;macromolecule metabolic process;metabolic process;mRNA metabolic process;mRNA pro
26.3541	26.2178	25.8839	26.0119	1:Experimental evidence at protein level	cellular component organization;cellular component organization at cellular level;cellular component organization or biogenesis;cellular component organization or biogenesis at cellular level;cellular macromolecule metabolic
#NUM!	25.0183	23.911	24.4925	1:Experimental evidence at protein level	biological regulation;biosynthetic process;cellular biosynthetic process;cellular macromolecule biosynthetic process;cellular macromolecule metabolic process;cellular metabolic process;cellular nitrogen compound metabolic
27.138	27.0867	27.148	27.1096	1:Experimental evidence at protein level	cellular macromolecule metabolic process;cellular metabolic process;cellular process;cellular protein metabolic process;cullin deneddylation;macromolecule metabolic process;macromolecule modification;metabolic process
26.9297	25.9818	26.5247	26.2907	1:Experimental evidence at protein level	
24.9204	25.5615	25.1174	25.2122	1:Experimental evidence at protein level	
25.5106	25.5082	25.6366	25.6651	1:Experimental evidence at protein level	
25.2496	#NUM!	25.6567	#NUM!	1:Experimental evidence at protein level	alcohol metabolic process;amine metabolic process;amine transport;biological regulation;biosynthetic process;cation transport;cellular amine metabolic process;cellular biogenic amine metabolic process;cellular biosynthetic
26.9574	27.0592	27.0568	26.9375	1:Experimental evidence at protein level	abscission;adipose tissue development;anatomical structure development;biological regulation;cell division;cellular chemical homeostasis;cellular component organization;cellular component organization at cellular level;cellu
26.498	26.4972	26.9976	26.4766	1:Experimental evidence at protein level	biological regulation;biosynthetic process;cell fate commitment;cell fate commitment involved in formation of primary germ layers;cell surface receptor linked signaling pathway;cellular biosynthetic process;cellular componer
26.7381	26.7085	26.3813	26.7592	1:Experimental evidence at protein level	biological regulation;cellular component organization;cellular component organization at cellular level;cellular component organization or biogenesis;cellular component organization or biogenesis at cellular level;cellular proce
26.6838	26.2583	26.2794	26.7318	1:Experimental evidence at protein level	
25.1342	26.2135	#NUM!	25.23	1:Experimental evidence at protein level	alcohol metabolic process;amino sugar metabolic process;biosynthetic process;carbohydrate biosynthetic process;carbohydrate metabolic process;carboxylic acid metabolic process;cellular carbohydrate metabolic process
25.648	#NUM!	#NUM!	#NUM!	1:Experimental evidence at protein level	cellular component organization;cellular component organization at cellular level;cellular component organization or biogenesis;cellular component organization or biogenesis at cellular level;cellular process;establishment of l
26.0597	26.3704	26.2523	26.1003	1:Experimental evidence at protein level	
27.8415	28.799	27.8398	27.9685	1:Experimental evidence at protein level	developmental process;multicellular organismal development;multicellular organismal process
27.5644	27.3613	27.4242	27.4396	1:Experimental evidence at protein level	catabolic process;cellular catabolic process;cellular macromolecule catabolic process;cellular macromolecule metabolic process;cellular metabolic process;cellular process;ER-associated protein catabolic process;macromo
26.0809	#NUM!	#NUM!	26.0748	1:Experimental evidence at protein level	biological adhesion;biological regulation;cell adhesion;cellular macromolecule metabolic process;cellular metabolic process;cellular nitrogen compound metabolic process;cellular process;macromolecule metabolic process;r
25.5608	25.2636	25.4967	24.9924	1:Experimental evidence at protein level	biological regulation;calcium ion homeostasis;calcium ion transmembrane transport;calcium ion transport;cation homeostasis;cation transport;cell cycle arrest;cell cycle process;cell proliferation;cellular calcium ion homeosta
25.6145	#NUM!	24.0413	25.2006	1:Experimental evidence at protein level	ATP biosynthetic process;ATP hydrolysis coupled proton transport;ATP metabolic process;ATP synthesis coupled proton transport;biological regulation;biosynthetic process;cation homeostasis;cation transport;cell surface re
25.4471	24.9058	25.284	25.309	1:Experimental evidence at protein level	biological regulation;induction of apoptosis;induction of apoptosis by intracellular signals;induction of apoptosis by oxidative stress;induction of programmed cell death;positive regulation of apoptosis;positive regulation of bi
#NUM!	25.1959	25.313	25.1014	1:Experimental evidence at protein level	activation of immune response;activation of innate immune response;anatomical structure formation involved in morphogenesis;angiogenesis;biological regulation;canonical Wnt receptor signaling pathway;cell surface recept
26.2945	26.2447	26.6092	26.3902	1:Experimental evidence at protein level	
26.0722	25.734	26.2751	25.4449	1:Experimental evidence at protein level	
28.0823	28.8387	28.2692	28.467	1:Experimental evidence at protein level	anatomical structure development;biological regulation;cell differentiation;cell proliferation;cellular developmental process;cellular process;developmental process;gamete generation;male gamete generation;maternal placent
#NUM!	#NUM!	#NUM!	27.5397	1:Experimental evidence at protein level	catabolic process;cellular catabolic process;cellular lipid metabolic process;cellular macromolecule catabolic process;cellular macromolecule metabolic process;cellular metabolic process;cellular nitrogen compound metabo
26.8129	26.5132	26.6686	26.2541	1:Experimental evidence at protein level	biological regulation;biosynthetic process;cellular biosynthetic process;cellular macromolecule biosynthetic process;cellular macromolecule metabolic process;cellular metabolic process;cellular nitrogen compound metabolic
27.8574	28.4396	28.5399	28.6144	1:Experimental evidence at protein level	biological regulation;cell activation;cell activation involved in immune response;cellular process;cytokine secretion;establishment of localization;establishment of localization in cell;establishment of protein localization;exocyto
25.4762	25.2643	25.5239	25.4386	1:Experimental evidence at protein level	cellular component assembly;cellular component assembly at cellular level;cellular component organization;cellular component organization at cellular level;cellular component organization or biogenesis;cellular component c
27.5269	27.6729	27.2294	27.5521	1:Experimental evidence at protein level	acute inflammatory response;acute-phase response;alcohol metabolic process;cellular macromolecule metabolic process;cellular metabolic process;cellular process;cholesterol metabolic process;defense response;developr
27.0377	27.1931	27.0881	27.1562	1:Experimental evidence at protein level	alcohol catabolic process;alcohol metabolic process;carbohydrate catabolic process;carbohydrate metabolic process;catabolic process;cellular carbohydrate catabolic process;cellular carbohydrate metabolic process;cellula
27.294	27.668	27.6469	27.7292	1:Experimental evidence at protein level	ATP-dependent chromatin remodeling;cellular component organization;cellular component organization at cellular level;cellular component organization or biogenesis;cellular component organization or biogenesis at cellular l
26.2584	26.6556	26.249	26.8364	1:Experimental evidence at protein level	
25.5787	#NUM!	25.1884	25.4947	1:Experimental evidence at protein level	biological regulation;cellular process;cellular response to abiotic stimulus;cellular response to acidity;cellular response to pH;cellular response to stimulus;establishment of localization;establishment of localization in cell;exoc
25.475	#NUM!	25.6136	25.8448	1:Experimental evidence at protein level	7-methylguanosine biosynthetic process;7-methylguanosine metabolic process;biosynthetic process;cellular biosynthetic process;cellular macromolecule metabolic process;cellular metabolic process;cellular nitrogen compo
25.9179	25.4831	25.7739	#NUM!	1:Experimental evidence at protein level	4-hydroxyproline metabolic process;amine metabolic process;anatomical structure development;anatomical structure formation involved in morphogenesis;anatomical structure morphogenesis;biological regulation;carboxylic
#NUM!	#NUM!	25.7506	24.8538	1:Experimental evidence at protein level	biological regulation;development of secondary female sexual characteristics;development of secondary sexual characteristics;developmental process;developmental process involved in reproduction;negative regulation of b
26.0532	25.6173	26.1348	25.8428	1:Experimental evidence at protein level	biosynthetic process;cellular biosynthetic process;cellular component disassembly;cellular component disassembly at cellular level;cellular component organization;cellular component organization at cellular level;cellular cor
28.3408	28.8617	29.0065	29.1547	1:Experimental evidence at protein level	
26.905	26.7617	27.0037	26.8382	1:Experimental evidence at protein level	biosynthetic process;establishment of localization;lipid biosynthetic process;lipid metabolic process;metabolic process;primary metabolic process;steroid biosynthetic process;steroid metabolic process;transport
25.5467	25.8935	26.2249	25.9922	1:Experimental evidence at protein level	biological regulation;cellular component organization;cellular component organization or biogenesis;cellular process;cellular response to stimulus;intracellular signal transduction;positive regulation of actin filament polymeriza
26.2119	25.9772	25.9982	25.9265	1:Experimental evidence at protein level	establishment of localization;lipid transport;organic substance transport;transport
26.6333	26.9902	26.2303	26.0581	1:Experimental evidence at protein level	acidic amino acid transport;amine transport;amino acid transport;anion transport;carboxylic acid transport;establishment of localization;ion transport;L-glutamate transport;nitrogen compound transport;organic acid transpor
25.8273	25.9894	26.1202	26.1739	1:Experimental evidence at protein level	amine metabolic process;biosynthetic process;carboxylic acid metabolic process;cellular amine metabolic process;cellular amino acid metabolic process;cellular biosynthetic process;cellular ketone metabolic process;cellula
26.1536	26.2808	26.3816	26.3609	1:Experimental evidence at protein level	biological regulation;biosynthetic process;carbohydrate homeostasis;cellular biosynthetic process;cellular macromolecule biosynthetic process;cellular macromolecule metabolic process;cellular metabolic process;cellular nit
26.3551	25.6135	26.1311	25.6879	1:Experimental evidence at protein level	anatomical structure development;biosynthetic process;blastocyst development;cellular biosynthetic process;cellular macromolecule biosynthetic process;cellular macromolecule metabolic process;cellular metabolic process

25.9347	26.2574	#NUM!	26.3261	1:Experimental evidence at protein level	apoptosis;biological regulation;biosynthetic process;cell death;cell differentiation;cell proliferation;cellular biosynthetic process;cellular developmental process;cellular macromolecule biosynthetic process;cellular macromolec
26.9177	27.0287	26.7215	26.851	1:Experimental evidence at protein level	ameboidal cell migration;anatomical structure development;associative learning;basement membrane organization;behavior;biological adhesion;biological regulation;cAMP-mediated signaling;cell adhesion;cell migration;cell
26.2096	#NUM!	26.2008	25.887	1:Experimental evidence at protein level	biosynthetic process;cellular biosynthetic process;cellular component disassembly;cellular component disassembly at cellular level;cellular component organization;cellular component organization at cellular level;cellular cor
27.0257	26.8711	27.1298	27.1368	1:Experimental evidence at protein level	
25.0831	24.9025	24.9742	24.599	1:Experimental evidence at protein level	defense response;defense response to virus;immune effector process;immune system process;multi-organism process;response to biotic stimulus;response to other organism;response to stimulus;response to stress;respons
25.856	25.6155	25.4441	25.7699	1:Experimental evidence at protein level	anatomical structure development;biological regulation;cell proliferation;developmental process;negative regulation of biological process;negative regulation of cell communication;negative regulation of cellular process;negat
25.495	#NUM!	24.3324	25.0322	1:Experimental evidence at protein level	biological regulation;cellular component organization;cellular component organization at cellular level;cellular component organization or biogenesis;cellular component organization or biogenesis at cellular level;cellular proce
26.3125	26.3232	26.7401	26.8916	1:Experimental evidence at protein level	biological regulation;biosynthetic process;cellular biosynthetic process;cellular macromolecule biosynthetic process;cellular macromolecule metabolic process;cellular metabolic process;cellular nitrogen compound metabolic
26.3775	#NUM!	26.5753	26.4826	1:Experimental evidence at protein level	cellular macromolecule metabolic process;cellular metabolic process;cellular nitrogen compound metabolic process;cellular process;gene expression;macromolecule metabolic process;metabolic process;mRNA metabolic p
25.5812	#NUM!	25.5978	25.7454	1:Experimental evidence at protein level	
27.1808	27.0655	26.875	27.0545	1:Experimental evidence at protein level	activation of JUN kinase activity;activation of MAPK activity;biological regulation;biosynthetic process;carboxylic acid biosynthetic process;carboxylic acid metabolic process;cellular biosynthetic process;cellular ketone meta
28.0461	27.3706	27.6954	27.9404	1:Experimental evidence at protein level	biological regulation;cell cycle;cell differentiation;cellular developmental process;cellular macromolecule metabolic process;cellular metabolic process;cellular nitrogen compound metabolic process;cellular process;cellular re
26.4558	#NUM!	26.1417	25.7397	1:Experimental evidence at protein level	alcohol metabolic process;anatomical structure development;biological regulation;biosynthetic process;blood vessel development;cell differentiation;cellular developmental process;cellular process;cholesterol biosynthetic pr
26.6386	26.0558	26.7585	26.5406	1:Experimental evidence at protein level	alcohol biosynthetic process;alcohol metabolic process;amine catabolic process;amine metabolic process;biosynthetic process;carbohydrate biosynthetic process;carbohydrate metabolic process;carboxylic acid catabolic p
26.7592	26.6851	26.6876	26.8001	1:Experimental evidence at protein level	biological regulation;cell differentiation;cellular developmental process;cellular process;cellular response to stimulus;developmental process;gamete generation;male gamete generation;multicellular organismal process;multic
28.9046	28.7035	29.2348	29.1216	1:Experimental evidence at protein level	biological regulation;cellular macromolecule metabolic process;cellular metabolic process;cellular process;cellular protein metabolic process;'de novo' posttranslational protein folding;'de novo' protein folding;macromolecule
26.7075	26.6885	26.3884	26.4022	1:Experimental evidence at protein level	ATP hydrolysis coupled proton transport;biological regulation;cation homeostasis;cation transport;cell surface receptor linked signaling pathway;cellular cation homeostasis;cellular chemical homeostasis;cellular component c
25.5045	25.5969	25.5463	25.5476	1:Experimental evidence at protein level	cellular macromolecule metabolic process;cellular metabolic process;cellular nitrogen compound metabolic process;cellular process;macromolecule metabolic process;metabolic process;mRNA metabolic process;mRNA pro
25.6926	25.1438	25.5458	25.7309	1:Experimental evidence at protein level	cellular component organization;cellular component organization at cellular level;cellular component organization or biogenesis;cellular component organization or biogenesis at cellular level;cellular macromolecule metabolic
25.9925	26.2745	26.396	26.2863	1:Experimental evidence at protein level	biosynthetic process;cellular biosynthetic process;cellular component disassembly;cellular component disassembly at cellular level;cellular component organization;cellular component organization at cellular level;cellular cor
25.9014	26.3032	#NUM!	26.4182	1:Experimental evidence at protein level	cellular macromolecule metabolic process;cellular metabolic process;cellular nitrogen compound metabolic process;cellular process;macromolecule metabolic process;metabolic process;ncRNA metabolic process;ncRNA pr
24.8959	#NUM!	#NUM!	25.4198	1:Experimental evidence at protein level	biological regulation;blood vessel endothelial cell migration;blood vessel endothelial cell migration involved in intussusceptive angiogenesis;cell differentiation;cell migration;cell motility;cellular component movement;cellular c
28.0127	29.0774	29.0709	29.1921	1:Experimental evidence at protein level	biological regulation;cellular macromolecule metabolic process;cellular metabolic process;cellular nitrogen compound metabolic process;cellular process;cellular response to stimulus;intracellular signal transduction;macromo
26.3052	26.0106	25.8994	25.9471	1:Experimental evidence at protein level	
26.601	25.5131	26.5476	26.4514	1:Experimental evidence at protein level	cellular component biogenesis;cellular component biogenesis at cellular level;cellular component organization or biogenesis;cellular component organization or biogenesis at cellular level;cellular process;ribonucleoprotein co
26.7833	26.9456	26.5749	26.8615	1:Experimental evidence at protein level	cellular macromolecule metabolic process;cellular metabolic process;cellular process;cellular protein metabolic process;cellular response to stimulus;cellular response to stress;macromolecule metabolic process;macromolec
27.2532	26.868	27.4903	27.286	1:Experimental evidence at protein level	biological regulation;cell surface receptor linked signaling pathway;cellular process;cellular response to stimulus;regulation of biological process;regulation of cellular process;response to stimulus;signal transduction;Wnt rece
27.337	26.7469	27.0267	26.9796	1:Experimental evidence at protein level	cellular metabolic process;cellular process;electron transport chain;generation of precursor metabolites and energy;metabolic process;mitochondrial electron transport, NADH to ubiquinone;multicellular organismal process;n
26.4956	27.2006	26.831	26.8824	1:Experimental evidence at protein level	carbohydrate metabolic process;cellular macromolecule metabolic process;cellular metabolic process;cellular process;cellular protein metabolic process;dephosphorylation;macromolecule metabolic process;macromolecule
30.0576	30.1588	30.0062	30.4091	1:Experimental evidence at protein level	biological regulation;biosynthetic process;cellular biosynthetic process;cellular macromolecule biosynthetic process;cellular macromolecule metabolic process;cellular metabolic process;cellular nitrogen compound metabolic
29.3306	29.3839	29.6918	29.5657	1:Experimental evidence at protein level	
27.5336	27.2684	27.4892	27.7187	1:Experimental evidence at protein level	biological regulation;cell surface receptor linked signaling pathway;cellular component assembly;cellular component organization;cellular component organization or biogenesis;cellular process;cellular response to chemical s
25.9223	25.8332	25.8816	26.0563	1:Experimental evidence at protein level	anatomical structure development;anatomical structure formation involved in morphogenesis;androgen receptor signaling pathway;ATP-dependent chromatin remodeling;biological regulation;biosynthetic process;blood vess
27.0862	26.9229	27.5741	27.2387	1:Experimental evidence at protein level	acid secretion;actin cytoskeleton organization;actin filament-based process;activation of adenylate cyclase activity;activation of adenylate cyclase activity by dopamine receptor signaling pathway;activation of adenylate cycla
#NUM!	#NUM!	#NUM!	#NUM!	1:Experimental evidence at protein level	aging;anatomical structure homeostasis;biological regulation;biosynthetic process;cell aging;cellular biosynthetic process;cellular component organization;cellular component organization at cellular level;cellular component c
26.7157	26.658	26.8307	26.7102	1:Experimental evidence at protein level	biological regulation;cell surface receptor linked signaling pathway;cellular process;cellular response to chemical stimulus;cellular response to cytokine stimulus;cellular response to interferon-alpha;cellular response to organi
27.7491	27.6951	26.9276	27.4905	1:Experimental evidence at protein level	anatomical structure development;cell development;cellular developmental process;cellular metabolic process;cellular process;central nervous system neuron development;cerebellar Purkinje cell layer development;developm
25.9197	26.0717	26.259	25.9708	1:Experimental evidence at protein level	cellular component organization;cellular component organization at cellular level;cellular component organization or biogenesis;cellular component organization or biogenesis at cellular level;cellular membrane fusion;cellular r
27.2931	27.6639	27.7714	27.5754	1:Experimental evidence at protein level	carbohydrate metabolic process;cellular aldehyde metabolic process;cellular metabolic process;cellular process;metabolic process;primary metabolic process;small molecule metabolic process;xenobiotic metabolic process
26.1902	26.4372	26.5453	26.4447	1:Experimental evidence at protein level	biological regulation;cell cycle phase;cell cycle process;cellular component organization;cellular component organization at cellular level;cellular component organization or biogenesis;cellular component organization or bioge
26.9331	#NUM!	26.9297	27.0429	1:Experimental evidence at protein level	establishment of localization;establishment of localization in cell;establishment of RNA localization;intracellular transport;mRNA export from nucleus;mRNA transport;nuclear export;nuclear transport;nucleic acid transport;nuc
25.3485	25.2091	25.0695	24.7174	1:Experimental evidence at protein level	anatomical structure maturation;biological regulation;blood vessel maturation;defense response;defense response to bacterium;developmental maturation;developmental process;immune response;immune system process;ir
27.3874	27.563	27.7241	27.8455	1:Experimental evidence at protein level	biological regulation;cation homeostasis;cation transport;cell surface receptor linked signaling pathway;cellular cation homeostasis;cellular chemical homeostasis;cellular component organization;cellular component organizat
26.4848	26.3308	#NUM!	26.4744	1:Experimental evidence at protein level	anatomical structure development;biological regulation;cellular metabolic process;cellular process;developmental process;electron transport chain;generation of precursor metabolites and energy;metabolic process;mitochon
25.3814	#NUM!	25.4426	25.3092	1:Experimental evidence at protein level	cellular component biogenesis;cellular component biogenesis at cellular level;cellular component organization or biogenesis;cellular component organization or biogenesis at cellular level;cellular process;ribonucleoprotein co
27.0116	27.299	27.0586	27.4028	1:Experimental evidence at protein level	catabolic process;cellular catabolic process;cellular ketone metabolic process;cellular metabolic process;cellular process;coenzyme catabolic process;coenzyme metabolic process;cofactor catabolic process;cofactor metab
27.8036	27.895	27.6821	27.7682	1:Experimental evidence at protein level	CAAX-box protein processing;catabolic process;cellular catabolic process;cellular component organization;cellular component organization or biogenesis;cellular macromolecule catabolic process;cellular macromolecule me
26.6185	26.4839	26.4519	26.3501	1:Experimental evidence at protein level	antigen processing and presentation;antigen processing and presentation of exogenous antigen;antigen processing and presentation of exogenous peptide antigen;antigen processing and presentation of exogenous peptide
28.2326	27.8297	28.4518	28.3061	1:Experimental evidence at protein level	biological regulation;cell redox homeostasis;cellular homeostasis;cellular process;homeostatic process;negative regulation of biological process;negative regulation of cardiac muscle hypertrophy;negative regulation of multic
25.8723	25.4208	26.0826	26.0594	1:Experimental evidence at protein level	anatomical structure development;biological regulation;biosynthetic process;cellular biosynthetic process;cellular macromolecule biosynthetic process;cellular macromolecule metabolic process;cellular metabolic process;ce
27.7887	27.8923	28.0378	27.9638	1:Experimental evidence at protein level	alcohol catabolic process;alcohol metabolic process;carbohydrate catabolic process;carbohydrate metabolic process;catabolic process;cellular carbohydrate catabolic process;cellular carbohydrate metabolic process;cellula
25.5096	25.4716	25.6192	#NUM!	1:Experimental evidence at protein level	anatomical structure development;anatomical structure formation involved in morphogenesis;anatomical structure morphogenesis;antigen processing and presentation;antigen processing and presentation of exogenous anti
26.9359	27.7737	27.8796	28.1654	1:Experimental evidence at protein level	amine catabolic process;amine metabolic process;carboxylic acid catabolic process;carboxylic acid metabolic process;catabolic process;cellular amine metabolic process;cellular amino acid catabolic process;cellular amino
27.4964	27.3849	27.5075	27.3463	1:Experimental evidence at protein level	anatomical structure development;ATP-dependent chromatin remodeling;biological regulation;biosynthetic process;cellular biosynthetic process;cellular component organization;cellular component organization at cellular lev
#NUM!	27.607	#NUM!	#NUM!	1:Experimental evidence at protein level	acute inflammatory response;acute-phase response;biological regulation;blood coagulation;blood coagulation, intrinsic pathway;calcium ion homeostasis;cation homeostasis;cell activation;cell migration;cell motility;cell surfa
#NUM!	#NUM!	#NUM!	#NUM!	1:Experimental evidence at protein level	biological regulation;blood coagulation;coagulation;hemostasis;multicellular organismal process;negative regulation of biological process;negative regulation of catalytic activity;negative regulation of defense response;negati
27.1213	27.2443	27.3648	27.388	1:Experimental evidence at protein level	activation of immune response;activation of innate immune response;activation of MAPKK activity;activation of protein kinase activity;axon guidance;behavior;behavioral interaction between organisms;biological regulation;bl
26.1582	#NUM!	27.3556	29.4889	1:Experimental evidence at protein level	biological regulation;cellular process;cellular response to cadmium ion;cellular response to chemical stimulus;cellular response to inorganic substance;cellular response to metal ion;cellular response to stimulus;cellular respo
29.476	29.4656	30.0146	29.7614	1:Experimental evidence at protein level	biosynthetic process;catabolic process;cellular biosynthetic process;cellular catabolic process;cellular component disassembly;cellular component disassembly at cellular level;cellular component organization;cellular compo
26.8044	26.912	27.1992	27.1889	1:Experimental evidence at protein level	anatomical structure development;anatomical structure morphogenesis;axon guidance;cellular component disassembly;cellular component disassembly at cellular level;cellular component organization;cellular component org
27.514	27.8224	27.9311	#NUM!	1:Experimental evidence at protein level	adenine biosynthetic process;adenine metabolic process;adenine salvage;AMP biosynthetic process;AMP metabolic process;AMP salvage;behavior;biological regulation;biosynthetic process;body fluid secretion;cellular aron
28.5183	28.5392	28.3864	28.2256	1:Experimental evidence at protein level	biological regulation;cellular macromolecule metabolic process;cellular metabolic process;cellular nitrogen compound metabolic process;cellular process;gene expression;macromolecule metabolic process;metabolic proces
28.7958	30.6257	30.3843	31.0147	1:Experimental evidence at protein level	
28.5076	28.4736	28.3966	28.1909	1:Experimental evidence at protein level	biological regulation;biosynthetic process;carbohydrate biosynthetic process;carbohydrate metabolic process;carbohydrate transmembrane transport;carbohydrate transport;carboxylic acid metabolic process;cation transpo
28.4522	28.1835	28.4127	28.3772	1:Experimental evidence at protein level	actin cytoskeleton organization;actin cytoskeleton reorganization;actin filament-based process;biological regulation;cell surface receptor linked signaling pathway;cellular component organization;cellular component organizat
28.7696	29.3286	29.2556	29.3441	1:Experimental evidence at protein level	autophagic cell death;autophagy;biological regulation;catabolic process;cell death;cellular catabolic process;cellular metabolic process;cellular process;death;establishment of localization;establishment of localization in cell;e
26.4026	26.1341	26.0901	25.8645	1:Experimental evidence at protein level	alcohol metabolic process;anatomical structure development;biosynthetic process;carbohydrate biosynthetic process;carbohydrate metabolic process;cellular biosynthetic process;cellular carbohydrate biosynthetic process;
27.187	27.2431	27.2448	27.4951	1:Experimental evidence at protein level	aerobic respiration;cellular metabolic process;cellular process;cellular respiration;electron transport chain;energy derivation by oxidation of organic compounds;generation of precursor metabolites and energy;metabolic proce
28.3852	28.7156	28.6974	28.8921	1:Experimental evidence at protein level	bile acid biosynthetic process;bile acid metabolic process;biological regulation;biosynthetic process;C21-steroid hormone biosynthetic process;C21-steroid hormone metabolic process;carboxylic acid biosynthetic process;c
26.8824	26.9925	26.9059	26.6553	1:Experimental evidence at protein level	anaphase-promoting complex-dependent proteasomal ubiquitin-dependent protein catabolic process;biological regulation;biosynthetic process;blood coagulation;catabolic process;cation transport;cell cycle;cell cycle check
26.1199	26.2802	26.5613	26.4266	1:Experimental evidence at protein level	amine catabolic process;amine metabolic process;branched chain family amino acid catabolic process;branched chain family amino acid metabolic process;carboxylic acid catabolic process;carboxylic acid metabolic proces
25.3902	#NUM!	25.3757	25.3732	1:Experimental evidence at protein level	anatomical structure development;anatomical structure formation involved in morphogenesis;anatomical structure morphogenesis;angiogenesis;axon guidance;axonal fasciculation;axonogenesis;behavior;biological regulatio
27.5835	27.9755	27.9126	28.0142	1:Experimental evidence at protein level	anatomical structure development;anatomical structure morphogenesis;appendage morphogenesis;biological regulation;biosynthetic process;carboxylic acid metabolic process;cellular biosynthetic process;cellular hormone
28.5372	28.7267	29.1159	28.9402	1:Experimental evidence at protein level	biological regulation;biosynthetic process;catabolic process;cellular biosynthetic process;cellular catabolic process;cellular macromolecule metabolic process;cellular metabolic process;cellular nitrogen compound metabolic
27.8609	29.1721	29.1958	29.4052	1:Experimental evidence at protein level	biosynthetic process;cellular biosynthetic process;cellular metabolic process;cellular process;melanin biosynthetic process;melanin metabolic process;metabolic process;pigment biosynthetic process;pigment metabolic proc
26.2968	#NUM!	26.4655	#NUM!	1:Experimental evidence at protein level	actin cytoskeleton organization;actin filament organization;actin filament-based process;anatomical structure homeostasis;biological adhesion;biological regulation;calcium ion transport;cation transport;cell activation;cell act
27.0583	26.7217	26.744	26.7586	1:Experimental evidence at protein level	alcohol metabolic process;biological regulation;cell differentiation;cellular component assembly;cellular component organization;cellular component organization or biogenesis;cellular developmental process;cellular lipid met
#NUM!	#NUM!	#NUM!	#NUM!	1:Experimental evidence at protein level	biological regulation;cellular process;cellular response to stimulus;regulation of biological process;regulation of cellular process;response to stimulus;signal transduction
27.1829	26.8057	26.8311	26.7846	1:Experimental evidence at protein level	anatomical structure homeostasis;base-excision repair;biological regulation;biosynthetic process;cell cycle;cellular biosynthetic process;cellular component organization;cellular component organization at cellular level;cellula
25.5529	25.6068	25.6431	25.4343	1:Experimental evidence at protein level	activation of immune response;adherens junction organization;anatomical structure development;anatomical structure morphogenesis;antigen receptor-mediated signaling pathway;biological regulation;blood coagulation;bra
27.253	27.2475	27.3144	27.3653	1:Experimental evidence at protein level	activation of MAPKK activity;activation of protein kinase activity;biological regulation;blood coagulation;cell activation;cell surface receptor linked signaling pathway;cellular macromolecule metabolic process;cellular metaboli
27.6493	28.0811	27.9869	27.9911	1:Experimental evidence at protein level	alcohol metabolic process;amine metabolic process;biosynthetic process;CDP-choline pathway;cellular amine metabolic process;cellular biogenic amine metabolic process;cellular biosynthetic process;cellular lipid metaboli
27.6453	27.845	27.6548	27.4809	1:Experimental evidence at protein level	actin cytoskeleton organization;actin filament-based process;biological regulation;cell chemotaxis;cell junction assembly;cell junction organization;cell migration;cell motility;cell projection organization;cell-cell junction assem
27.8796	28.5592	28.0182	28.6164	1:Experimental evidence at protein level	biological regulation;catabolic process;cellular catabolic process;cellular component organization;cellular component organization at cellular level;cellular component organization or biogenesis;cellular component organizatio
26.7073	#NUM!	26.4411	26.3829	1:Experimental evidence at protein level	cellular component organization;cellular component organization at cellular level;cellular component organization or biogenesis;cellular component organization or biogenesis at cellular level;cellular process;extracellular matr
26.1288	26.4501	26.2082	26.187	1:Experimental evidence at protein level	biological regulation;biosynthetic process;cellular biosynthetic process;cellular macromolecule biosynthetic process;cellular macromolecule metabolic process;cellular metabolic process;cellular process;macromolecule biosy
27.7811	27.6899	28.0117	28.0125	1:Experimental evidence at protein level	anatomical structure development;behavior;biological regulation;cellular macromolecule metabolic process;cellular metabolic process;cellular process;cellular protein metabolic process;cellular response to stimulus;cognition
28.895	28.8222	28.6965	28.8387	1:Experimental evidence at protein level	activation of MAPKK activity;activation of protein kinase activity;anatomical structure development;biological regulation;blood coagulation;cell activation;cell development;cell surface receptor linked signaling pathway;cellular
29.0161	28.5224	29.2229	29.6209	1:Experimental evidence at protein level	activation of immune response;activation of innate immune response;anaphase-promoting complex-dependent proteasomal ubiquitin-dependent protein catabolic process;biological regulation;catabolic process;cell cycle;ce
28.4451	28.7863	29.0319	28.8028	1:Experimental evidence at protein level	anatomical structure development;biological regulation;brain development;catabolic process;developmental process;gamete generation;lipid catabolic process;lipid metabolic process;male gamete generation;metabolic proc
28.4681	28.7663	28.6441	28.7067	1:Experimental evidence at protein level	biosynthetic process;cellular biosynthetic process;cellular macromolecule biosynthetic process;cellular macromolecule metabolic process;cellular metabolic process;cellular nitrogen compound metabolic process;cellular pro
27.6345	27.5873	27.7885	27.8602	1:Experimental evidence at protein level	acetaldehyde metabolic process;activation of immune response;activation of innate immune response;aging;anatomical structure development;anatomical structure morphogenesis;antigen receptor-mediated signaling pathw
27.01	28.5385	27.9698	28.2536	1:Experimental evidence at protein level	anatomical structure morphogenesis;biological regulation;biosynthetic process;cell part morphogenesis;cellular biosynthetic process;cellular component morphogenesis;cellular component organization;cellular component or
27.554	27.0609	27.524	27.4688	1:Experimental evidence at protein level	anatomical structure morphogenesis;anterior/posterior pattern specification;cellular macromolecule metabolic process;cellular metabolic process;cellular nitrogen compound metabolic process;cellular process;developmenta
26.3517	27.1863	27.2641	27.0686	1:Experimental evidence at protein level	establishment of localization;establishment of protein localization;protein transport;transport
25.4313	25.0496	25.0928	25.2339	1:Experimental evidence at protein level	behavior;biological regulation;biosynthetic process;catabolic process;cellular biosynthetic process;cellular catabolic process;cellular component organization;cellular component organization at cellular level;cellular componer
25.6387	26.4428	26.5811	25.8603	1:Experimental evidence at protein level	activation of immune response;activation of innate immune response;antigen receptor-mediated signaling pathway;biological regulation;cell differentiation;cell surface receptor linked signaling pathway;cellular developmental
27.8466	28.5346	28.6674	28.5799	1:Experimental evidence at protein level	biological regulation;cell cycle;cell surface receptor linked signaling pathway;cellular macromolecule metabolic process;cellular metabolic process;cellular process;cellular protein metabolic process;cellular response to chemi
26.5456	24.8687	27.5871	26.8769	1:Experimental evidence at protein level	biological regulation;catabolic process;cellular catabolic process;cellular localization;cellular macromolecule catabolic process;cellular macromolecule localization;cellular macromolecule metabolic process;cellular metabolic
26.0393	25.7432	25.8604	25.735	1:Experimental evidence at protein level	biological regulation;biosynthetic process;cellular biosynthetic process;cellular component organization;cellular component organization at cellular level;cellular component organization or biogenesis;cellular component orga
26.108	26.1444	25.8008	25.7769	1:Experimental evidence at protein level	biological regulation;cell redox homeostasis;cellular homeostasis;cellular macromolecule metabolic process;cellular metabolic process;cellular process;cellular protein metabolic process;cellular response to chemical stimulus
27.0383	26.6984	26.5913	26.5371	1:Experimental evidence at protein level	activation of immune response;activation of innate immune response;alcohol metabolic process;anatomical structure development;biological regulation;carbohydrate metabolic process;cell surface receptor linked signaling p
#NUM!	#NUM!	#NUM!	#NUM!	1:Experimental evidence at protein level	biological regulation;cell surface receptor linked signaling pathway;cellular component organization;cellular component organization at cellular level;cellular component organization or biogenesis;cellular component organizat
25.7455	24.6646	24.7863	24.7925	1:Experimental evidence at protein level	cellular component assembly;cellular component assembly at cellular level;cellular component organization;cellular component organization at cellular level;cellular component organization or biogenesis;cellular component c
26.1211	25.8448	25.8575	26.1118	1:Experimental evidence at protein level	actin cytoskeleton organization;actin filament-based process;anatomical structure morphogenesis;axonogenesis;biological regulation;cell part morphogenesis;cell projection morphogenesis;cell projection organization;cellula
26.4628	26.2938	26.5792	26.3578	1:Experimental evidence at protein level	biological regulation;cell cycle process;cell proliferation;cellular component movement;cellular metabolic process;cellular process;chromosome movement towards spindle pole;dephosphorylation;establishment of chromoso
27.2432	27.1542	26.9911	27.3413	1:Experimental evidence at protein level	biological regulation;cell junction assembly;cell junction organization;cellular component assembly;cellular component assembly at cellular level;cellular component organization;cellular component organization at cellular leve
26.5233	26.6632	26.9614	26.9368	1:Experimental evidence at protein level	anatomical structure development;biological adhesion;biological regulation;cell adhesion;cellular process;cellular response to chemical stimulus;cellular response to endogenous stimulus;cellular response to hormone stimulu
25.9742	25.4203	25.6456	24.9489	1:Experimental evidence at protein level	cation transport;cellular metabolic process;cellular process;electron transport chain;establishment of localization;generation of precursor metabolites and energy;ion transport;metabolic process;metal ion transport;mitochond
26.35	26.5301	26.2215	26.3302	1:Experimental evidence at protein level	alcohol metabolic process;biosynthetic process;cellular metabolic process;cellular process;cholesterol biosynthetic process;cholesterol biosynthetic process via 24,25-dihydrolanosterol;cholesterol metabolic process;demeth
26.572	#NUM!	26.5688	26.3761	1:Experimental evidence at protein level	cellular component organization;cellular component organization at cellular level;cellular component organization or biogenesis;cellular component organization or biogenesis at cellular level;cellular process;endosome organi
26.8016	27.0447	26.8856	26.9719	1:Experimental evidence at protein level	amine biosynthetic process;amine metabolic process;biosynthetic process;carboxylic acid biosynthetic process;carboxylic acid metabolic process;cellular amine metabolic process;cellular amino acid biosynthetic process;ce
25.6407	#NUM!	#NUM!	#NUM!	1:Experimental evidence at protein level	amine metabolic process;amino acid activation;carboxylic acid metabolic process;cellular amine metabolic process;cellular amino acid metabolic process;cellular ketone metabolic process;cellular macromolecule metabolic p
26.4525	#NUM!	27.5576	27.2469	1:Experimental evidence at protein level	
27.381	27.198	27.2769	27.3963	1:Experimental evidence at protein level	anatomical structure development;biological regulation;cell migration;cell motility;cellular component movement;cellular process;developmental process;locomotion;lung alveolus development;positive regulation of biological
27.778	27.91	27.8886	27.5873	1:Experimental evidence at protein level	amine catabolic process;amine metabolic process;branched chain family amino acid catabolic process;branched chain family amino acid metabolic process;carboxylic acid catabolic process;carboxylic acid metabolic proces
26.0814	27.2434	27.181	27.4434	1:Experimental evidence at protein level	alkaloid metabolic process;biosynthetic process;cellular amide metabolic process;cellular biosynthetic process;cellular metabolic compound salvage;cellular metabolic process;cellular nitrogen compound biosynthetic proces
25.4287	#NUM!	25.4574	25.5918	1:Experimental evidence at protein level	activation of protein kinase activity;autophagy;biological regulation;catabolic process;cell migration;cell motility;cell surface receptor linked signaling pathway;cellular catabolic process;cellular component movement;cellular l
25.9728	25.4789	26.1352	25.9884	1:Experimental evidence at protein level	biological regulation;cellular macromolecule metabolic process;cellular metabolic process;cellular nitrogen compound metabolic process;cellular process;macromolecule metabolic process;macromolecule methylation;macro
24.6884	24.9627	24.8134	24.6911	1:Experimental evidence at protein level	cell cycle process;cell division;cellular component assembly;cellular component assembly at cellular level;cellular component organization;cellular component organization at cellular level;cellular component organization or b
25.0374	26.1784	26.1981	26.0706	1:Experimental evidence at protein level	metabolic process;nitrogen compound metabolic process
25.3406	25.4922	25.3552	25.2119	1:Experimental evidence at protein level	cell projection organization;cellular component movement;cellular component organization;cellular component organization at cellular level;cellular component organization or biogenesis;cellular component organization or bio
26.1968	26.1243	26.3608	26.3085	1:Experimental evidence at protein level	anatomical structure development;biological regulation;calcium ion homeostasis;calcium ion transport;calcium ion transport into cytosol;cation homeostasis;cation transport;cellular calcium ion homeostasis;cellular cation ho
25.8755	25.9946	25.8001	25.7114	1:Experimental evidence at protein level	cellular component assembly;cellular component assembly at cellular level;cellular component disassembly;cellular component disassembly at cellular level;cellular component organization;cellular component organization at
25.3581	#NUM!	25.2612	25.131	1:Experimental evidence at protein level	cellular macromolecule metabolic process;cellular metabolic process;cellular nitrogen compound metabolic process;cellular process;macromolecule metabolic process;macromolecule methylation;macromolecule modificatio
26.2966	#NUM!	26.6408	26.1949	1:Experimental evidence at protein level	acylglycerol biosynthetic process;acylglycerol metabolic process;anatomical structure development;anatomical structure homeostasis;biological regulation;biosynthetic process;carboxylic acid biosynthetic process;carboxylic
25.7742	#NUM!	25.7276	25.9651	1:Experimental evidence at protein level	biological regulation;positive regulation of biological process;positive regulation of biosynthetic process;positive regulation of cellular biosynthetic process;positive regulation of cellular metabolic process;positive regulation of
27.4807	27.5411	27.5727	27.4937	1:Experimental evidence at protein level	biological regulation;cellular process;cellular response to stimulus;positive regulation of biological process;positive regulation of cell communication;positive regulation of cellular process;positive regulation of I-kappaB kinase
26.5699	26.7892	26.5841	26.4778	1:Experimental evidence at protein level	alcohol biosynthetic process;alcohol metabolic process;biosynthetic process;carbohydrate biosynthetic process;carbohydrate metabolic process;cellular biosynthetic process;cellular carbohydrate biosynthetic process;cellul

26.9973	26.7439	26.9206	26.6448	1:Experimental evidence at protein level	anatomical structure development;biological regulation;brain development;cell cycle checkpoint;cell cycle process;cell proliferation;cellular macromolecule metabolic process;cellular metabolic process;cellular process;cellula
26.7779	27.313	26.8527	26.9935	1:Experimental evidence at protein level	biological regulation;cell redox homeostasis;cellular homeostasis;cellular macromolecule metabolic process;cellular metabolic process;cellular process;cellular protein metabolic process;cellular response to stimulus;cellular r
25.549	26.0167	25.6291	25.9413	1:Experimental evidence at protein level	actin cytoskeleton organization;actin cytoskeleton reorganization;actin filament organization;actin filament reorganization involved in cell cycle;actin filament-based process;biological regulation;cell cycle process;cellular com
25.9132	25.9498	25.971	25.8184	1:Experimental evidence at protein level	anatomical structure development;biological regulation;cell cycle arrest;cell cycle process;cell differentiation;cell migration;cell motility;cell projection assembly;cell projection organization;cellular component assembly;cellular
26.5584	26.2228	26.4269	26.4798	1:Experimental evidence at protein level	biological regulation;biosynthetic process;cellular biosynthetic process;cellular component organization;cellular component organization at cellular level;cellular component organization or biogenesis;cellular component orga
27.1122	27.6356	27.7115	27.8989	1:Experimental evidence at protein level	cellular macromolecule metabolic process;cellular metabolic process;cellular process;cellular protein metabolic process;macromolecule metabolic process;metabolic process;primary metabolic process;protein folding;protein
27.9947	28.0981	28.1222	28.1145	1:Experimental evidence at protein level	amine catabolic process;amine metabolic process;branched chain family amino acid catabolic process;branched chain family amino acid metabolic process;carboxylic acid catabolic process;carboxylic acid metabolic proces
26.7451	26.8049	26.9222	27.0281	1:Experimental evidence at protein level	biological regulation;cellular component assembly;cellular component organization;cellular component organization or biogenesis;cellular process;establishment of localization;establishment of localization in cell;establishmer
27.61	27.6694	27.4262	27.3767	1:Experimental evidence at protein level	establishment of localization;transport
26.1114	25.7618	25.5646	25.7451	1:Experimental evidence at protein level	cellular component movement;cellular localization;cellular process;cytoskeleton-dependent intracellular transport;establishment of localization;establishment of localization in cell;intracellular transport;localization;microtubule
27.7821	27.5074	27.6038	27.3905	1:Experimental evidence at protein level	biological regulation;cellular macromolecule metabolic process;cellular metabolic process;cellular process;cellular protein metabolic process;cytokine production;defense response;defense response to virus;immune effector
25.74	25.714	25.743	26.0746	1:Experimental evidence at protein level	biological regulation;posttranscriptional regulation of gene expression;regulation of biological process;regulation of biosynthetic process;regulation of cellular biosynthetic process;regulation of cellular macromolecule biosynth
25.5854	24.7283	25.5741	#NUM!	1:Experimental evidence at protein level	biological regulation;cellular component organization;cellular component organization at cellular level;cellular component organization or biogenesis;cellular component organization or biogenesis at cellular level;cellular proce
26.9351	27.0554	27.0577	27.0461	1:Experimental evidence at protein level	anatomical structure formation involved in morphogenesis;angiogenesis;biological regulation;cell migration;cell motility;cellular component assembly;cellular component assembly at cellular level;cellular component movemer
26.7926	27.1181	27.4448	27.3042	1:Experimental evidence at protein level	
26.7723	27.1769	27.0844	27.3388	1:Experimental evidence at protein level	
26.9847	26.4013	26.6887	26.7697	1:Experimental evidence at protein level	biological regulation;establishment of localization;establishment of localization in cell;establishment of RNA localization;intracellular transport;mRNA export from nucleus;mRNA transport;nuclear export;nuclear transport;nucle
26.9729	26.7109	26.9096	26.8668	1:Experimental evidence at protein level	biological regulation;cell differentiation;cellular developmental process;cellular localization;cellular macromolecule localization;cellular process;cellular protein localization;developmental process;establishment of localization;fa
25.616	25.5807	#NUM!	25.7611	1:Experimental evidence at protein level	biological regulation;regulation of biological process;regulation of biosynthetic process;regulation of cellular biosynthetic process;regulation of cellular macromolecule biosynthetic process;regulation of cellular metabolic proc
#NUM!	#NUM!	#NUM!	#NUM!	2:Experimental evidence at transcript level	anatomical structure development;biological regulation;cell communication;cell development;cell differentiation;cell fate commitment;cell surface receptor linked signaling pathway;cellular developmental process;cellular proc
31.0464	31.8904	31.7785	31.9226	1:Experimental evidence at protein level	biological regulation;cell redox homeostasis;cellular component organization;cellular component organization or biogenesis;cellular homeostasis;cellular process;homeostatic process;positive regulation of catalytic activity;po
27.3974	26.8523	26.4662	26.7069	1:Experimental evidence at protein level	biological regulation;negative regulation of catalytic activity;negative regulation of hydrolase activity;negative regulation of molecular function;negative regulation of phosphatase activity;regulation of biological process;regulat
26.9917	27.6709	27.5968	27.7312	1:Experimental evidence at protein level	behavior;exploration behavior;locomotory behavior;locomotory exploration behavior;pigmentation;response to stimulus
27.6633	28.5899	28.3451	28.4725	1:Experimental evidence at protein level	
27.8528	28.0151	28.1106	28.0374	1:Experimental evidence at protein level	cellular component organization;cellular component organization or biogenesis;cellular process;endosome transport;establishment of localization;establishment of localization in cell;establishment of protein localization;intrac
27.237	28.2113	27.3254	#NUM!	1:Experimental evidence at protein level	adenosine metabolic process;cellular macromolecule metabolic process;cellular metabolic process;cellular nitrogen compound metabolic process;cellular process;heterocycle metabolic process;macromolecule metabolic pro
26.2887	#NUM!	#NUM!	26.0116	2:Experimental evidence at transcript level	
25.3819	25.4129	25.2414	25.4053	1:Experimental evidence at protein level	biological regulation;biosynthetic process;cellular biosynthetic process;cellular component organization;cellular component organization at cellular level;cellular component organization or biogenesis;cellular component orga
26.9101	27.8872	27.9895	28.1653	1:Experimental evidence at protein level	biological regulation;cellular macromolecule metabolic process;cellular metabolic process;cellular process;cellular protein metabolic process;dephosphorylation;macromolecule metabolic process;macromolecule modification
26.6298	26.8118	26.7056	26.3674	1:Experimental evidence at protein level	antigen processing and presentation;antigen processing and presentation of exogenous antigen;antigen processing and presentation of exogenous peptide antigen;antigen processing and presentation of exogenous peptide
26.3524	26.078	26.2663	26.2299	1:Experimental evidence at protein level	actin cytoskeleton organization;actin filament-based process;biological regulation;cellular component organization;cellular component organization at cellular level;cellular component organization or biogenesis;cellular compo
25.0227	25.503	#NUM!	24.5492	1:Experimental evidence at protein level	biological regulation;cell cycle;cellular macromolecule metabolic process;cellular metabolic process;cellular nitrogen compound metabolic process;cellular process;cellular response to stimulus;cellular response to stress;DN
26.7085	26.3143	26.5425	26.7371	1:Experimental evidence at protein level	biological regulation;cellular macromolecule metabolic process;cellular metabolic process;cellular nitrogen compound metabolic process;cellular process;cellular response to chemical stimulus;cellular response to drug;cellula
26.7751	26.5447	26.7455	26.8031	1:Experimental evidence at protein level	
25.288	#NUM!	24.9445	#NUM!	1:Experimental evidence at protein level	cellular macromolecule metabolic process;cellular metabolic process;cellular nitrogen compound metabolic process;cellular process;macromolecule metabolic process;maturation of SSU-rRNA;maturation of SSU-rRNA from
26.0876	25.3368	25.4953	25.7611	1:Experimental evidence at protein level	
26.878	26.4081	26.367	26.4144	1:Experimental evidence at protein level	anaphase-promoting complex-dependent proteasomal ubiquitin-dependent protein catabolic process;biological regulation;catabolic process;cell cycle;cell cycle checkpoint;cell cycle phase;cell cycle process;cell division;cel
26.2978	26.158	26.3563	25.8466	1:Experimental evidence at protein level	biosynthetic process;cellular biosynthetic process;cellular macromolecule biosynthetic process;cellular macromolecule metabolic process;cellular metabolic process;cellular nitrogen compound metabolic process;cellular pro
29.1102	29.7273	29.4686	29.6628	1:Experimental evidence at protein level	cell proliferation
26.6045	26.595	26.2926	26.5656	1:Experimental evidence at protein level	anatomical structure morphogenesis;cell part morphogenesis;cellular component morphogenesis;cellular component organization;cellular component organization at cellular level;cellular component organization or biogenesi
27.2688	27.3998	27.1323	27.1145	1:Experimental evidence at protein level	biological regulation;biosynthetic process;cellular biosynthetic process;cellular macromolecule biosynthetic process;cellular macromolecule metabolic process;cellular metabolic process;cellular process;cellular protein metab
25.6553	25.5718	25.1972	25.3969	1:Experimental evidence at protein level	biological regulation;cell cycle;cellular process;positive regulation of catalytic activity;positive regulation of GTPase activity;positive regulation of hydrolase activity;positive regulation of molecular function;regulation of biologic
28.3969	27.9884	29.1306	27.9471	1:Experimental evidence at protein level	biological regulation;biosynthetic process;cellular biosynthetic process;cellular macromolecule biosynthetic process;cellular macromolecule metabolic process;cellular metabolic process;cellular nitrogen compound metabolic
25.7859	26.3122	25.9103	25.9768	1:Experimental evidence at protein level	alcohol metabolic process;anatomical structure development;biological regulation;brain development;carbohydrate homeostasis;carbohydrate metabolic process;cell proliferation;cell surface receptor linked signaling pathwa
26.0709	26.0452	26.0813	#NUM!	1:Experimental evidence at protein level	cellular macromolecule metabolic process;cellular metabolic process;cellular nitrogen compound metabolic process;cellular process;macromolecule metabolic process;metabolic process;ncRNA metabolic process;ncRNA pr
25.4894	26.1235	25.5864	25.5361	1:Experimental evidence at protein level	establishment of localization;establishment of localization in cell;establishment of protein localization;intracellular protein transport;intracellular transport;nuclear import;nuclear transport;nucleocytoplasmic transport;protein in
24.6685	24.2073	24.9057	24.6888	1:Experimental evidence at protein level	biological regulation;biosynthetic process;cell surface receptor linked signaling pathway;cellular biosynthetic process;cellular component organization;cellular component organization at cellular level;cellular component orgar
25.1683	26.4755	25.7565	25.6292	1:Experimental evidence at protein level	amine metabolic process;aromatic compound biosynthetic process;biosynthetic process;carboxylic acid metabolic process;cellular amine metabolic process;cellular amino acid metabolic process;cellular aromatic compound
25.2035	24.7154	24.6099	24.5967	1:Experimental evidence at protein level	biological regulation;cellular component organization;cellular component organization at cellular level;cellular component organization or biogenesis;cellular component organization or biogenesis at cellular level;cellular meml
25.1959	#NUM!	25.3248	25.2094	1:Experimental evidence at protein level	biological regulation;cell cycle;cell cycle checkpoint;cell cycle phase;cell cycle process;cell division;cellular component organization;cellular component organization at cellular level;cellular component organization or biogene
26.5039	#NUM!	26.4747	26.5045	1:Experimental evidence at protein level	carboxylic acid metabolic process;cellular ketone metabolic process;cellular lipid metabolic process;cellular metabolic process;cellular process;fatty acid metabolic process;lipid metabolic process;metabolic process;monoca
26.912	27.4472	26.8999	27.2124	1:Experimental evidence at protein level	biological regulation;cellular process;cellular response to stimulus;immune response;immune system process;metabolic process;positive regulation of biological process;positive regulation of catabolic process;positive regula
27.6873	27.7229	27.7862	27.7763	1:Experimental evidence at protein level	cellular process;establishment of localization;establishment of localization in cell;establishment of protein localization;Golgi vesicle transport;intracellular transport;post-Golgi vesicle-mediated transport;protein transport;resp
24.7957	#NUM!	25.2212	24.8703	1:Experimental evidence at protein level	biological adhesion;biological regulation;calcium ion import;calcium ion transmembrane transport;calcium ion transport;cation transport;cell adhesion;cell communication;cell-cell signaling;cellular component organization;cel
26.3059	25.9756	25.854	25.8573	1:Experimental evidence at protein level	alcohol biosynthetic process;alcohol metabolic process;amine metabolic process;biosynthetic process;cellular biosynthetic process;cellular lipid metabolic process;cellular metabolic process;cellular nitrogen compound bios
27.6501	28.0005	27.6912	27.5985	1:Experimental evidence at protein level	biological regulation;cell cycle;cell cycle checkpoint;cell cycle phase;cell cycle process;cell division;cellular component assembly;cellular component organization;cellular component organization at cellular level;cellular comp
25.9938	26.5568	26.8427	27.1154	1:Experimental evidence at protein level	cap-independent translational initiation;cellular component assembly;cellular component assembly at cellular level;cellular component disassembly;cellular component disassembly at cellular level;cellular component organiza
28.0643	27.7908	28.1412	27.9201	1:Experimental evidence at protein level	anion transport;biological regulation;cellular localization;cellular macromolecule localization;cellular macromolecule metabolic process;cellular metabolic process;cellular process;cellular protein localization;cellular protein me
25.8918	26.4743	26.1244	25.9691	1:Experimental evidence at protein level	biological regulation;biosynthetic process;cellular biosynthetic process;cellular macromolecule biosynthetic process;cellular macromolecule metabolic process;cellular metabolic process;cellular nitrogen compound metabolic
26.1656	25.8307	25.8449	25.8974	1:Experimental evidence at protein level	apoptosis;biological regulation;cell cycle;cell cycle phase;cell cycle process;cell death;cell division;cellular component organization;cellular component organization at cellular level;cellular component organization or biogenes
25.3408	24.8306	25.4007	#NUM!	1:Experimental evidence at protein level	anterograde axon cargo transport;apoptosis;axon cargo transport;cell communication;cell death;cell-cell signaling;cellular component movement;cellular process;cytoskeleton-dependent intracellular transport;death;establish
#NUM!	24.7548	24.6628	24.8484	1:Experimental evidence at protein level	apoptosis;biological regulation;cell death;cell differentiation;cellular developmental process;cellular process;death;developmental process;inner ear receptor cell differentiation;mechanoreceptor differentiation;multicellular org
29.3216	29.0341	29.5107	29.4497	1:Experimental evidence at protein level	anatomical structure development;anatomical structure formation involved in morphogenesis;anatomical structure morphogenesis;apoptosis;appendage development;appendage morphogenesis;biological regulation;blood v
24.3568	#NUM!	23.8229	24.3859	1:Experimental evidence at protein level	apoptosis;cell death;cellular component organization;cellular component organization or biogenesis;cellular membrane organization;cellular process;clathrin-mediated endocytosis;death;endocytosis;establishment of localiza
27.5377	27.2113	27.5022	27.0094	1:Experimental evidence at protein level	assembly of spliceosomal tri-snRNP;biological regulation;cellular component assembly;cellular component assembly at cellular level;cellular component organization;cellular component organization at cellular level;cellular co
27.1467	27.1704	26.4905	27.0093	1:Experimental evidence at protein level	carboxylic acid catabolic process;carboxylic acid metabolic process;catabolic process;cellular catabolic process;cellular ketone metabolic process;cellular lipid catabolic process;cellular lipid metabolic process;cellular metab
28.442	28.5865	28.7174	28.7319	1:Experimental evidence at protein level	cell cycle;cell cycle process;cellular component disassembly;cellular component disassembly at cellular level;cellular component organization;cellular component organization at cellular level;cellular component organization o
27.0656	26.855	27.1121	27.1741	1:Experimental evidence at protein level	cellular macromolecule metabolic process;cellular metabolic process;cellular nitrogen compound metabolic process;cellular process;gene expression;macromolecule metabolic process;metabolic process;mRNA metabolic p
25.1058	#NUM!	25.4024	25.7051	1:Experimental evidence at protein level	anatomical structure development;anatomical structure morphogenesis;apoptosis;apoptosis involved in luteolysis;axon extension;axon extension involved in axon guidance;axon guidance;biological adhesion;biological regula
26.0764	25.9583	25.8545	25.8165	1:Experimental evidence at protein level	ameboidal cell migration;anatomical structure development;anatomical structure morphogenesis;androgen metabolic process;biological regulation;biosynthetic process;carboxylic acid metabolic process;catabolic process;ce
25.8338	25.8877	25.9811	25.3492	1:Experimental evidence at protein level	biological regulation;multicellular organismal process;negative regulation of biological process;negative regulation of cellular component organization;negative regulation of cellular process;negative regulation of cytoskeleton
28.2115	28.3967	28.6264	28.5996	1:Experimental evidence at protein level	adenine biosynthetic process;adenine metabolic process;adenine salvage;alcohol metabolic process;amine metabolic process;anatomical structure development;anatomical structure morphogenesis;behavior;benzene-conta
#NUM!	#NUM!	#NUM!	26.9292	1:Experimental evidence at protein level	actin cytoskeleton organization;actin filament-based process;activation of immune response;activation of innate immune response;activation of MAPKK activity;activation of protein kinase activity;anatomical structure morpho
30.1768	30.2009	30.1507	29.9779	1:Experimental evidence at protein level	antigen processing and presentation;antigen processing and presentation of exogenous antigen;antigen processing and presentation of exogenous peptide antigen;antigen processing and presentation of exogenous peptide
29.0719	28.0707	28.117	28.2542	1:Experimental evidence at protein level	apoptosis;catabolic process;cell death;cellular catabolic process;cellular component assembly;cellular component assembly at cellular level;cellular component disassembly;cellular component disassembly at cellular level;ce
27.4048	27.6579	28.2921	28.1544	1:Experimental evidence at protein level	aerobic respiration;cellular metabolic process;cellular process;cellular respiration;electron transport chain;energy derivation by oxidation of organic compounds;generation of precursor metabolites and energy;metabolic proce
29.273	29.0857	29.4563	29.122	1:Experimental evidence at protein level	cellular macromolecule metabolic process;cellular metabolic process;cellular nitrogen compound metabolic process;cellular process;gene expression;macromolecule metabolic process;metabolic process;mRNA metabolic p
29.0173	29.0653	29.0376	29.0436	1:Experimental evidence at protein level	biological regulation;biosynthetic process;cellular biosynthetic process;cellular component assembly;cellular component assembly at cellular level;cellular component organization;cellular component organization at cellular le
26.5317	26.8442	26.918	26.8229	1:Experimental evidence at protein level	catabolic process;cellular catabolic process;cellular macromolecule catabolic process;cellular macromolecule metabolic process;cellular metabolic process;cellular process;cellular protein metabolic process;macromolecule c
25.839	26.5995	26.6193	26.7304	1:Experimental evidence at protein level	amine catabolic process;amine metabolic process;arginine catabolic process;arginine metabolic process;aromatic amino acid family catabolic process;aromatic amino acid family metabolic process;aromatic compound catab
28.3745	28.115	28.2681	28.1036	1:Experimental evidence at protein level	antigen processing and presentation;biological regulation;cellular macromolecule metabolic process;cellular metabolic process;cellular process;cellular protein metabolic process;cellular response to stimulus;establishment o
26.6636	26.6017	26.496	26.6053	1:Experimental evidence at protein level	antigen processing and presentation;biological regulation;cellular component movement;cellular localization;cellular macromolecule localization;cellular macromolecule metabolic process;cellular metabolic process;cellular pr
27.7493	27.6189	27.6911	27.5023	1:Experimental evidence at protein level	cell cycle;cell cycle process;cellular component organization;cellular component organization at cellular level;cellular component organization or biogenesis;cellular component organization or biogenesis at cellular level;cellula
25.394	#NUM!	25.855	25.2259	1:Experimental evidence at protein level	anatomical structure homeostasis;biological regulation;biosynthetic process;cell surface receptor linked signaling pathway;cellular biosynthetic process;cellular component organization;cellular component organization at cell
29.3024	29.7946	29.5801	29.6188	1:Experimental evidence at protein level	activation of immune response;activation of innate immune response;anaphase-promoting complex-dependent proteasomal ubiquitin-dependent protein catabolic process;antigen processing and presentation;antigen proces
28.2353	29.0124	29.0056	28.9769	1:Experimental evidence at protein level	catabolic process;cellular catabolic process;cellular metabolic process;cellular nitrogen compound catabolic process;cellular nitrogen compound metabolic process;cellular process;cofactor catabolic process;cofactor metab
27.2258	27.3884	27.1391	27.0751	1:Experimental evidence at protein level	biological regulation;catabolic process;cation homeostasis;cellular catabolic process;cellular cation homeostasis;cellular chemical homeostasis;cellular homeostasis;cellular ion homeostasis;cellular iron ion homeostasis;cellu
27.0708	27.6767	27.5101	27.9066	1:Experimental evidence at protein level	anatomical structure development;biological regulation;calcium ion transport;cation transport;cellular chemical homeostasis;cellular homeostasis;cellular ion homeostasis;cellular process;cellular response to stimulus;chemica
26.5139	26.5129	26.848	26.1949	1:Experimental evidence at protein level	anatomical structure homeostasis;base-excision repair;biological regulation;biosynthetic process;cell cycle;cellular biosynthetic process;cellular component organization;cellular component organization at cellular level;cellula
26.279	26.1414	26.2772	25.8673	1:Experimental evidence at protein level	anatomical structure morphogenesis;biological regulation;biosynthetic process;cell fate commitment;cell proliferation;cell surface receptor linked signaling pathway;cellular biosynthetic process;cellular developmental process
28.3272	28.9629	29.0264	29.1132	1:Experimental evidence at protein level	aging;biological regulation;cell aging;cell cycle;cell cycle arrest;cell cycle process;cellular component assembly;cellular component assembly at cellular level;cellular component organization;cellular component organization a
29.3321	29.1902	28.4182	29.2422	1:Experimental evidence at protein level	acyl-CoA metabolic process;carboxylic acid metabolic process;cellular ketone metabolic process;cellular lipid metabolic process;cellular metabolic process;cellular process;coenzyme metabolic process;cofactor metabolic p
27.1794	27.2546	27.0673	26.9481	1:Experimental evidence at protein level	anatomical structure development;anatomical structure formation involved in morphogenesis;anatomical structure morphogenesis;axon guidance;biological regulation;canonical Wnt receptor signaling pathway;canonical Wnt
29.7894	29.903	29.7794	29.8461	1:Experimental evidence at protein level	biological regulation;cellular process;cellular response to stimulus;establishment of localization;establishment of localization in cell;establishment of protein localization;intracellular protein transport;intracellular signal transdu
27.1922	27.1094	27.0472	27.0615	1:Experimental evidence at protein level	actin cytoskeleton organization;actin filament-based process;biological regulation;calcium ion homeostasis;cation homeostasis;cellular calcium ion homeostasis;cellular cation homeostasis;cellular chemical homeostasis;cellu
27.2447	27.3426	27.7376	27.6201	1:Experimental evidence at protein level	biological regulation;biosynthetic process;cellular biosynthetic process;cellular component disassembly;cellular component disassembly at cellular level;cellular component organization;cellular component organization at cell
25.3808	#NUM!	26.5232	26.3091	1:Experimental evidence at protein level	anatomical structure development;biological regulation;developmental process;hemopoiesis;hemopoietic or lymphoid organ development;organ development;positive regulation of biological process;positive regulation of cell
27.1168	27.2324	27.2119	27.0391	1:Experimental evidence at protein level	anatomical structure development;apical protein localization;asymmetric protein localization;biological regulation;brain development;cell communication;cell differentiation;cell-cell signaling;cellular component disassembly;ce
26.5051	26.1323	26.1585	26.3302	1:Experimental evidence at protein level	biological regulation;cellular process;cellular response to stimulus;regulation of biological process;regulation of cellular process;response to stimulus;signal transduction
26.2772	26.2904	26.0329	25.9222	1:Experimental evidence at protein level	cellular macromolecule metabolic process;cellular metabolic process;cellular nitrogen compound metabolic process;cellular process;macromolecule metabolic process;metabolic process;ncRNA metabolic process;ncRNA pr
27.429	27.6831	27.9812	27.9046	1:Experimental evidence at protein level	biological regulation;cellular metabolic process;cellular process;dephosphorylation;metabolic process;phosphate-containing compound metabolic process;phosphorus metabolic process;regulation of biological process;regu
28.3732	28.7564	28.2752	28.6716	1:Experimental evidence at protein level	activation of immune response;activation of innate immune response;biological regulation;cell surface receptor linked signaling pathway;cellular macromolecule metabolic process;cellular metabolic process;cellular process;c
#NUM!	26.683	26.4668	26.4558	1:Experimental evidence at protein level	alcohol metabolic process;biological regulation;chemical homeostasis;cholesterol efflux;cholesterol homeostasis;cholesterol metabolic process;cholesterol transport;establishment of localization;establishment of localization
26.4314	28.6265	28.079	29.2958	1:Experimental evidence at protein level	anatomical structure formation involved in morphogenesis;anatomical structure morphogenesis;biological regulation;calcium ion transmembrane transport;calcium ion transport;cardiac muscle tissue morphogenesis;cation tr
27.391	27.4005	27.5607	27.7626	1:Experimental evidence at protein level	aging;anatomical structure formation involved in morphogenesis;anatomical structure morphogenesis;axon guidance;biological regulation;blood coagulation;branching involved in embryonic placenta morphogenesis;cell activ
27.9268	28.3784	28.1798	28.1013	1:Experimental evidence at protein level	biological regulation;biosynthetic process;catabolic process;cell proliferation;cellular biosynthetic process;cellular catabolic process;cellular macromolecule biosynthetic process;cellular macromolecule catabolic process;cellu
#NUM!	#NUM!	#NUM!	#NUM!	1:Experimental evidence at protein level	autophagy;biological adhesion;biological regulation;catabolic process;cell adhesion;cellular catabolic process;cellular component assembly;cellular component assembly at cellular level;cellular component organization;cellul
#NUM!	26.9452	#NUM!	27.1031	1:Experimental evidence at protein level	biological regulation;cation homeostasis;cellular cation homeostasis;cellular chemical homeostasis;cellular divalent inorganic cation homeostasis;cellular homeostasis;cellular ion homeostasis;cellular process;cellular respons
30.2807	30.7985	30.94	31.0657	1:Experimental evidence at protein level	biological regulation;biosynthetic process;cellular biosynthetic process;cellular macromolecule biosynthetic process;cellular macromolecule metabolic process;cellular metabolic process;cellular nitrogen compound metabolic
27.4172	27.5196	27.2735	27.5438	1:Experimental evidence at protein level	anatomical structure development;biological regulation;biosynthetic process;cell differentiation;cellular biosynthetic process;cellular developmental process;cellular macromolecule biosynthetic process;cellular macromolecule
27.2965	27.4589	27.0219	27.2528	1:Experimental evidence at protein level	
24.277	24.1699	#NUM!	24.8448	1:Experimental evidence at protein level	anatomical structure development;biological adhesion;cell adhesion;cell differentiation;cellular component disassembly;cellular component disassembly at cellular level;cellular component organization;cellular component org
29.0723	28.827	28.7284	28.58	1:Experimental evidence at protein level	acylglycerol metabolic process;anatomical structure development;anatomical structure formation involved in morphogenesis;angiogenesis;biological regulation;blood coagulation;body fluid secretion;calcium ion homeostasis
26.5633	26.5911	26.5472	26.537	1:Experimental evidence at protein level	attachment of GPI anchor to protein;biological regulation;blood coagulation;cellular component movement;cellular macromolecule metabolic process;cellular metabolic process;cellular process;cellular protein metabolic proc
28.2691	28.0628	28.2168	27.9796	1:Experimental evidence at protein level	cellular macromolecule metabolic process;cellular metabolic process;cellular process;cellular protein metabolic process;'de novo' cotranslational protein folding;'de novo' protein folding;macromolecule metabolic process;me
25.4533	25.1749	25.4551	#NUM!	1:Experimental evidence at protein level	actin cytoskeleton organization;actin filament-based process;anatomical structure development;anatomical structure morphogenesis;biological regulation;brain development;cell surface receptor linked signaling pathway;cell
26.4944	26.0661	26.2474	26.1283	1:Experimental evidence at protein level	
27.0595	26.8974	27.0224	26.9107	1:Experimental evidence at protein level	carbohydrate metabolic process;cellular carbohydrate metabolic process;cellular macromolecule metabolic process;cellular metabolic process;cellular process;cellular protein metabolic process;glycosylation;macromolecule
26.3473	26.075	26.1539	26.2043	1:Experimental evidence at protein level	activation of immune response;activation of innate immune response;antigen processing and presentation;antigen receptor-mediated signaling pathway;B cell receptor signaling pathway;biological regulation;cell surface rece
25.7448	25.3083	25.015	25.1438	1:Experimental evidence at protein level	biological regulation;posttranscriptional regulation of gene expression;regulation of biological process;regulation of biosynthetic process;regulation of cellular biosynthetic process;regulation of cellular macromolecule biosynth
27.5498	27.1554	27.1007	27.2474	1:Experimental evidence at protein level	biological regulation;cell cycle phase;cell cycle process;cell division;cell proliferation;cellular component organization;cellular component organization at cellular level;cellular component organization or biogenesis;cellular com
27.5096	27.8222	27.8112	27.7538	1:Experimental evidence at protein level	cellular macromolecule metabolic process;cellular metabolic process;cellular nitrogen compound metabolic process;cellular process;DNA metabolic process;DNA recombination;gene expression;macromolecule metabolic pr
28.1656	27.4078	27.7585	27.4215	1:Experimental evidence at protein level	anatomical structure development;biosynthetic process;cell cycle phase;cell cycle process;cell development;cell maturation;cellular biosynthetic process;cellular component assembly;cellular component assembly at cellular
27.8073	27.6126	27.6992	27.5562	1:Experimental evidence at protein level	3'-UTR-mediated mRNA stabilization;activation of immune response;activation of innate immune response;activation of MAPK activity;aging;alcohol metabolic process;anatomical structure development;anatomical structure
26.9772	27.1328	27.4741	27.284	1:Experimental evidence at protein level	cellular component assembly;cellular component organization;cellular component organization or biogenesis;cellular macromolecule metabolic process;cellular metabolic process;cellular nitrogen compound metabolic proce
25.3464	25.2378	25.4444	25.0332	1:Experimental evidence at protein level	
25.6836	25.6757	25.0756	25.1813	1:Experimental evidence at protein level	biological regulation;biosynthetic process;carbohydrate metabolic process;carbohydrate transport;cell cycle;cell cycle process;cell surface receptor linked signaling pathway;cellular biosynthetic process;cellular component c
32.9897	30.8618	31.5045	31.9293	1:Experimental evidence at protein level	
25.5159	25.3472	25.4135	25.1748	1:Experimental evidence at protein level	biological regulation;cell cycle;cellular process;cellular response to stimulus;cellular response to stress;developmental process;negative regulation of biological process;negative regulation of cell differentiation;negative regula

25.5239	25.2982	25.2969	25.4745	1:Experimental evidence at protein level	
25.6526	25.5327	25.3183	25.7148	1:Experimental evidence at protein level	biological regulation;cell cycle checkpoint;cellular macromolecule metabolic process;cellular metabolic process;cellular nitrogen compound metabolic process;cellular process;cellular response to stimulus;cellular response to
26.6663	26.5613	#NUM!	26.7823	1:Experimental evidence at protein level	biological regulation;biosynthetic process;cellular biosynthetic process;cellular component organization;cellular component organization at cellular level;cellular component organization or biogenesis;cellular component orga
27.4191	27.055	27.5659	27.781	1:Experimental evidence at protein level	biological regulation;biosynthetic process;cellular biosynthetic process;cellular macromolecule biosynthetic process;cellular macromolecule metabolic process;cellular metabolic process;cellular nitrogen compound metaboli
26.4739	#NUM!	26.4211	26.181	1:Experimental evidence at protein level	biological regulation;biosynthetic process;carbohydrate metabolic process;carbohydrate transport;cell cycle;cell cycle process;cell surface receptor linked signaling pathway;cellular biosynthetic process;cellular component c
25.0624	25.1189	25.1863	25.256	1:Experimental evidence at protein level	biological adhesion;biological regulation;cell adhesion;cell junction assembly;cell junction organization;cell-cell adhesion;cell-cell junction organization;cellular component assembly;cellular component assembly at cellular lev
27.7043	27.1771	27.42	27.5635	1:Experimental evidence at protein level	biological regulation;cellular process;cellular response to stimulus;establishment of localization;establishment of protein localization;positive regulation of biological process;positive regulation of cell communication;positive r
25.1409	#NUM!	24.7913	24.6512	1:Experimental evidence at protein level	activation of MAPK activity;biological regulation;cell cycle process;cellular component organization;cellular component organization or biogenesis;cellular macromolecule metabolic process;cellular metabolic process;cellular
#NUM!	#NUM!	#NUM!	24.4553	1:Experimental evidence at protein level	cell differentiation;cell envelope organization;cellular component organization;cellular component organization or biogenesis;cellular developmental process;cellular process;developmental process;external encapsulating stru
26.619	26.7716	26.7266	26.9425	1:Experimental evidence at protein level	
26.4441	#NUM!	#NUM!	#NUM!	1:Experimental evidence at protein level	
26.0284	25.9402	25.7039	25.8081	1:Experimental evidence at protein level	apoptosis;apoptosis in response to endoplasmic reticulum stress;biological regulation;catabolic process;cell death;cell redox homeostasis;cellular catabolic process;cellular homeostasis;cellular macromolecule catabolic proc
26.3826	26.2535	26.2912	26.4497	1:Experimental evidence at protein level	biological regulation;cellular component movement;cellular homeostasis;cellular process;cellular response to stimulus;cytoskeleton-dependent intracellular transport;establishment of localization;establishment of localization
26.077	#NUM!	25.4323	25.2007	1:Experimental evidence at protein level	biological regulation;biosynthetic process;cellular biosynthetic process;cellular macromolecule biosynthetic process;cellular macromolecule metabolic process;cellular metabolic process;cellular nitrogen compound metaboli
25.2207	25.1136	24.9917	25.3726	1:Experimental evidence at protein level	
25.7573	24.9711	25.6631	25.3973	1:Experimental evidence at protein level	cell projection organization;cellular component organization;cellular component organization at cellular level;cellular component organization or biogenesis;cellular component organization or biogenesis at cellular level;cellula
26.3297	#NUM!	26.0302	26.1108	1:Experimental evidence at protein level	
27.2377	27.2497	27.2933	27.2701	1:Experimental evidence at protein level	biological regulation;cellular component organization;cellular component organization at cellular level;cellular component organization or biogenesis;cellular component organization or biogenesis at cellular level;cellular proce
25.4598	25.1224	24.862	25.2476	1:Experimental evidence at protein level	amine metabolic process;aminoglycan catabolic process;aminoglycan metabolic process;biological regulation;carbohydrate catabolic process;carbohydrate metabolic process;catabolic process;glycosaminoglycan catabolic
25.8477	25.8828	25.4079	25.7283	1:Experimental evidence at protein level	ATP-dependent chromatin remodeling;biological regulation;biosynthetic process;cellular biosynthetic process;cellular component disassembly;cellular component disassembly at cellular level;cellular component organization
25.7734	25.4084	#NUM!	25.7042	1:Experimental evidence at protein level	apoptosis;attachment of GPI anchor to protein;cell death;cell differentiation;cellular developmental process;cellular macromolecule metabolic process;cellular metabolic process;cellular process;cellular protein metabolic proc
27.016	26.6673	26.7414	26.5781	1:Experimental evidence at protein level	cellular process;ER to Golgi vesicle-mediated transport;establishment of localization;establishment of localization in cell;Golgi vesicle transport;intracellular transport;transport;vesicle-mediated transport
27.8384	27.6053	27.8161	27.6956	1:Experimental evidence at protein level	
27.2243	27.3487	27.168	27.426	1:Experimental evidence at protein level	cellular macromolecule metabolic process;cellular metabolic process;cellular nitrogen compound metabolic process;cellular process;gene expression;macromolecule metabolic process;metabolic process;mRNA metabolic p
25.8216	24.6554	25.8194	25.2941	1:Experimental evidence at protein level	biosynthetic process;cellular biosynthetic process;cellular component disassembly;cellular component disassembly at cellular level;cellular component organization;cellular component organization at cellular level;cellular co
26.4071	25.9841	26.1593	25.795	1:Experimental evidence at protein level	biological regulation;biosynthetic process;cellular biosynthetic process;cellular component disassembly;cellular component disassembly at cellular level;cellular component organization;cellular component organization at cel
26.1498	26.2188	26.3053	26.1765	1:Experimental evidence at protein level	alternative nuclear mRNA splicing, via spliceosome;cellular macromolecule metabolic process;cellular metabolic process;cellular nitrogen compound metabolic process;cellular process;macromolecule metabolic process;me
26.0601	27.1343	28.0993	26.197	1:Experimental evidence at protein level	cell cycle phase;cell cycle process;cellular component assembly;cellular component assembly at cellular level;cellular component organization;cellular component organization at cellular level;cellular component organization
26.5349	26.3597	26.5994	26.4513	1:Experimental evidence at protein level	base-excision repair;biological regulation;catabolic process;cellular catabolic process;cellular macromolecule catabolic process;cellular macromolecule metabolic process;cellular metabolic process;cellular nitrogen compou
27.4599	27.8462	27.5842	27.8562	1:Experimental evidence at protein level	biological regulation;cell cycle phase;cell cycle process;cellular component organization;cellular component organization at cellular level;cellular component organization or biogenesis;cellular component organization or bioge
26.632	26.7768	26.9991	26.7934	1:Experimental evidence at protein level	cell differentiation;cellular developmental process;cellular process;developmental process;establishment of localization;establishment of protein localization;gamete generation;gene expression;macromolecule metabolic proc
27.0439	26.855	26.7475	26.3891	1:Experimental evidence at protein level	catabolic process;cell cycle;cell division;cell proliferation;cellular catabolic process;cellular component organization;cellular component organization at cellular level;cellular component organization or biogenesis;cellular comp
26.0712	26.2938	25.7767	26.0883	1:Experimental evidence at protein level	amine biosynthetic process;amine metabolic process;amino acid salvage;aspartate family amino acid biosynthetic process;aspartate family amino acid metabolic process;biosynthetic process;carboxylic acid biosynthetic pro
26.414	26.6752	26.6227	26.6525	1:Experimental evidence at protein level	
27.8591	#NUM!	#NUM!	#NUM!	1:Experimental evidence at protein level	biological regulation;positive regulation of catalytic activity;positive regulation of GTPase activity;positive regulation of hydrolase activity;positive regulation of molecular function;regulation of biological process;regulation of ca
25.4187	25.5463	#NUM!	25.7906	1:Experimental evidence at protein level	biosynthetic process;cellular biosynthetic process;cellular component disassembly;cellular component disassembly at cellular level;cellular component organization;cellular component organization at cellular level;cellular co
26.2772	25.7894	25.9337	25.8821	1:Experimental evidence at protein level	biological regulation;biosynthetic process;cellular biosynthetic process;cellular component assembly;cellular component assembly at cellular level;cellular component organization;cellular component organization at cellular le
26.8196	26.7496	26.5805	26.5555	1:Experimental evidence at protein level	actin cytoskeleton organization;actin filament-based process;actomyosin structure organization;anion transport;antigen processing and presentation;antigen processing and presentation of exogenous antigen;antigen process
26.5401	26.2477	26.0959	26.2444	1:Experimental evidence at protein level	asymmetric Golgi ribbon formation;biological adhesion;biological regulation;biosynthetic process;cell adhesion;cell adhesion molecule production;cell migration;cell motility;cell projection assembly;cell projection organization
25.4531	24.7214	25.0626	24.8648	1:Experimental evidence at protein level	cellular macromolecule metabolic process;cellular metabolic process;cellular nitrogen compound metabolic process;cellular process;establishment of localization;establishment of localization in cell;establishment of RNA loca
25.5507	25.0596	25.4374	25.3527	1:Experimental evidence at protein level	cellular macromolecule metabolic process;cellular metabolic process;cellular nitrogen compound metabolic process;cellular process;macromolecule metabolic process;metabolic process;nitrogen compound metabolic proce
26.6085	26.7466	26.9928	26.5087	1:Experimental evidence at protein level	apical protein localization;asymmetric protein localization;biological regulation;cellular component assembly;cellular component organization;cellular component organization at cellular level;cellular component organization or
29.9171	29.9743	29.664	29.9283	1:Experimental evidence at protein level	anatomical structure formation involved in morphogenesis;anatomical structure morphogenesis;angiogenesis;apoptosis;axonal fasciculation;biological regulation;cardiac epithelial to mesenchymal transition;cell death;cell mig
24.6307	24.6772	24.7856	24.4949	1:Experimental evidence at protein level	biological regulation;cell activation;cellular component movement;cellular process;cellular response to stimulus;establishment of localization;establishment of localization in cell;establishment of protein localization;immune sy
28.1476	28.3895	28.1497	28.1971	1:Experimental evidence at protein level	anatomical structure development;biological regulation;biosynthetic process;cell development;cellular biosynthetic process;cellular developmental process;cellular macromolecule biosynthetic process;cellular macromolecule
#NUM!	#NUM!	#NUM!	#NUM!	2:Experimental evidence at transcript level	developmental process;multicellular organismal development;multicellular organismal process
25.3257	#NUM!	#NUM!	25.0826	1:Experimental evidence at protein level	biological regulation;cell surface receptor linked signaling pathway;cellular process;cellular response to stimulus;establishment of localization;establishment of localization in cell;establishment of protein localization;glutamate
26.8734	26.6319	26.7135	26.6095	1:Experimental evidence at protein level	biological regulation;biosynthetic process;cellular biosynthetic process;cellular macromolecule biosynthetic process;cellular macromolecule metabolic process;cellular metabolic process;cellular nitrogen compound metaboli
28.2362	28.3853	28.0085	28.323	1:Experimental evidence at protein level	
25.8456	26.0445	25.4143	25.6968	1:Experimental evidence at protein level	cellular macromolecule metabolic process;cellular metabolic process;cellular nitrogen compound metabolic process;cellular process;macromolecule metabolic process;metabolic process;mRNA metabolic process;mRNA pro
26.4124	25.8199	25.9946	26.1379	1:Experimental evidence at protein level	biological regulation;cellular component organization;cellular component organization at cellular level;cellular component organization or biogenesis;cellular component organization or biogenesis at cellular level;cellular locali
25.4307	24.9603	25.2409	25.0882	1:Experimental evidence at protein level	aggresome assembly;autophagy;biological regulation;biosynthetic process;catabolic process;cell communication;cellular biosynthetic process;cellular catabolic process;cellular component assembly;cellular component asse
26.8884	26.72	26.7084	26.4653	1:Experimental evidence at protein level	aging;anatomical structure development;biological regulation;cellular macromolecule metabolic process;cellular metabolic process;cellular process;cellular protein metabolic process;cellular response to chemical stimulus;ce
26.848	26.6552	27.0119	26.37	1:Experimental evidence at protein level	alcohol metabolic process;biosynthetic process;lipid biosynthetic process;lipid metabolic process;metabolic process;primary metabolic process;small molecule metabolic process;steroid biosynthetic process;steroid metabo
27.1645	27.3884	27.3277	27.2933	1:Experimental evidence at protein level	alcohol catabolic process;alcohol metabolic process;amino sugar catabolic process;amino sugar metabolic process;carbohydrate catabolic process;carbohydrate metabolic process;carbohydrate phosphorylation;carboxylic
26.351	28.609	28.3349	28.9684	1:Experimental evidence at protein level	developmental process
26.8984	27.3231	27.3822	27.4196	1:Experimental evidence at protein level	biological regulation;biosynthetic process;cap-independent translational initiation;cell cycle;cellular biosynthetic process;cellular component assembly;cellular component assembly at cellular level;cellular component disasse
26.0482	#NUM!	25.2846	24.9932	1:Experimental evidence at protein level	cellular macromolecule metabolic process;cellular metabolic process;cellular nitrogen compound metabolic process;cellular process;macromolecule metabolic process;maturation of SSU-rRNA;maturation of SSU-rRNA from
25.8336	#NUM!	25.7555	25.6574	1:Experimental evidence at protein level	alcohol biosynthetic process;alcohol metabolic process;amino sugar biosynthetic process;amino sugar metabolic process;biological adhesion;biosynthetic process;carbohydrate biosynthetic process;carbohydrate metabolic
28.3904	28.3917	28.4637	28.5037	1:Experimental evidence at protein level	biological regulation;cell cycle phase;cell cycle process;cellular component organization;cellular component organization at cellular level;cellular component organization or biogenesis;cellular component organization or bioge
27.8064	27.4816	27.8002	27.5511	1:Experimental evidence at protein level	biological regulation;biosynthetic process;cellular biosynthetic process;cellular macromolecule biosynthetic process;cellular macromolecule metabolic process;cellular metabolic process;cellular process;cellular protein metab
27.4378	27.7947	27.4316	27.6819	1:Experimental evidence at protein level	biological regulation;cell cycle cytokinesis;cell cycle phase;cell cycle process;cellular component assembly;cellular component assembly at cellular level;cellular component organization;cellular component organization at cel
27.5275	27.1489	27.2219	27.2506	1:Experimental evidence at protein level	cellular localization;cellular macromolecule localization;cellular process;cellular protein localization;localization;macromolecule localization;protein localization;protein localization in mitochondrion;protein localization to organe
28.9832	28.9132	28.9099	28.8685	1:Experimental evidence at protein level	biological regulation;biosynthetic process;cellular biosynthetic process;cellular component assembly;cellular component assembly at cellular level;cellular component organization;cellular component organization at cellular le
25.858	25.4304	25.4284	25.3558	1:Experimental evidence at protein level	biological regulation;carboxylic acid metabolic process;cellular ketone metabolic process;cellular metabolic process;cellular process;metabolic process;monocarboxylic acid metabolic process;organic acid metabolic process
27.0031	26.9769	27.1452	26.8442	1:Experimental evidence at protein level	establishment of localization;transport
25.5149	25.1627	25.3891	25.0368	1:Experimental evidence at protein level	cellular component organization;cellular component organization at cellular level;cellular component organization or biogenesis;cellular component organization or biogenesis at cellular level;cellular macromolecule metabolic
28.9676	28.4889	28.7136	28.6358	1:Experimental evidence at protein level	activation of immune response;activation of innate immune response;anaphase-promoting complex-dependent proteasomal ubiquitin-dependent protein catabolic process;antigen processing and presentation;antigen proces
27.1492	27.3332	26.8026	27.2735	1:Experimental evidence at protein level	biological regulation;cell surface receptor linked signaling pathway;cellular process;cellular response to chemical stimulus;cellular response to cytokine stimulus;cellular response to organic substance;cellular response to stim
28.8466	28.588	28.8258	28.6232	1:Experimental evidence at protein level	cellular component organization;cellular component organization at cellular level;cellular component organization or biogenesis;cellular component organization or biogenesis at cellular level;cellular process;COPI coating of G
29.6658	30.1992	29.7916	30.1651	1:Experimental evidence at protein level	axon guidance;biological regulation;cell surface receptor linked signaling pathway;cellular process;cellular response to stimulus;chemotaxis;enzyme linked receptor protein signaling pathway;ephrin receptor signaling pathwa
27.0127	27.1898	27.3746	27.3889	1:Experimental evidence at protein level	cellular component assembly;cellular component assembly at cellular level;cellular component organization;cellular component organization at cellular level;cellular component organization or biogenesis;cellular component c
27.2492	26.913	26.7558	26.8842	1:Experimental evidence at protein level	alcohol biosynthetic process;alcohol metabolic process;amine metabolic process;biosynthetic process;cellular biosynthetic process;cellular lipid metabolic process;cellular metabolic process;cellular nitrogen compound biosy
28.4099	28.4108	28.1719	28.1973	1:Experimental evidence at protein level	anion transport;biological regulation;blood coagulation;carboxylic acid metabolic process;carboxylic acid transport;cell migration;cell motility;cellular component movement;cellular ketone metabolic process;cellular metaboli
24.8397	25.8122	25.3589	24.839	1:Experimental evidence at protein level	biosynthetic process;cellular biosynthetic process;cellular macromolecule biosynthetic process;cellular macromolecule metabolic process;cellular metabolic process;cellular nitrogen compound metabolic process;cellular pro
29.3132	29.4672	29.5034	29.6582	1:Experimental evidence at protein level	biological regulation;cell redox homeostasis;cellular homeostasis;cellular macromolecule metabolic process;cellular metabolic process;cellular process;cellular protein metabolic process;cellular response to chemical stimulus
28.0768	27.975	28.5042	28.1844	1:Experimental evidence at protein level	biological regulation;biosynthetic process;cation transport;cellular biosynthetic process;cellular component organization;cellular component organization at cellular level;cellular component organization or biogenesis;cellular c
28.0045	28.1784	28.0893	27.8432	1:Experimental evidence at protein level	biological regulation;establishment of localization;establishment of localization in cell;establishment of protein localization;intracellular protein transport;intracellular transport;maternal process involved in female pregnancy;mu
26.8964	26.8776	26.8563	26.779	1:Experimental evidence at protein level	biological regulation;biosynthetic process;cell cycle process;cell proliferation;cellular biosynthetic process;cellular component organization;cellular component organization at cellular level;cellular component organization or b
27.8687	27.9536	28.2481	28.3568	1:Experimental evidence at protein level	actin cytoskeleton organization;actin filament-based process;anatomical structure development;B cell activation;biological regulation;cell activation;cell cycle;cellular component organization;cellular component organization a
28.8412	29.1164	28.6905	29.0258	1:Experimental evidence at protein level	amine biosynthetic process;amine metabolic process;apoptotic mitochondrial changes;biosynthetic process;carboxylic acid biosynthetic process;carboxylic acid metabolic process;cellular amine metabolic process;cellular a
27.6986	27.5169	27.7351	27.6782	1:Experimental evidence at protein level	biological regulation;cell cycle checkpoint;cellular process;cellular response to stimulus;cellular response to stress;DNA damage checkpoint;DNA integrity checkpoint;negative regulation of catalytic activity;negative regulation
25.8305	26.0479	25.8249	25.7689	1:Experimental evidence at protein level	anatomical structure morphogenesis;biological regulation;biosynthetic process;cell morphogenesis;cellular biosynthetic process;cellular component morphogenesis;cellular component organization;cellular component organi
25.9235	25.4035	#NUM!	25.6125	1:Experimental evidence at protein level	biological regulation;biosynthetic process;cellular biosynthetic process;cellular macromolecule biosynthetic process;cellular macromolecule metabolic process;cellular metabolic process;cellular nitrogen compound metaboli
27.4667	27.5229	27.3634	27.4038	1:Experimental evidence at protein level	biological regulation;cellular macromolecule metabolic process;cellular metabolic process;cellular nitrogen compound metabolic process;cellular process;macromolecule metabolic process;metabolic process;mRNA metabol
28.073	28.0648	28.2117	28.0227	1:Experimental evidence at protein level	cellular macromolecule metabolic process;cellular metabolic process;cellular process;cellular protein metabolic process;chaperone-mediated protein folding;macromolecule metabolic process;macromolecule modification;me
27.0149	26.9135	#NUM!	#NUM!	1:Experimental evidence at protein level	biological regulation;cellular component organization;cellular component organization at cellular level;cellular component organization or biogenesis;cellular component organization or biogenesis at cellular level;cellular macr
27.0116	27.0348	27.0585	26.9885	1:Experimental evidence at protein level	antigen processing and presentation;antigen processing and presentation of exogenous antigen;antigen processing and presentation of exogenous peptide antigen;antigen processing and presentation of exogenous peptide
26.7915	26.702	27.0158	26.9461	1:Experimental evidence at protein level	autophagic vacuole fusion;cellular component assembly;cellular component assembly at cellular level;cellular component organization;cellular component organization at cellular level;cellular component organization or bioge
30.1811	30.2521	30.4379	30.3584	1:Experimental evidence at protein level	establishment of localization;fat-soluble vitamin metabolic process;lipid metabolic process;metabolic process;primary metabolic process;small molecule metabolic process;steroid metabolic process;transport;vitamin D meta
28.2642	28.0217	28.1691	27.9666	1:Experimental evidence at protein level	anatomical structure development;biological regulation;biosynthetic process;catabolic process;cell cycle process;cell surface receptor linked signaling pathway;cellular biosynthetic process;cellular catabolic process;cellular
26.5055	26.6961	26.584	26.6207	1:Experimental evidence at protein level	amine metabolic process;aminoglycan catabolic process;aminoglycan metabolic process;carbohydrate catabolic process;carbohydrate metabolic process;catabolic process;cellular catabolic process;cellular lipid metabolic p
30.3624	31.2627	31.7513	31.6501	1:Experimental evidence at protein level	activation of protein kinase activity;activation of protein kinase B activity;biological regulation;biosynthetic process;cell communication;cell proliferation;cell redox homeostasis;cell-cell signaling;cellular biosynthetic process;c
27.9647	#NUM!	#NUM!	27.8385	1:Experimental evidence at protein level	aging;base-excision repair;biological regulation;biosynthetic process;cell aging;cellular biosynthetic process;cellular component assembly;cellular component assembly at cellular level;cellular component disassembly;cellular
28.0794	28.2423	28.2079	27.9132	1:Experimental evidence at protein level	apoptosis;axon guidance;biological regulation;biosynthetic process;cell cycle;cell cycle checkpoint;cell death;cell surface receptor linked signaling pathway;cellular biosynthetic process;cellular macromolecule biosynthetic p
26.5945	27.1922	27.1215	27.2159	1:Experimental evidence at protein level	anatomical structure morphogenesis;anterior/posterior pattern specification;biological regulation;camera-type eye morphogenesis;cell differentiation;cellular component organization;cellular component organization at cellula
27.4662	27.5113	27.5838	27.7203	1:Experimental evidence at protein level	biological regulation;cellular macromolecule metabolic process;cellular metabolic process;cellular process;cellular protein metabolic process;cellular response to heat;cellular response to stimulus;cellular response to stress;c
28.782	28.4384	28.8024	28.9182	1:Experimental evidence at protein level	anatomical structure morphogenesis;biological regulation;cell morphogenesis;cell morphogenesis involved in differentiation;cellular component morphogenesis;cellular component organization;cellular component organizatio
#NUM!	#NUM!	25.9674	25.6569	1:Experimental evidence at protein level	biological regulation;cell activation;cell migration;cell motility;cellular component movement;cellular process;endothelial cell migration;entry into cell of other organism involved in symbiotic interaction;entry into host;entry into
26.3663	25.9797	26.352	26.1194	1:Experimental evidence at protein level	activation-induced cell death of T cells;aging;alcohol metabolic process;amine metabolic process;aminoglycan metabolic process;anatomical structure development;apoptosis;apoptotic mitochondrial changes;biological regu
29.0072	29.9059	29.7582	29.7966	1:Experimental evidence at protein level	2'-deoxyribonucleotide biosynthetic process;2'-deoxyribonucleotide metabolic process;biosynthetic process;catabolic process;cellular aromatic compound metabolic process;cellular biosynthetic process;cellular catabolic p
30.3621	29.6737	30.1142	30.0513	1:Experimental evidence at protein level	alpha-beta T cell activation;alpha-beta T cell differentiation;biosynthetic process;catabolic process;cell activation;cell differentiation;cellular biosynthetic process;cellular catabolic process;cellular component disassembly;cell
27.6898	27.3095	27.1798	27.2758	1:Experimental evidence at protein level	biological regulation;cellular macromolecule metabolic process;cellular metabolic process;cellular nitrogen compound metabolic process;cellular process;macromolecule metabolic process;metabolic process;mRNA metabol
30.249	30.1465	30.4194	30.2109	1:Experimental evidence at protein level	biosynthetic process;catabolic process;cellular biosynthetic process;cellular catabolic process;cellular component disassembly;cellular component disassembly at cellular level;cellular component organization;cellular compo
27.3579	27.1801	26.972	26.9503	1:Experimental evidence at protein level	anatomical structure formation involved in morphogenesis;anatomical structure morphogenesis;angiogenesis;angiogenesis involved in wound healing;biological adhesion;biological regulation;cell adhesion;cellular process;de
28.0442	27.7626	28.1211	28.1219	1:Experimental evidence at protein level	cellular metabolic process;cellular process;electron transport chain;generation of precursor metabolites and energy;metabolic process;oxidation-reduction process;respiratory electron transport chain;response to antibiotic;re
25.0494	#NUM!	25.3814	25.1505	1:Experimental evidence at protein level	biosynthetic process;carbohydrate metabolic process;carboxylic acid metabolic process;cellular biosynthetic process;cellular component assembly;cellular component organization;cellular component organization or biogen
29.1751	#NUM!	29.0485	29.5054	1:Experimental evidence at protein level	biological adhesion;cell activation;cell adhesion;cell junction assembly;cell junction organization;cell migration;cell motility;cell proliferation;cell-substrate junction assembly;cellular component assembly;cellular component as
26.0291	26.1248	26.6024	26.5668	1:Experimental evidence at protein level	aging;amine biosynthetic process;amine metabolic process;anatomical structure development;biosynthetic process;carboxylic acid biosynthetic process;carboxylic acid metabolic process;cellular amine metabolic process;ce
26.2803	26.331	26.0906	26.2643	1:Experimental evidence at protein level	biosynthetic process;cellular biosynthetic process;cellular component disassembly;cellular component disassembly at cellular level;cellular component organization;cellular component organization at cellular level;cellular co
27.1281	26.765	26.8755	26.8354	1:Experimental evidence at protein level	biological regulation;negative regulation of biological process;negative regulation of biosynthetic process;negative regulation of cellular biosynthetic process;negative regulation of cellular macromolecule biosynthetic process
28.4097	28.2064	28.2233	28.0175	1:Experimental evidence at protein level	activation of immune response;activation of innate immune response;anaphase-promoting complex-dependent proteasomal ubiquitin-dependent protein catabolic process;antigen processing and presentation;antigen proces
27.4198	28.0383	27.5769	27.6968	1:Experimental evidence at protein level	apoptosis;biological regulation;catabolic process;cell death;cell surface receptor linked signaling pathway;cellular catabolic process;cellular component disassembly;cellular component disassembly at cellular level;cellular co
27.6931	27.6415	27.9135	27.862	1:Experimental evidence at protein level	activation of caspase activity;adaptive immune response;adaptive immune response based on somatic recombination of immune receptors built from immunoglobulin superfamily domains;aging;biological regulation;cell killin
28.5385	27.8482	27.1927	27.4321	1:Experimental evidence at protein level	biological regulation;cellular component assembly;cellular component assembly at cellular level;cellular component organization;cellular component organization at cellular level;cellular component organization or biogenesis;
28.015	27.9552	27.9082	27.8627	1:Experimental evidence at protein level	biological regulation;cell cycle;cellular component organization;cellular component organization at cellular level;cellular component organization or biogenesis;cellular component organization or biogenesis at cellular level;cell
#NUM!	#NUM!	#NUM!	#NUM!	1:Experimental evidence at protein level	anatomical structure development;biological regulation;blood coagulation;cell activation;cell development;cell proliferation;cellular developmental process;cellular process;cellular response to cAMP;cellular response to chem
29.1054	28.965	28.8882	28.8836	1:Experimental evidence at protein level	biological regulation;biosynthetic process;catabolic process;cellular biosynthetic process;cellular catabolic process;cellular macromolecule biosynthetic process;cellular macromolecule catabolic process;cellular macromolec
29.1623	28.8589	28.917	28.9255	1:Experimental evidence at protein level	antigen processing and presentation;antigen processing and presentation of exogenous antigen;antigen processing and presentation of exogenous peptide antigen;antigen processing and presentation of exogenous peptide
29.8654	29.8332	30.0869	29.8224	1:Experimental evidence at protein level	biosynthetic process;cellular biosynthetic process;cellular component assembly;cellular component assembly at cellular level;cellular component organization;cellular component organization at cellular level;cellular compone
29.7124	30.1605	29.6769	29.79	1:Experimental evidence at protein level	actin cytoskeleton organization;actin filament organization;actin filament polymerization;actin filament-based process;actin polymerization or depolymerization;anatomical structure arrangement;anatomical structure homeost
28.5463	28.5682	28.6205	28.4432	1:Experimental evidence at protein level	biological regulation;biosynthetic process;cellular biosynthetic process;cellular macromolecule biosynthetic process;cellular macromolecule metabolic process;cellular metabolic process;cellular process;cellular protein metab
27.5295	27.7474	27.9162	27.8682	1:Experimental evidence at protein level	biological regulation;biosynthetic process;carbohydrate metabolic process;carbohydrate transport;cell cycle;cell cycle process;cell surface receptor linked signaling pathway;cellular biosynthetic process;cellular component c
27.5827	27.7919	27.9632	27.6652	1:Experimental evidence at protein level	activation of JUN kinase activity;activation of MAPK activity;anatomical structure development;anatomical structure morphogenesis;apoptosis;biological regulation;cell death;cell differentiation;cell morphogenesis;cell morpho
28.2399	28.6847	28.7499	28.6935	1:Experimental evidence at protein level	cellular process;establishment of localization;establishment of localization in cell;exocytosis;secretion;secretion by cell;transport;vesicle-mediated transport
26.2545	25.9903	26.0192	25.8614	1:Experimental evidence at protein level	biosynthetic process;cellular biosynthetic process;cellular macromolecule biosynthetic process;cellular macromolecule metabolic process;cellular metabolic process;cellular nitrogen compound metabolic process;cellular pro
26.9609	26.7881	26.7635	26.8231	1:Experimental evidence at protein level	amine metabolic process;biosynthetic process;carboxylic acid metabolic process;cellular amine metabolic process;cellular amino acid metabolic process;cellular biosynthetic process;cellular ketone metabolic process;cellula
28.1965	27.8402	27.8039	27.7221	1:Experimental evidence at protein level	amine metabolic process;amino acid activation;apoptosis;biological regulation;carboxylic acid metabolic process;cell death;cell differentiation;cellular amine metabolic process;cellular amino acid metabolic process;cellular d
27.81	27.894	27.6901	27.6523	1:Experimental evidence at protein level	biological regulation;biosynthetic process;cellular biosynthetic process;cellular component assembly;cellular component assembly at cellular level;cellular component organization;cellular component organization at cellular le

26.9485	27.0278	26.853	27.0663	1:Experimental evidence at protein level	biological regulation;cell differentiation;cellular component organization;cellular component organization at cellular level;cellular component organization or biogenesis;cellular component organization or biogenesis at cellular
27.2868	27.1116	27.1353	27.0575	1:Experimental evidence at protein level	activation of MAPKK activity;activation of protein kinase activity;biological adhesion;biological regulation;cell activation;cell adhesion;cell adhesion mediated by integrin;cell differentiation;cell surface receptor linked signaling
29.1646	28.9893	29.3712	29.1846	1:Experimental evidence at protein level	autophagy;biological regulation;catabolic process;cell communication;cell differentiation;cell surface receptor linked signaling pathway;cellular catabolic process;cellular component assembly;cellular component organization;
26.6283	26.2185	26.2876	26.3241	1:Experimental evidence at protein level	activation of caspase activity;activation of immune response;activation of innate immune response;activation of JUN kinase activity;activation of MAPK activity;apoptosis;biological regulation;catabolic process;cell death;cell
26.7266	26.2146	26.3771	26.4282	1:Experimental evidence at protein level	biological adhesion;cell adhesion;cell differentiation;cell proliferation;cellular developmental process;cellular macromolecule metabolic process;cellular metabolic process;cellular nitrogen compound metabolic process;cellula
26.6304	#NUM!	24.795	25.8147	1:Experimental evidence at protein level	anatomical structure development;biological adhesion;biological regulation;cell adhesion;cellular component assembly;cellular component assembly at cellular level;cellular component organization;cellular component organi
26.6081	26.6187	26.4601	26.3835	1:Experimental evidence at protein level	alcohol catabolic process;alcohol metabolic process;carbohydrate catabolic process;carbohydrate metabolic process;catabolic process;cellular carbohydrate catabolic process;cellular carbohydrate metabolic process;cellula
26.7815	26.5111	26.7719	26.5455	1:Experimental evidence at protein level	biological regulation;cellular component organization;cellular component organization or biogenesis;cellular process;establishment of localization;establishment of localization in cell;Golgi vesicle transport;intracellular transpo
27.0446	27.3708	27.2375	26.956	1:Experimental evidence at protein level	alcohol metabolic process;amyloid precursor protein catabolic process;amyloid precursor protein metabolic process;anatomical structure development;apoptosis;biological regulation;biosynthetic process;catabolic process;
28.2737	27.6717	28.0941	28.0426	1:Experimental evidence at protein level	cellular component assembly;cellular component assembly at cellular level;cellular component organization;cellular component organization at cellular level;cellular component organization or biogenesis;cellular component c
28.8436	30.0912	29.4867	29.8222	1:Experimental evidence at protein level	biological regulation;catabolic process;cell proliferation;cellular catabolic process;cellular macromolecule catabolic process;cellular macromolecule metabolic process;cellular metabolic process;cellular nitrogen compound m
27.875	27.8992	27.5477	27.8757	1:Experimental evidence at protein level	ameboidal cell migration;apical junction assembly;apoptosis;biological adhesion;biological regulation;biosynthetic process;cell adhesion;cell cycle;cell death;cell division;cell junction assembly;cell junction organization;cell m
27.9251	28.0274	28.541	28.3956	1:Experimental evidence at protein level	cell differentiation;cellular developmental process;cellular process;developmental process;multicellular organismal development;multicellular organismal process
27.2034	26.8649	26.9727	27.0781	1:Experimental evidence at protein level	
25.9217	26.0378	25.6105	25.8267	1:Experimental evidence at protein level	aging;apoptosis;biological regulation;cell aging;cell death;cellular process;death;developmental process;negative regulation of biological process;negative regulation of biosynthetic process;negative regulation of catalytic act
#NUM!	#NUM!	#NUM!	#NUM!	1:Experimental evidence at protein level	biological adhesion;cell adhesion;cellular process
27.3812	27.1609	27.4434	27.5701	1:Experimental evidence at protein level	anatomical structure development;apoptosis;biological regulation;biosynthetic process;cell death;cellular biosynthetic process;cellular metabolic process;cellular process;cofactor biosynthetic process;cofactor metabolic pro
25.1049	#NUM!	25.3017	25.303	1:Experimental evidence at protein level	
25.7172	#NUM!	26.042	26.5127	1:Experimental evidence at protein level	axon extension;biological adhesion;biological regulation;cell adhesion;cell growth;cell-matrix adhesion;cell-substrate adhesion;cellular process;cellular response to stimulus;developmental cell growth;developmental growth;d
25.8313	25.6573	25.7503	25.5514	1:Experimental evidence at protein level	biological regulation;carboxylic acid metabolic process;cellular ketone metabolic process;cellular metabolic process;cellular process;metabolic process;monocarboxylic acid metabolic process;organic acid metabolic process
27.7837	27.5818	27.6856	27.4369	1:Experimental evidence at protein level	cellular component assembly;cellular component organization;cellular component organization or biogenesis;cellular metabolic process;cellular nitrogen compound metabolic process;cellular process;coenzyme metabolic pro
25.4349	24.361	24.5106	24.8101	1:Experimental evidence at protein level	cellular macromolecule metabolic process;cellular metabolic process;cellular nitrogen compound metabolic process;cellular process;establishment of localization;establishment of localization in cell;establishment of RNA loca
26.6788	26.1864	26.4011	25.8826	1:Experimental evidence at protein level	adaptation of rhodopsin mediated signaling;adaptation of signaling pathway;apocarotenoid metabolic process;biological regulation;cellular aldehyde metabolic process;cellular hormone metabolic process;cellular lipid metab
26.8169	25.7855	#NUM!	26.2046	1:Experimental evidence at protein level	biological regulation;biosynthetic process;cellular biosynthetic process;cellular macromolecule biosynthetic process;cellular macromolecule metabolic process;cellular metabolic process;cellular nitrogen compound metabolic
28.0343	28.2319	28.2896	28.1701	1:Experimental evidence at protein level	behavior;behavioral interaction between organisms;multi-organism process;response to stimulus;social behavior
25.947	#NUM!	26.164	25.6881	1:Experimental evidence at protein level	biological regulation;cell cycle;cellular component assembly;cellular component assembly at cellular level;cellular component organization;cellular component organization at cellular level;cellular component organization or bi
26.9714	26.7992	27.0572	27.739	1:Experimental evidence at protein level	anatomical structure development;ATP-dependent chromatin remodeling;biological regulation;cellular component disassembly;cellular component disassembly at cellular level;cellular component organization;cellular compon
26.2893	26.1444	25.7703	26.0095	1:Experimental evidence at protein level	cell cycle;cell cycle process;cellular component organization;cellular component organization at cellular level;cellular component organization or biogenesis;cellular component organization or biogenesis at cellular level;cellula
26.802	27.2391	26.9974	27.0001	1:Experimental evidence at protein level	biological regulation;biosynthetic process;cell differentiation;cell proliferation;cellular biosynthetic process;cellular developmental process;cellular macromolecule biosynthetic process;cellular macromolecule metabolic proces
27.2671	27.2888	27.1768	27.0042	1:Experimental evidence at protein level	antigen processing and presentation;antigen processing and presentation of exogenous antigen;antigen processing and presentation of exogenous peptide antigen;antigen processing and presentation of exogenous peptide
28.7111	28.787	29.1331	28.8249	1:Experimental evidence at protein level	biological regulation;cell surface receptor linked signaling pathway;cellular process;cellular response to chemical stimulus;cellular response to cytokine stimulus;cellular response to organic substance;cellular response to stim
29.5806	29.6238	29.6078	29.6096	1:Experimental evidence at protein level	biological regulation;cellular component organization;cellular component organization at cellular level;cellular component organization or biogenesis;cellular component organization or biogenesis at cellular level;cellular proce
26.5638	26.2318	26.2555	26.1868	1:Experimental evidence at protein level	biological regulation;cation homeostasis;chemical homeostasis;homeostatic process;ion homeostasis;iron ion homeostasis;regulation of biological quality
26.401	26.7885	#NUM!	26.8817	1:Experimental evidence at protein level	
25.7972	#NUM!	25.6407	25.481	1:Experimental evidence at protein level	biological regulation;biosynthetic process;cellular biosynthetic process;cellular macromolecule biosynthetic process;cellular macromolecule metabolic process;cellular metabolic process;cellular nitrogen compound metabolic
26.1707	25.6053	25.9256	25.8208	1:Experimental evidence at protein level	
27.3903	27.3077	27.3341	27.3932	1:Experimental evidence at protein level	cellular metabolic process;cellular process;metabolic process;phosphate-containing compound metabolic process;phosphorus metabolic process;phosphorylation
25.1453	25.0391	25.2598	25.4677	1:Experimental evidence at protein level	amine catabolic process;amine metabolic process;biotin metabolic process;branched chain family amino acid catabolic process;branched chain family amino acid metabolic process;carboxylic acid catabolic process;carbox
27.4419	27.395	27.3373	27.4378	1:Experimental evidence at protein level	biological regulation;cellular process;establishment of localization;establishment of localization in cell;establishment of protein localization;intracellular protein transport;intracellular transport;positive regulation of catalytic acti
24.9449	24.9907	25.0016	24.8758	1:Experimental evidence at protein level	amine metabolic process;amino acid activation;carboxylic acid metabolic process;cellular amine metabolic process;cellular amino acid metabolic process;cellular ketone metabolic process;cellular macromolecule metabolic p
26.8475	27.3845	27.5912	27.6084	1:Experimental evidence at protein level	biological regulation;cellular component assembly;cellular component assembly at cellular level;cellular component organization;cellular component organization at cellular level;cellular component organization or biogenesis;
26.3156	26.165	26.315	26.3139	1:Experimental evidence at protein level	biological regulation;biosynthetic process;cellular biosynthetic process;cellular macromolecule biosynthetic process;cellular macromolecule metabolic process;cellular metabolic process;cellular nitrogen compound metabolic
27.5751	27.8155	27.7569	27.9742	1:Experimental evidence at protein level	alternative nuclear mRNA splicing, via spliceosome;anatomical structure development;anatomical structure morphogenesis;appendage morphogenesis;biological regulation;cell differentiation;cellular component assembly;ce
28.3134	27.9024	27.8032	27.8027	1:Experimental evidence at protein level	actin cytoskeleton organization;actin cytoskeleton reorganization;actin filament-based process;actin-mediated cell contraction;anatomical structure formation involved in morphogenesis;anatomical structure morphogenesis;a
24.159	23.3777	23.9881	23.8667	1:Experimental evidence at protein level	
27.3259	27.1783	27.0987	27.0869	1:Experimental evidence at protein level	anatomical structure development;cell projection organization;cellular component organization;cellular component organization at cellular level;cellular component organization or biogenesis;cellular component organization o
28.2825	28.6545	28.5025	28.5065	1:Experimental evidence at protein level	carboxylic acid metabolic process;cellular aldehyde metabolic process;cellular component assembly;cellular component organization;cellular component organization or biogenesis;cellular ketone metabolic process;cellular r
28.883	29.027	29.1287	29.3805	1:Experimental evidence at protein level	anatomical structure morphogenesis;angiotensin maturation;biological regulation;branching morphogenesis of a tube;cellular macromolecule metabolic process;cellular metabolic process;cellular process;cellular protein meta
26.1921	26.4293	26.3019	26.2355	1:Experimental evidence at protein level	establishment of localization;establishment of localization in cell;establishment of RNA localization;gene expression;intracellular transport;macromolecule metabolic process;metabolic process;mRNA export from nucleus;mR
26.1973	26.5094	26.4041	26.5768	1:Experimental evidence at protein level	ADP biosynthetic process;ADP metabolic process;AMP metabolic process;biological regulation;biosynthetic process;blood coagulation;cellular biosynthetic process;cellular metabolic process;cellular nitrogen compound bios
26.5245	26.6051	26.5621	26.7735	1:Experimental evidence at protein level	multicellular organismal process;neurological system process;sensory perception;sensory perception of light stimulus;system process;visual perception
25.8633	26.1785	26.1166	26.0841	1:Experimental evidence at protein level	
26.4133	26.7302	26.9117	26.8815	1:Experimental evidence at protein level	biological regulation;cellular component organization;cellular component organization at cellular level;cellular component organization or biogenesis;cellular component organization or biogenesis at cellular level;cellular memb
25.9165	26.3152	25.7847	26.0487	1:Experimental evidence at protein level	anion transport;biological regulation;cation transport;cell volume homeostasis;cellular homeostasis;cellular process;chloride transport;establishment of localization;homeostatic process;inorganic anion transport;ion transport;
27.3711	27.8728	28.1775	28.3539	1:Experimental evidence at protein level	biological regulation;negative regulation of biological process;negative regulation of gene expression;negative regulation of macromolecule metabolic process;negative regulation of metabolic process;positive regulation of bio
26.7087	27.3298	27.4077	27.5262	1:Experimental evidence at protein level	cell differentiation;cellular developmental process;cellular macromolecule metabolic process;cellular metabolic process;cellular process;cellular protein metabolic process;developmental process;gamete generation;macromo
25.6865	25.9972	25.7813	25.8203	1:Experimental evidence at protein level	biosynthetic process;cellular biosynthetic process;cellular macromolecule biosynthetic process;cellular macromolecule metabolic process;cellular metabolic process;cellular nitrogen compound metabolic process;cellular pro
26.3063	26.416	#NUM!	26.1755	1:Experimental evidence at protein level	adipose tissue development;anatomical structure development;biological regulation;bone development;cell differentiation;cellular component assembly;cellular component disassembly;cellular component disassembly at cell
26.8877	26.844	27.2092	26.8856	1:Experimental evidence at protein level	anatomical structure development;biological adhesion;biological regulation;blood coagulation;brain development;carbohydrate transport;cell activation;cell activation involved in immune response;cell adhesion;cell-cell adhes
28.3152	28.0551	28.3476	28.1368	1:Experimental evidence at protein level	axon guidance;chemotaxis;locomotion;response to chemical stimulus;response to external stimulus;response to stimulus;taxis
26.8456	26.8958	26.6645	26.2358	1:Experimental evidence at protein level	activation of caspase activity;apoptosis;biological regulation;biosynthetic process;cell death;cell differentiation;cellular biosynthetic process;cellular component assembly;cellular component assembly at cellular level;cellular c
27.0414	27.2528	27.5419	27.52	1:Experimental evidence at protein level	biosynthetic process;cell proliferation;cellular biosynthetic process;cellular metabolic compound salvage;cellular metabolic process;cellular process;coenzyme biosynthetic process;coenzyme metabolic process;cofactor bios
29.3494	28.9748	28.8813	28.7597	1:Experimental evidence at protein level	actin cytoskeleton organization;actin filament organization;actin filament-based process;actin nucleation;Arp2/3 complex-mediated actin nucleation;axon guidance;biological regulation;cell surface receptor linked signaling pa
26.5588	26.8434	26.5833	26.5512	1:Experimental evidence at protein level	biological regulation;cell cycle process;cellular component assembly;cellular component assembly at cellular level;cellular component organization;cellular component organization at cellular level;cellular component organiza
28.7425	28.3814	28.6361	28.2564	1:Experimental evidence at protein level	catabolic process;cellular catabolic process;cellular macromolecule catabolic process;cellular macromolecule metabolic process;cellular metabolic process;cellular process;ER-associated protein catabolic process;macromo
28.4599	28.5022	28.6538	28.6105	1:Experimental evidence at protein level	ATP biosynthetic process;ATP metabolic process;ATP synthesis coupled proton transport;biosynthetic process;cation transport;cellular biosynthetic process;cellular metabolic process;cellular nitrogen compound biosynthetic
26.98	26.2937	27.1738	26.7114	1:Experimental evidence at protein level	cellular component assembly;cellular component assembly at cellular level;cellular component organization;cellular component organization at cellular level;cellular component organization or biogenesis;cellular component o
26.4137	26.4164	26.3	26.2892	1:Experimental evidence at protein level	anatomical structure morphogenesis;biological regulation;cell maturation;cellular developmental process;cellular macromolecule metabolic process;cellular metabolic process;cellular nitrogen compound metabolic process;c
26.3797	26.8175	26.7471	27.0624	1:Experimental evidence at protein level	amine catabolic process;amine metabolic process;arginine catabolic process;arginine metabolic process;biological regulation;biosynthetic process;carboxylic acid catabolic process;carboxylic acid metabolic process;catabo
30.0775	31.5905	31.5248	32.829	1:Experimental evidence at protein level	biological regulation;cation homeostasis;cell surface receptor linked signaling pathway;cellular cation homeostasis;cellular chemical homeostasis;cellular copper ion homeostasis;cellular homeostasis;cellular ion homeostasis
27.3565	27.2451	27.1841	27.403	1:Experimental evidence at protein level	anatomical structure development;anatomical structure morphogenesis;behavior;biological regulation;blood coagulation;cellular component organization;cellular component organization at cellular level;cellular component or
28.442	28.4608	28.3361	28.3477	1:Experimental evidence at protein level	cellular metabolic process;cellular process;electron transport chain;generation of precursor metabolites and energy;metabolic process;oxidation-reduction process;respiratory electron transport chain;response to chemical sti
27.2889	27.1628	27.3588	27.2998	1:Experimental evidence at protein level	anatomical structure morphogenesis;axon guidance;biological regulation;cell communication;cell differentiation;cell-cell signaling;cellular developmental process;cellular process;cellular response to stimulus;chemotaxis;deve
28.8868	28.8394	28.9319	28.6403	1:Experimental evidence at protein level	antigen processing and presentation;antigen processing and presentation of exogenous antigen;antigen processing and presentation of exogenous peptide antigen;antigen processing and presentation of exogenous peptide
25.5679	25.3189	25.7202	25.8783	1:Experimental evidence at protein level	amine catabolic process;amine metabolic process;branched chain family amino acid catabolic process;branched chain family amino acid metabolic process;carboxylic acid catabolic process;carboxylic acid metabolic proces
26.5304	27.1138	26.9456	27.1376	1:Experimental evidence at protein level	alcohol metabolic process;aldehyde catabolic process;biosynthetic process;carbohydrate catabolic process;carbohydrate metabolic process;carboxylic acid biosynthetic process;carboxylic acid catabolic process;carboxylic
27.354	27.0797	27.4731	27.1254	1:Experimental evidence at protein level	anatomical structure homeostasis;base-excision repair;biological regulation;biosynthetic process;catabolic process;cell cycle;cell cycle checkpoint;cell cycle process;cellular biosynthetic process;cellular catabolic process;ce
27.732	27.6234	27.5356	27.4051	1:Experimental evidence at protein level	activation of immune response;activation of innate immune response;activation of phospholipase C activity;activation of protein kinase A activity;activation of protein kinase activity;acylglycerol catabolic process;acylglycerol
28.4132	28.5257	28.7037	28.7703	1:Experimental evidence at protein level	anatomical structure development;biological regulation;cell chemotaxis;cell differentiation;cell migration;cell motility;cellular component movement;cellular component organization;cellular component organization at cellular l
28.2378	28.2288	28.3145	28.4155	1:Experimental evidence at protein level	biological regulation;cell cycle phase;cell cycle process;cell division;cellular component assembly;cellular component assembly at cellular level;cellular component organization;cellular component organization at cellular level
27.6094	28.0771	27.7633	27.8457	1:Experimental evidence at protein level	biological regulation;cellular component organization;cellular component organization or biogenesis;cellular membrane organization;cellular process;cellular response to stimulus;endocytosis;endosome to lysosome transport
25.8098	#NUM!	25.6242	25.7458	1:Experimental evidence at protein level	biosynthetic process;cell activation;cell proliferation;cellular aromatic compound metabolic process;cellular biosynthetic process;cellular metabolic process;cellular nitrogen compound biosynthetic process;cellular nitrogen co
25.9029	25.5016	25.1891	24.9837	1:Experimental evidence at protein level	anatomical structure morphogenesis;axon guidance;biological regulation;blood vessel morphogenesis;cell cycle cytokinesis;cell cycle process;cell differentiation;cell surface receptor linked signaling pathway;cellular compon
28.2004	28.101	28.0057	28.1894	1:Experimental evidence at protein level	ATP hydrolysis coupled proton transport;ATP metabolic process;biological regulation;cation homeostasis;cation transport;cell surface receptor linked signaling pathway;cellular cation homeostasis;cellular chemical homeosta
28.9688	29.6985	29.6685	29.6511	1:Experimental evidence at protein level	cellular macromolecule metabolic process;cellular metabolic process;cellular process;cellular protein metabolic process;macromolecule metabolic process;macromolecule methylation;macromolecule modification;metabolic p
25.277	25.5098	25.4186	25.3746	1:Experimental evidence at protein level	alcohol metabolic process;biological regulation;biosynthetic process;C21-steroid hormone biosynthetic process;C21-steroid hormone metabolic process;cellular biosynthetic process;cellular hormone metabolic process;cellu
26.8617	26.8281	26.8287	26.655	1:Experimental evidence at protein level	carboxylic acid metabolic process;cellular ketone metabolic process;cellular metabolic process;cellular process;dicarboxylic acid metabolic process;malate metabolic process;metabolic process;organic acid metabolic proce
29.9722	30.3087	30.3536	30.6054	1:Experimental evidence at protein level	activation of immune response;activation of innate immune response;anaphase-promoting complex-dependent proteasomal ubiquitin-dependent protein catabolic process;antigen processing and presentation;antigen proces
27.3088	27.0024	27.3319	26.9233	1:Experimental evidence at protein level	apoptosis;biological regulation;cell death;cell surface receptor linked signaling pathway;cellular macromolecule metabolic process;cellular metabolic process;cellular process;cellular protein metabolic process;cellular respons
26.4288	26.5934	26.5392	26.7396	1:Experimental evidence at protein level	amine biosynthetic process;amine metabolic process;biological regulation;biosynthetic process;carboxylic acid biosynthetic process;carboxylic acid metabolic process;cellular amine metabolic process;cellular amino acid bio
26.7931	27.0333	27.3699	27.5173	1:Experimental evidence at protein level	biosynthetic process;cellular biosynthetic process;cellular macromolecule biosynthetic process;cellular macromolecule metabolic process;cellular metabolic process;cellular nitrogen compound metabolic process;cellular pro
25.6417	25.8155	25.2357	25.6284	1:Experimental evidence at protein level	arachidonic acid metabolic process;carboxylic acid metabolic process;cellular alkene metabolic process;cellular ketone metabolic process;cellular lipid metabolic process;cellular metabolic process;cellular process;cobalami
26.4849	26.2684	26.271	26.2088	1:Experimental evidence at protein level	activation of JUN kinase activity;activation of MAPK activity;activation of protein kinase activity;activation of protein kinase B activity;ameboidal cell migration;anatomical structure development;anatomical structure formation
27.6723	27.7465	27.7957	27.6922	1:Experimental evidence at protein level	cellular component organization;cellular component organization at cellular level;cellular component organization or biogenesis;cellular component organization or biogenesis at cellular level;cellular macromolecule metabolic
26.9842	27.0508	26.9663	26.7688	1:Experimental evidence at protein level	anatomical structure development;anatomical structure morphogenesis;biological regulation;bradykinin catabolic process;calcitonin catabolic process;catabolic process;cellular catabolic process;cellular hormone metabolic p
27.7553	27.7256	27.7532	27.6755	1:Experimental evidence at protein level	biological regulation;biosynthetic process;cellular biosynthetic process;cellular component organization;cellular component organization at cellular level;cellular component organization or biogenesis;cellular component orga
26.9505	27.3634	27.4441	27.3811	1:Experimental evidence at protein level	alcohol catabolic process;alcohol metabolic process;amino sugar catabolic process;amino sugar metabolic process;carbohydrate catabolic process;carbohydrate metabolic process;catabolic process;cellular carbohydrate c
27.757	28.5004	28.369	28.6115	1:Experimental evidence at protein level	anatomical structure development;anatomical structure morphogenesis;biological regulation;biosynthetic process;cell differentiation;cell morphogenesis;cell proliferation;cellular biosynthetic process;cellular component morp
28.3364	28.4371	28.2453	28.4531	1:Experimental evidence at protein level	ATP biosynthetic process;ATP metabolic process;ATP synthesis coupled proton transport;biosynthetic process;cation transport;cellular biosynthetic process;cellular metabolic process;cellular nitrogen compound biosynthetic
27.3849	27.2533	27.1122	26.8516	1:Experimental evidence at protein level	biological regulation;cell cycle phase;cell cycle process;cell division;cell surface receptor linked signaling pathway;cellular component organization;cellular component organization at cellular level;cellular component organiza
27.3987	26.9936	27.1599	27.2578	1:Experimental evidence at protein level	biological adhesion;biological regulation;cell adhesion;cell communication;cell surface receptor linked signaling pathway;cell-cell signaling;cellular component movement;cellular process;cellular response to stimulus;defense
28.5473	28.6983	29.1082	28.7837	1:Experimental evidence at protein level	biological regulation;biosynthetic process;cellular biosynthetic process;cellular macromolecule biosynthetic process;cellular macromolecule metabolic process;cellular metabolic process;cellular process;cellular protein metab
28.3487	28.0019	28.3829	27.7759	1:Experimental evidence at protein level	biological regulation;cell cycle;cell cycle checkpoint;cell cycle phase;cell cycle process;cell division;cellular component assembly;cellular component organization;cellular component organization at cellular level;cellular comp
26.3717	26.3614	26.6306	26.4621	1:Experimental evidence at protein level	biological adhesion;biological regulation;cell adhesion;cell-matrix adhesion;cell-substrate adhesion;cellular process;cellular response to stimulus;regulation of biological process;regulation of cellular process;response to stimu
26.8681	27.0471	27.0773	26.9794	1:Experimental evidence at protein level	acetyl-CoA catabolic process;acetyl-CoA metabolic process;carboxylic acid metabolic process;catabolic process;cellular catabolic process;cellular ketone metabolic process;cellular metabolic process;cellular process;coenz
28.8813	28.4867	28.6053	28.8127	1:Experimental evidence at protein level	biological regulation;blood coagulation;cation homeostasis;cation transport;cell migration;cell motility;cellular cation homeostasis;cellular chemical homeostasis;cellular component movement;cellular component organization
29.0408	28.5303	29.0605	29.0405	1:Experimental evidence at protein level	activation of immune response;activation of innate immune response;anaphase-promoting complex-dependent proteasomal ubiquitin-dependent protein catabolic process;antigen processing and presentation;antigen proces
28.7303	28.8176	29.0481	28.7595	1:Experimental evidence at protein level	antigen processing and presentation;antigen processing and presentation of exogenous antigen;antigen processing and presentation of exogenous peptide antigen;antigen processing and presentation of exogenous peptide
31.0166	30.705	30.7999	30.922	1:Experimental evidence at protein level	biosynthetic process;catabolic process;cellular biosynthetic process;cellular catabolic process;cellular component disassembly;cellular component disassembly at cellular level;cellular component organization;cellular compo
27.3109	27.5304	27.3933	27.0609	1:Experimental evidence at protein level	biological regulation;cell differentiation;cellular developmental process;cellular process;cellular response to stimulus;developmental process;intracellular signal transduction;metabolic process;osteoblast differentiation;positive
30.9793	30.7633	30.9837	30.9864	1:Experimental evidence at protein level	biological regulation;biosynthetic process;catabolic process;cell differentiation;cellular biosynthetic process;cellular catabolic process;cellular component assembly;cellular component assembly at cellular level;cellular compo
30.1236	29.6771	29.6917	30.0634	1:Experimental evidence at protein level	biosynthetic process;catabolic process;cellular biosynthetic process;cellular catabolic process;cellular component disassembly;cellular component disassembly at cellular level;cellular component organization;cellular compo
29.8965	29.2802	29.8526	29.5932	1:Experimental evidence at protein level	biosynthetic process;catabolic process;cell proliferation;cellular biosynthetic process;cellular catabolic process;cellular component disassembly;cellular component disassembly at cellular level;cellular component organizatio
28.598	29.2626	28.9475	29.0591	1:Experimental evidence at protein level	activation of immune response;activation of innate immune response;activation of MAPK activity;alcohol metabolic process;anaphase-promoting complex-dependent proteasomal ubiquitin-dependent protein catabolic proce
25.8199	26.0038	26.1117	26.0719	1:Experimental evidence at protein level	amine biosynthetic process;amine metabolic process;biosynthetic process;carboxylic acid biosynthetic process;carboxylic acid metabolic process;cellular amine metabolic process;cellular amino acid biosynthetic process;ce
31.0938	30.6395	30.8558	30.868	1:Experimental evidence at protein level	biological regulation;cellular component organization;cellular component organization at cellular level;cellular component organization or biogenesis;cellular component organization or biogenesis at cellular level;cellular proce
25.9664	26.2249	26.3964	26.5661	1:Experimental evidence at protein level	biosynthetic process;cellular biosynthetic process;cellular component disassembly;cellular component disassembly at cellular level;cellular component organization;cellular component organization at cellular level;cellular cor
26.6875	26.261	26.4994	26.2853	1:Experimental evidence at protein level	biosynthetic process;cellular biosynthetic process;cellular component disassembly;cellular component disassembly at cellular level;cellular component organization;cellular component organization at cellular level;cellular cor
25.8378	25.7428	25.7375	25.6902	1:Experimental evidence at protein level	biosynthetic process;cellular biosynthetic process;cellular component disassembly;cellular component disassembly at cellular level;cellular component organization;cellular component organization at cellular level;cellular cor
26.6595	27.8606	25.814	#NUM!	1:Experimental evidence at protein level	alcohol metabolic process;anatomical structure development;biosynthetic process;brain development;cellular biosynthetic process;cellular lipid metabolic process;cellular metabolic process;cellular process;cellular response
27.6369	27.4337	28.0758	27.7867	1:Experimental evidence at protein level	amine metabolic process;aminoglycan metabolic process;biological regulation;carbohydrate metabolic process;glycosaminoglycan metabolic process;hyaluronan metabolic process;macromolecule metabolic process;metab
25.1898	#NUM!	#NUM!	25.2655	1:Experimental evidence at protein level	cell differentiation;cellular developmental process;cellular process;developmental process
27.8894	27.614	28.3231	28.0885	1:Experimental evidence at protein level	biological regulation;BMP signaling pathway;cell surface receptor linked signaling pathway;cellular process;cellular response to stimulus;enzyme linked receptor protein signaling pathway;regulation of biological process;regu
27.4311	27.228	27.3347	27.2965	1:Experimental evidence at protein level	biological regulation;calcium ion homeostasis;calcium ion transport;calcium-mediated signaling;cation homeostasis;cation transport;cell communication;cell cycle process;cell growth;cell growth involved in cardiac muscle ce
27.2607	27.0008	27.2855	27.0488	1:Experimental evidence at protein level	apoptosis;biological regulation;cell cycle checkpoint;cell death;cellular macromolecule metabolic process;cellular metabolic process;cellular process;cellular protein metabolic process;cellular response to stimulus;death;DNA
28.0968	27.633	28.0084	27.7294	1:Experimental evidence at protein level	biological adhesion;cell adhesion;cellular process;establishment of localization;establishment of localization in cell;establishment of protein localization;establishment of protein localization to organelle;establishment of protei
25.7386	25.4987	25.7265	25.7037	1:Experimental evidence at protein level	biological regulation;biosynthetic process;cellular biosynthetic process;cellular macromolecule biosynthetic process;cellular macromolecule metabolic process;cellular metabolic process;cellular nitrogen compound metabolic
25.6122	25.4209	25.8965	25.7648	1:Experimental evidence at protein level	biological regulation;carboxylic acid catabolic process;carboxylic acid metabolic process;catabolic process;cellular catabolic process;cellular component organization;cellular component organization at cellular level;cellular c
25.7146	26.2052	26.07	26.1137	1:Experimental evidence at protein level	acetyl-CoA metabolic process;alcohol metabolic process;biosynthetic process;cellular biosynthetic process;cellular lipid metabolic process;cellular metabolic process;cellular process;cholesterol biosynthetic process;cholest

27.1028	27.2378	26.8449	26.8606	1:Experimental evidence at protein level	
26.6147	26.4517	26.4823	26.2827	1:Experimental evidence at protein level	apoptosis;biological regulation;biosynthetic process;cell death;cellular biosynthetic process;cellular macromolecule biosynthetic process;cellular macromolecule metabolic process;cellular metabolic process;cellular nitrogen
27.0083	26.5968	26.703	26.3462	1:Experimental evidence at protein level	biological regulation;biosynthetic process;cell fate commitment;cell fate commitment involved in formation of primary germ layers;cell surface receptor linked signaling pathway;cellular biosynthetic process;cellular componer
26.305	25.8821	25.9841	26.2864	1:Experimental evidence at protein level	biological regulation;cellular process;cellular response to stimulus;intracellular signal transduction;regulation of biological process;regulation of cellular process;response to stimulus;signal transduction
26.9886	27.6811	27.6238	27.2362	1:Experimental evidence at protein level	
25.1448	24.5092	24.9556	25.2279	1:Experimental evidence at protein level	alcohol metabolic process;amine metabolic process;anatomical structure formation involved in morphogenesis;anatomical structure morphogenesis;angiogenesis;catabolic process;cellular amine metabolic process;cellular b
26.3907	26.0896	26.1373	26.1337	1:Experimental evidence at protein level	cellular macromolecule metabolic process;cellular metabolic process;cellular nitrogen compound metabolic process;cellular process;enzyme-directed rRNA 2'-O-methylation;macromolecule metabolic process;macromolecul
27.267	27.1959	27.1895	27.3747	1:Experimental evidence at protein level	activation of immune response;activation of innate immune response;biological regulation;cell cycle;cell surface receptor linked signaling pathway;cellular macromolecule metabolic process;cellular metabolic process;cellular
26.0864	26.0201	25.9698	25.7955	1:Experimental evidence at protein level	biological regulation;cellular macromolecule metabolic process;cellular metabolic process;cellular process;cellular protein metabolic process;cellular response to stimulus;dephosphorylation;macromolecule metabolic process
27.0478	27.0784	27.1995	27.2794	1:Experimental evidence at protein level	biological regulation;cell surface receptor linked signaling pathway;cellular process;cellular response to chemical stimulus;cellular response to cytokine stimulus;cellular response to organic substance;cellular response to stim
26.1018	26.3061	26.3412	26.3318	1:Experimental evidence at protein level	biological regulation;biosynthetic process;cell fate commitment;cell fate commitment involved in formation of primary germ layers;cell surface receptor linked signaling pathway;cellular biosynthetic process;cellular componer
27.9594	27.9659	27.8925	28.065	1:Experimental evidence at protein level	biosynthetic process;cellular biosynthetic process;cellular macromolecule biosynthetic process;cellular macromolecule metabolic process;cellular metabolic process;cellular nitrogen compound metabolic process;cellular pro
23.7378	#NUM!	#NUM!	24.607	1:Experimental evidence at protein level	
27.1292	27.3649	27.3584	27.1328	1:Experimental evidence at protein level	response to calcium ion;response to chemical stimulus;response to inorganic substance;response to metal ion;response to stimulus
26.9181	27.539	27.2414	27.4442	1:Experimental evidence at protein level	biological regulation;cellular macromolecule metabolic process;cellular metabolic process;cellular nitrogen compound metabolic process;cellular process;cellular protein metabolic process;cellular response to stimulus;cellula
26.6212	26.5392	26.4323	26.6102	1:Experimental evidence at protein level	asymmetric protein localization;basal protein localization;biological adhesion;biological regulation;cell adhesion;cell cycle;cell growth;cell surface receptor linked signaling pathway;cellular component organization;cellular com
27.0749	27.0299	27.0429	26.8205	1:Experimental evidence at protein level	multicellular organismal process;neurological system process;sensory perception;sensory perception of pain;system process
26.907	26.8266	27.0294	26.8571	1:Experimental evidence at protein level	cell cycle;cell cycle process;cell division;cellular component organization;cellular component organization at cellular level;cellular component organization or biogenesis;cellular component organization or biogenesis at cellula
26.5442	26.3054	26.2452	26.4897	1:Experimental evidence at protein level	adherens junction assembly;adherens junction organization;biological regulation;cell junction assembly;cell junction organization;cell-cell junction assembly;cell-cell junction organization;cellular component assembly;cellular
#NUM!	25.7204	25.1237	25.8259	1:Experimental evidence at protein level	alcohol catabolic process;alcohol metabolic process;biological regulation;biosynthetic process;carbohydrate biosynthetic process;carbohydrate catabolic process;carbohydrate metabolic process;catabolic process;cell migra
26.013	25.8349	25.6356	25.9299	1:Experimental evidence at protein level	biosynthetic process;cellular biosynthetic process;cellular component disassembly;cellular component disassembly at cellular level;cellular component organization;cellular component organization at cellular level;cellular cor
26.9709	#NUM!	27.1209	27.0021	1:Experimental evidence at protein level	biological regulation;cell proliferation;cell surface receptor linked signaling pathway;cellular component biogenesis;cellular component biogenesis at cellular level;cellular component organization or biogenesis;cellular compor
26.2505	#NUM!	26.2846	26.392	1:Experimental evidence at protein level	cellular macromolecule metabolic process;cellular metabolic process;cellular nitrogen compound metabolic process;cellular process;macromolecule metabolic process;metabolic process;mRNA metabolic process;mRNA pro
28.3024	28.7016	28.8273	28.8206	1:Experimental evidence at protein level	biological regulation;cell cycle process;cellular component assembly;cellular component organization;cellular component organization at cellular level;cellular component organization or biogenesis;cellular component organiz
27.7544	27.6701	27.8596	27.5963	1:Experimental evidence at protein level	alcohol metabolic process;anatomical structure development;anatomical structure formation involved in morphogenesis;angiogenesis;anterior/posterior pattern specification;biological regulation;carbohydrate metabolic proce
25.5653	25.6817	25.1586	25.517	1:Experimental evidence at protein level	anatomical structure development;biological regulation;cell development;cellular developmental process;cellular macromolecule metabolic process;cellular metabolic process;cellular process;cellular protein metabolic proces
26.3521	26.1624	#NUM!	26.1679	1:Experimental evidence at protein level	cation transport;establishment of localization;ion transport;transport
27.3298	27.1117	26.985	26.8665	1:Experimental evidence at protein level	biological regulation;cellular macromolecule metabolic process;cellular metabolic process;cellular process;cellular protein metabolic process;macromolecule metabolic process;metabolic process;negative regulation of cataly
26.5292	26.8516	27.0253	26.8005	1:Experimental evidence at protein level	amine biosynthetic process;amine metabolic process;biological regulation;biosynthetic process;carboxylic acid biosynthetic process;carboxylic acid metabolic process;cellular amine metabolic process;cellular amino acid bic
26.5663	26.8444	26.5286	26.5979	1:Experimental evidence at protein level	cell cycle;cell division;cellular process
26.4452	26.303	25.6574	25.7078	1:Experimental evidence at protein level	anatomical structure development;axon ensheathment;biological regulation;biosynthetic process;cell development;cellular biosynthetic process;cellular developmental process;cellular macromolecule biosynthetic process;ce
27.8417	27.6697	28.0177	27.7308	1:Experimental evidence at protein level	activation of immune response;alpha-beta T cell activation;antigen receptor-mediated signaling pathway;ATP biosynthetic process;ATP metabolic process;ATP synthesis coupled proton transport;biological regulation;biosynth
26.703	26.1683	26.7171	26.4987	1:Experimental evidence at protein level	biological regulation;biosynthetic process;carbohydrate metabolic process;carbohydrate transport;cell cycle;cell cycle process;cell surface receptor linked signaling pathway;cellular biosynthetic process;cellular component c
25.9932	25.5264	26.1376	25.7698	1:Experimental evidence at protein level	activation of protein kinase activity;apoptosis;biological regulation;cell cycle phase;cell cycle process;cell death;cell division;cell proliferation;cellular component organization;cellular component organization at cellular level;ce
26.5408	26.5961	26.7453	26.7176	1:Experimental evidence at protein level	anatomical structure development;anatomical structure morphogenesis;biological regulation;biosynthetic process;catabolic process;cell surface receptor linked signaling pathway;cellular biosynthetic process;cellular cataboli
26.05	26.3753	26.6126	26.5822	1:Experimental evidence at protein level	actin cytoskeleton organization;actin filament-based movement;actin filament-based process;ameboidal cell migration;anatomical structure development;anatomical structure formation involved in morphogenesis;anatomical
25.7237	26.6564	25.8605	26.3239	1:Experimental evidence at protein level	axon guidance;biological regulation;cell activation;cell communication;cell surface receptor linked signaling pathway;cell-cell signaling;cellular component disassembly;cellular component disassembly at cellular level;cellular
26.3621	26.1913	26.3086	26.2764	1:Experimental evidence at protein level	anatomical structure arrangement;anatomical structure development;anatomical structure formation involved in morphogenesis;anatomical structure morphogenesis;angiogenesis;angiogenesis involved in coronary vascular r
26.9672	26.4719	26.8799	26.5241	1:Experimental evidence at protein level	adult behavior;adult locomotory behavior;adult walking behavior;alcohol metabolic process;autophagy;behavior;bile acid metabolic process;biological regulation;carbohydrate metabolic process;carboxylic acid metabolic pro
29.7062	29.4068	29.7342	29.615	1:Experimental evidence at protein level	actin cytoskeleton organization;actin filament organization;actin filament-based process;actin nucleation;Arp2/3 complex-mediated actin nucleation;axon guidance;biological regulation;cell migration;cell motility;cell surface re
26.5327	26.532	26.6633	26.5363	1:Experimental evidence at protein level	biological regulation;cell surface receptor linked signaling pathway;cellular process;cellular response to stimulus;G-protein coupled receptor protein signaling pathway;regulation of biological process;regulation of cellular proc
26.4798	25.9061	25.8813	25.3756	1:Experimental evidence at protein level	cellular macromolecule metabolic process;cellular metabolic process;cellular nitrogen compound metabolic process;cellular process;macromolecule metabolic process;metabolic process;mRNA metabolic process;mRNA pro
25.804	#NUM!	25.8339	25.7716	1:Experimental evidence at protein level	axon guidance;biological regulation;cell cycle;cell cycle phase;cell cycle process;cell division;cellular component organization;cellular component organization at cellular level;cellular component organization or biogenesis;ce
27.3046	27.1611	27.1464	26.9461	1:Experimental evidence at protein level	cellular metabolic process;cellular process;electron transport chain;generation of precursor metabolites and energy;metabolic process;mitochondrial electron transport, NADH to ubiquinone;oxidation-reduction process;respi
27.6956	27.9584	27.9598	27.8714	1:Experimental evidence at protein level	cellular process;endosome transport;establishment of localization;establishment of localization in cell;establishment of protein localization;intracellular protein transport;intracellular transport;protein transport;retrograde trans
27.5484	26.7004	27.5377	27.0894	1:Experimental evidence at protein level	catabolic process;cellular catabolic process;cellular macromolecule catabolic process;cellular macromolecule metabolic process;cellular metabolic process;cellular process;ER-associated protein catabolic process;macromo
26.6328	26.7222	26.8435	26.6783	1:Experimental evidence at protein level	anterograde axon cargo transport;antigen processing and presentation;antigen processing and presentation of exogenous antigen;antigen processing and presentation of exogenous peptide antigen;antigen processing and p
28.988	29.0576	29.0246	28.8803	1:Experimental evidence at protein level	alcohol metabolic process;biosynthetic process;blood circulation;carboxylic acid metabolic process;cellular ketone metabolic process;cellular metabolic process;cellular process;cholesterol biosynthetic process;cholesterol m
31.9683	29.3258	30.4809	30.6572	1:Experimental evidence at protein level	biological regulation;negative regulation of biological process;negative regulation of cell proliferation;negative regulation of cellular process;regulation of biological process;regulation of cell proliferation;regulation of cellular pr
31.654	32.0393	32.3475	32.1606	1:Experimental evidence at protein level	anatomical structure morphogenesis;axonogenesis;biological regulation;cell part morphogenesis;cell projection morphogenesis;cell projection organization;cellular component morphogenesis;cellular component organization
25.9234	25.8645	26.0202	25.9583	1:Experimental evidence at protein level	anatomical structure homeostasis;biological regulation;biosynthetic process;cell cycle;cell cycle process;cell proliferation;cellular biosynthetic process;cellular component organization;cellular component organization at cellu
25.4081	24.9099	25.5814	#NUM!	1:Experimental evidence at protein level	activation of immune response;biological regulation;complement activation;complement activation, classical pathway;defense response;humoral immune response;immune effector process;immune response;immune system
33.9323	31.6909	32.5956	33.1868	1:Experimental evidence at protein level	
#NUM!	#NUM!	#NUM!	#NUM!	1:Experimental evidence at protein level	biological regulation;blood circulation;blood coagulation;cell activation;cellular process;circulatory system process;coagulation;establishment of localization;establishment of localization in cell;exocytosis;hemostasis;multicell
29.2044	29.1926	29.3118	28.9611	1:Experimental evidence at protein level	biosynthetic process;cellular biosynthetic process;cellular macromolecule metabolic process;cellular metabolic process;cellular nitrogen compound metabolic process;cellular process;electron transport chain;gene expressio
#NUM!	26.7581	26.4104	26.8362	1:Experimental evidence at protein level	3'-UTR-mediated mRNA stabilization;anatomical structure development;biological regulation;cellular hyperosmotic response;cellular macromolecule metabolic process;cellular metabolic process;cellular nitrogen compound r
26.46	26.2014	26.113	25.9816	1:Experimental evidence at protein level	activation of immune response;activation of innate immune response;antigen receptor-mediated signaling pathway;apoptosis;biological regulation;biosynthetic process;cell death;cell surface receptor linked signaling pathway
29.197	29.3583	29.6765	29.4713	1:Experimental evidence at protein level	activation of immune response;activation of innate immune response;anaphase-promoting complex-dependent proteasomal ubiquitin-dependent protein catabolic process;antigen processing and presentation;antigen proces
26.4697	26.3934	26.508	26.4944	1:Experimental evidence at protein level	biosynthetic process;catabolic process;cellular biosynthetic process;cellular catabolic process;cellular metabolic process;cellular nitrogen compound catabolic process;cellular nitrogen compound metabolic process;cellular p
26.7751	26.7805	26.8317	26.4769	1:Experimental evidence at protein level	carboxylic acid catabolic process;carboxylic acid metabolic process;catabolic process;cellular catabolic process;cellular component organization;cellular component organization at cellular level;cellular component organizati
25.6542	26.6019	26.3486	26.4063	1:Experimental evidence at protein level	macromolecule metabolic process;metabolic process;primary metabolic process;protein metabolic process;proteolysis
26.2572	26.837	27.0511	27.117	1:Experimental evidence at protein level	alcohol biosynthetic process;alcohol metabolic process;biological regulation;biosynthetic process;carbohydrate biosynthetic process;carbohydrate metabolic process;catabolic process;cellular biosynthetic process;cellular ca
26.6831	26.9207	26.9087	27.0885	1:Experimental evidence at protein level	activation of caspase activity;apoptosis;biological regulation;cell death;cellular chemical homeostasis;cellular homeostasis;cellular ion homeostasis;cellular process;cellular response to abiotic stimulus;cellular response to ch
28.8225	28.77	28.9917	28.8818	1:Experimental evidence at protein level	activation of MAPK activity;biological regulation;cellular macromolecule metabolic process;cellular metabolic process;cellular process;cellular protein metabolic process;establishment of localization;establishment of localizati
27.7542	28.9589	28.8078	29.1012	1:Experimental evidence at protein level	apoptotic mitochondrial changes;biological regulation;cell death;cellular component organization;cellular component organization at cellular level;cellular component organization or biogenesis;cellular component organization
25.2812	25.207	25.162	#NUM!	1:Experimental evidence at protein level	anatomical structure development;axon guidance;biological adhesion;biological regulation;blood coagulation;cell adhesion;cell adhesion mediated by integrin;cell migration;cell motility;cell surface receptor linked signaling pa
27.6235	27.8642	27.5464	27.7655	1:Experimental evidence at protein level	biological regulation;biosynthetic process;cellular biosynthetic process;cellular macromolecule biosynthetic process;cellular macromolecule metabolic process;cellular metabolic process;cellular nitrogen compound metabolic
29.6561	29.7754	29.78	29.819	1:Experimental evidence at protein level	anatomical structure development;anatomical structure morphogenesis;biological regulation;blood coagulation;carboxylic acid metabolic process;cell migration;cell motility;cell surface receptor linked signaling pathway;cellul
27.796	#NUM!	28.0735	27.8638	1:Experimental evidence at protein level	acylglycerol catabolic process;acylglycerol metabolic process;biological regulation;carbohydrate metabolic process;catabolic process;cell cycle;cell differentiation;cell division;cell surface receptor linked signaling pathway;ce
27.8103	27.7417	27.7139	27.6676	1:Experimental evidence at protein level	biological regulation;blood coagulation;coagulation;hemostasis;multicellular organismal process;negative regulation of biological process;negative regulation of biosynthetic process;negative regulation of cellular biosynthetic
30.8103	30.9152	30.6508	31.0962	1:Experimental evidence at protein level	biosynthetic process;catabolic process;cellular biosynthetic process;cellular catabolic process;cellular component disassembly;cellular component disassembly at cellular level;cellular component organization;cellular compo
28.0524	28.1189	28.3486	28.4152	1:Experimental evidence at protein level	biosynthetic process;cellular biosynthetic process;cellular macromolecule biosynthetic process;cellular macromolecule metabolic process;cellular metabolic process;cellular process;cellular protein metabolic process;gene ex
26.143	26.3466	26.2208	26.0069	1:Experimental evidence at protein level	anatomical structure development;axon ensheathment;biological regulation;biosynthetic process;cell development;cellular biosynthetic process;cellular developmental process;cellular macromolecule biosynthetic process;ce
27.1157	27.12	27.071	27.0677	1:Experimental evidence at protein level	adenosine metabolic process;catabolic process;cellular aromatic compound metabolic process;cellular catabolic process;cellular metabolic process;cellular nitrogen compound catabolic process;cellular nitrogen compound
26.7562	26.5634	26.9659	26.2529	1:Experimental evidence at protein level	biological regulation;negative regulation of catalytic activity;negative regulation of endopeptidase activity;negative regulation of hydrolase activity;negative regulation of molecular function;negative regulation of peptidase acti
25.71	26.2543	26.5296	26.1257	1:Experimental evidence at protein level	
28.5823	28.3496	28.357	28.4031	1:Experimental evidence at protein level	anatomical structure development;developmental process;multicellular organismal process;muscle contraction;muscle organ development;muscle structure development;muscle system process;organ development;smooth r
27.6401	28.0751	28.0434	28.1629	1:Experimental evidence at protein level	2'-deoxyribonucleotide biosynthetic process;2'-deoxyribonucleotide metabolic process;adenosine metabolic process;AMP biosynthetic process;AMP metabolic process;AMP salvage;biological regulation;biosynthetic process
26.2114	26.055	26.2696	26.2282	1:Experimental evidence at protein level	anatomical structure development;astrocyte development;axon extension;axon extension involved in development;axon extension involved in regeneration;axon guidance;biological adhesion;cell adhesion;cell development;c
30.0015	30.2408	29.938	30.2487	1:Experimental evidence at protein level	actin cytoskeleton organization;actin filament branching;actin filament bundle assembly;actin filament organization;actin filament-based process;adherens junction organization;anatomical structure development;anatomical s
29.1642	29.319	29.4622	29.2661	1:Experimental evidence at protein level	cellular component assembly;cellular component assembly at cellular level;cellular component organization;cellular component organization at cellular level;cellular component organization or biogenesis;cellular component c
26.9584	27.0777	27.5353	27.3848	1:Experimental evidence at protein level	
25.8968	25.4618	25.6907	25.5092	1:Experimental evidence at protein level	biosynthetic process;cellular biosynthetic process;cellular component disassembly;cellular component disassembly at cellular level;cellular component organization;cellular component organization at cellular level;cellular cor
30.0744	30.12	30.1241	29.9513	1:Experimental evidence at protein level	biosynthetic process;cellular biosynthetic process;cellular macromolecule biosynthetic process;cellular macromolecule metabolic process;cellular metabolic process;cellular nitrogen compound metabolic process;cellular pro
29.6694	29.7803	29.6722	29.3702	1:Experimental evidence at protein level	biosynthetic process;cellular biosynthetic process;cellular macromolecule biosynthetic process;cellular macromolecule metabolic process;cellular metabolic process;cellular nitrogen compound metabolic process;cellular pro
27.0252	26.8213	26.7011	26.7803	1:Experimental evidence at protein level	antigen processing and presentation;antigen processing and presentation of exogenous antigen;antigen processing and presentation of exogenous peptide antigen;antigen processing and presentation of exogenous peptide
28.0693	28.2662	28.1741	28.3923	1:Experimental evidence at protein level	biological regulation;catabolic process;cellular catabolic process;cellular macromolecule catabolic process;cellular macromolecule metabolic process;cellular metabolic process;cellular process;macromolecule catabolic proc
28.6372	29.3661	30.0185	29.8548	1:Experimental evidence at protein level	amine metabolic process;biological regulation;carbohydrate metabolic process;carboxylic acid metabolic process;cell differentiation;cellular aldehyde metabolic process;cellular amine metabolic process;cellular amino acid m
26.2913	26.039	26.2511	26.2028	1:Experimental evidence at protein level	cellular process;establishment of localization;establishment of localization in cell;Golgi vesicle transport;intracellular transport;intra-Golgi vesicle-mediated transport;transport;vesicle-mediated transport
29.4465	29.5094	29.8449	29.6478	1:Experimental evidence at protein level	biological regulation;biosynthetic process;cellular biosynthetic process;cellular component organization;cellular component organization at cellular level;cellular component organization or biogenesis;cellular component orga
27.5024	27.2706	27.0735	27.2597	1:Experimental evidence at protein level	anatomical structure development;anatomical structure morphogenesis;axonogenesis;biological regulation;cargo loading into vesicle;cation homeostasis;cell part morphogenesis;cell projection morphogenesis;cell projection
27.5764	27.3505	27.4227	27.1789	1:Experimental evidence at protein level	anatomical structure morphogenesis;biological regulation;cell part morphogenesis;cell projection morphogenesis;cell projection organization;cellular component morphogenesis;cellular component organization;cellular compo
28.2693	28.3623	28.0174	28.1379	1:Experimental evidence at protein level	axon guidance;biological adhesion;biological regulation;cell adhesion;cellular process;cellular response to stimulus;chemotaxis;locomotion;motor axon guidance;regulation of biological process;regulation of cellular process;r
#NUM!	#NUM!	#NUM!	#NUM!	1:Experimental evidence at protein level	basement membrane organization;biological adhesion;cell adhesion;cell-matrix adhesion;cell-substrate adhesion;cellular component organization;cellular component organization at cellular level;cellular component organizat
26.591	26.087	26.3413	26.402	1:Experimental evidence at protein level	biological regulation;cell cycle checkpoint;cellular macromolecule metabolic process;cellular metabolic process;cellular nitrogen compound metabolic process;cellular process;cellular protein metabolic process;cellular respo
25.9056	25.7755	25.3616	25.037	1:Experimental evidence at protein level	cellular component assembly;cellular component assembly at cellular level;cellular component organization;cellular component organization at cellular level;cellular component organization or biogenesis;cellular component c
27.1348	27.0792	27.1251	27.0735	1:Experimental evidence at protein level	autophagy;biological regulation;biosynthetic process;catabolic process;cellular biosynthetic process;cellular catabolic process;cellular macromolecule biosynthetic process;cellular macromolecule catabolic process;cellular m
27.9609	28.4008	28.4476	28.5965	1:Experimental evidence at protein level	arachidonic acid metabolic process;carboxylic acid metabolic process;cellular alkene metabolic process;cellular ketone metabolic process;cellular lipid metabolic process;cellular metabolic process;cellular process;cyclooxyg
27.2342	27.6906	27.5527	27.7648	1:Experimental evidence at protein level	anatomical structure development;brain development;catabolic process;developmental process;gamete generation;lipid catabolic process;lipid metabolic process;male gamete generation;metabolic process;multicellular orga
28.7327	29.0806	29.4748	29.3704	1:Experimental evidence at protein level	anatomical structure development;camera-type eye development;chordate embryonic development;developmental process;embryo development;embryo development ending in birth or egg hatching;eye development;in uter
26.3131	26.3213	26.6003	26.4007	1:Experimental evidence at protein level	establishment of localization;establishment of localization in cell;establishment of protein localization;establishment of protein localization in mitochondrion;establishment of protein localization to organelle;intracellular protein
27.8135	27.1321	27.3848	27.2513	1:Experimental evidence at protein level	
26.9754	26.9332	26.9467	26.6578	1:Experimental evidence at protein level	catabolic process;cell cycle;cell division;cellular catabolic process;cellular component assembly;cellular component assembly at cellular level;cellular component organization;cellular component organization at cellular level;c
26.9314	27.0424	26.7755	26.5724	1:Experimental evidence at protein level	alanyl-tRNA aminoacylation;amine metabolic process;amino acid activation;carboxylic acid metabolic process;cellular amine metabolic process;cellular amino acid metabolic process;cellular component assembly;cellular cor
25.7822	25.8144	25.9889	25.8393	1:Experimental evidence at protein level	anatomical structure development;developmental process;embryonic skeletal system development;skeletal system development;system development
27.5853	27.1891	26.9272	27.3565	1:Experimental evidence at protein level	cellular macromolecule metabolic process;cellular metabolic process;cellular nitrogen compound metabolic process;cellular process;macromolecule metabolic process;metabolic process;mRNA metabolic process;mRNA pro
25.5389	25.1591	25.4678	25.211	1:Experimental evidence at protein level	biological regulation;cellular hormone metabolic process;cellular lipid metabolic process;cellular metabolic process;cellular process;diterpenoid metabolic process;fat-soluble vitamin metabolic process;hormone metabolic pr
27.1059	26.3463	26.8448	26.4761	1:Experimental evidence at protein level	biological regulation;biosynthetic process;cell cycle;cell fate commitment;cell fate commitment involved in formation of primary germ layers;cell surface receptor linked signaling pathway;cellular biosynthetic process;cellular c
26.2401	25.8846	25.3967	25.8712	1:Experimental evidence at protein level	biological regulation;biosynthetic process;cellular biosynthetic process;cellular macromolecule biosynthetic process;cellular macromolecule metabolic process;cellular metabolic process;cellular nitrogen compound metabolic
28.1583	28.2126	28.1496	28.5345	1:Experimental evidence at protein level	cellular component organization;cellular component organization at cellular level;cellular component organization or biogenesis;cellular component organization or biogenesis at cellular level;cellular macromolecule metabolic
27.6605	27.4146	27.3386	27.2846	1:Experimental evidence at protein level	biological regulation;cellular component assembly;cellular component assembly at cellular level;cellular component organization;cellular component organization at cellular level;cellular component organization or biogenesis;
27.0803	27.4185	27.1429	27.2909	1:Experimental evidence at protein level	biological regulation;cell cycle process;cell division;cellular component organization;cellular component organization at cellular level;cellular component organization or biogenesis;cellular component organization or biogenes
26.6375	26.664	26.9567	26.7557	1:Experimental evidence at protein level	
26.3613	25.9255	26.3219	25.9095	1:Experimental evidence at protein level	
27.2199	27.2959	27.5957	27.0954	1:Experimental evidence at protein level	anatomical structure morphogenesis;biological regulation;biosynthetic process;bone morphogenesis;cellular biosynthetic process;cellular component organization;cellular component organization at cellular level;cellular comp
28.1732	#NUM!	28.0092	27.997	1:Experimental evidence at protein level	biosynthetic process;cellular biosynthetic process;cellular macromolecule biosynthetic process;cellular macromolecule metabolic process;cellular metabolic process;cellular nitrogen compound metabolic process;cellular pro
26.2308	26.4806	26.3904	26.649	1:Experimental evidence at protein level	actin cytoskeleton organization;actin filament organization;actin filament-based process;actin polymerization or depolymerization;anatomical structure development;anatomical structure formation involved in morphogenesis;a
26.6595	26.6612	26.5702	26.5434	1:Experimental evidence at protein level	cellular process;establishment of localization;establishment of protein localization;protein transport;transport;vesicle-mediated transport
26.6165	26.8845	26.9852	27.094	1:Experimental evidence at protein level	catabolic process;lipid catabolic process;lipid metabolic process;metabolic process;primary metabolic process
25.3705	24.8195	24.8429	24.8287	1:Experimental evidence at protein level	anatomical structure homeostasis;biological regulation;cellular macromolecule metabolic process;cellular metabolic process;cellular nitrogen compound metabolic process;cellular process;homeostatic process;macromolecu
28.1625	27.7486	27.9152	27.6344	1:Experimental evidence at protein level	biological regulation;gamete generation;male gamete generation;multicellular organismal process;multicellular organismal reproductive process;positive regulation of biological process;positive regulation of cell proliferation;p
29.1302	28.9131	28.9504	28.7266	1:Experimental evidence at protein level	anatomical structure morphogenesis;biological regulation;cell morphogenesis;cellular component morphogenesis;cellular component organization;cellular component organization or biogenesis;cellular developmental proces
26.9673	26.3782	26.6766	26.4	1:Experimental evidence at protein level	biological regulation;biosynthetic process;cellular biosynthetic process;cellular macromolecule biosynthetic process;cellular macromolecule metabolic process;cellular metabolic process;cellular nitrogen compound metabolic
26.4549	26.5756	26.36	26.3187	1:Experimental evidence at protein level	apoptosis;biological regulation;cell death;cellular macromolecule metabolic process;cellular metabolic process;cellular nitrogen compound metabolic process;cellular process;death;gene expression;immune system process;
27.6407	27.6345	27.449	27.4011	1:Experimental evidence at protein level	biological regulation;posttranscriptional regulation of gene expression;protein destabilization;regulation of biological process;regulation of biological quality;regulation of cellular component biogenesis;regulation of cellular cor
28.5339	28.274	28.5022	28.3291	1:Experimental evidence at protein level	amine biosynthetic process;amine metabolic process;biosynthetic process;carboxylic acid biosynthetic process;carboxylic acid metabolic process;cellular amine metabolic process;cellular amino acid biosynthetic process;ce
26.7561	26.6655	27.1343	27.0833	1:Experimental evidence at protein level	autophagy;biological regulation;catabolic process;cell communication;cell cycle;cell cycle process;cell death;cellular catabolic process;cellular component organization;cellular component organization at cellular level;cellular
28.7237	28.9282	29.0722	29.1341	1:Experimental evidence at protein level	
26.1641	26.0524	26.0299	25.9218	1:Experimental evidence at protein level	biosynthetic process;cellular biosynthetic process;cellular component disassembly;cellular component disassembly at cellular level;cellular component organization;cellular component organization at cellular level;cellular cor

26.6149	26.8679	26.4463	26.6238	1:Experimental evidence at protein level	attachment of spindle microtubules to chromosome;attachment of spindle microtubules to kinetochore;attachment of spindle microtubules to kinetochore involved in mitotic sister chromatid segregation;biological regulation;
27.3581	27.1794	27.0992	27.0468	1:Experimental evidence at protein level	biological regulation;cell death;cellular metabolic process;cellular process;cellular response to stimulus;death;dephosphorylation;intracellular signal transduction;metabolic process;necroptosis;necrotic cell death;phosphate-c
27.5888	27.2558	27.4399	27.7329	1:Experimental evidence at protein level	biological regulation;catabolic process;cellular catabolic process;cellular macromolecule catabolic process;cellular macromolecule metabolic process;cellular metabolic process;cellular nitrogen compound metabolic process
27.9198	28.0913	28.0298	27.9964	1:Experimental evidence at protein level	biosynthetic process;cell cycle phase;cell cycle process;cell division;cellular biosynthetic process;cellular component organization;cellular component organization at cellular level;cellular component organization or biogenesi
28.1329	28.2081	27.7786	28.1398	1:Experimental evidence at protein level	cellular component organization;cellular component organization or biogenesis;cellular membrane organization;cellular process;membrane organization;nuclear membrane organization
27.5194	27.4908	27.398	27.3583	1:Experimental evidence at protein level	antigen processing and presentation;antigen processing and presentation of exogenous antigen;antigen processing and presentation of exogenous peptide antigen;antigen processing and presentation of exogenous peptide
25.7301	25.2068	25.1429	24.7577	1:Experimental evidence at protein level	cellular macromolecule metabolic process;cellular metabolic process;cellular nitrogen compound metabolic process;cellular process;macromolecule metabolic process;metabolic process;nitrogen compound metabolic proce
#NUM!	#NUM!	#NUM!	#NUM!	1:Experimental evidence at protein level	cellular component assembly;cellular component assembly at cellular level;cellular component organization;cellular component organization at cellular level;cellular component organization or biogenesis;cellular component c
27.7875	28.063	27.8317	28.0644	1:Experimental evidence at protein level	biological regulation;biosynthetic process;carbon catabolite regulation of transcription;carbon catabolite repression of transcription;cell communication;cell cycle process;cell division;cellular biosynthetic process;cellular com
26.6081	26.3486	26.5654	26.683	1:Experimental evidence at protein level	
27.1237	26.9837	27.0002	26.8584	1:Experimental evidence at protein level	amine catabolic process;amine metabolic process;carboxylic acid catabolic process;carboxylic acid metabolic process;catabolic process;cellular amine metabolic process;cellular amino acid catabolic process;cellular amino
27.76	27.7961	28.0135	27.7991	1:Experimental evidence at protein level	apoptosis;autophagic cell death;biological regulation;catabolic process;cell communication;cell death;cellular catabolic process;cellular component assembly;cellular component organization;cellular component organization
27.0154	26.9153	27.0736	26.8001	1:Experimental evidence at protein level	biological regulation;biosynthetic process;cellular biosynthetic process;cellular macromolecule biosynthetic process;cellular macromolecule metabolic process;cellular metabolic process;cellular process;cellular protein metab
27.2125	27.1125	27.1057	26.9805	1:Experimental evidence at protein level	cellular component organization;cellular component organization at cellular level;cellular component organization or biogenesis;cellular component organization or biogenesis at cellular level;cellular macromolecule metabolic
26.7282	27.1847	27.6292	27.694	5:Protein uncertain	
26.672	26.5216	26.8338	26.8439	1:Experimental evidence at protein level	biological regulation;cell differentiation;cell proliferation;cellular component assembly;cellular component assembly at cellular level;cellular component organization;cellular component organization at cellular level;cellular com
28.1325	28.0633	28.2654	28.0421	1:Experimental evidence at protein level	biological regulation;cellular component assembly;cellular component assembly at cellular level;cellular component organization;cellular component organization at cellular level;cellular component organization or biogenesis;
25.4779	25.279	25.1568	24.7859	1:Experimental evidence at protein level	anatomical structure development;anatomical structure morphogenesis;biological regulation;cell cycle;cell development;cell differentiation;cellular component disassembly;cellular component disassembly at cellular level;cell
26.5962	26.8428	26.8032	26.5899	1:Experimental evidence at protein level	3'-phosphoadenosine 5'-phosphosulfate biosynthetic process;3'-phosphoadenosine 5'-phosphosulfate metabolic process;amine metabolic process;aminoglycan metabolic process;anatomical structure development;biosynt
26.3046	26.0899	26.3035	26.0947	1:Experimental evidence at protein level	cellular macromolecule metabolic process;cellular metabolic process;cellular nitrogen compound metabolic process;cellular process;macromolecule metabolic process;metabolic process;ncRNA metabolic process;ncRNA pr
#NUM!	#NUM!	#NUM!	#NUM!	1:Experimental evidence at protein level	biological adhesion;biological regulation;cell adhesion;cellular process;developmental process;multicellular organismal development;multicellular organismal process;positive regulation of biological process;positive regulation
25.3071	25.8043	#NUM!	25.2379	1:Experimental evidence at protein level	alcohol biosynthetic process;alcohol metabolic process;biological regulation;biosynthetic process;carbohydrate biosynthetic process;carbohydrate metabolic process;cell surface receptor linked signaling pathway;cellular bio
28.8018	28.2997	28.7178	28.6401	1:Experimental evidence at protein level	biological regulation;biosynthetic process;cell differentiation;cellular biosynthetic process;cellular developmental process;cellular macromolecule biosynthetic process;cellular macromolecule metabolic process;cellular metab
27.3408	26.9831	27.4393	27.3776	1:Experimental evidence at protein level	biological regulation;cellular macromolecule metabolic process;cellular metabolic process;cellular process;cellular protein metabolic process;macromolecule metabolic process;metabolic process;positive regulation of biologi
26.0247	26.3947	26.0735	25.8005	1:Experimental evidence at protein level	actin filament severing;actin filament-based process;anatomical structure development;anatomical structure morphogenesis;axon guidance;biological adhesion;biological regulation;cell adhesion;cell morphogenesis;cell morp
26.3657	26.7064	27.1006	26.8428	1:Experimental evidence at protein level	anatomical structure development;biological regulation;biosynthetic process;Cajal body organization;cell cycle process;cell proliferation;cellular biosynthetic process;cellular component organization;cellular component organ
28.9668	29.0402	28.9565	28.7364	1:Experimental evidence at protein level	biological regulation;cellular process;ER to Golgi vesicle-mediated transport;establishment of localization;establishment of localization in cell;establishment of protein localization;Golgi vesicle transport;intracellular transport;p
27.5595	27.4849	27.5724	27.313	1:Experimental evidence at protein level	antigen processing and presentation;antigen processing and presentation of exogenous antigen;antigen processing and presentation of exogenous peptide antigen;antigen processing and presentation of exogenous peptide
28.362	27.9025	28.0747	27.9717	1:Experimental evidence at protein level	activation of signaling protein activity involved in unfolded protein response;biological regulation;biosynthetic process;calcium ion homeostasis;cation homeostasis;cellular biosynthetic process;cellular calcium ion homeostas
27.2247	27.3722	26.6255	26.7098	1:Experimental evidence at protein level	cellular metabolic process;cellular process;electron transport chain;generation of precursor metabolites and energy;metabolic process;mitochondrial electron transport, NADH to ubiquinone;oxidation-reduction process;respi
26.2675	26.5624	26.9488	26.8443	1:Experimental evidence at protein level	amine metabolic process;biological regulation;biosynthetic process;carboxylic acid metabolic process;cell redox homeostasis;cellular amine metabolic process;cellular amino acid metabolic process;cellular biosynthetic proc
30.3248	30.9723	31.3742	31.2918	1:Experimental evidence at protein level	activation of MAPK activity;aging;amine metabolic process;anatomical structure development;anatomical structure homeostasis;anterograde axon cargo transport;auditory receptor cell stereocilium organization;axon cargo tr
25.5842	25.7562	26.266	#NUM!	1:Experimental evidence at protein level	alcohol catabolic process;alcohol metabolic process;biological regulation;biosynthetic process;carbohydrate metabolic process;catabolic process;cell communication;cell-cell signaling;cellular biosynthetic process;cellular m
29.197	29.5096	29.0148	29.3158	1:Experimental evidence at protein level	apical junction assembly;axon guidance;biological regulation;cell junction assembly;cell junction organization;cell migration;cell motility;cell-cell junction assembly;cell-cell junction organization;cellular component assembly;c
30.0789	31.4845	31.0352	30.994	1:Experimental evidence at protein level	amine metabolic process;anatomical structure development;biological regulation;carboxylic acid metabolic process;cell proliferation;cellular amine metabolic process;cellular amino acid metabolic process;cellular ketone met
29.3775	29.1966	29.5128	29.5553	1:Experimental evidence at protein level	antigen processing and presentation;antigen processing and presentation of exogenous antigen;antigen processing and presentation of exogenous peptide antigen;antigen processing and presentation of exogenous peptide
#NUM!	26.0324	25.9034	26.4695	1:Experimental evidence at protein level	alcohol metabolic process;anatomical structure development;anatomical structure homeostasis;anatomical structure morphogenesis;behavior;biological regulation;carbohydrate catabolic process;carbohydrate metabolic pro
27.3214	26.8222	26.7417	26.7762	1:Experimental evidence at protein level	acetyl-CoA catabolic process;acetyl-CoA metabolic process;alcohol metabolic process;biological regulation;carbohydrate metabolic process;carboxylic acid metabolic process;catabolic process;cellular carbohydrate metabo
25.9651	25.8288	25.2442	25.515	1:Experimental evidence at protein level	anatomical structure development;anatomical structure morphogenesis;B cell activation;B cell differentiation;biological adhesion;biological regulation;blood coagulation;blood vessel remodeling;cell activation;cell adhesion;ce
28.2908	28.5572	28.5764	28.4543	1:Experimental evidence at protein level	amine biosynthetic process;amine metabolic process;biosynthetic process;cellular amine metabolic process;cellular biogenic amine biosynthetic process;cellular biogenic amine metabolic process;cellular biosynthetic proces
28.9469	29.0868	29.2168	29.1486	1:Experimental evidence at protein level	amine metabolic process;aminoglycan metabolic process;biological regulation;carbohydrate metabolic process;glycosaminoglycan metabolic process;hyaluronan metabolic process;macromolecule metabolic process;metabo
26.4454	26.1968	25.9578	26.0264	1:Experimental evidence at protein level	apoptosis;biological regulation;cell death;cell surface receptor linked signaling pathway;cellular process;cellular response to chemical stimulus;cellular response to cytokine stimulus;cellular response to organic substance;cel
30.644	30.824	31.0329	31.0335	1:Experimental evidence at protein level	biosynthetic process;cellular biosynthetic process;cellular macromolecule biosynthetic process;cellular macromolecule metabolic process;cellular metabolic process;cellular process;cellular protein metabolic process;gene ex
27.3852	27.3434	27.2881	27.1866	1:Experimental evidence at protein level	biological regulation;defense response;immune effector process;immune response;immune system process;inflammatory response;innate immune response;multi-organism process;negative regulation of biological process;n
29.2104	29.4553	29.7042	29.4546	1:Experimental evidence at protein level	biological regulation;biosynthetic process;cellular biosynthetic process;cellular macromolecule biosynthetic process;cellular macromolecule metabolic process;cellular metabolic process;cellular nitrogen compound metabolic
27.8969	28.9911	29.3147	29.3595	1:Experimental evidence at protein level	
27.8147	27.7158	27.463	27.7173	1:Experimental evidence at protein level	apoptosis;apoptosis involved in morphogenesis;cell death;cellular developmental process;cellular process;death;developmental process;developmental programmed cell death;programmed cell death
28.0199	28.0456	27.858	27.8964	1:Experimental evidence at protein level	biological regulation;biosynthetic process;cell cycle;cell cycle process;cellular biosynthetic process;cellular component assembly;cellular component organization;cellular component organization or biogenesis;cellular macror
30.6279	31.5688	31.5899	31.688	1:Experimental evidence at protein level	biological regulation;cellular process;cellular response to stimulus;negative regulation of biological process;negative regulation of biosynthetic process;negative regulation of cell proliferation;negative regulation of cellular bios
27.3202	27.1437	27.2729	27.0921	1:Experimental evidence at protein level	anatomical structure homeostasis;base-excision repair;biological regulation;biosynthetic process;cell cycle;cellular biosynthetic process;cellular component organization;cellular component organization at cellular level;cellula
26.2009	25.9838	25.964	25.6515	1:Experimental evidence at protein level	anatomical structure homeostasis;base-excision repair;biological regulation;biosynthetic process;cell cycle;cellular biosynthetic process;cellular component organization;cellular component organization at cellular level;cellula
26.8081	26.5481	26.6362	26.5655	1:Experimental evidence at protein level	anatomical structure homeostasis;base-excision repair;biological regulation;biosynthetic process;cell cycle;cellular biosynthetic process;cellular component organization;cellular component organization at cellular level;cellula
30.5767	30.8071	31.1485	31.0314	1:Experimental evidence at protein level	biological regulation;cellular process;cellular response to stimulus;establishment of localization;establishment of localization in cell;intracellular transport;regulation of biological process;regulation of catalytic activity;regulation
27.661	27.4782	27.537	27.5996	1:Experimental evidence at protein level	anatomical structure development;apoptosis;apoptosis in response to endoplasmic reticulum stress;biological regulation;brain development;catabolic process;cell cycle process;cell death;cell differentiation;cellular catabolic
28.9863	28.7322	29.0829	29.0234	1:Experimental evidence at protein level	activation of immune response;activation of innate immune response;anaphase-promoting complex-dependent proteasomal ubiquitin-dependent protein catabolic process;antigen processing and presentation;antigen proces
27.3301	27.3814	27.4512	27.5058	1:Experimental evidence at protein level	anatomical structure homeostasis;base-excision repair;biological regulation;biosynthetic process;cell cycle checkpoint;cell cycle process;cell proliferation;cellular biosynthetic process;cellular component organization;cellular
27.0354	27.3118	27.6085	27.5237	1:Experimental evidence at protein level	biological regulation;biosynthetic process;cell cycle phase;cell cycle process;cellular biosynthetic process;cellular component assembly;cellular component organization;cellular component organization at cellular level;cellula
27.9764	27.6083	27.9139	27.7271	1:Experimental evidence at protein level	biological regulation;biosynthetic process;cell differentiation;cell proliferation;cellular biosynthetic process;cellular developmental process;cellular macromolecule biosynthetic process;cellular macromolecule metabolic proces
30.0872	29.9672	29.7243	30.0287	1:Experimental evidence at protein level	biosynthetic process;catabolic process;cellular biosynthetic process;cellular catabolic process;cellular component disassembly;cellular component disassembly at cellular level;cellular component organization;cellular compo
26.8945	26.9355	27.215	26.9403	1:Experimental evidence at protein level	biosynthetic process;cellular biosynthetic process;cellular macromolecule biosynthetic process;cellular macromolecule metabolic process;cellular metabolic process;cellular nitrogen compound metabolic process;cellular pro
27.6367	27.5386	27.4951	27.8245	1:Experimental evidence at protein level	apoptosis;biological regulation;cell death;cellular component assembly;cellular component organization;cellular component organization at cellular level;cellular component organization or biogenesis;cellular component orga
28.6639	28.4252	28.289	28.3682	1:Experimental evidence at protein level	4-hydroxyproline metabolic process;amine metabolic process;anatomical structure development;biological regulation;carboxylic acid metabolic process;cell redox homeostasis;cellular amine metabolic process;cellular aminc
26.3317	26.1446	26.3392	26.2674	1:Experimental evidence at protein level	cellular macromolecule metabolic process;cellular metabolic process;cellular nitrogen compound metabolic process;cellular process;cellular protein metabolic process;macromolecule metabolic process;macromolecule modi
26.5512	26.7285	26.5219	26.4751	1:Experimental evidence at protein level	alcohol biosynthetic process;alcohol metabolic process;biological adhesion;biosynthetic process;carbohydrate biosynthetic process;carbohydrate metabolic process;cell adhesion;cell death;cell-cell adhesion;cellular biosynt
25.9956	25.5407	25.5311	25.6705	1:Experimental evidence at protein level	cellular component biogenesis;cellular component biogenesis at cellular level;cellular component organization or biogenesis;cellular component organization or biogenesis at cellular level;cellular process;metabolic process;ri
27.443	27.6038	27.5902	27.3314	1:Experimental evidence at protein level	biological regulation;biosynthetic process;cellular biosynthetic process;cellular macromolecule biosynthetic process;cellular macromolecule metabolic process;cellular metabolic process;cellular nitrogen compound metabolic
30.1088	30.0462	30.2565	30.0093	1:Experimental evidence at protein level	biological regulation;biosynthetic process;cellular biosynthetic process;cellular macromolecule biosynthetic process;cellular macromolecule metabolic process;cellular metabolic process;cellular nitrogen compound metabolic
28.507	28.6058	28.5652	28.5887	1:Experimental evidence at protein level	antigen processing and presentation;biological regulation;cell projection organization;cellular component organization;cellular component organization at cellular level;cellular component organization or biogenesis;cellular co
26.758	26.601	27.0984	26.9082	1:Experimental evidence at protein level	
25.5198	25.6222	25.6055	25.4716	1:Experimental evidence at protein level	activation of JUN kinase activity;activation of MAPK activity;ameboidal cell migration;biological regulation;biosynthetic process;cell migration;cell motility;cellular biosynthetic process;cellular component movement;cellular co
27.7127	27.5067	27.575	27.5964	1:Experimental evidence at protein level	cellular component assembly;cellular component assembly at cellular level;cellular component organization;cellular component organization at cellular level;cellular component organization or biogenesis;cellular component c
26.5675	27.1066	27.1186	26.6559	1:Experimental evidence at protein level	
26.5851	26.7538	26.9877	26.6779	1:Experimental evidence at protein level	biological regulation;cellular component organization;cellular component organization or biogenesis;cellular macromolecule metabolic process;cellular membrane organization;cellular metabolic process;cellular process;cellul
26.7887	26.4629	27.0792	26.7637	1:Experimental evidence at protein level	cellular process;endosome transport;establishment of localization;establishment of localization in cell;establishment of protein localization;intracellular transport;protein transport;retrograde transport, endosome to Golgi;trans
27.4222	27.4897	27.5976	27.4179	1:Experimental evidence at protein level	response to abiotic stimulus;response to ionizing radiation;response to radiation;response to stimulus;response to X-ray
25.6764	25.842	25.6961	25.6645	1:Experimental evidence at protein level	biological regulation;cell cycle;cell cycle phase;cell cycle process;cell division;cellular component organization;cellular component organization at cellular level;cellular component organization or biogenesis;cellular componer
26.5322	26.3812	26.3942	26.5573	1:Experimental evidence at protein level	biological regulation;cellular component organization;cellular component organization at cellular level;cellular component organization or biogenesis;cellular component organization or biogenesis at cellular level;cellular macro
26.2153	26.3078	26.5084	26.0467	1:Experimental evidence at protein level	biological regulation;cell differentiation;cellular developmental process;cellular process;developmental process;muscle cell differentiation;negative regulation of biological process;negative regulation of cell communication;neg
25.7155	#NUM!	25.3957	24.6782	1:Experimental evidence at protein level	cell differentiation;cellular developmental process;cellular process;developmental process;fat cell differentiation
26.8981	26.6221	26.6187	26.4013	1:Experimental evidence at protein level	assembly of spliceosomal tri-snRNP;cellular component assembly;cellular component assembly at cellular level;cellular component organization;cellular component organization at cellular level;cellular component organizatic
27.3583	27.3365	27.1595	27.1198	1:Experimental evidence at protein level	adherens junction organization;antigen processing and presentation;biological regulation;cell junction organization;cell-cell junction organization;cellular component organization;cellular component organization at cellular leve
26.3506	#NUM!	26.8581	26.0911	1:Experimental evidence at protein level	biological regulation;cell cycle arrest;cell cycle process;cellular metabolic process;cellular process;cellular respiration;energy derivation by oxidation of organic compounds;generation of precursor metabolites and energy;met
27.7636	27.7579	27.708	27.6477	1:Experimental evidence at protein level	biological regulation;cellular component organization;cellular component organization at cellular level;cellular component organization or biogenesis;cellular component organization or biogenesis at cellular level;cellular proce
25.7154	25.3514	25.719	25.7606	1:Experimental evidence at protein level	
26.565	25.6897	26.0656	25.9656	1:Experimental evidence at protein level	cellular macromolecule metabolic process;cellular metabolic process;cellular nitrogen compound metabolic process;cellular process;macromolecule metabolic process;metabolic process;nitrogen compound metabolic proce
25.9783	#NUM!	26.1156	26.1085	1:Experimental evidence at protein level	cellular component assembly;cellular component assembly at cellular level;cellular component organization;cellular component organization at cellular level;cellular component organization or biogenesis;cellular component c
25.8746	25.946	25.7232	25.3557	1:Experimental evidence at protein level	cellular macromolecule metabolic process;cellular metabolic process;cellular nitrogen compound metabolic process;cellular process;macromolecule metabolic process;macromolecule modification;metabolic process;ncRNA
27.3695	27.6742	27.495	27.3888	1:Experimental evidence at protein level	anatomical structure morphogenesis;biological regulation;cell differentiation;cell differentiation in hindbrain;cell part morphogenesis;cell projection morphogenesis;cell projection organization;cellular component assembly;cell
25.0589	#NUM!	24.6917	24.8328	1:Experimental evidence at protein level	amine biosynthetic process;amine metabolic process;anatomical structure development;aspartate family amino acid biosynthetic process;aspartate family amino acid metabolic process;biosynthetic process;carboxylic acid b
27.1072	26.3586	26.7545	26.6992	1:Experimental evidence at protein level	cellular component biogenesis;cellular component biogenesis at cellular level;cellular component organization or biogenesis;cellular component organization or biogenesis at cellular level;cellular process;ribonucleoprotein co
25.9853	25.9473	25.8894	26.018	1:Experimental evidence at protein level	cellular component organization;cellular component organization at cellular level;cellular component organization or biogenesis;cellular component organization or biogenesis at cellular level;cellular macromolecule metabolic
27.4236	26.5823	26.8927	26.8904	1:Experimental evidence at protein level	biological regulation;cell proliferation;cellular component biogenesis;cellular component biogenesis at cellular level;cellular component organization or biogenesis;cellular component organization or biogenesis at cellular level
28.5172	28.5296	28.3468	28.6941	1:Experimental evidence at protein level	
25.5129	#NUM!	25.4615	25.7716	1:Experimental evidence at protein level	actin cytoskeleton organization;actin filament-based process;cell communication;cell-cell signaling;cellular component organization;cellular component organization at cellular level;cellular component organization or biogene
28.2122	28.151	28.0603	28.1062	1:Experimental evidence at protein level	apoptosis;cell death;cellular macromolecule metabolic process;cellular metabolic process;cellular process;cellular protein metabolic process;death;macromolecule metabolic process;macromolecule modification;metabolic p
27.4682	27.5112	27.6567	27.6925	1:Experimental evidence at protein level	cellular macromolecule metabolic process;cellular metabolic process;cellular process;cellular protein metabolic process;establishment of localization;establishment of localization in cell;establishment of protein localization;es
25.2132	24.9681	24.8641	24.7978	1:Experimental evidence at protein level	anatomical structure development;apoptosis;biological regulation;cell cycle phase;cell cycle process;cell death;cell division;cellular component organization;cellular component organization at cellular level;cellular component
26.299	26.457	26.3845	26.6339	1:Experimental evidence at protein level	
25.9107	25.8058	25.5213	25.4294	1:Experimental evidence at protein level	
26.546	26.7293	26.6179	26.3071	1:Experimental evidence at protein level	activation of JUN kinase activity;activation of MAPK activity;activation of MAPKK activity;activation of protein kinase activity;biological regulation;cell cycle arrest;cell cycle checkpoint;cell cycle process;cell death;cell differen
27.3418	26.8811	26.7641	26.6155	1:Experimental evidence at protein level	anatomical structure formation involved in morphogenesis;angiogenesis;antigen processing and presentation;antigen processing and presentation of endogenous antigen;antigen processing and presentation of endogenous
28.5304	28.3418	28.108	28.12	1:Experimental evidence at protein level	cellular component biogenesis;cellular component biogenesis at cellular level;cellular component organization or biogenesis;cellular component organization or biogenesis at cellular level;cellular process;ribonucleoprotein co
26.9027	26.9705	27.4332	27.1185	1:Experimental evidence at protein level	anatomical structure homeostasis;biological regulation;cell cycle process;cellular component organization;cellular component organization at cellular level;cellular component organization or biogenesis;cellular component org
28.3113	28.3811	28.7245	28.5958	1:Experimental evidence at protein level	actin cytoskeleton organization;actin filament depolymerization;actin filament organization;actin filament-based process;actin polymerization or depolymerization;actomyosin structure organization;anatomical structure homec
25.711	25.3872	25.5033	25.7333	1:Experimental evidence at protein level	apoptosis;biological regulation;biosynthetic process;cell death;cellular biosynthetic process;cellular component assembly;cellular component assembly at cellular level;cellular component organization;cellular component orga
24.2462	#NUM!	24.0564	23.6763	1:Experimental evidence at protein level	biological regulation;biosynthetic process;cell cycle checkpoint;cellular biosynthetic process;cellular component organization;cellular component organization at cellular level;cellular component organization or biogenesis;cel
27.0026	26.9051	26.9245	26.7531	1:Experimental evidence at protein level	cellular component organization;cellular component organization at cellular level;cellular component organization or biogenesis;cellular component organization or biogenesis at cellular level;cellular membrane organization;ce
25.2015	25.0336	25.2764	24.9964	1:Experimental evidence at protein level	biological regulation;cellular macromolecule metabolic process;cellular metabolic process;cellular nitrogen compound metabolic process;cellular process;macromolecule metabolic process;macromolecule modification;metab
26.8316	26.6674	26.9901	26.7361	1:Experimental evidence at protein level	anatomical structure development;astral microtubule organization;biological regulation;cell differentiation;cell proliferation;cellular component organization;cellular component organization at cellular level;cellular component o
27.917	27.8553	27.8593	27.7163	1:Experimental evidence at protein level	acrosome assembly;anatomical structure formation involved in morphogenesis;cellular component assembly;cellular component assembly at cellular level;cellular component assembly involved in morphogenesis;cellular com
27.1266	27.0694	27.5363	27.577	1:Experimental evidence at protein level	apoptosis;biological regulation;catabolic process;cell death;cellular catabolic process;cellular component disassembly;cellular component disassembly at cellular level;cellular component disassembly involved in apoptosis;c
26.3277	26.2044	26.4167	26.1131	1:Experimental evidence at protein level	anatomical structure development;biological regulation;cell cycle phase;cell cycle process;cell differentiation;cell projection organization;cellular component organization;cellular component organization at cellular level;cellula
27.7212	27.8218	27.6466	27.6719	1:Experimental evidence at protein level	biological regulation;cell surface receptor linked signaling pathway;cellular component organization;cellular component organization or biogenesis;cellular localization;cellular macromolecule localization;cellular membrane org
29.2152	29.2444	29.4023	29.4507	1:Experimental evidence at protein level	biological regulation;regulation of biological process;regulation of cell proliferation;regulation of cellular process
28.2536	28.5387	28.4975	28.5242	1:Experimental evidence at protein level	anaphase-promoting complex-dependent proteasomal ubiquitin-dependent protein catabolic process;attachment of spindle microtubules to chromosome;attachment of spindle microtubules to kinetochore;biological regulati
27.0671	26.7292	26.9297	26.598	1:Experimental evidence at protein level	anatomical structure development;biological regulation;biosynthetic process;bone development;cellular biosynthetic process;cellular metabolic process;cellular nitrogen compound biosynthetic process;cellular nitrogen comp
27.148	26.9838	27.0253	27.0601	1:Experimental evidence at protein level	activation of JUN kinase activity;activation of MAPK activity;biological regulation;cell differentiation;cellular component assembly;cellular component organization;cellular component organization or biogenesis;cellular develop
25.7226	25.4423	25.5105	25.3225	1:Experimental evidence at protein level	axon guidance;biological regulation;cell surface receptor linked signaling pathway;cellular process;cellular response to stimulus;chemotaxis;enzyme linked receptor protein signaling pathway;intracellular signal transduction;lc
26.7974	26.5195	26.5922	26.4022	1:Experimental evidence at protein level	actin cytoskeleton organization;actin filament-based process;activation of protein kinase activity;apoptosis;biological regulation;cell cycle;cell death;cell growth;cell migration;cell motility;cell proliferation;cellular component m
24.808	25.1239	#NUM!	25.4344	1:Experimental evidence at protein level	alcohol metabolic process;biological regulation;catabolic process;cellular catabolic process;cellular component organization;cellular component organization or biogenesis;cellular lipid metabolic process;cellular macromolec
28.31	28.7506	28.5788	28.4249	1:Experimental evidence at protein level	biological regulation;blood coagulation;coagulation;fibrinolysis;hemostasis;multicellular organismal process;negative regulation of apoptosis;negative regulation of biological process;negative regulation of blood coagulation;n
27.5009	27.0113	27.1356	27.0155	1:Experimental evidence at protein level	cellular macromolecule metabolic process;cellular metabolic process;cellular nitrogen compound metabolic process;cellular process;gene expression;macromolecule metabolic process;metabolic process;mRNA metabolic p
29.0184	29.0949	29.2466	29.2696	1:Experimental evidence at protein level	actin cytoskeleton organization;actin filament-based process;activation of phospholipase C activity;activation of protein kinase A activity;activation of protein kinase activity;actomyosin structure organization;anatomical struc
29.6283	29.476	29.5515	29.5699	1:Experimental evidence at protein level	acetyl-CoA biosynthetic process;acetyl-CoA biosynthetic process from pyruvate;acetyl-CoA catabolic process;acetyl-CoA metabolic process;alcohol metabolic process;biological regulation;biosynthetic process;carbohydrate
30.9123	30.9628	31.4738	31.2199	1:Experimental evidence at protein level	biological regulation;calcium ion homeostasis;calcium ion transport;cation homeostasis;cation transport;cellular calcium ion homeostasis;cellular cation homeostasis;cellular chemical homeostasis;cellular divalent inorganic c
25.7399	#NUM!	25.5409	24.8258	1:Experimental evidence at protein level	biological regulation;cell cycle;cell cycle checkpoint;cellular component assembly;cellular component assembly at cellular level;cellular component organization;cellular component organization at cellular level;cellular compor
27.134	26.9641	26.7885	26.8252	1:Experimental evidence at protein level	ATP hydrolysis coupled proton transport;biological regulation;cation homeostasis;cation transport;cell surface receptor linked signaling pathway;cellular cation homeostasis;cellular chemical homeostasis;cellular component c
28.2215	27.7498	27.6373	27.8223	1:Experimental evidence at protein level	box C/D snoRNA 3'-end processing;box C/D snoRNA metabolic process;box C/D snoRNA processing;cell differentiation;cellular developmental process;cellular macromolecule metabolic process;cellular metabolic process;c
28.4476	28.2893	28.0305	28.1005	1:Experimental evidence at protein level	biological regulation;catabolic process;cellular catabolic process;cellular component assembly;cellular component assembly at cellular level;cellular component organization;cellular component organization at cellular level;ce

28.3086	28.3678	28.5239	28.6322	1:Experimental evidence at protein level	biological regulation;blastocyst hatching;cellular process;cellular response to stimulus;developmental process;embryo implantation;hatching;organism emergence from protective structure;positive regulation of biological proc
28.3304	28.3053	28.7591	28.3287	1:Experimental evidence at protein level	carboxylic acid catabolic process;carboxylic acid metabolic process;catabolic process;cellular catabolic process;cellular ketone metabolic process;cellular lipid catabolic process;cellular lipid metabolic process;cellular metab
27.4719	27.7987	27.5789	27.4774	1:Experimental evidence at protein level	biological regulation;cell surface receptor linked signaling pathway;cellular process;cellular response to chemical stimulus;cellular response to cytokine stimulus;cellular response to interferon-gamma;cellular response to orga
27.0634	27.2405	27.0414	27.0988	1:Experimental evidence at protein level	amine biosynthetic process;amine catabolic process;amine metabolic process;biosynthetic process;carboxylic acid biosynthetic process;carboxylic acid catabolic process;carboxylic acid metabolic process;catabolic process
27.07	27.3596	27.0778	27.2817	1:Experimental evidence at protein level	adaptive immune response;adaptive immune response based on somatic recombination of immune receptors built from immunoglobulin superfamily domains;biological regulation;cellular component biogenesis;cellular comp
29.8697	29.4596	29.6751	29.5233	1:Experimental evidence at protein level	actin filament capping;barbed-end actin filament capping;biological regulation;blood coagulation;cellular component assembly;cellular component movement;cellular component organization;cellular component organization
28.907	28.9039	29.0006	28.6859	1:Experimental evidence at protein level	anatomical structure development;cargo loading into vesicle;cellular component assembly;cellular component assembly at cellular level;cellular component organization;cellular component organization at cellular level;cellula
28.1953	30.1827	29.394	30.25	1:Experimental evidence at protein level	biological regulation;biosynthetic process;catabolic process;cellular biosynthetic process;cellular catabolic process;cellular macromolecule biosynthetic process;cellular macromolecule metabolic process;cellular metabolic p
29.96	29.9204	29.8836	29.8531	1:Experimental evidence at protein level	biological regulation;cell surface receptor linked signaling pathway;cellular macromolecule metabolic process;cellular metabolic process;cellular nitrogen compound metabolic process;cellular process;cellular response to che
25.5449	25.2982	25.4784	25.1479	1:Experimental evidence at protein level	anatomical structure formation involved in morphogenesis;angiogenesis;axon guidance;biological regulation;blood coagulation;cell activation;cell migration;cell motility;cell projection assembly;cell projection organization;cell
28.0429	28.0054	28.2697	27.923	1:Experimental evidence at protein level	amine biosynthetic process;amine metabolic process;aspartate family amino acid metabolic process;biosynthetic process;carboxylic acid metabolic process;cellular amine metabolic process;cellular amino acid metabolic pro
28.2906	28.9337	28.475	28.6009	1:Experimental evidence at protein level	biological regulation;cellular process;cellular response to stimulus;regulation of biological process;regulation of biosynthetic process;regulation of cellular biosynthetic process;regulation of cellular macromolecule biosynthetic
27.8846	27.9889	27.7046	27.9126	1:Experimental evidence at protein level	biological regulation;biosynthetic process;cell differentiation;cellular biosynthetic process;cellular developmental process;cellular macromolecule biosynthetic process;cellular macromolecule metabolic process;cellular metab
29.6149	29.2111	29.3924	29.2152	1:Experimental evidence at protein level	antigen processing and presentation;antigen processing and presentation of exogenous antigen;antigen processing and presentation of exogenous peptide antigen;antigen processing and presentation of exogenous peptide
28.0045	27.9357	28.022	27.9132	1:Experimental evidence at protein level	activation of immune response;activation of innate immune response;anaphase-promoting complex-dependent proteasomal ubiquitin-dependent protein catabolic process;antigen processing and presentation;antigen proces
31.1374	31.2656	31.0102	31.1108	1:Experimental evidence at protein level	actin cytoskeleton organization;actin filament-based process;anatomical structure development;anatomical structure morphogenesis;apical junction assembly;apolipoprotein A-I-mediated signaling pathway;axon guidance;bi
28.8696	#NUM!	29.3086	29.3288	1:Experimental evidence at protein level	biosynthetic process;catabolic process;cellular biosynthetic process;cellular catabolic process;cellular component disassembly;cellular component disassembly at cellular level;cellular component organization;cellular compo
29.6721	29.6401	29.7681	29.916	1:Experimental evidence at protein level	acylglycerol catabolic process;acylglycerol metabolic process;anatomical structure development;anatomical structure morphogenesis;biological regulation;branching morphogenesis of a tube;carbohydrate metabolic process
28.7252	28.7959	28.6833	28.7413	1:Experimental evidence at protein level	acylglycerol catabolic process;acylglycerol metabolic process;biological regulation;carbohydrate metabolic process;catabolic process;cell cycle;cell cycle process;cell division;cell surface receptor linked signaling pathway;ce
30.7465	30.2253	30.4899	30.2056	1:Experimental evidence at protein level	biological regulation;biosynthetic process;catabolic process;cellular biosynthetic process;cellular catabolic process;cellular component disassembly;cellular component disassembly at cellular level;cellular component organiz
28.5503	28.6075	28.7684	28.3883	1:Experimental evidence at protein level	biological regulation;biosynthetic process;catabolic process;cellular biosynthetic process;cellular catabolic process;cellular component disassembly;cellular component disassembly at cellular level;cellular component organiz
30.2284	30.0741	30.5132	30.5109	1:Experimental evidence at protein level	biosynthetic process;catabolic process;cellular biosynthetic process;cellular catabolic process;cellular component biogenesis;cellular component biogenesis at cellular level;cellular component disassembly;cellular componen
30.8273	30.6268	30.9996	31.0962	1:Experimental evidence at protein level	biosynthetic process;catabolic process;cellular biosynthetic process;cellular catabolic process;cellular component biogenesis;cellular component biogenesis at cellular level;cellular component disassembly;cellular componen
26.7869	26.8311	27.0452	26.8646	1:Experimental evidence at protein level	anatomical structure development;anatomical structure morphogenesis;apoptosis;appendage morphogenesis;atrial septum morphogenesis;biological regulation;biosynthetic process;blood vessel development;bone remodel
26.3725	26.2395	26.2074	#NUM!	1:Experimental evidence at protein level	amine catabolic process;amine metabolic process;anatomical structure development;anatomical structure morphogenesis;biological regulation;biosynthetic process;body fluid secretion;carboxylic acid catabolic process;carb
28.6771	28.8401	28.9437	28.8433	1:Experimental evidence at protein level	activation of immune response;activation of innate immune response;anaphase-promoting complex-dependent proteasomal ubiquitin-dependent protein catabolic process;antigen processing and presentation;antigen proces
26.2413	26.2392	26.2857	26.2746	1:Experimental evidence at protein level	anatomical structure development;biological regulation;central nervous system development;developmental process;establishment of localization;establishment of RNA localization;mRNA transport;negative regulation of biol
27.4374	27.8252	27.2503	27.2113	1:Experimental evidence at protein level	biological regulation;biosynthetic process;cell differentiation;cellular biosynthetic process;cellular developmental process;cellular macromolecule biosynthetic process;cellular macromolecule metabolic process;cellular metab
25.6778	24.9553	25.322	25.4733	1:Experimental evidence at protein level	biosynthetic process;cellular biosynthetic process;cellular macromolecule biosynthetic process;cellular macromolecule metabolic process;cellular metabolic process;cellular nitrogen compound metabolic process;cellular pro
26.0804	25.8632	25.8307	25.5749	1:Experimental evidence at protein level	cellular process;endosome transport;establishment of localization;establishment of localization in cell;establishment of protein localization;intracellular transport;protein transport;transport;vesicle-mediated transport
26.2732	25.5417	26.1507	25.9641	1:Experimental evidence at protein level	biological regulation;cell surface receptor linked signaling pathway;cellular macromolecule metabolic process;cellular metabolic process;cellular process;cellular protein metabolic process;cellular response to stimulus;enzym
25.4941	#NUM!	25.7486	25.4556	1:Experimental evidence at protein level	amine biosynthetic process;amine metabolic process;beta-alanine biosynthetic process;beta-alanine metabolic process;biosynthetic process;carboxylic acid biosynthetic process;carboxylic acid metabolic process;catabolic
27.7283	27.0415	27.3355	26.9699	1:Experimental evidence at protein level	biological regulation;biosynthetic process;cellular biosynthetic process;cellular macromolecule biosynthetic process;cellular macromolecule metabolic process;cellular metabolic process;cellular nitrogen compound metabolic
30.1775	29.7439	29.9564	29.9114	1:Experimental evidence at protein level	biological regulation;biosynthetic process;cellular biosynthetic process;cellular macromolecule biosynthetic process;cellular macromolecule metabolic process;cellular metabolic process;cellular nitrogen compound metabolic
27.3786	27.9518	28.36	28.1476	1:Experimental evidence at protein level	amine biosynthetic process;amine metabolic process;amino acid salvage;aspartate family amino acid biosynthetic process;aspartate family amino acid metabolic process;biosynthetic process;carboxylic acid biosynthetic pro
25.7167	26.4686	26.2964	25.8161	1:Experimental evidence at protein level	actin filament-based movement;actin filament-based process;anatomical structure morphogenesis;biological regulation;cell chemotaxis;cell migration;cell motility;cell part morphogenesis;cell projection morphogenesis;cell pr
25.8288	25.3039	25.5402	25.6012	1:Experimental evidence at protein level	cellular macromolecule metabolic process;cellular metabolic process;cellular nitrogen compound metabolic process;cellular process;macromolecule metabolic process;metabolic process;mRNA metabolic process;mRNA pro
26.5985	26.6694	26.8806	26.8409	1:Experimental evidence at protein level	anatomical structure development;anatomical structure morphogenesis;biological regulation;cell differentiation;cellular developmental process;cellular process;developmental process;muscle organ development;muscle struc
28.2933	28.5894	28.8802	28.6896	1:Experimental evidence at protein level	biological regulation;cellular component assembly;cellular component assembly at cellular level;cellular component organization;cellular component organization at cellular level;cellular component organization or biogenesis;
25.8824	25.5747	25.3514	25.5482	1:Experimental evidence at protein level	biological regulation;cell proliferation;cellular component biogenesis;cellular component biogenesis at cellular level;cellular component organization or biogenesis;cellular component organization or biogenesis at cellular level
27.2813	27.0839	27.3959	27.1569	1:Experimental evidence at protein level	cellular component assembly;cellular component assembly at cellular level;cellular component organization;cellular component organization at cellular level;cellular component organization or biogenesis;cellular component c
25.8227	25.8486	25.6321	25.2565	1:Experimental evidence at protein level	
28.344	28.5423	28.5327	28.2538	1:Experimental evidence at protein level	biological regulation;biosynthetic process;cellular biosynthetic process;cellular macromolecule biosynthetic process;cellular macromolecule metabolic process;cellular metabolic process;cellular process;cellular protein metab
26.5759	#NUM!	26.0036	25.8249	1:Experimental evidence at protein level	anatomical structure development;biological adhesion;biological regulation;cell adhesion;cellular component organization;cellular component organization at cellular level;cellular component organization or biogenesis;cellular
30.6088	30.9445	31.0744	31.1531	1:Experimental evidence at protein level	anatomical structure homeostasis;arachidonic acid metabolic process;biological regulation;biosynthetic process;carboxylic acid biosynthetic process;carboxylic acid metabolic process;cellular biosynthetic process;cellular co
30.3048	30.5801	30.6292	30.6219	1:Experimental evidence at protein level	actin cytoskeleton organization;actin filament-based process;actomyosin structure organization;cell differentiation;cellular component organization;cellular component organization at cellular level;cellular component organiza
28.5803	28.7591	28.883	28.7965	1:Experimental evidence at protein level	biological regulation;biosynthetic process;cell differentiation;cellular biosynthetic process;cellular component assembly;cellular component assembly at cellular level;cellular component organization;cellular component organi
26.415	26.5978	26.4434	26.4359	1:Experimental evidence at protein level	actin cytoskeleton organization;actin filament-based process;biological regulation;cell communication;cellular component organization;cellular component organization at cellular level;cellular component organization or bioge
25.7036	#NUM!	25.6239	25.2078	1:Experimental evidence at protein level	anatomical structure development;biosynthetic process;cardiac septum development;cellular biosynthetic process;cellular component organization;cellular component organization at cellular level;cellular component organiza
28.1075	28.4951	28.4596	28.6586	1:Experimental evidence at protein level	biological regulation;biosynthetic process;cell junction assembly;cell junction organization;cell-substrate junction assembly;cellular biosynthetic process;cellular component assembly;cellular component assembly at cellular l
29.2231	28.6525	28.8769	28.8971	1:Experimental evidence at protein level	biological regulation;biosynthetic process;cellular biosynthetic process;cellular macromolecule biosynthetic process;cellular macromolecule metabolic process;cellular metabolic process;cellular nitrogen compound metabolic
28.1677	27.8629	28.0028	27.8973	1:Experimental evidence at protein level	anatomical structure development;biological regulation;bone development;cell growth;cellular component organization;cellular component organization at cellular level;cellular component organization or biogenesis;cellular co
24.953	25.0621	24.6485	25.0102	1:Experimental evidence at protein level	alcohol metabolic process;alditol metabolic process;biological regulation;carbohydrate metabolic process;carbohydrate phosphorylation;cellular carbohydrate metabolic process;cellular metabolic process;cellular process;de
26.6909	26.5796	26.261	26.6218	1:Experimental evidence at protein level	apoptotic mitochondrial changes;cellular component organization;cellular component organization at cellular level;cellular component organization or biogenesis;cellular component organization or biogenesis at cellular level;
26.516	25.6479	25.8623	26.4014	1:Experimental evidence at protein level	biological regulation;cellular macromolecule metabolic process;cellular metabolic process;cellular nitrogen compound metabolic process;cellular process;macromolecule metabolic process;metabolic process;mRNA metabol
27.2982	27.1749	27.1469	27.1614	1:Experimental evidence at protein level	carboxylic acid metabolic process;cellular ketone metabolic process;cellular metabolic process;cellular process;metabolic process;organic acid metabolic process;oxoacid metabolic process;small molecule metabolic proces
26.3348	26.2751	25.4263	25.8945	1:Experimental evidence at protein level	biological regulation;biosynthetic process;cellular biosynthetic process;cellular component organization;cellular component organization at cellular level;cellular component organization or biogenesis;cellular component orga
26.3172	26.2265	26.6356	26.1524	1:Experimental evidence at protein level	biological adhesion;cell adhesion;cellular process
27.7629	28.0184	27.8967	27.9323	1:Experimental evidence at protein level	biosynthetic process;cellular biosynthetic process;cellular component assembly;cellular component organization;cellular component organization or biogenesis;cellular macromolecule biosynthetic process;cellular macromole
25.6138	25.5464	25.314	25.6226	1:Experimental evidence at protein level	anatomical structure development;anatomical structure morphogenesis;apoptosis;biological regulation;biosynthetic process;camera-type eye development;cardiac chamber morphogenesis;cardiac muscle contraction;cardia
25.9145	25.5933	25.4748	25.6416	1:Experimental evidence at protein level	ATP-dependent chromatin remodeling;biosynthetic process;cellular biosynthetic process;cellular component organization;cellular component organization at cellular level;cellular component organization or biogenesis;cellular
26.3296	26.1034	26.1352	25.9285	1:Experimental evidence at protein level	biosynthetic process;cellular biosynthetic process;cellular component disassembly;cellular component disassembly at cellular level;cellular component organization;cellular component organization at cellular level;cellular com
27.3987	27.1674	27.1926	27.0411	1:Experimental evidence at protein level	anatomical structure development;anatomical structure formation involved in morphogenesis;anatomical structure morphogenesis;ATP-dependent chromatin remodeling;biological regulation;biosynthetic process;blood coagu
27.2663	27.1771	27.5566	27.4376	1:Experimental evidence at protein level	anatomical structure development;biological regulation;cell development;cellular component assembly;cellular component assembly at cellular level;cellular component organization;cellular component organization at cellular
27.9261	27.8516	27.8128	28.2784	1:Experimental evidence at protein level	actin cytoskeleton organization;actin filament-based process;biological regulation;cellular component organization;cellular component organization at cellular level;cellular component organization or biogenesis;cellular compo
25.8558	26.0021	26.1116	25.6195	1:Experimental evidence at protein level	anatomical structure development;biological regulation;cell proliferation;cellular component movement;cellular process;developmental process;fertilization;muscle tissue development;nervous system development;regulation
25.6643	25.6408	25.6319	25.6615	1:Experimental evidence at protein level	cellular component organization;cellular component organization at cellular level;cellular component organization or biogenesis;cellular component organization or biogenesis at cellular level;cellular macromolecule metabolic
27.176	27.6385	27.6999	27.4245	1:Experimental evidence at protein level	cell cycle;cell division;cellular process
27.164	27.1265	27.3465	26.9262	1:Experimental evidence at protein level	immune system process
26.1952	25.9091	25.8465	25.9934	1:Experimental evidence at protein level	biological regulation;cellular component organization;cellular component organization at cellular level;cellular component organization or biogenesis;cellular component organization or biogenesis at cellular level;cellular macro
28.672	28.8565	29.0394	28.8866	1:Experimental evidence at protein level	anatomical structure development;cell differentiation;cellular developmental process;cellular macromolecule metabolic process;cellular metabolic process;cellular process;cellular protein metabolic process;'de novo' posttran
29.5485	29.7253	29.6055	29.4272	1:Experimental evidence at protein level	biological regulation;negative regulation of biological process;negative regulation of cellular component organization;negative regulation of cellular process;negative regulation of developmental process;negative regulation of
28.9229	29.0531	28.8087	28.9538	1:Experimental evidence at protein level	biological regulation;biosynthetic process;cellular biosynthetic process;cellular lipid metabolic process;cellular metabolic process;cellular process;cellular response to calcium ion;cellular response to chemical stimulus;cellula
26.8119	26.9036	27.2552	27.1566	1:Experimental evidence at protein level	anatomical structure development;calcium ion-dependent exocytosis;cell differentiation;cellular developmental process;cellular process;cerebellum development;developmental process;establishment of localization;establish
26.5969	26.3123	26.4688	26.3883	1:Experimental evidence at protein level	biological regulation;biosynthetic process;carbohydrate metabolic process;carbohydrate transport;cell chemotaxis;cell cycle;cell cycle process;cell migration;cell motility;cell projection assembly;cell projection organization;ce
26.0091	26.0373	25.8553	25.7725	1:Experimental evidence at protein level	biological regulation;cellular macromolecule metabolic process;cellular metabolic process;cellular process;cellular protein metabolic process;endosome transport;establishment of localization;establishment of localization in c
26.3058	25.9665	26.2698	25.9409	1:Experimental evidence at protein level	cellular process;cellular response to heat;cellular response to stimulus;cellular response to stress;response to abiotic stimulus;response to heat;response to stimulus;response to stress;response to temperature stimulus
27.1696	27.0481	27.0188	27.2514	1:Experimental evidence at protein level	biological regulation;cellular macromolecule metabolic process;cellular metabolic process;cellular process;cellular protein metabolic process;dephosphorylation;macromolecule metabolic process;macromolecule modification
25.2814	#NUM!	26.2846	25.6059	1:Experimental evidence at protein level	biological regulation;biosynthetic process;carboxylic acid biosynthetic process;carboxylic acid metabolic process;cell redox homeostasis;cellular biosynthetic process;cellular homeostasis;cellular ketone metabolic process;c
26.64	26.9678	26.8839	27.0332	1:Experimental evidence at protein level	amine metabolic process;asparagine metabolic process;aspartate family amino acid metabolic process;carboxylic acid metabolic process;cellular amine metabolic process;cellular amino acid metabolic process;cellular ketor
24.9657	25.036	24.9451	#NUM!	1:Experimental evidence at protein level	acetate biosynthetic process;acetate metabolic process;acetyl-CoA biosynthetic process;acetyl-CoA biosynthetic process from acetate;acetyl-CoA metabolic process;alcohol metabolic process;biosynthetic process;carboxy
27.3112	27.5471	27.3368	27.3828	1:Experimental evidence at protein level	
29.1577	28.9331	29.317	29.1259	1:Experimental evidence at protein level	actin cytoskeleton organization;actin filament capping;actin filament organization;actin filament-based process;actomyosin structure organization;anatomical structure development;anatomical structure formation involved in
28.0331	27.6957	27.6111	27.6863	1:Experimental evidence at protein level	acyl-CoA biosynthetic process;acyl-CoA metabolic process;acylglycerol biosynthetic process;acylglycerol metabolic process;biosynthetic process;carboxylic acid biosynthetic process;carboxylic acid metabolic process;cellu
26.1965	#NUM!	25.8946	25.9748	1:Experimental evidence at protein level	biological regulation;cellular macromolecule metabolic process;cellular metabolic process;cellular nitrogen compound metabolic process;cellular process;CRD-mediated mRNA stabilization;establishment of localization;estab
28.1607	28.014	28.1366	27.922	1:Experimental evidence at protein level	actin filament-based movement;actin filament-based process;biological regulation;cell cycle process;cellular component movement;cellular component organization;cellular component organization at cellular level;cellular co
26.4159	26.5842	26.1653	26.1754	1:Experimental evidence at protein level	biological regulation;cellular lipid metabolic process;cellular metabolic process;cellular process;cellular response to stimulus;defense response;inflammatory response;lipid metabolic process;metabolic process;organophosph
27.8058	27.7116	27.4029	27.6309	1:Experimental evidence at protein level	biosynthetic process;cellular biosynthetic process;cellular macromolecule biosynthetic process;cellular macromolecule metabolic process;cellular metabolic process;cellular nitrogen compound metabolic process;cellular pro
28.1379	28.0938	27.9108	28.1547	1:Experimental evidence at protein level	amine metabolic process;carboxylic acid metabolic process;cell differentiation;cellular amine metabolic process;cellular amino acid metabolic process;cellular developmental process;cellular ketone metabolic process;cellula
27.6302	26.682	27.1422	27.0203	1:Experimental evidence at protein level	cell growth;cellular macromolecule metabolic process;cellular metabolic process;cellular nitrogen compound metabolic process;cellular process;establishment of localization;establishment of localization in cell;establishment
28.4631	28.3895	28.7567	28.6718	1:Experimental evidence at protein level	anatomical structure homeostasis;biological regulation;cell surface receptor linked signaling pathway;cellular component assembly;cellular component assembly at cellular level;cellular component organization;cellular compo
25.7695	26.0674	26.2726	26.289	1:Experimental evidence at protein level	biological regulation;cell cycle phase;cell cycle process;cell division;cell projection assembly;cell projection organization;cellular component assembly;cellular component assembly at cellular level;cellular component organiza
26.4053	26.6415	26.1321	26.1983	1:Experimental evidence at protein level	
27.1888	26.9749	27.2826	26.8556	1:Experimental evidence at protein level	cellular macromolecule metabolic process;cellular metabolic process;cellular nitrogen compound metabolic process;cellular process;macromolecule metabolic process;metabolic process;nitrogen compound metabolic proce
28.9368	28.601	28.871	28.8229	1:Experimental evidence at protein level	biological regulation;biosynthetic process;cellular biosynthetic process;cellular component assembly;cellular component assembly at cellular level;cellular component organization;cellular component organization at cellular le
26.3236	26.6553	26.28	26.4477	1:Experimental evidence at protein level	cellular macromolecule metabolic process;cellular metabolic process;cellular process;cellular protein metabolic process;establishment of localization;establishment of localization in cell;establishment of protein localization;es
26.6928	26.4451	26.5792	26.4875	1:Experimental evidence at protein level	2-oxoglutarate metabolic process;acetyl-CoA catabolic process;acetyl-CoA metabolic process;carboxylic acid metabolic process;catabolic process;cellular catabolic process;cellular ketone metabolic process;cellular metabo
26.6447	26.2607	26.5818	26.2287	1:Experimental evidence at protein level	2'-deoxyribonucleotide metabolic process;aging;alcohol metabolic process;amino sugar metabolic process;biological regulation;carbohydrate metabolic process;catabolic process;cell death;cellular carbohydrate metabolic p
27.7897	28.0397	27.9235	27.8309	1:Experimental evidence at protein level	anatomical structure morphogenesis;cell part morphogenesis;cell projection morphogenesis;cell projection organization;cellular component assembly;cellular component morphogenesis;cellular component organization;cellu
26.2536	26.258	26.3105	26.423	1:Experimental evidence at protein level	catabolic process;cellular catabolic process;cellular component organization;cellular component organization at cellular level;cellular component organization or biogenesis;cellular component organization or biogenesis at ce
26.6028	27.0246	27.006	27.0564	1:Experimental evidence at protein level	biological regulation;catabolic process;cation transport;cell cycle process;cellular catabolic process;cellular component disassembly;cellular component disassembly at cellular level;cellular component organization;cellular co
27.2385	27.2197	27.0718	27.0948	1:Experimental evidence at protein level	cellular macromolecule metabolic process;cellular metabolic process;cellular process;cellular protein metabolic process;establishment of localization;establishment of localization in cell;establishment of protein localization;es
29.5401	29.0662	29.2592	29.4056	1:Experimental evidence at protein level	biological regulation;biosynthetic process;cellular biosynthetic process;cellular component assembly;cellular component assembly at cellular level;cellular component organization;cellular component organization at cellular le
28.2228	28.1214	28.1467	28.0962	1:Experimental evidence at protein level	alcohol biosynthetic process;alcohol metabolic process;amino sugar biosynthetic process;amino sugar metabolic process;anatomical structure development;biosynthetic process;carbohydrate biosynthetic process;carbohyd
28.0248	28.0873	28.0212	28.0046	1:Experimental evidence at protein level	biological regulation;positive regulation of ATPase activity;positive regulation of catalytic activity;positive regulation of hydrolase activity;positive regulation of molecular function;regulation of ATPase activity;regulation of biolo
27.6087	27.2475	27.2546	27.6686	1:Experimental evidence at protein level	biological regulation;cellular localization;cellular macromolecule localization;cellular process;chemical homeostasis;homeostatic process;interaction with host;interspecies interaction between organisms;intracellular mRNA loc
26.0992	25.5311	#NUM!	26.0785	1:Experimental evidence at protein level	
25.4787	26.0123	26.2335	26.1535	1:Experimental evidence at protein level	biological regulation;biosynthetic process;catabolic process;cellular aromatic compound metabolic process;cellular biosynthetic process;cellular catabolic process;cellular metabolic compound salvage;cellular metabolic proc
25.479	25.5106	25.5024	25.5279	1:Experimental evidence at protein level	biological regulation;blood coagulation;cell migration;cell motility;cellular component movement;cellular process;cellular response to stimulus;chemotaxis;coagulation;fibrinolysis;hemostasis;locomotion;macromolecule metab
29.1933	29.0959	29.0043	28.8517	1:Experimental evidence at protein level	aging;biosynthetic process;carbohydrate metabolic process;cellular biosynthetic process;cellular carbohydrate metabolic process;cellular macromolecule biosynthetic process;cellular macromolecule metabolic process;cellul
25.6912	25.2853	#NUM!	#NUM!	1:Experimental evidence at protein level	aging;anatomical structure formation involved in morphogenesis;angiogenesis;biological regulation;biosynthetic process;blood coagulation;cell activation;cell aging;cell surface receptor linked signaling pathway;cellular biosy
25.6798	25.4868	25.9225	25.5921	1:Experimental evidence at protein level	activation of immune response;adaptive immune response;adaptive immune response based on somatic recombination of immune receptors built from immunoglobulin superfamily domains;biological regulation;calcium ion h
30.1258	30.228	30.4785	30.2158	1:Experimental evidence at protein level	adult behavior;adult locomotory behavior;adult walking behavior;anatomical structure development;axon cargo transport;axon target recognition;axon transport of mitochondrion;behavior;biological regulation;catabolic proce
26.8739	26.4423	26.4785	26.2896	1:Experimental evidence at protein level	basement membrane organization;biological adhesion;biological regulation;cell adhesion;cell-matrix adhesion;cell-substrate adhesion;cellular component disassembly;cellular component disassembly at cellular level;cellular c
27.7644	27.7673	27.5282	27.9411	1:Experimental evidence at protein level	anatomical structure development;biological regulation;biosynthetic process;carbohydrate homeostasis;cellular biosynthetic process;cellular chemical homeostasis;cellular component organization;cellular component organiz
31.1614	31.1477	31.1539	30.9338	1:Experimental evidence at protein level	amine metabolic process;aminoglycan catabolic process;aminoglycan metabolic process;anatomical structure development;anatomical structure morphogenesis;biological adhesion;biological regulation;blood coagulation;br
32.7399	33.2547	33.1215	33.4633	1:Experimental evidence at protein level	biological adhesion;biological regulation;biosynthetic process;cell adhesion;cell surface receptor linked signaling pathway;cellular biosynthetic process;cellular macromolecule biosynthetic process;cellular macromolecule me
25.5889	25.4038	25.3288	25.5771	1:Experimental evidence at protein level	amine transport;amino acid transmembrane transport;amino acid transport;betaine transport;carboxylic acid catabolic process;carboxylic acid metabolic process;carboxylic acid transport;carnitine shuttle;carnitine transport;
27.0511	27.0734	27.1787	27.1752	1:Experimental evidence at protein level	2'-deoxyribonucleotide biosynthetic process;2'-deoxyribonucleotide metabolic process;biosynthetic process;cell cycle;cell differentiation;cell proliferation;cellular biosynthetic process;cellular developmental process;cellular r
28.2815	27.947	28.0948	28.1259	1:Experimental evidence at protein level	biological regulation;cellular component assembly;cellular component organization;cellular component organization or biogenesis;macromolecular complex assembly;macromolecular complex subunit organization;positive re
26.6147	27.0467	26.5789	26.8997	1:Experimental evidence at protein level	activation of immune response;activation of innate immune response;activation of MAPK activity;activation of MAPKK activity;activation of protein kinase activity;anatomical structure development;anatomical structure morph
28.2652	28.4706	28.6735	28.4895	1:Experimental evidence at protein level	anatomical structure development;biological regulation;catabolic process;cell differentiation;cellular catabolic process;cellular chemical homeostasis;cellular component organization;cellular component organization at cellular
30.1805	30.3392	30.068	30.0528	1:Experimental evidence at protein level	biosynthetic process;catabolic process;cellular biosynthetic process;cellular catabolic process;cellular component disassembly;cellular component disassembly at cellular level;cellular component organization;cellular compo
26.8968	26.8774	27.218	27.4385	1:Experimental evidence at protein level	biological regulation;cellular component organization;cellular component organization or biogenesis;cellular macromolecule metabolic process;cellular membrane organization;cellular metabolic process;cellular process;cellula
26.2478	26.3801	26.4396	26.4921	1:Experimental evidence at protein level	biosynthetic process;cellular aromatic compound metabolic process;cellular biosynthetic process;cellular metabolic process;cellular nitrogen compound biosynthetic process;cellular nitrogen compound metabolic process;ce
29.7399	29.5717	29.6175	29.4902	1:Experimental evidence at protein level	biosynthetic process;carbohydrate metabolic process;cell activation;cellular biosynthetic process;cellular carbohydrate metabolic process;cellular macromolecule biosynthetic process;cellular macromolecule metabolic proce
28.8719	29.4117	29.1294	29.5083	1:Experimental evidence at protein level	biosynthetic process;catabolic process;cellular biosynthetic process;cellular catabolic process;cellular component disassembly;cellular component disassembly at cellular level;cellular component organization;cellular compo
29.3928	29.4897	29.3278	29.3325	1:Experimental evidence at protein level	anatomical structure homeostasis;antigen processing and presentation;antigen processing and presentation of exogenous antigen;antigen processing and presentation of exogenous peptide antigen;antigen processing and p
24.7044	25.163	26.0594	24.5469	1:Experimental evidence at protein level	alcohol metabolic process;anatomical structure development;cellular aldehyde metabolic process;cellular lipid metabolic process;cellular metabolic process;cellular process;central nervous system development;development

26.0565	26.3547	26.7165	26.509	1:Experimental evidence at protein level	actin cytoskeleton organization;actin filament-based process;biological regulation;cellular component movement;cellular component organization;cellular component organization at cellular level;cellular component organizati
27.6856	27.7408	27.622	27.5924	1:Experimental evidence at protein level	antigen processing and presentation;antigen processing and presentation of exogenous antigen;antigen processing and presentation of exogenous peptide antigen;antigen processing and presentation of exogenous peptide
27.9757	27.5648	27.6762	27.6891	1:Experimental evidence at protein level	acyl-CoA biosynthetic process;acyl-CoA metabolic process;acylglycerol biosynthetic process;acylglycerol metabolic process;alcohol biosynthetic process;alcohol metabolic process;anion transport;biosynthetic process;carb
27.4741	27.6814	27.588	27.6764	1:Experimental evidence at protein level	biological adhesion;biological regulation;cell adhesion;cell surface receptor linked signaling pathway;cell-cell adhesion;cellular macromolecule metabolic process;cellular metabolic process;cellular process;cellular protein me
30.2197	30.3577	30.7778	30.5252	1:Experimental evidence at protein level	biological regulation;biosynthetic process;cellular biosynthetic process;cellular component assembly;cellular component assembly at cellular level;cellular component organization;cellular component organization at cellular le
31.6966	31.5917	31.9215	31.9323	1:Experimental evidence at protein level	actin filament-based movement;actin filament-based process;actin-mediated cell contraction;actin-myosin filament sliding;anatomical structure development;axon guidance;biological regulation;cell surface receptor linked si
27.6365	27.6994	27.9705	28.0968	1:Experimental evidence at protein level	cellular macromolecule metabolic process;cellular metabolic process;cellular process;cellular protein metabolic process;'de novo' posttranslational protein folding;'de novo' protein folding;macromolecule metabolic process;
27.2856	27.1489	27.293	27.3803	1:Experimental evidence at protein level	biological regulation;biosynthetic process;cellular biosynthetic process;cellular component assembly;cellular component assembly at cellular level;cellular component organization;cellular component organization at cellular le
26.8601	27.1255	26.8008	27.0689	1:Experimental evidence at protein level	biological regulation;biosynthetic process;cellular aromatic compound metabolic process;cellular biosynthetic process;cellular metabolic compound salvage;cellular metabolic process;cellular nitrogen compound biosynthetic
26.9705	26.8811	26.4987	26.6652	1:Experimental evidence at protein level	biological regulation;biosynthetic process;cellular biosynthetic process;cellular macromolecule biosynthetic process;cellular macromolecule metabolic process;cellular metabolic process;cellular nitrogen compound metabolic
25.8002	#NUM!	25.0405	25.2744	1:Experimental evidence at protein level	biological regulation;cell surface receptor linked signaling pathway;cellular macromolecule metabolic process;cellular metabolic process;cellular nitrogen compound metabolic process;cellular process;cellular response to stir
28.6351	28.6457	28.5611	28.5258	1:Experimental evidence at protein level	biological regulation;cellular component organization;cellular component organization or biogenesis;maintenance of location;maintenance of protein location;negative regulation of actin filament polymerization;negative regula
28.5991	28.7193	28.5317	28.5027	1:Experimental evidence at protein level	alcohol metabolic process;amine metabolic process;amino acid activation;anatomical structure formation involved in morphogenesis;angiogenesis;apoptosis;biological adhesion;biological regulation;carbohydrate metabolic
28.0178	28.0599	28.1252	28.0941	1:Experimental evidence at protein level	3'-UTR-mediated mRNA stabilization;biological regulation;biosynthetic process;cellular biosynthetic process;cellular component organization;cellular component organization or biogenesis;cellular macromolecule biosyntheti
27.5458	27.2035	27.7897	27.6211	1:Experimental evidence at protein level	biological regulation;biosynthetic process;cellular biosynthetic process;cellular component assembly;cellular component assembly at cellular level;cellular component organization;cellular component organization at cellular le
28.2246	28.0942	28.6201	28.4192	1:Experimental evidence at protein level	biological regulation;cell proliferation;cellular process;cellular response to stimulus;regulation of biological process;regulation of cellular process;response to stimulus;signal transduction
29.1175	28.8574	28.8018	28.7402	1:Experimental evidence at protein level	anatomical structure development;anatomical structure formation involved in morphogenesis;anatomical structure morphogenesis;ATP-dependent chromatin remodeling;biological regulation;biosynthetic process;blood coagu
30.5974	30.9076	30.6804	30.7536	1:Experimental evidence at protein level	alkene biosynthetic process;biosynthetic process;carboxylic acid biosynthetic process;carboxylic acid metabolic process;cellular alkene metabolic process;cellular biosynthetic process;cellular ketone metabolic process;cellu
28.0297	27.7911	27.9744	28.0342	1:Experimental evidence at protein level	alcohol metabolic process;biosynthetic process;cholesterol biosynthetic process;cholesterol metabolic process;lipid biosynthetic process;lipid metabolic process;metabolic process;primary metabolic process;small molecule
26.1854	25.7614	25.9992	25.9557	1:Experimental evidence at protein level	anatomical structure formation involved in morphogenesis;biological regulation;cell cycle;cell cycle process;cell projection assembly;cell projection organization;cellular component assembly;cellular component assembly at c
26.8134	26.6753	26.3977	26.5985	1:Experimental evidence at protein level	adult behavior;adult locomotory behavior;anatomical structure morphogenesis;axonogenesis;behavior;cell part morphogenesis;cell projection morphogenesis;cell projection organization;cellular component morphogenesis;ce
25.1825	24.4673	24.579	24.4314	1:Experimental evidence at protein level	actin cytoskeleton organization;actin cytoskeleton reorganization;actin filament-based process;biological regulation;cell surface receptor linked signaling pathway;cellular component organization;cellular component organizat
25.5624	#NUM!	26.0635	#NUM!	1:Experimental evidence at protein level	anatomical structure development;anatomical structure formation involved in morphogenesis;biological regulation;cellular process;developmental process;epidermis development;hair cycle process;hair follicle development;
26.568	26.2903	26.3447	26.1617	1:Experimental evidence at protein level	biological regulation;biosynthetic process;cellular biosynthetic process;cellular component organization;cellular component organization at cellular level;cellular component organization or biogenesis;cellular component orga
25.5545	25.6433	25.5098	25.5609	1:Experimental evidence at protein level	cellular component assembly;cellular component assembly at cellular level;cellular component organization;cellular component organization at cellular level;cellular component organization or biogenesis;cellular component c
27.1334	26.9028	26.7471	26.7869	1:Experimental evidence at protein level	
27.3155	27.4711	27.4249	27.6525	1:Experimental evidence at protein level	biological adhesion;biological regulation;cell adhesion;cell junction assembly;cell junction organization;cell-cell adhesion;cellular component assembly;cellular component assembly at cellular level;cellular component organiza
24.3085	#NUM!	24.8159	24.5702	1:Experimental evidence at protein level	biosynthetic process;cellular biosynthetic process;cellular macromolecule biosynthetic process;cellular macromolecule metabolic process;cellular metabolic process;cellular nitrogen compound metabolic process;cellular pro
27.458	27.1024	27.462	27.2788	1:Experimental evidence at protein level	
29.0338	28.8574	29.1862	28.9975	1:Experimental evidence at protein level	activation of caspase activity;anatomical structure development;biological regulation;cellular component assembly;cellular component assembly at cellular level;cellular component organization;cellular component organizatio
29.5584	29.5795	29.6932	29.7584	1:Experimental evidence at protein level	cellular macromolecule metabolic process;cellular metabolic process;cellular process;cellular protein metabolic process;chaperone-mediated protein folding;macromolecule metabolic process;macromolecule modification;me
27.9851	28.0865	27.9481	27.8848	1:Experimental evidence at protein level	biological regulation;biosynthetic process;cellular biosynthetic process;cellular macromolecule biosynthetic process;cellular macromolecule metabolic process;cellular metabolic process;cellular nitrogen compound metabolic
26.4943	26.3996	26.5244	25.7916	1:Experimental evidence at protein level	activation of immune response;activation of innate immune response;aging;biological regulation;biosynthetic process;blood coagulation;carbohydrate homeostasis;cell differentiation;cellular biosynthetic process;cellular cher
27.7286	27.611	27.5969	27.4026	1:Experimental evidence at protein level	biological regulation;biosynthetic process;carbohydrate metabolic process;carbohydrate transport;cell cycle;cell cycle process;cell surface receptor linked signaling pathway;cellular biosynthetic process;cellular component c
28.9899	28.6797	28.6574	28.651	1:Experimental evidence at protein level	biological regulation;cell projection organization;cell surface receptor linked signaling pathway;cellular component organization;cellular component organization at cellular level;cellular component organization or biogenesis;c
28.6243	28.6072	28.8986	28.9728	1:Experimental evidence at protein level	anatomical structure development;biological regulation;cellular component organization;cellular component organization or biogenesis;cellular membrane organization;cellular process;cellular response to stimulus;central nerv
26.5236	26.368	26.1937	26.1372	1:Experimental evidence at protein level	cellular macromolecule metabolic process;cellular metabolic process;cellular nitrogen compound metabolic process;cellular process;macromolecule metabolic process;metabolic process;nitrogen compound metabolic proce
27.9846	27.7214	27.7438	27.5566	1:Experimental evidence at protein level	cellular macromolecule metabolic process;cellular metabolic process;cellular process;cellular protein metabolic process;cullin deneddylation;macromolecule metabolic process;macromolecule modification;metabolic process
25.2011	#NUM!	25.2591	25.1042	1:Experimental evidence at protein level	amine metabolic process;amino acid activation;carboxylic acid metabolic process;cellular amine metabolic process;cellular amino acid metabolic process;cellular ketone metabolic process;cellular macromolecule metabolic
27.703	27.1823	27.55	27.3671	1:Experimental evidence at protein level	actin cytoskeleton organization;actin filament capping;actin filament-based process;actin modification;barbed-end actin filament capping;biological regulation;cell cycle phase;cell cycle process;cell division;cellular compone
26.4296	26.025	26.1237	25.8804	1:Experimental evidence at protein level	anatomical structure morphogenesis;biological regulation;catabolic process;cell part morphogenesis;cell projection morphogenesis;cell projection organization;cellular catabolic process;cellular component morphogenesis;ce
27.2032	27.0356	27.158	27.2607	1:Experimental evidence at protein level	cellular component assembly;cellular component assembly at cellular level;cellular component biogenesis;cellular component organization;cellular component organization at cellular level;cellular component organization or b
26.9316	26.8067	26.7005	26.5158	1:Experimental evidence at protein level	biosynthetic process;cellular biosynthetic process;cellular lipid metabolic process;cellular metabolic process;cellular process;dephosphorylation;glycerolipid biosynthetic process;glycerolipid metabolic process;glycerophosph
27.3473	27.5739	27.2818	27.4315	1:Experimental evidence at protein level	
27.8863	#NUM!	28.5961	28.2746	1:Experimental evidence at protein level	anatomical structure formation involved in morphogenesis;angiogenesis;biological regulation;biosynthetic process;cell differentiation;cellular biosynthetic process;cellular chemical homeostasis;cellular component assembly;
26.1532	25.628	26.0891	25.8073	1:Experimental evidence at protein level	acidic amino acid transport;alcohol biosynthetic process;alcohol metabolic process;amine transport;amino acid transport;anion transport;aspartate transport;ATP biosynthetic process;ATP metabolic process;biosynthetic pro
26.3479	25.9629	26.337	26.076	1:Experimental evidence at protein level	cell death;cellular macromolecule metabolic process;cellular metabolic process;cellular nitrogen compound metabolic process;cellular process;cellular protein metabolic process;cellular response to stimulus;cellular response
25.84	#NUM!	25.4592	25.2864	1:Experimental evidence at protein level	5S class rRNA transcription from RNA polymerase III type 1 promoter;biological regulation;biosynthetic process;cellular biosynthetic process;cellular component organization;cellular component organization at cellular level;c
26.0996	25.7418	26.2731	26.6449	1:Experimental evidence at protein level	biological regulation;cellular component organization;cellular component organization at cellular level;cellular component organization or biogenesis;cellular component organization or biogenesis at cellular level;cellular mem
28.7076	28.6056	29.0421	28.8661	1:Experimental evidence at protein level	biological regulation;cellular process;cellular response to stimulus;cytoplasmic sequestering of NF-kappaB;cytoplasmic sequestering of protein;cytoplasmic sequestering of transcription factor;establishment of localization;es
26.7696	27.2355	26.8307	26.8193	1:Experimental evidence at protein level	biological regulation;catabolic process;cell surface receptor linked signaling pathway;cellular catabolic process;cellular macromolecule catabolic process;cellular macromolecule metabolic process;cellular metabolic process;
27.96	27.8719	27.8832	27.8449	1:Experimental evidence at protein level	biological regulation;catabolic process;cellular catabolic process;cellular macromolecule catabolic process;cellular macromolecule metabolic process;cellular metabolic process;cellular process;cellular protein metabolic proc
29.8886	29.7468	29.8492	29.5444	1:Experimental evidence at protein level	cellular macromolecule metabolic process;cellular metabolic process;cellular nitrogen compound metabolic process;cellular process;macromolecule metabolic process;metabolic process;mRNA metabolic process;mRNA pro
30.3195	30.1117	30.0686	30.1923	1:Experimental evidence at protein level	biosynthetic process;catabolic process;cellular biosynthetic process;cellular catabolic process;cellular component disassembly;cellular component disassembly at cellular level;cellular component organization;cellular compo
27.5758	27.6955	27.5278	27.4337	1:Experimental evidence at protein level	biological regulation;cellular macromolecule metabolic process;cellular metabolic process;cellular process;cellular protein metabolic process;demethylation;macromolecule metabolic process;macromolecule modification;me
27.867	27.7916	27.8977	27.7489	1:Experimental evidence at protein level	biological regulation;cell cycle phase;cell cycle process;cell division;cellular component movement;cellular component organization;cellular component organization at cellular level;cellular component organization or biogene
26.8496	27.2129	27.001	27.2645	1:Experimental evidence at protein level	biological regulation;cellular macromolecule metabolic process;cellular metabolic process;cellular process;cellular protein metabolic process;cellular response to stimulus;establishment of localization;establishment of localiza
26.0694	#NUM!	25.7371	25.4279	1:Experimental evidence at protein level	establishment of localization;establishment of localization in cell;establishment of protein localization;establishment of RNA localization;intracellular protein transport;intracellular transport;nuclear export;nuclear transport;nuc
26.548	26.0445	26.0486	26.0413	1:Experimental evidence at protein level	anatomical structure homeostasis;biological regulation;biosynthetic process;box H/ACA snoRNA 3'-end processing;box H/ACA snoRNA metabolic process;box H/ACA snoRNA processing;cell proliferation;cellular biosynthet
27.2648	27.241	27.6956	27.2895	1:Experimental evidence at protein level	cellular macromolecule metabolic process;cellular metabolic process;cellular nitrogen compound metabolic process;cellular process;gene expression;macromolecule metabolic process;metabolic process;mRNA metabolic p
26.8869	26.7398	26.6333	26.4872	1:Experimental evidence at protein level	biological regulation;cell differentiation;cellular developmental process;cellular macromolecule metabolic process;cellular metabolic process;cellular process;cellular protein metabolic process;cellular response to stimulus;cell
#NUM!	25.2842	24.9307	25.3186	1:Experimental evidence at protein level	alcohol catabolic process;alcohol metabolic process;carbohydrate catabolic process;carbohydrate metabolic process;catabolic process;cellular carbohydrate catabolic process;cellular carbohydrate metabolic process;cellula
27.8951	27.8861	28.2846	28.3484	1:Experimental evidence at protein level	cellular macromolecule metabolic process;cellular metabolic process;cellular process;cellular protein metabolic process;cellular response to heat;cellular response to stimulus;cellular response to stress;macromolecule metab
27.2227	27.5274	27.5501	27.5332	1:Experimental evidence at protein level	anatomical structure development;biological regulation;brain development;cellular macromolecule metabolic process;cellular metabolic process;cellular process;cellular protein metabolic process;cellular response to abiotic s
28.361	28.2416	28.4118	28.3478	1:Experimental evidence at protein level	aging;anatomical structure development;anatomical structure formation involved in morphogenesis;anatomical structure morphogenesis;anterior/posterior pattern specification;apoptosis;apoptotic mitochondrial changes;B c
25.5675	#NUM!	26.0462	25.69	1:Experimental evidence at protein level	biological adhesion;biological regulation;blood coagulation;calcium ion homeostasis;calcium-mediated signaling;cardiac muscle contraction;cation homeostasis;cation transport;cell adhesion;cell migration;cell motility;cellula
26.736	26.5458	26.5162	26.6445	1:Experimental evidence at protein level	amine biosynthetic process;amine metabolic process;anatomical structure development;asparagine biosynthetic process;asparagine metabolic process;aspartate family amino acid biosynthetic process;aspartate family amin
33.816	33.9303	34.2803	34.2173	1:Experimental evidence at protein level	apoptosis;B cell activation;B cell activation involved in immune response;B cell differentiation;biological regulation;carbohydrate homeostasis;cell activation;cell activation involved in immune response;cell death;cell differenti
27.7901	27.9047	27.7306	27.6286	1:Experimental evidence at protein level	actin cytoskeleton organization;actin filament-based process;actomyosin structure organization;adrenal gland development;amine metabolic process;aminoglycan biosynthetic process;aminoglycan metabolic process;anator
26.2285	26.0542	26.1729	26.0061	1:Experimental evidence at protein level	anatomical structure development;anatomical structure morphogenesis;biological regulation;biosynthetic process;cell part morphogenesis;cell projection morphogenesis;cell projection organization;cell surface receptor linked
30.9021	31.2151	31.1505	31.22	1:Experimental evidence at protein level	anatomical structure development;anatomical structure homeostasis;base-excision repair;biological regulation;biosynthetic process;cell cycle;cell cycle process;cell differentiation;cell proliferation;cellular biosynthetic process
27.6011	26.9792	27.1297	27.0169	1:Experimental evidence at protein level	biological regulation;biosynthetic process;cellular biosynthetic process;cellular component organization;cellular component organization at cellular level;cellular component organization or biogenesis;cellular component orga
24.9524	25.2942	24.8222	24.9845	1:Experimental evidence at protein level	activation of protein kinase activity;activation of protein kinase C activity by G-protein coupled receptor protein signaling pathway;biological regulation;blood coagulation;cell activation;cell surface receptor linked signaling pa
30.558	29.971	30.5915	30.3581	1:Experimental evidence at protein level	biosynthetic process;catabolic process;cellular biosynthetic process;cellular catabolic process;cellular component disassembly;cellular component disassembly at cellular level;cellular component organization;cellular compo
28.4023	28.4687	28.6619	28.4821	1:Experimental evidence at protein level	2'-deoxyribonucleotide biosynthetic process;2'-deoxyribonucleotide metabolic process;benzene-containing compound metabolic process;biosynthetic process;carboxylic acid metabolic process;CDP biosynthetic process;C
27.0983	27.2674	27.1783	27.3526	1:Experimental evidence at protein level	acetyl-CoA catabolic process;acetyl-CoA metabolic process;amine catabolic process;amine metabolic process;aspartate family amino acid catabolic process;aspartate family amino acid metabolic process;carboxylic acid ca
28.671	29.1541	28.821	28.8339	1:Experimental evidence at protein level	biosynthetic process;cellular biosynthetic process;cellular macromolecule biosynthetic process;cellular macromolecule metabolic process;cellular metabolic process;cellular process;cellular protein metabolic process;cotrans
30.2042	29.4205	29.7954	29.7142	1:Experimental evidence at protein level	anion transport;biological regulation;establishment of localization;ion transport;negative regulation of biological process;negative regulation of cellular component organization;negative regulation of cellular process;negative r
28.0271	28.2263	27.9907	28.0907	1:Experimental evidence at protein level	2-oxoglutarate metabolic process;acetyl-CoA catabolic process;acetyl-CoA metabolic process;carbohydrate metabolic process;carboxylic acid metabolic process;catabolic process;cellular aldehyde metabolic process;cellul
27.6222	27.1425	27.5395	27.0844	1:Experimental evidence at protein level	cellular metabolic process;cellular process;electron transport chain;generation of precursor metabolites and energy;metabolic process;mitochondrial electron transport, NADH to ubiquinone;oxidation-reduction process;respi
27.1181	26.4238	26.504	26.3897	1:Experimental evidence at protein level	anatomical structure development;anatomical structure formation involved in morphogenesis;anatomical structure morphogenesis;angiogenesis;astrocyte cell migration;biological regulation;branching morphogenesis of a tub
30.0673	30.2006	30.4269	30.2343	1:Experimental evidence at protein level	biological regulation;cell surface receptor linked signaling pathway;cellular component movement;cellular process;cellular response to stimulus;enzyme linked receptor protein signaling pathway;intracellular signal transductio
28.3454	28.3878	28.1567	27.9852	1:Experimental evidence at protein level	actin cytoskeleton organization;actin filament organization;actin filament polymerization;actin filament-based process;actin nucleation;actin polymerization or depolymerization;Arp2/3 complex-mediated actin nucleation;axon
27.9597	28.3022	28.1227	27.9519	1:Experimental evidence at protein level	anatomical structure formation involved in morphogenesis;anatomical structure morphogenesis;axonogenesis;biological regulation;cell cycle;cell cycle process;cell part morphogenesis;cell projection assembly;cell projection
27.5065	27.3811	27.388	27.3073	1:Experimental evidence at protein level	biosynthetic process;cellular biosynthetic process;cellular component assembly;cellular component assembly at cellular level;cellular component organization;cellular component organization at cellular level;cellular compone
28.8008	28.7147	28.667	28.5936	1:Experimental evidence at protein level	anatomical structure morphogenesis;antigen processing and presentation;axonogenesis;biological regulation;cell differentiation;cell part morphogenesis;cell projection morphogenesis;cell projection organization;cellular comp
28.7927	28.9659	28.9966	28.8524	1:Experimental evidence at protein level	biological regulation;cell surface receptor linked signaling pathway;cellular component assembly;cellular component assembly at cellular level;cellular component organization;cellular component organization at cellular level;
26.9094	26.7399	26.6831	26.6759	1:Experimental evidence at protein level	axon target recognition;biological adhesion;biological regulation;cell adhesion;cell communication;cell-cell adhesion;cell-cell signaling;cellular component organization;cellular component organization at cellular level;cellular c
29.0936	29.5742	29.9261	29.6427	1:Experimental evidence at protein level	alcohol metabolic process;biological regulation;biosynthetic process;cellular biosynthetic process;cellular macromolecule biosynthetic process;cellular macromolecule metabolic process;cellular metabolic process;cellular nit
29.9183	29.7755	29.8089	29.7466	1:Experimental evidence at protein level	biosynthetic process;catabolic process;cellular biosynthetic process;cellular catabolic process;cellular component disassembly;cellular component disassembly at cellular level;cellular component organization;cellular compo
29.5208	29.1172	29.2768	29.3859	1:Experimental evidence at protein level	biological regulation;biosynthetic process;catabolic process;cellular biosynthetic process;cellular catabolic process;cellular component biogenesis;cellular component biogenesis at cellular level;cellular component disassemb
30.782	30.3832	30.5618	30.5488	1:Experimental evidence at protein level	biosynthetic process;catabolic process;cellular biosynthetic process;cellular catabolic process;cellular component disassembly;cellular component disassembly at cellular level;cellular component organization;cellular compo
26.7234	26.6803	27.026	26.3251	1:Experimental evidence at protein level	biological regulation;catabolic process;cell cycle;cell cycle process;cellular catabolic process;cellular component organization;cellular component organization or biogenesis;cellular macromolecule catabolic process;cellular r
29.4256	29.1693	29.3826	29.2692	1:Experimental evidence at protein level	axon guidance;biological regulation;biosynthetic process;cell cycle;cell cycle checkpoint;cell surface receptor linked signaling pathway;cellular biosynthetic process;cellular macromolecule biosynthetic process;cellular macro
28.1421	27.846	28.1063	28.1426	1:Experimental evidence at protein level	cellular macromolecule metabolic process;cellular metabolic process;cellular process;cellular protein metabolic process;chaperone-mediated protein folding;macromolecule metabolic process;macromolecule modification;me
26.4818	26.082	26.2974	26.5443	1:Experimental evidence at protein level	amine metabolic process;carboxylic acid metabolic process;cellular amine metabolic process;cellular amino acid metabolic process;cellular ketone metabolic process;cellular metabolic process;cellular nitrogen compound m
25.8187	25.7759	25.7354	25.5469	1:Experimental evidence at protein level	cellular macromolecule metabolic process;cellular metabolic process;cellular process;cellular protein metabolic process;dephosphorylation;macromolecule metabolic process;macromolecule modification;metabolic process;
26.8841	26.7963	27.2023	27.0533	1:Experimental evidence at protein level	activation of immune response;activation of innate immune response;anatomical structure development;biological regulation;calcineurin-NFAT signaling pathway;calcium ion transport;calcium-mediated signaling;carbohydrat
26.5688	26.4165	26.5847	26.4622	1:Experimental evidence at protein level	activation of MAPK activity;alcohol metabolic process;autophagy;biological regulation;biosynthetic process;carbohydrate homeostasis;carbohydrate metabolic process;carboxylic acid biosynthetic process;carboxylic acid me
26.258	25.7985	26.0159	25.8428	1:Experimental evidence at protein level	cellular macromolecule metabolic process;cellular metabolic process;cellular nitrogen compound metabolic process;cellular process;cellular protein metabolic process;macromolecule metabolic process;macromolecule modi
27.9649	27.57	28.0001	27.8884	1:Experimental evidence at protein level	biological regulation;biosynthetic process;cell communication;cell surface receptor linked signaling pathway;cellular biosynthetic process;cellular macromolecule biosynthetic process;cellular macromolecule metabolic proces
27.5653	27.2531	27.1986	27.3436	1:Experimental evidence at protein level	activation of immune response;activation of innate immune response;anaphase-promoting complex-dependent proteasomal ubiquitin-dependent protein catabolic process;anatomical structure morphogenesis;biological regu
28.8535	29.0614	29.12	28.9744	1:Experimental evidence at protein level	actin crosslink formation;actin cytoskeleton organization;actin filament bundle assembly;actin filament organization;actin filament-based process;axon guidance;biological regulation;cellular component assembly;cellular com
26.202	26.5229	26.4449	26.1118	1:Experimental evidence at protein level	biological regulation;cellular macromolecule metabolic process;cellular metabolic process;cellular nitrogen compound metabolic process;cellular process;macromolecule metabolic process;metabolic process;ncRNA metabo
26.1838	25.8837	25.9535	26.0152	1:Experimental evidence at protein level	cell cycle process;cell differentiation;cellular component biogenesis;cellular component biogenesis at cellular level;cellular component organization or biogenesis;cellular component organization or biogenesis at cellular level;
29.6842	29.6962	30.0285	30.2261	1:Experimental evidence at protein level	biological regulation;biosynthetic process;cellular biosynthetic process;cellular macromolecule biosynthetic process;cellular macromolecule metabolic process;cellular metabolic process;cellular process;cellular protein metab
28.0065	27.7928	27.8796	27.754	1:Experimental evidence at protein level	cell differentiation;cellular developmental process;cellular macromolecule metabolic process;cellular metabolic process;cellular process;cellular protein metabolic process;developmental process;gamete generation;macromo
27.8312	27.8886	27.9446	27.7368	1:Experimental evidence at protein level	actin filament capping;barbed-end actin filament capping;biological regulation;cell communication;cell projection organization;cellular component organization;cellular component organization at cellular level;cellular compone
27.5151	27.673	27.4919	27.5793	1:Experimental evidence at protein level	biological regulation;biosynthetic process;cellular biosynthetic process;cellular macromolecule biosynthetic process;cellular macromolecule metabolic process;cellular metabolic process;cellular nitrogen compound metabolic
26.1552	26.1066	25.9959	25.8644	1:Experimental evidence at protein level	
25.6968	25.2106	25.213	25.7485	1:Experimental evidence at protein level	anatomical structure development;biological regulation;biosynthetic process;cell cycle phase;cell cycle process;cellular biosynthetic process;cellular component organization;cellular component organization at cellular level;c
26.8338	26.8559	27.0156	26.9019	1:Experimental evidence at protein level	biological regulation;biosynthetic process;cellular biosynthetic process;cellular macromolecule biosynthetic process;cellular macromolecule metabolic process;cellular metabolic process;cellular nitrogen compound metabolic
27.249	27.123	27.0406	27.0611	1:Experimental evidence at protein level	
27.298	27.2255	27.1873	27.0035	1:Experimental evidence at protein level	ATP-dependent chromatin remodeling;biological regulation;biosynthetic process;cellular biosynthetic process;cellular component disassembly;cellular component disassembly at cellular level;cellular component organization
28.7264	28.204	28.1606	28.2427	1:Experimental evidence at protein level	cellular component assembly;cellular component assembly at cellular level;cellular component organization;cellular component organization at cellular level;cellular component organization or biogenesis;cellular component c
27.887	28.2617	28.4923	28.1381	1:Experimental evidence at protein level	biological regulation;positive regulation of biological process;positive regulation of biosynthetic process;positive regulation of cellular biosynthetic process;positive regulation of cellular metabolic process;positive regulation of
26.9848	27.2087	27.057	26.7693	1:Experimental evidence at protein level	
27.5718	27.978	27.9023	28.1106	1:Experimental evidence at protein level	
26.6177	26.5915	26.6835	26.5731	1:Experimental evidence at protein level	biosynthetic process;cellular biosynthetic process;cellular component organization;cellular component organization at cellular level;cellular component organization or biogenesis;cellular component organization or biogenesis
26.0273	25.7435	25.7313	25.8265	1:Experimental evidence at protein level	anatomical structure development;anatomical structure morphogenesis;biological regulation;blood vessel development;branching morphogenesis of a tube;cardiac septum morphogenesis;developmental process;hemopoieti
28.3165	28.2409	28.4271	28.5134	1:Experimental evidence at protein level	biological regulation;cap-independent translational initiation;cell differentiation;cellular developmental process;cellular macromolecule metabolic process;cellular metabolic process;cellular nitrogen compound metabolic proce
26.1872	26.3356	26.2757	26.0962	1:Experimental evidence at protein level	
26.1366	26.1818	25.8843	25.9732	1:Experimental evidence at protein level	biological regulation;biosynthetic process;cellular biosynthetic process;cellular macromolecule biosynthetic process;cellular macromolecule metabolic process;cellular metabolic process;cellular nitrogen compound metabolic
25.3912	25.4589	25.0511	25.1507	1:Experimental evidence at protein level	
27.714	28.6109	28.5789	29.1177	1:Experimental evidence at protein level	
27.3377	27.1511	26.9908	26.9434	1:Experimental evidence at protein level	biological regulation;cell cycle phase;cell cycle process;cellular component organization;cellular component organization at cellular level;cellular component organization or biogenesis;cellular component organization or bioge
28.4912	28.2982	28.3991	28.3728	1:Experimental evidence at protein level	biosynthetic process;metabolic process
27.7761	28.0499	27.8922	28.0331	1:Experimental evidence at protein level	biological regulation;cellular macromolecule metabolic process;cellular metabolic process;cellular nitrogen compound metabolic process;cellular process;cellular protein metabolic process;cellular response to chemical stimu
26.8097	26.7802	26.8962	26.8021	1:Experimental evidence at protein level	apoptosis;biological regulation;cell cycle;cell death;cell proliferation;cell surface receptor linked signaling pathway;cellular process;cellular response to chemical stimulus;cellular response to endogenous stimulus;cellular resp

30.8615	30.6552	30.782	30.8102	1:Experimental evidence at protein level	activation of immune response;activation of innate immune response;anaphase-promoting complex-dependent proteasomal ubiquitin-dependent protein catabolic process;antigen processing and presentation;antigen proces
28.1231	28.1415	28.0729	27.9275	1:Experimental evidence at protein level	cellular macromolecule metabolic process;cellular metabolic process;cellular nitrogen compound metabolic process;cellular process;establishment of localization;establishment of localization in cell;establishment of protein lo
28.3388	28.5764	28.5373	28.7915	1:Experimental evidence at protein level	
27.0955	27.1451	26.9259	27.0421	1:Experimental evidence at protein level	anatomical structure development;cell differentiation;cellular developmental process;cellular process;developmental process;nervous system development;system development
27.591	27.2915	27.3672	27.397	1:Experimental evidence at protein level	anterograde axon cargo transport;anterograde synaptic vesicle transport;antigen processing and presentation;antigen processing and presentation of exogenous antigen;antigen processing and presentation of lipid antigen v
27.7712	27.886	27.5624	27.4361	1:Experimental evidence at protein level	antigen processing and presentation;antigen processing and presentation of exogenous antigen;antigen processing and presentation of exogenous peptide antigen;antigen processing and presentation of exogenous peptide
26.3961	26.3343	26.5278	26.1081	1:Experimental evidence at protein level	biological regulation;cell migration;cell motility;cell proliferation;cellular component movement;cellular component organization;cellular component organization or biogenesis;cellular process;locomotion;macromolecule metab
26.0585	25.8522	25.788	25.7681	1:Experimental evidence at protein level	biological regulation;cell differentiation;cellular component organization;cellular component organization at cellular level;cellular component organization or biogenesis;cellular component organization or biogenesis at cellular
29.1291	29.8923	30.2219	30.1296	1:Experimental evidence at protein level	cellular component assembly;cellular component assembly at cellular level;cellular component organization;cellular component organization at cellular level;cellular component organization or biogenesis;cellular component c
27.5782	24.2171	25.8091	26.1382	1:Experimental evidence at protein level	biological regulation;cellular component assembly;cellular component assembly at cellular level;cellular component organization;cellular component organization at cellular level;cellular component organization or biogenesis;
29.2041	29.4385	29.286	29.1811	1:Experimental evidence at protein level	acetyl-CoA catabolic process;acetyl-CoA metabolic process;carbohydrate metabolic process;catabolic process;cellular carbohydrate metabolic process;cellular catabolic process;cellular metabolic process;cellular process;c
27.7473	27.7364	27.6631	27.5113	1:Experimental evidence at protein level	biological regulation;cellular component maintenance;cellular component maintenance at cellular level;cellular component organization;cellular component organization at cellular level;cellular component organization or biog
26.5414	26.3865	26.3674	26.53	1:Experimental evidence at protein level	biological regulation;biosynthetic process;cellular biosynthetic process;cellular component assembly;cellular component organization;cellular component organization at cellular level;cellular component organization or bioger
27.0296	26.931	27.2506	26.7948	1:Experimental evidence at protein level	biological regulation;blood coagulation;cell migration;cell motility;cellular component disassembly;cellular component disassembly at cellular level;cellular component movement;cellular component organization;cellular comp
28.9821	28.9202	29.2693	28.8994	1:Experimental evidence at protein level	biological regulation;cellular component disassembly;cellular component disassembly at cellular level;cellular component organization;cellular component organization at cellular level;cellular component organization or bioge
32.7038	32.4722	33.056	33.1086	1:Experimental evidence at protein level	anatomical structure homeostasis;biological adhesion;biological regulation;cell adhesion;cell surface receptor linked signaling pathway;cell-cell adhesion;cellular component movement;cellular process;cellular response to ch
28.3677	28.9336	29.1523	29.1293	1:Experimental evidence at protein level	biological regulation;blood coagulation;cell activation;cell differentiation;cellular developmental process;cellular lipid metabolic process;cellular metabolic process;cellular process;cellular process involved in reproduction;cellu
28.1538	27.8304	27.8108	27.5914	1:Experimental evidence at protein level	alcohol biosynthetic process;alcohol metabolic process;biosynthetic process;cellular biosynthetic process;cellular lipid metabolic process;cellular metabolic process;cellular process;cholesterol biosynthetic process;choleste
30.5386	30.668	30.5878	30.5156	1:Experimental evidence at protein level	aging;biological regulation;biosynthetic process;cell communication;cell-cell signaling;cellular biosynthetic process;cellular metabolic process;cellular nitrogen compound biosynthetic process;cellular nitrogen compound met
26.6243	26.738	26.863	26.7593	1:Experimental evidence at protein level	activation of adenylate cyclase activity;activation of phospholipase C activity;anatomical structure formation involved in morphogenesis;angiogenesis;biological adhesion;biological regulation;blood coagulation;calcium ion ho
27.0485	27.0123	26.7044	26.8785	1:Experimental evidence at protein level	actin cytoskeleton organization;actin cytoskeleton reorganization;actin filament-based process;activation of signaling protein activity involved in unfolded protein response;biological regulation;blood coagulation;cell activation
27.7823	27.5904	27.4905	27.6038	1:Experimental evidence at protein level	biological regulation;cellular component organization;cellular component organization at cellular level;cellular component organization or biogenesis;cellular component organization or biogenesis at cellular level;cellular macro
27.413	27.1271	27.3969	27.4139	1:Experimental evidence at protein level	anatomical structure development;anatomical structure morphogenesis;axon guidance;biological adhesion;biological regulation;biosynthetic process;blood vessel development;cell adhesion;cell migration;cell motility;cellular
28.7197	28.6947	28.6444	28.4814	1:Experimental evidence at protein level	anatomical structure development;ATP biosynthetic process;ATP metabolic process;ATP synthesis coupled proton transport;biosynthetic process;cation transport;cellular biosynthetic process;cellular metabolic process;cellu
29.5514	29.2676	29.6826	29.5885	1:Experimental evidence at protein level	activation of immune response;activation of innate immune response;anaphase-promoting complex-dependent proteasomal ubiquitin-dependent protein catabolic process;antigen processing and presentation;antigen proces
29.0286	28.899	29.2687	29.1595	1:Experimental evidence at protein level	cell differentiation;cellular developmental process;cellular macromolecule metabolic process;cellular metabolic process;cellular nitrogen compound metabolic process;cellular process;developmental process;epithelial cell diff
26.0915	26.3022	25.8438	25.8445	1:Experimental evidence at protein level	alcohol metabolic process;biosynthetic process;cellular biosynthetic process;cellular lipid metabolic process;cellular metabolic process;cellular process;cholesterol biosynthetic process;cholesterol metabolic process;farnesyl
27.2096	27.7597	27.9654	27.6917	1:Experimental evidence at protein level	actin filament capping;barbed-end actin filament capping;biological regulation;cell projection assembly;cell projection organization;cellular component assembly;cellular component assembly at cellular level;cellular compone
28.078	27.766	27.825	27.6293	1:Experimental evidence at protein level	carbohydrate metabolic process;cellular carbohydrate metabolic process;cellular macromolecule metabolic process;cellular metabolic process;cellular process;cellular protein metabolic process;co-translational protein modif
27.2111	27.2258	27.0526	27.0143	1:Experimental evidence at protein level	alcohol metabolic process;biosynthetic process;cholesterol biosynthetic process;cholesterol metabolic process;lipid biosynthetic process;lipid metabolic process;metabolic process;primary metabolic process;small molecule
27.2212	27.933	27.4818	27.6478	1:Experimental evidence at protein level	biosynthetic process;cellular biosynthetic process;cellular lipid metabolic process;cellular metabolic process;cellular process;chordate embryonic development;developmental process;embryo development;embryo developm
27.4653	27.7782	27.6052	27.5115	1:Experimental evidence at protein level	acylglycerol metabolic process;alcohol metabolic process;amine metabolic process;amine transport;amino acid transmembrane transport;amino acid transport;behavior;betaine metabolic process;betaine transport;biological
26.9961	26.7274	26.8074	26.6911	1:Experimental evidence at protein level	biological regulation;cellular process;cellular response to chemical stimulus;cellular response to endogenous stimulus;cellular response to estrogen stimulus;cellular response to hormone stimulus;cellular response to organic
27.501	27.5428	27.7743	27.7327	1:Experimental evidence at protein level	biological regulation;cell surface receptor linked signaling pathway;cellular macromolecule metabolic process;cellular metabolic process;cellular process;cellular protein metabolic process;cellular response to stimulus;detecti
26.6313	26.8157	26.7559	26.8148	1:Experimental evidence at protein level	alcohol catabolic process;alcohol metabolic process;alditol metabolic process;carbohydrate catabolic process;carbohydrate metabolic process;catabolic process;cellular carbohydrate catabolic process;cellular carbohydrate
27.4586	27.07	27.0865	27.0359	1:Experimental evidence at protein level	biological regulation;biosynthetic process;carbohydrate metabolic process;carbohydrate transport;cell cycle;cell cycle process;cell surface receptor linked signaling pathway;cellular biosynthetic process;cellular component a
32.3665	32.6668	32.6539	32.768	1:Experimental evidence at protein level	amine biosynthetic process;amine metabolic process;apoptosis;biological regulation;biosynthetic process;carboxylic acid biosynthetic process;carboxylic acid metabolic process;cell death;cellular amine metabolic process;c
30.4036	30.4303	30.6989	30.8448	1:Experimental evidence at protein level	biological regulation;biosynthetic process;cellular biosynthetic process;cellular macromolecule biosynthetic process;cellular macromolecule metabolic process;cellular metabolic process;cellular nitrogen compound metabolic
28.6864	28.3926	28.5912	28.6961	1:Experimental evidence at protein level	biological regulation;biosynthetic process;cellular biosynthetic process;cellular macromolecule biosynthetic process;cellular macromolecule metabolic process;cellular metabolic process;cellular nitrogen compound metabolic
27.3829	27.4726	27.338	27.0532	1:Experimental evidence at protein level	alcohol biosynthetic process;alcohol metabolic process;alpha-ketoglutarate transport;anion transport;biosynthetic process;carbohydrate biosynthetic process;carbohydrate metabolic process;carboxylic acid transport;cellula
24.9776	#NUM!	25.148	24.7827	1:Experimental evidence at protein level	biological adhesion;biological regulation;cell adhesion;cellular component organization;cellular component organization or biogenesis;cellular defense response;cellular membrane organization;cellular process;cellular respons
26.7742	27.1524	27.0163	26.9049	1:Experimental evidence at protein level	cellular macromolecule metabolic process;cellular metabolic process;cellular process;cellular protein metabolic process;establishment of localization;establishment of localization in cell;establishment of protein localization;es
#NUM!	23.9298	23.9426	23.2536	1:Experimental evidence at protein level	biological regulation;biosynthetic process;cellular biosynthetic process;cellular component organization;cellular component organization at cellular level;cellular component organization or biogenesis;cellular component orga
29.2368	29.2036	29.0464	28.9518	1:Experimental evidence at protein level	biological regulation;cellular process;establishment of localization;establishment of localization in cell;establishment of protein localization;Golgi vesicle transport;intracellular transport;positive regulation of biological process;
29.8465	29.7494	29.5717	29.7329	1:Experimental evidence at protein level	alternative nuclear mRNA splicing, via spliceosome;biological regulation;biosynthetic process;cellular biosynthetic process;cellular component assembly;cellular component assembly at cellular level;cellular component orga
25.6109	25.2737	25.829	25.5168	1:Experimental evidence at protein level	biological regulation;cellular process;establishment of localization;establishment of localization in cell;establishment of protein localization;establishment of protein localization in Golgi;establishment of protein localization to c
27.7125	27.6303	27.5416	27.5264	1:Experimental evidence at protein level	actin cytoskeleton organization;actin filament bundle assembly;actin filament organization;actin filament-based process;anatomical structure morphogenesis;biological regulation;catabolic process;cell cycle arrest;cell cycle p
27.7403	27.6529	27.576	27.6107	1:Experimental evidence at protein level	carbohydrate metabolic process;cellular carbohydrate metabolic process;cellular macromolecule metabolic process;cellular metabolic process;cellular process;cellular protein metabolic process;glycosylation;macromolecule
25.9784	26.3923	26.1434	26.3841	1:Experimental evidence at protein level	biological regulation;cellular macromolecule metabolic process;cellular metabolic process;cellular nitrogen compound metabolic process;cellular process;cellular protein metabolic process;cellular response to stimulus;cellula
29.4912	29.5036	29.6134	29.5624	1:Experimental evidence at protein level	biological regulation;cell cycle;cell cycle phase;cell cycle process;cell division;cell proliferation;cellular component organization;cellular component organization at cellular level;cellular component organization or biogenesis;c
26.2869	26.3308	26.356	26.2101	1:Experimental evidence at protein level	alcohol metabolic process;anatomical structure development;biological regulation;biosynthetic process;blood vessel development;cell surface receptor linked signaling pathway;cellular process;cellular response to stimulus;c
27.604	27.4542	27.473	27.4164	1:Experimental evidence at protein level	activation of immune response;activation of innate immune response;anaphase-promoting complex-dependent proteasomal ubiquitin-dependent protein catabolic process;antigen processing and presentation;antigen proces
28.0996	28.1327	27.9835	28.0009	1:Experimental evidence at protein level	biological regulation;cellular component organization;cellular component organization or biogenesis;cellular localization;cellular macromolecule localization;cellular membrane organization;cellular process;cellular protein local
29.8771	28.5739	28.6983	28.8341	1:Experimental evidence at protein level	biological regulation;blood coagulation;cellular component assembly;cellular component assembly at cellular level;cellular component organization;cellular component organization at cellular level;cellular component organiza
26.001	25.8062	25.9957	25.7318	1:Experimental evidence at protein level	axon guidance;biological regulation;cell cycle;cell cycle process;cell division;cellular component organization;cellular component organization at cellular level;cellular component organization or biogenesis;cellular component
29.3226	29.1925	29.576	29.5351	1:Experimental evidence at protein level	biological regulation;biosynthetic process;cell differentiation;cellular biosynthetic process;cellular developmental process;cellular macromolecule biosynthetic process;cellular macromolecule metabolic process;cellular metab
28.0329	28.0671	28.2263	28.1407	1:Experimental evidence at protein level	
25.7697	25.8067	25.5738	#NUM!	1:Experimental evidence at protein level	actin cytoskeleton organization;actin cytoskeleton reorganization;actin filament-based process;activation of protein kinase activity;anatomical structure morphogenesis;biological regulation;cell communication;cell part morph
27.3379	27.2017	27.1387	27.01	1:Experimental evidence at protein level	carbohydrate metabolic process;catabolic process;cellular carbohydrate metabolic process;cellular catabolic process;cellular macromolecule catabolic process;cellular macromolecule metabolic process;cellular metabolic pr
27.3663	27.4095	27.5724	27.6617	1:Experimental evidence at protein level	cellular component assembly;cellular component assembly at cellular level;cellular component organization;cellular component organization at cellular level;cellular component organization or biogenesis;cellular component c
27.7378	28.2809	28.0989	28.3243	1:Experimental evidence at protein level	catabolic process;cell cycle;cellular catabolic process;cellular macromolecule catabolic process;cellular macromolecule metabolic process;cellular metabolic process;cellular process;cellular protein metabolic process;macro
27.9528	27.306	27.5352	27.622	1:Experimental evidence at protein level	cellular component assembly;cellular component assembly at cellular level;cellular component organization;cellular component organization at cellular level;cellular component organization or biogenesis;cellular component c
27.6756	28.0013	28.356	28.1216	1:Experimental evidence at protein level	amine biosynthetic process;amine metabolic process;biosynthetic process;carboxylic acid biosynthetic process;carboxylic acid metabolic process;cellular amine metabolic process;cellular amino acid biosynthetic process;ce
28.4704	28.4455	28.3374	28.404	1:Experimental evidence at protein level	apoptosis;biological regulation;cell death;cellular process;death;negative regulation of apoptosis;negative regulation of biological process;negative regulation of cell death;negative regulation of cellular process;negative regula
27.0443	#NUM!	26.8059	27.2693	1:Experimental evidence at protein level	anatomical structure development;cellular metabolic process;cellular process;cellular response to chemical stimulus;cellular response to endogenous stimulus;cellular response to glucagon stimulus;cellular response to horm
#NUM!	#NUM!	#NUM!	#NUM!	1:Experimental evidence at protein level	biological regulation;blood coagulation;cellular component assembly;cellular component organization;cellular component organization or biogenesis;cellular process;coagulation;endocytic recycling;endosome transport;estab
26.5589	26.2604	26.5649	26.25	1:Experimental evidence at protein level	cellular process;establishment of localization;establishment of localization in cell;establishment of protein localization;Golgi vesicle transport;intracellular protein transport;intracellular transport;intra-Golgi vesicle-mediated tra
26.7905	26.7932	26.8683	26.6703	1:Experimental evidence at protein level	cell differentiation;cellular component organization;cellular component organization or biogenesis;cellular developmental process;cellular membrane organization;cellular process;collagen catabolic process;collagen metabolic
27.0618	27.1425	26.9475	27.2582	1:Experimental evidence at protein level	biological regulation;cellular macromolecule metabolic process;cellular metabolic process;cellular process;cellular protein metabolic process;chaperone-mediated protein folding;macromolecule metabolic process;metabolic
25.1947	25.5412	25.3337	25.2001	1:Experimental evidence at protein level	biological regulation;catabolic process;cellular catabolic process;cellular macromolecule catabolic process;cellular macromolecule metabolic process;cellular metabolic process;cellular process;cellular protein metabolic proc
28.3898	27.5522	28.1053	27.8614	1:Experimental evidence at protein level	adenine transport;cellular process;establishment of localization;nitrogen compound transport;nucleobase transport;nucleobase-containing compound transport;purine base transport;purine-containing compound transmemb
27.0528	26.9225	27.0231	26.7653	1:Experimental evidence at protein level	activation of protein kinase activity;biological regulation;cell cycle arrest;cell cycle process;cell surface receptor linked signaling pathway;cellular component assembly;cellular component organization;cellular component orga
27.9053	28.0402	28.0709	27.7969	1:Experimental evidence at protein level	anatomical structure development;biological regulation;biomineral tissue development;bone marrow development;bone mineralization;cell chemotaxis;cell cycle process;cell migration;cell motility;cell proliferation;cellular com
29.8115	30.1402	29.8979	30.0584	1:Experimental evidence at protein level	anatomical structure formation involved in morphogenesis;anatomical structure morphogenesis;angiogenesis;anion transport;apoptosis;biological regulation;branching morphogenesis of a tube;cation homeostasis;cell death;
27.2382	27.0791	27.0982	27.0698	1:Experimental evidence at protein level	biological regulation;biosynthetic process;cell activation;cell proliferation;cellular biosynthetic process;cellular component assembly;cellular component assembly at cellular level;cellular component organization;cellular comp
27.3167	27.1205	27.5279	27.3503	1:Experimental evidence at protein level	biological regulation;cell cycle arrest;cell cycle process;cellular component assembly;cellular component assembly at cellular level;cellular component organization;cellular component organization at cellular level;cellular com
27.0258	26.4633	26.7187	26.5864	1:Experimental evidence at protein level	biological regulation;cell cycle;cell division;cell migration;cell motility;cellular component movement;cellular component organization;cellular component organization at cellular level;cellular component organization or biogene
26.4153	25.5764	25.6621	25.942	1:Experimental evidence at protein level	anatomical structure morphogenesis;axonogenesis;biological regulation;cell part morphogenesis;cell projection morphogenesis;cell projection organization;cellular component assembly;cellular component assembly at cellula
28.1124	28.0048	28.1562	27.9207	1:Experimental evidence at protein level	amine biosynthetic process;amine catabolic process;amine metabolic process;behavior;behavioral interaction between organisms;biological regulation;biosynthetic process;carboxylic acid biosynthetic process;carboxylic aci
30.78	31.9857	31.6841	31.5231	1:Experimental evidence at protein level	biological regulation;blood coagulation;blood coagulation, intrinsic pathway;cell activation;cell differentiation;cellular component disassembly;cellular component disassembly at cellular level;cellular component organization;c
25.8458	#NUM!	26.0327	25.5815	1:Experimental evidence at protein level	activation of protein kinase activity;anatomical structure development;anatomical structure formation involved in morphogenesis;anatomical structure morphogenesis;angiogenesis;angiogenesis involved in wound healing;apo
27.8679	28.1169	28.211	28.1073	1:Experimental evidence at protein level	acute inflammatory response;acute inflammatory response to antigenic stimulus;adhesion to host;adhesion to other organism involved in symbiotic interaction;aging;anatomical structure development;antigen processing and
26.1105	26.5714	26.3492	26.13	1:Experimental evidence at protein level	aging;amine metabolic process;anatomical structure development;angiotensin maturation;beta-amyloid metabolic process;biological regulation;carboxylic acid metabolic process;cell aging;cellular amide metabolic process;c
27.9309	28.181	28.3021	28.564	1:Experimental evidence at protein level	cellular component organization;cellular component organization or biogenesis;cellular membrane organization;cellular process;clathrin-mediated endocytosis;endocytosis;establishment of localization;establishment of localiz
27.6661	27.5988	27.5416	27.5677	1:Experimental evidence at protein level	biological regulation;biosynthetic process;body fluid secretion;cellular aromatic compound metabolic process;cellular biosynthetic process;cellular metabolic process;cellular nitrogen compound biosynthetic process;cellular
#NUM!	#NUM!	23.0818	#NUM!	1:Experimental evidence at protein level	actin cytoskeleton organization;actin filament bundle assembly;actin filament organization;actin filament-based process;anatomical structure morphogenesis;biological regulation;cell activation;cell activation involved in immu
28.4288	28.4895	28.5276	28.5328	1:Experimental evidence at protein level	activation of phospholipase C activity;activation of protein kinase A activity;activation of protein kinase activity;biological regulation;blood coagulation;cell surface receptor linked signaling pathway;cellular metabolic process;
27.5151	27.6439	27.6474	27.5625	1:Experimental evidence at protein level	acetyl-CoA catabolic process;acetyl-CoA metabolic process;aerobic respiration;carboxylic acid metabolic process;catabolic process;cellular catabolic process;cellular ketone metabolic process;cellular metabolic process;cel
29.4753	29.3099	29.5087	29.5175	1:Experimental evidence at protein level	androgen receptor signaling pathway;biological regulation;cell motility;cellular component movement;cellular localization;cellular macromolecule localization;cellular macromolecule metabolic process;cellular metabolic proce
30.0266	30.2512	30.4013	30.2805	1:Experimental evidence at protein level	cellular macromolecule metabolic process;cellular metabolic process;cellular nitrogen compound metabolic process;cellular process;gene expression;macromolecule metabolic process;metabolic process;mRNA metabolic p
28.7873	28.7242	28.9857	29.0773	1:Experimental evidence at protein level	biological regulation;biosynthetic process;cellular biosynthetic process;cellular macromolecule biosynthetic process;cellular macromolecule metabolic process;cellular metabolic process;cellular nitrogen compound metabolic
28.6175	28.6251	28.9368	28.714	1:Experimental evidence at protein level	activation of immune response;activation of innate immune response;anaphase-promoting complex-dependent proteasomal ubiquitin-dependent protein catabolic process;anatomical structure development;antigen processi
26.2252	26.3548	26.4314	26.1203	1:Experimental evidence at protein level	biological regulation;regulation of biological process;regulation of cellular component organization;regulation of cellular metabolic process;regulation of cellular process;regulation of chromosome organization;regulation of DN
28.9337	29.1842	29.0559	28.9428	1:Experimental evidence at protein level	biological regulation;biosynthetic process;cellular biosynthetic process;cellular macromolecule biosynthetic process;cellular macromolecule metabolic process;cellular metabolic process;cellular process;cellular protein metab
28.4252	28.6093	28.8543	28.8609	1:Experimental evidence at protein level	biological regulation;circulatory system process;multicellular organismal process;positive regulation of blood pressure;positive regulation of systemic arterial blood pressure;regulation of anatomical structure size;regulation of
#NUM!	#NUM!	#NUM!	27.245	1:Experimental evidence at protein level	astral microtubule organization;biological regulation;cell cycle process;cell projection organization;cellular component assembly;cellular component assembly at cellular level;cellular component organization;cellular compone
29.2922	28.9254	29.0855	29.1679	1:Experimental evidence at protein level	activation of immune response;activation of innate immune response;activation of MAPK activity;alcohol metabolic process;anaphase-promoting complex-dependent proteasomal ubiquitin-dependent protein catabolic proce
29.2456	29.2981	29.2022	29.4368	1:Experimental evidence at protein level	anatomical structure development;anatomical structure morphogenesis;anterior/posterior pattern specification;biological regulation;cell differentiation;cell differentiation involved in kidney development;cell morphogenesis;cel
29.7578	30.0378	29.6604	29.9136	1:Experimental evidence at protein level	biological regulation;biosynthetic process;cell cycle arrest;cell cycle process;cell proliferation;cell surface receptor linked signaling pathway;cellular biosynthetic process;cellular macromolecule biosynthetic process;cellular m
28.1793	28.1709	28.1642	28.1907	1:Experimental evidence at protein level	biological regulation;cellular process;cellular response to stimulus;intracellular signal transduction;positive regulation of biological process;positive regulation of catalytic activity;positive regulation of cell communication;positi
26.1811	25.8164	27.0369	25.4249	1:Experimental evidence at protein level	biological regulation;biosynthetic process;carbohydrate metabolic process;carbohydrate transport;cell cycle;cell cycle process;cell surface receptor linked signaling pathway;cellular biosynthetic process;cellular component c
29.0297	29.0229	29.0965	29.0094	1:Experimental evidence at protein level	antigen processing and presentation;antigen processing and presentation of exogenous antigen;antigen processing and presentation of exogenous peptide antigen;antigen processing and presentation of exogenous peptide
26.6283	26.3554	26.5546	26.5423	1:Experimental evidence at protein level	cellular process;ER to Golgi vesicle-mediated transport;establishment of localization;establishment of localization in cell;establishment of protein localization;Golgi vesicle transport;intracellular protein transport;intracellular tra
27.8149	28.0824	28.118	28.3659	1:Experimental evidence at protein level	biological regulation;cell activation;cell activation involved in immune response;cell killing;cellular process;establishment of localization;establishment of localization in cell;establishment of protein localization;exocytosis;immu
27.7935	27.7789	27.6901	27.6209	1:Experimental evidence at protein level	carboxylic acid catabolic process;carboxylic acid metabolic process;catabolic process;cellular catabolic process;cellular component assembly;cellular component organization;cellular component organization or biogenesis;c
27.2673	27.1607	27.2367	27.0538	1:Experimental evidence at protein level	actin cytoskeleton organization;actin filament-based process;biological regulation;cellular component organization;cellular component organization at cellular level;cellular component organization or biogenesis;cellular compo
26.8465	27.1139	26.5118	26.7309	1:Experimental evidence at protein level	anatomical structure development;biological regulation;cell development;cellular component organization;cellular component organization at cellular level;cellular component organization or biogenesis;cellular component org
27.3093	26.9662	26.9497	27.2886	1:Experimental evidence at protein level	cellular component assembly;cellular component organization;cellular component organization at cellular level;cellular component organization or biogenesis;cellular component organization or biogenesis at cellular level;cellu
26.8035	26.7226	26.8247	27.0346	1:Experimental evidence at protein level	
26.6076	26.1159	26.228	26.3658	1:Experimental evidence at protein level	biological regulation;cellular macromolecule metabolic process;cellular metabolic process;cellular nitrogen compound metabolic process;cellular process;macromolecule metabolic process;metabolic process;mRNA metaboli
27.0316	27.1285	26.8773	26.9297	1:Experimental evidence at protein level	adult behavior;adult locomotory behavior;anatomical structure homeostasis;behavior;biological regulation;cell cycle checkpoint;cell differentiation;cell differentiation in spinal cord;cell surface receptor linked signaling pathway
25.9756	25.5026	25.6086	25.4643	1:Experimental evidence at protein level	biological regulation;cell cycle;cell cycle phase;cell cycle process;cell division;cellular component organization;cellular component organization at cellular level;cellular component organization or biogenesis;cellular componen
26.8201	26.708	26.791	26.5943	1:Experimental evidence at protein level	biological regulation;cell activation;cell activation involved in immune response;cell chemotaxis;cell migration;cell motility;cellular component disassembly;cellular component disassembly at cellular level;cellular component m
26.9123	27.2541	27.4397	27.565	1:Experimental evidence at protein level	biological adhesion;cell adhesion;cellular process
28.4947	#NUM!	29.3043	28.1597	1:Experimental evidence at protein level	biosynthetic process;cellular biosynthetic process;cellular macromolecule biosynthetic process;cellular macromolecule metabolic process;cellular metabolic process;cellular process;cellular protein metabolic process;macron
25.5096	25.4215	25.7141	25.6288	1:Experimental evidence at protein level	apoptosis;biological regulation;cell communication;cell death;cell migration;cell motility;cell surface receptor linked signaling pathway;cell-cell signaling;cellular component movement;cellular component organization;cellular
27.9204	28.4085	28.4231	28.4109	1:Experimental evidence at protein level	biological regulation;cell redox homeostasis;cellular homeostasis;cellular macromolecule metabolic process;cellular metabolic process;cellular process;cellular protein metabolic process;cellular response to stimulus;cellular r
27.2564	27.0166	27.0453	26.995	1:Experimental evidence at protein level	biological regulation;cell differentiation;cellular component biogenesis;cellular component biogenesis at cellular level;cellular component organization or biogenesis;cellular component organization or biogenesis at cellular lev
27.4492	27.5371	27.1838	27.3561	1:Experimental evidence at protein level	biological regulation;cellular process;cellular response to stimulus;cellular response to stress;negative regulation of biological process;negative regulation of cellular metabolic process;negative regulation of cellular process;ne
25.8291	#NUM!	26.5777	25.6736	1:Experimental evidence at protein level	amine metabolic process;amino acid activation;carboxylic acid metabolic process;cellular amine metabolic process;cellular amino acid metabolic process;cellular ketone metabolic process;cellular macromolecule metabolic p
26.4054	#NUM!	26.1811	26.0666	1:Experimental evidence at protein level	cellular component assembly;cellular component assembly at cellular level;cellular component organization;cellular component organization at cellular level;cellular component organization or biogenesis;cellular component c
28.888	28.5804	28.6156	28.5802	1:Experimental evidence at protein level	biological regulation;cellular component organization;cellular component organization at cellular level;cellular component organization or biogenesis;cellular component organization or biogenesis at cellular level;cellular memb
26.2673	25.9345	25.8442	25.9399	1:Experimental evidence at protein level	anatomical structure formation involved in morphogenesis;cellular macromolecule metabolic process;cellular metabolic process;cellular process;cellular protein metabolic process;developmental process;macromolecule meta
27.8437	27.9245	27.924	27.8526	1:Experimental evidence at protein level	aging;anatomical structure formation involved in morphogenesis;anatomical structure morphogenesis;angiogenesis;biological adhesion;biological regulation;biosynthetic process;cell adhesion;cell migration;cell morphogenes
27.0889	26.8297	27.0742	26.867	1:Experimental evidence at protein level	biological regulation;cell communication;cellular process;cellular response to chemical stimulus;cellular response to external stimulus;cellular response to extracellular stimulus;cellular response to glucose starvation;cellular r
28.1613	27.8672	27.8792	27.7754	1:Experimental evidence at protein level	anatomical structure morphogenesis;biological regulation;developmental process;establishment of localization;establishment of RNA localization;gene expression;macromolecule metabolic process;metabolic process;mRNA
27.2963	27.2147	27.2765	27.2703	1:Experimental evidence at protein level	anatomical structure morphogenesis;biological regulation;biosynthetic process;cellular biosynthetic process;cellular macromolecule biosynthetic process;cellular macromolecule metabolic process;cellular metabolic process;
28.3598	28.7352	28.4488	28.4067	1:Experimental evidence at protein level	biological regulation;cell cycle arrest;cell cycle process;cellular macromolecule metabolic process;cellular metabolic process;cellular process;cellular protein metabolic process;dephosphorylation;macromolecule metabolic pr
26.8741	26.9342	26.632	26.7479	1:Experimental evidence at protein level	antigen processing and presentation;antigen processing and presentation of exogenous antigen;antigen processing and presentation of exogenous peptide antigen;antigen processing and presentation of exogenous peptide

27.3559	27.2077	27.2703	27.4562	1:Experimental evidence at protein level	assembly of spliceosomal tri-snRNP;cellular component assembly;cellular component assembly at cellular level;cellular component organization;cellular component organization at cellular level;cellular component organizatic
28.9052	28.9506	29.0615	28.7607	1:Experimental evidence at protein level	amine metabolic process;biological regulation;carboxylic acid metabolic process;cellular amine metabolic process;cellular amino acid metabolic process;cellular ketone metabolic process;cellular macromolecule metabolic pr
25.7412	25.569	25.7453	25.117	1:Experimental evidence at protein level	biological regulation;cellular localization;cellular process;localization;negative regulation of catalytic activity;negative regulation of hydrolase activity;negative regulation of molecular function;negative regulation of phosphatase
26.7984	26.3485	26.7816	26.4938	1:Experimental evidence at protein level	cellular component organization;cellular component organization at cellular level;cellular component organization or biogenesis;cellular component organization or biogenesis at cellular level;cellular membrane organization;ce
29.2609	29.9478	29.8829	29.6815	1:Experimental evidence at protein level	antigen processing and presentation;antigen processing and presentation of exogenous antigen;antigen processing and presentation of exogenous peptide antigen;antigen processing and presentation of exogenous peptide
29.8754	29.9025	29.8361	29.825	1:Experimental evidence at protein level	activation of immune response;activation of innate immune response;activation of MAPK activity;activation of MAPKK activity;activation of protein kinase activity;aging;anaphase-promoting complex-dependent proteasomal u
27.735	28.0228	27.747	27.8308	1:Experimental evidence at protein level	cellular metabolic process;cellular process;electron transport chain;generation of precursor metabolites and energy;metabolic process;oxidation-reduction process;respiratory electron transport chain;small molecule metaboli
26.9142	26.8314	26.9861	26.8303	1:Experimental evidence at protein level	biological regulation;establishment of localization;lipid transport;modulation of growth of symbiont involved in interaction with host;organic substance transport;positive regulation of biological process;positive regulation of gr
27.3834	27.7094	27.4866	27.6308	1:Experimental evidence at protein level	amine metabolic process;AMP biosynthetic process;AMP metabolic process;aspartate family amino acid metabolic process;aspartate metabolic process;biosynthetic process;carboxylic acid metabolic process;cellular amine
28.4783	28.5476	28.557	28.5395	1:Experimental evidence at protein level	acrosome assembly;actin cytoskeleton organization;actin filament-based process;adult behavior;adult locomotory behavior;alcohol metabolic process;ameboidal cell migration;amine metabolic process;anatomical structure c
30.9	30.5396	30.7141	30.5657	1:Experimental evidence at protein level	biosynthetic process;catabolic process;cellular biosynthetic process;cellular catabolic process;cellular component disassembly;cellular component disassembly at cellular level;cellular component organization;cellular compo
26.3085	26.1563	26.4474	26.3403	1:Experimental evidence at protein level	
28.8587	28.7232	28.709	28.5563	1:Experimental evidence at protein level	biological regulation;blood coagulation;carbohydrate metabolic process;cellular carbohydrate metabolic process;cellular component organization;cellular component organization at cellular level;cellular component organizati
27.8936	27.726	27.8396	27.8424	1:Experimental evidence at protein level	acetyl-CoA catabolic process;acetyl-CoA metabolic process;carbohydrate metabolic process;catabolic process;cellular catabolic process;cellular metabolic process;cellular process;coenzyme catabolic process;coenzyme m
27.1672	27.1022	27.0376	27.0286	1:Experimental evidence at protein level	catabolic process;cellular catabolic process;cellular metabolic process;cellular nitrogen compound catabolic process;cellular nitrogen compound metabolic process;cellular process;cofactor catabolic process;cofactor metab
28.8646	28.7604	28.6723	28.6997	1:Experimental evidence at protein level	antigen processing and presentation;antigen processing and presentation of exogenous antigen;antigen processing and presentation of exogenous peptide antigen;antigen processing and presentation of exogenous peptide
31.506	31.0863	31.3577	31.4395	1:Experimental evidence at protein level	biosynthetic process;catabolic process;cellular biosynthetic process;cellular catabolic process;cellular component disassembly;cellular component disassembly at cellular level;cellular component organization;cellular compo
31.7124	29.7597	30.0677	30.514	1:Experimental evidence at protein level	anatomical structure homeostasis;ATP-dependent chromatin remodeling;biological regulation;cell cycle;cellular component assembly;cellular component assembly at cellular level;cellular component organization;cellular com
28.8929	29.2195	29.1936	29.3564	1:Experimental evidence at protein level	biological regulation;calcium-mediated signaling;carboxylic acid metabolic process;catabolic process;cellular catabolic process;cellular ketone metabolic process;cellular metabolic process;cellular process;cellular response
30.5198	30.5867	30.3149	30.6204	1:Experimental evidence at protein level	biosynthetic process;catabolic process;cellular biosynthetic process;cellular catabolic process;cellular component disassembly;cellular component disassembly at cellular level;cellular component organization;cellular compo
25.9718	25.9064	25.8836	25.8595	1:Experimental evidence at protein level	activation of immune response;activation of innate immune response;adaptive immune response;adaptive immune response based on somatic recombination of immune receptors built from immunoglobulin superfamily dom
25.7865	26.1082	26.0187	25.982	1:Experimental evidence at protein level	alcohol biosynthetic process;alcohol catabolic process;alcohol metabolic process;alditol catabolic process;alditol metabolic process;biosynthetic process;carbohydrate biosynthetic process;carbohydrate catabolic process;c
30.676	30.1193	30.2644	30.4415	1:Experimental evidence at protein level	biosynthetic process;catabolic process;cellular biosynthetic process;cellular catabolic process;cellular component disassembly;cellular component disassembly at cellular level;cellular component organization;cellular compo
29.0933	29.1359	29.343	29.0798	1:Experimental evidence at protein level	cell communication;cell-cell signaling;cellular process;immune response;immune system process;response to stimulus;signaling
27.9466	28.0886	27.6826	27.8482	1:Experimental evidence at protein level	biological regulation;cellular macromolecule metabolic process;cellular metabolic process;cellular process;cellular protein metabolic process;cellular response to chemical stimulus;cellular response to organic substance;cellu
26.0934	26.5883	26.3575	26.4194	1:Experimental evidence at protein level	biological regulation;cell cycle arrest;cell cycle process;cellular macromolecule metabolic process;cellular metabolic process;cellular process;cellular protein metabolic process;cellular response to chemical stimulus;cellular re
27.9347	27.8409	28.0339	28.0175	1:Experimental evidence at protein level	gamete generation;male gamete generation;multicellular organismal process;multicellular organismal reproductive process;reproductive process;spermatogenesis
30.1167	30.5724	30.2377	30.4506	1:Experimental evidence at protein level	biological regulation;catabolic process;cellular catabolic process;cellular macromolecule catabolic process;cellular macromolecule metabolic process;cellular metabolic process;cellular nitrogen compound metabolic process
29.6695	29.5698	29.57	29.6261	1:Experimental evidence at protein level	biological regulation;cation transport;cellular macromolecule metabolic process;cellular metabolic process;cellular process;cellular response to abiotic stimulus;cellular response to acidity;cellular response to pH;cellular respo
26.9303	26.9473	27.1247	26.9517	1:Experimental evidence at protein level	biological regulation;biosynthetic process;cellular biosynthetic process;cellular macromolecule biosynthetic process;cellular macromolecule metabolic process;cellular metabolic process;cellular nitrogen compound metabolic
27.3283	26.965	27.3766	27.1045	1:Experimental evidence at protein level	catabolic process;cellular catabolic process;cellular macromolecule catabolic process;cellular macromolecule metabolic process;cellular metabolic process;cellular nitrogen compound metabolic process;cellular process;exo
27.0887	26.9882	27.0143	26.9545	1:Experimental evidence at protein level	amine metabolic process;amino acid activation;asparaginyl-tRNA aminoacylation;carboxylic acid metabolic process;cellular amine metabolic process;cellular amino acid metabolic process;cellular ketone metabolic process;c
28.0072	27.726	27.7619	27.8107	1:Experimental evidence at protein level	biological regulation;cell junction assembly;cell junction organization;cell surface receptor linked signaling pathway;cell-cell junction assembly;cell-cell junction organization;cellular component assembly;cellular component as
27.2487	27.2906	27.4252	27.3299	1:Experimental evidence at protein level	actin cytoskeleton organization;actin filament organization;actin filament-based process;actin polymerization or depolymerization;activation of immune response;anatomical structure formation involved in morphogenesis;anti
28.7347	28.7834	28.7176	28.6436	1:Experimental evidence at protein level	cellular component organization;cellular component organization at cellular level;cellular component organization or biogenesis;cellular component organization or biogenesis at cellular level;cellular process;establishment of l
28.2215	27.9944	28.0228	28.1483	1:Experimental evidence at protein level	biological regulation;calcium ion homeostasis;cation homeostasis;cell differentiation;cellular developmental process;cellular process;cellular response to biotic stimulus;cellular response to stimulus;cellular response to stress
26.4845	25.9047	26.2882	26.0997	1:Experimental evidence at protein level	developmental process;multicellular organismal development;multicellular organismal process
26.0802	25.8369	25.7081	25.6937	1:Experimental evidence at protein level	biological regulation;cellular component organization;cellular component organization or biogenesis;cellular membrane organization;cellular process;endocytosis;establishment of localization;membrane invagination;membrar
29.1729	29.528	29.6132	29.6447	1:Experimental evidence at protein level	cellular component assembly;cellular component assembly at cellular level;cellular component organization;cellular component organization at cellular level;cellular component organization or biogenesis;cellular component c
27.8259	28.4013	28.4672	28.3424	1:Experimental evidence at protein level	lipid metabolic process;metabolic process;primary metabolic process
27.8273	27.6658	27.5653	27.8564	1:Experimental evidence at protein level	biological regulation;cellular process;cellular response to stimulus;cellular response to stress;DNA damage response, signal transduction resulting in induction of apoptosis;induction of apoptosis;induction of apoptosis by int
26.1512	26.1159	26.2905	26.0811	1:Experimental evidence at protein level	antigen processing and presentation;antigen processing and presentation of exogenous antigen;antigen processing and presentation of exogenous peptide antigen;antigen processing and presentation of exogenous peptide
28.0826	28.026	28.1581	27.9771	1:Experimental evidence at protein level	biological regulation;cellular component assembly;cellular component organization;cellular component organization or biogenesis;cellular membrane organization;cellular process;cellular response to chemical stimulus;cellula
28.0865	28.0756	27.9517	27.9961	1:Experimental evidence at protein level	biological regulation;cation homeostasis;cation transport;cell differentiation;cellular developmental process;cellular process;chemical homeostasis;developmental process;erythrocyte differentiation;establishment of localizatic
26.9662	26.5223	26.6648	26.6708	1:Experimental evidence at protein level	biological regulation;biosynthetic process;cell surface receptor linked signaling pathway;cellular biosynthetic process;cellular macromolecule biosynthetic process;cellular macromolecule metabolic process;cellular metabolic
28.6462	28.7178	28.9953	28.8638	1:Experimental evidence at protein level	biosynthetic process;carbohydrate biosynthetic process;carbohydrate metabolic process;cellular biosynthetic process;cellular carbohydrate biosynthetic process;cellular carbohydrate metabolic process;cellular lipid metabol
28.6697	28.8931	28.9752	28.8301	1:Experimental evidence at protein level	
25.9826	25.7056	25.446	25.4436	1:Experimental evidence at protein level	cellular macromolecule metabolic process;cellular metabolic process;cellular nitrogen compound metabolic process;cellular process;macromolecule metabolic process;metabolic process;mRNA metabolic process;mRNA pro
26.0735	26.4322	25.9848	26.3131	1:Experimental evidence at protein level	apoptosis;biological regulation;biosynthetic process;cell death;cellular biosynthetic process;cellular macromolecule biosynthetic process;cellular macromolecule metabolic process;cellular metabolic process;cellular nitrogen
26.6248	26.4111	26.4744	26.4737	1:Experimental evidence at protein level	establishment of localization;establishment of localization in cell;establishment of protein localization;establishment of RNA localization;intracellular protein transport;intracellular transport;mRNA transport;nuclear export;nucle
29.2079	29.2143	29.4526	29.3612	1:Experimental evidence at protein level	activation of JUN kinase activity;activation of MAPK activity;anatomical structure morphogenesis;apoptosis;biological regulation;cell death;cell part morphogenesis;cell projection morphogenesis;cell projection organization;c
28.3412	28.2289	28.6536	28.2691	1:Experimental evidence at protein level	autophagic vacuole assembly;biological regulation;catabolic process;cellular catabolic process;cellular component assembly;cellular component assembly at cellular level;cellular component organization;cellular component
28.428	28.2258	28.2864	28.0746	1:Experimental evidence at protein level	biological regulation;cellular component assembly;cellular component assembly at cellular level;cellular component organization;cellular component organization at cellular level;cellular component organization or biogenesis;
27.0637	26.7923	26.9532	26.7663	1:Experimental evidence at protein level	biological regulation;catabolic process;cellular catabolic process;cellular macromolecule catabolic process;cellular macromolecule metabolic process;cellular metabolic process;cellular nitrogen compound metabolic process
27.2888	26.6555	26.8032	26.633	1:Experimental evidence at protein level	biological regulation;cellular component organization;cellular component organization at cellular level;cellular component organization or biogenesis;cellular component organization or biogenesis at cellular level;cellular proce
27.9594	28.1826	28.1721	28.2461	1:Experimental evidence at protein level	biosynthetic process;carboxylic acid biosynthetic process;carboxylic acid catabolic process;carboxylic acid metabolic process;catabolic process;cellular biosynthetic process;cellular catabolic process;cellular ketone metabo
27.4929	27.4706	27.5142	27.1988	1:Experimental evidence at protein level	anterograde axon cargo transport;anterograde synaptic vesicle transport;antigen processing and presentation;antigen processing and presentation of exogenous antigen;antigen processing and presentation of lipid antigen v
27.5648	27.1957	27.8797	27.9411	1:Experimental evidence at protein level	actin cytoskeleton organization;actin filament-based process;axon guidance;biological regulation;cell communication;cell projection assembly;cell projection organization;cell surface receptor linked signaling pathway;cell-cel
28.2566	28.5085	29.0208	28.7219	1:Experimental evidence at protein level	biological regulation;cellular process;cellular response to chemical stimulus;cellular response to endogenous stimulus;cellular response to growth factor stimulus;cellular response to organic substance;cellular response to sti
30.4488	30.0723	30.8062	30.5605	1:Experimental evidence at protein level	biological regulation;biosynthetic process;cellular biosynthetic process;cellular macromolecule biosynthetic process;cellular macromolecule metabolic process;cellular metabolic process;cellular nitrogen compound metabolic
29.5568	29.5897	29.4745	29.3958	1:Experimental evidence at protein level	biological regulation;biosynthetic process;cellular biosynthetic process;cellular component assembly;cellular component assembly at cellular level;cellular component organization;cellular component organization at cellular le
30.5414	30.8527	31.1164	31.0908	1:Experimental evidence at protein level	biological regulation;blood coagulation;cell activation;cellular process;coagulation;establishment of localization;establishment of localization in cell;exocytosis;hemostasis;multicellular organismal process;platelet activation;pla
26.0783	25.9741	26.1651	25.8191	1:Experimental evidence at protein level	biological regulation;catabolic process;cell proliferation;cellular catabolic process;cellular macromolecule catabolic process;cellular macromolecule metabolic process;cellular metabolic process;cellular nitrogen compound m
26.9808	27.9304	27.716	27.8719	1:Experimental evidence at protein level	3'-phosphoadenosine 5'-phosphosulfate metabolic process;anatomical structure development;cellular lipid metabolic process;cellular metabolic process;cellular nitrogen compound metabolic process;cellular process;depho
30.6909	30.5221	30.587	30.4335	1:Experimental evidence at protein level	biosynthetic process;catabolic process;cellular biosynthetic process;cellular catabolic process;cellular component biogenesis;cellular component biogenesis at cellular level;cellular component disassembly;cellular componer
26.5058	26.6553	26.3184	25.9866	1:Experimental evidence at protein level	aromatic compound catabolic process;catabolic process;cellular aromatic compound metabolic process;cellular catabolic process;cellular metabolic process;cellular process;metabolic process;response to chemical stimulus
30.5836	30.8561	31.1655	31.0152	1:Experimental evidence at protein level	activation of immune response;activation of innate immune response;anatomical structure development;biological regulation;cell differentiation;cellular component disassembly;cellular component disassembly at cellular leve
28.2422	28.2535	28.2371	28.036	1:Experimental evidence at protein level	biological regulation;biosynthetic process;cellular biosynthetic process;cellular macromolecule biosynthetic process;cellular macromolecule metabolic process;cellular metabolic process;cellular process;cellular protein metab
30.5543	30.7757	30.6198	30.576	1:Experimental evidence at protein level	biological regulation;biosynthetic process;catabolic process;cellular biosynthetic process;cellular catabolic process;cellular component assembly;cellular component assembly at cellular level;cellular component biogenesis;c
31.8324	31.7963	32.1029	32.0058	1:Experimental evidence at protein level	biological adhesion;biosynthetic process;catabolic process;cell adhesion;cellular biosynthetic process;cellular catabolic process;cellular component assembly;cellular component assembly at cellular level;cellular component
28.8166	29.2926	29.0596	29.1233	1:Experimental evidence at protein level	aldehyde catabolic process;catabolic process;cellular aldehyde metabolic process;cellular catabolic process;cellular metabolic process;cellular process;formaldehyde catabolic process;formaldehyde metabolic process;meta
26.6257	27.0557	27.0864	26.9506	1:Experimental evidence at protein level	amine biosynthetic process;amine metabolic process;betaine biosynthetic process;betaine metabolic process;biosynthetic process;carboxylic acid biosynthetic process;carboxylic acid catabolic process;carboxylic acid meta
26.9261	27.5491	27.4729	27.5009	1:Experimental evidence at protein level	beta-amyloid metabolic process;cellular macromolecule metabolic process;cellular metabolic process;cellular process;glycoprotein metabolic process;macromolecule metabolic process;metabolic process;primary metabolic
26.5592	26.6591	26.5525	27.0206	1:Experimental evidence at protein level	amine biosynthetic process;amine catabolic process;amine metabolic process;biological regulation;biosynthetic process;carboxylic acid biosynthetic process;carboxylic acid catabolic process;carboxylic acid metabolic proc
26.0722	26.8193	26.1998	26.1427	1:Experimental evidence at protein level	actin cytoskeleton organization;actin filament bundle assembly;actin filament organization;actin filament-based process;antigen processing and presentation;antigen processing and presentation of exogenous antigen;antiger
28.0997	28.1525	28.0773	27.9494	1:Experimental evidence at protein level	anatomical structure development;biosynthetic process;cell proliferation;cellular biosynthetic process;cellular macromolecule biosynthetic process;cellular macromolecule metabolic process;cellular metabolic process;cellular
28.0553	28.277	27.9937	28.0379	1:Experimental evidence at protein level	amine metabolic process;anatomical structure development;benzene-containing compound metabolic process;carboxylic acid metabolic process;catabolic process;cell development;cellular amine metabolic process;cellular
28.2124	27.5585	27.6423	27.6614	1:Experimental evidence at protein level	biosynthetic process;catabolic process;cellular biosynthetic process;cellular catabolic process;cellular component disassembly;cellular component disassembly at cellular level;cellular component organization;cellular compo
29.3757	29.2252	29.7045	29.5826	1:Experimental evidence at protein level	activation of immune response;activation of innate immune response;anaphase-promoting complex-dependent proteasomal ubiquitin-dependent protein catabolic process;antigen processing and presentation;antigen proces
28.3394	28.0999	27.817	28.0076	1:Experimental evidence at protein level	anatomical structure development;anatomical structure homeostasis;base-excision repair;biological regulation;biosynthetic process;catabolic process;cell cycle;cell cycle checkpoint;cell cycle phase;cell cycle process;cellula
29.0048	29.2056	29.2808	29.301	1:Experimental evidence at protein level	biological regulation;biosynthetic process;catabolic process;cellular biosynthetic process;cellular catabolic process;cellular macromolecule metabolic process;cellular metabolic process;cellular nitrogen compound metabolic
29.8815	29.2009	29.2514	29.3087	1:Experimental evidence at protein level	biosynthetic process;catabolic process;cellular biosynthetic process;cellular catabolic process;cellular component disassembly;cellular component disassembly at cellular level;cellular component organization;cellular compo
28.0334	28.1024	28.07	27.8974	1:Experimental evidence at protein level	biological regulation;cellular process;cellular response to abiotic stimulus;cellular response to osmotic stress;cellular response to stimulus;cellular response to stress;multicellular organismal process;negative regulation of cata
25.3027	25.6506	25.8344	25.1261	1:Experimental evidence at protein level	alcohol metabolic process;biosynthetic process;carbohydrate biosynthetic process;carbohydrate catabolic process;carbohydrate metabolic process;catabolic process;cellular biosynthetic process;cellular carbohydrate biosy
27.1329	26.8932	26.7776	26.843	1:Experimental evidence at protein level	biological regulation;biosynthetic process;carbohydrate metabolic process;carbohydrate transport;cell cycle;cell cycle process;cell surface receptor linked signaling pathway;cellular biosynthetic process;cellular component c
27.9847	27.739	27.7764	27.8566	1:Experimental evidence at protein level	cellular metabolic process;cellular process;electron transport chain;generation of precursor metabolites and energy;metabolic process;oxidation-reduction process;respiratory electron transport chain;small molecule metaboli
30.0437	29.9038	29.8622	29.8011	1:Experimental evidence at protein level	anatomical structure morphogenesis;cellular metabolic process;cellular process;developmental process;metabolic process;methylation;one-carbon metabolic process;organ morphogenesis;organ regeneration;regeneration;re
25.4561	25.587	#NUM!	25.5592	1:Experimental evidence at protein level	alcohol catabolic process;alcohol metabolic process;anatomical structure development;androgen biosynthetic process;androgen metabolic process;apocarotenoid metabolic process;arachidonic acid metabolic process;biolo
31.2287	30.7976	30.9521	31.0048	1:Experimental evidence at protein level	biosynthetic process;catabolic process;cellular biosynthetic process;cellular catabolic process;cellular component disassembly;cellular component disassembly at cellular level;cellular component organization;cellular compo
29.289	29.0782	29.1471	29.0522	1:Experimental evidence at protein level	antigen processing and presentation;antigen processing and presentation of peptide antigen;antigen processing and presentation of peptide antigen via MHC class I;apoptosis;biological regulation;calcium ion homeostasis;ca
31.5269	31.1393	31.1873	31.4925	1:Experimental evidence at protein level	biosynthetic process;catabolic process;cellular biosynthetic process;cellular catabolic process;cellular component assembly;cellular component assembly at cellular level;cellular component disassembly;cellular component c
27.6758	28.127	27.6913	27.9102	1:Experimental evidence at protein level	activation of adenylate cyclase activity;activation of adenylate cyclase activity by adrenergic receptor signaling pathway;activation of adenylate cyclase activity by dopamine receptor signaling pathway;activation of adenylate
26.9255	26.8425	26.7203	26.686	1:Experimental evidence at protein level	biological regulation;blood coagulation;cAMP-mediated signaling;cell activation;cell communication;cell cycle;cell division;cell surface receptor linked signaling pathway;cell-cell signaling;cellular process;cellular response to s
26.8497	26.9893	27.2426	27.1617	1:Experimental evidence at protein level	cellular macromolecule metabolic process;cellular metabolic process;cellular nitrogen compound metabolic process;cellular process;macromolecule metabolic process;metabolic process;mRNA metabolic process;mRNA pro
28.1545	28.7189	28.8657	28.9081	1:Experimental evidence at protein level	acylglycerol catabolic process;acylglycerol metabolic process;alcohol metabolic process;amine metabolic process;anatomical structure development;biosynthetic process;carbohydrate metabolic process;carbohydrate trans
27.5814	27.2499	27.4051	27.4213	1:Experimental evidence at protein level	biological regulation;catabolic process;cellular catabolic process;cellular localization;cellular macromolecule catabolic process;cellular macromolecule localization;cellular macromolecule metabolic process;cellular metabolic
27.4586	27.4322	27.4411	27.4917	1:Experimental evidence at protein level	biological regulation;catabolic process;cell communication;cell-cell signaling;cellular process;cellular response to stimulus;inositol phosphate metabolic process;intracellular signal transduction;lipid catabolic process;lipid me
31.8757	31.8162	31.8424	31.9332	1:Experimental evidence at protein level	cellular component assembly;cellular component assembly at cellular level;cellular component organization;cellular component organization at cellular level;cellular component organization or biogenesis;cellular component c
28.0655	27.6202	27.8239	27.7293	1:Experimental evidence at protein level	activation of immune response;activation of innate immune response;activation of MAPK activity;activation of MAPKK activity;activation of protein kinase activity;aging;anatomical structure development;axon guidance;biolog
27.3476	27.3912	27.2714	27.2448	1:Experimental evidence at protein level	abortive mitotic cell cycle;activation of MAPK activity;acylglycerol metabolic process;anatomical structure development;anatomical structure formation involved in morphogenesis;anatomical structure morphogenesis;axon gu
27.4271	27.3871	27.398	27.2717	1:Experimental evidence at protein level	biological regulation;cell cycle;cellular macromolecule metabolic process;cellular metabolic process;cellular process;cellular protein metabolic process;cellular response to stimulus;cellular response to stress;cullin deneddyla
25.6264	25.664	25.7317	25.2309	1:Experimental evidence at protein level	biological adhesion;biological regulation;cell adhesion;cell communication;cell-cell signaling;cell-matrix adhesion;cell-substrate adhesion;cellular process;cellular response to stimulus;establishment of localization;establishme
28.4799	27.9657	28.1423	28.1155	1:Experimental evidence at protein level	actin cytoskeleton organization;actin cytoskeleton reorganization;actin filament-based process;anatomical structure development;anatomical structure formation involved in morphogenesis;anatomical structure morphogenes
28.1382	28.0995	28.2734	28.019	1:Experimental evidence at protein level	cell cycle;cell cycle process;cell division;cellular component organization;cellular component organization at cellular level;cellular component organization or biogenesis;cellular component organization or biogenesis at cellula
26.8144	26.5085	26.2686	26.444	1:Experimental evidence at protein level	biological adhesion;biological regulation;cell adhesion;cellular component organization;cellular component organization at cellular level;cellular component organization or biogenesis;cellular component organization or bioger
28.5408	28.1322	28.4295	28.3696	1:Experimental evidence at protein level	actin cytoskeleton organization;actin filament organization;actin filament-based process;biological regulation;cell communication;cell communication by chemical coupling;cell communication by electrical coupling;cell prolife
27.4064	27.2997	27.4146	27.2375	1:Experimental evidence at protein level	cellular macromolecule metabolic process;cellular metabolic process;cellular nitrogen compound metabolic process;cellular process;localization;macromolecule localization;macromolecule metabolic process;metabolic proce
26.9831	27.3913	27.0637	27.1919	1:Experimental evidence at protein level	biosynthetic process;catabolic process;cellular biosynthetic process;cellular catabolic process;cellular component organization;cellular component organization at cellular level;cellular component organization or biogenesis;c
28.1891	28.5331	28.3137	28.2046	1:Experimental evidence at protein level	actin cytoskeleton organization;actin filament organization;actin filament-based process;actin nucleation;Arp2/3 complex-mediated actin nucleation;axon guidance;biological regulation;cell surface receptor linked signaling pa
27.3472	27.3801	27.5216	27.4728	1:Experimental evidence at protein level	acetyl-CoA catabolic process;acetyl-CoA metabolic process;carboxylic acid metabolic process;catabolic process;cellular catabolic process;cellular ketone metabolic process;cellular metabolic process;cellular process;coenz
27.0978	26.5766	26.8883	26.5874	1:Experimental evidence at protein level	biological regulation;cell cycle;cell cycle process;cell division;cell proliferation;cellular component organization;cellular component organization at cellular level;cellular component organization or biogenesis;cellular componer
26.4832	26.3992	26.3252	26.2768	1:Experimental evidence at protein level	biological regulation;cell surface receptor linked signaling pathway;cellular process;cellular response to stimulus;G-protein coupled receptor protein signaling pathway;opioid receptor signaling pathway;regulation of biologica
27.1105	27.3149	27.1511	26.8841	1:Experimental evidence at protein level	acyl-CoA metabolic process;cellular metabolic process;cellular process;coenzyme metabolic process;cofactor metabolic process;metabolic process;thioester metabolic process
26.8875	26.3911	26.5994	26.4056	1:Experimental evidence at protein level	biological regulation;cell proliferation;cellular component biogenesis;cellular component biogenesis at cellular level;cellular component organization;cellular component organization at cellular level;cellular component organiza
27.2845	26.6932	26.8116	26.7378	1:Experimental evidence at protein level	cellular macromolecule metabolic process;cellular metabolic process;cellular nitrogen compound metabolic process;cellular process;macromolecule metabolic process;macromolecule modification;metabolic process;ncRNA
28.8931	28.722	28.8365	28.8325	1:Experimental evidence at protein level	biological regulation;biosynthetic process;cellular biosynthetic process;cellular component assembly;cellular component assembly at cellular level;cellular component organization;cellular component organization at cellular le
29.0245	29.2877	29.3907	29.531	1:Experimental evidence at protein level	antigen processing and presentation;antigen processing and presentation of exogenous antigen;antigen processing and presentation of exogenous peptide antigen;antigen processing and presentation of exogenous peptide
28.5686	28.4252	28.5961	28.5423	1:Experimental evidence at protein level	amine metabolic process;aminoglycan catabolic process;aminoglycan metabolic process;anatomical structure development;astrocyte cell migration;axon ensheathment;behavior;biological regulation;biosynthetic process;cal
31.8685	32.8494	32.7412	32.9945	1:Experimental evidence at protein level	actin cytoskeleton organization;actin filament-based process;anatomical structure formation involved in morphogenesis;biological regulation;blood coagulation;cell activation;cellular component organization;cellular compone
28.2163	27.9845	28.4332	28.3212	1:Experimental evidence at protein level	anatomical structure development;anatomical structure morphogenesis;biological regulation;blood coagulation;blood vessel development;cell activation;cell migration;cell motility;cell surface receptor linked signaling pathwa
29.6016	29.4466	29.3827	29.3091	1:Experimental evidence at protein level	anatomical structure formation involved in morphogenesis;angiogenesis;axon guidance;behavior;biological adhesion;biological regulation;blood coagulation;cell adhesion;cell adhesion mediated by integrin;cell differentiation;
28.5681	28.7604	28.5568	28.6018	1:Experimental evidence at protein level	aging;alcohol catabolic process;alcohol metabolic process;aldehyde catabolic process;biological regulation;catabolic process;cellular aldehyde metabolic process;cellular catabolic process;cellular lipid metabolic process;cel
26.3514	26.9779	26.6635	26.6378	1:Experimental evidence at protein level	5-phosphoribose 1-diphosphate biosynthetic process;5-phosphoribose 1-diphosphate metabolic process;alcohol biosynthetic process;alcohol metabolic process;AMP biosynthetic process;AMP metabolic process;anatomic
30.0908	30.0702	29.4434	29.9214	1:Experimental evidence at protein level	biosynthetic process;catabolic process;cellular biosynthetic process;cellular catabolic process;cellular component biogenesis;cellular component biogenesis at cellular level;cellular component disassembly;cellular componer
28.682	28.3618	28.5409	28.3163	1:Experimental evidence at protein level	aerobic respiration;cellular metabolic process;cellular process;cellular respiration;electron transport chain;energy derivation by oxidation of organic compounds;generation of precursor metabolites and energy;metabolic proce
29.0901	29.1785	29.0032	29.1067	1:Experimental evidence at protein level	aging;anatomical structure homeostasis;base-excision repair;biological regulation;biosynthetic process;cell cycle;cell redox homeostasis;cellular biosynthetic process;cellular component organization;cellular component orga
29.1142	29.2187	29.321	29.3211	1:Experimental evidence at protein level	activation of immune response;activation of innate immune response;activation of MAPK activity;activation of MAPKK activity;activation of protein kinase activity;anatomical structure development;anatomical structure morph
27.64	28.031	27.8493	28.0287	1:Experimental evidence at protein level	aerobic respiration;AMP biosynthetic process;AMP metabolic process;biosynthetic process;cellular aromatic compound metabolic process;cellular biosynthetic process;cellular component assembly;cellular component orga
25.8355	26.3975	26.1293	26.1285	1:Experimental evidence at protein level	alcohol catabolic process;alcohol metabolic process;carbohydrate metabolic process;catabolic process;ethanol catabolic process;ethanol metabolic process;metabolic process;primary alcohol catabolic process;primary alco

25.5608	25.5163	25.3093	25.3862	1:Experimental evidence at protein level	biological adhesion;biological regulation;cell adhesion;cellular process;negative regulation of angiogenesis;negative regulation of biological process;negative regulation of developmental process;positive regulation of biologic
27.2996	27.0159	26.9584	26.968	1:Experimental evidence at protein level	cellular macromolecule metabolic process;cellular metabolic process;cellular nitrogen compound metabolic process;cellular process;macromolecule metabolic process;maturation of 5.8S rRNA;metabolic process;mRNA met
27.423	27.7382	27.6267	27.737	1:Experimental evidence at protein level	biological regulation;carboxylic acid catabolic process;carboxylic acid metabolic process;catabolic process;cell differentiation;cellular catabolic process;cellular developmental process;cellular ketone metabolic process;cellul
26.6206	26.5399	26.2397	26.3955	1:Experimental evidence at protein level	activation of immune response;activation of innate immune response;anatomical structure development;axon guidance;biological regulation;cell communication;cell cycle;cell surface receptor linked signaling pathway;cell-ce
27.7923	28.714	28.597	28.6316	1:Experimental evidence at protein level	2'-deoxyribonucleotide metabolic process;ADP biosynthetic process;ADP metabolic process;AMP metabolic process;anatomical structure development;biosynthetic process;brain development;cellular biosynthetic process;c
28.7719	29.0962	28.9846	29.0574	1:Experimental evidence at protein level	actin cytoskeleton organization;actin filament depolymerization;actin filament organization;actin filament severing;actin filament-based process;actin polymerization or depolymerization;biological regulation;cellular componen
30.8243	30.4206	30.6569	30.8081	1:Experimental evidence at protein level	biosynthetic process;catabolic process;cellular biosynthetic process;cellular catabolic process;cellular component disassembly;cellular component disassembly at cellular level;cellular component organization;cellular compo
30.355	29.9827	29.9431	30.1896	1:Experimental evidence at protein level	biosynthetic process;catabolic process;cellular biosynthetic process;cellular catabolic process;cellular component disassembly;cellular component disassembly at cellular level;cellular component organization;cellular compo
30.6046	30.078	30.3565	30.4378	1:Experimental evidence at protein level	biosynthetic process;catabolic process;cellular biosynthetic process;cellular catabolic process;cellular component disassembly;cellular component disassembly at cellular level;cellular component organization;cellular compo
29.5005	29.0137	29.1017	29.2025	1:Experimental evidence at protein level	biological regulation;cellular macromolecule metabolic process;cellular metabolic process;cellular nitrogen compound metabolic process;cellular process;macromolecule metabolic process;metabolic process;mRNA metabol
30.1301	29.9668	29.9264	29.9093	1:Experimental evidence at protein level	anion transport;cellular metabolic process;cellular process;establishment of localization;generation of precursor metabolites and energy;inorganic anion transport;ion transmembrane transport;ion transport;metabolic process
29.8783	30.1914	30.2823	30.1036	1:Experimental evidence at protein level	anatomical structure development;cell differentiation;cellular developmental process;cellular process;developmental process;epithelial cell differentiation;muscle organ development;muscle structure development;organ devel
27.6871	27.4566	27.283	27.3778	1:Experimental evidence at protein level	activation of immune response;activation of innate immune response;anaphase-promoting complex-dependent proteasomal ubiquitin-dependent protein catabolic process;antigen processing and presentation;antigen proces
28.9101	29.0118	29.1214	29.1688	1:Experimental evidence at protein level	anatomical structure formation involved in morphogenesis;biological regulation;cell cycle phase;cell cycle process;cell division;cell projection assembly;cell projection organization;cell surface receptor linked signaling pathwa
26.3963	26.4518	26.3253	26.4524	1:Experimental evidence at protein level	anatomical structure development;anatomical structure morphogenesis;biological regulation;brain development;camera-type eye development;cellular chemical homeostasis;cellular component organization;cellular compone
31.3538	31.3635	31.3845	31.3616	1:Experimental evidence at protein level	cellular macromolecule metabolic process;cellular metabolic process;cellular nitrogen compound metabolic process;cellular process;gene expression;macromolecule metabolic process;metabolic process;mRNA metabolic p
28.942	28.7092	28.8538	28.7575	1:Experimental evidence at protein level	3'-UTR-mediated mRNA stabilization;biological regulation;cellular macromolecule metabolic process;cellular metabolic process;cellular nitrogen compound metabolic process;cellular process;developmental process;gene ex
26.9155	26.8876	27.0379	26.9281	1:Experimental evidence at protein level	cellular component organization;cellular component organization at cellular level;cellular component organization or biogenesis;cellular component organization or biogenesis at cellular level;cellular process;cytoskeleton orga
27.6295	27.6369	27.2693	27.4806	1:Experimental evidence at protein level	adenine nucleotide transport;ATP transport;biological regulation;cellular process;cellular response to calcium ion;cellular response to chemical stimulus;cellular response to inorganic substance;cellular response to metal ion;c
26.161	26.1688	26.2241	26.3077	1:Experimental evidence at protein level	aromatic compound biosynthetic process;biosynthetic process;carboxylic acid metabolic process;cellular aromatic compound metabolic process;cellular biosynthetic process;cellular ketone metabolic process;cellular metab
26.8412	26.2379	26.5054	26.4126	1:Experimental evidence at protein level	
26.8713	26.534	26.7441	26.7251	1:Experimental evidence at protein level	biological regulation;cellular macromolecule metabolic process;cellular metabolic process;cellular process;cellular protein metabolic process;developmental process;endosome transport;establishment of localization;establish
26.2259	26.1483	26.1714	26.2135	1:Experimental evidence at protein level	anatomical structure morphogenesis;biological regulation;catabolic process;cell cycle arrest;cell cycle process;cell part morphogenesis;cellular catabolic process;cellular component assembly;cellular component morphogene
26.6759	26.5776	26.42	26.657	1:Experimental evidence at protein level	anatomical structure development;biological regulation;biosynthetic process;carbohydrate metabolic process;carbohydrate transport;cell cycle;cell cycle process;cell surface receptor linked signaling pathway;cellular biosynt
30.1515	30.1844	30.3417	30.3555	1:Experimental evidence at protein level	actin cytoskeleton organization;actin filament-based process;actomyosin structure organization;anatomical structure development;biological regulation;cellular component organization;cellular component organization at cellu
29.1961	28.5258	29.1499	29.0457	1:Experimental evidence at protein level	anatomical structure morphogenesis;biological regulation;cell morphogenesis;cell morphogenesis involved in differentiation;cellular component morphogenesis;cellular component organization;cellular component organizatio
26.8868	26.7122	26.5598	26.5572	1:Experimental evidence at protein level	biological regulation;establishment of localization;establishment of localization in cell;establishment of protein localization;establishment of protein localization in endoplasmic reticulum membrane;establishment of protein loc
27.0914	26.9642	26.7828	26.7313	1:Experimental evidence at protein level	establishment of localization;establishment of localization in cell;establishment of protein localization;establishment of RNA localization;gene expression;intracellular protein transport;intracellular transport;macromolecule met
28.836	28.9592	28.8705	28.8813	1:Experimental evidence at protein level	aging;biological regulation;cardiac cell differentiation;cardiac muscle cell differentiation;cell differentiation;cellular developmental process;cellular process;cellular response to calcium ion;cellular response to chemical stimulus
27.0548	26.7664	27.0231	26.9507	1:Experimental evidence at protein level	acetyl-CoA catabolic process;acetyl-CoA metabolic process;biosynthetic process;carboxylic acid metabolic process;catabolic process;cellular biosynthetic process;cellular catabolic process;cellular ketone metabolic process
26.9156	26.7884	26.7673	26.6213	1:Experimental evidence at protein level	biosynthetic process;cellular biosynthetic process;cellular lipid metabolic process;cellular metabolic process;cellular process;ether lipid biosynthetic process;ether lipid metabolic process;glycerol ether biosynthetic process;g
28.8744	29.2505	29.595	29.4852	1:Experimental evidence at protein level	biological regulation;regulation of biological process;regulation of biosynthetic process;regulation of cellular biosynthetic process;regulation of cellular macromolecule biosynthetic process;regulation of cellular metabolic proc
27.0761	26.9692	26.838	26.6744	1:Experimental evidence at protein level	biological regulation;biosynthetic process;cell cycle;cellular biosynthetic process;cellular component organization;cellular component organization at cellular level;cellular component organization or biogenesis;cellular compo
28.6894	28.7862	28.7663	28.6705	1:Experimental evidence at protein level	actin cytoskeleton organization;actin filament organization;actin filament-based process;actin nucleation;Arp2/3 complex-mediated actin nucleation;axon guidance;biological regulation;cell surface receptor linked signaling pa
27.2123	26.9546	27.1786	26.7729	1:Experimental evidence at protein level	biosynthetic process;cellular biosynthetic process;cellular macromolecule biosynthetic process;cellular macromolecule metabolic process;cellular metabolic process;cellular process;cellular protein metabolic process;cotrans
27.2467	27.2351	26.9458	26.7377	1:Experimental evidence at protein level	biological regulation;blood coagulation;cAMP-mediated signaling;cell activation;cell communication;cell cycle;cell division;cell surface receptor linked signaling pathway;cell-cell signaling;cellular component organization;cellu
29.6478	30.3986	30.1913	30.381	1:Experimental evidence at protein level	biological adhesion;cell adhesion;cell-cell adhesion;cellular process;homotypic cell-cell adhesion;platelet aggregation
27.5649	27.5354	27.0814	27.3347	1:Experimental evidence at protein level	anatomical structure development;behavior;biological adhesion;biological regulation;blood coagulation;cell adhesion;cell differentiation;cell migration;cell motility;cell surface receptor linked signaling pathway;cell-matrix adhe
26.7146	26.6187	26.6325	26.784	1:Experimental evidence at protein level	biological regulation;cell cycle;cell cycle phase;cell cycle process;cellular component organization;cellular component organization at cellular level;cellular component organization or biogenesis;cellular component organizatio
31.1118	28.7029	30.2292	30.996	1:Experimental evidence at protein level	cellular component assembly;cellular component assembly at cellular level;cellular component organization;cellular component organization at cellular level;cellular component organization or biogenesis;cellular component o
28.2857	28.3893	28.3131	28.069	1:Experimental evidence at protein level	ATP biosynthetic process;ATP metabolic process;ATP synthesis coupled proton transport;biosynthetic process;cation transport;cellular biosynthetic process;cellular metabolic process;cellular nitrogen compound biosyntheti
32.4066	32.7444	32.6993	32.7007	1:Experimental evidence at protein level	cell differentiation;cellular developmental process;cellular process;developmental process;epithelial cell differentiation
27.7283	27.4387	27.4399	27.4476	1:Experimental evidence at protein level	ATP hydrolysis coupled proton transport;ATP metabolic process;biological regulation;cation homeostasis;cation transport;cell surface receptor linked signaling pathway;cellular cation homeostasis;cellular chemical homeosta
28.1222	27.8065	27.7539	27.7135	1:Experimental evidence at protein level	acute inflammatory response;acute-phase response;anatomical structure development;astrocyte differentiation;behavior;biological regulation;carbohydrate homeostasis;cell differentiation;cell proliferation;cell surface recepto
26.6585	26.8258	26.7937	26.8616	1:Experimental evidence at protein level	alcohol metabolic process;biosynthetic process;carboxylic acid metabolic process;cellular ketone metabolic process;cellular lipid metabolic process;cellular metabolic process;cellular process;cellular response to chemical st
28.8048	28.6035	28.6167	28.4918	1:Experimental evidence at protein level	biological regulation;cell cycle;cellular process;cellular response to stimulus;intracellular signal transduction;mitotic cell cycle;negative regulation of biological process;negative regulation of intracellular protein transport;negat
27.5468	27.6515	27.8795	27.8215	1:Experimental evidence at protein level	activation of MAPK activity;anatomical structure morphogenesis;biological adhesion;biological regulation;branching morphogenesis of a tube;cell adhesion;cell junction assembly;cell junction organization;cell morphogenesis
29.0406	28.6906	28.8477	28.7343	1:Experimental evidence at protein level	anatomical structure development;apoptosis;biological regulation;cell death;cell differentiation;cellular developmental process;cellular process;death;developmental process;muscle organ development;muscle structure devel
28.8201	28.7347	28.9568	28.7751	1:Experimental evidence at protein level	biological regulation;cellular macromolecule metabolic process;cellular metabolic process;cellular nitrogen compound metabolic process;cellular process;gene expression;macromolecule metabolic process;metabolic proces
31.0896	31.204	31.2523	31.0897	1:Experimental evidence at protein level	actin filament capping;barbed-end actin filament capping;biological regulation;blood coagulation;cellular component assembly;cellular component movement;cellular component organization;cellular component organization
27.4623	27.2359	27.3383	27.3567	1:Experimental evidence at protein level	biological regulation;biosynthetic process;carbohydrate metabolic process;carbohydrate transport;cell cycle;cell cycle process;cell surface receptor linked signaling pathway;cellular biosynthetic process;cellular component a
28.3703	28.4724	28.4756	28.4407	1:Experimental evidence at protein level	biological regulation;catabolic process;cell communication;cell-cell signaling;cellular catabolic process;cellular macromolecule catabolic process;cellular macromolecule metabolic process;cellular metabolic process;cellular p
33.6674	31.5357	32.4627	32.9192	1:Experimental evidence at protein level	anatomical structure development;antibacterial humoral response;antimicrobial humoral response;defense response;defense response to bacterium;defense response to Gram-positive bacterium;developmental process;hum
28.3712	28.3926	28.4545	28.1691	1:Experimental evidence at protein level	5-phosphoribose 1-diphosphate biosynthetic process;5-phosphoribose 1-diphosphate metabolic process;alcohol biosynthetic process;alcohol metabolic process;AMP biosynthetic process;AMP metabolic process;anatomic
29.2108	29.6979	29.4395	29.308	1:Experimental evidence at protein level	anatomical structure morphogenesis;autophagic vacuole assembly;autophagy;biological adhesion;biological regulation;cargo loading into COPII-coated vesicle;cargo loading into vesicle;catabolic process;cell adhesion;cell c
30.0202	30.1781	30.0321	29.9196	1:Experimental evidence at protein level	biological regulation;cell communication;cell surface receptor linked signaling pathway;cell-cell signaling;cellular metabolic process;cellular process;cellular response to chemical stimulus;cellular response to endogenous stin
27.7571	27.708	27.6663	27.721	1:Experimental evidence at protein level	
30.0249	30.177	30.378	30.3639	1:Experimental evidence at protein level	biological regulation;biosynthetic process;cell cycle;cellular biosynthetic process;cellular component assembly;cellular component assembly at cellular level;cellular component disassembly;cellular component disassembly a
25.9039	#NUM!	25.5455	25.2332	1:Experimental evidence at protein level	axon guidance;biological regulation;cell surface receptor linked signaling pathway;cellular component organization;cellular component organization at cellular level;cellular component organization or biogenesis;cellular comp
27.9787	28.0506	27.6285	27.6907	1:Experimental evidence at protein level	anatomical structure development;anatomical structure formation involved in morphogenesis;anatomical structure morphogenesis;angiogenesis;apoptosis;axon guidance;axonogenesis;biological regulation;blood coagulation
25.6231	25.8025	26.0068	25.6544	1:Experimental evidence at protein level	anatomical structure formation involved in morphogenesis;angiogenesis;biological adhesion;biological regulation;cell adhesion;cell recognition;cellular component organization;cellular component organization or biogenesis;c
26.6435	26.8958	26.4695	26.5171	1:Experimental evidence at protein level	carboxylic acid catabolic process;carboxylic acid metabolic process;catabolic process;cellular catabolic process;cellular ketone metabolic process;cellular lipid catabolic process;cellular lipid metabolic process;cellular metab
28.8703	28.8897	28.5915	28.7143	1:Experimental evidence at protein level	3'-UTR-mediated mRNA stabilization;biological regulation;cellular macromolecule metabolic process;cellular metabolic process;cellular nitrogen compound metabolic process;cellular process;defense response;gene express
28.9954	28.678	28.7727	28.6982	1:Experimental evidence at protein level	actin cytoskeleton organization;actin filament-based process;activation of immune response;activation of innate immune response;activation of protein kinase activity;antigen receptor-mediated signaling pathway;apoptosis;a
29.3157	29.4574	29.4929	29.4411	1:Experimental evidence at protein level	biological regulation;biosynthetic process;cellular biosynthetic process;cellular component assembly;cellular component assembly at cellular level;cellular component organization;cellular component organization at cellular le
29.3317	30.0572	30.0209	30.0819	1:Experimental evidence at protein level	amine metabolic process;amino acid activation;carboxylic acid metabolic process;cellular amine metabolic process;cellular amino acid metabolic process;cellular ketone metabolic process;cellular macromolecule metabolic p
26.3617	25.9759	26.1504	25.5256	1:Experimental evidence at protein level	activation of immune response;activation of innate immune response;axon guidance;biological regulation;cell communication;cell cycle;cell proliferation;cell surface receptor linked signaling pathway;cell-cell signaling;cellular
30.2975	30.5898	30.6845	30.6921	1:Experimental evidence at protein level	actin cytoskeleton organization;actin filament bundle assembly;actin filament organization;actin filament-based process;biological adhesion;biological regulation;cell adhesion;cell communication;cell surface receptor linked s
27.5695	27.6266	27.7101	27.6384	1:Experimental evidence at protein level	biological regulation;carboxylic acid catabolic process;carboxylic acid metabolic process;catabolic process;cellular catabolic process;cellular ketone metabolic process;cellular lipid catabolic process;cellular lipid metabolic p
28.198	27.9967	28.4134	28.3907	1:Experimental evidence at protein level	biological regulation;biosynthetic process;cellular biosynthetic process;cellular macromolecule biosynthetic process;cellular macromolecule metabolic process;cellular metabolic process;cellular nitrogen compound metabolic
26.7674	26.4951	26.5978	26.6335	1:Experimental evidence at protein level	anatomical structure development;anatomical structure morphogenesis;artery development;blood vessel development;cardiac septum development;developmental process;embryonic morphogenesis;morphogenesis of an ep
26.9832	26.5099	26.8288	26.6744	1:Experimental evidence at protein level	apoptosis;biological regulation;biosynthetic process;cell cycle;cell death;cellular biosynthetic process;cellular macromolecule biosynthetic process;cellular macromolecule metabolic process;cellular metabolic process;cellula
28.1517	28.177	28.3909	28.3527	1:Experimental evidence at protein level	biological regulation;cell activation;cellular component organization;cellular component organization or biogenesis;cellular membrane organization;cellular process;cellular response to chemical stimulus;cellular response to hy
25.9381	25.9774	25.7761	25.4735	1:Experimental evidence at protein level	anatomical structure morphogenesis;axonogenesis;biological regulation;cell differentiation;cell part morphogenesis;cell projection morphogenesis;cell projection organization;cellular component morphogenesis;cellular comp
27.2232	27.5567	27.252	27.3358	1:Experimental evidence at protein level	establishment of localization;establishment of localization in cell;establishment of protein localization;intracellular protein transport;intracellular transport;nuclear import;nuclear transport;nucleocytoplasmic transport;protein in
30.2018	30.4301	30.4053	30.5923	1:Experimental evidence at protein level	activation of protein kinase activity;activation of protein kinase B activity;adult behavior;adult locomotory behavior;alcohol biosynthetic process;alcohol metabolic process;aldehyde catabolic process;amine transport;autopha
#NUM!	#NUM!	#NUM!	#NUM!	2:Experimental evidence at transcript level	ameboidal cell migration;anatomical structure development;anatomical structure formation involved in morphogenesis;anatomical structure morphogenesis;anterior/posterior pattern specification;axon guidance;blood vessel
27.8096	27.8026	27.6991	27.7656	1:Experimental evidence at protein level	actin cytoskeleton organization;actin filament branching;actin filament bundle assembly;actin filament organization;actin filament-based process;biological regulation;cell chemotaxis;cell migration;cell motility;cell projection o
27.9596	27.8252	27.7365	27.7347	1:Experimental evidence at protein level	biological regulation;cellular component assembly;cellular component assembly at cellular level;cellular component organization;cellular component organization at cellular level;cellular component organization or biogenesis;
26.9603	27.1332	26.9249	26.9175	1:Experimental evidence at protein level	bradykinin catabolic process;catabolic process;cellular catabolic process;cellular metabolic process;cellular process;macromolecule metabolic process;metabolic process;peptide catabolic process;peptide metabolic proces
29.0289	29.3649	29.5295	29.3371	1:Experimental evidence at protein level	cell cycle;cell division;cellular component assembly;cellular component organization;cellular component organization or biogenesis;cellular process;macromolecular complex assembly;macromolecular complex subunit organ
27.3833	27.525	27.2916	27.3171	1:Experimental evidence at protein level	macromolecule metabolic process;metabolic process;primary metabolic process;protein metabolic process;proteolysis
28.4881	28.4031	28.3609	28.4502	1:Experimental evidence at protein level	biological regulation;cellular macromolecule metabolic process;cellular metabolic process;cellular process;cellular protein metabolic process;macromolecule metabolic process;macromolecule modification;metabolic process
27.8186	27.6412	27.7406	27.7143	1:Experimental evidence at protein level	actin cytoskeleton organization;actin filament-based process;anatomical structure morphogenesis;biological regulation;caveola assembly;caveolin-mediated endocytosis;cell part morphogenesis;cell projection morphogenesi
28.0894	28.045	28.1162	27.9241	1:Experimental evidence at protein level	biological regulation;cellular component organization;cellular component organization at cellular level;cellular component organization or biogenesis;cellular component organization or biogenesis at cellular level;cellular meml
28.8621	28.6194	28.542	28.5071	1:Experimental evidence at protein level	activation of immune response;activation of innate immune response;anaphase-promoting complex-dependent proteasomal ubiquitin-dependent protein catabolic process;antigen processing and presentation;antigen proces
27.3257	27.1354	27.0685	26.9609	1:Experimental evidence at protein level	biological regulation;biosynthetic process;catabolic process;cellular biosynthetic process;cellular catabolic process;cellular macromolecule biosynthetic process;cellular macromolecule catabolic process;cellular macromolec
30.5667	31.1896	30.9893	30.9766	1:Experimental evidence at protein level	anion transport;biological adhesion;biological regulation;cell adhesion;cell-cell adhesion;cellular chemical homeostasis;cellular homeostasis;cellular ion homeostasis;cellular process;cellular response to stimulus;chemical hor
28.9409	29.0678	29.3273	29.2831	1:Experimental evidence at protein level	activation of immune response;activation of innate immune response;anaphase-promoting complex-dependent proteasomal ubiquitin-dependent protein catabolic process;antigen processing and presentation;antigen proces
25.4728	25.5279	25.2051	25.2762	1:Experimental evidence at protein level	actin cytoskeleton organization;actin filament organization;actin filament-based process;alcohol metabolic process;biological adhesion;biological regulation;biosynthetic process;carbohydrate metabolic process;cell adhesion
27.1528	27.3256	27.4554	27.4674	1:Experimental evidence at protein level	biosynthetic process;cellular biosynthetic process;cellular lipid metabolic process;cellular macromolecule metabolic process;cellular metabolic process;cellular process;cellular protein metabolic process;cellular response to c
26.3295	#NUM!	25.5552	25.7652	1:Experimental evidence at protein level	biological regulation;cellular macromolecule metabolic process;cellular metabolic process;cellular nitrogen compound metabolic process;cellular process;cleavage involved in rRNA processing;endonucleolytic cleavage in 5'-
26.7773	26.6753	26.957	26.7925	1:Experimental evidence at protein level	activation of protein kinase activity;biological adhesion;biological regulation;cell adhesion;cell cycle;cell-cell adhesion;cellular macromolecule metabolic process;cellular metabolic process;cellular process;cellular protein meta
27.7379	27.5194	27.6201	27.5278	1:Experimental evidence at protein level	cellular macromolecule metabolic process;cellular metabolic process;cellular process;cellular protein metabolic process;establishment of localization;establishment of localization in cell;establishment of protein localization;es
28.9219	29.2648	28.9866	29.018	1:Experimental evidence at protein level	3'-phosphoadenosine 5'-phosphosulfate biosynthetic process;3'-phosphoadenosine 5'-phosphosulfate metabolic process;amine metabolic process;aminoglycan metabolic process;anatomical structure development;biologi
26.9417	27.4678	27.6252	27.2485	1:Experimental evidence at protein level	activation of immune response;biological regulation;carboxylic acid metabolic process;cell surface receptor linked signaling pathway;cellular ketone metabolic process;cellular lipid metabolic process;cellular metabolic proces
28.6044	29.8213	29.4829	29.7644	1:Experimental evidence at protein level	alcohol biosynthetic process;alcohol metabolic process;biological regulation;biosynthetic process;C21-steroid hormone biosynthetic process;C21-steroid hormone metabolic process;carbohydrate biosynthetic process;carbo
28.4007	29.4282	29.5168	29.6281	1:Experimental evidence at protein level	arachidonic acid metabolic process;carboxylic acid metabolic process;cell differentiation;cellular developmental process;cellular ketone metabolic process;cellular lipid metabolic process;cellular metabolic process;cellular pr
27.46	27.7538	27.3459	27.3923	1:Experimental evidence at protein level	amine metabolic process;betaine metabolic process;biological regulation;carboxylic acid metabolic process;carnitine metabolic process;catabolic process;cellular amine metabolic process;cellular amino acid metabolic proce
27.9484	28.0554	28.0873	27.74	1:Experimental evidence at protein level	biological regulation;negative regulation of catalytic activity;negative regulation of endopeptidase activity;negative regulation of hydrolase activity;negative regulation of molecular function;negative regulation of peptidase acti
28.832	28.8612	28.8451	28.8188	1:Experimental evidence at protein level	biological regulation;biosynthetic process;cell differentiation;cellular biosynthetic process;cellular component organization;cellular component organization at cellular level;cellular component organization or biogenesis;cellula
29.2467	28.9859	29.186	28.8213	1:Experimental evidence at protein level	biological regulation;biosynthetic process;cellular biosynthetic process;cellular component organization;cellular component organization at cellular level;cellular component organization or biogenesis;cellular component orga
28.4702	28.2398	28.7281	28.7369	1:Experimental evidence at protein level	B cell activation;cell activation;cell proliferation;cellular process;establishment of localization;establishment of localization in cell;exocytosis;immune system process;leukocyte activation;lymphocyte activation;secretion;secret
27.049	27.2203	27.632	27.4139	1:Experimental evidence at protein level	aging;biological regulation;cell aging;cell junction assembly;cell junction organization;cellular component assembly;cellular component assembly at cellular level;cellular component organization;cellular component organizatio
28.3396	28.4097	28.444	28.2658	1:Experimental evidence at protein level	amine biosynthetic process;amine metabolic process;anatomical structure development;betaine biosynthetic process;betaine metabolic process;biological regulation;biosynthetic process;carboxylic acid biosynthetic process
29.0384	28.9541	28.9808	28.9271	1:Experimental evidence at protein level	biological regulation;biosynthetic process;catabolic process;cellular biosynthetic process;cellular catabolic process;cellular component assembly;cellular component assembly at cellular level;cellular component organization;
30.4985	29.9782	30.1294	29.9799	1:Experimental evidence at protein level	biological regulation;biosynthetic process;catabolic process;cellular biosynthetic process;cellular catabolic process;cellular component disassembly;cellular component disassembly at cellular level;cellular component organiza
30.4175	30.5764	30.5403	30.2907	1:Experimental evidence at protein level	activation of adenylate cyclase activity;activation of adenylate cyclase activity by dopamine receptor signaling pathway;activation of adenylate cyclase activity by G-protein signaling pathway;activation of phospholipase C ac
27.3149	27.3419	27.528	27.3923	1:Experimental evidence at protein level	
30.4242	30.2852	30.7319	30.5167	1:Experimental evidence at protein level	antigen processing and presentation;antigen processing and presentation of exogenous antigen;antigen processing and presentation of exogenous peptide antigen;antigen processing and presentation of exogenous peptide
27.5933	27.6927	27.4716	27.4549	1:Experimental evidence at protein level	cell cycle;cell cycle process;cell division;cellular component organization;cellular component organization at cellular level;cellular component organization or biogenesis;cellular component organization or biogenesis at cellula
26.4884	26.5973	26.4357	26.1652	1:Experimental evidence at protein level	catabolic process;cellular catabolic process;cellular macromolecule catabolic process;cellular macromolecule metabolic process;cellular metabolic process;cellular nitrogen compound metabolic process;cellular process;mac
27.4353	27.3904	27.5211	27.2654	1:Experimental evidence at protein level	cellular macromolecule metabolic process;cellular metabolic process;cellular process;cellular protein metabolic process;cellular response to heat;cellular response to stimulus;cellular response to stress;chaperone cofactor-d
30.1897	30.2396	30.0606	30.0422	1:Experimental evidence at protein level	autophagy;biological regulation;catabolic process;cellular catabolic process;cellular component assembly;cellular component assembly at cellular level;cellular component organization;cellular component organization at cellu
27.8755	27.2915	27.3992	27.1731	1:Experimental evidence at protein level	cellular macromolecule metabolic process;cellular metabolic process;cellular nitrogen compound metabolic process;cellular process;macromolecule metabolic process;metabolic process;nitrogen compound metabolic proce
28.5101	28.6596	28.538	28.653	1:Experimental evidence at protein level	establishment of localization;transport
28.6534	28.1774	28.1806	28.2786	1:Experimental evidence at protein level	cellular macromolecule metabolic process;cellular metabolic process;cellular nitrogen compound metabolic process;cellular process;macromolecule metabolic process;metabolic process;mRNA metabolic process;mRNA pro
27.7493	28.1269	28.2888	28.2805	1:Experimental evidence at protein level	amine biosynthetic process;amine metabolic process;biosynthetic process;carboxylic acid biosynthetic process;carboxylic acid metabolic process;cellular amine metabolic process;cellular amino acid biosynthetic process;ce
28.3066	27.8678	28.1019	27.8962	1:Experimental evidence at protein level	biological regulation;cellular macromolecule metabolic process;cellular metabolic process;cellular nitrogen compound metabolic process;cellular process;cellular response to stimulus;cellular response to stress;establishment
25.8981	25.9813	25.9812	26.0363	1:Experimental evidence at protein level	biological regulation;cell surface receptor linked signaling pathway;cellular process;cellular response to chemical stimulus;cellular response to cytokine stimulus;cellular response to organic substance;cellular response to stim
26.5314	26.3228	26.5674	26.1843	1:Experimental evidence at protein level	cellular macromolecule metabolic process;cellular metabolic process;cellular nitrogen compound metabolic process;cellular process;macromolecule metabolic process;metabolic process;mRNA metabolic process;mRNA pro
27.7002	28.1904	28.0894	28.0231	1:Experimental evidence at protein level	ADP biosynthetic process;ADP metabolic process;AMP metabolic process;ATP metabolic process;biological regulation;biosynthetic process;cell cycle arrest;cell cycle process;cellular biosynthetic process;cellular metabolic p
28.2801	28.2991	28.4243	28.3075	1:Experimental evidence at protein level	amine biosynthetic process;amine metabolic process;biosynthetic process;carboxylic acid biosynthetic process;carboxylic acid metabolic process;cellular amine metabolic process;cellular amino acid biosynthetic process;ce
26.5134	26.5344	26.2947	26.26	1:Experimental evidence at protein level	beta-amyloid metabolic process;biological regulation;bradykinin catabolic process;catabolic process;cell surface receptor linked signaling pathway;cellular catabolic process;cellular component assembly;cellular component
32.8678	32.5503	32.5569	32.6709	1:Experimental evidence at protein level	biological regulation;cellular component assembly;cellular component assembly at cellular level;cellular component organization;cellular component organization at cellular level;cellular component organization or biogenesis;
28.1209	28.319	28.4024	28.4719	1:Experimental evidence at protein level	2-oxoglutarate metabolic process;alcohol biosynthetic process;alcohol metabolic process;alditol biosynthetic process;alditol metabolic process;amine biosynthetic process;amine catabolic process;amine metabolic process;
28.0627	28.113	28.248	28.351	1:Experimental evidence at protein level	biological regulation;negative regulation of apoptosis;negative regulation of biological process;negative regulation of catalytic activity;negative regulation of cell death;negative regulation of cellular process;negative regulation
31.7223	32.0843	32.3269	32.3219	1:Experimental evidence at protein level	actin cytoskeleton organization;actin filament depolymerization;actin filament organization;actin filament-based process;actin polymerization or depolymerization;ameboidal cell migration;anatomical structure morphogenesis;
30.5248	30.4049	30.3378	30.41	1:Experimental evidence at protein level	anatomical structure development;ATP-dependent chromatin remodeling;biological regulation;biosynthetic process;brain development;cell differentiation;cellular biosynthetic process;cellular component organization;cellular c

27.9046	28.3007	28.0708	27.9355	1:Experimental evidence at protein level	biological regulation;cellular process;cellular response to stimulus;establishment of localization;establishment of protein localization;intracellular signal transduction;negative regulation of axonogenesis;negative regulation of b
27.1625	27.3382	27.1194	27.1627	1:Experimental evidence at protein level	activation of immune response;activation of innate immune response;activation of MAPK activity;activation of MAPKK activity;activation of protein kinase activity;axon guidance;biological regulation;cell surface receptor linke
26.7953	26.2269	26.4614	26.4413	1:Experimental evidence at protein level	biological regulation;cellular macromolecule metabolic process;cellular metabolic process;cellular nitrogen compound metabolic process;cellular process;macromolecule metabolic process;metabolic process;ncRNA metabo
31.0729	31.0027	30.7311	30.971	1:Experimental evidence at protein level	biosynthetic process;catabolic process;cellular biosynthetic process;cellular catabolic process;cellular component disassembly;cellular component disassembly at cellular level;cellular component organization;cellular compo
26.2127	26.4823	26.1992	25.8823	1:Experimental evidence at protein level	adherens junction organization;biological adhesion;biological regulation;cell adhesion;cell communication;cell junction assembly;cell junction organization;cell-cell junction organization;cell-cell signaling;cellular component as
26.7498	27.0254	27.1654	27.0657	1:Experimental evidence at protein level	adipose tissue development;anatomical structure development;biological regulation;brain development;catabolic process;cellular catabolic process;cellular ketone body metabolic process;cellular ketone metabolic process;c
26.4718	26.1127	26.1968	26.1994	1:Experimental evidence at protein level	activation of MAPK activity;axon guidance;biological adhesion;biological regulation;blood coagulation;cell adhesion;cell chemotaxis;cell migration;cell motility;cell surface receptor linked signaling pathway;cell-matrix adhesio
31.5208	31.5042	31.5075	31.5534	1:Experimental evidence at protein level	biosynthetic process;catabolic process;cellular biosynthetic process;cellular catabolic process;cellular component disassembly;cellular component disassembly at cellular level;cellular component organization;cellular compo
26.2229	26.5015	26.6281	26.45	1:Experimental evidence at protein level	biological regulation;cell cycle;cellular process;mitotic cell cycle;negative regulation of binding;negative regulation of molecular function;negative regulation of protein binding;regulation of binding;regulation of molecular funct
28.7679	28.9962	29.2326	29.1439	1:Experimental evidence at protein level	ATP-dependent chromatin remodeling;biological regulation;biosynthetic process;cell cycle;cell cycle process;cellular biosynthetic process;cellular component assembly;cellular component assembly at cellular level;cellular co
28.2233	27.982	28.3178	28.0877	1:Experimental evidence at protein level	cellular component assembly;cellular component assembly at cellular level;cellular component organization;cellular component organization at cellular level;cellular component organization or biogenesis;cellular component c
28.8122	28.6782	28.9729	28.8612	1:Experimental evidence at protein level	biological regulation;negative regulation of biological process;negative regulation of biosynthetic process;negative regulation of cellular biosynthetic process;negative regulation of cellular macromolecule biosynthetic process
26.6535	26.7316	26.84	26.7151	1:Experimental evidence at protein level	biological regulation;cellular component organization;cellular component organization or biogenesis;cellular membrane organization;cellular process;cellular response to stimulus;endocytosis;establishment of localization;mer
28.566	28.3298	28.1882	28.4631	1:Experimental evidence at protein level	alcohol biosynthetic process;alcohol metabolic process;biological regulation;biosynthetic process;carbohydrate biosynthetic process;carbohydrate homeostasis;carbohydrate metabolic process;carboxylic acid metabolic pro
29.9746	29.6812	29.737	29.5959	1:Experimental evidence at protein level	biological regulation;biosynthetic process;cellular biosynthetic process;cellular macromolecule biosynthetic process;cellular macromolecule metabolic process;cellular metabolic process;cellular nitrogen compound metabolic
26.8621	26.7125	26.8812	26.7501	1:Experimental evidence at protein level	biological regulation;catabolic process;cellular catabolic process;cellular component organization;cellular component organization at cellular level;cellular component organization or biogenesis;cellular component organizatio
28.3887	28.1631	28.1505	28.2635	1:Experimental evidence at protein level	anatomical structure morphogenesis;biological adhesion;biological regulation;cell adhesion;cell junction assembly;cell junction organization;cell morphogenesis;cell morphogenesis involved in differentiation;cell surface recep
28.6731	28.5762	28.5508	28.6805	1:Experimental evidence at protein level	biological regulation;biosynthetic process;cellular biosynthetic process;cellular component organization;cellular component organization at cellular level;cellular component organization or biogenesis;cellular component orga
28.5924	28.6285	28.7901	28.8277	1:Experimental evidence at protein level	actin cytoskeleton organization;actin filament-based process;biological regulation;cell differentiation;cellular component organization;cellular component organization at cellular level;cellular component organization or biogen
28.3029	28.4337	28.3219	28.1714	1:Experimental evidence at protein level	amine metabolic process;amino acid activation;biosynthetic process;carboxylic acid metabolic process;cellular amine metabolic process;cellular amino acid metabolic process;cellular biosynthetic process;cellular ketone me
27.343	26.9546	27.1151	27.2038	1:Experimental evidence at protein level	
28.0648	27.6835	27.7091	27.7091	1:Experimental evidence at protein level	B cell activation;B cell activation involved in immune response;cell activation;cell activation involved in immune response;cellular macromolecule metabolic process;cellular metabolic process;cellular nitrogen compound meta
29.3174	29.0418	28.9964	29.0971	1:Experimental evidence at protein level	apoptosis;biological regulation;biosynthetic process;cell death;cellular biosynthetic process;cellular macromolecule biosynthetic process;cellular macromolecule metabolic process;cellular metabolic process;cellular nitrogen
28.7142	28.8238	28.7063	28.9103	1:Experimental evidence at protein level	anatomical structure development;cellular macromolecule metabolic process;cellular metabolic process;cellular nitrogen compound metabolic process;cellular process;chordate embryonic development;developmental proce
28.5477	28.3924	28.2593	28.1238	1:Experimental evidence at protein level	activation of immune response;activation of innate immune response;anaphase-promoting complex-dependent proteasomal ubiquitin-dependent protein catabolic process;antigen processing and presentation;antigen proces
27.6555	27.8873	27.8154	27.6728	1:Experimental evidence at protein level	anatomical structure development;anatomical structure formation involved in morphogenesis;anatomical structure morphogenesis;angiogenesis;apoptosis;apoptosis involved in heart morphogenesis;apoptosis involved in mo
28.2951	28.5153	28.3499	28.5059	1:Experimental evidence at protein level	biological regulation;cell cycle;cell cycle phase;cell cycle process;cellular component organization;cellular component organization at cellular level;cellular component organization or biogenesis;cellular component organizatio
26.6379	26.5966	26.5201	26.5109	1:Experimental evidence at protein level	anatomical structure development;anatomical structure formation involved in morphogenesis;biological regulation;brain development;cell proliferation;cell surface receptor linked signaling pathway;cellular process;cellular res
29.0307	28.7581	28.8965	28.5955	1:Experimental evidence at protein level	actin cytoskeleton organization;actin filament organization;actin filament-based process;actin nucleation;Arp2/3 complex-mediated actin nucleation;biological regulation;cellular component movement;cellular component orga
27.909	27.614	28.1585	28.0488	1:Experimental evidence at protein level	biological regulation;cellular component organization;cellular component organization at cellular level;cellular component organization or biogenesis;cellular component organization or biogenesis at cellular level;cellular macro
26.884	27.9214	27.2594	27.2944	1:Experimental evidence at protein level	2-oxoglutarate metabolic process;4-hydroxyproline catabolic process;4-hydroxyproline metabolic process;alcohol biosynthetic process;alcohol metabolic process;amine biosynthetic process;amine catabolic process;amine r
26.5639	27.1784	27.5451	27.5239	1:Experimental evidence at protein level	amine metabolic process;anatomical structure development;anion homeostasis;biological regulation;carboxylic acid metabolic process;cellular amine metabolic process;cellular amino acid metabolic process;cellular anion ho
30.4241	30.4258	30.6324	30.5814	1:Experimental evidence at protein level	biological regulation;carbohydrate metabolic process;cellular carbohydrate metabolic process;cellular macromolecule metabolic process;cellular metabolic process;cellular process;cellular protein metabolic process;cellular r
28.476	28.2317	28.4642	28.3487	1:Experimental evidence at protein level	autophagy;behavior;behavioral interaction between organisms;biological regulation;calcium ion homeostasis;catabolic process;cation homeostasis;cell differentiation;cell proliferation;cell volume homeostasis;cellular calcium
31.1621	31.3314	31.4727	31.5058	1:Experimental evidence at protein level	anatomical structure development;biological regulation;bone development;cellular component organization;cellular component organization at cellular level;cellular component organization or biogenesis;cellular component o
29.4165	29.4151	29.5359	29.3785	1:Experimental evidence at protein level	aerobic respiration;cellular metabolic process;cellular process;cellular respiration;electron transport chain;energy derivation by oxidation of organic compounds;generation of precursor metabolites and energy;metabolic proce
25.706	25.5919	25.6561	25.7088	1:Experimental evidence at protein level	acyl-CoA biosynthetic process;acyl-CoA metabolic process;acylglycerol biosynthetic process;acylglycerol metabolic process;adiponectin-mediated signaling pathway;biological regulation;biosynthetic process;carboxylic aci
30.2685	30.5013	30.165	30.4142	1:Experimental evidence at protein level	acetyl-CoA catabolic process;acetyl-CoA metabolic process;alcohol biosynthetic process;alcohol metabolic process;biological regulation;biosynthetic process;carbohydrate biosynthetic process;carbohydrate metabolic proc
28.991	28.9695	28.8519	28.8319	1:Experimental evidence at protein level	alcohol metabolic process;amine biosynthetic process;amine catabolic process;amine metabolic process;aspartate family amino acid catabolic process;aspartate family amino acid metabolic process;betaine biosynthetic pro
29.4922	29.6577	29.5813	29.7429	1:Experimental evidence at protein level	biological regulation;cellular component assembly;cellular component organization;cellular component organization or biogenesis;cellular macromolecule metabolic process;cellular metabolic process;cellular process;cellular
26.9949	26.8806	26.661	26.7049	1:Experimental evidence at protein level	biological regulation;biosynthetic process;cell cycle;cellular biosynthetic process;cellular component organization;cellular component organization at cellular level;cellular component organization or biogenesis;cellular compo
28.8358	29.2097	29.3884	29.3061	1:Experimental evidence at protein level	biological regulation;cell proliferation;cellular macromolecule metabolic process;cellular metabolic process;cellular process;cellular protein metabolic process;cellular response to chemical stimulus;cellular response to organic
28.8178	28.9925	29.2145	29.1807	1:Experimental evidence at protein level	activation of immune response;activation of innate immune response;anaphase-promoting complex-dependent proteasomal ubiquitin-dependent protein catabolic process;antigen processing and presentation;antigen proces
29.7157	30.0998	30.3761	30.3417	1:Experimental evidence at protein level	activation of caspase activity;biological regulation;cell differentiation;cellular developmental process;cellular macromolecule metabolic process;cellular metabolic process;cellular process;cellular protein metabolic process;de
31.3412	30.8071	30.8808	30.8687	1:Experimental evidence at protein level	biosynthetic process;catabolic process;cellular biosynthetic process;cellular catabolic process;cellular component biogenesis;cellular component biogenesis at cellular level;cellular component disassembly;cellular componen
31.2019	30.5947	30.8366	30.7561	1:Experimental evidence at protein level	activation of caspase activity;anatomical structure morphogenesis;biological regulation;carbohydrate homeostasis;cell cycle;cell differentiation;cellular chemical homeostasis;cellular developmental process;cellular glucose ho
29.2252	29.5071	29.5044	29.4192	1:Experimental evidence at protein level	anatomical structure development;apoptosis;biological regulation;catabolic process;cell cycle;cell cycle process;cell death;cell surface receptor linked signaling pathway;cellular catabolic process;cellular component assembl
27.4738	27.3395	27.2602	26.9537	1:Experimental evidence at protein level	biological regulation;biosynthetic process;catabolic process;cellular biosynthetic process;cellular catabolic process;cellular component assembly;cellular component assembly at cellular level;cellular component organization;
29.4824	29.5234	29.7453	29.5645	1:Experimental evidence at protein level	biological regulation;cellular macromolecule metabolic process;cellular metabolic process;cellular nitrogen compound metabolic process;cellular process;cellular response to stimulus;DNA conformation change;DNA duplex u
26.448	25.3881	26.302	26.0691	1:Experimental evidence at protein level	actin cytoskeleton organization;actin filament-based process;actin-mediated cell contraction;apical constriction;apoptosis;axon guidance;biological adhesion;biological regulation;bleb assembly;cell adhesion;cell death;cell m
27.1407	27.0298	27.1971	26.9817	1:Experimental evidence at protein level	biological regulation;biosynthetic process;catabolic process;cell surface receptor linked signaling pathway;cellular biosynthetic process;cellular catabolic process;cellular macromolecule biosynthetic process;cellular macrom
27.8915	28.0719	28.1429	27.9511	1:Experimental evidence at protein level	alcohol biosynthetic process;alcohol metabolic process;amino sugar biosynthetic process;amino sugar metabolic process;biosynthetic process;carbohydrate biosynthetic process;carbohydrate metabolic process;cellular bio
26.2646	26.2191	#NUM!	#NUM!	1:Experimental evidence at protein level	10-formyltetrahydrofolate catabolic process;10-formyltetrahydrofolate metabolic process;aromatic compound catabolic process;biosynthetic process;catabolic process;cellular aromatic compound metabolic process;cellular
29.689	29.8659	30.0291	29.9824	1:Experimental evidence at protein level	apoptotic cell clearance;biological regulation;cell redox homeostasis;cellular component organization;cellular component organization or biogenesis;cellular homeostasis;cellular macromolecule metabolic process;cellular me
26.4198	26.2359	26.303	26.2334	1:Experimental evidence at protein level	cellular component assembly;cellular component assembly at cellular level;cellular component organization;cellular component organization at cellular level;cellular component organization or biogenesis;cellular component c
26.3407	25.5447	25.7622	25.449	1:Experimental evidence at protein level	actin cytoskeleton organization;actin filament-based process;actomyosin structure organization;asymmetric Golgi ribbon formation;biological regulation;cell migration;cell motility;cellular component movement;cellular compo
27.0494	26.7992	26.9269	26.9794	1:Experimental evidence at protein level	anatomical structure development;anatomical structure morphogenesis;ATP-dependent chromatin remodeling;biological regulation;biosynthetic process;cell surface receptor linked signaling pathway;cellular biosynthetic proc
28.785	28.761	28.7173	28.6354	1:Experimental evidence at protein level	antigen processing and presentation;antigen processing and presentation of exogenous antigen;antigen processing and presentation of exogenous peptide antigen;antigen processing and presentation of exogenous peptide
27.6184	27.8107	27.7375	27.647	1:Experimental evidence at protein level	biological regulation;biosynthetic process;cell cycle;cell proliferation;cellular biosynthetic process;cellular component organization;cellular component organization at cellular level;cellular component organization or biogenesi
26.856	26.7805	26.9232	26.7377	1:Experimental evidence at protein level	
28.9758	28.9266	28.9691	28.9246	1:Experimental evidence at protein level	biological regulation;biosynthetic process;cell death;cell projection organization;cellular biosynthetic process;cellular component organization;cellular component organization at cellular level;cellular component organization o
27.8173	27.5027	27.5808	27.4302	1:Experimental evidence at protein level	amine metabolic process;amino acid activation;carboxylic acid metabolic process;cellular amine metabolic process;cellular amino acid metabolic process;cellular ketone metabolic process;cellular macromolecule metabolic p
28.2333	27.9474	28.1644	28.0966	1:Experimental evidence at protein level	cellular component assembly;cellular component assembly at cellular level;cellular component organization;cellular component organization at cellular level;cellular component organization or biogenesis;cellular component c
26.092	25.8161	26.0055	25.6739	1:Experimental evidence at protein level	biological adhesion;biological regulation;cell adhesion;cell junction assembly;cell junction organization;cell-cell junction assembly;cell-cell junction organization;cellular component assembly;cellular component assembly at c
26.5159	27.2328	26.9934	26.8263	1:Experimental evidence at protein level	anatomical structure development;anatomical structure morphogenesis;axon ensheathment;axonogenesis;biological regulation;cell development;cell part morphogenesis;cell projection morphogenesis;cell projection organiza
27.6258	27.7963	27.5704	27.6337	1:Experimental evidence at protein level	catabolic process;cellular catabolic process;cellular macromolecule catabolic process;cellular macromolecule metabolic process;cellular metabolic process;cellular process;cellular protein metabolic process;macromolecule c
25.8279	25.7258	25.5864	25.7672	1:Experimental evidence at protein level	cellular component organization;cellular component organization or biogenesis;cellular membrane organization;cellular process;endocytosis;establishment of localization;lipid transport;membrane invagination;membrane orga
27.0878	27.0368	26.8159	26.9856	1:Experimental evidence at protein level	biological regulation;biosynthetic process;cellular biosynthetic process;cellular macromolecule biosynthetic process;cellular macromolecule metabolic process;cellular metabolic process;cellular nitrogen compound metabolic
28.5398	28.6588	28.5156	28.437	1:Experimental evidence at protein level	amine metabolic process;amino acid activation;asparaginyl-tRNA aminoacylation;carboxylic acid metabolic process;cellular amine metabolic process;cellular amino acid metabolic process;cellular ketone metabolic process;
25.9987	25.5127	25.3842	25.0233	1:Experimental evidence at protein level	
29.2564	29.3139	29.0496	29.106	1:Experimental evidence at protein level	activation of MAPKK activity;activation of protein kinase activity;adenosine receptor signaling pathway;biological regulation;blood coagulation;cAMP-mediated signaling;cell activation;cell communication;cell cycle;cell divisio
28.1826	28.0145	28.1193	27.9157	1:Experimental evidence at protein level	adult behavior;adult locomotory behavior;aging;anatomical structure development;anatomical structure morphogenesis;axonogenesis;behavior;biological regulation;catabolic process;cell communication;cell differentiation;ce
28.9759	29.0505	29.0494	29.081	1:Experimental evidence at protein level	catabolic process;cell cycle process;cellular catabolic process;cellular component disassembly;cellular component disassembly at cellular level;cellular component organization;cellular component organization at cellular leve
27.9522	27.9323	27.7244	27.7874	1:Experimental evidence at protein level	alcohol catabolic process;alcohol metabolic process;biological regulation;carbohydrate catabolic process;carbohydrate metabolic process;carbohydrate phosphorylation;catabolic process;cellular carbohydrate catabolic proc
26.22	25.3182	25.7346	25.7945	1:Experimental evidence at protein level	biological regulation;biosynthetic process;cellular biosynthetic process;cellular macromolecule biosynthetic process;cellular macromolecule metabolic process;cellular metabolic process;cellular nitrogen compound metabolic
27.1237	27.111	27.1135	27.0408	1:Experimental evidence at protein level	anatomical structure homeostasis;base-excision repair;base-excision repair, gap-filling;biological regulation;biosynthetic process;cell cycle;cellular biosynthetic process;cellular component organization;cellular component org
27.1826	27.2914	27.2157	27.3779	1:Experimental evidence at protein level	activation of caspase activity;aging;anatomical structure morphogenesis;apoptosis;apoptosis in response to endoplasmic reticulum stress;biological regulation;biosynthetic process;branching involved in mammary gland duc
29.0675	29.3223	29.3599	29.2513	1:Experimental evidence at protein level	amine biosynthetic process;amine metabolic process;biosynthetic process;carboxylic acid biosynthetic process;carboxylic acid metabolic process;cellular amine metabolic process;cellular amino acid biosynthetic process;ce
27.8934	27.2856	27.9053	27.6294	1:Experimental evidence at protein level	anatomical structure formation involved in morphogenesis;anatomical structure morphogenesis;appendage morphogenesis;biological regulation;bone trabecula formation;cellular component disassembly;cellular component
28.461	28.3942	28.3309	28.2323	1:Experimental evidence at protein level	anatomical structure development;apoptosis;biological regulation;biosynthetic process;blood circulation;cell death;cell differentiation;cell differentiation involved in kidney development;cell differentiation involved in metaneph
27.8882	27.8602	27.6875	27.7081	1:Experimental evidence at protein level	ATP metabolic process;biological regulation;cation transport;cell communication;cell-cell signaling;cellular component organization;cellular component organization or biogenesis;cellular membrane fusion;cellular membrane
30.4125	30.0718	30.1968	30.398	1:Experimental evidence at protein level	biosynthetic process;catabolic process;cellular biosynthetic process;cellular catabolic process;cellular component disassembly;cellular component disassembly at cellular level;cellular component organization;cellular compo
30.2663	30.1611	30.1038	30.1744	1:Experimental evidence at protein level	actin cytoskeleton organization;actin filament capping;actin filament-based process;barbed-end actin filament capping;biological regulation;blood coagulation;cellular component movement;cellular component organization;c
28.7544	29.1745	28.7563	28.7158	1:Experimental evidence at protein level	actin cytoskeleton organization;actin filament organization;actin filament-based process;actin polymerization or depolymerization;activation of immune response;anatomical structure formation involved in morphogenesis;anti
27.1357	26.6819	26.8424	26.6521	1:Experimental evidence at protein level	anatomical structure development;anatomical structure formation involved in morphogenesis;anatomical structure morphogenesis;aortic smooth muscle cell differentiation;appendage morphogenesis;ATP-dependent chroma
29.2206	29.2076	29.2288	29.148	1:Experimental evidence at protein level	activation of immune response;activation of innate immune response;anaphase-promoting complex-dependent proteasomal ubiquitin-dependent protein catabolic process;antigen processing and presentation;antigen proces
32.0495	32.3518	32.3459	32.2276	1:Experimental evidence at protein level	actin cytoskeleton organization;actin filament-based process;anatomical structure development;androgen receptor signaling pathway;biological regulation;cell cycle phase;cell cycle process;cell development;cell division;cell
31.131	30.7675	30.7981	30.8873	1:Experimental evidence at protein level	biosynthetic process;catabolic process;cellular biosynthetic process;cellular catabolic process;cellular component biogenesis;cellular component biogenesis at cellular level;cellular component disassembly;cellular componen
31.8383	33.6628	33.3652	33.9176	1:Experimental evidence at protein level	biological regulation;biosynthetic process;blood coagulation;cell activation;cell migration;cell motility;cellular biosynthetic process;cellular component assembly;cellular component assembly at cellular level;cellular componen
28.1391	28.0259	28.1199	28.1104	1:Experimental evidence at protein level	antigen processing and presentation;antigen processing and presentation of exogenous antigen;antigen processing and presentation of exogenous peptide antigen;antigen processing and presentation of exogenous peptide
27.971	27.9543	27.8824	28.0247	1:Experimental evidence at protein level	biosynthetic process;carbohydrate metabolic process;cellular carbohydrate metabolic process;cellular macromolecule metabolic process;cellular metabolic process;cellular process;cellular protein metabolic process;glycosyl
#NUM!	25.0785	#NUM!	#NUM!	1:Experimental evidence at protein level	anatomical structure formation involved in morphogenesis;angiogenesis;biological adhesion;biological regulation;cell adhesion;cell cycle arrest;cell cycle process;cell migration;cell motility;cellular component movement;cellu
27.5641	27.3815	27.5585	27.3845	1:Experimental evidence at protein level	biological regulation;biosynthetic process;blood coagulation;catabolic process;cellular biosynthetic process;cellular catabolic process;cellular macromolecule biosynthetic process;cellular macromolecule catabolic process;ce
25.7029	25.5415	25.5009	25.3461	1:Experimental evidence at protein level	biological regulation;biosynthetic process;cell proliferation;cellular biosynthetic process;cellular macromolecule biosynthetic process;cellular macromolecule metabolic process;cellular metabolic process;cellular nitrogen com
27.3479	27.5534	27.3237	27.2047	1:Experimental evidence at protein level	biological regulation;cell proliferation;cell surface receptor linked signaling pathway;cellular process;cellular response to stimulus;enzyme linked receptor protein signaling pathway;intracellular signal transduction;negative reg
29.8946	30.1089	29.8803	29.8665	1:Experimental evidence at protein level	
27.5243	27.6452	27.689	27.8216	1:Experimental evidence at protein level	alcohol catabolic process;alcohol metabolic process;biosynthetic process;carbohydrate biosynthetic process;carbohydrate catabolic process;carbohydrate metabolic process;catabolic process;cellular biosynthetic process;c
27.407	27.4335	27.2923	27.3652	1:Experimental evidence at protein level	4-hydroxyproline metabolic process;amine metabolic process;apoptosis;apoptosis in response to endoplasmic reticulum stress;biological regulation;brown fat cell differentiation;calcium ion homeostasis;calcium ion transport
25.8153	25.5454	25.704	25.5094	1:Experimental evidence at protein level	biosynthetic process;cellular biosynthetic process;cellular macromolecule biosynthetic process;cellular macromolecule metabolic process;cellular metabolic process;cellular nitrogen compound metabolic process;cellular pro
28.022	28.0218	27.8091	27.8186	1:Experimental evidence at protein level	biological regulation;biosynthetic process;catabolic process;cell growth;cellular biosynthetic process;cellular catabolic process;cellular macromolecule catabolic process;cellular macromolecule metabolic process;cellular met
24.792	26.3825	25.0951	24.4636	1:Experimental evidence at protein level	cellular component organization;cellular component organization at cellular level;cellular component organization or biogenesis;cellular component organization or biogenesis at cellular level;cellular process;lipid particle orga
28.8051	28.5344	28.5577	28.6101	1:Experimental evidence at protein level	assembly of spliceosomal tri-snRNP;biological regulation;biosynthetic process;cell proliferation;cellular component assembly;cellular component assembly at cellular level;cellular component organization;cellular component
27.8721	27.5829	27.5692	27.4933	1:Experimental evidence at protein level	antigen processing and presentation;antigen processing and presentation of exogenous antigen;antigen processing and presentation of exogenous peptide antigen;antigen processing and presentation of exogenous peptide
26.6038	26.7615	26.9344	26.4841	1:Experimental evidence at protein level	alcohol catabolic process;alcohol metabolic process;anatomical structure homeostasis;biological regulation;carbohydrate catabolic process;carbohydrate homeostasis;carbohydrate metabolic process;carbohydrate phospho
27.696	27.5614	27.6009	27.6492	1:Experimental evidence at protein level	acetyl-CoA biosynthetic process;acetyl-CoA biosynthetic process from pyruvate;acetyl-CoA catabolic process;acetyl-CoA metabolic process;alcohol catabolic process;alcohol metabolic process;biological regulation;biosynt
28.3544	29.0434	29.0627	29.1414	1:Experimental evidence at protein level	alcohol biosynthetic process;alcohol metabolic process;biosynthetic process;carbohydrate biosynthetic process;carbohydrate metabolic process;cellular biosynthetic process;cellular carbohydrate biosynthetic process;cellul
31.118	30.7291	30.8193	30.806	1:Experimental evidence at protein level	biological regulation;biosynthetic process;catabolic process;cellular biosynthetic process;cellular catabolic process;cellular component disassembly;cellular component disassembly at cellular level;cellular component organi
27.6753	27.9802	28.0243	27.8755	1:Experimental evidence at protein level	biological regulation;biosynthetic process;cell differentiation;cellular biosynthetic process;cellular developmental process;cellular macromolecule biosynthetic process;cellular macromolecule metabolic process;cellular metab
29.4956	29.6504	29.9394	29.7483	1:Experimental evidence at protein level	anatomical structure development;apoptosis;base-excision repair, DNA ligation;biological regulation;catabolic process;cell chemotaxis;cell death;cell migration;cell motility;cellular catabolic process;cellular component assem
27.4204	26.8859	27.0645	26.9595	1:Experimental evidence at protein level	antigen processing and presentation;antigen processing and presentation of peptide antigen;antigen processing and presentation of peptide antigen via MHC class I;cellular macromolecule metabolic process;cellular metabo
27.6769	27.7585	27.5434	27.761	1:Experimental evidence at protein level	adherens junction assembly;adherens junction organization;anatomical structure development;anatomical structure formation involved in morphogenesis;anatomical structure homeostasis;anatomical structure morphogenesi
29.4262	29.1006	29.2655	29.2225	1:Experimental evidence at protein level	
27.4136	27.2024	27.1735	27.1291	1:Experimental evidence at protein level	adaptive immune response;adaptive immune response based on somatic recombination of immune receptors built from immunoglobulin superfamily domains;anatomical structure development;B cell activation;B cell activati
27.2553	27.2461	27.3016	27.1368	1:Experimental evidence at protein level	biological regulation;cellular macromolecule metabolic process;cellular metabolic process;cellular nitrogen compound metabolic process;cellular process;macromolecule metabolic process;metabolic process;mRNA metabol
27.7772	27.8387	27.9882	27.835	1:Experimental evidence at protein level	cell cycle;cell division;cellular component organization;cellular component organization or biogenesis;cellular membrane organization;cellular process;endocytosis;establishment of localization;membrane invagination;membra
25.9288	25.8983	25.8359	25.6133	1:Experimental evidence at protein level	anatomical structure development;biological regulation;biosynthetic process;catabolic process;cell cycle arrest;cell cycle process;cellular biosynthetic process;cellular catabolic process;cellular macromolecule biosynthetic p
27.9688	28.0728	28.2813	28.2639	1:Experimental evidence at protein level	binding of sperm to zona pellucida;cell recognition;cell-cell recognition;cellular process;cellular process involved in reproduction;reproductive process;sperm-egg recognition
28.7375	28.714	29.1608	29.0314	1:Experimental evidence at protein level	anatomical structure development;anatomical structure formation involved in morphogenesis;androgen receptor signaling pathway;atrial cardiac muscle cell development;biological regulation;biosynthetic process;cardiac cel
27.5222	27.4262	27.6432	27.4095	1:Experimental evidence at protein level	biological regulation;biosynthetic process;cell surface receptor linked signaling pathway;cellular biosynthetic process;cellular macromolecule biosynthetic process;cellular macromolecule metabolic process;cellular metabolic
27.3452	27.1318	27.0999	27.0943	1:Experimental evidence at protein level	actin cytoskeleton organization;actin filament organization;actin filament-based process;anatomical structure morphogenesis;aorta smooth muscle tissue morphogenesis;biological regulation;bleb assembly;cell projection ass
28.3633	28.6019	28.4345	28.4932	1:Experimental evidence at protein level	apoptotic mitochondrial changes;biological regulation;cell cycle;cellular component organization;cellular component organization at cellular level;cellular component organization or biogenesis;cellular component organization
30.0431	29.7934	30.0817	30.2264	1:Experimental evidence at protein level	biological regulation;cellular macromolecule metabolic process;cellular metabolic process;cellular nitrogen compound metabolic process;cellular process;macromolecule metabolic process;metabolic process;mRNA metabol
28.6891	28.442	28.4502	28.5249	1:Experimental evidence at protein level	activation of immune response;activation of innate immune response;anaphase-promoting complex-dependent proteasomal ubiquitin-dependent protein catabolic process;antigen processing and presentation;antigen proces
27.3118	27.1688	27.0559	27.2159	1:Experimental evidence at protein level	biological adhesion;biological regulation;biosynthetic process;cell adhesion;cellular biosynthetic process;cellular macromolecule biosynthetic process;cellular macromolecule metabolic process;cellular metabolic process;cell
26.7078	26.9304	27.0214	26.8406	1:Experimental evidence at protein level	
27.4114	27.3384	27.2411	27.1594	1:Experimental evidence at protein level	biosynthetic process;cellular biosynthetic process;cellular macromolecule biosynthetic process;cellular macromolecule metabolic process;cellular metabolic process;cellular process;cellular protein metabolic process;cotrans
28.7951	28.6291	28.6615	28.5234	1:Experimental evidence at protein level	activation of immune response;activation of innate immune response;anaphase-promoting complex-dependent proteasomal ubiquitin-dependent protein catabolic process;antigen processing and presentation;antigen proces
28.5116	28.4264	28.577	28.4552	1:Experimental evidence at protein level	anatomical structure development;anatomical structure morphogenesis;basement membrane assembly;basement membrane organization;biological regulation;cell morphogenesis;cell morphogenesis involved in differentiatio
27.4941	27.8294	27.8841	27.9746	1:Experimental evidence at protein level	acylglycerol metabolic process;aerobic respiration;aging;alcohol metabolic process;anatomical structure development;biological regulation;catabolic process;cell differentiation;cellular aromatic compound metabolic process;

31.8134	31.7597	31.6326	31.6769	1:Experimental evidence at protein level	carbohydrate metabolic process;carboxylic acid metabolic process;cellular ketone metabolic process;cellular metabolic process;cellular nitrogen compound metabolic process;cellular process;coenzyme metabolic process;c
27.6678	27.8189	27.6622	27.7316	1:Experimental evidence at protein level	amine transport;amino acid import;amino acid transport;aromatic amino acid transport;biological regulation;blood coagulation;branched-chain aliphatic amino acid transport;calcium ion transport;carbohydrate metabolic proc
#NUM!	#NUM!	#NUM!	#NUM!	1:Experimental evidence at protein level	aging;anatomical structure development;anatomical structure morphogenesis;biological regulation;cell differentiation;cell migration;cell motility;cell proliferation;cellular component movement;cellular component organization;
28.0355	27.9272	28.1342	28.0273	1:Experimental evidence at protein level	4-hydroxyproline metabolic process;amine metabolic process;carboxylic acid metabolic process;cellular amine metabolic process;cellular amino acid metabolic process;cellular component organization;cellular component or
25.7797	24.6757	25.9603	26.37	1:Experimental evidence at protein level	adherens junction assembly;adherens junction organization;anatomical structure development;biological adhesion;biological regulation;cell adhesion;cell junction assembly;cell junction organization;cell migration;cell motility;
28.9469	29.0834	29.1181	28.9302	1:Experimental evidence at protein level	amine metabolic process;amino acid activation;anatomical structure formation involved in morphogenesis;angiogenesis;biological regulation;biosynthetic process;carboxylic acid metabolic process;cellular amine metabolic p
28.9543	28.883	28.6743	28.9383	1:Experimental evidence at protein level	biological regulation;blood coagulation;calcium ion homeostasis;calcium ion transmembrane transport;calcium ion transport;cation homeostasis;cation transport;cellular calcium ion homeostasis;cellular cation homeostasis;c
27.9986	28.348	28.2822	28.3825	1:Experimental evidence at protein level	adipose tissue development;amine catabolic process;amine metabolic process;anatomical structure development;biosynthetic process;brain development;branched chain family amino acid catabolic process;branched chain
30.7738	31.2116	31.3136	31.2036	1:Experimental evidence at protein level	biological regulation;biosynthetic process;cellular biosynthetic process;cellular macromolecule biosynthetic process;cellular macromolecule metabolic process;cellular metabolic process;cellular nitrogen compound metabolic
29.763	29.8683	29.8265	29.8972	1:Experimental evidence at protein level	alcohol biosynthetic process;alcohol catabolic process;alcohol metabolic process;biosynthetic process;carbohydrate biosynthetic process;carbohydrate catabolic process;carbohydrate metabolic process;catabolic process;c
29.1386	28.8593	28.8864	28.8355	1:Experimental evidence at protein level	anatomical structure morphogenesis;biological regulation;catabolic process;cell surface receptor linked signaling pathway;cellular catabolic process;cellular macromolecule catabolic process;cellular macromolecule metaboli
29.4048	29.4082	29.3418	29.2306	1:Experimental evidence at protein level	adult behavior;adult locomotory behavior;anatomical structure maturation;behavior;cellular component organization;cellular component organization at cellular level;cellular component organization or biogenesis;cellular comp
28.0245	28.2272	28.258	28.0147	1:Experimental evidence at protein level	amine metabolic process;amino acid activation;carboxylic acid metabolic process;cellular amine metabolic process;cellular amino acid metabolic process;cellular ketone metabolic process;cellular macromolecule metabolic p
24.3665	23.8223	24.3695	24.0653	1:Experimental evidence at protein level	cellular process;endosome transport;establishment of localization;establishment of localization in cell;intracellular transport;transport;vesicle-mediated transport
27.5355	27.8682	27.68	27.9364	1:Experimental evidence at protein level	biological regulation;cellular metabolic process;cellular process;cellular response to stimulus;intracellular signal transduction;metabolic process;peptide metabolic process;regulation of biological process;regulation of cellular
31.049	30.7304	30.8318	31.0292	1:Experimental evidence at protein level	biosynthetic process;catabolic process;cellular biosynthetic process;cellular catabolic process;cellular component biogenesis;cellular component biogenesis at cellular level;cellular component disassembly;cellular componer
31.0873	31.0482	30.8306	30.9519	1:Experimental evidence at protein level	biosynthetic process;catabolic process;cellular biosynthetic process;cellular catabolic process;cellular component disassembly;cellular component disassembly at cellular level;cellular component organization;cellular compo
28.3358	28.1233	28.2681	28.2361	1:Experimental evidence at protein level	activation of immune response;activation of innate immune response;anaphase-promoting complex-dependent proteasomal ubiquitin-dependent protein catabolic process;antigen processing and presentation;antigen proces
28.6066	28.3185	28.443	28.3456	1:Experimental evidence at protein level	amine biosynthetic process;amine metabolic process;anatomical structure development;aspartate family amino acid biosynthetic process;aspartate family amino acid metabolic process;biosynthetic process;carboxylic acid b
26.5753	26.4629	26.4335	26.5625	1:Experimental evidence at protein level	cellular macromolecule metabolic process;cellular metabolic process;cellular process;cellular protein metabolic process;macromolecule metabolic process;macromolecule modification;metabolic process;primary metabolic p
29.0544	29.0539	29.1122	28.9972	1:Experimental evidence at protein level	biological regulation;cellular macromolecule metabolic process;cellular metabolic process;cellular process;cellular protein metabolic process;establishment of localization;establishment of localization in cell;establishment of p
26.7153	27.139	26.8939	26.9232	1:Experimental evidence at protein level	
27.5716	27.2686	27.7758	27.6174	1:Experimental evidence at protein level	biological regulation;biosynthetic process;cellular biosynthetic process;cellular macromolecule biosynthetic process;cellular macromolecule metabolic process;cellular metabolic process;cellular nitrogen compound metabolic
28.2356	28.1046	28.1511	28.2663	1:Experimental evidence at protein level	biological regulation;regulation of anatomical structure morphogenesis;regulation of biological process;regulation of cell development;regulation of cell differentiation;regulation of cell morphogenesis;regulation of cell morpho
29.3359	28.8715	29.0193	28.8926	1:Experimental evidence at protein level	actin cytoskeleton organization;actin filament-based process;biological regulation;blood coagulation;cellular component organization;cellular component organization at cellular level;cellular component organization or biogen
27.9846	27.8471	27.6645	27.5751	1:Experimental evidence at protein level	
29.3583	29.2536	29.4255	29.4021	1:Experimental evidence at protein level	biological regulation;cell cycle;cell cycle phase;cell cycle process;cell division;cell proliferation;cellular component organization;cellular component organization at cellular level;cellular component organization or biogenesis;c
27.7327	27.4566	27.6495	27.3483	1:Experimental evidence at protein level	actin cytoskeleton organization;actin filament-based process;ameboidal cell migration;anatomical structure development;anatomical structure formation involved in morphogenesis;anatomical structure morphogenesis;anteri
27.1009	26.9813	26.9045	26.9532	1:Experimental evidence at protein level	actin cytoskeleton organization;actin cytoskeleton reorganization;actin filament-based process;actomyosin structure organization;biological regulation;cell migration;cell motility;cellular component movement;cellular compon
27.5727	27.6614	27.4603	27.5304	1:Experimental evidence at protein level	actin cytoskeleton organization;actin filament-based process;cellular component organization;cellular component organization at cellular level;cellular component organization or biogenesis;cellular component organization or
27.2887	27.5255	27.4494	27.2409	1:Experimental evidence at protein level	2-oxoglutarate metabolic process;acetyl-CoA catabolic process;acetyl-CoA metabolic process;amine metabolic process;anatomical structure development;carbohydrate metabolic process;carboxylic acid metabolic process
26.4007	26.2289	26.3396	26.2353	1:Experimental evidence at protein level	activation of protein kinase activity;anatomical structure morphogenesis;biological regulation;cell part morphogenesis;cell projection morphogenesis;cell projection organization;cellular component morphogenesis;cellular con
27.56	27.5738	27.4637	27.4157	1:Experimental evidence at protein level	activation of phospholipase A2 activity;activation of phospholipase A2 activity by calcium-mediated signaling;activation of phospholipase C activity;alkanesulfonate metabolic process;anatomical structure development;anato
28.7911	28.5347	28.5405	28.4429	1:Experimental evidence at protein level	biological regulation;biosynthetic process;cellular biosynthetic process;cellular macromolecule biosynthetic process;cellular macromolecule metabolic process;cellular metabolic process;cellular process;cellular protein metab
27.6142	27.6314	27.8348	27.9049	1:Experimental evidence at protein level	acetyl-CoA catabolic process;acetyl-CoA metabolic process;amine catabolic process;amine metabolic process;anatomical structure morphogenesis;aspartate family amino acid catabolic process;aspartate family amino acid
32.3722	31.999	31.826	32.0489	1:Experimental evidence at protein level	cellular component assembly;cellular component assembly at cellular level;cellular component organization;cellular component organization at cellular level;cellular component organization or biogenesis;cellular component c
28.3954	28.1777	28.0724	28.0202	1:Experimental evidence at protein level	amine metabolic process;B cell activation;B cell proliferation;biosynthetic process;carboxylic acid metabolic process;cell activation;cell proliferation;cellular amine metabolic process;cellular amino acid metabolic process;cell
27.1337	26.253	26.5444	26.6338	1:Experimental evidence at protein level	adenosine biosynthetic process;adenosine metabolic process;AMP catabolic process;AMP metabolic process;biological adhesion;biological regulation;biosynthetic process;catabolic process;cell adhesion;cell-cell adhesion;
27.9746	27.5749	27.7536	27.4691	1:Experimental evidence at protein level	biosynthetic process;cellular biosynthetic process;cellular macromolecule biosynthetic process;cellular macromolecule metabolic process;cellular metabolic process;cellular nitrogen compound metabolic process;cellular pro
29.8723	29.9571	29.5443	29.8572	1:Experimental evidence at protein level	biological regulation;biosynthetic process;catabolic process;cellular biosynthetic process;cellular catabolic process;cellular component disassembly;cellular component disassembly at cellular level;cellular component organiz
26.6509	26.8847	26.8718	26.8451	1:Experimental evidence at protein level	macromolecule metabolic process;metabolic process;primary metabolic process;protein metabolic process;proteolysis
29.1847	29.3796	29.3653	29.4316	1:Experimental evidence at protein level	anatomical structure development;ATP-dependent chromatin remodeling;biosynthetic process;blastocyst development;cell cycle;cell proliferation;cellular biosynthetic process;cellular component assembly;cellular componen
26.6419	26.4158	26.535	26.5008	1:Experimental evidence at protein level	biological regulation;biosynthetic process;carbohydrate metabolic process;carbohydrate transport;cell cycle;cell cycle process;cell surface receptor linked signaling pathway;cellular biosynthetic process;cellular component a
30.4259	30.5527	30.5929	30.449	1:Experimental evidence at protein level	anatomical structure morphogenesis;antigen processing and presentation;antigen processing and presentation of exogenous antigen;antigen processing and presentation of exogenous peptide antigen;antigen processing an
30.7398	30.4889	30.6187	30.453	1:Experimental evidence at protein level	biosynthetic process;catabolic process;cellular biosynthetic process;cellular catabolic process;cellular component biogenesis;cellular component biogenesis at cellular level;cellular component disassembly;cellular componer
29.8986	29.7463	29.8073	29.8228	1:Experimental evidence at protein level	alcohol biosynthetic process;alcohol catabolic process;alcohol metabolic process;aldonic acid metabolic process;biosynthetic process;carbohydrate biosynthetic process;carbohydrate catabolic process;carbohydrate metab
27.0405	27.1257	26.9573	26.8	1:Experimental evidence at protein level	B cell activation;B cell activation involved in immune response;biological regulation;cell activation;cell activation involved in immune response;cell cycle process;cellular macromolecule metabolic process;cellular metabolic pr
27.4521	27.1151	27.6256	27.376	1:Experimental evidence at protein level	cellular macromolecule metabolic process;cellular metabolic process;cellular nitrogen compound metabolic process;cellular process;gene expression;macromolecule metabolic process;metabolic process;mRNA metabolic p
28.2973	28.3418	28.4822	28.2684	1:Experimental evidence at protein level	activation of immune response;activation of innate immune response;anaphase-promoting complex-dependent proteasomal ubiquitin-dependent protein catabolic process;antigen processing and presentation;antigen proces
31.1845	30.6788	30.672	30.6626	1:Experimental evidence at protein level	biosynthetic process;catabolic process;cellular biosynthetic process;cellular catabolic process;cellular component biogenesis;cellular component biogenesis at cellular level;cellular component disassembly;cellular componer
29.9366	29.7914	29.7768	29.7994	1:Experimental evidence at protein level	anatomical structure development;axon guidance;biological regulation;biosynthetic process;catabolic process;cell cycle checkpoint;cell cycle process;cellular biosynthetic process;cellular catabolic process;cellular compone
28.8089	28.6037	28.7028	28.6738	1:Experimental evidence at protein level	alcohol biosynthetic process;alcohol metabolic process;amine metabolic process;amino sugar biosynthetic process;amino sugar metabolic process;biological regulation;biosynthetic process;carbohydrate biosynthetic proces
27.6951	27.6139	27.4158	27.4343	1:Experimental evidence at protein level	actin cytoskeleton organization;actin filament severing;actin filament-based process;biological regulation;biosynthetic process;cellular biosynthetic process;cellular component organization;cellular component organization at
27.4012	26.9874	27.0709	26.9389	1:Experimental evidence at protein level	anatomical structure morphogenesis;assembly of spliceosomal tri-snRNP;biological regulation;cell morphogenesis;cell proliferation;cellular component assembly;cellular component assembly at cellular level;cellular compone
28.6146	28.3177	28.4363	28.2636	1:Experimental evidence at protein level	amine metabolic process;amino acid activation;biosynthetic process;bis(5'-nucleosidyl) oligophosphate biosynthetic process;bis(5'-nucleosidyl) oligophosphate metabolic process;carboxylic acid metabolic process;cellular a
30.521	30.764	30.7223	30.5662	1:Experimental evidence at protein level	apoptotic cell clearance;biological regulation;cell redox homeostasis;cellular component organization;cellular component organization or biogenesis;cellular homeostasis;cellular macromolecule metabolic process;cellular me
28.9143	29.1616	29.486	29.4208	1:Experimental evidence at protein level	ATP-dependent chromatin remodeling;biological regulation;biosynthetic process;cell proliferation;cellular biosynthetic process;cellular component assembly;cellular component assembly at cellular level;cellular component or
25.329	24.995	24.686	#NUM!	1:Experimental evidence at protein level	cellular macromolecule metabolic process;cellular metabolic process;cellular process;cellular protein metabolic process;macromolecule metabolic process;macromolecule modification;metabolic process;primary metabolic p
27.0886	26.6954	26.6426	26.6087	1:Experimental evidence at protein level	cellular macromolecule metabolic process;cellular metabolic process;cellular nitrogen compound metabolic process;cellular process;macromolecule metabolic process;metabolic process;nitrogen compound metabolic proce
26.9708	26.8087	26.7175	26.4583	1:Experimental evidence at protein level	
28.2071	28.2747	28.3669	28.3615	1:Experimental evidence at protein level	biological regulation;cellular macromolecule metabolic process;cellular metabolic process;cellular process;cellular protein metabolic process;macromolecule metabolic process;macromolecule modification;metabolic process
25.8679	25.554	25.5168	25.315	1:Experimental evidence at protein level	amine metabolic process;aminoglycan biosynthetic process;aminoglycan catabolic process;aminoglycan metabolic process;axon guidance;biological regulation;biosynthetic process;carbohydrate biosynthetic process;carbo
28.9847	28.8679	28.9959	29.0241	1:Experimental evidence at protein level	4-hydroxyproline metabolic process;amine metabolic process;carboxylic acid metabolic process;cellular amine metabolic process;cellular amino acid metabolic process;cellular ketone metabolic process;cellular macromolec
27.7136	27.487	27.6168	27.4744	1:Experimental evidence at protein level	acyl-CoA biosynthetic process;acyl-CoA metabolic process;acylglycerol biosynthetic process;acylglycerol metabolic process;anatomical structure development;biological regulation;biosynthetic process;carboxylic acid meta
28.9335	28.7164	28.6984	28.5025	1:Experimental evidence at protein level	biological regulation;biosynthetic process;cellular biosynthetic process;cellular macromolecule biosynthetic process;cellular macromolecule metabolic process;cellular metabolic process;cellular process;cellular protein metab
26.8548	26.9267	26.9802	26.7403	1:Experimental evidence at protein level	activation of caspase activity;apoptosis;apoptosis in response to endoplasmic reticulum stress;biological regulation;catabolic process;cell death;cell differentiation;cell redox homeostasis;cellular catabolic process;cellular co
31.2075	31.0425	#NUM!	31.3336	1:Experimental evidence at protein level	cellular component assembly;cellular component assembly at cellular level;cellular component organization;cellular component organization at cellular level;cellular component organization or biogenesis;cellular component c
30.2615	30.0714	30.1309	30.1964	1:Experimental evidence at protein level	biosynthetic process;catabolic process;cellular biosynthetic process;cellular catabolic process;cellular component disassembly;cellular component disassembly at cellular level;cellular component organization;cellular compo
25.5645	25.2384	25.1354	25.1037	1:Experimental evidence at protein level	3'-phosphoadenosine 5'-phosphosulfate metabolic process;anatomical structure development;anion homeostasis;anion transport;biological regulation;biomineral tissue development;bone remodeling;catabolic process;cellul
29.0737	29.2571	29.491	29.6743	1:Experimental evidence at protein level	activation of immune response;activation of innate immune response;anaphase-promoting complex-dependent proteasomal ubiquitin-dependent protein catabolic process;antigen processing and presentation;antigen proces
32.5741	32.6808	32.9827	32.8448	1:Experimental evidence at protein level	actin cytoskeleton organization;actin filament-based process;aging;antigen processing and presentation;antigen processing and presentation of exogenous antigen;antigen processing and presentation of exogenous peptide
31.1038	31.5307	31.3535	31.2993	1:Experimental evidence at protein level	catabolic process;cellular catabolic process;cellular lipid catabolic process;cellular lipid metabolic process;cellular metabolic process;cellular process;cellular response to chemical stimulus;cellular response to hydrogen pero
29.764	30.0916	29.8624	29.8324	1:Experimental evidence at protein level	apoptosis;biological regulation;catabolic process;cell cycle;cell cycle process;cell death;cell surface receptor linked signaling pathway;cellular catabolic process;cellular component assembly;cellular component organization;
29.1083	28.944	28.9793	28.939	1:Experimental evidence at protein level	activation of immune response;activation of innate immune response;anaphase-promoting complex-dependent proteasomal ubiquitin-dependent protein catabolic process;antigen processing and presentation;antigen proces
30.0161	29.6439	29.7326	29.5533	1:Experimental evidence at protein level	biological regulation;biosynthetic process;catabolic process;cellular biosynthetic process;cellular catabolic process;cellular component disassembly;cellular component disassembly at cellular level;cellular component organiz
27.9708	27.6705	27.7386	27.6628	1:Experimental evidence at protein level	anatomical structure development;androgen metabolic process;bile acid biosynthetic process;bile acid metabolic process;biological regulation;biosynthetic process;carboxylic acid biosynthetic process;carboxylic acid catab
29.1437	28.9786	28.9061	28.7867	1:Experimental evidence at protein level	cellular component organization;cellular component organization at cellular level;cellular component organization or biogenesis;cellular component organization or biogenesis at cellular level;cellular process;COPI coating of G
31.7887	32.4937	33.0673	32.7094	1:Experimental evidence at protein level	alcohol biosynthetic process;alcohol catabolic process;alcohol metabolic process;biosynthetic process;carbohydrate biosynthetic process;carbohydrate catabolic process;carbohydrate metabolic process;catabolic process;c
29.0782	28.9698	29.1247	28.8572	1:Experimental evidence at protein level	actin cytoskeleton organization;actin filament organization;actin filament-based process;actin nucleation;anatomical structure formation involved in morphogenesis;Arp2/3 complex-mediated actin nucleation;asymmetric cell
30.5077	30.2994	30.1011	30.1861	1:Experimental evidence at protein level	biosynthetic process;catabolic process;cellular biosynthetic process;cellular catabolic process;cellular component disassembly;cellular component disassembly at cellular level;cellular component organization;cellular compo
31.7482	31.3176	31.5692	31.485	1:Experimental evidence at protein level	activation-induced cell death of T cells;anatomical structure development;anatomical structure morphogenesis;apoptosis;biological regulation;biosynthetic process;carbohydrate homeostasis;catabolic process;cell activation
31.121	30.7167	30.9347	30.7465	1:Experimental evidence at protein level	anatomical structure morphogenesis;biosynthetic process;catabolic process;cellular biosynthetic process;cellular catabolic process;cellular component disassembly;cellular component disassembly at cellular level;cellular co
28.9771	28.9708	28.8478	28.838	1:Experimental evidence at protein level	antigen processing and presentation;antigen processing and presentation of exogenous antigen;antigen processing and presentation of exogenous peptide antigen;antigen processing and presentation of exogenous peptide
31.181	31.2224	31.0676	31.0737	1:Experimental evidence at protein level	anatomical structure development;apoptosis;biological regulation;biosynthetic process;C21-steroid hormone catabolic process;C21-steroid hormone metabolic process;catabolic process;cell death;cellular biosynthetic proce
32.2727	32.6662	32.6115	32.5695	1:Experimental evidence at protein level	anatomical structure development;anatomical structure homeostasis;biological regulation;biosynthetic process;catabolic process;cell killing;cell proliferation;cellular biosynthetic process;cellular catabolic process;cellular mac
29.4693	29.1223	29.5114	29.3202	1:Experimental evidence at protein level	biological regulation;biosynthetic process;cardiac muscle contraction;cellular biosynthetic process;cellular component assembly;cellular component assembly at cellular level;cellular component organization;cellular compone
27.9255	27.565	27.6608	27.6082	1:Experimental evidence at protein level	antigen processing and presentation;antigen processing and presentation of exogenous antigen;antigen processing and presentation of exogenous peptide antigen;antigen processing and presentation of exogenous peptide
28.3583	28.2421	28.1886	27.9767	1:Experimental evidence at protein level	aging;ameboidal cell migration;anatomical structure development;anatomical structure formation involved in morphogenesis;anatomical structure morphogenesis;axon ensheathment;biological adhesion;biological regulation;
26.2636	26.3787	26.5998	26.4333	1:Experimental evidence at protein level	biological regulation;biosynthetic process;cellular biosynthetic process;cellular macromolecule biosynthetic process;cellular macromolecule metabolic process;cellular metabolic process;cellular nitrogen compound metabolic
26.3476	26.0055	25.9707	26.1391	1:Experimental evidence at protein level	anatomical structure development;anatomical structure formation involved in morphogenesis;anatomical structure morphogenesis;apoptosis;apoptosis involved in morphogenesis;astrocyte cell migration;asymmetric protein l
29.6287	30.3349	30.5349	30.4051	1:Experimental evidence at protein level	biological regulation;establishment of localization;ion transport;positive regulation of biological process;positive regulation of cell communication;positive regulation of cellular process;positive regulation of response to stimulu
27.5539	27.2126	27.1036	27.007	1:Experimental evidence at protein level	cell cycle;cell cycle phase;cell cycle process;cellular component organization;cellular component organization at cellular level;cellular component organization or biogenesis;cellular component organization or biogenesis at ce
29.4725	29.322	29.2167	29.2677	1:Experimental evidence at protein level	antigen processing and presentation;antigen processing and presentation of exogenous antigen;antigen processing and presentation of exogenous peptide antigen;antigen processing and presentation of exogenous peptide
29.3914	29.6703	29.7946	29.6672	1:Experimental evidence at protein level	anatomical structure development;axon guidance;biological regulation;cellular component organization;cellular component organization at cellular level;cellular component organization or biogenesis;cellular component orgar
27.8547	28.0668	28.1031	28.0658	1:Experimental evidence at protein level	biological regulation;cellular component assembly;cellular component assembly at cellular level;cellular component organization;cellular component organization at cellular level;cellular component organization or biogenesis;
28.9039	28.8653	28.9068	28.7925	1:Experimental evidence at protein level	ATP-dependent chromatin remodeling;biological regulation;biosynthetic process;cell cycle phase;cell cycle process;cell division;cellular biosynthetic process;cellular component assembly;cellular component assembly at cell
30.155	30.2741	30.5134	30.3799	1:Experimental evidence at protein level	cellular process;establishment of localization;transport;vesicle-mediated transport
28.0671	27.8976	27.7533	27.6543	1:Experimental evidence at protein level	cell cycle;cellular component organization;cellular component organization at cellular level;cellular component organization or biogenesis;cellular component organization or biogenesis at cellular level;cellular membrane fusio
27.1753	26.8398	26.8885	26.6819	1:Experimental evidence at protein level	biological regulation;biosynthetic process;carbohydrate metabolic process;carbohydrate transport;cell cycle;cell cycle process;cell surface receptor linked signaling pathway;cellular biosynthetic process;cellular component c
27.558	27.4148	27.5697	27.3084	1:Experimental evidence at protein level	acyl-CoA biosynthetic process;acyl-CoA metabolic process;acylglycerol biosynthetic process;acylglycerol metabolic process;anatomical structure development;biological regulation;biosynthetic process;brain development;c
30.0292	30.3868	30.336	30.7655	1:Experimental evidence at protein level	actin filament-based movement;actin filament-based process;actin-mediated cell contraction;actin-myosin filament sliding;cellular component movement;cellular process;multicellular organismal process;muscle contraction;
29.0626	29.3656	29.3374	29.4604	1:Experimental evidence at protein level	acetyl-CoA catabolic process;acetyl-CoA metabolic process;anatomical structure homeostasis;biological regulation;carboxylic acid metabolic process;catabolic process;cellular catabolic process;cellular ketone metabolic pr
28.5121	28.318	28.5685	28.7147	1:Experimental evidence at protein level	biological regulation;cellular macromolecule metabolic process;cellular metabolic process;cellular nitrogen compound metabolic process;cellular process;gene expression;macromolecule metabolic process;metabolic proces
27.5851	27.5561	27.6273	27.5701	1:Experimental evidence at protein level	biological regulation;cell differentiation;cellular developmental process;cellular process;cellular response to stimulus;developmental process;epithelial cell differentiation;negative regulation of apoptosis;negative regulation of b
29.7357	29.261	29.4658	29.4914	1:Experimental evidence at protein level	anion transport;apoptosis;behavior;behavioral defense response;behavioral fear response;cell communication;cell death;cell differentiation;cell-cell signaling;cellular developmental process;cellular process;cognition;death;de
30.8803	30.4905	30.5116	30.5543	1:Experimental evidence at protein level	apoptosis;biological regulation;biosynthetic process;catabolic process;cell cycle phase;cell cycle process;cell death;cell division;cellular biosynthetic process;cellular catabolic process;cellular component disassembly;cellula
28.7147	28.8813	28.8385	28.7802	1:Experimental evidence at protein level	amine metabolic process;carboxylic acid metabolic process;cellular amine metabolic process;cellular amino acid metabolic process;cellular ketone metabolic process;cellular metabolic process;cellular modified amino acid m
31.0515	30.729	30.7545	30.7621	1:Experimental evidence at protein level	biosynthetic process;catabolic process;cellular biosynthetic process;cellular catabolic process;cellular component disassembly;cellular component disassembly at cellular level;cellular component organization;cellular compo
27.0211	27.2466	27.1056	27.0588	1:Experimental evidence at protein level	apoptotic mitochondrial changes;ATP metabolic process;biological regulation;cellular chemical homeostasis;cellular component organization;cellular component organization at cellular level;cellular component organization o
27.2311	27.228	27.3069	27.1434	1:Experimental evidence at protein level	anatomical structure development;anatomical structure formation involved in morphogenesis;anatomical structure morphogenesis;angiogenesis;axial mesoderm formation;axon guidance;biological adhesion;biological regulat
28.8923	28.7258	29.0663	29.0432	1:Experimental evidence at protein level	biological regulation;regulation of biological process;regulation of biosynthetic process;regulation of cellular biosynthetic process;regulation of cellular macromolecule biosynthetic process;regulation of cellular metabolic proc
28.2938	28.4267	28.3683	28.4093	1:Experimental evidence at protein level	biological regulation;catabolic process;cellular catabolic process;cellular macromolecule catabolic process;cellular macromolecule metabolic process;cellular metabolic process;cellular nitrogen compound metabolic process
30.5661	30.1478	30.4341	30.3568	1:Experimental evidence at protein level	biosynthetic process;catabolic process;cellular biosynthetic process;cellular catabolic process;cellular component biogenesis;cellular component biogenesis at cellular level;cellular component disassembly;cellular componer
29.8322	29.474	29.5431	29.4971	1:Experimental evidence at protein level	biosynthetic process;catabolic process;cellular biosynthetic process;cellular catabolic process;cellular component disassembly;cellular component disassembly at cellular level;cellular component organization;cellular compo
29.237	28.9876	29.0196	28.9711	1:Experimental evidence at protein level	activation of immune response;activation of innate immune response;anaphase-promoting complex-dependent proteasomal ubiquitin-dependent protein catabolic process;antigen processing and presentation;antigen proces
26.9846	26.9145	26.6538	26.7002	1:Experimental evidence at protein level	acetyl-CoA catabolic process;acetyl-CoA metabolic process;biological regulation;catabolic process;cation transport;cell redox homeostasis;cellular catabolic process;cellular homeostasis;cellular metabolic process;cellular p
30.0086	29.8865	29.9108	29.7812	1:Experimental evidence at protein level	biological regulation;cellular component assembly;cellular component assembly at cellular level;cellular component organization;cellular component organization at cellular level;cellular component organization or biogenesis;
30.6506	30.7326	30.9239	30.7771	1:Experimental evidence at protein level	biological regulation;biosynthetic process;catabolic process;cellular biosynthetic process;cellular catabolic process;cellular macromolecule biosynthetic process;cellular macromolecule catabolic process;cellular macromolec
28.3892	28.3821	28.5302	28.4383	1:Experimental evidence at protein level	biological regulation;cellular component assembly;cellular component assembly at cellular level;cellular component organization;cellular component organization at cellular level;cellular component organization or biogenesis;
28.9749	28.9658	28.8891	28.8369	1:Experimental evidence at protein level	alcohol metabolic process;biosynthetic process;carbohydrate biosynthetic process;carbohydrate metabolic process;carboxylic acid biosynthetic process;carboxylic acid metabolic process;cellular biosynthetic process;cellula
28.7113	28.9001	29.0374	28.9714	1:Experimental evidence at protein level	anatomical structure formation involved in morphogenesis;biological regulation;biosynthetic process;cell proliferation;cell redox homeostasis;cellular biosynthetic process;cellular homeostasis;cellular lipid metabolic process;
30.3167	30.3487	30.5895	30.77	1:Experimental evidence at protein level	biological regulation;biosynthetic process;cellular biosynthetic process;cellular macromolecule biosynthetic process;cellular macromolecule metabolic process;cellular metabolic process;cellular nitrogen compound metabolic
27.9722	27.8951	27.9759	27.7476	1:Experimental evidence at protein level	biological regulation;biosynthetic process;cellular biosynthetic process;cellular macromolecule biosynthetic process;cellular macromolecule metabolic process;cellular metabolic process;cellular process;cellular protein metab
27.2655	26.6101	27.0249	26.6707	1:Experimental evidence at protein level	biological regulation;biosynthetic process;carbohydrate metabolic process;carbohydrate transport;cell cycle;cell cycle process;cell surface receptor linked signaling pathway;cellular biosynthetic process;cellular component a
27.4584	27.9334	27.9757	28.1432	1:Experimental evidence at protein level	biological regulation;biosynthetic process;cellular biosynthetic process;cellular macromolecule biosynthetic process;cellular macromolecule metabolic process;cellular metabolic process;cellular nitrogen compound metabolic
27.3715	27.2366	27.1718	27.1253	1:Experimental evidence at protein level	anatomical structure development;cell differentiation;cellular developmental process;cellular macromolecule metabolic process;cellular metabolic process;cellular process;cellular protein metabolic process;chaperone-mediat
28.0172	28.0348	27.9496	27.8105	1:Experimental evidence at protein level	biological regulation;cell cycle;cell cycle phase;cell cycle process;cell division;cell junction assembly;cell junction organization;cell surface receptor linked signaling pathway;cell-substrate junction assembly;cellular componer
30.4952	30.1984	30.0439	30.1449	1:Experimental evidence at protein level	actin cytoskeleton organization;actin filament-based process;ameboidal cell migration;biological regulation;cell migration;cell motility;cellular component movement;cellular component organization;cellular component organiz
27.4587	27.2586	27.2365	27.1034	1:Experimental evidence at protein level	amine catabolic process;amine metabolic process;carboxylic acid catabolic process;carboxylic acid metabolic process;catabolic process;cellular amine metabolic process;cellular amino acid catabolic process;cellular amino

26.4551	26.3068	26.3976	26.3815	1:Experimental evidence at protein level	aging;anatomical structure formation involved in morphogenesis;apoptosis;axon cargo transport;axon transport of mitochondrion;biological regulation;cell aging;cell death;cellular component movement;cellular component o
26.9431	26.7851	26.973	26.4838	1:Experimental evidence at protein level	actin cytoskeleton organization;actin filament-based process;anatomical structure formation involved in morphogenesis;anatomical structure morphogenesis;axon guidance;biological regulation;cell part morphogenesis;cell p
27.525	26.7691	27.0076	27.1415	1:Experimental evidence at protein level	biological regulation;cell differentiation;cellular developmental process;cellular process;developmental process;osteoblast differentiation;regulation of apoptosis;regulation of biological process;regulation of cell aging;regulatio
27.9455	27.8662	27.8485	27.8249	1:Experimental evidence at protein level	ATP-dependent chromatin remodeling;biological regulation;cellular component organization;cellular component organization at cellular level;cellular component organization or biogenesis;cellular component organization or b
26.9833	26.8485	26.8414	26.8954	1:Experimental evidence at protein level	anatomical structure formation involved in morphogenesis;anatomical structure morphogenesis;angiogenesis;antigen processing and presentation;antigen processing and presentation of exogenous antigen;antigen processin
30.4245	30.5059	30.5797	30.4442	1:Experimental evidence at protein level	biological regulation;catabolic process;cellular catabolic process;cellular macromolecule catabolic process;cellular macromolecule metabolic process;cellular metabolic process;cellular nitrogen compound metabolic process
28.1588	28.1358	27.9619	27.9339	1:Experimental evidence at protein level	anatomical structure development;biosynthetic process;cell cycle;cell proliferation;cell proliferation in forebrain;cellular aromatic compound metabolic process;cellular biosynthetic process;cellular component assembly;cellula
30.0043	29.8718	29.9089	29.9061	1:Experimental evidence at protein level	alternative nuclear mRNA splicing, via spliceosome;biological regulation;cellular macromolecule metabolic process;cellular metabolic process;cellular nitrogen compound metabolic process;cellular process;gene expression;r
29.0765	29.5436	29.5461	29.616	1:Experimental evidence at protein level	anatomical structure development;anatomical structure morphogenesis;aromatic compound biosynthetic process;biosynthetic process;brainstem development;cellular aromatic compound metabolic process;cellular biosynth
27.5165	27.3175	27.4457	27.3613	1:Experimental evidence at protein level	alcohol biosynthetic process;alcohol catabolic process;alcohol metabolic process;alditol catabolic process;alditol metabolic process;anatomical structure development;biosynthetic process;camera-type eye development;car
28.0729	27.0736	27.6425	27.598	1:Experimental evidence at protein level	anatomical structure development;anatomical structure morphogenesis;biological regulation;brain development;camera-type eye development;cell cycle process;cell part morphogenesis;cell projection morphogenesis;cell pr
29.6451	29.6474	29.7002	29.6541	1:Experimental evidence at protein level	biosynthetic process;cellular biosynthetic process;cellular component organization;cellular component organization at cellular level;cellular component organization or biogenesis;cellular component organization or biogenesis
27.0254	26.4714	26.5392	26.7875	1:Experimental evidence at protein level	alcohol catabolic process;alcohol metabolic process;apoptotic mitochondrial changes;biological regulation;body fluid secretion;carbohydrate catabolic process;carbohydrate homeostasis;carbohydrate metabolic process;car
30.7521	30.8737	30.9125	30.8765	1:Experimental evidence at protein level	anatomical structure development;apoptosis;biological regulation;biosynthetic process;cell cycle;cell cycle process;cell death;cell migration;cell motility;cell surface receptor linked signaling pathway;cellular biosynthetic proc
30.0361	29.4526	29.5967	29.833	1:Experimental evidence at protein level	biosynthetic process;catabolic process;cellular biosynthetic process;cellular catabolic process;cellular component disassembly;cellular component disassembly at cellular level;cellular component organization;cellular compo
28.3464	28.2058	28.1084	28.0962	1:Experimental evidence at protein level	biological regulation;biosynthetic process;cellular biosynthetic process;cellular component organization;cellular component organization at cellular level;cellular component organization or biogenesis;cellular component orga
27.3139	27.4686	27.4456	27.3151	1:Experimental evidence at protein level	actin filament-based movement;actin filament-based process;anatomical structure development;biological regulation;cell development;cell differentiation;cell surface receptor linked signaling pathway;cellular component mov
26.4732	26.1695	26.18	26.057	1:Experimental evidence at protein level	cellular component organization;cellular component organization at cellular level;cellular component organization or biogenesis;cellular component organization or biogenesis at cellular level;cellular process;Golgi organization
26.7244	26.629	26.4516	26.5024	1:Experimental evidence at protein level	biological regulation;cell cycle;cell cycle process;cell division;cellular component organization;cellular component organization at cellular level;cellular component organization or biogenesis;cellular component organization or
28.5689	28.3936	28.1924	28.2736	1:Experimental evidence at protein level	biological regulation;cellular component assembly;cellular component assembly at cellular level;cellular component organization;cellular component organization at cellular level;cellular component organization or biogenesis;
26.7802	26.9386	26.6695	26.7795	1:Experimental evidence at protein level	
28.4163	28.1307	28.132	27.9503	1:Experimental evidence at protein level	cellular component organization;cellular component organization at cellular level;cellular component organization or biogenesis;cellular component organization or biogenesis at cellular level;cellular process;extracellular matr
27.1469	26.9053	27.0101	27.0438	1:Experimental evidence at protein level	biological regulation;biosynthetic process;cellular biosynthetic process;cellular macromolecule biosynthetic process;cellular macromolecule metabolic process;cellular metabolic process;cellular nitrogen compound metabolic
30.7915	30.9732	30.8396	30.7486	1:Experimental evidence at protein level	cellular component assembly;cellular component assembly at cellular level;cellular component organization;cellular component organization at cellular level;cellular component organization or biogenesis;cellular component o
26.9194	26.728	26.6405	26.5064	1:Experimental evidence at protein level	anatomical structure morphogenesis;biological regulation;biosynthetic process;cellular biosynthetic process;cellular component organization;cellular component organization at cellular level;cellular component organization o
25.7327	25.4177	25.5548	25.5429	1:Experimental evidence at protein level	biological adhesion;biological regulation;cell adhesion;cellular component organization;cellular component organization at cellular level;cellular component organization or biogenesis;cellular component organization or bioger
28.392	28.3155	28.1927	28.1679	1:Experimental evidence at protein level	biological regulation;cell cycle;cell surface receptor linked signaling pathway;cellular localization;cellular macromolecule localization;cellular process;cellular protein localization;cellular response to stimulus;enzyme linked rece
28.3834	28.1714	28.145	28.0565	1:Experimental evidence at protein level	activation of immune response;activation of innate immune response;anaphase-promoting complex-dependent proteasomal ubiquitin-dependent protein catabolic process;antigen processing and presentation;antigen proces
29.0504	29.084	29.0891	28.9291	1:Experimental evidence at protein level	alcohol metabolic process;amine metabolic process;aminoglycan biosynthetic process;aminoglycan metabolic process;anatomical structure morphogenesis;biosynthetic process;carbohydrate biosynthetic process;carbohyd
29.1047	29.381	29.2694	29.2061	1:Experimental evidence at protein level	cellular macromolecule metabolic process;cellular metabolic process;cellular nitrogen compound metabolic process;cellular process;histone mRNA metabolic process;macromolecule metabolic process;macromolecule modi
27.9073	28.1104	27.8236	27.9686	1:Experimental evidence at protein level	alkene biosynthetic process;arachidonic acid metabolic process;biosynthetic process;carboxylic acid biosynthetic process;carboxylic acid metabolic process;catabolic process;cellular alkene metabolic process;cellular biosy
27.8342	27.1382	27.2241	27.1316	1:Experimental evidence at protein level	apoptotic chromosome condensation;biological regulation;cell cycle;cell cycle process;cell differentiation;cell division;cellular component organization;cellular component organization at cellular level;cellular component orga
28.945	28.4226	28.6459	28.5801	1:Experimental evidence at protein level	axon guidance;biological adhesion;cell adhesion;cell differentiation;cellular component assembly;cellular component disassembly;cellular component disassembly at cellular level;cellular component organization;cellular com
31.4316	31.5025	32.1784	31.9405	1:Experimental evidence at protein level	anatomical structure development;anatomical structure morphogenesis;axonogenesis;biological regulation;brain development;cell cycle process;cell part morphogenesis;cell projection morphogenesis;cell projection organiza
29.2225	29.5068	29.6587	29.4932	1:Experimental evidence at protein level	activation of immune response;activation of innate immune response;anaphase-promoting complex-dependent proteasomal ubiquitin-dependent protein catabolic process;anatomical structure development;antigen processi
31.9894	32.1856	31.8449	31.926	1:Experimental evidence at protein level	anatomical structure development;apoptosis;biological regulation;biosynthetic process;cell death;cellular biosynthetic process;cellular component organization;cellular component organization or biogenesis;cellular macromo
31.8204	31.6495	31.6932	31.8208	1:Experimental evidence at protein level	biological regulation;biosynthetic process;catabolic process;cell chemotaxis;cell differentiation;cell migration;cell motility;cellular biosynthetic process;cellular catabolic process;cellular component assembly;cellular compone
29.1017	29.0158	28.9686	28.9102	1:Experimental evidence at protein level	anatomical structure homeostasis;base-excision repair;behavior;biological regulation;biosynthetic process;catabolic process;cell cycle;cellular biosynthetic process;cellular catabolic process;cellular component organization;
27.9987	28.0437	27.88	27.9044	1:Experimental evidence at protein level	amine metabolic process;amino acid activation;biosynthetic process;carboxylic acid metabolic process;cellular amine metabolic process;cellular amino acid metabolic process;cellular biosynthetic process;cellular ketone me
28.8458	28.9737	28.819	28.938	1:Experimental evidence at protein level	amine metabolic process;biosynthetic process;carboxylic acid metabolic process;cellular amine metabolic process;cellular amino acid metabolic process;cellular aromatic compound metabolic process;cellular biosynthetic p
31.6129	31.5301	31.5097	31.3723	1:Experimental evidence at protein level	anatomical structure development;biological regulation;biosynthetic process;cell development;cellular component organization;cellular component organization at cellular level;cellular component organization or biogenesis;
29.9568	29.5918	29.656	29.8621	1:Experimental evidence at protein level	cellular macromolecule metabolic process;cellular metabolic process;cellular nitrogen compound metabolic process;cellular process;gene expression;macromolecule metabolic process;metabolic process;mRNA metabolic p
28.091	27.8312	27.7905	27.7146	1:Experimental evidence at protein level	amine biosynthetic process;amine metabolic process;biosynthetic process;carboxylic acid biosynthetic process;carboxylic acid metabolic process;cellular amine metabolic process;cellular amino acid biosynthetic process;ce
29.4601	29.3588	29.3707	29.1592	1:Experimental evidence at protein level	actin cytoskeleton organization;actin filament organization;actin filament-based process;actin nucleation;anatomical structure morphogenesis;Arp2/3 complex-mediated actin nucleation;asymmetric cell division;axon guidanc
27.6988	27.6158	27.7961	27.7611	1:Experimental evidence at protein level	anatomical structure formation involved in morphogenesis;apoptosis;biological regulation;blastocyst formation;carbohydrate homeostasis;cell death;cell junction assembly;cell junction organization;cell-cell junction assembly
28.781	28.6464	28.8904	28.5044	1:Experimental evidence at protein level	anatomical structure morphogenesis;biological regulation;cell cycle phase;cell cycle process;cell part morphogenesis;cell projection morphogenesis;cell projection organization;cellular component assembly;cellular compone
26.8098	26.5295	26.5033	26.5391	1:Experimental evidence at protein level	
27.6166	27.3821	27.2347	27.372	1:Experimental evidence at protein level	
28.6099	28.6958	28.4205	28.6046	1:Experimental evidence at protein level	actin cytoskeleton organization;actin filament organization;actin filament-based process;anatomical structure morphogenesis;biological regulation;cell cycle phase;cell cycle process;cell division;cell morphogenesis;cell surfac
28.2549	28.2328	28.1327	27.9367	1:Experimental evidence at protein level	cellular process;endosome transport;establishment of localization;establishment of localization in cell;establishment of protein localization;intracellular protein transport;intracellular transport;protein transport;retrograde transp
28.8598	28.6311	28.6442	28.6222	1:Experimental evidence at protein level	establishment of localization;lipid transport;organic substance transport;transport
26.6391	26.3848	26.6394	26.7661	1:Experimental evidence at protein level	
26.8067	24.5967	24.819	24.4265	1:Experimental evidence at protein level	actin cytoskeleton organization;actin filament-based process;actomyosin structure organization;cell differentiation;cell differentiation involved in embryonic placenta development;cellular component organization;cellular comp
30.9437	30.8627	31.2546	31.0282	1:Experimental evidence at protein level	apoptosis;base-excision repair, DNA ligation;biological regulation;catabolic process;cell activation;cell chemotaxis;cell death;cell migration;cell motility;cell projection organization;cellular catabolic process;cellular component
27.9637	28.6188	28.1728	28.4191	1:Experimental evidence at protein level	aging;alcohol biosynthetic process;alcohol metabolic process;anatomical structure morphogenesis;apoptosis;biosynthetic process;carbohydrate biosynthetic process;carbohydrate metabolic process;cell death;cell differentia
27.0705	29.3635	29.9535	29.1681	1:Experimental evidence at protein level	cell differentiation;cellular developmental process;cellular process;cellular response to calcium ion;cellular response to chemical stimulus;cellular response to inorganic substance;cellular response to metal ion;cellular respons
28.212	28.0666	27.6936	27.7715	1:Experimental evidence at protein level	biological regulation;blood coagulation;cellular process;coagulation;establishment of localization;hemostasis;ion transmembrane transport;ion transport;multicellular organismal process;regulation of biological quality;regulatio
25.9245	25.9878	25.8498	25.824	1:Experimental evidence at protein level	anatomical structure development;biological adhesion;biological regulation;blood coagulation;brown fat cell differentiation;cell adhesion;cell adhesion mediated by integrin;cell communication;cell differentiation;cell junction a
31.1329	31.0188	31.0043	31.1905	1:Experimental evidence at protein level	biosynthetic process;catabolic process;cellular biosynthetic process;cellular catabolic process;cellular component disassembly;cellular component disassembly at cellular level;cellular component organization;cellular compo
27.0874	26.8068	26.6935	26.6212	1:Experimental evidence at protein level	biological regulation;biosynthetic process;cellular biosynthetic process;cellular macromolecule biosynthetic process;cellular macromolecule metabolic process;cellular metabolic process;cellular nitrogen compound metabolic
30.3438	30.54	30.4289	30.448	1:Experimental evidence at protein level	activation of MAPKK activity;activation of protein kinase activity;apoptosis;axon guidance;biological regulation;biosynthetic process;cell death;cell surface receptor linked signaling pathway;cellular biosynthetic process;cellula
28.5931	28.3601	28.2914	28.3279	1:Experimental evidence at protein level	biosynthetic process;carboxylic acid catabolic process;carboxylic acid metabolic process;cardiolipin acyl-chain remodeling;cardiolipin metabolic process;catabolic process;cellular biosynthetic process;cellular catabolic proc
28.812	28.7846	28.7608	28.5714	1:Experimental evidence at protein level	2-oxoglutarate metabolic process;acetyl-CoA catabolic process;acetyl-CoA metabolic process;alcohol catabolic process;alcohol metabolic process;amine catabolic process;amine metabolic process;anatomical structure de
27.6176	27.351	27.4299	27.3943	1:Experimental evidence at protein level	biological regulation;biosynthetic process;carbohydrate metabolic process;carbohydrate transport;cell cycle;cell cycle process;cell surface receptor linked signaling pathway;cellular biosynthetic process;cellular component a
28.0468	28.009	28.1447	28.1443	1:Experimental evidence at protein level	cellular component organization;cellular component organization at cellular level;cellular component organization or biogenesis;cellular component organization or biogenesis at cellular level;cellular process;cytoskeleton orga
28.1837	28.2182	28.2386	28.2107	1:Experimental evidence at protein level	acetyl-CoA catabolic process;acetyl-CoA metabolic process;carboxylic acid metabolic process;catabolic process;cellular catabolic process;cellular ketone metabolic process;cellular metabolic process;cellular process;citrate
28.1305	28.0223	28.0038	27.8892	1:Experimental evidence at protein level	biological regulation;blood coagulation;cell projection organization;cellular component assembly;cellular component organization;cellular component organization at cellular level;cellular component organization or biogenesis
26.9542	26.9391	27.048	27.0525	1:Experimental evidence at protein level	biological regulation;negative regulation of biological process;negative regulation of cell proliferation;negative regulation of cellular process;regulation of biological process;regulation of cell proliferation;regulation of cellular pr
29.1446	29.0691	28.9712	28.8517	1:Experimental evidence at protein level	cellular component movement;cellular component organization;cellular component organization at cellular level;cellular component organization or biogenesis;cellular component organization or biogenesis at cellular level;cel
28.4789	28.2956	28.4022	28.3121	1:Experimental evidence at protein level	biological regulation;cellular process;cellular response to stimulus;defense response;establishment of localization;establishment of localization in cell;establishment of protein localization;immune response;immune system pro
31.9193	32.4311	32.3151	32.3994	1:Experimental evidence at protein level	alcohol biosynthetic process;alcohol catabolic process;alcohol metabolic process;biological regulation;biosynthetic process;carbohydrate biosynthetic process;carbohydrate catabolic process;carbohydrate metabolic proces
30.6784	30.8563	30.7166	30.5893	1:Experimental evidence at protein level	aging;antigen processing and presentation;antigen processing and presentation of exogenous antigen;antigen processing and presentation of exogenous peptide antigen;antigen processing and presentation of exogenous pe
25.8386	28.1155	27.7557	27.1657	1:Experimental evidence at protein level	anatomical structure development;cellular component organization;cellular component organization at cellular level;cellular component organization or biogenesis;cellular component organization or biogenesis at cellular level
30.0657	30.3887	30.778	30.6793	1:Experimental evidence at protein level	acetyl-CoA catabolic process;acetyl-CoA metabolic process;alcohol biosynthetic process;alcohol metabolic process;biosynthetic process;carbohydrate biosynthetic process;carbohydrate metabolic process;carboxylic acid r
28.3566	28.5685	28.4071	28.4383	1:Experimental evidence at protein level	biological regulation;catabolic process;cellular catabolic process;cellular macromolecule catabolic process;cellular macromolecule metabolic process;cellular metabolic process;cellular process;cellular protein metabolic proc
29.5488	29.7252	29.7407	29.7752	1:Experimental evidence at protein level	anatomical structure development;androgen receptor signaling pathway;biological regulation;cation transport;cellular component assembly;cellular component assembly at cellular level;cellular component organization;cellula
31.9385	31.8376	31.7386	31.8184	1:Experimental evidence at protein level	biological regulation;biosynthetic process;catabolic process;cellular biosynthetic process;cellular catabolic process;cellular component disassembly;cellular component disassembly at cellular level;cellular component organiz
28.0634	28.1026	28.1486	27.9063	1:Experimental evidence at protein level	alcohol metabolic process;biosynthetic process;carbohydrate biosynthetic process;carbohydrate metabolic process;cellular biosynthetic process;cellular carbohydrate biosynthetic process;cellular carbohydrate metabolic pro
28.7249	28.4834	28.3605	28.5989	1:Experimental evidence at protein level	activation of store-operated calcium channel activity;anatomical structure development;anatomical structure morphogenesis;appendage morphogenesis;biological regulation;calcium ion transmembrane transport;calcium ion
28.571	28.55	28.3726	28.341	1:Experimental evidence at protein level	activation of immune response;activation of innate immune response;anatomical structure development;autophagy;biological regulation;catabolic process;cell communication;cell differentiation;cell proliferation;cellular catabo
26.6649	26.8341	26.9257	26.7765	1:Experimental evidence at protein level	biological regulation;macromolecule metabolic process;metabolic process;positive regulation of catalytic activity;positive regulation of molecular function;primary metabolic process;protein metabolic process;proteolysis;regu
27.3126	27.0425	27.3014	26.9949	1:Experimental evidence at protein level	biological regulation;cell proliferation;cellular process;cellular response to stimulus;intracellular signal transduction;positive regulation of autophagy;positive regulation of biological process;positive regulation of biosynthetic p
27.5547	27.4777	27.435	27.3737	1:Experimental evidence at protein level	biological regulation;defense response;defense response to virus;immune effector process;immune response;immune system process;innate immune response;multi-organism process;negative regulation of biological proces
27.7952	27.6218	27.774	27.8149	1:Experimental evidence at protein level	biological regulation;cellular macromolecule metabolic process;cellular metabolic process;cellular process;cellular protein metabolic process;macromolecule metabolic process;macromolecule modification;metabolic process
29.001	28.8083	28.8605	28.8027	1:Experimental evidence at protein level	biological regulation;biosynthetic process;catabolic process;cellular biosynthetic process;cellular catabolic process;cellular macromolecule biosynthetic process;cellular macromolecule catabolic process;cellular macromolec
28.0216	28.373	28.185	28.1708	1:Experimental evidence at protein level	alkene biosynthetic process;biosynthetic process;carboxylic acid biosynthetic process;carboxylic acid metabolic process;cellular alkene metabolic process;cellular biosynthetic process;cellular ketone metabolic process;cellu
29.316	29.3846	29.3509	29.3117	1:Experimental evidence at protein level	biological regulation;cell cycle cytokinesis;cell cycle phase;cell cycle process;cell differentiation;cellular component assembly;cellular component assembly at cellular level;cellular component organization;cellular component
26.8943	26.948	26.8873	27.201	1:Experimental evidence at protein level	anatomical structure development;cellular component organization;cellular component organization at cellular level;cellular component organization or biogenesis;cellular component organization or biogenesis at cellular level
29.0229	28.9119	28.7754	28.8391	1:Experimental evidence at protein level	antigen processing and presentation;antigen processing and presentation of exogenous antigen;antigen processing and presentation of exogenous peptide antigen;antigen processing and presentation of exogenous peptide
29.0413	29.1275	28.9996	28.9067	1:Experimental evidence at protein level	anatomical structure development;bone development;developmental process
27.804	28.1243	28.0832	27.9978	1:Experimental evidence at protein level	acetyl-CoA catabolic process;acetyl-CoA metabolic process;anatomical structure development;carboxylic acid metabolic process;catabolic process;cellular catabolic process;cellular ketone metabolic process;cellular metab
29.1082	29.2562	29.1829	29.1397	1:Experimental evidence at protein level	amine biosynthetic process;amine metabolic process;biological regulation;biosynthetic process;carboxylic acid biosynthetic process;carboxylic acid metabolic process;cellular amine metabolic process;cellular amino acid bio
28.7321	28.7431	28.7217	28.6733	1:Experimental evidence at protein level	biosynthetic process;cellular biosynthetic process;cellular macromolecule biosynthetic process;cellular macromolecule metabolic process;cellular metabolic process;cellular process;cellular protein metabolic process;establis
25.0094	25.1394	24.8823	24.6026	1:Experimental evidence at protein level	anatomical structure development;biological regulation;biosynthetic process;catabolic process;cell communication;cell cycle arrest;cell cycle process;cell development;cell growth;cell projection organization;cell surface rece
30.9153	30.5452	30.4297	30.4712	1:Experimental evidence at protein level	biological regulation;biosynthetic process;catabolic process;cellular biosynthetic process;cellular catabolic process;cellular component disassembly;cellular component disassembly at cellular level;cellular component organiz
33.1871	33.4981	33.3664	33.2881	1:Experimental evidence at protein level	amine transport;apoptosis;biological regulation;biosynthetic process;blood coagulation;cell activation;cell death;cellular biosynthetic process;cellular component assembly;cellular component assembly at cellular level;cellula
28.3341	28.0663	28.0983	27.8496	1:Experimental evidence at protein level	biological regulation;cell cycle arrest;cell cycle process;cell death;cell surface receptor linked signaling pathway;cellular process;cellular response to chemical stimulus;cellular response to cytokine stimulus;cellular response t
28.5525	28.1098	28.0004	27.992	1:Experimental evidence at protein level	biological adhesion;biological regulation;cell adhesion;cell cycle arrest;cell cycle process;cell junction assembly;cell junction organization;cell motility;cell surface receptor linked signaling pathway;cell-substrate junction asse
28.9946	28.7178	29.0641	28.8951	1:Experimental evidence at protein level	cellular macromolecule metabolic process;cellular metabolic process;cellular nitrogen compound metabolic process;cellular process;gene expression;macromolecule metabolic process;metabolic process;mRNA metabolic p
28.3172	28.0243	28.1367	27.9752	1:Experimental evidence at protein level	biological regulation;biosynthetic process;cell cycle;cellular biosynthetic process;cellular component organization;cellular component organization at cellular level;cellular component organization or biogenesis;cellular compo
29.8305	29.5678	29.6486	29.5841	1:Experimental evidence at protein level	anatomical structure development;anatomical structure morphogenesis;biological regulation;biosynthetic process;branching involved in mammary gland duct morphogenesis;branching morphogenesis of a tube;cellular biosy
27.9068	27.7034	27.7366	27.633	1:Experimental evidence at protein level	apoptosis;biological regulation;biosynthetic process;cell death;cellular biosynthetic process;cellular macromolecule biosynthetic process;cellular macromolecule metabolic process;cellular metabolic process;cellular nitrogen
30.2099	30.082	30.437	30.1947	1:Experimental evidence at protein level	biological regulation;biosynthetic process;cell cycle arrest;cell cycle process;cell proliferation;cellular biosynthetic process;cellular macromolecule biosynthetic process;cellular macromolecule metabolic process;cellular meta
28.4149	28.5237	28.3605	28.1864	1:Experimental evidence at protein level	androgen receptor signaling pathway;biological regulation;biosynthetic process;catabolic process;cellular biosynthetic process;cellular catabolic process;cellular macromolecule catabolic process;cellular macromolecule meta
29.089	29.5237	29.0361	29.2467	1:Experimental evidence at protein level	amine biosynthetic process;amine metabolic process;anatomical structure development;aspartate family amino acid metabolic process;biological regulation;biosynthetic process;brain development;carboxylic acid biosynthet
29.6866	29.5686	29.5959	29.5336	1:Experimental evidence at protein level	cellular macromolecule metabolic process;cellular metabolic process;cellular nitrogen compound metabolic process;cellular process;gene expression;macromolecule metabolic process;metabolic process;mRNA metabolic p
26.6658	26.6507	26.6224	26.6824	1:Experimental evidence at protein level	cellular component organization;cellular component organization at cellular level;cellular component organization or biogenesis;cellular component organization or biogenesis at cellular level;cellular localization;cellular proces
28.1125	28.0052	28.1061	28.1636	1:Experimental evidence at protein level	anatomical structure development;biological regulation;catabolic process;cellular catabolic process;cellular macromolecule catabolic process;cellular macromolecule metabolic process;cellular metabolic process;cellular nitro
32.6332	33.0135	33.0984	33.2222	1:Experimental evidence at protein level	aging;ATP-dependent chromatin remodeling;biological regulation;cell aging;cell cycle process;cellular component assembly;cellular component assembly at cellular level;cellular component organization;cellular component o
28.5959	28.4819	28.5118	28.4338	1:Experimental evidence at protein level	biological regulation;catabolic process;cellular catabolic process;cellular component disassembly;cellular component disassembly at cellular level;cellular component organization;cellular component organization at cellular le
28.1352	27.7861	28.147	27.8193	1:Experimental evidence at protein level	axon guidance;biological adhesion;cell adhesion;cellular component assembly;cellular component disassembly;cellular component disassembly at cellular level;cellular component organization;cellular component organizatio
29.5885	29.4341	29.7584	29.647	1:Experimental evidence at protein level	anatomical structure development;biosynthetic process;cell activation;cell proliferation;cellular aromatic compound metabolic process;cellular biosynthetic process;cellular component assembly;cellular component organizati
27.7409	27.7416	27.7303	27.7185	1:Experimental evidence at protein level	acetyl-CoA catabolic process;acetyl-CoA metabolic process;biological regulation;carboxylic acid metabolic process;catabolic process;cation homeostasis;cellular catabolic process;cellular cation homeostasis;cellular chemic
29.2574	29.3435	29.2162	29.2405	1:Experimental evidence at protein level	biosynthetic process;cellular aromatic compound metabolic process;cellular biosynthetic process;cellular metabolic process;cellular nitrogen compound biosynthetic process;cellular nitrogen compound metabolic process;ce
30.7003	30.7134	30.6613	30.5155	1:Experimental evidence at protein level	biosynthetic process;cellular biosynthetic process;cellular macromolecule biosynthetic process;cellular macromolecule metabolic process;cellular metabolic process;cellular process;cellular protein metabolic process;gene ex
29.9305	29.6411	29.9222	29.7171	1:Experimental evidence at protein level	biological regulation;cellular macromolecule metabolic process;cellular metabolic process;cellular nitrogen compound metabolic process;cellular process;gene expression;macromolecule metabolic process;metabolic proces
26.7054	26.9245	26.9444	27.0514	1:Experimental evidence at protein level	alcohol biosynthetic process;alcohol catabolic process;alcohol metabolic process;biosynthetic process;carbohydrate biosynthetic process;carbohydrate catabolic process;carbohydrate metabolic process;catabolic process;
28.2141	28.0818	27.9953	27.9475	1:Experimental evidence at protein level	amine metabolic process;amino acid activation;arginyl-tRNA aminoacylation;carboxylic acid metabolic process;cellular amine metabolic process;cellular amino acid metabolic process;cellular ketone metabolic process;cellula
30.5197	30.2275	30.4169	30.5036	1:Experimental evidence at protein level	biosynthetic process;catabolic process;cellular biosynthetic process;cellular catabolic process;cellular component disassembly;cellular component disassembly at cellular level;cellular component organization;cellular compo
28.5775	28.8678	28.7839	28.7469	1:Experimental evidence at protein level	axon guidance;cell cycle phase;cell cycle process;cellular component assembly;cellular component assembly at cellular level;cellular component organization;cellular component organization at cellular level;cellular compone
29.9639	30.0562	30.0961	29.9611	1:Experimental evidence at protein level	cellular component assembly;cellular component assembly at cellular level;cellular component organization;cellular component organization at cellular level;cellular component organization or biogenesis;cellular component o
27.1037	26.8921	26.9396	26.8599	1:Experimental evidence at protein level	cellular component assembly;cellular component assembly at cellular level;cellular component organization;cellular component organization at cellular level;cellular component organization or biogenesis;cellular component o
27.8008	27.7515	27.6918	27.7567	1:Experimental evidence at protein level	anatomical structure formation involved in morphogenesis;angiogenesis;biological regulation;biosynthetic process;cell differentiation;cellular biosynthetic process;cellular developmental process;cellular macromolecule biosyn
25.9867	25.5648	25.8236	25.6631	1:Experimental evidence at protein level	actin filament-based movement;actin filament-based process;anatomical structure development;anatomical structure morphogenesis;auditory receptor cell differentiation;behavior;biological regulation;cell communication;cel
31.5575	31.7566	31.7066	31.6546	1:Experimental evidence at protein level	anatomical structure formation involved in morphogenesis;angiogenesis;ATP biosynthetic process;ATP hydrolysis coupled proton transport;ATP metabolic process;ATP synthesis coupled proton transport;biological regulation
31.5289	30.8108	31.5296	31.2079	1:Experimental evidence at protein level	3'-UTR-mediated mRNA stabilization;ATP-dependent chromatin remodeling;biological regulation;cell differentiation;cellular component organization;cellular component organization at cellular level;cellular component organiz
30.9013	30.7207	31.3093	31.1198	1:Experimental evidence at protein level	actin cytoskeleton organization;actin filament-based movement;actin filament-based process;actin-mediated cell contraction;actin-myosin filament sliding;actomyosin structure organization;anatomical structure morphogene
27.6377	27.385	27.5104	27.4771	1:Experimental evidence at protein level	amine metabolic process;amino acid activation;biosynthetic process;carboxylic acid metabolic process;cellular amine metabolic process;cellular amino acid metabolic process;cellular biosynthetic process;cellular componen
30.1249	29.6623	29.9265	29.926	1:Experimental evidence at protein level	cellular macromolecule metabolic process;cellular metabolic process;cellular nitrogen compound metabolic process;cellular process;gene expression;macromolecule metabolic process;metabolic process;mRNA metabolic p

26.4268	26.4114	26.3675	26.1553	1:Experimental evidence at protein level	anatomical structure development;anatomical structure morphogenesis;axon guidance;biological adhesion;biological regulation;blood coagulation;cell adhesion;cell adhesion mediated by integrin;cell differentiation;cell migra
26.2487	28.3351	28.6999	28.1389	1:Experimental evidence at protein level	anatomical structure development;cell activation;cell migration;cell motility;cell proliferation;cellular component movement;cellular process;developmental process;epidermis development;keratinization;keratinocyte activation
30.4246	30.3105	30.2022	30.3273	1:Experimental evidence at protein level	binding of sperm to zona pellucida;cell recognition;cell-cell recognition;cellular macromolecule metabolic process;cellular metabolic process;cellular process;cellular process involved in reproduction;cellular protein metabolic
31.0814	30.6698	30.721	30.7443	1:Experimental evidence at protein level	biological regulation;biosynthetic process;catabolic process;cellular biosynthetic process;cellular catabolic process;cellular component disassembly;cellular component disassembly at cellular level;cellular component organiz
30.9824	30.9382	31.0947	31.0138	1:Experimental evidence at protein level	biological regulation;cellular macromolecule metabolic process;cellular metabolic process;cellular nitrogen compound metabolic process;cellular process;cellular response to stimulus;gene expression;macromolecule metabo
32.205	32.4619	32.3328	32.1981	1:Experimental evidence at protein level	apoptosis;biological regulation;biosynthetic process;cell cycle;cell cycle process;cell death;cellular biosynthetic process;cellular component organization;cellular component organization at cellular level;cellular component org
34.003	33.6998	33.7575	33.7642	1:Experimental evidence at protein level	actin cytoskeleton organization;actin filament organization;actin filament-based movement;actin filament-based process;actin-mediated cell contraction;actin-myosin filament sliding;actomyosin structure organization;anatom
27.8236	27.5389	27.6501	27.5445	1:Experimental evidence at protein level	anatomical structure development;antigen processing and presentation;antigen processing and presentation of exogenous antigen;antigen processing and presentation of exogenous peptide antigen;antigen processing and p
27.4689	27.4013	27.2879	27.1818	1:Experimental evidence at protein level	catabolic process;cellular catabolic process;cellular macromolecule catabolic process;cellular macromolecule metabolic process;cellular metabolic process;cellular process;ER-associated protein catabolic process;macromo
28.3436	28.5011	28.3548	28.5319	1:Experimental evidence at protein level	biological regulation;biosynthetic process;cellular biosynthetic process;cellular component assembly;cellular component assembly at cellular level;cellular component organization;cellular component organization at cellular le
28.5776	28.2507	28.5251	28.5482	1:Experimental evidence at protein level	biological regulation;biosynthetic process;cellular biosynthetic process;cellular macromolecule biosynthetic process;cellular macromolecule metabolic process;cellular metabolic process;cellular nitrogen compound metabolic
28.0676	27.9482	28.0269	28.1194	1:Experimental evidence at protein level	biological regulation;biosynthetic process;cell proliferation;cellular biosynthetic process;cellular macromolecule biosynthetic process;cellular macromolecule metabolic process;cellular metabolic process;cellular nitrogen com
27.1299	27.0351	26.9478	27.0336	1:Experimental evidence at protein level	anatomical structure morphogenesis;apoptosis;apoptotic mitochondrial changes;biological regulation;cell death;cell part morphogenesis;cellular component assembly;cellular component assembly at cellular level;cellular cor
27.7823	27.9952	27.946	27.9204	1:Experimental evidence at protein level	amine metabolic process;biosynthetic process;carboxylic acid metabolic process;cellular amine metabolic process;cellular amino acid metabolic process;cellular aromatic compound metabolic process;cellular biosynthetic p
28.7085	28.2914	28.556	28.5932	1:Experimental evidence at protein level	anatomical structure development;axon extension;biological regulation;cell growth;cell projection organization;cellular component movement;cellular component organization;cellular component organization at cellular level;c
28.5051	28.4736	28.3883	28.3943	1:Experimental evidence at protein level	amine metabolic process;amino acid activation;anatomical structure development;brain development;carboxylic acid metabolic process;cellular amine metabolic process;cellular amino acid metabolic process;cellular ketone
31.3514	30.9178	30.9292	30.9897	1:Experimental evidence at protein level	biosynthetic process;catabolic process;cellular biosynthetic process;cellular catabolic process;cellular component biogenesis;cellular component biogenesis at cellular level;cellular component disassembly;cellular componer
27.5047	27.2884	27.2941	27.1943	1:Experimental evidence at protein level	biological regulation;cellular macromolecule metabolic process;cellular metabolic process;cellular process;cellular protein metabolic process;chaperone-mediated protein folding;macromolecule metabolic process;metabolic
27.3342	27.1138	27.2098	27.0108	1:Experimental evidence at protein level	anatomical structure arrangement;anatomical structure development;axon extension;biological regulation;biosynthetic process;BMP signaling pathway;catabolic process;cell cycle phase;cell cycle process;cell division;cell gr
29.1044	29.1307	29.0926	29.094	1:Experimental evidence at protein level	cellular component organization;cellular component organization at cellular level;cellular component organization or biogenesis;cellular component organization or biogenesis at cellular level;cellular macromolecule metabolic
30.3238	30.2762	30.3092	30.3763	1:Experimental evidence at protein level	biological regulation;cell differentiation;cellular developmental process;cellular macromolecule metabolic process;cellular metabolic process;cellular nitrogen compound metabolic process;cellular process;cellular response to
28.6972	28.5611	28.5168	28.537	1:Experimental evidence at protein level	antigen processing and presentation;antigen processing and presentation of exogenous antigen;antigen processing and presentation of exogenous peptide antigen;antigen processing and presentation of exogenous peptide
29.685	29.2059	29.4605	29.4804	1:Experimental evidence at protein level	anatomical structure development;anatomical structure formation involved in morphogenesis;anatomical structure morphogenesis;biological regulation;biomineral tissue development;biosynthetic process;blood coagulation;b
31.4237	31.657	31.448	31.4266	1:Experimental evidence at protein level	adenine transport;biological regulation;cellular metabolic process;cellular process;chromosome segregation;energy derivation by oxidation of organic compounds;energy reserve metabolic process;establishment of localizatio
30.7516	30.7125	30.7749	30.5295	1:Experimental evidence at protein level	actin cytoskeleton organization;actin filament-based process;actomyosin structure organization;anatomical structure homeostasis;anatomical structure morphogenesis;associative learning;axon extension;axon guidance;B ce
29.2722	29.2667	29.2018	29.1818	1:Experimental evidence at protein level	anatomical structure development;anatomical structure formation involved in morphogenesis;biological regulation;blastocyst development;cellular process;cellular response to acid;cellular response to amine stimulus;cellular
28.8543	28.8309	29.1992	29.0716	1:Experimental evidence at protein level	anatomical structure development;biological regulation;cell cycle phase;cell cycle process;cell development;cellular component disassembly;cellular component disassembly at cellular level;cellular component organization;c
29.532	29.1764	29.2885	29.3225	1:Experimental evidence at protein level	biological regulation;biosynthetic process;cellular biosynthetic process;cellular macromolecule biosynthetic process;cellular macromolecule metabolic process;cellular metabolic process;cellular nitrogen compound metabolic
28.3844	28.0922	28.171	28.0308	1:Experimental evidence at protein level	cellular macromolecule metabolic process;cellular metabolic process;cellular nitrogen compound metabolic process;cellular process;gene expression;macromolecule metabolic process;metabolic process;mRNA metabolic p
26.3913	26.1822	26.6023	26.3549	1:Experimental evidence at protein level	anatomical structure formation involved in morphogenesis;angiogenesis;biological adhesion;biological regulation;cell adhesion;cell differentiation;cell proliferation;cellular component organization;cellular component organizat
28.3475	28.267	28.1622	28.0631	1:Experimental evidence at protein level	axon extension;biological regulation;cell growth;cell projection assembly;cell projection organization;cell surface receptor linked signaling pathway;cellular component assembly;cellular component assembly at cellular level;c
27.3107	27.2646	27.4148	27.3172	1:Experimental evidence at protein level	cell differentiation;cellular developmental process;cellular process;developmental process;hemopoietic progenitor cell differentiation
29.6731	29.726	29.5296	29.5436	1:Experimental evidence at protein level	biological regulation;cellular process;cellular response to acid;cellular response to amine stimulus;cellular response to amino acid stimulus;cellular response to chemical stimulus;cellular response to endogenous stimulus;cell
28.1005	27.1237	27.6182	27.6491	1:Experimental evidence at protein level	anatomical structure morphogenesis;biological regulation;cell cycle;cell surface receptor linked signaling pathway;cellular component organization;cellular component organization at cellular level;cellular component organiza
28.5834	28.3559	28.5325	28.4501	1:Experimental evidence at protein level	anatomical structure development;anatomical structure morphogenesis;axon guidance;biological adhesion;biological regulation;cell adhesion;cell junction assembly;cell junction organization;cell migration;cell morphogenesis
28.0952	28.6565	28.4726	28.4766	1:Experimental evidence at protein level	active induction of host immune response by virus;adenine nucleotide transport;ADP transport;apoptosis;ATP transport;biological regulation;cell death;cellular macromolecule metabolic process;cellular metabolic process;cel
28.4599	28.2863	28.3187	28.2386	1:Experimental evidence at protein level	amine metabolic process;amino acid activation;aspartyl-tRNA aminoacylation;biosynthetic process;carboxylic acid metabolic process;cellular amine metabolic process;cellular amino acid metabolic process;cellular biosynthe
27.4922	27.37	27.2512	27.0643	1:Experimental evidence at protein level	aging;anatomical structure development;anatomical structure morphogenesis;biological regulation;cell differentiation;cell part morphogenesis;cell projection morphogenesis;cell projection organization;cellular component mo
28.9337	28.8805	28.9796	28.9894	1:Experimental evidence at protein level	
26.7741	26.7694	26.9777	26.822	1:Experimental evidence at protein level	activation of protein kinase activity;apoptosis;biological regulation;cell death;cellular macromolecule metabolic process;cellular metabolic process;cellular process;cellular protein metabolic process;cellular response to stimul
29.2255	29.1235	29.0489	28.9938	1:Experimental evidence at protein level	biological regulation;cell growth;cellular process;growth;negative regulation of apoptosis;negative regulation of biological process;negative regulation of cell death;negative regulation of cellular process;negative regulation of
28.131	28.1188	28.199	27.9969	1:Experimental evidence at protein level	biological regulation;biosynthetic process;cellular biosynthetic process;cellular component organization;cellular component organization at cellular level;cellular component organization or biogenesis;cellular component orga
27.2734	27.1642	27.0673	27.0927	1:Experimental evidence at protein level	assembly of spliceosomal tri-snRNP;biological regulation;cellular component assembly;cellular component assembly at cellular level;cellular component organization;cellular component organization at cellular level;cellular co
29.2606	29.2641	29.6024	29.3949	1:Experimental evidence at protein level	actin cytoskeleton organization;actin filament capping;actin filament organization;actin filament polymerization;actin filament severing;actin filament-based process;actin nucleation;actin polymerization or depolymerization;ag
31.0324	31.3883	31.8418	31.5008	1:Experimental evidence at protein level	actin filament-based movement;actin filament-based process;actin-mediated cell contraction;actin-myosin filament sliding;biological regulation;cellular component movement;cellular process;multicellular organismal process
#NUM!	26.6285	27.1165	#NUM!	1:Experimental evidence at protein level	anatomical structure development;cell junction assembly;cell junction organization;cell-substrate junction assembly;cellular component assembly;cellular component assembly at cellular level;cellular component organization
29.0402	28.91	29.0399	28.8572	1:Experimental evidence at protein level	actin cytoskeleton organization;actin filament organization;actin filament-based process;adherens junction organization;aging;anatomical structure development;anatomical structure morphogenesis;apical junction assembly;
27.1208	26.8541	27.082	26.959	1:Experimental evidence at protein level	anatomical structure development;anatomical structure morphogenesis;biological regulation;cellular component disassembly;cellular component disassembly at cellular level;cellular component organization;cellular compone
28.7522	28.6494	28.5798	28.5796	1:Experimental evidence at protein level	amine metabolic process;amino acid activation;carboxylic acid metabolic process;cellular amine metabolic process;cellular amino acid metabolic process;cellular ketone metabolic process;cellular macromolecule metabolic
27.25	27.1952	27.2211	27.0944	1:Experimental evidence at protein level	biological regulation;biosynthetic process;calcium ion homeostasis;cation homeostasis;cellular biosynthetic process;cellular calcium ion homeostasis;cellular cation homeostasis;cellular chemical homeostasis;cellular divalen
31.3415	31.2378	31.1989	31.1263	1:Experimental evidence at protein level	apoptosis;biological regulation;catabolic process;cell death;cell surface receptor linked signaling pathway;cellular catabolic process;cellular component disassembly;cellular component disassembly at cellular level;cellular co
27.5851	27.4953	27.5027	27.4422	1:Experimental evidence at protein level	biological regulation;cellular macromolecule metabolic process;cellular metabolic process;cellular nitrogen compound metabolic process;cellular process;macromolecule metabolic process;metabolic process;mRNA metabol
28.6912	29.1603	29.5192	29.4676	1:Experimental evidence at protein level	biological regulation;biosynthetic process;cellular biosynthetic process;cellular macromolecule biosynthetic process;cellular macromolecule metabolic process;cellular metabolic process;cellular nitrogen compound metabolic
27.4355	27.1079	27.2088	27.0809	1:Experimental evidence at protein level	ATP-dependent chromatin remodeling;biological regulation;biosynthetic process;cellular biosynthetic process;cellular component assembly;cellular component assembly at cellular level;cellular component organization;cellula
28.0555	27.8138	27.8826	27.7246	1:Experimental evidence at protein level	cell cycle;cell cycle process;cell division;cellular component organization;cellular component organization at cellular level;cellular component organization or biogenesis;cellular component organization or biogenesis at cellula
#NUM!	29.2905	29.5772	28.9075	1:Experimental evidence at protein level	aging;anatomical structure development;cell differentiation;cell junction assembly;cell junction organization;cell-substrate junction assembly;cellular component assembly;cellular component assembly at cellular level;cellular
29.7401	29.7085	29.7153	29.6729	1:Experimental evidence at protein level	amine metabolic process;anatomical structure development;aromatic compound biosynthetic process;biosynthetic process;brainstem development;carboxylic acid metabolic process;cellular amine metabolic process;cellula
27.6867	27.8452	27.7692	27.619	1:Experimental evidence at protein level	aging;biological regulation;catabolic process;cellular catabolic process;cellular component assembly;cellular component assembly at cellular level;cellular component organization;cellular component organization at cellular le
29.8729	29.6415	29.6796	29.5328	1:Experimental evidence at protein level	biological regulation;biosynthetic process;cellular biosynthetic process;cellular component assembly;cellular component assembly at cellular level;cellular component organization;cellular component organization at cellular le
27.013	26.8826	27.3118	27.0565	1:Experimental evidence at protein level	cellular component organization;cellular component organization at cellular level;cellular component organization or biogenesis;cellular component organization or biogenesis at cellular level;cellular membrane fusion;cellular
28.4291	28.275	28.5164	28.4454	1:Experimental evidence at protein level	biological regulation;biosynthetic process;cellular biosynthetic process;cellular component organization;cellular component organization at cellular level;cellular component organization or biogenesis;cellular component orga
29.165	28.9739	28.9173	28.8228	1:Experimental evidence at protein level	biological regulation;negative regulation of apoptosis;negative regulation of biological process;negative regulation of cell death;negative regulation of cellular process;negative regulation of programmed cell death;regulation o
31.5154	31.5967	31.7707	31.5878	1:Experimental evidence at protein level	alternative nuclear mRNA splicing, via spliceosome;cellular macromolecule metabolic process;cellular metabolic process;cellular nitrogen compound metabolic process;cellular process;establishment of localization;establishm
29.5568	29.2892	29.2931	29.4059	1:Experimental evidence at protein level	biological regulation;biosynthetic process;catabolic process;cellular biosynthetic process;cellular catabolic process;cellular macromolecule biosynthetic process;cellular macromolecule catabolic process;cellular macromolec
30.2925	30.2812	30.1657	30.2091	1:Experimental evidence at protein level	actin cytoskeleton organization;actin filament-based process;activation of adenylate cyclase activity;ameboidal cell migration;axon guidance;biological regulation;blood coagulation;cell activation;cell migration;cell motility;cel
29.8596	29.9433	30.2139	30.1793	1:Experimental evidence at protein level	cellular component movement;cellular process;multicellular organismal process;muscle contraction;muscle system process;system process
25.8556	25.9758	25.6524	25.7227	1:Experimental evidence at protein level	acetyl-CoA metabolic process;acyl-CoA biosynthetic process;acyl-CoA metabolic process;acylglycerol biosynthetic process;acylglycerol metabolic process;amine transport;amino acid transmembrane transport;amino acid t
28.6903	28.3094	28.4348	28.2658	1:Experimental evidence at protein level	biological regulation;cellular component assembly;cellular component organization;cellular component organization or biogenesis;cellular macromolecule metabolic process;cellular metabolic process;cellular nitrogen compo
27.1578	26.9986	27.3112	27.2289	1:Experimental evidence at protein level	apoptosis;biological regulation;cell death;cellular component disassembly;cellular component disassembly at cellular level;cellular component disassembly involved in apoptosis;cellular component organization;cellular comp
28.64	28.7736	28.7717	28.7214	1:Experimental evidence at protein level	biological regulation;cellular component organization;cellular component organization or biogenesis;cellular macromolecule metabolic process;cellular membrane organization;cellular metabolic process;cellular process;cellula
28.3747	28.306	28.2228	28.0333	1:Experimental evidence at protein level	cellular macromolecule metabolic process;cellular metabolic process;cellular nitrogen compound metabolic process;cellular process;macromolecule metabolic process;metabolic process;mRNA metabolic process;mRNA pro
25.2986	25.2746	25.5569	24.8087	1:Experimental evidence at protein level	cellular macromolecule metabolic process;cellular metabolic process;cellular process;cellular protein metabolic process;macromolecule metabolic process;metabolic process;primary metabolic process;protein folding;protein
28.984	29.1124	28.9449	29.0212	1:Experimental evidence at protein level	amine biosynthetic process;amine catabolic process;amine metabolic process;anatomical structure development;biological regulation;biosynthetic process;carboxylic acid biosynthetic process;carboxylic acid catabolic proc
28.4286	28.5167	28.2974	28.2292	1:Experimental evidence at protein level	biological regulation;cation homeostasis;cation transport;cell differentiation;cellular cation homeostasis;cellular chemical homeostasis;cellular developmental process;cellular homeostasis;cellular ion homeostasis;cellular iron
25.7246	26.3689	25.968	25.9895	1:Experimental evidence at protein level	biological regulation;cell activation;cell activation involved in immune response;cellular component organization;cellular component organization or biogenesis;cellular membrane organization;cellular process;defense respons
31.3179	30.7886	30.9995	30.876	1:Experimental evidence at protein level	biosynthetic process;catabolic process;cellular biosynthetic process;cellular catabolic process;cellular component biogenesis;cellular component biogenesis at cellular level;cellular component disassembly;cellular componer
28.9256	28.7609	28.6792	28.779	1:Experimental evidence at protein level	biosynthetic process;cell cycle;cell cycle process;cellular biosynthetic process;cellular macromolecule biosynthetic process;cellular macromolecule metabolic process;cellular metabolic process;cellular nitrogen compound m
30.7905	30.3895	30.5998	30.6467	1:Experimental evidence at protein level	biosynthetic process;catabolic process;cellular biosynthetic process;cellular catabolic process;cellular component biogenesis;cellular component biogenesis at cellular level;cellular component disassembly;cellular componer
29.495	29.4549	29.4199	29.3911	1:Experimental evidence at protein level	apoptosis;cell death;cell proliferation;cellular process;death;establishment of localization;establishment of localization in cell;establishment of protein localization;intracellular protein transport;intracellular transport;nuclear exp
27.7527	27.6822	27.542	27.4485	1:Experimental evidence at protein level	adenosine to inosine editing;anatomical structure development;base conversion or substitution editing;biological regulation;cell differentiation;cell surface receptor linked signaling pathway;cellular component assembly;cellul
32.5021	32.4598	32.4189	32.3035	1:Experimental evidence at protein level	anatomical structure morphogenesis;biological regulation;biosynthetic process;catabolic process;cell surface receptor linked signaling pathway;cellular biosynthetic process;cellular catabolic process;cellular macromolecule b
33.1534	33.3493	33.8496	33.7822	1:Experimental evidence at protein level	actin filament-based movement;actin filament-based process;actin-mediated cell contraction;actin-myosin filament sliding;cell differentiation;cellular component movement;cellular developmental process;cellular process;dev
27.4277	27.4631	27.2702	27.2659	1:Experimental evidence at protein level	cell cycle phase;cell cycle process;cell division;cellular component organization;cellular component organization at cellular level;cellular component organization or biogenesis;cellular component organization or biogenesis at
26.6201	26.702	26.447	26.731	1:Experimental evidence at protein level	cellular macromolecule metabolic process;cellular metabolic process;cellular process;cellular protein metabolic process;chaperone-mediated protein folding;macromolecule metabolic process;macromolecule modification;me
27.6111	27.4216	27.4771	27.2924	1:Experimental evidence at protein level	carbohydrate metabolic process;cellular carbohydrate metabolic process;cellular macromolecule metabolic process;cellular metabolic process;cellular process;cellular protein metabolic process;'de novo' posttranslational pr
27.2058	26.9281	27.1237	27.0668	1:Experimental evidence at protein level	anatomical structure morphogenesis;axon guidance;biological adhesion;biological regulation;cell adhesion;cell differentiation;cell morphogenesis;cell morphogenesis involved in differentiation;cell projection organization;cell-
28.0897	27.4327	27.6236	27.4104	1:Experimental evidence at protein level	biological regulation;biosynthetic process;cell death;cell division;cellular biosynthetic process;cellular component organization;cellular component organization at cellular level;cellular component organization or biogenesis;ce
29.6102	29.5968	29.562	29.4043	1:Experimental evidence at protein level	alcohol biosynthetic process;alcohol catabolic process;alcohol metabolic process;amine metabolic process;anatomical structure development;biological regulation;biosynthetic process;carbohydrate biosynthetic process;car
25.9606	25.6676	25.4173	25.6809	1:Experimental evidence at protein level	alcohol biosynthetic process;alcohol metabolic process;biological regulation;biosynthetic process;biotin metabolic process;carbohydrate biosynthetic process;carbohydrate metabolic process;carboxylic acid metabolic proce
28.4215	27.7407	27.8331	27.8335	1:Experimental evidence at protein level	anatomical structure formation involved in morphogenesis;angiogenesis;angiotensin maturation;biological regulation;cell differentiation;cellular developmental process;cellular macromolecule metabolic process;cellular metab
28.6777	28.6496	28.6133	28.606	1:Experimental evidence at protein level	amine metabolic process;amino acid activation;biosynthetic process;bis(5'-nucleosidyl) oligophosphate biosynthetic process;bis(5'-nucleosidyl) oligophosphate metabolic process;carboxylic acid metabolic process;cellular a
30.5361	30.5926	30.3827	30.3592	1:Experimental evidence at protein level	binding of sperm to zona pellucida;cell recognition;cell-cell recognition;cellular component assembly;cellular component assembly at cellular level;cellular component organization;cellular component organization at cellular le
27.3483	27.2148	27.2048	27.0611	1:Experimental evidence at protein level	5-phosphoribose 1-diphosphate biosynthetic process;5-phosphoribose 1-diphosphate metabolic process;alcohol biosynthetic process;alcohol metabolic process;biological regulation;biosynthetic process;carbohydrate biosy
30.2039	29.9816	30.0501	29.8952	1:Experimental evidence at protein level	binding of sperm to zona pellucida;biological regulation;cell recognition;cell-cell recognition;cellular component assembly;cellular component assembly at cellular level;cellular component organization;cellular component orga
30.0824	30.058	29.9852	29.9601	1:Experimental evidence at protein level	biological regulation;posttranscriptional regulation of gene expression;regulation of biological process;regulation of gene expression;regulation of macromolecule metabolic process;regulation of metabolic process
32.9122	33.0312	32.9281	32.6684	1:Experimental evidence at protein level	biological regulation;blood coagulation;cell activation;cell cycle;cell cycle process;cellular component assembly;cellular component assembly at cellular level;cellular component organization;cellular component organization a
32.051	32.4642	32.4072	32.1502	1:Experimental evidence at protein level	biological regulation;cell cycle;cell cycle process;cell killing;cellular component assembly;cellular component assembly at cellular level;cellular component movement;cellular component organization;cellular component orga
27.3512	27.2201	27.6586	27.4988	1:Experimental evidence at protein level	anatomical structure homeostasis;biological regulation;biosynthetic process;catabolic process;cellular biosynthetic process;cellular catabolic process;cellular component organization;cellular component organization at cellula
30.0606	30.0149	30.1563	30.2266	1:Experimental evidence at protein level	actin cytoskeleton organization;actin filament bundle assembly;actin filament organization;actin filament-based process;biological regulation;cell projection organization;cellular component assembly;cellular component asser
27.012	29.5292	29.9256	29.0588	1:Experimental evidence at protein level	activation of immune response;anatomical structure homeostasis;biological regulation;complement activation;complement activation, lectin pathway;defense response;fibrinolysis;homeostatic process;humoral immune respo
34.6864	34.9419	34.8376	34.7998	1:Experimental evidence at protein level	biological regulation;cell cycle;cell cycle process;cell division;cell killing;cellular component assembly;cellular component assembly at cellular level;cellular component movement;cellular component organization;cellular comp
30.9814	30.9041	30.8354	30.7451	1:Experimental evidence at protein level	anatomical structure development;anatomical structure homeostasis;biological regulation;biosynthetic process;brain development;cellular biosynthetic process;cellular component organization;cellular component organizatio
29.9728	29.8251	29.7345	29.7021	1:Experimental evidence at protein level	anatomical structure development;anatomical structure homeostasis;biological regulation;biosynthetic process;brain development;cell differentiation;cell proliferation;cellular biosynthetic process;cellular component organizat
30.9205	31.1643	31.091	31.1871	1:Experimental evidence at protein level	biological regulation;cellular macromolecule metabolic process;cellular metabolic process;cellular nitrogen compound metabolic process;cellular process;establishment of localization;establishment of RNA localization;gene e
28.1041	28.1144	27.9792	28.1885	1:Experimental evidence at protein level	biological regulation;biosynthetic process;cellular biosynthetic process;cellular component organization;cellular component organization at cellular level;cellular component organization or biogenesis;cellular component orga
29.6767	29.5949	29.6792	29.6065	1:Experimental evidence at protein level	alkaloid metabolic process;biological regulation;biosynthetic process;cell communication;cell-cell signaling;cellular amide metabolic process;cellular biosynthetic process;cellular metabolic process;cellular nitrogen compoun
28.4556	27.9494	28.1006	27.9124	1:Experimental evidence at protein level	biosynthetic process;cell cycle;cell cycle process;cellular biosynthetic process;cellular component assembly;cellular component assembly at cellular level;cellular component organization;cellular component organization at c
30.1889	30.272	30.136	30.1259	1:Experimental evidence at protein level	binding of sperm to zona pellucida;cell recognition;cell-cell recognition;cellular macromolecule metabolic process;cellular metabolic process;cellular process;cellular process involved in reproduction;cellular protein metabolic
29.3788	29.4541	29.39	29.2148	1:Experimental evidence at protein level	alcohol metabolic process;carbohydrate metabolic process;carbohydrate phosphorylation;cellular carbohydrate metabolic process;cellular metabolic process;cellular process;fructose 6-phosphate metabolic process;fructose
27.243	27.1389	27.5093	27.2993	1:Experimental evidence at protein level	biological regulation;cell surface receptor linked signaling pathway;cellular process;cellular response to stimulus;establishment of localization;establishment of localization in cell;establishment of protein localization;G-protein
28.2397	28.0618	28.0467	27.9064	1:Experimental evidence at protein level	biological regulation;biosynthetic process;catabolic process;cell surface receptor linked signaling pathway;cellular biosynthetic process;cellular catabolic process;cellular component organization;cellular component organizat
28.0819	27.9221	27.9925	27.8312	1:Experimental evidence at protein level	anatomical structure morphogenesis;biological regulation;blood coagulation;bone morphogenesis;cell migration;cell motility;cellular component movement;cellular process;coagulation;developmental process;hemostasis;imr
27.4766	27.0599	27.3745	27.2836	1:Experimental evidence at protein level	apoptosis;apoptotic chromosome condensation;biological regulation;cell death;cell differentiation;cellular component disassembly;cellular component disassembly at cellular level;cellular component disassembly involved in
28.0021	28.2269	28.0268	28.0502	1:Experimental evidence at protein level	adherens junction organization;anatomical structure development;biological adhesion;biological regulation;biosynthetic process;brain development;cell adhesion;cell junction assembly;cell junction organization;cell surface re
29.4598	29.2212	29.6293	29.5803	1:Experimental evidence at protein level	biological regulation;biosynthetic process;catabolic process;cell surface receptor linked signaling pathway;cellular biosynthetic process;cellular catabolic process;cellular macromolecule biosynthetic process;cellular macrom
28.795	28.8597	28.8896	28.6421	1:Experimental evidence at protein level	alanyl-tRNA aminoacylation;amine metabolic process;amino acid activation;anatomical structure development;biological regulation;carboxylic acid metabolic process;cellular amine metabolic process;cellular amino acid met
28.2008	28.1003	28.0173	28.0133	1:Experimental evidence at protein level	antigen processing and presentation;antigen processing and presentation of peptide antigen;antigen processing and presentation of peptide antigen via MHC class I;cellular macromolecule metabolic process;cellular metabo
29.3033	28.6209	28.7667	28.7735	1:Experimental evidence at protein level	biological regulation;cell proliferation;establishment of localization;establishment of protein localization;establishment of RNA localization;macromolecule metabolic process;metabolic process;mRNA transport;negative regula
28.4485	28.2406	28.1495	28.1213	1:Experimental evidence at protein level	ATP-dependent chromatin remodeling;biological regulation;biosynthetic process;cellular biosynthetic process;cellular component assembly;cellular component assembly at cellular level;cellular component organization;cellula
26.6911	26.5407	26.5649	26.5181	1:Experimental evidence at protein level	anatomical structure homeostasis;biological regulation;biosynthetic process;cell cycle process;cellular biosynthetic process;cellular component organization;cellular component organization at cellular level;cellular componen
34.9699	35.1733	35.0422	34.9501	1:Experimental evidence at protein level	cell division;cellular component assembly;cellular component assembly at cellular level;cellular component organization;cellular component organization at cellular level;cellular component organization or biogenesis;cellular c
28.2678	28.6331	29.1878	28.5825	1:Experimental evidence at protein level	anatomical structure morphogenesis;biological regulation;cell differentiation;cellular developmental process;cellular process;developmental process;morphogenesis of an epithelium;positive regulation of biological process;po
30.9503	31.2364	31.1444	30.8554	1:Experimental evidence at protein level	activation of MAPK activity;anatomical structure development;anatomical structure formation involved in morphogenesis;anatomical structure morphogenesis;angiogenesis;behavior;behavioral response to pain;biological adh
27.5123	26.7753	26.791	26.9625	1:Experimental evidence at protein level	viral reproduction
30.1631	30.1698	30.1189	30.0806	1:Experimental evidence at protein level	biological regulation;cellular process;cellular response to stimulus;establishment of localization;establishment of protein localization;intracellular signal transduction;protein transport;regulation of biological process;regulation
28.4195	28.2266	27.9993	28.0665	1:Experimental evidence at protein level	biosynthetic process;cell cycle;cell cycle process;cellular biosynthetic process;cellular macromolecule biosynthetic process;cellular macromolecule metabolic process;cellular metabolic process;cellular nitrogen compound m
28.6276	28.7344	28.5238	28.4365	1:Experimental evidence at protein level	biological regulation;cell differentiation;cellular component assembly;cellular component assembly at cellular level;cellular component organization;cellular component organization at cellular level;cellular component organiza
29.9914	30.0183	30.0064	29.9267	1:Experimental evidence at protein level	binding of sperm to zona pellucida;cell recognition;cell-cell recognition;cellular macromolecule metabolic process;cellular metabolic process;cellular process;cellular process involved in reproduction;cellular protein metabolic
28.7477	28.3223	28.5347	28.4614	1:Experimental evidence at protein level	cell cycle;cell cycle process;cell division;cellular component organization;cellular component organization at cellular level;cellular component organization or biogenesis;cellular component organization or biogenesis at cellula

29.777	29.6775	29.6187	29.5007	1:Experimental evidence at protein level	biosynthetic process;carbohydrate metabolic process;cellular biosynthetic process;cellular carbohydrate metabolic process;cellular macromolecule biosynthetic process;cellular macromolecule metabolic process;cellular met
31.9411	32.0541	32.039	31.9045	1:Experimental evidence at protein level	biological regulation;blood coagulation;cellular process;cellular response to stimulus;coagulation;hemostasis;multicellular organismal process;negative regulation of apoptosis;negative regulation of biological process;negative
29.0665	28.8003	28.7932	28.6333	1:Experimental evidence at protein level	biosynthetic process;cell cycle;cell cycle process;cellular biosynthetic process;cellular macromolecule biosynthetic process;cellular macromolecule metabolic process;cellular metabolic process;cellular nitrogen compound m
28.8697	28.9826	28.7923	28.869	1:Experimental evidence at protein level	amine metabolic process;amino acid activation;apoptosis;biological regulation;carboxylic acid metabolic process;cell death;cellular amine metabolic process;cellular amino acid metabolic process;cellular ketone metabolic pr
27.092	28.1959	28.0006	28.5866	1:Experimental evidence at protein level	anatomical structure development;anatomical structure morphogenesis;biological regulation;cellular component organization;cellular component organization at cellular level;cellular component organization or biogenesis;cell
29.5538	30.1014	29.622	29.7121	1:Experimental evidence at protein level	actin cytoskeleton organization;actin filament bundle assembly;actin filament organization;actin filament-based process;cell migration;cell motility;cell proliferation;cellular component assembly;cellular component assembly a
29.5626	29.9279	30.0975	30.0469	1:Experimental evidence at protein level	biological regulation;biosynthetic process;catabolic process;cellular biosynthetic process;cellular catabolic process;cellular macromolecule biosynthetic process;cellular macromolecule catabolic process;cellular macromolec
30.0168	29.6596	29.76	29.445	1:Experimental evidence at protein level	anatomical structure development;biological adhesion;cell adhesion;cell differentiation;cellular component disassembly;cellular component disassembly at cellular level;cellular component organization;cellular component org
28.9434	28.4603	28.5112	28.5142	1:Experimental evidence at protein level	biosynthetic process;cell differentiation;cellular biosynthetic process;cellular developmental process;cellular macromolecule biosynthetic process;cellular macromolecule metabolic process;cellular metabolic process;cellular
27.7623	27.7041	27.6122	27.7039	1:Experimental evidence at protein level	amine metabolic process;amino acid activation;biosynthetic process;carboxylic acid metabolic process;cellular amine metabolic process;cellular amino acid metabolic process;cellular biosynthetic process;cellular componen
29.2066	29.0633	28.9615	29.0061	1:Experimental evidence at protein level	biological regulation;biosynthetic process;cellular biosynthetic process;cellular component disassembly;cellular component disassembly at cellular level;cellular component organization;cellular component organization at cell
28.7617	28.8515	28.7244	28.694	1:Experimental evidence at protein level	biological regulation;biosynthetic process;cellular biosynthetic process;cellular component assembly;cellular component assembly at cellular level;cellular component organization;cellular component organization at cellular le
32.9514	33.1976	32.9447	32.8818	1:Experimental evidence at protein level	alcohol catabolic process;alcohol metabolic process;anatomical structure development;carbohydrate catabolic process;carbohydrate metabolic process;carboxylic acid metabolic process;catabolic process;cell communicati
27.3763	27.4041	27.216	27.2916	1:Experimental evidence at protein level	acute inflammatory response;acute-phase response;aging;amide biosynthetic process;amine biosynthetic process;amine metabolic process;anatomical structure development;arginine biosynthetic process;arginine metabolic
29.1702	29.2022	28.9676	28.9127	1:Experimental evidence at protein level	anatomical structure development;biological adhesion;biological regulation;blood coagulation;calcium ion homeostasis;calcium ion transmembrane transport;calcium ion transport;calcium-mediated signaling;cation homeost
30.3549	30.2999	30.447	30.3465	1:Experimental evidence at protein level	binding of sperm to zona pellucida;cell recognition;cell-cell recognition;cellular component assembly;cellular component assembly at cellular level;cellular component organization;cellular component organization at cellular le
29.8269	29.0911	29.5158	29.4567	1:Experimental evidence at protein level	
28.5762	28.5229	28.4404	28.5008	1:Experimental evidence at protein level	apoptosis;biological regulation;cell cycle;cell death;cellular process;cytokinetic cell separation;cytokinetic process;death;establishment of localization;establishment of protein localization;programmed cell death;protein transp
28.6172	28.3991	28.3202	28.2391	1:Experimental evidence at protein level	biological regulation;biosynthetic process;cellular biosynthetic process;cellular component assembly;cellular component assembly at cellular level;cellular component organization;cellular component organization at cellular le
27.5893	27.3398	27.2012	27.139	1:Experimental evidence at protein level	actin cytoskeleton organization;actin filament organization;actin filament polymerization;actin filament-based process;actin polymerization or depolymerization;biological regulation;cell projection organization;cellular compone
32.8734	33.1008	33.0756	33.0969	1:Experimental evidence at protein level	actin cytoskeleton organization;actin filament organization;actin filament-based process;alcohol biosynthetic process;alcohol catabolic process;alcohol metabolic process;anatomical structure homeostasis;ATP biosynthetic p
28.1947	28.0886	28.358	28.3927	1:Experimental evidence at protein level	actin filament capping;anatomical structure development;apical protein localization;asymmetric protein localization;biological regulation;cell development;cell projection assembly;cell projection organization;cellular compone
29.2048	29.1115	29.0229	29.0123	1:Experimental evidence at protein level	cellular component organization;cellular component organization at cellular level;cellular component organization or biogenesis;cellular component organization or biogenesis at cellular level;cellular process;COPI coating of G
30.0759	30.224	30.2616	30.1988	1:Experimental evidence at protein level	binding of sperm to zona pellucida;cell recognition;cell-cell recognition;cellular macromolecule metabolic process;cellular metabolic process;cellular process;cellular process involved in reproduction;cellular protein metabolic
30.1727	30.1443	30.1883	30.1642	1:Experimental evidence at protein level	anatomical structure morphogenesis;biological regulation;biosynthetic process;cell morphogenesis;cell morphogenesis involved in differentiation;cellular biosynthetic process;cellular component assembly;cellular component
27.5112	27.3768	27.5575	27.0784	1:Experimental evidence at protein level	biological regulation;biosynthetic process;cell communication;cell differentiation;cellular biosynthetic process;cellular developmental process;cellular macromolecule biosynthetic process;cellular macromolecule metabolic pro
28.432	28.3999	28.1976	28.1218	1:Experimental evidence at protein level	biosynthetic process;carboxylic acid catabolic process;carboxylic acid metabolic process;cardiolipin acyl-chain remodeling;cardiolipin metabolic process;catabolic process;cellular biosynthetic process;cellular catabolic proc
36.3358	36.6328	36.4478	36.5915	1:Experimental evidence at protein level	adherens junction organization;anatomical structure development;anatomical structure homeostasis;ATP-dependent chromatin remodeling;axon guidance;biological adhesion;biological regulation;blood coagulation;cell adhes
29.7499	29.5787	29.9117	29.8378	1:Experimental evidence at protein level	actin cytoskeleton organization;actin cytoskeleton reorganization;actin filament organization;actin filament polymerization;actin filament-based process;actin polymerization or depolymerization;anatomical structure morphoge
29.4601	29.5428	29.5964	29.4507	1:Experimental evidence at protein level	biological regulation;cellular component organization;cellular component organization or biogenesis;cellular macromolecule metabolic process;cellular membrane organization;cellular metabolic process;cellular process;cellula
27.6783	27.3589	27.4364	27.3678	1:Experimental evidence at protein level	biological regulation;cell cycle;cell cycle phase;cell cycle process;cell division;cellular component organization;cellular component organization at cellular level;cellular component organization or biogenesis;cellular componer
26.5338	26.3871	26.7631	26.7897	1:Experimental evidence at protein level	anatomical structure formation involved in morphogenesis;anatomical structure morphogenesis;apoptotic cell clearance;biological regulation;blood vessel remodeling;branching involved in salivary gland morphogenesis;calci
28.4171	28.1545	28.2819	28.1353	1:Experimental evidence at protein level	biological regulation;biosynthetic process;cell cycle;cell cycle process;cell proliferation;cellular biosynthetic process;cellular macromolecule biosynthetic process;cellular macromolecule metabolic process;cellular metabolic p
28.125	27.8792	27.9622	27.8914	1:Experimental evidence at protein level	biological regulation;cell cycle;cell cycle phase;cell cycle process;cell division;cellular component organization;cellular component organization at cellular level;cellular component organization or biogenesis;cellular componer
29.1364	29.1672	29.1707	29.0702	1:Experimental evidence at protein level	cell cycle;cell division;cellular component assembly;cellular component organization;cellular component organization or biogenesis;cellular process;macromolecular complex assembly;macromolecular complex subunit organ
28.7891	28.2102	28.368	28.1987	1:Experimental evidence at protein level	alcohol catabolic process;alcohol metabolic process;biological regulation;carbohydrate catabolic process;carbohydrate homeostasis;carbohydrate metabolic process;carbohydrate phosphorylation;carbohydrate transport;ca
28.816	28.5956	28.5992	28.6064	1:Experimental evidence at protein level	biosynthetic process;cell cycle;cell cycle process;cellular biosynthetic process;cellular macromolecule biosynthetic process;cellular macromolecule metabolic process;cellular metabolic process;cellular nitrogen compound m
27.9564	27.2567	27.4711	27.3916	1:Experimental evidence at protein level	activation of immune response;activation of innate immune response;activation of phospholipase C activity;behavior;biological regulation;blood coagulation;calcium ion homeostasis;calcium ion transport;calcium ion transpo
29.0143	29.092	28.9574	28.9122	1:Experimental evidence at protein level	activation of MAPK activity;amine metabolic process;aminoglycan biosynthetic process;aminoglycan catabolic process;aminoglycan metabolic process;anatomical structure formation involved in morphogenesis;angiogenesis
30.7192	30.8107	30.7038	30.7061	1:Experimental evidence at protein level	biological regulation;blood coagulation;cell activation;cellular process;coagulation;establishment of localization;establishment of localization in cell;exocytosis;hemostasis;multicellular organismal process;neurological system
31.6516	31.649	31.5931	31.5703	1:Experimental evidence at protein level	acid secretion;alpha-beta T cell activation;alpha-beta T cell differentiation;anatomical structure development;arachidonic acid secretion;biological regulation;carboxylic acid transport;cell activation;cell differentiation;cell surfa
34.0916	34.5939	34.6794	34.6187	1:Experimental evidence at protein level	alcohol biosynthetic process;alcohol metabolic process;apoptosis;biological regulation;biosynthetic process;carbohydrate biosynthetic process;carbohydrate metabolic process;cell death;cellular biosynthetic process;cellular
29.7353	29.8652	29.7632	29.8443	1:Experimental evidence at protein level	biological regulation;cell redox homeostasis;cellular homeostasis;cellular macromolecule metabolic process;cellular metabolic process;cellular process;cellular protein metabolic process;cellular response to stimulus;cellular r
29.2651	28.4709	28.7776	28.7618	1:Experimental evidence at protein level	biological regulation;biosynthetic process;cell differentiation;cellular biosynthetic process;cellular component assembly;cellular component assembly at cellular level;cellular component organization;cellular component organi
27.639	27.3755	27.1959	27.2781	1:Experimental evidence at protein level	anatomical structure development;biosynthetic process;cellular biosynthetic process;cellular macromolecule biosynthetic process;cellular macromolecule metabolic process;cellular metabolic process;cellular nitrogen compo
27.701	27.2791	27.4971	27.413	1:Experimental evidence at protein level	biological regulation;cell cycle;cell cycle checkpoint;cell cycle phase;cell cycle process;cell division;cellular component organization;cellular component organization at cellular level;cellular component organization or biogene
30.3133	30.4507	30.3002	30.3313	1:Experimental evidence at protein level	binding of sperm to zona pellucida;cell recognition;cell-cell recognition;cellular component assembly;cellular component assembly at cellular level;cellular component organization;cellular component organization at cellular le
29.3735	29.5524	29.3657	29.3446	1:Experimental evidence at protein level	catabolic process;cellular catabolic process;cellular macromolecule catabolic process;cellular macromolecule metabolic process;cellular metabolic process;cellular process;cellular protein metabolic process;cellular response
28.2938	28.3367	28.3608	28.2795	1:Experimental evidence at protein level	amine metabolic process;amino acid activation;biosynthetic process;carboxylic acid metabolic process;cellular amine metabolic process;cellular amino acid metabolic process;cellular biosynthetic process;cellular ketone me
31.2641	30.7997	31.0851	30.9814	1:Experimental evidence at protein level	biological regulation;biosynthetic process;catabolic process;cell differentiation;cellular biosynthetic process;cellular catabolic process;cellular component disassembly;cellular component disassembly at cellular level;cellular c
26.7118	29.2755	29.7166	29.6992	1:Experimental evidence at protein level	actin cytoskeleton organization;actin filament-based process;actomyosin structure organization;biological regulation;cell differentiation;cell differentiation involved in embryonic placenta development;cell surface receptor link
29.8798	29.8429	29.8116	30.0049	1:Experimental evidence at protein level	cell cycle process;cell division;cellular component movement;cellular component organization;cellular component organization at cellular level;cellular component organization or biogenesis;cellular component organization o
34.4746	34.7702	34.5852	34.6	1:Experimental evidence at protein level	biological regulation;biosynthetic process;cellular biosynthetic process;cellular macromolecule biosynthetic process;cellular macromolecule metabolic process;cellular metabolic process;cellular nitrogen compound metabolic
27.9156	27.2281	27.5828	27.3077	1:Experimental evidence at protein level	biological regulation;calcium ion homeostasis;cation homeostasis;cellular calcium ion homeostasis;cellular cation homeostasis;cellular chemical homeostasis;cellular divalent inorganic cation homeostasis;cellular homeostasi
29.4031	29.1188	29.1167	29.1528	1:Experimental evidence at protein level	biological regulation;biosynthetic process;cell growth;cellular biosynthetic process;cellular macromolecule biosynthetic process;cellular macromolecule metabolic process;cellular metabolic process;cellular nitrogen compoun
29.3446	29.4074	29.3159	29.2679	1:Experimental evidence at protein level	acyl-CoA biosynthetic process;acyl-CoA metabolic process;acylglycerol biosynthetic process;acylglycerol metabolic process;biological regulation;biosynthetic process;carbohydrate metabolic process;carboxylic acid metabo
26.9861	26.8935	26.8203	26.8352	1:Experimental evidence at protein level	cellular macromolecule metabolic process;cellular metabolic process;cellular process;cellular protein metabolic process;macromolecule metabolic process;macromolecule modification;metabolic process;primary metabolic p
28.0273	27.8735	27.7902	27.8036	1:Experimental evidence at protein level	actin cytoskeleton organization;actin filament bundle assembly;actin filament organization;actin filament-based movement;actin filament-based process;cellular component assembly;cellular component assembly at cellular le
29.4683	29.6275	29.3972	29.3654	1:Experimental evidence at protein level	biological regulation;cardiac muscle contraction;cation homeostasis;cation transport;cellular cation homeostasis;cellular chemical homeostasis;cellular homeostasis;cellular ion homeostasis;cellular metal ion homeostasis;cel
29.6611	29.8177	29.6734	29.6814	1:Experimental evidence at protein level	10-formyltetrahydrofolate biosynthetic process;10-formyltetrahydrofolate metabolic process;amine biosynthetic process;amine metabolic process;anatomical structure development;anatomical structure formation involved in
28.4043	28.299	28.423	28.2818	1:Experimental evidence at protein level	axon guidance;biological regulation;cellular chemical homeostasis;cellular component disassembly;cellular component disassembly at cellular level;cellular component movement;cellular component organization;cellular com
30.6322	30.5203	30.7574	30.5562	1:Experimental evidence at protein level	
29.937	29.6724	29.6128	29.7682	1:Experimental evidence at protein level	anatomical structure development;cell development;cell junction assembly;cell junction organization;cellular component assembly;cellular component assembly at cellular level;cellular component organization;cellular compo
30.3689	30.4603	30.912	30.7237	1:Experimental evidence at protein level	
31.4642	31.4886	31.366	31.306	1:Experimental evidence at protein level	ATP biosynthetic process;ATP hydrolysis coupled proton transport;ATP metabolic process;ATP synthesis coupled proton transport;biological regulation;biosynthetic process;cation transport;cellular biosynthetic process;cellu
31.0529	30.8107	30.7692	30.9125	1:Experimental evidence at protein level	biosynthetic process;catabolic process;cellular biosynthetic process;cellular catabolic process;cellular component disassembly;cellular component disassembly at cellular level;cellular component organization;cellular compo
30.1799	30.0945	30.0505	30.0204	1:Experimental evidence at protein level	carbohydrate metabolic process;cellular carbohydrate metabolic process;cellular macromolecule metabolic process;cellular metabolic process;cellular process;cellular protein metabolic process;glycosylation;macromolecule
29.7875	29.532	29.7146	29.4218	1:Experimental evidence at protein level	biological regulation;biosynthetic process;cellular biosynthetic process;cellular macromolecule biosynthetic process;cellular macromolecule metabolic process;cellular metabolic process;cellular nitrogen compound metabolic
29.5187	29.4222	29.5159	29.4679	1:Experimental evidence at protein level	biological regulation;biosynthetic process;cellular biosynthetic process;cellular macromolecule biosynthetic process;cellular macromolecule metabolic process;cellular metabolic process;cellular nitrogen compound metabolic
27.7242	27.3892	27.436	27.3583	1:Experimental evidence at protein level	amine metabolic process;amino acid activation;carboxylic acid metabolic process;cellular amine metabolic process;cellular amino acid metabolic process;cellular ketone metabolic process;cellular macromolecule metabolic p
28.4449	28.2634	28.326	28.247	1:Experimental evidence at protein level	cellular macromolecule metabolic process;cellular metabolic process;cellular nitrogen compound metabolic process;cellular process;macromolecule metabolic process;metabolic process;mRNA metabolic process;mRNA pro
29.7668	29.4998	30.3863	29.6387	1:Experimental evidence at protein level	alcohol biosynthetic process;alcohol catabolic process;alcohol metabolic process;aldehyde biosynthetic process;biological regulation;biosynthetic process;carbohydrate biosynthetic process;carbohydrate catabolic process;
31.6549	31.7931	31.8771	31.8788	1:Experimental evidence at protein level	antigen processing and presentation;antigen processing and presentation of exogenous antigen;antigen processing and presentation of exogenous peptide antigen;antigen processing and presentation of exogenous peptide
28.8444	28.6694	28.4651	28.5184	1:Experimental evidence at protein level	biological regulation;cellular component organization;cellular component organization or biogenesis;defense response;establishment of localization;establishment of localization in cell;establishment of protein localization;esta
26.0466	26.0176	25.6276	25.9558	1:Experimental evidence at protein level	amine metabolic process;aminoglycan biosynthetic process;aminoglycan catabolic process;aminoglycan metabolic process;anatomical structure development;anatomical structure formation involved in morphogenesis;anato
30.9155	31.5268	31.7311	31.7489	1:Experimental evidence at protein level	alcohol biosynthetic process;alcohol metabolic process;biosynthetic process;carbohydrate biosynthetic process;carbohydrate metabolic process;cell differentiation;cellular biosynthetic process;cellular carbohydrate biosynth
27.7523	27.7375	27.5847	27.6056	1:Experimental evidence at protein level	amine metabolic process;amino acid activation;carboxylic acid metabolic process;cellular amine metabolic process;cellular amino acid metabolic process;cellular ketone metabolic process;cellular macromolecule metabolic p
28.268	27.93	27.8973	27.8915	1:Experimental evidence at protein level	amide biosynthetic process;amine biosynthetic process;amine catabolic process;amine metabolic process;anatomical structure development;anatomical structure morphogenesis;arginine biosynthetic process;arginine metab
28.7555	28.4797	28.4915	28.2541	1:Experimental evidence at protein level	amine metabolic process;amino acid activation;carboxylic acid metabolic process;cell differentiation;cellular amine metabolic process;cellular amino acid metabolic process;cellular developmental process;cellular ketone met
28.6003	28.3053	28.2875	28.2534	1:Experimental evidence at protein level	anterior/posterior pattern specification;cellular macromolecule metabolic process;cellular metabolic process;cellular nitrogen compound metabolic process;cellular process;developmental process;gene expression;macromol
30.9555	31.0687	31.1665	31.0839	1:Experimental evidence at protein level	4-hydroxyproline metabolic process;amine metabolic process;biological regulation;carboxylic acid metabolic process;cell redox homeostasis;cellular amine metabolic process;cellular amino acid metabolic process;cellular co
29.9808	30.1178	30.1287	30.0374	1:Experimental evidence at protein level	actin cytoskeleton organization;actin cytoskeleton reorganization;actin filament bundle assembly;actin filament organization;actin filament-based process;anatomical structure development;axon guidance;biological adhesion
26.0991	25.9339	26.3157	25.8562	1:Experimental evidence at protein level	anatomical structure morphogenesis;axis elongation;axon regeneration;axonogenesis;biological adhesion;biological regulation;branch elongation of an epithelium;bud outgrowth involved in lung branching;cell adhesion;cell c
28.4445	28.0321	28.1253	28.0424	1:Experimental evidence at protein level	cell differentiation;cellular component assembly;cellular component assembly at cellular level;cellular component organization;cellular component organization at cellular level;cellular component organization or biogenesis;ce
30.1422	30.4016	30.4227	30.4175	1:Experimental evidence at protein level	alcohol biosynthetic process;alcohol metabolic process;aldehyde biosynthetic process;aldehyde catabolic process;anatomical structure formation involved in morphogenesis;angiogenesis;behavior;biological regulation;biosy
30.1571	29.4317	29.9305	29.8606	1:Experimental evidence at protein level	apoptosis;cell death;cellular component disassembly;cellular component disassembly at cellular level;cellular component disassembly involved in apoptosis;cellular component organization;cellular component organization a
30.1889	30.474	30.5955	30.539	1:Experimental evidence at protein level	biological regulation;cellular macromolecule metabolic process;cellular metabolic process;cellular process;cellular protein metabolic process;establishment of localization;establishment of localization in cell;establishment of p
30.912	31.0494	30.9774	30.8889	1:Experimental evidence at protein level	biological regulation;cell differentiation;cellular developmental process;cellular macromolecule metabolic process;cellular metabolic process;cellular nitrogen compound metabolic process;cellular process;circadian regulation
30.5124	30.4811	30.5544	30.4819	1:Experimental evidence at protein level	alternative nuclear mRNA splicing, via spliceosome;biological regulation;biosynthetic process;cellular biosynthetic process;cellular component organization;cellular component organization at cellular level;cellular component
28.313	28.256	28.1697	28.2211	1:Experimental evidence at protein level	anatomical structure development;anatomical structure morphogenesis;biological regulation;cell surface receptor linked signaling pathway;cellular component organization;cellular component organization or biogenesis;cellu
30.3214	30.3904	30.5583	30.5009	1:Experimental evidence at protein level	activation of caspase activity;aggresome assembly;biological regulation;biosynthetic process;carbohydrate metabolic process;catabolic process;cellular biosynthetic process;cellular carbohydrate metabolic process;cellular c
30.1359	30.0718	29.9402	29.8987	1:Experimental evidence at protein level	biological regulation;biosynthetic process;catabolic process;cell surface receptor linked signaling pathway;cellular biosynthetic process;cellular catabolic process;cellular macromolecule biosynthetic process;cellular macrom
26.205	25.3583	25.5341	25.6518	1:Experimental evidence at protein level	
32.5601	32.101	32.2073	32.0503	1:Experimental evidence at protein level	anatomical structure formation involved in morphogenesis;angiogenesis;biological regulation;body fluid secretion;cellular component assembly;cellular component assembly at cellular level;cellular component organization;ce
32.6932	32.9916	33.1397	33.2093	1:Experimental evidence at protein level	axon guidance;biological regulation;biosynthetic process;cell communication;cell-cell signaling;cellular biosynthetic process;cellular component disassembly;cellular component disassembly at cellular level;cellular componer
31.7103	31.7138	31.671	31.5719	1:Experimental evidence at protein level	anatomical structure formation involved in morphogenesis;angiogenesis;developmental process
27.8892	27.5015	27.7517	27.5657	1:Experimental evidence at protein level	cell cycle phase;cell cycle process;cell proliferation;cellular process;meiosis
31.3556	31.4732	31.6865	31.785	1:Experimental evidence at protein level	activation of signaling protein activity involved in unfolded protein response;anatomical structure arrangement;anatomical structure development;biological regulation;blood coagulation;catabolic process;cell activation;cell co
27.7629	27.6572	27.6386	27.7042	1:Experimental evidence at protein level	alcohol metabolic process;carbohydrate catabolic process;carbohydrate metabolic process;catabolic process;cellular carbohydrate catabolic process;cellular carbohydrate metabolic process;cellular glucan metabolic proces
29.7355	29.9251	30.0078	30.056	1:Experimental evidence at protein level	response to stimulus;response to stress
30.1113	29.8996	29.9256	29.7784	1:Experimental evidence at protein level	biological regulation;cell differentiation;cellular developmental process;cellular macromolecule metabolic process;cellular metabolic process;cellular nitrogen compound metabolic process;cellular process;cellular response to
31.1067	30.8321	30.8313	30.9406	1:Experimental evidence at protein level	biosynthetic process;catabolic process;cellular biosynthetic process;cellular catabolic process;cellular component disassembly;cellular component disassembly at cellular level;cellular component organization;cellular compo
28.6966	28.502	28.4117	28.4413	1:Experimental evidence at protein level	biological regulation;biosynthetic process;cellular biosynthetic process;cellular component movement;cellular macromolecule biosynthetic process;cellular macromolecule metabolic process;cellular metabolic process;cellula
26.103	26.569	26.2207	27.2115	1:Experimental evidence at protein level	actin filament-based movement;actin filament-based process;ameboidal cell migration;biological regulation;cell migration;cell motility;cellular component movement;cellular component organization;cellular component organi
32.7315	33.6432	33.591	33.8356	1:Experimental evidence at protein level	alcohol biosynthetic process;alcohol metabolic process;biological regulation;biosynthetic process;carbohydrate biosynthetic process;carbohydrate metabolic process;cellular biosynthetic process;cellular carbohydrate biosyn
31.5601	31.4329	31.4962	31.403	1:Experimental evidence at protein level	actin crosslink formation;actin cytoskeleton organization;actin filament bundle assembly;actin filament organization;actin filament-based process;biological regulation;blood coagulation;cell activation;cell junction assembly;ce
28.1171	27.6988	28.0263	27.7651	1:Experimental evidence at protein level	actin filament capping;apoptosis;axon guidance;biological regulation;cell death;cellular component disassembly;cellular component disassembly at cellular level;cellular component disassembly involved in apoptosis;cellular
30.0504	29.7452	29.8062	29.7482	1:Experimental evidence at protein level	biological regulation;biosynthetic process;cell differentiation;cellular biosynthetic process;cellular developmental process;cellular macromolecule biosynthetic process;cellular macromolecule metabolic process;cellular metab
26.7575	26.7919	26.7319	26.8933	1:Experimental evidence at protein level	biological regulation;catabolic process;cellular catabolic process;cellular component organization;cellular component organization at cellular level;cellular component organization or biogenesis;cellular component organizatio
31.4618	31.5834	31.7079	31.5429	1:Experimental evidence at protein level	activation of caspase activity;activation of immune response;activation of innate immune response;adaptive immune response;adaptive immune response based on somatic recombination of immune receptors built from imm
29.0273	28.7195	28.7993	28.7011	1:Experimental evidence at protein level	activation of protein kinase activity;activation of protein kinase C activity by G-protein coupled receptor protein signaling pathway;aging;alcohol metabolic process;anatomical structure morphogenesis;aorta morphogenesis;a
29.7209	29.0441	29.2415	29.0837	1:Experimental evidence at protein level	biological regulation;calcium ion transport;cation transport;cellular component assembly;cellular component organization;cellular component organization or biogenesis;cellular process;divalent inorganic cation transport;diva
29.9959	29.9056	29.8767	29.8503	1:Experimental evidence at protein level	adherens junction assembly;adherens junction organization;anatomical structure morphogenesis;apical junction assembly;axon extension;biological adhesion;biological regulation;blood coagulation;cell activation;cell adhesic
29.4298	29.3448	29.2251	29.207	1:Experimental evidence at protein level	biological regulation;body fluid secretion;cellular component organization;cellular component organization at cellular level;cellular component organization or biogenesis;cellular component organization or biogenesis at cellula
27.9314	27.4657	27.5581	27.4294	1:Experimental evidence at protein level	assembly of spliceosomal tri-snRNP;cellular component assembly;cellular component assembly at cellular level;cellular component organization;cellular component organization at cellular level;cellular component organizatio
29.2695	29.0213	29.1277	29.0426	1:Experimental evidence at protein level	alcohol metabolic process;cholesterol metabolic process;establishment of localization;lipid metabolic process;lipid transport;metabolic process;organic substance transport;primary metabolic process;small molecule metabo
29.133	29.0184	28.9499	28.8407	1:Experimental evidence at protein level	anatomical structure homeostasis;base-excision repair;biological regulation;biosynthetic process;cell differentiation;cell surface receptor linked signaling pathway;cellular biosynthetic process;cellular component organization
32.1824	32.1989	32.2171	32.0459	1:Experimental evidence at protein level	actin cytoskeleton organization;actin filament organization;actin filament-based process;actin rod assembly;activation of immune response;activation of innate immune response;biological regulation;catabolic process;cellular
28.7815	28.4455	28.7062	28.5465	1:Experimental evidence at protein level	biological regulation;cellular component movement;cellular process;cellular response to stimulus;intracellular signal transduction;microtubule-based movement;microtubule-based process;regulation of biological process;regu
31.9492	31.9314	32.0226	31.8563	1:Experimental evidence at protein level	anatomical structure development;biological adhesion;biological regulation;cell adhesion;cell development;cell migration;cell motility;cell-cell adhesion;cellular component movement;cellular developmental process;cellular pr
29.2773	29.3655	29.2873	29.2615	1:Experimental evidence at protein level	cellular component assembly;cellular component assembly at cellular level;cellular component organization;cellular component organization at cellular level;cellular component organization or biogenesis;cellular component c
32.3263	32.4683	32.2696	32.2839	1:Experimental evidence at protein level	biological regulation;biosynthetic process;cell differentiation;cellular biosynthetic process;cellular developmental process;cellular macromolecule biosynthetic process;cellular macromolecule metabolic process;cellular metab
27.0322	26.6186	27.0047	26.7607	1:Experimental evidence at protein level	acute inflammatory response;acute-phase response;anatomical structure formation involved in morphogenesis;anatomical structure morphogenesis;angiogenesis;biological adhesion;biological regulation;blood coagulation;ca
33.8512	34.1674	33.9947	33.9051	1:Experimental evidence at protein level	alcohol metabolic process;carbohydrate metabolic process;cell death;cellular carbohydrate metabolic process;cellular metabolic process;cellular process;death;glucose metabolic process;hexose metabolic process;metaboli
30.8998	31.0729	30.9667	30.9647	1:Experimental evidence at protein level	alternative nuclear mRNA splicing, via spliceosome;cellular macromolecule metabolic process;cellular metabolic process;cellular nitrogen compound metabolic process;cellular process;gene expression;macromolecule metab
32.4357	32.5273	32.4314	32.3847	1:Experimental evidence at protein level	apoptosis;axon guidance;biological regulation;cardiac muscle cell apoptosis;cell cycle;cell cycle process;cell death;cell migration;cell motility;cell surface receptor linked signaling pathway;cellular component assembly;cellula
27.8052	27.5165	27.6415	27.386	1:Experimental evidence at protein level	biological regulation;biosynthetic process;carbohydrate metabolic process;carbohydrate transport;cell cycle;cell cycle process;cell surface receptor linked signaling pathway;cellular biosynthetic process;cellular component c
36.1741	35.2508	35.7348	35.7223	1:Experimental evidence at protein level	actin filament-based movement;actin filament-based process;actin-mediated cell contraction;actin-myosin filament sliding;anatomical structure development;apoptosis;astrocyte development;astrocyte differentiation;Bergma
29.6786	29.4171	29.376	29.3377	1:Experimental evidence at protein level	anatomical structure development;biological regulation;cell development;cell surface receptor linked signaling pathway;cellular developmental process;cellular metabolic process;cellular process;cellular response to calcium i

30.8823	30.7169	30.7075	30.7956	1:Experimental evidence at protein level	actin cytoskeleton organization;actin filament bundle assembly;actin filament organization;actin filament-based process;biological regulation;blood coagulation;cell activation;cell junction assembly;cell junction organization;c
27.0041	27.0348	27.06	27.0808	1:Experimental evidence at protein level	anatomical structure formation involved in morphogenesis;biological regulation;cell cycle arrest;cell cycle process;cell surface receptor linked signaling pathway;cellular process;cellular response to stimulus;developmental pr
28.4025	27.845	28.0534	27.8731	1:Experimental evidence at protein level	actin filament capping;axon guidance;biological regulation;cell cycle cytokinesis;cell cycle process;cellular component assembly;cellular component assembly at cellular level;cellular component organization;cellular compone
31.7384	31.6119	31.7544	31.7293	1:Experimental evidence at protein level	anatomical structure development;apoptosis;biological regulation;cardiac cell development;cardiac muscle cell development;cell cycle;cell cycle process;cell death;cell development;cellular component disassembly;cellular c
32.9676	33.1179	32.8996	32.8925	1:Experimental evidence at protein level	anatomical structure development;axon guidance;biological regulation;cellular macromolecule metabolic process;cellular metabolic process;cellular process;cellular protein metabolic process;cellular response to chemical sti
30.3891	30.095	30.1672	30.0654	1:Experimental evidence at protein level	biological regulation;biosynthetic process;cellular biosynthetic process;cellular component assembly;cellular component assembly at cellular level;cellular component organization;cellular component organization at cellular le
27.8851	27.607	27.7141	27.6443	1:Experimental evidence at protein level	biological regulation;biosynthetic process;cellular biosynthetic process;cellular macromolecule biosynthetic process;cellular macromolecule metabolic process;cellular metabolic process;cellular process;cellular protein metab
27.2077	27.2547	27.1182	27.0281	1:Experimental evidence at protein level	base-excision repair;cell differentiation;cellular component organization;cellular component organization at cellular level;cellular component organization or biogenesis;cellular component organization or biogenesis at cellular
28.79	28.6128	28.5983	28.4982	1:Experimental evidence at protein level	anatomical structure formation involved in morphogenesis;biological regulation;blood circulation;cellular component organization;cellular component organization or biogenesis;cellular membrane organization;cellular process
29.2768	29.1245	29.098	29.0364	1:Experimental evidence at protein level	amine metabolic process;amino acid activation;biological regulation;carboxylic acid metabolic process;cellular amine metabolic process;cellular amino acid metabolic process;cellular component assembly;cellular componen
29.4276	28.9133	29.2385	29.003	1:Experimental evidence at protein level	biosynthetic process;cell differentiation;cellular biosynthetic process;cellular developmental process;cellular macromolecule biosynthetic process;cellular macromolecule metabolic process;cellular metabolic process;cellular p
28.2998	27.8773	28.0945	27.8148	1:Experimental evidence at protein level	actin cytoskeleton organization;actin filament-based movement;actin filament-based process;actomyosin structure organization;adult heart development;anatomical structure development;anatomical structure formation invo
29.0725	28.6002	28.7473	28.6548	1:Experimental evidence at protein level	cell cycle;cell cycle phase;cell cycle process;cell differentiation;cell division;cellular component organization;cellular component organization at cellular level;cellular component organization or biogenesis;cellular component c
29.073	28.5797	28.9566	28.7932	1:Experimental evidence at protein level	biological regulation;biosynthetic process;carbohydrate metabolic process;carbohydrate transport;cell cycle;cell cycle checkpoint;cell cycle phase;cell cycle process;cell division;cell surface receptor linked signaling pathway
30.6949	30.4237	30.4989	30.3767	1:Experimental evidence at protein level	antigen processing and presentation;antigen processing and presentation of exogenous antigen;antigen processing and presentation of exogenous peptide antigen;antigen processing and presentation of exogenous peptide
28.6092	28.2416	28.2915	28.1544	1:Experimental evidence at protein level	anatomical structure development;axon guidance;biological adhesion;cell adhesion;cellular component disassembly;cellular component disassembly at cellular level;cellular component organization;cellular component organ
27.6221	27.2963	26.7954	27.2203	1:Experimental evidence at protein level	acylglycerol catabolic process;acylglycerol metabolic process;amide biosynthetic process;amine biosynthetic process;amine catabolic process;amine metabolic process;anatomical structure development;anion homeostasis;
31.6705	31.5633	31.4446	31.4579	1:Experimental evidence at protein level	actin crosslink formation;actin cytoskeleton organization;actin cytoskeleton reorganization;actin filament organization;actin filament-based process;anatomical structure formation involved in morphogenesis;anatomical struct
29.1776	28.7704	28.9809	28.8602	1:Experimental evidence at protein level	actin cytoskeleton organization;actin filament-based process;anatomical structure development;biological regulation;cell differentiation;cell surface receptor linked signaling pathway;cellular component organization;cellular c
29.3563	29.392	29.3128	29.2131	1:Experimental evidence at protein level	acetyl-CoA metabolic process;acyl-CoA biosynthetic process;acyl-CoA metabolic process;acylglycerol biosynthetic process;acylglycerol metabolic process;amine metabolic process;biological regulation;biosynthetic process
30.4753	30.419	30.3715	30.3304	1:Experimental evidence at protein level	actin cytoskeleton organization;actin filament-based process;axon guidance;biological adhesion;biological regulation;blood coagulation;cell activation;cell adhesion;cell junction assembly;cell junction organization;cell-cell ad
30.0409	29.7984	29.8203	29.6898	1:Experimental evidence at protein level	antigen processing and presentation;antigen processing and presentation of exogenous antigen;antigen processing and presentation of exogenous peptide antigen;antigen processing and presentation of exogenous peptide
29.7094	29.5058	29.4991	29.3865	1:Experimental evidence at protein level	anatomical structure development;anatomical structure formation involved in morphogenesis;anatomical structure homeostasis;anterior/posterior pattern specification;B cell lineage commitment;biological regulation;brain de
32.2794	32.0055	32.0687	31.9136	1:Experimental evidence at protein level	actin cytoskeleton organization;actin cytoskeleton reorganization;actin filament-based movement;actin filament-based process;actomyosin structure organization;anatomical structure formation involved in morphogenesis;an
31.1972	30.6811	30.9727	30.9663	1:Experimental evidence at protein level	biological regulation;cellular component assembly;cellular component organization;cellular component organization or biogenesis;macromolecular complex assembly;macromolecular complex subunit organization;protein co
30.3754	29.1932	29.6261	29.451	1:Experimental evidence at protein level	apoptosis;cell death;cell junction assembly;cell junction organization;cell-substrate junction assembly;cellular component assembly;cellular component assembly at cellular level;cellular component disassembly;cellular comp

C: GOMF name
binding;cation binding;enzyme activator activity;enzyme regulator activity;GTPase activator activity;GTPase regulator activity;ion binding;metal ion binding;nucleoside-triphosphatase regulator activity;transition metal ion bind
binding;G-protein coupled receptor activity;molecular transducer activity;odorant binding;olfactory receptor activity;receptor activity;signal transducer activity;signaling receptor activity;transmembrane signaling receptor activ
catalytic activity;hydrolase activity;peptidase activity
binding;nucleic acid binding;RNA binding;RNA cap binding
cation transmembrane transporter activity;hydrogen ion transmembrane transporter activity;inorganic cation transmembrane transporter activity;ion transmembrane transporter activity;monovalent inorganic cation transmemb
binding;cation binding;heme binding;ion binding;iron ion binding;metal ion binding;oxygen binding;oxygen transporter activity;substrate-specific transporter activity;tetrapyrrole binding;transition metal ion binding;transporter
binding;endopeptidase inhibitor activity;endopeptidase regulator activity;enzyme inhibitor activity;enzyme regulator activity;hormone binding;peptidase inhibitor activity;peptidase regulator activity;serine-type endopeptidase i
binding;endopeptidase inhibitor activity;endopeptidase regulator activity;enzyme binding;enzyme inhibitor activity;enzyme regulator activity;identical protein binding;peptidase inhibitor activity;peptidase regulator activity;prot
molecular transducer activity;receptor signaling protein activity;signal transducer activity
cyclin-dependent protein kinase regulator activity;enzyme regulator activity;kinase regulator activity;protein kinase regulator activity
binding;catalytic activity;enzyme binding;hydrolase activity;hydrolase activity, acting on ester bonds;kinase binding;MAP kinase phosphatase activity;phosphatase activity;phosphoprotein phosphatase activity;phosphoric est
binding;lipid binding
binding;endoribonuclease inhibitor activity;enzyme binding;enzyme inhibitor activity;enzyme regulator activity;protein binding;ribonuclease inhibitor activity;ubiquitin protein ligase binding
binding;enzyme binding;protein binding
molecular transducer activity;receptor signaling protein activity;signal transducer activity
binding;cation binding;copper chaperone activity;copper ion binding;ion binding;metal ion binding;metallochaperone activity;transition metal ion binding
binding;chromatin binding
antigen binding;binding;peptide antigen binding;peptide binding;protein binding;receptor binding
binding;catalytic activity;cation binding;ferric-chelate reductase activity;ion binding;metal ion binding;oxidoreductase activity;oxidoreductase activity, oxidizing metal ions;oxidoreductase activity, oxidizing metal ions, NAD or
binding;lipid binding;phosphatidylinositol binding;phosphatidylinositol-3,5-bisphosphate binding;phosphatidylinositol-3-phosphate binding;phospholipid binding
binding;calcium ion binding;catalytic activity;cation binding;endopeptidase activity;hydrolase activity;integrin binding;ion binding;metal ion binding;metalloendopeptidase activity;metallopeptidase activity;peptidase activity;pe
G-protein coupled receptor activity;molecular transducer activity;pheromone receptor activity;receptor activity;signal transducer activity;signaling receptor activity;transmembrane signaling receptor activity
binding;identical protein binding;lipid binding;phosphatidylinositol binding;phospholipid binding;protein binding;protein dimerization activity;protein domain specific binding;protein homodimerization activity
binding;bioactive lipid receptor activity;G-protein coupled receptor activity;lipid binding;lysophosphatidic acid receptor activity;molecular transducer activity;phospholipid binding;receptor activity;signal transducer activity;sig
acid-amino acid ligase activity;binding;catalytic activity;cation binding;ion binding;ligase activity;ligase activity, forming carbon-nitrogen bonds;metal ion binding;NF-kappaB binding;protein binding;small conjugating protein li
protein binding transcription factor activity;RNA polymerase II transcription cofactor activity;RNA polymerase II transcription factor binding transcription factor activity;transcription cofactor activity;transcription factor binding
binding;molecular transducer activity;protein binding;protein domain specific binding;signal transducer activity;WW domain binding
binding;DNA binding;nucleic acid binding;nucleic acid binding transcription factor activity;sequence-specific DNA binding;sequence-specific DNA binding transcription factor activity
binding;calcium ion binding;cation binding;ion binding;metal ion binding
catalytic activity;diphthine synthase activity;methyltransferase activity;S-adenosylmethionine-dependent methyltransferase activity;transferase activity;transferase activity, transferring one-carbon groups
binding;identical protein binding;protein binding;protein dimerization activity;protein homodimerization activity
acyl-CoA hydrolase activity;catalytic activity;CoA hydrolase activity;hydrolase activity;hydrolase activity, acting on ester bonds;thiolester hydrolase activity
binding;cell adhesion molecule binding;identical protein binding;protein binding;protein dimerization activity;protein homodimerization activity
active transmembrane transporter activity;amine transmembrane transporter activity;amino acid transmembrane transporter activity;carboxylic acid transmembrane transporter activity;L-amino acid transmembrane transport
acid-amino acid ligase activity;binding;catalytic activity;cation binding;ion binding;ligase activity;ligase activity, forming carbon-nitrogen bonds;metal ion binding;small conjugating protein ligase activity;transition metal ion bin
binding;carbohydrate binding;catalytic activity;cation binding;endopeptidase activity;glycosaminoglycan binding;heparin binding;hydrolase activity;ion binding;metal ion binding;metalloendopeptidase activity;metallopeptidas
acidic amino acid transmembrane transporter activity;active transmembrane transporter activity;amine transmembrane transporter activity;amino acid transmembrane transporter activity;antiporter activity;carboxylic acid tran
alcohol binding;binding;carbohydrate binding;catalytic activity;cation binding;CDP-alcohol phosphatidyltransferase activity;CDP-diacylglycerol-inositol 3-phosphatidyltransferase activity;diacylglycerol binding;ion binding;lipid
binding;core promoter proximal region DNA binding;core promoter proximal region sequence-specific DNA binding;DNA binding;nucleic acid binding;nucleic acid binding transcription factor activity;protein binding;protein bin
antigen binding;binding;integrin binding;protein binding;protein complex binding;receptor binding
acid-amino acid ligase activity;catalytic activity;ISG15 ligase activity;ligase activity;ligase activity, forming carbon-nitrogen bonds;small conjugating protein ligase activity;ubiquitin-protein ligase activity
acid-amino acid ligase activity;binding;caspase inhibitor activity;caspase regulator activity;catalytic activity;cation binding;chaperone binding;cobalt ion binding;cofactor binding;cysteine-type endopeptidase inhibitor activity;
binding;cation binding;ion binding;metal ion binding;transition metal ion binding;zinc ion binding
adenyl nucleotide binding;adenyl ribonucleotide binding;ATP binding;binding;CARD domain binding;catalytic activity;identical protein binding;kinase activity;LIM domain binding;molecular transducer activity;non-membrane s
molecular transducer activity;signal transducer activity
aspartic-type endopeptidase activity;aspartic-type peptidase activity;catalytic activity;endopeptidase activity;hydrolase activity;peptidase activity;peptidase activity, acting on L-amino acid peptides;receptor activity
binding;cation binding;enzyme binding;heme binding;identical protein binding;ion binding;iron ion binding;metal ion binding;protein binding;protein dimerization activity;protein homodimerization activity;tetrapyrrole binding;tr
binding;epidermal growth factor receptor binding;growth factor receptor binding;insulin receptor binding;lipid binding;phosphatidylinositol binding;phospholipid binding;protein binding;protein complex binding;receptor bindir
binding;cation binding;cytokine activity;endopeptidase inhibitor activity;endopeptidase regulator activity;enzyme binding;enzyme inhibitor activity;enzyme regulator activity;ion binding;metal ion binding;metalloendopeptidase
binding;chromatin binding;DNA binding;nucleic acid binding
binding;identical protein binding;protein binding
binding;DNA binding;double-stranded DNA binding;nucleic acid binding;nucleic acid binding transcription factor activity;regulatory region DNA binding;regulatory region nucleic acid binding;RNA polymerase II regulatory regi
binding;catalytic activity;collagen binding;cysteine-type endopeptidase activity;cysteine-type peptidase activity;endopeptidase activity;extracellular matrix binding;fibronectin binding;glycoprotein binding;hydrolase activity;lar
binding;cation binding;enzyme regulator activity;GTPase regulator activity;guanyl-nucleotide exchange factor activity;ion binding;metal ion binding;nucleoside-triphosphatase regulator activity;transition metal ion binding;zinc
binding;catalytic activity;GDP binding;GTP binding;GTPase activity;guanyl nucleotide binding;guanyl ribonucleotide binding;hydrolase activity;hydrolase activity, acting on acid anhydrides;hydrolase activity, acting on acid an
binding;protein binding;protein dimerization activity;protein heterodimerization activity
transmembrane transporter activity;transporter activity
catalytic activity;dolichyl-diphosphooligosaccharide-protein glycotransferase activity;oligosaccharyl transferase activity;transferase activity;transferase activity, transferring glycosyl groups;transferase activity, transferring hex
catalytic activity;endopeptidase activity;hydrolase activity;peptidase activity;peptidase activity, acting on L-amino acid peptides;serine hydrolase activity;serine-type endopeptidase activity;serine-type peptidase activity
molecular transducer activity;receptor activity;signal transducer activity;signaling receptor activity;thrombospondin receptor activity
binding;enzyme binding;protein binding;protein C-terminus binding
catalytic activity;guanidinoacetate N-methyltransferase activity;methyltransferase activity;S-adenosylmethionine-dependent methyltransferase activity;transferase activity;transferase activity, transferring one-carbon groups
cation transmembrane transporter activity;divalent inorganic cation transmembrane transporter activity;ferrous iron transmembrane transporter activity;inorganic cation transmembrane transporter activity;ion transmembrane
C-8 sterol isomerase activity;catalytic activity;cholestenol delta-isomerase activity;drug transmembrane transporter activity;intramolecular oxidoreductase activity;intramolecular oxidoreductase activity, transposing C=C bon
binding;catalytic activity;chromatin binding;chromatin DNA binding;core promoter binding;DNA binding;histone methyltransferase activity;histone methyltransferase activity (H3-K27 specific);histone-lysine N-methyltransferas
enzyme activator activity;enzyme regulator activity;GTPase activator activity;GTPase regulator activity;nucleoside-triphosphatase regulator activity
binding;cation binding;enzyme activator activity;enzyme regulator activity;GTPase activator activity;GTPase regulator activity;ion binding;metal ion binding;nucleoside-triphosphatase regulator activity;PDZ domain binding;pro
molecular transducer activity;signal transducer activity
binding;catalytic activity;cation binding;ion binding;ligase activity;metal ion binding;transition metal ion binding;zinc ion binding
binding;protein binding;receptor binding
binding;catalytic activity;cation binding;enzyme binding;hydrolase activity;hydrolase activity, acting on ester bonds;ion binding;kinase binding;metal ion binding;phosphatase activity;phosphoprotein phosphatase activity;phos
binding;nucleic acid binding;RNA binding
binding;calcium ion binding;cation binding;collagen binding;enzyme binding;ion binding;metal ion binding;protease binding;protein binding
binding;CARD domain binding;enzyme binding;kinase binding;molecular transducer activity;protein binding;protein domain specific binding;protein kinase binding;signal transducer activity
binding;calcium ion binding;carbohydrate binding;catalytic activity;cation binding;cytokine binding;glycosaminoglycan binding;growth factor binding;integrin binding;ion binding;kinase activity;metal ion binding;molecular tran
cation transmembrane transporter activity;ion transmembrane transporter activity;substrate-specific transmembrane transporter activity;substrate-specific transporter activity;transmembrane transporter activity;transporter a
binding;enzyme binding;GTPase binding;GTP-Rho binding;lipid binding;phosphatidylinositol binding;phosphatidylinositol-4,5-bisphosphate binding;phospholipid binding;protein binding;Ras GTPase binding;Rho GTPase bin
cation transmembrane transporter activity;ion transmembrane transporter activity;metal ion transmembrane transporter activity;substrate-specific transmembrane transporter activity;substrate-specific transporter activity;tran
binding;enzyme binding;enzyme inhibitor activity;enzyme regulator activity;growth factor receptor binding;hepatocyte growth factor receptor binding;ligase inhibitor activity;ligase regulator activity;protein binding;receptor bin
binding;mRNA binding;nucleic acid binding;nucleotide binding;poly(A) RNA binding;poly-purine tract binding;protein binding transcription factor activity;RNA binding;single-stranded RNA binding;transcription coactivator acti
enzyme regulator activity;GTPase regulator activity;guanyl-nucleotide exchange factor activity;nucleoside-triphosphatase regulator activity;Ras guanyl-nucleotide exchange factor activity;Rho guanyl-nucleotide exchange fac
binding;cytoskeletal protein binding;myosin binding;protein binding
bHLH transcription factor binding;binding;core promoter proximal region DNA binding;core promoter proximal region sequence-specific DNA binding;DNA binding;E-box binding;enhancer binding;identical protein binding;nu
beta-1,4-mannosyltransferase activity;catalytic activity;chitobiosyldiphosphodolichol beta-mannosyltransferase activity;mannosyltransferase activity;transferase activity;transferase activity, transferring glycosyl groups;transfe
binding;nucleic acid binding;RNA binding;rRNA binding;structural constituent of ribosome;structural molecule activity
anion channel activity;anion transmembrane transporter activity;channel activity;chloride channel activity;gated channel activity;intracellular calcium activated chloride channel activity;ion channel activity;ion gated channel ac
binding;identical protein binding;protein binding;transcription factor binding
binding;catalytic activity;cation binding;histone methyltransferase activity;histone-lysine N-methyltransferase activity;ion binding;lysine N-methyltransferase activity;metal ion binding;methyltransferase activity;N-methyltransfe
binding;cation binding;DNA binding;ion binding;metal ion binding;nucleic acid binding;transition metal ion binding;zinc ion binding
binding;enzyme activator activity;enzyme regulator activity;GTPase activator activity;GTPase regulator activity;guanyl-nucleotide exchange factor activity;identical protein binding;molecular transducer activity;nucleoside-trip

binding;catalytic activity;GTP binding;GTPase activity;guanyl nucleotide binding;guanyl ribonucleotide binding;hydrolase activity;hydrolase activity, acting on acid anhydrides;hydrolase activity, acting on acid anhydrides, in pl
binding;nucleic acid binding;RNA binding;snoRNA binding
binding;binding, bridging;cytoskeletal adaptor activity;cytoskeletal protein binding;protein binding;protein binding, bridging
binding;catalytic activity;DNA binding;DNA polymerase activity;DNA-directed DNA polymerase activity;nucleic acid binding;nucleotidyltransferase activity;sequence-specific DNA binding;transferase activity;transferase activit
binding;catalytic activity;DNA binding;DNA polymerase activity;DNA-directed DNA polymerase activity;nucleic acid binding;nucleotidyltransferase activity;sequence-specific DNA binding;transferase activity;transferase activit
binding;cation binding;ion binding;metal ion binding;transition metal ion binding;zinc ion binding
binding;catalytic activity;cis-trans isomerase activity;drug binding;FK506 binding;isomerase activity;macrolide binding;peptidyl-prolyl cis-trans isomerase activity
binding;DNA binding;nucleic acid binding;nucleic acid binding transcription factor activity;sequence-specific DNA binding;sequence-specific DNA binding RNA polymerase II transcription factor activity;sequence-specific DN
adenyl nucleotide binding;adenyl ribonucleotide binding;adenylate kinase activity;ATP binding;ATPase activity;binding;catalytic activity;hydrolase activity;hydrolase activity, acting on acid anhydrides;hydrolase activity, acting
nucleic acid binding transcription factor activity;sequence-specific DNA binding transcription factor activity
binding;nucleotide binding
binding;calcium ion binding;catalytic activity;cation binding;cis-trans isomerase activity;drug binding;FK506 binding;ion binding;isomerase activity;macrolide binding;metal ion binding;peptidyl-prolyl cis-trans isomerase activi
binding;cytoskeletal protein binding;microtubule binding;protein binding;tubulin binding
binding;identical protein binding;protein binding;protein dimerization activity;protein homodimerization activity;structural constituent of eye lens;structural molecule activity
catalytic activity;electron carrier activity;oxidoreductase activity;oxidoreductase activity, acting on paired donors, with incorporation or reduction of molecular oxygen;sphingolipid delta-4 desaturase activity
structural constituent of ribosome;structural molecule activity
binding;catalytic activity;DNA-directed RNA polymerase activity;nucleotide binding;nucleotidyltransferase activity;protein binding;RNA polymerase activity;transferase activity;transferase activity, transferring phosphorus-cont
molecular transducer activity;protein binding transcription factor activity;signal transducer activity;transcription cofactor activity;transcription corepressor activity;transcription factor binding transcription factor activity
binding;enzyme binding;nucleic acid binding;protein binding;RNA binding;RNA cap binding;translation factor activity, nucleic acid binding;translation initiation factor activity;ubiquitin protein ligase binding
binding;carbohydrate binding;glycosaminoglycan binding;hyaluronic acid binding;pattern binding;polysaccharide binding
beta-tubulin binding;binding;cytoskeletal protein binding;microtubule binding;protein binding;protein binding transcription factor activity;protein N-terminus binding;receptor binding;transcription cofactor activity;transcription
binding;identical protein binding;protein binding;structural molecule activity
binding;cation binding;copper ion binding;ion binding;metal ion binding;transition metal ion binding
catalytic activity;hydrolase activity
binding;chromatin binding;enzyme inhibitor activity;enzyme regulator activity;phosphatase inhibitor activity;phosphatase regulator activity;protein binding transcription factor activity;protein phosphatase inhibitor activity;prote
binding;enzyme binding;kinase binding;mitogen-activated protein kinase kinase kinase binding;protein binding;protein complex scaffold;protein C-terminus binding;protein kinase binding;structural molecule activity
binding;catalytic activity;GPI anchor binding;hydrolase activity;hydrolase activity, acting on carbon-nitrogen (but not peptide) bonds;hydrolase activity, acting on carbon-nitrogen (but not peptide) bonds, in linear amides;lipid b
binding;nucleic acid binding;RNA binding;snRNA binding;U6 snRNA 3'-end binding;U6 snRNA binding
cysteine-type endopeptidase inhibitor activity;endopeptidase inhibitor activity;endopeptidase regulator activity;enzyme inhibitor activity;enzyme regulator activity;kinase inhibitor activity;kinase regulator activity;peptidase inhil
binding;cation binding;cation transmembrane transporter activity;ferric iron binding;ferric iron transmembrane transporter activity;inorganic cation transmembrane transporter activity;ion binding;ion transmembrane transporte
aminopeptidase activity;binding;carbohydrate binding;catalytic activity;endopeptidase activity;exopeptidase activity;glycosaminoglycan binding;heparin binding;hydrolase activity;pattern binding;peptidase activity;peptidase a
binding;enzyme activator activity;enzyme regulator activity;GPI anchor binding;GTPase activator activity;GTPase regulator activity;integrin binding;lipid binding;nucleoside-triphosphatase regulator activity;phosphatidylinosito
binding;calcium ion binding;carbohydrate binding;cation binding;glycosaminoglycan binding;heparin binding;ion binding;metal ion binding;pattern binding;polysaccharide binding
acid-amino acid ligase activity;catalytic activity;ligase activity;ligase activity, forming carbon-nitrogen bonds;small conjugating protein ligase activity
channel activity;gap junction channel activity;ion transmembrane transporter activity;molecular transducer activity;passive transmembrane transporter activity;signal transducer activity;substrate-specific transmembrane trans
catalytic activity;NADH dehydrogenase (quinone) activity;NADH dehydrogenase (ubiquinone) activity;NADH dehydrogenase activity;oxidoreductase activity;oxidoreductase activity, acting on NADH or NADPH;oxidoreductase
binding;catalytic activity;DNA binding;DNA-directed RNA polymerase activity;nucleic acid binding;nucleotidyltransferase activity;RNA polymerase activity;transferase activity;transferase activity, transferring phosphorus-conta
binding;carbohydrate binding;glycosaminoglycan binding;growth factor binding;heparin binding;insulin-like growth factor binding;integrin binding;pattern binding;polysaccharide binding;protein binding;protein complex bindir
androgen binding;benzodiazepine receptor activity;binding;cholesterol binding;hormone binding;lipid binding;molecular transducer activity;neurotransmitter binding;neurotransmitter receptor activity;receptor activity;signal tra
binding;cation binding;identical protein binding;ion binding;metal ion binding;protein binding;structural molecule activity
enzyme inhibitor activity;enzyme regulator activity;phosphatase inhibitor activity;phosphatase regulator activity;protein phosphatase inhibitor activity;protein phosphatase regulator activity;protein serine/threonine phosphatas
binding;nucleic acid binding;RNA binding;translation factor activity, nucleic acid binding;translation initiation factor activity
binding;vitamin binding;vitamin E binding
calmodulin-dependent protein kinase activity;catalytic activity;hydrolase activity;hydrolase activity, acting on glycosyl bonds;hydrolase activity, hydrolyzing O-glycosyl compounds;kinase activity;phosphorylase kinase activity;
binding;chemokine activity;chemokine receptor binding;CXCR chemokine receptor binding;cytokine activity;cytokine receptor binding;G-protein-coupled receptor binding;protein binding;receptor binding
5'-deoxyribose-5-phosphate lyase activity;AT DNA binding;binding;C2H2 zinc finger domain binding;cAMP response element binding;carbon-oxygen lyase activity;catalytic activity;chromatin binding;chromatin DNA binding;c
binding;catalytic activity;cation binding;enzyme binding;GTPase binding;ion binding;metal ion binding;prenyltransferase activity;protein binding;protein geranylgeranyltransferase activity;protein prenyltransferase activity;Rab g
protein transporter activity;substrate-specific transporter activity;transporter activity
receptor activity
catalytic activity;NADH dehydrogenase (quinone) activity;NADH dehydrogenase (ubiquinone) activity;NADH dehydrogenase activity;oxidoreductase activity;oxidoreductase activity, acting on NADH or NADPH;oxidoreductase
acid-amino acid ligase activity;adenyl nucleotide binding;adenyl ribonucleotide binding;ATP binding;binding;catalytic activity;enzyme binding;ligase activity;ligase activity, forming carbon-nitrogen bonds;nucleotide binding;pro
cyclin-dependent protein kinase regulator activity;enzyme regulator activity;kinase regulator activity;protein kinase regulator activity
binding;catalytic activity;GTP binding;GTPase activity;guanyl nucleotide binding;guanyl ribonucleotide binding;hydrolase activity;hydrolase activity, acting on acid anhydrides;hydrolase activity, acting on acid anhydrides, in pl
binding;nucleic acid binding;RNA binding
binding;nucleic acid binding;RNA binding;structural constituent of ribosome;structural molecule activity
binding;catalytic activity;GTPase activity;hydrolase activity;hydrolase activity, acting on acid anhydrides;hydrolase activity, acting on acid anhydrides, in phosphorus-containing anhydrides;molecular transducer activity;nucleo
structural constituent of ribosome;structural molecule activity
aldehyde dehydrogenase (NAD) activity;binding;carboxylic acid binding;catalytic activity;coenzyme binding;cofactor binding;fatty acid binding;fatty-acyl-CoA binding;lipid binding;malonate-semialdehyde dehydrogenase (ace
catalytic activity;hydrolase activity;hydrolase activity, acting on glycosyl bonds;hydrolase activity, hydrolyzing N-glycosyl compounds;lyase activity;NAD(P)+ nucleosidase activity;NAD+ nucleosidase activity;phosphorus-oxyg
binding;carbohydrate binding
binding;carbohydrate binding;catalytic activity;endopeptidase activity;glycosaminoglycan binding;hydrolase activity;pattern binding;peptidase activity;peptidase activity, acting on L-amino acid peptides;polysaccharide bindir
binding;DNA binding;identical protein binding;nucleic acid binding;nucleic acid binding transcription factor activity;protein binding;protein binding transcription factor activity;protein dimerization activity;protein homodimeriza
acid-amino acid ligase activity;adenyl nucleotide binding;adenyl ribonucleotide binding;ATP binding;binding;catalytic activity;enzyme binding;ligase activity;ligase activity, forming carbon-nitrogen bonds;nucleotide binding;pro
adenyl nucleotide binding;adenyl ribonucleotide binding;AP-2 adaptor complex binding;ATP binding;binding;catalytic activity;kinase activity;Notch binding;nucleotide binding;phosphotransferase activity, alcohol group as acc
binding;carbohydrate binding;cargo receptor activity;cation binding;galactose binding;ion binding;lipoprotein particle binding;low-density lipoprotein particle binding;metal ion binding;molecular transducer activity;monosacch
binding;cation binding;DNA binding;ion binding;metal ion binding;nucleic acid binding;polyubiquitin binding;protein binding;small conjugating protein binding;transition metal ion binding;ubiquitin binding;zinc ion binding
catalytic activity;cis-trans isomerase activity;isomerase activity;peptidyl-prolyl cis-trans isomerase activity
binding;catalytic activity;GTP binding;GTPase activity;guanyl nucleotide binding;guanyl ribonucleotide binding;hydrolase activity;hydrolase activity, acting on acid anhydrides;hydrolase activity, acting on acid anhydrides, in pl
actin binding;binding;cytoskeletal protein binding;protein binding
binding;cation binding;ion binding;metal ion binding
2 iron, 2 sulfur cluster binding;binding;catalytic activity;cation binding;disulfide oxidoreductase activity;electron carrier activity;ion binding;iron-sulfur cluster binding;metal cluster binding;metal ion binding;oxidoreductase acti
catalytic activity;hydrolase activity
enzyme regulator activity;GDP-dissociation inhibitor activity;GTPase regulator activity;nucleoside-triphosphatase regulator activity;small GTPase regulator activity
enzyme activator activity;enzyme regulator activity;GTPase activator activity;GTPase regulator activity;nucleoside-triphosphatase regulator activity
binding;calcium ion binding;cation binding;ion binding;metal ion binding;protein binding;protein dimerization activity;protein heterodimerization activity
catalytic activity;NADH dehydrogenase (quinone) activity;NADH dehydrogenase (ubiquinone) activity;NADH dehydrogenase activity;oxidoreductase activity;oxidoreductase activity, acting on NADH or NADPH;oxidoreductase
cation transmembrane transporter activity;divalent inorganic cation transmembrane transporter activity;inorganic cation transmembrane transporter activity;ion transmembrane transporter activity;magnesium ion transmembra
anion binding;binding;catalytic activity;ion binding;N-acetylgalactosamine 4-O-sulfotransferase activity;phosphate ion binding;sulfotransferase activity;transferase activity;transferase activity, transferring sulfur-containing grou
G-protein coupled receptor activity;molecular transducer activity;receptor activity;signal transducer activity;signaling receptor activity;transmembrane signaling receptor activity
adenyl nucleotide binding;adenyl ribonucleotide binding;ATP binding;ATPase activity;ATPase activity, coupled;ATP-dependent helicase activity;ATP-dependent RNA helicase activity;binding;catalytic activity;helicase activity;h
acid-amino acid ligase activity;binding;catalytic activity;cation binding;ion binding;ligase activity;ligase activity, forming carbon-nitrogen bonds;metal ion binding;small conjugating protein ligase activity;transition metal ion bin
3'-phosphoadenosine 5'-phosphosulfate transmembrane transporter activity;molecular transducer activity;nucleobase-containing compound transmembrane transporter activity;nucleoside transmembrane transporter activity
binding;catalytic activity;histone methyltransferase activity;histone-lysine N-methyltransferase activity;lysine N-methyltransferase activity;methyltransferase activity;N-methyltransferase activity;p53 binding;protein binding;pro
catalytic activity;exopeptidase activity;gamma-glutamyl-peptidase activity;hydrolase activity;omega peptidase activity;peptidase activity;peptidase activity, acting on L-amino acid peptides
catalytic activity
catalytic activity;methyltransferase activity;RNA methyltransferase activity;S-adenosylmethionine-dependent methyltransferase activity;transferase activity;transferase activity, transferring one-carbon groups;tRNA (adenine) m
binding;cation binding;ion binding;metal ion binding;transition metal ion binding;zinc ion binding
active transmembrane transporter activity;amine transmembrane transporter activity;amino acid transmembrane transporter activity;carboxylic acid transmembrane transporter activity;organic acid transmembrane transporte
catalytic activity;cysteine-type endopeptidase activity;cysteine-type peptidase activity;endopeptidase activity;hydrolase activity;peptidase activity;peptidase activity, acting on L-amino acid peptides
channel inhibitor activity;channel regulator activity;ion channel inhibitor activity;potassium channel inhibitor activity;potassium channel regulator activity
acylglycerol lipase activity;binding;carboxylic ester hydrolase activity;catalytic activity;hydrolase activity;hydrolase activity, acting on ester bonds;identical protein binding;lipase activity;lipid binding;lysophospholipase activity;
binding;DNA binding;nucleic acid binding;nucleic acid binding transcription factor activity;sequence-specific DNA binding transcription factor activity
binding;identical protein binding;protein binding;protein dimerization activity;protein homodimerization activity;protein N-terminus binding;structural molecule activity
binding;catalytic activity;coenzyme binding;cofactor binding;DNA binding;electron-transferring-flavoprotein dehydrogenase activity;flavin adenine dinucleotide binding;NADH dehydrogenase (quinone) activity;NADH dehydrog
3-keto sterol reductase activity;alcohol dehydrogenase [NAD(P)+] activity;binding;carbonyl reductase (NADPH) activity;catalytic activity;oxidoreductase activity;oxidoreductase activity, acting on CH-OH group of donors;oxido
binding;catalytic activity;cation binding;ion binding;ligase activity;metal ion binding;transition metal ion binding;zinc ion binding
adenyl nucleotide binding;adenyl ribonucleotide binding;ATP binding;binding;catalytic activity;kinase activity;nucleotide binding;phosphotransferase activity, alcohol group as acceptor;protein kinase activity;protein serine/thre
binding;catalytic activity;GTP binding;GTPase activity;guanyl nucleotide binding;guanyl ribonucleotide binding;hydrolase activity;hydrolase activity, acting on acid anhydrides;hydrolase activity, acting on acid anhydrides, in pl
binding;carbohydrate binding;catalytic activity;chitin binding;chitinase activity;hydrolase activity;hydrolase activity, acting on glycosyl bonds;hydrolase activity, hydrolyzing O-glycosyl compounds;oligosaccharide binding;patt
binding;mRNA binding;nucleic acid binding;RNA binding
binding;catalytic activity;cation binding;GDP binding;GTP binding;GTPase activity;guanyl nucleotide binding;guanyl ribonucleotide binding;hydrolase activity;hydrolase activity, acting on acid anhydrides;hydrolase activity, act
binding;DNA binding;nucleic acid binding
catalytic activity;CTD phosphatase activity;hydrolase activity;hydrolase activity, acting on ester bonds;phosphatase activity;phosphoprotein phosphatase activity;phosphoric ester hydrolase activity;protein serine/threonine ph
armadillo repeat domain binding;binding;calmodulin binding;enzyme binding;phosphatase binding;protein binding;protein complex binding;protein domain specific binding;protein phosphatase 2A binding;protein phosphatas
binding;catalytic activity;cation binding;hydrolase activity;hydrolase activity, acting on carbon-nitrogen (but not peptide) bonds;hydrolase activity, acting on carbon-nitrogen (but not peptide) bonds, in cyclic amidines;ion bindi

[myelin basic protein]-arginine N-methyltransferase activity;arginine N-methyltransferase activity;binding;catalytic activity;histone binding;histone methyltransferase activity;histone methyltransferase activity (H4-R3 specific);h
adenyl nucleotide binding;adenyl ribonucleotide binding;ATP binding;binding;nucleotide binding;purine nucleotide binding;purine ribonucleoside triphosphate binding;purine ribonucleotide binding;ribonucleotide binding
binding;cytoskeletal protein binding;protein binding;tropomyosin binding
binding;PDZ domain binding;protein binding;protein domain specific binding;protein self-association
binding;protein binding;SNAP receptor activity
acid-amino acid ligase activity;binding;catalytic activity;cation binding;copper ion binding;ion binding;ligase activity;ligase activity, forming carbon-nitrogen bonds;metal ion binding;NEDD8 ligase activity;small conjugating pro
binding;calcium ion binding;calcium-dependent cysteine-type endopeptidase activity;catalytic activity;cation binding;cysteine-type endopeptidase activity;cysteine-type peptidase activity;endopeptidase activity;hydrolase act
catalytic activity;electron carrier activity;NADH dehydrogenase (quinone) activity;NADH dehydrogenase (ubiquinone) activity;NADH dehydrogenase activity;oxidoreductase activity;oxidoreductase activity, acting on NADH or N
binding;identical protein binding;protein binding
aminopeptidase activity;binding;catalytic activity;cation binding;exopeptidase activity;hydrolase activity;ion binding;metal ion binding;metallopeptidase activity;peptidase activity;peptidase activity, acting on L-amino acid pep
catalytic activity;methyltransferase activity;N-methyltransferase activity;RNA methyltransferase activity;rRNA (adenine) methyltransferase activity;rRNA (adenine-N6,N6-)-dimethyltransferase activity;rRNA methyltransferase ac
binding;coenzyme binding;cofactor binding;flavin adenine dinucleotide binding
protein binding transcription factor activity;transcription coactivator activity;transcription cofactor activity;transcription factor binding transcription factor activity
4 iron, 4 sulfur cluster binding;adenyl nucleotide binding;adenyl ribonucleotide binding;ATP binding;binding;cation binding;ion binding;iron-sulfur cluster binding;metal cluster binding;metal ion binding;nucleotide binding;purin
binding;catalytic activity;hydrolase activity;nucleic acid binding;RNA binding;snoRNA binding
binding;nucleic acid binding;RNA binding
binding;catalytic activity;cation binding;endopeptidase activity;exopeptidase activity;hydrolase activity;ion binding;metal ion binding;peptidase activity;peptidase activity, acting on L-amino acid peptides;peptide binding;serin
antioxidant activity;catalytic activity;glutathione peroxidase activity;glutathione transferase activity;oxidoreductase activity;oxidoreductase activity, acting on peroxide as acceptor;peroxidase activity;transferase activity;transfe
binding;cytoskeletal protein binding;enzyme regulator activity;GTPase regulator activity;guanyl-nucleotide exchange factor activity;kinesin binding;nucleoside-triphosphatase regulator activity;protein binding;Ras guanyl-nucle
binding;calcium ion binding;cation binding;CD4 receptor binding;DNA binding;enzyme binding;epidermal growth factor receptor binding;growth factor receptor binding;ion binding;lipid transporter activity;metal ion binding;nu
catalytic activity;DNA-directed RNA polymerase activity;nucleotidyltransferase activity;RNA polymerase activity;transferase activity;transferase activity, transferring phosphorus-containing groups
active transmembrane transporter activity;antigen binding;ATPase activity;ATPase activity, coupled;ATPase activity, coupled to movement of substances;ATPase activity, coupled to transmembrane movement of substances;b
androgen receptor binding;binding;cation binding;hormone receptor binding;ion binding;I-SMAD binding;metal ion binding;nuclear hormone receptor binding;protein binding;protein binding transcription factor activity;recepto
catalytic activity;NADH dehydrogenase (quinone) activity;NADH dehydrogenase (ubiquinone) activity;NADH dehydrogenase activity;oxidoreductase activity;oxidoreductase activity, acting on NADH or NADPH;oxidoreductase
binding;cation binding;ion binding;metal ion binding;protein binding transcription factor activity;transcription cofactor activity;transcription factor binding transcription factor activity;transition metal ion binding;zinc ion binding
binding;enzyme binding;protein binding;small conjugating protein binding;transcription factor binding;ubiquitin binding;ubiquitin protein ligase binding
catalytic activity;endopeptidase activity;hydrolase activity;peptidase activity;peptidase activity, acting on L-amino acid peptides;serine hydrolase activity;serine-type endopeptidase activity;serine-type peptidase activity
catalytic activity;NADH dehydrogenase (quinone) activity;NADH dehydrogenase (ubiquinone) activity;NADH dehydrogenase activity;oxidoreductase activity;oxidoreductase activity, acting on NADH or NADPH;oxidoreductase
binding;protein binding;SNAP receptor activity
structural molecule activity
binding;ribonucleoprotein binding
binding;catalytic activity;cation binding;coenzyme binding;cofactor binding;ion binding;metal ion binding;NADP binding;NADPH:quinone reductase activity;nucleotide binding;oxidoreductase activity;oxidoreductase activity, a
binding;catalytic activity;cation binding;diphosphoinositol-polyphosphate diphosphatase activity;hydrolase activity;hydrolase activity, acting on acid anhydrides;hydrolase activity, acting on acid anhydrides, in phosphorus-co
binding;catalytic activity;cation binding;ion binding;metal ion binding;protein-glutamine gamma-glutamyltransferase activity;transferase activity;transferase activity, transferring acyl groups;transferase activity, transferring ami
binding;calcium ion binding;catalytic activity;cation binding;endopeptidase activity;hydrolase activity;ion binding;lipid binding;metal ion binding;peptidase activity;peptidase activity, acting on L-amino acid peptides;phospholi
catalytic activity;endopeptidase activity;hydrolase activity;peptidase activity;peptidase activity, acting on L-amino acid peptides;serine hydrolase activity;serine-type endopeptidase activity;serine-type peptidase activity
binding;carbohydrate binding;enzyme activator activity;enzyme regulator activity;glycoprotein binding;glycosaminoglycan binding;heparin binding;identical protein binding;lipase activator activity;lipid binding;lipoprotein lipase
protein tag
binding;carbohydrate binding;endopeptidase inhibitor activity;endopeptidase regulator activity;enzyme inhibitor activity;enzyme regulator activity;glycosaminoglycan binding;heparin binding;pattern binding;peptidase inhibitor
binding;carbohydrate binding;chemoattractant activity;cytokine activity;fibroblast growth factor receptor binding;glycosaminoglycan binding;growth factor activity;growth factor receptor binding;heparin binding;ligand-depend
carboxypeptidase activity;catalytic activity;enzyme activator activity;enzyme regulator activity;exopeptidase activity;hydrolase activity;peptidase activity;peptidase activity, acting on L-amino acid peptides;serine hydrolase ac
binding;chaperone binding;enzyme binding;misfolded protein binding;protein binding;ubiquitin protein ligase binding
binding;cytokine binding;cytokine receptor activity;enzyme binding;lipid binding;molecular transducer activity;phospholipid binding;protease binding;protein binding;receptor activity;signal transducer activity;signaling recepto
alpha-tubulin binding;beta-N-acetylglucosaminylglycopeptide beta-1,4-galactosyltransferase activity;beta-tubulin binding;binding;catalytic activity;cation binding;cytoskeletal protein binding;galactosyltransferase activity;iden
binding;cation binding;endopeptidase inhibitor activity;endopeptidase regulator activity;enzyme activator activity;enzyme binding;enzyme inhibitor activity;enzyme regulator activity;ion binding;metal ion binding;metalloendope
binding;DNA binding;nucleic acid binding;nucleic acid binding transcription factor activity;regulatory region DNA binding;regulatory region nucleic acid binding;RNA polymerase II regulatory region DNA binding;RNA polymera
alpha-catenin binding;beta-catenin binding;binding;calcium ion binding;cation binding;gamma-catenin binding;ion binding;metal ion binding;protein binding
adenyl nucleotide binding;adenyl ribonucleotide binding;ATP binding;binding;catalytic activity;cytokine receptor binding;enzyme binding;growth hormone receptor binding;hormone receptor binding;kinase activity;non-membr
catalytic activity;endopeptidase activity;hydrolase activity;peptidase activity;peptidase activity, acting on L-amino acid peptides;threonine-type endopeptidase activity;threonine-type peptidase activity
active transmembrane transporter activity;ATPase activity;ATPase activity, coupled;ATPase activity, coupled to movement of substances;ATPase activity, coupled to transmembrane movement of ions;ATPase activity, couplec
binding;catalytic activity;cation binding;enzyme activator activity;enzyme regulator activity;GTP binding;GTPase activity;guanyl nucleotide binding;guanyl ribonucleotide binding;hydrolase activity;hydrolase activity, acting on a
binding;carbohydrate binding;catalytic activity;cation binding;glucose binding;glucosyltransferase activity;glycogenin glucosyltransferase activity;ion binding;metal ion binding;monosaccharide binding;sugar binding;transfera
adenyl nucleotide binding;adenyl ribonucleotide binding;ATP binding;binding;nucleotide binding;purine nucleotide binding;purine ribonucleoside triphosphate binding;purine ribonucleotide binding;ribonucleotide binding
catalytic activity;deoxyhypusine synthase activity;transferase activity;transferase activity, transferring alkyl or aryl (other than methyl) groups
binding;calcium-dependent protein serine/threonine phosphatase activity;calmodulin-dependent protein phosphatase activity;catalytic activity;cation binding;hydrolase activity;hydrolase activity, acting on ester bonds;ion bin
binding;bis(5'-nucleosyl)-tetraphosphatase (asymmetrical) activity;bis(5'-nucleosyl)-tetraphosphatase (symmetrical) activity;bis(5'-nucleosyl)-tetraphosphatase activity;catalytic activity;GTP binding;guanyl nucleotide binding;g
binding;cation binding;ion binding;metal ion binding;protein binding;protein N-terminus binding;transition metal ion binding;zinc ion binding
adenyl nucleotide binding;adenyl ribonucleotide binding;anaphase-promoting complex binding;ATP binding;binding;catalytic activity;cytoskeletal protein binding;enzyme binding;kinase activity;kinase binding;microtubule binc
adiponectin binding;binding;cadherin binding;calcium ion binding;cation binding;cell adhesion molecule binding;hormone binding;ion binding;lipoprotein particle binding;low-density lipoprotein particle binding;metal ion bindi
binding;channel regulator activity;enzyme binding;enzyme inhibitor activity;enzyme regulator activity;phosphatase binding;phosphatase inhibitor activity;phosphatase regulator activity;potassium channel regulator activity;prot
binding;enzyme binding;kinase binding;molecular transducer activity;protein binding;protein kinase binding;signal transducer activity
acid-amino acid ligase activity;adenyl nucleotide binding;adenyl ribonucleotide binding;ATP binding;binding;catalytic activity;ligase activity;ligase activity, forming carbon-nitrogen bonds;nucleotide binding;purine nucleotide b
active transmembrane transporter activity;ATPase activity;ATPase activity, coupled;ATPase activity, coupled to movement of substances;ATPase activity, coupled to transmembrane movement of ions;ATPase activity, couplec
binding;cation binding;chaperone binding;identical protein binding;ion binding;metal ion binding;protein binding;protein dimerization activity;protein homodimerization activity;protein transporter activity;substrate-specific tran
binding;nucleic acid binding;RNA binding
binding;nucleic acid binding;RNA binding
binding;nucleic acid binding;RNA binding;structural constituent of ribosome;structural molecule activity
acid-amino acid ligase activity;binding;catalytic activity;cation binding;enzyme binding;ion binding;ligase activity;ligase activity, forming carbon-nitrogen bonds;metal ion binding;NEDD8 ligase activity;protein binding;small co
binding;cation binding;ion binding;metal ion binding;nucleic acid binding transcription factor activity;protein binding;protein dimerization activity;protein heterodimerization activity;sequence-specific DNA binding transcription
binding;DNA binding;nucleic acid binding;nucleotide binding;RNA binding
catalytic activity;hydrolase activity;peptidase activity
binding;DNA binding;nucleic acid binding;protein binding transcription factor activity;transcription cofactor activity;transcription factor binding transcription factor activity
adenyl nucleotide binding;adenyl ribonucleotide binding;ATP binding;ATPase activity;binding;catalytic activity;hydrolase activity;hydrolase activity, acting on acid anhydrides;hydrolase activity, acting on acid anhydrides, in ph
binding;cation binding;chromatin binding;core promoter binding;core promoter proximal region DNA binding;core promoter proximal region sequence-specific DNA binding;core promoter sequence-specific DNA binding;DNA
acid-amino acid ligase activity;binding;catalytic activity;cation binding;ion binding;ligase activity;ligase activity, forming carbon-nitrogen bonds;metal ion binding;protein binding transcription factor activity;small conjugating p
binding;endopeptidase inhibitor activity;endopeptidase regulator activity;enzyme inhibitor activity;enzyme regulator activity;identical protein binding;metalloendopeptidase inhibitor activity;metalloenzyme inhibitor activity;meta
binding;calcium ion binding;catalytic activity;cation binding;epidermal growth factor receptor binding;epidermal growth factor-activated receptor activity;growth factor receptor binding;ion binding;kinase activity;metal ion binc
catalytic activity;hydrolase activity;hydrolase activity, acting on ester bonds;phosphatase activity;phosphoprotein phosphatase activity;phosphoric ester hydrolase activity;prenylated protein tyrosine phosphatase activity;prote
receptor activity
binding;carbohydrate binding;glycoprotein binding;protein binding
binding;eukaryotic initiation factor 4E binding;protein binding;translation initiation factor binding;translation regulator activity;translation repressor activity
acid-amino acid ligase activity;catalytic activity;ligase activity;ligase activity, forming carbon-nitrogen bonds;small conjugating protein ligase activity;ubiquitin-protein ligase activity;ubiquitin-ubiquitin ligase activity
binding;protein binding;transcription factor binding
endopeptidase inhibitor activity;endopeptidase regulator activity;enzyme inhibitor activity;enzyme regulator activity;peptidase inhibitor activity;peptidase regulator activity;serine-type endopeptidase inhibitor activity
binding;enzyme activator activity;enzyme regulator activity;GTPase activator activity;GTPase regulator activity;identical protein binding;nucleoside-triphosphatase regulator activity;protein binding;protein dimerization activity;
protein binding transcription factor activity;RNA polymerase II transcription cofactor activity;RNA polymerase II transcription factor binding transcription factor activity;transcription cofactor activity;transcription factor binding
binding;enzyme binding;extracellular matrix binding;laminin binding;molecular transducer activity;protease binding;protein binding;protein C-terminus binding;signal transducer activity
binding;catalytic activity;GTP binding;GTPase activity;guanyl nucleotide binding;guanyl ribonucleotide binding;hydrolase activity;hydrolase activity, acting on acid anhydrides;hydrolase activity, acting on acid anhydrides, in ph
adenyl nucleotide binding;adenyl ribonucleotide binding;ATP binding;binding;catalytic activity;kinase activity;nucleic acid binding;nucleobase-containing compound kinase activity;nucleotide binding;phosphotransferase activ
binding;lipid binding
catalytic activity;cytochrome-b5 reductase activity;oxidoreductase activity;oxidoreductase activity, acting on NADH or NADPH;oxidoreductase activity, acting on NADH or NADPH, heme protein as acceptor
binding;cation binding;DNA binding;ion binding;metal ion binding;nucleic acid binding
antioxidant activity;catalytic activity;oxidoreductase activity;oxidoreductase activity, acting on a sulfur group of donors;oxidoreductase activity, acting on a sulfur group of donors, NAD or NADP as acceptor;oxidoreductase ac
binding;catalytic activity;cation binding;copper ion binding;ion binding;metal ion binding;monooxygenase activity;oxidoreductase activity;oxidoreductase activity, acting on paired donors, with incorporation or reduction of mo
binding;catalytic activity;endopeptidase activity;enzyme binding;hydrolase activity;metalloendopeptidase activity;metallopeptidase activity;peptidase activity;peptidase activity, acting on L-amino acid peptides;protease bindi
adenyl nucleotide binding;adenyl ribonucleotide binding;ATP binding;binding;nucleic acid binding;nucleotide binding;purine nucleotide binding;purine ribonucleoside triphosphate binding;purine ribonucleotide binding;ribonuc
binding;cyclic nucleotide binding;cyclic-di-GMP binding;enzyme binding;guanyl nucleotide binding;guanyl ribonucleotide binding;identical protein binding;kinase binding;nucleotide binding;protein binding;protein dimerization
binding;cation binding;ion binding;metal ion binding

catalytic activity;glucosyltransferase activity;protein xylosyltransferase activity;transferase activity;transferase activity, transferring glycosyl groups;transferase activity, transferring hexosyl groups;transferase activity, transferri
catalytic activity;oxidoreductase activity
1-phosphatidylinositol binding;apolipoprotein A-I binding;apolipoprotein binding;binding;cargo receptor activity;high-density lipoprotein particle binding;high-density lipoprotein particle receptor activity;lipid binding;lipopolysa
binding;identical protein binding;protein binding;protein dimerization activity;protein domain specific binding;protein homodimerization activity
AU-rich element binding;binding;nucleic acid binding;RNA binding
aldose 1-epimerase activity;binding;carbohydrate binding;catalytic activity;isomerase activity;racemase and epimerase activity;racemase and epimerase activity, acting on carbohydrates and derivatives
binding;catalytic activity;hydrolase activity;hydrolase activity, acting on acid anhydrides;hydrolase activity, acting on acid anhydrides, in phosphorus-containing anhydrides;m7G(5')pppN diphosphatase activity;nucleic acid bi
binding;enzyme binding;kinetochore binding;protein binding
enzyme regulator activity;kinase regulator activity;phosphorylase kinase regulator activity;protein kinase regulator activity
structural constituent of ribosome;structural molecule activity
adenyl nucleotide binding;adenyl ribonucleotide binding;ATP binding;binding;catalytic activity;cation binding;histone kinase activity;histone serine kinase activity;ion binding;kinase activity;metal ion binding;nucleotide binding
binding;histone binding;protein binding;protein binding transcription factor activity;transcription cofactor activity;transcription corepressor activity;transcription factor binding transcription factor activity
binding;cation binding;ion binding;lipid binding;metal ion binding;phosphatidylinositol binding;phosphatidylinositol-3-phosphate binding;phospholipid binding;transition metal ion binding;zinc ion binding
catalytic activity;oxidoreductase activity
binding;lipid binding;phosphatidylinositol binding;phosphatidylinositol-4,5-bisphosphate binding;phospholipid binding
binding;calcium ion binding;cation binding;ion binding;metal ion binding
binding;enzyme binding;GTPase binding;protein binding;Ran GTPase binding;Ras GTPase binding;small GTPase binding
protein binding transcription factor activity;transcription coactivator activity;transcription cofactor activity;transcription factor binding transcription factor activity
binding;nucleic acid binding;RNA binding
nucleobase-containing compound transmembrane transporter activity;nucleoside transmembrane transporter activity;substrate-specific transmembrane transporter activity;substrate-specific transporter activity;transmembra
binding;identical protein binding;protein binding;protein dimerization activity;protein homodimerization activity
5S rRNA binding;binding;nucleic acid binding;RNA binding;rRNA binding;structural constituent of ribosome;structural molecule activity
adenyl nucleotide binding;adenyl ribonucleotide binding;ATP binding;binding;catalytic activity;enzyme inhibitor activity;enzyme regulator activity;kinase activity;kinase inhibitor activity;kinase regulator activity;MAP kinase kina
catalytic activity;oxidoreductase activity
acid-amino acid ligase activity;binding;catalytic activity;histone binding;ligase activity;ligase activity, forming carbon-nitrogen bonds;methylated histone residue binding;nucleic acid binding;protein binding;RNA binding;single
aminopeptidase activity;binding;catalytic activity;cation binding;exopeptidase activity;hydrolase activity;ion binding;manganese ion binding;metal ion binding;metallopeptidase activity;peptidase activity;peptidase activity, acti
binding;calcium ion binding;calcium-dependent protein binding;cation binding;ion binding;lipid transporter activity;metal ion binding;phospholipid scramblase activity;phospholipid transporter activity;protein binding;substrate
binding;cytoskeletal protein binding;L27 domain binding;protein binding;protein domain specific binding
binding;catalytic activity;endopeptidase inhibitor activity;endopeptidase regulator activity;enzyme inhibitor activity;enzyme regulator activity;insulin-like growth factor-activated receptor activity;kinase activity;molecular transd
binding;catalytic activity;cation binding;ion binding;ligase activity;metal ion binding;transition metal ion binding;zinc ion binding
binding;nucleic acid binding;ribonucleoprotein binding;ribosome binding;RNA binding;translation factor activity, nucleic acid binding;translation initiation factor activity
catalytic activity;cation transmembrane transporter activity;hydrogen ion transmembrane transporter activity;inorganic cation transmembrane transporter activity;ion transmembrane transporter activity;monovalent inorganic c
catalytic activity;oxidoreductase activity;oxidoreductase activity, acting on a sulfur group of donors;oxidoreductase activity, acting on a sulfur group of donors, disulfide as acceptor;peptide-methionine-(S)-S-oxide reductase
binding;calcium ion binding;cation binding;ion binding;metal ion binding
binding;cation binding;ion binding;metal ion binding;transition metal ion binding;zinc ion binding
binding;cation binding;ion binding;metal ion binding;transition metal ion binding;zinc ion binding
binding;catalytic activity;GTP binding;guanyl nucleotide binding;guanyl ribonucleotide binding;guanylyltransferase activity;mannose-1-phosphate guanylyltransferase activity;mannose-phosphate guanylyltransferase activity;r
binding;identical protein binding;lipid binding;phosphatidylinositol binding;phospholipid binding;protein binding
binding;histone binding;methylated histone residue binding;protein binding
binding;lipid binding
binding;catalytic activity;deoxyribonuclease activity;deoxyribonuclease II activity;DNA binding;endodeoxyribonuclease activity;endodeoxyribonuclease activity, producing 3'-phosphomonoesters;endonuclease activity;endonu
acid-amino acid ligase activity;adenyl nucleotide binding;adenyl ribonucleotide binding;ATP binding;binding;catalytic activity;enzyme binding;ligase activity;ligase activity, forming carbon-nitrogen bonds;nucleotide binding;pr
adenyl nucleotide binding;adenyl ribonucleotide binding;ATP binding;ATPase activity;binding;catalytic activity;hydrolase activity;hydrolase activity, acting on acid anhydrides;hydrolase activity, acting on acid anhydrides, in ph
actin binding;binding;cytoskeletal protein binding;identical protein binding;myosin binding;protein binding;protein dimerization activity;protein homodimerization activity;receptor binding
binding;calcium ion binding;cation binding;ion binding;metal ion binding;transition metal ion binding;zinc ion binding
binding;cation binding;heme binding;ion binding;iron ion binding;metal ion binding;tetrapyrrole binding;transition metal ion binding
binding;fibroblast growth factor binding;growth factor binding;protein binding
catalytic activity;NADH dehydrogenase (quinone) activity;NADH dehydrogenase (ubiquinone) activity;NADH dehydrogenase activity;oxidoreductase activity;oxidoreductase activity, acting on NADH or NADPH;oxidoreductase
binding;cation binding;ion binding;metal ion binding
binding;DNA binding;nucleic acid binding;protein binding transcription factor activity;ribonucleoprotein binding;transcription coactivator activity;transcription cofactor activity;transcription factor binding transcription factor ac
catalytic activity;endonuclease activity;endonuclease activity, active with either ribo- or deoxyribonucleic acids and producing 5'-phosphomonoesters;endoribonuclease activity;endoribonuclease activity, producing 5'-phosph
cation transmembrane transporter activity;hydrogen ion transmembrane transporter activity;inorganic cation transmembrane transporter activity;ion transmembrane transporter activity;monovalent inorganic cation transmemb
binding;nucleic acid binding;protein transporter activity;RNA binding;RNA cap binding;substrate-specific transporter activity;transporter activity
catalytic activity;NADH dehydrogenase (quinone) activity;NADH dehydrogenase (ubiquinone) activity;NADH dehydrogenase activity;oxidoreductase activity;oxidoreductase activity, acting on NADH or NADPH;oxidoreductase
binding;catalytic activity;cation binding;cysteine-type peptidase activity;hydrolase activity;ion binding;metal ion binding;metallopeptidase activity;peptidase activity;peptidase activity, acting on L-amino acid peptides;protein b
binding;chromatin binding;DNA binding;methyl-CpG binding;nucleic acid binding;nucleotide binding;sequence-specific DNA binding
aldo-keto reductase (NADP) activity;binding;catalytic activity;cation binding;cation transmembrane transporter activity;cytochrome-c oxidase activity;enzyme binding;heme binding;heme-copper terminal oxidase activity;hydr
binding;catalytic activity;coenzyme binding;cofactor binding;dihydrofolate reductase activity;drug binding;mRNA binding;NADP binding;nucleic acid binding;nucleotide binding;oxidoreductase activity;oxidoreductase activity,
binding;isoprenoid binding;lipid binding;retinal binding;retinoid binding;retinol binding;retinol transporter activity;transmembrane transporter activity;transporter activity;vitamin binding;vitamin transporter activity
binding;cysteine-type endopeptidase inhibitor activity;endopeptidase inhibitor activity;endopeptidase regulator activity;enzyme binding;enzyme inhibitor activity;enzyme regulator activity;peptidase inhibitor activity;peptidase
alpha-galactosidase activity;binding;carbohydrate binding;catalytic activity;galactosidase activity;galactoside binding;hydrolase activity;hydrolase activity, acting on glycosyl bonds;hydrolase activity, hydrolyzing O-glycosyl co
binding;catalytic activity;collagen binding;cysteine-type endopeptidase activity;cysteine-type peptidase activity;endopeptidase activity;fibronectin binding;glycoprotein binding;histone binding;hydrolase activity;peptidase acti
binding;catalytic activity;cation binding;endopeptidase activity;hydrolase activity;ion binding;metal ion binding;metalloendopeptidase activity;metallopeptidase activity;peptidase activity;peptidase activity, acting on L-amino a
6,7-dihydropteridine reductase activity;binding;catalytic activity;coenzyme binding;cofactor binding;electron carrier activity;NAD binding;NADH binding;NADP binding;NADPH binding;nucleotide binding;oxidoreductase activi
catalytic activity;cation transmembrane transporter activity;cytochrome-c oxidase activity;heme-copper terminal oxidase activity;hydrogen ion transmembrane transporter activity;inorganic cation transmembrane transporter a
binding;catalytic activity;DNA binding;DNA-directed RNA polymerase activity;nucleic acid binding;nucleotidyltransferase activity;RNA polymerase activity;transferase activity;transferase activity, transferring phosphorus-conta
3'-5' DNA helicase activity;adenyl deoxyribonucleotide binding;adenyl nucleotide binding;adenyl ribonucleotide binding;ATP binding;ATPase activity;ATPase activity, coupled;ATP-dependent DNA helicase activity;ATP-depend
binding;catalytic activity;cis-trans isomerase activity;cyclosporin A binding;drug binding;isomerase activity;peptide binding;peptidyl-prolyl cis-trans isomerase activity
binding;damaged DNA binding;DNA binding;nucleic acid binding;single-stranded DNA binding;structure-specific DNA binding
binding;catalytic activity;cation binding;cytoskeletal protein binding;GDP binding;GTP binding;GTPase activity;guanyl nucleotide binding;guanyl ribonucleotide binding;hydrolase activity;hydrolase activity, acting on acid anhy
endopeptidase inhibitor activity;endopeptidase regulator activity;enzyme inhibitor activity;enzyme regulator activity;peptidase inhibitor activity;peptidase regulator activity;serine-type endopeptidase inhibitor activity
amine binding;amino acid binding;antioxidant activity;binding;carboxylic acid binding;catalytic activity;glutathione binding;glutathione peroxidase activity;modified amino acid binding;oxidoreductase activity;oxidoreductase a
binding;chromatin binding;hormone receptor binding;nuclear hormone receptor binding;protein binding;protein complex scaffold;protein domain specific binding;receptor binding;receptor signaling complex scaffold activity;S
carboxypeptidase activity;catalytic activity;exopeptidase activity;hydrolase activity;peptidase activity;peptidase activity, acting on L-amino acid peptides;serine hydrolase activity;serine-type carboxypeptidase activity;serine-t
binding;catalytic activity;cis-trans isomerase activity;cyclosporin A binding;drug binding;isomerase activity;peptide binding;peptidyl-prolyl cis-trans isomerase activity
adenyl nucleotide binding;adenyl ribonucleotide binding;apoptotic protease activator activity;ATP binding;binding;caspase activator activity;caspase regulator activity;catalytic activity;enzyme activator activity;enzyme regulat
binding;catalytic activity;cation binding;cysteine-type peptidase activity;enzyme regulator activity;hydrolase activity;ion binding;metal ion binding;metallopeptidase activity;peptidase activity;peptidase activity, acting on L-ami
acid-amino acid ligase activity;binding;catalytic activity;enzyme binding;enzyme regulator activity;glutamate-cysteine ligase activity;glutamate-cysteine ligase catalytic subunit binding;ligase activity;ligase activity, forming carb
AT DNA binding;binding;cation binding;DNA bending activity;DNA binding;ion binding;metal ion binding;nucleic acid binding;peptide binding;sequence-specific DNA binding;transition metal ion binding;zinc ion binding
adenyl nucleotide binding;adenyl ribonucleotide binding;androgen receptor binding;ATP binding;ATPase activity;ATPase activity, coupled;binding;catalytic activity;cyclin-dependent protein kinase activity;DNA-dependent ATP
acyl-CoA thioesterase activity;catalytic activity;CoA hydrolase activity;hydrolase activity;hydrolase activity, acting on ester bonds;palmitoyl-(protein) hydrolase activity;palmitoyl-CoA hydrolase activity;thiolester hydrolase activ
binding;death domain binding;protein binding;protein domain specific binding
adenyl nucleotide binding;adenyl ribonucleotide binding;ATP binding;binding;catalytic activity;cation binding;coenzyme binding;cofactor binding;ion binding;isocitrate dehydrogenase (NAD+) activity;isocitrate dehydrogenase
binding;calcium channel activity;calcium channel regulator activity;cation binding;cation channel activity;cation transmembrane transporter activity;channel activity;channel regulator activity;gated channel activity;ion binding;i
active transmembrane transporter activity;ATPase activity;ATPase activity, coupled;ATPase activity, coupled to movement of substances;ATPase activity, coupled to transmembrane movement of ions;ATPase activity, couplec
antigen binding;binding;MHC class II protein complex binding;MHC protein complex binding
binding;enzyme binding;GTPase binding;protein binding;Ran GTPase binding;Ras GTPase binding;small GTPase binding;transporter activity
binding;nucleic acid binding;RNA binding;structural constituent of ribosome;structural molecule activity
binding;cation binding;chromatin binding;enzyme regulator activity;identical protein binding;ion binding;kinase regulator activity;metal ion binding;protein binding;protein domain specific binding;protein kinase regulator activit
binding;binding, bridging;protein binding;protein binding, bridging;protein domain specific binding;receptor activity;SH3 domain binding;SH3/SH2 adaptor activity;signaling adaptor activity
catalytic activity;hydrolase activity
binding;enzyme binding;GTPase binding;GTP-Rho binding;protein binding;Ras GTPase binding;Rho GTPase binding;small GTPase binding

binding;catalytic activity;coenzyme binding;cofactor binding;dihydroorotate dehydrogenase activity;drug binding;FMN binding;nucleotide binding;oxidoreductase activity;oxidoreductase activity, acting on the CH-CH group o
active transmembrane transporter activity;adenyl nucleotide binding;adenyl ribonucleotide binding;ADP binding;antigen binding;ATP binding;ATPase activity;ATPase activity, coupled;ATPase activity, coupled to movement of s
acid-amino acid ligase activity;adenyl nucleotide binding;adenyl ribonucleotide binding;ATP binding;binding;catalytic activity;ligase activity;ligase activity, forming carbon-nitrogen bonds;nucleotide binding;purine nucleotide b
actin binding;actin filament binding;binding;calmodulin-dependent protein kinase activity;catalytic activity;cytoskeletal protein binding;kinase activity;phosphotransferase activity, alcohol group as acceptor;protein binding;pro
binding;enzyme activator activity;enzyme regulator activity;lipase activator activity;phospholipase activator activity;protein binding;SNAP receptor activity;SNARE binding;sphingomyelin phosphodiesterase activator activity
actin binding;binding;binding, bridging;cytoskeletal protein binding;enzyme binding;GTPase binding;protein binding;protein binding, bridging;Rac GTPase binding;Ras GTPase binding;Rho GTPase binding;SH3/SH2 adaptor
binding;nucleic acid binding;RNA binding;single-stranded RNA binding;tRNA binding
binding;enzyme regulator activity;GDP binding;GTP binding;guanyl nucleotide binding;guanyl ribonucleotide binding;nucleic acid binding;nucleotide binding;purine nucleotide binding;purine ribonucleoside triphosphate bindi
binding;G-protein coupled receptor activity;molecular transducer activity;PDZ domain binding;protein binding;protein domain specific binding;receptor activity;signal transducer activity;signaling receptor activity;transmembra
catalytic activity
binding;DNA binding;histone binding;methylated histone residue binding;nucleic acid binding;protein binding;regulatory region DNA binding;regulatory region nucleic acid binding;transcription regulatory region DNA binding
ATPase activity;ATPase activity, coupled;ATPase activity, coupled to movement of substances;binding;catalytic activity;enzyme activator activity;enzyme binding;enzyme regulator activity;GTPase activator activity;GTPase bir
binding;nucleic acid binding;nucleotide binding;RNA binding
adenyl nucleotide binding;adenyl ribonucleotide binding;ATP binding;binding;catalytic activity;DNA binding;double-stranded RNA binding;hormone receptor binding;nuclear hormone receptor binding;nucleic acid binding;nuc
activating transcription factor binding;binding;cation binding;chromatin binding;core promoter proximal region DNA binding;core promoter proximal region sequence-specific DNA binding;co-SMAD binding;cytokine receptor
binding;carbohydrate binding;catalytic activity;estradiol 17-beta-dehydrogenase activity;glycosaminoglycan binding;heparin binding;oxidoreductase activity;oxidoreductase activity, acting on CH-OH group of donors;oxidored
binding;cytokine binding;growth factor binding;protein binding;transforming growth factor beta binding
binding;calcium ion binding;cation binding;ion binding;metal ion binding
binding;carbon-sulfur lyase activity;catalytic activity;cofactor binding;cysteine-S-conjugate beta-lyase activity;kynurenine-glyoxylate transaminase activity;kynurenine-oxoglutarate transaminase activity;lyase activity;pyridoxal
1-acylglycerol-3-phosphate O-acyltransferase activity;1-acylglycerophosphocholine O-acyltransferase activity;1-alkylglycerophosphocholine O-acetyltransferase activity;acetyltransferase activity;acylglycerol O-acyltransferas
ARF guanyl-nucleotide exchange factor activity;enzyme regulator activity;GTPase regulator activity;guanyl-nucleotide exchange factor activity;nucleoside-triphosphatase regulator activity;small GTPase regulator activity
binding;histone binding;methylated histone residue binding;protein binding
3'-5' exonuclease activity;3'-5'-exoribonuclease activity;binding;catalytic activity;cation binding;exonuclease activity;exonuclease activity, active with either ribo- or deoxyribonucleic acids and producing 5'-phosphomonoeste
binding;nucleotide binding
binding;nucleotide binding
catalytic activity;intramolecular transferase activity;isomerase activity;pseudouridine synthase activity
14-3-3 protein binding;5S rDNA binding;binding;DNA binding;enzyme binding;kinase binding;nucleic acid binding;protein binding;protein complex binding;protein kinase binding;rDNA binding;regulatory region DNA binding;re
binding;catalytic activity;cis-trans isomerase activity;isomerase activity;nucleotide binding;peptidyl-prolyl cis-trans isomerase activity
binding;protein binding;protein complex binding
catalytic activity;endopeptidase activity;hydrolase activity;peptidase activity;peptidase activity, acting on L-amino acid peptides
binding;cell adhesion molecule binding;coreceptor activity;identical protein binding;molecular transducer activity;protein binding;protein dimerization activity;protein homodimerization activity;receptor activity;signal transduce
binding;cation binding;core promoter proximal region DNA binding;core promoter proximal region sequence-specific DNA binding;DNA binding;ion binding;metal ion binding;nucleic acid binding;regulatory region DNA bindin
catalytic activity;hyaluronan synthase activity;transferase activity;transferase activity, transferring glycosyl groups;transferase activity, transferring hexosyl groups;UDP-glycosyltransferase activity
bHLH transcription factor binding;binding;catalytic activity;cation binding;coenzyme binding;cofactor binding;core promoter binding;core promoter sequence-specific DNA binding;deacetylase activity;DNA binding;enzyme bi
adenyl nucleotide binding;adenyl ribonucleotide binding;ATP binding;binding;cation binding;chaperone binding;cytokine receptor binding;enzyme binding;interferon-gamma receptor binding;ion binding;kinase binding;metal i
binding;enzyme binding;GTPase binding;kinase binding;protein binding;protein kinase binding;Ral GTPase binding;Ras GTPase binding;small GTPase binding
binding;cation binding;DNA binding;ion binding;metal ion binding;nucleic acid binding;transition metal ion binding;zinc ion binding
protein transporter activity;receptor activity;substrate-specific transporter activity;transporter activity
acid-amino acid ligase activity;binding;catalytic activity;cation binding;chromatin binding;ion binding;ligase activity;ligase activity, forming carbon-nitrogen bonds;metal ion binding;protein binding;protein domain specific bind
binding;DNA binding;nucleic acid binding;nucleic acid binding transcription factor activity;protein binding;RNA polymerase II transcription factor binding;sequence-specific DNA binding transcription factor activity;transcriptio
binding;calcium-dependent protein binding;DNA binding;enzyme binding;identical protein binding;nucleic acid binding;protein binding;protein binding transcription factor activity;protein dimerization activity;protein homodime
1-acylglycerol-3-phosphate O-acyltransferase activity;acylglycerol O-acyltransferase activity;catalytic activity;O-acyltransferase activity;transferase activity;transferase activity, transferring acyl groups;transferase activity, tran
3-hydroxybutyrate dehydrogenase activity;binding;catalytic activity;coenzyme binding;cofactor binding;NAD binding;nucleotide binding;oxidoreductase activity;oxidoreductase activity, acting on CH-OH group of donors;oxido
catalytic activity;methyltransferase activity;N-terminal protein N-methyltransferase activity;protein methyltransferase activity;transferase activity;transferase activity, transferring one-carbon groups
structural constituent of ribosome;structural molecule activity
binding;catalytic activity;enzyme binding;GTP binding;GTPase activity;GTPase binding;guanyl nucleotide binding;guanyl ribonucleotide binding;hydrolase activity;hydrolase activity, acting on acid anhydrides;hydrolase activit
beta-catenin binding;binding;DNA binding;histone binding;nucleic acid binding;protein binding;protein binding transcription factor activity;protein N-terminus binding;regulatory region DNA binding;regulatory region nucleic ac
protein tag
adenyl nucleotide binding;adenyl ribonucleotide binding;ATP binding;binding;catalytic activity;cation binding;ion binding;kinase activity;metal ion binding;nucleotide binding;phosphotransferase activity, alcohol group as acce
catalytic activity;disulfide oxidoreductase activity;intramolecular oxidoreductase activity;intramolecular oxidoreductase activity, interconverting keto- and enol-groups;intramolecular oxidoreductase activity, transposing S-S bo
alpha-1,3-mannosyltransferase activity;alpha-1,6-mannosyltransferase activity;binding;calcium-dependent protein binding;catalytic activity;glycolipid 3-alpha-mannosyltransferase activity;glycolipid 6-alpha-mannosyltransfera
binding;nucleic acid binding;RNA binding
binding;cation binding;ion binding;metal ion binding
binding;calcium ion binding;carbohydrate binding;cation binding;extracellular matrix binding;ion binding;metal ion binding
catalytic activity;hydrolase activity;hydrolase activity, acting on acid anhydrides;hydrolase activity, acting on acid anhydrides, in phosphorus-containing anhydrides;microtubule motor activity;motor activity;nucleoside-triphosp
binding;carbohydrate binding
3'-5' exonuclease activity;3'-5'-exoribonuclease activity;AU-rich element binding;binding;catalytic activity;exonuclease activity;exonuclease activity, active with either ribo- or deoxyribonucleic acids and producing 5'-phosph
3'-5' exonuclease activity;3'-5'-exoribonuclease activity;binding;catalytic activity;exonuclease activity;exonuclease activity, active with either ribo- or deoxyribonucleic acids and producing 5'-phosphomonoesters;exoribonucl
adenyl nucleotide binding;adenyl ribonucleotide binding;ATP binding;binding;catalytic activity;CTP synthase activity;ligase activity;ligase activity, forming carbon-nitrogen bonds;nucleotide binding;purine nucleotide binding;p
binding;cation binding;ion binding;metal ion binding
binding;nucleic acid binding;RNA binding;snoRNA binding
binding;catalytic activity;cation binding;hydrolase activity;hydrolase activity, acting on glycosyl bonds;hydrolase activity, hydrolyzing O-glycosyl compounds;ion binding;metal ion binding;poly(ADP-ribose) glycohydrolase activ
3'(2'),5'-bisphosphate nucleotidase activity;3'-nucleotidase activity;binding;catalytic activity;cation binding;hydrolase activity;hydrolase activity, acting on ester bonds;inositol monophosphate phosphatase activity;inositol or p
binding;catalytic activity;coenzyme binding;cofactor binding;double-stranded RNA binding;enzyme inhibitor activity;enzyme regulator activity;flavin adenine dinucleotide binding;kinase inhibitor activity;kinase regulator activity
cation channel activity;cation transmembrane transporter activity;channel activity;ion channel activity;ion transmembrane transporter activity;passive transmembrane transporter activity;substrate-specific channel activity;sub
binding;DNA binding;nucleic acid binding
binding;enzyme binding;enzyme regulator activity;GDP-dissociation inhibitor activity;GTPase binding;GTPase regulator activity;identical protein binding;nucleoside-triphosphatase regulator activity;protein binding;Ras GTPas
binding;DNA binding;nucleic acid binding;nucleotide binding
adenyl nucleotide binding;adenyl ribonucleotide binding;ATP binding;binding;catalytic activity;identical protein binding;kinase activity;nucleotide binding;phosphotransferase activity, alcohol group as acceptor;protein binding;
acireductone synthase activity;binding;catalytic activity;cation binding;hydrolase activity;hydrolase activity, acting on ester bonds;ion binding;magnesium ion binding;metal ion binding;phosphatase activity;phosphoric ester h
protein transporter activity;substrate-specific transporter activity;transporter activity
catalytic activity;methyltransferase activity;protein methyltransferase activity;transferase activity;transferase activity, transferring one-carbon groups
binding;catalytic activity;GDP binding;GTP binding;GTPase activity;guanyl nucleotide binding;guanyl ribonucleotide binding;hydrolase activity;hydrolase activity, acting on acid anhydrides;hydrolase activity, acting on acid anh
binding;DNA binding;nucleic acid binding;nucleic acid binding transcription factor activity;sequence-specific DNA binding;sequence-specific DNA binding transcription factor activity
receptor activity
acid-amino acid ligase activity;binding;catalytic activity;cation binding;co-SMAD binding;DNA binding;ion binding;ligase activity;ligase activity, forming carbon-nitrogen bonds;metal ion binding;nucleic acid binding;protein bin
calcium activated cation channel activity;calcium-activated potassium channel activity;catalytic activity;cation channel activity;cation transmembrane transporter activity;channel activity;gated channel activity;hydrolase activi
binding;enzyme binding;GTPase binding;nucleic acid binding;protein binding;Ral GTPase binding;Ras GTPase binding;RNA binding;small GTPase binding;snRNA binding;U6 snRNA binding
binding;nucleic acid binding;RNA binding
alcohol binding;amine binding;binding;cation binding;identical protein binding;ion binding;lipid binding;phosphatidylcholine binding;phospholipid binding;protein binding;protein dimerization activity;protein homodimerization
binding;DNA binding;nucleic acid binding;protein binding;sequence-specific DNA binding;transcription factor binding
adenyl nucleotide binding;adenyl ribonucleotide binding;ATP binding;binding;catalytic activity;cyclase activity;ligase activity;ligase activity, forming phosphoric ester bonds;nucleic acid binding;nucleotide binding;purine nucle
binding;identical protein binding;protein binding;structural constituent of cytoskeleton;structural molecule activity
binding;calcium ion binding;cation binding;ion binding;metal ion binding;protein binding;unfolded protein binding
binding;channel inhibitor activity;channel regulator activity;chloride channel inhibitor activity;chloride channel regulator activity;ion channel inhibitor activity;protein binding;SNAP receptor activity;SNARE binding;syntaxin bind
adenyl nucleotide binding;adenyl ribonucleotide binding;ATP binding;binding;catalytic activity;cation binding;ion binding;kinase activity;magnesium ion binding;MAP kinase kinase activity;MAP kinase kinase kinase activity;me
binding;channel inhibitor activity;channel regulator activity;enzyme binding;enzyme inhibitor activity;enzyme regulator activity;ion channel inhibitor activity;phosphatase binding;phosphatase inhibitor activity;phosphatase regu
catalytic activity;NADH dehydrogenase (quinone) activity;NADH dehydrogenase (ubiquinone) activity;NADH dehydrogenase activity;oxidoreductase activity;oxidoreductase activity, acting on NADH or NADPH;oxidoreductase
alcohol binding;binding;carbohydrate binding;catalytic activity;dolichyl-phosphate beta-D-mannosyltransferase activity;dolichyl-phosphate-mannose-protein mannosyltransferase activity;mannose binding;mannosyltransferas
binding;double-stranded RNA binding;enzyme activator activity;enzyme binding;enzyme regulator activity;identical protein binding;nucleic acid binding;protein binding;protein dimerization activity;protein homodimerization ac
carboxylic ester hydrolase activity;catalytic activity;hydrolase activity;hydrolase activity, acting on ester bonds;lipase activity;lysophospholipase activity;palmitoyl-(protein) hydrolase activity;phospholipase activity;thiolester hy
binding;catalytic activity;cation binding;hydrolase activity;hydrolase activity, acting on ester bonds;ion binding;magnesium ion binding;manganese ion binding;metal ion binding;phosphatase activity;phosphoprotein phosphat
binding;protein binding;transcription factor binding
binding;cofactor binding;pyridoxal phosphate binding;vitamin B6 binding;vitamin binding

binding;cation binding;DNA binding;ion binding;metal ion binding;nucleic acid binding;transition metal ion binding;zinc ion binding
catalytic activity;NADH dehydrogenase (quinone) activity;NADH dehydrogenase (ubiquinone) activity;NADH dehydrogenase activity;oxidoreductase activity;oxidoreductase activity, acting on NADH or NADPH;oxidoreductase
adenyl nucleotide binding;adenyl ribonucleotide binding;ATP binding;ATPase activity;binding;catalytic activity;enzyme binding;hydrolase activity;hydrolase activity, acting on acid anhydrides;hydrolase activity, acting on acid a
acid-amino acid ligase activity;binding;catalytic activity;cation binding;ion binding;ligase activity;ligase activity, forming carbon-nitrogen bonds;metal ion binding;nucleic acid binding;small conjugating protein ligase activity;tra
catalytic activity;oxidoreductase activity;oxidoreductase activity, acting on a sulfur group of donors;oxidoreductase activity, acting on a sulfur group of donors, disulfide as acceptor;protein-disulfide reductase (glutathione) act
binding;calcium ion binding;cation binding;extracellular matrix structural constituent;ion binding;metal ion binding;structural molecule activity
binding;calcium ion binding;catalytic activity;cation binding;endopeptidase activity;hydrolase activity;ion binding;metal ion binding;peptidase activity;peptidase activity, acting on L-amino acid peptides;serine hydrolase activit
apolipoprotein binding;binding;catalytic activity;endopeptidase activity;hydrolase activity;peptidase activity;peptidase activity, acting on L-amino acid peptides;protein binding;protein domain specific binding;receptor binding
binding;chaperone binding;protein binding;structural molecule activity
adenyl nucleotide binding;adenyl ribonucleotide binding;ATP binding;binding;catalytic activity;cation binding;identical protein binding;ion binding;kinase activity;MAP kinase kinase kinase activity;metal ion binding;molecular t
binding;catalytic activity;ceramidase activity;glucosylceramidase activity;hydrolase activity;hydrolase activity, acting on carbon-nitrogen (but not peptide) bonds;hydrolase activity, acting on carbon-nitrogen (but not peptide) b
adenyl nucleotide binding;adenyl ribonucleotide binding;ATP binding;binding;catalytic activity;cation binding;deoxynucleoside kinase activity;identical protein binding;ion binding;kinase activity;metal ion binding;nucleobase-c
5,10-methylenetetrahydrofolate-dependent methyltransferase activity;amine binding;amino acid binding;binding;carboxylic acid binding;catalytic activity;cofactor binding;drug binding;folic acid binding;methyltransferase acti
catalytic activity;hydroxymethylbilane synthase activity;transferase activity;transferase activity, transferring alkyl or aryl (other than methyl) groups
binding;cation binding;identical protein binding;ion binding;metal ion binding;mRNA binding;nucleic acid binding;protein binding;protein dimerization activity;protein homodimerization activity;RNA binding;single-stranded RN
binding;calcium ion binding;cation binding;collagen binding;extracellular matrix binding;ion binding;metal ion binding;protein binding
binding;catalytic activity;cation binding;cation transmembrane transporter activity;cytochrome-c oxidase activity;heme-copper terminal oxidase activity;hydrogen ion transmembrane transporter activity;inorganic cation trans
endopeptidase inhibitor activity;endopeptidase regulator activity;enzyme inhibitor activity;enzyme regulator activity;peptidase inhibitor activity;peptidase regulator activity;serine-type endopeptidase inhibitor activity
acid phosphatase activity;catalytic activity;hydrolase activity;hydrolase activity, acting on ester bonds;phosphatase activity;phosphoric ester hydrolase activity
active transmembrane transporter activity;carbohydrate transmembrane transporter activity;cation transmembrane transporter activity;cation:sugar symporter activity;glucose transmembrane transporter activity;hexose trans
binding;binding, bridging;cation binding;extracellular matrix binding;extracellular matrix structural constituent;ion binding;metal ion binding;protein binding;protein binding, bridging;structural molecule activity
binding;enzyme binding;protein binding
binding;complement binding;protein binding
activin binding;binding;carbohydrate binding;catalytic activity;cytokine binding;cytokine receptor binding;galactose binding;glycosaminoglycan binding;growth factor binding;identical protein binding;kinase activity;molecular
binding;nucleic acid binding;nucleotide binding;RNA binding
cation transmembrane transporter activity;hydrogen ion transmembrane transporter activity;inorganic cation transmembrane transporter activity;ion transmembrane transporter activity;monovalent inorganic cation transmemb
adenyl nucleotide binding;adenyl ribonucleotide binding;ATP binding;binding;catalytic activity;double-stranded RNA binding;enzyme regulator activity;eukaryotic translation initiation factor 2alpha kinase activity;kinase activity
antioxidant activity;binding;catalytic activity;cation binding;dioxygenase activity;heme binding;ion binding;iron ion binding;metal ion binding;oxidoreductase activity;oxidoreductase activity, acting on paired donors, with incorp
binding;chromatin binding;histone acetyl-lysine binding;histone binding;protein binding
adenyl nucleotide binding;adenyl ribonucleotide binding;ATP binding;binding;catalytic activity;deoxycytidine kinase activity;deoxynucleoside kinase activity;drug binding;identical protein binding;kinase activity;nucleobase-co
catalytic activity;endopeptidase activity;hydrolase activity;peptidase activity;peptidase activity, acting on L-amino acid peptides;threonine-type endopeptidase activity;threonine-type peptidase activity
binding;catalytic activity;cation binding;copper ion binding;ion binding;metal ion binding;oxidoreductase activity;oxidoreductase activity, acting on the CH-NH2 group of donors;oxidoreductase activity, acting on the CH-NH2
adenyl nucleotide binding;adenyl ribonucleotide binding;ATP binding;binding;catalytic activity;kinase activity;lipid binding;molecular transducer activity;nucleotide binding;phosphatidylserine binding;phospholipid binding;pho
active transmembrane transporter activity;amine transmembrane transporter activity;amino acid transmembrane transporter activity;antiporter activity;arginine transmembrane transporter activity;basic amino acid transmemb
adenyl nucleotide binding;adenyl ribonucleotide binding;ATP binding;binding;catalytic activity;kinase activity;nucleotide binding;phosphotransferase activity, alcohol group as acceptor;protein kinase activity;protein serine/thre
actin binding;actin monomer binding;adenyl nucleotide binding;adenyl-nucleotide exchange factor activity;ATPase regulator activity;binding;cytoskeletal protein binding;enzyme regulator activity;lipid binding;nucleoside-triph
binding;carbon-carbon lyase activity;carboxylic acid binding;catalytic activity;cation binding;coenzyme binding;cofactor binding;fatty acid binding;fatty-acyl-CoA binding;hydroxymethylglutaryl-CoA lyase activity;identical pro
catalytic activity;endopeptidase activity;hydrolase activity;peptidase activity;peptidase activity, acting on L-amino acid peptides;threonine-type endopeptidase activity;threonine-type peptidase activity
aspartic-type endopeptidase activity;aspartic-type peptidase activity;catalytic activity;cyclin-dependent protein kinase inhibitor activity;cyclin-dependent protein kinase regulator activity;cysteine-type endopeptidase activity;c
binding;catalytic activity;cation binding;DNA primase activity;DNA-directed RNA polymerase activity;ion binding;metal ion binding;nucleotidyltransferase activity;RNA polymerase activity;transferase activity;transferase activity
catalytic activity;cysteine-type endopeptidase activity;cysteine-type peptidase activity;endopeptidase activity;hydrolase activity;peptidase activity;peptidase activity, acting on L-amino acid peptides
binding;calcium ion binding;carbohydrate binding;cation binding;collagen binding;enzyme binding;extracellular matrix structural constituent;glycoprotein binding;glycosaminoglycan binding;heparan sulfate proteoglycan bind
binding;catalytic activity;coenzyme binding;cofactor binding;flavin adenine dinucleotide binding;oxidoreductase activity;oxidoreductase activity, acting on the CH-CH group of donors;oxidoreductase activity, acting on the CH
binding;catalytic activity;GDP binding;GTP binding;GTPase activity;guanyl nucleotide binding;guanyl ribonucleotide binding;hydrolase activity;hydrolase activity, acting on acid anhydrides;hydrolase activity, acting on acid anh
catalytic activity;methyltransferase activity;S-adenosylmethionine-dependent methyltransferase activity;S-methyltransferase activity;thiopurine S-methyltransferase activity;transferase activity;transferase activity, transferring c
binding;collagen binding;extracellular matrix structural constituent;protein binding;structural molecule activity
catalytic activity;cyclin-dependent protein kinase regulator activity;enzyme regulator activity;kinase activity;kinase regulator activity;protein kinase regulator activity;transferase activity;transferase activity, transferring phospho
binding;DNA binding;identical protein binding;molecular transducer activity;nucleic acid binding;nucleic acid binding transcription factor activity;protein binding;sequence-specific DNA binding transcription factor activity;sign
binding;enzyme binding;GTP binding;GTPase binding;GTP-dependent protein binding;guanyl nucleotide binding;guanyl ribonucleotide binding;lipid binding;nucleotide binding;phosphatidylinositol binding;phosphatidylinosito
binding;protein binding;protein complex binding;protein domain specific binding
binding;calcium ion binding;cation binding;ion binding;metal ion binding
binding;cytokine receptor binding;death receptor binding;protein binding;receptor binding;tumor necrosis factor receptor superfamily binding
cation transmembrane transporter activity;hydrogen ion transmembrane transporter activity;inorganic cation transmembrane transporter activity;ion transmembrane transporter activity;monovalent inorganic cation transmemb
binding;nucleic acid binding;ribonucleoprotein binding;ribosomal large subunit binding;ribosome binding;RNA binding;translation factor activity, nucleic acid binding;translation initiation factor activity
structural constituent of nuclear pore;structural molecule activity
binding;epidermal growth factor binding;growth factor binding;hormone binding;protein binding
binding;catalytic activity;GDP binding;GTP binding;GTPase activity;GTP-dependent protein binding;guanyl nucleotide binding;guanyl ribonucleotide binding;hydrolase activity;hydrolase activity, acting on acid anhydrides;hyd
binding;mRNA binding;nucleic acid binding;RNA binding;structural constituent of ribosome;structural molecule activity
binding;enzyme binding;enzyme regulator activity;phosphatase binding;phosphatase regulator activity;protein binding;protein phosphatase 2A binding;protein phosphatase binding;protein phosphatase regulator activity;prote
binding;chromatin binding;enzyme binding;protein binding
AU-rich element binding;binding;nucleic acid binding;nucleotide binding;RNA binding
active transmembrane transporter activity;amine transmembrane transporter activity;amino acid transmembrane transporter activity;antigen binding;antiporter activity;aromatic amino acid transmembrane transporter activity;l
catalytic activity;cysteine-type peptidase activity;hydrolase activity;peptidase activity;peptidase activity, acting on L-amino acid peptides;small conjugating protein-specific protease activity;ubiquitin-specific protease activity
binding;catalytic activity;cation binding;coenzyme binding;cofactor binding;ion binding;metal ion binding;mRNA 3'-UTR binding;mRNA binding;NADP binding;NADPH binding;NADPH:quinone reductase activity;nucleic acid b
receptor activity
binding;protein binding;protein N-terminus binding;SNAP receptor activity;SNARE binding
binding;carbohydrate binding;glycosaminoglycan binding;heparin binding;pattern binding;polysaccharide binding
binding;DNA binding;nucleic acid binding;nucleic acid binding transcription factor activity;protein binding transcription factor activity;sequence-specific DNA binding transcription factor activity;transcription coactivator activit
aminoacyl-tRNA hydrolase activity;binding;carboxylic ester hydrolase activity;catalytic activity;hydrolase activity;hydrolase activity, acting on ester bonds;nucleic acid binding;RNA binding;translation factor activity, nucleic aci
binding;calcium ion binding;cation binding;ion binding;metal ion binding
binding;mRNA 3'-UTR binding;mRNA binding;nucleic acid binding;nucleotide binding;RNA binding
binding;catalytic activity;hydrolase activity;hydrolase activity, acting on ester bonds;phosphatase activity;phosphoprotein phosphatase activity;phosphoric ester hydrolase activity;protein binding;protein binding transcription f
binding;enzyme binding;protein binding;ubiquitin protein ligase binding
catalytic activity;NADH dehydrogenase (quinone) activity;NADH dehydrogenase (ubiquinone) activity;NADH dehydrogenase activity;oxidoreductase activity;oxidoreductase activity, acting on NADH or NADPH;oxidoreductase
adenyl nucleotide binding;adenyl ribonucleotide binding;ATP binding;binding;catalytic activity;guanylate kinase activity;kinase activity;nucleobase-containing compound kinase activity;nucleotide binding;nucleotide kinase ac
adenyl nucleotide binding;adenyl ribonucleotide binding;ATP binding;binding;nucleotide binding;purine nucleotide binding;purine ribonucleoside triphosphate binding;purine ribonucleotide binding;ribonucleotide binding
acid-amino acid ligase activity;binding;catalytic activity;cation binding;chromatin binding;enzyme binding;histone binding;ion binding;ligase activity;ligase activity, forming carbon-nitrogen bonds;metal ion binding;mRNA 3'-U
catalytic activity
catalytic activity;oxidoreductase activity
binding;catalytic activity;cation binding;demethylase activity;ion binding;metal ion binding;oxidative RNA demethylase activity;oxidoreductase activity;oxidoreductase activity, acting on paired donors, with incorporation or rec
acid phosphatase activity;adenyl nucleotide binding;adenyl ribonucleotide binding;ATP binding;binding;catalytic activity;diphosphoinositol-pentakisphosphate kinase activity;hydrolase activity;hydrolase activity, acting on este
binding;nucleic acid binding;nucleotide binding;RNA binding
binding;integrin binding;protein binding;protein complex binding;receptor binding
binding;cytoskeletal protein binding;identical protein binding;microtubule binding;protein binding;tubulin binding
binding;carbohydrate binding;catalytic activity;cysteine-type endopeptidase activity;cysteine-type peptidase activity;drug binding;endopeptidase activity;frizzled binding;glycosaminoglycan binding;G-protein-coupled recepto
catalytic activity;cysteine-type peptidase activity;hydrolase activity;peptidase activity;peptidase activity, acting on L-amino acid peptides
adenyl nucleotide binding;adenyl ribonucleotide binding;ATP binding;binding;nucleotide binding;purine nucleotide binding;purine ribonucleoside triphosphate binding;purine ribonucleotide binding;ribonucleotide binding
adenyl nucleotide binding;adenyl ribonucleotide binding;ATP binding;binding;catalytic activity;cation binding;enzyme binding;ion binding;kinase activity;kinase binding;metal ion binding;nucleotide binding;phosphotransferase
acid-amino acid ligase activity;binding;catalytic activity;cation binding;histone binding;ion binding;ligase activity;ligase activity, forming carbon-nitrogen bonds;metal ion binding;protein binding;small conjugating protein ligase
antioxidant activity;catalytic activity;glutathione peroxidase activity;oxidoreductase activity;oxidoreductase activity, acting on peroxide as acceptor;peroxidase activity
catalytic activity;hydrolase activity;hydrolase activity, acting on ester bonds;phosphatase activity;phosphoprotein phosphatase activity;phosphoric ester hydrolase activity
active transmembrane transporter activity;amine transmembrane transporter activity;choline transmembrane transporter activity;substrate-specific transmembrane transporter activity;substrate-specific transporter activity;tra
catalytic activity;endopeptidase activity;hydrolase activity;peptidase activity;peptidase activity, acting on L-amino acid peptides;serine hydrolase activity;serine-type endopeptidase activity;serine-type peptidase activity
acetyltransferase activity;activating transcription factor binding;bHLH transcription factor binding;binding;catalytic activity;cation binding;chromatin binding;core promoter proximal region DNA binding;core promoter proximal
catalytic activity;cysteine-type peptidase activity;hydrolase activity;peptidase activity;peptidase activity, acting on L-amino acid peptides;small conjugating protein-specific protease activity;ubiquitin-specific protease activity
binding;catalytic activity;GTP binding;GTPase activity;guanyl nucleotide binding;guanyl ribonucleotide binding;hydrolase activity;hydrolase activity, acting on acid anhydrides;hydrolase activity, acting on acid anhydrides, in p
enzyme inhibitor activity;enzyme regulator activity;phosphatase inhibitor activity;phosphatase regulator activity;protein phosphatase inhibitor activity;protein phosphatase regulator activity
binding;DNA binding;nucleic acid binding;RNA binding
binding;cation binding;ion binding;metal ion binding;nucleic acid binding
catalytic activity;hydrolase activity
binding;cytokine activity;cytokine receptor binding;protein binding;receptor binding;transforming growth factor beta receptor binding
catalytic activity;deacetylase activity;hydrolase activity;hydrolase activity, acting on glycosyl bonds
ligand-dependent nuclear receptor transcription coactivator activity;protein binding transcription factor activity;transcription coactivator activity;transcription cofactor activity;transcription factor binding transcription factor act
adenyl nucleotide binding;adenyl ribonucleotide binding;ATP binding;binding;catalytic activity;hydrolase activity;hydrolase activity, acting on acid anhydrides;hydrolase activity, acting on acid anhydrides, in phosphorus-conta
binding;catalytic activity;hydrolase activity;nucleotide binding

binding;catalytic activity;cation binding;dITP diphosphatase activity;hydrolase activity;hydrolase activity, acting on acid anhydrides;hydrolase activity, acting on acid anhydrides, in phosphorus-containing anhydrides;ion bindi
structural constituent of ribosome;structural molecule activity
binding;nucleic acid binding;RNA binding
adenyl nucleotide binding;adenyl ribonucleotide binding;ATP binding;binding;catalytic activity;kinase activity;nucleobase-containing compound kinase activity;nucleoside kinase activity;nucleotide binding;purine nucleotide bi
binding;identical protein binding;protein binding;protein dimerization activity;protein homodimerization activity
cation transmembrane transporter activity;divalent inorganic cation transmembrane transporter activity;inorganic cation transmembrane transporter activity;ion transmembrane transporter activity;magnesium ion transmembra
2 iron, 2 sulfur cluster binding;4 iron, 4 sulfur cluster binding;binding;cation binding;ferrous iron binding;ion binding;iron ion binding;iron-sulfur cluster binding;metal cluster binding;metal ion binding;protein binding;protein co
binding;cation binding;ion binding;metal ion binding;nucleotide binding;protein binding;SNARE binding;syntaxin binding;transition metal ion binding;zinc ion binding
catalytic activity;cis-trans isomerase activity;isomerase activity;peptidyl-prolyl cis-trans isomerase activity
catalytic activity;cysteine-type peptidase activity;hydrolase activity;peptidase activity;peptidase activity, acting on L-amino acid peptides
binding;enzyme binding;GTPase binding;protein binding;Ran GTPase binding;Ras GTPase binding;R-SMAD binding;SMAD binding;small GTPase binding
structural constituent of ribosome;structural molecule activity
binding;nucleotide binding;purine nucleotide binding
adenyl nucleotide binding;adenyl ribonucleotide binding;ATP binding;binding;catalytic activity;DNA binding;helicase activity;hydrolase activity;hydrolase activity, acting on acid anhydrides;hydrolase activity, acting on acid anh
carbon-carbon lyase activity;carboxy-lyase activity;catalytic activity;lyase activity;methylmalonyl-CoA decarboxylase activity
2,4-dienoyl-CoA reductase (NADPH) activity;binding;catalytic activity;oxidoreductase activity;oxidoreductase activity, acting on the CH-CH group of donors;oxidoreductase activity, acting on the CH-CH group of donors, NAD
catalytic activity;hydrolase activity;hydrolase activity, acting on glycosyl bonds;hydrolase activity, hydrolyzing O-glycosyl compounds
binding;nucleic acid binding;p53 binding;protein binding;RNA binding
binding;enzyme binding;phosphatase binding;protein binding;protein phosphatase 2A binding;protein phosphatase binding
binding;cation binding;ion binding;metal ion binding;transition metal ion binding;zinc ion binding
binding;cation binding;ion binding;metal ion binding;protein binding transcription factor activity;transcription cofactor activity;transcription corepressor activity;transcription factor binding transcription factor activity;transition
binding;cation binding;ion binding;metal ion binding
acid-amino acid ligase activity;binding;catalytic activity;cation binding;DNA binding;enzyme binding;enzyme regulator activity;GTPase binding;GTPase regulator activity;guanyl-nucleotide exchange factor activity;ion binding;
binding;protein binding;protein complex binding;small conjugating protein binding;ubiquitin binding
binding;nucleic acid binding;RNA binding;snRNA binding;U6 snRNA binding
binding;catalytic activity;GDP binding;GTP binding;GTPase activity;guanyl nucleotide binding;guanyl ribonucleotide binding;hydrolase activity;hydrolase activity, acting on acid anhydrides;hydrolase activity, acting on acid anh
binding;catalytic activity;GTP binding;GTPase activity;guanyl nucleotide binding;guanyl ribonucleotide binding;hydrolase activity;hydrolase activity, acting on acid anhydrides;hydrolase activity, acting on acid anhydrides, in ph
binding;enzyme binding;phosphatase binding;protein binding;protein phosphatase binding
binding;nucleotide binding
basal RNA polymerase II transcription machinery binding;basal transcription machinery binding;binding;enzyme binding;identical protein binding;nucleic acid binding;protein binding;RNA binding;RNA polymerase binding;RN
3'-5' exonuclease activity;3'-5'-exoribonuclease activity;binding;catalytic activity;exonuclease activity;exonuclease activity, active with either ribo- or deoxyribonucleic acids and producing 5'-phosphomonoesters;exoribonucl
structural constituent of ribosome;structural molecule activity
adenyl nucleotide binding;adenyl ribonucleotide binding;ATP binding;binding;calcium ion binding;catalytic activity;cation binding;enzyme binding;GTPase binding;hydrolase activity;hydrolase activity, acting on acid anhydride
binding;protein binding;protein complex binding
structural constituent of ribosome;structural molecule activity
binding;cation binding;cation transmembrane transporter activity;copper chaperone activity;copper ion binding;copper ion transmembrane transporter activity;copper-dependent protein binding;inorganic cation transmembra
1-phosphatidylinositol-3-kinase activity;adenyl nucleotide binding;adenyl ribonucleotide binding;ATP binding;binding;catalytic activity;inositol or phosphatidylinositol kinase activity;kinase activity;lipid binding;lipid kinase activ
binding;catalytic activity;NADH dehydrogenase (quinone) activity;NADH dehydrogenase (ubiquinone) activity;NADH dehydrogenase activity;oxidoreductase activity;oxidoreductase activity, acting on NADH or NADPH;oxidored
antioxidant activity;binding;catalytic activity;cation binding;cation transmembrane transporter activity;copper ion binding;copper ion transmembrane transporter activity;disulfide oxidoreductase activity;inorganic cation transm
amine binding;amino acid binding;binding;carboxylic acid binding;catalytic activity;glutathione binding;intramolecular oxidoreductase activity;isomerase activity;modified amino acid binding;peptide binding;prostaglandin-E s
binding;protein binding;receptor activity;TRAIL binding
adenyl nucleotide binding;adenyl ribonucleotide binding;ATP binding;binding;catalytic activity;enzyme binding;histone kinase activity;histone serine kinase activity;kinase activity;kinase binding;nucleotide binding;phosphotra
calcium-dependent cysteine-type endopeptidase activity;catalytic activity;cysteine-type endopeptidase activity;cysteine-type peptidase activity;endopeptidase activity;hydrolase activity;peptidase activity;peptidase activity, a
adenyl nucleotide binding;adenyl ribonucleotide binding;ATP binding;binding;calmodulin-dependent protein kinase activity;cAMP response element binding protein binding;catalytic activity;enzyme binding;GTPase binding;id
enzyme activator activity;enzyme regulator activity;GTPase activator activity;GTPase regulator activity;nucleoside-triphosphatase regulator activity
binding;carbohydrate binding;cation binding;cytoskeletal protein binding;DNA binding;glycosaminoglycan binding;heparin binding;ion binding;metal ion binding;microtubule binding;nucleic acid binding;nucleic acid binding tr
AU-rich element binding;binding;catalytic activity;enzyme binding;histone deacetylase binding;hydrolase activity;identical protein binding;mRNA binding;nucleic acid binding;protein binding;protein dimerization activity;protei
androgen receptor binding;bHLH transcription factor binding;binding;catalytic activity;chromatin binding;coenzyme binding;cofactor binding;demethylase activity;DNA binding;enzyme binding;flavin adenine dinucleotide bind
binding;protein binding;receptor binding
binding;protein binding;protein complex binding
binding;growth factor activity;lipoprotein particle receptor binding;low-density lipoprotein particle receptor binding;molecular transducer activity;protein binding;receptor antagonist activity;receptor binding;receptor inhibitor a
catalytic activity;hydrolase activity
binding;enzyme binding;kinase binding;NF-kappaB binding;protease binding;protein binding;protein binding transcription factor activity;protein C-terminus binding;protein kinase B binding;protein kinase binding;protein self-
binding;chemokine activity;chemokine receptor binding;cytokine activity;cytokine receptor binding;endopeptidase inhibitor activity;endopeptidase regulator activity;enzyme inhibitor activity;enzyme regulator activity;G-protein
antigen binding;beta-2-microglobulin binding;binding;peptide antigen binding;peptide binding;protein binding
binding;cation binding;extracellular matrix structural constituent;growth factor binding;integrin binding;ion binding;metal ion binding;platelet-derived growth factor binding;protein binding;protein complex binding;receptor bin
binding;carbohydrate binding;cargo receptor activity;extracellular matrix binding;glycosaminoglycan binding;heparin binding;integrin binding;pattern binding;polysaccharide binding;protein binding;protein complex binding;re
alpha-L-fucosidase activity;binding;carbohydrate binding;catalytic activity;fucose binding;fucosidase activity;hydrolase activity;hydrolase activity, acting on glycosyl bonds;hydrolase activity, hydrolyzing O-glycosyl compoun
binding;carbohydrate binding;cholesterol transporter activity;enzyme binding;glycosaminoglycan binding;heparin binding;lipase binding;lipid binding;lipid transporter activity;lipoprotein particle receptor binding;low-density li
acetyl-CoA C-acyltransferase activity;acyl-CoA oxidase activity;C-acyltransferase activity;catalytic activity;oxidoreductase activity;oxidoreductase activity, acting on the CH-CH group of donors;oxidoreductase activity, acting
7S RNA binding;binding;nucleic acid binding;RNA binding
catalytic activity;cation transmembrane transporter activity;cytochrome-c oxidase activity;heme-copper terminal oxidase activity;hydrogen ion transmembrane transporter activity;inorganic cation transmembrane transporter
binding;nucleic acid binding;RNA binding
binding;cell adhesion molecule binding;protein binding;receptor activity
binding;core promoter proximal region DNA binding;core promoter proximal region sequence-specific DNA binding;DNA binding;double-stranded DNA binding;nucleic acid binding;nucleic acid binding transcription factor act
beta-N-acetylgalactosaminidase activity;beta-N-acetylhexosaminidase activity;catalytic activity;enzyme activator activity;enzyme regulator activity;hexosaminidase activity;hydrolase activity;hydrolase activity, acting on glyco
adenyl nucleotide binding;adenyl ribonucleotide binding;ATP binding;binding;catalytic activity;cation binding;DNA binding;DNA ligase (ATP) activity;DNA ligase activity;ion binding;ligase activity;ligase activity, forming phosph
binding;catalytic activity;GDP binding;GTP binding;GTPase activity;guanyl nucleotide binding;guanyl ribonucleotide binding;hydrolase activity;hydrolase activity, acting on acid anhydrides;hydrolase activity, acting on acid anh
adenyl nucleotide binding;adenyl ribonucleotide binding;ATP binding;binding;catalytic activity;cyclin-dependent protein kinase activity;kinase activity;nucleotide binding;phosphotransferase activity, alcohol group as acceptor
acid phosphatase activity;catalytic activity;hydrolase activity;hydrolase activity, acting on ester bonds;non-membrane spanning protein tyrosine phosphatase activity;phosphatase activity;phosphoprotein phosphatase activity
binding;catalytic activity;endopeptidase activity;hydrolase activity;lipopolysaccharide binding;peptidase activity;peptidase activity, acting on L-amino acid peptides;threonine-type endopeptidase activity;threonine-type peptic
catalytic activity;endopeptidase activity;hydrolase activity;peptidase activity;peptidase activity, acting on L-amino acid peptides;threonine-type endopeptidase activity;threonine-type peptidase activity
alkylbase DNA N-glycosylase activity;binding;catalytic activity;damaged DNA binding;DNA binding;DNA N-glycosylase activity;DNA-3-methyladenine glycosylase I activity;DNA-7-methylguanine glycosylase activity;hydrolase
3-hydroxyisobutyrate dehydrogenase activity;binding;catalytic activity;coenzyme binding;cofactor binding;NAD binding;nucleotide binding;oxidoreductase activity;oxidoreductase activity, acting on CH-OH group of donors;o
binding;calcium ion binding;carbohydrate binding;cation binding;glycosaminoglycan binding;heparin binding;integrin binding;ion binding;metal ion binding;pattern binding;polysaccharide binding;protein binding;protein comp
binding;nucleic acid binding;receptor activity;RNA binding;translation factor activity, nucleic acid binding;translation initiation factor activity
adenyl nucleotide binding;adenyl ribonucleotide binding;ATP binding;binding;catalytic activity;cation binding;ion binding;kinase activity;lipid binding;metal ion binding;nucleotide binding;phospholipid binding;phosphotransfer
catalytic activity;endopeptidase activity;hydrolase activity;peptidase activity;peptidase activity, acting on L-amino acid peptides;threonine-type endopeptidase activity;threonine-type peptidase activity
adenyl nucleotide binding;adenyl ribonucleotide binding;ATP binding;binding;catalytic activity;enzyme binding;kinase activity;kinase binding;nucleotide binding;phosphotransferase activity, alcohol group as acceptor;protein b
adenyl nucleotide binding;adenyl ribonucleotide binding;adenylyltransferase activity;ATP binding;binding;catalytic activity;cation binding;ion binding;magnesium ion binding;manganese ion binding;metal ion binding;nucleic a
binding;cation binding;DNA binding;ion binding;metal ion binding;nucleic acid binding;RNA binding;transition metal ion binding;zinc ion binding
active transmembrane transporter activity;carboxylic acid transmembrane transporter activity;lactate transmembrane transporter activity;mevalonate transmembrane transporter activity;monocarboxylic acid transmembrane t
catalytic activity;cysteine-type endopeptidase activity;cysteine-type peptidase activity;endopeptidase activity;hydrolase activity;peptidase activity;peptidase activity, acting on L-amino acid peptides
binding;identical protein binding;protein binding
binding;DNA binding;nucleic acid binding;structural constituent of ribosome;structural molecule activity
acid-amino acid ligase activity;binding;catalytic activity;enzyme binding;ligase activity;ligase activity, forming carbon-nitrogen bonds;protein binding;small conjugating protein ligase activity;SUMO ligase activity;transcription f
binding;cation binding;heme binding;ion binding;iron ion binding;metal ion binding;oxygen binding;oxygen transporter activity;substrate-specific transporter activity;tetrapyrrole binding;transition metal ion binding;transporter
binding;catalytic activity;GTP binding;GTPase activity;guanyl nucleotide binding;guanyl ribonucleotide binding;hydrolase activity;hydrolase activity, acting on acid anhydrides;hydrolase activity, acting on acid anhydrides, in ph
active transmembrane transporter activity;adenyl nucleotide binding;adenyl ribonucleotide binding;ATP binding;ATPase activity;ATPase activity, coupled;ATPase activity, coupled to movement of substances;ATPase activity, c
binding;catalytic activity;DNA binding;endonuclease activity;enzyme inhibitor activity;enzyme regulator activity;hydrolase activity;hydrolase activity, acting on ester bonds;nuclease activity;nucleic acid binding;phosphatase in
adenyl nucleotide binding;adenyl ribonucleotide binding;ATP binding;binding;catalytic activity;cation binding;enzyme activator activity;enzyme regulator activity;identical protein binding;ion binding;kinase activator activity;kin
binding;identical protein binding;protein binding;protein dimerization activity;protein homodimerization activity
AP-1 adaptor complex binding;AP-3 adaptor complex binding;binding;catalytic activity;GTP binding;GTPase activity;GTP-dependent protein binding;guanyl nucleotide binding;guanyl ribonucleotide binding;hydrolase activity
binding;catalytic activity;cation binding;cis-trans isomerase activity;drug binding;FK506 binding;identical protein binding;ion binding;isomerase activity;macrolide binding;metal ion binding;peptidyl-prolyl cis-trans isomerase a
binding;catalytic activity;cysteine-type endopeptidase activity;cysteine-type endopeptidase activity involved in apoptotic process;cysteine-type peptidase activity;death effector domain binding;endopeptidase activity;enzyme
binding;polyubiquitin binding;protein binding;small conjugating protein binding;ubiquitin binding
binding;carbohydrate binding;collagen binding;enzyme activator activity;enzyme regulator activity;glycosaminoglycan binding;heparin binding;pattern binding;peptidase activator activity;peptidase regulator activity;polysacch
binding;catalytic activity;cation binding;GDP binding;GTP binding;GTPase activity;guanyl nucleotide binding;guanyl ribonucleotide binding;hydrolase activity;hydrolase activity, acting on acid anhydrides;hydrolase activity, act
binding;protein binding;SNAP receptor activity;SNARE binding
adenyl nucleotide binding;adenyl ribonucleotide binding;ATP binding;binding;catalytic activity;DNA binding;helicase activity;hydrolase activity;hydrolase activity, acting on acid anhydrides;hydrolase activity, acting on acid anh
actin binding;binding;cytoskeletal protein binding;enzyme binding;identical protein binding;kinase binding;lipid binding;microtubule binding;phosphatidylinositol binding;phospholipid binding;protein binding;protein dimerizati
binding;catalytic activity;coenzyme binding;cofactor binding;DNA binding;histone binding;methylated histone residue binding;NAD binding;nucleic acid binding;nucleotide binding;oxidoreductase activity;oxidoreductase activ
binding;nucleotide binding
binding;calcium ion binding;cation binding;enzyme activator activity;enzyme regulator activity;GTPase activator activity;GTPase regulator activity;ion binding;metal ion binding;nucleoside-triphosphatase regulator activity
actin binding;actin filament binding;beta-tubulin binding;binding;cytoskeletal protein binding;DNA binding;microtubule binding;nucleic acid binding;protein binding;tubulin binding
catalytic activity;flavin-linked sulfhydryl oxidase activity;intramolecular oxidoreductase activity;intramolecular oxidoreductase activity, interconverting keto- and enol-groups;intramolecular oxidoreductase activity, transposing
structural molecule activity

binding;nucleic acid binding;poly(G) RNA binding;poly(U) RNA binding;poly-purine tract binding;poly-pyrimidine tract binding;RNA binding;single-stranded RNA binding
catalytic activity;cysteine-type endopeptidase activity;cysteine-type peptidase activity;endopeptidase activity;hydrolase activity;peptidase activity;peptidase activity, acting on L-amino acid peptides;small conjugating protein-
acetoacetate-CoA ligase activity;acid-thiol ligase activity;adenyl nucleotide binding;adenyl ribonucleotide binding;ATP binding;binding;butyrate-CoA ligase activity;catalytic activity;ligase activity;ligase activity, forming carbon
binding;protein binding;SNAP receptor activity;SNARE binding
binding;carboxylic acid binding;catalytic activity;cation binding;ion binding;iron ion binding;L-ascorbic acid binding;metal ion binding;oxidoreductase activity;oxidoreductase activity, acting on paired donors, with incorporatio
acylglycerol lipase activity;carboxylic ester hydrolase activity;catalytic activity;hydrolase activity;hydrolase activity, acting on ester bonds;lipase activity
enzyme activator activity;enzyme regulator activity;GTPase activator activity;GTPase regulator activity;nucleoside-triphosphatase regulator activity
binding;carboxylic acid binding;catalytic activity;cation binding;ion binding;iron ion binding;L-ascorbic acid binding;metal ion binding;oxidoreductase activity;oxidoreductase activity, acting on paired donors, with incorporatio
2 iron, 2 sulfur cluster binding;binding;cation binding;identical protein binding;ion binding;iron-sulfur cluster binding;metal cluster binding;metal ion binding;protein binding;protein dimerization activity;protein homodimerizatio
binding;cation binding;ion binding;metal ion binding
active transmembrane transporter activity;calcium channel activity;cation channel activity;cation transmembrane transporter activity;channel activity;ion channel activity;ion transmembrane transporter activity;passive transm
catalytic activity;hydrolase activity
enzyme activator activity;enzyme regulator activity;GTPase activator activity;GTPase regulator activity;nucleoside-triphosphatase regulator activity
adenyl nucleotide binding;adenyl ribonucleotide binding;ATP binding;binding;catalytic activity;cation binding;ion binding;kinase activity;metal ion binding;nucleotide binding;phosphotransferase activity, alcohol group as acce
actin binding;binding;catalytic activity;cation binding;coenzyme binding;cofactor binding;cytoskeletal protein binding;FAD binding;flavin adenine dinucleotide binding;ion binding;metal ion binding;monooxygenase activity;oxi
binding;endopeptidase inhibitor activity;endopeptidase regulator activity;enzyme inhibitor activity;enzyme regulator activity;peptidase inhibitor activity;peptidase regulator activity;proteasome binding;protein binding;protein co
AP-2 adaptor complex binding;binding;enzyme activator activity;enzyme regulator activity;GTPase activator activity;GTPase regulator activity;nucleoside-triphosphatase regulator activity;protein binding;protein complex bind
binding;catalytic activity;cation binding;identical protein binding;ion binding;isomerase activity;metal ion binding;protein binding;protein dimerization activity;protein homodimerization activity;racemase and epimerase activity;
binding;binding, bridging;protein binding;protein binding, bridging;ribonucleoprotein binding;ribosomal large subunit binding
binding;catalytic activity;DNA binding;N-acyltransferase activity;nucleic acid binding;sphingosine N-acyltransferase activity;transferase activity;transferase activity, transferring acyl groups;transferase activity, transferring acyl
actin binding;binding;cytoskeletal protein binding;enzyme binding;leucine zipper domain binding;LRR domain binding;protein binding;protein binding transcription factor activity;protein domain specific binding;transcription c
binding;protein binding;receptor binding;receptor tyrosine kinase binding
binding;C2H2 zinc finger domain binding;catalytic activity;cation binding;histone methyltransferase activity;histone methyltransferase activity (H3-K27 specific);histone methyltransferase activity (H3-K9 specific);histone-lysine
binding;lipid binding;phosphatidylinositol binding;phosphatidylinositol-3-phosphate binding;phospholipid binding
catalytic activity;lysophospholipid acyltransferase activity;transferase activity;transferase activity, transferring acyl groups;transferase activity, transferring acyl groups other than amino-acyl groups
catalytic activity;methyltransferase activity;transferase activity;transferase activity, transferring one-carbon groups
binding;mRNA binding;nucleic acid binding;RNA binding
protein transporter activity;substrate-specific transporter activity;transporter activity
binding;binding, bridging;estrogen receptor binding;hormone receptor binding;nuclear hormone receptor binding;protein binding;protein binding transcription factor activity;protein binding, bridging;protein complex scaffold;r
catalytic activity;dolichyl-phosphate-mannose-protein mannosyltransferase activity;mannosyltransferase activity;transferase activity;transferase activity, transferring glycosyl groups;transferase activity, transferring hexosyl gro
1-phosphatidylinositol 4-kinase activity;adenyl nucleotide binding;adenyl ribonucleotide binding;AP-3 adaptor complex binding;ATP binding;binding;catalytic activity;cation binding;inositol or phosphatidylinositol kinase activi
catalytic activity;oxidoreductase activity;protein binding transcription factor activity;transcription coactivator activity;transcription cofactor activity;transcription factor binding transcription factor activity
acireductone dioxygenase [iron(II)-requiring] activity;binding;catalytic activity;cation binding;dioxygenase activity;ion binding;iron ion binding;metal ion binding;oxidoreductase activity;oxidoreductase activity, acting on single
binding;catalytic activity;cation binding;ion binding;metal ion binding;oxidoreductase activity;oxidoreductase activity, acting on the CH-CH group of donors;oxidoreductase activity, acting on the CH-CH group of donors, NAD
binding;cholesterol binding;lipid binding;steroid binding;sterol binding
structural constituent of ribosome;structural molecule activity
binding;nucleotide binding
acetyltransferase activity;catalytic activity;H4 histone acetyltransferase activity;histone acetyltransferase activity;lysine N-acetyltransferase activity;N-acetyltransferase activity;N-acyltransferase activity;peptide alpha-N-acetyl
adenyl nucleotide binding;adenyl ribonucleotide binding;ATP binding;ATPase activity;ATPase activity, coupled;ATP-dependent helicase activity;ATP-dependent RNA helicase activity;binding;catalytic activity;helicase activity;h
binding;catalytic activity;cation binding;hydrolase activity;hydrolase activity, acting on ester bonds;ion binding;metal ion binding;transition metal ion binding;zinc ion binding
binding;catalytic activity;cation binding;dCTP diphosphatase activity;deoxyribonucleotide binding;hydrolase activity;hydrolase activity, acting on acid anhydrides;hydrolase activity, acting on acid anhydrides, in phosphorus-c
binding;nucleic acid binding;RNA binding;toxin binding
binding;cation binding;ion binding;metal ion binding
binding;nucleic acid binding;RNA binding
adenyl nucleotide binding;adenyl ribonucleotide binding;ATP binding;ATPase activity;ATPase activity, coupled;beta-catenin binding;binding;catalytic activity;chromatin binding;DNA binding;DNA helicase activity;DNA-depend
structural constituent of ribosome;structural molecule activity
binding;identical protein binding;protein binding;protein complex binding
binding;identical protein binding;polyubiquitin binding;protein binding;small conjugating protein binding;ubiquitin binding
binding;cation binding;heme binding;ion binding;iron ion binding;metal ion binding;tetrapyrrole binding;transition metal ion binding
binding;DNA binding;enzyme activator activity;enzyme regulator activity;GTP binding;GTPase activator activity;GTPase regulator activity;guanyl nucleotide binding;guanyl ribonucleotide binding;nucleic acid binding;nucleosi
alpha-tubulin binding;beta-tubulin binding;binding;catalytic activity;cytoskeletal protein binding;GDP binding;GTP binding;GTPase activity;guanyl nucleotide binding;guanyl ribonucleotide binding;hydrolase activity;hydrolase
binding;DNA binding;nucleic acid binding;nucleic acid binding transcription factor activity;protein binding transcription factor activity;sequence-specific DNA binding;sequence-specific DNA binding transcription factor activit
adenyl nucleotide binding;adenyl ribonucleotide binding;ATP binding;binding;catalytic activity;NADH dehydrogenase (quinone) activity;NADH dehydrogenase (ubiquinone) activity;NADH dehydrogenase activity;nucleotide bin
binding;catalytic activity;cation binding;GMP reductase activity;ion binding;metal ion binding;oxidoreductase activity;oxidoreductase activity, acting on NADH or NADPH;oxidoreductase activity, acting on NADH or NADPH, ni
binding;chromatin binding;protein binding;protein N-terminus binding
adenyl nucleotide binding;adenyl ribonucleotide binding;ATP binding;binding;catalytic activity;kinase activity;nucleic acid binding;nucleotide binding;phosphoprotein binding;phosphotransferase activity, alcohol group as acce
binding;protein binding;protein N-terminus binding
ADP-ribose diphosphatase activity;ADP-sugar diphosphatase activity;binding;catalytic activity;cation binding;hydrolase activity;hydrolase activity, acting on acid anhydrides;hydrolase activity, acting on acid anhydrides, in ph
binding;cation binding;enzyme activator activity;enzyme regulator activity;GTPase activator activity;GTPase regulator activity;ion binding;lipid binding;metal ion binding;nucleoside-triphosphatase regulator activity;phosphatid
binding;enzyme binding;enzyme regulator activity;heat shock protein binding;kinase binding;kinase regulator activity;NF-kappaB binding;protein binding;protein kinase binding;protein kinase regulator activity;small conjugatin
bent DNA binding;binding;catalytic activity;cis-trans isomerase activity;DNA binding;double-stranded DNA binding;isomerase activity;nucleic acid binding;peptidyl-prolyl cis-trans isomerase activity;structure-specific DNA bi
binding;nucleotide binding
catalytic activity;cis-trans isomerase activity;isomerase activity;peptidyl-prolyl cis-trans isomerase activity
aminoacyl-tRNA hydrolase activity;carboxylic ester hydrolase activity;catalytic activity;hydrolase activity;hydrolase activity, acting on ester bonds
binding;catalytic activity;cysteine-type endopeptidase activity;cysteine-type peptidase activity;cytokine receptor binding;endopeptidase activity;hydrolase activity;identical protein binding;peptidase activity;peptidase activity,
adenyl nucleotide binding;adenyl ribonucleotide binding;ATP binding;binding;catalytic activity;kinase activity;MAP kinase kinase kinase kinase activity;molecular transducer activity;nucleotide binding;phosphotransferase acti
binding;mRNA binding;nucleic acid binding;nucleotide binding;RNA binding
binding;catalytic activity;cell adhesion molecule binding;fibroblast growth factor receptor binding;growth factor receptor binding;kinase activity;molecular transducer activity;phosphotransferase activity, alcohol group as acce
adenyl nucleotide binding;adenyl ribonucleotide binding;ATP binding;binding;catalytic activity;cation binding;ion binding;kinase activity;metal ion binding;nucleotide binding;phosphotransferase activity, alcohol group as acce
protein binding transcription factor activity;transcription cofactor activity;transcription corepressor activity;transcription factor binding transcription factor activity
binding;catalytic activity;cation binding;hydrolase activity;hydrolase activity, acting on ester bonds;ion binding;metal ion binding;phosphatase activity;phosphoprotein phosphatase activity;phosphoric ester hydrolase activity;p
binding;cation binding;heme binding;ion binding;iron ion binding;ligand-dependent nuclear receptor activity;lipid binding;metal ion binding;molecular transducer activity;receptor activity;signal transducer activity;signaling rec
catalytic activity;deoxyribonucleoside 5'-monophosphate N-glycosidase activity;hydrolase activity;hydrolase activity, acting on glycosyl bonds;hydrolase activity, hydrolyzing N-glycosyl compounds;nucleoside deoxyribosyltra
binding;damaged DNA binding;DNA binding;nucleic acid binding;protein binding;protein N-terminus binding;transcription factor binding
active transmembrane transporter activity;adenyl nucleotide binding;adenyl ribonucleotide binding;ATP binding;ATPase activity;ATPase activity, coupled;ATPase activity, coupled to movement of substances;ATPase activity, c
binding;nucleic acid binding;RNA binding;translation factor activity, nucleic acid binding;translation initiation factor activity
binding;nucleotide binding
catalytic activity;NADH dehydrogenase (quinone) activity;NADH dehydrogenase (ubiquinone) activity;NADH dehydrogenase activity;oxidoreductase activity;oxidoreductase activity, acting on NADH or NADPH;oxidoreductase
binding;catalytic activity;cation binding;cation transmembrane transporter activity;copper ion binding;cytochrome-c oxidase activity;heme-copper terminal oxidase activity;hydrogen ion transmembrane transporter activity;inc
adenosine deaminase activity;binding;catalytic activity;cation binding;deaminase activity;hydrolase activity;hydrolase activity, acting on carbon-nitrogen (but not peptide) bonds;hydrolase activity, acting on carbon-nitrogen (b
binding;carbohydrate binding;catalytic activity;cation binding;DNA binding;endopeptidase activity;enzyme activator activity;enzyme regulator activity;glycosaminoglycan binding;heparin binding;hydrolase activity;ion binding;i
amidine-lyase activity;argininosuccinate lyase activity;carbon-nitrogen lyase activity;catalytic activity;lyase activity
binding;DNA binding;nucleic acid binding
carbon-carbon lyase activity;carboxy-lyase activity;catalytic activity;lyase activity;uroporphyrinogen decarboxylase activity
amine binding;amino acid binding;antioxidant activity;binding;carboxylic acid binding;catalytic activity;glutathione binding;glutathione peroxidase activity;modified amino acid binding;oxidoreductase activity;oxidoreductase a
adenyl nucleotide binding;adenyl ribonucleotide binding;ATP binding;binding;catalytic activity;DNA binding;helicase activity;hydrolase activity;hydrolase activity, acting on acid anhydrides;hydrolase activity, acting on acid anh
anion binding;binding;catalytic activity;cation binding;cyclohydrolase activity;hydrolase activity;hydrolase activity, acting on carbon-nitrogen (but not peptide) bonds;hydrolase activity, acting on carbon-nitrogen (but not pepti
binding;catalytic activity;chemoattractant activity;cytokine activity;cytokine receptor binding;dopachrome isomerase activity;intramolecular oxidoreductase activity;intramolecular oxidoreductase activity, interconverting keto-
binding;catalytic activity;enzyme binding;histone kinase activity;kinase activity;kinase binding;patched binding;phosphotransferase activity, alcohol group as acceptor;protein binding;protein kinase activity;protein kinase bind
catalytic activity;cation transmembrane transporter activity;cytochrome-c oxidase activity;heme-copper terminal oxidase activity;hydrogen ion transmembrane transporter activity;inorganic cation transmembrane transporter a
binding;catalytic activity;cation binding;hydrolase activity;hydrolase activity, acting on ester bonds;ion binding;metal ion binding;N-acetylglucosamine-6-sulfatase activity;sulfuric ester hydrolase activity
binding;calcium ion binding;catalytic activity;cation binding;cytokine receptor binding;enzyme binding;hydrolase activity;hydrolase activity, acting on ester bonds;ion binding;kinase binding;lipase activity;metal ion binding;mo
binding;catalytic activity;cation binding;cation transmembrane transporter activity;cytochrome-c oxidase activity;electron carrier activity;heme-copper terminal oxidase activity;hydrogen ion transmembrane transporter activity
endopeptidase inhibitor activity;endopeptidase regulator activity;enzyme inhibitor activity;enzyme regulator activity;peptidase inhibitor activity;peptidase regulator activity;serine-type endopeptidase inhibitor activity
amine binding;amino acid binding;binding;carboxylic acid binding;catalytic activity;cation binding;cobalamin binding;intramolecular transferase activity;ion binding;isomerase activity;metal ion binding;methylmalonyl-CoA mut
2 iron, 2 sulfur cluster binding;binding;catalytic activity;cation binding;ferrochelatase activity;ferrous iron binding;heme binding;ion binding;iron ion binding;iron-responsive element binding;iron-sulfur cluster binding;lyase activ
binding;calcium ion binding;cation binding;enzyme activator activity;enzyme regulator activity;extracellular matrix structural constituent;ion binding;metal ion binding;peptidase activator activity;peptidase regulator activity;stru
binding;catalytic activity;cis-trans isomerase activity;drug binding;FK506 binding;isomerase activity;macrolide binding;peptidyl-prolyl cis-trans isomerase activity
binding;cation binding;DNA binding;enhancer binding;enhancer sequence-specific DNA binding;ion binding;ligand-dependent nuclear receptor activity;metal ion binding;molecular transducer activity;nucleic acid binding;nucl
adenyl nucleotide binding;adenyl ribonucleotide binding;ATP binding;binding;catalytic activity;kinase activity;nucleotide binding;phosphotransferase activity, alcohol group as acceptor;protein kinase activity;protein serine/thre
binding;catalytic activity;cation binding;intramolecular oxidoreductase activity;intramolecular oxidoreductase activity, interconverting aldoses and ketoses;ion binding;isomerase activity;mannose-6-phosphate isomerase activ
binding;cation binding;copper ion binding;extracellular matrix binding;fibroblast growth factor binding;growth factor binding;ion binding;laminin binding;metal ion binding;protein binding;transition metal ion binding
binding;catalytic activity;disulfide oxidoreductase activity;electron carrier activity;glutathione disulfide oxidoreductase activity;oxidoreductase activity;oxidoreductase activity, acting on a sulfur group of donors;peptide disulfid
binding;calcium-dependent protein serine/threonine phosphatase activity;calmodulin-dependent protein phosphatase activity;catalytic activity;cation binding;hydrolase activity;hydrolase activity, acting on ester bonds;ion bin
catalytic activity;dodecenoyl-CoA delta-isomerase activity;intramolecular oxidoreductase activity;intramolecular oxidoreductase activity, transposing C=C bonds;isomerase activity
endopeptidase inhibitor activity;endopeptidase regulator activity;enzyme inhibitor activity;enzyme regulator activity;extracellular matrix structural constituent;peptidase inhibitor activity;peptidase regulator activity;serine-type

adenyl nucleotide binding;adenyl ribonucleotide binding;ATP binding;binding;calcium-dependent protein kinase activity;calcium-dependent protein serine/threonine kinase activity;calmodulin binding;calmodulin-dependent p
binding;chromatin binding;dynein binding;identical protein binding;protein binding;protein C-terminus binding;protein dimerization activity;protein homodimerization activity;transcription factor binding
4 iron, 4 sulfur cluster binding;binding;catalytic activity;cation binding;DNA binding;DNA primase activity;DNA-directed RNA polymerase activity;ion binding;iron-sulfur cluster binding;metal cluster binding;metal ion binding;nu
adenyl nucleotide binding;adenyl ribonucleotide binding;ATP binding;binding;catalytic activity;GTP binding;guanyl nucleotide binding;guanyl ribonucleotide binding;identical protein binding;kinase activity;nucleotide binding;p
binding;nucleic acid binding;nucleotide binding;RNA 7-methylguanosine cap binding;RNA binding;RNA cap binding
binding;lipid binding;phosphatidylinositol binding;phosphatidylinositol-4-phosphate binding;phospholipid binding
4 iron, 4 sulfur cluster binding;adenyl nucleotide binding;adenyl ribonucleotide binding;ATP binding;binding;cation binding;ion binding;iron-sulfur cluster binding;metal cluster binding;metal ion binding;nucleotide binding;purin
binding;box C/D snoRNA binding;nucleic acid binding;RNA binding;snoRNA binding;snRNA binding;U3 snoRNA binding;U4 snRNA binding
catalytic activity;hydrolase activity;peptidase activity
acid-amino acid ligase activity;adenyl nucleotide binding;adenyl ribonucleotide binding;ATP binding;binding;catalytic activity;enzyme binding;ligase activity;ligase activity, forming carbon-nitrogen bonds;nucleotide binding;pro
binding;catalytic activity;GTP binding;GTPase activity;guanyl nucleotide binding;guanyl ribonucleotide binding;hydrolase activity;hydrolase activity, acting on acid anhydrides;hydrolase activity, acting on acid anhydrides, in ph
acid-amino acid ligase activity;binding;catalytic activity;enzyme binding;ligase activity;ligase activity, forming carbon-nitrogen bonds;protein binding;small conjugating protein ligase activity;SUMO ligase activity;ubiquitin prot
catalytic activity;enzyme regulator activity;hydrolase activity;hydrolase activity, acting on acid anhydrides;hydrolase activity, acting on acid anhydrides, in phosphorus-containing anhydrides;motor activity;nitric-oxide synthase
binding;catalytic activity;GTP binding;GTPase activity;guanyl nucleotide binding;guanyl ribonucleotide binding;hydrolase activity;hydrolase activity, acting on acid anhydrides;hydrolase activity, acting on acid anhydrides, in ph
binding;DNA binding;nucleic acid binding;nucleic acid binding transcription factor activity;regulatory region DNA binding;regulatory region nucleic acid binding;RNA polymerase II regulatory region DNA binding;RNA polymera
binding;cation binding;identical protein binding;ion binding;metal ion binding;nucleic acid binding;nucleotide binding;protein binding;RNA binding;transition metal ion binding;zinc ion binding
binding;nucleotide binding
adenyl nucleotide binding;adenyl ribonucleotide binding;ATP binding;binding;calcium-independent protein kinase C activity;catalytic activity;cation binding;enzyme activator activity;enzyme binding;enzyme regulator activity;i
3'-5' exonuclease activity;3'-5'-exoribonuclease activity;AU-rich element binding;binding;catalytic activity;exonuclease activity;exonuclease activity, active with either ribo- or deoxyribonucleic acids and producing 5'-phospho
acid-amino acid ligase activity;binding;catalytic activity;cation binding;CD40 receptor binding;cytokine receptor binding;enzyme binding;identical protein binding;ion binding;kinase binding;ligase activity;ligase activity, forming
binding;cation binding;ion binding;metal ion binding;transition metal ion binding;zinc ion binding
aminopeptidase activity;carboxypeptidase activity;catalytic activity;cysteine-type endopeptidase activity;cysteine-type peptidase activity;endopeptidase activity;exopeptidase activity;hydrolase activity;peptidase activity;pept
binding;enzyme binding;enzyme regulator activity;GTPase regulator activity;guanyl-nucleotide exchange factor activity;kinase binding;nucleoside-triphosphatase regulator activity;protein binding;protein kinase binding;Ras gu
binding;cation binding;enzyme activator activity;enzyme regulator activity;GTPase activator activity;GTPase regulator activity;ion binding;metal ion binding;nucleoside-triphosphatase regulator activity;transition metal ion bind
protein transporter activity;substrate-specific transporter activity;transporter activity
binding;enzyme activator activity;enzyme regulator activity;histone acetyl-lysine binding;histone binding;peptidase activator activity;peptidase regulator activity;protein binding
binding;catalytic activity;chaperone binding;GTPase activity;hydrolase activity;hydrolase activity, acting on acid anhydrides;hydrolase activity, acting on acid anhydrides, in phosphorus-containing anhydrides;nucleoside-triph
binding;endopeptidase activator activity;endopeptidase regulator activity;enzyme activator activity;enzyme binding;enzyme regulator activity;peptidase activator activity;peptidase regulator activity;protease binding;proteaso
binding;carbohydrate binding;catalytic activity;cation binding;hydrolase activity;hydrolase activity, acting on glycosyl bonds;hydrolase activity, hydrolyzing N-glycosyl compounds;hydrolase activity, hydrolyzing O-glycosyl con
5S rRNA binding;binding;catalytic activity;nucleic acid binding;RNA binding;rRNA binding;sulfurtransferase activity;thiosulfate sulfurtransferase activity;transferase activity;transferase activity, transferring sulfur-containing gro
binding;catalytic activity;cation binding;hydrolase activity;hydrolase activity, acting on ester bonds;hydroxyacylglutathione hydrolase activity;ion binding;metal ion binding;thiolester hydrolase activity;transition metal ion bindin
catalytic activity;transferase activity;transferase activity, transferring glycosyl groups;transferase activity, transferring pentosyl groups;uridine phosphorylase activity
binding;catalytic activity;cation binding;hydrolase activity;ion binding;metal ion binding
binding;enzyme binding;phosphatase binding;protein binding;protein phosphatase binding
5'-nucleotidase activity;binding;catalytic activity;cation binding;hydrolase activity;hydrolase activity, acting on ester bonds;ion binding;metal ion binding;nucleotidase activity;phosphatase activity;phosphoric ester hydrolase a
enzyme activator activity;enzyme regulator activity;GTPase activator activity;GTPase regulator activity;nucleoside-triphosphatase regulator activity
binding;cation binding;ion binding;metal ion binding;transition metal ion binding;zinc ion binding
structural constituent of ribosome;structural molecule activity
carboxylic ester hydrolase activity;catalytic activity;hydrolase activity;hydrolase activity, acting on ester bonds
catalytic activity;L-xylulose reductase (NADP+) activity;oxidoreductase activity;oxidoreductase activity, acting on CH-OH group of donors;oxidoreductase activity, acting on NADH or NADPH;oxidoreductase activity, acting on
binding;catalytic activity;GTP binding;GTPase activity;guanyl nucleotide binding;guanyl ribonucleotide binding;hydrolase activity;hydrolase activity, acting on acid anhydrides;hydrolase activity, acting on acid anhydrides, in ph
binding;catalytic activity;cation binding;ion binding;ligase activity;metal ion binding
catalytic activity;hydrolase activity;hydrolase activity, acting on ester bonds;lipase activity;NAPE-specific phospholipase D activity;phospholipase activity;phospholipase D activity;phosphoric diester hydrolase activity;phosph
binding;cytoskeletal protein binding;microtubule binding;protein binding;structural molecule activity;tubulin binding
binding;catalytic activity;cation binding;hydrolase activity;ion binding;metal ion binding;metallopeptidase activity;peptidase activity;peptidase activity, acting on L-amino acid peptides;transition metal ion binding;zinc ion bind
catalytic activity;methyltransferase activity;transferase activity;transferase activity, transferring one-carbon groups
catalytic activity;estradiol 17-beta-dehydrogenase activity;oxidoreductase activity;oxidoreductase activity, acting on CH-OH group of donors;oxidoreductase activity, acting on the CH-OH group of donors, NAD or NADP as a
adenyl nucleotide binding;adenyl ribonucleotide binding;ATP binding;ATPase activity;ATPase activity, coupled;binding;catalytic activity;hydrolase activity;hydrolase activity, acting on acid anhydrides;hydrolase activity, acting o
14-3-3 protein binding;activating transcription factor binding;binding;catalytic activity;cation binding;chromatin binding;deacetylase activity;enzyme binding;histone deacetylase activity;histone deacetylase activity (H3-K14 sp
binding;GTP binding;guanyl nucleotide binding;guanyl ribonucleotide binding;nucleotide binding;purine nucleotide binding;purine ribonucleoside triphosphate binding;purine ribonucleotide binding;ribonucleotide binding
binding;DNA binding;nucleic acid binding;single-stranded DNA binding;structure-specific DNA binding
binding;catalytic activity;cation binding;cysteine-type peptidase activity;hydrolase activity;ion binding;metal ion binding;metallopeptidase activity;nucleic acid binding;peptidase activity;peptidase activity, acting on L-amino ac
adenyl nucleotide binding;adenyl ribonucleotide binding;ATP binding;binding;catalytic activity;ligase activity;nucleotide binding;purine nucleotide binding;purine ribonucleoside triphosphate binding;purine ribonucleotide bindi
binding;cytoskeletal protein binding;kinesin binding;protein binding
binding;identical protein binding;nucleic acid binding;protein binding;RNA binding
ammonia-lyase activity;binding;carbon-nitrogen lyase activity;catalytic activity;cofactor binding;L-serine ammonia-lyase activity;L-threonine ammonia-lyase activity;lyase activity;pyridoxal phosphate binding;vitamin B6 bindin
binding;cation binding;ion binding;metal ion binding;nucleic acid binding
acid-amino acid ligase activity;binding;catalytic activity;cation binding;ion binding;ligase activity;ligase activity, forming carbon-nitrogen bonds;metal ion binding;nucleic acid binding;small conjugating protein ligase activity;ub
catalytic activity;endopeptidase activity;hydrolase activity;peptidase activity;peptidase activity, acting on L-amino acid peptides;threonine-type endopeptidase activity;threonine-type peptidase activity
catalytic activity;cytidylyltransferase activity;ethanolamine-phosphate cytidylyltransferase activity;nucleotidyltransferase activity;transferase activity;transferase activity, transferring phosphorus-containing groups
protein binding transcription factor activity;transcription cofactor activity;transcription corepressor activity;transcription factor binding transcription factor activity
adenyl nucleotide binding;adenyl ribonucleotide binding;ATP binding;binding;catalytic activity;chromatin binding;enzyme binding;histone binding;histone kinase activity;histone kinase activity (H3-S10 specific);histone kinase
binding;calcium ion binding;cation binding;identical protein binding;ion binding;metal ion binding;protein binding;protein dimerization activity;protein heterodimerization activity
binding;DNA binding;nucleic acid binding
binding;catalytic activity;cation binding;demethylase activity;DNA demethylase activity;DNA-N1-methyladenine dioxygenase activity;ferrous iron binding;ion binding;iron ion binding;metal ion binding;oxidative DNA demethyla
binding;nucleic acid binding;RNA binding;translation activator activity;translation regulator activity;translation regulator activity, nucleic acid binding
acetyltransferase activity;binding;catalytic activity;cation binding;enzyme regulator activity;H3 histone acetyltransferase activity;H4 histone acetyltransferase activity;histone acetyltransferase activity;ion binding;iron-sulfur clu
binding;catalytic activity;GDP binding;GTP binding;GTPase activity;guanyl nucleotide binding;guanyl ribonucleotide binding;hydrolase activity;hydrolase activity, acting on acid anhydrides;hydrolase activity, acting on acid anh
carbohydrate phosphatase activity;catalytic activity;fructose-2,6-bisphosphate 2-phosphatase activity;hydrolase activity;hydrolase activity, acting on ester bonds;phosphatase activity;phosphoric ester hydrolase activity;suga
binding;GTP binding;guanyl nucleotide binding;guanyl ribonucleotide binding;nucleotide binding;purine nucleotide binding;purine ribonucleoside triphosphate binding;purine ribonucleotide binding;ribonucleotide binding
acid-amino acid ligase activity;Atg12 ligase activity;Atg8 ligase activity;binding;catalytic activity;enzyme binding;ligase activity;ligase activity, forming carbon-nitrogen bonds;protein binding;small conjugating protein ligase ac
enzyme activator activity;enzyme regulator activity;GTPase activator activity;GTPase regulator activity;nucleoside-triphosphatase regulator activity
binding;catalytic activity;cation binding;hydrolase activity;hydrolase activity, acting on ester bonds;ion binding;lipase activity;metal ion binding;phospholipase activity;phosphoric diester hydrolase activity;phosphoric ester hyd
binding;binding, bridging;cytoskeletal adaptor activity;cytoskeletal protein binding;DNA binding;enzyme binding;kinase binding;nucleic acid binding;proline-rich region binding;protein binding;protein binding, bridging;protein
2 iron, 2 sulfur cluster binding;binding;cation binding;ion binding;iron-sulfur cluster binding;metal cluster binding;metal ion binding
structural constituent of ribosome;structural molecule activity
binding;nucleic acid binding;RNA binding
binding;calcium ion binding;cation binding;ion binding;metal ion binding
anion binding;binding;ion binding;molecular transducer activity;PDZ domain binding;phosphate ion binding;protein binding;protein domain specific binding;signal transducer activity
structural constituent of cytoskeleton;structural molecule activity
binding;carbon-carbon lyase activity;catalytic activity;cation binding;coenzyme binding;cofactor binding;identical protein binding;ion binding;lyase activity;magnesium ion binding;metal ion binding;protein binding;receptor bin
acid-amino acid ligase activity;catalytic activity;ligase activity;ligase activity, forming carbon-nitrogen bonds;small conjugating protein ligase activity;ubiquitin-protein ligase activity
binding;identical protein binding;protein binding;protein complex binding;protein dimerization activity;protein homodimerization activity
binding;enzyme binding;GTPase binding;protein binding;Rab GTPase binding;Ras GTPase binding;small GTPase binding
structural constituent of ribosome;structural molecule activity
binding;catalytic activity;cation binding;deoxyribonucleotide binding;dGTP binding;dGTPase activity;guanyl deoxyribonucleotide binding;guanyl nucleotide binding;hydrolase activity;hydrolase activity, acting on ester bonds;io
enzyme regulator activity;GTPase regulator activity;guanyl-nucleotide exchange factor activity;nucleoside-triphosphatase regulator activity;Rac guanyl-nucleotide exchange factor activity;Ras guanyl-nucleotide exchange fact
active transmembrane transporter activity;ATPase activity;ATPase activity, coupled;ATPase activity, coupled to movement of substances;ATPase activity, coupled to transmembrane movement of substances;catalytic activity;
catalytic activity;dolichyl-phosphate beta-glucosyltransferase activity;glucosyltransferase activity;oligosaccharyl transferase activity;transferase activity;transferase activity, transferring glycosyl groups;transferase activity, tran
binding;cation binding;enzyme binding;identical protein binding;ion binding;K63-linked polyubiquitin binding;metal ion binding;molecular transducer activity;polyubiquitin binding;protein binding;protein dimerization activity;pr
4 iron, 4 sulfur cluster binding;binding;catalytic activity;cation binding;ion binding;iron-sulfur cluster binding;metal cluster binding;metal ion binding;NADH dehydrogenase (quinone) activity;NADH dehydrogenase (ubiquinone)
bHLH transcription factor binding;binding;protein binding;protein binding transcription factor activity;transcription coactivator activity;transcription cofactor activity;transcription factor binding;transcription factor binding trans
alpha-mannosidase activity;binding;carbohydrate binding;catalytic activity;cation binding;hydrolase activity;hydrolase activity, acting on glycosyl bonds;hydrolase activity, hydrolyzing O-glycosyl compounds;ion binding;mann
acetylglucosaminyltransferase activity;binding;catalytic activity;enzyme activator activity;enzyme regulator activity;lipid binding;phosphatidylinositol binding;phosphatidylinositol-3,4,5-trisphosphate binding;phospholipid bindi
branched-chain-amino-acid transaminase activity;catalytic activity;L-isoleucine transaminase activity;L-leucine transaminase activity;L-valine transaminase activity;transaminase activity;transferase activity;transferase activity
binding;nucleic acid binding;RNA binding;snRNA binding;U4 snRNA binding;U6 snRNA binding
binding;catalytic activity;cis-trans isomerase activity;cyclosporin A binding;drug binding;isomerase activity;peptide binding;peptidyl-prolyl cis-trans isomerase activity;ribonucleoprotein binding
binding;cytoskeletal protein binding;enzyme binding;identical protein binding;kinase binding;kinesin binding;protein binding;protein kinase binding
binding;clathrin binding;protein binding

binding;binding, bridging;calcium ion binding;calcium-dependent cysteine-type endopeptidase activity;calcium-dependent protein binding;catalytic activity;cation binding;cysteine-type endopeptidase activity;cysteine-type p
amine binding;amino acid binding;binding;carboxylic acid binding;catalytic activity;cation binding;dimethylargininase activity;hydrolase activity;hydrolase activity, acting on carbon-nitrogen (but not peptide) bonds;hydrolase a
binding;carbohydrate binding;catalytic activity;glutamine-fructose-6-phosphate transaminase (isomerizing) activity;transaminase activity;transferase activity;transferase activity, transferring nitrogenous groups
acid-amino acid ligase activity;binding;catalytic activity;cation binding;ion binding;ligase activity;ligase activity, forming carbon-nitrogen bonds;metal ion binding;small conjugating protein ligase activity;transition metal ion bin
binding;catalytic activity;enzyme binding;GTP binding;GTPase activity;guanyl nucleotide binding;guanyl ribonucleotide binding;hydrolase activity;hydrolase activity, acting on acid anhydrides;hydrolase activity, acting on acid
acid-amino acid ligase activity;binding;catalytic activity;enzyme binding;ligase activity;ligase activity, forming carbon-nitrogen bonds;protein binding;small conjugating protein ligase activity;ubiquitin-protein ligase activity;ubic
APG12 activating enzyme activity;APG8 activating enzyme activity;binding;catalytic activity;identical protein binding;protein binding;protein dimerization activity;protein homodimerization activity;small protein activating enzym
catalytic activity;methyltransferase activity;O-methyltransferase activity;transferase activity;transferase activity, transferring one-carbon groups
binding;catalytic activity;cytoskeletal protein binding;GTP binding;GTPase activity;guanyl nucleotide binding;guanyl ribonucleotide binding;hydrolase activity;hydrolase activity, acting on acid anhydrides;hydrolase activity, act
adenyl nucleotide binding;adenyl ribonucleotide binding;ATP binding;binding;catalytic activity;cation binding;ion binding;kinase activity;magnesium ion binding;metal ion binding;molecular transducer activity;nucleotide bindir
channel activity;macromolecule transmembrane transporter activity;passive transmembrane transporter activity;porin activity;protein transmembrane transporter activity;protein transporter activity;substrate-specific transmen
antioxidant activity;beta-amyloid binding;binding;carbohydrate binding;cation binding;cholesterol binding;cholesterol transporter activity;cytoskeletal protein binding;enzyme activator activity;enzyme regulator activity;glycosa
binding;cation binding;ion binding;metal ion binding
antioxidant activity;binding;catalytic activity;cation binding;DNA binding;identical protein binding;ion binding;manganese ion binding;metal ion binding;nucleic acid binding;oxidoreductase activity;oxidoreductase activity, actir
acetylcholine receptor binding;binding;carbohydrate binding;cation binding;DNA binding;endopeptidase inhibitor activity;endopeptidase regulator activity;enzyme activator activity;enzyme binding;enzyme inhibitor activity;enz
adenyl nucleotide binding;adenyl ribonucleotide binding;ATP binding;binding;catalytic activity;enzyme binding;ion channel binding;kinase activity;non-membrane spanning protein tyrosine kinase activity;nucleotide binding;ph
binding;nucleic acid binding;RNA binding;structural constituent of ribosome;structural molecule activity
binding;chromatin binding;chromatin DNA binding;core promoter binding;core promoter proximal region DNA binding;core promoter proximal region sequence-specific DNA binding;core promoter sequence-specific DNA bin
amine binding;amino acid binding;antioxidant activity;binding;carboxylic acid binding;catalytic activity;glutathione binding;glutathione peroxidase activity;glutathione transferase activity;modified amino acid binding;oxidoredu
ATPase binding;binding;catalytic activity;enzyme binding;GDP binding;GTP binding;GTPase activity;guanyl nucleotide binding;guanyl ribonucleotide binding;hydrolase activity;hydrolase activity, acting on acid anhydrides;hyd
adenyl nucleotide binding;adenyl ribonucleotide binding;ATP binding;binding;catalytic activity;cyclin binding;cyclin-dependent protein kinase activity;cyclin-dependent protein kinase regulator activity;enzyme regulator activity
aminopeptidase activity;binding;carboxypeptidase activity;catalytic activity;cation binding;dipeptidase activity;exopeptidase activity;hydrolase activity;ion binding;manganese ion binding;metal ion binding;metallocarboxypept
binding;calcium ion binding;catalytic activity;cation binding;endopeptidase activity;hydrolase activity;ion binding;metal ion binding;metalloendopeptidase activity;metallopeptidase activity;peptidase activity;peptidase activity,
binding;binding, bridging;cytoskeletal adaptor activity;cytoskeletal protein binding;enzyme inhibitor activity;enzyme regulator activity;eukaryotic initiation factor eIF2 binding;kinase inhibitor activity;kinase regulator activity;pro
binding;catalytic activity;epidermal growth factor receptor binding;growth factor receptor binding;GTP binding;GTPase activity;guanyl nucleotide binding;guanyl ribonucleotide binding;hydrolase activity;hydrolase activity, acti
binding;damaged DNA binding;DNA binding;enzyme binding;nucleic acid binding;protein binding
binding;calcium ion binding;cation binding;endopeptidase inhibitor activity;endopeptidase regulator activity;enzyme inhibitor activity;enzyme regulator activity;ion binding;metal ion binding;peptidase inhibitor activity;peptidas
activin binding;binding;molecular transducer activity;protein binding;protein complex binding;signal transducer activity
binding;catalytic activity;catechol O-methyltransferase activity;cation binding;ion binding;magnesium ion binding;metal ion binding;methyltransferase activity;O-methyltransferase activity;S-adenosylmethionine-dependent me
acid-amino acid ligase activity;binding;calcium ion binding;catalytic activity;cation binding;ephrin receptor binding;ion binding;ligase activity;ligase activity, forming carbon-nitrogen bonds;metal ion binding;molecular transduc
binding;fibronectin binding;growth factor binding;insulin-like growth factor binding;insulin-like growth factor I binding;insulin-like growth factor II binding;protein binding
binding;calcium ion binding;cation binding;ion binding;metal ion binding;structural constituent of muscle;structural molecule activity
catalytic activity;endopeptidase activity;hydrolase activity;peptidase activity;peptidase activity, acting on L-amino acid peptides;threonine-type endopeptidase activity;threonine-type peptidase activity
actin binding;actin filament binding;binding;calmodulin binding;cytoskeletal protein binding;protein binding
binding;catalytic activity;cation binding;G-protein beta/gamma-subunit complex binding;G-protein-coupled receptor binding;GTP binding;GTPase activity;guanyl nucleotide binding;guanyl ribonucleotide binding;hydrolase ac
binding;catalytic activity;cation binding;dCMP deaminase activity;deaminase activity;hydrolase activity;hydrolase activity, acting on carbon-nitrogen (but not peptide) bonds;hydrolase activity, acting on carbon-nitrogen (but n
aldehyde-lyase activity;amine binding;amino acid binding;binding;carbon-carbon lyase activity;carboxylic acid binding;catalytic activity;cofactor binding;glycine hydroxymethyltransferase activity;hydroxymethyl-, formyl- and
antioxidant activity;arachidonate 15-lipoxygenase activity;binding;catalytic activity;cation binding;dioxygenase activity;enzyme binding;heme binding;ion binding;iron ion binding;lipid binding;metal ion binding;oxidoreductase
actin binding;actin filament binding;binding;cytoskeletal protein binding;identical protein binding;protein binding;protein dimerization activity;protein heterodimerization activity;protein homodimerization activity;spectrin bindin
binding;cation binding;endopeptidase inhibitor activity;endopeptidase regulator activity;enzyme binding;enzyme inhibitor activity;enzyme regulator activity;ion binding;metal ion binding;metalloendopeptidase inhibitor activity;
active transmembrane transporter activity;ATPase activity;ATPase activity, coupled;ATPase activity, coupled to movement of substances;ATPase activity, coupled to transmembrane movement of ions;ATPase activity, couplec
binding;cation binding;DNA binding;ion binding;metal ion binding;nucleic acid binding;structural constituent of ribosome;structural molecule activity;transition metal ion binding;zinc ion binding
beta-tubulin binding;binding;cytoskeletal protein binding;diazepam binding;drug binding;dynactin binding;dynein binding;dynein intermediate chain binding;identical protein binding;ion channel binding;p53 binding;profilin bin
adenyl nucleotide binding;adenyl ribonucleotide binding;ATP binding;binding;catalytic activity;enzyme binding;kinase activity;kinase binding;MAP kinase kinase activity;nucleotide binding;phosphotransferase activity, alcohol
binding;catalytic activity;DNA binding;DNA polymerase activity;DNA-directed DNA polymerase activity;nucleic acid binding;nucleotidyltransferase activity;transferase activity;transferase activity, transferring phosphorus-conta
binding;cation binding;chromatin binding;chromatin DNA binding;chromatin insulator sequence binding;core promoter proximal region DNA binding;core promoter proximal region sequence-specific DNA binding;DNA binding
actin binding;beta-tubulin binding;binding;cytoskeletal protein binding;protein binding;tubulin binding
binding;catalytic activity;NADH dehydrogenase (quinone) activity;NADH dehydrogenase (ubiquinone) activity;NADH dehydrogenase activity;oxidoreductase activity;oxidoreductase activity, acting on NADH or NADPH;oxidored
binding;carbon-sulfur lyase activity;catalytic activity;cation binding;holocytochrome-c synthase activity;ion binding;lyase activity;metal ion binding
actin binding;binding;cytoskeletal protein binding;protein binding
acid-amino acid ligase activity;adenyl nucleotide binding;adenyl ribonucleotide binding;ATP binding;binding;catalytic activity;enzyme binding;ligase activity;ligase activity, forming carbon-nitrogen bonds;nucleotide binding;pr
binding;catalytic activity;enzyme binding;GTP binding;GTPase activity;guanyl nucleotide binding;guanyl ribonucleotide binding;hydrolase activity;hydrolase activity, acting on acid anhydrides;hydrolase activity, acting on acid
catalytic activity;endonuclease activity;endonuclease activity, active with either ribo- or deoxyribonucleic acids and producing 5'-phosphomonoesters;endoribonuclease activity;endoribonuclease activity, producing 5'-phosph
binding;carbohydrate binding;cation binding;DNA binding;endopeptidase inhibitor activity;endopeptidase regulator activity;enzyme inhibitor activity;enzyme regulator activity;glycosaminoglycan binding;heparin binding;identi
binding;catalytic activity;cation binding;hydrolase activity;intramolecular oxidoreductase activity;intramolecular oxidoreductase activity, transposing C=C bonds;ion binding;isomerase activity;isopentenyl-diphosphate delta-is
angiotensin receptor binding;binding;catalytic activity;cation binding;D5 dopamine receptor binding;dopamine receptor binding;G-protein beta/gamma-subunit complex binding;G-protein-coupled receptor binding;GTP bindi
binding;catalytic activity;cation binding;diphosphotransferase activity;enzyme inhibitor activity;enzyme regulator activity;ion binding;magnesium ion binding;metal ion binding;ribose phosphate diphosphokinase activity;transfe
3'-5' exonuclease activity;3'-5'-exoribonuclease activity;binding;catalytic activity;exonuclease activity;exonuclease activity, active with either ribo- or deoxyribonucleic acids and producing 5'-phosphomonoesters;exoribonucl
adenyl nucleotide binding;adenyl ribonucleotide binding;ATP binding;binding;catalytic activity;cis-trans isomerase activity;enzyme activator activity;enzyme binding;enzyme regulator activity;identical protein binding;isomeras
beta-catenin binding;binding;catalytic activity;enzyme binding;gamma-catenin binding;hydrolase activity;hydrolase activity, acting on ester bonds;kinase binding;molecular transducer activity;phosphatase activity;phosphopro
binding;enzyme binding;protein binding;ubiquitin protein ligase binding
enzyme regulator activity;phosphatase regulator activity;protein phosphatase regulator activity;protein phosphatase type 1 regulator activity
active transmembrane transporter activity;amine transmembrane transporter activity;amino acid transmembrane transporter activity;anion transmembrane transporter activity;anion:cation symporter activity;carboxylic acid tra
acetylglucosaminyltransferase activity;binding;catalytic activity;cation binding;glucuronosyl-N-acetylglucosaminyl-proteoglycan 4-alpha-N-acetylglucosaminyltransferase activity;glucuronosyltransferase activity;heparan sulfa
catalytic activity;nucleotidyltransferase activity;transferase activity;transferase activity, transferring phosphorus-containing groups;uridylyltransferase activity
anion channel activity;anion transmembrane transporter activity;calcium activated cation channel activity;cation channel activity;cation transmembrane transporter activity;channel activity;chloride channel activity;gated chann
binding;nucleic acid binding;RNA binding;rRNA binding;snoRNA binding;U3 snoRNA binding
binding;chromatin binding
binding;cation binding;DNA binding;ion binding;metal ion binding;nucleic acid binding;nucleic acid binding transcription factor activity;regulatory region DNA binding;regulatory region nucleic acid binding;RNA polymerase II c
active transmembrane transporter activity;amine transmembrane transporter activity;choline transmembrane transporter activity;molecular transducer activity;signal transducer activity;substrate-specific transmembrane trans
binding;enzyme binding;protein binding;ubiquitin protein ligase binding
basal RNA polymerase II transcription machinery binding;basal transcription machinery binding;binding;chromatin binding;enzyme binding;protein binding;RNA polymerase binding;RNA polymerase core enzyme binding;RNA
binding;DNA binding;nucleic acid binding
catalytic activity;cytidylyltransferase activity;N-acylneuraminate cytidylyltransferase activity;nucleotidyltransferase activity;transferase activity;transferase activity, transferring phosphorus-containing groups
binding;enzyme binding;GTPase binding;identical protein binding;protein binding;protein dimerization activity;protein homodimerization activity;Rab GTPase binding;Ras GTPase binding;small GTPase binding
binding;cytokine activity;protein binding;receptor binding
binding;calcium channel activity;cation channel activity;cation transmembrane transporter activity;channel activity;enzyme binding;ion channel activity;ion transmembrane transporter activity;passive transmembrane transport
active transmembrane transporter activity;ATPase activity;ATPase activity, coupled;ATPase activity, coupled to movement of substances;ATPase activity, coupled to transmembrane movement of ions;ATPase activity, couplec
binding;cation binding;ion binding;metal ion binding;transition metal ion binding;zinc ion binding
catalytic activity;cysteine-type peptidase activity;hydrolase activity;peptidase activity;peptidase activity, acting on L-amino acid peptides;small conjugating protein-specific protease activity;ubiquitin-specific protease activity
binding;nucleic acid binding;nucleotide binding;RNA binding;RNA stem-loop binding
adenyl nucleotide binding;adenyl ribonucleotide binding;ATP binding;binding;catalytic activity;cation binding;ion binding;kinase activity;metal ion binding;nucleotide binding;phosphotransferase activity, alcohol group as accep
binding;DNA binding;nucleic acid binding
binding;calcium ion binding;cation binding;copper ion binding;fibroblast growth factor binding;growth factor binding;identical protein binding;ion binding;lipid binding;metal ion binding;protein binding;protein dimerization acti
binding;cation binding;ion binding;metal ion binding;transition metal ion binding;zinc ion binding
binding;high-density lipoprotein particle binding;lipid binding;lipoprotein particle binding;protein binding;protein-lipid complex binding;receptor binding
ADP-specific glucokinase activity;binding;catalytic activity;cation binding;ion binding;kinase activity;metal ion binding;phosphotransferase activity, alcohol group as acceptor;transferase activity;transferase activity, transferring
binding;enzyme inhibitor activity;enzyme regulator activity;histone binding;phosphatase inhibitor activity;phosphatase regulator activity;protein binding
binding;cytoskeletal protein binding;enzyme binding;gamma-tubulin binding;GTPase binding;protein binding;Rab GTPase binding;Ras GTPase binding;small GTPase binding;tubulin binding
binding;catalytic activity;cation binding;ion binding;metal ion binding;queuine tRNA-ribosyltransferase activity;transferase activity;transferase activity, transferring glycosyl groups;transferase activity, transferring pentosyl grou
binding;carboxylic acid binding;catalytic activity;cation binding;enzyme binding;ion binding;iron ion binding;L-ascorbic acid binding;metal ion binding;oxidoreductase activity;oxidoreductase activity, acting on paired donors, v
binding;cation binding;ion binding;metal ion binding
catalytic activity;hydrolase activity
binding;carboxylic acid binding;coenzyme binding;cofactor binding;fatty acid binding;fatty-acyl-CoA binding;lipid binding;monocarboxylic acid binding
binding;enzyme binding;GTPase binding;GTP-Rho binding;protein binding;Ras GTPase binding;Rho GTPase binding;small GTPase binding
binding;cholesterol binding;lipid binding;steroid binding;sterol binding
acidic amino acid transmembrane transporter activity;active transmembrane transporter activity;amine transmembrane transporter activity;amino acid transmembrane transporter activity;carboxylic acid transmembrane trans
acid-amino acid ligase activity;catalytic activity;ligase activity;ligase activity, forming carbon-nitrogen bonds;phosphopantothenate--cysteine ligase activity
binding;chromatin binding

ligand-dependent nuclear receptor transcription coactivator activity;protein binding transcription factor activity;transcription coactivator activity;transcription cofactor activity;transcription factor binding transcription factor act
binding;enzyme activator activity;enzyme regulator activity;G-protein alpha-subunit binding;GTPase activator activity;GTPase regulator activity;guanyl-nucleotide exchange factor activity;nucleoside-triphosphatase regulator a
binding;protein binding;protein domain specific binding;structural constituent of ribosome;structural molecule activity
binding;nucleotide binding
adenyl nucleotide binding;adenyl ribonucleotide binding;ATP binding;ATPase activity;binding;catalytic activity;hydrolase activity;hydrolase activity, acting on acid anhydrides;hydrolase activity, acting on acid anhydrides, in ph
binding;cytoskeletal protein binding;protein binding
binding;cation binding;ion binding;metal ion binding;protein binding transcription factor activity;transcription cofactor activity;transcription corepressor activity;transcription factor binding transcription factor activity;transition
binding;nucleic acid binding;RNA binding
3-hydroxyacyl-CoA dehydratase activity;binding;carbon-oxygen lyase activity;catalytic activity;enzyme activator activity;enzyme binding;enzyme regulator activity;GTPase activator activity;GTPase regulator activity;hydro-lyas
enzyme regulator activity;kinase regulator activity
7-dehydrocholesterol reductase activity;catalytic activity;oxidoreductase activity;oxidoreductase activity, acting on the CH-CH group of donors;oxidoreductase activity, acting on the CH-CH group of donors, NAD or NADP as
carboxylic acid transmembrane transporter activity;dicarboxylic acid transmembrane transporter activity;organic acid transmembrane transporter activity;substrate-specific transmembrane transporter activity;substrate-speci
binding;protein binding;protein binding involved in protein folding;unfolded protein binding
active transmembrane transporter activity;ATPase activity;ATPase activity, coupled;ATPase activity, coupled to movement of substances;ATPase activity, coupled to transmembrane movement of ions;ATPase activity, couplec
binding;catalytic activity;cation binding;endonuclease activity;endonuclease activity, active with either ribo- or deoxyribonucleic acids and producing 5'-phosphomonoesters;endoribonuclease activity;endoribonuclease activit
adenyl nucleotide binding;adenyl ribonucleotide binding;ATP binding;ATPase activity;ATPase activity, coupled;ATP-dependent helicase activity;ATP-dependent RNA helicase activity;binding;catalytic activity;helicase activity;h
binding;DNA binding;nucleic acid binding;nucleotide binding
binding;DNA binding;enzyme binding;mRNA 3'-UTR binding;mRNA binding;nucleic acid binding;phosphatase binding;protein binding;RNA binding
acid-amino acid ligase activity;catalytic activity;ligase activity;ligase activity, forming carbon-nitrogen bonds;small conjugating protein ligase activity;UFM1 conjugating enzyme activity
binding;LRR domain binding;protein binding;protein domain specific binding
catalytic activity;NADH dehydrogenase (quinone) activity;NADH dehydrogenase (ubiquinone) activity;NADH dehydrogenase activity;oxidoreductase activity;oxidoreductase activity, acting on NADH or NADPH;oxidoreductase
binding;catalytic activity;cation binding;hydrolase activity;hydrolase activity, acting on ester bonds;ion binding;magnesium ion binding;metal ion binding;phosphatase activity;phosphoglycolate phosphatase activity;phosphop
binding;DNA binding;nucleic acid binding
binding;nuclear localization sequence binding;peptide binding;protein transporter activity;signal sequence binding;substrate-specific transporter activity;transporter activity
binding;DNA binding;ligand-dependent nuclear receptor binding;nucleic acid binding;protein binding;protein binding transcription factor activity;receptor binding;transcription coactivator activity;transcription cofactor activity;
adrenergic receptor binding;beta-2 adrenergic receptor binding;beta-catenin binding;binding;channel regulator activity;chloride channel regulator activity;enzyme binding;G-protein-coupled receptor binding;growth factor rece
binding;catalytic activity;cation binding;DNA binding;DNA polymerase activity;identical protein binding;ion binding;metal ion binding;nucleic acid binding;nucleotidyltransferase activity;protein binding;protein dimerization acti
binding;identical protein binding;protein binding
catalytic activity;cation transmembrane transporter activity;hydrogen ion transmembrane transporter activity;inorganic cation transmembrane transporter activity;ion transmembrane transporter activity;monovalent inorganic c
binding;catalytic activity;palmitoyltransferase activity;protein binding;protein-cysteine S-acyltransferase activity;protein-cysteine S-palmitoleyltransferase activity;S-acyltransferase activity;SNAP receptor activity;SNARE bindi
alcohol dehydrogenase (NADP+) activity;alditol:NADP+ 1-oxidoreductase activity;aldo-keto reductase (NADP) activity;catalytic activity;electron carrier activity;epoxide hydrolase activity;ether hydrolase activity;hydrolase activ
binding;cation binding;DNA binding;double-stranded DNA binding;ion binding;metal ion binding;nucleic acid binding;structure-specific DNA binding;transition metal ion binding;zinc ion binding
binding;cation binding;ion binding;metal ion binding
binding;chromatin binding
active transmembrane transporter activity;ATPase activity;ATPase activity, coupled;ATPase activity, coupled to movement of substances;ATPase activity, coupled to transmembrane movement of ions;ATPase activity, couplec
catalytic activity;electron carrier activity;NADH dehydrogenase (quinone) activity;NADH dehydrogenase (ubiquinone) activity;NADH dehydrogenase activity;oxidoreductase activity;oxidoreductase activity, acting on NADH or N
binding;DNA binding;nucleic acid binding;RNA binding
3-keto sterol reductase activity;binding;carbonyl reductase (NADPH) activity;catalytic activity;coenzyme binding;cofactor binding;NADP binding;NADPH binding;nucleotide binding;oxidoreductase activity;oxidoreductase acti
binding;catalytic activity;cation binding;endopeptidase activity;exopeptidase activity;hydrolase activity;ion binding;metal ion binding;metalloendopeptidase activity;metalloexopeptidase activity;metallopeptidase activity;peptic
structural molecule activity
binding;catalytic activity;cation binding;disulfide oxidoreductase activity;electron carrier activity;ion binding;iron-sulfur cluster binding;metal cluster binding;metal ion binding;oxidoreductase activity;oxidoreductase activity, ac
binding;cyclin-dependent protein kinase inhibitor activity;cyclin-dependent protein kinase regulator activity;enzyme inhibitor activity;enzyme regulator activity;kinase inhibitor activity;kinase regulator activity;nucleic acid bindi
6-phosphogluconolactonase activity;binding;carbohydrate binding;carboxylic ester hydrolase activity;catalytic activity;hydrolase activity;hydrolase activity, acting on ester bonds;monosaccharide binding;sugar binding
binding;cation binding;ion binding;metal ion binding;transition metal ion binding;transporter activity;zinc ion binding
binding;catalytic activity;cation binding;dioxygenase activity;ion binding;iron ion binding;metal ion binding;oxidoreductase activity;oxidoreductase activity, acting on single donors with incorporation of molecular oxygen;oxido
binding;chromatin binding;protein binding transcription factor activity;transcription coactivator activity;transcription cofactor activity;transcription factor binding transcription factor activity
binding;calcium ion binding;catalytic activity;cation binding;endopeptidase activity;growth factor activity;hydrolase activity;ion binding;metal ion binding;molecular transducer activity;peptidase activity;peptidase activity, actin
binding;carbohydrate binding;endopeptidase inhibitor activity;endopeptidase regulator activity;enzyme binding;enzyme inhibitor activity;enzyme regulator activity;glycosaminoglycan binding;heparin binding;pattern binding;pe
binding;GDP binding;GMP binding;GTP binding;guanyl nucleotide binding;guanyl ribonucleotide binding;nucleotide binding;protein binding;protein complex binding;purine nucleotide binding;purine ribonucleoside triphospha
binding;cation binding;ion binding;metal ion binding;transition metal ion binding;zinc ion binding
binding;nucleic acid binding;RNA binding;structural constituent of ribosome;structural molecule activity
binding;cation binding;extracellular matrix structural constituent;ion binding;metal ion binding;structural molecule activity
adenine binding;adenine phosphoribosyltransferase activity;adenyl nucleotide binding;adenyl ribonucleotide binding;AMP binding;binding;catalytic activity;nucleobase binding;nucleotide binding;purine base binding;purine nu
binding;nucleic acid binding;nucleotide binding;RNA binding;snRNA binding
active transmembrane transporter activity;binding;carbohydrate transmembrane transporter activity;cation transmembrane transporter activity;cation:sugar symporter activity;dehydroascorbic acid transporter activity;D-gluco
ATPase binding;binding;catalytic activity;cytoskeletal protein binding;Edg-2 lysophosphatidic acid receptor binding;endothelial differentiation G-protein coupled receptor binding;enzyme binding;GDP binding;G-protein-coupl
binding;enzyme binding;protein binding
binding;carbohydrate binding;catalytic activity;enzyme binding;glucose binding;glucosyltransferase activity;glycogen (starch) synthase activity;kinase binding;monosaccharide binding;protein binding;protein kinase binding;su
binding;C-acyltransferase activity;carboxylic acid binding;catalytic activity;cholesterol binding;coenzyme binding;cofactor binding;fatty acid binding;fatty-acyl-CoA binding;lipid binding;lipid transporter activity;monocarboxyli
adenyl nucleotide binding;adenyl ribonucleotide binding;ATP binding;binding;catalytic activity;cation binding;cyclin binding;cyclin-dependent protein kinase activity;ion binding;kinase activity;metal ion binding;nucleotide bind
acyl-CoA dehydrogenase activity;binding;catalytic activity;coenzyme binding;cofactor binding;flavin adenine dinucleotide binding;isovaleryl-CoA dehydrogenase activity;oxidoreductase activity;oxidoreductase activity, acting
adenyl nucleotide binding;adenyl ribonucleotide binding;ATP binding;axon guidance receptor activity;binding;catalytic activity;ephrin receptor activity;kinase activity;molecular transducer activity;nucleotide binding;phosphotr
binding;carboxylic acid binding;isoprenoid binding;lipid binding;monocarboxylic acid binding;retinal binding;retinoic acid binding;retinoid binding;retinol binding;transporter activity;vitamin binding
antioxidant activity;binding;caspase inhibitor activity;caspase regulator activity;catalytic activity;cysteine-type endopeptidase inhibitor activity;DNA binding;endopeptidase inhibitor activity;endopeptidase regulator activity;enz
carbon-carbon lyase activity;carboxy-lyase activity;catalytic activity;D-dopachrome decarboxylase activity;dopachrome isomerase activity;intramolecular oxidoreductase activity;intramolecular oxidoreductase activity, transpo
actin binding;actin filament binding;actin monomer binding;binding;cytoskeletal protein binding;enzyme binding;identical protein binding;kinase binding;myosin binding;myosin heavy chain binding;phosphatidylinositol 3-kina
binding;carboxylic acid binding;catalytic activity;cholesterol binding;cholesterol O-acyltransferase activity;coenzyme binding;cofactor binding;fatty acid binding;fatty-acyl-CoA binding;lipid binding;monocarboxylic acid bindin
binding;calcium ion binding;cation binding;ion binding;metal ion binding
adenyl nucleotide binding;adenyl ribonucleotide binding;ATP binding;binding;DNA binding;enzyme binding;nucleic acid binding;nucleotide binding;protein binding;purine nucleotide binding;purine ribonucleoside triphosphate
adenyl nucleotide binding;adenyl ribonucleotide binding;ATP binding;binding;catalytic activity;identical protein binding;kinase activity;non-membrane spanning protein tyrosine kinase activity;nucleotide binding;phosphotrans
binding;ephrin receptor binding;protein binding;protein domain specific binding;receptor binding;SH2 domain binding
binding;catalytic activity;choline-phosphate cytidylyltransferase activity;cytidylyltransferase activity;lipid binding;nucleotidyltransferase activity;transferase activity;transferase activity, transferring phosphorus-containing group
binding;catalytic activity;GTP binding;GTPase activity;guanyl nucleotide binding;guanyl ribonucleotide binding;hydrolase activity;hydrolase activity, acting on acid anhydrides;hydrolase activity, acting on acid anhydrides, in ph
binding;damaged DNA binding;DNA binding;nucleic acid binding;polyubiquitin binding;protein binding;single-stranded DNA binding;small conjugating protein binding;structure-specific DNA binding;ubiquitin binding
binding;catalytic activity;GTP binding;GTPase activity;guanyl nucleotide binding;guanyl ribonucleotide binding;hydrolase activity;hydrolase activity, acting on acid anhydrides;hydrolase activity, acting on acid anhydrides, in ph
enzyme activator activity;enzyme inhibitor activity;enzyme regulator activity;kinase inhibitor activity;kinase regulator activity;molecular transducer activity;protein kinase inhibitor activity;protein kinase regulator activity;signal tr
binding;catalytic activity;enzyme regulator activity;GTP binding;GTPase activity;GTPase regulator activity;guanyl nucleotide binding;guanyl ribonucleotide binding;guanyl-nucleotide exchange factor activity;hydrolase activity;
acid-amino acid ligase activity;catalytic activity;ligase activity;ligase activity, forming carbon-nitrogen bonds;small conjugating protein ligase activity;ubiquitin-protein ligase activity
1-alkyl-2-acetylglycerophosphocholine esterase activity;acetyltransferase activity;carboxylic ester hydrolase activity;catalytic activity;hydrolase activity;hydrolase activity, acting on ester bonds;platelet-activating factor acetylt
binding;cation binding;ion binding;metal ion binding;nucleotide binding
activating transcription factor binding;anion binding;binding;chromatin binding;core promoter proximal region DNA binding;core promoter proximal region sequence-specific DNA binding;DNA binding;enhancer binding;enhar
binding;chromatin binding;DNA binding;nucleic acid binding;single-stranded DNA binding;structure-specific DNA binding
binding;mRNA binding;nucleic acid binding;nucleotide binding;RNA binding
binding;selenium binding
binding;cation binding;chromatin binding;core promoter binding;core promoter sequence-specific DNA binding;DNA binding;ion binding;metal ion binding;nucleic acid binding;nucleic acid binding transcription factor activity;
acid-amino acid ligase activity;binding;catalytic activity;enzyme binding;ligase activity;ligase activity, forming carbon-nitrogen bonds;protein binding;small conjugating protein ligase activity;ubiquitin protein ligase binding
amine binding;amino acid binding;binding;carboxylic acid binding;catalytic activity;cis-trans isomerase activity;enzyme binding;GTPase activating protein binding;isomerase activity;kinase binding;mitogen-activated protein k
3'-5' exonuclease activity;3'-5'-exoribonuclease activity;7S RNA binding;binding;catalytic activity;exonuclease activity;exonuclease activity, active with either ribo- or deoxyribonucleic acids and producing 5'-phosphomonoes
adenyl nucleotide binding;adenyl ribonucleotide binding;ATP binding;ATPase activity;binding;catalytic activity;cation binding;DNA binding;helicase activity;hydrolase activity;hydrolase activity, acting on acid anhydrides;hydrol
catalytic activity;intramolecular oxidoreductase activity;intramolecular oxidoreductase activity, interconverting keto- and enol-groups;intramolecular oxidoreductase activity, transposing S-S bonds;isomerase activity;oxidored
catalytic activity;enzyme regulator activity;hydrolase activity;hydrolase activity, acting on ester bonds;phosphatase activity;phosphatase regulator activity;phosphoprotein phosphatase activity;phosphoric ester hydrolase activ
binding;calcium ion binding;catalytic activity;cation binding;cytokine binding;growth factor binding;ion binding;kinase activity;metal ion binding;microfibril binding;molecular transducer activity;phosphotransferase activity, alco
binding;nucleic acid binding;RNA binding;snoRNA binding
binding;enzyme activator activity;enzyme binding;enzyme regulator activity;GTPase activator activity;GTPase regulator activity;kinase binding;nucleoside-triphosphatase regulator activity;protein binding;protein kinase binding
catalytic activity;hydrolase activity;hydrolase activity, acting on ester bonds;phosphatase activity;phosphoprotein phosphatase activity;phosphoric ester hydrolase activity
binding;catalytic activity;coenzyme binding;cofactor binding;NADH dehydrogenase (quinone) activity;NADH dehydrogenase (ubiquinone) activity;NADH dehydrogenase activity;oxidoreductase activity;oxidoreductase activity,
binding;catalytic activity;cation binding;demethylase activity;heme binding;ion binding;iron ion binding;metal ion binding;monooxygenase activity;oxidoreductase activity;oxidoreductase activity, acting on paired donors, with
catalytic activity;oxidoreductase activity;oxidoreductase activity, acting on the CH-NH group of donors;oxidoreductase activity, acting on the CH-NH group of donors, NAD or NADP as acceptor;pyrroline-5-carboxylate reduc
adenyl nucleotide binding;adenyl ribonucleotide binding;aminoacyl-tRNA ligase activity;ATP binding;binding;catalytic activity;glutamate-tRNA ligase activity;glutamate-tRNA(Gln) ligase activity;ligase activity;ligase activity, for
binding;cation binding;ion binding;metal ion binding
3-hydroxyisobutyryl-CoA hydrolase activity;catalytic activity;CoA hydrolase activity;hydrolase activity;hydrolase activity, acting on ester bonds;thiolester hydrolase activity
catalytic activity;ligase activity;nicotinate phosphoribosyltransferase activity;nicotinate-nucleotide diphosphorylase (carboxylating) activity;transferase activity;transferase activity, transferring glycosyl groups;transferase activit
adenyl nucleotide binding;adenyl ribonucleotide binding;ATP binding;binding;catalytic activity;cation binding;enzyme activator activity;enzyme regulator activity;ion binding;kinase activator activity;kinase activity;kinase regula
catalytic activity;methyltransferase activity;RNA methyltransferase activity;S-adenosylmethionine-dependent methyltransferase activity;transferase activity;transferase activity, transferring one-carbon groups
binding;cation binding;DNA binding;ion binding;metal ion binding;nucleic acid binding
catalytic activity;hydrolase activity;hydrolase activity, acting on carbon-nitrogen (but not peptide) bonds;hydrolase activity, acting on carbon-nitrogen (but not peptide) bonds, in nitriles;nitrilase activity
binding;dynein binding;dynein intermediate chain binding;protein binding
binding;ion channel binding;protein binding
binding;enzyme binding;GTPase binding;protein binding;Ral GTPase binding;Ras GTPase binding;small GTPase binding
binding;catalytic activity;methyltransferase activity;mRNA (nucleoside-2'-O-)-methyltransferase activity;mRNA methyltransferase activity;nucleic acid binding;O-methyltransferase activity;RNA methyltransferase activity;S-ade
1-acylglycerophosphocholine O-acyltransferase activity;1-alkenylglycerophosphocholine O-acyltransferase activity;1-alkylglycerophosphocholine O-acetyltransferase activity;1-alkylglycerophosphocholine O-acyltransferase a
molecular transducer activity;signal transducer activity
catalytic activity;nucleotidyltransferase activity;transferase activity;transferase activity, transferring phosphorus-containing groups

binding;cyclin binding;enzyme binding;kinase binding;NF-kappaB binding;protein binding;protein kinase binding;transcription factor binding
catalytic activity;intramolecular oxidoreductase activity;intramolecular oxidoreductase activity, interconverting keto- and enol-groups;intramolecular oxidoreductase activity, transposing S-S bonds;isomerase activity;protein d
binding;cation binding;enzyme activator activity;enzyme regulator activity;GTPase activator activity;GTPase regulator activity;ion binding;lipid binding;metal ion binding;nucleoside-triphosphatase regulator activity;phosphatid
binding;enzyme binding;enzyme inhibitor activity;enzyme regulator activity;phosphatase binding;phosphatase inhibitor activity;phosphatase regulator activity;protein binding;protein phosphatase 1 binding;protein phosphatas
binding;chromatin binding;DNA binding;heat shock protein binding;histone binding;Hsp70 protein binding;nucleic acid binding;protein binding;small conjugating protein binding;ubiquitin binding
binding;protein binding;unfolded protein binding
3-hydroxy-2-methylbutyryl-CoA dehydrogenase activity;3-hydroxyacyl-CoA dehydrogenase activity;catalytic activity;cholate 7-alpha-dehydrogenase activity;oxidoreductase activity;oxidoreductase activity, acting on CH-OH g
binding;protein binding;SNARE binding;soluble NSF attachment protein activity;syntaxin binding
binding;cation binding;ion binding;metal ion binding
acid-amino acid ligase activity;binding;catalytic activity;cation binding;ion binding;ligase activity;ligase activity, forming carbon-nitrogen bonds;metal ion binding;small conjugating protein ligase activity;transition metal ion bin
binding;cation binding;chromatin binding;DNA binding;ion binding;metal ion binding;nucleic acid binding;nucleic acid binding transcription factor activity;sequence-specific DNA binding;sequence-specific DNA binding transc
binding;enzyme binding;identical protein binding;kinase binding;protein binding;protein dimerization activity;protein homodimerization activity;protein kinase binding;protein N-terminus binding
binding;nucleotide binding
binding;cholesterol binding;lipid binding;steroid binding;sterol binding
catalytic activity;exonuclease activity;hydrolase activity;hydrolase activity, acting on ester bonds;nuclease activity;nucleic acid binding transcription factor activity;sequence-specific DNA binding transcription factor activity
binding;frizzled binding;G-protein-coupled receptor binding;protein binding;receptor binding
binding;catalytic activity;disulfide oxidoreductase activity;electron carrier activity;enzyme activator activity;enzyme regulator activity;GTPase activator activity;GTPase regulator activity;nucleoside-triphosphatase regulator acti
binding;enzyme binding;phosphatase binding;protein binding
binding;protein binding;protein C-terminus binding
binding;catalytic activity;cation binding;ion binding;metal ion binding;transferase activity;transferase activity, transferring acyl groups
binding;cation binding;ion binding;metal ion binding;transition metal ion binding;zinc ion binding
binding;calcium channel inhibitor activity;calcium channel regulator activity;catalytic activity;channel inhibitor activity;channel regulator activity;hydrolase activity;hydrolase activity, acting on ester bonds;ion channel binding;io
adenyl nucleotide binding;adenyl ribonucleotide binding;ATP binding;ATPase activity;binding;catalytic activity;DNA binding;hydrolase activity;hydrolase activity, acting on acid anhydrides;hydrolase activity, acting on acid anhy
binding;DNA binding;DNA polymerase binding;enzyme binding;nucleic acid binding;protein binding
binding;calcium-dependent protein binding;cation binding;ion binding;metal ion binding;nucleic acid binding;nucleocytoplasmic transporter activity;nucleotide binding;pre-mRNA binding;protein binding;RNA binding;snRNA b
binding;nucleic acid binding;RNA binding;snoRNA binding
catalytic activity;dipeptidyl-peptidase activity;exopeptidase activity;hydrolase activity;peptidase activity;peptidase activity, acting on L-amino acid peptides;serine hydrolase activity;serine-type peptidase activity
binding;enzyme binding;phosphatase binding;protein binding;protein phosphatase binding
5'-3' exonuclease activity;binding;catalytic activity;cation binding;endonuclease activity;endoribonuclease activity;exonuclease activity;hydrolase activity;hydrolase activity, acting on ester bonds;ion binding;metal ion binding;
binding;DNA binding;nucleic acid binding
structural constituent of ribosome;structural molecule activity
binding;cytoskeletal protein binding;DNA binding;enzyme activator activity;enzyme binding;enzyme regulator activity;GTPase activator activity;GTPase binding;GTPase regulator activity;nucleic acid binding;nucleoside-tripho
binding;enzyme binding;insulin receptor binding;kinase binding;molecular transducer activity;phosphatidylinositol 3-kinase binding;protein binding;protein complex binding;receptor binding;signal transducer activity
receptor activity
binding;chromatin binding;DNA binding;enzyme binding;histone deacetylase binding;Notch binding;nucleic acid binding;protein binding;protein binding transcription factor activity;protein N-terminus binding;receptor binding;
binding;catalytic activity;cation binding;cofactor binding;cysteine desulfurase activity;identical protein binding;ion binding;iron-sulfur cluster binding;metal cluster binding;metal ion binding;protein binding;protein dimerization
ARF guanyl-nucleotide exchange factor activity;binding;cytoskeletal protein binding;enzyme regulator activity;GTPase regulator activity;guanyl-nucleotide exchange factor activity;myosin binding;nucleoside-triphosphatase re
binding;cation binding;ion binding;metal ion binding;transition metal ion binding;zinc ion binding
binding;catalytic activity;GTP binding;GTPase activity;guanyl nucleotide binding;guanyl ribonucleotide binding;hydrolase activity;hydrolase activity, acting on acid anhydrides;hydrolase activity, acting on acid anhydrides, in ph
binding;enzyme binding;protein binding;ubiquitin protein ligase binding
adenyl nucleotide binding;adenyl ribonucleotide binding;ATP binding;binding;catalytic activity;guanylate kinase activity;kinase activity;nucleobase-containing compound kinase activity;nucleotide binding;nucleotide kinase act
binding;C-acyltransferase activity;catalytic activity;cofactor binding;C-palmitoyltransferase activity;palmitoyltransferase activity;pyridoxal phosphate binding;serine C-palmitoyltransferase activity;transferase activity;transferas
binding;centromeric DNA binding;DNA binding;nucleic acid binding;sequence-specific DNA binding
binding;nucleic acid binding;RNA binding;translation factor activity, nucleic acid binding;translation initiation factor activity
adenyl nucleotide binding;adenyl ribonucleotide binding;anion transmembrane transporter activity;arsenite transmembrane transporter activity;ATP binding;ATPase activity;binding;catalytic activity;cation binding;hydrolase ac
binding;nucleotide binding
adenyl nucleotide binding;adenyl ribonucleotide binding;ATP binding;ATPase activity;binding;catalytic activity;cytoskeletal protein binding;hydrolase activity;hydrolase activity, acting on acid anhydrides;hydrolase activity, acti
binding;cytokine receptor binding;enzyme activator activity;enzyme regulator activity;growth factor receptor binding;kinase activator activity;kinase regulator activity;morphogen activity;protein binding;protein kinase activator
binding;lipid binding;phosphatidylinositol binding;phospholipid binding
binding;nucleic acid binding;nucleotide binding;protein binding;protein domain specific binding;RNA binding;RS domain binding;unfolded protein binding
binding;carboxylic acid binding;catalytic activity;coenzyme binding;cofactor binding;dodecenoyl-CoA delta-isomerase activity;fatty acid binding;fatty-acyl-CoA binding;intramolecular oxidoreductase activity;intramolecular ox
binding;DNA binding;nucleic acid binding
binding;calcium ion binding;carbohydrate binding;cation binding;chemorepellent activity;enzyme inhibitor activity;enzyme regulator activity;extracellular matrix binding;glycoprotein binding;glycosaminoglycan binding;GTPase
aldehyde-lyase activity;binding;carbon-carbon lyase activity;carboxy-lyase activity;catalytic activity;cofactor binding;lyase activity;pyridoxal phosphate binding;sphinganine-1-phosphate aldolase activity;vitamin B6 binding;vi
binding;cytoskeletal protein binding;microtubule binding;protein binding;tubulin binding
binding;catalytic activity;cation binding;guanine phosphoribosyltransferase activity;hypoxanthine phosphoribosyltransferase activity;identical protein binding;ion binding;magnesium ion binding;metal ion binding;nucleotide bi
binding;GTP binding;guanyl nucleotide binding;guanyl ribonucleotide binding;nucleotide binding;protein binding;protein complex binding;purine nucleotide binding;purine ribonucleoside triphosphate binding;purine ribonucle
antigen binding;binding;peptide antigen binding;peptide binding;protein binding;receptor binding
binding;chromatin binding;chromatin DNA binding;DNA binding;nucleic acid binding;structure-specific DNA binding
catalytic activity;cation transmembrane transporter activity;hydrogen ion transmembrane transporter activity;inorganic cation transmembrane transporter activity;ion transmembrane transporter activity;monovalent inorganic c
binding;nucleic acid binding;nucleotide binding;RNA binding;snRNA binding
binding;histone pre-mRNA DCP binding;nucleic acid binding;RNA binding
binding;catalytic activity;cysteine-type peptidase activity;hydrolase activity;peptidase activity;peptidase activity, acting on L-amino acid peptides;protein binding;small conjugating protein binding;small conjugating protein-sp
binding;catalytic activity;cation binding;fumarylacetoacetase activity;hydrolase activity;hydrolase activity, acting on acid carbon-carbon bonds;hydrolase activity, acting on acid carbon-carbon bonds, in ketonic substances;io
binding;catalytic activity;cytoskeletal protein binding;GDP binding;GTP binding;GTPase activity;guanyl nucleotide binding;guanyl ribonucleotide binding;hydrolase activity;hydrolase activity, acting on acid anhydrides;hydrolas
binding;catalytic activity;cytoskeletal protein binding;GTP binding;GTPase activity;guanyl nucleotide binding;guanyl ribonucleotide binding;hydrolase activity;hydrolase activity, acting on acid anhydrides;hydrolase activity, act
binding;catalytic activity;GTP binding;GTPase activity;guanyl nucleotide binding;guanyl ribonucleotide binding;hydrolase activity;hydrolase activity, acting on acid anhydrides;hydrolase activity, acting on acid anhydrides, in ph
binding;chromatin binding;chromo shadow domain binding;DNA binding;enzyme binding;identical protein binding;kinase binding;nucleic acid binding;protein binding;protein binding transcription factor activity;protein dimeriz
catalytic activity;endopeptidase activity;hydrolase activity;peptidase activity;peptidase activity, acting on L-amino acid peptides;threonine-type endopeptidase activity;threonine-type peptidase activity
biliverdin reductase activity;catalytic activity;flavin reductase activity;oxidoreductase activity;oxidoreductase activity, acting on the CH-CH group of donors;oxidoreductase activity, acting on the CH-CH group of donors, NAD
binding;catalytic activity;cation binding;heme oxygenase (decyclizing) activity;ion binding;metal ion binding;oxidoreductase activity;oxidoreductase activity, acting on paired donors, with incorporation or reduction of molecula
binding;calcium channel regulator activity;calcium ion binding;calcium-dependent cysteine-type endopeptidase activity;catalytic activity;cation binding;channel regulator activity;cysteine-type endopeptidase activity;cysteine-
adenyl nucleotide binding;adenyl ribonucleotide binding;ATP binding;binding;DNA binding;nucleic acid binding;nucleotide binding;purine nucleotide binding;purine ribonucleoside triphosphate binding;purine ribonucleotide bi
binding;core promoter binding;core promoter sequence-specific DNA binding;DNA binding;enzyme binding;identical protein binding;molecular transducer activity;nucleic acid binding;nucleic acid binding transcription factor a
binding;cyclin-dependent protein kinase inhibitor activity;cyclin-dependent protein kinase regulator activity;enzyme binding;enzyme inhibitor activity;enzyme regulator activity;kinase binding;kinase inhibitor activity;kinase regu
acyl-CoA hydrolase activity;acyl-CoA thioesterase activity;binding;carboxylic ester hydrolase activity;catalytic activity;CoA hydrolase activity;hydrolase activity;hydrolase activity, acting on ester bonds;palmitoyl-CoA hydrolase
adenyl nucleotide binding;adenyl ribonucleotide binding;ATP binding;beta-catenin binding;binding;catalytic activity;enzyme binding;kinase activity;kinase binding;NF-kappaB binding;nucleotide binding;p53 binding;phosphotr
binding;catalytic activity;GDP binding;GTP binding;GTPase activity;guanyl nucleotide binding;guanyl ribonucleotide binding;hydrolase activity;hydrolase activity, acting on acid anhydrides;hydrolase activity, acting on acid anh
enzyme activator activity;enzyme regulator activity;GTPase activator activity;GTPase regulator activity;nucleoside-triphosphatase regulator activity
binding;nucleic acid binding;RNA binding;structural constituent of ribosome;structural molecule activity
binding;cation binding;ion binding;metal ion binding;transition metal ion binding;zinc ion binding
binding;protein binding;SNARE binding;soluble NSF attachment protein activity;syntaxin binding
binding;GTP binding;guanyl nucleotide binding;guanyl ribonucleotide binding;nucleotide binding;purine nucleotide binding;purine ribonucleoside triphosphate binding;purine ribonucleotide binding;ribonucleotide binding
binding;catalytic activity;cation binding;hydrolase activity;hydrolase activity, acting on ester bonds;ion binding;kinase activity;metal ion binding;molecular transducer activity;NF-kappaB-inducing kinase activity;phosphatase a
acid-amino acid ligase activity;adenyl nucleotide binding;adenyl ribonucleotide binding;ATP binding;binding;catalytic activity;ligase activity;ligase activity, forming carbon-nitrogen bonds;NEDD8 ligase activity;nucleotide bindi
binding;cholesterol binding;enzyme binding;lipid binding;protein binding;steroid binding;sterol binding
activin binding;binding;catalytic activity;cis-trans isomerase activity;cytokine receptor binding;drug binding;FK506 binding;ion channel binding;isomerase activity;macrolide binding;molecular transducer activity;peptidyl-prolyl
binding;binding, bridging;cytokine receptor binding;enzyme binding;ephrin receptor binding;epidermal growth factor receptor binding;growth factor receptor binding;identical protein binding;insulin receptor substrate binding;
acid-amino acid ligase activity;adenyl nucleotide binding;adenyl ribonucleotide binding;ATP binding;binding;catalytic activity;enzyme binding;enzyme regulator activity;ligase activity;ligase activity, forming carbon-nitrogen bo
binding;cation binding;ion binding;metal ion binding;transition metal ion binding;zinc ion binding
binding;nucleic acid binding;nucleotide binding;ribonucleoprotein binding;ribosomal large subunit binding;RNA binding
binding;DNA binding;double-stranded DNA binding;double-stranded telomeric DNA binding;nucleic acid binding;nucleic acid binding transcription factor activity;protein binding;purine-rich negative regulatory element binding
endopeptidase inhibitor activity;endopeptidase regulator activity;enzyme inhibitor activity;enzyme regulator activity;peptidase inhibitor activity;peptidase regulator activity;serine-type endopeptidase inhibitor activity
binding;channel inhibitor activity;channel regulator activity;cholesterol binding;enzyme activator activity;enzyme binding;enzyme regulator activity;identical protein binding;inward rectifier potassium channel inhibitor activity;io
binding;enzyme binding;molecular transducer activity;protein binding;protein domain specific binding;receptor activity;receptor binding;signal transducer activity;signaling receptor activity;U-plasminogen activator receptor ac
adenyl nucleotide binding;adenyl ribonucleotide binding;ATP binding;binding;nucleotide binding;purine nucleotide binding;purine ribonucleoside triphosphate binding;purine ribonucleotide binding;ribonucleotide binding
enzyme activator activity;enzyme regulator activity;GTPase activator activity;GTPase regulator activity;guanyl-nucleotide exchange factor activity;nucleoside-triphosphatase regulator activity;Ras guanyl-nucleotide exchange
binding;carbohydrate binding;enzyme binding;protein binding
binding;double-stranded RNA binding;nucleic acid binding;RNA binding
binding;DNA binding;mRNA binding;nucleic acid binding;RNA binding
binding;catalytic activity;DNA binding;endonuclease activity;endoribonuclease activity;hydrolase activity;hydrolase activity, acting on ester bonds;mRNA binding;nuclease activity;nucleic acid binding;ribonuclease activity;RN
adenyl nucleotide binding;adenyl ribonucleotide binding;ATP binding;binding;identical protein binding;nucleotide binding;protein binding;protein binding transcription factor activity;purine nucleotide binding;purine ribonucleo
adenyl nucleotide binding;adenyl ribonucleotide binding;ATP binding;binding;catalytic activity;kinase activity;MAP kinase activity;MAP kinase kinase activity;molecular transducer activity;NFAT protein binding;nucleotide bindi
binding;mRNA binding;nucleic acid binding;nucleotide binding;RNA binding
binding;DNA binding;nucleic acid binding

binding;DNA binding;nucleic acid binding;nucleic acid binding transcription factor activity;sequence-specific DNA binding transcription factor activity
nucleocytoplasmic transporter activity;transporter activity
binding;cation binding;ion binding;metal ion binding;transition metal ion binding;zinc ion binding
molecular transducer activity;signal transducer activity
binding;calmodulin binding;cytoskeletal protein binding;myosin binding;myosin light chain binding;myosin VI binding;myosin VI light chain binding;protein binding
binding;calcium ion binding;cation binding;ion binding;metal ion binding
binding;enzyme binding;protein binding;ubiquitin protein ligase binding
ATPase activator activity;ATPase binding;ATPase regulator activity;binding;catalytic activity;chaperone binding;disulfide oxidoreductase activity;enzyme activator activity;enzyme binding;enzyme regulator activity;heat shock p
binding;calcium ion binding;catalytic activity;cation binding;GTP binding;GTPase activity;guanyl nucleotide binding;guanyl ribonucleotide binding;hydrolase activity;hydrolase activity, acting on acid anhydrides;hydrolase activ
binding;chromatin binding;protein binding;transcription factor binding
binding;cation binding;enzyme binding;GTPase binding;identical protein binding;ion binding;lipid binding;metal ion binding;phosphatidic acid binding;phospholipid binding;protein binding;Rab GTPase binding;Ras GTPase bi
ATPase activator activity;ATPase binding;ATPase regulator activity;binding;enzyme activator activity;enzyme binding;enzyme regulator activity;nucleoside-triphosphatase regulator activity;protein binding
binding;carbohydrate binding;catalytic activity;clathrin binding;clathrin heavy chain binding;ER retention sequence binding;glycosaminoglycan binding;hexosaminidase activity;hyaluronic acid binding;hyaluronoglucosaminid
protein binding transcription factor activity;transcription coactivator activity;transcription cofactor activity;transcription factor binding transcription factor activity
catalytic activity;GPI-anchor transamidase activity;hydrolase activity
binding;nucleic acid binding;ribonucleoprotein binding;ribosomal small subunit binding;RNA binding;rRNA binding
binding;nucleic acid binding;nucleotide binding;RNA binding
catalytic activity;cysteine-type peptidase activity;hydrolase activity;peptidase activity;peptidase activity, acting on L-amino acid peptides;small conjugating protein-specific protease activity;ubiquitin-specific protease activity
binding;catalytic activity;cysteine-type endopeptidase activity;cysteine-type peptidase activity;endopeptidase activity;hydrolase activity;peptidase activity;peptidase activity, acting on L-amino acid peptides;protein binding;pr
adenyl nucleotide binding;adenyl ribonucleotide binding;ATP binding;binding;catalytic activity;kinase activity;nucleotide binding;phosphotransferase activity, alcohol group as acceptor;protein kinase activity;protein serine/thre
binding;catalytic activity;cation binding;DNA binding;endonuclease activity;endoribonuclease activity;hydrolase activity;hydrolase activity, acting on ester bonds;ion binding;metal ion binding;nuclease activity;nucleic acid binc
binding;catalytic activity;cation binding;endonuclease activity;hydrolase activity;hydrolase activity, acting on ester bonds;ion binding;metal ion binding;nuclease activity
binding;catalytic activity;identical protein binding;intramolecular oxidoreductase activity;intramolecular oxidoreductase activity, interconverting aldoses and ketoses;isomerase activity;protein binding;S-methyl-5-thioribose-1-
binding;catalytic activity;methyltransferase activity;nucleic acid binding;RNA binding;transferase activity;transferase activity, transferring one-carbon groups
binding;cadherin binding;cell adhesion molecule binding;enzyme activator activity;enzyme regulator activity;GTPase activator activity;GTPase regulator activity;nucleoside-triphosphatase regulator activity;protein binding
structural constituent of ribosome;structural molecule activity
binding;catalytic activity;DNA binding;DNA-directed RNA polymerase activity;nucleic acid binding;nucleotidyltransferase activity;RNA polymerase activity;transferase activity;transferase activity, transferring phosphorus-conta
alpha-tubulin binding;beta-tubulin binding;binding;cation binding;cytoskeletal protein binding;enzyme activator activity;enzyme binding;enzyme regulator activity;gamma-tubulin binding;GTPase activator activity;GTPase regu
binding;enzyme binding;lipid binding;phosphatidylinositol binding;phosphatidylinositol-4-phosphate binding;phospholipid binding;protein binding
adenyl nucleotide binding;adenyl ribonucleotide binding;ATP binding;ATPase activity;ATPase activity, coupled;ATP-dependent helicase activity;ATP-dependent RNA helicase activity;binding;catalytic activity;enzyme binding;h
binding;ion channel binding;protein binding
14-3-3 protein binding;adenyl nucleotide binding;adenyl ribonucleotide binding;ATP binding;binding;catalytic activity;enzyme binding;hydrolase activity;hydrolase activity, acting on acid anhydrides;hydrolase activity, acting o
binding;catalytic activity;enzyme regulator activity;GTPase regulator activity;guanyl-nucleotide exchange factor activity;nucleic acid binding;nucleoside-triphosphatase regulator activity;nucleotidyltransferase activity;RNA binc
binding;glutamate receptor binding;G-protein-coupled receptor binding;metabotropic glutamate receptor binding;protein binding;receptor binding
binding;cation binding;ion binding;metal ion binding;nucleotide binding
binding;cation binding;enzyme binding;GTPase binding;ion binding;metal ion binding;protein binding;Rab GTPase binding;Ras GTPase binding;small GTPase binding
alpha-tubulin binding;beta-catenin binding;binding;catalytic activity;cation binding;core promoter binding;cytoskeletal protein binding;deacetylase activity;DNA binding;dynein complex binding;enzyme binding;heat shock pro
protein transporter activity;receptor activity;substrate-specific transporter activity;transporter activity
catalytic activity;cytochrome-b5 reductase activity;oxidoreductase activity;oxidoreductase activity, acting on NADH or NADPH;oxidoreductase activity, acting on NADH or NADPH, heme protein as acceptor
adenyl nucleotide binding;adenyl ribonucleotide binding;ATP binding;binding;carbohydrate kinase activity;catalytic activity;kinase activity;N-acetylglucosamine kinase activity;N-acylmannosamine kinase activity;nucleotide bir
binding;nucleic acid binding;RNA binding;translation factor activity, nucleic acid binding;translation initiation factor activity
binding;nucleic acid binding;RNA binding;snoRNA binding
adenyl nucleotide binding;adenyl ribonucleotide binding;ATP binding;binding;carbohydrate kinase activity;catalytic activity;cation binding;hydrolase activity;hydrolase activity, acting on glycosyl bonds;hydrolase activity, hydro
binding;GTP binding;guanyl nucleotide binding;guanyl ribonucleotide binding;nucleotide binding;purine nucleotide binding;purine ribonucleoside triphosphate binding;purine ribonucleotide binding;ribonucleotide binding
1-phosphatidylinositol binding;Arp2/3 complex binding;binding;enzyme binding;identical protein binding;lipid binding;phosphatidylinositol binding;phospholipid binding;protein binding;protein complex binding;protein dimeriz
binding;catalytic activity;cysteine-type peptidase activity;hydrolase activity;nucleic acid binding;peptidase activity;peptidase activity, acting on L-amino acid peptides;RNA binding;small conjugating protein-specific protease a
catalytic activity;transferase activity;transferase activity, transferring acyl groups
binding;enzyme binding;GTPase binding;protein binding;Ral GTPase binding;Ras GTPase binding;small GTPase binding
binding;catalytic activity;cation binding;endopeptidase activator activity;endopeptidase regulator activity;enzyme activator activity;enzyme regulator activity;hydrolase activity;ion binding;metal ion binding;metallopeptidase ac
protein transporter activity;substrate-specific transporter activity;transporter activity
structural molecule activity
binding;calcium ion binding;cation binding;ion binding;metal ion binding
binding;chaperone binding;protein binding;unfolded protein binding
binding;C-acyltransferase activity;catalytic activity;cofactor binding;C-palmitoyltransferase activity;palmitoyltransferase activity;pyridoxal phosphate binding;serine C-palmitoyltransferase activity;transferase activity;transferas
active transmembrane transporter activity;carboxylic acid transmembrane transporter activity;lactate transmembrane transporter activity;monocarboxylic acid transmembrane transporter activity;organic acid transmembrane
binding;catalytic activity;methyltransferase activity;mRNA (guanine-N7-)-methyltransferase activity;mRNA methyltransferase activity;N-methyltransferase activity;nucleic acid binding;RNA binding;RNA methyltransferase activ
catalytic activity;disulfide oxidoreductase activity;oxidoreductase activity;oxidoreductase activity, acting on a sulfur group of donors;oxidoreductase activity, acting on a sulfur group of donors, disulfide as acceptor;protein dis
binding;enzyme binding;lipid binding;phosphatase binding;phosphatidylinositol binding;phosphatidylinositol-3,5-bisphosphate binding;phosphatidylinositol-3-phosphate binding;phosphatidylinositol-4-phosphate binding;pho
protein transporter activity;substrate-specific transporter activity;transporter activity
binding;chromatin binding;DNA binding;histone acetyl-lysine binding;histone binding;nucleic acid binding;p53 binding;protein binding
binding;nucleic acid binding transcription factor activity;protein binding;protein binding involved in protein folding;sequence-specific DNA binding transcription factor activity
binding;catalytic activity;gamma-glutamylcyclotransferase activity;identical protein binding;protein binding;protein dimerization activity;protein homodimerization activity;transferase activity;transferase activity, transferring acy
adenyl nucleotide binding;adenyl ribonucleotide binding;ATP binding;binding;catalytic activity;enzyme activator activity;enzyme regulator activity;GTP binding;GTPase activator activity;GTPase regulator activity;guanyl nucleo
binding;cation binding;ion binding;metal ion binding;nucleic acid binding;nucleic acid binding transcription factor activity;RNA binding;sequence-specific DNA binding transcription factor activity;transition metal ion binding;zi
binding;calcium ion binding;catalytic activity;cation binding;cis-trans isomerase activity;drug binding;FK506 binding;ion binding;isomerase activity;macrolide binding;metal ion binding;peptidyl-prolyl cis-trans isomerase activi
binding;cation binding;cytokine binding;DNA binding;growth factor binding;interleukin-1 binding;ion binding;metal ion binding;NACHT domain binding;nucleic acid binding;protein binding;protein domain specific binding;trans
binding;cation binding;ion binding;metal ion binding;transition metal ion binding;zinc ion binding
binding;protein binding;SNAP receptor activity;SNARE binding;syntaxin binding
actin binding;binding;cytoskeletal protein binding;lipid binding;protein binding;steroid binding;transmembrane transporter activity;transporter activity;vitamin binding;vitamin D binding;vitamin transporter activity
binding;nucleic acid binding;RNA binding;RNA cap binding;translation factor activity, nucleic acid binding;translation initiation factor activity
beta-N-acetylhexosaminidase activity;binding;catalytic activity;hexosaminidase activity;hydrolase activity;hydrolase activity, acting on glycosyl bonds;hydrolase activity, hydrolyzing O-glycosyl compounds;protein binding;pro
catalytic activity;disulfide oxidoreductase activity;oxidoreductase activity;oxidoreductase activity, acting on a sulfur group of donors;oxidoreductase activity, acting on a sulfur group of donors, disulfide as acceptor;peptide di
5'-deoxyribose-5-phosphate lyase activity;AT DNA binding;binding;carbon-oxygen lyase activity;catalytic activity;DNA binding;DNA-(apurinic or apyrimidinic site) lyase activity;enzyme binding;hormone receptor binding;ligand
adenyl nucleotide binding;adenyl ribonucleotide binding;ATP binding;binding;catalytic activity;kinase activity;nucleotide binding;phosphotransferase activity, alcohol group as acceptor;protein binding;protein kinase activity;pr
binding;cation binding;DNA binding;DNA secondary structure binding;enhancer binding;enhancer sequence-specific DNA binding;four-way junction DNA binding;ion binding;metal ion binding;nucleic acid binding;nucleic acid
ATPase activator activity;ATPase binding;ATPase regulator activity;binding;chaperone binding;enzyme activator activity;enzyme binding;enzyme regulator activity;heat shock protein binding;Hsp70 protein binding;nucleoside-
binding;calcium ion binding;cation binding;ion binding;metal ion binding;protein binding;RAGE receptor binding;receptor binding
binding;catalytic activity;dipeptidyl-peptidase activity;endopeptidase activity;enzyme binding;exopeptidase activity;hydrolase activity;identical protein binding;peptidase activity;peptidase activity, acting on L-amino acid pept
14-3-3 protein binding;adenyl nucleotide binding;adenyl ribonucleotide binding;ATP binding;binding;catalytic activity;enzyme binding;enzyme regulator activity;identical protein binding;kinase activity;lipid binding;nitric-oxide
binding;catalytic activity;cation binding;dUTP diphosphatase activity;hydrolase activity;hydrolase activity, acting on acid anhydrides;hydrolase activity, acting on acid anhydrides, in phosphorus-containing anhydrides;ion bind
binding;carbohydrate binding;glycosaminoglycan binding;heparin binding;nucleic acid binding;pattern binding;polysaccharide binding;RNA binding;structural constituent of ribosome;structural molecule activity
nucleic acid binding transcription factor activity;sequence-specific DNA binding transcription factor activity
binding;mRNA binding;nucleic acid binding;RNA binding;structural constituent of ribosome;structural molecule activity
2 iron, 2 sulfur cluster binding;binding;catalytic activity;cation binding;cation transmembrane transporter activity;hydrogen ion transmembrane transporter activity;inorganic cation transmembrane transporter activity;ion bindir
adenyl nucleotide binding;adenyl ribonucleotide binding;ADP binding;binding;carbon-carbon lyase activity;carboxy-lyase activity;catalytic activity;cation binding;coenzyme binding;cofactor binding;electron carrier activity;ion
binding;integrin binding;protein binding;protein complex binding;receptor binding
acid-amino acid ligase activity;adenyl nucleotide binding;adenyl ribonucleotide binding;amine binding;amino acid binding;ATP binding;binding;carboxylic acid binding;catalytic activity;cation binding;glutathione binding;glutat
structural constituent of ribosome;structural molecule activity
binding;identical protein binding;mRNA binding;nucleic acid binding;protein binding;RNA binding
binding;identical protein binding;protein binding;protein dimerization activity;protein homodimerization activity
binding;nuclear localization sequence binding;peptide binding;protein transporter activity;signal sequence binding;substrate-specific transporter activity;transporter activity
anion binding;apoptotic protease activator activity;binding;caspase activator activity;caspase regulator activity;catalytic activity;chaperone binding;chloride ion binding;cysteine-type endopeptidase activity;cysteine-type pept
binding;calcium ion binding;cation binding;identical protein binding;ion binding;ion channel binding;metal ion binding;protein binding;protein dimerization activity;protein homodimerization activity
binding;catalytic activity;GDP binding;GTP binding;GTPase activity;guanyl nucleotide binding;guanyl ribonucleotide binding;hydrolase activity;hydrolase activity, acting on acid anhydrides;hydrolase activity, acting on acid anh
binding;catalytic activity;GDP binding;GTP binding;GTPase activity;guanyl nucleotide binding;guanyl ribonucleotide binding;hydrolase activity;hydrolase activity, acting on acid anhydrides;hydrolase activity, acting on acid anh
binding;ribonucleoprotein binding;ribosome binding
binding;enzyme binding;histone pre-mRNA DCP binding;nucleic acid binding;protein binding;RNA binding
binding;catalytic activity;enzyme binding;GDP-dissociation inhibitor binding;GTP binding;GTPase activity;guanyl nucleotide binding;guanyl ribonucleotide binding;hydrolase activity;hydrolase activity, acting on acid anhydride
catalytic activity;hydrolase activity;peptidase activity;peptidase activity, acting on L-amino acid peptides;serine hydrolase activity;serine-type peptidase activity
binding;cytoskeletal protein binding;microtubule binding;nucleic acid binding;protein binding;RNA binding;tubulin binding
binding;binding, bridging;cargo receptor activity;clathrin adaptor activity;clathrin binding;lipid binding;phosphatidylinositol binding;phosphatidylinositol-4,5-bisphosphate binding;phospholipid binding;protein binding;protein b
catalytic activity;dipeptidase activity;exopeptidase activity;hydrolase activity;peptidase activity;peptidase activity, acting on L-amino acid peptides
binding;mRNA binding;nucleic acid binding;RNA binding
adenyl nucleotide binding;adenyl ribonucleotide binding;adenylyltransferase activity;ATP binding;binding;catalytic activity;dephospho-CoA kinase activity;kinase activity;nucleotide binding;nucleotidyltransferase activity;pante
binding;nucleotide binding

binding;catalytic activity;coenzyme binding;cofactor binding;NAD binding;nucleic acid binding transcription factor activity;nucleotide binding;oxidoreductase activity;oxidoreductase activity, acting on CH-OH group of donors
binding;catalytic activity;cation binding;collagen binding;endopeptidase activity;enzyme binding;extracellular matrix binding;hydrolase activity;integrin binding;ion binding;kinase binding;laminin binding;metal ion binding;meta
binding;catalytic activity;cation binding;enzyme binding;identical protein binding;ion binding;kinase activity;kinase binding;metal ion binding;phosphotransferase activity, alcohol group as acceptor;protein binding;protein dom
adenyl nucleotide binding;adenyl ribonucleotide binding;ATP binding;binding;catalytic activity;cytokine receptor binding;death domain binding;death receptor binding;enzyme binding;identical protein binding;kinase activity;nu
binding;catalytic activity;coenzyme binding;cofactor binding;identical protein binding;isomerase activity;protein binding;protein dimerization activity;protein homodimerization activity;racemase and epimerase activity;racemas
antigen binding;binding;catalytic activity;coenzyme binding;cofactor binding;delta24(24-1) sterol reductase activity;delta24-sterol reductase activity;enzyme binding;flavin adenine dinucleotide binding;oxidoreductase activity;
binding;cation binding;ion binding;metal ion binding;transition metal ion binding;zinc ion binding
acid-amino acid ligase activity;binding;catalytic activity;enzyme binding;ligase activity;ligase activity, forming carbon-nitrogen bonds;protein binding;small conjugating protein ligase activity;ubiquitin protein ligase binding
adenyl nucleotide binding;adenyl ribonucleotide binding;ATP binding;binding;catalytic activity;enzyme binding;histone deacetylase binding;kinase activity;nucleotide binding;phosphotransferase activity, alcohol group as acce
binding;cation binding;ion binding;metal ion binding;transition metal ion binding;zinc ion binding
binding;nucleic acid binding;RNA binding
2 iron, 2 sulfur cluster binding;binding;cation binding;electron carrier activity;ion binding;iron-sulfur cluster binding;metal cluster binding;metal ion binding
catalytic activity;oxidoreductase activity
binding;cation binding;ion binding;metal ion binding;nucleotide binding
binding;nucleic acid binding;RNA binding
alcohol dehydrogenase (NADP+) activity;aldo-keto reductase (NADP) activity;catalytic activity;NADP-retinol dehydrogenase activity;oxidoreductase activity;oxidoreductase activity, acting on CH-OH group of donors;oxidoredu
adenyl nucleotide binding;adenyl ribonucleotide binding;ATP binding;ATPase activity;ATPase activity, coupled;ATP-dependent helicase activity;ATP-dependent RNA helicase activity;binding;catalytic activity;estrogen receptor
binding;DNA binding;nucleic acid binding;nucleic acid binding transcription factor activity;sequence-specific DNA binding transcription factor activity
acetyltransferase activity;binding;catalytic activity;chromatin binding;DNA binding;ligand-dependent nuclear receptor binding;N-acetyltransferase activity;N-acyltransferase activity;nucleic acid binding;protein binding;protein
binding;cytoskeletal protein binding;gamma-tubulin binding;protein binding;structural constituent of cytoskeleton;structural molecule activity;tubulin binding
binding;DNA binding;double-stranded DNA binding;mRNA binding;nucleic acid binding;nucleic acid binding transcription factor activity;protein binding;RNA binding;sequence-specific DNA binding transcription factor activity
adenyl nucleotide binding;adenyl ribonucleotide binding;ATP binding;ATPase activity;binding;catalytic activity;centromeric DNA binding;cytoskeletal protein binding;DNA binding;hydrolase activity;hydrolase activity, acting on
antioxidant activity;catalytic activity;electron carrier activity;oxidoreductase activity;oxidoreductase activity, acting on a sulfur group of donors;oxidoreductase activity, acting on a sulfur group of donors, NAD or NADP as acce
binding;protein binding;SNARE binding;syntaxin binding
ion transmembrane transporter activity;substrate-specific transmembrane transporter activity;substrate-specific transporter activity;transmembrane transporter activity;transporter activity
binding;cation binding;chromatin binding;DNA binding;ion binding;metal ion binding;nucleic acid binding
catalytic activity;kinase activity;transferase activity;transferase activity, transferring phosphorus-containing groups
adenyl nucleotide binding;adenyl ribonucleotide binding;ATP binding;binding;catalytic activity;CoA carboxylase activity;ligase activity;ligase activity, forming carbon-carbon bonds;methylcrotonoyl-CoA carboxylase activity;nu
binding;cation binding;enzyme activator activity;enzyme regulator activity;GTPase activator activity;GTPase regulator activity;ion binding;metal ion binding;nucleoside-triphosphatase regulator activity;protein transporter activ
adenyl nucleotide binding;adenyl ribonucleotide binding;aminoacyl-tRNA ligase activity;ATP binding;binding;catalytic activity;ligase activity;ligase activity, forming aminoacyl-tRNA and related compounds;ligase activity, formi
binding;identical protein binding;mRNA binding;nucleic acid binding;protein binding;RNA binding
basal RNA polymerase II transcription machinery binding;basal transcription machinery binding;binding;enzyme binding;protein binding;RNA polymerase binding;RNA polymerase core enzyme binding;RNA polymerase II core
binding;cation binding;double-stranded RNA binding;ion binding;metal ion binding;nucleic acid binding;RNA binding
catalytic activity
binding;carboxylic acid binding;catalytic activity;coenzyme binding;cofactor binding;glycerate dehydrogenase activity;glyoxylate reductase (NADP) activity;hydroxypyruvate reductase activity;identical protein binding;NAD bin
catalytic activity;cysteine-type endopeptidase activity;cysteine-type peptidase activity;endopeptidase activity;hydrolase activity;peptidase activity;peptidase activity, acting on L-amino acid peptides
binding;mRNA binding;nucleic acid binding;nucleocytoplasmic transporter activity;nucleotide binding;RNA binding;transporter activity
adenyl nucleotide binding;adenyl ribonucleotide binding;ATP binding;binding;catalytic activity;GTP binding;guanyl nucleotide binding;guanyl ribonucleotide binding;kinase activity;nucleobase-containing compound kinase act
binding;cation binding;ion binding;metal ion binding
binding;cation binding;DEAD/H-box RNA helicase binding;DNA binding;enzyme binding;ion binding;metal ion binding;nucleic acid binding;protein binding
binding;enzyme binding;lipid binding;phosphatidylinositol binding;phospholipid binding;protein binding
active transmembrane transporter activity;anion transmembrane transporter activity;anion:cation symporter activity;binding;cation transmembrane transporter activity;cation:chloride symporter activity;chloride transmembran
binding;catalytic activity;cation binding;ion binding;ligase activity;metal ion binding;transition metal ion binding;zinc ion binding
binding;lipid binding;phosphatidylinositol binding;phosphatidylinositol-3,5-bisphosphate binding;phosphatidylinositol-3-phosphate binding;phosphatidylinositol-4-phosphate binding;phosphatidylinositol-5-phosphate binding
binding;protein binding;SNARE binding;syntaxin binding
binding;cation binding;heme binding;ion binding;iron ion binding;lipid binding;metal ion binding;steroid binding;tetrapyrrole binding;transition metal ion binding
binding;clathrin binding;lipid binding;phosphatidylinositol binding;phospholipid binding;protein binding;structural constituent of cytoskeleton;structural molecule activity
adenyl nucleotide binding;adenyl ribonucleotide binding;alkali metal ion binding;ATP binding;binding;catalytic activity;cation binding;cofactor binding;identical protein binding;ion binding;kinase activity;lithium ion binding;mag
structural constituent of cytoskeleton;structural molecule activity
alcohol binding;amine binding;binding;cation binding;ion binding;lipid binding;phosphatidylcholine binding;phospholipid binding;protein binding;protein domain specific binding;quaternary ammonium group binding
cation transmembrane transporter activity;hydrogen ion transmembrane transporter activity;inorganic cation transmembrane transporter activity;ion transmembrane transporter activity;monovalent inorganic cation transmemb
binding;DNA binding;mRNA binding;nucleic acid binding;RNA binding
binding;nucleic acid binding;nucleotide binding;RNA binding
amine binding;amino acid binding;binding;carboxylic acid binding;catalytic activity;dimethylargininase activity;hydrolase activity;hydrolase activity, acting on carbon-nitrogen (but not peptide) bonds;hydrolase activity, acting o
binding;cation binding;drug binding;ion binding;metal ion binding;transition metal ion binding;zinc ion binding
binding;carbohydrate binding;endopeptidase inhibitor activity;endopeptidase regulator activity;enzyme inhibitor activity;enzyme regulator activity;glycosaminoglycan binding;heparin binding;pattern binding;peptidase inhibitor
binding;cation binding;electron carrier activity;electron transporter, transferring electrons from CoQH2-cytochrome c reductase complex and cytochrome c oxidase complex activity;heme binding;ion binding;iron ion binding;r
binding;catalytic activity;GDP binding;GTP binding;GTPase activity;guanyl nucleotide binding;guanyl ribonucleotide binding;hydrolase activity;hydrolase activity, acting on acid anhydrides;hydrolase activity, acting on acid anh
antigen binding;binding;peptide antigen binding;peptide binding
3-methyl-2-oxobutanoate dehydrogenase (2-methylpropanoyl-transferring) activity;alpha-ketoacid dehydrogenase activity;binding;carbon-carbon lyase activity;carboxy-lyase activity;catalytic activity;cation binding;ion bindin
alcohol dehydrogenase (NADP+) activity;alditol:NADP+ 1-oxidoreductase activity;aldo-keto reductase (NADP) activity;catalytic activity;electron carrier activity;L-glucuronate reductase activity;oxidoreductase activity;oxidored
binding;damaged DNA binding;DNA binding;enzyme binding;nucleic acid binding;phosphatase binding;protein binding;protein phosphatase binding;single-stranded DNA binding;structure-specific DNA binding;ubiquitin prote
adenyl nucleotide binding;adenyl ribonucleotide binding;ATP binding;binding;cAMP-dependent protein kinase activity;catalytic activity;cyclic nucleotide-dependent protein kinase activity;enzyme binding;kinase activity;kinase
binding;carbohydrate binding;chemoattractant activity;extracellular matrix binding;IgE binding;immunoglobulin binding;laminin binding;protein binding;protein complex binding
binding;chromatin binding;chromatin DNA binding;DNA binding;enzyme regulator activity;GTPase regulator activity;guanyl-nucleotide exchange factor activity;histone binding;nucleic acid binding;nucleoside-triphosphatase r
binding;carbohydrate binding;carbohydrate transmembrane transporter activity;hexose transmembrane transporter activity;mannose binding;mannose transmembrane transporter activity;molecular transducer activity;monos
binding;catalytic activity;cation binding;DNA binding;IMP dehydrogenase activity;ion binding;metal ion binding;nucleic acid binding;nucleotide binding;oxidoreductase activity;oxidoreductase activity, acting on CH-OH group
binding;channel inhibitor activity;channel regulator activity;enzyme activator activity;enzyme binding;enzyme regulator activity;glycoprotein binding;GTPase activator activity;GTPase binding;GTPase regulator activity;ion chan
active transmembrane transporter activity;adenyl nucleotide binding;adenyl ribonucleotide binding;ATP binding;ATPase activity;ATPase activity, coupled;ATPase activity, coupled to movement of substances;ATPase activity, c
carboxyl-O-methyltransferase activity;catalytic activity;methyltransferase activity;O-methyltransferase activity;protein carboxyl O-methyltransferase activity;protein methyltransferase activity;protein-L-isoaspartate (D-aspartat
catalytic activity;ferredoxin-NADP+ reductase activity;NADPH-adrenodoxin reductase activity;oxidoreductase activity;oxidoreductase activity, acting on iron-sulfur proteins as donors;oxidoreductase activity, acting on iron-sul
binding;carbon-carbon lyase activity;carboxy-lyase activity;catalytic activity;cation binding;coenzyme binding;cofactor binding;electron carrier activity;ion binding;lyase activity;malate dehydrogenase (decarboxylating) activity
catalytic activity;endopeptidase activity;hydrolase activity;peptidase activity;peptidase activity, acting on L-amino acid peptides;threonine-type endopeptidase activity;threonine-type peptidase activity
catalytic activity;glycylpeptide N-tetradecanoyltransferase activity;myristoyltransferase activity;N-acyltransferase activity;transferase activity;transferase activity, transferring acyl groups;transferase activity, transferring acyl gr
binding;catalytic activity;identical protein binding;oxidoreductase activity;oxidoreductase activity, acting on the CH-NH group of donors;oxidoreductase activity, acting on the CH-NH group of donors, NAD or NADP as accept
binding;nucleic acid binding;nucleotide binding;RNA binding
active transmembrane transporter activity;adenyl nucleotide binding;adenyl ribonucleotide binding;ATP binding;ATPase activity;ATPase activity, coupled;ATPase activity, coupled to movement of substances;ATPase activity, c
binding;DNA binding;frizzled binding;G-protein-coupled receptor binding;nucleic acid binding;nucleic acid binding transcription factor activity;protein binding;receptor activator activity;receptor agonist activity;receptor bindin
acetyltransferase activity;catalytic activity;N-acetyltransferase activity;N-acyltransferase activity;transferase activity;transferase activity, transferring acyl groups;transferase activity, transferring acyl groups other than amino-ac
binding;catalytic activity;cation binding;endopeptidase activity;hormone binding;hydrolase activity;identical protein binding;ion binding;metal ion binding;metalloendopeptidase activity;metallopeptidase activity;peptidase acti
binding;nucleic acid binding;RNA binding;translation elongation factor activity;translation factor activity, nucleic acid binding
catalytic activity;deaminase activity;glucosamine-6-phosphate deaminase activity;hydrolase activity;intramolecular oxidoreductase activity;intramolecular oxidoreductase activity, interconverting aldoses and ketoses;isomeras
binding;chromatin binding;core promoter binding;core promoter sequence-specific DNA binding;DNA binding;nucleic acid binding;protein binding transcription factor activity;regulatory region DNA binding;regulatory region n
binding;cation transmembrane transporter activity;drug binding;hydrogen ion transmembrane transporter activity;hydrogen ion transporting ATP synthase activity, rotational mechanism;inorganic cation transmembrane transp
adenyl nucleotide binding;adenyl ribonucleotide binding;ATP binding;binding;catalytic activity;kinase activity;nucleotide binding;phosphotransferase activity, alcohol group as acceptor;protein kinase activity;protein serine/thre
binding;calcium ion binding;cation binding;G-protein coupled receptor activity;ion binding;metal ion binding;molecular transducer activity;receptor activity;signal transducer activity;signaling receptor activity;transmembrane s
7S RNA binding;binding;nucleic acid binding;ribonucleoprotein binding;RNA binding;signal recognition particle binding
binding;extracellular matrix binding;laminin binding;laminin receptor activity;molecular transducer activity;protein binding;protein C-terminus binding;receptor activity;signal transducer activity;signaling receptor activity;transm
acid-thiol ligase activity;ATP citrate synthase activity;binding;catalytic activity;CoA-ligase activity;cofactor binding;GDP binding;GTP binding;guanyl nucleotide binding;guanyl ribonucleotide binding;ligase activity;ligase activit
active transmembrane transporter activity;ATPase activator activity;ATPase activity;ATPase activity, coupled;ATPase activity, coupled to movement of substances;ATPase activity, coupled to transmembrane movement of ions
binding;identical protein binding;protein binding
binding;cation binding;ion binding;metal ion binding;structural constituent of ribosome;structural molecule activity
binding;catalytic activity;GTP binding;GTPase activity;guanyl nucleotide binding;guanyl ribonucleotide binding;hydrolase activity;hydrolase activity, acting on acid anhydrides;hydrolase activity, acting on acid anhydrides, in pl
binding;mRNA 5'-UTR binding;mRNA binding;nucleic acid binding;RNA binding;structural constituent of ribosome;structural molecule activity;translation regulator activity
binding;cation binding;ion binding;metal ion binding;structural constituent of ribosome;structural molecule activity;transition metal ion binding;zinc ion binding
binding;nucleic acid binding;nucleotide binding;RNA binding;rRNA binding;structural constituent of ribosome;structural molecule activity
structural constituent of ribosome;structural molecule activity
binding;calcium ion binding;catalytic activity;cation binding;hydrolase activity;hydrolase activity, acting on ester bonds;identical protein binding;ion binding;magnesium ion binding;metal ion binding;phosphatase activity;phos
structural molecule activity
structural constituent of ribosome;structural molecule activity
structural constituent of ribosome;structural molecule activity
binding;catalytic activity;drug binding;hydroxymethylglutaryl-CoA synthase activity;isomerase activity;organic acid binding;transferase activity;transferase activity, transferring acyl groups;transferase activity, transferring acyl g
endopeptidase inhibitor activity;endopeptidase regulator activity;enzyme inhibitor activity;enzyme regulator activity;peptidase inhibitor activity;peptidase regulator activity;serine-type endopeptidase inhibitor activity
adenyl nucleotide binding;adenyl ribonucleotide binding;ATP binding;binding;nucleotide binding;purine nucleotide binding;purine ribonucleoside triphosphate binding;purine ribonucleotide binding;ribonucleotide binding
binding;calcium ion binding;carbohydrate binding;cation binding;glycosaminoglycan binding;heparin binding;ion binding;metal ion binding;pattern binding;polysaccharide binding
adenyl nucleotide binding;adenyl ribonucleotide binding;ATP binding;binding;calmodulin binding;calmodulin-dependent protein kinase activity;catalytic activity;channel inhibitor activity;channel regulator activity;cytoskeletal p
binding;catalytic activity;enzyme binding;NEDD8 activating enzyme activity;protein binding;protein dimerization activity;protein heterodimerization activity;small protein activating enzyme activity;ubiquitin protein ligase binding
binding;enzyme binding;protein binding;receptor activity
binding;DNA binding;nucleic acid binding
acyl-CoA dehydrogenase activity;acyl-CoA oxidase activity;binding;carboxylic acid binding;catalytic activity;coenzyme binding;cofactor binding;FAD binding;fatty acid binding;flavin adenine dinucleotide binding;lipid binding;
adenyl nucleotide binding;adenyl ribonucleotide binding;ATP binding;binding;catalytic activity;kinase activity;nucleotide binding;phosphomevalonate kinase activity;phosphotransferase activity, phosphate group as acceptor;p

basal RNA polymerase II transcription machinery binding;basal transcription machinery binding;binding;enzyme binding;protein binding;RNA polymerase binding;RNA polymerase core enzyme binding;RNA polymerase II core
enzyme regulator activity;GTPase regulator activity;guanyl-nucleotide exchange factor activity;nucleoside-triphosphatase regulator activity;Ras guanyl-nucleotide exchange factor activity;Rho guanyl-nucleotide exchange fac
catalytic activity;oxidoreductase activity
carboxylic ester hydrolase activity;catalytic activity;hydrolase activity;hydrolase activity, acting on ester bonds;lipase activity;lysophospholipase activity;phospholipase activity
catalytic activity;methyltransferase activity;RNA methyltransferase activity;rRNA methyltransferase activity;S-adenosylmethionine-dependent methyltransferase activity;transferase activity;transferase activity, transferring one-
acid-amino acid ligase activity;adenyl nucleotide binding;adenyl ribonucleotide binding;ATP binding;binding;catalytic activity;ligase activity;ligase activity, forming carbon-nitrogen bonds;NEDD8 activating enzyme activity;nuc
enzyme regulator activity;phosphatase regulator activity;protein phosphatase regulator activity;protein phosphatase type 4 regulator activity
binding;cation binding;ion binding;metal ion binding
adenyl nucleotide binding;adenyl ribonucleotide binding;ATP binding;ATPase activity;ATPase activity, coupled;ATP-dependent helicase activity;ATP-dependent RNA helicase activity;binding;catalytic activity;helicase activity;h
catalytic activity;hydrolase activity
binding;calcium ion binding;cation binding;identical protein binding;ion binding;metal ion binding;protein binding;protein dimerization activity;protein homodimerization activity
binding;catalytic activity;cysteine-type peptidase activity;enzyme binding;hydrolase activity;NEDD8-specific protease activity;peptidase activity;peptidase activity, acting on L-amino acid peptides;protein binding;small conjug
binding;ErbB-2 class receptor binding;integrin binding;protein binding;protein complex binding;receptor binding;structural constituent of cytoskeleton;structural molecule activity
binding;neurotransmitter binding
beta-tubulin binding;binding;chaperone binding;cytoskeletal protein binding;enzyme activator activity;enzyme regulator activity;GTPase activator activity;GTPase regulator activity;nucleoside-triphosphatase regulator activity;
alpha(1,6)-fucosyltransferase activity;catalytic activity;fucosyltransferase activity;glycoprotein 6-alpha-L-fucosyltransferase activity;transferase activity;transferase activity, transferring glycosyl groups;transferase activity, trans
binding;ribonucleoprotein binding
binding;nucleotide binding
binding;identical protein binding;protein binding;protein dimerization activity;protein homodimerization activity
catalytic activity;fucosyltransferase activity;peptide-O-fucosyltransferase activity;transferase activity;transferase activity, transferring glycosyl groups;transferase activity, transferring hexosyl groups
adenyl nucleotide binding;adenyl ribonucleotide binding;ATP binding;binding;catalytic activity;channel inhibitor activity;channel regulator activity;chloride channel inhibitor activity;chloride channel regulator activity;enzyme bir
adenyl nucleotide binding;adenyl ribonucleotide binding;ATP binding;ATPase activity;binding;catalytic activity;cation binding;hydrolase activity;hydrolase activity, acting on acid anhydrides;hydrolase activity, acting on acid an
enzyme inhibitor activity;enzyme regulator activity
binding;enzyme binding;enzyme regulator activity;methionine adenosyltransferase regulator activity;protein binding
binding;GTP binding;guanyl nucleotide binding;guanyl ribonucleotide binding;nucleotide binding;purine nucleotide binding;purine ribonucleoside triphosphate binding;purine ribonucleotide binding;ribonucleotide binding
binding;nucleic acid binding;protein binding;RNA binding;translation factor activity, nucleic acid binding;translation initiation factor activity;translation initiation factor binding
binding;enzyme binding;GTPase binding;protein binding;receptor binding
adenyl nucleotide binding;adenyl ribonucleotide binding;ATP binding;binding;enzyme binding;GTP binding;guanyl nucleotide binding;guanyl ribonucleotide binding;kinase binding;nucleotide binding;protein binding;protein kir
binding;catalytic activity;cysteine-type peptidase activity;endopeptidase inhibitor activity;endopeptidase regulator activity;enzyme inhibitor activity;enzyme regulator activity;exopeptidase activity;hydrolase activity;omega pep
actin binding;binding;cytoskeletal protein binding;protein binding;protein complex binding
binding;catalytic activity;cation binding;endopeptidase activity;enzyme binding;hydrolase activity;identical protein binding;integrin binding;ion binding;kinase binding;metal ion binding;metalloendopeptidase activity;metallope
binding;carbohydrate binding;catalytic activity;cation binding;coreceptor activity;cytokine binding;glycosaminoglycan binding;growth factor binding;heparin binding;ion binding;kinase activity;metal ion binding;molecular trans
binding;cholesterol binding;hedgehog receptor activity;lipid binding;lipid transporter activity;molecular transducer activity;receptor activity;signal transducer activity;signaling receptor activity;steroid binding;sterol binding;ster
structural constituent of cytoskeleton;structural molecule activity
amine binding;amino acid binding;binding;carboxylic acid binding;catalytic activity;cation binding;glutathione binding;G-protein coupled receptor activity;ion binding;lipoprotein particle receptor binding;low-density lipoprotei
alpha(1,2)-fucosyltransferase activity;binding;catalytic activity;cytoskeletal protein binding;fucosyltransferase activity;galactoside 2-alpha-L-fucosyltransferase activity;microtubule binding;microtubule plus-end binding;protein
4 iron, 4 sulfur cluster binding;binding;catalytic activity;cation binding;coenzyme binding;cofactor binding;electron carrier activity;enzyme binding;ion binding;iron-sulfur cluster binding;metal cluster binding;metal ion binding;
protein transporter activity;substrate-specific transporter activity;transporter activity
adenyl nucleotide binding;adenyl ribonucleotide binding;ATP binding;ATPase activity;binding;catalytic activity;DNA binding;hydrolase activity;hydrolase activity, acting on acid anhydrides;hydrolase activity, acting on acid anh
adenyl nucleotide binding;adenyl ribonucleotide binding;ADP binding;AMP binding;binding;catalytic activity;coenzyme binding;cofactor binding;cytochrome-b5 reductase activity;FAD binding;flavin adenine dinucleotide bindi
binding;DNA binding;nucleic acid binding
binding;calcium ion binding;calcium-dependent protein binding;cation binding;cytoskeletal protein binding;identical protein binding;ion binding;ion transmembrane transporter activity;metal ion binding;protein binding;protein
3'-5' exonuclease activity;4 iron, 4 sulfur cluster binding;binding;catalytic activity;cation binding;chromatin binding;DNA binding;DNA polymerase activity;DNA-directed DNA polymerase activity;enzyme binding;exonuclease a
binding;complement binding;complement component C1q binding;endopeptidase inhibitor activity;endopeptidase regulator activity;enzyme inhibitor activity;enzyme regulator activity;opsonin binding;peptidase inhibitor activ
binding;DNA binding;enzyme binding;nucleic acid binding;protein binding
binding;catalytic activity;cation binding;copper ion binding;endopeptidase activity;hydrolase activity;ion binding;metal ion binding;peptidase activity;peptidase activity, acting on L-amino acid peptides;serine hydrolase activity
catalytic activity;cation transmembrane transporter activity;cytochrome-c oxidase activity;heme-copper terminal oxidase activity;hydrogen ion transmembrane transporter activity;inorganic cation transmembrane transporter a
binding;DNA binding;double-stranded DNA binding;enzyme binding;GTPase binding;mRNA 3'-UTR binding;mRNA binding;nucleic acid binding;nucleic acid binding transcription factor activity;protein binding;protein binding
binding;chromatin binding;DNA binding;double-stranded DNA binding;enhancer binding;enhancer sequence-specific DNA binding;identical protein binding;nucleic acid binding;nucleic acid binding transcription factor activity
catalytic activity;endopeptidase activity;hydrolase activity;peptidase activity;peptidase activity, acting on L-amino acid peptides;threonine-type endopeptidase activity;threonine-type peptidase activity
3-mercaptopyruvate sulfurtransferase activity;catalytic activity;sulfurtransferase activity;thiosulfate sulfurtransferase activity;transferase activity;transferase activity, transferring sulfur-containing groups
active transmembrane transporter activity;adenyl nucleotide binding;adenyl ribonucleotide binding;ATP binding;ATPase activity;ATPase activity, coupled;ATPase activity, coupled to movement of substances;ATPase activity, c
aminopeptidase activity;binding;catalytic activity;cation binding;exopeptidase activity;hydrolase activity;ion binding;manganese ion binding;metal ion binding;metalloexopeptidase activity;metallopeptidase activity;peptidase a
alkali metal ion binding;binding;catalytic activity;cation binding;hydrolase activity;hydrolase activity, acting on ester bonds;identical protein binding;inositol monophosphate phosphatase activity;inositol or phosphatidylinositol
apoptotic protease activator activity;caspase activator activity;caspase regulator activity;catalytic activity;cysteine-type endopeptidase activity;cysteine-type peptidase activity;endopeptidase activity;enzyme activator activity
binding;chaperone binding;identical protein binding;protein binding;protein dimerization activity;protein homodimerization activity
binding;catalytic activity;cis-trans isomerase activity;cyclosporin A binding;drug binding;isomerase activity;peptide binding;peptidyl-prolyl cis-trans isomerase activity
binding;carbohydrate binding;monosaccharide binding;sialic acid binding;sugar binding
binding;catalytic activity;DNA binding;enzyme activator activity;enzyme regulator activity;nucleic acid binding;phosphatase activator activity;phosphatase regulator activity;protein binding;protein binding transcription factor a
binding;carbohydrate binding;mannose binding;monosaccharide binding;sugar binding
binding;catalytic activity;cation binding;enzyme binding;hydrolase activity;hydrolase activity, acting on ester bonds;ion binding;kinase binding;metal ion binding;phosphatase activity;phosphoprotein phosphatase activity;phos
binding;binding, bridging;chromatin binding;histone binding;methylated histone residue binding;protein binding;protein binding, bridging;repressing transcription factor binding;transcription factor binding
binding;nucleic acid binding;RNA binding;structural constituent of ribosome;structural molecule activity
binding;nucleic acid binding;RNA binding;translation factor activity, nucleic acid binding;translation initiation factor activity
adenyl nucleotide binding;adenyl ribonucleotide binding;ATP binding;binding;GTP binding;guanyl nucleotide binding;guanyl ribonucleotide binding;nucleic acid binding;nucleotide binding;purine nucleotide binding;purine ribo
5'-nucleotidase activity;binding;catalytic activity;cation binding;hydrolase activity;hydrolase activity, acting on ester bonds;ion binding;kinase activity;metal ion binding;nucleobase-containing compound kinase activity;nucleo
endopeptidase inhibitor activity;endopeptidase regulator activity;enzyme inhibitor activity;enzyme regulator activity;peptidase inhibitor activity;peptidase regulator activity;serine-type endopeptidase inhibitor activity
binding;cation binding;ion binding;metal ion binding;transition metal ion binding;zinc ion binding
actin binding;binding;cytoskeletal protein binding;protein binding;structural constituent of muscle;structural molecule activity
adenosine kinase activity;adenyl nucleotide binding;adenyl ribonucleotide binding;ATP binding;binding;catalytic activity;cation binding;ion binding;kinase activity;metal ion binding;nucleobase-containing compound kinase ac
structural molecule activity
apolipoprotein A-I receptor binding;apolipoprotein receptor binding;binding;catalytic activity;enzyme binding;GTP binding;GTPase activity;guanyl nucleotide binding;guanyl ribonucleotide binding;hydrolase activity;hydrolase
binding;calcium ion binding;cation binding;DNA binding;ion binding;metal ion binding;nucleic acid binding
structural constituent of ribosome;structural molecule activity
binding;nucleic acid binding;nucleotide binding;RNA binding
binding;nucleotide binding;protein binding transcription factor activity;transcription cofactor activity;transcription corepressor activity;transcription factor binding transcription factor activity
adenyl nucleotide binding;adenyl ribonucleotide binding;ATP binding;ATPase activity;binding;catalytic activity;cytoskeletal protein binding;hydrolase activity;hydrolase activity, acting on acid anhydrides;hydrolase activity, acti
ATPase binding;binding;enzyme binding;K6-linked polyubiquitin binding;polyubiquitin binding;protein binding;small conjugating protein binding;ubiquitin binding;ubiquitin protein ligase binding
binding;carbon-sulfur lyase activity;catalytic activity;cation binding;ion binding;lactoylglutathione lyase activity;lyase activity;metal ion binding;transition metal ion binding;zinc ion binding
transporter activity
binding;enzyme binding;identical protein binding;protein binding;protein domain specific binding
1-phosphatidylinositol binding;binding;binding, bridging;clathrin adaptor activity;clathrin binding;clathrin heavy chain binding;lipid binding;phosphatidylinositol binding;phospholipid binding;protein binding;protein binding, bri
binding;epidermal growth factor receptor binding;growth factor receptor binding;identical protein binding;insulin receptor binding;lipid binding;phosphatidylinositol binding;phospholipid binding;protein binding;protein comple
binding;protein binding;receptor binding
binding;calcium ion binding;cation binding;collagen binding;ion binding;metal ion binding;protein binding
binding;FHA domain binding;protein binding;protein C-terminus binding;protein domain specific binding
adenyl nucleotide binding;adenyl ribonucleotide binding;ATP binding;binding;nucleotide binding;purine nucleotide binding;purine ribonucleoside triphosphate binding;purine ribonucleotide binding;ribonucleotide binding
binding;catalytic activity;cysteine-type endopeptidase activity;cysteine-type peptidase activity;endopeptidase activity;hydrolase activity;ion channel binding;p53 binding;peptidase activity;peptidase activity, acting on L-amino
15-oxoprostaglandin 13-oxidase activity;2-alkenal reductase [NAD(P)] activity;binding;catalytic activity;cation binding;ion binding;metal ion binding;oxidoreductase activity;oxidoreductase activity, acting on the CH-CH group
1-alkyl-2-acetylglycerophosphocholine esterase activity;acetyltransferase activity;binding;carboxylic ester hydrolase activity;catalytic activity;hydrolase activity;hydrolase activity, acting on ester bonds;identical protein binding
binding;calcium ion binding;cation binding;ion binding;metal ion binding
binding;heat shock protein binding;protein binding
binding;cation binding;ion binding;metal ion binding;transition metal ion binding;zinc ion binding
adenyl nucleotide binding;adenyl ribonucleotide binding;alanine-tRNA ligase activity;aminoacyl-tRNA ligase activity;ATP binding;binding;catalytic activity;cation binding;ion binding;ligase activity;ligase activity, forming aminoa
3'-5' exonuclease activity;3'-5'-exoribonuclease activity;binding;catalytic activity;cation binding;exonuclease activity;exonuclease activity, active with either ribo- or deoxyribonucleic acids and producing 5'-phosphomonoeste
all-trans-retinol 13,14-reductase activity;catalytic activity;oxidoreductase activity;oxidoreductase activity, acting on the CH-CH group of donors
basal RNA polymerase II transcription machinery binding;basal transcription machinery binding;binding;enzyme binding;protein binding;RNA polymerase binding;RNA polymerase core enzyme binding;RNA polymerase II core
binding;core promoter proximal region DNA binding;core promoter proximal region sequence-specific DNA binding;DNA binding;molecular transducer activity;nucleic acid binding;nucleic acid binding transcription factor acti
catalytic activity;methyltransferase activity;transferase activity;transferase activity, transferring one-carbon groups
binding;nucleic acid binding;RNA binding;translation factor activity, nucleic acid binding;translation initiation factor activity
binding;protein binding;protein domain specific binding
binding;nucleic acid binding;RNA binding
binding;cation binding;ion binding;metal ion binding
arginine N-methyltransferase activity;beta-catenin binding;binding;catalytic activity;DNA binding;histone acetyl-lysine binding;histone binding;histone methyltransferase activity;histone methyltransferase activity (H3-R17 spec
binding;DNA binding;nucleic acid binding
binding;cytoskeletal protein binding;enzyme activator activity;enzyme regulator activity;kinase activator activity;kinase regulator activity;protein binding;protein complex binding;protein kinase activator activity;protein kinase r
binding;cation binding;enzyme activator activity;enzyme regulator activity;GTPase activator activity;GTPase regulator activity;ion binding;metal ion binding;nucleoside-triphosphatase regulator activity;transition metal ion bind
catalytic activity;hydrolase activity
binding;protein binding;SNARE binding;syntaxin binding
binding;enzyme binding;protein binding
catalytic activity;oxidoreductase activity;oxidoreductase activity, acting on the CH-NH group of donors;oxidoreductase activity, acting on the CH-NH group of donors, NAD or NADP as acceptor;pyrroline-5-carboxylate reduc
binding;enzyme binding;GTPase binding;identical protein binding;K63-linked polyubiquitin binding;polyubiquitin binding;protein binding;protein C-terminus binding;Rab GTPase binding;Ras GTPase binding;small conjugating
binding;calcium ion binding;cation binding;ion binding;metal ion binding

binding;catalytic activity;enzyme activator activity;enzyme regulator activity;GTPase activator activity;GTPase regulator activity;hydrolase activity;hydrolase activity, acting on ester bonds;molecular transducer activity;nucleos
binding;ribonucleoprotein binding;ribosome binding
binding;chromatin binding
adenyl nucleotide binding;adenyl ribonucleotide binding;ATP binding;ATPase activity;ATPase activity, coupled;ATP-dependent helicase activity;ATP-dependent RNA helicase activity;binding;catalytic activity;helicase activity;h
binding;catalytic activity;GTP binding;GTPase activity;guanyl nucleotide binding;guanyl ribonucleotide binding;hydrolase activity;hydrolase activity, acting on acid anhydrides;hydrolase activity, acting on acid anhydrides, in pl
binding;catalytic activity;cation binding;hydrolase activity;identical protein binding;ion binding;metal ion binding;metallopeptidase activity;peptidase activity;peptidase activity, acting on L-amino acid peptides;protein binding;p
binding;enzyme binding;protein binding
active transmembrane transporter activity;anion transmembrane transporter activity;ATPase activity;ATPase activity, coupled;ATPase activity, coupled to movement of substances;ATPase activity, coupled to transmembrane r
binding;identical protein binding;lipid binding;protein binding;protein dimerization activity;protein homodimerization activity
binding;catalytic activity;GTP binding;GTPase activity;guanyl nucleotide binding;guanyl ribonucleotide binding;hydrolase activity;hydrolase activity, acting on acid anhydrides;hydrolase activity, acting on acid anhydrides, in pl
binding;cytoskeletal protein binding;enzyme binding;identical protein binding;protein binding;RNA polymerase binding;tau protein binding
acetyltransferase activity;catalytic activity;H4 histone acetyltransferase activity;histone acetyltransferase activity;lysine N-acetyltransferase activity;N-acetyltransferase activity;N-acyltransferase activity;transferase activity;tran
adenyl nucleotide binding;adenyl ribonucleotide binding;ATP binding;binding;catalytic activity;kinase activity;nucleotide binding;phosphotransferase activity, alcohol group as acceptor;protein kinase activity;protein serine/thre
adenyl nucleotide binding;adenyl ribonucleotide binding;adenylylsulfate kinase activity;adenylyltransferase activity;ATP binding;binding;catalytic activity;kinase activity;nucleotide binding;nucleotidyltransferase activity;phosph
binding;nucleic acid binding;RNA binding;snoRNA binding
binding;calcium ion binding;cation binding;integrin binding;ion binding;metal ion binding;protein binding;protein complex binding;receptor binding
binding;carbon-oxygen lyase activity;catalytic activity;coenzyme binding;cofactor binding;GDP-mannose 4,6-dehydratase activity;hydro-lyase activity;lyase activity;NADP binding;NADP+ binding;nucleotide binding
binding;DNA binding;nucleic acid binding;protein binding transcription factor activity;sequence-specific DNA binding;transcription coactivator activity;transcription cofactor activity;transcription factor binding transcription fac
adenyl nucleotide binding;adenyl ribonucleotide binding;ATP binding;binding;cation binding;chaperone binding;ion binding;metal ion binding;nucleotide binding;protein binding;purine nucleotide binding;purine ribonucleoside
binding;enzyme activator activity;enzyme binding;enzyme regulator activity;GTPase activator activity;GTPase binding;GTPase regulator activity;identical protein binding;nucleoside-triphosphatase regulator activity;protein bin
binding;cation binding;ion binding;metal ion binding;protein binding;transition metal ion binding;translation initiation factor binding;zinc ion binding
binding;cation binding;ion binding;metal ion binding;transition metal ion binding;zinc ion binding
beta-tubulin binding;binding;cytoskeletal protein binding;enzyme binding;FFAT motif binding;identical protein binding;microtubule binding;protein binding;protein dimerization activity;protein domain specific binding;protein he
adenyl nucleotide binding;adenyl ribonucleotide binding;ATP binding;binding;catalytic activity;kinase activity;NADH dehydrogenase (quinone) activity;NADH dehydrogenase (ubiquinone) activity;NADH dehydrogenase activity;
antioxidant activity;binding;catalytic activity;coenzyme binding;cofactor binding;disulfide oxidoreductase activity;electron carrier activity;flavin adenine dinucleotide binding;glutathione disulfide oxidoreductase activity;glutathi
antioxidant activity;binding;catalytic activity;cation binding;chaperone binding;copper ion binding;enzyme binding;GTPase binding;identical protein binding;ion binding;metal ion binding;oxidoreductase activity;oxidoreductas
aldehyde dehydrogenase (NAD) activity;aldehyde dehydrogenase [NAD(P)+] activity;catalytic activity;electron carrier activity;oxidoreductase activity;oxidoreductase activity, acting on the aldehyde or oxo group of donors;oxid
binding;GTP binding;guanyl nucleotide binding;guanyl ribonucleotide binding;molecular transducer activity;nucleotide binding;purine nucleotide binding;purine ribonucleoside triphosphate binding;purine ribonucleotide bindir
binding;catalytic activity;dinitrosyl-iron complex binding;enzyme binding;enzyme regulator activity;glutathione transferase activity;JUN kinase binding;kinase binding;kinase regulator activity;nitric oxide binding;protein binding
binding;clathrin binding;clathrin heavy chain binding;peptide binding;protein binding;structural molecule activity
alpha-glucosidase activity;binding;carbohydrate binding;catalytic activity;glucosidase activity;hydrolase activity;hydrolase activity, acting on glycosyl bonds;hydrolase activity, hydrolyzing O-glycosyl compounds;maltose alpha
acetyltransferase activity;catalytic activity;dihydrolipoamide S-acyltransferase activity;dihydrolipoyllysine-residue acetyltransferase activity;S-acetyltransferase activity;S-acyltransferase activity;transferase activity;transferase
binding;cation binding;cell adhesion molecule binding;ion binding;metal ion binding;protein binding
binding;catalytic activity;identical protein binding;protein binding;protein dimerization activity;protein homodimerization activity;spermidine synthase activity;transferase activity;transferase activity, transferring alkyl or aryl (oth
endopeptidase inhibitor activity;endopeptidase regulator activity;enzyme inhibitor activity;enzyme regulator activity;peptidase inhibitor activity;peptidase regulator activity;serine-type endopeptidase inhibitor activity
binding;catalytic activity;GTP binding;GTPase activity;guanyl nucleotide binding;guanyl ribonucleotide binding;hydrolase activity;hydrolase activity, acting on acid anhydrides;hydrolase activity, acting on acid anhydrides, in pl
binding;nucleic acid binding;RNA binding;translation elongation factor activity;translation factor activity, nucleic acid binding
1,3-beta-D-glucan binding;binding;carbohydrate binding;complement binding;complement component C1q binding;opsonin binding;pattern binding;polysaccharide binding;protein binding;virion binding
binding;enzyme binding;nucleotide binding;protein binding
adenyl nucleotide binding;adenyl ribonucleotide binding;ATP binding;binding;endopeptidase inhibitor activity;endopeptidase regulator activity;enzyme binding;enzyme inhibitor activity;enzyme regulator activity;kinase binding
binding;catalytic activity;cation binding;ion binding;metal ion binding;oxidoreductase activity;oxidoreductase activity, acting on CH or CH2 groups;oxidoreductase activity, acting on CH or CH2 groups, disulfide as acceptor;ri
binding;calcium ion binding;calcium-dependent protein binding;cation binding;identical protein binding;ion binding;metal ion binding;protein binding;protein dimerization activity;protein homodimerization activity
adenyl nucleotide binding;adenyl ribonucleotide binding;ATP binding;binding;DNA binding;nucleic acid binding;nucleotide binding;purine nucleotide binding;purine ribonucleoside triphosphate binding;purine ribonucleotide bi
adenyl nucleotide binding;adenyl ribonucleotide binding;ATP binding;ATPase activity;ATPase activity, coupled;binding;catalytic activity;DNA binding;DNA clamp loader activity;DNA-dependent ATPase activity;double-stranded
ATPase activity;ATPase activity, coupled;binding;catalytic activity;DNA binding;DNA clamp loader activity;DNA-dependent ATPase activity;hydrolase activity;hydrolase activity, acting on acid anhydrides;hydrolase activity, acti
binding;enzyme activator activity;enzyme binding;enzyme regulator activity;GDP-dissociation inhibitor activity;GTPase activator activity;GTPase binding;GTPase regulator activity;nucleoside-triphosphatase regulator activity;p
binding;enzyme binding;polyubiquitin binding;proteasome binding;protein binding;protein complex binding;ribonucleoprotein binding;ribosome binding;small conjugating protein binding;ubiquitin binding;ubiquitin protein ligas
catalytic activity;endopeptidase activity;hydrolase activity;peptidase activity;peptidase activity, acting on L-amino acid peptides;threonine-type endopeptidase activity;threonine-type peptidase activity
3'-5' exonuclease activity;binding;catalytic activity;cation binding;deoxyribonuclease activity;DNA binding;double-stranded DNA binding;endodeoxyribonuclease activity;endonuclease activity;exonuclease activity;hydrolase a
binding;catalytic activity;cation binding;hydrolase activity;hydrolase activity, acting on ester bonds;identical protein binding;ion binding;lipid binding;metal ion binding;molecular transducer activity;nucleic acid binding;phosph
molecular transducer activity;protein binding transcription factor activity;signal transducer activity;transcription cofactor activity;transcription corepressor activity;transcription factor binding transcription factor activity
structural constituent of ribosome;structural molecule activity
binding;nucleic acid binding;RNA binding
binding;catalytic activity;cis-trans isomerase activity;cyclosporin A binding;drug binding;estrogen receptor binding;heat shock protein binding;hormone receptor binding;Hsp70 protein binding;Hsp90 protein binding;isomeras
antioxidant activity;catalytic activity;oxidoreductase activity;oxidoreductase activity, acting on paired donors, with incorporation or reduction of molecular oxygen;oxidoreductase activity, acting on peroxide as acceptor;perox
binding;catalytic activity;cis-trans isomerase activity;cyclosporin A binding;drug binding;isomerase activity;peptide binding;peptidyl-prolyl cis-trans isomerase activity
binding;catalytic activity;coenzyme binding;cofactor binding;electron carrier activity;GDP-4-dehydro-D-rhamnose reductase activity;GDP-L-fucose synthase activity;isomerase activity;oxidoreductase activity;oxidoreductase a
binding;catalytic activity;GTP binding;GTPase activity;guanyl nucleotide binding;guanyl ribonucleotide binding;hydrolase activity;hydrolase activity, acting on acid anhydrides;hydrolase activity, acting on acid anhydrides, in pl
binding;nucleotide binding
binding;cation binding;DNA binding;double-stranded DNA binding;ion binding;metal ion binding;nucleic acid binding;nucleotide binding;RNA binding;structure-specific DNA binding;transition metal ion binding;zinc ion bindin
binding;catalytic activity;GDP binding;GTP binding;GTPase activity;guanyl nucleotide binding;guanyl ribonucleotide binding;hydrolase activity;hydrolase activity, acting on acid anhydrides;hydrolase activity, acting on acid anh
structural constituent of cytoskeleton;structural molecule activity
adenyl nucleotide binding;adenyl ribonucleotide binding;androgen receptor binding;ATP binding;binding;catalytic activity;chromatin binding;enzyme binding;GTPase binding;GTP-Rho binding;histone binding;histone deacetyl
binding;lipid binding
binding;enzyme binding;enzyme regulator activity;phosphatase binding;phosphatase regulator activity;protein binding;protein phosphatase 2A binding;protein phosphatase binding;protein phosphatase regulator activity;prote
binding;cation binding;enzyme activator activity;enzyme regulator activity;GTPase activator activity;GTPase regulator activity;ion binding;metal ion binding;nucleoside-triphosphatase regulator activity;transition metal ion bind
binding;chromatin binding
binding;ribonucleoprotein binding;snRNP binding
binding;catalytic activity;GDP binding;GTP binding;GTPase activity;guanyl nucleotide binding;guanyl ribonucleotide binding;hydrolase activity;hydrolase activity, acting on acid anhydrides;hydrolase activity, acting on acid anh
catalytic activity;kinase activity;phosphotransferase activity, alcohol group as acceptor;protein kinase activity;transferase activity;transferase activity, transferring phosphorus-containing groups
binding;enzyme binding;enzyme inhibitor activity;enzyme regulator activity;lipase binding;lipase inhibitor activity;protein binding;small conjugating protein binding;ubiquitin binding;ubiquitin protein ligase binding
binding;catalytic activity;cation binding;coenzyme binding;cofactor binding;flavin adenine dinucleotide binding;ion binding;metal ion binding;oxidoreductase activity;oxidoreductase activity, acting on the CH-CH group of dono
adenyl nucleotide binding;adenyl ribonucleotide binding;ATP binding;ATPase activity;ATPase activity, coupled;ATP-dependent helicase activity;ATP-dependent RNA helicase activity;binding;catalytic activity;helicase activity;h
binding;nucleic acid binding;RNA binding
binding;catalytic activity;enzyme binding;intramolecular transferase activity;isomerase activity;protein binding;pseudouridine synthase activity
binding;epidermal growth factor receptor binding;growth factor receptor binding;nucleic acid binding;protein binding;protein C-terminus binding;receptor binding;RNA binding
5-methyltetrahydrofolate-dependent methyltransferase activity;binding;catalytic activity;cation binding;cobalamin binding;homocysteine S-methyltransferase activity;ion binding;metal ion binding;methionine synthase activity;
3'-tRNA processing endoribonuclease activity;binding;catalytic activity;cation binding;endonuclease activity;endonuclease activity, active with either ribo- or deoxyribonucleic acids and producing 5'-phosphomonoesters;end
binding;catalytic activity;GTP binding;GTPase activity;guanyl nucleotide binding;guanyl ribonucleotide binding;hydrolase activity;hydrolase activity, acting on acid anhydrides;hydrolase activity, acting on acid anhydrides, in pl
structural molecule activity
acid-amino acid ligase activity;adenyl nucleotide binding;adenyl ribonucleotide binding;ATP binding;binding;catalytic activity;enzyme binding;ligase activity;ligase activity, forming carbon-nitrogen bonds;nucleotide binding;pro
adenyl nucleotide binding;adenyl-nucleotide exchange factor activity;ATPase regulator activity;binding;enzyme regulator activity;nucleoside-triphosphatase regulator activity;nucleotide binding;protein binding;purine nucleotic
acid-amino acid ligase activity;catalytic activity;cysteine-type endopeptidase inhibitor activity;endopeptidase inhibitor activity;endopeptidase regulator activity;enzyme inhibitor activity;enzyme regulator activity;ligase activity;l
binding;catalytic activity;cation binding;ion binding;metal ion binding;methyltransferase activity;N-methyltransferase activity;nucleic acid binding;RNA binding;RNA methyltransferase activity;S-adenosylmethionine-dependent
adenyl nucleotide binding;adenyl ribonucleotide binding;ATP binding;binding;catalytic activity;cation binding;ion binding;kinase activity;magnesium ion binding;MAP kinase kinase kinase activity;metal ion binding;molecular tr
aminopeptidase activity;binding;catalytic activity;cation binding;cytokine receptor binding;exopeptidase activity;growth factor receptor binding;hydrolase activity;interleukin-1 receptor binding;interleukin-1, Type II receptor bir
binding;protein binding;protein domain specific binding
binding;chromatin binding;histone binding;nucleosome binding;protein binding;protein binding transcription factor activity;repressing transcription factor binding;transcription cofactor activity;transcription corepressor activity
catalytic activity;kinase activity;phosphotransferase activity, alcohol group as acceptor;protein binding transcription factor activity;transcription coactivator activity;transcription cofactor activity;transcription factor binding tran
binding;cytoskeletal protein binding;dynactin binding;lipid binding;phosphatidylinositol binding;phospholipid binding;protein binding
carbon-oxygen lyase activity;catalytic activity;hydro-lyase activity;intramolecular transferase activity;isomerase activity;ligand-dependent nuclear receptor transcription coactivator activity;lyase activity;protein binding transcri
binding;cation binding;ion binding;metal ion binding
adenyl nucleotide binding;adenyl ribonucleotide binding;ATP binding;binding;catalytic activity;cation binding;ion binding;kinase activity;metal ion binding;nucleotide binding;PDZ domain binding;phosphotransferase activity, a
binding;cation binding;ion binding;metal ion binding;protein binding;protein domain specific binding
binding;identical protein binding;protein binding;protein dimerization activity;protein heterodimerization activity;protein homodimerization activity
binding;catalytic activity;cation binding;diphosphotransferase activity;enzyme inhibitor activity;enzyme regulator activity;ion binding;magnesium ion binding;metal ion binding;ribose phosphate diphosphokinase activity;transfe
binding;binding, bridging;cytoskeletal protein binding;enzyme binding;JUN kinase binding;kinase binding;kinesin binding;MAP-kinase scaffold activity;protein binding;protein complex scaffold;protein kinase binding;receptor s
adenyl nucleotide binding;adenyl ribonucleotide binding;ATP binding;binding;catalytic activity;enzyme regulator activity;GTPase regulator activity;guanyl-nucleotide exchange factor activity;kinase activity;nucleoside-triphospl
adenyl nucleotide binding;adenyl ribonucleotide binding;ATP binding;binding;catalytic activity;enzyme binding;GTPase binding;kinase activity;nucleotide binding;phosphotransferase activity, alcohol group as acceptor;protein
binding;calcium ion binding;cargo receptor activity;cation binding;clathrin binding;clathrin heavy chain binding;glycoprotein binding;ion binding;lipoprotein particle binding;lipoprotein particle receptor activity;low-density lipop
endopeptidase inhibitor activity;endopeptidase regulator activity;enzyme inhibitor activity;enzyme regulator activity;peptidase inhibitor activity;peptidase regulator activity;serine-type endopeptidase inhibitor activity
adenyl nucleotide binding;adenyl ribonucleotide binding;AMP binding;binding;cAMP binding;cAMP-dependent protein kinase inhibitor activity;cAMP-dependent protein kinase regulator activity;cyclic nucleotide binding;enzyr
catalytic activity;oxidoreductase activity;oxidoreductase activity, acting on the aldehyde or oxo group of donors;oxidoreductase activity, acting on the aldehyde or oxo group of donors, disulfide as acceptor;pyruvate dehydrog
binding;calcium ion binding;cation binding;ion binding;metal ion binding
binding;damaged DNA binding;DNA binding;enzyme binding;histone binding;nucleic acid binding;protein binding
active transmembrane transporter activity;ATPase activity;ATPase activity, coupled;ATPase activity, coupled to movement of substances;ATPase activity, coupled to transmembrane movement of ions;ATPase activity, couplec
binding;catalytic activity;methyltransferase activity;nucleic acid binding;RNA binding;RNA methyltransferase activity;rRNA methyltransferase activity;S-adenosylmethionine-dependent methyltransferase activity;transferase ac
adenyl nucleotide binding;adenyl ribonucleotide binding;ATP binding;ATPase activity;ATPase activity, coupled;ATP-dependent helicase activity;ATP-dependent RNA helicase activity;binding;catalytic activity;helicase activity;h

binding;growth factor activity;protein binding;receptor binding
carbon-oxygen lyase activity;catalytic activity;enoyl-CoA hydratase activity;hydro-lyase activity;lyase activity
actin binding;binding;catalytic activity;cytokine binding;cytoskeletal protein binding;enzyme binding;GTP binding;GTPase activity;guanyl nucleotide binding;guanyl ribonucleotide binding;heat shock protein binding;Hsp90 pro
amine binding;amino acid binding;binding;carbon monoxide binding;carbon-oxygen lyase activity;carboxylic acid binding;catalytic activity;cation binding;cofactor binding;cystathionine beta-synthase activity;enzyme binding;
binding;catalytic activity;GTP binding;GTPase activity;guanyl nucleotide binding;guanyl ribonucleotide binding;hydrolase activity;hydrolase activity, acting on acid anhydrides;hydrolase activity, acting on acid anhydrides, in pl
binding;protein binding;SNARE binding;syntaxin binding
binding;catalytic activity;enzyme binding;hydrolase activity;kinase binding;nucleotide binding;protein binding;protein kinase binding;protein kinase C binding
binding;enzyme binding;histone deacetylase binding;nuclear localization sequence binding;peptide binding;protein binding;protein transporter activity;signal sequence binding;substrate-specific transporter activity;transporte
binding;cation binding;enzyme regulator activity;GTPase regulator activity;guanyl-nucleotide exchange factor activity;ion binding;metal ion binding;nucleoside-triphosphatase regulator activity;Ras guanyl-nucleotide exchange
catalytic activity;spermine synthase activity;transferase activity;transferase activity, transferring alkyl or aryl (other than methyl) groups
binding;DNA binding;nucleic acid binding;protein binding transcription factor activity;single-stranded DNA binding;structure-specific DNA binding;transcription coactivator activity;transcription cofactor activity;transcription fa
binding;catalytic activity;chromatin binding;coenzyme binding;cofactor binding;NAD binding;nucleotide binding;oxidoreductase activity;oxidoreductase activity, acting on CH-OH group of donors;oxidoreductase activity, actin
adenyl nucleotide binding;adenyl ribonucleotide binding;ATP binding;binding;nucleotide binding;purine nucleotide binding;purine ribonucleoside triphosphate binding;purine ribonucleotide binding;ribonucleotide binding
binding;endopeptidase activator activity;endopeptidase regulator activity;enzyme activator activity;enzyme regulator activity;identical protein binding;p53 binding;peptidase activator activity;peptidase regulator activity;protein
binding;catalytic activity;cytoskeletal protein binding;GTP binding;GTPase activity;guanyl nucleotide binding;guanyl ribonucleotide binding;hydrolase activity;hydrolase activity, acting on acid anhydrides;hydrolase activity, act
binding;cation binding;ion binding;metal ion binding;nucleic acid binding;RNA binding;rRNA binding;structural constituent of ribosome;structural molecule activity
binding;catalytic activity;cation binding;hydrolase activity;hydrolase activity, acting on ester bonds;ion binding;metal ion binding;phosphatase activity;phosphoprotein phosphatase activity;phosphoric ester hydrolase activity;p
binding;catalytic activity;cation binding;enzyme binding;hydrolase activity;hydrolase activity, acting on ester bonds;ion binding;kinase binding;metal ion binding;myosin phosphatase activity;myosin-light-chain-phosphatase a
binding;nucleic acid binding;RNA binding;structural constituent of ribosome;structural molecule activity
binding;nucleic acid binding;ribonucleoprotein binding;ribosome binding;RNA binding;translation factor activity, nucleic acid binding;translation release factor activity;translation release factor activity, codon specific;translatio
structural constituent of ribosome;structural molecule activity
binding;protein binding;protein N-terminus binding;structural constituent of ribosome;structural molecule activity
binding;calcium ion binding;cation binding;ion binding;metal ion binding;receptor activity
4 iron, 4 sulfur cluster binding;amidophosphoribosyltransferase activity;binding;catalytic activity;cation binding;ion binding;iron-sulfur cluster binding;metal cluster binding;metal ion binding;transferase activity;transferase acti
endopeptidase activator activity;endopeptidase regulator activity;enzyme activator activity;enzyme regulator activity;peptidase activator activity;peptidase regulator activity
binding;mRNA binding;nucleic acid binding;RNA binding
binding;nucleotide binding
binding;mRNA 3'-UTR binding;mRNA binding;nucleic acid binding;RNA binding
binding;cAMP-dependent protein kinase activity;catalytic activity;cation binding;cyclic nucleotide-dependent protein kinase activity;enzyme regulator activity;GTPase regulator activity;guanyl-nucleotide exchange factor activ
4 iron, 4 sulfur cluster binding;binding;catalytic activity;cation binding;coenzyme binding;cofactor binding;dihydroorotate dehydrogenase activity;dihydropyrimidine dehydrogenase (NADP+) activity;flavin adenine dinucleotide
activating transcription factor binding;binding;damaged DNA binding;DNA binding;histone binding;methylated histone residue binding;nucleic acid binding;p53 binding;protein binding;protein binding transcription factor activ
adenyl nucleotide binding;adenyl ribonucleotide binding;ATP binding;binding;catalytic activity;DNA binding;double-stranded RNA binding;nucleic acid binding;nucleotide binding;purine nucleotide binding;purine ribonucleosid
catalytic activity;phosphorylase activity;purine-nucleoside phosphorylase activity;S-methyl-5-thioadenosine phosphorylase activity;transferase activity;transferase activity, transferring glycosyl groups;transferase activity, trans
actin binding;actin-dependent ATPase activity;adenyl nucleotide binding;adenyl ribonucleotide binding;ADP binding;ATP binding;ATPase activity;ATPase activity, coupled;binding;calmodulin binding;catalytic activity;cation bin
binding;nucleotide binding
binding;cation binding;ion binding;ion channel binding;metal ion binding;protein binding;transition metal ion binding;zinc ion binding
binding;mRNA 3'-UTR binding;mRNA binding;nucleic acid binding;nucleotide binding;RNA binding;rRNA binding;SSU rRNA binding;translation regulator activity;translation repressor activity
binding;ribonucleoprotein binding
binding;clathrin binding;lipid binding;protein binding
catalytic activity;hydrolase activity;peptidase activity
binding;carbohydrate binding;cell adhesion molecule binding;glycosaminoglycan binding;heparin binding;pattern binding;polysaccharide binding;protein binding
binding;catalytic activity;DNA polymerase activity;intramolecular oxidoreductase activity;isomerase activity;nucleotidyltransferase activity;prostaglandin-E synthase activity;protein binding;RNA-directed DNA polymerase activ
binding;cation binding;ion binding;metal ion binding;nucleic acid binding;protein binding transcription factor activity;RNA binding;transcription cofactor activity;transcription corepressor activity;transcription factor binding tran
binding;identical protein binding;lipid binding;protein binding
protein binding transcription factor activity;transcription coactivator activity;transcription cofactor activity;transcription factor binding transcription factor activity
binding;cation binding;cytokine receptor binding;enzyme binding;growth factor receptor binding;hormone receptor binding;interleukin-1 receptor binding;ion binding;kinase binding;metal ion binding;nuclear hormone recepto
binding;cation binding;ion binding;metal ion binding;nucleotide binding;transition metal ion binding;zinc ion binding
binding;carboxylic acid binding;catalytic activity;cation binding;ion binding;iron ion binding;L-ascorbic acid binding;metal ion binding;oxidoreductase activity;oxidoreductase activity, acting on paired donors, with incorporatio
adenyl nucleotide binding;adenyl ribonucleotide binding;ATP binding;binding;catalytic activity;cation binding;cyclase activity;FAD-AMP lyase (cyclizing) activity;glycerone kinase activity;ion binding;kinase activity;lyase activity;
binding;catalytic activity;cytokine receptor binding;growth factor receptor binding;hydrolase activity;hydrolase activity, acting on ester bonds;interleukin-2 receptor binding;nucleic acid binding;phosphatase activity;phosphop
binding;cation binding;ion binding;metal ion binding;nucleotide binding
binding;carbon-carbon lyase activity;carboxy-lyase activity;catalytic activity;cofactor binding;lyase activity;pyridoxal phosphate binding;vitamin B6 binding;vitamin binding
binding;catalytic activity;cation binding;dioxygenase activity;ion binding;metal ion binding;oxidoreductase activity
binding;nucleotide binding
binding;identical protein binding;protein binding;protein binding transcription factor activity;transcription cofactor activity;transcription corepressor activity;transcription factor binding;transcription factor binding transcription
binding;cation binding;DNA binding;ion binding;metal ion binding;nucleic acid binding;nucleic acid binding transcription factor activity;sequence-specific DNA binding;sequence-specific DNA binding transcription factor activ
binding;catalytic activity;chromatin binding;chromatin DNA binding;core promoter binding;deacetylase activity;DNA binding;enzyme binding;histone deacetylase activity;histone deacetylase activity (H3-K14 specific);histone d
binding;BRE binding;mRNA binding;nucleic acid binding;nucleotide binding;RNA binding;translation regulator activity;translation regulator activity, nucleic acid binding;translation repressor activity;translation repressor activity
binding;enzyme binding;kinase binding;protein binding;protein kinase binding;protein kinase C binding
binding;enzyme binding;GTPase binding;protein binding;protein N-terminus binding;Ral GTPase binding;Ras GTPase binding;small GTPase binding
adenyl nucleotide binding;adenyl ribonucleotide binding;ATP binding;binding;catalytic activity;cation binding;ion binding;kinase activity;magnesium ion binding;metal ion binding;nucleotide binding;phosphotransferase activity
binding;catalytic activity;cation binding;ion binding;metal ion binding;oxidoreductase activity;transition metal ion binding;zinc ion binding
binding;calcium ion binding;calcium-dependent phospholipid binding;catalytic activity;cation binding;endopeptidase activity;hydrolase activity;identical protein binding;ion binding;lipid binding;metal ion binding;NF-kappaB b
binding;calcium ion binding;cation binding;identical protein binding;ion binding;metal ion binding;protein binding
acid-amino acid ligase activity;adenyl nucleotide binding;adenyl ribonucleotide binding;ATP binding;binding;catalytic activity;enzyme binding;ligase activity;ligase activity, forming carbon-nitrogen bonds;nucleotide binding;pr
adenyl nucleotide binding;adenyl ribonucleotide binding;ATP binding;binding;nucleotide binding;purine nucleotide binding;purine ribonucleoside triphosphate binding;purine ribonucleotide binding;ribonucleotide binding
binding;catalytic activity;cation binding;hydrolase activity;hydrolase activity, acting on ester bonds;ion binding;metal ion binding;phosphatase activity;phosphoprotein phosphatase activity;phosphoric ester hydrolase activity;p
amine binding;amino acid binding;binding;carboxylic acid binding;catalytic activity;cation binding;disulfide oxidoreductase activity;DNA binding;electron carrier activity;glutathione binding;heme binding;intramolecular oxidore
catalytic activity;hydrolase activity;hydrolase activity, acting on carbon-nitrogen (but not peptide) bonds;hydrolase activity, acting on carbon-nitrogen (but not peptide) bonds, in linear amides;omega-amidase activity
acetate-CoA ligase activity;acid-thiol ligase activity;adenyl nucleotide binding;adenyl ribonucleotide binding;AMP binding;ATP binding;binding;catalytic activity;CoA-ligase activity;ligase activity;ligase activity, forming carbon-
binding;cytoskeletal protein binding;protein binding;tropomyosin binding
catalytic activity;oxidoreductase activity;oxidoreductase activity, acting on the CH-CH group of donors
binding;mRNA 3'-UTR binding;mRNA 5'-UTR binding;mRNA binding;nucleic acid binding;nucleotide binding;RNA binding;translation regulator activity
binding;cytoskeletal protein binding;identical protein binding;lamin binding;microtubule binding;protein binding;tubulin binding
enzyme activator activity;enzyme regulator activity;lipase activator activity;phospholipase A2 activator activity;phospholipase activator activity
binding;GTP binding;guanyl nucleotide binding;guanyl ribonucleotide binding;identical protein binding;nucleotide binding;protein binding;purine nucleotide binding;purine ribonucleoside triphosphate binding;purine ribonucle
antioxidant activity;binding;catalytic activity;disulfide oxidoreductase activity;glutathione peroxidase activity;glutathione transferase activity;oxidoreductase activity;oxidoreductase activity, acting on a sulfur group of donors;o
binding;nucleic acid binding;RNA binding;snoRNA binding
binding;protein binding;protein domain specific binding;SH3 domain binding;structural constituent of cytoskeleton;structural molecule activity
binding;catalytic activity;cation binding;coenzyme binding;cofactor binding;ion binding;magnesium ion binding;metal ion binding;thiamine pyrophosphate binding;transferase activity;vitamin binding
binding;nucleotide binding
binding;nucleic acid binding;RNA binding;translation factor activity, nucleic acid binding;translation initiation factor activity
active transmembrane transporter activity;adenyl nucleotide binding;adenyl ribonucleotide binding;ATP binding;binding;chaperone binding;macromolecule transmembrane transporter activity;nucleotide binding;P-P-bond-hy
binding;catalytic activity;cation binding;coenzyme binding;cofactor binding;electron carrier activity;ion binding;isocitrate dehydrogenase (NAD+) activity;isocitrate dehydrogenase activity;magnesium ion binding;metal ion binc
acetyltransferase activity;beta-N-acetylglucosaminidase activity;beta-N-acetylhexosaminidase activity;catalytic activity;hexosaminidase activity;histone acetyltransferase activity;hyalurononglucosaminidase activity;hydrolase
binding;epidermal growth factor receptor binding;growth factor receptor binding;insulin receptor binding;lipid binding;phosphatidylinositol binding;phospholipid binding;protein binding;protein complex binding;receptor bindin
acid-amino acid ligase activity;binding;catalytic activity;cation binding;enzyme binding;identical protein binding;ion binding;ligase activity;ligase activity, forming carbon-nitrogen bonds;metal ion binding;mRNA 3'-UTR binding
adenyl nucleotide binding;adenyl ribonucleotide binding;ATP binding;ATPase activity;ATPase activity, coupled;binding;catalytic activity;hydrolase activity;hydrolase activity, acting on acid anhydrides;hydrolase activity, acting
binding;catalytic activity;cation binding;endopeptidase activity;hydrolase activity;ion binding;metal ion binding;metalloendopeptidase activity;metallopeptidase activity;peptidase activity;peptidase activity, acting on L-amino a
binding;nucleic acid binding;nucleotide binding;RNA binding;translation factor activity, nucleic acid binding;translation initiation factor activity
binding;catalytic activity;cation binding;intramolecular transferase activity;intramolecular transferase activity, phosphotransferases;ion binding;isomerase activity;magnesium ion binding;metal ion binding;phosphoacetylgluco
ATPase activator activity;ATPase regulator activity;binding;chaperone binding;enzyme activator activity;enzyme regulator activity;nucleoside-triphosphatase regulator activity;protein binding
binding;double-stranded RNA binding;nucleic acid binding;RNA binding
binding;enzyme binding;kinase binding;lipid binding;phosphatidylserine binding;phospholipid binding;protein binding;protein kinase binding;protein kinase C binding
anion binding;binding;catalytic activity;drug binding;ion binding;nucleobase binding;nucleoside binding;phosphate ion binding;purine base binding;purine-nucleoside phosphorylase activity;transferase activity;transferase act
catalytic activity;endopeptidase activity;hydrolase activity;peptidase activity;peptidase activity, acting on L-amino acid peptides;serine hydrolase activity;serine-type endopeptidase activity;serine-type peptidase activity
binding;catalytic activity;ribonucleoprotein binding;ribosome binding;transferase activity;transferase activity, transferring glycosyl groups
binding;endopeptidase inhibitor activity;endopeptidase regulator activity;enzyme binding;enzyme inhibitor activity;enzyme regulator activity;peptidase inhibitor activity;peptidase regulator activity;protease binding;protein binc
binding;lipid binding;receptor activity;viral receptor activity
adrenergic receptor binding;alpha-2A adrenergic receptor binding;binding;catalytic activity;cysteine-type endopeptidase activity;cysteine-type peptidase activity;endopeptidase activity;enzyme binding;exopeptidase activity;C
binding;calcium ion binding;cation binding;collagen binding;extracellular matrix binding;glycoprotein binding;ion binding;laminin binding;metal ion binding;protein binding;proteoglycan binding
adenyl nucleotide binding;adenyl ribonucleotide binding;ATP binding;binding;catalytic activity;cation binding;deoxyribonuclease activity;DNA binding;GTP binding;guanyl nucleotide binding;guanyl ribonucleotide binding;hydr
binding;carbohydrate binding;catalytic activity;collagen binding;glycosaminoglycan binding;hexosaminidase activity;hyaluronic acid binding;hyalurononglucosaminidase activity;hydrolase activity;hydrolase activity, acting on g
adenyl nucleotide binding;adenyl ribonucleotide binding;ATP binding;binding;catalytic activity;cation binding;DNA binding;ion binding;kinase activity;metal ion binding;nucleic acid binding;nucleic acid binding transcription fac
carnitine O-acyltransferase activity;carnitine O-palmitoyltransferase activity;catalytic activity;O-acyltransferase activity;O-palmitoyltransferase activity;palmitoyltransferase activity;transferase activity;transferase activity, transf
adenyl nucleotide binding;adenyl ribonucleotide binding;ATP binding;binding;catalytic activity;kinase activity;nucleobase-containing compound kinase activity;nucleoside phosphate kinase activity;nucleotide binding;nucleotic
binding;enzyme binding;protein binding;RNA polymerase binding
adenyl nucleotide binding;adenyl ribonucleotide binding;ATP binding;binding;catalytic activity;enzyme binding;kinase activity;MAP kinase activity;molecular transducer activity;nucleotide binding;phosphatase binding;phosph
alkyl hydroperoxide reductase activity;antioxidant activity;binding;caspase inhibitor activity;caspase regulator activity;catalytic activity;cysteine-type endopeptidase inhibitor activity;endopeptidase inhibitor activity;endopeptic
structural constituent of ribosome;structural molecule activity
asialoglycoprotein receptor activity;binding;carbohydrate binding;cargo receptor activity;glycosaminoglycan binding;heparin binding;lipoprotein particle receptor binding;low-density lipoprotein particle receptor binding;patter
binding;catalytic activity;coproporphyrinogen oxidase activity;identical protein binding;oxidoreductase activity;oxidoreductase activity, acting on the CH-CH group of donors;oxidoreductase activity, acting on the CH-CH grou
catalytic activity;dolichyl-diphosphooligosaccharide-protein glycotransferase activity;oligosaccharyl transferase activity;transferase activity;transferase activity, transferring glycosyl groups;transferase activity, transferring hex
binding;carbohydrate binding;glycosaminoglycan binding;heparin binding;nucleic acid binding;pattern binding;polysaccharide binding;RNA binding;structural constituent of ribosome;structural molecule activity
binding;catalytic activity;GDP binding;GTP binding;GTPase activity;guanyl nucleotide binding;guanyl ribonucleotide binding;hydrolase activity;hydrolase activity, acting on acid anhydrides;hydrolase activity, acting on acid anh
3-chloroallyl aldehyde dehydrogenase activity;aldehyde dehydrogenase (NAD) activity;aldehyde dehydrogenase [NAD(P)+] activity;catalytic activity;long-chain-alcohol oxidase activity;long-chain-aldehyde dehydrogenase acti

catalytic activity;enzyme activator activity;enzyme regulator activity;GDP-dissociation inhibitor activity;GTPase activator activity;GTPase activity;GTPase regulator activity;hydrolase activity;hydrolase activity, acting on acid an
adenyl nucleotide binding;adenyl ribonucleotide binding;ATP binding;ATPase activity;binding;catalytic activity;enzyme binding;hydrolase activity;hydrolase activity, acting on acid anhydrides;hydrolase activity, acting on acid a
carboxylic acid transmembrane transporter activity;citrate transmembrane transporter activity;organic acid transmembrane transporter activity;substrate-specific transmembrane transporter activity;substrate-specific transpo
aminopeptidase activity;binding;catalytic activity;cation binding;exopeptidase activity;hydrolase activity;ion binding;metal ion binding;metalloaminopeptidase activity;metalloexopeptidase activity;metallopeptidase activity;pep
actin-dependent ATPase activity;ATPase activity;ATPase activity, coupled;binding;calcium ion binding;catalytic activity;cation binding;hydrolase activity;hydrolase activity, acting on acid anhydrides;hydrolase activity, acting o
binding;chromatin binding;core promoter proximal region DNA binding;core promoter proximal region sequence-specific DNA binding;DNA bending activity;DNA binding;mitochondrial light strand promoter sense binding;nuc
AMP deaminase activity;binding;catalytic activity;cation binding;deaminase activity;hydrolase activity;hydrolase activity, acting on carbon-nitrogen (but not peptide) bonds;hydrolase activity, acting on carbon-nitrogen (but not
binding;core promoter proximal region DNA binding;core promoter proximal region sequence-specific DNA binding;DNA binding;nucleic acid binding;nucleic acid binding transcription factor activity;regulatory region DNA bin
binding;molecular transducer activity;nucleic acid binding;receptor signaling protein activity;RNA binding;signal transducer activity;snoRNA binding
actin binding;actin monomer binding;adenyl nucleotide binding;adenyl ribonucleotide binding;ATP binding;binding;cytoskeletal protein binding;lipid binding;nucleotide binding;phosphatidylinositol binding;phosphatidylinositol
binding;cytokine activity;enzyme binding;GTPase binding;identical protein binding;nucleic acid binding;protein binding;protein dimerization activity;protein homodimerization activity;receptor binding;RNA binding;tRNA bindin
binding;DNA binding;double-stranded DNA binding;identical protein binding;mRNA 3'-UTR binding;mRNA binding;nucleic acid binding;nucleic acid binding transcription factor activity;nucleotide binding;protein binding;RNA
binding;nucleotide binding
activating transcription factor binding;binding;catalytic activity;core promoter binding;deacetylase activity;DNA binding;enzyme binding;histone deacetylase activity;histone deacetylase activity (H3-K14 specific);histone deace
actin binding;binding;cytoskeletal protein binding;enzyme binding;protein binding
binding;catalytic activity;chromo shadow domain binding;DNA binding;lamin binding;nucleic acid binding;oxidoreductase activity;oxidoreductase activity, acting on the CH-CH group of donors;oxidoreductase activity, acting
binding;identical protein binding;protein binding
binding;chaperone binding;protein binding
binding;enzyme binding;kinase binding;protein binding;protein kinase binding;ribonucleoprotein binding;ribosome binding
binding;cytokine binding;endopeptidase inhibitor activity;endopeptidase regulator activity;enzyme inhibitor activity;enzyme regulator activity;growth factor binding;peptidase inhibitor activity;peptidase regulator activity;protein
binding;DNA binding;histone binding;nucleic acid binding;nucleic acid binding transcription factor activity;protein binding;sequence-specific DNA binding transcription factor activity
binding;catalytic activity;GTP binding;GTPase activity;guanyl nucleotide binding;guanyl ribonucleotide binding;hydrolase activity;hydrolase activity, acting on acid anhydrides;hydrolase activity, acting on acid anhydrides, in pl
binding;cation binding;cytoskeletal protein binding;filamin binding;ion binding;metal ion binding;protein binding;transition metal ion binding;zinc ion binding
binding;cation binding;ion binding;metal ion binding;transition metal ion binding;zinc ion binding
binding;GTP binding;guanyl nucleotide binding;guanyl ribonucleotide binding;nucleotide binding;purine nucleotide binding;purine ribonucleoside triphosphate binding;purine ribonucleotide binding;ribonucleotide binding
binding;enzyme binding;histone binding;protein binding;RNA polymerase binding
binding;calcium ion binding;catalytic activity;cation binding;cis-trans isomerase activity;drug binding;FK506 binding;ion binding;isomerase activity;macrolide binding;metal ion binding;peptidyl-prolyl cis-trans isomerase activi
adenyl nucleotide binding;adenyl ribonucleotide binding;ATP binding;binding;catalytic activity;DNA binding;kinase activity;nucleic acid binding;nucleotide binding;phosphotransferase activity, alcohol group as acceptor;protei
activating transcription factor binding;binding;catalytic activity;chromatin binding;deacetylase activity;DNA binding;hydrolase activity;nucleic acid binding;nucleic acid binding transcription factor activity;protein binding;protei
transporter activity
arginine N-methyltransferase activity;binding;catalytic activity;histone methyltransferase activity;histone methyltransferase activity (H4-R3 specific);histone-arginine N-methyltransferase activity;identical protein binding;methyl
binding;identical protein binding;lipid binding;protein binding
adenyl nucleotide binding;adenyl ribonucleotide binding;ATP binding;ATPase activity;ATPase activity, coupled;ATP-dependent helicase activity;ATP-dependent RNA helicase activity;binding;catalytic activity;helicase activity;h
adenyl nucleotide binding;adenyl ribonucleotide binding;aminoacyl-tRNA ligase activity;ATP binding;binding;catalytic activity;ligase activity;ligase activity, forming aminoacyl-tRNA and related compounds;ligase activity, formi
actin binding;actin filament binding;binding;cytoskeletal protein binding;enzyme binding;GTPase binding;GTP-Rho binding;myosin binding;myosin II binding;protein binding;Ras GTPase binding;Rho GTPase binding;small GT
binding;catalytic activity;enzyme binding;hydrolase activity;hydrolase activity, acting on ester bonds;kinase binding;phosphatase activity;phosphoprotein phosphatase activity;phosphoric ester hydrolase activity;protein bindir
acyl-CoA dehydrogenase activity;binding;catalytic activity;coenzyme binding;cofactor binding;flavin adenine dinucleotide binding;oxidoreductase activity;oxidoreductase activity, acting on the CH-CH group of donors
catalytic activity;hydrolase activity;hydrolase activity, acting on ester bonds;inositol or phosphatidylinositol phosphatase activity;lipid phosphatase activity;phosphatase activity;phosphatidylinositol bisphosphate phosphatase
angiostatin binding;ATPase binding;ATPase inhibitor activity;ATPase regulator activity;binding;calmodulin binding;enzyme binding;enzyme inhibitor activity;enzyme regulator activity;identical protein binding;nucleoside-triphos
acidic amino acid transmembrane transporter activity;active transmembrane transporter activity;amine transmembrane transporter activity;amino acid transmembrane transporter activity;binding;calcium ion binding;carboxyli
binding;catalytic activity;DNA binding;enzyme binding;NAD+ ADP-ribosyltransferase activity;nucleic acid binding;protein binding;transferase activity;transferase activity, transferring glycosyl groups;transferase activity, transfe
acetyltransferase activity;binding;catalytic activity;DNA binding;enzyme activator activity;enzyme regulator activity;histone acetyltransferase activity;lysine N-acetyltransferase activity;N-acetyltransferase activity;N-acyltransfe
binding;calcium channel inhibitor activity;calcium channel regulator activity;channel inhibitor activity;channel regulator activity;cytoskeletal protein binding;ion channel inhibitor activity;lipid binding;phospholipid binding;protei
binding;nucleotide binding;protein binding;protein complex scaffold;receptor signaling complex scaffold activity;structural molecule activity
acid-amino acid ligase activity;binding;binding, bridging;catalytic activity;enzyme binding;G-protein-coupled receptor binding;heat shock protein binding;Hsp70 protein binding;Hsp90 protein binding;identical protein binding;
binding;DNA binding;enzyme regulator activity;nucleic acid binding;phosphatase regulator activity;protein binding;protein domain specific binding;protein phosphatase regulator activity;protein phosphatase type 1 regulator a
structural constituent of ribosome;structural molecule activity
binding;carboxylic ester hydrolase activity;catalytic activity;enzyme binding;enzyme inhibitor activity;enzyme regulator activity;hydrolase activity;hydrolase activity, acting on ester bonds;phosphatase binding;phosphatase inh
adenyl nucleotide binding;adenyl ribonucleotide binding;ATP binding;binding;catalytic activity;hydrolase activity;hydrolase activity, acting on acid anhydrides;hydrolase activity, acting on acid anhydrides, in phosphorus-conta
binding;GAF domain binding;molecular transducer activity;protein binding;protein binding transcription factor activity;protein domain specific binding;signal transducer activity;transcription coactivator activity;transcription co
binding;nucleic acid binding;RNA binding;tRNA binding
binding;catalytic activity;DNA polymerase activity;intramolecular transferase activity;isomerase activity;nucleic acid binding;nucleotidyltransferase activity;pseudouridine synthase activity;RNA binding;RNA-directed DNA poly
acid-amino acid ligase activity;catalytic activity;ligase activity;ligase activity, forming carbon-nitrogen bonds;small conjugating protein ligase activity;UFM1 conjugating enzyme activity
6-phosphogluconolactonase activity;binding;carboxylic ester hydrolase activity;catalytic activity;coenzyme binding;cofactor binding;glucose 1-dehydrogenase [NAD(P)] activity;glucose-6-phosphate dehydrogenase activity;hy
binding;identical protein binding;protein binding
adenyl nucleotide binding;adenyl ribonucleotide binding;ATP binding;binding;cation binding;chaperone binding;chromatin binding;copper ion binding;core promoter binding;core promoter sequence-specific DNA binding;dan
active transmembrane transporter activity;antigen binding;ATPase activator activity;ATPase activity;ATPase activity, coupled;ATPase activity, coupled to movement of substances;ATPase activity, coupled to transmembrane m
adenyl nucleotide binding;adenyl ribonucleotide binding;asparagine synthase (glutamine-hydrolyzing) activity;ATP binding;binding;carbon-nitrogen ligase activity, with glutamine as amido-N-donor;catalytic activity;cofactor bi
binding;carbohydrate binding;disaccharide binding;galactoside binding;lactose binding;molecular transducer activity;monosaccharide binding;signal transducer activity;sugar binding
adenyl nucleotide binding;adenyl ribonucleotide binding;ATP binding;binding;catalytic activity;enzyme binding;G-protein coupled receptor activity;growth factor binding;growth factor receptor binding;kinase activity;kinase bi
binding;cation binding;ion binding;metal ion binding;nucleic acid binding;RNA binding;snRNA binding;U2 snRNA binding
binding;catalytic activity;chromatin binding;damaged DNA binding;dinucleotide insertion or deletion binding;DNA binding;DNA insertion or deletion binding;DNA N-glycosylase activity;DNA polymerase binding;DNA polymera
binding;chromatin binding;DNA binding;nucleic acid binding
adenyl nucleotide binding;adenyl ribonucleotide binding;ATP binding;binding;calcium ion binding;catalytic activity;cation binding;diacylglycerol kinase activity;ion binding;kinase activity;lipid binding;metal ion binding;NAD+ ki
structural constituent of ribosome;structural molecule activity
adenyl nucleotide binding;adenyl ribonucleotide binding;ATP binding;binding;catalytic activity;cytidylate kinase activity;kinase activity;nucleobase-containing compound kinase activity;nucleoside diphosphate kinase activity;r
catalytic activity;dihydrolipoyllysine-residue succinyltransferase activity;S-acyltransferase activity;S-succinyltransferase activity;succinyltransferase activity;transferase activity;transferase activity, transferring acyl groups;trans
7S RNA binding;binding;endoplasmic reticulum signal peptide binding;nucleic acid binding;peptide binding;RNA binding;signal sequence binding
anion channel activity;anion transmembrane transporter activity;binding;channel activity;gated channel activity;ion channel activity;ion transmembrane transporter activity;nucleotide binding;passive transmembrane transporte
binding;catalytic activity;cation binding;coenzyme binding;cofactor binding;ion binding;isocitrate dehydrogenase (NADP+) activity;isocitrate dehydrogenase activity;magnesium ion binding;metal ion binding;NAD binding;nucle
4 iron, 4 sulfur cluster binding;binding;catalytic activity;cation binding;coenzyme binding;cofactor binding;FMN binding;ion binding;iron-sulfur cluster binding;metal cluster binding;metal ion binding;NAD binding;NADH dehydr
binding;calcium ion binding;catalytic activity;cation binding;endopeptidase activity;enzyme activator activity;enzyme regulator activity;hydrolase activity;ion binding;metal ion binding;metalloendopeptidase activity;metallopep
enzyme activator activity;enzyme regulator activity;GDP-dissociation inhibitor activity;GTPase activator activity;GTPase regulator activity;nucleoside-triphosphatase regulator activity;Rho GDP-dissociation inhibitor activity;sm
binding;enzyme binding;protein binding;structural constituent of cytoskeleton;structural molecule activity
binding;catalytic activity;cytoskeletal protein binding;enzyme binding;GDP binding;GTP binding;GTPase activity;GTPase binding;guanyl nucleotide binding;guanyl ribonucleotide binding;hydrolase activity;hydrolase activity, a
7S RNA binding;binding;catalytic activity;drug binding;endoplasmic reticulum signal peptide binding;GDP binding;GTP binding;GTPase activity;guanyl nucleotide binding;guanyl ribonucleotide binding;hydrolase activity;hydro
binding;catalytic activity;cytoskeletal protein binding;GDP binding;GTP binding;GTPase activity;guanyl nucleotide binding;guanyl ribonucleotide binding;hydrolase activity;hydrolase activity, acting on acid anhydrides;hydrolas
binding;catalytic activity;cytoskeletal protein binding;GDP binding;GTP binding;GTPase activity;guanyl nucleotide binding;guanyl ribonucleotide binding;hydrolase activity;hydrolase activity, acting on acid anhydrides;hydrolas
binding;identical protein binding;protein binding;SNARE binding;syntaxin binding;syntaxin-1 binding
binding;cation binding;DNA binding;ion binding;metal ion binding;nucleic acid binding;nucleic acid binding transcription factor activity;RNA binding;sequence-specific DNA binding transcription factor activity;single-stranded
structural constituent of ribosome;structural molecule activity
binding;nucleotide binding;protein binding;structural constituent of ribosome;structural molecule activity;translation initiation factor binding
binding;nucleic acid binding;RNA binding;structural constituent of ribosome;structural molecule activity
catalytic activity;enzyme regulator activity;hydrolase activity;hydrolase activity, acting on ester bonds;phosphatase activity;phosphatase regulator activity;phosphoprotein phosphatase activity;phosphoric ester hydrolase activ
adenyl nucleotide binding;adenyl ribonucleotide binding;ATP binding;binding;catalytic activity;heat shock protein binding;Hsp90 protein binding;kinase activity;nucleotide binding;phosphotransferase activity, alcohol group as
binding;catalytic activity;cis-trans isomerase activity;drug binding;FK506 binding;isomerase activity;macrolide binding;peptidyl-prolyl cis-trans isomerase activity;receptor activity
aminoacylase activity;binding;catalytic activity;cation binding;hydrolase activity;hydrolase activity, acting on carbon-nitrogen (but not peptide) bonds;hydrolase activity, acting on carbon-nitrogen (but not peptide) bonds, in lin
binding;catalytic activity;hydrolase activity;hydrolase activity, acting on ester bonds;non-membrane spanning protein tyrosine phosphatase activity;phosphatase activity;phosphoprotein phosphatase activity;phosphoric ester
binding;calcium ion binding;calcium-dependent protein serine/threonine phosphatase activity;calmodulin binding;calmodulin-dependent protein phosphatase activity;catalytic activity;cation binding;drug binding;enzyme bind
[acetyl-CoA carboxylase] kinase activity;[hydroxymethylglutaryl-CoA reductase (NADPH)] kinase activity;adenyl nucleotide binding;adenyl ribonucleotide binding;AMP-activated protein kinase activity;ATP binding;binding;cAM
adenyl nucleotide binding;adenyl ribonucleotide binding;ATP binding;binding;catalytic activity;kinase activity;nucleotide binding;phosphotransferase activity, alcohol group as acceptor;protein kinase activity;protein serine/thre
binding;hormone receptor binding;Notch binding;nuclear hormone receptor binding;protein binding;protein binding transcription factor activity;receptor binding;retinoic acid receptor binding;SMAD binding;transcription coact
binding;carbohydrate binding;catalytic activity;chondroitin sulfate binding;glycosaminoglycan binding;hydrolase activity;hydrolase activity, acting on carbon-nitrogen (but not peptide) bonds;pattern binding;polysaccharide bir
binding;nucleic acid binding;nucleotide binding;RNA binding;translation factor activity, nucleic acid binding;translation initiation factor activity
acid-amino acid ligase activity;catalytic activity;ligase activity;ligase activity, forming carbon-nitrogen bonds;small conjugating protein ligase activity;ubiquitin-protein ligase activity
actin binding;actin monomer binding;adenyl nucleotide binding;adenyl ribonucleotide binding;ATP binding;binding;cytoskeletal protein binding;enzyme binding;kinase binding;lipid binding;nucleotide binding;phosphatidylinos
binding;chromatin binding;DNA binding;nucleic acid binding
aminopeptidase activity;binding;catalytic activity;exopeptidase activity;hydrolase activity;identical protein binding;peptidase activity;peptidase activity, acting on L-amino acid peptides;protein binding;serine hydrolase activity
binding;chromatin binding;DNA binding;nucleic acid binding
binding;cation binding;cytoskeletal protein binding;DNA binding;enzyme binding;histone binding;histone deacetylase binding;ion binding;metal ion binding;nucleic acid binding;phosphoprotein binding;protein binding;ribonuc
aldehyde dehydrogenase (NAD) activity;catalytic activity;oxidoreductase activity;oxidoreductase activity, acting on the aldehyde or oxo group of donors;oxidoreductase activity, acting on the aldehyde or oxo group of donors,
binding;chromatin binding;DNA binding;nucleic acid binding;protein binding transcription factor activity;transcription coactivator activity;transcription cofactor activity;transcription factor binding transcription factor activity
binding;cation binding;DNA binding;ion binding;metal ion binding;nucleic acid binding;nucleotide binding;transition metal ion binding;zinc ion binding
binding;nucleic acid binding
binding;calcium ion binding;cation binding;ion binding;metal ion binding
binding;catalytic activity;GTP binding;GTPase activity;guanyl nucleotide binding;guanyl ribonucleotide binding;hydrolase activity;hydrolase activity, acting on acid anhydrides;hydrolase activity, acting on acid anhydrides, in pl
binding;nucleotide binding
binding;cation binding;ion binding;metal ion binding;miRNA binding;mRNA 3'-UTR binding;mRNA binding;nucleic acid binding;nucleotide binding;RNA binding;transition metal ion binding;zinc ion binding
binding;catalytic activity;cation binding;endopeptidase activity;hydrolase activity;ion binding;metal ion binding;metalloendopeptidase activity;metallopeptidase activity;peptidase activity;peptidase activity, acting on L-amino a
adenyl nucleotide binding;adenyl ribonucleotide binding;ATP binding;ATPase activity;ATPase activity, coupled;ATP-dependent helicase activity;ATP-dependent RNA helicase activity;binding;catalytic activity;core promoter bin
amine-lyase activity;arylesterase activity;carbon-nitrogen lyase activity;carboxylic ester hydrolase activity;catalytic activity;hydrolase activity;hydrolase activity, acting on ester bonds;lyase activity;strictosidine synthase activity
binding;enzyme binding;identical protein binding;kinase binding;protein binding;protein kinase B binding;protein kinase binding

adenyl nucleotide binding;adenyl ribonucleotide binding;ATP binding;ATPase activity;ATPase activity, coupled;ATP-dependent helicase activity;ATP-dependent RNA helicase activity;binding;catalytic activity;helicase activity;h
transporter activity
binding;cytoskeletal protein binding;enzyme binding;GTPase binding;GTP-dependent protein binding;kinesin binding;protein binding;protein transporter activity;Rab GTPase binding;Ras GTPase binding;small GTPase bindin
binding;catalytic activity;cation binding;endopeptidase activity;epidermal growth factor binding;growth factor binding;hormone binding;hydrolase activity;ion binding;metal ion binding;metalloendopeptidase activity;metallope
binding;chaperone binding;protein binding
binding;chromatin binding;chromatin DNA binding;core promoter binding;core promoter sequence-specific DNA binding;DNA binding;double-stranded DNA binding;double-stranded methylated DNA binding;enzyme binding;
catalytic activity;citrate (Si)-synthase activity;transferase activity;transferase activity, transferring acyl groups;transferase activity, transferring acyl groups, acyl groups converted into alkyl on transfer
binding;chromatin binding;DNA binding;nucleic acid binding;RNA binding
enzyme regulator activity;kinase regulator activity;molecular transducer activity;phosphorylase kinase regulator activity;protein kinase regulator activity;signal transducer activity
binding;calcium ion binding;catalytic activity;cation binding;endopeptidase activity;hydrolase activity;ion binding;metal ion binding;metalloendopeptidase activity;metallopeptidase activity;peptidase activity;peptidase activity,
binding;calcium ion binding;calcium-dependent cysteine-type endopeptidase activity;catalytic activity;cation binding;cysteine-type endopeptidase activity;cysteine-type peptidase activity;endopeptidase activity;hydrolase ac
binding;enzyme binding;enzyme inhibitor activity;enzyme regulator activity;identical protein binding;kinase binding;kinase inhibitor activity;kinase regulator activity;protein binding;protein kinase binding;protein kinase C bindin
binding;enzyme activator activity;enzyme regulator activity;lipid binding
binding;catalytic activity;cation binding;dimethylallyltranstransferase activity;geranyltranstransferase activity;ion binding;metal ion binding;prenyltransferase activity;transferase activity;transferase activity, transferring alkyl or a
antioxidant activity;binding;catalytic activity;cytochrome-b5 reductase activity;identical protein binding;NAD(P)H dehydrogenase (quinone) activity;oxidoreductase activity;oxidoreductase activity, acting on NADH or NADPH;o
adenyl nucleotide binding;adenyl ribonucleotide binding;ATP binding;binding;calcium-dependent protein kinase activity;calcium-dependent protein kinase C activity;calcium-dependent protein serine/threonine kinase activity;
binding;catalytic activity;cation binding;enzyme binding;ephrin receptor binding;hydrolase activity;hydrolase activity, acting on ester bonds;ion binding;kinase binding;metal ion binding;phosphatase activity;phosphoprotein ph
binding;DNA binding;nucleic acid binding;RNA binding
binding;carbohydrate binding;cation binding;extracellular matrix structural constituent;glycoprotein binding;glycosaminoglycan binding;growth factor binding;heparin binding;integrin binding;ion binding;metal ion binding;patt
cation transmembrane transporter activity;hydrogen ion transmembrane transporter activity;inorganic cation transmembrane transporter activity;ion transmembrane transporter activity;monovalent inorganic cation transmemb
catalytic activity;endopeptidase activity;hydrolase activity;peptidase activity;peptidase activity, acting on L-amino acid peptides;threonine-type endopeptidase activity;threonine-type peptidase activity
binding;nucleic acid binding;nucleotide binding;RNA binding
catalytic activity;farnesyl-diphosphate farnesyltransferase activity;farnesyltranstransferase activity;oxidoreductase activity;prenyltransferase activity;squalene synthase activity;transferase activity;transferase activity, transferri
binding;protein binding;protein complex binding;protein domain specific binding
catalytic activity;dolichyl-diphosphooligosaccharide-protein glycotransferase activity;oligosaccharyl transferase activity;transferase activity;transferase activity, transferring glycosyl groups;transferase activity, transferring hex
catalytic activity;intramolecular transferase activity;isomerase activity;lanosterol synthase activity;oxidosqualene cyclase activity
binding;lipid binding
carnitine O-acyltransferase activity;carnitine O-palmitoyltransferase activity;catalytic activity;O-acyltransferase activity;O-palmitoyltransferase activity;palmitoyltransferase activity;transferase activity;transferase activity, transf
binding;caspase inhibitor activity;caspase regulator activity;cysteine-type endopeptidase inhibitor activity;endopeptidase inhibitor activity;endopeptidase regulator activity;enzyme binding;enzyme inhibitor activity;enzyme reg
aminopeptidase activity;binding;catalytic activity;cation binding;exopeptidase activity;hydrolase activity;ion binding;metal ion binding;metalloaminopeptidase activity;metalloexopeptidase activity;metallopeptidase activity;pep
adenyl nucleotide binding;adenyl ribonucleotide binding;ATP binding;binding;carbohydrate binding;carbohydrate kinase activity;catalytic activity;galactokinase activity;galactose binding;kinase activity;monosaccharide bindin
nucleocytoplasmic transporter activity;structural constituent of nuclear pore;structural molecule activity;transporter activity
binding;nucleic acid binding;protein binding;protein N-terminus binding;ribonucleoprotein binding;ribosome binding;RNA binding;snRNA binding;translation elongation factor activity;translation factor activity, nucleic acid binc
binding;core promoter proximal region DNA binding;core promoter proximal region sequence-specific DNA binding;DNA binding;double-stranded DNA binding;enzyme binding;GTPase binding;nucleic acid binding;nucleic ac
binding;DNA binding;nucleic acid binding
active transmembrane transporter activity;anion transmembrane transporter activity;anion:anion antiporter activity;antiporter activity;C4-dicarboxylate transmembrane transporter activity;carboxylic acid transmembrane trans
cargo receptor activity;receptor activity;scavenger receptor activity
binding;catalytic activity;cation binding;endopeptidase activity;hydrolase activity;ion binding;metal ion binding;metalloendopeptidase activity;metallopeptidase activity;peptidase activity;peptidase activity, acting on L-amino a
adenyl nucleotide binding;adenyl ribonucleotide binding;ATP binding;ATPase activity;ATPase activity, coupled;ATP-dependent DNA helicase activity;ATP-dependent helicase activity;binding;catalytic activity;cation binding;DN
binding;carbohydrate binding;cation binding;glycoprotein binding;heat shock protein binding;ion binding;mannose binding;metal ion binding;monosaccharide binding;protein binding;sugar binding
binding;nucleic acid binding;nucleotide binding;pre-mRNA binding;RNA binding
binding;enzyme binding;GTPase binding;protein binding
binding;POZ domain binding;protein binding;protein domain specific binding
catalytic activity;glucosidase activity;hydrolase activity;hydrolase activity, acting on glycosyl bonds;hydrolase activity, hydrolyzing O-glycosyl compounds;mannosyl-oligosaccharide glucosidase activity
acid-amino acid ligase activity;binding;catalytic activity;hormone receptor binding;ligase activity;ligase activity, forming carbon-nitrogen bonds;nuclear hormone receptor binding;protein binding;receptor binding;small conjuga
binding;cytoskeletal protein binding;microtubule binding;microtubule plus-end binding;protein binding;protein C-terminus binding;tubulin binding
3-beta-hydroxy-delta5-steroid dehydrogenase activity;catalytic activity;oxidoreductase activity;oxidoreductase activity, acting on CH-OH group of donors;oxidoreductase activity, acting on the CH-OH group of donors, NAD o
ATPase activator activity;ATPase binding;ATPase regulator activity;binding;cytoskeletal protein binding;enzyme activator activity;enzyme binding;enzyme regulator activity;nucleoside-triphosphatase regulator activity;protein b
binding;DNA binding;histone binding;nucleic acid binding;protein binding
binding;cytoskeletal protein binding;kinetochore binding;microtubule binding;microtubule plus-end binding;protein binding;tubulin binding
binding;nucleotide binding
adenyl nucleotide binding;adenyl ribonucleotide binding;ATP binding;binding;catalytic activity;kinase activity;nucleotide binding;phosphotransferase activity, alcohol group as acceptor;protein kinase activity;protein serine/thre
catalytic activity;dolichyl-diphosphooligosaccharide-protein glycotransferase activity;oligosaccharyl transferase activity;transferase activity;transferase activity, transferring glycosyl groups;transferase activity, transferring hex
binding;DNA binding;nucleic acid binding
binding;carboxypeptidase activity;catalytic activity;cation binding;dipeptidase activity;exopeptidase activity;hydrolase activity;ion binding;metal ion binding;metallopeptidase activity;peptidase activity;peptidase activity, acting
binding;fibroblast growth factor binding;growth factor binding;protein binding
binding;molecular transducer activity;protein binding;protein complex binding;signal transducer activity
adenyl nucleotide binding;adenyl ribonucleotide binding;ATP binding;binding;calcium ion binding;cation binding;GTP binding;guanyl nucleotide binding;guanyl ribonucleotide binding;ion binding;metal ion binding;nucleic acid
structural molecule activity
binding;carbohydrate binding;collagen binding;protein binding
binding;cation binding;heat shock protein binding;Hsp90 protein binding;ion binding;metal ion binding;protein binding;transition metal ion binding;zinc ion binding
catalytic activity;cysteine-type endopeptidase activity;cysteine-type peptidase activity;endopeptidase activity;hydrolase activity;peptidase activity;peptidase activity, acting on L-amino acid peptides;small conjugating protein-
anion channel activity;anion transmembrane transporter activity;binding;channel activity;gated channel activity;ion channel activity;ion transmembrane transporter activity;nucleotide binding;passive transmembrane transport
binding;catalytic activity;enzyme activator activity;enzyme binding;enzyme regulator activity;kinase activator activity;kinase activity;kinase binding;kinase regulator activity;phosphotransferase activity, alcohol group as accept
binding;cytoskeletal protein binding;microtubule binding;nucleic acid binding;protein binding;ribonucleoprotein binding;ribosome binding;RNA binding;rRNA binding;tubulin binding
anion channel activity;anion transmembrane transporter activity;channel activity;chloride channel activity;gated channel activity;ion channel activity;ion transmembrane transporter activity;passive transmembrane transporter
arginine N-methyltransferase activity;binding;catalytic activity;chromatin binding;core promoter binding;core promoter sequence-specific DNA binding;DNA binding;histone methyltransferase activity;histone-arginine N-methy
binding;enzyme binding;protease binding;protein binding
catalytic activity;glutaminase activity;hydrolase activity;hydrolase activity, acting on carbon-nitrogen (but not peptide) bonds;hydrolase activity, acting on carbon-nitrogen (but not peptide) bonds, in linear amides
binding;calcium-dependent protein binding;chemokine binding;C-X-C chemokine binding;cytokine binding;endopeptidase inhibitor activity;endopeptidase regulator activity;enzyme binding;enzyme inhibitor activity;enzyme re
binding;catalytic activity;cell adhesion molecule binding;cytokine receptor binding;enzyme binding;extracellular matrix binding;fibronectin binding;growth factor receptor binding;identical protein binding;intramolecular oxidore
binding;integrin binding;molecular transducer activity;protein binding;protein complex binding;receptor activity;receptor binding;signal transducer activity;signaling receptor activity;transmembrane signaling receptor activity;v
binding;catalytic activity;cation binding;endopeptidase activity;exopeptidase activity;hydrolase activity;ion binding;metal ion binding;metalloendopeptidase activity;metallopeptidase activity;peptidase activity;peptidase activit
binding;clathrin binding;clathrin heavy chain binding;peptide binding;protein binding;structural molecule activity
carbon-carbon lyase activity;carboxy-lyase activity;catalytic activity;lyase activity;orotate phosphoribosyltransferase activity;orotidine-5'-phosphate decarboxylase activity;transferase activity;transferase activity, transferring g
actin binding;actin filament binding;binding;calcium ion binding;cation binding;cytoskeletal protein binding;enzyme binding;GTPase binding;ion binding;metal ion binding;protein binding
adenyl nucleotide binding;adenyl ribonucleotide binding;AMP binding;binding;cAMP binding;cAMP-dependent protein kinase inhibitor activity;cAMP-dependent protein kinase regulator activity;cyclic nucleotide binding;enzym
2 iron, 2 sulfur cluster binding;3 iron, 4 sulfur cluster binding;4 iron, 4 sulfur cluster binding;binding;catalytic activity;cation binding;cofactor binding;electron carrier activity;ion binding;iron-sulfur cluster binding;metal cluster b
adenyl nucleotide binding;adenyl ribonucleotide binding;ATP binding;binding;C3HC4-type RING finger domain binding;cation binding;chaperone binding;enzyme binding;G-protein-coupled receptor binding;heat shock protei
binding;cation binding;DNA binding;identical protein binding;ion binding;metal ion binding;nucleic acid binding;nucleotide binding;protein binding;protein binding transcription factor activity;RNA binding;transcription coactiva
adenyl nucleotide binding;adenyl ribonucleotide binding;ATP binding;ATPase activity;binding;catalytic activity;hydrolase activity;hydrolase activity, acting on acid anhydrides;hydrolase activity, acting on acid anhydrides, in ph
nucleic acid binding transcription factor activity;RNA polymerase II core promoter proximal region sequence-specific DNA binding transcription factor activity;RNA polymerase II core promoter proximal region sequence-spec
binding;catalytic activity;GTP binding;GTPase activity;guanyl nucleotide binding;guanyl ribonucleotide binding;hydrolase activity;hydrolase activity, acting on acid anhydrides;hydrolase activity, acting on acid anhydrides, in p
binding;catalytic activity;cytoskeletal protein binding;GTP binding;GTPase activity;guanyl nucleotide binding;guanyl ribonucleotide binding;hydrolase activity;hydrolase activity, acting on acid anhydrides;hydrolase activity, act
binding;cation binding;ion binding;metal ion binding;structural constituent of ribosome;structural molecule activity
binding;DNA binding;nucleic acid binding;protein binding;protein binding transcription factor activity;protein domain specific binding;regulatory region DNA binding;regulatory region nucleic acid binding;transcription cofactor
binding;binding, bridging;DNA binding;identical protein binding;mRNA binding;nucleic acid binding;poly(A) RNA binding;poly(U) RNA binding;poly-purine tract binding;poly-pyrimidine tract binding;protein binding;protein bind
binding;binding, bridging;enzyme activator activity;enzyme regulator activity;GTPase activator activity;GTPase regulator activity;nucleoside-triphosphatase regulator activity;protein binding;protein binding, bridging;SH3/SH2
nucleocytoplasmic transporter activity;transporter activity
catalytic activity;hydrolase activity;hydrolase activity, acting on acid anhydrides;hydrolase activity, acting on acid anhydrides, in phosphorus-containing anhydrides;microtubule motor activity;motor activity;nucleoside-triphos
binding;cation binding;ion binding;metal ion binding;transition metal ion binding;zinc ion binding
binding;protein binding;SNARE binding;syntaxin binding;syntaxin-3 binding
2,4-dienoyl-CoA reductase (NADPH) activity;binding;catalytic activity;coenzyme binding;cofactor binding;NADP binding;NADPH binding;nucleotide binding;oxidoreductase activity;oxidoreductase activity, acting on NADH or
binding;catalytic activity;GTP binding;GTPase activity;guanyl nucleotide binding;guanyl ribonucleotide binding;hydrolase activity;hydrolase activity, acting on acid anhydrides;hydrolase activity, acting on acid anhydrides, in p
binding;cation binding;ion binding;metal ion binding;mRNA binding;nucleic acid binding;poly(A) RNA binding;poly-purine tract binding;RNA binding;single-stranded RNA binding
binding;chromatin binding
ARF guanyl-nucleotide exchange factor activity;binding;enzyme regulator activity;GTPase regulator activity;guanyl-nucleotide exchange factor activity;lipid binding;nucleoside-triphosphatase regulator activity;phosphatidylino
binding;cation binding;ion binding;metal ion binding;transition metal ion binding;zinc ion binding
structural constituent of ribosome;structural molecule activity
catalytic activity;intramolecular oxidoreductase activity;intramolecular oxidoreductase activity, interconverting keto- and enol-groups;intramolecular oxidoreductase activity, transposing S-S bonds;isomerase activity;protein d
binding;catalytic activity;GTP binding;GTPase activity;guanyl nucleotide binding;guanyl ribonucleotide binding;hydrolase activity;hydrolase activity, acting on acid anhydrides;hydrolase activity, acting on acid anhydrides, in p
binding;catalytic activity;cation binding;hydrolase activity;hydrolase activity, acting on acid anhydrides;hydrolase activity, acting on acid anhydrides, in phosphorus-containing anhydrides;hydrolase activity, acting on ester bor
binding;nucleic acid binding;RNA binding;rRNA binding
binding;enzyme binding;phosphatase binding;protein binding;protein complex scaffold;structural molecule activity
acid-amino acid ligase activity;binding;catalytic activity;cation binding;ion binding;ligase activity;ligase activity, forming carbon-nitrogen bonds;metal ion binding;small conjugating protein ligase activity;ubiquitin-protein ligase
binding;carbohydrate binding;cargo receptor activity;catalytic activity;cation binding;chromatin binding;copper ion binding;electron carrier activity;histone binding;ion binding;metal ion binding;methylated histone residue binc
binding;enzyme binding;kinase binding;phosphoprotein binding;protein binding;protein kinase binding;translation initiation factor binding
binding;mRNA 3'-UTR binding;mRNA 5'-UTR binding;mRNA binding;nucleic acid binding;nucleotide binding;RNA binding;translation regulator activity
binding;mRNA 3'-UTR binding;mRNA 5'-UTR binding;mRNA binding;nucleic acid binding;nucleotide binding;RNA binding;translation regulator activity
binding;catalytic activity;cation binding;hydrolase activity;hydrolase activity, acting on ester bonds;ion binding;metal ion binding;phosphatase activity;phosphoprotein phosphatase activity;phosphoric ester hydrolase activity;p
adenyl nucleotide binding;adenyl ribonucleotide binding;ATP binding;binding;catalytic activity;hydrolase activity;hydrolase activity, acting on acid anhydrides;hydrolase activity, acting on acid anhydrides, in phosphorus-conta

binding;identical protein binding;protein binding
catalytic activity;hydrolase activity
actin binding;actin filament binding;actin-dependent ATPase activity;adenyl nucleotide binding;adenyl ribonucleotide binding;ATP binding;ATPase activity;ATPase activity, coupled;binding;calmodulin binding;catalytic activity;
binding;calcium ion binding;cation binding;ion binding;metal ion binding
antigen binding;beta-2-microglobulin binding;binding;peptide antigen binding;peptide binding;protein binding;TAP binding
adenyl nucleotide binding;adenyl ribonucleotide binding;ATP binding;binding;catalytic activity;cyclin-dependent protein kinase activity;histone kinase activity;kinase activity;nucleotide binding;phosphotransferase activity, alco
binding;catalytic activity;coenzyme binding;cofactor binding;electron carrier activity;flavin adenine dinucleotide binding;oxidoreductase activity
binding;lipid binding;oxysterol binding;protein binding;protein domain specific binding;steroid binding;sterol binding
adenylosuccinate synthase activity;anion binding;binding;catalytic activity;cation binding;GTP binding;guanyl nucleotide binding;guanyl ribonucleotide binding;ion binding;ligase activity;ligase activity, forming carbon-nitrogen
binding;carbohydrate binding;cytoskeletal protein binding;dynactin binding;dynein binding;enzyme binding;glycosaminoglycan binding;heparin binding;identical protein binding;microtubule binding;pattern binding;phospholip
binding;carbohydrate binding;cation binding;ion binding;mannose binding;metal ion binding;monosaccharide binding;protein binding;sugar binding;unfolded protein binding
binding;catalytic activity;cation binding;coenzyme binding;cofactor binding;ion binding;isocitrate dehydrogenase (NAD+) activity;isocitrate dehydrogenase activity;magnesium ion binding;metal ion binding;NAD binding;nucle
biliverdin reductase activity;binding;catalytic activity;cation binding;ion binding;metal ion binding;oxidoreductase activity;oxidoreductase activity, acting on the CH-CH group of donors;oxidoreductase activity, acting on the C
binding;cation binding;ion binding;metal ion binding;transition metal ion binding;zinc ion binding
structural constituent of ribosome;structural molecule activity
binding;catalytic activity;demethylase activity;DNA binding;histone binding;histone demethylase activity;histone demethylase activity (H4-K20 specific);nucleic acid binding;protein binding
antioxidant activity;catalytic activity;disulfide oxidoreductase activity;glutathione dehydrogenase (ascorbate) activity;glutathione disulfide oxidoreductase activity;glutathione transferase activity;methylarsonate reductase activ
structural constituent of ribosome;structural molecule activity
binding;chromatin binding;core promoter proximal region DNA binding;core promoter proximal region sequence-specific DNA binding;DNA binding;nucleic acid binding;nucleic acid binding transcription factor activity;protein
binding;carbohydrate binding;catalytic activity;cation binding;coenzyme binding;cofactor binding;ion binding;L-iditol 2-dehydrogenase activity;metal ion binding;NAD binding;nucleotide binding;oxidoreductase activity;oxidor
binding;nucleic acid binding;RNA binding;structural constituent of ribosome;structural molecule activity
binding;identical protein binding;protein binding
enzyme inhibitor activity;enzyme regulator activity;kinase inhibitor activity;kinase regulator activity;protein kinase inhibitor activity;protein kinase regulator activity
binding;enzyme binding;protein binding;ubiquitin protein ligase binding
binding;DNA binding;enzyme binding;nucleic acid binding;protein binding;RNA binding;ubiquitin protein ligase binding
binding;catalytic activity;cytoskeletal protein binding;GDP binding;GTP binding;GTPase activity;guanyl nucleotide binding;guanyl ribonucleotide binding;hydrolase activity;hydrolase activity, acting on acid anhydrides;hydrolas
basal RNA polymerase II transcription machinery binding;basal transcription machinery binding;binding;cation binding;DNA binding;enzyme binding;ion binding;metal ion binding;nucleic acid binding;protein binding;RNA poly
adenyl nucleotide binding;adenyl ribonucleotide binding;aminoacyl-tRNA ligase activity;aspartate-tRNA ligase activity;aspartate-tRNA(Asn) ligase activity;ATP binding;binding;catalytic activity;identical protein binding;ligase a
binding;double-stranded RNA binding;NF-kappaB binding;nucleic acid binding;protein binding;protein binding transcription factor activity;RNA binding;RNA polymerase II transcription factor binding;transcription coactivator
binding;protein binding;protein domain specific binding;WW domain binding
binding;nuclear localization sequence binding;peptide binding;signal sequence binding
binding;calcium ion binding;cation binding;ion binding;metal ion binding
binding;cation binding;DNA binding;ion binding;metal ion binding;nucleic acid binding;transition metal ion binding;zinc ion binding
binding;cation binding;ion binding;lipid binding;metal ion binding;protein transporter activity;substrate-specific transporter activity;transition metal ion binding;transporter activity;zinc ion binding
binding;protein binding;unfolded protein binding
acetyl-CoA C-acetyltransferase activity;acetyl-CoA C-acyltransferase activity;acetyltransferase activity;C-acetyltransferase activity;C-acyltransferase activity;catalytic activity;transferase activity;transferase activity, transferrin
binding;catalytic activity;cytoskeletal protein binding;hydrolase activity;hydrolase activity, acting on acid anhydrides;hydrolase activity, acting on acid anhydrides, in phosphorus-containing anhydrides;kinesin binding;microtu
adenyl nucleotide binding;adenyl ribonucleotide binding;ATP binding;binding;calcium ion binding;cation binding;GTP binding;guanyl nucleotide binding;guanyl ribonucleotide binding;ion binding;metal ion binding;nucleic acid
ion transmembrane transporter activity;substrate-specific transmembrane transporter activity;substrate-specific transporter activity;transmembrane transporter activity;transporter activity
adenyl nucleotide binding;adenyl ribonucleotide binding;ATP binding;binding;catalytic activity;helicase activity;hydrolase activity;hydrolase activity, acting on acid anhydrides;hydrolase activity, acting on acid anhydrides, in p
catalytic activity;cytidylyltransferase activity;N-acetylneuraminate synthase activity;N-acylneuraminate cytidylyltransferase activity;N-acylneuraminate-9-phosphate synthase activity;nucleotidyltransferase activity;transferase
adenyl nucleotide binding;adenyl ribonucleotide binding;ATP binding;ATPase activity;binding;catalytic activity;cation binding;GTP binding;guanyl nucleotide binding;guanyl ribonucleotide binding;hydrolase activity;hydrolase
binding;nucleotide binding
binding;nucleotide binding
binding;nuclear export signal receptor activity;protein binding
actin binding;actin filament binding;binding;cytoskeletal protein binding;enzyme activator activity;enzyme regulator activity;protein binding
binding;enzyme regulator activity;lipid binding;phosphatase regulator activity;protein binding;protein phosphatase regulator activity;protein phosphatase type 1 regulator activity;small conjugating protein binding;ubiquitin bin
binding;nucleic acid binding;RNA binding;translation factor activity, nucleic acid binding;translation initiation factor activity
3'-5' exonuclease activity;3'-5'-exoribonuclease activity;binding;catalytic activity;endonuclease activity;enzyme regulator activity;exonuclease activity;exonuclease activity, active with either ribo- or deoxyribonucleic acids an
adenyl nucleotide binding;adenyl ribonucleotide binding;ATP binding;binding;nucleotide binding;purine nucleotide binding;purine ribonucleoside triphosphate binding;purine ribonucleotide binding;ribonucleotide binding
acyl-CoA thioesterase activity;binding;carboxylic acid binding;carboxylic ester hydrolase activity;catalytic activity;CoA hydrolase activity;coenzyme binding;cofactor binding;fatty acid binding;fatty-acyl-CoA binding;hydrolase
binding;enzyme binding;GTP-dependent protein binding;phosphatase binding;protein binding;protein phosphatase binding
binding;binding, bridging;cytokine receptor binding;cytoskeletal adaptor activity;cytoskeletal protein binding;frizzled binding;G-protein-coupled receptor binding;growth factor receptor binding;identical protein binding;interleu
acetyltransferase activator activity;beta-tubulin binding;binding;carbohydrate binding;cytoskeletal protein binding;DNA binding;enzyme activator activity;enzyme regulator activity;glycosaminoglycan binding;heparin binding;
binding;DNA binding;double-stranded DNA binding;mRNA binding;nucleic acid binding;nucleotide binding;poly(A) RNA binding;poly(G) RNA binding;poly-purine tract binding;RNA binding;single-stranded DNA binding;single-
binding;nucleic acid binding;RNA binding;translation factor activity, nucleic acid binding;translation initiation factor activity
binding;calcium ion binding;cation binding;ion binding;metal ion binding
acid-amino acid ligase activity;binding;catalytic activity;cation binding;ion binding;ligase activity;ligase activity, forming carbon-nitrogen bonds;metal ion binding;nucleic acid binding;RNA binding;small conjugating protein lig
3'(2'),5'-bisphosphate nucleotidase activity;binding;catalytic activity;cation binding;hydrolase activity;hydrolase activity, acting on ester bonds;inositol bisphosphate phosphatase activity;inositol or phosphatidylinositol phosp
binding;nucleic acid binding;RNA binding;structural constituent of ribosome;structural molecule activity
catalytic activity;cis-stilbene-oxide hydrolase activity;epoxide hydrolase activity;ether hydrolase activity;hydrolase activity;hydrolase activity, acting on ether bonds
binding;catalytic activity;collagen binding;cysteine-type endopeptidase activity;cysteine-type peptidase activity;endopeptidase activity;glycoprotein binding;hydrolase activity;peptidase activity;peptidase activity, acting on L-
binding;catalytic activity;GTP binding;GTPase activity;guanyl nucleotide binding;guanyl ribonucleotide binding;hydrolase activity;hydrolase activity, acting on acid anhydrides;hydrolase activity, acting on acid anhydrides, in p
structural constituent of ribosome;structural molecule activity
binding;laminin receptor activity;receptor activity;ribonucleoprotein binding;ribosome binding;structural constituent of ribosome;structural molecule activity
carboxylic ester hydrolase activity;catalytic activity;deacetylase activity;hydrolase activity;hydrolase activity, acting on ester bonds;methylumbelliferyl-acetate deacetylase activity;S-formylglutathione hydrolase activity;thioles
acyl-CoA dehydrogenase activity;binding;catalytic activity;coenzyme binding;cofactor binding;flavin adenine dinucleotide binding;identical protein binding;medium-chain-acyl-CoA dehydrogenase activity;oxidoreductase acti
catalytic activity;endopeptidase activity;exopeptidase activity;hydrolase activity;omega peptidase activity;peptidase activity;peptidase activity, acting on L-amino acid peptides;serine hydrolase activity;serine-type endopeptic
acid-ammonia (or amide) ligase activity;adenyl nucleotide binding;adenyl ribonucleotide binding;amine binding;amino acid binding;ammonia ligase activity;ATP binding;binding;carbon-carbon lyase activity;carboxylic acid bin
receptor activity
binding;cation binding;ion binding;metal ion binding;nucleic acid binding;RNA binding;translation factor activity, nucleic acid binding;translation initiation factor activity
amine binding;amino acid binding;binding;carboxylic acid binding;catalytic activity;enzyme binding;glutathione binding;glutathione transferase activity;identical protein binding;modified amino acid binding;peptide binding;pro
binding;nucleic acid binding;RNA binding;rRNA binding;structural constituent of ribosome;structural molecule activity
catalytic activity;endopeptidase activity;hydrolase activity;peptidase activity;peptidase activity, acting on L-amino acid peptides;threonine-type endopeptidase activity;threonine-type peptidase activity
binding;cation binding;chromatin binding;damaged DNA binding;DNA binding;ion binding;metal ion binding;nucleic acid binding;single-stranded DNA binding;structure-specific DNA binding
antioxidant activity;catalytic activity;oxidoreductase activity;oxidoreductase activity, acting on paired donors, with incorporation or reduction of molecular oxygen;oxidoreductase activity, acting on peroxide as acceptor;perox
binding;nucleic acid binding;RNA binding;rRNA binding;structural constituent of ribosome;structural molecule activity
binding;endopeptidase inhibitor activity;endopeptidase regulator activity;enzyme binding;enzyme inhibitor activity;enzyme regulator activity;peptidase inhibitor activity;peptidase regulator activity;protease binding;protein bin
4-alpha-glucanotransferase activity;alpha-glucosidase activity;amylo-alpha-1,6-glucosidase activity;binding;carbohydrate binding;catalytic activity;glucosidase activity;glycogen debranching enzyme activity;hydrolase activity
nucleocytoplasmic transporter activity;transporter activity
electron carrier activity
catalytic activity;methyltransferase activity;nicotinamide N-methyltransferase activity;N-methyltransferase activity;S-adenosylmethionine-dependent methyltransferase activity;transferase activity;transferase activity, transferri
15-hydroxyprostaglandin-D dehydrogenase (NADP+) activity;alcohol dehydrogenase (NADP+) activity;alditol:NADP+ 1-oxidoreductase activity;aldo-keto reductase (NADP) activity;androsterone dehydrogenase activity;catalyt
binding;nucleic acid binding;RNA binding;structural constituent of ribosome;structural molecule activity
binding;nucleic acid binding;RNA binding;structural constituent of ribosome;structural molecule activity
adenylate cyclase activity;binding;catalytic activity;cation binding;cyclase activity;GTP binding;GTPase activity;guanyl nucleotide binding;guanyl ribonucleotide binding;hydrolase activity;hydrolase activity, acting on acid anhy
binding;catalytic activity;cation binding;G-protein beta/gamma-subunit complex binding;G-protein-coupled receptor binding;GTP binding;GTPase activity;guanyl nucleotide binding;guanyl ribonucleotide binding;hydrolase ac
binding;cation binding;identical protein binding;ion binding;metal ion binding;nucleotide binding;protein binding;protein complex binding;transition metal ion binding;zinc ion binding
binding;carboxylic acid binding;fatty acid binding;lipid binding;monocarboxylic acid binding;transporter activity
3'-5' exonuclease activity;binding;catalytic activity;exonuclease activity;exoribonuclease activity;hydrolase activity;hydrolase activity, acting on ester bonds;nuclease activity;nucleotide binding;ribonuclease activity
binding;calcium ion binding;calmodulin binding;catalytic activity;cation binding;hydrolase activity;hydrolase activity, acting on ester bonds;ion binding;lipase activity;metal ion binding;molecular transducer activity;phosphatid
binding;chromatin binding;chromatin DNA binding;DNA binding;nucleic acid binding;structure-specific DNA binding
adenyl nucleotide binding;adenyl ribonucleotide binding;ATP binding;binding;catalytic activity;enzyme activator activity;enzyme regulator activity;hydrolase activity;hydrolase activity, acting on ester bonds;kinase activator act
binding;binding, bridging;catalytic activity;hydrolase activity;hydrolase activity, acting on ester bonds;insulin receptor binding;non-membrane spanning protein tyrosine phosphatase activity;phosphatase activity;phosphoprot
enzyme inhibitor activity;enzyme regulator activity;GTPase inhibitor activity;GTPase regulator activity;nucleoside-triphosphatase regulator activity
molecular transducer activity;signal transducer activity
adenyl nucleotide binding;adenyl ribonucleotide binding;ATP binding;binding;catalytic activity;kinase activity;nucleotide binding;phosphotransferase activity, alcohol group as acceptor;protein kinase activity;protein tyrosine k
extracellular matrix structural constituent;structural molecule activity
actin binding;binding;cytoskeletal protein binding;profilin binding;protein binding
adenyl nucleotide binding;adenyl ribonucleotide binding;ATP binding;ATPase activity;ATPase activity, coupled;ATP-dependent helicase activity;ATP-dependent RNA helicase activity;binding;catalytic activity;helicase activity;h
binding;cation binding;enzyme binding;ion binding;metal ion binding;protein binding;small conjugating protein binding;transition metal ion binding;ubiquitin binding;ubiquitin protein ligase binding;zinc ion binding
actin binding;actin filament binding;binding;cytoskeletal protein binding;protein binding
acid-thiol ligase activity;adenyl nucleotide binding;adenyl ribonucleotide binding;ATP binding;binding;catalytic activity;CoA-ligase activity;GDP binding;GTP binding;guanyl nucleotide binding;guanyl ribonucleotide binding;lig
adenyl nucleotide binding;adenyl ribonucleotide binding;ATP binding;binding;DNA binding;nucleic acid binding;nucleotide binding;purine nucleotide binding;purine ribonucleoside triphosphate binding;purine ribonucleotide b
binding;G-protein coupled receptor activity;molecular transducer activity;opioid receptor activity;peptide binding;peptide receptor activity;peptide receptor activity, G-protein coupled;receptor activity;signal transducer activity
acetyl-CoA hydrolase activity;carboxylic ester hydrolase activity;catalytic activity;CoA hydrolase activity;hydrolase activity;hydrolase activity, acting on ester bonds;thiolester hydrolase activity
binding;ribonucleoprotein binding
binding;nucleic acid binding;RNA binding;snoRNA binding
activating transcription factor binding;binding;chromatin binding;DNA binding;nucleic acid binding;protein binding;protein binding transcription factor activity;RNA polymerase II transcription coactivator activity;RNA polymera
aspartic-type endopeptidase activity;aspartic-type peptidase activity;catalytic activity;endopeptidase activity;hydrolase activity;peptidase activity;peptidase activity, acting on L-amino acid peptides
beta-N-acetylhexosaminidase activity;binding;catalytic activity;hexosaminidase activity;hydrolase activity;hydrolase activity, acting on glycosyl bonds;hydrolase activity, hydrolyzing O-glycosyl compounds;identical protein bi
actin binding;actin monomer binding;adenyl nucleotide binding;adenyl-nucleotide exchange factor activity;ATPase regulator activity;binding;cytoskeletal protein binding;enzyme regulator activity;lipid binding;nucleoside-triph
binding;binding, bridging;cation binding;extracellular matrix structural constituent;growth factor binding;identical protein binding;ion binding;metal ion binding;platelet-derived growth factor binding;protein binding;protein bin
binding;cation binding;cytokine receptor binding;growth factor receptor binding;ion binding;metal ion binding;platelet-derived growth factor receptor binding;protein binding;receptor binding;vascular endothelial growth facto
alcohol dehydrogenase (NAD) activity;binding;carboxylic acid binding;catalytic activity;cation binding;electron carrier activity;fatty acid binding;formaldehyde dehydrogenase activity;ion binding;lipid binding;metal ion binding;
adenyl nucleotide binding;adenyl ribonucleotide binding;ADP binding;AMP binding;ATP binding;binding;carbohydrate binding;catalytic activity;cation binding;diphosphotransferase activity;GDP binding;guanyl nucleotide bin
binding;nucleic acid binding;RNA binding;structural constituent of ribosome;structural molecule activity;tRNA binding
binding;catalytic activity;cation binding;endopeptidase activity;hydrolase activity;ion binding;metal ion binding;metalloendopeptidase activity;metallopeptidase activity;peptidase activity;peptidase activity, acting on L-amino a
3'-5' exonuclease activity;3'-5'-exodeoxyribonuclease activity;binding;carbon-oxygen lyase activity;catalytic activity;cation binding;chromatin binding;chromatin DNA binding;damaged DNA binding;deoxyribonuclease activit
adenyl nucleotide binding;adenyl ribonucleotide binding;ATP binding;binding;catalytic activity;DNA binding;enzyme binding;kinase activity;MAP kinase activity;molecular transducer activity;nucleic acid binding;nucleotide bin
(S)-2-(5-amino-1-(5-phospho-D-ribosyl)imidazole-4-carboxamido)succinate AMP-lyase (fumarate-forming) activity;amidine-lyase activity;carbon-nitrogen lyase activity;catalytic activity;lyase activity;N6-(1,2-dicarboxyethyl)AM
aldehyde dehydrogenase (NAD) activity;catalytic activity;oxidoreductase activity;oxidoreductase activity, acting on the aldehyde or oxo group of donors;oxidoreductase activity, acting on the aldehyde or oxo group of donors,

binding;calcium ion binding;carbohydrate binding;cation binding;glycosaminoglycan binding;heparin binding;ion binding;metal ion binding;pattern binding;polysaccharide binding
adenyl nucleotide binding;adenyl ribonucleotide binding;ATP binding;binding;catalytic activity;helicase activity;hydrolase activity;hydrolase activity, acting on acid anhydrides;hydrolase activity, acting on acid anhydrides, in ph
acyl-CoA dehydrogenase activity;binding;catalytic activity;coenzyme binding;cofactor binding;flavin adenine dinucleotide binding;long-chain-acyl-CoA dehydrogenase activity;oxidoreductase activity;oxidoreductase activity, a
adenyl nucleotide binding;adenyl ribonucleotide binding;ATP binding;binding;caspase inhibitor activity;caspase regulator activity;catalytic activity;cation binding;cysteine-type endopeptidase inhibitor activity;endopeptidase in
adenyl nucleotide binding;adenyl ribonucleotide binding;adenylate kinase activity;ATP binding;binding;catalytic activity;kinase activity;nucleobase-containing compound kinase activity;nucleotide binding;nucleotide kinase ac
structural constituent of ribosome;structural molecule activity
binding;nucleic acid binding;RNA binding;structural constituent of ribosome;structural molecule activity
structural constituent of ribosome;structural molecule activity
binding;mRNA binding;nucleic acid binding;nucleotide binding;RNA binding
active transmembrane transporter activity;anion transmembrane transporter activity;binding;inorganic anion transmembrane transporter activity;ion transmembrane transporter activity;phosphate ion carrier activity;phosphate
ATPase activity;catalytic activity;hydrolase activity;hydrolase activity, acting on acid anhydrides;hydrolase activity, acting on acid anhydrides, in phosphorus-containing anhydrides;nucleoside-triphosphatase activity;pyrophos
binding;enzyme regulator activity;GTP binding;guanyl nucleotide binding;guanyl ribonucleotide binding;nucleotide binding;purine nucleotide binding;purine ribonucleoside triphosphate binding;purine ribonucleotide binding;ri
binding;enzyme activator activity;enzyme binding;enzyme regulator activity;GTPase activator activity;GTPase binding;GTPase regulator activity;nucleoside-triphosphatase regulator activity;protein binding;Rab GTPase binding
binding;DNA binding;nucleic acid binding;RNA binding;single-stranded DNA binding;structure-specific DNA binding
AU-rich element binding;binding;double-stranded RNA binding;enzyme binding;kinase binding;mRNA 3'-UTR AU-rich region binding;mRNA 3'-UTR binding;mRNA binding;nucleic acid binding;nucleotide binding;protein bind
structural molecule activity
adenine nucleotide transmembrane transporter activity;ATP transmembrane transporter activity;binding;calcium ion binding;cation binding;ion binding;metal ion binding;nucleobase-containing compound transmembrane tran
adenyl nucleotide binding;adenyl ribonucleotide binding;ATP binding;binding;catalytic activity;formate-tetrahydrofolate ligase activity;identical protein binding;ligase activity;ligase activity, forming carbon-nitrogen bonds;nucle
binding;enzyme binding;GTPase binding;leucine zipper domain binding;LRR domain binding;PDZ domain binding;protein binding;protein domain specific binding;Rab GTPase binding;Ras GTPase binding;small GTPase bind
3'-5' exonuclease activity;3'-5'-exoribonuclease activity;binding;catalytic activity;exonuclease activity;exonuclease activity, active with either ribo- or deoxyribonucleic acids and producing 5'-phosphomonoesters;exoribonucl
nucleocytoplasmic transporter activity;transporter activity
actin binding;binding;cytoskeletal protein binding;protein binding
binding;mRNA binding;nucleic acid binding;nucleotide binding;RNA binding
binding;enzyme activator activity;enzyme binding;enzyme regulator activity;GTPase activator activity;GTPase binding;GTPase regulator activity;nucleoside-triphosphatase regulator activity;protein binding;protein dimerization
binding;enzyme binding;GTPase binding;mRNA binding;nucleic acid binding;pre-miRNA binding;protein binding;protein transporter activity;Ran GTPase binding;Ras GTPase binding;RNA binding;small GTPase binding;subst
binding;identical protein binding;protein binding;protein dimerization activity;protein homodimerization activity
acid-thiol ligase activity;adenyl nucleotide binding;adenyl ribonucleotide binding;ATP binding;binding;catalytic activity;cation binding;CoA-ligase activity;ion binding;ligase activity;ligase activity, forming carbon-sulfur bonds;m
alkylglycerone-phosphate synthase activity;binding;catalytic activity;coenzyme binding;cofactor binding;FAD binding;flavin adenine dinucleotide binding;oxidoreductase activity;oxidoreductase activity, acting on CH-OH grou
binding;cation binding;ion binding;metal ion binding;protein binding transcription factor activity;transcription coactivator activity;transcription cofactor activity;transcription factor binding transcription factor activity;transition r
binding;chromatin binding;enzyme binding;protein binding;protein dimerization activity;protein heterodimerization activity
structural constituent of cytoskeleton;structural molecule activity
7S RNA binding;binding;nucleic acid binding;ribonucleoprotein binding;RNA binding;signal recognition particle binding
binding;catalytic activity;cation binding;G-protein beta/gamma-subunit complex binding;G-protein-coupled receptor binding;GTP binding;GTPase activity;guanyl nucleotide binding;guanyl ribonucleotide binding;hydrolase ac
binding;cation binding;ion binding;metal ion binding;transition metal ion binding;zinc ion binding
binding;cation binding;enzyme binding;glycoprotein binding;ion binding;metal ion binding;protease binding;protein binding;protein dimerization activity;protein heterodimerization activity
binding;cation binding;cytoskeletal protein binding;identical protein binding;ion binding;metal ion binding;microtubule binding;microtubule plus-end binding;nucleic acid binding;protein binding;protein dimerization activity;pro
binding;DNA binding;nucleic acid binding
active transmembrane transporter activity;ATPase activity;ATPase activity, coupled;ATPase activity, coupled to movement of substances;ATPase activity, coupled to transmembrane movement of ions;ATPase activity, couplec
active transmembrane transporter activity;adenyl nucleotide binding;adenyl ribonucleotide binding;ATP binding;ATPase activity;ATPase activity, coupled;ATPase activity, coupled to movement of substances;ATPase activity, c
binding;core promoter proximal region DNA binding;core promoter proximal region sequence-specific DNA binding;DNA binding;enzyme binding;kinase binding;ligand-dependent nuclear receptor activity;molecular transduce
acetyl-CoA C-acyltransferase activity;C-acyltransferase activity;catalytic activity;transferase activity;transferase activity, transferring acyl groups;transferase activity, transferring acyl groups other than amino-acyl groups
enzyme activator activity;enzyme regulator activity;GTPase activator activity;GTPase regulator activity;nucleoside-triphosphatase regulator activity
beta-catenin binding;binding;cation binding;cytoskeletal protein binding;ion binding;metal ion binding;protein binding;transition metal ion binding;vinculin binding;zinc ion binding
binding;G-quadruplex RNA binding;mRNA 3'-UTR binding;mRNA binding;nucleic acid binding;RNA binding
binding;nucleic acid binding;nucleotide binding;RNA binding;single-stranded RNA binding
actin binding;binding;cytoskeletal protein binding;protein binding
binding;peptide binding;structural constituent of nuclear pore;structural molecule activity;transporter activity
binding;catalytic activity;cysteine-type endopeptidase activity;cysteine-type peptidase activity;endopeptidase activity;endopeptidase inhibitor activity;endopeptidase regulator activity;enzyme inhibitor activity;enzyme regulato
binding;DNA binding;nucleic acid binding
adenyl nucleotide binding;adenyl ribonucleotide binding;ADP binding;AMP binding;ATP binding;binding;carbohydrate binding;catalytic activity;cation binding;diphosphotransferase activity;GDP binding;guanyl nucleotide binc
binding;catalytic activity;GTP binding;GTPase activity;guanyl nucleotide binding;guanyl ribonucleotide binding;hydrolase activity;hydrolase activity, acting on acid anhydrides;hydrolase activity, acting on acid anhydrides, in pl
binding;calcium channel regulator activity;catalytic activity;channel regulator activity;enzyme binding;GTPase activity;GTPase binding;hydrolase activity;hydrolase activity, acting on acid anhydrides;hydrolase activity, acting o
binding;carbohydrate binding
binding;DNA binding;enzyme inhibitor activity;enzyme regulator activity;histone binding;nucleic acid binding;phosphatase inhibitor activity;phosphatase regulator activity;protein binding;protein phosphatase inhibitor activity;p
binding;catalytic activity;enzyme binding;GTP binding;GTPase activity;guanyl nucleotide binding;guanyl ribonucleotide binding;hydrolase activity;hydrolase activity, acting on acid anhydrides;hydrolase activity, acting on acid
actin binding;adenyl nucleotide binding;adenyl ribonucleotide binding;ATP binding;binding;catalytic activity;cytoskeletal protein binding;enzyme binding;JUN kinase binding;kinase activity;kinase binding;molecular transducer
binding;lipid binding;phosphatidylethanolamine binding;phosphatidylserine binding;phospholipid binding
binding;catalytic activity;isomerase activity;protein binding;receptor binding
AU-rich element binding;binding;enzyme binding;kinase binding;nucleic acid binding;nucleotide binding;protein binding;protein kinase binding;RNA binding
adenyl nucleotide binding;adenyl ribonucleotide binding;ATP binding;binding;catalytic activity;enzyme activator activity;enzyme binding;enzyme regulator activity;GTPase binding;identical protein binding;kinase activator activ
binding;nucleic acid binding;RNA binding;translation factor activity, nucleic acid binding;translation initiation factor activity
binding;catalytic activity;cation binding;hydrolase activity;hydrolase activity, acting on acid anhydrides;hydrolase activity, acting on acid anhydrides, in phosphorus-containing anhydrides;inorganic diphosphatase activity;ion b
adenyl nucleotide binding;adenyl ribonucleotide binding;ATP binding;binding;caspase inhibitor activity;caspase regulator activity;catalytic activity;cation binding;cysteine-type endopeptidase inhibitor activity;endopeptidase ir
binding;cation binding;ion binding;metal ion binding;transition metal ion binding;zinc ion binding
3-hydroxyacyl-CoA dehydrogenase activity;binding;catalytic activity;coenzyme binding;cofactor binding;NAD binding;NAD+ binding;nucleotide binding;oxidoreductase activity;oxidoreductase activity, acting on CH-OH group
binding;DNA binding;double-stranded RNA binding;identical protein binding;nucleic acid binding;protein binding;protein dimerization activity;protein homodimerization activity;RNA binding
ligand-dependent nuclear receptor transcription coactivator activity;protein binding transcription factor activity;transcription coactivator activity;transcription cofactor activity;transcription factor binding transcription factor act
binding;cadherin binding;cell adhesion molecule binding;cytoskeletal protein binding;enzyme binding;gamma-tubulin binding;GTPase binding;microtubule binding;protein binding;Rab GTPase binding;Ras GTPase binding;sm
binding;enzyme binding;enzyme regulator activity;GTPase binding;GTPase regulator activity;guanyl-nucleotide exchange factor activity;nucleoside-triphosphatase regulator activity;protein binding;Rac GTPase binding;Ras G
binding;histone binding;protein binding;protein transporter activity;substrate-specific transporter activity;transporter activity
androgen receptor binding;binding;catalytic activity;cation binding;copper chaperone activity;copper ion binding;cytokine binding;enzyme binding;glyoxalase III activity;hormone receptor binding;hydrolase activity;identical pr
receptor activity
actin binding;actin filament binding;Arp2/3 complex binding;binding;cytoskeletal protein binding;identical protein binding;protein binding;protein complex binding
binding;nucleic acid binding;ribonucleoprotein binding;ribosome binding;RNA binding;translation factor activity, nucleic acid binding;translation initiation factor activity;tRNA binding
aminopeptidase activity;binding;catalytic activity;cation binding;exopeptidase activity;hydrolase activity;identical protein binding;ion binding;manganese ion binding;metal ion binding;metalloaminopeptidase activity;metalloex
binding;GTP binding;guanyl nucleotide binding;guanyl ribonucleotide binding;nucleotide binding;purine nucleotide binding;purine ribonucleoside triphosphate binding;purine ribonucleotide binding;ribonucleotide binding
binding;catalytic activity;cation binding;dipeptidyl-peptidase activity;exopeptidase activity;hydrolase activity;ion binding;metal ion binding;metallopeptidase activity;peptidase activity;peptidase activity, acting on L-amino acid
ATP-dependent protein binding;binding;catalytic activity;enzyme activator activity;enzyme regulator activity;protein binding;protein C-terminus binding;protein dimerization activity;protein heterodimerization activity;small prot
binding;cytoskeletal protein binding;identical protein binding;lipid binding;phosphatidic acid binding;phospholipid binding;protein binding;transporter activity
binding;cytoskeletal protein binding;dynactin binding;identical protein binding;lipid binding;phosphatidylinositol binding;phospholipid binding;protein binding;protein dimerization activity;protein homodimerization activity
armadillo repeat domain binding;binding;estrogen receptor binding;hormone receptor binding;nuclear hormone receptor binding;protein binding;protein complex scaffold;protein domain specific binding;receptor binding;retin
anion channel activity;anion transmembrane transporter activity;channel activity;chloride channel activity;gated channel activity;ion channel activity;ion transmembrane transporter activity;passive transmembrane transporter
binding;catalytic activity;endopeptidase activity;hydrolase activity;identical protein binding;peptidase activity;peptidase activity, acting on L-amino acid peptides;protein binding;threonine-type endopeptidase activity;threonin
binding;catalytic activity;hydrolase activity;protein binding;protein domain specific binding;SH2 domain binding
binding;calcium-dependent phospholipid binding;catalytic activity;kinase activity;lipid binding;phospholipid binding;phosphotransferase activity, alcohol group as acceptor;protein kinase activity;protein serine/threonine kinas
adenyl nucleotide binding;adenyl ribonucleotide binding;ATP binding;binding;catalytic activity;identical protein binding;kinase activity;molecular transducer activity;nucleotide binding;phosphotransferase activity, alcohol grou
macromolecule transmembrane transporter activity;protein transmembrane transporter activity;protein transporter activity;substrate-specific transmembrane transporter activity;substrate-specific transporter activity;transmer
adenyl nucleotide binding;adenyl ribonucleotide binding;adenylylsulfate kinase activity;adenylyltransferase activity;ATP binding;binding;catalytic activity;kinase activity;nucleotide binding;nucleotidyltransferase activity;phosph
binding;C5a anaphylatoxin chemotactic receptor binding;C5L2 anaphylatoxin chemotactic receptor binding;endopeptidase inhibitor activity;endopeptidase regulator activity;enzyme inhibitor activity;enzyme regulator activity;
alcohol dehydrogenase (NADP+) activity;alditol:NADP+ 1-oxidoreductase activity;aldo-keto reductase (NADP) activity;catalytic activity;electron carrier activity;glyceraldehyde oxidoreductase activity;oxidoreductase activity;ox
15-hydroxyprostaglandin dehydrogenase (NADP+) activity;carbonyl reductase (NADPH) activity;catalytic activity;oxidoreductase activity;oxidoreductase activity, acting on CH-OH group of donors;oxidoreductase activity, acti
binding;catalytic activity;cation binding;coenzyme binding;cofactor binding;cytochrome-b5 reductase activity;dioxygenase activity;electron carrier activity;flavin adenine dinucleotide binding;FMN binding;hydrolase activity;ion
endopeptidase inhibitor activity;endopeptidase regulator activity;enzyme inhibitor activity;enzyme regulator activity;peptidase inhibitor activity;peptidase regulator activity;serine-type endopeptidase inhibitor activity
binding;complement binding;complement component C3a binding;complement component C3b binding;DNA binding;enzyme binding;G-protein-coupled receptor binding;histone deacetylase binding;nucleic acid binding;nu
binding;DNA binding;histone binding;nucleic acid binding;protein binding
binding;cytokine activity;growth factor receptor binding;high molecular weight B cell growth factor receptor binding;protein binding;receptor binding
binding;cation binding;enzyme binding;ion binding;kinase binding;metal ion binding;protein binding;protein kinase binding;transition metal ion binding;zinc ion binding
1-pyrroline dehydrogenase activity;3-chloroallyl aldehyde dehydrogenase activity;4-trimethylammoniobutyraldehyde dehydrogenase activity;aldehyde dehydrogenase (NAD) activity;amine binding;aminobutyraldehyde dehydr
binding;nucleic acid binding;protein binding;protein N-terminus binding;RNA binding;translation factor activity, nucleic acid binding;translation initiation factor activity
binding;mRNA binding;nucleic acid binding;RNA binding;rRNA binding;SSU rRNA binding;structural constituent of ribosome;structural molecule activity
binding;catalytic activity;enzyme binding;GTPase activity;GTPase binding;hydrolase activity;hydrolase activity, acting on acid anhydrides;hydrolase activity, acting on acid anhydrides, in phosphorus-containing anhydrides;mo
binding;calcium ion binding;cation binding;DNA binding;ion binding;metal ion binding;nucleic acid binding
binding;catalytic activity;cytoskeletal protein binding;hydrolase activity;hydrolase activity, acting on acid anhydrides;hydrolase activity, acting on acid anhydrides, in phosphorus-containing anhydrides;motor activity;nucleosid
binding;histone binding;protein binding
catalytic activity;endonuclease activity;endonuclease activity, active with either ribo- or deoxyribonucleic acids and producing 5'-phosphomonoesters;endoribonuclease activity;endoribonuclease activity, producing 5'-phosph
binding;heat shock protein binding;protein binding
binding;catalytic activity;GTP binding;GTPase activity;guanyl nucleotide binding;guanyl ribonucleotide binding;hydrolase activity;hydrolase activity, acting on acid anhydrides;hydrolase activity, acting on acid anhydrides, in pl
adenyl nucleotide binding;adenyl ribonucleotide binding;ATP binding;ATPase activity;ATPase activity, coupled;ATP-dependent helicase activity;ATP-dependent RNA helicase activity;binding;catalytic activity;helicase activity;h
adenyl nucleotide binding;adenyl ribonucleotide binding;ATP binding;ATPase activity;binding;catalytic activity;hydrolase activity;hydrolase activity, acting on acid anhydrides;hydrolase activity, acting on acid anhydrides, in ph
binding;nucleic acid binding;nucleotide binding;RNA binding
binding;catalytic activity;cofactor binding;O-phospho-L-serine:2-oxoglutarate aminotransferase activity;pyridoxal phosphate binding;transaminase activity;transferase activity;transferase activity, transferring nitrogenous grou
adenyl nucleotide binding;adenyl ribonucleotide binding;ATP binding;ATPase activity;ATPase activity, coupled;ATP-dependent helicase activity;ATP-dependent RNA helicase activity;binding;catalytic activity;helicase activity;h
binding;nucleic acid binding;RNA binding;RNA cap binding;translation factor activity, nucleic acid binding;translation initiation factor activity
adenyl nucleotide binding;adenyl ribonucleotide binding;ATP binding;ATPase activity;ATPase activity, coupled;ATP-dependent helicase activity;ATP-dependent RNA helicase activity;binding;catalytic activity;helicase activity;h
adenyl nucleotide binding;adenyl ribonucleotide binding;adenylate kinase activity;ATP binding;binding;catalytic activity;kinase activity;nucleobase-containing compound kinase activity;nucleoside diphosphate kinase activity;n
binding;catalytic activity;cofactor binding;ornithine-oxo-acid transaminase activity;pyridoxal phosphate binding;transaminase activity;transferase activity;transferase activity, transferring nitrogenous groups;vitamin B6 binding
adenyl nucleotide binding;adenyl ribonucleotide binding;ATP binding;ATPase activity;beta-amyloid binding;beta-endorphin binding;binding;catalytic activity;cation binding;endopeptidase activity;glycoprotein binding;hormone
binding;chromatin binding;chromatin DNA binding;DNA binding;enzyme binding;histone deacetylase binding;nucleic acid binding;protein binding;structure-specific DNA binding
binding;carbon-carbon lyase activity;carboxylic acid binding;carboxy-lyase activity;catalytic activity;cofactor binding;L-aspartate:2-oxoglutarate aminotransferase activity;L-cysteine:2-oxoglutarate aminotransferase activity;L
cysteine-type endopeptidase inhibitor activity;endopeptidase inhibitor activity;endopeptidase regulator activity;enzyme inhibitor activity;enzyme regulator activity;peptidase inhibitor activity;peptidase regulator activity
adenyl nucleotide binding;adenyl ribonucleotide binding;ATP binding;ATPase activity;binding;catalytic activity;chromatin binding;DNA binding;helicase activity;hydrolase activity;hydrolase activity, acting on acid anhydrides;hy

enzyme activator activity;enzyme regulator activity;GDP-dissociation inhibitor activity;GTPase activator activity;GTPase regulator activity;nucleoside-triphosphatase regulator activity;Rab GDP-dissociation inhibitor activity;sm
adenyl nucleotide binding;adenyl ribonucleotide binding;ATP binding;binding;catalytic activity;enzyme activator activity;enzyme regulator activity;kinase activator activity;kinase activity;kinase regulator activity;MAP kinase kin
catalytic activity;methyltransferase activity;S-adenosylmethionine-dependent methyltransferase activity;transferase activity;transferase activity, transferring one-carbon groups
binding;nucleic acid binding;RNA binding;structural constituent of ribosome;structural molecule activity
binding;enzyme binding;GTPase binding;protein binding;protein C-terminus binding;Ras GTPase binding;small GTPase binding
3-oxoacid CoA-transferase activity;binding;catalytic activity;CoA-transferase activity;identical protein binding;protein binding;protein dimerization activity;protein homodimerization activity;transferase activity;transferase activ
binding;cation binding;collagen binding;enzyme binding;ion binding;metal ion binding;phosphatase binding;protein binding;protein phosphatase binding
structural constituent of ribosome;structural molecule activity
binding;enzyme binding;kinase binding;protein binding;protein kinase binding;SNARE binding;syntaxin binding
binding;enzyme binding;histone binding;histone deacetylase binding;protein binding
binding;cation binding;ion binding;metal ion binding;transition metal ion binding;zinc ion binding
binding;nucleic acid binding;RNA binding
binding;enzyme activator activity;enzyme regulator activity;GTPase activating protein binding;GTPase activator activity;GTPase regulator activity;guanyl-nucleotide exchange factor activity;nucleoside-triphosphatase regulato
binding;carbon-carbon lyase activity;carboxy-lyase activity;catalytic activity;cation binding;GTP binding;guanyl nucleotide binding;guanyl ribonucleotide binding;ion binding;lyase activity;metal ion binding;nucleotide binding;p
binding;catalytic activity;cysteine-type endopeptidase activity;cysteine-type peptidase activity;endopeptidase activity;enzyme binding;hydrolase activity;p53 binding;peptidase activity;peptidase activity, acting on L-amino aci
binding;lipid binding;phosphatidylinositol binding;phosphatidylinositol-3,4,5-trisphosphate binding;phospholipid binding
binding;binding, bridging;ligand-dependent nuclear receptor transcription coactivator activity;nucleic acid binding;nucleotide binding;protein binding;protein binding transcription factor activity;protein binding, bridging;RNA b
binding;cation binding;ion binding;metal ion binding;transition metal ion binding;zinc ion binding
adenyl nucleotide binding;adenyl ribonucleotide binding;aminoacyl-tRNA ligase activity;ATP binding;binding;catalytic activity;cation binding;ion binding;ligase activity;ligase activity, forming aminoacyl-tRNA and related comp
adenyl nucleotide binding;adenyl ribonucleotide binding;ATP binding;binding;calcium ion binding;cation binding;DNA binding;ion binding;metal ion binding;nucleic acid binding;nucleotide binding;purine nucleotide binding;pu
binding;DNA binding;identical protein binding;nucleic acid binding;nucleotide binding;protein binding
adenyl nucleotide binding;adenyl ribonucleotide binding;ATP binding;binding;catalytic activity;cation binding;cytoskeletal protein binding;ion binding;ligase activity;ligase activity, forming phosphoric ester bonds;metal ion bin
binding;carbohydrate binding;extracellular matrix binding;glycosaminoglycan binding;heparin binding;integrin binding;pattern binding;polysaccharide binding;protein binding;protein complex binding;receptor binding
14-3-3 protein binding;binding;enzyme binding;enzyme inhibitor activity;enzyme regulator activity;kinase binding;molecular transducer activity;phosphatase regulator activity;protein binding;protein kinase binding;signal trans
molecular transducer activity;receptor activity;semaphorin receptor activity;signal transducer activity;signaling receptor activity;transmembrane signaling receptor activity
structural constituent of cytoskeleton;structural molecule activity
binding;chromatin binding;DNA bending activity;DNA binding;double-stranded DNA binding;nucleic acid binding;structure-specific DNA binding
binding;catalytic activity;cofactor binding;kynurenine-oxoglutarate transaminase activity;L-aspartate:2-oxoglutarate aminotransferase activity;L-phenylalanine aminotransferase activity;L-phenylalanine:2-oxoglutarate aminotra
adenyl nucleotide binding;adenyl ribonucleotide binding;ATP binding;binding;catalytic activity;creatine kinase activity;enzyme binding;kinase activity;nucleotide binding;phosphotransferase activity, nitrogenous group as acce
binding;calcium ion binding;cation binding;enzyme binding;ion binding;ion channel binding;kinase binding;metal ion binding;phosphoprotein binding;protein binding;protein kinase binding;protein kinase C binding
binding;calcium ion binding;calcium-dependent phospholipid binding;calcium-dependent protein binding;cation binding;integrin binding;ion binding;lipid binding;metal ion binding;phospholipid binding;protein binding;protein
binding;catalytic activity;cis-trans isomerase activity;enzyme binding;isomerase activity;peptide binding;peptidyl-prolyl cis-trans isomerase activity;protein binding;protein complex binding;RNA polymerase binding;unfolded p
binding;catalytic activity;cation binding;cation transmembrane transporter activity;enzyme binding;hydrogen ion transmembrane transporter activity;inorganic cation transmembrane transporter activity;ion binding;ion transme
adenyl nucleotide binding;adenyl ribonucleotide binding;ATP binding;binding;catalytic activity;fatty acid ligase activity;ligase activity;ligase activity, forming carbon-sulfur bonds;long-chain fatty acid-CoA ligase activity;nucleot
binding;catalytic activity;coenzyme binding;cofactor binding;diiodophenylpyruvate reductase activity;L-malate dehydrogenase activity;malate dehydrogenase activity;malic enzyme activity;NAD binding;nucleotide binding;oxi
aldehyde dehydrogenase (NAD) activity;betaine-aldehyde dehydrogenase activity;catalytic activity;L-aminoadipate-semialdehyde dehydrogenase activity;oxidoreductase activity;oxidoreductase activity, acting on the aldehyd
adenyl deoxyribonucleotide binding;adenyl nucleotide binding;binding;binding, bridging;dATP binding;deoxyribonucleotide binding;nucleotide binding;protein binding;protein binding, bridging;purine deoxyribonucleotide bind
binding;chromatin binding;identical protein binding;nucleic acid binding transcription factor activity;protein binding;protein binding transcription factor activity;sequence-specific DNA binding transcription factor activity;transc
binding;carbohydrate binding;DNA binding;glycosaminoglycan binding;heparin binding;nucleic acid binding;nucleotide binding;pattern binding;polysaccharide binding;protein binding transcription factor activity;RNA polymer
binding;catalytic activity;endopeptidase activity;hydrolase activity;NF-kappaB binding;nucleic acid binding;nucleotide binding;peptidase activity;peptidase activity, acting on L-amino acid peptides;protein binding;purine nucle
adenyl nucleotide binding;adenyl ribonucleotide binding;ATP binding;binding;chaperone binding;nucleotide binding;protein binding;purine nucleotide binding;purine ribonucleoside triphosphate binding;purine ribonucleotide b
binding;nucleic acid binding;RNA binding;structural constituent of ribosome;structural molecule activity
apoptotic protease activator activity;binding;binding, bridging;caspase activator activity;caspase regulator activity;channel inhibitor activity;channel regulator activity;enzyme activator activity;enzyme binding;enzyme inhibitor
binding;catalytic activity;cation binding;hydrolase activity;hydrolase activity, acting on ester bonds;ion binding;metal ion binding;phosphatase activity;phosphoprotein phosphatase activity;phosphoric ester hydrolase activity;p
binding;nucleic acid binding;RNA binding;RNA cap binding
adenyl nucleotide binding;adenyl ribonucleotide binding;ATP binding;ATPase activity;ATPase activity, coupled;ATP-dependent DNA helicase activity;ATP-dependent helicase activity;ATP-dependent RNA helicase activity;bind
adenyl nucleotide binding;adenyl ribonucleotide binding;ATP binding;binding;catalytic activity;cation binding;ion binding;kinase activity;metal ion binding;nucleotide binding;phosphotransferase activity, alcohol group as acce
adenyl nucleotide binding;adenyl ribonucleotide binding;ATP binding;ATPase activity;ATPase activity, coupled;ATP-dependent helicase activity;ATP-dependent RNA helicase activity;binding;catalytic activity;helicase activity;h
binding;carbohydrate binding;catalytic activity;identical protein binding;nucleotidyltransferase activity;protein binding;transferase activity;transferase activity, transferring phosphorus-containing groups;UDP-N-acetylglucosan
aldehyde dehydrogenase (NAD) activity;catalytic activity;formyltetrahydrofolate dehydrogenase activity;hydroxymethyl-, formyl- and related transferase activity;methyltransferase activity;oxidoreductase activity;oxidoreductas
catalytic activity;intramolecular oxidoreductase activity;intramolecular oxidoreductase activity, interconverting keto- and enol-groups;intramolecular oxidoreductase activity, transposing S-S bonds;isomerase activity;protein d
binding;nucleic acid binding;RNA binding;snRNA binding
actin binding;actin filament binding;adenyl nucleotide binding;adenyl ribonucleotide binding;ADP binding;ATP binding;binding;catalytic activity;cytoskeletal protein binding;DNA binding;hydrolase activity;hydrolase activity, ac
binding;chromatin binding;DNA binding;nucleic acid binding;protein binding;protein binding transcription factor activity;protein N-terminus binding;transcription coactivator activity;transcription cofactor activity;transcription f
binding;lipid binding;lipoprotein particle receptor binding;low-density lipoprotein particle receptor binding;peptide binding;protein binding;receptor binding;signal sequence binding;transporter activity
acid-amino acid ligase activity;binding;catalytic activity;cation binding;chromatin binding;core promoter proximal region DNA binding;core promoter proximal region sequence-specific DNA binding;DNA binding;histone bindi
binding;cytoskeletal protein binding;FFAT motif binding;microtubule binding;molecular transducer activity;protein binding;protein dimerization activity;protein domain specific binding;protein heterodimerization activity;signal t
adenyl nucleotide binding;adenyl ribonucleotide binding;aminoacyl-tRNA ligase activity;ATP binding;binding;catalytic activity;ligase activity;ligase activity, forming aminoacyl-tRNA and related compounds;ligase activity, formi
binding;enzyme binding;mRNA binding;nucleic acid binding;protein binding;RNA binding
actin binding;binding;cytoskeletal protein binding;protein binding;structural constituent of cytoskeleton;structural molecule activity
adenyl nucleotide binding;adenyl ribonucleotide binding;ATP binding;binding;catalytic activity;cation binding;endopeptidase activity;hydrolase activity;ion binding;metal ion binding;metalloendopeptidase activity;metallopeptic
adenyl nucleotide binding;adenyl ribonucleotide binding;ATP binding;binding;catalytic activity;FAT10 activating enzyme activity;nucleotide binding;purine nucleotide binding;purine ribonucleoside triphosphate binding;purine r
alcohol binding;amine binding;binding;calcium ion binding;calcium-dependent phospholipid binding;cation binding;identical protein binding;ion binding;lipid binding;metal ion binding;phosphatidylcholine binding;phosphatidy
binding;protein binding;protein binding transcription factor activity;repressing transcription factor binding;RNA polymerase II repressing transcription factor binding;RNA polymerase II transcription cofactor activity;RNA polym
adenyl nucleotide binding;adenyl ribonucleotide binding;aminoacyl-tRNA ligase activity;asparagine-tRNA ligase activity;ATP binding;binding;catalytic activity;ligase activity;ligase activity, forming aminoacyl-tRNA and related c
binding;carboxypeptidase activity;catalytic activity;cation binding;exopeptidase activity;hydrolase activity;ion binding;metal ion binding;metallocarboxypeptidase activity;metalloexopeptidase activity;metallopeptidase activity;
binding;catalytic activity;cation binding;G-protein beta/gamma-subunit complex binding;G-protein-coupled receptor binding;GTP binding;GTPase activity;guanyl nucleotide binding;guanyl ribonucleotide binding;hydrolase ac
2',3'-cyclic-nucleotide 3'-phosphodiesterase activity;binding;catalytic activity;cyclic nucleotide binding;cyclic-nucleotide phosphodiesterase activity;hydrolase activity;hydrolase activity, acting on ester bonds;nucleic acid bin
binding;catalytic activity;GTP binding;GTPase activity;guanyl nucleotide binding;guanyl ribonucleotide binding;hydrolase activity;hydrolase activity, acting on acid anhydrides;hydrolase activity, acting on acid anhydrides, in ph
6-phosphofructokinase activity;adenyl nucleotide binding;adenyl ribonucleotide binding;ATP binding;binding;carbohydrate binding;carbohydrate kinase activity;catalytic activity;cation binding;enzyme binding;fructose binding
binding;catalytic activity;cation binding;DNA binding;DNA-directed RNA polymerase activity;enzyme binding;ion binding;metal ion binding;nucleic acid binding;nucleotidyltransferase activity;protein binding;RNA polymerase a
3'-5' exonuclease activity;3'-5'-exodeoxyribonuclease activity;4 iron, 4 sulfur cluster binding;binding;catalytic activity;cation binding;chromatin binding;damaged DNA binding;deoxyribonuclease activity;DNA binding;DNA pol
binding;cation binding;cobalt ion binding;DNA binding;enzyme binding;identical protein binding;ion binding;metal ion binding;nucleic acid binding;protein binding;protein binding transcription factor activity;protein dimerizatio
adenyl nucleotide binding;adenyl ribonucleotide binding;ATP binding;binding;catalytic activity;cation binding;ion binding;metal ion binding;methionine adenosyltransferase activity;nucleotide binding;purine nucleotide binding;
binding;calcium ion binding;cation binding;extracellular matrix structural constituent;ion binding;metal ion binding;structural molecule activity
binding;core promoter binding;core promoter proximal region DNA binding;core promoter proximal region sequence-specific DNA binding;core promoter sequence-specific DNA binding;cytokine receptor binding;DNA bindin
adenyl nucleotide binding;adenyl ribonucleotide binding;ATP binding;ATPase activity;ATPase activity, coupled;binding;catalytic activity;cation binding;enzyme binding;GTPase binding;hydrolase activity;hydrolase activity, acti
binding;nucleic acid binding;RNA binding;structural constituent of ribosome;structural molecule activity
actin binding;actin filament binding;binding;cytoskeletal protein binding;protein binding
binding;profilin binding;protein binding
adenyl nucleotide binding;adenyl ribonucleotide binding;androgen receptor binding;ATP binding;ATPase activity;ATPase activity, coupled;binding;catalytic activity;chromatin binding;DNA binding;DNA polymerase binding;DN
adenyl nucleotide binding;adenyl ribonucleotide binding;ATP binding;ATPase activity;binding;catalytic activity;hydrolase activity;hydrolase activity, acting on acid anhydrides;hydrolase activity, acting on acid anhydrides, in ph
androgen receptor binding;binding;catalytic activity;chromatin binding;GDP binding;GTP binding;GTPase activity;guanyl nucleotide binding;guanyl ribonucleotide binding;hormone receptor binding;hydrolase activity;hydrolas
binding;nucleic acid binding;RNA binding;rRNA binding;structural constituent of ribosome;structural molecule activity
binding;catalytic activity;cis-trans isomerase activity;isomerase activity;peptide binding;peptidyl-prolyl cis-trans isomerase activity;protein binding;unfolded protein binding;virion binding
catalytic activity;hydrolase activity;hydrolase activity, acting on acid anhydrides;hydrolase activity, acting on acid anhydrides, in phosphorus-containing anhydrides;microtubule motor activity;motor activity;nucleoside-triphosp
acetylgalactosaminyltransferase activity;binding;carbohydrate binding;catalytic activity;cation binding;ion binding;manganese ion binding;metal ion binding;polypeptide N-acetylgalactosaminyltransferase activity;transferase a
binding;catalytic activity;dipeptidyl-peptidase activity;endopeptidase activity;enzyme binding;exopeptidase activity;hydrolase activity;identical protein binding;integrin binding;metalloendopeptidase activity;metallopeptidase a
binding;mRNA binding;nucleic acid binding;nucleotide binding;poly(A) RNA binding;poly(U) RNA binding;poly-purine tract binding;poly-pyrimidine tract binding;RNA binding;single-stranded RNA binding
3'-5' DNA helicase activity;adenyl nucleotide binding;adenyl ribonucleotide binding;ATP binding;ATPase activity;ATPase activity, coupled;ATP-dependent 3'-5' DNA helicase activity;ATP-dependent DNA helicase activity;ATP-
enzyme activator activity;enzyme regulator activity;GTPase activator activity;GTPase regulator activity;guanyl-nucleotide exchange factor activity;nucleoside-triphosphatase regulator activity;Ras guanyl-nucleotide exchange
binding;catalytic activity;cation binding;intramolecular transferase activity;intramolecular transferase activity, phosphotransferases;ion binding;isomerase activity;magnesium ion binding;metal ion binding;phosphoglucomutas
catalytic activity;disulfide oxidoreductase activity;intramolecular oxidoreductase activity;intramolecular oxidoreductase activity, interconverting keto- and enol-groups;intramolecular oxidoreductase activity, transposing S-S bo
binding;cation binding;ion binding;metal ion binding;transition metal ion binding;zinc ion binding
5'-3' exonuclease activity;5'-3' exoribonuclease activity;binding;catalytic activity;cation binding;DNA binding;exonuclease activity;exonuclease activity, active with either ribo- or deoxyribonucleic acids and producing 5'-phos
acid-amino acid ligase activity;binding;catalytic activity;identical protein binding;ligase activity;ligase activity, forming carbon-nitrogen bonds;protein binding;small conjugating protein ligase activity;ubiquitin-protein ligase acti
binding;enzyme binding;kinase binding;lipid binding;protein binding;protein kinase binding;protein transporter activity;substrate-specific transporter activity;transporter activity
6-phosphofructokinase activity;adenyl nucleotide binding;adenyl ribonucleotide binding;ATP binding;binding;carbohydrate binding;carbohydrate kinase activity;catalytic activity;cation binding;enzyme binding;fructose binding
catalytic activity;oxidoreductase activity;oxidoreductase activity, acting on the aldehyde or oxo group of donors;oxidoreductase activity, acting on the aldehyde or oxo group of donors, disulfide as acceptor;pyruvate dehydro
binding;carbon-oxygen lyase activity;catalytic activity;cation binding;hydro-lyase activity;ion binding;lyase activity;magnesium ion binding;metal ion binding;phosphopyruvate hydratase activity
binding;enzyme binding;fibroblast growth factor binding;growth factor binding;mRNA binding;nucleic acid binding;protein binding;RNA binding;structural constituent of ribosome;structural molecule activity
binding;cation binding;DNA binding;ion binding;metal ion binding;nucleic acid binding;transition metal ion binding;zinc ion binding
binding;chemoattractant activity;chromatin binding;damaged DNA binding;DNA bending activity;DNA binding;double-stranded DNA binding;nucleic acid binding;nucleic acid binding transcription factor activity;protein bindin
aminopeptidase activity;binding;catalytic activity;endopeptidase activity;exopeptidase activity;hydrolase activity;peptidase activity;peptidase activity, acting on L-amino acid peptides;peptide binding;serine hydrolase activity;
activating transcription factor binding;alpha-catenin binding;androgen receptor binding;binding;cadherin binding;cell adhesion molecule binding;DNA binding;double-stranded DNA binding;enzyme binding;estrogen receptor
binding;DNA binding;lamin binding;nucleic acid binding;protein binding
adenyl nucleotide binding;adenyl ribonucleotide binding;ATP binding;ATPase activity;ATPase activity, coupled;binding;catalytic activity;centromeric DNA binding;damaged DNA binding;DNA binding;DNA insertion or deletion
binding;mRNA binding;nucleic acid binding;nucleotide binding;RNA binding
antigen binding;binding;calcium ion binding;calcium-dependent phospholipid binding;calcium-dependent protein binding;cation binding;ion binding;lipid binding;metal ion binding;MHC class II protein complex binding;MHC
androgen receptor binding;binding;cation binding;hormone receptor binding;identical protein binding;ion binding;metal ion binding;nuclear hormone receptor binding;protein binding;protein binding transcription factor activity
acid-amino acid ligase activity;binding;catalytic activity;cation binding;ion binding;ligase activity;ligase activity, forming carbon-nitrogen bonds;metal ion binding;nucleic acid binding transcription factor activity;sequence-spec
adenyl nucleotide binding;adenyl ribonucleotide binding;ATP binding;binding;calmodulin-dependent protein kinase activity;catalytic activity;cation binding;ion binding;kinase activity;metal ion binding;myosin light chain kinase
basal RNA polymerase II transcription machinery binding;basal transcription machinery binding;binding;enzyme binding;enzyme inhibitor activity;enzyme regulator activity;protein binding;RNA polymerase binding;RNA polym
binding;mRNA 3'-UTR binding;mRNA binding;nucleic acid binding;RNA binding
enzyme regulator activity
binding;DNA binding;nucleic acid binding;structural molecule activity
binding;cation binding;ion binding;metal ion binding
7S RNA binding;binding;endoplasmic reticulum signal peptide binding;nucleic acid binding;peptide binding;ribonucleoprotein binding;RNA binding;signal recognition particle binding;signal sequence binding
binding;carboxylic acid binding;catalytic activity;cation binding;galactosyltransferase activity;glucosyltransferase activity;ion binding;iron ion binding;L-ascorbic acid binding;metal ion binding;oxidoreductase activity;oxidored
aminoacylase activity;antioxidant activity;binding;catalase activity;catalytic activity;cation binding;coenzyme binding;cofactor binding;enzyme binding;heme binding;hydrolase activity;hydrolase activity, acting on carbon-nitro

binding;catalytic activity;coenzyme binding;cofactor binding;lactate dehydrogenase activity;L-lactate dehydrogenase activity;NAD binding;nucleotide binding;oxidoreductase activity;oxidoreductase activity, acting on CH-OH
active transmembrane transporter activity;amine transmembrane transporter activity;amino acid transmembrane transporter activity;antiporter activity;binding;calcium ion transmembrane transporter activity;calcium:cation an
structural constituent of cytoskeleton;structural molecule activity
binding;carboxylic acid binding;catalytic activity;cation binding;dioxygenase activity;ion binding;iron ion binding;L-ascorbic acid binding;metal ion binding;oxidoreductase activity;oxidoreductase activity, acting on paired don
alpha-catenin binding;binding;cadherin binding;cell adhesion molecule binding;enzyme binding;identical protein binding;molecular transducer activity;phosphatase binding;protein binding;protein binding transcription factor a
adenyl nucleotide binding;adenyl ribonucleotide binding;aminoacyl-tRNA ligase activity;ATP binding;binding;catalytic activity;ligase activity;ligase activity, forming aminoacyl-tRNA and related compounds;ligase activity, formi
active transmembrane transporter activity;adenyl nucleotide binding;adenyl ribonucleotide binding;ATP binding;ATPase activity;ATPase activity, coupled;ATPase activity, coupled to movement of substances;ATPase activity, c
acetyl-CoA C-acetyltransferase activity;acetyl-CoA C-acyltransferase activity;acetyltransferase activity;binding;C-acetyltransferase activity;C-acyltransferase activity;catalytic activity;cation binding;coenzyme binding;cofactor
activating transcription factor binding;binding;DNA binding;heat shock protein binding;molecular transducer activity;nucleic acid binding;protein binding;RNA binding;signal transducer activity;transcription factor binding;trans
catalytic activity;sedoheptulose-7-phosphate:D-glyceraldehyde-3-phosphate glyceronetransferase activity;transferase activity;transferase activity, transferring aldehyde or ketonic groups
adenyl nucleotide binding;adenyl ribonucleotide binding;ATP binding;ATPase activity;ATPase activity, coupled;ATP-dependent helicase activity;ATP-dependent RNA helicase activity;binding;catalytic activity;helicase activity;h
adenyl nucleotide binding;adenyl ribonucleotide binding;aminoacyl-tRNA ligase activity;ATP binding;binding;catalytic activity;cation binding;cysteine-tRNA ligase activity;identical protein binding;ion binding;ligase activity;liga
binding;lipid binding;phospholipid binding
binding;catalytic activity;cation binding;endopeptidase activity;hydrolase activity;ion binding;metal ion binding;metalloendopeptidase activity;metallopeptidase activity;peptidase activity;peptidase activity, acting on L-amino a
binding;nucleic acid binding;RNA binding;structural constituent of ribosome;structural molecule activity
binding;nucleic acid binding;RNA binding;structural constituent of ribosome;structural molecule activity
adenyl nucleotide binding;adenyl ribonucleotide binding;ATP binding;ATPase activity;binding;binding, bridging;catalytic activity;hydrolase activity;hydrolase activity, acting on acid anhydrides;hydrolase activity, acting on acid
binding;carboxylic acid binding;catalytic activity;cation binding;identical protein binding;ion binding;iron ion binding;L-ascorbic acid binding;metal ion binding;oxidoreductase activity;oxidoreductase activity, acting on paired
binding;chaperone binding;enzyme binding;heat shock protein binding;kinase binding;protein binding;unfolded protein binding
binding;core promoter binding;DNA binding;nucleic acid binding;nucleotide binding;regulatory region DNA binding;regulatory region nucleic acid binding;transcription regulatory region DNA binding
actin binding;actinin binding;binding;cation binding;cytoskeletal protein binding;enzyme binding;ion binding;kinase binding;metal ion binding;protein binding;protein kinase binding;protein kinase C binding;transition metal ion
adenyl nucleotide binding;adenyl ribonucleotide binding;ATP binding;binding;calcium ion binding;catalytic activity;cation binding;GTP binding;guanyl nucleotide binding;guanyl ribonucleotide binding;hydrolase activity;ion bin
binding;protein binding;protein complex binding
adenyl nucleotide binding;adenyl ribonucleotide binding;ATP binding;binding;catalytic activity;cation binding;ion binding;kinase activity;magnesium ion binding;metal ion binding;nucleotide binding;phosphotransferase activity
structural molecule activity
binding;catalytic activity;cation binding;coenzyme binding;cofactor binding;identical protein binding;ion binding;isocitrate dehydrogenase (NADP+) activity;isocitrate dehydrogenase activity;magnesium ion binding;metal ion b
adenyl nucleotide binding;adenyl ribonucleotide binding;ATP binding;binding;catalytic activity;creatine kinase activity;kinase activity;nucleotide binding;phosphotransferase activity, alcohol group as acceptor;phosphotransfer
actin binding;actin filament binding;adenyl nucleotide binding;adenyl ribonucleotide binding;ATP binding;binding;catalytic activity;chromatin binding;cytoskeletal protein binding;DNA binding;double-stranded DNA binding;enz
binding;nucleic acid binding;ribonucleoprotein binding;ribosome binding;RNA binding;translation factor activity, nucleic acid binding;translation initiation factor activity
binding;catalytic activity;coenzyme binding;cofactor binding;dihydrolipoyl dehydrogenase activity;flavin adenine dinucleotide binding;oxidoreductase activity;oxidoreductase activity, acting on a sulfur group of donors;oxidore
binding;chromatin binding;chromatin DNA binding;DNA binding;nucleic acid binding;structure-specific DNA binding
adenyl nucleotide binding;adenyl ribonucleotide binding;ATP binding;binding;catalytic activity;CTP synthase activity;ligase activity;ligase activity, forming carbon-nitrogen bonds;nucleotide binding;purine nucleotide binding;p
5'-nucleotidase activity;binding;catalytic activity;cation binding;hydrolase activity;hydrolase activity, acting on ester bonds;ion binding;metal ion binding;nucleotidase activity;nucleotide binding;phosphatase activity;phosphori
3'-5' DNA helicase activity;adenyl nucleotide binding;adenyl ribonucleotide binding;ATP binding;ATPase activity;ATPase activity, coupled;ATP-dependent 3'-5' DNA helicase activity;ATP-dependent DNA helicase activity;ATP-
binding;mRNA binding;nucleic acid binding;RNA binding;structural constituent of ribosome;structural molecule activity
catalytic activity;endopeptidase activity;exopeptidase activity;hydrolase activity;peptidase activity;peptidase activity, acting on L-amino acid peptides;serine hydrolase activity;serine-type endopeptidase activity;serine-type ex
binding;heat shock protein binding;Hsp90 protein binding;protein binding;protein complex binding
binding;cation binding;DNA binding;identical protein binding;ion binding;metal ion binding;nucleic acid binding;nucleocytoplasmic transporter activity;protein anchor;protein binding;structural constituent of nuclear pore;struc
binding;catalytic activity;cytoskeletal protein binding;enzyme binding;GTP binding;GTPase activity;guanyl nucleotide binding;guanyl ribonucleotide binding;hydrolase activity;hydrolase activity, acting on acid anhydrides;hydro
binding;nucleic acid binding;RNA binding;structural constituent of ribosome;structural molecule activity
binding;catalytic activity;coenzyme binding;cofactor binding;NADP binding;nucleotide binding;oxidoreductase activity;oxidoreductase activity, acting on CH-OH group of donors;oxidoreductase activity, acting on the CH-OH
adenyl nucleotide binding;adenyl ribonucleotide binding;ATP binding;ATPase activity;ATPase activity, coupled;binding;catalytic activity;chromatin binding;damaged DNA binding;DNA binding;DNA-dependent ATPase activity;
binding;nucleic acid binding;nucleotide binding;RNA binding
adenyl nucleotide binding;adenyl ribonucleotide binding;ATP binding;ATPase activity;binding;catalytic activity;G-protein-coupled receptor binding;hormone receptor binding;hydrolase activity;hydrolase activity, acting on acid
binding;nucleic acid binding;RNA binding;rRNA binding;structural constituent of ribosome;structural molecule activity
binding;nucleic acid binding;RNA binding;structural constituent of ribosome;structural molecule activity
amine binding;amino acid binding;binding;carbohydrate binding;carboxylic acid binding;catalytic activity;glutamine-fructose-6-phosphate transaminase (isomerizing) activity;transaminase activity;transferase activity;transfera
actin binding;binding;cytoskeletal protein binding;protein binding
binding;histone binding;nucleic acid binding;nucleotide binding;protein binding;RNA binding;snRNA binding;U4 snRNA binding;U6 snRNA binding;U6atac snRNA binding
adenyl nucleotide binding;adenyl ribonucleotide binding;amine binding;amino acid binding;aminoacyl-tRNA ligase activity;ATP binding;binding;carboxylic acid binding;catalytic activity;cation binding;ion binding;ligase activity
catalytic activity;intramolecular oxidoreductase activity;intramolecular oxidoreductase activity, interconverting keto- and enol-groups;intramolecular oxidoreductase activity, transposing S-S bonds;isomerase activity;protein d
binding;nucleic acid binding;RNA binding
acid-amino acid ligase activity;ATPase activity;binding;catalytic activity;cation binding;hydrolase activity;hydrolase activity, acting on acid anhydrides;hydrolase activity, acting on acid anhydrides, in phosphorus-containing an
adenyl nucleotide binding;adenyl ribonucleotide binding;ATP binding;ATPase activity;ATPase activity, coupled;ATP-dependent helicase activity;ATP-dependent RNA helicase activity;binding;catalytic activity;chromatin binding
acetyltransferase activity;adenyl nucleotide binding;adenyl ribonucleotide binding;ATP binding;binding;catalytic activity;N-acetyltransferase activity;N-acyltransferase activity;nucleotide binding;purine nucleotide binding;purin
adenyl nucleotide binding;adenyl ribonucleotide binding;ATP binding;binding;catalytic activity;cation binding;enzyme activator activity;enzyme regulator activity;ion binding;metal ion binding;nucleotide binding;protein binding
acetylcholine receptor regulator activity;binding;calcium ion binding;carbohydrate binding;cation binding;chondroitin sulfate binding;dystroglycan binding;extracellular matrix binding;glycoprotein binding;glycosaminoglycan b
binding;carboxylic acid binding;catalytic activity;cation binding;dioxygenase activity;electron carrier activity;ion binding;iron ion binding;L-ascorbic acid binding;metal ion binding;oxidoreductase activity;oxidoreductase activit
acid-thiol ligase activity;adenyl nucleotide binding;adenyl ribonucleotide binding;arachidonate-CoA ligase activity;ATP binding;binding;catalytic activity;fatty acid ligase activity;ligase activity;ligase activity, forming carbon-sulf
binding;catalytic activity;GTP binding;GTPase activity;guanyl nucleotide binding;guanyl ribonucleotide binding;hydrolase activity;hydrolase activity, acting on acid anhydrides;hydrolase activity, acting on acid anhydrides, in ph
binding;catalytic activity;coenzyme binding;cofactor binding;DNA binding;electron carrier activity;FAD binding;flavin adenine dinucleotide binding;NAD(P)H oxidase activity;nucleic acid binding;oxidoreductase activity;oxidore
binding;chromatin binding;chromatin DNA binding;DNA binding;nucleic acid binding;structure-specific DNA binding
structural constituent of ribosome;structural molecule activity
3'-phosphoadenosine 5'-phosphosulfate binding;adenyl nucleotide binding;adenyl ribonucleotide binding;ATP binding;binding;calcium ion binding;carbohydrate binding;cargo receptor activity;catalytic activity;cation binding;
binding;catalytic activity;endopeptidase activity;hydrolase activity;nucleic acid binding;peptidase activity;peptidase activity, acting on L-amino acid peptides;RNA binding;threonine-type endopeptidase activity;threonine-type
androgen receptor binding;binding;calcium ion binding;carbohydrate binding;cation binding;chaperone binding;complement binding;complement component C1q binding;DNA binding;enzyme binding;glycoprotein binding;ho
antioxidant activity;binding;carboxylic ester hydrolase activity;catalytic activity;enzyme binding;glutathione peroxidase activity;hydrolase activity;hydrolase activity, acting on ester bonds;lipase activity;oxidoreductase activity;
antigen binding;binding;catalytic activity;enzyme regulator activity;hydrolase activity;hydrolase activity, acting on ester bonds;phosphatase activity;phosphatase regulator activity;phosphoprotein phosphatase activity;phospho
adenyl nucleotide binding;adenyl ribonucleotide binding;ATP binding;ATPase activity;binding;catalytic activity;hydrolase activity;hydrolase activity, acting on acid anhydrides;hydrolase activity, acting on acid anhydrides, in ph
structural constituent of ribosome;structural molecule activity
3alpha,7alpha,12alpha-trihydroxy-5beta-cholest-24-enoyl-CoA hydratase activity;3-hydroxyacyl-CoA dehydrogenase activity;binding;carbon-oxygen lyase activity;catalytic activity;hydro-lyase activity;identical protein binding
structural molecule activity
binding;catalytic activity;enzyme binding;intramolecular oxidoreductase activity;intramolecular oxidoreductase activity, interconverting aldoses and ketoses;isomerase activity;protein binding;triose-phosphate isomerase activ
adenyl nucleotide binding;adenyl ribonucleotide binding;ATP binding;binding;nucleotide binding;purine nucleotide binding;purine ribonucleoside triphosphate binding;purine ribonucleotide binding;ribonucleotide binding;struc
binding;nucleic acid binding;RNA binding;rRNA binding;structural constituent of ribosome;structural molecule activity
binding;enzyme binding;kinase binding;protein binding;protein kinase binding;structural constituent of ribosome;structural molecule activity
structural constituent of ribosome;structural molecule activity
binding;clathrin binding;peptide binding;protein binding;protein transporter activity;signal sequence binding;substrate-specific transporter activity;transporter activity
binding;channel regulator activity;enzyme binding;glucocorticoid receptor binding;hormone receptor binding;insulin-like growth factor receptor binding;ion channel binding;nuclear hormone receptor binding;protein binding;pr
antioxidant activity;binding;catalytic activity;cation binding;heme binding;ion binding;iron ion binding;metal ion binding;oxidoreductase activity;oxidoreductase activity, acting on paired donors, with incorporation or reduction
binding;mRNA binding;nucleic acid binding;nucleotide binding;RNA binding
binding;enzyme binding;kinase binding;protein binding;protein kinase binding;protein transporter activity;substrate-specific transporter activity;transporter activity
adenyl nucleotide binding;adenyl ribonucleotide binding;ATP binding;binding;catalytic activity;enzyme binding;kinase activity;kinase binding;molecular transducer activity;nucleotide binding;phosphotransferase activity, alcoh
binding;DNA binding;identical protein binding;nucleic acid binding;nucleotide binding;protein binding
binding;binding, bridging;cation binding;ion binding;ion transmembrane transporter activity;metal ion binding;protein binding;protein binding, bridging;SH3/SH2 adaptor activity;signaling adaptor activity;substrate-specific tra
adenyl nucleotide binding;adenyl ribonucleotide binding;ATP binding;binding;GTP binding;guanyl nucleotide binding;guanyl ribonucleotide binding;nucleotide binding;purine nucleotide binding;purine ribonucleoside triphosph
binding;cation binding;ion binding;metal ion binding;transition metal ion binding;zinc ion binding
catalytic activity;dihydropyrimidinase activity;hydrolase activity;hydrolase activity, acting on carbon-nitrogen (but not peptide) bonds;hydrolase activity, acting on carbon-nitrogen (but not peptide) bonds, in cyclic amides
adenyl nucleotide binding;adenyl ribonucleotide binding;ATP binding;binding;catalytic activity;DNA helicase activity;helicase activity;hydrolase activity;hydrolase activity, acting on acid anhydrides;hydrolase activity, acting on
protein transporter activity;substrate-specific transporter activity;transporter activity
structural constituent of nuclear pore;structural molecule activity;transporter activity
adenyl nucleotide binding;adenyl ribonucleotide binding;ATP binding;binding;catalytic activity;enzyme binding;fatty acid ligase activity;kinase binding;ligase activity;ligase activity, forming carbon-sulfur bonds;long-chain fatty
carbon-oxygen lyase activity;catalytic activity;fumarate hydratase activity;hydro-lyase activity;lyase activity
binding;nucleic acid binding;nucleotide binding;RNA binding
binding;calcium ion binding;calcium-dependent phospholipid binding;calcium-dependent protein binding;cation binding;enzyme inhibitor activity;enzyme regulator activity;identical protein binding;ion binding;lipase inhibitor a
anion channel activity;anion transmembrane transporter activity;binding;channel activity;enzyme binding;gated channel activity;ion channel activity;ion transmembrane transporter activity;kinase binding;passive transmembra
binding;carbon-oxygen lyase activity;catalytic activity;damaged DNA binding;DNA binding;DNA N-glycosylase activity;DNA-(apurinic or apyrimidinic site) lyase activity;enzyme binding;hydrolase activity;hydrolase activity, acti
adenosylhomocysteinase activity;catalytic activity;hydrolase activity;hydrolase activity, acting on ether bonds;trialkylsulfonium hydrolase activity
structural constituent of ribosome;structural molecule activity
2 iron, 2 sulfur cluster binding;4 iron, 4 sulfur cluster binding;binding;catalytic activity;cation binding;electron carrier activity;ion binding;iron-sulfur cluster binding;metal cluster binding;metal ion binding;NADH dehydrogenase
adenyl nucleotide binding;adenyl ribonucleotide binding;ATP binding;binding;catalytic activity;ephrin receptor activity;kinase activity;molecular transducer activity;nucleotide binding;phosphotransferase activity, alcohol group
binding;DNA binding;lamin binding;nucleic acid binding;protein binding
adenyl nucleotide binding;adenyl ribonucleotide binding;ATP binding;ATPase activity;binding;catalytic activity;enzyme inhibitor activity;enzyme regulator activity;hydrolase activity;hydrolase activity, acting on acid anhydrides;
binding;nucleic acid binding;RNA binding;structural constituent of ribosome;structural molecule activity
binding;nucleic acid binding;RNA binding;structural constituent of ribosome;structural molecule activity
enzyme regulator activity
binding;catalytic activity;cation transmembrane transporter activity;coenzyme binding;cofactor binding;hydrogen ion transmembrane transporter activity;inorganic cation transmembrane transporter activity;ion transmembran
adenyl nucleotide binding;adenyl ribonucleotide binding;ATP binding;ATPase activity;ATPase activity, coupled;ATP-dependent helicase activity;ATP-dependent protein binding;ATP-dependent RNA helicase activity;binding;ca
binding;DNA binding;nucleic acid binding;nucleotide binding;RNA binding;sequence-specific DNA binding;telomeric DNA binding
binding;nucleic acid binding;RNA binding
binding;carbohydrate binding;catalytic activity;cation binding;glucose binding;identical protein binding;ion binding;metal ion binding;monosaccharide binding;nucleotide binding;nucleotidyltransferase activity;protein binding;
antioxidant activity;binding;catalytic activity;coenzyme binding;cofactor binding;disulfide oxidoreductase activity;electron carrier activity;flavin adenine dinucleotide binding;oxidoreductase activity;oxidoreductase activity, acti
binding;nucleic acid binding;RNA binding;rRNA binding;rRNA primary transcript binding
adenyl nucleotide binding;adenyl ribonucleotide binding;ATP binding;ATPase activity;binding;catalytic activity;hydrolase activity;hydrolase activity, acting on acid anhydrides;hydrolase activity, acting on acid anhydrides, in ph
structural constituent of nuclear pore;structural molecule activity
binding;enzyme binding;nucleic acid binding;protein binding;RNA binding
binding;cytoskeletal protein binding;enzyme binding;GTPase binding;kinase binding;microtubule binding;protein binding;protein domain specific binding;protein kinase binding;Rac GTPase binding;Ras GTPase binding;Rho G
actin binding;actin filament binding;binding;cytoskeletal protein binding;protein binding
binding;catalytic activity;cofactor binding;oxidoreductase activity;oxidoreductase activity, acting on a sulfur group of donors;oxidoreductase activity, acting on a sulfur group of donors, quinone or similar compound as accept

binding;catalytic activity;cation binding;GTP binding;GTPase activity;guanyl nucleotide binding;guanyl ribonucleotide binding;hydrolase activity;hydrolase activity, acting on acid anhydrides;hydrolase activity, acting on acid a
adenyl nucleotide binding;adenyl ribonucleotide binding;ATP binding;binding;catalytic activity;cation binding;ion binding;kinase activity;metal ion binding;nucleotide binding;phosphotransferase activity, alcohol group as acce
binding;catalytic activity;cation binding;chromatin binding;deacetylase activity;DNA binding;histone deacetylase activity;hydrolase activity;hydrolase activity, acting on carbon-nitrogen (but not peptide) bonds;hydrolase activit
binding;calcium channel activity;cation binding;cation channel activity;cation transmembrane transporter activity;channel activity;cytokine binding;enzyme binding;extracellular matrix binding;fibronectin binding;gated channe
enzyme inhibitor activity;enzyme regulator activity;ribonuclease inhibitor activity
adenyl nucleotide binding;adenyl ribonucleotide binding;ATP binding;binding;catalytic activity;nucleotide binding;oxidoreductase activity;oxidoreductase activity, acting on CH or CH2 groups;oxidoreductase activity, acting o
binding;nucleic acid binding;nucleotide binding;poly-pyrimidine tract binding;pre-mRNA binding;RNA binding;single-stranded RNA binding
binding;catalytic activity;cyclohydrolase activity;hydrolase activity;hydrolase activity, acting on carbon-nitrogen (but not peptide) bonds;hydrolase activity, acting on carbon-nitrogen (but not peptide) bonds, in cyclic amidines;
binding;calcium ion binding;catalytic activity;cation binding;glycerol-3-phosphate dehydrogenase activity;ion binding;metal ion binding;oxidoreductase activity;oxidoreductase activity, acting on CH-OH group of donors;oxido
binding;intermediate filament binding;protein binding;protein complex binding;structural molecule activity
binding;catalytic activity;GTP binding;GTPase activity;guanyl nucleotide binding;guanyl ribonucleotide binding;hydrolase activity;hydrolase activity, acting on acid anhydrides;hydrolase activity, acting on acid anhydrides, in p
adenyl nucleotide binding;adenyl ribonucleotide binding;ATP binding;binding;carbohydrate binding;carbohydrate kinase activity;catalytic activity;fructokinase activity;glucokinase activity;glucose binding;hexokinase activity;k
amine binding;amino acid binding;antigen binding;binding;carboxylic acid binding;channel regulator activity;enzyme binding;histone deacetylase binding;ion channel binding;MHC class II protein complex binding;MHC protei
binding;nucleic acid binding;RNA binding;structural constituent of ribosome;structural molecule activity
binding;chromatin binding;DNA binding;nucleic acid binding
actin binding;actin filament binding;adenyl nucleotide binding;adenyl ribonucleotide binding;ATP binding;ATPase activity;ATPase activity, coupled;binding;calmodulin binding;catalytic activity;cytoskeletal protein binding;hydro
binding;chromatin binding;DNA binding;nucleic acid binding;nucleosome binding
catalytic activity;galactosyltransferase activity;procollagen galactosyltransferase activity;transferase activity;transferase activity, transferring glycosyl groups;transferase activity, transferring hexosyl groups;UDP-galactosyltran
binding;chromatin binding;DNA binding;nucleic acid binding;protein binding;protein domain specific binding;WD40-repeat domain binding
binding;catalytic activity;GTP binding;GTPase activity;guanyl nucleotide binding;guanyl ribonucleotide binding;hydrolase activity;hydrolase activity, acting on acid anhydrides;hydrolase activity, acting on acid anhydrides, in p
acetylcholine receptor regulator activity;adenyl nucleotide binding;adenyl ribonucleotide binding;ATP binding;binding;catalytic activity;cation binding;chromatin binding;histone acetyl-lysine binding;histone binding;histone kin
extracellular matrix constituent conferring elasticity;extracellular matrix structural constituent;structural molecule activity
binding;nucleic acid binding;nucleocytoplasmic transporter activity;protein transporter activity;RNA binding;substrate-specific transporter activity;transporter activity
enzyme regulator activity
binding;catalytic activity;coenzyme binding;cofactor binding;electron carrier activity;NAD binding;nucleotide binding;oxidoreductase activity;oxidoreductase activity, acting on CH-OH group of donors;oxidoreductase activity,
binding;mRNA binding;nucleic acid binding;nucleotide binding;RNA binding;tRNA binding
aminopeptidase activity;binding;catalytic activity;cation binding;epoxide hydrolase activity;ether hydrolase activity;exopeptidase activity;hydrolase activity;hydrolase activity, acting on ether bonds;ion binding;leukotriene-A4 h
adenyl nucleotide binding;adenyl ribonucleotide binding;ATP binding;ATPase activity;ATPase activity, coupled;binding;catalytic activity;cation binding;chromatin binding;DNA bending activity;DNA binding;DNA topoisomerase
binding;growth factor binding;platelet-derived growth factor binding;protein binding
binding;cytoskeletal protein binding;molecular transducer activity;protein binding;signal transducer activity;tubulin binding
adenyl nucleotide binding;adenyl ribonucleotide binding;ATP binding;ATPase activity;binding;catalytic activity;hydrolase activity;hydrolase activity, acting on acid anhydrides;hydrolase activity, acting on acid anhydrides, in ph
binding;protein binding;protein N-terminus binding
binding;enzyme binding;fibroblast growth factor binding;growth factor binding;identical protein binding;kinase binding;protein binding;protein dimerization activity;protein homodimerization activity;protein kinase binding;struc
5'-3' exonuclease activity;5'-flap endonuclease activity;binding;catalytic activity;cation binding;damaged DNA binding;deoxyribonuclease activity;DNA binding;double-stranded DNA binding;double-stranded DNA specific ex
adenyl nucleotide binding;adenyl ribonucleotide binding;aminoacyl-tRNA ligase activity;ATP binding;binding;catalytic activity;ligase activity;ligase activity, forming aminoacyl-tRNA and related compounds;ligase activity, formi
adenyl nucleotide binding;adenyl ribonucleotide binding;ATP binding;binding;carbon-nitrogen ligase activity, with glutamine as amido-N-donor;catalytic activity;GMP synthase (glutamine-hydrolyzing) activity;GMP synthase a
binding;collagen binding;endopeptidase inhibitor activity;endopeptidase regulator activity;enzyme inhibitor activity;enzyme regulator activity;peptidase inhibitor activity;peptidase regulator activity;protein binding;serine-type e
binding;nucleic acid binding;nucleotide binding;RNA binding
adenyl nucleotide binding;adenyl ribonucleotide binding;amino acid kinase activity;ATP binding;binding;catalytic activity;coenzyme binding;cofactor binding;glutamate 5-kinase activity;glutamate-5-semialdehyde dehydrogen
adenyl nucleotide binding;adenyl ribonucleotide binding;ATP binding;binding;nucleotide binding;purine nucleotide binding;purine ribonucleoside triphosphate binding;purine ribonucleotide binding;ribonucleotide binding;struc
binding;GTP binding;guanyl nucleotide binding;guanyl ribonucleotide binding;identical protein binding;nucleotide binding;protein binding;purine nucleotide binding;purine ribonucleoside triphosphate binding;purine ribonucle
adenyl nucleotide binding;adenyl ribonucleotide binding;ATP binding;binding;nucleotide binding;purine nucleotide binding;purine ribonucleoside triphosphate binding;purine ribonucleotide binding;ribonucleotide binding
binding;cation binding;cytoskeletal protein binding;enzyme binding;enzyme regulator activity;GTPase binding;GTPase regulator activity;guanyl-nucleotide exchange factor activity;ion binding;metal ion binding;microtubule bi
protein transporter activity;substrate-specific transporter activity;transporter activity
binding;cation binding;ion binding;lipid binding;metal ion binding;phospholipid binding
binding;cation binding;ion binding;metal ion binding;transition metal ion binding;zinc ion binding
structural constituent of cytoskeleton;structural constituent of muscle;structural molecule activity
binding;chemoattractant activity;chromatin binding;cytokine activity;damaged DNA binding;DNA bending activity;DNA binding;double-stranded DNA binding;nucleic acid binding;nucleic acid binding transcription factor activi
aldehyde-lyase activity;binding;carbon-carbon lyase activity;catalytic activity;cytoskeletal protein binding;fructose-bisphosphate aldolase activity;lyase activity;protein binding
structural constituent of epidermis;structural molecule activity
active transmembrane transporter activity;adenyl nucleotide binding;adenyl ribonucleotide binding;ATP binding;ATPase activity;ATPase activity, coupled;ATPase activity, coupled to movement of substances;ATPase activity, c
binding;cation binding;ion binding;metal ion binding
binding;nucleic acid binding;RNA binding;structural constituent of ribosome;structural molecule activity
binding;catalytic activity;cation binding;chromatin binding;DNA binding;DNA-directed RNA polymerase activity;ion binding;metal ion binding;nucleic acid binding;nucleoside binding;nucleotidyltransferase activity;ribonucleosi
amine binding;amino acid binding;binding;carboxylic acid binding;enzyme binding;histone deacetylase binding;phosphoprotein binding;phosphoserine binding;protein binding;protein binding transcription factor activity;prote
3-hydroxyacyl-CoA dehydrogenase activity;acetyl-CoA C-acyltransferase activity;binding;C-acyltransferase activity;carbon-oxygen lyase activity;carboxylic acid binding;catalytic activity;coenzyme binding;cofactor binding;e
binding;catalytic activity;cation binding;coenzyme binding;cofactor binding;ion binding;metal ion binding;oxidoreductase activity;oxidoreductase activity, acting on the aldehyde or oxo group of donors;oxidoreductase activity
structural constituent of nuclear pore;structural molecule activity
actinin binding;alpha-actinin binding;binding;cytoskeletal protein binding;muscle alpha-actinin binding;protein binding
3 iron, 4 sulfur cluster binding;4 iron, 4 sulfur cluster binding;aconitate hydratase activity;binding;carbon-oxygen lyase activity;catalytic activity;cation binding;hydro-lyase activity;ion binding;iron ion binding;iron-sulfur cluster
adenyl nucleotide binding;adenyl ribonucleotide binding;ATP binding;binding;calcium ion binding;cation binding;enzyme binding;GTP binding;GTPase binding;guanyl nucleotide binding;guanyl ribonucleotide binding;ion bindi
binding;cation binding;ion binding;metal ion binding;transition metal ion binding;zinc ion binding
structural molecule activity
binding;enzyme binding;enzyme regulator activity;GTPase binding;GTPase regulator activity;nucleoside-triphosphatase regulator activity;protein binding;Ran GTPase binding;Ras GTPase binding;small GTPase binding;trans
binding;bisphosphoglycerate 2-phosphatase activity;bisphosphoglycerate mutase activity;bisphosphoglycerate phosphatase activity;catalytic activity;enzyme binding;hydrolase activity;hydrolase activity, acting on ester bon
binding;calcium ion binding;carbohydrate binding;cation binding;ion binding;metal ion binding
structural constituent of cytoskeleton;structural molecule activity
catalytic activity;L-malate dehydrogenase activity;malate dehydrogenase (NADP+) activity;malate dehydrogenase activity;oxidoreductase activity;oxidoreductase activity, acting on CH-OH group of donors;oxidoreductase act
binding;catalytic activity;cation binding;cysteine-type endopeptidase activity;cysteine-type peptidase activity;endopeptidase activity;exopeptidase activity;hydrolase activity;ion binding;metal ion binding;omega peptidase act
adenyl nucleotide binding;adenyl ribonucleotide binding;ATP binding;binding;binding, bridging;catalytic activity;cis-trans isomerase activity;drug binding;FK506 binding;GTP binding;guanyl nucleotide binding;guanyl ribonucle
binding;DNA binding;nucleic acid binding;RNA binding;structural constituent of ribosome;structural molecule activity
1,4-alpha-glucan branching enzyme activity;binding;catalytic activity;cation binding;hydrolase activity;hydrolase activity, acting on glycosyl bonds;hydrolase activity, hydrolyzing O-glycosyl compounds;ion binding;transferase
binding;calcium ion binding;catalytic activity;cation binding;electron carrier activity;ion binding;ion channel binding;metal ion binding;oxidoreductase activity;oxidoreductase activity, acting on paired donors, with incorporatio
binding;core promoter binding;core promoter proximal region DNA binding;core promoter proximal region sequence-specific DNA binding;DNA binding;double-stranded DNA binding;identical protein binding;nucleic acid bin
binding;catalytic activity;cation binding;endopeptidase activity;enzyme activator activity;enzyme regulator activity;hydrolase activity;ion binding;metal ion binding;metalloendopeptidase activity;metallopeptidase activity;pepti
binding;eukaryotic initiation factor 4E binding;mRNA 3'-UTR binding;mRNA 5'-UTR binding;mRNA binding;nucleic acid binding;protein binding;RNA binding;RNA cap binding;translation activator activity;translation initiation f
binding;catalytic activity;cation binding;ion binding;metal ion binding;NAD+ ADP-ribosyltransferase activity;transferase activity;transferase activity, transferring glycosyl groups;transferase activity, transferring pentosyl groups
acid-amino acid ligase activity;binding;catalytic activity;cation binding;ion binding;ligase activity;ligase activity, forming carbon-nitrogen bonds;metal ion binding;small conjugating protein ligase activity;transition metal ion bin
adenyl nucleotide binding;adenyl ribonucleotide binding;ATP binding;ATPase activity;ATPase activity, coupled;ATP-dependent helicase activity;ATP-dependent RNA helicase activity;binding;catalytic activity;cation binding;ch
aminopeptidase activity;binding;catalytic activity;cation binding;epoxide hydrolase activity;ether hydrolase activity;exopeptidase activity;hydrolase activity;hydrolase activity, acting on ether bonds;ion binding;metal ion bindin
actin binding;binding;cytoskeletal protein binding;protein binding
binding;enzyme binding;kinase binding;protein binding;protein kinase binding;protein transporter activity;substrate-specific transporter activity;transporter activity
binding;calcium ion binding;cation binding;ion binding;metal ion binding
binding;catalytic activity;coenzyme binding;cofactor binding;flavin adenine dinucleotide binding;oxidoreductase activity;oxidoreductase activity, acting on the CH-CH group of donors;oxidoreductase activity, acting on the CH
aldehyde-lyase activity;amine binding;amino acid binding;binding;carbon-carbon lyase activity;carboxylic acid binding;catalytic activity;chromatin binding;cofactor binding;glycine hydroxymethyltransferase activity;hydroxym
binding;catalytic activity;GTP binding;GTPase activity;guanyl nucleotide binding;guanyl ribonucleotide binding;hydrolase activity;hydrolase activity, acting on acid anhydrides;hydrolase activity, acting on acid anhydrides, in p
5S rDNA binding;adenyl nucleotide binding;adenyl ribonucleotide binding;ATP binding;binding;catalytic activity;DNA binding;drug binding;kinase activity;nucleic acid binding;nucleotide binding;phosphoprotein binding;phosp
binding;nucleic acid binding;RNA binding;rRNA binding;structural constituent of ribosome;structural molecule activity
binding;enzyme binding;identical protein binding;kinase binding;protein binding;protein kinase binding;transcription factor binding;ubiquitin protein ligase binding
binding;nucleic acid binding;RNA binding;translation factor activity, nucleic acid binding;translation initiation factor activity
binding;calcium ion binding;cation binding;cytoskeletal protein binding;identical protein binding;integrin binding;ion binding;metal ion binding;microtubule binding;microtubule plus-end binding;protein binding;protein comple
binding;estrogen receptor binding;hormone receptor binding;nuclear hormone receptor binding;protein binding;protein N-terminus binding;receptor binding;steroid hormone receptor binding
binding;DNA binding;nucleic acid binding
binding;DNA binding;enzyme binding;nucleic acid binding;nucleic acid binding transcription factor activity;protein binding;sequence-specific DNA binding transcription factor activity;ubiquitin protein ligase binding
adenyl nucleotide binding;adenyl ribonucleotide binding;ATP binding;binding;core promoter binding;core promoter sequence-specific DNA binding;DNA binding;hormone receptor binding;ligand-dependent nuclear receptor t
binding;catalytic activity;coenzyme binding;cofactor binding;electron carrier activity;NAD binding;nucleotide binding;oxidoreductase activity;oxidoreductase activity, acting on CH-OH group of donors;oxidoreductase activity,
binding;nucleic acid binding;nucleotide binding;RNA binding
binding;mRNA binding;nucleic acid binding;RNA binding
binding;DNA binding;nucleic acid binding
binding;enzyme binding;enzyme inhibitor activity;enzyme regulator activity;histone binding;identical protein binding;kinase binding;kinase inhibitor activity;kinase regulator activity;NF-kappaB binding;nucleic acid binding;prot
binding;calcium ion binding;calcium-dependent cysteine-type endopeptidase activity;catalytic activity;cation binding;cysteine-type endopeptidase activity;cysteine-type peptidase activity;endopeptidase activity;hydrolase ac
binding;catalytic activity;cation binding;DNA binding;IMP dehydrogenase activity;ion binding;metal ion binding;nucleic acid binding;nucleotide binding;oxidoreductase activity;oxidoreductase activity, acting on CH-OH group
4 iron, 4 sulfur cluster binding;aconitate hydratase activity;binding;carbon-oxygen lyase activity;catalytic activity;cation binding;hydro-lyase activity;ion binding;iron-responsive element binding;iron-sulfur cluster binding;lyase
acid-amino acid ligase activity;adenyl nucleotide binding;adenyl ribonucleotide binding;ATP binding;binding;carbon-carbon lyase activity;carboxy-lyase activity;catalytic activity;identical protein binding;ligase activity;ligase a
binding;nucleic acid binding;RNA binding;translation elongation factor activity;translation factor activity, nucleic acid binding
binding;nucleic acid binding;nucleotide binding;poly(U) RNA binding;poly-pyrimidine tract binding;RNA binding;single-stranded RNA binding
binding;catalytic activity;cation binding;intramolecular transferase activity;intramolecular transferase activity, phosphotransferases;ion binding;isomerase activity;magnesium ion binding;metal ion binding;phosphoglucomuta
adenyl nucleotide binding;adenyl ribonucleotide binding;amine binding;amino acid binding;aminoacyl-tRNA ligase activity;arginine binding;arginine-tRNA ligase activity;ATP binding;binding;carboxylic acid binding;catalytic ac
binding;nucleic acid binding;RNA binding;rRNA binding;structural constituent of ribosome;structural molecule activity
binding;catalytic activity;GTP binding;GTPase activity;guanyl nucleotide binding;guanyl ribonucleotide binding;hydrolase activity;hydrolase activity, acting on acid anhydrides;hydrolase activity, acting on acid anhydrides, in p
binding;catalytic activity;GTP binding;GTPase activity;guanyl nucleotide binding;guanyl ribonucleotide binding;hydrolase activity;hydrolase activity, acting on acid anhydrides;hydrolase activity, acting on acid anhydrides, in p
adenyl nucleotide binding;adenyl ribonucleotide binding;ATP binding;ATPase activity;ATPase activity, coupled;ATP-dependent helicase activity;ATP-dependent RNA helicase activity;binding;catalytic activity;helicase activity;h
acetyltransferase activity;binding;catalytic activity;N-acetyltransferase activity;N-acyltransferase activity;ribonucleoprotein binding;ribosome binding;transferase activity;transferase activity, transferring acyl groups;transferase
actin binding;actin filament binding;adenyl nucleotide binding;adenyl ribonucleotide binding;ADP binding;ATP binding;binding;calmodulin binding;catalytic activity;cytoskeletal protein binding;hydrolase activity;hydrolase activ
active transmembrane transporter activity;adenyl nucleotide binding;adenyl ribonucleotide binding;ATP binding;ATPase activity;ATPase activity, coupled;ATPase activity, coupled to movement of substances;ATPase activity, c
binding;chromatin binding;chromatin DNA binding;core promoter proximal region DNA binding;core promoter proximal region sequence-specific DNA binding;DNA binding;enhancer binding;enhancer sequence-specific DNA
actin binding;binding;cytoskeletal protein binding;protein binding;structural constituent of cytoskeleton;structural constituent of muscle;structural molecule activity
adenyl nucleotide binding;adenyl ribonucleotide binding;aminoacyl-tRNA ligase activity;ATP binding;binding;catalytic activity;histidine-tRNA ligase activity;ligase activity;ligase activity, forming aminoacyl-tRNA and related co
binding;DNA binding;nucleic acid binding;nucleotide binding;regulatory region DNA binding;regulatory region nucleic acid binding;RNA binding;transcription regulatory region DNA binding

binding;cation binding;collagen binding;ion binding;metal ion binding;protein binding;receptor activity;viral receptor activity
structural constituent of cytoskeleton;structural molecule activity
adenyl nucleotide binding;adenyl ribonucleotide binding;ATP binding;binding;nucleotide binding;protein binding;purine nucleotide binding;purine ribonucleoside triphosphate binding;purine ribonucleotide binding;ribonucleoti
binding;nucleic acid binding;RNA binding;rRNA binding;structural constituent of ribosome;structural molecule activity;translation regulator activity
binding;core promoter proximal region DNA binding;core promoter proximal region sequence-specific DNA binding;DNA binding;nucleic acid binding;nucleic acid binding transcription factor activity;regulatory region DNA bin
binding;enzyme binding;enzyme inhibitor activity;enzyme regulator activity;insulin-like growth factor receptor binding;kinase binding;kinase inhibitor activity;kinase regulator activity;protein binding;protein kinase binding;prote
adenyl nucleotide binding;adenyl ribonucleotide binding;ATP binding;ATPase activity;binding;catalytic activity;cytoskeletal protein binding;hydrolase activity;hydrolase activity, acting on acid anhydrides;hydrolase activity, acti
catalytic activity;hydrolase activity;hydrolase activity, acting on acid anhydrides;hydrolase activity, acting on acid anhydrides, in phosphorus-containing anhydrides;motor activity;nucleoside-triphosphatase activity;pyrophosp
adenyl nucleotide binding;adenyl ribonucleotide binding;ATP binding;ATPase activity;ATPase activity, coupled;ATP-dependent helicase activity;ATP-dependent RNA helicase activity;binding;catalytic activity;chromatin binding
binding;core promoter proximal region DNA binding;core promoter proximal region sequence-specific DNA binding;DNA binding;nucleic acid binding;nucleic acid binding transcription factor activity;regulatory region DNA bin
binding;DNA binding;nucleic acid binding
binding;catalytic activity;enzyme binding;GTP binding;GTPase activity;guanyl nucleotide binding;guanyl ribonucleotide binding;hydrolase activity;hydrolase activity, acting on acid anhydrides;hydrolase activity, acting on acid
adenyl nucleotide binding;adenyl ribonucleotide binding;ATP binding;binding;carbon-nitrogen ligase activity, with glutamine as amido-N-donor;catalytic activity;cation binding;ion binding;ligase activity;ligase activity, forming
structural molecule activity
adenyl nucleotide binding;adenyl ribonucleotide binding;aminoacyl-tRNA ligase activity;ATP binding;binding;catalytic activity;glutamine-tRNA ligase activity;ligase activity;ligase activity, forming aminoacyl-tRNA and related co
binding;nucleic acid binding;RNA binding;structural constituent of ribosome;structural molecule activity
adenyl nucleotide binding;adenyl ribonucleotide binding;ATP binding;binding;cytokine receptor binding;enzyme binding;kinase binding;nucleotide binding;protein binding;protein kinase binding;purine nucleotide binding;purin
binding;catalytic activity;co-SMAD binding;cysteine-type endopeptidase activity;cysteine-type peptidase activity;endopeptidase activity;hydrolase activity;peptidase activity;peptidase activity, acting on L-amino acid peptides
binding;carboxylic acid binding;catalytic activity;cation binding;ion binding;iron ion binding;L-ascorbic acid binding;metal ion binding;oxidoreductase activity;oxidoreductase activity, acting on paired donors, with incorporatio
binding;mRNA binding;nucleic acid binding;nucleotide binding;poly(A) RNA binding;poly-purine tract binding;RNA binding;single-stranded RNA binding
binding;calcium-dependent protein binding;protein binding
binding;cation binding;extracellular matrix structural constituent;growth factor binding;identical protein binding;ion binding;metal ion binding;platelet-derived growth factor binding;protein binding;structural molecule activity
adenine transmembrane transporter activity;binding;enzyme binding;nucleobase transmembrane transporter activity;nucleobase-containing compound transmembrane transporter activity;protein binding;purine base transme
actin binding;binding;cation binding;cell adhesion molecule binding;cytoskeletal protein binding;enzyme binding;ion binding;metal ion binding;peptide binding;protease binding;protein binding;protein dimerization activity;pro
binding;calcium ion binding;calcium-dependent cysteine-type endopeptidase activity;catalytic activity;cation binding;cysteine-type endopeptidase activity;cysteine-type peptidase activity;cytoskeletal protein binding;endope
adenyl nucleotide binding;adenyl ribonucleotide binding;ATP binding;binding;enzyme binding;glycolipid binding;lipid binding;nucleotide binding;protein binding;purine nucleotide binding;purine ribonucleoside triphosphate bi
binding;DNA binding;double-stranded RNA binding;nucleic acid binding;RNA binding
binding;catalytic activity;GTP binding;GTPase activity;guanyl nucleotide binding;guanyl ribonucleotide binding;hydrolase activity;hydrolase activity, acting on acid anhydrides;hydrolase activity, acting on acid anhydrides, in pl
binding;collagen binding;extracellular matrix binding;integrin binding;protein binding;protein complex binding;receptor binding
actin binding;actin filament binding;binding;cytoskeletal protein binding;enzyme binding;GTPase binding;protein binding;protein complex binding;Rac GTPase binding;Ras GTPase binding;Rho GTPase binding;small GTPase
binding;protein binding;protein C-terminus binding
binding;enzyme binding;enzyme inhibitor activity;enzyme regulator activity;GTPase binding;GTPase inhibitor activity;GTPase regulator activity;nucleoside-triphosphatase regulator activity;protein binding;protein transporter a
binding;protein binding;scaffold protein binding;structural molecule activity
binding;extracellular matrix structural constituent;glycolipid binding;glycosphingolipid binding;lipid binding;sphingolipid binding;structural molecule activity
active transmembrane transporter activity;antiporter activity;ATP:ADP antiporter activity;secondary active transmembrane transporter activity;solute:solute antiporter activity;transmembrane transporter activity;transporter act
adenyl nucleotide binding;adenyl ribonucleotide binding;aminoacylase activity;aminoacyl-tRNA ligase activity;aspartate-tRNA ligase activity;ATP binding;binding;catalytic activity;hydrolase activity;hydrolase activity, acting on
actin binding;binding;cation binding;cytoskeletal protein binding;enzyme binding;integrin binding;ion binding;kinase binding;metal ion binding;protein binding;protein complex binding;protein kinase binding;receptor binding;t
adenyl nucleotide binding;adenyl ribonucleotide binding;ATP binding;binding;catalytic activity;identical protein binding;kinase activity;nucleotide binding;phosphotransferase activity, alcohol group as acceptor;protein binding
adenyl nucleotide binding;adenyl ribonucleotide binding;ATP binding;binding;nucleotide binding;purine nucleotide binding;purine ribonucleoside triphosphate binding;purine ribonucleotide binding;ribonucleotide binding
5'-3' DNA helicase activity;adenyl nucleotide binding;adenyl ribonucleotide binding;ATP binding;ATPase activity;ATPase activity, coupled;ATP-dependent 5'-3' DNA helicase activity;ATP-dependent DNA helicase activity;ATP-
androgen receptor binding;binding;hormone receptor binding;nuclear hormone receptor binding;nucleic acid binding;protein binding;protein binding transcription factor activity;receptor binding;ribonucleoprotein binding;RNA
actin binding;binding;calcium ion binding;cation binding;cytoskeletal protein binding;ion binding;metal ion binding;myosin binding;myosin II binding;protein binding;protein domain specific binding
actin binding;binding;cytoskeletal protein binding;protein binding;structural constituent of muscle;structural molecule activity
binding;protein binding;scaffold protein binding;structural constituent of cytoskeleton;structural molecule activity
beta-catenin binding;binding;cadherin binding;cell adhesion molecule binding;cytoskeletal protein binding;gamma-catenin binding;protein binding;structural molecule activity;vinculin binding
binding;calcium ion binding;cation binding;extracellular matrix structural constituent;ion binding;metal ion binding;structural molecule activity
adenyl nucleotide binding;adenyl ribonucleotide binding;aminoacyl-tRNA ligase activity;ATP binding;binding;catalytic activity;ligase activity;ligase activity, forming aminoacyl-tRNA and related compounds;ligase activity, formi
binding;DNA binding;nucleic acid binding;nucleic acid binding transcription factor activity;sequence-specific DNA binding transcription factor activity
binding;cation binding;enzyme binding;ion binding;metal ion binding;nuclear localization sequence binding;peptide binding;protein binding;protein domain specific binding;protein transporter activity;signal sequence binding;
adenyl nucleotide binding;adenyl ribonucleotide binding;ATP binding;ATPase activity;ATPase activity, coupled;ATP-dependent helicase activity;ATP-dependent RNA helicase activity;binding;catalytic activity;helicase activity;h
binding;DNA binding;nucleic acid binding;nucleic acid binding transcription factor activity;sequence-specific DNA binding transcription factor activity;single-stranded DNA binding;structure-specific DNA binding
adenyl nucleotide binding;adenyl ribonucleotide binding;ATP binding;ATPase activity;binding;catalytic activity;chromatin binding;DNA binding;helicase activity;hydrolase activity;hydrolase activity, acting on acid anhydrides;hy
adenyl nucleotide binding;adenyl ribonucleotide binding;ATP binding;binding;nucleotide binding;protein binding;protein dimerization activity;protein heterodimerization activity;purine nucleotide binding;purine ribonucleoside t
structural constituent of cytoskeleton;structural molecule activity
adenyl nucleotide binding;adenyl ribonucleotide binding;ATP binding;binding;catalytic activity;cation binding;cyclo-ligase activity;hydroxymethyl-, formyl- and related transferase activity;ion binding;ligase activity;ligase activit
adenyl nucleotide binding;adenyl ribonucleotide binding;ADP binding;ATP binding;ATPase activity;ATPase activity, coupled;ATP-dependent peptidase activity;binding;catalytic activity;DNA binding;DNA polymerase binding;er
binding;nucleic acid binding;nucleotide binding;protein binding;protein complex scaffold;RNA binding;structural molecule activity;translation factor activity, nucleic acid binding;translation initiation factor activity
1-phosphatidylinositol binding;binding;calmodulin binding;cation binding;GTP-dependent protein binding;identical protein binding;ion binding;lipid binding;metal ion binding;phosphatidylinositol binding;phospholipid binding;
binding;chromatin binding;core promoter binding;DNA binding;double-stranded DNA binding;nucleic acid binding;nucleotide binding;regulatory region DNA binding;regulatory region nucleic acid binding;structure-specific DN
binding;DNA binding;nucleic acid binding;nucleotide binding;RNA binding;single-stranded DNA binding;single-stranded RNA binding;structure-specific DNA binding
binding;mRNA binding;nucleic acid binding;nucleotide binding;poly(A) RNA binding;poly(U) RNA binding;poly-purine tract binding;poly-pyrimidine tract binding;protein binding;protein C-terminus binding;RNA binding;single-s
actin binding;binding;calmodulin binding;cytoskeletal protein binding;protein binding;tropomyosin binding
acetyl-CoA carboxylase activity;adenyl nucleotide binding;adenyl ribonucleotide binding;ATP binding;binding;biotin carboxylase activity;catalytic activity;cation binding;CoA carboxylase activity;ion binding;ligase activity;ligas
binding;nucleic acid binding;RNA binding
binding;binding, bridging;catalytic activity;guanylate kinase activity;kinase activity;nucleobase-containing compound kinase activity;nucleotide kinase activity;phosphotransferase activity, phosphate group as acceptor;protein
adenyl nucleotide binding;adenyl ribonucleotide binding;ATP binding;binding;chaperone binding;nucleotide binding;protein binding;purine nucleotide binding;purine ribonucleoside triphosphate binding;purine ribonucleotide b
adenyl nucleotide binding;adenyl ribonucleotide binding;ATP binding;ATPase activity;ATPase activity, coupled;ATP-dependent helicase activity;ATP-dependent RNA helicase activity;binding;catalytic activity;double-stranded R
adenyl nucleotide binding;adenyl ribonucleotide binding;ATP binding;binding;nucleotide binding;purine nucleotide binding;purine ribonucleoside triphosphate binding;purine ribonucleotide binding;ribonucleotide binding
adenyl nucleotide binding;adenyl ribonucleotide binding;ADP binding;amine binding;amino acid binding;ATP binding;binding;carboxylic acid binding;catalytic activity;coenzyme binding;cofactor binding;glutamate dehydroger
binding;cargo receptor activity;double-stranded RNA binding;identical protein binding;nucleic acid binding;protein binding;receptor activity;RNA binding;transferrin receptor activity
binding;calcium ion binding;calcium-dependent phospholipid binding;calcium-dependent protein binding;cation binding;enzyme inhibitor activity;enzyme regulator activity;ion binding;lipase inhibitor activity;lipid binding;meta
binding;DNA binding;identical protein binding;mRNA binding;nucleic acid binding;protein binding;protein dimerization activity;protein homodimerization activity;RNA binding;structural constituent of ribosome;structural molec
adenyl nucleotide binding;adenyl ribonucleotide binding;ATP binding;binding;catalytic activity;DNA binding;DNA helicase activity;helicase activity;hydrolase activity;hydrolase activity, acting on acid anhydrides;hydrolase activ
5S rRNA binding;binding;nucleic acid binding;RNA binding;rRNA binding;structural constituent of ribosome;structural molecule activity
binding;importin-alpha export receptor activity;nuclear export signal receptor activity;protein binding;protein transporter activity;substrate-specific transporter activity;transporter activity
adenosine deaminase activity;binding;catalytic activity;cation binding;deaminase activity;DNA binding;double-stranded RNA adenosine deaminase activity;hydrolase activity;hydrolase activity, acting on carbon-nitrogen (but r
adenyl nucleotide binding;adenyl ribonucleotide binding;ATP binding;ATPase activity;ATPase activity, coupled;ATP-dependent helicase activity;ATP-dependent RNA helicase activity;binding;catalytic activity;double-stranded R
binding;calcium ion binding;cation binding;ion binding;metal ion binding;structural constituent of muscle;structural molecule activity
binding;catalytic activity;methyltransferase activity;nucleic acid binding;RNA binding;RNA methyltransferase activity;S-adenosylmethionine-dependent methyltransferase activity;transferase activity;transferase activity, transfe
binding;catalytic activity;cis-trans isomerase activity;drug binding;FK506 binding;heat shock protein binding;isomerase activity;macrolide binding;peptidyl-prolyl cis-trans isomerase activity;protein binding
binding;catalytic activity;glucosyltransferase activity;protein binding;transferase activity;transferase activity, transferring glycosyl groups;transferase activity, transferring hexosyl groups;UDP-glucose:glycoprotein glucosyltrans
binding;extracellular matrix structural constituent;glycolipid binding;glycosphingolipid binding;lipid binding;sphingolipid binding;structural molecule activity
ATPase activity;ATPase activity, coupled;binding;catalytic activity;chromatin binding;core promoter binding;core promoter sequence-specific DNA binding;DNA binding;DNA topoisomerase (ATP-hydrolyzing) activity;DNA topo
binding;carbohydrate binding;catalytic activity;coenzyme binding;cofactor binding;glucose binding;glucose-6-phosphate dehydrogenase activity;identical protein binding;monosaccharide binding;NADP binding;nucleotide bi
adenyl nucleotide binding;adenyl ribonucleotide binding;ATP binding;binding;biotin binding;biotin carboxylase activity;carboxylic acid binding;catalytic activity;cation binding;DNA binding;ion binding;ligase activity;ligase activ
aminopeptidase activity;binding;catalytic activity;cation binding;exopeptidase activity;hydrolase activity;ion binding;metal ion binding;metallopeptidase activity;peptidase activity;peptidase activity, acting on L-amino acid pep
adenyl nucleotide binding;adenyl ribonucleotide binding;aminoacyl-tRNA ligase activity;ATP binding;binding;catalytic activity;glycine-tRNA ligase activity;ligase activity;ligase activity, forming aminoacyl-tRNA and related com
adenyl nucleotide binding;adenyl ribonucleotide binding;ATP binding;binding;nucleotide binding;protein binding;purine nucleotide binding;purine ribonucleoside triphosphate binding;purine ribonucleotide binding;ribonucleoti
adenyl nucleotide binding;adenyl ribonucleotide binding;AMP binding;ATP binding;bile acid binding;binding;carbohydrate binding;carboxylic acid binding;catalytic activity;cofactor binding;drug binding;glucose binding;glycog
adenyl nucleotide binding;adenyl ribonucleotide binding;ATP binding;binding;enzyme binding;nucleotide binding;protein binding;purine nucleotide binding;purine ribonucleoside triphosphate binding;purine ribonucleotide bind
binding;cation binding;ion binding;metal ion binding;nucleotide binding;structural molecule activity;transition metal ion binding;zinc ion binding
binding;catalytic activity;GTP binding;GTPase activity;guanyl nucleotide binding;guanyl ribonucleotide binding;hydrolase activity;hydrolase activity, acting on acid anhydrides;hydrolase activity, acting on acid anhydrides, in pl
binding;catalytic activity;double-stranded RNA binding;GTP binding;GTPase activity;guanyl nucleotide binding;guanyl ribonucleotide binding;hydrolase activity;hydrolase activity, acting on acid anhydrides;hydrolase activity, a
ankyrin binding;binding;cytoskeletal protein binding;enzyme binding;protein binding
actin binding;actin filament binding;actin monomer binding;binding;cation binding;cytoskeletal protein binding;ion binding;metal ion binding;protein binding;transition metal ion binding;zinc ion binding
binding;carbohydrate binding;receptor activity;structural molecule activity
binding;catalytic activity;enzyme binding;GTP binding;GTPase activity;guanyl nucleotide binding;guanyl ribonucleotide binding;hydrolase activity;hydrolase activity, acting on acid anhydrides;hydrolase activity, acting on acid
5'-deoxyribose-5-phosphate lyase activity;adenyl nucleotide binding;adenyl ribonucleotide binding;ATP binding;ATPase activity;ATPase activity, coupled;ATP-dependent DNA helicase activity;ATP-dependent helicase activity;
adenyl nucleotide binding;adenyl ribonucleotide binding;ATP binding;ATPase activity;ATPase activity, coupled;ATP-dependent DNA helicase activity;ATP-dependent helicase activity;binding;catalytic activity;damaged DNA bi
binding;DNA binding;nucleic acid binding;nucleotide binding;RNA binding;sequence-specific DNA binding;single-stranded DNA binding;single-stranded telomeric DNA binding;structure-specific DNA binding;telomeric DNA b
binding;catalytic activity;cation binding;chromatin binding;DNA (cytosine-5-)-methyltransferase activity;DNA binding;DNA-methyltransferase activity;ion binding;metal ion binding;methyl-CpG binding;methyltransferase activity
binding;catalytic activity;cytokine activity;drug binding;nicotinamide phosphoribosyltransferase activity;nicotinate-nucleotide diphosphorylase (carboxylating) activity;protein binding;receptor binding;transferase activity;transf
adenyl nucleotide binding;adenyl ribonucleotide binding;ATP binding;binding;catalytic activity;cation binding;DNA binding;DNA helicase activity;DNA replication origin binding;helicase activity;hydrolase activity;hydrolase acti
adenyl nucleotide binding;adenyl ribonucleotide binding;ATP binding;binding;nucleotide binding;protein binding;purine nucleotide binding;purine ribonucleoside triphosphate binding;purine ribonucleotide binding;ribonucleoti
6-phosphofructokinase activity;adenyl nucleotide binding;adenyl ribonucleotide binding;ATP binding;binding;carbohydrate kinase activity;catalytic activity;cation binding;ion binding;kinase activity;metal ion binding;nucleotide
adenylate cyclase binding;binding;enzyme binding;protein binding;protein kinase A binding
binding;damaged DNA binding;DNA binding;nucleic acid binding
binding;protein binding;receptor binding
ATPase activity;binding;catalytic activity;enzyme binding;hydrolase activity;hydrolase activity, acting on acid anhydrides;hydrolase activity, acting on acid anhydrides, in phosphorus-containing anhydrides;nucleic acid binding
binding;cadherin binding;cell adhesion molecule binding;protein binding;receptor binding
binding;nucleic acid binding;nucleotide binding;RNA binding;translation factor activity, nucleic acid binding;translation initiation factor activity
adenyl nucleotide binding;adenyl ribonucleotide binding;alanine-tRNA ligase activity;amine binding;amino acid binding;aminoacyl-tRNA editing activity;aminoacyl-tRNA ligase activity;ATP binding;binding;carboxylic acid bindi
aminopeptidase activity;binding;catalytic activity;cation binding;exopeptidase activity;hydrolase activity;ion binding;metal ion binding;metallopeptidase activity;peptidase activity;peptidase activity, acting on L-amino acid pep
binding;enzyme binding;kinase binding;phosphatase binding;protein binding;protein kinase binding;protein phosphatase binding
adenyl nucleotide binding;adenyl ribonucleotide binding;ATP binding;ATPase activity;ATPase activity, coupled;ATP-dependent DNA helicase activity;ATP-dependent helicase activity;binding;catalytic activity;cation binding;DN
adenyl nucleotide binding;adenyl ribonucleotide binding;ATP binding;ATPase activity;binding;binding, bridging;catalytic activity;cation binding;DNA binding;hydrolase activity;hydrolase activity, acting on acid anhydrides;hydro
binding;catalytic activity;GTP binding;GTPase activity;guanyl nucleotide binding;guanyl ribonucleotide binding;hydrolase activity;hydrolase activity, acting on acid anhydrides;hydrolase activity, acting on acid anhydrides, in pl
structural constituent of cytoskeleton;structural molecule activity
binding;calcium ion binding;carbohydrate binding;cation binding;collagen binding;collagen V binding;cytokine binding;extracellular matrix binding;fibrinogen binding;fibroblast growth factor binding;fibronectin binding;glycopr
structural molecule activity
enzyme activator activity;enzyme regulator activity;GDP-dissociation inhibitor activity;GTPase activator activity;GTPase regulator activity;nucleoside-triphosphatase regulator activity;Rab GDP-dissociation inhibitor activity;sm
adenyl nucleotide binding;adenyl ribonucleotide binding;ATP binding;binding;catalytic activity;DNA binding;DNA helicase activity;helicase activity;hydrolase activity;hydrolase activity, acting on acid anhydrides;hydrolase activ
adenyl nucleotide binding;adenyl ribonucleotide binding;ATP binding;binding;nucleotide binding;protein binding;purine nucleotide binding;purine ribonucleoside triphosphate binding;purine ribonucleotide binding;ribonucleoti
adenyl nucleotide binding;adenyl ribonucleotide binding;ATP binding;binding;nucleotide binding;protein binding;protein dimerization activity;protein heterodimerization activity;purine nucleotide binding;purine ribonucleoside t

catalytic activity;dolichyl-diphosphooligosaccharide-protein glycotransferase activity;oligosaccharyl transferase activity;transferase activity;transferase activity, transferring glycosyl groups;transferase activity, transferring hex
binding;calcium ion binding;calcium-dependent phospholipid binding;cation binding;enzyme inhibitor activity;enzyme regulator activity;ion binding;lipase inhibitor activity;lipid binding;metal ion binding;phospholipase inhibitor
adenyl nucleotide binding;adenyl ribonucleotide binding;ATP binding;binding;catalytic activity;chromatin binding;DNA binding;DNA helicase activity;DNA replication origin binding;helicase activity;hydrolase activity;hydrolase
adenyl nucleotide binding;adenyl ribonucleotide binding;aminoacyl-tRNA ligase activity;ATP binding;binding;catalytic activity;chemokine receptor binding;CXCR chemokine receptor binding;cytokine receptor binding;G-protei
binding;MHC class II protein binding;MHC class II receptor activity;MHC protein binding;molecular transducer activity;protein binding;receptor activity;receptor binding;signal transducer activity;signaling receptor activity;stru
actin binding;actin filament binding;binding;cytoskeletal protein binding;drug binding;protein binding
binding;DNA binding;mRNA binding;nucleic acid binding;RNA binding
extracellular matrix structural constituent;extracellular matrix structural constituent conferring tensile strength;structural molecule activity
adenyl nucleotide binding;adenyl ribonucleotide binding;ATP binding;ATPase activity;ATPase activity, coupled;ATP-dependent helicase activity;ATP-dependent RNA helicase activity;binding;catalytic activity;double-stranded
adenyl nucleotide binding;adenyl ribonucleotide binding;aminoacyl-tRNA editing activity;aminoacyl-tRNA ligase activity;ATP binding;binding;carboxylic ester hydrolase activity;catalytic activity;hydrolase activity;hydrolase act
adenyl nucleotide binding;adenyl ribonucleotide binding;ATP binding;ATPase activity;ATPase activity, coupled;ATP-dependent DNA helicase activity;ATP-dependent helicase activity;ATP-dependent RNA helicase activity;bind
catalytic activity;lactate dehydrogenase activity;L-lactate dehydrogenase activity;oxidoreductase activity;oxidoreductase activity, acting on CH-OH group of donors;oxidoreductase activity, acting on the CH-OH group of don
adenyl nucleotide binding;adenyl ribonucleotide binding;amine binding;amino acid binding;argininosuccinate synthase activity;ATP binding;binding;carboxylic acid binding;catalytic activity;identical protein binding;ligase activ
active transmembrane transporter activity;adenyl nucleotide binding;adenyl ribonucleotide binding;ATP binding;ATPase activity;ATPase activity, coupled;ATPase activity, coupled to movement of substances;ATPase activity, c
adenyl nucleotide binding;adenyl ribonucleotide binding;ATP binding;binding;enzyme binding;nucleotide binding;protein binding;purine nucleotide binding;purine ribonucleoside triphosphate binding;purine ribonucleotide bin
structural molecule activity
binding;calcium-dependent protein binding;G-protein-coupled receptor binding;identical protein binding;protein binding;protein dimerization activity;protein homodimerization activity;proteinase activated receptor binding;rec
binding;nucleic acid binding;RNA binding;translation factor activity, nucleic acid binding;translation initiation factor activity
binding;ion channel binding;protein binding;receptor binding
actin binding;aldehyde-lyase activity;binding;carbohydrate binding;carbon-carbon lyase activity;catalytic activity;cytoskeletal protein binding;fructose binding;fructose-bisphosphate aldolase activity;identical protein binding;l
structural molecule activity
adenyl nucleotide binding;adenyl ribonucleotide binding;ATP binding;beta-tubulin binding;binding;cytoskeletal protein binding;G-protein beta-subunit binding;nucleotide binding;protein binding;purine nucleotide binding;purin
acid-amino acid ligase activity;binding;catalytic activity;cation binding;chromo shadow domain binding;DNA binding;enzyme binding;ion binding;kinase activity;Krueppel-associated box domain binding;ligase activity;ligase a
binding;catalytic activity;core promoter binding;DNA binding;DNA polymerase activity;DNA-directed DNA polymerase activity;nucleic acid binding;nucleotidyltransferase activity;protein binding;protein binding transcription fa
3-hydroxyacyl-CoA dehydrogenase activity;acetyl-CoA C-acetyltransferase activity;acetyl-CoA C-acyltransferase activity;acetyltransferase activity;binding;C-acetyltransferase activity;C-acyltransferase activity;carbon-oxyge
adenyl nucleotide binding;adenyl ribonucleotide binding;ATP binding;binding;cytoskeletal protein binding;enzyme binding;identical protein binding;kinesin binding;nitric-oxide synthase binding;nucleotide binding;protein bindi
binding;profilin binding;protein binding
adenyl nucleotide binding;adenyl ribonucleotide binding;ATP binding;binding;nucleotide binding;purine nucleotide binding;purine ribonucleoside triphosphate binding;purine ribonucleotide binding;ribonucleotide binding
adenyl nucleotide binding;adenyl ribonucleotide binding;ATP binding;binding;catalytic activity;chromatin binding;dynein binding;hydrolase activity;hydrolase activity, acting on acid anhydrides;hydrolase activity, acting on acid
binding;catalytic activity;cation binding;GTP binding;guanyl nucleotide binding;guanyl ribonucleotide binding;ion binding;metal ion binding;nucleotide binding;protein-glutamine gamma-glutamyltransferase activity;purine nuc
adenyl nucleotide binding;adenyl ribonucleotide binding;ATP binding;binding;catalytic activity;DNA binding;DNA helicase activity;helicase activity;hydrolase activity;hydrolase activity, acting on acid anhydrides;hydrolase activ
binding;catalytic activity;GTP binding;GTPase activity;guanyl nucleotide binding;guanyl ribonucleotide binding;hydrolase activity;hydrolase activity, acting on acid anhydrides;hydrolase activity, acting on acid anhydrides, in p
adenyl nucleotide binding;adenyl ribonucleotide binding;ATP binding;binding;carbohydrate kinase activity;catalytic activity;fructokinase activity;glucokinase activity;hexokinase activity;kinase activity;mannokinase activity;nuc
adenyl nucleotide binding;adenyl ribonucleotide binding;ATP binding;binding;catalytic activity;DNA binding;DNA helicase activity;helicase activity;hydrolase activity;hydrolase activity, acting on acid anhydrides;hydrolase activ
alcohol binding;binding;calcium channel activity;calcium-release channel activity;cation channel activity;cation transmembrane transporter activity;channel activity;gated channel activity;inositol 1,3,4,5 tetrakisphosphate bind
binding;enzyme binding;kinase binding;molecular transducer activity;protein binding;protein kinase binding;signal transducer activity
ATPase activity;ATPase activity, coupled;binding;binding, bridging;calcium ion binding;calcium-dependent phospholipid binding;calcium-dependent protein binding;catalytic activity;cation binding;DNA binding;DNA-depende
binding;catalytic activity;coenzyme binding;cofactor binding;cytoskeletal protein binding;glyceraldehyde-3-phosphate dehydrogenase (NAD+) (phosphorylating) activity;identical protein binding;microtubule binding;NAD bindi
catalytic activity;intramolecular oxidoreductase activity;intramolecular oxidoreductase activity, interconverting keto- and enol-groups;intramolecular oxidoreductase activity, transposing S-S bonds;isomerase activity;protein d
binding;chromatin binding;core promoter binding;DNA binding;mRNA binding;nucleic acid binding;nucleotide binding;regulatory region DNA binding;regulatory region nucleic acid binding;RNA binding;single-stranded RNA bi
transporter activity
adenyl nucleotide binding;adenyl ribonucleotide binding;ATP binding;binding;catalytic activity;chromatin binding;hydrolase activity;hydrolase activity, acting on acid anhydrides;hydrolase activity, acting on acid anhydrides, in
adenyl nucleotide binding;adenyl ribonucleotide binding;ATP binding;ATPase activity;ATPase activity, coupled;binding;catalytic activity;hydrolase activity;hydrolase activity, acting on acid anhydrides;hydrolase activity, acting
acid-amino acid ligase activity;adenyl nucleotide binding;adenyl ribonucleotide binding;ATP binding;binding;catalytic activity;ligase activity;ligase activity, forming carbon-nitrogen bonds;nucleotide binding;purine nucleotide b
adenyl nucleotide binding;adenyl ribonucleotide binding;aminoacyl-tRNA ligase activity;ATP binding;binding;catalytic activity;identical protein binding;ligase activity;ligase activity, forming aminoacyl-tRNA and related compou
binding;nucleic acid binding;RNA binding;structural constituent of ribosome;structural molecule activity
binding;protein binding;scaffold protein binding;structural molecule activity
structural molecule activity
binding;catalytic activity;enzyme binding;GTP binding;GTPase activity;guanyl nucleotide binding;guanyl ribonucleotide binding;hydrolase activity;hydrolase activity, acting on acid anhydrides;hydrolase activity, acting on acid
adenyl nucleotide binding;adenyl ribonucleotide binding;androgen receptor binding;ATP binding;ATPase activity;ATPase activity, coupled;ATP-dependent helicase activity;ATP-dependent RNA helicase activity;binding;catalyti
acid-thiol ligase activity;adenyl nucleotide binding;adenyl ribonucleotide binding;ATP binding;ATP citrate synthase activity;binding;catalytic activity;cation binding;CoA-ligase activity;cofactor binding;ion binding;ligase activity
acid-amino acid ligase activity;binding;catalytic activity;cation binding;ion binding;ligase activity;ligase activity, forming carbon-nitrogen bonds;metal ion binding;small conjugating protein ligase activity;transition metal ion bin
actin binding;actin filament binding;adenyl nucleotide binding;adenyl ribonucleotide binding;ATP binding;binding;catalytic activity;cytoskeletal protein binding;hydrolase activity;hydrolase activity, acting on acid anhydrides;hy
active transmembrane transporter activity;adenyl nucleotide binding;adenyl ribonucleotide binding;ADP binding;alkali metal ion binding;ATP binding;ATPase activity;ATPase activity, coupled;ATPase activity, coupled to moven
adenyl nucleotide binding;adenyl ribonucleotide binding;ATP binding;binding;catalytic activity;cyclohydrolase activity;formate-tetrahydrofolate ligase activity;hydrolase activity;hydrolase activity, acting on carbon-nitrogen (but
adenyl nucleotide binding;adenyl ribonucleotide binding;ATP binding;ATPase activity;binding;catalytic activity;cytoskeletal protein binding;hydrolase activity;hydrolase activity, acting on acid anhydrides;hydrolase activity, acti
ankyrin binding;binding;cytoskeletal protein binding;protein binding
active transmembrane transporter activity;adenyl nucleotide binding;adenyl ribonucleotide binding;ATP binding;ATPase activity;ATPase activity, coupled;ATPase activity, coupled to movement of substances;ATPase activity, c
binding;nucleic acid binding;RNA binding;structural constituent of ribosome;structural molecule activity
alpha-glucosidase activity;binding;carbohydrate binding;catalytic activity;glucan 1,3-alpha-glucosidase activity;glucosidase activity;hydrolase activity;hydrolase activity, acting on glycosyl bonds;hydrolase activity, hydrolyzing
binding;core promoter binding;DNA binding;identical protein binding;nucleic acid binding;nucleotide binding;protein binding;regulatory region DNA binding;regulatory region nucleic acid binding;transcription regulatory region
adenyl nucleotide binding;adenyl ribonucleotide binding;ATP binding;ATPase activity;ATPase activity, coupled;ATP-dependent helicase activity;ATP-dependent RNA helicase activity;binding;catalytic activity;estrogen receptor
adenyl nucleotide binding;adenyl ribonucleotide binding;aminoacyl-tRNA editing activity;aminoacyl-tRNA ligase activity;ATP binding;binding;carboxylic ester hydrolase activity;catalytic activity;hydrolase activity;hydrolase act
binding;C2H2 zinc finger domain binding;protein binding;protein domain specific binding;protein N-terminus binding
binding;catalytic activity;cation binding;cofactor binding;identical protein binding;ion binding;metal ion binding;protein binding;protein dimerization activity;protein homodimerization activity;transferase activity;transferase acti
catalytic activity;cysteine-type endopeptidase activity;cysteine-type peptidase activity;disulfide oxidoreductase activity;endopeptidase activity;hydrolase activity;hydrolase activity, acting on ester bonds;intramolecular oxidore
actin-dependent ATPase activity;adenyl nucleotide binding;adenyl ribonucleotide binding;ATP binding;ATPase activity;ATPase activity, coupled;binding;catalytic activity;enzyme binding;GTPase binding;hydrolase activity;hydr
binding;cation binding;ion binding;metal ion binding;protein binding;protein C-terminus binding
adenyl nucleotide binding;adenyl ribonucleotide binding;ATP binding;binding;catalytic activity;kinase activity;nucleotide binding;phosphoglycerate kinase activity;phosphotransferase activity, carboxyl group as acceptor;purin
adenyl nucleotide binding;adenyl ribonucleotide binding;aminoacyl-tRNA editing activity;aminoacyl-tRNA ligase activity;ATP binding;binding;carboxylic ester hydrolase activity;catalytic activity;hydrolase activity;hydrolase act
adenyl nucleotide binding;adenyl ribonucleotide binding;amine binding;amino acid binding;aspartate binding;aspartate carbamoyltransferase activity;ATP binding;binding;carbamoyl-phosphate synthase (ammonia) activity;ca
adenyl nucleotide binding;adenyl ribonucleotide binding;aminoacyl-tRNA editing activity;aminoacyl-tRNA ligase activity;ATP binding;binding;carboxylic ester hydrolase activity;catalytic activity;enzyme binding;GTPase binding
binding;chromatin binding
catalytic activity;endopeptidase activity;hydrolase activity;intramolecular oxidoreductase activity;intramolecular oxidoreductase activity, interconverting keto- and enol-groups;intramolecular oxidoreductase activity, transposin
actin binding;actin filament binding;binding;cell adhesion molecule binding;cytoskeletal protein binding;protein binding
binding;protein binding;syndecan binding
adenyl nucleotide binding;adenyl ribonucleotide binding;ATP binding;ATPase activity;ATPase activity, coupled;ATP-dependent helicase activity;ATP-dependent RNA helicase activity;binding;catalytic activity;helicase activity;h
binding;carbohydrate binding;catalytic activity;glucose-6-phosphate isomerase activity;intramolecular oxidoreductase activity;intramolecular oxidoreductase activity, interconverting aldoses and ketoses;intramolecular transfe
structural molecule activity
adenyl nucleotide binding;adenyl ribonucleotide binding;ATP binding;binding;enzyme binding;nucleotide binding;protein binding;purine nucleotide binding;purine ribonucleoside triphosphate binding;purine ribonucleotide bin
adenyl nucleotide binding;adenyl ribonucleotide binding;ATP binding;binding;core promoter binding;DNA binding;nucleic acid binding;nucleotide binding;purine nucleotide binding;purine ribonucleoside triphosphate binding;
binding;core promoter binding;DNA binding;enzyme binding;histone deacetylase binding;nucleic acid binding;nucleotide binding;protein binding;regulatory region DNA binding;regulatory region nucleic acid binding;sequence
binding;carbohydrate binding;carboxylic acid binding;catalytic activity;glycoprotein binding;G-protein coupled receptor activity;identical protein binding;insulin-like growth factor-activated receptor activity;isoprenoid binding;
adenyl nucleotide binding;adenyl ribonucleotide binding;ADP binding;ATP binding;ATPase activity;binding;catalytic activity;enzyme activator activity;enzyme binding;enzyme regulator activity;hydrolase activity;hydrolase activ
binding;nucleic acid binding;RNA binding;translation factor activity, nucleic acid binding;translation initiation factor activity
binding;calcium ion binding;calcium-dependent phospholipid binding;calcium-dependent protein binding;cation binding;enzyme inhibitor activity;enzyme regulator activity;ion binding;lipase inhibitor activity;lipid binding;meta
adenyl nucleotide binding;adenyl ribonucleotide binding;antigen binding;ATP binding;ATPase activity;ATPase activity, coupled;binding;C3HC4-type RING finger domain binding;catalytic activity;enzyme binding;G-protein-cou
binding;DNA binding;identical protein binding;nucleic acid binding;nucleotide binding;protein binding;protein C-terminus binding;RNA binding;sequence-specific DNA binding;telomeric DNA binding
adenyl nucleotide binding;adenyl ribonucleotide binding;ATP binding;binding;nucleotide binding;protein binding;protein C-terminus binding;purine nucleotide binding;purine ribonucleoside triphosphate binding;purine ribonuc
adenyl nucleotide binding;adenyl ribonucleotide binding;ATP binding;ATPase activity;binding;calcium ion binding;catalytic activity;cation binding;chaperone binding;enzyme binding;glycoprotein binding;hydrolase activity;hyd
binding;catalytic activity;cofactor binding;glycogen phosphorylase activity;phosphorylase activity;pyridoxal phosphate binding;transferase activity;transferase activity, transferring glycosyl groups;transferase activity, transferri
adenyl nucleotide binding;adenyl ribonucleotide binding;ATP binding;ATPase activity;ATPase activity, coupled;ATP-dependent DNA helicase activity;ATP-dependent helicase activity;ATP-dependent RNA helicase activity;bind
binding;nucleic acid binding;RNA binding;structural constituent of ribosome;structural molecule activity
beta-tubulin binding;binding;cytoskeletal protein binding;DNA binding;enzyme binding;microtubule binding;nucleic acid binding;protein binding;RNA binding;single-stranded DNA binding;structure-specific DNA binding;tubuli
actin binding;binding;cytoskeletal protein binding;protein binding
binding;carbon-oxygen lyase activity;catalytic activity;cation binding;DNA binding;enzyme binding;GTPase binding;hydro-lyase activity;ion binding;lyase activity;magnesium ion binding;metal ion binding;nucleic acid binding;
binding;calcium ion binding;cation binding;cytoskeletal protein binding;double-stranded RNA binding;integrin binding;ion binding;ion channel binding;ligand-dependent nuclear receptor transcription coactivator activity;metal
actin binding;binding;calcium ion binding;cation binding;cytoskeletal protein binding;ion binding;metal ion binding;protein binding;structural constituent of cytoskeleton;structural molecule activity
catalytic activity;hydrolase activity;hydrolase activity, acting on ester bonds;nuclease activity;protein binding transcription factor activity;transcription cofactor activity;transcription factor binding transcription factor activity
antioxidant activity;binding;catalytic activity;cation binding;cytokine receptor binding;extracellular matrix structural constituent;heme binding;interleukin-1 receptor antagonist activity;ion binding;iron ion binding;metal ion bin
adenyl nucleotide binding;adenyl ribonucleotide binding;ATP binding;ATPase activity;binding;catalytic activity;chaperone binding;DNA binding;DNA replication origin binding;double-stranded RNA binding;enzyme binding;hy
apolipoprotein binding;binding;calcium ion binding;cation binding;ion binding;lipoprotein particle receptor binding;lipoprotein transporter activity;metal ion binding;protein binding;protein complex binding;protein transporter a
binding;calcium ion binding;calcium-dependent phospholipid binding;calcium-dependent protein binding;cation binding;channel activity;cholesterol binding;gated channel activity;GTP binding;guanyl nucleotide binding;guan
actin binding;alpha-catenin binding;beta-catenin binding;binding;cadherin binding;cell adhesion molecule binding;cytoskeletal protein binding;dystroglycan binding;enzyme binding;glycoprotein binding;protein binding;struct
structural molecule activity
binding;catalytic activity;K63-linked polyubiquitin binding;nucleic acid binding;polyubiquitin binding;protein binding;RNA binding;second spliceosomal transesterification activity;small conjugating protein binding;snRNA bindi
binding;lipid binding
binding;catalytic activity;cation binding;coenzyme binding;cofactor binding;DNA binding;enzyme binding;identical protein binding;ion binding;kinase binding;metal ion binding;NAD binding;NAD+ ADP-ribosyltransferase activ
adenyl nucleotide binding;adenyl ribonucleotide binding;ATP binding;binding;calcium ion binding;cation binding;enzyme binding;ion binding;lipoprotein particle receptor binding;low-density lipoprotein particle receptor bindin
binding;cell adhesion molecule binding;double-stranded RNA binding;enzyme binding;kinase binding;nucleic acid binding;protein binding;protein kinase binding;receptor binding;RNA binding;structural constituent of cytoske
adenyl nucleotide binding;adenyl ribonucleotide binding;ATP binding;binding;nucleotide binding;purine nucleotide binding;purine ribonucleoside triphosphate binding;purine ribonucleotide binding;ribonucleotide binding
binding;catalytic activity;enzyme binding;GTP binding;GTPase activity;guanyl nucleotide binding;guanyl ribonucleotide binding;hydrolase activity;hydrolase activity, acting on acid anhydrides;hydrolase activity, acting on acid
binding;carbohydrate binding;collagen binding;enzyme activator activity;enzyme binding;enzyme regulator activity;glycosaminoglycan binding;heparin binding;integrin binding;pattern binding;peptidase activator activity;pepti
adenyl nucleotide binding;adenyl ribonucleotide binding;alkali metal ion binding;antigen binding;ATP binding;binding;catalytic activity;cation binding;ion binding;kinase activity;magnesium ion binding;metal ion binding;MHC c
binding;nucleic acid binding;nucleotide binding;protein binding;protein domain specific binding;RNA binding
adenyl deoxyribonucleotide binding;adenyl nucleotide binding;adenyl ribonucleotide binding;antigen binding;ATP binding;ATPase activity;binding;catalytic activity;CTP binding;dATP binding;deoxyribonucleotide binding;enzy
binding;catalytic activity;cation binding;cis-trans isomerase activity;enzyme binding;GTPase binding;ion binding;isomerase activity;ligase activity;metal ion binding;nucleic acid binding;peptidyl-prolyl cis-trans isomerase activ
binding;double-stranded RNA binding;glycoprotein binding;identical protein binding;nucleic acid binding;protein binding;protein C-terminus binding;RNA binding;scaffold protein binding;structural constituent of cytoskeleton;
binding;calcium ion binding;calmodulin binding;cation binding;enzyme activator activity;enzyme binding;enzyme inhibitor activity;enzyme regulator activity;GTPase activator activity;GTPase inhibitor activity;GTPase regulator

actin binding;actin filament binding;binding;calcium ion binding;cation binding;cytoskeletal protein binding;hormone receptor binding;identical protein binding;integrin binding;ion binding;ligand-dependent nuclear receptor tra
actin binding;actin filament binding;ATPase activity;binding;calcium ion binding;catalytic activity;cation binding;cytoskeletal protein binding;hydrolase activity;hydrolase activity, acting on acid anhydrides;hydrolase activity, ac
actin binding;ankyrin binding;binding;cytoskeletal protein binding;enzyme binding;GTPase binding;lipid binding;phospholipid binding;protein binding;structural constituent of cytoskeleton;structural molecule activity
structural molecule activity
adenyl nucleotide binding;adenyl ribonucleotide binding;antigen binding;ATP binding;binding;double-stranded RNA binding;enzyme binding;enzyme regulator activity;kinase binding;MHC class II protein complex binding;MH
binding;nucleic acid binding;RNA binding;structural molecule activity;translation factor activity, nucleic acid binding;translation initiation factor activity
binding;nucleic acid binding;RNA binding;translation factor activity, nucleic acid binding
acid-amino acid ligase activity;binding;catalytic activity;DNA binding;ligase activity;ligase activity, forming carbon-nitrogen bonds;nucleic acid binding;small conjugating protein ligase activity;ubiquitin-protein ligase activity
binding;lipid binding;phospholipid binding
adenyl nucleotide binding;adenyl ribonucleotide binding;aminoacyl-tRNA ligase activity;ATP binding;binding;catalytic activity;enzyme binding;glutamate-tRNA ligase activity;GTPase binding;ligase activity;ligase activity, formi
receptor activity
actin binding;actin filament binding;actin-dependent ATPase activity;adenyl nucleotide binding;adenyl ribonucleotide binding;ADP binding;ATP binding;ATPase activity;ATPase activity, coupled;binding;catalytic activity;cytosk
structural molecule activity
binding;chromatin binding;cytoskeletal protein binding;dynein complex binding;enzyme binding;heat shock protein binding;identical protein binding;kinase binding;mitogen-activated protein kinase binding;mRNA binding;nuc
binding;clathrin binding;clathrin light chain binding;double-stranded RNA binding;enzyme binding;kinase binding;nucleic acid binding;protein binding;protein kinase binding;RNA binding;structural molecule activity
endopeptidase inhibitor activity;endopeptidase regulator activity;enzyme inhibitor activity;enzyme regulator activity;peptidase inhibitor activity;peptidase regulator activity;serine-type endopeptidase inhibitor activity
adenyl nucleotide binding;adenyl ribonucleotide binding;amine binding;amino acid binding;ATP binding;binding;calcium ion binding;carbamoyl-phosphate synthase (ammonia) activity;carbamoyl-phosphate synthase (glutami
actin binding;actin filament binding;binding;cytoskeletal protein binding;enzyme binding;Fc-gamma receptor I complex binding;glycoprotein binding;GTPase binding;identical protein binding;immunoglobulin receptor binding;
actin binding;binding;cytoskeletal protein binding;identical protein binding;protein binding
[acyl-carrier-protein] S-acetyltransferase activity;[acyl-carrier-protein] S-malonyltransferase activity;3-hydroxyacyl-[acyl-carrier-protein] dehydratase activity;3-hydroxyoctanoyl-[acyl-carrier-protein] dehydratase activity;3-hydr
binding;cytoskeletal protein binding;integrin binding;LIM domain binding;protein binding;protein complex binding;protein domain specific binding;receptor binding;structural constituent of cytoskeleton;structural molecule act
adenyl nucleotide binding;adenyl ribonucleotide binding;ATP binding;ATPase activity;binding;catalytic activity;hydrolase activity;hydrolase activity, acting on acid anhydrides;hydrolase activity, acting on acid anhydrides, in ph
adenyl nucleotide binding;adenyl ribonucleotide binding;ATP binding;binding;catalytic activity;DNA binding;DNA-dependent protein kinase activity;kinase activity;nucleic acid binding;nucleotide binding;phosphotransferase a
actin binding;actin filament binding;actin-dependent ATPase activity;adenyl nucleotide binding;adenyl ribonucleotide binding;ADP binding;ATP binding;ATPase activity;ATPase activity, coupled;binding;catalytic activity;cytosk
ankyrin binding;binding;cytoskeletal protein binding;protein binding;structural constituent of muscle;structural molecule activity

C: GOCC name	C: KEGG name	C: Organism	N: Protein Probability	N: Combined Total Peptides
cell part;cytoplasmic part;endosomal part;endosome membrane;intracellular organelle part;intracellular part;membrane;organelle membrane;organelle part;recycling endosome membrane	Endocytosis	Homo sapiens	0.9999	1
cell part;cytoplasmic part;endoplasmic reticulum;integral to membrane;intracellular membrane-bounded organelle;intracellular organelle;intracellular part;intrinsic to membrane;membrane part;membrane-bounded organelle;organelle		Homo sapiens	0.926	1
cell part;integral to membrane;integral to plasma membrane;intrinsic to membrane;intrinsic to plasma membrane;membrane;membrane part;plasma membrane;plasma membrane part	Olfactory transduction	Homo sapiens	0.9165	1
cell part;cytoplasmic part;endoplasmic reticulum membrane;endoplasmic reticulum part;integral to endoplasmic reticulum membrane;integral to membrane;integral to organelle membrane;intracellular organelle part;intracellu	Protein export	Homo sapiens	0.9999	1
cell part;cytoplasm;cytoplasmic mRNA processing body;cytoplasmic part;cytosol;intracellular membrane-bounded organelle;intracellular non-membrane-bounded organelle;intracellular organelle;intracellular part;macromolec	RNA degradation	Homo sapiens	0.9999	2
adherens junction;anchoring junction;cell junction;cell part;cell-substrate adherens junction;cell-substrate junction;focal adhesion;integral to membrane;intrinsic to membrane;membrane;membrane part;plasma membrane		Homo sapiens	0.9997	2
cell part;cytoplasmic part;extracellular membrane-bounded organelle;extracellular organelle;extracellular region part;extracellular vesicular exosome;integral to membrane;intracellular organelle part;intracellular part;intrinsic to	Alzheimer's disease;Huntington's disease;Oxidative phosphorylation;Parkinson's disease	Homo sapiens	0.9999	2
blood microparticle;cell part;cytoplasmic part;cytosol;cytosolic part;extracellular region part;hemoglobin complex;intracellular part;macromolecular complex;protein complex	African trypanosomiasis;Malaria	Homo sapiens	1	2
extracellular membrane-bounded organelle;extracellular organelle;extracellular region;extracellular region part;extracellular space;extracellular vesicular exosome;membrane-bounded organelle;membrane-bounded vesicle;organelle;vesicle		Homo sapiens	0.9991	2
blood microparticle;cell part;cell surface;cytoplasmic membrane-bounded vesicle lumen;cytoplasmic part;cytoplasmic vesicle part;extracellular membrane-bounded organelle;extracellular organelle;extracellular region;extrace	Complement and coagulation cascades	Homo sapiens	0.9999	2
cell part;integral to membrane;intrinsic to membrane;membrane;membrane part;plasma membrane		Homo sapiens	0.9999	2
		Homo sapiens	0.9999	2
cell part;cytoplasmic part;endoplasmic reticulum;endoplasmic reticulum membrane;endoplasmic reticulum part;integral to membrane;intracellular membrane-bounded organelle;intracellular organelle;intracellular organelle pa	Protein processing in endoplasmic reticulum	Homo sapiens	0.9999	2
cell part;cytoplasm;cytoplasmic part;cytosol;extracellular membrane-bounded organelle;extracellular organelle;extracellular region part;extracellular vesicular exosome;immunological synapse;intracellular membrane-bounded	MAPK signaling pathway	Homo sapiens	0.9998	2
extracellular region;extracellular region part;extracellular space	PPAR signaling pathway	Homo sapiens	0.9969	2
cell part;cytoplasm;cytoplasmic part;endoplasmic reticulum;endoplasmic reticulum membrane;endoplasmic reticulum part;integral to membrane;integral to plasma membrane;intracellular membrane-bounded organelle;intracellular organelle;intracellular organelle part;intracellular part;intrinsic to membrane;intrinsic to plasma mem		Homo sapiens	0.997	2
cell part;integral to membrane;intrinsic to membrane;membrane part		Homo sapiens	1	2
cell part;integral to membrane;intrinsic to membrane;membrane;membrane part;plasma membrane		Homo sapiens	0.9998	2
cell part;cytoplasm;intracellular part		Homo sapiens	0.9999	2
cell part;cytoplasm;cytoplasmic part;intracellular membrane-bounded organelle;intracellular organelle;intracellular organelle part;intracellular part;membrane-bounded organelle;membrane-enclosed lumen;mitochondrial inter	Oxidative phosphorylation	Homo sapiens	1	2
cell part;cytoplasm;cytoplasmic part;intracellular membrane-bounded organelle;intracellular organelle;intracellular organelle part;intracellular part;membrane-bounded organelle;nuclear part;nucleoplasm;nucleus;organelle;organelle part;perinuclear region of cytoplasm		Homo sapiens	1	2
apical part of cell;basal part of cell;cell part;cytoplasmic part;cytoplasmic vesicle;early endosome;endosome;external side of plasma membrane;integral to membrane;integral to plasma membrane;intracellular membrane-bounded organelle;intracellular organelle;intracellular part;intrinsic to membrane;intrinsic to plasma membra		Homo sapiens	0.9999	2
brush border membrane;cell part;cell projection membrane;cell projection part;extracellular membrane-bounded organelle;extracellular organelle;extracellular region part;extracellular vesicular exosome;integral to membrane;	Mineral absorption	Homo sapiens	0.9997	2
cell part;integral to membrane;intrinsic to membrane;membrane part		Homo sapiens	1	2
cell part;cytoplasmic part;cytosol;extrinsic to membrane;intracellular part;membrane;membrane part;pre-autophagosomal structure membrane		Homo sapiens	0.9999	2
cell part;cell surface;cytoplasmic part;endoplasmic reticulum lumen;endoplasmic reticulum part;extracellular matrix;extracellular region part;extracellular space;intracellular organelle lumen;intracellular organelle part;intracellular part;membrane-enclosed lumen;organelle lumen;organelle part;proteinaceous extracellular matrix		Homo sapiens	0.9998	2
extracellular membrane-bounded organelle;extracellular organelle;extracellular region part;extracellular vesicular exosome;membrane-bounded organelle;membrane-bounded vesicle;organelle;vesicle		Homo sapiens	0.9999	2
cell part;integral to membrane;intrinsic to membrane;membrane;membrane part;plasma membrane		Homo sapiens	0.9865	2
		Homo sapiens	0.9999	2
cell part;cytoplasmic part;cytoplasmic vesicle;extracellular membrane-bounded organelle;extracellular organelle;extracellular region part;extracellular vesicular exosome;intracellular organelle;intracellular part;membrane;membrane-bounded organelle;membrane-bounded vesicle;organelle;plasma membrane;vesicle		Homo sapiens	0.9886	2
		Homo sapiens	0.9999	2
cell part;cytoplasmic part;endosomal part;endosome membrane;extracellular membrane-bounded organelle;extracellular organelle;extracellular region part;extracellular vesicular exosome;extrinsic to membrane;intracellular membrane-bounded organelle;intracellular organelle;intracellular organelle part;intracellular part;late endo		Homo sapiens	0.9999	2
cell part;cell projection;cell projection part;cell surface;cytoplasm;dendritic shaft;dendritic spine;integral to membrane;integral to plasma membrane;intracellular part;intrinsic to membrane;intrinsic to plasma membrane;memb	Gap junction;Neuroactive ligand-receptor interaction	Homo sapiens	0.9963	2
cell part;cytoplasmic part;intracellular membrane-bounded organelle;intracellular organelle;intracellular organelle part;intracellular part;macromolecular complex;membrane part;membrane-bounded organelle;mitochondrial membrane part;mitochondrial outer membrane translocase complex;mitochondrial part;mitochondrion;orga		Homo sapiens	0.9999	2
cell part;cytoplasmic part;cytosol;intracellular membrane-bounded organelle;intracellular organelle;intracellular part;membrane-bounded organelle;nucleus;organelle		Homo sapiens	0.9999	2
cell part;cytoplasm;intracellular membrane-bounded organelle;intracellular organelle;intracellular organelle part;intracellular part;macromolecular complex;mediator complex;membrane;membrane-bounded organelle;nuclear part;nucleoplasm;nucleoplasm part;nucleus;organelle;organelle part;protein complex		Homo sapiens	1	2
cell part;cytoplasm;cytoplasmic part;Golgi apparatus;intracellular membrane-bounded organelle;intracellular organelle;intracellular organelle part;intracellular part;lysosomal membrane;membrane;membrane-bounded organelle;nuclear part;nucleoplasm;organelle;organelle membrane;organelle part;plasma membrane;vacuolar m		Homo sapiens	0.9977	2
cell part;intracellular membrane-bounded organelle;intracellular organelle;intracellular part;membrane-bounded organelle;nucleus;organelle		Homo sapiens	0.9966	2
extracellular membrane-bounded organelle;extracellular organelle;extracellular region part;extracellular space;extracellular vesicular exosome;membrane-bounded organelle;membrane-bounded vesicle;organelle;vesicle		Homo sapiens	0.9999	2
cell part;cell projection;integral to membrane;intracellular part;intrinsic to membrane;membrane;membrane part;microvillus;organelle part;plasma membrane;stereocilium		Homo sapiens	0.9929	2
cell part;cytoplasmic part;cytosol;intracellular part		Homo sapiens	0.9998	2
cell part;cytoplasmic part;extracellular membrane-bounded organelle;extracellular organelle;extracellular region part;extracellular vesicular exosome;Golgi apparatus part;Golgi membrane;intracellular organelle part;intracellular part;membrane;membrane-bounded organelle;membrane-bounded vesicle;organelle;organelle membr		Homo sapiens	0.9999	2
cell part;cytoplasmic part;cytoskeletal part;cytosol;extracellular membrane-bounded organelle;extracellular organelle;extracellular region part;extracellular vesicular exosome;intracellular membrane-bounded organelle;intracellular non-membrane-bounded organelle;intracellular organelle;intracellular organelle part;intracellular par		Homo sapiens	0.9999	2
adherens junction;anchoring junction;apical junction complex;cell junction;cell part;cell-cell adherens junction;cell-cell junction;integral to membrane;intrinsic to membrane;membrane;membrane part;plasma membrane;posts	Adherens junction;Cell adhesion molecules (CAMs)	Homo sapiens	0.9996	2
cell part;cytoplasmic part;endoplasmic reticulum membrane;endoplasmic reticulum part;extracellular membrane-bounded organelle;extracellular organelle;extracellular region part;extracellular vesicular exosome;integral to membrane;intracellular organelle part;intracellular part;intrinsic to membrane;membrane;membrane part;m		Homo sapiens	1	2
cell part;cytoplasm;intracellular membrane-bounded organelle;intracellular organelle;intracellular part;membrane-bounded organelle;nucleus;organelle		Homo sapiens	0.9999	2
		Homo sapiens	1	2
basement membrane;cell part;cytoplasm;cytoplasmic part;cytoplasmic vesicle;extracellular matrix part;extracellular region part;intracellular organelle;intracellular part;organelle;vesicle		Homo sapiens	0.9975	2
cell part;cell surface;cytoplasmic part;cytoskeleton;endoplasmic reticulum;integral to membrane;integral to plasma membrane;intracellular membrane-bounded organelle;intracellular non-membrane-bounded organelle;intracellular organelle;intracellular part;intrinsic to membrane;intrinsic to plasma membrane;membrane;membra		Homo sapiens	0.9985	2
cell part;integral to membrane;intrinsic to membrane;membrane part		Homo sapiens	1	3
cell part;cytoplasmic part;endoplasmic reticulum membrane;endoplasmic reticulum part;Golgi apparatus;Golgi apparatus part;Golgi membrane;integral to membrane;intracellular membrane-bounded organelle;intracellular or	Glycerophospholipid metabolism;Inositol phosphate metabolism;Phosphatidylinositol signaling system	Homo sapiens	1	3
cell part;intracellular membrane-bounded organelle;intracellular organelle;intracellular part;membrane-bounded organelle;nucleus;organelle		Homo sapiens	1	3
adherens junction;anchoring junction;cell junction;cell part;cell-substrate adherens junction;cell-substrate junction;focal adhesion;integral to membrane;intrinsic to membrane;membrane;membrane part;organelle;plasma membrane;vesicle		Homo sapiens	0.9995	3
cell part;cytoplasmic part;cytosol;intracellular organelle part;intracellular part;nuclear part;nucleoplasm;organelle part	Parkinson's disease;Ubiquitin mediated proteolysis	Homo sapiens	1	3
cell part;centriole;chromosomal part;chromosome;chromosome passenger complex;chromosome, centromeric region;condensed chromosome kinetochore;cytoplasm;cytoplasmic microtubule;cytoplasmic part;cytoskeletal p	Colorectal cancer;Pathways in cancer	Homo sapiens	1	3
		Homo sapiens	1	3
cell part;cytoplasm;cytoplasmic part;cytoskeleton;cytosol;intracellular non-membrane-bounded organelle;intracellular organelle;intracellular part;macromolecular complex;non-membrane-bounded organelle;organelle;protein	ko05152;Neurotrophin signaling pathway;NOD-like receptor signaling pathway;Shigellosis	Homo sapiens	1	3
cell part;extracellular membrane-bounded organelle;extracellular organelle;extracellular region part;extracellular vesicular exosome;integral to membrane;intrinsic to membrane;membrane part;membrane-bounded organelle;membrane-bounded vesicle;organelle;vesicle		Homo sapiens	1	3
cell body;cell part;cell projection;external side of plasma membrane;extracellular membrane-bounded organelle;extracellular organelle;extracellular region part;extracellular vesicular exosome;integral to membrane;intrinsic to membrane;membrane;membrane part;membrane-bounded organelle;membrane-bounded vesicle;neuro		Homo sapiens	1	3
cell part;cytoplasmic part;endoplasmic reticulum;extracellular region part;extracellular space;Golgi apparatus;intracellular membrane-bounded organelle;intracellular organelle;intracellular part;membrane-bounded organelle;organelle;perinuclear region of cytoplasm		Homo sapiens	0.9998	3
cell part;cytoplasm;cytoplasmic dynein complex;cytoplasmic part;cytoskeletal part;dynein complex;early endosome membrane;endosomal part;endosome membrane;extrinsic to membrane;intracellular organelle part;intracellular part;macromolecular complex;membrane;membrane part;microtubule associated complex;organelle		Homo sapiens	1	3
basement membrane;cell part;cytoplasmic membrane-bounded vesicle lumen;cytoplasmic part;cytoplasmic vesicle part;extracellular matrix;extracellular matrix part;extracellular membrane-bounded organelle;extracellular organelle;extracellular region;extracellular region part;extracellular space;extracellular vesicular exosome;in		Homo sapiens	1	3
blood microparticle;cell part;cytoplasm;extracellular membrane-bounded organelle;extracellular organelle;extracellular region;extracellular region part;extracellular vesicular exosome;integral to membrane;integral to plasma n	Amoebiasis;Complement and coagulation cascades;Prion diseases;Systemic lupus erythematosus	Homo sapiens	1	3
blood microparticle;extracellular membrane-bounded organelle;extracellular organelle;extracellular region;extracellular region part;extracellular space;extracellular vesicular exosome;membrane-bounded organelle;membrane-bounded vesicle;organelle;vesicle		Homo sapiens	0.9948	3
cell part;chromatin;chromosomal part;cytoplasm;intracellular membrane-bounded organelle;intracellular organelle;intracellular organelle part;intracellular part;membrane-bounded organelle;nucleus;organelle;organelle part		Homo sapiens	1	3
anchored to membrane;cell part;cell surface;cytoplasmic part;external side of plasma membrane;extracellular membrane-bounded organelle;extracellular organelle;extracellular region part;extracellular vesicular exosome;Gol	Cell adhesion molecules (CAMs);Prion diseases	Homo sapiens	0.9998	3
cell part;intracellular membrane-bounded organelle;intracellular organelle;intracellular organelle part;intracellular part;membrane-bounded organelle;nuclear part;nucleoplasm;nucleus;organelle;organelle part	Osteoclast differentiation	Homo sapiens	1	3
cell part;cytoplasmic part;extracellular region;extracellular region part;extracellular space;intracellular membrane-bounded organelle;intracellular organelle;intracellular organelle lumen;intracellular organelle part;intracellular pa	Antigen processing and presentation;ko05152;Lysosome;Phagosome	Homo sapiens	1	3
		Homo sapiens	1	3
cell part;cytoplasmic membrane-bounded vesicle;cytoplasmic part;cytoplasmic vesicle;cytoplasmic vesicle membrane;cytoplasmic vesicle part;endocytic vesicle;endocytic vesicle membrane;endoplasmic reticulum membrar	Measles	Homo sapiens	1	3
cell part;cytoplasm;cytoplasmic part;cytoskeleton;cytosol;intracellular membrane-bounded organelle;intracellular non-membrane-bounded organelle;intracellular organelle;intracellular organelle part;intracellular part;macromo	RNA transport	Homo sapiens	1	3
cell part;cytoplasmic part;extracellular membrane-bounded organelle;extracellular organelle;extracellular region part;extracellular vesicular exosome;integral to membrane;intracellular membrane-bounded organelle;intracellul	Oxidative phosphorylation	Homo sapiens	1	3
cell part;cytoplasmic part;endoplasmic reticulum membrane;endoplasmic reticulum part;extracellular membrane-bounded organelle;extracellular organelle;extracellular region part;extracellular vesicular exosome;integral to m	N-Glycan biosynthesis;Protein processing in endoplasmic reticulum;Various types of N-glycan biosynthe	Homo sapiens	1	3
extracellular region;extracellular region part;extracellular space		Homo sapiens	1	3
cell part;extracellular membrane-bounded organelle;extracellular organelle;extracellular region part;extracellular vesicular exosome;integral to membrane;integral to plasma membrane;intrinsic to membrane;intrinsic to plasma	ECM-receptor interaction	Homo sapiens	1	3
cell part;centrosome;cytoplasm;cytoplasmic part;cytoskeletal part;cytosol;intracellular membrane-bounded organelle;intracellular non-membrane-bounded organelle;intracellular organelle;intracellular organelle part;intracellul	Cell cycle;Cell cycle - yeast;Meiosis - yeast;Oocyte meiosis;Ubiquitin mediated proteolysis	Homo sapiens	1	3
cell part;cytoplasmic part;cytosol;extracellular membrane-bounded organelle;extracellular organelle;extracellular region part;extracellular vesicular exosome;intracellular part;membrane-bounded organelle;membrane-bounde	Arginine and proline metabolism;Glycine, serine and threonine metabolism	Homo sapiens	1	3
cell part;cell projection;cytoplasmic part;endoplasmic reticulum;Golgi apparatus;integral to membrane;integral to plasma membrane;intracellular membrane-bounded organelle;intracellular organelle;intracellular part;intrinsic to membrane;intrinsic to plasma membrane;lamellipodium;membrane;membrane part;membrane-bounde		Homo sapiens	1	3
cell part;cytoplasmic part;endoplasmic reticulum;endoplasmic reticulum membrane;endoplasmic reticulum part;integral to membrane;integral to plasma membrane;intracellular membrane-bounded organelle;intracellular orga	Steroid biosynthesis	Homo sapiens	1	3
cell part;chromatin;chromosomal part;cytoplasm;ESC/E(Z) complex;histone methyltransferase complex;intracellular membrane-bounded organelle;intracellular organelle;intracellular organelle part;intracellular part;macromolecular complex;membrane-bounded organelle;methyltransferase complex;nuclear chromatin;nuclear chro		Homo sapiens	1	3
cell part;cytoplasm;intracellular part;membrane;plasma membrane		Homo sapiens	1	3
cell part;cytoplasmic part;cytosol;intracellular part		Homo sapiens	1	3
cell part;cell projection cytoplasm;cell projection membrane;cell projection part;cytoplasmic part;cytoplasmic vesicle membrane;cytoplasmic vesicle part;dendrite cytoplasm;dendrite membrane;early endosome membrane;endosomal part;endosome membrane;Golgi apparatus part;Golgi membrane;integral to membrane;intrace		Homo sapiens	1	3
cell part;cytoplasm;cytoplasmic part;intracellular part;macromolecular complex;NatC complex;N-terminal protein acetyltransferase complex;protein acetyltransferase complex;protein complex		Homo sapiens	1	3
cell part;cytoplasm;intracellular part		Homo sapiens	1	3
cell part;external side of plasma membrane;extracellular membrane-bounded organelle;extracellular organelle;extracellular region part;extracellular vesicular exosome;integral to membrane;intrinsic to membrane;membrane p	Cell adhesion molecules (CAMs)	Homo sapiens	1	3
adherens junction;anchoring junction;cell junction;cell part;cell-substrate adherens junction;cell-substrate junction;focal adhesion;membrane;plasma membrane		Homo sapiens	0.9985	3
cell part;cytoplasm;intracellular membrane-bounded organelle;intracellular organelle;intracellular organelle part;intracellular part;macromolecular complex;membrane-bounded organelle;nuclear body;nuclear part;nuclear spec	RNA transport	Homo sapiens	1	3
collagen;extracellular matrix;extracellular matrix part;extracellular region part;extracellular space;proteinaceous extracellular matrix		Homo sapiens	1	3
		Homo sapiens	1	3
cell part;cytoplasmic part;integral to membrane;intracellular membrane-bounded organelle;intracellular organelle;intracellular organelle part;intracellular part;intrinsic to membrane;membrane;membrane part;membrane-bounc	Cytosolic DNA-sensing pathway;Hepatitis C;Measles;RIG-I-like receptor signaling pathway	Homo sapiens	1	3
cell part;extracellular membrane-bounded organelle;extracellular organelle;extracellular region part;extracellular vesicular exosome;intracellular organelle part;intracellular part;membrane-bounded organelle;membrane-bounded vesicle;nuclear part;nucleoplasm;organelle;organelle part;vesicle		Homo sapiens	1	3
cell part;cytoplasmic part;endoplasmic reticulum;Golgi apparatus;Golgi apparatus part;intracellular membrane-bounded organelle;intracellular organelle;intracellular organelle part;intracellular part;macromolecular complex;membrane-bounded organelle;organelle;organelle part;protein complex;TRAPP complex		Homo sapiens	1	3
cell part;intracellular organelle part;intracellular part;macromolecular complex;nuclear part;nucleoplasm;organelle part;ribonucleoprotein complex;spliceosomal complex		Homo sapiens	1	3
cell part;cytoplasmic part;endosome;intracellular membrane-bounded organelle;intracellular organelle;intracellular part;late endosome;membrane;membrane-bounded organelle;organelle;plasma membrane		Homo sapiens	1	3
cell part;cytoplasmic part;endoplasmic reticulum;envelope;intracellular membrane-bounded organelle;intracellular organelle;intracellular organelle part;intracellular part;membrane;membrane-bounded organelle;nuclear envelope;nuclear part;organelle;organelle envelope;organelle part		Homo sapiens	1	3
extracellular matrix;extracellular membrane-bounded organelle;extracellular organelle;extracellular region;extracellular region part;extracellular space;extracellular vesicular exosome;membrane-bounded organelle;membrane-bounded vesicle;organelle;proteinaceous extracellular matrix;vesicle		Homo sapiens	0.9999	3
cell part;cytoplasmic part;endoplasmic reticulum;integral to membrane;intracellular membrane-bounded organelle;intracellular organelle;intracellular part;intrinsic to membrane;membrane part;membrane-bounded organelle;organelle		Homo sapiens	1	3
cell part;cytoplasm;cytoplasmic membrane-bounded vesicle;cytoplasmic part;cytoplasmic vesicle;extracellular membrane-bounded organelle;extracellular organelle;extracellular region part;extracellular vesicular exosome;Golgi apparatus;integral to membrane;intracellular membrane-bounded organelle;intracellular organelle;intr		Homo sapiens	1	3
cell part;centrosome;cytoplasm;cytoplasmic part;cytoskeletal part;endosomal part;endosome membrane;ESCRT I complex;intracellular membrane-bounded organelle;intracellular non-membrane-bounded organelle;intracellu	Endocytosis	Homo sapiens	1	3
cell cortex part;cell part;cytoplasmic part;exocyst;integral to membrane;intracellular part;intrinsic to membrane;macromolecular complex;membrane;membrane part;plasma membrane;protein complex		Homo sapiens	1	3
AP-type membrane coat adaptor complex;cell junction;cell part;cell projection;clathrin adaptor complex;clathrin-coated vesicle;coated vesicle;cytoplasm;cytoplasmic membrane-bounded vesicle;cytoplasmic part;cytoplasmic vesicle;intracellular membrane-bounded organelle;intracellular non-membrane-bounded organelle;intra		Homo sapiens	1	3
cell part;cytoplasmic part;endoplasmic reticulum;endoplasmic reticulum membrane;endoplasmic reticulum part;Golgi apparatus;integral to membrane;intracellular membrane-bounded organelle;intracellular organelle;intracellular organelle part;intracellular part;intrinsic to membrane;membrane;membrane part;membrane-bounde		Homo sapiens	1	3
cell part;Cul2-RING ubiquitin ligase complex;Cul3-RING ubiquitin ligase complex;CUL4 RING ubiquitin ligase complex;Cul4A-RING ubiquitin ligase complex;cullin-RING ubiquitin ligase complex;intracellular;intracellular part;macromolecular complex;protein complex;ubiquitin ligase complex		Homo sapiens	1	3
cell part;cytoplasm;intracellular organelle part;intracellular part;nuclear part;nucleoplasm;organelle part		Homo sapiens	1	3
cell part;extracellular membrane-bounded organelle;extracellular organelle;extracellular region part;extracellular vesicular exosome;membrane;membrane-bounded organelle;membrane-bounded vesicle;organelle;vesicle		Homo sapiens	0.9999	3
cell part;cytoplasmic part;intracellular non-membrane-bounded organelle;intracellular organelle;intracellular organelle part;intracellular part;macromolecular complex;membrane;mitochondrial inner membrane;mitochondrial membrane;mitochondrial part;mitochondrial ribosome;non-membrane-bounded organelle;organellar ribos		Homo sapiens	1	3
autophagic vacuole membrane;cell part;cytoplasmic part;endoplasmic reticulum;endoplasmic reticulum-Golgi intermediate compartment membrane;integral to membrane;intracellular membrane-bounded organelle;intracellular organelle;intracellular organelle part;intracellular part;intrinsic to membrane;membrane;membrane par		Homo sapiens	1	3
cell junction;cell part;cell projection membrane;cell projection part;cell-cell junction;cytoplasmic part;dendritic shaft;extracellular membrane-bounded organelle;extracellular organelle;extracellular region part;extracellular vesicular exosome;integral to membrane;intracellular part;intrinsic to membrane;membrane;membrane part;m		Homo sapiens	1	3
cell junction;cell part;cytoplasm;cytoskeletal part;intracellular organelle part;intracellular part;membrane;organelle part;postsynaptic density;postsynaptic membrane;synapse part;synaptic membrane		Homo sapiens	0.9941	3
		Homo sapiens	1	3
cell part;integral to membrane;intrinsic to membrane;membrane part		Homo sapiens	1	3
cell part;chromatin;chromosomal part;cytoplasm;intracellular membrane-bounded organelle;intracellular organelle;intracellular organelle part;intracellular part;membrane-bounded organelle;nuclear chromatin;nuclear chromosome part;nuclear part;nucleus;organelle;organelle part		Homo sapiens	0.9999	3
cell part;cytoplasm;cytoplasmic part;extracellular membrane-bounded organelle;extracellular organelle;extracellular region part;extracellular vesicular exosome;intracellular membrane-bounded organelle;intracellular organelle;intracellular organelle part;intracellular part;macromolecular complex;membrane-bounded organelle;me		Homo sapiens	1	3
cell part;cytoplasmic part;endoplasmic reticulum;endoplasmic reticulum membrane;endoplasmic reticulum part;integral to membrane;intracellular membrane-bounded organelle;intracellular organelle;intracellular organelle pa	N-Glycan biosynthesis;Various types of N-glycan biosynthesis	Homo sapiens	1	3
cell division site part;cell part;cleavage furrow;cytoplasm;cytoplasmic part;cytoskeletal part;intracellular membrane-bounded organelle;intracellular organelle;intracellular organelle part;intracellular part;macromolecular complex;membrane-bounded organelle;microtubule;microtubule organizing center;nucleus;organelle;organelle p		Homo sapiens	1	3
cell part;cytoplasmic part;intracellular non-membrane-bounded organelle;intracellular organelle;intracellular organelle part;intracellular part;macromolecular complex;membrane;mitochondrial inner membrane;mitochondrial membrane;mitochondrial part;mitochondrial ribosome;non-membrane-bounded organelle;organellar ribos		Homo sapiens	1	3
cell part;cell projection;centriole;centrosome;cilium;cytoplasmic part;cytoskeletal part;cytosol;intracellular non-membrane-bounded organelle;intracellular organelle;intracellular organelle part;intracellular part;membrane;microtubule organizing center;microtubule organizing center part;non-membrane-bounded organelle;organelle		Homo sapiens	1	3
		Homo sapiens	1	3
cell part;chloride channel complex;extracellular membrane-bounded organelle;extracellular organelle;extracellular region part;extracellular vesicular exosome;ion channel complex;macromolecular complex;membrane;membrane part;membrane-bounded organelle;membrane-bounded vesicle;organelle;plasma membrane;protein		Homo sapiens	1	3
cell part;cytoplasm;cytoplasmic part;intracellular membrane-bounded organelle;intracellular organelle;intracellular part;membrane-bounded organelle;mitochondrion;nucleus;organelle		Homo sapiens	1	3
cell part;integral to membrane;intrinsic to membrane;membrane part		Homo sapiens	1	3
cell part;cytoplasm;intracellular organelle part;intracellular part;nuclear part;nucleoplasm;organelle part		Homo sapiens	1	3
		Homo sapiens	0.9998	3
cell division site part;cell junction;cell part;cell-cell junction;centralspindlin complex;cleavage furrow;cytoplasm;cytoplasmic part;cytoskeletal part;cytosol;intracellular membrane-bounded organelle;intracellular non-membrane-bounded organelle;intracellular organelle;intracellular organelle part;intracellular part;macromolecular co		Homo sapiens	1	3
basement membrane;cell part;extracellular matrix part;extracellular region part;membrane;plasma membrane	Axon guidance	Homo sapiens	1	3

cell part;cytoplasm;intracellular membrane-bounded organelle;intracellular organelle;intracellular part;membrane-bounded organelle;nucleus;organelle		Homo sapiens	1	3
box H/ACA RNP complex;Cajal body;cell part;intracellular non-membrane-bounded organelle;intracellular organelle;intracellular organelle part;intracellular part;macromolecular complex;non-membrane-bounded organelle;nu	Ribosome biogenesis in eukaryotes	Homo sapiens	1	3
actin filament;cell part;cytoplasm;cytoskeletal part;intracellular organelle part;intracellular part;macromolecular complex;organelle part;protein complex		Homo sapiens	1	3
Ada2/Gcn5/Ada3 transcription activator complex;cell part;chromosomal part;DNA polymerase complex;epsilon DNA polymerase complex;histone acetyltransferase complex;intracellular membrane-bounded organelle;intrace	Base excision repair;DNA replication;Nucleotide excision repair;Purine metabolism;Pyrimidine metabolis	Homo sapiens	1	3
cell part;chromatin accessibility complex;chromatin remodeling complex;chromosomal part;DNA polymerase complex;epsilon DNA polymerase complex;intracellular organelle part;intracellular part;macromolecular complex;nuclear chromosome part;nuclear part;organelle part;protein complex		Homo sapiens	1	3
		Homo sapiens	1	3
cell part;integral to membrane;intrinsic to membrane;membrane part		Homo sapiens	1	3
cell part;intracellular organelle part;intracellular part;macromolecular complex;mediator complex;nuclear part;nucleoplasm;nucleoplasm part;organelle part;protein complex		Homo sapiens	1	3
cell part;cytoplasmic part;endoplasmic reticulum membrane;endoplasmic reticulum part;integral to membrane;intracellular organelle part;intracellular part;intrinsic to membrane;membrane;membrane part;organelle membrane;organelle part		Homo sapiens	1	3
cell part;cytoplasmic membrane-bounded vesicle;cytoplasmic part;cytoplasmic vesicle;intracellular membrane-bounded organelle;intracellular organelle;intracellular part;membrane-bounded organelle;membrane-bounded vesicle;nucleus;organelle;vesicle		Homo sapiens	1	3
cell body;cell part;cell projection;cytoplasmic part;cytosol;dendrite;intracellular part;membrane;neuron projection;neuronal cell body		Homo sapiens	1	3
cell part;coated pit;cytoplasmic part;endosomal part;endosome lumen;Golgi apparatus part;Golgi membrane;intracellular organelle lumen;intracellular organelle part;intracellular part;macromolecular complex;membrane;mem	Lysosome	Homo sapiens	0.9998	3
cell part;intracellular membrane-bounded organelle;intracellular organelle;intracellular part;membrane-bounded organelle;nucleus;organelle		Homo sapiens	1	3
Cajal body;cell part;intracellular organelle part;intracellular part;membrane;nuclear body;nuclear part;nucleoplasm;nucleoplasm part;organelle part		Homo sapiens	1	3
cell part;intracellular membrane-bounded organelle;intracellular organelle;intracellular part;membrane-bounded organelle;nucleus;organelle		Homo sapiens	1	3
cell part;intracellular membrane-bounded organelle;intracellular organelle;intracellular organelle part;intracellular part;membrane-bounded organelle;nuclear part;nucleoplasm;nucleus;organelle;organelle part		Homo sapiens	1	3
cell part;cytoplasmic part;intracellular membrane-bounded organelle;intracellular organelle;intracellular organelle part;intracellular part;membrane-bounded organelle;nuclear part;nucleoplasm;nucleus;organelle;organelle part;perinuclear region of cytoplasm		Homo sapiens	1	3
cell part;cytoplasmic part;endoplasmic reticulum lumen;endoplasmic reticulum membrane;endoplasmic reticulum part;intracellular organelle lumen;intracellular organelle part;intracellular part;membrane;membrane part;membrane-enclosed lumen;organelle lumen;organelle membrane;organelle part		Homo sapiens	1	3
autophagic vacuole membrane;cell part;cytoplasmic part;cytoplasmic vesicle;cytoskeletal part;cytosol;endomembrane system;extrinsic to membrane;intracellular organelle;intracellular organelle part;intracellular part;macromolecular complex;membrane;membrane part;microtubule;organelle;organelle membrane;organelle part;p		Homo sapiens	1	4
cell part;cytoplasm;intracellular membrane-bounded organelle;intracellular organelle;intracellular part;membrane-bounded organelle;nucleus;organelle		Homo sapiens	1	4
cell part;cytoplasmic part;endoplasmic reticulum;endoplasmic reticulum membrane;endoplasmic reticulum part;integral to membrane;integral to plasma membrane;intracellular membrane-bounded organelle;intracellular orga	Sphingolipid metabolism	Homo sapiens	1	4
cell part;cytoplasmic part;intracellular non-membrane-bounded organelle;intracellular organelle;intracellular organelle part;intracellular part;macromolecular complex;membrane;mitochondrial inner membrane;mitochondrial membrane;mitochondrial part;mitochondrial ribosome;non-membrane-bounded organelle;organellar ribos		Homo sapiens	1	4
cell part;cytoplasmic mRNA processing body;cytoplasmic part;DNA-directed RNA polymerase complex;DNA-directed RNA polymerase II, core complex;intracellular membrane-bounded organelle;intracellular non-membrane	Huntington's disease;Purine metabolism;Pyrimidine metabolism;RNA polymerase	Homo sapiens	1	4
cell part;cytoplasmic membrane-bounded vesicle;cytoplasmic part;cytoplasmic vesicle;Golgi apparatus;Golgi apparatus part;integral to membrane;intracellular membrane-bounded organelle;intracellular organelle;intracellular organelle part;intracellular part;intrinsic to membrane;membrane;membrane part;membrane-bounded o		Homo sapiens	1	4
catalytic step 2 spliceosome;cell part;intracellular membrane-bounded organelle;intracellular organelle;intracellular organelle part;intracellular part;macromolecular complex;membrane-bounded organelle;nuclear body;nuclea	Spliceosome	Homo sapiens	1	4
		Homo sapiens	1	4
cell part;cytoplasmic part;cytosol;intracellular part;macromolecular complex;mRNA cap binding complex;protein complex;RNA cap binding complex	Insulin signaling pathway;mTOR signaling pathway;RNA transport	Homo sapiens	1	4
cell part;cell surface;cytoplasm;intracellular part;membrane;plasma membrane	ECM-receptor interaction	Homo sapiens	1	4
cell part;cytoplasmic part;integral to membrane;intracellular;intracellular membrane-bounded organelle;intracellular non-membrane-bounded organelle;intracellular organelle;intracellular organelle part;intracellular part;intrinsic	Peroxisome	Homo sapiens	1	4
basal part of cell;cell junction;cell part;cell-cell junction;extracellular membrane-bounded organelle;extracellular organelle;extracellular region part;extracellular vesicular exosome;integral to membrane;intrinsic to membrane;n	Cell adhesion molecules (CAMs);Hepatitis C;Leukocyte transendothelial migration;Tight junction	Homo sapiens	1	4
cell part;contractile fiber;cytoplasmic part;intracellular membrane-bounded organelle;intracellular non-membrane-bounded organelle;intracellular organelle;intracellular organelle part;intracellular part;membrane;membrane-bounded organelle;mitochondrial inner membrane;mitochondrial membrane;mitochondrial part;mitochondr		Homo sapiens	1	4
cell part;cytoplasm;extracellular membrane-bounded organelle;extracellular organelle;extracellular region part;extracellular vesicular exosome;intracellular membrane-bounded organelle;intracellular non-membrane-bounded organelle;intracellular organelle;intracellular organelle part;intracellular part;membrane-bounded organelle		Homo sapiens	1	4
cell part;cell projection;cytoplasm;cytoplasmic part;dendrite;DNA-directed RNA polymerase complex;DNA-directed RNA polymerase II, core complex;intracellular membrane-bounded organelle;intracellular organelle;intracellular organelle part;intracellular part;macromolecular complex;membrane-bounded organelle;mitochondri		Homo sapiens	1	4
cell part;macromolecular complex;membrane;protein complex		Homo sapiens	1	4
anchored to membrane;cell part;extracellular membrane-bounded organelle;extracellular organelle;extracellular region part;extracellular vesicular exosome;integral to membrane;intrinsic to membrane;membrane;membrane p	Pantothenate and CoA biosynthesis	Homo sapiens	1	4
cell part;intracellular organelle part;intracellular part;macromolecular complex;nuclear part;organelle part;ribonucleoprotein complex;small nuclear ribonucleoprotein complex;spliceosomal complex;U4/U6 x U5 tri-snRNP com	RNA degradation;Spliceosome	Homo sapiens	1	4
blood microparticle;extracellular membrane-bounded organelle;extracellular organelle;extracellular region;extracellular region part;extracellular space;extracellular vesicular exosome;membrane-bounded organelle;membrane-bounded vesicle;organelle;vesicle		Homo sapiens	1	4
apical plasma membrane;basal part of cell;basal plasma membrane;blood microparticle;cell part;cell surface;coated pit;cytoplasmic membrane-bounded vesicle;cytoplasmic membrane-bounded vesicle lumen;cytoplasmic p	Mineral absorption	Homo sapiens	1	4
cell part;extracellular membrane-bounded organelle;extracellular organelle;extracellular region;extracellular region part;extracellular space;extracellular vesicular exosome;membrane;membrane-bounded organelle;membrane	Complement and coagulation cascades	Homo sapiens	0.9999	4
adherens junction;anchored to external side of plasma membrane;anchored to membrane;anchored to plasma membrane;anchoring junction;apical plasma membrane;cell junction;cell part;cell projection;cell projection part;c	Leukocyte transendothelial migration	Homo sapiens	1	4
cell part;cytoplasm;extracellular membrane-bounded organelle;extracellular organelle;extracellular region;extracellular region part;extracellular space;extracellular vesicular exosome;granular component;intracellular organelle part;intracellular part;membrane-bounded organelle;membrane-bounded vesicle;nuclear part;nucleolar p		Homo sapiens	1	4
BAT3 complex;cell part;cytoplasmic part;cytosol;cytosolic part;ER membrane insertion complex;intracellular part;macromolecular complex;protein complex		Homo sapiens	1	4
adherens junction;anchoring junction;cell junction;cell part;cell-substrate adherens junction;cell-substrate junction;cytoplasm;focal adhesion;integral to membrane;integral to plasma membrane;intracellular part;intrinsic to me	Cell adhesion molecules (CAMs);Leukocyte transendothelial migration	Homo sapiens	1	4
adherens junction;anchoring junction;apical plasma membrane;cell junction;cell part;cell-cell adherens junction;cell-cell contact zone;cell-cell junction;cell-substrate adherens junction;cell-substrate junction;connexon comple	Arrhythmogenic right ventricular cardiomyopathy (ARVC);Gap junction	Homo sapiens	1	4
cell part;cytoplasmic part;intracellular membrane-bounded organelle;intracellular organelle;intracellular organelle part;intracellular part;macromolecular complex;membrane;membrane part;membrane-bounded organelle;mem	Alzheimer's disease;Huntington's disease;Oxidative phosphorylation;Parkinson's disease	Homo sapiens	1	4
cell part;cytoplasm;cytoskeleton;DNA-directed RNA polymerase complex;DNA-directed RNA polymerase II, core complex;intracellular membrane-bounded organelle;intracellular non-membrane-bounded organelle;intracellul	Huntington's disease;Purine metabolism;Pyrimidine metabolism;RNA polymerase	Homo sapiens	1	4
cell part;cytoplasmic part;extracellular matrix;extracellular membrane-bounded organelle;extracellular organelle;extracellular region;extracellular region part;extracellular space;extracellular vesicular exosome;Golgi apparatus part;Golgi lumen;intracellular organelle lumen;intracellular organelle part;intracellular part;lysosomal lume		Homo sapiens	1	4
cell cortex;cell part;cis-Golgi network;cytoplasmic part;cytosol;extracellular matrix;extracellular region;extracellular region part;extracellular space;Golgi apparatus;Golgi apparatus part;intracellular membrane-bounded organelle;intracellular organelle;intracellular organelle part;intracellular part;membrane;membrane-bounded org		Homo sapiens	1	4
cell part;cytoplasm;cytoplasmic part;extracellular membrane-bounded organelle;extracellular organelle;extracellular region part;extracellular vesicular exosome;integral to membrane;intracellular membrane-bounded organelle	Neuroactive ligand-receptor interaction	Homo sapiens	1	4
basement membrane;cell part;collagen;cytoplasmic part;endoplasmic reticulum lumen;endoplasmic reticulum part;extracellular matrix;extracellular matrix part;extracellular membrane-bounded organelle;extracellular organell	Protein digestion and absorption	Homo sapiens	1	4
		Homo sapiens	1	4
cell part;cytoplasm;intracellular membrane-bounded organelle;intracellular organelle;intracellular part;membrane-bounded organelle;nucleus;organelle	RNA transport	Homo sapiens	1	4
blood microparticle;extracellular membrane-bounded organelle;extracellular organelle;extracellular region;extracellular region part;extracellular space;extracellular vesicular exosome;membrane-bounded organelle;membrane-bounded vesicle;organelle;vesicle		Homo sapiens	1	4
cell part;cytoplasmic part;cytosol;intracellular part;macromolecular complex;membrane;phosphorylase kinase complex;plasma membrane;protein complex	Calcium signaling pathway;Insulin signaling pathway	Homo sapiens	0.9979	4
cell part;external side of plasma membrane;extracellular membrane-bounded organelle;extracellular organelle;extracellular region;extracellular region part;extracellular space;extracellular vesicular exosome;membrane part;m	Chemokine signaling pathway;Cytokine-cytokine receptor interaction;Intestinal immune network for IgA	Homo sapiens	1	4
cell part;chromatin;chromosomal part;chromosome;heterochromatin;intracellular membrane-bounded organelle;intracellular non-membrane-bounded organelle;intracellular organelle;intracellular organelle part;intracellular part;macromolecular complex;membrane-bounded organelle;non-membrane-bounded organelle;nuclear ch		Homo sapiens	1	4
cell part;cytoplasmic part;intracellular part;macromolecular complex;protein complex;Rab-protein geranylgeranyltransferase complex		Homo sapiens	1	4
AP-2 adaptor complex;AP-type membrane coat adaptor complex;cell part;clathrin adaptor complex;clathrin coated vesicle membrane;clathrin-coated endocytic vesicle membrane;coated vesicle membrane;cytoplasmic part;	Endocrine and other factor-regulated calcium reabsorption;Endocytosis;Huntington's disease	Homo sapiens	1	4
cell part;cytoplasm;extracellular membrane-bounded organelle;extracellular organelle;extracellular region part;extracellular vesicular exosome;integral to membrane;intracellular membrane-bounded organelle;intracellular organelle;intracellular part;intrinsic to membrane;membrane;membrane part;membrane-bounded organelle;m		Homo sapiens	1	4
cell part;cytoplasmic part;intracellular organelle part;intracellular part;macromolecular complex;membrane;membrane part;mitochondrial inner membrane;mitochondrial membrane;mitochondrial membrane part;mitochondrial	Alzheimer's disease;Huntington's disease;Oxidative phosphorylation;Parkinson's disease	Homo sapiens	1	4
cell part;cytoplasmic part;integral to membrane;intracellular membrane-bounded organelle;intracellular organelle;intracellular organelle part;intracellular part;intrinsic to membrane;membrane;membrane part;membrane-bounded organelle;mitochondrial inner membrane;mitochondrial membrane;mitochondrial part;mitochondrion;		Homo sapiens	1	4
cell part;cytoplasmic part;cytosol;endoplasmic reticulum;intracellular membrane-bounded organelle;intracellular organelle;intracellular part;membrane-bounded organelle;organelle	Parkinson's disease;Protein processing in endoplasmic reticulum;Ubiquitin mediated proteolysis	Homo sapiens	1	4
cell part;intracellular organelle part;intracellular part;nuclear part;nucleoplasm;organelle part	Cell cycle - yeast;Pathways in cancer;Small cell lung cancer	Homo sapiens	1	4
adherens junction;anchoring junction;cell junction;cell part;cell-substrate adherens junction;cell-substrate junction;cytoplasmic part;extracellular membrane-bounded organelle;extracellular organelle;extracellular region part;extracellular vesicular exosome;focal adhesion;Golgi apparatus part;Golgi membrane;intracellular organell		Homo sapiens	1	4
catalytic step 2 spliceosome;cell part;cytoplasmic part;cytosol;extracellular membrane-bounded organelle;extracellular organelle;extracellular region part;extracellular vesicular exosome;intracellular membrane-bounded orga	Spliceosome	Homo sapiens	1	4
cell part;cytoplasmic part;cytosol;cytosolic large ribosomal subunit;extracellular region part;extracellular space;intracellular organelle part;intracellular part;large ribosomal subunit;macromolecular complex;organelle part;ribor	Ribosome	Homo sapiens	1	4
cell part;extracellular membrane-bounded organelle;extracellular organelle;extracellular region part;extracellular vesicular exosome;extrinsic to internal side of plasma membrane;extrinsic to membrane;extrinsic to plasma me	Chemokine signaling pathway	Homo sapiens	1	4
cell part;cytoplasmic part;intracellular membrane-bounded organelle;intracellular organelle;intracellular organelle part;intracellular part;macromolecular complex;membrane;membrane-bounded organelle;mitochondrial inner membrane;mitochondrial membrane;mitochondrial part;mitochondrial small ribosomal subunit;mitochond		Homo sapiens	1	4
cell part;cytoplasmic part;extracellular membrane-bounded organelle;extracellular organelle;extracellular region part;extracellular vesicular exosome;intracellular membrane-bounded organelle;intracellular organelle;intracellul	Inositol phosphate metabolism;Propanoate metabolism;Valine, leucine and isoleucine degradation	Homo sapiens	1	4
cell part;cytoplasmic part;extracellular matrix;extracellular region;extracellular region part;extracellular space;Golgi apparatus part;Golgi lumen;intracellular organelle lumen;intracellular organelle part;intracellular part;lysosomal lumen;membrane-enclosed lumen;organelle lumen;organelle part;proteinaceous extracellular matrix;va		Homo sapiens	1	4
anchored to membrane;cell part;extracellular membrane-bounded organelle;extracellular organelle;extracellular region part;extracellular vesicular exosome;extrinsic to membrane;intrinsic to membrane;membrane;membrane	Calcium signaling pathway;Nicotinate and nicotinamide metabolism;Pancreatic secretion;Salivary secre	Homo sapiens	1	4
anchored to membrane;cell part;extracellular membrane-bounded organelle;extracellular organelle;extracellular region part;extracellular vesicular exosome;intrinsic to membrane;membrane;membrane part;membrane-bounde	Cell adhesion molecules (CAMs)	Homo sapiens	1	4
extracellular region;extracellular region part;extracellular space		Homo sapiens	1	4
cell part;cytoplasm;cytoplasmic part;cytosol;intracellular membrane-bounded organelle;intracellular organelle;intracellular organelle part;intracellular part;membrane-bounded organelle;nuclear part;nucleoplasm;nucleus;orgar	Cytosolic DNA-sensing pathway;Hepatitis C;Measles;RIG-I-like receptor signaling pathway;Toll-like rece	Homo sapiens	1	4
anaphase-promoting complex;cell part;cullin-RING ubiquitin ligase complex;cytoplasm;intracellular organelle part;intracellular part;macromolecular complex;nuclear part;nuclear ubiquitin ligase complex;organelle part;protein	Ubiquitin mediated proteolysis	Homo sapiens	1	4
axon part;cell leading edge;cell part;cell projection part;clathrin-coated vesicle;coated pit;coated vesicle;cytoplasmic membrane-bounded vesicle;cytoplasmic part;cytoplasmic vesicle;extrinsic to membrane;extrinsic to plasma membrane;intracellular membrane-bounded organelle;intracellular organelle;intracellular part;membrar		Homo sapiens	1	4
cell part;cytoplasmic part;extrinsic to membrane;intracellular part;lipid particle;membrane;membrane part;pre-autophagosomal structure;pre-autophagosomal structure membrane		Homo sapiens	1	4
cell part;intracellular membrane-bounded organelle;intracellular organelle;intracellular part;membrane-bounded organelle;nucleus;organelle		Homo sapiens	1	4
cell part;collagen;cytoplasmic part;cytoplasmic vesicle membrane;cytoplasmic vesicle part;endocytic vesicle membrane;extracellular matrix part;extracellular membrane-bounded organelle;extracellular organelle;extracellular	Phagosome	Homo sapiens	1	4
cell part;cytoplasmic part;intracellular membrane-bounded organelle;intracellular organelle;intracellular part;membrane-bounded organelle;mitochondrion;organelle		Homo sapiens	1	4
cell part;cytoplasm;intracellular part		Homo sapiens	1	4
cell part;extracellular membrane-bounded organelle;extracellular organelle;extracellular region part;extracellular vesicular exosome;integral to membrane;intrinsic to membrane;membrane part;membrane-bounded organelle;membrane-bounded vesicle;organelle;vesicle		Homo sapiens	1	4
catalytic step 2 spliceosome;cell part;intracellular organelle part;intracellular part;macromolecular complex;nuclear part;organelle part;ribonucleoprotein complex;spliceosomal complex		Homo sapiens	1	4
cell part;cytoplasm;cytoplasmic part;EGO complex;Golgi apparatus;intracellular membrane-bounded organelle;intracellular organelle;intracellular organelle part;intracellular part;lysosomal membrane;lysosome;lytic vacuole;macromolecular complex;membrane;membrane part;membrane-bounded organelle;nucleus;organelle;orga		Homo sapiens	1	4
actomyosin contractile ring;cell cortex part;cell division site part;cell part;contractile ring;cytoplasm;cytoplasmic part;cytoskeletal part;cytoskeleton;integral to membrane;integral to plasma membrane;intracellular membrane-bounded organelle;intracellular non-membrane-bounded organelle;intracellular organelle;intracellular orga		Homo sapiens	1	4
		Homo sapiens	1	4
cell part;cytoplasmic part;extracellular membrane-bounded organelle;extracellular organelle;extracellular region part;extracellular vesicular exosome;Golgi apparatus part;Golgi stack;intracellular organelle part;intracellular part;intrinsic to Golgi membrane;intrinsic to membrane;intrinsic to organelle membrane;macromolecular con		Homo sapiens	1	4
cell part;cytoplasmic part;intracellular membrane-bounded organelle;intracellular organelle;intracellular part;membrane-bounded organelle;mitochondrion;organelle		Homo sapiens	1	4
cell part;cytoplasmic part;intracellular membrane-bounded organelle;intracellular organelle;intracellular part;membrane-bounded organelle;mitochondrion;nucleus;organelle		Homo sapiens	1	4
cell part;cytoplasmic part;endosomal part;endosome membrane;integral to membrane;intracellular organelle part;intracellular part;intrinsic to membrane;late endosome membrane;lysosomal membrane;membrane;membrane part;organelle membrane;organelle part;vacuolar membrane;vacuolar part		Homo sapiens	1	4
cell part;cytoplasmic part;cytosol;endoplasmic reticulum membrane;endoplasmic reticulum part;Golgi apparatus;Golgi apparatus part;Golgi membrane;intracellular membrane-bounded organelle;intracellular organelle;intracellular organelle part;intracellular part;membrane;membrane part;membrane-bounded organelle;nuclear pa		Homo sapiens	1	4
cell part;membrane;plasma membrane		Homo sapiens	1	4
cell part;cytoplasmic part;cytosol;intracellular part		Homo sapiens	1	4
calcium channel complex;cation channel complex;cell part;cytoplasmic part;intracellular membrane-bounded organelle;intracellular organelle;intracellular organelle part;intracellular part;ion channel complex;macromolecular complex;membrane part;membrane-bounded organelle;membrane-enclosed lumen;mitochondrial interm		Homo sapiens	1	4
cell part;cytoplasmic part;intracellular membrane-bounded organelle;intracellular organelle;intracellular part;membrane-bounded organelle;mitochondrion;organelle		Homo sapiens	1	4
cell part;cytoplasmic part;early endosome;early endosome membrane;endosomal part;endosome;endosome membrane;ER membrane protein complex;Golgi apparatus;Golgi apparatus part;Golgi membrane;integral to membrane;intracellular membrane-bounded organelle;intracellular organelle;intracellular organelle part;intrace		Homo sapiens	1	4
cell part;cytoplasmic part;extracellular membrane-bounded organelle;extracellular organelle;extracellular region part;extracellular vesicular exosome;Golgi apparatus part;Golgi membrane;integral to membrane;intracellular or	Glycosaminoglycan biosynthesis - chondroitin sulfate	Homo sapiens	0.9999	4
cell part;cytoplasmic part;cytoplasmic vesicle membrane;cytoplasmic vesicle part;extracellular membrane-bounded organelle;extracellular organelle;extracellular region part;extracellular vesicular exosome;integral to membrane;integral to plasma membrane;intracellular membrane-bounded organelle;intracellular organelle;intrace		Homo sapiens	1	4
cell part;cytoplasm;intracellular non-membrane-bounded organelle;intracellular organelle;intracellular organelle part;intracellular part;membrane;non-membrane-bounded organelle;nuclear part;nucleolus;organelle;organelle part		Homo sapiens	1	4
		Homo sapiens	1	4
cell part;cytoplasmic part;Golgi apparatus;Golgi apparatus part;Golgi membrane;integral to membrane;intracellular membrane-bounded organelle;intracellular organelle;intracellular organelle part;intracellular part;intrinsic to membrane;membrane;membrane part;membrane-bounded organelle;organelle;organelle membrane;orgar		Homo sapiens	1	4
cell part;chromosome;intracellular non-membrane-bounded organelle;intracellular organelle;intracellular organelle part;intracellular part;non-membrane-bounded organelle;nuclear part;nucleolus;nucleoplasm;organelle;organe	Lysine degradation	Homo sapiens	1	4
cell part;cytoplasmic membrane-bounded vesicle;cytoplasmic part;cytoplasmic vesicle;cytosol;extracellular membrane-bounded organelle;extracellular organelle;extracellular region part;extracellular space;extracellular vesic	Folate biosynthesis	Homo sapiens	1	4
cell part;cytoplasm;cytoplasmic part;intracellular membrane-bounded organelle;intracellular organelle;intracellular part;membrane-bounded organelle;mitochondrion;nucleus;organelle		Homo sapiens	1	4
cell part;cytoplasmic part;intracellular membrane-bounded organelle;intracellular organelle;intracellular part;macromolecular complex;membrane-bounded organelle;methyltransferase complex;nucleus;organelle;protein complex;tRNA (m1A) methyltransferase complex;tRNA methyltransferase complex		Homo sapiens	1	4
cell part;cytoplasm;intracellular organelle part;intracellular part;nuclear part;nucleoplasm;organelle part		Homo sapiens	1	4
actin cytoskeleton;actin filament bundle;actomyosin;adherens junction;anchoring junction;cell cortex part;cell junction;cell part;cell-substrate adherens junction;cell-substrate junction;cortical actin cytoskeleton;cortical cytoskeleton;cytoplasmic part;cytoskeletal part;cytoskeleton;extracellular membrane-bounded organelle;extra		Homo sapiens	1	4
cell part;integral to membrane;integral to plasma membrane;intrinsic to membrane;intrinsic to plasma membrane;membrane;membrane part;plasma membrane;plasma membrane part	Protein digestion and absorption	Homo sapiens	1	4
apical part of cell;cell part;cytoplasmic part;endosome;extracellular membrane-bounded organelle;extracellular organelle;extracellular region part;extracellular vesicular exosome;intracellular membrane-bounded organelle;int	Antigen processing and presentation;Lysosome	Homo sapiens	1	4
cell part;intracellular membrane-bounded organelle;intracellular organelle;intracellular organelle part;intracellular part;macromolecular complex;membrane-bounded organelle;nuclear body;nuclear part;nuclear speck;nucleopl	Spliceosome	Homo sapiens	1	4
axon part;cell part;cell projection part;cytoplasmic part;cytosol;endoplasmic reticulum membrane;endoplasmic reticulum part;extrinsic to membrane;intracellular organelle part;intracellular part;membrane;membrane part;nuc	Glycerolipid metabolism	Homo sapiens	1	4
cell part;intracellular membrane-bounded organelle;intracellular organelle;intracellular part;membrane-bounded organelle;nucleus;organelle		Homo sapiens	1	4
cell part;cytoplasm;cytoplasmic part;cytosol;endosomal part;endosome membrane;ESCRT II complex;extracellular membrane-bounded organelle;extracellular organelle;extracellular region part;extracellular vesicular exosom	Endocytosis	Homo sapiens	1	4
cell part;intracellular part;macromolecular complex;proteasome complex;protein complex		Homo sapiens	1	4
cell part;cytoplasm;cytoplasmic part;cytosol;extracellular region part;extracellular space;integral to membrane;intracellular membrane-bounded organelle;intracellular organelle;intracellular organelle part;intracellular part;intrinsic to membrane;lipid particle;membrane;membrane part;membrane-bounded organelle;mitochondrial m		Homo sapiens	1	4
cell part;intracellular organelle part;intracellular part;nuclear part;nucleoplasm;organelle part		Homo sapiens	1	4
cell part;cytoplasmic part;endoplasmic reticulum membrane;endoplasmic reticulum part;extracellular membrane-bounded organelle;extracellular organelle;extracellular region part;extracellular vesicular exosome;intracellular	Peroxisome;Retinol metabolism	Homo sapiens	1	4
cell part;cytoplasm;intracellular membrane-bounded organelle;intracellular organelle;intracellular part;membrane-bounded organelle;nucleus;organelle		Homo sapiens	1	4
	Ribosome biogenesis in eukaryotes	Homo sapiens	1	4
axoneme part;cell part;cell projection;cell projection part;centrosome;cilium;cytoplasmic part;cytoskeletal part;endomembrane system;intracellular non-membrane-bounded organelle;intracellular organelle;intracellular organelle part;intracellular part;intraflagellar transport particle B;macromolecular complex;microtubule organizin		Homo sapiens	1	4
cell part;cytoplasmic part;endosome;extracellular membrane-bounded organelle;extracellular organelle;extracellular region part;extracellular space;extracellular vesicular exosome;Golgi apparatus part;intracellular membrane-bounded organelle;intracellular organelle;intracellular organelle part;intracellular part;late endosome;lyso		Homo sapiens	1	4
cell part;intracellular part;macromolecular complex;ribonucleoprotein complex		Homo sapiens	1	4
cell part;intracellular membrane-bounded organelle;intracellular organelle;intracellular organelle part;intracellular part;macromolecular complex;membrane-bounded organelle;nuclear body;nuclear part;nuclear speck;nucleoplasm part;nucleus;organelle;organelle part;ribonucleoprotein complex		Homo sapiens	1	4
Atg12-Atg5-Atg16 complex;autophagic vacuole;axoneme;cell part;cell projection part;cytoplasm;cytoplasmic part;endoplasmic reticulum part;ER-mitochondrion membrane contact site;intracellular membrane-bounded orgar	Regulation of autophagy;RIG-I-like receptor signaling pathway;Shigellosis	Homo sapiens	1	4
cell part;cytoplasm;cytoplasmic part;EGO complex;intracellular membrane-bounded organelle;intracellular organelle;intracellular organelle part;intracellular part;lysosome;lytic vacuole;macromolecular complex;membrane part;membrane-bounded organelle;nuclear part;nucleoplasm;nucleus;organelle;organelle part;protein comp		Homo sapiens	1	4
cell part;cytoplasmic part;endoplasmic reticulum membrane;endoplasmic reticulum part;Golgi apparatus part;Golgi membrane;integral to membrane;intracellular membrane-bounded organelle;intracellular organelle;intracellul	Protein processing in endoplasmic reticulum	Homo sapiens	1	4
cell part;cytoplasm;intracellular membrane-bounded organelle;intracellular organelle;intracellular part;membrane-bounded organelle;nucleus;organelle		Homo sapiens	1	4
cell part;cell projection;cell projection part;cytoplasm;dendritic spine;intracellular part;macromolecular complex;membrane;neuron projection;neuron spine;protein complex		Homo sapiens	0.9978	4
cell part;cytoplasm;intracellular membrane-bounded organelle;intracellular organelle;intracellular part;membrane-bounded organelle;nucleus;organelle	Atrazine degradation	Homo sapiens	1	4

apical plasma membrane;cell part;cytoplasmic part;cytoplasmic vesicle membrane;cytoplasmic vesicle part;endoplasmic reticulum;Golgi apparatus;integral to membrane;intracellular membrane-bounded organelle;intracellular organelle;intracellular organelle part;intracellular part;intrinsic to membrane;membrane;membrane part;		Homo sapiens	1	4
cell part;cytoplasmic part;cytosol;intracellular membrane-bounded organelle;intracellular non-membrane-bounded organelle;intracellular organelle;intracellular organelle part;intracellular part;membrane;membrane-bounded organelle;non-membrane-bounded organelle;nuclear part;nucleolus;nucleoplasm;nucleus;organelle;organ		Homo sapiens	1	4
cell part;intracellular part;macromolecular complex;pre-snoRNP complex;ribonucleoprotein complex		Homo sapiens	1	4
cell part;cytoplasm;intracellular membrane-bounded organelle;intracellular organelle;intracellular part;membrane-bounded organelle;nucleus;organelle		Homo sapiens	1	4
cell part;integral to membrane;intrinsic to membrane;membrane part		Homo sapiens	1	4
cell part;cell projection part;contractile fiber part;cytoplasmic part;cytoskeletal part;growth cone;intracellular organelle part;intracellular part;organelle part;site of polarized growth;striated muscle thin filament		Homo sapiens	0.9998	4
cell part;cytoplasm;intracellular part		Homo sapiens	0.9992	4
cell part;cytoplasmic part;endoplasmic reticulum;endoplasmic reticulum membrane;endoplasmic reticulum part;Golgi apparatus part;Golgi membrane;integral to membrane;intracellular membrane-bounded organelle;intracell	Phagosome;SNARE interactions in vesicular transport	Homo sapiens	1	4
cell part;Cul2-RING ubiquitin ligase complex;Cul3-RING ubiquitin ligase complex;CUL4 RING ubiquitin ligase complex;Cul5-RING ubiquitin ligase complex;Cul7-RING ubiquitin ligase complex;cullin-RING ubiquitin ligase com	Ubiquitin mediated proteolysis	Homo sapiens	1	4
cell part;cytoplasm;extracellular membrane-bounded organelle;extracellular organelle;extracellular region part;extracellular vesicular exosome;intracellular part;membrane;membrane-bounded organelle;membrane-bounded vesicle;organelle;vesicle		Homo sapiens	1	4
cell part;cytoplasm;cytoplasmic part;intracellular membrane-bounded organelle;intracellular organelle;intracellular organelle part;intracellular part;macromolecular complex;membrane;membrane part;membrane-bounded orga	Alzheimer's disease;Huntington's disease;Oxidative phosphorylation;Parkinson's disease	Homo sapiens	1	4
cell junction;cell part;clathrin-coated vesicle;coated vesicle;cytoplasmic membrane-bounded vesicle;cytoplasmic part;cytoplasmic vesicle;Golgi apparatus;integral to membrane;intracellular membrane-bounded organelle;intracellular organelle;intracellular part;intrinsic to membrane;membrane;membrane part;membrane-bounde		Homo sapiens	1	4
blood microparticle;cell part;cytoplasm;extracellular region part;intracellular membrane-bounded organelle;intracellular organelle;intracellular organelle part;intracellular part;membrane-bounded organelle;nuclear part;nucleoplasm;nucleus;organelle;organelle part		Homo sapiens	1	4
cell part;cytoplasm;intracellular membrane-bounded organelle;intracellular non-membrane-bounded organelle;intracellular organelle;intracellular organelle part;intracellular part;membrane-bounded organelle;non-membrane-bounded organelle;nuclear part;nucleolus;nucleus;organelle;organelle part		Homo sapiens	1	4
apical plasma membrane;cell junction;cell part;cell-cell junction;cytoplasmic part;cytoplasmic vesicle;cytosol;endosome;intracellular membrane-bounded organelle;intracellular organelle;intracellular part;membrane part;membrane-bounded organelle;occluding junction;organelle;plasma membrane part;recycling endosome;tight		Homo sapiens	1	4
cell part;cytoplasmic part;integral to membrane;integral to mitochondrial inner membrane;integral to mitochondrial membrane;integral to organelle membrane;intracellular membrane-bounded organelle;intracellular organelle;intracellular organelle part;intracellular part;intrinsic to membrane;intrinsic to mitochondrial inner membra		Homo sapiens	1	4
cell part;cytoplasmic part;intracellular membrane-bounded organelle;intracellular organelle;intracellular part;membrane-bounded organelle;mitochondrion;organelle		Homo sapiens	1	4
cell part;integral to membrane;intrinsic to membrane;membrane part		Homo sapiens	1	4
cell part;chromatin;chromosomal part;cytoplasm;intracellular membrane-bounded organelle;intracellular organelle;intracellular organelle part;intracellular part;macromolecular complex;membrane;membrane-bounded organel	Neurotrophin signaling pathway	Homo sapiens	1	4
cell part;centrosome;cytoplasm;cytoplasmic part;cytoskeletal part;intracellular membrane-bounded organelle;intracellular non-membrane-bounded organelle;intracellular organelle;intracellular organelle part;intracellular part;membrane-bounded organelle;microtubule organizing center;non-membrane-bounded organelle;nucleus;		Homo sapiens	1	4
extracellular membrane-bounded organelle;extracellular organelle;extracellular region part;extracellular vesicular exosome;membrane-bounded organelle;membrane-bounded vesicle;organelle;vesicle		Homo sapiens	1	4
		Homo sapiens	1	5
cell part;cytoplasmic part;endoplasmic reticulum membrane;endoplasmic reticulum part;intracellular organelle part;intracellular part;macromolecular complex;membrane;membrane part;nuclear membrane;nuclear part;nuclear pore;organelle membrane;organelle part;pore complex;protein complex		Homo sapiens	1	5
cell part;intracellular organelle part;intracellular part;macromolecular complex;nuclear part;organelle part;ribonucleoprotein complex;small nucleolar ribonucleoprotein complex;spliceosomal complex		Homo sapiens	1	5
		Homo sapiens	1	5
		Homo sapiens	1	5
cell part;cytoplasmic membrane-bounded vesicle;cytoplasmic part;cytoplasmic vesicle;extracellular membrane-bounded organelle;extracellular organelle;extracellular region part;extracellular vesicular exosome;intracellular m	Lysosome	Homo sapiens	1	5
cell part;cytoplasmic part;endoplasmic reticulum membrane;endoplasmic reticulum part;envelope;extracellular membrane-bounded organelle;extracellular organelle;extracellular region part;extracellular vesicular exosome;int	Drug metabolism - cytochrome P450;Glutathione metabolism;Metabolism of xenobiotics by cytochrome	Homo sapiens	1	5
cell part;centrosome;cytoplasmic part;cytoskeletal part;cytosol;intracellular non-membrane-bounded organelle;intracellular organelle;intracellular organelle part;intracellular part;microtubule organizing center;non-membrane-bounded organelle;organelle;organelle part		Homo sapiens	1	5
cell part;cytoplasmic part;cytosol;extracellular matrix;extracellular membrane-bounded organelle;extracellular organelle;extracellular region part;extracellular vesicular exosome;Golgi apparatus;integral to membrane;integral to plasma membrane;intracellular membrane-bounded organelle;intracellular non-membrane-bounded or		Homo sapiens	1	5
cell part;chromosome;cytoplasm;cytoplasmic part;DNA-directed RNA polymerase complex;DNA-directed RNA polymerase I complex;intracellular membrane-bounded organelle;intracellular non-membrane-bounded organelle;intracellular organelle;intracellular organelle part;intracellular part;macromolecular complex;membrane-		Homo sapiens	1	5
cell part;cytoplasmic part;endoplasmic reticulum;endoplasmic reticulum membrane;endoplasmic reticulum part;Golgi apparatus part;Golgi membrane;integral to endoplasmic reticulum membrane;integral to lumenal side of e	Antigen processing and presentation	Homo sapiens	1	5
adherens junction;anchoring junction;cell junction;cell part;cell-substrate adherens junction;cell-substrate junction;cytoplasm;cytoskeleton;focal adhesion;intracellular;intracellular non-membrane-bounded organelle;intracellular organelle;intracellular organelle part;intracellular part;non-membrane-bounded organelle;nuclear matri		Homo sapiens	1	5
cell part;cytoplasmic part;intracellular organelle part;intracellular part;macromolecular complex;membrane;membrane part;mitochondrial inner membrane;mitochondrial membrane;mitochondrial membrane part;mitochondrial	Alzheimer's disease;Huntington's disease;Oxidative phosphorylation;Parkinson's disease	Homo sapiens	1	5
adherens junction;anchoring junction;cell junction;cell part;cell projection;cell-substrate adherens junction;cell-substrate junction;cytoplasm;cytoplasmic part;cytoskeletal part;focal adhesion;intracellular membrane-bounded organelle;intracellular non-membrane-bounded organelle;intracellular organelle;intracellular organelle par		Homo sapiens	1	5
cell part;cytoplasmic part;endosomal part;endosome membrane;integral to membrane;intracellular organelle part;intracellular part;intrinsic to membrane;membrane;membrane part;organelle membrane;organelle part		Homo sapiens	1	5
Cajal body;cell part;cytoplasm;cytoskeleton;intermediate filament cytoskeleton;intracellular membrane-bounded organelle;intracellular non-membrane-bounded organelle;intracellular organelle;intracellular organelle part;intra	Spliceosome	Homo sapiens	1	5
Cdc48p-Npl4p-Ufd1p AAA ATPase complex;cell part;cytoplasmic part;endoplasmic reticulum part;intracellular organelle part;intracellular part;macromolecular complex;membrane part;nuclear part;nucleoplasm;organelle part;protein complex		Homo sapiens	1	5
cell part;centrosome;cytoplasmic part;cytoskeletal part;cytoskeleton;cytosol;intracellular non-membrane-bounded organelle;intracellular organelle;intracellular organelle part;intracellular part;membrane;microtubule cytoskelet	Cell cycle;p53 signaling pathway;Progesterone-mediated oocyte maturation	Homo sapiens	1	5
cell part;extracellular membrane-bounded organelle;extracellular organelle;extracellular region part;extracellular vesicular exosome;intracellular membrane-bounded organelle;intracellular organelle;intracellular part;membrane-bounded organelle;membrane-bounded vesicle;nucleus;organelle;vesicle		Homo sapiens	1	5
cell part;cytoplasmic part;integral to membrane;intracellular membrane-bounded organelle;intracellular organelle;intracellular organelle part;intracellular part;intrinsic to membrane;macromolecular complex;membrane;membra	Alzheimer's disease;Huntington's disease;Oxidative phosphorylation;Parkinson's disease	Homo sapiens	1	5
cell part;cis-Golgi network;cytoplasmic part;Golgi apparatus;Golgi apparatus part;Golgi membrane;integral to membrane;intracellular membrane-bounded organelle;intracellular organelle;intracellular organelle part;intracellula	SNARE interactions in vesicular transport	Homo sapiens	1	5
adherens junction;anchoring junction;cell junction;cell part;cell surface;cell-substrate adherens junction;cell-substrate junction;focal adhesion;integral to membrane;integral to plasma membrane;intrinsic to membrane;intrinsic	Cell adhesion molecules (CAMs)	Homo sapiens	1	5
cell part;cytoplasm;intracellular membrane-bounded organelle;intracellular organelle;intracellular organelle part;intracellular part;macromolecular complex;membrane-bounded organelle;nuclear part;nucleoplasm;nucleus;organelle;organelle part;ribonucleoprotein complex;small nuclear ribonucleoprotein complex;U5 snRNP		Homo sapiens	1	5
cell part;cytoplasmic part;cytosol;intracellular part		Homo sapiens	1	5
cell part;cytoplasmic part;cytosol;extracellular membrane-bounded organelle;extracellular organelle;extracellular region part;extracellular vesicular exosome;intracellular part;membrane-bounded organelle;membrane-bounded vesicle;organelle;vesicle		Homo sapiens	1	5
blood microparticle;cell part;cytoplasmic membrane-bounded vesicle lumen;cytoplasmic part;cytoplasmic vesicle part;extracellular region;extracellular region part;intracellular organelle part;intracellular part;membrane-enclo	Complement and coagulation cascades	Homo sapiens	1	5
cell part;cytoplasmic part;endoplasmic reticulum lumen;endoplasmic reticulum part;extracellular region;Golgi apparatus part;Golgi lumen;intracellular organelle lumen;intracellular organelle part;intracellular part;intrinsic to ext	Complement and coagulation cascades	Homo sapiens	1	5
apical part of cell;cell part;cell surface;cytoplasm;cytoplasmic membrane-bounded vesicle;cytoplasmic part;cytoplasmic vesicle;extracellular membrane-bounded organelle;extracellular organelle;extracellular region;extracellu	Complement and coagulation cascades	Homo sapiens	1	5
cell part;cell surface;chylomicron;extracellular membrane-bounded organelle;extracellular organelle;extracellular region part;extracellular space;extracellular vesicular exosome;high-density lipoprotein particle;macromolecular complex;membrane-bounded organelle;membrane-bounded vesicle;organelle;plasma lipoprotein partic		Homo sapiens	1	5
cell part;cytoplasmic part;cytosol;extracellular region;intracellular organelle part;intracellular part;nuclear part;nucleoplasm;organelle part	RIG-I-like receptor signaling pathway	Homo sapiens	1	5
extracellular membrane-bounded organelle;extracellular organelle;extracellular region;extracellular region part;extracellular space;extracellular vesicular exosome;membrane-bounded organelle;membrane-bounded vesicle;or	Complement and coagulation cascades	Homo sapiens	1	5
cell part;extracellular region;extracellular region part;extracellular space;intracellular membrane-bounded organelle;intracellular organelle;intracellular part;membrane-bounded organelle;nucleus;organelle	MAPK signaling pathway;Melanoma;Pathways in cancer;Regulation of actin cytoskeleton	Homo sapiens	1	5
cell part;cytoplasm;cytoplasmic part;cytosol;endoplasmic reticulum;intracellular membrane-bounded organelle;intracellular organelle;intracellular part;membrane-bounded organelle;organelle		Homo sapiens	1	5
cell part;cytoplasmic part;endoplasmic reticulum;extracellular membrane-bounded organelle;extracellular organelle;extracellular region part;extracellular vesicular exosome;intracellular membrane-bounded organelle;intracellu	Lysosome;Renin-angiotensin system	Homo sapiens	1	5
blood microparticle;cell part;chromaffin granule;cytoplasm;cytoplasmic membrane-bounded vesicle;cytoplasmic membrane-bounded vesicle lumen;cytoplasmic part;cytoplasmic vesicle;cytoplasmic vesicle part;cytosol;endoplasmic reticulum;extracellular matrix;extracellular membrane-bounded organelle;extracellular organelle;		Homo sapiens	1	5
cell part;cell surface;extracellular matrix;extracellular membrane-bounded organelle;extracellular organelle;extracellular region part;extracellular space;extracellular vesicular exosome;integral to membrane;intrinsic to external	Complement and coagulation cascades	Homo sapiens	1	5
anchoring junction;basolateral plasma membrane;brush border membrane;cell junction;cell part;cell projection;cell projection membrane;cell projection part;cell-cell junction;cytoplasmic part;desmosome;external encapsulati	Galactose metabolism;Glycosaminoglycan biosynthesis - keratan sulfate;Glycosphingolipid biosynthesi	Homo sapiens	1	5
basement membrane;cell body;cell part;cell projection part;cell surface;extracellular matrix;extracellular matrix part;extracellular membrane-bounded organelle;extracellular organelle;extracellular region;extracellular region part;extracellular space;extracellular vesicular exosome;growth cone;membrane-bounded organelle;membr		Homo sapiens	1	5
cell part;intracellular membrane-bounded organelle;intracellular organelle;intracellular organelle part;intracellular part;membrane-bounded organelle;nuclear part;nucleoplasm;nucleus;organelle;organelle part		Homo sapiens	0.9999	5
adherens junction;anchoring junction;apical plasma membrane;catenin complex;cell junction;cell part;cell projection;cell-cell adherens junction;cell-cell contact zone;cell-cell junction;cell-substrate adherens junction;cell-subs	Arrhythmogenic right ventricular cardiomyopathy (ARVC);Cell adhesion molecules (CAMs)	Homo sapiens	1	5
adherens junction;anchoring junction;cell junction;cell part;cell-substrate adherens junction;cell-substrate junction;cytoplasm;cytoplasmic part;cytoskeleton;cytosol;endomembrane system;extrinsic to internal side of plasma	Hepatitis C;Jak-STAT signaling pathway;ko05152;Leishmaniasis;Measles;Osteoclast differentiation;Panc	Homo sapiens	1	5
cell part;cytoplasm;cytoplasmic part;cytosol;extracellular membrane-bounded organelle;extracellular organelle;extracellular region part;extracellular vesicular exosome;intracellular membrane-bounded organelle;intracellular o	Proteasome	Homo sapiens	1	5
cell part;cytoplasmic part;intracellular membrane-bounded organelle;intracellular organelle;intracellular organelle lumen;intracellular organelle part;intracellular part;macromolecular complex;membrane;membrane part;membr	Alzheimer's disease;Huntington's disease;Oxidative phosphorylation;Parkinson's disease	Homo sapiens	1	5
cell part;cytoplasm;cytoplasmic part;extracellular membrane-bounded organelle;extracellular organelle;extracellular region part;extracellular vesicular exosome;Golgi apparatus;Golgi apparatus part;Golgi membrane;intracellular membrane-bounded organelle;intracellular organelle;intracellular organelle part;intracellular part;mem		Homo sapiens	1	5
cell part;cytoplasmic part;cytosol;extracellular membrane-bounded organelle;extracellular organelle;extracellular region part;extracellular vesicular exosome;intracellular organelle lumen;intracellular organelle part;intracellular part;lysosomal lumen;membrane;membrane-bounded organelle;membrane-bounded vesicle;membrane-		Homo sapiens	1	5
cell part;cytoplasmic part;endoplasmic reticulum;extracellular membrane-bounded organelle;extracellular organelle;extracellular region part;extracellular vesicular exosome;intracellular membrane-bounded organelle;intracellular organelle;intracellular part;membrane-bounded organelle;membrane-bounded vesicle;organelle;vesic		Homo sapiens	1	5
cell part;cytoplasmic part;cytosol;intracellular part		Homo sapiens	1	5
cell part;cytoplasmic part;cytosol;intracellular part;macromolecular complex;perinuclear region of cytoplasm;protein complex		Homo sapiens	1	5
cell part;cytoplasmic part;intracellular membrane-bounded organelle;intracellular organelle;intracellular part;membrane-bounded organelle;mitochondrion;organelle	Purine metabolism;Pyrimidine metabolism	Homo sapiens	1	5
cell part;cytoplasm;holo TFIIH complex;intracellular organelle part;intracellular part;macromolecular complex;nuclear part;nucleoplasm;nucleoplasm part;organelle part;protein complex;transcription factor complex	Basal transcription factors;Nucleotide excision repair	Homo sapiens	1	5
apical plasma membrane;cell part;cytoplasm;extracellular region part;extracellular space;intracellular membrane-bounded organelle;intracellular organelle;intracellular part;membrane part;membrane-bounded organelle;nucleus;organelle;plasma membrane part		Homo sapiens	1	5
cell part;centrosome;chromosomal part;condensed chromosome outer kinetochore;condensed nuclear chromosome outer kinetochore;cytoplasm;cytoplasmic part;cytoskeletal part;cytoskeleton;cytosol;intracellular membrar	Cell cycle;Oocyte meiosis;Progesterone-mediated oocyte maturation	Homo sapiens	1	5
adherens junction;anchored to membrane;anchoring junction;caveola;cell junction;cell part;cell projection;cell-substrate adherens junction;cell-substrate junction;cytoplasm;external side of plasma membrane;extracellular membrane-bounded organelle;extracellular organelle;extracellular region part;extracellular space;extracellul		Homo sapiens	1	5
cell part;cytoplasm;intracellular organelle part;intracellular part;nuclear part;nucleoplasm;organelle part		Homo sapiens	1	5
actin cytoskeleton;adherens junction;anchoring junction;cell junction;cell part;cell projection;cell-substrate adherens junction;cell-substrate junction;cytoplasm;cytoplasmic part;cytoskeleton;cytosol;focal adhesion;intracellula	Bacterial invasion of epithelial cells;Chemokine signaling pathway;Focal adhesion;Leukocyte transendot	Homo sapiens	1	5
cell part;cytoplasmic part;integral to membrane;intracellular membrane-bounded organelle;intracellular organelle;intracellular organelle part;intracellular part;intrinsic to membrane;membrane;membrane part;membrane-bounded organelle;mitochondrial membrane;mitochondrial outer membrane;mitochondrial part;mitochondrion;		Homo sapiens	1	5
cell part;cytoplasmic part;cytosol;endosomal part;endosome membrane;extracellular membrane-bounded organelle;extracellular organelle;extracellular region part;extracellular vesicular exosome;intracellular organelle part;in	Protein processing in endoplasmic reticulum;Ubiquitin mediated proteolysis	Homo sapiens	1	5
apical plasma membrane;axon part;axon terminus;cell part;cell projection part;clathrin-coated vesicle;coated vesicle;cytoplasmic membrane-bounded vesicle;cytoplasmic part;cytoplasmic vesicle;cytoplasmic vesicle membr	Collecting duct acid secretion;Epithelial cell signaling in Helicobacter pylori infection;ko05152;Lysosome	Homo sapiens	1	5
cell part;CUL4 RING ubiquitin ligase complex;cullin-RING ubiquitin ligase complex;cytoplasm;intracellular organelle part;intracellular part;macromolecular complex;nuclear matrix;nuclear part;nucleoplasm;organelle part;protein complex;ubiquitin ligase complex		Homo sapiens	1	5
Ada2/Gcn5/Ada3 transcription activator complex;cell part;chromatin remodeling complex;histone acetyltransferase complex;histone methyltransferase complex;intracellular membrane-bounded organelle;intracellular organelle;intracellular organelle part;intracellular part;macromolecular complex;membrane-bounded organelle;m		Homo sapiens	1	5
cell part;cytoplasmic part;intracellular membrane-bounded organelle;intracellular organelle;intracellular organelle part;intracellular part;macromolecular complex;membrane;membrane part;membrane-bounded organelle;membrane-enclosed lumen;mitochondrial inner membrane;mitochondrial inner membrane presequence trans		Homo sapiens	1	5
catalytic step 2 spliceosome;cell part;cytoplasmic part;cytosol;intracellular organelle part;intracellular part;macromolecular complex;methylosome;methyltransferase complex;nuclear part;nucleoplasm;organelle part;pICln-Sm	Spliceosome	Homo sapiens	1	5
catalytic step 2 spliceosome;cell part;cytoplasmic part;cytosol;intracellular membrane-bounded organelle;intracellular organelle;intracellular organelle part;intracellular part;macromolecular complex;membrane-bounded orga	Spliceosome;Systemic lupus erythematosus	Homo sapiens	1	5
cell part;cytoplasmic part;cytosol;cytosolic small ribosomal subunit;extracellular region part;extracellular space;intracellular organelle part;intracellular part;macromolecular complex;organelle part;ribonucleoprotein complex;small ribosomal subunit		Homo sapiens	1	5
cell part;Cul2-RING ubiquitin ligase complex;Cul3-RING ubiquitin ligase complex;CUL4 RING ubiquitin ligase complex;Cul4A-RING ubiquitin ligase complex;Cul4B-RING ubiquitin ligase complex;Cul5-RING ubiquitin ligase c	Cell cycle;Cell cycle - yeast;Circadian rhythm - mammal;Nucleotide excision repair;Oocyte meiosis;Path	Homo sapiens	1	5
cell part;DSIF complex;intracellular membrane-bounded organelle;intracellular organelle;intracellular organelle part;intracellular part;macromolecular complex;membrane-bounded organelle;nuclear part;nucleoplasm;nucleoplasm part;nucleus;organelle;organelle part;protein complex;transcription elongation factor complex		Homo sapiens	1	5
cell part;intracellular membrane-bounded organelle;intracellular organelle;intracellular part;membrane-bounded organelle;nucleus;organelle		Homo sapiens	1	5
extracellular membrane-bounded organelle;extracellular organelle;extracellular region;extracellular region part;extracellular vesicular exosome;membrane-bounded organelle;membrane-bounded vesicle;organelle;vesicle		Homo sapiens	1	5
cell part;cytoplasmic part;endoplasmic reticulum;Golgi apparatus;Golgi apparatus part;Golgi membrane;intracellular membrane-bounded organelle;intracellular organelle;intracellular organelle part;intracellular part;membrane;membrane-bounded organelle;nucleus;organelle;organelle membrane;organelle part		Homo sapiens	1	5
cell part;chromosomal part;chromosome;chromosome, centromeric region;condensed chromosome kinetochore;condensed chromosome outer kinetochore;condensed chromosome, centromeric region;condensed nuclear chromosome kinetochore;cytoplasm;cytoplasmic part;cytoskeletal part;cytoskeleton;cytosol;intracellular r		Homo sapiens	0.9999	5
cell part;cytoplasm;cytoplasmic part;Golgi apparatus;intracellular membrane-bounded organelle;intracellular organelle;intracellular organelle part;intracellular part;macromolecular complex;membrane;membrane-bounded organelle;nuclear body;nuclear part;nucleoplasm;nucleoplasm part;nucleus;organelle;organelle part;plasma		Homo sapiens	1	5
cell part;cytoplasm;cytoplasmic part;cytosol;intracellular membrane-bounded organelle;intracellular organelle;intracellular part;macromolecular complex;membrane-bounded organelle;nucleus;organelle;proteasome complex;	Ubiquitin mediated proteolysis	Homo sapiens	1	5
anchored to membrane;apical plasma membrane;cell part;cell surface;cytoplasm;cytoplasmic part;endosome;extracellular membrane-bounded organelle;extracellular organelle;extracellular region part;extracellular vesicular exosome;Golgi apparatus;integral to membrane;integral to plasma membrane;intracellular membrane-bo		Homo sapiens	1	5
extracellular matrix;extracellular membrane-bounded organelle;extracellular organelle;extracellular region;extracellular region part;extracellular space;extracellular vesicular exosome;membrane-bounded organelle;membrane-bounded vesicle;organelle;proteinaceous extracellular matrix;vesicle		Homo sapiens	1	5
cell part;cytoplasm;cytoplasmic part;early endosome;endosome;extracellular membrane-bounded organelle;extracellular organelle;extracellular region part;extracellular vesicular exosome;intracellular membrane-bounded organelle;intracellular organelle;intracellular part;membrane;membrane-bounded organelle;membrane-boun		Homo sapiens	1	5
cell part;cytoplasmic part;endoplasmic reticulum;extracellular membrane-bounded organelle;extracellular organelle;extracellular region part;extracellular vesicular exosome;intracellular membrane-bounded organelle;intracellu	Axon guidance	Homo sapiens	1	5
cell part;cytoplasmic part;cytoplasmic ubiquitin ligase complex;endoplasmic reticulum lumen;endoplasmic reticulum part;endoplasmic reticulum quality control compartment;ER ubiquitin ligase complex;Hrd1p ubiquitin ligase	Protein processing in endoplasmic reticulum	Homo sapiens	1	5
cell part;cytoplasm;cytoplasmic part;cytosol;intracellular organelle part;intracellular part;macromolecular complex;nuclear part;nucleoplasm;organelle part;protein complex	Acute myeloid leukemia;ErbB signaling pathway;Insulin signaling pathway;mTOR signaling pathway;RN	Homo sapiens	1	5
cell part;cytoplasm;intracellular part;macromolecular complex;protein complex;ubiquitin ligase complex	Ubiquitin mediated proteolysis	Homo sapiens	1	5
cell part;Cul3-RING ubiquitin ligase complex;cullin-RING ubiquitin ligase complex;cytoplasm;cytoplasmic part;cytoskeletal part;cytosol;endoplasmic reticulum;intracellular membrane-bounded organelle;intracellular organelle	Ubiquitin mediated proteolysis	Homo sapiens	1	5
Cajal body;catalytic step 2 spliceosome;cell part;contractile fiber part;cytoplasmic part;intracellular non-membrane-bounded organelle;intracellular organelle;intracellular organelle part;intracellular part;macromolecular complex;non-membrane-bounded organelle;nuclear body;nuclear part;nucleolus;nucleoplasm part;organelle;or		Homo sapiens	1	5
blood microparticle;cell part;cytoplasm;extracellular membrane-bounded organelle;extracellular organelle;extracellular region;extracellular region part;extracellular vesicular exosome;intracellular part;membrane;membrane-bounded organelle;membrane-bounded vesicle;organelle;plasma membrane;vesicle		Homo sapiens	1	5
cell part;intracellular non-membrane-bounded organelle;intracellular organelle;intracellular organelle part;intracellular part;non-membrane-bounded organelle;nuclear part;nucleolus;nucleoplasm;organelle;organelle part		Homo sapiens	1	5
cell part;cytoplasmic membrane-bounded vesicle;cytoplasmic part;cytoplasmic vesicle;early endosome;endocytic vesicle;endosome;intracellular membrane-bounded organelle;intracellular organelle;intracellular part;membra	Endocytosis	Homo sapiens	1	5
cell part;cytoplasm;intracellular organelle part;intracellular part;macromolecular complex;mediator complex;nuclear part;nucleoplasm part;organelle part;protein complex		Homo sapiens	1	5
extracellular matrix;extracellular membrane-bounded organelle;extracellular organelle;extracellular region part;extracellular space;extracellular vesicular exosome;membrane-bounded organelle;membrane-bounded vesicle;organelle;proteinaceous extracellular matrix;vesicle		Homo sapiens	1	5
cell part;cytoplasmic part;endoplasmic reticulum;integral to membrane;intracellular membrane-bounded organelle;intracellular organelle;intracellular part;intrinsic to membrane;membrane part;membrane-bounded organelle;organelle		Homo sapiens	1	5
cell part;cytoplasmic part;intracellular membrane-bounded organelle;intracellular organelle;intracellular part;membrane-bounded organelle;mitochondrion;organelle		Homo sapiens	1	5
cell part;endomembrane system		Homo sapiens	1	5
cell part;intracellular non-membrane-bounded organelle;intracellular organelle;intracellular organelle part;intracellular part;membrane;non-membrane-bounded organelle;nuclear part;nucleolus;organelle;organelle part		Homo sapiens	1	5
cell cortex;cell part;cytoplasm;cytoplasmic membrane-bounded vesicle;cytoplasmic part;cytoplasmic vesicle;cytoskeleton;extracellular membrane-bounded organelle;extracellular organelle;extracellular region part;extracellular vesicular exosome;intracellular membrane-bounded organelle;intracellular non-membrane-bounded o		Homo sapiens	1	5
		Homo sapiens	1	5
cell part;cytoplasm;extracellular membrane-bounded organelle;extracellular organelle;extracellular region part;extracellular vesicular exosome;intracellular part;membrane-bounded organelle;membrane-bounded vesicle;organelle;vesicle		Homo sapiens	1	5
cell part;cytoplasm;extracellular membrane-bounded organelle;extracellular organelle;extracellular region part;extracellular vesicular exosome;intracellular membrane-bounded organelle;intracellular organelle;intracellular part;membrane-bounded organelle;membrane-bounded vesicle;nucleus;organelle;vesicle		Homo sapiens	1	5
cell part;cytoplasmic part;intracellular membrane-bounded organelle;intracellular organelle;intracellular part;membrane;membrane-bounded organelle;mitochondrion;nucleus;organelle	Amino sugar and nucleotide sugar metabolism	Homo sapiens	1	5
cell part;intracellular membrane-bounded organelle;intracellular organelle;intracellular part;membrane-bounded organelle;nucleus;organelle		Homo sapiens	1	5
cell part;cytoplasmic part;intracellular non-membrane-bounded organelle;intracellular organelle;intracellular part;macromolecular complex;non-membrane-bounded organelle;organelle;ribonucleoprotein complex;ribosome		Homo sapiens	1	5
cell part;cytoplasmic part;cytosol;intracellular membrane-bounded organelle;intracellular organelle;intracellular part;membrane-bounded organelle;nucleus;organelle		Homo sapiens	1	5
cell part;cytoplasmic part;endoplasmic reticulum membrane;endoplasmic reticulum part;integral to membrane;intracellular organelle part;intracellular part;intrinsic to membrane;membrane;membrane part;organelle membrane;organelle part		Homo sapiens	1	5
cell part;cytoplasmic part;endoplasmic reticulum lumen;endoplasmic reticulum part;extracellular matrix;extracellular region part;interstitial matrix;intracellular organelle lumen;intracellular organelle part;intracellular part;membrane-enclosed lumen;organelle lumen;organelle part;proteinaceous extracellular matrix		Homo sapiens	1	5
cell part;cytoplasm;extracellular membrane-bounded organelle;extracellular organelle;extracellular region part;extracellular vesicular exosome;intracellular part;membrane-bounded organelle;membrane-bounded vesicle;organelle;vesicle		Homo sapiens	1	5
cell part;extracellular membrane-bounded organelle;extracellular organelle;extracellular region part;extracellular vesicular exosome;integral to membrane;integral to plasma membrane;intrinsic to membrane;intrinsic to plasma membrane;membrane part;membrane-bounded organelle;membrane-bounded vesicle;organelle;plasma		Homo sapiens	1	5
aggresome;cell part;cytoplasm;inclusion body;intracellular membrane-bounded organelle;intracellular organelle;intracellular organelle part;intracellular part;membrane-bounded organelle;nuclear part;nucleoplasm;nucleus;organelle;organelle part		Homo sapiens	1	5
extracellular membrane-bounded organelle;extracellular organelle;extracellular region part;extracellular vesicular exosome;membrane-bounded organelle;membrane-bounded vesicle;organelle;vesicle		Homo sapiens	1	5
cell part;cytoplasmic part;cytoplasmic vesicle membrane;cytoplasmic vesicle part;endoplasmic reticulum membrane;endoplasmic reticulum part;Golgi apparatus;integral to membrane;intracellular membrane-bounded organe	Cytosolic DNA-sensing pathway;RIG-I-like receptor signaling pathway	Homo sapiens	1	5
cell part;intracellular membrane-bounded organelle;intracellular organelle;intracellular part;membrane-bounded organelle;nucleus;organelle		Homo sapiens	1	5
cell part;intracellular organelle part;intracellular part;nuclear part;nucleoplasm;organelle part		Homo sapiens	1	5
cell part;integral to membrane;intrinsic to membrane;membrane;membrane part		Homo sapiens	1	5

		Homo sapiens	1	5
cell part;cytoplasmic part;endoplasmic reticulum lumen;endoplasmic reticulum part;extracellular membrane-bounded organelle;extracellular organelle;extracellular region part;extracellular vesicular exosome;intracellular orga	Other types of O-glycan biosynthesis	Homo sapiens	1	5
cell part;chromosomal part;condensed chromosome kinetochore;cytoplasmic part;cytosol;intracellular membrane-bounded organelle;intracellular non-membrane-bounded organelle;intracellular organelle;intracellular organelle part;intracellular part;kinetochore;macromolecular complex;membrane-bounded organelle;Ndc80 com		Homo sapiens	1	5
cell part;cytoplasmic part;intracellular membrane-bounded organelle;intracellular organelle;intracellular part;lipid particle;membrane;membrane-bounded organelle;midbody;mitochondrion;nucleus;organelle		Homo sapiens	1	5
		Homo sapiens	1	5
cell part;chromosomal part;condensed chromosome kinetochore;cytoplasmic part;cytosol;envelope;intracellular non-membrane-bounded organelle;intracellular organelle;intracellular organelle part;intracellular part;kinetocho	RNA transport	Homo sapiens	1	5
caveola;cell part;cell projection membrane;cell projection part;cell surface;cytoplasmic part;cytoplasmic vesicle membrane;cytoplasmic vesicle part;endocytic vesicle membrane;extracellular membrane-bounded organelle;ex	Bile secretion;Fat digestion and absorption;Hepatitis C;Phagosome;Vitamin digestion and absorption	Homo sapiens	1	5
CCR4-NOT complex;cell part;cytoplasmic mRNA processing body;cytoplasmic part;cytosol;intracellular membrane-bounded organelle;intracellular non-membrane-bounded organelle;intracellular organelle;intracellular organe	RNA degradation	Homo sapiens	1	5
cell part;cytoplasm;cytoplasmic part;cytosol;exosome (RNase complex);intracellular membrane-bounded organelle;intracellular non-membrane-bounded organelle;intracellular organelle;intracellular organelle part;intracellular	RNA degradation	Homo sapiens	1	5
cell part;cytoplasm;extracellular membrane-bounded organelle;extracellular organelle;extracellular region part;extracellular vesicular exosome;intracellular part;membrane-bounded organelle;membrane-bounded vesicle;orga	Glycolysis / Gluconeogenesis	Homo sapiens	1	5
cell part;cytoplasm;cytoplasmic part;cytosol;intracellular membrane-bounded organelle;intracellular organelle;intracellular organelle part;intracellular part;membrane-bounded organelle;mitochondrion;nuclear part;nucleoplasr	RNA degradation	Homo sapiens	1	5
cell part;chromosomal part;condensed chromosome outer kinetochore;cytoplasmic part;cytoskeletal part;cytosol;intracellular membrane-bounded organelle;intracellular organelle;intracellular organelle part;intracellular part;macromolecular complex;membrane-bounded organelle;microtubule organizing center;nucleus;organelle;c		Homo sapiens	1	5
cell part;cytoplasm;Elongator holoenzyme complex;histone acetyltransferase complex;intracellular organelle part;intracellular part;macromolecular complex;nuclear part;nucleoplasm;nucleoplasm part;organelle part;protein complex;transcription elongation factor complex		Homo sapiens	1	5
cell part;cytoplasmic part;intracellular organelle part;intracellular part;large ribosomal subunit;macromolecular complex;membrane;mitochondrial inner membrane;mitochondrial large ribosomal subunit;mitochondrial membrane;mitochondrial part;mitochondrial small ribosomal subunit;organellar large ribosomal subunit;organellar		Homo sapiens	1	5
cell part;cytoplasmic part;intracellular non-membrane-bounded organelle;intracellular organelle;intracellular organelle part;intracellular part;macromolecular complex;membrane;mitochondrial inner membrane;mitochondrial membrane;mitochondrial part;non-membrane-bounded organelle;organelle;organelle inner membrane;orga		Homo sapiens	1	5
cell part;centrosome;chromocenter;chromosomal part;chromosome passenger complex;chromosome, centromeric region;condensed chromosome, centromeric region;condensed nuclear chromosome, centromeric region;cytoplasmic part;cytoskeletal part;cytosol;intracellular membrane-bounded organelle;intracellular non-mer		Homo sapiens	1	5
cell part;chromosomal part;cytoplasm;intracellular organelle part;intracellular part;nuclear chromosome part;nuclear part;nuclear replication fork;nucleoplasm;organelle part;replication fork		Homo sapiens	1	5
cell division site part;cell part;centrosome;cleavage furrow;cytoplasm;cytoplasmic part;cytoskeletal part;intracellular non-membrane-bounded organelle;intracellular organelle;intracellular organelle part;intracellular part;microtubule organizing center;midbody;non-membrane-bounded organelle;organelle;organelle part		Homo sapiens	1	5
cell part;cytoplasmic part;endoplasmic reticulum;intracellular membrane-bounded organelle;intracellular organelle;intracellular organelle part;intracellular part;membrane;membrane-bounded organelle;mitochondrial inner membrane;mitochondrial membrane;mitochondrial part;organelle;organelle inner membrane;organelle memb		Homo sapiens	1	5
		Homo sapiens	1	5
cell body;cell part;cell projection part;cytoplasmic membrane-bounded vesicle;cytoplasmic part;cytoplasmic vesicle;cytoplasmic vesicle membrane;cytoplasmic vesicle part;endosomal part;endosome;endosome membrane;extracellular membrane-bounded organelle;extracellular organelle;extracellular region part;extracellular ve		Homo sapiens	1	5
basement membrane;cell cortex;cell junction;cell part;cytoplasmic part;extracellular matrix part;extracellular membrane-bounded organelle;extracellular organelle;extracellular region part;extracellular vesicular exosome;intracellular part;membrane-bounded organelle;membrane-bounded vesicle;organelle;vesicle		Homo sapiens	1	5
cell part;cytoplasmic part;cytoskeletal part;cytosol;intracellular membrane-bounded organelle;intracellular organelle;intracellular organelle part;intracellular part;macromolecular complex;membrane-bounded organelle;microtubule associated complex;nucleus;organelle;organelle part;protein complex		Homo sapiens	1	5
cell part;centrosome;cytoplasm;cytoplasmic part;cytoskeletal part;intracellular membrane-bounded organelle;intracellular non-membrane-bounded organelle;intracellular organelle;intracellular organelle part;intracellular part;membrane-bounded organelle;microtubule organizing center;mitochondrion;non-membrane-bounded org		Homo sapiens	1	5
cell part;cytoplasm;exosome (RNase complex);intracellular membrane-bounded organelle;intracellular non-membrane-bounded organelle;intracellular organelle;intracellular organelle part;intracellular part;macromolecular con	RNA degradation	Homo sapiens	1	5
apical plasma membrane;basolateral plasma membrane;cell part;integral to membrane;integral to plasma membrane;intrinsic to membrane;intrinsic to plasma membrane;membrane;membrane part;plasma membrane;plasma membrane part		Homo sapiens	1	5
cell part;cytoplasmic part;intracellular membrane-bounded organelle;intracellular non-membrane-bounded organelle;intracellular organelle;intracellular organelle lumen;intracellular organelle part;intracellular part;membrane;membrane-bounded organelle;membrane-enclosed lumen;mitochondrial inner membrane;mitochondrial ir		Homo sapiens	1	5
cell part;centrosome;cytoplasm;cytoplasmic part;cytoskeletal part;HAUS complex;intracellular non-membrane-bounded organelle;intracellular organelle;intracellular organelle part;intracellular part;macromolecular complex;microtubule;microtubule associated complex;microtubule organizing center;non-membrane-bounded orga		Homo sapiens	1	5
cell part;cytoplasmic part;extracellular region part;extracellular space;high-density lipoprotein particle;integral to membrane;intracellular membrane-bounded organelle;intracellular organelle;intracellular part;intrinsic to membrane;low-density lipoprotein particle;macromolecular complex;membrane part;membrane-bounded orgar		Homo sapiens	1	5
cell part;cytoplasmic part;endoplasmic reticulum membrane;endoplasmic reticulum part;extracellular membrane-bounded organelle;extracellular organelle;extracellular region part;extracellular vesicular exosome;integral to membrane;intracellular organelle part;intracellular part;intrinsic to membrane;membrane;membrane part;m		Homo sapiens	1	5
cell part;intracellular membrane-bounded organelle;intracellular organelle;intracellular organelle part;intracellular part;macromolecular complex;membrane-bounded organelle;nuclear part;nucleoplasm;nucleus;organelle;organ	Spliceosome	Homo sapiens	1	5
cell part;collagen;extracellular matrix part;extracellular membrane-bounded organelle;extracellular organelle;extracellular region part;extracellular space;extracellular vesicular exosome;membrane;membrane-bounded organelle;membrane-bounded vesicle;organelle;vesicle		Homo sapiens	1	5
cell part;cytoplasmic membrane-bounded vesicle;cytoplasmic part;cytoplasmic vesicle;Golgi apparatus;integral to membrane;intracellular membrane-bounded organelle;intracellular organelle;intracellular organelle part;intracellular part;intrinsic to membrane;membrane;membrane part;membrane-bounded organelle;membrane-b		Homo sapiens	1	5
cell junction;cell part;clathrin-coated vesicle;coated vesicle;cytoplasmic membrane-bounded vesicle;cytoplasmic part;cytoplasmic vesicle;integral to membrane;integral to mitochondrial membrane;integral to organelle membrane;intracellular membrane-bounded organelle;intracellular organelle;intracellular organelle part;intracell		Homo sapiens	1	5
cell part;cytoplasmic part;extracellular region part;extracellular space;intracellular membrane-bounded organelle;intracellular non-membrane-bounded organelle;intracellular organelle;intracellular organelle part;intracellular part;macromolecular complex;membrane;membrane-bounded organelle;mitochondrial inner membrane;mi		Homo sapiens	1	5
cell part;cytoplasm;intracellular organelle part;intracellular part;nuclear part;nucleoplasm;organelle part		Homo sapiens	1	5
cell part;cytoplasm;intracellular membrane-bounded organelle;intracellular organelle;intracellular part;membrane;membrane-bounded organelle;nucleus;organelle;plasma membrane	MAPK signaling pathway	Homo sapiens	1	5
cell part;chromosomal part;cytoplasm;cytoplasmic part;cytosol;intracellular membrane-bounded organelle;intracellular organelle;intracellular organelle part;intracellular part;macromolecular complex;membrane-bounded organelle;MIS12/MIND type complex;nucleus;organelle;organelle part;protein complex		Homo sapiens	1	5
cell part;cytoplasm;intracellular part		Homo sapiens	1	5
cell part;cytoplasm;intracellular organelle part;intracellular part;macromolecular complex;nuclear part;nucleoplasm;organelle part;protein complex;signalosome		Homo sapiens	1	5
cell part;cytoplasmic part;endoplasmic reticulum;endoplasmic reticulum membrane;endoplasmic reticulum part;intracellular membrane-bounded organelle;intracellular organelle;intracellular organelle part;intracellular part;lysosomal membrane;membrane;membrane part;membrane-bounded organelle;mitochondrion;nucleus;orga		Homo sapiens	1	5
cell part;cytoplasmic part;early endosome membrane;endosomal part;endosome membrane;Golgi apparatus;Golgi apparatus part;Golgi membrane;integral to membrane;intracellular membrane-bounded organelle;intracellular organelle;intracellular organelle part;intracellular part;intrinsic to membrane;late endosome membrane;l		Homo sapiens	1	5
cell part;chromatin;chromosomal part;heterochromatin;intracellular membrane-bounded organelle;intracellular organelle;intracellular organelle part;intracellular part;macromolecular complex;membrane-bounded organelle;nuclear chromatin;nuclear chromosome part;nuclear part;nucleoplasm;nucleus;organelle;organelle part;PcG		Homo sapiens	1	5
cell part;cytoplasm;intracellular part;membrane;plasma membrane		Homo sapiens	1	5
cell part;cytoplasmic part;extracellular membrane-bounded organelle;extracellular organelle;extracellular region part;extracellular vesicular exosome;intracellular membrane-bounded organelle;intracellular organelle;intracellular part;membrane-bounded organelle;membrane-bounded vesicle;mitochondrion;organelle;vesicle		Homo sapiens	1	5
cell part;cytoplasmic part;integral to membrane;intracellular organelle part;intracellular part;intrinsic to membrane;membrane;membrane part;mitochondrial membrane;mitochondrial part;organelle membrane;organelle part;plasma membrane		Homo sapiens	1	5
basolateral plasma membrane;cell junction;cell part;cell projection;cell-cell junction;cytoplasm;cytoskeletal part;extracellular membrane-bounded organelle;extracellular organelle;extracellular region part;extracellular vesicular exosome;intracellular organelle part;intracellular part;macromolecular complex;membrane;membrane pa		Homo sapiens	1	5
cell part;extracellular membrane-bounded organelle;extracellular organelle;extracellular region part;extracellular vesicular exosome;integral to membrane;intrinsic to membrane;membrane;membrane part;membrane-bounded organelle;membrane-bounded vesicle;organelle;plasma membrane;vesicle		Homo sapiens	1	5
		Homo sapiens	1	5
cell part;extracellular membrane-bounded organelle;extracellular organelle;extracellular region part;extracellular vesicular exosome;membrane;membrane-bounded organelle;membrane-bounded vesicle;organelle;vesicle		Homo sapiens	1	5
cell part;cytoplasm;cytoplasmic part;cytosol;eukaryotic 43S preinitiation complex;eukaryotic 48S preinitiation complex;eukaryotic translation initiation factor 3 complex;extracellular membrane-bounded organelle;extracellular organelle;extracellular region part;extracellular vesicular exosome;intracellular membrane-bounded orga		Homo sapiens	1	5
cell part;cytoplasmic part;extracellular membrane-bounded organelle;extracellular organelle;extracellular region part;extracellular vesicular exosome;intracellular organelle part;intracellular part;macromolecular complex;memt	Alzheimer's disease;Cardiac muscle contraction;Huntington's disease;Oxidative phosphorylation;Parkin	Homo sapiens	1	5
cell part;cytoplasmic part;cytoplasmic vesicle part;GARP complex;Golgi apparatus;Golgi apparatus part;integral to membrane;intracellular membrane-bounded organelle;intracellular organelle;intracellular organelle part;intracellular part;intrinsic to membrane;macromolecular complex;membrane part;membrane-bounded organel		Homo sapiens	1	5
actin cytoskeleton;cell part;cytoplasm;cytoplasmic part;cytoskeleton;extracellular membrane-bounded organelle;extracellular organelle;extracellular region part;extracellular vesicular exosome;intracellular membrane-bounded organelle;intracellular non-membrane-bounded organelle;intracellular organelle;intracellular organelle pa		Homo sapiens	1	5
cell junction;cell part;cell-cell junction;external side of plasma membrane;extracellular region;integral to membrane;intrinsic to membrane;membrane part;plasma membrane part		Homo sapiens	1	5
catalytic step 2 spliceosome;cell part;intracellular organelle part;intracellular part;macromolecular complex;nuclear part;organelle part;ribonucleoprotein complex;spliceosomal complex	Spliceosome	Homo sapiens	1	5
		Homo sapiens	1	5
cell part;cytoplasmic part;extracellular region part;extracellular space;intracellular organelle part;intracellular part;macromolecular complex;membrane;mitochondrial inner membrane;mitochondrial intermembrane space protein transporter complex;mitochondrial membrane;mitochondrial part;organelle inner membrane;organelle		Homo sapiens	1	5
cell part;cytoplasmic part;intracellular membrane-bounded organelle;intracellular non-membrane-bounded organelle;intracellular organelle;intracellular organelle part;intracellular part;macromolecular complex;membrane;membrane-bounded organelle;mitochondrial inner membrane;mitochondrial intermembrane space protein tra		Homo sapiens	1	5
cell part;cytoplasm;cytoplasmic part;extracellular membrane-bounded organelle;extracellular organelle;extracellular region part;extracellular vesicular exosome;intracellular membrane-bounded organelle;intracellular organelle	Amino sugar and nucleotide sugar metabolism;Fructose and mannose metabolism	Homo sapiens	1	5
cell part;cytoplasmic part;early endosome membrane;endosomal part;endosome membrane;extrinsic to membrane;intracellular membrane-bounded organelle;intracellular organelle;intracellular organelle part;intracellular part;membrane;membrane part;membrane-bounded organelle;organelle;organelle membrane;organelle part		Homo sapiens	1	5
cell part;cytoskeletal part;intracellular membrane-bounded organelle;intracellular non-membrane-bounded organelle;intracellular organelle;intracellular organelle part;intracellular part;membrane-bounded organelle;non-membrane-bounded organelle;nuclear part;nucleolus;nucleus;organelle;organelle part;spindle		Homo sapiens	1	5
cell part;coated pit;cytoplasm;intracellular membrane-bounded organelle;intracellular organelle;intracellular part;membrane;membrane part;membrane-bounded organelle;nucleus;organelle;plasma membrane	Endocytosis	Homo sapiens	1	5
cell part;cytoplasmic part;extracellular membrane-bounded organelle;extracellular organelle;extracellular region part;extracellular vesicular exosome;intracellular;intracellular membrane-bounded organelle;intracellular organel	Lysosome	Homo sapiens	1	6
anaphase-promoting complex;cell part;cullin-RING ubiquitin ligase complex;cytoplasm;cytoplasmic part;cytosol;intracellular organelle part;intracellular part;macromolecular complex;nuclear part;nuclear ubiquitin ligase comp	Ubiquitin mediated proteolysis	Homo sapiens	1	6
cell part;cytoplasmic part;endoplasmic reticulum;endoplasmic reticulum lumen;endoplasmic reticulum part;envelope;extracellular membrane-bounded organelle;extracellular organelle;extracellular region part;extracellular vesicular exosome;intracellular membrane-bounded organelle;intracellular organelle;intracellular organelle lu		Homo sapiens	1	6
brush border;cell cortex;cell part;cell projection;cell projection part;clathrin-coated vesicle;coated vesicle;cytoplasm;cytoplasmic membrane-bounded vesicle;cytoplasmic part;cytoplasmic vesicle;cytosol;dendritic shaft;dendritic spine;endocytic vesicle;extracellular membrane-bounded organelle;extracellular organelle;extracellul		Homo sapiens	1	6
cell part;cytoplasmic membrane-bounded vesicle;cytoplasmic part;cytoplasmic vesicle;endosomal part;endosome membrane;extracellular membrane-bounded organelle;extracellular organelle;extracellular region part;extracellular vesicular exosome;Golgi apparatus;Golgi apparatus part;Golgi membrane;integral to membrane;in		Homo sapiens	1	6
cell part;cell surface;cytoplasmic part;endoplasmic reticulum-Golgi intermediate compartment;Golgi apparatus;Golgi apparatus part;integral to Golgi membrane;integral to membrane;integral to organelle membrane;intracellular membrane-bounded organelle;intracellular organelle;intracellular organelle part;intracellular part;intrins		Homo sapiens	1	6
		Homo sapiens	1	6
cell part;cytoplasmic part;integral to membrane;intracellular organelle part;intracellular part;intrinsic to membrane;membrane;membrane part;mitochondrial membrane;mitochondrial outer membrane;mitochondrial part;organelle membrane;organelle outer membrane;organelle part;outer membrane		Homo sapiens	1	6
cell part;cytoplasmic part;endomembrane system;extracellular membrane-bounded organelle;extracellular organelle;extracellular region part;extracellular vesicular exosome;intracellular membrane-bounded organelle;intracellular organelle;intracellular organelle part;intracellular part;membrane;membrane-bounded organelle;mem		Homo sapiens	1	6
cell part;cytoplasmic part;endoplasmic reticulum;Golgi apparatus;intracellular membrane-bounded organelle;intracellular organelle;intracellular part;membrane-bounded organelle;organelle		Homo sapiens	1	6
cell part;cytoplasmic part;integral to membrane;intracellular membrane-bounded organelle;intracellular organelle;intracellular organelle part;intracellular part;intrinsic to membrane;macromolecular complex;membrane;membra	Alzheimer's disease;Huntington's disease;Oxidative phosphorylation;Parkinson's disease	Homo sapiens	1	6
cell part;cytoplasmic part;intracellular membrane-bounded organelle;intracellular organelle;intracellular organelle part;intracellular part;membrane;membrane-bounded organelle;membrane-enclosed lumen;mitochondrial inner membrane;mitochondrial intermembrane space;mitochondrial membrane;mitochondrial part;mitochond		Homo sapiens	1	6
cell part;cell projection part;cytoplasm;cytoplasmic part;intracellular membrane-bounded organelle;intracellular non-membrane-bounded organelle;intracellular organelle;intracellular organelle part;intracellular part;macromole	Spliceosome	Homo sapiens	1	6
cell part;cytoplasm;intracellular part		Homo sapiens	1	6
cell part;cytoplasmic part;intracellular membrane-bounded organelle;intracellular organelle;intracellular organelle part;intracellular part;macromolecular complex;membrane-bounded organelle;mitochondrial part;mitochondria	Ribosome biogenesis in eukaryotes;RNA transport	Homo sapiens	1	6
cell part;cytoplasmic part;extracellular membrane-bounded organelle;extracellular organelle;extracellular region part;extracellular vesicular exosome;intracellular membrane-bounded organelle;intracellular organelle;intracellula	Oxidative phosphorylation	Homo sapiens	1	6
cell part;cytoplasmic part;cytosol;extracellular membrane-bounded organelle;extracellular organelle;extracellular region part;extracellular vesicular exosome;intracellular organelle part;intracellular part;macromolecular comple	RNA transport	Homo sapiens	1	6
cell part;cytoplasmic part;endoplasmic reticulum;integral to membrane;intracellular membrane-bounded organelle;intracellular organelle;intracellular organelle part;intracellular part;intrinsic to membrane;macromolecular com	Alzheimer's disease;Huntington's disease;Oxidative phosphorylation;Parkinson's disease	Homo sapiens	1	6
cell part;intracellular membrane-bounded organelle;intracellular non-membrane-bounded organelle;intracellular organelle;intracellular organelle part;intracellular part;macromolecular complex;membrane-bounded organelle;non-membrane-bounded organelle;nuclear part;nucleolus;nucleus;organelle;organelle part;ribonucleoprote		Homo sapiens	1	6
cell division site part;cell part;cleavage furrow;cytoplasm;cytoplasmic part;early endosome;endosome;extracellular membrane-bounded organelle;extracellular organelle;extracellular region part;extracellular vesicular exosome	Endocytosis	Homo sapiens	1	6
		Homo sapiens	1	6
cell part;chromatin;chromatin remodeling complex;chromosomal part;cytoplasm;heterochromatin;histone deacetylase complex;intracellular organelle part;intracellular part;macromolecular complex;nuclear chromatin;nuclear chromosome part;nuclear part;nucleoplasm;nucleoplasm part;NuRD complex;organelle part;protein com		Homo sapiens	1	6
cell part;cytoplasmic part;endoplasmic reticulum membrane;endoplasmic reticulum part;extracellular membrane-bounded organelle;extracellular organelle;extracellular region part;extracellular vesicular exosome;integral to membrane;intracellular organelle part;intracellular part;intrinsic to membrane;membrane;membrane part;m		Homo sapiens	1	6
cell part;cytoplasmic part;cytosol;intracellular organelle part;intracellular part;nuclear part;nucleoplasm;organelle part	Folate biosynthesis;One carbon pool by folate	Homo sapiens	1	6
cell part;cytoplasmic part;cytosol;extracellular membrane-bounded organelle;extracellular organelle;extracellular region;extracellular region part;extracellular space;extracellular vesicular exosome;intracellular part;macromolecular complex;membrane-bounded organelle;membrane-bounded vesicle;organelle;protein complex;ves		Homo sapiens	1	6
cell part;cytoplasm;extracellular membrane-bounded organelle;extracellular organelle;extracellular region part;extracellular space;extracellular vesicular exosome;intracellular non-membrane-bounded organelle;intracellular organelle;intracellular organelle part;intracellular part;membrane-bounded organelle;membrane-bounded v		Homo sapiens	1	6
cell part;cytoplasm;cytoplasmic part;extracellular membrane-bounded organelle;extracellular organelle;extracellular region;extracellular region part;extracellular vesicular exosome;Golgi apparatus;intracellular membrane-bou	Galactose metabolism;Glycerolipid metabolism;Glycosphingolipid biosynthesis - globo series;Lysosome	Homo sapiens	1	6
cell part;cytoplasmic part;extracellular membrane-bounded organelle;extracellular organelle;extracellular region;extracellular region part;extracellular space;extracellular vesicular exosome;intracellular membrane-bounded org	Antigen processing and presentation;Lysosome;Phagosome;Rheumatoid arthritis	Homo sapiens	1	6
cell part;contractile fiber part;cytoplasmic part;extracellular matrix;extracellular region;extracellular region part;extracellular space;intracellular membrane-bounded organelle;intracellular organelle;intracellular part;membrane;r	Bladder cancer;GnRH signaling pathway;Leukocyte transendothelial migration;Pathways in cancer	Homo sapiens	1	6
cell part;cell surface;cytoplasmic membrane-bounded vesicle;cytoplasmic part;cytoplasmic vesicle;cytoplasmic vesicle membrane;cytoplasmic vesicle part;endosomal part;endosome lumen;endosome membrane;extracellula	Lysosome	Homo sapiens	1	6
cell part;cell projection;cytoplasm;cytoplasmic part;cytosol;extracellular membrane-bounded organelle;extracellular organelle;extracellular region part;extracellular vesicular exosome;intracellular membrane-bounded organell	Folate biosynthesis	Homo sapiens	1	6
cell part;cytoplasmic part;integral to membrane;intracellular membrane-bounded organelle;intracellular organelle;intracellular organelle part;intracellular part;intrinsic to membrane;membrane;membrane part;membrane-bound	Alzheimer's disease;Cardiac muscle contraction;Huntington's disease;Oxidative phosphorylation;Parkin	Homo sapiens	1	6
cell part;cytoplasmic part;cytosol;DNA-directed RNA polymerase complex;DNA-directed RNA polymerase I complex;DNA-directed RNA polymerase II, core complex;DNA-directed RNA polymerase III complex;intracellular me	Cytosolic DNA-sensing pathway;Huntington's disease;Purine metabolism;Pyrimidine metabolism;RNA p	Homo sapiens	1	6
carboxy-terminal domain protein kinase complex;cell part;core TFIIH complex;holo TFIIH complex;intracellular membrane-bounded organelle;intracellular organelle;intracellular organelle part;intracellular part;macromolecular	Basal transcription factors;Nucleotide excision repair	Homo sapiens	1	6
cell part;extracellular membrane-bounded organelle;extracellular organelle;extracellular region part;extracellular vesicular exosome;integral to membrane;integral to plasma membrane;intrinsic to membrane;intrinsic to plasma	p53 signaling pathway	Homo sapiens	1	6
cell part;membrane		Homo sapiens	1	6
cell part;chromosomal part;DNA replication factor A complex;intracellular organelle part;intracellular part;macromolecular complex;nuclear chromosome part;nuclear part;nucleoplasm;organelle part;protein complex	DNA replication;Homologous recombination;Mismatch repair;Nucleotide excision repair	Homo sapiens	1	6
cell part;cell projection;centrosome;cilium;cytoplasmic microtubule;cytoplasmic part;cytoskeletal part;extracellular membrane-bounded organelle;extracellular organelle;extracellular region part;extracellular vesicular exosome;Golgi apparatus;Golgi apparatus part;Golgi membrane;intracellular membrane-bounded organelle;intrac		Homo sapiens	1	6
axon hillock;axon part;basement membrane;cell part;cell projection part;cytoplasmic membrane-bounded vesicle;cytoplasmic part;cytoplasmic vesicle;extracellular matrix part;extracellular membrane-bounded organelle;extracellular organelle;extracellular region;extracellular region part;extracellular space;extracellular vesicular e		Homo sapiens	1	6
cell part;cytoplasmic part;cytosol;extracellular membrane-bounded organelle;extracellular organelle;extracellular region part;extracellular vesicular exosome;intracellular membrane-bounded organelle;intracellular organelle;int	Glutathione metabolism	Homo sapiens	1	6
cell part;cytoplasm;cytoskeletal part;envelope;intracellular membrane-bounded organelle;intracellular organelle;intracellular organelle part;intracellular part;macromolecular complex;membrane;membrane part;membrane-bou	RNA transport	Homo sapiens	1	6
basal part of cell;cell part;cytoplasmic part;extracellular membrane-bounded organelle;extracellular organelle;extracellular region part;extracellular vesicular exosome;intracellular membrane-bounded organelle;intracellular org	Protein digestion and absorption	Homo sapiens	1	6
cell part;cytoplasm;extracellular membrane-bounded organelle;extracellular organelle;extracellular region part;extracellular vesicular exosome;intracellular part;membrane-bounded organelle;membrane-bounded vesicle;organelle;vesicle		Homo sapiens	1	6
cell part;cytoplasmic part;cytosol;intracellular membrane-bounded organelle;intracellular organelle;intracellular organelle part;intracellular part;membrane-bounded organelle;mitochondrion;nuclear part;nucleoplasm;organelle	Adipocytokine signaling pathway;Chagas disease (American trypanosomiasis);Colorectal cancer;Epithel	Homo sapiens	1	6
BRCA1-A complex;BRISC complex;cell part;intracellular membrane-bounded organelle;intracellular organelle;intracellular organelle part;intracellular part;macromolecular complex;membrane-bounded organelle;nuclear part;nuclear ubiquitin ligase complex;nucleoplasm;nucleus;organelle;organelle part;protein complex;ubiquitin l		Homo sapiens	1	6
cell part;cytoplasmic part;cytosol;glutamate-cysteine ligase complex;intracellular part;macromolecular complex;protein complex	Glutathione metabolism	Homo sapiens	1	6
cell part;cytoplasm;intracellular part		Homo sapiens	1	6
cell part;cytoplasm;cytoplasmic part;holo TFIIH complex;intracellular membrane-bounded organelle;intracellular organelle;intracellular organelle part;intracellular part;macromolecular complex;membrane-bounded organelle;n	Basal transcription factors;Cell cycle;Nucleotide excision repair	Homo sapiens	1	6
axon;cell body;cell part;cell projection;clathrin-coated vesicle;coated vesicle;cytoplasmic membrane-bounded vesicle;cytoplasmic part;cytoplasmic vesicle;cytosol;dendrite;extracellular membrane-bounded organelle;extrace	Fatty acid elongation in mitochondria;Lysosome	Homo sapiens	1	6
		Homo sapiens	1	6
cell part;cytoplasmic part;intracellular membrane-bounded organelle;intracellular non-membrane-bounded organelle;intracellular organelle;intracellular organelle lumen;intracellular organelle part;intracellular part;membrane-b	Citrate cycle (TCA cycle)	Homo sapiens	1	6
calcium channel complex;cation channel complex;cell part;cytoplasmic part;endoplasmic reticulum;extracellular membrane-bounded organelle;extracellular organelle;extracellular region part;extracellular vesicular exosome;ir	Arrhythmogenic right ventricular cardiomyopathy (ARVC);Cardiac muscle contraction;Dilated cardiomyo	Homo sapiens	1	6
cell part;cytoplasmic part;intracellular organelle lumen;intracellular organelle part;intracellular part;macromolecular complex;membrane part;membrane-enclosed lumen;mitochondrial matrix;mitochondrial membrane part;mitc	Alzheimer's disease;Huntington's disease;Oxidative phosphorylation;Parkinson's disease	Homo sapiens	1	6
cell part;cytoplasm;cytoplasmic part;cytosol;extracellular membrane-bounded organelle;extracellular organelle;extracellular region part;extracellular vesicular exosome;intracellular non-membrane-bounded organelle;intracellu	RNA transport	Homo sapiens	1	6
axon;cell part;cell projection;cytoplasmic part;cytoskeletal part;cytosol;extracellular membrane-bounded organelle;extracellular organelle;extracellular region part;extracellular vesicular exosome;F-actin capping protein complex;intracellular membrane-bounded organelle;intracellular organelle;intracellular organelle part;intracellul		Homo sapiens	1	6
adherens junction;anchoring junction;cell junction;cell part;cell-substrate adherens junction;cell-substrate junction;extracellular membrane-bounded organelle;extracellular organelle;extracellular region part;extracellular vesicu	B cell receptor signaling pathway;Hepatitis C;Malaria	Homo sapiens	1	6
cell part;COPI vesicle coat;cytoplasmic part;cytoplasmic vesicle part;cytosol;Golgi apparatus part;intracellular organelle part;intracellular part;macromolecular complex;membrane coat;membrane part;organelle part;protein complex;vesicle coat		Homo sapiens	1	6
cell part;cytoplasmic part;cytosol;extracellular membrane-bounded organelle;extracellular organelle;extracellular region part;extracellular vesicular exosome;intracellular organelle part;intracellular part;macromolecular complex;membrane part;membrane-bounded organelle;membrane-bounded vesicle;nuclear part;nuclear pore;c		Homo sapiens	1	6
catalytic step 2 spliceosome;cell part;cytoplasmic part;cytosol;intracellular membrane-bounded organelle;intracellular organelle;intracellular organelle part;intracellular part;macromolecular complex;membrane-bounded orgar	RNA degradation;Spliceosome	Homo sapiens	1	6
cell part;cytoplasmic mRNA processing body;cytoplasmic part;cytosol;extracellular membrane-bounded organelle;extracellular organelle;extracellular region part;extracellular vesicular exosome;intracellular non-membrane-b	RNA degradation;Spliceosome	Homo sapiens	1	6
adherens junction;anchoring junction;cell junction;cell part;cell-substrate adherens junction;cell-substrate junction;cytoplasmic part;cytosol;cytosolic large ribosomal subunit;eukaryotic 80S initiation complex;focal adhesion;ir	Ribosome	Homo sapiens	1	6
cell part;cytoplasm;cytoplasmic part;cytosol;extracellular membrane-bounded organelle;extracellular organelle;extracellular region part;extracellular vesicular exosome;intracellular membrane-bounded organelle;intracellular c	Adherens junction;Circadian rhythm - plant;Measles;Ribosome biogenesis in eukaryotes;Tight junction;\	Homo sapiens	1	6
axon part;cation channel complex;cell part;cell projection part;integral to membrane;integral to plasma membrane;intrinsic to membrane;intrinsic to plasma membrane;ion channel complex;macromolecular complex;membrar	Cell adhesion molecules (CAMs)	Homo sapiens	1	6
cell part;cytoplasmic part;intracellular membrane-bounded organelle;intracellular non-membrane-bounded organelle;intracellular organelle;intracellular organelle part;intracellular part;macromolecular complex;membrane;membrane-bounded organelle;mitochondrial inner membrane;mitochondrial membrane;mitochondrial part;m		Homo sapiens	1	6
cell part;cytoplasm;cytoplasmic part;intracellular membrane-bounded organelle;intracellular organelle;intracellular part;membrane-bounded organelle;mitochondrion;organelle		Homo sapiens	1	6
actin cytoskeleton;adherens junction;anchoring junction;cell junction;cell part;cell-substrate adherens junction;cell-substrate junction;cytoplasm;cytoplasmic part;cytoskeleton;focal adhesion;Golgi apparatus;intracellular membrane-bounded organelle;intracellular non-membrane-bounded organelle;intracellular organelle;intracellu		Homo sapiens	1	6

cell body;cell part;cytoplasmic part;integral to membrane;intracellular membrane-bounded organelle;intracellular organelle;intracellular organelle part;intracellular part;intrinsic to membrane;intrinsic to mitochondrial inner men	Pyrimidine metabolism	Homo sapiens	1	6
cell part;cytoplasmic part;endoplasmic reticulum membrane;endoplasmic reticulum part;integral to endoplasmic reticulum membrane;integral to membrane;integral to organelle membrane;intracellular membrane-bounded or	ABC transporters;Antigen processing and presentation;Phagosome;Primary immunodeficiency	Homo sapiens	1	6
cell part;cytoplasm;cytoplasmic part;cytosol;intracellular membrane-bounded organelle;intracellular organelle;intracellular organelle lumen;intracellular organelle part;intracellular part;membrane;membrane-bounded organelle	Folate biosynthesis	Homo sapiens	1	6
adherens junction;anchoring junction;cell junction;cell part;cell-substrate adherens junction;cell-substrate junction;contractile fiber part;cytoplasmic part;cytoskeleton;focal adhesion;intracellular non-membrane-bounded organelle;intracellular organelle;intracellular part;non-membrane-bounded organelle;organelle;organelle part;Z		Homo sapiens	1	6
basolateral plasma membrane;cell part;cell projection;cell projection part;cell surface;clathrin-coated vesicle;coated vesicle;cytoplasmic membrane-bounded vesicle;cytoplasmic part;cytoplasmic vesicle;cytosol;dendritic spi	SNARE interactions in vesicular transport;Vasopressin-regulated water reabsorption	Homo sapiens	1	6
cell cortex;cell junction;cell part;cell projection;cell projection membrane;cell projection part;cytoplasmic part;cytoskeletal part;extracellular membrane-bounded organelle;extracellular organelle;extracellular region part;extracellular vesicular exosome;growth cone;intracellular organelle part;intracellular part;ionotropic glutamate re		Homo sapiens	1	6
actin cytoskeleton;apical part of cell;cell part;cell projection membrane;cell projection part;cytoskeleton;intracellular non-membrane-bounded organelle;intracellular organelle;intracellular part;leading edge membrane;membrane part;non-membrane-bounded organelle;organelle;plasma membrane part;ruffle membrane		Homo sapiens	1	6
cell part;cytoplasm;cytoplasmic part;cytosol;eukaryotic translation initiation factor 2B complex;intracellular part;macromolecular complex;membrane;plasma membrane;protein complex	RNA transport	Homo sapiens	1	6
adherens junction;anchoring junction;cell junction;cell part;cell-substrate adherens junction;cell-substrate junction;clathrin coated vesicle membrane;clathrin-coated endocytic vesicle membrane;coated vesicle membrane;cyt	Basal cell carcinoma;Melanogenesis;Pathways in cancer;Wnt signaling pathway	Homo sapiens	0.9999	6
cell part;cell surface;intracellular membrane-bounded organelle;intracellular organelle;intracellular part;membrane-bounded organelle;nucleus;organelle		Homo sapiens	1	6
cell part;intracellular membrane-bounded organelle;intracellular organelle;intracellular organelle part;intracellular part;membrane;membrane-bounded organelle;nuclear body;nuclear membrane;nuclear part;nuclear speck;nucleoplasm;nucleoplasm part;nucleus;organelle;organelle membrane;organelle part		Homo sapiens	1	6
cell part;chromatin remodeling complex;histone methyltransferase complex;intracellular membrane-bounded organelle;intracellular non-membrane-bounded organelle;intracellular organelle;intracellular organelle part;intracellular part;macromolecular complex;membrane-bounded organelle;methyltransferase complex;MLL1 comp		Homo sapiens	1	6
cell part;cytoplasmic part;cytosol;intracellular part;membrane	Pancreatic cancer;Pathways in cancer	Homo sapiens	1	6
cell part;intracellular membrane-bounded organelle;intracellular organelle;intracellular part;membrane-bounded organelle;nucleus;organelle		Homo sapiens	1	6
cell part;cytoplasm;cytoplasmic part;cytosol;intracellular non-membrane-bounded organelle;intracellular organelle;intracellular organelle part;intracellular part;membrane;non-membrane-bounded organelle;nuclear part;nucleolus;organelle;organelle part		Homo sapiens	1	6
activin responsive factor complex;cell part;chromatin;chromosomal part;cytoplasm;cytoplasmic part;cytosol;intracellular membrane-bounded organelle;intracellular organelle;intracellular organelle part;intracellular part;macro	Adherens junction;Cell cycle;Chagas disease (American trypanosomiasis);Colorectal cancer;Endocytosi	Homo sapiens	1	6
		Homo sapiens	1	6
cell part;cytoplasm;intracellular membrane-bounded organelle;intracellular organelle;intracellular part;membrane;membrane-bounded organelle;nucleus;organelle		Homo sapiens	1	6
cell part;cytoplasmic part;endoplasmic reticulum membrane;endoplasmic reticulum part;extracellular matrix;extracellular region part;integral to membrane;intracellular organelle part;intracellular part;intrinsic to membrane;me	Biosynthesis of unsaturated fatty acids;Steroid hormone biosynthesis	Homo sapiens	1	6
cell part;intracellular membrane-bounded organelle;intracellular non-membrane-bounded organelle;intracellular organelle;intracellular organelle part;intracellular part;membrane-bounded organelle;non-membrane-bounded organelle;nuclear part;nucleolus;nucleus;organelle;organelle part		Homo sapiens	1	6
adherens junction;anchoring junction;cell junction;cell part;cell-substrate adherens junction;cell-substrate junction;focal adhesion;integral to membrane;intrinsic to membrane;membrane part		Homo sapiens	1	6
cell part;intracellular organelle part;intracellular part;nuclear body;nuclear part;nuclear speck;nucleoplasm part;organelle part		Homo sapiens	1	6
cell part;cell surface;cytoplasmic part;extracellular membrane-bounded organelle;extracellular organelle;extracellular region part;extracellular space;extracellular vesicular exosome;integral to membrane;intracellular membrane-bounded organelle;intracellular organelle;intracellular organelle part;intracellular part;intrinsic to membr		Homo sapiens	1	6
cell part;cytoplasmic part;cytoskeletal part;endoplasmic reticulum membrane;endoplasmic reticulum part;integral to membrane;intracellular organelle part;intracellular part;intrinsic to membrane;macromolecular complex;membrane;membrane part;microtubule;organelle membrane;organelle part;protein complex		Homo sapiens	1	6
cell part;cytoplasmic part;endoplasmic reticulum;extracellular region part;extracellular space;Golgi apparatus;intracellular membrane-bounded organelle;intracellular organelle;intracellular part;membrane-bounded organelle;organelle		Homo sapiens	1	6
cell part;cytoplasmic part;integral to membrane;intracellular organelle part;intracellular part;intrinsic to membrane;membrane;membrane part;mitochondrial inner membrane;mitochondrial membrane;mitochondrial part;organelle inner membrane;organelle membrane;organelle part		Homo sapiens	1	6
cell part;cytoplasmic part;intracellular membrane-bounded organelle;intracellular organelle;intracellular part;membrane-bounded organelle;mitochondrion;organelle	Selenocompound metabolism;Tryptophan metabolism	Homo sapiens	1	6
cell part;cytoplasmic part;endoplasmic reticulum;endoplasmic reticulum membrane;endoplasmic reticulum part;Golgi apparatus part;Golgi membrane;Golgi stack;integral to membrane;intracellular membrane-bounded organ	Ether lipid metabolism;Glycerophospholipid metabolism	Homo sapiens	1	6
cell part;cytoplasmic part;extracellular membrane-bounded organelle;extracellular organelle;extracellular region part;extracellular vesicular exosome;Golgi apparatus part;intracellular organelle part;intracellular part;membrane-bounded organelle;membrane-bounded vesicle;organelle;organelle part;trans-Golgi network;vesicle		Homo sapiens	1	6
cell part;cytoplasmic part;endoplasmic reticulum lumen;endoplasmic reticulum part;intracellular organelle lumen;intracellular organelle part;intracellular part;membrane-enclosed lumen;organelle lumen;organelle part		Homo sapiens	1	6
		Homo sapiens	1	6
cell part;intracellular membrane-bounded organelle;intracellular organelle;intracellular organelle part;intracellular part;membrane;membrane-bounded organelle;nuclear part;nucleoplasm;nucleus;organelle;organelle part		Homo sapiens	1	6
cell part;cytoplasm;histone pre-mRNA 3'end processing complex;intracellular membrane-bounded organelle;intracellular non-membrane-bounded organelle;intracellular organelle;intracellular organelle part;intracellular part;macromolecular complex;membrane-bounded organelle;non-membrane-bounded organelle;nuclear part;n		Homo sapiens	1	6
cell part;intracellular organelle part;intracellular part;macromolecular complex;membrane;NELF complex;nuclear part;nucleoplasm;nucleoplasm part;organelle part;protein complex;transcription elongation factor complex		Homo sapiens	1	6
		Homo sapiens	1	6
cell part;cytoplasm;intracellular membrane-bounded organelle;intracellular organelle;intracellular part;membrane-bounded organelle;nucleus;organelle		Homo sapiens	1	6
		Homo sapiens	1	6
cell body;cell part;cell projection;cytoplasm;cytoplasmic part;cytosol;dendrite;intracellular membrane-bounded organelle;intracellular organelle;intracellular organelle part;intracellular part;lysosomal membrane;lysosome;lytic	Insulin signaling pathway;mTOR signaling pathway	Homo sapiens	1	6
actin cytoskeleton;adherens junction;anchoring junction;cell junction;cell part;cell-substrate adherens junction;cell-substrate junction;cytoplasm;cytoskeleton;focal adhesion;intracellular non-membrane-bounded organelle;intracellular organelle;intracellular part;non-membrane-bounded organelle;organelle		Homo sapiens	1	6
apical part of cell;cell part;centriole;centrosome;cytoplasmic part;cytoskeletal part;cytosol;intracellular non-membrane-bounded organelle;intracellular organelle;intracellular organelle part;intracellular part;membrane;microtubule organizing center;microtubule organizing center part;non-membrane-bounded organelle;nuclear part		Homo sapiens	1	6
cell part;intracellular membrane-bounded organelle;intracellular organelle;intracellular part;membrane-bounded organelle;nucleus;organelle		Homo sapiens	1	6
cell part;cell projection;cytoplasmic part;cytoskeleton;cytosol;extracellular membrane-bounded organelle;extracellular organelle;extracellular region part;extracellular vesicular exosome;intracellular non-membrane-bounded o	Regulation of actin cytoskeleton	Homo sapiens	1	6
cell part;centrosome;cytoplasm;cytoplasmic microtubule;cytoplasmic part;cytoskeletal part;cytoskeleton;intracellular non-membrane-bounded organelle;intracellular organelle;intracellular organelle part;intracellular part;macromolecular complex;microtubule;microtubule cytoskeleton;microtubule organizing center;non-membrane		Homo sapiens	1	6
adherens junction;anchoring junction;cell junction;cell part;cell-substrate adherens junction;cell-substrate junction;cytoplasmic membrane-bounded vesicle;cytoplasmic part;cytoplasmic vesicle;endoplasmic reticulum;extrac	Alzheimer's disease;Notch signaling pathway	Homo sapiens	1	6
adherens junction;anchoring junction;cell junction;cell part;cell surface;cell-cell adherens junction;cell-cell junction;cell-substrate adherens junction;cell-substrate junction;extracellular membrane-bounded organelle;extracellu	Adherens junction;Cell adhesion molecules (CAMs)	Homo sapiens	1	6
cell part;centrosome;chromatin;chromosomal part;cytoplasm;cytoplasmic part;cytoskeletal part;intracellular membrane-bounded organelle;intracellular non-membrane-bounded organelle;intracellular organelle;intracellular organelle part;intracellular part;membrane-bounded organelle;microtubule organizing center;non-membran		Homo sapiens	1	6
cell part;integral to membrane;integral to plasma membrane;intrinsic to membrane;intrinsic to plasma membrane;membrane part;plasma membrane part		Homo sapiens	1	6
cell part;cytoplasm;intracellular part		Homo sapiens	1	6
cell part;chromatin;chromatin remodeling complex;chromatin silencing complex;chromosomal part;cytoplasm;cytoplasmic part;envelope;euchromatin;heterochromatin;intracellular membrane-bounded organelle;intracellular non-membrane-bounded organelle;intracellular organelle;intracellular organelle part;intracellular part;mac		Homo sapiens	1	6
cell part;cytoplasmic part;integral to membrane;intracellular membrane-bounded organelle;intracellular organelle;intracellular organelle part;intracellular part;intrinsic to membrane;membrane part;membrane-bounded organelle;mitochondrion;nuclear part;nucleoplasm;organelle;organelle part		Homo sapiens	1	6
cell junction;cell part;cytoplasm;cytoplasmic part;cytosol;extrinsic to membrane;extrinsic to plasma membrane;intracellular membrane-bounded organelle;intracellular non-membrane-bounded organelle;intracellular organelle;intracellular organelle lumen;intracellular organelle part;intracellular part;membrane;membrane part;mem		Homo sapiens	1	6
		Homo sapiens	1	6
cell cortex part;cell part;cytoplasmic part;cytosol;exocyst;intracellular membrane-bounded organelle;intracellular organelle;intracellular part;macromolecular complex;membrane;membrane-bounded organelle;organelle;plasma membrane;protein complex		Homo sapiens	1	6
cell part;intracellular membrane-bounded organelle;intracellular organelle;intracellular part;membrane-bounded organelle;nucleus;organelle	Spliceosome	Homo sapiens	1	6
aggresome;cell part;cytoplasmic part;cytoskeleton;cytosol;endoplasmic reticulum;endoplasmic reticulum membrane;endoplasmic reticulum part;inclusion body;integral to membrane;intermediate filament cytoskeleton;intrace	Protein export;Protein processing in endoplasmic reticulum	Homo sapiens	1	6
cell part;chromatin;chromosomal part;euchromatin;heterochromatin;histone methyltransferase complex;intracellular membrane-bounded organelle;intracellular organelle;intracellular organelle part;intracellular part;macromolecular complex;membrane-bounded organelle;methyltransferase complex;MLL1 complex;nuclear body;nu		Homo sapiens	1	6
cell part;cytoplasmic part;cytosol;endoplasmic reticulum;extracellular region;intracellular membrane-bounded organelle;intracellular organelle;intracellular part;lipid particle;membrane;membrane-bounded organelle;nucleus;organelle;plasma membrane		Homo sapiens	1	6
cell part;intracellular organelle part;intracellular part;macromolecular complex;nuclear part;nucleoplasm;nucleoplasm part;organelle part;protein complex;transcription factor complex		Homo sapiens	0.9999	6
cell part;cytoplasm;cytoplasmic part;early endosome;endosomal part;endosome;endosome membrane;ESCRT I complex;extracellular membrane-bounded organelle;extracellular organelle;extracellular region part;extracellula	Endocytosis	Homo sapiens	1	6
cell part;cytoplasmic part;endoplasmic reticulum;endoplasmic reticulum membrane;endoplasmic reticulum part;integral to membrane;intracellular membrane-bounded organelle;intracellular organelle;intracellular organelle pa	Fat digestion and absorption;Glycerolipid metabolism;Glycerophospholipid metabolism	Homo sapiens	1	6
		Homo sapiens	1	6
cell part;cytoplasmic part;endoplasmic reticulum lumen;endoplasmic reticulum part;intracellular organelle lumen;intracellular organelle part;intracellular part;membrane-enclosed lumen;organelle lumen;organelle part		Homo sapiens	1	6
		Homo sapiens	1	6
cell part;cytoplasm;extracellular membrane-bounded organelle;extracellular organelle;extracellular region part;extracellular vesicular exosome;intracellular part;membrane-bounded organelle;membrane-bounded vesicle;orga	Butanoate metabolism;Synthesis and degradation of ketone bodies	Homo sapiens	1	6
cell part;cytoplasm;intracellular membrane-bounded organelle;intracellular organelle;intracellular organelle part;intracellular part;membrane-bounded organelle;nuclear part;nucleoplasm;nucleus;organelle;organelle part		Homo sapiens	1	6
cell part;cytoplasmic part;integral to membrane;intracellular membrane-bounded organelle;intracellular non-membrane-bounded organelle;intracellular organelle;intracellular organelle part;intracellular part;intrinsic to membrane;macromolecular complex;membrane;membrane part;membrane-bounded organelle;Noc complex;No		Homo sapiens	1	6
cell part;cytoplasmic part;cytosolic part;cytosolic ribosome;intracellular non-membrane-bounded organelle;intracellular organelle;intracellular organelle part;intracellular part;macromolecular complex;membrane;mitochondrial inner membrane;mitochondrial membrane;mitochondrial part;non-membrane-bounded organelle;organe		Homo sapiens	1	6
cell part;cytoplasmic membrane-bounded vesicle;cytoplasmic part;cytoplasmic vesicle;cytoplasmic vesicle membrane;cytoplasmic vesicle part;endocytic vesicle;endocytic vesicle membrane;extracellular membrane-bounded organelle;extracellular organelle;extracellular region part;extracellular vesicular exosome;Golgi apparatu		Homo sapiens	1	6
cell part;cytoskeletal part;intracellular membrane-bounded organelle;intracellular organelle;intracellular organelle part;intracellular part;macromolecular complex;membrane-bounded organelle;microtubule;nuclear part;nucleo	Wnt signaling pathway	Homo sapiens	1	6
cell part;cytoplasm;intracellular membrane-bounded organelle;intracellular organelle;intracellular part;membrane-bounded organelle;nucleus;organelle		Homo sapiens	1	6
cell part;cytoplasm;cytoplasmic part;Golgi apparatus;intracellular membrane-bounded organelle;intracellular organelle;intracellular organelle part;intracellular part;membrane;membrane-bounded organelle;nuclear part;nucleoplasm;nucleus;organelle;organelle part;plasma membrane		Homo sapiens	1	6
cell part;cytoplasm;cytoplasmic part;cytoskeletal part;extracellular membrane-bounded organelle;extracellular organelle;extracellular region part;extracellular vesicular exosome;intracellular membrane-bounded organelle;intra	Protein processing in endoplasmic reticulum	Homo sapiens	1	6
cell part;cytoplasmic part;integral to membrane;intracellular membrane-bounded organelle;intracellular organelle;intracellular organelle part;intracellular part;intrinsic to membrane;membrane;membrane part;membrane-bounded organelle;mitochondrial inner membrane;mitochondrial membrane;mitochondrial part;mitochondrion;		Homo sapiens	1	6
cell part;cytoplasmic part;endoplasmic reticulum;endoplasmic reticulum membrane;endoplasmic reticulum part;integral to membrane;intracellular membrane-bounded organelle;intracellular non-membrane-bounded organelle;intracellular organelle;intracellular organelle part;intracellular part;intrinsic to membrane;membrane;men		Homo sapiens	1	6
cell part;cytoplasm;cytoplasmic part;endoplasmic reticulum membrane;endoplasmic reticulum part;integral to membrane;intracellular membrane-bounded organelle;intracellular organelle;intracellular organelle part;intracellula	N-Glycan biosynthesis;Various types of N-glycan biosynthesis	Homo sapiens	1	6
cell part;intracellular membrane-bounded organelle;intracellular organelle;intracellular part;membrane-bounded organelle;nucleus;organelle		Homo sapiens	1	6
cell part;cytoplasmic part;cytosol;extracellular membrane-bounded organelle;extracellular organelle;extracellular region part;extracellular vesicular exosome;intracellular part;membrane-bounded organelle;membrane-bounde	Cell cycle - yeast	Homo sapiens	0.9999	6
cell part;cytoplasm;extracellular matrix;extracellular membrane-bounded organelle;extracellular organelle;extracellular region part;extracellular vesicular exosome;integral to membrane;intracellular part;intrinsic to membrane;membrane part;membrane-bounded organelle;membrane-bounded vesicle;organelle;proteinaceous extra		Homo sapiens	1	6
cell part;cell projection;centrosome;cilium;cytoplasm;cytoplasmic dynein complex;cytoplasmic part;cytoskeletal part;dynein complex;intracellular non-membrane-bounded organelle;intracellular organelle;intracellular organelle part;intracellular part;macromolecular complex;membrane;microtubule;microtubule associated comple		Homo sapiens	1	6
cell part;ER membrane protein complex;integral to membrane;intrinsic to membrane;macromolecular complex;membrane part;protein complex		Homo sapiens	1	6
cell part;chromatin;chromosomal part;cytoplasm;cytoplasmic exosome (RNase complex);cytoplasmic part;cytosol;exosome (RNase complex);intracellular membrane-bounded organelle;intracellular non-membrane-bounded	RNA degradation	Homo sapiens	1	6
cell part;chromatin;chromosomal part;cytoplasm;cytoplasmic exosome (RNase complex);cytoplasmic part;cytosol;exosome (RNase complex);intracellular membrane-bounded organelle;intracellular non-membrane-bounded	RNA degradation	Homo sapiens	1	6
CD40 receptor complex;cell part;cytoplasmic part;cytosol;internal side of plasma membrane;intracellular membrane-bounded organelle;intracellular organelle;intracellular organelle part;intracellular part;macromolecular complex;membrane part;membrane-bounded organelle;membrane-enclosed lumen;mitochondrial intermembr		Homo sapiens	1	6
cell part;cytoplasmic part;cytosol;intracellular membrane-bounded organelle;intracellular organelle;intracellular part;membrane-bounded organelle;mitochondrion;organelle	Pyrimidine metabolism	Homo sapiens	1	6
cell part;centrosome;cytoplasm;cytoplasmic part;cytoskeletal part;envelope;intracellular membrane-bounded organelle;intracellular non-membrane-bounded organelle;intracellular organelle;intracellular organelle part;intracell	RNA transport	Homo sapiens	1	6
cell part;cytoplasmic part;intracellular membrane-bounded organelle;intracellular organelle;intracellular part;membrane-bounded organelle;mitochondrion;organelle		Homo sapiens	1	6
Cajal body;cell part;cytoplasm;intracellular non-membrane-bounded organelle;intracellular organelle;intracellular organelle part;intracellular part;macromolecular complex;non-membrane-bounded organelle;nuclear body;nucl	Ribosome biogenesis in eukaryotes	Homo sapiens	1	6
cell part;cytoplasmic part;intracellular organelle lumen;intracellular organelle part;intracellular part;membrane-enclosed lumen;mitochondrial matrix;mitochondrial part;nuclear part;nucleoplasm;organelle lumen;organelle part		Homo sapiens	1	6
cell part;cytoplasmic part;Golgi apparatus;integral to membrane;intracellular membrane-bounded organelle;intracellular organelle;intracellular part;intrinsic to membrane;membrane;membrane part;membrane-bounded organelle;organelle		Homo sapiens	1	6
cell part;cytoplasm;cytoplasmic part;endoplasmic reticulum;intracellular membrane-bounded organelle;intracellular organelle;intracellular part;membrane-bounded organelle;mitochondrion;organelle		Homo sapiens	1	6
box H/ACA RNP complex;box H/ACA snoRNP complex;Cajal body;cell part;intracellular membrane-bounded organelle;intracellular non-membrane-bounded organelle;intracellular organelle;intracellular organelle part;intracell	Ribosome biogenesis in eukaryotes	Homo sapiens	1	6
cell part;chromosomal part;chromosome;chromosome, telomeric region;cytoplasm;envelope;intracellular membrane-bounded organelle;intracellular non-membrane-bounded organelle;intracellular organelle;intracellular organelle part;intracellular part;macromolecular complex;membrane-bounded organelle;non-membrane-boun		Homo sapiens	1	6
cell part;clathrin-coated vesicle;coated pit;coated vesicle;cytoplasm;cytoplasmic membrane-bounded vesicle;cytoplasmic part;cytoplasmic vesicle;extracellular membrane-bounded organelle;extracellular organelle;extracellular region part;extracellular vesicular exosome;intracellular membrane-bounded organelle;intracellular or		Homo sapiens	1	6
cell part;cytoplasm;intracellular membrane-bounded organelle;intracellular organelle;intracellular organelle part;intracellular part;membrane-bounded organelle;nuclear part;nucleoplasm;nucleus;organelle;organelle part		Homo sapiens	1	6
cell part;cytoplasmic part;integral to membrane;integral to mitochondrial membrane;integral to mitochondrial outer membrane;integral to organelle membrane;intracellular organelle part;intracellular part;intrinsic to membrane;intrinsic to mitochondrial outer membrane;intrinsic to organelle membrane;membrane part;mitochondrial		Homo sapiens	1	6
cell part;cytoplasm;intracellular membrane-bounded organelle;intracellular organelle;intracellular part;membrane;membrane-bounded organelle;nucleus;organelle;plasma membrane	Protein processing in endoplasmic reticulum	Homo sapiens	1	6
cell cortex;cell part;cell projection;cytoplasmic part;endomembrane system;intracellular organelle part;intracellular part;lamellipodium;membrane;nuclear part;nucleoplasm;organelle part;perinuclear region of cytoplasm		Homo sapiens	1	6
cell part;cytoplasmic part;cytosol;extracellular membrane-bounded organelle;extracellular organelle;extracellular region part;extracellular vesicular exosome;intracellular membrane-bounded organelle;intracellular organelle;int	Cysteine and methionine metabolism	Homo sapiens	1	6
cell part;cytoplasm;intracellular membrane-bounded organelle;intracellular organelle;intracellular part;membrane-bounded organelle;nucleus;organelle		Homo sapiens	1	6
extracellular membrane-bounded organelle;extracellular organelle;extracellular region part;extracellular vesicular exosome;macromolecular complex;membrane-bounded organelle;membrane-bounded vesicle;organelle;protein complex;vesicle		Homo sapiens	1	6
actin cytoskeleton;cell part;cell projection;cytoplasmic membrane-bounded vesicle;cytoplasmic part;cytoplasmic vesicle;cytoplasmic vesicle membrane;cytoplasmic vesicle part;cytoskeleton;early endosome;endocytic vesicl	Endocytosis	Homo sapiens	1	6
cell part;intracellular organelle part;intracellular part;nuclear part;nucleoplasm;organelle part		Homo sapiens	0.9999	6
adherens junction;anchoring junction;cell junction;cell part;cell surface;cell-substrate adherens junction;cell-substrate junction;centrosome;cytoplasmic part;cytoskeletal part;extracellular membrane-bounded organelle;extracellular organelle;extracellular region;extracellular region part;extracellular vesicular exosome;focal adhesi		Homo sapiens	1	6
cell part;intracellular membrane-bounded organelle;intracellular organelle;intracellular organelle part;intracellular part;membrane-bounded organelle;nuclear part;nucleoplasm;nucleus;organelle;organelle part		Homo sapiens	1	6
cell part;cytoplasm;cytoplasmic part;cytosol;envelope;intracellular organelle part;intracellular part;nuclear envelope;nuclear part;organelle envelope;organelle part	Fructose and mannose metabolism	Homo sapiens	1	6
cell part;cytoplasmic part;endoplasmic reticulum membrane;endoplasmic reticulum part;endoplasmic reticulum-Golgi intermediate compartment membrane;Golgi apparatus;integral to membrane;intracellular membrane-bounded organelle;intracellular organelle;intracellular organelle part;intracellular part;intrinsic to membrane;m		Homo sapiens	1	6
catalytic step 2 spliceosome;cell part;cytoplasmic part;cytosol;intracellular organelle part;intracellular part;macromolecular complex;nuclear part;nucleoplasm;organelle part;ribonucleoprotein complex;spliceosomal complex	RNA degradation;Spliceosome	Homo sapiens	1	6
cell part;cytoplasm;cytoplasmic part;cytosol;exosome (RNase complex);intracellular membrane-bounded organelle;intracellular non-membrane-bounded organelle;intracellular organelle;intracellular organelle part;intracellular	RNA degradation	Homo sapiens	1	6
cell part;cytoplasmic membrane-bounded vesicle;cytoplasmic part;cytoplasmic vesicle;cytosol;endosomal part;endosome membrane;ESCRT III complex;extracellular membrane-bounded organelle;extracellular organelle;ext	Endocytosis	Homo sapiens	1	6
cell part;cytoplasmic mRNA processing body;cytoplasmic part;intracellular non-membrane-bounded organelle;intracellular organelle;intracellular part;macromolecular complex;non-membrane-bounded organelle;organelle;ribonucleoprotein complex;RNA granule		Homo sapiens	1	6
cell part;cytoplasmic part;endoplasmic reticulum membrane;endoplasmic reticulum part;extracellular membrane-bounded organelle;extracellular organelle;extracellular region part;extracellular vesicular exosome;integral to membrane;intracellular organelle part;intracellular part;intrinsic to membrane;membrane;membrane part;m		Homo sapiens	1	6
cell part;cytoplasmic part;intracellular membrane-bounded organelle;intracellular organelle;intracellular part;membrane-bounded organelle;mitochondrion;nucleus;organelle		Homo sapiens	1	6
cell part;intracellular membrane-bounded organelle;intracellular organelle;intracellular organelle part;intracellular part;membrane-bounded organelle;nuclear part;nucleoplasm;nucleus;organelle;organelle part		Homo sapiens	1	7
cell part;chromosome;intracellular non-membrane-bounded organelle;intracellular organelle;intracellular organelle part;intracellular part;macromolecular complex;Mpp10 complex;non-membrane-bounded organelle;nuclear p	Ribosome biogenesis in eukaryotes	Homo sapiens	1	7
cell part;chromosomal part;chromosome, centromeric region;condensed chromosome kinetochore;condensed chromosome outer kinetochore;condensed nuclear chromosome outer kinetochore;cytoplasmic part;cytosol;intracellular membrane-bounded organelle;intracellular non-membrane-bounded organelle;intracellular orga		Homo sapiens	1	7
cell part;cytoplasmic part;endoplasmic reticulum;endoplasmic reticulum membrane;endoplasmic reticulum part;envelope;integral to membrane;intracellular membrane-bounded organelle;intracellular organelle;intracellular organelle part;intracellular part;intrinsic to membrane;membrane;membrane part;membrane-bounded orga		Homo sapiens	1	7
azurophil granule;cell part;cytoplasmic membrane-bounded vesicle;cytoplasmic part;cytoplasmic vesicle;early endosome;early endosome membrane;endocytic vesicle;endomembrane system;endosomal part;endosome;end	Phagosome;SNARE interactions in vesicular transport	Homo sapiens	1	7
Ada2/Gcn5/Ada3 transcription activator complex;cell part;cytoplasm;cytoplasmic part;cytosol;endosomal part;endosome membrane;histone acetyltransferase complex;intracellular membrane-bounded organelle;intracellular	Adherens junction;Leishmaniasis;MAPK signaling pathway;Measles;NOD-like receptor signaling pathwa	Homo sapiens	1	7
cell part;cytoplasm;cytoplasmic part;ER membrane protein complex;intracellular membrane-bounded organelle;intracellular organelle;intracellular part;macromolecular complex;membrane;membrane-bounded organelle;mitochondrion;nucleus;organelle;protein complex		Homo sapiens	1	7
cell part;cytoplasm;intracellular organelle part;intracellular part;nuclear part;nucleoplasm;organelle part		Homo sapiens	1	7
cell part;cytoplasmic part;intracellular membrane-bounded organelle;intracellular organelle;intracellular organelle part;intracellular part;macromolecular complex;membrane;membrane part;membrane-bounded organelle;mem	Alzheimer's disease;Huntington's disease;Oxidative phosphorylation;Parkinson's disease	Homo sapiens	1	7
cell part;cytoplasm;intracellular membrane-bounded organelle;intracellular organelle;intracellular part;membrane-bounded organelle;nucleus;organelle		Homo sapiens	1	7
cell part;cytoplasmic part;dolichol-phosphate-mannose synthase complex;endoplasmic reticulum;endoplasmic reticulum membrane;endoplasmic reticulum part;intracellular membrane-bounded organelle;intracellular organel	N-Glycan biosynthesis	Homo sapiens	1	7
cell part;cytoplasmic part;endosome;intracellular membrane-bounded organelle;intracellular organelle;intracellular part;membrane-bounded organelle;organelle		Homo sapiens	1	7
cell part;cytoplasm;cytoplasmic part;cytosol;intracellular organelle part;intracellular part;membrane;nuclear part;nucleoplasm;organelle part;perinuclear region of cytoplasm		Homo sapiens	1	7
cell part;cytoplasmic part;cytosol;extracellular membrane-bounded organelle;extracellular organelle;extracellular region part;extracellular vesicular exosome;intracellular membrane-bounded organelle;intracellular organelle;int	Glycerophospholipid metabolism	Homo sapiens	1	7
cell part;cytoplasmic part;cytosol;intracellular part;membrane	MAPK signaling pathway	Homo sapiens	1	7
cell part;cytoplasm;cytoplasmic part;cytoskeleton;cytosol;intermediate filament cytoskeleton;intracellular membrane-bounded organelle;intracellular non-membrane-bounded organelle;intracellular organelle;intracellular organelle part;intracellular part;macromolecular complex;membrane-bounded organelle;non-membrane-boun		Homo sapiens	1	7
cell part;cytoplasm;cytoplasmic part;extracellular membrane-bounded organelle;extracellular organelle;extracellular region part;extracellular vesicular exosome;intracellular;intracellular membrane-bounded organelle;intracellular organelle;intracellular part;membrane-bounded organelle;membrane-bounded vesicle;mitochondrion;		Homo sapiens	1	7

cell part;cytoplasmic part;Golgi apparatus;integral to membrane;intracellular membrane-bounded organelle;intracellular organelle;intracellular part;intrinsic to membrane;membrane part;membrane-bounded organelle;nucleus;organelle		Homo sapiens	1	7
cell part;cytoplasmic part;extracellular membrane-bounded organelle;extracellular organelle;extracellular region part;extracellular vesicular exosome;integral to membrane;intracellular membrane-bounded organelle;intracellula	Alzheimer's disease;Huntington's disease;Oxidative phosphorylation;Parkinson's disease	Homo sapiens	1	7
cell part;cytoplasmic part;cytoskeletal part;Golgi apparatus;intracellular membrane-bounded organelle;intracellular non-membrane-bounded organelle;intracellular organelle;intracellular organelle part;intracellular part;kinesin complex;macromolecular complex;membrane-bounded organelle;microtubule;microtubule associated co		Homo sapiens	1	7
cell part;cytoplasm;intracellular membrane-bounded organelle;intracellular organelle;intracellular part;membrane-bounded organelle;nucleus;organelle		Homo sapiens	1	7
cell part;cytoplasm;cytoplasmic part;endoplasmic reticulum;intracellular membrane-bounded organelle;intracellular organelle;intracellular part;membrane-bounded organelle;nucleus;organelle		Homo sapiens	1	7
cell part;cytoplasmic part;endoplasmic reticulum lumen;endoplasmic reticulum part;intracellular organelle lumen;intracellular organelle part;intracellular part;membrane-enclosed lumen;organelle lumen;organelle part	Glutathione metabolism	Homo sapiens	1	7
basement membrane;extracellular matrix part;extracellular membrane-bounded organelle;extracellular organelle;extracellular region;extracellular region part;extracellular vesicular exosome;membrane-bounded organelle;membrane-bounded vesicle;organelle;vesicle		Homo sapiens	1	7
blood microparticle;extracellular membrane-bounded organelle;extracellular organelle;extracellular region;extracellular region part;extracellular vesicular exosome;membrane-bounded organelle;membrane-bounded vesicle;o	Complement and coagulation cascades;Phagosome;Staphylococcus aureus infection;Systemic lupus e	Homo sapiens	1	7
blood microparticle;cell part;cell surface;cytoplasmic membrane-bounded vesicle lumen;cytoplasmic part;cytoplasmic vesicle part;extracellular membrane-bounded organelle;extracellular organelle;extracellular region;extrac	Complement and coagulation cascades;Neuroactive ligand-receptor interaction;Staphylococcus aureus	Homo sapiens	1	7
blood microparticle;cell cortex;cell part;cell surface;cytoplasmic membrane-bounded vesicle;cytoplasmic membrane-bounded vesicle lumen;cytoplasmic part;cytoplasmic vesicle;cytoplasmic vesicle part;external side of plas	Complement and coagulation cascades	Homo sapiens	1	7
cell part;cell projection;cytoplasm;cytoplasmic part;cytosol;Golgi apparatus;intracellular membrane-bounded organelle;intracellular organelle;intracellular organelle part;intracellular part;membrane;membrane-bounded organe	Acute myeloid leukemia;B cell receptor signaling pathway;Bladder cancer;Chemokine signaling pathway	Homo sapiens	1	7
cell part;cytoplasmic part;extracellular membrane-bounded organelle;extracellular organelle;extracellular region part;extracellular vesicular exosome;intracellular organelle lumen;intracellular organelle part;intracellular part;lys	Lysosome;Other glycan degradation;Sphingolipid metabolism	Homo sapiens	1	7
cell part;cytoplasmic part;cytosol;intracellular part	Drug metabolism - other enzymes;Pyrimidine metabolism	Homo sapiens	1	7
cell part;cytoplasm;cytoplasmic part;cytosol;intracellular membrane-bounded organelle;intracellular non-membrane-bounded organelle;intracellular organelle;intracellular organelle lumen;intracellular organelle part;intracellula	One carbon pool by folate;Pyrimidine metabolism	Homo sapiens	1	7
cell part;cytoplasm;extracellular membrane-bounded organelle;extracellular organelle;extracellular region part;extracellular vesicular exosome;intracellular membrane-bounded organelle;intracellular organelle;intracellular organelle part;intracellular part;membrane-bounded organelle;membrane-bounded vesicle;nuclear part;nucle		Homo sapiens	1	7
cell part;cytoplasmic part;cytosol;intracellular part	Porphyrin and chlorophyll metabolism	Homo sapiens	1	7
Cajal body;cell part;commitment complex;intracellular organelle part;intracellular part;macromolecular complex;nuclear body;nuclear part;nucleoplasm;nucleoplasm part;organelle part;prespliceosome;ribonucleoprotein comp	Spliceosome	Homo sapiens	1	7
basement membrane;cell part;cell surface;cytoplasm;cytoplasmic membrane-bounded vesicle;cytoplasmic membrane-bounded vesicle lumen;cytoplasmic part;cytoplasmic vesicle;cytoplasmic vesicle membrane;cytoplasmic vesicle part;endocytic vesicle lumen;extracellular matrix part;extracellular region;extracellular region pa		Homo sapiens	1	7
		Homo sapiens	1	7
cell part;cytoplasmic part;extracellular membrane-bounded organelle;extracellular organelle;extracellular region part;extracellular vesicular exosome;intracellular membrane-bounded organelle;intracellular organelle;intracellula	Alzheimer's disease;Cardiac muscle contraction;Huntington's disease;Oxidative phosphorylation;Parkin	Homo sapiens	1	7
cell part;extracellular membrane-bounded organelle;extracellular organelle;extracellular region;extracellular region part;extracellular vesicular exosome;macromolecular complex;membrane attack complex;membrane part;me	Complement and coagulation cascades;Prion diseases;Systemic lupus erythematosus	Homo sapiens	1	7
anchored to membrane;cell part;cell surface;cytoplasmic part;endoplasmic reticulum;extracellular region;extracellular region part;extracellular space;intracellular membrane-bounded organelle;intracellular organelle;intracellul	Complement and coagulation cascades	Homo sapiens	1	7
cell part;cytoplasmic part;extracellular membrane-bounded organelle;extracellular organelle;extracellular region part;extracellular vesicular exosome;integral to membrane;intracellular membrane-bounded organelle;intracellula	Riboflavin metabolism	Homo sapiens	1	7
cell part;extracellular membrane-bounded organelle;extracellular organelle;extracellular region part;extracellular vesicular exosome;integral to membrane;integral to plasma membrane;intrinsic to membrane;intrinsic to plasma membrane;membrane;membrane part;membrane-bounded organelle;membrane-bounded vesicle;organ		Homo sapiens	1	7
cell part;collagen;collagen type XI;cytoplasmic part;endoplasmic reticulum lumen;endoplasmic reticulum part;extracellular matrix part;extracellular region;extracellular region part;fibrillar collagen;intracellular organelle lumen;i	Amoebiasis;ECM-receptor interaction;Focal adhesion;Protein digestion and absorption	Homo sapiens	1	7
cell part;cytoplasmic part;cytoplasmic vesicle membrane;cytoplasmic vesicle part;endocytic vesicle membrane;endosomal part;endosome;endosome membrane;extracellular membrane-bounded organelle;extracellular orga	ko05152;Lysosome;Phagosome	Homo sapiens	1	7
adherens junction;anchored to external side of plasma membrane;anchored to membrane;anchored to plasma membrane;anchoring junction;cell junction;cell part;cell surface;cell-substrate adherens junction;cell-substrate ju	Complement and coagulation cascades;Hematopoietic cell lineage	Homo sapiens	1	7
adherens junction;anchoring junction;blood microparticle;cell junction;cell part;cell surface;cell-substrate adherens junction;cell-substrate junction;cytoplasm;endothelial microparticle;external side of plasma membrane;extracellular region part;extracellular space;focal adhesion;intracellular organelle part;intracellular part;macrom		Homo sapiens	1	7
cell part;cytoplasmic part;intracellular membrane-bounded organelle;intracellular organelle;intracellular organelle part;intracellular part;macromolecular complex;membrane-bounded organelle;mitochondrion;NELF complex;nuclear part;nucleoplasm;nucleoplasm part;nucleus;organelle;organelle part;protein complex;transcription		Homo sapiens	1	7
cell part;cytoplasmic part;intracellular membrane-bounded organelle;intracellular organelle;intracellular organelle part;intracellular part;macromolecular complex;membrane;membrane part;membrane-bounded organelle;mitoc	Alzheimer's disease;Huntington's disease;Oxidative phosphorylation;Parkinson's disease	Homo sapiens	1	7
cell part;cytoplasm;cytoplasmic part;cytosol;intracellular membrane-bounded organelle;intracellular non-membrane-bounded organelle;intracellular organelle;intracellular part;macromolecular complex;membrane;membrane-	Hepatitis C;Measles;Protein processing in endoplasmic reticulum	Homo sapiens	1	7
adherens junction;anchoring junction;apical plasma membrane;cell junction;cell part;cell-substrate adherens junction;cell-substrate junction;clathrin coated vesicle membrane;clathrin-coated endocytic vesicle membrane;coa	Hematopoietic cell lineage	Homo sapiens	1	7
cell part;cell projection;cilium;cytoplasm;cytoplasmic part;endoplasmic reticulum membrane;endoplasmic reticulum part;extracellular membrane-bounded organelle;extracellular organelle;extracellular region part;extracellular	Arachidonic acid metabolism	Homo sapiens	1	7
cell part;cytoplasm;intracellular membrane-bounded organelle;intracellular organelle;intracellular part;membrane-bounded organelle;nucleus;organelle		Homo sapiens	1	7
cell part;cytoplasmic part;cytosol;intracellular membrane-bounded organelle;intracellular organelle;intracellular part;membrane-bounded organelle;nucleus;organelle	Purine metabolism;Pyrimidine metabolism	Homo sapiens	1	7
cell part;cytoplasm;cytoplasmic part;cytosol;extracellular membrane-bounded organelle;extracellular organelle;extracellular region part;extracellular vesicular exosome;intracellular membrane-bounded organelle;intracellular o	Proteasome	Homo sapiens	1	7
cell part;collagen;extracellular matrix;extracellular matrix part;extracellular region;extracellular region part;extracellular space;intracellular membrane-bounded organelle;intracellular organelle;intracellular part;membrane-bounded organelle;nucleus;organelle;proteinaceous extracellular matrix		Homo sapiens	1	7
cell part;cell surface;extracellular membrane-bounded organelle;extracellular organelle;extracellular region part;extracellular space;extracellular vesicular exosome;host cell part;host cell surface;integral to membrane;integral to plasma membrane;intrinsic to membrane;intrinsic to plasma membrane;membrane;membrane part;me		Homo sapiens	1	7
cell part;integral to membrane;integral to plasma membrane;intrinsic to membrane;intrinsic to plasma membrane;membrane;membrane part;plasma membrane;plasma membrane part		Homo sapiens	1	7
cell part;cytoskeletal part;intracellular non-membrane-bounded organelle;intracellular organelle;intracellular organelle part;intracellular part;membrane;non-membrane-bounded organelle;organelle;organelle part;spindle	Cell cycle;Cell cycle - yeast	Homo sapiens	1	7
axon part;cell part;cell projection part;cytoplasm;cytoskeleton;extracellular membrane-bounded organelle;extracellular organelle;extracellular region part;extracellular vesicular exosome;intracellular non-membrane-bounded o	Regulation of actin cytoskeleton;Shigellosis	Homo sapiens	1	7
cell part;cytoplasmic part;intracellular membrane-bounded organelle;intracellular organelle;intracellular organelle lumen;intracellular organelle part;intracellular part;membrane;membrane-bounded organelle;membrane-enclos	Butanoate metabolism;Geraniol degradation;Peroxisome;Synthesis and degradation of ketone bodies;V	Homo sapiens	1	7
cell part;cytoplasm;cytoplasmic part;cytosol;intracellular membrane-bounded organelle;intracellular organelle;intracellular organelle part;intracellular part;macromolecular complex;membrane-bounded organelle;nuclear part;n	Proteasome	Homo sapiens	1	7
cell part;cytoplasmic part;cytosol;intracellular membrane-bounded organelle;intracellular organelle;intracellular organelle part;intracellular part;membrane;membrane-bounded organelle;nuclear part;nucleoplasm;nucleus;orga	Alzheimer's disease;Amoebiasis;Amyotrophic lateral sclerosis (ALS);Apoptosis;Colorectal cancer;Epithe	Homo sapiens	1	7
cell part;intracellular organelle part;intracellular part;membrane;nuclear part;nucleoplasm;organelle part	DNA replication;Purine metabolism;Pyrimidine metabolism	Homo sapiens	1	7
cell part;cytoplasm;cytoplasmic part;endoplasmic reticulum;endoplasmic reticulum membrane;endoplasmic reticulum part;intracellular membrane-bounded organelle;intracellular organelle;intracellular organelle part;intracellular part;IPAF inflammasome complex;macromolecular complex;membrane;membrane part;membrane-bo		Homo sapiens	1	7
extracellular matrix;extracellular membrane-bounded organelle;extracellular organelle;extracellular region;extracellular region part;extracellular space;extracellular vesicular exosome;membrane-bounded organelle;membrane-	ECM-receptor interaction;Focal adhesion;Malaria;Phagosome;TGF-beta signaling pathway	Homo sapiens	1	7
cell part;cytoplasmic part;integral to membrane;integral to mitochondrial inner membrane;integral to mitochondrial membrane;integral to organelle membrane;intracellular organelle part;intracellular part;intrinsic to membrane;	Porphyrin and chlorophyll metabolism	Homo sapiens	1	7
apical plasma membrane;cell part;cell projection;cilium;cytoplasmic membrane-bounded vesicle;cytoplasmic part;cytoplasmic vesicle;cytoplasmic vesicle membrane;cytoplasmic vesicle part;dendrite;endosomal part;endosome;endosome membrane;exocytic vesicle;extracellular membrane-bounded organelle;extracellular orga		Homo sapiens	1	7
cell part;cytoplasmic part;cytosol;extracellular membrane-bounded organelle;extracellular organelle;extracellular region part;extracellular vesicular exosome;intracellular part;membrane-bounded organelle;membrane-bounde	Drug metabolism - other enzymes	Homo sapiens	1	7
cell part;collagen;cytoplasmic part;extracellular matrix;extracellular matrix part;extracellular membrane-bounded organelle;extracellular organelle;extracellular region;extracellular region part;extracellular space;extracellular vesicular exosome;fibrillar collagen;Golgi apparatus part;Golgi lumen;intracellular organelle lumen;intracellu		Homo sapiens	1	7
cell part;cyclin-dependent protein kinase activating kinase holoenzyme complex;holo TFIIH complex;intracellular membrane-bounded organelle;intracellular organelle;intracellular organelle part;intracellular part;macromolecul	Basal transcription factors;Cell cycle;Nucleotide excision repair	Homo sapiens	1	7
cell part;cytoplasm;cytoplasmic part;cytosol;intracellular organelle part;intracellular part;membrane;nuclear part;nucleoplasm;organelle part;plasma membrane	Chemokine signaling pathway;Hepatitis C;Jak-STAT signaling pathway;ko05152;Measles;Osteoclast dif	Homo sapiens	1	7
cell cortex;cell part;cell projection;cytoplasm;cytoplasmic part;Golgi apparatus part;Golgi membrane;intracellular organelle part;intracellular part;membrane;organelle membrane;organelle part;plasma membrane;ruffle;trans-Golgi network membrane		Homo sapiens	1	7
cell part;centrosome;cytoplasmic membrane-bounded vesicle;cytoplasmic part;cytoplasmic vesicle;cytoskeletal part;cytosol;endoplasmic reticulum-Golgi intermediate compartment;extracellular membrane-bounded organelle;extracellular organelle;extracellular region part;extracellular vesicular exosome;intracellular membrane-		Homo sapiens	1	7
cell part;cytoplasm;extracellular membrane-bounded organelle;extracellular organelle;extracellular region part;extracellular vesicular exosome;integral to membrane;intracellular part;intrinsic to membrane;membrane;membrane part;membrane-bounded organelle;membrane-bounded vesicle;organelle;plasma membrane;vesicle		Homo sapiens	1	7
cell part;cytoplasmic part;cytosol;extracellular membrane-bounded organelle;extracellular organelle;extracellular region part;extracellular vesicular exosome;integral to membrane;integral to mitochondrial membrane;integral t	Alzheimer's disease;Amyotrophic lateral sclerosis (ALS);Apoptosis;ko05152;Natural killer cell mediated c	Homo sapiens	1	7
cell part;cytoplasmic part;intracellular membrane-bounded organelle;intracellular organelle;intracellular organelle part;intracellular part;macromolecular complex;membrane;membrane part;membrane-bounded organelle;mitoc	Oxidative phosphorylation	Homo sapiens	1	7
cell part;cytoplasm;cytoskeletal part;extracellular membrane-bounded organelle;extracellular organelle;extracellular region part;extracellular vesicular exosome;intermediate filament;intracellular membrane-bounded organelle	Ribosome biogenesis in eukaryotes	Homo sapiens	1	7
cell part;cytoplasmic membrane-bounded vesicle;cytoplasmic part;cytoplasmic vesicle;endoplasmic reticulum;extracellular membrane-bounded organelle;extracellular organelle;extracellular region part;extracellular vesicular exosome;intracellular membrane-bounded organelle;intracellular organelle;intracellular organelle part;intr		Homo sapiens	1	7
cell part;cytoplasmic part;cytosol;endoplasmic reticulum;endoplasmic reticulum membrane;endoplasmic reticulum part;endoplasmic reticulum Sec complex;integral to membrane;intracellular membrane-bounded organelle;in	Phagosome;Protein export;Protein processing in endoplasmic reticulum;Vibrio cholerae infection	Homo sapiens	1	7
cell part;cytoplasmic membrane-bounded vesicle;cytoplasmic part;cytoplasmic vesicle;early endosome;early endosome membrane;endocytic vesicle;endosomal part;endosome;endosome membrane;extracellular membrane	Amoebiasis;Endocytosis;ko05152;Phagosome;Vasopressin-regulated water reabsorption	Homo sapiens	1	7
cell part;cytoplasm;cytoplasmic part;cytosol;cytosolic small ribosomal subunit;extracellular membrane-bounded organelle;extracellular organelle;extracellular region part;extracellular vesicular exosome;intracellular non-meml	Ribosome	Homo sapiens	1	7
cell part;cytoplasm;cytoplasmic part;cytoskeletal part;cytosol;intracellular organelle part;intracellular part;macromolecular complex;microtubule;organelle part;protein complex		Homo sapiens	1	7
cell part;centromeric heterochromatin;chromatin;chromocenter;chromosomal part;chromosome, centromeric region;cytoskeletal part;female pronucleus;heterochromatin;intracellular membrane-bounded organelle;intracellular non-membrane-bounded organelle;intracellular organelle;intracellular organelle part;intracellular part;ma		Homo sapiens	1	7
cell part;cytoplasm;cytoplasmic part;extracellular membrane-bounded organelle;extracellular organelle;extracellular region part;extracellular vesicular exosome;intracellular membrane-bounded organelle;intracellular non-membrane-bounded organelle;intracellular organelle;intracellular part;lysosome;lytic vacuole;macromolecular		Homo sapiens	1	7
apical plasma membrane;cell part;cytoplasm;cytoplasmic part;cytosol;extracellular membrane-bounded organelle;extracellular organelle;extracellular region part;extracellular vesicular exosome;integral to membrane;integral to plasma membrane;intracellular membrane-bounded organelle;intracellular non-membrane-bounded o		Homo sapiens	1	7
		Homo sapiens	1	7
cell part;intracellular membrane-bounded organelle;intracellular organelle;intracellular organelle part;intracellular part;membrane-bounded organelle;nuclear part;nucleoplasm;nucleus;organelle;organelle part		Homo sapiens	1	7
cell part;cytoplasm;cytoplasmic part;cytosol;extracellular membrane-bounded organelle;extracellular organelle;extracellular region part;extracellular vesicular exosome;Golgi apparatus;intracellular membrane-bounded organelle;intracellular organelle;intracellular part;membrane-bounded organelle;membrane-bounded vesicle;org		Homo sapiens	1	7
cell part;cytoplasmic part;early endosome membrane;endoplasmic reticulum;endosomal part;endosome membrane;Golgi apparatus part;Golgi membrane;intracellular membrane-bounded organelle;intracellular organelle;intracellular organelle part;intracellular part;late endosome membrane;macromolecular complex;membrane;r		Homo sapiens	1	7
cell part;cytoplasmic part;endoplasmic reticulum;endoplasmic reticulum-Golgi intermediate compartment membrane;Golgi apparatus;Golgi apparatus part;Golgi membrane;integral to membrane;intracellular membrane-boun	SNARE interactions in vesicular transport	Homo sapiens	1	7
cell part;cell surface;cytoplasmic part;endoplasmic reticulum;integral to membrane;integral to plasma membrane;intracellular membrane-bounded organelle;intracellular organelle;intracellular part;intrinsic to membrane;intrinsic to plasma membrane;membrane part;membrane-bounded organelle;organelle;plasma membrane part		Homo sapiens	1	7
cell part;cell surface;cytoplasm;intracellular part;membrane;plasma membrane		Homo sapiens	1	7
cell part;intracellular membrane-bounded organelle;intracellular organelle;intracellular part;membrane;membrane-bounded organelle;nucleus;organelle		Homo sapiens	1	7
cell part;cytoplasmic part;intracellular membrane-bounded organelle;intracellular organelle;intracellular organelle lumen;intracellular organelle part;intracellular part;large ribosomal subunit;macromolecular complex;membrane;membrane-bounded organelle;membrane-enclosed lumen;mitochondrial inner membrane;mitochondria		Homo sapiens	1	7
cell part;cytoplasmic part;endoplasmic reticulum;endoplasmic reticulum lumen;endoplasmic reticulum part;intracellular membrane-bounded organelle;intracellular non-membrane-bounded organelle;intracellular organelle;intracellular organelle lumen;intracellular organelle part;intracellular part;membrane-bounded organelle;mem		Homo sapiens	1	7
cell part;cytoplasmic part;endoplasmic reticulum;intracellular membrane-bounded organelle;intracellular organelle;intracellular part;membrane;membrane-bounded organelle;organelle;plasma membrane		Homo sapiens	1	7
cell part;cytoplasm;cytoplasmic part;cytosol;intracellular membrane-bounded organelle;intracellular organelle;intracellular organelle part;intracellular part;membrane-bounded organelle;nuclear body;nuclear part;nuclear spec	mRNA surveillance pathway;RNA transport	Homo sapiens	1	7
cell part;coated vesicle;coated vesicle membrane;COPI coated vesicle membrane;COPI-coated vesicle;cytoplasmic membrane-bounded vesicle;cytoplasmic part;cytoplasmic vesicle;cytoplasmic vesicle membrane;cytoplasmic vesicle part;endoplasmic reticulum;endoplasmic reticulum membrane;endoplasmic reticulum part;en		Homo sapiens	1	7
cell part;cytoplasm;cytoskeleton;intracellular membrane-bounded organelle;intracellular non-membrane-bounded organelle;intracellular organelle;intracellular organelle part;intracellular part;membrane-bounded organelle;non-membrane-bounded organelle;nuclear part;nucleoplasm;nucleus;organelle;organelle part		Homo sapiens	1	7
cell part;cytoplasmic part;cytosol;extracellular membrane-bounded organelle;extracellular organelle;extracellular region part;extracellular vesicular exosome;intracellular membrane-bounded organelle;intracellular organelle;intracellular part;membrane-bounded organelle;membrane-bounded vesicle;nucleus;organelle;vesicle		Homo sapiens	1	7
cell part;cytoplasmic part;intracellular organelle part;intracellular part;macromolecular complex;membrane;membrane part;mitochondrial inner membrane;mitochondrial membrane;mitochondrial membrane part;mitochondrial	Alzheimer's disease;Huntington's disease;Oxidative phosphorylation;Parkinson's disease	Homo sapiens	1	7
cell part;cytoplasmic part;cytosol;intracellular part	Purine metabolism	Homo sapiens	1	7
cell part;intracellular organelle part;intracellular part;nuclear part;nucleoplasm;organelle part		Homo sapiens	1	7
cell part;cytoplasm;intracellular part		Homo sapiens	1	7
		Homo sapiens	1	7
cell part;chromosomal part;HULC complex;intracellular membrane-bounded organelle;intracellular non-membrane-bounded organelle;intracellular organelle;intracellular organelle part;intracellular part;macromolecular complex;membrane-bounded organelle;non-membrane-bounded organelle;nuclear part;nucleolus;nucleoplasm;		Homo sapiens	1	7
		Homo sapiens	1	7
		Homo sapiens	1	7
cell part;cytoplasmic part;endoplasmic reticulum membrane;endoplasmic reticulum part;integral to membrane;intracellular organelle part;intracellular part;intrinsic to membrane;membrane;membrane part;organelle membrane;organelle part		Homo sapiens	1	7
cell part;intracellular membrane-bounded organelle;intracellular organelle;intracellular organelle part;intracellular part;membrane-bounded organelle;nuclear body;nuclear part;nuclear speck;nucleoplasm part;nucleus;organelle;organelle part		Homo sapiens	1	7
cell part;cytoplasm;cytoplasmic part;cytosol;intracellular organelle part;intracellular part;membrane;nuclear part;nucleoplasm;organelle part;plasma membrane		Homo sapiens	1	7
cell part;intracellular non-membrane-bounded organelle;intracellular organelle;intracellular organelle part;intracellular part;non-membrane-bounded organelle;nuclear part;nucleolus;organelle;organelle part		Homo sapiens	1	7
cell part;integral to membrane;intrinsic to membrane;membrane part		Homo sapiens	1	7
cell part;cytoplasm;intracellular membrane-bounded organelle;intracellular organelle;intracellular organelle part;intracellular part;macromolecular complex;membrane-bounded organelle;nuclear part;nucleoplasm;nucleus;orga	mRNA surveillance pathway	Homo sapiens	1	7
cell junction;cell part;cell projection;cytoskeletal part;extracellular membrane-bounded organelle;extracellular organelle;extracellular region part;extracellular vesicular exosome;intracellular non-membrane-bounded organelle;intracellular organelle;intracellular organelle part;intracellular part;macromolecular complex;membrane;m		Homo sapiens	1	7
cell part;centriolar satellite;centrosome;cis-Golgi network;cytoplasm;cytoplasmic part;cytoskeletal part;FHF complex;Golgi apparatus;Golgi apparatus part;intracellular membrane-bounded organelle;intracellular non-membrane-bounded organelle;intracellular organelle;intracellular organelle part;intracellular part;macromolecular		Homo sapiens	1	7
cell part;intracellular membrane-bounded organelle;intracellular organelle;intracellular part;membrane-bounded organelle;nucleus;organelle		Homo sapiens	1	7
cell part;cell surface;cytoplasmic part;cytosol;extracellular matrix;extracellular membrane-bounded organelle;extracellular organelle;extracellular region;extracellular region part;extracellular space;extracellular vesicular exoso	Wnt signaling pathway	Homo sapiens	1	7
		Homo sapiens	1	7
cell part;cytoplasm;cytoplasmic part;intracellular membrane-bounded organelle;intracellular organelle;intracellular part;membrane-bounded organelle;mitochondrion;organelle	Ribosome biogenesis in eukaryotes	Homo sapiens	1	7
cell part;cytoplasm;cytoplasmic part;Golgi apparatus;intracellular membrane-bounded organelle;intracellular organelle;intracellular organelle part;intracellular part;membrane-bounded organelle;nuclear part;nucleoplasm;organelle;organelle part		Homo sapiens	1	7
cell part;cytoskeleton;envelope;intermediate filament cytoskeleton;intracellular membrane-bounded organelle;intracellular non-membrane-bounded organelle;intracellular organelle;intracellular organelle part;intracellular part;r	RNA transport	Homo sapiens	1	7
cell part;cytoplasmic part;cytosol;intracellular membrane-bounded organelle;intracellular organelle;intracellular part;membrane-bounded organelle;nucleus;organelle		Homo sapiens	1	7
cell part;cytoplasm;intracellular membrane-bounded organelle;intracellular organelle;intracellular organelle part;intracellular part;membrane-bounded organelle;nuclear part;nucleoplasm;nucleus;organelle;organelle part	Notch signaling pathway	Homo sapiens	1	7
cell part;cytoplasmic part;endoplasmic reticulum lumen;endoplasmic reticulum part;integral to membrane;intracellular organelle lumen;intracellular organelle part;intracellular part;intrinsic to membrane;membrane part;membrane-enclosed lumen;organelle lumen;organelle part		Homo sapiens	1	7
cell part;cytoplasmic part;cytoplasmic vesicle membrane;cytoplasmic vesicle part;intracellular non-membrane-bounded organelle;intracellular organelle;intracellular organelle part;intracellular part;membrane;non-membrane-bounded organelle;nuclear part;nucleolus;nucleoplasm;organelle;organelle membrane;organelle part;plas		Homo sapiens	1	7
cell part;intracellular membrane-bounded organelle;intracellular non-membrane-bounded organelle;intracellular organelle;intracellular organelle part;intracellular part;membrane-bounded organelle;non-membrane-bounded organelle;nuclear part;nucleolus;nucleus;organelle;organelle part		Homo sapiens	1	7
cell part;cytoplasmic part;extracellular membrane-bounded organelle;extracellular organelle;extracellular region part;extracellular vesicular exosome;integral to membrane;intracellular organelle part;intracellular part;intrinsic to membrane;membrane;membrane part;membrane-bounded organelle;membrane-bounded vesicle;mitoc		Homo sapiens	1	7
cell part;cytoplasmic part;cytosol;extracellular matrix;extracellular membrane-bounded organelle;extracellular organelle;extracellular region part;extracellular space;extracellular vesicular exosome;intracellular part;membrane-bounded organelle;membrane-bounded vesicle;organelle;vesicle		Homo sapiens	1	7
cell part;chromatin;chromosomal part;condensed chromosome outer kinetochore;cytoplasm;histone acetyltransferase complex;intracellular membrane-bounded organelle;intracellular organelle;intracellular organelle part;intra	Adherens junction;Cell cycle;Huntington's disease;Jak-STAT signaling pathway;ko05152;Long-term pot	Homo sapiens	1	7
cell part;cytoplasmic part;cytosol;intracellular membrane-bounded organelle;intracellular organelle;intracellular organelle part;intracellular part;membrane-bounded organelle;nuclear part;nucleoplasm;nucleus;organelle;organe	Protein processing in endoplasmic reticulum	Homo sapiens	1	7
cell part;cytoplasmic part;intracellular organelle lumen;intracellular organelle part;intracellular part;membrane-enclosed lumen;mitochondrial matrix;mitochondrial part;organelle lumen;organelle part		Homo sapiens	1	7
cell part;intracellular membrane-bounded organelle;intracellular organelle;intracellular part;membrane-bounded organelle;nucleus;organelle		Homo sapiens	1	7
cell part;cytoplasm;intracellular part		Homo sapiens	1	7
cell part;cytoplasm;extracellular region part;intercellular bridge;intracellular membrane-bounded organelle;intracellular organelle;intracellular organelle part;intracellular part;macromolecular complex;membrane-bounded orga	RNA transport;Spliceosome	Homo sapiens	1	7
cell part;intracellular membrane-bounded organelle;intracellular non-membrane-bounded organelle;intracellular organelle;intracellular organelle part;intracellular part;membrane-bounded organelle;non-membrane-bounded organelle;nuclear body;nuclear part;nuclear speck;nucleolus;nucleoplasm;nucleoplasm part;nucleus;organ		Homo sapiens	1	7
		Homo sapiens	1	7
cell part;cytoplasmic part;endoplasmic reticulum;endoplasmic reticulum membrane;endoplasmic reticulum part;intracellular membrane-bounded organelle;intracellular organelle;intracellular organelle part;intracellular part;membrane;membrane part;membrane-bounded organelle;organelle;organelle membrane;organelle part		Homo sapiens	1	7
cell part;cytoplasm;cytoplasmic part;cytosol;extracellular membrane-bounded organelle;extracellular organelle;extracellular region part;extracellular vesicular exosome;intracellular membrane-bounded organelle;intracellular non-membrane-bounded organelle;intracellular organelle;intracellular organelle part;intracellular part;mem		Homo sapiens	1	7
cell part;chromosomal part;condensed chromosome kinetochore;cytoplasm;cytoplasmic part;cytoskeletal part;intracellular membrane-bounded organelle;intracellular non-membrane-bounded organelle;intracellular organelle;intracellular organelle part;intracellular part;kinetochore;macromolecular complex;membrane-bounded o		Homo sapiens	1	7
extracellular membrane-bounded organelle;extracellular organelle;extracellular region;extracellular region part;extracellular space;extracellular vesicular exosome;membrane-bounded organelle;membrane-bounded vesicle;organelle;vesicle		Homo sapiens	1	7
cell part;cytoplasm;intracellular membrane-bounded organelle;intracellular non-membrane-bounded organelle;intracellular organelle;intracellular organelle part;intracellular part;membrane-bounded organelle;non-membrane-bounded organelle;nuclear part;nucleolus;nucleus;organelle;organelle part		Homo sapiens	1	7
cell part;cytoplasmic part;intracellular membrane-bounded organelle;intracellular organelle;intracellular part;membrane-bounded organelle;mitochondrion;nucleus;organelle		Homo sapiens	1	7
cell part;cytoplasm;cytoplasmic part;cytosol;Golgi apparatus;intracellular membrane-bounded organelle;intracellular organelle;intracellular organelle part;intracellular part;macromolecular complex;membrane-bounded organelle;methylosome;methyltransferase complex;nuclear part;nucleoplasm;nucleus;organelle;organelle part;p		Homo sapiens	1	7
cell part;extracellular membrane-bounded organelle;extracellular organelle;extracellular region part;extracellular vesicular exosome;membrane;membrane-bounded organelle;membrane-bounded vesicle;organelle;vesicle	Purine metabolism;Thiamine metabolism	Homo sapiens	1	7
cell part;cytoplasmic part;endoplasmic reticulum membrane;endoplasmic reticulum part;integral to membrane;intracellular organelle part;intracellular part;intrinsic to membrane;membrane;membrane part;organelle membrane;organelle part		Homo sapiens	1	7
cell part;cytoplasmic part;intracellular membrane-bounded organelle;intracellular non-membrane-bounded organelle;intracellular organelle;intracellular organelle part;intracellular part;membrane-bounded organelle;mitochondrion;non-membrane-bounded organelle;nuclear part;nucleolus;organelle;organelle part		Homo sapiens	1	7

cell part;cytoplasm;cytoplasmic part;cytosol;intracellular part	Drug metabolism - other enzymes;Purine metabolism;Pyrimidine metabolism	Homo sapiens	1	7
cell part;cytoplasmic part;intracellular non-membrane-bounded organelle;intracellular organelle;intracellular organelle part;intracellular part;large ribosomal subunit;macromolecular complex;membrane;mitochondrial inner me	Ribosome	Homo sapiens	1	7
catalytic step 2 spliceosome;cell part;cytoplasm;intracellular organelle part;intracellular part;macromolecular complex;nuclear body;nuclear part;nuclear speck;nucleoplasm part;organelle part;ribonucleoprotein complex;splic	Spliceosome	Homo sapiens	1	7
cell part;cytoplasmic part;cytosol;intracellular membrane-bounded organelle;intracellular organelle;intracellular part;membrane-bounded organelle;organelle	Drug metabolism - other enzymes;Pyrimidine metabolism	Homo sapiens	1	7
cell part;chromatin remodeling complex;cytoplasmic part;Golgi apparatus;Golgi apparatus part;histone methyltransferase complex;intracellular membrane-bounded organelle;intracellular organelle;intracellular organelle part;intracellular part;macromolecular complex;membrane-bounded organelle;methyltransferase complex;nuc		Homo sapiens	1	7
cell part;cytoplasmic part;endoplasmic reticulum part;integral to membrane;integral to plasma membrane;intracellular organelle part;intracellular part;intrinsic to membrane;intrinsic to plasma membrane;macromolecular complex;membrane;membrane part;oligosaccharyltransferase complex;organelle part;plasma membrane;plas		Homo sapiens	1	7
cell part;cytoplasm;cytoplasmic part;cytosol;intracellular membrane-bounded organelle;intracellular organelle;intracellular organelle lumen;intracellular organelle part;intracellular part;membrane-bounded organelle;membrane-enclosed lumen;mitochondrial matrix;mitochondrial part;mitochondrion;nucleus;organelle;organelle lume		Homo sapiens	1	7
cell part;cytoplasm;cytoplasmic membrane-bounded vesicle;cytoplasmic part;cytoplasmic vesicle;endosomal part;endosome;endosome membrane;intracellular membrane-bounded organelle;intracellular organelle;intracellular organelle part;intracellular part;late endosome;late endosome membrane;lysosomal membrane;lysoso		Homo sapiens	1	7
cell part;cytoplasmic membrane-bounded vesicle;cytoplasmic part;cytoplasmic vesicle;endocytic vesicle;endosomal part;endosome;endosome membrane;HOPS complex;intracellular membrane-bounded organelle;intracellular organelle;intracellular organelle part;intracellular part;late endosome;late endosome membrane;lysoso		Homo sapiens	1	7
catalytic step 2 spliceosome;cell part;intracellular organelle part;intracellular part;macromolecular complex;nuclear part;organelle part;ribonucleoprotein complex;spliceosomal complex		Homo sapiens	1	7
cell part;cytoplasm;histone methyltransferase complex;intracellular membrane-bounded organelle;intracellular non-membrane-bounded organelle;intracellular organelle;intracellular organelle part;intracellular part;macromolecular complex;membrane-bounded organelle;methyltransferase complex;MLL1 complex;non-membrane-		Homo sapiens	1	7
cell part;cytoplasm;intracellular membrane-bounded organelle;intracellular organelle;intracellular organelle part;intracellular part;membrane-bounded organelle;nuclear part;nucleoplasm;nucleus;organelle;organelle part		Homo sapiens	1	7
cell part;cytoplasmic part;Golgi apparatus part;Golgi membrane;Golgi transport complex;intracellular organelle part;intracellular part;macromolecular complex;membrane;organelle membrane;organelle part;protein complex		Homo sapiens	1	7
cell part;cytoplasmic part;endoplasmic reticulum lumen;endoplasmic reticulum part;intracellular organelle lumen;intracellular organelle part;intracellular part;membrane;membrane-enclosed lumen;organelle lumen;organelle part		Homo sapiens	1	7
cell part;integral to membrane;intrinsic to membrane;membrane part		Homo sapiens	1	7
cell part;cytoplasmic part;intracellular non-membrane-bounded organelle;intracellular organelle;intracellular organelle part;intracellular part;macromolecular complex;membrane;mitochondrial inner membrane;mitochondrial membrane;mitochondrial part;non-membrane-bounded organelle;organelle;organelle inner membrane;orga		Homo sapiens	1	7
		Homo sapiens	1	7
cell part;centromeric heterochromatin;chromatin;chromosomal part;chromosome, centromeric region;heterochromatin;intracellular membrane-bounded organelle;intracellular organelle;intracellular organelle part;intracellular part;membrane-bounded organelle;nucleus;organelle;organelle part		Homo sapiens	1	7
cell part;cytoplasmic part;cytosol;extracellular membrane-bounded organelle;extracellular organelle;extracellular region part;extracellular vesicular exosome;intracellular part;membrane-bounded organelle;membrane-bounded vesicle;organelle;vesicle		Homo sapiens	1	7
cell part;cytoplasmic part;intracellular membrane-bounded organelle;intracellular organelle;intracellular organelle part;intracellular part;membrane;membrane-bounded organelle;microbody;microbody membrane;microbody p	Peroxisome	Homo sapiens	1	7
cell part;cytoplasmic part;cytosol;intracellular membrane-bounded organelle;intracellular organelle;intracellular part;membrane-bounded organelle;mitochondrion;organelle		Homo sapiens	1	7
		Homo sapiens	1	7
cell part;clathrin coat;clathrin vesicle coat;coated pit;cytoplasmic part;cytoplasmic vesicle part;intracellular;intracellular organelle part;intracellular part;macromolecular complex;membrane;membrane coat;membrane part;organelle part;plasma membrane;protein complex;vesicle coat		Homo sapiens	1	7
cell part;cytoplasm;granular component;intracellular membrane-bounded organelle;intracellular non-membrane-bounded organelle;intracellular organelle;intracellular organelle part;intracellular part;membrane-bounded organelle;non-membrane-bounded organelle;nuclear part;nucleolar part;nucleolus;nucleoplasm;nucleus;organ		Homo sapiens	1	7
cell part;centrosome;cytoplasm;cytoplasmic part;cytoskeletal part;cytoskeleton;intracellular non-membrane-bounded organelle;intracellular organelle;intracellular organelle part;intracellular part;microtubule cytoskeleton;microtubule organizing center;non-membrane-bounded organelle;nuclear part;nucleolus;nucleoplasm;organe		Homo sapiens	1	7
actin cytoskeleton;cell part;cytoplasm;cytoskeleton;intracellular non-membrane-bounded organelle;intracellular organelle;intracellular part;non-membrane-bounded organelle;organelle		Homo sapiens	1	7
cell part;cytoplasm;cytoplasmic part;cytosol;early endosome;endosomal part;endosome;endosome membrane;extracellular membrane-bounded organelle;extracellular organelle;extracellular region part;extracellular vesicular exosome;intracellular membrane-bounded organelle;intracellular organelle;intracellular organelle part;int		Homo sapiens	1	7
adherens junction;anchoring junction;cell junction;cell part;cell-substrate adherens junction;cell-substrate junction;cytoplasm;cytoplasmic mRNA processing body;cytoplasmic part;focal adhesion;intracellular membrane-bounded organelle;intracellular non-membrane-bounded organelle;intracellular organelle;intracellular part;ma		Homo sapiens	1	7
cell part;intracellular membrane-bounded organelle;intracellular organelle;intracellular part;membrane-bounded organelle;nucleus;organelle		Homo sapiens	1	7
cell part;cytoplasmic part;early endosome;endosome;intracellular membrane-bounded organelle;intracellular organelle;intracellular part;membrane-bounded organelle;organelle;recycling endosome;vesicle		Homo sapiens	1	7
cell part;cytoplasmic part;intracellular membrane-bounded organelle;intracellular organelle;intracellular part;macromolecular complex;membrane-bounded organelle;methyltransferase complex;nucleus;organelle;protein complex;tRNA (m1A) methyltransferase complex;tRNA methyltransferase complex		Homo sapiens	1	7
cell part;cytoplasm;cytoplasmic part;cytosol;early endosome;endosomal part;endosome;endosome membrane;ESCRT I complex;extracellular membrane-bounded organelle;extracellular organelle;extracellular region part;ext	Endocytosis	Homo sapiens	1	7
cell part;cytoplasmic part;cytosol;intracellular organelle part;intracellular part;macromolecular complex;nuclear part;organelle part;ribonucleoprotein complex;small nucleolar ribonucleoprotein complex;spliceosomal complex	RNA degradation;Spliceosome	Homo sapiens	1	7
adherens junction;anchoring junction;cell division site part;cell junction;cell part;cell-substrate adherens junction;cell-substrate junction;cleavage furrow;cytoplasmic part;cytoplasmic vesicle membrane;cytoplasmic vesicle part;endoplasmic reticulum membrane;endoplasmic reticulum part;endosome;extracellular membrane-bou		Homo sapiens	1	7
autophagic vacuole;cell part;cytoplasm;cytoplasmic membrane-bounded vesicle;cytoplasmic part;cytoplasmic vesicle;cytoplasmic vesicle membrane;cytoplasmic vesicle part;endocytic vesicle;endocytic vesicle membrane;e	Hedgehog signaling pathway	Homo sapiens	1	7
cell part;cytoplasmic part;endoplasmic reticulum part;integral to endoplasmic reticulum membrane;integral to membrane;integral to organelle membrane;intracellular organelle part;intracellular part;intrinsic to endoplasmic ret	Protein processing in endoplasmic reticulum	Homo sapiens	1	7
		Homo sapiens	1	7
cell part;cytoplasm;intracellular part		Homo sapiens	1	7
cell part;cytoplasmic mRNA processing body;cytoplasmic part;cytosol;intracellular non-membrane-bounded organelle;intracellular organelle;intracellular part;macromolecular complex;non-membrane-bounded organelle;organelle;ribonucleoprotein complex;RNA granule		Homo sapiens	1	7
cell part;cytoplasm;cytoplasmic part;cytoskeletal part;cytosol;intracellular membrane-bounded organelle;intracellular organelle;intracellular organelle part;intracellular part;macromolecular complex;membrane-bounded organelle;microtubule organizing center;nuclear part;nucleoplasm;nucleus;organelle;organelle part;perinuclear		Homo sapiens	1	7
adherens junction;anchoring junction;cell junction;cell part;cell-substrate adherens junction;cell-substrate junction;cytoplasmic part;focal adhesion;intracellular membrane-bounded organelle;intracellular non-membrane-bour	Ribosome biogenesis in eukaryotes	Homo sapiens	1	7
cell part;cytoplasmic part;cytoskeleton;intermediate filament cytoskeleton;intracellular membrane-bounded organelle;intracellular non-membrane-bounded organelle;intracellular organelle;intracellular organelle part;intracellular part;macromolecular complex;membrane;membrane-bounded organelle;mitochondrial inner membran		Homo sapiens	1	7
actomyosin;cell body;cell part;cell projection;cell projection part;cilium;cytoplasm;cytoplasmic membrane-bounded vesicle;cytoplasmic part;cytoplasmic vesicle;cytoskeletal part;cytosol;extracellular membrane-bounded organelle;extracellular organelle;extracellular region part;extracellular vesicular exosome;filopodium tip;Golgi		Homo sapiens	1	7
cell part;chromosomal part;chromosome, telomeric region;cytoplasm;intracellular;intracellular membrane-bounded organelle;intracellular organelle;intracellular organelle part;intracellular part;macromolecular complex;membrane;membrane-bounded organelle;nucleus;organelle;organelle part;protein complex;TORC1 complex;TO		Homo sapiens	1	7
cell junction;cell part;cytoplasmic part;intracellular membrane-bounded organelle;intracellular organelle;intracellular organelle part;intracellular part;macromolecular complex;membrane;membrane-bounded organelle;mitochondrial inner membrane;mitochondrial membrane;mitochondrial part;mitochondrial small ribosomal subun		Homo sapiens	1	7
cell part;cytoplasmic part;cytosol;intracellular part	Mineral absorption	Homo sapiens	1	8
cell part;clathrin-coated vesicle;coated vesicle;cytoplasm;cytoplasmic membrane-bounded vesicle;cytoplasmic part;cytoplasmic vesicle;cytosol;cytosolic part;extracellular membrane-bounded organelle;extracellular organell	Inositol phosphate metabolism;Phosphatidylinositol signaling system	Homo sapiens	1	8
cell part;cytoplasmic part;extracellular membrane-bounded organelle;extracellular organelle;extracellular region part;extracellular vesicular exosome;intracellular membrane-bounded organelle;intracellular organelle;intracellula	Alzheimer's disease;Huntington's disease;Oxidative phosphorylation;Parkinson's disease	Homo sapiens	1	8
apical plasma membrane;cell body;cell part;cell projection;cell projection membrane;cell projection part;cytoplasm;extracellular membrane-bounded organelle;extracellular organelle;extracellular region part;extracellular space;extracellular vesicular exosome;filopodium;integral to membrane;integral to plasma membrane;intracell		Homo sapiens	1	8
cell part;cytoplasm;cytoplasmic part;cytosol;intracellular membrane-bounded organelle;intracellular organelle;intracellular part;membrane-bounded organelle;nucleus;organelle	Amyotrophic lateral sclerosis (ALS)	Homo sapiens	1	8
cell part;cytoplasmic part;endoplasmic reticulum membrane;endoplasmic reticulum part;integral to membrane;intracellular organelle part;intracellular part;intrinsic to membrane;membrane;membrane part;membrane-enclose	Arachidonic acid metabolism	Homo sapiens	1	8
cell part;cell surface;integral to membrane;intrinsic to membrane;membrane;membrane part;plasma membrane	Apoptosis;Cytokine-cytokine receptor interaction;Measles;Natural killer cell mediated cytotoxicity	Homo sapiens	1	8
axon hillock;axon part;cell part;cell projection part;centrosome;chromosomal part;chromosome passenger complex;chromosome, centromeric region;condensed chromosome, centromeric region;condensed nuclear chromos	Oocyte meiosis	Homo sapiens	1	8
adherens junction;anchoring junction;cell junction;cell part;cell surface;cell-substrate adherens junction;cell-substrate junction;cytoplasm;extracellular membrane-bounded organelle;extracellular organelle;extracellular region part;extracellular vesicular exosome;focal adhesion;intracellular part;membrane-bounded organelle;mem		Homo sapiens	1	8
cell part;cytoplasm;intracellular membrane-bounded organelle;intracellular organelle;intracellular organelle part;intracellular part;membrane part;membrane raft;membrane-bounded organelle;nuclear body;nuclear part;nucleo	Bladder cancer;Pathways in cancer	Homo sapiens	1	8
axon part;axon terminus;cell body;cell part;cell projection;cell projection part;cytoplasm;dendritic spine;intracellular membrane-bounded organelle;intracellular organelle;intracellular part;membrane;membrane-bounded organelle;neuron projection;neuron projection terminus;neuron spine;neuronal cell body;nucleus;organelle;plas		Homo sapiens	1	8
cell part;chromosomal part;condensed chromosome kinetochore;cytoskeletal part;intracellular membrane-bounded organelle;intracellular non-membrane-bounded organelle;intracellular organelle;intracellular organelle part;intracellular part;kinetochore;macromolecular complex;membrane-bounded organelle;microtubule;non-me		Homo sapiens	1	8
cell part;cytoplasm;intracellular part		Homo sapiens	1	8
cell part;centrosome;cytoplasmic part;cytoskeletal part;intracellular membrane-bounded organelle;intracellular non-membrane-bounded organelle;intracellular organelle;intracellular organelle part;intracellular part;macromolec	mRNA surveillance pathway	Homo sapiens	1	8
cell part;chromatin;chromosomal part;intracellular membrane-bounded organelle;intracellular organelle;intracellular organelle part;intracellular part;macromolecular complex;membrane-bounded organelle;nuclear chromatin;nuclear chromosome part;nuclear part;nucleoplasm;nucleoplasm part;nucleus;organelle;organelle part;pro		Homo sapiens	1	8
catalytic step 2 spliceosome;cell part;intracellular organelle part;intracellular part;macromolecular complex;nuclear part;nucleoplasm;organelle part;ribonucleoprotein complex;spliceosomal complex	Spliceosome	Homo sapiens	1	8
cell part;cytoplasmic part;cytosol;early endosome;endosome;Golgi apparatus;intracellular membrane-bounded organelle;intracellular non-membrane-bounded organelle;intracellular organelle;intracellular organelle part;intrac	Regulation of actin cytoskeleton	Homo sapiens	1	8
cell part;cytoplasmic part;endoplasmic reticulum;endoplasmic reticulum lumen;endoplasmic reticulum part;extracellular matrix;extracellular region part;extracellular space;intracellular membrane-bounded organelle;intracellular organelle;intracellular organelle lumen;intracellular organelle part;intracellular part;macromolecular com		Homo sapiens	1	8
cell part;cytoskeletal part;intracellular organelle part;intracellular part;macromolecular complex;MMXD complex;organelle part;protein complex		Homo sapiens	1	8
cell part;cytoplasmic part;early endosome membrane;endosomal part;endosome membrane;extracellular region;extracellular region part;extracellular space;intracellular organelle part;intracellular part;membrane;organelle me	Wnt signaling pathway	Homo sapiens	1	8
cell part;cytoplasm;extracellular membrane-bounded organelle;extracellular organelle;extracellular region part;extracellular vesicular exosome;intracellular part;membrane-bounded organelle;membrane-bounded vesicle;orga	Glycerophospholipid metabolism	Homo sapiens	1	8
CBM complex;cell part;cytoplasm;cytoplasmic microtubule;cytoplasmic part;cytoskeletal part;cytosol;immunological synapse;intracellular membrane-bounded organelle;intracellular organelle;intracellular organelle part;intrac	B cell receptor signaling pathway;ko05152;T cell receptor signaling pathway	Homo sapiens	1	8
cell part;extracellular membrane-bounded organelle;extracellular organelle;extracellular region;extracellular region part;extracellular space;extracellular vesicular exosome;macromolecular complex;membrane attack complex;	Complement and coagulation cascades;Prion diseases;Staphylococcus aureus infection;Systemic lupus	Homo sapiens	1	8
cell part;cell surface;integral to membrane;integral to plasma membrane;intrinsic to membrane;intrinsic to plasma membrane;macromolecular complex;membrane part;MHC class I protein complex;MHC protein complex;plasma membrane part;protein complex		Homo sapiens	0.9949	8
cell part;collagen;collagen type III;cytoplasmic part;endoplasmic reticulum lumen;endoplasmic reticulum part;extracellular matrix;extracellular matrix part;extracellular region;extracellular region part;extracellular space;fibrillar	Amoebiasis;ECM-receptor interaction;Focal adhesion;Protein digestion and absorption	Homo sapiens	1	8
alphav-beta3 integrin-vitronectin complex;blood microparticle;cell part;extracellular matrix;extracellular membrane-bounded organelle;extracellular organelle;extracellular region;extracellular region part;extracellular space;ext	ECM-receptor interaction;Focal adhesion	Homo sapiens	1	8
cell part;cytoplasm;cytoplasmic part;extracellular membrane-bounded organelle;extracellular organelle;extracellular region part;extracellular vesicular exosome;intracellular membrane-bounded organelle;intracellular organelle	Other glycan degradation	Homo sapiens	1	8
actin cytoskeleton;cell body;cell part;chylomicron;chylomicron remnant;clathrin coated vesicle membrane;clathrin-coated endocytic vesicle membrane;coated vesicle membrane;cytoplasm;cytoplasmic membrane-bounded v	Fat digestion and absorption;Vitamin digestion and absorption	Homo sapiens	1	8
cell part;cytoplasmic part;intracellular membrane-bounded organelle;intracellular organelle;intracellular organelle lumen;intracellular organelle part;intracellular part;membrane;membrane-bounded organelle;membrane-enclos	Benzoate degradation;Biosynthesis of unsaturated fatty acids;Fatty acid metabolism;Peroxisome;PPAR	Homo sapiens	1	8
cell part;cytoplasm;cytoplasmic part;cytosol;intracellular membrane-bounded organelle;intracellular non-membrane-bounded organelle;intracellular organelle;intracellular organelle part;intracellular part;macromolecular comp	Protein export	Homo sapiens	1	8
cell part;cytoplasmic part;integral to membrane;intracellular membrane-bounded organelle;intracellular organelle;intracellular organelle part;intracellular part;intrinsic to membrane;membrane;membrane part;membrane-bounc	Alzheimer's disease;Cardiac muscle contraction;Huntington's disease;Oxidative phosphorylation;Parkin	Homo sapiens	1	8
cell part;cytoplasm;cytoplasmic part;cytosol;intracellular part	Hepatitis C	Homo sapiens	1	8
adherens junction;anchoring junction;cell junction;cell part;cell surface;cell-substrate adherens junction;cell-substrate junction;cytoplasm;extracellular membrane-bounded organelle;extracellular organelle;extracellular region	Cell adhesion molecules (CAMs)	Homo sapiens	1	8
cell part;chromatin;chromosomal part;intracellular organelle part;intracellular part;macromolecular complex;nuclear chromatin;nuclear chromosome part;nuclear part;nucleoplasm;nucleoplasm part;organelle part;protein comp	Osteoclast differentiation	Homo sapiens	1	8
apical cortex;cell cortex part;cell part;cytoplasmic part;extracellular membrane-bounded organelle;extracellular organelle;extracellular region part;extracellular vesicular exosome;hydrogen:potassium-exchanging ATPase com	Lysosome	Homo sapiens	1	8
cell part;cytoplasmic part;Golgi apparatus;intracellular membrane-bounded organelle;intracellular organelle;intracellular organelle part;intracellular part;membrane-bounded organelle;mitochondrion;nuclear part;nucleoplasm;r	Base excision repair;DNA replication;Mismatch repair;Nucleotide excision repair	Homo sapiens	1	8
actin cytoskeleton;axon;axon part;axon terminus;cell body;cell part;cell projection;cell projection part;clathrin-coated vesicle;coated vesicle;cytoplasm;cytoplasmic membrane-bounded vesicle;cytoplasmic part;cytoplasmic v	Amoebiasis;Amyotrophic lateral sclerosis (ALS);Endocytosis;ko05152;Phagosome;Vasopressin-regulate	Homo sapiens	1	8
cell part;intracellular membrane-bounded organelle;intracellular organelle;intracellular part;membrane-bounded organelle;nucleus;organelle		Homo sapiens	1	8
cell part;cytoplasm;intracellular membrane-bounded organelle;intracellular organelle;intracellular part;membrane-bounded organelle;nucleus;organelle		Homo sapiens	1	8
cell part;cytoplasm;extracellular membrane-bounded organelle;extracellular organelle;extracellular region part;extracellular vesicular exosome;internal side of plasma membrane;intracellular part;membrane part;membrane-bo	Riboflavin metabolism	Homo sapiens	1	8
cell part;cytoplasm;cytoplasmic part;cytosol;extracellular membrane-bounded organelle;extracellular organelle;extracellular region part;extracellular vesicular exosome;intracellular membrane-bounded organelle;intracellular c	Proteasome	Homo sapiens	1	8
cell part;cytoplasm;cytoplasmic part;cytosol;extracellular membrane-bounded organelle;extracellular organelle;extracellular region part;extracellular vesicular exosome;Golgi apparatus;intracellular membrane-bounded organe	Proteasome	Homo sapiens	1	8
cell part;cytoplasmic part;intracellular non-membrane-bounded organelle;intracellular organelle;intracellular organelle part;intracellular part;mitochondrial nucleoid;mitochondrial part;non-membrane-bounded organelle;nuclea	Base excision repair	Homo sapiens	1	8
cell part;cytoplasmic part;intracellular organelle lumen;intracellular organelle part;intracellular part;membrane-enclosed lumen;mitochondrial matrix;mitochondrial part;organelle lumen;organelle part	Valine, leucine and isoleucine degradation	Homo sapiens	1	8
basement membrane;cell part;cytoplasmic part;endoplasmic reticulum;extracellular matrix;extracellular matrix part;extracellular membrane-bounded organelle;extracellular organelle;extracellular region;extracellular region pa	ECM-receptor interaction;Focal adhesion;Malaria;Phagosome;TGF-beta signaling pathway	Homo sapiens	1	8
cell part;cytoplasm;intracellular membrane-bounded organelle;intracellular organelle;intracellular organelle part;intracellular part;membrane-bounded organelle;nuclear part;nucleoplasm;nucleus;organelle;organelle part		Homo sapiens	1	8
apical plasma membrane;cell junction;cell leading edge;cell part;cell-cell junction;cytoplasm;cytoplasmic part;cytoskeleton;cytosol;endosome;extracellular membrane-bounded organelle;extracellular organelle;extracellular re	Endocytosis;Insulin signaling pathway;Tight junction	Homo sapiens	1	8
actin cytoskeleton;cell part;cytoplasm;cytoplasmic part;cytoskeleton;cytosol;extracellular membrane-bounded organelle;extracellular organelle;extracellular region part;extracellular vesicular exosome;Golgi apparatus;intracel	Proteasome	Homo sapiens	1	8
beta-catenin destruction complex;cell part;cytoplasmic part;cytosol;intracellular part;macromolecular complex;protein complex	Chemokine signaling pathway	Homo sapiens	1	8
cell part;cytoplasm;intracellular membrane-bounded organelle;intracellular organelle;intracellular organelle part;intracellular part;membrane-bounded organelle;nuclear part;nucleoplasm;nucleus;organelle;organelle part	mRNA surveillance pathway	Homo sapiens	1	8
cell body;cell part;cell projection;cytoplasmic membrane-bounded vesicle;cytoplasmic part;cytoplasmic vesicle;intracellular membrane-bounded organelle;intracellular organelle;intracellular organelle part;intracellular part;macromolecular complex;membrane part;membrane-bounded organelle;membrane-bounded vesicle;neuro		Homo sapiens	1	8
cell part;centrosome;cytoplasmic part;cytoskeletal part;extracellular membrane-bounded organelle;extracellular organelle;extracellular region part;extracellular vesicular exosome;integral to membrane;integral to plasma membrane;intracellular membrane-bounded organelle;intracellular non-membrane-bounded organelle;intrace		Homo sapiens	1	8
cell part;cytoplasm;cytoplasmic part;cytosol;intracellular membrane-bounded organelle;intracellular organelle;intracellular organelle part;intracellular part;membrane-bounded organelle;nuclear part;nucleoplasm;nucleus;orga	Alzheimer's disease;Apoptosis	Homo sapiens	1	8
adherens junction;anchoring junction;cell junction;cell part;cell-substrate adherens junction;cell-substrate junction;coated vesicle membrane;cytoplasm;cytoplasmic part;cytoplasmic vesicle membrane;cytoplasmic vesicle pa	Antigen processing and presentation	Homo sapiens	1	8
adherens junction;anchoring junction;cell junction;cell part;cell-substrate adherens junction;cell-substrate junction;cytoplasm;cytoplasmic part;cytosol;cytosolic small ribosomal subunit;focal adhesion;intracellular membrane-	Ribosome	Homo sapiens	1	8
cell part;cell projection;cytoplasm;dendrite;fibrillar center;intracellular membrane-bounded organelle;intracellular non-membrane-bounded organelle;intracellular organelle;intracellular organelle part;intracellular part;macromol	RNA transport	Homo sapiens	1	8
blood microparticle;cell part;cytoplasmic membrane-bounded vesicle lumen;cytoplasmic part;cytoplasmic vesicle part;cytosol;cytosolic part;cytosolic small ribosomal subunit;endocytic vesicle lumen;extracellular membrane-	African trypanosomiasis;Malaria	Homo sapiens	1	8
cell part;cytoplasmic part;extracellular membrane-bounded organelle;extracellular organelle;extracellular region part;extracellular vesicular exosome;Golgi apparatus;intracellular membrane-bounded organelle;intracellular organelle;intracellular part;membrane;membrane-bounded organelle;membrane-bounded vesicle;organelle;		Homo sapiens	1	8
cell part;cytoplasmic part;endoplasmic reticulum membrane;endoplasmic reticulum part;integral to endoplasmic reticulum membrane;integral to membrane;integral to organelle membrane;intracellular organelle part;intracellu	ABC transporters;Antigen processing and presentation;Phagosome;Primary immunodeficiency	Homo sapiens	1	8
cell part;cytoplasm;intracellular membrane-bounded organelle;intracellular organelle;intracellular organelle part;intracellular part;macromolecular complex;membrane-bounded organelle;nuclear body;nuclear part;nuclear speck;nucleoplasm;nucleoplasm part;nucleus;organelle;organelle part;ribonucleoprotein complex;spliceosom		Homo sapiens	1	8
cell part;cytoplasm;cytoplasmic part;cytosol;intracellular membrane-bounded organelle;intracellular organelle;intracellular organelle part;intracellular part;membrane-bounded organelle;nuclear part;nucleoplasm;nucleus;orga	MAPK signaling pathway;Non-small cell lung cancer;Pathways in cancer	Homo sapiens	1	8
cell part;chromosomal part;condensed chromosome kinetochore;cytoplasmic part;cytoskeletal part;cytosol;intracellular membrane-bounded organelle;intracellular non-membrane-bounded organelle;intracellular organelle;int	Cell cycle;Cell cycle - yeast;Meiosis - yeast;Oocyte meiosis;Progesterone-mediated oocyte maturation	Homo sapiens	1	8
cell part;cytoplasmic part;integral to membrane;intracellular organelle part;intracellular part;intrinsic to membrane;membrane;membrane part;mitochondrial membrane;mitochondrial outer membrane;mitochondrial part;organelle membrane;organelle outer membrane;organelle part;outer membrane		Homo sapiens	1	8
cell part;cytoplasmic membrane-bounded vesicle;cytoplasmic part;cytoplasmic vesicle;cytoplasmic vesicle membrane;cytoplasmic vesicle part;early endosome;endocytic vesicle;endocytic vesicle membrane;endosome;Golgi apparatus part;intracellular membrane-bounded organelle;intracellular organelle;intracellular organelle p		Homo sapiens	1	8
cell part;cytoplasmic part;endoplasmic reticulum membrane;endoplasmic reticulum part;integral to endoplasmic reticulum membrane;integral to membrane;integral to organelle membrane;intracellular organelle part;intracellular part;intrinsic to endoplasmic reticulum membrane;intrinsic to membrane;intrinsic to organelle membra		Homo sapiens	1	8
CD95 death-inducing signaling complex;cell body;cell part;cell projection;cytoplasm;cytoplasmic part;cytoskeletal part;cytoskeleton;cytosol;death-inducing signaling complex;intracellular membrane-bounded organelle;intrac	Alzheimer's disease;Apoptosis;Chagas disease (American trypanosomiasis);Huntington's disease;ko05	Homo sapiens	1	8
BRISC complex;cell part;cytoplasm;intracellular organelle part;intracellular part;macromolecular complex;nuclear part;organelle part;protein complex		Homo sapiens	1	8
cell part;cytoplasmic part;cytosol;endoplasmic reticulum part;integral to membrane;intracellular organelle part;intracellular part;intrinsic to membrane;macromolecular complex;membrane;membrane part;organelle part;protein complex;Sec61 translocon complex;translocon complex		Homo sapiens	1	8
extracellular membrane-bounded organelle;extracellular organelle;extracellular region part;extracellular space;extracellular vesicular exosome;membrane-bounded organelle;membrane-bounded vesicle;organelle;vesicle		Homo sapiens	1	8
cell body;cell part;cell projection;cytoplasmic part;cytosol;dendrite;endoplasmic reticulum membrane;endoplasmic reticulum part;extracellular membrane-bounded organelle;extracellular organelle;extracellular region part;ext	Insulin signaling pathway;mTOR signaling pathway	Homo sapiens	1	8
apical plasma membrane;cell junction;cell part;cell projection;cell surface;clathrin coated vesicle membrane;clathrin-coated vesicle;coated vesicle;coated vesicle membrane;cytoplasmic membrane-bounded vesicle;cytoplasr	Phagosome;SNARE interactions in vesicular transport	Homo sapiens	1	8
cell part;centrosome;chromosomal part;condensed chromosome kinetochore;cytoplasm;cytoplasmic part;cytoskeletal part;cytosol;intracellular non-membrane-bounded organelle;intracellular organelle;intracellular organelle part;intracellular part;kinetochore;macromolecular complex;membrane;microtubule organizing center;non		Homo sapiens	1	8
cell part;cell projection;cytoplasmic membrane-bounded vesicle;cytoplasmic part;cytoplasmic vesicle;cytosol;endoplasmic reticulum;Golgi apparatus;intracellular membrane-bounded organelle;intracellular organelle;intracellular part;lamellipodium;membrane;membrane-bounded organelle;membrane-bounded vesicle;organelle;p		Homo sapiens	1	8
cell part;cytoplasm;cytoplasmic part;Golgi apparatus;intracellular membrane-bounded organelle;intracellular organelle;intracellular organelle part;intracellular part;membrane-bounded organelle;nuclear part;nucleoplasm;organelle;organelle part		Homo sapiens	1	8
cell division site part;cell part;centriole;centrosome;cleavage furrow;cytoplasmic part;cytoskeletal part;extracellular region part;intercellular bridge;intracellular non-membrane-bounded organelle;intracellular organelle;intracellular organelle part;intracellular part;membrane;microtubule organizing center;microtubule organizing cent		Homo sapiens	1	8
		Homo sapiens	1	8
cell part;cytoplasmic part;early endosome;endosome;intracellular membrane-bounded organelle;intracellular organelle;intracellular part;membrane-bounded organelle;organelle		Homo sapiens	1	8
cell part;integral to membrane;intrinsic to membrane;membrane part		Homo sapiens	1	8
cell body;cell junction;cell part;cell projection;cytoplasm;cytoplasmic part;cytoskeletal part;cytosol;dendrite;intracellular membrane-bounded organelle;intracellular non-membrane-bounded organelle;intracellular organelle;intracellular organelle part;intracellular part;macromolecular complex;membrane-bounded organelle;microtu		Homo sapiens	1	8
cell part;cytoplasm;cytoplasmic microtubule;cytoplasmic part;cytoskeletal part;endoplasmic reticulum;endoplasmic reticulum membrane;endoplasmic reticulum part;Golgi apparatus;integral to membrane;intracellular membrane-bounded organelle;intracellular organelle;intracellular organelle part;intracellular part;intrinsic to mem		Homo sapiens	1	8
		Homo sapiens	1	8
cell part;cytoplasmic part;extracellular region part;extracellular space;Golgi apparatus part;integral to Golgi membrane;integral to membrane;integral to organelle membrane;intracellular organelle part;intracellular part;intrinsic to Golgi membrane;intrinsic to membrane;intrinsic to organelle membrane;membrane;membrane part;nu		Homo sapiens	1	8
cell part;cytoplasm;cytoskeletal part;extracellular membrane-bounded organelle;extracellular organelle;extracellular region part;extracellular vesicular exosome;intermediate filament;intracellular organelle part;intracellular part;macromolecular complex;membrane-bounded organelle;membrane-bounded vesicle;organelle;organell		Homo sapiens	1	8
cell part;cell projection;lamellipodium		Homo sapiens	1	8

cell part;cytoplasmic mRNA processing body;cytoplasmic part;cytosol;intracellular membrane-bounded organelle;intracellular non-membrane-bounded organelle;intracellular organelle;intracellular organelle part;intracellular p	RNA degradation	Homo sapiens	1	8
cell part;cytoplasm;cytoplasmic part;intracellular membrane-bounded organelle;intracellular organelle;intracellular organelle part;intracellular part;membrane-bounded organelle;mitochondrion;nuclear part;nucleoplasm;organelle;organelle part		Homo sapiens	1	8
cell part;cytoplasmic part;cytosol;intracellular part	Butanoate metabolism	Homo sapiens	1	8
cell part;cytoplasmic membrane-bounded vesicle;cytoplasmic part;cytoplasmic vesicle;endocytic vesicle;endomembrane system;endosomal part;endosome membrane;extracellular membrane-bounded organelle;extracellula	Phagosome	Homo sapiens	1	8
cell part;cytoplasmic part;endoplasmic reticulum lumen;endoplasmic reticulum part;intracellular organelle lumen;intracellular organelle part;intracellular part;membrane-enclosed lumen;organelle lumen;organelle part		Homo sapiens	1	8
alpha-amino-3-hydroxy-5-methyl-4-isoxazolepropionic acid selective glutamate receptor complex;cell part;integral to membrane;intrinsic to membrane;ionotropic glutamate receptor complex;macromolecular complex;membrane part;plasma membrane part;protein complex;receptor complex		Homo sapiens	1	8
cell part;cytoplasmic part;cytosol;intracellular part		Homo sapiens	1	8
cell part;cytoplasm;cytoplasmic part;intracellular membrane-bounded organelle;intracellular non-membrane-bounded organelle;intracellular organelle;intracellular part;macromolecular complex;membrane-bounded organelle;non-membrane-bounded organelle;nucleus;organelle;ribonucleoprotein complex;RNA granule;stress gra		Homo sapiens	1	8
cell part;cytoplasmic part;endoplasmic reticulum;endoplasmic reticulum membrane;endoplasmic reticulum part;integral to membrane;intracellular membrane-bounded organelle;intracellular organelle;intracellular organelle part;intracellular part;intrinsic to membrane;macromolecular complex;membrane;membrane part;membrane		Homo sapiens	1	8
cell part;integral to membrane;intrinsic to membrane;membrane part		Homo sapiens	1	8
cell part;cytoplasmic part;endoplasmic reticulum lumen;endoplasmic reticulum part;intracellular organelle lumen;intracellular organelle part;intracellular part;membrane-enclosed lumen;organelle lumen;organelle part		Homo sapiens	1	8
calcium channel complex;cation channel complex;cell part;cytoplasmic part;integral to membrane;integral to mitochondrial inner membrane;integral to mitochondrial membrane;integral to organelle membrane;intracellular membrane-bounded organelle;intracellular organelle;intracellular organelle part;intracellular part;intrinsic to		Homo sapiens	1	8
cell part;cytoplasmic part;intracellular membrane-bounded organelle;intracellular organelle;intracellular part;membrane-bounded organelle;mitochondrion;organelle		Homo sapiens	1	8
cell part;cytoplasm;cytoplasmic part;extracellular membrane-bounded organelle;extracellular organelle;extracellular region part;extracellular vesicular exosome;intracellular membrane-bounded organelle;intracellular organelle;intracellular part;membrane-bounded organelle;membrane-bounded vesicle;mitochondrion;organelle;ve		Homo sapiens	1	8
cell part;cytoplasm;cytoplasmic part;cytoskeletal part;intracellular organelle part;intracellular part;macromolecular complex;microtubule;microtubule organizing center;nuclear part;nucleoplasm;organelle part;protein complex;spindle pole		Homo sapiens	1	8
cell part;cytoplasm;cytoskeletal part;intermediate filament;intracellular organelle part;intracellular part;macromolecular complex;organelle part;protein complex		Homo sapiens	1	8
cell part;chromosomal part;condensed chromosome kinetochore;cytoplasm;cytoplasmic part;cytoskeletal part;cytoskeleton;extracellular membrane-bounded organelle;extracellular organelle;extracellular region part;extracellular vesicular exosome;intracellular;intracellular non-membrane-bounded organelle;intracellular organelle		Homo sapiens	1	8
cell part;cytoplasm;cytoplasmic part;cytosol;endoplasmic reticulum;intracellular membrane-bounded organelle;intracellular organelle;intracellular organelle part;intracellular part;macromolecular complex;membrane;membran	Proteasome	Homo sapiens	1	8
cell part;integral to membrane;intrinsic to membrane;membrane part		Homo sapiens	1	8
autophagic vacuole;cell part;cytoplasmic part;cytoplasmic vesicle;endosomal part;endosome membrane;intracellular membrane-bounded organelle;intracellular organelle;intracellular organelle part;intracellular part;macromolecular complex;membrane;membrane part;membrane-bounded organelle;organelle;organelle membrane		Homo sapiens	1	8
cell part;cytoplasm;extracellular membrane-bounded organelle;extracellular organelle;extracellular region part;extracellular vesicular exosome;intracellular;intracellular part;membrane-bounded organelle;membrane-bounded vesicle;organelle;vesicle		Homo sapiens	1	8
cell part;cytoplasmic part;cytosol;extracellular membrane-bounded organelle;extracellular organelle;extracellular region part;extracellular vesicular exosome;intracellular part;membrane-bounded organelle;membrane-bounde	Carbon fixation in photosynthetic organisms;Pentose and glucuronate interconversions;Pentose phosph	Homo sapiens	1	8
cell part;cytoplasmic part;early endosome;endosomal part;endosome;endosome membrane;intracellular membrane-bounded organelle;intracellular organelle;intracellular organelle part;intracellular part;late endosome;late endosome membrane;lysosomal membrane;membrane;membrane-bounded organelle;organelle;organelle r		Homo sapiens	1	8
cell part;cytoplasm;intracellular membrane-bounded organelle;intracellular non-membrane-bounded organelle;intracellular organelle;intracellular organelle part;intracellular part;membrane;membrane-bounded organelle;non-r	Ribosome biogenesis in eukaryotes;RNA transport	Homo sapiens	1	8
cell part;cytoplasmic part;endoplasmic reticulum;endoplasmic reticulum membrane;endoplasmic reticulum part;integral to membrane;intracellular membrane-bounded organelle;intracellular organelle;intracellular organelle part;intracellular part;intrinsic to membrane;membrane;membrane part;membrane-bounded organelle;nucle		Homo sapiens	1	8
actin cytoskeleton;cell part;cytoplasm;cytoskeleton;intracellular membrane-bounded organelle;intracellular non-membrane-bounded organelle;intracellular organelle;intracellular part;membrane;membrane-bounded organelle;non-membrane-bounded organelle;nucleus;organelle;plasma membrane		Homo sapiens	1	8
cell part;cytoplasm;cytoplasmic part;cytoskeleton;cytosol;intracellular non-membrane-bounded organelle;intracellular organelle;intracellular part;membrane;non-membrane-bounded organelle;organelle		Homo sapiens	1	8
cell part;chromatin;chromosomal part;cytoskeleton;intermediate filament cytoskeleton;intracellular membrane-bounded organelle;intracellular non-membrane-bounded organelle;intracellular organelle;intracellular organelle pa	Lysine degradation	Homo sapiens	1	8
cell part;cytoplasm;cytoplasmic part;cytosol;early endosome;early endosome membrane;endosomal part;endosome;endosome membrane;immunological synapse;intracellular membrane-bounded organelle;intracellular organelle;intracellular organelle part;intracellular part;membrane;membrane part;membrane-bounded organel		Homo sapiens	1	8
cell part;cytoplasmic part;Golgi apparatus;intracellular membrane-bounded organelle;intracellular organelle;intracellular part;membrane-bounded organelle;organelle		Homo sapiens	1	8
cell part;cytoplasmic part;endoplasmic reticulum membrane;endoplasmic reticulum part;integral to membrane;intracellular organelle part;intracellular part;intrinsic to membrane;membrane;membrane part;organelle membrane	Glycerophospholipid metabolism	Homo sapiens	1	8
cell part;intracellular membrane-bounded organelle;intracellular non-membrane-bounded organelle;intracellular organelle;intracellular organelle part;intracellular part;membrane-bounded organelle;non-membrane-bounded organelle;nuclear part;nucleolus;nucleus;organelle;organelle part		Homo sapiens	1	8
cell part;cytoplasm;intracellular membrane-bounded organelle;intracellular organelle;intracellular part;membrane-bounded organelle;nucleus;organelle		Homo sapiens	1	8
cell part;cytoplasm;intracellular membrane-bounded organelle;intracellular non-membrane-bounded organelle;intracellular organelle;intracellular organelle part;intracellular part;membrane;membrane-bounded organelle;non-membrane-bounded organelle;nuclear part;nucleolus;nucleoplasm;nucleus;organelle;organelle part;plasm		Homo sapiens	1	8
cell part;cytoplasm;cytoskeletal part;holo TFIIH complex;intracellular membrane-bounded organelle;intracellular non-membrane-bounded organelle;intracellular organelle;intracellular organelle part;intracellular part;macromolecular complex;membrane;membrane-bounded organelle;MMXD complex;non-membrane-bounded orga		Homo sapiens	1	8
cell part;extracellular region part;extracellular space;membrane		Homo sapiens	1	8
		Homo sapiens	1	8
cell part;centrosome;cytoplasmic microtubule;cytoplasmic part;cytoskeletal part;cytosol;intracellular non-membrane-bounded organelle;intracellular organelle;intracellular organelle part;intracellular part;macromolecular complex;membrane;microtubule;microtubule organizing center;non-membrane-bounded organelle;nuclear pa		Homo sapiens	1	8
cell body;cell junction;cell part;cell pole;cell projection;cell tip;cytoplasmic membrane-bounded vesicle;cytoplasmic part;cytoplasmic vesicle;cytosol;dendrite;endosome;extracellular region part;Golgi apparatus;growing cell ti	Inositol phosphate metabolism;Phosphatidylinositol signaling system	Homo sapiens	1	8
cell part;cytoplasm;envelope;intracellular membrane-bounded organelle;intracellular organelle;intracellular organelle part;intracellular part;membrane;membrane-bounded organelle;nuclear envelope;nuclear part;nucleus;organelle;organelle envelope;organelle part		Homo sapiens	1	8
cell part;cytoplasm;cytoplasmic part;cytosol;Golgi apparatus;intracellular membrane-bounded organelle;intracellular organelle;intracellular organelle part;intracellular part;membrane;membrane-bounded organelle;nuclear par	Cysteine and methionine metabolism	Homo sapiens	1	8
cell part;cytoplasmic part;intracellular membrane-bounded organelle;intracellular organelle;intracellular part;membrane-bounded organelle;mitochondrion;nucleus;organelle	Fatty acid elongation in mitochondria	Homo sapiens	1	8
		Homo sapiens	1	8
cell part;cytoplasmic part;intracellular membrane-bounded organelle;intracellular non-membrane-bounded organelle;intracellular organelle;intracellular organelle part;intracellular part;macromolecular complex;membrane;membrane-bounded organelle;mitochondrial inner membrane;mitochondrial membrane;mitochondrial part;m		Homo sapiens	1	8
cell part;chromosomal part;chromosome, centromeric region;condensed chromosome kinetochore;cytoplasmic part;cytosol;intracellular membrane-bounded organelle;intracellular non-membrane-bounded organelle;intracellular organelle;intracellular organelle part;intracellular part;kinetochore;macromolecular complex;membrar		Homo sapiens	1	8
Cdc73/Paf1 complex;cell part;chromatin;chromosomal part;cytoplasm;cytoplasmic part;intracellular membrane-bounded organelle;intracellular organelle;intracellular organelle part;intracellular part;macromolecular complex;r	RNA degradation	Homo sapiens	1	8
acrosomal vesicle;cell part;cytoplasmic membrane-bounded vesicle;cytoplasmic part;cytoplasmic vesicle;intracellular membrane-bounded organelle;intracellular organelle;intracellular part;macromolecular complex;membrane-bounded organelle;membrane-bounded vesicle;mitochondrion;nucleus;organelle;perinuclear region of		Homo sapiens	1	8
cell part;cytoplasm;extracellular membrane-bounded organelle;extracellular organelle;extracellular region part;extracellular vesicular exosome;intracellular part;membrane-bounded organelle;membrane-bounded vesicle;organelle;vesicle		Homo sapiens	1	8
cell part;cytoplasm;intracellular non-membrane-bounded organelle;intracellular organelle;intracellular organelle part;intracellular part;membrane;non-membrane-bounded organelle;nuclear part;nucleolus;organelle;organelle part		Homo sapiens	1	8
cell part;extracellular membrane-bounded organelle;extracellular organelle;extracellular region part;extracellular vesicular exosome;intracellular membrane-bounded organelle;intracellular organelle;intracellular part;membrane-bounded organelle;membrane-bounded vesicle;nucleus;organelle;vesicle		Homo sapiens	1	8
cell part;cytoplasmic part;cytosol;intracellular part		Homo sapiens	1	8
Cajal body;cell body;cell part;cytoplasmic part;cytosol;intracellular membrane-bounded organelle;intracellular non-membrane-bounded organelle;intracellular organelle;intracellular organelle part;intracellular part;membrane-b	RNA transport	Homo sapiens	1	8
cell part;membrane		Homo sapiens	1	8
cell part;cytoplasmic part;Golgi apparatus;Golgi apparatus part;Golgi membrane;intracellular membrane-bounded organelle;intracellular organelle;intracellular organelle part;intracellular part;membrane;membrane-bounded organelle;organelle;organelle membrane;organelle part		Homo sapiens	1	8
catalytic step 2 spliceosome;cell part;cytoplasm;cytoplasmic part;Golgi apparatus;intracellular membrane-bounded organelle;intracellular organelle;intracellular organelle part;intracellular part;macromolecular complex;membrane-bounded organelle;nuclear body;nuclear part;nuclear speck;nucleoplasm;nucleoplasm part;organell		Homo sapiens	1	8
cell part;histone methyltransferase complex;intracellular membrane-bounded organelle;intracellular organelle;intracellular organelle part;intracellular part;macromolecular complex;membrane-bounded organelle;methyltransfe	Wnt signaling pathway	Homo sapiens	1	8
cell part;cytoplasmic part;intracellular membrane-bounded organelle;intracellular organelle;intracellular organelle part;intracellular part;large ribosomal subunit;macromolecular complex;membrane;membrane-bounded organelle;mitochondrial inner membrane;mitochondrial large ribosomal subunit;mitochondrial membrane;mitoch		Homo sapiens	1	8
BBSome;cell part;cytoplasmic part;cytosol;intracellular part;macromolecular complex;protein complex		Homo sapiens	1	8
cell part;cytoplasm;cytoplasmic part;cytosol;endoplasmic reticulum membrane;endoplasmic reticulum part;intracellular membrane-bounded organelle;intracellular organelle;intracellular organelle part;intracellular part;membra	Protein processing in endoplasmic reticulum	Homo sapiens	1	8
cell part;cytoplasmic part;cytosol;extracellular membrane-bounded organelle;extracellular organelle;extracellular region;extracellular region part;extracellular vesicular exosome;intracellular membrane-bounded organelle;intracellular organelle;intracellular part;membrane-bounded organelle;membrane-bounded vesicle;mitochond		Homo sapiens	1	8
actin cytoskeleton;cell part;cytoplasmic part;cytoskeleton;cytosol;intracellular membrane-bounded organelle;intracellular non-membrane-bounded organelle;intracellular organelle;intracellular part;membrane-bounded organe	Focal adhesion;Leukocyte transendothelial migration;Regulation of actin cytoskeleton	Homo sapiens	1	8
cell part;cytoplasm;cytoplasmic part;cytoskeletal part;endosomal part;endosome membrane;extracellular membrane-bounded organelle;extracellular organelle;extracellular region part;extracellular vesicular exosome;intracellular organelle part;intracellular part;late endosome membrane;lysosomal membrane;membrane;membrar		Homo sapiens	1	8
BRCA1-A complex;BRISC complex;cell part;cytoplasm;intracellular membrane-bounded organelle;intracellular organelle;intracellular organelle part;intracellular part;macromolecular complex;membrane-bounded organelle;nuclear part;nucleoplasm;nucleus;organelle;organelle part;protein complex		Homo sapiens	1	8
cell part;integral to membrane;intrinsic to membrane;membrane;membrane part	Protein processing in endoplasmic reticulum	Homo sapiens	1	8
cell part;cytoplasm;intracellular membrane-bounded organelle;intracellular organelle;intracellular organelle part;intracellular part;membrane-bounded organelle;nuclear part;nucleoplasm;nucleus;organelle;organelle part		Homo sapiens	1	8
cell part;cytoplasmic part;cytosol;endosomal part;endosome membrane;extracellular membrane-bounded organelle;extracellular organelle;extracellular region part;extracellular vesicular exosome;intracellular membrane-bour	Endocytosis	Homo sapiens	1	8
cell part;cytoplasm;cytoplasmic part;extracellular membrane-bounded organelle;extracellular organelle;extracellular region part;extracellular vesicular exosome;integral to membrane;intracellular membrane-bounded organelle	Alzheimer's disease;Huntington's disease;Oxidative phosphorylation;Parkinson's disease	Homo sapiens	1	8
cell part;cytoplasmic part;cytosol;intracellular part	Purine metabolism	Homo sapiens	1	8
cell part;chromatin remodeling complex;chromosomal part;H4/H2A histone acetyltransferase complex;histone acetyltransferase complex;histone deacetylase complex;intracellular organelle part;intracellular part;macromolecular complex;NuA4 histone acetyltransferase complex;nuclear chromosome part;nuclear part;nucleoplasr		Homo sapiens	1	8
cell part;cytoplasmic part;cytoplasmic ubiquitin ligase complex;endoplasmic reticulum membrane;endoplasmic reticulum part;ER ubiquitin ligase complex;Hrd1p ubiquitin ligase complex;integral to membrane;intracellular org	Protein processing in endoplasmic reticulum	Homo sapiens	1	8
cell part;cytoplasm;cytoplasmic part;cytosol;endosomal part;endosome membrane;intracellular organelle part;intracellular part;membrane;organelle membrane;organelle part	Cytosolic DNA-sensing pathway;Hepatitis C;Measles;RIG-I-like receptor signaling pathway;Toll-like rece	Homo sapiens	1	8
cell part;centrosome;cytoplasm;cytoplasmic dynein complex;cytoplasmic part;cytoskeletal part;cytosol;dynactin complex;dynein complex;intracellular membrane-bounded organelle;intracellular non-membrane-bounded org	Huntington's disease;Vasopressin-regulated water reabsorption	Homo sapiens	1	8
cell part;cytoplasmic part;cytosol;extracellular membrane-bounded organelle;extracellular organelle;extracellular region part;extracellular vesicular exosome;intracellular;intracellular membrane-bounded organelle;intracellular	Purine metabolism	Homo sapiens	1	8
cell part;cytoplasmic part;cytosol;intracellular part;membrane	Endocytosis;Fc gamma R-mediated phagocytosis	Homo sapiens	1	8
CD95 death-inducing signaling complex;Cdc48p-Npl4p-Ufd1p AAA ATPase complex;cell part;cytoplasmic part;cytosol;death-inducing signaling complex;endoplasmic reticulum part;envelope;intracellular membrane-bounded organelle;intracellular organelle;intracellular organelle part;intracellular part;macromolecular complex;m		Homo sapiens	1	8
cell part;cytoplasm;cytoplasmic part;cytoskeletal part;extracellular membrane-bounded organelle;extracellular organelle;extracellular region part;extracellular vesicular exosome;intracellular membrane-bounded organelle;intracellular non-membrane-bounded organelle;intracellular organelle;intracellular organelle lumen;intracellula		Homo sapiens	1	8
cell part;intracellular organelle part;intracellular part;macromolecular complex;nuclear part;nucleoplasm;organelle part;ribonucleoprotein complex;spliceosomal complex;U12-type spliceosomal complex	Spliceosome	Homo sapiens	1	8
catalytic step 2 spliceosome;cell part;extracellular membrane-bounded organelle;extracellular organelle;extracellular region part;extracellular vesicular exosome;intracellular organelle part;intracellular part;macromolecular cor	Spliceosome	Homo sapiens	1	8
cell part;cytoplasmic part;cytosol;intracellular membrane-bounded organelle;intracellular organelle;intracellular part;membrane;membrane-bounded organelle;mitochondrion;organelle		Homo sapiens	1	8
cell part;cytoplasm;intracellular membrane-bounded organelle;intracellular organelle;intracellular part;membrane-bounded organelle;nucleus;organelle		Homo sapiens	1	8
cell part;cytoplasm;intracellular part;membrane;plasma membrane		Homo sapiens	1	8
cell part;cytoplasmic part;cytosol;intracellular organelle part;intracellular part;macromolecular complex;nuclear part;organelle part;ribonucleoprotein complex;small nuclear ribonucleoprotein complex;spliceosomal complex;U	RNA degradation;Spliceosome	Homo sapiens	1	8
catalytic step 2 spliceosome;cell part;cytoplasm;cytoplasmic part;cytosol;exon-exon junction complex;intracellular membrane-bounded organelle;intracellular organelle;intracellular organelle part;intracellular part;macromolec	mRNA surveillance pathway;RNA transport;Spliceosome	Homo sapiens	1	8
cell part;cell surface;integral to membrane;intrinsic to membrane;membrane;membrane part;presynaptic membrane;synapse part;synaptic membrane		Homo sapiens	1	8
cell part;cytoplasm;cytoplasmic part;cytosol;extracellular membrane-bounded organelle;extracellular organelle;extracellular region part;extracellular vesicular exosome;intracellular membrane-bounded organelle;intracellular non-membrane-bounded organelle;intracellular organelle;intracellular organelle part;intracellular part;mem		Homo sapiens	1	8
cell part;chromatin remodeling complex;cytoplasm;histone deacetylase complex;intracellular organelle part;intracellular part;macromolecular complex;nuclear body;nuclear part;nuclear speck;nucleoplasm;nucleoplasm part;c	mRNA surveillance pathway;RNA transport	Homo sapiens	1	9
cell part;cytoplasmic part;cytosol;intracellular part		Homo sapiens	1	9
cell part;clathrin coated vesicle membrane;clathrin-coated vesicle;coated vesicle;coated vesicle membrane;cytoplasmic membrane-bounded vesicle;cytoplasmic part;cytoplasmic vesicle;cytoplasmic vesicle membrane;cytoplasmic vesicle part;endosomal part;endosome membrane;Golgi apparatus part;integral to membrane;int		Homo sapiens	1	9
cell part;endomembrane system;integral to membrane;intrinsic to membrane;membrane;membrane part		Homo sapiens	1	9
cell part;cytoplasm;cytoplasmic part;cytosol;extracellular membrane-bounded organelle;extracellular organelle;extracellular region part;extracellular vesicular exosome;intracellular membrane-bounded organelle;intracellular organelle;intracellular part;membrane-bounded organelle;membrane-bounded vesicle;nucleus;organelle;v		Homo sapiens	1	9
cell part;cytoplasm;extracellular membrane-bounded organelle;extracellular organelle;extracellular region part;extracellular vesicular exosome;intracellular membrane-bounded organelle;intracellular organelle;intracellular organelle part;intracellular part;membrane-bounded organelle;membrane-bounded vesicle;nuclear part;nucle		Homo sapiens	1	9
cell part;chromosomal part;chromosome, telomeric region;inclusion body;intracellular membrane-bounded organelle;intracellular non-membrane-bounded organelle;intracellular organelle;intracellular organelle part;intracellula	Homologous recombination	Homo sapiens	1	9
cell part;cytoplasmic part;integral to membrane;intracellular membrane-bounded organelle;intracellular organelle;intracellular organelle part;intracellular part;intrinsic to membrane;membrane;membrane part;membrane-bounc	ABC transporters	Homo sapiens	1	9
cell part;cytoplasmic part;integral to membrane;integral to plasma membrane;intracellular membrane-bounded organelle;intracellular organelle;intracellular part;intrinsic to membrane;intrinsic to plasma membrane;membrane;membrane part;membrane-bounded organelle;mitochondrion;organelle;plasma membrane part		Homo sapiens	1	9
cell part;cytoplasmic part;cytosol;eukaryotic 43S preinitiation complex;eukaryotic 48S preinitiation complex;eukaryotic translation initiation factor 3 complex;intracellular part;macromolecular complex;protein complex;ribonuc	RNA transport	Homo sapiens	1	9
cell part;intracellular organelle part;intracellular part;nuclear part;nucleoplasm;organelle part		Homo sapiens	1	9
cell part;cytoplasmic part;endoplasmic reticulum;endoplasmic reticulum membrane;endoplasmic reticulum part;extracellular membrane-bounded organelle;extracellular organelle;extracellular region part;extracellular space;extracellular vesicular exosome;Golgi apparatus part;Golgi membrane;integral to membrane;intracellular m		Homo sapiens	1	9
cell part;cytoplasmic part;extracellular membrane-bounded organelle;extracellular organelle;extracellular region part;extracellular vesicular exosome;intracellular organelle part;intracellular part;macromolecular complex;memb	Alzheimer's disease;Huntington's disease;Oxidative phosphorylation;Parkinson's disease	Homo sapiens	1	9
cell part;external side of plasma membrane;integral to membrane;integral to plasma membrane;intrinsic to membrane;intrinsic to plasma membrane;membrane;membrane part;plasma membrane part		Homo sapiens	1	9
cell part;cytoplasmic part;extracellular membrane-bounded organelle;extracellular organelle;extracellular region part;extracellular vesicular exosome;integral to membrane;intracellular membrane-bounded organelle;intracellula	Alzheimer's disease;Cardiac muscle contraction;Huntington's disease;Oxidative phosphorylation;Parkin	Homo sapiens	1	9
cell body;cell junction;cell part;cell projection cytoplasm;cell projection part;cell surface;cytoplasm;cytoplasmic membrane-bounded vesicle lumen;cytoplasmic part;cytoplasmic vesicle part;cytosol;dendrite cytoplasm;extern	Primary immunodeficiency;Purine metabolism	Homo sapiens	1	9
cell part;cytoplasm;cytoplasmic membrane-bounded vesicle;cytoplasmic membrane-bounded vesicle lumen;cytoplasmic part;cytoplasmic vesicle;cytoplasmic vesicle part;endocytic vesicle lumen;extracellular membrane-bounded organelle;extracellular organelle;extracellular region;extracellular region part;extracellular space;ex		Homo sapiens	1	9
cell part;cytoplasm;cytoplasmic part;cytosol;extracellular membrane-bounded organelle;extracellular organelle;extracellular region part;extracellular vesicular exosome;intracellular part;membrane-bounded organelle;membra	Alanine, aspartate and glutamate metabolism;Arginine and proline metabolism	Homo sapiens	1	9
cell part;chromatin;chromosomal part;cytoplasm;intracellular membrane-bounded organelle;intracellular organelle;intracellular organelle part;intracellular part;membrane-bounded organelle;nucleus;organelle;organelle part		Homo sapiens	1	9
cell part;cytoplasm;cytoplasmic part;cytosol;intracellular organelle part;intracellular part;nuclear part;nucleoplasm;organelle part	Porphyrin and chlorophyll metabolism	Homo sapiens	1	9
cell part;cytoplasm;cytoplasmic part;cytosol;extracellular membrane-bounded organelle;extracellular organelle;extracellular region part;extracellular vesicular exosome;intracellular membrane-bounded organelle;intracellular c	Arachidonic acid metabolism;Glutathione metabolism	Homo sapiens	1	9
cell part;cytoskeleton;intracellular membrane-bounded organelle;intracellular non-membrane-bounded organelle;intracellular organelle;intracellular organelle part;intracellular part;macromolecular complex;membrane-bounde	Basal transcription factors	Homo sapiens	1	9
cell part;cytoplasmic part;extracellular region part;extracellular space;intracellular membrane-bounded organelle;intracellular organelle;intracellular part;membrane-bounded organelle;mitochondrion;organelle	One carbon pool by folate	Homo sapiens	1	9
cell part;cell surface;cytoplasm;extracellular membrane-bounded organelle;extracellular organelle;extracellular region;extracellular region part;extracellular space;extracellular vesicular exosome;intracellular organelle part;intra	Phenylalanine metabolism;Tyrosine metabolism	Homo sapiens	1	9
cell part;centrosome;chromosomal part;condensed chromosome outer kinetochore;condensed nuclear chromosome outer kinetochore;cytoplasm;cytoplasmic part;cytoskeletal part;cytosol;intracellular membrane-bounded c	Cell cycle;p53 signaling pathway;Progesterone-mediated oocyte maturation	Homo sapiens	1	9
cell part;cytoplasmic part;intracellular membrane-bounded organelle;intracellular organelle;intracellular organelle part;intracellular part;membrane;membrane-bounded organelle;membrane-enclosed lumen;mitochondrial inne	Alzheimer's disease;Cardiac muscle contraction;Huntington's disease;Oxidative phosphorylation;Parkin	Homo sapiens	1	9
cell part;cytoplasmic part;extracellular membrane-bounded organelle;extracellular organelle;extracellular region part;extracellular vesicular exosome;intracellular organelle lumen;intracellular organelle part;intracellular part;lys	Glycosaminoglycan degradation;Lysosome	Homo sapiens	1	9
cell junction;cell part;cell projection;cell-cell junction;cytoplasm;cytoplasmic part;cytosol;intracellular organelle part;intracellular part;lamellipodium;macromolecular complex;membrane;nuclear part;organelle part;plasma mem	Calcium signaling pathway;Epithelial cell signaling in Helicobacter pylori infection;ErbB signaling pathwa	Homo sapiens	1	9
cell part;cytoplasmic part;extracellular membrane-bounded organelle;extracellular organelle;extracellular region part;extracellular vesicular exosome;intracellular organelle part;intracellular part;membrane;membrane-boundec	Alzheimer's disease;Cardiac muscle contraction;Huntington's disease;Oxidative phosphorylation;Parkin	Homo sapiens	1	9
blood microparticle;extracellular membrane-bounded organelle;extracellular organelle;extracellular region;extracellular region part;extracellular vesicular exosome;membrane-bounded organelle;membrane-bounded vesicle;organelle;vesicle		Homo sapiens	1	9
cell part;cytoplasmic part;intracellular membrane-bounded organelle;intracellular organelle;intracellular organelle lumen;intracellular organelle part;intracellular part;membrane-bounded organelle;membrane-enclosed lumen;m	Carbon fixation pathways in prokaryotes;Glyoxylate and dicarboxylate metabolism;Propanoate metabol	Homo sapiens	1	9
cell part;cytoplasmic part;intracellular membrane-bounded organelle;intracellular organelle;intracellular organelle lumen;intracellular organelle part;intracellular part;membrane;membrane-bounded organelle;membrane-enclos	Porphyrin and chlorophyll metabolism	Homo sapiens	1	9
basement membrane;extracellular matrix;extracellular matrix part;extracellular membrane-bounded organelle;extracellular organelle;extracellular region;extracellular region part;extracellular space;extracellular vesicular exosome;membrane-bounded organelle;membrane-bounded vesicle;organelle;proteinaceous extracellular mat		Homo sapiens	1	9
cell part;cytoplasmic part;endoplasmic reticulum;endoplasmic reticulum membrane;endoplasmic reticulum part;extracellular membrane-bounded organelle;extracellular organelle;extracellular region part;extracellular vesicular exosome;intracellular membrane-bounded organelle;intracellular organelle;intracellular organelle part;int		Homo sapiens	1	9
cell part;intracellular membrane-bounded organelle;intracellular organelle;intracellular organelle part;intracellular part;membrane-bounded organelle;nuclear part;nucleoplasm;nucleus;organelle;organelle part	Adipocytokine signaling pathway;Non-small cell lung cancer;Pathways in cancer;PPAR signaling pathwa	Homo sapiens	1	9
cell cortex;cell part;cell projection membrane;cell projection part;cytoplasmic part;cytosol;intracellular membrane-bounded organelle;intracellular organelle;intracellular organelle part;intracellular part;leading edge membrane;	Acute myeloid leukemia;Adipocytokine signaling pathway;Apoptosis;B cell receptor signaling pathway;(	Homo sapiens	1	9
cell part;cytoplasmic part;cytosol;extracellular membrane-bounded organelle;extracellular organelle;extracellular region part;extracellular vesicular exosome;intracellular part;membrane-bounded organelle;membrane-bounde	Amino sugar and nucleotide sugar metabolism;Fructose and mannose metabolism	Homo sapiens	1	9
anchored to membrane;cell part;cytoplasmic part;endosome;extracellular matrix;extracellular membrane-bounded organelle;extracellular organelle;extracellular region part;extracellular space;extracellular vesicular exosome;Golgi apparatus part;Golgi lumen;integral to membrane;integral to plasma membrane;intracellular membr		Homo sapiens	1	9
cell part;cytoplasmic part;cytosol;extracellular membrane-bounded organelle;extracellular organelle;extracellular region part;extracellular vesicular exosome;intracellular membrane-bounded organelle;intracellular organelle;intracellular part;membrane-bounded organelle;membrane-bounded vesicle;mitochondrion;nucleus;organe		Homo sapiens	1	9
calcium channel complex;cation channel complex;cell part;cell projection;cytoplasmic part;cytosol;intracellular membrane-bounded organelle;intracellular organelle;intracellular organelle part;intracellular part;ion channel com	MAPK signaling pathway	Homo sapiens	1	9
cell part;membrane;plasma membrane		Homo sapiens	1	9
cell part;cytoplasmic part;extracellular membrane-bounded organelle;extracellular organelle;extracellular region part;extracellular vesicular exosome;intracellular membrane-bounded organelle;intracellular organelle;intracellula	Fatty acid metabolism	Homo sapiens	1	9
cell part;extracellular matrix;extracellular region part;intracellular membrane-bounded organelle;intracellular organelle;intracellular part;membrane-bounded organelle;nucleus;organelle;proteinaceous extracellular matrix		Homo sapiens	1	9
cell part;cytoplasm;extracellular membrane-bounded organelle;extracellular organelle;extracellular region part;extracellular vesicular exosome;intracellular part;membrane;membrane-bounded organelle;membrane-bounded v	Fc gamma R-mediated phagocytosis;Leishmaniasis	Homo sapiens	1	9

cell part;centrosome;cytoplasm;cytoplasmic part;cytoskeletal part;cytosol;extracellular membrane-bounded organelle;extracellular organelle;extracellular region part;extracellular vesicular exosome;intracellular membrane-bo	MAPK signaling pathway;Neurotrophin signaling pathway;VEGF signaling pathway	Homo sapiens		1	9
cell part;chromosomal part;chromosome, centromeric region;condensed chromosome outer kinetochore;cytoplasm;cytoplasmic part;cytoskeletal part;cytosol;envelope;intracellular membrane-bounded organelle;intracellular non-membrane-bounded organelle;intracellular organelle;intracellular organelle part;intracellular part;kine		Homo sapiens		1	9
cell part;intracellular organelle part;intracellular part;nuclear part;nucleoplasm;organelle part	DNA replication;Purine metabolism;Pyrimidine metabolism	Homo sapiens		1	9
cell part;cytoplasm;intracellular organelle part;intracellular part;membrane;nuclear membrane;nuclear part;organelle membrane;organelle part;plasma membrane	Selenocompound metabolism	Homo sapiens		1	9
cell part;cytoplasm;cytoplasmic part;cytosol;intracellular membrane-bounded organelle;intracellular organelle;intracellular organelle part;intracellular part;macromolecular complex;membrane-bounded organelle;mRNA cap bi	mRNA surveillance pathway;RNA transport;Spliceosome	Homo sapiens		1	9
cell part;cytoplasmic part;cytosol;Golgi apparatus part;Golgi membrane;intracellular organelle part;intracellular part;membrane;organelle membrane;organelle part;trans-Golgi network membrane		Homo sapiens		1	9
cell part;cytoplasm;cytoplasmic part;cytosol;extracellular membrane-bounded organelle;extracellular organelle;extracellular region part;extracellular vesicular exosome;intracellular part;membrane;membrane-bounded organelle;membrane-bounded vesicle;organelle;plasma membrane;vesicle		Homo sapiens		1	9
box C/D snoRNP complex;cell part;intracellular membrane-bounded organelle;intracellular non-membrane-bounded organelle;intracellular organelle;intracellular organelle part;intracellular part;macromolecular complex;memb	Ribosome biogenesis in eukaryotes;Spliceosome	Homo sapiens		1	9
cell part;cytoplasmic part;endoplasmic reticulum;endoplasmic reticulum membrane;endoplasmic reticulum part;integral to membrane;intracellular membrane-bounded organelle;intracellular organelle;intracellular organelle pa	Protein export	Homo sapiens		1	9
actin cytoskeleton;cell part;cytoplasm;cytoskeleton;extracellular membrane-bounded organelle;extracellular organelle;extracellular region part;extracellular vesicular exosome;intermediate filament cytoskeleton;intracellular m	Ubiquitin mediated proteolysis	Homo sapiens		1	9
cell part;cytoplasmic part;extracellular membrane-bounded organelle;extracellular organelle;extracellular region part;extracellular vesicular exosome;Golgi apparatus part;Golgi membrane;intracellular organelle part;intracellular part;membrane;membrane-bounded organelle;membrane-bounded vesicle;organelle;organelle membr		Homo sapiens		1	9
cell part;intracellular membrane-bounded organelle;intracellular organelle;intracellular organelle part;intracellular part;membrane-bounded organelle;nuclear body;nuclear part;nucleoplasm;nucleoplasm part;nucleus;organelle;	RNA transport	Homo sapiens		1	9
cell part;cytoplasmic membrane-bounded vesicle lumen;cytoplasmic part;cytoplasmic vesicle part;cytoskeleton;cytosol;extracellular region;intracellular membrane-bounded organelle;intracellular non-membrane-bounded org	Regulation of actin cytoskeleton	Homo sapiens		1	9
cell part;cell projection;centrosome;chromosomal part;cilium;cytoplasm;cytoplasmic dynein complex;cytoplasmic part;cytoskeletal part;cytosol;dynein complex;extracellular membrane-bounded organelle;extracellular organe	Vasopressin-regulated water reabsorption	Homo sapiens		1	9
adherens junction;anchoring junction;cell junction;cell part;cell-substrate adherens junction;cell-substrate junction;cytoplasmic part;cytosol;endoplasmic reticulum membrane;endoplasmic reticulum part;extracellular membra	Bacterial invasion of epithelial cells;Shigellosis	Homo sapiens		1	9
cell part;cytoplasmic part;cytosol;intracellular membrane-bounded organelle;intracellular organelle;intracellular part;membrane-bounded organelle;nucleus;organelle		Homo sapiens		1	9
cell part;intracellular membrane-bounded organelle;intracellular non-membrane-bounded organelle;intracellular organelle;intracellular organelle part;intracellular part;membrane-bounded organelle;non-membrane-bounded organelle;nuclear part;nucleolus;nucleoplasm;nucleus;organelle;organelle part		Homo sapiens		1	9
cell part;cytoplasm;intracellular membrane-bounded organelle;intracellular non-membrane-bounded organelle;intracellular organelle;intracellular organelle part;intracellular part;membrane;membrane-bounded organelle;non-membrane-bounded organelle;nuclear part;nucleolus;nucleoplasm;nucleus;organelle;organelle part;plasm		Homo sapiens		1	9
cell part;intracellular membrane-bounded organelle;intracellular organelle;intracellular organelle part;intracellular part;membrane-bounded organelle;nuclear part;nucleoplasm;nucleus;organelle;organelle part		Homo sapiens		1	9
cell junction;cell part;cell-cell junction;cytoplasm;cytoplasmic part;cytosol;endoplasmic reticulum;extracellular membrane-bounded organelle;extracellular organelle;extracellular region part;extracellular vesicular exosome;intr	Tight junction;Vascular smooth muscle contraction	Homo sapiens		1	9
cell part;chromosome;cytoplasm;cytoplasmic part;cytoskeleton;cytosol;exosome (RNase complex);extracellular membrane-bounded organelle;extracellular organelle;extracellular region part;extracellular vesicular exosome;ir	RNA degradation	Homo sapiens		1	9
CD40 receptor complex;cell cortex;cell part;cytoplasm;cytoplasmic part;cytosol;internal side of plasma membrane;intracellular part;macromolecular complex;membrane;membrane part;membrane raft;organelle membrane;or	Adipocytokine signaling pathway;Apoptosis;Hepatitis C;MAPK signaling pathway;Osteoclast differentiat	Homo sapiens		1	9
cell part;cytoplasm;extracellular region part;intercellular bridge;intracellular membrane-bounded organelle;intracellular non-membrane-bounded organelle;intracellular organelle;intracellular organelle part;intracellular part;macromolecular complex;membrane;membrane-bounded organelle;non-membrane-bounded organelle;nucle		Homo sapiens		1	9
actin filament bundle;actomyosin;adherens junction;anchoring junction;cell junction;cell part;cell-substrate adherens junction;cell-substrate junction;contractile fiber part;cytoplasmic part;cytoskeletal part;focal adhesion;intracellular membrane-bounded organelle;intracellular organelle;intracellular organelle part;intracellular part;n		Homo sapiens		1	9
cell part;cytoplasm;cytoplasmic part;cytosol;extracellular membrane-bounded organelle;extracellular organelle;extracellular region part;extracellular vesicular exosome;intracellular membrane-bounded organelle;intracellular organelle;intracellular organelle part;intracellular part;membrane-bounded organelle;membrane-bounded		Homo sapiens		1	9
adherens junction;anchoring junction;cell body;cell cortex;cell junction;cell part;cell projection;cell-substrate adherens junction;cell-substrate junction;cytoplasmic part;cytosol;focal adhesion;intracellular part;lamellipodium;m	Regulation of actin cytoskeleton	Homo sapiens		1	9
adherens junction;anchoring junction;cell junction;cell part;cell-substrate adherens junction;cell-substrate junction;focal adhesion;intracellular organelle part;intracellular part;nuclear part;nucleoplasm;organelle part	Endocytosis	Homo sapiens		1	9
cell part;cytoplasmic part;cytosol;Golgi apparatus part;Golgi membrane;Golgi stack;Golgi transport complex;intracellular organelle part;intracellular part;macromolecular complex;membrane;organelle membrane;organelle part;protein complex		Homo sapiens		1	9
cell part;cytoplasmic part;cytosol;intracellular membrane-bounded organelle;intracellular organelle;intracellular organelle part;intracellular part;membrane-bounded organelle;nuclear body;nuclear part;nuclear speck;nucleopla	Proteasome	Homo sapiens		1	9
cell part;cytoplasm;cytoplasmic part;endoplasmic reticulum;ER membrane protein complex;intracellular membrane-bounded organelle;intracellular non-membrane-bounded organelle;intracellular organelle;intracellular organelle part;intracellular part;macromolecular complex;membrane-bounded organelle;mitochondrion;non-me		Homo sapiens		1	9
cell part;cell projection;cilium;cytoplasm;cytoskeletal part;cytoskeleton;intracellular non-membrane-bounded organelle;intracellular organelle;intracellular organelle part;intracellular part;macromolecular complex;microtubule;non-membrane-bounded organelle;nonmotile primary cilium;organelle;organelle part;photoreceptor conne		Homo sapiens		1	9
cell part;cytoplasm;integral to membrane;integral to plasma membrane;intracellular organelle part;intracellular part;intrinsic to membrane;intrinsic to plasma membrane;macromolecular complex;membrane;membrane part;nuclear part;nucleoplasm;organelle part;plasma membrane;plasma membrane part;proteasome complex;p		Homo sapiens		1	9
cell part;cis-Golgi network;cytoplasmic part;extracellular membrane-bounded organelle;extracellular organelle;extracellular region part;extracellular vesicular exosome;Golgi apparatus part;Golgi membrane;integral to membra	N-Glycan biosynthesis;Various types of N-glycan biosynthesis	Homo sapiens		1	9
cell part;cytoplasmic part;extracellular membrane-bounded organelle;extracellular organelle;extracellular region part;extracellular space;extracellular vesicular exosome;intracellular membrane-bounded organelle;intracellular	Cysteine and methionine metabolism;Sulfur relay system	Homo sapiens		1	9
cell part;cytoplasm;cytoplasmic part;extracellular membrane-bounded organelle;extracellular organelle;extracellular region part;extracellular vesicular exosome;intracellular organelle lumen;intracellular organelle part;intracellu	Pyruvate metabolism	Homo sapiens		1	9
cell part;cytoplasmic part;cytosol;intracellular part	Drug metabolism - other enzymes;Pyrimidine metabolism	Homo sapiens		1	9
cell part;cytoplasmic part;intracellular membrane-bounded organelle;intracellular organelle;intracellular part;membrane-bounded organelle;mitochondrion;organelle		Homo sapiens		1	9
cell part;cytoplasm;intracellular organelle part;intracellular part;membrane;nuclear part;nucleoplasm;organelle part;plasma membrane		Homo sapiens		1	9
cell part;cytoplasmic exosome (RNase complex);cytoplasmic part;cytosol;exosome (RNase complex);intracellular non-membrane-bounded organelle;intracellular organelle;intracellular organelle part;intracellular part;macromo	RNA degradation	Homo sapiens		1	9
		Homo sapiens		1	9
		Homo sapiens		1	9
cell part;intracellular membrane-bounded organelle;intracellular non-membrane-bounded organelle;intracellular organelle;intracellular organelle part;intracellular part;membrane-bounded organelle;non-membrane-bounded organelle;nuclear body;nuclear part;nuclear speck;nucleolus;nucleoplasm part;nucleus;organelle;organelle		Homo sapiens		1	9
cell junction;cell part;cell-cell junction;cytoplasmic part;cytosol;intracellular part;membrane;occluding junction;plasma membrane;tight junction		Homo sapiens		1	9
catalytic step 2 spliceosome;cell part;intracellular membrane-bounded organelle;intracellular organelle;intracellular organelle part;intracellular part;macromolecular complex;membrane-bounded organelle;nuclear part;nucleoplasm;nucleus;organelle;organelle part;ribonucleoprotein complex;spliceosomal complex		Homo sapiens		1	9
cell part;cytoplasmic part;intracellular non-membrane-bounded organelle;intracellular organelle;intracellular part;macromolecular complex;non-membrane-bounded organelle;organelle;ribonucleoprotein complex;ribosome	Ribosome	Homo sapiens		1	9
cell part;cytoplasmic part;endoplasmic reticulum;integral to membrane;intracellular membrane-bounded organelle;intracellular organelle;intracellular part;intrinsic to membrane;membrane;membrane part;membrane-bounded	Bile secretion	Homo sapiens		1	9
cell part;membrane		Homo sapiens		1	9
brush border;cell part;cell projection;cytoplasmic microtubule;cytoplasmic part;cytoskeletal part;extracellular membrane-bounded organelle;extracellular organelle;extracellular region part;extracellular vesicular exosome;intra	Pentose and glucuronate interconversions	Homo sapiens		1	9
cell part;cytoplasmic membrane-bounded vesicle;cytoplasmic part;cytoplasmic vesicle;cytoplasmic vesicle membrane;cytoplasmic vesicle part;endocytic vesicle;endocytic vesicle membrane;endosome;extracellular membrane-bounded organelle;extracellular organelle;extracellular region part;extracellular vesicular exosome;Go		Homo sapiens		1	9
aggresome;cell part;inclusion body;intracellular membrane-bounded organelle;intracellular organelle;intracellular organelle part;intracellular part;membrane-bounded organelle;nuclear part;nucleoplasm;organelle;organelle part		Homo sapiens		1	9
cell part;cytoplasmic part;endoplasmic reticulum membrane;endoplasmic reticulum part;extracellular membrane-bounded organelle;extracellular organelle;extracellular region part;extracellular vesicular exosome;integral to membrane;intracellular organelle part;intracellular part;intrinsic to membrane;membrane;membrane part;m		Homo sapiens		1	9
actin cytoskeleton;cell part;centrosome;cytoplasm;cytoplasmic part;cytoskeletal part;cytoskeleton;intracellular membrane-bounded organelle;intracellular non-membrane-bounded organelle;intracellular organelle;intracellular organelle part;intracellular part;macromolecular complex;membrane;membrane-bounded organelle;micro		Homo sapiens		1	9
cell part;cytoplasmic part;endoplasmic reticulum lumen;endoplasmic reticulum part;extracellular matrix;extracellular region part;intracellular organelle lumen;intracellular organelle part;intracellular part;membrane-enclosed lumen;organelle lumen;organelle part;proteinaceous extracellular matrix		Homo sapiens		1	9
		Homo sapiens		1	9
		Homo sapiens		1	9
cell part;cytoplasm;cytoplasmic part;extracellular region;intracellular part;lipid particle		Homo sapiens		1	9
cell junction;cell part;cytoplasmic part;intracellular membrane-bounded organelle;intracellular organelle;intracellular organelle part;intracellular part;membrane;membrane-bounded organelle;microbody;microbody membrane;microbody part;mitochondrion;nucleus;organelle;organelle membrane;organelle part;peroxisomal membr		Homo sapiens		1	9
cell part;chromosomal part;condensed chromosome kinetochore;cytoplasmic part;cytosol;envelope;intracellular membrane-bounded organelle;intracellular non-membrane-bounded organelle;intracellular organelle;intracellula	RNA transport	Homo sapiens		1	9
cell part;chromatin remodeling complex;cytoplasm;histone deacetylase complex;intracellular membrane-bounded organelle;intracellular organelle;intracellular organelle part;intracellular part;macromolecular complex;membrane-bounded organelle;nuclear part;nucleoplasm;nucleoplasm part;nucleus;organelle;organelle part;prote		Homo sapiens		1	9
		Homo sapiens		1	9
Cdc73/Paf1 complex;cell part;intracellular non-membrane-bounded organelle;intracellular organelle;intracellular organelle part;intracellular part;macromolecular complex;non-membrane-bounded organelle;nuclear part;nucleolus;nucleoplasm part;organelle;organelle part;protein complex;transcription elongation factor complex		Homo sapiens		1	9
cell junction;cell part;clathrin-coated vesicle;coated vesicle;cytoplasm;cytoplasmic membrane-bounded vesicle;cytoplasmic part;cytoplasmic vesicle;eukaryotic translation initiation factor 3 complex;intracellular membrane-bounded organelle;intracellular organelle;intracellular organelle part;intracellular part;macromolecular com		Homo sapiens		1	9
cell part;cytoplasmic part;endoplasmic reticulum lumen;endoplasmic reticulum part;endoplasmic reticulum-Golgi intermediate compartment;extracellular membrane-bounded organelle;extracellular organelle;extracellular region part;extracellular space;extracellular vesicular exosome;intracellular membrane-bounded organelle;int		Homo sapiens		1	9
cell part;cytoplasm;cytoplasmic part;extracellular membrane-bounded organelle;extracellular organelle;extracellular region part;extracellular vesicular exosome;integral to membrane;intracellular membrane-bounded organelle;intracellular organelle;intracellular part;intrinsic to membrane;membrane part;membrane-bounded orga		Homo sapiens		1	9
cell part;cytoplasmic part;intracellular organelle part;intracellular part;membrane-enclosed lumen;mitochondrial intermembrane space;mitochondrial part;organelle envelope lumen;organelle part		Homo sapiens		1	9
cell part;cytoplasm;cytoplasmic part;intracellular membrane-bounded organelle;intracellular organelle;intracellular part;membrane-bounded organelle;mitochondrion;organelle		Homo sapiens		1	9
cell part;cytoplasmic part;intracellular membrane-bounded organelle;intracellular organelle;intracellular part;membrane-bounded organelle;mitochondrion;organelle		Homo sapiens		1	9
cell part;cytoplasmic part;cytoskeleton;intracellular membrane-bounded organelle;intracellular non-membrane-bounded organelle;intracellular organelle;intracellular part;membrane-bounded organelle;mitochondrion;non-membrane-bounded organelle;organelle		Homo sapiens		1	9
cell part;cytoplasmic mRNA processing body;cytoplasmic part;cytosol;intracellular non-membrane-bounded organelle;intracellular organelle;intracellular part;macromolecular complex;membrane;non-membrane-bounded org	RNA degradation	Homo sapiens		1	9
cell part;cytoplasm;cytoplasmic part;extracellular membrane-bounded organelle;extracellular organelle;extracellular region part;extracellular vesicular exosome;intracellular membrane-bounded organelle;intracellular organelle;intracellular part;membrane-bounded organelle;membrane-bounded vesicle;mitochondrion;organelle;ve		Homo sapiens		1	9
cell part;chromosomal part;chromosome;condensed chromosome;condensed chromosome kinetochore;cytoplasm;cytoskeletal part;intracellular membrane-bounded organelle;intracellular non-membrane-bounded organelle;intracellular organelle;intracellular organelle part;intracellular part;kinetochore;macromolecular complex;r		Homo sapiens		1	9
cell part;intracellular membrane-bounded organelle;intracellular organelle;intracellular part;membrane-bounded organelle;nucleus;organelle		Homo sapiens		1	9
cell part;cytoplasm;cytoplasmic part;cytosol;extracellular membrane-bounded organelle;extracellular organelle;extracellular region part;extracellular vesicular exosome;intracellular membrane-bounded organelle;intracellular c	Proteasome	Homo sapiens		1	9
cell part;cytoplasmic part;endoplasmic reticulum membrane;endoplasmic reticulum part;intracellular organelle part;intracellular part;membrane;membrane part;organelle membrane;organelle part	Glycerophospholipid metabolism;Phosphonate and phosphinate metabolism	Homo sapiens		1	9
cell part;cytoplasm;cytoplasmic part;cytosolic part;intracellular membrane-bounded organelle;intracellular organelle;intracellular part;macromolecular complex;membrane-bounded organelle;nucleus;organelle;prefoldin complex;protein complex		Homo sapiens		1	9
cell part;cytoplasm;cytoplasmic part;cytoskeletal part;cytosol;Golgi apparatus part;Golgi stack;intracellular membrane-bounded organelle;intracellular non-membrane-bounded organelle;intracellular organelle;intracellular organelle part;intracellular part;membrane-bounded organelle;non-membrane-bounded organelle;nuclear pa		Homo sapiens		1	9
calcium channel complex;cation channel complex;cell part;cytoplasmic part;integral to membrane;integral to mitochondrial membrane;integral to organelle membrane;intracellular;intracellular membrane-bounded organelle;intracellular organelle;intracellular organelle part;intracellular part;intrinsic to membrane;intrinsic to organel		Homo sapiens		1	9
cell part;cytoplasmic part;intracellular membrane-bounded organelle;intracellular non-membrane-bounded organelle;intracellular organelle;intracellular organelle part;intracellular part;macromolecular complex;membrane;membrane-bounded organelle;mitochondrial inner membrane;mitochondrial membrane;mitochondrial part;m		Homo sapiens		1	9
cell part;cytoplasm;intracellular organelle part;intracellular part;nuclear part;nucleoplasm;organelle part		Homo sapiens		1	9
cell part;cytoplasm;intracellular membrane-bounded organelle;intracellular organelle;intracellular organelle part;intracellular part;membrane;membrane-bounded organelle;nuclear part;nucleoplasm;nucleus;organelle;organelle part;plasma membrane		Homo sapiens		1	9
cell part;chromosome;cytoplasm;cytoskeletal part;intracellular membrane-bounded organelle;intracellular non-membrane-bounded organelle;intracellular organelle;intracellular organelle part;intracellular part;macromolecular complex;membrane-bounded organelle;microtubule;non-membrane-bounded organelle;nuclear part;nuc		Homo sapiens		1	9
cell part;intracellular membrane-bounded organelle;intracellular non-membrane-bounded organelle;intracellular organelle;intracellular organelle part;intracellular part;membrane-bounded organelle;non-membrane-bounded organelle;nuclear part;nucleolus;organelle;organelle part		Homo sapiens		1	9
cell part;intracellular membrane-bounded organelle;intracellular organelle;intracellular organelle part;intracellular part;membrane-bounded organelle;nuclear body;nuclear part;nuclear speck;nucleoplasm part;nucleus;organelle;organelle part		Homo sapiens		1	9
cell part;intracellular membrane-bounded organelle;intracellular organelle;intracellular part;membrane-bounded organelle;nucleus;organelle		Homo sapiens		1	9
cell part;cytoplasm;cytoplasmic part;cytosol;intracellular part	RNA transport	Homo sapiens		1	9
cell part;intracellular membrane-bounded organelle;intracellular non-membrane-bounded organelle;intracellular organelle;intracellular organelle part;intracellular part;membrane-bounded organelle;non-membrane-bounded organelle;nuclear part;nucleolus;nucleoplasm;nucleus;organelle;organelle part		Homo sapiens		1	9
		Homo sapiens		1	9
		Homo sapiens		1	9
cell part;membrane		Homo sapiens		1	9
cell part;cytoplasm;Elongator holoenzyme complex;histone acetyltransferase complex;intracellular non-membrane-bounded organelle;intracellular organelle;intracellular organelle part;intracellular part;macromolecular complex;non-membrane-bounded organelle;nuclear part;nucleolus;nucleoplasm part;organelle;organelle part;pr		Homo sapiens		1	9
cell part;cytoplasmic part;endoplasmic reticulum part;endoplasmic reticulum tubular network;extracellular membrane-bounded organelle;extracellular organelle;extracellular region part;extracellular vesicular exosome;Golgi apparatus;intracellular;intracellular membrane-bounded organelle;intracellular organelle;intracellular organe		Homo sapiens		1	9
cell part;cytoplasmic part;cytosol;intracellular;intracellular part	Fructose and mannose metabolism	Homo sapiens		1	9
cell part;cytoplasmic part;endoplasmic reticulum;extracellular membrane-bounded organelle;extracellular organelle;extracellular region part;extracellular vesicular exosome;Golgi apparatus;intracellular membrane-bounded or	Protein processing in endoplasmic reticulum	Homo sapiens		1	9
cell part;intracellular membrane-bounded organelle;intracellular non-membrane-bounded organelle;intracellular organelle;intracellular organelle part;intracellular part;membrane-bounded organelle;non-membrane-bounded organelle;nuclear part;nucleolus;nucleus;organelle;organelle part		Homo sapiens		1	9
cell part;cytoplasmic part;cytoplasmic ubiquitin ligase complex;cytosol;intracellular part;macromolecular complex;protein complex;ubiquitin ligase complex	Regulation of autophagy	Homo sapiens		1	9
		Homo sapiens		1	9
extracellular membrane-bounded organelle;extracellular organelle;extracellular region part;extracellular vesicular exosome;membrane-bounded organelle;membrane-bounded vesicle;organelle;vesicle		Homo sapiens		1	9
cell part;cytoplasmic part;endoplasmic reticulum;endoplasmic reticulum membrane;endoplasmic reticulum part;Golgi apparatus;Golgi apparatus part;Golgi membrane;integral to membrane;intracellular membrane-bounded c	Sphingolipid metabolism	Homo sapiens		1	9
cell part;cell projection;cytoplasm;cytoplasmic part;cytoskeleton;cytosol;filopodium;intracellular non-membrane-bounded organelle;intracellular organelle;intracellular part;lamellipodium;macromolecular complex;non-membra	Regulation of actin cytoskeleton	Homo sapiens		1	9
cell part;cytoplasmic part;extracellular membrane-bounded organelle;extracellular organelle;extracellular region part;extracellular vesicular exosome;integral to membrane;intracellular membrane-bounded organelle;intracellular organelle;intracellular organelle part;intracellular part;intrinsic to membrane;membrane;membrane part		Homo sapiens		1	9
cell part;cytoplasmic part;integral to membrane;intracellular;intracellular membrane-bounded organelle;intracellular organelle;intracellular organelle part;intracellular part;intrinsic to membrane;membrane;membrane part;membrane-bounded organelle;mitochondrial inner membrane;mitochondrial membrane;mitochondrial part;mit		Homo sapiens		1	9
cell part;cytoplasmic part;intracellular membrane-bounded organelle;intracellular organelle;intracellular organelle part;intracellular part;large ribosomal subunit;macromolecular complex;membrane;membrane-bounded organelle;mitochondrial inner membrane;mitochondrial large ribosomal subunit;mitochondrial membrane;mitoch		Homo sapiens		1	9
cell part;intracellular organelle part;intracellular part;macromolecular complex;membrane;mRNA cleavage and polyadenylation specificity factor complex;mRNA cleavage factor complex;nuclear part;nucleoplasm;nucleoplasn	mRNA surveillance pathway	Homo sapiens		1	9
cell part;clathrin coat;clathrin coat of coated pit;cytoplasmic part;intracellular membrane-bounded organelle;intracellular organelle;intracellular part;macromolecular complex;membrane;membrane coat;membrane part;membrane-bounded organelle;nucleus;organelle;plasma membrane;plasma membrane part;protein complex		Homo sapiens		1	9
actin filament;cell part;cytoskeletal part;extracellular membrane-bounded organelle;extracellular organelle;extracellular region part;extracellular vesicular exosome;extrinsic to internal side of plasma membrane;extrinsic to me	Chemokine signaling pathway;MAPK signaling pathway;Regulation of actin cytoskeleton	Homo sapiens		1	9
cell cortex;cell junction;cell part;cell-cell junction;chromosome;condensed chromosome;condensed nuclear chromosome;cytoplasm;cytoplasmic part;cytoskeleton;cytosol;intracellular non-membrane-bounded organelle;intracellular organelle;intracellular organelle part;intracellular part;membrane;non-membrane-bounded organe		Homo sapiens		1	9
cell part;cytoplasmic part;endoplasmic reticulum membrane;endoplasmic reticulum part;integral to membrane;intracellular organelle part;intracellular part;intrinsic to membrane;membrane;membrane part;organelle membrane;organelle part		Homo sapiens		1	9
cell part;cytoplasmic part;intracellular membrane-bounded organelle;intracellular organelle;intracellular organelle lumen;intracellular organelle part;intracellular part;membrane-bounded organelle;membrane-enclosed lumen;m	Peroxisome	Homo sapiens		1	9
anaphase-promoting complex;cell part;cullin-RING ubiquitin ligase complex;cytoplasmic part;cytosol;intracellular;intracellular organelle part;intracellular part;macromolecular complex;nuclear part;nuclear ubiquitin ligase com	Cell cycle;Cell cycle - yeast;Meiosis - yeast;Oocyte meiosis;Progesterone-mediated oocyte maturation;	Homo sapiens		1	9
cell part;cytoplasmic part;endoplasmic reticulum;endoplasmic reticulum membrane;endoplasmic reticulum part;Golgi apparatus part;Golgi membrane;integral to membrane;intracellular membrane-bounded organelle;intracellular organelle;intracellular organelle part;intracellular part;intrinsic to membrane;membrane;membrane pa		Homo sapiens		1	9
acrosomal vesicle;cell part;cell projection;centriolar satellite;centriole;centrosome;cytoplasmic membrane-bounded vesicle;cytoplasmic part;cytoplasmic vesicle;cytoskeletal part;cytoskeleton;cytosol;extracellular region part;intercellular bridge;intracellular membrane-bounded organelle;intracellular non-membrane-bounded orga		Homo sapiens		1	9
AP-type membrane coat adaptor complex;cell part;clathrin adaptor complex;cytoplasmic part;cytoplasmic vesicle membrane;cytoplasmic vesicle part;Golgi apparatus;intracellular membrane-bounded organelle;intracellular c	Lysosome	Homo sapiens		1	9
cell part;cytoplasm;cytoplasmic part;cytosol;Golgi apparatus part;Golgi membrane;intracellular membrane-bounded organelle;intracellular organelle;intracellular organelle part;intracellular part;membrane;membrane-bounded organelle;nuclear part;nucleoplasm;nucleus;organelle;organelle membrane;organelle part		Homo sapiens		1	9
cell part;COPI vesicle coat;COPII vesicle coat;cytoplasmic part;cytoplasmic vesicle part;endoplasmic reticulum;endoplasmic reticulum membrane;endoplasmic reticulum part;endoplasmic reticulum-Golgi intermediate compartment;endoplasmic reticulum-Golgi intermediate compartment membrane;extracellular membrane-bour		Homo sapiens		1	9
cell part;cytoplasmic part;intracellular non-membrane-bounded organelle;intracellular organelle;intracellular organelle part;intracellular part;macromolecular complex;membrane;mitochondrial inner membrane;mitochondrial membrane;mitochondrial part;mitochondrial ribosome;non-membrane-bounded organelle;organellar ribos		Homo sapiens		1	9
cell part;intracellular;intracellular membrane-bounded organelle;intracellular organelle;intracellular organelle part;intracellular part;membrane;membrane-bounded organelle;nuclear part;nucleoplasm;nucleus;organelle;organelle part;plasma membrane		Homo sapiens		1	9
cell junction;cell part;cell projection;cell projection part;cytoplasm;cytoplasmic part;cytoskeleton;cytosol;dendrite;dendritic spine;extrinsic to internal side of plasma membrane;extrinsic to membrane;extrinsic to plasma membrane;filopodium;intracellular non-membrane-bounded organelle;intracellular organelle;intracellular part;n		Homo sapiens		1	9
cell part;cytoplasmic part;cytosol;extracellular membrane-bounded organelle;extracellular organelle;extracellular region part;extracellular vesicular exosome;intracellular organelle part;intracellular part;lysosomal membrane;m	Collecting duct acid secretion;Epithelial cell signaling in Helicobacter pylori infection;Oxidative phospho	Homo sapiens		1	9
cell part;cytoplasmic part;endoplasmic reticulum membrane;endoplasmic reticulum part;integral to membrane;intracellular organelle part;intracellular part;intrinsic to membrane;membrane;membrane part;organelle membrane	N-Glycan biosynthesis	Homo sapiens		1	9
cell part;cytoplasm;cytoplasmic part;cytoskeletal part;cytosol;cytosolic part;IkappaB kinase complex;intracellular;intracellular membrane-bounded organelle;intracellular non-membrane-bounded organelle;intracellular organe	Acute myeloid leukemia;Adipocytokine signaling pathway;Apoptosis;B cell receptor signaling pathway;C	Homo sapiens		1	9
cell part;cytoplasmic part;intracellular membrane-bounded organelle;intracellular organelle;intracellular organelle part;intracellular part;macromolecular complex;membrane;membrane part;membrane-bounded organelle;mitoc	Alzheimer's disease;Huntington's disease;Oxidative phosphorylation;Parkinson's disease	Homo sapiens		1	10
cell part;cytoplasm;cytoplasmic part;cytosol;intracellular membrane-bounded organelle;intracellular organelle;intracellular organelle part;intracellular part;macromolecular complex;membrane-bounded organelle;nuclear part;nucleoplasm;nucleus;organelle;organelle part;proteasome regulatory particle;protein complex		Homo sapiens		1	10
cell part;cytoplasmic part;extracellular membrane-bounded organelle;extracellular organelle;extracellular region part;extracellular vesicular exosome;intracellular membrane-bounded organelle;intracellular organelle;intracellula	Lysosome;Other glycan degradation	Homo sapiens		1	10
cell part;cytoplasm;cytoplasmic part;cytoskeletal part;cytosol;histone acetyltransferase complex;histone methyltransferase complex;intracellular membrane-bounded organelle;intracellular organelle;intracellular organelle part	Other types of O-glycan biosynthesis	Homo sapiens		1	10
cell part;cytoplasmic part;intracellular membrane-bounded organelle;intracellular organelle;intracellular organelle lumen;intracellular organelle part;intracellular part;membrane-bounded organelle;membrane-enclosed lumen;m	Pantothenate and CoA biosynthesis;Valine, leucine and isoleucine biosynthesis;Valine, leucine and isole	Homo sapiens		1	10
Cajal body;cell part;intracellular membrane-bounded organelle;intracellular organelle;intracellular organelle part;intracellular part;macromolecular complex;membrane-bounded organelle;nuclear body;nuclear part;nuclear spec	Spliceosome	Homo sapiens		1	10
cell part;cytoplasm;intracellular organelle part;intracellular part;macromolecular complex;nuclear body;nuclear part;nuclear speck;nucleoplasm part;organelle part;ribonucleoprotein complex;small nuclear ribonucleoprotein cc	Spliceosome	Homo sapiens		1	10
cell part;cytoplasm;cytoplasmic part;cytoskeletal part;cytoskeleton;cytosol;intracellular membrane-bounded organelle;intracellular non-membrane-bounded organelle;intracellular organelle;intracellular organelle part;intracellular part;macromolecular complex;membrane;membrane-bounded organelle;microtubule;microtubule cyt		Homo sapiens		1	10
cell part;cytoplasm;cytoplasmic part;cytosol;early endosome;endosome;extracellular membrane-bounded organelle;extracellular organelle;extracellular region part;extracellular vesicular exosome;intracellular membrane-bounded organelle;intracellular organelle;intracellular part;membrane;membrane-bounded organelle;membrai		Homo sapiens		1	10

CCR4-NOT complex;cell part;cytoplasmic mRNA processing body;cytoplasmic part;cytosol;intracellular membrane-bounded organelle;intracellular non-membrane-bounded organelle;intracellular organelle;intracellular organe	RNA degradation	Homo sapiens	1	10
cell part;cytoplasm;cytoplasmic part;cytoplasmic vesicle;endoplasmic reticulum;endoplasmic reticulum exit site;endoplasmic reticulum membrane;endoplasmic reticulum part;endosome;extracellular membrane-bounded organelle;extracellular organelle;extracellular region part;extracellular vesicular exosome;intracellular membr		Homo sapiens	1	10
cell part;cytoplasm;cytoplasmic part;cytoskeletal part;cytoskeleton;intermediate filament cytoskeleton;intracellular membrane-bounded organelle;intracellular non-membrane-bounded organelle;intracellular organelle;intracellular organelle part;intracellular part;membrane-bounded organelle;microtubule cytoskeleton;microtubule		Homo sapiens	1	10
cell part;cytoplasmic part;intracellular organelle part;intracellular part;membrane;mitochondrial membrane;mitochondrial outer membrane;mitochondrial part;organelle membrane;organelle outer membrane;organelle part;outer membrane		Homo sapiens	1	10
cell part;cytoplasmic part;endoplasmic reticulum;endoplasmic reticulum membrane;endoplasmic reticulum part;extracellular membrane-bounded organelle;extracellular organelle;extracellular region part;extracellular vesicular exosome;integral to membrane;intracellular membrane-bounded organelle;intracellular organelle;intrace		Homo sapiens	1	10
cell part;cytoplasmic part;extracellular membrane-bounded organelle;extracellular organelle;extracellular region part;extracellular vesicular exosome;intracellular membrane-bounded organelle;intracellular organelle;intracellular part;membrane-bounded organelle;membrane-bounded vesicle;mitochondrion;organelle;vesicle		Homo sapiens	1	10
cell part;cytoplasmic part;cytosol;intracellular part	Alanine, aspartate and glutamate metabolism;Amino sugar and nucleotide sugar metabolism	Homo sapiens	1	10
		Homo sapiens	1	10
cell part;cytoplasmic part;cytoskeleton;cytosol;integral to membrane;intracellular membrane-bounded organelle;intracellular non-membrane-bounded organelle;intracellular organelle;intracellular organelle part;intracellular part;intrinsic to membrane;intrinsic to mitochondrial outer membrane;intrinsic to organelle membrane;memb		Homo sapiens	1	10
cell part;cytoplasm;intracellular membrane-bounded organelle;intracellular organelle;intracellular part;macromolecular complex;membrane-bounded organelle;nucleus;organelle;protein complex;ubiquitin ligase complex	Protein processing in endoplasmic reticulum;Ubiquitin mediated proteolysis	Homo sapiens	1	10
axoneme;cell part;cell projection part;cytoplasm;cytoplasmic part;cytosol;intracellular part;organelle part;pre-autophagosomal structure	Regulation of autophagy	Homo sapiens	1	10
cell part;cytoplasm;intracellular part		Homo sapiens	1	10
cell part;clathrin-coated vesicle;coated vesicle;cytoplasmic membrane-bounded vesicle;cytoplasmic part;cytoplasmic vesicle;cytoplasmic vesicle membrane;cytoplasmic vesicle part;endosome;extracellular membrane-bound	Pancreatic secretion	Homo sapiens	1	10
cell part;cytoplasm;extracellular membrane-bounded organelle;extracellular organelle;extracellular region part;extracellular vesicular exosome;intracellular part;membrane-bounded organelle;membrane-bounded vesicle;organelle;vesicle		Homo sapiens	1	10
cell part;cytoplasm;cytoplasmic part;extracellular membrane-bounded organelle;extracellular organelle;extracellular region part;extracellular vesicular exosome;integral to membrane;integral to mitochondrial membrane;integr	Amyotrophic lateral sclerosis (ALS)	Homo sapiens	1	10
blood microparticle;cell body;cell part;cell projection;chylomicron;cytoplasm;cytoplasmic membrane-bounded vesicle lumen;cytoplasmic part;cytoplasmic vesicle part;cytoskeletal part;dendrite;discoidal high-density lipoprot	Alzheimer's disease	Homo sapiens	1	10
cell part;cytoplasm;extracellular region part;extracellular space;intracellular part		Homo sapiens	1	10
cell part;cytoplasmic part;extracellular membrane-bounded organelle;extracellular organelle;extracellular region part;extracellular vesicular exosome;intracellular membrane-bounded organelle;intracellular non-membrane-bou	Huntington's disease;Peroxisome	Homo sapiens	1	10
apical part of cell;axon;cell junction;cell part;cell projection;cell projection part;cell surface;cell-cell junction;ciliary rootlet;cilium part;coated pit;coated vesicle;cytoplasm;cytoplasmic membrane-bounded vesicle;cytoplasmic r	Alzheimer's disease	Homo sapiens	1	10
actin filament;adherens junction;anchoring junction;cell junction;cell part;cell-substrate adherens junction;cell-substrate junction;cytoplasm;cytoplasmic part;cytoskeletal part;cytosol;extracellular membrane-bounded organel	Adherens junction;Tight junction	Homo sapiens	1	10
cell part;cytoplasmic part;intracellular organelle part;intracellular part;large ribosomal subunit;macromolecular complex;membrane;mitochondrial inner membrane;mitochondrial large ribosomal subunit;mitochondrial membrane;mitochondrial part;organellar large ribosomal subunit;organelle inner membrane;organelle membrane;c		Homo sapiens	1	10
cell part;chromatin;chromosomal part;euchromatin;extracellular membrane-bounded organelle;extracellular organelle;extracellular region part;extracellular vesicular exosome;heterochromatin;intracellular membrane-bounded	Systemic lupus erythematosus	Homo sapiens	1	10
apical part of cell;cell part;cytoplasmic part;endoplasmic reticulum;endoplasmic reticulum membrane;endoplasmic reticulum part;integral to membrane;intracellular membrane-bounded organelle;intracellular organelle;intrace	Drug metabolism - cytochrome P450;Glutathione metabolism;Metabolism of xenobiotics by cytochrome	Homo sapiens	1	10
cell part;extracellular membrane-bounded organelle;extracellular organelle;extracellular region part;extracellular vesicular exosome;membrane;membrane-bounded organelle;membrane-bounded vesicle;midbody;organelle;pl	Pancreatic cancer;Pathways in cancer	Homo sapiens	1	10
cell junction;cell part;cell-cell junction;chromatin;chromosomal part;cyclin-dependent protein kinase holoenzyme complex;cytoplasmic part;cytosol;intracellular membrane-bounded organelle;intracellular non-membrane-bou	Bladder cancer;Cell cycle;Chronic myeloid leukemia;Glioma;Measles;Melanoma;Non-small cell lung car	Homo sapiens	1	10
extracellular membrane-bounded organelle;extracellular organelle;extracellular region part;extracellular vesicular exosome;membrane-bounded organelle;membrane-bounded vesicle;organelle;vesicle		Homo sapiens	1	10
cell part;cytoplasmic part;extracellular matrix;extracellular region;extracellular region part;extracellular space;Golgi apparatus;intracellular membrane-bounded organelle;intracellular organelle;intracellular part;membrane-bounded organelle;membrane-bounded vesicle;organelle;proteinaceous extracellular matrix;vesicle		Homo sapiens	1	10
cell junction;cell part;cell-cell junction;cytoplasm;cytoplasmic part;cytosol;endoplasmic reticulum;intracellular membrane-bounded organelle;intracellular non-membrane-bounded organelle;intracellular organelle;intracellular p	ErbB signaling pathway;Pathogenic Escherichia coli infection;T cell receptor signaling pathway	Homo sapiens	1	10
cell part;cell projection membrane;cell projection part;cytoplasmic part;cytosol;extracellular membrane-bounded organelle;extracellular organelle;extracellular region part;extracellular vesicular exosome;Golgi apparatus;intracellular membrane-bounded organelle;intracellular organelle;intracellular part;leading edge membrane;me		Homo sapiens	1	10
cell part;intracellular membrane-bounded organelle;intracellular organelle;intracellular organelle part;intracellular part;membrane-bounded organelle;nuclear part;nucleoplasm;nucleus;organelle;organelle part	Base excision repair	Homo sapiens	1	10
blood microparticle;extracellular membrane-bounded organelle;extracellular organelle;extracellular region;extracellular region part;extracellular vesicular exosome;membrane-bounded organelle;membrane-bounded vesicle;organelle;vesicle		Homo sapiens	1	10
cell part;cytoplasm;extracellular region;intracellular membrane-bounded organelle;intracellular organelle;intracellular part;membrane-bounded organelle;nucleus;organelle	TGF-beta signaling pathway	Homo sapiens	1	10
cell part;cytoplasm;intracellular membrane-bounded organelle;intracellular organelle;intracellular part;membrane-bounded organelle;nucleus;organelle		Homo sapiens	1	10
axon;cell body;cell part;cell projection;cell projection part;cytoplasmic part;cytosol;dendritic spine;extracellular membrane-bounded organelle;extracellular organelle;extracellular region part;extracellular vesicular exosome;int	Betalain biosynthesis;Steroid hormone biosynthesis;Tyrosine metabolism	Homo sapiens	1	10
cell part;cytoplasm;cytoplasmic part;cytosol;flotillin complex;intracellular membrane-bounded organelle;intracellular organelle;intracellular part;macromolecular complex;membrane;membrane part;membrane-bounded organe	Bacterial invasion of epithelial cells;Chronic myeloid leukemia;Endocytosis;ErbB signaling pathway;Insu	Homo sapiens	1	10
extracellular region;extracellular region part;extracellular space;insulin-like growth factor binding protein complex;macromolecular complex;protein complex		Homo sapiens	1	10
actin filament bundle;actomyosin;cell part;contractile fiber part;cytoplasmic part;cytoskeletal part;cytosol;intracellular organelle part;intracellular part;macromolecular complex;muscle myosin complex;myosin complex;myosir	Focal adhesion;Leukocyte transendothelial migration;Regulation of actin cytoskeleton;Tight junction;Vas	Homo sapiens	1	10
cell part;centrosome;cytoplasm;cytoplasmic part;cytoskeletal part;cytosol;extracellular membrane-bounded organelle;extracellular organelle;extracellular region part;extracellular vesicular exosome;intracellular membrane-bo	Proteasome	Homo sapiens	1	10
actin cytoskeleton;adherens junction;anchoring junction;cell cortex;cell junction;cell part;cell-substrate adherens junction;cell-substrate junction;centrosome;cytoplasmic part;cytoskeletal part;cytoskeleton;extracellular memb	Fc gamma R-mediated phagocytosis	Homo sapiens	1	10
cell part;cytoplasm;cytoplasmic part;extracellular membrane-bounded organelle;extracellular organelle;extracellular region part;extracellular vesicular exosome;extrinsic to internal side of plasma membrane;extrinsic to memb	Amoebiasis;Calcium signaling pathway;Chagas disease (American trypanosomiasis);Gap junction;GnRH	Homo sapiens	1	10
cell part;cytoplasmic part;cytosol;extracellular membrane-bounded organelle;extracellular organelle;extracellular region part;extracellular vesicular exosome;intracellular part;membrane-bounded organelle;membrane-bounde	Pyrimidine metabolism	Homo sapiens	1	10
cell part;cytoplasm;cytoplasmic part;cytosol;extracellular membrane-bounded organelle;extracellular organelle;extracellular region part;extracellular vesicular exosome;intracellular membrane-bounded organelle;intracellular o	Cyanoamino acid metabolism;Glycine, serine and threonine metabolism;Methane metabolism;One carb	Homo sapiens	1	10
caveola;cell part;cell projection;cytoplasm;cytoplasmic part;endoplasmic reticulum membrane;endoplasmic reticulum part;intracellular membrane-bounded organelle;intracellular organelle;intracellular organelle part;intracellul	Arachidonic acid metabolism;Leishmaniasis;Pathways in cancer;Small cell lung cancer;VEGF signaling p	Homo sapiens	1	10
adherens junction;anchoring junction;cell junction;cell part;cell projection;cell projection part;cell-substrate adherens junction;cell-substrate junction;cytoplasmic part;cytoskeletal part;cytoskeleton;cytosol;dendritic spine;F-actin capping protein complex;focal adhesion;intermediate filament cytoskeleton;intracellular membrane-b		Homo sapiens	1	10
basement membrane;cell part;extracellular matrix;extracellular matrix part;extracellular membrane-bounded organelle;extracellular organelle;extracellular region part;extracellular space;extracellular vesicular exosome;intracellular membrane-bounded organelle;intracellular organelle;intracellular part;membrane-bounded organelle		Homo sapiens	1	10
apical plasma membrane;cell part;cell projection;cytoplasmic part;cytosol;endosome;extracellular membrane-bounded organelle;extracellular organelle;extracellular region part;extracellular vesicular exosome;intracellular me	Collecting duct acid secretion;Epithelial cell signaling in Helicobacter pylori infection;Oxidative phospho	Homo sapiens	1	10
cell part;cytoplasmic part;cytosol;cytosolic small ribosomal subunit;intracellular membrane-bounded organelle;intracellular non-membrane-bounded organelle;intracellular organelle;intracellular organelle part;intracellular part	Ribosome	Homo sapiens	1	10
autophagic vacuole;axon;cell part;cell projection;cytoplasm;cytoplasmic part;cytoplasmic vesicle membrane;cytoplasmic vesicle part;cytosol;dendrite;endoplasmic reticulum;endosome;Golgi apparatus;inclusion body;intrace	Huntington's disease	Homo sapiens	1	10
cell part;cytoplasm;cytoplasmic part;cytosol;intracellular organelle part;intracellular part;membrane;nuclear part;nucleoplasm;organelle part	Amyotrophic lateral sclerosis (ALS);Fc epsilon RI signaling pathway;GnRH signaling pathway;MAPK sigr	Homo sapiens	1	10
cell part;cytoplasmic part;cytosol;extracellular membrane-bounded organelle;extracellular organelle;extracellular region part;extracellular vesicular exosome;intracellular membrane-bounded organelle;intracellular organelle;int	Proteasome	Homo sapiens	1	10
cell part;intracellular membrane-bounded organelle;intracellular organelle;intracellular organelle part;intracellular part;membrane-bounded organelle;nuclear part;nucleoplasm;nucleus;organelle;organelle part	Base excision repair;DNA replication;Homologous recombination;Mismatch repair;Nucleotide excision r	Homo sapiens	1	10
cell part;chromosomal part;chromosome;chromosome, centromeric region;condensed chromosome;intracellular membrane-bounded organelle;intracellular non-membrane-bounded organelle;intracellular organelle;intracellular organelle part;intracellular part;membrane-bounded organelle;non-membrane-bounded organelle;nucl		Homo sapiens	1	10
adherens junction;anchoring junction;apical part of cell;basolateral plasma membrane;cell junction;cell part;cell-substrate adherens junction;cell-substrate junction;clathrin-coated vesicle;coated vesicle;cytoplasmic membrar	Notch signaling pathway	Homo sapiens	1	10
cell part;cytoplasmic part;cytoskeletal part;endoplasmic reticulum;envelope;integral to membrane;intracellular membrane-bounded organelle;intracellular organelle;intracellular organelle part;intracellular part;intrinsic to memb	Arrhythmogenic right ventricular cardiomyopathy (ARVC);Dilated cardiomyopathy;Hypertrophic cardiom	Homo sapiens	1	10
cell part;cytoplasmic part;endoplasmic reticulum part;extracellular membrane-bounded organelle;extracellular organelle;extracellular region part;extracellular vesicular exosome;integral to membrane;intracellular organelle par	Protein processing in endoplasmic reticulum	Homo sapiens	1	10
cell part;cytoplasmic part;intracellular membrane-bounded organelle;intracellular organelle;intracellular organelle part;intracellular part;macromolecular complex;membrane;membrane part;membrane-bounded organelle;mem	Alzheimer's disease;Huntington's disease;Oxidative phosphorylation;Parkinson's disease	Homo sapiens	1	10
cell part;cytoplasmic part;intracellular membrane-bounded organelle;intracellular organelle;intracellular organelle part;intracellular part;membrane;membrane-bounded organelle;mitochondrial inner membrane;mitochondrial m	Porphyrin and chlorophyll metabolism	Homo sapiens	1	10
cell part;cytoplasmic membrane-bounded vesicle;cytoplasmic part;cytoplasmic vesicle;cytosol;Golgi apparatus;Golgi apparatus part;Golgi membrane;integral to membrane;intracellular membrane-bounded organelle;intracellular organelle;intracellular organelle part;intracellular part;intrinsic to membrane;membrane;membrane pa		Homo sapiens	1	10
cell part;cytoplasm;cytoplasmic part;cytosol;extracellular membrane-bounded organelle;extracellular organelle;extracellular region part;extracellular vesicular exosome;intracellular membrane-bounded organelle;intracellular o	Ubiquitin mediated proteolysis	Homo sapiens	1	10
adherens junction;anchoring junction;cell cortex;cell division site part;cell junction;cell part;cell projection;cell projection membrane;cell projection part;cell-substrate adherens junction;cell-substrate junction;cleavage furrow;c	Endocytosis;Fc gamma R-mediated phagocytosis	Homo sapiens	1	10
cell part;cytoplasm;cytoskeleton;intracellular non-membrane-bounded organelle;intracellular organelle;intracellular part;non-membrane-bounded organelle;organelle		Homo sapiens	1	10
cell part;intracellular membrane-bounded organelle;intracellular organelle;intracellular organelle part;intracellular part;macromolecular complex;membrane-bounded organelle;multimeric ribonuclease P complex;nuclear part;n	Ribosome biogenesis in eukaryotes;RNA transport	Homo sapiens	1	10
cell part;membrane;midbody		Homo sapiens	1	10
cell part;extracellular membrane-bounded organelle;extracellular organelle;extracellular region part;extracellular vesicular exosome;integral to membrane;intracellular membrane-bounded organelle;intracellular organelle;intracellular part;intrinsic to membrane;membrane;membrane part;membrane-bounded organelle;membrane-b		Homo sapiens	1	10
cell part;cytoplasmic part;Golgi apparatus;intracellular membrane-bounded organelle;intracellular non-membrane-bounded organelle;intracellular organelle;intracellular organelle part;intracellular part;membrane-bounded organelle;non-membrane-bounded organelle;nuclear part;nucleolus;nucleus;organelle;organelle part		Homo sapiens	1	10
cell part;cytoplasmic part;cytosol;intracellular membrane-bounded organelle;intracellular organelle;intracellular part;membrane-bounded organelle;microbody;mitochondrion;organelle;peroxisome	Terpenoid backbone biosynthesis	Homo sapiens	1	10
adherens junction;anchoring junction;brush border membrane;cell junction;cell part;cell projection membrane;cell projection part;cell-substrate adherens junction;cell-substrate junction;cytoplasmic membrane-bounded vesic	Long-term depression;Regulation of actin cytoskeleton;Vascular smooth muscle contraction	Homo sapiens	1	10
macromolecular complex;protein complex;ribose phosphate diphosphokinase complex		Homo sapiens	1	10
cell part;cytoplasmic part;cytosol;exosome (RNase complex);intracellular membrane-bounded organelle;intracellular non-membrane-bounded organelle;intracellular organelle;intracellular organelle part;intracellular part;macrc	RNA degradation	Homo sapiens	1	10
cell part;cytoplasm;cytoskeletal part;intracellular organelle part;intracellular part;macromolecular complex;microtubule associated complex;organelle part;protein complex		Homo sapiens	1	10
calcium channel complex;cation channel complex;cell part;cytoplasm;extracellular membrane-bounded organelle;extracellular organelle;extracellular region part;extracellular vesicular exosome;intracellular membrane-bounded organelle;intracellular organelle;intracellular organelle part;intracellular part;ion channel complex;macrc		Homo sapiens	1	10
adherens junction;anchoring junction;axon;cell body;cell junction;cell part;cell projection;cell surface;cell-cell junction;cilium;dendrite;integral to membrane;integral to plasma membrane;intrinsic to membrane;intrinsic to plasma membrane;leading edge membrane;membrane part;neuron projection;neuronal cell body;nonmotile pı		Homo sapiens	1	10
cell part;cytoplasmic part;cytosol;elongin complex;intracellular organelle part;intracellular part;macromolecular complex;nuclear part;nucleoplasm;nucleoplasm part;organelle part;protein complex;transcription elongation fact	Pathways in cancer;Renal cell carcinoma;Ubiquitin mediated proteolysis	Homo sapiens	1	10
cell part;Cul2-RING ubiquitin ligase complex;Cul5-RING ubiquitin ligase complex;cullin-RING ubiquitin ligase complex;cytoplasmic part;cytoplasmic ubiquitin ligase complex;cytosol;elongin complex;extracellular membrane-l	Pathways in cancer;Renal cell carcinoma;Ubiquitin mediated proteolysis	Homo sapiens	1	10
cell part;chromosome;cytoplasm;extracellular membrane-bounded organelle;extracellular organelle;extracellular region part;extracellular vesicular exosome;intracellular membrane-bounded organelle;intracellular non-membrane-bounded organelle;intracellular organelle;intracellular part;membrane-bounded organelle;membrane-		Homo sapiens	1	10
cell part;cytoplasmic membrane-bounded vesicle;cytoplasmic part;cytoplasmic vesicle;extracellular membrane-bounded organelle;extracellular organelle;extracellular region part;extracellular vesicular exosome;Golgi apparat	Protein digestion and absorption	Homo sapiens	1	10
cell part;cytoplasmic part;endoplasmic reticulum;endoplasmic reticulum membrane;endoplasmic reticulum part;Golgi apparatus;Golgi apparatus part;Golgi membrane;integral to endoplasmic reticulum membrane;integral to	Glycosaminoglycan biosynthesis - heparan sulfate	Homo sapiens	1	10
	Amino sugar and nucleotide sugar metabolism	Homo sapiens	1	10
cell part;cell surface;chloride channel complex;extracellular membrane-bounded organelle;extracellular organelle;extracellular region part;extracellular vesicular exosome;intracellular;ion channel complex;macromolecular complex;membrane;membrane part;membrane-bounded organelle;membrane-bounded vesicle;organelle;pl		Homo sapiens	1	10
cell part;extracellular membrane-bounded organelle;extracellular organelle;extracellular region part;extracellular vesicular exosome;membrane;membrane-bounded organelle;membrane-bounded vesicle;organelle;vesicle		Homo sapiens	1	10
cell part;intracellular non-membrane-bounded organelle;intracellular organelle;intracellular organelle part;intracellular part;macromolecular complex;non-membrane-bounded organelle;nuclear part;nucleolus;organelle;organelle part;preribosome;ribonucleoprotein complex;small-subunit processome		Homo sapiens	1	10
		Homo sapiens	1	10
cell part;cytoplasmic part;intracellular organelle part;intracellular part;lysosomal membrane;membrane;organelle membrane;organelle part;vacuolar membrane;vacuolar part		Homo sapiens	1	10
cell part;intracellular organelle part;intracellular part;macromolecular complex;mRNA cleavage and polyadenylation specificity factor complex;mRNA cleavage factor complex;nuclear part;nucleoplasm;nucleoplasm part;orgar	mRNA surveillance pathway	Homo sapiens	1	10
cell part;chromatin;chromatin remodeling complex;chromosomal part;histone methyltransferase complex;intracellular organelle part;intracellular part;macromolecular complex;methyltransferase complex;nuclear part;nucleoplasm part;organelle part;protein complex;protein serine/threonine phosphatase complex;PTW/PP1 phosp		Homo sapiens	1	10
cell part;integral to membrane;intracellular membrane-bounded organelle;intracellular organelle;intracellular part;intrinsic to membrane;membrane;membrane part;membrane-bounded organelle;nucleus;organelle		Homo sapiens	1	10
cell part;cytoplasm;extracellular membrane-bounded organelle;extracellular organelle;extracellular region part;extracellular vesicular exosome;intracellular organelle part;intracellular part;macromolecular complex;membrane-bounded organelle;membrane-bounded vesicle;nuclear part;nucleoplasm;organelle;organelle part;protein		Homo sapiens	1	10
		Homo sapiens	1	10
cell part;cytoplasm;cytoskeleton;intermediate filament cytoskeleton;intracellular non-membrane-bounded organelle;intracellular organelle;intracellular part;membrane;non-membrane-bounded organelle;organelle;plasma membrane		Homo sapiens	1	10
cell part;cytoplasm;intracellular part		Homo sapiens	1	10
cell part;cytoplasmic part;extracellular membrane-bounded organelle;extracellular organelle;extracellular region part;extracellular vesicular exosome;integral to membrane;intracellular organelle part;intracellular part;intrinsic to membrane;lysosomal membrane;membrane;membrane part;membrane-bounded organelle;membrane-		Homo sapiens	1	10
cell part;cytoplasm;cytoplasmic part;intracellular organelle part;intracellular part;lipid particle;membrane;midbody;mitochondrial membrane;mitochondrial outer membrane;mitochondrial part;organelle membrane;organelle outer membrane;organelle part;outer membrane;plasma membrane;synapse		Homo sapiens	1	10
Cdc73/Paf1 complex;cell part;cytoplasm;intracellular organelle part;intracellular part;macromolecular complex;membrane;nuclear part;nucleoplasm part;organelle part;protein complex;transcription elongation factor complex		Homo sapiens	1	10
cell part;cytoplasmic part;extracellular membrane-bounded organelle;extracellular organelle;extracellular region part;extracellular space;extracellular vesicular exosome;Golgi apparatus;integral to membrane;integral to plasma membrane;intracellular membrane-bounded organelle;intracellular organelle;intracellular part;intrinsic to		Homo sapiens	1	10
cell part;intracellular organelle part;intracellular part;nuclear part;nucleoplasm;organelle part		Homo sapiens	1	10
cell part;intracellular membrane-bounded organelle;intracellular organelle;intracellular organelle part;intracellular part;membrane;membrane-bounded organelle;nuclear part;nucleoplasm;nucleus;organelle;organelle part	Amino sugar and nucleotide sugar metabolism	Homo sapiens	1	10
cell part;cis-Golgi network;cytoplasmic part;Golgi apparatus;Golgi apparatus part;Golgi cisterna;Golgi membrane;integral to membrane;intracellular membrane-bounded organelle;intracellular organelle;intracellular organelle part;intracellular part;intrinsic to membrane;membrane;membrane part;membrane-bounded organelle;org		Homo sapiens	1	10
extracellular region		Homo sapiens	1	10
cell part;cytoplasmic membrane-bounded vesicle;cytoplasmic part;cytoplasmic vesicle;extracellular membrane-bounded organelle;extracellular organelle;extracellular region;extracellular region part;extracellular vesicular exosome;Golgi apparatus;intracellular membrane-bounded organelle;intracellular organelle;intracellular part;		Homo sapiens	1	10
cell part;cytoplasm;cytoplasmic part;endoplasmic reticulum;endoplasmic reticulum membrane;endoplasmic reticulum part;envelope;intracellular membrane-bounded organelle;intracellular organelle;intracellular organelle part;intracellular part;membrane;membrane part;membrane-bounded organelle;nuclear envelope;nuclear par		Homo sapiens	1	10
cell junction;cell part;cell-cell junction;cytoplasm;cytoskeleton;intracellular non-membrane-bounded organelle;intracellular organelle;intracellular organelle part;intracellular part;membrane;non-membrane-bounded organelle;n	mRNA surveillance pathway;Tight junction	Homo sapiens	1	10
cell part;Cul5-RING ubiquitin ligase complex;cullin-RING ubiquitin ligase complex;cytoplasmic part;cytosol;intracellular membrane-bounded organelle;intracellular organelle;intracellular part;macromolecular complex;membra	Ubiquitin mediated proteolysis	Homo sapiens	1	10
cell part;cytoplasm;cytoplasmic membrane-bounded vesicle;cytoplasmic part;cytoplasmic vesicle;cytoplasmic vesicle membrane;cytoplasmic vesicle part;endocytic vesicle membrane;endosomal part;endosome membrane;	Collecting duct acid secretion;Epithelial cell signaling in Helicobacter pylori infection;ko05152;Lysosome	Homo sapiens	1	10
cell part;cytoplasm;cytoplasmic part;Golgi apparatus;intracellular membrane-bounded organelle;intracellular non-membrane-bounded organelle;intracellular organelle;intracellular organelle part;intracellular part;membrane-bounded organelle;non-membrane-bounded organelle;nuclear part;nucleolus;organelle;organelle part		Homo sapiens	1	10
cell part;cytoplasm;intracellular part;LUBAC complex;macromolecular complex;protein complex;ubiquitin ligase complex		Homo sapiens	1	10
		Homo sapiens	1	10
cell part;centrosome;cytoplasmic part;cytoskeletal part;intracellular membrane-bounded organelle;intracellular non-membrane-bounded organelle;intracellular organelle;intracellular organelle part;intracellular part;macromolecular complex;membrane-bounded organelle;microtubule;microtubule organizing center;mitochondrion;n		Homo sapiens	1	10
cell part;cytoplasm;intracellular organelle part;intracellular part;nuclear part;nucleoplasm;organelle part	mRNA surveillance pathway	Homo sapiens	1	10
cell part;cytoplasm;cytoplasmic part;cytosol;intracellular membrane-bounded organelle;intracellular organelle;intracellular part;membrane-bounded organelle;nucleus;organelle	mRNA surveillance pathway	Homo sapiens	1	10
cell part;cytoplasm;intracellular membrane-bounded organelle;intracellular organelle;intracellular organelle part;intracellular part;membrane-bounded organelle;nuclear part;nucleoplasm;nucleus;organelle;organelle part		Homo sapiens	1	10
cell part;cytoplasm;cytoplasmic part;cytosol;extracellular membrane-bounded organelle;extracellular organelle;extracellular region part;extracellular space;extracellular vesicular exosome;intracellular membrane-bounded organelle;intracellular organelle;intracellular part;lysosome;lytic vacuole;mast cell granule;membrane-bounde		Homo sapiens	1	10
cell part;intracellular membrane-bounded organelle;intracellular non-membrane-bounded organelle;intracellular organelle;intracellular organelle part;intracellular part;membrane-bounded organelle;non-membrane-bounded organelle;nuclear part;nucleolus;nucleoplasm;nucleus;organelle;organelle part		Homo sapiens	1	10
cell part;cytoplasmic part;endoplasmic reticulum membrane;endoplasmic reticulum part;extracellular region;intracellular organelle part;intracellular part;membrane;membrane part;organelle membrane;organelle part		Homo sapiens	1	10
cell part;cytoplasmic part;endoplasmic reticulum;extracellular region;intracellular membrane-bounded organelle;intracellular organelle;intracellular part;membrane;membrane-bounded organelle;organelle	Glycolysis / Gluconeogenesis	Homo sapiens	1	10
cell part;chromatin remodeling complex;chromosomal part;cytoplasmic membrane-bounded vesicle;cytoplasmic part;cytoplasmic vesicle;intracellular membrane-bounded organelle;intracellular organelle;intracellular organelle part;intracellular part;macromolecular complex;membrane-bounded organelle;membrane-bounded ves		Homo sapiens	1	10
		Homo sapiens	1	10
cell part;cytoplasmic membrane-bounded vesicle;cytoplasmic part;cytoplasmic vesicle;cytoplasmic vesicle membrane;cytoplasmic vesicle part;cytoskeletal part;early endosome;early endosome membrane;endosomal part;e	Endocytosis	Homo sapiens	1	10
cell part;cytoplasmic part;intracellular membrane-bounded organelle;intracellular organelle;intracellular part;membrane-bounded organelle;mitochondrion;nucleus;organelle		Homo sapiens	1	10
cell part;cytoplasm;cytoplasmic part;cytosol;intracellular membrane-bounded organelle;intracellular organelle;intracellular part;membrane-bounded organelle;nucleus;organelle	Pathways in cancer;Renal cell carcinoma	Homo sapiens	1	10
cell part;extracellular region part;extracellular space;intracellular membrane-bounded organelle;intracellular organelle;intracellular organelle part;intracellular part;membrane-bounded organelle;nuclear part;nucleoplasm;nucleus;organelle;organelle part		Homo sapiens	1	10
cell junction;cell part;cytoplasmic part;intracellular membrane-bounded organelle;intracellular non-membrane-bounded organelle;intracellular organelle;intracellular organelle part;intracellular part;macromolecular complex;membrane;membrane-bounded organelle;mitochondrial inner membrane;mitochondrial membrane;mitocho		Homo sapiens	1	10
extracellular membrane-bounded organelle;extracellular organelle;extracellular region part;extracellular vesicular exosome;membrane-bounded organelle;membrane-bounded vesicle;organelle;vesicle		Homo sapiens	1	10
cell part;cytoplasmic part;Golgi apparatus;Golgi apparatus part;Golgi membrane;integral to membrane;intracellular membrane-bounded organelle;intracellular organelle;intracellular organelle part;intracellular part;intrinsic to membrane;membrane;membrane part;membrane-bounded organelle;mitochondrion;organelle;organelle m		Homo sapiens	1	10
actin cytoskeleton;cell part;cytoplasm;cytoskeleton;endomembrane system;intracellular non-membrane-bounded organelle;intracellular organelle;intracellular part;membrane;microtubule cytoskeleton;non-membrane-bounded organelle;organelle;plasma membrane		Homo sapiens	1	10
cell part;cytoplasmic part;cytosol;endoplasmic reticulum part;intracellular organelle part;intracellular part;membrane;nuclear membrane;nuclear part;organelle membrane;organelle part;perinuclear endoplasmic reticulum;plasma membrane		Homo sapiens	1	10
cell part;cytoplasmic part;integral to membrane;intracellular membrane-bounded organelle;intracellular organelle;intracellular organelle part;intracellular part;intrinsic to membrane;membrane;membrane part;membrane-bounded organelle;mitochondrial inner membrane;mitochondrial membrane;mitochondrial part;mitochondrion;		Homo sapiens	1	10
cell part;cytoplasmic part;cytosol;extracellular membrane-bounded organelle;extracellular organelle;extracellular region part;extracellular vesicular exosome;intracellular part;membrane-bounded organelle;membrane-bounde	Pantothenate and CoA biosynthesis	Homo sapiens	1	10
actin cytoskeleton;cell part;cytoskeleton;extracellular region part;extracellular space;intracellular membrane-bounded organelle;intracellular non-membrane-bounded organelle;intracellular organelle;intracellular organelle part;intracellular part;membrane-bounded organelle;non-membrane-bounded organelle;nuclear part;nucleop		Homo sapiens	1	10
catalytic step 2 spliceosome;cell part;intracellular membrane-bounded organelle;intracellular organelle;intracellular organelle part;intracellular part;macromolecular complex;membrane;membrane-bounded organelle;nuclear p	Spliceosome	Homo sapiens	1	10

cell part;cytoplasm;cytoskeleton;extracellular region part;intercellular bridge;intracellular membrane-bounded organelle;intracellular non-membrane-bounded organelle;intracellular organelle;intracellular organelle part;intracellular part;macromolecular complex;membrane;membrane-bounded organelle;microtubule cytoskeleton;n		Homo sapiens	1	10
cell part;cytoplasm;intracellular part;membrane;plasma membrane		Homo sapiens	1	10
cell part;cytoplasmic part;intracellular organelle part;intracellular part;large ribosomal subunit;macromolecular complex;membrane;mitochondrial inner membrane;mitochondrial large ribosomal subunit;mitochondrial membrane;mitochondrial part;organellar large ribosomal subunit;organelle inner membrane;organelle membrane;o		Homo sapiens	1	10
cell part;intracellular organelle part;intracellular part;nuclear part;nucleoplasm;organelle part		Homo sapiens	1	10
cell part;membrane		Homo sapiens	1	10
cell part;intracellular non-membrane-bounded organelle;intracellular organelle;intracellular organelle part;intracellular part;membrane;non-membrane-bounded organelle;nuclear part;nucleolus;nucleoplasm;organelle;organelle part;plasma membrane		Homo sapiens	1	10
cell part;cytoplasmic part;cytoskeletal part;cytosol;intermediate filament;intracellular membrane-bounded organelle;intracellular organelle;intracellular organelle part;intracellular part;macromolecular complex;membrane-bounded organelle;nucleus;organelle;organelle part;protein complex		Homo sapiens	1	10
cell part;cytoplasm;extracellular region part;extracellular space;intracellular membrane-bounded organelle;intracellular organelle;intracellular part;membrane-bounded organelle;nucleus;organelle		Homo sapiens	1	10
catalytic step 2 spliceosome;cell part;intracellular membrane-bounded organelle;intracellular organelle;intracellular organelle part;intracellular part;macromolecular complex;membrane-bounded organelle;nuclear part;nucleop	Spliceosome	Homo sapiens	1	10
cell part;cytoplasm;intracellular membrane-bounded organelle;intracellular non-membrane-bounded organelle;intracellular organelle;intracellular organelle part;intracellular part;membrane-bounded organelle;non-membrane-bounded organelle;nuclear part;nucleolus;nucleus;organelle;organelle part		Homo sapiens	1	10
adherens junction;anchoring junction;cell junction;cell part;cell-substrate adherens junction;cell-substrate junction;cytoplasmic part;endoplasmic reticulum;endoplasmic reticulum membrane;endoplasmic reticulum part;focal adhesion;integral to membrane;intracellular membrane-bounded organelle;intracellular organelle;intracell		Homo sapiens	1	10
cell part;cyclin-dependent protein kinase holoenzyme complex;intracellular membrane-bounded organelle;intracellular organelle;intracellular organelle part;intracellular part;macromolecular complex;membrane-bounded organelle;nuclear cyclin-dependent protein kinase holoenzyme complex;nuclear part;nucleus;organelle;organe		Homo sapiens	1	10
cell part;cytoplasmic part;endoplasmic reticulum;endoplasmic reticulum membrane;endoplasmic reticulum part;integral to membrane;intracellular membrane-bounded organelle;intracellular organelle;intracellular organelle pa	Steroid biosynthesis	Homo sapiens	1	10
cell part;cytoplasmic part;integral to membrane;intracellular membrane-bounded organelle;intracellular organelle;intracellular organelle part;intracellular part;intrinsic to membrane;membrane;membrane part;membrane-bounc	Proximal tubule bicarbonate reclamation	Homo sapiens	1	10
cell part;cytoplasm;extracellular membrane-bounded organelle;extracellular organelle;extracellular region part;extracellular vesicular exosome;intracellular part;membrane-bounded organelle;membrane-bounded vesicle;organelle;vesicle		Homo sapiens	1	10
cell part;cytoplasm;cytoplasmic part;cytosolic part;extracellular membrane-bounded organelle;extracellular organelle;extracellular region part;extracellular vesicular exosome;intracellular membrane-bounded organelle;intracellular non-membrane-bounded organelle;intracellular organelle;intracellular organelle part;intracellular pa		Homo sapiens	1	10
cell part;cytoplasmic part;cytosol;extracellular membrane-bounded organelle;extracellular organelle;extracellular region part;extracellular vesicular exosome;intracellular organelle part;intracellular part;lysosomal membrane;m	Epithelial cell signaling in Helicobacter pylori infection;ko05152;Lysosome;Oxidative phosphorylation;Ph	Homo sapiens	1	10
cell part;intracellular membrane-bounded organelle;intracellular organelle;intracellular part;membrane-bounded organelle;nucleus;organelle		Homo sapiens	1	10
cell cortex part;cell part;cell projection part;centriolar satellite;cytoplasmic part;cytoskeletal part;cytosol;exocyst;growth cone membrane;intracellular organelle part;intracellular part;macromolecular complex;membrane;memb	Insulin signaling pathway	Homo sapiens	1	10
cell part;cytoplasmic part;intracellular membrane-bounded organelle;intracellular organelle;intracellular organelle part;intracellular part;macromolecular complex;membrane;membrane-bounded organelle;mitochondrial inner membrane;mitochondrial membrane;mitochondrial part;mitochondrial small ribosomal subunit;mitochond		Homo sapiens	1	10
cell part;cytoplasm;intracellular membrane-bounded organelle;intracellular non-membrane-bounded organelle;intracellular organelle;intracellular organelle part;intracellular part;membrane;membrane-bounded organelle;non-membrane-bounded organelle;nuclear part;nucleolus;nucleus;organelle;organelle part		Homo sapiens	1	10
cell part;integral to membrane;integral to nuclear inner membrane;integral to organelle membrane;intracellular organelle part;intracellular part;intrinsic to membrane;intrinsic to nuclear inner membrane;intrinsic to organelle membrane;membrane;membrane part;nuclear inner membrane;nuclear membrane;nuclear membrane part;r		Homo sapiens	1	10
cell part;cytoplasmic mRNA processing body;cytoplasmic part;cytosol;extracellular membrane-bounded organelle;extracellular organelle;extracellular region part;extracellular vesicular exosome;intracellular non-membrane-bounded organelle;intracellular organelle;intracellular part;macromolecular complex;membrane-bounded c		Homo sapiens	1	10
cell part;cytoplasmic part;intracellular membrane-bounded organelle;intracellular non-membrane-bounded organelle;intracellular organelle;intracellular organelle part;intracellular part;membrane-bounded organelle;mitochondrion;non-membrane-bounded organelle;nuclear part;nucleolus;nucleus;organelle;organelle part		Homo sapiens	1	10
cell part;intracellular membrane-bounded organelle;intracellular non-membrane-bounded organelle;intracellular organelle;intracellular organelle part;intracellular part;membrane-bounded organelle;non-membrane-bounded organelle;nuclear part;nucleolus;nucleus;organelle;organelle part		Homo sapiens	1	10
extracellular membrane-bounded organelle;extracellular organelle;extracellular region part;extracellular vesicular exosome;membrane-bounded organelle;membrane-bounded vesicle;organelle;vesicle		Homo sapiens	1	10
		Homo sapiens	1	10
cell part;cytoplasmic part;extracellular membrane-bounded organelle;extracellular organelle;extracellular region part;extracellular vesicular exosome;intracellular membrane-bounded organelle;intracellular organelle;intracellula	Alzheimer's disease;Huntington's disease;Oxidative phosphorylation;Parkinson's disease	Homo sapiens	1	10
	Glyoxylate and dicarboxylate metabolism	Homo sapiens	1	11
cell part;cytoplasm;intracellular membrane-bounded organelle;intracellular organelle;intracellular part;membrane-bounded organelle;nucleus;organelle		Homo sapiens	1	11
		Homo sapiens	1	11
cell part;cytoplasmic part;cytosol;intracellular organelle part;intracellular part;macromolecular complex;membrane part;nuclear part;nuclear pore;nucleoplasm;organelle part;pore complex;protein complex		Homo sapiens	1	11
cell part;chromatin;chromatin remodeling complex;chromosomal part;intracellular membrane-bounded organelle;intracellular organelle;intracellular organelle part;intracellular part;macromolecular complex;membrane-bounded organelle;nBAF complex;npBAF complex;nuclear chromatin;nuclear chromosome part;nuclear part;nuc		Homo sapiens	1	11
actin cytoskeleton;apical plasma membrane;brush border membrane;cell part;cell periphery;cell projection;cell projection membrane;cell projection part;centrosome;cytoplasm;cytoplasmic part;cytoskeletal part;cytoskeleton;endomembrane system;extracellular membrane-bounded organelle;extracellular organelle;extracellular r		Homo sapiens	1	11
cell part;chromosomal part;chromosome, telomeric region;cytoplasmic part;intracellular non-membrane-bounded organelle;intracellular organelle;intracellular organelle part;intracellular part;macromolecular complex;mitochondrial nucleoid;mitochondrial part;non-membrane-bounded organelle;nuclear body;nuclear chromosome		Homo sapiens	1	11
cell part;cytoplasm;cytoplasmic part;cytosol;intracellular membrane-bounded organelle;intracellular organelle;intracellular part;membrane-bounded organelle;mitochondrion;organelle		Homo sapiens	1	11
cell part;cytoplasmic part;intracellular membrane-bounded organelle;intracellular organelle;intracellular organelle part;intracellular part;membrane;membrane part;membrane-bounded organelle;mitochondrial inner membrane	Alzheimer's disease;Cardiac muscle contraction;Huntington's disease;Oxidative phosphorylation;Parkin	Homo sapiens	1	11
cell part;cytoplasm;cytoplasmic part;cytoplasmic vesicle membrane;cytoplasmic vesicle part;cytosol;endoplasmic reticulum;endosome;extracellular membrane-bounded organelle;extracellular organelle;extracellular region pa	SNARE interactions in vesicular transport	Homo sapiens	1	11
cell part;cytoplasmic part;cytosol;extracellular membrane-bounded organelle;extracellular organelle;extracellular region part;extracellular vesicular exosome;Golgi apparatus;intracellular membrane-bounded organelle;intracellular organelle;intracellular part;membrane-bounded organelle;membrane-bounded vesicle;mitochondrio		Homo sapiens	1	11
cell part;chromosome;condensed chromosome;cytoplasmic part;female pronucleus;Golgi apparatus;intracellular membrane-bounded organelle;intracellular non-membrane-bounded organelle;intracellular organelle;intracellular organelle part;intracellular part;membrane;membrane-bounded organelle;mitochondrion;non-membra		Homo sapiens	1	11
		Homo sapiens	1	11
cell part;chromatin;chromosomal part;cytoplasm;intracellular membrane-bounded organelle;intracellular organelle;intracellular organelle part;intracellular part;membrane;membrane-bounded organelle;nucleus;organelle;organelle part		Homo sapiens	1	11
cell part;cytoplasmic part;cytosol;extracellular membrane-bounded organelle;extracellular organelle;extracellular region part;extracellular vesicular exosome;intracellular organelle part;intracellular part;lysosomal membrane;m	Collecting duct acid secretion;Epithelial cell signaling in Helicobacter pylori infection;Oxidative phospho	Homo sapiens	1	11
cell part;cytoplasmic part;intracellular membrane-bounded organelle;intracellular organelle;intracellular organelle part;intracellular part;macromolecular complex;membrane;membrane part;membrane-bounded organelle;mitoc	Alzheimer's disease;Huntington's disease;Oxidative phosphorylation;Parkinson's disease	Homo sapiens	1	11
cell part;granular component;intracellular non-membrane-bounded organelle;intracellular organelle;intracellular organelle part;intracellular part;non-membrane-bounded organelle;nuclear part;nucleolar part;nucleolus;nucleoplasm;organelle;organelle part		Homo sapiens	1	11
cell part;cytoplasm;cytoplasmic part;cytosol;extracellular region part;extracellular space;intracellular organelle part;intracellular part;nuclear part;nucleoplasm;organelle part	Arachidonic acid metabolism	Homo sapiens	1	11
cell part;cytoplasmic part;endoplasmic reticulum membrane;endoplasmic reticulum part;extracellular membrane-bounded organelle;extracellular organelle;extracellular region part;extracellular vesicular exosome;integral to membrane;intracellular organelle part;intracellular part;intrinsic to membrane;membrane;membrane part;m		Homo sapiens	1	11
cell division site part;cell part;centrosome;chromosomal part;cleavage furrow;condensed chromosome kinetochore;cytoplasmic part;cytoskeletal part;cytosol;dynactin complex;intracellular non-membrane-bounded organelle;intracellular organelle;intracellular organelle part;intracellular part;kinetochore;macromolecular complex;r		Homo sapiens	1	11
cell cortex;cell part;contractile fiber part;cytoplasmic part;extracellular membrane-bounded organelle;extracellular organelle;extracellular region part;extracellular vesicular exosome;intracellular part;membrane-bounded organelle;membrane-bounded vesicle;organelle;organelle part;vesicle;Z disc		Homo sapiens	1	11
cell part;cytoplasm;intracellular membrane-bounded organelle;intracellular organelle;intracellular organelle part;intracellular part;membrane-bounded organelle;nuclear part;nucleoplasm;nucleus;organelle;organelle part		Homo sapiens	1	11
cell part;cytoplasmic part;cytosol;extracellular membrane-bounded organelle;extracellular organelle;extracellular region part;extracellular vesicular exosome;intracellular part;membrane-bounded organelle;membrane-bounde	Pentose phosphate pathway	Homo sapiens	1	11
cell part;coated vesicle membrane;COPII vesicle coat;cytoplasmic part;cytoplasmic vesicle membrane;cytoplasmic vesicle part;cytosol;endoplasmic reticulum membrane;endoplasmic reticulum part;ER to Golgi transport ves	Protein processing in endoplasmic reticulum	Homo sapiens	1	11
cell part;cytoplasm;cytoplasmic part;intracellular membrane-bounded organelle;intracellular organelle;intracellular organelle lumen;intracellular organelle part;intracellular part;membrane-bounded organelle;membrane-enclosed lumen;mitochondrial matrix;mitochondrial part;mitochondrion;nuclear part;nucleoplasm;organelle;orga		Homo sapiens	1	11
cell part;chromatin;chromatin remodeling complex;chromosomal part;DNA helicase complex;H4/H2A histone acetyltransferase complex;histone acetyltransferase complex;Ino80 complex;intracellular membrane-bounded organelle;intracellular organelle;intracellular organelle part;intracellular part;macromolecular complex;membr		Homo sapiens	1	11
blood microparticle;cell part;cytoplasmic part;endoplasmic reticulum lumen;endoplasmic reticulum part;extracellular membrane-bounded organelle;extracellular organelle;extracellular region;extracellular region part;extracellu	Complement and coagulation cascades;Neuroactive ligand-receptor interaction;Regulation of actin cytc	Homo sapiens	1	11
blood microparticle;cell part;extracellular membrane-bounded organelle;extracellular organelle;extracellular region;extracellular region part;extracellular space;extracellular vesicular exosome;membrane;membrane-bounded c	Complement and coagulation cascades	Homo sapiens	1	11
adherens junction;anchoring junction;cell junction;cell part;cell-substrate adherens junction;cell-substrate junction;cytoplasm;cytoplasmic part;cytosol;extrinsic to internal side of plasma membrane;extrinsic to membrane;ext	Acute myeloid leukemia;Aldosterone-regulated sodium reabsorption;Axon guidance;B cell receptor sign	Homo sapiens	1	11
cell part;cytoplasm;cytoplasmic part;intracellular membrane-bounded organelle;intracellular organelle;intracellular part;membrane-bounded organelle;nucleus;organelle;perinuclear region of cytoplasm	Mineral absorption	Homo sapiens	1	11
adherens junction;anchoring junction;cell junction;cell part;cell-substrate adherens junction;cell-substrate junction;cytoplasmic part;cytosol;cytosolic large ribosomal subunit;extracellular membrane-bounded organelle;extrac	Ribosome	Homo sapiens	1	11
cell part;collagen;collagen type V;cytoplasmic part;endoplasmic reticulum lumen;endoplasmic reticulum part;extracellular matrix;extracellular matrix part;extracellular region;extracellular region part;fibrillar collagen;intracellula	Amoebiasis;ECM-receptor interaction;Focal adhesion;Protein digestion and absorption	Homo sapiens	1	11
cell part;cytoplasm;cytoplasmic part;cytosol;extracellular membrane-bounded organelle;extracellular organelle;extracellular region part;extracellular vesicular exosome;intracellular organelle part;intracellular part;membrane-b	Purine metabolism	Homo sapiens	1	11
catalytic step 2 spliceosome;cell part;intracellular non-membrane-bounded organelle;intracellular organelle;intracellular organelle part;intracellular part;macromolecular complex;non-membrane-bounded organelle;nuclear par	Spliceosome	Homo sapiens	1	11
		Homo sapiens	1	11
basolateral plasma membrane;blood microparticle;caveola;cell cortex part;cell junction;cell part;cell-cell junction;cortical actin cytoskeleton;cortical cytoskeleton;cytoplasmic membrane-bounded vesicle;cytoplasmic part;cyto	Adipocytokine signaling pathway;Bile secretion;Pathways in cancer;Renal cell carcinoma	Homo sapiens	1	11
adherens junction;anchoring junction;cell division site part;cell junction;cell part;cell surface;cell-substrate adherens junction;cell-substrate junction;cleavage furrow;cytoplasmic part;cytoplasmic vesicle membrane;cytoplasm	Pancreatic cancer;Pathways in cancer	Homo sapiens	1	11
cell body;cell part;cell projection;cytolytic granule;cytoplasm;cytoplasmic membrane-bounded vesicle;cytoplasmic part;cytoplasmic vesicle;dendrite;endosomal part;endosome;endosome membrane;external side of plasma r	ko05152;Lysosome;Phagosome	Homo sapiens	1	11
cell part;cytoplasm;cytoplasmic part;cytosol;inclusion body;intracellular part;membrane	Insulin signaling pathway;Starch and sucrose metabolism	Homo sapiens	1	11
cell part;cytoplasmic part;intracellular organelle part;intracellular part;macromolecular complex;membrane;membrane part;mitochondrial inner membrane;mitochondrial membrane;mitochondrial membrane part;mitochondrial	Alzheimer's disease;Cardiac muscle contraction;Huntington's disease;Oxidative phosphorylation;Parkin	Homo sapiens	1	11
cell part;cytoplasm;cytoplasmic part;extracellular membrane-bounded organelle;extracellular organelle;extracellular region part;extracellular vesicular exosome;intracellular membrane-bounded organelle;intracellular organelle	Peroxisome;PPAR signaling pathway;Primary bile acid biosynthesis	Homo sapiens	1	11
Cajal body;cell part;centrosome;chromosomal part;chromosome;chromosome, telomeric region;condensed chromosome;cyclin-dependent protein kinase holoenzyme complex;cytoplasm;cytoplasmic part;cytoskeletal part;c	Cell cycle;Measles;Oocyte meiosis;p53 signaling pathway;Pathways in cancer;Progesterone-mediated c	Homo sapiens	1	11
cell part;cytoplasmic part;intracellular membrane-bounded organelle;intracellular organelle;intracellular organelle lumen;intracellular organelle part;intracellular part;membrane-bounded organelle;membrane-enclosed lumen;m	Valine, leucine and isoleucine degradation	Homo sapiens	1	11
axon;cell body;cell part;cell projection;cytoplasmic part;cytosol;dendrite;extracellular region;integral to membrane;integral to plasma membrane;intracellular part;intrinsic to membrane;intrinsic to plasma membrane;membrane	Axon guidance	Homo sapiens	1	11
cell part;cytoplasm;cytoplasmic part;cytosol;endoplasmic reticulum;extracellular membrane-bounded organelle;extracellular organelle;extracellular region part;extracellular vesicular exosome;intracellular membrane-bounded organelle;intracellular organelle;intracellular organelle part;intracellular part;membrane-bounded organell		Homo sapiens	1	11
cell part;cytoplasm;cytoplasmic part;cytoplasmic vesicle;cytosol;extracellular membrane-bounded organelle;extracellular organelle;extracellular region part;extracellular space;extracellular vesicular exosome;intracellular men	Peroxisome	Homo sapiens	1	11
cell part;cytoplasm;extracellular membrane-bounded organelle;extracellular organelle;extracellular region part;extracellular vesicular exosome;intracellular part;membrane-bounded organelle;membrane-bounded vesicle;organelle;vesicle		Homo sapiens	1	11
actin filament;axon;cell cortex part;cell junction;cell part;cell projection;cell-cell junction;cortical actin cytoskeleton;cortical cytoskeleton;cytoplasm;cytoplasmic membrane-bounded vesicle;cytoplasmic part;cytoplasmic vesic	ko05152;Phagosome	Homo sapiens	1	11
cell part;cytoplasmic part;endoplasmic reticulum;endoplasmic reticulum membrane;endoplasmic reticulum part;integral to membrane;intracellular membrane-bounded organelle;intracellular organelle;intracellular organelle pa	Steroid biosynthesis	Homo sapiens	1	11
extracellular membrane-bounded organelle;extracellular organelle;extracellular region part;extracellular vesicular exosome;membrane-bounded organelle;membrane-bounded vesicle;organelle;vesicle		Homo sapiens	1	11
cell part;chromosomal part;DNA replication factor C complex;intracellular membrane-bounded organelle;intracellular organelle;intracellular organelle part;intracellular part;macromolecular complex;membrane-bounded organe	DNA replication;Mismatch repair;Nucleotide excision repair	Homo sapiens	1	11
cell junction;cell part;cell-cell junction;cytoplasm;cytoplasmic part;cytosol;extracellular membrane-bounded organelle;extracellular organelle;extracellular region part;extracellular vesicular exosome;extrinsic to internal side of	Chemokine signaling pathway;Epithelial cell signaling in Helicobacter pylori infection;Neurotrophin signa	Homo sapiens	1	11
actin cytoskeleton;cell part;cytoplasm;cytoplasmic part;cytoskeleton;cytosol;extracellular membrane-bounded organelle;extracellular organelle;extracellular region part;extracellular vesicular exosome;intracellular membrane-	Bacterial invasion of epithelial cells;Chemokine signaling pathway;Chronic myeloid leukemia;ErbB signa	Homo sapiens	1	11
cell part;cytoplasmic part;cytosol;endoplasmic reticulum membrane;endoplasmic reticulum part;glycogen granule;intracellular organelle part;intracellular part;membrane;membrane part;organelle membrane;organelle part	Glycerophospholipid metabolism;Phosphonate and phosphinate metabolism	Homo sapiens	1	11
cell junction;cell part;cell projection;cell-cell junction;cytoplasm;cytoplasmic membrane-bounded vesicle;cytoplasmic part;cytoplasmic vesicle;cytoplasmic vesicle membrane;cytoplasmic vesicle part;endocytic vesicle;endos	Tight junction	Homo sapiens	1	11
cell part;cytoplasm;intracellular membrane-bounded organelle;intracellular organelle;intracellular organelle part;intracellular part;macromolecular complex;membrane-bounded organelle;nuclear part;nucleoplasm;nucleotide-e	Nucleotide excision repair;Protein processing in endoplasmic reticulum	Homo sapiens	1	11
extracellular matrix part;extracellular region;extracellular region part;fibril;microfibril		Homo sapiens	1	11
cell part;cytoplasmic part;intracellular membrane-bounded organelle;intracellular organelle;intracellular part;macromolecular complex;membrane-bounded organelle;mitochondrion;nucleus;organelle;ribonucleoprotein complex		Homo sapiens	1	11
cell part;intracellular		Homo sapiens	1	11
cell junction;cell part;cell projection;cytoplasm;cytoplasmic part;cytosol;early endosome;endosome;extracellular membrane-bounded organelle;extracellular organelle;extracellular region part;extracellular vesicular exosome;g	Chemokine signaling pathway;Focal adhesion;Leukocyte transendothelial migration;Long-term potentia	Homo sapiens	1	11
cell part;Cul7-RING ubiquitin ligase complex;cullin-RING ubiquitin ligase complex;cytoplasmic part;cytosol;extracellular membrane-bounded organelle;extracellular organelle;extracellular region part;extracellular vesicular exo	Cell cycle;Cell cycle - yeast;Circadian rhythm - mammal;Oocyte meiosis;Protein processing in endoplas	Homo sapiens	1	11
cell part;cytoplasm;cytoplasmic part;cytosol;extracellular membrane-bounded organelle;extracellular organelle;extracellular region part;extracellular vesicular exosome;intracellular non-membrane-bounded organelle;intracellu	Ether lipid metabolism	Homo sapiens	1	11
Cajal body;catalytic step 2 spliceosome;cell part;intracellular organelle part;intracellular part;macromolecular complex;nuclear body;nuclear part;nuclear speck;nucleoplasm;nucleoplasm part;organelle part;ribonucleoprotein c	Shigellosis;Spliceosome	Homo sapiens	1	11
cell part;cytoplasm;cytoplasmic part;cytosol;I-kappaB/NF-kappaB complex;intracellular membrane-bounded organelle;intracellular organelle;intracellular organelle part;intracellular part;macromolecular complex;membrane-b	Acute myeloid leukemia;Adipocytokine signaling pathway;Amoebiasis;Apoptosis;B cell receptor signalir	Homo sapiens	1	11
cell part;cytoplasmic part;extracellular membrane-bounded organelle;extracellular organelle;extracellular region part;extracellular vesicular exosome;intracellular membrane-bounded organelle;intracellular non-membrane-bou	DNA replication;Homologous recombination;Mismatch repair	Homo sapiens	1	11
cell part;cytoplasm;cytoplasmic part;intracellular membrane-bounded organelle;intracellular non-membrane-bounded organelle;intracellular organelle;intracellular organelle part;intracellular part;membrane-bounded organelle;mitochondrial nucleoid;mitochondrial part;mitochondrion;non-membrane-bounded organelle;nucleoid;or		Homo sapiens	1	11
cell part;cytoplasmic part;cytosol;extracellular membrane-bounded organelle;extracellular organelle;extracellular region part;extracellular space;extracellular vesicular exosome;intracellular non-membrane-bounded organelle;intracellular organelle;intracellular organelle part;intracellular part;membrane;membrane-bounded organe		Homo sapiens	1	11
cell part;chromatin remodeling complex;cytoplasm;cytoskeletal part;envelope;histone deacetylase complex;intracellular membrane-bounded organelle;intracellular organelle;intracellular organelle part;intracellular part;macromolecular complex;membrane-bounded organelle;microtubule;nuclear envelope;nuclear part;nucleoplasm		Homo sapiens	1	11
cell part;cytoplasm;cytoplasmic part;cytosol;extracellular membrane-bounded organelle;extracellular organelle;extracellular region part;extracellular vesicular exosome;intracellular membrane-bounded organelle;intracellular organelle;intracellular part;macromolecular complex;membrane-bounded organelle;membrane-bounded v		Homo sapiens	1	11
cell part;cytoplasm;intracellular membrane-bounded organelle;intracellular organelle;intracellular organelle part;intracellular part;membrane-bounded organelle;midbody;nuclear body;nuclear part;nuclear speck;nucleoplasm;r	RIG-I-like receptor signaling pathway	Homo sapiens	1	11
cell part;cytoplasm;cytoplasmic exosome (RNase complex);cytoplasmic part;cytosol;exosome (RNase complex);intracellular membrane-bounded organelle;intracellular non-membrane-bounded organelle;intracellular organell	RNA degradation	Homo sapiens	1	11
cell part;cytoplasm;intracellular membrane-bounded organelle;intracellular non-membrane-bounded organelle;intracellular organelle;intracellular organelle part;intracellular part;membrane;membrane-bounded organelle;non-membrane-bounded organelle;nuclear part;nucleolus;nucleoplasm;nucleus;organelle;organelle part		Homo sapiens	1	11
cell part;cytoplasmic part;endoplasmic reticulum;endoplasmic reticulum lumen;endoplasmic reticulum membrane;endoplasmic reticulum part;intracellular membrane-bounded organelle;intracellular organelle;intracellular organelle lumen;intracellular organelle part;intracellular part;membrane;membrane part;membrane-bounded c		Homo sapiens	1	11
cell part;cytoplasmic part;cytosol;intracellular membrane-bounded organelle;intracellular organelle;intracellular organelle part;intracellular part;macromolecular complex;membrane-bounded organelle;nuclear part;nucleoplasm	Meiosis - yeast;mRNA surveillance pathway;Oocyte meiosis;Wnt signaling pathway	Homo sapiens	1	11
extracellular matrix;extracellular matrix part;extracellular region;extracellular region part;fibril;macromolecular complex;microfibril;protein complex;proteinaceous extracellular matrix		Homo sapiens	1	11
cell part;cytoplasm;intracellular non-membrane-bounded organelle;intracellular organelle;intracellular organelle part;intracellular part;macromolecular complex;non-membrane-bounded organelle;nuclear part;nucleolar part;nu	Ribosome biogenesis in eukaryotes	Homo sapiens	1	11
axon;cell cortex part;cell part;cell projection;cortical actin cytoskeleton;cortical cytoskeleton;cytoplasm;cytoplasmic part;cytoskeletal part;cytoskeleton;early endosome membrane;endosomal part;endosome membrane;Golg	Tight junction	Homo sapiens	1	11
cell part;centrosome;cytoplasm;cytoplasmic part;cytoskeletal part;intracellular membrane-bounded organelle;intracellular non-membrane-bounded organelle;intracellular organelle;intracellular organelle part;intracellular part;membrane-bounded organelle;microtubule organizing center;non-membrane-bounded organelle;nucleus;		Homo sapiens	1	11
adherens junction;anchoring junction;cell junction;cell part;cell-substrate adherens junction;cell-substrate junction;cytoplasmic part;cytosol;extracellular membrane-bounded organelle;extracellular organelle;extracellular region part;extracellular vesicular exosome;focal adhesion;intracellular part;membrane-bounded organelle;me		Homo sapiens	1	11
extracellular membrane-bounded organelle;extracellular organelle;extracellular region;extracellular region part;extracellular space;extracellular vesicular exosome;membrane-bounded organelle;membrane-bounded vesicle;organelle;vesicle		Homo sapiens	1	11
cell part;cytoplasmic part;intracellular membrane-bounded organelle;intracellular organelle;intracellular organelle lumen;intracellular organelle part;intracellular part;macromolecular complex;membrane;membrane part;membr	Alzheimer's disease;Huntington's disease;Oxidative phosphorylation;Parkinson's disease	Homo sapiens	1	11
cell part;cytoplasmic part;endoplasmic reticulum;endoplasmic reticulum membrane;endoplasmic reticulum part;integral to membrane;intracellular membrane-bounded organelle;intracellular organelle;intracellular organelle pa	Steroid biosynthesis	Homo sapiens	1	11
cell part;cytoplasmic part;early endosome;endosome;intracellular membrane-bounded organelle;intracellular organelle;intracellular organelle part;intracellular part;macromolecular complex;membrane-bounded organelle;nuclear part;nucleoplasm;organelle;organelle part;protein complex;WASH complex		Homo sapiens	1	11
	Arginine and proline metabolism	Homo sapiens	1	11
cell part;cytoplasmic part;intracellular membrane-bounded organelle;intracellular organelle;intracellular organelle lumen;intracellular organelle part;intracellular part;membrane-bounded organelle;membrane-enclosed lumen;m	Aminoacyl-tRNA biosynthesis;Porphyrin and chlorophyll metabolism	Homo sapiens	1	11
		Homo sapiens	1	11
adherens junction;anchoring junction;cell junction;cell part;cell-substrate adherens junction;cell-substrate junction;cytoplasm;focal adhesion;intracellular part		Homo sapiens	1	11
cell part;cytoplasmic part;extracellular membrane-bounded organelle;extracellular organelle;extracellular region part;extracellular vesicular exosome;intracellular organelle lumen;intracellular organelle part;intracellular part;me	beta-Alanine metabolism;Propanoate metabolism;Valine, leucine and isoleucine degradation	Homo sapiens	1	11
cell part;cytoplasm;cytoplasmic part;cytosol;extracellular membrane-bounded organelle;extracellular organelle;extracellular region part;extracellular vesicular exosome;Golgi apparatus;intracellular membrane-bounded organe	Nicotinate and nicotinamide metabolism	Homo sapiens	1	11
actin filament;basal cortex;cell cortex part;cell part;cytoplasmic part;cytoskeletal part;intracellular membrane-bounded organelle;intracellular organelle;intracellular organelle part;intracellular part;lateral plasma membrane;macromolecular complex;membrane;membrane part;membrane-bounded organelle;mitochondrion;nucleus;		Homo sapiens	1	11
		Homo sapiens	1	11
cell part;chromatin;chromosomal part;cytoplasm;intracellular membrane-bounded organelle;intracellular organelle;intracellular organelle part;intracellular part;membrane-bounded organelle;nuclear chromatin;nuclear chromosome part;nuclear part;nucleoplasm;nucleus;organelle;organelle part		Homo sapiens	1	11
cell part;cytoplasmic part;extracellular membrane-bounded organelle;extracellular organelle;extracellular region part;extracellular vesicular exosome;intracellular membrane-bounded organelle;intracellular organelle;intracellular part;membrane-bounded organelle;membrane-bounded vesicle;mitochondrion;nucleus;organelle;vesic		Homo sapiens	1	11
cell part;cytoplasm;intracellular part		Homo sapiens	1	11
cell part;cytoplasm;cytoplasmic part;endoplasmic reticulum membrane;endoplasmic reticulum part;intracellular organelle part;intracellular part;membrane;membrane part;organelle membrane;organelle part;perinuclear regior	Spliceosome	Homo sapiens	1	11
cell cortex part;cell part;cell projection part;cytoplasm;cytoplasmic part;cytosol;endosome;exocyst;growth cone;intracellular membrane-bounded organelle;intracellular organelle;intracellular part;late endosome;macromolecular complex;membrane;membrane-bounded organelle;organelle;plasma membrane;protein complex;site		Homo sapiens	1	11
cell part;cytoplasm;intracellular membrane-bounded organelle;intracellular organelle;intracellular organelle part;intracellular part;membrane-bounded organelle;nuclear part;nucleoplasm;nucleus;organelle;organelle part		Homo sapiens	1	11
cell part;cytoplasmic part;endoplasmic reticulum;endoplasmic reticulum membrane;endoplasmic reticulum part;Golgi apparatus;Golgi apparatus part;Golgi membrane;integral to membrane;intracellular membrane-bounded c	Ether lipid metabolism;Glycerophospholipid metabolism	Homo sapiens	1	11
cell part;cytoplasm;cytoplasmic part;cytosol;intracellular non-membrane-bounded organelle;intracellular organelle;intracellular organelle part;intracellular part;macromolecular complex;membrane;non-membrane-bounded organelle;nuclear part;nucleolus;organelle;organelle part;polysomal ribosome;ribonucleoprotein complex;rib		Homo sapiens	1	11
cell part;cytoplasm;extracellular membrane-bounded organelle;extracellular organelle;extracellular region part;extracellular vesicular exosome;intracellular part;membrane-bounded organelle;membrane-bounded vesicle;orga	Pathways in cancer;Thyroid cancer	Homo sapiens	1	11
cell part;cytoplasm;extracellular membrane-bounded organelle;extracellular organelle;extracellular region part;extracellular vesicular exosome;intracellular part;membrane-bounded organelle;membrane-bounded vesicle;orga	Amino sugar and nucleotide sugar metabolism;Fructose and mannose metabolism	Homo sapiens	1	11

cell part;centrosome;cytoplasm;cytoplasmic part;cytoskeletal part;endomembrane system;intracellular membrane-bounded organelle;intracellular non-membrane-bounded organelle;intracellular organelle;intracellular organelle part;intracellular part;macromolecular complex;membrane;membrane-bounded organelle;microtubule		Homo sapiens	1	11
cell part;cell surface;cytoplasmic part;endoplasmic reticulum;endoplasmic reticulum membrane;endoplasmic reticulum part;integral to membrane;intracellular membrane-bounded organelle;intracellular organelle;intracellular organelle part;intracellular part;intrinsic to membrane;membrane;membrane part;membrane-bounded or		Homo sapiens	1	11
cell part;cytoplasmic part;cytoplasmic vesicle;cytosol;Golgi apparatus;Golgi apparatus part;Golgi cisterna membrane;Golgi membrane;intracellular membrane-bounded organelle;intracellular organelle;intracellular organelle p	Endocytosis	Homo sapiens	1	11
adherens junction;anchoring junction;cell junction;cell part;cell projection;cell projection membrane;cell projection part;cytoplasm;cytoplasmic part;cytoskeleton;dendritic spine;filopodium;intracellular non-membrane-bounded organelle;intracellular organelle;intracellular organelle part;intracellular part;lamellipodium;leading edge		Homo sapiens	1	11
cell part;cytoplasm;cytoplasmic part;cytosol;intracellular membrane-bounded organelle;intracellular organelle;intracellular organelle part;intracellular part;membrane;membrane-bounded organelle;nuclear membrane;nuclear part;nucleoplasm;nucleus;organelle;organelle membrane;organelle part		Homo sapiens	1	11
cell part;cytoplasm;cytoplasmic part;intracellular membrane-bounded organelle;intracellular organelle;intracellular organelle part;intracellular part;membrane;membrane-bounded organelle;microbody;microbody membrane;microbody part;organelle;organelle membrane;organelle part;peroxisomal membrane;peroxisomal part		Homo sapiens	1	11
cell part;cytoplasm;cytoplasmic part;endoplasmic reticulum;intracellular membrane-bounded organelle;intracellular organelle;intracellular organelle lumen;intracellular organelle part;intracellular part;membrane;membrane-bou	Alzheimer's disease;Valine, leucine and isoleucine degradation	Homo sapiens	1	11
cell part;cytoplasmic part;extracellular membrane-bounded organelle;extracellular organelle;extracellular region part;extracellular vesicular exosome;intracellular membrane-bounded organelle;intracellular organelle;intracellular organelle part;intracellular part;lysosomal membrane;macromolecular complex;membrane;membrane		Homo sapiens	1	11
cell part;cytoplasmic part;endosomal part;endosome;endosome membrane;extracellular membrane-bounded organelle;extracellular organelle;extracellular region part;extracellular vesicular exosome;integral to membrane;integral to plasma membrane;intracellular membrane-bounded organelle;intracellular organelle;intracellular		Homo sapiens	1	11
autophagic vacuole;cell part;cytoplasmic part;cytoplasmic vesicle;endosome;integral to membrane;intracellular membrane-bounded organelle;intracellular organelle;intracellular part;intrinsic to membrane;late endosome;lysosome;lytic vacuole;membrane;membrane part;membrane-bounded organelle;organelle;vacuole;vesicle		Homo sapiens	1	11
cell part;cytoplasm;cytoplasmic part;cytosol;intracellular part		Homo sapiens	1	11
cell part;cytoplasmic part;intracellular membrane-bounded organelle;intracellular organelle;intracellular organelle part;intracellular part;membrane-bounded organelle;mitochondrion;nuclear part;nucleoplasm;nucleus;organelle;organelle part		Homo sapiens	1	11
cell part;intracellular membrane-bounded organelle;intracellular organelle;intracellular organelle part;intracellular part;membrane-bounded organelle;nuclear part;nucleoplasm;nucleus;organelle;organelle part		Homo sapiens	1	11
cell part;cytoplasm;cytoplasmic part;cytosol;extracellular membrane-bounded organelle;extracellular organelle;extracellular region part;extracellular vesicular exosome;Golgi apparatus;Golgi apparatus part;Golgi membrane;intracellular membrane-bounded organelle;intracellular organelle;intracellular organelle part;intracellular pa		Homo sapiens	1	11
		Homo sapiens	1	11
		Homo sapiens	1	11
cell part;cytoplasm;intracellular membrane-bounded organelle;intracellular organelle;intracellular organelle part;intracellular part;membrane-bounded organelle;nuclear body;nuclear part;nuclear speck;nucleoplasm;nucleoplasm part;organelle;organelle part		Homo sapiens	1	11
cell part;intracellular organelle part;intracellular part;membrane;nuclear membrane;nuclear part;organelle membrane;organelle part		Homo sapiens	1	11
cell part;intracellular membrane-bounded organelle;intracellular non-membrane-bounded organelle;intracellular organelle;intracellular organelle part;intracellular part;membrane-bounded organelle;non-membrane-bounded organelle;nuclear part;nucleolus;nucleus;organelle;organelle part		Homo sapiens	1	11
cell part;cell surface;cytoplasmic part;endoplasmic reticulum lumen;endoplasmic reticulum part;extracellular matrix;extracellular membrane-bounded organelle;extracellular organelle;extracellular region;extracellular region pa	Basal cell carcinoma;Hedgehog signaling pathway;Melanogenesis;Pathways in cancer;Wnt signaling pa	Homo sapiens	1	11
cell part;cell projection;cytoplasm;extracellular membrane-bounded organelle;extracellular organelle;extracellular region part;extracellular vesicular exosome;intracellular membrane-bounded organelle;intracellular organelle;intracellular part;lamellipodium;membrane-bounded organelle;membrane-bounded vesicle;nucleus;organe		Homo sapiens	1	11
cell part;cytoplasm;intracellular part		Homo sapiens	1	11
cell part;cytoplasmic part;extracellular membrane-bounded organelle;extracellular organelle;extracellular region part;extracellular vesicular exosome;intracellular membrane-bounded organelle;intracellular organelle;intracellular organelle part;intracellular part;membrane-bounded organelle;membrane-bounded vesicle;mitochondr		Homo sapiens	1	11
cell part;cytoplasmic part;extracellular membrane-bounded organelle;extracellular organelle;extracellular region part;extracellular vesicular exosome;intracellular membrane-bounded organelle;intracellular organelle;intracellular part;membrane-bounded organelle;membrane-bounded vesicle;mitochondrion;organelle;vesicle		Homo sapiens	1	11
cell part;cytoplasmic part;cytosol;endosomal part;endosome membrane;extracellular membrane-bounded organelle;extracellular organelle;extracellular region part;extracellular vesicular exosome;intracellular organelle part;in	Endocytosis	Homo sapiens	1	11
cell part;cytoplasm;EKC/KEOPS complex;intracellular membrane-bounded organelle;intracellular organelle;intracellular part;macromolecular complex;membrane-bounded organelle;nucleus;organelle;protein complex		Homo sapiens	1	11
cell part;intracellular membrane-bounded organelle;intracellular non-membrane-bounded organelle;intracellular organelle;intracellular organelle part;intracellular part;membrane-bounded organelle;non-membrane-bounded organelle;nuclear part;nucleolus;nucleus;organelle;organelle part		Homo sapiens	1	11
ACF complex;cell part;chromatin accessibility complex;chromatin assembly complex;chromatin remodeling complex;chromosome;intracellular non-membrane-bounded organelle;intracellular organelle;intracellular organelle part;intracellular part;macromolecular complex;non-membrane-bounded organelle;nuclear chromosome;		Homo sapiens	1	11
cell part;cytoplasmic part;cytosol;extracellular membrane-bounded organelle;extracellular organelle;extracellular region part;extracellular vesicular exosome;intracellular part;membrane-bounded organelle;membrane-bounde	Fructose and mannose metabolism	Homo sapiens	1	11
cell part;centrosome;cytoplasmic part;cytoskeletal part;cytosol;intracellular non-membrane-bounded organelle;intracellular organelle;intracellular organelle part;intracellular part;kinesin complex;macromolecular complex;membrane;microtubule;microtubule associated complex;microtubule organizing center;non-membrane-boun		Homo sapiens	1	11
cell part;cytoplasmic part;cytosol;intracellular membrane-bounded organelle;intracellular organelle;intracellular part;membrane-bounded organelle;nucleus;organelle	Regulation of actin cytoskeleton	Homo sapiens	1	11
cell part;cytoplasm;intracellular membrane-bounded organelle;intracellular organelle;intracellular organelle part;intracellular part;membrane;membrane-bounded organelle;nuclear part;nucleoplasm;nucleus;organelle;organelle part;plasma membrane		Homo sapiens	1	11
catalytic step 2 spliceosome;cell part;cytoplasm;intracellular membrane-bounded organelle;intracellular organelle;intracellular organelle part;intracellular part;macromolecular complex;membrane-bounded organelle;nuclear p	Spliceosome	Homo sapiens	1	11
		Homo sapiens	1	11
cell part;intracellular non-membrane-bounded organelle;intracellular organelle;intracellular organelle part;intracellular part;macromolecular complex;non-membrane-bounded organelle;nuclear part;nucleolar part;nucleolus;org	Ribosome biogenesis in eukaryotes	Homo sapiens	1	11
cell part;cytoplasmic membrane-bounded vesicle;cytoplasmic part;cytoplasmic vesicle;cytosol;extracellular membrane-bounded organelle;extracellular organelle;extracellular region part;extracellular vesicular exosome;Golgi apparatus;intracellular membrane-bounded organelle;intracellular organelle;intracellular part;lysosome;ly		Homo sapiens	1	11
anaphase-promoting complex;cell part;cullin-RING ubiquitin ligase complex;cytoplasmic part;cytosol;intracellular membrane-bounded organelle;intracellular organelle;intracellular organelle part;intracellular part;macromolecu	Cell cycle;Cell cycle - yeast;Meiosis - yeast;Oocyte meiosis;Progesterone-mediated oocyte maturation;U	Homo sapiens	1	11
cell part;intracellular organelle part;intracellular part;macromolecular complex;mRNA cleavage and polyadenylation specificity factor complex;mRNA cleavage factor complex;nuclear part;nucleoplasm;nucleoplasm part;orgar	mRNA surveillance pathway	Homo sapiens	1	11
cell part;cytoplasm;intracellular non-membrane-bounded organelle;intracellular organelle;intracellular organelle part;intracellular part;membrane;non-membrane-bounded organelle;nuclear part;nucleolus;organelle;organelle part;plasma membrane		Homo sapiens	1	11
cell part;cytoplasmic part;intracellular membrane-bounded organelle;intracellular non-membrane-bounded organelle;intracellular organelle;intracellular organelle part;intracellular part;membrane-bounded organelle;mitochondrial nucleoid;mitochondrial part;mitochondrion;non-membrane-bounded organelle;nucleoid;nucleus;orga		Homo sapiens	1	11
cell part;cytoplasmic part;intracellular membrane-bounded organelle;intracellular organelle;intracellular organelle part;intracellular part;macromolecular complex;membrane-bounded organelle;mitochondrial part;mitochondrial small ribosomal subunit;mitochondrion;organellar small ribosomal subunit;organelle;organelle part;ribon		Homo sapiens	1	11
cell part;centrosome;cytoplasmic part;cytoskeletal part;cytosol;intracellular non-membrane-bounded organelle;intracellular organelle;intracellular organelle part;intracellular part;macromolecular complex;microtubule associated complex;microtubule organizing center;non-membrane-bounded organelle;organelle;organelle part;pr		Homo sapiens	1	11
cell part;intracellular membrane-bounded organelle;intracellular non-membrane-bounded organelle;intracellular organelle;intracellular organelle part;intracellular part;macromolecular complex;membrane-bounded organelle;non-membrane-bounded organelle;nuclear body;nuclear part;nuclear speck;nucleolus;nucleoplasm;nucle		Homo sapiens	1	11
cell part;cytoplasmic part;cytosol;intracellular part;membrane;plasma membrane	Adipocytokine signaling pathway;Aldosterone-regulated sodium reabsorption;Insulin signaling pathway;	Homo sapiens	1	11
cell part;cytoplasm;cytoplasmic part;cytoskeletal part;histone methyltransferase complex;intracellular non-membrane-bounded organelle;intracellular organelle;intracellular organelle part;intracellular part;macromolecular complex;membrane;methyltransferase complex;microtubule organizing center;MLL1 complex;non-membran		Homo sapiens	1	11
cell part;cytoplasm;intracellular membrane-bounded organelle;intracellular organelle;intracellular part;membrane-bounded organelle;nucleus;organelle		Homo sapiens	1	11
cell part;intracellular membrane-bounded organelle;intracellular organelle;intracellular organelle part;intracellular part;macromolecular complex;membrane;membrane-bounded organelle;nuclear body;nuclear matrix;nuclear pa	Notch signaling pathway	Homo sapiens	1	11
cell part;cytoplasm;cytoplasmic part;cytosol;intracellular membrane-bounded organelle;intracellular non-membrane-bounded organelle;intracellular organelle;intracellular organelle lumen;intracellular organelle part;intracellula	Sulfur relay system;Thiamine metabolism	Homo sapiens	1	11
cell part;cytoplasm;cytoplasmic part;cytosol;Golgi apparatus;Golgi apparatus part;Golgi membrane;intracellular membrane-bounded organelle;intracellular non-membrane-bounded organelle;intracellular organelle;intracellular organelle part;intracellular part;macromolecular complex;membrane;membrane-bounded organelle;non		Homo sapiens	1	11
actin cytoskeleton;cell part;centrosome;chromosomal part;condensed chromosome kinetochore;cytoplasm;cytoplasmic part;cytoskeletal part;cytoskeleton;cytosol;intracellular membrane-bounded organelle;intracellular non-	Cell cycle;Progesterone-mediated oocyte maturation	Homo sapiens	1	11
cell part;cytoplasm;cytoplasmic part;cytosol;intracellular membrane-bounded organelle;intracellular organelle;intracellular organelle part;intracellular part;membrane-bounded organelle;nuclear part;nucleoplasm;nucleus;organelle;organelle part		Homo sapiens	1	11
cell part;cytoplasmic exosome (RNase complex);cytoplasmic part;cytosol;exosome (RNase complex);intracellular part;macromolecular complex;membrane;protein complex		Homo sapiens	1	12
cell part;cytoplasmic part;extracellular membrane-bounded organelle;extracellular organelle;extracellular region part;extracellular vesicular exosome;Golgi apparatus part;Golgi membrane;integral to membrane;intracellular organelle part;intracellular part;intrinsic to membrane;membrane;membrane part;membrane-bounded orga		Homo sapiens	1	12
actin cytoskeleton;adherens junction;anchoring junction;basement membrane;basolateral plasma membrane;cell junction;cell part;cell projection membrane;cell projection part;cell-cell junction;cell-substrate adherens junctio	Tight junction	Homo sapiens	1	12
cell part;cytoplasmic part;endoplasmic reticulum membrane;endoplasmic reticulum palmitoyltransferase complex;endoplasmic reticulum part;integral to membrane;intracellular membrane-bounded organelle;intracellular orga	Sphingolipid metabolism	Homo sapiens	1	12
cell part;chromosomal part;condensed chromosome kinetochore;cytoplasm;cytoplasmic part;cytoskeletal part;cytosol;endoplasmic reticulum;endoplasmic reticulum membrane;endoplasmic reticulum part;intracellular membrane-bounded organelle;intracellular non-membrane-bounded organelle;intracellular organelle;intracellula		Homo sapiens	1	12
		Homo sapiens	1	12
cell part;cytoplasm;cytoplasmic part;endoplasmic reticulum membrane;endoplasmic reticulum part;extracellular membrane-bounded organelle;extracellular organelle;extracellular region part;extracellular vesicular exosome;intracellular membrane-bounded organelle;intracellular non-membrane-bounded organelle;intracellular or		Homo sapiens	1	12
cell part;intracellular membrane-bounded organelle;intracellular organelle;intracellular organelle part;intracellular part;membrane-bounded organelle;nuclear part;nucleoplasm;nucleus;organelle;organelle part		Homo sapiens	1	12
cell part;chromatin;chromosomal part;chromosome;chromosome, centromeric region;cohesin complex;cytoplasmic part;cytosol;intracellular non-membrane-bounded organelle;intracellular organelle;intracellular organelle par	Cell cycle;Cell cycle - yeast	Homo sapiens	1	12
cell part;cell projection;cytoplasmic part;cytoplasmic vesicle;cytoplasmic vesicle membrane;cytoplasmic vesicle part;cytoskeletal part;intracellular membrane-bounded organelle;intracellular organelle;intracellular organelle part;intracellular part;kinesin complex;macromolecular complex;membrane;membrane-bounded organelle;		Homo sapiens	1	12
cell part;cytoplasm;intracellular part		Homo sapiens	1	12
cell part;cell surface;extracellular region part;extracellular space		Homo sapiens	1	12
cell part;clathrin coated vesicle membrane;clathrin-coated vesicle;coated pit;coated vesicle;coated vesicle membrane;cytoplasm;cytoplasmic membrane-bounded vesicle;cytoplasmic part;cytoplasmic vesicle;cytoplasmic vesicle membrane;cytoplasmic vesicle part;cytoskeleton;intracellular membrane-bounded organelle;intrace		Homo sapiens	1	12
cell part;cytoplasm;intracellular membrane-bounded organelle;intracellular organelle;intracellular organelle part;intracellular part;membrane-bounded organelle;nuclear body;nuclear part;nuclear speck;nucleoplasm;nucleoplas	Spliceosome	Homo sapiens	1	12
cell part;cytoplasmic part;intracellular membrane-bounded organelle;intracellular organelle;intracellular organelle lumen;intracellular organelle part;intracellular part;membrane;membrane-bounded organelle;membrane-enclos	Fatty acid metabolism;Peroxisome	Homo sapiens	1	12
cell part;chromosome;cytoplasm;cytoplasmic part;cytosol;extracellular membrane-bounded organelle;extracellular organelle;extracellular region part;extracellular vesicular exosome;intracellular membrane-bounded organelle;intracellular non-membrane-bounded organelle;intracellular organelle;intracellular organelle part;intracell		Homo sapiens	1	12
cell junction;cell part;intracellular membrane-bounded organelle;intracellular non-membrane-bounded organelle;intracellular organelle;intracellular organelle part;intracellular part;macromolecular complex;membrane-boundec	Spliceosome	Homo sapiens	1	12
cell part;cell surface;cytoplasm;extracellular membrane-bounded organelle;extracellular organelle;extracellular region;extracellular region part;extracellular space;extracellular vesicular exosome;intracellular part;membrane;m	Axon guidance	Homo sapiens	1	12
cell part;cytoplasmic part;endoplasmic reticulum;endoplasmic reticulum membrane;endoplasmic reticulum part;integral to endoplasmic reticulum membrane;integral to membrane;integral to organelle membrane;intracellular	Sphingolipid metabolism	Homo sapiens	1	12
cell part;cytoplasm;cytoskeletal part;intracellular non-membrane-bounded organelle;intracellular organelle;intracellular organelle part;intracellular part;macromolecular complex;microtubule;microtubule associated complex;non-membrane-bounded organelle;organelle;organelle part;protein complex;spindle		Homo sapiens	1	12
cell part;cytoplasm;cytoplasmic part;cytosol;extracellular membrane-bounded organelle;extracellular organelle;extracellular region part;extracellular vesicular exosome;intracellular part;membrane-bounded organelle;membra	Drug metabolism - other enzymes;Purine metabolism	Homo sapiens	1	12
cell part;cytoplasmic part;extracellular membrane-bounded organelle;extracellular organelle;extracellular region part;extracellular vesicular exosome;Golgi apparatus;Golgi apparatus part;Golgi membrane;intracellular membra	Acute myeloid leukemia;Axon guidance;B cell receptor signaling pathway;Bladder cancer;Chemokine si	Homo sapiens	1	12
cell part;cell surface;coated vesicle membrane;cytoplasmic part;cytoplasmic vesicle membrane;cytoplasmic vesicle part;early endosome membrane;endocytic vesicle membrane;endoplasmic reticulum;endoplasmic reticulun	Allograft rejection;Antigen processing and presentation;Autoimmune thyroid disease;Cell adhesion mole	Homo sapiens	1	12
actin cytoskeleton;cell part;chromatin;chromosomal part;cytoplasmic part;cytoskeleton;euchromatin;Golgi apparatus;intracellular membrane-bounded organelle;intracellular non-membrane-bounded organelle;intracellular organelle;intracellular organelle part;intracellular part;macromolecular complex;membrane-bounded organel		Homo sapiens	1	12
cell part;cytoplasmic part;intracellular membrane-bounded organelle;intracellular organelle;intracellular organelle part;intracellular part;membrane;membrane part;membrane-bounded organelle;mitochondrial inner membrane	Alzheimer's disease;Cardiac muscle contraction;Huntington's disease;Oxidative phosphorylation;Parkin	Homo sapiens	1	12
cell part;cytoplasm;intracellular non-membrane-bounded organelle;intracellular organelle;intracellular organelle part;intracellular part;macromolecular complex;non-membrane-bounded organelle;nuclear part;nucleolus;nucleo	Spliceosome	Homo sapiens	1	12
catalytic step 2 spliceosome;cell part;cytoplasmic part;cytosol;extracellular membrane-bounded organelle;extracellular organelle;extracellular region part;extracellular vesicular exosome;histone pre-mRNA 3'end processing c	Spliceosome;Systemic lupus erythematosus	Homo sapiens	1	12
cell part;cytoplasm;extracellular membrane-bounded organelle;extracellular organelle;extracellular region part;extracellular vesicular exosome;intracellular membrane-bounded organelle;intracellular organelle;intracellular organelle part;intracellular part;membrane-bounded organelle;membrane-bounded vesicle;nuclear part;nucle		Homo sapiens	1	12
cell part;cytoplasmic part;cytosol;extracellular membrane-bounded organelle;extracellular organelle;extracellular region part;extracellular vesicular exosome;intracellular part;membrane-bounded organelle;membrane-bounde	Styrene degradation;Tyrosine metabolism	Homo sapiens	1	12
cell part;clathrin-coated vesicle;coated vesicle;cytoplasm;cytoplasmic membrane-bounded vesicle;cytoplasmic part;cytoplasmic vesicle;cytoplasmic vesicle membrane;cytoplasmic vesicle part;cytosol;endosome;extracellula	Tight junction	Homo sapiens	1	12
cell part;cytoplasmic part;cytoplasmic vesicle;cytosol;extracellular membrane-bounded organelle;extracellular organelle;extracellular region part;extracellular vesicular exosome;Golgi apparatus;Golgi apparatus part;Golgi membrane;intracellular membrane-bounded organelle;intracellular organelle;intracellular organelle part;intra		Homo sapiens	1	12
cell part;centrosome;chromosome;condensed chromosome;condensed nuclear chromosome;cytoplasm;cytoplasmic part;cytoskeletal part;cytosol;endosome;gamma-tubulin complex;intracellular membrane-bounded organelle;intracellular non-membrane-bounded organelle;intracellular organelle;intracellular organelle part;intra		Homo sapiens	1	12
cell part;cytoplasm;intracellular membrane-bounded organelle;intracellular non-membrane-bounded organelle;intracellular organelle;intracellular organelle part;intracellular part;membrane-bounded organelle;non-membrane-bounded organelle;nuclear body;nuclear part;nuclear periphery;nucleolus;nucleoplasm;nucleoplasm part;		Homo sapiens	1	12
cell part;cytoplasm;cytoplasmic mRNA processing body;cytoplasmic part;cytosol;extracellular membrane-bounded organelle;extracellular organelle;extracellular region part;extracellular vesicular exosome;intracellular memb	Proteasome	Homo sapiens	1	12
cell part;cytoplasm;cytoplasmic part;cytosol;extracellular membrane-bounded organelle;extracellular organelle;extracellular region part;extracellular vesicular exosome;intracellular organelle part;intracellular part;membrane;m	Porphyrin and chlorophyll metabolism;Riboflavin metabolism	Homo sapiens	1	12
cell part;cytoplasmic part;endoplasmic reticulum membrane;endoplasmic reticulum part;intracellular organelle part;intracellular part;membrane;membrane part;organelle membrane;organelle part;plasma membrane	Mineral absorption;Porphyrin and chlorophyll metabolism	Homo sapiens	1	12
axon part;axon terminus;cell part;cell projection part;contractile fiber part;cytoplasm;cytoplasmic part;cytosol;dendritic spine neck;endoplasmic reticulum;endoplasmic reticulum membrane;endoplasmic reticulum part;extracellular membrane-bounded organelle;extracellular organelle;extracellular region part;extracellular vesicula		Homo sapiens	1	12
cell part;chromosomal part;cytoplasmic part;DNA replication factor C complex;Golgi apparatus;intracellular membrane-bounded organelle;intracellular organelle;intracellular organelle part;intracellular part;macromolecular co	DNA replication;Mismatch repair;Nucleotide excision repair	Homo sapiens	1	12
cell part;chromatin;chromosomal part;cytoplasm;cytoplasmic part;cytosol;intracellular organelle part;intracellular part;membrane part;membrane raft;nuclear chromatin;nuclear chromosome part;nuclear part;nucleoplasm;org	Jak-STAT signaling pathway;ko05152	Homo sapiens	1	12
cell part;chromatin;chromosomal part;cytoplasm;cytoplasmic part;cytosol;heterochromatin;intracellular membrane-bounded organelle;intracellular organelle;intracellular organelle part;intracellular part;membrane-bounded or	Bladder cancer;Cell cycle;Chronic myeloid leukemia;Glioma;Melanoma;Non-small cell lung cancer;p53	Homo sapiens	1	12
cell part;cytoplasmic part;extracellular membrane-bounded organelle;extracellular organelle;extracellular region part;extracellular vesicular exosome;intracellular membrane-bounded organelle;intracellular organelle;intracellul	Biosynthesis of unsaturated fatty acids	Homo sapiens	1	12
beta-catenin destruction complex;cell body;cell part;cell projection;cell projection part;centrosome;cytoplasm;cytoplasmic part;cytoskeletal part;cytosol;dendritic shaft;dendritic spine;growth cone;intracellular membrane-bou	Alzheimer's disease;Axon guidance;B cell receptor signaling pathway;Basal cell carcinoma;Cell cycle;Cl	Homo sapiens	1	12
cell part;cytoplasmic membrane-bounded vesicle;cytoplasmic part;cytoplasmic vesicle;early endosome;early endosome membrane;endocytic vesicle;endosomal part;endosome;endosome membrane;extracellular membrane	Amoebiasis;Endocytosis;ko05152;Phagosome;Vasopressin-regulated water reabsorption	Homo sapiens	1	12
cell part;cytoplasmic part;cytosol;endoplasmic reticulum;extracellular membrane-bounded organelle;extracellular organelle;extracellular region part;extracellular vesicular exosome;intracellular membrane-bounded organelle;intracellular organelle;intracellular part;membrane-bounded organelle;membrane-bounded vesicle;mitoch		Homo sapiens	1	12
cell part;cytoplasmic part;intracellular membrane-bounded organelle;intracellular organelle;intracellular organelle part;intracellular part;large ribosomal subunit;macromolecular complex;membrane;membrane-bounded organelle;mitochondrial inner membrane;mitochondrial large ribosomal subunit;mitochondrial membrane;mitoch		Homo sapiens	1	12
cell cortex;cell part;cytoplasmic part;intracellular part		Homo sapiens	1	12
cell part;cytoplasmic part;cytosol;extracellular membrane-bounded organelle;extracellular organelle;extracellular region part;extracellular vesicular exosome;intracellular organelle part;intracellular part;macromolecular complex;membrane;membrane part;membrane-bounded organelle;membrane-bounded vesicle;organelle;organ		Homo sapiens	1	12
cell part;cytoplasm;cytoplasmic part;intracellular membrane-bounded organelle;intracellular organelle;intracellular part;membrane;membrane-bounded organelle;mitochondrion;organelle		Homo sapiens	1	12
cell part;intracellular membrane-bounded organelle;intracellular non-membrane-bounded organelle;intracellular organelle;intracellular organelle part;intracellular part;macromolecular complex;membrane-bounded organelle;non-membrane-bounded organelle;nuclear part;nucleolus;nucleus;organelle;organelle part;preribosome;pr		Homo sapiens	1	12
cell part;cytoplasm;cytoplasmic part;cytoskeletal part;intracellular membrane-bounded organelle;intracellular organelle;intracellular organelle part;intracellular part;macromolecular complex;membrane;membrane-bounded organelle;microtubule organizing center;nuclear part;nucleoplasm;nucleus;organelle;organelle part;plasma		Homo sapiens	1	12
cell part;cytoplasm;cytoplasmic part;cytosol;extracellular membrane-bounded organelle;extracellular organelle;extracellular region part;extracellular vesicular exosome;intracellular part;membrane-bounded organelle;membra	Ubiquitin mediated proteolysis	Homo sapiens	1	12
cell part;cytoplasmic part;endoplasmic reticulum;extracellular membrane-bounded organelle;extracellular organelle;extracellular region part;extracellular vesicular exosome;intracellular membrane-bounded organelle;intracellu	Lysosome	Homo sapiens	1	12
cell part;contractile fiber part;cytoplasm;cytoplasmic part;cytosol;endoplasmic reticulum membrane;endoplasmic reticulum part;extracellular membrane-bounded organelle;extracellular organelle;extracellular region part;extracellular vesicular exosome;intracellular organelle part;intracellular part;membrane;membrane part;memb		Homo sapiens	1	12
cell junction;cell part;cell-cell junction;cytoplasm;cytoplasmic part;cytosol;endosome;extracellular membrane-bounded organelle;extracellular organelle;extracellular region part;extracellular vesicular exosome;Golgi apparatus	Acute myeloid leukemia;B cell receptor signaling pathway;Chemokine signaling pathway;Chronic myelo	Homo sapiens	1	12
cell part;cytoplasm;extracellular membrane-bounded organelle;extracellular organelle;extracellular region part;extracellular vesicular exosome;intracellular membrane-bounded organelle;intracellular organelle;intracellular part	Parkinson's disease;Ubiquitin mediated proteolysis	Homo sapiens	1	12
autophagic vacuole;cell part;cell surface;cytoplasmic part;endoplasmic reticulum;intracellular membrane-bounded organelle;intracellular organelle;intracellular part;membrane;membrane-bounded organelle;organelle;vacuole		Homo sapiens	1	12
cell part;cytoplasm;cytoplasmic part;intracellular membrane-bounded organelle;intracellular organelle;intracellular part;membrane-bounded organelle;nucleus;organelle;perinuclear region of cytoplasm	Mineral absorption	Homo sapiens	1	12
cell part;cell projection;cytoplasm;dendrite;intracellular membrane-bounded organelle;intracellular organelle;intracellular part;membrane-bounded organelle;neuron projection;nucleus;organelle		Homo sapiens	1	12
cell body;cell part;cell projection;chromosomal part;chromosome, telomeric region;cytoplasm;dendrite;DNA replication factor A complex;intracellular membrane-bounded organelle;intracellular organelle;intracellular organelle part;intracellular part;macromolecular complex;membrane-bounded organelle;neuron projection;neurona		Homo sapiens	1	12
cell part;extracellular membrane-bounded organelle;extracellular organelle;extracellular region part;extracellular vesicular exosome;integral to membrane;intrinsic to membrane;membrane part;membrane-bounded organelle;membrane-bounded vesicle;organelle;vesicle		Homo sapiens	1	12
anchoring collagen;basement membrane;cell part;collagen;collagen type VII;cytoplasmic part;endoplasmic reticulum lumen;endoplasmic reticulum part;extracellular matrix part;extracellular region;extracellular region part;extracellular space;intracellular organelle lumen;intracellular organelle part;intracellular part;membrane-enclo		Homo sapiens	1	12
acrosomal membrane;adherens junction;anchoring junction;apical plasma membrane;basolateral plasma membrane;caveola;cell cortex;cell junction;cell part;cell projection;cell-substrate adherens junction;cell-substrate junc	Bacterial invasion of epithelial cells;Endocytosis;Focal adhesion;Viral myocarditis	Homo sapiens	1	12
adherens junction;anchored to membrane;anchoring junction;cell junction;cell part;cell projection membrane;cell projection part;cell-substrate adherens junction;cell-substrate junction;cytoplasmic part;endoplasmic reticulum	Complement and coagulation cascades	Homo sapiens	1	12
cell part;cytoplasmic part;cytosol;intracellular part;membrane		Homo sapiens	1	12
cell part;cytoplasmic part;cytosol;intracellular part;membrane;plasma membrane		Homo sapiens	1	12
adherens junction;anchoring junction;cell junction;cell part;cell-substrate adherens junction;cell-substrate junction;cytoplasm;cytoplasmic part;cytoplasmic vesicle membrane;cytoplasmic vesicle part;cytoskeletal part;dystrophin-associated glycoprotein complex;extracellular membrane-bounded organelle;extracellular organelle;		Homo sapiens	1	12
cell part;cytoplasmic part;endoplasmic reticulum;endoplasmic reticulum membrane;endoplasmic reticulum part;integral to membrane;intracellular membrane-bounded organelle;intracellular organelle;intracellular organelle part;intracellular part;intrinsic to membrane;membrane;membrane part;membrane-bounded organelle;orga		Homo sapiens	1	12
cell part;cytoplasm;cytoplasmic part;endosome;extracellular membrane-bounded organelle;extracellular organelle;extracellular region part;extracellular vesicular exosome;intracellular membrane-bounded organelle;intracellular organelle;intracellular part;macromolecular complex;membrane;membrane part;membrane raft;memb		Homo sapiens	1	12
cell part;cytoplasmic part;endoplasmic reticulum;intracellular membrane-bounded organelle;intracellular non-membrane-bounded organelle;intracellular organelle;intracellular organelle part;intracellular part;membrane-bounded organelle;non-membrane-bounded organelle;nuclear part;nucleolus;organelle;organelle part		Homo sapiens	1	12
cell part;cytoplasm;cytoskeletal part;cytoskeleton;intracellular non-membrane-bounded organelle;intracellular organelle;intracellular organelle part;intracellular part;microtubule cytoskeleton;microtubule plus end;non-membrane-bounded organelle;organelle;organelle part		Homo sapiens	1	12
cell part;cytoplasm;cytoplasmic part;cytosol;intracellular membrane-bounded organelle;intracellular organelle;intracellular part;membrane-bounded organelle;nucleus;organelle		Homo sapiens	1	12
cell part;germ cell nucleus;intracellular membrane-bounded organelle;intracellular organelle;intracellular part;male germ cell nucleus;membrane-bounded organelle;nucleus;organelle		Homo sapiens	1	12
cell part;cytoplasm;cytoplasmic part;cytoskeletal part;cytosol;extracellular membrane-bounded organelle;extracellular organelle;extracellular region part;extracellular vesicular exosome;intracellular membrane-bounded organ	Amyotrophic lateral sclerosis (ALS);Chagas disease (American trypanosomiasis);Epithelial cell signaling	Homo sapiens	1	12
cell part;intracellular membrane-bounded organelle;intracellular organelle;intracellular organelle part;intracellular part;macromolecular complex;membrane;membrane-bounded organelle;mRNA cleavage factor complex;nucle	mRNA surveillance pathway	Homo sapiens	1	12
		Homo sapiens	1	12
cell part;envelope;intracellular organelle part;intracellular part;macromolecular complex;membrane;membrane part;nuclear envelope;nuclear part;nuclear pore;organelle envelope;organelle part;pore complex;protein complex	RNA transport	Homo sapiens	1	12
cell part;chromosomal part;intracellular membrane-bounded organelle;intracellular organelle;intracellular organelle part;intracellular part;macromolecular complex;membrane-bounded organelle;nucleosome;nucleus;organelle;organelle part;protein-DNA complex		Homo sapiens	1	12
cell part;chromosomal part;chromosome, telomeric region;cytoplasm;cytoskeletal part;female pronucleus;intracellular membrane-bounded organelle;intracellular non-membrane-bounded organelle;intracellular organelle;intracellular organelle part;intracellular part;male pronucleus;membrane;membrane-bounded organelle;non-m		Homo sapiens	1	12

		Homo sapiens	1	12
cell part;integrator complex;intracellular membrane-bounded organelle;intracellular organelle;intracellular organelle part;intracellular part;macromolecular complex;membrane-bounded organelle;nuclear part;nucleoplasm part;nucleus;organelle;organelle part;protein complex;SOSS complex		Homo sapiens	1	12
cell part;cytoplasm;Elongator holoenzyme complex;histone acetyltransferase complex;intracellular organelle part;intracellular part;macromolecular complex;nuclear part;nucleoplasm part;organelle part;protein complex;transcription elongation factor complex		Homo sapiens	1	12
cell part;intracellular organelle part;intracellular part;macromolecular complex;nuclear body;nuclear matrix;nuclear part;nuclear speck;nucleoplasm;nucleoplasm part;organelle part;ribonucleoprotein complex;small nuclear rib	Spliceosome	Homo sapiens	1	12
cell part;cytoplasm;envelope;intracellular organelle part;intracellular part;macromolecular complex;membrane;membrane part;nuclear envelope;nuclear membrane;nuclear part;nuclear pore;nucleoplasm;organelle envelope;or	RNA transport	Homo sapiens	1	12
adherens junction;anchoring junction;cell junction;cell part;cell-substrate adherens junction;cell-substrate junction;cytoplasmic part;cytosol;focal adhesion;intracellular membrane-bounded organelle;intracellular organelle;intracellular part;membrane;membrane-bounded organelle;nucleus;organelle;plasma membrane		Homo sapiens	1	12
cell part;cytoplasmic part;endoplasmic reticulum membrane;endoplasmic reticulum part;extracellular membrane-bounded organelle;extracellular organelle;extracellular region part;extracellular vesicular exosome;integral to membrane;intracellular organelle part;intracellular part;intrinsic to membrane;membrane;membrane part;m		Homo sapiens	1	12
cell junction;cell part;cell-cell junction;cytoplasmic part;cytosol;intracellular part;lateral plasma membrane;membrane part;plasma membrane part	Regulation of actin cytoskeleton	Homo sapiens	1	12
cell envelope;cell part;cornified envelope;cytoplasm;cytoplasmic part;cytoskeleton;envelope;extracellular membrane-bounded organelle;extracellular organelle;extracellular region part;extracellular vesicular exosome;intracellular membrane-bounded organelle;intracellular non-membrane-bounded organelle;intracellular organelle;		Homo sapiens	1	12
cell part;cytoplasm;intracellular part		Homo sapiens	1	12
cell part;cytoplasm;intracellular part		Homo sapiens	1	12
cell part;cytoplasmic part;endoplasmic reticulum;endoplasmic reticulum chaperone complex;endoplasmic reticulum lumen;endoplasmic reticulum part;intracellular membrane-bounded organelle;intracellular organelle;intracel	Protein processing in endoplasmic reticulum	Homo sapiens	1	12
cell part;cytoplasmic part;cytosol;extracellular membrane-bounded organelle;extracellular organelle;extracellular region part;extracellular vesicular exosome;integral to membrane;integral to mitochondrial membrane;integral to mitochondrial outer membrane;integral to organelle membrane;intracellular organelle part;intracellular p		Homo sapiens	1	12
cell part;chromatin;chromosomal part;cytoplasm;histone methyltransferase complex;intracellular membrane-bounded organelle;intracellular non-membrane-bounded organelle;intracellular organelle;intracellular organelle part;intracellular part;macromolecular complex;membrane;membrane-bounded organelle;methyltransferase		Homo sapiens	1	12
cell part;membrane		Homo sapiens	1	12
cell part;cytoplasmic part;endosomal part;endosome;endosome membrane;extrinsic to membrane;intracellular membrane-bounded organelle;intracellular organelle;intracellular organelle part;intracellular part;late endosome;late endosome membrane;membrane;membrane part;membrane-bounded organelle;organelle;organelle		Homo sapiens	1	12
cell part;intracellular part;macromolecular complex;protein complex;tRNA-splicing ligase complex		Homo sapiens	1	12
cell part;cytoplasm;cytoplasmic part;endoplasmic reticulum;endoplasmic reticulum membrane;endoplasmic reticulum part;integral to membrane;intracellular membrane-bounded organelle;intracellular organelle;intracellular organelle part;intracellular part;intrinsic to membrane;membrane;membrane part;membrane-bounded orga		Homo sapiens	1	12
cell part;clathrin coated vesicle membrane;coated pit;coated vesicle membrane;cytoplasm;cytoplasmic part;cytoplasmic vesicle membrane;cytoplasmic vesicle part;endoplasmic reticulum;extracellular region;intracellular membrane-bounded organelle;intracellular organelle;intracellular organelle part;intracellular part;membrane;		Homo sapiens	1	12
cell part;chromatin;chromatin remodeling complex;chromosomal part;intracellular membrane-bounded organelle;intracellular organelle;intracellular organelle part;intracellular part;macromolecular complex;membrane-bounded organelle;nuclear chromatin;nuclear chromosome part;nuclear part;nucleus;organelle;organelle part;pro		Homo sapiens	1	12
cell part;cytoplasmic membrane-bounded vesicle;cytoplasmic part;cytoplasmic vesicle;endoplasmic reticulum membrane;endoplasmic reticulum part;GPI-anchor transamidase complex;integral to endoplasmic reticulum mer	Glycosylphosphatidylinositol(GPI)-anchor biosynthesis	Homo sapiens	1	12
cell part;cytoplasmic part;endoplasmic reticulum membrane;endoplasmic reticulum part;endoplasmic reticulum-Golgi intermediate compartment;endoplasmic reticulum-Golgi intermediate compartment membrane;Golgi apparatus part;Golgi membrane;integral to membrane;intracellular membrane-bounded organelle;intracellula		Homo sapiens	1	12
cell part;cytoplasm;cytoplasmic part;extracellular membrane-bounded organelle;extracellular organelle;extracellular region part;extracellular vesicular exosome;Golgi apparatus;intracellular membrane-bounded organelle;intracellular organelle;intracellular organelle part;intracellular part;membrane-bounded organelle;membrane-b		Homo sapiens	1	12
catalytic step 2 spliceosome;cell part;cytoplasm;intracellular organelle part;intracellular part;macromolecular complex;nuclear part;nucleoplasm;organelle part;ribonucleoprotein complex;small nuclear ribonucleoprotein comp	Spliceosome	Homo sapiens	1	12
cell part;cytoplasmic part;intracellular organelle lumen;intracellular organelle part;intracellular part;membrane-enclosed lumen;mitochondrial matrix;mitochondrial part;organelle lumen;organelle part		Homo sapiens	1	12
cell part;cytoplasmic part;intracellular membrane-bounded organelle;intracellular organelle;intracellular organelle part;intracellular part;membrane;membrane-bounded organelle;mitochondrial inner membrane;mitochondrial membrane;mitochondrial part;mitochondrion;organelle;organelle inner membrane;organelle membrane;org		Homo sapiens	1	12
cell part;intracellular organelle part;intracellular part;macromolecular complex;nuclear part;nucleoplasm;organelle part;ribonucleoprotein complex;spliceosomal complex	Spliceosome	Homo sapiens	1	12
cell part;cytoplasm;cytoplasmic part;endoplasmic reticulum;Golgi apparatus part;Golgi stack;intracellular membrane-bounded organelle;intracellular organelle;intracellular organelle part;intracellular part;membrane-bounded organelle;organelle;organelle part		Homo sapiens	1	12
cell part;cullin-RING ubiquitin ligase complex;cytoplasm;intracellular membrane-bounded organelle;intracellular organelle;intracellular part;macromolecular complex;membrane-bounded organelle;nucleus;organelle;protein complex;SCF ubiquitin ligase complex;ubiquitin ligase complex		Homo sapiens	1	12
		Homo sapiens	1	12
cell part;cytoplasm;cytoplasmic part;cytosol;Golgi apparatus;intracellular membrane-bounded organelle;intracellular organelle;intracellular part;membrane-bounded organelle;nucleus;organelle;perinuclear region of cytoplasm		Homo sapiens	1	12
cell part;cytoplasm;intracellular membrane-bounded organelle;intracellular organelle;intracellular part;membrane-bounded organelle;nucleus;organelle	mRNA surveillance pathway	Homo sapiens	1	12
cell part;cell projection;cytoplasm;cytoplasmic part;cytosol;intracellular non-membrane-bounded organelle;intracellular organelle;intracellular organelle part;intracellular part;non-membrane-bounded organelle;nuclear part;nu	Cysteine and methionine metabolism	Homo sapiens	1	12
cell part;cytoplasm;intracellular non-membrane-bounded organelle;intracellular organelle;intracellular organelle part;intracellular part;non-membrane-bounded organelle;nuclear part;nucleolus;organelle;organelle part		Homo sapiens	1	12
cell junction;cell part;cytoplasmic membrane-bounded vesicle;cytoplasmic part;cytoplasmic vesicle;intracellular membrane-bounded organelle;intracellular organelle;intracellular part;membrane-bounded organelle;membrane-bounded vesicle;organelle;vesicle		Homo sapiens	1	12
cell part;cytoplasmic part;intracellular membrane-bounded organelle;intracellular non-membrane-bounded organelle;intracellular organelle;intracellular organelle part;intracellular part;macromolecular complex;membrane;membrane-bounded organelle;mitochondrial inner membrane;mitochondrial membrane;mitochondrial part;m		Homo sapiens	1	12
cell part;DNA-directed RNA polymerase complex;DNA-directed RNA polymerase I complex;intracellular membrane-bounded organelle;intracellular non-membrane-bounded organelle;intracellular organelle;intracellular organe	Purine metabolism;Pyrimidine metabolism;RNA polymerase	Homo sapiens	1	12
acrosomal vesicle;cell division site part;cell part;centralspindlin complex;cleavage furrow;cytoplasmic membrane-bounded vesicle;cytoplasmic part;cytoplasmic vesicle;cytoskeletal part;cytosol;extracellular membrane-bounded organelle;extracellular organelle;extracellular region part;extracellular vesicular exosome;extrinsic to i		Homo sapiens	1	12
cell part;cytoplasmic part;cytosol;endosome;Golgi apparatus;Golgi apparatus part;Golgi cisterna;Golgi cisterna membrane;Golgi membrane;intracellular membrane-bounded organelle;intracellular organelle;intracellular organelle part;intracellular part;membrane;membrane-bounded organelle;membrane-enclosed lumen;mitochor		Homo sapiens	1	12
cell part;chromosome;condensed chromosome;condensed nuclear chromosome;CURI complex;cytoplasmic part;intracellular membrane-bounded organelle;intracellular non-membrane-bounded organelle;intracellular organe	Ribosome biogenesis in eukaryotes	Homo sapiens	1	12
cell part;cytoplasmic part;endoplasmic reticulum;intracellular membrane-bounded organelle;intracellular organelle;intracellular part;membrane-bounded organelle;nucleus;organelle		Homo sapiens	1	12
cell junction;cell part;cell projection;clathrin-coated vesicle;coated vesicle;cytoplasm;cytoplasmic membrane-bounded vesicle;cytoplasmic part;cytoplasmic vesicle;cytoplasmic vesicle membrane;cytoplasmic vesicle part;cytoskeletal part;dendrite;Golgi apparatus;Golgi apparatus part;Golgi membrane;Golgi-associated vesicle;G		Homo sapiens	1	12
cell body;cell part;cell projection;cytoplasmic part;endoplasmic reticulum;endoplasmic reticulum part;envelope;extracellular membrane-bounded organelle;extracellular organelle;extracellular region part;extracellular vesicular exosome;integral to endoplasmic reticulum membrane;integral to membrane;integral to organelle memb		Homo sapiens	1	12
axon;cell part;cell projection;cytoplasm;cytoskeletal part;intracellular organelle part;intracellular part;kinesin complex;macromolecular complex;microtubule;microtubule associated complex;neuron projection;organelle part;protein complex		Homo sapiens	1	12
cell part;cytoplasm;cytoplasmic part;cytosol;eukaryotic translation initiation factor 2B complex;intracellular part;macromolecular complex;protein complex	RNA transport	Homo sapiens	1	12
extracellular region;organelle;vesicle		Homo sapiens	1	12
basal part of cell;cell junction;cell part;cytoplasm;cytoplasmic part;cytosol;intracellular part;membrane;plasma membrane;postsynaptic membrane;synapse part;synaptic membrane		Homo sapiens	1	12
cell part;cytoplasmic part;intracellular membrane-bounded organelle;intracellular organelle;intracellular organelle part;intracellular part;membrane-bounded organelle;mitochondrion;nuclear part;nucleoplasm;organelle;organelle part		Homo sapiens	1	12
cell part;cytoplasmic part;endosome;intracellular membrane-bounded organelle;intracellular organelle;intracellular part;membrane;membrane-bounded organelle;mitochondrion;organelle		Homo sapiens	1	12
cell part;cytoplasm;intracellular organelle part;intracellular part;nuclear body;nuclear part;nuclear speck;nucleoplasm;nucleoplasm part;organelle part		Homo sapiens	1	12
cell part;cytoplasmic part;early endosome;endosomal part;endosome;endosome membrane;extracellular membrane-bounded organelle;extracellular organelle;extracellular region part;extracellular vesicular exosome;intracellular membrane-bounded organelle;intracellular organelle;intracellular organelle part;intracellular part;lysos		Homo sapiens	1	12
aggresome;axon;caveola;cell leading edge;cell part;cell projection;chromatin remodeling complex;cytoplasm;cytoplasmic microtubule;cytoplasmic part;cytoskeletal part;cytosol;dendrite;histone deacetylase complex;inclusion body;intracellular membrane-bounded organelle;intracellular organelle;intracellular organelle part;intrac		Homo sapiens	1	12
cell part;cytoplasmic part;cytosol;endoplasmic reticulum;endoplasmic reticulum membrane;endoplasmic reticulum part;integral to membrane;intracellular membrane-bounded organelle;intracellular organelle;intracellular orga	Protein export;Protein processing in endoplasmic reticulum	Homo sapiens	1	12
cell part;cytoplasmic part;extracellular membrane-bounded organelle;extracellular organelle;extracellular region part;extracellular vesicular exosome;integral to membrane;intracellular membrane-bounded organelle;intracellul	Amino sugar and nucleotide sugar metabolism	Homo sapiens	1	12
extracellular membrane-bounded organelle;extracellular organelle;extracellular region part;extracellular vesicular exosome;membrane-bounded organelle;membrane-bounded vesicle;organelle;vesicle	Amino sugar and nucleotide sugar metabolism	Homo sapiens	1	12
cell part;intracellular membrane-bounded organelle;intracellular organelle;intracellular part;membrane-bounded organelle;nucleus;organelle		Homo sapiens	1	12
cell part;cytoplasm;intracellular part;membrane;plasma membrane		Homo sapiens	1	12
cell part;intracellular membrane-bounded organelle;intracellular non-membrane-bounded organelle;intracellular organelle;intracellular organelle part;intracellular part;macromolecular complex;membrane;membrane-bounded	Ribosome biogenesis in eukaryotes	Homo sapiens	1	12
cell part;cytoplasm;cytoplasmic part;cytosol;intracellular part	Amino sugar and nucleotide sugar metabolism	Homo sapiens	1	12
cell part;chromosomal part;cytoplasm;cytoplasmic part;cytosol;intracellular membrane-bounded organelle;intracellular non-membrane-bounded organelle;intracellular organelle;intracellular organelle part;intracellular part;kine	NOD-like receptor signaling pathway;Plant-pathogen interaction	Homo sapiens	1	12
cell part;cytoplasm;cytoplasmic microtubule;cytoplasmic part;cytoskeletal part;endoplasmic reticulum membrane;endoplasmic reticulum part;integral to membrane;intracellular organelle part;intracellular part;intrinsic to mem	Protein export	Homo sapiens	1	12
cell part;cell projection;clathrin-coated vesicle;coated vesicle;cytoplasm;cytoplasmic membrane-bounded vesicle;cytoplasmic part;cytoplasmic vesicle;cytoplasmic vesicle membrane;cytoplasmic vesicle part;endosome;extracellular membrane-bounded organelle;extracellular organelle;extracellular region part;extracellular vesic		Homo sapiens	1	12
cell part;cytoplasmic part;extracellular membrane-bounded organelle;extracellular organelle;extracellular region part;extracellular vesicular exosome;integral to membrane;intracellular membrane-bounded organelle;intracellular organelle;intracellular organelle part;intracellular part;intrinsic to membrane;membrane;membrane part		Homo sapiens	1	12
cell part;cytoplasmic part;cytosol;eukaryotic 43S preinitiation complex;eukaryotic 48S preinitiation complex;eukaryotic translation initiation factor 3 complex;intracellular part;macromolecular complex;membrane;protein comp	RNA transport	Homo sapiens	1	13
cell part;cytoplasmic part;intracellular organelle lumen;intracellular organelle part;intracellular part;membrane-enclosed lumen;mitochondrial matrix;mitochondrial part;organelle lumen;organelle part	Citrate cycle (TCA cycle);Glycolysis / Gluconeogenesis;Pyruvate metabolism	Homo sapiens	1	13
cell part;cis-Golgi network;cytoplasmic membrane-bounded vesicle;cytoplasmic part;cytoplasmic vesicle;endocytic vesicle;endosomal part;endosome membrane;Golgi apparatus;Golgi apparatus part;Golgi cisterna membrane;Golgi lumen;Golgi membrane;integral to membrane;intracellular membrane-bounded organelle;intrace		Homo sapiens	1	13
cell part;cytoplasm;cytoplasmic part;cytosol;intracellular part		Homo sapiens	1	13
cell part;cytoplasmic part;cytosol;extracellular membrane-bounded organelle;extracellular organelle;extracellular region part;extracellular vesicular exosome;intracellular membrane-bounded organelle;intracellular organelle;int	Proteasome	Homo sapiens	1	13
cell part;cytoplasmic part;cytosol;extracellular membrane-bounded organelle;extracellular organelle;extracellular region part;extracellular vesicular exosome;intracellular organelle part;intracellular part;membrane-bounded organelle;membrane-bounded vesicle;nuclear part;nucleoplasm;organelle;organelle part;vesicle		Homo sapiens	1	13
cell part;COPI vesicle coat;cytoplasmic part;cytoplasmic vesicle part;cytosol;Golgi apparatus;Golgi apparatus part;intracellular membrane-bounded organelle;intracellular organelle;intracellular organelle part;intracellular part;macromolecular complex;membrane coat;membrane part;membrane-bounded organelle;nuclear part;nu		Homo sapiens	1	13
actin filament bundle;actomyosin;apical part of cell;cell part;contractile fiber part;cytoplasmic part;cytoskeletal part;cytosol;extracellular membrane-bounded organelle;extracellular organelle;extracellular region part;extracellu	Focal adhesion;Leukocyte transendothelial migration;Regulation of actin cytoskeleton;Tight junction	Homo sapiens	1	13
cell part;cytoplasm;cytoplasmic part;cytosolic part;intracellular part;macromolecular complex;prefoldin complex;protein complex		Homo sapiens	1	13
cell part;cytoplasmic part;endoplasmic reticulum membrane;endoplasmic reticulum palmitoyltransferase complex;endoplasmic reticulum part;integral to membrane;intracellular organelle part;intracellular part;intrinsic to mem	Sphingolipid metabolism	Homo sapiens	1	13
actin cytoskeleton;cell part;cytoskeleton;integral to membrane;integral to plasma membrane;intracellular non-membrane-bounded organelle;intracellular organelle;intracellular organelle part;intracellular part;intrinsic to membrane;intrinsic to plasma membrane;membrane;membrane part;non-membrane-bounded organelle;nuclea		Homo sapiens	1	13
cell part;intracellular membrane-bounded organelle;intracellular non-membrane-bounded organelle;intracellular organelle;intracellular organelle part;intracellular part;macromolecular complex;membrane-bounded organelle;m	mRNA surveillance pathway	Homo sapiens	1	13
cell part;cytoplasm;extracellular membrane-bounded organelle;extracellular organelle;extracellular region part;extracellular vesicular exosome;intracellular membrane-bounded organelle;intracellular organelle;intracellular part;macromolecular complex;membrane-bounded organelle;membrane-bounded vesicle;nucleus;organelle;		Homo sapiens	1	13
cell part;clathrin-coated vesicle;coated vesicle;cytoplasm;cytoplasmic membrane-bounded vesicle;cytoplasmic part;cytoplasmic vesicle;cytosol;early endosome;early endosome membrane;early phagosome;endocytic vesicle;endosomal part;endosome;endosome membrane;extracellular membrane-bounded organelle;extracellu		Homo sapiens	1	13
cell part;cytoplasm;intracellular membrane-bounded organelle;intracellular organelle;intracellular part;membrane;membrane-bounded organelle;nucleus;organelle		Homo sapiens	1	13
carboxy-terminal domain protein kinase complex;cell part;chromatin;chromosomal part;chromosome;condensed chromosome;condensed nuclear chromosome;cytoplasm;intracellular membrane-bounded organelle;intracellular non-membrane-bounded organelle;intracellular organelle;intracellular organelle part;intracellular part;		Homo sapiens	1	13
cell part;cytoplasmic part;cytosolic part;intracellular part;macromolecular complex;prefoldin complex;protein complex		Homo sapiens	1	13
cell part;cytoplasmic part;cytosol;extracellular membrane-bounded organelle;extracellular organelle;extracellular region part;extracellular vesicular exosome;intracellular part;membrane-bounded organelle;membrane-bounde	Glutathione metabolism	Homo sapiens	1	13
cell part;cytoplasm;intracellular membrane-bounded organelle;intracellular organelle;intracellular part;membrane-bounded organelle;organelle		Homo sapiens	1	13
cell part;cell projection;centriole;cilium;cytoplasm;cytoplasmic part;cytoplasmic vesicle;cytoskeletal part;extracellular membrane-bounded organelle;extracellular organelle;extracellular region part;extracellular vesicular exosome;Golgi apparatus;intracellular membrane-bounded organelle;intracellular non-membrane-bounded org		Homo sapiens	1	13
		Homo sapiens	1	13
cell part;intracellular membrane-bounded organelle;intracellular organelle;intracellular organelle part;intracellular part;membrane-bounded organelle;nuclear part;nucleoplasm;nucleus;organelle;organelle part		Homo sapiens	1	13
cell part;cytoplasmic part;endoplasmic reticulum;endoplasmic reticulum membrane;endoplasmic reticulum part;intracellular membrane-bounded organelle;intracellular organelle;intracellular organelle part;intracellular part;membrane;membrane part;membrane-bounded organelle;organelle;organelle membrane;organelle part		Homo sapiens	1	13
cell part;cytoplasm;intracellular organelle part;intracellular part;nuclear body;nuclear part;nucleoplasm part;organelle part;PML body		Homo sapiens	1	13
cell part;coated vesicle membrane;COPII vesicle coat;cytoplasmic part;cytoplasmic vesicle membrane;cytoplasmic vesicle part;cytosol;endoplasmic reticulum membrane;endoplasmic reticulum part;ER to Golgi transport ves	Protein processing in endoplasmic reticulum	Homo sapiens	1	13
cell junction;cell part;cell projection;centrosome;cytoplasm;cytoplasmic part;cytoskeletal part;intracellular non-membrane-bounded organelle;intracellular organelle;intracellular organelle part;intracellular part;macromolecular	SNARE interactions in vesicular transport	Homo sapiens	1	13
blood microparticle;cell part;cytoplasmic part;cytosol;extracellular membrane-bounded organelle;extracellular organelle;extracellular region;extracellular region part;extracellular space;extracellular vesicular exosome;intracellular organelle lumen;intracellular organelle part;intracellular part;lysosomal lumen;membrane-bounded or		Homo sapiens	1	13
cell part;chromatoid body;cytoplasm;cytoplasmic mRNA processing body;cytoplasmic part;cytosol;eukaryotic translation initiation factor 4F complex;extracellular membrane-bounded organelle;extracellular organelle;extrace	Insulin signaling pathway;mTOR signaling pathway;RNA transport	Homo sapiens	1	13
cell part;cytoplasmic part;extracellular membrane-bounded organelle;extracellular organelle;extracellular region part;extracellular vesicular exosome;intracellular organelle lumen;intracellular organelle part;intracellular part;lys	Amino sugar and nucleotide sugar metabolism;Glycosaminoglycan degradation;Glycosphingolipid biosy	Homo sapiens	1	13
cell part;cytoplasm;cytoplasmic part;cytosol;extracellular membrane-bounded organelle;extracellular organelle;extracellular region part;extracellular vesicular exosome;intracellular membrane-bounded organelle;intracellular organelle;intracellular organelle part;intracellular part;membrane-bounded organelle;membrane-bounded		Homo sapiens	1	13
adherens junction;anchoring junction;cell junction;cell part;cell-substrate adherens junction;cell-substrate junction;chromatin;chromosomal part;cytoplasmic part;cytosol;focal adhesion;heterochromatin;intracellular membrane-bounded organelle;intracellular organelle;intracellular organelle part;intracellular part;macromolecular c		Homo sapiens	1	13
cell part;cytoplasmic part;cytosol;intracellular membrane-bounded organelle;intracellular organelle;intracellular part;membrane-bounded organelle;nucleus;organelle	Adherens junction;Circadian rhythm - plant;Measles;Ribosome biogenesis in eukaryotes;Tight junction;	Homo sapiens	1	13
cell part;chromatin remodeling complex;chromosomal part;DNA helicase complex;Ino80 complex;intracellular membrane-bounded organelle;intracellular organelle;intracellular organelle part;intracellular part;macromolecular complex;membrane-bounded organelle;nuclear chromosome part;nuclear matrix;nuclear part;nucleoplas		Homo sapiens	1	13
cell part;cytoplasmic part;cytosol;extracellular membrane-bounded organelle;extracellular organelle;extracellular region part;extracellular vesicular exosome;intracellular membrane-bounded organelle;intracellular non-membr	Protein processing in endoplasmic reticulum	Homo sapiens	1	13
cell part;cell projection;cytoplasmic part;extracellular membrane-bounded organelle;extracellular organelle;extracellular region part;extracellular space;extracellular vesicular exosome;intracellular membrane-bounded organelle;intracellular organelle;intracellular part;membrane-bounded organelle;membrane-bounded vesicle;neu		Homo sapiens	1	13
adherens junction;anchoring junction;apical plasma membrane;cell junction;cell part;cell projection;cell projection membrane;cell projection part;cell surface;cell-cell junction;cell-substrate adherens junction;cell-substrate jun	Protein digestion and absorption	Homo sapiens	1	13
cell junction;cell part;cell-cell junction;cytoplasm;cytoplasmic part;cytoskeletal part;cytoskeleton;cytosol;intracellular membrane-bounded organelle;intracellular non-membrane-bounded organelle;intracellular organelle;intrac	Acute myeloid leukemia;Adipocytokine signaling pathway;Apoptosis;B cell receptor signaling pathway;	Homo sapiens	1	13
cell part;cytoplasmic part;extracellular membrane-bounded organelle;extracellular organelle;extracellular region part;extracellular vesicular exosome;intracellular membrane-bounded organelle;intracellular organelle;intracellul	Pyrimidine metabolism	Homo sapiens	1	13
adherens junction;anchoring junction;cell junction;cell part;cell-substrate adherens junction;cell-substrate junction;cytoplasm;cytoplasmic part;cytosol;cytosolic large ribosomal subunit;extracellular membrane-bounded orgar	Ribosome	Homo sapiens	1	13
cell part;intracellular membrane-bounded organelle;intracellular organelle;intracellular organelle part;intracellular part;macromolecular complex;membrane-bounded organelle;nuclear part;nucleus;organelle;organelle part;ribor	Spliceosome	Homo sapiens	1	13
cell part;cytoplasm;cytoplasmic part;cytosol;cytosolic large ribosomal subunit;intracellular non-membrane-bounded organelle;intracellular organelle;intracellular organelle part;intracellular part;large ribosomal subunit;macrom	Ribosome	Homo sapiens	1	13
adherens junction;anchoring junction;cell junction;cell part;cell-substrate adherens junction;cell-substrate junction;external side of plasma membrane;extracellular region part;extracellular space;focal adhesion;integral to membrane;intracellular membrane-bounded organelle;intracellular organelle;intracellular part;intrinsic to mem		Homo sapiens	1	13
cell part;cytoplasmic part;intracellular membrane-bounded organelle;intracellular organelle;intracellular organelle part;intracellular part;macromolecular complex;membrane;membrane part;membrane-bounded organelle;mito	Alzheimer's disease;Cardiac muscle contraction;Huntington's disease;Oxidative phosphorylation;Parkin	Homo sapiens	1	13
cell part;cytoplasmic part;cytosol;intracellular membrane-bounded organelle;intracellular organelle;intracellular part;membrane-bounded organelle;mitochondrion;organelle	Carbon fixation in photosynthetic organisms;Pyruvate metabolism	Homo sapiens	1	13
adherens junction;anchoring junction;basement membrane;cell junction;cell part;cell-substrate adherens junction;cell-substrate junction;cytoplasmic part;cytosol;extracellular matrix part;extracellular region part;focal adhesion;integral to membrane;integral to plasma membrane;intracellular part;intrinsic to membrane;intrinsic to p		Homo sapiens	1	13
cell part;cytoplasmic part;cytosol;extracellular membrane-bounded organelle;extracellular organelle;extracellular region part;extracellular vesicular exosome;intracellular part;membrane-bounded organelle;membrane-bounde	Glutathione metabolism	Homo sapiens	1	13
cell part;cytoplasmic part;intracellular membrane-bounded organelle;intracellular non-membrane-bounded organelle;intracellular organelle;intracellular organelle part;intracellular part;macromolecular complex;membrane;membrane-bounded organelle;mitochondrial inner membrane;mitochondrial membrane;mitochondrial part;nu		Homo sapiens	1	13
cell part;cytoplasm;cytosolic large ribosomal subunit;extracellular membrane-bounded organelle;extracellular organelle;extracellular region part;extracellular vesicular exosome;intracellular membrane-bounded organelle;intracellular organelle;intracellular organelle part;intracellular part;large ribosomal subunit;macromolecular cor		Homo sapiens	1	13
cell part;cytoplasmic part;cytosol;extracellular membrane-bounded organelle;extracellular organelle;extracellular region part;extracellular vesicular exosome;intracellular membrane-bounded organelle;intracellular organelle;int	Proteasome	Homo sapiens	1	13
cell part;cell projection;cytoplasm;cytoplasmic part;cytosol;dendrite;intracellular membrane-bounded organelle;intracellular organelle;intracellular organelle part;intracellular part;macromolecular complex;membrane part;membrane-bounded organelle;neuron projection;nuclear part;nuclear pore;nucleoplasm;nucleus;organelle;org		Homo sapiens	1	13
cell part;cytoplasmic part;endoplasmic reticulum;extracellular membrane-bounded organelle;extracellular organelle;extracellular region part;extracellular space;extracellular vesicular exosome;Golgi apparatus;intracellular mer	Lysosome	Homo sapiens	1	13
cell part;extracellular membrane-bounded organelle;extracellular organelle;extracellular region part;extracellular vesicular exosome;extrinsic to membrane;extrinsic to plasma membrane;membrane part;membrane-bounded organelle;membrane-bounded vesicle;organelle;plasma membrane part;vesicle		Homo sapiens	1	13
cell part;cytoplasmic membrane-bounded vesicle;cytoplasmic part;cytoplasmic vesicle;endoplasmic reticulum membrane;endoplasmic reticulum part;endoplasmic reticulum-Golgi intermediate compartment membrane;extracellular membrane-bounded organelle;extracellular organelle;extracellular region part;extracellular vesicu		Homo sapiens	1	13
cell junction;cell part;cell-cell junction;cytoplasmic part;cytosol;extracellular membrane-bounded organelle;extracellular organelle;extracellular region part;extracellular vesicular exosome;intracellular;intracellular part;lipid part	Chemokine signaling pathway;Focal adhesion;Leukocyte transendothelial migration;Long-term potentia	Homo sapiens	1	13
catalytic step 2 spliceosome;cell part;cytoplasmic part;cytosol;exon-exon junction complex;intracellular membrane-bounded organelle;intracellular organelle;intracellular organelle part;intracellular part;macromolecular compl	mRNA surveillance pathway;RNA transport;Spliceosome	Homo sapiens	1	13
cell part;cytoplasmic part;cytosol;endoplasmic reticulum;endoplasmic reticulum part;integral to endoplasmic reticulum membrane;integral to membrane;integral to organelle membrane;intracellular membrane-bounded organ	Phagosome;Protein export;Protein processing in endoplasmic reticulum;Vibrio cholerae infection	Homo sapiens	1	13
catalytic step 2 spliceosome;cell part;cytoplasm;cytoplasmic part;cytosol;extracellular membrane-bounded organelle;extracellular organelle;extracellular region part;extracellular vesicular exosome;intracellular organelle part;i	Spliceosome;Systemic lupus erythematosus	Homo sapiens	1	13
adherens junction;anchoring junction;cell junction;cell part;cell projection;cell projection membrane;cell projection part;cell-substrate adherens junction;cell-substrate junction;cytoplasm;cytoplasmic membrane-bounded vesi	Adherens junction;Amyotrophic lateral sclerosis (ALS);Axon guidance;B cell receptor signaling pathway;	Homo sapiens	1	13
cell part;cytoplasmic part;endoplasmic reticulum membrane;endoplasmic reticulum part;extracellular membrane-bounded organelle;extracellular organelle;extracellular region part;extracellular vesicular exosome;integral to m	Protein export	Homo sapiens	1	13
cell part;cytoplasm;cytoskeleton;envelope;intracellular membrane-bounded organelle;intracellular non-membrane-bounded organelle;intracellular organelle;intracellular organelle part;intracellular part;macromolecular complex	RNA transport	Homo sapiens	1	13
adherens junction;anchoring junction;apical plasma membrane;cell junction;cell part;cell-substrate adherens junction;cell-substrate junction;clathrin coat;clathrin coat of coated pit;clathrin coated vesicle membrane;clathrin-c	Endocytosis	Homo sapiens	1	13
cell part;cytoplasm;intracellular membrane-bounded organelle;intracellular organelle;intracellular organelle part;intracellular part;membrane;membrane-bounded organelle;nuclear membrane;nuclear part;nucleus;organelle;organelle membrane;organelle part		Homo sapiens	1	13
cell part;intracellular membrane-bounded organelle;intracellular organelle;intracellular organelle part;intracellular part;membrane-bounded organelle;nuclear part;nucleoplasm;nucleus;organelle;organelle part	mRNA surveillance pathway	Homo sapiens	1	13
cell part;cytoplasm;cytoplasmic part;extracellular membrane-bounded organelle;extracellular organelle;extracellular region part;extracellular vesicular exosome;intracellular organelle lumen;intracellular organelle part;intracellu	Pantothenate and CoA biosynthesis	Homo sapiens	1	13
cell part;cytoplasmic part;cytosol;intracellular membrane-bounded organelle;intracellular organelle;intracellular part;membrane;membrane-bounded organelle;nucleus;organelle		Homo sapiens	1	13
cell part;cytoplasm;intracellular membrane-bounded organelle;intracellular non-membrane-bounded organelle;intracellular organelle;intracellular organelle part;intracellular part;membrane-bounded organelle;non-membrane-l	Spliceosome	Homo sapiens	1	13

cell part;cytoplasmic part;cytosol;intracellular membrane-bounded organelle;intracellular organelle;intracellular organelle part;intracellular part;macromolecular complex;membrane-bounded organelle;nuclear part;nucleoplasn	Chronic myeloid leukemia;Notch signaling pathway;Pathways in cancer;Wnt signaling pathway	Homo sapiens	1	13
adherens junction;anchoring junction;basolateral plasma membrane;cell junction;cell part;cell surface;cell-substrate adherens junction;cell-substrate junction;cytoplasm;extracellular membrane-bounded organelle;extracellular organelle;extracellular region part;extracellular space;extracellular vesicular exosome;focal adhesion;int		Homo sapiens	1	13
aggresome;autophagic vacuole;cell part;cytoplasm;cytoplasmic mRNA processing body;cytoplasmic part;cytoplasmic vesicle;cytosol;endoplasmic reticulum;endosome;extracellular membrane-bounded organelle;extracellula	Osteoclast differentiation	Homo sapiens	1	13
cell part;cytoplasm;cytoplasmic part;cytosol;death-inducing signaling complex;intracellular membrane-bounded organelle;intracellular organelle;intracellular part;macromolecular complex;membrane part;membrane raft;mem	Apoptosis;Cytosolic DNA-sensing pathway;Hepatitis C;RIG-I-like receptor signaling pathway;Toll-like re	Homo sapiens	1	13
apical part of cell;cell part;cytoplasm;intracellular membrane-bounded organelle;intracellular non-membrane-bounded organelle;intracellular organelle;intracellular organelle part;intracellular part;membrane;membrane-bounded organelle;non-membrane-bounded organelle;nuclear part;nucleolus;nucleus;organelle;organelle part		Homo sapiens	1	13
acrosomal membrane;adherens junction;anchoring junction;caveola;cell junction;cell part;cell-substrate adherens junction;cell-substrate junction;cytoplasmic membrane-bounded vesicle;cytoplasmic part;cytoplasmic vesicle	Insulin signaling pathway	Homo sapiens	1	13
cell part;cytoplasmic part;cytosol;extracellular membrane-bounded organelle;extracellular organelle;extracellular region part;extracellular vesicular exosome;intracellular part;membrane-bounded organelle;membrane-bounde	Amino sugar and nucleotide sugar metabolism;Galactose metabolism	Homo sapiens	1	13
cell part;cytoplasmic part;cytosol;intracellular part		Homo sapiens	1	13
cell part;cytoplasmic part;cytoskeleton;cytosol;endoplasmic reticulum;endoplasmic reticulum membrane;endoplasmic reticulum part;Golgi apparatus part;Golgi membrane;integral to membrane;intracellular membrane-bounc	Steroid biosynthesis	Homo sapiens	1	13
catalytic step 2 spliceosome;cell part;intracellular organelle part;intracellular part;macromolecular complex;nuclear part;nucleoplasm;organelle part;ribonucleoprotein complex;small nuclear ribonucleoprotein complex;spliceos	Spliceosome	Homo sapiens	1	13
cell part;cytoplasm;extracellular membrane-bounded organelle;extracellular organelle;extracellular region part;extracellular vesicular exosome;intracellular membrane-bounded organelle;intracellular organelle;intracellular part;macromolecular complex;membrane-bounded organelle;membrane-bounded vesicle;nucleus;organelle;		Homo sapiens	1	13
apical junction complex;cell division site part;cell junction;cell part;cell projection;cell-cell junction;centrosome;cleavage furrow;cytoplasm;cytoplasmic part;cytoskeletal part;cytoskeleton;cytosol;intermediate filament cytoskeleton;intracellular membrane-bounded organelle;intracellular non-membrane-bounded organelle;intracellu		Homo sapiens	1	13
adherens junction;anchoring junction;cell junction;cell part;cell-substrate adherens junction;cell-substrate junction;focal adhesion;intracellular membrane-bounded organelle;intracellular organelle;intracellular part;membrane-bounded organelle;nucleus;organelle		Homo sapiens	1	13
cell part;cytoplasm;intracellular membrane-bounded organelle;intracellular organelle;intracellular part;macromolecular complex;membrane-bounded organelle;nucleus;organelle;protein complex;tRNA-splicing ligase complex		Homo sapiens	1	13
cell part;cytoplasm;cytoplasmic part;cytosol;intracellular membrane-bounded organelle;intracellular organelle;intracellular organelle part;intracellular part;membrane-bounded organelle;nuclear part;nucleoplasm;nucleus;organelle;organelle part		Homo sapiens	1	13
cell part;intracellular membrane-bounded organelle;intracellular organelle;intracellular part;membrane-bounded organelle;nucleus;organelle		Homo sapiens	1	13
cell part;cytoplasm;cytoplasmic part;intracellular non-membrane-bounded organelle;intracellular organelle;intracellular organelle part;intracellular part;membrane-enclosed lumen;mitochondrial intermembrane space;mitochondrial part;non-membrane-bounded organelle;nuclear part;nucleolus;nucleoplasm;organelle;organelle env		Homo sapiens	1	13
cell part;chromatin;chromosomal part;cytoplasm;cytoplasmic part;intracellular membrane-bounded organelle;intracellular organelle;intracellular organelle part;intracellular part;macromolecular complex;membrane-bounded o	RNA degradation	Homo sapiens	1	13
cell part;cell projection;cytoplasm;cytoskeleton;filopodium;intracellular non-membrane-bounded organelle;intracellular organelle;intracellular part;lamellipodium;membrane;non-membrane-bounded organelle;organelle;plasma membrane		Homo sapiens	1	13
cell part;cytoplasm;cytoplasmic part;intracellular organelle lumen;intracellular organelle part;intracellular part;membrane-enclosed lumen;mitochondrial matrix;mitochondrial part;organelle lumen;organelle part		Homo sapiens	1	13
cell body;cell part;cell projection;cilium;cytoplasm;cytoplasmic part;extracellular membrane-bounded organelle;extracellular organelle;extracellular region;extracellular region part;extracellular space;extracellular vesicular exosome;intracellular membrane-bounded organelle;intracellular organelle;intracellular organelle part;intracel		Homo sapiens	1	13
cell part;intracellular organelle part;intracellular part;macromolecular complex;nuclear body;nuclear part;nuclear speck;nucleoplasm;nucleoplasm part;organelle part;protein complex;THO complex;THO complex part of transc	RNA transport;Spliceosome	Homo sapiens	1	13
cell part;cytoplasmic part;endoplasmic reticulum membrane;endoplasmic reticulum part;integral to membrane;intracellular;intracellular organelle part;intracellular part;intrinsic to membrane;membrane;membrane part;organel	Retinol metabolism	Homo sapiens	1	13
cell part;cytoplasm;intracellular membrane-bounded organelle;intracellular non-membrane-bounded organelle;intracellular organelle;intracellular organelle part;intracellular part;membrane;membrane-bounded organelle;non-membrane-bounded organelle;nuclear part;nucleolus;nucleus;organelle;organelle part		Homo sapiens	1	13
cell part;intracellular membrane-bounded organelle;intracellular organelle;intracellular part;membrane-bounded organelle;nucleus;organelle		Homo sapiens	1	13
cell part;chromosomal part;condensed chromosome kinetochore;cytoplasmic part;cytosol;extracellular membrane-bounded organelle;extracellular organelle;extracellular region part;extracellular vesicular exosome;intracellular membrane-bounded organelle;intracellular non-membrane-bounded organelle;intracellular organelle;in		Homo sapiens	1	13
cell part;chromatin;chromatin remodeling complex;chromosomal part;chromosome;intracellular membrane-bounded organelle;intracellular non-membrane-bounded organelle;intracellular organelle;intracellular organelle part;intracellular part;macromolecular complex;membrane-bounded organelle;nBAF complex;non-membrane-		Homo sapiens	1	13
cell part;centriole;centrosome;cytoplasm;cytoplasmic part;cytoskeletal part;cytosol;intracellular non-membrane-bounded organelle;intracellular organelle;intracellular organelle part;intracellular part;macromolecular complex;membrane;microtubule;microtubule organizing center;microtubule organizing center part;non-membrane-		Homo sapiens	1	13
cell part;chromosomal part;DNA replication factor A complex;intracellular membrane-bounded organelle;intracellular organelle;intracellular organelle part;intracellular part;macromolecular complex;membrane-bounded organelle;nuclear chromosome part;nuclear part;nucleoplasm;nucleus;organelle;organelle part;protein complex		Homo sapiens	1	13
cell part;chromosomal part;chromosome, centromeric region;condensed chromosome kinetochore;cytoplasmic part;cytoskeletal part;cytoskeleton;cytosol;intracellular membrane-bounded organelle;intracellular non-membrane-bounded organelle;intracellular organelle;intracellular organelle part;intracellular part;kinesin complex;		Homo sapiens	1	13
cell part;cytoplasmic part;cytosol;extracellular membrane-bounded organelle;extracellular organelle;extracellular region part;extracellular vesicular exosome;intracellular part;membrane-bounded organelle;membrane-bounded vesicle;organelle;vesicle		Homo sapiens	1	13
cell part;clathrin-coated vesicle;coated vesicle;cytoplasmic membrane-bounded vesicle;cytoplasmic part;cytoplasmic vesicle;endoplasmic reticulum;endoplasmic reticulum membrane;endoplasmic reticulum part;endoplasmic reticulum-Golgi intermediate compartment;endoplasmic reticulum-Golgi intermediate compartment me		Homo sapiens	1	13
cell part;cytoplasmic part;integral to membrane;intracellular membrane-bounded organelle;intracellular organelle;intracellular organelle part;intracellular part;intrinsic to membrane;membrane;membrane part;membrane-bounded organelle;mitochondrial membrane;mitochondrial part;mitochondrion;organelle;organelle membrane;		Homo sapiens	1	13
cell part;cytoplasmic part;intracellular membrane-bounded organelle;intracellular organelle;intracellular part;membrane-bounded organelle;mitochondrion;organelle		Homo sapiens	1	13
cell part;extracellular region part;extracellular space;intracellular membrane-bounded organelle;intracellular organelle;intracellular part;membrane-bounded organelle;nucleus;organelle		Homo sapiens	1	13
cell part;cytoplasm;intracellular organelle part;intracellular part;nuclear part;nucleoplasm;organelle part		Homo sapiens	1	13
		Homo sapiens	1	13
cell part;cytoplasmic part;cytosol;intracellular membrane-bounded organelle;intracellular organelle;intracellular organelle lumen;intracellular organelle part;intracellular part;membrane-bounded organelle;membrane-enclosed l	Valine, leucine and isoleucine degradation	Homo sapiens	1	13
cell part;cytoplasm;cytoplasmic part;cytosol;Golgi apparatus;Golgi apparatus part;Golgi membrane;intracellular membrane-bounded organelle;intracellular organelle;intracellular organelle part;intracellular part;membrane;mer	Endocytosis	Homo sapiens	1	13
cell part;cytoplasmic part;intracellular membrane-bounded organelle;intracellular organelle;intracellular organelle lumen;intracellular organelle part;intracellular part;membrane-bounded organelle;membrane-enclosed lumen;m	Aminoacyl-tRNA biosynthesis	Homo sapiens	1	13
cell part;intracellular organelle part;intracellular part;macromolecular complex;nuclear part;organelle part;ribonucleoprotein complex;small nuclear ribonucleoprotein complex;U1 snRNP		Homo sapiens	1	13
cell part;DNA-directed RNA polymerase II, holoenzyme;intracellular membrane-bounded organelle;intracellular organelle;intracellular organelle part;intracellular part;macromolecular complex;membrane-bounded organelle;nuclear part;nucleoplasm part;nucleus;organelle;organelle part;protein complex		Homo sapiens	1	13
cell part;centrosome;cytoplasm;cytoplasmic part;cytoskeletal part;intracellular membrane-bounded organelle;intracellular non-membrane-bounded organelle;intracellular organelle;intracellular organelle part;intracellular part;macromolecular complex;membrane-bounded organelle;microtubule organizing center;non-membrane-b		Homo sapiens	1	13
actin cytoskeleton;adherens junction;anchoring junction;cell junction;cell part;cell-substrate adherens junction;cell-substrate junction;contractile fiber part;cytoplasm;cytoplasmic part;cytoskeleton;cytosol;focal adhesion;intra	Focal adhesion	Homo sapiens	1	13
cell part;cytoplasm;extracellular membrane-bounded organelle;extracellular organelle;extracellular region part;extracellular vesicular exosome;intracellular membrane-bounded organelle;intracellular organelle;intracellular part;membrane;membrane-bounded organelle;membrane-bounded vesicle;nucleus;organelle;vesicle		Homo sapiens	1	13
cell body;cell part;cell projection;cytoplasm;cytoplasmic part;cytosol;dendrite;extracellular region part;extracellular space;intracellular part;membrane;neuron projection;neuronal cell body;perinuclear region of cytoplasm;plasma membrane		Homo sapiens	1	13
cell part;cytoplasm;cytoplasmic part;cytosol;extracellular membrane-bounded organelle;extracellular organelle;extracellular region part;extracellular vesicular exosome;intracellular organelle lumen;intracellular organelle part;i	Glyoxylate and dicarboxylate metabolism;Pyruvate metabolism	Homo sapiens	1	13
cell part;cytoplasmic part;endoplasmic reticulum;extracellular membrane-bounded organelle;extracellular organelle;extracellular region part;extracellular space;extracellular vesicular exosome;intracellular membrane-boundec	Lysosome	Homo sapiens	1	13
cell part;cytoplasmic part;cytosol;inclusion body;intracellular organelle part;intracellular part;macromolecular complex;membrane part;nuclear body;nuclear inclusion body;nuclear part;nuclear pore;nuclear speck;nucleoplasn	mRNA surveillance pathway;Ribosome biogenesis in eukaryotes;RNA transport	Homo sapiens	1	13
cell part;cytoplasmic part;intracellular membrane-bounded organelle;intracellular organelle;intracellular organelle lumen;intracellular organelle part;intracellular part;membrane-bounded organelle;membrane-enclosed lumen;m	Pyrimidine metabolism	Homo sapiens	1	13
cell part;intracellular membrane-bounded organelle;intracellular organelle;intracellular part;membrane-bounded organelle;nucleus;organelle	Ribosome biogenesis in eukaryotes	Homo sapiens	1	13
cell part;cytoplasm;intracellular membrane-bounded organelle;intracellular organelle;intracellular organelle part;intracellular part;membrane-bounded organelle;nuclear matrix;nuclear part;nucleoplasm;nucleus;organelle;organelle part		Homo sapiens	1	13
cell part;cytoplasmic part;early endosome;endosome;extracellular membrane-bounded organelle;extracellular organelle;extracellular region part;extracellular vesicular exosome;extrinsic to membrane;intracellular membrane-bounded organelle;intracellular organelle;intracellular part;membrane part;membrane-bounded organelle;		Homo sapiens	1	13
cell part;cytoplasmic part;integral to membrane;integral to plasma membrane;intracellular organelle part;intracellular part;intrinsic to membrane;intrinsic to plasma membrane;lysosomal membrane;membrane;membrane part;organelle membrane;organelle part;plasma membrane;plasma membrane part;vacuolar membrane;vacuc		Homo sapiens	1	13
cell part;cytoplasmic part;endoplasmic reticulum;integral to membrane;integral to plasma membrane;intracellular membrane-bounded organelle;intracellular organelle;intracellular part;intrinsic to membrane;intrinsic to plasma membrane;membrane part;membrane-bounded organelle;organelle;plasma membrane part		Homo sapiens	1	13
cell part;cytoplasm;intracellular;intracellular membrane-bounded organelle;intracellular organelle;intracellular part;membrane-bounded organelle;nucleus;organelle		Homo sapiens	1	13
actin cytoskeleton;cell part;cytoplasm;cytoskeleton;intracellular non-membrane-bounded organelle;intracellular organelle;intracellular organelle part;intracellular part;macromolecular complex;non-membrane-bounded organelle;nuclear part;nucleoplasm;nucleoplasm part;organelle;organelle part;protein complex;ribonucleoprotei		Homo sapiens	1	13
cell junction;cell part;cell projection;cytoplasm;cytoskeletal part;intracellular non-membrane-bounded organelle;intracellular organelle;intracellular organelle part;intracellular part;macromolecular complex;non-membrane-bounded organelle;organelle;organelle part;podosome;protein complex		Homo sapiens	1	14
apical plasma membrane;basolateral plasma membrane;cell part;cytoplasmic membrane-bounded vesicle;cytoplasmic part;cytoplasmic vesicle;cytosol;extracellular membrane-bounded organelle;extracellular organelle;extracellular region part;extracellular vesicular exosome;intracellular membrane-bounded organelle;intracellula		Homo sapiens	1	14
cell part;cytoplasmic part;endoplasmic reticulum;endoplasmic reticulum membrane;endoplasmic reticulum part;extracellular membrane-bounded organelle;extracellular organelle;extracellular region part;extracellular vesicular exosome;integral to membrane;intracellular membrane-bounded organelle;intracellular non-membrane-		Homo sapiens	1	14
cell part;clathrin coated vesicle membrane;clathrin-coated vesicle;coated vesicle;coated vesicle membrane;cytoplasm;cytoplasmic membrane-bounded vesicle;cytoplasmic part;cytoplasmic vesicle;cytoplasmic vesicle meml	Huntington's disease	Homo sapiens	1	14
cell part;cytoplasm;cytoplasmic part;cytosol;extracellular membrane-bounded organelle;extracellular organelle;extracellular region part;extracellular vesicular exosome;intracellular membrane-bounded organelle;intracellular c	Vitamin B6 metabolism	Homo sapiens	1	14
actin cytoskeleton;adherens junction;anchoring junction;Arp2/3 protein complex;cell junction;cell part;cell projection;cell-substrate adherens junction;cell-substrate junction;cytoplasmic part;cytoskeletal part;cytoskeleton;cyt	Bacterial invasion of epithelial cells;Fc gamma R-mediated phagocytosis;Pathogenic Escherichia coli inf	Homo sapiens	1	14
cell part;cytoplasmic part;cytosol;endosomal part;endosome membrane;extracellular membrane-bounded organelle;extracellular organelle;extracellular region part;extracellular vesicular exosome;intracellular organelle part;in	Endocytosis	Homo sapiens	1	14
cell part;cytoplasmic part;endoplasmic reticulum;endoplasmic reticulum membrane;endoplasmic reticulum part;integral to membrane;intracellular membrane-bounded organelle;intracellular organelle;intracellular organelle part;intracellular part;intrinsic to membrane;macromolecular complex;membrane;membrane part;membrane		Homo sapiens	1	14
cell part;cytoplasm;cytoplasmic part;extracellular membrane-bounded organelle;extracellular organelle;extracellular region part;extracellular vesicular exosome;intracellular membrane-bounded organelle;intracellular organelle	Alzheimer's disease;Huntington's disease;Oxidative phosphorylation;Parkinson's disease	Homo sapiens	1	14
cell part;intracellular membrane-bounded organelle;intracellular organelle;intracellular organelle part;intracellular part;macromolecular complex;membrane-bounded organelle;nuclear body;nuclear part;nuclear speck;nucleoplasm;nucleoplasm part;nucleus;organelle;organelle part;ribonucleoprotein complex;small nuclear ribonucl		Homo sapiens	1	14
cell part;intracellular membrane-bounded organelle;intracellular organelle;intracellular part;membrane-bounded organelle;nucleus;organelle		Homo sapiens	1	14
cell part;centrosome;cytoplasm;cytoplasmic part;cytoskeletal part;extracellular membrane-bounded organelle;extracellular organelle;extracellular region part;extracellular vesicular exosome;intracellular membrane-bounded organelle;intracellular non-membrane-bounded organelle;intracellular organelle;intracellular organelle part		Homo sapiens	1	14
cell part;cytoplasm;cytoplasmic part;cytosol;intracellular membrane-bounded organelle;intracellular organelle;intracellular part;membrane-bounded organelle;nucleus;organelle;perinuclear region of cytoplasm	Mineral absorption	Homo sapiens	1	14
cell part;cytoplasmic membrane-bounded vesicle;cytoplasmic part;cytoplasmic vesicle;cytosol;extracellular matrix;extracellular region;extracellular region part;extracellular space;extrinsic to external side of plasma membrane;extrinsic to membrane;extrinsic to plasma membrane;intracellular membrane-bounded organelle;intrac		Homo sapiens	1	14
cell part;cytoplasmic part;integral to membrane;intracellular membrane-bounded organelle;intracellular organelle;intracellular organelle part;intracellular part;intrinsic to membrane;membrane;membrane part;membrane-bounc	Alzheimer's disease;Cardiac muscle contraction;Huntington's disease;Oxidative phosphorylation;Parkin	Homo sapiens	1	14
adherens junction;anchoring junction;cell junction;cell part;cell-substrate adherens junction;cell-substrate junction;extracellular membrane-bounded organelle;extracellular organelle;extracellular region part;extracellular vesicu	MAPK signaling pathway;Regulation of actin cytoskeleton;Tight junction	Homo sapiens	1	14
cell part;cell surface;coated vesicle membrane;cytoplasmic part;cytoplasmic vesicle membrane;cytoplasmic vesicle part;early endosome membrane;endocytic vesicle membrane;endoplasmic reticulum;endoplasmic reticulun	Allograft rejection;Antigen processing and presentation;Autoimmune thyroid disease;Cell adhesion mole	Homo sapiens	1	14
cell part;cytoplasmic part;dihydrolipoyl dehydrogenase complex;intracellular membrane-bounded organelle;intracellular organelle;intracellular organelle lumen;intracellular organelle part;intracellular part;macromolecular comp	Valine, leucine and isoleucine degradation	Homo sapiens	1	14
apical plasma membrane;cell part;cytoplasmic part;cytosol;extracellular membrane-bounded organelle;extracellular organelle;extracellular region part;extracellular space;extracellular vesicular exosome;intracellular part;mem	Caprolactam degradation;Glycerolipid metabolism;Glycolysis / Gluconeogenesis	Homo sapiens	1	14
cell part;chromatin;chromosomal part;DNA replication factor A complex;intracellular membrane-bounded organelle;intracellular organelle;intracellular organelle part;intracellular part;macromolecular complex;membrane-boun	DNA replication;Homologous recombination;Mismatch repair;Nucleotide excision repair	Homo sapiens	1	14
AMP-activated protein kinase complex;calcium channel complex;cAMP-dependent protein kinase complex;cation channel complex;cell part;cell projection;centrosome;cilium;cytoplasmic part;cytoskeletal part;cytosol;extrac	Amoebiasis;Apoptosis;Bile secretion;Calcium signaling pathway;Chemokine signaling pathway;Dilated c	Homo sapiens	1	14
cell part;cytoplasm;cytoplasmic part;external side of plasma membrane;extracellular matrix;extracellular membrane-bounded organelle;extracellular organelle;extracellular region part;extracellular space;extracellular vesicular exosome;immunological synapse;intracellular membrane-bounded organelle;intracellular organelle;intrac		Homo sapiens	1	14
cell part;chromatin;chromosomal part;chromosome;condensed chromosome;condensed nuclear chromosome;cytoplasm;intracellular membrane-bounded organelle;intracellular non-membrane-bounded organelle;intracellular organelle;intracellular organelle part;intracellular part;membrane;membrane-bounded organelle;non-me		Homo sapiens	1	14
cell part;cytoplasmic part;endosome;integral to membrane;integral to plasma membrane;intracellular membrane-bounded organelle;intracellular organelle;intracellular organelle part;intracellular part;intrinsic to membrane;intri	Lysosome;Phagosome	Homo sapiens	1	14
cell junction;cell part;cytoplasm;cytoplasmic part;cytosol;intracellular membrane-bounded organelle;intracellular organelle;intracellular organelle part;intracellular part;membrane-bounded organelle;nuclear part;nucleoplasm;r	Drug metabolism - other enzymes;Purine metabolism	Homo sapiens	1	14
cell part;cell projection;cytoplasm;cytoplasmic part;cytosol;intracellular part;intrinsic to internal side of plasma membrane;intrinsic to membrane;intrinsic to plasma membrane;membrane part;plasma membrane part;ruffle	Axon guidance;MAPK signaling pathway	Homo sapiens	1	14
cell part;cell projection;cytoplasmic membrane-bounded vesicle;cytoplasmic part;cytoplasmic vesicle;cytosol;endomembrane system;extracellular membrane-bounded organelle;extracellular organelle;extracellular region par	Collecting duct acid secretion;Epithelial cell signaling in Helicobacter pylori infection;Oxidative phospho	Homo sapiens	1	14
cell part;cytoplasm;cytoplasmic part;endoplasmic reticulum;extracellular membrane-bounded organelle;extracellular organelle;extracellular region part;extracellular vesicular exosome;intracellular membrane-bounded organelle;intracellular organelle;intracellular part;membrane-bounded organelle;membrane-bounded vesicle;orga		Homo sapiens	1	14
cell part;cytoplasmic part;intracellular membrane-bounded organelle;intracellular organelle;intracellular organelle lumen;intracellular organelle part;intracellular part;membrane-bounded organelle;membrane-enclosed lumen;mitochondrial matrix;mitochondrial part;mitochondrion;organelle;organelle lumen;organelle part		Homo sapiens	1	14
cell part;cytoplasmic part;intracellular membrane-bounded organelle;intracellular organelle;intracellular organelle lumen;intracellular organelle part;intracellular part;membrane-bounded organelle;membrane-enclosed lumen;m	Pyruvate metabolism;Two-component system	Homo sapiens	1	14
cell part;cytoplasm;cytoplasmic part;cytosol;extracellular membrane-bounded organelle;extracellular organelle;extracellular region part;extracellular vesicular exosome;intracellular membrane-bounded organelle;intracellular c	Proteasome	Homo sapiens	1	14
actin cytoskeleton;cell junction;cell part;cytoplasm;cytoplasmic part;cytoskeleton;cytosol;extrinsic to membrane;intracellular non-membrane-bounded organelle;intracellular organelle;intracellular part;membrane;membrane part;non-membrane-bounded organelle;organelle;plasma membrane		Homo sapiens	1	14
cell part;cytoplasmic part;intracellular membrane-bounded organelle;intracellular organelle;intracellular organelle lumen;intracellular organelle part;intracellular part;membrane-bounded organelle;membrane-enclosed lumen;m	Arginine and proline metabolism	Homo sapiens	1	14
cell part;cleavage body;intracellular membrane-bounded organelle;intracellular organelle;intracellular organelle part;intracellular part;macromolecular complex;membrane-bounded organelle;mRNA cleavage and polyadenylati	mRNA surveillance pathway	Homo sapiens	1	14
basolateral plasma membrane;cell part;extracellular membrane-bounded organelle;extracellular organelle;extracellular region part;extracellular vesicular exosome;integral to membrane;integral to plasma membrane;intrinsic t	ABC transporters;Vitamin digestion and absorption	Homo sapiens	1	14
cell part;cell surface;clathrin coated vesicle membrane;clathrin-coated endocytic vesicle membrane;coated vesicle membrane;cytoplasmic part;cytoplasmic vesicle membrane;cytoplasmic vesicle part;endocytic vesicle mem	Basal cell carcinoma;Hedgehog signaling pathway;Melanogenesis;Pathways in cancer;Wnt signaling pa	Homo sapiens	1	14
cell part;cytoplasm;cytoplasmic part;cytosolic part;cytosolic ribosome;intracellular;intracellular membrane-bounded organelle;intracellular non-membrane-bounded organelle;intracellular organelle;intracellular organelle part;intracellular part;macromolecular complex;membrane;membrane-bounded organelle;NatA complex;non-m		Homo sapiens	1	14
cell part;clathrin-coated vesicle;coated vesicle;cytoplasmic membrane-bounded vesicle;cytoplasmic part;cytoplasmic vesicle;early endosome;endosomal part;endosome;external side of plasma membrane;extracellular membrane-bounded organelle;extracellular organelle;extracellular region part;extracellular vesicular exosome;		Homo sapiens	1	14
cell part;cytoplasmic part;intracellular membrane-bounded organelle;intracellular organelle;intracellular organelle part;intracellular part;membrane-bounded organelle;mitochondrion;nuclear part;nucleoplasm;nucleus;organelle;organelle part		Homo sapiens	1	14
cell part;cytoplasm;extracellular membrane-bounded organelle;extracellular organelle;extracellular region part;extracellular vesicular exosome;intracellular part;membrane-bounded organelle;membrane-bounded vesicle;orgai	Amino sugar and nucleotide sugar metabolism	Homo sapiens	1	14
cell junction;cell part;cytoplasm;cytoplasmic part;cytosol;intracellular membrane-bounded organelle;intracellular organelle;intracellular organelle part;intracellular part;macromolecular complex;membrane;membrane-bounded organelle;nuclear part;nucleoplasm;nucleoplasm part;nucleus;organelle;organelle part;protein complex;t		Homo sapiens	1	14
cell part;cytoplasmic part;extracellular membrane-bounded organelle;extracellular organelle;extracellular region part;extracellular vesicular exosome;intracellular membrane-bounded organelle;intracellular organelle;intracellula	Alzheimer's disease;Huntington's disease;Oxidative phosphorylation;Parkinson's disease	Homo sapiens	1	14
cell part;cell projection;centrosome;chromosomal part;cilium;condensed chromosome kinetochore;cytoplasm;cytoplasmic part;cytoskeletal part;cytosol;intracellular non-membrane-bounded organelle;intracellular organelle;ir	Hedgehog signaling pathway;Wnt signaling pathway	Homo sapiens	1	14
adherens junction;anchoring junction;cell junction;cell part;cell-substrate adherens junction;cell-substrate junction;extracellular membrane-bounded organelle;extracellular organelle;extracellular region part;extracellular space;extracellular vesicular exosome;focal adhesion;integral to membrane;integral to plasma membrane;intrir		Homo sapiens	1	14
cell part;cytoplasmic part;cytosol;endoplasmic reticulum part;extracellular membrane-bounded organelle;extracellular organelle;extracellular region part;extracellular vesicular exosome;intracellular organelle part;intracellular	Protein export	Homo sapiens	1	14
actin cytoskeleton;cell part;chromosomal part;condensed chromosome kinetochore;cytoplasm;cytoplasmic part;cytoskeletal part;cytoskeleton;cytosol;intracellular membrane-bounded organelle;intracellular non-membrane-bounded organelle;intracellular organelle;intracellular organelle part;intracellular part;kinetochore;kinetoch		Homo sapiens	1	14
cell part;external side of plasma membrane;extracellular membrane-bounded organelle;extracellular organelle;extracellular region part;extracellular vesicular exosome;integral to membrane;integral to plasma membrane;intrinsic to membrane;intrinsic to plasma membrane;membrane;membrane part;membrane-bounded organelle		Homo sapiens	1	14
cell part;cytoplasm;cytoplasmic part;extracellular membrane-bounded organelle;extracellular organelle;extracellular region part;extracellular vesicular exosome;intracellular membrane-bounded organelle;intracellular organelle	Citrate cycle (TCA cycle);Propanoate metabolism	Homo sapiens	1	14
caveola;cell part;cytoplasmic membrane-bounded vesicle;cytoplasmic part;cytoplasmic vesicle;extracellular membrane-bounded organelle;extracellular organelle;extracellular region part;extracellular vesicular exosome;intrac	Aldosterone-regulated sodium reabsorption;Bile secretion;Carbohydrate digestion and absorption;Cardi	Homo sapiens	1	14
cell part;cytoplasm;cytoplasmic part;cytosol;intracellular organelle part;intracellular part;macromolecular complex;nuclear part;nucleoplasm;organelle part;proteasome accessory complex;proteasome complex;proteasome re	Proteasome	Homo sapiens	1	14
cell part;coated vesicle membrane;cytoplasmic part;cytoplasmic vesicle membrane;cytoplasmic vesicle part;cytosol;endoplasmic reticulum membrane;endoplasmic reticulum part;envelope;ER to Golgi transport vesicle mem	Protein processing in endoplasmic reticulum;RNA transport	Homo sapiens	1	14
adherens junction;anchoring junction;cell junction;cell part;cell-substrate adherens junction;cell-substrate junction;cytoplasmic part;cytosol;cytosolic large ribosomal subunit;extracellular membrane-bounded organelle;extrac	Ribosome	Homo sapiens	1	14
adherens junction;anchoring junction;cell junction;cell part;cell-substrate adherens junction;cell-substrate junction;cytoplasmic part;endoplasmic reticulum;extracellular membrane-bounded organelle;extracellular organelle;ex	MAPK signaling pathway;Regulation of actin cytoskeleton;Tight junction	Homo sapiens	1	14
adherens junction;anchoring junction;cell junction;cell part;cell-substrate adherens junction;cell-substrate junction;cytoplasm;cytoplasmic part;cytosol;cytosolic small ribosomal subunit;extracellular membrane-bounded orgai	Ribosome	Homo sapiens	1	14
adherens junction;anchoring junction;cell junction;cell part;cell-substrate adherens junction;cell-substrate junction;cytoplasm;cytoplasmic part;cytosol;cytosolic small ribosomal subunit;extracellular membrane-bounded orgai	Ribosome	Homo sapiens	1	14
cell part;cytoplasm;cytoplasmic part;cytosol;cytosolic large ribosomal subunit;extracellular membrane-bounded organelle;extracellular organelle;extracellular region part;extracellular vesicular exosome;intracellular membrane	Ribosome	Homo sapiens	1	14
cell part;cytoplasmic part;cytoplasmic vesicle membrane;cytoplasmic vesicle part;cytosol;cytosolic large ribosomal subunit;endocytic vesicle membrane;endosomal part;endosome membrane;extracellular membrane-bounde	Ribosome	Homo sapiens	1	14
cell part;cell projection;cytoplasm;cytoplasmic part;cytosol;intracellular part;neuron projection	Glycine, serine and threonine metabolism;Methane metabolism	Homo sapiens	1	14
cell part;cytoplasmic part;cytoskeletal part;cytosol;intracellular organelle part;intracellular part;macromolecular complex;microtubule;microtubule associated complex;organelle part;protein complex		Homo sapiens	1	14
cell part;cytoplasmic part;intracellular membrane-bounded organelle;intracellular non-membrane-bounded organelle;intracellular organelle;intracellular organelle part;intracellular part;macromolecular complex;membrane;membrane-bounded organelle;mitochondrial inner membrane;mitochondrial membrane;mitochondrial part;m		Homo sapiens	1	14
cell part;cytoplasmic part;intracellular membrane-bounded organelle;intracellular organelle;intracellular organelle part;intracellular part;macromolecular complex;membrane;membrane-bounded organelle;mitochondrial inner membrane;mitochondrial membrane;mitochondrial part;mitochondrial small ribosomal subunit;mitochond		Homo sapiens	1	14
cell part;cytoplasmic part;intracellular membrane-bounded organelle;intracellular non-membrane-bounded organelle;intracellular organelle;intracellular organelle part;intracellular part;macromolecular complex;membrane;membrane-bounded organelle;mitochondrial inner membrane;mitochondrial membrane;mitochondrial part;m		Homo sapiens	1	14
cell part;cytoplasm;cytoplasmic part;cytosol;intracellular membrane-bounded organelle;intracellular organelle;intracellular organelle part;intracellular part;membrane;membrane-bounded organelle;nuclear part;nucleoplasm;nu	Butanoate metabolism;Synthesis and degradation of ketone bodies;Terpenoid backbone biosynthesis;V	Homo sapiens	1	14
extracellular membrane-bounded organelle;extracellular organelle;extracellular region;extracellular region part;extracellular vesicular exosome;membrane-bounded organelle;membrane-bounded vesicle;organelle;vesicle		Homo sapiens	1	14
cell part;intracellular membrane-bounded organelle;intracellular organelle;intracellular part;membrane-bounded organelle;nucleus;organelle		Homo sapiens	1	14
extracellular membrane-bounded organelle;extracellular organelle;extracellular region;extracellular region part;extracellular space;extracellular vesicular exosome;membrane-bounded organelle;membrane-bounded vesicle;organelle;vesicle		Homo sapiens	1	14
axon part;calcium channel complex;cation channel complex;cell body;cell junction;cell part;cell projection part;cell-cell contact zone;cell-cell junction;cytoplasm;cytoplasmic part;cytoplasmic vesicle membrane;cytoplasmic v	Calcium signaling pathway;ErbB signaling pathway;Gastric acid secretion;Glioma;GnRH signaling pathv	Homo sapiens	1	14
cell part;cytoplasm;cytoplasmic part;cytosol;intracellular membrane-bounded organelle;intracellular organelle;intracellular part;membrane;membrane-bounded organelle;nucleus;organelle;plasma membrane	Alzheimer's disease	Homo sapiens	1	14
adherens junction;anchoring junction;cell junction;cell part;cell-substrate adherens junction;cell-substrate junction;cytoplasmic part;extracellular membrane-bounded organelle;extracellular organelle;extracellular region part;e	Lysosome	Homo sapiens	1	14
cell part;cytoplasm;elongin complex;extracellular region part;extracellular space;integral to membrane;intracellular membrane-bounded organelle;intracellular organelle;intracellular organelle part;intracellular part;intrinsic to membrane;macromolecular complex;membrane part;membrane-bounded organelle;nuclear part;nucleopla		Homo sapiens	1	14
cell part;cytoplasmic part;intracellular membrane-bounded organelle;intracellular non-membrane-bounded organelle;intracellular organelle;intracellular organelle lumen;intracellular organelle part;intracellular part;membrane;m	alpha-Linolenic acid metabolism;Biosynthesis of unsaturated fatty acids;Fatty acid metabolism;Peroxisc	Homo sapiens	1	14
cell part;cytoplasmic part;cytosol;extracellular membrane-bounded organelle;extracellular organelle;extracellular region part;extracellular vesicular exosome;intracellular membrane-bounded organelle;intracellular organelle;int	Peroxisome;Terpenoid backbone biosynthesis	Homo sapiens	1	14

cell part;integral to membrane;intrinsic to membrane;membrane part		Homo sapiens	1	14
cell part;cytoplasm;cytoplasmic part;intracellular membrane-bounded organelle;intracellular organelle;intracellular part;membrane-bounded organelle;mitochondrion;organelle		Homo sapiens	1	14
Cdc73/Paf1 complex;cell part;chromatin;chromosomal part;intracellular organelle part;intracellular part;macromolecular complex;nuclear body;nuclear part;nuclear speck;nucleoplasm part;organelle part;protein complex;transcription elongation factor complex;transcriptionally active chromatin		Homo sapiens	1	14
cell junction;cell part;cytoplasmic part;cytoskeleton;Golgi apparatus part;Golgi stack;intracellular non-membrane-bounded organelle;intracellular organelle;intracellular organelle part;intracellular part;non-membrane-bounded organelle;organelle;organelle part;synapse		Homo sapiens	1	14
cell part;cytoplasmic part;intracellular membrane-bounded organelle;intracellular organelle;intracellular part;membrane;membrane-bounded organelle;microbody;mitochondrion;organelle;peroxisome		Homo sapiens	1	14
cell part;cytoplasmic part;endoplasmic reticulum;endoplasmic reticulum membrane;endoplasmic reticulum part;integral to membrane;intracellular membrane-bounded organelle;intracellular organelle;intracellular organelle pa	Glycerophospholipid metabolism	Homo sapiens	1	14
cell part;intracellular membrane-bounded organelle;intracellular non-membrane-bounded organelle;intracellular organelle;intracellular organelle part;intracellular part;macromolecular complex;membrane-bounded organelle;non-membrane-bounded organelle;nuclear part;nucleolus;nucleus;organelle;organelle part;preribosome;pr		Homo sapiens	1	14
cell part;cytoplasmic part;cytosol;intracellular membrane-bounded organelle;intracellular organelle;intracellular part;membrane-bounded organelle;nucleus;organelle	Ubiquitin mediated proteolysis	Homo sapiens	1	14
cell part;intracellular part;macromolecular complex;protein complex;protein phosphatase 4 complex;protein serine/threonine phosphatase complex		Homo sapiens	1	14
cell part;cytoplasm;intracellular non-membrane-bounded organelle;intracellular organelle;intracellular organelle part;intracellular part;non-membrane-bounded organelle;nuclear part;nucleolus;organelle;organelle part		Homo sapiens	1	14
Cdc73/Paf1 complex;cell part;intracellular non-membrane-bounded organelle;intracellular organelle;intracellular organelle part;intracellular part;macromolecular complex;non-membrane-bounded organelle;nuclear part;nucleolus;nucleoplasm;nucleoplasm part;organelle;organelle part;protein complex;transcription elongation fact		Homo sapiens	1	14
catalytic step 2 spliceosome;cell part;cytoplasm;intracellular membrane-bounded organelle;intracellular organelle;intracellular organelle part;intracellular part;macromolecular complex;membrane;membrane-bounded organell	Spliceosome	Homo sapiens	1	14
cell part;cytoplasmic part;cytosol;extracellular membrane-bounded organelle;extracellular organelle;extracellular region part;extracellular vesicular exosome;intracellular part;membrane-bounded organelle;membrane-bounde	Chlorocyclohexane and chlorobenzene degradation;Fluorobenzoate degradation;Toluene degradation	Homo sapiens	1	14
cell part;cytoplasm;cytoplasmic part;cytosol;extracellular membrane-bounded organelle;extracellular organelle;extracellular region part;extracellular vesicular exosome;intracellular non-membrane-bounded organelle;intracellular organelle;intracellular organelle part;intracellular part;membrane;membrane-bounded organelle;meml		Homo sapiens	1	14
cell part;cytoplasm;extracellular membrane-bounded organelle;extracellular organelle;extracellular region part;extracellular vesicular exosome;intracellular part;membrane-bounded organelle;membrane-bounded vesicle;organelle;vesicle		Homo sapiens	1	14
basal plasma membrane;basement membrane;cell junction;cell part;cell-substrate junction;cytoplasm;extracellular matrix part;extracellular region part;hemidesmosome;intracellular membrane-bounded organelle;intracellular	NOD-like receptor signaling pathway	Homo sapiens	1	14
cell part;cytoplasmic part;intracellular membrane-bounded organelle;intracellular organelle;intracellular organelle part;intracellular part;membrane;membrane-bounded organelle;mitochondrial inner membrane;mitochondrial membrane;mitochondrial part;mitochondrion;organelle;organelle inner membrane;organelle membrane;org		Homo sapiens	1	14
actin cytoskeleton;cell part;centrosome;chromosomal part;condensin complex;cytoplasm;cytoplasmic part;cytoskeletal part;cytoskeleton;cytosol;intracellular membrane-bounded organelle;intracellular non-membrane-bounc	Cell cycle - yeast	Homo sapiens	1	14
adherens junction;anchoring junction;cell junction;cell part;cell-cell junction;cytoplasm;cytoskeletal part;intracellular organelle part;intracellular part;lateral plasma membrane;macromolecular complex;membrane part;microtubule;occluding junction;organelle part;plasma membrane part;protein complex;tight junction		Homo sapiens	1	14
cell part;cytoplasm;cytoplasmic part;extracellular membrane-bounded organelle;extracellular organelle;extracellular region part;extracellular vesicular exosome;Golgi apparatus;Golgi apparatus part;Golgi cisterna membrane;	Glycosaminoglycan biosynthesis - keratan sulfate;N-Glycan biosynthesis;Various types of N-glycan bios	Homo sapiens	1	14
cell part;cytoplasmic part;intracellular membrane-bounded organelle;intracellular organelle;intracellular organelle part;intracellular part;macromolecular complex;membrane;membrane-bounded organelle;mitochondrial inner membrane;mitochondrial membrane;mitochondrial part;mitochondrial small ribosomal subunit;mitochond		Homo sapiens	1	14
cell part;intracellular non-membrane-bounded organelle;intracellular organelle;intracellular organelle part;intracellular part;macromolecular complex;non-membrane-bounded organelle;nuclear part;nucleolar part;nucleolus;nucleoplasm;organelle;organelle part;PeBoW complex;preribosome;preribosome, large subunit precursor;pr		Homo sapiens	1	14
cell part;intracellular;intracellular membrane-bounded organelle;intracellular organelle;intracellular part;membrane-bounded organelle;nucleus;organelle	mRNA surveillance pathway	Homo sapiens	1	14
cell part;cytoplasm;cytoplasmic part;cytosol;endosomal part;endosome;endosome membrane;ESCRT III complex;extracellular membrane-bounded organelle;extracellular organelle;extracellular region part;extracellular vesicu	Endocytosis	Homo sapiens	1	14
cell part;cytoplasmic part;endoplasmic reticulum;intracellular membrane-bounded organelle;intracellular organelle;intracellular part;membrane;membrane-bounded organelle;organelle	Other types of O-glycan biosynthesis	Homo sapiens	1	14
cell part;cytoplasm;intracellular part		Homo sapiens	1	14
cell part;cytoplasmic part;endoplasmic reticulum membrane;endoplasmic reticulum part;integral to membrane;intracellular organelle part;intracellular part;intrinsic to membrane;membrane;membrane part;organelle membrane;organelle part		Homo sapiens	1	14
	Protein processing in endoplasmic reticulum	Homo sapiens	1	14
cell part;cytoplasmic part;cytosol;extracellular membrane-bounded organelle;extracellular organelle;extracellular region part;extracellular vesicular exosome;intracellular;intracellular membrane-bounded organelle;intracellular	Cysteine and methionine metabolism	Homo sapiens	1	14
cell part;cytoplasm;cytoskeleton;intracellular non-membrane-bounded organelle;intracellular organelle;intracellular part;non-membrane-bounded organelle;organelle		Homo sapiens	1	14
cell part;cytoplasm;cytoplasmic part;cytosol;eukaryotic translation initiation factor 2B complex;intracellular part;macromolecular complex;protein complex	RNA transport	Homo sapiens	1	14
cell part;cytoplasmic part;cytoskeleton;extrinsic to membrane;extrinsic to plasma membrane;intracellular non-membrane-bounded organelle;intracellular organelle;intracellular organelle part;intracellular part;membrane;membrane part;membrane raft;membrane-enclosed lumen;mitochondrial inner membrane;mitochondrial intern		Homo sapiens	1	14
cell part;cytoplasm;intracellular organelle part;intracellular part;macromolecular complex;membrane;membrane part;nuclear membrane;nuclear part;nuclear pore;nucleoplasm;organelle membrane;organelle part;pore complex	RNA transport	Homo sapiens	1	14
axon hillock;axon part;cell part;cell projection part;cytoplasm;cytoskeletal part;cytoskeleton;intracellular membrane-bounded organelle;intracellular non-membrane-bounded organelle;intracellular organelle;intracellular organelle part;intracellular part;macromolecular complex;membrane-bounded organelle;microtubule;microtubul		Homo sapiens	1	14
cell part;chromatin remodeling complex;chromosomal part;cytoplasmic part;cytosol;DNA helicase complex;Ino80 complex;intracellular membrane-bounded organelle;intracellular organelle;intracellular organelle part;intracellular part;macromolecular complex;membrane-bounded organelle;nuclear chromosome part;nuclear part;r		Homo sapiens	1	14
actin cytoskeleton;cell junction;cell part;cell projection;cell-cell junction;cytoplasmic part;cytoskeleton;cytosol;early endosome;endosome;extracellular membrane-bounded organelle;extracellular organelle;extracellular region	Adherens junction;Bacterial invasion of epithelial cells;Fc gamma R-mediated phagocytosis;Regulation (	Homo sapiens	1	14
adherens junction;anchoring junction;cell junction;cell part;cell surface;cell-substrate adherens junction;cell-substrate junction;cytoplasm;cytoplasmic membrane-bounded vesicle;cytoplasmic part;cytoplasmic vesicle;cytoske	Alzheimer's disease;Epithelial cell signaling in Helicobacter pylori infection	Homo sapiens	1	15
adherens junction;anchoring junction;axon;axon part;cell body;cell junction;cell part;cell projection;cell projection part;cell surface;cell-substrate adherens junction;cell-substrate junction;cytoplasmic part;cytoplasmic vesicle;	Axon guidance	Homo sapiens	1	15
cell part;cytoplasmic part;endoplasmic reticulum;endosomal part;endosome membrane;envelope;extracellular membrane-bounded organelle;extracellular organelle;extracellular region;extracellular region part;extracellular ve	Lysosome	Homo sapiens	1	15
actin cytoskeleton;adherens junction;anchoring junction;Arp2/3 protein complex;cell junction;cell part;cell projection;cell-substrate adherens junction;cell-substrate junction;cytoplasm;cytoplasmic part;cytoskeletal part;cytosl	Bacterial invasion of epithelial cells;Fc gamma R-mediated phagocytosis;Pathogenic Escherichia coli inf	Homo sapiens	1	15
cell part;cytoplasm;extracellular membrane-bounded organelle;extracellular organelle;extracellular region part;extracellular vesicular exosome;integral to membrane;integral to plasma membrane;intracellular membrane-bounded organelle;intracellular organelle;intracellular part;intrinsic to membrane;intrinsic to plasma membrane		Homo sapiens	1	15
catalytic step 2 spliceosome;cell part;intracellular membrane-bounded organelle;intracellular organelle;intracellular organelle part;intracellular part;macromolecular complex;membrane;membrane-bounded organelle;nuclear p	Spliceosome	Homo sapiens	1	15
cell cortex;cell part;cell projection membrane;cell projection part;chromosomal part;condensed chromosome kinetochore;cytoplasm;cytoplasmic microtubule;cytoplasmic part;cytoskeletal part;cytosol;Golgi apparatus;Golgi apparatus part;intracellular membrane-bounded organelle;intracellular non-membrane-bounded organelle		Homo sapiens	1	15
cell part;cytoplasmic part;intracellular membrane-bounded organelle;intracellular organelle;intracellular organelle part;intracellular part;macromolecular complex;membrane;membrane part;membrane-bounded organelle;mitoc	Alzheimer's disease;Huntington's disease;Oxidative phosphorylation;Parkinson's disease	Homo sapiens	1	15
cell part;cytoplasmic part;cytosol;early endosome;endosomal part;endosome;endosome membrane;extracellular membrane-bounded organelle;extracellular organelle;extracellular region part;extracellular vesicular exosome;intracellular membrane-bounded organelle;intracellular organelle;intracellular organelle part;intracellular p		Homo sapiens	1	15
cell part;cytoplasm;cytoplasmic part;endoplasmic reticulum;endoplasmic reticulum membrane;endoplasmic reticulum part;extracellular membrane-bounded organelle;extracellular organelle;extracellular region part;extracellular vesicular exosome;integral to membrane;intracellular membrane-bounded organelle;intracellular orgar		Homo sapiens	1	15
cell part;chromosome;cytoplasm;cytoplasmic part;cytoskeletal part;cytosol;intracellular non-membrane-bounded organelle;intracellular organelle;intracellular organelle part;intracellular part;kinesin complex;macromolecular complex;membrane;microtubule;microtubule associated complex;midbody;non-membrane-bounded orga		Homo sapiens	1	15
cell part;cytoplasm;cytoplasmic part;cytosolic part;endoplasmic reticulum;endoplasmic reticulum membrane;endoplasmic reticulum part;extracellular membrane-bounded organelle;extracellular organelle;extracellular region (	Amino sugar and nucleotide sugar metabolism	Homo sapiens	1	15
cell part;chromosomal part;extracellular membrane-bounded organelle;extracellular organelle;extracellular region part;extracellular vesicular exosome;intracellular membrane-bounded organelle;intracellular organelle;intracellu	Systemic lupus erythematosus	Homo sapiens	1	15
cell part;cell projection;cytoplasm;cytoplasmic part;cytosol;envelope;extracellular membrane-bounded organelle;extracellular organelle;extracellular region part;extracellular vesicular exosome;extrinsic to internal side of plasma membrane;extrinsic to membrane;extrinsic to plasma membrane;intracellular membrane-bounded org		Homo sapiens	1	15
alpha DNA polymerase:primase complex;cell part;chromosomal part;cytoplasm;envelope;intracellular membrane-bounded organelle;intracellular non-membrane-bounded organelle;intracellular organelle;intracellular organelle	DNA replication;Purine metabolism;Pyrimidine metabolism	Homo sapiens	1	15
blood microparticle;cell part;extracellular membrane-bounded organelle;extracellular organelle;extracellular region;extracellular region part;extracellular vesicular exosome;membrane;membrane-bounded organelle;membrane	Complement and coagulation cascades;Staphylococcus aureus infection;Systemic lupus erythematosu	Homo sapiens	1	15
cell part;chromosomal part;extracellular membrane-bounded organelle;extracellular organelle;extracellular region part;extracellular vesicular exosome;intracellular membrane-bounded organelle;intracellular organelle;intracellu	Systemic lupus erythematosus	Homo sapiens	1	15
cell part;cytoplasmic membrane-bounded vesicle lumen;cytoplasmic part;cytoplasmic vesicle part;endoplasmic reticulum;extracellular region;extracellular region part;extracellular space;Golgi apparatus;intracellular membrar	Complement and coagulation cascades	Homo sapiens	1	15
cell part;cytoplasmic part;extracellular membrane-bounded organelle;extracellular organelle;extracellular region part;extracellular vesicular exosome;intracellular membrane-bounded organelle;intracellular organelle;intracellula	Alzheimer's disease;Cardiac muscle contraction;Huntington's disease;Oxidative phosphorylation;Parkin	Homo sapiens	1	15
cell junction;cell part;cell-cell junction;cytoplasm;cytoplasmic part;intracellular membrane-bounded organelle;intracellular organelle;intracellular part;macromolecular complex;membrane-bounded organelle;nucleus;occluding	Tight junction	Homo sapiens	1	15
cell part;cytoplasm;cytoplasmic part;cytosol;I-kappaB/NF-kappaB complex;intracellular membrane-bounded organelle;intracellular organelle;intracellular organelle part;intracellular part;macromolecular complex;membrane-b	Acute myeloid leukemia;Adipocytokine signaling pathway;Amoebiasis;Apoptosis;B cell receptor signalir	Homo sapiens	1	15
cell part;cytoplasm;cytoplasmic part;cytosol;extracellular membrane-bounded organelle;extracellular organelle;extracellular region part;extracellular vesicular exosome;intracellular membrane-bounded organelle;intracellular c	Proteasome	Homo sapiens	1	15
cell junction;cell part;cell projection;cytoplasmic part;extracellular membrane-bounded organelle;extracellular organelle;extracellular region part;extracellular vesicular exosome;intracellular organelle part;intracellular part;mem	Cysteine and methionine metabolism;Sulfur relay system	Homo sapiens	1	15
cell part;cytoplasmic part;cytosol;integral to membrane;intracellular membrane-bounded organelle;intracellular organelle;intracellular organelle lumen;intracellular organelle part;intracellular part;intrinsic to membrane;membra	ABC transporters;Peroxisome	Homo sapiens	1	15
adherens junction;anchoring junction;cell junction;cell part;cell-substrate adherens junction;cell-substrate junction;cytoplasm;cytoplasmic part;extracellular membrane-bounded organelle;extracellular organelle;extracellular re	Arginine and proline metabolism;Glutathione metabolism	Homo sapiens	1	15
cell part;cytoplasm;cytoplasmic part;cytosol;extracellular membrane-bounded organelle;extracellular organelle;extracellular region part;extracellular vesicular exosome;intracellular part;membrane-bounded organelle;membra	Inositol phosphate metabolism;Phosphatidylinositol signaling system;Streptomycin biosynthesis	Homo sapiens	1	15
cell part;cytoplasm;cytoplasmic part;cytosol;extracellular region;intracellular part;IPAF inflammasome complex;macromolecular complex;NALP1 inflammasome complex;NALP3 inflammasome complex;protein complex	Amyotrophic lateral sclerosis (ALS);Cytosolic DNA-sensing pathway;NOD-like receptor signaling pathwa	Homo sapiens	1	15
cell part;cell surface;cytoplasmic membrane-bounded vesicle;cytoplasmic part;cytoplasmic vesicle;endoplasmic reticulum;endoplasmic reticulum lumen;endoplasmic reticulum part;extracellular membrane-bounded organelle	Protein processing in endoplasmic reticulum	Homo sapiens	1	15
cell part;cytoplasmic part;intracellular organelle lumen;intracellular organelle part;intracellular part;membrane;membrane-enclosed lumen;mitochondrial inner membrane;mitochondrial matrix;mitochondrial membrane;mitocho	Toxoplasmosis	Homo sapiens	1	15
adherens junction;anchoring junction;axon part;cell junction;cell part;cell projection part;cell surface;cell-substrate adherens junction;cell-substrate junction;external side of plasma membrane;focal adhesion;integral to memb	Axon guidance;Cell adhesion molecules (CAMs)	Homo sapiens	1	15
cell junction;cell part;intracellular membrane-bounded organelle;intracellular organelle;intracellular organelle part;intracellular part;macromolecular complex;membrane-bounded organelle;nuclear part;nucleoplasm;nucleoplas	Basal transcription factors	Homo sapiens	1	15
acrosomal membrane;adherens junction;anchoring junction;cell junction;cell part;cell-substrate adherens junction;cell-substrate junction;cytoplasmic membrane-bounded vesicle;cytoplasmic part;cytoplasmic vesicle;cytoplasmic vesicle membrane;cytoplasmic vesicle part;extracellular membrane-bounded organelle;extracellular		Homo sapiens	1	15
adherens junction;anchoring junction;cell division site part;cell junction;cell part;cell projection;cell projection part;cell-substrate adherens junction;cell-substrate junction;chromosomal part;cleavage furrow;condensed chromo	Focal adhesion;Insulin signaling pathway;Long-term potentiation;Meiosis - yeast;Oocyte meiosis;Regula	Homo sapiens	1	15
cell part;centromeric heterochromatin;chromatin;chromatin remodeling complex;chromocenter;chromosomal part;envelope;heterochromatin;histone deacetylase complex;histone methyltransferase complex;intracellular membrane-bounded organelle;intracellular non-membrane-bounded organelle;intracellular organelle;intracellu		Homo sapiens	1	15
cell part;cytoplasmic part;cytosol;cytosolic large ribosomal subunit;intracellular organelle part;intracellular part;large ribosomal subunit;macromolecular complex;membrane;organelle part;ribonucleoprotein complex	Ribosome	Homo sapiens	1	15
cell part;cytoplasmic part;cytosol;intracellular part	RNA transport	Homo sapiens	1	15
cell part;cytoplasm;cytoplasmic part;cytosol;eukaryotic translation initiation factor 2B complex;intracellular part;macromolecular complex;protein complex	RNA transport	Homo sapiens	1	15
cell part;cytoplasmic part;cytosol;intracellular part	Nicotinate and nicotinamide metabolism;Purine metabolism;Pyrimidine metabolism	Homo sapiens	1	15
cell part;cytoplasmic part;cytosol;extracellular membrane-bounded organelle;extracellular organelle;extracellular region part;extracellular space;extracellular vesicular exosome;intracellular part;membrane-bounded organelle;membrane-bounded vesicle;organelle;vesicle		Homo sapiens	1	15
		Homo sapiens	1	15
actin cytoskeleton;cell part;cytoplasm;cytoskeleton;intracellular non-membrane-bounded organelle;intracellular organelle;intracellular organelle part;intracellular part;non-membrane-bounded organelle;nuclear part;nucleoplasm;organelle;organelle part		Homo sapiens	1	15
cell part;cytoplasm;cytoplasmic part;cytosol;intracellular organelle part;intracellular part;nuclear part;nucleoplasm;organelle part	Purine metabolism	Homo sapiens	1	15
basal lamina;basement membrane;extracellular matrix part;extracellular membrane-bounded organelle;extracellular organelle;extracellular region;extracellular region part;extracellular vesicular exosome;laminin complex;lamir	Amoebiasis;ECM-receptor interaction;Focal adhesion;Pathways in cancer;Small cell lung cancer;Toxopl	Homo sapiens	1	15
adherens junction;anchoring junction;apical part of cell;cell body;cell junction;cell part;cell projection;cell-cell junction;cell-substrate adherens junction;cell-substrate junction;cytoplasm;cytoplasmic membrane-bounded vesic	Adherens junction;Axon guidance;Bacterial invasion of epithelial cells;Chemokine signaling pathway;Enc	Homo sapiens	1	15
catalytic step 2 spliceosome;cell part;cytoplasmic part;cytosol;extracellular membrane-bounded organelle;extracellular organelle;extracellular region part;extracellular vesicular exosome;intracellular organelle part;intracellular	Spliceosome	Homo sapiens	1	15
cell part;cytoplasmic part;cytosol;endoplasmic reticulum;endoplasmic reticulum-Golgi intermediate compartment;envelope;extracellular membrane-bounded organelle;extracellular organelle;extracellular region part;extracellular space;extracellular vesicular exosome;Golgi apparatus;intracellular membrane-bounded organelle;int		Homo sapiens	1	15
cell part;cytoplasmic part;intracellular membrane-bounded organelle;intracellular non-membrane-bounded organelle;intracellular organelle;intracellular organelle part;intracellular part;macromolecular complex;membrane;membrane-bounded organelle;mitochondrial inner membrane;mitochondrial membrane;mitochondrial part;m		Homo sapiens	1	15
cell part;cytoplasm;intracellular organelle part;intracellular part;nuclear part;nucleoplasm;organelle part	Spliceosome	Homo sapiens	1	15
cell part;cytoplasm;extracellular membrane-bounded organelle;extracellular organelle;extracellular region part;extracellular vesicular exosome;intracellular membrane-bounded organelle;intracellular organelle;intracellular orga	Spliceosome	Homo sapiens	1	15
adherens junction;anchoring junction;cell junction;cell part;cell-substrate adherens junction;cell-substrate junction;centralspindlin complex;centrosome;cytoplasmic part;cytoskeletal part;cytosol;extracellular region part;focal adhesion;intercellular bridge;intracellular membrane-bounded organelle;intracellular non-membrane-bou		Homo sapiens	1	15
Cdc48p-Npl4p-Ufd1p AAA ATPase complex;cell body;cell part;cell projection;cytoplasm;cytoplasmic part;dendrite;endoplasmic reticulum part;intracellular membrane-bounded organelle;intracellular organelle;intracellular organelle part;intracellular part;macromolecular complex;membrane part;membrane-bounded organelle;neu		Homo sapiens	1	15
cell part;cytoplasm;extracellular membrane-bounded organelle;extracellular organelle;extracellular region part;extracellular vesicular exosome;intracellular part;membrane-bounded organelle;membrane-bounded vesicle;orgai	MAPK signaling pathway - yeast;Pyruvate metabolism	Homo sapiens	1	15
cell part;cytoplasmic part;cytosol;endoplasmic reticulum-Golgi intermediate compartment;Golgi apparatus;Golgi apparatus part;Golgi cisterna membrane;Golgi membrane;Golgi transport complex;intracellular membrane-bounded organelle;intracellular non-membrane-bounded organelle;intracellular organelle;intracellular organe		Homo sapiens	1	15
cell part;centromeric heterochromatin;chromatin;chromosomal part;chromosome, centromeric region;condensed chromosome, centromeric region;cytoskeletal part;envelope;euchromatin;heterochromatin;intracellular membrane-bounded organelle;intracellular non-membrane-bounded organelle;intracellular organelle;intracellula		Homo sapiens	1	15
cell body;cell part;clathrin-coated vesicle;coated pit;coated vesicle;cytoplasmic membrane-bounded vesicle;cytoplasmic part;cytoplasmic vesicle;Golgi apparatus;intracellular membrane-bounded organelle;intracellular organelle;intracellular part;membrane;membrane part;membrane-bounded organelle;membrane-bounded ves		Homo sapiens	1	15
cell part;cell projection;cytoplasm;cytoplasmic part;cytosol;early endosome membrane;endosomal part;endosome membrane;extrinsic to membrane;Golgi apparatus;intracellular membrane-bounded organelle;intracellular organelle;intracellular organelle part;intracellular part;lamellipodium;macromolecular complex;membrane;m		Homo sapiens	1	15
adherens junction;anchoring junction;axon;cell body;cell junction;cell part;cell projection;cell-substrate adherens junction;cell-substrate junction;external side of plasma membrane;extracellular membrane-bounded organelle;	Cell adhesion molecules (CAMs)	Homo sapiens	1	15
basement membrane;cell part;cell surface;extracellular matrix;extracellular matrix part;extracellular membrane-bounded organelle;extracellular organelle;extracellular region;extracellular region part;extracellular vesicular exosome;membrane-bounded organelle;membrane-bounded vesicle;organelle;vesicle		Homo sapiens	1	15
adherens junction;anchoring junction;cell junction;cell part;cell-substrate adherens junction;cell-substrate junction;chromosome;focal adhesion;intracellular membrane-bounded organelle;intracellular non-membrane-bounded organelle;intracellular organelle;intracellular organelle part;intracellular part;membrane-bounded organel		Homo sapiens	1	15
cell part;intracellular membrane-bounded organelle;intracellular non-membrane-bounded organelle;intracellular organelle;intracellular organelle part;intracellular part;membrane-bounded organelle;non-membrane-bounded or	Ribosome biogenesis in eukaryotes	Homo sapiens	1	15
cell part;cytoplasm;cytoplasmic part;early endosome;endosome;intracellular membrane-bounded organelle;intracellular organelle;intracellular organelle part;intracellular part;membrane-bounded organelle;nuclear part;nucleoplasm;nucleus;organelle;organelle part		Homo sapiens	1	15
cell part;cytoplasm;extracellular membrane-bounded organelle;extracellular organelle;extracellular region part;extracellular vesicular exosome;intracellular part;membrane-bounded organelle;membrane-bounded vesicle;organelle;vesicle		Homo sapiens	1	15
cell part;cytoplasm;cytoplasmic part;cytosol;extracellular membrane-bounded organelle;extracellular organelle;extracellular region part;extracellular vesicular exosome;intracellular part;membrane;membrane-bounded organe	Ether lipid metabolism	Homo sapiens	1	15
cell part;cytoplasmic part;endoplasmic reticulum;endoplasmic reticulum lumen;endoplasmic reticulum part;intracellular membrane-bounded organelle;intracellular organelle;intracellular organelle lumen;intracellular organelle part;intracellular part;membrane-bounded organelle;membrane-enclosed lumen;organelle;organelle lumer		Homo sapiens	1	15
cell part;cytoplasm;cytoplasmic part;integral to membrane;intracellular membrane-bounded organelle;intracellular organelle;intracellular organelle part;intracellular part;intrinsic to membrane;membrane;membrane part;membrane-bounded organelle;mitochondrial membrane;mitochondrial outer membrane;mitochondrial part;nuc		Homo sapiens	1	15
cell part;cytoplasm;intracellular membrane-bounded organelle;intracellular organelle;intracellular part;membrane-bounded organelle;nucleus;organelle		Homo sapiens	1	15
cell part;intracellular organelle part;intracellular part;macromolecular complex;nuclear part;organelle part;ribonucleoprotein complex;spliceosomal complex	Spliceosome	Homo sapiens	1	15
cell part;cytoplasmic part;intracellular membrane-bounded organelle;intracellular organelle;intracellular part;membrane-bounded organelle;mitochondrion;organelle	Aminoacyl-tRNA biosynthesis	Homo sapiens	1	15
cell part;cytoplasm;intracellular part		Homo sapiens	1	15
cell part;cytoplasmic part;intracellular organelle lumen;intracellular organelle part;intracellular part;membrane-enclosed lumen;mitochondrial matrix;mitochondrial part;organelle lumen;organelle part		Homo sapiens	1	15
cell part;cytoplasmic part;endoplasmic reticulum membrane;endoplasmic reticulum part;intracellular organelle part;intracellular part;membrane;membrane part;nuclear membrane;nuclear outer membrane;nuclear part;organe	Retinol metabolism	Homo sapiens	1	15
Cdc73/Paf1 complex;cell part;intracellular membrane-bounded organelle;intracellular organelle;intracellular organelle part;intracellular part;macromolecular complex;membrane-bounded organelle;nuclear part;nucleoplasm;nucleoplasm part;nucleus;organelle;organelle part;protein complex;transcription elongation factor complex		Homo sapiens	1	15
cell part;cytoplasm;cytoplasmic part;cytoskeletal part;extracellular membrane-bounded organelle;extracellular organelle;extracellular region part;extracellular vesicular exosome;intracellular membrane-bounded organelle;intracellular organelle;intracellular organelle part;intracellular part;membrane;membrane-bounded organelle;r		Homo sapiens	1	15
cell part;cytoplasmic part;intracellular membrane-bounded organelle;intracellular non-membrane-bounded organelle;intracellular organelle;intracellular organelle part;intracellular part;membrane-bounded organelle;mitochondrial nucleoid;mitochondrial part;mitochondrion;non-membrane-bounded organelle;nuclear part;nucleoid;		Homo sapiens	1	15
cell part;cytoplasmic part;eukaryotic 43S preinitiation complex;eukaryotic 48S preinitiation complex;eukaryotic translation initiation factor 3 complex;eukaryotic translation initiation factor 3 complex, eIF3m;intracellular part;macromolecular complex;protein complex;ribonucleoprotein complex;translation preinitiation complex		Homo sapiens	1	15
cell part;cytoplasmic part;cytosol;endosomal part;endosome membrane;ESCRT III complex;extracellular membrane-bounded organelle;extracellular organelle;extracellular region part;extracellular vesicular exosome;intracellu	Endocytosis	Homo sapiens	1	15
cell part;cytoplasm;cytoplasmic part;intracellular membrane-bounded organelle;intracellular non-membrane-bounded organelle;intracellular organelle;intracellular part;macromolecular complex;membrane;membrane-bounded organelle;non-membrane-bounded organelle;nucleus;organelle;polysomal ribosome;ribonucleoprotein (		Homo sapiens	1	15
cell part;intracellular organelle part;intracellular part;nuclear part;nucleoplasm;organelle part		Homo sapiens	1	15
cell part;cytoplasm;cytoplasmic part;cytosol;intracellular membrane-bounded organelle;intracellular organelle;intracellular organelle part;intracellular part;membrane-bounded organelle;nuclear part;nucleoplasm;nucleus;organelle;organelle part		Homo sapiens	1	15
catalytic step 2 spliceosome;cell part;cytoplasmic part;cytosol;intracellular membrane-bounded organelle;intracellular organelle;intracellular organelle part;intracellular part;macromolecular complex;membrane-bounded orgai	mRNA surveillance pathway;RNA transport	Homo sapiens	1	15
cell junction;cell part;cell projection;cell projection part;cytoplasmic part;cytoskeletal part;cytoskeleton;cytosol;endoplasmic reticulum;extracellular membrane-bounded organelle;extracellular organelle;extracellular region part;extracellular vesicular exosome;filopodium;growth cone;intracellular;intracellular membrane-bounded o		Homo sapiens	1	15
cell part;cytoplasm;cytoplasmic part;Golgi apparatus;Golgi apparatus part;Golgi membrane;intracellular membrane-bounded organelle;intracellular non-membrane-bounded organelle;intracellular organelle;intracellular organe	Endocytosis	Homo sapiens	1	15
cell part;cytoplasmic part;extracellular membrane-bounded organelle;extracellular organelle;extracellular region part;extracellular vesicular exosome;intracellular organelle lumen;intracellular organelle part;intracellular part;lysosomal lumen;membrane-bounded organelle;membrane-bounded vesicle;membrane-enclosed lumen;org		Homo sapiens	1	15
cell part;intracellular non-membrane-bounded organelle;intracellular organelle;intracellular organelle part;intracellular part;macromolecular complex;non-membrane-bounded organelle;nuclear part;nucleolar part;nucleolus;org	Ribosome biogenesis in eukaryotes	Homo sapiens	1	15
cell part;cytoplasm;integral to membrane;intracellular part;intrinsic to membrane;membrane;membrane part;plasma membrane		Homo sapiens	1	15
cell part;cis-Golgi network;cytoplasmic membrane-bounded vesicle;cytoplasmic part;cytoplasmic vesicle;endoplasmic reticulum membrane;endoplasmic reticulum part;Golgi apparatus part;Golgi cisterna membrane;Golgi membrane;Golgi transport complex;Golgi-associated vesicle;intracellular membrane-bounded organelle;in		Homo sapiens	1	15
cell part;cytoplasm;intracellular organelle part;intracellular part;macromolecular complex;NELF complex;nuclear part;nucleoplasm;nucleoplasm part;organelle part;protein complex;transcription elongation factor complex		Homo sapiens	1	15
cell part;cytoplasm;intracellular membrane-bounded organelle;intracellular organelle;intracellular organelle part;intracellular part;macromolecular complex;membrane;membrane-bounded organelle;nuclear part;nucleoplasm;n	Spliceosome	Homo sapiens	1	15
cell part;cytoplasmic part;endoplasmic reticulum membrane;endoplasmic reticulum part;integral to membrane;intracellular organelle part;intracellular part;intrinsic to membrane;membrane;membrane part;organelle membrane;organelle part		Homo sapiens	1	15
cell part;cytoplasm;intracellular part	Arginine and proline metabolism	Homo sapiens	1	15
autophagic vacuole;cell part;cytoplasm;cytoplasmic membrane-bounded vesicle;cytoplasmic part;cytoplasmic vesicle;cytosol;endosome;Golgi apparatus;Golgi apparatus part;Golgi membrane;intracellular membrane-bounded organelle;intracellular organelle;intracellular organelle part;intracellular part;membrane;membrane-bou		Homo sapiens	1	15
cell part;cytoplasmic part;endoplasmic reticulum lumen;endoplasmic reticulum part;intracellular organelle lumen;intracellular organelle part;intracellular part;membrane-enclosed lumen;organelle lumen;organelle part		Homo sapiens	1	15
cell part;cytoplasmic part;intracellular non-membrane-bounded organelle;intracellular organelle;intracellular organelle part;intracellular part;macromolecular complex;membrane;mitochondrial inner membrane;mitochondrial membrane;mitochondrial part;non-membrane-bounded organelle;organelle;organelle inner membrane;orga		Homo sapiens	1	15

cell part;chromosomal part;condensed chromosome kinetochore;cytoplasmic part;cytosol;envelope;intracellular non-membrane-bounded organelle;intracellular organelle;intracellular organelle part;intracellular part;kinetocho	RNA transport	Homo sapiens	1	15
cell part;cytoplasmic part;integral to membrane;intracellular membrane-bounded organelle;intracellular organelle;intracellular organelle part;intracellular part;intrinsic to membrane;membrane;membrane part;membrane-bounded organelle;mitochondrial membrane;mitochondrial outer membrane;mitochondrial part;mitochondrion;		Homo sapiens	1	15
cell part;cytoplasm;exon-exon junction complex;intracellular non-membrane-bounded organelle;intracellular organelle;intracellular organelle part;intracellular part;macromolecular complex;non-membrane-bounded organelle;	mRNA surveillance pathway;RNA transport	Homo sapiens	1	15
cell part;cytoplasm;intracellular membrane-bounded organelle;intracellular organelle;intracellular organelle part;intracellular part;membrane;membrane-bounded organelle;nuclear part;nucleoplasm;nucleus;organelle;organelle part;plasma membrane		Homo sapiens	1	15
cell part;cytoplasmic part;endoplasmic reticulum lumen;endoplasmic reticulum part;Golgi apparatus;integral to membrane;integral to nuclear inner membrane;integral to organelle membrane;intracellular membrane-bounded organelle;intracellular organelle;intracellular organelle lumen;intracellular organelle part;intracellular part;i		Homo sapiens	1	15
AP-type membrane coat adaptor complex;cell part;clathrin adaptor complex;clathrin coated vesicle membrane;clathrin-coated endocytic vesicle membrane;coated vesicle membrane;cytoplasmic part;cytoplasmic vesicle me	Lysosome	Homo sapiens	1	15
cell part;cytoplasm;intracellular non-membrane-bounded organelle;intracellular organelle;intracellular organelle part;intracellular part;membrane;non-membrane-bounded organelle;nuclear part;nucleolus;organelle;organelle part		Homo sapiens	1	15
cell part;cytoplasm;cytoskeletal part;extracellular membrane-bounded organelle;extracellular organelle;extracellular region part;extracellular vesicular exosome;intracellular organelle part;intracellular part;macromolecular com	Gap junction;Pathogenic Escherichia coli infection;Phagosome	Homo sapiens	1	15
cell part;chromosome;condensed chromosome;condensed nuclear chromosome;cytoplasmic part;cytoskeletal part;early endosome;endomembrane system;endosomal part;endosome;ESCRT III complex;extracellular membrane-bounded organelle;extracellular organelle;extracellular region part;extracellular vesicular exosome;ir		Homo sapiens	1	15
		Homo sapiens	1	15
cell part;cytoplasmic part;extracellular membrane-bounded organelle;extracellular organelle;extracellular region part;extracellular vesicular exosome;intracellular membrane-bounded organelle;intracellular organelle;intracellular organelle part;intracellular part;lysosome;lytic vacuole;macromolecular complex;membrane;membrane		Homo sapiens	1	15
autophagic vacuole membrane;cell part;cytoplasm;cytoplasmic part;extracellular membrane-bounded organelle;extracellular organelle;extracellular region part;extracellular vesicular exosome;Golgi apparatus part;Golgi mem	Endocytosis	Homo sapiens	1	15
cell part;extracellular membrane-bounded organelle;extracellular organelle;extracellular region part;extracellular vesicular exosome;membrane;membrane-bounded organelle;membrane-bounded vesicle;organelle;vesicle	mRNA surveillance pathway	Homo sapiens	1	15
cell part;cytoplasmic part;extracellular membrane-bounded organelle;extracellular organelle;extracellular region part;extracellular vesicular exosome;integral to membrane;intracellular membrane-bounded organelle;intracellular organelle;intracellular organelle part;intracellular part;intrinsic to membrane;macromolecular complex;r		Homo sapiens	1	15
		Homo sapiens	1	16
actin cytoskeleton;axon;axon part;axon terminus;cell part;cell projection;cell projection part;cerebellar mossy fiber;clathrin-coated vesicle;coated vesicle;contractile fiber part;cytoplasm;cytoplasmic membrane-bounded vesicle;cytoplasmic part;cytoplasmic vesicle;cytoskeleton;I band;initial segment;intracellular membrane-boun		Homo sapiens	1	16
cell part;chromatin;chromosomal part;cytoplasm;intracellular membrane-bounded organelle;intracellular organelle;intracellular organelle part;intracellular part;macromolecular complex;membrane-bounded organelle;nuclear chromatin;nuclear chromosome part;nuclear matrix;nuclear part;nucleoplasm;nucleus;organelle;organelle		Homo sapiens	1	16
adherens junction;anchoring junction;cell junction;cell part;cell-substrate adherens junction;cell-substrate junction;cytoplasmic part;focal adhesion;Golgi apparatus;intracellular membrane-bounded organelle;intracellular organelle;intracellular part;membrane;membrane-bounded organelle;organelle;perinuclear region of cytoplasr		Homo sapiens	1	16
cell part;cytoplasmic part;cytosol;intracellular part	Purine metabolism;Selenocompound metabolism;Sulfur metabolism	Homo sapiens	1	16
box C/D snoRNP complex;cell part;intracellular membrane-bounded organelle;intracellular non-membrane-bounded organelle;intracellular organelle;intracellular organelle part;intracellular part;macromolecular complex;membrane-bounded organelle;non-membrane-bounded organelle;nuclear part;nucleolar part;nucleolus;nucleu		Homo sapiens	1	16
extracellular membrane-bounded organelle;extracellular organelle;extracellular region part;extracellular vesicular exosome;membrane-bounded organelle;membrane-bounded vesicle;organelle;vesicle		Homo sapiens	1	16
cell part;cytoplasm;extracellular membrane-bounded organelle;extracellular organelle;extracellular region part;extracellular vesicular exosome;intracellular part;membrane-bounded organelle;membrane-bounded vesicle;orga	Amino sugar and nucleotide sugar metabolism;Fructose and mannose metabolism	Homo sapiens	1	16
cell part;cytoplasm;extracellular membrane-bounded organelle;extracellular organelle;extracellular region part;extracellular vesicular exosome;intracellular;intracellular membrane-bounded organelle;intracellular non-membrane-bounded organelle;intracellular organelle;intracellular organelle part;intracellular part;membrane-bounc		Homo sapiens	1	16
cell part;cytoplasmic part;cytosol;extracellular membrane-bounded organelle;extracellular organelle;extracellular region part;extracellular vesicular exosome;intracellular part;membrane;membrane-bounded organelle;membra	Protein processing in endoplasmic reticulum	Homo sapiens	1	16
cell junction;cell part;cell projection;cell projection part;cytoplasm;cytoplasmic membrane-bounded vesicle;cytoplasmic part;cytoplasmic vesicle;cytoskeletal part;cytosol;dendritic spine head;endocytic vesicle;intracellular m	Axon guidance	Homo sapiens	1	16
axon;Cajal body;cell body;cell part;cell projection;cell projection part;cytoplasm;cytoplasmic part;growth cone;intracellular membrane-bounded organelle;intracellular non-membrane-bounded organelle;intracellular organelle;intracellular organelle part;intracellular part;macromolecular complex;membrane-bounded organelle;neur		Homo sapiens	1	16
cell part;cytoplasmic membrane-bounded vesicle;cytoplasmic part;cytoplasmic vesicle;endoplasmic reticulum membrane;endoplasmic reticulum part;endoplasmic reticulum-Golgi intermediate compartment;endoplasmic reti	Phagosome;SNARE interactions in vesicular transport	Homo sapiens	1	16
cell part;coated vesicle membrane;COPII vesicle coat;cytoplasmic part;cytoplasmic vesicle membrane;cytoplasmic vesicle part;cytosol;endoplasmic reticulum membrane;endoplasmic reticulum part;ER to Golgi transport ves	Protein processing in endoplasmic reticulum	Homo sapiens	1	16
cell part;cytoplasmic part;endoplasmic reticulum;endoplasmic reticulum membrane;endoplasmic reticulum part;Golgi apparatus;Golgi apparatus part;Golgi membrane;integral to membrane;intracellular membrane-bounded organelle;intracellular organelle;intracellular organelle part;intracellular part;intrinsic to membrane;membra		Homo sapiens	1	16
cell part;cytoplasmic part;intracellular organelle lumen;intracellular organelle part;intracellular part;macromolecular complex;membrane;membrane part;membrane-enclosed lumen;mitochondrial inner membrane;mitochondria	Alzheimer's disease;Huntington's disease;Oxidative phosphorylation;Parkinson's disease	Homo sapiens	1	16
cell part;cytoplasmic part;cytosol;external side of plasma membrane;extracellular membrane-bounded organelle;extracellular organelle;extracellular region part;extracellular vesicular exosome;intracellular organelle lumen;intr	Glutathione metabolism	Homo sapiens	1	16
cell body;cell part;cell projection cytoplasm;cell projection part;cytoplasm;cytoplasmic membrane-bounded vesicle;cytoplasmic part;cytoplasmic vesicle;cytosol;dendrite cytoplasm;dense core granule;extracellular matrix;ext	Amyotrophic lateral sclerosis (ALS);Huntington's disease;Peroxisome;Prion diseases	Homo sapiens	1	16
cell part;cytoplasmic part;extracellular membrane-bounded organelle;extracellular organelle;extracellular region part;extracellular vesicular exosome;intracellular organelle lumen;intracellular organelle part;intracellular part;me	Arginine and proline metabolism;Ascorbate and aldarate metabolism;beta-Alanine metabolism;Chloroall	Homo sapiens	1	16
cell division site part;cell part;cleavage furrow;cytoplasmic part;cytosol;extracellular membrane-bounded organelle;extracellular organelle;extracellular region part;extracellular vesicular exosome;intracellular membrane-bounded organelle;intracellular organelle;intracellular part;membrane;membrane-bounded organelle;membrane		Homo sapiens	1	16
cell part;cytoplasm;cytoplasmic part;cytosol;extracellular membrane-bounded organelle;extracellular organelle;extracellular region part;extracellular space;extracellular vesicular exosome;intracellular;intracellular membrane-b	Drug metabolism - cytochrome P450;Glutathione metabolism;Metabolism of xenobiotics by cytochrome	Homo sapiens	1	16
cell part;clathrin coat;clathrin coat of coated pit;clathrin coat of trans-Golgi network vesicle;clathrin coated vesicle membrane;clathrin complex;clathrin vesicle coat;clathrin-coated endocytic vesicle membrane;coated vesicle	Bacterial invasion of epithelial cells;Endocrine and other factor-regulated calcium reabsorption;Endocyto	Homo sapiens	1	16
cell part;cytoplasmic part;extracellular membrane-bounded organelle;extracellular organelle;extracellular region part;extracellular vesicular exosome;intracellular membrane-bounded organelle;intracellular organelle;intracellula	Galactose metabolism;Lysosome;Starch and sucrose metabolism	Homo sapiens	1	16
cell part;cytoplasmic part;intracellular membrane-bounded organelle;intracellular organelle;intracellular organelle lumen;intracellular organelle part;intracellular part;macromolecular complex;membrane-bounded organelle;mer	Citrate cycle (TCA cycle);Glycolysis / Gluconeogenesis;Pyruvate metabolism	Homo sapiens	1	16
adherens junction;alpha4-beta7 integrin complex;anchoring junction;cell junction;cell part;cell surface;cell-cell junction;cell-substrate adherens junction;cell-substrate junction;external side of plasma membrane;extracellular r	Arrhythmogenic right ventricular cardiomyopathy (ARVC);Cell adhesion molecules (CAMs);Dilated cardic	Homo sapiens	1	16
cell part;cytoplasmic part;cytosol;intracellular part	Arginine and proline metabolism;beta-Alanine metabolism;Cysteine and methionine metabolism;Glutath	Homo sapiens	1	16
blood microparticle;extracellular membrane-bounded organelle;extracellular organelle;extracellular region;extracellular region part;extracellular vesicular exosome;membrane-bounded organelle;membrane-bounded vesicle;organelle;vesicle		Homo sapiens	1	16
cell part;cytoplasm;cytoplasmic part;cytosol;endoplasmic reticulum membrane;endoplasmic reticulum part;intracellular organelle part;intracellular part;membrane;membrane part;nuclear membrane;nuclear part;organelle mer	Measles	Homo sapiens	1	16
cell part;cytoplasm;cytoplasmic part;cytosol;eukaryotic translation elongation factor 1 complex;intracellular part;macromolecular complex;protein complex		Homo sapiens	1	16
extracellular region;extracellular region part;extracellular space		Homo sapiens	1	16
cell part;intracellular organelle part;intracellular part;macromolecular complex;nuclear body;nuclear part;nuclear speck;nucleoplasm;nucleoplasm part;organelle part;ribonucleoprotein complex;spliceosomal complex	Spliceosome	Homo sapiens	1	16
cell part;cytoplasm;extracellular membrane-bounded organelle;extracellular organelle;extracellular region part;extracellular vesicular exosome;intracellular membrane-bounded organelle;intracellular organelle;intracellular part;membrane-bounded organelle;membrane-bounded vesicle;nucleus;organelle;vesicle		Homo sapiens	1	16
cell part;extracellular membrane-bounded organelle;extracellular organelle;extracellular region part;extracellular vesicular exosome;membrane part;membrane raft;membrane-bounded organelle;membrane-bounded vesicle;o	Cell cycle - yeast;Chagas disease (American trypanosomiasis);Hepatitis C;Long-term depression;Meiosi	Homo sapiens	1	16
cell part;cytoplasm;cytoplasmic part;cytosol;intracellular membrane-bounded organelle;intracellular organelle;intracellular organelle part;intracellular part;membrane-bounded organelle;nuclear part;nucleoplasm;nucleus;orgai	Glutathione metabolism;p53 signaling pathway;Purine metabolism;Pyrimidine metabolism	Homo sapiens	1	16
cell part;cell projection;cytoplasm;extracellular membrane-bounded organelle;extracellular organelle;extracellular region part;extracellular space;extracellular vesicular exosome;intracellular membrane-bounded organelle;intracellular organelle;intracellular organelle part;intracellular part;membrane-bounded organelle;membrane-b		Homo sapiens	1	16
cell part;chromosomal part;cytoplasm;DNA replication factor C complex;intracellular membrane-bounded organelle;intracellular organelle;intracellular organelle part;intracellular part;macromolecular complex;membrane-bour	DNA replication;Mismatch repair;Nucleotide excision repair	Homo sapiens	1	16
cell junction;cell part;chromosomal part;cytoplasmic part;DNA replication factor C complex;extracellular membrane-bounded organelle;extracellular organelle;extracellular region part;extracellular vesicular exosome;Golgi app	DNA replication;Mismatch repair;Nucleotide excision repair	Homo sapiens	1	16
cell part;chromosomal part;DNA replication factor C complex;intracellular membrane-bounded organelle;intracellular organelle;intracellular organelle part;intracellular part;macromolecular complex;membrane-bounded organe	DNA replication;Mismatch repair;Nucleotide excision repair	Homo sapiens	1	16
cell part;cytoplasm;envelope;intracellular membrane-bounded organelle;intracellular organelle;intracellular organelle part;intracellular part;membrane-bounded organelle;nuclear envelope;nuclear part;nucleus;organelle;organelle envelope;organelle part		Homo sapiens	1	16
BAT3 complex;cell part;cytoplasm;cytoplasmic part;cytosol;cytosolic part;ER membrane insertion complex;intracellular membrane-bounded organelle;intracellular organelle;intracellular organelle part;intracellular part;macromolecular complex;membrane-bounded organelle;nuclear part;nucleoplasm;nucleus;organelle;organelle p		Homo sapiens	1	16
cell part;cytoplasm;cytoplasmic part;cytosol;extracellular membrane-bounded organelle;extracellular organelle;extracellular region part;extracellular vesicular exosome;intracellular membrane-bounded organelle;intracellular c	Proteasome	Homo sapiens	1	16
cell part;chromatin;chromosomal part;cytoplasmic part;cytosol;intracellular membrane-bounded organelle;intracellular organelle;intracellular organelle part;intracellular part;macromolecular complex;membrane-bounded orga	Homologous recombination;Non-homologous end-joining	Homo sapiens	1	16
cell body;cell part;cell projection;cytoplasm;cytoplasmic part;cytosol;intracellular membrane-bounded organelle;intracellular organelle;intracellular organelle part;intracellular part;membrane;membrane-bounded organelle;neu	MAPK signaling pathway	Homo sapiens	1	16
cell part;cytoplasm;intracellular organelle part;intracellular part;macromolecular complex;nuclear part;organelle part;protein complex;signalosome		Homo sapiens	1	16
cell part;cytoplasm;cytoplasmic part;cytosol;cytosolic small ribosomal subunit;intracellular non-membrane-bounded organelle;intracellular organelle;intracellular organelle part;intracellular part;macromolecular complex;memb	Ribosome	Homo sapiens	1	16
cell part;cytoplasm;intracellular membrane-bounded organelle;intracellular organelle;intracellular organelle part;intracellular part;membrane-bounded organelle;nuclear part;nucleoplasm;nucleus;organelle;organelle part	mRNA surveillance pathway	Homo sapiens	1	16
cell part;cytoplasm;intracellular membrane-bounded organelle;intracellular non-membrane-bounded organelle;intracellular organelle;intracellular organelle part;intracellular part;membrane-bounded organelle;non-membrane-l	Calcium signaling pathway;Huntington's disease;Parkinson's disease	Homo sapiens	1	16
cell part;cytoplasmic part;cytosol;endoplasmic reticulum;extracellular membrane-bounded organelle;extracellular organelle;extracellular region part;extracellular space;extracellular vesicular exosome;intracellular membrane-bounded organelle;intracellular organelle;intracellular part;membrane-bounded organelle;membrane-bour		Homo sapiens	1	16
cell part;cytoplasm;intracellular membrane-bounded organelle;intracellular organelle;intracellular organelle part;intracellular part;membrane-bounded organelle;nuclear body;nuclear matrix;nuclear part;nuclear speck;nucleoplasm;nucleoplasm part;nucleus;organelle;organelle part		Homo sapiens	1	16
cell part;cytoplasm;extracellular membrane-bounded organelle;extracellular organelle;extracellular region part;extracellular vesicular exosome;intracellular part;membrane-bounded organelle;membrane-bounded vesicle;orga	Amino sugar and nucleotide sugar metabolism;Fructose and mannose metabolism	Homo sapiens	1	16
cell part;intracellular membrane-bounded organelle;intracellular non-membrane-bounded organelle;intracellular organelle;intracellular organelle part;intracellular part;membrane;membrane-bounded organelle;non-membrane-	Ribosome biogenesis in eukaryotes	Homo sapiens	1	16
cell part;cytoplasmic part;cytoskeletal part;cytoskeleton;intracellular non-membrane-bounded organelle;intracellular organelle;intracellular organelle part;intracellular part;microtubule cytoskeleton;microtubule organizing center;non-membrane-bounded organelle;nuclear body;nuclear part;nuclear speck;nucleoplasm;nucleoplasm		Homo sapiens	1	16
cell part;cytoplasm;intracellular membrane-bounded organelle;intracellular organelle;intracellular organelle part;intracellular part;membrane-bounded organelle;nuclear body;nuclear part;nuclear speck;nucleoplasm;nucleoplasm part;organelle;organelle part		Homo sapiens	1	16
cell part;cell projection membrane;cell projection part;clathrin-coated endocytic vesicle;clathrin-coated vesicle;coated pit;coated vesicle;cytoplasmic membrane-bounded vesicle;cytoplasmic part;cytoplasmic vesicle;endocytic vesicle;endosomal part;endosome membrane;extracellular membrane-bounded organelle;extracellular		Homo sapiens	1	16
cell part;cytoplasm;cytoskeleton;intracellular non-membrane-bounded organelle;intracellular organelle;intracellular part;non-membrane-bounded organelle;organelle	Pathways in cancer;Thyroid cancer	Homo sapiens	1	16
cell division site part;cell part;cleavage furrow;cytoplasm;cytoplasmic membrane-bounded vesicle;cytoplasmic part;cytoplasmic vesicle;cytosol;endosome;intracellular membrane-bounded organelle;intracellular organelle;intracellular organelle part;intracellular part;membrane;membrane-bounded organelle;membrane-bounded v		Homo sapiens	1	16
cell part;intracellular non-membrane-bounded organelle;intracellular organelle;intracellular organelle part;intracellular part;non-membrane-bounded organelle;nuclear part;nucleolus;organelle;organelle part		Homo sapiens	1	16
blood microparticle;cell part;cytoplasmic part;early endosome;endosome;extracellular region part;intracellular membrane-bounded organelle;intracellular organelle;intracellular part;membrane-bounded organelle;organelle		Homo sapiens	1	16
actin filament;axon;cell part;cell projection;cell projection part;cytoplasmic part;cytoskeletal part;early endosome;endosome;growth cone;intracellular membrane-bounded organelle;intracellular organelle;intracellular organelle part;intracellular part;macromolecular complex;membrane;membrane-bounded organelle;neuron projec		Homo sapiens	1	16
cell part;cytoplasmic part;early endosome;early endosome membrane;endosomal part;endosome;endosome membrane;intracellular membrane-bounded organelle;intracellular organelle;intracellular organelle part;intracellular part;macromolecular complex;membrane;membrane-bounded organelle;organelle;organelle membrane;		Homo sapiens	1	16
cell part;cytoplasmic part;endoplasmic reticulum;endoplasmic reticulum membrane;endoplasmic reticulum part;integral to membrane;intracellular membrane-bounded organelle;intracellular organelle;intracellular organelle part;intracellular part;intrinsic to membrane;membrane;membrane part;membrane-bounded organelle;orgar		Homo sapiens	1	16
cell part;cytoplasmic part;endoplasmic reticulum membrane;endoplasmic reticulum part;integral to endoplasmic reticulum membrane;integral to membrane;integral to organelle membrane;intracellular organelle part;intracellular part;intrinsic to endoplasmic reticulum membrane;intrinsic to membrane;intrinsic to organelle membra		Homo sapiens	1	16
cell part;cytoplasmic part;cytoplasmic vesicle membrane;cytoplasmic vesicle part;cytosol;Golgi apparatus part;Golgi membrane;Golgi-associated vesicle membrane;intracellular organelle part;intracellular part;membrane;orga	Endocytosis	Homo sapiens	1	16
cell part;integral to membrane;integral to nuclear inner membrane;integral to organelle membrane;intracellular organelle part;intracellular part;intrinsic to membrane;intrinsic to nuclear inner membrane;intrinsic to organelle membrane;membrane;membrane part;nuclear membrane;nuclear membrane part;nuclear part;organelle me		Homo sapiens	1	16
cell part;intracellular membrane-bounded organelle;intracellular non-membrane-bounded organelle;intracellular organelle;intracellular organelle part;intracellular part;membrane-bounded organelle;non-membrane-bounded organelle;nuclear body;nuclear part;nuclear speck;nucleolus;nucleoplasm part;nucleus;organelle;organelle		Homo sapiens	1	16
Cajal body;cell part;histone methyltransferase complex;intracellular membrane-bounded organelle;intracellular organelle;intracellular organelle part;intracellular part;macromolecular complex;membrane-bounded organelle;me	Spliceosome	Homo sapiens	1	16
cell part;cell pole;cell tip;clathrin-coated vesicle;coated vesicle;cytoplasmic membrane-bounded vesicle;cytoplasmic part;cytoplasmic vesicle;cytoplasmic vesicle membrane;cytoplasmic vesicle part;endocytic vesicle;endocytic vesicle membrane;endosome;extracellular membrane-bounded organelle;extracellular organelle;extra		Homo sapiens	1	16
cell part;cytoplasmic part;intracellular membrane-bounded organelle;intracellular organelle;intracellular part;membrane-bounded organelle;mitochondrion;organelle		Homo sapiens	1	16
Cdc48p-Npl4p-Ufd1p AAA ATPase complex;cell part;cytoplasmic part;endoplasmic reticulum;endoplasmic reticulum part;intracellular membrane-bounded organelle;intracellular organelle;intracellular organelle part;intracellular part;lipid particle;macromolecular complex;membrane part;membrane-bounded organelle;organelle;or		Homo sapiens	1	16
		Homo sapiens	1	16
cell part;cytoplasm;intracellular non-membrane-bounded organelle;intracellular organelle;intracellular organelle part;intracellular part;non-membrane-bounded organelle;nuclear part;nucleolus;organelle;organelle part		Homo sapiens	1	16
cell part;intracellular membrane-bounded organelle;intracellular organelle;intracellular organelle part;intracellular part;membrane-bounded organelle;nuclear body;nuclear part;nucleoplasm part;nucleus;organelle;organelle part		Homo sapiens	1	16
		Homo sapiens	1	16
cell part;cytoplasm;cytoplasmic part;Golgi apparatus;Golgi apparatus part;intracellular membrane-bounded organelle;intracellular non-membrane-bounded organelle;intracellular organelle;intracellular organelle part;intracellular part;macromolecular complex;membrane;membrane-bounded organelle;non-membrane-bounded org		Homo sapiens	1	16
cell part;cytoplasm;cytoplasmic part;cytosol;intracellular part	Cysteine and methionine metabolism;One carbon pool by folate;Selenocompound metabolism	Homo sapiens	1	16
cell part;intracellular membrane-bounded organelle;intracellular non-membrane-bounded organelle;intracellular organelle;intracellular organelle part;intracellular part;membrane;membrane-bounded organelle;non-membrane-bounded organelle;nuclear part;nucleolus;nucleus;organelle;organelle part		Homo sapiens	1	16
cell part;cytoplasmic part;intracellular membrane-bounded organelle;intracellular organelle;intracellular organelle part;intracellular part;membrane-bounded organelle;mitochondrion;nuclear part;nucleoplasm;nucleus;organelle	RNA transport	Homo sapiens	1	16
cell part;extracellular region part;extracellular space;intracellular membrane-bounded organelle;intracellular non-membrane-bounded organelle;intracellular organelle;intracellular organelle part;intracellular part;membrane;mer	Ribosome biogenesis in eukaryotes	Homo sapiens	1	16
cell part;cytoplasm;intracellular membrane-bounded organelle;intracellular organelle;intracellular part;membrane-bounded organelle;nucleus;organelle		Homo sapiens	1	16
cell part;cytoplasmic part;cytoskeleton;cytosol;extrinsic to membrane;intracellular non-membrane-bounded organelle;intracellular organelle;intracellular part;membrane;membrane part;non-membrane-bounded organelle;orga	Tight junction	Homo sapiens	1	16
cell part;cytoplasm;intracellular organelle part;intracellular part;nuclear part;nucleoplasm;organelle part	Ubiquitin mediated proteolysis	Homo sapiens	1	16
cell part;cytoplasmic part;intracellular membrane-bounded organelle;intracellular organelle;intracellular organelle lumen;intracellular organelle part;intracellular part;membrane-bounded organelle;membrane-enclosed lumen;mitochondrial matrix;mitochondrial part;mitochondrion;organelle;organelle lumen;organelle part		Homo sapiens	1	16
cell part;cytoplasmic part;cytoskeletal part;endosome;Golgi apparatus part;intracellular membrane-bounded organelle;intracellular organelle;intracellular organelle part;intracellular part;membrane;membrane-bounded organe	Ubiquitin mediated proteolysis	Homo sapiens	1	16
cell part;cytoplasm;intracellular membrane-bounded organelle;intracellular organelle;intracellular organelle part;intracellular part;membrane-bounded organelle;nuclear part;nucleoplasm;nucleus;organelle;organelle part	Endocytosis	Homo sapiens	1	16
cell part;cytoplasmic part;intracellular membrane-bounded organelle;intracellular organelle;intracellular part;membrane-bounded organelle;mitochondrion;nucleus;organelle		Homo sapiens	1	16
cell part;cytoplasm;intracellular membrane-bounded organelle;intracellular organelle;intracellular part;membrane-bounded organelle;nucleus;organelle	MAPK signaling pathway;Tight junction	Homo sapiens	1	16
cell part;cytoplasmic part;cytosol;endoplasmic reticulum;endoplasmic reticulum lumen;endoplasmic reticulum membrane;endoplasmic reticulum part;extracellular membrane-bounded organelle;extracellular organelle;extracellular region;extracellular region part;extracellular space;extracellular vesicular exosome;integral to memb		Homo sapiens	1	16
cell part;cytoplasm;intracellular membrane-bounded organelle;intracellular non-membrane-bounded organelle;intracellular organelle;intracellular organelle part;intracellular part;membrane;membrane-bounded organelle;non-membrane-bounded organelle;nuclear membrane;nuclear part;nucleolus;nucleoplasm;nucleus;organelle;o		Homo sapiens	1	16
cell part;cytoplasm;cytoplasmic part;cytosol;endosomal part;endosome membrane;ESCRT III complex;extracellular membrane-bounded organelle;extracellular organelle;extracellular region part;extracellular vesicular exosom	Endocytosis	Homo sapiens	1	16
actin cytoskeleton;cell part;contractile fiber part;cytoplasmic part;cytoskeleton;extracellular membrane-bounded organelle;extracellular organelle;extracellular region part;extracellular space;extracellular vesicular exosome;I b	Axon guidance;Fc gamma R-mediated phagocytosis;Regulation of actin cytoskeleton	Homo sapiens	1	16
cell part;intracellular membrane-bounded organelle;intracellular non-membrane-bounded organelle;intracellular organelle;intracellular organelle part;intracellular part;membrane-bounded organelle;non-membrane-bounded organelle;nuclear part;nucleolus;nucleoplasm;nucleus;organelle;organelle part		Homo sapiens	1	16
cell part;chromatin remodeling complex;chromosomal part;cytoplasmic part;Golgi apparatus;H4/H2A histone acetyltransferase complex;histone acetyltransferase complex;intracellular membrane-bounded organelle;intracellular organelle;intracellular organelle part;intracellular part;macromolecular complex;membrane-bounded c		Homo sapiens	1	16
cell part;cell projection;cytoplasmic part;cytoplasmic vesicle membrane;cytoplasmic vesicle part;early endosome membrane;endosomal part;endosome membrane;extrinsic to endosome membrane;extrinsic to internal side of plasma membrane;extrinsic to membrane;extrinsic to organelle membrane;extrinsic to plasma membra		Homo sapiens	1	16
cell part;chromatin;chromosomal part;cytoplasmic part;intracellular membrane-bounded organelle;intracellular non-membrane-bounded organelle;intracellular organelle;intracellular organelle part;intracellular part;macromolecular complex;membrane-bounded organelle;mitochondrion;non-membrane-bounded organelle;nuclear c		Homo sapiens	1	16
cell part;centrosome;cytoplasm;cytoplasmic part;cytoskeletal part;intracellular non-membrane-bounded organelle;intracellular organelle;intracellular organelle part;intracellular part;microtubule organizing center;non-membrar	RNA transport	Homo sapiens	1	16
cell part;cis-Golgi network;coated vesicle membrane;cytoplasm;cytoplasmic part;cytoplasmic vesicle membrane;cytoplasmic vesicle part;endoplasmic reticulum exit site;endoplasmic reticulum part;endoplasmic reticulum-Golgi intermediate compartment;ER to Golgi transport vesicle membrane;Golgi apparatus part;intracellular		Homo sapiens	1	16
cell part;chromatin;chromosomal part;cytoplasm;cytoplasmic part;cytosol;intracellular membrane-bounded organelle;intracellular organelle;intracellular organelle part;intracellular part;membrane-bounded organelle;nuclear ch	Apoptosis	Homo sapiens	1	17
actin cytoskeleton;cell body;cell part;cell projection;cytoplasmic part;cytoskeleton;cytosol;Golgi apparatus part;Golgi cisterna;intracellular membrane-bounded organelle;intracellular non-membrane-bounded organelle;intracellular organelle;intracellular organelle part;intracellular part;membrane;membrane-bounded organelle;neu		Homo sapiens	1	17
cell part;cytoplasm;cytoplasmic membrane-bounded vesicle;cytoplasmic part;cytoplasmic vesicle;cytosol;early endosome;early endosome membrane;endosomal part;endosome;endosome membrane;extracellular membran	Endocytosis;Phagosome	Homo sapiens	1	17
cell part;cytoplasm;cytoplasmic part;intracellular part;perinuclear region of cytoplasm		Homo sapiens	1	17
cell part;chromosomal part;condensed chromosome kinetochore;cytoplasmic part;cytosol;intracellular non-membrane-bounded organelle;intracellular organelle;intracellular organelle part;intracellular part;kinetochore;macron	Cell cycle;Cell cycle - yeast	Homo sapiens	1	17
macromolecular complex;protein complex;ribose phosphate diphosphokinase complex		Homo sapiens	1	17
acrosomal vesicle;cell part;cytoplasm;cytoplasmic membrane-bounded vesicle;cytoplasmic part;cytoplasmic vesicle;cytosol;extracellular membrane-bounded organelle;extracellular organelle;extracellular region part;extracellular vesicular exosome;integral to membrane;intracellular membrane-bounded organelle;intracellular org		Homo sapiens	1	17
cell part;cytoplasmic part;cytosol;intracellular part		Homo sapiens	1	17
adherens junction;anchoring junction;cell junction;cell part;cell-substrate adherens junction;cell-substrate junction;cytoplasmic part;focal adhesion;Golgi apparatus;intracellular membrane-bounded organelle;intracellular orga	Axon guidance;ErbB signaling pathway;Focal adhesion;Regulation of actin cytoskeleton;Renal cell carci	Homo sapiens	1	17
apical part of cell;basolateral plasma membrane;cell part;cell surface;clathrin coated vesicle membrane;clathrin-coated endocytic vesicle membrane;coated pit;coated vesicle membrane;cytoplasmic part;cytoplasmic vesicle	Bile secretion;Endocytosis;Hepatitis C;Toxoplasmosis	Homo sapiens	1	17
cell part;cytoplasm;extracellular region;extracellular region part;extracellular space;intracellular part;membrane;plasma membrane	Amoebiasis	Homo sapiens	1	17
catalytic step 2 spliceosome;cell part;intracellular organelle part;intracellular part;macromolecular complex;nuclear part;nucleoplasm;organelle part;ribonucleoprotein complex;small nuclear ribonucleoprotein complex;spliceos	Spliceosome	Homo sapiens	1	17
AMP-activated protein kinase complex;cAMP-dependent protein kinase complex;cell part;cytoplasmic part;cytosol;intracellular part;macromolecular complex;membrane;neuromuscular junction;plasma membrane;protein cor	Apoptosis;Insulin signaling pathway	Homo sapiens	1	17
cell part;cytoplasmic part;extracellular membrane-bounded organelle;extracellular organelle;extracellular region part;extracellular vesicular exosome;intracellular membrane-bounded organelle;intracellular organelle;intracellula	Butanoate metabolism;Citrate cycle (TCA cycle);Glycolysis / Gluconeogenesis;Pyruvate metabolism;Vali	Homo sapiens	1	17
cell part;cytoplasm;cytoplasmic part;endosome;extracellular membrane-bounded organelle;extracellular organelle;extracellular region part;extracellular space;extracellular vesicular exosome;intracellular membrane-bounded organelle;intracellular organelle;intracellular part;late endosome;membrane-bounded organelle;membran		Homo sapiens	1	17
cell part;chromatin;chromosomal part;chromosome;condensed chromosome;condensed nuclear chromosome;extracellular membrane-bounded organelle;extracellular organelle;extracellular region part;extracellular vesicular	Systemic lupus erythematosus	Homo sapiens	1	17
apical part of cell;cell part;cytoplasmic part;cytoplasmic vesicle;cytosol;extracellular membrane-bounded organelle;extracellular organelle;extracellular region part;extracellular vesicular exosome;intracellular organelle;intracel	Collecting duct acid secretion;Epithelial cell signaling in Helicobacter pylori infection;Oxidative phospho	Homo sapiens	1	17
box C/D snoRNP complex;Cajal body;cell part;extracellular membrane-bounded organelle;extracellular organelle;extracellular region part;extracellular vesicular exosome;granular component;intracellular membrane-bounded	Ribosome biogenesis in eukaryotes	Homo sapiens	1	17
cell part;cytoplasm;cytoplasmic mRNA processing body;cytoplasmic part;cytosol;intracellular membrane-bounded organelle;intracellular non-membrane-bounded organelle;intracellular organelle;intracellular organelle part;int	RNA degradation	Homo sapiens	1	17

cell part;cytoplasmic part;extracellular membrane-bounded organelle;extracellular organelle;extracellular region part;extracellular space;extracellular vesicular exosome;intracellular membrane-bounded organelle;intracellular organelle;intracellular part;membrane-bounded organelle;membrane-bounded vesicle;mitochondrion;org		Homo sapiens	1	17
cell part;cytoplasmic part;extracellular membrane-bounded organelle;extracellular organelle;extracellular region part;extracellular vesicular exosome;intracellular membrane-bounded organelle;intracellular organelle;intracellul	Aminobenzoate degradation;Benzoate degradation;beta-Alanine metabolism;Butanoate metabolism;Ca	Homo sapiens	1	17
cell part;cytoplasmic part;cytosol;extracellular region;Golgi apparatus part;Golgi membrane;intracellular organelle part;intracellular part;membrane;organelle membrane;organelle part		Homo sapiens	1	17
cell part;cytoplasmic part;cytosol;intracellular membrane-bounded organelle;intracellular non-membrane-bounded organelle;intracellular organelle;intracellular organelle part;intracellular part;membrane-bounded organelle;no	Cysteine and methionine metabolism;Glycine, serine and threonine metabolism	Homo sapiens	1	17
cell part;extracellular region part;extracellular space;intracellular membrane-bounded organelle;intracellular organelle;intracellular part;membrane-bounded organelle;nucleus;organelle		Homo sapiens	1	17
actin cytoskeleton;cell cortex part;cell part;cortical cytoskeleton;cytoplasmic part;cytoskeletal part;cytoskeleton;cytosol;extracellular membrane-bounded organelle;extracellular organelle;extracellular region;extracellular region part;extracellular vesicular exosome;F-actin capping protein complex;intracellular non-membrane-bou		Homo sapiens	1	17
cell part;cis-Golgi network;clathrin-coated vesicle;coated vesicle;COPI-coated vesicle;cytoplasmic membrane-bounded vesicle;cytoplasmic part;cytoplasmic vesicle;cytoplasmic vesicle membrane;cytoplasmic vesicle part;endoplasmic reticulum;endoplasmic reticulum membrane;endoplasmic reticulum part;endoplasmic reticulu		Homo sapiens	1	17
cell part;chromatin remodeling complex;cytoplasm;cytoskeleton;extracellular membrane-bounded organelle;extracellular organelle;extracellular region part;extracellular vesicular exosome;histone deacetylase complex;intracellular membrane-bounded organelle;intracellular non-membrane-bounded organelle;intracellular organell		Homo sapiens	1	17
cell part;cytoplasm;cytoplasmic part;cytosol;intracellular organelle part;intracellular part;membrane;nuclear part;nucleoplasm;organelle part		Homo sapiens	1	17
cell part;cytoplasmic part;cytosol;intracellular part;membrane;plasma membrane	B cell receptor signaling pathway;Chemokine signaling pathway;Fc epsilon RI signaling pathway;Fc gan	Homo sapiens	1	17
cell part;cytoplasmic part;cytosol;extracellular membrane-bounded organelle;extracellular organelle;extracellular region part;extracellular vesicular exosome;intracellular part;membrane-bounded organelle;membrane-bounde	Arginine and proline metabolism;beta-Alanine metabolism;Cysteine and methionine metabolism;Glutath	Homo sapiens	1	17
cell part;extracellular membrane-bounded organelle;extracellular organelle;extracellular region part;extracellular vesicular exosome;intracellular membrane-bounded organelle;intracellular non-membrane-bounded organelle;intracellular organelle;intracellular organelle part;intracellular part;macromolecular complex;membrane-bou		Homo sapiens	1	17
cell junction;cell part;intracellular membrane-bounded organelle;intracellular organelle;intracellular organelle part;intracellular part;macromolecular complex;membrane-bounded organelle;nuclear part;nucleoplasm part;nucleu	Chronic myeloid leukemia;Notch signaling pathway;Pathways in cancer;Wnt signaling pathway	Homo sapiens	1	17
cell part;centrosome;cytoplasm;cytoplasmic part;cytoskeletal part;cytosol;dynactin complex;extracellular membrane-bounded organelle;extracellular organelle;extracellular region part;extracellular vesicular exosome;intracellular non-membrane-bounded organelle;intracellular organelle;intracellular organelle part;intracellular part;		Homo sapiens	1	17
cell part;cytoplasm;cytoplasmic part;cytosol;intracellular membrane-bounded organelle;intracellular organelle;intracellular organelle part;intracellular part;macromolecular complex;membrane;membrane-bounded organelle;nu	Antigen processing and presentation;Hepatitis C;Proteasome	Homo sapiens	1	17
adherens junction;anchoring junction;apical junction complex;cell cortex;cell division site part;cell junction;cell part;cell projection;cell-cell junction;cell-substrate adherens junction;cell-substrate junction;cleavage furrow;cyto	Adherens junction;Axon guidance;Bacterial invasion of epithelial cells;Chemokine signaling pathway;Co	Homo sapiens	1	17
cell part;cytoplasmic part;cytosol;cytosolic large ribosomal subunit;intracellular organelle part;intracellular part;large ribosomal subunit;macromolecular complex;organelle part;ribonucleoprotein complex	Ribosome	Homo sapiens	1	17
cell part;cell projection;cell projection part;cytoplasm;cytoplasmic part;cytosol;dendritic spine;extracellular membrane-bounded organelle;extracellular organelle;extracellular region part;extracellular vesicular exosome;glycog	Focal adhesion;Insulin signaling pathway;Long-term potentiation;Meiosis - yeast;Oocyte meiosis;Regula	Homo sapiens	1	17
adherens junction;anchoring junction;cell junction;cell part;cell-substrate adherens junction;cell-substrate junction;cytoplasmic part;cytosol;extracellular membrane-bounded organelle;extracellular organelle;extracellular regio	Focal adhesion;Insulin signaling pathway;Long-term potentiation;Meiosis - yeast;Oocyte meiosis;Regula	Homo sapiens	1	17
cell part;cytoplasm;cytoplasmic part;cytosol;cytosolic small ribosomal subunit;extracellular membrane-bounded organelle;extracellular organelle;extracellular region part;extracellular vesicular exosome;intracellular organelle	Ribosome	Homo sapiens	1	17
cell part;cytoplasm;cytoplasmic part;cytosol;intracellular part;macromolecular complex;protein complex;translation release factor complex	mRNA surveillance pathway	Homo sapiens	1	17
cell part;cytoplasm;cytoplasmic part;cytosol;cytosolic small ribosomal subunit;extracellular membrane-bounded organelle;extracellular organelle;extracellular region part;extracellular vesicular exosome;intracellular organelle	Ribosome	Homo sapiens	1	17
cell part;cytoplasm;cytoplasmic part;cytosol;cytosolic small ribosomal subunit;intracellular organelle part;intracellular part;macromolecular complex;organelle part;ribonucleoprotein complex;small ribosomal subunit	Ribosome	Homo sapiens	1	17
cell part;cell projection;cell surface;cilium;cytoplasmic part;endoplasmic reticulum membrane;endoplasmic reticulum part;extracellular region;Golgi apparatus part;Golgi membrane;integral to membrane;integral to plasma me	Dorso-ventral axis formation;Notch signaling pathway	Homo sapiens	1	17
cell part;cytoplasmic part;cytosol;intracellular part	Alanine, aspartate and glutamate metabolism;Purine metabolism	Homo sapiens	1	17
cell part;cytoplasm;cytoplasmic part;cytosol;extracellular membrane-bounded organelle;extracellular organelle;extracellular region part;extracellular vesicular exosome;intracellular organelle part;intracellular part;macromolec	Antigen processing and presentation;Proteasome	Homo sapiens	1	17
cell body;cell part;cell projection;cell projection part;cytoplasm;cytoplasmic part;cytoskeletal part;dendritic shaft;dendritic spine;intracellular non-membrane-bounded organelle;intracellular organelle;intracellular organelle part;intracellular part;macromolecular complex;membrane;microtubule;mRNA cap binding complex;neuron p		Homo sapiens	1	17
cell part;intracellular membrane-bounded organelle;intracellular organelle;intracellular organelle part;intracellular part;membrane-bounded organelle;nuclear body;nuclear part;nuclear speck;nucleoplasm;nucleoplasm part;nuc	Spliceosome	Homo sapiens	1	17
cell part;intracellular organelle part;intracellular part;macromolecular complex;mRNA cleavage and polyadenylation specificity factor complex;mRNA cleavage factor complex;nuclear part;nucleoplasm;nucleoplasm part;organ	mRNA surveillance pathway	Homo sapiens	1	17
cell part;cytoplasmic part;cytosol;early endosome;endoplasmic reticulum;endosome;intracellular membrane-bounded organelle;intracellular organelle;intracellular part;macromolecular complex;membrane-bounded organelle;organelle;protein complex;WASH complex		Homo sapiens	1	17
cell part;cytoplasm;cytoplasmic part;cytosol;intracellular membrane-bounded organelle;intracellular organelle;intracellular part;membrane;membrane-bounded organelle;nucleus;organelle;perinuclear region of cytoplasm		Homo sapiens	1	17
cell part;cytoplasm;cytoplasmic part;cytosol;intracellular part	beta-Alanine metabolism;Drug metabolism - other enzymes;Pantothenate and CoA biosynthesis;Pyrimic	Homo sapiens	1	17
cell part;chromosomal part;condensed chromosome kinetochore;cytoplasm;intracellular membrane-bounded organelle;intracellular non-membrane-bounded organelle;intracellular organelle;intracellular organelle part;intracellular part;kinetochore;macromolecular complex;membrane-bounded organelle;non-membrane-bounded		Homo sapiens	1	17
cell part;cytoplasm;intracellular membrane-bounded organelle;intracellular non-membrane-bounded organelle;intracellular organelle;intracellular organelle part;intracellular part;macromolecular complex;membrane;membrane-bounded organelle;non-membrane-bounded organelle;nuclear part;nucleolus;nucleoplasm;nucleus;orga		Homo sapiens	1	17
cell part;cytoplasm;cytoplasmic part;cytosol;extracellular membrane-bounded organelle;extracellular organelle;extracellular region part;extracellular vesicular exosome;intracellular membrane-bounded organelle;intracellular c	Cysteine and methionine metabolism	Homo sapiens	1	17
actin cytoskeleton;cell cortex;cell part;cell projection;cell projection part;cytoplasm;cytoplasmic part;cytoskeletal part;cytoskeleton;cytosol;filamentous actin;filopodium tip;intracellular non-membrane-bounded organelle;intracellular organelle;intracellular organelle part;intracellular part;lamellipodium;macromolecular complex;me		Homo sapiens	1	17
cell part;intracellular membrane-bounded organelle;intracellular non-membrane-bounded organelle;intracellular organelle;intracellular organelle part;intracellular part;membrane-bounded organelle;non-membrane-bounded or	Spliceosome	Homo sapiens	1	17
adherens junction;anchoring junction;cell junction;cell part;cell-substrate adherens junction;cell-substrate junction;cytoplasm;cytoplasmic part;cytosol;focal adhesion;intracellular membrane-bounded organelle;intracellular organelle;intracellular part;membrane;membrane-bounded organelle;nucleus;organelle;plasma membrane		Homo sapiens	1	17
cell part;cytoplasm;cytoplasmic part;intracellular membrane-bounded organelle;intracellular non-membrane-bounded organelle;intracellular organelle;intracellular organelle part;intracellular part;macromolecular complex;membrane-bounded organelle;non-membrane-bounded organelle;nuclear part;nucleoplasm;nucleus;organell		Homo sapiens	1	17
cell part;intracellular organelle part;intracellular part;macromolecular complex;nuclear part;nucleolar part;nucleoplasm;organelle part;PeBoW complex;preribosome;preribosome, large subunit precursor;protein complex;ribonucleoprotein complex		Homo sapiens	1	17
cell part;clathrin-coated vesicle;coated vesicle;cytoplasmic membrane-bounded vesicle;cytoplasmic part;cytoplasmic vesicle;cytosol;Golgi apparatus;intracellular membrane-bounded organelle;intracellular organelle;intracellular organelle part;intracellular part;membrane;membrane-bounded organelle;membrane-bounded vesicl		Homo sapiens	1	17
cell part;cytoplasm;cytoplasmic part;Golgi apparatus;intracellular membrane-bounded organelle;intracellular organelle;intracellular organelle part;intracellular part;membrane-bounded organelle;nuclear part;nucleoplasm;organelle;organelle part		Homo sapiens	1	17
cell part;cytoplasmic part;endoplasmic reticulum membrane;endoplasmic reticulum part;integral to membrane;intracellular organelle part;intracellular part;intrinsic to membrane;macromolecular complex;membrane;membran	Protein export	Homo sapiens	1	17
cell part;cytoplasmic part;extracellular matrix;extracellular region part;extracellular space;Golgi apparatus part;intracellular organelle part;intracellular part;organelle part;proteinaceous extracellular matrix;trans-Golgi network		Homo sapiens	1	17
cell part;chromosomal part;chromosome, telomeric region;cytoplasm;cytoplasmic part;cytosol;extracellular membrane-bounded organelle;extracellular organelle;extracellular region part;extracellular vesicular exosome;intracellular membrane-bounded organelle;intracellular organelle;intracellular organelle part;intracellular part;ma		Homo sapiens	1	17
actin cytoskeleton;adherens junction;anchoring junction;cell junction;cell part;cell-substrate adherens junction;cell-substrate junction;cytoplasm;cytoskeleton;focal adhesion;intracellular non-membrane-bounded organelle;intracellular organelle;intracellular part;non-membrane-bounded organelle;organelle		Homo sapiens	1	17
cell part;cytoplasmic part;intracellular membrane-bounded organelle;intracellular non-membrane-bounded organelle;intracellular organelle;intracellular organelle part;intracellular part;macromolecular complex;membrane-bounded organelle;non-membrane-bounded organelle;nuclear part;nucleoplasm;nucleus;organelle;organelle		Homo sapiens	1	17
cell cortex;cell part;cell projection;cytoplasm;cytoplasmic part;cytoskeleton;cytosol;extracellular membrane-bounded organelle;extracellular organelle;extracellular region part;extracellular vesicular exosome;Golgi apparatus;i	Insulin signaling pathway	Homo sapiens	1	17
cell part;cis-Golgi network;cytoplasmic part;cytoplasmic vesicle part;cytoskeleton;Golgi apparatus;Golgi apparatus part;Golgi membrane;inner acrosomal membrane;intracellular membrane-bounded organelle;intracellular non-membrane-bounded organelle;intracellular organelle;intracellular organelle part;intracellular part;memb		Homo sapiens	1	17
adherens junction;anchoring junction;cell junction;cell part;cell-substrate adherens junction;cell-substrate junction;cytoplasm;cytoskeleton;focal adhesion;intracellular membrane-bounded organelle;intracellular non-membran	NOD-like receptor signaling pathway	Homo sapiens	1	17
cell part;cytoplasm;extracellular membrane-bounded organelle;extracellular organelle;extracellular region part;extracellular vesicular exosome;intracellular membrane-bounded organelle;intracellular organelle;intracellular orga	Spliceosome	Homo sapiens	1	17
cell part;cytoplasmic part;endoplasmic reticulum;endoplasmic reticulum lumen;endoplasmic reticulum part;extracellular matrix;extracellular membrane-bounded organelle;extracellular organelle;extracellular region part;extracellular vesicular exosome;intracellular membrane-bounded organelle;intracellular organelle;intracellular o		Homo sapiens	1	17
cell part;cytoplasmic part;cytosol;extracellular membrane-bounded organelle;extracellular organelle;extracellular region part;extracellular vesicular exosome;intracellular membrane-bounded organelle;intracellular organelle;int	Glycerolipid metabolism;Methane metabolism;RIG-I-like receptor signaling pathway	Homo sapiens	1	17
cell part;cytoplasmic part;integral to membrane;intracellular membrane-bounded organelle;intracellular organelle;intracellular organelle part;intracellular part;intrinsic to membrane;macromolecular complex;membrane;membrane part;membrane-bounded organelle;mitochondrial inner membrane;mitochondrial inner membrane pr		Homo sapiens	1	17
		Homo sapiens	1	17
cell part;cytoplasmic part;Golgi apparatus;intracellular membrane-bounded organelle;intracellular organelle;intracellular part;membrane-bounded organelle;organelle		Homo sapiens	1	17
cell part;intracellular organelle part;intracellular part;nuclear part;nucleoplasm;organelle part		Homo sapiens	1	17
adherens junction;anchoring junction;cell junction;cell part;cell-substrate adherens junction;cell-substrate junction;focal adhesion;membrane;plasma membrane		Homo sapiens	1	17
cell part;intracellular membrane-bounded organelle;intracellular organelle;intracellular organelle part;intracellular part;macromolecular complex;membrane;membrane-bounded organelle;mRNA cleavage factor complex;nucle	mRNA surveillance pathway	Homo sapiens	1	17
cell junction;cell part;cytoplasm;extracellular region part;intercellular bridge;intracellular membrane-bounded organelle;intracellular organelle;intracellular part;membrane-bounded organelle;nucleus;organelle		Homo sapiens	1	17
cell part;chromatin;chromosomal part;intracellular organelle part;intracellular part;macromolecular complex;nuclear body;nuclear chromatin;nuclear chromosome part;nuclear part;nuclear speck;nucleoplasm;nucleoplasm part;organelle part;protein complex		Homo sapiens	1	17
cell part;cytoplasmic part;intracellular non-membrane-bounded organelle;intracellular organelle;intracellular organelle part;intracellular part;macromolecular complex;membrane;mitochondrial inner membrane;mitochondrial membrane;mitochondrial part;non-membrane-bounded organelle;organelle;organelle inner membrane;orga		Homo sapiens	1	17
cell part;chromatin;chromatin remodeling complex;chromosomal part;cytoplasm;ESC/E(Z) complex;heterochromatin;histone deacetylase complex;histone methyltransferase complex;intracellular membrane-bounded organell	Cell cycle;Chronic myeloid leukemia;Huntington's disease;Notch signaling pathway;Pathways in cancer	Homo sapiens	1	17
cell part;cytoplasm;intracellular membrane-bounded organelle;intracellular organelle;intracellular organelle part;intracellular part;macromolecular complex;membrane;membrane-bounded organelle;nuclear part;nucleoplasm;nucleus;organelle;organelle part;ribonucleoprotein complex		Homo sapiens	1	17
caveola;cell part;cytoplasm;intracellular part;macromolecular complex;membrane part;membrane raft;plasma membrane part;protein complex		Homo sapiens	1	17
axon;cell part;cell projection;extracellular membrane-bounded organelle;extracellular organelle;extracellular region part;extracellular vesicular exosome;integral to membrane;intrinsic to membrane;membrane;membrane part;membrane-bounded organelle;membrane-bounded vesicle;neuron projection;organelle;plasma membran		Homo sapiens	1	17
cell cortex part;cell part;cell projection part;cytoplasm;cytoplasmic part;cytosol;exocyst;growth cone membrane;intracellular part;macromolecular complex;membrane;membrane part;myelin sheath abaxonal region;plasma m	Tight junction	Homo sapiens	1	17
cell part;cytoplasm;cytoplasmic part;cytoskeletal part;intracellular organelle part;intracellular part;membrane;microtubule organizing center;midbody;organelle part;plasma membrane		Homo sapiens	1	17
cell part;cytoplasm;intracellular membrane-bounded organelle;intracellular organelle;intracellular organelle part;intracellular part;membrane-bounded organelle;nuclear part;nucleoplasm;nucleus;organelle;organelle part		Homo sapiens	1	17
cell part;cytoplasm;cytoplasmic part;endoplasmic reticulum;intracellular membrane-bounded organelle;intracellular organelle;intracellular organelle part;intracellular part;membrane;membrane-bounded organelle;nuclear matrix;nuclear part;nucleoplasm;nucleus;organelle;organelle part;plasma membrane		Homo sapiens	1	17
cell part;cytoplasm;cytoskeletal part;cytoskeleton;intracellular non-membrane-bounded organelle;intracellular organelle;intracellular organelle part;intracellular part;macromolecular complex;microtubule;microtubule cytoskeleton;non-membrane-bounded organelle;nuclear part;nucleoplasm;organelle;organelle part;protein comple		Homo sapiens	1	17
cell part;cytoplasmic part;extracellular membrane-bounded organelle;extracellular organelle;extracellular region part;extracellular vesicular exosome;integral to membrane;intracellular organelle part;intracellular part;intrinsic to membrane;membrane;membrane part;membrane-bounded organelle;membrane-bounded vesicle;mito		Homo sapiens	1	17
cell part;cytoplasm;cytoplasmic part;cytosol;extracellular membrane-bounded organelle;extracellular organelle;extracellular region part;extracellular vesicular exosome;intracellular membrane-bounded organelle;intracellular organelle;intracellular part;membrane;membrane-bounded organelle;membrane-bounded vesicle;nucleus		Homo sapiens	1	17
bleb;cell part;cell projection;cytoplasm;cytoplasmic part;endosome;extracellular membrane-bounded organelle;extracellular organelle;extracellular region part;extracellular vesicular exosome;Golgi apparatus;Golgi apparatus part;Golgi lumen;intracellular membrane-bounded organelle;intracellular organelle;intracellular organelle l		Homo sapiens	1	17
cell part;chromosomal part;condensed chromosome kinetochore;cytoplasmic part;cytoskeletal part;cytosol;envelope;intracellular non-membrane-bounded organelle;intracellular organelle;intracellular organelle part;intracellul	RNA transport	Homo sapiens	1	17
cell part;cytoplasm;intracellular membrane-bounded organelle;intracellular organelle;intracellular organelle part;intracellular part;membrane-bounded organelle;nuclear part;nucleoplasm;nucleus;organelle;organelle part	Ubiquitin mediated proteolysis	Homo sapiens	1	17
cell part;cytoplasmic part;intracellular membrane-bounded organelle;intracellular organelle;intracellular part;membrane-bounded organelle;mitochondrion;organelle		Homo sapiens	1	17
cell part;cytoplasm;intracellular part		Homo sapiens	1	17
cell part;cytoplasmic part;cytosol;Golgi apparatus part;Golgi membrane;integral to membrane;intracellular membrane-bounded organelle;intracellular organelle;intracellular organelle part;intracellular part;intrinsic to membran	Arachidonic acid metabolism	Homo sapiens	1	17
cell part;centrosome;cytoplasm;cytoplasmic part;cytoskeletal part;extracellular membrane-bounded organelle;extracellular organelle;extracellular region part;extracellular vesicular exosome;intracellular membrane-bounded o	Alanine, aspartate and glutamate metabolism	Homo sapiens	1	17
cell part;cytoplasm;cytoplasmic part;cytosol;intracellular membrane-bounded organelle;intracellular organelle;intracellular organelle part;intracellular part;membrane-bounded organelle;nuclear part;nucleoplasm;organelle;orga	Carbon fixation pathways in prokaryotes;Glycolysis / Gluconeogenesis;Methane metabolism;Propanoat	Homo sapiens	1	17
cell part;cytoplasm;intracellular membrane-bounded organelle;intracellular non-membrane-bounded organelle;intracellular organelle;intracellular organelle part;intracellular part;macromolecular complex;membrane-bounded organelle;non-membrane-bounded organelle;nuclear part;nucleolus;nucleus;organelle;organelle part;prot		Homo sapiens	1	17
cell part;contractile fiber part;cytoplasmic part;cytoskeletal part;intracellular organelle part;intracellular part;organelle part;striated muscle thin filament		Homo sapiens	1	17
cell part;cytoplasmic part;endoplasmic reticulum;endoplasmic reticulum membrane;endoplasmic reticulum part;integral to endoplasmic reticulum membrane;integral to membrane;integral to organelle membrane;intracellular	Biosynthesis of unsaturated fatty acids	Homo sapiens	1	17
axon;cell part;cell projection;cell projection part;CRD-mediated mRNA stability complex;cytoplasm;cytoplasmic part;cytosol;dendritic spine;filopodium;growth cone;intracellular membrane-bounded organelle;intracellular non-membrane-bounded organelle;intracellular organelle;intracellular part;lamellipodium;macromolecular cor		Homo sapiens	1	17
cell part;chromosomal part;chromosome;chromosome, telomeric region;condensed chromosome;condensed nuclear chromosome;cytoplasmic part;endosomal part;endosome membrane;envelope;integral to membrane;integral to nuclear inner membrane;integral to organelle membrane;intracellular non-membrane-bounded org		Homo sapiens	1	17
extracellular membrane-bounded organelle;extracellular organelle;extracellular region part;extracellular vesicular exosome;membrane-bounded organelle;membrane-bounded vesicle;organelle;vesicle	Protein processing in endoplasmic reticulum	Homo sapiens	1	17
cell part;cytoplasm;intracellular part;macromolecular complex;membrane;polysome;ribonucleoprotein complex		Homo sapiens	1	17
cell part;cytoplasmic part;extracellular membrane-bounded organelle;extracellular organelle;extracellular region part;extracellular vesicular exosome;intracellular;intracellular membrane-bounded organelle;intracellular organel	Drug metabolism - cytochrome P450;Glutathione metabolism;Metabolism of xenobiotics by cytochrome	Homo sapiens	1	17
box C/D snoRNP complex;Cajal body;cell part;cytoplasm;intracellular membrane-bounded organelle;intracellular non-membrane-bounded organelle;intracellular organelle;intracellular organelle part;intracellular part;macromo	Ribosome biogenesis in eukaryotes	Homo sapiens	1	17
cell part;cytoplasm;cytoplasmic part;cytosol;intracellular membrane-bounded organelle;intracellular organelle;intracellular organelle part;intracellular part;macromolecular complex;membrane;membrane-bounded organelle;nu	RNA transport	Homo sapiens	1	17
actin cytoskeleton;cell junction;cell part;cell projection;cell-cell junction;cytoplasm;cytoskeletal part;cytoskeleton;extracellular membrane-bounded organelle;extracellular organelle;extracellular region part;extracellular vesicul	Bacterial invasion of epithelial cells	Homo sapiens	1	17
cell part;integral to membrane;intrinsic to membrane;membrane;membrane part		Homo sapiens	1	18
cell part;intracellular membrane-bounded organelle;intracellular organelle;intracellular organelle part;intracellular part;membrane-bounded organelle;nuclear part;nucleoplasm;nucleus;organelle;organelle part	Spliceosome	Homo sapiens	1	18
cell part;cytoplasmic part;cytosol;eukaryotic 43S preinitiation complex;eukaryotic 48S preinitiation complex;eukaryotic translation initiation factor 3 complex;eukaryotic translation initiation factor 3 complex, eIF3m;extracellula	Measles;RNA transport	Homo sapiens	1	18
cell part;cytoplasmic part;intracellular membrane-bounded organelle;intracellular organelle;intracellular organelle lumen;intracellular organelle part;intracellular part;membrane;membrane-bounded organelle;membrane-enclosed lumen;mitochondrial inner membrane;mitochondrial matrix;mitochondrial membrane;mitochondrial pa		Homo sapiens	1	18
cell part;cytoplasmic part;intracellular membrane-bounded organelle;intracellular organelle;intracellular organelle lumen;intracellular organelle part;intracellular part;membrane-bounded organelle;membrane-enclosed lumen;m	Citrate cycle (TCA cycle)	Homo sapiens	1	18
cell part;cytoplasmic part;cytosol;intracellular membrane-bounded organelle;intracellular organelle;intracellular part;membrane;membrane-bounded organelle;nucleus;organelle		Homo sapiens	1	18
cell part;cell projection;cytoplasm;cytoplasmic part;early endosome membrane;endosomal part;endosome membrane;extracellular membrane-bounded organelle;extracellular organelle;extracellular region part;extracellular vesicular exosome;extrinsic to membrane;intracellular membrane-bounded organelle;intracellular organelle		Homo sapiens	1	18
cell part;cell projection;chromosomal part;HULC complex;intracellular membrane-bounded organelle;intracellular organelle;intracellular organelle part;intracellular part;macromolecular complex;membrane;membrane-bounded organelle;neuron projection;nuclear part;nucleoplasm;nucleus;organelle;organelle part;protein complex;		Homo sapiens	1	18
cell part;centrosome;cytoplasm;cytoplasmic part;cytoskeletal part;cytosol;endosomal part;endosome;endosome membrane;extracellular membrane-bounded organelle;extracellular organelle;extracellular region part;extracell	Endocytosis	Homo sapiens	1	18
cell part;cytoplasmic part;intracellular organelle lumen;intracellular organelle part;intracellular part;membrane;membrane-enclosed lumen;mitochondrial inner membrane;mitochondrial matrix;mitochondrial membrane;mitochondrial part;organelle inner membrane;organelle lumen;organelle membrane;organelle part		Homo sapiens	1	18
cell part;cytoplasm;cytoplasmic part;cytosol;eukaryotic 43S preinitiation complex;eukaryotic 48S preinitiation complex;eukaryotic translation initiation factor 3 complex;intracellular part;macromolecular complex;perinuclear re	RNA transport	Homo sapiens	1	18
cell part;cytoplasmic part;cytosol;intracellular part	Amino sugar and nucleotide sugar metabolism	Homo sapiens	1	18
cell part;cytoplasm;cytoplasmic part;cytosol;endoplasmic reticulum;extracellular membrane-bounded organelle;extracellular organelle;extracellular region part;extracellular vesicular exosome;intracellular membrane-bounded organelle;intracellular organelle;intracellular part;membrane-bounded organelle;membrane-bounded ves		Homo sapiens	1	18
cell body;cell part;cell projection;cytoplasm;cytoplasmic part;cytoskeletal part;dendrite;endoplasmic reticulum;extracellular membrane-bounded organelle;extracellular organelle;extracellular region part;extracellular vesicular exosome;intracellular membrane-bounded organelle;intracellular non-membrane-bounded organelle;intra		Homo sapiens	1	18
caveola;cell part;cytoplasm;cytoplasmic part;cytosol;intracellular part;membrane part;membrane raft;plasma membrane part		Homo sapiens	1	18
cell part;cytoplasm;cytoplasmic part;cytoskeleton;cytosol;extracellular membrane-bounded organelle;extracellular organelle;extracellular region part;extracellular vesicular exosome;intracellular;intracellular non-membrane-bo	Nicotinate and nicotinamide metabolism;Purine metabolism;Pyrimidine metabolism	Homo sapiens	1	18
adherens junction;anchoring junction;cell junction;cell part;cell surface;cell-substrate adherens junction;cell-substrate junction;extracellular membrane-bounded organelle;extracellular organelle;extracellular region;extracellula	Complement and coagulation cascades	Homo sapiens	1	18
autophagic vacuole membrane;cell part;cytoplasmic part;endoplasmic reticulum;endoplasmic reticulum membrane;endoplasmic reticulum part;integral to membrane;intracellular membrane-bounded organelle;intracellular org	N-Glycan biosynthesis;Protein processing in endoplasmic reticulum;Various types of N-glycan biosynthe	Homo sapiens	1	18
cell part;cytoplasmic membrane-bounded vesicle lumen;cytoplasmic part;cytoplasmic vesicle part;extracellular matrix;extracellular membrane-bounded organelle;extracellular organelle;extracellular region;extracellular region	Chagas disease (American trypanosomiasis);Complement and coagulation cascades;p53 signaling path	Homo sapiens	1	18
anchored to membrane;cell part;cell surface;extracellular membrane-bounded organelle;extracellular organelle;extracellular region;extracellular region part;extracellular vesicular exosome;integral to membrane;integral to plas	Complement and coagulation cascades;Hematopoietic cell lineage;Viral myocarditis	Homo sapiens	1	18
axon;cell body;cell part;cell projection;cytoplasm;cytoplasmic part;cytosol;endoplasmic reticulum membrane;endoplasmic reticulum part;extracellular membrane-bounded organelle;extracellular organelle;extracellular region	Parkinson's disease	Homo sapiens	1	18
basal lamina;basement membrane;cell part;cell periphery;extracellular matrix;extracellular matrix part;extracellular membrane-bounded organelle;extracellular organelle;extracellular region;extracellular region part;extracellular vesicular exosome;membrane-bounded organelle;membrane-bounded vesicle;organelle;vesicle		Homo sapiens	1	18
cell part;cell projection membrane;cell projection part;centrosome;cytoplasm;cytoplasmic part;cytoskeletal part;cytosol;extracellular membrane-bounded organelle;extracellular organelle;extracellular region part;extracellular	Purine metabolism;Pyrimidine metabolism	Homo sapiens	1	18
adherens junction;anchoring junction;basolateral plasma membrane;cell junction;cell part;cell surface;cell-substrate adherens junction;cell-substrate junction;cytoplasm;cytoplasmic part;external side of plasma membrane;ex	ECM-receptor interaction;Hematopoietic cell lineage;Shigellosis	Homo sapiens	1	18
cell part;cell periphery;cell projection;cytoplasm;cytoplasmic part;cytosol;extracellular membrane-bounded organelle;extracellular organelle;extracellular region part;extracellular vesicular exosome;intracellular membrane-bou	Purine metabolism;Pyrimidine metabolism	Homo sapiens	1	18
cell part;cytoplasmic part;intracellular membrane-bounded organelle;intracellular non-membrane-bounded organelle;intracellular organelle;intracellular organelle part;intracellular part;membrane;membrane-bounded organelle	Fatty acid metabolism;PPAR signaling pathway	Homo sapiens	1	18
cell part;cytoplasmic part;cytosol;intracellular membrane-bounded organelle;intracellular organelle;intracellular organelle lumen;intracellular organelle part;intracellular part;membrane-bounded organelle;membrane-enclosed l	Pyrimidine metabolism	Homo sapiens	1	18
blood microparticle;cell part;cytoplasm;cytoplasmic membrane-bounded vesicle;cytoplasmic part;cytoplasmic vesicle;cytoskeleton;endoplasmic reticulum;extracellular membrane-bounded organelle;extracellular organelle;extracellular region part;extracellular space;extracellular vesicular exosome;integral to membrane;integral t		Homo sapiens	1	18
adherens junction;anchoring junction;caveola;cell junction;cell part;cell projection;cell-substrate adherens junction;cell-substrate junction;cytoplasmic part;cytoskeleton;cytosol;early endosome;endosome;envelope;extracellu	Acute myeloid leukemia;Adherens junction;Aldosterone-regulated sodium reabsorption;Alzheimer's dise	Homo sapiens	1	18
cell part;cytoplasm;cytoplasmic part;cytosol;early endosome;endosome;extracellular membrane-bounded organelle;extracellular organelle;extracellular region part;extracellular vesicular exosome;intracellular membrane-bounded organelle;intracellular organelle;intracellular organelle lumen;intracellular organelle part;intracellular p		Homo sapiens	1	18
adherens junction;anchoring junction;cell junction;cell part;cell-substrate adherens junction;cell-substrate junction;cytoplasmic part;cytosol;cytosolic large ribosomal subunit;extracellular membrane-bounded organelle;extrac	Ribosome	Homo sapiens	1	18
cell part;cell surface;cytoplasmic part;endoplasmic reticulum;endoplasmic reticulum lumen;endoplasmic reticulum part;extracellular region;integral to membrane;intracellular membrane-bounded organelle;intracellular organelle;intracellular organelle lumen;intracellular organelle part;intracellular part;intrinsic to membrane;membra		Homo sapiens	1	18
cell part;cytoplasm;cytoplasmic part;intracellular membrane-bounded organelle;intracellular organelle;intracellular organelle part;intracellular part;membrane;membrane-bounded organelle;membrane-enclosed lumen;mitocho	Porphyrin and chlorophyll metabolism	Homo sapiens	1	18
cell part;cytoplasmic part;endoplasmic reticulum;endoplasmic reticulum membrane;endoplasmic reticulum part;integral to membrane;intracellular membrane-bounded organelle;intracellular organelle;intracellular organelle pa	N-Glycan biosynthesis;Protein processing in endoplasmic reticulum;Various types of N-glycan biosynthe	Homo sapiens	1	18
cell part;cytoplasmic part;cytosol;cytosolic large ribosomal subunit;intracellular organelle part;intracellular part;large ribosomal subunit;macromolecular complex;membrane;organelle part;ribonucleoprotein complex	Ribosome	Homo sapiens	1	18
cell part;cytoplasmic membrane-bounded vesicle;cytoplasmic part;cytoplasmic vesicle;cytoplasmic vesicle membrane;cytoplasmic vesicle part;endocytic vesicle;endocytic vesicle membrane;endosome;extracellular membra	Amoebiasis;Endocytosis;ko05152;Phagosome	Homo sapiens	1	18
cell part;cytoplasmic part;endoplasmic reticulum membrane;endoplasmic reticulum part;extracellular membrane-bounded organelle;extracellular organelle;extracellular region part;extracellular vesicular exosome;integral to m	Arginine and proline metabolism;Ascorbate and aldarate metabolism;beta-Alanine metabolism;Chloroall	Homo sapiens	1	18

cell part;cytoplasm;cytoplasmic membrane-bounded vesicle;cytoplasmic part;cytoplasmic vesicle;cytoskeleton;cytosol;extracellular membrane-bounded organelle;extracellular organelle;extracellular region part;extracellular	Neurotrophin signaling pathway;Vasopressin-regulated water reabsorption	Homo sapiens		1	18
cell part;cytoplasm;cytoplasmic part;cytoskeletal part;cytosol;intracellular non-membrane-bounded organelle;intracellular organelle;intracellular organelle part;intracellular part;kinesin complex;macromolecular complex;membrane;microtubule;microtubule associated complex;non-membrane-bounded organelle;organelle;organell		Homo sapiens		1	18
cell part;cytoplasmic part;extracellular membrane-bounded organelle;extracellular organelle;extracellular region part;extracellular vesicular exosome;integral to membrane;intracellular membrane-bounded organelle;intracellular organelle;intracellular organelle part;intracellular part;intrinsic to membrane;membrane;membrane part		Homo sapiens		1	18
cell part;cytoplasm;cytoplasmic part;cytosol;cytosolic part;cytosolic ribosome;intracellular membrane-bounded organelle;intracellular non-membrane-bounded organelle;intracellular organelle;intracellular part;macromolecular complex;membrane-bounded organelle;non-membrane-bounded organelle;nucleus;organelle;ribonucle		Homo sapiens		1	18
cell part;cytoplasmic membrane-bounded vesicle;cytoplasmic part;cytoplasmic vesicle;intracellular membrane-bounded organelle;intracellular organelle;intracellular part;melanosome;membrane;membrane-bounded organelle;membrane-bounded vesicle;nucleus;organelle;pigment granule;vesicle		Homo sapiens		1	18
brush border;cell part;cell projection;cytoplasmic part;cytoskeletal part;cytosol;extracellular membrane-bounded organelle;extracellular organelle;extracellular region part;extracellular vesicular exosome;intracellular organelle	Vascular smooth muscle contraction	Homo sapiens		1	18
cell part;cytoplasm;cytoplasmic part;cytosolic part;intracellular membrane-bounded organelle;intracellular organelle;intracellular part;macromolecular complex;membrane-bounded organelle;nucleus;organelle;prefoldin complex;protein complex		Homo sapiens		1	18
cell part;cytoplasmic part;cytosol;intracellular membrane-bounded organelle;intracellular non-membrane-bounded organelle;intracellular organelle;intracellular organelle lumen;intracellular organelle part;intracellular part;mem	Huntington's disease	Homo sapiens		1	18
cell part;cytoplasmic part;cytosol;intracellular part	Purine metabolism	Homo sapiens		1	18
cell part;intracellular membrane-bounded organelle;intracellular organelle;intracellular part;membrane-bounded organelle;nucleus;organelle		Homo sapiens		1	18
cell part;intracellular membrane-bounded organelle;intracellular non-membrane-bounded organelle;intracellular organelle;intracellular organelle part;intracellular part;macromolecular complex;membrane-bounded organelle;nc	Ribosome biogenesis in eukaryotes	Homo sapiens		1	18
actin cytoskeleton;adherens junction;anchoring junction;cell junction;cell part;cell projection;cell-cell junction;cell-substrate adherens junction;cell-substrate junction;contractile fiber;cytoplasm;cytoplasmic part;cytoskeleton;filopodium;focal adhesion;intracellular non-membrane-bounded organelle;intracellular organelle;intracellu		Homo sapiens		1	18
aminoacyl-tRNA synthetase multienzyme complex;cell part;cell surface;cytoplasm;cytoplasmic membrane-bounded vesicle;cytoplasmic part;cytoplasmic vesicle;cytosol;endoplasmic reticulum;extracellular region part;extracellular space;Golgi apparatus;intracellular membrane-bounded organelle;intracellular organelle;intracellula		Homo sapiens		1	18
cell part;intracellular membrane-bounded organelle;intracellular organelle;intracellular organelle part;intracellular part;membrane-bounded organelle;nuclear part;nucleoplasm;nucleus;organelle;organelle part		Homo sapiens		1	18
cell part;intracellular non-membrane-bounded organelle;intracellular organelle;intracellular organelle part;intracellular part;non-membrane-bounded organelle;nuclear part;nucleolus;nucleoplasm;organelle;organelle part	Spliceosome	Homo sapiens		1	18
		Homo sapiens		1	18
cell part;chromatin;chromatin remodeling complex;chromosomal part;cytoplasm;cytoplasmic part;cytosol;histone deacetylase complex;intracellular membrane-bounded organelle;intracellular organelle;intracellular organelle p	Cell cycle;Chronic myeloid leukemia;Huntington's disease;Notch signaling pathway;Pathways in cancer	Homo sapiens		1	18
cell part;cytoplasm;cytoskeleton;extracellular membrane-bounded organelle;extracellular organelle;extracellular region part;extracellular vesicular exosome;intracellular non-membrane-bounded organelle;intracellular organelle;intracellular organelle part;intracellular part;membrane;membrane-bounded organelle;membrane-bounc		Homo sapiens		1	18
cell part;envelope;integral to membrane;integral to nuclear inner membrane;integral to organelle membrane;intracellular organelle part;intracellular part;intrinsic to membrane;intrinsic to nuclear inner membrane;intrinsic to organelle membrane;membrane;membrane part;nuclear envelope;nuclear membrane;nuclear membrane pa		Homo sapiens		1	18
cell part;centriolar satellite;centrosome;cytoplasm;cytoplasmic part;cytoskeletal part;cytosol;intracellular non-membrane-bounded organelle;intracellular organelle;intracellular organelle part;intracellular part;macromolecular complex;membrane;microtubule organizing center;microtubule organizing center part;non-membrane-bou		Homo sapiens		1	18
cell part;cytoplasm;cytoskeletal part;intracellular organelle part;intracellular part;macromolecular complex;microtubule;organelle part;protein complex		Homo sapiens		1	18
cell part;cytoplasmic part;cytosol;intracellular part;macromolecular complex;protein complex;TORC2 complex	mTOR signaling pathway	Homo sapiens		1	18
anchored to membrane;cell part;cell surface;extracellular region part;extracellular space;intrinsic to membrane;membrane;membrane part;plasma membrane		Homo sapiens		1	18
cell part;intracellular membrane-bounded organelle;intracellular organelle;intracellular part;membrane-bounded organelle;nucleus;organelle		Homo sapiens		1	18
	Ribosome biogenesis in eukaryotes	Homo sapiens		1	18
cell part;ER membrane protein complex;integral to membrane;intrinsic to membrane;macromolecular complex;membrane part;protein complex		Homo sapiens		1	18
actin filament bundle;actomyosin;adherens junction;anchoring junction;cell cortex;cell junction;cell part;cell-substrate adherens junction;cell-substrate junction;cytoplasmic part;cytoskeletal part;cytosol;focal adhesion;intracellular organelle part;intracellular part;organelle part;stress fiber		Homo sapiens		1	18
cell part;intracellular membrane-bounded organelle;intracellular organelle;intracellular part;membrane-bounded organelle;nucleus;organelle		Homo sapiens		1	18
cell part;cytoplasm;cytoskeleton;intracellular non-membrane-bounded organelle;intracellular organelle;intracellular part;non-membrane-bounded organelle;organelle		Homo sapiens		1	18
cell part;cytoplasm;extracellular membrane-bounded organelle;extracellular organelle;extracellular region part;extracellular vesicular exosome;intracellular membrane-bounded organelle;intracellular organelle;intracellular part;membrane-bounded organelle;membrane-bounded vesicle;nucleus;organelle;vesicle		Homo sapiens		1	18
cell part;cytoplasmic part;endoplasmic reticulum lumen;endoplasmic reticulum membrane;endoplasmic reticulum part;intracellular organelle lumen;intracellular organelle part;intracellular part;membrane;membrane part;membrane-enclosed lumen;organelle lumen;organelle membrane;organelle part		Homo sapiens		1	18
cell part;cis-Golgi network;COPI vesicle coat;cytoplasm;cytoplasmic part;cytoplasmic vesicle part;cytoskeletal part;endoplasmic reticulum-Golgi intermediate compartment;Golgi apparatus;Golgi apparatus part;intracellular membrane-bounded organelle;intracellular organelle;intracellular organelle part;intracellular part;macromo		Homo sapiens		1	18
cell part;chromatin remodeling complex;chromosomal part;cytoplasm;extracellular region part;histone deacetylase complex;intercellular bridge;intracellular membrane-bounded organelle;intracellular non-membrane-boundec	Huntington's disease	Homo sapiens		1	18
cell part;cytoplasmic part;cytosol;intracellular organelle part;intracellular part;macromolecular complex;membrane part;nuclear part;nuclear pore;nucleoplasm;organelle part;pore complex;protein complex	RNA transport	Homo sapiens		1	18
cell part;cytoplasm;cytoplasmic part;cytosol;intracellular membrane-bounded organelle;intracellular organelle;intracellular organelle part;intracellular part;membrane-bounded organelle;nuclear part;nucleoplasm;nucleus;organelle;organelle part		Homo sapiens		1	18
cell junction;cell part;cell projection;cytoplasm;cytoplasmic part;cytoskeletal part;early endosome membrane;endosomal part;endosome membrane;intracellular non-membrane-bounded organelle;intracellular organelle;intrac	Endocytosis	Homo sapiens		1	18
cell part;cytoplasm;intracellular non-membrane-bounded organelle;intracellular organelle;intracellular organelle part;intracellular part;membrane;non-membrane-bounded organelle;nuclear part;nucleolus;organelle;organelle part;plasma membrane		Homo sapiens		1	18
cell junction;cell part;clathrin-coated vesicle;coated vesicle;cytoplasmic membrane-bounded vesicle;cytoplasmic part;cytoplasmic vesicle;extracellular membrane-bounded organelle;extracellular organelle;extracellular region part;extracellular vesicular exosome;intracellular membrane-bounded organelle;intracellular organelle;in		Homo sapiens		1	18
cell part;cytoplasmic part;intracellular organelle lumen;intracellular organelle part;intracellular part;membrane-enclosed lumen;mitochondrial matrix;mitochondrial part;organelle lumen;organelle part	Aminoacyl-tRNA biosynthesis	Homo sapiens		1	18
actin cytoskeleton;adherens junction;anchoring junction;cell junction;cell part;cell-substrate adherens junction;cell-substrate junction;cytoplasm;cytoplasmic part;cytoskeletal part;cytoskeleton;focal adhesion;intracellular membrane-bounded organelle;intracellular non-membrane-bounded organelle;intracellular organelle;intracell		Homo sapiens		1	18
cell part;cytoplasm;cytoplasmic membrane-bounded vesicle;cytoplasmic part;cytoplasmic vesicle;early endosome;endosome;extracellular membrane-bounded organelle;extracellular organelle;extracellular region part;extracellular vesicular exosome;intracellular membrane-bounded organelle;intracellular organelle;intracellular or		Homo sapiens		1	18
cell part;cell projection;cytoplasmic part;dendrite;intracellular membrane-bounded organelle;intracellular organelle;intracellular part;membrane-bounded organelle;mitochondrion;neuron projection;nucleus;organelle	Geraniol degradation;Naphthalene degradation	Homo sapiens		1	18
alpha-amino-3-hydroxy-5-methyl-4-isoxazolepropionic acid selective glutamate receptor complex;cell part;cytoplasmic part;endoplasmic reticulum membrane;endoplasmic reticulum part;Golgi apparatus;Golgi apparatus part;Golgi membrane;integral to endoplasmic reticulum membrane;integral to membrane;integral to organel		Homo sapiens		1	18
cell part;cell projection;cilium;extracellular membrane-bounded organelle;extracellular organelle;extracellular region part;extracellular vesicular exosome;membrane-bounded organelle;membrane-bounded vesicle;organelle;vesicle		Homo sapiens		1	18
cell part;cell surface;cytoplasmic part;intracellular membrane-bounded organelle;intracellular organelle;intracellular part;membrane-bounded organelle;mitochondrion;organelle		Homo sapiens		1	18
cell part;cytoplasmic part;integral to membrane;integral to plasma membrane;intracellular membrane-bounded organelle;intracellular organelle;intracellular organelle part;intracellular part;intrinsic to membrane;intrinsic to plasma membrane;membrane;membrane part;membrane-bounded organelle;mitochondrial inner membrane		Homo sapiens		1	18
cell part;cytoplasm;cytoskeletal part;extracellular membrane-bounded organelle;extracellular organelle;extracellular region part;extracellular vesicular exosome;intracellular membrane-bounded organelle;intracellular organelle	Base excision repair	Homo sapiens		1	18
cell part;intracellular organelle part;intracellular part;macromolecular complex;nuclear part;nucleoplasm;nucleoplasm part;organelle part;protein complex;transcription factor complex;transcription factor TFIIIC complex		Homo sapiens		1	18
cell part;cytoplasm;cytoplasmic part;endosome;extracellular membrane-bounded organelle;extracellular organelle;extracellular region part;extracellular vesicular exosome;intracellular membrane-bounded organelle;intracellular organelle;intracellular organelle part;intracellular part;membrane;membrane-bounded organelle;membr		Homo sapiens		1	18
cell part;cytoplasm;intracellular part		Homo sapiens		1	18
cell part;cytoplasm;cytoplasmic part;cytoskeleton;cytosol;endoplasmic reticulum;extracellular membrane-bounded organelle;extracellular organelle;extracellular region part;extracellular vesicular exosome;inclusion body;inter	Protein processing in endoplasmic reticulum;Ubiquitin mediated proteolysis	Homo sapiens		1	18
cell part;cytoplasm;intracellular membrane-bounded organelle;intracellular organelle;intracellular organelle part;intracellular part;macromolecular complex;membrane-bounded organelle;nuclear part;nucleoplasm;nucleus;organelle;organelle part;protein complex;signalosome		Homo sapiens		1	18
cell part;cytoplasm;intracellular membrane-bounded organelle;intracellular organelle;intracellular organelle part;intracellular part;membrane-bounded organelle;nuclear body;nuclear part;nuclear speck;nucleoplasm;nucleoplas	Spliceosome	Homo sapiens		1	18
cell part;cytoplasm;cytoplasmic part;cytosol;cytosolic large ribosomal subunit;intracellular non-membrane-bounded organelle;intracellular organelle;intracellular organelle part;intracellular part;large ribosomal subunit;macrom	Ribosome	Homo sapiens		1	18
		Homo sapiens		1	18
cell part;centrosome;chromosomal part;condensed chromosome kinetochore;cytoplasm;cytoplasmic dynein complex;cytoplasmic part;cytoskeletal part;dynein complex;intracellular non-membrane-bounded organelle;intrac	Phagosome;Vasopressin-regulated water reabsorption	Homo sapiens		1	18
cell part;cytoplasm;cytoplasmic part;cytosol;intracellular organelle part;intracellular part;membrane;nuclear part;nucleoplasm;organelle part		Homo sapiens		1	19
cell part;cytoplasm;intracellular organelle part;intracellular part;nuclear part;nucleoplasm;organelle part	RNA transport	Homo sapiens		1	19
box H/ACA RNP complex;box H/ACA snoRNP complex;Cajal body;cell part;cytoplasm;intracellular membrane-bounded organelle;intracellular non-membrane-bounded organelle;intracellular organelle;intracellular organelle p	Ribosome biogenesis in eukaryotes	Homo sapiens		1	19
cell part;intracellular organelle part;intracellular part;nuclear part;nucleoplasm;organelle part		Homo sapiens		1	19
cell part;cytoplasm;cytoplasmic part;cytosol;endoplasmic reticulum;endoplasmic reticulum membrane;endoplasmic reticulum part;intracellular membrane-bounded organelle;intracellular organelle;intracellular organelle part;intracellular part;macromolecular complex;membrane;membrane part;membrane-bounded organelle;nucle		Homo sapiens		1	19
cell part;cytoplasmic part;endoplasmic reticulum lumen;endoplasmic reticulum part;intracellular organelle lumen;intracellular organelle part;intracellular part;membrane-enclosed lumen;organelle lumen;organelle part	Pentose phosphate pathway	Homo sapiens		1	19
cell part;cytoplasmic part;cytosol;intracellular part	Protein processing in endoplasmic reticulum	Homo sapiens		1	19
cell part;cell projection;contractile fiber part;cytoplasm;cytoplasmic part;cytosol;intracellular part;membrane;neuron projection;organelle part;plasma membrane;Z disc		Homo sapiens		1	19
cell part;chromatin;chromosomal part;cytoplasm;cytoplasmic part;cytosol;endoplasmic reticulum;intracellular membrane-bounded organelle;intracellular non-membrane-bounded organelle;intracellular organelle;intracellular c	Amyotrophic lateral sclerosis (ALS);Apoptosis;Basal cell carcinoma;Bladder cancer;Cell cycle;Chronic m	Homo sapiens		1	19
apical plasma membrane;basolateral plasma membrane;caveola;cell junction;cell part;cell-cell contact zone;cell-cell junction;extracellular membrane-bounded organelle;extracellular organelle;extracellular region part;extracel	Aldosterone-regulated sodium reabsorption;Bile secretion;Carbohydrate digestion and absorption;Cardi	Homo sapiens		1	19
cell part;cytoplasmic part;cytosol;intracellular part	Alanine, aspartate and glutamate metabolism;Nitrogen metabolism	Homo sapiens		1	19
cell part;cell surface;cytoplasm;extracellular matrix;extracellular membrane-bounded organelle;extracellular organelle;extracellular region part;extracellular space;extracellular vesicular exosome;intracellular;intracellular membrane-bounded organelle;intracellular organelle;intracellular part;membrane-bounded organelle;membrane		Homo sapiens		1	19
adherens junction;anchoring junction;apical plasma membrane;cell junction;cell part;cell surface;cell-substrate adherens junction;cell-substrate junction;cytoplasm;cytoplasmic membrane-bounded vesicle;cytoplasmic part;c	Calcium signaling pathway;Cytokine-cytokine receptor interaction;Focal adhesion;Gap junction;Glioma;	Homo sapiens		1	19
cell part;cytoplasm;intracellular organelle part;intracellular part;macromolecular complex;nuclear part;nucleoplasm;organelle part;ribonucleoprotein complex	Systemic lupus erythematosus	Homo sapiens		1	19
cell part;centrosome;chromosomal part;cytoplasm;cytoplasmic part;cytoskeletal part;DNA replication factor C complex;extracellular membrane-bounded organelle;extracellular organelle;extracellular region part;extracellular	Base excision repair;Cell cycle;DNA replication;Mismatch repair;Nucleotide excision repair	Homo sapiens		1	19
cell part;intracellular non-membrane-bounded organelle;intracellular organelle;intracellular organelle part;intracellular part;non-membrane-bounded organelle;nuclear part;nucleolus;nucleoplasm;organelle;organelle part		Homo sapiens		1	19
cell part;cytoplasmic part;cytosol;intracellular part;membrane;plasma membrane	Glycerolipid metabolism;Glycerophospholipid metabolism;Phosphatidylinositol signaling system	Homo sapiens		1	19
cell part;cytoplasmic part;cytosol;cytosolic small ribosomal subunit;intracellular organelle part;intracellular part;macromolecular complex;membrane;organelle part;ribonucleoprotein complex;small ribosomal subunit	Ribosome	Homo sapiens		1	19
cell part;cytoplasm;cytoplasmic part;cytosol;extracellular membrane-bounded organelle;extracellular organelle;extracellular region part;extracellular vesicular exosome;intracellular membrane-bounded organelle;intracellular r	Pyrimidine metabolism	Homo sapiens		1	19
cell part;cytoplasmic part;extracellular membrane-bounded organelle;extracellular organelle;extracellular region part;extracellular vesicular exosome;intracellular membrane-bounded organelle;intracellular organelle;intracellula	Citrate cycle (TCA cycle);Lysine degradation	Homo sapiens		1	19
cell part;cytoplasm;cytoplasmic part;cytosol;extracellular membrane-bounded organelle;extracellular organelle;extracellular region part;extracellular vesicular exosome;intercellular bridge;intracellular membrane-bounded orga	Protein export	Homo sapiens		1	19
cell part;cytoplasmic part;extracellular membrane-bounded organelle;extracellular organelle;extracellular region part;extracellular vesicular exosome;intracellular membrane-bounded organelle;intracellular non-membrane-bou	Calcium signaling pathway;Huntington's disease;Parkinson's disease	Homo sapiens		1	19
cell part;cytoplasmic part;extracellular membrane-bounded organelle;extracellular organelle;extracellular region part;extracellular vesicular exosome;intracellular membrane-bounded organelle;intracellular organelle;intracellula	Carbon fixation pathways in prokaryotes;Citrate cycle (TCA cycle);Glutathione metabolism;Peroxisome	Homo sapiens		1	19
cell part;cytoplasmic part;intracellular organelle part;intracellular part;macromolecular complex;membrane;membrane part;mitochondrial inner membrane;mitochondrial membrane;mitochondrial membrane part;mitochondrial	Alzheimer's disease;Huntington's disease;Oxidative phosphorylation;Parkinson's disease	Homo sapiens		1	19
adherens junction;anchoring junction;cell junction;cell part;cell-substrate adherens junction;cell-substrate junction;cytoplasmic membrane-bounded vesicle;cytoplasmic part;cytoplasmic vesicle;extracellular matrix;extracellula	GnRH signaling pathway	Homo sapiens		1	19
cell part;cytoplasmic part;cytoskeleton;cytosol;extracellular membrane-bounded organelle;extracellular organelle;extracellular region part;extracellular vesicular exosome;immunological synapse;intracellular non-membrane-b	Neurotrophin signaling pathway;Vasopressin-regulated water reabsorption	Homo sapiens		1	19
Arp2/3 protein complex;cell part;cell projection;cytoplasmic part;cytoskeletal part;cytosol;extracellular membrane-bounded organelle;extracellular organelle;extracellular region part;extracellular vesicular exosome;intracellula	Bacterial invasion of epithelial cells;Fc gamma R-mediated phagocytosis;Pathogenic Escherichia coli inf	Homo sapiens		1	19
cell body;cell part;cell projection;centrosome;cilium;clathrin-coated vesicle;coated vesicle;cytoplasmic membrane-bounded vesicle;cytoplasmic part;cytoplasmic vesicle;cytoplasmic vesicle membrane;cytoplasmic vesicle pa	Pancreatic secretion	Homo sapiens		1	19
cell part;cytoplasm;cytoplasmic part;cytosol;intracellular membrane-bounded organelle;intracellular non-membrane-bounded organelle;intracellular organelle;intracellular organelle part;intracellular part;macromolecular comp	Bacterial secretion system;Protein export	Homo sapiens		1	19
adherens junction;anchoring junction;cell junction;cell part;cell projection;cell-substrate adherens junction;cell-substrate junction;cilium;cytoplasmic membrane-bounded vesicle;cytoplasmic part;cytoplasmic vesicle;cytoplasmic vesicle membrane;cytoplasmic vesicle part;endocytic vesicle membrane;endoplasmic reticulum mem		Homo sapiens		1	19
cell part;clathrin-coated vesicle;coated vesicle;cytoplasmic membrane-bounded vesicle;cytoplasmic part;cytoplasmic vesicle;cytoplasmic vesicle membrane;cytoplasmic vesicle part;cytosol;early endosome;early endosome membrane;endocytic vesicle;endocytic vesicle membrane;endoplasmic reticulum;endosomal part;endos		Homo sapiens		1	19
cell part;cytoplasm;cytoplasmic membrane-bounded vesicle;cytoplasmic part;cytoplasmic vesicle;cytosol;extracellular membrane-bounded organelle;extracellular organelle;extracellular region part;extracellular vesicular exosome;intracellular membrane-bounded organelle;intracellular organelle;intracellular organelle part;intracel		Homo sapiens		1	19
cell part;cytoplasmic part;cytosol;endoplasmic reticulum;intracellular membrane-bounded organelle;intracellular organelle;intracellular part;membrane-bounded organelle;nucleus;organelle		Homo sapiens		1	19
adherens junction;anchoring junction;cell junction;cell part;cell-substrate adherens junction;cell-substrate junction;cytoplasm;cytoplasmic part;cytosol;cytosolic large ribosomal subunit;extracellular membrane-bounded orgar	Ribosome	Homo sapiens		1	19
cell part;cytoplasm;cytoplasmic part;cytosol;cytosolic small ribosomal subunit;intracellular membrane-bounded organelle;intracellular non-membrane-bounded organelle;intracellular organelle;intracellular organelle part;intrac	Ribosome	Homo sapiens		1	19
adherens junction;anchoring junction;cell junction;cell part;cell-substrate adherens junction;cell-substrate junction;cytoplasm;cytoplasmic part;cytosol;cytosolic large ribosomal subunit;extracellular membrane-bounded orgar	Ribosome	Homo sapiens		1	19
cell part;cytoplasmic part;cytosol;intracellular organelle part;intracellular part;macromolecular complex;nuclear part;nucleoplasm;organelle part;protein complex;protein phosphatase type 2A complex;protein serine/threonine	Cell cycle - yeast;Chagas disease (American trypanosomiasis);Hepatitis C;mRNA surveillance pathway;	Homo sapiens		1	19
cell part;chromatin remodeling complex;chromosomal part;cytoplasmic part;cytosol;histone deacetylase complex;intracellular membrane-bounded organelle;intracellular organelle;intracellular organelle part;intracellular part;n	Adherens junction;Circadian rhythm - plant;Measles;Ribosome biogenesis in eukaryotes;Tight junction;	Homo sapiens		1	19
cell part;cytoplasmic part;endoplasmic reticulum membrane;endoplasmic reticulum part;intracellular membrane-bounded organelle;intracellular organelle;intracellular organelle part;intracellular part;membrane;membrane part;membrane-bounded organelle;nucleus;organelle;organelle membrane;organelle part		Homo sapiens		1	19
cell part;cytoplasmic part;cytosol;extracellular membrane-bounded organelle;extracellular organelle;extracellular region part;extracellular vesicular exosome;intracellular part;membrane-bounded organelle;membrane-bounde	Arginine and proline metabolism	Homo sapiens		1	19
cell junction;cell part;cell projection;cytoplasm;cytoplasmic part;cytoskeletal part;cytosol;intracellular non-membrane-bounded organelle;intracellular organelle;intracellular organelle part;intracellular part;macromolecular complex;membrane;non-membrane-bounded organelle;organelle;organelle part;plasma membrane;podosom		Homo sapiens		1	19
calcineurin complex;cell part;contractile fiber part;cytoplasm;cytoplasmic part;cytosol;intracellular membrane-bounded organelle;intracellular organelle;intracellular organelle part;intracellular part;macromolecular complex;me	Alzheimer's disease;Amyotrophic lateral sclerosis (ALS);Apoptosis;Axon guidance;B cell receptor signall	Homo sapiens		1	19
AMP-activated protein kinase complex;apical plasma membrane;cell part;cytoplasm;cytoplasmic part;cytosol;intracellular;intracellular membrane-bounded organelle;intracellular organelle;intracellular organelle part;intracellul	Adipocytokine signaling pathway;Hypertrophic cardiomyopathy (HCM);Insulin signaling pathway;mTOR	Homo sapiens		1	19
catalytic step 2 spliceosome;cell part;chromosome;intracellular non-membrane-bounded organelle;intracellular organelle;intracellular organelle part;intracellular part;macromolecular complex;non-membrane-bounded organelle;nuclear part;nucleoplasm;organelle;organelle part;ribonucleoprotein complex;spliceosomal complex		Homo sapiens		1	19
catalytic step 2 spliceosome;cell part;chromatin;chromosomal part;intracellular membrane-bounded organelle;intracellular organelle;intracellular organelle part;intracellular part;macromolecular complex;membrane-bounded c	Notch signaling pathway;Spliceosome	Homo sapiens		1	19
cell part;cullin-RING ubiquitin ligase complex;cytoplasmic part;cytosol;intracellular organelle part;intracellular part;macromolecular complex;nuclear part;nucleoplasm;organelle part;protein complex;SCF ubiquitin ligase comp	Cell cycle;Cell cycle - yeast;Circadian rhythm - mammal;Oocyte meiosis;Protein processing in endoplas	Homo sapiens		1	19
cell body;cell part;cell projection;cell projection part;cytoplasmic membrane-bounded vesicle;cytoplasmic part;cytoplasmic vesicle;cytoskeletal part;cytosol;exocytic vesicle;extracellular region part;extracellular space;filamentous actin;growth cone;intracellular membrane-bounded organelle;intracellular organelle;intracellular orga		Homo sapiens		1	19
cell part;chromatin;chromosomal part;cytoplasmic part;cytosol;euchromatin;heterochromatin;intracellular membrane-bounded organelle;intracellular non-membrane-bounded organelle;intracellular organelle;intracellular organelle part;intracellular part;macromolecular complex;membrane-bounded organelle;non-membrane-boun		Homo sapiens		1	19
cell part;chromosome;condensed chromosome;condensed nuclear chromosome;cytoplasmic part;endoplasmic reticulum;intracellular membrane-bounded organelle;intracellular non-membrane-bounded organelle;intracellular organelle;intracellular organelle part;intracellular part;membrane-bounded organelle;non-membrane-bo		Homo sapiens		1	19
cell part;cytoplasmic part;cytosol;eukaryotic translation initiation factor 4F complex;intracellular part;macromolecular complex;membrane;perinuclear region of cytoplasm;protein complex		Homo sapiens		1	19
cell part;cytoplasm;cytoplasmic part;cytosol;intracellular membrane-bounded organelle;intracellular organelle;intracellular part;membrane-bounded organelle;nucleus;organelle	Ubiquitin mediated proteolysis	Homo sapiens		1	19
cell part;cell projection;cell projection part;contractile fiber;cytoplasm;cytoplasmic part;cytoskeleton;extracellular membrane-bounded organelle;extracellular organelle;extracellular region part;extracellular vesicular exosome;filopodium;growth cone;intracellular non-membrane-bounded organelle;intracellular organelle;intracellula		Homo sapiens		1	19
cell part;cytoplasmic part;intracellular membrane-bounded organelle;intracellular organelle;intracellular organelle part;intracellular part;membrane-bounded organelle;mitochondrion;nuclear part;nucleoplasm;nucleus;organelle;organelle part		Homo sapiens		1	19
cell part;cytoplasm;cytoplasmic part;cytosol;intracellular membrane-bounded organelle;intracellular organelle;intracellular part;membrane;membrane-bounded organelle;nucleus;organelle		Homo sapiens		1	19
cell part;chromosomal part;chromosome;intracellular non-membrane-bounded organelle;intracellular organelle;intracellular organelle part;intracellular part;kinetochore;macromolecular complex;non-membrane-bounded organelle;nuclear chromosome;nuclear part;organelle;organelle part;protein complex		Homo sapiens		1	19
cell part;chromosomal part;condensed chromosome kinetochore;intracellular membrane-bounded organelle;intracellular non-membrane-bounded organelle;intracellular organelle;intracellular organelle part;intracellular part;kinetochore;macromolecular complex;membrane-bounded organelle;non-membrane-bounded organelle;n		Homo sapiens		1	19
cell part;extracellular membrane-bounded organelle;extracellular organelle;extracellular region part;extracellular vesicular exosome;membrane;membrane-bounded organelle;membrane-bounded vesicle;organelle;vesicle		Homo sapiens		1	19
cell part;chromatin;chromatin remodeling complex;chromosomal part;intracellular organelle part;intracellular part;macromolecular complex;nBAF complex;npBAF complex;nuclear chromatin;nuclear chromosome part;nuclear part;nucleoplasm;organelle part;protein complex;SWI/SNF complex;SWI/SNF-type complex		Homo sapiens		1	19
cell part;intracellular membrane-bounded organelle;intracellular non-membrane-bounded organelle;intracellular organelle;intracellular organelle part;intracellular part;membrane-bounded organelle;non-membrane-bounded organelle;nuclear part;nucleolus;nucleus;organelle;organelle part		Homo sapiens		1	19
cell part;cytoplasm;intracellular membrane-bounded organelle;intracellular organelle;intracellular organelle part;intracellular part;membrane-bounded organelle;nuclear part;nucleoplasm;nucleus;organelle;organelle part	Basal transcription factors	Homo sapiens		1	19
cell part;intracellular organelle part;intracellular part;nuclear part;nucleoplasm;organelle part		Homo sapiens		1	19
cell part;membrane part;membrane raft		Homo sapiens		1	19
cell part;cytoplasmic part;intracellular organelle lumen;intracellular organelle part;intracellular part;membrane-enclosed lumen;mitochondrial matrix;mitochondrial part;organelle lumen;organelle part		Homo sapiens		1	19
cell part;intracellular organelle part;intracellular part;membrane;nuclear membrane;nuclear part;nucleoplasm;organelle membrane;organelle part		Homo sapiens		1	19
cell part;cytoplasm;cytoplasmic part;intracellular membrane-bounded organelle;intracellular non-membrane-bounded organelle;intracellular organelle;intracellular organelle part;intracellular part;macromolecular complex;membrane-bounded organelle;non-membrane-bounded organelle;nuclear body;nuclear part;nuclear speck;n		Homo sapiens		1	19
cell part;cytoplasmic part;intracellular organelle part;intracellular part;membrane;membrane-enclosed lumen;mitochondrial intermembrane space;mitochondrial part;organelle envelope lumen;organelle part;plasma membrane	Renin-angiotensin system	Homo sapiens		1	19
cell part;chromosomal part;chromosome, telomeric region;cytoplasm;cytoplasmic part;cytosol;extracellular membrane-bounded organelle;extracellular organelle;extracellular region part;extracellular vesicular exosome;intrac	RNA degradation	Homo sapiens		1	19
cell part;extracellular region;integral to membrane;intrinsic to membrane;membrane;membrane part;plasma membrane		Homo sapiens		1	19
cell part;cytoplasm;intracellular membrane-bounded organelle;intracellular organelle;intracellular part;membrane;membrane-bounded organelle;nucleus;organelle;plasma membrane		Homo sapiens		1	19
cell part;cytoplasm;cytoskeletal part;intracellular non-membrane-bounded organelle;intracellular organelle;intracellular organelle part;intracellular part;macromolecular complex;membrane;microtubule;mitotic spindle;non-membrane-bounded organelle;organelle;organelle part;protein complex;spindle		Homo sapiens		1	19
cell part;cell surface;extracellular membrane-bounded organelle;extracellular organelle;extracellular region part;extracellular vesicular exosome;integral to membrane;intrinsic to membrane;membrane;membrane part;membrane-bounded organelle;membrane-bounded vesicle;organelle;vesicle		Homo sapiens		1	19
cell part;cytoplasmic part;endoplasmic reticulum;endoplasmic reticulum lumen;endoplasmic reticulum part;intracellular membrane-bounded organelle;intracellular organelle;intracellular organelle lumen;intracellular organelle p	Protein processing in endoplasmic reticulum	Homo sapiens		1	19
cell part;cytoplasm;cytoplasmic part;cytosol;early endosome membrane;endosomal part;endosome membrane;extracellular membrane-bounded organelle;extracellular organelle;extracellular region part;extracellular vesicula	Colorectal cancer;Pathways in cancer	Homo sapiens		1	19

cell part;cytoplasmic part;cytosol;extracellular membrane-bounded organelle;extracellular organelle;extracellular region part;extracellular vesicular exosome;intracellular organelle part;intracellular part;macromolecular comple	Antigen processing and presentation;Proteasome	Homo sapiens	1	19
cell part;cytoplasm;envelope;extracellular membrane-bounded organelle;extracellular organelle;extracellular region part;extracellular vesicular exosome;intracellular non-membrane-bounded organelle;intracellular organelle;intracellular organelle part;intracellular part;macromolecular complex;membrane;membrane part;membrane		Homo sapiens	1	19
		Homo sapiens	1	19
cell part;membrane		Homo sapiens	1	19
axon part;cell part;cell projection part;cytoplasmic part;endosomal part;endosome membrane;Golgi apparatus;Golgi apparatus part;Golgi membrane;intracellular membrane-bounded organelle;intracellular organelle;intracellu	Lysosome	Homo sapiens	1	20
AP-type membrane coat adaptor complex;cell part;clathrin adaptor complex;clathrin coated vesicle membrane;clathrin-coated endocytic vesicle membrane;clathrin-coated vesicle;coated vesicle;coated vesicle membrane;cy	Lysosome	Homo sapiens	1	20
cell part;cell surface;cytoplasmic part;cytosol;intracellular part		Homo sapiens	1	20
cell part;cytoplasmic part;early endosome membrane;endosomal part;endosome membrane;extracellular membrane-bounded organelle;extracellular organelle;extracellular region part;extracellular vesicular exosome;intracellular organelle part;intracellular part;lysosomal membrane;membrane;membrane-bounded organelle;mem		Homo sapiens	1	20
cell part;cytoplasm;cytoskeletal part;cytoskeleton;extracellular membrane-bounded organelle;extracellular organelle;extracellular region part;extracellular vesicular exosome;intracellular non-membrane-bounded organelle;intracellular organelle;intracellular organelle part;intracellular part;macromolecular complex;membrane-boun		Homo sapiens	1	20
Barr body;cell part;centromeric heterochromatin;chromatin;chromosomal part;chromosome;condensed chromosome;extracellular membrane-bounded organelle;extracellular organelle;extracellular region part;extracellular ve	Systemic lupus erythematosus	Homo sapiens	1	20
cell part;cytoplasmic part;extracellular membrane-bounded organelle;extracellular organelle;extracellular region part;extracellular vesicular exosome;intracellular membrane-bounded organelle;intracellular organelle;intracellul	Citrate cycle (TCA cycle);Glyoxylate and dicarboxylate metabolism	Homo sapiens	1	20
cell part;chromosomal part;chromosome, centromeric region;cytoplasm;intracellular organelle part;intracellular part;nuclear part;nucleoplasm;organelle part		Homo sapiens	1	20
cell part;cytoplasm;Elongator holoenzyme complex;histone acetyltransferase complex;intracellular non-membrane-bounded organelle;intracellular organelle;intracellular organelle part;intracellular part;macromolecular complex;non-membrane-bounded organelle;nuclear part;nucleolus;nucleoplasm part;organelle;organelle part;pr		Homo sapiens	1	20
extracellular matrix;extracellular region;extracellular region part;proteinaceous extracellular matrix	Bladder cancer;Pathways in cancer;PPAR signaling pathway;Rheumatoid arthritis	Homo sapiens	1	20
cell part;cytoplasm;cytoplasmic part;cytosol;extracellular membrane-bounded organelle;extracellular organelle;extracellular region part;extracellular vesicular exosome;intracellular part;membrane;membrane-bounded organelle;membrane-bounded vesicle;organelle;plasma membrane;vesicle		Homo sapiens	1	20
adherens junction;anchoring junction;cell junction;cell part;cell-substrate adherens junction;cell-substrate junction;contractile fiber part;cytoplasm;cytoplasmic part;cytoskeletal part;cytoskeleton;cytosol;extracellular membra	Amoebiasis;MAPK signaling pathway;VEGF signaling pathway	Homo sapiens	1	20
cell part;cytoplasmic part;extracellular membrane-bounded organelle;extracellular organelle;extracellular region;extracellular region part;extracellular space;extracellular vesicular exosome;Golgi apparatus;integral to membra	Lysosome	Homo sapiens	1	20
cell part;cytoplasm;cytoplasmic part;cytosol;intracellular membrane-bounded organelle;intracellular organelle;intracellular organelle part;intracellular part;membrane-bounded organelle;mitochondrion;nuclear part;nucleoplasr	Terpenoid backbone biosynthesis	Homo sapiens	1	20
cell body;cell part;cytoplasm;cytoplasmic part;cytosol;extracellular membrane-bounded organelle;extracellular organelle;extracellular region part;extracellular vesicular exosome;intracellular part;membrane-bounded organelle;membrane-bounded vesicle;neuronal cell body;organelle;vesicle		Homo sapiens	1	20
apical part of cell;cell body;cell part;cell projection;cilium;cytoplasm;cytoplasmic part;cytosol;dendrite;endoplasmic reticulum;extracellular membrane-bounded organelle;extracellular organelle;extracellular region part;extrace	African trypanosomiasis;Aldosterone-regulated sodium reabsorption;Amoebiasis;Calcium signaling path	Homo sapiens	1	20
cell part;cytoplasmic part;cytoplasmic vesicle;cytosol;early endosome;endoplasmic reticulum;endosome;intracellular membrane-bounded organelle;intracellular organelle;intracellular part;membrane-bounded organelle;organ	Adherens junction;Insulin signaling pathway	Homo sapiens	1	20
cell part;intracellular organelle part;intracellular part;nuclear body;nuclear part;nuclear speck;nucleoplasm;nucleoplasm part;organelle part		Homo sapiens	1	20
basement membrane;cell part;collagen;collagen type V;cytoplasmic part;endoplasmic reticulum lumen;endoplasmic reticulum part;extracellular matrix;extracellular matrix part;extracellular membrane-bounded organelle;extra	Amoebiasis;ECM-receptor interaction;Focal adhesion;Protein digestion and absorption	Homo sapiens	1	20
cell part;cytoplasmic part;extracellular membrane-bounded organelle;extracellular organelle;extracellular region part;extracellular vesicular exosome;intracellular membrane-bounded organelle;intracellular organelle;intracellul	Alzheimer's disease;Huntington's disease;Oxidative phosphorylation;Parkinson's disease	Homo sapiens	1	20
cell part;cytoplasm;cytoplasmic mRNA processing body;cytoplasmic part;cytosol;extracellular membrane-bounded organelle;extracellular organelle;extracellular region part;extracellular vesicular exosome;intracellular membr	Proteasome	Homo sapiens	1	20
cell part;intracellular membrane-bounded organelle;intracellular organelle;intracellular organelle part;intracellular part;macromolecular complex;membrane-bounded organelle;nuclear part;nucleoplasm;nucleus;organelle;organelle part;ribonucleoprotein complex;spliceosomal complex		Homo sapiens	1	20
cell part;cytoplasmic part;endoplasmic reticulum;endoplasmic reticulum membrane;endoplasmic reticulum part;integral to membrane;intracellular membrane-bounded organelle;intracellular organelle;intracellular organelle pa	Steroid biosynthesis	Homo sapiens	1	20
cell part;centriole;cytoplasm;cytoplasmic membrane-bounded vesicle;cytoplasmic part;cytoplasmic vesicle;cytoskeletal part;extracellular membrane-bounded organelle;extracellular organelle;extracellular region part;extracellular vesicular exosome;F-actin capping protein complex;intracellular membrane-bounded organelle;intra		Homo sapiens	1	20
cell part;cytoplasmic part;endoplasmic reticulum membrane;endoplasmic reticulum part;integral to membrane;intracellular organelle part;intracellular part;intrinsic to membrane;macromolecular complex;membrane;membran	N-Glycan biosynthesis;Protein processing in endoplasmic reticulum;Various types of N-glycan biosynthe	Homo sapiens	1	20
cell part;cytoplasmic part;endoplasmic reticulum membrane;endoplasmic reticulum part;intracellular organelle part;intracellular part;lipid particle;membrane;membrane part;organelle membrane;organelle part	Steroid biosynthesis	Homo sapiens	1	20
cell part;cytoplasmic part;endoplasmic reticulum membrane;endoplasmic reticulum part;extracellular membrane-bounded organelle;extracellular organelle;extracellular region part;extracellular vesicular exosome;Golgi apparatus part;Golgi membrane;intracellular organelle part;intracellular part;membrane;membrane part;membra		Homo sapiens	1	20
cell part;cytoplasmic part;integral to membrane;integral to mitochondrial membrane;integral to mitochondrial outer membrane;integral to organelle membrane;intracellular membrane-bounded organelle;intracellular organelle;	Adipocytokine signaling pathway;Fatty acid metabolism;PPAR signaling pathway	Homo sapiens	1	20
cell part;cytoplasm;cytoplasmic part;cytosol;extracellular membrane-bounded organelle;extracellular organelle;extracellular region part;extracellular space;extracellular vesicular exosome;intracellular;intracellular membrane-b	Amoebiasis	Homo sapiens	1	20
cell part;cytoplasm;cytoplasmic part;cytosol;intracellular part;membrane;plasma membrane		Homo sapiens	1	20
cell part;cytoplasm;cytoplasmic part;cytosol;extracellular membrane-bounded organelle;extracellular organelle;extracellular region part;extracellular vesicular exosome;intracellular part;membrane;membrane-bounded organe	Amino sugar and nucleotide sugar metabolism;Galactose metabolism	Homo sapiens	1	20
cell part;centrosome;chromosomal part;condensed chromosome kinetochore;cytoplasmic part;cytoskeletal part;cytosol;envelope;intracellular non-membrane-bounded organelle;intracellular organelle;intracellular organelle pa	RNA transport	Homo sapiens	1	20
annulate lamellae;cell part;cytoplasm;cytoplasmic part;cytosol;endoplasmic reticulum membrane;endoplasmic reticulum part;extracellular membrane-bounded organelle;extracellular organelle;extracellular region part;extracellular vesicular exosome;intracellular membrane-bounded organelle;intracellular organelle;intracellular org		Homo sapiens	1	20
cell part;CRD-mediated mRNA stability complex;cytoplasm;cytoplasmic part;extracellular membrane-bounded organelle;extracellular organelle;extracellular region part;extracellular vesicular exosome;histone pre-mRNA 3'end processing complex;intracellular membrane-bounded organelle;intracellular non-membrane-bounded c		Homo sapiens	1	20
cell part;intracellular membrane-bounded organelle;intracellular organelle;intracellular organelle part;intracellular part;macromolecular complex;membrane-bounded organelle;nuclear body;nuclear part;nuclear speck;nucleoplasm;nucleoplasm part;nucleus;organelle;organelle part;protein complex;transcription export complex		Homo sapiens	1	20
cell part;cytoplasmic part;integral to membrane;integral to plasma membrane;intracellular membrane-bounded organelle;intracellular organelle;intracellular organelle part;intracellular part;intrinsic to membrane;intrinsic to plasma membrane;membrane;membrane part;membrane-bounded organelle;mitochondrial inner membrane		Homo sapiens	1	20
blood microparticle;cell part;extracellular matrix;extracellular membrane-bounded organelle;extracellular organelle;extracellular region part;extracellular space;extracellular vesicular exosome;membrane;membrane-bounded organelle;membrane-bounded vesicle;organelle;proteinaceous extracellular matrix;vesicle		Homo sapiens	1	20
cell part;cytoplasmic part;extracellular region part;extracellular space;intracellular membrane-bounded organelle;intracellular organelle;intracellular organelle lumen;intracellular organelle part;intracellular part;membrane;membrane-bounded organelle;membrane-enclosed lumen;mitochondrial inner membrane;mitochondrial matri		Homo sapiens	1	20
cell part;centrosome;chromatin remodeling complex;cytoplasm;cytoplasmic part;cytoskeletal part;cytoskeleton;histone deacetylase complex;intermediate filament cytoskeleton;intracellular membrane-bounded organelle;intracellular non-membrane-bounded organelle;intracellular organelle;intracellular organelle part;intracellular		Homo sapiens	1	20
cell part;cell surface;cytoplasmic part;endoplasmic reticulum membrane;endoplasmic reticulum part;endoplasmic reticulum-Golgi intermediate compartment;endoplasmic reticulum-Golgi intermediate compartment membran	Protein processing in endoplasmic reticulum	Homo sapiens	1	20
cell part;intracellular organelle part;intracellular part;nuclear body;nuclear part;nuclear speck;nucleoplasm;nucleoplasm part;organelle part	Spliceosome	Homo sapiens	1	20
cell part;cytoplasm;cytoplasmic part;extracellular membrane-bounded organelle;extracellular organelle;extracellular region part;extracellular vesicular exosome;Golgi apparatus;Golgi apparatus part;Golgi membrane;intracellular membrane-bounded organelle;intracellular organelle;intracellular organelle part;intracellular part;mem		Homo sapiens	1	20
cell part;Cul3-RING ubiquitin ligase complex;cullin-RING ubiquitin ligase complex;cytoplasmic part;cytoskeletal part;cytosol;extracellular membrane-bounded organelle;extracellular organelle;extracellular region part;extracell	Ubiquitin mediated proteolysis	Homo sapiens	1	20
cell part;cytoplasmic part;endoplasmic reticulum;endoplasmic reticulum membrane;endoplasmic reticulum part;extracellular membrane-bounded organelle;extracellular organelle;extracellular region part;extracellular vesicula	N-Glycan biosynthesis;Protein processing in endoplasmic reticulum	Homo sapiens	1	20
cell part;cytoplasm;intracellular membrane-bounded organelle;intracellular organelle;intracellular organelle part;intracellular part;membrane-bounded organelle;nuclear part;nucleoplasm;nucleus;organelle;organelle part	Ubiquitin mediated proteolysis	Homo sapiens	1	20
cell cortex part;cell part;cell projection membrane;cell projection part;centrosome;cortical cytoskeleton;cortical microtubule cytoskeleton;cytoplasm;cytoplasmic microtubule;cytoplasmic part;cytoskeletal part;cytoskeleton;cytosol;Golgi apparatus;intracellular membrane-bounded organelle;intracellular non-membrane-bounded o		Homo sapiens	1	20
cell part;cytoplasmic part;endoplasmic reticulum;endoplasmic reticulum membrane;endoplasmic reticulum part;integral to membrane;intracellular membrane-bounded organelle;intracellular organelle;intracellular organelle pa	Steroid biosynthesis	Homo sapiens	1	20
cell part;cytoplasmic part;cytosol;intracellular organelle part;intracellular part;macromolecular complex;nuclear part;nucleoplasm;organelle part;proteasome accessory complex;proteasome complex;protein complex		Homo sapiens	1	20
cell part;integral to membrane;intracellular membrane-bounded organelle;intracellular organelle;intracellular organelle part;intracellular part;intrinsic to membrane;membrane;membrane part;membrane-bounded organelle;nuclear inner membrane;nuclear membrane;nuclear part;nucleus;organelle;organelle inner membrane;organe		Homo sapiens	1	20
cell part;chromosomal part;extracellular membrane-bounded organelle;extracellular organelle;extracellular region;extracellular region part;extracellular vesicular exosome;intracellular membrane-bounded organelle;intracellula	Systemic lupus erythematosus	Homo sapiens	1	20
cell cortex;cell cortex part;cell part;centrosomal corona;centrosome;chromosomal part;condensed chromosome kinetochore;cortical cytoskeleton;cortical microtubule cytoskeleton;cytoplasmic microtubule;cytoplasmic part;cytoskeletal part;cytoskeleton;cytosol;extracellular membrane-bounded organelle;extracellular organelle;e		Homo sapiens	1	20
catalytic step 2 spliceosome;cell part;cytoplasmic part;cytosol;extracellular membrane-bounded organelle;extracellular organelle;extracellular region part;extracellular vesicular exosome;intracellular membrane-bounded orga	mRNA surveillance pathway;RNA transport;Spliceosome	Homo sapiens	1	20
cell part;cytoplasm;intracellular part		Homo sapiens	1	20
axon;cell junction;cell part;cell projection;cytoplasm;cytoplasmic part;cytoskeletal part;cytosol;dendrite;extracellular membrane-bounded organelle;extracellular organelle;extracellular region part;extracellular vesicular exosome;Golgi apparatus;intracellular membrane-bounded organelle;intracellular organelle;intracellular organell		Homo sapiens	1	20
cell part;cytoplasmic part;endoplasmic reticulum;endoplasmic reticulum part;integral to membrane;intracellular membrane-bounded organelle;intracellular organelle;intracellular organelle part;intracellular part;intrinsic to mem	N-Glycan biosynthesis;Protein processing in endoplasmic reticulum;Various types of N-glycan biosynthe	Homo sapiens	1	20
cell part;chromatin;chromosomal part;cytoplasm;intracellular organelle part;intracellular part;macromolecular complex;membrane;nuclear chromatin;nuclear chromosome part;nuclear part;organelle part;protein complex		Homo sapiens	1	20
cell part;intracellular membrane-bounded organelle;intracellular organelle;intracellular organelle part;intracellular part;membrane-bounded organelle;nuclear part;nucleoplasm;nucleus;organelle;organelle part		Homo sapiens	1	20
cell part;chromosomal part;intracellular membrane-bounded organelle;intracellular non-membrane-bounded organelle;intracellular organelle;intracellular organelle part;intracellular part;macromolecular complex;membrane-bounded organelle;non-membrane-bounded organelle;nuclear part;nucleolus;nucleosome;nucleus;organell		Homo sapiens	1	20
cell part;cytoplasmic part;cytosol;extracellular membrane-bounded organelle;extracellular organelle;extracellular region part;extracellular vesicular exosome;intracellular part;membrane-bounded organelle;membrane-bounded vesicle;organelle;vesicle		Homo sapiens	1	20
cell part;cytoplasm;intracellular membrane-bounded organelle;intracellular organelle;intracellular organelle part;intracellular part;macromolecular complex;membrane;membrane-bounded organelle;nuclear part;nucleoplasm;nucleus;organelle;organelle part;ribonucleoprotein complex;spliceosomal complex		Homo sapiens	1	20
cell part;cytoplasmic part;extracellular membrane-bounded organelle;extracellular organelle;extracellular region part;extracellular vesicular exosome;intracellular organelle part;intracellular part;lysosomal membrane;membran	Chemokine signaling pathway	Homo sapiens	1	20
adherens junction;anchoring junction;cell junction;cell part;cell-substrate adherens junction;cell-substrate junction;cytoplasm;cytoplasmic membrane-bounded vesicle;cytoplasmic part;cytoplasmic vesicle;endocytic vesicle;e	Endocytosis	Homo sapiens	1	20
cell part;COPI vesicle coat;cytoplasmic part;cytoplasmic vesicle part;cytosol;Golgi apparatus part;intracellular organelle part;intracellular part;macromolecular complex;membrane coat;membrane part;organelle part;protein complex;vesicle coat		Homo sapiens	1	20
adherens junction;anchoring junction;cell junction;cell part;cell-substrate adherens junction;cell-substrate junction;focal adhesion;integral to membrane;intrinsic to membrane;membrane;membrane part	ko05152;Phagosome	Homo sapiens	1	20
		Homo sapiens	1	20
		Homo sapiens	1	20
cell part;cytoplasmic part;extracellular membrane-bounded organelle;extracellular organelle;extracellular region part;extracellular vesicular exosome;intracellular membrane-bounded organelle;intracellular organelle;intracellul	Calcium signaling pathway;Huntington's disease;Parkinson's disease	Homo sapiens	1	20
cell part;cytoplasmic part;cytosol;extracellular membrane-bounded organelle;extracellular organelle;extracellular region part;extracellular vesicular exosome;intracellular part;membrane-bounded organelle;membrane-bounde	mTOR signaling pathway	Homo sapiens	1	20
cell part;cytoplasm;cytoskeletal part;intracellular membrane-bounded organelle;intracellular non-membrane-bounded organelle;intracellular organelle;intracellular organelle part;intracellular part;membrane-bounded organelle	Ribosome biogenesis in eukaryotes	Homo sapiens	1	20
actin cytoskeleton;apical part of cell;cell junction;cell part;cell projection;cell surface;cell-cell junction;centrosome;chloride channel complex;cytoplasm;cytoplasmic part;cytoplasmic vesicle membrane;cytoplasmic vesicle part;cytoskeletal part;cytoskeleton;cytosol;extracellular membrane-bounded organelle;extracellular organell		Homo sapiens	1	20
cell part;cytoplasm;cytoplasmic part;cytosol;intracellular membrane-bounded organelle;intracellular organelle;intracellular organelle part;intracellular part;macromolecular complex;membrane-bounded organelle;methylosome	Cell cycle - yeast;RNA transport	Homo sapiens	1	21
Cajal body;catalytic step 2 spliceosome;cell part;cytoplasm;cytoplasmic part;cytosol;Golgi apparatus;intracellular membrane-bounded organelle;intracellular organelle;intracellular organelle part;intracellular part;macromolecu	Spliceosome	Homo sapiens	1	21
cell part;cytoplasm;intracellular organelle part;intracellular part;membrane;nuclear body;nuclear matrix;nuclear part;nuclear speck;nucleoplasm;nucleoplasm part;organelle part	Spliceosome	Homo sapiens	1	21
adherens junction;anchoring junction;caveola;cell junction;cell part;cell-substrate adherens junction;cell-substrate junction;centriolar satellite;cytoplasmic membrane-bounded vesicle;cytoplasmic part;cytoplasmic vesicle;cyto	Insulin signaling pathway	Homo sapiens	1	21
cell part;cytoplasmic part;cytosol;intracellular membrane-bounded organelle;intracellular organelle;intracellular organelle lumen;intracellular organelle part;intracellular part;membrane-bounded organelle;membrane-enclosed l	Alanine, aspartate and glutamate metabolism;Arginine and proline metabolism;D-Glutamine and D-gluta	Homo sapiens	1	21
blood microparticle;cell part;cytoplasmic membrane-bounded vesicle lumen;cytoplasmic part;cytoplasmic vesicle part;cytosol;extracellular membrane-bounded organelle;extracellular organelle;extracellular region;extracellula	Complement and coagulation cascades	Homo sapiens	1	21
adherens junction;alphav-beta3 integrin complex;alphav-beta3 integrin-vitronectin complex;anchoring junction;cell junction;cell part;cell projection membrane;cell projection part;cell surface;cell-substrate adherens junction;c	Arrhythmogenic right ventricular cardiomyopathy (ARVC);Dilated cardiomyopathy;ECM-receptor interact	Homo sapiens	1	21
adherens junction;anchoring junction;cell junction;cell part;cell surface;cell-substrate adherens junction;cell-substrate junction;external side of plasma membrane;extracellular membrane-bounded organelle;extracellular orga	African trypanosomiasis;Cell adhesion molecules (CAMs);Leukocyte transendothelial migration;Malaria;I	Homo sapiens	1	21
adherens junction;anchoring junction;axon;brush border;cell junction;cell part;cell projection;cell projection part;cell-substrate adherens junction;cell-substrate junction;clathrin-coated vesicle;coated vesicle;cytoplasm;cytopla	Alzheimer's disease;Hematopoietic cell lineage;Protein digestion and absorption;Renin-angiotensin syst	Homo sapiens	1	21
cell part;cell projection membrane;cell projection part;cilium membrane;cilium part;clathrin coat;clathrin coat of coated pit;clathrin coat of trans-Golgi network vesicle;clathrin vesicle coat;cytoplasm;cytoplasmic part;cytoplas	Bacterial invasion of epithelial cells;Endocrine and other factor-regulated calcium reabsorption;Endocyto	Homo sapiens	1	21
cell part;cytoplasm;cytoplasmic part;cytosol;intracellular membrane-bounded organelle;intracellular organelle;intracellular part;membrane-bounded organelle;nucleus;organelle	Drug metabolism - other enzymes;Pyrimidine metabolism	Homo sapiens	1	21
actin cytoskeleton;actin filament;actin filament bundle;adherens junction;anchoring junction;cell junction;cell part;cell projection;cell projection membrane;cell projection part;cell-substrate adherens junction;cell-substrate junction;cytoplasm;cytoplasmic part;cytoskeletal part;cytoskeleton;cytosol;extracellular membrane-boundec		Homo sapiens	1	21
adherens junction;AMP-activated protein kinase complex;anchoring junction;cAMP-dependent protein kinase complex;cell junction;cell part;cell-substrate adherens junction;cell-substrate junction;centrosome;cytoplasm;cyto	Apoptosis;Insulin signaling pathway	Homo sapiens	1	21
cell part;cytoplasmic part;extracellular membrane-bounded organelle;extracellular organelle;extracellular region part;extracellular vesicular exosome;fumarate reductase complex;intracellular membrane-bounded organelle;int	Alzheimer's disease;Citrate cycle (TCA cycle);Huntington's disease;Oxidative phosphorylation;Parkinsor	Homo sapiens	1	21
cell part;cytoplasmic part;cytosol;extracellular membrane-bounded organelle;extracellular organelle;extracellular region part;extracellular vesicular exosome;intracellular membrane-bounded organelle;intracellular organelle;int	Protein processing in endoplasmic reticulum	Homo sapiens	1	21
cell part;cytoplasm;intracellular membrane-bounded organelle;intracellular organelle;intracellular organelle part;intracellular part;membrane-bounded organelle;nuclear part;nucleoplasm;nucleus;organelle;organelle part		Homo sapiens	1	21
cell part;cytoplasm;cytoplasmic part;endoplasmic reticulum;intracellular membrane-bounded organelle;intracellular organelle;intracellular organelle part;intracellular part;membrane-bounded organelle;nuclear part;nucleoplasm;nucleus;organelle;organelle part;perinuclear region of cytoplasm		Homo sapiens	1	21
cell part;cytoplasm;cytoplasmic part;cytosol;cytosolic part;cytosolic proteasome complex;inclusion body;intracellular membrane-bounded organelle;intracellular organelle;intracellular organelle part;intracellular part;macromo	Proteasome	Homo sapiens	1	21
cell part;cytoplasm;intracellular membrane-bounded organelle;intracellular organelle;intracellular organelle part;intracellular part;membrane-bounded organelle;nuclear body;nuclear part;nuclear speck;nucleoplasm;nucleoplasm part;nucleus;organelle;organelle part		Homo sapiens	1	21
cell part;cytoplasm;cytoplasmic part;cytosol;intracellular part;membrane;plasma membrane	RNA transport	Homo sapiens	1	21
cell part;cytoplasmic part;endoplasmic reticulum;extracellular region part;extracellular space;intracellular membrane-bounded organelle;intracellular organelle;intracellular part;membrane-bounded organelle;nucleus;organelle;perinuclear region of cytoplasm		Homo sapiens	1	21
axon;cell division site part;cell part;cell projection;cleavage furrow;cytoplasmic membrane-bounded vesicle;cytoplasmic part;cytoplasmic vesicle;cytoplasmic vesicle membrane;cytoplasmic vesicle part;cytoskeletal part;cyto	Endocrine and other factor-regulated calcium reabsorption;Endocytosis;Pancreatic secretion;Vasopress	Homo sapiens	0.9992	21
cell part;cytoplasm;cytoplasmic part;cytoplasmic vesicle membrane;cytoplasmic vesicle part;cytosol;cytosolic small ribosomal subunit;endocytic vesicle membrane;endosomal part;endosome membrane;extracellular membr	Ribosome	Homo sapiens	1	21
cell junction;cell part;cell projection part;cytoplasm;cytoskeleton;extracellular membrane-bounded organelle;extracellular organelle;extracellular region part;extracellular vesicular exosome;growth cone;intracellular membrane-bounded organelle;intracellular non-membrane-bounded organelle;intracellular organelle;intracellular org		Homo sapiens	1	21
cell part;cytoplasm;Grb2-Sos complex;intracellular membrane-bounded organelle;intracellular organelle;intracellular organelle part;intracellular part;macromolecular complex;membrane;membrane part;membrane-bounded organelle;nuclear part;nucleoplasm;nucleus;organelle;organelle part;plasma membrane part;protein compl		Homo sapiens	1	21
cell part;cell projection;cytoplasm;cytoplasmic part;cytosol;extracellular membrane-bounded organelle;extracellular organelle;extracellular region part;extracellular vesicular exosome;intracellular part;membrane;membrane-bounded organelle;membrane-bounded vesicle;organelle;plasma membrane;ruffle;vesicle		Homo sapiens	1	21
cell part;cytoplasmic part;cytosol;envelope;intracellular organelle part;intracellular part;macromolecular complex;membrane part;nuclear envelope;nuclear part;nuclear pore;Nup107-160 complex;organelle envelope;organelle	RNA transport	Homo sapiens	1	21
cell part;centrosome;cytoplasm;cytoplasmic dynein complex;cytoplasmic part;cytoskeletal part;cytosol;dynein complex;intracellular non-membrane-bounded organelle;intracellular organelle;intracellular organelle part;intracel	Phagosome;Vasopressin-regulated water reabsorption	Homo sapiens	1	21
cell part;COPII vesicle coat;cytoplasmic part;cytoplasmic vesicle part;endomembrane system;endoplasmic reticulum membrane;endoplasmic reticulum part;endoplasmic reticulum-Golgi intermediate compartment membrane	Protein processing in endoplasmic reticulum	Homo sapiens	1	21
apical plasma membrane;azurophil granule;cell part;cytolytic granule;cytoplasmic membrane-bounded vesicle;cytoplasmic part;cytoplasmic vesicle;cytosol;extracellular membrane-bounded organelle;extracellular organelle;extracellular region part;extracellular vesicular exosome;intracellular membrane-bounded organelle;intrace		Homo sapiens	1	21
cell part;cytoplasm;cytoplasmic part;extracellular membrane-bounded organelle;extracellular organelle;extracellular region part;extracellular vesicular exosome;intracellular membrane-bounded organelle;intracellular organelle;intracellular organelle lumen;intracellular organelle part;intracellular part;membrane-bounded organelle;r		Homo sapiens	1	21
cell part;cytoplasm;cytoplasmic part;endoplasmic reticulum;intracellular membrane-bounded organelle;intracellular organelle;intracellular part;membrane-bounded organelle;nucleus;organelle;perinuclear region of cytoplasm		Homo sapiens	1	21
cell part;cytoplasmic part;endoplasmic reticulum membrane;endoplasmic reticulum part;integral to membrane;intracellular membrane-bounded organelle;intracellular organelle;intracellular organelle part;intracellular part;intrinsic to membrane;membrane;membrane part;membrane-bounded organelle;organelle;organelle membrar		Homo sapiens	1	21
cell part;cytoplasmic part;endoplasmic reticulum;endoplasmic reticulum membrane;endoplasmic reticulum part;integral to membrane;intracellular membrane-bounded organelle;intracellular organelle;intracellular organelle part;intracellular part;intrinsic to membrane;membrane;membrane part;membrane-bounded organelle;organ		Homo sapiens	1	21
cell part;cytoplasm;cytoskeleton;intracellular non-membrane-bounded organelle;intracellular organelle;intracellular part;non-membrane-bounded organelle;organelle		Homo sapiens	1	21
cell part;cytoplasm;intracellular membrane-bounded organelle;intracellular non-membrane-bounded organelle;intracellular organelle;intracellular organelle part;intracellular part;membrane-bounded organelle;non-membrane-bounded organelle;nuclear body;nuclear part;nuclear speck;nucleolus;nucleoplasm;nucleoplasm part;nuc		Homo sapiens	1	21
cell part;cytoplasm;intracellular part;membrane		Homo sapiens	1	21
actin cytoskeleton;cell part;chromatin;chromosomal part;chromosome;chromosome, centromeric region;cytoplasmic part;cytoskeleton;cytosol;intermediate filament cytoskeleton;intracellular membrane-bounded organelle;in	Cell cycle;Cell cycle - yeast;Meiosis - yeast	Homo sapiens	1	21
cell leading edge;cell part;cis-Golgi network;cytoplasmic part;endoplasmic reticulum lumen;endoplasmic reticulum part;endoplasmic reticulum-Golgi intermediate compartment;Golgi apparatus;Golgi apparatus part;Golgi membrane;Golgi stack;intracellular membrane-bounded organelle;intracellular organelle;intracellular organel		Homo sapiens	1	21
adherens junction;anchoring junction;cell junction;cell part;cell-substrate adherens junction;cell-substrate junction;cytoplasm;focal adhesion;intracellular membrane-bounded organelle;intracellular organelle;intracellular part;membrane;membrane-bounded organelle;nucleus;organelle;plasma membrane		Homo sapiens	1	21
cell part;cytosolic large ribosomal subunit;intracellular organelle part;intracellular part;large ribosomal subunit;macromolecular complex;organelle part;ribonucleoprotein complex	Ribosome	Homo sapiens	1	21
adherens junction;anchoring junction;cell junction;cell part;cell projection;cell-cell junction;cell-substrate adherens junction;cell-substrate junction;cytoplasmic part;cytoplasmic vesicle membrane;cytoplasmic vesicle part;cyto	Endocytosis	Homo sapiens	1	21
cell part;cell surface;cytoplasmic part;endoplasmic reticulum lumen;endoplasmic reticulum membrane;endoplasmic reticulum part;endoplasmic reticulum-Golgi intermediate compartment;extracellular membrane-bounded organelle;extracellular organelle;extracellular region part;extracellular vesicular exosome;intracellular memb		Homo sapiens	1	21
cell part;cytoplasm;cytoplasmic part;Golgi apparatus;intracellular membrane-bounded organelle;intracellular non-membrane-bounded organelle;intracellular organelle;intracellular organelle part;intracellular part;membrane;me	Ribosome biogenesis in eukaryotes	Homo sapiens	1	21
cell part;cytoplasm;extracellular membrane-bounded organelle;extracellular organelle;extracellular region part;extracellular vesicular exosome;intracellular part;membrane;membrane-bounded organelle;membrane-bounded vesicle;organelle;plasma membrane;vesicle		Homo sapiens	1	21
cell part;cytoplasmic part;extracellular membrane-bounded organelle;extracellular organelle;extracellular region part;extracellular vesicular exosome;intracellular organelle lumen;intracellular organelle part;intracellular part;me	Oxidative phosphorylation	Homo sapiens	1	21
cell part;intracellular membrane-bounded organelle;intracellular non-membrane-bounded organelle;intracellular organelle;intracellular organelle part;intracellular part;membrane-bounded organelle;non-membrane-bounded organelle;nuclear part;nucleolus;nucleus;organelle;organelle part		Homo sapiens	1	21
cell part;cytoplasm;cytoplasmic part;extracellular membrane-bounded organelle;extracellular organelle;extracellular region part;extracellular vesicular exosome;intracellular membrane-bounded organelle;intracellular organelle;intracellular organelle part;intracellular part;membrane;membrane-bounded organelle;membrane-bound		Homo sapiens	1	21
cell part;cytoplasm;intracellular membrane-bounded organelle;intracellular organelle;intracellular part;membrane-bounded organelle;nucleus;organelle		Homo sapiens	1	21
basement membrane;cell part;chromosome;extracellular matrix part;extracellular region part;extracellular space;intracellular membrane-bounded organelle;intracellular non-membrane-bounded organelle;intracellular organelle;intracellular organelle part;intracellular part;membrane;membrane-bounded organelle;non-membrane-b		Homo sapiens	1	21
cell part;cytoplasmic part;endoplasmic reticulum;endoplasmic reticulum part;integral to endoplasmic reticulum membrane;integral to membrane;integral to organelle membrane;intracellular membrane-bounded organelle;intracellular organelle;intracellular organelle part;intracellular part;intrinsic to endoplasmic reticulum membrar		Homo sapiens	1	21
cell part;cytoplasm;cytoplasmic part;cytoskeleton;cytosol;intracellular membrane-bounded organelle;intracellular non-membrane-bounded organelle;intracellular organelle;intracellular part;membrane-bounded organelle;non-membrane-bounded organelle;nucleus;organelle		Homo sapiens	1	21
cell part;cytoplasm;cytoplasmic part;cytosol;intracellular membrane-bounded organelle;intracellular organelle;intracellular part;membrane-bounded organelle;nucleus;organelle		Homo sapiens	1	22
cell part;cytoplasm;intracellular membrane-bounded organelle;intracellular organelle;intracellular organelle part;intracellular part;membrane-bounded organelle;nuclear part;nucleoplasm;nucleus;organelle;organelle part		Homo sapiens	1	22
cell part;centrosome;cytoplasmic dynein complex;cytoplasmic part;cytoskeletal part;cytosol;dynein complex;intracellular non-membrane-bounded organelle;intracellular organelle;intracellular organelle part;intracellular part;m	Phagosome;Vasopressin-regulated water reabsorption	Homo sapiens	1	22

Cajal body;cell part;cytoplasm;intracellular membrane-bounded organelle;intracellular organelle;intracellular organelle part;intracellular part;macromolecular complex;membrane-bounded organelle;nuclear body;nuclear part;	Spliceosome	Homo sapiens	1	22
cell part;cytoplasmic part;cytosol;endoplasmic reticulum;extracellular membrane-bounded organelle;extracellular organelle;extracellular region part;extracellular vesicular exosome;intracellular membrane-bounded organelle;	Cysteine and methionine metabolism	Homo sapiens	1	22
axolemma;axon;axon part;basolateral plasma membrane;brush border;cell body;cell part;cell projection;cell projection membrane;cell projection part;cytoplasmic part;cytoplasmic vesicle;cytoskeletal part;endoplasmic reticulum;endosome;extracellular membrane-bounded organelle;extracellular organelle;extracellular region par		Homo sapiens	1	22
cell part;cytoplasmic part;integral to membrane;intracellular membrane-bounded organelle;intracellular organelle;intracellular organelle part;intracellular part;intrinsic to membrane;membrane;membrane part;membrane-bounded organelle;mitochondrial inner membrane;mitochondrial membrane;mitochondrial part;mitochondrion;		Homo sapiens	1	22
cell part;cell surface;coated vesicle membrane;cytoplasmic part;cytoplasmic vesicle membrane;cytoplasmic vesicle part;early endosome membrane;endocytic vesicle membrane;endoplasmic reticulum;endoplasmic reticulum	Allograft rejection;Antigen processing and presentation;Autoimmune thyroid disease;Cell adhesion mole	Homo sapiens	1	22
cell part;centrosome;cytoplasm;cytoplasmic part;cytoskeletal part;cytosol;extracellular membrane-bounded organelle;extracellular organelle;extracellular region part;extracellular vesicular exosome;intracellular membrane-bo	Cell cycle;Gap junction;Oocyte meiosis;p53 signaling pathway;Progesterone-mediated oocyte maturati	Homo sapiens	1	22
cell part;cytoplasmic part;extracellular membrane-bounded organelle;extracellular organelle;extracellular region part;extracellular vesicular exosome;intracellular membrane-bounded organelle;intracellular organelle;intracellular organelle lumen;intracellular organelle part;intracellular part;membrane-bounded organelle;membrane-		Homo sapiens	1	22
cell junction;cell part;cytoplasm;cytoplasmic part;Golgi apparatus;Golgi apparatus part;Golgi membrane;intracellular membrane-bounded organelle;intracellular non-membrane-bounded organelle;intracellular organelle;intracellular organelle part;intracellular part;membrane;membrane-bounded organelle;non-membrane-bounded		Homo sapiens	1	22
cell part;cytoplasm;cytoplasmic part;cytosol;extracellular membrane-bounded organelle;extracellular organelle;extracellular region part;extracellular vesicular exosome;intracellular part;membrane;membrane-bounded organe	Alanine, aspartate and glutamate metabolism;Purine metabolism	Homo sapiens	1	22
astral microtubule;axon;cell body;cell cortex;cell leading edge;cell part;cell projection;cell projection part;centrosome;chromosomal part;cilium;cytoplasmic microtubule;cytoplasmic part;cytoskeletal part;cytosol;envelope;ext	Ether lipid metabolism	Homo sapiens	1	22
adherens junction;anchoring junction;cell junction;cell part;cell-substrate adherens junction;cell-substrate junction;cytoplasm;cytoplasmic part;cytosol;cytosolic small ribosomal subunit;extracellular membrane-bounded orga	Ribosome	Homo sapiens	1	22
cell part;cytoplasm;extracellular membrane-bounded organelle;extracellular organelle;extracellular region part;extracellular vesicular exosome;intracellular membrane-bounded organelle;intracellular organelle;intracellular part;membrane;membrane-bounded organelle;membrane-bounded vesicle;nucleus;organelle;vesicle		Homo sapiens	1	22
cell part;coated vesicle membrane;contractile fiber part;cytoplasmic part;cytoplasmic vesicle membrane;cytoplasmic vesicle part;endoplasmic reticulum membrane;endoplasmic reticulum part;endoplasmic reticulum-Golgi in	Protein processing in endoplasmic reticulum	Homo sapiens	1	22
cell part;cytoplasmic part;intracellular membrane-bounded organelle;intracellular organelle;intracellular organelle lumen;intracellular organelle part;intracellular part;membrane-bounded organelle;membrane-enclosed lumen;	Citrate cycle (TCA cycle)	Homo sapiens	1	22
cell part;cytoplasmic part;cytosol;extracellular membrane-bounded organelle;extracellular organelle;extracellular region part;extracellular vesicular exosome;intracellular part;membrane-bounded organelle;membrane-bounde	Porphyrin and chlorophyll metabolism	Homo sapiens	1	22
cell part;coated vesicle membrane;COPII vesicle coat;cytoplasmic part;cytoplasmic vesicle membrane;cytoplasmic vesicle part;cytosol;endoplasmic reticulum membrane;endoplasmic reticulum part;ER to Golgi transport ves	Protein processing in endoplasmic reticulum	Homo sapiens	1	22
cell part;cytoplasm;cytoplasmic part;cytosol;cytosolic small ribosomal subunit;extracellular membrane-bounded organelle;extracellular organelle;extracellular region part;extracellular vesicular exosome;intracellular organelle	Ribosome	Homo sapiens	1	22
cell part;chromosomal part;chromosome;extracellular membrane-bounded organelle;extracellular organelle;extracellular region;extracellular region part;extracellular vesicular exosome;intracellular membrane-bounded organe	Systemic lupus erythematosus	Homo sapiens	1	22
cell part;cytoplasm;cytoplasmic part;cytosol;extracellular membrane-bounded organelle;extracellular organelle;extracellular region part;extracellular vesicular exosome;intracellular part;membrane-bounded organelle;membra	Drug metabolism - cytochrome P450;Glutathione metabolism;Metabolism of xenobiotics by cytochrome	Homo sapiens	1	22
cell part;cytoplasmic part;cytosol;cytosolic large ribosomal subunit;intracellular non-membrane-bounded organelle;intracellular organelle;intracellular organelle part;intracellular part;large ribosomal subunit;macromolecular co	Ribosome	Homo sapiens	1	22
Bcl3/NF-kappaB2 complex;cell part;cytoplasm;cytoplasmic part;cytosol;I-kappaB/NF-kappaB complex;intracellular membrane-bounded organelle;intracellular organelle;intracellular organelle part;intracellular part;macromole	MAPK signaling pathway;Osteoclast differentiation;Pathways in cancer	Homo sapiens	1	22
cell part;cell projection;cilium;cytoplasmic part;cytosol;extracellular membrane-bounded organelle;extracellular organelle;extracellular region part;extracellular space;extracellular vesicular exosome;intracellular organelle part;	Fructose and mannose metabolism	Homo sapiens	1	22
adherens junction;anchoring junction;cell junction;cell part;cell-substrate adherens junction;cell-substrate junction;cytoplasm;cytoplasmic part;cytosol;cytosolic large ribosomal subunit;focal adhesion;intracellular membrane-	Ribosome	Homo sapiens	1	22
cell part;extracellular region part;extracellular space;intracellular non-membrane-bounded organelle;intracellular organelle;intracellular organelle part;intracellular part;non-membrane-bounded organelle;nuclear part;nucleolus;nucleoplasm;organelle;organelle part		Homo sapiens	1	22
cell part;cytoplasm;cytoplasmic part;endoplasmic reticulum;endoplasmic reticulum lumen;endoplasmic reticulum part;endoplasmic reticulum Sec complex;extracellular membrane-bounded organelle;extracellular organelle;ex	Protein processing in endoplasmic reticulum	Homo sapiens	1	22
cell part;Cul2-RING ubiquitin ligase complex;cullin-RING ubiquitin ligase complex;cytoplasmic part;cytosol;intracellular organelle part;intracellular part;macromolecular complex;nuclear part;nucleoplasm;organelle part;protein	Pathways in cancer;Renal cell carcinoma;Ubiquitin mediated proteolysis	Homo sapiens	1	22
cell part;intracellular membrane-bounded organelle;intracellular organelle;intracellular organelle part;intracellular part;membrane-bounded organelle;nuclear part;nucleoplasm;nucleus;organelle;organelle part		Homo sapiens	1	22
adherens junction;anchoring junction;cell junction;cell part;cell-substrate adherens junction;cell-substrate junction;cytoplasm;cytoplasmic part;cytosol;extracellular membrane-bounded organelle;extracellular organelle;extracellular region part;extracellular vesicular exosome;focal adhesion;intracellular membrane-bounded organe		Homo sapiens	1	22
cell junction;cell part;clathrin coated vesicle membrane;clathrin-coated vesicle;coated vesicle;coated vesicle membrane;cytoplasmic membrane-bounded vesicle;cytoplasmic part;cytoplasmic vesicle;cytoplasmic vesicle mer	Endocytosis;Vasopressin-regulated water reabsorption	Homo sapiens	1	22
cell part;intracellular membrane-bounded organelle;intracellular organelle;intracellular organelle part;intracellular part;membrane-bounded organelle;nuclear matrix;nuclear part;nucleoplasm;organelle;organelle part		Homo sapiens	1	22
cell part;cytoplasm;cytoplasmic mRNA processing body;cytoplasmic part;cytosol;intracellular membrane-bounded organelle;intracellular non-membrane-bounded organelle;intracellular organelle;intracellular organelle part;int	RNA degradation	Homo sapiens	1	22
cell part;cytoplasmic part;intracellular membrane-bounded organelle;intracellular organelle;intracellular organelle lumen;intracellular organelle part;intracellular part;membrane-bounded organelle;membrane-enclosed lumen;	Aminoacyl-tRNA biosynthesis	Homo sapiens	1	22
apical plasma membrane;cell junction;cell part;cell-cell junction;cytoplasm;cytoplasmic part;endoplasmic reticulum;endoplasmic reticulum membrane;endoplasmic reticulum part;integral to membrane;intercellular canaliculus;intracellular membrane-bounded organelle;intracellular non-membrane-bounded organelle;intracellular		Homo sapiens	1	22
actin filament bundle;actomyosin;adherens junction;anchoring junction;cell junction;cell part;cell projection;cell-substrate adherens junction;cell-substrate junction;cytoplasm;cytoplasmic part;cytoskeletal part;cytosol;filopodi	Regulation of actin cytoskeleton	Homo sapiens	1	22
cell part;cell projection;cilium;cytoplasm;cytoplasmic part;cytosol;extracellular membrane-bounded organelle;extracellular organelle;extracellular region part;extracellular vesicular exosome;intracellular membrane-bounded organelle;intracellular organelle;intracellular part;membrane-bounded organelle;membrane-bounded vesic		Homo sapiens	1	22
cell part;cytoplasmic part;endoplasmic reticulum;integral to membrane;intracellular membrane-bounded organelle;intracellular organelle;intracellular part;intrinsic to membrane;membrane;membrane part;membrane-bounded organelle;organelle		Homo sapiens	1	22
cell part;chromosome;cytoplasm;intracellular non-membrane-bounded organelle;intracellular organelle;intracellular organelle part;intracellular part;non-membrane-bounded organelle;nuclear part;nucleoplasm;organelle;organelle part		Homo sapiens	1	22
cell part;cytoplasm;cytoplasmic part;early endosome membrane;endosomal part;endosome membrane;intracellular membrane-bounded organelle;intracellular organelle;intracellular organelle part;intracellular part;membrane;	Endocytosis	Homo sapiens	1	22
cell part;cytoplasm;intracellular membrane-bounded organelle;intracellular organelle;intracellular part;membrane-bounded organelle;nucleus;organelle		Homo sapiens	1	22
cell part;cytoplasm;extracellular membrane-bounded organelle;extracellular organelle;extracellular region part;extracellular vesicular exosome;intracellular membrane-bounded organelle;intracellular non-membrane-bounded	Benzoate degradation;Butanoate metabolism;Carbon fixation pathways in prokaryotes;Fatty acid metab	Homo sapiens	1	22
apoptosome;cell part;cytoplasm;cytoplasmic part;cytoskeleton;cytosolic part;envelope;extracellular membrane-bounded organelle;extracellular organelle;extracellular region;extracellular region part;extracellular vesicular exosome;intracellular membrane-bounded organelle;intracellular non-membrane-bounded organelle;intracel		Homo sapiens	1	22
cell part;cell projection;cell projection part;ciliary rootlet;cilium part;cytoplasmic part;cytoskeletal part;cytosol;intracellular organelle part;intracellular part;kinesin complex;kinesin I complex;macromolecular complex;membrane;microtubule;microtubule associated complex;neuron projection;organelle part;protein complex		Homo sapiens	1	22
cell part;cytoplasmic part;early endosome membrane;endoplasmic reticulum;endosomal part;endosome membrane;extracellular membrane-bounded organelle;extracellular organelle;extracellular region part;extracellular vesi	Endocytosis	Homo sapiens	1	22
cell part;cytoplasmic part;integral to membrane;intracellular membrane-bounded organelle;intracellular organelle;intracellular organelle part;intracellular part;intrinsic to membrane;membrane;membrane part;membrane-bounded organelle;mitochondrial inner membrane;mitochondrial membrane;mitochondrial part;mitochondrion;		Homo sapiens	1	22
cell part;cytoplasmic mRNA processing body;cytoplasmic part;cytosol;extracellular region part;extracellular space;intracellular non-membrane-bounded organelle;intracellular organelle;intracellular part;macromolecular complex;non-membrane-bounded organelle;organelle;ribonucleoprotein complex;RNA granule		Homo sapiens	1	22
cell part;cytoplasm;cytoplasmic part;cytosol;extracellular membrane-bounded organelle;extracellular organelle;extracellular region part;extracellular vesicular exosome;intracellular part;membrane-bounded organelle;membra	Amino sugar and nucleotide sugar metabolism	Homo sapiens	1	22
cell part;centrosome;cytoplasm;cytoplasmic part;cytoskeletal part;extracellular membrane-bounded organelle;extracellular organelle;extracellular region part;extracellular vesicular exosome;intracellular non-membrane-bounded organelle;intracellular organelle;intracellular organelle part;intracellular part;membrane;membrane-bo		Homo sapiens	1	22
cell part;intracellular non-membrane-bounded organelle;intracellular organelle;intracellular organelle part;intracellular part;macromolecular complex;non-membrane-bounded organelle;nuclear part;nucleolus;organelle;organell	Ribosome biogenesis in eukaryotes	Homo sapiens	1	22
cell part;intracellular organelle part;intracellular part;nuclear part;nucleoplasm;organelle part		Homo sapiens	1	22
cell part;cytoplasm;intracellular membrane-bounded organelle;intracellular organelle;intracellular organelle part;intracellular part;macromolecular complex;membrane part;membrane-bounded organelle;nuclear part;nuclear pore;nucleus;organelle;organelle part;pore complex;protein complex		Homo sapiens	1	22
cell cortex;cell junction;cell part;cell projection;clathrin coated vesicle membrane;coated vesicle membrane;cytoplasm;cytoplasmic part;cytoplasmic vesicle membrane;cytoplasmic vesicle part;cytoskeletal part;cytosol;dendrite;extracellular membrane-bounded organelle;extracellular organelle;extracellular region part;extracellula		Homo sapiens	1	22
cell part;chromosome;cytoplasm;cytoplasmic part;cytoskeleton;cytosol;Golgi apparatus part;Golgi stack;intermediate filament cytoskeleton;intracellular non-membrane-bounded organelle;intracellular organelle;intracellular o	Protein processing in endoplasmic reticulum	Homo sapiens	1	22
cell part;cytoplasmic part;eukaryotic 43S preinitiation complex;eukaryotic 48S preinitiation complex;eukaryotic translation initiation factor 3 complex;fibrillar center;intracellular organelle part;intracellular part;macromolecular complex;membrane;nuclear part;nucleolar part;nucleoplasm;organelle part;protein complex;ribonucleopr		Homo sapiens	1	22
cell part;cytoplasmic exosome (RNase complex);cytoplasmic part;cytosol;exosome (RNase complex);intracellular organelle part;intracellular part;macromolecular complex;membrane;nuclear exosome (RNase complex);nucle	RNA degradation	Homo sapiens	1	22
Barr body;cell part;chromosome;intracellular non-membrane-bounded organelle;intracellular organelle;intracellular organelle part;intracellular part;non-membrane-bounded organelle;nuclear chromosome;nuclear part;organelle;organelle part;sex chromosome;X chromosome		Homo sapiens	1	23
cell body;cell part;cell projection;cytoplasm;cytoplasmic part;cytosol;extracellular membrane-bounded organelle;extracellular organelle;extracellular region part;extracellular vesicular exosome;intracellular membrane-bounde	Biosynthesis of unsaturated fatty acids	Homo sapiens	1	23
AP-3 adaptor complex;AP-type membrane coat adaptor complex;cell part;clathrin coated vesicle membrane;coated vesicle membrane;cytoplasmic part;cytoplasmic vesicle membrane;cytoplasmic vesicle part;Golgi apparatu	Lysosome	Homo sapiens	1	23
adherens junction;anchoring junction;blood microparticle;cell junction;cell part;cell-substrate adherens junction;cell-substrate junction;cytoplasm;cytoplasmic membrane-bounded vesicle;cytoplasmic part;cytoplasmic vesicle;cytoskeleton;cytosol;endoplasmic reticulum membrane;endoplasmic reticulum part;extracellular membr		Homo sapiens	1	23
cell part;cytoplasm;extracellular membrane-bounded organelle;extracellular organelle;extracellular region part;extracellular vesicular exosome;intracellular membrane-bounded organelle;intracellular organelle;intracellular part;membrane-bounded organelle;membrane-bounded vesicle;nucleus;organelle;vesicle		Homo sapiens	1	23
cell part;cytoplasm;extracellular membrane-bounded organelle;extracellular organelle;extracellular region part;extracellular vesicular exosome;intracellular membrane-bounded organelle;intracellular organelle;intracellular organelle part;intracellular part;macromolecular complex;membrane-bounded organelle;membrane-bounded		Homo sapiens	1	23
cell part;cytoplasmic part;cytosol;eukaryotic 43S preinitiation complex;eukaryotic 48S preinitiation complex;eukaryotic translation initiation factor 3 complex;eukaryotic translation initiation factor 3 complex, eIF3m;intracellula	RNA transport	Homo sapiens	1	23
cell part;cytoplasmic membrane-bounded vesicle;cytoplasmic part;cytoplasmic vesicle;endoplasmic reticulum;endoplasmic reticulum lumen;endoplasmic reticulum part;extracellular region;Golgi apparatus;intracellular membrane-bounded organelle;intracellular organelle;intracellular organelle lumen;intracellular organelle part;int		Homo sapiens	1	23
cell part;cytoplasm;intracellular membrane-bounded organelle;intracellular organelle;intracellular organelle part;intracellular part;membrane;membrane-bounded organelle;nuclear part;nucleoplasm;nucleus;organelle;organelle	Ubiquitin mediated proteolysis	Homo sapiens	1	23
cell part;cytoplasmic part;cytosol;extracellular membrane-bounded organelle;extracellular organelle;extracellular region part;extracellular vesicular exosome;intracellular part;membrane-bounded organelle;membrane-bounde	Sulfur metabolism	Homo sapiens	1	23
adherens junction;anchoring junction;cell junction;cell part;cell-substrate adherens junction;cell-substrate junction;cytoplasm;cytoplasmic part;cytosol;cytosolic large ribosomal subunit;extracellular membrane-bounded orga	Ribosome	Homo sapiens	1	23
cell part;cytoplasmic part;endoplasmic reticulum membrane;endoplasmic reticulum part;integral to membrane;intracellular organelle part;intracellular part;intrinsic to membrane;membrane;membrane part;organelle membrane	Bile secretion;Metabolism of xenobiotics by cytochrome P450	Homo sapiens	1	23
cell part;cytoplasmic membrane-bounded vesicle;cytoplasmic part;cytoplasmic vesicle;extracellular membrane-bounded organelle;extracellular organelle;extracellular region;extracellular region part;extracellular space;extrac	Antigen processing and presentation;Lysosome	Homo sapiens	1	23
cell part;cytoplasmic part;endoplasmic reticulum;endoplasmic reticulum membrane;endoplasmic reticulum part;extracellular membrane-bounded organelle;extracellular organelle;extracellular region part;extracellular vesicula	Protein export	Homo sapiens	1	23
adherens junction;anchoring junction;cell junction;cell part;cell-substrate adherens junction;cell-substrate junction;cytoplasmic part;cytosol;cytosolic small ribosomal subunit;extracellular membrane-bounded organelle;extrac	Ribosome	Homo sapiens	1	23
90S preribosome;cell part;cytoplasm;cytoplasmic part;cytosol;cytosolic small ribosomal subunit;extracellular membrane-bounded organelle;extracellular organelle;extracellular region part;extracellular vesicular exosome;intra	Ribosome	Homo sapiens	1	23
cell part;cytoplasm;cytoplasmic membrane-bounded vesicle;cytoplasmic part;cytoplasmic vesicle;extracellular membrane-bounded organelle;extracellular organelle;extracellular region part;extracellular vesicular exosome;G	Methane metabolism	Homo sapiens	1	23
axon;cell part;cell projection;cytoplasmic part;extracellular membrane-bounded organelle;extracellular organelle;extracellular region part;extracellular vesicular exosome;intracellular membrane-bounded organelle;intracellular	beta-Alanine metabolism;Fatty acid metabolism;PPAR signaling pathway;Propanoate metabolism;Valine	Homo sapiens	1	23
cell part;cytoplasm;extracellular membrane-bounded organelle;extracellular organelle;extracellular region part;extracellular vesicular exosome;intracellular organelle part;intracellular part;membrane;membrane-bounded organelle;membrane-bounded vesicle;nuclear membrane;nuclear part;organelle;organelle membrane;organelle		Homo sapiens	1	23
axon part;axon terminus;cell part;cell projection part;cytoplasm;cytoplasmic part;cytosol;endoplasmic reticulum;extracellular membrane-bounded organelle;extracellular organelle;extracellular region part;extracellular vesicula	Alanine, aspartate and glutamate metabolism;Arginine and proline metabolism;Nitrogen metabolism;Tw	Homo sapiens	1	23
adherens junction;alphav-beta5 integrin complex;anchoring junction;cell junction;cell leading edge;cell part;cell surface;cell-substrate adherens junction;cell-substrate junction;cytoplasmic membrane-bounded vesicle;cytopla	Arrhythmogenic right ventricular cardiomyopathy (ARVC);Dilated cardiomyopathy;ECM-receptor interac	Homo sapiens	1	23
cell part;cytoplasm;cytoplasmic part;cytosol;eukaryotic translation initiation factor 2 complex;intracellular part;macromolecular complex;protein complex	RNA transport	Homo sapiens	1	23
cell part;cell projection part;cytoplasm;cytoplasmic part;cytosol;extracellular membrane-bounded organelle;extracellular organelle;extracellular region part;extracellular vesicular exosome;flagellar fibrous sheath;flagellum part	Drug metabolism - cytochrome P450;Glutathione metabolism;Metabolism of xenobiotics by cytochrome	Homo sapiens	1	23
cell part;cytoplasmic part;cytosol;cytosolic small ribosomal subunit;intracellular membrane-bounded organelle;intracellular organelle;intracellular organelle part;intracellular part;macromolecular complex;membrane;membrane	Ribosome	Homo sapiens	1	23
cell part;cytoplasm;cytoplasmic part;cytosol;extracellular membrane-bounded organelle;extracellular organelle;extracellular region part;extracellular vesicular exosome;intracellular membrane-bounded organelle;intracellular	Proteasome	Homo sapiens	1	23
cell part;chromosomal part;DNA replication factor A complex;germ cell nucleus;intracellular membrane-bounded organelle;intracellular organelle;intracellular organelle part;intracellular part;lateral element;macromolecular co	DNA replication;Homologous recombination;Mismatch repair;Nucleotide excision repair	Homo sapiens	1	23
cell part;cytoplasm;cytoplasmic part;cytosol;extracellular membrane-bounded organelle;extracellular organelle;extracellular region part;extracellular vesicular exosome;intracellular part;membrane-bounded organelle;membrane-bounded vesicle;organelle;vesicle		Homo sapiens	1	23
adherens junction;anchoring junction;cell junction;cell part;cell-substrate adherens junction;cell-substrate junction;cytoplasm;cytoplasmic part;cytosol;cytosolic large ribosomal subunit;focal adhesion;intracellular membrane-	Ribosome	Homo sapiens	1	23
cell part;cytoplasm;cytoplasmic part;cytosol;extracellular membrane-bounded organelle;extracellular organelle;extracellular region part;extracellular space;extracellular vesicular exosome;intracellular part;macromolecular co	Amoebiasis	Homo sapiens	1	23
cell part;cytoplasm;cytoplasmic part;cytosol;endoplasmic reticulum;inclusion body;intracellular membrane-bounded organelle;intracellular organelle;intracellular organelle part;intracellular part;isoamylase complex;macromole	Starch and sucrose metabolism	Homo sapiens	1	23
adherens junction;anchoring junction;cell junction;cell part;cell-substrate adherens junction;cell-substrate junction;cytoplasmic part;cytosol;focal adhesion;intracellular membrane-bounded organelle;intracellular organelle;intr	RNA transport	Homo sapiens	1	23
cell part;cytoplasmic part;extracellular membrane-bounded organelle;extracellular organelle;extracellular region part;extracellular vesicular exosome;intracellular membrane-bounded organelle;intracellular organelle;intracellular organelle lumen;intracellular organelle part;intracellular part;membrane-bounded organelle;membrane-		Homo sapiens	1	23
cell part;cytoplasmic part;cytosol;intracellular part	Nicotinate and nicotinamide metabolism	Homo sapiens	1	23
cell part;cytoplasm;cytoplasmic part;cytosol;extracellular membrane-bounded organelle;extracellular organelle;extracellular region part;extracellular vesicular exosome;intracellular;intracellular membrane-bounded organelle;	Arachidonic acid metabolism;Metabolism of xenobiotics by cytochrome P450;Steroid hormone biosynt	Homo sapiens	1	23
cell part;cytoplasm;cytoplasmic part;cytosol;cytosolic large ribosomal subunit;extracellular membrane-bounded organelle;extracellular organelle;extracellular region part;extracellular vesicular exosome;intracellular non-mem	Ribosome	Homo sapiens	1	23
cell part;clathrin-coated vesicle;coated vesicle;cytoplasmic membrane-bounded vesicle;cytoplasmic part;cytoplasmic vesicle;cytosol;endoplasmic reticulum;endoplasmic reticulum membrane;endoplasmic reticulum part;end	Protein processing in endoplasmic reticulum	Homo sapiens	1	23
cell part;cytoplasm;cytoplasmic part;cytosol;cytosolic small ribosomal subunit;extracellular membrane-bounded organelle;extracellular organelle;extracellular region part;extracellular vesicular exosome;intracellular membrane	Ribosome	Homo sapiens	1	23
cell part;cytoplasm;cytoplasmic part;cytosol;extracellular membrane-bounded organelle;extracellular organelle;extracellular region part;extracellular vesicular exosome;extrinsic to internal side of plasma membrane;extrinsic t	Amoebiasis;Bile secretion;Calcium signaling pathway;Chagas disease (American trypanosomiasis);Dilat	Homo sapiens	1	23
cell part;centrosome;cytoplasm;cytoplasmic part;cytoskeletal part;extracellular membrane-bounded organelle;extracellular organelle;extracellular region part;extracellular vesicular exosome;extrinsic to internal side of plasma	Axon guidance;Chagas disease (American trypanosomiasis);Chemokine signaling pathway;Gap junctio	Homo sapiens	1	23
cell part;intracellular membrane-bounded organelle;intracellular organelle;intracellular organelle part;intracellular part;membrane-bounded organelle;nuclear part;nucleoplasm;nucleus;organelle;organelle part		Homo sapiens	1	23
cell part;cytoplasm;cytoplasmic part;cytosol;extracellular membrane-bounded organelle;extracellular organelle;extracellular region part;extracellular vesicular exosome;intracellular organelle part;intracellular part;membrane-b	PPAR signaling pathway	Homo sapiens	1	23
cell part;chromatin;chromosomal part;cytoplasm;exosome (RNase complex);intracellular membrane-bounded organelle;intracellular non-membrane-bounded organelle;intracellular organelle;intracellular organelle part;intracel	RNA degradation	Homo sapiens	1	23
cell part;cytoplasmic part;cytosol;intracellular organelle part;intracellular part;macromolecular complex;membrane;nuclear part;nucleoplasm;organelle part;protein complex	African trypanosomiasis;Alzheimer's disease;Amoebiasis;Calcium signaling pathway;Chagas disease (A	Homo sapiens	1	23
cell part;chromatin;chromosomal part;euchromatin;intracellular organelle part;intracellular part;macromolecular complex;nuclear chromatin;nuclear chromosome part;nuclear euchromatin;nuclear part;nucleosome;organelle part;protein-DNA complex		Homo sapiens	1	23
adherens junction;anchoring junction;axon;cell cortex;cell junction;cell part;cell projection;cell projection cytoplasm;cell projection part;cell-substrate adherens junction;cell-substrate junction;cytoplasm;cytoplasmic part;cyto	Acute myeloid leukemia;B cell receptor signaling pathway;Bladder cancer;Chemokine signaling pathwa	Homo sapiens	1	23
cell part;cytoplasm;cytoplasmic part;cytosol;intracellular membrane-bounded organelle;intracellular organelle;intracellular part;macromolecular complex;membrane-bounded organelle;mitochondrion;nucleus;organelle;protei	Adipocytokine signaling pathway;Chronic myeloid leukemia;Epithelial cell signaling in Helicobacter pylo	Homo sapiens	1	23
cell part;cytoplasm;intracellular membrane-bounded organelle;intracellular organelle;intracellular organelle part;intracellular part;macromolecular complex;membrane-bounded organelle;nuclear part;nucleus;organelle;organelle part;protein complex;signalosome		Homo sapiens	1	23
adherens junction;anchoring junction;cell junction;cell part;cell-substrate adherens junction;cell-substrate junction;cytoplasm;cytoplasmic part;cytosol;focal adhesion;intracellular part;presynaptic active zone;synapse part		Homo sapiens	1	23
adherens junction;anchoring junction;cell junction;cell part;cell-cell junction;cell-substrate adherens junction;cell-substrate junction;focal adhesion;integral to membrane;integral to plasma membrane;intrinsic to membrane;intrinsic to plasma membrane;membrane part;plasma membrane part		Homo sapiens	1	23
cell part;chromosomal part;condensin complex;cytoplasm;cytoplasmic part;cytosol;intracellular membrane-bounded organelle;intracellular organelle;intracellular organelle part;intracellular part;macromolecular complex;mem	Cell cycle - yeast	Homo sapiens	1	23
basal lamina;basement membrane;extracellular matrix part;extracellular membrane-bounded organelle;extracellular organelle;extracellular region;extracellular region part;extracellular vesicular exosome;laminin complex;lami	African trypanosomiasis;Amoebiasis;ECM-receptor interaction;Focal adhesion;Pathways in cancer;Sma	Homo sapiens	1	23
actin cytoskeleton;actomyosin;cell cortex;cell junction;cell part;cell projection;cell-cell junction;cytoplasm;cytoplasmic part;cytoskeletal part;cytoskeleton;dendrite;gap junction;intracellular non-membrane-bounded organelle;intracellular organelle;intracellular organelle part;intracellular part;membrane;neuron projection;non-mem		Homo sapiens	1	23
Cajal body;cell part;cytoplasm;intracellular membrane-bounded organelle;intracellular non-membrane-bounded organelle;intracellular organelle;intracellular organelle part;intracellular part;membrane;membrane-bounded orga	Spliceosome	Homo sapiens	1	23
cell part;cytoplasmic part;cytosol;endoplasmic reticulum;intracellular membrane-bounded organelle;intracellular organelle;intracellular organelle part;intracellular part;membrane;membrane part;membrane-bounded organelle;	Protein processing in endoplasmic reticulum	Homo sapiens	1	23
actin cytoskeleton;Arp2/3 protein complex;cell part;cytoplasmic part;cytoskeletal part;cytoskeleton;cytosol;extracellular membrane-bounded organelle;extracellular organelle;extracellular region part;extracellular vesicular ex	Bacterial invasion of epithelial cells;Fc gamma R-mediated phagocytosis;Pathogenic Escherichia coli in	Homo sapiens	1	23
cell part;cytoplasmic part;intracellular membrane-bounded organelle;intracellular organelle;intracellular organelle lumen;intracellular organelle part;intracellular part;macromolecular complex;membrane;membrane-bounded or	Citrate cycle (TCA cycle);Propanoate metabolism	Homo sapiens	1	23
cell part;chromatin;chromosomal part;chromosome;chromosome, centromeric region;cytoplasmic part;cytosol;intracellular membrane-bounded organelle;intracellular non-membrane-bounded organelle;intracellular organelle;intracellular organelle part;intracellular part;membrane-bounded organelle;non-membrane-bounded orga		Homo sapiens	1	23
cell part;cytoplasm;intracellular membrane-bounded organelle;intracellular organelle;intracellular part;membrane;membrane-bounded organelle;nucleus;organelle		Homo sapiens	1	23
cell part;cytoplasmic part;intracellular membrane-bounded organelle;intracellular organelle;intracellular part;membrane-bounded organelle;mitochondrion;organelle		Homo sapiens	1	23
cell part;chromosome;condensed chromosome;cytoplasm;intracellular membrane-bounded organelle;intracellular non-membrane-bounded organelle;intracellular organelle;intracellular organelle part;intracellular part;macromolecular complex;membrane;membrane-bounded organelle;non-membrane-bounded organelle;nuclear		Homo sapiens	1	24
box C/D snoRNP complex;cell part;cytoplasm;intracellular non-membrane-bounded organelle;intracellular organelle;intracellular organelle part;intracellular part;macromolecular complex;membrane;non-membrane-bounded	Ribosome biogenesis in eukaryotes	Homo sapiens	1	24
cell part;chromatin;chromosomal part;cytoplasmic part;cytosol;heterochromatin;intracellular membrane-bounded organelle;intracellular organelle;intracellular organelle part;intracellular part;membrane-bounded organelle;nuclear chromatin;nuclear chromosome part;nuclear heterochromatin;nuclear part;nuclear periphery;nucleop		Homo sapiens	1	24
cell part;cytoplasmic membrane-bounded vesicle;cytoplasmic part;cytoplasmic vesicle;extracellular membrane-bounded organelle;extracellular organelle;extracellular region;extracellular region part;extracellular space;extrac	ko05152;Lysosome	Homo sapiens	1	24
acrosomal vesicle;cell part;cytoplasmic membrane-bounded vesicle;cytoplasmic part;cytoplasmic vesicle;extracellular membrane-bounded organelle;extracellular organelle;extracellular region part;extracellular vesicular exos	Amino sugar and nucleotide sugar metabolism;Glycosaminoglycan degradation;Glycosphingolipid biosy	Homo sapiens	1	24
adherens junction;anchoring junction;blood microparticle;cell cortex;cell junction;cell part;cell projection;cell-substrate adherens junction;cell-substrate junction;cytoplasm;cytoplasmic part;cytoskeleton;cytosol;extracellular	Regulation of actin cytoskeleton;Shigellosis	Homo sapiens	1	24
cell part;collagen;collagen type I;cytoplasmic part;endoplasmic reticulum lumen;endoplasmic reticulum part;extracellular matrix;extracellular matrix part;extracellular membrane-bounded organelle;extracellular organelle;extra	Amoebiasis;ECM-receptor interaction;Focal adhesion;Protein digestion and absorption	Homo sapiens	1	24
adherens junction;alphav-beta3 integrin-vitronectin complex;anchoring junction;cell junction;cell part;cell projection;cell surface;cell-substrate adherens junction;cell-substrate junction;cytoplasm;external side of plasma mem	Arrhythmogenic right ventricular cardiomyopathy (ARVC);Bacterial invasion of epithelial cells;Dilated car	Homo sapiens	1	24
cell part;cytoplasmic part;extracellular membrane-bounded organelle;extracellular organelle;extracellular region part;extracellular vesicular exosome;intracellular membrane-bounded organelle;intracellular organelle;intracellula	Chloroalkane and chloroalkene degradation;Drug metabolism - cytochrome P450;Fatty acid metabolism	Homo sapiens	1	24
extracellular membrane-bounded organelle;extracellular organelle;extracellular region part;extracellular vesicular exosome;macromolecular complex;membrane-bounded organelle;membrane-bounded vesicle;organelle;prote	Pentose phosphate pathway;Purine metabolism	Homo sapiens	1	24
cell part;cytoplasmic part;cytosol;cytosolic large ribosomal subunit;extracellular membrane-bounded organelle;extracellular organelle;extracellular region part;extracellular vesicular exosome;intracellular membrane-bounded	Ribosome	Homo sapiens	1	24
cell part;cytoplasmic part;extracellular membrane-bounded organelle;extracellular organelle;extracellular region part;extracellular vesicular exosome;intracellular membrane-bounded organelle;intracellular organelle;intracellula	Alzheimer's disease;Cardiac muscle contraction;Huntington's disease;Oxidative phosphorylation;Parkin	Homo sapiens	1	24
cell part;centrosome;cytoplasm;cytoplasmic part;cytoskeletal part;endoplasmic reticulum;intracellular membrane-bounded organelle;intracellular non-membrane-bounded organelle;intracellular organelle;intracellular organelle	Base excision repair	Homo sapiens	1	24
adherens junction;anchoring junction;axon;caveola;cell junction;cell part;cell projection;cell projection cytoplasm;cell projection part;cell-substrate adherens junction;cell-substrate junction;cytoplasm;cytoplasmic part;cytoske	Acute myeloid leukemia;Adherens junction;Aldosterone-regulated sodium reabsorption;Alzheimer's dise	Homo sapiens	1	24
cell part;cytoplasmic part;cytosol;intracellular membrane-bounded organelle;intracellular organelle;intracellular part;membrane-bounded organelle;mitochondrion;organelle	Alanine, aspartate and glutamate metabolism;Purine metabolism	Homo sapiens	1	24
cell part;cytoplasmic part;intracellular membrane-bounded organelle;intracellular organelle;intracellular organelle lumen;intracellular organelle part;intracellular part;membrane-bounded organelle;membrane-enclosed lumen;	Arginine and proline metabolism;Ascorbate and aldarate metabolism;beta-Alanine metabolism;Chloroal	Homo sapiens	1	24

basement membrane;cell part;cytoplasmic membrane-bounded vesicle;cytoplasmic part;cytoplasmic vesicle;extracellular matrix part;extracellular region;extracellular region part;intracellular membrane-bounded organelle;int	ECM-receptor interaction;Focal adhesion;Malaria;Phagosome;TGF-beta signaling pathway	Homo sapiens	1	24
catalytic step 2 spliceosome;cell part;intracellular membrane-bounded organelle;intracellular non-membrane-bounded organelle;intracellular organelle;intracellular organelle part;intracellular part;macromolecular complex;mer	RNA degradation	Homo sapiens	1	24
cell part;cytoplasm;cytoplasmic part;intracellular membrane-bounded organelle;intracellular non-membrane-bounded organelle;intracellular organelle;intracellular organelle lumen;intracellular organelle part;intracellular part;m	Fatty acid metabolism	Homo sapiens	1	24
cell part;cytoplasm;cytoplasmic part;cytosol;intracellular membrane-bounded organelle;intracellular organelle;intracellular organelle part;intracellular part;membrane-bounded organelle;nuclear part;nucleoplasm;nucleus;orga	Long-term potentiation;MAPK signaling pathway;mTOR signaling pathway;Neurotrophin signaling pathw	Homo sapiens	1	24
cell part;cytoplasmic part;cytosol;extracellular membrane-bounded organelle;extracellular organelle;extracellular region part;extracellular vesicular exosome;intracellular organelle part;intracellular part;membrane;membrane-b	Purine metabolism	Homo sapiens	1	24
actin cytoskeleton;cell cortex part;cell part;cortical actin cytoskeleton;cortical cytoskeleton;cytoplasmic part;cytoskeletal part;cytoskeleton;extracellular membrane-bounded organelle;extracellular organelle;extracellular region part;extracellular vesicular exosome;intracellular non-membrane-bounded organelle;intracellular organe		Homo sapiens	1	24
adherens junction;anchoring junction;cell junction;cell part;cell-substrate adherens junction;cell-substrate junction;cytoplasmic part;cytosol;cytosolic large ribosomal subunit;extracellular membrane-bounded organelle;extrac	Ribosome	Homo sapiens	1	24
adherens junction;anchoring junction;cell junction;cell part;cell-substrate adherens junction;cell-substrate junction;cytoplasmic part;cytosol;cytosolic large ribosomal subunit;extracellular membrane-bounded organelle;extrac	Ribosome	Homo sapiens	1	24
cell part;centrosome;cytoplasm;cytoplasmic part;cytoskeletal part;cytosol;cytosolic large ribosomal subunit;intracellular non-membrane-bounded organelle;intracellular organelle;intracellular organelle part;intracellular part;lar	Ribosome	Homo sapiens	1	24
cell part;intracellular membrane-bounded organelle;intracellular organelle;intracellular part;membrane-bounded organelle;nucleus;organelle	Spliceosome	Homo sapiens	1	24
cell part;cytoplasmic part;extracellular membrane-bounded organelle;extracellular organelle;extracellular region part;extracellular vesicular exosome;integral to membrane;integral to plasma membrane;intracellular membrane-bounded organelle;intracellular organelle;intracellular organelle part;intracellular part;intrinsic to membra		Homo sapiens	1	24
cell part;cytoplasm;intracellular part		Homo sapiens	1	24
cell part;cytoplasmic part;cytosol;extracellular membrane-bounded organelle;extracellular organelle;extracellular region part;extracellular vesicular exosome;intracellular organelle part;intracellular part;macromolecular comple	Proteasome	Homo sapiens	1	24
actin cytoskeleton;cell cortex part;cell division site part;cell part;cell projection membrane;cell projection part;chromosomal part;ciliary transition zone;cilium membrane;cilium part;cleavage furrow;condensed chromosome kinetochore;cytoplasm;cytoplasmic part;cytoskeletal part;cytoskeleton;exocyst;extracellular membrane-bo		Homo sapiens	1	24
cell part;cytoplasm;cytoplasmic part;endoplasmic reticulum part;endoplasmic reticulum tubular network;extracellular membrane-bounded organelle;extracellular organelle;extracellular region part;extracellular vesicular exosome;intracellular organelle part;intracellular part;macromolecular complex;membrane-bounded organelle;r		Homo sapiens	1	24
cell part;cytoplasm;extracellular membrane-bounded organelle;extracellular organelle;extracellular region part;extracellular vesicular exosome;intracellular membrane-bounded organelle;intracellular organelle;intracellular orga	Spliceosome	Homo sapiens	1	24
cell part;cytoplasm;cytoplasmic part;cytosol;intracellular membrane-bounded organelle;intracellular organelle;intracellular organelle part;intracellular part;membrane;membrane-bounded organelle;nuclear part;nucleoplasm;nucleus;organelle;organelle part		Homo sapiens	1	24
cell part;cytoplasm;cytoskeletal part;intracellular non-membrane-bounded organelle;intracellular organelle;intracellular organelle part;intracellular part;macromolecular complex;microtubule;non-membrane-bounded organelle;organelle;organelle part;protein complex;spindle		Homo sapiens	1	24
cell part;cytoplasm;cytoplasmic part;integral to membrane;intracellular membrane-bounded organelle;intracellular organelle;intracellular organelle part;intracellular part;intrinsic to membrane;membrane;membrane part;membrane-bounded organelle;mitochondrial inner membrane;mitochondrial membrane;mitochondrial part;mitc		Homo sapiens	1	24
cell part;cytoplasmic part;intracellular membrane-bounded organelle;intracellular organelle;intracellular part;membrane;membrane-bounded organelle;mitochondrion;organelle	One carbon pool by folate	Homo sapiens	1	24
actin cytoskeleton;adherens junction;anchoring junction;cell junction;cell part;cell-substrate adherens junction;cell-substrate junction;cytoplasm;cytoskeleton;focal adhesion;intracellular non-membrane-bounded organelle;intracellular organelle;intracellular part;non-membrane-bounded organelle;organelle		Homo sapiens	1	24
cell part;cytoplasm;cytoplasmic part;cytosolic part;Golgi apparatus part;Golgi membrane;IkappaB kinase complex;intracellular organelle part;intracellular part;macromolecular complex;membrane;organelle membrane;organelle part;presynaptic active zone;presynaptic membrane;protein complex;synapse;synapse part;synaptic r		Homo sapiens	1	24
cell part;cytoplasm;cytoplasmic exosome (RNase complex);cytoplasmic part;exosome (RNase complex);intracellular membrane-bounded organelle;intracellular organelle;intracellular organelle part;intracellular part;macromole	Purine metabolism;Pyrimidine metabolism;RNA degradation	Homo sapiens	1	24
cell part;chromosomal part;condensed chromosome kinetochore;cytoplasmic part;cytosol;envelope;intracellular non-membrane-bounded organelle;intracellular organelle;intracellular organelle part;intracellular part;kinetocho	RNA transport	Homo sapiens	1	24
actin filament bundle;actomyosin;adherens junction;anchoring junction;cell junction;cell part;cell-cell junction;cell-substrate adherens junction;cell-substrate junction;cytoskeletal part;cytoskeleton;extracellular membrane-bounded organelle;extracellular organelle;extracellular region part;extracellular vesicular exosome;focal adhe		Homo sapiens	1	24
cell part;cytoplasm;intracellular membrane-bounded organelle;intracellular organelle;intracellular organelle part;intracellular part;macromolecular complex;membrane-bounded organelle;nuclear part;nucleoplasm;nucleus;organelle;organelle part;ribonucleoprotein complex		Homo sapiens	1	24
cell part;cytoplasm;intracellular part;macromolecular complex;membrane;plasma membrane;protein complex		Homo sapiens	1	24
cell part;cytoplasm;cytoplasmic part;cytosol;intracellular membrane-bounded organelle;intracellular organelle;intracellular organelle part;intracellular part;macromolecular complex;membrane-bounded organelle;nuclear part;r	RNA transport	Homo sapiens	1	24
beta-catenin destruction complex;cell body;cell part;cell projection;cytoplasm;cytoplasmic part;extracellular membrane-bounded organelle;extracellular organelle;extracellular region part;extracellular vesicular exosome;intrac	Wnt signaling pathway	Homo sapiens	1	24
cell part;cytoplasmic part;extracellular membrane-bounded organelle;extracellular organelle;extracellular region part;extracellular vesicular exosome;intracellular membrane-bounded organelle;intracellular organelle;intracellula	Citrate cycle (TCA cycle);Propanoate metabolism	Homo sapiens	1	24
cell part;cytoplasmic part;intracellular membrane-bounded organelle;intracellular non-membrane-bounded organelle;intracellular organelle;intracellular organelle lumen;intracellular organelle part;intracellular part;membrane;m	Ether lipid metabolism;Peroxisome	Homo sapiens	1	25
adherens junction;anchoring junction;cell junction;cell part;cell-substrate adherens junction;cell-substrate junction;cytoplasm;cytoskeleton;focal adhesion;intracellular non-membrane-bounded organelle;intracellular organelle;intracellular organelle part;intracellular part;macromolecular complex;non-membrane-bounded organelle		Homo sapiens	1	25
cell part;DSIF complex;intracellular membrane-bounded organelle;intracellular organelle;intracellular organelle part;intracellular part;macromolecular complex;membrane-bounded organelle;nuclear part;nucleoplasm;nucleoplasm part;nucleus;organelle;organelle part;protein complex;transcription elongation factor complex		Homo sapiens	1	25
actin cytoskeleton;adherens junction;anchoring junction;Arp2/3 protein complex;cell junction;cell leading edge;cell part;cell projection;cell-substrate adherens junction;cell-substrate junction;cytoplasm;cytoplasmic part;cytos	Bacterial invasion of epithelial cells;Fc gamma R-mediated phagocytosis;Pathogenic Escherichia coli inf	Homo sapiens	1	25
cell part;cytoplasm;cytoplasmic part;cytosol;endoplasmic reticulum;intracellular membrane-bounded organelle;intracellular non-membrane-bounded organelle;intracellular organelle;intracellular organelle part;intracellular part	Protein export	Homo sapiens	1	25
cell part;centrosome;cytoplasm;cytoplasmic membrane-bounded vesicle;cytoplasmic part;cytoplasmic vesicle;cytoskeletal part;extracellular membrane-bounded organelle;extracellular organelle;extracellular region part;extra	Axon guidance;Chagas disease (American trypanosomiasis);Chemokine signaling pathway;Gap junction	Homo sapiens	1	25
adherens junction;anchoring junction;cell junction;cell part;cell-substrate adherens junction;cell-substrate junction;extracellular membrane-bounded organelle;extracellular organelle;extracellular region part;extracellular vesicular exosome;focal adhesion;intracellular membrane-bounded organelle;intracellular organelle;intracellula		Homo sapiens	1	25
adherens junction;alpha3-beta1 integrin complex;anchoring junction;basolateral plasma membrane;cell junction;cell part;cell periphery;cell projection membrane;cell projection part;cell surface;cell-substrate adherens junctio	Arrhythmogenic right ventricular cardiomyopathy (ARVC);Dilated cardiomyopathy;ECM-receptor interact	Homo sapiens	1	25
cell part;cell projection;centrosome;chromosomal part;cytoplasm;cytoplasmic microtubule;cytoplasmic part;cytoplasmic vesicle membrane;cytoplasmic vesicle part;cytoskeletal part;cytoskeleton;cytosol;endosome;intermediate filament;intracellular membrane-bounded organelle;intracellular non-membrane-bounded organelle;ir		Homo sapiens	1	25
cell part;chromosomal part;intracellular membrane-bounded organelle;intracellular organelle;intracellular organelle part;intracellular part;macromolecular complex;membrane-bounded organelle;nuclear chromosome part;nucl	Systemic lupus erythematosus	Homo sapiens	1	25
cell part;cytoplasmic part;extracellular membrane-bounded organelle;extracellular organelle;extracellular region part;extracellular vesicular exosome;intracellular membrane-bounded organelle;intracellular organelle;intracellula	Alzheimer's disease;Huntington's disease;Oxidative phosphorylation;Parkinson's disease	Homo sapiens	1	25
extracellular membrane-bounded organelle;extracellular organelle;extracellular region part;extracellular vesicular exosome;membrane-bounded organelle;membrane-bounded vesicle;organelle;vesicle		Homo sapiens	1	25
apical plasma membrane;cell part;cell projection;cytoplasmic part;cytosol;extracellular membrane-bounded organelle;extracellular organelle;extracellular region part;extracellular vesicular exosome;integral to membrane;integ	Collecting duct acid secretion;Epithelial cell signaling in Helicobacter pylori infection;Oxidative phospho	Homo sapiens	1	25
cell part;chromatin;chromosomal part;cytoplasm;cytoplasmic part;cytosol;intracellular membrane-bounded organelle;intracellular organelle;intracellular organelle part;intracellular part;membrane;membrane-bounded organell	Acute myeloid leukemia;Adipocytokine signaling pathway;Chemokine signaling pathway;Hepatitis C;Jak	Homo sapiens	1	25
cell part;cytoplasmic part;extracellular membrane-bounded organelle;extracellular organelle;extracellular region part;extracellular vesicular exosome;intracellular membrane-bounded organelle;intracellular organelle;intracellula	Benzoate degradation;Fatty acid elongation in mitochondria;Fatty acid metabolism;Valine, leucine and is	Homo sapiens	1	25
cell part;chromosomal part;condensed chromosome kinetochore;cytoplasm;cytoplasmic part;cytoskeletal part;cytosol;intracellular non-membrane-bounded organelle;intracellular organelle;intracellular organelle part;intracellu	RNA transport	Homo sapiens	1	25
actin filament bundle;actomyosin;adherens junction;anchoring junction;cell cortex;cell junction;cell part;cell projection;cell-substrate adherens junction;cell-substrate junction;cytoplasm;cytoplasmic part;cytoskeletal part;cyto	Bacterial invasion of epithelial cells;Chemokine signaling pathway;Focal adhesion;Leukocyte transendot	Homo sapiens	1	25
cell part;contractile fiber part;costamere;cytoplasm;cytoplasmic part;intracellular non-membrane-bounded organelle;intracellular organelle;intracellular organelle part;intracellular part;macromolecular complex;membrane;non-membrane-bounded organelle;nuclear part;nucleolus;organelle;organelle part;polysome;ribonucleoprote		Homo sapiens	1	25
catalytic step 2 spliceosome;cell part;intracellular membrane-bounded organelle;intracellular organelle;intracellular organelle part;intracellular part;macromolecular complex;membrane;membrane-bounded organelle;nuclear part;nucleoplasm;nucleus;organelle;organelle part;ribonucleoprotein complex;spliceosomal complex		Homo sapiens	1	25
actin cytoskeleton;cell part;cytoplasmic part;cytoskeletal part;cytoskeleton;cytosol;extracellular membrane-bounded organelle;extracellular organelle;extracellular region;extracellular region part;extracellular vesicular exosome;F-actin capping protein complex;intracellular non-membrane-bounded organelle;intracellular organelle;		Homo sapiens	1	25
cell part;cytoplasmic part;cytosol;envelope;inclusion body;intracellular organelle part;intracellular part;macromolecular complex;membrane;membrane part;nuclear envelope;nuclear inclusion body;nuclear membrane;nuclear	RNA transport	Homo sapiens	1	25
cell part;cell surface;cytoplasm;cytoplasmic membrane-bounded vesicle;cytoplasmic part;cytoplasmic vesicle;extracellular membrane-bounded organelle;extracellular organelle;extracellular region part;extracellular vesicular exosome;intracellular membrane-bounded organelle;intracellular organelle;intracellular part;macromolecu		Homo sapiens	1	25
cell part;chromosomal part;extracellular region part;extracellular space;intracellular membrane-bounded organelle;intracellular organelle;intracellular organelle part;intracellular part;macromolecular complex;membrane-bounded organelle;nucleosome;nucleus;organelle;organelle part;protein-DNA complex		Homo sapiens	1	25
cell part;cytoplasmic part;cytosol;intracellular part;macromolecular complex;protein complex;ribose phosphate diphosphokinase complex	Pentose phosphate pathway;Purine metabolism	Homo sapiens	1	25
cell part;cytoplasmic membrane-bounded vesicle;cytoplasmic part;cytoplasmic vesicle;cytosol;early endosome;endoplasmic reticulum membrane;endoplasmic reticulum part;endosome;extracellular membrane-bounded organelle;extracellular organelle;extracellular region part;extracellular vesicular exosome;Golgi apparatus;Go		Homo sapiens	1	25
adherens junction;anchoring junction;cell body;cell junction;cell part;cell-substrate adherens junction;cell-substrate junction;cytoplasmic part;extracellular membrane-bounded organelle;extracellular organelle;extracellular reg	Chemokine signaling pathway	Homo sapiens	1	25
cell part;integral to membrane;intrinsic to membrane;membrane part		Homo sapiens	1	25
cell part;cytoplasm;cytoplasmic part;cytosol;endoplasmic reticulum;intracellular membrane-bounded organelle;intracellular organelle;intracellular organelle part;intracellular part;macromolecular complex;membrane-bounded organelle;nuclear part;nucleoplasm;nucleus;organelle;organelle part;perinuclear region of cytoplasm;prot		Homo sapiens	1	25
cell part;cytoplasmic part;cytoskeletal part;extracellular membrane-bounded organelle;extracellular organelle;extracellular region part;extracellular vesicular exosome;intracellular organelle part;intracellular part;macromolecula	Bacterial invasion of epithelial cells;Endocrine and other factor-regulated calcium reabsorption;Endocyto	Homo sapiens	1	25
actin filament bundle;actomyosin;adherens junction;anchoring junction;apical plasma membrane;cell cortex;cell junction;cell part;cell projection;cell-substrate adherens junction;cell-substrate junction;cytoplasm;cytoplasmic	Amoebiasis;Axon guidance;Bacterial invasion of epithelial cells;Chemokine signaling pathway;ErbB sign	Homo sapiens	1	25
cell part;external side of plasma membrane;extracellular membrane-bounded organelle;extracellular organelle;extracellular region;extracellular region part;extracellular space;extracellular vesicular exosome;extrinsic to membrane;extrinsic to plasma membrane;membrane;membrane part;membrane-bounded organelle;membrane		Homo sapiens	1	25
cell part;cytoplasmic part;extracellular membrane-bounded organelle;extracellular organelle;extracellular region part;extracellular vesicular exosome;intracellular membrane-bounded organelle;intracellular organelle;intracellula	Peroxisome	Homo sapiens	1	25
cell part;intracellular membrane-bounded organelle;intracellular organelle;intracellular organelle part;intracellular part;macromolecular complex;membrane-bounded organelle;nuclear part;nucleoplasm;nucleus;organelle;organelle part;ribonucleoprotein complex		Homo sapiens	1	25
cell part;cytoplasm;cytoplasmic part;cytosol;intracellular organelle part;intracellular part;membrane;nuclear part;nucleoplasm;organelle part;perinuclear region of cytoplasm;plasma membrane	Axon guidance;ErbB signaling pathway;Focal adhesion;MAPK signaling pathway;Regulation of actin cyt	Homo sapiens	1	25
cell part;cytoplasmic part;cytosol;eukaryotic 43S preinitiation complex;eukaryotic 48S preinitiation complex;eukaryotic translation initiation factor 3 complex;eukaryotic translation initiation factor 3 complex, eIF3m;extracellula	RNA transport	Homo sapiens	1	25
cell part;cytoplasm;cytoplasmic part;cytosol;extracellular membrane-bounded organelle;extracellular organelle;extracellular region part;extracellular vesicular exosome;intracellular part;membrane-bounded organelle;membra	Oxidative phosphorylation	Homo sapiens	1	25
cell part;cytoplasm;cytoplasmic part;cytoskeletal part;cytosol;intracellular membrane-bounded organelle;intracellular non-membrane-bounded organelle;intracellular organelle;intracellular organelle part;intracellular part;meml	Long-term potentiation;MAPK signaling pathway;mTOR signaling pathway;Neurotrophin signaling pathw	Homo sapiens	1	25
actin filament bundle;actomyosin;adherens junction;anchoring junction;cell junction;cell part;cell-cell adherens junction;cell-cell junction;cell-substrate adherens junction;cell-substrate junction;cytoplasm;cytoskeletal part;foc	Focal adhesion	Homo sapiens	1	25
cell part;cytoplasm;cytoplasmic part;intracellular membrane-bounded organelle;intracellular organelle;intracellular organelle lumen;intracellular organelle part;intracellular part;membrane;membrane-bounded organelle;membr	Butanoate metabolism;Caprolactam degradation;Fatty acid elongation in mitochondria;Fatty acid metab	Homo sapiens	1	25
cell part;cytoplasm;cytoplasmic part;cytoskeleton;cytosol;intracellular membrane-bounded organelle;intracellular non-membrane-bounded organelle;intracellular organelle;intracellular part;membrane;membrane-bounded organelle;non-membrane-bounded organelle;nucleus;organelle;plasma membrane		Homo sapiens	1	25
cell part;extracellular membrane-bounded organelle;extracellular organelle;extracellular region part;extracellular vesicular exosome;intracellular membrane-bounded organelle;intracellular organelle;intracellular part;membrane;membrane-bounded organelle;membrane-bounded vesicle;nucleus;organelle;vesicle		Homo sapiens	1	25
cell part;cytoplasmic part;intracellular organelle part;intracellular part;nuclear part;nucleoplasm;organelle part;perinuclear region of cytoplasm		Homo sapiens	1	25
adherens junction;anchoring junction;cell junction;cell part;cell-cell adherens junction;cell-cell junction;centrosome;cytoplasm;cytoplasmic part;cytoskeletal part;cytoskeleton;cytosol;endosomal part;endosome membrane;extracellular membrane-bounded organelle;extracellular organelle;extracellular region part;extracellular vesi		Homo sapiens	1	25
adherens junction;anchoring junction;axon;basal part of cell;cell junction;cell part;cell projection;cell projection part;cell-substrate adherens junction;cell-substrate junction;focal adhesion;growth cone;neuron projection;site of polarized growth		Homo sapiens	1	25
cell part;cytoplasm;intracellular membrane-bounded organelle;intracellular organelle;intracellular part;membrane;membrane-bounded organelle;nucleus;organelle		Homo sapiens	1	25
axon;cell body;cell part;cell projection;chromatin;chromosomal part;cytoplasm;cytoplasmic part;cytosol;extracellular membrane-bounded organelle;extracellular organelle;extracellular region part;extracellular vesicular exoso	Parkinson's disease	Homo sapiens	1	25
cell part;extracellular membrane-bounded organelle;extracellular organelle;extracellular region part;extracellular space;extracellular vesicular exosome;membrane;membrane-bounded organelle;membrane-bounded vesicle;or	Axon guidance	Homo sapiens	1	25
actin cytoskeleton;actin filament;actin filament bundle;actomyosin;adherens junction;anchoring junction;cell junction;cell leading edge;cell part;cell periphery;cell projection;cell-substrate adherens junction;cell-substrate junction;cytoplasm;cytoplasmic part;cytoskeletal part;cytoskeleton;cytosol;extracellular membrane-bounded		Homo sapiens	1	25
blood microparticle;cell part;cytoplasm;cytoplasmic part;eukaryotic translation initiation factor 2 complex;extracellular region part;extracellular space;intracellular part;macromolecular complex;protein complex		Homo sapiens	1	25
cell part;cytoplasm;cytoplasmic part;cytosol;extracellular membrane-bounded organelle;extracellular organelle;extracellular region part;extracellular vesicular exosome;intracellular part;membrane-bounded organelle;membrane-bounded vesicle;organelle;vesicle		Homo sapiens	1	25
actin filament bundle;actomyosin;axon;cell junction;cell part;cell projection;cell projection part;cytoplasm;cytoskeletal part;dendritic spine;extracellular membrane-bounded organelle;extracellular organelle;extracellular region part;extracellular vesicular exosome;intracellular organelle part;intracellular part;membrane-bounded org		Homo sapiens	1	25
cell part;cytoplasm;extracellular membrane-bounded organelle;extracellular organelle;extracellular region part;extracellular vesicular exosome;intracellular organelle part;intracellular part;membrane;membrane-bounded organelle;membrane-bounded vesicle;nuclear part;nucleoplasm;organelle;organelle part;plasma membrane;ve		Homo sapiens	1	25
cell part;cytoplasmic part;cytosol;intracellular membrane-bounded organelle;intracellular organelle;intracellular organelle part;intracellular part;macromolecular complex;membrane-bounded organelle;nuclear part;nucleoplasm	Ubiquitin mediated proteolysis	Homo sapiens	1	25
adherens junction;anchoring junction;caveola;cell junction;cell part;cell projection membrane;cell projection part;cell-cell junction;cell-substrate adherens junction;cell-substrate junction;cytoplasm;cytoplasmic part;cytoplasmic vesicle membrane;cytoplasmic vesicle part;cytoskeleton;cytosol;early endosome;endosomal part;endo		Homo sapiens	1	25
cell part;cytoplasm;cytoplasmic membrane-bounded vesicle;cytoplasmic part;cytoplasmic vesicle;early endosome membrane;endosomal part;endosome membrane;extrinsic to membrane;intracellular;intracellular membrane-bounded organelle;intracellular organelle;intracellular organelle part;intracellular part;macromolecular co		Homo sapiens	1	25
cell part;cytoplasmic part;cytosol;extracellular membrane-bounded organelle;extracellular organelle;extracellular region part;extracellular vesicular exosome;intracellular membrane-bounded organelle;intracellular organelle;int	Proteasome	Homo sapiens	1	25
CCR4-NOT complex;cell part;cytoplasmic mRNA processing body;cytoplasmic part;cytosol;extracellular region part;extracellular space;intracellular membrane-bounded organelle;intracellular non-membrane-bounded organe	RNA degradation	Homo sapiens	1	26
blood microparticle;brush border;cell part;cell projection;chloride channel complex;cytoplasm;cytoplasmic part;envelope;extracellular membrane-bounded organelle;extracellular organelle;extracellular region part;extracellular space;extracellular vesicular exosome;intracellular membrane-bounded organelle;intracellular organelle;		Homo sapiens	1	26
cell part;cytoplasm;cytoplasmic part;cytosol;extracellular membrane-bounded organelle;extracellular organelle;extracellular region part;extracellular vesicular exosome;intracellular membrane-bounded organelle;intracellular c	Proteasome	Homo sapiens	1	26
cell part;cell projection;cytoplasm;cytoplasmic part;cytoskeleton;cytosol;filopodium;Golgi apparatus;intracellular membrane-bounded organelle;intracellular non-membrane-bounded organelle;intracellular organelle;intracellula	Inositol phosphate metabolism;Phosphatidylinositol signaling system	Homo sapiens	1	26
cell part;cytoplasm;cytoplasmic part;cytosol;extracellular membrane-bounded organelle;extracellular organelle;extracellular region part;extracellular vesicular exosome;intracellular membrane-bounded organelle;intracellular organelle;intracellular part;membrane;membrane-bounded organelle;membrane-bounded vesicle;nucleus		Homo sapiens	1	26
90S preribosome;cell part;cytoplasm;intracellular non-membrane-bounded organelle;intracellular organelle;intracellular organelle part;intracellular part;macromolecular complex;non-membrane-bounded organelle;nuclear part;nucleolus;nucleoplasm;organelle;organelle part;preribosome;preribosome, small subunit precursor;ribor		Homo sapiens	1	26
cell part;cytoplasm;extracellular membrane-bounded organelle;extracellular organelle;extracellular region part;extracellular vesicular exosome;intracellular part;membrane;membrane-bounded organelle;membrane-bounded vesicle;organelle;plasma membrane;vesicle		Homo sapiens	1	26
cell part;cytoplasmic part;extracellular membrane-bounded organelle;extracellular organelle;extracellular region part;extracellular vesicular exosome;integral to membrane;intracellular membrane-bounded organelle;intracellular organelle;intracellular organelle part;intracellular part;intrinsic to membrane;macromolecular complex;r		Homo sapiens	1	26
cell part;cytoplasmic part;cytosol;intracellular part	Purine metabolism;Selenocompound metabolism;Sulfur metabolism	Homo sapiens	1	26
blood microparticle;cell part;extracellular membrane-bounded organelle;extracellular organelle;extracellular region;extracellular region part;extracellular space;extracellular vesicular exosome;membrane;membrane-bounded c	Chagas disease (American trypanosomiasis);Complement and coagulation cascades;ko05152;Leishmar	Homo sapiens	1	26
cell part;cytoplasm;cytoplasmic part;cytosol;extracellular membrane-bounded organelle;extracellular organelle;extracellular region part;extracellular space;extracellular vesicular exosome;intracellular organelle part;intracellula	Fructose and mannose metabolism;Galactose metabolism;Glycerolipid metabolism;Pentose and glucur	Homo sapiens	1	26
cell part;cytoplasmic part;cytosol;extracellular membrane-bounded organelle;extracellular organelle;extracellular region part;extracellular vesicular exosome;intracellular part;membrane-bounded organelle;membrane-bounde	Arachidonic acid metabolism	Homo sapiens	1	26
cell part;cytoplasmic part;endoplasmic reticulum membrane;endoplasmic reticulum part;intracellular membrane-bounded organelle;intracellular organelle;intracellular organelle part;intracellular part;membrane;membrane part;membrane-bounded organelle;mitochondrion;organelle;organelle membrane;organelle part		Homo sapiens	1	26
cell part;cytoplasm;extracellular membrane-bounded organelle;extracellular organelle;extracellular region part;extracellular space;extracellular vesicular exosome;intracellular part;membrane;membrane-bounded organelle;me	Amoebiasis	Homo sapiens	1	26
cell part;cell surface;cytoplasm;cytoplasmic part;early endosome;endosome;extracellular membrane-bounded organelle;extracellular organelle;extracellular region part;extracellular vesicular exosome;integral to membrane;integral to plasma membrane;intracellular membrane-bounded organelle;intracellular organelle;intracellular		Homo sapiens	1	26
cell part;contractile fiber;cytoplasmic part;intracellular membrane-bounded organelle;intracellular non-membrane-bounded organelle;intracellular organelle;intracellular part;membrane-bounded organelle;non-membrane-bounded organelle;nucleus;organelle		Homo sapiens	1	26
cell part;cytoplasm;extracellular region;intracellular part;membrane		Homo sapiens	1	26
adherens junction;anchoring junction;cell junction;cell part;cell-cell junction;cell-substrate adherens junction;cell-substrate junction;cytoplasmic part;cytosol;focal adhesion;intracellular part;membrane;perinuclear region of cytoplasm;plasma membrane		Homo sapiens	1	26
cell part;cytoplasm;cytoplasmic part;cytosol;extracellular membrane-bounded organelle;extracellular organelle;extracellular region part;extracellular vesicular exosome;intracellular membrane-bounded organelle;intracellular c	Arginine and proline metabolism;Ascorbate and aldarate metabolism;beta-Alanine metabolism;Fatty aci	Homo sapiens	1	26
cell part;cytoplasm;cytoplasmic part;cytosol;eukaryotic 43S preinitiation complex;eukaryotic 48S preinitiation complex;eukaryotic translation initiation factor 3 complex;extracellular membrane-bounded organelle;extracellular	Hepatitis C;RNA transport	Homo sapiens	1	26
adherens junction;anchoring junction;cell junction;cell part;cell-substrate adherens junction;cell-substrate junction;cytoplasmic part;cytosol;cytosolic small ribosomal subunit;extracellular membrane-bounded organelle;extrac	Ribosome	Homo sapiens	1	26
cell body;cell part;cell projection;cell projection part;cilium part;cytoplasmic part;dendrite;extracellular membrane-bounded organelle;extracellular organelle;extracellular region part;extracellular vesicular exosome;extrinsic to	Chemokine signaling pathway;Phototransduction;Taste transduction	Homo sapiens	1	26
cell part;cytoplasmic part;cytoskeleton;endoplasmic reticulum-Golgi intermediate compartment;extracellular membrane-bounded organelle;extracellular organelle;extracellular region part;extracellular space;extracellular vesicular exosome;Golgi apparatus;intracellular membrane-bounded organelle;intracellular non-membrane-bo		Homo sapiens	1	26
cell part;cell projection part;centrosome;chromosomal part;cytoplasm;cytoplasmic part;cytoskeletal part;cytosol;dynactin complex;dynein complex;extracellular membrane-bounded organelle;extracellular organelle;extracellu	Huntington's disease;Vasopressin-regulated water reabsorption	Homo sapiens	1	26
cell part;chromosomal part;chromosome;condensed chromosome;condensin complex;condensin core heterodimer;cytoplasm;cytoplasmic part;cytosol;intracellular membrane-bounded organelle;intracellular non-membrane-	Cell cycle - yeast	Homo sapiens	1	26
cell part;extracellular region part;extracellular space;intracellular organelle part;intracellular part;macromolecular complex;multimeric ribonuclease P complex;nuclear part;nucleolar part;nucleolar ribonuclease P complex;orga	Ribosome biogenesis in eukaryotes;RNA transport	Homo sapiens	1	26
cell part;cytoplasm;cytoplasmic part;cytoskeleton;cytosol;extracellular membrane-bounded organelle;extracellular organelle;extracellular region part;extracellular vesicular exosome;intracellular non-membrane-bounded organelle;intracellular organelle;intracellular organelle part;intracellular part;membrane;membrane-bounded or		Homo sapiens	1	26
cell part;cytoplasmic part;endoplasmic reticulum membrane;endoplasmic reticulum part;endoplasmic reticulum-Golgi intermediate compartment;extracellular membrane-bounded organelle;extracellular organelle;extracellular region part;extracellular vesicular exosome;Golgi apparatus;Golgi apparatus part;Golgi membrane;intrac		Homo sapiens	1	26
cell part;cytoplasm;intracellular non-membrane-bounded organelle;intracellular organelle;intracellular organelle part;intracellular part;membrane;non-membrane-bounded organelle;nuclear part;nucleolus;organelle;organelle part		Homo sapiens	1	26
ATP-binding cassette (ABC) transporter complex;cell part;cytoplasmic part;envelope;intracellular organelle part;intracellular part;macromolecular complex;membrane;membrane part;mitochondrial envelope;mitochondrial part;organelle envelope;organelle part;protein complex		Homo sapiens	1	26
catalytic step 2 spliceosome;cell part;intracellular membrane-bounded organelle;intracellular organelle;intracellular organelle part;intracellular part;macromolecular complex;membrane-bounded organelle;nuclear part;nucleus;organelle;organelle part;ribonucleoprotein complex;spliceosomal complex		Homo sapiens	1	26
cell part;cytoplasm;cytoplasmic part;cytosol;extracellular membrane-bounded organelle;extracellular organelle;extracellular region part;extracellular vesicular exosome;intracellular part;membrane-bounded organelle;membra	Glycine, serine and threonine metabolism;Methane metabolism;Vitamin B6 metabolism	Homo sapiens	1	26
cell part;cytoplasm;intracellular membrane-bounded organelle;intracellular non-membrane-bounded organelle;intracellular organelle;intracellular organelle part;intracellular part;macromolecular complex;membrane;membrane-bounded organelle;non-membrane-bounded organelle;nuclear part;nucleolus;nucleus;organelle;organel		Homo sapiens	1	27
cell part;cytoplasmic part;cytosol;eukaryotic translation initiation factor 4F complex;intracellular part;macromolecular complex;protein complex	RNA transport;Viral myocarditis	Homo sapiens	1	27
cell part;cytoplasm;intracellular membrane-bounded organelle;intracellular organelle;intracellular organelle part;intracellular part;macromolecular complex;membrane-bounded organelle;nuclear part;nucleoplasm;nucleus;orga	Spliceosome	Homo sapiens	1	27
axoneme part;cell part;cell projection part;cytoplasm;cytoplasmic part;cytosol;extracellular membrane-bounded organelle;extracellular organelle;extracellular region part;extracellular vesicular exosome;intracellular part;memb	Purine metabolism	Homo sapiens	1	27
cell part;cytoplasmic part;intracellular membrane-bounded organelle;intracellular organelle;intracellular organelle lumen;intracellular organelle part;intracellular part;membrane-bounded organelle;membrane-enclosed lumen;m	Arginine and proline metabolism	Homo sapiens	1	27
cell part;cell surface;cytoplasm;cytoplasmic part;cytosol;cytosolic part;cytosolic proteasome complex;extracellular region part;extracellular space;intracellular membrane-bounded organelle;intracellular organelle;intracellular	Alzheimer's disease	Homo sapiens	1	27
cell part;chromatin;chromosomal part;extracellular membrane-bounded organelle;extracellular organelle;extracellular region part;extracellular vesicular exosome;heterochromatin;intracellular organelle part;intracellular part;macromolecular complex;membrane-bounded organelle;membrane-bounded vesicle;nuclear chromatin;nu		Homo sapiens	1	27
axon part;axon terminus;cell part;cell projection part;cytoplasm;cytoplasmic part;cytosol;extracellular membrane-bounded organelle;extracellular organelle;extracellular region part;extracellular vesicular exosome;intracellular	Alanine, aspartate and glutamate metabolism;Arginine and proline metabolism;Cysteine and methionine	Homo sapiens	1	27
cell part;cytoplasm;cytoplasmic part;cytosol;intracellular part;membrane		Homo sapiens	1	27
adherens junction;anchoring junction;cell cortex part;cell junction;cell part;cell projection membrane;cell projection part;cell-cell junction;cell-substrate adherens junction;cell-substrate junction;cortical actin cytoskeleton;corti	Axon guidance;Fc gamma R-mediated phagocytosis;Regulation of actin cytoskeleton	Homo sapiens	1	27
cell part;chromatin remodeling complex;intracellular membrane-bounded organelle;intracellular organelle;intracellular organelle part;intracellular part;ISWI complex;macromolecular complex;membrane-bounded organelle;nuclear part;nucleus;NURF complex;organelle;organelle part;protein complex		Homo sapiens	1	27

cell part;cell projection;cytoplasm;cytoplasmic part;cytosol;Golgi apparatus;intracellular membrane-bounded organelle;intracellular organelle;intracellular part;macromolecular complex;membrane-bounded organelle;midbody;neuron projection;organelle;protein complex		Homo sapiens	1	27
adherens junction;anchoring junction;cell cortex;cell junction;cell part;cell-cell junction;cell-substrate adherens junction;cell-substrate junction;cytoplasm;cytoplasmic part;cytoskeletal part;cytosol;early endosome;endoplasm	Acute myeloid leukemia;B cell receptor signaling pathway;Bladder cancer;Chronic myeloid leukemia;En	Homo sapiens	1	27
cell part;intracellular non-membrane-bounded organelle;intracellular organelle;intracellular organelle part;intracellular part;non-membrane-bounded organelle;nuclear part;nucleolus;organelle;organelle part		Homo sapiens	1	27
cell body;cell part;cell projection;cytoplasm;cytoplasmic part;cytosol;cytosolic large ribosomal subunit;dendrite;extracellular membrane-bounded organelle;extracellular organelle;extracellular region part;extracellular vesicula	Ribosome	Homo sapiens	1	27
adherens junction;anchoring junction;apical part of cell;cell junction;cell part;cell-cell adherens junction;cell-cell junction;cytoplasm;cytoplasmic part;cytosol;intracellular organelle part;intracellular part;membrane;nuclear part;	Adherens junction;Leukocyte transendothelial migration;Tight junction	Homo sapiens	1	27
cell part;cytoplasmic part;intracellular membrane-bounded organelle;intracellular organelle;intracellular organelle lumen;intracellular organelle part;intracellular part;membrane-bounded organelle;membrane-enclosed lumen;m	Butanoate metabolism;Synthesis and degradation of ketone bodies;Valine, leucine and isoleucine degra	Homo sapiens	1	27
acrosomal vesicle;adherens junction;alpha1-beta1 integrin complex;anchoring junction;basal part of cell;cell junction;cell part;cell projection;cell surface;cell-substrate adherens junction;cell-substrate junction;cytoplasmic me	Arrhythmogenic right ventricular cardiomyopathy (ARVC);Dilated cardiomyopathy;ECM-receptor interact	Homo sapiens	1	27
adherens junction;anchoring junction;cell junction;cell part;cell-substrate adherens junction;cell-substrate junction;cytoplasm;cytoplasmic part;cytosol;cytosolic small ribosomal subunit;extracellular membrane-bounded orga	Ribosome	Homo sapiens	1	27
cell part;cis-Golgi network;coated vesicle;cytoplasmic membrane-bounded vesicle;cytoplasmic part;cytoplasmic vesicle;ER to Golgi transport vesicle;Golgi apparatus;Golgi apparatus part;Golgi cis cisterna;Golgi cisterna;Golgi cisterna membrane;Golgi membrane;intracellular membrane-bounded organelle;intracellular organelle		Homo sapiens	1	27
CAF-1 complex;cell part;chromatin;chromatin assembly complex;chromatin remodeling complex;chromosomal part;ESC/E(Z) complex;histone deacetylase complex;histone methyltransferase complex;intracellular membrane-bounded organelle;intracellular organelle;intracellular organelle part;intracellular part;ISWI complex;mac		Homo sapiens	1	27
catalytic step 2 spliceosome;cell part;intracellular organelle part;intracellular part;macromolecular complex;nuclear body;nuclear part;nuclear speck;nucleoplasm;nucleoplasm part;organelle part;ribonucleoprotein complex;sp	Spliceosome	Homo sapiens	1	27
cell part;cell projection;cytoplasm;cytoplasmic mRNA processing body;cytoplasmic part;cytosol;dendrite;integral to membrane;integral to plasma membrane;intracellular non-membrane-bounded organelle;intracellular organelle;intracellular part;intrinsic to membrane;intrinsic to plasma membrane;macromolecular complex;meml		Homo sapiens	1	27
cell part;cytoplasmic part;cytosol;endosome;intracellular membrane-bounded organelle;intracellular organelle;intracellular part;membrane;membrane-bounded organelle;organelle		Homo sapiens	1	27
cell part;cytoplasmic part;extracellular membrane-bounded organelle;extracellular organelle;extracellular region part;extracellular vesicular exosome;intracellular membrane-bounded organelle;intracellular organelle;intracellul	Adipocytokine signaling pathway;Citrate cycle (TCA cycle);Glycolysis / Gluconeogenesis;Insulin signalin	Homo sapiens	1	27
cell part;cytoplasm;intracellular part;membrane		Homo sapiens	1	27
cell part;cytoplasmic part;cytosol;intracellular membrane-bounded organelle;intracellular organelle;intracellular organelle part;intracellular part;membrane-bounded organelle;nuclear body;nuclear part;nucleoplasm part;nucleus;organelle;organelle part;PML body		Homo sapiens	1	27
actin filament bundle;actomyosin;adherens junction;anchoring junction;cell cortex;cell junction;cell part;cell projection membrane;cell projection part;cell surface;cell-substrate adherens junction;cell-substrate junction;contractile fiber part;cytoplasm;cytoplasmic part;cytoskeletal part;cytosol;extrinsic to internal side of plasma me		Homo sapiens	1	27
cell part;intracellular non-membrane-bounded organelle;intracellular organelle;intracellular organelle part;intracellular part;macromolecular complex;mediator complex;non-membrane-bounded organelle;nuclear part;nucleolus;nucleoplasm;nucleoplasm part;organelle;organelle part;protein complex;ribonucleoprotein complex;tran		Homo sapiens	1	27
actin cytoskeleton;actin filament bundle;actomyosin;adherens junction;anchoring junction;cell junction;cell part;cell projection;cell-substrate adherens junction;cell-substrate junction;cytoplasm;cytoskeletal part;cytoskeleton;focal adhesion;intracellular membrane-bounded organelle;intracellular non-membrane-bounded organelle		Homo sapiens	1	27
cell part;cytoplasm;cytoplasmic part;cytosol;intracellular part;macromolecular complex;membrane;phenylalanine-tRNA ligase complex;protein complex	Aminoacyl-tRNA biosynthesis	Homo sapiens	1	27
cell part;cytoplasm;intracellular part;membrane;plasma membrane		Homo sapiens	1	27
cell part;cell projection;cytoplasm;intracellular membrane-bounded organelle;intracellular organelle;intracellular part;lamellipodium;membrane;membrane-bounded organelle;nucleus;organelle;plasma membrane		Homo sapiens	1	27
cell part;cytoplasmic part;Golgi apparatus;intracellular membrane-bounded organelle;intracellular organelle;intracellular organelle part;intracellular part;macromolecular complex;membrane-bounded organelle;mitochondrion;r	Spliceosome	Homo sapiens	1	27
cell part;cytoplasm;cytoplasmic part;endoplasmic reticulum membrane;endoplasmic reticulum part;envelope;intracellular membrane-bounded organelle;intracellular organelle;intracellular organelle part;intracellular part;macromolecular complex;membrane;membrane part;membrane-bounded organelle;nuclear envelope;nuclear		Homo sapiens	1	27
cell part;cytoplasmic part;cytosol;extracellular membrane-bounded organelle;extracellular organelle;extracellular region part;extracellular vesicular exosome;intracellular organelle part;intracellular part;macromolecular comple	Proteasome	Homo sapiens	1	28
extracellular matrix;extracellular region part;proteinaceous extracellular matrix		Homo sapiens	1	28
actin cytoskeleton;adherens junction;anchoring junction;cell junction;cell part;cell-substrate adherens junction;cell-substrate junction;centrosome;chromosomal part;contractile fiber;cytoplasm;cytoplasmic part;cytoskeletal pa	Focal adhesion;Long-term potentiation;Regulation of actin cytoskeleton;Vascular smooth muscle contra	Homo sapiens	1	28
cell part;cell surface;extracellular membrane-bounded organelle;extracellular organelle;extracellular region part;extracellular vesicular exosome;integral to membrane;integral to plasma membrane;intracellular;intrinsic to mem	Axon guidance	Homo sapiens	1	28
actin cytoskeleton;adherens junction;anchoring junction;Arp2/3 protein complex;cell junction;cell part;cell-substrate adherens junction;cell-substrate junction;cytoplasm;cytoskeletal part;cytoskeleton;extracellular membrane-	Bacterial invasion of epithelial cells;Fc gamma R-mediated phagocytosis;Pathogenic Escherichia coli in	Homo sapiens	1	28
cell part;chromosome;intracellular membrane-bounded organelle;intracellular non-membrane-bounded organelle;intracellular organelle;intracellular part;membrane-bounded organelle;non-membrane-bounded organelle;nucleus;organelle		Homo sapiens	1	28
cell part;cytoplasmic part;extracellular membrane-bounded organelle;extracellular organelle;extracellular region part;extracellular vesicular exosome;intracellular membrane-bounded organelle;intracellular organelle;intracellul	Alanine, aspartate and glutamate metabolism;Arginine and proline metabolism;Cysteine and methionine	Homo sapiens	1	28
cell part;cytoplasmic part;cytosol;extracellular membrane-bounded organelle;extracellular organelle;extracellular region part;extracellular vesicular exosome;intracellular membrane-bounded organelle;intracellular organelle;in	Arginine and proline metabolism	Homo sapiens	1	28
cell part;cytoplasmic part;endoplasmic reticulum;endoplasmic reticulum lumen;endoplasmic reticulum part;intracellular;intracellular membrane-bounded organelle;intracellular organelle;intracellular organelle lumen;intracellula	Protein processing in endoplasmic reticulum	Homo sapiens	1	28
cell part;cytoplasmic part;cytosol;endoplasmic reticulum membrane;endoplasmic reticulum part;envelope;extracellular membrane-bounded organelle;extracellular organelle;extracellular region part;extracellular vesicular exosome;intracellular membrane-bounded organelle;intracellular organelle;intracellular organelle part;intracell		Homo sapiens	1	28
adherens junction;anchoring junction;cell junction;cell part;cell-substrate adherens junction;cell-substrate junction;cytoplasmic membrane-bounded vesicle;cytoplasmic part;cytoplasmic vesicle;endoplasmic reticulum;endoplasmic reticulum lumen;endoplasmic reticulum part;extracellular membrane-bounded organelle;extracellu		Homo sapiens	1	28
cell part;cytoplasmic part;intracellular membrane-bounded organelle;intracellular organelle;intracellular organelle part;intracellular part;macromolecular complex;membrane;membrane part;membrane-bounded organelle;mito	Alzheimer's disease;Cardiac muscle contraction;Huntington's disease;Oxidative phosphorylation;Parkin	Homo sapiens	1	28
cell part;cytoplasmic part;endoplasmic reticulum membrane;endoplasmic reticulum part;integral to membrane;intracellular membrane-bounded organelle;intracellular organelle;intracellular organelle part;intracellular part;intrir	Adipocytokine signaling pathway;Fatty acid metabolism;Peroxisome;PPAR signaling pathway	Homo sapiens	1	28
cell part;centrosome;cytoplasm;cytoplasmic part;cytoskeletal part;cytosol;extracellular membrane-bounded organelle;extracellular organelle;extracellular region part;extracellular space;extracellular vesicular exosome;intrace	Carbon fixation in photosynthetic organisms;Citrate cycle (TCA cycle);Glyoxylate and dicarboxylate met	Homo sapiens	1	28
cell part;cytoplasm;cytoplasmic part;cytosol;extracellular membrane-bounded organelle;extracellular organelle;extracellular region part;extracellular vesicular exosome;intracellular membrane-bounded organelle;intracellular c	Arginine and proline metabolism;Ascorbate and aldarate metabolism;beta-Alanine metabolism;Fatty aci	Homo sapiens	1	28
cell part;cytoplasm;cytoplasmic part;cytosol;extracellular membrane-bounded organelle;extracellular organelle;extracellular region part;extracellular vesicular exosome;intracellular part;macromolecular complex;membrane-bounded organelle;membrane-bounded vesicle;organelle;protein complex;vesicle		Homo sapiens	1	28
cell body;cell part;chromatin remodeling complex;cytoplasm;cytoplasmic part;histone acetyltransferase complex;histone methyltransferase complex;intracellular membrane-bounded organelle;intracellular organelle;intracellular organelle part;intracellular part;macromolecular complex;membrane;membrane-bounded organelle;me		Homo sapiens	1	28
cell part;cytoplasm;extracellular region part;extracellular space;intracellular organelle part;intracellular part;macromolecular complex;nuclear part;nucleoplasm;nucleoplasm part;organelle part;protein complex;transcriptional repressor complex		Homo sapiens	1	28
cell part;contractile fiber;contractile fiber part;cytoplasm;cytoplasmic mRNA processing body;cytoplasmic part;cytosol;extracellular membrane-bounded organelle;extracellular organelle;extracellular region part;extracellular v	Proteasome	Homo sapiens	1	28
cell part;cytoplasmic part;extracellular membrane-bounded organelle;extracellular organelle;extracellular region part;extracellular vesicular exosome;intracellular membrane-bounded organelle;intracellular organelle;intracellular organelle lumen;intracellular organelle part;intracellular part;membrane;membrane-bounded organelle;		Homo sapiens	1	28
adherens junction;anchoring junction;cell junction;cell part;cell-substrate adherens junction;cell-substrate junction;cytoplasmic part;cytosol;cytosolic small ribosomal subunit;extracellular membrane-bounded organelle;extrac	Ribosome	Homo sapiens	1	28
cell body;cell part;cell projection;cytoplasm;cytoplasmic part;cytoskeleton;cytosol;dendrite;extracellular membrane-bounded organelle;extracellular organelle;extracellular region part;extracellular vesicular exosome;intracellul	Measles	Homo sapiens	1	28
cell part;chromosomal part;chromosome, centromeric region;cytoplasmic part;cytoskeletal part;cytoskeleton;cytosol;extracellular membrane-bounded organelle;extracellular organelle;extracellular region part;extracellular ves	Cell cycle - yeast;Chagas disease (American trypanosomiasis);Hepatitis C;Long-term depression;Meios	Homo sapiens	1	28
cell part;cytoplasm;cytoplasmic part;cytosol;intracellular membrane-bounded organelle;intracellular organelle;intracellular organelle part;intracellular part;macromolecular complex;membrane-bounded organelle;mitochondrio	mRNA surveillance pathway;RNA transport;Spliceosome	Homo sapiens	1	28
adherens junction;anchoring junction;cell junction;cell part;cell-substrate adherens junction;cell-substrate junction;cytoplasm;cytoplasmic part;cytosol;focal adhesion;intracellular membrane-bounded organelle;intracellular non-membrane-bounded organelle;intracellular organelle;intracellular part;macromolecular complex;memb		Homo sapiens	1	28
bleb;cell part;cell projection;centriole;cytoplasmic part;cytoskeletal part;cytoskeleton;cytosol;Golgi apparatus part;Golgi membrane;intracellular non-membrane-bounded organelle;intracellular organelle;intracellular organelle	Axon guidance;Chemokine signaling pathway;Focal adhesion;Leukocyte transendothelial migration;Path	Homo sapiens	1	28
cell part;cytoplasmic part;cytosol;eukaryotic translation initiation factor 4F complex;intracellular non-membrane-bounded organelle;intracellular organelle;intracellular organelle part;intracellular part;macromolecular complex;r	RNA transport	Homo sapiens	1	28
cell part;cytoplasm;cytoplasmic part;cytosol;intracellular organelle part;intracellular part;membrane;nuclear part;nucleoplasm;organelle part;plasma membrane	Amino sugar and nucleotide sugar metabolism	Homo sapiens	1	28
cell part;cytoplasmic part;extracellular membrane-bounded organelle;extracellular organelle;extracellular region part;extracellular vesicular exosome;intracellular membrane-bounded organelle;intracellular organelle;intracellula	One carbon pool by folate	Homo sapiens	1	28
cell part;cytoplasmic part;endoplasmic reticulum;endoplasmic reticulum lumen;endoplasmic reticulum part;extracellular membrane-bounded organelle;extracellular organelle;extracellular region part;extracellular vesicular exo	Protein processing in endoplasmic reticulum	Homo sapiens	1	28
cell part;cytoplasm;cytoplasmic part;cytosol;intracellular membrane-bounded organelle;intracellular organelle;intracellular organelle part;intracellular part;macromolecular complex;membrane;membrane-bounded organelle;nu	RNA transport	Homo sapiens	1	28
actomyosin;cell part;cytoplasmic part;cytoskeletal part;endoplasmic reticulum-Golgi intermediate compartment;Golgi apparatus part;Golgi membrane;intracellular membrane-bounded organelle;intracellular organelle;intracellular organelle part;intracellular part;macromolecular complex;membrane;membrane-bounded organelle;r		Homo sapiens	1	28
cell part;chromatin;chromatin remodeling complex;chromosomal part;chromosome;intracellular non-membrane-bounded organelle;intracellular organelle;intracellular organelle part;intracellular part;macromolecular complex;nBAF complex;non-membrane-bounded organelle;npBAF complex;nuclear chromatin;nuclear chromoson		Homo sapiens	1	28
AP-2 adaptor complex;AP-type membrane coat adaptor complex;cell part;clathrin adaptor complex;clathrin coated vesicle membrane;clathrin-coated endocytic vesicle membrane;coated vesicle membrane;cytoplasmic part;	Endocrine and other factor-regulated calcium reabsorption;Endocytosis;Huntington's disease	Homo sapiens	1	28
cell part;chromatin;chromosomal part;euchromatin;heterochromatin;intracellular membrane-bounded organelle;intracellular organelle;intracellular organelle part;intracellular part;membrane-bounded organelle;nuclear chromatin;nuclear chromosome part;nuclear heterochromatin;nuclear matrix;nuclear part;nucleus;organelle;orgai		Homo sapiens	1	28
actin cytoskeleton;cell cortex;cell part;cytoplasm;cytoplasmic part;cytoskeleton;intracellular membrane-bounded organelle;intracellular non-membrane-bounded organelle;intracellular organelle;intracellular organelle part;intracellular part;membrane-bounded organelle;mitochondrion;non-membrane-bounded organelle;nuclear pa		Homo sapiens	1	28
cell part;cytoplasmic part;cytoskeleton;endoplasmic reticulum;endoplasmic reticulum membrane;endoplasmic reticulum part;Golgi apparatus part;Golgi membrane;integral to membrane;intracellular membrane-bounded orga	Tight junction	Homo sapiens	1	28
cell part;cytoplasm;cytoplasmic part;cytosol;intracellular part;macromolecular complex;membrane;phenylalanine-tRNA ligase complex;protein complex	Aminoacyl-tRNA biosynthesis	Homo sapiens	1	28
cell part;intracellular organelle part;intracellular part;macromolecular complex;nuclear body;nuclear part;nuclear speck;nucleoplasm part;organelle part;ribonucleoprotein complex;small nuclear ribonucleoprotein complex;U1 snRNP		Homo sapiens	1	28
actin cytoskeleton;adherens junction;anchoring junction;cell junction;cell part;cell projection;cell-cell adherens junction;cell-cell junction;cell-substrate adherens junction;cell-substrate junction;cytoplasm;cytoskeleton;fascia a	Focal adhesion	Homo sapiens	1	28
cell part;cytoplasmic part;integral to membrane;intracellular membrane-bounded organelle;intracellular organelle;intracellular organelle part;intracellular part;intrinsic to membrane;membrane;membrane part;membrane-bounded organelle;mitochondrial inner membrane;mitochondrial membrane;mitochondrial part;mitochondrion;		Homo sapiens	1	28
cell part;cytoplasm;cytoplasmic part;cytosol;intracellular part	Ubiquitin mediated proteolysis	Homo sapiens	1	29
cell part;cytoplasmic part;endoplasmic reticulum part;extrinsic to internal side of plasma membrane;extrinsic to membrane;extrinsic to plasma membrane;integral to membrane;integral to plasma membrane;intracellular organelle part;intracellular part;intrinsic to endoplasmic reticulum membrane;intrinsic to membrane;intrinsic to		Homo sapiens	1	29
actin cytoskeleton;cell part;cytoskeleton;intracellular membrane-bounded organelle;intracellular non-membrane-bounded organelle;intracellular organelle;intracellular organelle part;intracellular part;membrane-bounded orgar	Spliceosome	Homo sapiens	1	29
cell part;cytoplasm;cytoplasmic part;cytosol;extracellular membrane-bounded organelle;extracellular organelle;extracellular region part;extracellular vesicular exosome;intracellular membrane-bounded organelle;intracellular c	Aminoacyl-tRNA biosynthesis	Homo sapiens	1	29
cell part;extracellular membrane-bounded organelle;extracellular organelle;extracellular region part;extracellular vesicular exosome;integral to membrane;intrinsic to membrane;membrane;membrane part;membrane-bounded organelle;membrane-bounded vesicle;organelle;vesicle		Homo sapiens	1	29
cell part;centrosome;cytoplasm;cytoplasmic part;cytoskeletal part;cytosol;extracellular membrane-bounded organelle;extracellular organelle;extracellular region part;extracellular vesicular exosome;extrinsic to internal side of	Axon guidance;Chagas disease (American trypanosomiasis);Chemokine signaling pathway;Gap junctior	Homo sapiens	1	29
cell part;cell projection;cytoplasm;cytoplasmic membrane-bounded vesicle;cytoplasmic part;cytoplasmic vesicle;cytoskeletal part;extracellular membrane-bounded organelle;extracellular organelle;extracellular region part;extracellular space;extracellular vesicular exosome;intracellular membrane-bounded organelle;intracellular c		Homo sapiens	1	29
cell part;intracellular	mRNA surveillance pathway	Homo sapiens	1	29
6-phosphofructokinase complex;cell part;cytoplasmic part;cytosol;cytosolic part;extracellular membrane-bounded organelle;extracellular organelle;extracellular region part;extracellular vesicular exosome;intracellular part;ma	Fructose and mannose metabolism;Galactose metabolism;Glycolysis / Gluconeogenesis;Methane meta	Homo sapiens	1	29
cell part;DNA-directed RNA polymerase complex;DNA-directed RNA polymerase II, core complex;intracellular membrane-bounded organelle;intracellular organelle;intracellular organelle part;intracellular part;macromolecular c	Huntington's disease;Purine metabolism;Pyrimidine metabolism;RNA polymerase	Homo sapiens	1	29
aggresome;cell part;chromosomal part;cytoplasm;delta DNA polymerase complex;DNA polymerase complex;inclusion body;intracellular membrane-bounded organelle;intracellular organelle;intracellular organelle part;intracel	Base excision repair;DNA replication;Homologous recombination;Mismatch repair;Nucleotide excision r	Homo sapiens	1	29
cell part;cytoplasm;cytoplasmic part;cytosol;early endosome membrane;endoplasmic reticulum part;endosomal part;endosome membrane;extrinsic to endoplasmic reticulum membrane;extrinsic to membrane;extrinsic to org	Acute myeloid leukemia;Endocytosis;Pathways in cancer;Ubiquitin mediated proteolysis	Homo sapiens	1	29
cell part;cytoplasmic part;cytosol;intracellular part;macromolecular complex;methionine adenosyltransferase complex;protein complex	Cysteine and methionine metabolism	Homo sapiens	1	29
extracellular matrix;extracellular matrix part;extracellular region;extracellular region part;fibril;microfibril;proteinaceous extracellular matrix		Homo sapiens	1	29
axon;cell part;cell projection;chromatin;chromosomal part;cytoplasm;cytoplasmic part;cytosol;dendrite;intracellular membrane-bounded organelle;intracellular non-membrane-bounded organelle;intracellular organelle;intracel	Chemokine signaling pathway;Hepatitis C;Jak-STAT signaling pathway;ko05152;Leishmaniasis;Measles	Homo sapiens	1	29
cell part;cell projection part;cytoplasm;cytoplasmic part;cytoskeletal part;cytosol;dendritic shaft;extracellular membrane-bounded organelle;extracellular organelle;extracellular region part;extracellular vesicular exosome;Golg	Vasopressin-regulated water reabsorption	Homo sapiens	1	29
cell part;cytoplasm;cytoplasmic part;cytosol;cytosolic large ribosomal subunit;intracellular non-membrane-bounded organelle;intracellular organelle;intracellular organelle part;intracellular part;large ribosomal subunit;macrom	Ribosome	Homo sapiens	1	29
actin cytoskeleton;actin filament;cell part;contractile fiber part;cytoplasmic part;cytoskeletal part;cytoskeleton;cytosol;extracellular membrane-bounded organelle;extracellular organelle;extracellular region part;extracellular vesicular exosome;F-actin capping protein complex;intracellular non-membrane-bounded organelle;intrace		Homo sapiens	1	29
actin cytoskeleton;adherens junction;anchoring junction;cell junction;cell part;cell projection membrane;cell projection part;cell-cell junction;cell-substrate adherens junction;cell-substrate junction;cytoplasm;cytoplasmic part	Fc gamma R-mediated phagocytosis;Focal adhesion;Leukocyte transendothelial migration	Homo sapiens	1	29
cell part;chromatin;chromatin remodeling complex;chromosomal part;euchromatin;extracellular region part;extracellular space;heterochromatin;intracellular membrane-bounded organelle;intracellular non-membrane-bounded organelle;intracellular organelle;intracellular organelle part;intracellular part;macromolecular complex;me		Homo sapiens	1	29
cell junction;cell part;cytoplasm;cytoplasmic part;cytosol;cytosolic part;cytosolic proteasome complex;intracellular membrane-bounded organelle;intracellular organelle;intracellular organelle part;intracellular part;macromolec	Proteasome	Homo sapiens	1	29
cell part;centriole;cytoplasm;cytoplasmic membrane-bounded vesicle;cytoplasmic part;cytoplasmic vesicle;cytoskeletal part;cytosol;endosome;extracellular membrane-bounded organelle;extracellular organelle;extracellular r	Ribosome biogenesis in eukaryotes;RNA transport	Homo sapiens	1	29
cell part;cytoplasm;cytoplasmic part;cytosol;cytosolic large ribosomal subunit;extracellular membrane-bounded organelle;extracellular organelle;extracellular region part;extracellular vesicular exosome;intracellular non-memt	Ribosome	Homo sapiens	1	29
adherens junction;anchoring junction;cell junction;cell part;cell-substrate adherens junction;cell-substrate junction;cytoplasmic part;cytosol;extracellular membrane-bounded organelle;extracellular organelle;extracellular region;extracellular region part;extracellular space;extracellular vesicular exosome;focal adhesion;intracellular		Homo sapiens	1	29
cell part;cell projection part;cytoplasmic membrane-bounded vesicle;cytoplasmic part;cytoplasmic vesicle;cytoskeletal part;cytosol;growth cone;intracellular membrane-bounded organelle;intracellular organelle;intracellular organelle part;intracellular part;kinesin complex;macromolecular complex;membrane;membrane-bounded		Homo sapiens	1	29
cell part;cytoplasmic part;extracellular membrane-bounded organelle;extracellular organelle;extracellular region part;extracellular vesicular exosome;Golgi apparatus;Golgi apparatus part;Golgi cisterna membrane;Golgi meml	Mucin type O-Glycan biosynthesis	Homo sapiens	1	29
adherens junction;anchoring junction;apical part of cell;basal part of cell;cell junction;cell part;cell projection;cell projection membrane;cell projection part;cell surface;cell-substrate adherens junction;cell-substrate junction;cytoplasm;extracellular region part;extracellular space;focal adhesion;integral to membrane;intracellular pa		Homo sapiens	1	29
cell part;cytoplasm;cytoplasmic part;intracellular membrane-bounded organelle;intracellular non-membrane-bounded organelle;intracellular organelle;intracellular part;macromolecular complex;membrane-bounded organelle;	mRNA surveillance pathway;RNA degradation;RNA transport	Homo sapiens	1	29
cell part;cytoplasm;cytoplasmic part;Golgi apparatus;intracellular;intracellular membrane-bounded organelle;intracellular organelle;intracellular organelle part;intracellular part;membrane;membrane-bounded organelle;nuclear part;nucleoplasm;nucleus;organelle;organelle part		Homo sapiens	1	29
cell part;cytoplasm;cytoplasmic part;cytosol;intracellular part;membrane;plasma membrane	ko05152;Regulation of actin cytoskeleton;Vascular smooth muscle contraction	Homo sapiens	1	29
cell part;cytoplasmic part;endoplasmic reticulum;endoplasmic reticulum membrane;endoplasmic reticulum part;envelope;integral to membrane;intracellular membrane-bounded organelle;intracellular non-membrane-bounded organelle;intracellular organelle;intracellular organelle part;intracellular part;intrinsic to membrane;memb		Homo sapiens	1	29
cell part;cytoplasmic part;cytosol;extracellular membrane-bounded organelle;extracellular organelle;extracellular region part;extracellular vesicular exosome;intracellular part;membrane-bounded organelle;membrane-bounde	Amino sugar and nucleotide sugar metabolism;Galactose metabolism;Glycolysis / Gluconeogenesis;Pe	Homo sapiens	1	29
cell part;cytoplasmic part;endoplasmic reticulum;endoplasmic reticulum lumen;endoplasmic reticulum membrane;endoplasmic reticulum part;intracellular membrane-bounded organelle;intracellular organelle;intracellular orga	Protein processing in endoplasmic reticulum;Vibrio cholerae infection	Homo sapiens	1	29
cell part;cytoplasm;cytoskeletal part;intracellular membrane-bounded organelle;intracellular non-membrane-bounded organelle;intracellular organelle;intracellular organelle part;intracellular part;membrane-bounded organelle;non-membrane-bounded organelle;nucleus;organelle;organelle part;spindle		Homo sapiens	1	29
aggresome;cell part;inclusion body;intracellular membrane-bounded organelle;intracellular non-membrane-bounded organelle;intracellular organelle;intracellular organelle part;intracellular part;membrane;membrane-bounded	Ribosome biogenesis in eukaryotes;RNA degradation	Homo sapiens	1	29
cell part;cell surface;cytoplasmic part;endoplasmic reticulum membrane;endoplasmic reticulum part;Golgi apparatus;integral to membrane;intracellular membrane-bounded organelle;intracellular organelle;intracellular organelle part;intracellular part;intrinsic to membrane;membrane;membrane part;membrane-bounded organelle;		Homo sapiens	1	29
catalytic step 1 spliceosome;catalytic step 2 spliceosome;cell part;chromosomal part;cytoplasm;cytoplasmic part;cytoskeletal part;intracellular membrane-bounded organelle;intracellular non-membrane-bounded organelle;ir	Spliceosome;Ubiquitin mediated proteolysis	Homo sapiens	1	29
AP-2 adaptor complex;AP-type membrane coat adaptor complex;cell part;clathrin adaptor complex;clathrin coated vesicle membrane;clathrin-coated endocytic vesicle membrane;coated vesicle membrane;cytoplasmic men	Endocrine and other factor-regulated calcium reabsorption;Endocytosis;Huntington's disease	Homo sapiens	1	30
6-phosphofructokinase complex;apical plasma membrane;cell part;cytoplasmic part;cytosol;cytosolic part;extracellular membrane-bounded organelle;extracellular organelle;extracellular region part;extracellular vesicular exo	Fructose and mannose metabolism;Galactose metabolism;Glycolysis / Gluconeogenesis;Methane meta	Homo sapiens	1	30
cell part;cytoplasmic part;intracellular membrane-bounded organelle;intracellular organelle;intracellular organelle lumen;intracellular organelle part;intracellular part;macromolecular complex;membrane-bounded organelle;mer	Butanoate metabolism;Citrate cycle (TCA cycle);Glycolysis / Gluconeogenesis;Pyruvate metabolism;Vali	Homo sapiens	1	30
cell part;cytoplasmic part;cytosol;cytosolic part;extracellular membrane-bounded organelle;extracellular organelle;extracellular region part;extracellular space;extracellular vesicular exosome;intracellular part;macromolecular	Glycolysis / Gluconeogenesis;Methane metabolism;RNA degradation	Homo sapiens	1	30
adherens junction;anchoring junction;cell junction;cell part;cell-substrate adherens junction;cell-substrate junction;cytoplasm;cytoplasmic part;cytosol;cytosolic small ribosomal subunit;extracellular membrane-bounded orgai	Ribosome	Homo sapiens	1	30
cell part;intracellular membrane-bounded organelle;intracellular non-membrane-bounded organelle;intracellular organelle;intracellular organelle part;intracellular part;membrane-bounded organelle;non-membrane-bounded organelle;nuclear part;nucleolus;nucleoplasm;nucleus;organelle;organelle part		Homo sapiens	1	30
cell part;chromosome;condensed chromosome;cytoplasm;cytoplasmic part;extracellular region part;extracellular space;intracellular membrane-bounded organelle;intracellular non-membrane-bounded organelle;intracellular organelle;intracellular organelle part;intracellular part;macromolecular complex;membrane-bounded orga		Homo sapiens	1	30
cell part;cytoplasm;cytoplasmic part;cytosol;intracellular membrane-bounded organelle;intracellular organelle;intracellular organelle part;intracellular part;membrane-bounded organelle;nuclear part;nucleoplasm;nucleus;organelle;organelle part		Homo sapiens	1	30
adherens junction;anchoring junction;apical part of cell;basolateral plasma membrane;beta-catenin destruction complex;beta-catenin-TCF7L2 complex;catenin complex;catenin-TCF7L2 complex;cell cortex;cell junction;cell p	Adherens junction;Arrhythmogenic right ventricular cardiomyopathy (ARVC);Bacterial invasion of epithel	Homo sapiens	1	30
cell part;cytoplasm;envelope;integral to membrane;intracellular membrane-bounded organelle;intracellular organelle;intracellular organelle part;intracellular part;intrinsic to membrane;membrane;membrane part;membrane-bounded organelle;nuclear envelope;nuclear inner membrane;nuclear membrane;nuclear part;nucleus;orga		Homo sapiens	1	30
cell part;chromosome;intracellular non-membrane-bounded organelle;intracellular organelle;intracellular organelle part;intracellular part;macromolecular complex;membrane;mismatch repair complex;MutSalpha complex;Mut	Colorectal cancer;Mismatch repair;Pathways in cancer	Homo sapiens	1	30
cell part;cytoplasm;intracellular organelle part;intracellular part;macromolecular complex;nuclear body;nuclear part;nuclear speck;nucleoplasm;nucleoplasm part;organelle part;ribonucleoprotein complex;spliceosomal comple	Spliceosome	Homo sapiens	1	30
azurophil granule;cell part;cytoplasm;cytoplasmic membrane-bounded vesicle;cytoplasmic part;cytoplasmic vesicle;cytoskeletal part;endocytic vesicle;envelope;extracellular membrane-bounded organelle;extracellular organelle;extracellular region part;extracellular vesicular exosome;intracellular membrane-bounded organelle;ir		Homo sapiens	1	30
cell part;CUL4 RING ubiquitin ligase complex;Cul4A-RING ubiquitin ligase complex;cullin-RING ubiquitin ligase complex;intracellular organelle part;intracellular part;macromolecular complex;nuclear part;nucleoplasm;organel	Nucleotide excision repair;Ubiquitin mediated proteolysis	Homo sapiens	1	30
cell part;intracellular organelle part;intracellular part;macromolecular complex;nuclear part;organelle part;PcG protein complex;protein complex		Homo sapiens	1	30
actin cytoskeleton;adherens junction;anchoring junction;cell junction;cell part;cell-substrate adherens junction;cell-substrate junction;contractile fiber part;cytoplasmic part;cytoskeleton;focal adhesion;intracellular membrane-	Osteoclast differentiation	Homo sapiens	1	30
cell part;cytoplasm;cytoplasmic part;cytosol;intracellular organelle part;intracellular part;nuclear part;nucleoplasm;organelle part	RIG-I-like receptor signaling pathway	Homo sapiens	1	30
actin filament bundle;actomyosin;cell division site part;cell junction;cell part;cell projection;cell-cell junction;cleavage furrow;cytoplasm;cytoplasmic part;cytoskeletal part;cytosol;extracellular membrane-bounded organelle;ex	Calcium signaling pathway;Focal adhesion;Gastric acid secretion;Regulation of actin cytoskeleton;Vasc	Homo sapiens	1	30
cell part;chromatin;chromosomal part;cytoplasm;cytoplasmic part;intracellular membrane-bounded organelle;intracellular organelle;intracellular organelle lumen;intracellular organelle part;intracellular part;membrane-bounded organelle;membrane-enclosed lumen;mitochondrial matrix;mitochondrial part;nuclear chromatin;nuclea		Homo sapiens	1	30
cell part;cytoplasm;cytoplasmic part;extracellular membrane-bounded organelle;extracellular organelle;extracellular region part;extracellular vesicular exosome;intracellular membrane-bounded organelle;intracellular organelle;intracellular part;membrane;membrane-bounded organelle;membrane-bounded vesicle;nucleus;organel		Homo sapiens	1	30
cell part;cytoplasmic part;cytosol;extracellular membrane-bounded organelle;extracellular organelle;extracellular region part;extracellular vesicular exosome;intracellular organelle part;intracellular part;macromolecular comple	Proteasome	Homo sapiens	1	30
anchoring junction;catalytic step 2 spliceosome;cell junction;cell part;cell-cell junction;cytoplasm;cytoskeletal part;desmosome;intermediate filament;intracellular organelle part;intracellular part;macromolecular complex;mem	mRNA surveillance pathway;RNA transport	Homo sapiens	1	30
cell part;intracellular membrane-bounded organelle;intracellular non-membrane-bounded organelle;intracellular organelle;intracellular organelle part;intracellular part;membrane-bounded organelle;non-membrane-bounded organelle;nuclear part;nucleolus;nucleus;organelle;organelle part		Homo sapiens	1	30
adherens junction;anchoring junction;cell junction;cell part;cell-substrate adherens junction;cell-substrate junction;cytoplasm;cytoplasmic part;cytosol;endoplasmic reticulum;focal adhesion;intracellular membrane-bounded c	Protein export	Homo sapiens	1	30
cell part;cytoplasmic part;cytosol;extracellular membrane-bounded organelle;extracellular organelle;extracellular region part;extracellular vesicular exosome;intracellular membrane-bounded organelle;intracellular organelle;in	Proteasome	Homo sapiens	1	31
cell part;cytoplasmic part;endoplasmic reticulum;endoplasmic reticulum membrane;endoplasmic reticulum part;extracellular membrane-bounded organelle;extracellular organelle;extracellular region part;extracellular vesicula	Lysine degradation;Other types of O-glycan biosynthesis	Homo sapiens	1	31
adherens junction;anchoring junction;cell junction;cell part;cell-substrate adherens junction;cell-substrate junction;cytoplasmic part;cytosol;endoplasmic reticulum;extracellular membrane-bounded organelle;extracellular orga	Amyotrophic lateral sclerosis (ALS);Methane metabolism;Peroxisome;Tryptophan metabolism	Homo sapiens	1	31

cell part;cytoplasm;cytoplasmic part;cytosol;extracellular membrane-bounded organelle;extracellular organelle;extracellular region part;extracellular vesicular exosome;intracellular membrane-bounded organelle;intracellular c	Cysteine and methionine metabolism;Glycolysis / Gluconeogenesis;Propanoate metabolism;Pyruvate m	Homo sapiens	1	31
apical plasma membrane;cell part;cell surface;cytoplasm;cytoplasmic membrane-bounded vesicle;cytoplasmic part;cytoplasmic vesicle;extracellular membrane-bounded organelle;extracellular organelle;extracellular region p	Protein digestion and absorption	Homo sapiens	1	31
cell part;cytoskeletal part;cytoskeleton;extracellular membrane-bounded organelle;extracellular organelle;extracellular region part;extracellular vesicular exosome;intermediate filament;intracellular membrane-bounded organelle;intracellular non-membrane-bounded organelle;intracellular organelle;intracellular organelle part;intrac		Homo sapiens	1	31
cell part;cytoplasmic part;endoplasmic reticulum;endoplasmic reticulum lumen;endoplasmic reticulum part;intracellular membrane-bounded organelle;intracellular organelle;intracellular organelle lumen;intracellular organelle p	Arginine and proline metabolism	Homo sapiens	1	31
actin cytoskeleton;adherens junction;anchoring junction;apicolateral plasma membrane;catenin complex;catenin-TCF7L2 complex;cell junction;cell part;cell-cell adherens junction;cell-cell contact zone;cell-cell junction;cell-s	Acute myeloid leukemia;Arrhythmogenic right ventricular cardiomyopathy (ARVC);Pathways in cancer	Homo sapiens	1	31
cell part;cytoplasm;cytoplasmic part;cytosol;extracellular membrane-bounded organelle;extracellular organelle;extracellular region part;extracellular vesicular exosome;intracellular membrane-bounded organelle;intracellular c	Aminoacyl-tRNA biosynthesis;Tryptophan metabolism	Homo sapiens	1	31
caveola;cell part;contractile fiber part;cytoplasmic part;integral to membrane;integral to plasma membrane;intracellular part;intrinsic to membrane;intrinsic to plasma membrane;macromolecular complex;membrane;membran	Calcium signaling pathway;Pancreatic secretion;Salivary secretion	Homo sapiens	1	31
cell part;cytoplasmic part;extracellular membrane-bounded organelle;extracellular organelle;extracellular region part;extracellular vesicular exosome;intracellular membrane-bounded organelle;intracellular organelle;intracellula	Benzoate degradation;Butanoate metabolism;Carbon fixation pathways in prokaryotes;Fatty acid metab	Homo sapiens	1	31
cell part;cytoplasm;cytoplasmic part;cytosol;eukaryotic translation elongation factor 1 complex;intracellular membrane-bounded organelle;intracellular non-membrane-bounded organelle;intracellular organelle;intracellular organelle part;intracellular part;macromolecular complex;membrane-bounded organelle;non-membrane-bou		Homo sapiens	1	31
cell part;cytoplasm;cytoplasmic part;cytosol;extracellular membrane-bounded organelle;extracellular organelle;extracellular region part;extracellular vesicular exosome;intracellular membrane-bounded organelle;intracellular c	Pentose phosphate pathway	Homo sapiens	1	31
catalytic step 2 spliceosome;cell part;cytoplasm;cytoplasmic part;cytosol;exon-exon junction complex;intracellular non-membrane-bounded organelle;intracellular organelle;intracellular organelle part;intracellular part;macron	mRNA surveillance pathway;RNA transport;Spliceosome	Homo sapiens	1	31
AP-type membrane coat adaptor complex;cell part;clathrin adaptor complex;COPI vesicle coat;cytoplasm;cytoplasmic part;cytoplasmic vesicle part;cytosol;endoplasmic reticulum;Golgi apparatus;Golgi apparatus part;intracellular membrane-bounded organelle;intracellular organelle;intracellular organelle part;intracellular part;m		Homo sapiens	1	31
cell part;cytoplasm;cytoplasmic part;cytosol;intracellular part	Aminoacyl-tRNA biosynthesis	Homo sapiens	1	31
cell part;cytoplasmic membrane-bounded vesicle;cytoplasmic part;cytoplasmic vesicle;endomembrane system;endoplasmic reticulum;extrinsic to membrane;Golgi apparatus;integral to membrane;intracellular membrane-bounded organelle;intracellular organelle;intracellular part;intrinsic to membrane;lysosome;lytic vacuole;mer		Homo sapiens	1	31
cell part;cytoplasm;intracellular part	African trypanosomiasis;Renin-angiotensin system	Homo sapiens	1	31
cell part;cytoplasmic part;cytosol;cytosolic large ribosomal subunit;extracellular membrane-bounded organelle;extracellular organelle;extracellular region part;extracellular vesicular exosome;intracellular organelle part;intracel	Ribosome	Homo sapiens	1	31
cell part;cytoplasmic part;cytosol;cytosolic large ribosomal subunit;extracellular membrane-bounded organelle;extracellular organelle;extracellular region part;extracellular vesicular exosome;intracellular membrane-bounded	Ribosome	Homo sapiens	1	31
cell part;cytoplasmic part;cytosol;cytosolic part;cytosolic proteasome complex;extracellular membrane-bounded organelle;extracellular organelle;extracellular region part;extracellular vesicular exosome;intracellular membran	Proteasome	Homo sapiens	1	31
cell part;cytoplasmic part;endoplasmic reticulum membrane;endoplasmic reticulum part;extracellular membrane-bounded organelle;extracellular organelle;extracellular region part;extracellular vesicular exosome;intracellular	Lysine degradation	Homo sapiens	1	31
		Homo sapiens	1	31
cell part;cell projection membrane;cell projection part;cytoplasm;cytoplasmic part;cytosol;extracellular membrane-bounded organelle;extracellular organelle;extracellular region part;extracellular vesicular exosome;intracellular part;leading edge membrane;macromolecular complex;membrane part;membrane-bounded organelle;r		Homo sapiens	1	31
		Homo sapiens	1	31
cell part;cytoplasm;intracellular non-membrane-bounded organelle;intracellular organelle;intracellular organelle part;intracellular part;non-membrane-bounded organelle;nuclear body;nuclear matrix;nuclear part;nuclear speck;nucleolus;nucleoplasm;nucleoplasm part;organelle;organelle part;paraspeckles		Homo sapiens	1	31
actin cytoskeleton;cell junction;cell part;cell projection;contractile fiber part;cytoplasmic part;cytoskeletal part;cytoskeleton;cytosol;intracellular non-membrane-bounded organelle;intracellular organelle;intracellular organelle part;intracellular part;membrane;neuron projection;non-membrane-bounded organelle;organelle;organelle		Homo sapiens	1	31
caveola;cell part;cytoplasmic part;endosomal part;endosome membrane;extracellular membrane-bounded organelle;extracellular organelle;extracellular region part;extracellular vesicular exosome;intracellular membrane-bou	Endocytosis	Homo sapiens	1	31
cell part;membrane		Homo sapiens	1	31
cell part;cytoplasm;cytoplasmic part;cytoskeletal part;cytosol;intracellular organelle part;intracellular part;macromolecular complex;microtubule;nuclear part;nucleoplasm;organelle part;protein complex		Homo sapiens	1	31
adherens junction;anchoring junction;cell junction;cell part;cell projection membrane;cell projection part;cell-substrate adherens junction;cell-substrate junction;cytoplasmic part;cytosol;extracellular membrane-bounded orga	Regulation of actin cytoskeleton	Homo sapiens	1	31
actomyosin;cell junction;cell leading edge;cell part;cell-cell junction;cytoplasm;cytoskeletal part;cytoskeleton;extracellular membrane-bounded organelle;extracellular organelle;extracellular region part;extracellular vesicular exosome;intracellular non-membrane-bounded organelle;intracellular organelle;intracellular organelle part;		Homo sapiens	1	31
adherens junction;anchoring junction;cell cortex part;cell junction;cell part;cell-substrate adherens junction;cell-substrate junction;cytoplasmic part;cytoskeletal part;extracellular membrane-bounded organelle;extracellular or	Tight junction	Homo sapiens	1	32
cell part;cytoplasm;cytoplasmic part;cytosol;extracellular membrane-bounded organelle;extracellular organelle;extracellular region part;extracellular vesicular exosome;intracellular membrane-bounded organelle;intracellular c	Carbon fixation pathways in prokaryotes;Citrate cycle (TCA cycle);Glutathione metabolism;Peroxisome	Homo sapiens	1	32
cell part;cytoplasm;intracellular part	MAPK signaling pathway	Homo sapiens	1	32
adherens junction;anchoring junction;apical plasma membrane;basolateral plasma membrane;cell junction;cell part;cell surface;cell-substrate adherens junction;cell-substrate junction;cytoplasm;cytoplasmic membrane-bour	Adherens junction;Bladder cancer;Calcium signaling pathway;Cytokine-cytokine receptor interaction;Dc	Homo sapiens	1	32
cell part;cytoplasmic part;cytosol;eukaryotic 48S preinitiation complex;eukaryotic translation initiation factor 2 complex;extracellular membrane-bounded organelle;extracellular organelle;extracellular region part;extracellular v	Hepatitis C;Measles;Protein processing in endoplasmic reticulum;RNA transport	Homo sapiens	1	32
acrosomal matrix;cell part;cell projection;cilium;cytoplasmic part;cytoplasmic vesicle part;intracellular membrane-bounded organelle;intracellular organelle;intracellular organelle lumen;intracellular organelle part;intracellular p	Citrate cycle (TCA cycle);Glycine, serine and threonine metabolism;Glycolysis / Gluconeogenesis;Pyruva	Homo sapiens	1	32
cell part;chromatin;chromosomal part;extracellular membrane-bounded organelle;extracellular organelle;extracellular region part;extracellular vesicular exosome;heterochromatin;intracellular membrane-bounded organelle;intracellular organelle;intracellular organelle part;intracellular part;macromolecular complex;membrane-bour		Homo sapiens	1	32
cell part;cytoplasmic part;cytosol;intracellular part;membrane	Pyrimidine metabolism	Homo sapiens	1	32
anchored to membrane;cell part;cell surface;cytoplasm;extracellular membrane-bounded organelle;extracellular organelle;extracellular region part;extracellular vesicular exosome;intracellular part;intrinsic to membrane;memb	Nicotinate and nicotinamide metabolism;Purine metabolism;Pyrimidine metabolism	Homo sapiens	1	32
cell part;intracellular membrane-bounded organelle;intracellular organelle;intracellular organelle part;intracellular part;membrane;membrane-bounded organelle;nuclear part;nucleoplasm;nucleus;organelle;organelle part		Homo sapiens	1	32
adherens junction;anchoring junction;cell junction;cell part;cell-substrate adherens junction;cell-substrate junction;cytoplasmic part;cytosol;cytosolic small ribosomal subunit;extracellular membrane-bounded organelle;extrac	Ribosome	Homo sapiens	1	32
cell part;cytoplasm;intracellular membrane-bounded organelle;intracellular organelle;intracellular part;membrane;membrane-bounded organelle;nucleus;organelle		Homo sapiens	1	32
cell part;chromatin;chromosomal part;cytoplasm;intracellular membrane-bounded organelle;intracellular organelle;intracellular organelle part;intracellular part;macromolecular complex;membrane-bounded organelle;nuclear chromatin;nuclear chromosome part;nuclear part;nucleoplasm;nucleus;organelle;organelle part;protein co		Homo sapiens	1	32
cell part;cytoplasm;inclusion body;intracellular non-membrane-bounded organelle;intracellular organelle;intracellular organelle part;intracellular part;macromolecular complex;membrane;membrane part;non-membrane-bounc	RNA transport	Homo sapiens	1	32
adherens junction;anchoring junction;cell junction;cell part;cell-substrate adherens junction;cell-substrate junction;coated pit;cytoplasm;cytoplasmic part;cytoplasmic vesicle membrane;cytoplasmic vesicle part;cytoskeletal p	Bacterial invasion of epithelial cells;Endocrine and other factor-regulated calcium reabsorption;Endocyto	Homo sapiens	1	32
cell part;cytoplasm;cytoplasmic part;cytosol;cytosolic large ribosomal subunit;extracellular membrane-bounded organelle;extracellular organelle;extracellular region part;extracellular vesicular exosome;intracellular organelle p	Ribosome	Homo sapiens	1	32
cell part;cytoplasmic part;cytosol;extracellular membrane-bounded organelle;extracellular organelle;extracellular region part;extracellular vesicular exosome;intracellular membrane-bounded organelle;intracellular organelle;int	Glutathione metabolism;Pentose phosphate pathway	Homo sapiens	1	32
cell part;chromatin;chromosomal part;chromosome;cytoplasm;cytoplasmic part;Golgi apparatus;intracellular membrane-bounded organelle;intracellular non-membrane-bounded organelle;intracellular organelle;intracellular o	Colorectal cancer;Mismatch repair;Pathways in cancer	Homo sapiens	1	32
cell part;intracellular membrane-bounded organelle;intracellular organelle;intracellular organelle part;intracellular part;macromolecular complex;membrane;membrane-bounded organelle;nuclear part;nucleoplasm;nucleus;organelle;organelle part;ribonucleoprotein complex		Homo sapiens	1	32
blood microparticle;cell part;cytoplasm;cytoplasmic part;cytoplasmic vesicle;cytosol;cytosolic part;cytosolic proteasome complex;extracellular membrane-bounded organelle;extracellular organelle;extracellular region part;ex	Proteasome	Homo sapiens	1	32
adherens junction;anchoring junction;cell junction;cell part;cell-substrate adherens junction;cell-substrate junction;cytoplasm;cytoplasmic part;cytosol;cytosolic small ribosomal subunit;extracellular membrane-bounded orga	Ribosome	Homo sapiens	1	32
cell part;cytoplasmic part;cytosol;cytosolic large ribosomal subunit;extracellular membrane-bounded organelle;extracellular organelle;extracellular region part;extracellular vesicular exosome;intracellular organelle part;intracel	Ribosome	Homo sapiens	1	32
cell part;cytoplasmic part;cytosol;extracellular membrane-bounded organelle;extracellular organelle;extracellular region part;extracellular vesicular exosome;intracellular part;membrane-bounded organelle;membrane-bounde	Alanine, aspartate and glutamate metabolism;Amino sugar and nucleotide sugar metabolism	Homo sapiens	1	32
adherens junction;anchoring junction;cell junction;cell part;cell-substrate adherens junction;cell-substrate junction;cytoplasm;cytoplasmic part;cytoskeletal part;focal adhesion;intracellular organelle part;intracellular part;microtubule organizing center;nuclear part;nucleoplasm;organelle part		Homo sapiens	1	32
Cajal body;cell part;cytoplasm;intracellular membrane-bounded organelle;intracellular organelle;intracellular organelle part;intracellular part;membrane-bounded organelle;nuclear body;nuclear part;nuclear speck;nucleoplasm;nucleoplasm part;nucleus;organelle;organelle part		Homo sapiens	1	32
aminoacyl-tRNA synthetase multienzyme complex;cell part;cytoplasm;cytoplasmic part;cytoskeleton;cytosol;extracellular region;intracellular membrane-bounded organelle;intracellular non-membrane-bounded organelle;intra	Aminoacyl-tRNA biosynthesis	Homo sapiens	1	32
cell part;cytoplasmic membrane-bounded vesicle;cytoplasmic part;cytoplasmic vesicle;endoplasmic reticulum;endoplasmic reticulum chaperone complex;endoplasmic reticulum lumen;endoplasmic reticulum membrane;end	Protein processing in endoplasmic reticulum	Homo sapiens	1	32
cell part;chromatin remodeling complex;ESC/E(Z) complex;histone deacetylase complex;histone methyltransferase complex;intracellular membrane-bounded organelle;intracellular organelle;intracellular organelle part;intracellular part;macromolecular complex;membrane-bounded organelle;methyltransferase complex;nuclear pa		Homo sapiens	1	32
cell part;cytoplasm;intracellular non-membrane-bounded organelle;intracellular organelle;intracellular organelle part;intracellular part;membrane;non-membrane-bounded organelle;nuclear part;nucleolus;organelle;organelle part		Homo sapiens	1	32
cell part;cytoplasm;cytoplasmic part;intracellular membrane-bounded organelle;intracellular non-membrane-bounded organelle;intracellular organelle;intracellular organelle part;intracellular part;membrane-bounded organelle;mitochondrial nucleoid;mitochondrial part;mitochondrion;non-membrane-bounded organelle;nucleoid;nu		Homo sapiens	1	32
cell part;intracellular membrane-bounded organelle;intracellular non-membrane-bounded organelle;intracellular organelle;intracellular organelle part;intracellular part;membrane;membrane-bounded organelle;non-membrane-	Ribosome biogenesis in eukaryotes	Homo sapiens	1	32
cell part;cytoplasmic part;cytosol;intracellular membrane-bounded organelle;intracellular organelle;intracellular organelle part;intracellular part;macromolecular complex;membrane-bounded organelle;nuclear part;nucleoplasn	Ubiquitin mediated proteolysis	Homo sapiens	1	32
basal lamina;cell junction;cell part;cell surface;cytoplasm;cytoplasmic part;extracellular matrix;extracellular matrix part;extracellular membrane-bounded organelle;extracellular organelle;extracellular region;extracellular region	ECM-receptor interaction	Homo sapiens	1	33
cell part;cytoplasm;cytoplasmic part;endoplasmic reticulum;endoplasmic reticulum lumen;endoplasmic reticulum part;intracellular membrane-bounded organelle;intracellular organelle;intracellular organelle lumen;intracellular	Arginine and proline metabolism	Homo sapiens	1	33
cell body;cell part;cytoplasm;cytoplasmic part;endoplasmic reticulum membrane;endoplasmic reticulum part;ER-mitochondrion membrane contact site;extracellular membrane-bounded organelle;extracellular organelle;extra	Adipocytokine signaling pathway;Fatty acid metabolism;Peroxisome;PPAR signaling pathway	Homo sapiens	1	33
cell part;cytoplasm;cytoplasmic part;cytosol;intracellular part	RNA transport	Homo sapiens	1	33
cell part;cytoplasmic part;cytosol;intracellular membrane-bounded organelle;intracellular organelle;intracellular organelle part;intracellular part;membrane;membrane-bounded organelle;membrane-enclosed lumen;mitochond	Apoptosis	Homo sapiens	1	33
cell part;chromatin;chromosomal part;euchromatin;intracellular membrane-bounded organelle;intracellular organelle;intracellular organelle part;intracellular part;macromolecular complex;membrane-bounded organelle;nuclear chromatin;nuclear chromosome part;nuclear euchromatin;nuclear part;nucleosome;nucleus;organelle;or		Homo sapiens	1	33
cell part;cytoplasmic part;cytosol;cytosolic large ribosomal subunit;intracellular membrane-bounded organelle;intracellular organelle;intracellular organelle part;intracellular part;large ribosomal subunit;macromolecular comple	Ribosome	Homo sapiens	1	33
basolateral plasma membrane;cell part;cell surface;cytoplasmic part;extracellular region part;extracellular space;integral to membrane;integral to plasma membrane;intracellular organelle part;intracellular part;intrinsic to mem	Nicotinate and nicotinamide metabolism;Pantothenate and CoA biosynthesis;Purine metabolism;Ribofla	Homo sapiens	1	33
cell part;centrosome;cytoplasm;cytoplasmic part;cytoskeletal part;cytosol;extracellular membrane-bounded organelle;extracellular organelle;extracellular region part;extracellular vesicular exosome;intracellular membrane-bo	Proteasome	Homo sapiens	1	33
acrosomal vesicle;adherens junction;anchoring junction;cell junction;cell part;cell surface;cell-substrate adherens junction;cell-substrate junction;cytoplasm;cytoplasmic membrane-bounded vesicle;cytoplasmic membrane-b	Antigen processing and presentation;Chagas disease (American trypanosomiasis);Phagosome;Protein p	Homo sapiens	1	33
cell part;cytoplasm;cytoplasmic membrane-bounded vesicle;cytoplasmic part;cytoplasmic vesicle;cytosol;extracellular membrane-bounded organelle;extracellular organelle;extracellular region part;extracellular space;extrace	Methane metabolism;Phenylalanine metabolism;Phenylpropanoid biosynthesis	Homo sapiens	1	33
cell part;chromosomal part;chromosome, centromeric region;cytoplasmic part;cytoskeleton;cytosol;extracellular membrane-bounded organelle;extracellular organelle;extracellular region part;extracellular vesicular exosome;ir	Cell cycle - yeast;Chagas disease (American trypanosomiasis);Hepatitis C;Long-term depression;Meios	Homo sapiens	1	33
cell part;cytoplasm;cytoplasmic mRNA processing body;cytoplasmic part;cytosol;cytosolic part;cytosolic proteasome complex;intracellular membrane-bounded organelle;intracellular non-membrane-bounded organelle;intra	Proteasome	Homo sapiens	1	33
adherens junction;anchoring junction;cell junction;cell part;cell-substrate adherens junction;cell-substrate junction;cytoplasm;cytoplasmic part;cytosol;cytosolic large ribosomal subunit;focal adhesion;intracellular membrane-	Ribosome	Homo sapiens	1	33
cell part;cytoplasmic part;intracellular membrane-bounded organelle;intracellular organelle;intracellular organelle lumen;intracellular organelle part;intracellular part;membrane;membrane-bounded organelle;membrane-enclos	Peroxisome;Primary bile acid biosynthesis	Homo sapiens	1	33
cell part;COPI vesicle coat;cytoplasm;cytoplasmic membrane-bounded vesicle;cytoplasmic part;cytoplasmic vesicle;cytoplasmic vesicle part;cytosol;endoplasmic reticulum-Golgi intermediate compartment;Golgi apparatus;Golgi apparatus part;Golgi membrane;Golgi-associated vesicle;intracellular membrane-bounded organell		Homo sapiens	1	33
cell part;cytoplasmic part;cytosol;extracellular membrane-bounded organelle;extracellular organelle;extracellular region part;extracellular space;extracellular vesicular exosome;intracellular membrane-bounded organelle;intra	Carbon fixation in photosynthetic organisms;Fructose and mannose metabolism;Glycolysis / Gluconeog	Homo sapiens	1	33
actin cap;actin cytoskeleton;adherens junction;anchoring junction;Arp2/3 protein complex;cell cortex part;cell junction;cell part;cell projection;cell-substrate adherens junction;cell-substrate junction;cytoplasm;cytoplasmic part;cytoskeletal part;cytoskeleton;cytosol;extracellular membrane-bounded organelle;extracellular organel		Homo sapiens	1	33
adherens junction;anchoring junction;cell junction;cell part;cell-substrate adherens junction;cell-substrate junction;cytoplasm;cytoplasmic part;cytosol;cytosolic small ribosomal subunit;extracellular membrane-bounded orga	Ribosome	Homo sapiens	1	33
cell body;cell part;cell projection;cytoplasm;cytoplasmic part;cytosol;cytosolic small ribosomal subunit;dendrite;intracellular membrane-bounded organelle;intracellular non-membrane-bounded organelle;intracellular organelle	Insulin signaling pathway;mTOR signaling pathway;Ribosome	Homo sapiens	1	33
adherens junction;anchoring junction;cell junction;cell part;cell-substrate adherens junction;cell-substrate junction;cytoplasm;cytoplasmic part;cytosol;cytosolic large ribosomal subunit;extracellular membrane-bounded orga	Ribosome	Homo sapiens	1	33
AP-2 adaptor complex;AP-type membrane coat adaptor complex;cell part;clathrin adaptor complex;clathrin coated vesicle membrane;clathrin-coated endocytic vesicle membrane;coated vesicle membrane;cytoplasmic part;	Endocrine and other factor-regulated calcium reabsorption;Endocytosis;Huntington's disease	Homo sapiens	1	33
cell junction;cell part;cell-cell contact zone;cell-cell junction;cytoplasm;cytoplasmic part;cytoplasmic vesicle membrane;cytoplasmic vesicle part;cytosol;extracellular membrane-bounded organelle;extracellular organelle;extra	Cell cycle;Neurotrophin signaling pathway;Oocyte meiosis	Homo sapiens	1	33
cell part;chromatin;chromosomal part;cytoplasm;cytoplasmic membrane-bounded vesicle;cytoplasmic part;cytoplasmic vesicle;cytosol;euchromatin;extracellular membrane-bounded organelle;extracellular organelle;extracel	Peroxisome	Homo sapiens	1	33
catalytic step 2 spliceosome;cell part;cytoplasm;extracellular membrane-bounded organelle;extracellular organelle;extracellular region part;extracellular vesicular exosome;intracellular membrane-bounded organelle;intracellu	Spliceosome	Homo sapiens	1	33
AP-type membrane coat adaptor complex;cell part;clathrin adaptor complex;clathrin coated vesicle membrane;clathrin-coated endocytic vesicle membrane;coated vesicle membrane;cytoplasmic part;cytoplasmic vesicle me	Lysosome	Homo sapiens	1	33
actin filament bundle;actomyosin;adherens junction;anchoring junction;axon part;cell body;cell junction;cell part;cell projection;cell projection part;cell-cell junction;cell-substrate adherens junction;cell-substrate junction;cont	Bacterial invasion of epithelial cells;Endometrial cancer;Focal adhesion;PPAR signaling pathway	Homo sapiens	1	33
cell part;cytoplasm;extracellular membrane-bounded organelle;extracellular organelle;extracellular region part;extracellular vesicular exosome;intracellular membrane-bounded organelle;intracellular organelle;intracellular organelle part;intracellular part;membrane-bounded organelle;membrane-bounded vesicle;nuclear part;nucle		Homo sapiens	1	33
adherens junction;anchoring junction;basolateral plasma membrane;cell junction;cell part;cell projection;cell-cell adherens junction;cell-cell junction;cytoplasmic part;extracellular membrane-bounded organelle;extracellular organelle;extracellular region part;extracellular vesicular exosome;intracellular organelle part;intracellular p		Homo sapiens	1	33
adherens junction;anchoring junction;cell cortex part;cell junction;cell part;cell-substrate adherens junction;cell-substrate junction;cortical actin cytoskeleton;cortical cytoskeleton;cytoplasmic part;cytoskeletal part;cytoskeleton;extracellular membrane-bounded organelle;extracellular organelle;extracellular region part;extracellula		Homo sapiens	1	33
Cajal body;cell part;cytoplasm;intracellular non-membrane-bounded organelle;intracellular organelle;intracellular organelle part;intracellular part;non-membrane-bounded organelle;nuclear body;nuclear part;nucleolus;nucleoplasm;nucleoplasm part;organelle;organelle part		Homo sapiens	1	33
cell part;coated vesicle membrane;COPII vesicle coat;cytoplasmic part;cytoplasmic vesicle membrane;cytoplasmic vesicle part;cytosol;endoplasmic reticulum;endoplasmic reticulum membrane;endoplasmic reticulum part;El	Protein processing in endoplasmic reticulum	Homo sapiens	1	33
axon part;cell body;cell part;cell projection;cell projection part;cytoplasmic part;cytoskeleton;cytosol;dendrite;extracellular membrane-bounded organelle;extracellular organelle;extracellular region part;extracellular vesicular e	Axon guidance	Homo sapiens	1	33
cell part;cytoplasm;cytoplasmic part;intracellular non-membrane-bounded organelle;intracellular organelle;intracellular organelle part;intracellular part;macromolecular complex;membrane;non-membrane-bounded organelle;nuclear body;nuclear part;nuclear speck;nucleoplasm part;organelle;organelle part;ribonucleoprotein com		Homo sapiens	1	33
cell part;chromatin remodeling complex;chromosomal part;cytoplasm;cytoplasmic part;cytoskeletal part;DNA helicase complex;extracellular membrane-bounded organelle;extracellular organelle;extracellular region part;extra	Wnt signaling pathway	Homo sapiens	1	33
cell part;cytoplasm;cytoplasmic part;endosomal part;endosome;endosome membrane;Golgi apparatus;intracellular membrane-bounded organelle;intracellular organelle;intracellular organelle part;intracellular part;lipid particle;membrane;membrane-bounded organelle;organelle;organelle membrane;organelle part		Homo sapiens	1	34
cell part;coated vesicle membrane;cytoplasmic part;cytoplasmic vesicle membrane;cytoplasmic vesicle part;cytosol;endoplasmic reticulum;ER to Golgi transport vesicle membrane;Golgi apparatus;Golgi apparatus part;Golgi membrane;Golgi stack;intracellular membrane-bounded organelle;intracellular non-membrane-bounded		Homo sapiens	1	34
cell part;envelope;intracellular organelle part;intracellular part;macromolecular complex;membrane;membrane part;nuclear envelope;nuclear membrane;nuclear part;nuclear pore;organelle envelope;organelle membrane;orgar	RNA transport	Homo sapiens	1	34
cell part;cytoplasmic part;endoplasmic reticulum;endoplasmic reticulum membrane;endoplasmic reticulum part;Golgi apparatus;integral to membrane;intracellular membrane-bounded organelle;intracellular organelle;intracell	Adipocytokine signaling pathway;Fatty acid metabolism;Peroxisome;PPAR signaling pathway	Homo sapiens	1	34
actin filament bundle;actomyosin;cell part;contractile fiber part;cytoplasmic part;cytoskeletal part;cytoskeleton;cytosol;extracellular membrane-bounded organelle;extracellular organelle;extracellular region part;extracellular v	Cardiac muscle contraction;Dilated cardiomyopathy;Hypertrophic cardiomyopathy (HCM);Pathways in c	Homo sapiens	1	34
cell part;cytoplasm;cytoplasmic part;extracellular membrane-bounded organelle;extracellular organelle;extracellular region part;extracellular vesicular exosome;intracellular membrane-bounded organelle;intracellular organelle	Carbon fixation pathways in prokaryotes;Citrate cycle (TCA cycle);Pathways in cancer;Renal cell carcinc	Homo sapiens	1	34
cell part;intracellular membrane-bounded organelle;intracellular organelle;intracellular organelle part;intracellular part;macromolecular complex;membrane-bounded organelle;nuclear body;nuclear part;nuclear speck;nucleopl	Spliceosome	Homo sapiens	1	34
cell part;cell surface;cytoplasm;cytoplasmic part;extracellular membrane-bounded organelle;extracellular organelle;extracellular region part;extracellular vesicular exosome;intracellular membrane-bounded organelle;intracellular organelle;intracellular organelle part;intracellular part;membrane;membrane-bounded organelle;memb		Homo sapiens	1	34
cell part;cytoplasmic part;extracellular membrane-bounded organelle;extracellular organelle;extracellular region part;extracellular vesicular exosome;intracellular membrane-bounded organelle;intracellular non-membrane-bou	Calcium signaling pathway;Huntington's disease;Parkinson's disease	Homo sapiens	1	34
adherens junction;anchoring junction;cell junction;cell part;cell projection membrane;cell projection part;cell-substrate adherens junction;cell-substrate junction;cytoplasm;cytoplasmic part;cytoskeletal part;cytosol;cytosolic s	Ribosome	Homo sapiens	1	34
cell part;cytoplasm;cytoplasmic membrane-bounded vesicle;cytoplasmic part;cytoplasmic vesicle;cytosol;extracellular membrane-bounded organelle;extracellular organelle;extracellular region part;extracellular vesicular exos	Cysteine and methionine metabolism	Homo sapiens	1	34
cell part;cytoplasmic part;cytosol;cytosolic large ribosomal subunit;endoplasmic reticulum;intracellular membrane-bounded organelle;intracellular organelle;intracellular organelle part;intracellular part;large ribosomal subunit;r	Ribosome	Homo sapiens	1	34
cell part;cytoplasmic part;intracellular membrane-bounded organelle;intracellular organelle;intracellular organelle part;intracellular part;macromolecular complex;membrane;membrane part;membrane-bounded organelle;mem	Alzheimer's disease;Huntington's disease;Oxidative phosphorylation;Parkinson's disease	Homo sapiens	1	34
adherens junction;anchoring junction;cell junction;cell part;cell projection membrane;cell projection part;cell surface;cell-substrate adherens junction;cell-substrate junction;focal adhesion;integral to membrane;integral to plas	Axon guidance	Homo sapiens	1	34
cell part;chromatin;chromosomal part;envelope;intracellular membrane-bounded organelle;intracellular organelle;intracellular organelle part;intracellular part;membrane;membrane-bounded organelle;nuclear envelope;nuclear inner membrane;nuclear membrane;nuclear part;nucleus;organelle;organelle envelope;organelle inner m		Homo sapiens	1	34
cell part;cytoplasm;cytoplasmic part;intracellular membrane-bounded organelle;intracellular organelle;intracellular organelle lumen;intracellular organelle part;intracellular part;membrane;membrane-bounded organelle;membrane-enclosed lumen;mitochondrial matrix;mitochondrial part;mitochondrion;organelle;organelle lumen;or		Homo sapiens	1	34
90S preribosome;adherens junction;anchoring junction;cell junction;cell part;cell-substrate adherens junction;cell-substrate junction;cytoplasm;cytoplasmic part;cytoskeletal part;cytosol;cytosolic small ribosomal subunit;extr	Ribosome	Homo sapiens	1	34
cell part;cytoplasmic part;cytosol;cytosolic large ribosomal subunit;intracellular organelle part;intracellular part;large ribosomal subunit;macromolecular complex;membrane;organelle part;ribonucleoprotein complex	Ribosome	Homo sapiens	1	34
cell part;cytoplasmic part;cytosol;extracellular membrane-bounded organelle;extracellular organelle;extracellular region part;extracellular vesicular exosome;intracellular organelle part;intracellular part;macromolecular comple	Proteasome	Homo sapiens	1	34
cell part;cytoplasmic part;integral to membrane;intracellular membrane-bounded organelle;intracellular organelle;intracellular organelle part;intracellular part;intrinsic to membrane;membrane;membrane part;membrane-bounc	Nicotinate and nicotinamide metabolism	Homo sapiens	1	34
cell part;cytoplasm;intracellular membrane-bounded organelle;intracellular non-membrane-bounded organelle;intracellular organelle;intracellular organelle part;intracellular part;macromolecular complex;membrane-bounded c	mRNA surveillance pathway;RNA transport;Spliceosome	Homo sapiens	1	34
cell part;cytoplasmic part;cytosol;extracellular membrane-bounded organelle;extracellular organelle;extracellular region part;extracellular vesicular exosome;intracellular membrane-bounded organelle;intracellular organelle;intracellular organelle part;intracellular part;macromolecular complex;membrane-bounded organelle;memb		Homo sapiens	1	34
catalytic step 2 spliceosome;cell part;intracellular organelle part;intracellular part;macromolecular complex;nuclear part;nucleoplasm;organelle part;prespliceosome;ribonucleoprotein complex;small nuclear ribonucleoprotein	Spliceosome	Homo sapiens	1	34
cell part;cytoplasmic part;cytosol;extracellular membrane-bounded organelle;extracellular organelle;extracellular region part;extracellular vesicular exosome;intracellular membrane-bounded organelle;intracellular organelle;int	Amino sugar and nucleotide sugar metabolism;Galactose metabolism;Pentose and glucuronate intercor	Homo sapiens	1	34
cell part;cytoplasmic part;cytosol;extracellular membrane-bounded organelle;extracellular organelle;extracellular region part;extracellular vesicular exosome;intracellular membrane-bounded organelle;intracellular non-membr	Pyrimidine metabolism;Selenocompound metabolism	Homo sapiens	1	34
caveola;cell part;cytoplasm;cytoplasmic part;cytosol;endoplasmic reticulum;intracellular membrane-bounded organelle;intracellular organelle;intracellular organelle part;intracellular part;macromolecular complex;membrane;membrane part;membrane raft;membrane-bounded organelle;mitochondrion;nuclear part;nucleoplasm;nu		Homo sapiens	1	34
cell part;cytoplasm;cytoplasmic part;cytosol;envelope;intracellular non-membrane-bounded organelle;intracellular organelle;intracellular organelle part;intracellular part;macromolecular complex;membrane;non-membrane-bounded organelle;nuclear envelope;nuclear part;nucleoplasm;organelle;organelle envelope;organelle part;		Homo sapiens	1	34
cell part;cytoplasm;envelope;intracellular organelle part;intracellular part;macromolecular complex;membrane;membrane part;nuclear envelope;nuclear membrane;nuclear part;nuclear periphery;nuclear pore;nucleoplasm;org	RNA transport	Homo sapiens	1	34
cell part;intracellular membrane-bounded organelle;intracellular organelle;intracellular organelle part;intracellular part;macromolecular complex;membrane-bounded organelle;nuclear part;nucleoplasm;nucleus;organelle;organelle part;ribonucleoprotein complex		Homo sapiens	1	34
cell part;cytoplasm;cytoplasmic part;Golgi apparatus;intracellular membrane-bounded organelle;intracellular organelle;intracellular organelle part;intracellular part;membrane-bounded organelle;nuclear part;nucleoplasm;organelle;organelle part;perinuclear region of cytoplasm		Homo sapiens	1	34
cell part;chromosomal part;chromosome, centromeric core region;cytoplasmic part;cytoskeletal part;cytosol;intracellular non-membrane-bounded organelle;intracellular organelle;intracellular organelle part;intracellular part;macromolecular complex;microtubule;midbody;non-membrane-bounded organelle;nuclear part;nucleolus;		Homo sapiens	1	34
actin cytoskeleton;adherens junction;anchoring junction;cell junction;cell part;cell projection;cell-substrate adherens junction;cell-substrate junction;cytoplasm;cytoskeleton;flotillin complex;focal adhesion;intracellular non-membrane-bounded organelle;intracellular organelle;intracellular part;lamellipodium;lateral plasma membra		Homo sapiens	1	34
cell part;cytoplasmic part;intracellular organelle part;intracellular part;membrane;mitochondrial inner membrane;mitochondrial membrane;mitochondrial part;organelle inner membrane;organelle membrane;organelle part		Homo sapiens	1	34

cell part;cell projection;cytoplasm;cytoplasmic part;dendrite;integral to membrane;intracellular membrane-bounded organelle;intracellular organelle;intracellular organelle part;intracellular part;intrinsic to membrane;membrane;membrane part;membrane-bounded organelle;membrane-enclosed lumen;mitochondrial crista;mitocho		Homo sapiens	1	35
cell part;centrosome;cytoplasmic part;cytoskeletal part;cytosol;intracellular membrane-bounded organelle;intracellular non-membrane-bounded organelle;intracellular organelle;intracellular organelle part;intracellular part;mer	Axon guidance;Chemokine signaling pathway;Focal adhesion;Leukocyte transendothelial migration;Path	Homo sapiens	1	35
cell part;intracellular non-membrane-bounded organelle;intracellular organelle;intracellular organelle part;intracellular part;membrane;non-membrane-bounded organelle;nuclear part;nucleolus;organelle;organelle part		Homo sapiens	1	35
cell part;chromatin;chromatin remodeling complex;chromosomal part;histone deacetylase complex;intracellular organelle part;intracellular part;macromolecular complex;membrane;nuclear chromatin;nuclear chromosome part;nuclear part;nucleoplasm;nucleoplasm part;NuRD complex;organelle part;protein complex;transcriptio		Homo sapiens	1	35
adherens junction;alphav-beta3 integrin complex;alphav-beta3 integrin-IGF-1-IGF1R complex;alphav-beta5 integrin complex;alphav-beta8 integrin complex;anchoring junction;cell junction;cell part;cell projection membrane;c	Arrhythmogenic right ventricular cardiomyopathy (ARVC);Cell adhesion molecules (CAMs);Dilated cardic	Homo sapiens	1	35
angiogenin-PRI complex;cell part;cytoplasm;extracellular membrane-bounded organelle;extracellular organelle;extracellular region part;extracellular vesicular exosome;intracellular part;macromolecular complex;membrane-bounded organelle;membrane-bounded vesicle;organelle;protein complex;vesicle		Homo sapiens	1	35
cell body;cell part;cell projection;cytoplasm;cytoplasmic part;cytosol;envelope;extracellular membrane-bounded organelle;extracellular organelle;extracellular region part;extracellular vesicular exosome;intracellular organelle	Glutathione metabolism;Purine metabolism;Pyrimidine metabolism	Homo sapiens	1	35
cell part;extracellular membrane-bounded organelle;extracellular organelle;extracellular region part;extracellular vesicular exosome;intracellular non-membrane-bounded organelle;intracellular organelle;intracellular organelle part;intracellular part;membrane;membrane-bounded organelle;membrane-bounded vesicle;non-membra		Homo sapiens	1	35
cell part;cytoplasmic part;cytosol;extracellular membrane-bounded organelle;extracellular organelle;extracellular region part;extracellular vesicular exosome;intracellular membrane-bounded organelle;intracellular organelle;int	One carbon pool by folate;Purine metabolism	Homo sapiens	1	35
cell part;cytoplasmic part;glycerol-3-phosphate dehydrogenase complex;intracellular organelle part;intracellular part;macromolecular complex;membrane;mitochondrial inner membrane;mitochondrial membrane;mitochondri	Glycerophospholipid metabolism	Homo sapiens	1	35
cell part;cytoplasm;cytoskeletal part;cytoskeleton;intermediate filament;intermediate filament cytoskeleton;intracellular non-membrane-bounded organelle;intracellular organelle;intracellular organelle part;intracellular part;macromolecular complex;non-membrane-bounded organelle;organelle;organelle part;protein complex		Homo sapiens	1	35
cell part;cytoplasmic part;extracellular membrane-bounded organelle;extracellular organelle;extracellular region part;extracellular vesicular exosome;intracellular membrane-bounded organelle;intracellular non-membrane-bou	Plant-pathogen interaction	Homo sapiens	1	35
cell part;cytoplasmic part;cytosol;intracellular organelle part;intracellular part;membrane;mitochondrial membrane;mitochondrial outer membrane;mitochondrial part;organelle membrane;organelle outer membrane;organelle p	Amino sugar and nucleotide sugar metabolism;Butirosin and neomycin biosynthesis;Carbohydrate diges	Homo sapiens	1	35
adherens junction;anchoring junction;axon;cell junction;cell part;cell projection;cell-substrate adherens junction;cell-substrate junction;cytoplasmic membrane-bounded vesicle;cytoplasmic part;cytoplasmic vesicle;cytoplasm	Cell cycle;Neurotrophin signaling pathway;Oocyte meiosis	Homo sapiens	1	35
adherens junction;anchoring junction;cell junction;cell part;cell-substrate adherens junction;cell-substrate junction;cytoplasm;cytoplasmic part;cytosol;cytosolic large ribosomal subunit;focal adhesion;intracellular non-membr	Ribosome	Homo sapiens	1	35
cell part;chromosome;cytoplasm;intracellular membrane-bounded organelle;intracellular non-membrane-bounded organelle;intracellular organelle;intracellular organelle part;intracellular part;membrane-bounded organelle;non-membrane-bounded organelle;nuclear part;nucleolus;nucleoplasm;nucleus;organelle;organelle part		Homo sapiens	1	35
actin cytoskeleton;adherens junction;anchoring junction;cell junction;cell part;cell-cell junction;clathrin-coated vesicle;coated vesicle;cytoplasm;cytoplasmic membrane-bounded vesicle;cytoplasmic part;cytoplasmic vesicle;cytoskeletal part;cytoskeleton;extracellular membrane-bounded organelle;extracellular organelle;extracell		Homo sapiens	1	35
cell part;cis-Golgi network;cytoplasmic part;endoplasmic reticulum-Golgi intermediate compartment;Golgi apparatus;Golgi apparatus part;Golgi membrane;Golgi stack;integral to membrane;intracellular membrane-bounded organelle;intracellular organelle;intracellular organelle part;intracellular part;intrinsic to membrane;membra		Homo sapiens	1	35
cell part;chromatin;chromosomal part;chromosome;chromosome, centromeric region;cytoplasmic part;cytosol;intracellular non-membrane-bounded organelle;intracellular organelle;intracellular organelle part;intracellular part;membrane;non-membrane-bounded organelle;nuclear part;nucleoplasm;organelle;organelle part;plasma		Homo sapiens	1	35
cell part;chromosomal part;chromosome;intracellular membrane-bounded organelle;intracellular non-membrane-bounded organelle;intracellular organelle;intracellular organelle part;intracellular part;macromolecular complex;membrane-bounded organelle;non-membrane-bounded organelle;nucleosome;nucleus;organelle;organe		Homo sapiens	1	35
cell part;centriole;centrosome;cytoplasm;cytoplasmic part;cytoskeletal part;intracellular non-membrane-bounded organelle;intracellular organelle;intracellular organelle part;intracellular part;macromolecular complex;membrane;microtubule;microtubule organizing center;microtubule organizing center part;non-membrane-bounde		Homo sapiens	1	35
cell part;cytoplasmic part;endoplasmic reticulum lumen;endoplasmic reticulum part;intracellular organelle lumen;intracellular organelle part;intracellular part;membrane;membrane-enclosed lumen;organelle lumen;organelle pa	Lysine degradation;Other types of O-glycan biosynthesis	Homo sapiens	1	35
catalytic step 2 spliceosome;cell part;cytoplasm;intracellular membrane-bounded organelle;intracellular non-membrane-bounded organelle;intracellular organelle;intracellular organelle part;intracellular part;macromolecular co	Spliceosome	Homo sapiens	1	35
cell part;cytoplasm;cytoskeletal part;extracellular membrane-bounded organelle;extracellular organelle;extracellular region part;extracellular vesicular exosome;intracellular membrane-bounded organelle;intracellular organelle	Gap junction;Pathogenic Escherichia coli infection;Phagosome	Homo sapiens	1	35
cell part;centromeric heterochromatin;chromatin;chromosomal part;chromosome;condensed chromosome;heterochromatin;intracellular membrane-bounded organelle;intracellular non-membrane-bounded organelle;intracellular organelle;intracellular organelle part;intracellular part;membrane-bounded organelle;non-membrane-b		Homo sapiens	1	35
collagen;extracellular matrix;extracellular matrix part;extracellular membrane-bounded organelle;extracellular organelle;extracellular region;extracellular region part;extracellular vesicular exosome;membrane-bounded organelle;membrane-bounded vesicle;organelle;proteinaceous extracellular matrix;vesicle		Homo sapiens	1	35
annulate lamellae;Cajal body;cell part;chromosomal part;cytoplasm;cytoplasmic part;cytosol;envelope;intracellular membrane-bounded organelle;intracellular non-membrane-bounded organelle;intracellular organelle;intracell	Ribosome biogenesis in eukaryotes;RNA transport	Homo sapiens	1	36
cell part;cytoplasm;cytoplasmic part;cytosol;extracellular membrane-bounded organelle;extracellular organelle;extracellular region part;extracellular vesicular exosome;intracellular membrane-bounded organelle;intracellular o	Proteasome	Homo sapiens	1	36
cell part;cytoplasmic part;cytosol;extracellular membrane-bounded organelle;extracellular organelle;extracellular region part;extracellular vesicular exosome;intracellular organelle part;intracellular part;membrane-bounded or	Amino sugar and nucleotide sugar metabolism;Ascorbate and aldarate metabolism;Pentose and glucuro	Homo sapiens	1	36
cell part;intracellular membrane-bounded organelle;intracellular organelle;intracellular part;macromolecular complex;membrane-bounded organelle;nucleus;organelle;ribonucleoprotein complex	Systemic lupus erythematosus	Homo sapiens	1	36
cell part;cytoplasm;cytoplasmic part;cytosol;extracellular membrane-bounded organelle;extracellular organelle;extracellular region part;extracellular vesicular exosome;intracellular organelle part;intracellular part;membrane-b	Arachidonic acid metabolism	Homo sapiens	1	36
cell part;chromosome;condensed chromosome;cytoplasm;DNA topoisomerase complex (ATP-hydrolyzing);intracellular membrane-bounded organelle;intracellular non-membrane-bounded organelle;intracellular organelle;intracellular organelle part;intracellular part;macromolecular complex;membrane-bounded organelle;non-me		Homo sapiens	1	36
anchoring collagen;cell part;collagen;collagen type VI;cytoplasmic part;endoplasmic reticulum lumen;endoplasmic reticulum part;extracellular matrix;extracellular matrix part;extracellular membrane-bounded organelle;extrac	ECM-receptor interaction;Focal adhesion;Protein digestion and absorption	Homo sapiens	1	36
cell part;cell projection;cytoplasm;cytoplasmic part;cytoskeletal part;cytosol;extracellular membrane-bounded organelle;extracellular organelle;extracellular region part;extracellular vesicular exosome;intracellular;intracellular	MAPK signaling pathway	Homo sapiens	1	36
cell part;cytoplasmic mRNA processing body;cytoplasmic part;cytosol;cytosolic part;cytosolic proteasome complex;intracellular membrane-bounded organelle;intracellular non-membrane-bounded organelle;intracellular orga	Proteasome	Homo sapiens	1	36
adherens junction;anchoring junction;cell junction;cell part;cell-substrate adherens junction;cell-substrate junction;cytoplasm;cytoplasmic part;cytoplasmic vesicle membrane;cytoplasmic vesicle part;cytosol;extracellular mer	Cell cycle;Neurotrophin signaling pathway;Oocyte meiosis	Homo sapiens	1	36
adherens junction;anchoring junction;cell junction;cell part;cell-substrate adherens junction;cell-substrate junction;cytoplasm;cytoplasmic part;cytosol;cytosolic small ribosomal subunit;extracellular membrane-bounded orga	Ribosome	Homo sapiens	1	36
cell part;cytoplasmic part;intracellular membrane-bounded organelle;intracellular non-membrane-bounded organelle;intracellular organelle;intracellular organelle part;intracellular part;membrane;membrane-bounded organelle	Base excision repair;DNA replication;Non-homologous end-joining	Homo sapiens	1	36
cell part;cytoplasm;cytoplasmic part;cytosol;extracellular membrane-bounded organelle;extracellular organelle;extracellular region part;extracellular vesicular exosome;intracellular membrane-bounded organelle;intracellular c	Aminoacyl-tRNA biosynthesis	Homo sapiens	1	36
cell part;cytoplasm;cytoplasmic part;cytosol;intracellular part	Drug metabolism - other enzymes;Purine metabolism	Homo sapiens	1	36
cell part;cytoplasmic part;endoplasmic reticulum;endoplasmic reticulum lumen;endoplasmic reticulum part;endoplasmic reticulum-Golgi intermediate compartment;extracellular membrane-bounded organelle;extracellular organelle;extracellular region part;extracellular vesicular exosome;intracellular membrane-bounded organelle		Homo sapiens	1	36
catalytic step 2 spliceosome;cell part;intracellular membrane-bounded organelle;intracellular organelle;intracellular organelle part;intracellular part;macromolecular complex;membrane-bounded organelle;nuclear part;nucleop	Spliceosome	Homo sapiens	1	36
cell part;cytoplasm;cytoplasmic part;intracellular membrane-bounded organelle;intracellular organelle;intracellular organelle part;intracellular part;membrane;membrane-bounded organelle;mitochondrial inner membrane;mito	Arginine and proline metabolism	Homo sapiens	1	36
actin cytoskeleton;actin filament;adherens junction;anchoring junction;Arp2/3 protein complex;cell junction;cell part;cell projection;cell-cell junction;cell-substrate adherens junction;cell-substrate junction;cytoplasmic part;cytoskeletal part;cytoskeleton;cytosol;excitatory synapse;extracellular membrane-bounded organelle;extrac		Homo sapiens	1	36
adherens junction;anchoring junction;apical junction complex;apical part of cell;apical plasma membrane;apicolateral plasma membrane;basolateral plasma membrane;cell junction;cell part;cell-cell adherens junction;cell-cel	Adherens junction;Epithelial cell signaling in Helicobacter pylori infection;Gap junction;Tight junction;Vib	Homo sapiens	1	36
actin cytoskeleton;actin filament bundle;actomyosin;axon part;axon terminus;axoneme;cell cortex part;cell division site part;cell part;cell projection part;chromosomal part;cleavage furrow;condensed chromosome kinetochore;cytoplasm;cytoplasmic part;cytoskeletal part;cytoskeleton;extracellular membrane-bounded organelle;		Homo sapiens	1	36
cell part;integral to membrane;intracellular non-membrane-bounded organelle;intracellular organelle;intracellular organelle part;intracellular part;intrinsic to membrane;membrane;membrane part;non-membrane-bounded organelle;nuclear membrane;nuclear part;nucleolus;organelle;organelle membrane;organelle part		Homo sapiens	1	36
cell part;cytoplasmic part;intracellular organelle part;intracellular part;membrane;mitochondrial inner membrane;mitochondrial membrane;mitochondrial part;organelle inner membrane;organelle membrane;organelle part		Homo sapiens	1	36
adherens junction;anchoring junction;cell body;cell junction;cell part;cell projection membrane;cell projection part;cell-cell junction;cell-substrate adherens junction;cell-substrate junction;cytoplasm;cytoplasmic membrane-bo	Pathogenic Escherichia coli infection	Homo sapiens	1	36
cell part;cytoplasmic part;cytosol;early endosome;endosome;extracellular membrane-bounded organelle;extracellular organelle;extracellular region part;extracellular vesicular exosome;intracellular membrane-bounded organelle;intracellular organelle;intracellular organelle part;intracellular part;late endosome;lysosomal membran		Homo sapiens	1	36
cell part;cytoplasmic part;endoplasmic reticulum part;integral to endoplasmic reticulum membrane;integral to membrane;integral to organelle membrane;intracellular organelle part;intracellular part;intrinsic to endoplasmic reticulum membrane;intrinsic to membrane;intrinsic to organelle membrane;membrane;membrane part;orga		Homo sapiens	1	36
actin cytoskeleton;adherens junction;anchoring junction;cell junction;cell part;cell-substrate adherens junction;cell-substrate junction;cytoplasm;cytoskeleton;focal adhesion;intracellular non-membrane-bounded organelle;intracellular organelle;intracellular part;membrane;non-membrane-bounded organelle;organelle;plasma mer		Homo sapiens	1	37
cell part;cell periphery;contractile fiber part;costamere;cytoplasmic part;cytoskeletal part;dystrophin-associated glycoprotein complex;extracellular membrane-bounded organelle;extracellular organelle;extracellular region part;extracellular vesicular exosome;intermediate filament;intracellular organelle part;intracellular part;macro		Homo sapiens	1	37
cell part;cell surface;chromosome;condensed chromosome;extracellular region;extracellular region part;extracellular space;intracellular membrane-bounded organelle;intracellular non-membrane-bounded organelle;intracellu	Base excision repair	Homo sapiens	1	37
axon;cell part;cell projection;cytoplasmic part;cytoskeleton;cytosol;extracellular membrane-bounded organelle;extracellular organelle;extracellular region part;extracellular vesicular exosome;intracellular membrane-bounded c	Carbon fixation in photosynthetic organisms;Fructose and mannose metabolism;Glycolysis / Gluconeog	Homo sapiens	1	37
cell part;cytoplasm;cytoskeletal part;extracellular membrane-bounded organelle;extracellular organelle;extracellular region part;extracellular space;extracellular vesicular exosome;intermediate filament;intracellular membrane-bounded organelle;intracellular organelle;intracellular organelle part;intracellular part;keratin filament;ma		Homo sapiens	1	37
cell part;extracellular membrane-bounded organelle;extracellular organelle;extracellular region part;extracellular vesicular exosome;integral to membrane;integral to plasma membrane;intracellular membrane-bounded organe	Calcium signaling pathway;Pancreatic secretion;Salivary secretion	Homo sapiens	1	37
adherens junction;alpha6-beta4 integrin complex;anchoring junction;basal plasma membrane;basement membrane;cell junction;cell part;cell projection;cell surface;cell-cell adherens junction;cell-cell junction;cell-substrate a	Arrhythmogenic right ventricular cardiomyopathy (ARVC);Cell adhesion molecules (CAMs);Dilated cardic	Homo sapiens	1	37
cell part;cytoplasm;cytoplasmic part;cytosol;cytosolic large ribosomal subunit;cytosolic part;cytosolic ribosome;intracellular membrane-bounded organelle;intracellular non-membrane-bounded organelle;intracellular organelle	Ribosome	Homo sapiens	1	37
cell part;DNA-directed RNA polymerase complex;DNA-directed RNA polymerase II, core complex;intracellular membrane-bounded organelle;intracellular organelle;intracellular organelle part;intracellular part;macromolecular c	Huntington's disease;Purine metabolism;Pyrimidine metabolism;RNA polymerase	Homo sapiens	1	37
adherens junction;anchoring junction;cell junction;cell part;cell-substrate adherens junction;cell-substrate junction;cytoplasm;cytoplasmic membrane-bounded vesicle;cytoplasmic part;cytoplasmic vesicle;cytoplasmic vesicle	Cell cycle;Neurotrophin signaling pathway;Oocyte meiosis	Homo sapiens	1	37
cell part;cytoplasmic part;endoplasmic reticulum;envelope;extracellular membrane-bounded organelle;extracellular organelle;extracellular region part;extracellular vesicular exosome;fatty acid beta-oxidation multienzyme com	Benzoate degradation;Fatty acid elongation in mitochondria;Fatty acid metabolism;Valine, leucine and is	Homo sapiens	1	37
cell part;cytoplasmic part;intracellular membrane-bounded organelle;intracellular organelle;intracellular organelle lumen;intracellular organelle part;intracellular part;macromolecular complex;membrane;membrane-bounded or	Citrate cycle (TCA cycle);Lysine degradation;Tryptophan metabolism	Homo sapiens	1	37
cell part;envelope;intracellular organelle part;intracellular part;macromolecular complex;membrane;membrane part;nuclear envelope;nuclear membrane;nuclear part;nuclear periphery;nuclear pore;organelle envelope;organell	RNA transport	Homo sapiens	1	37
actin cytoskeleton;actin filament;adherens junction;anchoring junction;cell junction;cell part;cell projection;cell-substrate adherens junction;cell-substrate junction;contractile fiber part;cytoplasm;cytoplasmic part;cytoskeletal part;cytoskeleton;focal adhesion;intracellular membrane-bounded organelle;intracellular non-membrane-		Homo sapiens	1	37
cell part;cytoplasmic part;intracellular membrane-bounded organelle;intracellular organelle;intracellular organelle lumen;intracellular organelle part;intracellular part;membrane-bounded organelle;membrane-enclosed lumen;m	Carbon fixation pathways in prokaryotes;Citrate cycle (TCA cycle);Glyoxylate and dicarboxylate metabo	Homo sapiens	1	37
cell part;cytoplasmic part;early endosome membrane;endosomal part;endosome membrane;extracellular membrane-bounded organelle;extracellular organelle;extracellular region part;extracellular vesicular exosome;intracell	Endocytosis	Homo sapiens	1	37
adherens junction;anchoring junction;cell junction;cell part;cell-substrate adherens junction;cell-substrate junction;cytoplasm;focal adhesion;intracellular membrane-bounded organelle;intracellular organelle;intracellular part;macromolecular complex;membrane;membrane-bounded organelle;nucleus;organelle;plasma membrane;		Homo sapiens	1	37
cell part;COPI vesicle coat;cytoplasmic part;cytoplasmic vesicle part;cytosol;Golgi apparatus part;Golgi membrane;intracellular organelle part;intracellular part;macromolecular complex;membrane;membrane coat;membrane part;organelle membrane;organelle part;protein complex;vesicle coat		Homo sapiens	1	37
cell part;cytoplasm;intracellular organelle part;intracellular part;macromolecular complex;membrane;membrane part;nuclear part;nuclear pore;nucleoplasm;organelle part;pore complex;protein complex		Homo sapiens	1	38
cell part;cytoplasm;cytoplasmic part;cytosol;extracellular membrane-bounded organelle;extracellular organelle;extracellular region part;extracellular vesicular exosome;intracellular part;membrane;membrane-bounded organe	Glycolysis / Gluconeogenesis;Methane metabolism	Homo sapiens	1	38
axon;cell body;cell part;cell projection;cell projection cytoplasm;cell projection part;cytoplasmic membrane-bounded vesicle;cytoplasmic part;cytoplasmic vesicle;dendrite cytoplasm;dendritic spine;endoplasmic reticulum;en	Antigen processing and presentation;Phagosome;Protein processing in endoplasmic reticulum	Homo sapiens	1	38
cell part;cytoskeletal part;extracellular membrane-bounded organelle;extracellular organelle;extracellular region part;extracellular space;extracellular vesicular exosome;intermediate filament;intracellular membrane-bounded organelle;intracellular organelle;intracellular organelle part;intracellular part;macromolecular complex;mem		Homo sapiens	1	38
cell part;cytoplasmic part;extracellular membrane-bounded organelle;extracellular organelle;extracellular region part;extracellular vesicular exosome;intracellular membrane-bounded organelle;intracellular organelle;intracellula	Carbon fixation in photosynthetic organisms;Citrate cycle (TCA cycle);Glyoxylate and dicarboxylate met	Homo sapiens	1	38
cell part;cytoplasmic part;intracellular membrane-bounded organelle;intracellular organelle;intracellular part;lysosome;lytic vacuole;membrane-bounded organelle;organelle;vacuole		Homo sapiens	1	38
axon part;axonal growth cone;cell body;cell part;cell projection part;cytoplasm;cytoplasmic part;cytoskeletal part;cytosol;endoplasmic reticulum membrane;endoplasmic reticulum part;extracellular membrane-bounded organelle;extracellular organelle;extracellular region part;extracellular vesicular exosome;growth cone;intracellu		Homo sapiens	1	38
adherens junction;anchoring junction;cell junction;cell part;cell-substrate adherens junction;cell-substrate junction;cytoplasmic part;cytosol;cytosolic large ribosomal subunit;focal adhesion;intracellular membrane-bounded o	Ribosome	Homo sapiens	1	38
cell part;cytoplasmic part;cytosol;extracellular membrane-bounded organelle;extracellular organelle;extracellular region part;extracellular vesicular exosome;intracellular part;membrane-bounded organelle;membrane-bounde	Starch and sucrose metabolism	Homo sapiens	1	38
calcium channel complex;cation channel complex;cell cortex part;cell part;cortical endoplasmic reticulum;cytoplasmic part;endoplasmic reticulum;endoplasmic reticulum lumen;endoplasmic reticulum membrane;endoplasmic reticulum part;integral to endoplasmic reticulum membrane;integral to membrane;integral to organelle r		Homo sapiens	1	38
cell part;cytoplasm;cytoplasmic part;cytosol;intracellular membrane-bounded organelle;intracellular non-membrane-bounded organelle;intracellular organelle;intracellular organelle part;intracellular part;membrane;membrane-bounded organelle;non-membrane-bounded organelle;nuclear body;nuclear part;nuclear speck;nucleolu		Homo sapiens	1	38
cell part;cytoplasmic part;intracellular membrane-bounded organelle;intracellular organelle;intracellular organelle lumen;intracellular organelle part;intracellular part;membrane-bounded organelle;membrane-enclosed lumen;mitochondrial matrix;mitochondrial part;mitochondrion;organelle;organelle lumen;organelle part		Homo sapiens	1	38
cell part;cytoplasm;intracellular organelle part;intracellular part;membrane;nuclear part;nucleoplasm;organelle part		Homo sapiens	1	38
cell part;cytoplasm;cytoplasmic part;Golgi apparatus;intracellular membrane-bounded organelle;intracellular organelle;intracellular part;membrane-bounded organelle;nucleus;organelle		Homo sapiens	1	38
adherens junction;anchoring junction;cell junction;cell part;cell-substrate adherens junction;cell-substrate junction;cytoplasm;focal adhesion;intracellular membrane-bounded organelle;intracellular organelle;intracellular part;r	Adherens junction	Homo sapiens	1	38
cell part;chromatin;chromosomal part;cytoplasm;cytoplasmic mRNA processing body;cytoplasmic part;cytosol;exon-exon junction complex;intracellular membrane-bounded organelle;intracellular non-membrane-bounded o	mRNA surveillance pathway;RNA transport	Homo sapiens	1	38
cell part;extracellular membrane-bounded organelle;extracellular organelle;extracellular region;extracellular region part;extracellular space;extracellular vesicular exosome;membrane;membrane-bounded organelle;membrane-bounded vesicle;organelle;plasma membrane;vesicle		Homo sapiens	1	38
actin cytoskeleton;actomyosin contractile ring;cell cortex part;cell division site part;cell part;contractile ring;cytoplasmic part;cytoskeletal part;cytoskeleton;intracellular membrane-bounded organelle;intracellular non-membrane-bounded organelle;intracellular organelle;intracellular organelle part;intracellular part;membrane-boun		Homo sapiens	1	38
cell part;cytoplasm;cytoplasmic part;cytosol;endoplasmic reticulum membrane;endoplasmic reticulum part;Golgi apparatus;Golgi apparatus part;Golgi membrane;intracellular membrane-bounded organelle;intracellular organelle;intracellular organelle part;intracellular part;membrane;membrane part;membrane-bounded organelle		Homo sapiens	1	39
AP-2 adaptor complex;apical plasma membrane;AP-type membrane coat adaptor complex;basolateral plasma membrane;cell part;clathrin adaptor complex;clathrin coat;clathrin coat of trans-Golgi network vesicle;clathrin co	Endocrine and other factor-regulated calcium reabsorption;Endocytosis;Huntington's disease	Homo sapiens	1	39
cell part;cytoplasm;intracellular part		Homo sapiens	1	39
cell part;cytoplasmic part;fumarate reductase complex;intracellular membrane-bounded organelle;intracellular organelle;intracellular organelle part;intracellular part;macromolecular complex;membrane;membrane part;memb	Alzheimer's disease;Citrate cycle (TCA cycle);Huntington's disease;Oxidative phosphorylation;Parkinsor	Homo sapiens	1	39
cell part;cytoplasmic part;cytoskeleton;extracellular membrane-bounded organelle;extracellular organelle;extracellular region part;extracellular vesicular exosome;intracellular membrane-bounded organelle;intracellular non-m	Cyanoamino acid metabolism;Glycine, serine and threonine metabolism;Methane metabolism;One carb	Homo sapiens	1	39
cell part;cytoplasm;cytoplasmic part;cytosol;extracellular membrane-bounded organelle;extracellular organelle;extracellular region part;extracellular vesicular exosome;intracellular part;membrane-bounded organelle;membra	RNA transport	Homo sapiens	1	39
cell body;cell part;cell projection;cytoplasm;cytoplasmic part;cytosol;cytosolic part;dendrite;endomembrane system;endoplasmic reticulum membrane;endoplasmic reticulum part;Golgi apparatus;Golgi membrane;intrac	Acute myeloid leukemia;Adipocytokine signaling pathway;ErbB signaling pathway;Glioma;Insulin signali	Homo sapiens	1	39
adherens junction;anchoring junction;cell junction;cell part;cell-substrate adherens junction;cell-substrate junction;cytoplasmic part;cytosol;cytosolic small ribosomal subunit;extracellular membrane-bounded organelle;extrac	Ribosome	Homo sapiens	1	39
adherens junction;anchoring junction;blood microparticle;cell junction;cell leading edge;cell part;cell-substrate adherens junction;cell-substrate junction;cytoplasm;cytoplasmic membrane-bounded vesicle;cytoplasmic part;cy	Cell cycle;Neurotrophin signaling pathway;Oocyte meiosis	Homo sapiens	1	39
cell part;cytoplasmic part;cytosol;eukaryotic translation initiation factor 4F complex;intracellular part;macromolecular complex;membrane;protein complex	RNA transport;Viral myocarditis	Homo sapiens	1	39
actin cytoskeleton;adherens junction;anchoring junction;axon;basal plasma membrane;basement membrane;cell cortex;cell junction;cell leading edge;cell part;cell projection;cell-substrate adherens junction;cell-substrate junction;contractile fiber part;cytoplasm;cytoplasmic membrane-bounded vesicle;cytoplasmic part;cytoplas		Homo sapiens	1	39
catalytic step 2 spliceosome;cell part;intracellular organelle part;intracellular part;macromolecular complex;nuclear part;nucleoplasm;organelle part;ribonucleoprotein complex;spliceosomal complex;U12-type spliceosomal co	Spliceosome	Homo sapiens	1	39
cell part;CUL4 RING ubiquitin ligase complex;Cul4B-RING ubiquitin ligase complex;cullin-RING ubiquitin ligase complex;cytoplasm;extracellular membrane-bounded organelle;extracellular organelle;extracellular region part;e	Nucleotide excision repair;Ubiquitin mediated proteolysis	Homo sapiens	1	39
cell part;cell surface;cytoplasmic part;extracellular membrane-bounded organelle;extracellular organelle;extracellular region part;extracellular vesicular exosome;intracellular membrane-bounded organelle;intracellular organelle;intracellular organelle part;intracellular part;macromolecular complex;membrane;membrane-bounded c		Homo sapiens	1	39
cell part;cytoplasm;intracellular membrane-bounded organelle;intracellular non-membrane-bounded organelle;intracellular organelle;intracellular organelle part;intracellular part;membrane-bounded organelle;non-membrane-bounded organelle;nuclear part;nucleolus;nucleoplasm;nucleus;organelle;organelle part		Homo sapiens	1	39
cell part;cytoplasm;extracellular membrane-bounded organelle;extracellular organelle;extracellular region part;extracellular vesicular exosome;intracellular membrane-bounded organelle;intracellular non-membrane-bounded organelle;intracellular organelle;intracellular organelle part;intracellular part;macromolecular complex;mer		Homo sapiens	1	39
cell part;extracellular membrane-bounded organelle;extracellular organelle;extracellular region part;extracellular vesicular exosome;intracellular membrane-bounded organelle;intracellular organelle;intracellular organelle part;intracellular part;macromolecular complex;mediator complex;membrane-bounded organelle;membrane-b		Homo sapiens	1	39
cell part;cytoplasmic part;cytosol;extracellular membrane-bounded organelle;extracellular organelle;extracellular region part;extracellular vesicular exosome;intracellular part;membrane-bounded organelle;membrane-bounde	Glycine, serine and threonine metabolism;Methane metabolism	Homo sapiens	1	40
catalytic step 2 spliceosome;cell part;cytoplasm;intracellular organelle part;intracellular part;macromolecular complex;nuclear part;nucleoplasm;organelle part;ribonucleoprotein complex;spliceosomal complex		Homo sapiens	1	40
cell part;cytoplasm;intracellular part		Homo sapiens	1	40
cell part;CRD-mediated mRNA stability complex;cytoplasm;cytoplasmic part;Golgi apparatus;intracellular membrane-bounded organelle;intracellular organelle;intracellular organelle part;intracellular part;macromolecular complex;membrane;membrane-bounded organelle;mitochondrial inner membrane;mitochondrial membrane;r		Homo sapiens	1	40
adherens junction;anchoring junction;cell junction;cell part;cell-substrate adherens junction;cell-substrate junction;centrosome;cytoplasm;cytoplasmic part;cytoskeletal part;cytosol;focal adhesion;intracellular membrane-bounded organelle;intracellular non-membrane-bounded organelle;intracellular organelle;intracellular organel		Homo sapiens	1	40
adherens junction;anchoring junction;cell junction;cell part;cell-substrate adherens junction;cell-substrate junction;cytoplasm;cytoplasmic part;cytosol;extracellular membrane-bounded organelle;extracellular organelle;extrac	Alzheimer's disease;Apoptosis;Protein processing in endoplasmic reticulum	Homo sapiens	1	40
cell part;collagen;cytoplasmic part;endoplasmic reticulum lumen;endoplasmic reticulum part;extracellular matrix;extracellular matrix part;extracellular membrane-bounded organelle;extracellular organelle;extracellular region;e	ECM-receptor interaction;Focal adhesion;Protein digestion and absorption	Homo sapiens	1	40
cell part;cytoplasm;cytoplasmic part;cytosol;extracellular membrane-bounded organelle;extracellular organelle;extracellular region part;extracellular vesicular exosome;intracellular membrane-bounded organelle;intracellular c	Drug metabolism - other enzymes;Purine metabolism	Homo sapiens	1	40
cell part;cytoplasm;cytoplasmic part;cytosol;endoplasmic reticulum;extracellular membrane-bounded organelle;extracellular organelle;extracellular region part;extracellular vesicular exosome;Golgi apparatus;intracellular mer	Carbon fixation pathways in prokaryotes;Citrate cycle (TCA cycle);Glyoxylate and dicarboxylate metabo	Homo sapiens	1	40
cell part;cytoplasmic part;cytosol;extracellular membrane-bounded organelle;extracellular organelle;extracellular region part;extracellular vesicular exosome;intracellular part;membrane;membrane-bounded organelle;membra	Purine metabolism	Homo sapiens	1	40
cell part;cytoplasm;cytoplasmic part;cytosol;extracellular membrane-bounded organelle;extracellular organelle;extracellular region part;extracellular vesicular exosome;intracellular membrane-bounded organelle;intracellular organelle;intracellular part;membrane;membrane-bounded organelle;membrane-bounded vesicle;nucleus		Homo sapiens	1	40
catalytic step 2 spliceosome;cell part;intracellular membrane-bounded organelle;intracellular organelle;intracellular organelle part;intracellular part;macromolecular complex;membrane;membrane-bounded organelle;nuclear part;nucleoplasm;nucleus;organelle;organelle part;ribonucleoprotein complex;spliceosomal complex		Homo sapiens	1	40
actin cytoskeleton;cell part;cytoplasm;cytoplasmic part;cytoskeleton;cytosol;extracellular membrane-bounded organelle;extracellular organelle;extracellular region part;extracellular vesicular exosome;intracellular non-membr	Amino sugar and nucleotide sugar metabolism;Galactose metabolism;Glycolysis / Gluconeogenesis;Per	Homo sapiens	1	40
aminoacyl-tRNA synthetase multienzyme complex;cell part;cytoplasm;cytoplasmic part;cytosol;extracellular membrane-bounded organelle;extracellular organelle;extracellular region part;extracellular vesicular exosome;intrac	Aminoacyl-tRNA biosynthesis	Homo sapiens	1	40
adherens junction;anchoring junction;cell junction;cell part;cell-substrate adherens junction;cell-substrate junction;cytoplasm;cytoplasmic part;cytosol;cytosolic large ribosomal subunit;focal adhesion;intracellular non-membr	Ribosome	Homo sapiens	1	40
axon;cell body;cell part;cell periphery;cell projection;cytoplasm;cytoskeletal part;dendrite;extracellular membrane-bounded organelle;extracellular organelle;extracellular region part;extracellular vesicular exosome;intracellular	Gap junction;Pathogenic Escherichia coli infection;Phagosome	Homo sapiens	1	40
cell part;cytoplasm;cytoskeletal part;extracellular membrane-bounded organelle;extracellular organelle;extracellular region part;extracellular vesicular exosome;intracellular membrane-bounded organelle;intracellular organelle	Gap junction;Pathogenic Escherichia coli infection;Phagosome	Homo sapiens	1	40
catalytic step 2 spliceosome;cell part;cytoplasmic part;extracellular membrane-bounded organelle;extracellular organelle;extracellular region part;extracellular vesicular exosome;intracellular membrane-bounded organelle;int	Spliceosome	Homo sapiens	1	40
cell part;cytoplasm;cytoplasmic part;intracellular membrane-bounded organelle;intracellular organelle;intracellular organelle part;intracellular part;macromolecular complex;membrane;membrane-bounded organelle;NatA complex;N-terminal protein acetyltransferase complex;nuclear part;nucleoplasm part;nucleus;organelle;orgar		Homo sapiens	1	40
apical part of cell;axon;cell body;cell cortex;cell part;cell projection;cell projection membrane;cell projection part;clathrin coated vesicle membrane;coated pit;coated vesicle membrane;cytoplasm;cytoplasmic membrane-bounded vesicle;cytoplasmic part;cytoplasmic vesicle;cytoplasmic vesicle membrane;cytoplasmic vesicle pa		Homo sapiens	1	40
cell part;cell surface;cytoplasmic part;extracellular membrane-bounded organelle;extracellular organelle;extracellular region part;extracellular vesicular exosome;intracellular membrane-bounded organelle;intracellular non-me	Alzheimer's disease;Huntington's disease;Oxidative phosphorylation;Parkinson's disease	Homo sapiens	1	41
catalytic step 2 spliceosome;cell part;chromatin;chromosomal part;cytoplasmic part;cytosol;extracellular membrane-bounded organelle;extracellular organelle;extracellular region part;extracellular vesicular exosome;intracellu	Spliceosome	Homo sapiens	1	41
actin filament bundle;actomyosin;bleb;cell part;cell projection;cell projection membrane;cell projection part;contractile fiber part;cytoplasmic part;cytoskeletal part;cytoskeleton;cytosol;filamentous actin;intracellular non-mem	Cardiac muscle contraction;Dilated cardiomyopathy;Hypertrophic cardiomyopathy (HCM)	Homo sapiens	1	41
cell part;cytoplasm;cytoplasmic part;cytosol;intracellular membrane-bounded organelle;intracellular organelle;intracellular part;membrane-bounded organelle;mitochondrion;organelle	Aminoacyl-tRNA biosynthesis	Homo sapiens	1	41
cell part;cytoplasm;extracellular membrane-bounded organelle;extracellular organelle;extracellular region part;extracellular vesicular exosome;intracellular membrane-bounded organelle;intracellular organelle;intracellular organelle part;intracellular part;macromolecular complex;membrane;membrane-bounded organelle;membran		Homo sapiens	1	41

adherens junction;alpha2-beta1 integrin complex;anchoring junction;axon part;axon terminus;basal part of cell;cell junction;cell outer membrane;cell part;cell projection part;cell surface;cell-substrate adherens junction;cell-su	Arrhythmogenic right ventricular cardiomyopathy (ARVC);Dilated cardiomyopathy;ECM-receptor interac	Homo sapiens	1	41
cell part;cytoplasm;cytoplasmic part;cytoskeletal part;cytoskeleton;extracellular membrane-bounded organelle;extracellular organelle;extracellular region part;extracellular space;extracellular vesicular exosome;Golgi apparatus;intermediate filament;intermediate filament cytoskeleton;intracellular membrane-bounded organelle;in		Homo sapiens	1	41
acrosomal vesicle;cell body;cell part;chaperonin-containing T-complex;cytoplasm;cytoplasmic membrane-bounded vesicle;cytoplasmic part;cytoplasmic vesicle;cytoskeletal part;cytosol;cytosolic part;extracellular membrane-bounded organelle;extracellular organelle;extracellular region part;extracellular vesicular exosome;intrac		Homo sapiens	1	41
adherens junction;anchoring junction;cell junction;cell part;cell-substrate adherens junction;cell-substrate junction;cytoplasm;cytoplasmic part;cytosol;cytosolic small ribosomal subunit;extracellular membrane-bounded orga	Ribosome	Homo sapiens	1	41
adherens junction;anchoring junction;catalytic step 2 spliceosome;cell junction;cell part;cell projection;cell-substrate adherens junction;cell-substrate junction;chromatin;chromosomal part;cytoplasm;cytoskeletal part;extrace	Spliceosome	Homo sapiens	1	41
adherens junction;anchoring junction;cell junction;cell part;cell-substrate adherens junction;cell-substrate junction;cytoplasmic part;cytoplasmic vesicle membrane;cytoplasmic vesicle part;cytosol;extracellular membrane-bo	Cell cycle;Neurotrophin signaling pathway;Oocyte meiosis	Homo sapiens	1	41
actin filament;actomyosin, actin part;adherens junction;anchoring junction;blood microparticle;cell junction;cell part;cell-substrate adherens junction;cell-substrate junction;contractile fiber part;cytoplasm;cytoplasmic part;cyt	Cardiac muscle contraction;Dilated cardiomyopathy;Hypertrophic cardiomyopathy (HCM)	Homo sapiens	1	41
cell leading edge;cell part;centrosome;chromosomal part;cytoplasm;cytoplasmic part;cytoskeletal part;cytosol;dynactin complex;dynein complex;intracellular non-membrane-bounded organelle;intracellular organelle;intracell	Huntington's disease;Vasopressin-regulated water reabsorption	Homo sapiens	1	41
cell part;centrosome;coated vesicle;cytoplasmic membrane-bounded vesicle;cytoplasmic part;cytoplasmic vesicle;cytoskeletal part;early endosome;endocytic vesicle;endoplasmic reticulum;endoplasmic reticulum-Golgi intermediate compartment;endosome;ER to Golgi transport vesicle;intracellular membrane-bounded organel		Homo sapiens	1	41
cell part;cleavage body;cytoplasm;cytoplasmic part;intracellular membrane-bounded organelle;intracellular non-membrane-bounded organelle;intracellular organelle;intracellular organelle part;intracellular part;macromolecular complex;membrane;membrane-bounded organelle;non-membrane-bounded organelle;nuclear body;nu		Homo sapiens	1	41
cell part;cytoplasm;intracellular membrane-bounded organelle;intracellular organelle;intracellular part;membrane;membrane-bounded organelle;nucleus;organelle		Homo sapiens	1	41
cell part;cytoplasm;intracellular membrane-bounded organelle;intracellular organelle;intracellular organelle part;intracellular part;membrane-bounded organelle;nuclear part;nucleoplasm;nucleus;organelle;organelle part		Homo sapiens	1	41
cell junction;cell part;clathrin coated vesicle membrane;coated pit;coated vesicle membrane;cytoplasm;cytoplasmic part;cytoplasmic vesicle membrane;cytoplasmic vesicle part;cytoskeletal part;cytosol;Golgi apparatus;intra	Bacterial invasion of epithelial cells;Endocrine and other factor-regulated calcium reabsorption;Endocyto	Homo sapiens	1	42
cell part;cytoplasm;cytoplasmic part;cytosol;extracellular membrane-bounded organelle;extracellular organelle;extracellular region part;extracellular vesicular exosome;intracellular part;membrane-bounded organelle;membra	Purine metabolism	Homo sapiens	1	42
cell junction;cell part;cell projection;cell projection part;cytoplasm;cytoplasmic part;cytoskeletal part;cytosol;dendritic spine;intracellular organelle part;intracellular part;macromolecular complex;membrane;microtubule;microtubule associated complex;neuron projection;neuron spine;organelle part;plasma membrane;postsynaptic		Homo sapiens	1	42
cell part;cytoplasm;cytoplasmic part;cytosol;intracellular organelle lumen;intracellular organelle part;intracellular part;membrane-enclosed lumen;mitochondrial matrix;mitochondrial part;organelle lumen;organelle part	Aminoacyl-tRNA biosynthesis	Homo sapiens	1	42
adherens junction;anchoring junction;cell junction;cell part;cell-substrate adherens junction;cell-substrate junction;cytoplasm;cytoplasmic part;cytosol;cytosolic large ribosomal subunit;extracellular membrane-bounded orgar	Ribosome	Homo sapiens	1	42
cell part;cytoplasmic part;extracellular membrane-bounded organelle;extracellular organelle;extracellular region part;extracellular vesicular exosome;intracellular membrane-bounded organelle;intracellular organelle;intracellular organelle lumen;intracellular organelle part;intracellular part;lipid particle;membrane;membrane-bounde		Homo sapiens	1	42
apical part of cell;cell part;cell projection part;cytoplasm;cytoplasmic part;cytosol;growth cone;intracellular part;membrane;site of polarized growth		Homo sapiens	1	42
cell part;cytoplasmic part;endoplasmic reticulum;endoplasmic reticulum membrane;endoplasmic reticulum part;extracellular membrane-bounded organelle;extracellular organelle;extracellular region part;extracellular vesicula	Lysine degradation	Homo sapiens	1	43
catalytic step 2 spliceosome;cell part;CRD-mediated mRNA stability complex;cytoplasmic part;endoplasmic reticulum;histone pre-mRNA 3'end processing complex;intracellular membrane-bounded organelle;intracellular organelle;intracellular organelle part;intracellular part;macromolecular complex;membrane;membrane-bound		Homo sapiens	1	43
cell part;coated vesicle;coated vesicle membrane;COPII vesicle coat;cytoplasm;cytoplasmic membrane-bounded vesicle;cytoplasmic part;cytoplasmic vesicle;cytoplasmic vesicle membrane;cytoplasmic vesicle part;cytosol;	Protein processing in endoplasmic reticulum	Homo sapiens	1	43
cell part;collagen;collagen type I;cytoplasmic membrane-bounded vesicle;cytoplasmic part;cytoplasmic vesicle;endoplasmic reticulum lumen;endoplasmic reticulum part;extracellular matrix;extracellular matrix part;extracellu	Amoebiasis;ECM-receptor interaction;Focal adhesion;Protein digestion and absorption	Homo sapiens	1	43
cell part;cytoplasmic part;cytoskeletal part;extracellular membrane-bounded organelle;extracellular organelle;extracellular region part;extracellular vesicular exosome;integral to membrane;integral to plasma membrane;intrac	Calcium signaling pathway;Huntington's disease;Parkinson's disease	Homo sapiens	1	43
acrosomal vesicle;adherens junction;alpha10-beta1 integrin complex;alpha11-beta1 integrin complex;alpha1-beta1 integrin complex;alpha2-beta1 integrin complex;alpha3-beta1 integrin complex;alpha7-beta1 integrin comp	Arrhythmogenic right ventricular cardiomyopathy (ARVC);Axon guidance;Bacterial invasion of epithelial	Homo sapiens	1	43
adherens junction;anchoring junction;cell cortex part;cell junction;cell part;cell projection;cell-substrate adherens junction;cell-substrate junction;chromatin;chromosomal part;cortical actin cytoskeleton;cortical cytoskeleton;c	Alzheimer's disease;Apoptosis;Focal adhesion;Protein processing in endoplasmic reticulum	Homo sapiens	1	43
blood microparticle;cell part;cell surface;chromosomal part;cytoplasmic part;cytosol;extracellular membrane-bounded organelle;extracellular organelle;extracellular region part;extracellular vesicular exosome;germ cell nucleu	Antigen processing and presentation;Endocytosis;MAPK signaling pathway;Measles;Protein processing	Homo sapiens	1	43
cell part;cytoplasm;cytoplasmic part;intracellular membrane-bounded organelle;intracellular non-membrane-bounded organelle;intracellular organelle;intracellular organelle part;intracellular part;macromolecular complex;membrane;membrane-bounded organelle;mitochondrion;non-membrane-bounded organelle;nuclear part;nuc		Homo sapiens	1	43
Cajal body;catalytic step 2 spliceosome;cell part;cytoplasm;intracellular organelle part;intracellular part;macromolecular complex;membrane;nuclear body;nuclear part;nuclear speck;nucleoplasm;nucleoplasm part;organelle p	Spliceosome	Homo sapiens	1	43
basement membrane;cell part;cytoplasmic part;extracellular matrix;extracellular matrix part;extracellular membrane-bounded organelle;extracellular organelle;extracellular region;extracellular region part;extracellular space;extracellular vesicular exosome;Golgi apparatus part;intracellular organelle part;intracellular part;membrane		Homo sapiens	1	43
adherens junction;anchoring junction;cell junction;cell part;cell projection;cell-substrate adherens junction;cell-substrate junction;cytoplasmic part;cytosol;extracellular membrane-bounded organelle;extracellular organelle;ext	Regulation of actin cytoskeleton	Homo sapiens	1	43
cell part;membrane		Homo sapiens	1	43
cell part;cytoplasm;cytoplasmic part;Golgi apparatus;intracellular membrane-bounded organelle;intracellular non-membrane-bounded organelle;intracellular organelle;intracellular organelle part;intracellular part;macromolecular complex;membrane;membrane part;membrane-bounded organelle;non-membrane-bounded organelle		Homo sapiens	1	44
cell part;cell periphery;centriolar satellite;cytoplasm;cytoplasmic part;cytoskeletal part;extracellular membrane-bounded organelle;extracellular organelle;extracellular region part;extracellular vesicular exosome;intermediate filament;intracellular non-membrane-bounded organelle;intracellular organelle;intracellular organelle part;ir		Homo sapiens	1	44
basement membrane;extracellular matrix;extracellular matrix part;extracellular membrane-bounded organelle;extracellular organelle;extracellular region;extracellular region part;extracellular space;extracellular vesicular exoso	Amoebiasis;ECM-receptor interaction;Focal adhesion;Pathways in cancer;Prion diseases;Small cell lung	Homo sapiens	1	44
cell part;cytoplasmic part;integral to membrane;intracellular membrane-bounded organelle;intracellular organelle;intracellular organelle part;intracellular part;intrinsic to membrane;macromolecular complex;membrane;membra	Calcium signaling pathway;Huntington's disease;Parkinson's disease	Homo sapiens	1	44
cell part;cytoplasm;cytoplasmic part;cytosol;extracellular membrane-bounded organelle;extracellular organelle;extracellular region part;extracellular vesicular exosome;intracellular part;membrane;membrane-bounded organe	Aminoacyl-tRNA biosynthesis	Homo sapiens	1	44
cell junction;cell part;cell projection;cell projection membrane;cell projection part;contractile fiber;cytoplasm;cytoplasmic part;cytoskeleton;dystrophin-associated glycoprotein complex;extracellular membrane-bounded organelle;extracellular organelle;extracellular region part;extracellular vesicular exosome;filopodium;filopodium		Homo sapiens	1	44
cell part;intracellular membrane-bounded organelle;intracellular organelle;intracellular part;membrane;membrane-bounded organelle;nucleus;organelle		Homo sapiens	1	44
cell part;cytoplasm;extracellular membrane-bounded organelle;extracellular organelle;extracellular region part;extracellular vesicular exosome;intracellular part;membrane-bounded organelle;membrane-bounded vesicle;orga	Oocyte meiosis	Homo sapiens	1	44
cell part;cytoplasmic part;integral to membrane;intracellular membrane-bounded organelle;intracellular non-membrane-bounded organelle;intracellular organelle;intracellular organelle part;intracellular part;intrinsic to membrane;membrane;membrane part;membrane-bounded organelle;mitochondrial inner membrane;mitochondri		Homo sapiens	1	44
cell part;chromatin;chromatin remodeling complex;chromosomal part;cytoplasm;DNA helicase complex;euchromatin;extracellular membrane-bounded organelle;extracellular organelle;extracellular region part;extracellular vesicular exosome;H4/H2A histone acetyltransferase complex;histone acetyltransferase complex;histone m		Homo sapiens	1	44
catalytic step 2 spliceosome;cell part;intracellular membrane-bounded organelle;intracellular organelle;intracellular organelle part;intracellular part;macromolecular complex;membrane;membrane-bounded organelle;nuclear b	Spliceosome	Homo sapiens	1	45
actin cap;actin cytoskeleton;adherens junction;anchoring junction;blood microparticle;cell cortex part;cell junction;cell part;cell projection;cell-substrate adherens junction;cell-substrate junction;cortical actin cytoskeleton;cor	Fc gamma R-mediated phagocytosis;Regulation of actin cytoskeleton	Homo sapiens	1	45
cell part;contractile fiber part;cytoplasmic part;cytoskeletal part;cytosol;intracellular organelle part;intracellular part;muscle thin filament tropomyosin;organelle part	Cardiac muscle contraction;Dilated cardiomyopathy;Hypertrophic cardiomyopathy (HCM)	Homo sapiens	1	45
cell part;cytoplasm;cytoplasmic part;cytoskeletal part;cytosol;extracellular membrane-bounded organelle;extracellular organelle;extracellular region part;extracellular vesicular exosome;intermediate filament;intracellular membrane-bounded organelle;intracellular organelle;intracellular organelle part;intracellular part;keratin filame		Homo sapiens	1	45
acrosomal vesicle;actin cytoskeleton;adherens junction;anchoring junction;catenin complex;cell junction;cell part;cell projection;cell-cell adherens junction;cell-cell contact zone;cell-cell junction;cell-substrate adherens juncti	Adherens junction;Arrhythmogenic right ventricular cardiomyopathy (ARVC);Bacterial invasion of epithel	Homo sapiens	1	45
basement membrane;extracellular matrix;extracellular matrix part;extracellular membrane-bounded organelle;extracellular organelle;extracellular region;extracellular region part;extracellular space;extracellular vesicular exosome;fibril;membrane-bounded organelle;membrane-bounded vesicle;microfibril;organelle;proteinaceous e		Homo sapiens	1	45
cell part;cytoplasm;cytoplasmic part;cytosol;extracellular membrane-bounded organelle;extracellular organelle;extracellular region part;extracellular vesicular exosome;intracellular membrane-bounded organelle;intracellular c	Aminoacyl-tRNA biosynthesis;Selenocompound metabolism	Homo sapiens	1	45
cell body;cell part;cell projection;cytoplasm;intracellular membrane-bounded organelle;intracellular organelle;intracellular organelle part;intracellular part;membrane;membrane-bounded organelle;neuronal cell body;nuclear pa	Basal transcription factors	Homo sapiens	1	45
cell part;cytoplasm;cytoplasmic part;cytosol;endoplasmic reticulum part;endoplasmic reticulum tubular network;envelope;extracellular membrane-bounded organelle;extracellular organelle;extracellular region part;extracellula	RNA transport	Homo sapiens	1	45
Cajal body;cell part;cytoplasm;intracellular membrane-bounded organelle;intracellular non-membrane-bounded organelle;intracellular organelle;intracellular organelle part;intracellular part;membrane-bounded organelle;non-r	Spliceosome	Homo sapiens	1	45
cell part;intracellular membrane-bounded organelle;intracellular organelle;intracellular organelle part;intracellular part;membrane-bounded organelle;nuclear part;nucleoplasm;nucleus;organelle;organelle part		Homo sapiens	1	45
cell part;chromatin remodeling complex;chromatin silencing complex;chromosome;condensed chromosome;intracellular membrane-bounded organelle;intracellular non-membrane-bounded organelle;intracellular organelle;intracellular organelle part;intracellular part;ISWI complex;macromolecular complex;membrane-bounded o		Homo sapiens	1	46
cell part;chromosomal part;chromosome;condensed chromosome;condensin complex;cytoplasm;cytoplasmic part;cytosol;extracellular membrane-bounded organelle;extracellular organelle;extracellular region part;extracellu	Cell cycle - yeast	Homo sapiens	1	46
cell part;cell periphery;cytoplasm;cytoplasmic part;cytoskeletal part;cytosol;extracellular membrane-bounded organelle;extracellular organelle;extracellular region part;extracellular vesicular exosome;intermediate filament;intracellular membrane-bounded organelle;intracellular organelle;intracellular organelle part;intracellular part		Homo sapiens	1	46
cell part;cytoplasmic part;cytosol;extracellular membrane-bounded organelle;extracellular organelle;extracellular region part;extracellular vesicular exosome;intracellular part;membrane-bounded organelle;membrane-bounde	One carbon pool by folate;Purine metabolism	Homo sapiens	1	46
cell part;cytoplasm;cytoplasmic part;intracellular membrane-bounded organelle;intracellular non-membrane-bounded organelle;intracellular organelle;intracellular organelle lumen;intracellular organelle part;intracellular part;membrane;membrane-bounded organelle;membrane-enclosed lumen;mitochondrial matrix;mitochondrial r		Homo sapiens	1	46
cell part;cytoplasm;cytoplasmic part;cytosol;eukaryotic 43S preinitiation complex;eukaryotic 48S preinitiation complex;eukaryotic translation initiation factor 3 complex;eukaryotic translation initiation factor 3 complex, eIF3m	RNA transport	Homo sapiens	1	46
axon part;axonal spine;cell part;cell projection;cell projection part;cytoplasm;cytoplasmic part;cytoplasmic vesicle;cytosol;early endosome;early endosome membrane;endosomal part;endosome;endosome membrane;extrac	Endocytosis;ko05152;Phagosome	Homo sapiens	1	46
cell part;intracellular membrane-bounded organelle;intracellular organelle;intracellular organelle part;intracellular part;membrane-bounded organelle;nuclear part;nucleoplasm;nucleus;organelle;organelle part		Homo sapiens	1	46
actin cytoskeleton;adherens junction;anchoring junction;cell junction;cell part;cytoplasm;cytoplasmic part;cytoskeleton;cytosol;extracellular membrane-bounded organelle;extracellular organelle;extracellular region part;extracellular vesicular exosome;intracellular membrane-bounded organelle;intracellular non-membrane-bound		Homo sapiens	1	46
catalytic step 2 spliceosome;cell part;cytoplasm;cytoskeleton;extracellular membrane-bounded organelle;extracellular organelle;extracellular region part;extracellular vesicular exosome;intermediate filament cytoskeleton;intra	Spliceosome	Homo sapiens	1	47
adherens junction;anchoring junction;catalytic step 2 spliceosome;cell junction;cell part;cell-substrate adherens junction;cell-substrate junction;cytoplasm;cytoplasmic part;cytosol;extracellular membrane-bounded organelle;	mRNA surveillance pathway;RNA degradation;RNA transport	Homo sapiens	1	47
adherens junction;anchoring junction;cell cortex part;cell junction;cell part;cell-substrate adherens junction;cell-substrate junction;cortical actin cytoskeleton;cortical cytoskeleton;cytoplasmic part;cytoskeletal part;cytoskeleton;extracellular membrane-bounded organelle;extracellular organelle;extracellular region part;extracellula		Homo sapiens	1	47
actin cap;actin cytoskeleton;cell cortex part;cell part;contractile fiber;cytoplasmic part;cytoskeletal part;cytoskeleton;cytosol;intracellular non-membrane-bounded organelle;intracellular organelle;intracellular organelle part;int	Vascular smooth muscle contraction	Homo sapiens	1	47
actin cytoskeleton;cell part;cytoplasm;cytoplasmic part;cytoskeleton;cytosol;extracellular membrane-bounded organelle;extracellular organelle;extracellular region part;extracellular vesicular exosome;intracellular membrane-	Fatty acid biosynthesis;Insulin signaling pathway;Propanoate metabolism;Pyruvate metabolism	Homo sapiens	1	47
catalytic step 2 spliceosome;cell part;intracellular organelle part;intracellular part;macromolecular complex;nuclear part;nucleoplasm;organelle part;ribonucleoprotein complex;small nuclear ribonucleoprotein complex;spliceo	Spliceosome	Homo sapiens	1	47
adherens junction;anchoring junction;cell junction;cell part;cell-cell junction;cytoplasm;cytoplasmic part;cytosol;intracellular organelle part;intracellular part;membrane;nuclear part;nucleoplasm;occluding junction;organelle pa	Tight junction;Vibrio cholerae infection	Homo sapiens	1	47
adherens junction;anchoring junction;cell junction;cell part;cell-substrate adherens junction;cell-substrate junction;cytoplasmic membrane-bounded vesicle lumen;cytoplasmic part;cytoplasmic vesicle part;endocytic vesicle l	Protein processing in endoplasmic reticulum	Homo sapiens	1	47
cell part;cytoplasm;intracellular membrane-bounded organelle;intracellular non-membrane-bounded organelle;intracellular organelle;intracellular organelle part;intracellular part;macromolecular complex;membrane-bounded c	Spliceosome	Homo sapiens	1	48
cell part;cytoplasm;cytoplasmic part;cytosol;intracellular membrane-bounded organelle;intracellular organelle;intracellular part;membrane-bounded organelle;nucleus;organelle	Protein processing in endoplasmic reticulum	Homo sapiens	1	48
cell part;cytoplasm;cytoplasmic part;intracellular membrane-bounded organelle;intracellular organelle;intracellular organelle lumen;intracellular organelle part;intracellular part;membrane-bounded organelle;membrane-enclose	Alanine, aspartate and glutamate metabolism;Arginine and proline metabolism;D-Glutamine and D-gluta	Homo sapiens	1	48
blood microparticle;cell part;cell surface;coated pit;cytoplasmic membrane-bounded vesicle;cytoplasmic part;cytoplasmic vesicle;endosome;external side of plasma membrane;extracellular membrane-bounded organelle;ext	Endocytosis;Hematopoietic cell lineage;Phagosome	Homo sapiens	1	48
cell part;cytoplasm;cytoplasmic membrane-bounded vesicle;cytoplasmic part;cytoplasmic vesicle;cytoplasmic vesicle membrane;cytoplasmic vesicle part;endocytic vesicle membrane;extracellular membrane-bounded organelle;extracellular organelle;extracellular region part;extracellular vesicular exosome;intracellular membrar		Homo sapiens	1	48
adherens junction;anchoring junction;cell junction;cell part;cell-substrate adherens junction;cell-substrate junction;cytoplasm;cytoplasmic part;cytosol;cytosolic large ribosomal subunit;extracellular membrane-bounded orgar	Ribosome	Homo sapiens	1	48
alpha DNA polymerase:primase complex;cell part;centrosome;chromosomal part;cytoplasmic part;cytoskeletal part;intracellular membrane-bounded organelle;intracellular non-membrane-bounded organelle;intracellular orga	Cell cycle;Cell cycle - yeast;DNA replication;Meiosis - yeast	Homo sapiens	1	48
adherens junction;anchoring junction;cell junction;cell part;cell-substrate adherens junction;cell-substrate junction;cytoplasm;cytoplasmic part;cytosol;cytosolic large ribosomal subunit;extracellular membrane-bounded orgar	Ribosome	Homo sapiens	1	48
cell part;cytoplasm;extracellular membrane-bounded organelle;extracellular organelle;extracellular region part;extracellular vesicular exosome;intracellular membrane-bounded organelle;intracellular organelle;intracellular organelle part;intracellular part;membrane;membrane-bounded organelle;membrane-bounded vesicle;nuclea		Homo sapiens	1	48
cell part;cytoplasm;intracellular membrane-bounded organelle;intracellular non-membrane-bounded organelle;intracellular organelle;intracellular organelle part;intracellular part;membrane;membrane-bounded organelle;non-r	Cytosolic DNA-sensing pathway;Measles	Homo sapiens	1	48
cell part;cytoplasm;cytoplasmic part;cytosol;eukaryotic translation initiation factor 4F complex;extracellular membrane-bounded organelle;extracellular organelle;extracellular region part;extracellular vesicular exosome;intrace	RNA transport	Homo sapiens	1	48
actin filament bundle;actomyosin;adherens junction;anchoring junction;cell cortex part;cell junction;cell part;cell-substrate adherens junction;cell-substrate junction;contractile fiber part;cortical cytoskeleton;cytoplasmic part;	Cardiac muscle contraction;Dilated cardiomyopathy;Hypertrophic cardiomyopathy (HCM)	Homo sapiens	1	48
cell part;cytoplasm;cytoskeletal part;intracellular membrane-bounded organelle;intracellular non-membrane-bounded organelle;intracellular organelle;intracellular organelle part;intracellular part;membrane-bounded organelle;non-membrane-bounded organelle;nuclear part;nucleolus;nucleus;organelle;organelle part;spindle		Homo sapiens	1	48
cell part;cytoplasmic part;endoplasmic reticulum membrane;endoplasmic reticulum part;extracellular membrane-bounded organelle;extracellular organelle;extracellular region part;extracellular vesicular exosome;intracellular organelle part;intracellular part;membrane;membrane part;membrane-bounded organelle;membrane-bou		Homo sapiens	1	48
cell part;cytoplasmic part;endoplasmic reticulum;endoplasmic reticulum lumen;endoplasmic reticulum part;endoplasmic reticulum-Golgi intermediate compartment;extracellular membrane-bounded organelle;extracellular org	Protein processing in endoplasmic reticulum	Homo sapiens	1	48
basement membrane;cell part;cytoplasmic part;extracellular matrix;extracellular matrix part;extracellular membrane-bounded organelle;extracellular organelle;extracellular region;extracellular region part;extracellular space;ex	Amoebiasis;ECM-receptor interaction;Focal adhesion;Pathways in cancer;Small cell lung cancer;Toxopl	Homo sapiens	1	49
cell part;chromosomal part;cytoplasmic mRNA processing body;cytoplasmic part;intracellular membrane-bounded organelle;intracellular non-membrane-bounded organelle;intracellular organelle;intracellular organelle part;intracellular part;macromolecular complex;membrane-bounded organelle;non-membrane-bounded organe		Homo sapiens	1	49
cell part;centrosome;cytoplasm;cytoplasmic part;cytoskeletal part;cytosol;extracellular membrane-bounded organelle;extracellular organelle;extracellular region part;extracellular vesicular exosome;internal side of plasma me	Glutathione metabolism;Pentose phosphate pathway	Homo sapiens	1	49
cell part;cytoplasm;cytoplasmic part;cytosol;intracellular membrane-bounded organelle;intracellular organelle;intracellular organelle lumen;intracellular organelle part;intracellular part;membrane;membrane-bounded organelle	Citrate cycle (TCA cycle);Pyruvate metabolism	Homo sapiens	1	49
cell part;cytoplasmic part;cytosol;endoplasmic reticulum-Golgi intermediate compartment;external side of plasma membrane;extracellular membrane-bounded organelle;extracellular organelle;extracellular region part;extrace	Glutathione metabolism;Hematopoietic cell lineage;Renin-angiotensin system	Homo sapiens	1	49
axon;cell part;cell projection;cytoplasm;cytoplasmic membrane-bounded vesicle;cytoplasmic part;cytoplasmic vesicle;cytosol;extracellular membrane-bounded organelle;extracellular organelle;extracellular region part;extrac	Aminoacyl-tRNA biosynthesis	Homo sapiens	1	49
cell body;cell part;chaperonin-containing T-complex;cytoplasm;cytoplasmic part;cytoskeletal part;cytoskeleton;cytosol;cytosolic part;extracellular membrane-bounded organelle;extracellular organelle;extracellular region part;extracellular vesicular exosome;intracellular non-membrane-bounded organelle;intracellular organelle;int		Homo sapiens	1	49
cell part;cytoplasm;cytoplasmic part;cytosol;extracellular membrane-bounded organelle;extracellular organelle;extracellular region part;extracellular vesicular exosome;intracellular part;membrane;membrane-bounded organe	Insulin signaling pathway;Starch and sucrose metabolism	Homo sapiens	1	50
acrosomal vesicle;cell body;cell part;centrosome;chaperonin-containing T-complex;chromatin;chromosomal part;cytoplasmic membrane-bounded vesicle;cytoplasmic part;cytoplasmic vesicle;cytoskeletal part;cytosol;cytosolic part;extracellular membrane-bounded organelle;extracellular organelle;extracellular region part;extrac		Homo sapiens	1	50
cell part;intracellular organelle part;intracellular part;membrane;nuclear inner membrane;nuclear matrix;nuclear membrane;nuclear part;nucleoplasm;organelle inner membrane;organelle membrane;organelle part		Homo sapiens	1	50
cell part;cytoplasmic part;cytoskeletal part;cytosol;extracellular membrane-bounded organelle;extracellular organelle;extracellular region;extracellular region part;extracellular vesicular exosome;intracellular organelle part;intra	Gap junction;Pathogenic Escherichia coli infection;Phagosome	Homo sapiens	1	50
cell part;cytoplasmic part;cytoskeletal part;cytoskeleton;cytosol;extracellular membrane-bounded organelle;extracellular organelle;extracellular region part;extracellular vesicular exosome;intracellular membrane-bounded org	Gap junction;Pathogenic Escherichia coli infection;Phagosome	Homo sapiens	1	50
CCR4-NOT complex;cell part;chromatin;chromosomal part;cytoplasm;cytoplasmic part;cytoskeleton;cytosol;heterochromatin;intracellular membrane-bounded organelle;intracellular non-membrane-bounded organelle;intracellular organelle;intracellular organelle part;intracellular part;macromolecular complex;membrane;membra		Homo sapiens	1	50
actin cytoskeleton;actin filament bundle;actomyosin;adherens junction;anchoring junction;cell division site part;cell junction;cell part;cell-substrate adherens junction;cell-substrate junction;cleavage furrow;cytoplasm;cytoskeletal part;cytoskeleton;focal adhesion;intracellular non-membrane-bounded organelle;intracellular organe		Homo sapiens	1	50
blood microparticle;cell part;cytoskeletal part;cytoskeleton;extracellular matrix;extracellular membrane-bounded organelle;extracellular organelle;extracellular region part;extracellular space;extracellular vesicular exosome;intermediate filament;intracellular membrane-bounded organelle;intracellular non-membrane-bounded orga		Homo sapiens	1	51
cell body;cell part;cytoplasmic part;cytoskeletal part;cytoskeleton;cytosol;extracellular membrane-bounded organelle;extracellular organelle;extracellular region part;extracellular vesicular exosome;intracellular membrane-bo	Gap junction;Pathogenic Escherichia coli infection;Phagosome	Homo sapiens	1	51
cell part;chromosomal part;cytoplasmic part;cytosol;intracellular membrane-bounded organelle;intracellular non-membrane-bounded organelle;intracellular organelle;intracellular organelle part;intracellular part;Ku70:Ku80 co	Non-homologous end-joining	Homo sapiens	1	51
cell part;chromosomal part;chromosome, telomeric region;cytoplasmic part;cytosol;intracellular membrane-bounded organelle;intracellular non-membrane-bounded organelle;intracellular organelle;intracellular organelle part;	Non-homologous end-joining	Homo sapiens	1	51
catalytic step 2 spliceosome;cell part;cytoplasm;intracellular membrane-bounded organelle;intracellular organelle;intracellular organelle part;intracellular part;macromolecular complex;membrane;membrane-bounded organelle;nuclear part;nucleoplasm;nucleus;organelle;organelle part;ribonucleoprotein complex;spliceosomal co		Homo sapiens	1	51
cell part;centromeric heterochromatin;chromatin;chromosomal part;heterochromatin;intracellular membrane-bounded organelle;intracellular organelle;intracellular organelle part;intracellular part;membrane-bounded organelle	Cysteine and methionine metabolism	Homo sapiens	1	51
cell part;cytoplasmic part;cytosol;extracellular membrane-bounded organelle;extracellular organelle;extracellular region part;extracellular space;extracellular vesicular exosome;intracellular membrane-bounded organelle;intra	Nicotinate and nicotinamide metabolism	Homo sapiens	1	51
cell part;chromatin;chromosomal part;cytoplasm;cytoskeleton;intracellular membrane-bounded organelle;intracellular non-membrane-bounded organelle;intracellular organelle;intracellular organelle part;intracellular part;mac	Cell cycle;Cell cycle - yeast;DNA replication;Meiosis - yeast	Homo sapiens	1	51
cell body;cell part;centrosome;chaperonin-containing T-complex;cytoplasm;cytoplasmic membrane-bounded vesicle;cytoplasmic part;cytoplasmic vesicle;cytoskeletal part;cytosol;cytosolic part;extracellular membrane-bounded organelle;extracellular organelle;extracellular region part;extracellular vesicular exosome;intracellular		Homo sapiens	1	51
cell part;cytoplasmic part;cytosol;extracellular membrane-bounded organelle;extracellular organelle;extracellular region part;extracellular vesicular exosome;intracellular membrane-bounded organelle;intracellular organelle;in	Fructose and mannose metabolism;Galactose metabolism;Glycolysis / Gluconeogenesis;Methane meta	Homo sapiens	1	51
adherens junction;anchoring junction;cell cortex;cell junction;cell part;cell-substrate adherens junction;cell-substrate junction;cytoplasm;cytoplasmic part;cytoskeleton;focal adhesion;intracellular non-membrane-bounded organelle;intracellular organelle;intracellular part;membrane;non-membrane-bounded organelle;organelle;pla		Homo sapiens	1	51
cell part;CUL4 RING ubiquitin ligase complex;Cul4A-RING ubiquitin ligase complex;Cul4B-RING ubiquitin ligase complex;cullin-RING ubiquitin ligase complex;cytoplasm;extracellular membrane-bounded organelle;extracellu	Nucleotide excision repair;Ubiquitin mediated proteolysis	Homo sapiens	1	51
cell part;cytoplasmic part;extracellular matrix;extracellular membrane-bounded organelle;extracellular organelle;extracellular region part;extracellular vesicular exosome;Golgi apparatus;Golgi apparatus part;Golgi membrane;	Cell adhesion molecules (CAMs)	Homo sapiens	1	51
cell part;cytoplasm;cytoplasmic part;cytosol;intracellular membrane-bounded organelle;intracellular non-membrane-bounded organelle;intracellular organelle;intracellular organelle part;intracellular part;membrane;membrane-	mRNA surveillance pathway;RNA transport;Spliceosome	Homo sapiens	1	51
adherens junction;anchoring junction;cell junction;cell part;cell projection;cell projection part;cell-cell adherens junction;cell-cell junction;cytoplasm;cytoplasmic part;cytosol;dendritic spine;extracellular membrane-bounded o	Adherens junction;Leukocyte transendothelial migration	Homo sapiens	1	52
cell part;cytoplasmic part;cytosol;eukaryotic translation initiation factor 4F complex;intracellular part;macromolecular complex;protein complex	mTOR signaling pathway;RNA transport	Homo sapiens	1	52
cell part;cytoplasm;cytoplasmic part;cytosol;extracellular membrane-bounded organelle;extracellular organelle;extracellular region part;extracellular vesicular exosome;intracellular part;membrane;membrane-bounded organe	Aminoacyl-tRNA biosynthesis	Homo sapiens	1	52
cell part;cytoplasmic part;cytosol;extracellular membrane-bounded organelle;extracellular organelle;extracellular region part;extracellular vesicular exosome;intracellular membrane-bounded organelle;intracellular organelle;intracellular part;membrane-bounded organelle;membrane-bounded vesicle;nucleus;organelle;vesicle		Homo sapiens	1	52
cell part;cytoplasm;cytoplasmic part;cytoskeleton;extracellular membrane-bounded organelle;extracellular organelle;extracellular region part;extracellular vesicular exosome;intracellular membrane-bounded organelle;intracellular non-membrane-bounded organelle;intracellular organelle;intracellular organelle part;intracellular part		Homo sapiens	1	52
cell part;centrosome;chromatin;chromatin remodeling complex;chromosomal part;cytoplasm;cytoplasmic part;cytoskeletal part;histone deacetylase complex;intracellular membrane-bounded organelle;intracellular non-membrane-bounded organelle;intracellular organelle;intracellular organelle part;intracellular part;macromolecul		Homo sapiens	1	52
cell part;chromosomal part;chromosome, telomeric region;intracellular membrane-bounded organelle;intracellular organelle;intracellular organelle part;intracellular part;macromolecular complex;membrane;membrane-bounde	Homologous recombination;Non-homologous end-joining	Homo sapiens	1	52
cell part;cytoplasmic microtubule;cytoplasmic part;cytoskeletal part;intracellular membrane-bounded organelle;intracellular organelle;intracellular organelle part;intracellular part;macromolecular complex;membrane-bounded	Gap junction;Pathogenic Escherichia coli infection;Phagosome	Homo sapiens	1	52
cell part;cytoskeletal part;extracellular membrane-bounded organelle;extracellular organelle;extracellular region part;extracellular vesicular exosome;intermediate filament;intracellular membrane-bounded organelle;intracellular organelle;intracellular organelle part;intracellular part;keratin filament;macromolecular complex;membra		Homo sapiens	1	53
cell part;cell surface;cytoplasmic membrane-bounded vesicle;cytoplasmic membrane-bounded vesicle lumen;cytoplasmic part;cytoplasmic vesicle;cytoplasmic vesicle part;endoplasmic reticulum;endoplasmic reticulum lum	ECM-receptor interaction;Focal adhesion;Malaria;Phagosome;TGF-beta signaling pathway	Homo sapiens	1	53
cell part;cytoplasm;cytoskeletal part;extracellular membrane-bounded organelle;extracellular organelle;extracellular region part;extracellular vesicular exosome;intermediate filament;intracellular membrane-bounded organelle;intracellular organelle;intracellular organelle part;intracellular part;keratin filament;macromolecular compl		Homo sapiens	1	53
adherens junction;anchoring junction;cell junction;cell part;cell-substrate adherens junction;cell-substrate junction;cytoplasm;cytoplasmic part;cytosol;extracellular membrane-bounded organelle;extracellular organelle;extracellular region part;extracellular vesicular exosome;focal adhesion;Golgi apparatus;intracellular membrane		Homo sapiens	1	53
cell part;chromosomal part;intracellular membrane-bounded organelle;intracellular organelle;intracellular organelle part;intracellular part;macromolecular complex;MCM complex;membrane-bounded organelle;nuclear chromo	Cell cycle;Cell cycle - yeast;DNA replication;Meiosis - yeast	Homo sapiens	1	53
cell part;cullin-RING ubiquitin ligase complex;cytoplasm;extracellular membrane-bounded organelle;extracellular organelle;extracellular region part;extracellular vesicular exosome;intracellular membrane-bounded organelle;intracellular organelle;intracellular organelle part;intracellular part;macromolecular complex;membrane;me		Homo sapiens	1	53
cell body;cell part;chaperonin-containing T-complex;cytoplasm;cytoplasmic part;cytoskeletal part;cytosol;cytosolic part;extracellular membrane-bounded organelle;extracellular organelle;extracellular region part;extracellular vesicular exosome;intracellular membrane-bounded organelle;intracellular organelle;intracellular organell		Homo sapiens	1	53
cell part;chromosomal part;condensin complex;cytoplasm;cytoplasmic part;cytosol;intracellular membrane-bounded organelle;intracellular organelle;intracellular organelle part;intracellular part;macromolecular complex;mem	Cell cycle - yeast	Homo sapiens	1	53

cell part;cytoplasmic membrane-bounded vesicle;cytoplasmic part;cytoplasmic vesicle;endoplasmic reticulum;endoplasmic reticulum membrane;endoplasmic reticulum part;integral to membrane;intracellular membrane-bou	N-Glycan biosynthesis;Protein processing in endoplasmic reticulum;Various types of N-glycan biosynthe	Homo sapiens	1	54
adherens junction;anchoring junction;blood microparticle;cell junction;cell part;cell-substrate adherens junction;cell-substrate junction;cytoplasm;endothelial microparticle;external side of plasma membrane;extracellular membrane-bounded organelle;extracellular organelle;extracellular region part;extracellular vesicular exosome		Homo sapiens	1	54
cell part;chromosomal part;intracellular membrane-bounded organelle;intracellular organelle;intracellular organelle part;intracellular part;macromolecular complex;MCM complex;membrane;membrane-bounded organelle;nuc	Cell cycle;Cell cycle - yeast;DNA replication;Meiosis - yeast	Homo sapiens	1	54
cell part;cytoplasm;cytoplasmic part;cytosol;extracellular region part;extracellular space;intracellular membrane-bounded organelle;intracellular organelle;intracellular part;membrane-bounded organelle;nucleus;organelle	Aminoacyl-tRNA biosynthesis	Homo sapiens	1	54
cell part;cell periphery;cytoplasm;cytoskeletal part;extracellular membrane-bounded organelle;extracellular organelle;extracellular region part;extracellular vesicular exosome;intermediate filament;intracellular organelle part;intracellular part;macromolecular complex;membrane-bounded organelle;membrane-bounded vesicle;orga		Homo sapiens	1	54
actin cytoskeleton;actin filament bundle;actomyosin;cell junction;cell part;cell projection;cytoplasm;cytoplasmic part;cytoskeletal part;cytoskeleton;cytosol;extracellular membrane-bounded organelle;extracellular organelle;extracellular region part;extracellular vesicular exosome;filopodium;intracellular non-membrane-bounded o		Homo sapiens	1	54
cell part;cytoplasmic part;cytosol;intracellular non-membrane-bounded organelle;intracellular organelle;intracellular organelle part;intracellular part;macromolecular complex;membrane;non-membrane-bounded organelle;nuclear part;nucleoplasm;organelle;organelle part;ribonucleoprotein complex;RNA granule;stress granule		Homo sapiens	1	54
anchoring collagen;cell part;collagen;collagen type XII;cytoplasmic part;endoplasmic reticulum lumen;endoplasmic reticulum part;extracellular matrix;extracellular matrix part;extracellular membrane-bounded organelle;extrac	Protein digestion and absorption	Homo sapiens	1	54
cell part;cytoplasm;intracellular membrane-bounded organelle;intracellular non-membrane-bounded organelle;intracellular organelle;intracellular organelle part;intracellular part;membrane;membrane-bounded organelle;non-membrane-bounded organelle;nuclear part;nucleolus;nucleoplasm;nucleus;organelle;organelle part		Homo sapiens	1	54
cell part;cytoplasm;cytoplasmic part;cytosol;intracellular membrane-bounded organelle;intracellular organelle;intracellular organelle lumen;intracellular organelle part;intracellular part;membrane-bounded organelle;membrane	Aminoacyl-tRNA biosynthesis;Valine, leucine and isoleucine biosynthesis	Homo sapiens	1	54
cell part;chromosome;intracellular membrane-bounded organelle;intracellular non-membrane-bounded organelle;intracellular organelle;intracellular organelle part;intracellular part;membrane-bounded organelle;non-membrane-bounded organelle;nuclear part;nucleoplasm;nucleus;organelle;organelle part		Homo sapiens	1	54
cell part;cytoplasm;cytoplasmic part;extracellular membrane-bounded organelle;extracellular organelle;extracellular region part;extracellular vesicular exosome;intracellular membrane-bounded organelle;intracellular non-mem	RIG-I-like receptor signaling pathway	Homo sapiens	1	55
cell part;cell projection part;cytoplasmic part;cytosol;extracellular membrane-bounded organelle;extracellular organelle;extracellular region part;extracellular vesicular exosome;flagellar fibrous sheath;flagellum part;intracellula	Cysteine and methionine metabolism;Glycolysis / Gluconeogenesis;Propanoate metabolism;Pyruvate m	Homo sapiens	1	55
cell body fiber;cell part;cell projection;cytoplasm;cytoplasmic part;cytosol;endoplasmic reticulum;extracellular membrane-bounded organelle;extracellular organelle;extracellular region part;extracellular vesicular exosome;intr	Alanine, aspartate and glutamate metabolism;Arginine and proline metabolism	Homo sapiens	1	55
cell part;cytoplasmic part;endoplasmic reticulum;endoplasmic reticulum membrane;endoplasmic reticulum part;integral to membrane;integral to plasma membrane;intracellular membrane-bounded organelle;intracellular orga	Alzheimer's disease;Calcium signaling pathway;Pancreatic secretion	Homo sapiens	1	55
cell body;cell part;chaperonin-containing T-complex;cytoplasm;cytoplasmic part;cytoskeletal part;cytosol;cytosolic part;extracellular membrane-bounded organelle;extracellular organelle;extracellular region part;extracellular vesicular exosome;intracellular organelle part;intracellular part;macromolecular complex;membrane-bour		Homo sapiens	1	55
cell part;cytoskeletal part;intermediate filament;intracellular organelle part;intracellular part;lamin filament;macromolecular complex;membrane;nuclear inner membrane;nuclear membrane;nuclear part;organelle inner membrane;organelle membrane;organelle part;protein complex		Homo sapiens	1	55
adherens junction;anchoring junction;cell junction;cell part;cell-substrate adherens junction;cell-substrate junction;cytoplasm;cytoplasmic membrane-bounded vesicle;cytoplasmic part;cytoplasmic vesicle;cytoskeletal part;cy	Endocytosis	Homo sapiens	1	55
cell part;cytoplasmic part;cytosol;eukaryotic 43S preinitiation complex;eukaryotic 48S preinitiation complex;eukaryotic translation initiation factor 3 complex;intracellular part;macromolecular complex;protein complex;ribonuc	RNA transport	Homo sapiens	1	55
cell part;cell projection;cell projection membrane;cell projection part;cytoplasmic part;cytoskeletal part;cytosol;intracellular non-membrane-bounded organelle;intracellular organelle;intracellular organelle part;intracellular part	Focal adhesion;Regulation of actin cytoskeleton;Shigellosis	Homo sapiens	1	56
actin cytoskeleton;cell part;contractile fiber part;cytoplasmic membrane-bounded vesicle lumen;cytoplasmic part;cytoplasmic vesicle part;cytoskeleton;cytosol;extracellular membrane-bounded organelle;extracellular organe	Carbon fixation in photosynthetic organisms;Fructose and mannose metabolism;Glycolysis / Gluconeog	Homo sapiens	1	56
adherens junction;anchoring junction;apical part of cell;cell division site part;cell junction;cell part;cell periphery;cell pole;cell projection;cell tip;cell-substrate adherens junction;cell-substrate junction;cleavage furrow;cytoplasi	Regulation of actin cytoskeleton	Homo sapiens	1	56
cell part;COPI vesicle coat;cytoplasmic part;cytoplasmic vesicle part;cytosol;Golgi apparatus part;intracellular organelle part;intracellular part;macromolecular complex;membrane coat;membrane part;organelle part;protein complex;vesicle coat		Homo sapiens	1	56
cell body;cell part;centrosome;chaperonin-containing T-complex;cytoplasm;cytoplasmic part;cytoskeletal part;cytosol;cytosolic part;extracellular membrane-bounded organelle;extracellular organelle;extracellular region part;extracellular vesicular exosome;intracellular non-membrane-bounded organelle;intracellular organelle;intr		Homo sapiens	1	56
cell part;chromatin;chromosomal part;euchromatin;heterochromatin;intracellular membrane-bounded organelle;intracellular organelle;intracellular organelle part;intracellular part;membrane-bounded organelle;nuclear chromatin;nuclear chromosome part;nuclear euchromatin;nuclear heterochromatin;nuclear part;nucleoplasm;nuc		Homo sapiens	1	56
cell part;cytoplasm;intracellular membrane-bounded organelle;intracellular non-membrane-bounded organelle;intracellular organelle;intracellular organelle part;intracellular part;macromolecular complex;membrane;membrane-bounded organelle;NLS-dependent protein nuclear import complex;non-membrane-bounded organelle;		Homo sapiens	1	56
cell part;cytoplasmic part;fatty acid beta-oxidation multienzyme complex;intracellular membrane-bounded organelle;intracellular non-membrane-bounded organelle;intracellular organelle;intracellular organelle part;intracellula	Aminobenzoate degradation;Benzoate degradation;beta-Alanine metabolism;Biosynthesis of unsaturate	Homo sapiens	1	57
adherens junction;anchoring junction;axon;blood microparticle;cell cortex part;cell junction;cell part;cell projection;cell-substrate adherens junction;cell-substrate junction;chromatin;chromosomal part;cortical cytoskeleton;cy	Adherens junction;Arrhythmogenic right ventricular cardiomyopathy (ARVC);Bacterial invasion of epithel	Homo sapiens	1	57
adherens junction;anchoring junction;cell cortex;cell cortex part;cell junction;cell part;cell projection;cell projection part;cell-substrate adherens junction;cell-substrate junction;coated pit;cortical cytoskeleton;cytoplasm;cytop	Bacterial invasion of epithelial cells;Pathogenic Escherichia coli infection;Shigellosis;Tight junction	Homo sapiens	1	57
cell part;cytoplasm;cytoplasmic membrane-bounded vesicle lumen;cytoplasmic part;cytoplasmic vesicle part;cytoskeletal part;cytosol;endocytic vesicle lumen;extracellular membrane-bounded organelle;extracellular organel	Protein processing in endoplasmic reticulum	Homo sapiens	1	57
basement membrane;cell part;chromatin;chromosomal part;chromosome;chromosome, centromeric region;cohesin complex;cohesin core heterodimer;cytoplasm;cytoplasmic part;cytoskeletal part;cytosol;extracellular matrix	Cell cycle;Cell cycle - yeast;Meiosis - yeast;Oocyte meiosis	Homo sapiens	1	57
adherens junction;anchoring junction;cell junction;cell part;cell-substrate adherens junction;cell-substrate junction;cytoplasmic part;cytosol;extracellular membrane-bounded organelle;extracellular organelle;extracellular regio	Huntington's disease	Homo sapiens	1	58
cell part;chromatin;chromosomal part;cytoplasm;cytoplasmic part;cytosol;intracellular organelle part;intracellular part;macromolecular complex;MCM complex;membrane;nuclear chromosome part;nuclear part;nucleoplasm;r	Cell cycle;Cell cycle - yeast;DNA replication;Meiosis - yeast	Homo sapiens	1	58
cell part;centrosome;cytoplasmic part;cytoskeletal part;cytosol;intracellular non-membrane-bounded organelle;intracellular organelle;intracellular organelle part;intracellular part;macromolecular complex;membrane;microtubule organizing center;non-membrane-bounded organelle;organelle;organelle part;protein complex;spindle		Homo sapiens	1	58
actin cytoskeleton;actin filament bundle;actomyosin;cell part;cytoplasm;cytoplasmic part;cytoskeletal part;cytoskeleton;intracellular non-membrane-bounded organelle;intracellular organelle;intracellular organelle part;intracellular part;macromolecular complex;microtubule;non-membrane-bounded organelle;organelle;organelle p		Homo sapiens	1	58
cell part;cytoplasmic part;cytosol;intracellular membrane-bounded organelle;intracellular organelle;intracellular organelle part;intracellular part;membrane;membrane part;membrane raft;membrane-bounded organelle;mitocho	Amino sugar and nucleotide sugar metabolism;Butirosin and neomycin biosynthesis;Carbohydrate diges	Homo sapiens	1	59
cell part;chromosomal part;intracellular membrane-bounded organelle;intracellular organelle;intracellular organelle part;intracellular part;macromolecular complex;MCM complex;membrane;membrane-bounded organelle;nuc	Cell cycle;Cell cycle - yeast;DNA replication;Meiosis - yeast	Homo sapiens	1	59
apical part of cell;brush border;cell body;cell part;cell projection;cytoplasm;cytoplasmic part;endoplasmic reticulum;endoplasmic reticulum membrane;endoplasmic reticulum part;integral to membrane;integral to plasma mem	Alzheimer's disease;Calcium signaling pathway;Gap junction;Gastric acid secretion;GnRH signaling pat	Homo sapiens	1	59
adherens junction;anchoring junction;apical plasma membrane;cell junction;cell part;cell projection membrane;cell projection part;cell surface;cell-substrate adherens junction;cell-substrate junction;cytoplasmic part;extracellular membrane-bounded organelle;extracellular organelle;extracellular region;extracellular region part;ext		Homo sapiens	1	59
actin filament;cell junction;cell part;cell projection;cytoplasm;cytoplasmic part;cytoskeletal part;cytosol;extracellular membrane-bounded organelle;extracellular organelle;extracellular region;extracellular region part;extracellular vesicular exosome;intracellular non-membrane-bounded organelle;intracellular organelle;intracellular c		Homo sapiens	1	60
adherens junction;anchoring junction;basolateral plasma membrane;cell junction;cell part;cell projection;cell surface;cell-substrate adherens junction;cell-substrate junction;cilium;cornified envelope;cytoplasm;cytoplasmic part;cytoskeleton;endosome;extracellular membrane-bounded organelle;extracellular organelle;extracellula		Homo sapiens	1	60
cell part;cytoplasm;cytoplasmic part;cytoskeleton;cytosol;extracellular membrane-bounded organelle;extracellular organelle;extracellular region part;extracellular vesicular exosome;intracellular membrane-bounded organelle	Alzheimer's disease;Glycolysis / Gluconeogenesis	Homo sapiens	1	60
cell part;cell surface;cytoplasmic membrane-bounded vesicle;cytoplasmic part;cytoplasmic vesicle;endoplasmic reticulum;endoplasmic reticulum lumen;endoplasmic reticulum part;intracellular membrane-bounded organelle	Protein processing in endoplasmic reticulum;Vibrio cholerae infection	Homo sapiens	1	60
catalytic step 2 spliceosome;cell part;chromatin;chromosomal part;euchromatin;extracellular membrane-bounded organelle;extracellular organelle;extracellular region part;extracellular space;extracellular vesicular exosome;ir	Spliceosome	Homo sapiens	1	60
cell part;cytoplasm;intracellular membrane-bounded organelle;intracellular non-membrane-bounded organelle;intracellular organelle;intracellular organelle part;intracellular part;membrane-bounded organelle;non-membrane-l	Ribosome biogenesis in eukaryotes	Homo sapiens	1	60
cell part;chromosomal part;chromosome;chromosome, centromeric region;cohesin complex;cohesin core heterodimer;condensed chromosome;condensed chromosome kinetochore;condensed nuclear chromosome;cytopla	Cell cycle;Cell cycle - yeast;Meiosis - yeast;Oocyte meiosis	Homo sapiens	1	60
aggresome;cell body;cell part;centrosome;chaperonin-containing T-complex;cytoplasm;cytoplasmic part;cytoskeletal part;cytoskeleton;cytosol;cytosolic part;extracellular membrane-bounded organelle;extracellular organelle;extracellular region part;extracellular vesicular exosome;inclusion body;intermediate filament cytoskeleto		Homo sapiens	1	61
cell part;cytoplasm;cytoplasmic part;cytosol;extracellular membrane-bounded organelle;extracellular organelle;extracellular region part;extracellular vesicular exosome;intracellular membrane-bounded organelle;intracellular c	Parkinson's disease;Ubiquitin mediated proteolysis	Homo sapiens	1	62
actin cytoskeleton;cell part;cytoplasm;cytoplasmic part;cytoskeleton;cytosol;extracellular membrane-bounded organelle;extracellular organelle;extracellular region part;extracellular vesicular exosome;intracellular non-membr	Aminoacyl-tRNA biosynthesis	Homo sapiens	1	62
adherens junction;anchoring junction;cell junction;cell part;cell-substrate adherens junction;cell-substrate junction;cytoplasm;cytoplasmic part;cytosol;cytosolic small ribosomal subunit;extracellular membrane-bounded orga	Ribosome	Homo sapiens	1	62
cell junction;cell part;cell-cell junction;contractile fiber part;costamere;cytoplasm;cytoplasmic part;cytoskeletal part;cytoskeleton;dystrophin-associated glycoprotein complex;extracellular membrane-bounded organelle;extracellular organelle;extracellular region part;extracellular vesicular exosome;intermediate filament;intermedia		Homo sapiens	1	63
cell part;cytoplasm;cytoskeletal part;cytoskeleton;extracellular membrane-bounded organelle;extracellular organelle;extracellular region part;extracellular vesicular exosome;intracellular non-membrane-bounded organelle;intracellular organelle;intracellular organelle part;intracellular part;macromolecular complex;membrane;mem		Homo sapiens	1	63
cell cortex part;cell part;cell projection membrane;cell projection part;cortical actin cytoskeleton;cortical cytoskeleton;cytoplasm;cytoplasmic part;cytoskeletal part;cytoskeleton;cytosol;eukaryotic translation elongation factor	RNA transport	Homo sapiens	1	63
cell part;cytoplasmic part;integral to membrane;intracellular membrane-bounded organelle;intracellular organelle;intracellular organelle part;intracellular part;intrinsic to membrane;membrane;membrane part;membrane-bounded organelle;mitochondrial inner membrane;mitochondrial membrane;mitochondrial part;mitochondrion;		Homo sapiens	1	63
catalytic step 2 spliceosome;cell part;cytoplasm;extracellular membrane-bounded organelle;extracellular organelle;extracellular region part;extracellular vesicular exosome;intracellular membrane-bounded organelle;intracellu	Spliceosome	Homo sapiens	1	64
cell part;citrate lyase complex;cytoplasm;cytoplasmic part;cytosol;extracellular membrane-bounded organelle;extracellular organelle;extracellular region part;extracellular vesicular exosome;intracellular membrane-bounded c	Carbon fixation pathways in prokaryotes;Citrate cycle (TCA cycle)	Homo sapiens	1	64
cell part;centrosome;cytoplasm;cytoplasmic part;cytoskeletal part;integral to membrane;intracellular non-membrane-bounded organelle;intracellular organelle;intracellular organelle part;intracellular part;intrinsic to membrane;membrane;membrane part;microtubule organizing center;non-membrane-bounded organelle;nuclear pa		Homo sapiens	1	64
actin filament;brush border;cell part;cell periphery;cell projection;cytoplasm;cytoplasmic part;cytoskeletal part;early endosome;endosomal part;endosome;endosome membrane;extracellular membrane-bounded organelle;extracellular organelle;extracellular region part;extracellular vesicular exosome;filopodium;intracellular mem		Homo sapiens	1	65
apical plasma membrane;basolateral plasma membrane;caveola;cell part;cytoplasmic membrane-bounded vesicle;cytoplasmic part;cytoplasmic vesicle;endoplasmic reticulum;endosome;extracellular membrane-bounded or	Aldosterone-regulated sodium reabsorption;Bile secretion;Carbohydrate digestion and absorption;Cardi	Homo sapiens	1	65
cell part;cytoplasmic part;cytosol;extracellular membrane-bounded organelle;extracellular organelle;extracellular region part;extracellular vesicular exosome;intracellular membrane-bounded organelle;intracellular organelle;int	One carbon pool by folate	Homo sapiens	1	65
cell part;cell projection;cell projection part;ciliary rootlet;cilium part;cytoplasm;cytoplasmic membrane-bounded vesicle;cytoplasmic part;cytoplasmic vesicle;cytoskeletal part;endocytic vesicle;intracellular membrane-bounded organelle;intracellular organelle;intracellular organelle part;intracellular part;kinesin complex;macromole		Homo sapiens	1	65
cell part;cytoplasmic part;cytoskeleton;endoplasmic reticulum;endoplasmic reticulum membrane;endoplasmic reticulum part;extracellular membrane-bounded organelle;extracellular organelle;extracellular region part;extrace	Protein processing in endoplasmic reticulum	Homo sapiens	1	65
adherens junction;anchoring junction;cell junction;cell part;cell-substrate adherens junction;cell-substrate junction;contractile fiber part;costamere;cytoplasm;cytoplasmic part;cytoskeleton;cytosol;focal adhesion;intracellular	Focal adhesion;MAPK signaling pathway	Homo sapiens	1	65
		Homo sapiens	1	66
cell part;cytoplasmic part;extracellular membrane-bounded organelle;extracellular organelle;extracellular region part;extracellular vesicular exosome;intracellular membrane-bounded organelle;intracellular organelle;intracellula	Alzheimer's disease;Huntington's disease;Oxidative phosphorylation;Parkinson's disease	Homo sapiens	1	66
adherens junction;anchoring junction;cell junction;cell part;cell-substrate adherens junction;cell-substrate junction;cytoplasm;cytoplasmic part;cytosol;cytosolic large ribosomal subunit;extracellular membrane-bounded orga	Ribosome	Homo sapiens	1	66
cell part;cytoplasmic membrane-bounded vesicle;cytoplasmic part;cytoplasmic vesicle;endoplasmic reticulum lumen;endoplasmic reticulum part;extracellular membrane-bounded organelle;extracellular organelle;extracellula	N-Glycan biosynthesis;Protein processing in endoplasmic reticulum	Homo sapiens	1	66
cell part;intracellular membrane-bounded organelle;intracellular non-membrane-bounded organelle;intracellular organelle;intracellular organelle part;intracellular part;membrane;membrane-bounded organelle;non-membrane-bounded organelle;nuclear body;nuclear matrix;nuclear part;nuclear speck;nucleolus;nucleoplasm;nucle		Homo sapiens	1	66
cell part;cytoplasm;intracellular membrane-bounded organelle;intracellular non-membrane-bounded organelle;intracellular organelle;intracellular organelle part;intracellular part;membrane;membrane-bounded organelle;non-membrane-bounded organelle;nuclear part;nucleolus;nucleoplasm;nucleus;organelle;organelle part		Homo sapiens	1	66
cell part;cytoplasm;cytoplasmic part;cytosol;intracellular membrane-bounded organelle;intracellular organelle;intracellular part;membrane-bounded organelle;nucleus;organelle	Aminoacyl-tRNA biosynthesis;Valine, leucine and isoleucine biosynthesis	Homo sapiens	1	66
Cajal body;catalytic step 2 spliceosome;cell part;intracellular organelle part;intracellular part;macromolecular complex;nuclear body;nuclear part;nuclear speck;nucleoplasm;nucleoplasm part;organelle part;ribonucleoprotein complex;spliceosomal complex		Homo sapiens	1	66
cell part;cytoplasmic part;cytosol;extracellular membrane-bounded organelle;extracellular organelle;extracellular region part;extracellular vesicular exosome;intracellular membrane-bounded organelle;intracellular organelle;int	Biosynthesis of ansamycins;Carbon fixation in photosynthetic organisms;Pentose phosphate pathway	Homo sapiens	1	67
adherens junction;anchoring junction;cell junction;cell part;cell surface;cell-substrate adherens junction;cell-substrate junction;cytoplasmic membrane-bounded vesicle;cytoplasmic part;cytoplasmic vesicle;endoplasmic retic	Antigen processing and presentation;Protein processing in endoplasmic reticulum	Homo sapiens	1	67
basal plasma membrane;brush border;cell part;cell projection;cell projection membrane;cell projection part;cytoplasm;cytoplasmic membrane-bounded vesicle;cytoplasmic part;cytoplasmic vesicle;cytoskeletal part;cytosol;extracellular membrane-bounded organelle;extracellular organelle;extracellular region part;extracellular ve		Homo sapiens	1	68
adherens junction;anchoring junction;basal lamina;cell junction;cell part;cell-substrate adherens junction;cell-substrate junction;cytoplasmic part;extracellular matrix;extracellular matrix part;extracellular membrane-bounded c	ECM-receptor interaction	Homo sapiens	1	68
cell part;cytoplasmic part;cytosol;extracellular membrane-bounded organelle;extracellular organelle;extracellular region part;extracellular vesicular exosome;intracellular part;membrane;membrane part;membrane raft;membra	Carbon fixation in photosynthetic organisms;Glycolysis / Gluconeogenesis	Homo sapiens	1	69
cell part;cytoplasmic part;cytosol;intracellular membrane-bounded organelle;intracellular organelle;intracellular part;membrane-bounded organelle;mitochondrion;organelle	Aminoacyl-tRNA biosynthesis;Valine, leucine and isoleucine biosynthesis	Homo sapiens	1	69
axon part;cell body;cell part;cell projection;cell projection part;cytoplasmic part;cytosol;extracellular membrane-bounded organelle;extracellular organelle;extracellular region part;extracellular vesicular exosome;intracellular n	Alanine, aspartate and glutamate metabolism;Pyrimidine metabolism	Homo sapiens	1	69
cell part;cytoplasm;cytoplasmic part;cytosol;extracellular membrane-bounded organelle;extracellular organelle;extracellular region part;extracellular vesicular exosome;intracellular organelle part;intracellular part;membrane;n	Aminoacyl-tRNA biosynthesis;Valine, leucine and isoleucine biosynthesis	Homo sapiens	1	69
catalytic step 2 spliceosome;cell part;intracellular membrane-bounded organelle;intracellular organelle;intracellular organelle part;intracellular part;macromolecular complex;membrane-bounded organelle;nuclear body;nuclea	Spliceosome	Homo sapiens	1	70
adherens junction;anchoring junction;cell junction;cell part;cell-substrate adherens junction;cell-substrate junction;cytoplasmic membrane-bounded vesicle;cytoplasmic part;cytoplasmic vesicle;endoplasmic reticulum;endopl	Protein processing in endoplasmic reticulum	Homo sapiens	1	70
actin cytoskeleton;actin filament;adherens junction;anchoring junction;apical part of cell;apical plasma membrane;basolateral plasma membrane;brush border;cell body;cell cortex part;cell junction;cell part;cell periphery;cell	Gastric acid secretion;Leukocyte transendothelial migration;Pathogenic Escherichia coli infection;Regul	Homo sapiens	1	70
adherens junction;anchoring junction;basement membrane;cell junction;cell part;cell-substrate adherens junction;cell-substrate junction;extracellular matrix;extracellular matrix part;extracellular region;extracellular region part	ECM-receptor interaction;Focal adhesion	Homo sapiens	1	70
catalytic step 2 spliceosome;cell part;intracellular membrane-bounded organelle;intracellular organelle;intracellular organelle part;intracellular part;macromolecular complex;membrane;membrane-bounded organelle;nuclear p	Spliceosome	Homo sapiens	1	71
cell part;cell projection;cytoplasm;cytoplasmic part;cytosol;extracellular membrane-bounded organelle;extracellular organelle;extracellular region part;extracellular space;extracellular vesicular exosome;intracellular organelle	Amino sugar and nucleotide sugar metabolism;Glycolysis / Gluconeogenesis;Pentose phosphate pathw	Homo sapiens	1	71
cell part;cytoskeletal part;envelope;intermediate filament;intracellular organelle part;intracellular part;lamin filament;macromolecular complex;membrane;nuclear envelope;nuclear inner membrane;nuclear membrane;nuclear part;nucleoplasm;organelle envelope;organelle inner membrane;organelle membrane;organelle part;protei		Homo sapiens	1	72
adherens junction;anchoring junction;cell junction;cell part;cell-substrate adherens junction;cell-substrate junction;cytoplasm;cytoplasmic part;extracellular membrane-bounded organelle;extracellular organelle;extracellular re	ko05152;RNA degradation	Homo sapiens	1	72
catalytic step 2 spliceosome;cell part;cell surface;CRD-mediated mRNA stability complex;cytoplasmic part;intracellular membrane-bounded organelle;intracellular organelle;intracellular organelle part;intracellular part;macrom	Spliceosome	Homo sapiens	1	72
cell part;chromatin;chromosomal part;cytoplasm;intracellular membrane-bounded organelle;intracellular organelle;intracellular organelle part;intracellular part;membrane-bounded organelle;nuclear body;nuclear matrix;nuclear part;nucleoplasm;nucleoplasm part;nucleus;organelle;organelle part;paraspeckles		Homo sapiens	1	73
adherens junction;anchoring junction;cell junction;cell part;cell surface;cell-substrate adherens junction;cell-substrate junction;clathrin coat;clathrin-coated vesicle;coated vesicle;cytoplasmic membrane-bounded vesicle;cyto	Lysosome	Homo sapiens	1	74
cell part;chromosomal part;cytoplasm;cytoplasmic part;cytoplasmic ubiquitin ligase complex;cytosol;endoplasmic reticulum;endoplasmic reticulum membrane;endoplasmic reticulum part;ER ubiquitin ligase complex;extrace	Protein processing in endoplasmic reticulum	Homo sapiens	1	74
cell part;cytoplasmic part;cytosol;eukaryotic translation initiation factor 4F complex;intracellular part;macromolecular complex;membrane;protein complex	RNA transport;Viral myocarditis	Homo sapiens	1	74
cell part;contractile fiber part;costamere;cytoplasm;cytoplasmic part;cytoplasmic vesicle membrane;cytoplasmic vesicle part;intracellular membrane-bounded organelle;intracellular organelle;intracellular organelle part;intracellular part;membrane;membrane part;membrane-bounded organelle;nucleus;organelle;organelle membra		Homo sapiens	1	74
basement membrane;basolateral plasma membrane;cell cortex;cell part;cell projection;cell surface;cytoplasmic membrane-bounded vesicle;cytoplasmic part;cytoplasmic vesicle;early endosome;endosomal part;endosome;endosome membrane;extracellular matrix part;extracellular membrane-bounded organelle;extracellular or		Homo sapiens	1	75
adherens junction;anchoring junction;blood microparticle;cell junction;cell part;cell-substrate adherens junction;cell-substrate junction;clathrin coated vesicle membrane;clathrin sculpted gamma-aminobutyric acid transport v	Antigen processing and presentation;Endocytosis;MAPK signaling pathway;Measles;Protein processing	Homo sapiens	1	75
cell cortex;cell part;cytoplasmic part;extracellular membrane-bounded organelle;extracellular organelle;extracellular region part;extracellular vesicular exosome;intracellular membrane-bounded organelle;intracellular non-mer	Pathogenic Escherichia coli infection	Homo sapiens	1	75
cell part;chromosomal part;chromosome;chromosome, centromeric region;condensed chromosome;cytoplasm;intracellular membrane-bounded organelle;intracellular non-membrane-bounded organelle;intracellular organelle;intracellular organelle part;intracellular part;membrane;membrane-bounded organelle;non-membrane-b		Homo sapiens	1	76
adherens junction;anchoring junction;cell junction;cell part;cell surface;cell-substrate adherens junction;cell-substrate junction;cytoplasmic membrane-bounded vesicle;cytoplasmic part;cytoplasmic vesicle;endoplasmic retic	Prion diseases;Protein export;Protein processing in endoplasmic reticulum	Homo sapiens	1	77
cell part;cytoplasm;extracellular membrane-bounded organelle;extracellular organelle;extracellular region part;extracellular vesicular exosome;intracellular part;membrane;membrane-bounded organelle;membrane-bounded v	Insulin signaling pathway;Starch and sucrose metabolism	Homo sapiens	1	77
cell part;cytoplasmic part;Golgi apparatus;intracellular membrane-bounded organelle;intracellular organelle;intracellular part;membrane-bounded organelle;nucleus;organelle	Prion diseases	Homo sapiens	1	77
cell part;centrosome;CRD-mediated mRNA stability complex;cytoplasm;cytoplasmic part;cytoskeletal part;cytosol;intracellular membrane-bounded organelle;intracellular non-membrane-bounded organelle;intracellular organelle;intracellular organelle part;intracellular part;macromolecular complex;membrane;membrane-boundec		Homo sapiens	1	77
adherens junction;anchoring junction;cell junction;cell part;cell-substrate adherens junction;cell-substrate junction;cytoplasm;cytoplasmic part;cytosol;cytosolic large ribosomal subunit;extracellular membrane-bounded orga	Ribosome	Homo sapiens	1	78
cell part;chromosome;condensed chromosome;condensed nuclear chromosome;cytoplasmic part;cytoskeletal part;cytoskeleton;intracellular membrane-bounded organelle;intracellular non-membrane-bounded organelle;intracellular organelle;intracellular organelle part;intracellular part;macromolecular complex;membrane;mem		Homo sapiens	1	78
adherens junction;aggresome;anchoring junction;cell junction;cell part;cell projection membrane;cell projection part;cell-substrate adherens junction;cell-substrate junction;contractile fiber part;cytoplasm;cytoplasmic part;cytoskeleton;endoplasmic reticulum;endoplasmic reticulum membrane;endoplasmic reticulum part;envelop		Homo sapiens	1	78
cell part;contractile fiber part;cytoplasm;cytoplasmic part;cytosol;cytosolic part;extracellular membrane-bounded organelle;extracellular organelle;extracellular region part;extracellular space;extracellular vesicular exosome;in	Glycolysis / Gluconeogenesis;Methane metabolism;RNA degradation	Homo sapiens	1	79
actin filament bundle;actomyosin;adherens junction;anchoring junction;cell cortex part;cell junction;cell part;cell projection;cell projection part;cell-cell adherens junction;cell-cell junction;cell-substrate adherens junction;cell-s	Adherens junction;Amoebiasis;Arrhythmogenic right ventricular cardiomyopathy (ARVC);Focal adhesion	Homo sapiens	1	79
adherens junction;anchoring junction;cell cortex part;cell junction;cell part;cell-cell adherens junction;cell-cell junction;contractile fiber part;cuticular plate;cytoplasmic part;cytoskeletal part;cytoskeleton;cytosol;extracellular membrane-bounded organelle;extracellular organelle;extracellular region part;extracellular vesicular exoso		Homo sapiens	1	79
cell part;cytoplasm;cytoplasmic membrane-bounded vesicle;cytoplasmic part;cytoplasmic vesicle;extracellular membrane-bounded organelle;extracellular organelle;extracellular region part;extracellular vesicular exosome;intracellular membrane-bounded organelle;intracellular organelle;intracellular part;macromolecular complex		Homo sapiens	1	79
cell part;cytoplasmic part;endoplasmic reticulum;extracellular matrix;extracellular membrane-bounded organelle;extracellular organelle;extracellular region part;extracellular space;extracellular vesicular exosome;intracellular membrane-bounded organelle;intracellular organelle;intracellular part;membrane-bounded organelle;mem		Homo sapiens	1	79
cell part;cell surface;coated pit;coated vesicle;cytoplasm;cytoplasmic membrane-bounded vesicle;cytoplasmic part;cytoplasmic vesicle;cytosol;early endosome;endosome;extracellular membrane-bounded organelle;extrace	ko05152;RNA degradation;Type I diabetes mellitus	Homo sapiens	1	80
adherens junction;anchoring junction;cell body;cell junction;cell part;cell projection;cell-substrate adherens junction;cell-substrate junction;clathrin-coated vesicle;coated pit;coated vesicle;cytoplasm;cytoplasmic membrane-	Alzheimer's disease;Malaria	Homo sapiens	1	80
adherens junction;anchoring junction;cell junction;cell part;cell-substrate adherens junction;cell-substrate junction;cytoplasmic membrane-bounded vesicle;cytoplasmic part;cytoplasmic vesicle;endosomal part;endosome membrane;extracellular membrane-bounded organelle;extracellular organelle;extracellular region part;extra		Homo sapiens	1	81
actin cytoskeleton;adherens junction;anchoring junction;cell junction;cell part;cell-cell adherens junction;cell-cell junction;cell-substrate adherens junction;cell-substrate junction;contractile fiber part;costamere;cytoplasmic pa	Adherens junction;Amoebiasis;Bacterial invasion of epithelial cells;Focal adhesion;Leukocyte transendo	Homo sapiens	1	82
cell part;COPI vesicle coat;cytoplasm;cytoplasmic part;cytoplasmic vesicle part;cytosol;extracellular membrane-bounded organelle;extracellular organelle;extracellular region part;extracellular space;extracellular vesicular exo	Neuroactive ligand-receptor interaction	Homo sapiens	1	82
catalytic step 2 spliceosome;cell part;intracellular membrane-bounded organelle;intracellular organelle;intracellular organelle part;intracellular part;macromolecular complex;membrane;membrane-bounded organelle;nuclear b	Spliceosome	Homo sapiens	1	84
cell part;cytoplasm;extracellular region part;high-density lipoprotein particle;intracellular membrane-bounded organelle;intracellular organelle;intracellular part;macromolecular complex;membrane;membrane-bounded organelle;nucleus;organelle;plasma lipoprotein particle;plasma membrane;protein-lipid complex		Homo sapiens	1	85
cell part;envelope;intracellular membrane-bounded organelle;intracellular non-membrane-bounded organelle;intracellular organelle;intracellular organelle part;intracellular part;macromolecular complex;membrane;membrane-	Base excision repair	Homo sapiens	1	86
adherens junction;anchoring junction;cell junction;cell part;cell-substrate adherens junction;cell-substrate junction;cytoplasmic membrane-bounded vesicle;cytoplasmic membrane-bounded vesicle lumen;cytoplasmic part;cy	NOD-like receptor signaling pathway;Pathways in cancer;Plant-pathogen interaction;Prostate cancer;Pr	Homo sapiens	1	86
cell part;cytoplasmic part;endoplasmic reticulum;endoplasmic reticulum membrane;endoplasmic reticulum part;integral to membrane;integral to plasma membrane;intracellular membrane-bounded organelle;intracellular organelle;intracellular organelle part;intracellular part;intrinsic to membrane;intrinsic to plasma membrane;me		Homo sapiens	1	86
adherens junction;anchoring junction;apical part of cell;apical plasma membrane;basolateral plasma membrane;blood microparticle;cell junction;cell part;cell periphery;cell projection;cell projection membrane;cell projection p	Leukocyte transendothelial migration;Measles;Regulation of actin cytoskeleton	Homo sapiens	1	88
cell part;cytoplasmic part;cytosol;extracellular membrane-bounded organelle;extracellular organelle;extracellular region part;extracellular vesicular exosome;intracellular membrane-bounded organelle;intracellular organelle;int	Antigen processing and presentation	Homo sapiens	1	88
aggresome;cell part;cytoplasm;cytoplasmic part;cytosol;extracellular membrane-bounded organelle;extracellular organelle;extracellular region part;extracellular vesicular exosome;inclusion body;intracellular membrane-bounded organelle;intracellular organelle;intracellular part;macromolecular complex;membrane;membrane-bo		Homo sapiens	1	89
apical plasma membrane;basal lamina;blood microparticle;cell part;cytoplasmic membrane-bounded vesicle lumen;cytoplasmic part;cytoplasmic vesicle part;endoplasmic reticulum-Golgi intermediate compartment;extracell	Amoebiasis;Bacterial invasion of epithelial cells;ECM-receptor interaction;Focal adhesion;Pathways in c	Homo sapiens	1	95
cell part;cell projection;cilium;cytoplasm;cytoplasmic part;cytosol;extracellular matrix;extracellular membrane-bounded organelle;extracellular organelle;extracellular region part;extracellular vesicular exosome;intracellular me	Carbon fixation in photosynthetic organisms;Glycolysis / Gluconeogenesis;Purine metabolism;Pyruvate	Homo sapiens	1	95
catalytic step 2 spliceosome;cell part;extracellular matrix;extracellular membrane-bounded organelle;extracellular organelle;extracellular region part;extracellular vesicular exosome;integral to membrane;integral to plasma me	Spliceosome	Homo sapiens	1	95
apical plasma membrane;basolateral plasma membrane;brush border membrane;cell body;cell part;cell projection;cell projection membrane;cell projection part;cell surface;cytoplasm;cytoplasmic membrane-bounded vesicle	Antigen processing and presentation;NOD-like receptor signaling pathway;Pathways in cancer;Plant-pa	Homo sapiens	1	98
cell part;cytoplasmic part;cytosol;inclusion body;intracellular membrane-bounded organelle;intracellular organelle;intracellular organelle part;intracellular part;macromolecular complex;membrane;membrane part;membrane-b	RNA transport	Homo sapiens	1	98
adherens junction;anchoring junction;cell junction;cell leading edge;cell part;cell projection;cell-substrate adherens junction;cell-substrate junction;cytoplasm;cytoplasmic part;cytoskeletal part;cytoskeleton;cytosol;extracellular membrane-bounded organelle;extracellular organelle;extracellular region part;extracellular vesicular ex		Homo sapiens	1	99
actin filament;adherens junction;anchoring junction;axon;cell junction;cell leading edge;cell part;cell projection;cell projection part;cell-substrate adherens junction;cell-substrate junction;cytoplasm;cytoplasmic part;cytoskele	Regulation of actin cytoskeleton	Homo sapiens	1	99

actin filament bundle;actomyosin;adherens junction;anchoring junction;cell cortex part;cell junction;cell part;cell projection;cell-cell junction;cell-substrate adherens junction;cell-substrate junction;contractile fiber part;cortical	Adherens junction;Amoebiasis;Arrhythmogenic right ventricular cardiomyopathy (ARVC);Focal adhesion	Homo sapiens	1	100
actin cytoskeleton;cell part;cell projection membrane;cell projection part;cytoplasm;cytoplasmic part;cytoskeletal part;cytoskeleton;Golgi apparatus;intracellular membrane-bounded organelle;intracellular non-membrane-bounded organelle;intracellular organelle;intracellular organelle part;intracellular part;leading edge membrane		Homo sapiens	1	100
axolemma;axon part;cell cortex part;cell part;cell projection membrane;cell projection part;contractile fiber part;cuticular plate;cytoplasm;cytoplasmic part;cytoskeletal part;cytoskeleton;cytosol;extracellular membrane-bounded organelle;extracellular organelle;extracellular region part;extracellular vesicular exosome;intracellular		Homo sapiens	1	103
cell part;cytoplasm;cytoplasmic part;cytoskeletal part;cytosol;envelope;intermediate filament;intracellular membrane-bounded organelle;intracellular organelle;intracellular organelle part;intracellular part;lamin filament;macron	Arrhythmogenic right ventricular cardiomyopathy (ARVC);Dilated cardiomyopathy;Hypertrophic cardiom	Homo sapiens	1	106
cell part;cytoplasm;cytoplasmic membrane-bounded vesicle;cytoplasmic part;cytoplasmic vesicle;cytosol;extracellular membrane-bounded organelle;extracellular organelle;extracellular region part;extracellular vesicular exos	Antigen processing and presentation;NOD-like receptor signaling pathway;Pathways in cancer;Plant-pa	Homo sapiens	1	109
cell part;cytoplasm;cytoplasmic part;cytosol;eukaryotic 43S preinitiation complex;eukaryotic 48S preinitiation complex;eukaryotic translation initiation factor 3 complex;eukaryotic translation initiation factor 3 complex, eIF3m	RNA transport	Homo sapiens	1	109
cell part;cytoplasm;cytoplasmic part;intracellular non-membrane-bounded organelle;intracellular organelle;intracellular part;macromolecular complex;membrane;non-membrane-bounded organelle;organelle;ribonucleoprotein complex;ribosome		Homo sapiens	1	113
cell part;cytoplasm;extracellular membrane-bounded organelle;extracellular organelle;extracellular region part;extracellular vesicular exosome;intracellular membrane-bounded organelle;intracellular organelle;intracellular orga	Ubiquitin mediated proteolysis	Homo sapiens	1	115
caveola;cell part;cytoplasmic part;cytoplasmic vesicle;cytoplasmic vesicle membrane;cytoplasmic vesicle part;envelope;extracellular membrane-bounded organelle;extracellular organelle;extracellular region part;extracellular vesicular exosome;integral to membrane;intracellular membrane-bounded organelle;intracellular organel		Homo sapiens	1	115
aminoacyl-tRNA synthetase multienzyme complex;cell part;cytoplasm;cytoplasmic part;cytosol;intracellular part;macromolecular complex;membrane;protein complex;ribonucleoprotein complex	Aminoacyl-tRNA biosynthesis;Porphyrin and chlorophyll metabolism	Homo sapiens	1	117
cell part;cytoplasmic part;endoplasmic reticulum;endoplasmic reticulum part;integral to endoplasmic reticulum membrane;integral to membrane;integral to organelle membrane;intracellular membrane-bounded organelle;intra	Protein processing in endoplasmic reticulum	Homo sapiens	1	118
actin cytoskeleton;actin filament bundle;actomyosin;axon;cell body;cell cortex;cell division site part;cell part;cell projection;cell projection part;cleavage furrow;cytoplasm;cytoplasmic part;cytoskeletal part;cytoskeleton;cytos	Tight junction;Viral myocarditis	Homo sapiens	1	129
apical part of cell;cell body;cell part;cell projection;chromosome;cytoplasm;cytoplasmic part;cytoskeletal part;cytosol;dendrite;extracellular membrane-bounded organelle;extracellular organelle;extracellular region part;extracellular vesicular exosome;Golgi apparatus part;Golgi membrane;intracellular membrane-bounded organe		Homo sapiens	1	132
cell part;chromosomal part;cytoplasm;cytoplasmic dynein complex;cytoplasmic part;cytoskeletal part;dynein complex;envelope;extrinsic to membrane;inclusion body;intracellular membrane-bounded organelle;intracellular n	MAPK signaling pathway - yeast;Pathways in cancer;RNA transport;Thyroid cancer	Homo sapiens	1	133
adherens junction;anchoring junction;cell junction;cell part;cell-substrate adherens junction;cell-substrate junction;clathrin coat;clathrin coat of coated pit;clathrin coat of trans-Golgi network vesicle;clathrin coated vesicle me	Bacterial invasion of epithelial cells;Endocrine and other factor-regulated calcium reabsorption;Endocyt	Homo sapiens	1	135
anchoring collagen;cell part;collagen;collagen type VI;cytoplasmic part;endoplasmic reticulum lumen;endoplasmic reticulum part;extracellular matrix;extracellular matrix part;extracellular membrane-bounded organelle;extrac	ECM-receptor interaction;Focal adhesion;Protein digestion and absorption	Homo sapiens	1	136
cell part;cytoplasmic part;intracellular non-membrane-bounded organelle;intracellular organelle;intracellular organelle lumen;intracellular organelle part;intracellular part;macromolecular complex;membrane;membrane-enclos	Alanine, aspartate and glutamate metabolism;Arginine and proline metabolism;Nitrogen metabolism	Homo sapiens	1	138
actin cytoskeleton;actin filament;adherens junction;anchoring junction;cell body;cell cortex part;cell junction;cell part;cell projection part;cell-substrate adherens junction;cell-substrate junction;chromosomal part;cortical cyto	Focal adhesion;MAPK signaling pathway	Homo sapiens	1	156
actin cytoskeleton;actin filament bundle;actomyosin;adherens junction;anchoring junction;cell cortex;cell junction;cell part;cell-substrate adherens junction;cell-substrate junction;contractile fiber part;cytoplasm;cytoplasmic p	Focal adhesion;MAPK signaling pathway	Homo sapiens	1	171
cell part;cytoplasm;cytoplasmic membrane-bounded vesicle;cytoplasmic part;cytoplasmic vesicle;cytosol;extracellular membrane-bounded organelle;extracellular organelle;extracellular region part;extracellular vesicular exos	Fatty acid biosynthesis;Insulin signaling pathway	Homo sapiens	1	178
adherens junction;anchoring junction;cell junction;cell part;cell projection;cell projection membrane;cell projection part;cell surface;cell-cell junction;cell-substrate adherens junction;cell-substrate junction;cytoplasm;cytoplasr	Focal adhesion	Homo sapiens	1	183
cell part;cell projection;centrosome;cytoplasmic dynein complex;cytoplasmic part;cytoskeletal part;cytosol;dynein complex;extracellular membrane-bounded organelle;extracellular organelle;extracellular region part;extracellu	Phagosome;Vasopressin-regulated water reabsorption	Homo sapiens	1	267
cell part;cytoplasmic part;cytosol;DNA-dependent protein kinase-DNA ligase 4 complex;intracellular non-membrane-bounded organelle;intracellular organelle;intracellular organelle part;intracellular part;macromolecular comp	Cell cycle;Non-homologous end-joining	Homo sapiens	1	274
actin cytoskeleton;actin filament bundle;actomyosin;actomyosin contractile ring;adherens junction;anchoring junction;cell cortex part;cell division site part;cell junction;cell leading edge;cell part;cell projection;cell-cell adhere	Tight junction;Viral myocarditis	Homo sapiens	1	281
actin cytoskeleton;adherens junction;anchoring junction;cell junction;cell part;cell-cell contact zone;cell-cell junction;cell-substrate adherens junction;cell-substrate junction;contractile fiber part;costamere;cytoplasm;cytoplasmic part;cytoskeleton;cytosol;extracellular membrane-bounded organelle;extracellular organelle;extracel		Homo sapiens	1	324
adherens junction;anchoring junction;cell junction;cell part;cell-substrate adherens junction;cell-substrate junction;contractile fiber part;costamere;cytoplasm;cytoplasmic part;cytoskeleton;cytosol;extracellular membrane-bounded organelle;extracellular organelle;extracellular region part;extracellular vesicular exosome;focal adh		Homo sapiens	1	512

N: Combined Spectral Count	N: Combined Unique Spectral Count	N: Combined Total Spectral Count	N: Top Peptide Probability	N: Protein Length	T: Protein	T: Gene	T: Protein ID	T: Description	T: Entry Name	T: Indistinguishable Proteins	T: Gene name
113	113	113	0.9989	740	sp|Q15027|ACAP1_HUMAN	ACAP1	Q15027	Arf-GAP with coiled-coil, ANK repeat and PH domain-containing protein 1	ACAP1_HUMAN		ACAP1
77	77	77	0.998	380	sp|Q6YI46|TMM64_HUMAN	TMEM64	Q6YI46	Transmembrane protein 64	TMM64_HUMAN		TMEM64
49	49	49	0.9977	309	sp|Q9NZP5|O5AC2_HUMAN	OR5AC2	Q9NZP5	Olfactory receptor 5AC2	O5AC2_HUMAN		OR5AC2
92	92	92	0.999	169	sp|Q9Y6A9|SPCS1_HUMAN	SPCS1	Q9Y6A9	Signal peptidase complex subunit 1	SPCS1_HUMAN		SPCS1
104	104	104	0.999	133	sp|O15116|LSM1_HUMAN	LSM 1.00	O15116	U6 snRNA-associated Sm-like protein LSm1	LSM1_HUMAN		LSM 1.00
3	3	3	0.9985	239	sp|O75954|TSN9_HUMAN	TSPAN9	O75954	Tetraspanin-9	TSN9_HUMAN		TSPAN9
54	54	54	0.999	226	sp|P00846|ATP6_HUMAN	MT-ATP6	P00846	ATP synthase subunit a	ATP6_HUMAN		MT-ATP6
27	0	27	0.9973	147	sp|P02042|HBD_HUMAN	HBD	P02042	Hemoglobin subunit delta	HBD_HUMAN	sp|P68871|HBB_HUMAN	HBD
16	16	16	0.9989	415	sp|P05543|THBG_HUMAN	SERPINA7	P05543	Thyroxine-binding globulin	THBG_HUMAN		SERPINA7
22	22	22	0.999	491	sp|P08697|A2AP_HUMAN	SERPINF2	P08697	Alpha-2-antiplasmin	A2AP_HUMAN		SERPINF2
21	14	21	0.999	125	sp|P13164|IFM1_HUMAN	IFITM1	P13164	Interferon-induced transmembrane protein 1	IFM1_HUMAN		IFITM1
13	13	13	0.999	79	sp|P33552|CKS2_HUMAN	CKS2	P33552	Cyclin-dependent kinases regulatory subunit 2	CKS2_HUMAN		CKS2
84	84	84	0.999	286	sp|P43307|SSRA_HUMAN	SSR1	P43307	Translocon-associated protein subunit alpha	SSRA_HUMAN		SSR1
56	56	56	0.999	185	sp|P51452|DUS3_HUMAN	DUSP3	P51452	Dual specificity protein phosphatase 3	DUS3_HUMAN		DUSP3
10	10	10	0.9989	493	sp|P55058|PLTP_HUMAN	PLTP	P55058	Phospholipid transfer protein	PLTP_HUMAN		PLTP
24	24	24	0.999	237	sp|P55061|BI1_HUMAN	TMBIM6	P55061	Bax inhibitor 1	BI1_HUMAN		TMBIM6
76	76	76	0.999	79	sp|P61165|TM258_HUMAN	TMEM258	P61165	Transmembrane protein 258	TM258_HUMAN		TMEM258
5	5	5	0.999	268	sp|P62079|TSN5_HUMAN	TSPAN5	P62079	Tetraspanin-5	TSN5_HUMAN		TSPAN5
43	43	43	0.999	301	sp|Q13371|PHLP_HUMAN	PDCL	Q13371	Phosducin-like protein	PHLP_HUMAN		PDCL
88	88	88	0.999	63	sp|Q14061|COX17_HUMAN	COX17	Q14061	Cytochrome c oxidase copper chaperone	COX17_HUMAN		COX17
20	20	20	0.999	111	sp|Q15004|PAF15_HUMAN	PCLAF	Q15004	PCNA-associated factor	PAF15_HUMAN		KIAA0101
42	42	42	0.999	348	sp|Q30201|HFE_HUMAN	HFE	Q30201	Hereditary hemochromatosis protein	HFE_HUMAN		HFE
8	8	8	0.999	286	sp|Q53TN4|CYBR1_HUMAN	CYBRD1	Q53TN4	Plasma membrane ascorbate-dependent reductase CYBRD1	CYBR1_HUMAN		CYBRD1
17	17	17	0.999	314	sp|Q5BJH7|YIF1B_HUMAN	YIF1B	Q5BJH7	Protein YIF1B	YIF1B_HUMAN		YIF1B
70	70	70	0.999	344	sp|Q5MNZ6|WIPI3_HUMAN	WDR45B	Q5MNZ6	WD repeat domain phosphoinositide-interacting protein 3	WIPI3_HUMAN		WDR45B
9	9	9	0.9987	1427	sp|Q76LX8|ATS13_HUMAN	ADAMTS13	Q76LX8	A disintegrin and metalloproteinase with thrombospondin motifs 13	ATS13_HUMAN		ADAMTS13
30	30	30	0.999	191	sp|Q7Z4R8|CF120_HUMAN	C6orf120	Q7Z4R8	UPF0669 protein C6orf120	CF120_HUMAN		C6orf120
111	111	111	0.9957	357	sp|Q7Z5H4|VN1R5_HUMAN	VN1R5	Q7Z5H4	Vomeronasal type-1 receptor 5	VN1R5_HUMAN		VN1R5
65	65	65	0.999	194	sp|Q8IXQ3|CI040_HUMAN	C9orf40	Q8IXQ3	Uncharacterized protein C9orf40	CI040_HUMAN		C9orf40
4	4	4	0.9946	433	sp|Q8N5I2|ARRD1_HUMAN	ARRDC1	Q8N5I2	Arrestin domain-containing protein 1	ARRD1_HUMAN		ARRDC1
12	12	12	0.999	63	sp|Q8TAD7|OCC1_HUMAN	OCC1	Q8TAD7	Overexpressed in colon carcinoma 1 protein	OCC1_HUMAN		OCC1
8	8	8	0.999	249	sp|Q8WV92|MITD1_HUMAN	MITD1	Q8WV92	MIT domain-containing protein 1	MITD1_HUMAN		MITD1
3	3	3	0.9947	364	sp|Q92633|LPAR1_HUMAN	LPAR1	Q92633	Lysophosphatidic acid receptor 1	LPAR1_HUMAN		LPAR1
73	73	73	0.999	74	sp|Q96B49|TOM6_HUMAN	TOMM6	Q96B49	Mitochondrial import receptor subunit TOM6 homolog	TOM6_HUMAN		TOMM6
23	23	23	0.999	459	sp|Q96BH1|RNF25_HUMAN	RNF25	Q96BH1	E3 ubiquitin-protein ligase RNF25	RNF25_HUMAN		RNF25
70	70	70	0.999	788	sp|Q96RN5|MED15_HUMAN	MED15	Q96RN5	Mediator of RNA polymerase II transcription subunit 15	MED15_HUMAN		MED15
38	38	38	0.9987	161	sp|Q99732|LITAF_HUMAN	LITAF	Q99732	Lipopolysaccharide-induced tumor necrosis factor-alpha factor	LITAF_HUMAN		LITAF
10	10	10	0.999	253	sp|Q99811|PRRX2_HUMAN	PRRX2	Q99811	Paired mesoderm homeobox protein 2	PRRX2_HUMAN		PRRX2
10	10	10	0.9988	223	sp|Q9GZX9|TWSG1_HUMAN	TWSG1	Q9GZX9	Twisted gastrulation protein homolog 1	TWSG1_HUMAN		TWSG1
32	32	32	0.9966	3354	sp|Q9H251|CAD23_HUMAN	CDH23	Q9H251	Cadherin-23	CAD23_HUMAN		CDH23
69	69	69	0.999	285	sp|Q9H2P9|DPH5_HUMAN	DPH5	Q9H2P9	Diphthine methyl ester synthase	DPH5_HUMAN		DPH5
13	13	13	0.999	154	sp|Q9H4G4|GAPR1_HUMAN	GLIPR2	Q9H4G4	Golgi-associated plant pathogenesis-related protein 1	GAPR1_HUMAN		GLIPR2
103	103	103	0.999	140	sp|Q9NPJ3|ACO13_HUMAN	ACOT13	Q9NPJ3	Acyl-coenzyme A thioesterase 13	ACO13_HUMAN		ACOT13
9	9	9	0.999	549	sp|Q9NQS3|NECT3_HUMAN	NECTIN3	Q9NQS3	Nectin-3	NECT3_HUMAN		PVRL3
65	65	65	0.999	453	sp|Q9NRX5|SERC1_HUMAN	SERINC1	Q9NRX5	Serine incorporator 1	SERC1_HUMAN		SERINC1
61	61	61	0.999	183	sp|Q9NX08|COMD8_HUMAN	COMMD8	Q9NX08	COMM domain-containing protein 8	COMD8_HUMAN		COMMD8
18	18	18	0.999	153	sp|Q9P0P0|RN181_HUMAN	RNF181	Q9P0P0	E3 ubiquitin-protein ligase RNF181	RN181_HUMAN		RNF181
2	2	2	0.9988	967	sp|Q9UHI8|ATS1_HUMAN	ADAMTS1	Q9UHI8	A disintegrin and metalloproteinase with thrombospondin motifs 1	ATS1_HUMAN		ADAMTS1
32	32	32	0.999	501	sp|Q9UPY5|XCT_HUMAN	SLC7A11	Q9UPY5	Cystine/glutamate transporter	XCT_HUMAN		SLC7A11
59	59	59	0.999	444	sp|O14524|NEMP1_HUMAN	NEMP1	O14524	Nuclear envelope integral membrane protein 1	NEMP1_HUMAN		TMEM194A
39	39	39	0.999	213	sp|O14735|CDIPT_HUMAN	CDIPT	O14735	CDP-diacylglycerol--inositol 3-phosphatidyltransferase	CDIPT_HUMAN		CDIPT
13	1	13	0.999	477	sp|O14770|MEIS2_HUMAN	MEIS2	O14770	Homeobox protein Meis2	MEIS2_HUMAN		MEIS2
7	7	7	0.9956	238	sp|O14817|TSN4_HUMAN	TSPAN4	O14817	Tetraspanin-4	TSN4_HUMAN		TSPAN4
75	75	75	0.999	153	sp|O14933|UB2L6_HUMAN	UBE2L6	O14933	Ubiquitin/ISG15-conjugating enzyme E2 L6	UB2L6_HUMAN		UBE2L6
11	11	11	0.999	142	sp|O15392|BIRC5_HUMAN	BIRC5	O15392	Baculoviral IAP repeat-containing protein 5	BIRC5_HUMAN		BIRC5
47	47	47	0.999	343	sp|O15541|R113A_HUMAN	RNF113A	O15541	E3 ubiquitin-protein ligase RNF113A	R113A_HUMAN		RNF113A
33	30	33	0.999	540	sp|O43353|RIPK2_HUMAN	RIPK2	O43353	Receptor-interacting serine/threonine-protein kinase 2	RIPK2_HUMAN		RIPK2
77	77	77	0.999	245	sp|O43657|TSN6_HUMAN	TSPAN6	O43657	Tetraspanin-6	TSN6_HUMAN		TSPAN6
55	55	55	0.999	350	sp|O75787|RENR_HUMAN	ATP6AP2	O75787	Renin receptor	RENR_HUMAN		ATP6AP2
8	8	8	0.999	302	sp|O76061|STC2_HUMAN	STC2	O76061	Stanniocalcin-2	STC2_HUMAN		STC2
99	99	99	0.999	450	sp|O95219|SNX4_HUMAN	SNX4	O95219	Sorting nexin-4	SNX4_HUMAN		SNX4
44	44	44	0.999	207	sp|P01033|TIMP1_HUMAN	TIMP1	P01033	Metalloproteinase inhibitor 1	TIMP1_HUMAN		TIMP1
18	18	18	0.999	559	sp|P02748|CO9_HUMAN	C9	P02748	Complement component C9	CO9_HUMAN		C9
47	47	47	0.9957	495	sp|P04217|A1BG_HUMAN	A1BG	P04217	Alpha-1B-glycoprotein	A1BG_HUMAN		A1BG
72	72	72	0.999	100	sp|P05114|HMGN1_HUMAN	HMGN1	P05114	Non-histone chromosomal protein HMG-14	HMGN1_HUMAN		HMGN1
9	9	9	0.999	858	sp|P13591|NCAM1_HUMAN	NCAM1	P13591	Neural cell adhesion molecule 1	NCAM1_HUMAN		NCAM1
61	61	61	0.999	326	sp|P15408|FOSL2_HUMAN	FOSL2	P15408	Fos-related antigen 2	FOSL2_HUMAN		FOSL2
15	15	15	0.999	331	sp|P25774|CATS_HUMAN	CTSS	P25774	Cathepsin S	CATS_HUMAN		CTSS
52	52	52	0.999	123	sp|P47224|MSS4_HUMAN	RABIF	P47224	Guanine nucleotide exchange factor MSS4	MSS4_HUMAN		RABIF
60	32	60	0.999	201	sp|P51151|RAB9A_HUMAN	RAB9A	P51151	Ras-related protein Rab-9A	RAB9A_HUMAN		RAB9A
55	55	55	0.999	237	sp|P54105|ICLN_HUMAN	CLNS1A	P54105	Methylosome subunit pICln	ICLN_HUMAN		CLNS1A
97	97	97	0.999	94	sp|P56134|ATPK_HUMAN	ATP5MF	P56134	ATP synthase subunit f, mitochondrial	ATPK_HUMAN		ATP5J2
56	56	56	0.999	113	sp|P61803|DAD1_HUMAN	DAD1	P61803	Dolichyl-diphosphooligosaccharide--protein glycosyltransferase subunit DA	DAD1_HUMAN		DAD1
11	11	11	0.999	655	sp|Q04756|HGFA_HUMAN	HGFAC	Q04756	Hepatocyte growth factor activator	HGFA_HUMAN		HGFAC
99	99	99	0.999	323	sp|Q08722|CD47_HUMAN	CD47	Q08722	Leukocyte surface antigen CD47	CD47_HUMAN		CD47
28	28	28	0.999	499	sp|Q12834|CDC20_HUMAN	CDC20	Q12834	Cell division cycle protein 20 homolog	CDC20_HUMAN		CDC20
67	67	67	0.999	236	sp|Q14353|GAMT_HUMAN	GAMT	Q14353	Guanidinoacetate N-methyltransferase	GAMT_HUMAN		GAMT
22	22	22	0.999	492	sp|Q15043|S39AE_HUMAN	SLC39A14	Q15043	Metal cation symporter ZIP14	S39AE_HUMAN		SLC39A14
189	189	189	0.999	230	sp|Q15125|EBP_HUMAN	EBP	Q15125	3-beta-hydroxysteroid-Delta(8),Delta(7)-isomerase	EBP_HUMAN		EBP
44	44	44	0.999	746	sp|Q15910|EZH2_HUMAN	EZH2	Q15910	Histone-lysine N-methyltransferase EZH2	EZH2_HUMAN		EZH2
33	33	33	0.999	808	sp|Q4KMP7|TB10B_HUMAN	TBC1D10B	Q4KMP7	TBC1 domain family member 10B	TB10B_HUMAN		TBC1D10B
29	29	29	0.999	1261	sp|Q52LW3|RHG29_HUMAN	ARHGAP29	Q52LW3	Rho GTPase-activating protein 29	RHG29_HUMAN		ARHGAP29
47	47	47	0.999	541	sp|Q5T9L3|WLS_HUMAN	WLS	Q5T9L3	Protein wntless homolog	WLS_HUMAN		WLS
98	98	98	0.999	725	sp|Q5VZE5|NAA35_HUMAN	NAA35	Q5VZE5	N-alpha-acetyltransferase 35, NatC auxiliary subunit	NAA35_HUMAN		NAA35
59	59	59	0.999	1314	sp|Q5XPI4|RN123_HUMAN	RNF123	Q5XPI4	E3 ubiquitin-protein ligase RNF123	RN123_HUMAN		RNF123
39	39	39	0.999	534	sp|Q5ZPR3|CD276_HUMAN	CD276	Q5ZPR3	CD276 antigen	CD276_HUMAN		CD276
25	25	25	0.999	1409	sp|Q63HR2|TENS2_HUMAN	TNS2	Q63HR2	Tensin-2	TENS2_HUMAN		TNS2
35	35	35	0.999	204	sp|Q6I9Y2|THOC7_HUMAN	THOC7	Q6I9Y2	THO complex subunit 7 homolog	THOC7_HUMAN		THOC7
3	3	3	0.999	406	sp|Q6UXH8|CCBE1_HUMAN	CCBE1	Q6UXH8	Collagen and calcium-binding EGF domain-containing protein 1	CCBE1_HUMAN		CCBE1
54	54	54	0.999	355	sp|Q6ZSR9|YJ005_HUMAN		Q6ZSR9	Uncharacterized protein FLJ45252	YJ005_HUMAN		
47	47	47	0.999	540	sp|Q7Z434|MAVS_HUMAN	MAVS	Q7Z434	Mitochondrial antiviral-signaling protein	MAVS_HUMAN		MAVS
77	77	77	0.999	139	sp|Q86WQ0|NR2CA_HUMAN	NR2C2AP	Q86WQ0	Nuclear receptor 2C2-associated protein	NR2CA_HUMAN		NR2C2AP
82	82	82	0.999	188	sp|Q8IUR0|TPPC5_HUMAN	TRAPPC5	Q8IUR0	Trafficking protein particle complex subunit 5	TPPC5_HUMAN		TRAPPC5
10	10	10	0.999	645	sp|Q8IWZ8|SUGP1_HUMAN	SUGP1	Q8IWZ8	SURP and G-patch domain-containing protein 1	SUGP1_HUMAN		SUGP1
11	11	11	0.999	590	sp|Q8IZ07|AN13A_HUMAN	ANKRD13A	Q8IZ07	Ankyrin repeat domain-containing protein 13A	AN13A_HUMAN		ANKRD13A
31	31	31	0.999	530	sp|Q8N2G8|GHDC_HUMAN	GHDC	Q8N2G8	GH3 domain-containing protein	GHDC_HUMAN		GHDC
6	6	6	0.999	1624	sp|Q8N2S1|LTBP4_HUMAN	LTBP4	Q8N2S1	Latent-transforming growth factor beta-binding protein 4	LTBP4_HUMAN		LTBP4
76	76	76	0.999	344	sp|Q8NBM4|UBAC2_HUMAN	UBAC2	Q8NBM4	Ubiquitin-associated domain-containing protein 2	UBAC2_HUMAN		UBAC2
105	105	105	0.999	376	sp|Q8NEW0|ZNT7_HUMAN	SLC30A7	Q8NEW0	Zinc transporter 7	ZNT7_HUMAN		SLC30A7
53	53	53	0.999	397	sp|Q8NEZ2|VP37A_HUMAN	VPS37A	Q8NEZ2	Vacuolar protein sorting-associated protein 37A	VP37A_HUMAN		VPS37A
9	9	9	0.999	210	sp|Q8NFX7|STXB6_HUMAN	STXBP6	Q8NFX7	Syntaxin-binding protein 6	STXB6_HUMAN		STXBP6
108	108	108	0.999	905	sp|Q8WXE9|STON2_HUMAN	STON2	Q8WXE9	Stonin-2	STON2_HUMAN		STON2
71	71	71	0.999	469	sp|Q92504|S39A7_HUMAN	SLC39A7	Q92504	Zinc transporter SLC39A7	S39A7_HUMAN		SLC39A7
40	40	40	0.999	594	sp|Q92990|GLMN_HUMAN	GLMN	Q92990	Glomulin	GLMN_HUMAN		GLMN
4	2	4	0.999	196	sp|Q93062|RBPMS_HUMAN	RBPMS	Q93062	RNA-binding protein with multiple splicing	RBPMS_HUMAN		RBPMS
8	8	8	0.999	2186	sp|Q96BY6|DOC10_HUMAN	DOCK10	Q96BY6	Dedicator of cytokinesis protein 10	DOC10_HUMAN		DOCK10
66	66	66	0.999	212	sp|Q96GC5|RM48_HUMAN	MRPL48	Q96GC5	Large ribosomal subunit protein mL48	RM48_HUMAN		MRPL48
176	176	176	0.999	406	sp|Q96GC9|VMP1_HUMAN	VMP1	Q96GC9	Vacuole membrane protein 1	VMP1_HUMAN		VMP1
10	10	10	0.999	757	sp|Q96J84|KIRR1_HUMAN	KIRREL1	Q96J84	Kin of IRRE-like protein 1	KIRR1_HUMAN		KIRREL
32	32	34	0.9948	1537	sp|Q96NW7|LRRC7_HUMAN	LRRC7	Q96NW7	Leucine-rich repeat-containing protein 7	LRRC7_HUMAN		LRRC7
25	25	25	0.999	204	sp|Q96S19|MTL26_HUMAN	METTL26	Q96S19	Methyltransferase-like 26	MTL26_HUMAN		C16orf13
52	52	52	0.999	561	sp|Q96SK2|TM209_HUMAN	TMEM209	Q96SK2	Transmembrane protein 209	TM209_HUMAN		TMEM209
43	41	43	0.999	682	sp|Q99081|HTF4_HUMAN	TCF12	Q99081	Transcription factor 12	HTF4_HUMAN		TCF12
123	123	123	0.999	209	sp|Q99627|CSN8_HUMAN	COPS8	Q99627	COP9 signalosome complex subunit 8	CSN8_HUMAN		COPS8
70	70	70	0.999	464	sp|Q9BT22|ALG1_HUMAN	ALG1	Q9BT22	Chitobiosyldiphosphodolichol beta-mannosyltransferase	ALG1_HUMAN		ALG1
33	33	33	0.999	496	sp|Q9BTV5|FSD1_HUMAN	FSD1	Q9BTV5	Fibronectin type III and SPRY domain-containing protein 1	FSD1_HUMAN		FSD1
118	118	118	0.999	149	sp|Q9BYC9|RM20_HUMAN	MRPL20	Q9BYC9	Large ribosomal subunit protein bL20m	RM20_HUMAN		MRPL20
39	39	39	0.999	373	sp|Q9BYV8|CEP41_HUMAN	CEP41	Q9BYV8	Centrosomal protein of 41 kDa	CEP41_HUMAN		CEP41
56	56	56	0.999	744	sp|Q9C0D3|ZY11B_HUMAN	ZYG11B	Q9C0D3	Protein zyg-11 homolog B	ZY11B_HUMAN		ZYG11B
29	29	29	0.999	523	sp|Q9C0H2|TTYH3_HUMAN	TTYH3	Q9C0H2	Protein tweety homolog 3	TTYH3_HUMAN		TTYH3
145	91	145	0.999	377	sp|Q9GZT8|NIF3L_HUMAN	NIF3L1	Q9GZT8	NIF3-like protein 1	NIF3L_HUMAN		NIF3L1
86	86	86	0.999	879	sp|Q9H330|TM245_HUMAN	TMEM245	Q9H330	Transmembrane protein 245	TM245_HUMAN		TMEM245
62	62	62	0.999	428	sp|Q9H7B4|SMYD3_HUMAN	SMYD3	Q9H7B4	Histone-lysine N-methyltransferase SMYD3	SMYD3_HUMAN		SMYD3
18	18	18	0.999	227	sp|Q9H8U3|ZFAN3_HUMAN	ZFAND3	Q9H8U3	AN1-type zinc finger protein 3	ZFAN3_HUMAN		ZFAND3
35	35	35	0.999	914	sp|Q9H8V3|ECT2_HUMAN	ECT2	Q9H8V3	Protein ECT2	ECT2_HUMAN		ECT2
15	15	15	0.999	628	sp|Q9HB63|NET4_HUMAN	NTN4	Q9HB63	Netrin-4	NET4_HUMAN		NTN4

75	75	75	0.999	374	sp|Q9HCN4|GPN1_HUMAN	GPN1	Q9HCN4	GPN-loop GTPase 1	GPN1_HUMAN		GPN1
100	100	100	0.999	64	sp|Q9NPE3|NOP10_HUMAN	NOP10	Q9NPE3	H/ACA ribonucleoprotein complex subunit 3	NOP10_HUMAN		NOP10
24	24	24	0.999	253	sp|Q9NQY0|BIN3_HUMAN	BIN3	Q9NQY0	Bridging integrator 3	BIN3_HUMAN		BIN3
60	60	60	0.999	147	sp|Q9NRF9|DPOE3_HUMAN	POLE3	Q9NRF9	DNA polymerase epsilon subunit 3	DPOE3_HUMAN		POLE3
77	77	77	0.999	131	sp|Q9NRG0|CHRC1_HUMAN	CHRAC1	Q9NRG0	Chromatin accessibility complex protein 1	CHRC1_HUMAN		CHRAC1
41	41	41	0.999	403	sp|Q9NUD5|ZCHC3_HUMAN	ZCCHC3	Q9NUD5	Zinc finger CCHC domain-containing protein 3	ZCHC3_HUMAN		ZCCHC3
14	11	14	0.999	165	sp|Q9NVM1|EVA1B_HUMAN	EVA1B	Q9NVM1	Protein eva-1 homolog B	EVA1B_HUMAN		EVA1B
82	82	82	0.999	200	sp|Q9NX70|MED29_HUMAN	MED29	Q9NX70	Mediator of RNA polymerase II transcription subunit 29	MED29_HUMAN		MED29
9	9	9	0.999	201	sp|Q9NYL4|FKB11_HUMAN	FKBP11	Q9NYL4	Peptidyl-prolyl cis-trans isomerase FKBP11	FKB11_HUMAN		FKBP11
27	27	27	0.999	198	sp|Q9P000|COMD9_HUMAN	COMMD9	Q9P000	COMM domain-containing protein 9	COMD9_HUMAN		COMMD9
66	66	66	0.999	729	sp|Q9UBB6|NCDN_HUMAN	NCDN	Q9UBB6	Neurochondrin	NCDN_HUMAN		NCDN
27	27	27	0.999	1137	sp|Q9UPM8|AP4E1_HUMAN	AP4E1	Q9UPM8	AP-4 complex subunit epsilon-1	AP4E1_HUMAN		AP4E1
38	26	38	0.999	622	sp|Q9UPW0|FOXJ3_HUMAN	FOXJ3	Q9UPW0	Forkhead box protein J3	FOXJ3_HUMAN		FOXJ3
60	60	60	0.999	172	sp|Q9Y3D8|KAD6_HUMAN	AK6	Q9Y3D8	Adenylate kinase isoenzyme 6	KAD6_HUMAN		AK6
92	92	92	0.999	395	sp|Q9Y3Q8|T22D4_HUMAN	TSC22D4	Q9Y3Q8	TSC22 domain family protein 4	T22D4_HUMAN		TSC22D4
55	55	55	0.999	266	sp|Q9Y580|RBM7_HUMAN	RBM7	Q9Y580	RNA-binding protein 7	RBM7_HUMAN		RBM7
48	47	48	0.999	127	sp|Q9Y605|MOFA1_HUMAN	MRFAP1	Q9Y605	MORF4 family-associated protein 1	MOFA1_HUMAN		MRFAP1
28	28	28	0.999	222	sp|Q9Y680|FKBP7_HUMAN	FKBP7	Q9Y680	Peptidyl-prolyl cis-trans isomerase FKBP7	FKBP7_HUMAN		FKBP7
45	0	45	0.999	125	sp|A6NCE7|MP3B2_HUMAN	MAP1LC3B2	A6NCE7	Microtubule-associated proteins 1A/1B light chain 3 beta 2	MP3B2_HUMAN	sp|Q9GZQ8|MLP3B_HUMAN	MAP1LC3B2
11	11	11	0.999	160	sp|O14558|HSPB6_HUMAN	HSPB6	O14558	Heat shock protein beta-6	HSPB6_HUMAN		HSPB6
44	44	44	0.999	323	sp|O15121|DEGS1_HUMAN	DEGS1	O15121	Sphingolipid delta(4)-desaturase DES1	DEGS1_HUMAN		DEGS1
125	125	125	0.999	138	sp|O15235|RT12_HUMAN	MRPS12	O15235	Small ribosomal subunit protein uS12m	RT12_HUMAN		MRPS12
22	22	22	0.999	142	sp|O15514|RPB4_HUMAN	POLR2D	O15514	DNA-directed RNA polymerase II subunit RPB4	RPB4_HUMAN		POLR2D
23	23	23	0.999	437	sp|O43493|TGON2_HUMAN	TGOLN2	O43493	Trans-Golgi network integral membrane protein 2	TGON2_HUMAN		TGOLN2
106	106	106	0.999	514	sp|O43660|PLRG1_HUMAN	PLRG1	O43660	Pleiotropic regulator 1	PLRG1_HUMAN		PLRG1
91	91	91	0.999	199	sp|O60232|ZNRD2_HUMAN	ZNRD2	O60232	Protein ZNRD2	ZNRD2_HUMAN		SSSCA1
111	111	111	0.999	245	sp|O60573|IF4E2_HUMAN	EIF4E2	O60573	Eukaryotic translation initiation factor 4E type 2	IF4E2_HUMAN		EIF4E2
18	18	18	0.999	724	sp|O75330|HMMR_HUMAN	HMMR	O75330	Hyaluronan mediated motility receptor	HMMR_HUMAN		HMMR
82	82	82	0.999	377	sp|O75381|PEX14_HUMAN	PEX14	O75381	Peroxisomal membrane protein PEX14	PEX14_HUMAN		PEX14
26	26	26	0.999	207	sp|O75508|CLD11_HUMAN	CLDN11	O75508	Claudin-11	CLD11_HUMAN		CLDN11
167	167	167	0.999	301	sp|O75880|SCO1_HUMAN	SCO1	O75880	Protein SCO1 homolog, mitochondrial	SCO1_HUMAN		SCO1
93	93	93	0.999	186	sp|O75884|RBBP9_HUMAN	RBBP9	O75884	Serine hydrolase RBBP9	RBBP9_HUMAN		RBBP9
61	61	61	0.999	535	sp|O94763|RMP_HUMAN	URI1	O94763	Unconventional prefoldin RPB5 interactor 1	RMP_HUMAN		URI1
8	8	8	0.9987	1247	sp|O94885|SASH1_HUMAN	SASH1	O94885	SAM and SH3 domain-containing protein 1	SASH1_HUMAN		SASH1
20	20	20	0.999	513	sp|O95497|VNN1_HUMAN	VNN 1.00	O95497	Pantetheinase	VNN1_HUMAN		VNN 1.00
163	163	163	0.999	96	sp|O95777|LSM8_HUMAN	LSM 8.00	O95777	U6 snRNA-associated Sm-like protein LSm8	LSM8_HUMAN		LSM 8.00
338	338	338	0.999	367	sp|P02765|FETUA_HUMAN	AHSG	P02765	Alpha-2-HS-glycoprotein	FETUA_HUMAN		AHSG
143	143	143	0.999	698	sp|P02787|TRFE_HUMAN	TF	P02787	Serotransferrin	TRFE_HUMAN		TF
10	10	10	0.999	625	sp|P03951|FA11_HUMAN	F11	P03951	Coagulation factor XI	FA11_HUMAN		F11
16	16	16	0.999	161	sp|P04216|THY1_HUMAN	THY1	P04216	Thy-1 membrane glycoprotein	THY1_HUMAN		THY1
29	29	29	0.999	202	sp|P05452|TETN_HUMAN	CLEC3B	P05452	Tetranectin	TETN_HUMAN		CLEC3B
73	73	73	0.999	157	sp|P11441|UBL4A_HUMAN	UBL4A	P11441	Ubiquitin-like protein 4A	UBL4A_HUMAN		UBL4A
156	156	156	0.999	185	sp|P14209|CD99_HUMAN	CD99	P14209	CD99 antigen	CD99_HUMAN		CD99
16	16	16	0.999	382	sp|P17302|CXA1_HUMAN	GJA1	P17302	Gap junction alpha-1 protein	CXA1_HUMAN		GJA1
67	67	67	0.999	137	sp|P17568|NDUB7_HUMAN	NDUFB7	P17568	NADH dehydrogenase [ubiquinone] 1 beta subcomplex subunit 7	NDUB7_HUMAN		NDUFB7
76	76	76	0.999	275	sp|P19387|RPB3_HUMAN	POLR2C	P19387	DNA-directed RNA polymerase II subunit RPB3	RPB3_HUMAN		POLR2C
14	14	14	0.9961	298	sp|P20774|MIME_HUMAN	OGN	P20774	Mimecan	MIME_HUMAN		OGN
5	5	5	0.9983	349	sp|P29279|CCN2_HUMAN	CCN2	P29279	CCN family member 2	CCN2_HUMAN		CTGF
132	132	132	0.9989	169	sp|P30536|TSPO_HUMAN	TSPO	P30536	Translocator protein	TSPO_HUMAN		TSPO
14	14	14	0.9987	1754	sp|P39060|COIA1_HUMAN	COL18A1	P39060	Collagen alpha-1(XVIII) chain	COIA1_HUMAN		COL18A1
115	80	115	0.999	205	sp|P41236|IPP2_HUMAN	PPP1R2	P41236	Protein phosphatase inhibitor 2	IPP2_HUMAN		PPP1R2
209	35	209	0.999	113	sp|P41567|EIF1_HUMAN	EIF1	P41567	Eukaryotic translation initiation factor 1	EIF1_HUMAN		EIF1
42	42	42	0.998	599	sp|P43652|AFAM_HUMAN	AFM	P43652	Afamin	AFAM_HUMAN		AFM
31	5	31	0.999	1223	sp|P46020|KPB1_HUMAN	PHKA1	P46020	Phosphorylase b kinase regulatory subunit alpha, skeletal muscle isoform	KPB1_HUMAN		PHKA1
15	15	15	0.9983	93	sp|P48061|SDF1_HUMAN	CXCL12	P48061	Stromal cell-derived factor 1	SDF1_HUMAN		CXCL12
55	55	55	0.999	109	sp|P52926|HMGA2_HUMAN	HMGA2	P52926	High mobility group protein HMGI-C	HMGA2_HUMAN		HMGA2
103	103	103	0.999	331	sp|P53611|PGTB2_HUMAN	RABGGTB	P53611	Geranylgeranyl transferase type-2 subunit beta	PGTB2_HUMAN		RABGGTB
101	101	101	0.999	142	sp|P53680|AP2S1_HUMAN	AP2S1	P53680	AP-2 complex subunit sigma	AP2S1_HUMAN		AP2S1
71	71	71	0.999	180	sp|P53801|PTTG_HUMAN	PTTG1IP	P53801	Pituitary tumor-transforming gene 1 protein-interacting protein	PTTG_HUMAN		PTTG1IP
63	63	63	0.999	128	sp|P56556|NDUA6_HUMAN	NDUFA6	P56556	NADH dehydrogenase [ubiquinone] 1 alpha subcomplex subunit 6	NDUA6_HUMAN		NDUFA6
123	123	123	0.999	79	sp|P60602|ROMO1_HUMAN	ROMO1	P60602	Reactive oxygen species modulator 1	ROMO1_HUMAN		ROMO1
79	79	79	0.999	165	sp|P60604|UB2G2_HUMAN	UBE2G2	P60604	Ubiquitin-conjugating enzyme E2 G2	UB2G2_HUMAN		UBE2G2
103	103	103	0.999	79	sp|P61024|CKS1_HUMAN	CKS1B	P61024	Cyclin-dependent kinases regulatory subunit 1	CKS1_HUMAN		CKS1B
12	12	12	0.999	244	sp|P61587|RND3_HUMAN	RND3	P61587	Rho-related GTP-binding protein RhoE	RND3_HUMAN		RND3
132	132	132	0.999	92	sp|P62304|RUXE_HUMAN	SNRPE	P62304	Small nuclear ribonucleoprotein E	RUXE_HUMAN		SNRPE
79	1	79	0.999	51	sp|P62891|RL39_HUMAN	RPL39	P62891	Large ribosomal subunit protein eL39	RL39_HUMAN		RPL39
143	104	143	0.999	68	sp|P63218|GBG5_HUMAN	GNG5	P63218	Guanine nucleotide-binding protein G(I)/G(S)/G(O) subunit gamma-5	GBG5_HUMAN		GNG5
125	125	125	0.999	194	sp|P82912|RT11_HUMAN	MRPS11	P82912	Small ribosomal subunit protein uS11m	RT11_HUMAN		MRPS11
59	59	59	0.999	535	sp|Q02252|MMSA_HUMAN	ALDH6A1	Q02252	Methylmalonate-semialdehyde dehydrogenase [acylating], mitochondrial	MMSA_HUMAN		ALDH6A1
17	17	17	0.999	376	sp|Q06828|FMOD_HUMAN	FMOD	Q06828	Fibromodulin	FMOD_HUMAN		FMOD
10	10	10	0.9977	318	sp|Q10588|BST1_HUMAN	BST1	Q10588	ADP-ribosyl cyclase/cyclic ADP-ribose hydrolase 2	BST1_HUMAN		BST1
16	16	16	0.999	1018	sp|Q12860|CNTN1_HUMAN	CNTN1	Q12860	Contactin-1	CNTN1_HUMAN		CNTN1
14	14	14	0.999	560	sp|Q14520|HABP2_HUMAN	HABP2	Q14520	Hyaluronan-binding protein 2	HABP2_HUMAN		HABP2
101	101	101	0.999	427	sp|Q14653|IRF3_HUMAN	IRF3	Q14653	Interferon regulatory factor 3	IRF3_HUMAN		IRF3
7	7	7	0.999	222	sp|Q16763|UBE2S_HUMAN	UBE2S	Q16763	Ubiquitin-conjugating enzyme E2 S	UBE2S_HUMAN		UBE2S
122	117	122	0.999	961	sp|Q2M2I8|AAK1_HUMAN	AAK1	Q2M2I8	AP2-associated protein kinase 1	AAK1_HUMAN		AAK1
35	35	35	0.999	1938	sp|Q2TAZ0|ATG2A_HUMAN	ATG2A	Q2TAZ0	Autophagy-related protein 2 homolog A	ATG2A_HUMAN		ATG2A
69	0	69	0.999	206	sp|Q5H9L2|TCEAL5_HUMAN	TCEAL5	Q5H9L2	Transcription elongation factor A protein-like 5	TCAL5_HUMAN	sp|Q969E4|TCAL3_HUMAN	TCEAL5
13	13	13	0.999	742	sp|Q5KU26|COL12_HUMAN	COLEC12	Q5KU26	Collectin-12	COL12_HUMAN		COLEC12
84	84	84	0.999	328	sp|Q5TC12|ATPF1_HUMAN	ATPAF1	Q5TC12	ATP synthase mitochondrial F1 complex assembly factor 1	ATPF1_HUMAN		ATPAF1
33	33	33	0.999	208	sp|Q6FIF0|ZFAN6_HUMAN	ZFAND6	Q6FIF0	AN1-type zinc finger protein 6	ZFAN6_HUMAN		ZFAND6
93	93	93	0.999	189	sp|Q6UW68|TM205_HUMAN	TMEM205	Q6UW68	Transmembrane protein 205	TM205_HUMAN		TMEM205
46	46	46	0.999	472	sp|Q6UX04|CWC27_HUMAN	CWC27	Q6UX04	Spliceosome-associated protein CWC27 homolog	CWC27_HUMAN		CWC27
48	1	48	0.999	313	sp|Q7L523|RRAGA_HUMAN	RRAGA	Q7L523	Ras-related GTP-binding protein A	RRAGA_HUMAN		RRAGA
21	21	21	0.999	396	sp|Q7L5Y9|MAEA_HUMAN	MAEA	Q7L5Y9	E3 ubiquitin-protein transferase MAEA	MAEA_HUMAN		MAEA
65	65	65	0.999	152	sp|Q7Z422|SZRD1_HUMAN	SZRD1	Q7Z422	SUZ domain-containing protein 1	SZRD1_HUMAN		SZRD1
19	19	19	0.999	137	sp|Q7Z5G4|GOGA7_HUMAN	GOLGA7	Q7Z5G4	Golgin subfamily A member 7	GOGA7_HUMAN		GOLGA7
48	48	48	0.999	106	sp|Q7Z7K0|COXM1_HUMAN	CMC1	Q7Z7K0	COX assembly mitochondrial protein homolog	COXM1_HUMAN		CMC1
125	125	125	0.999	157	sp|Q86SX6|GLRX5_HUMAN	GLRX5	Q86SX6	Glutaredoxin-related protein 5, mitochondrial	GLRX5_HUMAN		GLRX5
70	69	70	0.999	277	sp|Q86T03|PP4P1_HUMAN	PIP4P1	Q86T03	Type 1 phosphatidylinositol 4,5-bisphosphate 4-phosphatase	PP4P1_HUMAN		TMEM55B
55	51	55	0.999	675	sp|Q86YR5|GPSM1_HUMAN	GPSM1	Q86YR5	G-protein-signaling modulator 1	GPSM1_HUMAN		GPSM1
47	47	47	0.999	547	sp|Q8IWB1|IPRI_HUMAN	ITPRIP	Q8IWB1	Inositol 1,4,5-trisphosphate receptor-interacting protein	IPRI_HUMAN		ITPRIP
45	45	45	0.999	846	sp|Q8IWW6|RHG12_HUMAN	ARHGAP12	Q8IWW6	Rho GTPase-activating protein 12	RHG12_HUMAN		ARHGAP12
70	70	70	0.999	434	sp|Q8IYU8|MICU2_HUMAN	MICU2	Q8IYU8	Calcium uptake protein 2, mitochondrial	MICU2_HUMAN		MICU2
97	97	97	0.999	169	sp|Q8N183|NDUF2_HUMAN	NDUFAF2	Q8N183	NADH dehydrogenase [ubiquinone] 1 alpha subcomplex assembly factor 2	NDUF2_HUMAN		NDUFAF2
126	126	126	0.999	131	sp|Q8N4V1|EMC5_HUMAN	MMGT1	Q8N4V1	ER membrane protein complex subunit 5	EMC5_HUMAN		MMGT1
23	23	23	0.9967	376	sp|Q8NCH0|CHSTE_HUMAN	CHST14	Q8NCH0	Carbohydrate sulfotransferase 14	CHSTE_HUMAN		CHST14
17	17	17	0.9989	357	sp|Q8NFJ5|RAI3_HUMAN	GPRC5A	Q8NFJ5	Retinoic acid-induced protein 3	RAI3_HUMAN		GPRC5A
37	37	37	0.999	600	sp|Q8NHQ9|DDX55_HUMAN	DDX55	Q8NHQ9	ATP-dependent RNA helicase DDX55	DDX55_HUMAN		DDX55
62	62	62	0.999	745	sp|Q8TB52|FBX30_HUMAN	FBXO30	Q8TB52	F-box only protein 30	FBX30_HUMAN		FBXO30
56	56	56	0.999	432	sp|Q8TB61|S35B2_HUMAN	SLC35B2	Q8TB61	Adenosine 3'-phospho 5'-phosphosulfate transporter 1	S35B2_HUMAN		SLC35B2
96	96	96	0.999	366	sp|Q8WTS6|SETD7_HUMAN	SETD7	Q8WTS6	Histone-lysine N-methyltransferase SETD7	SETD7_HUMAN		SETD7
171	171	171	0.999	318	sp|Q92820|GGH_HUMAN	GGH	Q92820	Gamma-glutamyl hydrolase	GGH_HUMAN		GGH
114	114	114	0.999	205	sp|Q96AB3|ISOC2_HUMAN	ISOC2	Q96AB3	Isochorismatase domain-containing protein 2	ISOC2_HUMAN		ISOC2
91	91	91	0.999	289	sp|Q96FX7|TRM61_HUMAN	TRMT61A	Q96FX7	tRNA (adenine(58)-N(1))-methyltransferase catalytic subunit TRMT61A	TRM61_HUMAN		TRMT61A
49	49	49	0.999	475	sp|Q96GA3|LTV1_HUMAN	LTV1	Q96GA3	Protein LTV1 homolog	LTV1_HUMAN		LTV1
46	46	46	0.999	352	sp|Q96JY6|PDLI2_HUMAN	PDLIM2	Q96JY6	PDZ and LIM domain protein 2	PDLI2_HUMAN		PDLIM2
50	50	50	0.999	506	sp|Q96QD8|S38A2_HUMAN	SLC38A2	Q96QD8	Sodium-coupled neutral amino acid symporter 2	S38A2_HUMAN		SLC38A2
92	92	92	0.999	433	sp|Q99538|LGMN_HUMAN	LGMN	Q99538	Legumain	LGMN_HUMAN		LGMN
11	11	11	0.999	342	sp|Q99633|PRP18_HUMAN	PRPF18	Q99633	Pre-mRNA-splicing factor 18	PRP18_HUMAN		PRPF18
42	42	42	0.999	303	sp|Q99685|MGLL_HUMAN	MGLL	Q99685	Monoglyceride lipase	MGLL_HUMAN		MGLL
89	89	89	0.999	676	sp|Q9BQ70|TCF25_HUMAN	TCF25	Q9BQ70	Ribosome quality control complex subunit TCF25	TCF25_HUMAN		TCF25
73	73	73	0.999	176	sp|Q9BRG1|VPS25_HUMAN	VPS25	Q9BRG1	Vacuolar protein-sorting-associated protein 25	VPS25_HUMAN		VPS25
44	44	44	0.999	392	sp|Q9BRP4|PAAF1_HUMAN	PAAF1	Q9BRP4	Proteasomal ATPase-associated factor 1	PAAF1_HUMAN		PAAF1
46	46	46	0.999	373	sp|Q9BRQ8|FSP1_HUMAN	AIFM2	Q9BRQ8	Ferroptosis suppressor protein 1	FSP1_HUMAN		AIFM2
50	50	50	0.999	216	sp|Q9BRX5|PSF3_HUMAN	GINS3	Q9BRX5	DNA replication complex GINS protein PSF3	PSF3_HUMAN		GINS3
54	52	54	0.999	278	sp|Q9BTZ2|DHRS4_HUMAN	DHRS4	Q9BTZ2	Dehydrogenase/reductase SDR family member 4	DHRS4_HUMAN		DHRS4
109	109	109	0.999	311	sp|Q9BV68|RN126_HUMAN	RNF126	Q9BV68	E3 ubiquitin-protein ligase RNF126	RN126_HUMAN		RNF126
31	31	31	0.999	552	sp|Q9BVS4|RIOK2_HUMAN	RIOK2	Q9BVS4	Serine/threonine-protein kinase RIO2	RIOK2_HUMAN		RIOK2
31	31	31	0.999	186	sp|Q9BW83|IFT27_HUMAN	IFT27	Q9BW83	Intraflagellar transport protein 27 homolog	IFT27_HUMAN		IFT27
63	63	63	0.999	393	sp|Q9BWS9|CHID1_HUMAN	CHID1	Q9BWS9	Chitinase domain-containing protein 1	CHID1_HUMAN		CHID1
18	18	18	0.999	385	sp|Q9BX40|LS14B_HUMAN	LSM14B	Q9BX40	Protein LSM14 homolog B	LS14B_HUMAN		LSM14B
49	49	49	0.999	558	sp|Q9H0G5|NSRP1_HUMAN	NSRP1	Q9H0G5	Nuclear speckle splicing regulatory protein 1	NSRP1_HUMAN		NSRP1
127	127	127	0.999	275	sp|Q9H1Y0|ATG5_HUMAN	ATG5	Q9H1Y0	Autophagy protein 5	ATG5_HUMAN		ATG5
43	10	43	0.999	399	sp|Q9HB90|RRAGC_HUMAN	RRAGC	Q9HB90	Ras-related GTP-binding protein C	RRAGC_HUMAN		RRAGC
76	76	76	0.999	417	sp|Q9HCU5|PREB_HUMAN	PREB	Q9HCU5	Prolactin regulatory element-binding protein	PREB_HUMAN		PREB
60	60	60	0.999	194	sp|Q9NP77|SSU72_HUMAN	SSU72	Q9NP77	RNA polymerase II subunit A C-terminal domain phosphatase SSU72	SSU72_HUMAN		SSU72
65	44	65	0.999	753	sp|Q9NRL3|STRN4_HUMAN	STRN4	Q9NRL3	Striatin-4	STRN4_HUMAN		STRN4
108	74	108	0.999	190	sp|Q9NRW3|ABC3C_HUMAN	APOBEC3C	Q9NRW3	DNA dC->dU-editing enzyme APOBEC-3C	ABC3C_HUMAN		APOBEC3C

57	12	57	0.999	361	sp|Q9NV96|CC50A_HUMAN	TMEM30A	Q9NV96	Cell cycle control protein 50A	CC50A_HUMAN		TMEM30A
55	55	55	0.999	692	sp|Q9NVM4|ANM7_HUMAN	PRMT7	Q9NVM4	Protein arginine N-methyltransferase 7	ANM7_HUMAN		PRMT7
73	73	73	0.999	290	sp|Q9NWS0|PIHD1_HUMAN	PIH1D1	Q9NWS0	PIH1 domain-containing protein 1	PIHD1_HUMAN		PIH1D1
233	233	233	0.999	121	sp|Q9NX55|HYPK_HUMAN	HYPK	Q9NX55	Huntingtin-interacting protein K	HYPK_HUMAN		HYPK
26	26	26	0.999	423	sp|Q9NXH8|TOR4A_HUMAN	TOR4A	Q9NXH8	Torsin-4A	TOR4A_HUMAN		TOR4A
82	82	126	0.9971	351	sp|Q9NZR1|TMOD2_HUMAN	TMOD2	Q9NZR1	Tropomodulin-2	TMOD2_HUMAN		TMOD2
20	20	20	0.999	2028	sp|Q9P219|DAPLE_HUMAN	CCDC88C	Q9P219	Protein Daple	DAPLE_HUMAN		CCDC88C
102	102	102	0.999	335	sp|Q9P2W9|STX18_HUMAN	STX18	Q9P2W9	Syntaxin-18	STX18_HUMAN		STX18
111	111	111	0.999	113	sp|Q9UBF6|RBX2_HUMAN	RNF7	Q9UBF6	RING-box protein 2	RBX2_HUMAN		RNF7
76	76	76	0.999	284	sp|Q9UBV8|PEF1_HUMAN	PEF1	Q9UBV8	Peflin	PEF1_HUMAN		PEF1
76	76	76	0.999	145	sp|Q9UI09|NDUAC_HUMAN	NDUFA12	Q9UI09	NADH dehydrogenase [ubiquinone] 1 alpha subcomplex subunit 12	NDUAC_HUMAN		NDUFA12
56	56	56	0.999	185	sp|Q9UI14|PRAF1_HUMAN	RABAC1	Q9UI14	Prenylated Rab acceptor protein 1	PRAF1_HUMAN		RABAC1
60	60	60	0.999	485	sp|Q9ULA0|DNPEP_HUMAN	DNPEP	Q9ULA0	Aspartyl aminopeptidase	DNPEP_HUMAN		DNPEP
21	21	21	0.999	313	sp|Q9UNQ2|DIM1_HUMAN	DIMT1	Q9UNQ2	Probable dimethyladenosine transferase	DIM1_HUMAN		DIMT1
8	8	8	0.999	779	sp|Q9Y2J4|AMOL2_HUMAN	AMOTL2	Q9Y2J4	Angiomotin-like protein 2	AMOL2_HUMAN		AMOTL2
123	123	123	0.999	106	sp|Q9Y2R0|COA3_HUMAN	COA3	Q9Y2R0	Cytochrome c oxidase assembly factor 3 homolog, mitochondrial	COA3_HUMAN		COA3
21	21	21	0.999	717	sp|Q9Y2Z2|MTO1_HUMAN	MTO1	Q9Y2Z2	Protein MTO1 homolog, mitochondrial	MTO1_HUMAN		MTO1
131	131	131	0.999	296	sp|Q9Y320|TMX2_HUMAN	TMX2	Q9Y320	Thioredoxin-related transmembrane protein 2	TMX2_HUMAN		TMX2
10	10	10	0.999	778	sp|Q9Y5V3|MAGD1_HUMAN	MAGED1	Q9Y5V3	Melanoma-associated antigen D1	MAGD1_HUMAN		MAGED1
130	130	130	0.999	271	sp|Q9Y5Y2|NUBP2_HUMAN	NUBP2	Q9Y5Y2	Cytosolic Fe-S cluster assembly factor NUBP2	NUBP2_HUMAN		NUBP2
72	72	72	0.999	313	sp|Q9Y6I9|TX264_HUMAN	TEX264	Q9Y6I9	Testis-expressed protein 264	TX264_HUMAN		TEX264
55	0	55	0.999	181	sp|A0A024RBG1|NUD4B_HUM	NUDT4B	A0A024RBG1	Diphosphoinositol polyphosphate phosphohydrolase NUDT4B	NUD4B_HUMAN	sp|Q9NZJ9|NUDT4_HUMAN	NUDT4
120	4	120	0.999	1229	sp|A8CG34|P121C_HUMAN	POM121C	A8CG34	Nuclear envelope pore membrane protein POM 121C	P121C_HUMAN		POM121C
104	0	104	0.999	76	sp|A8MWD9|RUXGL_HUMAN	SNRPGP15	A8MWD9	Putative small nuclear ribonucleoprotein G-like protein 15	RUXGL_HUMAN	sp|P62308|RUXG_HUMAN	SNRPGP15
107	107	107	0.999	123	sp|E9PRG8|CK098_HUMAN	C11orf98	E9PRG8	Uncharacterized protein C11orf98	CK098_HUMAN		C11orf98
73	73	73	0.999	103	sp|L0R6Q1|S35U4_HUMAN	SLC35A4	L0R6Q1	SLC35A4 upstream open reading frame protein	S35U4_HUMAN		SLC35A4
115	115	115	0.999	563	sp|O14773|TPP1_HUMAN	TPP1	O14773	Tripeptidyl-peptidase 1	TPP1_HUMAN		TPP1
300	300	300	0.999	152	sp|O14880|MGST3_HUMAN	MGST3	O14880	Glutathione S-transferase 3, mitochondrial	MGST3_HUMAN		MGST3
95	95	95	0.999	1369	sp|O15013|ARHGA_HUMAN	ARHGEF10	O15013	Rho guanine nucleotide exchange factor 10	ARHGA_HUMAN		ARHGEF10
17	17	17	0.9987	318	sp|O15162|PLS1_HUMAN	PLSCR1	O15162	Phospholipid scramblase 1	PLS1_HUMAN		PLSCR1
94	94	94	0.999	510	sp|O15446|RPA34_HUMAN	POLR1G	O15446	DNA-directed RNA polymerase I subunit RPA34	RPA34_HUMAN		CD3EAP
152	152	152	0.999	448	sp|O15533|TPSN_HUMAN	TAPBP	O15533	Tapasin	TPSN_HUMAN		TAPBP
29	29	29	0.9989	461	sp|O43294|TGFI1_HUMAN	TGFB1I1	O43294	Transforming growth factor beta-1-induced transcript 1 protein	TGFI1_HUMAN		TGFB1I1
98	98	98	0.999	99	sp|O43678|NDUA2_HUMAN	NDUFA2	O43678	NADH dehydrogenase [ubiquinone] 1 alpha subcomplex subunit 2	NDUA2_HUMAN		NDUFA2
55	55	55	0.999	386	sp|O60711|LPXN_HUMAN	LPXN	O60711	Leupaxin	LPXN_HUMAN		LPXN
85	85	85	0.999	178	sp|O60831|PRAF2_HUMAN	PRAF2	O60831	PRA1 family protein 2	PRAF2_HUMAN		PRAF2
129	129	129	0.999	238	sp|O75940|SPF30_HUMAN	SMNDC1	O75940	Survival of motor neuron-related-splicing factor 30	SPF30_HUMAN		SMNDC1
124	124	124	0.999	489	sp|O94888|UBXN7_HUMAN	UBXN7	O94888	UBX domain-containing protein 7	UBXN7_HUMAN		UBXN7
19	19	19	0.999	398	sp|O95067|CCNB2_HUMAN	CCNB2	O95067	G2/mitotic-specific cyclin-B2	CCNB2_HUMAN		CCNB2
13	13	13	0.9975	383	sp|O95084|PRS23_HUMAN	PRSS23	O95084	Serine protease 23	PRS23_HUMAN		PRSS23
94	94	94	0.999	128	sp|O95139|NDUB6_HUMAN	NDUFB6	O95139	NADH dehydrogenase [ubiquinone] 1 beta subcomplex subunit 6	NDUB6_HUMAN		NDUFB6
62	62	62	0.999	250	sp|O95249|GOSR1_HUMAN	GOSR1	O95249	Golgi SNAP receptor complex member 1	GOSR1_HUMAN		GOSR1
78	78	78	0.999	269	sp|O95297|MPZL1_HUMAN	MPZL1	O95297	Myelin protein zero-like protein 1	MPZL1_HUMAN		MPZL1
91	91	91	0.999	341	sp|O95400|CD2B2_HUMAN	CD2BP2	O95400	CD2 antigen cytoplasmic tail-binding protein 2	CD2B2_HUMAN		CD2BP2
132	132	132	0.999	349	sp|O95825|QORL1_HUMAN	CRYZL1	O95825	Quinone oxidoreductase-like protein 1	QORL1_HUMAN		CRYZL1
41	41	54	0.999	172	sp|O95989|NUDT3_HUMAN	NUDT3	O95989	Diphosphoinositol polyphosphate phosphohydrolase 1	NUDT3_HUMAN		NUDT3
15	13	15	0.9947	732	sp|P00488|F13A_HUMAN	F13A1	P00488	Coagulation factor XIII A chain	F13A_HUMAN		F13A1
26	26	26	0.9987	488	sp|P00742|FA10_HUMAN	F10	P00742	Coagulation factor X	FA10_HUMAN		F10
66	66	66	0.999	562	sp|P00750|TPA_HUMAN	PLAT	P00750	Tissue-type plasminogen activator	TPA_HUMAN		PLAT
71	71	71	0.999	345	sp|P02749|APOH_HUMAN	APOH	P02749	Beta-2-glycoprotein 1	APOH_HUMAN		APOH
180	180	180	0.999	165	sp|P05161|ISG15_HUMAN	ISG15	P05161	Ubiquitin-like protein ISG15	ISG15_HUMAN		ISG15
54	54	54	0.999	499	sp|P05546|HEP2_HUMAN	SERPIND1	P05546	Heparin cofactor 2	HEP2_HUMAN		SERPIND1
49	49	49	0.999	288	sp|P09038|FGF2_HUMAN	FGF2	P09038	Fibroblast growth factor 2	FGF2_HUMAN		FGF2
32	32	34	0.999	472	sp|P09913|IFIT2_HUMAN	IFIT2	P09913	Interferon-induced protein with tetratricopeptide repeats 2	IFIT2_HUMAN		IFIT2
87	87	87	0.999	480	sp|P10619|PPGB_HUMAN	CTSA	P10619	Lysosomal protective protein	PPGB_HUMAN		CTSA
14	14	14	0.9989	449	sp|P10909|CLUS_HUMAN	CLU	P10909	Clusterin	CLUS_HUMAN		CLU
47	47	47	0.999	295	sp|P13726|TF_HUMAN	F3	P13726	Tissue factor	TF_HUMAN		F3
91	62	91	0.999	398	sp|P15291|B4GT1_HUMAN	B4GALT1	P15291	Beta-1,4-galactosyltransferase 1	B4GT1_HUMAN		B4GALT1
24	24	24	0.999	220	sp|P16035|TIMP2_HUMAN	TIMP2	P16035	Metalloproteinase inhibitor 2	TIMP2_HUMAN		TIMP2
111	23	111	0.999	271	sp|P18846|ATF1_HUMAN	ATF1	P18846	Cyclic AMP-dependent transcription factor ATF-1	ATF1_HUMAN		ATF1
34	34	34	0.999	906	sp|P19022|CADH2_HUMAN	CDH2	P19022	Cadherin-2	CADH2_HUMAN		CDH2
23	23	23	0.999	1154	sp|P23458|JAK1_HUMAN	JAK1	P23458	Tyrosine-protein kinase JAK1	JAK1_HUMAN		JAK1
125	125	125	0.999	276	sp|P28062|PSB8_HUMAN	PSMB8	P28062	Proteasome subunit beta type-8	PSB8_HUMAN		PSMB8
156	156	156	0.999	168	sp|P30049|ATPD_HUMAN	ATP5F1D	P30049	ATP synthase subunit delta, mitochondrial	ATPD_HUMAN		ATP5D
88	88	88	0.999	181	sp|P40616|ARL1_HUMAN	ARL 1.00	P40616	ADP-ribosylation factor-like protein 1	ARL1_HUMAN		ARL 1.00
48	48	48	0.999	350	sp|P46976|GLYG_HUMAN	GYG1	P46976	Glycogenin-1	GLYG_HUMAN		GYG1
21	21	21	0.999	471	sp|P48723|HSP13_HUMAN	HSPA13	P48723	Heat shock 70 kDa protein 13	HSP13_HUMAN		HSPA13
106	106	106	0.999	369	sp|P49366|DHYS_HUMAN	DHPS	P49366	Deoxyhypusine synthase	DHYS_HUMAN		DHPS
111	111	111	0.999	454	sp|P49593|PPM1F_HUMAN	PPM1F	P49593	Protein phosphatase 1F	PPM1F_HUMAN		PPM1F
121	121	121	0.999	147	sp|P50583|AP4A_HUMAN	NUDT2	P50583	Bis(5'-nucleosyl)-tetraphosphatase [asymmetrical]	AP4A_HUMAN		NUDT2
90	90	90	0.999	309	sp|P51948|MAT1_HUMAN	MNAT1	P51948	CDK-activating kinase assembly factor MAT1	MAT1_HUMAN		MNAT1
36	36	36	0.999	247	sp|P52823|STC1_HUMAN	STC1	P52823	Stanniocalcin-1	STC1_HUMAN		STC1
18	18	18	0.999	603	sp|P53350|PLK1_HUMAN	PLK1	P53350	Serine/threonine-protein kinase PLK1	PLK1_HUMAN		PLK1
60	60	60	0.999	713	sp|P55290|CAD13_HUMAN	CDH13	P55290	Cadherin-13	CAD13_HUMAN		CDH13
88	88	180	0.999	112	sp|P56211|ARP19_HUMAN	ARPP19	P56211	cAMP-regulated phosphoprotein 19	ARP19_HUMAN		ARPP19
85	66	85	0.999	870	sp|P56945|BCAR1_HUMAN	BCAR1	P56945	Breast cancer anti-estrogen resistance protein 1	BCAR1_HUMAN		BCAR1
163	163	163	0.999	145	sp|P57105|SYJ2B_HUMAN	SYNJ2BP	P57105	Synaptojanin-2-binding protein	SYJ2B_HUMAN		SYNJ2BP
379	40	379	0.999	147	sp|P61077|UB2D3_HUMAN	UBE2D3	P61077	Ubiquitin-conjugating enzyme E2 D3	UB2D3_HUMAN		UBE2D3
59	57	59	0.999	351	sp|P61421|VA0D1_HUMAN	ATP6V0D1	P61421	V-type proton ATPase subunit d 1	VA0D1_HUMAN		ATP6V0D1
98	98	98	0.999	342	sp|P61962|DCAF7_HUMAN	DCAF7	P61962	DDB1- and CUL4-associated factor 7	DCAF7_HUMAN		DCAF7
58	58	58	0.999	334	sp|P61964|WDR5_HUMAN	WDR5	P61964	WD repeat-containing protein 5	WDR5_HUMAN		WDR5
90	90	90	0.999	90	sp|P62072|TIM10_HUMAN	TIMM10	P62072	Mitochondrial import inner membrane translocase subunit Tim10	TIM10_HUMAN		TIMM10
72	72	72	0.999	86	sp|P62306|RUXF_HUMAN	SNRPF	P62306	Small nuclear ribonucleoprotein F	RUXF_HUMAN		SNRPF
149	149	149	0.999	119	sp|P62314|SMD1_HUMAN	SNRPD1	P62314	Small nuclear ribonucleoprotein Sm D1	SMD1_HUMAN		SNRPD1
217	217	217	0.999	133	sp|P62861|RS30_HUMAN	FAU	P62861	Ubiquitin-like FUBI-ribosomal protein eS30 fusion protein	RS30_HUMAN		FAU
132	132	132	0.999	108	sp|P62877|RBX1_HUMAN	RBX1	P62877	E3 ubiquitin-protein ligase RBX1	RBX1_HUMAN		RBX1
120	120	120	0.999	117	sp|P63272|SPT4H_HUMAN	SUPT4H1	P63272	Transcription elongation factor SPT4	SPT4H_HUMAN		SUPT4H1
37	37	47	0.999	1123	sp|P78332|RBM6_HUMAN	RBM6	P78332	RNA-binding protein 6	RBM6_HUMAN		RBM6
63	63	63	0.999	110	sp|P81605|DCD_HUMAN	DCD	P81605	Dermcidin	DCD_HUMAN		DCD
18	18	18	0.999	1093	sp|P82094|TMF1_HUMAN	TMF1	P82094	TATA element modulatory factor	TMF1_HUMAN		TMF1
43	43	43	0.9953	2701	sp|Q02224|CENPE_HUMAN	CENPE	Q02224	Centromere-associated protein E	CENPE_HUMAN		CENPE
117	57	117	0.999	781	sp|Q02447|SP3_HUMAN	SP3	Q02447	Transcription factor Sp3	SP3_HUMAN		SP3
48	48	48	0.999	875	sp|Q05086|UBE3A_HUMAN	UBE3A	Q05086	Ubiquitin-protein ligase E3A	UBE3A_HUMAN		UBE3A
54	54	54	0.999	180	sp|Q10589|BST2_HUMAN	BST2	Q10589	Bone marrow stromal antigen 2	BST2_HUMAN		BST2
14	14	14	0.999	493	sp|Q12805|FBLN3_HUMAN	EFEMP1	Q12805	EGF-containing fibulin-like extracellular matrix protein 1	FBLN3_HUMAN		EFEMP1
85	15	85	0.999	167	sp|Q12974|TP4A2_HUMAN	PTP4A2	Q12974	Protein tyrosine phosphatase type IVA 2	TP4A2_HUMAN		PTP4A2
13	13	13	0.9981	749	sp|Q13214|SEM3B_HUMAN	SEMA3B	Q13214	Semaphorin-3B	SEM3B_HUMAN		SEMA3B
79	79	79	0.999	667	sp|Q13438|OS9_HUMAN	OS9	Q13438	Protein OS-9	OS9_HUMAN		OS9
114	114	114	0.999	118	sp|Q13541|4EBP1_HUMAN	EIF4EBP1	Q13541	Eukaryotic translation initiation factor 4E-binding protein 1	4EBP1_HUMAN		EIF4EBP1
67	67	67	0.999	1066	sp|Q14139|UBE4A_HUMAN	UBE4A	Q14139	Ubiquitin conjugation factor E4 A	UBE4A_HUMAN		UBE4A
120	120	120	0.999	624	sp|Q14145|KEAP1_HUMAN	KEAP1	Q14145	Kelch-like ECH-associated protein 1	KEAP1_HUMAN		KEAP1
37	37	37	0.999	258	sp|Q14331|FRG1_HUMAN	FRG1	Q14331	Protein FRG1	FRG1_HUMAN		FRG1
39	39	39	0.999	930	sp|Q14624|ITIH4_HUMAN	ITIH4	Q14624	Inter-alpha-trypsin inhibitor heavy chain H4	ITIH4_HUMAN		ITIH4
81	81	81	0.999	288	sp|Q15014|MO4L2_HUMAN	MORF4L2	Q15014	Mortality factor 4-like protein 2	MO4L2_HUMAN		MORF4L2
68	68	68	0.999	862	sp|Q15276|RABE1_HUMAN	RABEP1	Q15276	Rab GTPase-binding effector protein 1	RABE1_HUMAN		RABEP1
76	76	76	0.999	200	sp|Q15528|MED22_HUMAN	MED22	Q15528	Mediator of RNA polymerase II transcription subunit 22	MED22_HUMAN		MED22
23	23	23	0.999	540	sp|Q16610|ECM1_HUMAN	ECM1	Q16610	Extracellular matrix protein 1	ECM1_HUMAN		ECM1
17	17	17	0.999	283	sp|Q4V9L6|TM119_HUMAN	TMEM119	Q4V9L6	Transmembrane protein 119	TM119_HUMAN		TMEM119
296	296	296	0.999	144	sp|Q4VC31|MIX23_HUMAN	MIX23	Q4VC31	Protein MIX23	MIX23_HUMAN		CCDC58
134	134	134	0.999	236	sp|Q5HYI8|RABL3_HUMAN	RABL3	Q5HYI8	Rab-like protein 3	RABL3_HUMAN		RABL3
110	110	110	0.999	702	sp|Q5SY16|NOL9_HUMAN	NOL9	Q5SY16	Polynucleotide 5'-hydroxyl-kinase NOL9	NOL9_HUMAN		NOL9
70	70	70	0.999	605	sp|Q5T0N5|FBP1L_HUMAN	FNBP1L	Q5T0N5	Formin-binding protein 1-like	FBP1L_HUMAN		FNBP1L
111	111	111	0.999	1119	sp|Q5T6F2|UBAP2_HUMAN	UBAP2	Q5T6F2	Ubiquitin-associated protein 2	UBAP2_HUMAN		UBAP2
7	7	7	0.9988	579	sp|Q5T749|KPRP_HUMAN	KPRP	Q5T749	Keratinocyte proline-rich protein	KPRP_HUMAN		KPRP
29	11	29	0.999	837	sp|Q5VSL9|STRP1_HUMAN	STRIP1	Q5VSL9	Striatin-interacting protein 1	STRP1_HUMAN		STRIP1
18	18	18	0.999	276	sp|Q6BCY4|NB5R2_HUMAN	CYB5R2	Q6BCY4	NADH-cytochrome b5 reductase 2	NB5R2_HUMAN		CYB5R2
116	116	116	0.999	382	sp|Q6DD87|ZN787_HUMAN	ZNF787	Q6DD87	Zinc finger protein 787	ZN787_HUMAN		ZNF787
62	62	62	0.999	255	sp|Q6DKI1|RL7L_HUMAN	RPL7L1	Q6DKI1	Ribosomal protein uL30-like	RL7L_HUMAN		RPL7L1
70	70	70	0.999	435	sp|Q6DKJ4|NXN_HUMAN	NXN	Q6DKJ4	Nucleoredoxin	NXN_HUMAN		NXN
14	14	14	0.999	613	sp|Q6UVY6|MOXD1_HUMAN	MOXD1	Q6UVY6	DBH-like monooxygenase protein 1	MOXD1_HUMAN		MOXD1
5	5	5	0.999	1074	sp|Q6UY14|ATL4_HUMAN	ADAMTSL4	Q6UY14	ADAMTS-like protein 4	ATL4_HUMAN		ADAMTSL4
11	11	11	0.999	1223	sp|Q6ZS17|RIPR1_HUMAN	RIPOR1	Q6ZS17	Rho family-interacting cell polarization regulator 1	RIPR1_HUMAN		FAM65A
23	18	23	0.999	527	sp|Q7KYR7|BT2A1_HUMAN	BTN2A1	Q7KYR7	Butyrophilin subfamily 2 member A1	BT2A1_HUMAN		BTN2A1
8	7	8	0.9976	901	sp|Q7Z7L1|SLN11_HUMAN	SLFN11	Q7Z7L1	Schlafen family member 11	SLN11_HUMAN		SLFN11
30	30	30	0.999	208	sp|Q86WR0|CCD25_HUMAN	CCDC25	Q86WR0	Coiled-coil domain-containing protein 25	CCD25_HUMAN		CCDC25
50	50	50	0.999	379	sp|Q86WV6|STING_HUMAN	STING1	Q86WV6	Stimulator of interferon genes protein	STING_HUMAN		TMEM173
88	88	88	0.999	971	sp|Q8IWR0|Z3H7A_HUMAN	ZC3H7A	Q8IWR0	Zinc finger CCCH domain-containing protein 7A	Z3H7A_HUMAN		ZC3H7A
38	38	38	0.999	1082	sp|Q8IX01|SUGP2_HUMAN	SUGP2	Q8IX01	SURP and G-patch domain-containing protein 2	SUGP2_HUMAN		SUGP2
43	43	43	0.999	634	sp|Q8IYS2|K2013_HUMAN	KIAA2013	Q8IYS2	Uncharacterized protein KIAA2013	K2013_HUMAN		KIAA2013

97	97	97	0.999	171	sp|Q8N5M4|TTC9C_HUMAN	TTC9C	Q8N5M4	Tetratricopeptide repeat protein 9C	TTC9C_HUMAN		TTC9C
82	82	82	0.999	392	sp|Q8NBL1|PGLT1_HUMAN	POGLUT1	Q8NBL1	Protein O-glucosyltransferase 1	PGLT1_HUMAN		POGLUT1
56	56	56	0.999	197	sp|Q8NBT2|SPC24_HUMAN	SPC24	Q8NBT2	Kinetochore protein Spc24	SPC24_HUMAN		SPC24
92	92	92	0.999	429	sp|Q8NBX0|SCPDL_HUMAN	SCCPDH	Q8NBX0	Saccharopine dehydrogenase-like oxidoreductase	SCPDL_HUMAN		SCCPDH
28	28	28	0.999	672	sp|Q8NEM2|SHCBP_HUMAN	SHCBP1	Q8NEM2	SHC SH2 domain-binding protein 1	SHCBP_HUMAN		SHCBP1
67	67	67	0.999	380	sp|Q8NFH3|NUP43_HUMAN	NUP43	Q8NFH3	Nucleoporin Nup43	NUP43_HUMAN		NUP43
71	71	71	0.999	552	sp|Q8WTV0|SCRB1_HUMAN	SCARB1	Q8WTV0	Scavenger receptor class B member 1	SCRB1_HUMAN		SCARB1
123	123	123	0.999	299	sp|Q92600|CNOT9_HUMAN	CNOT9	Q92600	CCR4-NOT transcription complex subunit 9	CNOT9_HUMAN		RQCD1
82	82	82	0.999	276	sp|Q96B26|EXOS8_HUMAN	EXOSC8	Q96B26	Exosome complex component RRP43	EXOS8_HUMAN		EXOSC8
64	64	64	0.999	342	sp|Q96C23|GALM_HUMAN	GALM	Q96C23	Galactose mutarotase	GALM_HUMAN		GALM
184	184	184	0.999	337	sp|Q96C86|DCPS_HUMAN	DCPS	Q96C86	m7GpppX diphosphatase	DCPS_HUMAN		DCPS
6	6	6	0.999	605	sp|Q96EA4|SPDLY_HUMAN	SPDL1	Q96EA4	Protein Spindly	SPDLY_HUMAN		SPDL1
73	73	73	0.999	424	sp|Q96EB1|ELP4_HUMAN	ELP4	Q96EB1	Elongator complex protein 4	ELP4_HUMAN		ELP4
116	116	116	0.999	167	sp|Q96EL2|RT24_HUMAN	MRPS24	Q96EL2	Small ribosomal subunit protein uS3m	RT24_HUMAN		MRPS24
116	116	116	0.999	112	sp|Q96EL3|RM53_HUMAN	MRPL53	Q96EL3	Large ribosomal subunit protein mL53	RM53_HUMAN		MRPL53
35	35	35	0.999	344	sp|Q96GD4|AURKB_HUMAN	AURKB	Q96GD4	Aurora kinase B	AURKB_HUMAN		AURKB
50	50	50	0.999	1378	sp|Q96HA7|TONSL_HUMAN	TONSL	Q96HA7	Tonsoku-like protein	TONSL_HUMAN		TONSL
64	64	101	0.999	471	sp|Q96K21|ANCHR_HUMAN	ZFYVE19	Q96K21	Abscission/NoCut checkpoint regulator	ANCHR_HUMAN		ZFYVE19
37	37	37	0.999	313	sp|Q96LJ7|DHRS1_HUMAN	DHRS1	Q96LJ7	Dehydrogenase/reductase SDR family member 1	DHRS1_HUMAN		DHRS1
177	177	177	0.999	224	sp|Q96MW1|CCD43_HUMAN	CCDC43	Q96MW1	Coiled-coil domain-containing protein 43	CCD43_HUMAN		CCDC43
8	8	8	0.999	628	sp|Q96RF0|SNX18_HUMAN	SNX18	Q96RF0	Sorting nexin-18	SNX18_HUMAN		SNX18
55	55	55	0.997	5635	sp|Q96RW7|HMCN1_HUMAN	HMCN1	Q96RW7	Hemicentin-1	HMCN1_HUMAN		HMCN1
41	41	41	0.999	729	sp|Q96S59|RANB9_HUMAN	RANBP9	Q96S59	Ran-binding protein 9	RANB9_HUMAN		RANBP9
215	215	215	0.999	103	sp|Q99417|MYCBP_HUMAN	MYCBP	Q99417	c-Myc-binding protein	MYCBP_HUMAN		MYCBP
52	52	52	0.999	160	sp|Q99547|MPH6_HUMAN	MPHOSPH6	Q99547	M-phase phosphoprotein 6	MPH6_HUMAN		MPHOSPH6
96	96	96	0.999	456	sp|Q99808|S29A1_HUMAN	SLC29A1	Q99808	Equilibrative nucleoside transporter 1	S29A1_HUMAN		SLC29A1
117	117	117	0.999	126	sp|Q9BRT2|UQCC2_HUMAN	UQCC2	Q9BRT2	Ubiquinol-cytochrome-c reductase complex assembly factor 2	UQCC2_HUMAN		UQCC2
111	111	111	0.999	410	sp|Q9BT25|HAUS8_HUMAN	HAUS8	Q9BT25	HAUS augmin-like complex subunit 8	HAUS8_HUMAN		HAUS8
111	111	111	0.999	198	sp|Q9BUR5|MIC26_HUMAN	APOO	Q9BUR5	MICOS complex subunit MIC26	MIC26_HUMAN		APOO
179	179	179	0.999	243	sp|Q9BVC6|TM109_HUMAN	TMEM109	Q9BVC6	Transmembrane protein 109	TM109_HUMAN		TMEM109
155	155	155	0.999	86	sp|Q9BWJ5|SF3B5_HUMAN	SF3B5	Q9BWJ5	Splicing factor 3B subunit 5	SF3B5_HUMAN		SF3B5
13	13	13	0.999	246	sp|Q9BXJ4|C1QT3_HUMAN	C1QTNF3	Q9BXJ4	Complement C1q tumor necrosis factor-related protein 3	C1QT3_HUMAN		C1QTNF3
42	42	42	0.999	350	sp|Q9GZM5|YIPF3_HUMAN	YIPF3	Q9GZM5	Protein YIPF3	YIPF3_HUMAN		YIPF3
122	122	122	0.999	342	sp|Q9GZY8|MFF_HUMAN	MFF	Q9GZY8	Mitochondrial fission factor	MFF_HUMAN		MFF
114	114	114	0.999	180	sp|Q9H0U6|RM18_HUMAN	MRPL18	Q9H0U6	Large ribosomal subunit protein uL18m	RM18_HUMAN		MRPL18
27	27	27	0.999	485	sp|Q9H1Z4|WDR13_HUMAN	WDR13	Q9H1Z4	WD repeat-containing protein 13	WDR13_HUMAN		WDR13
55	55	55	0.999	898	sp|Q9H2K8|TAOK3_HUMAN	TAOK3	Q9H2K8	Serine/threonine-protein kinase TAO3	TAOK3_HUMAN		TAOK3
41	41	41	0.999	356	sp|Q9H410|DSN1_HUMAN	DSN1	Q9H410	Kinetochore-associated protein DSN1 homolog	DSN1_HUMAN		DSN1
20	20	20	0.999	327	sp|Q9H425|CA198_HUMAN	C1orf198	Q9H425	Uncharacterized protein C1orf198	CA198_HUMAN		C1orf198
83	83	83	0.999	264	sp|Q9H9Q2|CSN7B_HUMAN	COPS7B	Q9H9Q2	COP9 signalosome complex subunit 7b	CSN7B_HUMAN		COPS7B
70	70	70	0.999	336	sp|Q9HBH5|RDH14_HUMAN	RDH14	Q9HBH5	Retinol dehydrogenase 14	RDH14_HUMAN		RDH14
69	69	69	0.999	324	sp|Q9HC07|TM165_HUMAN	TMEM165	Q9HC07	Transmembrane protein 165	TM165_HUMAN		TMEM165
77	77	77	0.999	389	sp|Q9HC52|CBX8_HUMAN	CBX8	Q9HC52	Chromobox protein homolog 8	CBX8_HUMAN		CBX8
76	76	76	0.999	1121	sp|Q9NNW5|WDR6_HUMAN	WDR6	Q9NNW5	WD repeat-containing protein 6	WDR6_HUMAN		WDR6
52	52	56	0.999	507	sp|Q9NQH7|XPP3_HUMAN	XPNPEP3	Q9NQH7	Xaa-Pro aminopeptidase 3	XPP3_HUMAN		XPNPEP3
143	143	143	0.999	295	sp|Q9NRY6|PLS3_HUMAN	PLSCR3	Q9NRY6	Phospholipid scramblase 3	PLS3_HUMAN		PLSCR3
41	39	41	0.999	197	sp|Q9NUP9|LIN7C_HUMAN	LIN7C	Q9NUP9	Protein lin-7 homolog C	LIN7C_HUMAN		LIN7C
18	18	18	0.999	1036	sp|Q9NZV1|CRIM1_HUMAN	CRIM1	Q9NZV1	Cysteine-rich motor neuron 1 protein	CRIM1_HUMAN		CRIM1
111	111	111	0.999	381	sp|Q9P0J7|KCMF1_HUMAN	KCMF1	Q9P0J7	E3 ubiquitin-protein ligase KCMF1	KCMF1_HUMAN		KCMF1
26	26	26	0.999	2071	sp|Q9P2D3|HTR5B_HUMAN	HEATR5B	Q9P2D3	HEAT repeat-containing protein 5B	HTR5B_HUMAN		HEATR5B
158	158	158	0.999	218	sp|Q9UBQ5|EIF3K_HUMAN	EIF3K	Q9UBQ5	Eukaryotic translation initiation factor 3 subunit K	EIF3K_HUMAN		EIF3K
195	195	195	0.999	63	sp|Q9UDW1|QCR9_HUMAN	UQCR10	Q9UDW1	Cytochrome b-c1 complex subunit 9	QCR9_HUMAN		UQCR10
61	61	61	0.999	782	sp|Q9UID3|VPS51_HUMAN	VPS51	Q9UID3	Vacuolar protein sorting-associated protein 51 homolog	VPS51_HUMAN		VPS51
71	71	71	0.999	235	sp|Q9UJ68|MSRA_HUMAN	MSRA	Q9UJ68	Mitochondrial peptide methionine sulfoxide reductase	MSRA_HUMAN		MSRA
14	14	14	0.999	1381	sp|Q9ULI3|HEG1_HUMAN	HEG1	Q9ULI3	Protein HEG homolog 1	HEG1_HUMAN		HEG1
35	35	35	0.999	285	sp|Q9ULR0|ISY1_HUMAN	ISY1	Q9ULR0	Pre-mRNA-splicing factor ISY1 homolog	ISY1_HUMAN		ISY1
130	115	130	0.999	64	sp|Q9Y2S6|TMA7_HUMAN	TMA7	Q9Y2S6	Translation machinery-associated protein 7	TMA7_HUMAN		TMA7
96	96	96	0.999	83	sp|Q9Y5J9|TIM8B_HUMAN	TIMM8B	Q9Y5J9	Mitochondrial import inner membrane translocase subunit Tim8 B	TIM8B_HUMAN		TIMM8B
185	185	185	0.999	95	sp|Q9Y5L4|TIM13_HUMAN	TIMM13	Q9Y5L4	Mitochondrial import inner membrane translocase subunit Tim13	TIM13_HUMAN		TIMM13
47	47	47	0.999	360	sp|Q9Y5P6|GMPPB_HUMAN	GMPPB	Q9Y5P6	Mannose-1-phosphate guanyltransferase beta	GMPPB_HUMAN		GMPPB
60	60	60	0.999	465	sp|Q9Y5X2|SNX8_HUMAN	SNX8	Q9Y5X2	Sorting nexin-8	SNX8_HUMAN		SNX8
66	66	66	0.999	262	sp|Q9Y657|SPIN1_HUMAN	SPIN1	Q9Y657	Spindlin-1	SPIN1_HUMAN		SPIN1
76	65	76	0.999	576	sp|Q9Y6I3|EPN1_HUMAN	EPN1	Q9Y6I3	Epsin-1	EPN1_HUMAN		EPN1
138	138	138	0.999	360	sp|O00115|DNS2A_HUMAN	DNASE2	O00115	Deoxyribonuclease-2-alpha	DNS2A_HUMAN		DNASE2
69	69	69	0.999	179	sp|O00762|UBE2C_HUMAN	UBE2C	O00762	Ubiquitin-conjugating enzyme E2 C	UBE2C_HUMAN		UBE2C
31	31	31	0.999	336	sp|O14657|TOR1B_HUMAN	TOR1B	O14657	Torsin-1B	TOR1B_HUMAN		TOR1B
124	124	124	0.999	333	sp|O14908|GIPC1_HUMAN	GIPC1	O14908	PDZ domain-containing protein GIPC1	GIPC1_HUMAN		GIPC1
126	126	126	0.999	329	sp|O15127|SCAM2_HUMAN	SCAMP2	O15127	Secretory carrier-associated membrane protein 2	SCAM2_HUMAN		SCAMP2
223	223	223	0.999	196	sp|O15258|RER1_HUMAN	RER1	O15258	Protein RER1	RER1_HUMAN		RER1
49	49	49	0.999	2961	sp|O43149|ZZEF1_HUMAN	ZZEF1	O43149	Zinc finger ZZ-type and EF-hand domain-containing protein 1	ZZEF1_HUMAN		ZZEF1
154	154	154	0.999	150	sp|O43169|CYB5B_HUMAN	CYB5B	O43169	Cytochrome b5 type B	CYB5B_HUMAN		CYB5B
36	36	36	0.999	364	sp|O43427|FIBP_HUMAN	FIBP	O43427	Acidic fibroblast growth factor intracellular-binding protein	FIBP_HUMAN		FIBP
191	191	191	0.999	180	sp|O43617|TPPC3_HUMAN	TRAPPC3	O43617	Trafficking protein particle complex subunit 3	TPPC3_HUMAN		TRAPPC3
169	169	169	0.999	189	sp|O43674|NDUB5_HUMAN	NDUFB5	O43674	NADH dehydrogenase [ubiquinone] 1 beta subcomplex subunit 5, mitochor	NDUB5_HUMAN		NDUFB5
222	222	222	0.999	97	sp|O60220|TIM8A_HUMAN	TIMM8A	O60220	Mitochondrial import inner membrane translocase subunit Tim8 A	TIM8A_HUMAN		TIMM8A
166	166	166	0.999	265	sp|O60828|PQBP1_HUMAN	PQBP1	O60828	Polyglutamine-binding protein 1	PQBP1_HUMAN		PQBP1
50	50	50	0.999	336	sp|O75794|CD123_HUMAN	CDC123	O75794	Cell division cycle protein 123 homolog	CD123_HUMAN		CDC123
88	88	88	0.999	140	sp|O75817|POP7_HUMAN	POP7	O75817	Ribonuclease P protein subunit p20	POP7_HUMAN		POP7
153	145	153	0.999	103	sp|O75964|ATP5L_HUMAN	ATP5MG	O75964	ATP synthase subunit g, mitochondrial	ATP5L_HUMAN		ATP5L
47	47	47	0.999	360	sp|O95149|SPN1_HUMAN	SNUPN	O95149	Snurportin-1	SPN1_HUMAN		SNUPN
202	202	202	0.999	186	sp|O95169|NDUB8_HUMAN	NDUFB8	O95169	NADH dehydrogenase [ubiquinone] 1 beta subcomplex subunit 8, mitochor	NDUB8_HUMAN		NDUFB8
62	62	62	0.999	260	sp|O95478|NSA2_HUMAN	NSA2	O95478	Ribosome biogenesis protein NSA2 homolog	NSA2_HUMAN		NSA2
117	117	117	0.999	424	sp|O95630|STABP_HUMAN	STAMBP	O95630	STAM-binding protein	STABP_HUMAN		STAMBP
109	109	109	0.999	387	sp|O95801|TTC4_HUMAN	TTC4	O95801	Tetratricopeptide repeat protein 4	TTC4_HUMAN		TTC4
88	85	88	0.999	291	sp|O95983|MBD3_HUMAN	MBD3	O95983	Methyl-CpG-binding domain protein 3	MBD3_HUMAN		MBD3
165	165	165	0.999	134	sp|P00167|CYB5_HUMAN	CYB5A	P00167	Cytochrome b5	CYB5_HUMAN		CYB5A
127	69	127	0.999	187	sp|P00374|DYR_HUMAN	DHFR	P00374	Dihydrofolate reductase	DYR_HUMAN		DHFR
81	81	81	0.999	201	sp|P02753|RET4_HUMAN	RBP4	P02753	Retinol-binding protein 4	RET4_HUMAN		RBP4
172	172	172	0.999	98	sp|P04080|CYTB_HUMAN	CSTB	P04080	Cystatin-B	CYTB_HUMAN		CSTB
51	51	51	0.999	429	sp|P06280|AGAL_HUMAN	GLA	P06280	Alpha-galactosidase A	AGAL_HUMAN		GLA
46	8	46	0.999	333	sp|P07711|CATL1_HUMAN	CTSL	P07711	Procathepsin L	CATL1_HUMAN		CTSL
9	9	9	0.9989	660	sp|P08253|MMP2_HUMAN	MMP2	P08253	72 kDa type IV collagenase	MMP2_HUMAN		MMP2
107	107	107	0.999	238	sp|P08962|CD63_HUMAN	CD63	P08962	CD63 antigen	CD63_HUMAN		CD63
208	208	208	0.999	244	sp|P09417|DHPR_HUMAN	QDPR	P09417	Dihydropteridine reductase	DHPR_HUMAN		QDPR
187	187	187	0.999	63	sp|P15954|COX7C_HUMAN	COX7C	P15954	Cytochrome c oxidase subunit 7C, mitochondrial	COX7C_HUMAN		COX7C
99	99	99	0.999	210	sp|P19388|RPAB1_HUMAN	POLR2E	P19388	DNA-directed RNA polymerases I, II, and III subunit RPABC1	RPAB1_HUMAN		POLR2E
90	90	90	0.999	782	sp|P19447|ERCC3_HUMAN	ERCC3	P19447	General transcription and DNA repair factor IIH helicase subunit XPB	ERCC3_HUMAN		ERCC3
33	33	33	0.999	267	sp|P27701|CD82_HUMAN	CD82	P27701	CD82 antigen	CD82_HUMAN		CD82
109	109	109	0.999	1462	sp|P30414|NKTR_HUMAN	NKTR	P30414	NK-tumor recognition protein	NKTR_HUMAN		NKTR
124	124	124	0.999	121	sp|P35244|RFA3_HUMAN	RPA3	P35244	Replication protein A 14 kDa subunit	RFA3_HUMAN		RPA3
134	134	134	0.999	182	sp|P36405|ARL3_HUMAN	ARL 3.00	P36405	ADP-ribosylation factor-like protein 3	ARL3_HUMAN		ARL 3.00
42	42	42	0.999	418	sp|P36955|PEDF_HUMAN	SERPINF1	P36955	Pigment epithelium-derived factor	PEDF_HUMAN		SERPINF1
117	117	117	0.999	197	sp|P36969|GPX4_HUMAN	GPX4	P36969	Phospholipid hydroperoxide glutathione peroxidase	GPX4_HUMAN		GPX4
124	124	124	0.999	522	sp|P37198|NUP62_HUMAN	NUP62	P37198	Nuclear pore glycoprotein p62	NUP62_HUMAN		NUP62
154	154	154	0.999	496	sp|P42785|PCP_HUMAN	PRCP	P42785	Lysosomal Pro-X carboxypeptidase	PCP_HUMAN		PRCP
117	117	117	0.999	212	sp|P45877|PPIC_HUMAN	PPIC	P45877	Peptidyl-prolyl cis-trans isomerase C	PPIC_HUMAN		PPIC
57	40	108	0.999	424	sp|P45984|MK09_HUMAN	MAPK9	P45984	Mitogen-activated protein kinase 9	MK09_HUMAN		MAPK9
90	90	90	0.999	316	sp|P46736|BRCC3_HUMAN	BRCC3	P46736	Lys-63-specific deubiquitinase BRCC36	BRCC3_HUMAN		BRCC3
230	230	230	0.999	274	sp|P48507|GSH0_HUMAN	GCLM	P48507	Glutamate--cysteine ligase regulatory subunit	GSH0_HUMAN		GCLM
149	149	149	0.999	77	sp|P50238|CRIP1_HUMAN	CRIP1	P50238	Cysteine-rich protein 1	CRIP1_HUMAN		CRIP1
66	53	66	0.999	346	sp|P50613|CDK7_HUMAN	CDK7	P50613	Cyclin-dependent kinase 7	CDK7_HUMAN		CDK7
239	239	239	0.999	306	sp|P50897|PPT1_HUMAN	PPT1	P50897	Palmitoyl-protein thioesterase 1	PPT1_HUMAN		PPT1
109	109	109	0.999	102	sp|P51397|DAP1_HUMAN	DAP	P51397	Death-associated protein 1	DAP1_HUMAN		DAP
144	144	144	0.999	393	sp|P51553|IDH3G_HUMAN	IDH3G	P51553	Isocitrate dehydrogenase [NAD] subunit gamma, mitochondrial	IDH3G_HUMAN		IDH3G
14	14	14	0.999	1103	sp|P54289|CA2D1_HUMAN	CACNA2D1	P54289	Voltage-dependent calcium channel subunit alpha-2/delta-1	CA2D1_HUMAN		CACNA2D1
195	31	195	0.999	51	sp|P56381|ATP5E_HUMAN	ATP5F1E	P56381	ATP synthase subunit epsilon, mitochondrial	ATP5E_HUMAN		ATP5E
89	17	89	0.999	1058	sp|P57678|GEMI4_HUMAN	GEMIN4	P57678	Gem-associated protein 4	GEMI4_HUMAN		GEMIN4
234	234	234	0.999	118	sp|P58546|MTPN_HUMAN	MTPN	P58546	Myotrophin	MTPN_HUMAN		MTPN
76	76	76	0.999	236	sp|P60033|CD81_HUMAN	CD81	P60033	CD81 antigen	CD81_HUMAN		CD81
154	154	154	0.999	177	sp|P61923|COPZ1_HUMAN	COPZ1	P61923	Coatomer subunit zeta-1	COPZ1_HUMAN		COPZ1
242	242	242	0.999	127	sp|P61970|NTF2_HUMAN	NUTF2	P61970	Nuclear transport factor 2	NTF2_HUMAN		NUTF2
150	150	150	0.999	102	sp|P62310|LSM3_HUMAN	LSM 3.00	P62310	U6 snRNA-associated Sm-like protein LSm3	LSM3_HUMAN		LSM 3.00
153	153	153	0.999	80	sp|P62312|LSM6_HUMAN	LSM 6.00	P62312	U6 snRNA-associated Sm-like protein LSm6	LSM6_HUMAN		LSM 6.00
407	407	407	0.999	70	sp|P63173|RL38_HUMAN	RPL38	P63173	Large ribosomal subunit protein eL38	RL38_HUMAN		RPL38
165	165	165	0.999	215	sp|P67870|CSK2B_HUMAN	CSNK2B	P67870	Casein kinase II subunit beta	CSK2B_HUMAN		CSNK2B
35	35	35	0.999	1384	sp|P78357|CNTP1_HUMAN	CNTNAP1	P78357	Contactin-associated protein 1	CNTP1_HUMAN		CNTNAP1
113	113	113	0.999	173	sp|P82663|RT25_HUMAN	MRPS25	P82663	Small ribosomal subunit protein mS25	RT25_HUMAN		MRPS25
84	84	84	0.999	547	sp|P83111|LACTB_HUMAN	LACTB	P83111	Serine beta-lactamase-like protein LACTB, mitochondrial	LACTB_HUMAN		LACTB
124	124	124	0.999	391	sp|Q00587|BORG5_HUMAN	CDC42EP1	Q00587	Cdc42 effector protein 1	BORG5_HUMAN		CDC42EP1

58	58	58	0.999	395	sp|Q02127|PYRD_HUMAN	DHODH	Q02127	Dihydroorotate dehydrogenase (quinone), mitochondrial	PYRD_HUMAN		DHODH
69	69	69	0.999	748	sp|Q03518|TAP1_HUMAN	TAP1	Q03518	Antigen peptide transporter 1	TAP1_HUMAN		TAP1
97	97	97	0.999	587	sp|Q05932|FOLC_HUMAN	FPGS	Q05932	Folylpolyglutamate synthase, mitochondrial	FOLC_HUMAN		FPGS
9	9	9	0.9987	675	sp|Q0ZGT2|NEXN_HUMAN	NEXN	Q0ZGT2	Nexilin	NEXN_HUMAN		NEXN
88	88	88	0.999	297	sp|Q12846|STX4_HUMAN	STX4	Q12846	Syntaxin-4	STX4_HUMAN		STX4
12	12	12	0.999	822	sp|Q12929|EPS8_HUMAN	EPS8	Q12929	Epidermal growth factor receptor kinase substrate 8	EPS8_HUMAN		EPS8
96	96	96	0.999	482	sp|Q13325|IFIT5_HUMAN	IFIT5	Q13325	Interferon-induced protein with tetratricopeptide repeats 5	IFIT5_HUMAN		IFIT5
140	140	140	0.999	305	sp|Q14232|EI2BA_HUMAN	EIF2B1	Q14232	Translation initiation factor eIF-2B subunit alpha	EI2BA_HUMAN		EIF2B1
38	4	38	0.9989	565	sp|Q14332|FZD2_HUMAN	FZD2	Q14332	Frizzled-2	FZD2_HUMAN		FZD2
42	38	42	0.999	1571	sp|Q14689|DIP2A_HUMAN	DIP2A	Q14689	Disco-interacting protein 2 homolog A	DIP2A_HUMAN		DIP2A
167	167	167	0.999	396	sp|Q15007|FL2D_HUMAN	WTAP	Q15007	Pre-mRNA-splicing regulator WTAP	FL2D_HUMAN		WTAP
100	100	100	0.999	538	sp|Q15291|RBBP5_HUMAN	RBBP5	Q15291	Retinoblastoma-binding protein 5	RBBP5_HUMAN		RBBP5
102	102	102	0.999	655	sp|Q15311|RBP1_HUMAN	RALBP1	Q15311	RalA-binding protein 1	RBP1_HUMAN		RALBP1
60	4	60	0.999	407	sp|Q15434|RBMS2_HUMAN	RBMS2	Q15434	RNA-binding motif, single-stranded-interacting protein 2	RBMS2_HUMAN		RBMS2
28	28	28	0.999	514	sp|Q15646|OASL_HUMAN	OASL	Q15646	2'-5'-oligoadenylate synthase-like protein	OASL_HUMAN		OASL
35	19	35	0.999	467	sp|Q15796|SMAD2_HUMAN	SMAD2	Q15796	Mothers against decapentaplegic homolog 2	SMAD2_HUMAN		SMAD2
17	17	17	0.999	195	sp|Q3MHD2|LSM12_HUMAN	LSM 12.00	Q3MHD2	Protein LSM12	LSM12_HUMAN		LSM 12.00
27	27	27	0.999	1135	sp|Q4L180|FIL1L_HUMAN	FILIP1L	Q4L180	Filamin A-interacting protein 1-like	FIL1L_HUMAN		FILIP1L
205	205	205	0.999	312	sp|Q53GQ0|DHB12_HUMAN	HSD17B12	Q53GQ0	Very-long-chain 3-oxoacyl-CoA reductase	DHB12_HUMAN		HSD17B12
49	49	49	0.999	860	sp|Q5C9Z4|NOM1_HUMAN	NOM1	Q5C9Z4	Nucleolar MIF4G domain-containing protein 1	NOM1_HUMAN		NOM1
62	62	62	0.999	1801	sp|Q5VW36|FOCAD_HUMAN	FOCAD	Q5VW36	Focadhesin	FOCAD_HUMAN		FOCAD
18	18	18	0.9989	1812	sp|Q69YN4|VIR_HUMAN	VIRMA	Q69YN4	Protein virilizer homolog	VIR_HUMAN		KIAA1429
53	53	53	0.999	673	sp|Q6EMK4|VASN_HUMAN	VASN	Q6EMK4	Vasorin	VASN_HUMAN		VASN
9	9	10	0.999	255	sp|Q6NUK4|REEP3_HUMAN	REEP3	Q6NUK4	Receptor expression-enhancing protein 3	REEP3_HUMAN		REEP3
77	77	77	0.999	353	sp|Q6UXH1|CREL2_HUMAN	CRELD2	Q6UXH1	Protein disulfide isomerase CRELD2	CREL2_HUMAN		CRELD2
123	123	123	0.999	268	sp|Q6UXV4|MIC27_HUMAN	APOOL	Q6UXV4	MICOS complex subunit MIC27	MIC27_HUMAN		APOOL
117	87	117	0.999	454	sp|Q6YP21|KAT3_HUMAN	KYAT3	Q6YP21	Kynurenine--oxoglutarate transaminase 3	KAT3_HUMAN		CCBL2
115	115	115	0.999	544	sp|Q7L5N7|PCAT2_HUMAN	LPCAT2	Q7L5N7	Lysophosphatidylcholine acyltransferase 2	PCAT2_HUMAN		LPCAT2
75	75	75	0.999	1717	sp|Q7Z3U7|MON2_HUMAN	MON2	Q7Z3U7	Protein MON2 homolog	MON2_HUMAN		MON2
56	56	56	0.999	507	sp|Q7Z4H8|PLGT3_HUMAN	POGLUT3	Q7Z4H8	Protein O-glucosyltransferase 3	PLGT3_HUMAN		KDELC2
84	84	84	0.999	680	sp|Q7Z4Q2|HEAT3_HUMAN	HEATR3	Q7Z4Q2	HEAT repeat-containing protein 3	HEAT3_HUMAN		HEATR3
42	42	42	0.999	1143	sp|Q86XI2|CNDG2_HUMAN	NCAPG2	Q86XI2	Condensin-2 complex subunit G2	CNDG2_HUMAN		NCAPG2
102	102	102	0.999	349	sp|Q8IV48|ERI1_HUMAN	ERI1	Q8IV48	3'-5' exoribonuclease 1	ERI1_HUMAN		ERI1
112	112	112	0.999	590	sp|Q8IXH7|NELFD_HUMAN	NELFCD	Q8IXH7	Negative elongation factor C/D	NELFD_HUMAN		NELFCD
109	109	109	0.999	1001	sp|Q8IXT5|RB12B_HUMAN	RBM12B	Q8IXT5	RNA-binding protein 12B	RB12B_HUMAN		RBM12B
122	122	122	0.999	606	sp|Q8IY67|RAVR1_HUMAN	RAVER1	Q8IY67	Ribonucleoprotein PTB-binding 1	RAVR1_HUMAN		RAVER1
85	85	85	0.999	545	sp|Q8IZ73|RUSD2_HUMAN	RPUSD2	Q8IZ73	Pseudouridylate synthase RPUSD2	RUSD2_HUMAN		RPUSD2
38	38	38	0.999	1335	sp|Q8N122|RPTOR_HUMAN	RPTOR	Q8N122	Regulatory-associated protein of mTOR	RPTOR_HUMAN		RPTOR
27	27	27	0.999	730	sp|Q8N556|AFAP1_HUMAN	AFAP1	Q8N556	Actin filament-associated protein 1	AFAP1_HUMAN		AFAP1
58	58	58	0.999	660	sp|Q8NHV4|NEDD1_HUMAN	NEDD1	Q8NHV4	Protein NEDD1	NEDD1_HUMAN		NEDD1
14	14	14	0.999	492	sp|Q8WUA2|PPIL4_HUMAN	PPIL4	Q8WUA2	Peptidyl-prolyl cis-trans isomerase-like 4	PPIL4_HUMAN		PPIL4
74	74	74	0.999	75	sp|Q8WUW1|BRK1_HUMAN	BRK1	Q8WUW1	Protein BRICK1	BRK1_HUMAN		BRK1
48	48	48	0.999	683	sp|Q8WWK9|CKAP2_HUMAN	CKAP2	Q8WWK9	Cytoskeleton-associated protein 2	CKAP2_HUMAN		CKAP2
94	94	94	0.999	709	sp|Q92542|NICA_HUMAN	NCSTN	Q92542	Nicastrin	NICA_HUMAN		NCSTN
96	96	96	0.999	538	sp|Q92692|NECT2_HUMAN	NECTIN2	Q92692	Nectin-2	NECT2_HUMAN		PVRL2
93	86	93	0.999	391	sp|Q92785|REQU_HUMAN	DPF2	Q92785	Zinc finger protein ubi-d4	REQU_HUMAN		DPF2
9	7	9	0.9975	552	sp|Q92819|HYAS2_HUMAN	HAS2	Q92819	Hyaluronan synthase 2	HYAS2_HUMAN		HAS2
69	69	69	0.999	306	sp|Q96BJ3|AIDA_HUMAN	AIDA	Q96BJ3	Axin interactor, dorsalization-associated protein	AIDA_HUMAN		AIDA
33	33	33	0.999	747	sp|Q96EB6|SIR1_HUMAN	SIRT1	Q96EB6	NAD-dependent protein deacetylase sirtuin-1	SIR1_HUMAN		SIRT1
82	82	82	0.999	411	sp|Q96ER9|MITOK_HUMAN	CCDC51	Q96ER9	Mitochondrial potassium channel	MITOK_HUMAN		CCDC51
51	51	51	0.999	480	sp|Q96EY1|DNJA3_HUMAN	DNAJA3	Q96EY1	DnaJ homolog subfamily A member 3, mitochondrial	DNJA3_HUMAN		DNAJA3
206	206	206	0.999	158	sp|Q96K17|BT3L4_HUMAN	BTF3L4	Q96K17	Transcription factor BTF3 homolog 4	BT3L4_HUMAN		BTF3L4
82	82	82	0.999	924	sp|Q96KP1|EXOC2_HUMAN	EXOC2	Q96KP1	Exocyst complex component 2	EXOC2_HUMAN		EXOC2
123	123	123	0.999	199	sp|Q96NC0|ZMAT2_HUMAN	ZMAT2	Q96NC0	Zinc finger matrin-type protein 2	ZMAT2_HUMAN		ZMAT2
173	173	173	0.999	399	sp|Q99442|SEC62_HUMAN	SEC62	Q99442	Translocation protein SEC62	SEC62_HUMAN		SEC62
200	42	200	0.999	336	sp|Q99496|RING2_HUMAN	RNF2	Q99496	E3 ubiquitin-protein ligase RING2	RING2_HUMAN		RNF2
68	68	68	0.999	437	sp|Q99541|PLIN2_HUMAN	PLIN2	Q99541	Perilipin-2	PLIN2_HUMAN		PLIN2
50	3	50	0.999	435	sp|Q99594|TEAD3_HUMAN	TEAD3	Q99594	Transcriptional enhancer factor TEF-5	TEAD3_HUMAN		TEAD3
49	49	49	0.999	390	sp|Q99816|TS101_HUMAN	TSG101	Q99816	Tumor susceptibility gene 101 protein	TS101_HUMAN		TSG101
84	84	84	0.999	283	sp|Q99943|PLCA_HUMAN	AGPAT1	Q99943	1-acyl-sn-glycerol-3-phosphate acyltransferase alpha	PLCA_HUMAN		AGPAT1
68	68	68	0.999	279	sp|Q9BQ75|CMS1_HUMAN	CMSS1	Q9BQ75	Protein CMSS1	CMS1_HUMAN		CMSS1
113	113	113	0.999	278	sp|Q9BT09|CNPY3_HUMAN	CNPY3	Q9BT09	Protein canopy homolog 3	CNPY3_HUMAN		CNPY3
97	97	97	0.999	263	sp|Q9BU76|MMTA2_HUMAN	MMTAG2	Q9BU76	Multiple myeloma tumor-associated protein 2	MMTA2_HUMAN		MMTAG2
32	32	32	0.999	245	sp|Q9BUT1|DHRS6_HUMAN	BDH2	Q9BUT1	Dehydrogenase/reductase SDR family member 6	DHRS6_HUMAN		BDH2
211	211	211	0.999	223	sp|Q9BV86|NTM1A_HUMAN	NTMT1	Q9BV86	N-terminal Xaa-Pro-Lys N-methyltransferase 1	NTM1A_HUMAN		NTMT1
171	171	171	0.999	516	sp|Q9BVI4|NOC4L_HUMAN	NOC4L	Q9BVI4	Nucleolar complex protein 4 homolog	NOC4L_HUMAN		NOC4L
91	91	91	0.999	311	sp|Q9BYD3|RM04_HUMAN	MRPL4	Q9BYD3	Large ribosomal subunit protein uL4m	RM04_HUMAN		MRPL4
117	117	117	0.999	259	sp|Q9BZG1|RAB34_HUMAN	RAB34	Q9BZG1	Ras-related protein Rab-34	RAB34_HUMAN		RAB34
60	47	60	0.999	514	sp|Q9BZK7|TBL1R_HUMAN	TBL1XR1	Q9BZK7	F-box-like/WD repeat-containing protein TBL1XR1	TBL1R_HUMAN		TBL1XR1
87	87	87	0.999	73	sp|Q9BZL1|UBL5_HUMAN	UBL5	Q9BZL1	Ubiquitin-like protein 5	UBL5_HUMAN		UBL5
71	35	71	0.999	878	sp|Q9BZL6|KPCD2_HUMAN	PRKD2	Q9BZL6	Serine/threonine-protein kinase D2	KPCD2_HUMAN		PRKD2
74	74	74	0.999	441	sp|Q9BZV1|UBXN6_HUMAN	UBXN6	Q9BZV1	UBX domain-containing protein 6	UBXN6_HUMAN		UBXN6
67	67	67	0.999	195	sp|Q9H061|T126A_HUMAN	TMEM126A	Q9H061	Transmembrane protein 126A	T126A_HUMAN		TMEM126A
118	118	118	0.999	280	sp|Q9H3N1|TMX1_HUMAN	TMX1	Q9H3N1	Thioredoxin-related transmembrane protein 1	TMX1_HUMAN		TMX1
47	47	47	0.999	416	sp|Q9H553|ALG2_HUMAN	ALG2	Q9H553	Alpha-1,3/1,6-mannosyltransferase ALG2	ALG2_HUMAN		ALG2
27	17	27	0.999	1312	sp|Q9H7N4|SFR19_HUMAN	SCAF1	Q9H7N4	Splicing factor, arginine/serine-rich 19	SFR19_HUMAN		SCAF1
188	49	188	0.999	216	sp|Q9H8S9|MOB1A_HUMAN	MOB1A	Q9H8S9	MOB kinase activator 1A	MOB1A_HUMAN		MOB1A
12	12	12	0.999	757	sp|Q9HCU0|CD248_HUMAN	CD248	Q9HCU0	Endosialin	CD248_HUMAN		CD248
224	97	224	0.999	96	sp|Q9NP97|DLRB1_HUMAN	DYNLRB1	Q9NP97	Dynein light chain roadblock-type 1	DLRB1_HUMAN		DYNLRB1
162	162	162	0.999	242	sp|Q9NPA0|EMC7_HUMAN	EMC7	Q9NPA0	ER membrane protein complex subunit 7	EMC7_HUMAN		EMC7
107	107	107	0.999	245	sp|Q9NPD3|EXOS4_HUMAN	EXOSC4	Q9NPD3	Exosome complex component RRP41	EXOS4_HUMAN		EXOSC4
100	100	100	0.999	235	sp|Q9NQT4|EXOS5_HUMAN	EXOSC5	Q9NQT4	Exosome complex component RRP46	EXOS5_HUMAN		EXOSC5
78	78	78	0.999	239	sp|Q9NR28|DBLOH_HUMAN	DIABLO	Q9NR28	Diablo IAP-binding mitochondrial protein	DBLOH_HUMAN		DIABLO
45	45	48	0.999	586	sp|Q9NRF8|PYRG2_HUMAN	CTPS2	Q9NRF8	CTP synthase 2	PYRG2_HUMAN		CTPS2
96	96	96	0.999	546	sp|Q9NRG9|AAAS_HUMAN	AAAS	Q9NRG9	Aladin	AAAS_HUMAN		AAAS
109	109	109	0.999	282	sp|Q9NVT9|ARMC1_HUMAN	ARMC1	Q9NVT9	Armadillo repeat-containing protein 1	ARMC1_HUMAN		ARMC1
188	188	188	0.999	153	sp|Q9NX24|NHP2_HUMAN	NHP2	Q9NX24	H/ACA ribonucleoprotein complex subunit 2	NHP2_HUMAN		NHP2
68	68	68	0.999	363	sp|Q9NX46|ADPRS_HUMAN	ADPRS	Q9NX46	ADP-ribosylhydrolase ARH3	ADPRS_HUMAN		ADPRHL2
104	104	104	0.999	359	sp|Q9NX62|IMPA3_HUMAN	BPNT2	Q9NX62	Golgi-resident adenosine 3',5'-bisphosphate 3'-phosphatase	IMPA3_HUMAN		IMPAD1
59	49	59	0.999	493	sp|Q9NX74|DUS2L_HUMAN	DUS2	Q9NX74	tRNA-dihydrouridine(20) synthase [NAD(P)+]-like	DUS2L_HUMAN		DUS2
160	160	160	0.999	217	sp|Q9NY12|GAR1_HUMAN	GAR1	Q9NY12	H/ACA ribonucleoprotein complex subunit 1	GAR1_HUMAN		GAR1
18	18	18	0.999	399	sp|Q9NYB0|TE2IP_HUMAN	TERF2IP	Q9NYB0	Telomeric repeat-binding factor 2-interacting protein 1	TE2IP_HUMAN		TERF2IP
17	17	17	0.999	963	sp|Q9P0V3|SH3B4_HUMAN	SH3BP4	Q9P0V3	SH3 domain-binding protein 4	SH3B4_HUMAN		SH3BP4
19	19	112	0.999	600	sp|Q9P2K5|MYEF2_HUMAN	MYEF2	Q9P2K5	Myelin expression factor 2	MYEF2_HUMAN		MYEF2
55	55	55	0.999	379	sp|Q9UH62|ARMX3_HUMAN	ARMCX3	Q9UH62	Armadillo repeat-containing X-linked protein 3	ARMX3_HUMAN		ARMCX3
206	23	206	0.999	624	sp|Q9UHD9|UBQL2_HUMAN	UBQLN2	Q9UHD9	Ubiquilin-2	UBQL2_HUMAN		UBQLN2
97	97	97	0.999	535	sp|Q9UHY1|NRBP_HUMAN	NRBP1	Q9UHY1	Nuclear receptor-binding protein	NRBP_HUMAN		NRBP1
175	175	175	0.999	261	sp|Q9UHY7|ENOPH_HUMAN	ENOPH1	Q9UHY7	Enolase-phosphatase E1	ENOPH_HUMAN		ENOPH1
123	123	123	0.999	975	sp|Q9UI26|IPO11_HUMAN	IPO11	Q9UI26	Importin-11	IPO11_HUMAN		IPO11
101	101	101	0.999	125	sp|Q9UI30|TR112_HUMAN	TRMT112	Q9UI30	Multifunctional methyltransferase subunit TRM112-like protein	TR112_HUMAN		TRMT112
76	69	76	0.999	194	sp|Q9UL26|RB22A_HUMAN	RAB22A	Q9UL26	Ras-related protein Rab-22A	RB22A_HUMAN		RAB22A
75	54	75	0.999	164	sp|Q9ULX9|MAFF_HUMAN	MAFF	Q9ULX9	Transcription factor MafF	MAFF_HUMAN		MAFF
117	117	117	0.999	238	sp|Q9UNN8|EPCR_HUMAN	PROCR	Q9UNN8	Endothelial protein C receptor	EPCR_HUMAN		PROCR
58	55	58	0.999	1127	sp|Q9UPN9|TRI33_HUMAN	TRIM33	Q9UPN9	E3 ubiquitin-protein ligase TRIM33	TRI33_HUMAN		TRIM33
24	21	24	0.999	621	sp|Q9Y217|MTMR6_HUMAN	MTMR6	Q9Y217	Myotubularin-related protein 6	MTMR6_HUMAN		MTMR6
114	114	114	0.999	383	sp|Q9Y282|ERGI3_HUMAN	ERGIC3	Q9Y282	Endoplasmic reticulum-Golgi intermediate compartment protein 3	ERGI3_HUMAN		ERGIC3
55	55	55	0.999	95	sp|Q9Y333|LSM2_HUMAN	LSM 2.00	Q9Y333	U6 snRNA-associated Sm-like protein LSm2	LSM2_HUMAN		LSM 2.00
73	73	73	0.999	195	sp|Q9Y3B2|EXOS1_HUMAN	EXOSC1	Q9Y3B2	Exosome complex component CSL4	EXOS1_HUMAN		EXOSC1
54	54	54	0.999	222	sp|Q9Y3E7|CHMP3_HUMAN	CHMP3	Q9Y3E7	Charged multivesicular body protein 3	CHMP3_HUMAN		CHMP3
72	72	89	0.999	579	sp|Q9Y5A9|YTHD2_HUMAN	YTHDF2	Q9Y5A9	YTH domain-containing family protein 2	YTHD2_HUMAN		YTHDF2
73	73	73	0.999	410	sp|Q9Y679|AUP1_HUMAN	AUP1	Q9Y679	Lipid droplet-regulating VLDL assembly factor AUP1	AUP1_HUMAN		AUP1
158	42	158	0.999	151	sp|Q9Y6H1|CHCH2_HUMAN	CHCHD2	Q9Y6H1	Coiled-coil-helix-coiled-coil-helix domain-containing protein 2	CHCH2_HUMAN		CHCHD2
64	64	64	0.999	366	sp|O00442|RTCA_HUMAN	RTCA	O00442	RNA 3'-terminal phosphate cyclase	RTCA_HUMAN		RTCA
99	99	99	0.999	681	sp|O00566|MPP10_HUMAN	MPHOSPH1	O00566	U3 small nucleolar ribonucleoprotein protein MPP10	MPP10_HUMAN		MPHOSPH10
47	47	47	0.999	642	sp|O14777|NDC80_HUMAN	NDC80	O14777	Kinetochore protein NDC80 homolog	NDC80_HUMAN		NDC80
74	74	102	0.999	610	sp|O14967|CLGN_HUMAN	CLGN	O14967	Calmegin	CLGN_HUMAN		CLGN
141	141	141	0.999	261	sp|O15400|STX7_HUMAN	STX7	O15400	Syntaxin-7	STX7_HUMAN		STX7
65	65	65	0.999	606	sp|O43318|M3K7_HUMAN	MAP3K7	O43318	Mitogen-activated protein kinase kinase kinase 7	M3K7_HUMAN		MAP3K7
142	142	142	0.999	210	sp|O43402|EMC8_HUMAN	EMC8	O43402	ER membrane protein complex subunit 8	EMC8_HUMAN		EMC8
187	95	187	0.999	121	sp|O43768|ENSA_HUMAN	ENSA	O43768	Alpha-endosulfine	ENSA_HUMAN		ENSA
148	148	148	0.999	106	sp|O43920|NDUS5_HUMAN	NDUFS5	O43920	NADH dehydrogenase [ubiquinone] iron-sulfur protein 5	NDUS5_HUMAN		NDUFS5
52	52	106	0.999	1105	sp|O60518|RNBP6_HUMAN	RANBP6	O60518	Ran-binding protein 6	RNBP6_HUMAN		RANBP6
184	184	184	0.999	260	sp|O60762|DPM1_HUMAN	DPM1	O60762	Dolichol-phosphate mannosyltransferase subunit 1	DPM1_HUMAN		DPM1
126	126	126	0.999	627	sp|O60826|CCD22_HUMAN	CCDC22	O60826	Coiled-coil domain-containing protein 22	CCD22_HUMAN		CCDC22
168	168	168	0.999	313	sp|O75569|PRKRA_HUMAN	PRKRA	O75569	Interferon-inducible double-stranded RNA-dependent protein kinase activa	PRKRA_HUMAN		PRKRA
243	243	243	0.999	230	sp|O75608|LYPA1_HUMAN	LYPLA1	O75608	Acyl-protein thioesterase 1	LYPA1_HUMAN		LYPLA1
68	68	127	0.999	479	sp|O75688|PPM1B_HUMAN	PPM1B	O75688	Protein phosphatase 1B	PPM1B_HUMAN		PPM1B
220	220	220	0.999	226	sp|O75832|PSD10_HUMAN	PSMD10	O75832	26S proteasome non-ATPase regulatory subunit 10	PSD10_HUMAN		PSMD10
53	53	53	0.999	275	sp|O94903|PLPHP_HUMAN	PLPBP	O94903	Pyridoxal phosphate homeostasis protein	PLPHP_HUMAN		PROSC

196	196	196	0.999	310	sp|O95159|ZFPL1_HUMAN	ZFPL1	O95159	Zinc finger protein-like 1	ZFPL1_HUMAN		ZFPL1
182	182	182	0.999	129	sp|O95168|NDUB4_HUMAN	NDUFB4	O95168	NADH dehydrogenase [ubiquinone] 1 beta subcomplex subunit 4	NDUB4_HUMAN		NDUFB4
16	16	16	0.999	890	sp|O95235|KI20A_HUMAN	KIF20A	O95235	Kinesin-like protein KIF20A	KI20A_HUMAN		KIF20A
103	103	103	0.999	493	sp|O95376|ARI2_HUMAN	ARIH2	O95376	E3 ubiquitin-protein ligase ARIH2	ARI2_HUMAN		ARIH2
99	99	99	0.999	288	sp|O95456|PSMG1_HUMAN	PSMG1	O95456	Proteasome assembly chaperone 1	PSMG1_HUMAN		PSMG1
348	348	348	0.999	172	sp|O95881|TXD12_HUMAN	TXNDC12	O95881	Thioredoxin domain-containing protein 12	TXD12_HUMAN		TXNDC12
10	10	10	0.9975	443	sp|O95967|FBLN4_HUMAN	EFEMP2	O95967	EGF-containing fibulin-like extracellular matrix protein 2	FBLN4_HUMAN		EFEMP2
14	13	14	0.999	705	sp|P00736|C1R_HUMAN	C1R	P00736	Complement C1r subcomponent	C1R_HUMAN		C1R
53	52	53	0.999	810	sp|P00747|PLMN_HUMAN	PLG	P00747	Plasminogen	PLMN_HUMAN		PLG
29	29	29	0.9989	491	sp|P02675|FIBB_HUMAN	FGB	P02675	Fibrinogen beta chain	FIBB_HUMAN		FGB
82	68	82	0.999	648	sp|P04049|RAF1_HUMAN	RAF1	P04049	RAF proto-oncogene serine/threonine-protein kinase	RAF1_HUMAN		RAF1
59	59	59	0.999	536	sp|P04062|GBA1_HUMAN	GBA1	P04062	Lysosomal acid glucosylceramidase	GBA1_HUMAN		GBA
142	142	142	0.999	234	sp|P04183|KITH_HUMAN	TK1	P04183	Thymidine kinase, cytosolic	KITH_HUMAN		TK1
102	102	102	0.999	313	sp|P04818|TYSY_HUMAN	TYMS	P04818	Thymidylate synthase	TYSY_HUMAN		TYMS
427	427	427	0.999	111	sp|P06454|PTMA_HUMAN	PTMA	P06454	Prothymosin alpha	PTMA_HUMAN		PTMA
103	103	103	0.999	361	sp|P08397|HEM3_HUMAN	HMBS	P08397	Porphobilinogen deaminase	HEM3_HUMAN		HMBS
256	256	256	0.999	159	sp|P09234|RU1C_HUMAN	SNRPC	P09234	U1 small nuclear ribonucleoprotein C	RU1C_HUMAN		SNRPC
42	42	42	0.999	303	sp|P09486|SPRC_HUMAN	SPARC	P09486	SPARC	SPRC_HUMAN		SPARC
34	0	34	0.999	511	sp|P0DTE7|AMY1B_HUMAN	AMY1B	P0DTE7	Alpha-amylase 1B	AMY1B_HUMAN	sp|P0DTE8|AMY1C_HUMAN, sp|P0DUB6|AMY1	
300	300	300	0.999	129	sp|P10606|COX5B_HUMAN	COX5B	P10606	Cytochrome c oxidase subunit 5B, mitochondrial	COX5B_HUMAN		COX5B
35	35	35	0.999	843	sp|P10643|CO7_HUMAN	C7	P10643	Complement component C7	CO7_HUMAN		C7
41	41	41	0.999	304	sp|P10646|TFPI1_HUMAN	TFPI	P10646	Tissue factor pathway inhibitor	TFPI1_HUMAN		TFPI
98	98	98	0.999	423	sp|P11117|PPAL_HUMAN	ACP2	P11117	Lysosomal acid phosphatase	PPAL_HUMAN		ACP2
100	17	100	0.999	496	sp|P11169|GTR3_HUMAN	SLC2A3	P11169	Solute carrier family 2, facilitated glucose transporter member 3	GTR3_HUMAN		SLC2A3
8	8	24	0.999	1806	sp|P12107|COBA1_HUMAN	COL11A1	P12107	Collagen alpha-1(XI) chain	COBA1_HUMAN		COL11A1
262	262	262	0.999	410	sp|P13473|LAMP2_HUMAN	LAMP2	P13473	Lysosome-associated membrane glycoprotein 2	LAMP2_HUMAN		LAMP2
469	469	469	0.999	128	sp|P13987|CD59_HUMAN	CD59	P13987	CD59 glycoprotein	CD59_HUMAN		CD59
102	102	102	0.999	658	sp|P17813|EGLN_HUMAN	ENG	P17813	Endoglin	EGLN_HUMAN		ENG
200	200	200	0.999	380	sp|P18615|NELFE_HUMAN	NELFE	P18615	Negative elongation factor E	NELFE_HUMAN		NELFE
167	167	167	0.999	108	sp|P18859|ATP5J_HUMAN	ATP5PF	P18859	ATP synthase-coupling factor 6, mitochondrial	ATP5J_HUMAN		ATP5J
132	132	132	0.999	551	sp|P19525|E2AK2_HUMAN	EIF2AK2	P19525	Interferon-induced, double-stranded RNA-activated protein kinase	E2AK2_HUMAN		EIF2AK2
112	112	112	0.999	228	sp|P21926|CD9_HUMAN	CD9	P21926	CD9 antigen	CD9_HUMAN		CD9
51	51	51	0.999	599	sp|P23219|PGH1_HUMAN	PTGS1	P23219	Prostaglandin G/H synthase 1	PGH1_HUMAN		PTGS1
86	86	97	0.999	801	sp|P25440|BRD2_HUMAN	BRD2	P25440	Bromodomain-containing protein 2	BRD2_HUMAN		BRD2
83	83	83	0.999	260	sp|P27707|DCK_HUMAN	DCK	P27707	Deoxycytidine kinase	DCK_HUMAN		DCK
67	67	67	0.999	219	sp|P28065|PSB9_HUMAN	PSMB9	P28065	Proteasome subunit beta type-9	PSB9_HUMAN		PSMB9
47	47	47	0.999	417	sp|P28300|LYOX_HUMAN	LOX	P28300	Protein-lysine 6-oxidase	LYOX_HUMAN		LOX
75	75	75	0.999	894	sp|P30530|UFO_HUMAN	AXL	P30530	Tyrosine-protein kinase receptor UFO	UFO_HUMAN		AXL
170	170	170	0.999	629	sp|P30825|CTR1_HUMAN	SLC7A1	P30825	High affinity cationic amino acid transporter 1	CTR1_HUMAN		SLC7A1
35	35	35	0.999	857	sp|P33981|TTK_HUMAN	TTK	P33981	Dual specificity protein kinase TTK	TTK_HUMAN		TTK
349	349	349	0.999	140	sp|P35080|PROF2_HUMAN	PFN2	P35080	Profilin-2	PROF2_HUMAN		PFN2
173	121	173	0.999	325	sp|P35914|HMGCL_HUMAN	HMGCL	P35914	Hydroxymethylglutaryl-CoA lyase, mitochondrial	HMGCL_HUMAN		HMGCL
48	48	48	0.999	273	sp|P40306|PSB10_HUMAN	PSMB10	P40306	Proteasome subunit beta type-10	PSB10_HUMAN		PSMB10
140	113	140	0.999	277	sp|P42574|CASP3_HUMAN	CASP3	P42574	Caspase-3	CASP3_HUMAN		CASP3
104	104	104	0.999	420	sp|P49642|PRI1_HUMAN	PRIM1	P49642	DNA primase small subunit	PRI1_HUMAN		PRIM1
66	65	66	0.999	377	sp|P49662|CASP4_HUMAN	CASP4	P49662	Caspase-4	CASP4_HUMAN		CASP4
24	24	39	0.999	757	sp|P49747|COMP_HUMAN	COMP	P49747	Cartilage oligomeric matrix protein	COMP_HUMAN		COMP
79	79	79	0.999	477	sp|P50336|PPOX_HUMAN	PPOX	P50336	Protoporphyrinogen oxidase	PPOX_HUMAN		PPOX
118	80	118	0.999	221	sp|P51159|RB27A_HUMAN	RAB27A	P51159	Ras-related protein Rab-27A	RB27A_HUMAN		RAB27A
120	120	120	0.999	245	sp|P51580|TPMT_HUMAN	TPMT	P51580	Thiopurine S-methyltransferase	TPMT_HUMAN		TPMT
54	54	54	0.9989	338	sp|P51884|LUM_HUMAN	LUM	P51884	Lumican	LUM_HUMAN		LUM
110	110	110	0.999	323	sp|P51946|CCNH_HUMAN	CCNH	P51946	Cyclin-H	CCNH_HUMAN		CCNH
14	13	14	0.999	851	sp|P52630|STAT2_HUMAN	STAT2	P52630	Signal transducer and activator of transcription 2	STAT2_HUMAN		STAT2
124	124	124	0.999	341	sp|P53365|ARFP2_HUMAN	ARFIP2	P53365	Arfaptin-2	ARFP2_HUMAN		ARFIP2
105	105	105	0.999	364	sp|P53990|IST1_HUMAN	IST1	P53990	IST1 homolog	IST1_HUMAN		IST1
34	34	34	0.999	796	sp|P55287|CAD11_HUMAN	CDH11	P55287	Cadherin-11	CAD11_HUMAN		CDH11
159	159	159	0.999	195	sp|P55957|BID_HUMAN	BID	P55957	BH3-interacting domain death agonist	BID_HUMAN		BID
173	173	173	0.999	69	sp|P56385|ATP5I_HUMAN	ATP5ME	P56385	ATP synthase subunit e, mitochondrial	ATP5I_HUMAN		ATP5I
295	295	295	0.999	245	sp|P56537|IF6_HUMAN	EIF6	P56537	Eukaryotic translation initiation factor 6	IF6_HUMAN		EIF6
185	185	185	0.999	247	sp|P57088|TMM33_HUMAN	TMEM33	P57088	Transmembrane protein 33	TMM33_HUMAN		TMEM33
267	267	267	0.999	96	sp|P60468|SC61B_HUMAN	SEC61B	P60468	Protein transport protein Sec61 subunit beta	SC61B_HUMAN		SEC61B
107	107	300	0.999	215	sp|P61020|RAB5B_HUMAN	RAB5B	P61020	Ras-related protein Rab-5B	RAB5B_HUMAN		RAB5B
126	49	126	0.999	115	sp|P62854|RS26_HUMAN	RPS26	P62854	Small ribosomal subunit protein eS26	RS26_HUMAN		RPS26
112	81	112	0.999	339	sp|P78318|IGBP1_HUMAN	IGBP1	P78318	Immunoglobulin-binding protein 1	IGBP1_HUMAN		IGBP1
252	252	360	0.999	185	sp|P83916|CBX1_HUMAN	CBX1	P83916	Chromobox protein homolog 1	CBX1_HUMAN		CBX1
189	59	189	0.999	375	sp|Q01085|TIAR_HUMAN	TIAL1	Q01085	Nucleolysin TIAR	TIAR_HUMAN		TIAL1
399	301	399	0.999	507	sp|Q01650|LAT1_HUMAN	SLC7A5	Q01650	Large neutral amino acids transporter small subunit 1	LAT1_HUMAN		SLC7A5
58	57	58	0.999	1114	sp|Q01804|OTUD4_HUMAN	OTUD4	Q01804	OTU domain-containing protein 4	OTUD4_HUMAN		OTUD4
76	24	76	0.999	772	sp|Q04726|TLE3_HUMAN	TLE3	Q04726	Transducin-like enhancer protein 3	TLE3_HUMAN		TLE3
121	121	121	0.999	329	sp|Q08257|QOR_HUMAN	CRYZ	Q08257	Quinone oxidoreductase	QOR_HUMAN		CRYZ
159	159	159	0.999	782	sp|Q08AM6|VAC14_HUMAN	VAC14	Q08AM6	Protein VAC14 homolog	VAC14_HUMAN		VAC14
56	56	56	0.999	355	sp|Q13190|STX5_HUMAN	STX5	Q13190	Syntaxin-5	STX5_HUMAN		STX5
50	50	50	0.999	420	sp|Q13641|TPBG_HUMAN	TPBG	Q13641	Trophoblast glycoprotein	TPBG_HUMAN		TPBG
83	83	83	0.999	434	sp|Q13685|AAMP_HUMAN	AAMP	Q13685	Angio-associated migratory cell protein	AAMP_HUMAN		AAMP
108	108	108	0.999	182	sp|Q13951|PEBB_HUMAN	CBFB	Q13951	Core-binding factor subunit beta	PEBB_HUMAN		CBFB
109	109	109	0.999	206	sp|Q14197|ICT1_HUMAN	MRPL58	Q14197	Large ribosomal subunit protein mL62	ICT1_HUMAN		ICT1
177	177	177	0.999	317	sp|Q14257|RCN2_HUMAN	RCN2	Q14257	Reticulocalbin-2	RCN2_HUMAN		RCN2
318	318	318	0.999	234	sp|Q14696|MESD_HUMAN	MESD	Q14696	LRP chaperone MESD	MESD_HUMAN		MESDC2
238	238	238	0.999	305	sp|Q15287|RNPS1_HUMAN	RNPS1	Q15287	RNA-binding protein with serine-rich domain 1	RNPS1_HUMAN		RNPS1
197	197	197	0.999	201	sp|Q15363|TMED2_HUMAN	TMED2	Q15363	Transmembrane emp24 domain-containing protein 2	TMED2_HUMAN		TMED2
84	84	84	0.999	1187	sp|Q15678|PTN14_HUMAN	PTPN14	Q15678	Tyrosine-protein phosphatase non-receptor type 14	PTN14_HUMAN		PTPN14
308	308	308	0.999	81	sp|Q15843|NEDD8_HUMAN	NEDD8	Q15843	NEDD8	NEDD8_HUMAN		NEDD8
109	109	109	0.999	116	sp|Q16718|NDUA5_HUMAN	NDUFA5	Q16718	NADH dehydrogenase [ubiquinone] 1 alpha subcomplex subunit 5	NDUA5_HUMAN		NDUFA5
211	211	211	0.999	197	sp|Q16774|KGUA_HUMAN	GUK1	Q16774	Guanylate kinase	KGUA_HUMAN		GUK1
97	97	97	0.999	776	sp|Q2TAL8|QRIC1_HUMAN	QRICH1	Q2TAL8	Transcriptional regulator QRICH1	QRIC1_HUMAN		QRICH1
83	83	500	0.999	418	sp|Q58FG1|HS904_HUMAN	HSP90AA4P	Q58FG1	Putative heat shock protein HSP 90-alpha A4	HS904_HUMAN		HSP90AA4P
90	90	90	0.999	399	sp|Q5JVF3|PCID2_HUMAN	PCID2	Q5JVF3	PCI domain-containing protein 2	PCID2_HUMAN		PCID2
62	61	62	0.999	975	sp|Q5VTR2|BRE1A_HUMAN	RNF20	Q5VTR2	E3 ubiquitin-protein ligase BRE1A	BRE1A_HUMAN		RNF20
51	43	51	0.999	765	sp|Q5W0V3|FHI2A_HUMAN	FHIP2A	Q5W0V3	FHF complex subunit HOOK interacting protein 2A	FHI2A_HUMAN		FAM160B1
93	93	93	0.999	538	sp|Q69YN2|C19L1_HUMAN	CWF19L1	Q69YN2	CWF19-like protein 1	C19L1_HUMAN		CWF19L1
84	84	84	0.999	325	sp|Q6IAN0|DRS7B_HUMAN	DHRS7B	Q6IAN0	Dehydrogenase/reductase SDR family member 7B	DRS7B_HUMAN		DHRS7B
111	111	111	0.999	394	sp|Q6P6C2|ALKB5_HUMAN	ALKBH5	Q6P6C2	RNA demethylase ALKBH5	ALKB5_HUMAN		ALKBH5
80	6	80	0.999	1433	sp|Q6PFW1|VIP1_HUMAN	PPIP5K1	Q6PFW1	Inositol hexakisphosphate and diphosphoinositol-pentakisphosphate kinas	VIP1_HUMAN		PPIP5K1
54	54	54	0.999	289	sp|Q6PK04|CC137_HUMAN	CCDC137	Q6PK04	Coiled-coil domain-containing protein 137	CC137_HUMAN		CCDC137
81	81	87	0.999	915	sp|Q6ZXV5|TMTC3_HUMAN	TMTC3	Q6ZXV5	Protein O-mannosyl-transferase TMTC3	TMTC3_HUMAN		TMTC3
334	245	334	0.999	306	sp|Q86U42|PABP2_HUMAN	PABPN1	Q86U42	Polyadenylate-binding protein 2	PABP2_HUMAN		PABPN1
18	18	18	0.9989	667	sp|Q86UX7|URP2_HUMAN	FERMT3	Q86UX7	Fermitin family homolog 3	URP2_HUMAN		FERMT3
59	59	59	0.999	718	sp|Q86VS8|HOOK3_HUMAN	HOOK3	Q86VS8	Protein Hook homolog 3	HOOK3_HUMAN		HOOK3
21	20	21	0.999	417	sp|Q86VY4|TSYL5_HUMAN	TSPYL5	Q86VY4	Testis-specific Y-encoded-like protein 5	TSYL5_HUMAN		TSPYL5
54	54	54	0.999	314	sp|Q8N474|SFRP1_HUMAN	SFRP1	Q8N474	Secreted frizzled-related protein 1	SFRP1_HUMAN		SFRP1
138	138	138	0.999	293	sp|Q8N6M0|OTU6B_HUMAN	OTUD6B	Q8N6M0	Deubiquitinase OTUD6B	OTU6B_HUMAN		OTUD6B
46	46	46	0.999	893	sp|Q8NB90|AFG2A_HUMAN	AFG2A	Q8NB90	ATPase family gene 2 protein homolog A	AFG2A_HUMAN		SPATA5
86	86	86	0.999	458	sp|Q8NEY8|PPHLN_HUMAN	PPHLN1	Q8NEY8	Periphilin-1	PPHLN_HUMAN		PPHLN1
143	143	143	0.999	326	sp|Q8NFH5|NUP35_HUMAN	NUP35	Q8NFH5	Nucleoporin NUP35	NUP35_HUMAN		NUP35
131	131	131	0.999	979	sp|Q8TD19|NEK9_HUMAN	NEK9	Q8TD19	Serine/threonine-protein kinase Nek9	NEK9_HUMAN		NEK9
97	97	97	0.999	740	sp|Q8TDB6|DTX3L_HUMAN	DTX3L	Q8TDB6	E3 ubiquitin-protein ligase DTX3L	DTX3L_HUMAN		DTX3L
46	46	46	0.999	209	sp|Q8TED1|GPX8_HUMAN	GPX8	Q8TED1	Probable glutathione peroxidase 8	GPX8_HUMAN		GPX8
40	39	40	0.999	401	sp|Q8WV24|PHLA1_HUMAN	PHLDA1	Q8WV24	Pleckstrin homology-like domain family A member 1	PHLA1_HUMAN		PHLDA1
78	78	78	0.999	318	sp|Q8WVY7|UBCP1_HUMAN	UBLCP1	Q8WVY7	Ubiquitin-like domain-containing CTD phosphatase 1	UBCP1_HUMAN		UBLCP1
45	45	45	0.999	657	sp|Q8WWI5|CTL1_HUMAN	SLC44A1	Q8WWI5	Choline transporter-like protein 1	CTL1_HUMAN		SLC44A1
18	18	18	0.9989	480	sp|Q92743|HTRA1_HUMAN	HTRA1	Q92743	Serine protease HTRA1	HTRA1_HUMAN		HTRA1
15	12	15	0.999	2442	sp|Q92793|CBP_HUMAN	CREBBP	Q92793	CREB-binding protein	CBP_HUMAN		CREBBP
158	158	158	0.999	307	sp|Q92890|UFD1_HUMAN	UFD1	Q92890	Ubiquitin recognition factor in ER-associated degradation protein 1	UFD1_HUMAN		UFD1L
77	77	77	0.999	779	sp|Q969S9|RRF2M_HUMAN	GFM2	Q969S9	Ribosome-releasing factor 2, mitochondrial	RRF2M_HUMAN		GFM2
149	149	149	0.999	264	sp|Q969U7|PSMG2_HUMAN	PSMG2	Q969U7	Proteasome assembly chaperone 2	PSMG2_HUMAN		PSMG2
217	172	217	0.999	147	sp|Q96C90|PP14B_HUMAN	PPP1R14B	Q96C90	Protein phosphatase 1 regulatory subunit 14B	PP14B_HUMAN		PPP1R14B
82	82	82	0.999	657	sp|Q96FV9|THOC1_HUMAN	THOC1	Q96FV9	THO complex subunit 1	THOC1_HUMAN		THOC1
167	167	167	0.999	510	sp|Q96GM8|TOE1_HUMAN	TOE1	Q96GM8	Target of EGR1 protein 1	TOE1_HUMAN		TOE1
100	100	100	0.999	300	sp|Q96H79|ZCCHL_HUMAN	ZC3HAV1L	Q96H79	Zinc finger CCCH-type antiviral protein 1-like	ZCCHL_HUMAN		ZC3HAV1L
91	91	92	0.999	314	sp|Q96HY6|DDRGK_HUMAN	DDRGK1	Q96HY6	DDRGK domain-containing protein 1	DDRGK_HUMAN		DDRGK1
264	264	264	0.999	210	sp|Q96IU4|ABHEB_HUMAN	ABHD14B	Q96IU4	Putative protein-lysine deacylase ABHD14B	ABHEB_HUMAN		ABHD14B
29	29	29	0.999	1193	sp|Q96R06|SPAG5_HUMAN	SPAG5	Q96R06	Sperm-associated antigen 5	SPAG5_HUMAN		SPAG5
37	37	37	0.999	308	sp|Q99988|GDF15_HUMAN	GDF15	Q99988	Growth/differentiation factor 15	GDF15_HUMAN		GDF15
286	286	286	0.999	446	sp|Q9BQ67|GRWD1_HUMAN	GRWD1	Q9BQ67	Glutamate-rich WD repeat-containing protein 1	GRWD1_HUMAN		GRWD1
135	135	135	0.999	325	sp|Q9BQ69|MACD1_HUMAN	MACROD1	Q9BQ69	ADP-ribose glycohydrolase MACROD1	MACD1_HUMAN		MACROD1
215	215	215	0.999	342	sp|Q9BQA1|MEP50_HUMAN	WDR77	Q9BQA1	Methylosome protein WDR77	MEP50_HUMAN		WDR77
142	142	142	0.999	190	sp|Q9BSD7|NTPCR_HUMAN	NTPCR	Q9BSD7	Cancer-related nucleoside-triphosphatase	NTPCR_HUMAN		NTPCR
210	210	210	0.999	707	sp|Q9BU23|LMF2_HUMAN	LMF2	Q9BU23	Lipase maturation factor 2	LMF2_HUMAN		LMF2
78	78	78	0.999	163	sp|Q9BX68|HINT2_HUMAN	HINT2	Q9BX68	Adenosine 5'-monophosphoramidase HINT2	HINT2_HUMAN		HINT2

108	108	108	0.999	194	sp|Q9BY32|ITPA_HUMAN	ITPA	Q9BY32	Inosine triphosphate pyrophosphatase	ITPA_HUMAN		ITPA
189	189	189	0.999	178	sp|Q9BYD1|RM13_HUMAN	MRPL13	Q9BYD1	Large ribosomal subunit protein uL13m	RM13_HUMAN		MRPL13
24	24	24	0.999	848	sp|Q9BZJ0|CRNL1_HUMAN	CRNKL1	Q9BZJ0	Crooked neck-like protein 1	CRNL1_HUMAN		CRNKL1
119	118	119	0.999	261	sp|Q9BZX2|UCK2_HUMAN	UCK2	Q9BZX2	Uridine-cytidine kinase 2	UCK2_HUMAN		UCK2
170	170	170	0.999	99	sp|Q9C005|DPY30_HUMAN	DPY30	Q9C005	Protein dpy-30 homolog	DPY30_HUMAN		DPY30
210	210	210	0.999	335	sp|Q9H0U3|MAGT1_HUMAN	MAGT1	Q9H0U3	Magnesium transporter protein 1	MAGT1_HUMAN		MAGT1
121	121	121	0.999	167	sp|Q9H1K1|ISCU_HUMAN	ISCU	Q9H1K1	Iron-sulfur cluster assembly enzyme ISCU	ISCU_HUMAN		ISCU
141	141	141	0.999	617	sp|Q9H267|VP33B_HUMAN	VPS33B	Q9H267	Vacuolar protein sorting-associated protein 33B	VP33B_HUMAN		VPS33B
65	65	65	0.999	941	sp|Q9H270|VPS11_HUMAN	VPS11	Q9H270	Vacuolar protein sorting-associated protein 11 homolog	VPS11_HUMAN		VPS11
77	77	77	0.999	161	sp|Q9H2H8|PPIL3_HUMAN	PPIL3	Q9H2H8	Peptidyl-prolyl cis-trans isomerase-like 3	PPIL3_HUMAN		PPIL3
69	69	69	0.999	574	sp|Q9H4L4|SENP3_HUMAN	SENP3	Q9H4L4	Sentrin-specific protease 3	SENP3_HUMAN		SENP3
47	47	47	0.999	567	sp|Q9H6Z4|RANB3_HUMAN	RANBP3	Q9H6Z4	Ran-binding protein 3	RANB3_HUMAN		RANBP3
60	60	60	0.999	785	sp|Q9H9E3|COG4_HUMAN	COG4	Q9H9E3	Conserved oligomeric Golgi complex subunit 4	COG4_HUMAN		COG4
233	233	233	0.999	221	sp|Q9HCN8|SDF2L_HUMAN	SDF2L1	Q9HCN8	Stromal cell-derived factor 2-like protein 1	SDF2L_HUMAN		SDF2L1
326	326	326	0.999	589	sp|Q9HD45|TM9S3_HUMAN	TM9SF3	Q9HD45	Transmembrane 9 superfamily member 3	TM9S3_HUMAN		TM9SF3
91	91	96	0.999	439	sp|Q9NP92|RT30_HUMAN	MRPS30	Q9NP92	Large ribosomal subunit protein mL65	RT30_HUMAN		MRPS30
37	37	37	0.999	505	sp|Q9NRY5|F1142_HUMAN	FAM114A2	Q9NRY5	Protein FAM114A2	F1142_HUMAN		FAM114A2
37	37	37	0.999	838	sp|Q9NRZ9|HELLS_HUMAN	HELLS	Q9NRZ9	Lymphoid-specific helicase	HELLS_HUMAN		HELLS
137	137	137	0.999	307	sp|Q9NTX5|ECHD1_HUMAN	ECHDC1	Q9NTX5	Ethylmalonyl-CoA decarboxylase	ECHD1_HUMAN		ECHDC1
129	129	129	0.999	292	sp|Q9NUI1|DECR2_HUMAN	DECR2	Q9NUI1	Peroxisomal 2,4-dienoyl-CoA reductase [(3E)-enoyl-CoA-producing]	DECR2_HUMAN		DECR2
232	232	232	0.999	306	sp|Q9NUJ1|ABHDA_HUMAN	ABHD10	Q9NUJ1	Palmitoyl-protein thioesterase ABHD10, mitochondrial	ABHDA_HUMAN		ABHD10
64	64	64	0.999	699	sp|Q9NUY8|TBC23_HUMAN	TBC1D23	Q9NUY8	TBC1 domain family member 23	TBC23_HUMAN		TBC1D23
102	102	102	0.999	263	sp|Q9NVZ3|NECP2_HUMAN	NECAP2	Q9NVZ3	Adaptin ear-binding coat-associated protein 2	NECP2_HUMAN		NECAP2
61	61	61	0.999	580	sp|Q9NXV6|CARF_HUMAN	CDKN2AIP	Q9NXV6	CDKN2A-interacting protein	CARF_HUMAN		CDKN2AIP
32	32	32	0.999	581	sp|Q9P270|SLAI2_HUMAN	SLAIN2	Q9P270	SLAIN motif-containing protein 2	SLAI2_HUMAN		SLAIN2
32	32	32	0.999	639	sp|Q9P2B4|CT2NL_HUMAN	CTTNBP2NL	Q9P2B4	CTTNBP2 N-terminal-like protein	CT2NL_HUMAN		CTTNBP2NL
250	250	250	0.999	182	sp|Q9UBQ0|VPS29_HUMAN	VPS29	Q9UBQ0	Vacuolar protein sorting-associated protein 29	VPS29_HUMAN		VPS29
34	34	34	0.999	676	sp|Q9UGP4|LIMD1_HUMAN	LIMD1	Q9UGP4	LIM domain-containing protein 1	LIMD1_HUMAN		LIMD1
28	28	28	0.999	977	sp|Q9UGR2|Z3H7B_HUMAN	ZC3H7B	Q9UGR2	Zinc finger CCCH domain-containing protein 7B	Z3H7B_HUMAN		ZC3H7B
22	22	22	0.999	491	sp|Q9UJ41|RABX5_HUMAN	RABGEF1	Q9UJ41	Rab5 GDP/GTP exchange factor	RABX5_HUMAN		RABGEF1
150	150	150	0.999	497	sp|Q9UJA5|TRM6_HUMAN	TRMT6	Q9UJA5	tRNA (adenine(58)-N(1))-methyltransferase non-catalytic subunit TRM6	TRM6_HUMAN		TRMT6
233	233	233	0.999	221	sp|Q9UK41|VPS28_HUMAN	VPS28	Q9UK41	Vacuolar protein sorting-associated protein 28 homolog	VPS28_HUMAN		VPS28
198	189	198	0.999	103	sp|Q9UK45|LSM7_HUMAN	LSM 7.00	Q9UK45	U6 snRNA-associated Sm-like protein LSm7	LSM7_HUMAN		LSM 7.00
207	207	207	0.999	225	sp|Q9UL25|RAB21_HUMAN	RAB21	Q9UL25	Ras-related protein Rab-21	RAB21_HUMAN		RAB21
57	57	57	0.999	237	sp|Q9ULC3|RAB23_HUMAN	RAB23	Q9ULC3	Ras-related protein Rab-23	RAB23_HUMAN		RAB23
146	146	146	0.999	185	sp|Q9UNL2|SSRG_HUMAN	SSR3	Q9UNL2	Translocon-associated protein subunit gamma	SSRG_HUMAN		SSR3
120	120	120	0.999	99	sp|Q9UNZ5|L10K_HUMAN	C19orf53	Q9UNZ5	Leydig cell tumor 10 kDa protein homolog	L10K_HUMAN		C19orf53
170	170	170	0.999	881	sp|Q9UPN7|PP6R1_HUMAN	PPP6R1	Q9UPN7	Serine/threonine-protein phosphatase 6 regulatory subunit 1	PP6R1_HUMAN		PPP6R1
53	53	53	0.999	1833	sp|Q9UPQ9|TNR6B_HUMAN	TNRC6B	Q9UPQ9	Trinucleotide repeat-containing gene 6B protein	TNR6B_HUMAN		TNRC6B
146	146	146	0.999	244	sp|Q9Y224|RTRAF_HUMAN	RTRAF	Q9Y224	RNA transcription, translation and transport factor protein	RTRAF_HUMAN		C14orf166
141	141	141	0.999	237	sp|Q9Y3B8|ORN_HUMAN	REXO2	Q9Y3B8	Oligoribonuclease, mitochondrial	ORN_HUMAN		REXO2
64	64	64	0.999	190	sp|Q9Y3D9|RT23_HUMAN	MRPS23	Q9Y3D9	Small ribosomal subunit protein mS23	RT23_HUMAN		MRPS23
46	46	76	0.999	1855	sp|Q9Y4I1|MYO5A_HUMAN	MYO5A	Q9Y4I1	Unconventional myosin-Va	MYO5A_HUMAN		MYO5A
91	91	91	0.999	837	sp|Q9Y4R8|TELO2_HUMAN	TELO2	Q9Y4R8	Telomere length regulation protein TEL2 homolog	TELO2_HUMAN		TELO2
98	98	98	0.999	258	sp|Q9Y676|RT18B_HUMAN	MRPS18B	Q9Y676	Small ribosomal subunit protein mS40	RT18B_HUMAN		MRPS18B
314	314	314	0.999	68	sp|O00244|ATOX1_HUMAN	ATOX1	O00244	Copper transport protein ATOX1	ATOX1_HUMAN		ATOX1
119	119	119	0.999	1686	sp|O00443|P3C2A_HUMAN	PIK3C2A	O00443	Phosphatidylinositol 4-phosphate 3-kinase C2 domain-containing subunit a	P3C2A_HUMAN		PIK3C2A
259	259	259	0.999	81	sp|O00483|NDUA4_HUMAN	NDUFA4	O00483	Cytochrome c oxidase subunit NDUFA4	NDUA4_HUMAN		NDUFA4
104	104	104	0.999	558	sp|O00592|PODXL_HUMAN	PODXL	O00592	Podocalyxin	PODXL_HUMAN		PODXL
291	291	291	0.999	274	sp|O14618|CCS_HUMAN	CCS	O14618	Copper chaperone for superoxide dismutase	CCS_HUMAN		CCS
259	259	259	0.999	152	sp|O14684|PTGES_HUMAN	PTGES	O14684	Prostaglandin E synthase	PTGES_HUMAN		PTGES
80	80	80	0.999	440	sp|O14763|TR10B_HUMAN	TNFRSF10B	O14763	Tumor necrosis factor receptor superfamily member 10B	TR10B_HUMAN		TNFRSF10B
31	31	31	0.999	403	sp|O14965|AURKA_HUMAN	AURKA	O14965	Aurora kinase A	AURKA_HUMAN		AURKA
41	41	41	0.999	640	sp|O15484|CAN5_HUMAN	CAPN5	O15484	Calpain-5	CAN5_HUMAN		CAPN5
68	68	68	0.999	454	sp|O43293|DAPK3_HUMAN	DAPK3	O43293	Death-associated protein kinase 3	DAPK3_HUMAN		DAPK3
76	76	76	0.999	181	sp|O43665|RGS10_HUMAN	RGS10	O43665	Regulator of G-protein signaling 10	RGS10_HUMAN		RGS10
199	199	199	0.999	478	sp|O43670|ZN207_HUMAN	ZNF207	O43670	BUB3-interacting and GLEBS motif-containing protein ZNF207	ZN207_HUMAN		ZNF207
184	184	184	0.999	313	sp|O43765|SGTA_HUMAN	SGTA	O43765	Small glutamine-rich tetratricopeptide repeat-containing protein alpha	SGTA_HUMAN		SGTA
342	342	342	0.999	227	sp|O43809|CPSF5_HUMAN	NUDT21	O43809	Cleavage and polyadenylation specificity factor subunit 5	CPSF5_HUMAN		NUDT21
131	131	131	0.999	852	sp|O60341|KDM1A_HUMAN	KDM1A	O60341	Lysine-specific histone demethylase 1A	KDM1A_HUMAN		KDM1A
68	68	68	0.999	579	sp|O60508|PRP17_HUMAN	CDC40	O60508	Pre-mRNA-processing factor 17	PRP17_HUMAN		CDC40
106	106	106	0.999	1101	sp|O60879|DIAP2_HUMAN	DIAPH2	O60879	Protein diaphanous homolog 2	DIAP2_HUMAN		DIAPH2
151	150	151	0.999	401	sp|O75718|CRTAP_HUMAN	CRTAP	O75718	Cartilage-associated protein	CRTAP_HUMAN		CRTAP
142	142	142	0.999	339	sp|O76071|CIAO1_HUMAN	CIAO1	O76071	Probable cytosolic iron-sulfur protein assembly protein CIAO1	CIAO1_HUMAN		CIAO1
39	32	39	0.999	266	sp|O94907|DKK1_HUMAN	DKK 1.00	O94907	Dickkopf-related protein 1	DKK1_HUMAN		DKK 1.00
202	202	202	0.999	231	sp|O95372|LYPA2_HUMAN	LYPLA2	O95372	Acyl-protein thioesterase 2	LYPA2_HUMAN		LYPLA2
55	55	55	0.999	233	sp|O95999|BCL10_HUMAN	BCL10	O95999	B-cell lymphoma/leukemia 10	BCL10_HUMAN		BCL10
41	41	41	0.9989	1676	sp|P01031|CO5_HUMAN	C5	P01031	Complement C5	CO5_HUMAN		C5
168	2	298	0.999	362	sp|P01893|HLAH_HUMAN	HLA-H	P01893	Putative HLA class I histocompatibility antigen, alpha chain H	HLAH_HUMAN		HLA-H
36	36	36	0.999	1466	sp|P02461|CO3A1_HUMAN	COL3A1	P02461	Collagen alpha-1(III) chain	CO3A1_HUMAN		COL3A1
102	102	102	0.999	478	sp|P04004|VTNC_HUMAN	VTN	P04004	Vitronectin	VTNC_HUMAN		VTN
76	76	76	0.999	466	sp|P04066|FUCO_HUMAN	FUCA1	P04066	Tissue alpha-L-fucosidase	FUCO_HUMAN		FUCA1
17	17	21	0.999	4563	sp|P04114|APOB_HUMAN	APOB	P04114	Apolipoprotein B-100	APOB_HUMAN		APOB
178	178	178	0.999	424	sp|P09110|THIK_HUMAN	ACAA1	P09110	3-ketoacyl-CoA thiolase, peroxisomal	THIK_HUMAN		ACAA1
112	112	112	0.999	144	sp|P09132|SRP19_HUMAN	SRP19	P09132	Signal recognition particle 19 kDa protein	SRP19_HUMAN		SRP19
325	325	325	0.999	75	sp|P09669|COX6C_HUMAN	COX6C	P09669	Cytochrome c oxidase subunit 6C	COX6C_HUMAN		COX6C
86	86	86	0.999	478	sp|P09914|IFIT1_HUMAN	IFIT1	P09914	Interferon-induced protein with tetratricopeptide repeats 1	IFIT1_HUMAN		IFIT1
99	99	99	0.999	417	sp|P15151|PVR_HUMAN	PVR	P15151	Poliovirus receptor	PVR_HUMAN		PVR
164	120	164	0.999	347	sp|P17275|JUNB_HUMAN	JUNB	P17275	Transcription factor JunB	JUNB_HUMAN		JUNB
182	182	182	0.999	193	sp|P17900|SAP3_HUMAN	GM2A	P17900	Ganglioside GM2 activator	SAP3_HUMAN		GM2A
59	59	59	0.999	919	sp|P18858|DNLI1_HUMAN	LIG1	P18858	DNA ligase 1	DNLI1_HUMAN		LIG1
134	134	355	0.999	215	sp|P20339|RAB5A_HUMAN	RAB5A	P20339	Ras-related protein Rab-5A	RAB5A_HUMAN		RAB5A
476	476	476	0.999	102	sp|P20962|PTMS_HUMAN	PTMS	P20962	Parathymosin	PTMS_HUMAN		PTMS
122	0	122	0.999	795	sp|P21127|CD11B_HUMAN	CDK11B	P21127	Cyclin-dependent kinase 11B	CD11B_HUMAN	sp|Q9UQ88|CD11A_HUMAN	CDK11B
208	208	208	0.999	158	sp|P24666|PPAC_HUMAN	ACP1	P24666	Low molecular weight phosphotyrosine protein phosphatase	PPAC_HUMAN		ACP1
254	254	254	0.999	264	sp|P28070|PSB4_HUMAN	PSMB4	P28070	Proteasome subunit beta type-4	PSB4_HUMAN		PSMB4
392	392	392	0.999	239	sp|P28072|PSB6_HUMAN	PSMB6	P28072	Proteasome subunit beta type-6	PSB6_HUMAN		PSMB6
103	103	103	0.999	298	sp|P29372|3MG_HUMAN	MPG	P29372	DNA-3-methyladenine glycosylase	3MG_HUMAN		MPG
121	121	121	0.999	336	sp|P31937|3HIDH_HUMAN	HIBADH	P31937	3-hydroxyisobutyrate dehydrogenase, mitochondrial	3HIDH_HUMAN		HIBADH
46	31	46	0.999	961	sp|P35443|TSP4_HUMAN	THBS4	P35443	Thrombospondin-4	TSP4_HUMAN		THBS4
75	75	75	0.999	584	sp|P41214|EIF2D_HUMAN	EIF2D	P41214	Eukaryotic translation initiation factor 2D	EIF2D_HUMAN		EIF2D
115	81	115	0.999	596	sp|P41743|KPCI_HUMAN	PRKCI	P41743	Protein kinase C iota type	KPCI_HUMAN		PRKCI
382	382	382	0.999	205	sp|P49720|PSB3_HUMAN	PSMB3	P49720	Proteasome subunit beta type-3	PSB3_HUMAN		PSMB3
89	89	270	0.999	483	sp|P49840|GSK3A_HUMAN	GSK3A	P49840	Glycogen synthase kinase-3 alpha	GSK3A_HUMAN		GSK3A
101	66	101	0.999	745	sp|P51003|PAPOA_HUMAN	PAPOLA	P51003	Poly(A) polymerase alpha	PAPOA_HUMAN		PAPOLA
126	126	126	0.999	562	sp|P52594|AGFG1_HUMAN	AGFG1	P52594	Arf-GAP domain and FG repeat-containing protein 1	AGFG1_HUMAN		AGFG1
280	280	280	0.999	500	sp|P53985|MOT1_HUMAN	SLC16A1	P53985	Monocarboxylate transporter 1	MOT1_HUMAN		SLC16A1
61	61	61	0.999	303	sp|P55210|CASP7_HUMAN	CASP7	P55210	Caspase-7	CASP7_HUMAN		CASP7
379	379	379	0.999	119	sp|P61769|B2MG_HUMAN	B2M	P61769	Beta-2-microglobulin	B2MG_HUMAN		B2M
231	231	231	0.999	145	sp|P62841|RS15_HUMAN	RPS15	P62841	Small ribosomal subunit protein uS19	RS15_HUMAN		RPS15
238	122	238	0.999	101	sp|P63165|SUMO1_HUMAN	SUMO1	P63165	Small ubiquitin-related modifier 1	SUMO1_HUMAN		SUMO1
355	288	355	0.999	142	sp|P69905|HBA_HUMAN	HBA2	P69905	Hemoglobin subunit alpha	HBA_HUMAN		HBA1
92	92	277	0.999	180	sp|P84085|ARF5_HUMAN	ARF5	P84085	ADP-ribosylation factor 5	ARF5_HUMAN		ARF5
114	114	114	0.999	686	sp|Q03519|TAP2_HUMAN	TAP2	Q03519	Antigen peptide transporter 2	TAP2_HUMAN		TAP2
98	98	98	0.999	351	sp|Q12972|PP1R8_HUMAN	PPP1R8	Q12972	Nuclear inhibitor of protein phosphatase 1	PP1R8_HUMAN		PPP1R8
80	70	80	0.999	487	sp|Q13043|STK4_HUMAN	STK4	Q13043	Serine/threonine-protein kinase 4	STK4_HUMAN		STK4
120	120	120	0.999	205	sp|Q13257|MD2L1_HUMAN	MAD2L1	Q13257	Mitotic spindle assembly checkpoint protein MAD2A	MD2L1_HUMAN		MAD2L1
141	141	141	0.999	466	sp|Q13505|MTX1_HUMAN	MTX1	Q13505	Metaxin-1	MTX1_HUMAN		MTX1
80	77	80	0.999	225	sp|Q13637|RAB32_HUMAN	RAB32	Q13637	Ras-related protein Rab-32	RAB32_HUMAN		RAB32
148	148	148	0.999	412	sp|Q14318|FKBP8_HUMAN	FKBP8	Q14318	Peptidyl-prolyl cis-trans isomerase FKBP8	FKBP8_HUMAN		FKBP8
110	110	110	0.999	479	sp|Q14790|CASP8_HUMAN	CASP8	Q14790	Caspase-8	CASP8_HUMAN		CASP8
67	67	67	0.999	415	sp|Q15018|ABRX2_HUMAN	ABRAXAS2	Q15018	BRISC complex subunit Abraxas 2	ABRX2_HUMAN		FAM175B
197	197	197	0.999	203	sp|Q15041|AR6P1_HUMAN	ARL6IP1	Q15041	ADP-ribosylation factor-like protein 6-interacting protein 1	AR6P1_HUMAN		ARL6IP1
64	64	64	0.999	449	sp|Q15113|PCOC1_HUMAN	PCOLCE	Q15113	Procollagen C-endopeptidase enhancer 1	PCOC1_HUMAN		PCOLCE
180	180	180	0.999	184	sp|Q15382|RHEB_HUMAN	RHEB	Q15382	GTP-binding protein Rheb	RHEB_HUMAN		RHEB
252	99	252	0.999	100	sp|Q15836|VAMP3_HUMAN	VAMP3	Q15836	Vesicle-associated membrane protein 3	VAMP3_HUMAN		VAMP3
63	63	63	0.999	1250	sp|Q2NKX8|ERC6L_HUMAN	ERCC6L	Q2NKX8	DNA excision repair protein ERCC-6-like	ERC6L_HUMAN		ERCC6L
16	11	16	0.999	1871	sp|Q3V6T2|GRDN_HUMAN	CCDC88A	Q3V6T2	Girdin	GRDN_HUMAN		CCDC88A
93	93	93	0.999	553	sp|Q49A26|GLYR1_HUMAN	GLYR1	Q49A26	Cytokine-like nuclear factor N-PAC	GLYR1_HUMAN		GLYR1
17	17	17	0.999	464	sp|Q53EZ4|CEP55_HUMAN	CEP55	Q53EZ4	Centrosomal protein of 55 kDa	CEP55_HUMAN		CEP55
30	30	30	0.999	620	sp|Q53F19|NCBP3_HUMAN	NCBP3	Q53F19	Nuclear cap-binding protein subunit 3	NCBP3_HUMAN		C17orf85
103	103	103	0.999	631	sp|Q567U6|CCD93_HUMAN	CCDC93	Q567U6	Coiled-coil domain-containing protein 93	CCD93_HUMAN		CCDC93
132	89	132	0.999	1250	sp|Q66K14|TBC9B_HUMAN	TBC1D9B	Q66K14	TBC1 domain family member 9B	TBC9B_HUMAN		TBC1D9B
101	101	101	0.999	1059	sp|Q66K74|MAP1S_HUMAN	MAP1S	Q66K74	Microtubule-associated protein 1S	MAP1S_HUMAN		MAP1S
127	127	127	0.999	689	sp|Q6NUQ4|TM214_HUMAN	TMEM214	Q6NUQ4	Transmembrane protein 214	TM214_HUMAN		TMEM214
127	127	127	0.999	501	sp|Q6NXE6|ARMC6_HUMAN	ARMC6	Q6NXE6	Armadillo repeat-containing protein 6	ARMC6_HUMAN		ARMC6
73	73	73	0.999	698	sp|Q6ZRP7|QSOX2_HUMAN	QSOX2	Q6ZRP7	Sulfhydryl oxidase 2	QSOX2_HUMAN		QSOX2
42	29	166	0.999	459	sp|Q7Z3Y8|K1C27_HUMAN	KRT27	Q7Z3Y8	Keratin, type I cytoskeletal 27	K1C27_HUMAN		KRT27
80	80	80	0.999	226	sp|Q7Z6K5|ARPIN_HUMAN	ARPIN	Q7Z6K5	Arpin	ARPIN_HUMAN		ARPIN

98	98	98	0.999	770	sp|Q86TB9|PATL1_HUMAN	PATL1	Q86TB9	Protein PAT1 homolog 1	PATL1_HUMAN		PATL1
63	63	63	0.999	1035	sp|Q86UV5|UBP48_HUMAN	USP48	Q86UV5	Ubiquitin carboxyl-terminal hydrolase 48	UBP48_HUMAN		USP48
156	156	156	0.999	672	sp|Q86V21|AACS_HUMAN	AACS	Q86V21	Acetoacetyl-CoA synthetase	AACS_HUMAN		AACS
120	120	120	0.999	276	sp|Q86Y82|STX12_HUMAN	STX12	Q86Y82	Syntaxin-12	STX12_HUMAN		STX12
18	18	18	0.999	736	sp|Q8IVL6|P3H3_HUMAN	P3H3	Q8IVL6	Prolyl 3-hydroxylase 3	P3H3_HUMAN		LEPREL2
73	73	73	0.999	398	sp|Q8N2K0|ABD12_HUMAN	ABHD12	Q8N2K0	Lysophosphatidylserine lipase ABHD12	ABD12_HUMAN		ABHD12
99	99	99	0.999	663	sp|Q8N392|RHG18_HUMAN	ARHGAP18	Q8N392	Rho GTPase-activating protein 18	RHG18_HUMAN		ARHGAP18
109	109	109	0.999	542	sp|Q8N543|OGFD1_HUMAN	OGFOD1	Q8N543	Prolyl 3-hydroxylase OGFOD1	OGFD1_HUMAN		OGFOD1
138	138	138	0.999	135	sp|Q8N5K1|CISD2_HUMAN	CISD2	Q8N5K1	CDGSH iron-sulfur domain-containing protein 2	CISD2_HUMAN		CISD2
141	141	141	0.999	491	sp|Q8NBI5|S43A3_HUMAN	SLC43A3	Q8NBI5	Equilibrative nucleobase transporter 1	S43A3_HUMAN		SLC43A3
112	112	112	0.999	301	sp|Q8NBJ7|SUMF2_HUMAN	SUMF2	Q8NBJ7	Inactive C-alpha-formylglycine-generating enzyme 2	SUMF2_HUMAN		SUMF2
141	141	141	0.999	351	sp|Q8NE86|MCU_HUMAN	MCU	Q8NE86	Calcium uniporter protein, mitochondrial	MCU_HUMAN		MCU
172	172	172	0.999	315	sp|Q8NFV4|ABHDB_HUMAN	ABHD11	Q8NFV4	Protein ABHD11	ABHDB_HUMAN		ABHD11
105	105	105	0.999	691	sp|Q8TC07|TBC15_HUMAN	TBC1D15	Q8TC07	TBC1 domain family member 15	TBC15_HUMAN		TBC1D15
102	90	102	0.999	302	sp|Q8TDX7|NEK7_HUMAN	NEK7	Q8TDX7	Serine/threonine-protein kinase Nek7	NEK7_HUMAN		NEK7
35	35	35	0.999	1067	sp|Q8TDZ2|MICA1_HUMAN	MICAL1	Q8TDZ2	[F-actin]-monooxygenase MICAL1	MICA1_HUMAN		MICAL1
98	98	98	0.999	157	sp|Q8WVJ2|NUDC2_HUMAN	NUDCD2	Q8WVJ2	NudC domain-containing protein 2	NUDC2_HUMAN		NUDCD2
118	118	118	0.999	271	sp|Q92530|PSMF1_HUMAN	PSMF1	Q92530	Proteasome inhibitor PI31 subunit	PSMF1_HUMAN		PSMF1
219	219	219	0.999	642	sp|Q92544|TM9S4_HUMAN	TM9SF4	Q92544	Transmembrane 9 superfamily member 4	TM9S4_HUMAN		TM9SF4
99	99	99	0.999	795	sp|Q92609|TBCD5_HUMAN	TBC1D5	Q92609	TBC1 domain family member 5	TBCD5_HUMAN		TBC1D5
125	125	125	0.999	214	sp|Q92882|OSTF1_HUMAN	OSTF1	Q92882	Osteoclast-stimulating factor 1	OSTF1_HUMAN		OSTF1
247	245	247	0.999	228	sp|Q96AT9|RPE_HUMAN	RPE	Q96AT9	Ribulose-phosphate 3-epimerase	RPE_HUMAN		RPE
112	112	112	0.999	596	sp|Q96AX1|VP33A_HUMAN	VPS33A	Q96AX1	Vacuolar protein sorting-associated protein 33A	VP33A_HUMAN		VPS33A
178	178	178	0.999	503	sp|Q96D46|NMD3_HUMAN	NMD3	Q96D46	60S ribosomal export protein NMD3	NMD3_HUMAN		NMD3
256	256	256	0.999	380	sp|Q96G23|CERS2_HUMAN	CERS2	Q96G23	Ceramide synthase 2	CERS2_HUMAN		CERS2
132	132	132	0.999	340	sp|Q96IZ0|PAWR_HUMAN	PAWR	Q96IZ0	PRKC apoptosis WT1 regulator protein	PAWR_HUMAN		PAWR
44	28	44	0.999	720	sp|Q96JJ3|ELMO2_HUMAN	ELMO2	Q96JJ3	Engulfment and cell motility protein 2	ELMO2_HUMAN		ELMO2
84	84	84	0.999	1210	sp|Q96KQ7|EHMT2_HUMAN	EHMT2	Q96KQ7	Histone-lysine N-methyltransferase EHMT2	EHMT2_HUMAN		EHMT2
235	235	235	0.999	541	sp|Q96L92|SNX27_HUMAN	SNX27	Q96L92	Sorting nexin-27	SNX27_HUMAN		SNX27
260	260	260	0.999	445	sp|Q96M27|PRRC1_HUMAN	PRRC1	Q96M27	Protein PRRC1	PRRC1_HUMAN		PRRC1
163	163	163	0.999	472	sp|Q96N66|MBOA7_HUMAN	MBOAT7	Q96N66	Lysophospholipid acyltransferase 7	MBOA7_HUMAN		MBOAT7
55	36	73	0.999	429	sp|Q96P11|NSUN5_HUMAN	NSUN5	Q96P11	28S rRNA (cytosine-C(5))-methyltransferase	NSUN5_HUMAN		NSUN5
165	165	165	0.999	341	sp|Q96PU8|QKI_HUMAN	QKI	Q96PU8	KH domain-containing RNA-binding protein QKI	QKI_HUMAN		QKI
57	57	57	0.999	1125	sp|Q96QU8|XPO6_HUMAN	XPO6	Q96QU8	Exportin-6	XPO6_HUMAN		XPO6
102	102	102	0.999	1030	sp|Q96T76|MMS19_HUMAN	MMS19	Q96T76	MMS19 nucleotide excision repair protein homolog	MMS19_HUMAN		MMS19
57	57	57	0.999	211	sp|Q99470|SDF2_HUMAN	SDF2	Q99470	Stromal cell-derived factor 2	SDF2_HUMAN		SDF2
197	197	197	0.999	176	sp|Q9BQ61|TRIR_HUMAN	TRIR	Q9BQ61	Telomerase RNA component interacting RNase	TRIR_HUMAN		C19orf43
131	131	131	0.999	902	sp|Q9BSJ2|GCP2_HUMAN	TUBGCP2	Q9BSJ2	Gamma-tubulin complex component 2	GCP2_HUMAN		TUBGCP2
126	126	126	0.999	479	sp|Q9BTU6|P4K2A_HUMAN	PI4K2A	Q9BTU6	Phosphatidylinositol 4-kinase type 2-alpha	P4K2A_HUMAN		PI4K2A
118	118	118	0.999	242	sp|Q9BUP3|HTAI2_HUMAN	HTATIP2	Q9BUP3	Oxidoreductase HTATIP2	HTAI2_HUMAN		HTATIP2
66	66	66	0.999	179	sp|Q9BV57|MTND_HUMAN	ADI1	Q9BV57	Acireductone dioxygenase	MTND_HUMAN		ADI1
47	47	47	0.999	373	sp|Q9BV79|MECR_HUMAN	MECR	Q9BV79	Enoyl-[acyl-carrier-protein] reductase, mitochondrial	MECR_HUMAN		MECR
175	116	175	0.999	764	sp|Q9BXB5|OSB10_HUMAN	OSBPL10	Q9BXB5	Oxysterol-binding protein-related protein 10	OSB10_HUMAN		OSBPL10
141	141	141	0.999	267	sp|Q9BYD2|RM09_HUMAN	MRPL9	Q9BYD2	Large ribosomal subunit protein bL9m	RM09_HUMAN		MRPL9
22	22	22	0.999	464	sp|Q9BZD4|NUF2_HUMAN	NUF2	Q9BZD4	Kinetochore protein Nuf2	NUF2_HUMAN		NUF2
161	161	161	0.999	305	sp|Q9GZS3|SKI8_HUMAN	SKIC8	Q9GZS3	Superkiller complex protein 8	SKI8_HUMAN		WDR61
244	244	244	0.999	109	sp|Q9GZT3|SLIRP_HUMAN	SLIRP	Q9GZT3	SRA stem-loop-interacting RNA-binding protein, mitochondrial	SLIRP_HUMAN		SLIRP
218	218	218	0.999	169	sp|Q9GZZ1|NAA50_HUMAN	NAA50	Q9GZZ1	N-alpha-acetyltransferase 50	NAA50_HUMAN		NAA50
165	165	165	0.999	455	sp|Q9H0S4|DDX47_HUMAN	DDX47	Q9H0S4	Probable ATP-dependent RNA helicase DDX47	DDX47_HUMAN		DDX47
150	150	150	0.999	315	sp|Q9H0W9|CK054_HUMAN	C11orf54	Q9H0W9	Ester hydrolase C11orf54	CK054_HUMAN		C11orf54
193	193	193	0.999	170	sp|Q9H773|DCTP1_HUMAN	DCTPP1	Q9H773	dCTP pyrophosphatase 1	DCTP1_HUMAN		DCTPP1
104	104	104	0.999	394	sp|Q9H814|PHAX_HUMAN	PHAX	Q9H814	Phosphorylated adapter RNA export protein	PHAX_HUMAN		PHAX
59	59	59	0.999	726	sp|Q9H8Y5|ANKZ1_HUMAN	ANKZF1	Q9H8Y5	tRNA endonuclease ANKZF1	ANKZ1_HUMAN		ANKZF1
210	210	210	0.999	452	sp|Q9H8Y8|GORS2_HUMAN	GORASP2	Q9H8Y8	Golgi reassembly-stacking protein 2	GORS2_HUMAN		GORASP2
98	98	98	0.999	908	sp|Q9HCG8|CWC22_HUMAN	CWC22	Q9HCG8	Pre-mRNA-splicing factor CWC22 homolog	CWC22_HUMAN		CWC22
97	89	97	0.999	2581	sp|Q9HCK8|CHD8_HUMAN	CHD8	Q9HCK8	Chromodomain-helicase-DNA-binding protein 8	CHD8_HUMAN		CHD8
178	178	178	0.999	250	sp|Q9HD33|RM47_HUMAN	MRPL47	Q9HD33	Large ribosomal subunit protein uL29m	RM47_HUMAN		MRPL47
139	139	139	0.999	299	sp|Q9NQ48|LZTL1_HUMAN	LZTFL1	Q9NQ48	Leucine zipper transcription factor-like protein 1	LZTL1_HUMAN		LZTFL1
84	46	143	0.999	601	sp|Q9NRR5|UBQL4_HUMAN	UBQLN4	Q9NRR5	Ubiquilin-4	UBQL4_HUMAN		UBQLN4
119	119	119	0.999	189	sp|Q9NRV9|HEBP1_HUMAN	HEBP1	Q9NRV9	Heme-binding protein 1	HEBP1_HUMAN		HEBP1
22	22	22	0.999	1499	sp|Q9NRY4|RHG35_HUMAN	ARHGAP35	Q9NRY4	Rho GTPase-activating protein 35	RHG35_HUMAN		ARHGAP35
129	71	129	0.999	186	sp|Q9NVJ2|ARL8B_HUMAN	ARL8B	Q9NVJ2	ADP-ribosylation factor-like protein 8B	ARL8B_HUMAN		ARL8B
185	185	185	0.999	329	sp|Q9NWV8|BABA1_HUMAN	BABAM1	Q9NWV8	BRISC and BRCA1-A complex member 1	BABA1_HUMAN		BABAM1
51	51	51	0.999	375	sp|Q9NXW2|DJB12_HUMAN	DNAJB12	Q9NXW2	DnaJ homolog subfamily B member 12	DJB12_HUMAN		DNAJB12
130	73	140	0.999	540	sp|Q9NZI7|UBIP1_HUMAN	UBP1	Q9NZI7	Upstream-binding protein 1	UBIP1_HUMAN		UBP1
92	92	92	0.999	219	sp|Q9NZZ3|CHMP5_HUMAN	CHMP5	Q9NZZ3	Charged multivesicular body protein 5	CHMP5_HUMAN		CHMP5
165	165	165	0.999	144	sp|Q9P0J0|NDUAD_HUMAN	NDUFA13	Q9P0J0	NADH dehydrogenase [ubiquinone] 1 alpha subcomplex subunit 13	NDUAD_HUMAN		NDUFA13
147	99	147	0.999	348	sp|Q9P2T1|GMPR2_HUMAN	GMPR2	Q9P2T1	GMP reductase 2	GMPR2_HUMAN		GMPR2
51	51	51	0.999	362	sp|Q9UBU8|MO4L1_HUMAN	MORF4L1	Q9UBU8	Mortality factor 4-like protein 1	MO4L1_HUMAN		MORF4L1
122	122	122	0.999	794	sp|Q9UBV2|SE1L1_HUMAN	SEL1L	Q9UBV2	Protein sel-1 homolog 1	SE1L1_HUMAN		SEL1L
127	103	127	0.999	729	sp|Q9UHD2|TBK1_HUMAN	TBK1	Q9UHD2	Serine/threonine-protein kinase TBK1	TBK1_HUMAN		TBK1
173	173	173	0.999	460	sp|Q9UJW0|DCTN4_HUMAN	DCTN4	Q9UJW0	Dynactin subunit 4	DCTN4_HUMAN		DCTN4
335	335	335	0.999	219	sp|Q9UKK9|NUDT5_HUMAN	NUDT5	Q9UKK9	ADP-sugar pyrophosphatase	NUDT5_HUMAN		NUDT5
44	44	44	0.999	1129	sp|Q9ULH1|ASAP1_HUMAN	ASAP1	Q9ULH1	Arf-GAP with SH3 domain, ANK repeat and PH domain-containing protein	ASAP1_HUMAN		ASAP1
90	90	90	0.999	650	sp|Q9UNN5|FAF1_HUMAN	FAF1	Q9UNN5	FAS-associated factor 1	FAF1_HUMAN		FAF1
239	239	239	0.999	131	sp|Q9Y237|PIN4_HUMAN	PIN4	Q9Y237	Peptidyl-prolyl cis-trans isomerase NIMA-interacting 4	PIN4_HUMAN		PIN4
216	216	216	0.999	125	sp|Q9Y3B4|SF3B6_HUMAN	SF3B6	Q9Y3B4	Splicing factor 3B subunit 6	SF3B6_HUMAN		SF3B6
319	319	319	0.999	166	sp|Q9Y3C6|PPIL1_HUMAN	PPIL1	Q9Y3C6	Peptidyl-prolyl cis-trans isomerase-like 1	PPIL1_HUMAN		PPIL1
212	212	212	0.999	179	sp|Q9Y3E5|PTH2_HUMAN	PTRH2	Q9Y3E5	Peptidyl-tRNA hydrolase 2, mitochondrial	PTH2_HUMAN		PTRH2
152	103	152	0.999	981	sp|Q9Y4E8|UBP15_HUMAN	USP15	Q9Y4E8	Ubiquitin carboxyl-terminal hydrolase 15	UBP15_HUMAN		USP15
58	58	58	0.999	846	sp|Q9Y4K4|M4K5_HUMAN	MAP4K5	Q9Y4K4	Mitogen-activated protein kinase kinase kinase kinase 5	M4K5_HUMAN		MAP4K5
322	322	322	0.999	139	sp|Q9Y4Z0|LSM4_HUMAN	LSM 4.00	Q9Y4Z0	U6 snRNA-associated Sm-like protein LSm4	LSM4_HUMAN		LSM 4.00
275	275	275	0.999	174	sp|Q9Y5S9|RBM8A_HUMAN	RBM8A	Q9Y5S9	RNA-binding protein 8A	RBM8A_HUMAN		RBM8A
250	250	250	0.999	398	sp|Q9Y639|NPTN_HUMAN	NPTN	Q9Y639	Neuroplastin	NPTN_HUMAN		NPTN
153	98	153	0.999	443	sp|Q9Y6E0|STK24_HUMAN	STK24	Q9Y6E0	Serine/threonine-protein kinase 24	STK24_HUMAN		STK24
230	230	230	0.999	153	sp|O00422|SAP18_HUMAN	SAP18	O00422	Histone deacetylase complex subunit SAP18	SAP18_HUMAN		SAP18
230	230	230	0.999	305	sp|O00743|PPP6_HUMAN	PPP6C	O00743	Serine/threonine-protein phosphatase 6 catalytic subunit	PPP6_HUMAN		PPP6C
90	90	93	0.999	338	sp|O15126|SCAM1_HUMAN	SCAMP1	O15126	Secretory carrier-associated membrane protein 1	SCAM1_HUMAN		SCAMP1
211	211	211	0.999	223	sp|O15173|PGRC2_HUMAN	PGRMC2	O15173	Membrane-associated progesterone receptor component 2	PGRC2_HUMAN		PGRMC2
354	354	354	0.999	174	sp|O43324|MCA3_HUMAN	EEF1E1	O43324	Eukaryotic translation elongation factor 1 epsilon-1	MCA3_HUMAN		EEF1E1
301	301	301	0.999	174	sp|O43598|DNPH1_HUMAN	DNPH1	O43598	2'-deoxynucleoside 5'-phosphate N-hydrolase 1	DNPH1_HUMAN		DNPH1
86	86	86	0.999	754	sp|O60934|NBN_HUMAN	NBN	O60934	Nibrin	NBN_HUMAN		NBN
121	121	121	0.999	752	sp|O75027|ABCB7_HUMAN	ABCB7	O75027	Iron-sulfur clusters transporter ABCB7, mitochondrial	ABCB7_HUMAN		ABCB7
222	222	283	0.999	286	sp|O75323|NIPS2_HUMAN	NIPSNAP2	O75323	Protein NipSnap homolog 2	NIPS2_HUMAN		GBAS
209	209	209	0.999	258	sp|O75822|EIF3J_HUMAN	EIF3J	O75822	Eukaryotic translation initiation factor 3 subunit J	EIF3J_HUMAN		EIF3J
98	83	98	0.999	1147	sp|O95104|SCAF4_HUMAN	SCAF4	O95104	SR-related and CTD-associated factor 4	SCAF4_HUMAN		SCAF4
186	143	186	0.999	1032	sp|O95197|RTN3_HUMAN	RTN3	O95197	Reticulon-3	RTN3_HUMAN		RTN3
236	236	236	0.999	172	sp|O96000|NDUBA_HUMAN	NDUFB10	O96000	NADH dehydrogenase [ubiquinone] 1 beta subcomplex subunit 10	NDUBA_HUMAN		NDUFB10
159	159	159	0.999	669	sp|O96005|CLPT1_HUMAN	CLPTM1	O96005	Putative lipid scramblase CLPTM1	CLPT1_HUMAN		CLPTM1
319	319	319	0.999	227	sp|P00403|COX2_HUMAN	MT-CO2	P00403	Cytochrome c oxidase subunit 2	COX2_HUMAN		MT-CO2
153	153	153	0.999	363	sp|P00813|ADA_HUMAN	ADA	P00813	Adenosine deaminase	ADA_HUMAN		ADA
211	211	211	0.999	710	sp|P02788|TRFL_HUMAN	LTF	P02788	Lactotransferrin	TRFL_HUMAN		LTF
96	96	96	0.999	464	sp|P04424|ARLY_HUMAN	ASL	P04424	Argininosuccinate lyase	ARLY_HUMAN		ASL
604	568	604	0.999	90	sp|P05204|HMGN2_HUMAN	HMGN2	P05204	Non-histone chromosomal protein HMG-17	HMGN2_HUMAN		HMGN2
206	206	206	0.999	367	sp|P06132|DCUP_HUMAN	UROD	P06132	Uroporphyrinogen decarboxylase	DCUP_HUMAN		UROD
296	296	296	0.999	203	sp|P07203|GPX1_HUMAN	GPX1	P07203	Glutathione peroxidase 1	GPX1_HUMAN		GPX1
216	216	216	0.999	249	sp|P13984|T2FB_HUMAN	GTF2F2	P13984	General transcription factor IIF subunit 2	T2FB_HUMAN		GTF2F2
244	244	244	0.999	350	sp|P13995|MTDC_HUMAN	MTHFD2	P13995	Bifunctional methylenetetrahydrofolate dehydrogenase/cyclohydrolase, mit	MTDC_HUMAN		MTHFD2
366	366	366	0.999	115	sp|P14174|MIF_HUMAN	MIF	P14174	Macrophage migration inhibitory factor	MIF_HUMAN		MIF
118	118	118	0.999	433	sp|P14635|CCNB1_HUMAN	CCNB1	P14635	G2/mitotic-specific cyclin-B1	CCNB1_HUMAN		CCNB1
229	229	229	0.999	86	sp|P14854|CX6B1_HUMAN	COX6B1	P14854	Cytochrome c oxidase subunit 6B1	CX6B1_HUMAN		COX6B1
204	204	204	0.999	552	sp|P15586|GNS_HUMAN	GNS	P15586	N-acetylglucosamine-6-sulfatase	GNS_HUMAN		GNS
191	191	191	0.999	1290	sp|P19174|PLCG1_HUMAN	PLCG1	P19174	1-phosphatidylinositol 4,5-bisphosphate phosphodiesterase gamma-1	PLCG1_HUMAN		PLCG1
388	388	388	0.999	150	sp|P20674|COX5A_HUMAN	COX5A	P20674	Cytochrome c oxidase subunit 5A, mitochondrial	COX5A_HUMAN		COX5A
28	28	170	0.999	1482	sp|P20742|PZP_HUMAN	PZP	P20742	Pregnancy zone protein	PZP_HUMAN		PZP
165	165	165	0.999	750	sp|P22033|MUTA_HUMAN	MMUT	P22033	Methylmalonyl-CoA mutase, mitochondrial	MUTA_HUMAN		MUT
180	180	180	0.999	423	sp|P22830|HEMH_HUMAN	FECH	P22830	Ferrochelatase, mitochondrial	HEMH_HUMAN		FECH
94	94	94	0.999	703	sp|P23142|FBLN1_HUMAN	FBLN1	P23142	Fibulin-1	FBLN1_HUMAN		FBLN1
272	272	272	0.999	142	sp|P26885|FKBP2_HUMAN	FKBP2	P26885	Peptidyl-prolyl cis-trans isomerase FKBP2	FKBP2_HUMAN		FKBP2
55	5	55	0.999	533	sp|P28702|RXRB_HUMAN	RXRB	P28702	Retinoic acid receptor RXR-beta	RXRB_HUMAN		RXRB
38	38	125	0.999	481	sp|P31751|AKT2_HUMAN	AKT2	P31751	RAC-beta serine/threonine-protein kinase	AKT2_HUMAN		AKT2
112	112	112	0.999	423	sp|P34949|MPI_HUMAN	MPI	P34949	Mannose-6-phosphate isomerase	MPI_HUMAN		MPI
80	80	80	0.999	558	sp|P35052|GPC1_HUMAN	GPC1	P35052	Glypican-1	GPC1_HUMAN		GPC1
125	125	125	0.999	106	sp|P35754|GLRX1_HUMAN	GLRX	P35754	Glutaredoxin-1	GLRX1_HUMAN		GLRX
145	86	145	0.999	382	sp|P35813|PPM1A_HUMAN	PPM1A	P35813	Protein phosphatase 1A	PPM1A_HUMAN		PPM1A
94	94	154	0.999	477	sp|P40123|CAP2_HUMAN	CAP2	P40123	Adenylyl cyclase-associated protein 2	CAP2_HUMAN		CAP2
221	221	221	0.999	302	sp|P42126|ECI1_HUMAN	ECI1	P42126	Enoyl-CoA delta isomerase 1, mitochondrial	ECI1_HUMAN		ECI1
45	45	45	0.999	235	sp|P48307|TFPI2_HUMAN	TFPI2	P48307	Tissue factor pathway inhibitor 2	TFPI2_HUMAN		TFPI2
171	171	172	0.999	195	sp|P49006|MRP_HUMAN	MARCKSL1	P49006	MARCKS-related protein	MRP_HUMAN		MARCKSL1

79	53	79	0.999	400	sp|P49137|MAPK2_HUMAN	MAPKAPK2	P49137	MAP kinase-activated protein kinase 2	MAPK2_HUMAN		MAPKAPK2
34	34	34	0.999	3114	sp|P49454|CENPF_HUMAN	CENPF	P49454	Centromere protein F	CENPF_HUMAN		CENPF
73	73	73	0.999	509	sp|P49643|PRI2_HUMAN	PRIM2	P49643	DNA primase large subunit	PRI2_HUMAN		PRIM2
256	256	256	0.999	392	sp|P49903|SPS1_HUMAN	SEPHS1	P49903	Selenide, water dikinase 1	SPS1_HUMAN		SEPHS1
178	136	178	0.999	156	sp|P52298|NCBP2_HUMAN	NCBP2	P52298	Nuclear cap-binding protein subunit 2	NCBP2_HUMAN		NCBP2
74	74	74	0.999	373	sp|P53367|ARFP1_HUMAN	ARFIP1	P53367	Arfaptin-1	ARFP1_HUMAN		ARFIP1
335	335	335	0.999	320	sp|P53384|NUBP1_HUMAN	NUBP1	P53384	Cytosolic Fe-S cluster assembly factor NUBP1	NUBP1_HUMAN		NUBP1
163	163	163	0.999	128	sp|P55769|NH2L1_HUMAN	SNU13	P55769	NHP2-like protein 1	NH2L1_HUMAN		NHP2L1
219	219	219	0.999	180	sp|P61009|SPCS3_HUMAN	SPCS3	P61009	Signal peptidase complex subunit 3	SPCS3_HUMAN		SPCS3
259	259	259	0.999	200	sp|P61086|UBE2K_HUMAN	UBE2K	P61086	Ubiquitin-conjugating enzyme E2 K	UBE2K_HUMAN		UBE2K
470	0	470	0.999	181	sp|P61204|ARF3_HUMAN	ARF3	P61204	ADP-ribosylation factor 3	ARF3_HUMAN	sp|P84077|ARF1_HUMAN	ARF3
284	195	284	0.999	95	sp|P61956|SUMO2_HUMAN	SUMO2	P61956	Small ubiquitin-related modifier 2	SUMO2_HUMAN		SUMO2
496	496	510	0.999	44	sp|P62328|TYB4_HUMAN	TMSB4X	P62328	Thymosin beta-4	TYB4_HUMAN		TMSB4X
360	127	360	0.999	89	sp|P63167|DYL1_HUMAN	DYNLL1	P63167	Dynein light chain 1, cytoplasmic	DYL1_HUMAN		DYNLL1
161	161	297	0.999	191	sp|P84095|RHOG_HUMAN	RHOG	P84095	Rho-related GTP-binding protein RhoG	RHOG_HUMAN		RHOG
90	90	90	0.999	59	sp|P84101|SERF2_HUMAN	SERF2	P84101	Small EDRK-rich factor 2	SERF2_HUMAN		SERF2
304	256	304	0.999	733	sp|P85037|FOXK1_HUMAN	FOXK1	P85037	Forkhead box protein K1	FOXK1_HUMAN		FOXK1
224	224	224	0.999	656	sp|Q01844|EWS_HUMAN	EWSR1	Q01844	RNA-binding protein EWS	EWS_HUMAN		EWSR1
298	298	298	0.999	484	sp|Q05519|SRS11_HUMAN	SRSF11	Q05519	Serine/arginine-rich splicing factor 11	SRS11_HUMAN		SRSF11
54	54	59	0.999	676	sp|Q05655|KPCD_HUMAN	PRKCD	Q05655	Protein kinase C delta type	KPCD_HUMAN		PRKCD
95	95	95	0.999	439	sp|Q06265|EXOS9_HUMAN	EXOSC9	Q06265	Exosome complex component RRP45	EXOS9_HUMAN		EXOSC9
73	73	73	0.999	501	sp|Q12933|TRAF2_HUMAN	TRAF2	Q12933	TNF receptor-associated factor 2	TRAF2_HUMAN		TRAF2
106	106	106	0.999	381	sp|Q13601|KRR1_HUMAN	KRR1	Q13601	KRR1 small subunit processome component homolog	KRR1_HUMAN		KRR1
207	207	207	0.999	280	sp|Q13643|FHL3_HUMAN	FHL3	Q13643	Four and a half LIM domains protein 3	FHL3_HUMAN		FHL3
122	122	122	0.999	455	sp|Q13867|BLMH_HUMAN	BLMH	Q13867	Bleomycin hydrolase	BLMH_HUMAN		BLMH
143	143	143	0.999	803	sp|Q14155|ARHG7_HUMAN	ARHGEF7	Q14155	Rho guanine nucleotide exchange factor 7	ARHG7_HUMAN		ARHGEF7
119	63	119	0.999	759	sp|Q14161|GIT2_HUMAN	GIT2	Q14161	ARF GTPase-activating protein GIT2	GIT2_HUMAN		GIT2
102	102	102	0.999	738	sp|Q14746|COG2_HUMAN	COG2	Q14746	Conserved oligomeric Golgi complex subunit 2	COG2_HUMAN		COG2
119	119	119	0.999	1843	sp|Q14997|PSME4_HUMAN	PSME4	Q14997	Proteasome activator complex subunit 4	PSME4_HUMAN		PSME4
167	167	167	0.999	297	sp|Q15006|EMC2_HUMAN	EMC2	Q15006	ER membrane protein complex subunit 2	EMC2_HUMAN		EMC2
95	95	97	0.999	346	sp|Q15814|TBCC_HUMAN	TBCC	Q15814	Tubulin-specific chaperone C	TBCC_HUMAN		TBCC
159	159	159	0.999	407	sp|Q16186|ADRM1_HUMAN	ADRM1	Q16186	Proteasomal ubiquitin receptor ADRM1	ADRM1_HUMAN		ADRM1
114	114	114	0.999	1144	sp|Q16706|MA2A1_HUMAN	MAN2A1	Q16706	Alpha-mannosidase 2	MA2A1_HUMAN		MAN2A1
216	213	216	0.999	297	sp|Q16762|THTR_HUMAN	TST	Q16762	Thiosulfate sulfurtransferase	THTR_HUMAN		TST
181	181	181	0.999	308	sp|Q16775|GLO2_HUMAN	HAGH	Q16775	Hydroxyacylglutathione hydrolase, mitochondrial	GLO2_HUMAN		HAGH
158	158	158	0.999	310	sp|Q16831|UPP1_HUMAN	UPP1	Q16831	Uridine phosphorylase 1	UPP1_HUMAN		UPP1
100	100	100	0.999	288	sp|Q53H82|LACB2_HUMAN	LACTB2	Q53H82	Endoribonuclease LACTB2	LACB2_HUMAN		LACTB2
190	189	190	0.999	873	sp|Q5H9R7|PP6R3_HUMAN	PPP6R3	Q5H9R7	Serine/threonine-protein phosphatase 6 regulatory subunit 3	PP6R3_HUMAN		PPP6R3
211	211	211	0.999	272	sp|Q5RKV6|EXOS6_HUMAN	EXOSC6	Q5RKV6	Exosome complex component MTR3	EXOS6_HUMAN		EXOSC6
116	116	116	0.999	341	sp|Q5T6V5|QNG1_HUMAN	QNG1	Q5T6V5	Queuosine 5'-phosphate N-glycosylase/hydrolase	QNG1_HUMAN		C9orf64
136	136	136	0.999	455	sp|Q5TFE4|NT5D1_HUMAN	NT5DC1	Q5TFE4	5'-nucleotidase domain-containing protein 1	NT5D1_HUMAN		NT5DC1
172	172	172	0.999	237	sp|Q66PJ3|AR6P4_HUMAN	ARL6IP4	Q66PJ3	ADP-ribosylation factor-like protein 6-interacting protein 4	AR6P4_HUMAN		ARL6IP4
84	60	84	0.999	881	sp|Q68EM7|RHG17_HUMAN	ARHGAP17	Q68EM7	Rho GTPase-activating protein 17	RHG17_HUMAN		ARHGAP17
160	160	160	0.999	707	sp|Q6NZY4|ZCHC8_HUMAN	ZCCHC8	Q6NZY4	Zinc finger CCHC domain-containing protein 8	ZCHC8_HUMAN		ZCCHC8
75	75	202	0.999	122	sp|Q6P5R6|RL22L_HUMAN	RPL22L1	Q6P5R6	Ribosomal protein eL22-like	RL22L_HUMAN		RPL22L1
198	198	198	0.999	408	sp|Q6PIU2|NCEH1_HUMAN	NCEH1	Q6PIU2	Neutral cholesterol ester hydrolase 1	NCEH1_HUMAN		NCEH1
30	30	46	0.999	724	sp|Q71RC2|LARP4_HUMAN	LARP4	Q71RC2	La-related protein 4	LARP4_HUMAN		LARP4
67	67	67	0.999	244	sp|Q7Z4W1|DCXR_HUMAN	DCXR	Q7Z4W1	L-xylulose reductase	DCXR_HUMAN		DCXR
62	62	187	0.999	212	sp|Q86YS6|RAB43_HUMAN	RAB43	Q86YS6	Ras-related protein Rab-43	RAB43_HUMAN		RAB43
49	49	50	0.999	584	sp|Q8IU81|I2BP1_HUMAN	IRF2BP1	Q8IU81	Interferon regulatory factor 2-binding protein 1	I2BP1_HUMAN		IRF2BP1
79	79	79	0.999	490	sp|Q8IV08|PLD3_HUMAN	PLD3	Q8IV08	5'-3' exonuclease PLD3	PLD3_HUMAN		PLD3
55	55	58	0.999	876	sp|Q8IWC1|MA7D3_HUMAN	MAP7D3	Q8IWC1	MAP7 domain-containing protein 3	MA7D3_HUMAN		MAP7D3
13	13	13	0.9981	1762	sp|Q8N6G6|ATL1_HUMAN	ADAMTSL1	Q8N6G6	ADAMTS-like protein 1	ATL1_HUMAN		ADAMTSL1
102	102	102	0.999	699	sp|Q8N6R0|EFNMT_HUMAN	METTL13	Q8N6R0	eEF1A lysine and N-terminal methyltransferase	EFNMT_HUMAN		METTL13
110	110	110	0.999	726	sp|Q8NBF2|NHLC2_HUMAN	NHLRC2	Q8NBF2	NHL repeat-containing protein 2	NHLC2_HUMAN		NHLRC2
23	23	23	0.999	300	sp|Q8NBQ5|DHB11_HUMAN	HSD17B11	Q8NBQ5	Estradiol 17-beta-dehydrogenase 11	DHB11_HUMAN		HSD17B11
192	192	192	0.999	361	sp|Q8NBU5|ATAD1_HUMAN	ATAD1	Q8NBU5	Outer mitochondrial transmembrane helix translocase	ATAD1_HUMAN		ATAD1
121	121	121	0.999	326	sp|Q8NFH4|NUP37_HUMAN	NUP37	Q8NFH4	Nucleoporin Nup37	NUP37_HUMAN		NUP37
66	62	66	0.999	952	sp|Q8WUI4|HDAC7_HUMAN	HDAC7	Q8WUI4	Histone deacetylase 7	HDAC7_HUMAN		HDAC7
128	128	128	0.999	623	sp|Q8WZA9|IRGQ_HUMAN	IRGQ	Q8WZA9	Immunity-related GTPase family Q protein	IRGQ_HUMAN		IRGQ
108	108	108	0.999	710	sp|Q92541|RTF1_HUMAN	RTF1	Q92541	RNA polymerase-associated protein RTF1 homolog	RTF1_HUMAN		RTF1
240	240	240	0.999	334	sp|Q92905|CSN5_HUMAN	COPS5	Q92905	COP9 signalosome complex subunit 5	CSN5_HUMAN		COPS5
384	384	384	0.999	173	sp|Q969H8|MYDGF_HUMAN	MYDGF	Q969H8	Myeloid-derived growth factor	MYDGF_HUMAN		MYDGF
339	339	339	0.999	154	sp|Q96A26|F162A_HUMAN	FAM162A	Q96A26	Protein FAM162A	F162A_HUMAN		FAM162A
186	186	186	0.999	231	sp|Q96BR5|COA7_HUMAN	COA7	Q96BR5	Cytochrome c oxidase assembly factor 7	COA7_HUMAN		COA7
273	273	273	0.999	138	sp|Q96C01|F136A_HUMAN	FAM136A	Q96C01	Protein FAM136A	F136A_HUMAN		FAM136A
109	109	109	0.999	615	sp|Q96CM8|ACSF2_HUMAN	ACSF2	Q96CM8	Medium-chain acyl-CoA ligase ACSF2, mitochondrial	ACSF2_HUMAN		ACSF2
98	98	98	0.999	621	sp|Q96EK5|KBP_HUMAN	KIFBP	Q96EK5	KIF-binding protein	KBP_HUMAN		KIAA1279
69	69	69	0.999	508	sp|Q96F86|EDC3_HUMAN	EDC3	Q96F86	Enhancer of mRNA-decapping protein 3	EDC3_HUMAN		EDC3
81	81	81	0.999	329	sp|Q96GA7|SDSL_HUMAN	SDSL	Q96GA7	Serine dehydratase-like	SDSL_HUMAN		SDSL
78	78	78	0.999	812	sp|Q96JM3|CHAP1_HUMAN	CHAMP1	Q96JM3	Chromosome alignment-maintaining phosphoprotein 1	CHAP1_HUMAN		CHAMP1
203	203	203	0.999	570	sp|Q96JP5|ZFP91_HUMAN	ZFP91	Q96JP5	E3 ubiquitin-protein ligase ZFP91	ZFP91_HUMAN		ZFP91
260	260	260	0.999	277	sp|Q99436|PSB7_HUMAN	PSMB7	Q99436	Proteasome subunit beta type-7	PSB7_HUMAN		PSMB7
81	81	81	0.999	389	sp|Q99447|PCY2_HUMAN	PCYT2	Q99447	Ethanolamine-phosphate cytidylyltransferase	PCY2_HUMAN		PCYT2
338	338	338	0.999	154	sp|Q99471|PFD5_HUMAN	PFDN5	Q99471	Prefoldin subunit 5	PFD5_HUMAN		PFDN5
180	180	180	0.999	396	sp|Q99986|VRK1_HUMAN	VRK1	Q99986	Serine/threonine-protein kinase VRK1	VRK1_HUMAN		VRK1
114	114	114	0.999	476	sp|Q9BPX6|MICU1_HUMAN	MICU1	Q9BPX6	Calcium uptake protein 1, mitochondrial	MICU1_HUMAN		MICU1
140	140	140	0.999	306	sp|Q9BRJ2|RM45_HUMAN	MRPL45	Q9BRJ2	Large ribosomal subunit protein mL45	RM45_HUMAN		MRPL45
214	214	214	0.999	432	sp|Q9BV38|WDR18_HUMAN	WDR18	Q9BV38	WD repeat-containing protein 18	WDR18_HUMAN		WDR18
97	97	97	0.999	796	sp|Q9BWU0|NADAP_HUMAN	SLC4A1AP	Q9BWU0	Kanadaptin	NADAP_HUMAN		SLC4A1AP
64	64	64	0.999	441	sp|Q9BXS6|NUSAP_HUMAN	NUSAP1	Q9BXS6	Nucleolar and spindle-associated protein 1	NUSAP_HUMAN		NUSAP1
119	119	119	0.999	300	sp|Q9BXY0|MAK16_HUMAN	MAK16	Q9BXY0	Protein MAK16 homolog	MAK16_HUMAN		MAK16
85	85	85	0.999	505	sp|Q9C0B1|FTO_HUMAN	FTO	Q9C0B1	Alpha-ketoglutarate-dependent dioxygenase FTO	FTO_HUMAN		FTO
168	168	168	0.999	254	sp|Q9GZU8|PIP30_HUMAN	PSME3IP1	Q9GZU8	PSME3-interacting protein	PIP30_HUMAN		FAM192A
188	188	188	0.999	479	sp|Q9H074|PAIP1_HUMAN	PAIP1	Q9H074	Polyadenylate-binding protein-interacting protein 1	PAIP1_HUMAN		PAIP1
93	93	93	0.999	360	sp|Q9H6F5|CCD86_HUMAN	CCDC86	Q9H6F5	Coiled-coil domain-containing protein 86	CCD86_HUMAN		CCDC86
88	88	88	0.999	665	sp|Q9H6T3|RPAP3_HUMAN	RPAP3	Q9H6T3	RNA polymerase II-associated protein 3	RPAP3_HUMAN		RPAP3
143	143	143	0.999	229	sp|Q9H7E9|CH033_HUMAN	C8orf33	Q9H7E9	UPF0488 protein C8orf33	CH033_HUMAN		C8orf33
49	49	49	0.999	602	sp|Q9H9A6|LRC40_HUMAN	LRRC40	Q9H9A6	Leucine-rich repeat-containing protein 40	LRC40_HUMAN		LRRC40
103	103	103	0.999	547	sp|Q9H9T3|ELP3_HUMAN	ELP3	Q9H9T3	Elongator complex protein 3	ELP3_HUMAN		ELP3
87	87	87	0.999	206	sp|Q9NP72|RAB18_HUMAN	RAB18	Q9NP72	Ras-related protein Rab-18	RAB18_HUMAN		RAB18
231	231	231	0.999	270	sp|Q9NQ88|TIGAR_HUMAN	TIGAR	Q9NQ88	Fructose-2,6-bisphosphatase TIGAR	TIGAR_HUMAN		TIGAR
203	135	203	0.999	198	sp|Q9NR31|SAR1A_HUMAN	SAR1A	Q9NR31	GTP-binding protein SAR1a	SAR1A_HUMAN		SAR1A
120	114	120	0.999	252	sp|Q9NRX1|PNO1_HUMAN	PNO1	Q9NRX1	RNA-binding protein PNO1	PNO1_HUMAN		PNO1
142	142	142	0.999	314	sp|Q9NT62|ATG3_HUMAN	ATG3	Q9NT62	Ubiquitin-like-conjugating enzyme ATG3	ATG3_HUMAN		ATG3
124	124	124	0.999	400	sp|Q9NVG8|TBC13_HUMAN	TBC1D13	Q9NVG8	TBC1 domain family member 13	TBC13_HUMAN		TBC1D13
222	222	222	0.999	160	sp|Q9NWV4|CZIB_HUMAN	CZIB	Q9NWV4	CXXC motif containing zinc binding protein	CZIB_HUMAN		C1orf123
135	135	135	0.999	866	sp|Q9NXE4|NSMA3_HUMAN	SMPD4	Q9NXE4	Sphingomyelin phosphodiesterase 4	NSMA3_HUMAN		SMPD4
91	91	151	0.999	513	sp|Q9NYB9|ABI2_HUMAN	ABI2	Q9NYB9	Abl interactor 2	ABI2_HUMAN		ABI2
125	125	125	0.999	108	sp|Q9NZ45|CISD1_HUMAN	CISD1	Q9NZ45	CDGSH iron-sulfur domain-containing protein 1	CISD1_HUMAN		CISD1
171	171	171	0.999	389	sp|Q9NZJ7|MTCH1_HUMAN	MTCH1	Q9NZJ7	Mitochondrial carrier homolog 1	MTCH1_HUMAN		MTCH1
81	81	81	0.999	296	sp|Q9P015|RM15_HUMAN	MRPL15	Q9P015	Large ribosomal subunit protein uL15m	RM15_HUMAN		MRPL15
168	168	168	0.999	782	sp|Q9P2I0|CPSF2_HUMAN	CPSF2	Q9P2I0	Cleavage and polyadenylation specificity factor subunit 2	CPSF2_HUMAN		CPSF2
143	143	143	0.999	864	sp|Q9UBC2|EP15R_HUMAN	EPS15L1	Q9UBC2	Epidermal growth factor receptor substrate 15-like 1	EP15R_HUMAN		EPS15L1
421	421	421	0.999	72	sp|Q9UBI6|GBG12_HUMAN	GNG12	Q9UBI6	Guanine nucleotide-binding protein G(I)/G(S)/G(O) subunit gamma-12	GBG12_HUMAN		GNG12
56	56	56	0.999	706	sp|Q9UEY8|ADDG_HUMAN	ADD3	Q9UEY8	Gamma-adducin	ADDG_HUMAN		ADD3
86	86	86	0.999	241	sp|Q9UHQ4|BAP29_HUMAN	BCAP29	Q9UHQ4	B-cell receptor-associated protein 29	BAP29_HUMAN		BCAP29
101	101	101	0.999	578	sp|Q9UJ83|HACL1_HUMAN	HACL1	Q9UJ83	2-hydroxyacyl-CoA lyase 1	HACL1_HUMAN		HACL1
112	112	112	0.999	597	sp|Q9UJX2|CDC23_HUMAN	CDC23	Q9UJX2	Cell division cycle protein 23 homolog	CDC23_HUMAN		CDC23
289	289	289	0.999	239	sp|Q9UM00|TMCO1_HUMAN	TMCO1	Q9UM00	Calcium load-activated calcium channel	TMCO1_HUMAN		TMCO1
23	23	23	0.9989	1083	sp|Q9UPN4|CP131_HUMAN	CEP131	Q9UPN4	Centrosomal protein of 131 kDa	CP131_HUMAN		CEP131
241	182	241	0.999	418	sp|Q9Y2T2|AP3M1_HUMAN	AP3M1	Q9Y2T2	AP-3 complex subunit mu-1	AP3M1_HUMAN		AP3M1
231	231	231	0.999	301	sp|Q9Y314|NOSIP_HUMAN	NOSIP	Q9Y314	Nitric oxide synthase-interacting protein	NOSIP_HUMAN		NOSIP
155	155	155	0.999	224	sp|Q9Y3B3|TMED7_HUMAN	TMED7	Q9Y3B3	Transmembrane emp24 domain-containing protein 7	TMED7_HUMAN		TMED7
163	163	163	0.999	192	sp|Q9Y3B7|RM11_HUMAN	MRPL11	Q9Y3B7	Large ribosomal subunit protein uL11m	RM11_HUMAN		MRPL11
98	98	98	0.999	626	sp|Q9Y3Z3|SAMH1_HUMAN	SAMHD1	Q9Y3Z3	Deoxynucleoside triphosphate triphosphohydrolase SAMHD1	SAMH1_HUMAN		SAMHD1
127	127	127	0.999	1045	sp|Q9Y4F1|FARP1_HUMAN	FARP1	Q9Y4F1	FERM, ARHGEF and pleckstrin domain-containing protein 1	FARP1_HUMAN		FARP1
132	132	132	0.999	247	sp|Q9Y5K8|VATD_HUMAN	ATP6V1D	Q9Y5K8	V-type proton ATPase subunit D	VATD_HUMAN		ATP6V1D
164	164	164	0.999	324	sp|Q9Y673|ALG5_HUMAN	ALG5	Q9Y673	Dolichyl-phosphate beta-glucosyltransferase	ALG5_HUMAN		ALG5
198	198	198	0.999	419	sp|Q9Y6K9|NEMO_HUMAN	IKBKG	Q9Y6K9	NF-kappa-B essential modulator	NEMO_HUMAN		IKBKG
136	136	136	0.999	210	sp|O00217|NDUS8_HUMAN	NDUFS8	O00217	NADH dehydrogenase [ubiquinone] iron-sulfur protein 8, mitochondrial	NDUS8_HUMAN		NDUFS8
333	333	333	0.999	223	sp|O00233|PSMD9_HUMAN	PSMD9	O00233	26S proteasome non-ATPase regulatory subunit 9	PSMD9_HUMAN		PSMD9
133	133	133	0.999	1011	sp|O00754|MA2B1_HUMAN	MAN2B1	O00754	Lysosomal alpha-mannosidase	MA2B1_HUMAN		MAN2B1
119	119	119	0.999	1046	sp|O15294|OGT1_HUMAN	OGT	O15294	UDP-N-acetylglucosamine--peptide N-acetylglucosaminyltransferase 110 I	OGT1_HUMAN		OGT
205	205	205	0.999	392	sp|O15382|BCAT2_HUMAN	BCAT2	O15382	Branched-chain-amino-acid aminotransferase, mitochondrial	BCAT2_HUMAN		BCAT2
298	298	298	0.999	522	sp|O43172|PRP4_HUMAN	PRPF4	O43172	U4/U6 small nuclear ribonucleoprotein Prp4	PRP4_HUMAN		PRPF4
128	128	128	0.999	177	sp|O43447|PPIH_HUMAN	PPIH	O43447	Peptidyl-prolyl cis-trans isomerase H	PPIH_HUMAN		PPIH
35	35	35	0.999	620	sp|O43663|PRC1_HUMAN	PRC1	O43663	Protein regulator of cytokinesis 1	PRC1_HUMAN		PRC1
101	101	101	0.999	492	sp|O60784|TOM1_HUMAN	TOM1	O60784	Target of Myb1 membrane trafficking protein	TOM1_HUMAN		TOM1

124	124	124	0.999	753	sp|O75175|CNOT3_HUMAN	CNOT3	O75175	CCR4-NOT transcription complex subunit 3	CNOT3_HUMAN		CNOT3
287	287	287	0.999	191	sp|O75340|PDCD6_HUMAN	PDCD6	O75340	Programmed cell death protein 6	PDCD6_HUMAN		PDCD6
102	102	103	0.999	805	sp|O75410|TACC1_HUMAN	TACC1	O75410	Transforming acidic coiled-coil-containing protein 1	TACC1_HUMAN		TACC1
159	159	159	0.999	263	sp|O75431|MTX2_HUMAN	MTX2	O75431	Metaxin-2	MTX2_HUMAN		MTX2
205	205	205	0.999	188	sp|O75915|PRAF3_HUMAN	ARL6IP5	O75915	PRA1 family protein 3	PRAF3_HUMAN		ARL6IP5
102	102	127	0.999	285	sp|O94760|DDAH1_HUMAN	DDAH1	O94760	N(G),N(G)-dimethylarginine dimethylaminohydrolase 1	DDAH1_HUMAN		DDAH1
102	102	199	0.999	682	sp|O94808|GFPT2_HUMAN	GFPT2	O94808	Glutamine--fructose-6-phosphate aminotransferase [isomerizing] 2	GFPT2_HUMAN		GFPT2
131	131	131	0.999	1766	sp|O94822|LTN1_HUMAN	LTN1	O94822	E3 ubiquitin-protein ligase listerin	LTN1_HUMAN		LTN1
84	84	84	0.999	757	sp|O95140|MFN2_HUMAN	MFN2	O95140	Mitofusin-2	MFN2_HUMAN		MFN2
149	149	149	0.999	1302	sp|O95155|UBE4B_HUMAN	UBE4B	O95155	Ubiquitin conjugation factor E4 B	UBE4B_HUMAN		UBE4B
96	96	96	0.999	703	sp|O95352|ATG7_HUMAN	ATG7	O95352	Ubiquitin-like modifier-activating enzyme ATG7	ATG7_HUMAN		ATG7
165	165	165	0.999	621	sp|O95671|ASML_HUMAN	ASMTL	O95671	Probable bifunctional dTTP/UTP pyrophosphatase/methyltransferase prote	ASML_HUMAN		ASMTL
46	10	173	0.999	219	sp|O95716|RAB3D_HUMAN	RAB3D	O95716	Ras-related protein Rab-3D	RAB3D_HUMAN		RAB3D
182	161	182	0.999	527	sp|O95747|OXSR1_HUMAN	OXSR1	O95747	Serine/threonine-protein kinase OSR1	OXSR1_HUMAN		OXSR1
219	219	219	0.999	361	sp|O96008|TOM40_HUMAN	TOMM40	O96008	Mitochondrial import receptor subunit TOM40 homolog	TOM40_HUMAN		TOMM40
57	57	57	0.999	317	sp|P02649|APOE_HUMAN	APOE	P02649	Apolipoprotein E	APOE_HUMAN		APOE
109	109	109	0.999	609	sp|P02771|FETA_HUMAN	AFP	P02771	Alpha-fetoprotein	FETA_HUMAN		AFP
206	206	206	0.999	222	sp|P04179|SODM_HUMAN	SOD2	P04179	Superoxide dismutase [Mn], mitochondrial	SODM_HUMAN		SOD2
51	51	68	0.999	770	sp|P05067|A4_HUMAN	APP	P05067	Amyloid-beta precursor protein	A4_HUMAN		APP
164	102	302	0.999	543	sp|P07947|YES_HUMAN	YES1	P07947	Tyrosine-protein kinase Yes	YES_HUMAN		YES1
252	252	252	0.999	348	sp|P09001|RM03_HUMAN	MRPL3	P09001	Large ribosomal subunit protein uL3m	RM03_HUMAN		MRPL3
196	0	435	0.999	128	sp|P0C0S5|H2AZ_HUMAN	H2AZ1	P0C0S5	Histone H2A.Z	H2AZ_HUMAN	sp|Q71UI9|H2AV_HUMAN	H2AFZ
242	242	242	0.999	155	sp|P10620|MGST1_HUMAN	MGST1	P10620	Microsomal glutathione S-transferase 1	MGST1_HUMAN		MGST1
84	84	245	0.999	206	sp|P11234|RALB_HUMAN	RALB	P11234	Ras-related protein Ral-B	RALB_HUMAN		RALB
158	158	296	0.999	303	sp|P11802|CDK4_HUMAN	CDK4	P11802	Cyclin-dependent kinase 4	CDK4_HUMAN		CDK4
158	158	158	0.999	493	sp|P12955|PEPD_HUMAN	PEPD	P12955	Xaa-Pro dipeptidase	PEPD_HUMAN		PEPD
19	11	19	0.999	986	sp|P13497|BMP1_HUMAN	BMP1	P13497	Bone morphogenetic protein 1	BMP1_HUMAN		BMP1
95	55	95	0.999	377	sp|P16333|NCK1_HUMAN	NCK1	P16333	Cytoplasmic protein NCK1	NCK1_HUMAN		NCK1
137	137	322	0.999	180	sp|P18085|ARF4_HUMAN	ARF4	P18085	ADP-ribosylation factor 4	ARF4_HUMAN		ARF4
140	140	140	0.999	633	sp|P18887|XRCC1_HUMAN	XRCC1	P18887	DNA repair protein XRCC1	XRCC1_HUMAN		XRCC1
62	62	64	0.999	911	sp|P19827|ITIH1_HUMAN	ITIH1	P19827	Inter-alpha-trypsin inhibitor heavy chain H1	ITIH1_HUMAN		ITIH1
22	22	22	0.999	344	sp|P19883|FST_HUMAN	FST	P19883	Follistatin	FST_HUMAN		FST
405	405	405	0.999	206	sp|P20290|BTF3_HUMAN	BTF3	P20290	Transcription factor BTF3	BTF3_HUMAN		BTF3
301	301	301	0.999	271	sp|P21964|COMT_HUMAN	COMT	P21964	Catechol O-methyltransferase	COMT_HUMAN		COMT
56	54	56	0.999	906	sp|P22681|CBL_HUMAN	CBL	P22681	E3 ubiquitin-protein ligase CBL	CBL_HUMAN		CBL
18	18	18	0.999	272	sp|P24593|IBP5_HUMAN	IGFBP5	P24593	Insulin-like growth factor-binding protein 5	IBP5_HUMAN		IGFBP5
66	66	369	0.999	172	sp|P24844|MYL9_HUMAN	MYL9	P24844	Myosin regulatory light polypeptide 9	MYL9_HUMAN		MYL9
434	434	434	0.999	263	sp|P28074|PSB5_HUMAN	PSMB5	P28074	Proteasome subunit beta type-5	PSB5_HUMAN		PSMB5
293	292	293	0.999	332	sp|P29966|MARCS_HUMAN	MARCKS	P29966	Myristoylated alanine-rich C-kinase substrate	MARCS_HUMAN		MARCKS
66	55	151	0.999	359	sp|P29992|GNA11_HUMAN	GNA11	P29992	Guanine nucleotide-binding protein subunit alpha-11	GNA11_HUMAN		GNA11
224	224	224	0.999	178	sp|P32321|DCTD_HUMAN	DCTD	P32321	Deoxycytidylate deaminase	DCTD_HUMAN		DCTD
182	182	183	0.999	483	sp|P34896|GLYC_HUMAN	SHMT1	P34896	Serine hydroxymethyltransferase, cytosolic	GLYC_HUMAN		SHMT1
58	58	58	0.999	604	sp|P35354|PGH2_HUMAN	PTGS2	P35354	Prostaglandin G/H synthase 2	PGH2_HUMAN		PTGS2
98	97	98	0.999	737	sp|P35611|ADDA_HUMAN	ADD1	P35611	Alpha-adducin	ADDA_HUMAN		ADD1
20	20	20	0.999	211	sp|P35625|TIMP3_HUMAN	TIMP3	P35625	Metalloproteinase inhibitor 3	TIMP3_HUMAN		TIMP3
178	114	178	0.999	226	sp|P36543|VATE1_HUMAN	ATP6V1E1	P36543	V-type proton ATPase subunit E 1	VATE1_HUMAN		ATP6V1E1
303	121	303	0.999	84	sp|P42677|RS27_HUMAN	RPS27	P42677	Small ribosomal subunit protein eS27	RS27_HUMAN		RPS27
95	95	95	0.999	3142	sp|P42858|HD_HUMAN	HTT	P42858	Huntingtin	HD_HUMAN		HTT
146	80	146	0.999	347	sp|P46734|MP2K3_HUMAN	MAP2K3	P46734	Dual specificity mitogen-activated protein kinase kinase 3	MP2K3_HUMAN		MAP2K3
304	304	304	0.999	350	sp|P48556|PSMD8_HUMAN	PSMD8	P48556	26S proteasome non-ATPase regulatory subunit 8	PSMD8_HUMAN		PSMD8
70	70	70	0.999	469	sp|P49005|DPOD2_HUMAN	POLD2	P49005	DNA polymerase delta subunit 2	DPOD2_HUMAN		POLD2
187	123	187	0.999	727	sp|P49711|CTCF_HUMAN	CTCF	P49711	Transcriptional repressor CTCF	CTCF_HUMAN		CTCF
104	72	104	0.999	651	sp|P49757|NUMB_HUMAN	NUMB	P49757	Protein numb homolog	NUMB_HUMAN		NUMB
311	311	311	0.999	254	sp|P50402|EMD_HUMAN	EMD	P50402	Emerin	EMD_HUMAN		EMD
347	347	347	0.999	173	sp|P51571|SSRD_HUMAN	SSR4	P51571	Translocon-associated protein subunit delta	SSRD_HUMAN		SSR4
197	197	197	0.999	172	sp|P51970|NDUA8_HUMAN	NDUFA8	P51970	NADH dehydrogenase [ubiquinone] 1 alpha subcomplex subunit 8	NDUA8_HUMAN		NDUFA8
257	257	257	0.999	268	sp|P53701|CCHL_HUMAN	HCCS	P53701	Holocytochrome c-type synthase	CCHL_HUMAN		HCCS
114	114	114	0.999	925	sp|P57737|CORO7_HUMAN	CORO7	P57737	Coronin-7	CORO7_HUMAN		CORO7
556	293	556	0.999	152	sp|P61088|UBE2N_HUMAN	UBE2N	P61088	Ubiquitin-conjugating enzyme E2 N	UBE2N_HUMAN		UBE2N
190	190	190	0.999	175	sp|P62330|ARF6_HUMAN	ARF6	P62330	ADP-ribosylation factor 6	ARF6_HUMAN		ARF6
644	630	644	0.999	44	sp|P63313|TYB10_HUMAN	TMSB10	P63313	Thymosin beta-10	TYB10_HUMAN		TMSB10
144	144	144	0.999	268	sp|P78346|RPP30_HUMAN	RPP30	P78346	Ribonuclease P protein subunit p30	RPP30_HUMAN		RPP30
503	503	503	0.999	104	sp|P84090|ERH_HUMAN	ERH	P84090	Enhancer of rudimentary homolog	ERH_HUMAN		ERH
101	84	101	0.999	763	sp|Q06481|APLP2_HUMAN	APLP2	Q06481	Amyloid beta precursor like protein 2	APLP2_HUMAN		APLP2
226	226	226	0.999	501	sp|Q13610|PWP1_HUMAN	PWP1	Q13610	Periodic tryptophan protein 1 homolog	PWP1_HUMAN		PWP1
169	130	169	0.999	227	sp|Q13907|IDI1_HUMAN	IDI1	Q13907	Isopentenyl-diphosphate Delta-isomerase 1	IDI1_HUMAN		IDI1
153	68	170	0.999	377	sp|Q14344|GNA13_HUMAN	GNA13	Q14344	Guanine nucleotide-binding protein subunit alpha-13	GNA13_HUMAN		GNA13
215	215	319	0.999	356	sp|Q14558|KPRA_HUMAN	PRPSAP1	Q14558	Phosphoribosyl pyrophosphate synthase-associated protein 1	KPRA_HUMAN		PRPSAP1
152	152	152	0.999	291	sp|Q15024|EXOS7_HUMAN	EXOSC7	Q15024	Exosome complex component RRP42	EXOS7_HUMAN		EXOSC7
194	194	194	0.999	130	sp|Q15121|PEA15_HUMAN	PEA15	Q15121	Astrocytic phosphoprotein PEA-15	PEA15_HUMAN		PEA15
205	205	205	0.999	358	sp|Q15257|PTPA_HUMAN	PTPA	Q15257	Serine/threonine-protein phosphatase 2A activator	PTPA_HUMAN		PPP2R4
112	111	112	0.999	1439	sp|Q15262|PTPRK_HUMAN	PTPRK	Q15262	Receptor-type tyrosine-protein phosphatase kappa	PTPRK_HUMAN		PTPRK
299	299	299	0.999	112	sp|Q15369|ELOC_HUMAN	ELOC	Q15369	Elongin-C	ELOC_HUMAN		TCEB1
361	361	361	0.999	118	sp|Q15370|ELOB_HUMAN	ELOB	Q15370	Elongin-B	ELOB_HUMAN		TCEB2
126	126	126	0.999	360	sp|Q15435|PP1R7_HUMAN	PPP1R7	Q15435	Protein phosphatase 1 regulatory subunit 7	PP1R7_HUMAN		PPP1R7
414	414	414	0.999	541	sp|Q15758|AAAT_HUMAN	SLC1A5	Q15758	Neutral amino acid transporter B(0)	AAAT_HUMAN		SLC1A5
12	12	12	0.999	746	sp|Q16394|EXT1_HUMAN	EXT1	Q16394	Exostosin-1	EXT1_HUMAN		EXT1
70	70	117	0.999	507	sp|Q3KQV9|UAP1L_HUMAN	UAP1L1	Q3KQV9	UDP-N-acetylhexosamine pyrophosphorylase-like protein 1	UAP1L_HUMAN		UAP1L1
127	127	127	0.999	910	sp|Q4KMQ2|ANO6_HUMAN	ANO6	Q4KMQ2	Anoctamin-6	ANO6_HUMAN		ANO6
176	176	176	0.999	411	sp|Q5VW32|BROX_HUMAN	BROX	Q5VW32	BRO1 domain-containing protein BROX	BROX_HUMAN		BROX
135	135	135	0.999	756	sp|Q68CQ4|UTP25_HUMAN	UTP25	Q68CQ4	U3 small nucleolar RNA-associated protein 25 homolog	UTP25_HUMAN		DIEXF
102	102	102	0.999	843	sp|Q6P3X3|TTC27_HUMAN	TTC27	Q6P3X3	Tetratricopeptide repeat protein 27	TTC27_HUMAN		TTC27
117	117	117	0.999	456	sp|Q6P9B6|MEAK7_HUMAN	MEAK7	Q6P9B6	MTOR-associated protein MEAK7	MEAK7_HUMAN		TLDC1
219	219	219	0.999	594	sp|Q6UN15|FIP1_HUMAN	FIP1L1	Q6UN15	Pre-mRNA 3'-end-processing factor FIP1	FIP1_HUMAN		FIP1L1
164	164	164	0.999	313	sp|Q6UXN9|WDR82_HUMAN	WDR82	Q6UXN9	WD repeat-containing protein 82	WDR82_HUMAN		WDR82
85	85	85	0.999	911	sp|Q6ZNB6|NFXL1_HUMAN	NFXL1	Q6ZNB6	NF-X1-type zinc finger protein NFXL1	NFXL1_HUMAN		NFXL1
239	239	239	0.999	327	sp|Q7L5N1|CSN6_HUMAN	COPS6	Q7L5N1	COP9 signalosome complex subunit 6	CSN6_HUMAN		COPS6
93	12	93	0.999	585	sp|Q7Z739|YTHD3_HUMAN	YTHDF3	Q7Z739	YTH domain-containing family protein 3	YTHD3_HUMAN		YTHDF3
37	37	37	0.999	1253	sp|Q86SQ0|PHLB2_HUMAN	PHLDB2	Q86SQ0	Pleckstrin homology-like domain family B member 2	PHLB2_HUMAN		PHLDB2
116	116	116	0.999	545	sp|Q86XZ4|SPAS2_HUMAN	SPATS2	Q86XZ4	Spermatogenesis-associated serine-rich protein 2	SPAS2_HUMAN		SPATS2
55	55	55	0.999	706	sp|Q8IWA5|CTL2_HUMAN	SLC44A2	Q8IWA5	Choline transporter-like protein 2	CTL2_HUMAN		SLC44A2
90	90	90	0.999	666	sp|Q8N0X7|SPART_HUMAN	SPART	Q8N0X7	Spartin	SPART_HUMAN		SPG20
101	101	101	0.999	531	sp|Q8N7H5|PAF1_HUMAN	PAF1	Q8N7H5	RNA polymerase II-associated factor 1 homolog	PAF1_HUMAN		PAF1
63	63	63	0.999	401	sp|Q8NBJ4|GOLM1_HUMAN	GOLM1	Q8NBJ4	Golgi membrane protein 1	GOLM1_HUMAN		GOLM1
85	35	85	0.999	3051	sp|Q8NFC6|BD1L1_HUMAN	BOD1L1	Q8NFC6	Biorientation of chromosomes in cell division protein 1-like 1	BD1L1_HUMAN		BOD1L1
112	112	112	0.999	434	sp|Q8NFW8|NEUA_HUMAN	CMAS	Q8NFW8	N-acylneuraminate cytidylyltransferase	NEUA_HUMAN		CMAS
35	35	35	0.999	731	sp|Q8TBA6|GOGA5_HUMAN	GOLGA5	Q8TBA6	Golgin subfamily A member 5	GOGA5_HUMAN		GOLGA5
198	198	198	0.999	208	sp|Q8WUY1|THEM6_HUMAN	THEM6	Q8WUY1	Protein THEM6	THEM6_HUMAN		THEM6
177	177	177	0.999	227	sp|Q92520|FAM3C_HUMAN	FAM3C	Q92520	Protein FAM3C	FAM3C_HUMAN		FAM3C
117	117	117	0.999	508	sp|Q92575|UBXN4_HUMAN	UBXN4	Q92575	UBX domain-containing protein 4	UBXN4_HUMAN		UBXN4
78	78	78	0.999	1274	sp|Q92797|SYMPK_HUMAN	SYMPK	Q92797	Symplekin	SYMPK_HUMAN		SYMPK
134	134	134	0.999	780	sp|Q93034|CUL5_HUMAN	CUL5	Q93034	Cullin-5	CUL5_HUMAN		CUL5
83	83	83	0.999	837	sp|Q93050|VPP1_HUMAN	ATP6V0A1	Q93050	V-type proton ATPase 116 kDa subunit a 1	VPP1_HUMAN		ATP6V0A1
163	163	163	0.999	477	sp|Q969S3|ZN622_HUMAN	ZNF622	Q969S3	Cytoplasmic 60S subunit biogenesis factor ZNF622	ZN622_HUMAN		ZNF622
100	100	100	0.999	352	sp|Q96BN8|OTUL_HUMAN	OTULIN	Q96BN8	Ubiquitin thioesterase otulin	OTUL_HUMAN		OTULIN
175	175	175	0.999	140	sp|Q96CP2|FWCH2_HUMAN	FLYWCH2	Q96CP2	FLYWCH family member 2	FWCH2_HUMAN		FLYWCH2
129	95	129	0.999	314	sp|Q96DB5|RMD1_HUMAN	RMDN1	Q96DB5	Regulator of microtubule dynamics protein 1	RMD1_HUMAN		RMDN1
259	259	259	0.999	407	sp|Q96EP5|DAZP1_HUMAN	DAZAP1	Q96EP5	DAZ-associated protein 1	DAZP1_HUMAN		DAZAP1
74	58	74	0.999	3661	sp|Q96Q15|SMG1_HUMAN	SMG1	Q96Q15	Serine/threonine-protein kinase SMG1	SMG1_HUMAN		SMG1
152	152	152	0.999	819	sp|Q96ST2|IWS1_HUMAN	IWS1	Q96ST2	Protein IWS1 homolog	IWS1_HUMAN		IWS1
257	257	257	0.999	98	sp|Q99584|S10AD_HUMAN	S100A13	Q99584	Protein S100-A13	S10AD_HUMAN		S100A13
198	198	198	0.999	1463	sp|Q99590|SCAFB_HUMAN	SCAF11	Q99590	Protein SCAF11	SCAFB_HUMAN		SCAF11
173	173	173	0.999	337	sp|Q9BQE5|APOL2_HUMAN	APOL2	Q9BQE5	Apolipoprotein L2	APOL2_HUMAN		APOL2
220	220	220	0.999	497	sp|Q9BRR6|ADPGK_HUMAN	ADPGK	Q9BRR6	ADP-dependent glucokinase	ADPGK_HUMAN		ADPGK
274	274	274	0.999	268	sp|Q9BTT0|AN32E_HUMAN	ANP32E	Q9BTT0	Acidic leucine-rich nuclear phosphoprotein 32 family member E	AN32E_HUMAN		ANP32E
75	75	75	0.999	390	sp|Q9BTY7|HGH1_HUMAN	HGH1	Q9BTY7	Protein HGH1 homolog	HGH1_HUMAN		HGH1
108	108	108	0.999	653	sp|Q9BXF6|RFIP5_HUMAN	RAB11FIP5	Q9BXF6	Rab11 family-interacting protein 5	RFIP5_HUMAN		RAB11FIP5
95	95	95	0.999	403	sp|Q9BXR0|TGT_HUMAN	QTRT1	Q9BXR0	Queuine tRNA-ribosyltransferase catalytic subunit 1	TGT_HUMAN		QTRT1
108	108	108	0.999	426	sp|Q9GZT9|EGLN1_HUMAN	EGLN1	Q9GZT9	Egl nine homolog 1	EGLN1_HUMAN		EGLN1
47	47	49	0.999	796	sp|Q9H1B7|I2BPL_HUMAN	IRF2BPL	Q9H1B7	Probable E3 ubiquitin-protein ligase IRF2BPL	I2BPL_HUMAN		IRF2BPL
301	301	301	0.999	279	sp|Q9H2W6|RM46_HUMAN	MRPL46	Q9H2W6	Large ribosomal subunit protein mL46	RM46_HUMAN		MRPL46
507	507	507	0.999	86	sp|Q9H3K6|BOLA2_HUMAN	BOLA2	Q9H3K6	BolA-like protein 2	BOLA2_HUMAN		BOLA2
203	203	206	0.999	528	sp|Q9H3P7|GCP60_HUMAN	ACBD3	Q9H3P7	Golgi resident protein GCP60	GCP60_HUMAN		ACBD3
163	163	163	0.999	356	sp|Q9H3Q1|BORG4_HUMAN	CDC42EP4	Q9H3Q1	Cdc42 effector protein 4	BORG4_HUMAN		CDC42EP4
128	101	128	0.999	887	sp|Q9H4L5|OSBL3_HUMAN	OSBPL3	Q9H4L5	Oxysterol-binding protein-related protein 3	OSBL3_HUMAN		OSBPL3
179	106	179	0.999	323	sp|Q9H936|GHC1_HUMAN	SLC25A22	Q9H936	Mitochondrial glutamate carrier 1	GHC1_HUMAN		SLC25A22
135	135	135	0.999	311	sp|Q9HAB8|PPCS_HUMAN	PPCS	Q9HAB8	Phosphopantothenate--cysteine ligase	PPCS_HUMAN		PPCS
163	163	163	0.999	579	sp|Q9HCD5|NCOA5_HUMAN	NCOA5	Q9HCD5	Nuclear receptor coactivator 5	NCOA5_HUMAN		NCOA5
147	147	147	0.999	855	sp|Q9HCS7|SYF1_HUMAN	XAB2	Q9HCS7	Pre-mRNA-splicing factor SYF1	SYF1_HUMAN		XAB2

95	95	95	0.999	224	sp|Q9HD15|SRA1_HUMAN	SRA1	Q9HD15	Steroid receptor RNA activator 1	SRA1_HUMAN		SRA1
107	106	107	0.999	531	sp|Q9NPQ8|RIC8A_HUMAN	RIC8A	Q9NPQ8	Synembryn-A	RIC8A_HUMAN		RIC8A
150	150	150	0.999	175	sp|Q9NRX2|RM17_HUMAN	MRPL17	Q9NRX2	Large ribosomal subunit protein bL17m	RM17_HUMAN		MRPL17
218	218	218	0.999	932	sp|Q9NTZ6|RBM12_HUMAN	RBM12	Q9NTZ6	RNA-binding protein 12	RBM12_HUMAN		RBM12
140	140	140	0.999	709	sp|Q9NUQ8|ABCF3_HUMAN	ABCF3	Q9NUQ8	ATP-binding cassette sub-family F member 3	ABCF3_HUMAN		ABCF3
146	146	146	0.999	392	sp|Q9NWT1|PK1IP_HUMAN	PAK1IP1	Q9NWT1	p21-activated protein kinase-interacting protein 1	PK1IP_HUMAN		PAK1IP1
103	103	103	0.999	722	sp|Q9NZQ3|SPN90_HUMAN	NCKIPSD	Q9NZQ3	NCK-interacting protein with SH3 domain	SPN90_HUMAN		NCKIPSD
37	37	37	0.999	365	sp|Q9NZU5|LMCD1_HUMAN	LMCD1	Q9NZU5	LIM and cysteine-rich domains protein 1	LMCD1_HUMAN		LMCD1
92	92	92	0.999	229	sp|Q9P013|CWC15_HUMAN	CWC15	Q9P013	Spliceosome-associated protein CWC15 homolog	CWC15_HUMAN		CWC15
147	147	147	0.999	225	sp|Q9P016|THYN1_HUMAN	THYN1	Q9P016	Thymocyte nuclear protein 1	THYN1_HUMAN		THYN1
128	128	128	0.999	362	sp|Q9P035|HACD3_HUMAN	HACD3	Q9P035	Very-long-chain (3R)-3-hydroxyacyl-CoA dehydratase 3	HACD3_HUMAN		HACD3
188	188	188	0.999	314	sp|Q9P287|BCCIP_HUMAN	BCCIP	Q9P287	BRCA2 and CDKN1A-interacting protein	BCCIP_HUMAN		BCCIP
198	198	198	0.999	475	sp|Q9UBM7|DHCR7_HUMAN	DHCR7	Q9UBM7	7-dehydrocholesterol reductase	DHCR7_HUMAN		DHCR7
189	189	189	0.999	287	sp|Q9UBX3|DIC_HUMAN	SLC25A10	Q9UBX3	Mitochondrial dicarboxylate carrier	DIC_HUMAN		SLC25A10
200	200	200	0.999	375	sp|Q9UGV2|NDRG3_HUMAN	NDRG3	Q9UGV2	Protein NDRG3	NDRG3_HUMAN		NDRG3
401	401	401	0.999	154	sp|Q9UHV9|PFD2_HUMAN	PFDN2	Q9UHV9	Prefoldin subunit 2	PFD2_HUMAN		PFDN2
238	238	238	0.999	483	sp|Q9UI12|VATH_HUMAN	ATP6V1H	Q9UI12	V-type proton ATPase subunit H	VATH_HUMAN		ATP6V1H
256	256	256	0.999	544	sp|Q9UK59|DBR1_HUMAN	DBR1	Q9UK59	Lariat debranching enzyme	DBR1_HUMAN		DBR1
73	73	73	0.999	735	sp|Q9UPT5|EXOC7_HUMAN	EXOC7	Q9UPT5	Exocyst complex component 7	EXOC7_HUMAN		EXOC7
265	265	265	0.999	187	sp|Q9Y2Q9|RT28_HUMAN	MRPS28	Q9Y2Q9	Small ribosomal subunit protein bS1m	RT28_HUMAN		MRPS28
89	89	89	0.999	599	sp|Q9Y2R4|DDX52_HUMAN	DDX52	Q9Y2R4	Probable ATP-dependent RNA helicase DDX52	DDX52_HUMAN		DDX52
135	135	135	0.999	911	sp|Q9Y2U8|MAN1_HUMAN	LEMD3	Q9Y2U8	Inner nuclear membrane protein Man1	MAN1_HUMAN		LEMD3
502	502	502	0.999	147	sp|Q9Y2V2|CHSP1_HUMAN	CARHSP1	Q9Y2V2	Calcium-regulated heat-stable protein 1	CHSP1_HUMAN		CARHSP1
261	261	261	0.999	282	sp|Q9Y3B9|RRP15_HUMAN	RRP15	Q9Y3B9	RRP15-like protein	RRP15_HUMAN		RRP15
177	177	177	0.999	178	sp|Q9Y3C1|NOP16_HUMAN	NOP16	Q9Y3C1	Nucleolar protein 16	NOP16_HUMAN		NOP16
260	260	260	0.999	167	sp|Q9Y3C8|UFC1_HUMAN	UFC1	Q9Y3C8	Ubiquitin-fold modifier-conjugating enzyme 1	UFC1_HUMAN		UFC1
86	86	86	0.999	721	sp|Q9Y608|LRRF2_HUMAN	LRRFIP2	Q9Y608	Leucine-rich repeat flightless-interacting protein 2	LRRF2_HUMAN		LRRFIP2
183	183	183	0.999	179	sp|Q9Y6M9|NDUB9_HUMAN	NDUFB9	Q9Y6M9	NADH dehydrogenase [ubiquinone] 1 beta subcomplex subunit 9	NDUB9_HUMAN		NDUFB9
292	292	292	0.999	321	sp|A6NDG6|PGP_HUMAN	PGP	A6NDG6	Glycerol-3-phosphate phosphatase	PGP_HUMAN		PGP
480	0	480	0.999	2078	sp|E9PAV3|NACAM_HUMAN	NACA	E9PAV3	Nascent polypeptide-associated complex subunit alpha, muscle-specific fo	NACAM_HUMAN	sp|Q13765|NACA_HUMAN	NACA
328	328	328	0.999	183	sp|O00193|SMAP_HUMAN	SMAP	O00193	Small acidic protein	SMAP_HUMAN		SMAP
277	162	277	0.999	521	sp|O00505|IMA4_HUMAN	KPNA3	O00505	Importin subunit alpha-4	IMA4_HUMAN		KPNA3
51	51	51	0.999	2285	sp|O14497|ARI1A_HUMAN	ARID1A	O14497	AT-rich interactive domain-containing protein 1A	ARI1A_HUMAN		ARID1A
257	257	257	0.999	358	sp|O14745|NHRF1_HUMAN	NHERF1	O14745	Na(+)/H(+) exchange regulatory cofactor NHE-RF1	NHRF1_HUMAN		SLC9A3R1
34	33	34	0.999	1132	sp|O14746|TERT_HUMAN	TERT	O14746	Telomerase reverse transcriptase	TERT_HUMAN		TERT
53	51	53	0.999	490	sp|O14879|IFIT3_HUMAN	IFIT3	O14879	Interferon-induced protein with tetratricopeptide repeats 3	IFIT3_HUMAN		IFIT3
358	358	358	0.999	82	sp|O14949|QCR8_HUMAN	UQCRQ	O14949	Cytochrome b-c1 complex subunit 8	QCR8_HUMAN		UQCRQ
129	129	129	0.999	198	sp|O15498|YKT6_HUMAN	YKT6	O15498	Synaptobrevin homolog YKT6	YKT6_HUMAN		YKT6
298	156	298	0.999	359	sp|O43488|ARK72_HUMAN	AKR7A2	O43488	Aflatoxin B1 aldehyde reductase member 2	ARK72_HUMAN		AKR7A2
272	272	272	0.999	692	sp|O43823|AKAP8_HUMAN	AKAP8	O43823	A-kinase anchor protein 8	AKAP8_HUMAN		AKAP8
131	25	131	0.999	810	sp|O75152|ZC11A_HUMAN	ZC3H11A	O75152	Zinc finger CCCH domain-containing protein 11A	ZC11A_HUMAN		ZC3H11A
119	105	119	0.999	2603	sp|O75179|ANR17_HUMAN	ANKRD17	O75179	Ankyrin repeat domain-containing protein 17	ANR17_HUMAN		ANKRD17
296	249	296	0.999	118	sp|O75348|VATG1_HUMAN	ATP6V1G1	O75348	V-type proton ATPase subunit G 1	VATG1_HUMAN		ATP6V1G1
213	213	213	0.999	264	sp|O75489|NDUS3_HUMAN	NDUFS3	O75489	NADH dehydrogenase [ubiquinone] iron-sulfur protein 3, mitochondrial	NDUS3_HUMAN		NDUFS3
119	119	119	0.999	361	sp|O75683|SURF6_HUMAN	SURF6	O75683	Surfeit locus protein 6	SURF6_HUMAN		SURF6
97	97	365	0.999	277	sp|O75828|CBR3_HUMAN	CBR3	O75828	Carbonyl reductase [NADPH] 3	CBR3_HUMAN		CBR3
353	353	353	0.999	475	sp|O75844|FACE1_HUMAN	ZMPSTE24	O75844	CAAX prenyl protease 1 homolog	FACE1_HUMAN		ZMPSTE24
145	145	145	0.999	186	sp|O75935|DCTN3_HUMAN	DCTN3	O75935	Dynactin subunit 3	DCTN3_HUMAN		DCTN3
251	251	251	0.999	335	sp|O76003|GLRX3_HUMAN	GLRX3	O76003	Glutaredoxin-3	GLRX3_HUMAN		GLRX3
175	173	175	0.999	359	sp|O94992|HEXI1_HUMAN	HEXIM1	O94992	Protein HEXIM1	HEXI1_HUMAN		HEXIM1
343	343	343	0.999	258	sp|O95336|6PGL_HUMAN	PGLS	O95336	6-phosphogluconolactonase	6PGL_HUMAN		PGLS
65	65	104	0.999	1268	sp|O95487|SC24B_HUMAN	SEC24B	O95487	Protein transport protein Sec24B	SC24B_HUMAN		SEC24B
481	481	481	0.999	254	sp|O95571|ETHE1_HUMAN	ETHE1	O95571	Persulfide dioxygenase ETHE1, mitochondrial	ETHE1_HUMAN		ETHE1
193	138	193	0.999	429	sp|O96019|ACL6A_HUMAN	ACTL6A	O96019	Actin-like protein 6A	ACL6A_HUMAN		ACTL6A
65	65	65	0.999	622	sp|P00734|THRB_HUMAN	F2	P00734	Prothrombin	THRB_HUMAN		F2
73	73	73	0.999	464	sp|P01008|ANT3_HUMAN	SERPINC1	P01008	Antithrombin-III	ANT3_HUMAN		SERPINC1
275	108	275	0.999	189	sp|P01116|RASK_HUMAN	KRAS	P01116	GTPase KRas	RASK_HUMAN		KRAS
254	81	516	0.999	61	sp|P04732|MT1E_HUMAN	MT1E	P04732	Metallothionein-1E	MT1E_HUMAN		MT1E
635	635	635	0.999	115	sp|P05387|RLA2_HUMAN	RPLP2	P05387	Large ribosomal subunit protein P2	RLA2_HUMAN		RPLP2
28	28	28	0.999	1499	sp|P05997|CO5A2_HUMAN	COL5A2	P05997	Collagen alpha-2(V) chain	CO5A2_HUMAN		COL5A2
246	246	246	0.999	180	sp|P07741|APT_HUMAN	APRT	P07741	Adenine phosphoribosyltransferase	APT_HUMAN		APRT
244	244	303	0.999	225	sp|P08579|RU2B_HUMAN	SNRPB2	P08579	U2 small nuclear ribonucleoprotein B''	RU2B_HUMAN		SNRPB2
367	0	367	0.999	149	sp|P0DP23|CALM1_HUMAN	CALM1	P0DP23	Calmodulin-1	CALM1_HUMAN	sp|P0DP24|CALM2_HUMAN, sp|P0DP25|CALM	
386	386	386	0.999	492	sp|P11166|GTR1_HUMAN	SLC2A1	P11166	Solute carrier family 2, facilitated glucose transporter member 1	GTR1_HUMAN		SLC2A1
452	291	452	0.999	206	sp|P11233|RALA_HUMAN	RALA	P11233	Ras-related protein Ral-A	RALA_HUMAN		RALA
365	365	365	0.999	417	sp|P11279|LAMP1_HUMAN	LAMP1	P11279	Lysosome-associated membrane glycoprotein 1	LAMP1_HUMAN		LAMP1
154	153	154	0.999	737	sp|P13807|GYS1_HUMAN	GYS1	P13807	Glycogen [starch] synthase, muscle	GYS1_HUMAN		GYS1
253	253	253	0.999	111	sp|P14927|QCR7_HUMAN	UQCRB	P14927	Cytochrome b-c1 complex subunit 7	QCR7_HUMAN		UQCRB
249	249	249	0.999	547	sp|P22307|SCP2_HUMAN	SCP2	P22307	Sterol carrier protein 2	SCP2_HUMAN		SCP2
164	163	302	0.999	298	sp|P24941|CDK2_HUMAN	CDK2	P24941	Cyclin-dependent kinase 2	CDK2_HUMAN		CDK2
200	200	200	0.999	426	sp|P26440|IVD_HUMAN	IVD	P26440	Isovaleryl-CoA dehydrogenase, mitochondrial	IVD_HUMAN		IVD
51	50	106	0.999	1055	sp|P29323|EPHB2_HUMAN	EPHB2	P29323	Ephrin type-B receptor 2	EPHB2_HUMAN		EPHB2
135	98	135	0.999	138	sp|P29373|RABP2_HUMAN	CRABP2	P29373	Cellular retinoic acid-binding protein 2	RABP2_HUMAN		CRABP2
443	443	443	0.999	214	sp|P30044|PRDX5_HUMAN	PRDX5	P30044	Peroxiredoxin-5, mitochondrial	PRDX5_HUMAN		PRDX5
519	10	519	0.999	118	sp|P30046|DOPD_HUMAN	DDT	P30046	D-dopachrome decarboxylase	DOPD_HUMAN		DDT
53	53	143	0.999	461	sp|P31146|COR1A_HUMAN	CORO1A	P31146	Coronin-1A	COR1A_HUMAN		CORO1A
170	170	170	0.999	550	sp|P35610|SOAT1_HUMAN	SOAT1	P35610	Sterol O-acyltransferase 1	SOAT1_HUMAN		SOAT1
117	22	117	0.999	193	sp|P37235|HPCL1_HUMAN	HPCAL1	P37235	Hippocalcin-like protein 1	HPCL1_HUMAN		HPCAL1
248	248	248	0.999	340	sp|P40937|RFC5_HUMAN	RFC5	P40937	Replication factor C subunit 5	RFC5_HUMAN		RFC5
125	113	125	0.999	450	sp|P41240|CSK_HUMAN	CSK	P41240	Tyrosine-protein kinase CSK	CSK_HUMAN		CSK
172	172	172	0.999	304	sp|P46108|CRK_HUMAN	CRK	P46108	Adapter molecule crk	CRK_HUMAN		CRK
210	209	210	0.999	367	sp|P49585|PCY1A_HUMAN	PCYT1A	P49585	Choline-phosphate cytidylyltransferase A	PCY1A_HUMAN		PCYT1A
295	295	494	0.999	203	sp|P51153|RAB13_HUMAN	RAB13	P51153	Ras-related protein Rab-13	RAB13_HUMAN		RAB13
249	196	249	0.999	409	sp|P54727|RD23B_HUMAN	RAD23B	P54727	UV excision repair protein RAD23 homolog B	RD23B_HUMAN		RAD23B
170	170	170	0.999	439	sp|P55081|MFAP1_HUMAN	MFAP1	P55081	Microfibrillar-associated protein 1	MFAP1_HUMAN		MFAP1
226	226	226	0.999	596	sp|P57772|SELB_HUMAN	EEFSEC	P57772	Selenocysteine-specific elongation factor	SELB_HUMAN		EEFSEC
281	208	281	0.999	142	sp|P60983|GMFB_HUMAN	GMFB	P60983	Glia maturation factor beta	GMFB_HUMAN		GMFB
348	67	348	0.999	184	sp|P62834|RAP1A_HUMAN	RAP1A	P62834	Ras-related protein Rap-1A	RAP1A_HUMAN		RAP1A
243	243	243	0.999	163	sp|P63208|SKP1_HUMAN	SKP1	P63208	S-phase kinase-associated protein 1	SKP1_HUMAN		SKP1
274	274	274	0.999	229	sp|P68402|PA1B2_HUMAN	PAFAH1B2	P68402	Platelet-activating factor acetylhydrolase IB subunit alpha2	PA1B2_HUMAN		PAFAH1B2
476	448	476	0.999	240	sp|Q01081|U2AF1_HUMAN	U2AF1	Q01081	Splicing factor U2AF 35 kDa subunit	U2AF1_HUMAN		U2AF1
280	280	280	0.999	551	sp|Q04206|TF65_HUMAN	RELA	Q04206	Transcription factor p65	TF65_HUMAN		RELA
223	223	223	0.999	148	sp|Q04837|SSBP_HUMAN	SSBP1	Q04837	Single-stranded DNA-binding protein, mitochondrial	SSBP_HUMAN		SSBP1
157	157	157	0.999	480	sp|Q12849|GRSF1_HUMAN	GRSF1	Q12849	G-rich sequence factor 1	GRSF1_HUMAN		GRSF1
118	118	118	0.999	472	sp|Q13228|SBP1_HUMAN	SELENBP1	Q13228	Methanethiol oxidase	SBP1_HUMAN		SELENBP1
103	86	246	0.999	715	sp|Q13330|MTA1_HUMAN	MTA1	Q13330	Metastasis-associated protein MTA1	MTA1_HUMAN		MTA1
117	117	319	0.999	147	sp|Q13404|UB2V1_HUMAN	UBE2V1	Q13404	Ubiquitin-conjugating enzyme E2 variant 1	UB2V1_HUMAN		UBE2V1
519	453	519	0.999	163	sp|Q13526|PIN1_HUMAN	PIN1	Q13526	Peptidyl-prolyl cis-trans isomerase NIMA-interacting 1	PIN1_HUMAN		PIN1
134	134	134	0.999	293	sp|Q13868|EXOS2_HUMAN	EXOSC2	Q13868	Exosome complex component RRP4	EXOS2_HUMAN		EXOSC2
192	192	192	0.999	1009	sp|Q14527|HLTF_HUMAN	HLTF	Q14527	Helicase-like transcription factor	HLTF_HUMAN		HLTF
226	226	226	0.999	519	sp|Q14554|PDIA5_HUMAN	PDIA5	Q14554	Protein disulfide-isomerase A5	PDIA5_HUMAN		PDIA5
111	101	151	0.999	602	sp|Q14738|2A5D_HUMAN	PPP2R5D	Q14738	Serine/threonine-protein phosphatase 2A 56 kDa regulatory subunit delta is	2A5D_HUMAN		PPP2R5D
28	26	28	0.999	1721	sp|Q14766|LTBP1_HUMAN	LTBP1	Q14766	Latent-transforming growth factor beta-binding protein 1	LTBP1_HUMAN		LTBP1
141	141	141	0.999	919	sp|Q15269|PWP2_HUMAN	PWP2	Q15269	Periodic tryptophan protein 2 homolog	PWP2_HUMAN		PWP2
194	194	194	0.999	1064	sp|Q15334|L2GL1_HUMAN	LLGL1	Q15334	Lethal(2) giant larvae protein homolog 1	L2GL1_HUMAN		LLGL1
98	98	98	0.999	846	sp|Q15398|DLGP5_HUMAN	DLGAP5	Q15398	Disks large-associated protein 5	DLGP5_HUMAN		DLGAP5
262	262	262	0.999	277	sp|Q15404|RSU1_HUMAN	RSU1	Q15404	Ras suppressor protein 1	RSU1_HUMAN		RSU1
156	149	156	0.999	282	sp|Q16270|IBP7_HUMAN	IGFBP7	Q16270	Insulin-like growth factor-binding protein 7	IBP7_HUMAN		IGFBP7
194	194	194	0.999	377	sp|Q16795|NDUA9_HUMAN	NDUFA9	Q16795	NADH dehydrogenase [ubiquinone] 1 alpha subcomplex subunit 9, mitocho	NDUA9_HUMAN		NDUFA9
151	151	151	0.999	509	sp|Q16850|CP51A_HUMAN	CYP51A1	Q16850	Lanosterol 14-alpha demethylase	CP51A_HUMAN		CYP51A1
174	174	174	0.999	1173	sp|Q2M389|WASC4_HUMAN	WASHC4	Q2M389	WASH complex subunit 4	WASC4_HUMAN		KIAA1033
124	124	124	0.999	274	sp|Q53H96|P5CR3_HUMAN	PYCR3	Q53H96	Pyrroline-5-carboxylate reductase 3	P5CR3_HUMAN		PYCRL
90	90	90	0.999	523	sp|Q5JPH6|SYEM_HUMAN	EARS2	Q5JPH6	Probable glutamate--tRNA ligase, mitochondrial	SYEM_HUMAN		EARS2
69	69	71	0.999	1668	sp|Q5T200|ZC3HD_HUMAN	ZC3H13	Q5T200	Zinc finger CCCH domain-containing protein 13	ZC3HD_HUMAN		ZC3H13
107	107	107	0.999	1445	sp|Q68CZ2|TENS3_HUMAN	TNS3	Q68CZ2	Tensin-3	TENS3_HUMAN		TNS3
335	335	335	0.999	386	sp|Q6NVY1|HIBCH_HUMAN	HIBCH	Q6NVY1	3-hydroxyisobutyryl-CoA hydrolase, mitochondrial	HIBCH_HUMAN		HIBCH
404	404	404	0.999	538	sp|Q6XQN6|PNCB_HUMAN	NAPRT	Q6XQN6	Nicotinate phosphoribosyltransferase	PNCB_HUMAN		NAPRT
160	94	160	0.999	788	sp|Q7KZI7|MARK2_HUMAN	MARK2	Q7KZI7	Serine/threonine-protein kinase MARK2	MARK2_HUMAN		MARK2
273	273	273	0.999	689	sp|Q7L2J0|MEPCE_HUMAN	MEPCE	Q7L2J0	7SK snRNA methylphosphate capping enzyme	MEPCE_HUMAN		MEPCE
101	101	101	0.999	1410	sp|Q7Z3K3|POGZ_HUMAN	POGZ	Q7Z3K3	Pogo transposable element with ZNF domain	POGZ_HUMAN		POGZ
186	186	186	0.999	327	sp|Q86X76|NIT1_HUMAN	NIT1	Q86X76	Deaminated glutathione amidase	NIT1_HUMAN		NIT1
175	175	175	0.999	855	sp|Q86Y56|DAAF5_HUMAN	DNAAF5	Q86Y56	Dynein axonemal assembly factor 5	DAAF5_HUMAN		DNAAF5
147	147	147	0.999	916	sp|Q8IWX8|CHERP_HUMAN	CHERP	Q8IWX8	Calcium homeostasis endoplasmic reticulum protein	CHERP_HUMAN		CHERP
138	138	138	0.999	725	sp|Q8IYI6|EXOC8_HUMAN	EXOC8	Q8IYI6	Exocyst complex component 8	EXOC8_HUMAN		EXOC8
128	128	128	0.999	835	sp|Q8N1G2|CMTR1_HUMAN	CMTR1	Q8N1G2	Cap-specific mRNA (nucleoside-2'-O-)-methyltransferase 1	CMTR1_HUMAN		CMTR1
171	171	171	0.999	534	sp|Q8NF37|PCAT1_HUMAN	LPCAT1	Q8NF37	Lysophosphatidylcholine acyltransferase 1	PCAT1_HUMAN		LPCAT1
83	67	83	0.999	738	sp|Q92615|LAR4B_HUMAN	LARP4B	Q92615	La-related protein 4B	LAR4B_HUMAN		LARP4B
244	244	244	0.999	400	sp|Q92734|TFG_HUMAN	TFG	Q92734	Protein TFG	TFG_HUMAN		TFG
131	131	131	0.999	420	sp|Q96IJ6|GMPPA_HUMAN	GMPPA	Q96IJ6	Mannose-1-phosphate guanyltransferase alpha	GMPPA_HUMAN		GMPPA

<table>
<tr><td>156</td><td>156</td><td>156</td><td>0.999</td><td>506</td><td>sp|Q96JB5|CK5P3_HUMAN</td><td>CDK5RAP3</td><td>Q96JB5</td><td>CDK5 regulatory subunit-associated protein 3</td><td>CK5P3_HUMAN</td><td></td><td>CDK5RAP3</td></tr>
<tr><td>245</td><td>245</td><td>245</td><td>0.999</td><td>454</td><td>sp|Q96JJ7|TMX3_HUMAN</td><td>TMX3</td><td>Q96JJ7</td><td>Protein disulfide-isomerase TMX3</td><td>TMX3_HUMAN</td><td></td><td>TMX3</td></tr>
<tr><td>115</td><td>115</td><td>115</td><td>0.999</td><td>1450</td><td>sp|Q96P48|ARAP1_HUMAN</td><td>ARAP1</td><td>Q96P48</td><td>Arf-GAP with Rho-GAP domain, ANK repeat and PH domain-containing pro</td><td>ARAP1_HUMAN</td><td></td><td>ARAP1</td></tr>
<tr><td>117</td><td>117</td><td>117</td><td>0.999</td><td>817</td><td>sp|Q96SB3|NEB2_HUMAN</td><td>PPP1R9B</td><td>Q96SB3</td><td>Neurabin-2</td><td>NEB2_HUMAN</td><td></td><td>PPP1R9B</td></tr>
<tr><td>211</td><td>211</td><td>211</td><td>0.999</td><td>621</td><td>sp|Q99543|DNJC2_HUMAN</td><td>DNAJC2</td><td>Q99543</td><td>DnaJ homolog subfamily C member 2</td><td>DNJC2_HUMAN</td><td></td><td>DNAJC2</td></tr>
<tr><td>266</td><td>266</td><td>266</td><td>0.999</td><td>292</td><td>sp|Q99614|TTC1_HUMAN</td><td>TTC1</td><td>Q99614</td><td>Tetratricopeptide repeat protein 1</td><td>TTC1_HUMAN</td><td></td><td>TTC1</td></tr>
<tr><td>396</td><td>396</td><td>396</td><td>0.999</td><td>261</td><td>sp|Q99714|HCD2_HUMAN</td><td>HSD17B10</td><td>Q99714</td><td>3-hydroxyacyl-CoA dehydrogenase type-2</td><td>HCD2_HUMAN</td><td></td><td>HSD17B10</td></tr>
<tr><td>82</td><td>82</td><td>82</td><td>0.999</td><td>312</td><td>sp|Q99747|SNAG_HUMAN</td><td>NAPG</td><td>Q99747</td><td>Gamma-soluble NSF attachment protein</td><td>SNAG_HUMAN</td><td></td><td>NAPG</td></tr>
<tr><td>218</td><td>218</td><td>218</td><td>0.999</td><td>663</td><td>sp|Q99805|TM9S2_HUMAN</td><td>TM9SF2</td><td>Q99805</td><td>Transmembrane 9 superfamily member 2</td><td>TM9S2_HUMAN</td><td></td><td>TM9SF2</td></tr>
<tr><td>73</td><td>73</td><td>73</td><td>0.999</td><td>1478</td><td>sp|Q9BQS8|FYCO1_HUMAN</td><td>FYCO1</td><td>Q9BQS8</td><td>FYVE and coiled-coil domain-containing protein 1</td><td>FYCO1_HUMAN</td><td></td><td>FYCO1</td></tr>
<tr><td>58</td><td>55</td><td>58</td><td>0.999</td><td>755</td><td>sp|Q9BRZ2|TRI56_HUMAN</td><td>TRIM56</td><td>Q9BRZ2</td><td>E3 ubiquitin-protein ligase TRIM56</td><td>TRI56_HUMAN</td><td></td><td>TRIM56</td></tr>
<tr><td>161</td><td>161</td><td>161</td><td>0.999</td><td>297</td><td>sp|Q9BSH4|TACO1_HUMAN</td><td>TACO1</td><td>Q9BSH4</td><td>Translational activator of cytochrome c oxidase 1</td><td>TACO1_HUMAN</td><td></td><td>TACO1</td></tr>
<tr><td>90</td><td>90</td><td>200</td><td>0.999</td><td>594</td><td>sp|Q9BTC8|MTA3_HUMAN</td><td>MTA3</td><td>Q9BTC8</td><td>Metastasis-associated protein MTA3</td><td>MTA3_HUMAN</td><td></td><td>MTA3</td></tr>
<tr><td>244</td><td>244</td><td>244</td><td>0.999</td><td>212</td><td>sp|Q9BUL8|PDC10_HUMAN</td><td>PDCD10</td><td>Q9BUL8</td><td>Programmed cell death protein 10</td><td>PDC10_HUMAN</td><td></td><td>PDCD10</td></tr>
<tr><td>134</td><td>134</td><td>134</td><td>0.999</td><td>233</td><td>sp|Q9BVG4|PBDC1_HUMAN</td><td>PBDC1</td><td>Q9BVG4</td><td>Protein PBDC1</td><td>PBDC1_HUMAN</td><td></td><td>PBDC1</td></tr>
<tr><td>115</td><td>115</td><td>115</td><td>0.999</td><td>306</td><td>sp|Q9BY42|RTF2_HUMAN</td><td>RTF2</td><td>Q9BY42</td><td>Replication termination factor 2</td><td>RTF2_HUMAN</td><td></td><td>RTFDC1</td></tr>
<tr><td>161</td><td>161</td><td>161</td><td>0.999</td><td>421</td><td>sp|Q9BY77|PDIP3_HUMAN</td><td>POLDIP3</td><td>Q9BY77</td><td>Polymerase delta-interacting protein 3</td><td>PDIP3_HUMAN</td><td></td><td>POLDIP3</td></tr>
<tr><td>160</td><td>160</td><td>160</td><td>0.999</td><td>889</td><td>sp|Q9BZF1|OSBL8_HUMAN</td><td>OSBPL8</td><td>Q9BZF1</td><td>Oxysterol-binding protein-related protein 8</td><td>OSBL8_HUMAN</td><td></td><td>OSBPL8</td></tr>
<tr><td>110</td><td>110</td><td>110</td><td>0.999</td><td>422</td><td>sp|Q9GZR2|REXO4_HUMAN</td><td>REXO4</td><td>Q9GZR2</td><td>RNA exonuclease 4</td><td>REXO4_HUMAN</td><td></td><td>REXO4</td></tr>
<tr><td>9</td><td>9</td><td>54</td><td>0.9989</td><td>359</td><td>sp|Q9H1J7|WNT5B_HUMAN</td><td>WNT5B</td><td>Q9H1J7</td><td>Protein Wnt-5b</td><td>WNT5B_HUMAN</td><td></td><td>WNT5B</td></tr>
<tr><td>453</td><td>453</td><td>453</td><td>0.999</td><td>93</td><td>sp|Q9H299|SH3L3_HUMAN</td><td>SH3BGRL3</td><td>Q9H299</td><td>SH3 domain-binding glutamic acid-rich-like protein 3</td><td>SH3L3_HUMAN</td><td></td><td>SH3BGRL3</td></tr>
<tr><td>136</td><td>119</td><td>136</td><td>0.999</td><td>454</td><td>sp|Q9H788|SH24A_HUMAN</td><td>SH2D4A</td><td>Q9H788</td><td>SH2 domain-containing protein 4A</td><td>SH24A_HUMAN</td><td></td><td>SH2D4A</td></tr>
<tr><td>641</td><td>641</td><td>641</td><td>0.999</td><td>376</td><td>sp|Q9HB07|MYG1_HUMAN</td><td>MYG1</td><td>Q9HB07</td><td>MYG1 exonuclease</td><td>MYG1_HUMAN</td><td></td><td>C12orf10</td></tr>
<tr><td>283</td><td>283</td><td>283</td><td>0.999</td><td>313</td><td>sp|Q9HC38|GLOD4_HUMAN</td><td>GLOD4</td><td>Q9HC38</td><td>Glyoxalase domain-containing protein 4</td><td>GLOD4_HUMAN</td><td></td><td>GLOD4</td></tr>
<tr><td>268</td><td>268</td><td>268</td><td>0.999</td><td>307</td><td>sp|Q9NP79|VTA1_HUMAN</td><td>VTA1</td><td>Q9NP79</td><td>Vacuolar protein sorting-associated protein VTA1 homolog</td><td>VTA1_HUMAN</td><td></td><td>VTA1</td></tr>
<tr><td>82</td><td>82</td><td>82</td><td>0.999</td><td>335</td><td>sp|Q9NPF4|OSGEP_HUMAN</td><td>OSGEP</td><td>Q9NPF4</td><td>tRNA N6-adenosine threonylcarbamoyltransferase</td><td>OSGEP_HUMAN</td><td></td><td>OSGEP</td></tr>
<tr><td>205</td><td>205</td><td>205</td><td>0.999</td><td>473</td><td>sp|Q9NQ55|SSF1_HUMAN</td><td>PPAN</td><td>Q9NQ55</td><td>Suppressor of SWI4 1 homolog</td><td>SSF1_HUMAN</td><td></td><td>PPAN</td></tr>
<tr><td>108</td><td>108</td><td>108</td><td>0.999</td><td>1556</td><td>sp|Q9NRL2|BAZ1A_HUMAN</td><td>BAZ1A</td><td>Q9NRL2</td><td>Bromodomain adjacent to zinc finger domain protein 1A</td><td>BAZ1A_HUMAN</td><td></td><td>BAZ1A</td></tr>
<tr><td>450</td><td>450</td><td>450</td><td>0.999</td><td>125</td><td>sp|Q9NRX4|PHP14_HUMAN</td><td>PHPT1</td><td>Q9NRX4</td><td>14 kDa phosphohistidine phosphatase</td><td>PHP14_HUMAN</td><td></td><td>PHPT1</td></tr>
<tr><td>32</td><td>32</td><td>32</td><td>0.999</td><td>1388</td><td>sp|Q9NS87|KIF15_HUMAN</td><td>KIF15</td><td>Q9NS87</td><td>Kinesin-like protein KIF15</td><td>KIF15_HUMAN</td><td></td><td>KIF15</td></tr>
<tr><td>26</td><td>26</td><td>31</td><td>0.999</td><td>1193</td><td>sp|Q9NSV4|DIAP3_HUMAN</td><td>DIAPH3</td><td>Q9NSV4</td><td>Protein diaphanous homolog 3</td><td>DIAP3_HUMAN</td><td></td><td>DIAPH3</td></tr>
<tr><td>101</td><td>101</td><td>101</td><td>0.999</td><td>1328</td><td>sp|Q9NVI1|FANCI_HUMAN</td><td>FANCI</td><td>Q9NVI1</td><td>Fanconi anemia group I protein</td><td>FANCI_HUMAN</td><td></td><td>FANCI</td></tr>
<tr><td>249</td><td>249</td><td>249</td><td>0.999</td><td>420</td><td>sp|Q9NW64|RBM22_HUMAN</td><td>RBM22</td><td>Q9NW64</td><td>Pre-mRNA-splicing factor RBM22</td><td>RBM22_HUMAN</td><td></td><td>RBM22</td></tr>
<tr><td>183</td><td>183</td><td>183</td><td>0.999</td><td>353</td><td>sp|Q9NXG2|THUM1_HUMAN</td><td>THUMPD1</td><td>Q9NXG2</td><td>THUMP domain-containing protein 1</td><td>THUM1_HUMAN</td><td></td><td>THUMPD1</td></tr>
<tr><td>102</td><td>102</td><td>102</td><td>0.999</td><td>597</td><td>sp|Q9NYH9|UTP6_HUMAN</td><td>UTP6</td><td>Q9NYH9</td><td>U3 small nucleolar RNA-associated protein 6 homolog</td><td>UTP6_HUMAN</td><td></td><td>UTP6</td></tr>
<tr><td>187</td><td>187</td><td>187</td><td>0.999</td><td>492</td><td>sp|Q9UHL4|DPP2_HUMAN</td><td>DPP7</td><td>Q9UHL4</td><td>Dipeptidyl peptidase 2</td><td>DPP2_HUMAN</td><td></td><td>DPP7</td></tr>
<tr><td>142</td><td>142</td><td>142</td><td>0.999</td><td>565</td><td>sp|Q9UJX3|APC7_HUMAN</td><td>ANAPC7</td><td>Q9UJX3</td><td>Anaphase-promoting complex subunit 7</td><td>APC7_HUMAN</td><td></td><td>ANAPC7</td></tr>
<tr><td>151</td><td>151</td><td>151</td><td>0.999</td><td>684</td><td>sp|Q9UKF6|CPSF3_HUMAN</td><td>CPSF3</td><td>Q9UKF6</td><td>Cleavage and polyadenylation specificity factor subunit 3</td><td>CPSF3_HUMAN</td><td></td><td>CPSF3</td></tr>
<tr><td>317</td><td>317</td><td>317</td><td>0.999</td><td>258</td><td>sp|Q9UKY7|CDV3_HUMAN</td><td>CDV3</td><td>Q9UKY7</td><td>Protein CDV3 homolog</td><td>CDV3_HUMAN</td><td></td><td>CDV3</td></tr>
<tr><td>140</td><td>140</td><td>140</td><td>0.999</td><td>368</td><td>sp|Q9Y2S7|PDIP2_HUMAN</td><td>POLDIP2</td><td>Q9Y2S7</td><td>Polymerase delta-interacting protein 2</td><td>PDIP2_HUMAN</td><td></td><td>POLDIP2</td></tr>
<tr><td>303</td><td>303</td><td>303</td><td>0.999</td><td>296</td><td>sp|Q9Y399|RT02_HUMAN</td><td>MRPS2</td><td>Q9Y399</td><td>Small ribosomal subunit protein uS2m</td><td>RT02_HUMAN</td><td></td><td>MRPS2</td></tr>
<tr><td>119</td><td>101</td><td>119</td><td>0.999</td><td>1069</td><td>sp|Q9Y3P9|RBGP1_HUMAN</td><td>RABGAP1</td><td>Q9Y3P9</td><td>Rab GTPase-activating protein 1</td><td>RBGP1_HUMAN</td><td></td><td>RABGAP1</td></tr>
<tr><td>224</td><td>224</td><td>224</td><td>0.999</td><td>248</td><td>sp|Q9Y3Y2|CHTOP_HUMAN</td><td>CHTOP</td><td>Q9Y3Y2</td><td>Chromatin target of PRMT1 protein</td><td>CHTOP_HUMAN</td><td></td><td>CHTOP</td></tr>
<tr><td>44</td><td>44</td><td>44</td><td>0.999</td><td>1338</td><td>sp|Q9Y4H2|IRS2_HUMAN</td><td>IRS2</td><td>Q9Y4H2</td><td>Insulin receptor substrate 2</td><td>IRS2_HUMAN</td><td></td><td>IRS2</td></tr>
<tr><td>96</td><td>96</td><td>96</td><td>0.999</td><td>734</td><td>sp|Q9Y4W2|LAS1L_HUMAN</td><td>LAS1L</td><td>Q9Y4W2</td><td>Ribosomal biogenesis protein LAS1L</td><td>LAS1L_HUMAN</td><td></td><td>LAS1L</td></tr>
<tr><td>109</td><td>109</td><td>109</td><td>0.999</td><td>923</td><td>sp|Q9Y5L0|TNPO3_HUMAN</td><td>TNPO3</td><td>Q9Y5L0</td><td>Transportin-3</td><td>TNPO3_HUMAN</td><td></td><td>TNPO3</td></tr>
<tr><td>78</td><td>78</td><td>78</td><td>0.999</td><td>2514</td><td>sp|Q9Y618|NCOR2_HUMAN</td><td>NCOR2</td><td>Q9Y618</td><td>Nuclear receptor corepressor 2</td><td>NCOR2_HUMAN</td><td></td><td>NCOR2</td></tr>
<tr><td>135</td><td>135</td><td>135</td><td>0.999</td><td>457</td><td>sp|Q9Y697|NFS1_HUMAN</td><td>NFS1</td><td>Q9Y697</td><td>Cysteine desulfurase</td><td>NFS1_HUMAN</td><td></td><td>NFS1</td></tr>
<tr><td>129</td><td>59</td><td>129</td><td>0.999</td><td>1849</td><td>sp|Q9Y6D6|BIG1_HUMAN</td><td>ARFGEF1</td><td>Q9Y6D6</td><td>Brefeldin A-inhibited guanine nucleotide-exchange protein 1</td><td>BIG1_HUMAN</td><td></td><td>ARFGEF1</td></tr>
<tr><td>40</td><td>40</td><td>40</td><td>0.999</td><td>718</td><td>sp|Q9Y6D9|MD1L1_HUMAN</td><td>MAD1L1</td><td>Q9Y6D9</td><td>Mitotic spindle assembly checkpoint protein MAD1</td><td>MD1L1_HUMAN</td><td></td><td>MAD1L1</td></tr>
<tr><td>160</td><td>159</td><td>160</td><td>0.999</td><td>1032</td><td>sp|Q9Y6X9|MORC2_HUMAN</td><td>MORC2</td><td>Q9Y6X9</td><td>ATPase MORC2</td><td>MORC2_HUMAN</td><td></td><td>MORC2</td></tr>
<tr><td>138</td><td>138</td><td>138</td><td>0.999</td><td>669</td><td>sp|O00178|GTPB1_HUMAN</td><td>GTPBP1</td><td>O00178</td><td>GTP-binding protein 1</td><td>GTPB1_HUMAN</td><td></td><td>GTPBP1</td></tr>
<tr><td>331</td><td>331</td><td>331</td><td>0.999</td><td>347</td><td>sp|O14828|SCAM3_HUMAN</td><td>SCAMP3</td><td>O14828</td><td>Secretory carrier-associated membrane protein 3</td><td>SCAM3_HUMAN</td><td></td><td>SCAMP3</td></tr>
<tr><td>172</td><td>172</td><td>172</td><td>0.999</td><td>926</td><td>sp|O14936|CSKP_HUMAN</td><td>CASK</td><td>O14936</td><td>Peripheral plasma membrane protein CASK</td><td>CSKP_HUMAN</td><td></td><td>CASK</td></tr>
<tr><td>126</td><td>80</td><td>126</td><td>0.999</td><td>562</td><td>sp|O15270|SPTC2_HUMAN</td><td>SPTLC2</td><td>O15270</td><td>Serine palmitoyltransferase 2</td><td>SPTC2_HUMAN</td><td></td><td>SPTLC2</td></tr>
<tr><td>139</td><td>139</td><td>139</td><td>0.999</td><td>779</td><td>sp|O43264|ZW10_HUMAN</td><td>ZW10</td><td>O43264</td><td>Centromere/kinetochore protein zw10 homolog</td><td>ZW10_HUMAN</td><td></td><td>ZW10</td></tr>
<tr><td>187</td><td>187</td><td>187</td><td>0.999</td><td>198</td><td>sp|O43583|DENR_HUMAN</td><td>DENR</td><td>O43583</td><td>Density-regulated protein</td><td>DENR_HUMAN</td><td></td><td>DENR</td></tr>
<tr><td>397</td><td>397</td><td>397</td><td>0.999</td><td>348</td><td>sp|O43681|GET3_HUMAN</td><td>GET3</td><td>O43681</td><td>ATPase GET3</td><td>GET3_HUMAN</td><td></td><td>ASNA1</td></tr>
<tr><td>208</td><td>208</td><td>208</td><td>0.999</td><td>755</td><td>sp|O43719|HTSF1_HUMAN</td><td>HTATSF1</td><td>O43719</td><td>HIV Tat-specific factor 1</td><td>HTSF1_HUMAN</td><td></td><td>HTATSF1</td></tr>
<tr><td>108</td><td>73</td><td>108</td><td>0.999</td><td>631</td><td>sp|O60216|RAD21_HUMAN</td><td>RAD21</td><td>O60216</td><td>Double-strand-break repair protein rad21 homolog</td><td>RAD21_HUMAN</td><td></td><td>RAD21</td></tr>
<tr><td>70</td><td>43</td><td>70</td><td>0.999</td><td>1816</td><td>sp|O60333|KIF1B_HUMAN</td><td>KIF1B</td><td>O60333</td><td>Kinesin-like protein KIF1B</td><td>KIF1B_HUMAN</td><td></td><td>KIF1B</td></tr>
<tr><td>175</td><td>175</td><td>175</td><td>0.999</td><td>496</td><td>sp|O60443|GSDME_HUMAN</td><td>GSDME</td><td>O60443</td><td>Gasdermin-E</td><td>GSDME_HUMAN</td><td></td><td>DFNA5</td></tr>
<tr><td>125</td><td>90</td><td>125</td><td>0.999</td><td>184</td><td>sp|O60565|GREM1_HUMAN</td><td>GREM1</td><td>O60565</td><td>Gremlin-1</td><td>GREM1_HUMAN</td><td></td><td>GREM1</td></tr>
<tr><td>88</td><td>88</td><td>88</td><td>0.999</td><td>1068</td><td>sp|O75146|HIP1R_HUMAN</td><td>HIP1R</td><td>O75146</td><td>Huntingtin-interacting protein 1-related protein</td><td>HIP1R_HUMAN</td><td></td><td>HIP1R</td></tr>
<tr><td>268</td><td>242</td><td>268</td><td>0.999</td><td>262</td><td>sp|O75494|SRS10_HUMAN</td><td>SRSF10</td><td>O75494</td><td>Serine/arginine-rich splicing factor 10</td><td>SRS10_HUMAN</td><td></td><td>SRSF10</td></tr>
<tr><td>331</td><td>331</td><td>331</td><td>0.999</td><td>394</td><td>sp|O75521|ECI2_HUMAN</td><td>ECI2</td><td>O75521</td><td>Enoyl-CoA delta isomerase 2</td><td>ECI2_HUMAN</td><td></td><td>ECI2</td></tr>
<tr><td>410</td><td>410</td><td>410</td><td>0.999</td><td>89</td><td>sp|O75531|BAF_HUMAN</td><td>BANF1</td><td>O75531</td><td>Barrier-to-autointegration factor</td><td>BAF_HUMAN</td><td></td><td>BANF1</td></tr>
<tr><td>196</td><td>196</td><td>196</td><td>0.999</td><td>225</td><td>sp|O75934|SPF27_HUMAN</td><td>BCAS2</td><td>O75934</td><td>Pre-mRNA-splicing factor SPF27</td><td>SPF27_HUMAN</td><td></td><td>BCAS2</td></tr>
<tr><td>32</td><td>28</td><td>32</td><td>0.999</td><td>1529</td><td>sp|O94813|SLIT2_HUMAN</td><td>SLIT2</td><td>O94813</td><td>Slit homolog 2 protein</td><td>SLIT2_HUMAN</td><td></td><td>SLIT2</td></tr>
<tr><td>217</td><td>217</td><td>217</td><td>0.999</td><td>568</td><td>sp|O95470|SGPL1_HUMAN</td><td>SGPL1</td><td>O95470</td><td>Sphingosine-1-phosphate lyase 1</td><td>SGPL1_HUMAN</td><td></td><td>SGPL1</td></tr>
<tr><td>258</td><td>258</td><td>258</td><td>0.999</td><td>649</td><td>sp|O95834|EMAL2_HUMAN</td><td>EML2</td><td>O95834</td><td>Echinoderm microtubule-associated protein-like 2</td><td>EMAL2_HUMAN</td><td></td><td>EML2</td></tr>
<tr><td>560</td><td>499</td><td>560</td><td>0.999</td><td>218</td><td>sp|P00492|HPRT_HUMAN</td><td>HPRT1</td><td>P00492</td><td>Hypoxanthine-guanine phosphoribosyltransferase</td><td>HPRT_HUMAN</td><td></td><td>HPRT1</td></tr>
<tr><td>50</td><td>14</td><td>160</td><td>0.999</td><td>189</td><td>sp|P01111|RASN_HUMAN</td><td>NRAS</td><td>P01111</td><td>GTPase NRas</td><td>RASN_HUMAN</td><td></td><td>NRAS</td></tr>
<tr><td>436</td><td>272</td><td>436</td><td>0.999</td><td>362</td><td>sp|P01889|HLAB_HUMAN</td><td>HLA-B</td><td>P01889</td><td>HLA class I histocompatibility antigen, B alpha chain</td><td>HLAB_HUMAN</td><td></td><td>HLA-B</td></tr>
<tr><td>407</td><td>407</td><td>407</td><td>0.999</td><td>194</td><td>sp|P07305|H10_HUMAN</td><td>H1-0</td><td>P07305</td><td>Histone H1.0</td><td>H10_HUMAN</td><td></td><td>H1F0</td></tr>
<tr><td>520</td><td>405</td><td>520</td><td>0.999</td><td>91</td><td>sp|P07919|QCR6_HUMAN</td><td>UQCRH</td><td>P07919</td><td>Cytochrome b-c1 complex subunit 6, mitochondrial</td><td>QCR6_HUMAN</td><td></td><td>UQCRH</td></tr>
<tr><td>358</td><td>299</td><td>358</td><td>0.999</td><td>282</td><td>sp|P09012|SNRPA_HUMAN</td><td>SNRPA</td><td>P09012</td><td>U1 small nuclear ribonucleoprotein A</td><td>SNRPA_HUMAN</td><td></td><td>SNRPA</td></tr>
<tr><td>319</td><td>17</td><td>319</td><td>0.999</td><td>240</td><td>sp|P14678|RSMB_HUMAN</td><td>SNRPB</td><td>P14678</td><td>Small nuclear ribonucleoprotein-associated proteins B and B'</td><td>RSMB_HUMAN</td><td></td><td>SNRPB</td></tr>
<tr><td>211</td><td>211</td><td>211</td><td>0.999</td><td>230</td><td>sp|P15374|UCHL3_HUMAN</td><td>UCHL3</td><td>P15374</td><td>Ubiquitin carboxyl-terminal hydrolase isozyme L3</td><td>UCHL3_HUMAN</td><td></td><td>UCHL3</td></tr>
<tr><td>235</td><td>235</td><td>235</td><td>0.999</td><td>419</td><td>sp|P16930|FAAA_HUMAN</td><td>FAH</td><td>P16930</td><td>Fumarylacetoacetase</td><td>FAAA_HUMAN</td><td></td><td>FAH</td></tr>
<tr><td>17</td><td>17</td><td>125</td><td>0.999</td><td>219</td><td>sp|P20337|RAB3B_HUMAN</td><td>RAB3B</td><td>P20337</td><td>Ras-related protein Rab-3B</td><td>RAB3B_HUMAN</td><td></td><td>RAB3B</td></tr>
<tr><td>122</td><td>10</td><td>227</td><td>0.999</td><td>208</td><td>sp|P20340|RAB6A_HUMAN</td><td>RAB6A</td><td>P20340</td><td>Ras-related protein Rab-6A</td><td>RAB6A_HUMAN</td><td></td><td>RAB6A</td></tr>
<tr><td>306</td><td>105</td><td>306</td><td>0.999</td><td>451</td><td>sp|P23258|TBG1_HUMAN</td><td>TUBG1</td><td>P23258</td><td>Tubulin gamma-1 chain</td><td>TBG1_HUMAN</td><td></td><td>TUBG1</td></tr>
<tr><td>25</td><td>25</td><td>425</td><td>0.999</td><td>879</td><td>sp|P23497|SP100_HUMAN</td><td>SP100</td><td>P23497</td><td>Nuclear autoantigen Sp-100</td><td>SP100_HUMAN</td><td></td><td>SP100</td></tr>
<tr><td>415</td><td>415</td><td>415</td><td>0.999</td><td>234</td><td>sp|P25787|PSA2_HUMAN</td><td>PSMA2</td><td>P25787</td><td>Proteasome subunit alpha type-2</td><td>PSA2_HUMAN</td><td></td><td>PSMA2</td></tr>
<tr><td>310</td><td>310</td><td>310</td><td>0.999</td><td>206</td><td>sp|P30043|BLVRB_HUMAN</td><td>BLVRB</td><td>P30043</td><td>Flavin reductase (NADPH)</td><td>BLVRB_HUMAN</td><td></td><td>BLVRB</td></tr>
<tr><td>256</td><td>256</td><td>256</td><td>0.999</td><td>316</td><td>sp|P30519|HMOX2_HUMAN</td><td>HMOX2</td><td>P30519</td><td>Heme oxygenase 2</td><td>HMOX2_HUMAN</td><td></td><td>HMOX2</td></tr>
<tr><td>538</td><td>538</td><td>538</td><td>0.999</td><td>198</td><td>sp|P30626|SORCN_HUMAN</td><td>SRI</td><td>P30626</td><td>Sorcin</td><td>SORCN_HUMAN</td><td></td><td>SRI</td></tr>
<tr><td>148</td><td>148</td><td>148</td><td>0.999</td><td>354</td><td>sp|P35250|RFC2_HUMAN</td><td>RFC2</td><td>P35250</td><td>Replication factor C subunit 2</td><td>RFC2_HUMAN</td><td></td><td>RFC2</td></tr>
<tr><td>119</td><td>119</td><td>119</td><td>0.999</td><td>847</td><td>sp|P42226|STAT6_HUMAN</td><td>STAT6</td><td>P42226</td><td>Signal transducer and activator of transcription 6</td><td>STAT6_HUMAN</td><td></td><td>STAT6</td></tr>
<tr><td>142</td><td>93</td><td>142</td><td>0.999</td><td>156</td><td>sp|P42771|CDN2A_HUMAN</td><td>CDKN2A</td><td>P42771</td><td>Cyclin-dependent kinase inhibitor 2A</td><td>CDN2A_HUMAN</td><td></td><td>CDKN2A</td></tr>
<tr><td>200</td><td>5</td><td>200</td><td>0.999</td><td>483</td><td>sp|P49753|ACOT2_HUMAN</td><td>ACOT2</td><td>P49753</td><td>Acyl-coenzyme A thioesterase 2, mitochondrial</td><td>ACOT2_HUMAN</td><td></td><td>ACOT2</td></tr>
<tr><td>317</td><td>136</td><td>317</td><td>0.999</td><td>420</td><td>sp|P49841|GSK3B_HUMAN</td><td>GSK3B</td><td>P49841</td><td>Glycogen synthase kinase-3 beta</td><td>GSK3B_HUMAN</td><td></td><td>GSK3B</td></tr>
<tr><td>522</td><td>301</td><td>522</td><td>0.999</td><td>216</td><td>sp|P51148|RAB5C_HUMAN</td><td>RAB5C</td><td>P51148</td><td>Ras-related protein Rab-5C</td><td>RAB5C_HUMAN</td><td></td><td>RAB5C</td></tr>
<tr><td>256</td><td>200</td><td>256</td><td>0.999</td><td>607</td><td>sp|P52306|GDS1_HUMAN</td><td>RAP1GDS1</td><td>P52306</td><td>Rap1 GTPase-GDP dissociation stimulator 1</td><td>GDS1_HUMAN</td><td></td><td>RAP1GDS1</td></tr>
<tr><td>303</td><td>303</td><td>303</td><td>0.999</td><td>198</td><td>sp|P52815|RM12_HUMAN</td><td>MRPL12</td><td>P52815</td><td>Large ribosomal subunit protein bL12m</td><td>RM12_HUMAN</td><td></td><td>MRPL12</td></tr>
<tr><td>207</td><td>207</td><td>207</td><td>0.999</td><td>208</td><td>sp|P52943|CRIP2_HUMAN</td><td>CRIP2</td><td>P52943</td><td>Cysteine-rich protein 2</td><td>CRIP2_HUMAN</td><td></td><td>CRIP2</td></tr>
<tr><td>249</td><td>226</td><td>249</td><td>0.999</td><td>295</td><td>sp|P54920|SNAA_HUMAN</td><td>NAPA</td><td>P54920</td><td>Alpha-soluble NSF attachment protein</td><td>SNAA_HUMAN</td><td></td><td>NAPA</td></tr>
<tr><td>166</td><td>166</td><td>232</td><td>0.999</td><td>364</td><td>sp|P55039|DRG2_HUMAN</td><td>DRG2</td><td>P55039</td><td>Developmentally-regulated GTP-binding protein 2</td><td>DRG2_HUMAN</td><td></td><td>DRG2</td></tr>
<tr><td>228</td><td>228</td><td>228</td><td>0.999</td><td>461</td><td>sp|P56182|RRP1_HUMAN</td><td>RRP1</td><td>P56182</td><td>Ribosomal RNA processing protein 1 homolog A</td><td>RRP1_HUMAN</td><td></td><td>RRP1</td></tr>
<tr><td>150</td><td>150</td><td>211</td><td>0.999</td><td>307</td><td>sp|P60510|PP4C_HUMAN</td><td>PPP4C</td><td>P60510</td><td>Serine/threonine-protein phosphatase 4 catalytic subunit</td><td>PP4C_HUMAN</td><td></td><td>PPP4C</td></tr>
<tr><td>256</td><td>256</td><td>256</td><td>0.999</td><td>183</td><td>sp|P61081|UBC12_HUMAN</td><td>UBE2M</td><td>P61081</td><td>NEDD8-conjugating enzyme Ubc12</td><td>UBC12_HUMAN</td><td></td><td>UBE2M</td></tr>
<tr><td>127</td><td>127</td><td>127</td><td>0.999</td><td>151</td><td>sp|P61916|NPC2_HUMAN</td><td>NPC2</td><td>P61916</td><td>NPC intracellular cholesterol transporter 2</td><td>NPC2_HUMAN</td><td></td><td>NPC2</td></tr>
<tr><td>280</td><td>214</td><td>280</td><td>0.999</td><td>108</td><td>sp|P62942|FKB1A_HUMAN</td><td>FKBP1A</td><td>P62942</td><td>Peptidyl-prolyl cis-trans isomerase FKBP1A</td><td>FKB1A_HUMAN</td><td></td><td>FKBP1A</td></tr>
<tr><td>221</td><td>221</td><td>221</td><td>0.999</td><td>217</td><td>sp|P62993|GRB2_HUMAN</td><td>GRB2</td><td>P62993</td><td>Growth factor receptor-bound protein 2</td><td>GRB2_HUMAN</td><td></td><td>GRB2</td></tr>
<tr><td>164</td><td>18</td><td>164</td><td>0.999</td><td>154</td><td>sp|P68036|UB2L3_HUMAN</td><td>UBE2L3</td><td>P68036</td><td>Ubiquitin-conjugating enzyme E2 L3</td><td>UB2L3_HUMAN</td><td></td><td>UBE2L3</td></tr>
<tr><td>19</td><td>19</td><td>19</td><td>0.999</td><td>464</td><td>sp|P78539|SRPX_HUMAN</td><td>SRPX</td><td>P78539</td><td>Sushi repeat-containing protein SRPX</td><td>SRPX_HUMAN</td><td></td><td>SRPX</td></tr>
<tr><td>239</td><td>239</td><td>679</td><td>0.999</td><td>61</td><td>sp|P80297|MT1X_HUMAN</td><td>MT1X</td><td>P80297</td><td>Metallothionein-1X</td><td>MT1X_HUMAN</td><td></td><td>MT1X</td></tr>
<tr><td>551</td><td>551</td><td>551</td><td>0.999</td><td>157</td><td>sp|P98179|RBM3_HUMAN</td><td>RBM3</td><td>P98179</td><td>RNA-binding protein 3</td><td>RBM3_HUMAN</td><td></td><td>RBM3</td></tr>
<tr><td>238</td><td>238</td><td>246</td><td>0.999</td><td>322</td><td>sp|Q00577|PURA_HUMAN</td><td>PURA</td><td>Q00577</td><td>Transcriptional activator protein Pur-alpha</td><td>PURA_HUMAN</td><td></td><td>PURA</td></tr>
<tr><td>347</td><td>347</td><td>347</td><td>0.999</td><td>189</td><td>sp|Q00765|REEP5_HUMAN</td><td>REEP5</td><td>Q00765</td><td>Receptor expression-enhancing protein 5</td><td>REEP5_HUMAN</td><td></td><td>REEP5</td></tr>
<tr><td>66</td><td>65</td><td>66</td><td>0.999</td><td>2944</td><td>sp|Q02388|CO7A1_HUMAN</td><td>COL7A1</td><td>Q02388</td><td>Collagen alpha-1(VII) chain</td><td>CO7A1_HUMAN</td><td></td><td>COL7A1</td></tr>
<tr><td>359</td><td>332</td><td>359</td><td>0.999</td><td>178</td><td>sp|Q03135|CAV1_HUMAN</td><td>CAV1</td><td>Q03135</td><td>Caveolin-1</td><td>CAV1_HUMAN</td><td></td><td>CAV1</td></tr>
<tr><td>213</td><td>213</td><td>213</td><td>0.999</td><td>335</td><td>sp|Q03405|UPAR_HUMAN</td><td>PLAUR</td><td>Q03405</td><td>Urokinase plasminogen activator surface receptor</td><td>UPAR_HUMAN</td><td></td><td>PLAUR</td></tr>
<tr><td>325</td><td>325</td><td>325</td><td>0.999</td><td>509</td><td>sp|Q0VDF9|HSP7E_HUMAN</td><td>HSPA14</td><td>Q0VDF9</td><td>Heat shock 70 kDa protein 14</td><td>HSP7E_HUMAN</td><td></td><td>HSPA14</td></tr>
<tr><td>57</td><td>40</td><td>57</td><td>0.999</td><td>859</td><td>sp|Q12979|ABR_HUMAN</td><td>ABR</td><td>Q12979</td><td>Active breakpoint cluster region-related protein</td><td>ABR_HUMAN</td><td></td><td>ABR</td></tr>
<tr><td>68</td><td>49</td><td>68</td><td>0.999</td><td>540</td><td>sp|Q13425|SNTB2_HUMAN</td><td>SNTB2</td><td>Q13425</td><td>Beta-2-syntrophin</td><td>SNTB2_HUMAN</td><td></td><td>SNTB2</td></tr>
<tr><td>176</td><td>176</td><td>176</td><td>0.999</td><td>292</td><td>sp|Q14165|MLEC_HUMAN</td><td>MLEC</td><td>Q14165</td><td>Malectin</td><td>MLEC_HUMAN</td><td></td><td>MLEC</td></tr>
<tr><td>129</td><td>129</td><td>129</td><td>0.999</td><td>578</td><td>sp|Q14699|RFTN1_HUMAN</td><td>RFTN1</td><td>Q14699</td><td>Raftlin</td><td>RFTN1_HUMAN</td><td></td><td>RFTN1</td></tr>
<tr><td>121</td><td>121</td><td>121</td><td>0.999</td><td>648</td><td>sp|Q15397|PUM3_HUMAN</td><td>PUM3</td><td>Q15397</td><td>Pumilio homolog 3</td><td>PUM3_HUMAN</td><td></td><td>KIAA0020</td></tr>
<tr><td>81</td><td>80</td><td>81</td><td>0.999</td><td>327</td><td>sp|Q15555|MARE2_HUMAN</td><td>MAPRE2</td><td>Q15555</td><td>Microtubule-associated protein RP/EB family member 2</td><td>MARE2_HUMAN</td><td></td><td>MAPRE2</td></tr>
<tr><td>358</td><td>358</td><td>358</td><td>0.999</td><td>228</td><td>sp|Q15631|TSN_HUMAN</td><td>TSN</td><td>Q15631</td><td>Translin</td><td>TSN_HUMAN</td><td></td><td>TSN</td></tr>
<tr><td>199</td><td>199</td><td>199</td><td>0.999</td><td>432</td><td>sp|Q15645|PCH2_HUMAN</td><td>TRIP13</td><td>Q15645</td><td>Pachytene checkpoint protein 2 homolog</td><td>PCH2_HUMAN</td><td></td><td>TRIP13</td></tr>
<tr><td>255</td><td>204</td><td>255</td><td>0.999</td><td>360</td><td>sp|Q16539|MK14_HUMAN</td><td>MAPK14</td><td>Q16539</td><td>Mitogen-activated protein kinase 14</td><td>MK14_HUMAN</td><td></td><td>MAPK14</td></tr>
<tr><td>295</td><td>295</td><td>295</td><td>0.999</td><td>551</td><td>sp|Q16630|CPSF6_HUMAN</td><td>CPSF6</td><td>Q16630</td><td>Cleavage and polyadenylation specificity factor subunit 6</td><td>CPSF6_HUMAN</td><td></td><td>CPSF6</td></tr>
<tr><td>69</td><td>69</td><td>131</td><td>0.999</td><td>2229</td><td>sp|Q5JSZ5|PRC2B_HUMAN</td><td>PRRC2B</td><td>Q5JSZ5</td><td>Protein PRRC2B</td><td>PRC2B_HUMAN</td><td></td><td>PRRC2B</td></tr>
<tr><td>76</td><td>76</td><td>76</td><td>0.999</td><td>1749</td><td>sp|Q5SRE5|NU188_HUMAN</td><td>NUP188</td><td>Q5SRE5</td><td>Nucleoporin NUP188</td><td>NU188_HUMAN</td><td></td><td>NUP188</td></tr>
<tr><td>468</td><td>15</td><td>468</td><td>0.999</td><td>136</td><td>sp|Q5TEC6|H37_HUMAN</td><td>H3-7</td><td>Q5TEC6</td><td>Histone H3-7</td><td>H37_HUMAN</td><td></td><td>HIST2H3PS2</td></tr>
<tr><td>69</td><td>69</td><td>69</td><td>0.999</td><td>2472</td><td>sp|Q5UIP0|RIF1_HUMAN</td><td>RIF1</td><td>Q5UIP0</td><td>Telomere-associated protein RIF1</td><td>RIF1_HUMAN</td><td></td><td>RIF1</td></tr>
</table>

118	118	118	0.999	838	sp|Q658Y4|F91A1_HUMAN	FAM91A1	Q658Y4	Protein FAM91A1	F91A1_HUMAN		FAM91A1
170	170	170	0.999	1043	sp|Q68E01|INT3_HUMAN	INTS3	Q68E01	Integrator complex subunit 3	INT3_HUMAN		INTS3
146	146	146	0.999	826	sp|Q6IA86|ELP2_HUMAN	ELP2	Q6IA86	Elongator complex protein 2	ELP2_HUMAN		ELP2
194	194	194	0.999	110	sp|Q7RTV0|PHF5A_HUMAN	PHF5A	Q7RTV0	PHD finger-like domain-containing protein 5A	PHF5A_HUMAN		PHF5A
104	104	104	0.999	507	sp|Q7Z3B4|NUP54_HUMAN	NUP54	Q7Z3B4	Nucleoporin p54	NUP54_HUMAN		NUP54
10	10	134	0.999	341	sp|Q7Z4I7|LIMS2_HUMAN	LIMS2	Q7Z4I7	LIM and senescent cell antigen-like-containing domain protein 2	LIMS2_HUMAN		LIMS2
208	208	265	0.999	227	sp|Q7Z7H5|TMED4_HUMAN	TMED4	Q7Z7H5	Transmembrane emp24 domain-containing protein 4	TMED4_HUMAN		TMED4
22	22	106	0.999	1631	sp|Q86VI3|IQGA3_HUMAN	IQGAP3	Q86VI3	Ras GTPase-activating-like protein IQGAP3	IQGA3_HUMAN		IQGAP3
96	96	96	0.999	2850	sp|Q86YZ3|HORN_HUMAN	HRNR	Q86YZ3	Hornerin	HORN_HUMAN		HRNR
135	135	135	0.999	306	sp|Q8IVM0|CCD50_HUMAN	CCDC50	Q8IVM0	Coiled-coil domain-containing protein 50	CCD50_HUMAN		CCDC50
57	57	71	0.999	2542	sp|Q8IWZ3|ANKH1_HUMAN	ANKHD1	Q8IWZ3	Ankyrin repeat and KH domain-containing protein 1	ANKH1_HUMAN		ANKHD1
255	255	255	0.999	793	sp|Q8IXB1|DJC10_HUMAN	DNAJC10	Q8IXB1	DnaJ homolog subfamily C member 10	DJC10_HUMAN		DNAJC10
220	199	220	0.999	618	sp|Q8IXI1|MIRO2_HUMAN	RHOT2	Q8IXI1	Mitochondrial Rho GTPase 2	MIRO2_HUMAN		RHOT2
131	131	131	0.999	1130	sp|Q8IZL8|PELP1_HUMAN	PELP1	Q8IZL8	Proline-, glutamic acid- and leucine-rich protein 1	PELP1_HUMAN		PELP1
110	109	110	0.999	1523	sp|Q8N3D4|EH1L1_HUMAN	EHBP1L1	Q8N3D4	EH domain-binding protein 1-like protein 1	EH1L1_HUMAN		EHBP1L1
149	149	149	0.999	863	sp|Q8N3F8|MILK1_HUMAN	MICALL1	Q8N3F8	MICAL-like protein 1	MILK1_HUMAN		MICALL1
74	74	159	0.999	518	sp|Q8NCA5|FA98A_HUMAN	FAM98A	Q8NCA5	Protein FAM98A	FA98A_HUMAN		FAM98A
294	294	294	0.999	470	sp|Q8NFQ8|TOIP2_HUMAN	TOR1AIP2	Q8NFQ8	Torsin-1A-interacting protein 2	TOIP2_HUMAN		TOR1AIP2
25	25	25	0.9989	1361	sp|Q8WUJ3|CEMIP_HUMAN	CEMIP	Q8WUJ3	Cell migration-inducing and hyaluronan-binding protein	CEMIP_HUMAN		CEMIP
190	167	190	0.999	531	sp|Q92925|SMRD2_HUMAN	SMARCD2	Q92925	SWI/SNF-related matrix-associated actin-dependent regulator of chromati	SMRD2_HUMAN		SMARCD2
91	91	91	0.999	578	sp|Q969N2|PIGT_HUMAN	PIGT	Q969N2	GPI transamidase component PIG-T	PIGT_HUMAN		PIGT
309	309	309	0.999	290	sp|Q969X5|ERGI1_HUMAN	ERGIC1	Q969X5	Endoplasmic reticulum-Golgi intermediate compartment protein 1	ERGI1_HUMAN		ERGIC1
209	209	209	0.999	352	sp|Q96A49|SYAP1_HUMAN	SYAP1	Q96A49	Synapse-associated protein 1	SYAP1_HUMAN		SYAP1
224	224	224	0.999	357	sp|Q96DI7|SNR40_HUMAN	SNRNP40	Q96DI7	U5 small nuclear ribonucleoprotein 40 kDa protein	SNR40_HUMAN		SNRNP40
194	194	194	0.999	262	sp|Q96E11|RRFM_HUMAN	MRRF	Q96E11	Ribosome-recycling factor, mitochondrial	RRFM_HUMAN		MRRF
178	178	178	0.999	689	sp|Q96EY7|PTCD3_HUMAN	PTCD3	Q96EY7	Small ribosomal subunit protein mS39	PTCD3_HUMAN		PTCD3
160	160	160	0.999	401	sp|Q96I25|SPF45_HUMAN	RBM17	Q96I25	Splicing factor 45	SPF45_HUMAN		RBM17
50	50	50	0.999	1222	sp|Q96JH7|VCIP1_HUMAN	VCPIP1	Q96JH7	Deubiquitinating protein VCPIP1	VCIP1_HUMAN		VCPIP1
159	159	159	0.999	1375	sp|Q96K76|UBP47_HUMAN	USP47	Q96K76	Ubiquitin carboxyl-terminal hydrolase 47	UBP47_HUMAN		USP47
178	178	178	0.999	322	sp|Q96KB5|TOPK_HUMAN	PBK	Q96KB5	Lymphokine-activated killer T-cell-originated protein kinase	TOPK_HUMAN		PBK
286	286	286	0.999	290	sp|Q99598|TSNAX_HUMAN	TSNAX	Q99598	Translin-associated protein X	TSNAX_HUMAN		TSNAX
262	262	262	0.999	385	sp|Q9BRX2|PELO_HUMAN	PELO	Q9BRX2	Protein pelota homolog	PELO_HUMAN		PELO
384	384	384	0.999	369	sp|Q9BV20|MTNA_HUMAN	MRI1	Q9BV20	Methylthioribose-1-phosphate isomerase	MTNA_HUMAN		MRI1
97	97	97	0.999	507	sp|Q9BV44|THUM3_HUMAN	THUMPD3	Q9BV44	tRNA (guanine(6)-N2)-methyltransferase THUMP3	THUM3_HUMAN		THUMPD3
162	162	162	0.999	928	sp|Q9BYX2|TBD2A_HUMAN	TBC1D2	Q9BYX2	TBC1 domain family member 2A	TBD2A_HUMAN		TBC1D2
186	186	186	0.999	423	sp|Q9BZE1|RM37_HUMAN	MRPL37	Q9BZE1	Large ribosomal subunit protein mL37	RM37_HUMAN		MRPL37
152	152	152	0.999	419	sp|Q9GZS1|RPA49_HUMAN	POLR1E	Q9GZS1	DNA-directed RNA polymerase I subunit RPA49	RPA49_HUMAN		POLR1E
67	67	67	0.999	632	sp|Q9H0H5|RGAP1_HUMAN	RACGAP1	Q9H0H5	Rac GTPase-activating protein 1	RGAP1_HUMAN		RACGAP1
204	204	204	0.999	298	sp|Q9H4A6|GOLP3_HUMAN	GOLPH3	Q9H4A6	Golgi phosphoprotein 3	GOLP3_HUMAN		GOLPH3
183	183	183	0.999	1146	sp|Q9H6R4|NOL6_HUMAN	NOL6	Q9H6R4	Nucleolar protein 6	NOL6_HUMAN		NOL6
157	157	157	0.999	1430	sp|Q9H6S0|YTDC2_HUMAN	YTHDC2	Q9H6S0	3'-5' RNA helicase YTHDC2	YTDC2_HUMAN		YTHDC2
113	113	113	0.999	462	sp|Q9HD26|GOPC_HUMAN	GOPC	Q9HD26	Golgi-associated PDZ and coiled-coil motif-containing protein	GOPC_HUMAN		GOPC
472	472	472	0.999	1192	sp|Q9NQC3|RTN4_HUMAN	RTN4	Q9NQC3	Reticulon-4	RTN4_HUMAN		RTN4
122	111	122	0.999	1826	sp|Q9NQT8|KI13B_HUMAN	KIF13B	Q9NQT8	Kinesin-like protein KIF13B	KI13B_HUMAN		KIF13B
258	258	258	0.999	452	sp|Q9NR50|EI2BG_HUMAN	EIF2B3	Q9NR50	Translation initiation factor eIF-2B subunit gamma	EI2BG_HUMAN		EIF2B3
43	43	43	0.9989	406	sp|Q9NRN5|OLFL3_HUMAN	OLFML3	Q9NRN5	Olfactomedin-like protein 3	OLFL3_HUMAN		OLFML3
60	57	60	0.999	361	sp|Q9NSC5|HOME3_HUMAN	HOMER3	Q9NSC5	Homer protein homolog 3	HOME3_HUMAN		HOMER3
166	166	166	0.999	273	sp|Q9NWB6|ARGL1_HUMAN	ARGLU1	Q9NWB6	Arginine and glutamate-rich protein 1	ARGL1_HUMAN		ARGLU1
282	282	282	0.999	245	sp|Q9NX40|OCAD1_HUMAN	OCIAD1	Q9NX40	OCIA domain-containing protein 1	OCAD1_HUMAN		OCIAD1
146	146	146	0.999	1060	sp|Q9P2N5|RBM27_HUMAN	RBM27	Q9P2N5	RNA-binding protein 27	RBM27_HUMAN		RBM27
163	163	163	0.999	1169	sp|Q9P2R3|ANFY1_HUMAN	ANKFY1	Q9P2R3	Rabankyrin-5	ANFY1_HUMAN		ANKFY1
99	99	99	0.999	1215	sp|Q9UBN7|HDAC6_HUMAN	HDAC6	Q9UBN7	Histone deacetylase 6	HDAC6_HUMAN		HDAC6
277	277	277	0.999	760	sp|Q9UGP8|SEC63_HUMAN	SEC63	Q9UGP8	Translocation protein SEC63 homolog	SEC63_HUMAN		SEC63
280	280	280	0.999	305	sp|Q9UHQ9|NB5R1_HUMAN	CYB5R1	Q9UHQ9	NADH-cytochrome b5 reductase 1	NB5R1_HUMAN		CYB5R1
152	152	152	0.999	344	sp|Q9UJ70|NAGK_HUMAN	NAGK	Q9UJ70	N-acetyl-D-glucosamine kinase	NAGK_HUMAN		NAGK
355	355	355	0.999	154	sp|Q9UK76|JUPI1_HUMAN	JPT1	Q9UK76	Jupiter microtubule associated homolog 1	JUPI1_HUMAN		HN1
261	115	261	0.999	181	sp|Q9ULC4|MCTS1_HUMAN	MCTS1	Q9ULC4	Malignant T-cell-amplified sequence 1	MCTS1_HUMAN		MCTS1
155	155	155	0.999	943	sp|Q9UNX4|WDR3_HUMAN	WDR3	Q9UNX4	WD repeat-containing protein 3	WDR3_HUMAN		WDR3
69	69	69	0.999	722	sp|Q9Y223|GLCNE_HUMAN	GNE	Q9Y223	Bifunctional UDP-N-acetylglucosamine 2-epimerase/N-acetylmannosamine	GLCNE_HUMAN		GNE
339	339	339	0.999	365	sp|Q9Y2Z0|SGT1_HUMAN	SUGT1	Q9Y2Z0	Protein SGT1 homolog	SGT1_HUMAN		SUGT1
400	400	400	0.999	271	sp|Q9Y5M8|SRPRB_HUMAN	SRPRB	Q9Y5M8	Signal recognition particle receptor subunit beta	SRPRB_HUMAN		SRPRB
131	131	131	0.999	595	sp|Q9Y5X1|SNX9_HUMAN	SNX9	Q9Y5X1	Sorting nexin-9	SNX9_HUMAN		SNX9
515	515	515	0.999	303	sp|Q9Y6C9|MTCH2_HUMAN	MTCH2	Q9Y6C9	Mitochondrial carrier homolog 2	MTCH2_HUMAN		MTCH2
519	519	519	0.999	357	sp|O00303|EIF3F_HUMAN	EIF3F	O00303	Eukaryotic translation initiation factor 3 subunit F	EIF3F_HUMAN		EIF3F
241	241	241	0.999	501	sp|O00330|ODPX_HUMAN	PDHX	O00330	Pyruvate dehydrogenase protein X component, mitochondrial	ODPX_HUMAN		PDHX
210	210	210	0.999	696	sp|O00461|GOLI4_HUMAN	GOLIM4	O00461	Golgi integral membrane protein 4	GOLI4_HUMAN		GOLIM4
130	130	130	0.999	708	sp|O00471|EXOC5_HUMAN	EXOC5	O00471	Exocyst complex component 5	EXOC5_HUMAN		EXOC5
284	284	284	0.999	310	sp|O00487|PSDE_HUMAN	PSMD14	O00487	26S proteasome non-ATPase regulatory subunit 14	PSDE_HUMAN		PSMD14
99	99	214	0.999	521	sp|O00629|IMA3_HUMAN	KPNA4	O00629	Importin subunit alpha-3	IMA3_HUMAN		KPNA4
530	530	530	0.999	308	sp|O14579|COPE_HUMAN	COPE	O14579	Coatomer subunit epsilon	COPE_HUMAN		COPE
500	0	500	0.999	172	sp|O14950|ML12B_HUMAN	MYL12B	O14950	Myosin regulatory light chain 12B	ML12B_HUMAN	sp|P19105|ML12A_HUMAN	MYL12B
416	416	416	0.999	129	sp|O15212|PFD6_HUMAN	PFDN6	O15212	Prefoldin subunit 6	PFD6_HUMAN		PFDN6
281	281	281	0.999	473	sp|O15269|SPTC1_HUMAN	SPTLC1	O15269	Serine palmitoyltransferase 1	SPTC1_HUMAN		SPTLC1
572	572	572	0.999	465	sp|O15427|MOT4_HUMAN	SLC16A3	O15427	Monocarboxylate transporter 4	MOT4_HUMAN		SLC16A3
110	110	110	0.999	476	sp|O43148|MCES_HUMAN	RNMT	O43148	mRNA cap guanine-N7 methyltransferase	MCES_HUMAN		RNMT
444	444	444	0.999	289	sp|O43396|TXNL1_HUMAN	TXNL1	O43396	Thioredoxin-like protein 1	TXNL1_HUMAN		TXNL1
266	264	266	0.999	162	sp|O60493|SNX3_HUMAN	SNX3	O60493	Sorting nexin-3	SNX3_HUMAN		SNX3
137	75	195	0.999	536	sp|O60684|IMA7_HUMAN	KPNA6	O60684	Importin subunit alpha-7	IMA7_HUMAN		KPNA6
201	116	201	0.999	1362	sp|O60885|BRD4_HUMAN	BRD4	O60885	Bromodomain-containing protein 4	BRD4_HUMAN		BRD4
421	421	421	0.999	122	sp|O60925|PFD1_HUMAN	PFDN1	O60925	Prefoldin subunit 1	PFD1_HUMAN		PFDN1
295	295	295	0.999	188	sp|O75223|GGCT_HUMAN	GGCT	O75223	Gamma-glutamylcyclotransferase	GGCT_HUMAN		GGCT
184	184	184	0.999	272	sp|O75663|TIPRL_HUMAN	TIPRL	O75663	TIP41-like protein	TIPRL_HUMAN		TIPRL
110	110	110	0.999	350	sp|O75695|XRP2_HUMAN	RP2	O75695	Protein XRP2	XRP2_HUMAN		RP2
73	73	73	0.999	3013	sp|O94915|FRYL_HUMAN	FRYL	O94915	Protein furry homolog-like	FRYL_HUMAN		FRYL
288	288	288	0.999	330	sp|O95218|ZRAB2_HUMAN	ZRANB2	O95218	Zinc finger Ran-binding domain-containing protein 2	ZRAB2_HUMAN		ZRANB2
214	164	216	0.999	570	sp|O95302|FKBP9_HUMAN	FKBP9	O95302	Peptidyl-prolyl cis-trans isomerase FKBP9	FKBP9_HUMAN		FKBP9
193	137	193	0.999	564	sp|O95361|TRI16_HUMAN	TRIM16	O95361	Tripartite motif-containing protein 16	TRI16_HUMAN		TRIM16
186	147	186	0.999	1093	sp|O95486|SC24A_HUMAN	SEC24A	O95486	Protein transport protein Sec24A	SC24A_HUMAN		SEC24A
245	245	245	0.999	258	sp|O95721|SNP29_HUMAN	SNAP29	O95721	Synaptosomal-associated protein 29	SNP29_HUMAN		SNAP29
251	251	251	0.999	474	sp|P02774|VTDB_HUMAN	GC	P02774	Vitamin D-binding protein	VTDB_HUMAN		GC
416	411	416	0.999	217	sp|P06730|IF4E_HUMAN	EIF4E	P06730	Eukaryotic translation initiation factor 4E	IF4E_HUMAN		EIF4E
114	114	154	0.999	529	sp|P06865|HEXA_HUMAN	HEXA	P06865	Beta-hexosaminidase subunit alpha	HEXA_HUMAN		HEXA
651	651	651	0.999	105	sp|P10599|THIO_HUMAN	TXN	P10599	Thioredoxin	THIO_HUMAN		TXN
307	307	307	0.999	107	sp|P17096|HMGA1_HUMAN	HMGA1	P17096	High mobility group protein HMG-I/HMG-Y	HMGA1_HUMAN		HMGA1
371	371	392	0.999	350	sp|P19784|CSK22_HUMAN	CSNK2A2	P19784	Casein kinase II subunit alpha'	CSK22_HUMAN		CSNK2A2
347	138	347	0.999	414	sp|P25490|TYY1_HUMAN	YY1	P25490	Transcriptional repressor protein YY1	TYY1_HUMAN		YY1
332	332	332	0.999	340	sp|P25685|DNJB1_HUMAN	DNAJB1	P25685	DnaJ homolog subfamily B member 1	DNJB1_HUMAN		DNAJB1
500	500	500	0.999	101	sp|P26447|S10A4_HUMAN	S100A4	P26447	Protein S100-A4	S10A4_HUMAN		S100A4
20	20	20	0.9989	766	sp|P27487|DPP4_HUMAN	DPP4	P27487	Dipeptidyl peptidase 4	DPP4_HUMAN		DPP4
175	88	175	0.999	480	sp|P31749|AKT1_HUMAN	AKT1	P31749	RAC-alpha serine/threonine-protein kinase	AKT1_HUMAN		AKT1
452	452	452	0.999	252	sp|P33316|DUT_HUMAN	DUT	P33316	Deoxyuridine 5'-triphosphate nucleotidohydrolase, mitochondrial	DUT_HUMAN		DUT
370	243	370	0.999	128	sp|P35268|RL22_HUMAN	RPL22	P35268	Large ribosomal subunit protein eL22	RL22_HUMAN		RPL22
203	203	203	0.999	144	sp|P41223|BUD31_HUMAN	BUD31	P41223	Protein BUD31 homolog	BUD31_HUMAN		BUD31
520	520	520	0.999	123	sp|P42766|RL35_HUMAN	RPL35	P42766	Large ribosomal subunit protein uL29	RL35_HUMAN		RPL35
63	63	63	0.999	646	sp|P43121|MUC18_HUMAN	MCAM	P43121	Cell surface glycoprotein MUC18	MUC18_HUMAN		MCAM
341	122	341	0.999	274	sp|P47985|UCRI_HUMAN	UQCRFS1	P47985	Cytochrome b-c1 complex subunit Rieske, mitochondrial	UCRI_HUMAN		UQCRFS1
185	185	185	0.999	572	sp|P48163|MAOX_HUMAN	ME1	P48163	NADP-dependent malic enzyme	MAOX_HUMAN		ME1
304	268	304	0.999	253	sp|P48509|CD151_HUMAN	CD151	P48509	CD151 antigen	CD151_HUMAN		CD151
126	126	126	0.999	474	sp|P48637|GSHB_HUMAN	GSS	P48637	Glutathione synthetase	GSHB_HUMAN		GSS
207	207	207	0.999	292	sp|P49406|RM19_HUMAN	MRPL19	P49406	Large ribosomal subunit protein bL19m	RM19_HUMAN		MRPL19
244	244	430	0.999	673	sp|P51116|FXR2_HUMAN	FXR2	P51116	RNA-binding protein FXR2	FXR2_HUMAN		FXR2
471	471	471	0.999	324	sp|P51665|PSMD7_HUMAN	PSMD7	P51665	26S proteasome non-ATPase regulatory subunit 7	PSMD7_HUMAN		PSMD7
313	212	313	0.999	538	sp|P52294|IMA5_HUMAN	KPNA1	P52294	Importin subunit alpha-5	IMA5_HUMAN		KPNA1
431	431	431	0.999	463	sp|P53634|CATC_HUMAN	CTSC	P53634	Dipeptidyl peptidase 1	CATC_HUMAN		CTSC
634	634	634	0.999	97	sp|P60903|S10AA_HUMAN	S100A10	P60903	Protein S100-A10	S10AA_HUMAN		S100A10
463	216	463	0.999	212	sp|P61019|RAB2A_HUMAN	RAB2A	P61019	Ras-related protein Rab-2A	RAB2A_HUMAN		RAB2A
91	0	372	0.999	184	sp|P61224|RAP1B_HUMAN	RAP1B	P61224	Ras-related protein Rap-1b	RAP1B_HUMAN		RAP1B
392	0	392	0.999	146	sp|P61326|MGN_HUMAN	MAGOH	P61326	Protein mago nashi homolog	MGN_HUMAN	sp|Q96A72|MGN2_HUMAN	MAGOH
493	252	493	0.999	476	sp|P61619|S61A1_HUMAN	SEC61A1	P61619	Protein transport protein Sec61 subunit alpha isoform 1	S61A1_HUMAN		SEC61A1
629	629	629	0.999	126	sp|P62318|SMD3_HUMAN	SNRPD3	P62318	Small nuclear ribonucleoprotein Sm D3	SMD3_HUMAN		SNRPD3
539	156	675	0.999	192	sp|P63000|RAC1_HUMAN	RAC1	P63000	Ras-related C3 botulinum toxin substrate 1	RAC1_HUMAN		RAC1
382	193	382	0.999	179	sp|P67812|SC11A_HUMAN	SEC11A	P67812	Signal peptidase complex catalytic subunit SEC11A	SC11A_HUMAN		SEC11A
250	250	250	0.999	368	sp|P78406|RAE1L_HUMAN	RAE1	P78406	mRNA export factor RAE1	RAE1L_HUMAN		RAE1
59	59	59	0.999	770	sp|P98082|DAB2_HUMAN	DAB2	P98082	Disabled homolog 2	DAB2_HUMAN		DAB2
454	454	454	0.999	414	sp|Q12765|SCRN1_HUMAN	SCRN1	Q12765	Secernin-1	SCRN1_HUMAN		SCRN1
224	224	224	0.999	717	sp|Q12996|CSTF3_HUMAN	CSTF3	Q12996	Cleavage stimulation factor subunit 3	CSTF3_HUMAN		CSTF3
299	299	299	0.999	564	sp|Q13057|COASY_HUMAN	COASY	Q13057	Bifunctional coenzyme A synthase	COASY_HUMAN		COASY
495	495	495	0.999	320	sp|Q13155|AIMP2_HUMAN	AIMP2	Q13155	Aminoacyl tRNA synthase complex-interacting multifunctional protein 2	AIMP2_HUMAN		AIMP2
169	169	234	0.999	272	sp|Q13243|SRSF5_HUMAN	SRSF5	Q13243	Serine/arginine-rich splicing factor 5	SRSF5_HUMAN		SRSF5

146	146	361	0.999	440	sp|Q13363|CTBP1_HUMAN	CTBP1	Q13363	C-terminal-binding protein 1	CTBP1_HUMAN		CTBP1
206	206	206	0.999	819	sp|Q13443|ADAM9_HUMAN	ADAM9	Q13443	Disintegrin and metalloproteinase domain-containing protein 9	ADAM9_HUMAN		ADAM9
465	465	465	0.999	440	sp|Q13501|SQSTM_HUMAN	SQSTM1	Q13501	Sequestosome-1	SQSTM_HUMAN		SQSTM1
123	123	123	0.999	671	sp|Q13546|RIPK1_HUMAN	RIPK1	Q13546	Receptor-interacting serine/threonine-protein kinase 1	RIPK1_HUMAN		RIPK1
195	195	195	0.999	437	sp|Q13895|BYST_HUMAN	BYSL	Q13895	Bystin	BYST_HUMAN		BYSL
159	159	159	0.999	428	sp|Q14254|FLOT2_HUMAN	FLOT2	Q14254	Flotillin-2	FLOT2_HUMAN		FLOT2
267	267	267	0.999	348	sp|Q14376|GALE_HUMAN	GALE	Q14376	UDP-glucose 4-epimerase	GALE_HUMAN		GALE
330	266	330	0.999	1186	sp|Q14671|PUM1_HUMAN	PUM1	Q14671	Pumilio homolog 1	PUM1_HUMAN		PUM1
379	379	379	0.999	516	sp|Q15392|DHC24_HUMAN	DHCR24	Q15392	Delta(24)-sterol reductase	DHC24_HUMAN		DHCR24
494	494	494	0.999	464	sp|Q15428|SF3A2_HUMAN	SF3A2	Q15428	Splicing factor 3A subunit 2	SF3A2_HUMAN		SF3A2
341	139	341	0.999	145	sp|Q15819|UB2V2_HUMAN	UBE2V2	Q15819	Ubiquitin-conjugating enzyme E2 variant 2	UB2V2_HUMAN		UBE2V2
227	223	227	0.999	984	sp|Q16513|PKN2_HUMAN	PKN2	Q16513	Serine/threonine-protein kinase N2	PKN2_HUMAN		PKN2
329	329	331	0.999	193	sp|Q16527|CSRP2_HUMAN	CSRP2	Q16527	Cysteine and glycine-rich protein 2	CSRP2_HUMAN		CSRP2
295	210	295	0.999	433	sp|Q52LJ0|FA98B_HUMAN	FAM98B	Q52LJ0	Protein FAM98B	FA98B_HUMAN		FAM98B
273	273	273	0.999	469	sp|Q53EL6|PDCD4_HUMAN	PDCD4	Q53EL6	Programmed cell death protein 4	PDCD4_HUMAN		PDCD4
119	119	121	0.999	1068	sp|Q5M775|CYTSB_HUMAN	SPECC1	Q5M775	Cytospin-B	CYTSB_HUMAN		SPECC1
307	307	307	0.999	312	sp|Q6FI81|CPIN1_HUMAN	CIAPIN1	Q6FI81	Anamorsin	CPIN1_HUMAN		CIAPIN1
110	110	110	0.999	1564	sp|Q6PGP7|SKI3_HUMAN	SKIC3	Q6PGP7	Superkiller complex protein 3	SKI3_HUMAN		TTC37
89	89	89	0.999	1250	sp|Q70E73|RAPH1_HUMAN	RAPH1	Q70E73	Ras-associated and pleckstrin homology domains-containing protein 1	RAPH1_HUMAN		RAPH1
156	156	156	0.999	879	sp|Q8NCN5|PDPR_HUMAN	PDPR	Q8NCN5	Pyruvate dehydrogenase phosphatase regulatory subunit, mitochondrial	PDPR_HUMAN		PDPR
436	436	436	0.999	288	sp|Q8NCW5|NNRE_HUMAN	NAXE	Q8NCW5	NAD(P)H-hydrate epimerase	NNRE_HUMAN		APOA1BP
212	212	212	0.999	1593	sp|Q8NI27|THOC2_HUMAN	THOC2	Q8NI27	THO complex subunit 2	THOC2_HUMAN		THOC2
173	148	173	0.999	318	sp|Q8TC12|RDH11_HUMAN	RDH11	Q8TC12	Retinol dehydrogenase 11	RDH11_HUMAN		RDH11
181	181	181	0.999	881	sp|Q8TDD1|DDX54_HUMAN	DDX54	Q8TDD1	ATP-dependent RNA helicase DDX54	DDX54_HUMAN		DDX54
248	248	248	0.999	260	sp|Q8WXX5|DNJC9_HUMAN	DNAJC9	Q8WXX5	DnaJ homolog subfamily C member 9	DNJC9_HUMAN		DNAJC9
147	147	147	0.999	2266	sp|Q8WYP5|ELYS_HUMAN	AHCTF1	Q8WYP5	Protein ELYS	ELYS_HUMAN		AHCTF1
183	183	183	0.999	411	sp|Q969G3|SMCE1_HUMAN	SMARCE1	Q969G3	SWI/SNF-related matrix-associated actin-dependent regulator of chromatir	SMCE1_HUMAN		SMARCE1
246	246	246	0.999	907	sp|Q96CW5|GCP3_HUMAN	TUBGCP3	Q96CW5	Gamma-tubulin complex component 3	GCP3_HUMAN		TUBGCP3
250	224	250	0.999	312	sp|Q96QR8|PURB_HUMAN	PURB	Q96QR8	Transcriptional activator protein Pur-beta	PURB_HUMAN		PURB
167	95	167	0.999	725	sp|Q99661|KIF2C_HUMAN	KIF2C	Q99661	Kinesin-like protein KIF2C	KIF2C_HUMAN		KIF2C
299	299	299	0.999	123	sp|Q9BRA2|TXD17_HUMAN	TXNDC17	Q9BRA2	Thioredoxin domain-containing protein 17	TXD17_HUMAN		TXNDC17
499	442	499	0.999	235	sp|Q9BVK6|TMED9_HUMAN	TMED9	Q9BVK6	Transmembrane emp24 domain-containing protein 9	TMED9_HUMAN		TMED9
172	172	250	0.999	321	sp|Q9BWM7|SFXN3_HUMAN	SFXN3	Q9BWM7	Sideroflexin-3	SFXN3_HUMAN		SFXN3
267	267	267	0.999	423	sp|Q9BXW7|HDHD5_HUMAN	HDHD5	Q9BXW7	Haloacid dehalogenase-like hydrolase domain-containing 5	HDHD5_HUMAN		CECR5
218	218	218	0.999	1102	sp|Q9H2P0|ADNP_HUMAN	ADNP	Q9H2P0	Activity-dependent neuroprotector homeobox protein	ADNP_HUMAN		ADNP
177	177	177	0.999	661	sp|Q9H7D7|WDR26_HUMAN	WDR26	Q9H7D7	WD repeat-containing protein 26	WDR26_HUMAN		WDR26
166	166	166	0.999	309	sp|Q9HA64|KT3K_HUMAN	FN3KRP	Q9HA64	Ketosamine-3-kinase	KT3K_HUMAN		FN3KRP
155	155	155	0.999	563	sp|Q9HCC0|MCCB_HUMAN	MCCC2	Q9HCC0	Methylcrotonoyl-CoA carboxylase beta chain, mitochondrial	MCCB_HUMAN		MCCC2
246	246	246	0.999	516	sp|Q9NP61|ARFG3_HUMAN	ARFGAP3	Q9NP61	ADP-ribosylation factor GTPase-activating protein 3	ARFG3_HUMAN		ARFGAP3
180	180	180	0.999	518	sp|Q9NP81|SYSM_HUMAN	SARS2	Q9NP81	Serine--tRNA ligase, mitochondrial	SYSM_HUMAN		SARS2
100	100	419	0.999	371	sp|Q9NQ29|LUC7L_HUMAN	LUC7L	Q9NQ29	Putative RNA-binding protein Luc7-like 1	LUC7L_HUMAN		LUC7L
193	166	193	0.999	326	sp|Q9NQG5|RPR1B_HUMAN	RPRD1B	Q9NQG5	Regulation of nuclear pre-mRNA domain-containing protein 1B	RPR1B_HUMAN		RPRD1B
328	143	328	0.999	388	sp|Q9NR56|MBNL1_HUMAN	MBNL1	Q9NR56	Muscleblind-like protein 1	MBNL1_HUMAN		MBNL1
291	211	291	0.999	372	sp|Q9NVD7|PARVA_HUMAN	PARVA	Q9NVD7	Alpha-parvin	PARVA_HUMAN		PARVA
133	132	133	0.999	1576	sp|Q9P265|DIP2B_HUMAN	DIP2B	Q9P265	Disco-interacting protein 2 homolog B	DIP2B_HUMAN		DIP2B
256	256	256	0.999	475	sp|Q9UBB4|ATX10_HUMAN	ATXN10	Q9UBB4	Ataxin-10	ATX10_HUMAN		ATXN10
436	436	436	0.999	328	sp|Q9UBQ7|GRHPR_HUMAN	GRHPR	Q9UBQ7	Glyoxylate reductase/hydroxypyruvate reductase	GRHPR_HUMAN		GRHPR
415	415	415	0.999	303	sp|Q9UBR2|CATZ_HUMAN	CTSZ	Q9UBR2	Cathepsin Z	CATZ_HUMAN		CTSZ
156	156	156	0.999	619	sp|Q9UBU9|NXF1_HUMAN	NXF1	Q9UBU9	Nuclear RNA export factor 1	NXF1_HUMAN		NXF1
238	238	238	0.999	227	sp|Q9UIJ7|KAD3_HUMAN	AK3	Q9UIJ7	GTP:AMP phosphotransferase AK3, mitochondrial	KAD3_HUMAN		AK3
297	297	297	0.999	412	sp|Q9ULX3|NOB1_HUMAN	NOB1	Q9ULX3	RNA-binding protein NOB1	NOB1_HUMAN		NOB1
203	203	203	0.999	646	sp|Q9ULX6|AKP8L_HUMAN	AKAP8L	Q9ULX6	A-kinase anchor protein 8-like	AKP8L_HUMAN		AKAP8L
180	180	182	0.999	162	sp|Q9UMY4|SNX12_HUMAN	SNX12	Q9UMY4	Sorting nexin-12	SNX12_HUMAN		SNX12
114	93	114	0.999	1085	sp|Q9UP95|S12A4_HUMAN	SLC12A4	Q9UP95	Solute carrier family 12 member 4	S12A4_HUMAN		SLC12A4
392	392	392	0.999	182	sp|Q9Y2B0|CNPY2_HUMAN	CNPY2	Q9Y2B0	Protein canopy homolog 2	CNPY2_HUMAN		CNPY2
184	184	184	0.999	228	sp|Q9Y508|RN114_HUMAN	RNF114	Q9Y508	E3 ubiquitin-protein ligase RNF114	RN114_HUMAN		RNF114
130	130	130	0.999	642	sp|Q9Y6Y0|NS1BP_HUMAN	IVNS1ABP	Q9Y6Y0	Influenza virus NS1A-binding protein	NS1BP_HUMAN		IVNS1ABP
139	125	139	0.999	911	sp|A1X283|SPD2B_HUMAN	SH3PXD2B	A1X283	SH3 and PX domain-containing protein 2B	SPD2B_HUMAN		SH3PXD2B
263	263	263	0.999	592	sp|O00186|STXB3_HUMAN	STXBP3	O00186	Syntaxin-binding protein 3	STXB3_HUMAN		STXBP3
322	322	322	0.999	195	sp|O00264|PGRC1_HUMAN	PGRMC1	O00264	Membrane-associated progesterone receptor component 1	PGRC1_HUMAN		PGRMC1
56	56	56	0.999	1037	sp|O00291|HIP1_HUMAN	HIP1	O00291	Huntingtin-interacting protein 1	HIP1_HUMAN		HIP1
341	341	341	0.999	312	sp|O00764|PDXK_HUMAN	PDXK	O00764	Pyridoxal kinase	PDXK_HUMAN		PDXK
487	487	487	0.999	178	sp|O15145|ARPC3_HUMAN	ARPC3	O15145	Actin-related protein 2/3 complex subunit 3	ARPC3_HUMAN		ARPC3
206	202	206	0.999	222	sp|O43633|CHM2A_HUMAN	CHMP2A	O43633	Charged multivesicular body protein 2a	CHM2A_HUMAN		CHMP2A
405	142	405	0.999	348	sp|O75477|ERLN1_HUMAN	ERLIN1	O75477	Erlin-1	ERLN1_HUMAN		ERLIN1
513	513	513	0.999	161	sp|O75947|ATP5H_HUMAN	ATP5PD	O75947	ATP synthase subunit d, mitochondrial	ATP5H_HUMAN		ATP5H
170	170	172	0.999	432	sp|O95232|LC7L3_HUMAN	LUC7L3	O95232	Luc7-like protein 3	LC7L3_HUMAN		LUC7L3
145	145	386	0.999	552	sp|O95758|PTBP3_HUMAN	PTBP3	O95758	Polypyrimidine tract-binding protein 3	PTBP3_HUMAN		PTBP3
147	122	147	0.999	285	sp|O95865|DDAH2_HUMAN	DDAH2	O95865	N(G),N(G)-dimethylarginine dimethylaminohydrolase 2	DDAH2_HUMAN		DDAH2
1019	579	1019	0.999	61	sp|P02795|MT2_HUMAN	MT2A	P02795	Metallothionein-2	MT2_HUMAN		MT2A
97	97	97	0.999	398	sp|P07093|GDN_HUMAN	SERPINE2	P07093	Glia-derived nexin	GDN_HUMAN		SERPINE2
419	419	419	0.999	325	sp|P08574|CY1_HUMAN	CYC1	P08574	Cytochrome c1, heme protein, mitochondrial	CY1_HUMAN		CYC1
146	146	164	0.999	218	sp|P10301|RRAS_HUMAN	RRAS	P10301	Ras-related protein R-Ras	RRAS_HUMAN		RRAS
104	104	383	0.999	366	sp|P10321|HLAC_HUMAN	HLA-C	P10321	HLA class I histocompatibility antigen, C alpha chain	HLAC_HUMAN		HLA-C
223	223	223	0.999	445	sp|P12694|ODBA_HUMAN	BCKDHA	P12694	2-oxoisovalerate dehydrogenase subunit alpha, mitochondrial	ODBA_HUMAN		BCKDHA
416	355	477	0.999	325	sp|P14550|AK1A1_HUMAN	AKR1A1	P14550	Aldo-keto reductase family 1 member A1	AK1A1_HUMAN		AKR1A1
261	261	261	0.999	270	sp|P15927|RFA2_HUMAN	RPA2	P15927	Replication protein A 32 kDa subunit	RFA2_HUMAN		RPA2
250	76	250	0.999	351	sp|P17612|KAPCA_HUMAN	PRKACA	P17612	cAMP-dependent protein kinase catalytic subunit alpha	KAPCA_HUMAN		PRKACA
458	458	458	0.999	250	sp|P17931|LEG3_HUMAN	LGALS3	P17931	Galectin-3	LEG3_HUMAN		LGALS3
399	399	399	0.999	421	sp|P18754|RCC1_HUMAN	RCC1	P18754	Regulator of chromosome condensation	RCC1_HUMAN		RCC1
310	310	310	0.999	277	sp|P20645|MPRD_HUMAN	M6PR	P20645	Cation-dependent mannose-6-phosphate receptor	MPRD_HUMAN		M6PR
60	60	180	0.999	514	sp|P20839|IMDH1_HUMAN	IMPDH1	P20839	Inosine-5'-monophosphate dehydrogenase 1	IMDH1_HUMAN		IMPDH1
114	114	114	0.999	1047	sp|P20936|RASA1_HUMAN	RASA1	P20936	Ras GTPase-activating protein 1	RASA1_HUMAN		RASA1
351	146	351	0.999	511	sp|P21281|VATB2_HUMAN	ATP6V1B2	P21281	V-type proton ATPase subunit B, brain isoform	VATB2_HUMAN		ATP6V1B2
386	386	386	0.999	227	sp|P22061|PIMT_HUMAN	PCMT1	P22061	Protein-L-isoaspartate(D-aspartate) O-methyltransferase	PIMT_HUMAN		PCMT1
207	207	207	0.999	491	sp|P22570|ADRO_HUMAN	FDXR	P22570	NADPH:adrenodoxin oxidoreductase, mitochondrial	ADRO_HUMAN		FDXR
306	275	306	0.999	584	sp|P23368|MAOM_HUMAN	ME2	P23368	NAD-dependent malic enzyme, mitochondrial	MAOM_HUMAN		ME2
512	512	512	0.999	241	sp|P28066|PSA5_HUMAN	PSMA5	P28066	Proteasome subunit alpha type-5	PSA5_HUMAN		PSMA5
420	382	420	0.999	496	sp|P30419|NMT1_HUMAN	NMT1	P30419	Glycylpeptide N-tetradecanoyltransferase 1	NMT1_HUMAN		NMT1
228	218	228	0.999	319	sp|P32322|P5CR1_HUMAN	PYCR1	P32322	Pyrroline-5-carboxylate reductase 1, mitochondrial	P5CR1_HUMAN		PYCR1
208	158	208	0.999	577	sp|P33240|CSTF2_HUMAN	CSTF2	P33240	Cleavage stimulation factor subunit 2	CSTF2_HUMAN		CSTF2
202	150	202	0.999	1531	sp|P33527|MRP1_HUMAN	ABCC1	P33527	Multidrug resistance-associated protein 1	MRP1_HUMAN		ABCC1
94	49	94	0.999	380	sp|P41221|WNT5A_HUMAN	WNT5A	P41221	Protein Wnt-5a	WNT5A_HUMAN		WNT5A
536	372	536	0.999	235	sp|P41227|NAA10_HUMAN	NAA10	P41227	N-alpha-acetyltransferase 10	NAA10_HUMAN		NAA10
100	100	100	0.999	770	sp|P42892|ECE1_HUMAN	ECE1	P42892	Endothelin-converting enzyme 1	ECE1_HUMAN		ECE1
300	300	300	0.999	325	sp|P43897|EFTS_HUMAN	TSFM	P43897	Elongation factor Ts, mitochondrial	EFTS_HUMAN		TSFM
464	426	464	0.999	289	sp|P46926|GNPI1_HUMAN	GNPDA1	P46926	Glucosamine-6-phosphate isomerase 1	GNPI1_HUMAN		GNPDA1
319	319	319	0.999	504	sp|P46937|YAP1_HUMAN	YAP1	P46937	Transcriptional coactivator YAP1	YAP1_HUMAN		YAP1
492	492	492	0.999	213	sp|P48047|ATPO_HUMAN	ATP5PO	P48047	ATP synthase subunit O, mitochondrial	ATPO_HUMAN		ATP5O
252	178	252	0.999	337	sp|P48729|KC1A_HUMAN	CSNK1A1	P48729	Casein kinase I isoform alpha	KC1A_HUMAN		CSNK1A1
114	99	114	0.999	835	sp|P48960|AGRE5_HUMAN	ADGRE5	P48960	Adhesion G protein-coupled receptor E5	AGRE5_HUMAN		CD97
423	423	423	0.999	86	sp|P49458|SRP09_HUMAN	SRP9	P49458	Signal recognition particle 9 kDa protein	SRP09_HUMAN		SRP9
91	91	91	0.999	2209	sp|P50748|KNTC1_HUMAN	KNTC1	P50748	Kinetochore-associated protein 1	KNTC1_HUMAN		KNTC1
151	151	151	0.999	628	sp|P50895|BCAM_HUMAN	BCAM	P50895	Basal cell adhesion molecule	BCAM_HUMAN		BCAM
317	317	317	0.999	346	sp|P53597|SUCA_HUMAN	SUCLG1	P53597	Succinate--CoA ligase [ADP/GDP-forming] subunit alpha, mitochondrial	SUCA_HUMAN		SUCLG1
239	239	239	0.999	279	sp|P54709|AT1B3_HUMAN	ATP1B3	P54709	Sodium/potassium-transporting ATPase subunit beta-3	AT1B3_HUMAN		ATP1B3
368	349	368	0.999	377	sp|P55036|PSMD4_HUMAN	PSMD4	P55036	26S proteasome non-ATPase regulatory subunit 4	PSMD4_HUMAN		PSMD4
459	459	459	0.999	322	sp|P55735|SEC13_HUMAN	SEC13	P55735	Protein SEC13 homolog	SEC13_HUMAN		SEC13
532	414	532	0.999	92	sp|P61513|RL37A_HUMAN	RPL37A	P61513	Large ribosomal subunit protein eL43	RL37A_HUMAN		RPL37A
174	156	174	0.999	204	sp|P62070|RRAS2_HUMAN	RRAS2	P62070	Ras-related protein R-Ras2	RRAS2_HUMAN		RRAS2
634	634	634	0.999	151	sp|P62263|RS14_HUMAN	RPS14	P62263	Small ribosomal subunit protein uS11	RS14_HUMAN		RPS14
338	338	338	0.999	56	sp|P62273|RS29_HUMAN	RPS29	P62273	Small ribosomal subunit protein uS14	RS29_HUMAN		RPS29
896	896	896	0.999	156	sp|P62750|RL23A_HUMAN	RPL23A	P62750	Large ribosomal subunit protein uL23	RL23A_HUMAN		RPL23A
281	281	619	0.999	128	sp|P62987|RL40_HUMAN	UBA52	P62987	Ubiquitin-ribosomal protein eL40 fusion protein	RL40_HUMAN		UBA52
239	239	239	0.999	225	sp|P78330|SERB_HUMAN	PSPH	P78330	Phosphoserine phosphatase	SERB_HUMAN		PSPH
69	69	84	0.999	2803	sp|P78559|MAP1A_HUMAN	MAP1A	P78559	Microtubule-associated protein 1A	MAP1A_HUMAN		MAP1A
142	142	142	0.999	360	sp|P82650|RT22_HUMAN	MRPS22	P82650	Small ribosomal subunit protein mS22	RT22_HUMAN		MRPS22
262	262	262	0.999	430	sp|P82675|RT05_HUMAN	MRPS5	P82675	Small ribosomal subunit protein uS5m	RT05_HUMAN		MRPS5
458	458	458	0.999	218	sp|P82930|RT34_HUMAN	MRPS34	P82930	Small ribosomal subunit protein mS34	RT34_HUMAN		MRPS34
139	136	139	0.999	520	sp|Q01581|HMCS1_HUMAN	HMGCS1	Q01581	Hydroxymethylglutaryl-CoA synthase, cytoplasmic	HMCS1_HUMAN		HMGCS1
153	153	153	0.999	890	sp|Q06033|ITIH3_HUMAN	ITIH3	Q06033	Inter-alpha-trypsin inhibitor heavy chain H3	ITIH3_HUMAN		ITIH3
168	168	168	0.999	891	sp|Q08AF3|SLFN5_HUMAN	SLFN5	Q08AF3	Schlafen family member 5	SLFN5_HUMAN		SLFN5
45	45	45	0.999	308	sp|Q12841|FSTL1_HUMAN	FSTL1	Q12841	Follistatin-related protein 1	FSTL1_HUMAN		FSTL1
133	106	133	0.999	499	sp|Q13557|KCC2D_HUMAN	CAMK2D	Q13557	Calcium/calmodulin-dependent protein kinase type II subunit delta	KCC2D_HUMAN		CAMK2D
247	247	247	0.999	534	sp|Q13564|ULA1_HUMAN	NAE1	Q13564	NEDD8-activating enzyme E1 regulatory subunit	ULA1_HUMAN		NAE1
272	272	272	0.999	478	sp|Q14108|SCRB2_HUMAN	SCARB2	Q14108	Lysosome membrane protein 2	SCRB2_HUMAN		SCARB2
100	100	100	0.999	798	sp|Q14241|ELOA1_HUMAN	ELOA	Q14241	Elongin-A	ELOA1_HUMAN		TCEB3
113	113	113	0.999	660	sp|Q15067|ACOX1_HUMAN	ACOX1	Q15067	Peroxisomal acyl-coenzyme A oxidase 1	ACOX1_HUMAN		ACOX1
339	339	339	0.999	192	sp|Q15126|PMVK_HUMAN	PMVK	Q15126	Phosphomevalonate kinase	PMVK_HUMAN		PMVK

175	175	175	0.999	1204	sp|Q53EP0|FND3B_HUMAN	FNDC3B	Q53EP0	Fibronectin type III domain-containing protein 3B	FND3B_HUMAN		FNDC3B
116	116	116	0.999	851	sp|Q63ZY3|KANK2_HUMAN	KANK2	Q63ZY3	KN motif and ankyrin repeat domain-containing protein 2	KANK2_HUMAN		KANK2
188	188	188	0.999	1173	sp|Q6PD62|CTR9_HUMAN	CTR9	Q6PD62	RNA polymerase-associated protein CTR9 homolog	CTR9_HUMAN		CTR9
72	72	72	0.999	1577	sp|Q6XZF7|DNMBP_HUMAN	DNMBP	Q6XZF7	Dynamin-binding protein	DNMBP_HUMAN		DNMBP
257	257	257	0.999	418	sp|Q6YN16|HSDL2_HUMAN	HSDL2	Q6YN16	Hydroxysteroid dehydrogenase-like protein 2	HSDL2_HUMAN		HSDL2
95	95	95	0.999	1375	sp|Q8IY17|PLPL6_HUMAN	PNPLA6	Q8IY17	Patatin-like phospholipase domain-containing protein 6	PLPL6_HUMAN		PNPLA6
213	213	213	0.999	847	sp|Q8IY81|SPB1_HUMAN	FTSJ3	Q8IY81	pre-rRNA 2'-O-ribose RNA methyltransferase FTSJ3	SPB1_HUMAN		FTSJ3
257	257	257	0.999	463	sp|Q8TBC4|UBA3_HUMAN	UBA3	Q8TBC4	NEDD8-activating enzyme E1 catalytic subunit	UBA3_HUMAN		UBA3
197	197	197	0.999	950	sp|Q8TF05|PP4R1_HUMAN	PPP4R1	Q8TF05	Serine/threonine-protein phosphatase 4 regulatory subunit 1	PP4R1_HUMAN		PPP4R1
322	322	322	0.999	426	sp|Q8WU90|ZC3HF_HUMAN	ZC3H15	Q8WU90	Zinc finger CCCH domain-containing protein 15	ZC3HF_HUMAN		ZC3H15
158	157	158	0.999	666	sp|Q8WVC0|LEO1_HUMAN	LEO1	Q8WVC0	RNA polymerase-associated protein LEO1	LEO1_HUMAN		LEO1
157	157	157	0.999	1227	sp|Q92620|PRP16_HUMAN	DHX38	Q92620	Pre-mRNA-splicing factor ATP-dependent RNA helicase PRP16	PRP16_HUMAN		DHX38
299	299	299	0.999	245	sp|Q96DG6|CMBL_HUMAN	CMBL	Q96DG6	Carboxymethylenebutenolidase homolog	CMBL_HUMAN		CMBL
516	516	516	0.999	103	sp|Q96FQ6|S10AG_HUMAN	S100A16	Q96FQ6	Protein S100-A16	S10AG_HUMAN		S100A16
508	508	508	0.999	271	sp|Q96FW1|OTUB1_HUMAN	OTUB1	Q96FW1	Ubiquitin thioesterase OTUB1	OTUB1_HUMAN		OTUB1
133	131	133	0.999	1412	sp|Q96RT1|ERBIN_HUMAN	ERBIN	Q96RT1	Erbin	ERBIN_HUMAN		ERBB2IP
412	351	412	0.999	284	sp|Q9BPW8|NIPS1_HUMAN	NIPSNAP1	Q9BPW8	Protein NipSnap homolog 1	NIPS1_HUMAN		NIPSNAP1
202	202	202	0.999	1015	sp|Q9BPX3|CND3_HUMAN	NCAPG	Q9BPX3	Condensin complex subunit 3	CND3_HUMAN		NCAPG
284	284	284	0.999	1192	sp|Q9BTW9|TBCD_HUMAN	TBCD	Q9BTW9	Tubulin-specific chaperone D	TBCD_HUMAN		TBCD
60	60	60	0.999	575	sp|Q9BYC5|FUT8_HUMAN	FUT8	Q9BYC5	Alpha-(1,6)-fucosyltransferase	FUT8_HUMAN		FUT8
321	321	321	0.999	205	sp|Q9BYN8|RT26_HUMAN	MRPS26	Q9BYN8	Small ribosomal subunit protein mS26	RT26_HUMAN		MRPS26
211	211	211	0.999	423	sp|Q9GZL7|WDR12_HUMAN	WDR12	Q9GZL7	Ribosome biogenesis protein WDR12	WDR12_HUMAN		WDR12
139	139	189	0.999	616	sp|Q9H0L4|CSTFT_HUMAN	CSTF2T	Q9H0L4	Cleavage stimulation factor subunit 2 tau variant	CSTFT_HUMAN		CSTF2T
512	458	512	0.999	224	sp|Q9H444|CHM4B_HUMAN	CHMP4B	Q9H444	Charged multivesicular body protein 4b	CHM4B_HUMAN		CHMP4B
240	240	240	0.999	388	sp|Q9H488|OFUT1_HUMAN	POFUT1	Q9H488	GDP-fucose protein O-fucosyltransferase 1	OFUT1_HUMAN		POFUT1
159	136	159	0.999	2382	sp|Q9H4A3|WNK1_HUMAN	WNK1	Q9H4A3	Serine/threonine-protein kinase WNK1	WNK1_HUMAN		WNK1
98	98	98	0.999	1204	sp|Q9HD20|AT131_HUMAN	ATP13A1	Q9HD20	Endoplasmic reticulum transmembrane helix translocase	AT131_HUMAN		ATP13A1
320	320	320	0.999	359	sp|Q9NZL4|HPBP1_HUMAN	HSPBP1	Q9NZL4	Hsp70-binding protein 1	HPBP1_HUMAN		HSPBP1
380	380	380	0.999	334	sp|Q9NZL9|MAT2B_HUMAN	MAT2B	Q9NZL9	Methionine adenosyltransferase 2 subunit beta	MAT2B_HUMAN		MAT2B
299	299	376	0.999	454	sp|Q9P0V9|SEP10_HUMAN	SEPTIN10	Q9P0V9	Septin-10	SEP10_HUMAN		SEPT10
193	193	193	0.999	523	sp|Q9UI10|EI2BD_HUMAN	EIF2B4	Q9UI10	Translation initiation factor eIF-2B subunit delta	EI2BD_HUMAN		EIF2B4
359	359	359	0.999	356	sp|Q9UJZ1|STML2_HUMAN	STOML2	Q9UJZ1	Stomatin-like protein 2, mitochondrial	STML2_HUMAN		STOML2
281	281	281	0.999	468	sp|Q9UKX7|NUP50_HUMAN	NUP50	Q9UKX7	Nuclear pore complex protein Nup50	NUP50_HUMAN		NUP50
56	56	56	0.999	747	sp|Q9ULW0|TPX2_HUMAN	TPX2	Q9ULW0	Targeting protein for Xklp2	TPX2_HUMAN		TPX2
356	356	356	0.999	329	sp|Q9Y5K5|UCHL5_HUMAN	UCHL5	Q9Y5K5	Ubiquitin carboxyl-terminal hydrolase isozyme L5	UCHL5_HUMAN		UCHL5
178	178	178	0.999	498	sp|Q9Y6W5|WASF2_HUMAN	WASF2	Q9Y6W5	Actin-binding protein WASF2	WASF2_HUMAN		WASF2
74	74	74	0.999	748	sp|O14672|ADA10_HUMAN	ADAM10	O14672	Disintegrin and metalloproteinase domain-containing protein 10	ADA10_HUMAN		ADAM10
73	73	73	0.999	923	sp|O14786|NRP1_HUMAN	NRP1	O14786	Neuropilin-1	NRP1_HUMAN		NRP1
169	169	169	0.999	1278	sp|O15118|NPC1_HUMAN	NPC1	O15118	NPC intracellular cholesterol transporter 1	NPC1_HUMAN		NPC1
384	376	384	0.999	151	sp|O15511|ARPC5_HUMAN	ARPC5	O15511	Actin-related protein 2/3 complex subunit 5	ARPC5_HUMAN		ARPC5
334	334	334	0.999	399	sp|O43813|LANC1_HUMAN	LANCL1	O43813	Glutathione S-transferase LANCL1	LANC1_HUMAN		LANCL1
59	59	60	0.999	1485	sp|O60306|AQR_HUMAN	AQR	O60306	RNA helicase aquarius	AQR_HUMAN		AQR
92	92	123	0.999	1294	sp|O75122|CLAP2_HUMAN	CLASP2	O75122	CLIP-associating protein 2	CLAP2_HUMAN		CLASP2
456	456	456	0.999	463	sp|O75306|NDUS2_HUMAN	NDUFS2	O75306	NADH dehydrogenase [ubiquinone] iron-sulfur protein 2, mitochondrial	NDUS2_HUMAN		NDUFS2
338	337	338	0.999	327	sp|O75436|VP26A_HUMAN	VPS26A	O75436	Vacuolar protein sorting-associated protein 26A	VP26A_HUMAN		VPS26A
129	129	392	0.999	339	sp|O94905|ERLN2_HUMAN	ERLIN2	O94905	Erlin-2	ERLN2_HUMAN		ERLIN2
108	48	108	0.999	1232	sp|O95239|KIF4A_HUMAN	KIF4A	O95239	Chromosome-associated kinesin KIF4A	KIF4A_HUMAN		KIF4A
507	507	507	0.999	301	sp|P00387|NB5R3_HUMAN	CYB5R3	P00387	NADH-cytochrome b5 reductase 3	NB5R3_HUMAN		CYB5R3
330	0	877	0.999	130	sp|P04908|H2A1B_HUMAN	H2AC4	P04908	Histone H2A type 1-B/E	H2A1B_HUMAN	sp|Q7L7L0|H2A3_HUMAN	HIST1H2AB
717	717	717	0.999	90	sp|P06703|S10A6_HUMAN	S100A6	P06703	Protein S100-A6	S10A6_HUMAN		S100A6
157	157	157	0.999	1462	sp|P09884|DPOLA_HUMAN	POLA1	P09884	DNA polymerase alpha catalytic subunit	DPOLA_HUMAN		POLA1
96	0	96	0.999	1744	sp|P0C0L4|CO4A_HUMAN	C4A	P0C0L4	Complement C4-A	CO4A_HUMAN	sp|P0C0L5|CO4B_HUMAN	C4A
1050	0	1050	0.999	130	sp|P0C0S8|H2A1_HUMAN	H2AC11	P0C0S8	Histone H2A type 1	H2A1_HUMAN	sp|Q6FI13|H2A2A_HUMAN, sp	HIST1H2AG
41	41	41	0.9987	2224	sp|P12259|FA5_HUMAN	F5	P12259	Coagulation factor V	FA5_HUMAN		F5
595	595	595	0.999	169	sp|P13073|COX41_HUMAN	COX4I1	P13073	Cytochrome c oxidase subunit 4 isoform 1, mitochondrial	COX41_HUMAN		COX4I1
130	130	568	0.999	372	sp|P16989|YBOX3_HUMAN	YBX3	P16989	Y-box-binding protein 3	YBOX3_HUMAN		YBX3
168	168	168	0.999	968	sp|P19838|NFKB1_HUMAN	NFKB1	P19838	Nuclear factor NF-kappa-B p105 subunit	NFKB1_HUMAN		NFKB1
554	554	554	0.999	241	sp|P20618|PSB1_HUMAN	PSMB1	P20618	Proteasome subunit beta type-1	PSB1_HUMAN		PSMB1
415	415	415	0.999	297	sp|P25325|THTM_HUMAN	MPST	P25325	3-mercaptopyruvate sulfurtransferase	THTM_HUMAN		MPST
370	370	370	0.999	659	sp|P28288|ABCD3_HUMAN	ABCD3	P28288	ATP-binding cassette sub-family D member 3	ABCD3_HUMAN		ABCD3
117	117	117	0.999	519	sp|P28838|AMPL_HUMAN	LAP3	P28838	Cytosol aminopeptidase	AMPL_HUMAN		LAP3
317	317	317	0.999	277	sp|P29218|IMPA1_HUMAN	IMPA1	P29218	Inositol monophosphatase 1	IMPA1_HUMAN		IMPA1
153	125	153	0.999	404	sp|P29466|CASP1_HUMAN	CASP1	P29466	Caspase-1	CASP1_HUMAN		CASP1
635	635	635	0.999	261	sp|P30040|ERP29_HUMAN	ERP29	P30040	Endoplasmic reticulum resident protein 29	ERP29_HUMAN		ERP29
427	409	456	0.999	207	sp|P30405|PPIF_HUMAN	PPIF	P30405	Peptidyl-prolyl cis-trans isomerase F, mitochondrial	PPIF_HUMAN		PPIF
100	100	100	0.999	1257	sp|P32004|L1CAM_HUMAN	L1CAM	P32004	Neural cell adhesion molecule L1	L1CAM_HUMAN		L1CAM
280	280	280	0.999	517	sp|P35269|T2FA_HUMAN	GTF2F1	P35269	General transcription factor IIF subunit 1	T2FA_HUMAN		GTF2F1
637	637	637	0.999	385	sp|P35613|BASI_HUMAN	BSG	P35613	Basigin	BASI_HUMAN		BSG
73	73	665	0.999	323	sp|P36873|PP1G_HUMAN	PPP1CC	P36873	Serine/threonine-protein phosphatase PP1-gamma catalytic subunit	PP1G_HUMAN		PPP1CC
386	386	482	0.999	191	sp|P45973|CBX5_HUMAN	CBX5	P45973	Chromobox protein homolog 5	CBX5_HUMAN		CBX5
556	556	556	0.999	148	sp|P46776|RL27A_HUMAN	RPL27A	P46776	Large ribosomal subunit protein uL15	RL27A_HUMAN		RPL27A
796	311	796	0.999	144	sp|P47813|IF1AX_HUMAN	EIF1AX	P47813	Eukaryotic translation initiation factor 1A, X-chromosomal	IF1AX_HUMAN		EIF1AX
189	189	189	0.999	351	sp|P49770|EI2BB_HUMAN	EIF2B2	P49770	Translation initiation factor eIF-2B subunit beta	EI2BB_HUMAN		EIF2B2
244	244	244	0.999	561	sp|P49902|5NTC_HUMAN	NT5C2	P49902	Cytosolic purine 5'-nucleotidase	5NTC_HUMAN		NT5C2
174	146	261	0.999	374	sp|P50452|SPB8_HUMAN	SERPINB8	P50452	Serpin B8	SPB8_HUMAN		SERPINB8
178	178	178	0.999	330	sp|P50479|PDLI4_HUMAN	PDLIM4	P50479	PDZ and LIM domain protein 4	PDLI4_HUMAN		PDLIM4
93	93	93	0.999	917	sp|P53814|SMTN_HUMAN	SMTN	P53814	Smoothelin	SMTN_HUMAN		SMTN
398	398	398	0.999	362	sp|P55263|ADK_HUMAN	ADK	P55263	Adenosine kinase	ADK_HUMAN		ADK
87	87	87	0.999	1798	sp|P55268|LAMB2_HUMAN	LAMB2	P55268	Laminin subunit beta-2	LAMB2_HUMAN		LAMB2
665	529	665	0.999	191	sp|P60953|CDC42_HUMAN	CDC42	P60953	Cell division control protein 42 homolog	CDC42_HUMAN		CDC42
431	431	431	0.999	118	sp|P62316|SMD2_HUMAN	SNRPD2	P62316	Small nuclear ribonucleoprotein Sm D2	SMD2_HUMAN		SNRPD2
244	244	264	0.999	420	sp|P80303|NUCB2_HUMAN	NUCB2	P80303	Nucleobindin-2	NUCB2_HUMAN		NUCB2
317	317	317	0.999	396	sp|P82933|RT09_HUMAN	MRPS9	P82933	Small ribosomal subunit protein uS9m	RT09_HUMAN		MRPS9
802	802	802	0.999	164	sp|P84103|SRSF3_HUMAN	SRSF3	P84103	Serine/arginine-rich splicing factor 3	SRSF3_HUMAN		SRSF3
741	594	741	0.999	221	sp|Q01130|SRSF2_HUMAN	SRSF2	Q01130	Serine/arginine-rich splicing factor 2	SRSF2_HUMAN		SRSF2
108	108	108	0.999	960	sp|Q02241|KIF23_HUMAN	KIF23	Q02241	Kinesin-like protein KIF23	KIF23_HUMAN		KIF23
399	399	399	0.999	297	sp|Q04323|UBXN1_HUMAN	UBXN1	Q04323	UBX domain-containing protein 1	UBXN1_HUMAN		UBXN1
644	644	644	0.999	184	sp|Q04760|LGUL_HUMAN	GLO1	Q04760	Lactoylglutathione lyase	LGUL_HUMAN		GLO1
150	150	150	0.999	1498	sp|Q08378|GOGA3_HUMAN	GOLGA3	Q08378	Golgin subfamily A member 3	GOGA3_HUMAN		GOLGA3
759	555	759	0.999	183	sp|Q13185|CBX3_HUMAN	CBX3	Q13185	Chromobox protein homolog 3	CBX3_HUMAN		CBX3
309	254	309	0.999	652	sp|Q13492|PICAL_HUMAN	PICALM	Q13492	Phosphatidylinositol-binding clathrin assembly protein	PICAL_HUMAN		PICALM
254	254	262	0.999	522	sp|Q13596|SNX1_HUMAN	SNX1	Q13596	Sorting nexin-1	SNX1_HUMAN		SNX1
287	287	287	0.999	583	sp|Q13740|CD166_HUMAN	ALCAM	Q13740	CD166 antigen	CD166_HUMAN		ALCAM
35	34	35	0.999	1375	sp|Q14112|NID2_HUMAN	NID2	Q14112	Nidogen-2	NID2_HUMAN		NID2
168	168	168	0.999	2089	sp|Q14676|MDC1_HUMAN	MDC 1.00	Q14676	Mediator of DNA damage checkpoint protein 1	MDC1_HUMAN		MDC 1.00
239	239	239	0.999	1282	sp|Q14692|BMS1_HUMAN	BMS1	Q14692	Ribosome biogenesis protein BMS1 homolog	BMS1_HUMAN		BMS1
192	192	192	0.999	798	sp|Q14694|UBP10_HUMAN	USP10	Q14694	Ubiquitin carboxyl-terminal hydrolase 10	UBP10_HUMAN		USP10
383	383	383	0.999	329	sp|Q14914|PTGR1_HUMAN	PTGR1	Q14914	Prostaglandin reductase 1	PTGR1_HUMAN		PTGR1
751	751	751	0.999	231	sp|Q15102|PA1B3_HUMAN	PAFAH1B3	Q15102	Platelet-activating factor acetylhydrolase IB subunit alpha1	PA1B3_HUMAN		PAFAH1B3
483	483	483	0.999	331	sp|Q15293|RCN1_HUMAN	RCN1	Q15293	Reticulocalbin-1	RCN1_HUMAN		RCN1
307	307	307	0.999	309	sp|Q15785|TOM34_HUMAN	TOMM34	Q15785	Mitochondrial import receptor subunit TOM34	TOM34_HUMAN		TOMM34
276	276	276	0.999	513	sp|Q2TAY7|SMU1_HUMAN	SMU1	Q2TAY7	WD40 repeat-containing protein SMU1	SMU1_HUMAN		SMU1
256	256	256	0.999	565	sp|Q53GS9|SNUT2_HUMAN	USP39	Q53GS9	U4/U6.U5 tri-snRNP-associated protein 2	SNUT2_HUMAN		USP39
268	268	268	0.999	985	sp|Q5JTZ9|SYAM_HUMAN	AARS2	Q5JTZ9	Alanine--tRNA ligase, mitochondrial	SYAM_HUMAN		AARS2
97	97	97	0.999	1943	sp|Q5T5P2|SKT_HUMAN	KIAA1217	Q5T5P2	Sickle tail protein homolog	SKT_HUMAN		KIAA1217
204	204	204	0.999	609	sp|Q6L8Q7|PDE12_HUMAN	PDE12	Q6L8Q7	2',5'-phosphodiesterase 12	PDE12_HUMAN		PDE12
259	259	259	0.999	610	sp|Q6NUM9|RETST_HUMAN	RETSAT	Q6NUM9	All-trans-retinol 13,14-reductase	RETST_HUMAN		RETSAT
190	190	190	0.999	531	sp|Q6P1J9|CDC73_HUMAN	CDC73	Q6P1J9	Parafibromin	CDC73_HUMAN		CDC73
137	137	137	0.999	951	sp|Q6P1N0|C2D1A_HUMAN	CC2D1A	Q6P1N0	Coiled-coil and C2 domain-containing protein 1A	C2D1A_HUMAN		CC2D1A
316	316	316	0.999	403	sp|Q7L0Y3|TM10C_HUMAN	TRMT10C	Q7L0Y3	tRNA methyltransferase 10 homolog C	TM10C_HUMAN		TRMT10C
377	377	377	0.999	374	sp|Q7L2H7|EIF3M_HUMAN	EIF3M	Q7L2H7	Eukaryotic translation initiation factor 3 subunit M	EIF3M_HUMAN		EIF3M
326	326	326	0.999	199	sp|Q7LBR1|CHM1B_HUMAN	CHMP1B	Q7LBR1	Charged multivesicular body protein 1b	CHM1B_HUMAN		CHMP1B
142	142	142	0.999	695	sp|Q7Z417|NUFP2_HUMAN	NUFIP2	Q7Z417	FMR1-interacting protein NUFIP2	NUFP2_HUMAN		NUFIP2
199	199	199	0.999	953	sp|Q86VM9|ZCH18_HUMAN	ZC3H18	Q86VM9	Zinc finger CCCH domain-containing protein 18	ZCH18_HUMAN		ZC3H18
362	362	362	0.999	608	sp|Q86X55|CARM1_HUMAN	CARM1	Q86X55	Histone-arginine methyltransferase CARM1	CARM1_HUMAN		CARM1
238	223	238	0.999	904	sp|Q8IYB3|SRRM1_HUMAN	SRRM1	Q8IYB3	Serine/arginine repetitive matrix protein 1	SRRM1_HUMAN		SRRM1
275	215	275	0.999	508	sp|Q8IZP0|ABI1_HUMAN	ABI1	Q8IZP0	Abl interactor 1	ABI1_HUMAN		ABI1
227	227	227	0.999	521	sp|Q8N6H7|ARFG2_HUMAN	ARFGAP2	Q8N6H7	ADP-ribosylation factor GTPase-activating protein 2	ARFG2_HUMAN		ARFGAP2
190	190	190	0.999	589	sp|Q8NHP8|PLBL2_HUMAN	PLBD2	Q8NHP8	Putative phospholipase B-like 2	PLBL2_HUMAN		PLBD2
194	194	194	0.999	951	sp|Q8NI36|WDR36_HUMAN	WDR36	Q8NI36	WD repeat-containing protein 36	WDR36_HUMAN		WDR36
128	128	128	0.999	905	sp|Q8TCG1|CIP2A_HUMAN	CIP2A	Q8TCG1	Protein CIP2A	CIP2A_HUMAN		KIAA1524
213	213	213	0.999	642	sp|Q8WVM8|SCFD1_HUMAN	SCFD1	Q8WVM8	Sec1 family domain-containing protein 1	SCFD1_HUMAN		SCFD1
253	253	253	0.999	580	sp|Q8WX92|NELFB_HUMAN	NELFB	Q8WX92	Negative elongation factor B	NELFB_HUMAN		NELFB
394	394	394	0.999	563	sp|Q8WYA6|CTBL1_HUMAN	CTNNBL1	Q8WYA6	Beta-catenin-like protein 1	CTBL1_HUMAN		CTNNBL1
447	447	447	0.999	563	sp|Q969V3|NCLN_HUMAN	NCLN	Q969V3	BOS complex subunit NCLN	NCLN_HUMAN		NCLN
228	228	238	0.999	320	sp|Q96C36|P5CR2_HUMAN	PYCR2	Q96C36	Pyrroline-5-carboxylate reductase 2	P5CR2_HUMAN		PYCR2
139	139	139	0.999	577	sp|Q96CV9|OPTN_HUMAN	OPTN	Q96CV9	Optineurin	OPTN_HUMAN		OPTN
89	89	89	0.999	328	sp|Q96D15|RCN3_HUMAN	RCN3	Q96D15	Reticulocalbin-3	RCN3_HUMAN		RCN3
198	198	198	0.999	380	sp|Q96DV4|RM38_HUMAN	MRPL38	Q96DV4	Large ribosomal subunit protein mL38	RM38_HUMAN		MRPL38

231	231	231	0.999	360	sp|Q96EE3|SEH1_HUMAN	SEH1L	Q96EE3	Nucleoporin SEH1	SEH1_HUMAN		SEH1L
492	435	492	0.999	289	sp|Q96HS1|PGAM5_HUMAN	PGAM5	Q96HS1	Serine/threonine-protein phosphatase PGAM5, mitochondrial	PGAM5_HUMAN		PGAM5
421	421	421	0.999	204	sp|Q9BRP8|PYM1_HUMAN	PYM1	Q9BRP8	Partner of Y14 and mago	PYM1_HUMAN		WIBG
305	305	305	0.999	642	sp|Q9BTE3|MCMBP_HUMAN	MCMBP	Q9BTE3	Mini-chromosome maintenance complex-binding protein	MCMBP_HUMAN		MCMBP
282	282	282	0.999	400	sp|Q9BTV4|TMM43_HUMAN	TMEM43	Q9BTV4	Transmembrane protein 43	TMM43_HUMAN		TMEM43
567	464	567	0.999	423	sp|Q9BXS5|AP1M1_HUMAN	AP1M1	Q9BXS5	AP-1 complex subunit mu-1	AP1M1_HUMAN		AP1M1
93	93	93	0.999	859	sp|Q9GZR7|DDX24_HUMAN	DDX24	Q9GZR7	ATP-dependent RNA helicase DDX24	DDX24_HUMAN		DDX24
58	58	821	0.999	451	sp|Q9H4B7|TBB1_HUMAN	TUBB1	Q9H4B7	Tubulin beta-1 chain	TBB1_HUMAN		TUBB1
293	293	293	0.999	196	sp|Q9HD42|CHM1A_HUMAN	CHMP1A	Q9HD42	Charged multivesicular body protein 1a	CHM1A_HUMAN		CHMP1A
252	252	252	0.999	654	sp|Q9NW82|WDR70_HUMAN	WDR70	Q9NW82	WD repeat-containing protein 70	WDR70_HUMAN		WDR70
183	183	183	0.999	505	sp|Q9UHG3|PCYOX_HUMAN	PCYOX1	Q9UHG3	Prenylcysteine oxidase 1	PCYOX_HUMAN		PCYOX1
200	199	200	0.999	365	sp|Q9Y371|SHLB1_HUMAN	SH3GLB1	Q9Y371	Endophilin-B1	SHLB1_HUMAN		SH3GLB1
308	308	308	0.999	684	sp|Q9Y450|HBS1L_HUMAN	HBS1L	Q9Y450	HBS1-like protein	HBS1L_HUMAN		HBS1L
156	156	156	0.999	469	sp|Q9Y512|SAM50_HUMAN	SAMM50	Q9Y512	Sorting and assembly machinery component 50 homolog	SAM50_HUMAN		SAMM50
371	0	371	0.999	268	sp|A0A0B4J2D5|GAL3B_HUM	GATD3B	A0A0B4J2D5	Putative glutamine amidotransferase-like class 1 domain-containing protei	GAL3B_HUMAN	sp|P0DPI2|GAL3A_HUMAN	
314	263	314	0.999	593	sp|O00499|BIN1_HUMAN	BIN1	O00499	Myc box-dependent-interacting protein 1	BIN1_HUMAN		BIN1
224	224	224	0.999	419	sp|O14929|HAT1_HUMAN	HAT1	O14929	Histone acetyltransferase type B catalytic subunit	HAT1_HUMAN		HAT1
95	95	95	0.999	1311	sp|O14976|GAK_HUMAN	GAK	O14976	Cyclin-G-associated kinase	GAK_HUMAN		GAK
356	331	356	0.999	624	sp|O43252|PAPS1_HUMAN	PAPSS1	O43252	Bifunctional 3'-phosphoadenosine 5'-phosphosulfate synthase 1	PAPS1_HUMAN		PAPSS1
208	208	208	0.999	475	sp|O43818|U3IP2_HUMAN	RRP9	O43818	U3 small nucleolar RNA-interacting protein 2	U3IP2_HUMAN		RRP9
57	57	57	0.999	480	sp|O43854|EDIL3_HUMAN	EDIL3	O43854	EGF-like repeat and discoidin I-like domain-containing protein 3	EDIL3_HUMAN		EDIL3
255	255	255	0.999	372	sp|O60547|GMDS_HUMAN	GMDS	O60547	GDP-mannose 4,6 dehydratase	GMDS_HUMAN		GMDS
374	374	374	0.999	148	sp|O60869|EDF1_HUMAN	EDF1	O60869	Endothelial differentiation-related factor 1	EDF1_HUMAN		EDF1
353	353	353	0.999	412	sp|O60884|DNJA2_HUMAN	DNAJA2	O60884	DnaJ homolog subfamily A member 2	DNJA2_HUMAN		DNAJA2
114	38	114	0.999	1071	sp|O75044|SRGP2_HUMAN	SRGAP2	O75044	SLIT-ROBO Rho GTPase-activating protein 2	SRGP2_HUMAN		SRGAP2
147	147	147	0.999	459	sp|O75312|ZPR1_HUMAN	ZPR1	O75312	Zinc finger protein ZPR1	ZPR1_HUMAN		ZPR1
647	647	647	0.999	215	sp|O75396|SC22B_HUMAN	SEC22B	O75396	Vesicle-trafficking protein SEC22b	SC22B_HUMAN		SEC22B
163	163	177	0.999	1032	sp|O94855|SC24D_HUMAN	SEC24D	O94855	Protein transport protein Sec24D	SC24D_HUMAN		SEC24D
398	398	399	0.999	243	sp|O95292|VAPB_HUMAN	VAPB	O95292	Vesicle-associated membrane protein-associated protein B/C	VAPB_HUMAN		VAPB
287	287	287	0.999	355	sp|O95299|NDUAA_HUMAN	NDUFA10	O95299	NADH dehydrogenase [ubiquinone] 1 alpha subcomplex subunit 10, mitoch	NDUAA_HUMAN		NDUFA10
356	356	356	0.999	522	sp|P00390|GSHR_HUMAN	GSR	P00390	Glutathione reductase, mitochondrial	GSHR_HUMAN		GSR
1067	1067	1067	0.999	154	sp|P00441|SODC_HUMAN	SOD1	P00441	Superoxide dismutase [Cu-Zn]	SODC_HUMAN		SOD1
84	76	145	0.999	517	sp|P05091|ALDH2_HUMAN	ALDH2	P05091	Aldehyde dehydrogenase, mitochondrial	ALDH2_HUMAN		ALDH2
292	217	676	0.999	193	sp|P08134|RHOC_HUMAN	RHOC	P08134	Rho-related GTP-binding protein RhoC	RHOC_HUMAN		RHOC
1157	1157	1157	0.999	210	sp|P09211|GSTP1_HUMAN	GSTP1	P09211	Glutathione S-transferase P	GSTP1_HUMAN		GSTP1
635	570	635	0.999	248	sp|P09496|CLCA_HUMAN	CLTA	P09496	Clathrin light chain A	CLCA_HUMAN		CLTA
202	202	202	0.999	952	sp|P10253|LYAG_HUMAN	GAA	P10253	Lysosomal alpha-glucosidase	LYAG_HUMAN		GAA
341	341	341	0.999	647	sp|P10515|ODP2_HUMAN	DLAT	P10515	Dihydrolipoyllysine-residue acetyltransferase component of pyruvate dehyc	ODP2_HUMAN		DLAT
53	53	53	0.999	1032	sp|P13612|ITA4_HUMAN	ITGA4	P13612	Integrin alpha-4	ITA4_HUMAN		ITGA4
588	588	588	0.999	302	sp|P19623|SPEE_HUMAN	SRM	P19623	Spermidine synthase	SPEE_HUMAN		SRM
245	245	245	0.999	946	sp|P19823|ITIH2_HUMAN	ITIH2	P19823	Inter-alpha-trypsin inhibitor heavy chain H2	ITIH2_HUMAN		ITIH2
28	23	77	0.999	662	sp|P20591|MX1_HUMAN	MX1	P20591	Interferon-induced GTP-binding protein Mx1	MX1_HUMAN		MX1
655	655	732	0.999	225	sp|P24534|EF1B_HUMAN	EEF1B2	P24534	Elongation factor 1-beta	EF1B_HUMAN		EEF1B2
63	63	63	0.999	381	sp|P26022|PTX3_HUMAN	PTX3	P26022	Pentraxin-related protein PTX3	PTX3_HUMAN		PTX3
516	516	516	0.999	475	sp|P26368|U2AF2_HUMAN	U2AF2	P26368	Splicing factor U2AF 65 kDa subunit	U2AF2_HUMAN		U2AF2
778	778	778	0.999	187	sp|P30086|PEBP1_HUMAN	PEBP1	P30086	Phosphatidylethanolamine-binding protein 1	PEBP1_HUMAN		PEBP1
146	146	303	0.999	601	sp|P30154|2AAB_HUMAN	PPP2R1B	P30154	Serine/threonine-protein phosphatase 2A 65 kDa regulatory subunit A beta	2AAB_HUMAN		PPP2R1B
201	188	201	0.999	389	sp|P31350|RIR2_HUMAN	RRM2	P31350	Ribonucleoside-diphosphate reductase subunit M2	RIR2_HUMAN		RRM2
762	762	762	0.999	105	sp|P31949|S10AB_HUMAN	S100A11	P31949	Protein S100-A11	S10AB_HUMAN		S100A11
337	337	337	0.999	363	sp|P35249|RFC4_HUMAN	RFC4	P35249	Replication factor C subunit 4	RFC4_HUMAN		RFC4
123	123	123	0.999	1148	sp|P35251|RFC1_HUMAN	RFC1	P35251	Replication factor C subunit 1	RFC1_HUMAN		RFC1
194	194	194	0.999	356	sp|P40938|RFC3_HUMAN	RFC3	P40938	Replication factor C subunit 3	RFC3_HUMAN		RFC3
1085	1085	1085	0.999	201	sp|P43487|RANG_HUMAN	RANBP1	P43487	Ran-specific GTPase-activating protein	RANG_HUMAN		RANBP1
261	261	261	0.999	1132	sp|P46379|BAG6_HUMAN	BAG6	P46379	Large proline-rich protein BAG6	BAG6_HUMAN		BAG6
576	576	576	0.999	201	sp|P49721|PSB2_HUMAN	PSMB2	P49721	Proteasome subunit beta type-2	PSB2_HUMAN		PSMB2
362	362	362	0.999	708	sp|P49959|MRE11_HUMAN	MRE11	P49959	Double-strand break repair protein MRE11	MRE11_HUMAN		MRE11A
332	332	332	0.999	499	sp|P53041|PPP5_HUMAN	PPP5C	P53041	Serine/threonine-protein phosphatase 5	PPP5_HUMAN		PPP5C
316	316	316	0.999	443	sp|P61201|CSN2_HUMAN	COPS2	P61201	COP9 signalosome complex subunit 2	CSN2_HUMAN		COPS2
668	668	668	0.999	143	sp|P62266|RS23_HUMAN	RPS23	P62266	Small ribosomal subunit protein uS12	RS23_HUMAN		RPS23
208	208	208	0.999	431	sp|Q05048|CSTF1_HUMAN	CSTF1	Q05048	Cleavage stimulation factor subunit 1	CSTF1_HUMAN		CSTF1
290	290	319	0.999	370	sp|Q08752|PPID_HUMAN	PPID	Q08752	Peptidyl-prolyl cis-trans isomerase D	PPID_HUMAN		PPID
537	537	851	0.999	271	sp|Q13162|PRDX4_HUMAN	PRDX4	Q13162	Peroxiredoxin-4	PRDX4_HUMAN		PRDX4
266	266	266	0.999	754	sp|Q13427|PPIG_HUMAN	PPIG	Q13427	Peptidyl-prolyl cis-trans isomerase G	PPIG_HUMAN		PPIG
362	362	362	0.999	321	sp|Q13630|FCL_HUMAN	GFUS	Q13630	GDP-L-fucose synthase	FCL_HUMAN		TSTA3
168	168	168	0.999	731	sp|Q13823|NOG2_HUMAN	GNL2	Q13823	Nucleolar GTP-binding protein 2	NOG2_HUMAN		GNL2
315	256	315	0.999	530	sp|Q14498|RBM39_HUMAN	RBM39	Q14498	RNA-binding protein 39	RBM39_HUMAN		RBM39
305	305	305	0.999	1978	sp|Q14966|ZN638_HUMAN	ZNF638	Q14966	Zinc finger protein 638	ZN638_HUMAN		ZNF638
256	214	459	0.999	201	sp|Q15286|RAB35_HUMAN	RAB35	Q15286	Ras-related protein Rab-35	RAB35_HUMAN		RAB35
440	440	440	0.999	474	sp|Q16204|CCDC6_HUMAN	CCDC6	Q16204	Coiled-coil domain-containing protein 6	CCDC6_HUMAN		CCDC6
86	86	90	0.999	942	sp|Q16512|PKN1_HUMAN	PKN1	Q16512	Serine/threonine-protein kinase N1	PKN1_HUMAN		PKN1
253	253	253	0.999	804	sp|Q2NL82|TSR1_HUMAN	TSR1	Q2NL82	Pre-rRNA-processing protein TSR1 homolog	TSR1_HUMAN		TSR1
167	167	167	0.999	841	sp|Q4V328|GRAP1_HUMAN	GRIPAP1	Q4V328	GRIP1-associated protein 1	GRAP1_HUMAN		GRIPAP1
204	204	204	0.999	1219	sp|Q5T1M5|FKB15_HUMAN	FKBP15	Q5T1M5	FK506-binding protein 15	FKB15_HUMAN		FKBP15
216	48	216	0.999	1341	sp|Q641Q2|WAC2A_HUMAN	WASHC2A	Q641Q2	WASH complex subunit 2A	WAC2A_HUMAN		FAM21A
224	224	224	0.999	350	sp|Q70UQ0|IKIP_HUMAN	IKBIP	Q70UQ0	Inhibitor of nuclear factor kappa-B kinase-interacting protein	IKIP_HUMAN		IKBIP
110	110	110	0.999	938	sp|Q86XL3|ANKL2_HUMAN	ANKLE2	Q86XL3	Ankyrin repeat and LEM domain-containing protein 2	ANKL2_HUMAN		ANKLE2
246	246	246	0.999	406	sp|Q8N6T3|ARFG1_HUMAN	ARFGAP1	Q8N6T3	ADP-ribosylation factor GTPase-activating protein 1	ARFG1_HUMAN		ARFGAP1
209	209	209	0.999	503	sp|Q8NC56|LEMD2_HUMAN	LEMD2	Q8NC56	LEM domain-containing protein 2	LEMD2_HUMAN		LEMD2
124	124	124	0.999	800	sp|Q8WTT2|NOC3L_HUMAN	NOC3L	Q8WTT2	Nucleolar complex protein 3 homolog	NOC3L_HUMAN		NOC3L
284	284	284	0.999	499	sp|Q8WWY3|PRP31_HUMAN	PRPF31	Q8WWY3	U4/U6 small nuclear ribonucleoprotein Prp31	PRP31_HUMAN		PRPF31
68	68	554	0.999	207	sp|Q92930|RAB8B_HUMAN	RAB8B	Q92930	Ras-related protein Rab-8B	RAB8B_HUMAN		RAB8B
233	233	233	0.999	631	sp|Q969Z0|FAKD4_HUMAN	TBRG4	Q969Z0	FAST kinase domain-containing protein 4	FAKD4_HUMAN		TBRG4
389	389	389	0.999	445	sp|Q96CS3|FAF2_HUMAN	FAF2	Q96CS3	FAS-associated factor 2	FAF2_HUMAN		FAF2
146	146	146	0.999	650	sp|Q96G46|DUS3L_HUMAN	DUS3L	Q96G46	tRNA-dihydrouridine(47) synthase [NAD(P)(+)]-like	DUS3L_HUMAN		DUS3L
217	217	217	0.999	796	sp|Q96GQ7|DDX27_HUMAN	DDX27	Q96GQ7	Probable ATP-dependent RNA helicase DDX27	DDX27_HUMAN		DDX27
240	240	240	0.999	727	sp|Q96MU7|YTDC1_HUMAN	YTHDC1	Q96MU7	YTH domain-containing protein 1	YTDC1_HUMAN		YTHDC1
164	164	164	0.999	661	sp|Q96PZ0|PUS7_HUMAN	PUS7	Q96PZ0	Pseudouridylate synthase 7 homolog	PUS7_HUMAN		PUS7
203	203	241	0.999	1313	sp|Q99700|ATX2_HUMAN	ATXN2	Q99700	Ataxin-2	ATX2_HUMAN		ATXN2
132	132	132	0.999	1265	sp|Q99707|METH_HUMAN	MTR	Q99707	Methionine synthase	METH_HUMAN		MTR
313	313	313	0.999	306	sp|Q99848|EBP2_HUMAN	EBNA1BP2	Q99848	Probable rRNA-processing protein EBP2	EBP2_HUMAN		EBNA1BP2
297	297	297	0.999	826	sp|Q9BQ52|RNZ2_HUMAN	ELAC2	Q9BQ52	Zinc phosphodiesterase ELAC protein 2	RNZ2_HUMAN		ELAC2
363	363	363	0.999	549	sp|Q9BVP2|GNL3_HUMAN	GNL3	Q9BVP2	Guanine nucleotide-binding protein-like 3	GNL3_HUMAN		GNL3
616	616	616	0.999	243	sp|Q9H1E3|NUCKS_HUMAN	NUCKS1	Q9H1E3	Nuclear ubiquitous casein and cyclin-dependent kinase substrate 1	NUCKS_HUMAN		NUCKS1
123	122	142	0.999	881	sp|Q9H4G0|E41L1_HUMAN	EPB41L1	Q9H4G0	Band 4.1-like protein 1	E41L1_HUMAN		EPB41L1
222	222	222	0.999	354	sp|Q9H832|UBE2Z_HUMAN	UBE2Z	Q9H832	Ubiquitin-conjugating enzyme E2 Z	UBE2Z_HUMAN		UBE2Z
479	479	479	0.999	217	sp|Q9HAV7|GRPE1_HUMAN	GRPEL1	Q9HAV7	GrpE protein homolog 1, mitochondrial	GRPE1_HUMAN		GRPEL1
95	91	95	0.999	4857	sp|Q9NR09|BIRC6_HUMAN	BIRC6	Q9NR09	Baculoviral IAP repeat-containing protein 6	BIRC6_HUMAN		BIRC6
157	157	158	0.999	395	sp|Q9NR46|SHLB2_HUMAN	SH3GLB2	Q9NR46	Endophilin-B2	SHLB2_HUMAN		SH3GLB2
250	250	250	0.999	659	sp|Q9NXH9|TRM1_HUMAN	TRMT1	Q9NXH9	tRNA (guanine(26)-N(2))-dimethyltransferase	TRM1_HUMAN		TRMT1
133	133	133	0.999	800	sp|Q9NYL2|M3K20_HUMAN	MAP3K20	Q9NYL2	Mitogen-activated protein kinase kinase kinase 20	M3K20_HUMAN		ZAK
131	131	131	0.999	941	sp|Q9NZ08|ERAP1_HUMAN	ERAP1	Q9NZ08	Endoplasmic reticulum aminopeptidase 1	ERAP1_HUMAN		ERAP1
392	392	392	0.999	239	sp|Q9UKD2|MRT4_HUMAN	MRTO4	Q9UKD2	mRNA turnover protein 4 homolog	MRT4_HUMAN		MRTO4
371	368	371	0.999	213	sp|Q9UQN3|CHM2B_HUMAN	CHMP2B	Q9UQN3	Charged multivesicular body protein 2b	CHM2B_HUMAN		CHMP2B
197	197	924	0.999	166	sp|Q9Y281|COF2_HUMAN	CFL2	Q9Y281	Cofilin-2	COF2_HUMAN		CFL2
97	97	97	0.999	749	sp|Q9Y3T9|NOC2L_HUMAN	NOC2L	Q9Y3T9	Nucleolar complex protein 2 homolog	NOC2L_HUMAN		NOC2L
74	74	74	0.999	3859	sp|Q9Y4A5|TRRAP_HUMAN	TRRAP	Q9Y4A5	Transformation/transcription domain-associated protein	TRRAP_HUMAN		TRRAP
361	361	361	0.999	404	sp|Q9Y5X3|SNX5_HUMAN	SNX5	Q9Y5X3	Sorting nexin-5	SNX5_HUMAN		SNX5
253	253	253	0.999	427	sp|Q9Y606|PUS1_HUMAN	PUS1	Q9Y606	Pseudouridylate synthase 1 homolog	PUS1_HUMAN		PUS1
88	88	88	0.999	838	sp|Q9Y6A5|TACC3_HUMAN	TACC3	Q9Y6A5	Transforming acidic coiled-coil-containing protein 3	TACC3_HUMAN		TACC3
403	403	403	0.999	1000	sp|Q9Y6Y8|S23IP_HUMAN	SEC23IP	Q9Y6Y8	SEC23-interacting protein	S23IP_HUMAN		SEC23IP
260	260	260	0.999	331	sp|O00273|DFFA_HUMAN	DFFA	O00273	DNA fragmentation factor subunit alpha	DFFA_HUMAN		DFFA
53	53	53	0.999	2027	sp|O14578|CTRO_HUMAN	CIT	O14578	Citron Rho-interacting kinase	CTRO_HUMAN		CIT
376	376	376	0.999	777	sp|O14964|HGS_HUMAN	HGS	O14964	Hepatocyte growth factor-regulated tyrosine kinase substrate	HGS_HUMAN		HGS
618	618	618	0.999	206	sp|O43399|TPD54_HUMAN	TPD52L2	O43399	Tumor protein D54	TPD54_HUMAN		TPD52L2
662	662	662	0.999	328	sp|O43684|BUB3_HUMAN	BUB3	O43684	Mitotic checkpoint protein BUB3	BUB3_HUMAN		BUB3
381	277	381	0.999	369	sp|O60256|KPRB_HUMAN	PRPSAP2	O60256	Phosphoribosyl pyrophosphate synthase-associated protein 2	KPRB_HUMAN		PRPSAP2
220	189	220	0.999	1321	sp|O60271|JIP4_HUMAN	SPAG9	O60271	C-Jun-amino-terminal kinase-interacting protein 4	JIP4_HUMAN		SPAG9
88	80	88	0.999	3097	sp|O75962|TRIO_HUMAN	TRIO	O75962	Triple functional domain protein	TRIO_HUMAN		TRIO
238	235	238	0.999	591	sp|O96013|PAK4_HUMAN	PAK4	O96013	Serine/threonine-protein kinase PAK 4	PAK4_HUMAN		PAK4
169	160	169	0.999	860	sp|P01130|LDLR_HUMAN	LDLR	P01130	Low-density lipoprotein receptor	LDLR_HUMAN		LDLR
108	108	108	0.999	415	sp|P05120|PAI2_HUMAN	SERPINB2	P05120	Plasminogen activator inhibitor 2	PAI2_HUMAN		SERPINB2
301	301	301	0.999	255	sp|P09661|RU2A_HUMAN	SNRPA1	P09661	U2 small nuclear ribonucleoprotein A'	RU2A_HUMAN		SNRPA1
299	229	299	0.999	381	sp|P10644|KAP0_HUMAN	PRKAR1A	P10644	cAMP-dependent protein kinase type I-alpha regulatory subunit	KAP0_HUMAN		PRKAR1A
765	765	765	0.999	359	sp|P11177|ODPB_HUMAN	PDHB	P11177	Pyruvate dehydrogenase E1 component subunit beta, mitochondrial	ODPB_HUMAN		PDHB
1085	716	1085	0.999	172	sp|P13693|TCTP_HUMAN	TPT1	P13693	Translationally-controlled tumor protein	TCTP_HUMAN		TPT1
50	49	493	0.999	143	sp|P16104|H2AX_HUMAN	H2AX	P16104	Histone H2AX	H2AX_HUMAN		H2AFX
311	311	311	0.999	382	sp|P21283|VATC1_HUMAN	ATP6V1C1	P21283	V-type proton ATPase subunit C 1	VATC1_HUMAN		ATP6V1C1
672	631	672	0.999	321	sp|P22087|FBRL_HUMAN	FBL	P22087	rRNA 2'-O-methyltransferase fibrillarin	FBRL_HUMAN		FBL
542	542	542	0.999	483	sp|P26196|DDX6_HUMAN	DDX6	P26196	Probable ATP-dependent RNA helicase DDX6	DDX6_HUMAN		DDX6

314	314	314	0.999	593	sp|P28799|GRN_HUMAN	GRN	P28799	Progranulin	GRN_HUMAN		GRN
427	427	427	0.999	290	sp|P30084|ECHM_HUMAN	ECHS1	P30084	Enoyl-CoA hydratase, mitochondrial	ECHM_HUMAN		ECHS1
243	156	243	0.999	592	sp|P32455|GBP1_HUMAN	GBP1	P32455	Guanylate-binding protein 1	GBP1_HUMAN		GBP1
220	220	220	0.999	551	sp|P35520|CBS_HUMAN	CBS	P35520	Cystathionine beta-synthase	CBS_HUMAN		CBS
406	406	406	0.999	607	sp|P36915|GNL1_HUMAN	GNL1	P36915	Guanine nucleotide-binding protein-like 1	GNL1_HUMAN		GNL1
379	379	656	0.999	286	sp|P47755|CAZA2_HUMAN	CAPZA2	P47755	F-actin-capping protein subunit alpha-2	CAZA2_HUMAN		CAPZA2
564	564	564	0.999	219	sp|P49755|TMEDA_HUMAN	TMED10	P49755	Transmembrane emp24 domain-containing protein 10	TMEDA_HUMAN		TMED10
1116	1116	1116	0.999	126	sp|P49773|HINT1_HUMAN	HINT1	P49773	Adenosine 5'-monophosphoramidase HINT1	HINT1_HUMAN		HINT1
350	350	350	0.999	529	sp|P52292|IMA1_HUMAN	KPNA2	P52292	Importin subunit alpha-1	IMA1_HUMAN		KPNA2
221	221	221	0.999	878	sp|P52735|VAV2_HUMAN	VAV2	P52735	Guanine nucleotide exchange factor VAV2	VAV2_HUMAN		VAV2
779	779	779	0.999	366	sp|P52788|SPSY_HUMAN	SMS	P52788	Spermine synthase	SPSY_HUMAN		SMS
512	512	512	0.999	127	sp|P53999|TCP4_HUMAN	SUB1	P53999	Activated RNA polymerase II transcriptional coactivator p15	TCP4_HUMAN		SUB1
389	174	389	0.999	445	sp|P56545|CTBP2_HUMAN	CTBP2	P56545	C-terminal-binding protein 2	CTBP2_HUMAN		CTBP2
627	333	627	0.999	376	sp|P61163|ACTZ_HUMAN	ACTR1A	P61163	Alpha-centractin	ACTZ_HUMAN		ACTR1A
545	545	545	0.999	254	sp|P61289|PSME3_HUMAN	PSME3	P61289	Proteasome activator complex subunit 3	PSME3_HUMAN		PSME3
697	313	772	0.999	193	sp|P61586|RHOA_HUMAN	RHOA	P61586	Transforming protein RhoA	RHOA_HUMAN		RHOA
329	329	329	0.999	97	sp|P61927|RL37_HUMAN	RPL37	P61927	Large ribosomal subunit protein eL37	RL37_HUMAN		RPL37
818	226	818	0.999	330	sp|P62136|PP1A_HUMAN	PPP1CA	P62136	Serine/threonine-protein phosphatase PP1-alpha catalytic subunit	PP1A_HUMAN		PPP1CA
174	174	627	0.999	327	sp|P62140|PP1B_HUMAN	PPP1CB	P62140	Serine/threonine-protein phosphatase PP1-beta catalytic subunit	PP1B_HUMAN		PPP1CB
978	978	978	0.999	130	sp|P62244|RS15A_HUMAN	RPS15A	P62244	Small ribosomal subunit protein uS8	RS15A_HUMAN		RPS15A
380	380	380	0.999	437	sp|P62495|ERF1_HUMAN	ETF1	P62495	Eukaryotic peptide chain release factor subunit 1	ERF1_HUMAN		ETF1
764	764	764	0.999	69	sp|P62857|RS28_HUMAN	RPS28	P62857	Small ribosomal subunit protein eS28	RS28_HUMAN		RPS28
864	864	864	0.999	83	sp|P63220|RS21_HUMAN	RPS21	P63220	Small ribosomal subunit protein eS21	RS21_HUMAN		RPS21
136	131	136	0.999	2471	sp|Q04721|NOTC2_HUMAN	NOTCH2	Q04721	Neurogenic locus notch homolog protein 2	NOTC2_HUMAN		NOTCH2
271	271	271	0.999	517	sp|Q06203|PUR1_HUMAN	PPAT	Q06203	Amidophosphoribosyltransferase	PUR1_HUMAN		PPAT
819	819	819	0.999	249	sp|Q06323|PSME1_HUMAN	PSME1	Q06323	Proteasome activator complex subunit 1	PSME1_HUMAN		PSME1
402	402	458	0.999	632	sp|Q06787|FMR1_HUMAN	FMR1	Q06787	Fragile X messenger ribonucleoprotein 1	FMR1_HUMAN		FMR1
278	278	437	0.999	494	sp|Q08170|SRSF4_HUMAN	SRSF4	Q08170	Serine/arginine-rich splicing factor 4	SRSF4_HUMAN		SRSF4
185	185	185	0.999	1443	sp|Q10570|CPSF1_HUMAN	CPSF1	Q10570	Cleavage and polyadenylation specificity factor subunit 1	CPSF1_HUMAN		CPSF1
214	214	214	0.999	1159	sp|Q12768|WASC5_HUMAN	WASHC5	Q12768	WASH complex subunit 5	WASC5_HUMAN		KIAA0196
144	144	144	0.999	2813	sp|Q12802|AKP13_HUMAN	AKAP13	Q12802	A-kinase anchor protein 13	AKP13_HUMAN		AKAP13
110	110	110	0.999	1025	sp|Q12882|DPYD_HUMAN	DPYD	Q12882	Dihydropyrimidine dehydrogenase [NADP(+)]	DPYD_HUMAN		DPYD
197	197	197	0.999	1972	sp|Q12888|TP53B_HUMAN	TP53BP1	Q12888	TP53-binding protein 1	TP53B_HUMAN		TP53BP1
704	704	704	0.999	390	sp|Q12905|ILF2_HUMAN	ILF2	Q12905	Interleukin enhancer-binding factor 2	ILF2_HUMAN		ILF2
268	268	268	0.999	283	sp|Q13126|MTAP_HUMAN	MTAP	Q13126	S-methyl-5'-thioadenosine phosphorylase	MTAP_HUMAN		MTAP
118	116	118	0.999	2157	sp|Q13459|MYO9B_HUMAN	MYO9B	Q13459	Unconventional myosin-IXb	MYO9B_HUMAN		MYO9B
173	173	253	0.999	282	sp|Q13595|TRA2A_HUMAN	TRA2A	Q13595	Transformer-2 protein homolog alpha	TRA2A_HUMAN		TRA2A
239	239	239	0.999	323	sp|Q13642|FHL1_HUMAN	FHL1	Q13642	Four and a half LIM domains protein 1	FHL1_HUMAN		FHL1
522	522	522	0.999	172	sp|Q14011|CIRBP_HUMAN	CIRBP	Q14011	Cold-inducible RNA-binding protein	CIRBP_HUMAN		CIRBP
296	296	296	0.999	746	sp|Q14137|BOP1_HUMAN	BOP 1.00	Q14137	Ribosome biogenesis protein BOP1	BOP1_HUMAN		BOP 1.00
459	446	459	0.999	625	sp|Q14677|EPN4_HUMAN	CLINT1	Q14677	Clathrin interactor 1	EPN4_HUMAN		CLINT1
228	228	228	0.999	972	sp|Q14CX7|NAA25_HUMAN	NAA25	Q14CX7	N-alpha-acetyltransferase 25, NatB auxiliary subunit	NAA25_HUMAN		NAA25
403	403	403	0.999	226	sp|Q15005|SPCS2_HUMAN	SPCS2	Q15005	Signal peptidase complex subunit 2	SPCS2_HUMAN		SPCS2
70	70	70	0.999	836	sp|Q15063|POSTN_HUMAN	POSTN	Q15063	Periostin	POSTN_HUMAN		POSTN
799	799	799	0.999	160	sp|Q15185|TEBP_HUMAN	PTGES3	Q15185	Prostaglandin E synthase 3	TEBP_HUMAN		PTGES3
187	187	223	0.999	329	sp|Q15417|CNN3_HUMAN	CNN3	Q15417	Calponin-3	CNN3_HUMAN		CNN3
557	557	557	0.999	639	sp|Q15637|SF01_HUMAN	SF1	Q15637	Splicing factor 1	SF01_HUMAN		SF1
350	350	350	0.999	601	sp|Q15642|CIP4_HUMAN	TRIP10	Q15642	Cdc42-interacting protein 4	CIP4_HUMAN		TRIP10
89	89	89	0.999	1979	sp|Q15643|TRIPB_HUMAN	TRIP11	Q15643	Thyroid receptor-interacting protein 11	TRIPB_HUMAN		TRIP11
534	534	534	0.999	476	sp|Q15654|TRIP6_HUMAN	TRIP6	Q15654	Thyroid receptor-interacting protein 6	TRIP6_HUMAN		TRIP6
529	529	529	0.999	238	sp|Q16629|SRSF7_HUMAN	SRSF7	Q16629	Serine/arginine-rich splicing factor 7	SRSF7_HUMAN		SRSF7
264	264	264	0.999	736	sp|Q32P28|P3H1_HUMAN	P3H1	Q32P28	Prolyl 3-hydroxylase 1	P3H1_HUMAN		LEPRE1
309	309	309	0.999	575	sp|Q3LXA3|TKFC_HUMAN	TKFC	Q3LXA3	Triokinase/FMN cyclase	TKFC_HUMAN		DAK
366	366	366	0.999	353	sp|Q3ZCQ8|TIM50_HUMAN	TIMM50	Q3ZCQ8	Mitochondrial import inner membrane translocase subunit TIM50	TIM50_HUMAN		TIMM50
158	158	158	0.999	1007	sp|Q5T8P6|RBM26_HUMAN	RBM26	Q5T8P6	RNA-binding protein 26	RBM26_HUMAN		RBM26
274	267	274	0.999	788	sp|Q6P996|PDXD1_HUMAN	PDXDC1	Q6P996	Pyridoxal-dependent decarboxylase domain-containing protein 1	PDXD1_HUMAN		PDXDC1
199	197	199	0.999	1761	sp|Q7LBC6|KDM3B_HUMAN	KDM3B	Q7LBC6	Lysine-specific demethylase 3B	KDM3B_HUMAN		KDM3B
160	158	160	0.999	1011	sp|Q86W92|LIPB1_HUMAN	PPFIBP1	Q86W92	Liprin-beta-1	LIPB1_HUMAN		PPFIBP1
393	393	393	0.999	471	sp|Q8N684|CPSF7_HUMAN	CPSF7	Q8N684	Cleavage and polyadenylation specificity factor subunit 7	CPSF7_HUMAN		CPSF7
301	301	301	0.999	828	sp|Q8WUF5|IASPP_HUMAN	PPP1R13L	Q8WUF5	RelA-associated inhibitor	IASPP_HUMAN		PPP1R13L
158	158	158	0.999	593	sp|Q8WXI9|P66B_HUMAN	GATAD2B	Q8WXI9	Transcriptional repressor p66-beta	P66B_HUMAN		GATAD2B
390	390	390	0.999	414	sp|Q92552|RT27_HUMAN	MRPS27	Q92552	Small ribosomal subunit protein mS27	RT27_HUMAN		MRPS27
260	260	438	0.999	488	sp|Q92769|HDAC2_HUMAN	HDAC2	Q92769	Histone deacetylase 2	HDAC2_HUMAN		HDAC2
515	406	515	0.999	486	sp|Q92879|CELF1_HUMAN	CELF1	Q92879	CUGBP Elav-like family member 1	CELF1_HUMAN		CELF1
203	203	203	0.999	261	sp|Q969G5|CAVN3_HUMAN	CAVIN3	Q969G5	Caveolae-associated protein 3	CAVN3_HUMAN		PRKCDBP
101	101	101	0.999	613	sp|Q969P0|IGSF8_HUMAN	IGSF8	Q969P0	Immunoglobulin superfamily member 8	IGSF8_HUMAN		IGSF8
168	168	168	0.999	974	sp|Q96A65|EXOC4_HUMAN	EXOC4	Q96A65	Exocyst complex component 4	EXOC4_HUMAN		EXOC4
381	381	381	0.999	223	sp|Q96CT7|CC124_HUMAN	CCDC124	Q96CT7	Coiled-coil domain-containing protein 124	CC124_HUMAN		CCDC124
318	318	318	0.999	583	sp|Q96RS6|NUDC1_HUMAN	NUDCD1	Q96RS6	NudC domain-containing protein 1	NUDC1_HUMAN		NUDCD1
262	200	262	0.999	655	sp|Q96SB4|SRPK1_HUMAN	SRPK1	Q96SB4	SRSF protein kinase 1	SRPK1_HUMAN		SRPK1
503	503	503	0.999	244	sp|Q99426|TBCB_HUMAN	TBCB	Q99426	Tubulin-folding cofactor B	TBCB_HUMAN		TBCB
499	499	499	0.999	393	sp|Q99536|VAT1_HUMAN	VAT1	Q99536	Synaptic vesicle membrane protein VAT-1 homolog	VAT1_HUMAN		VAT1
506	506	506	0.999	537	sp|Q99829|CPNE1_HUMAN	CPNE1	Q99829	Copine-1	CPNE1_HUMAN		CPNE1
219	219	219	0.999	362	sp|Q9BRK5|CAB45_HUMAN	SDF4	Q9BRK5	45 kDa calcium-binding protein	CAB45_HUMAN		SDF4
227	227	227	0.999	656	sp|Q9BW27|NUP85_HUMAN	NUP85	Q9BW27	Nuclear pore complex protein Nup85	NUP85_HUMAN		NUP85
191	191	191	0.999	1292	sp|Q9C0C9|UBE2O_HUMAN	UBE2O	Q9C0C9	(E3-independent) E2 ubiquitin-conjugating enzyme	UBE2O_HUMAN		UBE2O
314	314	314	0.999	707	sp|Q9H078|CLPB_HUMAN	CLPB	Q9H078	Mitochondrial disaggregase	CLPB_HUMAN		CLPB
174	174	174	0.999	392	sp|Q9H0C8|ILKAP_HUMAN	ILKAP	Q9H0C8	Integrin-linked kinase-associated serine/threonine phosphatase 2C	ILKAP_HUMAN		ILKAP
336	336	336	0.999	377	sp|Q9H7Z7|PGES2_HUMAN	PTGES2	Q9H7Z7	Prostaglandin E synthase 2	PGES2_HUMAN		PTGES2
282	282	282	0.999	276	sp|Q9NQR4|NIT2_HUMAN	NIT2	Q9NQR4	Omega-amidase NIT2	NIT2_HUMAN		NIT2
178	178	178	0.999	701	sp|Q9NR19|ACSA_HUMAN	ACSS2	Q9NR19	Acetyl-coenzyme A synthetase, cytoplasmic	ACSA_HUMAN		ACSS2
197	197	197	0.999	558	sp|Q9NUQ6|SPS2L_HUMAN	SPATS2L	Q9NUQ6	SPATS2-like protein	SPS2L_HUMAN		SPATS2L
602	558	602	0.999	352	sp|Q9NYL9|TMOD3_HUMAN	TMOD3	Q9NYL9	Tropomodulin-3	TMOD3_HUMAN		TMOD3
532	532	532	0.999	308	sp|Q9NZ01|TECR_HUMAN	TECR	Q9NZ01	Very-long-chain enoyl-CoA reductase	TECR_HUMAN		TECR
100	100	249	0.999	577	sp|Q9NZI8|IF2B1_HUMAN	IGF2BP1	Q9NZI8	Insulin-like growth factor 2 mRNA-binding protein 1	IF2B1_HUMAN		IGF2BP1
196	193	196	0.999	717	sp|Q9UH99|SUN2_HUMAN	SUN2	Q9UH99	SUN domain-containing protein 2	SUN2_HUMAN		SUN2
270	270	270	0.999	795	sp|Q9Y263|PLAP_HUMAN	PLAA	Q9Y263	Phospholipase A-2-activating protein	PLAP_HUMAN		PLAA
407	341	407	0.999	367	sp|Q9Y295|DRG1_HUMAN	DRG1	Q9Y295	Developmentally-regulated GTP-binding protein 1	DRG1_HUMAN		DRG1
482	482	482	0.999	226	sp|Q9Y2Q3|GSTK1_HUMAN	GSTK1	Q9Y2Q3	Glutathione S-transferase kappa 1	GSTK1_HUMAN		GSTK1
411	411	411	0.999	529	sp|Q9Y2X3|NOP58_HUMAN	NOP58	Q9Y2X3	Nucleolar protein 58	NOP58_HUMAN		NOP58
709	709	709	0.999	350	sp|Q9Y3F4|STRAP_HUMAN	STRAP	Q9Y3F4	Serine-threonine kinase receptor-associated protein	STRAP_HUMAN		STRAP
339	339	339	0.999	639	sp|Q9Y5K6|CD2AP_HUMAN	CD2AP	Q9Y5K6	CD2-associated protein	CD2AP_HUMAN		CD2AP
307	307	307	0.999	632	sp|A1L0T0|HACL2_HUMAN	ILVBL	A1L0T0	2-hydroxyacyl-CoA lyase 2	HACL2_HUMAN		ILVBL
434	434	434	0.999	1029	sp|O15042|SR140_HUMAN	U2SURP	O15042	U2 snRNP-associated SURP motif-containing protein	SR140_HUMAN		U2SURP
404	404	404	0.999	352	sp|O15372|EIF3H_HUMAN	EIF3H	O15372	Eukaryotic translation initiation factor 3 subunit H	EIF3H_HUMAN		EIF3H
325	325	325	0.999	452	sp|O43615|TIM44_HUMAN	TIMM44	O43615	Mitochondrial import inner membrane translocase subunit TIM44	TIM44_HUMAN		TIMM44
351	351	351	0.999	385	sp|O43837|IDH3B_HUMAN	IDH3B	O43837	Isocitrate dehydrogenase [NAD] subunit beta, mitochondrial	IDH3B_HUMAN		IDH3B
210	210	210	0.999	916	sp|O60502|OGA_HUMAN	OGA	O60502	Protein O-GlcNAcase	OGA_HUMAN		MGEA5
331	323	331	0.999	519	sp|O60749|SNX2_HUMAN	SNX2	O60749	Sorting nexin-2	SNX2_HUMAN		SNX2
283	283	283	0.999	1001	sp|O75150|BRE1B_HUMAN	RNF40	O75150	E3 ubiquitin-protein ligase BRE1B	BRE1B_HUMAN		RNF40
332	275	332	0.999	444	sp|O75351|VPS4B_HUMAN	VPS4B	O75351	Vacuolar protein sorting-associated protein 4B	VPS4B_HUMAN		VPS4B
398	398	490	0.999	489	sp|O75439|MPPB_HUMAN	PMPCB	O75439	Mitochondrial-processing peptidase subunit beta	MPPB_HUMAN		PMPCB
622	622	622	0.999	320	sp|O75821|EIF3G_HUMAN	EIF3G	O75821	Eukaryotic translation initiation factor 3 subunit G	EIF3G_HUMAN		EIF3G
447	447	447	0.999	542	sp|O95394|AGM1_HUMAN	PGM3	O95394	Phosphoacetylglucosamine mutase	AGM1_HUMAN		PGM3
546	546	546	0.999	338	sp|O95433|AHSA1_HUMAN	AHSA1	O95433	Activator of 90 kDa heat shock protein ATPase homolog 1	AHSA1_HUMAN		AHSA1
270	241	270	0.999	577	sp|O95793|STAU1_HUMAN	STAU1	O95793	Double-stranded RNA-binding protein Staufen homolog 1	STAU1_HUMAN		STAU1
211	211	211	0.999	425	sp|O95810|CAVN2_HUMAN	CAVIN2	O95810	Caveolae-associated protein 2	CAVN2_HUMAN		SDPR
672	672	672	0.999	289	sp|P00491|PNPH_HUMAN	PNP	P00491	Purine nucleoside phosphorylase	PNPH_HUMAN		PNP
98	98	98	0.999	431	sp|P00749|UROK_HUMAN	PLAU	P00749	Urokinase-type plasminogen activator	UROK_HUMAN		PLAU
631	631	631	0.999	631	sp|P04844|RPN2_HUMAN	RPN2	P04844	Dolichyl-diphosphooligosaccharide--protein glycosyltransferase subunit 2	RPN2_HUMAN		RPN2
117	117	117	0.999	402	sp|P05121|PAI1_HUMAN	SERPINE1	P05121	Plasminogen activator inhibitor 1	PAI1_HUMAN		SERPINE1
290	290	290	0.999	381	sp|P08174|DAF_HUMAN	CD55	P08174	Complement decay-accelerating factor	DAF_HUMAN		CD55
140	140	140	0.999	223	sp|P09936|UCHL1_HUMAN	UCHL1	P09936	Ubiquitin carboxyl-terminal hydrolase isozyme L1	UCHL1_HUMAN		UCHL1
88	88	88	0.999	1247	sp|P14543|NID1_HUMAN	NID1	P14543	Nidogen-1	NID1_HUMAN		NID1
208	208	895	0.999	152	sp|P15531|NDKA_HUMAN	NME1	P15531	Nucleoside diphosphate kinase A	NDKA_HUMAN		NME1
713	713	713	0.999	742	sp|P16070|CD44_HUMAN	CD44	P16070	CD44 antigen	CD44_HUMAN		CD44
1086	303	1086	0.999	152	sp|P22392|NDKB_HUMAN	NME2	P22392	Nucleoside diphosphate kinase B	NDKB_HUMAN		NME2
296	296	296	0.999	658	sp|P23786|CPT2_HUMAN	CPT2	P23786	Carnitine O-palmitoyltransferase 2, mitochondrial	CPT2_HUMAN		CPT2
339	339	339	0.999	212	sp|P23919|KTHY_HUMAN	DTYMK	P23919	Thymidylate kinase	KTHY_HUMAN		DTYMK
570	521	570	0.999	288	sp|P27105|STOM_HUMAN	STOM	P27105	Stomatin	STOM_HUMAN		STOM
105	89	261	0.999	379	sp|P27361|MK03_HUMAN	MAPK3	P27361	Mitogen-activated protein kinase 3	MK03_HUMAN		MAPK3
777	777	777	0.999	256	sp|P30048|PRDX3_HUMAN	PRDX3	P30048	Thioredoxin-dependent peroxide reductase, mitochondrial	PRDX3_HUMAN		PRDX3
820	820	820	0.999	165	sp|P30050|RL12_HUMAN	RPL12	P30050	Large ribosomal subunit protein uL11	RL12_HUMAN		RPL12
395	395	395	0.999	357	sp|P30533|AMRP_HUMAN	LRPAP1	P30533	Alpha-2-macroglobulin receptor-associated protein	AMRP_HUMAN		LRPAP1
274	274	274	0.999	454	sp|P36551|HEM6_HUMAN	CPOX	P36551	Oxygen-dependent coproporphyrinogen-III oxidase, mitochondrial	HEM6_HUMAN		CPOX
584	584	584	0.999	456	sp|P39656|OST48_HUMAN	DDOST	P39656	Dolichyl-diphosphooligosaccharide--protein glycosyltransferase 48 kDa su	OST48_HUMAN		DDOST
596	596	596	0.999	159	sp|P47914|RL29_HUMAN	RPL29	P47914	Large ribosomal subunit protein eL29	RL29_HUMAN		RPL29
627	627	627	0.999	207	sp|P51149|RAB7A_HUMAN	RAB7A	P51149	Ras-related protein Rab-7a	RAB7A_HUMAN		RAB7A
320	291	320	0.999	485	sp|P51648|AL3A2_HUMAN	ALDH3A2	P51648	Aldehyde dehydrogenase family 3 member A2	AL3A2_HUMAN		ALDH3A2

268	268	268	0.999	201	sp|P52566|GDIR2_HUMAN	ARHGDIB	P52566	Rho GDP-dissociation inhibitor 2	GDIR2_HUMAN		ARHGDIB
195	195	195	0.999	1056	sp|P52732|KIF11_HUMAN	KIF11	P52732	Kinesin-like protein KIF11	KIF11_HUMAN		KIF11
427	427	427	0.999	311	sp|P53007|TXTP_HUMAN	SLC25A1	P53007	Tricarboxylate transport protein, mitochondrial	TXTP_HUMAN		SLC25A1
496	496	496	0.999	386	sp|P53582|MAP11_HUMAN	METAP1	P53582	Methionine aminopeptidase 1	MAP11_HUMAN		METAP1
753	581	753	0.999	391	sp|P55209|NP1L1_HUMAN	NAP1L1	P55209	Nucleosome assembly protein 1-like 1	NP1L1_HUMAN		NAP1L1
1193	927	1193	0.999	151	sp|P60660|MYL6_HUMAN	MYL6	P60660	Myosin light polypeptide 6	MYL6_HUMAN		MYL6
337	337	337	0.999	197	sp|P61758|PFD3_HUMAN	VBP1	P61758	Prefoldin subunit 3	PFD3_HUMAN		VBP1
482	482	482	0.999	246	sp|Q00059|TFAM_HUMAN	TFAM	Q00059	Transcription factor A, mitochondrial	TFAM_HUMAN		TFAM
153	152	153	0.999	879	sp|Q01433|AMPD2_HUMAN	AMPD2	Q01433	AMP deaminase 2	AMPD2_HUMAN		AMPD2
250	250	250	0.999	1054	sp|Q03701|CEBPZ_HUMAN	CEBPZ	Q03701	CCAAT/enhancer-binding protein zeta	CEBPZ_HUMAN		CEBPZ
264	264	264	0.999	808	sp|Q12788|TBL3_HUMAN	TBL3	Q12788	Transducin beta-like protein 3	TBL3_HUMAN		TBL3
601	489	601	0.999	350	sp|Q12792|TWF1_HUMAN	TWF1	Q12792	Twinfilin-1	TWF1_HUMAN		TWF1
587	587	587	0.999	312	sp|Q12904|AIMP1_HUMAN	AIMP1	Q12904	Aminoacyl tRNA synthase complex-interacting multifunctional protein 1	AIMP1_HUMAN		AIMP1
514	514	514	0.999	414	sp|Q13148|TADBP_HUMAN	TARDBP	Q13148	TAR DNA-binding protein 43	TADBP_HUMAN		TARDBP
344	344	364	0.999	221	sp|Q13242|SRSF9_HUMAN	SRSF9	Q13242	Serine/arginine-rich splicing factor 9	SRSF9_HUMAN		SRSF9
581	581	581	0.999	181	sp|Q13442|HAP28_HUMAN	PDAP1	Q13442	28 kDa heat- and acid-stable phosphoprotein	HAP28_HUMAN		PDAP1
472	294	472	0.999	482	sp|Q13547|HDAC1_HUMAN	HDAC1	Q13547	Histone deacetylase 1	HDAC1_HUMAN		HDAC1
736	736	736	0.999	142	sp|Q14019|COTL1_HUMAN	COTL1	Q14019	Coactosin-like protein	COTL1_HUMAN		COTL1
543	543	543	0.999	615	sp|Q14739|LBR_HUMAN	LBR	Q14739	Delta(14)-sterol reductase LBR	LBR_HUMAN		LBR
186	186	209	0.999	2024	sp|Q15154|PCM1_HUMAN	PCM1	Q15154	Pericentriolar material 1 protein	PCM1_HUMAN		PCM1
303	251	303	0.999	527	sp|Q15813|TBCE_HUMAN	TBCE	Q15813	Tubulin-specific chaperone E	TBCE_HUMAN		TBCE
140	140	140	0.999	1708	sp|Q6R327|RICTR_HUMAN	RICTOR	Q6R327	Rapamycin-insensitive companion of mTOR	RICTR_HUMAN		RICTOR
212	212	212	0.999	1445	sp|Q6YHK3|CD109_HUMAN	CD109	Q6YHK3	CD109 antigen	CD109_HUMAN		CD109
269	269	269	0.999	1726	sp|Q7KZ85|SPT6H_HUMAN	SUPT6H	Q7KZ85	Transcription elongation factor SPT6	SPT6H_HUMAN		SUPT6H
299	299	299	0.999	1120	sp|Q7Z2Z2|EFL1_HUMAN	EFL1	Q7Z2Z2	Elongation factor-like GTPase 1	EFL1_HUMAN		EFTUD1
266	266	266	0.999	993	sp|Q8N766|EMC1_HUMAN	EMC1	Q8N766	ER membrane protein complex subunit 1	EMC1_HUMAN		EMC1
248	248	248	0.999	373	sp|Q8WUP2|FBLI1_HUMAN	FBLIM1	Q8WUP2	Filamin-binding LIM protein 1	FBLI1_HUMAN		FBLIM1
167	167	167	0.999	2039	sp|Q92576|PHF3_HUMAN	PHF3	Q92576	PHD finger protein 3	PHF3_HUMAN		PHF3
212	212	366	0.999	483	sp|Q92599|SEPT8_HUMAN	SEPTIN8	Q92599	Septin-8	SEPT8_HUMAN		SEPT8
415	415	442	0.999	251	sp|Q92688|AN32B_HUMAN	ANP32B	Q92688	Acidic leucine-rich nuclear phosphoprotein 32 family member B	AN32B_HUMAN		ANP32B
317	312	317	0.999	582	sp|Q96AY3|FKB10_HUMAN	FKBP10	Q96AY3	Peptidyl-prolyl cis-trans isomerase FKBP10	FKB10_HUMAN		FKBP10
267	267	267	0.999	808	sp|Q96KG9|SCYL1_HUMAN	SCYL1	Q96KG9	N-terminal kinase-like protein	SCYL1_HUMAN		SCYL1
121	121	121	0.999	1273	sp|Q96ST3|SIN3A_HUMAN	SIN3A	Q96ST3	Paired amphipathic helix protein Sin3a	SIN3A_HUMAN		SIN3A
216	216	216	0.999	741	sp|Q99567|NUP88_HUMAN	NUP88	Q99567	Nuclear pore complex protein Nup88	NUP88_HUMAN		NUP88
438	423	438	0.999	371	sp|Q99873|ANM1_HUMAN	PRMT1	Q99873	Protein arginine N-methyltransferase 1	ANM1_HUMAN		PRMT1
396	332	396	0.999	368	sp|Q99961|SH3G1_HUMAN	SH3GL1	Q99961	Endophilin-A2	SH3G1_HUMAN		SH3GL1
146	146	210	0.999	737	sp|Q9BQ39|DDX50_HUMAN	DDX50	Q9BQ39	ATP-dependent RNA helicase DDX50	DDX50_HUMAN		DDX50
361	361	361	0.999	406	sp|Q9BT78|CSN4_HUMAN	COPS4	Q9BT78	COP9 signalosome complex subunit 4	CSN4_HUMAN		COPS4
194	194	240	0.999	718	sp|Q9BW92|SYTM_HUMAN	TARS2	Q9BW92	Threonine--tRNA ligase, mitochondrial	SYTM_HUMAN		TARS2
149	149	149	0.999	2365	sp|Q9H2D6|TARA_HUMAN	TRIOBP	Q9H2D6	TRIO and F-actin-binding protein	TARA_HUMAN		TRIOBP
145	145	145	0.999	1636	sp|Q9H3S7|PTN23_HUMAN	PTPN23	Q9H3S7	Tyrosine-protein phosphatase non-receptor type 23	PTN23_HUMAN		PTPN23
432	432	432	0.999	621	sp|Q9H845|ACAD9_HUMAN	ACAD9	Q9H845	Complex I assembly factor ACAD9, mitochondrial	ACAD9_HUMAN		ACAD9
318	318	318	0.999	587	sp|Q9NTJ5|SAC1_HUMAN	SACM1L	Q9NTJ5	Phosphatidylinositol-3-phosphatase SAC1	SAC1_HUMAN		SACM1L
607	541	607	0.999	324	sp|Q9NUQ9|CYRIB_HUMAN	CYRIB	Q9NUQ9	CYFIP-related Rac1 interactor B	CYRIB_HUMAN		FAM49B
447	408	447	0.999	106	sp|Q9UII2|ATIF1_HUMAN	ATP5IF1	Q9UII2	ATPase inhibitor, mitochondrial	ATIF1_HUMAN		ATPIF1
559	501	559	0.999	675	sp|Q9UJS0|S2513_HUMAN	SLC25A13	Q9UJS0	Electrogenic aspartate/glutamate antiporter SLC25A13, mitochondrial	S2513_HUMAN		SLC25A13
171	171	171	0.999	1724	sp|Q9UKK3|PARP4_HUMAN	PARP4	Q9UKK3	Protein mono-ADP-ribosyltransferase PARP4	PARP4_HUMAN		PARP4
230	230	230	0.999	822	sp|Q9UKN8|TF3C4_HUMAN	GTF3C4	Q9UKN8	General transcription factor 3C polypeptide 4	TF3C4_HUMAN		GTF3C4
365	365	365	0.999	424	sp|Q9UKS6|PACN3_HUMAN	PACSIN3	Q9UKS6	Protein kinase C and casein kinase substrate in neurons protein 3	PACN3_HUMAN		PACSIN3
292	292	431	0.999	482	sp|Q9UN86|G3BP2_HUMAN	G3BP2	Q9UN86	Ras GTPase-activating protein-binding protein 2	G3BP2_HUMAN		G3BP2
362	362	362	0.999	303	sp|Q9UNE7|CHIP_HUMAN	STUB1	Q9UNE7	E3 ubiquitin-protein ligase CHIP	CHIP_HUMAN		STUB1
325	325	325	0.999	423	sp|Q9UNS2|CSN3_HUMAN	COPS3	Q9UNS2	COP9 signalosome complex subunit 3	CSN3_HUMAN		COPS3
219	219	219	0.999	641	sp|Q9Y2W2|WBP11_HUMAN	WBP11	Q9Y2W2	WW domain-binding protein 11	WBP11_HUMAN		WBP11
849	849	849	0.999	105	sp|Q9Y3U8|RL36_HUMAN	RPL36	Q9Y3U8	Large ribosomal subunit protein eL36	RL36_HUMAN		RPL36
438	438	438	0.999	386	sp|Q9Y570|PPME1_HUMAN	PPME1	Q9Y570	Protein phosphatase methylesterase 1	PPME1_HUMAN		PPME1
248	248	249	0.999	523	sp|Q9Y6G9|DC1L1_HUMAN	DYNC1LI1	Q9Y6G9	Cytoplasmic dynein 1 light intermediate chain 1	DC1L1_HUMAN		DYNC1LI1
514	514	514	0.999	330	sp|O00170|AIP_HUMAN	AIP	O00170	AH receptor-interacting protein	AIP_HUMAN		AIP
272	272	272	0.999	962	sp|O43592|XPOT_HUMAN	XPOT	O43592	Exportin-T	XPOT_HUMAN		XPOT
374	374	374	0.999	514	sp|O60832|DKC1_HUMAN	DKC1	O60832	H/ACA ribonucleoprotein complex subunit DKC1	DKC1_HUMAN		DKC1
386	386	386	0.999	253	sp|O75937|DNJC8_HUMAN	DNAJC8	O75937	DnaJ homolog subfamily C member 8	DNJC8_HUMAN		DNAJC8
299	299	299	0.999	794	sp|O94874|UFL1_HUMAN	UFL1	O94874	E3 UFM1-protein ligase 1	UFL1_HUMAN		UFL1
60	60	60	0.999	791	sp|O95479|G6PE_HUMAN	H6PD	O95479	GDH/6PGL endoplasmic bifunctional protein	G6PE_HUMAN		H6PD
355	355	355	0.999	211	sp|O95816|BAG2_HUMAN	BAG2	O95816	BAG family molecular chaperone regulator 2	BAG2_HUMAN		BAG2
227	227	227	0.999	575	sp|O95817|BAG3_HUMAN	BAG3	O95817	BAG family molecular chaperone regulator 3	BAG3_HUMAN		BAG3
108	108	108	0.999	393	sp|P04637|P53_HUMAN	TP53	P04637	Cellular tumor antigen p53	P53_HUMAN		TP53
247	247	247	0.999	303	sp|P05026|AT1B1_HUMAN	ATP1B1	P05026	Sodium/potassium-transporting ATPase subunit beta-1	AT1B1_HUMAN		ATP1B1
292	292	292	0.999	561	sp|P08243|ASNS_HUMAN	ASNS	P08243	Asparagine synthetase [glutamine-hydrolyzing]	ASNS_HUMAN		ASNS
1481	1481	1481	0.999	135	sp|P09382|LEG1_HUMAN	LGALS1	P09382	Galectin-1	LEG1_HUMAN		LGALS1
54	50	113	0.999	1106	sp|P09619|PGFRB_HUMAN	PDGFRB	P09619	Platelet-derived growth factor receptor beta	PGFRB_HUMAN		PDGFRB
342	342	342	0.999	538	sp|P10155|RO60_HUMAN	RO60	P10155	RNA-binding protein RO60	RO60_HUMAN		TROVE2
969	969	969	0.999	261	sp|P12004|PCNA_HUMAN	PCNA	P12004	Proliferating cell nuclear antigen	PCNA_HUMAN		PCNA
182	182	182	0.999	764	sp|P17480|UBF1_HUMAN	UBTF	P17480	Nucleolar transcription factor 1	UBF1_HUMAN		UBTF
267	267	267	0.999	735	sp|P23743|DGKA_HUMAN	DGKA	P23743	Diacylglycerol kinase alpha	DGKA_HUMAN		DGKA
960	960	960	0.999	132	sp|P25398|RS12_HUMAN	RPS12	P25398	Small ribosomal subunit protein eS12	RS12_HUMAN		RPS12
759	759	759	0.999	196	sp|P30085|KCY_HUMAN	CMPK1	P30085	UMP-CMP kinase	KCY_HUMAN		CMPK1
627	627	627	0.999	453	sp|P36957|ODO2_HUMAN	DLST	P36957	Dihydrolipoyllysine-residue succinyltransferase component of 2-oxoglutara	ODO2_HUMAN		DLST
782	782	782	0.999	136	sp|P37108|SRP14_HUMAN	SRP14	P37108	Signal recognition particle 14 kDa protein	SRP14_HUMAN		SRP14
1021	1021	1021	0.999	294	sp|P45880|VDAC2_HUMAN	VDAC2	P45880	Voltage-dependent anion-selective channel protein 2	VDAC2_HUMAN		VDAC2
371	371	484	0.999	452	sp|P48735|IDHP_HUMAN	IDH2	P48735	Isocitrate dehydrogenase [NADP], mitochondrial	IDHP_HUMAN		IDH2
333	333	333	0.999	464	sp|P49821|NDUV1_HUMAN	NDUFV1	P49821	NADH dehydrogenase [ubiquinone] flavoprotein 1, mitochondrial	NDUV1_HUMAN		NDUFV1
100	99	100	0.999	582	sp|P50281|MMP14_HUMAN	MMP14	P50281	Matrix metalloproteinase-14	MMP14_HUMAN		MMP14
1228	1228	1228	0.999	204	sp|P52565|GDIR1_HUMAN	ARHGDIA	P52565	Rho GDP-dissociation inhibitor 1	GDIR1_HUMAN		ARHGDIA
283	283	283	0.999	168	sp|P59998|ARPC4_HUMAN	ARPC4	P59998	Actin-related protein 2/3 complex subunit 4	ARPC4_HUMAN		ARPC4
417	262	791	0.999	207	sp|P61006|RAB8A_HUMAN	RAB8A	P61006	Ras-related protein Rab-8A	RAB8A_HUMAN		RAB8A
466	466	466	0.999	504	sp|P61011|SRP54_HUMAN	SRP54	P61011	Signal recognition particle subunit SRP54	SRP54_HUMAN		SRP54
596	427	722	0.999	200	sp|P61026|RAB10_HUMAN	RAB10	P61026	Ras-related protein Rab-10	RAB10_HUMAN		RAB10
468	468	635	0.999	215	sp|P61106|RAB14_HUMAN	RAB14	P61106	Ras-related protein Rab-14	RAB14_HUMAN		RAB14
166	166	168	0.999	594	sp|P61764|STXB1_HUMAN	STXBP1	P61764	Syntaxin-binding protein 1	STXB1_HUMAN		STXBP1
805	805	805	0.999	177	sp|P62633|CNBP_HUMAN	CNBP	P62633	CCHC-type zinc finger nucleic acid binding protein	CNBP_HUMAN		CNBP
785	785	785	0.999	140	sp|P62829|RL23_HUMAN	RPL23	P62829	Large ribosomal subunit protein uL14	RL23_HUMAN		RPL23
552	552	552	0.999	133	sp|P62847|RS24_HUMAN	RPS24	P62847	Small ribosomal subunit protein eS24	RS24_HUMAN		RPS24
1087	1087	1087	0.999	115	sp|P62888|RL30_HUMAN	RPL30	P62888	Large ribosomal subunit protein eL30	RL30_HUMAN		RPL30
438	186	438	0.999	447	sp|P63151|2ABA_HUMAN	PPP2R2A	P63151	Serine/threonine-protein phosphatase 2A 55 kDa regulatory subunit B alph	2ABA_HUMAN		PPP2R2A
651	113	651	0.999	391	sp|P68400|CSK21_HUMAN	CSNK2A1	P68400	Casein kinase II subunit alpha	CSK21_HUMAN		CSNK2A1
630	630	630	0.999	224	sp|Q00688|FKBP3_HUMAN	FKBP3	Q00688	Peptidyl-prolyl cis-trans isomerase FKBP3	FKBP3_HUMAN		FKBP3
382	382	382	0.999	408	sp|Q03154|ACY1_HUMAN	ACY1	Q03154	Aminoacylase-1	ACY1_HUMAN		ACY1
168	168	168	0.999	780	sp|Q05209|PTN12_HUMAN	PTPN12	Q05209	Tyrosine-protein phosphatase non-receptor type 12	PTN12_HUMAN		PTPN12
299	125	299	0.999	521	sp|Q08209|PP2BA_HUMAN	PPP3CA	Q08209	Protein phosphatase 3 catalytic subunit alpha	PP2BA_HUMAN		PPP3CA
186	179	186	0.999	559	sp|Q13131|AAPK1_HUMAN	PRKAA1	Q13131	5'-AMP-activated protein kinase catalytic subunit alpha-1	AAPK1_HUMAN		PRKAA1
353	353	353	0.999	1007	sp|Q13523|PRP4B_HUMAN	PRPF4B	Q13523	Serine/threonine-protein kinase PRP4 homolog	PRP4B_HUMAN		PRPF4B
366	366	366	0.999	536	sp|Q13573|SNW1_HUMAN	SNW1	Q13573	SNW domain-containing protein 1	SNW1_HUMAN		SNW1
392	392	392	0.999	776	sp|Q13616|CUL1_HUMAN	CUL1	Q13616	Cullin-1	CUL1_HUMAN		CUL1
221	203	428	0.999	570	sp|Q14195|DPYL3_HUMAN	DPYSL3	Q14195	Dihydropyrimidinase-related protein 3	DPYL3_HUMAN		DPYSL3
274	274	274	0.999	758	sp|Q14684|RRP1B_HUMAN	RRP1B	Q14684	Ribosomal RNA processing protein 1 homolog B	RRP1B_HUMAN		RRP1B
316	316	316	0.999	365	sp|Q15050|RRS1_HUMAN	RRS1	Q15050	Ribosome biogenesis regulatory protein homolog	RRS1_HUMAN		RRS1
933	933	933	0.999	248	sp|Q15056|IF4H_HUMAN	EIF4H	Q15056	Eukaryotic translation initiation factor 4H	IF4H_HUMAN		EIF4H
215	215	215	0.999	1057	sp|Q5GLZ8|HERC4_HUMAN	HERC4	Q5GLZ8	Probable E3 ubiquitin-protein ligase HERC4	HERC4_HUMAN		HERC4
392	392	504	0.999	349	sp|Q6IBS0|TWF2_HUMAN	TWF2	Q6IBS0	Twinfilin-2	TWF2_HUMAN		TWF2
279	279	296	0.999	671	sp|Q7Z4V5|HDGR2_HUMAN	HDGFL2	Q7Z4V5	Hepatoma-derived growth factor-related protein 2	HDGR2_HUMAN		HDGFRP2
175	175	175	0.999	863	sp|Q86TI2|DPP9_HUMAN	DPP9	Q86TI2	Dipeptidyl peptidase 9	DPP9_HUMAN		DPP9
204	204	204	0.999	1689	sp|Q86U86|PB1_HUMAN	PBRM1	Q86U86	Protein polybromo-1	PB1_HUMAN		PBRM1
375	375	375	0.999	365	sp|Q8IWS0|PHF6_HUMAN	PHF6	Q8IWS0	PHD finger protein 6	PHF6_HUMAN		PHF6
209	209	209	0.999	802	sp|Q8IZ83|A16A1_HUMAN	ALDH16A1	Q8IZ83	Aldehyde dehydrogenase family 16 member A1	A16A1_HUMAN		ALDH16A1
208	208	371	0.999	1214	sp|Q8TAQ2|SMRC2_HUMAN	SMARCC2	Q8TAQ2	SWI/SNF complex subunit SMARCC2	SMRC2_HUMAN		SMARCC2
468	468	468	0.999	353	sp|Q8TDN6|BRX1_HUMAN	BRIX1	Q8TDN6	Ribosome biogenesis protein BRX1 homolog	BRX1_HUMAN		BRIX1
558	558	628	0.999	592	sp|Q92804|RBP56_HUMAN	TAF15	Q92804	TATA-binding protein-associated factor 2N	RBP56_HUMAN		TAF15
204	204	204	0.999	476	sp|Q92917|GPKOW_HUMAN	GPKOW	Q92917	G-patch domain and KOW motifs-containing protein	GPKOW_HUMAN		GPKOW
488	457	488	0.999	240	sp|Q96C19|EFHD2_HUMAN	EFHD2	Q96C19	EF-hand domain-containing protein D2	EFHD2_HUMAN		EFHD2
391	391	391	0.999	751	sp|Q96RP9|EFGM_HUMAN	GFM1	Q96RP9	Elongation factor G, mitochondrial	EFGM_HUMAN		GFM1
296	245	296	0.999	977	sp|Q96T37|RBM15_HUMAN	RBM15	Q96T37	RNA-binding protein 15	RBM15_HUMAN		RBM15
321	131	321	0.999	364	sp|Q9BWF3|RBM4_HUMAN	RBM4	Q9BWF3	RNA-binding protein 4	RBM4_HUMAN		RBM4
160	160	160	0.999	704	sp|Q9BYT8|NEUL_HUMAN	NLN	Q9BYT8	Neurolysin, mitochondrial	NEUL_HUMAN		NLN
256	228	256	0.999	1008	sp|Q9H2U1|DHX36_HUMAN	DHX36	Q9H2U1	ATP-dependent DNA/RNA helicase DHX36	DHX36_HUMAN		DHX36
213	213	213	0.999	836	sp|Q9H5V8|CDCP1_HUMAN	CDCP1	Q9H5V8	CUB domain-containing protein 1	CDCP1_HUMAN		CDCP1
580	580	580	0.999	190	sp|Q9H910|JUPI2_HUMAN	JPT2	Q9H910	Jupiter microtubule associated homolog 2	JUPI2_HUMAN		HN1L
162	162	162	0.999	981	sp|Q9HC35|EMAL4_HUMAN	EML4	Q9HC35	Echinoderm microtubule-associated protein-like 4	EMAL4_HUMAN		EML4
572	572	572	0.999	416	sp|Q9HDC9|APMAP_HUMAN	APMAP	Q9HDC9	Adipocyte plasma membrane-associated protein	APMAP_HUMAN		APMAP
537	537	537	0.999	358	sp|Q9UBS4|DJB11_HUMAN	DNAJB11	Q9UBS4	DnaJ homolog subfamily B member 11	DJB11_HUMAN		DNAJB11
320	319	320	0.999	709	sp|Q9UKG1|DP13A_HUMAN	APPL1	Q9UKG1	DCC-interacting protein 13-alpha	DP13A_HUMAN		APPL1

672	672	672	0.999	239	sp|Q9UL46|PSME2_HUMAN	PSME2	Q9UL46	Proteasome activator complex subunit 2	PSME2_HUMAN		PSME2
399	43	399	0.999	479	sp|Q9UMR2|DD19B_HUMAN	DDX19B	Q9UMR2	ATP-dependent RNA helicase DDX19B	DD19B_HUMAN		DDX19B
235	235	235	0.999	859	sp|Q9Y2D5|AKAP2_HUMAN	AKAP2	Q9Y2D5	A-kinase anchor protein 2	AKAP2_HUMAN		AKAP2
320	320	377	0.999	419	sp|Q9Y6E2|5MP1_HUMAN	BZW2	Q9Y6E2	eIF5-mimic protein 1	5MP1_HUMAN		BZW2
251	251	251	0.999	1153	sp|O14617|AP3D1_HUMAN	AP3D1	O14617	AP-3 complex subunit delta-1	AP3D1_HUMAN		AP3D1
369	369	369	0.999	822	sp|O43747|AP1G1_HUMAN	AP1G1	O43747	AP-1 complex subunit gamma-1	AP1G1_HUMAN		AP1G1
274	274	274	0.999	1151	sp|O43847|NRDC_HUMAN	NRDC	O43847	Nardilysin	NRDC_HUMAN		NRD1
204	204	204	0.999	2243	sp|O75165|DJC13_HUMAN	DNAJC13	O75165	DnaJ homolog subfamily C member 13	DJC13_HUMAN		DNAJC13
870	870	870	0.999	108	sp|O75347|TBCA_HUMAN	TBCA	O75347	Tubulin-specific chaperone A	TBCA_HUMAN		TBCA
435	386	435	0.999	369	sp|O75367|H2AY_HUMAN	MACROH2A	O75367	Core histone macro-H2A.1	H2AY_HUMAN		H2AFY
529	529	529	0.999	466	sp|O75390|CISY_HUMAN	CS	O75390	Citrate synthase, mitochondrial	CISY_HUMAN		CS
193	193	193	0.999	1129	sp|O75717|WDHD1_HUMAN	WDHD1	O75717	WD repeat and HMG-box DNA-binding protein 1	WDHD1_HUMAN		WDHD1
316	316	316	0.999	1332	sp|O95163|ELP1_HUMAN	ELP1	O95163	Elongator complex protein 1	ELP1_HUMAN		IKBKAP
63	63	63	0.999	469	sp|P03956|MMP1_HUMAN	MMP1	P03956	Interstitial collagenase	MMP1_HUMAN		MMP1
614	582	614	0.999	268	sp|P04632|CPNS1_HUMAN	CAPNS1	P04632	Calpain small subunit 1	CPNS1_HUMAN		CAPNS1
1178	1178	1178	0.999	205	sp|P04792|HSPB1_HUMAN	HSPB1	P04792	Heat shock protein beta-1	HSPB1_HUMAN		HSPB1
648	648	648	0.999	524	sp|P07602|SAP_HUMAN	PSAP	P07602	Prosaposin	SAP_HUMAN		PSAP
711	711	711	0.999	419	sp|P14324|FPPS_HUMAN	FDPS	P14324	Farnesyl pyrophosphate synthase	FPPS_HUMAN		FDPS
901	901	901	0.999	274	sp|P15559|NQO1_HUMAN	NQO1	P15559	NAD(P)H dehydrogenase [quinone] 1	NQO1_HUMAN		NQO1
149	120	149	0.999	672	sp|P17252|KPCA_HUMAN	PRKCA	P17252	Protein kinase C alpha type	KPCA_HUMAN		PRKCA
500	500	500	0.999	435	sp|P18031|PTN1_HUMAN	PTPN1	P18031	Tyrosine-protein phosphatase non-receptor type 1	PTN1_HUMAN		PTPN1
368	368	368	0.999	2426	sp|P18583|SON_HUMAN	SON	P18583	Protein SON	SON_HUMAN		SON
186	171	187	0.999	1838	sp|P20908|CO5A1_HUMAN	COL5A1	P20908	Collagen alpha-1(V) chain	CO5A1_HUMAN		COL5A1
536	536	536	0.999	256	sp|P24539|AT5F1_HUMAN	ATP5PB	P24539	ATP synthase F(0) complex subunit B1, mitochondrial	AT5F1_HUMAN		ATP5F1
687	687	687	0.999	261	sp|P25789|PSA4_HUMAN	PSMA4	P25789	Proteasome subunit alpha type-4	PSA4_HUMAN		PSMA4
568	568	568	0.999	346	sp|P31942|HNRH3_HUMAN	HNRNPH3	P31942	Heterogeneous nuclear ribonucleoprotein H3	HNRH3_HUMAN		HNRNPH3
254	254	254	0.999	417	sp|P37268|FDFT_HUMAN	FDFT1	P37268	Squalene synthase	FDFT_HUMAN		FDFT1
641	641	641	0.999	348	sp|P40121|CAPG_HUMAN	CAPG	P40121	Macrophage-capping protein	CAPG_HUMAN		CAPG
478	478	478	0.999	705	sp|P46977|STT3A_HUMAN	STT3A	P46977	Dolichyl-diphosphooligosaccharide--protein glycosyltransferase subunit ST	STT3A_HUMAN		STT3A
356	356	356	0.999	732	sp|P48449|LSS_HUMAN	LSS	P48449	Lanosterol synthase	LSS_HUMAN		LSS
495	446	495	0.999	271	sp|P48739|PIPNB_HUMAN	PITPNB	P48739	Phosphatidylinositol transfer protein beta isoform	PIPNB_HUMAN		PITPNB
470	470	470	0.999	773	sp|P50416|CPT1A_HUMAN	CPT1A	P50416	Carnitine O-palmitoyltransferase 1, liver isoform	CPT1A_HUMAN		CPT1A
69	69	134	0.999	376	sp|P50453|SPB9_HUMAN	SERPINB9	P50453	Serpin B9	SPB9_HUMAN		SERPINB9
570	570	570	0.999	478	sp|P50579|MAP2_HUMAN	METAP2	P50579	Methionine aminopeptidase 2	MAP2_HUMAN		METAP2
230	230	230	0.999	392	sp|P51570|GALK1_HUMAN	GALK1	P51570	Galactokinase	GALK1_HUMAN		GALK1
533	533	533	0.999	925	sp|P57740|NU107_HUMAN	NUP107	P57740	Nuclear pore complex protein Nup107	NU107_HUMAN		NUP107
1605	75	1605	0.999	154	sp|P63241|IF5A1_HUMAN	EIF5A	P63241	Eukaryotic translation initiation factor 5A-1	IF5A1_HUMAN		EIF5A
1118	680	1118	0.999	324	sp|P67809|YBOX1_HUMAN	YBX1	P67809	Y-box-binding protein 1	YBOX1_HUMAN		YBX1
612	612	612	0.999	210	sp|P82979|SARNP_HUMAN	SARNP	P82979	SAP domain-containing ribonucleoprotein	SARNP_HUMAN		SARNP
398	398	398	0.999	314	sp|Q02978|M2OM_HUMAN	SLC25A11	Q02978	Mitochondrial 2-oxoglutarate/malate carrier protein	M2OM_HUMAN		SLC25A11
321	321	321	0.999	585	sp|Q08380|LG3BP_HUMAN	LGALS3BP	Q08380	Galectin-3-binding protein	LG3BP_HUMAN		LGALS3BP
686	686	686	0.999	525	sp|Q10713|MPPA_HUMAN	PMPCA	Q10713	Mitochondrial-processing peptidase subunit alpha	MPPA_HUMAN		PMPCA
33	32	341	0.999	2000	sp|Q12873|CHD3_HUMAN	CHD3	Q12873	Chromodomain-helicase-DNA-binding protein 3	CHD3_HUMAN		CHD3
548	548	548	0.999	356	sp|Q12907|LMAN2_HUMAN	LMAN2	Q12907	Vesicular integral-membrane protein VIP36	LMAN2_HUMAN		LMAN2
709	550	709	0.999	344	sp|Q13247|SRSF6_HUMAN	SRSF6	Q13247	Serine/arginine-rich splicing factor 6	SRSF6_HUMAN		SRSF6
83	83	83	0.999	2230	sp|Q13439|GOGA4_HUMAN	GOLGA4	Q13439	Golgin subfamily A member 4	GOGA4_HUMAN		GOLGA4
383	353	383	0.999	768	sp|Q13618|CUL3_HUMAN	CUL3	Q13618	Cullin-3	CUL3_HUMAN		CUL3
387	387	387	0.999	837	sp|Q13724|MOGS_HUMAN	MOGS	Q13724	Mannosyl-oligosaccharide glucosidase	MOGS_HUMAN		MOGS
210	210	210	0.999	1992	sp|Q14669|TRIPC_HUMAN	TRIP12	Q14669	E3 ubiquitin-protein ligase TRIP12	TRIPC_HUMAN		TRIP12
891	891	891	0.999	268	sp|Q15691|MARE1_HUMAN	MAPRE1	Q15691	Microtubule-associated protein RP/EB family member 1	MARE1_HUMAN		MAPRE1
278	278	278	0.999	373	sp|Q15738|NSDHL_HUMAN	NSDHL	Q15738	Sterol-4-alpha-carboxylate 3-dehydrogenase, decarboxylating	NSDHL_HUMAN		NSDHL
416	416	416	0.999	504	sp|Q16401|PSMD5_HUMAN	PSMD5	Q16401	26S proteasome non-ATPase regulatory subunit 5	PSMD5_HUMAN		PSMD5
567	567	567	0.999	583	sp|Q5JTV8|TOIP1_HUMAN	TOR1AIP1	Q5JTV8	Torsin-1A-interacting protein 1	TOIP1_HUMAN		TOR1AIP1
281	9	734	0.999	136	sp|Q71DI3|H32_HUMAN	H3C15	Q71DI3	Histone H3.2	H32_HUMAN		HIST2H3A
265	234	265	0.999	1538	sp|Q7Z460|CLAP1_HUMAN	CLASP1	Q7Z460	CLIP-associating protein 1	CLAP1_HUMAN		CLASP1
627	627	627	0.999	257	sp|Q86V81|THOC4_HUMAN	ALYREF	Q86V81	THO complex subunit 4	THOC4_HUMAN		ALYREF
191	191	191	0.999	563	sp|Q8IWE2|NXP20_HUMAN	FAM114A1	Q8IWE2	Protein NOXP20	NXP20_HUMAN		FAM114A1
107	107	177	0.999	1332	sp|Q8N4C8|MINK1_HUMAN	MINK1	Q8N4C8	Misshapen-like kinase 1	MINK1_HUMAN		MINK1
378	378	378	0.999	826	sp|Q8TCJ2|STT3B_HUMAN	STT3B	Q8TCJ2	Dolichyl-diphosphooligosaccharide--protein glycosyltransferase subunit ST	STT3B_HUMAN		STT3B
185	185	185	0.999	1081	sp|Q8TEX9|IPO4_HUMAN	IPO4	Q8TEX9	Importin-4	IPO4_HUMAN		IPO4
767	767	767	0.999	178	sp|Q8WW12|PCNP_HUMAN	PCNP	Q8WW12	PEST proteolytic signal-containing nuclear protein	PCNP_HUMAN		PCNP
357	357	357	0.999	213	sp|Q92522|H1X_HUMAN	H1-10	Q92522	Histone H1.10	H1X_HUMAN		H1FX
516	516	516	0.999	475	sp|Q96KP4|CNDP2_HUMAN	CNDP2	Q96KP4	Cytosolic non-specific dipeptidase	CNDP2_HUMAN		CNDP2
420	420	420	0.999	524	sp|Q9BZZ5|API5_HUMAN	API5	Q9BZZ5	Apoptosis inhibitor 5	API5_HUMAN		API5
130	130	448	0.999	340	sp|Q9HAV0|GBB4_HUMAN	GNB4	Q9HAV0	Guanine nucleotide-binding protein subunit beta-4	GBB4_HUMAN		GNB4
5	5	293	0.9987	535	sp|Q9NZN3|EHD3_HUMAN	EHD3	Q9NZN3	EH domain-containing protein 3	EHD3_HUMAN		EHD3
116	116	169	0.999	871	sp|Q9UBF2|COPG2_HUMAN	COPG2	Q9UBF2	Coatomer subunit gamma-2	COPG2_HUMAN		COPG2
116	116	116	0.999	1479	sp|Q9UBG0|MRC2_HUMAN	MRC2	Q9UBG0	C-type mannose receptor 2	MRC2_HUMAN		MRC2
358	358	358	0.999	332	sp|Q9UHD1|CHRD1_HUMAN	CHORDC1	Q9UHD1	Cysteine and histidine-rich domain-containing protein 1	CHRD1_HUMAN		CHORDC1
133	133	148	0.999	2620	sp|Q9UPU5|UBP24_HUMAN	USP24	Q9UPU5	Ubiquitin carboxyl-terminal hydrolase 24	UBP24_HUMAN		USP24
515	515	632	0.999	283	sp|Q9Y277|VDAC3_HUMAN	VDAC3	Q9Y277	Voltage-dependent anion-selective channel protein 3	VDAC3_HUMAN		VDAC3
343	300	343	0.999	341	sp|Q9Y376|CAB39_HUMAN	CAB39	Q9Y376	Calcium-binding protein 39	CAB39_HUMAN		CAB39
403	403	403	0.999	250	sp|Q9Y3A5|SBDS_HUMAN	SBDS	Q9Y3A5	Ribosome maturation protein SBDS	SBDS_HUMAN		SBDS
790	790	790	0.999	253	sp|Q9Y696|CLIC4_HUMAN	CLIC4	Q9Y696	Chloride intracellular channel protein 4	CLIC4_HUMAN		CLIC4
580	580	580	0.999	637	sp|O14744|ANM5_HUMAN	PRMT5	O14744	Protein arginine N-methyltransferase 5	ANM5_HUMAN		PRMT5
451	451	451	0.999	800	sp|O43290|SNUT1_HUMAN	SART1	O43290	U4/U6.U5 tri-snRNP-associated protein 1	SNUT1_HUMAN		SART1
438	420	438	0.999	957	sp|O75400|PR40A_HUMAN	PRPF40A	O75400	Pre-mRNA-processing factor 40 homolog A	PR40A_HUMAN		PRPF40A
209	209	209	0.999	427	sp|O75955|FLOT1_HUMAN	FLOT1	O75955	Flotillin-1	FLOT1_HUMAN		FLOT1
356	356	356	0.999	669	sp|O94925|GLSK_HUMAN	GLS	O94925	Glutaminase kidney isoform, mitochondrial	GLSK_HUMAN		GLS
510	368	510	0.999	1474	sp|P01023|A2MG_HUMAN	A2M	P01023	Alpha-2-macroglobulin	A2MG_HUMAN		A2M
104	104	104	0.999	788	sp|P05106|ITB3_HUMAN	ITGB3	P05106	Integrin beta-3	ITB3_HUMAN		ITGB3
296	296	296	0.999	532	sp|P05362|ICAM1_HUMAN	ICAM1	P05362	Intercellular adhesion molecule 1	ICAM1_HUMAN		ICAM1
76	76	76	0.999	750	sp|P08473|NEP_HUMAN	MME	P08473	Neprilysin	NEP_HUMAN		MME
474	474	474	0.999	229	sp|P09497|CLCB_HUMAN	CLTB	P09497	Clathrin light chain B	CLCB_HUMAN		CLTB
479	479	479	0.999	480	sp|P11172|UMPS_HUMAN	UMPS	P11172	Uridine 5'-monophosphate synthase	UMPS_HUMAN		UMPS
222	222	285	0.999	627	sp|P13796|PLSL_HUMAN	LCP1	P13796	Plastin-2	PLSL_HUMAN		LCP1
676	658	676	0.999	404	sp|P13861|KAP2_HUMAN	PRKAR2A	P13861	cAMP-dependent protein kinase type II-alpha regulatory subunit	KAP2_HUMAN		PRKAR2A
523	519	523	0.999	280	sp|P21912|SDHB_HUMAN	SDHB	P21912	Succinate dehydrogenase [ubiquinone] iron-sulfur subunit, mitochondrial	SDHB_HUMAN		SDHB
938	938	938	0.999	397	sp|P31689|DNJA1_HUMAN	DNAJA1	P31689	DnaJ homolog subfamily A member 1	DNJA1_HUMAN		DNAJA1
747	677	747	0.999	526	sp|P35637|FUS_HUMAN	FUS	P35637	RNA-binding protein FUS	FUS_HUMAN		FUS
819	740	819	0.999	249	sp|P39687|AN32A_HUMAN	ANP32A	P39687	Acidic leucine-rich nuclear phosphoprotein 32 family member A	AN32A_HUMAN		ANP32A
1004	895	1004	0.999	418	sp|P43686|PRS6B_HUMAN	PSMC4	P43686	26S proteasome regulatory subunit 6B	PRS6B_HUMAN		PSMC4
206	206	206	0.999	2146	sp|P49750|YLPM1_HUMAN	YLPM1	P49750	YLP motif-containing protein 1	YLPM1_HUMAN		YLPM1
242	242	242	0.999	431	sp|P55010|IF5_HUMAN	EIF5	P55010	Eukaryotic translation initiation factor 5	IF5_HUMAN		EIF5
435	435	435	0.999	182	sp|P55145|MANF_HUMAN	MANF	P55145	Mesencephalic astrocyte-derived neurotrophic factor	MANF_HUMAN		MANF
84	4	814	0.999	216	sp|P62491|RB11A_HUMAN	RAB11A	P62491	Ras-related protein Rab-11A	RB11A_HUMAN		RAB11A
733	395	733	0.999	156	sp|P62979|RS27A_HUMAN	RPS27A	P62979	Ubiquitin-40S ribosomal protein S27a	RS27A_HUMAN		RPS27A
729	729	729	0.999	227	sp|P80723|BASP1_HUMAN	BASP1	P80723	Brain acid soluble protein 1	BASP1_HUMAN		BASP1
659	462	659	0.999	443	sp|Q07666|KHDR1_HUMAN	KHDRBS1	Q07666	KH domain-containing, RNA-binding, signal transduction-associated protei	KHDR1_HUMAN		KHDRBS1
461	461	461	0.999	439	sp|Q07960|RHG01_HUMAN	ARHGAP1	Q07960	Rho GTPase-activating protein 1	RHG01_HUMAN		ARHGAP1
112	112	112	0.999	1436	sp|Q12769|NU160_HUMAN	NUP160	Q12769	Nuclear pore complex protein Nup160	NU160_HUMAN		NUP160
520	508	520	0.999	638	sp|Q13409|DC1I2_HUMAN	DYNC1I2	Q13409	Cytoplasmic dynein 1 intermediate chain 2	DC1I2_HUMAN		DYNC1I2
312	312	327	0.999	767	sp|Q15437|SC23B_HUMAN	SEC23B	Q15437	Protein transport protein Sec23B	SC23B_HUMAN		SEC23B
256	254	256	0.999	593	sp|Q15833|STXB2_HUMAN	STXBP2	Q15833	Syntaxin-binding protein 2	STXB2_HUMAN		STXBP2
544	544	544	0.999	335	sp|Q16698|DECR_HUMAN	DECR1	Q16698	2,4-dienoyl-CoA reductase [(3E)-enoyl-CoA-producing], mitochondrial	DECR_HUMAN		DECR1
358	358	358	0.999	1249	sp|Q27J81|INF2_HUMAN	INF2	Q27J81	Inverted formin-2	INF2_HUMAN		INF2
172	172	172	0.999	1907	sp|Q5JRA6|TGO1_HUMAN	MIA3	Q5JRA6	Transport and Golgi organization protein 1 homolog	TGO1_HUMAN		MIA3
609	608	609	0.999	541	sp|Q6DD88|ATLA3_HUMAN	ATL3	Q6DD88	Atlastin-3	ATLA3_HUMAN		ATL3
266	266	266	0.999	613	sp|Q6NYC8|PPR18_HUMAN	PPP1R18	Q6NYC8	Phostensin	PPR18_HUMAN		PPP1R18
251	251	251	0.999	736	sp|Q6PJT7|ZC3HE_HUMAN	ZC3H14	Q6PJT7	Zinc finger CCCH domain-containing protein 14	ZC3HE_HUMAN		ZC3H14
233	233	238	0.999	1299	sp|Q6Y7W6|GGYF2_HUMAN	GIGYF2	Q6Y7W6	GRB10-interacting GYF protein 2	GGYF2_HUMAN		GIGYF2
270	268	270	0.999	1231	sp|Q8N3U4|STAG2_HUMAN	STAG2	Q8N3U4	Cohesin subunit SA-2	STAG2_HUMAN		STAG2
176	176	176	0.999	1859	sp|Q92538|GBF1_HUMAN	GBF1	Q92538	Golgi-specific brefeldin A-resistance guanine nucleotide exchange factor 1	GBF1_HUMAN		GBF1
377	377	377	0.999	612	sp|Q93052|LPP_HUMAN	LPP	Q93052	Lipoma-preferred partner	LPP_HUMAN		LPP
126	126	866	0.999	106	sp|Q969Q0|RL36L_HUMAN	RPL36AL	Q969Q0	Ribosomal protein eL42-like	RL36L_HUMAN		RPL36AL
271	271	271	0.999	665	sp|Q96B97|SH3K1_HUMAN	SH3KBP1	Q96B97	SH3 domain-containing kinase-binding protein 1	SH3K1_HUMAN		SH3KBP1
708	571	708	0.999	406	sp|Q9BS26|ERP44_HUMAN	ERP44	Q9BS26	Endoplasmic reticulum resident protein 44	ERP44_HUMAN		ERP44
458	458	458	0.999	634	sp|Q9BZE4|GTPB4_HUMAN	GTPBP4	Q9BZE4	GTP-binding protein 4	GTPB4_HUMAN		GTPBP4
231	231	231	0.999	928	sp|Q9BZQ8|NIBA1_HUMAN	NIBAN1	Q9BZQ8	Protein Niban 1	NIBA1_HUMAN		FAM129A
354	354	419	0.999	334	sp|Q9H2U2|IPYR2_HUMAN	PPA2	Q9H2U2	Inorganic pyrophosphatase 2, mitochondrial	IPYR2_HUMAN		PPA2
281	281	281	0.999	306	sp|Q9H7B2|RPF2_HUMAN	RPF2	Q9H7B2	Ribosome production factor 2 homolog	RPF2_HUMAN		RPF2
575	575	575	0.999	227	sp|Q9NX63|MIC19_HUMAN	CHCHD3	Q9NX63	MICOS complex subunit MIC19	MIC19_HUMAN		CHCHD3
117	117	117	0.999	2610	sp|Q9ULT8|HECD1_HUMAN	HECTD1	Q9ULT8	E3 ubiquitin-protein ligase HECTD1	HECD1_HUMAN		HECTD1
168	168	168	0.999	774	sp|Q9Y4K0|LOXL2_HUMAN	LOXL2	Q9Y4K0	Lysyl oxidase homolog 2	LOXL2_HUMAN		LOXL2
381	381	381	0.999	447	sp|Q9Y4P3|TBL2_HUMAN	TBL2	Q9Y4P3	Transducin beta-like protein 2	TBL2_HUMAN		TBL2
330	330	381	0.999	599	sp|Q9Y6M1|IF2B2_HUMAN	IGF2BP2	Q9Y6M1	Insulin-like growth factor 2 mRNA-binding protein 2	IF2B2_HUMAN		IGF2BP2
606	457	606	0.999	579	sp|O00425|IF2B3_HUMAN	IGF2BP3	O00425	Insulin-like growth factor 2 mRNA-binding protein 3	IF2B3_HUMAN		IGF2BP3
710	710	710	0.999	546	sp|O15355|PPM1G_HUMAN	PPM1G	O15355	Protein phosphatase 1G	PPM1G_HUMAN		PPM1G
324	323	324	0.999	492	sp|O43237|DC1L2_HUMAN	DYNC1LI2	O43237	Cytoplasmic dynein 1 light intermediate chain 2	DC1L2_HUMAN		DYNC1LI2

352	352	352	0.999	683	sp|O43395|PRPF3_HUMAN	PRPF3	O43395	U4/U6 small nuclear ribonucleoprotein Prp3	PRPF3_HUMAN		PRPF3
281	121	350	0.999	530	sp|O43865|SAHH2_HUMAN	AHCYL1	O43865	S-adenosylhomocysteine hydrolase-like protein 1	SAHH2_HUMAN		AHCYL1
152	150	182	0.999	1006	sp|O94832|MYO1D_HUMAN	MYO1D	O94832	Unconventional myosin-Id	MYO1D_HUMAN		MYO1D
456	456	456	0.999	739	sp|O95202|LETM1_HUMAN	LETM1	O95202	Mitochondrial proton/calcium exchanger protein	LETM1_HUMAN		LETM1
366	289	654	0.999	365	sp|P04439|HLAA_HUMAN	HLA-A	P04439	HLA class I histocompatibility antigen, A alpha chain	HLAA_HUMAN		HLA-A
629	491	629	0.999	297	sp|P06493|CDK1_HUMAN	CDK1	P06493	Cyclin-dependent kinase 1	CDK1_HUMAN		CDK1
535	535	535	0.999	333	sp|P13804|ETFA_HUMAN	ETFA	P13804	Electron transfer flavoprotein subunit alpha, mitochondrial	ETFA_HUMAN		ETFA
417	417	417	0.999	807	sp|P22059|OSBP1_HUMAN	OSBP	P22059	Oxysterol-binding protein 1	OSBP1_HUMAN		OSBP
502	463	502	0.999	456	sp|P30520|PURA2_HUMAN	ADSS2	P30520	Adenylosuccinate synthetase isozyme 2	PURA2_HUMAN		ADSS
462	462	599	0.999	410	sp|P43034|LIS1_HUMAN	PAFAH1B1	P43034	Platelet-activating factor acetylhydrolase IB subunit beta	LIS1_HUMAN		PAFAH1B1
1248	1036	1249	0.999	165	sp|P46783|RS10_HUMAN	RPS10	P46783	Small ribosomal subunit protein eS10	RS10_HUMAN		RPS10
324	324	380	0.999	2157	sp|P48634|PRC2A_HUMAN	PRRC2A	P48634	Protein PRRC2A	PRC2A_HUMAN		PRRC2A
817	817	817	0.999	510	sp|P49257|LMAN1_HUMAN	LMAN1	P49257	Protein ERGIC-53	LMAN1_HUMAN		LMAN1
603	603	603	0.999	366	sp|P50213|IDH3A_HUMAN	IDH3A	P50213	Isocitrate dehydrogenase [NAD] subunit alpha, mitochondrial	IDH3A_HUMAN		IDH3A
427	427	427	0.999	296	sp|P53004|BIEA_HUMAN	BLVRA	P53004	Biliverdin reductase A	BIEA_HUMAN		BLVRA
410	396	410	0.999	1094	sp|P53992|SC24C_HUMAN	SEC24C	P53992	Protein transport protein Sec24C	SC24C_HUMAN		SEC24C
921	921	921	0.999	119	sp|P60866|RS20_HUMAN	RPS20	P60866	Small ribosomal subunit protein uS10	RS20_HUMAN		RPS20
1020	1019	1020	0.999	103	sp|P62805|H4_HUMAN	H4C1	P62805	Histone H4	H4_HUMAN		HIST1H4A
758	758	758	0.999	241	sp|P78417|GSTO1_HUMAN	GSTO1	P78417	Glutathione S-transferase omega-1	GSTO1_HUMAN		GSTO1
900	160	900	0.999	106	sp|P83881|RL36A_HUMAN	RPL36A	P83881	Large ribosomal subunit protein eL42	RL36A_HUMAN		RPL36A
243	243	243	0.999	900	sp|Q00653|NFKB2_HUMAN	NFKB2	Q00653	Nuclear factor NF-kappa-B p100 subunit	NFKB2_HUMAN		NFKB2
681	681	681	0.999	357	sp|Q00796|DHSO_HUMAN	SORD	Q00796	Sorbitol dehydrogenase	DHSO_HUMAN		SORD
794	794	794	0.999	188	sp|Q07020|RL18_HUMAN	RPL18	Q07020	Large ribosomal subunit protein eL18	RL18_HUMAN		RPL18
302	266	302	0.999	557	sp|Q13123|RED_HUMAN	IK	Q13123	Protein Red	RED_HUMAN		IK
302	302	302	0.999	504	sp|Q13217|DNJC3_HUMAN	DNAJC3	Q13217	DnaJ homolog subfamily C member 3	DNJC3_HUMAN		DNAJC3
268	268	268	0.999	745	sp|Q13617|CUL2_HUMAN	CUL2	Q13617	Cullin-2	CUL2_HUMAN		CUL2
354	306	354	0.999	339	sp|Q14320|FA50A_HUMAN	FAM50A	Q14320	Protein FAM50A	FA50A_HUMAN		FAM50A
530	512	902	0.999	365	sp|Q15366|PCBP2_HUMAN	PCBP2	Q15366	Poly(rC)-binding protein 2	PCBP2_HUMAN		PCBP2
739	9	819	0.999	218	sp|Q15907|RB11B_HUMAN	RAB11B	Q15907	Ras-related protein Rab-11B	RB11B_HUMAN		RAB11B
332	332	332	0.999	582	sp|Q5BKZ1|ZN326_HUMAN	ZNF326	Q5BKZ1	DBIRD complex subunit ZNF326	ZN326_HUMAN		ZNF326
442	442	442	0.999	1401	sp|Q6P2E9|EDC4_HUMAN	EDC4	Q6P2E9	Enhancer of mRNA-decapping protein 4	EDC4_HUMAN		EDC4
523	523	523	0.999	645	sp|Q6PI48|SYDM_HUMAN	DARS2	Q6PI48	Aspartate--tRNA ligase, mitochondrial	SYDM_HUMAN		DARS2
754	754	754	0.999	582	sp|Q86UE4|LYRIC_HUMAN	MTDH	Q86UE4	Protein LYRIC	LYRIC_HUMAN		MTDH
604	596	604	0.999	591	sp|Q8N8S7|ENAH_HUMAN	ENAH	Q8N8S7	Protein enabled homolog	ENAH_HUMAN		ENAH
736	465	736	0.999	898	sp|Q92973|TNPO1_HUMAN	TNPO1	Q92973	Transportin-1	TNPO1_HUMAN		TNPO1
535	535	535	0.999	483	sp|Q96A33|CCD47_HUMAN	CCDC47	Q96A33	PAT complex subunit CCDC47	CCD47_HUMAN		CCDC47
291	291	306	0.999	1074	sp|Q96KR1|ZFR_HUMAN	ZFR	Q96KR1	Zinc finger RNA-binding protein	ZFR_HUMAN		ZFR
205	203	205	0.999	708	sp|Q96T51|RUFY1_HUMAN	RUFY1	Q96T51	RUN and FYVE domain-containing protein 1	RUFY1_HUMAN		RUFY1
485	485	657	0.999	375	sp|Q99733|NP1L4_HUMAN	NAP1L4	Q99733	Nucleosome assembly protein 1-like 4	NP1L4_HUMAN		NAP1L4
422	422	422	0.999	397	sp|Q9BWD1|THIC_HUMAN	ACAT2	Q9BWD1	Acetyl-CoA acetyltransferase, cytosolic	THIC_HUMAN		ACAT2
73	73	73	0.999	1416	sp|Q9BZF9|UACA_HUMAN	UACA	Q9BZF9	Uveal autoantigen with coiled-coil domains and ankyrin repeats	UACA_HUMAN		UACA
152	152	383	0.999	622	sp|Q9H0B6|KLC2_HUMAN	KLC2	Q9H0B6	Kinesin light chain 2	KLC2_HUMAN		KLC2
356	285	413	0.999	541	sp|Q9H223|EHD4_HUMAN	EHD4	Q9H223	EH domain-containing protein 4	EHD4_HUMAN		EHD4
731	653	731	0.999	322	sp|Q9H9B4|SFXN1_HUMAN	SFXN1	Q9H9B4	Sideroflexin-1	SFXN1_HUMAN		SFXN1
288	288	288	0.999	1003	sp|Q9HCE1|MOV10_HUMAN	MOV10	Q9HCE1	Helicase MOV-10	MOV10_HUMAN		MOV10
733	733	733	0.999	359	sp|Q9NR45|SIAS_HUMAN	NANS	Q9NR45	Sialic acid synthase	SIAS_HUMAN		NANS
802	802	802	0.999	396	sp|Q9NTK5|OLA1_HUMAN	OLA1	Q9NTK5	Obg-like ATPase 1	OLA1_HUMAN		OLA1
225	225	225	0.999	759	sp|Q9NW13|RBM28_HUMAN	RBM28	Q9NW13	RNA-binding protein 28	RBM28_HUMAN		RBM28
293	293	299	0.999	1034	sp|Q9NWH9|SLTM_HUMAN	SLTM	Q9NWH9	SAFB-like transcription modulator	SLTM_HUMAN		SLTM
565	565	565	0.999	1087	sp|Q9UIA9|XPO7_HUMAN	XPO7	Q9UIA9	Exportin-7	XPO7_HUMAN		XPO7
729	729	729	0.999	430	sp|Q9UJU6|DBNL_HUMAN	DBNL	Q9UJU6	Drebrin-like protein	DBNL_HUMAN		DBNL
405	405	405	0.999	370	sp|Q9UNZ2|NSF1C_HUMAN	NSFL1C	Q9UNZ2	NSFL1 cofactor p47	NSF1C_HUMAN		NSFL1C
709	709	709	0.999	564	sp|Q9Y262|EIF3L_HUMAN	EIF3L	Q9Y262	Eukaryotic translation initiation factor 3 subunit L	EIF3L_HUMAN		EIF3L
361	361	361	0.999	958	sp|Q9Y2L1|RRP44_HUMAN	DIS3	Q9Y2L1	Exosome complex exonuclease RRP44	RRP44_HUMAN		DIS3
145	145	145	0.999	2005	sp|A6NHR9|SMHD1_HUMAN	SMCHD1	A6NHR9	Structural maintenance of chromosomes flexible hinge domain-containing	SMHD1_HUMAN		SMCHD1
456	368	456	0.999	380	sp|O00154|BACH_HUMAN	ACOT7	O00154	Cytosolic acyl coenzyme A thioester hydrolase	BACH_HUMAN		ACOT7
266	215	266	0.999	1094	sp|O00203|AP3B1_HUMAN	AP3B1	O00203	AP-3 complex subunit beta-1	AP3B1_HUMAN		AP3B1
324	324	324	0.999	298	sp|O00560|SDCB1_HUMAN	SDCBP	O00560	Syntenin-1	SDCB1_HUMAN		SDCBP
648	648	648	0.999	125	sp|O14737|PDCD5_HUMAN	PDCD5	O14737	Programmed cell death protein 5	PDCD5_HUMAN		PDCD5
770	770	1032	0.999	420	sp|O14979|HNRDL_HUMAN	HNRNPDL	O14979	Heterogeneous nuclear ribonucleoprotein D-like	HNRDL_HUMAN		HNRNPDL
962	962	962	0.999	548	sp|O15371|EIF3D_HUMAN	EIF3D	O15371	Eukaryotic translation initiation factor 3 subunit D	EIF3D_HUMAN		EIF3D
648	648	648	0.999	315	sp|O43852|CALU_HUMAN	CALU	O43852	Calumenin	CALU_HUMAN		CALU
220	220	220	0.999	2799	sp|O95071|UBR5_HUMAN	UBR5	O95071	E3 ubiquitin-protein ligase UBR5	UBR5_HUMAN		UBR5
724	724	724	0.999	308	sp|O95861|BPNT1_HUMAN	BPNT1	O95861	3'(2'),5'-bisphosphate nucleotidase 1	BPNT1_HUMAN		BPNT1
782	345	782	0.999	317	sp|P05388|RLA0_HUMAN	RPLP0	P05388	Large ribosomal subunit protein uL10	RLA0_HUMAN		RPLP0
736	736	736	0.999	455	sp|P07099|HYEP_HUMAN	EPHX1	P07099	Epoxide hydrolase 1	HYEP_HUMAN		EPHX1
760	760	760	0.999	339	sp|P07858|CATB_HUMAN	CTSB	P07858	Cathepsin B	CATB_HUMAN		CTSB
322	298	322	0.999	638	sp|P08240|SRPRA_HUMAN	SRPRA	P08240	Signal recognition particle receptor subunit alpha	SRPRA_HUMAN		SRPR
852	852	852	0.999	135	sp|P08708|RS17_HUMAN	RPS17	P08708	Small ribosomal subunit protein eS17	RS17_HUMAN		RPS17
1317	333	1317	0.999	295	sp|P08865|RSSA_HUMAN	RPSA	P08865	Small ribosomal subunit protein uS2	RSSA_HUMAN		RPSA
627	627	627	0.999	282	sp|P10768|ESTD_HUMAN	ESD	P10768	S-formylglutathione hydrolase	ESTD_HUMAN		ESD
557	557	557	0.999	421	sp|P11310|ACADM_HUMAN	ACADM	P11310	Medium-chain specific acyl-CoA dehydrogenase, mitochondrial	ACADM_HUMAN		ACADM
560	560	560	0.999	732	sp|P13798|ACPH_HUMAN	APEH	P13798	Acylamino-acid-releasing enzyme	ACPH_HUMAN		APEH
262	262	262	0.999	373	sp|P15104|GLNA_HUMAN	GLUL	P15104	Glutamine synthetase	GLNA_HUMAN		GLUL
180	180	180	0.999	799	sp|P18084|ITB5_HUMAN	ITGB5	P18084	Integrin beta-5	ITB5_HUMAN		ITGB5
591	591	591	0.999	333	sp|P20042|IF2B_HUMAN	EIF2S2	P20042	Eukaryotic translation initiation factor 2 subunit 2	IF2B_HUMAN		EIF2S2
394	337	394	0.999	225	sp|P21266|GSTM3_HUMAN	GSTM3	P21266	Glutathione S-transferase Mu 3	GSTM3_HUMAN		GSTM3
41	24	1326	0.999	263	sp|P22090|RS4Y1_HUMAN	RPS4Y1	P22090	Small ribosomal subunit protein eS4, Y isoform 1	RS4Y1_HUMAN		RPS4Y1
1040	1040	1040	0.999	255	sp|P25788|PSA3_HUMAN	PSMA3	P25788	Proteasome subunit alpha type-3	PSA3_HUMAN		PSMA3
425	425	425	0.999	616	sp|P27694|RFA1_HUMAN	RPA1	P27694	Replication protein A 70 kDa DNA-binding subunit	RFA1_HUMAN		RPA1
994	994	1090	0.999	198	sp|P32119|PRDX2_HUMAN	PRDX2	P32119	Peroxiredoxin-2	PRDX2_HUMAN		PRDX2
1002	1002	1002	0.999	192	sp|P32969|RL9_HUMAN	RPL9	P32969	Large ribosomal subunit protein uL6	RL9_HUMAN		RPL9
695	645	695	0.999	376	sp|P35237|SPB6_HUMAN	SERPINB6	P35237	Serpin B6	SPB6_HUMAN		SERPINB6
257	257	257	0.999	1532	sp|P35573|GDE_HUMAN	AGL	P35573	Glycogen debranching enzyme	GDE_HUMAN		AGL
329	329	329	0.999	2090	sp|P35658|NU214_HUMAN	NUP214	P35658	Nuclear pore complex protein Nup214	NU214_HUMAN		NUP214
754	754	754	0.999	255	sp|P38117|ETFB_HUMAN	ETFB	P38117	Electron transfer flavoprotein subunit beta	ETFB_HUMAN		ETFB
723	723	723	0.999	264	sp|P40261|NNMT_HUMAN	NNMT	P40261	Nicotinamide N-methyltransferase	NNMT_HUMAN		NNMT
522	116	522	0.999	323	sp|P42330|AK1C3_HUMAN	AKR1C3	P42330	Aldo-keto reductase family 1 member C3	AK1C3_HUMAN		AKR1C3
750	750	750	0.999	117	sp|P49207|RL34_HUMAN	RPL34	P49207	Large ribosomal subunit protein eL34	RL34_HUMAN		RPL34
646	646	646	0.999	246	sp|P51572|BAP31_HUMAN	BCAP31	P51572	B-cell receptor-associated protein 31	BAP31_HUMAN		BCAP31
819	819	819	0.999	125	sp|P62851|RS25_HUMAN	RPS25	P62851	Small ribosomal subunit protein eS25	RS25_HUMAN		RPS25
415	11	440	0.999	394	sp|P63092|GNAS2_HUMAN	GNAS	P63092	Guanine nucleotide-binding protein G(s) subunit alpha isoforms short	GNAS2_HUMAN		GNAS
81	81	484	0.999	354	sp|P63096|GNAI1_HUMAN	GNAI1	P63096	Guanine nucleotide-binding protein G(i) subunit alpha-1	GNAI1_HUMAN		GNAI1
289	248	289	0.999	930	sp|P98175|RBM10_HUMAN	RBM10	P98175	RNA-binding protein 10	RBM10_HUMAN		RBM10
1098	930	1098	0.999	135	sp|Q01469|FABP5_HUMAN	FABP5	Q01469	Fatty acid-binding protein 5	FABP5_HUMAN		FABP5
433	433	433	0.999	885	sp|Q01780|EXOSX_HUMAN	EXOSC10	Q01780	Exosome component 10	EXOSX_HUMAN		EXOSC10
408	408	408	0.999	1234	sp|Q01970|PLCB3_HUMAN	PLCB3	Q01970	1-phosphatidylinositol 4,5-bisphosphate phosphodiesterase beta-3	PLCB3_HUMAN		PLCB3
662	242	1177	0.999	215	sp|Q02539|H11_HUMAN	H1-1	Q02539	Histone H1.1	H11_HUMAN		HIST1H1A
776	524	776	0.999	393	sp|Q02750|MP2K1_HUMAN	MAP2K1	Q02750	Dual specificity mitogen-activated protein kinase kinase 1	MP2K1_HUMAN		MAP2K1
545	545	545	0.999	593	sp|Q06124|PTN11_HUMAN	PTPN11	Q06124	Tyrosine-protein phosphatase non-receptor type 11	PTN11_HUMAN		PTPN11
513	513	513	0.999	491	sp|Q13098|CSN1_HUMAN	GPS1	Q13098	COP9 signalosome complex subunit 1	CSN1_HUMAN		GPS1
156	151	156	0.999	1202	sp|Q13136|LIPA1_HUMAN	PPFIA1	Q13136	Liprin-alpha-1	LIPA1_HUMAN		PPFIA1
171	171	171	0.999	1070	sp|Q13308|PTK7_HUMAN	PTK7	Q13308	Inactive tyrosine-protein kinase 7	PTK7_HUMAN		PTK7
457	419	457	0.999	741	sp|Q15003|CND2_HUMAN	NCAPH	Q15003	Condensin complex subunit 2	CND2_HUMAN		NCAPH
76	76	76	0.999	1823	sp|Q16363|LAMA4_HUMAN	LAMA4	Q16363	Laminin subunit alpha-4	LAMA4_HUMAN		LAMA4
394	394	394	0.999	649	sp|Q16643|DREB_HUMAN	DBN1	Q16643	Drebrin	DREB_HUMAN		DBN1
500	500	500	0.999	938	sp|Q86XP3|DDX42_HUMAN	DDX42	Q86XP3	ATP-dependent RNA helicase DDX42	DDX42_HUMAN		DDX42
466	466	466	0.999	608	sp|Q8TAT6|NPL4_HUMAN	NPLOC4	Q8TAT6	Nuclear protein localization protein 4 homolog	NPL4_HUMAN		NPLOC4
578	578	583	0.999	370	sp|Q92747|ARC1A_HUMAN	ARPC1A	Q92747	Actin-related protein 2/3 complex subunit 1A	ARC1A_HUMAN		ARPC1A
450	450	450	0.999	432	sp|Q96I99|SUCB2_HUMAN	SUCLG2	Q96I99	Succinate--CoA ligase [GDP-forming] subunit beta, mitochondrial	SUCB2_HUMAN		SUCLG2
365	365	388	0.999	1447	sp|Q9NTI5|PDS5B_HUMAN	PDS5B	Q9NTI5	Sister chromatid cohesion protein PDS5 homolog B	PDS5B_HUMAN		PDS5B
369	369	369	0.999	677	sp|Q9NZT2|OGFR_HUMAN	OGFR	Q9NZT2	Opioid growth factor receptor	OGFR_HUMAN		OGFR
527	527	527	0.999	439	sp|Q9Y305|ACOT9_HUMAN	ACOT9	Q9Y305	Acyl-coenzyme A thioesterase 9, mitochondrial	ACOT9_HUMAN		ACOT9
530	530	530	0.999	588	sp|O00541|PESC_HUMAN	PES 1.00	O00541	Pescadillo homolog	PESC_HUMAN		PES 1.00
475	475	475	0.999	594	sp|O00567|NOP56_HUMAN	NOP56	O00567	Nucleolar protein 56	NOP56_HUMAN		NOP56
394	394	411	0.999	530	sp|O75475|PSIP1_HUMAN	PSIP1	O75475	PC4 and SFRS1-interacting protein	PSIP1_HUMAN		PSIP1
558	558	558	0.999	412	sp|P07339|CATD_HUMAN	CTSD	P07339	Cathepsin D	CATD_HUMAN		CTSD
489	449	489	0.999	556	sp|P07686|HEXB_HUMAN	HEXB	P07686	Beta-hexosaminidase subunit beta	HEXB_HUMAN		HEXB
2748	2748	2748	0.999	140	sp|P07737|PROF1_HUMAN	PFN1	P07737	Profilin-1	PROF1_HUMAN		PFN1
118	118	118	0.999	1366	sp|P08123|CO1A2_HUMAN	COL1A2	P08123	Collagen alpha-2(I) chain	CO1A2_HUMAN		COL1A2
567	567	567	0.999	1049	sp|P08648|ITA5_HUMAN	ITGA5	P08648	Integrin alpha-5	ITA5_HUMAN		ITGA5
704	704	704	0.999	374	sp|P11766|ADHX_HUMAN	ADH5	P11766	Alcohol dehydrogenase class-3	ADHX_HUMAN		ADH5
204	166	490	0.999	318	sp|P11908|PRPS2_HUMAN	PRPS2	P11908	Ribose-phosphate pyrophosphokinase 2	PRPS2_HUMAN		PRPS2
845	845	845	0.999	110	sp|P18077|RL35A_HUMAN	RPL35A	P18077	Large ribosomal subunit protein eL33	RL35A_HUMAN		RPL35A
900	900	900	0.999	453	sp|P22695|QCR2_HUMAN	UQCRC2	P22695	Cytochrome b-c1 complex subunit 2, mitochondrial	QCR2_HUMAN		UQCRC2
780	780	780	0.999	318	sp|P27695|APEX1_HUMAN	APEX1	P27695	DNA-(apurinic or apyrimidinic site) endonuclease	APEX1_HUMAN		APEX1
627	471	627	0.999	360	sp|P28482|MK01_HUMAN	MAPK1	P28482	Mitogen-activated protein kinase 1	MK01_HUMAN		MAPK1
603	603	603	0.999	484	sp|P30566|PUR8_HUMAN	ADSL	P30566	Adenylosuccinate lyase	PUR8_HUMAN		ADSL
452	349	452	0.999	517	sp|P30837|AL1B1_HUMAN	ALDH1B1	P30837	Aldehyde dehydrogenase X, mitochondrial	AL1B1_HUMAN		ALDH1B1

43	43	133	0.999	1172	sp|P35442|TSP2_HUMAN	THBS2	P35442	Thrombospondin-2	TSP2_HUMAN		THBS2
444	444	444	0.999	1042	sp|P42285|MTREX_HUMAN	MTREX	P42285	Exosome RNA helicase MTR4	MTREX_HUMAN		SKIV2L2
687	687	687	0.999	655	sp|P49748|ACADV_HUMAN	ACADVL	P49748	Very long-chain specific acyl-CoA dehydrogenase, mitochondrial	ACADV_HUMAN		ACADVL
257	256	362	0.999	740	sp|P51812|KS6A3_HUMAN	RPS6KA3	P51812	Ribosomal protein S6 kinase alpha-3	KS6A3_HUMAN		RPS6KA3
714	714	714	0.999	239	sp|P54819|KAD2_HUMAN	AK2	P54819	Adenylate kinase 2, mitochondrial	KAD2_HUMAN		AK2
795	795	1200	0.999	165	sp|P60981|DEST_HUMAN	DSTN	P60981	Destrin	DEST_HUMAN		DSTN
995	995	995	0.999	136	sp|P61353|RL27_HUMAN	RPL27	P61353	Large ribosomal subunit protein eL27	RL27_HUMAN		RPL27
744	744	744	0.999	125	sp|P62899|RL31_HUMAN	RPL31	P62899	Large ribosomal subunit protein eL31	RL31_HUMAN		RPL31
1045	1045	1045	0.999	135	sp|P62910|RL32_HUMAN	RPL32	P62910	Large ribosomal subunit protein eL32	RL32_HUMAN		RPL32
751	671	751	0.999	288	sp|P62995|TRA2B_HUMAN	TRA2B	P62995	Transformer-2 protein homolog beta	TRA2B_HUMAN		TRA2B
1201	1201	1201	0.999	362	sp|Q00325|S25A3_HUMAN	SLC25A3	Q00325	Solute carrier family 25 member 3	S25A3_HUMAN		SLC25A3
420	402	420	0.999	201	sp|Q01995|TAGL_HUMAN	TAGLN	Q01995	Transgelin	TAGL_HUMAN		TAGLN
503	503	503	0.999	389	sp|Q15008|PSMD6_HUMAN	PSMD6	Q15008	26S proteasome non-ATPase regulatory subunit 6	PSMD6_HUMAN		PSMD6
790	790	790	0.999	361	sp|Q15019|SEPT2_HUMAN	SEPTIN2	Q15019	Septin-2	SEPT2_HUMAN		SEPT2
416	416	416	0.999	981	sp|Q15042|RB3GP_HUMAN	RAB3GAP1	Q15042	Rab3 GTPase-activating protein catalytic subunit	RB3GP_HUMAN		RAB3GAP1
1027	655	1027	0.999	356	sp|Q15365|PCBP1_HUMAN	PCBP1	Q15365	Poly(rC)-binding protein 1	PCBP1_HUMAN		PCBP1
671	588	671	0.999	326	sp|Q15717|ELAV1_HUMAN	ELAVL1	Q15717	ELAV-like protein 1	ELAV1_HUMAN		ELAVL1
189	189	192	0.999	841	sp|Q3KQU3|MA7D1_HUMAN	MAP7D1	Q3KQU3	MAP7 domain-containing protein 1	MA7D1_HUMAN		MAP7D1
519	519	519	0.999	477	sp|Q6NUK1|SCMC1_HUMAN	SLC25A24	Q6NUK1	Mitochondrial adenyl nucleotide antiporter SLC25A24	SCMC1_HUMAN		SLC25A24
408	408	506	0.999	978	sp|Q6UB35|C1TM_HUMAN	MTHFD1L	Q6UB35	Monofunctional C1-tetrahydrofolate synthase, mitochondrial	C1TM_HUMAN		MTHFD1L
223	223	223	0.999	1025	sp|Q6WCQ1|MPRIP_HUMAN	MPRIP	Q6WCQ1	Myosin phosphatase Rho-interacting protein	MPRIP_HUMAN		MPRIP
295	286	295	0.999	1116	sp|Q8IUD2|RB6I2_HUMAN	ERC1	Q8IUD2	ELKS/Rab6-interacting/CAST family member 1	RB6I2_HUMAN		ERC1
313	313	313	0.999	783	sp|Q8TCS8|PNPT1_HUMAN	PNPT1	Q8TCS8	Polyribonucleotide nucleotidyltransferase 1, mitochondrial	PNPT1_HUMAN		PNPT1
264	264	264	0.999	1156	sp|Q8WUM0|NU133_HUMAN	NUP133	Q8WUM0	Nuclear pore complex protein Nup133	NU133_HUMAN		NUP133
729	693	729	0.999	309	sp|Q99439|CNN2_HUMAN	CNN2	Q99439	Calponin-2	CNN2_HUMAN		CNN2
875	875	995	0.999	332	sp|Q99729|ROAA_HUMAN	HNRNPAB	Q99729	Heterogeneous nuclear ribonucleoprotein A/B	ROAA_HUMAN		HNRNPAB
551	546	551	0.999	1393	sp|Q9H2M9|RBGPR_HUMAN	RAB3GAP2	Q9H2M9	Rab3 GTPase-activating protein non-catalytic subunit	RBGPR_HUMAN		RAB3GAP2
340	340	340	0.999	1204	sp|Q9HAV4|XPO5_HUMAN	XPO5	Q9HAV4	Exportin-5	XPO5_HUMAN		XPO5
921	921	921	0.999	228	sp|Q9HB71|CYBP_HUMAN	CACYBP	Q9HB71	Calcyclin-binding protein	CYBP_HUMAN		CACYBP
343	343	343	0.999	463	sp|Q9P2R7|SUCB1_HUMAN	SUCLA2	Q9P2R7	Succinate--CoA ligase [ADP-forming] subunit beta, mitochondrial	SUCB1_HUMAN		SUCLA2
448	448	448	0.999	658	sp|O00116|ADAS_HUMAN	AGPS	O00116	Alkyldihydroxyacetonephosphate synthase, peroxisomal	ADAS_HUMAN		AGPS
748	748	748	0.999	329	sp|O00151|PDLI1_HUMAN	PDLIM1	O00151	PDZ and LIM domain protein 1	PDLI1_HUMAN		PDLIM1
346	346	346	0.999	1087	sp|O00267|SPT5H_HUMAN	SUPT5H	O00267	Transcription elongation factor SPT5	SPT5H_HUMAN		SUPT5H
940	940	940	0.999	300	sp|O15144|ARPC2_HUMAN	ARPC2	O15144	Actin-related protein 2/3 complex subunit 2	ARPC2_HUMAN		ARPC2
300	300	300	0.999	671	sp|O76094|SRP72_HUMAN	SRP72	O76094	Signal recognition particle subunit SRP72	SRP72_HUMAN		SRP72
205	205	562	0.999	354	sp|P08754|GNAI3_HUMAN	GNAI3	P08754	Guanine nucleotide-binding protein G(i) subunit alpha-3	GNAI3_HUMAN		GNAI3
809	807	809	0.999	193	sp|P21291|CSRP1_HUMAN	CSRP1	P21291	Cysteine and glycine-rich protein 1	CSRP1_HUMAN		CSRP1
715	715	715	0.999	1051	sp|P26006|ITA3_HUMAN	ITGA3	P26006	Integrin alpha-3	ITA3_HUMAN		ITGA3
228	210	228	0.999	1438	sp|P30622|CLIP1_HUMAN	CLIP1	P30622	CAP-Gly domain-containing linker protein 1	CLIP1_HUMAN		CLIP1
611	0	1339	0.999	126	sp|P33778|H2B1B_HUMAN	H2BC3	P33778	Histone H2B type 1-B	H2B1B_HUMAN		HIST1H2BB
557	557	557	0.999	298	sp|P36542|ATPG_HUMAN	ATP5F1C	P36542	ATP synthase subunit gamma, mitochondrial	ATPG_HUMAN		ATP5C1
1243	1167	1243	0.999	199	sp|P37802|TAGL2_HUMAN	TAGLN2	P37802	Transgelin-2	TAGL2_HUMAN		TAGLN2
757	757	757	0.999	617	sp|P38606|VATA_HUMAN	ATP6V1A	P38606	V-type proton ATPase catalytic subunit A	VATA_HUMAN		ATP6V1A
653	653	653	0.999	770	sp|P40763|STAT3_HUMAN	STAT3	P40763	Signal transducer and activator of transcription 3	STAT3_HUMAN		STAT3
689	689	689	0.999	397	sp|P42765|THIM_HUMAN	ACAA2	P42765	3-ketoacyl-CoA thiolase, mitochondrial	THIM_HUMAN		ACAA2
696	696	696	0.999	587	sp|P46060|RAGP1_HUMAN	RANGAP1	P46060	Ran GTPase-activating protein 1	RAGP1_HUMAN		RANGAP1
764	764	764	0.999	591	sp|P49023|PAXI_HUMAN	PXN	P49023	Paxillin	PAXI_HUMAN		PXN
587	401	587	0.999	621	sp|P51114|FXR1_HUMAN	FXR1	P51114	RNA-binding protein FXR1	FXR1_HUMAN		FXR1
717	717	850	0.999	415	sp|P52597|HNRPF_HUMAN	HNRNPF	P52597	Heterogeneous nuclear ribonucleoprotein F	HNRPF_HUMAN		HNRNPF
994	717	994	0.999	286	sp|P52907|CAZA1_HUMAN	CAPZA1	P52907	F-actin-capping protein subunit alpha-1	CAZA1_HUMAN		CAPZA1
368	368	368	0.999	1817	sp|P52948|NUP98_HUMAN	NUP98	P52948	Nuclear pore complex protein Nup98-Nup96	NUP98_HUMAN		NUP98
617	617	617	0.999	494	sp|P54578|UBP14_HUMAN	USP14	P54578	Ubiquitin carboxyl-terminal hydrolase 14	UBP14_HUMAN		USP14
1324	2	1324	0.999	126	sp|P57053|H2BFS_HUMAN	H2BC12L	P57053	Histone H2B type F-S	H2BFS_HUMAN		H2BFS
519	163	519	0.999	318	sp|P60891|PRPS1_HUMAN	PRPS1	P60891	Ribose-phosphate pyrophosphokinase 1	PRPS1_HUMAN		PRPS1
365	365	995	0.999	205	sp|P62820|RAB1A_HUMAN	RAB1A	P62820	Ras-related protein Rab-1A	RAB1A_HUMAN		RAB1A
408	297	692	0.999	340	sp|P62879|GBB2_HUMAN	GNB2	P62879	Guanine nucleotide-binding protein G(I)/G(S)/G(T) subunit beta-2	GBB2_HUMAN		GNB2
583	0	583	0.999	1222	sp|P69849|NOMO3_HUMAN	NOMO3	P69849	BOS complex subunit NOMO3	NOMO3_HUMAN	sp|Q5JPE7|NOMO2_HUMAN	NOMO3
1127	542	1127	0.999	290	sp|Q01105|SET_HUMAN	SET	Q01105	Protein SET	SET_HUMAN		SET
199	181	524	0.999	864	sp|Q05193|DYN1_HUMAN	DNM1	Q05193	Dynamin-1	DYN1_HUMAN		DNM1
276	257	276	0.999	1052	sp|Q05397|FAK1_HUMAN	PTK2	Q05397	Focal adhesion kinase 1	FAK1_HUMAN		PTK2
205	205	205	0.999	387	sp|Q08431|MFGM_HUMAN	MFGE8	Q08431	Lactadherin	MFGM_HUMAN		MFGE8
714	714	714	0.999	328	sp|Q13011|ECH1_HUMAN	ECH1	Q13011	Delta(3,5)-Delta(2,4)-dienoyl-CoA isomerase, mitochondrial	ECH1_HUMAN		ECH1
597	597	764	0.999	305	sp|Q13151|ROA0_HUMAN	HNRNPA0	Q13151	Heterogeneous nuclear ribonucleoprotein A0	ROA0_HUMAN		HNRNPA0
690	422	690	0.999	524	sp|Q13177|PAK2_HUMAN	PAK2	Q13177	Serine/threonine-protein kinase PAK 2	PAK2_HUMAN		PAK2
873	873	873	0.999	325	sp|Q13347|EIF3I_HUMAN	EIF3I	Q13347	Eukaryotic translation initiation factor 3 subunit I	EIF3I_HUMAN		EIF3I
1191	1126	1191	0.999	289	sp|Q15181|IPYR_HUMAN	PPA1	Q15181	Inorganic pyrophosphatase	IPYR_HUMAN		PPA1
431	286	431	0.999	735	sp|Q15418|KS6A1_HUMAN	RPS6KA1	Q15418	Ribosomal protein S6 kinase alpha-1	KS6A1_HUMAN		RPS6KA1
774	774	774	0.999	572	sp|Q15942|ZYX_HUMAN	ZYX	Q15942	Zyxin	ZYX_HUMAN		ZYX
683	683	683	0.999	314	sp|Q16836|HCDH_HUMAN	HADH	Q16836	Hydroxyacyl-coenzyme A dehydrogenase, mitochondrial	HCDH_HUMAN		HADH
393	393	393	0.999	808	sp|Q32MZ4|LRRF1_HUMAN	LRRFIP1	Q32MZ4	Leucine-rich repeat flightless-interacting protein 1	LRRF1_HUMAN		LRRFIP1
236	236	236	0.999	1076	sp|Q86V48|LUZP1_HUMAN	LUZP1	Q86V48	Leucine zipper protein 1	LUZP1_HUMAN		LUZP1
585	582	585	0.999	1150	sp|Q8IX12|CCAR1_HUMAN	CCAR1	Q8IX12	Cell division cycle and apoptosis regulator protein 1	CCAR1_HUMAN		CCAR1
1262	1262	1262	0.999	394	sp|Q92597|NDRG1_HUMAN	NDRG1	Q92597	Protein NDRG1	NDRG1_HUMAN		NDRG1
209	176	209	0.999	2140	sp|Q96N67|DOCK7_HUMAN	DOCK7	Q96N67	Dedicator of cytokinesis protein 7	DOCK7_HUMAN		DOCK7
599	599	599	0.999	1041	sp|Q96P70|IPO9_HUMAN	IPO9	Q96P70	Importin-9	IPO9_HUMAN		IPO9
1567	1567	1567	0.999	189	sp|Q99497|PARK7_HUMAN	PARK7	Q99497	Parkinson disease protein 7	PARK7_HUMAN		PARK7
49	49	49	0.999	751	sp|Q99985|SEM3C_HUMAN	SEMA3C	Q99985	Semaphorin-3C	SEM3C_HUMAN		SEMA3C
620	529	626	0.999	489	sp|Q9BR76|COR1B_HUMAN	CORO1B	Q9BR76	Coronin-1B	COR1B_HUMAN		CORO1B
629	629	629	0.999	585	sp|Q9BY44|EIF2A_HUMAN	EIF2A	Q9BY44	Eukaryotic translation initiation factor 2A	EIF2A_HUMAN		EIF2A
686	634	686	0.999	623	sp|Q9NQW7|XPP1_HUMAN	XPNPEP1	Q9NQW7	Xaa-Pro aminopeptidase 1	XPP1_HUMAN		XPNPEP1
554	398	554	0.999	429	sp|Q9NVA2|SEP11_HUMAN	SEPTIN11	Q9NVA2	Septin-11	SEP11_HUMAN		SEPT11
727	727	727	0.999	737	sp|Q9NY33|DPP3_HUMAN	DPP3	Q9NY33	Dipeptidyl peptidase 3	DPP3_HUMAN		DPP3
800	800	800	0.999	346	sp|Q9UBE0|SAE1_HUMAN	SAE1	Q9UBE0	SUMO-activating enzyme subunit 1	SAE1_HUMAN		SAE1
500	500	500	0.999	486	sp|Q9UNF0|PACN2_HUMAN	PACSIN2	Q9UNF0	Protein kinase C and casein kinase substrate in neurons protein 2	PACN2_HUMAN		PACSIN2
588	554	588	0.999	406	sp|Q9UNH7|SNX6_HUMAN	SNX6	Q9UNH7	Sorting nexin-6	SNX6_HUMAN		SNX6
828	828	828	0.999	376	sp|Q9UNM6|PSD13_HUMAN	PSMD13	Q9UNM6	26S proteasome non-ATPase regulatory subunit 13	PSD13_HUMAN		PSMD13
439	439	439	0.999	2376	sp|A5YKK6|CNOT1_HUMAN	CNOT1	A5YKK6	CCR4-NOT transcription complex subunit 1	CNOT1_HUMAN		CNOT1
1057	1057	1057	0.999	241	sp|O00299|CLIC1_HUMAN	CLIC1	O00299	Chloride intracellular channel protein 1	CLIC1_HUMAN		CLIC1
786	517	786	0.999	248	sp|O14818|PSA7_HUMAN	PSMA7	O14818	Proteasome subunit alpha type-7	PSA7_HUMAN		PSMA7
274	243	274	0.999	1258	sp|O15357|SHIP2_HUMAN	INPPL1	O15357	Phosphatidylinositol 3,4,5-trisphosphate 5-phosphatase 2	SHIP2_HUMAN		INPPL1
580	551	580	0.999	537	sp|O75131|CPNE3_HUMAN	CPNE3	O75131	Copine-3	CPNE3_HUMAN		CPNE3
149	149	149	0.999	2785	sp|O75691|UTP20_HUMAN	UTP20	O75691	Small subunit processome component 20 homolog	UTP20_HUMAN		UTP20
331	331	385	0.999	968	sp|O94804|STK10_HUMAN	STK10	O94804	Serine/threonine-protein kinase 10	STK10_HUMAN		STK10
556	556	556	0.999	608	sp|O94826|TOM70_HUMAN	TOMM70	O94826	Mitochondrial import receptor subunit TOM70	TOM70_HUMAN		TOMM70A
285	285	310	0.999	614	sp|O95340|PAPS2_HUMAN	PAPSS2	O95340	Bifunctional 3'-phosphoadenosine 5'-phosphosulfate synthase 2	PAPS2_HUMAN		PAPSS2
278	278	278	0.999	1663	sp|P01024|CO3_HUMAN	C3	P01024	Complement C3	CO3_HUMAN		C3
1122	1002	1122	0.999	316	sp|P15121|ALDR_HUMAN	AKR1B1	P15121	Aldo-keto reductase family 1 member B1	ALDR_HUMAN		AKR1B1
1020	752	1020	0.999	277	sp|P16152|CBR1_HUMAN	CBR1	P16152	Carbonyl reductase [NADPH] 1	CBR1_HUMAN		CBR1
340	340	340	0.999	677	sp|P16435|NCPR_HUMAN	POR	P16435	NADPH--cytochrome P450 reductase	NCPR_HUMAN		POR
741	704	741	0.999	379	sp|P30740|ILEU_HUMAN	SERPINB1	P30740	Leukocyte elastase inhibitor	ILEU_HUMAN		SERPINB1
957	957	957	0.999	272	sp|P35232|PHB1_HUMAN	PHB1	P35232	Prohibitin 1	PHB1_HUMAN		PHB
758	758	758	0.999	375	sp|P35659|DEK_HUMAN	DEK	P35659	Protein DEK	DEK_HUMAN		DEK
689	626	689	0.999	546	sp|P40222|TXLNA_HUMAN	TXLNA	P40222	Alpha-taxilin	TXLNA_HUMAN		TXLNA
316	192	316	0.999	325	sp|P48059|LIMS1_HUMAN	LIMS1	P48059	LIM and senescent cell antigen-like-containing domain protein 1	LIMS1_HUMAN		LIMS1
808	808	808	0.999	494	sp|P49189|AL9A1_HUMAN	ALDH9A1	P49189	4-trimethylaminobutyraldehyde dehydrogenase	AL9A1_HUMAN		ALDH9A1
687	687	687	0.999	445	sp|P60228|EIF3E_HUMAN	EIF3E	P60228	Eukaryotic translation initiation factor 3 subunit E	EIF3E_HUMAN		EIF3E
1098	1098	1098	0.999	151	sp|P62277|RS13_HUMAN	RPS13	P62277	Small ribosomal subunit protein uS15	RS13_HUMAN		RPS13
722	438	722	0.999	340	sp|P62873|GBB1_HUMAN	GNB1	P62873	Guanine nucleotide-binding protein G(I)/G(S)/G(T) subunit beta-1	GBB1_HUMAN		GNB1
440	420	440	0.999	461	sp|Q02818|NUCB1_HUMAN	NUCB1	Q02818	Nucleobindin-1	NUCB1_HUMAN		NUCB1
639	639	639	0.999	401	sp|Q13561|DCTN2_HUMAN	DCTN2	Q13561	Dynactin subunit 2	DCTN2_HUMAN		DCTN2
388	387	388	0.999	1401	sp|Q15021|CND1_HUMAN	NCAPD2	Q15021	Condensin complex subunit 1	CND1_HUMAN		NCAPD2
433	433	433	0.999	1024	sp|Q99575|POP1_HUMAN	POP1	Q99575	Ribonucleases P/MRP protein subunit POP1	POP1_HUMAN		POP1
424	424	424	0.999	494	sp|Q99615|DNJC7_HUMAN	DNAJC7	Q99615	DnaJ homolog subfamily C member 7	DNJC7_HUMAN		DNAJC7
1054	230	1054	0.999	201	sp|Q9H0U4|RAB1B_HUMAN	RAB1B	Q9H0U4	Ras-related protein Rab-1B	RAB1B_HUMAN		RAB1B
533	533	533	0.999	670	sp|Q9NVP1|DDX18_HUMAN	DDX18	Q9NVP1	ATP-dependent RNA helicase DDX18	DDX18_HUMAN		DDX18
439	439	439	0.999	623	sp|Q9UG63|ABCF2_HUMAN	ABCF2	Q9UG63	ATP-binding cassette sub-family F member 2	ABCF2_HUMAN		ABCF2
655	557	655	0.999	306	sp|Q9UKM9|RALY_HUMAN	RALY	Q9UKM9	RNA-binding protein Raly	RALY_HUMAN		RALY
859	859	859	0.999	370	sp|Q9Y617|SERC_HUMAN	PSAT1	Q9Y617	Phosphoserine aminotransferase	SERC_HUMAN		PSAT1
363	363	983	0.999	427	sp|O00148|DX39A_HUMAN	DDX39A	O00148	ATP-dependent RNA helicase DDX39A	DX39A_HUMAN		DDX39A
164	147	621	0.999	1585	sp|O43432|IF4G3_HUMAN	EIF4G3	O43432	Eukaryotic translation initiation factor 4 gamma 3	IF4G3_HUMAN		EIF4G3
343	343	397	0.999	1041	sp|O60231|DHX16_HUMAN	DHX16	O60231	Pre-mRNA-splicing factor ATP-dependent RNA helicase DHX16	DHX16_HUMAN		DHX16
714	714	714	0.999	194	sp|P00568|KAD1_HUMAN	AK1	P00568	Adenylate kinase isoenzyme 1	KAD1_HUMAN		AK1
688	688	688	0.999	439	sp|P04181|OAT_HUMAN	OAT	P04181	Ornithine aminotransferase, mitochondrial	OAT_HUMAN		OAT
375	375	375	0.999	1019	sp|P14735|IDE_HUMAN	IDE	P14735	Insulin-degrading enzyme	IDE_HUMAN		IDE
1236	716	1236	0.999	226	sp|P16401|H15_HUMAN	H1-5	P16401	Histone H1.5	H15_HUMAN		HIST1H1B
595	595	595	0.999	413	sp|P17174|AATC_HUMAN	GOT1	P17174	Aspartate aminotransferase, cytoplasmic	AATC_HUMAN		GOT1
812	812	812	0.999	708	sp|P20810|ICAL_HUMAN	CAST	P20810	Calpastatin	ICAL_HUMAN		CAST
1935	1208	1935	0.999	166	sp|P23528|COF1_HUMAN	CFL1	P23528	Cofilin-1	COF1_HUMAN		CFL1
57	57	341	0.999	1054	sp|P28370|SMCA1_HUMAN	SMARCA1	P28370	Probable global transcription activator SNF2L1	SMCA1_HUMAN		SMARCA1

294	294	716	0.999	447	sp|P31150|GDIA_HUMAN	GDI1	P31150	Rab GDP dissociation inhibitor alpha	GDIA_HUMAN		GDI1
387	387	639	0.999	400	sp|P36507|MP2K2_HUMAN	MAP2K2	P36507	Dual specificity mitogen-activated protein kinase kinase 2	MP2K2_HUMAN		MAP2K2
690	690	690	0.999	812	sp|P46087|NOP2_HUMAN	NOP2	P46087	Probable 28S rRNA (cytosine(4447)-C(5))-methyltransferase	NOP2_HUMAN		NOP2
1462	1462	1462	0.999	137	sp|P46779|RL28_HUMAN	RPL28	P46779	Large ribosomal subunit protein eL28	RL28_HUMAN		RPL28
174	174	174	0.999	1824	sp|P55196|AFAD_HUMAN	AFDN	P55196	Afadin	AFAD_HUMAN		MLLT4
466	425	466	0.999	520	sp|P55809|SCOT1_HUMAN	OXCT1	P55809	Succinyl-CoA:3-ketoacid coenzyme A transferase 1, mitochondrial	SCOT1_HUMAN		OXCT1
292	292	292	0.999	1179	sp|P56199|ITA1_HUMAN	ITGA1	P56199	Integrin alpha-1	ITA1_HUMAN		ITGA1
1526	1526	1526	0.999	208	sp|P62241|RS8_HUMAN	RPS8	P62241	Small ribosomal subunit protein eS8	RS8_HUMAN		RPS8
262	262	262	0.999	1002	sp|Q08379|GOGA2_HUMAN	GOLGA2	Q08379	Golgin subfamily A member 2	GOGA2_HUMAN		GOLGA2
455	455	998	0.999	425	sp|Q09028|RBBP4_HUMAN	RBBP4	Q09028	Histone-binding protein RBBP4	RBBP4_HUMAN		RBBP4
743	743	743	0.999	501	sp|Q12874|SF3A3_HUMAN	SF3A3	Q12874	Splicing factor 3A subunit 3	SF3A3_HUMAN		SF3A3
948	948	948	0.999	709	sp|Q14444|CAPR1_HUMAN	CAPRIN1	Q14444	Caprin-1	CAPR1_HUMAN		CAPRIN1
524	524	524	0.999	1478	sp|Q14C86|GAPD1_HUMAN	GAPVD1	Q14C86	GTPase-activating protein and VPS9 domain-containing protein 1	GAPD1_HUMAN		GAPVD1
384	384	384	0.999	640	sp|Q16822|PCKGM_HUMAN	PCK2	Q16822	Phosphoenolpyruvate carboxykinase [GTP], mitochondrial	PCKGM_HUMAN		PCK2
884	827	884	0.999	419	sp|Q7L1Q6|5MP2_HUMAN	BZW1	Q7L1Q6	eIF5-mimic protein 2	5MP2_HUMAN		BZW1
561	561	561	0.999	1102	sp|Q93009|UBP7_HUMAN	USP7	Q93009	Ubiquitin carboxyl-terminal hydrolase 7	UBP7_HUMAN		USP7
365	359	365	0.999	680	sp|Q96AC1|FERM2_HUMAN	FERMT2	Q96AC1	Fermitin family homolog 2	FERM2_HUMAN		FERMT2
661	661	661	0.999	669	sp|Q96PK6|RBM14_HUMAN	RBM14	Q96PK6	RNA-binding protein 14	RBM14_HUMAN		RBM14
787	787	787	0.999	457	sp|Q9NR12|PDLI7_HUMAN	PDLIM7	Q9NR12	PDZ and LIM domain protein 7	PDLI7_HUMAN		PDLIM7
755	755	755	0.999	589	sp|Q9NSD9|SYFB_HUMAN	FARSB	Q9NSD9	Phenylalanine--tRNA ligase beta subunit	SYFB_HUMAN		FARSB
583	576	583	0.999	1118	sp|Q9NZB2|F120A_HUMAN	FAM120A	Q9NZB2	Constitutive coactivator of PPAR-gamma-like protein 1	F120A_HUMAN		FAM120A
414	412	414	0.999	585	sp|Q9UH65|SWP70_HUMAN	SWAP70	Q9UH65	Switch-associated protein 70	SWP70_HUMAN		SWAP70
800	800	800	0.999	559	sp|Q9UHX1|PUF60_HUMAN	PUF60	Q9UHX1	Poly(U)-binding-splicing factor PUF60	PUF60_HUMAN		PUF60
678	678	678	0.999	505	sp|Q9Y3I0|RTCB_HUMAN	RTCB	Q9Y3I0	RNA-splicing ligase RtcB homolog	RTCB_HUMAN		RTCB
839	839	839	0.999	456	sp|O00232|PSD12_HUMAN	PSMD12	O00232	26S proteasome non-ATPase regulatory subunit 12	PSD12_HUMAN		PSMD12
143	143	143	0.999	381	sp|O00622|CCN1_HUMAN	CCN1	O00622	CCN family member 1	CCN1_HUMAN		CYR61
455	454	455	0.999	1030	sp|O14974|MYPT1_HUMAN	PPP1R12A	O14974	Protein phosphatase 1 regulatory subunit 12A	MYPT1_HUMAN		PPP1R12A
302	301	302	0.999	1838	sp|O15031|PLXB2_HUMAN	PLXNB2	O15031	Plexin-B2	PLXB2_HUMAN		PLXNB2
1224	1219	1224	0.999	372	sp|O15143|ARC1B_HUMAN	ARPC1B	O15143	Actin-related protein 2/3 complex subunit 1B	ARC1B_HUMAN		ARPC1B
647	647	652	0.999	200	sp|O15347|HMGB3_HUMAN	HMGB3	O15347	High mobility group protein B3	HMGB3_HUMAN		HMGB3
584	584	584	0.999	430	sp|P00505|AATM_HUMAN	GOT2	P00505	Aspartate aminotransferase, mitochondrial	AATM_HUMAN		GOT2
582	565	582	0.999	381	sp|P12277|KCRB_HUMAN	CKB	P12277	Creatine kinase B-type	KCRB_HUMAN		CKB
1052	1052	1052	0.999	528	sp|P14314|GLU2B_HUMAN	PRKCSH	P14314	Glucosidase 2 subunit beta	GLU2B_HUMAN		PRKCSH
786	785	786	0.999	488	sp|P20073|ANXA7_HUMAN	ANXA7	P20073	Annexin A7	ANXA7_HUMAN		ANXA7
1412	1412	1412	0.999	216	sp|P23284|PPIB_HUMAN	PPIB	P23284	Peptidyl-prolyl cis-trans isomerase B	PPIB_HUMAN		PPIB
1285	1193	1285	0.999	480	sp|P31930|QCR1_HUMAN	UQCRC1	P31930	Cytochrome b-c1 complex subunit 1, mitochondrial	QCR1_HUMAN		UQCRC1
512	475	512	0.999	698	sp|P33121|ACSL1_HUMAN	ACSL1	P33121	Long-chain-fatty-acid--CoA ligase 1	ACSL1_HUMAN		ACSL1
1114	1114	1114	0.999	334	sp|P40925|MDHC_HUMAN	MDH1	P40925	Malate dehydrogenase, cytoplasmic	MDHC_HUMAN		MDH1
836	836	836	0.999	539	sp|P49419|AL7A1_HUMAN	ALDH7A1	P49419	Alpha-aminoadipic semialdehyde dehydrogenase	AL7A1_HUMAN		ALDH7A1
699	116	699	0.999	369	sp|P50502|F10A1_HUMAN	ST13	P50502	Hsc70-interacting protein	F10A1_HUMAN		ST13
413	411	413	0.999	2035	sp|P51610|HCFC1_HUMAN	HCFC1	P51610	Host cell factor 1	HCFC1_HUMAN		HCFC1
883	865	883	0.999	240	sp|P51858|HDGF_HUMAN	HDGF	P51858	Hepatoma-derived growth factor	HDGF_HUMAN		HDGF
1050	1050	1050	0.999	246	sp|P60900|PSA6_HUMAN	PSMA6	P60900	Proteasome subunit alpha type-6	PSA6_HUMAN		PSMA6
1402	1402	1402	0.999	102	sp|P61604|CH10_HUMAN	HSPE1	P61604	10 kDa heat shock protein, mitochondrial	CH10_HUMAN		HSPE1
1483	1483	1483	0.999	146	sp|P62249|RS16_HUMAN	RPS16	P62249	Small ribosomal subunit protein uS9	RS16_HUMAN		RPS16
1205	1205	1205	0.999	317	sp|P63244|RACK1_HUMAN	RACK1	P63244	Small ribosomal subunit protein RACK1	RACK1_HUMAN		GNB2L1
988	147	988	0.999	309	sp|P67775|PP2AA_HUMAN	PPP2CA	P67775	Serine/threonine-protein phosphatase 2A catalytic subunit alpha isoform	PP2AA_HUMAN		PPP2CA
625	625	625	0.999	790	sp|Q09161|NCBP1_HUMAN	NCBP1	Q09161	Nuclear cap-binding protein subunit 1	NCBP1_HUMAN		NCBP1
1401	1262	1401	0.999	466	sp|Q13283|G3BP1_HUMAN	G3BP1	Q13283	Ras GTPase-activating protein-binding protein 1	G3BP1_HUMAN		G3BP1
108	108	170	0.999	1354	sp|Q13464|ROCK1_HUMAN	ROCK1	Q13464	Rho-associated protein kinase 1	ROCK1_HUMAN		ROCK1
101	101	1205	0.999	407	sp|Q14240|IF4A2_HUMAN	EIF4A2	Q14240	Eukaryotic initiation factor 4A-II	IF4A2_HUMAN		EIF4A2
683	636	683	0.999	522	sp|Q16222|UAP1_HUMAN	UAP1	Q16222	UDP-N-acetylhexosamine pyrophosphorylase	UAP1_HUMAN		UAP1
339	272	339	0.999	923	sp|Q3SY69|AL1L2_HUMAN	ALDH1L2	Q3SY69	Mitochondrial 10-formyltetrahydrofolate dehydrogenase	AL1L2_HUMAN		ALDH1L2
959	959	959	0.999	432	sp|Q8NBS9|TXND5_HUMAN	TXNDC5	Q8NBS9	Thioredoxin domain-containing protein 5	TXND5_HUMAN		TXNDC5
416	414	416	0.999	1508	sp|Q8TEQ6|GEMI5_HUMAN	GEMIN5	Q8TEQ6	Gem-associated protein 5	GEMI5_HUMAN		GEMIN5
266	266	266	0.999	2054	sp|Q92614|MY18A_HUMAN	MYO18A	Q92614	Unconventional myosin-XVIIIa	MY18A_HUMAN		MYO18A
484	321	484	0.999	1105	sp|Q92922|SMRC1_HUMAN	SMARCC1	Q92922	SWI/SNF complex subunit SMARCC1	SMRC1_HUMAN		SMARCC1
741	741	741	0.999	435	sp|Q96CW1|AP2M1_HUMAN	AP2M1	Q96CW1	AP-2 complex subunit mu	AP2M1_HUMAN		AP2M1
472	418	472	0.999	793	sp|Q96T88|UHRF1_HUMAN	UHRF1	Q96T88	E3 ubiquitin-protein ligase UHRF1	UHRF1_HUMAN		UHRF1
272	271	272	0.999	980	sp|Q9P0K7|RAI14_HUMAN	RAI14	Q9P0K7	Ankycorbin	RAI14_HUMAN		RAI14
999	998	999	0.999	249	sp|Q9P0L0|VAPA_HUMAN	VAPA	Q9P0L0	Vesicle-associated membrane protein-associated protein A	VAPA_HUMAN		VAPA
591	591	591	0.999	508	sp|Q9Y285|SYFA_HUMAN	FARSA	Q9Y285	Phenylalanine--tRNA ligase alpha subunit	SYFA_HUMAN		FARSA
1037	718	1037	0.999	392	sp|Q9Y383|LC7L2_HUMAN	LUC7L2	Q9Y383	Putative RNA-binding protein Luc7-like 2	LC7L2_HUMAN		LUC7L2
16	16	723	0.999	2542	sp|Q9Y4G6|TLN2_HUMAN	TLN2	Q9Y4G6	Talin-2	TLN2_HUMAN		TLN2
437	437	437	0.999	797	sp|Q9Y4W6|AFG32_HUMAN	AFG3L2	Q9Y4W6	AFG3-like protein 2	AFG32_HUMAN		AFG3L2
791	791	791	0.999	1052	sp|A0AVT1|UBA6_HUMAN	UBA6	A0AVT1	Ubiquitin-like modifier-activating enzyme 6	UBA6_HUMAN		UBA6
400	400	400	0.999	921	sp|A0FGR8|ESYT2_HUMAN	ESYT2	A0FGR8	Extended synaptotagmin-2	ESYT2_HUMAN		ESYT2
565	557	565	0.999	1098	sp|O14776|TCRG1_HUMAN	TCERG1	O14776	Transcription elongation regulator 1	TCRG1_HUMAN		TCERG1
955	955	955	0.999	548	sp|O43776|SYNC_HUMAN	NARS1	O43776	Asparagine--tRNA ligase, cytoplasmic	SYNC_HUMAN		NARS
188	188	188	0.999	1380	sp|O75976|CBPD_HUMAN	CPD	O75976	Carboxypeptidase D	CBPD_HUMAN		CPD
936	533	936	0.999	355	sp|P04899|GNAI2_HUMAN	GNAI2	P04899	Guanine nucleotide-binding protein G(i) subunit alpha-2	GNAI2_HUMAN		GNAI2
610	610	610	0.999	421	sp|P09543|CN37_HUMAN	CNP	P09543	2',3'-cyclic-nucleotide 3'-phosphodiesterase	CN37_HUMAN		CNP
874	330	874	0.999	499	sp|P15170|ERF3A_HUMAN	GSPT1	P15170	Eukaryotic peptide chain release factor GTP-binding subunit ERF3A	ERF3A_HUMAN		GSPT1
556	551	771	0.999	780	sp|P17858|PFKAL_HUMAN	PFKL	P17858	ATP-dependent 6-phosphofructokinase, liver type	PFKAL_HUMAN		PFKL
144	144	144	0.999	1970	sp|P24928|RPB1_HUMAN	POLR2A	P24928	DNA-directed RNA polymerase II subunit RPB1	RPB1_HUMAN		POLR2A
369	369	422	0.999	1107	sp|P28340|DPOD1_HUMAN	POLD1	P28340	DNA polymerase delta catalytic subunit	DPOD1_HUMAN		POLD1
521	521	521	0.999	882	sp|P29590|PML_HUMAN	PML	P29590	Protein PML	PML_HUMAN		PML
806	582	806	0.999	395	sp|P31153|METK2_HUMAN	MAT2A	P31153	S-adenosylmethionine synthase isoform type-2	METK2_HUMAN		MAT2A
119	119	124	0.999	2912	sp|P35556|FBN2_HUMAN	FBN2	P35556	Fibrillin-2	FBN2_HUMAN		FBN2
485	485	485	0.999	750	sp|P42224|STAT1_HUMAN	STAT1	P42224	Signal transducer and activator of transcription 1-alpha/beta	STAT1_HUMAN		STAT1
491	491	491	0.999	744	sp|P46459|NSF_HUMAN	NSF	P46459	Vesicle-fusing ATPase	NSF_HUMAN		NSF
1388	1388	1388	0.999	160	sp|P46778|RL21_HUMAN	RPL21	P46778	Large ribosomal subunit protein eL21	RL21_HUMAN		RPL21
975	975	975	0.999	272	sp|P47756|CAPZB_HUMAN	CAPZB	P47756	F-actin-capping protein subunit beta	CAPZB_HUMAN		CAPZB
727	727	727	0.999	380	sp|P50552|VASP_HUMAN	VASP	P50552	Vasodilator-stimulated phosphoprotein	VASP_HUMAN		VASP
302	209	302	0.999	1647	sp|P51532|SMCA4_HUMAN	SMARCA4	P51532	Transcription activator BRG1	SMCA4_HUMAN		SMARCA4
850	850	959	0.999	440	sp|P62191|PRS4_HUMAN	PSMC1	P62191	26S proteasome regulatory subunit 4	PRS4_HUMAN		PSMC1
1606	1606	1606	0.999	216	sp|P62826|RAN_HUMAN	RAN	P62826	GTP-binding nuclear protein Ran	RAN_HUMAN		RAN
1364	1364	1364	0.999	178	sp|P62913|RL11_HUMAN	RPL11	P62913	Large ribosomal subunit protein uL5	RL11_HUMAN		RPL11
3233	2945	3233	0.999	165	sp|P62937|PPIA_HUMAN	PPIA	P62937	Peptidyl-prolyl cis-trans isomerase A	PPIA_HUMAN		PPIA
604	315	604	0.999	573	sp|Q07866|KLC1_HUMAN	KLC1	Q07866	Kinesin light chain 1	KLC1_HUMAN		KLC1
740	740	740	0.999	571	sp|Q10471|GALT2_HUMAN	GALNT2	Q10471	Polypeptide N-acetylgalactosaminyltransferase 2	GALT2_HUMAN		GALNT2
94	94	94	0.999	760	sp|Q12884|SEPR_HUMAN	FAP	Q12884	Prolyl endopeptidase FAP	SEPR_HUMAN		FAP
431	431	1001	0.999	644	sp|Q13310|PABP4_HUMAN	PABPC4	Q13310	Polyadenylate-binding protein 4	PABP4_HUMAN		PABPC4
403	403	403	0.999	2202	sp|Q8N3C0|ASCC3_HUMAN	ASCC3	Q8N3C0	Activating signal cointegrator 1 complex subunit 3	ASCC3_HUMAN		ASCC3
560	560	560	0.999	912	sp|Q92888|ARHG1_HUMAN	ARHGEF1	Q92888	Rho guanine nucleotide exchange factor 1	ARHG1_HUMAN		ARHGEF1
875	875	875	0.999	307	sp|Q96AG4|LRC59_HUMAN	LRRC59	Q96AG4	Leucine-rich repeat-containing protein 59	LRC59_HUMAN		LRRC59
628	627	628	0.999	612	sp|Q96G03|PGM2_HUMAN	PGM2	Q96G03	Phosphopentomutase	PGM2_HUMAN		PGM2
667	594	667	0.999	468	sp|Q96HE7|ERO1A_HUMAN	ERO1A	Q96HE7	ERO1-like protein alpha	ERO1A_HUMAN		ERO1L
296	296	296	0.999	2240	sp|Q9BTC0|DIDO1_HUMAN	DIDO1	Q9BTC0	Death-inducer obliterator 1	DIDO1_HUMAN		DIDO1
658	658	658	0.999	950	sp|Q9H0D6|XRN2_HUMAN	XRN2	Q9H0D6	5'-3' exoribonuclease 2	XRN2_HUMAN		XRN2
351	351	351	0.999	879	sp|Q9P2B2|FPRP_HUMAN	PTGFRN	Q9P2B2	Prostaglandin F2 receptor negative regulator	FPRP_HUMAN		PTGFRN
957	957	957	0.999	504	sp|Q9UMS4|PRP19_HUMAN	PRPF19	Q9UMS4	Pre-mRNA-processing factor 19	PRP19_HUMAN		PRPF19
265	265	642	0.999	939	sp|O94973|AP2A2_HUMAN	AP2A2	O94973	AP-2 complex subunit alpha-2	AP2A2_HUMAN		AP2A2
607	607	753	0.999	780	sp|P08237|PFKAM_HUMAN	PFKM	P08237	ATP-dependent 6-phosphofructokinase, muscle type	PFKAM_HUMAN		PFKM
791	637	791	0.999	390	sp|P08559|ODPA_HUMAN	PDHA1	P08559	Pyruvate dehydrogenase E1 component subunit alpha, somatic form, mitoc	ODPA_HUMAN		PDHA1
349	330	597	0.999	434	sp|P09104|ENOG_HUMAN	ENO2	P09104	Gamma-enolase	ENOG_HUMAN		ENO2
1110	1110	1110	0.999	293	sp|P15880|RS2_HUMAN	RPS2	P15880	Small ribosomal subunit protein uS5	RS2_HUMAN		RPS2
639	581	639	0.999	301	sp|P23193|TCEA1_HUMAN	TCEA1	P23193	Transcription elongation factor A protein 1	TCEA1_HUMAN		TCEA1
970	970	1307	0.999	209	sp|P26583|HMGB2_HUMAN	HMGB2	P26583	High mobility group protein B2	HMGB2_HUMAN		HMGB2
467	467	467	0.999	1249	sp|P29144|TPP2_HUMAN	TPP2	P29144	Tripeptidyl-peptidase 2	TPP2_HUMAN		TPP2
581	581	671	0.999	781	sp|P35222|CTNB1_HUMAN	CTNNB1	P35222	Catenin beta-1	CTNB1_HUMAN		CTNNB1
1122	605	1122	0.999	454	sp|P42167|LAP2B_HUMAN	TMPO	P42167	Lamina-associated polypeptide 2, isoforms beta/gamma	LAP2B_HUMAN		TMPO
438	438	438	0.999	934	sp|P43246|MSH2_HUMAN	MSH2	P43246	DNA mismatch repair protein Msh2	MSH2_HUMAN		MSH2
644	644	644	0.999	843	sp|P49756|RBM25_HUMAN	RBM25	P49756	RNA-binding protein 25	RBM25_HUMAN		RBM25
600	575	601	0.999	505	sp|P50995|ANX11_HUMAN	ANXA11	P50995	Annexin A11	ANX11_HUMAN		ANXA11
188	188	364	0.999	759	sp|Q13619|CUL4A_HUMAN	CUL4A	Q13619	Cullin-4A	CUL4A_HUMAN		CUL4A
973	973	973	0.999	1087	sp|Q14157|UBP2L_HUMAN	UBAP2L	Q14157	Ubiquitin-associated protein 2-like	UBP2L_HUMAN		UBAP2L
657	657	657	0.999	279	sp|Q14192|FHL2_HUMAN	FHL2	Q14192	Four and a half LIM domains protein 2	FHL2_HUMAN		FHL2
907	907	907	0.999	630	sp|Q14258|TRI25_HUMAN	TRIM25	Q14258	E3 ubiquitin/ISG15 ligase TRIM25	TRI25_HUMAN		TRIM25
111	111	162	0.999	1914	sp|Q15746|MYLK_HUMAN	MYLK	Q15746	Myosin light chain kinase, smooth muscle	MYLK_HUMAN		MYLK
727	727	727	0.999	923	sp|Q8N163|CCAR2_HUMAN	CCAR2	Q8N163	Cell cycle and apoptosis regulator protein 2	CCAR2_HUMAN		CCAR2
1724	1724	1724	0.999	408	sp|Q8NC51|SERB1_HUMAN	SERBP1	Q8NC51	SERPINE1 mRNA-binding protein 1	SERB1_HUMAN		SERBP1
771	771	771	0.999	953	sp|Q99460|PSMD1_HUMAN	PSMD1	Q99460	26S proteasome non-ATPase regulatory subunit 1	PSMD1_HUMAN		PSMD1
619	619	619	0.999	717	sp|Q9H307|PININ_HUMAN	PNN	Q9H307	Pinin	PININ_HUMAN		PNN
422	422	422	0.999	379	sp|Q9NX58|LYAR_HUMAN	LYAR	Q9NX58	Cell growth-regulating nucleolar protein	LYAR_HUMAN		LYAR
496	496	496	0.999	627	sp|Q9UHB9|SRP68_HUMAN	SRP68	Q9UHB9	Signal recognition particle subunit SRP68	SRP68_HUMAN		SRP68
1082	1082	1082	0.999	422	sp|O00231|PSD11_HUMAN	PSMD11	O00231	26S proteasome non-ATPase regulatory subunit 11	PSD11_HUMAN		PSMD11
602	602	602	0.999	738	sp|O60568|PLOD3_HUMAN	PLOD3	O60568	Multifunctional procollagen lysine hydroxylase and glycosyltransferase LH3	PLOD3_HUMAN		PLOD3
710	710	710	0.999	527	sp|P04040|CATA_HUMAN	CAT	P04040	Catalase	CATA_HUMAN		CAT

1552	1552	1851	0.999	334	sp|P07195|LDHB_HUMAN	LDHB	P07195	L-lactate dehydrogenase B chain	LDHB_HUMAN		LDHB
1451	1451	1451	0.999	630	sp|P08195|4F2_HUMAN	SLC3A2	P08195	4F2 cell-surface antigen heavy chain	4F2_HUMAN		SLC3A2
59	59	788	0.999	473	sp|P08779|K1C16_HUMAN	KRT16	P08779	Keratin, type I cytoskeletal 16	K1C16_HUMAN		KRT16
521	521	521	0.999	534	sp|P13674|P4HA1_HUMAN	P4HA1	P13674	Prolyl 4-hydroxylase subunit alpha-1	P4HA1_HUMAN		P4HA1
802	712	802	0.999	745	sp|P14923|PLAK_HUMAN	JUP	P14923	Junction plakoglobin	PLAK_HUMAN		JUP
924	924	924	0.999	471	sp|P23381|SYWC_HUMAN	WARS1	P23381	Tryptophan--tRNA ligase, cytoplasmic	SYWC_HUMAN		WARS
588	331	588	0.999	1241	sp|P23634|AT2B4_HUMAN	ATP2B4	P23634	Plasma membrane calcium-transporting ATPase 4	AT2B4_HUMAN		ATP2B4
771	771	771	0.999	427	sp|P24752|THIL_HUMAN	ACAT1	P24752	Acetyl-CoA acetyltransferase, mitochondrial	THIL_HUMAN		ACAT1
1399	1322	1399	0.999	281	sp|P29692|EF1D_HUMAN	EEF1D	P29692	Elongation factor 1-delta	EF1D_HUMAN		EEF1D
1586	1586	1586	0.999	337	sp|P37837|TALDO_HUMAN	TALDO1	P37837	Transaldolase	TALDO_HUMAN		TALDO1
859	859	1220	0.999	411	sp|P38919|IF4A3_HUMAN	EIF4A3	P38919	Eukaryotic initiation factor 4A-III	IF4A3_HUMAN		EIF4A3
837	837	874	0.999	511	sp|P48444|COPD_HUMAN	ARCN1	P48444	Coatomer subunit delta	COPD_HUMAN		ARCN1
704	704	704	0.999	748	sp|P49589|SYCC_HUMAN	CARS1	P49589	Cysteine--tRNA ligase, cytoplasmic	SYCC_HUMAN		CARS
436	436	436	0.999	2863	sp|P50851|LRBA_HUMAN	LRBA	P50851	Lipopolysaccharide-responsive and beige-like anchor protein	LRBA_HUMAN		LRBA
740	740	740	0.999	689	sp|P52888|THOP1_HUMAN	THOP1	P52888	Thimet oligopeptidase	THOP1_HUMAN		THOP1
1503	451	1503	0.999	145	sp|P61254|RL26_HUMAN	RPL26	P61254	Large ribosomal subunit protein uL24	RL26_HUMAN		RPL26
1303	1303	1303	0.999	204	sp|P61313|RL15_HUMAN	RPL15	P61313	Large ribosomal subunit protein eL15	RL15_HUMAN		RPL15
1003	1003	1003	0.999	389	sp|P62333|PRS10_HUMAN	PSMC6	P62333	26S proteasome regulatory subunit 10B	PRS10_HUMAN		PSMC6
789	761	789	0.999	727	sp|Q02809|PLOD1_HUMAN	PLOD1	Q02809	Procollagen-lysine,2-oxoglutarate 5-dioxygenase 1	PLOD1_HUMAN		PLOD1
651	651	651	0.999	644	sp|Q14166|TTL12_HUMAN	TTLL12	Q14166	Tubulin--tyrosine ligase-like protein 12	TTL12_HUMAN		TTLL12
864	864	864	0.999	378	sp|Q16543|CDC37_HUMAN	CDC37	Q16543	Hsp90 co-chaperone Cdc37	CDC37_HUMAN		CDC37
702	702	702	0.999	583	sp|Q8N1G4|LRC47_HUMAN	LRRC47	Q8N1G4	Leucine-rich repeat-containing protein 47	LRC47_HUMAN		LRRC47
401	401	420	0.999	523	sp|Q8WXF1|PSPC1_HUMAN	PSPC1	Q8WXF1	Paraspeckle component 1	PSPC1_HUMAN		PSPC1
491	491	491	0.999	596	sp|Q96HC4|PDLI5_HUMAN	PDLIM5	Q96HC4	PDZ and LIM domain protein 5	PDLI5_HUMAN		PDLIM5
500	480	562	0.999	543	sp|Q9NZN4|EHD2_HUMAN	EHD2	Q9NZN4	EH domain-containing protein 2	EHD2_HUMAN		EHD2
404	369	404	0.999	606	sp|Q9UNF1|MAGD2_HUMAN	MAGED2	Q9UNF1	Melanoma-associated antigen D2	MAGD2_HUMAN		MAGED2
819	819	819	0.999	331	sp|Q9Y266|NUDC_HUMAN	NUDC	Q9Y266	Nuclear migration protein nudC	NUDC_HUMAN		NUDC
498	497	498	0.999	1128	sp|Q9Y2A7|NCKP1_HUMAN	NCKAP1	Q9Y2A7	Nck-associated protein 1	NCKP1_HUMAN		NCKAP1
194	128	229	0.999	1711	sp|Q9Y5S2|MRCKB_HUMAN	CDC42BPB	Q9Y5S2	Serine/threonine-protein kinase MRCK beta	MRCKB_HUMAN		CDC42BPB
333	269	333	0.999	1005	sp|O43491|E41L2_HUMAN	EPB41L2	O43491	Band 4.1-like protein 2	E41L2_HUMAN		EPB41L2
931	818	931	0.999	414	sp|O75874|IDHC_HUMAN	IDH1	O75874	Isocitrate dehydrogenase [NADP] cytoplasmic	IDHC_HUMAN		IDH1
417	346	417	0.999	1239	sp|O95819|M4K4_HUMAN	MAP4K4	O95819	Mitogen-activated protein kinase kinase kinase kinase 4	M4K4_HUMAN		MAP4K4
734	734	734	0.999	1210	sp|P00533|EGFR_HUMAN	EGFR	P00533	Epidermal growth factor receptor	EGFR_HUMAN		EGFR
1046	1046	1046	0.999	315	sp|P05198|IF2A_HUMAN	EIF2S1	P05198	Eukaryotic translation initiation factor 2 subunit 1	IF2A_HUMAN		EIF2S1
1070	1070	1070	0.999	509	sp|P09622|DLDH_HUMAN	DLD	P09622	Dihydrolipoyl dehydrogenase, mitochondrial	DLDH_HUMAN		DLD
743	81	1683	0.999	219	sp|P10412|H14_HUMAN	H1-4	P10412	Histone H1.4	H14_HUMAN		HIST1H1E
673	670	673	0.999	591	sp|P17812|PYRG1_HUMAN	CTPS1	P17812	CTP synthase 1	PYRG1_HUMAN		CTPS1
389	389	389	0.999	574	sp|P21589|5NTD_HUMAN	NT5E	P21589	5'-nucleotidase	5NTD_HUMAN		NT5E
881	831	881	0.999	649	sp|P46063|RECQ1_HUMAN	RECQL	P46063	ATP-dependent DNA helicase Q1	RECQ1_HUMAN		RECQL
1141	1141	1141	0.999	204	sp|P46782|RS5_HUMAN	RPS5	P46782	Small ribosomal subunit protein uS7	RS5_HUMAN		RPS5
454	454	454	0.999	710	sp|P48147|PPCE_HUMAN	PREP	P48147	Prolyl endopeptidase	PPCE_HUMAN		PREP
1036	1036	1036	0.999	788	sp|P49321|NASP_HUMAN	NASP	P49321	Nuclear autoantigenic sperm protein	NASP_HUMAN		NASP
462	462	462	0.999	1475	sp|P49790|NU153_HUMAN	NUP153	P49790	Nuclear pore complex protein Nup153	NU153_HUMAN		NUP153
738	372	738	0.999	870	sp|P50570|DYN2_HUMAN	DNM2	P50570	Dynamin-2	DYN2_HUMAN		DNM2
1177	1177	1177	0.999	215	sp|P50914|RL14_HUMAN	RPL14	P50914	Large ribosomal subunit protein eL14	RL14_HUMAN		RPL14
1254	1254	1254	0.999	483	sp|P52209|6PGD_HUMAN	PGD	P52209	6-phosphogluconate dehydrogenase, decarboxylating	6PGD_HUMAN		PGD
444	441	444	0.999	1360	sp|P52701|MSH6_HUMAN	MSH6	P52701	DNA mismatch repair protein Msh6	MSH6_HUMAN		MSH6
325	325	947	0.999	449	sp|P55795|HNRH2_HUMAN	HNRNPH2	P55795	Heterogeneous nuclear ribonucleoprotein H2	HNRH2_HUMAN		HNRNPH2
878	878	987	0.999	406	sp|P62195|PRS8_HUMAN	PSMC5	P62195	26S proteasome regulatory subunit 8	PRS8_HUMAN		PSMC5
1388	1388	1388	0.999	152	sp|P62269|RS18_HUMAN	RPS18	P62269	Small ribosomal subunit protein uS13	RS18_HUMAN		RPS18
1095	1095	1095	0.999	157	sp|P83731|RL24_HUMAN	RPL24	P83731	Large ribosomal subunit protein eL24	RL24_HUMAN		RPL24
765	668	765	0.999	699	sp|Q06210|GFPT1_HUMAN	GFPT1	Q06210	Glutamine--fructose-6-phosphate aminotransferase [isomerizing] 1	GFPT1_HUMAN		GFPT1
496	496	496	0.999	1269	sp|Q13045|FLII_HUMAN	FLII	Q13045	Protein flightless-1 homolog	FLII_HUMAN		FLII
688	688	688	0.999	963	sp|Q15020|SART3_HUMAN	SART3	Q15020	Squamous cell carcinoma antigen recognized by T-cells 3	SART3_HUMAN		SART3
1099	1099	1099	0.999	597	sp|Q15046|SYK_HUMAN	KARS1	Q15046	Lysine--tRNA ligase	SYK_HUMAN		KARS
1410	1410	1410	0.999	440	sp|Q15084|PDIA6_HUMAN	PDIA6	Q15084	Protein disulfide-isomerase A6	PDIA6_HUMAN		PDIA6
1219	676	1219	0.999	425	sp|Q16576|RBBP7_HUMAN	RBBP7	Q16576	Histone-binding protein RBBP7	RBBP7_HUMAN		RBBP7
137	137	137	0.999	5207	sp|Q63HN8|RN213_HUMAN	RNF213	Q63HN8	E3 ubiquitin-protein ligase RNF213	RN213_HUMAN		RNF213
413	413	413	0.999	1194	sp|Q7L2E3|DHX30_HUMAN	DHX30	Q7L2E3	ATP-dependent RNA helicase DHX30	DHX30_HUMAN		DHX30
480	480	480	0.999	1025	sp|Q9H0A0|NAT10_HUMAN	NAT10	Q9H0A0	RNA cytidine acetyltransferase	NAT10_HUMAN		NAT10
779	779	779	0.999	640	sp|Q9UBT2|SAE2_HUMAN	UBA2	Q9UBT2	SUMO-activating enzyme subunit 2	SAE2_HUMAN		UBA2
347	347	347	0.999	2068	sp|O00468|AGRIN_HUMAN	AGRN	O00468	Agrin	AGRIN_HUMAN		AGRN
545	545	545	0.999	535	sp|O15460|P4HA2_HUMAN	P4HA2	O15460	Prolyl 4-hydroxylase subunit alpha-2	P4HA2_HUMAN		P4HA2
590	590	680	0.999	711	sp|O60488|ACSL4_HUMAN	ACSL4	O60488	Long-chain-fatty-acid--CoA ligase 4	ACSL4_HUMAN		ACSL4
739	739	739	0.999	1220	sp|O60841|IF2P_HUMAN	EIF5B	O60841	Eukaryotic translation initiation factor 5B	IF2P_HUMAN		EIF5B
787	787	787	0.999	613	sp|O95831|AIFM1_HUMAN	AIFM1	O95831	Apoptosis-inducing factor 1, mitochondrial	AIFM1_HUMAN		AIFM1
106	106	1611	0.999	213	sp|P16403|H12_HUMAN	H1-2	P16403	Histone H1.2	H12_HUMAN		HIST1H1C
1379	1330	1379	0.999	184	sp|P18621|RL17_HUMAN	RPL17	P18621	Large ribosomal subunit protein uL22	RL17_HUMAN		RPL17
133	133	133	0.999	925	sp|P22413|ENPP1_HUMAN	ENPP1	P22413	Ectonucleotide pyrophosphatase/phosphodiesterase family member 1	ENPP1_HUMAN		ENPP1
1453	1453	1453	0.999	263	sp|P25786|PSA1_HUMAN	PSMA1	P25786	Proteasome subunit alpha type-1	PSA1_HUMAN		PSMA1
1994	1994	1994	0.999	417	sp|P27797|CALR_HUMAN	CALR	P27797	Calreticulin	CALR_HUMAN		CALR
1432	1432	1432	0.999	224	sp|P30041|PRDX6_HUMAN	PRDX6	P30041	Peroxiredoxin-6	PRDX6_HUMAN		PRDX6
1112	955	1112	0.999	589	sp|P30153|2AAA_HUMAN	PPP2R1A	P30153	Serine/threonine-protein phosphatase 2A 65 kDa regulatory subunit A alpha	2AAA_HUMAN		PPP2R1A
824	824	933	0.999	433	sp|P35998|PRS7_HUMAN	PSMC2	P35998	26S proteasome regulatory subunit 7	PRS7_HUMAN		PSMC2
1135	1000	1135	0.999	203	sp|P40429|RL13A_HUMAN	RPL13A	P40429	Large ribosomal subunit protein uL13	RL13A_HUMAN		RPL13A
682	682	682	0.999	736	sp|P51659|DHB4_HUMAN	HSD17B4	P51659	Peroxisomal multifunctional enzyme type 2	DHB4_HUMAN		HSD17B4
881	881	881	0.999	953	sp|P53618|COPB_HUMAN	COPB1	P53618	Coatomer subunit beta	COPB_HUMAN		COPB1
1812	1812	1812	0.999	249	sp|P60174|TPIS_HUMAN	TPI1	P60174	Triosephosphate isomerase	TPIS_HUMAN		TPI1
1007	1007	1007	0.999	394	sp|P61160|ARP2_HUMAN	ACTR2	P61160	Actin-related protein 2	ARP2_HUMAN		ACTR2
1614	1614	1614	0.999	158	sp|P62280|RS11_HUMAN	RPS11	P62280	Small ribosomal subunit protein uS17	RS11_HUMAN		RPS11
1449	1449	1449	0.999	249	sp|P62753|RS6_HUMAN	RPS6	P62753	Small ribosomal subunit protein eS6	RS6_HUMAN		RPS6
1438	1438	1438	0.999	217	sp|P62906|RL10A_HUMAN	RPL10A	P62906	Large ribosomal subunit protein uL1	RL10A_HUMAN		RPL10A
1014	517	1014	0.999	937	sp|P63010|AP2B1_HUMAN	AP2B1	P63010	AP-2 complex subunit beta	AP2B1_HUMAN		AP2B1
958	958	1486	0.999	246	sp|Q04917|1433F_HUMAN	YWHAH	Q04917	14-3-3 protein eta	1433F_HUMAN		YWHAH
2394	1984	2394	0.999	199	sp|Q06830|PRDX1_HUMAN	PRDX1	Q06830	Peroxiredoxin-1	PRDX1_HUMAN		PRDX1
962	942	962	0.999	248	sp|Q07955|SRSF1_HUMAN	SRSF1	Q07955	Serine/arginine-rich splicing factor 1	SRSF1_HUMAN		SRSF1
379	379	876	0.999	949	sp|Q10567|AP1B1_HUMAN	AP1B1	Q10567	AP-1 complex subunit beta-1	AP1B1_HUMAN		AP1B1
621	621	621	0.999	452	sp|Q13418|ILK_HUMAN	ILK	Q13418	Integrin-linked protein kinase	ILK_HUMAN		ILK
209	209	459	0.999	953	sp|Q14151|SAFB2_HUMAN	SAFB2	Q14151	Scaffold attachment factor B2	SAFB2_HUMAN		SAFB2
560	560	560	0.999	1630	sp|Q14160|SCRIB_HUMAN	SCRIB	Q14160	Protein scribble homolog	SCRIB_HUMAN		SCRIB
1246	1246	1246	0.999	261	sp|Q14847|LASP1_HUMAN	LASP1	Q14847	LIM and SH3 domain protein 1	LASP1_HUMAN		LASP1
614	614	614	0.999	699	sp|Q14978|NOLC1_HUMAN	NOLC1	Q14978	Nucleolar and coiled-body phosphoprotein 1	NOLC1_HUMAN		NOLC1
665	650	665	0.999	765	sp|Q15436|SC23A_HUMAN	SEC23A	Q15436	Protein transport protein Sec23A	SC23A_HUMAN		SEC23A
1200	937	1200	0.999	572	sp|Q16555|DPYL2_HUMAN	DPYSL2	Q16555	Dihydropyrimidinase-related protein 2	DPYL2_HUMAN		DPYSL2
777	739	777	0.999	1075	sp|Q8WWM7|ATX2L_HUMAN	ATXN2L	Q8WWM7	Ataxin-2-like protein	ATX2L_HUMAN		ATXN2L
1159	1159	1159	0.999	456	sp|Q9Y265|RUVB1_HUMAN	RUVBL1	Q9Y265	RuvB-like 1	RUVB1_HUMAN		RUVBL1
1224	1224	1224	0.999	434	sp|O60664|PLIN3_HUMAN	PLIN3	O60664	Perilipin-3	PLIN3_HUMAN		PLIN3
792	792	792	0.999	962	sp|O60763|USO1_HUMAN	USO1	O60763	General vesicular transport factor p115	USO1_HUMAN		USO1
568	568	568	0.999	1391	sp|O75694|NU155_HUMAN	NUP155	O75694	Nuclear pore complex protein Nup155	NU155_HUMAN		NUP155
779	689	779	0.999	720	sp|O95573|ACSL3_HUMAN	ACSL3	O95573	Fatty acid CoA ligase Acsl3	ACSL3_HUMAN		ACSL3
623	553	2008	0.999	285	sp|P06753|TPM3_HUMAN	TPM3	P06753	Tropomyosin alpha-3 chain	TPM3_HUMAN		TPM3
1100	1100	1100	0.999	510	sp|P07954|FUMH_HUMAN	FH	P07954	Fumarate hydratase, mitochondrial	FUMH_HUMAN		FH
1050	1050	1050	0.999	437	sp|P08621|RU17_HUMAN	SNRNP70	P08621	U1 small nuclear ribonucleoprotein 70 kDa	RU17_HUMAN		SNRNP70
738	738	738	0.999	319	sp|P09525|ANXA4_HUMAN	ANXA4	P09525	Annexin A4	ANXA4_HUMAN		ANXA4
1747	1630	1747	0.999	283	sp|P21796|VDAC1_HUMAN	VDAC1	P21796	Voltage-dependent anion-selective channel protein 1	VDAC1_HUMAN		VDAC1
2070	2070	2070	0.999	243	sp|P23396|RS3_HUMAN	RPS3	P23396	Small ribosomal subunit protein uS3	RS3_HUMAN		RPS3
1977	1908	1977	0.999	432	sp|P23526|SAHH_HUMAN	AHCY	P23526	Adenosylhomocysteinase	SAHH_HUMAN		AHCY
1639	1034	1639	0.999	214	sp|P27635|RL10_HUMAN	RPL10	P27635	Large ribosomal subunit protein uL16	RL10_HUMAN		RPL10
591	591	591	0.999	727	sp|P28331|NDUS1_HUMAN	NDUFS1	P28331	NADH-ubiquinone oxidoreductase 75 kDa subunit, mitochondrial	NDUS1_HUMAN		NDUFS1
239	184	239	0.999	976	sp|P29317|EPHA2_HUMAN	EPHA2	P29317	Ephrin type-A receptor 2	EPHA2_HUMAN		EPHA2
425	425	942	0.999	694	sp|P42166|LAP2A_HUMAN	TMPO	P42166	Lamina-associated polypeptide 2, isoform alpha	LAP2A_HUMAN		TMPO
855	855	855	0.999	599	sp|P61221|ABCE1_HUMAN	ABCE1	P61221	ATP-binding cassette sub-family E member 1	ABCE1_HUMAN		ABCE1
1243	1243	1243	0.999	194	sp|P62081|RS7_HUMAN	RPS7	P62081	Small ribosomal subunit protein eS7	RS7_HUMAN		RPS7
1370	1370	1370	0.999	176	sp|Q02543|RL18A_HUMAN	RPL18A	Q02543	Large ribosomal subunit protein eL20	RL18A_HUMAN		RPL18A
995	995	995	0.999	908	sp|Q13200|PSMD2_HUMAN	PSMD2	Q13200	26S proteasome non-ATPase regulatory subunit 2	PSMD2_HUMAN		PSMD2
535	535	535	0.999	1086	sp|Q13423|NNTM_HUMAN	NNT	Q13423	NAD(P) transhydrogenase, mitochondrial	NNTM_HUMAN		NNT
1202	582	1202	0.999	428	sp|Q13838|DX39B_HUMAN	DDX39B	Q13838	Spliceosome RNA helicase DDX39B	DX39B_HUMAN		DDX39B
1384	1122	1384	0.999	355	sp|Q14103|HNRPD_HUMAN	HNRNPD	Q14103	Heterogeneous nuclear ribonucleoprotein D0	HNRPD_HUMAN		HNRNPD
780	779	780	0.999	793	sp|Q15459|SF3A1_HUMAN	SF3A1	Q15459	Splicing factor 3A subunit 1	SF3A1_HUMAN		SF3A1
788	788	788	0.999	508	sp|Q16851|UGPA_HUMAN	UGP2	Q16851	UTP--glucose-1-phosphate uridylyltransferase	UGPA_HUMAN		UGP2
793	782	793	0.999	649	sp|Q16881|TRXR1_HUMAN	TXNRD1	Q16881	Thioredoxin reductase 1, cytoplasmic	TRXR1_HUMAN		TXNRD1
636	636	636	0.999	390	sp|Q6NZI2|CAVN1_HUMAN	CAVIN1	Q6NZI2	Caveolae-associated protein 1	CAVN1_HUMAN		PTRF
680	674	680	0.999	845	sp|Q8NE71|ABCF1_HUMAN	ABCF1	Q8NE71	ATP-binding cassette sub-family F member 1	ABCF1_HUMAN		ABCF1
445	445	445	0.999	2012	sp|Q92621|NU205_HUMAN	NUP205	Q92621	Nuclear pore complex protein Nup205	NU205_HUMAN		NUP205
713	713	713	0.999	856	sp|Q9BUJ2|HNRL1_HUMAN	HNRNPUL1	Q9BUJ2	Heterogeneous nuclear ribonucleoprotein U-like protein 1	HNRL1_HUMAN		HNRNPUL1
572	572	572	0.999	944	sp|Q9H3U1|UN45A_HUMAN	UNC45A	Q9H3U1	Protein unc-45 homolog A	UN45A_HUMAN		UNC45A
891	891	891	0.999	522	sp|Q9P258|RCC2_HUMAN	RCC2	Q9P258	Protein RCC2	RCC2_HUMAN		RCC2
1027	894	1027	0.999	474	sp|Q9ULV4|COR1C_HUMAN	CORO1C	Q9ULV4	Coronin-1C	COR1C_HUMAN		CORO1C
778	778	778	0.999	450	sp|Q9Y6N5|SQOR_HUMAN	SQOR	Q9Y6N5	Sulfide:quinone oxidoreductase, mitochondrial	SQOR_HUMAN		SQRDL

661	661	661	0.999	960	sp|O60313|OPA1_HUMAN	OPA1	O60313	Dynamin-like 120 kDa protein, mitochondrial	OPA1_HUMAN		OPA1
319	257	319	0.999	1388	sp|O75116|ROCK2_HUMAN	ROCK2	O75116	Rho-associated protein kinase 2	ROCK2_HUMAN		ROCK2
838	838	838	0.999	490	sp|O76021|RL1D1_HUMAN	RSL1D1	O76021	Ribosomal L1 domain-containing protein 1	RL1D1_HUMAN		RSL1D1
861	661	861	0.999	668	sp|O94776|MTA2_HUMAN	MTA2	O94776	Metastasis-associated protein MTA2	MTA2_HUMAN		MTA2
598	598	598	0.999	1048	sp|P06756|ITAV_HUMAN	ITGAV	P06756	Integrin alpha-V	ITAV_HUMAN		ITGAV
1127	1127	1127	0.999	461	sp|P13489|RINI_HUMAN	RNH1	P13489	Ribonuclease inhibitor	RINI_HUMAN		RNH1
912	912	912	0.999	792	sp|P23921|RIR1_HUMAN	RRM1	P23921	Ribonucleoside-diphosphate reductase large subunit	RIR1_HUMAN		RRM1
1287	1046	1287	0.999	557	sp|P26599|PTBP1_HUMAN	PTBP1	P26599	Polypyrimidine tract-binding protein 1	PTBP1_HUMAN		PTBP1
1192	1192	1192	0.999	592	sp|P31939|PUR9_HUMAN	ATIC	P31939	Bifunctional purine biosynthesis protein ATIC	PUR9_HUMAN		ATIC
782	782	782	0.999	727	sp|P43304|GPDM_HUMAN	GPD2	P43304	Glycerol-3-phosphate dehydrogenase, mitochondrial	GPDM_HUMAN		GPD2
161	161	161	0.999	1621	sp|P48681|NEST_HUMAN	NES	P48681	Nestin	NEST_HUMAN		NES
1401	1401	1401	0.999	455	sp|P49411|EFTU_HUMAN	TUFM	P49411	Elongation factor Tu, mitochondrial	EFTU_HUMAN		TUFM
408	408	570	0.999	917	sp|P52789|HXK2_HUMAN	HK2	P52789	Hexokinase-2	HXK2_HUMAN		HK2
1477	1477	1625	0.999	255	sp|P62258|1433E_HUMAN	YWHAE	P62258	14-3-3 protein epsilon	1433E_HUMAN		YWHAE
1238	1238	1238	0.999	196	sp|P84098|RL19_HUMAN	RPL19	P84098	Large ribosomal subunit protein eL19	RL19_HUMAN		RPL19
946	946	946	0.999	709	sp|Q08945|SSRP1_HUMAN	SSRP1	Q08945	FACT complex subunit SSRP1	SSRP1_HUMAN		SSRP1
487	448	487	0.999	1108	sp|Q12965|MYO1E_HUMAN	MYO1E	Q12965	Unconventional myosin-Ie	MYO1E_HUMAN		MYO1E
254	254	254	0.999	3259	sp|Q14789|GOGB1_HUMAN	GOLGB1	Q14789	Golgin subfamily B member 1	GOGB1_HUMAN		GOLGB1
644	621	644	0.999	1337	sp|Q29RF7|PDS5A_HUMAN	PDS5A	Q29RF7	Sister chromatid cohesion protein PDS5 homolog A	PDS5A_HUMAN		PDS5A
763	763	763	0.999	553	sp|Q5SSJ5|HP1B3_HUMAN	HP1BP3	Q5SSJ5	Heterochromatin protein 1-binding protein 3	HP1B3_HUMAN		HP1BP3
517	446	517	0.999	1584	sp|Q5SW79|CE170_HUMAN	CEP170	Q5SW79	Centrosomal protein of 170 kDa	CE170_HUMAN		CEP170
789	759	789	0.999	622	sp|Q8NBJ5|GT251_HUMAN	COLGALT1	Q8NBJ5	Procollagen galactosyltransferase 1	GT251_HUMAN		COLGALT1
551	551	551	0.999	802	sp|Q99459|CDC5L_HUMAN	CDC5L	Q99459	Cell division cycle 5-like protein	CDC5L_HUMAN		CDC5L
1201	876	2571	0.999	446	sp|Q9BUF5|TBB6_HUMAN	TUBB6	Q9BUF5	Tubulin beta-6 chain	TBB6_HUMAN		TUBB6
345	345	345	0.999	1483	sp|Q9UIG0|BAZ1B_HUMAN	BAZ1B	Q9UIG0	Tyrosine-protein kinase BAZ1B	BAZ1B_HUMAN		BAZ1B
143	143	143	0.999	1016	sp|Q9Y6C2|EMIL1_HUMAN	EMILIN1	Q9Y6C2	EMILIN-1	EMIL1_HUMAN		EMILIN1
916	916	916	0.999	1071	sp|O14980|XPO1_HUMAN	XPO1	O14980	Exportin-1	XPO1_HUMAN		XPO1
1063	1063	1063	0.999	534	sp|O43242|PSMD3_HUMAN	PSMD3	O43242	26S proteasome non-ATPase regulatory subunit 3	PSMD3_HUMAN		PSMD3
767	767	767	0.999	494	sp|O60701|UGDH_HUMAN	UGDH	O60701	UDP-glucose 6-dehydrogenase	UGDH_HUMAN		UGDH
1251	1251	1251	0.999	408	sp|P05455|LA_HUMAN	SSB	P05455	Lupus La protein	LA_HUMAN		SSB
1334	1334	1334	0.999	611	sp|P09960|LKHA4_HUMAN	LTA4H	P09960	Leukotriene A-4 hydrolase	LKHA4_HUMAN		LTA4H
426	307	426	0.999	1531	sp|P11388|TOP2A_HUMAN	TOP2A	P11388	DNA topoisomerase 2-alpha	TOP2A_HUMAN		TOP2A
256	256	256	0.999	1028	sp|P12109|CO6A1_HUMAN	COL6A1	P12109	Collagen alpha-1(VI) chain	CO6A1_HUMAN		COL6A1
1746	1268	1746	0.999	149	sp|P16949|STMN1_HUMAN	STMN1	P16949	Stathmin	STMN1_HUMAN		STMN1
777	777	777	0.999	439	sp|P17980|PRS6A_HUMAN	PSMC3	P17980	26S proteasome regulatory subunit 6A	PRS6A_HUMAN		PSMC3
1059	1059	1575	0.999	245	sp|P27348|1433T_HUMAN	YWHAQ	P27348	14-3-3 protein theta	1433T_HUMAN		YWHAQ
1689	1689	1689	0.999	145	sp|P39019|RS19_HUMAN	RPS19	P39019	Small ribosomal subunit protein eS19	RS19_HUMAN		RPS19
907	907	907	0.999	380	sp|P39748|FEN1_HUMAN	FEN1	P39748	Flap endonuclease 1	FEN1_HUMAN		FEN1
691	691	691	0.999	514	sp|P49591|SYSC_HUMAN	SARS1	P49591	Serine--tRNA ligase, cytoplasmic	SYSC_HUMAN		SARS
1023	1023	1023	0.999	693	sp|P49915|GUAA_HUMAN	GMPS	P49915	GMP synthase [glutamine-hydrolyzing]	GUAA_HUMAN		GMPS
1305	1305	1305	0.999	418	sp|P50454|SERPH_HUMAN	SERPINH1	P50454	Serpin H1	SERPH_HUMAN		SERPINH1
1815	1815	2068	0.999	378	sp|P51991|ROA3_HUMAN	HNRNPA3	P51991	Heterogeneous nuclear ribonucleoprotein A3	ROA3_HUMAN		HNRNPA3
1380	1380	1380	0.999	795	sp|P54886|P5CS_HUMAN	ALDH18A1	P54886	Delta-1-pyrroline-5-carboxylate synthase	P5CS_HUMAN		ALDH18A1
1387	1225	1387	0.999	418	sp|P61158|ARP3_HUMAN	ACTR3	P61158	Actin-related protein 3	ARP3_HUMAN		ACTR3
453	453	503	0.999	1748	sp|Q07157|ZO1_HUMAN	TJP1	Q07157	Tight junction protein ZO-1	ZO1_HUMAN		TJP1
1217	1217	1217	0.999	437	sp|Q16181|SEPT7_HUMAN	SEPTIN7	Q16181	Septin-7	SEPT7_HUMAN		SEPT7
527	526	527	0.999	1297	sp|Q5JTH9|RRP12_HUMAN	RRP12	Q5JTH9	RRP12-like protein	RRP12_HUMAN		RRP12
110	75	991	0.999	648	sp|Q5T9A4|ATD3B_HUMAN	ATAD3B	Q5T9A4	ATPase family AAA domain-containing protein 3B	ATD3B_HUMAN		ATAD3B
517	517	517	0.999	986	sp|Q92974|ARHG2_HUMAN	ARHGEF2	Q92974	Rho guanine nucleotide exchange factor 2	ARHG2_HUMAN		ARHGEF2
1255	1255	1255	0.999	796	sp|Q96QK1|VPS35_HUMAN	VPS35	Q96QK1	Vacuolar protein sorting-associated protein 35	VPS35_HUMAN		VPS35
976	976	976	0.999	1104	sp|Q9BSJ8|ESYT1_HUMAN	ESYT1	Q9BSJ8	Extended synaptotagmin-1	ESYT1_HUMAN		ESYT1
422	422	422	0.999	689	sp|O15231|ZN185_HUMAN	ZNF185	O15231	Zinc finger protein 185	ZN185_HUMAN		ZNF185
184	134	503	0.999	400	sp|P08727|K1C19_HUMAN	KRT19	P08727	Keratin, type I cytoskeletal 19	K1C19_HUMAN		KRT19
2103	855	2103	0.999	215	sp|P09429|HMGB1_HUMAN	HMGB1	P09429	High mobility group protein B1	HMGB1_HUMAN		HMGB1
493	485	1132	0.999	364	sp|P09972|ALDOC_HUMAN	ALDOC	P09972	Fructose-bisphosphate aldolase C	ALDOC_HUMAN		ALDOC
641	526	958	0.999	584	sp|P13645|K1C10_HUMAN	KRT10	P13645	Keratin, type I cytoskeletal 10	K1C10_HUMAN		KRT10
319	288	570	0.999	1220	sp|P20020|AT2B1_HUMAN	ATP2B1	P20020	Plasma membrane calcium-transporting ATPase 1	AT2B1_HUMAN		ATP2B1
756	756	756	0.999	1130	sp|P23229|ITA6_HUMAN	ITGA6	P23229	Integrin alpha-6	ITA6_HUMAN		ITGA6
1518	1518	1518	0.999	211	sp|P26373|RL13_HUMAN	RPL13	P26373	Large ribosomal subunit protein eL13	RL13_HUMAN		RPL13
460	460	460	0.999	1174	sp|P30876|RPB2_HUMAN	POLR2B	P30876	DNA-directed RNA polymerase II subunit RPB2	RPB2_HUMAN		POLR2B
985	893	1714	0.999	246	sp|P31946|1433B_HUMAN	YWHAB	P31946	14-3-3 protein beta/alpha	1433B_HUMAN		YWHAB
985	985	985	0.999	474	sp|P55084|ECHB_HUMAN	HADHB	P55084	Trifunctional enzyme subunit beta, mitochondrial	ECHB_HUMAN		HADHB
884	732	884	0.999	1023	sp|Q02218|ODO1_HUMAN	OGDH	Q02218	2-oxoglutarate dehydrogenase complex component E1	ODO1_HUMAN		OGDH
823	823	823	0.999	819	sp|Q8N1F7|NUP93_HUMAN	NUP93	Q8N1F7	Nuclear pore complex protein Nup93	NUP93_HUMAN		NUP93
474	447	474	0.999	1383	sp|Q8WX93|PALLD_HUMAN	PALLD	Q8WX93	Palladin	PALLD_HUMAN		PALLD
737	737	737	0.999	780	sp|Q99798|ACON_HUMAN	ACO2	Q99798	Aconitate hydratase, mitochondrial	ACON_HUMAN		ACO2
736	522	736	0.999	534	sp|Q9H4M9|EHD1_HUMAN	EHD1	Q9H4M9	EH domain-containing protein 1	EHD1_HUMAN		EHD1
1400	1400	1400	0.999	421	sp|Q9UGI8|TES_HUMAN	TES	Q9UGI8	Testin	TES_HUMAN		TES
929	876	929	0.999	874	sp|Q9Y678|COPG1_HUMAN	COPG1	Q9Y678	Coatomer subunit gamma-1	COPG1_HUMAN		COPG1
754	754	754	0.999	1038	sp|O95373|IPO7_HUMAN	IPO7	O95373	Importin-7	IPO7_HUMAN		IPO7
2306	916	2306	0.999	254	sp|P18669|PGAM1_HUMAN	PGAM1	P18669	Phosphoglycerate mutase 1	PGAM1_HUMAN		PGAM1
1519	1491	1519	0.999	592	sp|P27824|CALX_HUMAN	CANX	P27824	Calnexin	CALX_HUMAN		CANX
590	590	646	0.999	623	sp|P35527|K1C9_HUMAN	KRT9	P35527	Keratin, type I cytoskeletal 9	K1C9_HUMAN		KRT9
1703	1703	1703	0.999	338	sp|P40926|MDHM_HUMAN	MDH2	P40926	Malate dehydrogenase, mitochondrial	MDHM_HUMAN		MDH2
786	786	786	0.999	858	sp|P45974|UBP5_HUMAN	USP5	P45974	Ubiquitin carboxyl-terminal hydrolase 5	UBP5_HUMAN		USP5
1595	1595	1595	0.999	459	sp|Q02790|FKBP4_HUMAN	FKBP4	Q02790	Peptidyl-prolyl cis-trans isomerase FKBP4	FKBP4_HUMAN		FKBP4
2121	2121	2121	0.999	288	sp|Q02878|RL6_HUMAN	RPL6	Q02878	Large ribosomal subunit protein eL6	RL6_HUMAN		RPL6
1046	1046	1046	0.999	702	sp|Q04446|GLGB_HUMAN	GBE1	Q04446	1,4-alpha-glucan-branching enzyme	GLGB_HUMAN		GBE1
870	870	870	0.999	758	sp|Q12797|ASPH_HUMAN	ASPH	Q12797	Aspartyl/asparaginyl beta-hydroxylase	ASPH_HUMAN		ASPH
748	693	748	0.999	785	sp|Q16666|IF16_HUMAN	IFI16	Q16666	Gamma-interferon-inducible protein 16	IF16_HUMAN		IFI16
512	512	512	0.999	1037	sp|Q5JRX3|PREP_HUMAN	PITRM1	Q5JRX3	Presequence protease, mitochondrial	PREP_HUMAN		PITRM1
527	527	527	0.999	1096	sp|Q6PKG0|LARP1_HUMAN	LARP1	Q6PKG0	La-related protein 1	LARP1_HUMAN		LARP1
595	595	595	0.999	902	sp|Q7Z2W4|ZCCHV_HUMAN	ZC3HAV1	Q7Z2W4	Zinc finger CCCH-type antiviral protein 1	ZCCHV_HUMAN		ZC3HAV1
325	325	325	0.999	1683	sp|Q8WWI1|LMO7_HUMAN	LMO7	Q8WWI1	LIM domain only protein 7	LMO7_HUMAN		LMO7
700	631	700	0.999	1129	sp|Q92900|RENT1_HUMAN	UPF1	Q92900	Regulator of nonsense transcripts 1	RENT1_HUMAN		UPF1
979	979	979	0.999	650	sp|Q9H4A4|AMPB_HUMAN	RNPEP	Q9H4A4	Aminopeptidase B	AMPB_HUMAN		RNPEP
374	374	374	0.999	1124	sp|Q9NQW6|ANLN_HUMAN	ANLN	Q9NQW6	Anillin	ANLN_HUMAN		ANLN
567	567	567	0.999	2357	sp|O15027|SC16A_HUMAN	SEC16A	O15027	Protein transport protein Sec16A	SC16A_HUMAN		SEC16A
814	437	814	0.999	977	sp|O95782|AP2A1_HUMAN	AP2A1	O95782	AP-2 complex subunit alpha-1	AP2A1_HUMAN		AP2A1
1126	1007	1126	0.999	630	sp|P13797|PLST_HUMAN	PLS3	P13797	Plastin-3	PLST_HUMAN		PLS3
1372	1372	1372	0.999	664	sp|P31040|SDHA_HUMAN	SDHA	P31040	Succinate dehydrogenase [ubiquinone] flavoprotein subunit, mitochondrial	SDHA_HUMAN		SDHA
1625	1624	1625	0.999	504	sp|P34897|GLYM_HUMAN	SHMT2	P34897	Serine hydroxymethyltransferase, mitochondrial	GLYM_HUMAN		SHMT2
1717	320	1717	0.999	472	sp|P41091|IF2G_HUMAN	EIF2S3	P41091	Eukaryotic translation initiation factor 2 subunit 3	IF2G_HUMAN		EIF2S3
471	471	471	0.999	2549	sp|P42345|MTOR_HUMAN	MTOR	P42345	Serine/threonine-protein kinase mTOR	MTOR_HUMAN		MTOR
2288	702	2288	0.999	263	sp|P62701|RS4X_HUMAN	RPS4X	P62701	Small ribosomal subunit protein eS4, X isoform	RS4X_HUMAN		RPS4X
2575	2003	2575	0.999	245	sp|P63104|1433Z_HUMAN	YWHAZ	P63104	14-3-3 protein zeta/delta	1433Z_HUMAN		YWHAZ
898	898	898	0.999	907	sp|P78344|IF4G2_HUMAN	EIF4G2	P78344	Eukaryotic translation initiation factor 4 gamma 2	IF4G2_HUMAN		EIF4G2
256	218	256	0.999	7570	sp|Q03001|DYST_HUMAN	DST	Q03001	Dystonin	DYST_HUMAN		DST
1416	1416	1416	0.999	895	sp|Q13435|SF3B2_HUMAN	SF3B2	Q13435	Splicing factor 3B subunit 2	SF3B2_HUMAN		SF3B2
654	478	654	0.999	913	sp|Q13620|CUL4B_HUMAN	CUL4B	Q13620	Cullin-4B	CUL4B_HUMAN		CUL4B
1438	1438	1438	0.999	299	sp|Q99623|PHB2_HUMAN	PHB2	Q99623	Prohibitin-2	PHB2_HUMAN		PHB2
904	904	904	0.999	920	sp|Q9NYF8|BCLF1_HUMAN	BCLAF1	Q9NYF8	Bcl-2-associated transcription factor 1	BCLF1_HUMAN		BCLAF1
1647	1647	1647	0.999	394	sp|Q9UQ80|PA2G4_HUMAN	PA2G4	Q9UQ80	Proliferation-associated protein 2G4	PA2G4_HUMAN		PA2G4
901	901	901	0.999	955	sp|Q9Y2W1|TR150_HUMAN	THRAP3	Q9Y2W1	Thyroid hormone receptor-associated protein 3	TR150_HUMAN		THRAP3
1487	1487	1487	0.999	533	sp|O43175|SERA_HUMAN	PHGDH	O43175	D-3-phosphoglycerate dehydrogenase	SERA_HUMAN		PHGDH
756	756	1579	0.999	633	sp|O43390|HNRPR_HUMAN	HNRNPR	O43390	Heterogeneous nuclear ribonucleoprotein R	HNRPR_HUMAN		HNRNPR
696	658	696	0.999	1309	sp|O75153|CLU_HUMAN	CLUH	O75153	Clustered mitochondria protein homolog	CLU_HUMAN		CLUH
839	839	839	0.999	798	sp|O75534|CSDE1_HUMAN	CSDE1	O75534	Cold shock domain-containing protein E1	CSDE1_HUMAN		CSDE1
2810	2755	2810	0.999	294	sp|P06748|NPM_HUMAN	NPM1	P06748	Nucleophosmin	NPM_HUMAN		NPM1
814	814	815	0.999	714	sp|P07384|CAN1_HUMAN	CAPN1	P07384	Calpain-1 catalytic subunit	CAN1_HUMAN		CAPN1
227	227	227	0.999	1019	sp|P12110|CO6A2_HUMAN	COL6A2	P12110	Collagen alpha-2(VI) chain	CO6A2_HUMAN		COL6A2
1289	1169	1289	0.999	514	sp|P12268|IMDH2_HUMAN	IMPDH2	P12268	Inosine-5'-monophosphate dehydrogenase 2	IMDH2_HUMAN		IMPDH2
895	888	895	0.999	889	sp|P21399|ACOHC_HUMAN	ACO1	P21399	Cytoplasmic aconitate hydratase	ACOHC_HUMAN		ACO1
1310	1310	1310	0.999	425	sp|P22234|PUR6_HUMAN	PAICS	P22234	Bifunctional phosphoribosylaminoimidazole carboxylase/phosphoribosylan	PUR6_HUMAN		PAICS
2080	2080	2080	0.999	437	sp|P26641|EF1G_HUMAN	EEF1G	P26641	Elongation factor 1-gamma	EF1G_HUMAN		EEF1G
1403	763	1403	0.999	449	sp|P31943|HNRH1_HUMAN	HNRNPH1	P31943	Heterogeneous nuclear ribonucleoprotein H	HNRH1_HUMAN		HNRNPH1
939	939	939	0.999	562	sp|P36871|PGM1_HUMAN	PGM1	P36871	Phosphoglucomutase-1	PGM1_HUMAN		PGM1
1298	1298	1298	0.999	660	sp|P54136|SYRC_HUMAN	RARS1	P54136	Arginine--tRNA ligase, cytoplasmic	SYRC_HUMAN		RARS
1769	1769	1769	0.999	257	sp|P62917|RL8_HUMAN	RPL8	P62917	Large ribosomal subunit protein uL2	RL8_HUMAN		RPL8
544	544	4186	0.999	450	sp|Q13509|TBB3_HUMAN	TUBB3	Q13509	Tubulin beta-3 chain	TBB3_HUMAN		TUBB3
241	59	5182	0.999	445	sp|Q13885|TBB2A_HUMAN	TUBB2A	Q13885	Tubulin beta-2A chain	TBB2A_HUMAN		TUBB2A
788	788	788	0.999	820	sp|Q9BUQ8|DDX23_HUMAN	DDX23	Q9BUQ8	Probable ATP-dependent RNA helicase DDX23	DDX23_HUMAN		DDX23
1029	911	1029	0.999	866	sp|Q9BXJ9|NAA15_HUMAN	NAA15	Q9BXJ9	N-alpha-acetyltransferase 15, NatA auxiliary subunit	NAA15_HUMAN		NAA15
268	268	268	0.999	1294	sp|Q9UM54|MYO6_HUMAN	MYO6	Q9UM54	Unconventional myosin-VI	MYO6_HUMAN		MYO6
2467	2467	2467	0.999	529	sp|P06576|ATPB_HUMAN	ATP5F1B	P06576	ATP synthase subunit beta, mitochondrial	ATPB_HUMAN		ATP5B
1650	1008	1650	0.999	306	sp|P07910|HNRPC_HUMAN	HNRNPC	P07910	Heterogeneous nuclear ribonucleoproteins C1/C2	HNRPC_HUMAN		HNRNPC
383	383	1679	0.999	284	sp|P09493|TPM1_HUMAN	TPM1	P09493	Tropomyosin alpha-1 chain	TPM1_HUMAN		TPM1
1118	871	1118	0.999	509	sp|P12081|HARS1_HUMAN	HARS1	P12081	Histidine--tRNA ligase, cytoplasmic	HARS1_HUMAN		HARS
1447	1351	1447	0.999	589	sp|P14866|HNRPL_HUMAN	HNRNPL	P14866	Heterogeneous nuclear ribonucleoprotein L	HNRPL_HUMAN		HNRNPL

809	809	809	0.999	1181	sp|P17301|ITA2_HUMAN	ITGA2	P17301	Integrin alpha-2	ITA2_HUMAN		ITGA2
460	444	705	0.999	639	sp|P35908|K22E_HUMAN	KRT2	P35908	Keratin, type II cytoskeletal 2 epidermal	K22E_HUMAN		KRT2
1985	1595	1985	0.999	531	sp|P40227|TCPZ_HUMAN	CCT6A	P40227	T-complex protein 1 subunit zeta	TCPZ_HUMAN		CCT6A
1726	1726	1726	0.999	194	sp|P46781|RS9_HUMAN	RPS9	P46781	Small ribosomal subunit protein uS4	RS9_HUMAN		RPS9
2255	2255	2255	0.999	463	sp|P61978|HNRPK_HUMAN	HNRNPK	P61978	Heterogeneous nuclear ribonucleoprotein K	HNRPK_HUMAN		HNRNPK
1530	1210	1929	0.999	247	sp|P61981|1433G_HUMAN	YWHAG	P61981	14-3-3 protein gamma	1433G_HUMAN		YWHAG
1001	0	5874	0.999	377	sp|P68032|ACTC_HUMAN	ACTC1	P68032	Actin, alpha cardiac muscle 1	ACTC_HUMAN		ACTC1
656	656	656	0.999	1278	sp|Q14203|DCTN1_HUMAN	DCTN1	Q14203	Dynactin subunit 1	DCTN1_HUMAN		DCTN1
838	838	838	0.999	1845	sp|Q5VYK3|ECM29_HUMAN	ECPAS	Q5VYK3	Proteasome adapter and scaffold protein ECM29	ECM29_HUMAN		ECM29
1109	1109	1109	0.999	740	sp|Q92499|DDX1_HUMAN	DDX1	Q92499	ATP-dependent RNA helicase DDX1	DDX1_HUMAN		DDX1
958	958	1239	0.999	572	sp|Q96I24|FUBP3_HUMAN	FUBP3	Q96I24	Far upstream element-binding protein 3	FUBP3_HUMAN		FUBP3
1230	1230	1230	0.999	876	sp|Q9BXP5|SRRT_HUMAN	SRRT	Q9BXP5	Serrate RNA effector molecule homolog	SRRT_HUMAN		SRRT
794	794	794	0.999	736	sp|O00429|DNM1L_HUMAN	DNM1L	O00429	Dynamin-1-like protein	DNM1L_HUMAN		DNM1L
1200	1200	1200	0.999	1338	sp|O15067|PUR4_HUMAN	PFAS	O15067	Phosphoribosylformylglycinamidine synthase	PUR4_HUMAN		PFAS
361	346	361	0.999	2468	sp|P46821|MAP1B_HUMAN	MAP1B	P46821	Microtubule-associated protein 1B	MAP1B_HUMAN		MAP1B
1189	1189	1189	0.999	775	sp|P47897|SYQ_HUMAN	QARS1	P47897	Glutamine--tRNA ligase	SYQ_HUMAN		QARS
1988	1988	1988	0.999	266	sp|P62424|RL7A_HUMAN	RPL7A	P62424	Large ribosomal subunit protein eL8	RL7A_HUMAN		RPL7A
892	892	953	0.999	704	sp|Q12931|TRAP1_HUMAN	TRAP1	Q12931	Heat shock protein 75 kDa, mitochondrial	TRAP1_HUMAN		TRAP1
662	447	662	0.999	2554	sp|Q93008|USP9X_HUMAN	USP9X	Q93008	Probable ubiquitin carboxyl-terminal hydrolase FAF-X	USP9X_HUMAN		USP9X
942	942	942	0.999	737	sp|O00469|PLOD2_HUMAN	PLOD2	O00469	Procollagen-lysine,2-oxoglutarate 5-dioxygenase 2	PLOD2_HUMAN		PLOD2
1922	1099	1922	0.999	623	sp|O60506|HNRPQ_HUMAN	SYNCRIP	O60506	Heterogeneous nuclear ribonucleoprotein Q	HNRPQ_HUMAN		SYNCRIP
1069	1031	1069	0.999	1220	sp|O94979|SC31A_HUMAN	SEC31A	O94979	Protein transport protein Sec31A	SC31A_HUMAN		SEC31A
280	277	280	0.999	1464	sp|P02452|CO1A1_HUMAN	COL1A1	P02452	Collagen alpha-1(I) chain	CO1A1_HUMAN		COL1A1
2018	525	2018	0.999	298	sp|P05141|ADT2_HUMAN	SLC25A5	P05141	ADP/ATP translocase 2	ADT2_HUMAN		SLC25A5
2016	1950	2016	0.999	798	sp|P05556|ITB1_HUMAN	ITGB1	P05556	Integrin beta-1	ITB1_HUMAN		ITGB1
1441	1440	1441	0.999	700	sp|P17655|CAN2_HUMAN	CAPN2	P17655	Calpain-2 catalytic subunit	CAN2_HUMAN		CAPN2
83	78	1235	0.999	639	sp|P54652|HSP72_HUMAN	HSPA2	P54652	Heat shock-related 70 kDa protein 2	HSP72_HUMAN		HSPA2
1705	1580	1705	0.999	894	sp|Q12906|ILF3_HUMAN	ILF3	Q12906	Interleukin enhancer-binding factor 3	ILF3_HUMAN		ILF3
1083	1083	1345	0.999	972	sp|Q15029|U5S1_HUMAN	EFTUD2	Q15029	116 kDa U5 small nuclear ribonucleoprotein component	U5S1_HUMAN		EFTUD2
482	482	482	0.999	683	sp|Q15582|BGH3_HUMAN	TGFBI	Q15582	Transforming growth factor-beta-induced protein ig-h3	BGH3_HUMAN		TGFBI
868	423	868	0.999	1253	sp|Q7L576|CYFP1_HUMAN	CYFIP1	Q7L576	Cytoplasmic FMR1-interacting protein 1	CYFP1_HUMAN		CYFIP1
635	579	635	0.999	2896	sp|Q9Y520|PRC2C_HUMAN	PRRC2C	Q9Y520	Protein PRRC2C	PRC2C_HUMAN		PRRC2C
1371	1317	1371	0.999	1097	sp|O00410|IPO5_HUMAN	IPO5	O00410	Importin-5	IPO5_HUMAN		IPO5
1221	1184	1288	0.999	430	sp|P05783|K1C18_HUMAN	KRT18	P05783	Keratin, type I cytoskeletal 18	K1C18_HUMAN		KRT18
693	693	693	0.999	1609	sp|P11047|LAMC1_HUMAN	LAMC1	P11047	Laminin subunit gamma-1	LAMC1_HUMAN		LAMC1
515	377	2006	0.999	298	sp|P12236|ADT3_HUMAN	SLC25A6	P12236	ADP/ATP translocase 3	ADT3_HUMAN		SLC25A6
1354	1354	1354	0.999	501	sp|P14868|SYDC_HUMAN	DARS1	P14868	Aspartate--tRNA ligase, cytoplasmic	SYDC_HUMAN		DARS
305	272	305	0.999	3433	sp|P46939|UTRN_HUMAN	UTRN	P46939	Utrophin	UTRN_HUMAN		UTRN
1372	1372	1372	0.999	747	sp|Q1KMD3|HNRL2_HUMAN	HNRNPUL2	Q1KMD3	Heterogeneous nuclear ribonucleoprotein U-like protein 2	HNRL2_HUMAN		HNRNPUL2
655	599	655	0.999	1235	sp|Q9H2G2|SLK_HUMAN	SLK	Q9H2G2	STE20-like serine/threonine-protein kinase	SLK_HUMAN		SLK
1208	292	1208	0.999	634	sp|Q9NVI7|ATD3A_HUMAN	ATAD3A	Q9NVI7	ATPase family AAA domain-containing protein 3A	ATD3A_HUMAN		ATAD3A
1050	1050	1050	0.999	463	sp|Q9Y230|RUVB2_HUMAN	RUVBL2	Q9Y230	RuvB-like 2	RUVB2_HUMAN		RUVBL2
678	678	678	0.999	941	sp|O94906|PRP6_HUMAN	PRPF6	O94906	Pre-mRNA-processing factor 6	PRP6_HUMAN		PRPF6
643	643	643	0.999	782	sp|P06396|GELS_HUMAN	GSN	P06396	Gelsolin	GELS_HUMAN		GSN
426	263	1960	0.999	284	sp|P07951|TPM2_HUMAN	TPM2	P07951	Tropomyosin beta chain	TPM2_HUMAN		TPM2
594	506	929	0.999	590	sp|P13647|K2C5_HUMAN	KRT5	P13647	Keratin, type II cytoskeletal 5	K2C5_HUMAN		KRT5
1238	940	1238	0.999	906	sp|P35221|CTNA1_HUMAN	CTNNA1	P35221	Catenin alpha-1	CTNA1_HUMAN		CTNNA1
193	188	193	0.999	2871	sp|P35555|FBN1_HUMAN	FBN1	P35555	Fibrillin-1	FBN1_HUMAN		FBN1
1466	1466	1466	0.999	900	sp|P56192|SYMC_HUMAN	MARS1	P56192	Methionine--tRNA ligase, cytoplasmic	SYMC_HUMAN		MARS
910	909	910	0.999	998	sp|P78347|GTF2I_HUMAN	GTF2I	P78347	General transcription factor II-I	GTF2I_HUMAN		GTF2I
1751	1751	1751	0.999	876	sp|Q14974|IMB1_HUMAN	KPNB1	Q14974	Importin subunit beta-1	IMB1_HUMAN		KPNB1
1021	1021	1021	0.999	1031	sp|Q7L014|DDX46_HUMAN	DDX46	Q7L014	Probable ATP-dependent RNA helicase DDX46	DDX46_HUMAN		DDX46
1177	1034	1390	0.999	644	sp|Q96AE4|FUBP1_HUMAN	FUBP1	Q96AE4	Far upstream element-binding protein 1	FUBP1_HUMAN		FUBP1
1047	763	1047	0.999	1052	sp|O60264|SMCA5_HUMAN	SMARCA5	O60264	SWI/SNF-related matrix-associated actin-dependent regulator of chromatir	SMCA5_HUMAN		SMARCA5
711	711	711	0.999	1197	sp|O95347|SMC2_HUMAN	SMC2	O95347	Structural maintenance of chromosomes protein 2	SMC2_HUMAN		SMC2
616	287	1328	0.999	472	sp|P02533|K1C14_HUMAN	KRT14	P02533	Keratin, type I cytoskeletal 14	K1C14_HUMAN		KRT14
1480	1480	1480	0.999	1010	sp|P22102|PUR2_HUMAN	GART	P22102	Trifunctional purine biosynthetic protein adenosine-3	PUR2_HUMAN		GART
1032	1032	1032	0.999	959	sp|P36776|LONM_HUMAN	LONP1	P36776	Lon protease homolog, mitochondrial	LONM_HUMAN		LONP1
1241	1241	1241	0.999	814	sp|P55884|EIF3B_HUMAN	EIF3B	P55884	Eukaryotic translation initiation factor 3 subunit B	EIF3B_HUMAN		EIF3B
570	570	570	0.999	1411	sp|Q15075|EEA1_HUMAN	EEA1	Q15075	Early endosome antigen 1	EEA1_HUMAN		EEA1
827	577	827	0.999	915	sp|Q15424|SAFB1_HUMAN	SAFB	Q15424	Scaffold attachment factor B1	SAFB1_HUMAN		SAFB
1406	1406	1406	0.999	746	sp|Q96TA1|NIBA2_HUMAN	NIBAN2	Q96TA1	Protein Niban 2	NIBA2_HUMAN		FAM129B
2253	467	2757	0.999	372	sp|P09651|ROA1_HUMAN	HNRNPA1	P09651	Heterogeneous nuclear ribonucleoprotein A1	ROA1_HUMAN		HNRNPA1
1629	327	1629	0.999	636	sp|P11940|PABP1_HUMAN	PABPC1	P11940	Polyadenylate-binding protein 1	PABP1_HUMAN		PABPC1
1595	1535	1595	0.999	475	sp|Q01518|CAP1_HUMAN	CAP1	Q01518	Adenylyl cyclase-associated protein 1	CAP1_HUMAN		CAP1
1232	1232	1232	0.999	793	sp|Q05682|CALD1_HUMAN	CALD1	Q05682	Caldesmon	CALD1_HUMAN		CALD1
746	676	746	0.999	2346	sp|Q13085|ACACA_HUMAN	ACACA	Q13085	Acetyl-CoA carboxylase 1	ACACA_HUMAN		ACACA
1279	1279	1279	0.999	1217	sp|Q15393|SF3B3_HUMAN	SF3B3	Q15393	Splicing factor 3B subunit 3	SF3B3_HUMAN		SF3B3
583	533	583	0.999	1190	sp|Q9UDY2|ZO2_HUMAN	TJP2	Q9UDY2	Tight junction protein ZO-2	ZO2_HUMAN		TJP2
1110	1098	1110	0.999	999	sp|Q9Y4L1|HYOU1_HUMAN	HYOU1	Q9Y4L1	Hypoxia up-regulated protein 1	HYOU1_HUMAN		HYOU1
1436	1382	1436	0.999	795	sp|O43143|DHX15_HUMAN	DHX15	O43143	ATP-dependent RNA helicase DHX15	DHX15_HUMAN		DHX15
890	890	1107	0.999	839	sp|O95757|HS74L_HUMAN	HSPA4L	O95757	Heat shock 70 kDa protein 4L	HS74L_HUMAN		HSPA4L
1671	277	1671	0.999	558	sp|P00367|DHE3_HUMAN	GLUD1	P00367	Glutamate dehydrogenase 1, mitochondrial	DHE3_HUMAN		GLUD1
1264	1181	1264	0.999	760	sp|P02786|TFR1_HUMAN	TFRC	P02786	Transferrin receptor protein 1	TFR1_HUMAN		TFRC
972	972	972	0.999	323	sp|P12429|ANXA3_HUMAN	ANXA3	P12429	Annexin A3	ANXA3_HUMAN		ANXA3
1822	1822	1822	0.999	248	sp|P18124|RL7_HUMAN	RPL7	P18124	Large ribosomal subunit protein uL30	RL7_HUMAN		RPL7
1089	1089	1089	0.999	808	sp|P25205|MCM3_HUMAN	MCM3	P25205	DNA replication licensing factor MCM3	MCM3_HUMAN		MCM3
2210	2210	2210	0.999	297	sp|P46777|RL5_HUMAN	RPL5	P46777	Large ribosomal subunit protein uL18	RL5_HUMAN		RPL5
1421	1421	1421	0.999	971	sp|P55060|XPO2_HUMAN	CSE1L	P55060	Exportin-2	XPO2_HUMAN		CSE1L
1205	1205	1205	0.999	1226	sp|P55265|DSRAD_HUMAN	ADAR	P55265	Double-stranded RNA-specific adenosine deaminase	DSRAD_HUMAN		ADAR
2018	914	2018	0.999	406	sp|P60842|IF4A1_HUMAN	EIF4A1	P60842	Eukaryotic initiation factor 4A-I	IF4A1_HUMAN		EIF4A1
2865	1067	2865	0.999	248	sp|P67936|TPM4_HUMAN	TPM4	P67936	Tropomyosin alpha-4 chain	TPM4_HUMAN		TPM4
1157	1157	1157	0.999	767	sp|Q08J23|NSUN2_HUMAN	NSUN2	Q08J23	RNA cytosine C(5)-methyltransferase NSUN2	NSUN2_HUMAN		NSUN2
1100	1100	1100	0.999	457	sp|Q13451|FKBP5_HUMAN	FKBP5	Q13451	Peptidyl-prolyl cis-trans isomerase FKBP5	FKBP5_HUMAN		FKBP5
750	727	750	0.999	1555	sp|Q9NYU2|UGGG1_HUMAN	UGGT1	Q9NYU2	UDP-glucose:glycoprotein glucosyltransferase 1	UGGG1_HUMAN		UGGT1
467	467	467	0.999	1786	sp|P07942|LAMB1_HUMAN	LAMB1	P07942	Laminin subunit beta-1	LAMB1_HUMAN		LAMB1
1209	1051	1209	0.999	765	sp|P11387|TOP1_HUMAN	TOP 1.00	P11387	DNA topoisomerase 1	TOP1_HUMAN		TOP 1.00
1413	1413	1413	0.999	515	sp|P11413|G6PD_HUMAN	G6PD	P11413	Glucose-6-phosphate 1-dehydrogenase	G6PD_HUMAN		G6PD
800	800	800	0.999	1178	sp|P11498|PYC_HUMAN	PC	P11498	Pyruvate carboxylase, mitochondrial	PYC_HUMAN		PC
1194	1194	1194	0.999	967	sp|P15144|AMPN_HUMAN	ANPEP	P15144	Aminopeptidase N	AMPN_HUMAN		ANPEP
1709	1709	1709	0.999	739	sp|P41250|GARS_HUMAN	GARS1	P41250	Glycine--tRNA ligase	GARS_HUMAN		GARS
2126	2116	2126	0.999	545	sp|P49368|TCPG_HUMAN	CCT3	P49368	T-complex protein 1 subunit gamma	TCPG_HUMAN		CCT3
996	996	1261	0.999	847	sp|P06737|PYGL_HUMAN	PYGL	P06737	Glycogen phosphorylase, liver form	PYGL_HUMAN		PYGL
2273	2273	2273	0.999	556	sp|P17987|TCPA_HUMAN	TCP1	P17987	T-complex protein 1 subunit alpha	TCPA_HUMAN		TCP1
1476	1476	1476	0.999	847	sp|P43243|MATR3_HUMAN	MATR3	P43243	Matrin-3	MATR3_HUMAN		MATR3
1964	481	4198	0.999	448	sp|P68366|TBA4A_HUMAN	TUBA4A	P68366	Tubulin alpha-4A chain	TBA4A_HUMAN		TUBA4A
1046	188	6641	0.999	445	sp|P68371|TBB4B_HUMAN	TUBB4B	P68371	Tubulin beta-4B chain	TBB4B_HUMAN		TUBB4B
894	894	894	0.999	1729	sp|Q9C0C2|TB182_HUMAN	TNKS1BP1	Q9C0C2	182 kDa tankyrase-1-binding protein	TB182_HUMAN		TNKS1BP1
756	715	756	0.999	759	sp|Q9UHB6|LIMA1_HUMAN	LIMA1	Q9UHB6	LIM domain and actin-binding protein 1	LIMA1_HUMAN		LIMA1
1118	1033	1206	0.999	644	sp|P04264|K2C1_HUMAN	KRT1	P04264	Keratin, type II cytoskeletal 1	K2C1_HUMAN		KRT1
6821	1074	6821	0.999	444	sp|P07437|TBB5_HUMAN	TUBB	P07437	Tubulin beta chain	TBB5_HUMAN		TUBB
2006	2006	2006	0.999	609	sp|P12956|XRCC6_HUMAN	XRCC6	P12956	X-ray repair cross-complementing protein 6	XRCC6_HUMAN		XRCC6
1795	1795	1795	0.999	732	sp|P13010|XRCC5_HUMAN	XRCC5	P13010	X-ray repair cross-complementing protein 5	XRCC5_HUMAN		XRCC5
3740	3236	3740	0.999	353	sp|P22626|ROA2_HUMAN	HNRNPA2B1	P22626	Heterogeneous nuclear ribonucleoproteins A2/B1	ROA2_HUMAN		HNRNPA2B1
735	735	735	0.999	1616	sp|P26358|DNMT1_HUMAN	DNMT1	P26358	DNA (cytosine-5)-methyltransferase 1	DNMT1_HUMAN		DNMT1
1200	1200	1200	0.999	491	sp|P43490|NAMPT_HUMAN	NAMPT	P43490	Nicotinamide phosphoribosyltransferase	NAMPT_HUMAN		NAMPT
1297	1297	1297	0.999	904	sp|P49736|MCM2_HUMAN	MCM2	P49736	DNA replication licensing factor MCM2	MCM2_HUMAN		MCM2
2234	2121	2234	0.999	539	sp|P50991|TCPD_HUMAN	CCT4	P50991	T-complex protein 1 subunit delta	TCPD_HUMAN		CCT4
2042	1813	2042	0.999	784	sp|Q01813|PFKAP_HUMAN	PFKP	Q01813	ATP-dependent 6-phosphofructokinase, platelet type	PFKAP_HUMAN		PFKP
666	666	666	0.999	1782	sp|Q02952|AKA12_HUMAN	AKAP12	Q02952	A-kinase anchor protein 12	AKA12_HUMAN		AKAP12
1495	1495	1495	0.999	1140	sp|Q16531|DDB1_HUMAN	DDB1	Q16531	DNA damage-binding protein 1	DDB1_HUMAN		DDB1
944	944	944	0.999	1179	sp|Q92896|GSLG1_HUMAN	GLG1	Q92896	Golgi apparatus protein 1	GSLG1_HUMAN		GLG1
1038	1038	1038	0.999	1341	sp|Q9UKV3|ACINU_HUMAN	ACIN1	Q9UKV3	Apoptotic chromatin condensation inducer in the nucleus	ACINU_HUMAN		ACIN1
1045	1040	1045	0.999	968	sp|O60716|CTND1_HUMAN	CTNND1	O60716	Catenin delta-1	CTND1_HUMAN		CTNND1
1248	1248	1248	0.999	611	sp|P23588|IF4B_HUMAN	EIF4B	P23588	Eukaryotic translation initiation factor 4B	IF4B_HUMAN		EIF4B
1625	1625	1625	0.999	968	sp|P49588|SYAC_HUMAN	AARS1	P49588	Alanine--tRNA ligase, cytoplasmic	SYAC_HUMAN		AARS
1654	1014	1654	0.999	919	sp|P55786|PSA_HUMAN	NPEPPS	P55786	Puromycin-sensitive aminopeptidase	PSA_HUMAN		NPEPPS
1272	1272	1272	0.999	893	sp|Q14764|MVP_HUMAN	MVP	Q14764	Major vault protein	MVP_HUMAN		MVP
1146	798	1146	0.999	1912	sp|Q14839|CHD4_HUMAN	CHD4	Q14839	Chromodomain-helicase-DNA-binding protein 4	CHD4_HUMAN		CHD4
873	873	873	0.999	1312	sp|Q92878|RAD50_HUMAN	RAD50	Q92878	DNA repair protein RAD50	RAD50_HUMAN		RAD50
4064	308	4064	0.999	449	sp|Q9BQE3|TBA1C_HUMAN	TUBA1C	Q9BQE3	Tubulin alpha-1C chain	TBA1C_HUMAN		TUBA1C
808	67	1050	0.999	564	sp|P02538|K2C6A_HUMAN	KRT6A	P02538	Keratin, type II cytoskeletal 6A	K2C6A_HUMAN		KRT6A
811	721	811	0.999	1170	sp|P07996|TSP1_HUMAN	THBS1	P07996	Thrombospondin-1	TSP1_HUMAN		THBS1
1309	1195	1632	0.999	469	sp|P08729|K2C7_HUMAN	KRT7	P08729	Keratin, type II cytoskeletal 7	K2C7_HUMAN		KRT7
1736	1314	1736	0.999	445	sp|P50395|GDIB_HUMAN	GDI2	P50395	Rab GDP dissociation inhibitor beta	GDIB_HUMAN		GDI2
1274	1274	1274	0.999	821	sp|Q14566|MCM6_HUMAN	MCM6	Q14566	DNA replication licensing factor MCM6	MCM6_HUMAN		MCM6
1458	1283	1458	0.999	1230	sp|Q86VP6|CAND1_HUMAN	CAND1	Q86VP6	Cullin-associated NEDD8-dissociated protein 1	CAND1_HUMAN		CAND1
1718	1718	1718	0.999	543	sp|Q99832|TCPH_HUMAN	CCT7	Q99832	T-complex protein 1 subunit eta	TCPH_HUMAN		CCT7
979	979	979	0.999	1288	sp|Q9NTJ3|SMC4_HUMAN	SMC4	Q9NTJ3	Structural maintenance of chromosomes protein 4	SMC4_HUMAN		SMC4

2010	2010	2010	0.999	607	sp|P04843|RPN1_HUMAN	RPN1	P04843	Dolichyl-diphosphooligosaccharide--protein glycosyltransferase subunit 1	RPN1_HUMAN		RPN1
1659	1659	1659	0.999	320	sp|P08758|ANXA5_HUMAN	ANXA5	P08758	Annexin A5	ANXA5_HUMAN		ANXA5
1344	1344	1344	0.999	734	sp|P33992|MCM5_HUMAN	MCM5	P33992	DNA replication licensing factor MCM5	MCM5_HUMAN		MCM5
1564	1564	1564	0.999	528	sp|P54577|SYYC_HUMAN	YARS1	P54577	Tyrosine--tRNA ligase, cytoplasmic	SYYC_HUMAN		YARS
1871	1079	1938	0.999	432	sp|Q04695|K1C17_HUMAN	KRT17	Q04695	Keratin, type I cytoskeletal 17	K1C17_HUMAN		KRT17
2371	2371	2371	0.999	493	sp|Q16658|FSCN1_HUMAN	FSCN1	Q16658	Fascin	FSCN1_HUMAN		FSCN1
1709	1496	1709	0.999	711	sp|Q92945|FUBP2_HUMAN	KHSRP	Q92945	Far upstream element-binding protein 2	FUBP2_HUMAN		KHSRP
178	176	178	0.999	3063	sp|Q99715|COCA1_HUMAN	COL12A1	Q99715	Collagen alpha-1(XII) chain	COCA1_HUMAN		COL12A1
1284	1220	1284	0.999	783	sp|Q9NR30|DDX21_HUMAN	DDX21	Q9NR30	Nucleolar RNA helicase 2	DDX21_HUMAN		DDX21
1106	1106	1106	0.999	1012	sp|Q9NSE4|SYIM_HUMAN	IARS2	Q9NSE4	Isoleucine--tRNA ligase, mitochondrial	SYIM_HUMAN		IARS2
1411	1411	1411	0.999	1047	sp|Q9Y5B9|SP16H_HUMAN	SUPT16H	Q9Y5B9	FACT complex subunit SPT16	SP16H_HUMAN		SUPT16H
1709	743	1768	0.999	662	sp|O00571|DDX3X_HUMAN	DDX3X	O00571	ATP-dependent RNA helicase DDX3X	DDX3X_HUMAN		DDX3X
3524	3017	3524	0.999	332	sp|P00338|LDHA_HUMAN	LDHA	P00338	L-lactate dehydrogenase A chain	LDHA_HUMAN		LDHA
1451	1414	1451	0.999	412	sp|P00966|ASSY_HUMAN	ASS1	P00966	Argininosuccinate synthase	ASSY_HUMAN		ASS1
1852	1517	1852	0.999	1042	sp|P16615|AT2A2_HUMAN	ATP2A2	P16615	Sarcoplasmic/endoplasmic reticulum calcium ATPase 2	AT2A2_HUMAN		ATP2A2
2660	2660	2660	0.999	535	sp|P78371|TCPB_HUMAN	CCT2	P78371	T-complex protein 1 subunit beta	TCPB_HUMAN		CCT2
936	936	1441	0.999	620	sp|Q03252|LMNB2_HUMAN	LMNB2	Q03252	Lamin-B2	LMNB2_HUMAN		LMNB2
1513	1513	1513	0.999	868	sp|Q8WUM4|PDC6I_HUMAN	PDCD6IP	Q8WUM4	Programmed cell death 6-interacting protein	PDC6I_HUMAN		PDCD6IP
1805	152	1805	0.999	913	sp|Q99613|EIF3C_HUMAN	EIF3C	Q99613	Eukaryotic translation initiation factor 3 subunit C	EIF3C_HUMAN		EIF3C
1030	1015	1030	0.999	1272	sp|O60610|DIAP1_HUMAN	DIAPH1	O60610	Protein diaphanous homolog 1	DIAP1_HUMAN		DIAPH1
3663	479	3663	0.999	364	sp|P04075|ALDOA_HUMAN	ALDOA	P04075	Fructose-bisphosphate aldolase A	ALDOA_HUMAN		ALDOA
684	683	1928	0.999	583	sp|P35241|RADI_HUMAN	RDX	P35241	Radixin	RADI_HUMAN		RDX
1195	1195	1195	0.999	906	sp|P35606|COPB2_HUMAN	COPB2	P35606	Coatomer subunit beta'	COPB2_HUMAN		COPB2
1843	1843	1956	0.999	541	sp|P48643|TCPE_HUMAN	CCT5	P48643	T-complex protein 1 subunit epsilon	TCPE_HUMAN		CCT5
2086	2086	2086	0.999	835	sp|Q13263|TIF1B_HUMAN	TRIM28	Q13263	Transcription intermediary factor 1-beta	TIF1B_HUMAN		TRIM28
1319	1319	1319	0.999	1328	sp|Q9BQG0|MBB1A_HUMAN	MYBBP1A	Q9BQG0	Myb-binding protein 1A	MBB1A_HUMAN		MYBBP1A
1251	1251	1251	0.999	763	sp|P40939|ECHA_HUMAN	HADHA	P40939	Trifunctional enzyme subunit alpha, mitochondrial	ECHA_HUMAN		HADHA
8145	24	8145	0.999	375	sp|P60709|ACTB_HUMAN	ACTB	P60709	Actin, cytoplasmic 1	ACTB_HUMAN		ACTB
1806	1806	1806	0.999	550	sp|Q14247|SRC8_HUMAN	CTTN	Q14247	Src substrate cortactin	SRC8_HUMAN		CTTN
1380	1245	1520	0.999	858	sp|Q92598|HS105_HUMAN	HSPH1	Q92598	Heat shock protein 105 kDa	HS105_HUMAN		HSPH1
833	833	833	0.999	1217	sp|Q9UQE7|SMC3_HUMAN	SMC3	Q9UQE7	Structural maintenance of chromosomes protein 3	SMC3_HUMAN		SMC3
827	809	827	0.999	687	sp|P21980|TGM2_HUMAN	TGM2	P21980	Protein-glutamine gamma-glutamyltransferase 2	TGM2_HUMAN		TGM2
1129	1129	1129	0.999	719	sp|P33993|MCM7_HUMAN	MCM7	P33993	DNA replication licensing factor MCM7	MCM7_HUMAN		MCM7
1069	1069	1069	0.999	2032	sp|Q14008|CKAP5_HUMAN	CKAP5	Q14008	Cytoskeleton-associated protein 5	CKAP5_HUMAN		CKAP5
1878	1878	1878	0.999	586	sp|Q9UHD8|SEPT9_HUMAN	SEPTIN9	Q9UHD8	Septin-9	SEPT9_HUMAN		SEPT9
1487	1261	1487	0.999	917	sp|P19367|HXK1_HUMAN	HK1	P19367	Hexokinase-1	HXK1_HUMAN		HK1
1335	1280	1335	0.999	863	sp|P33991|MCM4_HUMAN	MCM4	P33991	DNA replication licensing factor MCM4	MCM4_HUMAN		MCM4
858	798	858	0.999	2671	sp|Q14573|ITPR3_HUMAN	ITPR3	Q14573	Inositol 1,4,5-trisphosphate receptor type 3	ITPR3_HUMAN		ITPR3
563	563	563	0.999	2322	sp|Q6UVK1|CSPG4_HUMAN	CSPG4	Q6UVK1	Chondroitin sulfate proteoglycan 4	CSPG4_HUMAN		CSPG4
2457	2457	2457	0.999	606	sp|O75083|WDR1_HUMAN	WDR1	O75083	WD repeat-containing protein 1	WDR1_HUMAN		WDR1
2701	2680	2701	0.999	346	sp|P04083|ANXA1_HUMAN	ANXA1	P04083	Annexin A1	ANXA1_HUMAN		ANXA1
7618	7289	7618	0.999	335	sp|P04406|G3P_HUMAN	GAPDH	P04406	Glyceraldehyde-3-phosphate dehydrogenase	G3P_HUMAN		GAPDH
2131	2131	2131	0.999	645	sp|P13667|PDIA4_HUMAN	PDIA4	P13667	Protein disulfide-isomerase A4	PDIA4_HUMAN		PDIA4
2210	718	2210	0.999	391	sp|P38159|RBMX_HUMAN	RBMX	P38159	RNA-binding motif protein, X chromosome	RBMX_HUMAN		RBMX
1518	1518	1518	0.999	1488	sp|Q13428|TCOF_HUMAN	TCOF1	Q13428	Treacle protein	TCOF_HUMAN		TCOF1
942	923	942	0.999	1233	sp|Q14683|SMC1A_HUMAN	SMC1A	Q14683	Structural maintenance of chromosomes protein 1A	SMC1A_HUMAN		SMC1A
2787	2787	2787	0.999	548	sp|P50990|TCPQ_HUMAN	CCT8	P50990	T-complex protein 1 subunit theta	TCPQ_HUMAN		CCT8
2982	2982	2982	0.999	1058	sp|P22314|UBA1_HUMAN	UBA1	P22314	Ubiquitin-like modifier-activating enzyme 1	UBA1_HUMAN		UBA1
1874	1649	1874	0.999	723	sp|P26639|SYTC_HUMAN	TARS1	P26639	Threonine--tRNA ligase 1, cytoplasmic	SYTC_HUMAN		TARS
2997	2997	2997	0.999	264	sp|P61247|RS3A_HUMAN	RPS3A	P61247	Small ribosomal subunit protein eS1	RS3A_HUMAN		RPS3A
1553	1267	1698	0.999	483	sp|P05787|K2C8_HUMAN	KRT8	P05787	Keratin, type II cytoskeletal 8	K2C8_HUMAN		KRT8
1676	1675	1676	0.999	1152	sp|P27816|MAP4_HUMAN	MAP4	P27816	Microtubule-associated protein 4	MAP4_HUMAN		MAP4
6701	75	6701	0.999	462	sp|P68104|EF1A1_HUMAN	EEF1A1	P68104	Elongation factor 1-alpha 1	EF1A1_HUMAN		EEF1A1
1477	1477	1477	0.999	758	sp|Q16891|MIC60_HUMAN	IMMT	Q16891	MICOS complex subunit MIC60	MIC60_HUMAN		IMMT
1211	1211	1922	0.999	614	sp|P17844|DDX5_HUMAN	DDX5	P17844	Probable ATP-dependent RNA helicase DDX5	DDX5_HUMAN		DDX5
2742	2742	2742	0.999	1101	sp|P53396|ACLY_HUMAN	ACLY	P53396	ATP-citrate synthase	ACLY_HUMAN		ACLY
971	971	971	0.999	5183	sp|Q5T4S7|UBR4_HUMAN	UBR4	Q5T4S7	E3 ubiquitin-protein ligase UBR4	UBR4_HUMAN		UBR4
1345	1285	1375	0.999	1136	sp|O43795|MYO1B_HUMAN	MYO1B	O43795	Unconventional myosin-Ib	MYO1B_HUMAN		MYO1B
2279	1542	2279	0.999	1023	sp|P05023|AT1A1_HUMAN	ATP1A1	P05023	Sodium/potassium-transporting ATPase subunit alpha-1	AT1A1_HUMAN		ATP1A1
2687	2589	2687	0.999	935	sp|P11586|C1TC_HUMAN	MTHFD1	P11586	C-1-tetrahydrofolate synthase, cytoplasmic	C1TC_HUMAN		MTHFD1
1456	1217	1456	0.999	963	sp|P33176|KINH_HUMAN	KIF5B	P33176	Kinesin-1 heavy chain	KINH_HUMAN		KIF5B
2249	2212	2249	0.999	602	sp|Q07065|CKAP4_HUMAN	CKAP4	Q07065	Cytoskeleton-associated protein 4	CKAP4_HUMAN		CKAP4
476	476	1006	0.999	2725	sp|Q14315|FLNC_HUMAN	FLNC	Q14315	Filamin-C	FLNC_HUMAN		FLNC
2671	0	3190	0.999	641	sp|P0DMV8|HS71A_HUMAN	HSPA1A	P0DMV8	Heat shock 70 kDa protein 1A	HS71A_HUMAN	sp|P0DMV9|HS71B_HUMAN	HSPA1A
2478	2478	2478	0.999	553	sp|P25705|ATPA_HUMAN	ATP5F1A	P25705	ATP synthase subunit alpha, mitochondrial	ATPA_HUMAN		ATP5A1
2785	2785	2785	0.999	427	sp|P36578|RL4_HUMAN	RPL4	P36578	Large ribosomal subunit protein uL4	RL4_HUMAN		RPL4
2591	2591	2591	0.999	944	sp|Q14697|GANAB_HUMAN	GANAB	Q14697	Neutral alpha-glucosidase AB	GANAB_HUMAN		GANAB
2139	2136	2283	0.999	471	sp|Q15233|NONO_HUMAN	NONO	Q15233	Non-POU domain-containing octamer-binding protein	NONO_HUMAN		NONO
2238	1527	2238	0.999	729	sp|Q92841|DDX17_HUMAN	DDX17	Q92841	Probable ATP-dependent RNA helicase DDX17	DDX17_HUMAN		DDX17
1496	1496	1496	0.999	1176	sp|Q9P2J5|SYLC_HUMAN	LARS1	Q9P2J5	Leucine--tRNA ligase, cytoplasmic	SYLC_HUMAN		LARS
1887	1887	1887	0.999	2752	sp|Q9UQ35|SRRM2_HUMAN	SRRM2	Q9UQ35	Serine/arginine repetitive matrix protein 2	SRRM2_HUMAN		SRRM2
2857	2795	2857	0.999	623	sp|P29401|TKT_HUMAN	TKT	P29401	Transketolase	TKT_HUMAN		TKT
2947	2947	2947	0.999	505	sp|P30101|PDIA3_HUMAN	PDIA3	P30101	Protein disulfide-isomerase A3	PDIA3_HUMAN		PDIA3
1212	1180	1242	0.999	1063	sp|O00159|MYO1C_HUMAN	MYO1C	O00159	Unconventional myosin-Ic	MYO1C_HUMAN		MYO1C
414	414	414	0.999	4391	sp|P98160|PGBM_HUMAN	HSPG2	P98160	Basement membrane-specific heparan sulfate proteoglycan core protein	PGBM_HUMAN		HSPG2
4297	3892	4297	0.999	417	sp|P00558|PGK1_HUMAN	PGK 1.00	P00558	Phosphoglycerate kinase 1	PGK1_HUMAN		PGK 1.00
1998	1998	1998	0.999	1264	sp|P26640|SYVC_HUMAN	VARS1	P26640	Valine--tRNA ligase	SYVC_HUMAN		VARS
1510	1510	1611	0.999	2225	sp|P27708|PYR1_HUMAN	CAD	P27708	CAD protein	PYR1_HUMAN		CAD
1635	1635	1635	0.999	1262	sp|P41252|SYIC_HUMAN	IARS1	P41252	Isoleucine--tRNA ligase, cytoplasmic	SYIC_HUMAN		IARS
1780	1780	1780	0.999	1304	sp|O75533|SF3B1_HUMAN	SF3B1	O75533	Splicing factor 3B subunit 1	SF3B1_HUMAN		SF3B1
3344	3344	3344	0.999	508	sp|P07237|PDIA1_HUMAN	P4HB	P07237	Protein disulfide-isomerase	PDIA1_HUMAN		P4HB
2317	2086	3169	0.999	586	sp|P15311|EZRI_HUMAN	EZR	P15311	Ezrin	EZRI_HUMAN		EZR
312	312	312	0.999	2201	sp|P24821|TENA_HUMAN	TNC	P24821	Tenascin	TENA_HUMAN		TNC
1366	1356	1366	0.999	2136	sp|O75643|U520_HUMAN	SNRNP200	O75643	U5 small nuclear ribonucleoprotein 200 kDa helicase	U520_HUMAN		SNRNP200
2970	2911	2970	0.999	558	sp|P06744|G6PI_HUMAN	GPI	P06744	Glucose-6-phosphate isomerase	G6PI_HUMAN		GPI
2493	2041	2546	0.999	586	sp|P20700|LMNB1_HUMAN	LMNB1	P20700	Lamin-B1	LMNB1_HUMAN		LMNB1
2520	2520	2576	0.999	679	sp|P38646|GRP75_HUMAN	HSPA9	P38646	Stress-70 protein, mitochondrial	GRP75_HUMAN		HSPA9
4195	4195	4195	0.999	825	sp|Q00839|HNRPU_HUMAN	HNRNPU	Q00839	Heterogeneous nuclear ribonucleoprotein U	HNRPU_HUMAN		HNRNPU
2878	2734	2878	0.999	707	sp|P23246|SFPQ_HUMAN	SFPQ	P23246	Splicing factor, proline- and glutamine-rich	SFPQ_HUMAN		SFPQ
1058	1058	1058	0.999	2491	sp|P11717|MPRI_HUMAN	IGF2R	P11717	Cation-independent mannose-6-phosphate receptor	MPRI_HUMAN		IGF2R
2850	2850	2850	0.999	806	sp|P55072|TERA_HUMAN	VCP	P55072	Transitional endoplasmic reticulum ATPase	TERA_HUMAN		VCP
2303	1846	2303	0.999	1599	sp|Q04637|IF4G1_HUMAN	EIF4G1	Q04637	Eukaryotic translation initiation factor 4 gamma 1	IF4G1_HUMAN		EIF4G1
528	528	531	0.999	5795	sp|Q8IVF2|AHNK2_HUMAN	AHNAK2	Q8IVF2	Protein AHNAK2	AHNK2_HUMAN		AHNAK2
4421	1025	4421	0.999	339	sp|P07355|ANXA2_HUMAN	ANXA2	P07355	Annexin A2	ANXA2_HUMAN		ANXA2
4114	2837	4114	0.999	646	sp|P11142|HSP7C_HUMAN	HSPA8	P11142	Heat shock cognate 71 kDa protein	HSP7C_HUMAN		HSPA8
2793	2793	2793	0.999	710	sp|P19338|NUCL_HUMAN	NCL	P19338	Nucleolin	NUCL_HUMAN		NCL
852	852	852	0.999	3256	sp|P46013|KI67_HUMAN	MKI67	P46013	Proliferation marker protein Ki-67	KI67_HUMAN		MKI67
3373	3312	3538	0.999	654	sp|P11021|BIP_HUMAN	HSPA5	P11021	Endoplasmic reticulum chaperone BiP	BIP_HUMAN		HSPA5
1653	1272	1653	0.999	843	sp|P11216|PYGB_HUMAN	PYGB	P11216	Glycogen phosphorylase, brain form	PYGB_HUMAN		PYGB
2668	2668	2668	0.999	543	sp|P31948|STIP1_HUMAN	STIP1	P31948	Stress-induced-phosphoprotein 1	STIP1_HUMAN		STIP1
2853	2853	2853	0.999	1270	sp|Q08211|DHX9_HUMAN	DHX9	Q08211	ATP-dependent RNA helicase A	DHX9_HUMAN		DHX9
3229	2902	3229	0.999	403	sp|P39023|RL3_HUMAN	RPL3	P39023	Large ribosomal subunit protein uL3	RL3_HUMAN		RPL3
1897	1871	1897	0.999	1394	sp|P42704|LPPRC_HUMAN	LRPPRC	P42704	Leucine-rich PPR motif-containing protein, mitochondrial	LPPRC_HUMAN		LRPPRC
576	525	576	0.999	6885	sp|Q8WXH0|SYNE2_HUMAN	SYNE2	Q8WXH0	Nesprin-2	SYNE2_HUMAN		SYNE2
6854	6470	6854	0.999	434	sp|P06733|ENOA_HUMAN	ENO1	P06733	Alpha-enolase	ENOA_HUMAN		ENO1
1573	1570	2907	0.999	892	sp|P12814|ACTN1_HUMAN	ACTN1	P12814	Alpha-actinin-1	ACTN1_HUMAN		ACTN1
1239	1239	1239	0.999	2472	sp|Q13813|SPTN1_HUMAN	SPTAN1	Q13813	Spectrin alpha chain, non-erythrocytic 1	SPTN1_HUMAN		SPTAN1
3313	3313	3313	0.999	910	sp|Q7KZF4|SND1_HUMAN	SND1	Q7KZF4	Staphylococcal nuclease domain-containing protein 1	SND1_HUMAN		SND1
416	416	416	0.999	1479	sp|Q92626|PXDN_HUMAN	PXDN	Q92626	Peroxidasin homolog	PXDN_HUMAN		PXDN
4025	4025	4025	0.999	573	sp|P10809|CH60_HUMAN	HSPD1	P10809	60 kDa heat shock protein, mitochondrial	CH60_HUMAN		HSPD1
820	816	820	0.999	4544	sp|Q07954|LRP1_HUMAN	LRP1	Q07954	Prolow-density lipoprotein receptor-related protein 1	LRP1_HUMAN		LRP1
1328	1328	1328	0.999	673	sp|P08133|ANXA6_HUMAN	ANXA6	P08133	Annexin A6	ANXA6_HUMAN		ANXA6
2699	2699	2699	0.999	1134	sp|P18206|VINC_HUMAN	VCL	P18206	Vinculin	VINC_HUMAN		VCL
2381	2381	2381	0.999	1224	sp|P53621|COPA_HUMAN	COPA	P53621	Coatomer subunit alpha	COPA_HUMAN		COPA
2108	2108	2108	0.999	2335	sp|Q6P2Q9|PRP8_HUMAN	PRPF8	Q6P2Q9	Pre-mRNA-processing-splicing factor 8	PRP8_HUMAN		PRPF8
1314	1314	1314	0.999	1268	sp|Q00341|VIGLN_HUMAN	HDLBP	Q00341	Vigilin	VIGLN_HUMAN		HDLBP
3037	3037	3037	0.999	1014	sp|P09874|PARP1_HUMAN	PARP1	P09874	Poly [ADP-ribose] polymerase 1	PARP1_HUMAN		PARP1
4247	3596	4500	0.999	803	sp|P14625|ENPL_HUMAN	HSP90B1	P14625	Endoplasmin	ENPL_HUMAN		HSP90B1
1806	1806	1806	0.999	1357	sp|Q86UP2|KTN1_HUMAN	KTN1	Q86UP2	Kinectin	KTN1_HUMAN		KTN1
4464	3386	4464	0.999	577	sp|P26038|MOES_HUMAN	MSN	P26038	Moesin	MOES_HUMAN		MSN
2777	2637	2777	0.999	840	sp|P34932|HSP74_HUMAN	HSPA4	P34932	Heat shock 70 kDa protein 4	HSP74_HUMAN		HSPA4
5887	5625	5887	0.999	858	sp|P13639|EF2_HUMAN	EEF2	P13639	Elongation factor 2	EF2_HUMAN		EEF2
687	687	687	0.999	2477	sp|P02751|FINC_HUMAN	FN1	P02751	Fibronectin	FINC_HUMAN		FN1
6447	6231	6447	0.999	531	sp|P14618|KPYM_HUMAN	PKM	P14618	Pyruvate kinase PKM	KPYM_HUMAN		PKM
4335	4242	4335	0.999	730	sp|P52272|HNRPM_HUMAN	HNRNPM	P52272	Heterogeneous nuclear ribonucleoprotein M	HNRPM_HUMAN		HNRNPM
3979	3477	9117	0.999	732	sp|P07900|HS90A_HUMAN	HSP90AA1	P07900	Heat shock protein HSP 90-alpha	HS90A_HUMAN		HSP90AA1
1673	1144	1673	0.999	3224	sp|P49792|RBP2_HUMAN	RANBP2	P49792	E3 SUMO-protein ligase RanBP2	RBP2_HUMAN		RANBP2
4493	3941	4493	0.999	466	sp|P08670|VIME_HUMAN	VIM	P08670	Vimentin	VIME_HUMAN		VIM
2883	2697	2883	0.999	1657	sp|P46940|IQGA1_HUMAN	IQGAP1	P46940	Ras GTPase-activating-like protein IQGAP1	IQGA1_HUMAN		IQGAP1

3750	2337	3750	0.999	911	sp|O43707|ACTN4_HUMAN	ACTN4	O43707	Alpha-actinin-4	ACTN4_HUMAN		ACTN4
739	392	806	0.999	7388	sp|Q9UPN3|MACF1_HUMAN	MACF1	Q9UPN3	Microtubule-actin cross-linking factor 1, isoforms 1/2/3/4/5	MACF1_HUMAN		MACF1
2059	1766	2059	0.999	2364	sp|Q01082|SPTB2_HUMAN	SPTBN1	Q01082	Spectrin beta chain, non-erythrocytic 1	SPTB2_HUMAN		SPTBN1
4424	4371	4424	0.999	664	sp|P02545|LMNA_HUMAN	LMNA	P02545	Prelamin-A/C	LMNA_HUMAN		LMNA
9991	2932	10048	0.999	724	sp|P08238|HS90B_HUMAN	HSP90AB1	P08238	Heat shock protein HSP 90-beta	HS90B_HUMAN		HSP90AB1
4022	4019	4022	0.999	1382	sp|Q14152|EIF3A_HUMAN	EIF3A	Q14152	Eukaryotic translation initiation factor 3 subunit A	EIF3A_HUMAN		EIF3A
2427	2427	2427	0.999	2671	sp|Q92616|GCN1_HUMAN	GCN1	Q92616	Stalled ribosome sensor GCN1	GCN1_HUMAN		GCN1L1
1726	1726	1726	0.999	4374	sp|Q7Z6Z7|HUWE1_HUMAN	HUWE1	Q7Z6Z7	E3 ubiquitin-protein ligase HUWE1	HUWE1_HUMAN		HUWE1
2023	1990	2023	0.999	2061	sp|Q9NZM1|MYOF_HUMAN	MYOF	Q9NZM1	Myoferlin	MYOF_HUMAN		MYOF
4147	4147	4147	0.999	1512	sp|P07814|SYEP_HUMAN	EPRS1	P07814	Bifunctional glutamate/proline--tRNA ligase	SYEP_HUMAN		EPRS
2496	2456	2496	0.999	1410	sp|Q9P2E9|RRBP1_HUMAN	RRBP1	Q9P2E9	Ribosome-binding protein 1	RRBP1_HUMAN		RRBP1
1734	1652	3323	0.999	1976	sp|P35580|MYH10_HUMAN	MYH10	P35580	Myosin-10	MYH10_HUMAN		MYH10
2602	2600	2602	0.999	2115	sp|Q14980|NUMA1_HUMAN	NUMA1	Q14980	Nuclear mitotic apparatus protein 1	NUMA1_HUMAN		NUMA1
2847	2806	2847	0.999	2363	sp|P12270|TPR_HUMAN	TPR	P12270	Nucleoprotein TPR	TPR_HUMAN		TPR
4895	3655	4895	0.999	1675	sp|Q00610|CLH1_HUMAN	CLTC	Q00610	Clathrin heavy chain 1	CLH1_HUMAN		CLTC
896	896	896	0.999	3177	sp|P12111|CO6A3_HUMAN	COL6A3	P12111	Collagen alpha-3(VI) chain	CO6A3_HUMAN		COL6A3
2718	2617	2718	0.999	1500	sp|P31327|CPSM_HUMAN	CPS1	P31327	Carbamoyl-phosphate synthase [ammonia], mitochondrial	CPSM_HUMAN		CPS1
5366	4781	5366	0.999	2647	sp|P21333|FLNA_HUMAN	FLNA	P21333	Filamin-A	FLNA_HUMAN		FLNA
4011	3967	4490	0.999	2602	sp|O75369|FLNB_HUMAN	FLNB	O75369	Filamin-B	FLNB_HUMAN		FLNB
5910	5857	5910	0.999	2511	sp|P49327|FAS_HUMAN	FASN	P49327	Fatty acid synthase	FAS_HUMAN		FASN
5761	5103	5761	0.999	2541	sp|Q9Y490|TLN1_HUMAN	TLN1	Q9Y490	Talin-1	TLN1_HUMAN		TLN1
7478	7478	7478	0.999	4646	sp|Q14204|DYHC1_HUMAN	DYNC1H1	Q14204	Cytoplasmic dynein 1 heavy chain 1	DYHC1_HUMAN		DYNC1H1
7829	7829	7829	0.999	4128	sp|P78527|PRKDC_HUMAN	PRKDC	P78527	DNA-dependent protein kinase catalytic subunit	PRKDC_HUMAN		PRKDC
12000	9852	12000	0.999	1960	sp|P35579|MYH9_HUMAN	MYH9	P35579	Myosin-9	MYH9_HUMAN		MYH9
7199	7188	7199	0.999	5890	sp|Q09666|AHNK_HUMAN	AHNAK	Q09666	Neuroblast differentiation-associated protein AHNAK	AHNK_HUMAN		AHNAK
10698	10363	10698	0.999	4684	sp|Q15149|PLEC_HUMAN	PLEC	Q15149	Plectin	PLEC_HUMAN		PLEC
