# Supplementary material for: Extracellular Vesicles Bearing Vimentin Drive Epithelial–Mesenchymal Transition
Source: Mol Cell Proteomics. 2025 Jul 4;24(12):101028. doi: 10.1016/j.mcpro.2025.101028 (PMC12719745; doi:10.1016/j.mcpro.2025.101028)
Supplement: Supplemental Data 5 [file mmc8.pdf]

sheet 5.MCF7 clean data

MCF7_EV_KO_1 MaxLFQ Intensity	MCF7_EV_KO_2 MaxLFQ Intensity	MCF7_EV_KO_3 MaxLFQ Intensity	MCF7_EV_KO_4 MaxLFQ Intensity	MCF7_EV_WT_1 MaxLFQ Intensity	MCF7_EV_WT_2 MaxLFQ Intensity	MCF7_EV_WT_3 MaxLFQ Intensity	MCF7_EV_WT_4 MaxLFQ Intensity	C: Protein Existence
27.8316	28.1526	27.6177	27.4588	27.1424	27.8055	27.011	#NUM!	1:Experimental evidence at protein level
29.7201	29.8089	29.7483	29.8134	29.5841	29.5896	29.9244	30.0881	1:Experimental evidence at protein level
25.4856	25.5037	25.1349	25.9694	25.9959	25.6393	26.1616	26.5626	1:Experimental evidence at protein level
25.3737	25.5369	25.523	25.8204	24.4232	#NUM!	24.53	#NUM!	3:Protein inferred from homology
27.4785	27.516	27.9103	27.3975	27.5399	27.8289	27.3514	27.3147	1:Experimental evidence at protein level
26.2216	26.4342	26.8282	26.3798	26.3118	26.2482	26.2791	26.2276	1:Experimental evidence at protein level
#NUM!	29.4677	#NUM!	29.3718	29.3673	29.5018	29.5018	29.5227	1:Experimental evidence at protein level
26.3156	26.2513	26.4715	26.542	26.1135	26.231	26.0849	26.5185	1:Experimental evidence at protein level
25.7827	25.913	25.9242	26.044	26.142	26.1885	25.8577	26.256	1:Experimental evidence at protein level
26.0003	25.4061	25.6337	#NUM!	26.3468	26.3981	25.8514	26.6332	1:Experimental evidence at protein level
25.5742	25.7254	25.8311	25.6066	#NUM!	#NUM!	#NUM!	#NUM!	2:Experimental evidence at transcript level
24.1221	24.4235	24.7076	24.1309	24.6988	24.9028	24.8107	#NUM!	1:Experimental evidence at protein level
28.9722	29.0119	29.0216	28.655	29.1157	29.2725	29.0946	#NUM!	1:Experimental evidence at protein level
26.9394	26.6809	27.3825	27.2039	26.8741	26.9073	26.4703	#NUM!	1:Experimental evidence at protein level
29.2614	29.4482	29.2958	29.0415	29.125	29.0492	28.8767	#NUM!	1:Experimental evidence at protein level
26.4717	26.5984	26.7347	26.8034	26.6284	26.6946	26.6585	27.2153	1:Experimental evidence at protein level
26.9432	26.8069	26.0851	26.6392	26.6684	26.6506	26.4276	27.004	1:Experimental evidence at protein level
#NUM!	24.5513	#NUM!	#NUM!	25.1878	24.9276	24.9778	25.374	1:Experimental evidence at protein level
27.2289	27.2441	26.8844	27.5107	27.3416	27.2214	27.2737	27.6529	1:Experimental evidence at protein level
25.0867	25.1824	25.3859	25.4511	25.3301	25.5233	25.5853	25.7138	1:Experimental evidence at protein level
26.2748	25.8372	25.2611	25.8535	26.0981	26.4983	25.9914	25.3467	1:Experimental evidence at protein level
25.6444	25.9432	24.8599	24.9145	25.4918	#NUM!	#NUM!	#NUM!	1:Experimental evidence at protein level
25.1609	24.9292	25.0109	25.3576	#NUM!	#NUM!	25.1696	25.6373	2:Experimental evidence at transcript level
25.45	25.6113	26.0369	26.2524	24.4657	24.0967	24.4506	25.2665	1:Experimental evidence at protein level
24.7164	24.631	24.8656	24.4605	24.9484	#NUM!	#NUM!	#NUM!	1:Experimental evidence at protein level
24.6911	25.1675	24.8413	25.0787	#NUM!	24.2536	#NUM!	24.8337	1:Experimental evidence at protein level
30.3383	30.2891	30.2883	29.6767	28.9761	29.0233	28.7685	27.1791	2:Experimental evidence at transcript level
23.966	24.5773	24.5618	24.1211	24.9193	24.5399	24.7682	#NUM!	1:Experimental evidence at protein level
25.7904	25.7638	25.5154	#NUM!	25.8782	26.1023	26.0794	25.8274	1:Experimental evidence at protein level
25.6044	25.8241	25.6971	26.0525	25.5909	25.7252	26.1351	26.0696	1:Experimental evidence at protein level
#NUM!	#NUM!	25.6482	26.414	25.9109	25.7245	26.1039	27.0643	1:Experimental evidence at protein level
#NUM!	26.3298	26.2181	27.0654	26.7122	26.1174	26.4007	27.2235	1:Experimental evidence at protein level
24.8347	24.7087	#NUM!	24.9623	25.1462	25.097	25.0733	25.3117	1:Experimental evidence at protein level
26.9698	26.9762	26.4078	26.4945	27.106	27.1927	27.1015	27.178	1:Experimental evidence at protein level
24.1088	#NUM!	23.6226	24.1264	25.0456	24.9175	24.965	25.6245	1:Experimental evidence at protein level
25.8561	25.8498	25.8406	25.8034	25.4463	25.395	#NUM!	26.0671	1:Experimental evidence at protein level
27.8812	27.784	27.7518	27.8162	27.6372	27.6661	27.3805	#NUM!	1:Experimental evidence at protein level
27.5358	27.4187	27.5347	27.6624	#NUM!	26.349	#NUM!	27.5465	1:Experimental evidence at protein level
27.9379	28.0487	28.4151	28.2255	28.0054	27.8729	27.9329	27.9125	1:Experimental evidence at protein level
24.6673	24.6827	24.5741	24.8843	#NUM!	#NUM!	#NUM!	#NUM!	1:Experimental evidence at protein level
25.8307	25.8926	#NUM!	#NUM!	26.1603	26.0036	25.7308	26.3451	1:Experimental evidence at protein level
25.078	25.1861	25.3074	25.0839	#NUM!	24.5172	25.056	#NUM!	1:Experimental evidence at protein level
26.1622	26.0364	26.3554	25.948	26.3136	25.9922	26.1479	26.0487	1:Experimental evidence at protein level
25.2787	25.331	25.603	26.3449	24.7852	#NUM!	24.3244	26.3412	1:Experimental evidence at protein level
25.5029	25.4634	25.9166	26.2862	25.6716	25.138	25.5739	25.8344	1:Experimental evidence at protein level
25.7874	25.7472	25.7038	25.2899	25.3264	25.7753	25.1947	#NUM!	1:Experimental evidence at protein level
26.0682	26.4428	25.6868	26.2739	26.7916	27.0699	26.6122	#NUM!	1:Experimental evidence at protein level
25.6452	25.4998	25.5301	25.1397	26.2827	26.4584	25.9655	25.7017	1:Experimental evidence at protein level
26.1435	25.8832	25.9546	26.0861	26.1282	#NUM!	26.178	26.5598	1:Experimental evidence at protein level
25.6215	25.6628	25.6181	25.971	25.8778	#NUM!	25.9198	26.4483	2:Experimental evidence at transcript level
26.4895	26.8274	26.4614	26.6006	26.4918	26.4977	26.6687	26.7064	1:Experimental evidence at protein level
26.3615	26.6001	26.0971	26.2071	26.6032	26.7168	26.4929	25.8984	1:Experimental evidence at protein level
25.3974	#NUM!	25.4071	25.4044	25.5437	25.1225	25.6939	25.7166	1:Experimental evidence at protein level
25.4054	25.6038	25.8551	25.7602	24.9369	#NUM!	24.9132	24.9826	1:Experimental evidence at protein level
25.1537	25.1431	25.1948	25.5167	24.4257	24.3034	24.9503	#NUM!	1:Experimental evidence at protein level
26.0314	26.1765	26.1975	26.2271	26.4185	26.2387	26.6356	26.8745	1:Experimental evidence at protein level
27.0295	27.2476	27.1095	27.4521	27.1118	26.7532	27.1096	#NUM!	1:Experimental evidence at protein level
28.8657	28.9082	28.3128	28.3429	#NUM!	28.4749	28.4208	27.8999	1:Experimental evidence at protein level
25.4897	25.8486	25.7442	25.5963	25.4411	25.3523	25.5815	#NUM!	1:Experimental evidence at protein level
26.3997	26.6841	26.6007	26.2434	#NUM!	25.6753	#NUM!	#NUM!	1:Experimental evidence at protein level
25.8139	25.8334	25.8092	26.1849	26.1295	26.0418	25.8096	26.298	1:Experimental evidence at protein level
25.6728	25.5851	25.6479	24.8564	#NUM!	#NUM!	#NUM!	#NUM!	1:Experimental evidence at protein level
25.8186	25.5974	25.7192	25.6762	25.9353	25.9827	25.6256	#NUM!	1:Experimental evidence at protein level
25.842	25.9626	26.0025	#NUM!	27.0545	26.9322	26.9339	27.3536	1:Experimental evidence at protein level
26.4353	26.6735	26.9542	26.6869	26.5749	26.4958	26.7855	26.4619	1:Experimental evidence at protein level
27.2735	26.7105	27.0277	27.005	#NUM!	26.9062	27.1233	26.6696	1:Experimental evidence at protein level
27.4293	27.3893	26.4495	27.2645	27.8134	27.7614	27.6227	28.1022	1:Experimental evidence at protein level
27.4278	27.5528	27.8044	27.7148	27.1335	#NUM!	27.6242	28.1677	1:Experimental evidence at protein level
#NUM!	#NUM!	24.8083	24.1529	25.8356	25.9478	26.013	26.5047	1:Experimental evidence at protein level
27.1733	26.8281	26.6892	26.7459	26.9432	27.0813	26.6829	#NUM!	1:Experimental evidence at protein level
#NUM!	#NUM!	#NUM!	25.5234	26.3177	25.974	26.1721	27.4198	1:Experimental evidence at protein level
26.7535	26.5035	27.1937	27.0383	26.8492	26.7624	26.5594	#NUM!	1:Experimental evidence at protein level
25.5709	25.7214	25.7215	26.0495	#NUM!	#NUM!	25.1306	25.5322	1:Experimental evidence at protein level
24.5622	24.6425	24.5649	24.5979	25.3942	25.3456	25.4987	25.405	1:Experimental evidence at protein level
30.7155	30.5876	30.6312	30.5951	30.8174	30.804	30.7193	30.0921	1:Experimental evidence at protein level
25.1395	#NUM!	24.9131	24.7261	25.2827	25.0724	25.0359	25.2918	1:Experimental evidence at protein level
25.4062	25.3253	25.4939	#NUM!	25.5077	25.4286	25.2649	25.5081	1:Experimental evidence at protein level
26.5967	26.3028	25.519	25.9609	26.6373	26.6472	26.4668	27.1081	1:Experimental evidence at protein level
25.9683	25.935	25.8028	26.3557	25.8009	#NUM!	25.9112	#NUM!	1:Experimental evidence at protein level
26.3149	26.5028	#NUM!	26.6869	25.6562	26.1886	26.1919	26.7631	1:Experimental evidence at protein level
26.5398	26.4359	27.1142	26.8848	#NUM!	#NUM!	25.9885	#NUM!	1:Experimental evidence at protein level
24.554	24.3106	#NUM!	#NUM!	24.9803	24.9982	25.1681	24.9228	1:Experimental evidence at protein level
#NUM!	25.1851	25.5574	#NUM!	24.9145	25.6265	25.4536	26.2044	1:Experimental evidence at protein level
25.6396	25.741	25.771	25.5589	26.1647	26.083	#NUM!	26.6523	1:Experimental evidence at protein level
26.2455	26.0464	25.4911	25.6996	26.1883	26.7721	26.0647	25.6571	1:Experimental evidence at protein level
26.9236	26.978	26.9099	27.2448	27.0355	27.0543	27.1717	27.6597	1:Experimental evidence at protein level
26.3838	26.5368	26.42	26.5659	26.7853	27.3688	26.9617	27.8236	1:Experimental evidence at protein level
25.71	25.2359	25.397	25.4493	#NUM!	#NUM!	#NUM!	25.2254	1:Experimental evidence at protein level
26.2094	25.9963	26.2051	24.3233	25.3402	25.9718	#NUM!	#NUM!	1:Experimental evidence at protein level
26.1419	26.0764	26.1328	25.9856	26.1688	26.0653	25.7577	#NUM!	1:Experimental evidence at protein level
26.4319	26.5339	26.2266	26.3039	26.7541	26.7753	26.6212	26.6546	1:Experimental evidence at protein level
26.3002	26.4501	26.5127	26.1173	26.1325	#NUM!	#NUM!	#NUM!	1:Experimental evidence at protein level
30.04	29.8379	29.2866	28.6994	28.9482	29.1617	28.5278	26.8016	1:Experimental evidence at protein level
25.3157	25.3279	25.6551	25.2857	26.0183	25.9368	26.0751	26.2226	1:Experimental evidence at protein level
25.2036	25.36	24.9702	25.3604	#NUM!	#NUM!	#NUM!	#NUM!	1:Experimental evidence at protein level
24.7384	25.0677	24.9575	24.6062	#NUM!	24.6384	24.8701	#NUM!	1:Experimental evidence at protein level
23.1021	23.5538	23.0428	23.1449	23.9432	23.7333	#NUM!	24.4005	1:Experimental evidence at protein level
26.3662	26.5869	26.7324	26.824	26.2336	26.1729	26.6045	27.1463	1:Experimental evidence at protein level
26.4608	26.1721	25.9966	26.1689	25.6147	#NUM!	25.6599	25.975	1:Experimental evidence at protein level
#NUM!	26.521	26.4715	26.2533	27.3212	27.3209	27.1005	27.4485	1:Experimental evidence at protein level
25.961	26.1659	26.2073	26.2947	26.0851	26.1255	26.2544	26.3373	1:Experimental evidence at protein level
25.1993	25.5164	25.5378	25.4511	25.6874	26.0715	25.6198	25.8148	1:Experimental evidence at protein level
25.3547	25.7271	25.6055	25.7607	25.6898	26.1999	25.9411	26.2453	1:Experimental evidence at protein level
#NUM!	24.2811	24.5879	#NUM!	25.0277	24.8123	24.8938	25.2315	1:Experimental evidence at protein level
24.3486	24.7193	24.9423	25.1696	22.7556	#NUM!	#NUM!	#NUM!	1:Experimental evidence at protein level
25.1554	24.7358	24.8859	24.7713	25.0408	24.8991	24.8993	25.6557	1:Experimental evidence at protein level
26.9404	27.0178	26.9699	27.0426	25.0689	25.3546	25.0203	#NUM!	1:Experimental evidence at protein level
24.2081	24.2596	23.8466	24.0947	24.7161	#NUM!	24.3816	25.3208	1:Experimental evidence at protein level
26.3588	26.5752	26.7004	26.7328	25.5854	25.7449	#NUM!	25.5776	1:Experimental evidence at protein level
25.7309	25.3124	25.2135	25.3295	25.4772	25.2125	25.4328	#NUM!	1:Experimental evidence at protein level
26.693	26.7906	26.8793	26.8085	26.7965	26.9431	26.6864	26.6289	1:Experimental evidence at protein level
#NUM!	27.1531	#NUM!	26.446	27.9646	28.2541	27.4307	28.284	1:Experimental evidence at protein level
24.6843	24.7003	24.7435	24.6285	25.2163	24.9667	24.8316	25.5819	1:Experimental evidence at protein level
23.8279	23.8199	#NUM!	24.2488	25.5454	25.8767	25.9123	26.1057	1:Experimental evidence at protein level
26.2752	26.3236	26.5094	26.2241	26.5143	26.495	26.5265	27.0646	1:Experimental evidence at protein level
26.4668	26.5764	27.0531	26.6444	26.3435	26.4363	26.3566	26.2928	1:Experimental evidence at protein level
25.6633	25.9022	26.0823	26.2161	25.6769	25.8272	25.3396	26.1425	1:Experimental evidence at protein level
26.3547	26.5299	26.0345	26.5205	26.2624	26.1237	26.3062	26.8695	1:Experimental evidence at protein level
24.9022	25.2064	25.219	25.2124	25.5797	25.6593	25.5125	#NUM!	1:Experimental evidence at protein level
26.0864	26.052	26.01	26.0268	26.1883	25.9678	25.9712	#NUM!	1:Experimental evidence at protein level
27.0138	27.0185	26.768	27.0996	27.0253	26.842	26.943	27.3167	1:Experimental evidence at protein level
25.6829	25.6069	25.7995	25.9332	#NUM!	25.3209	25.2516	25.7414	1:Experimental evidence at protein level
26.1527	26.0864	25.9209	26.0048	26.4767	26.3924	26.1841	#NUM!	1:Experimental evidence at protein level
26.265	26.3651	26.1167	26.1435	26.2305	26.2144	26.2424	26.5452	1:Experimental evidence at protein level
26.7822	26.9545	27.1807	27.1754	27.1902	27.1345	27.0183	27.6129	1:Experimental evidence at protein level
27.8768	27.8534	27.8335	28.036	28.0871	28.0634	27.9962	28.3269	1:Experimental evidence at protein level

24.6211	24.6242	24.3325	24.7437	#NUM!	#NUM!	#NUM!	#NUM!	1:Experimental evidence at protein level
#NUM!	25.4686	25.5483	25.9799	25.5145	25.4633	25.199	25.963	1:Experimental evidence at protein level
24.8147	#NUM!	24.8458	25.5725	25.4554	24.9336	25.274	25.5411	1:Experimental evidence at protein level
24.9606	24.7078	24.7983	24.6556	25.3005	25.4664	24.9691	#NUM!	1:Experimental evidence at protein level
26.177	26.3671	26.1248	26.3413	24.8564	#NUM!	#NUM!	#NUM!	1:Experimental evidence at protein level
24.579	24.6744	24.7508	24.5734	#NUM!	24.5442	24.5836	#NUM!	1:Experimental evidence at protein level
25.44	25.5377	25.5036	25.3841	25.7894	25.6563	26.0556	26.4265	1:Experimental evidence at protein level
24.6139	24.9232	24.6808	24.1635	25.2997	25.4693	25.2697	24.0972	1:Experimental evidence at protein level
26.9982	27.0536	#NUM!	25.93	25.4078	25.5968	25.9013	25.956	1:Experimental evidence at protein level
25.4609	25.6375	25.0542	25.5905	25.2944	25.4799	25.0703	25.6557	1:Experimental evidence at protein level
25.5805	25.5939	25.5761	24.9888	25.8849	25.7852	25.4008	#NUM!	1:Experimental evidence at protein level
25.9091	26.2104	26.0679	26.3428	25.876	#NUM!	25.787	26.3772	1:Experimental evidence at protein level
#NUM!	#NUM!	26.2161	27.1086	27.0532	26.7036	26.6183	27.304	1:Experimental evidence at protein level
24.8179	24.7729	24.8427	24.822	24.9633	#NUM!	24.832	25.6537	1:Experimental evidence at protein level
26.3364	26.0837	26.1174	26.0878	26.5701	26.4672	26.4535	27.1013	1:Experimental evidence at protein level
26.6899	26.7746	#NUM!	#NUM!	26.9602	26.6888	26.7481	27.6851	1:Experimental evidence at protein level
#NUM!	25.79	#NUM!	24.6426	24.9018	24.5489	24.8658	25.4733	1:Experimental evidence at protein level
26.3617	26.3145	26.2785	26.7958	#NUM!	#NUM!	#NUM!	#NUM!	1:Experimental evidence at protein level
26.5093	26.7959	26.5617	26.604	26.3745	26.3345	26.9575	26.7339	1:Experimental evidence at protein level
28.1393	28.0161	28.0518	28.0237	27.8852	27.5933	27.8698	28.3688	1:Experimental evidence at protein level
24.1341	24.1754	23.8433	24.1864	23.7591	23.9758	23.5165	#NUM!	1:Experimental evidence at protein level
26.5076	#NUM!	26.3149	26.1741	26.0732	26.2617	25.9079	26.0142	1:Experimental evidence at protein level
25.8278	25.7887	#NUM!	26.1664	25.7664	25.893	25.9821	26.078	1:Experimental evidence at protein level
25.69	#NUM!	#NUM!	25.2839	25.6072	25.9315	25.7531	24.9422	1:Experimental evidence at protein level
#NUM!	23.9389	#NUM!	#NUM!	26.3159	26.0869	26.1716	26.9014	1:Experimental evidence at protein level
29.0902	29.1528	29.0545	29.3402	29.1797	29.065	29.2258	29.8442	1:Experimental evidence at protein level
#NUM!	26.4619	26.5497	26.4839	26.0839	26.1875	25.5988	26.3278	1:Experimental evidence at protein level
25.3637	25.2656	25.2481	25.0618	25.2642	24.892	25.1984	#NUM!	1:Experimental evidence at protein level
27.701	27.8512	27.6712	26.8924	27.6658	27.9328	27.5931	26.1399	1:Experimental evidence at protein level
25.1216	25.4848	#NUM!	25.1846	25.549	25.9152	25.5494	25.8712	1:Experimental evidence at protein level
32.4734	32.7397	33.0321	32.8002	33.0527	33.2507	33.3139	32.2334	1:Experimental evidence at protein level
26.3925	26.6376	26.2983	26.7815	27.103	27.1453	27.1602	27.5762	1:Experimental evidence at protein level
26.7738	26.7918	26.9921	26.8915	25.7191	26.4551	25.7967	25.6531	1:Experimental evidence at protein level
24.9861	25.4523	25.6094	25.212	25.0045	25.0865	25.0496	24.9649	1:Experimental evidence at protein level
27.1129	26.812	25.8207	25.9229	26.2999	26.3227	26.3441	26.6021	1:Experimental evidence at protein level
24.4395	24.5168	24.6205	24.9194	#NUM!	#NUM!	#NUM!	#NUM!	1:Experimental evidence at protein level
#NUM!	#NUM!	#NUM!	26.5713	27.1419	27.131	27.2705	26.9325	1:Experimental evidence at protein level
27.1622	26.7249	26.7966	27.0614	26.9965	26.9574	26.9732	26.0412	1:Experimental evidence at protein level
24.5326	24.5464	24.4267	24.5208	24.633	24.537	24.1029	24.7669	1:Experimental evidence at protein level
27.7079	27.5403	27.9367	28.019	27.9937	27.0778	27.9448	28.1933	1:Experimental evidence at protein level
25.6444	25.744	25.973	25.9268	25.8903	25.6683	26.2016	26.6297	1:Experimental evidence at protein level
25.9544	25.6566	25.9867	25.7801	#NUM!	26.6321	#NUM!	#NUM!	1:Experimental evidence at protein level
26.8009	26.3147	26.744	26.8281	26.8473	#NUM!	26.6542	#NUM!	1:Experimental evidence at protein level
27.5316	27.4955	27.4023	27.5624	27.2842	27.5533	27.3964	#NUM!	1:Experimental evidence at protein level
26.3766	26.6787	26.9446	26.4758	27.1017	27.2862	27.3281	26.562	1:Experimental evidence at protein level
27.3204	27.6983	26.9045	27.1589	27.2726	27.9083	27.0347	27.4887	1:Experimental evidence at protein level
28.8517	28.5572	28.5985	28.5875	28.9254	29.4601	28.9774	28.8615	1:Experimental evidence at protein level
27.8014	27.8069	27.8606	27.4691	27.4484	27.8088	27.483	26.6461	1:Experimental evidence at protein level
25.2429	25.4354	24.9217	25.0667	24.325	#NUM!	24.3294	25.1014	1:Experimental evidence at protein level
24.9975	25.0039	25.0363	24.7269	25.1633	25.1389	25.3511	#NUM!	1:Experimental evidence at protein level
25.9651	#NUM!	26.3021	#NUM!	26.235	26.4834	26.2516	26.1666	1:Experimental evidence at protein level
26.0545	26.3188	26.3058	26.2041	25.9701	#NUM!	26.1019	#NUM!	1:Experimental evidence at protein level
25.6706	25.8848	25.7592	25.6892	26.0704	#NUM!	25.832	#NUM!	1:Experimental evidence at protein level
24.79	24.5079	24.4615	24.8066	#NUM!	24.4201	24.6825	#NUM!	1:Experimental evidence at protein level
25.8204	25.8358	25.9584	25.6066	25.3337	24.9636	25.1457	25.7936	1:Experimental evidence at protein level
26.4004	26.3796	26.2885	26.3533	26.247	25.9571	26.0036	26.1378	1:Experimental evidence at protein level
25.1362	25.1659	24.621	25.4599	25.7926	25.531	25.7574	26.2815	1:Experimental evidence at protein level
26.4113	26.3307	26.463	26.6226	26.1941	26.2547	26.5327	26.565	1:Experimental evidence at protein level
27.241	27.1075	27.2761	27.1956	#NUM!	#NUM!	#NUM!	#NUM!	1:Experimental evidence at protein level
24.8562	24.959	24.7283	24.63	24.9583	25.0819	25.0358	23.9819	1:Experimental evidence at protein level
25.2014	25.2627	25.3744	25.3767	#NUM!	#NUM!	25.0897	25.1944	1:Experimental evidence at protein level
26.8308	26.3782	26.1261	27.0406	26.8948	26.7889	26.6952	27.2484	1:Experimental evidence at protein level
24.9073	25.1422	24.9976	25.3074	24.8569	25.0177	#NUM!	25.4634	1:Experimental evidence at protein level
25.1527	#NUM!	#NUM!	#NUM!	25.5852	25.9568	25.2795	24.6287	1:Experimental evidence at protein level
27.1511	27.2498	27.3442	27.1398	26.774	27.0875	#NUM!	26.5883	1:Experimental evidence at protein level
25.1008	25.0893	24.6709	25.1139	24.9858	25.2414	25.0844	#NUM!	1:Experimental evidence at protein level
25.9888	26.1235	26.4497	26.2235	26.4218	26.2167	25.7144	26.2881	1:Experimental evidence at protein level
26.5919	26.5974	25.9536	26.5604	27.1702	26.791	26.4536	27.3043	1:Experimental evidence at protein level
25.981	25.6546	25.7279	25.5959	26.2414	26.4844	26.1717	26.3691	1:Experimental evidence at protein level
25.684	25.5514	25.2551	25.8685	25.4901	25.7624	25.3993	26.1661	1:Experimental evidence at protein level
23.7156	23.5014	23.6787	#NUM!	25.1272	24.8288	24.7528	25.9309	1:Experimental evidence at protein level
25.498	25.5249	25.5228	25.4045	25.8661	25.6482	#NUM!	26.0641	1:Experimental evidence at protein level
26.5404	26.6294	26.7202	26.5165	26.8801	26.9396	26.8298	26.4572	1:Experimental evidence at protein level
26.0815	26.3364	25.9054	25.1156	26.2002	#NUM!	26.0423	26.528	1:Experimental evidence at protein level
27.1426	27.147	27.2631	27.1454	27.4298	26.9444	27.037	27.4586	1:Experimental evidence at protein level
24.5977	24.4172	24.3597	#NUM!	26.5792	26.6696	26.6179	26.8082	1:Experimental evidence at protein level
27.7368	27.059	26.5844	27.7403	27.123	26.8719	26.8624	#NUM!	1:Experimental evidence at protein level
27.1169	27.0182	26.9318	27.3222	27.072	26.8683	27.026	27.5913	1:Experimental evidence at protein level
24.5455	24.5431	24.1924	24.5818	25.6325	25.9598	25.4778	#NUM!	1:Experimental evidence at protein level
26.7677	26.059	26.5385	27.0475	26.6328	26.4512	#NUM!	26.5453	1:Experimental evidence at protein level
26.3446	26.4613	26.5443	26.4984	27.0396	26.6295	26.8993	26.9632	1:Experimental evidence at protein level
25.9875	25.7072	25.2783	25.7914	25.6766	25.6093	25.5165	26.0808	1:Experimental evidence at protein level
27.2367	27.2683	27.9766	27.7585	26.5758	26.7345	26.7372	26.9113	1:Experimental evidence at protein level
26.1655	25.4693	25.9542	25.8704	25.9636	26.0121	25.5617	#NUM!	1:Experimental evidence at protein level
27.5606	27.7672	27.6462	27.7801	27.7939	27.874	27.8115	28.0223	1:Experimental evidence at protein level
25.6504	25.5901	25.4543	24.9192	25.4759	#NUM!	#NUM!	25.9861	1:Experimental evidence at protein level
26.5868	26.3396	26.5619	26.3324	26.8299	26.4513	26.8174	26.3322	1:Experimental evidence at protein level
26.2222	25.919	26.1679	26.0325	26.0587	25.9604	25.6969	25.6349	1:Experimental evidence at protein level
26.307	26.5825	26.8949	26.9543	26.3444	26.7533	26.4221	26.8584	1:Experimental evidence at protein level
#NUM!	#NUM!	#NUM!	25.6869	26.5217	26.8061	26.7434	26.907	1:Experimental evidence at protein level
26.6461	26.8364	26.6762	26.9408	26.3761	26.2355	26.178	26.9438	1:Experimental evidence at protein level
27.9768	28.154	28.1537	28.1055	28.3123	28.0708	28.3282	28.0015	1:Experimental evidence at protein level
25.6872	25.1887	#NUM!	25.4322	26.1762	25.6705	25.5944	26.4434	1:Experimental evidence at protein level
25.7101	#NUM!	25.7184	#NUM!	25.8466	27.0223	26.8072	27.2479	1:Experimental evidence at protein level
26.5472	26.5949	26.6422	26.8568	26.2243	26.2316	26.378	26.8339	1:Experimental evidence at protein level
26.006	26.1041	25.5714	25.7806	26.2421	25.6772	25.9606	26.7813	1:Experimental evidence at protein level
27.0095	26.9313	27.1161	26.8922	26.6215	27.2241	26.7501	27.592	1:Experimental evidence at protein level
26.8053	26.8926	27.3495	27.0877	27.0245	26.7553	26.9115	26.907	1:Experimental evidence at protein level
26.2137	26.1966	26.4744	26.1632	#NUM!	#NUM!	26.5147	#NUM!	1:Experimental evidence at protein level
25.2782	25.2561	25.3971	24.9213	24.2664	24.6649	24.3781	24.7227	1:Experimental evidence at protein level
26.2113	25.9778	26.0353	26.0548	26.093	26.1405	26.0568	26.894	1:Experimental evidence at protein level
25.2325	25.581	25.8162	25.454	25.5863	25.7238	25.8214	#NUM!	1:Experimental evidence at protein level
28.3077	27.9724	28.1097	28.645	28.5428	27.8794	28.669	28.8352	1:Experimental evidence at protein level
24.4365	24.5068	24.5467	24.2207	24.5959	24.4592	24.2629	24.4773	1:Experimental evidence at protein level
24.9704	25.006	24.9098	25.2014	25.2862	24.7114	25.1189	26.02	1:Experimental evidence at protein level
25.7906	25.9198	25.7736	26.0326	25.8966	25.4871	25.9688	26.1842	1:Experimental evidence at protein level
#NUM!	#NUM!	28.1856	28.9385	29.5601	29.4241	29.0639	29.6792	1:Experimental evidence at protein level
26.1409	#NUM!	26.3175	26.4123	26.1912	26.1084	26.2949	26.5364	1:Experimental evidence at protein level
26.8563	26.71	26.6098	27.0805	25.9054	26.038	25.8953	#NUM!	1:Experimental evidence at protein level
26.1832	26.5452	26.3873	26.5436	26.4103	26.6988	26.8402	27.1391	1:Experimental evidence at protein level
25.9991	25.8959	25.9246	26.1317	26.2416	#NUM!	26.2115	#NUM!	1:Experimental evidence at protein level
25.4907	26.0964	25.9759	26.1352	25.8867	26.0316	26.1514	26.5123	1:Experimental evidence at protein level
25.0945	24.8444	25.7641	25.4981	25.5473	25.5076	25.6699	26.1537	1:Experimental evidence at protein level
25.4537	25.3905	25.6138	25.915	#NUM!	#NUM!	25.3441	26.4355	1:Experimental evidence at protein level
25.168	25.1801	25.3394	25.2511	25.2562	25.373	25.3832	#NUM!	1:Experimental evidence at protein level
#NUM!	25.0908	25.1343	#NUM!	25.1419	25.5174	25.1272	25.5211	1:Experimental evidence at protein level
26.8399	26.9725	27.4254	26.9667	27.4891	27.3145	27.4644	26.8925	1:Experimental evidence at protein level
24.725	#NUM!	23.9121	24.8435	24.6857	24.9972	24.4751	25.388	1:Experimental evidence at protein level
23.9906	23.8428	23.7083	24.4155	24.8183	24.4116	24.58	25.1596	1:Experimental evidence at protein level
25.69	25.4531	25.4536	25.7868	25.3551	#NUM!	#NUM!	25.8929	1:Experimental evidence at protein level
25.1176	25.2782	25.0795	25.3171	24.8362	#NUM!	25.3304	25.904	1:Experimental evidence at protein level
25.1007	25.1881	25.1294	25.2477	24.5763	24.7094	24.8819	#NUM!	1:Experimental evidence at protein level
24.5823	24.2605	#NUM!	#NUM!	25.2068	25.5991	24.6756	24.0797	1:Experimental evidence at protein level
25.1083	25.457	24.8407	25.1294	25.9921	26.0133	26.1317	26.6948	1:Experimental evidence at protein level
23.6383	23.3586	23.4986	23.5436	23.941	23.8587	24.1917	24.8073	1:Experimental evidence at protein level
24.9676	24.9841	24.9206	24.4276	#NUM!	#NUM!	#NUM!	#NUM!	1:Experimental evidence at protein level
25.6619	25.6624	25.7171	25.5905	25.4059	26.0828	25.6016	25.892	1:Experimental evidence at protein level
27.6251	27.5575	27.5726	28.0425	27.983	28.0001	27.8254	28.3198	1:Experimental evidence at protein level
26.6405	26.7832	26.7874	26.9474	27.0952	26.967	27.2119	27.5113	1:Experimental evidence at protein level

25.5715	25.7602	25.8166	26.0713	26.3509	26.195	26.1059	27.0244	1:Experimental evidence at protein level
26.6726	26.8718	26.6856	27.0034	#NUM!	#NUM!	#NUM!	#NUM!	1:Experimental evidence at protein level
27.2104	27.1673	27.2314	27.2783	27.4507	27.3046	27.2944	27.461	1:Experimental evidence at protein level
26.1304	26.1354	25.4516	25.4138	26.4558	26.8624	26.4768	26.7394	1:Experimental evidence at protein level
28.9941	29.0751	28.811	28.9899	28.5541	29.0415	28.7077	28.4757	5:Protein uncertain
26.9024	27.0711	27.0873	27.1185	27.3256	27.3443	27.32	27.4177	4:Protein predicted
24.589	24.6235	25.1311	25.1919	25.2169	25.0958	24.8818	25.9735	3:Protein inferred from homology
29.4963	29.4935	29.5075	29.9347	29.4102	29.1865	29.2827	29.4406	1:Experimental evidence at protein level
28.2439	28.0332	27.6352	27.5167	28.0449	28.0354	27.7904	27.9812	1:Experimental evidence at protein level
25.1431	25.2495	25.2112	25.2712	25.7321	25.6503	25.58	25.9538	1:Experimental evidence at protein level
26.8204	26.3036	#NUM!	26.6887	27.2853	27.086	26.5839	27.4602	1:Experimental evidence at protein level
27.6082	#NUM!	#NUM!	27.7507	27.5201	27.6743	27.8689	27.949	1:Experimental evidence at protein level
27.6305	27.7976	27.7959	27.6631	27.6044	27.7348	27.833	27.7993	1:Experimental evidence at protein level
26.1764	26.1431	26.3417	26.1953	26.2744	26.0818	26.3041	26.8131	1:Experimental evidence at protein level
24.5669	24.6804	24.76	24.8744	25.0773	25.0888	25.0422	25.1438	1:Experimental evidence at protein level
27.8606	27.8467	26.845	27.8376	28.0788	27.6992	27.9249	28.1727	1:Experimental evidence at protein level
27.3969	27.3571	#NUM!	27.4411	27.6645	27.1995	27.328	27.4346	1:Experimental evidence at protein level
28.5472	28.3389	28.5756	28.8011	27.7787	27.5151	27.6287	28.0307	1:Experimental evidence at protein level
27.6055	27.5367	27.4043	27.5739	27.7122	27.7128	27.4542	28.0448	1:Experimental evidence at protein level
26.6047	26.7808	26.9491	26.7574	26.2979	26.5155	26.4884	25.9394	1:Experimental evidence at protein level
25.6859	25.8716	25.7959	25.5867	25.627	#NUM!	25.3111	25.9789	1:Experimental evidence at protein level
25.8281	25.8695	25.6727	25.9027	26.0467	25.7799	#NUM!	26.107	1:Experimental evidence at protein level
25.829	25.7125	26.2434	25.87	25.6211	#NUM!	25.4071	26.4095	1:Experimental evidence at protein level
26.1665	26.2576	26.5436	26.3749	#NUM!	#NUM!	25.8653	26.0269	1:Experimental evidence at protein level
26.4976	#NUM!	27.0413	26.5899	27.223	26.3586	26.2345	26.24	1:Experimental evidence at protein level
25.0208	24.9623	24.748	25.2883	25.2682	#NUM!	25.3519	25.7124	1:Experimental evidence at protein level
24.3389	24.4672	24.3143	24.7171	24.235	#NUM!	23.9986	25.0614	1:Experimental evidence at protein level
26.9024	27.0032	26.9897	26.9018	27.2806	27.2511	27.2721	#NUM!	1:Experimental evidence at protein level
28.092	28.2083	28.2677	28.3465	25.7158	25.5763	25.7852	25.6808	1:Experimental evidence at protein level
28.8906	28.9907	29.0651	29.1397	29.6113	29.744	29.6795	29.3695	1:Experimental evidence at protein level
25.007	25.0171	24.8362	#NUM!	25.8177	25.9756	26.3216	26.0095	1:Experimental evidence at protein level
#NUM!	#NUM!	#NUM!	24.2198	25.3625	25.206	24.958	25.4274	1:Experimental evidence at protein level
#NUM!	26.6528	26.5746	#NUM!	26.3568	26.7578	26.5044	26.6212	1:Experimental evidence at protein level
25.7938	26.0951	26.1621	25.8798	26.8766	27.0451	26.827	26.3521	1:Experimental evidence at protein level
26.8132	26.8338	26.8358	26.9626	26.7833	26.9392	26.8497	26.5022	1:Experimental evidence at protein level
25.61	25.9273	25.9522	25.799	26.9991	#NUM!	26.6815	26.6818	1:Experimental evidence at protein level
27.4056	27.7075	27.7398	27.0709	27.4136	27.548	27.4958	27.1089	1:Experimental evidence at protein level
29.485	29.638	29.264	29.6279	29.8447	29.5704	29.8274	29.2144	1:Experimental evidence at protein level
24.9414	25.4571	24.8286	25.3275	26.7266	27.0789	26.636	#NUM!	1:Experimental evidence at protein level
25.2713	25.2802	25.5794	25.2669	25.2209	25.5345	25.256	24.9511	1:Experimental evidence at protein level
26.2214	26.4648	26.5842	26.5233	26.0561	25.8877	26.0526	26.3255	1:Experimental evidence at protein level
26.5919	26.4123	26.1471	26.2858	26.3736	26.3634	26.1922	26.7129	1:Experimental evidence at protein level
25.1153	25.0722	25.4959	25.6313	25.3514	24.9092	25.1518	24.987	1:Experimental evidence at protein level
26.0549	26.2462	26.2421	26.1271	26.0227	25.5709	25.9479	26.1768	1:Experimental evidence at protein level
26.4629	26.2944	26.18	25.8316	25.7933	#NUM!	#NUM!	24.9873	1:Experimental evidence at protein level
27.0197	27.3514	27.4289	27.3574	27.5701	27.4308	27.3794	27.1513	1:Experimental evidence at protein level
26.1028	25.9945	26.1174	25.9877	26.0373	26.1312	26.1021	25.6186	1:Experimental evidence at protein level
27.8969	27.9812	27.5685	27.9866	27.2635	27.4952	27.2749	27.5043	1:Experimental evidence at protein level
29.3274	29.0962	29.3145	29.6218	29.7923	29.769	29.4032	29.3281	1:Experimental evidence at protein level
26.4455	26.7098	26.5558	26.605	#NUM!	25.3727	26.1849	26.3355	1:Experimental evidence at protein level
26.6392	26.7474	26.5208	26.7135	26.5029	26.699	26.8543	26.7287	1:Experimental evidence at protein level
#NUM!	26.1849	26.6515	#NUM!	26.1589	26.4701	26.6325	26.5829	1:Experimental evidence at protein level
29.0392	28.9448	28.5688	28.2807	28.4801	28.4215	28.0514	27.0241	1:Experimental evidence at protein level
30.54	30.4125	29.9839	30.1845	30.5179	30.5181	30.4581	30.7788	1:Experimental evidence at protein level
27.0685	26.9686	27.0919	27.0322	26.9041	26.9768	26.8712	27.6581	1:Experimental evidence at protein level
26.817	26.6986	26.5905	26.2577	26.8223	26.8741	26.8266	26.6061	1:Experimental evidence at protein level
26.177	26.2712	26.3012	26.646	26.6484	26.3923	26.4487	26.6637	1:Experimental evidence at protein level
24.635	24.6767	24.7439	24.8781	#NUM!	#NUM!	25.1934	#NUM!	1:Experimental evidence at protein level
25.6796	25.7405	26.0423	26.1044	25.5857	25.4839	25.5013	26.2718	1:Experimental evidence at protein level
29.6196	29.9936	29.9305	30.0683	29.1338	#NUM!	29.3208	30.7164	1:Experimental evidence at protein level
26.1988	26.1459	26.0811	26.5273	25.9866	26.0089	26.0042	26.5061	1:Experimental evidence at protein level
26.2121	26.2477	26.3517	26.2313	26.414	25.9928	26.0428	25.8551	1:Experimental evidence at protein level
26.687	26.7648	26.5371	26.213	#NUM!	#NUM!	#NUM!	#NUM!	1:Experimental evidence at protein level
25.7924	25.7291	25.8438	25.7817	26.5203	26.5578	26.3223	25.7469	1:Experimental evidence at protein level
25.1463	25.0731	24.6361	25.13	25.5215	25.7762	#NUM!	25.0367	1:Experimental evidence at protein level
28.6033	28.637	28.6081	28.6665	28.4881	28.7148	28.3096	27.7299	1:Experimental evidence at protein level
25.0576	25.4442	25.0108	24.6953	25.8827	25.8173	25.6772	25.0578	1:Experimental evidence at protein level
31.2812	31.1483	30.6244	30.7865	29.9312	30.3462	30.0133	29.8485	1:Experimental evidence at protein level
26.0513	26.6468	26.4254	26.6359	25.7768	25.5995	26.0564	26.1087	1:Experimental evidence at protein level
27.1013	27.311	27.2012	27.442	27.3244	27.0083	27.2042	#NUM!	1:Experimental evidence at protein level
25.2355	25.0168	25.5856	24.9261	25.7517	#NUM!	24.8049	#NUM!	1:Experimental evidence at protein level
25.0422	24.963	24.7564	25.3335	24.8873	24.7911	24.8538	25.2799	1:Experimental evidence at protein level
25.1984	25.1781	25.5319	25.4257	#NUM!	#NUM!	#NUM!	#NUM!	1:Experimental evidence at protein level
26.3873	26.6281	27.1074	26.6958	26.4235	26.3727	26.4411	25.9756	1:Experimental evidence at protein level
25.6055	25.7236	25.6997	25.8327	25.177	25.3102	25.4277	25.5965	1:Experimental evidence at protein level
27.2791	27.4588	27.771	27.4363	27.3409	27.2727	27.4386	27.2232	1:Experimental evidence at protein level
25.5577	25.5788	25.8845	26.0094	25.6379	25.268	25.6108	26.0302	1:Experimental evidence at protein level
26.3082	25.9871	25.8122	26.0281	26.2678	26.3825	26.1086	26.5735	1:Experimental evidence at protein level
25.6462	25.9333	25.8125	25.5548	26.0446	25.5984	25.8984	26.0853	1:Experimental evidence at protein level
24.6906	25.2427	25.4449	24.97	25.1349	#NUM!	25.4336	#NUM!	1:Experimental evidence at protein level
25.4245	25.8285	26.0205	25.9926	25.9706	26.0148	26.3046	#NUM!	1:Experimental evidence at protein level
25.8888	26.0739	26.3891	26.2828	25.73	25.7312	26.0115	26.2089	1:Experimental evidence at protein level
#NUM!	24.4308	24.4153	25.0443	25.4531	26.0804	25.7538	26.2815	1:Experimental evidence at protein level
24.614	24.6059	24.425	24.2302	#NUM!	24.1973	24.2705	#NUM!	1:Experimental evidence at protein level
27.9186	27.8137	27.6568	27.7522	27.4731	27.3577	27.3701	27.5496	1:Experimental evidence at protein level
26.0594	26.1868	26.4044	26.054	25.5941	25.931	25.8427	25.3443	1:Experimental evidence at protein level
24.9736	25.0133	25.0619	25.263	25.1306	25.7286	25.1655	26.0163	1:Experimental evidence at protein level
25.9745	26.1442	25.8724	26.4623	#NUM!	#NUM!	25.7081	26.0619	1:Experimental evidence at protein level
26.1603	25.8686	25.8607	26.2168	25.9861	25.7663	25.4189	26.1754	1:Experimental evidence at protein level
27.1347	27.259	27.4944	27.2455	27.3507	27.1774	27.2985	27.4166	1:Experimental evidence at protein level
24.3152	24.0209	23.9286	23.9811	24.465	24.1911	24.2444	25.1393	1:Experimental evidence at protein level
26.0866	26.6432	26.4117	26.6521	26.3221	26.1918	26.3504	26.807	1:Experimental evidence at protein level
24.6173	24.5923	24.8418	24.3193	24.3344	24.9396	24.6867	#NUM!	1:Experimental evidence at protein level
27.1293	27.3573	27.8253	27.1459	27.4233	27.3051	27.0198	26.8974	1:Experimental evidence at protein level
25.529	25.3847	23.9194	25.4202	25.2849	25.0559	25.4326	25.8858	1:Experimental evidence at protein level
26.3221	26.5557	26.8536	26.7896	25.823	#NUM!	26.1818	26.325	1:Experimental evidence at protein level
#NUM!	27.2487	26.8912	27.0383	27.0246	27.3337	27.056	27.4093	1:Experimental evidence at protein level
26.0098	25.6944	25.9084	25.8385	26.5339	26.4079	#NUM!	26.7713	1:Experimental evidence at protein level
26.2046	26.0311	26.0492	25.975	25.7462	26.0284	25.9115	25.759	1:Experimental evidence at protein level
26.3117	26.4581	26.4571	26.6189	26.2338	26.1113	26.4787	26.9838	1:Experimental evidence at protein level
26.2768	26.8051	26.7781	26.859	26.5488	26.1273	26.8739	27.0446	1:Experimental evidence at protein level
26.4438	26.3839	25.6488	25.6133	26.7967	26.9172	26.7629	26.8493	1:Experimental evidence at protein level
25.5866	25.6885	25.9225	25.8535	25.5741	25.0601	25.3362	25.2156	1:Experimental evidence at protein level
27.5464	27.6893	27.7717	27.4628	27.5034	27.0771	27.3782	27.197	1:Experimental evidence at protein level
26.3966	26.2036	26.1982	26.13	26.3705	26.3665	26.1358	26.4025	1:Experimental evidence at protein level
26.3274	26.4004	26.1518	26.4419	26.9411	27.195	27.0195	27.2684	1:Experimental evidence at protein level
#NUM!	25.4523	#NUM!	#NUM!	26.0927	25.895	25.9487	26.0943	1:Experimental evidence at protein level
25.6648	25.4546	25.0822	25.2271	25.6142	25.5511	25.201	26.0733	1:Experimental evidence at protein level
25.3984	24.9729	25.6379	26.0323	25.453	#NUM!	25.5145	25.8838	1:Experimental evidence at protein level
25.6902	25.5683	25.3723	25.6968	25.8792	25.6236	25.9049	26.2781	1:Experimental evidence at protein level
24.8501	25.3471	25.0747	25.1224	24.8102	24.3929	25.0289	25.5038	1:Experimental evidence at protein level
25.6338	#NUM!	25.6571	25.5736	25.2953	25.253	25.1571	25.9728	1:Experimental evidence at protein level
26.6924	27.1436	27.142	27.2885	27.1333	27.1462	27.2484	27.4985	1:Experimental evidence at protein level
27.7304	27.7704	27.9288	27.8154	27.7019	27.8041	27.674	27.0731	1:Experimental evidence at protein level
25.8055	25.9675	25.7858	25.9526	25.7111	25.9205	25.7564	26.5782	1:Experimental evidence at protein level
25.2208	25.6949	25.6921	25.3102	26.183	26.2573	26.2615	26.2862	1:Experimental evidence at protein level
26.9501	26.686	25.928	26.2174	26.3489	26.4637	26.2569	26.0479	1:Experimental evidence at protein level
26.2824	26.1988	26.3168	26.2417	27.1172	27.1526	26.7183	26.6136	1:Experimental evidence at protein level
#NUM!	25.7638	25.2781	25.4107	27.1161	26.8737	26.2795	26.8177	1:Experimental evidence at protein level
25.2197	25.0403	25.0388	25.6256	25.2225	25.3183	25.5812	26.1365	1:Experimental evidence at protein level
26.0436	25.9282	25.9308	26.1244	26.3646	25.834	26.2457	26.8739	1:Experimental evidence at protein level
25.9482	26.168	25.665	25.9512	25.7997	26.18	25.7113	26.3855	1:Experimental evidence at protein level
26.1276	26.342	#NUM!	#NUM!	26.8452	27.2512	26.8781	27.2631	1:Experimental evidence at protein level
27.9118	27.9615	28.1155	27.993	27.974	27.868	27.9836	27.708	1:Experimental evidence at protein level
29.3848	29.334	29.3622	29.4008	29.2828	29.1397	29.3188	29.2842	1:Experimental evidence at protein level
25.0297	24.9923	24.9009	24.8744	24.9147	25.1852	24.8375	#NUM!	1:Experimental evidence at protein level
28.1672	28.4011	28.4023	28.0647	27.9459	28.049	27.8835	28.5816	1:Experimental evidence at protein level
25.4081	25.2071	25.4156	25.5948	25.7879	25.9498	26.1677	26.5555	1:Experimental evidence at protein level
25.801	25.9355	25.7685	25.8183	26.3558	26.5779	26.4177	26.2983	1:Experimental evidence at protein level

25.1949	24.9299	25.1415	25.2867	25.2012	25.3051	25.1402	25.6947	1:Experimental evidence at protein level
25.4	25.3594	25.0584	25.0984	#NUM!	#NUM!	25.3888	25.2302	1:Experimental evidence at protein level
25.93	26.0571	26.1434	26.2767	26.143	26.2565	26.2818	26.5287	1:Experimental evidence at protein level
25.7645	25.8444	25.9915	26.3332	25.72	25.7166	25.6436	26.1381	1:Experimental evidence at protein level
25.8071	25.5647	25.9905	26.046	25.7892	#NUM!	25.5984	25.3014	1:Experimental evidence at protein level
25.9342	25.9557	25.7483	25.4848	24.8254	25.1092	24.6583	24.6923	1:Experimental evidence at protein level
25.6547	25.5138	25.4452	25.6296	26.1041	26.2459	25.8677	26.0797	1:Experimental evidence at protein level
26.5461	26.6773	26.589	26.9968	26.6413	26.5059	26.6727	27.458	1:Experimental evidence at protein level
27.2111	26.9899	26.5539	26.8544	27.3198	27.1199	26.9118	26.9132	1:Experimental evidence at protein level
28.1122	28.4576	#NUM!	28.3804	28.945	28.9044	29.0415	29.5011	1:Experimental evidence at protein level
25.6601	25.5974	25.9377	25.8417	25.4988	25.3122	25.5953	#NUM!	1:Experimental evidence at protein level
27.6346	27.1064	27.3381	27.6347	28.0718	27.9513	27.7799	27.8195	1:Experimental evidence at protein level
27.2585	27.2732	27.2556	27.0555	27.7828	27.8275	27.1854	26.6246	1:Experimental evidence at protein level
25.2505	25.5971	25.3378	25.5578	25.5796	25.8259	25.6	26.1	1:Experimental evidence at protein level
27.6349	27.7371	27.7142	27.6172	27.5282	27.6762	27.5211	27.2018	1:Experimental evidence at protein level
26.1259	26.7057	26.8877	26.3489	26.5002	25.9965	26.422	26.595	1:Experimental evidence at protein level
26.0956	26.3086	26.4012	26.3012	26.6663	26.5908	26.8631	26.6492	1:Experimental evidence at protein level
27.2671	27.5158	27.73	27.4063	27.4058	27.3467	27.3673	27.1274	1:Experimental evidence at protein level
25.922	25.9298	25.8884	26.0615	25.7414	25.9748	26.1784	26.328	1:Experimental evidence at protein level
25.1947	24.9202	24.8867	25.095	#NUM!	24.9979	#NUM!	24.8324	1:Experimental evidence at protein level
26.3123	26.0077	25.8002	26.2659	26.292	#NUM!	26.1488	27.1916	1:Experimental evidence at protein level
26.8355	26.6599	26.2819	26.2497	26.9841	26.989	26.8232	27.4649	1:Experimental evidence at protein level
27.4015	27.7474	27.7566	27.9112	27.7174	27.521	27.7977	28.1523	1:Experimental evidence at protein level
26.7367	26.5799	26.951	26.7893	25.99	26.0313	#NUM!	26.8285	1:Experimental evidence at protein level
27.9632	28.2457	27.869	27.9295	26.9102	26.931	26.8458	27.4349	1:Experimental evidence at protein level
#NUM!	26.0376	#NUM!	26.057	27.335	27.4949	27.3127	27.501	1:Experimental evidence at protein level
27.0072	#NUM!	26.9917	27.4021	27.6101	27.4281	27.4594	27.8103	1:Experimental evidence at protein level
28.1978	28.0541	27.915	28.1883	27.9005	28.0143	27.995	27.7982	1:Experimental evidence at protein level
28.3436	28.5792	28.5409	28.6851	28.4774	28.547	28.3706	27.616	1:Experimental evidence at protein level
27.1591	27.1798	27.4842	27.5539	27.3681	27.231	27.4109	27.7105	1:Experimental evidence at protein level
26.1401	25.9389	26.1332	26.1971	26.6319	26.2513	26.5274	27.1626	1:Experimental evidence at protein level
25.7695	#NUM!	25.7499	25.9313	25.9902	25.7363	25.5372	26.1235	1:Experimental evidence at protein level
27.9846	28.0182	28.2716	28.3184	28.0844	27.8246	28.1868	28.1093	1:Experimental evidence at protein level
28.5635	28.5959	28.5127	28.5104	28.8485	28.7965	28.7985	28.5704	1:Experimental evidence at protein level
26.5843	26.6305	26.5846	26.6475	26.7899	26.8743	26.9166	26.5982	1:Experimental evidence at protein level
28.425	28.3482	28.3901	28.7508	28.4625	28.291	28.6892	28.8011	1:Experimental evidence at protein level
25.2014	25.2764	25.2306	25.3126	25.012	25.5824	25.3158	25.6328	1:Experimental evidence at protein level
26.4349	26.4728	26.2264	26.3461	26.8424	26.6948	26.6656	27.3351	1:Experimental evidence at protein level
25.6069	25.2644	24.5896	24.5153	26.6416	26.7997	26.5386	26.3649	1:Experimental evidence at protein level
26.1989	26.0344	26.1812	26.2965	25.8833	25.845	25.9841	26.1846	1:Experimental evidence at protein level
26.5277	26.8247	27.0951	26.977	26.6086	26.5592	26.3673	26.5807	1:Experimental evidence at protein level
26.135	26.2184	25.5577	26.3129	26.4793	26.609	26.3648	26.9718	1:Experimental evidence at protein level
25.7273	25.7319	25.7367	25.6058	25.0388	25.4698	#NUM!	#NUM!	1:Experimental evidence at protein level
27.5497	27.632	27.7327	27.6832	27.742	27.671	27.7652	27.6972	1:Experimental evidence at protein level
27.4858	27.4828	27.4936	27.4505	27.6234	27.6738	27.598	27.3667	1:Experimental evidence at protein level
25.721	25.9901	25.6343	26.0091	25.3416	#NUM!	25.9319	26.6165	1:Experimental evidence at protein level
26.4127	25.9437	26.4543	26.8833	26.4821	26.4027	26.9434	27.2592	1:Experimental evidence at protein level
25.7239	25.3659	26.238	25.7848	25.3402	25.8786	25.5558	25.3874	1:Experimental evidence at protein level
25.8052	25.8304	25.7993	25.7514	25.3519	25.9556	25.5008	24.8273	1:Experimental evidence at protein level
25.8965	25.6455	26.1748	26.0481	25.7035	#NUM!	25.931	26.3691	1:Experimental evidence at protein level
25.622	25.8404	26.2896	25.941	25.7776	25.8048	25.8519	26.2366	1:Experimental evidence at protein level
27.3351	26.8939	26.5519	26.5194	25.8159	25.643	25.7841	26.1848	1:Experimental evidence at protein level
27.9101	28.0395	27.4328	27.0764	26.921	27.8843	27.1784	26.8737	1:Experimental evidence at protein level
27.1963	27.3357	26.9256	26.6244	26.5935	26.7266	26.8445	27.0652	1:Experimental evidence at protein level
25.4028	25.3672	25.5708	25.5746	25.3006	25.6819	25.4827	25.5576	1:Experimental evidence at protein level
29.3273	29.1182	28.8494	28.5368	29.47	29.5835	29.0106	27.5959	1:Experimental evidence at protein level
28.3656	28.2922	27.7214	28.7821	28.6521	28.8962	28.8388	29.3241	1:Experimental evidence at protein level
27.7366	27.5537	27.7957	27.977	27.9696	27.0829	27.7874	28.5953	1:Experimental evidence at protein level
25.0014	25.0939	24.4995	25.1298	25.4335	24.2494	24.6434	25.4043	1:Experimental evidence at protein level
28.2544	28.3685	28.5821	28.4337	27.9877	27.7711	27.9943	28.0569	1:Experimental evidence at protein level
27.0581	27.1085	27.3524	27.5277	26.9428	26.8017	27.1714	27.0335	1:Experimental evidence at protein level
26.5463	26.552	26.5742	26.3757	25.9442	26.1024	25.8238	25.8828	1:Experimental evidence at protein level
27.2271	27.2988	27.3733	26.6188	27.1078	26.8546	27.0835	26.9199	1:Experimental evidence at protein level
25.6975	25.6872	26.3145	26.4451	26.0205	26.3254	26.1597	25.9966	1:Experimental evidence at protein level
26.3829	26.3447	26.5533	26.5741	26.3302	26.2664	26.6067	26.952	1:Experimental evidence at protein level
25.4123	25.4463	25.6975	25.2368	#NUM!	25.6699	25.4195	25.3625	1:Experimental evidence at protein level
25.9137	25.7833	26.1337	26.103	26.1621	25.8578	#NUM!	26.2156	1:Experimental evidence at protein level
26.9773	26.9573	26.8993	27.0084	27.2411	27.1855	27.1356	27.149	1:Experimental evidence at protein level
26.1261	25.8809	25.8394	26.0083	25.7912	25.8274	25.9263	26.2981	1:Experimental evidence at protein level
29.0012	29.2641	29.6285	29.3101	29.6216	29.5164	29.729	29.6384	1:Experimental evidence at protein level
25.9621	26.0152	25.9515	25.8135	25.7793	25.8146	25.6711	26.3527	1:Experimental evidence at protein level
28.6886	28.81	28.7854	29.08	28.4419	28.2767	28.7717	29.1397	1:Experimental evidence at protein level
28.1542	28.2387	28.1362	28.4147	28.5267	28.3858	28.6004	28.4673	1:Experimental evidence at protein level
26.6468	26.7372	26.6979	26.7647	26.8259	27.1038	26.9659	27.2878	1:Experimental evidence at protein level
26.3321	25.9456	26.4926	26.321	26.1978	26.0456	26.1432	25.1886	1:Experimental evidence at protein level
#NUM!	25.5614	25.5547	25.7727	25.259	25.2831	25.62	25.9893	1:Experimental evidence at protein level
27.8598	28.0019	27.8823	27.86	27.8916	27.8332	27.9789	27.9807	1:Experimental evidence at protein level
27.5912	27.3621	28.0393	27.8375	27.2971	27.4409	27.6421	27.5648	1:Experimental evidence at protein level
27.5153	27.3943	27.7793	27.635	27.7381	27.4809	27.4582	27.4727	1:Experimental evidence at protein level
29.3608	29.0787	29.2968	29.534	29.493	29.0708	29.3518	29.5292	1:Experimental evidence at protein level
27.9088	27.8703	27.6225	27.7623	27.653	27.4227	27.7258	28.0839	1:Experimental evidence at protein level
27.3386	27.7213	27.6069	27.5024	26.9783	27.0383	27.0384	26.4584	1:Experimental evidence at protein level
30.6102	30.3904	30.4315	30.3499	30.677	30.5299	30.6179	30.4219	1:Experimental evidence at protein level
27.4047	27.2973	27.3179	27.2485	27.1039	27.3363	27.2156	26.9556	1:Experimental evidence at protein level
24.9836	24.9818	24.9484	25.1148	#NUM!	#NUM!	#NUM!	25.148	1:Experimental evidence at protein level
25.1771	25.208	25.2027	25.1632	25.2627	25.1864	25.1906	25.5701	1:Experimental evidence at protein level
26.1084	26.0984	26.2326	26.1057	26.6501	26.6581	26.6175	26.4873	1:Experimental evidence at protein level
24.5616	24.339	24.6221	24.5155	24.4889	24.3687	24.5483	#NUM!	1:Experimental evidence at protein level
24.6103	25.0547	25.4095	25.3566	25.0164	24.7199	25.2193	25.6495	1:Experimental evidence at protein level
25.0084	25.1402	25.1187	24.9536	25.1877	24.9062	25.098	26.0361	1:Experimental evidence at protein level
26.9857	27.0126	27.0172	27.3258	27.4127	27.5552	27.3996	27.7347	1:Experimental evidence at protein level
26.4425	26.2579	26.6949	26.6086	26.7807	26.3932	26.4776	27.2227	1:Experimental evidence at protein level
27.9084	27.6134	#NUM!	27.5812	28.1024	28.3143	27.5496	27.8855	1:Experimental evidence at protein level
25.2882	#NUM!	25.233	25.2943	25.5686	25.1298	25.2953	25.4926	1:Experimental evidence at protein level
27.2717	27.169	26.8978	27.3457	26.2481	#NUM!	26.6646	27.075	1:Experimental evidence at protein level
25.2987	25.5519	25.4614	25.4945	26.1188	25.8076	26.2644	#NUM!	1:Experimental evidence at protein level
27.1911	27.1425	27.4749	27.2107	27.2934	27.252	27.2548	27.1527	1:Experimental evidence at protein level
26.5743	26.5709	26.5023	26.5428	26.1342	26.0032	26.1116	26.4051	1:Experimental evidence at protein level
25.6407	25.6361	25.7905	25.701	25.3981	25.6288	25.3091	25.0797	1:Experimental evidence at protein level
26.2139	26.3391	26.4451	26.1565	25.8313	26.1482	25.6799	25.8884	1:Experimental evidence at protein level
26.2836	26.1382	25.9708	25.9348	26.3089	26.313	26.2732	25.644	1:Experimental evidence at protein level
24.8215	24.9235	24.8034	25.0058	24.7763	24.9511	24.9158	25.608	1:Experimental evidence at protein level
26.0335	25.9254	25.5898	25.4932	26.3885	26.673	26.2749	26.8713	1:Experimental evidence at protein level
26.1365	26.3924	26.5506	26.6256	26.0558	#NUM!	26.2989	26.3454	1:Experimental evidence at protein level
23.9764	24.344	24.1143	23.6049	24.566	#NUM!	24.2415	#NUM!	1:Experimental evidence at protein level
26.5168	25.6935	26.6392	26.5764	25.5545	#NUM!	24.7637	#NUM!	1:Experimental evidence at protein level
24.8298	24.9106	24.5428	24.7638	24.7893	24.4983	24.6016	24.4262	1:Experimental evidence at protein level
26.2963	26.3822	26.5886	26.4664	26.5649	26.5223	26.3011	26.9348	1:Experimental evidence at protein level
#NUM!	26.4852	26.2017	#NUM!	26.4041	26.6263	26.5938	26.7022	1:Experimental evidence at protein level
27.3486	#NUM!	#NUM!	26.6871	27.0733	27.1375	26.9633	27.1075	1:Experimental evidence at protein level
24.7999	24.7962	24.8244	24.494	25.3455	#NUM!	25.0176	25.0624	1:Experimental evidence at protein level
26.6855	26.7373	#NUM!	26.9805	27.8712	27.7946	28.1449	28.2251	1:Experimental evidence at protein level
25.6224	25.8089	25.7301	25.3291	25.0649	25.5436	25.3388	#NUM!	1:Experimental evidence at protein level
25.2764	25.1698	24.6232	25.3448	24.8622	25.16	24.9364	25.546	1:Experimental evidence at protein level
26.21	#NUM!	#NUM!	26.3256	26.9304	27.5896	27.0679	27.6482	1:Experimental evidence at protein level
23.9405	#NUM!	23.9838	24.5238	25.1448	24.9517	24.8019	25.4941	1:Experimental evidence at protein level
25.8536	25.9597	25.9343	25.9734	25.5868	25.7615	25.615	25.5074	1:Experimental evidence at protein level
25.4115	25.5436	25.8996	25.4665	25.57	25.6162	25.5155	25.8937	1:Experimental evidence at protein level
25.8546	25.5794	25.6111	25.5741	25.8313	25.9542	25.9217	25.4354	1:Experimental evidence at protein level
25.6691	25.8479	26.0521	25.8902	25.7531	25.7886	25.9027	25.7685	1:Experimental evidence at protein level
26.2152	25.439	25.0138	25.3063	25.3792	25.8754	25.4271	26.1879	1:Experimental evidence at protein level
27.514	27.5796	27.6313	27.485	27.373	27.3088	27.2386	27.3701	1:Experimental evidence at protein level
27.8529	27.766	27.7481	27.958	27.7743	27.33	27.6028	27.8999	1:Experimental evidence at protein level
25.1995	25.3536	25.3991	25.2524	25.535	25.1814	25.4176	#NUM!	1:Experimental evidence at protein level
24.6636	24.8404	24.4906	24.7807	24.5362	#NUM!	24.6569	24.9117	1:Experimental evidence at protein level
27.308	26.715	26.819	27.1868	27.1314	26.9446	26.903	#NUM!	1:Experimental evidence at protein level
24.6983	24.5338	24.9026	24.6917	24.5771	#NUM!	#NUM!	#NUM!	1:Experimental evidence at protein level
25.0233	25.0321	25.0745	25.1902	25.0181	25.5736	25.1023	#NUM!	1:Experimental evidence at protein level
25.9771	25.9121	25.8751	25.6271	25.9737	26.116	25.6701	25.9412	1:Experimental evidence at protein level

25.7688	26.0736	25.3022	#NUM!	26.4032	26.5062	26.4472	26.984	1:Experimental evidence at protein level
25.3573	25.0978	25.1128	25.5613	25.4616	25.3231	25.7301	#NUM!	1:Experimental evidence at protein level
26.4476	26.5212	26.6667	26.4195	26.8313	27.1119	26.9203	26.1302	1:Experimental evidence at protein level
28.9096	29.0104	28.7134	29.0054	28.941	28.9037	29.1291	29.2553	1:Experimental evidence at protein level
24.5744	24.6464	24.623	24.752	24.7154	24.6818	24.3958	25.1659	1:Experimental evidence at protein level
25.7979	25.7426	25.762	25.6031	25.604	25.9127	25.8693	25.4443	1:Experimental evidence at protein level
25.7117	25.5835	25.9739	26.0037	25.4837	#NUM!	#NUM!	#NUM!	1:Experimental evidence at protein level
26.69	26.8246	26.9069	26.9061	26.8286	26.9175	27.1435	27.1804	1:Experimental evidence at protein level
25.0241	25.0878	24.9294	24.9996	25.3584	25.5898	25.5009	26.184	1:Experimental evidence at protein level
26.8819	26.7432	26.1139	27.0519	26.1969	26.3944	26.6016	26.9319	1:Experimental evidence at protein level
26.5978	25.9801	26.0735	26.9605	26.3364	26.2515	26.7761	26.3171	1:Experimental evidence at protein level
26.7081	26.5669	26.4752	26.6896	26.4888	26.3038	26.3435	26.8442	1:Experimental evidence at protein level
26.6419	26.7373	26.9607	26.8791	#NUM!	#NUM!	24.6419	25.1368	1:Experimental evidence at protein level
26.2255	26.2909	25.9057	26.0464	26.4119	26.443	26.3339	26.8363	1:Experimental evidence at protein level
26.2814	26.278	26.5216	#NUM!	26.0442	26.0582	26.4324	26.0883	1:Experimental evidence at protein level
26.1175	26.0263	26.3707	25.9573	26.0912	26.1704	26.0834	26.4511	1:Experimental evidence at protein level
26.3851	26.3685	26.9117	26.6465	26.411	26.3564	26.5418	26.4629	1:Experimental evidence at protein level
26.9205	27.0918	27.2182	27.1445	27.0355	26.706	26.8465	27.0014	1:Experimental evidence at protein level
25.5563	25.5688	25.6739	25.3521	25.1602	25.3005	25.0705	#NUM!	1:Experimental evidence at protein level
27.6568	27.9352	27.7133	27.7273	27.2052	27.4437	27.5859	27.157	1:Experimental evidence at protein level
26.8497	27.1016	27.2462	27.152	26.9716	26.9082	27.1916	27.5563	1:Experimental evidence at protein level
25.517	25.6732	25.3393	25.0112	25.6359	25.8666	25.5436	25.8417	1:Experimental evidence at protein level
25.3664	25.5439	25.1995	24.9164	25.9666	26.1688	25.6223	26.0066	1:Experimental evidence at protein level
25.1828	24.786	24.7799	24.5553	25.3075	25.1855	24.9369	25.5646	1:Experimental evidence at protein level
25.3468	25.3515	25.2692	25.0646	25.788	25.5548	25.5521	26.1346	1:Experimental evidence at protein level
25.3143	25.2108	25.2985	25.4502	25.6275	25.6271	25.4663	25.5817	1:Experimental evidence at protein level
26.4521	26.0172	26.3143	26.2906	26.4297	26.0177	26.0416	25.838	1:Experimental evidence at protein level
25.0338	24.9047	25.056	24.2654	25.0199	#NUM!	#NUM!	#NUM!	1:Experimental evidence at protein level
25.3046	25.0896	25.2195	25.342	25.0567	25.1043	25.1262	25.9214	1:Experimental evidence at protein level
26.5219	26.7529	26.3398	26.3086	26.8126	26.6886	26.5635	26.7835	1:Experimental evidence at protein level
29.1828	29.072	29.2031	29.3399	28.8844	28.8407	28.8612	29.1587	1:Experimental evidence at protein level
26.9229	26.9439	27.1919	27.1728	27.2466	27.3747	27.3448	27.467	1:Experimental evidence at protein level
24.7618	24.6061	24.8105	25.4621	25.9742	25.5315	25.8666	26.1678	1:Experimental evidence at protein level
#NUM!	25.1205	24.2578	#NUM!	25.4099	25.021	24.916	25.6562	1:Experimental evidence at protein level
26.1292	26.3309	26.3253	26.4671	26.3758	26.4055	26.4521	26.8123	1:Experimental evidence at protein level
28.448	28.3837	28.6034	28.2862	28.5915	28.2913	28.0473	28.6348	1:Experimental evidence at protein level
27.5268	27.546	27.3672	27.4872	27.6597	27.523	27.4951	27.8831	1:Experimental evidence at protein level
26.1318	26.0863	26.4257	26.324	26.2066	26.2012	26.5821	26.1326	1:Experimental evidence at protein level
26.949	27.0217	27.243	26.9774	26.7017	26.8074	27.0823	26.9631	1:Experimental evidence at protein level
26.5522	26.5394	26.7426	26.7052	26.3009	26.7742	26.2836	27.0118	1:Experimental evidence at protein level
26.9183	27.4433	25.866	#NUM!	27.4878	27.553	27.5879	28.3585	1:Experimental evidence at protein level
#NUM!	25.4835	#NUM!	26.1407	26.2479	26.2727	25.9172	25.9453	1:Experimental evidence at protein level
27.1688	27.0734	26.5272	27.2012	27.4234	27.1799	27.0236	27.4295	1:Experimental evidence at protein level
25.3092	25.3332	26.0415	26.2596	25.4678	#NUM!	25.5625	25.4748	1:Experimental evidence at protein level
25.8851	25.7799	25.6899	26.1841	25.1692	#NUM!	25.1136	25.9956	1:Experimental evidence at protein level
27.3927	27.2901	26.897	26.8417	27.3511	27.461	27.2352	27.2722	1:Experimental evidence at protein level
28.3088	28.5051	28.5848	28.7256	28.182	27.6616	28.0294	28.3751	1:Experimental evidence at protein level
#NUM!	24.1892	24.4864	24.6613	24.5205	24.1632	24.5269	24.9428	1:Experimental evidence at protein level
25.383	25.4634	25.5199	25.3647	25.7682	25.4929	25.5795	24.8461	1:Experimental evidence at protein level
24.8754	24.9062	24.9492	24.8766	24.3446	24.5641	24.7831	#NUM!	1:Experimental evidence at protein level
26.2511	26.4773	26.5212	25.8747	26.9073	26.8725	26.7802	27.2907	1:Experimental evidence at protein level
28.1014	28.2983	28.3899	28.4141	27.8168	28.0057	27.9927	28.2826	1:Experimental evidence at protein level
25.8485	25.8517	26.0398	26.1978	26.2595	25.9275	26.5274	27.0804	1:Experimental evidence at protein level
25.8656	25.9656	26.0753	26.2345	25.8856	26.1209	26.1289	26.2079	1:Experimental evidence at protein level
27.6192	27.6306	27.6707	27.3172	27.4176	27.3025	27.4127	27.5299	1:Experimental evidence at protein level
26.5542	26.9996	27.168	27.3735	#NUM!	#NUM!	#NUM!	#NUM!	1:Experimental evidence at protein level
25.6288	25.0977	25.3862	25.4743	#NUM!	25.4438	25.0925	25.2482	1:Experimental evidence at protein level
26.684	26.8782	26.8352	26.925	25.7779	25.6588	25.8575	25.9782	1:Experimental evidence at protein level
26.0351	25.7309	25.9725	25.8489	26.3477	26.1977	26.0188	26.1965	1:Experimental evidence at protein level
25.3706	24.8467	25.0634	24.7176	25.5828	25.9386	25.0882	25.11	1:Experimental evidence at protein level
25.861	26.001	25.3981	25.7968	26.0111	26.1569	#NUM!	26.332	1:Experimental evidence at protein level
26.2727	26.0439	26.2367	26.4118	25.8761	25.8414	26.0356	25.7899	1:Experimental evidence at protein level
25.9119	26.0787	26.2936	26.4335	26.0694	26.3345	26.2637	26.3109	1:Experimental evidence at protein level
25.4508	25.6459	25.7272	26.0082	25.2462	25.3125	25.6735	26.4686	1:Experimental evidence at protein level
26.7012	26.9993	27.0866	26.8524	26.9727	26.4996	27.1136	26.8631	1:Experimental evidence at protein level
24.6364	24.9064	24.9748	24.822	24.5795	25.3058	25.0332	25.2428	1:Experimental evidence at protein level
29.2108	29.0753	29.3232	29.2104	28.3701	28.9015	28.5519	28.6129	1:Experimental evidence at protein level
23.8639	24.4936	24.2496	24.6181	23.9558	23.8897	24.3205	24.8618	1:Experimental evidence at protein level
25.8719	25.9441	25.8446	25.7709	25.8037	25.5806	26.0933	25.581	1:Experimental evidence at protein level
24.789	24.8863	24.9749	25.1764	25.4522	25.7467	25.3905	25.9744	1:Experimental evidence at protein level
24.3339	24.0783	23.9077	23.6985	23.9021	24.3003	23.4705	#NUM!	1:Experimental evidence at protein level
26.4932	26.7777	26.3477	26.4691	27.0002	26.1373	27.1024	27.4331	1:Experimental evidence at protein level
27.0942	27.3695	27.3064	27.5649	27.2944	27.2441	27.4634	27.8606	1:Experimental evidence at protein level
29.0869	29.3483	29.3433	29.3693	29.3582	29.3733	29.3783	29.6304	1:Experimental evidence at protein level
25.3178	25.2259	#NUM!	25.4973	25.5442	25.4417	25.6652	26.1384	1:Experimental evidence at protein level
25.6118	26.0376	25.8271	26.1097	25.4719	#NUM!	25.3418	26.3077	1:Experimental evidence at protein level
27.5037	27.443	27.6865	27.6064	27.2667	27.3419	27.2439	27.4961	1:Experimental evidence at protein level
25.9052	25.8794	25.8283	26.1806	26.0449	26.0845	25.9887	26.4875	1:Experimental evidence at protein level
26.7188	26.7816	26.7183	26.8774	26.4636	26.8609	26.7072	27.5977	1:Experimental evidence at protein level
27.8579	28.4118	28.2793	27.8271	28.008	27.6383	27.728	27.9316	1:Experimental evidence at protein level
27.4294	27.5014	27.8993	28.026	27.1358	27.0928	27.2136	27.8106	1:Experimental evidence at protein level
24.607	24.2474	23.5209	23.1516	#NUM!	#NUM!	#NUM!	#NUM!	1:Experimental evidence at protein level
26.3149	26.2839	26.6922	26.3778	26.08	26.1126	26.2067	26.6112	1:Experimental evidence at protein level
26.8705	26.7097	26.6272	26.6646	27.2241	27.3993	27.4652	27.6538	1:Experimental evidence at protein level
26.652	26.7114	27.047	26.9963	26.8654	26.5619	26.7768	27.0446	1:Experimental evidence at protein level
#NUM!	#NUM!	26.2866	26.3761	28.4317	28.0964	26.8095	27.5679	1:Experimental evidence at protein level
26.5577	26.6752	26.7075	26.5812	26.9231	26.7915	27.1144	26.5884	1:Experimental evidence at protein level
28.6589	28.6964	28.7754	28.6958	28.0118	27.8863	28.0124	27.9259	1:Experimental evidence at protein level
26.4698	26.3205	26.1585	26.4204	26.6246	26.612	26.3706	26.8184	1:Experimental evidence at protein level
27.4322	27.4411	27.2859	27.5116	27.4072	27.3712	27.2283	27.457	1:Experimental evidence at protein level
25.6217	26.0433	25.5902	26.1979	26.508	26.4367	26.6069	27.3133	1:Experimental evidence at protein level
31.4905	31.87	31.792	31.5375	31.4446	31.8893	31.5223	29.8698	1:Experimental evidence at protein level
25.9187	25.9254	26.0615	25.8442	26.1028	26.184	26.0277	#NUM!	1:Experimental evidence at protein level
28.9035	28.87	28.4351	28.4671	28.7009	28.9031	28.5238	28.6716	1:Experimental evidence at protein level
25.2164	25.9644	26.0535	26.0775	#NUM!	#NUM!	26.1634	#NUM!	1:Experimental evidence at protein level
25.2288	25.4353	25.1494	25.4611	25.9746	26.278	25.9619	26.4712	1:Experimental evidence at protein level
29.6325	29.4353	29.3392	29.3187	29.2993	29.3914	29.6272	29.742	1:Experimental evidence at protein level
26.2767	25.7001	26.0395	25.9474	25.9488	#NUM!	#NUM!	26.1405	1:Experimental evidence at protein level
27.3995	27.5126	27.6386	27.3681	27.2615	27.0178	26.982	27.4124	1:Experimental evidence at protein level
26.6607	26.922	27.0196	26.8449	#NUM!	#NUM!	#NUM!	#NUM!	1:Experimental evidence at protein level
26.5868	26.5038	25.814	25.8027	27.0329	26.6481	27.2885	26.8071	1:Experimental evidence at protein level
27.5952	27.5031	27.5037	26.9278	27.3106	27.403	27.1307	26.1234	1:Experimental evidence at protein level
30.0598	30.0031	29.6857	29.7754	29.8238	30.0482	29.7512	30.0014	1:Experimental evidence at protein level
26.688	26.8547	26.8	26.9023	25.782	25.1194	25.5019	26.2832	1:Experimental evidence at protein level
25.4985	25.6141	25.382	26.0731	#NUM!	#NUM!	25.7223	26.7515	1:Experimental evidence at protein level
25.2054	24.8744	24.6643	25.0513	25.9875	25.7682	25.9381	26.2786	1:Experimental evidence at protein level
28.9399	29.0012	29.1959	29.4086	#NUM!	#NUM!	#NUM!	#NUM!	1:Experimental evidence at protein level
26.9534	26.9304	27.1955	27.2897	27.1693	27.0577	27.393	27.3562	1:Experimental evidence at protein level
27.5285	27.9964	28.2931	27.6845	27.184	27.1338	27.8552	27.4034	1:Experimental evidence at protein level
25.9893	25.7736	25.6367	25.6946	25.9297	#NUM!	25.8654	25.8383	1:Experimental evidence at protein level
26.1603	26.3252	26.1204	26.4098	25.1535	24.7714	24.9015	25.7448	1:Experimental evidence at protein level
25.0794	25.2326	25.1281	24.9882	25.3984	#NUM!	#NUM!	#NUM!	1:Experimental evidence at protein level
26.7507	27.0529	27.1711	27.059	26.4102	26.548	26.4928	26.4681	1:Experimental evidence at protein level
26.9937	27.1775	27.2793	27.1609	27.0737	27.0754	27.0921	26.6656	1:Experimental evidence at protein level
25.9748	25.6594	25.2398	25.5008	25.4396	25.785	25.4024	25.8098	1:Experimental evidence at protein level
27.0865	27.2377	27.2235	27.0605	#NUM!	#NUM!	#NUM!	#NUM!	1:Experimental evidence at protein level
24.8958	24.8046	24.9506	25.0539	25.3066	25.1347	25.5093	26.2904	1:Experimental evidence at protein level
26.6399	26.6911	27.0398	26.8273	27.2735	27.3251	27.4405	27.4867	1:Experimental evidence at protein level
29.0987	29.0639	29.317	29.4493	28.9645	28.6719	28.9857	29.3749	1:Experimental evidence at protein level
26.3233	26.6802	26.637	26.7945	26.1843	26.2196	26.4199	26.3971	1:Experimental evidence at protein level
25.7034	25.6164	25.6638	26.2306	26.2002	25.8012	26.3288	27.0175	1:Experimental evidence at protein level
25.1079	25.0217	24.8688	25.1134	24.2868	24.5957	24.791	24.3909	1:Experimental evidence at protein level
25.6304	25.6362	25.8813	25.7295	25.9106	25.9075	25.7257	26.0705	1:Experimental evidence at protein level
25.2817	25.3234	25.5714	25.2714	25.0676	25.0607	25.0368	24.931	1:Experimental evidence at protein level
25.2983	25.5303	25.9267	25.6215	25.2127	#NUM!	25.5643	25.4112	1:Experimental evidence at protein level
25.6662	25.7401	25.8695	25.7727	25.5236	25.7374	25.7063	26.0583	1:Experimental evidence at protein level
25.9312	26.2492	26.343	26.1488	26.0615	25.9126	26.1618	26.1338	1:Experimental evidence at protein level
26.5584	26.3426	26.6735	26.7406	26.8164	26.7342	26.6371	26.3705	1:Experimental evidence at protein level
29.1553	28.878	28.5595	28.8636	29.4679	29.7331	28.736	28.9715	1:Experimental evidence at protein level

29.4499	29.6558	30.0515	29.8753	29.4397	29.5401	29.6702	29.6545	1:Experimental evidence at protein level
27.2982	27.3432	27.4088	27.0895	27.5062	27.608	27.4501	27.5175	1:Experimental evidence at protein level
30.1524	30.1685	29.8648	30.2823	30.0031	30.0848	29.9319	29.9665	1:Experimental evidence at protein level
27.2506	26.9231	26.9186	27.3191	27.3286	27.5817	26.9619	27.4984	1:Experimental evidence at protein level
29.6302	29.6298	29.184	29.3448	30.1217	30.1508	30.052	30.2137	1:Experimental evidence at protein level
26.2462	26.3009	26.443	26.36	26.3533	26.5899	26.7033	26.5665	1:Experimental evidence at protein level
26.6247	26.6223	26.3795	26.2457	26.0484	25.9138	26.2532	26.6448	1:Experimental evidence at protein level
26.5012	26.8252	26.6483	26.6423	27.1558	27.2528	27.1446	27.8733	1:Experimental evidence at protein level
26.5086	26.6052	27.0779	26.8459	26.8844	27.039	27.0897	26.9402	1:Experimental evidence at protein level
30.7179	30.6954	30.618	30.8016	31.3078	31.2909	31.1905	31.0424	1:Experimental evidence at protein level
26.1846	26.1745	26.3815	25.6069	25.4662	25.1389	25.5706	#NUM!	1:Experimental evidence at protein level
25.9905	#NUM!	26.3758	26.4982	25.8504	26.2025	25.7304	25.7444	1:Experimental evidence at protein level
26.5155	26.6667	26.5676	26.5167	26.5423	26.5069	26.4627	27.0592	1:Experimental evidence at protein level
25.4905	25.3878	25.582	25.5553	25.3585	25.4219	25.6128	25.7292	1:Experimental evidence at protein level
26.3591	26.2727	26.2988	26.2894	25.9337	26.1223	25.9418	26.4891	1:Experimental evidence at protein level
#NUM!	26.8534	27.1987	#NUM!	26.4448	26.0056	25.9987	25.7741	1:Experimental evidence at protein level
27.0212	27	27.0462	27.3819	27.2894	27.144	27.357	27.6779	1:Experimental evidence at protein level
26.5645	26.2037	26.1996	25.6813	26.5447	26.465	26.2499	27.0474	1:Experimental evidence at protein level
27.2474	27.5524	27.814	27.4255	27.2005	27.3334	27.2833	27.7757	1:Experimental evidence at protein level
27.3112	27.3786	27.6057	27.328	27.1516	26.9727	27.0939	26.9	1:Experimental evidence at protein level
29.758	29.8432	29.5439	29.6288	29.7184	29.7755	29.7564	29.8866	1:Experimental evidence at protein level
28.8557	28.7345	28.5115	28.8244	28.775	28.9555	28.6231	27.8235	1:Experimental evidence at protein level
28.287	28.4141	28.5787	28.0902	28.0158	27.9106	28.3428	27.5257	1:Experimental evidence at protein level
26.4005	26.5406	26.7196	26.5746	25.8485	25.9599	26.0596	25.8887	1:Experimental evidence at protein level
26.1687	26.26	26.1855	25.7991	25.5306	25.5142	25.7321	25.3014	1:Experimental evidence at protein level
27.5751	28.4553	27.9647	27.3408	28.3653	27.9636	28.0147	29.5215	5:Protein uncertain
#NUM!	25.6145	25.5552	25.5578	25.8604	26.0196	26.0471	25.6096	1:Experimental evidence at protein level
26.6972	26.8347	26.7076	26.9061	26.8634	26.8871	26.9654	27.0623	1:Experimental evidence at protein level
25.1264	25.0723	25.108	25.3627	25.5497	#NUM!	#NUM!	25.8738	1:Experimental evidence at protein level
27.6538	27.5744	27.0677	27.8284	26.7288	26.8283	26.7165	27.5966	1:Experimental evidence at protein level
26.0949	26.4117	26.2577	26.2759	26.2611	26.2695	26.2403	26.0032	1:Experimental evidence at protein level
24.9686	25.1813	24.8807	25.1606	25.4746	25.9067	25.732	25.9946	1:Experimental evidence at protein level
25.7755	25.4381	25.3853	25.8271	#NUM!	25.2578	#NUM!	26.3163	1:Experimental evidence at protein level
25.4547	25.8508	25.6861	26.0638	26.2062	26.3513	26.1015	26.7262	1:Experimental evidence at protein level
28.3005	28.0745	28.4846	28.2217	28.1137	27.9877	28.0254	27.8532	1:Experimental evidence at protein level
#NUM!	25.3851	25.4225	25.2873	26.0581	25.9321	25.8176	26.1083	1:Experimental evidence at protein level
28.796	28.4023	28.4692	29.1038	28.6869	28.7545	28.6573	29.7645	1:Experimental evidence at protein level
27.0057	27.1674	27.4443	27.5466	27.2109	27.1271	27.5221	27.8365	1:Experimental evidence at protein level
25.3389	25.2564	25.7869	25.8437	25.2691	#NUM!	25.4136	25.726	1:Experimental evidence at protein level
25.5017	25.5684	25.8337	25.7436	25.4547	25.2366	25.4969	25.9274	1:Experimental evidence at protein level
24.9101	24.9879	25.453	25.4712	#NUM!	#NUM!	25.5049	25.7059	1:Experimental evidence at protein level
26.5716	26.4555	26.4287	26.0087	26.3783	26.858	26.2163	26.0663	1:Experimental evidence at protein level
25.4234	25.3735	25.6285	25.6655	25.3706	25.8096	25.5636	25.9204	1:Experimental evidence at protein level
25.4019	25.4317	25.36	25.3325	26.0251	26.3386	25.9841	26.4218	1:Experimental evidence at protein level
26.3655	26.3	25.9663	26.1165	26.7672	26.8062	27.0405	26.9376	1:Experimental evidence at protein level
25.664	25.7487	25.5711	25.9828	#NUM!	#NUM!	25.7779	26.0948	1:Experimental evidence at protein level
25.0121	24.9811	25.1985	25.253	#NUM!	24.7973	24.6842	24.9284	1:Experimental evidence at protein level
26.0725	25.9775	25.8997	26.1493	26.4031	25.8843	26.2473	27.0705	1:Experimental evidence at protein level
26.7588	26.8148	26.9451	27.111	27.2312	27.3402	26.9986	27.0298	1:Experimental evidence at protein level
29.3664	29.4292	29.6038	29.8282	29.5747	29.3979	29.3859	29.6371	1:Experimental evidence at protein level
25.8199	26.0946	26.322	26.3923	26.3719	26.2403	26.2765	27.1093	1:Experimental evidence at protein level
26.6433	26.7301	25.727	25.4121	25.8618	26.6743	26.3921	26.6675	1:Experimental evidence at protein level
26.7565	26.6388	26.9439	26.7694	26.6842	26.922	26.8703	26.5507	1:Experimental evidence at protein level
25.5557	24.9665	24.8621	25.3371	#NUM!	#NUM!	24.7243	#NUM!	1:Experimental evidence at protein level
27.5697	27.6758	28.2085	27.8707	27.7674	27.578	27.7492	27.7682	1:Experimental evidence at protein level
26.189	25.6154	25.0748	25.6938	26.2025	26.1587	26.2689	27.1019	1:Experimental evidence at protein level
25.8856	25.6472	25.9503	25.9324	25.6575	25.9554	26.0375	25.9907	1:Experimental evidence at protein level
26.8313	26.9135	27.1207	26.7587	26.835	26.4721	26.8298	27.1366	1:Experimental evidence at protein level
27.9351	27.5578	27.8817	27.9476	27.3656	27.3112	27.2707	27.518	1:Experimental evidence at protein level
26.7033	26.9058	27.2228	26.8993	26.8072	26.6456	27.0183	26.987	1:Experimental evidence at protein level
27.235	27.0772	26.9906	27.4512	26.541	26.5182	26.7175	27.1966	1:Experimental evidence at protein level
26.4265	26.6231	26.4546	26.9993	26.4955	26.4211	26.6014	27.1276	1:Experimental evidence at protein level
26.3073	26.5061	26.7919	26.3884	26.4207	26.4017	26.4495	26.614	1:Experimental evidence at protein level
27.4933	27.548	27.7679	27.6387	27.3808	27.3671	27.6522	27.8997	1:Experimental evidence at protein level
26.4306	26.569	26.748	26.6766	26.5945	26.5604	26.8117	26.6538	1:Experimental evidence at protein level
25.1096	24.873	25.0691	24.1616	25.7507	25.7799	25.533	25.942	1:Experimental evidence at protein level
25.3829	25.3395	25.6327	25.7416	25.6557	25.8988	25.5729	25.9116	1:Experimental evidence at protein level
27.6297	27.9783	27.845	27.9924	27.5304	27.733	27.7706	27.7891	1:Experimental evidence at protein level
27.9039	28.0514	28.1638	28.1303	27.8991	27.7118	28.065	28.3293	1:Experimental evidence at protein level
26.6597	26.5079	26.3399	26.9755	27.0999	26.6533	26.9868	27.4579	1:Experimental evidence at protein level
26.5932	26.6801	26.4464	26.7002	26.5777	26.6986	26.4923	27.2177	1:Experimental evidence at protein level
25.5022	25.5631	25.3217	25.4939	#NUM!	#NUM!	#NUM!	#NUM!	1:Experimental evidence at protein level
28.9259	29.108	29.2624	29.1282	28.6269	28.8007	28.6385	29.01	1:Experimental evidence at protein level
25.366	25.4497	25.3314	25.6133	25.279	25.6572	25.2105	25.3533	1:Experimental evidence at protein level
27.1028	27.3308	27.3408	27.066	26.7882	26.8385	27.2372	26.6355	1:Experimental evidence at protein level
25.5746	25.6002	25.7424	25.5946	26.2223	26.2417	26.0526	26.1	1:Experimental evidence at protein level
25.1353	24.492	24.3649	24.9084	25.0726	24.9055	24.6974	#NUM!	1:Experimental evidence at protein level
24.543	24.3406	24.6512	24.7116	24.2486	24.5782	24.5385	25.0528	1:Experimental evidence at protein level
27.849	27.8136	27.8467	27.7468	27.5262	27.5485	27.6466	28.0594	1:Experimental evidence at protein level
27.7437	27.5774	27.8007	27.9471	27.6494	27.4895	27.5752	28.0556	1:Experimental evidence at protein level
26.0044	25.2559	25.8024	25.8592	26.2596	26.2083	26.1305	26.7368	1:Experimental evidence at protein level
25.2562	25.3832	#NUM!	#NUM!	25.5704	25.6617	25.5894	26.2101	1:Experimental evidence at protein level
26.5316	26.5968	26.6544	26.8628	26.6445	26.846	26.8697	26.7209	1:Experimental evidence at protein level
26.5481	26.667	26.4014	26.5812	26.5475	27.0066	26.8006	27.1721	1:Experimental evidence at protein level
26.3453	26.6567	26.7365	26.4634	26.5162	27.0158	26.7896	26.595	1:Experimental evidence at protein level
25.0289	24.9432	24.9994	25.5511	25.1672	#NUM!	24.6385	25.5601	1:Experimental evidence at protein level
25.4316	25.8121	25.4812	25.6779	25.8557	25.6589	26.0285	26.0839	1:Experimental evidence at protein level
25.9152	25.7594	25.5964	25.9169	26.4471	26.413	26.261	#NUM!	1:Experimental evidence at protein level
26.8701	26.7675	26.9575	26.9185	27.0651	27.0374	27.0264	26.8414	1:Experimental evidence at protein level
27.0892	27.235	27.4316	27.3812	27.2353	27.2451	27.2277	27.4177	1:Experimental evidence at protein level
26.0223	26.3481	26.341	26.4439	26.3719	26.4431	26.4633	26.7094	1:Experimental evidence at protein level
27.1988	27.1914	27.3911	27.5489	27.2993	26.95	27.3762	27.8818	1:Experimental evidence at protein level
27.2858	27.614	27.6246	27.6931	27.1115	27.017	27.3649	27.4135	1:Experimental evidence at protein level
27.9821	27.9722	27.7186	27.6093	27.8817	27.8861	27.8124	#NUM!	1:Experimental evidence at protein level
26.3247	26.352	26.2867	26.3797	26.2338	25.9497	25.7362	26.4095	1:Experimental evidence at protein level
27.0075	27.0729	26.843	27.1029	27.2252	27.3637	27.1571	27.5918	1:Experimental evidence at protein level
25.8104	25.8319	25.8951	26.1241	25.8926	25.8907	26.0727	26.5194	1:Experimental evidence at protein level
25.0055	24.8756	24.9331	24.6564	#NUM!	#NUM!	#NUM!	25.0698	1:Experimental evidence at protein level
27.011	26.8741	26.8266	26.8133	27.0209	27.0824	26.534	26.9443	1:Experimental evidence at protein level
27.4312	27.8301	28.2147	28.0356	27.5596	27.7757	28.0163	27.3155	1:Experimental evidence at protein level
25.2651	25.6116	25.7141	25.7589	25.6821	26.0476	25.7985	26.0978	1:Experimental evidence at protein level
24.7627	24.2512	#NUM!	24.7914	24.9256	25.2309	25.5252	25.3179	1:Experimental evidence at protein level
26.3157	26.4408	26.2243	26.5753	27.0896	27.2958	27.1397	27.7766	1:Experimental evidence at protein level
26.4904	26.7367	#NUM!	26.5897	27.227	27.0249	26.9066	27.2932	1:Experimental evidence at protein level
25.0792	24.895	25.0141	24.8325	#NUM!	#NUM!	#NUM!	#NUM!	1:Experimental evidence at protein level
26.762	27.0632	27.1058	27.16	26.6817	26.8087	26.7919	27.2822	1:Experimental evidence at protein level
25.6878	25.6153	25.8501	#NUM!	26.1465	25.8158	26.1685	26.3925	1:Experimental evidence at protein level
27.1171	27.6139	27.6019	27.6966	27.6285	27.3699	27.7336	28.0107	1:Experimental evidence at protein level
24.8944	24.7036	25.1501	24.851	24.3883	24.8014	#NUM!	#NUM!	1:Experimental evidence at protein level
26.5954	26.5491	26.4288	26.6064	28.3375	28.1702	28.2356	27.5126	1:Experimental evidence at protein level
27.3897	27.3377	27.3152	27.4829	28.3707	28.3659	28.2333	27.669	1:Experimental evidence at protein level
28.0699	28.2528	28.261	28.446	27.3442	27.2842	27.3995	27.9875	1:Experimental evidence at protein level
28.7863	28.8501	28.7115	28.9983	29.0121	29.1348	29.1254	29.2404	1:Experimental evidence at protein level
24.4079	24.3922	24.5726	24.0759	24.3364	#NUM!	24.3056	#NUM!	1:Experimental evidence at protein level
26.5555	26.5068	26.3038	26.6493	26.0853	26.1061	26.2956	26.8084	1:Experimental evidence at protein level
26.0726	26.1303	25.5041	26.2986	26.3582	25.9763	26.4907	26.9299	1:Experimental evidence at protein level
25.0875	25.0266	25.4506	25.377	25.4784	25.525	25.4513	25.5786	1:Experimental evidence at protein level
28.1281	28.0618	28.2296	27.9163	27.8995	27.6269	27.9445	28.1245	1:Experimental evidence at protein level
26.9506	26.6476	26.9838	26.8486	26.7976	26.6455	26.8654	26.536	1:Experimental evidence at protein level
28.6054	28.6508	28.7184	28.2906	28.5977	28.6163	28.3087	27.7431	1:Experimental evidence at protein level
26.0592	26.1039	26.1496	26.1298	26.3917	26.1045	26.0612	26.4847	1:Experimental evidence at protein level
26.9949	26.7796	26.9419	26.556	26.4157	26.2551	26.1615	25.6489	1:Experimental evidence at protein level
25.947	25.662	25.2601	25.0328	25.9236	26.0205	25.5156	25.9746	1:Experimental evidence at protein level
26.0366	26.0884	26.3061	26.1527	#NUM!	#NUM!	25.2437	24.6738	1:Experimental evidence at protein level
27.6471	27.885	27.3104	27.582	26.7321	26.5967	26.5696	27.1593	1:Experimental evidence at protein level
26.3409	26.1419	26.3648	25.9425	26.2099	26.3553	25.8374	25.7429	1:Experimental evidence at protein level
26.3369	26.4078	26.2563	26.4321	26.458	26.6004	26.5124	26.085	1:Experimental evidence at protein level
26.6491	26.5064	26.4901	26.8301	27.0901	27.2004	27.0581	27.2704	1:Experimental evidence at protein level
25.7272	26.0157	25.7615	26.0082	25.1183	25.0963	25.0215	25.4401	1:Experimental evidence at protein level

28.9285	28.9735	28.7041	28.8062	29.0003	29.1307	28.7307	28.7882	1:Experimental evidence at protein level
30.1549	30.2329	29.9652	30.1431	29.7054	29.5893	29.8983	30.007	5:Protein uncertain
26.5051	26.4039	26.5411	26.5852	25.4662	#NUM!	25.5248	#NUM!	1:Experimental evidence at protein level
25.5928	26.4037	26.5447	26.2747	25.4315	25.1964	25.5801	25.8651	1:Experimental evidence at protein level
26.6976	26.675	26.3705	26.5468	27.2221	26.7658	26.7931	27.1923	1:Experimental evidence at protein level
27.0444	26.9239	27.0837	27.231	27.2889	27.1429	27.2452	27.6982	1:Experimental evidence at protein level
25.9627	25.825	25.7904	26.0046	26.1598	25.8395	26.3594	26.6952	1:Experimental evidence at protein level
29.1557	29.2605	29.0728	29.1139	29.3051	29.2217	29.2011	29.5688	1:Experimental evidence at protein level
24.5575	24.5513	24.4055	24.5213	25.6092	25.5765	25.4434	26.1879	1:Experimental evidence at protein level
25.4377	25.3109	25.2602	25.0369	25.475	25.4921	25.2252	25.5212	1:Experimental evidence at protein level
26.5514	26.7262	26.7218	26.8847	26.7267	26.8737	26.8311	26.7617	1:Experimental evidence at protein level
26.9324	27.0099	27.3875	27.1307	26.9779	26.8761	27.0747	27.3478	1:Experimental evidence at protein level
25.0964	25.1344	25.1276	25.3316	#NUM!	#NUM!	25.0873	#NUM!	1:Experimental evidence at protein level
26.977	27.111	26.9702	26.9836	26.8063	26.9443	27.0971	26.7867	1:Experimental evidence at protein level
29.9933	30.4881	30.8643	30.8044	30.1765	30.1421	30.4214	30.3939	1:Experimental evidence at protein level
25.9452	25.9649	25.9277	25.982	25.922	26.1427	26.1509	26.0265	1:Experimental evidence at protein level
27.6265	27.7551	27.8544	27.9515	27.645	28.0451	27.5225	28.1041	1:Experimental evidence at protein level
28.9515	29.1917	29.1135	28.9931	28.9434	28.9902	29.047	28.8884	1:Experimental evidence at protein level
29.2301	29.2654	29.5557	29.3042	29.0431	29.0013	29.1095	28.6099	1:Experimental evidence at protein level
27.6961	27.743	27.7465	27.9049	27.2021	27.1407	27.0763	27.6271	1:Experimental evidence at protein level
27.4078	27.2315	27.3846	27.3264	27.2927	26.8711	27.0249	27.1585	1:Experimental evidence at protein level
25.7004	25.668	25.1981	26.2056	#NUM!	#NUM!	25.9627	#NUM!	1:Experimental evidence at protein level
27.0185	27.2233	27.2301	27.0584	27.0045	#NUM!	27.1608	27.4432	1:Experimental evidence at protein level
#NUM!	#NUM!	25.499	25.5475	24.9587	25.155	25.0911	25.2816	1:Experimental evidence at protein level
26.4326	26.2539	26.445	26.5201	#NUM!	#NUM!	#NUM!	#NUM!	1:Experimental evidence at protein level
26.1598	26.2391	26.0218	26.1824	26.8258	26.7637	26.995	27.2126	1:Experimental evidence at protein level
26.1107	25.7055	25.3402	25.5273	#NUM!	25.6403	25.6465	#NUM!	1:Experimental evidence at protein level
27.9915	27.9609	28.2434	28.0993	28.0084	28.0608	27.7767	27.6795	1:Experimental evidence at protein level
26.7288	26.6425	26.8381	27.0397	27.3797	27.0098	27.1979	27.4177	1:Experimental evidence at protein level
25.248	25.4577	25.5276	25.948	25.8002	25.6463	25.6528	25.9044	1:Experimental evidence at protein level
26.0353	26.0132	26.2583	26.4908	25.6033	25.6413	25.4522	26.863	1:Experimental evidence at protein level
27.9702	27.9658	28.0817	28.1188	27.9457	27.8868	27.9034	27.8209	1:Experimental evidence at protein level
29.996	30.1599	30.3717	30.2158	30.0438	30.0705	30.1269	29.8734	1:Experimental evidence at protein level
29.8782	29.7126	29.7107	29.1726	29.9789	29.8559	29.8501	29.0103	1:Experimental evidence at protein level
28.5478	28.7424	28.763	28.8892	28.7032	28.5239	28.7772	29.1557	1:Experimental evidence at protein level
28.4513	28.6217	29.1642	29.0857	29.1611	29.0013	29.1429	29.3727	1:Experimental evidence at protein level
26.7666	26.6491	27.0141	27.5511	27.3142	#NUM!	27.3698	#NUM!	1:Experimental evidence at protein level
26.5777	26.6221	26.3729	26.6054	26.6889	26.6364	26.6137	26.8802	1:Experimental evidence at protein level
26.6252	26.801	26.6043	26.4358	26.6404	26.6663	26.6424	26.0932	1:Experimental evidence at protein level
25.7352	26.0584	26.1064	26.3016	#NUM!	#NUM!	#NUM!	#NUM!	1:Experimental evidence at protein level
27.0477	26.8531	26.5717	26.6404	27.5332	27.7559	27.2753	27.0717	1:Experimental evidence at protein level
25.0978	25.0226	25.0321	#NUM!	25.5224	25.7632	25.7846	26.482	1:Experimental evidence at protein level
26.7645	26.9028	26.8261	26.9045	27.1117	27.1677	26.7653	27.7351	1:Experimental evidence at protein level
24.9451	24.8902	24.8745	25.6804	25.2608	25.353	25.3791	25.5314	1:Experimental evidence at protein level
26.7396	26.6426	26.9606	27.0656	27.3952	27.27	27.1243	27.8463	1:Experimental evidence at protein level
25.52	25.3914	25.5727	25.3748	24.9397	#NUM!	25.0514	25.229	1:Experimental evidence at protein level
27.459	27.7096	27.772	27.6932	27.7017	27.651	27.6414	27.9107	1:Experimental evidence at protein level
26.27	26.2007	26.0255	26.323	26.7285	#NUM!	#NUM!	27.2563	1:Experimental evidence at protein level
28.7429	28.4718	28.5402	28.7903	28.4365	28.7493	28.2459	28.7903	1:Experimental evidence at protein level
25.7418	25.5718	25.4897	25.713	25.868	25.8698	25.5185	26.4219	1:Experimental evidence at protein level
25.735	25.5175	25.511	25.2801	#NUM!	#NUM!	25.8004	25.5039	1:Experimental evidence at protein level
26.3325	26.5256	26.4751	26.6031	25.5143	25.4035	25.6165	26.2406	1:Experimental evidence at protein level
25.6301	25.3522	25.4499	25.4075	24.2105	#NUM!	24.4728	25.5221	1:Experimental evidence at protein level
25.7492	25.8136	25.8248	26.4139	25.5622	25.3293	25.566	26.4705	1:Experimental evidence at protein level
26.5483	26.5477	26.5314	26.7138	26.6091	26.4221	26.6041	26.9088	1:Experimental evidence at protein level
25.269	25.542	25.7945	25.7208	25.8429	25.8529	25.9684	26.111	1:Experimental evidence at protein level
25.5739	25.8636	25.5619	25.1798	26.0003	26.0135	25.6571	26.4357	1:Experimental evidence at protein level
25.8602	25.9451	25.9162	26.2691	26.0332	25.8678	26.0374	26.5648	1:Experimental evidence at protein level
25.705	25.6153	25.4032	25.7492	26.1635	25.7811	26.1239	26.5719	1:Experimental evidence at protein level
25.7136	25.7249	26.1181	25.8367	25.6385	25.993	25.6198	25.6629	1:Experimental evidence at protein level
25.2118	25.5268	25.3451	25.2102	25.2886	#NUM!	25.3038	#NUM!	1:Experimental evidence at protein level
25.3656	25.5669	25.6993	25.9839	25.9136	25.7246	25.9813	26.637	1:Experimental evidence at protein level
26.6261	26.6453	26.6493	26.74	26.8871	26.889	27.0376	27.1546	1:Experimental evidence at protein level
26.015	26.0696	26.2894	26.4216	26.16	25.9809	26.1147	26.5224	1:Experimental evidence at protein level
26.7972	26.8142	26.7678	26.9169	24.991	25.0539	24.6949	25.7351	1:Experimental evidence at protein level
23.9295	23.9606	24.2279	24.0783	24.7264	24.5757	24.7762	24.8915	1:Experimental evidence at protein level
25.1932	#NUM!	25.2223	24.9864	25.9273	25.5579	25.7904	25.4353	1:Experimental evidence at protein level
27.5494	27.6187	27.4939	27.7619	27.6453	27.6659	27.6393	27.9911	1:Experimental evidence at protein level
26.2368	26.5519	26.5037	26.7268	26.6924	26.608	#NUM!	26.7887	1:Experimental evidence at protein level
27.4397	27.6512	27.6128	27.4862	27.4491	27.3887	27.3241	27.5622	1:Experimental evidence at protein level
26.3214	26.1072	26.0617	25.9806	26.3619	26.2605	26.3139	26.6744	1:Experimental evidence at protein level
25.5619	25.6808	25.4994	25.5272	25.6726	#NUM!	25.5263	25.7742	1:Experimental evidence at protein level
24.9021	24.7924	25.0452	25.0135	25.1145	25.3678	24.8944	25.3343	1:Experimental evidence at protein level
25.8724	25.7955	25.8334	26	25.8339	25.8149	25.6465	26.2165	1:Experimental evidence at protein level
26.3907	26.5071	26.9739	26.9049	28.4821	28.5475	28.5561	28.3631	1:Experimental evidence at protein level
25.8183	25.9779	26.2519	26.249	25.8288	#NUM!	#NUM!	25.4894	1:Experimental evidence at protein level
26.5617	26.4566	26.7518	26.5533	26.3847	26.3581	26.5219	26.4369	1:Experimental evidence at protein level
27.9749	27.644	27.0792	27.6864	28.0155	27.4454	27.5727	#NUM!	1:Experimental evidence at protein level
26.4377	26.1589	26.3464	26.4126	26.1439	26.2642	26.02	26.2439	1:Experimental evidence at protein level
26.9086	26.971	26.8456	26.9442	27.0741	27.1442	27.1875	27.562	1:Experimental evidence at protein level
25.85	25.907	26.0896	26.0548	26.2624	26.3156	26.2869	26.4848	1:Experimental evidence at protein level
27.9142	27.8067	27.7821	27.9224	27.7544	27.77	27.7936	27.8598	1:Experimental evidence at protein level
27.1449	27.2863	27.2074	27.5023	28.2636	28.1241	28.207	28.0709	1:Experimental evidence at protein level
26.191	26.1797	26.1578	26.3931	26.3563	26.4426	26.3371	26.587	1:Experimental evidence at protein level
25.0015	25.0553	25.3534	25.1924	24.8341	25.0407	24.7208	25.533	1:Experimental evidence at protein level
26.2144	26.1583	26.3664	26.2927	26.4449	26.3313	26.3627	26.7047	1:Experimental evidence at protein level
24.8176	24.7616	25.0267	25.2951	25.4404	24.7411	25.4685	25.6626	1:Experimental evidence at protein level
27.5289	27.5853	27.6484	27.5349	27.3417	27.3898	27.5523	27.4651	1:Experimental evidence at protein level
27.0865	27.2551	27.0034	27.1892	26.9744	27.1154	26.9556	27.6631	1:Experimental evidence at protein level
25.4373	25.1979	25.0803	25.4624	25.6993	25.2641	25.3327	25.9653	1:Experimental evidence at protein level
27.6075	27.5492	27.7346	27.6708	27.5326	27.45	27.4057	27.7947	1:Experimental evidence at protein level
26.9717	26.7752	26.6807	26.7587	26.2231	26.2194	26.1683	#NUM!	1:Experimental evidence at protein level
25.8888	25.9637	25.6773	25.7957	26.2113	26.3311	26.1475	#NUM!	1:Experimental evidence at protein level
26.3874	26.2341	25.974	26.0731	26.1438	25.9593	26.2823	26.2951	1:Experimental evidence at protein level
27.8693	28.0376	28.1453	28.2763	28.2581	28.229	28.4735	28.2815	1:Experimental evidence at protein level
25.9569	25.1989	25.64	25.7273	26.4112	25.4966	25.0605	25.5776	1:Experimental evidence at protein level
25.506	25.2777	25.7052	26.1704	25.8148	25.6929	25.9462	26.0548	1:Experimental evidence at protein level
26.0903	26.016	26.2752	26.3205	25.5314	26.1006	25.6988	26.0533	1:Experimental evidence at protein level
26.3907	26.5097	26.5896	26.7487	26.2966	26.441	26.3023	26.8448	1:Experimental evidence at protein level
25.3189	25.3441	25.1805	25.1854	25.9765	26.0024	26.0102	26.0763	1:Experimental evidence at protein level
27.3676	27.553	27.6068	27.3846	27.0757	27.2105	27.1977	26.6411	1:Experimental evidence at protein level
27.5647	27.6251	27.5492	27.4954	28.0337	27.7278	27.8601	27.5888	1:Experimental evidence at protein level
28.2279	28.4184	28.1413	28.4819	28.7088	28.6333	28.8041	29.1088	1:Experimental evidence at protein level
26.9199	26.956	26.8966	26.9014	26.6612	26.6088	26.4289	27.0095	1:Experimental evidence at protein level
26.3239	26.565	26.8054	26.5838	26.5003	26.5593	26.7055	26.5482	1:Experimental evidence at protein level
28.2643	28.3625	28.3231	28.3389	28.7793	28.5624	28.8507	29.195	1:Experimental evidence at protein level
26.7694	26.7685	26.6812	26.8234	26.7458	26.8478	27.0209	27.4926	1:Experimental evidence at protein level
26.188	26.116	26.4095	26.2578	25.091	25.3039	25.146	25.6863	1:Experimental evidence at protein level
26.7801	26.963	27.1585	26.9165	26.903	26.992	26.9915	26.7004	1:Experimental evidence at protein level
25.3539	25.0614	24.8055	25.1624	25.2034	24.7113	24.8851	25.6694	1:Experimental evidence at protein level
25.1279	25.1	24.8495	24.4402	25.0562	25.0839	25.0133	24.5799	1:Experimental evidence at protein level
25.0601	25.0389	24.8269	25.027	25.4467	#NUM!	25.1907	25.8875	1:Experimental evidence at protein level
26.4925	26.5429	26.4554	26.479	26.6803	26.4574	26.4828	26.914	1:Experimental evidence at protein level
26.1063	26.3865	27.3775	26.8675	26.2632	26.2814	26.066	25.8887	1:Experimental evidence at protein level
26.1857	26.5744	26.7481	26.578	26.8388	26.3838	26.1393	26.7403	1:Experimental evidence at protein level
27.3172	27.4932	27.3163	27.4534	26.8099	26.78	26.8371	28.1465	1:Experimental evidence at protein level
26.6686	26.3984	26.4111	26.5023	26.4237	26.7102	26.2838	26.0394	1:Experimental evidence at protein level
25.6904	25.9679	25.9147	25.7882	25.4321	25.0404	#NUM!	25.0273	1:Experimental evidence at protein level
27.0257	27.1382	27.5092	27.155	26.9657	26.6504	27.0365	27.1271	1:Experimental evidence at protein level
25.3745	25.0502	25.1712	25.1037	25.424	25.3923	25.403	#NUM!	1:Experimental evidence at protein level
26.4835	26.3362	26.657	26.493	26.7788	27.0006	26.9264	26.4988	1:Experimental evidence at protein level
27.7685	28.0109	28.0317	28.1798	27.7636	#NUM!	28.0356	28.1446	1:Experimental evidence at protein level
25.2091	25.1904	25.4879	25.6709	25.3088	25.0162	25.3886	25.3343	1:Experimental evidence at protein level
26.6441	26.9713	26.7921	26.7939	#NUM!	27.0364	#NUM!	27.0224	1:Experimental evidence at protein level
26.5209	26.5031	26.4776	26.6364	26.6202	26.3731	26.1353	27.008	1:Experimental evidence at protein level
26.0948	25.7615	25.8068	26.0395	26.6412	26.5518	26.2813	26.8817	1:Experimental evidence at protein level
25.8082	25.7259	25.9218	25.7096	25.8735	25.7492	25.6477	25.7587	1:Experimental evidence at protein level
24.2587	24.3538	24.7319	24.8314	25.1066	24.7199	24.9013	25.2362	1:Experimental evidence at protein level
26.4468	26.1442	26.3592	26.6103	25.8916	25.8354	25.9835	26.7286	1:Experimental evidence at protein level

24.6778	24.0409	25.0162	25.0782	23.8968	#NUM!	#NUM!	25.3103	1:Experimental evidence at protein level
25.6831	25.8055	25.6452	25.6427	25.6335	25.7422	25.4438	26.3215	1:Experimental evidence at protein level
#NUM!	24.394	#NUM!	#NUM!	24.5841	24.8717	24.7088	25.3754	1:Experimental evidence at protein level
25.9776	26.3097	25.9915	26.1471	26.3436	26.6407	26.4332	26.6301	1:Experimental evidence at protein level
28.6971	28.9201	28.9525	28.9274	28.9261	28.8647	29.1672	29.2571	1:Experimental evidence at protein level
26.2758	26.4712	26.5612	26.3181	25.849	#NUM!	25.7925	26.0427	1:Experimental evidence at protein level
24.8865	24.7303	24.703	24.7195	24.9555	25.0257	24.7242	#NUM!	1:Experimental evidence at protein level
26.6332	26.9556	26.6985	26.7461	26.9286	26.3858	27.116	#NUM!	1:Experimental evidence at protein level
27.047	27.7764	28.1854	28.0065	27.7678	27.2491	27.8929	27.7164	1:Experimental evidence at protein level
28.7727	28.605	28.4773	28.5598	28.6839	28.9487	28.5004	28.7209	1:Experimental evidence at protein level
29.2948	29.2691	29.4119	29.249	29.2518	29.139	29.3103	29.3221	1:Experimental evidence at protein level
27.1575	27.1312	27.2697	27.3665	27.2909	27.118	27.1365	27.2551	1:Experimental evidence at protein level
26.3465	26.3018	26.4941	26.5316	26.8597	26.9693	26.8572	27.1077	1:Experimental evidence at protein level
28.2773	28.3398	28.2238	28.0604	27.9454	28.0432	28.0622	27.8929	1:Experimental evidence at protein level
29.7816	29.9495	29.4968	29.7641	29.6505	29.9038	29.9229	29.9146	1:Experimental evidence at protein level
27.0995	27.234	27.2933	27.1295	27.0058	27.2262	27.0303	26.6482	1:Experimental evidence at protein level
24.5297	24.8317	25.2121	24.5265	24.9686	24.4159	#NUM!	#NUM!	1:Experimental evidence at protein level
27.946	27.9771	27.5011	27.4387	27.7098	27.935	27.6289	27.5585	1:Experimental evidence at protein level
27.4112	27.496	27.5376	27.6793	27.7125	27.7091	27.593	27.9733	1:Experimental evidence at protein level
26.089	25.9075	26.0785	26.0885	26.2737	26.0984	26.131	26.6311	1:Experimental evidence at protein level
25.8394	25.8053	25.9877	25.8859	25.7259	25.594	25.7875	#NUM!	1:Experimental evidence at protein level
26.6442	26.7876	27.1336	27.0189	26.6929	26.8264	26.7632	26.6158	1:Experimental evidence at protein level
28.5757	28.5926	28.4646	28.5367	28.7995	28.9004	28.8806	28.5716	1:Experimental evidence at protein level
28.4867	28.6909	28.7599	28.4989	28.4049	28.2472	28.4676	28.7332	1:Experimental evidence at protein level
25.8961	26.1508	25.8674	25.9794	25.9545	25.9944	26.0081	26.3888	1:Experimental evidence at protein level
26.6214	26.642	26.5881	26.261	26.5583	26.5866	26.5733	26.7987	1:Experimental evidence at protein level
26.3993	26.3126	26.2494	26.7465	#NUM!	#NUM!	25.6811	#NUM!	1:Experimental evidence at protein level
26.5756	26.5787	26.6953	26.5881	26.7305	26.5891	26.7813	27.0743	1:Experimental evidence at protein level
25.7519	25.8053	25.769	25.7636	25.3888	25.7285	25.8276	25.7887	1:Experimental evidence at protein level
28.3385	28.3481	28.3656	28.264	28.704	28.7749	28.5787	28.1574	1:Experimental evidence at protein level
25.3463	25.5461	25.1966	25.5996	25.8487	25.6868	25.717	26.06	1:Experimental evidence at protein level
26.2063	26.0835	26.2851	26.2196	26.6401	26.4063	26.4002	26.8298	1:Experimental evidence at protein level
26.562	26.4825	26.1251	26.7082	27.0522	27.174	26.995	27.4691	1:Experimental evidence at protein level
28.5456	28.5821	28.5341	28.3001	28.7957	28.7442	28.6138	28.1321	1:Experimental evidence at protein level
27.4184	27.9029	27.9511	28.1776	27.5282	27.2008	27.4393	27.6853	1:Experimental evidence at protein level
28.6365	29.0278	29.152	29.0005	29.2708	28.8938	29.0335	28.1477	1:Experimental evidence at protein level
25.8458	25.9015	25.5432	26.0915	25.5553	25.5792	25.4212	#NUM!	1:Experimental evidence at protein level
28.6291	28.6459	27.6152	26.9153	27.054	27.1403	27.5085	26.3952	1:Experimental evidence at protein level
27.5097	27.6197	27.5131	27.7065	27.6281	27.728	27.6879	26.9053	1:Experimental evidence at protein level
27.1953	27.4306	27.8026	27.2167	#NUM!	25.9628	26.465	26.72	1:Experimental evidence at protein level
25.686	25.6535	25.5883	25.7074	25.5335	25.8628	25.5721	25.8561	1:Experimental evidence at protein level
27.1795	27.2465	27.3606	27.0933	27.0955	27.2503	27.1138	26.9541	1:Experimental evidence at protein level
26.5518	26.3305	26.205	26.38	26.412	26.9639	27.1233	27.4873	1:Experimental evidence at protein level
32.8038	32.9808	32.3247	32.7276	32.4024	32.0865	32.281	32.4257	1:Experimental evidence at protein level
27.3166	27.0897	27.0817	26.8855	25.7319	25.5168	25.0153	25.9799	1:Experimental evidence at protein level
25.2378	25.4512	25.5462	25.4381	25.1957	24.6075	25.2308	25.4328	1:Experimental evidence at protein level
29.0237	29.1569	28.9682	29.0046	29.1452	28.9382	29.2171	29.6364	1:Experimental evidence at protein level
28.6546	28.7792	28.8491	28.8049	28.6282	28.2592	28.4428	28.5396	1:Experimental evidence at protein level
26.701	26.8493	27.3283	27.5785	26.3017	26.7528	25.9856	26.4852	1:Experimental evidence at protein level
26.7233	26.6789	26.7171	26.6831	26.4974	26.5189	26.6692	26.6949	1:Experimental evidence at protein level
29.4991	29.7307	29.8561	29.6965	29.801	29.5792	29.7138	29.9078	1:Experimental evidence at protein level
24.1878	24.4405	24.9022	25.0379	25.061	24.7281	24.8704	25.7306	1:Experimental evidence at protein level
26.0111	26.2838	26.359	26.389	25.8035	26.0961	26.3356	26.6472	1:Experimental evidence at protein level
26.1239	26.3075	25.8123	26.3195	26.8378	26.6755	26.8924	27.3944	1:Experimental evidence at protein level
27.9409	28.1233	28.1639	28.4362	27.9473	27.9226	28.1625	28.115	1:Experimental evidence at protein level
25.0344	25.1379	25.5034	25.2901	24.9357	25.1443	25.0493	25.2147	1:Experimental evidence at protein level
27.6319	27.556	27.8645	28.0142	26.5888	#NUM!	26.8145	27.3486	1:Experimental evidence at protein level
26.8465	26.7905	26.292	26.877	26.3758	26.1912	26.5347	27.041	1:Experimental evidence at protein level
27.0081	27.2647	27.7579	27.4547	#NUM!	#NUM!	26.1951	#NUM!	1:Experimental evidence at protein level
25.7998	25.9064	26.1041	25.5324	26.1856	26.1332	26.0111	26.4008	1:Experimental evidence at protein level
26.7571	27.0722	27.4369	27.2667	26.2118	26.205	26.2083	26.0524	1:Experimental evidence at protein level
26.1461	26.1386	26.1227	26.2138	26.7129	26.7827	26.9833	26.6248	1:Experimental evidence at protein level
27.2434	27.3634	27.4975	27.6625	27.8414	27.7222	27.6723	27.6882	1:Experimental evidence at protein level
26.4409	26.5612	26.6342	26.0991	25.6721	25.8195	25.6914	#NUM!	1:Experimental evidence at protein level
29.2563	29.3282	29.6044	29.5301	29.4078	29.3638	29.4743	29.3267	1:Experimental evidence at protein level
25.6778	25.916	25.4772	25.9021	25.9325	26.2426	26.2346	26.4699	1:Experimental evidence at protein level
26.1539	26.0894	26.4524	26.2723	26.4756	26.1248	26.5582	26.8322	1:Experimental evidence at protein level
27.0093	27.1535	27.2037	26.9124	27.1456	26.9044	26.85	27.0387	1:Experimental evidence at protein level
26.784	26.7806	26.794	26.5617	26.328	26.1625	26.4675	26.2494	1:Experimental evidence at protein level
26.8601	26.9014	27.0747	26.9674	27.0656	26.9452	26.9952	26.7896	1:Experimental evidence at protein level
27.7081	27.538	27.2132	27.3561	27.2047	27.474	27.6496	27.6322	1:Experimental evidence at protein level
28.2194	28.4078	28.2682	28.4353	28.1314	28.1491	28.168	28.4244	1:Experimental evidence at protein level
27.0684	27.1669	27.439	27.2974	27.4338	27.4313	27.2536	27.0668	1:Experimental evidence at protein level
30.0473	30.0986	30.4599	29.8958	30.0015	30.063	30.1735	29.4336	1:Experimental evidence at protein level
28.7936	29.0198	28.698	28.5504	29.0361	28.9244	29.0229	28.467	1:Experimental evidence at protein level
33.2385	33.6549	33.4092	33.0283	33.7991	34.0872	33.9674	31.9379	1:Experimental evidence at protein level
27.5719	27.5123	27.6663	27.8512	26.3683	26.2079	26.3272	26.6826	1:Experimental evidence at protein level
30.1725	30.4178	30.508	30.1329	29.9748	30.0499	30.2884	30.1093	1:Experimental evidence at protein level
27.4487	27.3948	27.2023	27.6669	27.8682	27.7026	27.8344	28.2696	1:Experimental evidence at protein level
27.0628	27.1686	26.8221	27.2647	27.1186	27.4753	27.2059	27.7575	1:Experimental evidence at protein level
26.9983	27.0091	27.0965	27.0062	26.9031	26.9351	26.8938	27.2881	1:Experimental evidence at protein level
28.342	28.105	28.2665	28.1191	28.3906	27.9827	27.879	27.5578	1:Experimental evidence at protein level
28.0141	27.7527	28.2392	28.0069	28.0796	27.8938	28.0508	27.8833	1:Experimental evidence at protein level
26.0389	25.9594	25.8796	26.166	25.5298	25.5161	25.5412	26.2632	1:Experimental evidence at protein level
26.6816	26.4797	26.5639	26.5578	26.7443	26.6104	26.8197	26.6757	1:Experimental evidence at protein level
27.2822	27.0748	26.7424	26.724	26.7807	26.885	26.8881	26.1603	1:Experimental evidence at protein level
25.9796	26.0592	25.9739	26.6984	26.5088	25.9506	26.3101	27.1189	1:Experimental evidence at protein level
24.3677	24.5948	24.9191	24.382	24.2693	#NUM!	24.1399	23.9783	1:Experimental evidence at protein level
26.0846	26.3856	26.3023	26.0446	27.0719	27.1469	27.159	26.5451	1:Experimental evidence at protein level
27.7018	28.0718	27.799	28.0084	27.3453	27.2384	27.6187	27.782	1:Experimental evidence at protein level
26.2619	26.2122	26.2973	26.3272	25.9581	26.2619	26.1567	26.3936	1:Experimental evidence at protein level
26.3321	26.2915	26.3954	26.6134	26.2318	26.0512	26.3247	27.1135	1:Experimental evidence at protein level
25.182	24.9289	24.5746	24.7016	25.3995	25.7115	25.475	25.9623	1:Experimental evidence at protein level
25.5938	25.0962	25.2287	25.5825	25.6946	25.8878	25.7374	26.0339	1:Experimental evidence at protein level
28.3144	28.4648	28.1811	28.2572	28.2662	28.2084	28.0862	28.4595	1:Experimental evidence at protein level
27.7656	27.6602	27.6353	27.5028	27.4337	27.68	27.602	27.0084	1:Experimental evidence at protein level
25.3821	25.3329	25.3903	25.1225	25.9177	25.7565	25.7816	26.298	1:Experimental evidence at protein level
27.3314	27.4252	27.2641	27.5124	27.5683	27.5535	27.7678	27.9788	1:Experimental evidence at protein level
27.6577	27.6936	27.8286	28.0231	27.4936	27.6239	27.6409	27.7792	1:Experimental evidence at protein level
26.5378	26.8156	27.0316	26.5212	26.3891	26.6905	26.5194	26.5771	1:Experimental evidence at protein level
25.9647	25.6421	25.9775	26.0613	26.6511	26.69	26.7044	#NUM!	1:Experimental evidence at protein level
26.6471	26.6136	26.8393	26.8703	26.8948	26.7134	27.0617	27.0476	1:Experimental evidence at protein level
27.6047	27.7424	27.5775	27.8125	27.9262	27.8057	27.761	28.1849	1:Experimental evidence at protein level
27.8987	28.0914	27.9343	27.8659	27.7903	28.1332	27.9602	27.9796	1:Experimental evidence at protein level
25.694	25.6462	26.1363	25.9797	26.1557	26.2117	26.1011	26.1703	1:Experimental evidence at protein level
27.0433	27.1391	27.171	27.4252	27.2688	27.2313	27.2555	27.6601	1:Experimental evidence at protein level
26.6722	26.6835	26.9206	26.8105	26.5032	26.4334	26.9352	26.4764	1:Experimental evidence at protein level
25.4825	25.8403	25.9092	25.8103	25.9223	26.4065	25.6913	#NUM!	1:Experimental evidence at protein level
26.1594	26.4217	25.9614	26.2812	26.5149	26.3239	26.3967	27.4802	1:Experimental evidence at protein level
25.2147	24.9224	25.085	25.4317	25.7322	25.5793	25.6687	25.6795	1:Experimental evidence at protein level
26.259	26.1229	26.1175	26.1129	26.3842	26.0983	26.0935	27.0514	1:Experimental evidence at protein level
26.4477	26.4335	26.6411	27.0419	26.6328	26.4314	26.5237	27.1872	1:Experimental evidence at protein level
26.5311	26.6591	26.8141	26.9078	26.7677	26.7872	26.8125	#NUM!	1:Experimental evidence at protein level
26.0201	26.3486	26.2924	26.0212	26.0017	26.2699	25.9746	26.182	1:Experimental evidence at protein level
26.0355	25.7914	26.2652	26.0828	#NUM!	#NUM!	#NUM!	#NUM!	1:Experimental evidence at protein level
26.6233	26.8943	26.9356	27.1461	26.6757	26.7262	26.8619	26.9	1:Experimental evidence at protein level
25.5944	26.0765	25.5865	25.4805	25.7077	25.9157	25.8803	26.2975	1:Experimental evidence at protein level
25.6229	25.8313	25.896	25.9411	25.8149	25.8971	25.9426	26.0024	1:Experimental evidence at protein level
26.875	26.8992	26.7985	27.2515	27.122	27.0566	27.1874	27.3922	1:Experimental evidence at protein level
26.6397	26.629	26.5373	26.1981	26.6492	26.4716	26.5199	26.0481	1:Experimental evidence at protein level
25.5653	25.4893	25.5614	25.8231	26.138	26.0501	26.2453	26.5792	1:Experimental evidence at protein level
27.046	26.9475	27.0543	26.6908	27.1629	27.0956	26.8528	26.4594	1:Experimental evidence at protein level
28.4651	28.6934	29.0155	28.7871	28.274	28.2168	28.4386	28.2093	1:Experimental evidence at protein level
29.0606	29.0412	29.2147	29.1728	27.9506	27.8531	27.7844	27.8357	1:Experimental evidence at protein level
26.5332	26.836	26.9551	26.8133	27.0709	26.9684	27.1741	27.3889	1:Experimental evidence at protein level
26.8798	27.218	27.2633	27.1606	27.3232	27.3975	27.3932	27.3608	1:Experimental evidence at protein level
#NUM!	26.249	26.4741	25.4619	26.6308	26.9843	26.775	26.6763	1:Experimental evidence at protein level
25.6867	26.2255	26.2895	26.0854	26.2236	26.4697	26.2392	26.3003	1:Experimental evidence at protein level
26.2515	26.3638	26.4439	26.3769	26.2119	26.335	26.261	26.0273	1:Experimental evidence at protein level

26.3039	26.3224	26.284	26.2073	25.9937	26.1117	#NUM!	#NUM!	1:Experimental evidence at protein level
25.8837	26.0708	25.9975	26.2073	26.0887	26.101	26.1404	26.1429	1:Experimental evidence at protein level
25.9343	#NUM!	25.739	26.2891	25.7058	25.834	25.9481	26.4006	1:Experimental evidence at protein level
29.3329	29.495	29.7098	29.5625	29.2877	29.0562	29.4878	29.331	1:Experimental evidence at protein level
27.6722	27.9418	28.2936	28.0419	27.8556	27.8364	28.0266	27.4149	1:Experimental evidence at protein level
26.2722	26.3148	26.2873	26.1535	26.2439	26.2484	26.3585	26.2879	1:Experimental evidence at protein level
24.9563	24.9722	24.9001	25.3696	25.3091	25.3566	25.1482	25.9503	1:Experimental evidence at protein level
26.3068	26.1341	25.9041	26.2356	26.7546	26.6263	26.8905	27.1847	1:Experimental evidence at protein level
26.1992	26.171	26.3583	26.2464	26.347	26.7159	26.1209	26.2689	1:Experimental evidence at protein level
27.1539	27.1081	27.0571	26.9585	26.9678	27.0948	26.7458	26.763	1:Experimental evidence at protein level
25.5912	25.6206	25.7024	25.81	26.4653	25.8321	26.099	26.0585	1:Experimental evidence at protein level
25.0817	25.4473	25.2975	25.609	24.9412	24.5332	24.6979	25.3779	1:Experimental evidence at protein level
27.1725	26.6843	26.5558	26.9375	27.6109	27.4068	27.0749	27.9612	1:Experimental evidence at protein level
25.6926	25.5139	#NUM!	#NUM!	26.186	26.2632	25.9195	25.2936	1:Experimental evidence at protein level
25.857	26.281	26.5045	26.4161	#NUM!	25.4789	25.8556	#NUM!	1:Experimental evidence at protein level
25.7353	25.8135	26.1092	26.0962	25.8142	25.4109	25.8317	25.7863	1:Experimental evidence at protein level
26.9686	27.0915	27.1577	27.1271	27.037	27.2649	27.1919	27.5792	1:Experimental evidence at protein level
25.959	26.056	25.7925	25.8589	28.0027	28.0473	28.0181	28.0562	1:Experimental evidence at protein level
26.6853	26.6835	26.3651	26.6061	#NUM!	#NUM!	#NUM!	#NUM!	1:Experimental evidence at protein level
24.4904	24.349	#NUM!	24.8004	24.4446	23.9624	24.3562	24.9096	1:Experimental evidence at protein level
26.6511	26.5618	26.0122	26.1216	26.2949	26.345	26.2209	26.3828	1:Experimental evidence at protein level
26.041	25.8197	26.1574	25.9355	25.8815	#NUM!	26.3758	26.1814	1:Experimental evidence at protein level
27.1018	27.1586	27.3519	27.1495	27.4117	27.2835	27.3165	27.44	1:Experimental evidence at protein level
25.1705	25.2864	24.8918	25.646	26.5333	26.6281	26.5684	27.1963	1:Experimental evidence at protein level
26.0172	25.2109	25.9593	26.1102	25.898	26.1751	26.1405	26.5475	1:Experimental evidence at protein level
25.5083	25.6646	25.6808	25.4063	25.5467	25.4792	25.5299	25.5676	1:Experimental evidence at protein level
25.7332	25.5415	25.3521	25.6444	25.7015	25.5494	25.6315	26.599	1:Experimental evidence at protein level
27.2804	27.305	27.4089	26.6016	26.8328	26.9232	26.8414	27.0908	1:Experimental evidence at protein level
27.5821	27.6543	28.0044	27.6043	27.4758	27.8766	27.9755	27.4886	1:Experimental evidence at protein level
26.065	26.1207	25.6626	25.5177	26.0802	26.2401	26.1569	25.7753	1:Experimental evidence at protein level
26.302	26.2374	26.4325	26.5127	26.6358	26.4255	26.6374	27.2462	1:Experimental evidence at protein level
26.5386	26.5168	26.5396	26.414	26.914	27.1363	27.093	26.5289	1:Experimental evidence at protein level
27.2502	27.4384	27.4322	27.2177	27.2262	27.0264	27.0592	27.0113	1:Experimental evidence at protein level
25.5214	25.6043	25.8209	25.9773	25.1729	25.9542	25.4685	25.9834	1:Experimental evidence at protein level
25.8631	25.8888	26.0473	#NUM!	25.5813	26.065	25.9324	25.8914	1:Experimental evidence at protein level
#NUM!	#NUM!	25.8391	25.9213	25.6977	25.5656	25.8776	25.7957	1:Experimental evidence at protein level
29.6645	29.561	29.8487	29.8044	29.6034	29.4153	29.5035	29.6942	1:Experimental evidence at protein level
27.1164	27.3105	26.8721	27.3216	27.7814	28.0932	27.8126	27.7871	1:Experimental evidence at protein level
26.4594	26.3196	25.967	26.276	26.8429	26.883	26.6027	27.5026	1:Experimental evidence at protein level
25.6681	25.9324	25.6885	25.9156	25.834	25.5873	25.4286	26.2746	1:Experimental evidence at protein level
26.7871	26.5833	26.4905	26.7653	26.7931	26.587	26.7471	27.3072	1:Experimental evidence at protein level
26.026	26.0846	25.8686	25.8027	26.1949	26.9696	26.0401	26.0137	1:Experimental evidence at protein level
27.9941	28.0594	28.0001	27.9834	28.1205	28.346	28.0586	28.0437	1:Experimental evidence at protein level
26.1647	25.9778	26.3005	26.4594	25.6715	26.0764	25.992	26.1037	1:Experimental evidence at protein level
25.6256	25.8105	25.7572	25.7461	25.4997	25.2718	25.4491	25.5769	1:Experimental evidence at protein level
28.4578	28.5578	28.6721	28.6638	28.4902	28.4394	28.628	28.6677	1:Experimental evidence at protein level
25.9029	26.1936	26.1552	25.8868	26.1578	26.1344	26.0885	25.7106	1:Experimental evidence at protein level
#NUM!	#NUM!	#NUM!	#NUM!	26.684	26.8991	26.418	25.9841	1:Experimental evidence at protein level
26.267	26.0379	26.1704	26.0084	26.3794	26.2588	26.3448	26.1198	1:Experimental evidence at protein level
26.4386	26.3355	26.4505	26.2632	26.6099	26.8667	26.8854	27.1203	1:Experimental evidence at protein level
26.8863	27.0064	26.9052	26.8649	26.319	26.6749	26.4037	26.322	1:Experimental evidence at protein level
26.9671	27.0545	26.6115	27.0469	27.6131	27.5171	27.365	27.7514	1:Experimental evidence at protein level
25.4337	25.1793	25.2331	25.2854	26.3875	26.2148	26.3184	26.7959	1:Experimental evidence at protein level
26.2105	26.0924	26.3513	26.3911	25.9228	26.0583	25.8393	26.0274	1:Experimental evidence at protein level
26.2958	25.9052	26.1889	#NUM!	26.2887	26.4805	25.9818	26.5002	1:Experimental evidence at protein level
25.9503	26.0267	25.976	26.0569	26.581	26.5935	26.421	26.7963	1:Experimental evidence at protein level
25.6098	25.8344	25.8851	25.8467	25.5745	25.0362	26.0699	26.224	1:Experimental evidence at protein level
26.2254	26.3166	26.448	26.4624	26.8603	26.6138	26.8389	26.5732	1:Experimental evidence at protein level
27.5121	27.648	27.7564	27.7056	27.6114	27.6258	27.8045	27.414	1:Experimental evidence at protein level
27.4644	27.4663	27.4392	27.3568	27.2457	27.2761	27.104	27.2957	1:Experimental evidence at protein level
26.4127	26.2173	25.947	26.0514	25.9374	25.857	25.7068	#NUM!	1:Experimental evidence at protein level
26.3079	26.3478	26.2994	26.3758	26.111	26.3639	26.3598	26.6591	1:Experimental evidence at protein level
26.5147	26.7306	26.9111	27.0183	26.6664	26.3385	26.6159	27.3421	1:Experimental evidence at protein level
27.9927	27.8621	27.7239	27.7343	27.5664	27.3104	27.3783	27.5501	1:Experimental evidence at protein level
27.2318	27.2581	27.3568	27.0208	27.0498	26.978	26.969	26.8277	1:Experimental evidence at protein level
27.1187	27.1049	26.9977	26.7484	26.8007	26.7239	26.7543	#NUM!	1:Experimental evidence at protein level
25.9491	26.0341	26.2059	26.3706	25.9094	25.588	26.1214	26.3674	1:Experimental evidence at protein level
25.9667	26.1448	25.8098	26.186	25.924	26.0191	26.3261	26.3789	1:Experimental evidence at protein level
29.1893	29.1528	29.1767	28.5665	29.3592	29.3506	29.191	28.6393	1:Experimental evidence at protein level
24.5883	24.3668	24.5475	24.4374	25.5069	25.3967	25.4645	25.9365	1:Experimental evidence at protein level
26.7961	26.8192	26.5322	26.5522	26.9638	26.8318	26.8399	26.704	1:Experimental evidence at protein level
26.9945	27.2245	27.0949	27.0182	27.0577	27.2215	27.0452	26.8011	1:Experimental evidence at protein level
27.0947	27.2538	27.3796	27.3978	27.6271	28.1779	28.0254	28.2338	1:Experimental evidence at protein level
29.3677	28.9743	29.1309	29.1919	29.0487	28.6441	28.7537	28.7429	1:Experimental evidence at protein level
25.8294	25.6309	25.3881	26.1792	25.4543	25.8002	25.5867	26.9392	1:Experimental evidence at protein level
24.2013	24.2876	24.1318	24.5106	24.7723	#NUM!	24.3243	#NUM!	1:Experimental evidence at protein level
26.0826	26.1323	26.0726	26.136	26.2286	26.0878	26.328	26.9878	1:Experimental evidence at protein level
25.6386	25.4951	25.5417	26.0723	25.7561	25.4683	25.527	26.4897	1:Experimental evidence at protein level
25.7611	25.6574	25.446	25.2917	25.6829	25.8194	25.4348	#NUM!	1:Experimental evidence at protein level
27.946	27.9625	28.0785	28.25	27.1268	27.2719	27.3076	27.7208	1:Experimental evidence at protein level
27.3196	27.3419	27.3144	27.5106	27.0537	27.2519	27.1451	27.0684	1:Experimental evidence at protein level
28.0592	28.4616	28.0361	27.9937	28.5203	28.7206	28.7025	28.6258	1:Experimental evidence at protein level
27.29	27.478	27.7033	27.8619	27.7695	27.3695	28.0834	28.1484	1:Experimental evidence at protein level
28.1582	28.252	28.3236	28.4589	27.8686	28.2995	27.6867	#NUM!	1:Experimental evidence at protein level
25.793	26.1243	26.4002	26.1084	26.4116	#NUM!	26.3316	26.4728	1:Experimental evidence at protein level
26.2399	26.2117	25.9982	26.1064	26.7126	26.7235	26.6398	26.8455	1:Experimental evidence at protein level
26.8599	26.9848	26.7324	26.6997	27.1322	27.0964	26.9924	26.7585	1:Experimental evidence at protein level
31.0871	30.5256	29.035	28.4685	28.3488	29.0134	28.034	27.7599	1:Experimental evidence at protein level
26.9514	27.0957	26.4743	27.141	27.0325	27.1543	27.1352	27.8281	1:Experimental evidence at protein level
26.6008	26.5877	26.579	26.5544	26.3323	26.2197	26.3879	26.6762	1:Experimental evidence at protein level
27.4318	27.5675	27.6617	27.5231	27.8719	27.8277	27.9316	27.8222	1:Experimental evidence at protein level
25.7556	26.0429	26.0802	25.9596	26.3483	26.6276	26.5212	26.2013	1:Experimental evidence at protein level
26.6965	26.3694	26.322	26.0667	26.4343	26.2718	#NUM!	25.1995	1:Experimental evidence at protein level
29.1789	29.0249	29.0826	29.0611	29.4081	29.5442	29.2054	28.1933	1:Experimental evidence at protein level
26.3256	26.4309	26.3214	26.3605	26.6993	26.6475	26.7737	26.957	1:Experimental evidence at protein level
29.5292	29.4959	29.5823	29.6374	30.0339	29.9338	29.8976	30.0519	1:Experimental evidence at protein level
25.0437	25.093	#NUM!	#NUM!	25.9348	26.0658	25.583	25.3414	1:Experimental evidence at protein level
28.3564	28.2262	28.3303	28.1542	28.5679	28.3917	28.4102	28.3647	1:Experimental evidence at protein level
26.3936	26.2824	26.6168	26.5921	26.6953	26.4988	26.6827	26.5293	1:Experimental evidence at protein level
27.9917	28.2535	28.259	27.9552	28.0562	28.1982	27.9835	27.9655	1:Experimental evidence at protein level
28.6736	28.7929	28.6091	28.2868	28.9895	29.0718	29.0129	27.7341	1:Experimental evidence at protein level
25.7083	25.6999	25.6355	25.7641	25.7507	25.6556	25.8391	26.2232	1:Experimental evidence at protein level
27.9039	28.1154	27.8881	28.0602	28.0543	28.2287	28.2826	28.4053	1:Experimental evidence at protein level
28.4227	28.4285	28.424	28.6442	#NUM!	#NUM!	#NUM!	#NUM!	1:Experimental evidence at protein level
25.9785	25.855	25.6059	25.7624	25.5904	25.8769	25.8182	26.2689	1:Experimental evidence at protein level
26.0916	25.8778	26.0951	25.6703	26.362	26.5713	26.0676	26.5116	1:Experimental evidence at protein level
31.498	31.4804	31.1169	31.2417	31.7381	31.8703	31.8301	31.7804	1:Experimental evidence at protein level
25.9609	25.9553	25.7184	25.9281	25.6384	25.7026	25.7969	26.2955	1:Experimental evidence at protein level
26.2893	26.3533	25.9353	26.3436	26.6858	26.6589	26.7814	26.2353	1:Experimental evidence at protein level
27.7014	27.7129	27.9575	27.8726	27.7397	28.0424	27.7007	27.5839	1:Experimental evidence at protein level
26.701	26.8381	26.8543	26.6753	26.6074	26.5652	26.4796	26.4466	1:Experimental evidence at protein level
26.2012	26.1366	26.0497	26.3607	#NUM!	26.2332	#NUM!	26.8666	1:Experimental evidence at protein level
26.1721	26.4805	26.1125	26.0313	25.9536	26.1322	26.1643	26.0319	1:Experimental evidence at protein level
25.4229	25.2532	25.3524	25.5167	25.5392	25.5321	25.351	26.1528	1:Experimental evidence at protein level
29.3314	29.4392	29.4823	29.5308	29.1352	29.0587	29.1765	29.2554	1:Experimental evidence at protein level
25.5954	25.763	25.8317	25.7755	25.4686	25.3756	25.5584	26.196	1:Experimental evidence at protein level
26.6465	25.7554	26.5777	26.6612	27.0968	#NUM!	27.0544	#NUM!	1:Experimental evidence at protein level
29.2237	29.1565	29.3867	29.5878	28.9927	28.8299	28.9738	29.3106	1:Experimental evidence at protein level
26.0576	26.0588	26.5239	26.3414	26.5508	25.9934	25.9418	25.7466	1:Experimental evidence at protein level
26.7743	26.9247	26.9188	27.0134	27.1145	27.0302	27.2683	27.558	1:Experimental evidence at protein level
28.8293	28.9175	28.7376	28.9458	29.3949	29.8338	29.2224	28.499	1:Experimental evidence at protein level
27.0326	27.2904	27.811	27.4904	26.5901	26.9733	26.9638	26.8789	1:Experimental evidence at protein level
32.7739	33.1201	33.2411	32.5318	32.5794	32.4964	32.9165	31.8033	1:Experimental evidence at protein level
26.9237	27.0776	27.4928	27.081	27.6904	27.7041	27.3785	27.8509	1:Experimental evidence at protein level
31.0335	30.9163	31.0899	30.8954	31.0074	31.0815	30.7415	30.4197	1:Experimental evidence at protein level
26.0121	26.3176	26.5942	26.4715	26.6497	26.4038	26.1151	27.0533	1:Experimental evidence at protein level
27.164	27.2497	27.2784	27.2342	27.0541	27.283	26.9944	#NUM!	1:Experimental evidence at protein level
26.7937	26.7748	27.0335	27.1411	27.1522	26.667	27.0712	27.3376	1:Experimental evidence at protein level
26.6187	26.6606	26.6755	26.6053	26.6274	26.6673	26.6968	26.8573	1:Experimental evidence at protein level

26.6072	26.9491	27.015	27.0025	26.8825	26.5905	27.1191	27.7866	1:Experimental evidence at protein level
25.191	25.4474	23.9942	24.6246	24.9692	#NUM!	25.1822	25.8266	1:Experimental evidence at protein level
27.8782	27.7957	27.7015	27.8056	28.031	27.7464	27.7654	27.9531	1:Experimental evidence at protein level
#NUM!	24.3706	24.3678	24.3966	25.2207	25.3192	25.4386	25.5868	1:Experimental evidence at protein level
28.2412	28.4915	28.7533	28.6754	28.4715	28.2439	28.6419	28.5221	1:Experimental evidence at protein level
28.035	27.9412	28.1352	28.0352	28.1432	28.0623	27.9427	27.3912	1:Experimental evidence at protein level
25.7394	25.5749	25.9746	25.8309	25.4573	25.2517	25.4586	26.2728	1:Experimental evidence at protein level
28.2769	28.2472	28.3022	28.7027	29.4237	29.53	29.4658	29.6037	1:Experimental evidence at protein level
26.4043	26.3613	26.7805	26.8242	27.1865	27.1769	26.8852	27.5571	1:Experimental evidence at protein level
25.2708	25.2082	25.3141	25.2964	25.4626	25.7105	25.532	25.8116	1:Experimental evidence at protein level
27.4582	27.7668	27.8411	27.9118	27.3936	27.5209	27.5355	27.6624	1:Experimental evidence at protein level
26.6363	26.7614	26.7206	26.5258	26.9207	27.0212	27.2822	27.4817	1:Experimental evidence at protein level
25.5869	25.5973	25.7306	25.6929	25.8188	25.8684	25.4692	26.0906	1:Experimental evidence at protein level
25.331	25.1682	25.2049	25.4836	25.6193	#NUM!	25.2745	25.4728	1:Experimental evidence at protein level
26.6137	26.477	26.4669	26.6883	26.8277	26.7471	26.8064	26.5336	1:Experimental evidence at protein level
26.3058	26.1433	26.1456	26.2618	26.2905	26.248	26.3967	26.1991	1:Experimental evidence at protein level
26.6435	26.2567	26.8314	26.6669	27.1137	26.3573	26.4617	26.8549	1:Experimental evidence at protein level
24.9113	24.7093	24.5704	24.7007	26.0088	25.9863	25.8456	26.7515	1:Experimental evidence at protein level
26.4061	26.4567	26.8135	26.4946	26.4219	26.7236	26.3382	26.5832	1:Experimental evidence at protein level
24.4646	24.2657	24.3703	24.4232	#NUM!	#NUM!	24.4593	24.5228	1:Experimental evidence at protein level
25.9416	26.1226	26.0186	26.5255	26.2852	26.6651	26.3457	27.2206	1:Experimental evidence at protein level
25.6868	25.8623	25.6232	25.958	25.909	25.7899	25.9834	26.3281	1:Experimental evidence at protein level
26.3286	26.6585	26.2672	26.4877	26.3206	26.2801	26.3162	26.8802	1:Experimental evidence at protein level
26.1923	26.1508	26.1858	26.59	26.2579	26.1586	25.8927	26.7739	1:Experimental evidence at protein level
25.8092	25.632	25.341	25.5548	25.5547	#NUM!	#NUM!	#NUM!	1:Experimental evidence at protein level
26.6206	26.4821	26.6451	26.6247	26.6814	26.4563	26.4386	26.9422	1:Experimental evidence at protein level
27.405	27.5894	27.3889	27.8921	27.5251	27.2647	27.6809	28.0694	1:Experimental evidence at protein level
26.2621	26.3378	26.1918	26.4218	26.1943	26.2291	26.2487	26.5222	1:Experimental evidence at protein level
26.3004	26.2698	26.0687	26.2805	26.7408	26.7878	26.8186	26.8429	1:Experimental evidence at protein level
27.6225	27.3663	27.5472	27.6739	27.6452	27.4424	27.489	27.7759	1:Experimental evidence at protein level
27.7484	27.813	28.0074	28.0117	27.6968	27.4523	27.6588	27.8072	1:Experimental evidence at protein level
28.1939	27.9529	28.4375	28.5035	27.9042	28.0759	27.9107	28.3503	1:Experimental evidence at protein level
26.4691	26.0501	26.1298	26.2163	26.7531	26.6932	26.2746	#NUM!	1:Experimental evidence at protein level
27.2643	27.073	27.111	27.3316	27.154	27.2136	27.4796	27.8418	1:Experimental evidence at protein level
26.6837	26.7777	26.8671	26.6265	26.8672	26.785	26.9235	26.8373	1:Experimental evidence at protein level
27.6958	27.6528	28.0523	27.1687	27.9965	28.0236	28.1462	27.593	1:Experimental evidence at protein level
25.9647	26.0526	26.052	26.0086	25.5516	25.5922	25.6222	25.8345	1:Experimental evidence at protein level
28.1251	28.2566	28.4856	28.5643	27.7123	27.7128	27.8733	27.5108	1:Experimental evidence at protein level
26.9369	27.0073	27.1245	27.1878	26.9024	26.7091	26.8567	27.0753	1:Experimental evidence at protein level
28.4892	28.5459	28.4673	28.7107	28.496	28.7807	28.6867	29.0943	1:Experimental evidence at protein level
26.1996	26.1516	25.9232	26.27	26.8105	26.7134	26.6068	27.1874	1:Experimental evidence at protein level
26.2653	26.4211	26.5172	26.8724	26.5877	26.8441	26.5363	26.6612	1:Experimental evidence at protein level
27.6327	27.6386	27.7656	27.4456	#NUM!	25.9057	25.446	25.8224	5:Protein uncertain
27.0908	27.0903	27.3416	27.1701	25.047	25.1522	25.1531	25.2644	1:Experimental evidence at protein level
26.5797	26.4839	26.8331	26.7215	26.5044	26.5723	26.7464	26.7197	1:Experimental evidence at protein level
27.9558	27.9185	28.0481	27.9435	27.5382	27.7262	27.568	27.6988	1:Experimental evidence at protein level
28.8794	29.0118	29.6554	29.3053	28.907	28.8475	29.136	29.0042	1:Experimental evidence at protein level
26.5307	26.8764	26.9441	26.7074	26.7498	26.6738	26.8096	26.9976	1:Experimental evidence at protein level
25.4136	25.3827	25.4805	25.628	25.1723	25.3851	25.0694	26.7037	1:Experimental evidence at protein level
26.6489	26.6945	26.9243	26.1581	26.3449	26.4357	26.4064	26.6878	1:Experimental evidence at protein level
#NUM!	#NUM!	#NUM!	#NUM!	25.8472	25.8896	25.9092	26.2428	1:Experimental evidence at protein level
26.6422	26.5367	26.6101	26.8021	26.5761	26.8231	26.5471	26.9068	1:Experimental evidence at protein level
24.4387	24.6165	24.3668	24.7965	24.7549	24.6473	24.6644	#NUM!	1:Experimental evidence at protein level
25.7922	25.8982	25.6568	26.0913	25.5325	25.6988	25.6608	26.1222	1:Experimental evidence at protein level
26.8813	27.0314	26.9528	26.854	27.095	26.9869	26.9845	26.8735	1:Experimental evidence at protein level
25.9092	26.0166	25.6677	25.8741	25.6526	25.6601	25.739	25.7589	1:Experimental evidence at protein level
25.2747	25.3777	25.5478	25.3864	25.977	26.0307	25.9342	#NUM!	1:Experimental evidence at protein level
25.1882	25.2972	25.6317	25.7123	25.809	25.9343	25.8294	25.7378	1:Experimental evidence at protein level
25.9173	26.2316	26.2095	26.1147	26.2698	26.4295	26.2418	26.4513	1:Experimental evidence at protein level
25.0631	24.2681	24.2808	25.0828	25.3602	25.0776	#NUM!	26.279	1:Experimental evidence at protein level
27.4225	27.2929	27.4096	27.0747	27.2073	27.1633	27.1586	26.3944	1:Experimental evidence at protein level
24.9005	24.8453	25.2604	25.1991	25.177	24.9129	24.9016	25.4576	1:Experimental evidence at protein level
26.9211	26.759	26.8452	26.7626	26.8422	26.8694	26.5525	26.5473	1:Experimental evidence at protein level
26.5034	26.5928	26.5671	26.3757	25.8664	25.8428	25.9307	26.2389	1:Experimental evidence at protein level
26.6529	27.0119	27.0509	26.8386	26.5904	26.709	26.7076	26.5146	1:Experimental evidence at protein level
25.9985	26.1176	25.823	26.031	26.0466	26.1564	25.8914	27.2862	1:Experimental evidence at protein level
27.0132	26.9329	26.8014	27.0796	26.7835	26.9047	26.7569	26.9623	1:Experimental evidence at protein level
27.926	27.8457	28.0484	27.9481	28.0109	28.0999	27.8236	27.4409	1:Experimental evidence at protein level
27.1505	27.1995	27.2045	27.2939	27.1148	27.1633	27.2829	27.7634	1:Experimental evidence at protein level
27.3412	27.5204	27.6837	27.4784	27.5235	27.5509	27.513	27.7093	1:Experimental evidence at protein level
26.7728	26.5616	26.6751	26.7916	26.8766	26.8422	26.9388	27.3133	1:Experimental evidence at protein level
28.7781	29.0554	29.4826	29.3337	29.0037	28.923	29.1765	29.2879	1:Experimental evidence at protein level
26.4362	26.4972	26.4706	26.6007	26.6169	26.6903	26.6498	26.862	1:Experimental evidence at protein level
26.0268	26.2565	26.3393	26.363	26.3773	26.3387	26.4124	26.4121	1:Experimental evidence at protein level
25.9832	26.1576	26.3229	26.2842	24.7443	24.7326	24.8705	25.4128	1:Experimental evidence at protein level
27.0623	26.7913	26.711	26.945	27.0464	26.8456	26.9829	27.1084	1:Experimental evidence at protein level
26.4763	26.8601	25.8243	26.366	27.27	27.8581	27.6152	27.4358	1:Experimental evidence at protein level
25.3918	25.6573	25.058	25.535	25.6861	25.3701	25.6425	26.2724	1:Experimental evidence at protein level
27.8074	27.9686	28.0462	28.1539	27.9867	28.014	27.9308	28.0292	1:Experimental evidence at protein level
26.526	26.6161	26.3523	26.6672	26.6387	26.7342	26.6948	26.7664	1:Experimental evidence at protein level
26.4746	26.2645	26.4127	26.2139	25.982	26.3082	26.4181	27.1138	1:Experimental evidence at protein level
27.1811	27.2076	27.3519	27.41	27.7184	27.7458	27.7053	27.9086	1:Experimental evidence at protein level
26.2718	26.3503	26.5085	26.1281	26.5973	26.6073	26.7772	26.0233	1:Experimental evidence at protein level
27.7389	27.6447	27.6299	27.8962	27.9132	27.9754	28.2058	28.1613	1:Experimental evidence at protein level
30.0111	29.8359	29.8448	29.5953	30.4459	30.2755	30.322	29.5848	1:Experimental evidence at protein level
29.1634	29.2049	29.4515	29.7055	29.3696	28.9457	29.2288	29.9673	1:Experimental evidence at protein level
26.3017	26.3432	26.1117	26.2091	26.6208	26.5099	26.6555	26.8404	1:Experimental evidence at protein level
27.7818	27.8351	27.9082	27.9617	27.9363	27.7317	27.9689	27.8302	1:Experimental evidence at protein level
25.3142	25.4438	#NUM!	25.2353	25.1997	25.4195	24.9609	25.8455	1:Experimental evidence at protein level
26.4058	26.8805	27.0377	26.7732	26.0112	26.056	26.261	26.1233	1:Experimental evidence at protein level
25.0535	25.1524	#NUM!	#NUM!	25.9392	25.9252	26.1407	25.6378	1:Experimental evidence at protein level
27.7685	28.0055	27.737	27.7563	28.0688	28.276	28.2012	28.4198	1:Experimental evidence at protein level
#NUM!	25.1918	25.2012	25.5912	25.9554	25.7779	25.8248	26.8277	1:Experimental evidence at protein level
25.2431	25.1588	25.4866	25.5482	25.5828	25.2513	25.2931	25.4957	1:Experimental evidence at protein level
27.5469	27.7732	27.612	27.8435	27.3625	27.366	27.4017	27.8467	1:Experimental evidence at protein level
26.2213	26.3857	26.5764	26.616	26.2828	25.8081	26.2713	26.9074	1:Experimental evidence at protein level
26.6896	26.6473	26.6234	26.8967	25.542	25.3373	25.0969	25.8826	1:Experimental evidence at protein level
27.5876	27.7411	27.4945	27.5276	27.9218	28.0656	27.8395	27.8243	1:Experimental evidence at protein level
24.1286	24.3543	24.6324	24.1802	24.3148	24.2589	24.2711	24.5398	1:Experimental evidence at protein level
27.4492	27.5207	27.256	27.3674	27.6841	27.6734	27.6224	28.0389	1:Experimental evidence at protein level
27.2616	27.1118	26.8101	26.5293	28.2252	27.9768	27.4547	27.4959	1:Experimental evidence at protein level
26.3264	26.3431	26.033	26.0321	26.1023	26.7076	26.2488	26.3984	1:Experimental evidence at protein level
27.6343	27.6858	27.7431	27.7852	28.1522	28.0554	27.7712	27.6984	1:Experimental evidence at protein level
25.7025	25.1605	#NUM!	25.6376	25.8683	25.9249	25.8816	26.2627	1:Experimental evidence at protein level
28.5866	28.1179	28.0768	27.8586	27.8906	28.1596	27.9785	27.8006	1:Experimental evidence at protein level
25.1255	25.3051	25.5654	25.1596	25.661	25.4295	25.6066	26.1179	1:Experimental evidence at protein level
26.008	25.9748	26.3044	26.1248	25.9349	25.8215	26.0214	26.2876	1:Experimental evidence at protein level
27.9625	27.9534	28.4229	28.1172	28.4175	28.2781	28.3517	28.3267	1:Experimental evidence at protein level
26.946	27.3012	27.2872	27.1266	27.2928	27.2021	27.3624	27.2852	1:Experimental evidence at protein level
27.7838	27.6995	27.6033	27.7104	27.7845	27.5058	27.6342	28.1606	1:Experimental evidence at protein level
27.1833	27.3223	27.4704	27.6726	27.4056	27.4809	27.4653	27.5995	1:Experimental evidence at protein level
29.4089	29.69	28.8753	29.8825	29.9371	29.7337	30.1718	30.5927	1:Experimental evidence at protein level
30.0306	29.7211	29.867	29.3975	30.0476	30.1365	29.7748	28.8141	1:Experimental evidence at protein level
28.6859	28.7664	29.0558	28.8139	28.828	28.4217	28.4546	29.0677	1:Experimental evidence at protein level
28.892	29.0059	28.8109	28.7611	28.7371	28.9646	28.8156	28.4585	1:Experimental evidence at protein level
30.0774	30.3765	30.3109	30.3406	31.8325	31.6998	30.8827	29.5664	1:Experimental evidence at protein level
30.2542	30.134	30.1028	30.3534	28.8214	28.6719	28.6811	29.2069	1:Experimental evidence at protein level
28.4422	28.4492	28.5272	28.4643	28.3017	28.328	28.3974	28.1162	1:Experimental evidence at protein level
29.49	29.4052	29.3714	29.4012	29.5445	29.4798	29.4814	29.2866	1:Experimental evidence at protein level
26.8369	26.6356	26.2804	26.1917	25.7901	25.791	25.3591	26.0881	1:Experimental evidence at protein level
27.9117	28.0214	27.6206	27.8678	28.0507	28.1433	28.0673	27.9628	1:Experimental evidence at protein level
27.2646	27.2424	27.1542	27.3037	27.1474	27.3314	27.0427	27.6389	1:Experimental evidence at protein level
28.2208	28.2456	28.6961	28.5604	28.2208	28.0508	28.0218	28.3562	1:Experimental evidence at protein level
25.2016	25.6434	25.6185	25.3938	#NUM!	#NUM!	#NUM!	#NUM!	1:Experimental evidence at protein level
26.9007	26.9799	27.2383	27.1741	27.3635	27.0921	27.2293	27.7414	1:Experimental evidence at protein level
26.2215	26.4947	26.5589	26.4694	26.4308	26.5202	26.4148	26.7535	1:Experimental evidence at protein level
24.0314	24.5418	24.4097	24.5079	25.2485	24.5747	25.4546	25.5671	1:Experimental evidence at protein level
27.8109	27.9084	28.1449	27.6464	26.3759	26.4899	26.3962	26.4401	1:Experimental evidence at protein level
31.0254	30.9744	31.1465	30.9969	30.7842	30.6898	30.7969	30.5438	1:Experimental evidence at protein level

29.977	30.0061	30.1323	30.1807	30.1715	30.2011	30.1489	30.3605	1:Experimental evidence at protein level
26.6627	26.8252	26.7373	26.9281	27.2101	27.3553	27.0725	27.9248	1:Experimental evidence at protein level
27.663	27.318	27.3633	27.2363	27.7501	27.7872	27.1441	26.8793	1:Experimental evidence at protein level
27.2105	27.1965	27.3718	27.0382	27.1856	27.3548	27.1631	27.1605	1:Experimental evidence at protein level
#NUM!	26.3967	26.7354	27.1047	26.616	26.5503	26.6747	26.9802	1:Experimental evidence at protein level
27.2831	27.258	26.668	26.8336	27.1584	27.1983	27.4625	27.7998	1:Experimental evidence at protein level
26.5949	26.8863	26.8038	26.8541	26.7618	26.7128	26.991	27.1671	1:Experimental evidence at protein level
28.9003	28.7271	28.4945	28.5487	28.847	28.8196	28.5231	26.866	1:Experimental evidence at protein level
26.8945	26.8833	26.6562	26.6303	26.5075	26.7204	26.6693	26.7904	1:Experimental evidence at protein level
28.5018	28.4811	28.9511	28.8135	28.1797	28.0134	28.3973	28.4748	1:Experimental evidence at protein level
26.7201	26.8158	26.8289	26.5455	26.6797	26.581	26.6441	26.9697	1:Experimental evidence at protein level
26.8945	27.2129	27.1855	26.8181	27.1309	27.1901	27.1776	26.7378	1:Experimental evidence at protein level
28.9014	28.81	28.8516	28.9667	29.0321	28.9427	28.9841	28.8725	1:Experimental evidence at protein level
29.043	29.1862	29.4885	29.3084	29.3528	29.3942	29.348	29.5266	1:Experimental evidence at protein level
27.9304	28.325	28.376	28.3466	28.2178	28.197	28.277	27.7071	1:Experimental evidence at protein level
28.8463	28.8591	28.5869	28.4718	28.8858	28.8456	28.8934	28.3929	1:Experimental evidence at protein level
27.7245	27.7974	28.0274	28.0804	27.2233	27.0538	27.316	27.1933	1:Experimental evidence at protein level
#NUM!	26.8764	26.6032	27.6462	27.6039	27.7142	27.939	28.3673	1:Experimental evidence at protein level
26.5314	26.5993	26.544	26.7042	27.1139	26.9197	27.0576	27.2859	1:Experimental evidence at protein level
27.9667	28.0806	28.2874	28.1227	27.6697	27.7945	27.6677	27.5807	1:Experimental evidence at protein level
25.6053	25.7215	25.2677	25.3504	25.4167	25.6104	25.3971	25.4671	1:Experimental evidence at protein level
26.6447	26.3976	26.2609	26.5741	27.0357	27.064	26.4606	25.5567	1:Experimental evidence at protein level
28.062	28.0796	28.2584	28.3918	28.406	28.213	28.2989	28.6666	1:Experimental evidence at protein level
27.2529	27.3014	27.5656	27.3075	27.3057	27.0345	27.3422	27.9195	1:Experimental evidence at protein level
26.054	25.9748	25.9592	26.0473	25.6986	25.5754	25.6882	26.2957	1:Experimental evidence at protein level
26.149	26.1536	26.371	26.2791	25.5527	25.3246	25.5397	25.9207	1:Experimental evidence at protein level
27.2205	27.2177	27.2529	27.4633	27.244	27.1636	27.2479	27.6314	1:Experimental evidence at protein level
26.2906	26.4468	26.41	26.1108	26.9177	26.6994	26.9386	27.2567	1:Experimental evidence at protein level
25.5008	25.6879	25.1825	25.4484	25.3454	25.5222	#NUM!	25.4856	1:Experimental evidence at protein level
26.5633	26.5783	26.6113	26.7168	26.6799	26.8369	26.5795	26.9211	1:Experimental evidence at protein level
26.3005	26.1729	26.2753	26.2596	26.7701	26.688	26.8922	26.4605	1:Experimental evidence at protein level
25.2815	25.2796	25.5615	25.4392	26.4849	26.5405	26.5736	26.7949	1:Experimental evidence at protein level
24.8211	25.0912	25.2902	24.7285	#NUM!	#NUM!	#NUM!	26.2471	1:Experimental evidence at protein level
25.8289	25.7174	25.6053	25.8184	25.6266	25.4547	25.915	26.2088	1:Experimental evidence at protein level
26.4934	26.588	26.7953	26.6972	26.7343	26.7285	26.9822	27.0879	1:Experimental evidence at protein level
27.2661	27.3276	27.246	27.4817	27.6011	27.7166	27.7313	28.052	1:Experimental evidence at protein level
25.564	25.4634	25.7506	25.7031	25.9458	25.8318	26	26.0479	1:Experimental evidence at protein level
27.3492	27.387	26.9192	27.3948	27.134	27.0403	27.0043	#NUM!	1:Experimental evidence at protein level
24.8593	24.9462	24.8542	24.7671	25.5902	25.5921	25.7	25.7779	1:Experimental evidence at protein level
28.0574	28.1981	28.2417	28.3238	28.1258	28.2985	28.2619	28.2943	1:Experimental evidence at protein level
28.224	28.4232	28.6406	28.6564	28.6908	28.6482	28.7682	28.8525	1:Experimental evidence at protein level
25.8895	25.8781	25.6969	26.0817	26.0632	25.9421	25.949	26.75	1:Experimental evidence at protein level
26.3064	26.3233	26.3941	26.4589	26.4031	26.3242	26.4408	26.5424	1:Experimental evidence at protein level
25.7613	25.5864	25.4573	25.5909	25.7966	25.6192	25.5321	26.0205	1:Experimental evidence at protein level
26.7651	26.9159	26.9475	26.9434	26.948	26.7903	26.7546	27.2272	1:Experimental evidence at protein level
25.6085	25.6338	25.4068	25.7268	25.962	25.8144	25.8826	26.5332	1:Experimental evidence at protein level
26.2621	26.0779	26.2235	26.2756	26.2942	26.1258	26.1512	26.1746	1:Experimental evidence at protein level
25.5841	25.599	25.6299	25.7564	25.7732	25.7095	25.6903	26.0948	1:Experimental evidence at protein level
25.8113	26.0243	25.4661	25.9187	25.965	25.5626	25.8064	26.2921	1:Experimental evidence at protein level
27.8996	28.0728	28.1225	28.2264	27.6103	27.4583	27.4421	27.8412	1:Experimental evidence at protein level
26.6413	26.7608	26.2026	26.7049	26.7713	26.7941	#NUM!	27.0675	1:Experimental evidence at protein level
27.0197	26.6568	25.0731	25.1329	#NUM!	25.8019	24.9355	25.5265	1:Experimental evidence at protein level
27.1147	26.3286	26.6904	26.4831	27.1981	27.3792	26.6574	25.6471	1:Experimental evidence at protein level
25.8765	26.2943	26.8323	26.2119	26.3468	26.4757	26.218	26.651	1:Experimental evidence at protein level
25.8556	25.8639	26.0828	26.1186	26.0118	25.8645	25.8697	25.8398	1:Experimental evidence at protein level
25.6374	25.9104	25.7645	25.8495	25.9492	26.1413	26.0317	26.2281	1:Experimental evidence at protein level
26.9713	26.6493	27.101	26.7351	27.5757	27.6085	27.0149	#NUM!	1:Experimental evidence at protein level
25.8488	26.1385	26.4533	26.2209	25.4961	25.7059	26.0234	#NUM!	1:Experimental evidence at protein level
25.9397	25.8516	25.7278	25.8906	26.3445	25.8863	25.8808	#NUM!	1:Experimental evidence at protein level
25.8313	26.0172	25.8255	26.0195	26.3145	26.1794	26.3101	26.907	1:Experimental evidence at protein level
26.3644	26.331	26.5389	26.6108	26.4895	26.4689	26.5354	26.9026	1:Experimental evidence at protein level
27.0921	27.3722	27.2174	27.3095	27.5204	27.5405	27.5249	27.7362	1:Experimental evidence at protein level
29.0588	28.8452	29.1025	29.1354	29.3253	29.3944	29.2237	29.3482	1:Experimental evidence at protein level
27.1941	27.1978	27.4323	27.1467	27.4368	27.3697	27.2743	27.2254	1:Experimental evidence at protein level
26.9627	26.8574	27.2599	27.6163	27.2994	#NUM!	27.314	27.7923	1:Experimental evidence at protein level
25.7382	25.4291	25.544	25.6656	25.8879	25.7231	25.8452	26.3485	1:Experimental evidence at protein level
#NUM!	25.2095	25.1627	25.2675	24.8449	25.3794	24.9524	25.582	1:Experimental evidence at protein level
27.2074	27.097	27.1908	27.3536	27.0408	26.9214	27.2099	27.5816	1:Experimental evidence at protein level
27.2649	27.4342	27.211	27.2653	27.6084	27.6854	27.5396	27.7072	1:Experimental evidence at protein level
26.5967	26.3022	26.7498	26.7115	26.7267	26.5566	26.6364	27.209	1:Experimental evidence at protein level
26.1756	26.0924	26.358	26.1945	25.9099	26.332	26.1317	26.0914	1:Experimental evidence at protein level
26.9538	27.0171	26.8139	27.1256	27.3994	27.2802	27.3532	27.8227	1:Experimental evidence at protein level
24.6184	24.9532	25.0166	24.6361	25.244	25.5636	25.3169	25.0932	1:Experimental evidence at protein level
29.8228	30.092	30.1803	30.4438	29.3982	29.3104	29.6884	30.2067	1:Experimental evidence at protein level
25.9507	25.9831	26.5877	26.0075	26.6314	26.5713	26.664	26.5561	1:Experimental evidence at protein level
27.6915	27.836	28.0407	28.0392	27.9295	27.6309	28.0075	28.3468	1:Experimental evidence at protein level
27.913	27.9646	28.2894	28.2489	28.2207	27.813	28.1735	27.9897	1:Experimental evidence at protein level
27.7072	27.7175	27.9221	28.0994	27.7741	27.7156	27.6951	28.2493	1:Experimental evidence at protein level
28.3654	28.0513	28.0614	28.2636	28.656	28.3057	28.5217	29.4376	1:Experimental evidence at protein level
27.0658	26.9107	26.8424	27.043	26.8283	26.9986	26.9121	27.0989	2:Experimental evidence at transcript level
23.7663	24.3169	23.8882	24.0688	#NUM!	25.1995	25.7658	25.486	1:Experimental evidence at protein level
28.9999	29.3844	29.683	29.4107	28.8901	28.8704	29.2927	28.9433	1:Experimental evidence at protein level
25.6988	25.6025	25.8254	25.5165	26.2903	26.1641	25.8208	25.679	1:Experimental evidence at protein level
27.0537	26.9854	26.6125	26.4038	26.7682	26.9371	26.7344	26.8528	1:Experimental evidence at protein level
27.1053	27.2886	27.0127	27.0104	26.8342	27.2171	27.187	27.213	1:Experimental evidence at protein level
27.9435	28.0571	28.2181	28.3135	27.2641	27.2571	27.4876	27.6635	1:Experimental evidence at protein level
26.5563	26.5577	26.4989	26.7924	26.4944	26.6631	26.6196	26.9294	1:Experimental evidence at protein level
26.3113	26.0578	26.3136	26.2705	26.3197	26.2039	26.2673	26.6199	1:Experimental evidence at protein level
29.1168	28.9957	29.1184	29.1124	29.6202	29.4159	29.4108	29.2697	1:Experimental evidence at protein level
25.9347	26.1304	26.1617	26.4069	26.4795	26.2327	26.2597	26.955	1:Experimental evidence at protein level
27.4988	27.4128	27.3609	27.4767	27.7314	27.8132	27.8131	27.8142	1:Experimental evidence at protein level
25.1868	25.1517	25.267	25.1284	25.9309	25.993	25.9706	26.2611	1:Experimental evidence at protein level
28.7242	28.6891	28.3417	28.5257	28.7034	28.796	28.6157	28.9989	1:Experimental evidence at protein level
25.2536	25.127	24.997	25.1477	24.1632	#NUM!	#NUM!	#NUM!	1:Experimental evidence at protein level
26.8302	26.5789	26.514	26.8064	26.3058	27.1861	26.6206	27.0935	1:Experimental evidence at protein level
25.7326	25.8532	26.023	26.0437	26.0464	26.204	26.1909	25.5817	1:Experimental evidence at protein level
25.1613	25.2377	25.3608	25.421	24.9356	24.6963	25.0626	25.2505	1:Experimental evidence at protein level
26.3181	26.2777	26.0733	25.9536	26.5163	26.6184	26.9063	27.1237	1:Experimental evidence at protein level
25.6461	25.3491	25.4847	25.703	25.4491	25.4939	25.1218	25.7016	1:Experimental evidence at protein level
24.8877	24.8728	24.9953	24.8387	24.8676	25.0228	24.6933	#NUM!	1:Experimental evidence at protein level
25.515	25.2811	25.5779	25.0956	25.288	#NUM!	24.8753	#NUM!	1:Experimental evidence at protein level
26.631	26.5316	26.4596	26.6588	26.7537	26.4898	26.4769	26.7944	1:Experimental evidence at protein level
25.6749	25.9552	25.9439	26.149	26.2076	26.1781	26.2475	26.4018	1:Experimental evidence at protein level
27.4227	27.4969	27.2863	27.2388	27.6564	27.5113	27.6039	28.2304	1:Experimental evidence at protein level
27.8122	27.7381	27.6354	27.9395	27.8754	27.8363	27.9252	28.0416	1:Experimental evidence at protein level
26.7312	26.7496	26.7342	27.2401	27.5636	26.9364	26.9901	28.0339	1:Experimental evidence at protein level
26.2514	26.3423	26.4088	26.7768	26.33	26.0542	26.2143	26.9041	1:Experimental evidence at protein level
24.5765	24.7996	24.607	24.7355	25.0284	24.6427	24.834	25.3822	1:Experimental evidence at protein level
26.3996	26.1621	26.1787	26.8674	26.6421	27.2795	26.7774	27.4054	1:Experimental evidence at protein level
27.8614	27.7581	27.5145	27.5105	28.0355	27.8341	27.4564	27.0835	1:Experimental evidence at protein level
27.4032	27.3947	27.5395	27.3869	27.747	27.609	27.739	27.7449	1:Experimental evidence at protein level
26.9782	26.7893	26.933	26.9529	27.1131	27.0059	27.1174	27.3167	1:Experimental evidence at protein level
26.6458	26.4642	26.3012	26.1356	26.2777	25.948	26.0226	25.9827	1:Experimental evidence at protein level
26.2703	26.4165	26.7466	26.5165	26.3507	26.3022	26.2187	26.307	1:Experimental evidence at protein level
25.2311	25.3952	24.7735	25.9543	25.6056	25.4433	25.6538	26.2233	1:Experimental evidence at protein level
27.9593	27.9748	28.049	27.6693	27.6741	27.7266	27.5227	27.2435	1:Experimental evidence at protein level
27.9856	27.7625	27.7427	27.8857	27.7349	27.7029	27.6526	28.1887	1:Experimental evidence at protein level
29.7776	29.7735	29.7501	29.244	29.2949	29.315	29.1467	28.4708	1:Experimental evidence at protein level
#NUM!	#NUM!	24.9487	24.2095	25.5795	25.5845	24.8656	25.5539	1:Experimental evidence at protein level
27.5927	27.272	27.1314	27.1027	27.4765	27.1624	27.2289	27.4687	1:Experimental evidence at protein level
26.0017	26.0175	26.1154	26.3605	26.4138	26.4061	26.3931	26.779	1:Experimental evidence at protein level
26.9455	27.0556	27.2794	27.1387	26.2906	25.9659	26.1499	26.9033	1:Experimental evidence at protein level
29.2556	29.4663	29.6452	29.3977	29.0888	29.3265	29.0359	28.3393	1:Experimental evidence at protein level
26.3076	26.6339	26.0983	26.4912	26.5705	26.1307	26.1876	26.7606	1:Experimental evidence at protein level
29.6099	29.5408	29.942	29.9545	29.5108	29.5111	29.5898	29.595	1:Experimental evidence at protein level
30.2376	29.9199	28.5944	28.5683	28.7651	29.0668	28.8405	28.6262	1:Experimental evidence at protein level
28.7542	28.8127	28.9489	28.8362	28.7812	28.8396	28.8908	28.7083	1:Experimental evidence at protein level
29.2287	28.7771	28.8114	29.1362	29.0558	29.0461	28.4359	27.477	1:Experimental evidence at protein level
27.9612	27.9293	27.644	27.422	27.3075	27.4815	26.7806	27.0142	1:Experimental evidence at protein level

28.9113	29.0366	28.5638	28.3242	28.5971	28.7756	28.5295	28.0714	1:Experimental evidence at protein level
27.0561	27.0278	27.0509	27.4928	27.2889	27.3008	27.2782	28.3767	1:Experimental evidence at protein level
27.2583	27.1967	27.2737	27.0981	27.232	27.1049	27.1053	27.2046	1:Experimental evidence at protein level
27.004	27.0279	27.1759	27.0673	26.754	26.8506	26.8385	26.7113	1:Experimental evidence at protein level
27.715	27.6053	27.5145	27.6227	27.5619	27.5973	27.627	27.8871	1:Experimental evidence at protein level
29.9488	29.9687	30.145	29.8651	29.7222	29.9841	29.6389	29.5695	1:Experimental evidence at protein level
30.8222	30.9342	31.1743	31.1531	30.8592	30.7508	30.9337	30.8776	1:Experimental evidence at protein level
27.4104	27.4323	27.3812	27.3285	27.4044	27.3989	27.2814	27.1675	1:Experimental evidence at protein level
29.2899	29.2687	29.6405	29.3926	29.2531	29.006	29.3807	29.5042	1:Experimental evidence at protein level
27.3126	27.1764	27.2022	27.2744	27.3537	27.045	27.2	27.0471	1:Experimental evidence at protein level
25.3172	24.9941	25.5379	25.5431	25.1866	24.993	25.1157	#NUM!	1:Experimental evidence at protein level
27.8261	27.9771	28.1375	27.971	28.6198	28.3069	28.5886	28.9087	1:Experimental evidence at protein level
29.1457	29.1332	29.2583	29.2376	29.9542	29.6422	29.7482	29.6426	1:Experimental evidence at protein level
26.5335	26.5976	26.6764	26.7213	26.8318	26.785	26.904	27.2507	1:Experimental evidence at protein level
29.4771	29.503	29.5377	29.4809	29.4967	29.7113	29.4918	29.152	1:Experimental evidence at protein level
25.2846	25.4701	25.767	25.4639	25.8293	26.002	25.9067	25.7934	1:Experimental evidence at protein level
27.3197	27.3786	27.5779	27.5725	27.4694	27.4568	27.5165	27.412	1:Experimental evidence at protein level
27.5928	27.626	27.3665	27.6364	27.8092	27.5392	27.7867	27.9166	1:Experimental evidence at protein level
25.917	26.2222	26.5567	25.9356	26.6363	26.6976	26.479	26.6327	1:Experimental evidence at protein level
25.4929	25.9304	25.8021	26.1478	26.2427	#NUM!	26.4158	26.5506	1:Experimental evidence at protein level
26.2748	26.3562	26.0778	26.3393	26.1833	26.449	26.2679	26.3775	1:Experimental evidence at protein level
27.3863	27.4818	27.5148	27.3741	28.0566	27.7403	27.3109	28.1513	1:Experimental evidence at protein level
29.0763	29.0515	28.986	29.2637	29.3771	29.4874	29.2371	29.3813	1:Experimental evidence at protein level
28.5183	28.7781	28.9229	28.2615	28.3673	28.3255	28.4827	27.709	1:Experimental evidence at protein level
26.9754	27.159	27.3328	27.1714	26.9758	27.0528	26.9148	26.2989	1:Experimental evidence at protein level
27.6358	27.3256	27.5493	27.7066	27.7935	28.0567	27.774	27.405	1:Experimental evidence at protein level
29.7434	29.8116	29.3145	29.9514	29.2385	29.444	29.0062	29.676	1:Experimental evidence at protein level
29.529	29.7019	29.902	29.9071	30.072	29.8661	30.1613	30.4115	1:Experimental evidence at protein level
27.0666	26.9292	27.1085	27.1255	27.2033	27.3847	27.31	27.624	1:Experimental evidence at protein level
26.4898	26.5019	26.5962	26.328	26.5732	26.6712	26.7579	26.4172	1:Experimental evidence at protein level
26.1497	26.2276	26.4009	26.5026	25.8684	25.5963	25.9093	26.2388	1:Experimental evidence at protein level
28.5243	28.4241	27.9929	28.3926	27.4172	27.5079	27.2174	27.7671	1:Experimental evidence at protein level
27.8942	28.014	28.0843	27.8996	27.8493	27.9959	27.7877	27.7616	1:Experimental evidence at protein level
#NUM!	25.5574	25.4566	#NUM!	25.7629	25.4759	25.7519	26.0769	1:Experimental evidence at protein level
25.3233	25.4417	25.4644	25.6991	25.2279	25.2698	25.3861	25.5	1:Experimental evidence at protein level
27.0582	26.9853	27.2235	26.9905	26.6158	26.7505	26.2491	26.4322	1:Experimental evidence at protein level
25.8561	25.8664	25.8785	25.9452	26.3461	26.4311	26.363	26.3725	1:Experimental evidence at protein level
24.3198	24.4599	24.8621	24.7535	24.7882	24.9852	24.8514	24.8909	1:Experimental evidence at protein level
25.8615	25.8543	25.4789	25.1688	25.8573	26.0265	25.5947	25.4162	1:Experimental evidence at protein level
26.1883	26.0532	26.3345	26.2668	26.1877	26.0067	26.4692	26.7032	1:Experimental evidence at protein level
26.8239	26.5946	26.8844	26.9495	27.4696	27.5161	27.3872	27.1763	1:Experimental evidence at protein level
28.6081	28.5212	28.6812	28.4528	28.577	28.4299	28.3086	27.9625	1:Experimental evidence at protein level
27.7041	27.8222	27.9032	27.867	28.0562	28.1551	28.12	28.0943	1:Experimental evidence at protein level
27.7395	27.3867	27.2527	27.5019	27.4961	27.3901	27.4144	27.5694	1:Experimental evidence at protein level
25.6899	25.5515	25.3337	25.2481	25.0865	25.5849	25.6393	25.7388	1:Experimental evidence at protein level
35.4422	35.1178	33.1137	33.1288	32.5093	33.2467	32.2632	32.2044	1:Experimental evidence at protein level
25.2608	25.6001	25.6398	25.3594	25.0996	25.4335	25.0538	25.7479	1:Experimental evidence at protein level
24.8444	25.0003	25.1628	24.9785	24.4929	24.8355	24.7566	#NUM!	1:Experimental evidence at protein level
25.3687	25.4941	25.4757	25.6294	25.4804	25.418	25.2843	25.7036	1:Experimental evidence at protein level
26.9862	26.9308	26.9382	27.0929	26.6059	26.9766	26.8137	27.1701	1:Experimental evidence at protein level
23.6467	23.7199	23.563	23.8953	#NUM!	#NUM!	#NUM!	#NUM!	1:Experimental evidence at protein level
27.8189	27.9947	27.7165	27.6429	27.7438	27.6142	27.5716	28.0674	1:Experimental evidence at protein level
25.7071	25.9247	25.8734	25.9351	25.1117	25.1274	25.0307	25.7994	1:Experimental evidence at protein level
26.975	27.0483	27.294	27.2937	26.6462	26.2384	26.4361	26.8599	1:Experimental evidence at protein level
28.1836	28.3008	28.3689	28.2971	27.6265	27.39	27.8051	27.7632	1:Experimental evidence at protein level
24.4894	24.7723	24.2036	23.8197	25.0729	25.6264	#NUM!	25.6338	1:Experimental evidence at protein level
26.2943	26.2636	26.3077	26.4527	25.9977	25.6416	26.1688	26.7492	1:Experimental evidence at protein level
26.322	26.2683	26.18	26.3068	26.3366	26.077	26.2621	26.7587	1:Experimental evidence at protein level
26.3811	26.2125	26.2155	26.1686	25.9789	26.3154	26.0696	26.0472	1:Experimental evidence at protein level
26.7171	27.0091	27.0714	27.1243	27.1456	27.2308	27.3914	27.1165	1:Experimental evidence at protein level
25.8817	25.8977	25.9149	26.0241	25.3536	24.8156	25.3355	25.2891	1:Experimental evidence at protein level
27.0514	27.1198	27.1473	27.1983	26.937	26.8994	26.7102	27.246	1:Experimental evidence at protein level
25.6078	25.7521	25.4745	25.4558	25.8666	25.8218	25.5276	26.1103	1:Experimental evidence at protein level
26.5059	26.5602	26.7134	26.297	26.338	26.3769	26.4149	26.3287	1:Experimental evidence at protein level
27.5074	27.4869	27.5447	27.517	27.2825	27.2888	27.1975	27.4134	1:Experimental evidence at protein level
27.6881	27.8596	28.0342	27.9489	28.1511	27.9821	28.1452	28.0787	1:Experimental evidence at protein level
28.2914	28.0823	27.7119	27.7332	27.795	27.8571	27.8041	27.3847	1:Experimental evidence at protein level
26.5137	26.6288	26.4928	26.2533	26.8946	27.0801	26.8732	25.8108	1:Experimental evidence at protein level
25.8851	26.1358	26.1044	25.9254	26.3722	26.0917	25.8854	26.0224	1:Experimental evidence at protein level
25.7276	25.9587	26.0047	26.0922	25.7567	25.716	25.8994	26.2632	1:Experimental evidence at protein level
27.3375	27.3603	27.4503	27.3877	27.2693	26.8675	26.8547	26.9875	1:Experimental evidence at protein level
26.4718	26.4737	26.4095	26.5695	26.7697	26.5471	26.9366	27.3866	1:Experimental evidence at protein level
26	26.1295	25.8703	26.0002	25.921	26.0291	25.8842	26.104	1:Experimental evidence at protein level
26.7559	26.5935	26.6317	26.8184	27.1565	26.8827	27.0111	27.4893	1:Experimental evidence at protein level
26.5672	26.5627	26.6132	26.8137	26.4488	26.2335	26.5404	26.9306	1:Experimental evidence at protein level
26.9472	26.7752	27.0198	26.8267	27.0065	27.0052	27.0072	26.8683	1:Experimental evidence at protein level
26.8424	26.965	26.8464	27.0256	27.2895	27.3673	27.2518	27.6387	1:Experimental evidence at protein level
26.1989	25.9168	25.696	26.0466	26.5252	25.5603	26.3518	27.0015	1:Experimental evidence at protein level
27.104	27.0041	26.8151	26.9648	27.3171	27.221	27.1332	27.5256	1:Experimental evidence at protein level
25.4899	25.5065	#NUM!	#NUM!	25.4417	25.5293	25.5294	26.0048	1:Experimental evidence at protein level
27.2288	27.2137	27.2395	27.0717	27.3081	27.2942	27.2377	26.9287	1:Experimental evidence at protein level
25.4155	25.5094	25.2352	25.1779	25.3494	25.1443	25.2537	25.3155	1:Experimental evidence at protein level
24.9691	25.0972	25.3456	25.6278	25.0364	25.2561	25.2462	25.5642	1:Experimental evidence at protein level
27.3608	27.5742	27.6456	27.6454	24.9352	25.2832	25.4463	25.5725	1:Experimental evidence at protein level
29.3051	29.046	29.1122	28.9904	29.6257	29.7256	29.566	28.9077	1:Experimental evidence at protein level
24.364	24.6483	24.2612	25.0504	24.8651	25.1303	24.6472	25.2591	1:Experimental evidence at protein level
28.4697	28.392	28.5	28.5438	28.4834	28.3527	28.4493	28.7786	1:Experimental evidence at protein level
26.1502	25.923	25.37	25.5674	25.9374	25.8984	25.6352	26.3736	1:Experimental evidence at protein level
#NUM!	25.2665	#NUM!	#NUM!	25.4342	25.3598	25.6569	25.5015	1:Experimental evidence at protein level
26.7695	26.8739	26.8955	26.9859	26.5331	26.5566	26.7863	26.4552	1:Experimental evidence at protein level
27.7104	27.5958	27.4774	27.5434	27.6869	28.013	28.0574	28.3338	1:Experimental evidence at protein level
25.9444	26.08	26.0034	25.9157	26.3705	26.3864	26.1467	26.6985	1:Experimental evidence at protein level
26.0699	26.1453	26.1968	26.2452	26.3669	26.4227	26.2444	26.683	1:Experimental evidence at protein level
25.9863	26.0208	26.1007	26.2966	26.0428	26.112	26.2594	26.6244	1:Experimental evidence at protein level
27.0791	26.8964	27.0934	27.2865	27.223	26.5873	26.6456	27.072	1:Experimental evidence at protein level
26.6248	26.8677	26.8995	26.6892	26.9187	26.9039	26.9578	27.5598	1:Experimental evidence at protein level
25.8423	25.9781	25.9323	26.05	26.3438	26.3346	26.4707	26.0477	1:Experimental evidence at protein level
28.2985	28.3512	28.1256	28.424	28.63	28.4574	28.6717	28.7677	1:Experimental evidence at protein level
27.9457	27.9657	28.0673	27.9943	27.8731	28.1244	27.8703	27.7721	1:Experimental evidence at protein level
26.0554	26.1928	26.1633	25.9196	26.199	26.1965	25.7931	26.1761	1:Experimental evidence at protein level
25.6727	25.8172	25.3302	25.8794	25.6906	#NUM!	25.7136	26.3557	1:Experimental evidence at protein level
26.9857	27.1921	27.3711	27.2664	27.1005	27.0726	27.1808	27.1254	1:Experimental evidence at protein level
27.2112	26.9492	27.2809	27.2863	27.0725	26.822	27.0617	27.0876	1:Experimental evidence at protein level
27.4169	27.365	27.3312	27.2551	26.9339	26.8603	27.0938	27.5499	1:Experimental evidence at protein level
29.073	29.1	29.0115	28.8223	29.2424	29.2961	29.2697	29.1279	1:Experimental evidence at protein level
28.3927	28.3763	28.4139	28.3646	28.6605	28.6386	28.6021	28.6308	1:Experimental evidence at protein level
25.5425	25.6166	25.6718	25.4596	#NUM!	25.2215	25.0874	25.7615	1:Experimental evidence at protein level
26.5519	26.586	26.6766	26.9102	27.0125	27.0494	27.1227	27.1758	1:Experimental evidence at protein level
24.47	24.4024	24.7251	24.5536	#NUM!	#NUM!	24.1937	25.5985	1:Experimental evidence at protein level
28.5318	28.5006	28.4556	28.2896	28.7594	28.6729	28.4813	28.6974	1:Experimental evidence at protein level
28.0338	28.0103	28.1063	28.151	27.9366	27.8432	28.023	28.4814	1:Experimental evidence at protein level
29.7078	29.4285	29.1837	28.9909	29.9065	29.8724	29.3771	27.2563	1:Experimental evidence at protein level
27.5976	27.9956	28.0578	27.8214	27.802	28.0437	27.8564	27.2027	1:Experimental evidence at protein level
26.0158	26.2352	26.1615	26.2767	26.5593	26.6801	26.4412	26.7454	1:Experimental evidence at protein level
30.1607	29.9783	29.9342	29.9408	29.3714	29.36	29.0293	28.9054	1:Experimental evidence at protein level
25.2518	25.2101	25.5564	25.8998	24.8134	24.9406	25.0178	26.0155	1:Experimental evidence at protein level
28.0925	28.3069	28.3124	28.4808	28.0119	28.1341	28.1899	28.419	1:Experimental evidence at protein level
25.4572	25.1814	24.9536	24.9137	#NUM!	24.8587	25.1048	#NUM!	1:Experimental evidence at protein level
26.7501	26.9466	27.2649	26.8756	27.041	27.0713	27.1105	27.0763	1:Experimental evidence at protein level
27.8342	27.7943	27.7751	28.0951	28.0301	27.7225	28.1119	28.5605	1:Experimental evidence at protein level
26.8657	26.7674	26.5922	26.605	26.4965	26.5855	26.5584	26.7185	1:Experimental evidence at protein level
28.3859	28.5231	28.5158	28.62	28.334	28.415	28.6526	28.5105	1:Experimental evidence at protein level
29.1871	29.1361	29.5431	29.5497	29.4376	29.1838	29.4348	29.6794	1:Experimental evidence at protein level
27.1981	27.2866	27.451	27.4742	27.3097	27.1375	27.478	27.6134	1:Experimental evidence at protein level
25.6816	25.6461	25.8903	25.9427	25.9325	26.0601	25.8586	26.0905	1:Experimental evidence at protein level
27.5862	27.6817	27.7509	27.8104	27.8125	27.696	27.8464	27.8677	1:Experimental evidence at protein level
27.5138	27.7884	27.7488	27.7201	27.2284	27.2266	27.4783	27.2976	1:Experimental evidence at protein level
27.0156	#NUM!	#NUM!	#NUM!	27.5991	27.1914	27.6091	28.4518	1:Experimental evidence at protein level
26.929	26.7715	26.7848	26.9655	26.296	26.0677	26.4041	26.9729	1:Experimental evidence at protein level

25.7385	25.7116	25.8987	25.7193	25.8649	25.7634	25.6651	25.5907	1:Experimental evidence at protein level
28.3207	28.5838	28.6868	28.7591	28.6155	28.1624	28.8059	28.3352	1:Experimental evidence at protein level
28.106	27.8731	28.1375	28.0149	27.8688	28.0787	27.5529	26.7035	1:Experimental evidence at protein level
27.4931	27.5355	27.7945	27.6243	27.6158	27.5047	27.5726	27.431	1:Experimental evidence at protein level
27.261	27.2646	27.6373	27.4473	26.6927	26.6752	26.728	27.4429	1:Experimental evidence at protein level
31.4638	31.6254	32.0482	31.5282	31.5262	31.4116	31.6994	30.5273	1:Experimental evidence at protein level
29.2904	29.3849	28.8029	28.9822	29.4174	29.5116	29.0681	29.4897	1:Experimental evidence at protein level
29.2041	28.9651	28.2631	28.2245	29.0897	29.2652	29.0159	28.7642	1:Experimental evidence at protein level
28.0088	27.8766	27.838	27.9119	28.0923	27.9815	27.9657	28.2149	1:Experimental evidence at protein level
27.4925	27.4832	27.4523	27.5263	27.4268	27.3187	27.3988	27.7003	1:Experimental evidence at protein level
27.5914	27.7455	27.6228	27.8126	27.8152	27.689	27.7736	28.152	1:Experimental evidence at protein level
30.12	30.4472	30.741	29.8918	30.8551	30.9019	31.2672	30.8441	1:Experimental evidence at protein level
25.7205	25.5287	25.5061	25.1775	24.6352	24.8725	24.9531	24.7368	1:Experimental evidence at protein level
28.9368	28.9381	29.1697	29.2801	29.5514	29.4885	29.5474	29.578	1:Experimental evidence at protein level
30.4148	30.2399	30.2594	30.1606	30.3837	30.0807	30.4679	30.9191	1:Experimental evidence at protein level
27.514	27.4026	27.0617	27.2549	27.8652	27.9143	27.9179	27.9554	1:Experimental evidence at protein level
30.6009	30.3279	29.9907	30.1025	30.8663	30.8021	30.7339	30.9755	1:Experimental evidence at protein level
25.9718	26.0165	26.0559	26.372	26.8712	26.3768	26.7955	27.0977	1:Experimental evidence at protein level
28.0008	28.2765	28.3352	28.1512	27.8305	27.9692	28.1316	28.0679	1:Experimental evidence at protein level
25.6618	25.7253	26.0301	25.7066	26.3012	26.2177	26.0477	26.4133	1:Experimental evidence at protein level
28.0073	27.8589	27.8572	28.0644	28.4316	28.6681	28.6312	28.5444	1:Experimental evidence at protein level
26.3104	26.4172	26.0727	26.1082	27.0087	26.9725	26.9707	26.9212	1:Experimental evidence at protein level
26.767	26.7797	26.7388	26.6406	26.9135	26.676	26.7729	26.7635	1:Experimental evidence at protein level
27.8372	27.728	27.8829	27.7317	27.8817	27.9401	27.6134	27.6794	1:Experimental evidence at protein level
26.9222	27.0067	26.9379	27.0906	27.3408	27.3928	27.346	27.4906	1:Experimental evidence at protein level
28.604	28.8201	29.1219	28.6259	28.3953	28.3737	28.3658	28.1477	1:Experimental evidence at protein level
28.7531	28.8406	26.9583	26.4585	29.2692	29.5454	29.3974	28.1452	1:Experimental evidence at protein level
27.3029	27.3517	27.6505	27.3469	27.6543	27.725	27.6273	27.0907	1:Experimental evidence at protein level
29.776	29.7428	29.5694	29.3577	29.6648	29.8746	29.6483	29.2154	1:Experimental evidence at protein level
28.4489	28.5692	28.3967	28.6215	28.479	28.5003	28.5051	28.5018	1:Experimental evidence at protein level
29.0563	29.1135	28.8168	28.805	28.8197	29.0038	28.6636	28.4369	1:Experimental evidence at protein level
29.4487	29.285	29.3601	29.5549	29.548	29.563	29.5202	29.8796	1:Experimental evidence at protein level
28.4771	28.4972	28.6936	28.6152	28.4709	28.2841	28.5011	28.693	1:Experimental evidence at protein level
28.7947	28.8372	28.7321	28.6306	29.0223	28.6759	28.9431	28.8928	1:Experimental evidence at protein level
27.8424	27.9964	28.1621	28.1478	28.1373	27.9404	28.2665	28.3473	1:Experimental evidence at protein level
25.482	25.4137	25.5405	25.6136	25.5971	25.5564	25.3383	25.8111	1:Experimental evidence at protein level
26.7473	26.7194	26.5367	26.5201	26.6381	26.7362	26.6147	26.3078	1:Experimental evidence at protein level
27.4642	27.528	27.6559	27.6656	27.6247	27.7388	27.6749	27.5218	1:Experimental evidence at protein level
28.4683	28.361	28.3924	28.5457	28.607	28.6221	28.5587	28.614	1:Experimental evidence at protein level
27.8225	27.6236	27.6029	28.3353	27.6409	27.4295	27.8218	28.067	1:Experimental evidence at protein level
26.6031	26.6561	26.7174	26.81	26.2646	25.9592	26.2938	26.7048	1:Experimental evidence at protein level
25.7822	25.8089	25.83	25.9358	26.4146	26.16	26.4731	26.7641	1:Experimental evidence at protein level
26.7714	26.8883	26.9604	26.9037	28.2748	28.1073	28.26	28.3477	1:Experimental evidence at protein level
26.2496	26.2515	25.7872	26.0557	26.5577	26.4058	26.623	26.88	1:Experimental evidence at protein level
26.8088	26.6931	25.505	25.0251	27.0508	27.3196	26.5309	26.2584	1:Experimental evidence at protein level
27.437	27.4406	27.5878	27.6063	26.9163	26.8456	26.7003	26.9486	1:Experimental evidence at protein level
26.2699	26.2728	26.2881	26.3348	26.3918	26.1926	26.247	26.0429	1:Experimental evidence at protein level
26.1732	26.243	26.247	26.618	26.4655	26.5753	26.4565	26.9111	1:Experimental evidence at protein level
28.4344	28.5656	28.3849	28.1926	28.4482	28.4124	28.3937	28.3723	1:Experimental evidence at protein level
29.6633	29.5988	29.7439	29.8859	29.8639	29.8273	29.7602	29.4794	1:Experimental evidence at protein level
28.1747	28.1935	28.2363	28.1838	28.2803	28.1301	28.2729	28.6098	1:Experimental evidence at protein level
27.5337	27.5576	27.8219	27.8383	26.9604	26.8472	27.0672	27.1986	1:Experimental evidence at protein level
27.5458	27.5586	27.4494	27.459	27.6191	27.6422	27.4941	27.7779	1:Experimental evidence at protein level
26.7245	26.6391	26.7397	26.8241	26.9564	27.0232	26.8762	27.1629	1:Experimental evidence at protein level
27.3432	27.292	#NUM!	27.4301	27.0655	27.4574	26.5382	27.2706	1:Experimental evidence at protein level
25.8035	26.0981	26.4621	26.0969	26.31	26.3506	26.4296	26.2243	1:Experimental evidence at protein level
27.7932	28.036	28.1016	28.1693	27.8467	27.7946	28.0817	28.0022	1:Experimental evidence at protein level
25.9334	25.7537	25.6911	26.0422	25.8695	25.8633	25.9139	26.2948	1:Experimental evidence at protein level
24.844	25.1446	25.8631	24.7789	24.7815	24.8794	25.0778	25.336	1:Experimental evidence at protein level
25.2672	25.2099	25.1898	25.3738	25.4471	24.9203	25.4113	25.924	1:Experimental evidence at protein level
28.4894	28.7744	29.031	28.9803	28.7119	28.8243	28.7995	28.9167	1:Experimental evidence at protein level
26.2092	26.1749	25.9643	25.7199	26.1464	26.3106	26.0441	24.971	1:Experimental evidence at protein level
26.4169	26.6011	26.5396	26.4876	26.869	26.7255	26.7408	26.2819	1:Experimental evidence at protein level
26.4909	#NUM!	26.1276	#NUM!	27.2833	27.3198	27.2838	26.9684	1:Experimental evidence at protein level
27.5476	27.6906	27.9473	27.9989	27.6555	27.4373	27.7084	27.894	1:Experimental evidence at protein level
25.9049	25.9413	25.8927	26.0244	25.9532	25.7543	25.9868	26.2053	1:Experimental evidence at protein level
27.3345	27.6451	27.5521	27.4269	27.3238	27.2377	27.726	27.4731	1:Experimental evidence at protein level
27.0514	26.9986	26.7388	27.0735	27.0333	27.2375	27.3233	27.6976	1:Experimental evidence at protein level
26.3939	26.3288	26.3821	26.4852	26.2462	26.3119	26.2891	26.8005	1:Experimental evidence at protein level
26.965	27.1168	27.2108	27.1397	26.9798	26.9554	26.9286	27.4051	1:Experimental evidence at protein level
26.2192	26.3779	26.3504	26.4452	26.2153	26.334	26.3297	26.3415	1:Experimental evidence at protein level
29.055	29.0148	29.7647	29.5441	28.8571	28.554	28.7047	29.175	1:Experimental evidence at protein level
29.3554	29.3637	29.2857	29.5252	28.9724	28.8711	29.0122	29.4304	1:Experimental evidence at protein level
26.4388	26.5458	26.4848	26.7113	25.5886	25.7483	25.8416	26.0249	1:Experimental evidence at protein level
26.3563	27.1369	26.5253	27.0174	26.9328	27.096	26.537	26.8068	1:Experimental evidence at protein level
#NUM!	#NUM!	#NUM!	26.5931	25.971	25.6708	25.7229	26.1156	1:Experimental evidence at protein level
25.309	25.3882	25.1763	25.5414	26.0774	26.2216	26.1436	26.4583	1:Experimental evidence at protein level
25.9654	25.8715	25.6016	25.4653	25.5391	25.6661	25.5401	25.8877	1:Experimental evidence at protein level
25.795	25.7869	25.7659	25.8714	25.3203	25.6493	25.673	25.7059	1:Experimental evidence at protein level
27.2646	27.3555	27.3476	27.4975	27.2913	27.3693	27.3606	27.5905	1:Experimental evidence at protein level
25.4624	25.1465	25.082	25.2379	25.9276	26.1194	26.0454	25.8806	1:Experimental evidence at protein level
26.412	26.4024	26.5077	26.3961	26.1244	26.0644	26.0945	26.4941	1:Experimental evidence at protein level
26.0614	26.2467	26.3561	26.1721	26.3292	26.5956	26.5819	26.5165	1:Experimental evidence at protein level
26.9281	27.1835	27.1673	27.8199	27.3679	27.633	27.3938	27.9109	1:Experimental evidence at protein level
27.0706	26.7468	26.8973	27.1096	26.5931	26.4035	26.5311	27.275	1:Experimental evidence at protein level
27.6208	27.284	27.3997	27.2916	27.1885	27.4346	27.3396	27.4061	1:Experimental evidence at protein level
26.4423	26.6311	26.8988	26.5651	27.0926	27.2392	27.2388	27.2952	1:Experimental evidence at protein level
25.8032	25.539	25.7056	25.9113	25.9347	25.882	25.8496	26.6676	1:Experimental evidence at protein level
24.598	24.6534	24.6577	24.8827	25.2814	25.4006	25.0191	25.0855	1:Experimental evidence at protein level
26.7311	26.7778	26.902	26.7986	27.0928	27.0276	27.0427	26.7674	1:Experimental evidence at protein level
28.4118	28.3967	28.5833	28.6104	28.7465	28.7821	28.6313	28.6973	1:Experimental evidence at protein level
28.8208	28.9961	28.8931	28.9032	28.5102	28.4773	28.5833	28.6081	1:Experimental evidence at protein level
26.5538	26.4671	26.3267	26.6849	26.4923	26.5005	26.5926	27.0096	1:Experimental evidence at protein level
26.5917	26.3375	26.7834	26.7864	26.7013	26.4072	26.4712	27.1965	1:Experimental evidence at protein level
26.5596	26.5577	26.5053	26.5914	27.1968	27.3044	27.249	27.5258	1:Experimental evidence at protein level
26.4119	26.4852	26.567	26.5907	26.4199	26.4782	26.696	27.2817	1:Experimental evidence at protein level
26.7032	26.8821	27.2381	27.033	27.0485	27.1354	27.0843	27.2055	1:Experimental evidence at protein level
25.3098	25.3895	25.5975	25.7458	25.5713	25.5004	25.6255	26.3425	1:Experimental evidence at protein level
28.7759	29.0975	29.2269	29.1278	28.7953	28.8867	28.9518	28.8412	1:Experimental evidence at protein level
26.964	27.2405	27.2848	27.1776	27.1593	27.0217	26.9878	27.6303	1:Experimental evidence at protein level
25.6966	25.7166	25.7101	25.935	26.2959	26.4716	26.0481	26.4414	1:Experimental evidence at protein level
26.4909	26.6191	26.62	26.7048	26.4137	26.1178	26.2641	26.5742	1:Experimental evidence at protein level
#NUM!	26.0287	25.7484	#NUM!	26.6084	26.9061	26.5109	27.4261	1:Experimental evidence at protein level
26.7049	26.779	26.86	26.9848	26.5426	26.7795	26.8426	27.0434	1:Experimental evidence at protein level
28.5695	28.6125	28.7098	28.952	28.349	28.3752	28.3658	28.0044	1:Experimental evidence at protein level
27.2344	27.6443	27.6303	27.474	27.9597	28.1798	28.0732	27.9032	1:Experimental evidence at protein level
28.1187	28.0954	27.9527	27.9012	27.8325	27.9752	27.9621	27.6931	1:Experimental evidence at protein level
27.258	27.3334	27.3002	27.5576	26.9408	27.0322	27.2051	27.2883	1:Experimental evidence at protein level
27.3881	27.5263	27.4855	27.5311	26.9546	26.8669	27.1005	27.0173	1:Experimental evidence at protein level
28.4111	28.3882	28.5324	28.1902	28.3177	28.223	28.2929	27.8161	1:Experimental evidence at protein level
26.8742	26.9501	27.0873	27.0503	27.0825	27.119	27.222	27.1314	1:Experimental evidence at protein level
26.8736	26.7959	26.7523	26.7679	26.5906	26.663	26.6523	27.4495	1:Experimental evidence at protein level
26.7035	26.5222	26.5602	26.7522	26.9292	26.9327	27.0957	27.2659	1:Experimental evidence at protein level
32.1989	32.498	31.9645	32.7519	32.4555	32.5438	32.828	33.1225	1:Experimental evidence at protein level
26.3299	26.0391	25.8769	26.0282	#NUM!	25.6552	25.2122	#NUM!	1:Experimental evidence at protein level
29.4644	29.196	29.3488	29.3849	29.4702	29.3451	29.2862	29.1993	1:Experimental evidence at protein level
26.207	26.114	26.3566	26.6325	26.3528	26.1167	26.1877	26.4517	1:Experimental evidence at protein level
28.9218	28.6592	29.1423	29.0652	28.4591	28.2617	28.5991	29.0433	1:Experimental evidence at protein level
27.0597	27.07	27.1319	27.232	26.7744	26.4951	26.7486	27.1022	1:Experimental evidence at protein level
27.732	27.9567	28.2716	28.1002	27.7072	27.6073	27.7794	27.7986	1:Experimental evidence at protein level
27.6468	27.5936	27.6999	27.5444	27.3272	26.9073	27.1799	27.0105	1:Experimental evidence at protein level
26.5602	26.6513	26.7451	26.5446	26.8431	26.9284	26.8458	26.8102	1:Experimental evidence at protein level
28.8951	28.8539	29.0023	28.9867	29.1252	28.9282	28.8391	28.5633	1:Experimental evidence at protein level
29.7292	29.7032	29.4092	29.2701	29.4165	29.5292	29.4698	29.2227	1:Experimental evidence at protein level
28.4388	28.2962	28.301	28.5465	28.3442	28.4273	28.2861	28.1796	1:Experimental evidence at protein level
27.7816	27.7173	27.7704	27.7092	27.9329	28.0822	27.8422	28.0135	1:Experimental evidence at protein level
29.2615	29.3051	29.5372	29.5171	29.3079	29.3189	29.2567	29.095	1:Experimental evidence at protein level
26.4905	26.8213	26.4826	26.5582	26.8428	26.7319	26.7712	27.088	1:Experimental evidence at protein level
25.996	25.9448	26.0454	26.3488	26.6713	26.2351	26.3057	26.9758	1:Experimental evidence at protein level

30.6143	30.6663	30.7029	30.761	30.3115	30.3966	30.6253	30.6534	1:Experimental evidence at protein level
27.2472	27.2194	27.47	27.3199	27.3347	27.3963	27.4044	27.2587	1:Experimental evidence at protein level
27.9326	28.0971	27.9157	28.1731	28.3598	28.4754	28.2563	28.668	1:Experimental evidence at protein level
27.7187	27.7658	27.7983	27.9446	27.6417	27.6448	27.7469	27.6688	1:Experimental evidence at protein level
26.2222	26.0689	26.1554	26.1756	25.9932	26.0115	26.1578	26.3816	1:Experimental evidence at protein level
27.3634	27.3301	27.5325	27.3983	27.4284	27.4653	27.4211	27.7125	1:Experimental evidence at protein level
25.8329	25.6664	25.9908	25.8749	26.2426	26.0106	26.0585	26.2359	1:Experimental evidence at protein level
27.7533	27.7597	27.9845	27.7201	27.9952	27.9546	27.9146	28.255	1:Experimental evidence at protein level
27.4276	27.533	27.5979	27.2657	27.8265	27.8893	27.7078	26.8667	1:Experimental evidence at protein level
26.9414	27.0243	27.0651	27.2295	27.1772	27.0939	27.1123	27.414	1:Experimental evidence at protein level
28.0948	28.1371	28.0883	28.2089	28.2817	28.2093	28.2339	28.1943	1:Experimental evidence at protein level
26.8883	26.6411	26.5633	27.0673	26.473	26.712	26.2785	27.1123	1:Experimental evidence at protein level
29.1534	29.352	29.5075	29.2582	28.9198	29.0984	28.9838	28.6608	1:Experimental evidence at protein level
26.4729	27.0331	27.1448	27.2337	26.6635	26.8195	26.7535	26.43	1:Experimental evidence at protein level
26.7261	26.6946	26.6272	26.9003	27.0167	26.9727	27.1702	27.5238	1:Experimental evidence at protein level
26.096	25.8758	26.0758	26.2618	26.3451	26.4222	26.2599	26.996	1:Experimental evidence at protein level
27.6289	27.5931	27.5726	27.5799	28.39	28.2639	28.3115	28.1128	1:Experimental evidence at protein level
28.2609	28.4307	28.5192	28.4114	28.561	28.4368	28.5328	28.3078	1:Experimental evidence at protein level
28.5384	28.3703	28.3388	28.4703	28.3386	28.3927	28.4509	28.7066	1:Experimental evidence at protein level
31.0154	31.0788	30.7865	30.8166	31.3036	31.3751	31.3245	31.5142	1:Experimental evidence at protein level
26.5097	26.0234	26.1302	26.6199	#NUM!	#NUM!	26.1664	#NUM!	1:Experimental evidence at protein level
31.1595	31.0653	30.9141	31.0942	31.2481	31.1583	31.2418	31.4584	1:Experimental evidence at protein level
31.1497	31.0752	30.3705	30.3153	31.2639	31.623	31.265	31.4788	1:Experimental evidence at protein level
29.7968	29.6568	29.617	29.5505	29.869	29.847	29.8588	29.7065	1:Experimental evidence at protein level
29.3176	29.3297	28.633	29.3259	29.3938	29.3455	29.6579	30.1915	1:Experimental evidence at protein level
26.9288	27.2936	27.3381	27.3357	28.2794	28.3325	28.3142	28.3164	1:Experimental evidence at protein level
26.4491	26.4525	26.4029	26.4934	26.7571	26.7182	26.7118	27.3009	1:Experimental evidence at protein level
26.7062	26.5871	26.6649	26.7047	27.0952	27.0284	27.0977	27.4862	1:Experimental evidence at protein level
25.971	25.9073	25.8329	25.7496	26.1194	26.1004	26.0356	26.7418	1:Experimental evidence at protein level
25.7082	26.1553	26.0589	26.4424	26.6589	26.482	26.9164	26.9302	1:Experimental evidence at protein level
#NUM!	#NUM!	24.6324	25.1555	25.0096	25.2035	25.4157	25.7268	1:Experimental evidence at protein level
27.4196	27.4179	27.3517	27.1564	27.134	26.7841	26.9252	27.3102	1:Experimental evidence at protein level
27.2078	27.0944	27.3243	27.3548	26.7941	#NUM!	26.6947	26.521	1:Experimental evidence at protein level
26.5364	26.5381	26.8354	26.9988	26.9814	26.4206	26.6174	27.1304	1:Experimental evidence at protein level
25.3251	25.1344	25.8169	26.0252	25.3019	24.8886	25.5472	26.4619	1:Experimental evidence at protein level
25.9134	26.1453	26.2578	25.9102	#NUM!	26.1077	25.6368	25.3144	1:Experimental evidence at protein level
27.0631	27.1066	27.1861	27.2834	27.2356	26.9876	27.0894	27.5388	1:Experimental evidence at protein level
30.1943	30.0847	29.9798	30.1884	26.3292	28.3643	27.9579	27.7634	1:Experimental evidence at protein level
25.454	25.3434	25.4733	25.5744	25.6684	25.7912	25.6661	25.723	1:Experimental evidence at protein level
24.7843	24.9621	25.055	25.0927	24.8688	24.4604	24.7571	#NUM!	1:Experimental evidence at protein level
26.9081	26.9206	26.8124	26.9839	26.7061	26.8182	26.8507	26.7154	1:Experimental evidence at protein level
28.6959	28.5751	28.6822	28.8232	28.7806	28.7272	28.7623	28.8982	1:Experimental evidence at protein level
26.5861	26.6396	26.6056	26.5686	26.0683	26.2139	26.0931	26.6787	1:Experimental evidence at protein level
25.6505	25.6015	25.3936	25.6668	26.0672	25.8517	25.5357	26.5618	1:Experimental evidence at protein level
26.6559	26.4228	26.1991	26.3273	26.2481	26.5212	26.2214	26.5622	1:Experimental evidence at protein level
25.6368	25.6577	25.7455	25.7561	25.8512	25.9071	25.7886	26.22	1:Experimental evidence at protein level
27.2632	27.4005	27.5841	27.2429	27.5243	27.4223	27.293	27.3071	1:Experimental evidence at protein level
26.8183	26.8958	27.0573	27.1073	26.9561	26.9469	26.9124	27.1083	1:Experimental evidence at protein level
26.2689	26.4439	26.4641	26.3152	26.3837	26.1836	26.3802	26.7596	1:Experimental evidence at protein level
27.8385	27.8923	27.7796	28.1431	27.7118	27.6265	27.9097	28.0803	1:Experimental evidence at protein level
27.9335	28.0358	28.217	28.3023	28.1619	28.1012	28.2393	28.2109	1:Experimental evidence at protein level
27.2639	27.1282	27.5362	27.604	26.9319	27.4251	27.3562	27.1749	1:Experimental evidence at protein level
27.784	27.8646	28.1237	28.0519	28.1137	27.9493	27.8364	27.9657	1:Experimental evidence at protein level
24.9899	24.9978	25.0853	25.2607	24.9606	24.8321	24.8881	25.2068	1:Experimental evidence at protein level
27.7753	27.531	27.8979	27.8913	27.5947	27.6592	27.7727	27.5871	1:Experimental evidence at protein level
26.2326	25.9535	25.8759	26.2922	26.7046	26.6686	26.394	26.5879	1:Experimental evidence at protein level
26.5304	26.6466	26.6673	26.8624	26.8876	27.0189	26.8839	27.3388	1:Experimental evidence at protein level
25.695	25.7846	25.7861	25.9533	25.9542	26.1186	26.0904	26.1821	1:Experimental evidence at protein level
27.4402	27.6591	27.2956	27.5955	27.7102	27.8963	27.8191	27.9137	1:Experimental evidence at protein level
26.6286	26.5902	26.4255	26.6735	26.2647	26.299	26.3925	27.0634	1:Experimental evidence at protein level
28.3378	28.6292	28.6555	28.6095	28.5848	28.6067	28.6867	28.6251	1:Experimental evidence at protein level
28.0017	27.9724	28.2003	27.9906	28.0724	28.0149	28.0132	28.0996	1:Experimental evidence at protein level
25.4328	25.5429	25.5461	25.5665	25.1004	25.0458	25.8732	#NUM!	1:Experimental evidence at protein level
25.6989	25.773	25.7964	25.7684	25.5925	25.8718	25.8987	#NUM!	1:Experimental evidence at protein level
#NUM!	#NUM!	24.3022	24.6654	24.735	25.2069	24.6074	26.1215	1:Experimental evidence at protein level
26.7402	26.6227	26.859	26.7987	26.8366	26.4302	26.7298	27.093	1:Experimental evidence at protein level
26.975	26.8624	27.0723	26.9326	26.9156	26.8563	26.8527	27.2357	1:Experimental evidence at protein level
27.1324	27.1812	27.1118	27.2336	26.9279	27.0768	27.0825	27.3233	1:Experimental evidence at protein level
26.4193	26.2213	26.4657	26.5389	26.573	26.4125	26.1305	27.3099	1:Experimental evidence at protein level
27.3577	27.3613	27.342	27.3946	27.5197	27.4125	27.321	27.5535	1:Experimental evidence at protein level
26.5087	26.5193	26.6655	26.4471	26.2416	26.3129	26.3067	26.2827	1:Experimental evidence at protein level
25.3408	25.2822	24.8389	24.9119	24.8868	#NUM!	25.0024	25.5829	1:Experimental evidence at protein level
26.4888	26.7078	26.475	26.4388	26.2347	26.4391	26.4501	26.0478	1:Experimental evidence at protein level
25.0206	25.2729	25.3061	25.5383	25.3266	#NUM!	25.6037	26.0098	1:Experimental evidence at protein level
24.776	#NUM!	24.9807	#NUM!	26.0802	25.9507	25.9644	25.9502	1:Experimental evidence at protein level
27.4469	27.3727	27.5681	27.4348	27.2771	27.2607	27.2747	27.5189	1:Experimental evidence at protein level
25.6322	25.4859	25.7238	25.9861	25.5291	25.5575	25.8491	26.6902	1:Experimental evidence at protein level
26.416	26.3411	26.3457	26.4625	25.9554	26.2367	26.2482	26.4927	1:Experimental evidence at protein level
26.1357	25.9641	26.084	#NUM!	25.925	26.036	25.9875	25.7264	1:Experimental evidence at protein level
28.6261	28.4456	28.348	28.4039	28.5301	28.5929	28.3657	28.1376	1:Experimental evidence at protein level
28.4095	28.4944	28.8709	28.7415	28.2287	28.2173	28.382	28.1832	1:Experimental evidence at protein level
25.6648	25.8462	25.6909	25.3711	25.1781	25.5327	25.9854	#NUM!	1:Experimental evidence at protein level
25.2156	25.4483	25.1344	25.6571	25.9817	26.3086	26.1124	26.3351	1:Experimental evidence at protein level
26.7293	27.2064	27.1906	27.0076	27.1168	27.2867	27.1123	26.8886	1:Experimental evidence at protein level
28.0691	27.8681	28.1357	27.9129	28.2693	27.9334	27.969	27.916	1:Experimental evidence at protein level
27.125	27.4081	27.5623	27.3592	27.6088	27.3965	27.4429	26.6889	1:Experimental evidence at protein level
27.2803	27.2693	27.1274	27.3383	26.8136	26.8848	26.6865	27.1791	1:Experimental evidence at protein level
25.4214	25.4614	25.2666	25.4841	26.0048	25.8806	25.9217	26.2911	1:Experimental evidence at protein level
28.3504	28.3398	28.3171	28.2795	28.343	28.2895	28.1292	28.296	1:Experimental evidence at protein level
33.6773	33.3432	31.8467	31.7294	30.8798	31.2281	30.5846	30.7873	1:Experimental evidence at protein level
31.0542	30.927	30.8909	30.6403	31.2818	31.3413	31.4487	30.487	1:Experimental evidence at protein level
26.0952	26.1177	26.3236	25.8154	26.8088	26.6203	26.2453	26.3957	1:Experimental evidence at protein level
36.0868	35.813	33.9562	33.8977	33.522	33.9277	33.2202	32.6496	1:Experimental evidence at protein level
29.471	29.4146	29.607	29.564	29.5215	29.495	29.4554	29.3163	1:Experimental evidence at protein level
27.8271	27.8542	27.446	27.3509	27.9792	28.0671	27.3813	#NUM!	1:Experimental evidence at protein level
29.5679	29.5813	29.8024	29.6846	29.4573	29.4271	29.2889	29.5696	1:Experimental evidence at protein level
26.8654	27.1294	27.1879	27.0627	27.4716	27.5038	27.4753	27.4045	1:Experimental evidence at protein level
27.1387	27.1533	27.1144	27.1193	27.3696	27.4531	27.3387	27.6247	1:Experimental evidence at protein level
26.9615	27.0323	26.9767	27.42	26.9737	27.0091	27.1092	27.7357	1:Experimental evidence at protein level
28.6224	28.8075	29.0511	28.6304	28.7413	28.6813	28.8172	28.0677	1:Experimental evidence at protein level
27.2055	27.2596	26.9613	27.2859	27.7116	27.9825	27.682	27.0177	1:Experimental evidence at protein level
26.6424	26.5824	26.5638	26.6835	26.158	26.2819	26.1388	27.002	1:Experimental evidence at protein level
27.6508	27.6215	27.6076	27.7681	27.8107	27.7652	27.7427	28.2183	1:Experimental evidence at protein level
31.285	31.2713	31.4891	31.2574	30.7564	30.6872	30.5455	30.2522	1:Experimental evidence at protein level
26.7028	26.8494	26.7063	26.8558	26.9737	#NUM!	27.198	27.4977	1:Experimental evidence at protein level
28.3543	28.1528	27.7893	27.9287	27.6069	27.5971	27.8526	27.9681	1:Experimental evidence at protein level
31.2049	31.0506	30.5336	30.8181	31.6641	31.6427	31.4646	31.7314	1:Experimental evidence at protein level
29.0935	29.1765	29.2117	28.9687	29.1642	29.2804	29.2168	29.1063	1:Experimental evidence at protein level
26.0289	25.7456	25.9123	26.2508	26.4913	26.1277	26.3888	26.9708	1:Experimental evidence at protein level
26.3622	26.5535	26.7089	26.7999	26.7665	26.8874	27.0321	27.3744	1:Experimental evidence at protein level
27.9613	28.0993	28.3596	28.0786	28.2316	28.1664	28.2002	28.3692	1:Experimental evidence at protein level
25.1215	25.224	25.2058	25.0469	#NUM!	#NUM!	#NUM!	#NUM!	1:Experimental evidence at protein level
29.7834	29.728	29.6723	29.8429	30.1463	30.1353	30.1686	29.8857	1:Experimental evidence at protein level
28.6393	28.788	28.5552	28.2633	28.3144	28.4398	28.4678	27.5961	1:Experimental evidence at protein level
26.8571	26.974	27.1204	26.8313	26.4119	26.3696	26.3896	26.4052	1:Experimental evidence at protein level
25.928	25.9171	25.8249	25.6251	26.4655	26.379	26.2512	26.1593	1:Experimental evidence at protein level
30.3402	30.3823	30.4071	30.4541	30.0516	30.465	30.3781	29.7491	1:Experimental evidence at protein level
29.6639	29.7221	29.8577	29.7173	29.414	29.5373	29.6246	29.2562	1:Experimental evidence at protein level
25.9749	26.1249	25.9602	25.9764	25.9521	26.0443	25.5861	25.7002	1:Experimental evidence at protein level
27.9175	28.1671	28.1706	28.3286	28.4987	28.3278	28.7312	28.7886	1:Experimental evidence at protein level
29.2286	29.5306	29.8491	29.5381	29.0881	29.268	29.0235	28.362	1:Experimental evidence at protein level
25.189	25.2605	25.2563	25.3151	24.8616	24.8188	24.9672	25.3027	1:Experimental evidence at protein level
25.885	26.0256	25.7798	#NUM!	26.3304	26.2899	25.7418	26.8554	1:Experimental evidence at protein level
29.6845	29.5819	29.4621	29.3997	29.2277	29.2201	29.3156	29.2301	1:Experimental evidence at protein level
27.5541	27.4249	27.5418	27.5297	26.6785	27.2708	27.3043	27.3554	1:Experimental evidence at protein level
27.1769	27.1965	27.2865	27.5036	27.1335	26.9543	27.2465	27.6368	1:Experimental evidence at protein level
26.1655	26.4901	26.2065	26.5486	26.8345	26.6268	27.0736	26.7153	1:Experimental evidence at protein level
26.3158	25.9407	26.1126	26.3822	27.1351	27.107	27.2926	27.8391	1:Experimental evidence at protein level
26.7718	26.8226	26.4901	26.5526	26.9477	26.7251	26.7152	27.2675	1:Experimental evidence at protein level

26.1292	26.0982	25.9052	25.9035	25.6609	25.7318	25.9	26.1771	1:Experimental evidence at protein level
26.6205	26.6721	26.8764	26.6051	26.6005	26.4943	26.6468	26.9123	1:Experimental evidence at protein level
29.5136	29.6699	29.8552	29.8403	29.7148	29.7366	29.7379	29.6909	1:Experimental evidence at protein level
26.0665	25.5288	25.6217	25.8274	25.1091	25.2914	25.167	24.8482	1:Experimental evidence at protein level
28.4381	28.4884	28.8382	28.8158	28.5572	28.6906	28.7209	28.7613	1:Experimental evidence at protein level
28.5118	28.71	28.7501	28.3751	28.65	28.5753	28.6055	28.0116	1:Experimental evidence at protein level
27.0801	27.1669	27.175	27.2375	27.185	27.2584	27.0924	27.6338	1:Experimental evidence at protein level
27.657	27.7608	27.4685	27.4598	27.4032	27.3098	27.432	27.4874	1:Experimental evidence at protein level
27.0029	26.9126	26.9237	26.7521	26.8875	26.9111	26.7368	26.5102	1:Experimental evidence at protein level
27.6999	27.601	27.9017	27.8413	27.9785	27.8499	27.9248	28.2053	1:Experimental evidence at protein level
27.1483	27.2374	27.3047	27.4793	27.2288	27.2513	27.3451	27.7378	1:Experimental evidence at protein level
26.6237	26.7508	26.6524	26.9603	26.7503	26.8216	26.8291	27.2494	1:Experimental evidence at protein level
25.8593	25.9607	25.7073	26.0028	26.3806	26.2593	26.2292	26.9657	1:Experimental evidence at protein level
28.7251	28.5645	28.9806	28.7285	28.8062	28.7915	28.6312	29.2195	1:Experimental evidence at protein level
27.324	27.2992	27.3689	26.8532	27.7555	27.6875	27.4979	26.8196	1:Experimental evidence at protein level
27.8905	27.9411	27.9636	28.0658	27.555	27.5872	27.6271	27.8776	1:Experimental evidence at protein level
26.2517	26.205	26.3553	26.3261	26.4468	26.6303	26.6588	26.4704	1:Experimental evidence at protein level
26.4349	26.513	26.5322	26.5356	26.4517	26.5631	26.4685	26.1715	1:Experimental evidence at protein level
26.3813	26.5898	26.9387	26.9838	26.7562	26.6039	26.7335	27.2858	1:Experimental evidence at protein level
28.3534	28.1105	27.8738	27.9876	28.0353	28.3261	28.1839	28.1472	1:Experimental evidence at protein level
25.8725	25.9819	25.8737	26.254	26.1839	26.1154	26.4485	26.8315	1:Experimental evidence at protein level
26.5352	26.6671	26.7487	26.6495	26.7351	26.7667	26.681	26.4972	1:Experimental evidence at protein level
26.8663	26.9131	27.261	27.0033	26.8046	26.7739	26.7067	27.164	1:Experimental evidence at protein level
25.5351	25.5821	25.3262	25.1854	25.6237	25.5399	25.1173	25.4425	1:Experimental evidence at protein level
26.0581	26.1498	#NUM!	26.0731	26.2021	26.8853	26.544	26.0961	1:Experimental evidence at protein level
28.511	28.4427	28.5608	28.9454	28.6351	28.3622	28.5414	28.9147	1:Experimental evidence at protein level
26.8203	27.0416	27.2792	27.2182	26.697	26.5651	26.7915	27.26	1:Experimental evidence at protein level
27.0905	26.9439	26.9701	26.9936	26.8953	26.7985	26.8135	27.2985	1:Experimental evidence at protein level
27.6	27.6896	27.6025	27.6282	27.4699	27.472	27.4037	27.8179	1:Experimental evidence at protein level
29.1146	29.1418	29.1418	29.2185	29.0185	28.92	28.8132	28.9626	1:Experimental evidence at protein level
25.9553	26.2556	26.0177	25.9325	26.3812	26.3542	26.203	26.0345	1:Experimental evidence at protein level
27.1422	27.0822	26.9939	27.1605	26.9684	27.1355	27.0901	27.3511	1:Experimental evidence at protein level
27.6289	27.5981	27.4514	27.4527	27.8359	28.0206	27.7316	27.3039	1:Experimental evidence at protein level
28.4121	28.505	28.3864	28.5549	28.4861	28.3626	28.5275	28.869	1:Experimental evidence at protein level
27.1731	27.235	27.3165	27.5843	27.2206	27.3007	27.2203	27.9431	1:Experimental evidence at protein level
27.3598	27.1485	27.1026	27.0006	27.1974	27.2502	26.7006	26.4368	1:Experimental evidence at protein level
27.1361	27.1865	27.2319	27.2148	27.3295	27.323	27.2308	27.5686	1:Experimental evidence at protein level
26.2222	25.9821	26.028	25.6463	25.9723	25.9977	25.9524	25.8447	1:Experimental evidence at protein level
25.2702	25.2773	24.9188	25.2392	24.8028	24.9928	24.5299	25.4473	1:Experimental evidence at protein level
26.6589	26.8784	26.7233	27.0699	26.4942	26.525	26.7149	27.2429	1:Experimental evidence at protein level
27.8062	27.8328	27.8308	28.021	27.7929	27.8272	27.9001	28.3012	1:Experimental evidence at protein level
27.0668	27.03	27.1946	26.9934	26.5722	26.3961	26.3764	26.0908	1:Experimental evidence at protein level
25.6354	25.704	26.3491	26.2211	#NUM!	25.7171	25.6965	27.1992	1:Experimental evidence at protein level
26.7582	26.67	26.7107	26.9301	26.7215	26.6464	26.5813	26.9373	1:Experimental evidence at protein level
26.9035	26.7846	26.4644	#NUM!	27.3413	27.0831	26.908	27.4724	1:Experimental evidence at protein level
27.4733	27.4958	27.2354	27.473	27.4117	27.2482	27.4765	27.8654	5:Protein uncertain
26.5025	26.5596	26.6313	26.6307	27.0214	27.0974	27.3434	27.024	1:Experimental evidence at protein level
28.1305	28.3124	28.3846	28.3282	28.7278	28.5484	28.4939	28.5601	1:Experimental evidence at protein level
26.7012	26.827	26.8407	26.8083	26.9322	27.0059	26.8509	27.0485	1:Experimental evidence at protein level
26.1621	26.007	26.0993	25.7905	25.7762	25.9815	25.9279	25.8977	1:Experimental evidence at protein level
27.203	27.137	27.5114	27.0609	27.0601	26.937	27.1469	27.4736	1:Experimental evidence at protein level
26.9758	26.9162	27.0801	27.2097	26.6273	26.9873	26.4668	26.7596	1:Experimental evidence at protein level
29.0822	29.203	29.2019	29.2166	29.4425	29.3904	29.4406	29.5311	1:Experimental evidence at protein level
26.2994	26.4244	26.775	26.6054	26.6709	26.5736	26.6392	26.6215	1:Experimental evidence at protein level
25.7671	25.8531	25.3118	26.005	25.8085	25.9042	25.748	26.2057	1:Experimental evidence at protein level
28.5584	28.5796	28.7781	28.7377	28.6856	28.4677	28.6039	28.8019	1:Experimental evidence at protein level
25.932	26.1111	26.2971	26.1578	26.0743	26.035	25.9615	#NUM!	1:Experimental evidence at protein level
28.7827	28.9368	29.0452	28.8641	28.8287	28.6786	28.7898	28.3713	1:Experimental evidence at protein level
25.9689	26.0901	26.1668	26.5214	26.7114	26.7084	26.5479	27.2737	1:Experimental evidence at protein level
24.6745	25.0746	25.0727	25.8611	26.2662	25.8992	26.2571	25.7768	1:Experimental evidence at protein level
30.6519	30.9914	31.2162	30.8135	30.8258	30.6279	31.0338	30.6098	1:Experimental evidence at protein level
26.503	26.2049	26.5808	26.583	26.154	26.4686	26.3524	26.7085	1:Experimental evidence at protein level
27.2351	27.4105	27.5367	27.4203	27.4329	27.3802	27.7248	27.3861	1:Experimental evidence at protein level
32.5542	32.4032	32.5843	32.8368	33.0026	32.7832	32.7693	32.9072	1:Experimental evidence at protein level
28.9201	28.8953	28.8769	28.691	28.6406	28.762	28.8413	28.7912	1:Experimental evidence at protein level
28.2046	28.1283	28.1803	28.2393	27.2951	27.1276	27.3424	27.7671	1:Experimental evidence at protein level
27.1301	26.6943	26.8934	26.9423	26.6832	26.8193	26.7047	26.6666	1:Experimental evidence at protein level
28.3186	28.2728	28.5612	28.539	28.4393	28.3509	28.4301	28.6789	1:Experimental evidence at protein level
26.884	27.0513	27.4134	27.2579	27.9014	27.8008	27.8424	27.9294	1:Experimental evidence at protein level
30.1893	30.2395	30.2786	30.2569	30.2552	30.1958	30.3529	30.3929	1:Experimental evidence at protein level
29.3117	29.323	29.2107	29.4326	29.3121	29.2336	29.4569	29.5707	1:Experimental evidence at protein level
26.4968	26.7503	26.8337	26.4701	27.2079	27.7021	27.162	26.2274	1:Experimental evidence at protein level
28.4884	28.2782	28.8563	28.4621	28.7603	28.664	28.4259	27.9993	1:Experimental evidence at protein level
27.7136	27.5432	27.4717	27.7506	27.7469	27.5522	27.8072	27.9716	1:Experimental evidence at protein level
26.6961	26.7016	26.8701	26.8386	27.1685	26.9956	26.9671	26.7682	1:Experimental evidence at protein level
31.057	31.4747	31.4289	31.2076	31.9753	31.6249	31.9259	31.0595	1:Experimental evidence at protein level
27.3043	27.4989	27.484	27.6211	27.5315	27.4512	27.4556	27.8939	1:Experimental evidence at protein level
25.2567	25.1913	25.1376	25.3034	25.2392	25.3145	25.4326	26.086	1:Experimental evidence at protein level
26.681	26.6178	26.7608	26.7158	26.6658	26.583	26.7666	26.9199	1:Experimental evidence at protein level
30.8086	30.9145	30.9293	30.8462	31.054	30.8031	30.9692	30.3477	1:Experimental evidence at protein level
26.9849	27.0312	26.9454	27.2044	27.0397	27.0076	27.1847	27.409	1:Experimental evidence at protein level
27.623	27.5625	27.5925	27.3509	27.3395	27.3606	27.1632	27.8199	1:Experimental evidence at protein level
26.1716	26.1168	26.0489	26.0764	26.5699	26.6047	26.1418	26.5407	1:Experimental evidence at protein level
29.249	29.3941	29.3222	29.1266	29.1714	29.2784	29.2411	28.7594	1:Experimental evidence at protein level
27.5419	27.6719	27.4663	27.7146	27.3028	27.176	27.1996	27.7068	1:Experimental evidence at protein level
26.7965	26.906	27.0905	27.2719	27.1369	27.1172	27.2758	27.4238	1:Experimental evidence at protein level
27.5372	27.4134	27.537	27.4275	27.5372	27.5204	27.3625	27.5071	1:Experimental evidence at protein level
30.2505	30.2881	29.8801	30.045	30.4796	30.4265	30.4786	30.7226	1:Experimental evidence at protein level
27.3826	27.6744	27.4723	27.4179	27.102	27.2247	27.317	27.582	1:Experimental evidence at protein level
27.2625	27.4295	27.572	27.3628	27.5666	27.6451	27.5994	27.0635	1:Experimental evidence at protein level
25.6975	26.0071	25.8995	25.6595	25.7626	25.8005	26.0648	25.7744	1:Experimental evidence at protein level
29.3192	29.4054	29.5395	29.55	29.0143	29.0527	29.0328	28.9275	1:Experimental evidence at protein level
27.0709	27.0288	26.9011	26.7586	26.7817	26.8365	26.9009	26.9515	1:Experimental evidence at protein level
27.167	27.5598	27.4461	27.4895	27.3447	27.4834	27.3459	27.4049	1:Experimental evidence at protein level
25.0684	25.3687	25.0452	24.9769	25.4616	25.4491	25.3272	25.9345	1:Experimental evidence at protein level
28.099	28.2715	28.1813	28.1737	28.3207	28.2953	28.0458	27.6413	1:Experimental evidence at protein level
30.1689	30.202	29.7383	29.9755	29.7758	29.9239	29.8201	30.0306	1:Experimental evidence at protein level
28.3549	28.633	28.5334	28.7298	28.5621	28.6091	28.6049	28.8686	1:Experimental evidence at protein level
26.5267	26.7754	26.7483	26.7527	26.9153	26.9418	26.7984	27.2239	1:Experimental evidence at protein level
24.8032	24.4572	24.4374	24.774	25.2714	25.1044	25.0835	25.5473	1:Experimental evidence at protein level
27.9999	27.9391	27.8367	28.0089	28.1184	27.8419	28.05	28.1646	1:Experimental evidence at protein level
26.6515	26.8314	26.9728	27.1945	26.3113	27.0799	27.1279	26.6767	1:Experimental evidence at protein level
26.6865	26.3138	26.2378	26.6459	26.179	25.915	26.4667	26.7504	1:Experimental evidence at protein level
25.7124	25.6664	25.9571	26.0472	25.2674	#NUM!	25.4154	25.9818	1:Experimental evidence at protein level
26.8084	27.0283	26.9457	27.1245	26.3389	26.5223	26.4701	26.8879	1:Experimental evidence at protein level
25.4308	25.3809	25.4473	25.4342	25.8581	25.991	26.1427	26.6234	1:Experimental evidence at protein level
26.4952	26.5345	26.7554	26.574	27.057	26.8632	26.9808	27.137	1:Experimental evidence at protein level
25.7452	25.9238	25.9318	25.8769	26.2518	26.0427	26.3566	26.23	1:Experimental evidence at protein level
26.3495	26.0885	25.649	25.5034	25.9134	25.9409	25.7155	26.3325	1:Experimental evidence at protein level
26.7939	26.7943	26.624	26.6848	26.2991	26.4987	26.3498	26.1627	1:Experimental evidence at protein level
26.2901	26.2502	26.5088	26.5387	25.7831	25.8436	25.8633	25.8659	1:Experimental evidence at protein level
26.5204	26.6582	26.8301	26.7833	27.0452	27.0804	27.1531	27.1536	1:Experimental evidence at protein level
27.4468	27.4813	27.624	27.5842	27.6665	27.7146	27.7599	27.9674	1:Experimental evidence at protein level
26.5804	26.8018	26.5834	26.7683	26.9319	26.8455	26.9443	27.2305	1:Experimental evidence at protein level
26.3135	26.4285	26.4572	26.346	26.5476	26.6054	26.315	26.5648	1:Experimental evidence at protein level
26.5012	26.4071	26.1367	26.3719	26.6855	26.5459	26.4931	26.8656	1:Experimental evidence at protein level
26.9073	26.9023	26.9051	27.0042	26.7412	26.5745	26.6932	27.3166	1:Experimental evidence at protein level
26.3091	26.4039	26.187	26.3927	26.7462	26.7939	26.7264	27.223	1:Experimental evidence at protein level
24.5145	24.8666	24.8586	24.9708	24.7035	24.7018	24.7551	25.4189	1:Experimental evidence at protein level
27.8138	27.8477	27.6577	27.8178	27.5865	27.6891	27.5243	27.7125	1:Experimental evidence at protein level
25.891	26.1395	26.1179	26.0353	26.4085	26.4569	26.4928	26.772	1:Experimental evidence at protein level
27.7542	27.7603	27.8624	27.8949	27.9242	27.7334	27.8175	27.9922	1:Experimental evidence at protein level
29.9073	29.8478	29.6056	29.8742	29.6497	29.7042	29.7229	29.8843	1:Experimental evidence at protein level
27.2508	27.3329	27.0095	27.2752	28.0542	28.0137	28.0262	28.0391	1:Experimental evidence at protein level
27.7921	27.8641	28.0332	28.0589	27.734	27.8847	27.9385	27.7375	1:Experimental evidence at protein level
27.4795	27.4893	27.6368	27.4754	26.8103	26.7115	26.8563	26.3216	1:Experimental evidence at protein level
26.9936	27.0834	27.2486	27.3169	27.0366	26.9463	26.807	27.1034	1:Experimental evidence at protein level
25.5274	25.3032	25.8222	25.7139	25.2119	25.1134	25.9846	26.6981	1:Experimental evidence at protein level
26.3211	26.473	26.4033	26.6447	26.7945	26.87	26.8158	26.9376	1:Experimental evidence at protein level

25.663	25.4337	25.6208	25.7704	25.6892	25.3693	25.4164	25.8341	1:Experimental evidence at protein level
25.8878	25.5085	26.3173	26.561	25.3203	#NUM!	#NUM!	#NUM!	1:Experimental evidence at protein level
28.5892	28.6102	28.4987	28.4237	28.5362	28.594	28.4401	28.5497	1:Experimental evidence at protein level
26.4631	26.5675	26.525	26.6388	26.3096	26.3426	26.4243	26.6563	1:Experimental evidence at protein level
27.2875	27.3897	27.4611	27.3837	27.7303	27.6124	27.7159	27.4548	1:Experimental evidence at protein level
26.9008	27.0716	27.1751	27.0995	27.0513	27.2224	27.0906	26.8836	1:Experimental evidence at protein level
26.3085	26.2806	26.2313	26.5044	26.1798	26.1604	26.3869	26.3793	1:Experimental evidence at protein level
24.5798	24.2886	24.1025	#NUM!	24.2057	24.1831	23.7231	24.4944	1:Experimental evidence at protein level
26.5935	26.7561	27.1334	26.861	26.9101	27.0435	26.8967	26.8297	1:Experimental evidence at protein level
25.9767	25.9757	25.9645	25.9809	26.4394	26.3754	26.3939	26.6508	1:Experimental evidence at protein level
27.0068	27.166	27.1863	27.3069	27.1284	27.1932	27.2684	27.7763	1:Experimental evidence at protein level
27.3527	27.6357	27.7745	27.3618	27.3593	27.431	27.5351	27.1834	1:Experimental evidence at protein level
26.9774	27.001	26.7554	27.0866	27.0154	27.0095	27.0773	27.1797	1:Experimental evidence at protein level
28.3548	28.3001	27.8352	28.0999	28.3397	28.4944	28.3471	28.4364	1:Experimental evidence at protein level
29.1784	29.2088	29.2222	29.5717	29.0327	29.1809	29.0625	29.2984	1:Experimental evidence at protein level
28.3585	28.2566	28.3639	28.3174	28.263	28.1952	28.3703	28.5429	1:Experimental evidence at protein level
27.4073	27.4616	27.4891	27.7795	27.165	27.2138	27.3117	27.6949	1:Experimental evidence at protein level
26.2912	26.2418	26.4972	26.5062	26.4423	26.4719	26.4817	26.9426	1:Experimental evidence at protein level
26.5755	26.6361	26.6833	26.6803	26.7151	26.638	26.6406	26.6922	1:Experimental evidence at protein level
27.8408	27.8199	27.5645	27.4904	27.6889	27.8004	27.5095	27.4678	1:Experimental evidence at protein level
27.2492	27.0788	26.9332	27.3193	28.2107	28.3398	27.9255	28.3447	1:Experimental evidence at protein level
30.312	30.2016	30.2044	30.2006	30.4823	30.193	30.2338	30.0355	1:Experimental evidence at protein level
29.1743	29.3512	29.564	29.5087	29.5908	29.4552	29.4608	29.2417	1:Experimental evidence at protein level
27.3973	27.1833	24.8542	25.5451	25.2627	25.41	24.0078	24.7744	1:Experimental evidence at protein level
26.3857	26.4886	26.326	26.3633	26.5106	26.3997	26.2109	26.8024	1:Experimental evidence at protein level
28.7573	28.5972	28.1495	28.2011	28.6325	28.8508	28.5134	28.5527	1:Experimental evidence at protein level
28.1555	27.9875	27.9877	27.9458	28.0077	27.9482	27.8913	27.5876	1:Experimental evidence at protein level
27.4513	27.4128	27.5824	27.506	27.6921	27.4933	27.4982	27.8341	1:Experimental evidence at protein level
28.4773	28.498	28.5526	28.8219	28.832	28.5384	28.6359	28.5507	1:Experimental evidence at protein level
25.8471	25.8366	25.8806	26.1854	26.4999	26.4127	26.567	26.9761	1:Experimental evidence at protein level
26.9493	26.8665	27.0291	27.1238	27.9268	28.094	28.0681	28.2524	1:Experimental evidence at protein level
27.186	27.206	27.1246	27.5026	27.1418	27.1098	27.3459	27.7765	1:Experimental evidence at protein level
29.1042	29.1977	29.2961	29.4013	29.1502	29.1173	29.1363	29.3106	1:Experimental evidence at protein level
28.3705	28.4964	28.6738	28.9602	28.3122	27.9874	28.3004	28.7892	1:Experimental evidence at protein level
30.5843	30.6742	30.7842	30.6416	30.8579	30.5363	30.8903	31.1131	1:Experimental evidence at protein level
27.2371	27.244	27.3092	27.4176	28.2448	28.2593	28.3081	28.4908	1:Experimental evidence at protein level
26.8975	27.0373	27.139	27.0824	27.254	27.359	27.2946	27.346	1:Experimental evidence at protein level
28.6372	28.6739	29.0352	28.8978	28.5843	28.4568	28.6199	28.4087	1:Experimental evidence at protein level
29.865	29.9288	29.8331	29.9637	30.3539	30.2614	30.0669	30.0804	1:Experimental evidence at protein level
27.4114	27.5643	27.4942	27.4911	27.662	27.2961	27.3565	27.7398	1:Experimental evidence at protein level
27.7907	27.809	27.8288	27.9635	28.038	27.9221	28.0404	27.7776	1:Experimental evidence at protein level
27.5701	27.5187	27.7418	27.7923	27.8306	27.791	27.7856	27.6967	1:Experimental evidence at protein level
30.245	30.2115	30.3776	30.5196	30.5053	30.6794	30.5502	30.3958	1:Experimental evidence at protein level
29.4373	29.2684	28.3945	28.9643	29.7798	29.9144	29.7001	30.1586	1:Experimental evidence at protein level
29.1021	29.2411	29.3883	29.381	29.334	29.1502	29.3569	29.5926	1:Experimental evidence at protein level
28.7208	28.8085	28.7597	28.7847	28.7881	28.8448	28.9418	29.0918	1:Experimental evidence at protein level
30.736	30.5276	30.5193	30.517	30.6997	30.6103	30.5006	30.5537	1:Experimental evidence at protein level
28.5261	28.5166	28.6688	28.5904	28.8907	28.7279	28.6711	28.4844	1:Experimental evidence at protein level
30.4846	30.5041	30.5158	30.3503	30.2198	30.255	30.3912	30.2536	1:Experimental evidence at protein level
30.9552	31.0251	30.8528	30.8125	30.8822	30.8972	30.943	31.1376	1:Experimental evidence at protein level
25.5868	25.5791	25.5769	25.6246	26.3194	26.1829	26.6086	27.0243	1:Experimental evidence at protein level
27.1655	26.8971	27.201	27.2591	27.0029	26.8518	26.9889	27.4034	1:Experimental evidence at protein level
28.6878	28.7054	28.8033	28.8778	28.814	28.6777	28.9269	28.3423	1:Experimental evidence at protein level
26.7045	26.708	26.6839	26.8432	26.7537	26.821	26.9244	27.1476	1:Experimental evidence at protein level
27.7109	27.6125	27.4201	27.6997	27.8659	27.6935	27.756	27.9602	1:Experimental evidence at protein level
25.9219	25.7833	25.8286	25.9032	25.966	25.7243	25.8209	26.2164	1:Experimental evidence at protein level
25.3943	25.1805	25.3705	25.2386	25.3063	25.1741	25.2416	25.7646	1:Experimental evidence at protein level
26.0246	25.7974	26.0048	25.9432	26.1339	26.4081	25.9975	26.2416	1:Experimental evidence at protein level
26.0757	26.5425	26.1381	26.3577	24.9754	#NUM!	25.3663	#NUM!	1:Experimental evidence at protein level
27.7825	27.7698	27.2442	27.076	27.079	27.3292	26.9833	26.5623	1:Experimental evidence at protein level
31.0246	30.8515	30.5656	30.3407	30.6537	30.7179	30.532	29.8987	1:Experimental evidence at protein level
29.162	29.1997	29.462	29.3126	29.2665	29.2189	29.2991	29.3194	1:Experimental evidence at protein level
26.2841	26.0646	25.6977	25.787	26.0759	26.1625	26.1467	26.326	1:Experimental evidence at protein level
26.0468	26.4436	26.2723	26.4946	26.376	25.8961	26.5192	27.093	1:Experimental evidence at protein level
28.2254	28.3682	28.3966	28.6843	28.555	28.4517	28.6611	29.0705	1:Experimental evidence at protein level
26.3776	26.0431	25.5974	26.0916	26.5244	26.5003	26.4336	27.0092	1:Experimental evidence at protein level
26.8994	26.7159	26.8282	27.1521	26.8011	27.0313	26.9447	27.3074	1:Experimental evidence at protein level
25.8992	25.8423	26.089	26.0886	26.1659	26.0155	25.9999	26.4398	1:Experimental evidence at protein level
28.5194	28.5849	28.7008	28.5573	28.6453	28.62	28.5507	28.571	1:Experimental evidence at protein level
30.7055	30.7745	31.0381	30.9833	30.7466	30.3541	30.6437	30.9578	1:Experimental evidence at protein level
27.7196	27.6527	27.7913	28.1022	27.9728	28.0693	28.0836	28.3622	1:Experimental evidence at protein level
28.7799	28.8717	28.9004	28.9805	28.7295	28.74	28.8055	28.9526	1:Experimental evidence at protein level
26.5312	26.6365	26.5221	26.5166	26.5274	26.4356	26.6704	26.9227	1:Experimental evidence at protein level
27.7757	27.9166	28.0473	27.9189	28.2633	28.1771	28.3288	28.7127	1:Experimental evidence at protein level
29.4376	29.3129	29.041	28.9495	29.2522	29.3129	29.1159	28.5256	1:Experimental evidence at protein level
27.4522	27.5152	27.476	27.5375	27.0727	27.0651	27.1074	27.0564	1:Experimental evidence at protein level
27.774	27.82	27.8195	27.9792	27.4483	27.4564	27.4616	27.4352	1:Experimental evidence at protein level
26.9241	27.1261	27.081	27.0384	27.418	27.4771	27.5609	27.4309	1:Experimental evidence at protein level
#NUM!	24.7257	24.7302	24.7583	25.8505	25.9895	25.9825	25.8952	1:Experimental evidence at protein level
26.9038	26.987	26.8267	27.0769	26.9947	26.9758	26.8812	27.3026	1:Experimental evidence at protein level
26.8906	27.1431	27.2451	27.1133	27.3631	27.2433	27.4912	27.48	1:Experimental evidence at protein level
26.2467	26.2632	26.5422	26.4897	25.8909	25.8282	25.935	26.3428	1:Experimental evidence at protein level
25.4866	25.45	25.5416	25.4798	25.1801	25.5767	25.3997	#NUM!	1:Experimental evidence at protein level
28.7133	28.6689	28.802	28.8013	28.8032	28.714	28.7699	28.8826	1:Experimental evidence at protein level
27.1513	27.2037	27.0139	27.3277	27.7258	27.6865	27.7913	28.1192	1:Experimental evidence at protein level
26.325	26.452	26.2039	25.9994	26.1551	26.431	26.198	25.3754	1:Experimental evidence at protein level
26.1493	26.1026	26.3718	26.3318	26.2705	26.2822	26.3944	26.7209	1:Experimental evidence at protein level
27.563	27.5126	27.2457	27.3063	27.3174	27.4445	27.1733	27.1848	1:Experimental evidence at protein level
27.8316	27.8025	27.9781	27.9893	27.5045	27.6826	27.6649	27.3776	1:Experimental evidence at protein level
26.3276	26.1773	26.3173	26.4023	24.825	24.9149	24.9919	#NUM!	1:Experimental evidence at protein level
25.0729	25.0189	24.7905	24.7708	25.421	25.5012	25.2597	26.0012	1:Experimental evidence at protein level
28.3248	28.2002	28.1794	28.4215	27.8866	27.7273	27.7984	28.1626	1:Experimental evidence at protein level
26.818	26.8887	27.0801	26.8097	26.7816	26.7999	26.5638	26.7499	1:Experimental evidence at protein level
26.5485	26.3737	26.0128	26.1563	26.6373	26.7894	26.6084	26.548	1:Experimental evidence at protein level
27.3165	27.6037	27.9212	27.8542	27.5002	27.6685	27.7081	27.7014	1:Experimental evidence at protein level
28.6592	28.5337	28.6681	28.6671	28.7913	28.6658	28.5476	28.7345	1:Experimental evidence at protein level
29.7857	29.8891	30.0139	29.8787	29.973	29.7605	29.9313	29.9662	1:Experimental evidence at protein level
26.5887	26.8269	27.1231	26.9205	26.4973	26.8294	26.4295	26.5485	1:Experimental evidence at protein level
26.9643	26.9569	26.8285	26.7369	27.0002	26.9535	26.6294	26.7152	1:Experimental evidence at protein level
26.096	25.9647	26.0952	26.2376	25.6561	25.669	25.5096	26.2434	1:Experimental evidence at protein level
26.4398	26.6475	26.7814	26.8237	26.9893	27.1654	26.9188	26.8146	1:Experimental evidence at protein level
26.5455	26.6298	26.5548	26.7535	26.6675	26.5122	26.7846	26.9165	1:Experimental evidence at protein level
27.0303	27.035	27.2369	27.0519	27.2471	27.3346	27.2875	27.0338	1:Experimental evidence at protein level
27.9073	27.7739	28.0939	28.0341	27.6751	27.4431	27.5792	28.1418	1:Experimental evidence at protein level
24.5238	24.7181	24.7741	24.5603	25.3373	25.0462	25.4969	26.1454	1:Experimental evidence at protein level
28.6184	28.6818	28.8371	28.8723	28.0979	28.1137	28.1821	28.2751	1:Experimental evidence at protein level
28.4603	28.4892	28.3332	28.2389	28.5924	28.6656	28.6079	27.9215	1:Experimental evidence at protein level
26.3826	26.5085	26.3176	26.2838	26.4365	26.6347	26.3956	25.9134	1:Experimental evidence at protein level
27.1007	27.1472	26.9622	26.9197	#NUM!	26.4512	26.9148	27.0016	1:Experimental evidence at protein level
#NUM!	24.9777	24.8567	25.0337	25.9588	26.048	26.1948	26.3687	1:Experimental evidence at protein level
26.461	26.469	26.6289	26.596	26.5967	26.4534	26.4898	26.7848	1:Experimental evidence at protein level
27.569	27.6043	27.5716	27.6671	27.6617	27.6467	27.6827	27.8791	1:Experimental evidence at protein level
28.7357	28.6147	28.7316	28.6704	29.0352	29.0678	28.839	28.7421	1:Experimental evidence at protein level
27.8208	27.6548	27.2851	27.2599	27.5655	27.7047	27.4026	27.8182	1:Experimental evidence at protein level
27.9477	28.0815	28.4459	28.1412	28.2238	28.0442	28.1494	28.0606	1:Experimental evidence at protein level
27.1098	27.0196	26.9253	27.1364	27.2133	27.2063	27.0147	27.4495	1:Experimental evidence at protein level
26.4431	26.5479	26.7747	26.4994	26.1714	26.2834	26.1195	26.9197	1:Experimental evidence at protein level
27.0179	26.789	27.0634	27.2439	27.2402	27.1773	27.0978	27.3679	1:Experimental evidence at protein level
28.2499	28.2784	28.2937	28.2741	28.334	28.3783	28.4192	28.338	1:Experimental evidence at protein level
26.53	26.7147	26.8586	26.8596	27.2516	27.2935	27.4408	27.5328	1:Experimental evidence at protein level
26.3127	26.3878	26.4521	26.3653	26.7647	26.9863	26.7758	26.8308	1:Experimental evidence at protein level
26.2331	26.1151	26.2757	26.4078	25.7836	25.6836	25.6802	26.1605	1:Experimental evidence at protein level
27.8347	27.6878	28.0201	28.0371	27.9786	27.8179	27.8823	28.2838	1:Experimental evidence at protein level
25.8982	25.6592	25.6028	25.7223	25.8336	25.6493	25.5624	26.5042	1:Experimental evidence at protein level
26.3229	26.0114	25.9471	26.4514	26.32	26.4509	26.3027	26.5865	1:Experimental evidence at protein level
27.459	27.368	27.5392	27.7746	27.5351	27.6062	27.6891	27.8084	1:Experimental evidence at protein level
28.8349	28.8525	28.9277	28.9387	29.1605	29.1394	29.2686	28.9436	1:Experimental evidence at protein level
27.5314	27.6135	27.8691	27.7942	27.3783	27.3593	27.5761	27.5567	1:Experimental evidence at protein level
27.2066	27.1916	27.4638	27.3919	27.3732	27.05	27.3055	27.3908	1:Experimental evidence at protein level

28.5047	28.583	28.6944	28.3217	28.5488	28.8505	28.6533	27.9175	1:Experimental evidence at protein level
29.2871	29.1989	29.3476	29.2305	29.2958	29.3274	29.0197	29.4486	1:Experimental evidence at protein level
29.6701	29.6892	29.5172	29.1069	28.2534	28.4159	28.2692	28.1084	1:Experimental evidence at protein level
29.3377	29.0052	29.0405	28.6803	29.4829	29.7646	28.9613	27.6512	1:Experimental evidence at protein level
29.6504	29.4823	29.5327	29.6774	29.3777	29.3583	29.5606	29.4522	1:Experimental evidence at protein level
33.3103	33.404	33.3979	33.4683	33.6521	33.8156	33.7694	33.3139	1:Experimental evidence at protein level
26.2276	26.3734	26.5598	26.6119	26.3128	26.3153	26.4738	26.2611	1:Experimental evidence at protein level
25.8419	25.8549	26.1542	26.2692	26.1823	26.1119	26.0394	26.9557	1:Experimental evidence at protein level
28.3274	28.1454	27.698	27.7603	28.5884	28.7162	28.5658	28.7029	1:Experimental evidence at protein level
26.4188	26.4811	26.3093	26.4762	26.2339	26.4136	26.0993	26.7585	1:Experimental evidence at protein level
28.4183	28.2765	28.6963	28.6302	28.5362	28.661	28.5314	28.203	1:Experimental evidence at protein level
30.5637	30.3126	30.0576	30.2347	30.6235	30.7607	30.4976	30.3435	1:Experimental evidence at protein level
27.4228	27.5311	27.5147	27.4956	26.8296	26.7551	26.8444	26.5503	1:Experimental evidence at protein level
25.743	26.0332	25.963	25.7043	24.9063	25.3845	25.306	25.6838	1:Experimental evidence at protein level
29.6663	29.6335	29.8542	29.5794	29.5238	29.5704	29.5676	29.3937	1:Experimental evidence at protein level
29.9102	29.8203	28.8733	29.6501	30.0762	30.1634	30.1301	30.6192	1:Experimental evidence at protein level
29.1985	29.1473	29.1453	29.2252	29.2622	29.3341	29.0632	28.4651	1:Experimental evidence at protein level
26.8576	26.8218	26.9523	26.8638	26.928	26.8964	26.9293	26.8984	1:Experimental evidence at protein level
25.3617	25.3185	25.6697	25.9317	25.8614	25.5192	25.8131	26.6526	1:Experimental evidence at protein level
27.7565	28.0007	27.9497	27.9492	27.7467	27.8833	27.8424	28.173	1:Experimental evidence at protein level
27.4621	27.5355	27.5285	27.4961	27.3871	27.312	27.4889	27.9682	1:Experimental evidence at protein level
29.6552	29.73	29.933	29.9111	30.0592	29.8843	29.9572	30.2	1:Experimental evidence at protein level
30.6063	30.5711	30.6809	30.5532	30.4888	30.5658	30.4218	29.9999	1:Experimental evidence at protein level
28.0427	28.2156	28.1585	28.3489	28.4251	28.2727	28.4772	28.6767	1:Experimental evidence at protein level
27.7507	27.8347	27.9992	27.9161	27.9915	28.0009	27.959	27.7518	1:Experimental evidence at protein level
27.4223	27.3437	27.2445	27.6116	26.6512	26.7333	26.6971	27.3031	1:Experimental evidence at protein level
27.2078	27.0015	26.9321	27.3186	26.6542	26.9247	26.6944	27.4438	1:Experimental evidence at protein level
26.1207	25.9014	25.7377	25.6237	25.7858	26.0876	25.8508	25.9393	1:Experimental evidence at protein level
28.6967	28.6734	28.5939	28.85	28.6284	28.4949	28.6244	28.9359	1:Experimental evidence at protein level
29.1049	28.8879	28.7105	28.8615	28.9794	28.9246	28.804	28.974	1:Experimental evidence at protein level
28.3186	28.4748	28.3751	28.573	28.1665	28.2438	28.2151	28.3099	1:Experimental evidence at protein level
27.641	27.721	27.7104	27.1257	27.0238	27.2012	27.2107	26.6743	1:Experimental evidence at protein level
28.0889	28.1249	28.2607	28.4728	28.1202	27.8289	28.2494	28.5894	1:Experimental evidence at protein level
29.2366	29.2524	29.1744	28.9997	29.1587	29.0366	28.9335	29.1297	1:Experimental evidence at protein level
30.4823	30.3528	30.4556	30.3365	30.7752	30.7143	30.6831	30.4689	1:Experimental evidence at protein level
28.0565	27.9284	27.9615	28.0604	27.938	27.8479	27.7924	28.0062	1:Experimental evidence at protein level
28.0776	28.2372	28.159	28.2191	28.3301	28.1427	28.339	28.2624	1:Experimental evidence at protein level
25.6551	25.5237	25.2456	25.4387	25.3282	25.4312	25.7545	25.8793	1:Experimental evidence at protein level
26.6467	26.4144	26.7328	26.6785	26.4404	26.5698	26.7826	26.8409	1:Experimental evidence at protein level
25.8705	25.8836	25.9996	25.8807	26.053	25.9405	25.9635	26.2886	1:Experimental evidence at protein level
26.2348	26.2029	25.8885	25.7252	26.0035	26.0291	25.7844	26.1655	1:Experimental evidence at protein level
25.6136	25.8124	25.8628	25.8896	25.7479	25.8295	25.9719	26.4272	1:Experimental evidence at protein level
26.6646	26.6701	26.7776	26.7528	26.6654	26.6608	26.7626	27.2617	1:Experimental evidence at protein level
24.9433	25.114	25.12	25.3497	24.5853	24.6539	24.6204	24.9873	1:Experimental evidence at protein level
26.5898	26.498	26.5895	26.7383	25.7487	26.0496	26.0021	26.1568	1:Experimental evidence at protein level
29.2332	29.3504	29.4596	29.5534	29.616	29.6565	29.4912	29.0749	1:Experimental evidence at protein level
29.1973	29.2632	29.2492	29.3103	28.5351	28.6163	28.6228	28.9211	1:Experimental evidence at protein level
27.7101	27.7432	27.6611	27.7653	27.7933	27.7978	27.7815	27.9903	1:Experimental evidence at protein level
26.054	26.3137	26.3409	26.771	25.9684	26.7742	26.484	26.3181	1:Experimental evidence at protein level
27.837	27.8523	27.7121	27.8217	27.793	27.6329	27.7626	28.0166	1:Experimental evidence at protein level
28.4747	28.4777	28.7434	28.3428	28.5839	28.5535	28.4713	28.238	1:Experimental evidence at protein level
28.0376	28.0533	28.1929	28.222	28.1305	27.9828	28.0746	28.5686	1:Experimental evidence at protein level
26.3886	26.5486	26.4456	26.3982	26.1721	26.1441	26.2392	26.2873	1:Experimental evidence at protein level
27.1129	27.0479	27.2334	27.326	27.0868	27.1312	27.233	27.0286	1:Experimental evidence at protein level
26.2132	26.3771	26.4728	26.4515	26.0592	26.0547	26.2505	26.4562	1:Experimental evidence at protein level
25.9222	26.0968	25.7743	26.0571	25.6502	25.9527	26.0002	#NUM!	1:Experimental evidence at protein level
25.492	25.5425	25.8213	25.4466	25.5466	25.554	25.5738	#NUM!	1:Experimental evidence at protein level
27.6414	27.5879	27.6607	27.6388	27.6864	27.6133	27.7334	27.8201	1:Experimental evidence at protein level
26.2495	26.2645	26.3729	26.485	26.382	26.5625	26.3923	26.7281	1:Experimental evidence at protein level
28.3643	28.3489	28.337	28.6776	28.5508	28.4673	28.5936	28.8988	1:Experimental evidence at protein level
27.3615	27.9396	28.5159	28.4549	28.1606	27.9699	28.4187	28.741	1:Experimental evidence at protein level
27.4325	27.3193	27.5958	27.2167	27.5336	27.5556	27.4463	27.6193	1:Experimental evidence at protein level
26.4125	26.2778	26.624	26.4922	26.4038	26.3846	26.4558	26.8531	1:Experimental evidence at protein level
28.2544	28.3936	28.2372	28.3037	28.5379	28.4517	28.5572	28.7714	1:Experimental evidence at protein level
27.8125	27.9519	28.1727	27.9796	28.3121	27.9587	28.2228	28.6806	1:Experimental evidence at protein level
26.913	26.8598	26.8491	26.9517	26.8566	26.4042	27.0233	27.016	1:Experimental evidence at protein level
27.4098	27.4624	27.6774	27.3963	27.4397	27.4194	27.5121	27.561	1:Experimental evidence at protein level
29.8809	29.9502	29.7703	29.926	29.8543	29.8096	29.825	30.0613	1:Experimental evidence at protein level
30.2907	30.1706	29.9174	30.1127	30.603	30.8185	30.5164	30.7767	1:Experimental evidence at protein level
28.3176	28.3256	28.5137	28.5638	28.0147	27.9954	28.136	28.3106	1:Experimental evidence at protein level
27.0109	27.0982	27.2127	26.9906	27.2686	27.3422	27.3615	27.3266	1:Experimental evidence at protein level
27.8656	27.9198	27.7472	27.9728	27.7143	27.7369	27.8038	27.8236	1:Experimental evidence at protein level
26.4699	26.6716	26.4176	26.4106	26.7896	26.6049	26.3967	26.7262	1:Experimental evidence at protein level
27.3527	27.1715	26.725	26.9509	27.2481	27.4442	27.1029	27.1554	1:Experimental evidence at protein level
26.8011	26.8694	27.0202	27.0315	26.9205	26.9023	27.146	27.4354	1:Experimental evidence at protein level
26.499	26.5649	26.408	26.6761	26.2467	25.9829	26.2751	26.9266	1:Experimental evidence at protein level
28.3093	28.1522	28.1944	28.0885	28.5016	28.5704	28.4753	28.4575	1:Experimental evidence at protein level
25.8435	25.6872	25.9878	26.2912	26.3383	25.5319	25.6369	27.0649	1:Experimental evidence at protein level
26.4905	26.7201	27.0099	26.7682	27.9589	27.8968	28.1177	28.2634	1:Experimental evidence at protein level
31.7608	31.8768	32.0274	31.8488	31.5898	31.4724	31.4973	31.2603	1:Experimental evidence at protein level
25.7488	25.8319	26.0915	26.1354	26.3979	26.2941	26.292	26.2097	1:Experimental evidence at protein level
31.2093	31.1857	31.3509	31.3913	31.4443	31.3586	31.463	31.4606	1:Experimental evidence at protein level
27.9085	27.4657	27.4048	27.6091	27.2527	27.2626	27.5257	27.5799	1:Experimental evidence at protein level
25.429	25.5899	25.3862	25.5182	25.316	24.6973	25.5579	26.2157	1:Experimental evidence at protein level
30.3469	30.388	30.4919	29.9902	30.1929	29.9724	30.3008	29.8257	1:Experimental evidence at protein level
26.8714	26.8218	26.7736	27.0466	26.9664	27.0117	26.9314	27.3534	1:Experimental evidence at protein level
28.5075	28.6741	28.9332	28.7917	28.634	28.4278	28.6293	28.3754	1:Experimental evidence at protein level
27.386	27.3443	27.2427	26.9944	27.4187	27.5187	27.4305	26.6813	1:Experimental evidence at protein level
29.0186	29.0963	29.0857	29.0506	28.8997	28.9466	28.9971	28.7427	1:Experimental evidence at protein level
30.8191	30.6391	30.436	30.2293	30.3812	30.3484	30.314	30.3127	1:Experimental evidence at protein level
27.8008	27.8205	28.0907	27.8211	27.912	27.8737	27.8521	27.5023	1:Experimental evidence at protein level
26.9982	26.9182	27.0238	26.926	27.1015	26.7791	27.154	27.5486	1:Experimental evidence at protein level
28.9358	28.9949	29.4963	29.1444	29.2193	29.2282	29.2209	28.7936	1:Experimental evidence at protein level
27.9714	27.4868	27.3216	27.8919	27.8302	27.4632	27.3336	27.738	1:Experimental evidence at protein level
27.7494	27.6004	27.7202	27.8684	27.8378	27.7933	27.8988	27.5685	1:Experimental evidence at protein level
26.724	26.8776	26.8152	26.8833	26.831	26.8915	26.8874	26.9178	1:Experimental evidence at protein level
28.2818	28.3639	28.483	28.4954	28.7148	28.8242	28.6955	28.3055	1:Experimental evidence at protein level
29.0497	29.2374	29.2695	29.2021	29.2694	29.3015	29.1699	29.2097	1:Experimental evidence at protein level
27.2079	27.2389	27.073	27.4008	27.1525	27.1377	27.3265	27.6572	1:Experimental evidence at protein level
29.4241	29.6328	29.7269	29.6513	29.8916	29.8451	29.9379	30.0752	1:Experimental evidence at protein level
30.1812	30.0143	29.6973	29.8791	30.411	30.4298	30.2589	30.2746	1:Experimental evidence at protein level
29.5076	29.4095	29.2011	29.2255	29.5429	29.6389	29.5235	29.9439	1:Experimental evidence at protein level
31.376	31.1256	30.8847	30.7488	31.381	31.1263	31.3277	31.4879	1:Experimental evidence at protein level
28.1513	28.0597	28.275	27.9735	28.077	28.117	28.0245	27.9672	1:Experimental evidence at protein level
28.8856	28.8716	28.9368	28.9073	28.9712	28.9277	28.9288	29.0962	1:Experimental evidence at protein level
28.4751	28.3235	28.3928	28.4202	28.5598	28.3004	28.2756	28.3294	1:Experimental evidence at protein level
27.7431	27.9061	28.0656	28.074	27.1565	26.6383	26.9726	27.4026	1:Experimental evidence at protein level
25.593	25.7918	25.8966	26.1522	25.9441	25.8722	26.072	26.3539	1:Experimental evidence at protein level
27.7771	27.9662	27.8369	27.6891	27.1484	27.1621	27.2824	27.5246	1:Experimental evidence at protein level
26.3801	26.3828	26.4487	26.4119	26.5101	26.4332	26.5436	26.7877	1:Experimental evidence at protein level
27.1429	27.0022	26.7144	26.7591	27.0304	26.9769	27.0088	27.5387	1:Experimental evidence at protein level
28.1137	27.9462	27.8803	27.7142	27.7613	27.6849	27.755	27.6461	1:Experimental evidence at protein level
27.3017	27.4164	27.2343	27.369	27.4491	27.3952	27.4434	27.6173	1:Experimental evidence at protein level
26.8493	26.7638	26.6542	26.9721	26.9252	26.9604	26.8658	27.2833	1:Experimental evidence at protein level
26.3097	26.3105	25.9499	25.9861	26.4899	26.7157	26.3309	26.3829	1:Experimental evidence at protein level
29.7044	29.8111	29.8312	29.8125	29.6752	29.7973	29.7565	29.797	1:Experimental evidence at protein level
25.3945	25.8863	25.6885	25.6519	25.3395	25.3857	25.5896	25.2498	1:Experimental evidence at protein level
27.3805	27.3146	27.3483	27.3878	27.5472	27.5704	27.4139	27.6684	1:Experimental evidence at protein level
27.2829	27.6757	27.4869	27.4498	27.4647	27.3173	27.6776	27.748	1:Experimental evidence at protein level
26.16	26.0167	25.6488	26.0082	25.8872	25.8692	25.8718	26.053	1:Experimental evidence at protein level
27.7319	27.6899	27.5727	27.662	27.61	27.5313	27.7283	27.7017	1:Experimental evidence at protein level
26.2642	26.1549	26.1469	26.2826	26.2925	26.4086	26.4146	26.6937	1:Experimental evidence at protein level
25.6779	25.9514	25.5652	25.9617	26.3412	26.2036	26.1457	26.6063	1:Experimental evidence at protein level
26.9612	27.0542	26.9906	27.0902	26.8122	26.7527	26.8878	27.3729	1:Experimental evidence at protein level
29.6069	29.6539	29.4612	29.4633	29.4379	29.4691	29.2814	29.4285	1:Experimental evidence at protein level
28.716	28.7178	28.5221	28.6229	28.661	28.6486	28.6075	28.7128	1:Experimental evidence at protein level
27.3632	27.421	27.3763	27.6463	27.479	27.2203	27.2801	27.7837	1:Experimental evidence at protein level
28.0343	28.0899	28.1763	28.0821	27.6853	27.8903	27.7822	27.387	1:Experimental evidence at protein level
26.6689	26.8147	26.7661	27.0761	27.0357	26.9664	27.143	27.5361	1:Experimental evidence at protein level

26.4608	26.4525	26.5745	26.378	26.1824	26.2809	26.2649	26.3841	1:Experimental evidence at protein level
28.6956	28.8233	28.7547	28.7301	28.309	28.32	28.4011	28.3341	1:Experimental evidence at protein level
25.8447	25.9225	26.221	25.8074	26.1447	26.0106	26.1684	26.1546	1:Experimental evidence at protein level
25.9549	25.9805	25.7944	26.0743	26.3239	26.5162	26.4173	26.803	1:Experimental evidence at protein level
25.8676	25.857	25.904	25.7642	24.8329	24.8408	24.3397	#NUM!	1:Experimental evidence at protein level
28.5406	28.7672	28.7895	28.9089	28.4897	28.4332	28.7205	28.981	1:Experimental evidence at protein level
26.1212	25.9724	26.1972	26.1384	25.9761	25.9644	26.1678	25.9716	1:Experimental evidence at protein level
27.2446	27.2671	27.4946	27.4011	27.2331	27.2132	27.2284	27.3035	1:Experimental evidence at protein level
27.9827	28.0261	27.9616	27.9416	28.0013	27.7946	27.9067	27.7811	1:Experimental evidence at protein level
26.7865	26.8644	26.9404	27.1048	27.1565	26.8697	27.0681	27.2632	1:Experimental evidence at protein level
30.6281	30.7304	30.7603	30.9094	30.681	30.738	30.8073	30.5811	1:Experimental evidence at protein level
27.6959	27.8504	27.969	27.7046	28.1129	28.0819	28.0166	27.9205	1:Experimental evidence at protein level
28.3972	28.374	28.4319	28.4461	28.365	28.3388	28.3949	28.076	1:Experimental evidence at protein level
26.8723	26.8513	26.8101	26.9271	27.0645	27.1204	27.1527	27.0122	1:Experimental evidence at protein level
25.9102	25.9308	25.8936	26.0075	25.8006	26.093	25.9382	25.9651	1:Experimental evidence at protein level
27.3211	27.4541	27.4567	27.6057	27.6516	27.6113	27.4869	27.807	1:Experimental evidence at protein level
25.7612	25.219	24.9103	25.3185	26.2818	26.0773	25.8895	25.8999	1:Experimental evidence at protein level
25.0847	25.0436	24.9718	24.9649	25.1959	25.1498	24.9958	25.7817	1:Experimental evidence at protein level
29.8267	29.8244	30.1999	30.3323	29.9279	29.7084	29.9752	29.9532	1:Experimental evidence at protein level
29.9128	29.4482	27.6045	27.3576	26.6653	27.3202	26.0578	26.2996	1:Experimental evidence at protein level
28.6971	28.6945	28.8658	28.9914	29.0986	29.1657	29.181	29.5088	1:Experimental evidence at protein level
27.2512	27.1739	27.2651	27.3973	27.15	27.0478	27.201	28.0424	1:Experimental evidence at protein level
26.808	26.7847	26.9531	26.7151	26.8568	26.9311	26.9592	26.769	1:Experimental evidence at protein level
27.4809	27.6246	27.9927	27.8243	27.7892	27.3729	27.7142	28.4855	1:Experimental evidence at protein level
31.963	32.0689	31.9878	32.2097	32.7587	32.6152	32.7431	32.4115	1:Experimental evidence at protein level
28.9556	29.1772	29.2333	29.0855	29.3953	29.4975	29.5148	29.0844	1:Experimental evidence at protein level
26.4542	26.2907	26.4557	26.6609	27.6616	27.5991	27.3617	27.8415	1:Experimental evidence at protein level
28.6885	28.7208	29.0692	29.0334	28.8991	28.7596	28.8034	29.0178	1:Experimental evidence at protein level
29.2784	29.3001	29.5576	29.5665	29.7873	29.7782	29.7394	29.9132	1:Experimental evidence at protein level
25.8185	25.4602	25.6888	25.7815	25.5088	25.3945	25.4051	26.1272	1:Experimental evidence at protein level
27.315	27.3301	27.3076	27.4086	27.2095	27.4576	27.2107	27.3766	1:Experimental evidence at protein level
27.8872	27.9158	27.5604	27.5002	27.8864	27.9015	27.9472	28.2289	1:Experimental evidence at protein level
28.0599	28.0624	28.1482	28.0103	25.7378	26.133	25.9859	26.5741	1:Experimental evidence at protein level
28.4656	28.3773	28.3657	28.4381	28.5306	28.4006	28.3691	28.2713	1:Experimental evidence at protein level
29.813	29.8699	30.0935	29.7527	30.0365	30.0894	29.8049	29.153	1:Experimental evidence at protein level
29.3049	29.3781	29.1781	28.7191	28.7444	29.0836	28.8258	28.4491	1:Experimental evidence at protein level
28.4682	28.3449	28.5937	28.5575	28.4273	28.5203	28.3912	28.3356	1:Experimental evidence at protein level
27.3439	27.427	27.4284	27.369	27.3165	27.5207	27.3012	27.2345	1:Experimental evidence at protein level
26.2031	26.1849	26.0511	26.431	26.5016	26.4962	26.3614	26.7935	1:Experimental evidence at protein level
27.5026	27.6064	27.5309	27.6231	27.7741	27.8871	27.7122	28.0877	1:Experimental evidence at protein level
27.5678	27.6786	27.6272	27.6173	28.1903	28.096	28.1213	28.4082	1:Experimental evidence at protein level
26.944	27.082	27.1312	27.2414	27.1106	27.0564	27.1163	27.0347	1:Experimental evidence at protein level
27.005	27.1036	27.074	27.3174	27.0881	26.9856	27.2651	27.3844	1:Experimental evidence at protein level
27.5506	27.5354	27.4882	27.5298	27.469	27.5602	27.4788	27.4743	1:Experimental evidence at protein level
33.1436	33.2279	33.4171	33.1926	33.2939	33.1365	33.3696	32.6664	1:Experimental evidence at protein level
29.8208	29.9512	29.8988	29.6581	30.0018	30.0555	30.1107	29.3076	1:Experimental evidence at protein level
29.1037	29.0127	29.0979	29.1738	29.0407	28.9452	29.1477	29.1904	1:Experimental evidence at protein level
27.9112	28.0121	28.0985	27.9265	27.9498	28.1022	27.9494	27.8429	1:Experimental evidence at protein level
26.0419	26.1781	26.5036	26.2506	26.4246	26.3174	26.2489	26.2893	1:Experimental evidence at protein level
27.1585	27.1413	27.4179	27.3621	27.4634	27.4625	27.4561	27.5333	1:Experimental evidence at protein level
29.7034	29.6801	29.8201	29.6185	29.245	29.2119	29.0946	28.7214	1:Experimental evidence at protein level
30.4215	30.3308	30.0381	30.3827	30.2249	30.1277	30.2467	30.4865	1:Experimental evidence at protein level
27.3985	27.3589	27.1821	27.4353	27.4286	27.333	27.4638	28.0184	1:Experimental evidence at protein level
27.551	27.4703	27.5105	27.591	27.2916	27.2535	27.1536	27.5577	1:Experimental evidence at protein level
25.1042	24.7815	24.3604	25.1121	24.9645	#NUM!	24.7547	25.4595	1:Experimental evidence at protein level
29.3385	29.2946	29.4854	29.5221	29.3168	29.3474	29.4069	29.2745	1:Experimental evidence at protein level
26.5737	26.7069	26.6713	26.6939	26.465	26.4751	26.4696	26.7838	1:Experimental evidence at protein level
28.3815	28.3182	28.4846	28.5447	28.5179	28.4584	28.5249	28.352	1:Experimental evidence at protein level
25.7596	25.811	25.6337	25.7378	26.7496	26.7111	26.7608	27.1806	1:Experimental evidence at protein level
28.5288	28.5391	28.6317	28.717	28.3614	28.4775	28.2365	28.0921	1:Experimental evidence at protein level
31.9325	31.6838	29.9325	30.1655	30.1598	30.7926	29.4484	29.9074	1:Experimental evidence at protein level
25.6282	25.6924	25.4814	25.5872	25.8577	26.0947	25.8377	25.9716	1:Experimental evidence at protein level
29.5605	29.4162	29.2109	29.3716	29.6385	29.6518	29.4129	29.9309	1:Experimental evidence at protein level
28.3688	28.0502	25.7705	26.5698	25.8032	26.2	25.3755	25.7248	1:Experimental evidence at protein level
27.6003	27.5562	27.5241	27.5196	27.5906	27.5374	27.4989	27.7358	1:Experimental evidence at protein level
26.8676	27.2393	27.5148	27.0107	26.9791	27.5834	27.4966	#NUM!	1:Experimental evidence at protein level
27.8892	28.0543	28.2759	28.4383	27.8226	27.6859	28.1653	28.4935	1:Experimental evidence at protein level
28.7323	28.515	28.2463	28.4556	28.2161	28.2894	28.4085	28.8955	1:Experimental evidence at protein level
27.1491	27.2471	27.4679	27.4556	26.6067	26.5483	26.9811	27.0757	1:Experimental evidence at protein level
28.728	28.7516	28.9542	28.8642	28.5222	28.5411	28.5867	28.4048	1:Experimental evidence at protein level
27.2827	27.4153	27.0365	26.9293	27.3765	27.695	27.2817	27.0939	1:Experimental evidence at protein level
26.4084	26.633	26.384	26.7492	26.321	26.064	26.3558	26.5249	1:Experimental evidence at protein level
27.2725	27.2682	27.0689	27.3285	28.0416	28.0121	27.6849	27.7045	1:Experimental evidence at protein level
25.3691	24.9851	25.4546	25.288	25.5184	24.537	#NUM!	25.7254	1:Experimental evidence at protein level
28.702	28.5822	28.2576	28.1631	28.3362	28.604	28.3532	28.6844	1:Experimental evidence at protein level
26.2954	26.6774	26.7748	26.8333	26.2218	26.2187	26.3658	27.3238	1:Experimental evidence at protein level
28.2122	28.2187	28.3082	28.2609	28.5423	28.4971	28.4792	28.7148	1:Experimental evidence at protein level
27.8542	27.8624	27.7873	28.1388	28.1573	28.0214	28.235	28.6964	1:Experimental evidence at protein level
27.4194	27.3837	27.5452	27.4387	27.6118	27.5828	27.6117	27.8006	1:Experimental evidence at protein level
27.6095	27.5211	27.4053	27.4122	27.1485	27.2145	27.2221	27.3105	1:Experimental evidence at protein level
26.7365	26.757	26.9576	26.5559	26.8801	26.8458	26.8295	26.5846	1:Experimental evidence at protein level
26.3857	26.0423	25.0956	25.2839	26.5392	26.9145	26.2716	26.4216	1:Experimental evidence at protein level
26.6338	26.735	26.6161	26.5313	27.2889	27.2823	27.395	27.4423	1:Experimental evidence at protein level
27.7129	27.1259	27.9449	28.4834	29.3628	29.2638	29.1165	29.173	1:Experimental evidence at protein level
27.9821	27.836	27.2661	27.2045	28.9919	29.6315	28.2976	28.0181	1:Experimental evidence at protein level
27.7411	27.7424	27.7773	27.983	26.9548	26.8884	27.0692	27.4047	1:Experimental evidence at protein level
28.2361	28.3108	27.881	28.023	28.0742	28.1702	28.0852	27.4886	1:Experimental evidence at protein level
27.5662	27.4657	27.6164	27.6566	27.8218	27.929	27.8661	27.8018	1:Experimental evidence at protein level
28.7429	28.7875	28.8848	28.9058	28.8123	28.6516	28.8501	28.9161	1:Experimental evidence at protein level
28.4216	28.5099	28.5878	28.4997	28.1744	28.1123	28.1562	28.3207	1:Experimental evidence at protein level
28.0925	28.4027	28.4021	28.3584	28.3605	28.2458	28.5483	28.4234	1:Experimental evidence at protein level
30.614	30.6908	30.7079	30.5733	30.5546	30.3733	30.5933	30.3429	1:Experimental evidence at protein level
30.3663	30.35	30.4683	30.5196	30.4128	30.3213	30.4565	30.4005	1:Experimental evidence at protein level
28.0609	28.1409	28.2226	28.1891	28.028	27.9721	28.1786	28.1764	1:Experimental evidence at protein level
26.1122	26.0865	26.1865	26.1515	25.948	25.9004	25.8635	26.4523	1:Experimental evidence at protein level
28.2228	28.1284	28.1313	28.5886	28.11	27.7569	28.0496	28.4134	1:Experimental evidence at protein level
27.8605	28.081	28.4392	28.2702	27.8336	27.6848	27.8945	28.1347	1:Experimental evidence at protein level
29.5955	29.3749	28.9488	29.1803	28.9726	29.1284	28.9532	29.1095	1:Experimental evidence at protein level
31.5607	31.6865	31.6434	31.3849	31.4664	31.5604	31.6422	30.3623	1:Experimental evidence at protein level
30.325	30.3169	29.9529	30.149	30.2332	30.267	30.2965	30.4582	1:Experimental evidence at protein level
27.844	27.9723	28.0302	28.0067	28.1459	28.0816	28.0344	28.2941	1:Experimental evidence at protein level
26.7813	26.694	26.6089	26.5827	26.7093	26.5948	26.5368	26.722	1:Experimental evidence at protein level
28.4245	28.4543	28.394	28.2976	28.5105	28.3908	28.504	28.6154	1:Experimental evidence at protein level
26.347	26.1908	26.0481	26.4281	25.5574	25.5794	25.3871	26.2168	1:Experimental evidence at protein level
26.239	26.2913	26.2902	26.4426	26.0108	26.0694	25.9979	26.5501	1:Experimental evidence at protein level
28.1881	27.9142	28.2514	28.1997	28.0633	28.3104	28.1142	29.1098	1:Experimental evidence at protein level
27.8078	27.6943	27.9005	28.0001	27.6071	27.6645	27.524	27.7137	1:Experimental evidence at protein level
26.3542	26.4781	26.5908	26.5784	26.925	26.7989	26.85	26.8561	1:Experimental evidence at protein level
26.6491	26.5663	26.7219	27.074	26.5771	26.4581	26.74	26.9407	1:Experimental evidence at protein level
27.272	27.2389	27.4075	27.3143	27.2672	27.4231	27.2346	27.0079	1:Experimental evidence at protein level
25.5711	25.5979	25.5765	25.5801	25.2593	25.3709	25.5646	25.1695	1:Experimental evidence at protein level
25.9504	26.0758	25.9579	26.2541	26.0193	26.1149	26.2761	26.3975	1:Experimental evidence at protein level
26.3907	26.4163	26.2534	26.2625	26.3319	26.3763	26.3188	26.2353	1:Experimental evidence at protein level
25.9822	26.2064	26.2013	26.3056	25.9562	25.9561	26.0294	26.122	1:Experimental evidence at protein level
26.4355	26.3889	26.3088	26.2918	26.0749	26.3499	26.0838	26.1132	1:Experimental evidence at protein level
26.5094	26.6297	26.6415	26.8337	26.6115	26.3607	26.5934	27.1563	1:Experimental evidence at protein level
28.3395	27.9433	27.5919	27.8371	28.4305	28.5307	28.701	28.4379	1:Experimental evidence at protein level
24.5172	24.7673	24.6758	24.7392	24.7442	25.2217	24.7626	25.1047	1:Experimental evidence at protein level
28.2379	28.3212	28.4282	28.3347	27.7815	27.5936	27.7462	28.2845	1:Experimental evidence at protein level
27.1351	27.0884	26.8561	26.9941	27.3036	27.4381	27.1127	27.5544	1:Experimental evidence at protein level
26.7474	26.607	26.6475	26.8535	27.7483	27.6732	27.7457	27.9813	1:Experimental evidence at protein level
26.948	27.0914	27.2507	27.014	27.0186	26.7789	26.7722	27.1137	1:Experimental evidence at protein level
26.8563	26.3709	26.1154	26.2091	26.6644	26.7041	26.3801	26.9625	1:Experimental evidence at protein level
28.932	28.9582	28.6652	28.7477	28.7119	28.7511	29.0098	28.7932	1:Experimental evidence at protein level
24.8208	24.8068	24.1993	24.9411	24.7361	25.1291	24.9159	25.4686	1:Experimental evidence at protein level
28.1348	27.8899	27.9489	27.9116	25.872	25.6621	25.7525	25.7513	1:Experimental evidence at protein level
26.9542	27.0232	27.1395	27.251	26.7768	26.4953	26.6424	27.0967	1:Experimental evidence at protein level
25.9369	25.7725	25.8286	25.8668	25.9032	26.2041	25.715	26.0771	1:Experimental evidence at protein level
28.1325	28.2196	28.1498	28.0577	27.9852	28.0279	27.9991	28.072	1:Experimental evidence at protein level

27.8156	27.8281	27.7441	27.9634	28.1373	28.0301	28.2135	28.3414	1:Experimental evidence at protein level
26.5604	26.4308	26.4181	26.6469	26.8722	26.807	26.8551	27.1736	1:Experimental evidence at protein level
27.942	27.7373	27.5312	27.6207	27.7102	27.7054	27.5753	27.8876	1:Experimental evidence at protein level
29.1147	29.3443	29.3212	29.2734	29.2595	29.4501	29.5064	29.3723	1:Experimental evidence at protein level
26.0948	26.1753	26.282	26.5044	26.1385	26.2965	26.1204	26.4642	1:Experimental evidence at protein level
30.0743	30.0393	30.2387	30.2064	30.2304	29.7368	29.7899	29.7466	1:Experimental evidence at protein level
29.2763	29.1467	29.3375	29.3296	29.5702	29.4809	29.4169	29.5853	1:Experimental evidence at protein level
29.0077	28.9055	28.9446	28.9444	29.0493	28.7212	29.0036	28.775	1:Experimental evidence at protein level
26.9958	26.86	26.9298	26.9464	26.8266	26.8454	26.8492	27.1287	1:Experimental evidence at protein level
27.0809	27.1832	27.1682	27.3824	27.5187	27.4078	27.5026	28.0018	1:Experimental evidence at protein level
28.1951	28.5382	28.4907	28.54	28.4576	28.5274	28.5337	28.1975	1:Experimental evidence at protein level
31.0804	31.0521	30.7568	30.7201	31.1076	31.2687	31.1637	31.2485	1:Experimental evidence at protein level
27.6219	27.5592	27.8038	27.9148	27.6546	27.5209	27.4791	28.0139	1:Experimental evidence at protein level
26.3604	26.4395	26.276	26.5522	26.6138	26.4767	26.5487	26.7099	1:Experimental evidence at protein level
28.4612	28.4364	28.681	28.7008	27.7873	27.8023	27.8613	28.0002	1:Experimental evidence at protein level
27.8602	28.0246	27.8998	28.0718	28.1997	28.1009	28.1752	28.4123	1:Experimental evidence at protein level
26.9856	26.7719	27.1596	27.1379	27.0708	26.8225	26.8574	27.0958	1:Experimental evidence at protein level
28.7739	28.8098	28.9187	28.8961	28.877	28.643	28.7052	28.7059	1:Experimental evidence at protein level
31.6542	31.6618	31.418	31.4652	31.7649	31.865	31.7801	31.8005	1:Experimental evidence at protein level
34.2417	33.684	32.2131	31.7139	31.489	32.0572	30.9213	30.5621	1:Experimental evidence at protein level
28.2715	28.4075	28.5296	28.2513	28.4172	28.3134	28.2244	27.4786	1:Experimental evidence at protein level
30.7705	30.6195	30.2381	30.4301	31.0115	31.0939	30.8807	31.0577	1:Experimental evidence at protein level
25.7518	25.6931	26.0324	26.0313	25.8006	25.7613	25.6572	26.0842	1:Experimental evidence at protein level
28.1576	28.201	28.2681	28.2179	28.7489	28.8381	28.6343	28.0159	1:Experimental evidence at protein level
30.817	30.7721	30.3529	30.4606	31.0798	31.0086	31.0211	31.2494	1:Experimental evidence at protein level
29.2889	28.9922	28.9598	28.9702	29.0956	29.1516	29.0753	29.1915	1:Experimental evidence at protein level
27.5412	27.3518	27.5024	27.4867	27.2579	26.962	27.3122	27.7661	1:Experimental evidence at protein level
26.0566	25.6447	25.6909	26.0469	26.1453	25.9354	26.3228	26.6995	1:Experimental evidence at protein level
27.4184	27.6722	27.8862	27.6798	28.0393	27.7519	28.1034	28.2594	1:Experimental evidence at protein level
30.3647	30.3313	30.3849	30.3675	30.4681	30.4336	30.4652	30.1292	1:Experimental evidence at protein level
29.5087	29.4974	29.6166	29.6784	29.7188	29.6459	29.7432	29.7878	1:Experimental evidence at protein level
27.1042	27.134	27.217	27.1896	26.9329	27.0023	27.1086	27.3441	1:Experimental evidence at protein level
26.988	27.0597	26.9028	27.1109	26.9303	27.0687	27.1298	27.1751	1:Experimental evidence at protein level
26.782	26.6295	26.8434	26.8881	27.47	27.2924	27.2138	27.8751	1:Experimental evidence at protein level
28.1058	28.0794	28.0849	28.0854	27.6778	27.6785	27.5976	27.7738	1:Experimental evidence at protein level
26.8807	26.853	26.9542	26.928	27.2541	27.1554	27.3856	27.1662	1:Experimental evidence at protein level
28.8973	28.99	29.0936	29.0531	29.2869	29.1661	29.2402	29.1829	1:Experimental evidence at protein level
28.079	27.8844	28.0045	28.0191	28.0134	27.9451	27.911	28.27	1:Experimental evidence at protein level
26.6402	26.5441	26.3401	26.4338	26.616	26.5949	26.5941	27.0236	1:Experimental evidence at protein level
29.6263	29.5855	29.721	29.8394	29.8067	29.791	29.6967	30.0336	1:Experimental evidence at protein level
28.032	28.0827	28.2151	28.0987	28.5172	28.6704	28.5819	28.595	1:Experimental evidence at protein level
26.7581	26.5559	26.8067	26.7067	27.1264	26.7259	26.7743	27.6246	1:Experimental evidence at protein level
28.4363	28.3467	28.4447	28.757	28.4484	28.3068	28.3483	28.6713	1:Experimental evidence at protein level
26.903	27.026	26.6095	26.6277	26.815	26.786	26.4914	27.0325	1:Experimental evidence at protein level
28.7924	28.9378	29.2416	29.0466	28.8567	28.711	28.9469	28.9727	1:Experimental evidence at protein level
29.2216	29.0632	29.1282	29.261	29.2741	29.2071	29.0886	28.8038	1:Experimental evidence at protein level
26.4548	26.5531	26.4014	26.4782	26.2451	25.9627	26.2753	26.5015	1:Experimental evidence at protein level
26.5113	26.554	26.424	26.7112	26.6688	26.7694	26.8098	27.2518	1:Experimental evidence at protein level
26.8276	26.7663	26.9845	26.8883	26.8045	26.7201	26.5384	26.7857	1:Experimental evidence at protein level
28.1917	28.305	28.5246	28.5206	28.162	28.092	28.3292	28.5914	1:Experimental evidence at protein level
27.3392	27.6404	27.825	27.4324	27.1928	27.1539	27.4124	26.7029	1:Experimental evidence at protein level
27.679	27.6402	27.7094	27.6306	27.9323	27.7303	27.7845	27.8256	1:Experimental evidence at protein level
27.1805	27.1671	27.169	27.2628	27.3426	27.3818	27.2393	27.4315	1:Experimental evidence at protein level
26.8245	26.8268	26.7645	26.9266	26.5497	26.4451	26.4346	26.6017	1:Experimental evidence at protein level
26.9968	26.9734	27.163	27.2672	27.2891	27.299	27.4102	27.4863	1:Experimental evidence at protein level
27.0152	26.9945	26.9701	27.1049	27.1848	27.0427	26.9467	27.4575	1:Experimental evidence at protein level
28.6441	28.7658	29.0245	29.0279	28.885	28.8424	28.928	28.7373	1:Experimental evidence at protein level
30.7156	30.5714	30.4499	30.4147	30.3561	30.2859	30.3215	30.5295	1:Experimental evidence at protein level
28.9476	29.0043	28.9191	29.0409	29.2769	29.1996	29.0859	29.3267	1:Experimental evidence at protein level
30.0155	30.0983	30.2821	30.0742	29.7936	29.8606	29.7616	29.2447	1:Experimental evidence at protein level
26.3985	26.3709	26.4979	26.5933	25.9809	25.8251	25.8391	26.4048	1:Experimental evidence at protein level
28.1955	28.3644	28.6217	28.5664	28.5372	28.7035	28.6722	28.6175	1:Experimental evidence at protein level
30.918	30.7423	30.5289	30.601	31.1126	31.1427	30.9887	30.2184	1:Experimental evidence at protein level
28.2345	28.2037	28.3993	28.0768	28.3521	28.4015	28.2657	28.2067	1:Experimental evidence at protein level
29.4781	29.6412	29.8195	29.6696	28.9339	28.9336	29.1508	28.8794	1:Experimental evidence at protein level
27.384	27.5716	27.6245	27.5826	27.453	27.2962	27.4312	27.7351	1:Experimental evidence at protein level
30.3938	30.3127	30.0445	29.7304	30.9294	30.9641	30.9169	31.176	1:Experimental evidence at protein level
32.215	32.2047	32.176	31.9466	32.136	32.0522	32.1405	32.0546	1:Experimental evidence at protein level
28.561	28.7021	28.7263	28.8114	28.4017	28.2477	28.4096	28.8086	1:Experimental evidence at protein level
27.1263	27.3561	27.4512	27.5054	27.1777	27.2122	27.3385	27.7622	1:Experimental evidence at protein level
28.0757	28.0774	28.0463	28.2432	28.2325	27.9982	28.1632	28.4456	1:Experimental evidence at protein level
#NUM!	#NUM!	#NUM!	#NUM!	25.1551	25.8281	24.925	26.0191	1:Experimental evidence at protein level
25.5019	25.7819	25.74	25.7432	25.4832	25.6052	25.654	25.9433	1:Experimental evidence at protein level
28.4841	28.3808	28.2969	28.5437	28.7402	28.7742	28.6258	28.9447	1:Experimental evidence at protein level
29.2607	29.2402	29.4106	29.2159	29.3867	29.312	29.3074	28.8892	1:Experimental evidence at protein level
29.4185	29.6436	29.837	29.6561	29.2301	29.2941	29.4393	29.4259	1:Experimental evidence at protein level
28.5261	28.6567	28.7212	28.6527	28.3243	28.3474	28.3219	28.0762	1:Experimental evidence at protein level
31.4953	31.4883	31.7739	31.7591	30.7283	30.4523	30.8445	30.572	1:Experimental evidence at protein level
29.9289	29.6508	29.4633	29.5609	30.021	29.9447	29.8513	30.1676	1:Experimental evidence at protein level
28.94	28.9713	29.2389	29.084	28.5637	28.5814	28.531	28.2989	1:Experimental evidence at protein level
26.4363	26.2906	25.8105	25.9393	25.9515	26.05	25.6534	26.3328	1:Experimental evidence at protein level
27.5164	27.5047	27.0715	27.2954	27.5758	27.6579	27.4967	27.8917	1:Experimental evidence at protein level
28.8271	28.8003	29.0706	29.0209	28.8522	28.9995	28.9375	28.6764	1:Experimental evidence at protein level
28.7145	28.7379	28.9835	28.8499	28.6558	28.7281	28.6118	28.2204	1:Experimental evidence at protein level
27.008	27.3462	27.292	27.1947	26.2913	26.6669	26.5736	26.5075	1:Experimental evidence at protein level
31.2657	31.1482	30.7274	31.0579	31.399	31.3979	31.4975	31.6769	1:Experimental evidence at protein level
29.4472	29.6364	29.7141	29.4323	29.113	29.1801	29.1701	28.8364	1:Experimental evidence at protein level
31.7191	31.7168	31.2686	31.0691	31.9483	32.0218	31.9095	32.0445	1:Experimental evidence at protein level
28.0327	28.1088	28.0786	28.3334	28.051	27.9287	28.2217	28.6681	1:Experimental evidence at protein level
26.6639	26.9451	26.9979	26.9537	26.2773	26.2038	26.3348	26.4386	1:Experimental evidence at protein level
27.0967	27.2864	27.2351	27.3054	27.1974	27.1391	27.2579	27.0608	1:Experimental evidence at protein level
29.4492	29.5953	29.7861	29.605	29.3779	29.2965	29.4251	29.2564	1:Experimental evidence at protein level
27.2473	27.2699	27.2027	27.558	27.3065	27.2927	27.3675	27.7013	1:Experimental evidence at protein level
27.7609	27.6429	27.7357	27.763	27.7247	27.7014	27.6145	28.0468	1:Experimental evidence at protein level
32.2893	31.9596	31.4534	31.7874	31.5721	31.3185	31.5614	31.7422	1:Experimental evidence at protein level
28.1926	28.2684	28.4052	28.3536	27.9415	27.9868	27.9627	27.9067	1:Experimental evidence at protein level
26.9431	26.9649	27.0838	27.0695	26.9983	26.895	27.0177	27.4747	1:Experimental evidence at protein level
26.8085	26.8229	26.7575	27.1022	27.1214	26.9519	26.9355	27.5144	1:Experimental evidence at protein level
25.9019	25.9756	25.9684	25.8673	26.1935	26.0812	25.8417	26.6484	1:Experimental evidence at protein level
26.4975	26.0033	25.9568	26.1137	26.3721	26.4578	26.4696	27.5129	1:Experimental evidence at protein level
27.0966	26.9788	27.0223	27.2055	27.3906	27.4694	27.4278	27.8981	1:Experimental evidence at protein level
26.613	26.545	26.2406	25.9891	26.5692	27.1423	26.6968	26.0998	1:Experimental evidence at protein level
27.5074	27.4332	27.6277	27.4692	27.4283	27.4667	27.3918	27.4342	1:Experimental evidence at protein level
26.6193	26.649	26.7593	26.8887	26.9612	26.8042	26.8618	27.4722	1:Experimental evidence at protein level
27.9014	27.8718	27.9153	27.9741	27.6095	27.879	27.6473	27.6673	1:Experimental evidence at protein level
26.356	26.4228	26.7442	26.4184	26.3546	26.3963	26.7579	26.2612	1:Experimental evidence at protein level
27.155	27.0523	27.1283	26.8649	26.9076	26.828	26.9028	26.7462	1:Experimental evidence at protein level
27.2952	27.3783	27.4318	27.612	27.07	27.125	27.223	27.7081	1:Experimental evidence at protein level
27.2105	27.2599	27.465	27.4727	27.52	27.6466	27.5818	27.4963	1:Experimental evidence at protein level
27.1776	27.2323	27.1178	27.0408	27.1201	27.3196	27.1104	27.167	1:Experimental evidence at protein level
27.6976	27.4554	27.1149	27.1608	27.3036	27.6232	27.2711	27.4062	1:Experimental evidence at protein level
28.7273	28.805	28.5809	28.4617	28.3135	28.3488	28.368	28.4737	1:Experimental evidence at protein level
28.7621	28.852	29.1616	28.7581	28.5872	28.6306	28.4144	28.6396	1:Experimental evidence at protein level
27.7154	27.7769	28.0441	27.9305	27.3424	27.2217	27.3822	27.7628	1:Experimental evidence at protein level
32.2671	32.3368	32.5397	32.4019	32.467	32.3325	32.4768	32.3973	1:Experimental evidence at protein level
29.4459	29.3845	29.5027	29.5621	28.603	28.4378	28.5263	28.5833	1:Experimental evidence at protein level
27.8045	27.7421	27.9732	27.9848	28.0417	27.7985	27.9929	27.9012	1:Experimental evidence at protein level
27.9716	28.0845	28.1547	27.9247	28.1553	28.2855	28.1085	27.4923	1:Experimental evidence at protein level
30.6505	30.3971	29.9378	29.9281	30.9695	31.1189	30.6862	30.5265	1:Experimental evidence at protein level
28.666	28.6079	28.8269	28.6185	28.7853	28.9049	28.7974	28.7147	1:Experimental evidence at protein level
29.7467	29.7715	29.9221	29.9711	29.864	29.6911	29.8908	29.8136	1:Experimental evidence at protein level
28.55	28.5383	28.6168	28.6585	28.5873	28.4455	28.4839	28.6798	1:Experimental evidence at protein level
27.9594	27.9651	27.9416	28.1484	28.4419	28.2902	28.2496	28.5622	1:Experimental evidence at protein level
26.5113	26.3827	26.4493	26.8166	26.789	26.8559	26.7749	27.1845	1:Experimental evidence at protein level
26.874	26.7718	26.885	26.8702	26.6944	26.7307	26.7631	27.0062	1:Experimental evidence at protein level
28.7797	28.4045	28.5134	28.8408	28.2472	28.4983	27.5184	27.1526	1:Experimental evidence at protein level
27.308	27.2699	27.267	27.1286	27.4294	27.5095	27.4147	27.3421	1:Experimental evidence at protein level
29.9909	30.085	30.3662	30.2337	29.9264	29.9651	29.9223	29.9291	1:Experimental evidence at protein level
27.9986	28.256	28.3918	28.275	28.624	28.5702	28.8272	28.7706	1:Experimental evidence at protein level

31.0815	31.0511	30.7573	30.4646	31.2447	31.3178	31.1999	30.9241	1:Experimental evidence at protein level
30.5614	30.2112	29.7339	30.2651	30.6781	30.8104	30.5614	30.8457	1:Experimental evidence at protein level
30.7323	30.6215	30.2076	30.2713	30.7829	30.8063	30.6899	30.9837	1:Experimental evidence at protein level
29.7997	29.7551	29.3054	29.2841	29.6642	29.8704	29.6256	29.2098	1:Experimental evidence at protein level
30.3066	30.2566	30.3486	30.3033	30.3097	30.3385	30.2993	30.5439	1:Experimental evidence at protein level
25.6978	25.7074	26.2072	26.2828	27.5722	27.3966	27.5147	27.6108	1:Experimental evidence at protein level
26.35	26.1948	25.5004	25.6053	25.0282	26.069	25.6459	#NUM!	1:Experimental evidence at protein level
27.1009	27.0582	27.0819	27.0008	27.0441	27.2636	26.9826	26.8118	1:Experimental evidence at protein level
28.5134	28.4906	28.4685	28.4969	28.3826	28.3331	28.3233	28.2901	1:Experimental evidence at protein level
26.1519	26.0759	26.1891	26.2588	26.4856	26.5328	26.4232	26.5552	1:Experimental evidence at protein level
31.1488	31.1354	31.1917	31.457	30.9109	30.7948	30.9613	31.1513	1:Experimental evidence at protein level
28.9379	28.9047	29.1472	29.0559	28.7845	29.0633	28.8041	27.8498	1:Experimental evidence at protein level
24.9967	24.8096	24.5233	24.6086	25.416	25.4486	25.3914	25.7483	1:Experimental evidence at protein level
26.9404	26.9437	26.9513	26.9529	27.1677	27.0004	27.0325	26.9801	1:Experimental evidence at protein level
26.6608	26.662	26.6609	26.7422	26.8363	26.8392	26.8751	27.113	1:Experimental evidence at protein level
25.7217	25.3253	25.3079	25.5483	25.5979	25.9403	25.8809	26.265	1:Experimental evidence at protein level
25.747	25.9534	26.0978	26.1839	25.8624	26.1408	25.8902	26.2183	1:Experimental evidence at protein level
26.5178	26.3193	26.5776	26.695	26.6402	26.7804	26.5579	26.9131	1:Experimental evidence at protein level
27.1824	27.0847	27.0034	26.9956	27.0757	27.1046	26.8964	27.3008	1:Experimental evidence at protein level
28.4637	28.4064	28.4948	28.5831	28.9914	29.0707	28.9535	28.7026	1:Experimental evidence at protein level
29.3995	29.114	29.073	29.4866	29.2305	29.1311	29.1735	29.6602	1:Experimental evidence at protein level
26.4424	26.5251	26.5725	26.6216	26.7024	26.6117	26.7337	27.0414	1:Experimental evidence at protein level
27.9906	27.8791	28.0156	27.968	27.8908	27.9072	27.9521	27.8066	1:Experimental evidence at protein level
28.9245	28.785	28.9245	28.9345	29.1512	29.0994	28.9049	28.3568	1:Experimental evidence at protein level
26.141	26.2045	26.3756	26.3336	26.375	26.4823	26.5943	26.679	1:Experimental evidence at protein level
27.6948	27.6721	27.7367	27.78	28.2332	28.2217	28.124	28.1246	1:Experimental evidence at protein level
27.3717	27.3272	27.4912	27.4806	27.7557	27.49	27.6902	27.8523	1:Experimental evidence at protein level
27.4902	27.2674	26.9683	27.2405	27.6196	27.6417	27.3381	27.5465	1:Experimental evidence at protein level
27.9497	27.969	27.9899	27.8239	27.9648	27.8829	27.9492	27.4184	1:Experimental evidence at protein level
26.7165	26.7253	26.8866	26.6571	26.5479	26.6496	26.5746	26.4811	1:Experimental evidence at protein level
27.3957	27.1819	27.2791	27.2296	27.2472	26.9647	27.3136	27.2842	1:Experimental evidence at protein level
29.4557	29.4685	29.5565	29.68	29.9625	29.8468	29.9106	30.0588	1:Experimental evidence at protein level
27.5375	27.6202	27.4893	27.6935	27.8616	27.5365	27.7832	28.0458	1:Experimental evidence at protein level
26.713	26.3929	26.3645	26.348	27.0581	26.8522	27.0956	26.3727	1:Experimental evidence at protein level
33.8638	33.4124	31.0865	31.4634	31.2427	31.8847	30.7492	31.2796	1:Experimental evidence at protein level
28.4077	28.2576	28.3471	28.2809	28.3695	28.4207	28.271	28.3736	1:Experimental evidence at protein level
31.9546	31.8854	32.1396	32.3899	31.9921	31.9789	31.9535	31.3792	1:Experimental evidence at protein level
27.9572	27.9274	27.9349	27.9383	28.1093	28.343	28.0336	27.8795	1:Experimental evidence at protein level
27.795	27.7859	27.8488	27.9397	27.8814	27.906	27.8825	27.8546	1:Experimental evidence at protein level
27.5239	27.4982	27.6102	27.5212	27.5175	27.393	27.443	27.6684	1:Experimental evidence at protein level
27.5489	27.6092	27.6537	27.6523	27.8318	27.8872	27.8022	27.8315	1:Experimental evidence at protein level
26.4524	26.5661	26.5503	26.4332	26.8719	26.8962	26.9624	26.9894	1:Experimental evidence at protein level
27.9457	28.0479	28.2094	28.2802	28.0727	27.9287	27.9994	28.346	1:Experimental evidence at protein level
29.157	29.0697	29.0428	28.8115	29.2138	29.2288	28.9776	28.5212	1:Experimental evidence at protein level
30.3796	30.3393	30.5284	30.5256	30.3667	30.4246	30.372	30.6981	1:Experimental evidence at protein level
27.7933	27.7654	27.7277	27.5232	27.6868	27.7378	27.67	27.5511	1:Experimental evidence at protein level
27.68	27.6428	27.6803	27.713	27.5391	27.5674	27.7303	27.5704	1:Experimental evidence at protein level
35.88	35.6085	33.5716	33.913	33.2117	33.9117	32.9805	33.0593	1:Experimental evidence at protein level
29.1314	28.927	26.4904	26.6605	26.1292	27.2685	#NUM!	#NUM!	1:Experimental evidence at protein level
29.0581	28.9992	29.1812	29.0179	29.242	29.3681	29.1371	28.7125	1:Experimental evidence at protein level
28.9811	29.0958	28.8382	29.3385	29.0347	29.2861	29.0227	29.4396	1:Experimental evidence at protein level
29.5715	29.5273	29.6596	29.8682	29.566	29.4555	29.495	29.9119	1:Experimental evidence at protein level
27.7031	27.7702	27.8065	27.678	27.5922	27.5745	27.5257	27.4419	1:Experimental evidence at protein level
30.9484	31.2397	31.3339	31.1969	31.0855	31.0585	31.3004	30.9436	1:Experimental evidence at protein level
27.2154	27.2017	27.4768	27.3701	27.481	27.2864	27.4366	27.7974	1:Experimental evidence at protein level
27.4264	27.7554	28.1394	27.5659	27.51	28.3553	27.6775	26.885	1:Experimental evidence at protein level
29.3012	29.3654	29.5455	29.5263	29.2602	29.2155	29.2523	29.2541	1:Experimental evidence at protein level
29.4445	29.392	29.2197	29.2279	29.4569	29.5562	29.5588	29.4146	1:Experimental evidence at protein level
27.9568	27.8725	28.2319	28.1586	28.1221	27.8662	28.1094	28.378	1:Experimental evidence at protein level
29.0418	29.0891	29.0301	29.0658	29.3069	29.1207	29.275	29.4909	1:Experimental evidence at protein level
30.3813	30.3866	30.5899	30.4727	30.6241	30.4343	30.4493	30.0456	1:Experimental evidence at protein level
26.9019	26.9996	27.0613	27.1049	26.9618	26.9997	27.0374	27.142	1:Experimental evidence at protein level
28.3565	28.5084	28.6422	28.6424	29.113	29.0042	29.1903	29.0833	1:Experimental evidence at protein level
29.7355	29.7547	30.1134	29.9052	29.3578	29.1137	29.2181	29.4611	1:Experimental evidence at protein level
28.0816	28.2543	28.4726	28.31	28.2588	28.5103	28.3581	28.304	1:Experimental evidence at protein level
27.9614	27.7056	27.7706	28.1402	27.7316	27.3817	27.4376	28.0627	1:Experimental evidence at protein level
29.7292	29.4172	27.1272	27.4676	26.7159	27.8366	26.3733	26.7466	1:Experimental evidence at protein level
25.5078	25.3851	25.4656	25.3892	25.8289	25.7115	26.0931	26.3753	1:Experimental evidence at protein level
27.7391	27.611	27.5586	27.5142	27.5188	27.3623	27.5388	27.5166	1:Experimental evidence at protein level
32.2751	32.3476	32.4772	32.5717	26.9714	26.8871	26.9349	27.3459	1:Experimental evidence at protein level
24.8299	24.3508	24.6187	24.8443	#NUM!	#NUM!	#NUM!	#NUM!	1:Experimental evidence at protein level
28.0207	27.9677	28.0056	27.8669	28.1717	28.2493	28.0737	27.5323	1:Experimental evidence at protein level
30.9887	31.0452	31.1201	30.9419	31.0016	31.0349	31.0078	30.2543	1:Experimental evidence at protein level
27.7984	27.7431	27.4225	27.4413	27.9804	27.951	27.8697	28.034	1:Experimental evidence at protein level
27.6203	27.4342	27.5339	27.4897	27.7922	27.6147	27.656	27.6344	1:Experimental evidence at protein level
26.8141	26.9816	27.054	27.0944	26.5598	26.4465	26.4738	26.7785	1:Experimental evidence at protein level
27.7823	27.8222	27.8476	27.8124	27.8303	27.8807	27.7385	27.5882	1:Experimental evidence at protein level
27.5462	27.6527	27.8519	28.1051	27.9945	27.8272	27.8604	28.1079	1:Experimental evidence at protein level
28.8001	28.8462	29.0812	29.0429	28.8157	28.8194	28.9234	28.7922	1:Experimental evidence at protein level
28.037	28.0102	28.1497	28.0275	27.8329	27.9232	27.9161	27.8391	1:Experimental evidence at protein level
27.873	27.9335	28.1783	27.9468	27.928	27.9829	27.9863	28.1477	1:Experimental evidence at protein level
28.3536	28.3191	28.5049	28.4168	28.4051	28.5958	28.3745	28.2065	1:Experimental evidence at protein level
27.0545	27.105	27.0904	27.1229	27.0515	27.1578	27.0363	27.2671	1:Experimental evidence at protein level
30.5857	30.5688	30.8064	30.9107	30.4451	30.1358	30.3912	30.6533	1:Experimental evidence at protein level
29.4444	29.5123	29.6118	29.5439	29.3403	29.3932	29.3661	29.4071	1:Experimental evidence at protein level
26.9301	26.9379	26.9743	27.0354	27.2388	27.1484	27.2359	27.4571	1:Experimental evidence at protein level
25.7832	25.9766	25.0847	24.8803	27.0912	27.0574	26.8923	27.1106	1:Experimental evidence at protein level
27.8984	27.6825	27.8419	27.8071	27.7871	27.9257	27.7498	27.7173	1:Experimental evidence at protein level
27.0935	27.0959	27.0227	27.3318	27.0746	27.1096	27.01	27.4938	1:Experimental evidence at protein level
25.8062	27.2366	26.0393	25.9821	25.7451	25.7392	#NUM!	#NUM!	1:Experimental evidence at protein level
26.5543	26.6177	26.3292	26.2575	26.5702	26.7324	26.4313	25.9482	1:Experimental evidence at protein level
30.2193	30.3254	30.5534	30.4376	30.4724	30.526	30.4349	30.2369	1:Experimental evidence at protein level
29.7558	29.7589	30.0835	30.0256	29.6519	29.6137	29.64	29.7483	1:Experimental evidence at protein level
27.8689	27.7507	27.9182	27.989	27.9658	28.0344	28.0036	28.2365	1:Experimental evidence at protein level
30.6403	30.6725	30.7991	30.7731	29.4714	29.4428	29.3556	29.3361	1:Experimental evidence at protein level
29.5127	29.2844	29.1067	29.0371	29.2247	29.2231	29.0396	29.0376	1:Experimental evidence at protein level
28.9995	29.1506	29.1405	29.0339	29.0984	29.0278	29.1193	29.1059	1:Experimental evidence at protein level
27.5646	27.6053	27.6563	27.6794	27.7432	27.5984	27.8405	28.1632	1:Experimental evidence at protein level
26.1573	26.3192	26.2896	26.3397	26.7264	26.6476	26.5882	27.009	1:Experimental evidence at protein level
28.3787	28.4488	28.5586	28.3915	28.4537	28.4797	28.5395	28.3651	1:Experimental evidence at protein level
28.4719	28.4375	28.3946	28.2244	28.5814	28.7176	28.5814	28.5167	1:Experimental evidence at protein level
30.627	30.4917	30.4387	30.4344	30.4255	30.5266	30.5079	30.6752	1:Experimental evidence at protein level
29.4403	29.6094	29.7314	29.6037	29.5094	29.6355	29.6005	29.3974	1:Experimental evidence at protein level
26.7558	26.9771	27.0916	26.9726	27.2759	27.0439	27.2492	27.3892	1:Experimental evidence at protein level
29.002	28.9466	29.0866	28.8312	29.3031	29.2097	29.1817	28.7961	1:Experimental evidence at protein level
26.9691	27.2131	27.0623	27.3546	27.9345	28.0818	28.053	28.2473	1:Experimental evidence at protein level
26.299	26.4019	26.328	26.4045	26.9905	26.7749	26.7824	27.0468	1:Experimental evidence at protein level
26.5646	26.7218	26.7205	26.5879	26.7594	26.7723	26.6976	26.9512	1:Experimental evidence at protein level
26.7201	27.0205	27.1409	27.0139	27.3218	27.1476	27.3992	27.4906	1:Experimental evidence at protein level
29.619	29.6379	29.8509	29.9038	29.7944	29.7921	29.8949	29.7314	1:Experimental evidence at protein level
28.0411	28.0636	27.9198	27.9176	27.6916	27.967	27.7682	27.9321	1:Experimental evidence at protein level
28.0643	28.0467	27.8801	27.8995	28.7259	28.7302	28.6518	29.0337	1:Experimental evidence at protein level
29.4667	29.3902	28.7785	28.6445	29.1494	29.338	29.043	29.0755	1:Experimental evidence at protein level
28.6405	28.9937	29.0948	28.8169	29.8253	29.7936	29.7847	29.1507	1:Experimental evidence at protein level
27.7698	27.8217	27.6619	27.8454	27.8192	27.8628	27.8399	28.1045	1:Experimental evidence at protein level
26.0192	25.9668	25.903	26.0582	25.983	26.2683	25.939	26.3984	1:Experimental evidence at protein level
26.6172	26.6678	26.8356	26.6426	26.7629	26.5279	26.6427	26.8014	1:Experimental evidence at protein level
26.127	26.4939	25.9408	26.1871	25.613	25.6638	25.9095	25.3016	1:Experimental evidence at protein level
28.8797	28.7225	29.0021	28.9728	28.7716	28.5949	28.659	29.055	1:Experimental evidence at protein level
26.9636	27.1306	27.2552	27.3837	26.7318	26.8465	27.0304	27.1504	1:Experimental evidence at protein level
25.9529	25.9559	26.0926	25.9565	26.4131	26.6167	26.3774	26.272	1:Experimental evidence at protein level
33.0059	32.7784	31.6249	31.9523	32.2239	32.4506	32.185	32.328	1:Experimental evidence at protein level
26.9414	26.9214	27.3133	27.2884	27.2634	27.2249	27.3173	27.4766	1:Experimental evidence at protein level
28.407	28.3607	28.3906	28.4004	28.1725	28.1789	28.2527	28.3718	1:Experimental evidence at protein level
31.7798	31.8296	31.9667	32.0681	31.9685	31.7491	32.0111	31.7446	1:Experimental evidence at protein level
28.568	28.5319	28.5603	28.5381	29.1117	#NUM!	28.8629	29.2904	1:Experimental evidence at protein level
27.7704	27.4678	27.8145	27.8804	27.6754	27.753	27.5135	28.014	1:Experimental evidence at protein level
27.064	27.1761	27.273	27.1404	27.512	27.5572	27.5643	27.2173	1:Experimental evidence at protein level
27.2781	27.1944	27.058	27.1069	27.2302	27.2192	27.1179	27.2526	1:Experimental evidence at protein level

31.2687	31.0724	30.6251	30.8311	31.5385	31.6543	31.4158	31.676	1:Experimental evidence at protein level
28.965	29.0903	29.4148	29.4157	29.2761	29.2119	29.3971	29.4846	1:Experimental evidence at protein level
26.7809	26.9564	26.8766	26.9054	27.3765	27.5837	27.5348	27.5214	1:Experimental evidence at protein level
31.8348	31.5977	31.3933	31.4122	31.8255	31.7119	31.6488	31.9377	1:Experimental evidence at protein level
26.1741	26.2542	26.1134	26.4579	26.4429	26.6007	26.3324	27.1646	1:Experimental evidence at protein level
29.0875	29.1745	29.1349	28.9775	29.4351	29.3072	29.2813	29.5658	1:Experimental evidence at protein level
28.6722	28.4979	28.4828	28.3797	28.3367	28.4353	28.2966	28.0609	1:Experimental evidence at protein level
28.2405	28.4323	28.4951	28.2046	28.4782	28.4067	28.5221	28.249	1:Experimental evidence at protein level
27.9466	27.7988	27.8665	28.001	28.0239	27.948	27.9799	28.3112	1:Experimental evidence at protein level
29.1187	29.0359	29.0884	29.3478	30.4284	30.2941	30.3714	30.6679	1:Experimental evidence at protein level
29.9856	30.1461	30.3729	30.1782	29.0411	29.1803	29.0785	28.8752	1:Experimental evidence at protein level
26.5107	26.4176	26.2497	26.3721	27.4396	27.3245	27.3841	27.6644	1:Experimental evidence at protein level
27.0185	27.0345	27.0938	27.0735	26.8711	26.9557	26.8225	26.8076	1:Experimental evidence at protein level
27.3089	27.4757	27.5174	27.4774	27.8258	27.8998	27.9113	28.1048	1:Experimental evidence at protein level
29.0411	29.1206	29.0294	29.1186	29.1098	28.9783	29.0373	29.1434	1:Experimental evidence at protein level
27.3095	27.2583	27.1034	27.2769	28.4509	28.3703	28.3241	28.6823	1:Experimental evidence at protein level
28.0513	27.9575	27.8727	28.0699	27.8266	27.6708	27.7641	28.2039	1:Experimental evidence at protein level
27.5901	27.5877	27.452	27.5413	27.3821	27.6046	27.3776	27.7686	1:Experimental evidence at protein level
26.9768	27.0106	27.1909	27.2768	27.2	27.2358	27.2717	27.3347	1:Experimental evidence at protein level
29.0565	28.9805	29.057	29.1397	29.0577	28.9455	29.0472	29.1406	1:Experimental evidence at protein level
28.7104	28.7303	28.7452	28.7059	28.7384	28.7428	28.7305	29.0795	1:Experimental evidence at protein level
28.12	28.0854	28.1207	28.0584	28.066	28.1256	27.979	28.0724	1:Experimental evidence at protein level
24.8569	24.8866	24.6745	24.7682	24.716	24.9635	24.442	25.3165	1:Experimental evidence at protein level
27.5642	27.7257	27.5446	27.6169	27.895	27.959	27.9346	28.2665	1:Experimental evidence at protein level
26.4456	26.4668	26.6294	26.5995	26.1962	25.9944	26.3664	26.6408	1:Experimental evidence at protein level
27.5334	27.7146	27.7529	27.4291	27.6575	27.6063	27.6259	27.638	1:Experimental evidence at protein level
28.3125	28.4823	28.6417	28.7405	28.3033	28.3046	28.695	28.8299	1:Experimental evidence at protein level
26.0341	25.9707	25.6223	25.2092	26.1123	26.3423	26.0741	26.5086	1:Experimental evidence at protein level
30.4701	30.5968	30.8429	30.7776	30.1796	30.1797	30.3859	30.4735	1:Experimental evidence at protein level
30.383	30.4315	30.633	30.6377	30.2212	30.0437	30.1982	30.1313	1:Experimental evidence at protein level
26.1664	26.1642	25.6926	25.7592	28.2892	28.2162	28.3458	28.1568	1:Experimental evidence at protein level
31.3553	31.4623	31.5734	31.4141	31.0904	31.1993	31.1472	31.0839	1:Experimental evidence at protein level
29.7046	29.7748	29.7472	29.7024	29.7033	29.5402	29.6484	29.8226	1:Experimental evidence at protein level
24.294	24.6536	24.6155	24.5375	25.3983	25.4611	25.0723	#NUM!	1:Experimental evidence at protein level
30.2435	30.2451	30.2265	30.3812	30.4414	30.3398	30.3685	30.3409	1:Experimental evidence at protein level
29.2147	29.1707	29.1783	29.2394	29.013	28.953	29.0418	28.7677	1:Experimental evidence at protein level
29.3616	29.4284	29.5551	29.3855	29.9225	29.7838	29.7304	29.993	1:Experimental evidence at protein level
27.2964	27.2775	27.3115	27.2967	27.1588	27.241	27.0975	27.3207	1:Experimental evidence at protein level
30.0086	30.1336	30.1977	30.0827	29.9707	29.7863	29.8839	29.561	1:Experimental evidence at protein level
29.1764	29.1993	29.3785	29.1797	29.002	28.9352	29.0385	28.9976	1:Experimental evidence at protein level
30.5965	30.6475	30.9948	30.6715	30.5418	30.6019	30.7017	30.3367	1:Experimental evidence at protein level
31.644	31.5374	31.2871	31.2658	31.6676	31.7798	31.5546	31.3578	1:Experimental evidence at protein level
31.0383	30.9582	30.9784	30.6237	30.9612	31.132	30.82	30.4627	1:Experimental evidence at protein level
28.9229	29.0931	29.2338	29.0845	29.1591	29.1904	29.2952	29.4065	1:Experimental evidence at protein level
28.0854	27.9742	28.0279	28.0301	27.9199	27.8804	27.8256	28.0949	1:Experimental evidence at protein level
29.0188	29.1095	29.2446	29.0357	29.4594	29.3247	29.3628	29.356	1:Experimental evidence at protein level
26.4924	26.6735	25.6359	25.7672	26.6457	26.9856	26.3646	27.0585	1:Experimental evidence at protein level
27.7982	27.7553	27.8893	27.8761	27.6261	27.5855	27.4822	28.1552	1:Experimental evidence at protein level
26.9301	27.0527	27.297	27.1272	26.7431	26.9339	26.8136	27.0711	1:Experimental evidence at protein level
29.4073	29.496	29.5208	29.6537	29.435	29.1894	29.6951	29.6321	1:Experimental evidence at protein level
26.2627	26.1948	26.3608	26.4079	26.1396	26.1595	26.1459	26.5526	1:Experimental evidence at protein level
25.9782	26.2136	26.1834	25.8741	25.8823	25.8684	25.9998	25.8444	1:Experimental evidence at protein level
27.1634	27.1608	27.0658	27.0519	27.17	27.1666	27.1647	27.2087	1:Experimental evidence at protein level
27.7211	27.7198	27.7176	27.7813	27.6961	27.5713	27.6598	28.0386	1:Experimental evidence at protein level
27.1343	27.0752	27.0286	27.2159	27.6047	27.3778	27.4907	27.9211	1:Experimental evidence at protein level
25.7326	25.711	25.8195	26.1041	25.8227	25.875	25.935	26.8447	1:Experimental evidence at protein level
28.5587	28.5364	28.6163	28.3684	28.4036	28.4041	28.3605	27.9269	1:Experimental evidence at protein level
27.1	26.9971	27.1403	27.017	26.8063	26.7705	26.8741	27.0837	1:Experimental evidence at protein level
28.7185	28.7839	28.8387	28.8074	29.0057	28.9648	28.9437	28.801	1:Experimental evidence at protein level
26.9966	27.0437	26.8686	27.1325	26.4503	26.557	26.5703	26.9996	1:Experimental evidence at protein level
27.2644	26.9974	27.0476	27.3755	27.2775	27.2255	27.1078	27.3481	1:Experimental evidence at protein level
27.4001	27.3898	27.4871	27.4208	27.3386	27.3699	27.3318	27.3817	1:Experimental evidence at protein level
26.8367	27.0004	27.1125	27.0692	26.7284	26.5669	26.7446	26.8368	1:Experimental evidence at protein level
27.5306	27.5421	27.5945	27.5038	27.4837	27.4908	27.5165	27.7466	1:Experimental evidence at protein level
28.1733	28.2698	28.2656	28.2964	28.57	28.5145	28.5899	28.7101	1:Experimental evidence at protein level
28.264	28.1821	28.2515	28.3201	27.9617	27.7861	27.8622	28.1449	1:Experimental evidence at protein level
27.3732	27.3526	27.3894	27.4786	27.5148	27.647	27.5351	27.6426	1:Experimental evidence at protein level
28.7377	28.6906	28.8277	28.7079	28.923	28.9748	29.0604	28.6927	1:Experimental evidence at protein level
27.9881	28.0965	28.1944	28.1743	27.6936	27.6666	27.362	27.5852	1:Experimental evidence at protein level
26.2955	26.339	25.3054	25.2312	25.1962	25.6127	25.3174	25.7797	1:Experimental evidence at protein level
27.4209	27.5194	27.5562	27.7038	27.4197	27.1855	27.2285	27.6141	1:Experimental evidence at protein level
27.2417	27.3653	27.2115	27.591	27.2786	27.1784	27.3049	27.7002	1:Experimental evidence at protein level
28.801	28.7038	28.851	28.8546	28.8257	28.7973	28.8885	29.0257	1:Experimental evidence at protein level
27.0328	26.8989	27.1174	26.9811	27.565	27.5403	27.4685	27.5671	1:Experimental evidence at protein level
27.5972	27.5836	27.7607	27.7667	27.6576	27.7846	27.6897	27.5945	1:Experimental evidence at protein level
30.5619	30.4789	30.0794	30.2741	30.8383	30.7771	30.7088	31.0341	1:Experimental evidence at protein level
29.6093	29.5049	29.7015	29.6067	29.6592	29.5688	29.6368	29.151	1:Experimental evidence at protein level
28.3058	28.2259	28.281	28.4081	28.3846	28.2421	28.3479	28.4681	1:Experimental evidence at protein level
27.5099	27.4665	27.1968	27.319	27.1815	27.1802	27.2213	27.7547	1:Experimental evidence at protein level
28.5409	28.5569	28.6158	28.6424	28.5451	28.5033	28.6051	28.6732	1:Experimental evidence at protein level
32.5264	32.5542	32.6843	32.6018	32.5933	32.607	32.6155	32.6385	1:Experimental evidence at protein level
31.4607	31.2006	30.796	30.983	31.6075	31.5917	31.5568	31.7382	1:Experimental evidence at protein level
34.0981	34.3868	34.4763	34.3654	34.0456	33.8793	34.0969	34.0233	1:Experimental evidence at protein level
26.5977	26.775	26.7064	26.7096	26.6715	26.6969	26.8835	26.9803	1:Experimental evidence at protein level
29.4199	29.4004	29.2908	29.6066	29.1166	28.9791	29.0877	29.3625	1:Experimental evidence at protein level
28.2654	28.1954	28.1812	27.657	28.1499	28.133	28.06	27.1519	1:Experimental evidence at protein level
25.8353	25.7097	25.6398	25.5897	25.721	25.5916	25.5955	26.2629	1:Experimental evidence at protein level
27.1662	27.4134	27.2556	27.3317	27.4918	27.4764	27.5737	27.644	1:Experimental evidence at protein level
29.3234	29.2756	29.2662	29.4389	29.3992	29.2074	29.2306	29.2995	1:Experimental evidence at protein level
27.6682	27.4848	27.6812	27.6918	27.3548	27.3335	27.355	27.8994	1:Experimental evidence at protein level
29.1512	29.2074	29.4115	29.2157	26.8637	26.9388	26.8328	26.766	1:Experimental evidence at protein level
27.324	27.2816	27.4327	27.3635	27.3679	27.1984	27.4338	27.5823	1:Experimental evidence at protein level
27.9042	27.8722	27.8959	27.876	27.9796	28.0907	27.8696	28.08	1:Experimental evidence at protein level
27.319	27.1696	27.3081	27.371	27.0602	26.8775	26.9915	27.2857	1:Experimental evidence at protein level
29.3782	29.2513	29.2065	29.2331	29.2181	29.1732	29.1759	29.3114	1:Experimental evidence at protein level
26.8233	26.6215	26.7751	26.695	26.8487	26.677	26.8558	27.0984	1:Experimental evidence at protein level
27.2906	27.2401	27.349	27.3618	27.4334	27.2626	27.2561	27.3618	1:Experimental evidence at protein level
28.3684	28.3755	28.3746	28.2913	28.5418	28.5769	28.6329	28.6731	1:Experimental evidence at protein level
29.8314	29.955	30.2265	30.3382	28.1306	27.7585	28.1506	28.7293	1:Experimental evidence at protein level
31.1474	31.082	30.9386	30.9648	31.3079	31.169	31.2579	31.4247	1:Experimental evidence at protein level
27.9519	27.8658	27.982	27.9893	28.3264	28.2288	28.345	28.4381	1:Experimental evidence at protein level
29.107	29.1797	29.5316	29.5045	29.1023	28.8525	29.2613	29.4097	1:Experimental evidence at protein level
26.8473	26.771	26.8968	26.7632	26.8568	26.7885	26.8864	26.6334	1:Experimental evidence at protein level
26.2602	26.106	25.9944	26.0067	26.1615	26.4709	26.0282	26.0952	1:Experimental evidence at protein level
29.5082	29.6101	29.6731	29.4998	29.3793	29.2951	29.4287	29.1418	1:Experimental evidence at protein level
27.2015	27.3447	27.4015	27.3234	27.1713	27.3084	27.132	27.2521	1:Experimental evidence at protein level
27.3606	27.3053	27.2707	27.21	27.3412	27.2967	27.286	27.1975	1:Experimental evidence at protein level
23.832	#NUM!	#NUM!	#NUM!	27.3885	27.2335	27.0997	27.4077	1:Experimental evidence at protein level
26.3943	26.4972	26.58	26.3318	26.1675	26.5298	26.348	26.1613	1:Experimental evidence at protein level
28.108	28.0935	28.1797	28.2334	28.0042	27.9107	27.9353	27.7559	1:Experimental evidence at protein level
27.4291	27.5111	27.6252	27.4515	28.3749	28.3188	28.4198	28.7222	1:Experimental evidence at protein level
27.3952	27.463	27.4633	27.429	27.6509	27.6059	27.543	27.8584	1:Experimental evidence at protein level
28.5519	28.4597	28.5042	28.6517	28.5816	28.2788	28.4669	28.8862	1:Experimental evidence at protein level
30.6261	30.7064	30.6285	30.6109	30.7997	30.6759	30.8263	30.5944	1:Experimental evidence at protein level
28.2327	28.2111	28.1742	28.1209	28.1502	28.2236	28.138	27.9513	1:Experimental evidence at protein level
27.5764	27.6326	27.2301	27.5514	27.5808	27.6584	27.6616	27.8883	1:Experimental evidence at protein level
27.1857	27.044	26.8809	27.0342	26.6973	26.6148	26.6455	27.0734	1:Experimental evidence at protein level
26.8702	26.7141	26.8781	26.9267	26.6362	26.6545	26.6558	26.9489	1:Experimental evidence at protein level
28.1405	28.0318	28.3376	28.2264	28.2316	28.2463	28.0166	28.1085	1:Experimental evidence at protein level
28.509	28.4357	28.4327	28.5598	28.0159	27.8041	27.9621	28.1789	1:Experimental evidence at protein level
29.228	29.3335	29.407	29.2944	28.8045	28.7874	28.9028	28.907	1:Experimental evidence at protein level
32.1513	32.232	32.2416	32.382	32.3106	32.3163	32.3666	32.2093	1:Experimental evidence at protein level
30.6995	30.7818	30.9842	30.8062	31.1841	31.0848	31.1499	30.9349	1:Experimental evidence at protein level
28.7386	28.8326	28.9487	28.7935	26.6398	26.4885	26.6548	26.4362	1:Experimental evidence at protein level
27.6613	27.569	27.1143	27.2248	27.8284	28.2119	27.6002	27.6006	1:Experimental evidence at protein level
30.2519	30.2337	30.4312	30.36	30.9394	30.8365	30.8372	31.0331	1:Experimental evidence at protein level
28.7424	28.7243	28.6008	28.8615	28.2543	28.4702	28.412	28.6583	1:Experimental evidence at protein level
27.638	27.8419	27.8488	27.7542	27.7104	27.8734	27.8156	28.0876	1:Experimental evidence at protein level
30.9908	30.9533	31.0764	31.0524	31.2174	31.1188	31.2684	31.164	1:Experimental evidence at protein level

30.0139	30.0486	30.2522	30.2737	29.9857	30.0712	30.0153	30.004	1:Experimental evidence at protein level
29.4312	29.486	29.4858	29.2446	29.3463	29.4609	29.3205	29.2924	1:Experimental evidence at protein level
28.5843	28.5363	28.6208	28.6809	28.6526	28.6453	28.6327	28.6039	1:Experimental evidence at protein level
27.9463	27.9365	28.0173	28.0733	28.5497	28.5292	28.5505	28.8835	1:Experimental evidence at protein level
25.5073	25.5589	25.6196	25.7054	25.3491	25.4133	25.375	25.6285	1:Experimental evidence at protein level
28.3014	28.3823	28.4355	28.5976	28.5413	28.4521	28.5872	28.782	1:Experimental evidence at protein level
31.0346	31.0189	30.697	30.8209	31.3421	31.4062	31.3755	31.5994	1:Experimental evidence at protein level
31.2566	31.1689	30.8145	30.8869	31.5979	31.5692	31.5435	31.6816	1:Experimental evidence at protein level
28.1407	28.1097	28.3444	28.2507	27.8826	27.8338	28.0051	28.1454	1:Experimental evidence at protein level
29.1245	29.1255	29.1991	29.1452	27.4721	27.5929	27.5272	27.4967	1:Experimental evidence at protein level
27.96	27.9295	27.9892	28.0481	27.7767	27.6961	27.768	27.7905	1:Experimental evidence at protein level
28.2541	28.331	28.4381	28.2555	28.4421	28.3163	28.3291	27.8354	1:Experimental evidence at protein level
27.2512	27.2374	27.3214	27.2737	27.2945	27.2681	27.3106	27.5559	1:Experimental evidence at protein level
27.3659	27.4178	27.6805	27.5743	26.8954	26.8109	27.1582	27.7169	1:Experimental evidence at protein level
27.7712	27.9326	27.9992	27.8165	27.8721	27.656	27.7631	28.0311	1:Experimental evidence at protein level
26.0594	26.1669	26.4071	26.3833	25.3599	24.9963	25.41	26.1601	1:Experimental evidence at protein level
27.8727	27.8641	27.9549	27.96	27.662	27.7616	27.8017	27.9545	1:Experimental evidence at protein level
28.9321	29.1504	29.2269	29.0584	29.416	29.4467	29.4234	28.6456	1:Experimental evidence at protein level
26.7104	26.7392	26.7396	26.8037	26.7893	26.8355	26.8119	27.0768	1:Experimental evidence at protein level
27.3149	27.1602	27.0431	27.0885	26.8552	26.6385	26.8314	27.3716	1:Experimental evidence at protein level
27.1101	27.1393	26.8685	27.2436	27.1228	27.057	27.0796	27.6788	1:Experimental evidence at protein level
28.0305	28.1338	28.266	28.2057	28.1833	28.1675	28.0671	28.1495	1:Experimental evidence at protein level
25.2823	25.3314	25.3076	25.4996	25.478	25.0938	25.3757	26.3864	1:Experimental evidence at protein level
27.1149	27.0933	27.2406	27.3188	27.3781	27.3037	27.2617	27.488	1:Experimental evidence at protein level
29.0043	29.0137	29.1041	29.0081	29.0129	28.9139	28.9373	28.9662	1:Experimental evidence at protein level
27.4956	27.5225	27.7284	27.6727	27.8048	27.689	27.7878	28.0045	1:Experimental evidence at protein level
33.351	33.138	32.3895	32.7294	32.8459	33.0828	32.9051	33.0773	1:Experimental evidence at protein level
27.5115	27.3915	27.3672	27.4479	28.0359	27.8774	27.7669	28.0486	1:Experimental evidence at protein level
27.7832	27.692	27.7844	27.586	28.438	28.3909	28.2743	28.2718	1:Experimental evidence at protein level
27.548	27.5139	27.5908	27.5428	27.2699	27.2789	27.2136	27.1965	1:Experimental evidence at protein level
30.1093	30.0039	29.7677	29.9585	30.2584	30.2456	30.2394	30.3117	1:Experimental evidence at protein level
27.2305	27.2605	27.4215	27.0678	27.286	27.3426	27.2043	27.4457	1:Experimental evidence at protein level
29.3425	29.3605	29.4605	29.412	29.355	29.3023	29.3187	29.4191	1:Experimental evidence at protein level
26.8618	26.8799	26.8821	26.8741	26.8791	26.8792	26.7889	26.8368	1:Experimental evidence at protein level
30.4165	30.3099	30.6082	30.5419	30.4657	30.4728	30.4567	30.6906	1:Experimental evidence at protein level
30.8846	30.6944	30.6359	30.7061	31.0877	30.9326	30.8841	31.3144	1:Experimental evidence at protein level
29.6663	29.7176	29.8573	29.8329	29.8425	29.8009	29.8578	29.937	1:Experimental evidence at protein level
26.8598	26.7822	26.9239	26.7952	26.6883	26.6715	26.7647	26.8611	1:Experimental evidence at protein level
28.3974	28.1259	28.4681	28.4926	27.9464	27.8746	27.833	28.3668	1:Experimental evidence at protein level
27.9067	27.9485	27.9955	28.1167	27.9465	27.8663	27.8655	28.3649	1:Experimental evidence at protein level
31.1012	31.0062	30.836	30.6582	31.1449	31.1286	31.1859	31.2354	1:Experimental evidence at protein level
30.2189	29.9538	29.7098	29.8404	30.4472	30.4667	30.2059	30.4607	1:Experimental evidence at protein level
27.1413	27.0065	27.1013	27.179	27.5316	27.397	27.385	27.5458	1:Experimental evidence at protein level
26.5163	26.4306	26.4081	26.4621	26.4568	26.4659	26.3494	26.8738	1:Experimental evidence at protein level
27.0267	26.8483	26.6122	26.6557	26.2958	26.4214	26.6264	26.6685	1:Experimental evidence at protein level
27.5332	27.5632	27.4967	27.484	27.6209	27.539	27.4703	27.478	1:Experimental evidence at protein level
28.7577	28.7734	28.7982	28.7404	28.6756	28.7945	28.7317	28.6244	1:Experimental evidence at protein level
29.9817	30.1001	30.2646	30.3082	29.4745	29.2721	29.6314	29.6736	1:Experimental evidence at protein level
29.8806	29.8742	29.9999	29.7387	30.1218	30.0808	29.9551	29.6045	1:Experimental evidence at protein level
25.4232	24.7903	24.9252	25.1594	25.3442	25.6454	24.9058	25.6667	1:Experimental evidence at protein level
26.8709	26.5702	26.5382	26.7586	27.3038	27.288	27.2006	27.4644	1:Experimental evidence at protein level
28.0253	27.9946	27.8405	27.9562	27.5998	27.4418	27.39	27.8308	1:Experimental evidence at protein level
28.9557	29.0604	29.0778	29.1568	28.9791	28.9916	29.096	29.1445	1:Experimental evidence at protein level
27.4703	27.4717	27.4403	27.417	26.5449	26.69	26.5496	26.8987	1:Experimental evidence at protein level
28.6415	28.8244	28.9897	28.7528	27.0376	27.0004	27.0549	27.2647	1:Experimental evidence at protein level
26.6529	26.7693	26.9441	26.9975	27.2112	27.205	27.2362	27.0903	1:Experimental evidence at protein level
28.8269	28.733	28.7809	28.8528	28.8563	28.6976	28.8845	28.8967	1:Experimental evidence at protein level
27.9036	27.8162	27.8261	28.1082	28.1805	28.414	28.0565	28.0428	1:Experimental evidence at protein level
33.01	32.592	31.6223	32.6601	32.39	32.5613	32.2751	32.6974	1:Experimental evidence at protein level
30.4932	30.3335	29.9574	30.123	30.5689	30.604	30.5582	30.5042	1:Experimental evidence at protein level
29.8458	29.9268	30.0236	29.8678	29.625	29.6959	29.593	29.694	1:Experimental evidence at protein level
31.9946	32.0708	32.3156	32.1471	31.8791	31.8966	31.8479	30.9519	1:Experimental evidence at protein level
30.7204	30.7243	30.7991	30.7146	30.9675	30.859	30.7388	30.0572	1:Experimental evidence at protein level
29.4894	29.39	29.4278	29.5317	29.6665	29.5034	29.5199	29.6785	1:Experimental evidence at protein level
28.1876	28.2697	28.3357	28.385	28.254	28.2267	28.2849	28.2324	1:Experimental evidence at protein level
29.9286	29.8259	29.5568	29.7158	30.1767	30.0279	29.9868	30.2706	1:Experimental evidence at protein level
28.5209	28.4872	28.5705	28.4768	28.3446	28.4123	28.3152	28.0922	1:Experimental evidence at protein level
28.1347	28.2446	28.2309	28.0948	28.244	28.3563	28.2701	27.9404	1:Experimental evidence at protein level
30.9602	31.9409	31.9309	31.8883	30.2987	30.2527	30.6121	30.1451	1:Experimental evidence at protein level
27.8601	27.9287	27.9552	28.0864	27.8528	27.7674	27.8411	28.2341	1:Experimental evidence at protein level
30.8408	30.783	30.5598	30.5407	30.9519	30.98	30.9632	31.065	1:Experimental evidence at protein level
31.6211	31.6187	31.3645	31.4382	31.6921	31.6412	31.6773	31.9241	1:Experimental evidence at protein level
31.1499	30.9463	30.7385	30.8747	31.2376	31.0883	31.1019	31.3762	1:Experimental evidence at protein level
28.4366	28.4796	28.3898	28.4282	28.4914	28.5923	28.4317	28.3826	1:Experimental evidence at protein level
29.945	29.8554	30.0061	30.2358	30.3059	30.4176	30.3926	30.3646	1:Experimental evidence at protein level
33.6158	33.6474	33.8669	33.7583	33.6789	33.4103	33.7094	33.47	1:Experimental evidence at protein level
29.7621	29.7965	29.6617	29.5304	29.6678	29.8308	29.8053	29.3407	1:Experimental evidence at protein level
27.1559	27.1657	27.2075	27.0249	27.2474	27.455	27.3434	27.2272	1:Experimental evidence at protein level
26.7964	26.8607	26.8846	26.8528	27.4206	27.3215	27.3995	27.3397	1:Experimental evidence at protein level
26.7738	26.9332	26.6257	26.7764	26.7729	26.6633	26.714	27.4967	1:Experimental evidence at protein level
26.5382	26.7093	26.6298	26.7754	26.614	26.5179	26.7556	26.9966	1:Experimental evidence at protein level
28.4818	28.5726	28.7284	28.6642	28.911	28.5689	28.8908	28.8814	1:Experimental evidence at protein level
27.4667	27.5124	27.2343	27.3856	27.5547	27.6018	27.525	27.8165	1:Experimental evidence at protein level
28.6636	28.7565	28.7862	28.6474	28.6449	28.6042	28.6473	28.5126	1:Experimental evidence at protein level
28.1929	28.1256	28.0999	28.3037	28.3032	28.0962	28.2364	28.6106	1:Experimental evidence at protein level
27.626	27.7346	27.6219	27.6274	27.845	27.9444	27.8641	28.0803	1:Experimental evidence at protein level
29.2467	29.1153	29.2133	29.2687	29.1546	29.0293	29.1063	29.134	1:Experimental evidence at protein level
29.4326	29.336	29.6892	29.7568	29.1192	28.9434	29.1862	28.7761	1:Experimental evidence at protein level
27.2304	27.1906	27.2883	27.1733	27.0024	27.0047	27.0306	26.6409	1:Experimental evidence at protein level
27.9977	28.0223	27.915	27.8814	27.9241	27.8477	27.9102	28.0289	1:Experimental evidence at protein level
26.6585	26.5828	26.599	26.7931	27.1432	27.0311	27.046	27.4471	1:Experimental evidence at protein level
30.5602	30.5331	30.428	30.2692	30.6168	30.592	30.5634	29.7115	1:Experimental evidence at protein level
29.5438	29.5973	29.8639	29.8854	29.9632	30.0578	30.2113	29.7163	1:Experimental evidence at protein level
28.8173	28.8665	28.5307	28.5379	28.6388	28.8469	28.7021	28.8323	1:Experimental evidence at protein level
24.6728	24.6672	#NUM!	#NUM!	28.366	28.2846	28.2468	28.5457	1:Experimental evidence at protein level
30.5442	30.3429	30.0983	29.8082	29.8977	30.0486	29.7663	29.6939	1:Experimental evidence at protein level
31.1918	31.0452	30.8917	30.8483	31.0119	31.0454	30.8438	30.5356	1:Experimental evidence at protein level
29.5456	29.6344	29.7988	29.6683	29.9383	29.9206	29.8996	29.7036	1:Experimental evidence at protein level
31.1636	31.0367	30.7006	30.6445	31.3129	31.3405	31.2927	31.6344	1:Experimental evidence at protein level
26.9256	26.9366	26.8329	26.9614	26.5494	26.5594	26.8084	27.1876	1:Experimental evidence at protein level
26.8238	26.1937	26.3984	25.8729	26.3391	26.2276	26.3873	26.5959	1:Experimental evidence at protein level
28.8721	28.7749	29.0089	29.072	28.7552	28.6151	28.8187	28.8867	1:Experimental evidence at protein level
28.2733	28.2136	28.3696	28.3814	28.3763	28.2375	28.3436	28.3792	1:Experimental evidence at protein level
30.5061	30.4452	30.2869	30.3474	30.5316	30.5178	30.5756	30.5486	1:Experimental evidence at protein level
29.7113	29.6376	29.2847	29.6122	29.9627	30.0158	29.8953	30.2092	1:Experimental evidence at protein level
28.2568	28.2285	28.2838	28.2424	28.2497	28.3197	28.1895	28.2687	1:Experimental evidence at protein level
27.9973	27.9334	27.9198	27.8932	28.2032	28.0333	28.0634	27.9595	1:Experimental evidence at protein level
30.6934	30.5369	30.4285	30.4022	30.5435	30.5018	30.4219	30.4759	1:Experimental evidence at protein level
30.8801	30.7363	30.9915	30.7821	30.8802	30.7358	30.6765	30.2018	1:Experimental evidence at protein level
28.8243	28.7435	28.6327	28.5676	28.5882	28.6823	28.5149	28.5382	1:Experimental evidence at protein level
28.3441	28.3923	28.6585	28.6044	28.19	28.1346	28.2708	28.2938	1:Experimental evidence at protein level
28.597	28.7402	28.7008	28.4351	28.5799	28.8054	28.6438	28.5136	1:Experimental evidence at protein level
26.8872	26.8369	26.5885	26.9099	26.7899	27.2185	27.1619	27.5778	1:Experimental evidence at protein level
28.3799	28.3618	28.4215	28.4763	28.3143	28.1809	28.3689	28.4488	1:Experimental evidence at protein level
27.5795	27.5066	27.3668	27.2778	27.2688	27.3759	27.2239	27.5091	1:Experimental evidence at protein level
28.4545	28.4831	28.2894	28.3582	28.5878	28.7643	28.5274	28.3511	1:Experimental evidence at protein level
26.4546	26.532	26.6005	26.6477	26.8633	26.8926	26.8621	27.0686	1:Experimental evidence at protein level
29.1439	29.1316	29.2257	29.3504	29.6078	29.5098	29.553	29.7074	1:Experimental evidence at protein level
28.8356	28.7408	28.3208	28.0884	28.1776	28.4548	28.0928	28.1108	1:Experimental evidence at protein level
26.1172	26.2318	26.3096	26.3194	26.2005	26.1605	26.0435	26.2371	1:Experimental evidence at protein level
26.4843	26.5273	26.588	26.5431	26.6855	26.6179	26.7624	26.9506	1:Experimental evidence at protein level
26.4502	26.2538	26.52	26.5414	26.6266	26.6254	26.659	26.8462	1:Experimental evidence at protein level
28.2553	28.1672	27.9428	27.9861	27.9169	28.1526	27.8291	28.1093	1:Experimental evidence at protein level
28.0455	28.056	27.8673	27.9792	28.1927	28.1122	28.0663	28.4283	1:Experimental evidence at protein level
26.3833	26.5117	26.548	26.478	26.8591	26.9773	26.9437	26.8137	1:Experimental evidence at protein level
28.7453	28.8647	28.9382	28.8563	28.6463	28.3977	28.6329	28.5522	1:Experimental evidence at protein level
28.553	28.4981	28.6846	28.4607	28.4695	28.35	28.3857	28.415	1:Experimental evidence at protein level
30.3896	30.318	30.2109	30.3277	30.2917	30.3997	30.2831	30.0763	1:Experimental evidence at protein level
29.3813	29.478	29.6929	29.4726	29.5501	29.617	29.5864	29.5503	1:Experimental evidence at protein level

27.6983	27.7794	27.7944	27.9561	27.7457	27.7763	27.8796	28.2833	1:Experimental evidence at protein level
26.2306	26.2576	25.6956	25.7677	26.3073	26.2075	26.2854	26.4947	1:Experimental evidence at protein level
29.3074	29.4103	29.5297	29.5361	29.6588	29.6508	29.7372	29.7179	1:Experimental evidence at protein level
28.7935	28.7691	28.61	28.6773	26.2061	26.436	26.1988	27.157	1:Experimental evidence at protein level
31.3503	31.4309	31.5186	31.4719	31.397	31.4388	31.3804	30.9419	1:Experimental evidence at protein level
30.2136	30.1758	29.8455	29.9462	30.4908	30.585	30.5193	30.9416	1:Experimental evidence at protein level
28.61	28.5711	28.5615	28.3012	28.4142	28.3501	28.3743	28.241	1:Experimental evidence at protein level
28.4939	28.536	28.5694	28.4375	28.4983	28.3659	28.4788	28.7664	1:Experimental evidence at protein level
25.7109	25.8727	25.6554	25.9226	25.7596	25.833	25.9779	25.6093	1:Experimental evidence at protein level
26.9376	27.0126	26.8531	26.7	26.9802	26.9678	27.0385	26.5466	1:Experimental evidence at protein level
28.7826	28.8708	28.6331	28.7789	28.6425	28.5795	28.5884	28.9243	1:Experimental evidence at protein level
26.3065	26.0487	26.0118	26.35	26.6163	26.767	26.5599	26.8949	1:Experimental evidence at protein level
28.4613	28.4431	28.4094	28.3767	28.0838	27.9972	27.8491	28.185	1:Experimental evidence at protein level
28.5727	28.5102	28.0999	27.9552	28.0662	28.221	28.0544	28.0107	1:Experimental evidence at protein level
27.5189	27.3891	27.2197	27.2238	27.484	27.5043	27.3375	27.5785	1:Experimental evidence at protein level
28.1389	28.0791	28.1442	28.2081	28.3523	28.2558	28.3003	28.4421	1:Experimental evidence at protein level
26.8659	26.7743	26.8046	26.6786	27.202	27.0491	27.1141	27.4705	1:Experimental evidence at protein level
28.4664	28.2907	28.4284	28.5121	28.4009	28.3063	28.2037	27.9109	1:Experimental evidence at protein level
27.934	27.8714	27.9091	27.9229	27.9394	27.8666	27.8236	28.1764	1:Experimental evidence at protein level
29.1373	29.1002	29.3033	29.2366	29.0491	28.9608	28.9489	29.0348	1:Experimental evidence at protein level
29.2625	29.1428	29.279	29.1317	29.4428	29.4942	29.186	28.3247	1:Experimental evidence at protein level
28.7543	28.8832	28.9833	28.7968	28.9985	28.8324	28.8567	28.7785	1:Experimental evidence at protein level
27.8243	27.6414	26.9992	26.7456	26.6992	27.0388	26.4553	26.3871	1:Experimental evidence at protein level
30.6467	30.8829	31.1957	30.9164	30.8459	30.8616	30.9521	30.3961	1:Experimental evidence at protein level
29.2362	29.1616	29.2254	29.2497	29.3238	29.1247	29.2193	29.3216	1:Experimental evidence at protein level
31.2736	31.356	31.3906	31.4606	31.4825	31.4638	31.4881	31.4307	1:Experimental evidence at protein level
30.9113	30.9197	30.965	31.1614	31.1007	31.011	31.0763	30.9747	1:Experimental evidence at protein level
32.6176	32.4403	32.0944	32.2063	32.6535	32.5385	32.5116	32.3636	1:Experimental evidence at protein level
29.83	29.8187	29.9488	30.0382	29.8842	29.9767	29.9124	29.9721	1:Experimental evidence at protein level
28.1313	28.1518	28.2789	28.2958	28.6436	28.6338	28.5911	28.7225	1:Experimental evidence at protein level
28.8109	28.746	28.9455	29.0257	28.8856	28.6128	28.8503	28.9452	1:Experimental evidence at protein level
30.7106	30.7459	30.9543	30.9771	30.6167	30.6676	30.6007	30.8251	1:Experimental evidence at protein level
31.0246	30.8675	30.5251	30.2806	30.6703	30.8879	30.5961	30.0121	1:Experimental evidence at protein level
28.3781	28.3558	28.1388	28.1881	28.016	28.0158	28.0113	28.1456	1:Experimental evidence at protein level
28.3295	28.3497	28.309	28.4287	28.2699	28.4209	28.2455	28.4286	1:Experimental evidence at protein level
26.947	26.7634	26.2826	26.9477	27.4276	27.2966	27.5781	28.0264	1:Experimental evidence at protein level
28.0539	27.8963	28.0864	28.225	27.9855	27.9213	27.8271	27.9358	1:Experimental evidence at protein level
27.4765	27.4082	27.2074	27.0607	27.4025	27.4045	27.2633	27.5271	1:Experimental evidence at protein level
27.0465	26.9968	27.0216	27.168	27.1798	28.0796	27.4345	27.2358	1:Experimental evidence at protein level
27.3155	27.1025	26.9596	27.3558	28.1437	28.1975	27.9781	28.574	1:Experimental evidence at protein level
28.1358	28.2238	28.3001	28.2299	28.2654	28.2471	28.2074	28.1988	1:Experimental evidence at protein level
27.952	27.927	28.0493	28.0483	28.0577	28.1098	28.0938	28.3816	1:Experimental evidence at protein level
26.2569	26.4102	26.5554	26.7166	25.9574	25.7518	25.9716	26.8683	1:Experimental evidence at protein level
25.0483	25.0311	25.3257	25.286	25.3753	25.3004	25.5379	25.0225	1:Experimental evidence at protein level
30.0402	30.0291	30.3203	30.3971	30.1457	30.0342	30.3092	30.2594	1:Experimental evidence at protein level
32.1562	32.1695	32.2958	32.2345	29.9293	29.8116	30.0211	30.4229	1:Experimental evidence at protein level
28.5422	27.981	27.4025	27.5224	27.6318	28.4706	27.3372	29.3068	1:Experimental evidence at protein level
27.568	27.537	27.4887	27.7171	27.1267	27.1618	27.3638	27.7489	1:Experimental evidence at protein level
26.3056	26.205	26.2603	26.0931	26.3031	26.1614	26.0747	26.9789	1:Experimental evidence at protein level
31.4514	31.3588	30.9963	31.2232	31.8144	31.8661	31.6471	31.8452	1:Experimental evidence at protein level
27.6513	27.2407	26.5138	26.6835	26.839	26.864	26.6749	27.0678	1:Experimental evidence at protein level
30.6381	30.6749	30.8318	31.0179	30.5927	30.5682	30.7215	30.7965	1:Experimental evidence at protein level
28.5458	28.6377	28.6114	28.4045	28.6848	28.5924	28.5715	28.6004	1:Experimental evidence at protein level
28.9756	28.9609	28.8861	28.9419	28.725	28.7385	28.5424	28.9439	1:Experimental evidence at protein level
27.6806	27.5163	27.4934	27.3836	27.5892	27.6507	27.6282	27.5062	1:Experimental evidence at protein level
27.3615	27.4029	27.5154	27.3677	27.6147	27.4777	27.7016	27.9557	1:Experimental evidence at protein level
27.777	27.8841	28.0813	27.9453	27.6693	27.625	27.5268	27.9454	1:Experimental evidence at protein level
27.0467	26.8201	26.9382	26.8643	27.3278	27.1803	27.2295	27.126	1:Experimental evidence at protein level
28.747	28.7745	28.9758	29.0022	28.9832	28.821	29.0238	29.1281	1:Experimental evidence at protein level
28.1652	28.1182	28.2562	28.2101	28.2802	28.361	28.33	28.3906	1:Experimental evidence at protein level
28.1339	28.133	28.2721	28.1894	28.5952	28.4955	28.3666	28.3555	1:Experimental evidence at protein level
32.2983	32.4775	32.6339	32.4842	31.9122	32.0407	31.8644	31.801	1:Experimental evidence at protein level
30.8117	30.8327	30.8664	30.8327	30.5312	30.3279	30.5202	30.3778	1:Experimental evidence at protein level
27.5778	26.8424	27.0755	26.5675	27.6022	28.1729	25.9981	28.5411	1:Experimental evidence at protein level
30.5713	30.6529	30.8984	30.7663	30.6661	30.5549	30.6988	30.7739	1:Experimental evidence at protein level
28.3657	28.5251	28.5685	28.5595	28.3655	28.3003	28.3791	28.4084	1:Experimental evidence at protein level
29.6262	29.6585	29.7125	29.6257	29.5408	29.5144	29.5531	29.1839	1:Experimental evidence at protein level
32.1333	32.0536	31.6959	31.8669	32.366	32.3784	32.2715	32.313	1:Experimental evidence at protein level
28.9187	29.0004	29.112	29.0663	27.6254	27.6033	27.6929	27.7532	1:Experimental evidence at protein level
28.5271	28.5095	28.4525	28.6123	27.9716	28.0058	28.0314	28.3886	1:Experimental evidence at protein level
28.2411	28.2582	28.4186	28.1684	28.4405	28.4528	28.3376	28.3866	1:Experimental evidence at protein level
25.9474	25.9689	26.0637	25.9648	#NUM!	#NUM!	25.8755	26.7545	1:Experimental evidence at protein level
27.4658	27.5065	27.3362	27.3409	27.3029	27.2201	27.321	27.5585	1:Experimental evidence at protein level
27.3488	27.4448	27.4229	27.5286	27.6311	27.5797	27.6001	27.9178	1:Experimental evidence at protein level
26.8541	26.9457	26.9253	27.0736	26.162	26.4192	26.103	26.7286	1:Experimental evidence at protein level
28.3486	28.3419	28.3236	28.3091	28.4291	28.445	28.5393	28.6577	1:Experimental evidence at protein level
28.2481	28.2763	28.376	28.5095	28.4331	28.2358	28.4425	28.3059	1:Experimental evidence at protein level
28.0669	28.1686	28.1486	28.2553	26.4987	26.6081	26.623	27.033	1:Experimental evidence at protein level
27.8067	27.7032	27.6013	27.8067	27.8021	27.6341	27.7813	28.2508	1:Experimental evidence at protein level
28.2254	28.1863	28.2233	28.1063	28.2173	28.2807	28.2396	28.0315	1:Experimental evidence at protein level
29.5816	29.4699	29.5322	29.6845	30.0992	29.9519	29.8975	29.7845	1:Experimental evidence at protein level
29.4728	29.5933	29.7764	29.6429	28.9537	28.9027	28.9755	29.0192	1:Experimental evidence at protein level
29.5229	29.5287	29.6146	29.5768	30.0023	30.0263	29.9696	29.8161	1:Experimental evidence at protein level
29.222	29.2531	29.3009	29.302	29.4532	29.4946	29.4264	29.5725	1:Experimental evidence at protein level
25.5005	25.5111	25.5447	25.5449	25.705	25.5874	25.7198	25.9785	1:Experimental evidence at protein level
31.0156	31.0076	30.9009	30.6769	31.1678	31.1049	31.1208	30.9511	1:Experimental evidence at protein level
32.9837	32.9844	33.095	33.249	33.0384	32.8529	32.9443	32.9926	1:Experimental evidence at protein level
27.5144	27.448	27.5228	27.5955	27.6565	27.6875	27.644	27.7609	1:Experimental evidence at protein level
27.387	27.1025	27.1811	27.5251	#NUM!	27.5066	27.2436	27.6257	1:Experimental evidence at protein level
29.1254	29.0557	28.9371	28.8973	28.8258	28.8099	28.8004	28.6717	1:Experimental evidence at protein level
27.6985	27.7495	27.6244	27.6399	27.4851	27.6133	27.5326	27.5161	1:Experimental evidence at protein level
30.1096	30.0285	29.9108	29.9835	29.8728	29.9962	29.8824	30.0166	1:Experimental evidence at protein level
28.2532	28.2773	28.2245	28.3439	28.3433	28.26	28.251	28.4636	1:Experimental evidence at protein level
30.8497	30.9152	30.9727	30.8082	30.7319	30.7326	30.7832	30.434	1:Experimental evidence at protein level
28.5763	28.5576	28.4929	28.6459	28.6111	28.5446	28.6316	28.7035	1:Experimental evidence at protein level
30.1782	30.1621	30.1425	30.3804	31.1405	31.0689	31.0743	31.0293	1:Experimental evidence at protein level
30.4238	30.3195	29.9601	29.941	30.0596	30.0917	30.0366	30.033	1:Experimental evidence at protein level
26.5575	26.5883	26.4032	26.3993	26.6807	26.6656	26.6072	27.08	1:Experimental evidence at protein level
26.8257	26.8188	26.9159	26.7571	27.1907	27.2602	27.2145	26.8432	1:Experimental evidence at protein level
33.6353	33.5424	33.5054	33.5262	33.3295	33.1806	33.3998	32.9798	1:Experimental evidence at protein level
27.9317	27.9896	27.9972	27.9888	28.1627	28.1011	28.0284	28.449	1:Experimental evidence at protein level
29.275	29.29	29.4007	29.3282	29.5067	29.4809	29.5	29.5167	1:Experimental evidence at protein level
27.766	27.8436	27.9221	27.9275	27.401	27.5171	27.485	27.3795	1:Experimental evidence at protein level
29.5688	29.5443	29.7259	29.7213	29.58	29.5323	29.5076	29.4784	1:Experimental evidence at protein level
30.8881	30.8667	30.9696	30.8439	31.1767	31.1849	31.1866	30.7308	1:Experimental evidence at protein level
30.1793	30.0535	30.0056	29.9841	29.9288	29.8405	29.8483	29.3513	1:Experimental evidence at protein level
28.8228	28.8851	29.0504	28.8982	28.5071	28.6189	28.4793	28.0806	1:Experimental evidence at protein level
28.1764	28.0794	28.2565	28.0835	28.1737	28.1664	27.9965	27.8233	1:Experimental evidence at protein level
30.6159	30.5761	30.1195	30.3232	30.9088	31.0697	30.8454	30.966	1:Experimental evidence at protein level
28.0301	27.7366	27.9584	28.0724	27.7141	27.6579	27.7138	27.9161	1:Experimental evidence at protein level
27.8678	28.1061	28.1691	28.301	28.3438	28.0348	28.1153	28.7118	1:Experimental evidence at protein level
27.5285	27.5392	27.4893	27.5123	27.2415	27.2152	27.2867	27.4932	1:Experimental evidence at protein level
27.2284	27.2922	27.344	27.3158	27.3258	27.3084	27.3931	27.7887	1:Experimental evidence at protein level
31.4855	31.3921	31.4717	31.5345	31.4561	31.2815	31.3803	31.4734	1:Experimental evidence at protein level
32.0476	31.8385	31.4476	31.2817	31.4165	31.6417	31.3355	30.9993	1:Experimental evidence at protein level
27.4183	27.6028	27.5851	27.6368	29.0557	29.0499	29.203	29.1548	1:Experimental evidence at protein level
27.4506	27.31	27.6104	27.6176	27.48	27.4763	27.504	27.6632	1:Experimental evidence at protein level
30.8237	30.6507	30.2551	30.1639	30.3605	30.5222	30.2777	29.8291	1:Experimental evidence at protein level
27.3568	27.3232	27.7477	27.6252	26.3013	26.139	26.2055	26.5192	1:Experimental evidence at protein level
28.183	27.6687	26.407	26.6731	26.945	27.8511	26.4093	28.3973	1:Experimental evidence at protein level
30.1276	30.0535	30.1217	30.2434	30.2161	30.1129	30.2549	30.3412	1:Experimental evidence at protein level
31.3191	31.2341	31.0194	31.0794	31.4042	31.3406	31.3286	31.3446	1:Experimental evidence at protein level
31.3997	31.3733	31.3881	31.1695	31.1524	31.1367	31.1161	30.8045	1:Experimental evidence at protein level
32.4349	32.574	32.7368	32.7924	32.2762	32.2065	32.3592	32.2468	1:Experimental evidence at protein level
32.9034	32.9438	33.2298	33.137	32.5335	32.7549	33.0388	32.6458	1:Experimental evidence at protein level
26.3034	26.2829	26.3113	26.5094	26.488	26.566	26.6715	26.8103	1:Experimental evidence at protein level
27.4363	27.4204	27.6312	27.4891	27.4073	27.4755	27.4499	27.7384	1:Experimental evidence at protein level
28.2657	28.3378	28.2205	28.2798	28.2791	28.1763	28.3036	28.5233	1:Experimental evidence at protein level
29.0471	29.0648	29.1987	29.0606	28.9322	28.8365	28.9346	28.6926	1:Experimental evidence at protein level

28.3634	28.3826	28.4522	28.3127	28.4389	28.3796	28.4108	28.3435	1:Experimental evidence at protein level
26.5243	26.3798	26.5828	26.5262	26.7578	26.6238	26.6114	27.1433	1:Experimental evidence at protein level
28.1987	28.2073	28.3469	28.3887	28.2912	28.1481	28.2318	28.4896	1:Experimental evidence at protein level
26.6914	26.4412	26.3139	26.2418	27.1759	27.3844	27.1083	27.4039	1:Experimental evidence at protein level
28.4351	28.3079	28.1389	28.3188	28.7629	28.6591	28.6601	29.0923	1:Experimental evidence at protein level
31.3444	31.2136	30.8984	31.0486	31.57	31.5722	31.4744	31.7247	1:Experimental evidence at protein level
31.4912	31.4321	31.3573	31.6298	32.0163	31.8615	31.891	31.9777	1:Experimental evidence at protein level
28.3917	28.3367	28.5531	28.4659	28.5177	28.511	28.4815	28.5049	1:Experimental evidence at protein level
27.1484	27.1161	27.1451	27.2595	27.0219	26.9804	26.837	27.2252	1:Experimental evidence at protein level
30.0871	30.1587	30.2812	30.2684	28.0811	28.0728	28.134	28.3624	1:Experimental evidence at protein level
30.7491	30.7401	30.696	30.5734	30.6545	30.6535	30.5646	30.1196	1:Experimental evidence at protein level
27.7246	27.6342	27.6871	27.599	27.6353	27.6012	27.5446	27.55	1:Experimental evidence at protein level
31.7215	31.6981	31.762	31.8587	32.0066	32.0569	32.0607	32.334	1:Experimental evidence at protein level
30.0227	30.0016	30.2065	30.2118	30.1122	29.9022	29.991	29.8597	1:Experimental evidence at protein level
28.5201	28.3699	28.3629	28.5713	28.2128	28.1867	28.1215	28.1966	1:Experimental evidence at protein level
29.9736	29.8942	29.5467	29.4934	29.6298	29.7707	29.5342	29.4704	1:Experimental evidence at protein level
28.8289	28.6526	28.5713	28.4249	28.5023	28.5568	28.4549	28.6381	1:Experimental evidence at protein level
24.5325	24.776	25.0485	24.964	24.7575	24.5738	24.692	24.9835	1:Experimental evidence at protein level
27.4662	27.5101	27.5334	27.5892	27.6168	27.6598	27.7285	27.8593	1:Experimental evidence at protein level
26.6599	26.7132	26.632	26.7705	27.2233	27.1483	27.0389	27.4779	1:Experimental evidence at protein level
28.8467	28.8604	28.9381	28.976	29.2356	29.2691	29.2574	29.1995	1:Experimental evidence at protein level
26.9454	26.8512	26.5996	26.7008	25.7682	25.8802	25.8194	26.2074	1:Experimental evidence at protein level
31.0729	31.07	31.0317	31.0092	32.2139	32.2495	32.1569	31.2844	1:Experimental evidence at protein level
27.9316	27.9539	27.9736	27.8703	27.5548	27.4368	27.5174	27.5087	1:Experimental evidence at protein level
28.5233	28.4112	28.4932	28.5598	28.789	28.8505	28.7627	29.0859	1:Experimental evidence at protein level
29.1272	29.1102	29.1243	28.9596	28.6653	28.8476	28.5642	28.4821	1:Experimental evidence at protein level
25.5888	25.3758	25.5466	25.4311	25.7911	25.6845	25.6342	26.9146	1:Experimental evidence at protein level
29.6087	29.4609	29.4506	29.4959	29.1333	29.0363	29.0455	29.3958	1:Experimental evidence at protein level
26.4376	26.1072	26.1073	26.3817	26.4376	26.2949	26.2919	26.9229	1:Experimental evidence at protein level
29.234	29.3228	29.0135	29.1234	29.7495	29.8513	29.7691	30.0116	1:Experimental evidence at protein level
28.4814	28.469	28.5889	28.5144	28.4058	28.3968	28.3847	28.6554	1:Experimental evidence at protein level
27.9475	27.9187	27.7417	27.6995	27.667	27.8243	27.6361	27.7808	1:Experimental evidence at protein level
27.7724	28.1166	27.9282	27.8478	28.0564	28.0181	28.124	27.9562	1:Experimental evidence at protein level
28.7781	28.8288	29.0537	28.8576	28.5391	28.9982	28.902	28.8121	1:Experimental evidence at protein level
28.3855	28.336	28.4321	28.4262	28.6565	28.6928	28.6151	28.5205	1:Experimental evidence at protein level
25.5338	25.4821	25.4125	25.5714	25.4863	25.5346	25.5846	26.0768	1:Experimental evidence at protein level
29.2007	29.0891	29.1058	29.187	29.4468	29.4007	29.3782	29.507	1:Experimental evidence at protein level
27.8408	27.7882	27.9686	27.875	27.8762	27.7926	27.9231	27.9841	1:Experimental evidence at protein level
31.0086	31.0368	31.1557	31.1124	31.2556	31.19	31.1958	31.2014	1:Experimental evidence at protein level
27.7369	27.8011	27.8687	27.9786	27.7819	27.6687	27.7873	27.7942	1:Experimental evidence at protein level
29.5034	29.4878	29.6687	29.6491	29.4489	29.3124	29.3724	29.125	1:Experimental evidence at protein level
27.5312	27.4543	27.2611	27.2529	27.5327	27.5914	27.3224	27.5316	1:Experimental evidence at protein level
26.9041	26.7627	26.8162	27.0073	27.0768	27.1076	27.1187	27.1991	1:Experimental evidence at protein level
28.5757	28.6922	27.9062	28.28	27.3109	29.9844	27.9959	28.9899	1:Experimental evidence at protein level
29.9933	30.0034	30.2141	30.2476	30.3215	30.2101	30.2632	30.3818	1:Experimental evidence at protein level
28.9589	28.9125	28.9178	29.0518	28.9624	28.9148	28.9216	28.9604	1:Experimental evidence at protein level
29.464	29.3378	29.4759	29.4073	29.4778	29.4506	29.4185	29.5234	1:Experimental evidence at protein level
25.7284	25.8561	25.8246	25.8466	25.7035	25.9606	25.8418	25.9926	1:Experimental evidence at protein level
29.0508	28.8633	28.666	28.8205	28.9569	28.8673	28.9462	28.9513	1:Experimental evidence at protein level
29.1695	29.1967	29.3782	29.3981	28.532	28.5006	28.5322	28.4936	1:Experimental evidence at protein level
31.9717	31.9518	31.9975	31.8604	31.8437	31.8036	31.7698	31.2195	1:Experimental evidence at protein level
29.1876	29.1558	29.1025	28.9225	29.2803	29.3009	29.2039	28.4034	1:Experimental evidence at protein level
28.8781	28.948	28.9947	28.9316	29.2801	29.2429	29.1615	28.9367	1:Experimental evidence at protein level
27.9248	27.9557	28.0078	28.159	28.8337	28.7865	28.7775	28.715	1:Experimental evidence at protein level
26.5599	26.6494	26.6327	26.7068	27.135	27.1164	27.0861	27.5543	1:Experimental evidence at protein level
28.7879	28.7312	28.6019	28.5046	28.5331	28.5152	28.5604	28.5584	1:Experimental evidence at protein level
25.6818	26.3276	25.4347	25.8788	27.0186	26.6386	26.3289	27.0905	1:Experimental evidence at protein level
28.0314	28.1262	28.1847	28.1221	28.0853	28.1074	28.1069	28.2197	1:Experimental evidence at protein level
28.5664	28.5292	28.4713	28.52	28.5112	28.5336	28.4739	28.5399	1:Experimental evidence at protein level
26.5831	26.7218	26.7432	26.8446	26.5683	26.6047	26.6524	26.8768	1:Experimental evidence at protein level
28.6666	28.6579	28.8481	28.7417	28.6179	28.6528	28.5957	28.854	1:Experimental evidence at protein level
27.6491	27.536	27.6063	27.5682	28.1576	28.2435	28.2065	28.0992	1:Experimental evidence at protein level
28.0196	28.0134	28.0829	27.9266	29.3054	29.23	29.1474	29.272	1:Experimental evidence at protein level
31.2228	31.0937	30.9108	30.9854	31.4655	31.521	31.3593	31.5443	1:Experimental evidence at protein level
29.9822	29.9528	29.7799	29.8324	30.1114	30.0941	30.06	29.9875	1:Experimental evidence at protein level
31.0945	31.0415	30.8358	30.809	31.2686	31.2992	31.1346	31.3039	1:Experimental evidence at protein level
29.9374	29.9362	30.0349	29.8815	29.9444	29.9399	29.9511	29.6185	1:Experimental evidence at protein level
28.4313	28.3371	28.3422	28.4009	28.2539	28.1756	28.1947	28.4818	1:Experimental evidence at protein level
31.9452	31.9517	32.0792	32.1088	32.3596	32.3529	32.3466	32.3042	1:Experimental evidence at protein level
32.2775	32.3926	32.5294	32.354	32.337	32.3264	32.4354	31.8283	1:Experimental evidence at protein level
29.4111	29.3858	29.4666	29.4351	29.4053	29.3192	29.4158	29.3035	1:Experimental evidence at protein level
27.9383	28.07	28.0965	28.0605	27.9787	27.9389	27.9461	27.991	1:Experimental evidence at protein level
27.6884	27.7786	27.8604	27.7436	27.4341	27.3982	27.3053	27.2606	1:Experimental evidence at protein level
26.6467	26.5076	26.5286	26.6887	27.0198	26.9413	26.861	27.276	1:Experimental evidence at protein level
28.3481	28.1224	27.7077	27.5954	28.2756	28.3633	28.1588	28.3171	1:Experimental evidence at protein level
29.027	29.0103	29.1594	29.1816	29.5832	29.4479	29.4372	29.4543	1:Experimental evidence at protein level
27.1247	27.193	27.2254	27.3151	27.4166	27.3633	27.4933	27.6323	1:Experimental evidence at protein level
25.7453	26.0068	25.8173	25.7299	25.6014	25.7801	25.4599	25.8884	1:Experimental evidence at protein level
28.2962	28.4087	28.5327	28.4228	29.0608	29.0065	29.0853	28.8421	1:Experimental evidence at protein level
30.3601	30.2786	30.3486	30.391	30.6285	30.584	30.5402	30.4077	1:Experimental evidence at protein level
28.2024	28.0641	28.1616	28.0519	27.5335	27.5242	27.439	27.7007	1:Experimental evidence at protein level
29.7528	29.757	29.9245	29.8649	29.9395	29.9019	29.9269	29.8816	1:Experimental evidence at protein level
30.2861	30.285	30.3383	30.2824	30.1019	30.0488	30.1453	30.1933	1:Experimental evidence at protein level
32.3995	32.3154	32.5913	32.5332	32.7973	32.6206	32.6285	32.6559	1:Experimental evidence at protein level
32.0647	32.084	32.1879	32.2652	32.1857	32.0081	31.9868	32.3507	1:Experimental evidence at protein level
27.146	27.1255	27.1461	27.1804	27.3196	27.1326	27.3413	27.417	1:Experimental evidence at protein level
27.596	27.6914	27.7045	27.6212	28.4174	28.4884	28.2769	28.5134	1:Experimental evidence at protein level
28.814	28.2174	27.9978	27.8879	28.644	29.1429	27.2825	29.6091	1:Experimental evidence at protein level
34.1824	34.1615	34.2819	34.3481	34.4222	34.2436	34.495	34.5242	1:Experimental evidence at protein level
30.8276	30.7646	30.7971	30.7581	30.7597	30.67	30.6432	30.7225	1:Experimental evidence at protein level
29.7943	29.7712	29.8533	29.7396	29.6427	29.5319	29.5939	29.5457	1:Experimental evidence at protein level
32.158	32.0416	31.7398	31.6631	31.7737	31.8233	31.5641	30.9449	1:Experimental evidence at protein level
27.3207	27.3268	27.3238	27.3639	27.7318	27.7744	27.7909	27.9855	1:Experimental evidence at protein level
30.2261	30.1939	30.2948	30.2391	29.2188	29.1468	29.1899	29.1119	1:Experimental evidence at protein level
29.2275	29.1881	29.1362	29.1093	29.2734	29.2901	29.2476	29.0937	1:Experimental evidence at protein level
29.8978	29.9302	29.962	30.067	30.0896	30.1127	30.1024	29.9745	1:Experimental evidence at protein level
29.7029	29.6996	29.785	29.8214	29.2912	29.2681	29.2347	29.3968	1:Experimental evidence at protein level
28.4373	28.5393	28.6087	28.293	28.1983	28.2097	28.2406	27.6525	1:Experimental evidence at protein level
28.5245	28.4792	28.5456	28.3705	28.2693	28.3679	28.1874	28.1234	1:Experimental evidence at protein level
27.5314	27.5634	27.5987	27.5671	26.834	26.77	26.9115	26.9618	1:Experimental evidence at protein level
28.2066	28.0565	27.6921	27.7497	28.0089	28.0206	28.0863	28.2804	1:Experimental evidence at protein level
27.3898	27.347	27.2376	27.4764	27.5018	27.4512	27.488	27.7246	1:Experimental evidence at protein level
29.4792	29.6329	29.6472	29.4968	30.0276	30.0029	30.1151	29.7155	1:Experimental evidence at protein level
28.9968	29.0323	29.2493	29.1191	29.8415	29.7442	29.8961	29.4673	1:Experimental evidence at protein level
28.4392	28.4986	28.6975	28.7371	28.4473	28.3992	28.3753	28.5935	1:Experimental evidence at protein level
27.198	27.1388	26.9913	27.0957	25.774	25.986	25.9099	26.2436	1:Experimental evidence at protein level
28.3869	28.3094	28.1144	28.1848	28.2872	28.3412	28.2317	28.5952	1:Experimental evidence at protein level
26.7316	26.7356	26.7484	26.8779	26.3179	26.2525	26.4233	26.6683	1:Experimental evidence at protein level
34.6047	34.4962	34.6614	34.6284	34.7988	34.6548	34.7418	34.4687	1:Experimental evidence at protein level
27.5329	27.7264	27.1609	#NUM!		26.9413	29.7435	27.1597	28.8134	1:Experimental evidence at protein level
30.2497	30.1212	30.0277	30.1333	31.1214	31.2192	30.7421	30.2429	1:Experimental evidence at protein level
29.394	29.2986	29.4956	29.5212	29.6156	29.5237	29.4602	29.7651	1:Experimental evidence at protein level
29.5281	29.457	29.3375	29.2772	29.6333	29.6898	29.5481	29.2302	1:Experimental evidence at protein level
29.2129	29.1394	29.2386	29.2513	29.2477	29.1788	29.1213	28.8352	1:Experimental evidence at protein level
29.6315	29.6125	29.6298	29.7334	29.7959	29.6847	29.7536	29.6723	1:Experimental evidence at protein level
27.5628	27.4978	27.5515	27.5612	27.8732	27.8526	27.8991	27.9132	1:Experimental evidence at protein level
29.6781	29.5919	29.7896	29.7907	29.4234	29.3722	29.3979	29.4341	1:Experimental evidence at protein level
28.0609	27.978	28.0857	27.8603	30.3085	30.2585	30.4201	30.5917	1:Experimental evidence at protein level
29.9622	29.9432	29.9167	29.8729	29.9755	30.0665	29.9651	29.8389	1:Experimental evidence at protein level
29.3475	29.3017	29.4463	29.3496	30.1144	30.0927	30.135	29.7332	1:Experimental evidence at protein level
31.0281	30.7395	30.7958	30.8426	31.97	32.107	31.395	30.8142	1:Experimental evidence at protein level
30.0321	30.1082	30.2537	30.2516	30.4082	30.3486	30.3681	30.3554	1:Experimental evidence at protein level
29.982	30.089	30.2298	30.176	29.834	29.8199	29.9105	29.7106	1:Experimental evidence at protein level
28.8275	28.6217	28.3476	28.2993	29.2926	29.3124	29.1005	29.0428	1:Experimental evidence at protein level
28.1644	28.2275	28.3696	28.3234	28.2767	28.2334	28.1395	28.2632	1:Experimental evidence at protein level
29.5442	29.5865	29.4708	29.4044	29.4548	29.5454	29.4058	29.476	1:Experimental evidence at protein level
28.9632	29.0126	29.1425	29.1429	29.1635	29.1582	29.2581	29.174	1:Experimental evidence at protein level
34.0682	34.0308	34.0958	34.282	33.2234	33.1815	33.1912	33.0956	1:Experimental evidence at protein level
31.7316	31.7122	31.8999	31.9178	32.1399	31.9868	32.0659	32.0454	1:Experimental evidence at protein level

29.3501	29.2493	29.2921	29.3428	29.0054	29.0218	29.037	29.0624	1:Experimental evidence at protein level
29.9584	29.9398	30.0994	30.0267	30.0558	29.9412	30.0071	30.0555	1:Experimental evidence at protein level
29.0847	29.0101	28.4641	28.1938	28.6875	28.869	28.7144	28.3622	1:Experimental evidence at protein level
28.0156	28.0783	28.2299	28.2369	28.0685	27.9364	27.9976	27.9685	1:Experimental evidence at protein level
27.6889	27.6078	27.6285	27.7623	27.8361	27.9231	27.8628	28.0441	1:Experimental evidence at protein level
27.2694	27.2749	27.398	27.2138	27.4532	27.6659	27.4508	27.4578	1:Experimental evidence at protein level
33.5424	33.5928	33.7981	33.8207	32.4184	32.3599	32.5342	32.3325	1:Experimental evidence at protein level
28.202	28.3425	28.4343	28.1618	28.4299	28.4699	28.473	28.0814	1:Experimental evidence at protein level
28.4924	28.4751	28.4929	28.3587	28.5707	28.6009	28.5081	28.3879	1:Experimental evidence at protein level
29.9582	29.8875	29.9731	29.9521	30.0118	29.8604	30.0372	29.8075	1:Experimental evidence at protein level
30.2942	30.3243	30.3373	30.3614	30.3828	30.4181	30.4102	30.5523	1:Experimental evidence at protein level
28.1199	28.0688	28.0836	28.0841	28.0829	28.139	28.034	28.065	1:Experimental evidence at protein level
28.2934	28.2502	28.4058	28.4963	28.2422	28.3051	28.2301	28.3491	1:Experimental evidence at protein level
35.494	35.5117	35.5554	35.5955	35.5377	35.5246	35.6157	35.4249	1:Experimental evidence at protein level
29.184	29.3134	29.4656	29.3172	29.0932	29.1285	29.3744	29.1137	1:Experimental evidence at protein level
27.3335	27.4362	27.5422	27.6671	27.8965	27.7256	27.787	28.0604	1:Experimental evidence at protein level
27.9068	27.836	27.6728	27.8287	27.4365	27.5665	27.5485	27.4784	1:Experimental evidence at protein level
27.6964	27.7248	27.9421	27.8987	27.7283	27.7804	27.618	27.8161	1:Experimental evidence at protein level
29.3391	29.228	29.2126	29.2461	29.383	29.4522	29.2981	29.3035	1:Experimental evidence at protein level
26.8893	26.8201	26.8677	26.8906	26.927	26.9622	26.8539	27.1313	1:Experimental evidence at protein level
28.554	28.6092	28.5929	28.7412	28.5546	28.4223	28.5023	28.7152	1:Experimental evidence at protein level
28.5194	28.3497	27.9371	27.9672	28.0215	28.1193	27.9264	28.0659	1:Experimental evidence at protein level
29.825	29.7801	29.6259	29.644	29.8701	29.8934	29.833	29.9301	1:Experimental evidence at protein level
29.0308	28.9787	29.066	29.0867	28.9308	28.9437	28.9111	29.0238	1:Experimental evidence at protein level
27.676	27.537	27.5059	27.7458	27.6949	27.5734	27.6308	27.9381	1:Experimental evidence at protein level
28.5906	28.6149	28.5951	28.5701	28.9637	28.9387	28.9171	28.9317	1:Experimental evidence at protein level
29.6211	29.4764	29.2696	28.7707	30.8342	30.9071	30.8247	30.6414	1:Experimental evidence at protein level
35.1085	35.1752	35.3052	35.3064	34.4628	34.3716	34.4709	34.2045	1:Experimental evidence at protein level
29.8273	29.9089	30.047	30.0444	29.2375	29.2389	29.3915	29.2838	1:Experimental evidence at protein level
29.5004	29.4824	29.0577	28.8604	28.9809	29.2671	29.1288	28.6559	1:Experimental evidence at protein level
28.3375	28.3666	28.3041	28.4996	28.356	28.3087	28.4494	28.3949	1:Experimental evidence at protein level
28.0426	27.9663	27.9289	27.8463	27.646	27.765	27.6494	27.7307	1:Experimental evidence at protein level
30.2084	30.1608	30.251	30.3065	30.2422	30.2087	30.2719	30.2026	1:Experimental evidence at protein level
30.2201	30.2331	30.3861	30.4416	30.2512	30.216	30.255	30.1439	1:Experimental evidence at protein level
29.7362	29.849	29.7704	29.8138	30.0868	29.963	30.1163	29.9293	1:Experimental evidence at protein level
31.2872	31.2179	31.1061	31.0458	31.3667	31.3757	31.2586	31.418	1:Experimental evidence at protein level
31.0331	30.8228	30.7045	30.7037	32.1822	32.1796	31.8692	31.0612	1:Experimental evidence at protein level
28.817	28.9145	28.8892	28.8507	29.081	29.12	29.1701	29.2944	1:Experimental evidence at protein level
34.0528	34.0636	34.0706	34.1644	34.5251	34.4189	34.5229	34.172	1:Experimental evidence at protein level
27.7522	27.723	27.543	27.424	27.5151	27.699	27.5847	27.3872	1:Experimental evidence at protein level
28.3432	28.3005	28.392	28.4294	28.828	28.8277	28.7239	28.7655	1:Experimental evidence at protein level
28.9951	29.0163	29.1383	29.2004	29.3073	29.2722	29.2838	29.4675	1:Experimental evidence at protein level
26.4232	26.3763	26.3287	26.5748	26.8021	26.6793	26.5653	27.0195	1:Experimental evidence at protein level
27.0342	26.9342	26.9487	26.9614	26.9233	26.7375	26.613	26.9306	1:Experimental evidence at protein level
28.7818	28.7553	28.7466	29.0304	29.2738	29.296	29.2814	29.621	1:Experimental evidence at protein level
29.9641	29.9872	29.9911	30.1359	30.3901	30.3087	30.381	30.5604	1:Experimental evidence at protein level
27.2021	27.1135	27.198	27.3897	27.2231	27.2044	27.2379	27.3489	1:Experimental evidence at protein level
29.7199	29.6834	29.7723	29.7729	29.1919	29.028	29.1326	28.8505	1:Experimental evidence at protein level
26.3525	26.4075	26.5437	26.4747	26.674	26.8567	26.587	26.6119	1:Experimental evidence at protein level
31.1299	31.1052	31.3213	31.2193	30.9074	30.7461	30.9252	30.6793	1:Experimental evidence at protein level
31.287	31.2469	31.2691	31.2334	31.3186	31.3435	31.2483	30.9076	1:Experimental evidence at protein level
31.1885	31.1111	30.7707	30.8478	31.4062	31.3708	31.3109	31.5412	1:Experimental evidence at protein level
30.5906	30.5705	30.7767	30.7236	30.368	30.262	30.3075	30.1373	1:Experimental evidence at protein level
29.7432	29.8079	29.9052	29.8492	29.6805	29.7243	29.7387	29.7174	1:Experimental evidence at protein level
30.2779	30.2652	30.3615	30.2929	30.1899	30.1234	30.1464	30.1431	1:Experimental evidence at protein level
28.4333	28.3368	28.3326	28.2622	28.5077	28.5344	28.4009	28.5207	1:Experimental evidence at protein level
29.0893	29.0185	28.7201	28.8401	28.8164	28.8605	28.8055	28.8763	1:Experimental evidence at protein level
31.9392	32.7843	31.9255	32.0632	32.3674	32.3228	32.6933	32.5565	1:Experimental evidence at protein level
31.983	32.0138	32.1663	32.2112	31.4353	31.1788	31.4037	31.5806	1:Experimental evidence at protein level
27.362	27.2245	27.2626	27.3262	27.7878	27.7499	27.8276	28.2142	1:Experimental evidence at protein level
27.0628	26.9969	26.7399	26.9151	26.2797	26.3215	26.1834	26.6542	1:Experimental evidence at protein level
33.729	33.8905	34.1741	34.0271	32.401	32.5009	32.3976	32.1748	1:Experimental evidence at protein level
28.5878	28.5142	28.5912	28.6533	28.6945	28.597	28.632	28.7893	1:Experimental evidence at protein level
28.2184	28.2733	28.2801	28.2406	28.6397	28.6231	28.6154	28.4757	1:Experimental evidence at protein level
29.2116	29.1712	29.2699	29.2345	29.3315	29.3677	29.3139	29.3652	1:Experimental evidence at protein level
28.9888	28.7914	28.6138	28.5867	28.6415	28.8084	28.5031	28.3823	1:Experimental evidence at protein level
30.8729	30.8948	31.0788	30.9088	30.3384	30.2582	30.3312	29.882	1:Experimental evidence at protein level
30.0955	30.1812	30.2611	30.2516	30.2717	30.2991	30.3043	30.1161	1:Experimental evidence at protein level
28.8329	28.7299	28.4968	28.4629	28.4526	28.529	28.3455	28.5722	1:Experimental evidence at protein level
32.7648	32.8164	33.0372	33.1295	31.3643	31.3843	31.3931	31.5729	1:Experimental evidence at protein level
30.7521	30.5953	30.049	29.9929	30.0592	30.2653	30.1112	29.6842	1:Experimental evidence at protein level
30.5447	30.6061	30.6696	30.8586	30.8315	30.679	30.9448	30.6952	1:Experimental evidence at protein level
31.6825	31.677	31.72	31.6996	31.5766	31.4997	31.6039	31.5205	1:Experimental evidence at protein level
30.7674	30.8058	30.7607	30.8067	30.7546	30.6059	30.724	30.7261	1:Experimental evidence at protein level
28.0316	27.9878	28.0593	28.1112	28.1252	28.0446	28.0711	28.473	1:Experimental evidence at protein level
29.8402	29.8248	29.8524	29.9002	29.9161	29.755	29.822	29.9261	1:Experimental evidence at protein level
29.726	29.702	29.7594	29.8651	29.9438	29.9333	29.9209	30.0681	1:Experimental evidence at protein level
25.9053	26.0067	25.2847	25.4965	26.2633	26.4727	26.0067	25.8741	1:Experimental evidence at protein level
29.4755	29.0175	27.3395	27.0924	31.859	32.0062	31.8336	31.9113	1:Experimental evidence at protein level
32.253	32.2719	32.1659	32.256	32.3264	32.1667	32.2601	31.787	1:Experimental evidence at protein level
31.9488	31.8861	31.8084	31.9047	31.6271	31.6532	31.7549	31.3199	1:Experimental evidence at protein level
27.0284	26.9504	26.9655	26.992	26.7501	26.6776	26.6734	26.9321	1:Experimental evidence at protein level
31.7225	31.699	31.893	31.7941	31.2574	31.1854	31.2659	30.9837	1:Experimental evidence at protein level
27.2796	27.2908	27.4212	27.3997	27.242	27.2671	26.9691	27.1909	1:Experimental evidence at protein level
30.0053	30.1184	30.2001	30.1188	30.059	29.9754	30.1628	29.8251	1:Experimental evidence at protein level
30.4286	30.4951	30.4127	30.3553	30.3579	30.4089	30.3267	30.3828	1:Experimental evidence at protein level
31.2708	31.1895	30.8662	31.0304	31.4818	31.4783	31.4752	31.6064	1:Experimental evidence at protein level
28.2376	28.1827	28.3447	28.0935	28.4924	28.5085	28.466	28.0392	1:Experimental evidence at protein level
27.3348	26.9362	27.0393	27.0445	26.6455	26.541	26.4697	26.3361	1:Experimental evidence at protein level
34.5297	34.579	34.6215	34.7506	34.1286	33.9838	33.9693	34.0277	1:Experimental evidence at protein level
29.1448	29.103	29.0678	28.915	29.4411	29.5089	29.2529	28.2782	1:Experimental evidence at protein level
27.755	27.6609	27.3923	27.2519	27.7013	27.6764	27.6646	27.507	1:Experimental evidence at protein level
29.62	29.5205	29.4572	29.512	29.6601	29.6008	29.619	29.7753	1:Experimental evidence at protein level
#NUM!	25.1751	24.9907	25.1033	25.9052	25.957	26.066	26.3162	1:Experimental evidence at protein level
31.7989	31.8045	32.063	32.1482	31.8667	31.7914	31.9682	31.8391	1:Experimental evidence at protein level
28.4941	28.5067	28.5343	28.549	27.2938	27.3446	27.2434	27.6431	1:Experimental evidence at protein level
26.0882	25.9647	24.0489	24.4893	28.547	28.5116	28.3887	28.1524	1:Experimental evidence at protein level
28.8527	28.7912	28.9072	29.0051	29.4222	29.425	29.3773	28.8703	1:Experimental evidence at protein level
28.6995	28.7148	28.6347	28.6525	28.7714	28.7606	28.721	28.9297	1:Experimental evidence at protein level
28.2403	28.1609	27.9097	27.7911	27.8448	28.0346	27.7102	27.9406	1:Experimental evidence at protein level
27.7123	27.7216	27.7031	27.7486	27.8964	27.8979	27.8332	28.0052	1:Experimental evidence at protein level
29.662	29.6582	29.5643	29.5404	29.7358	29.7117	29.7451	29.6871	1:Experimental evidence at protein level
32.0462	32.0187	32.2286	32.2877	31.1247	31.0396	31.2066	31.1898	1:Experimental evidence at protein level
28.9988	29.0037	29.0811	28.9519	28.8565	28.8562	28.858	28.4626	1:Experimental evidence at protein level
31.3037	31.4226	31.567	31.3533	31.4203	31.3898	31.4317	30.9862	1:Experimental evidence at protein level
28.9538	28.9524	29.034	29.138	29.1895	29.0348	29.209	29.0515	1:Experimental evidence at protein level
27.2425	27.1915	26.9866	27.0078	28.3203	28.3547	28.2522	28.3902	1:Experimental evidence at protein level
31.6663	31.6031	31.7267	31.7406	31.9391	31.8738	31.9385	31.9048	1:Experimental evidence at protein level
34.408	34.3497	34.3412	34.5906	34.2052	34.0417	34.058	34.2342	1:Experimental evidence at protein level
31.1139	31.1564	31.1039	31.276	31.1616	31.1361	31.2288	31.3854	1:Experimental evidence at protein level
27.2466	27.043	26.885	27.1361	27.1379	27.1188	27.0599	27.8299	1:Experimental evidence at protein level
31.9298	31.9044	32.11	32.1556	31.9158	31.8485	31.961	32.0264	1:Experimental evidence at protein level
28.1242	28.1089	28.0551	28.1372	28.0451	27.984	28.0485	28.125	1:Experimental evidence at protein level
31.914	31.7198	31.4169	31.3983	32.321	32.5078	32.0918	31.7686	1:Experimental evidence at protein level
29.7399	29.6943	29.6174	29.5956	29.645	29.6089	29.5092	29.4411	1:Experimental evidence at protein level
30.3853	30.3257	30.123	29.995	30.0946	30.2719	29.9641	29.2419	1:Experimental evidence at protein level
27.7491	27.6217	27.3818	27.5178	27.609	27.7109	27.6565	27.6743	1:Experimental evidence at protein level
31.8736	31.5307	31.0763	31.0465	31.2027	31.4162	30.9907	30.2834	1:Experimental evidence at protein level
33.0972	33.0751	33.1654	33.1847	33.0871	32.9892	33.0748	32.9132	1:Experimental evidence at protein level
29.4113	29.4719	29.4356	29.5613	29.5229	29.5159	29.6408	29.746	1:Experimental evidence at protein level
28.2572	28.2497	28.3306	28.3625	28.4022	28.3564	28.3984	28.6332	1:Experimental evidence at protein level
28.0136	28.0133	28.0354	28.1033	27.9818	27.9789	27.9236	28.1034	1:Experimental evidence at protein level
27.8422	27.7892	27.7762	27.8494	28.207	28.1614	28.194	28.6033	1:Experimental evidence at protein level
29.9381	29.8769	29.8494	29.8436	30.0771	29.9856	29.9618	30.0918	1:Experimental evidence at protein level
27.635	27.7254	27.5966	27.6204	26.9831	27.007	27.2805	27.3176	1:Experimental evidence at protein level
27.7015	27.6023	27.7991	27.7683	27.5173	27.5551	27.4887	27.6499	1:Experimental evidence at protein level
29.2352	29.2608	29.2519	29.1406	28.8573	28.9205	28.9036	28.7564	1:Experimental evidence at protein level
29.1115	29.1363	29.1725	29.0803	28.7246	28.8377	28.8625	28.7125	1:Experimental evidence at protein level
30.3899	30.1839	29.9991	30.0085	30.0688	30.1305	30.0214	29.9904	1:Experimental evidence at protein level

31.9648	31.9062	32.0518	32.1581	31.7844	31.7029	31.657	31.7741	1:Experimental evidence at protein level
30.9154	30.7861	30.7002	30.7532	31.3969	31.423	31.286	30.8466	1:Experimental evidence at protein level
28.7371	28.6162	28.6338	28.5385	28.8498	28.8173	28.676	28.2786	1:Experimental evidence at protein level
29.8481	29.7732	29.8906	29.948	30.2126	30.1333	30.173	30.2869	1:Experimental evidence at protein level
28.8801	28.8651	28.8919	28.894	28.9979	28.9472	28.9293	28.781	1:Experimental evidence at protein level
26.4993	26.6598	26.5601	26.7364	27.0323	27.2901	26.7804	27.3887	1:Experimental evidence at protein level
29.2591	29.222	29.2383	29.1759	29.3644	29.3452	29.2489	29.3915	1:Experimental evidence at protein level
30.5863	30.5644	30.6258	30.5384	30.6078	30.6104	30.5536	30.4539	1:Experimental evidence at protein level
30.3692	30.3211	30.4189	30.4615	30.4338	30.4768	30.4512	30.2266	1:Experimental evidence at protein level
29.2695	28.9305	27.9218	27.9914	30.0411	30.1053	29.8272	29.4289	1:Experimental evidence at protein level
28.7824	28.6395	28.3997	28.4841	29.4662	29.5525	29.472	29.4459	1:Experimental evidence at protein level

C: GOBP name
establishment of localization;establishment of protein localization;protein transport;transport
cellular ketone metabolic process;cellular macromolecule metabolic process;cellular metabolic process;cellular process;cellular protein metabolic process;cellular response to chemical stimulus;cellular response to oxidative
biological regulation;cell surface receptor linked signaling pathway;cellular process;cellular response to stimulus;detection of chemical stimulus;detection of chemical stimulus involved in sensory perception;detection of chem
biological regulation;biosynthetic process;cellular biosynthetic process;cellular macromolecule biosynthetic process;cellular macromolecule metabolic process;cellular metabolic process;cellular process;cellular protein metab
catabolic process;cellular catabolic process;cellular macromolecule catabolic process;cellular macromolecule metabolic process;cellular metabolic process;cellular nitrogen compound metabolic process;cellular process;dea
biological regulation;biosynthetic process;cellular biosynthetic process;cellular component organization;cellular component organization at cellular level;cellular component organization or biogenesis;cellular component orga
cellular macromolecule metabolic process;cellular metabolic process;cellular process;cellular protein metabolic process;establishment of localization;establishment of localization in cell;establishment of protein localization;es
amine catabolic process;amine metabolic process;aspartate family amino acid catabolic process;aspartate family amino acid metabolic process;biosynthetic process;carboxylic acid catabolic process;carboxylic acid metabo
cellular metabolic process;cellular process;electron transport chain;generation of precursor metabolites and energy;metabolic process;mitochondrial electron transport, NADH to ubiquinone;oxidation-reduction process;respi
aromatic compound biosynthetic process;biosynthetic process;cellular aromatic compound metabolic process;cellular biosynthetic process;cellular metabolic process;cellular nitrogen compound biosynthetic process;cellular
antigen processing and presentation;antigen processing and presentation of exogenous antigen;antigen processing and presentation of exogenous peptide antigen;antigen processing and presentation of exogenous peptide
biological regulation;biosynthetic process;cellular biosynthetic process;cellular component assembly;cellular component organization;cellular component organization or biogenesis;cellular metabolic process;cellular nitrogen
biological regulation;biosynthetic process;cellular biosynthetic process;cellular macromolecule biosynthetic process;cellular macromolecule metabolic process;cellular metabolic process;cellular process;cellular protein metab
activation of immune response;activation of innate immune response;biological regulation;cell surface receptor linked signaling pathway;cellular macromolecule metabolic process;cellular metabolic process;cellular process;c
anatomical structure morphogenesis;biological regulation;cellular localization;cellular macromolecule localization;cellular process;cellular protein localization;cellular response to stimulus;developmental process;embryonic mo
anatomical structure development;brain development;cation transport;cellular metabolic process;cellular process;copper ion transport;developmental process;establishment of localization;generation of precursor metabolites
biological regulation;regulation of biological process;regulation of biosynthetic process;regulation of cellular biosynthetic process;regulation of cellular macromolecule biosynthetic process;regulation of cellular metabolic proc
biological regulation;cellular macromolecule metabolic process;cellular metabolic process;cellular nitrogen compound metabolic process;cellular process;gene expression;macromolecule metabolic process;metabolic process
cellular component organization;cellular component organization at cellular level;cellular component organization or biogenesis;cellular component organization or biogenesis at cellular level;cellular process;chromatin modific
cellular component assembly;cellular component organization;cellular component organization or biogenesis;cellular macromolecule metabolic process;cellular metabolic process;cellular process;cellular protein metabolic pr
autophagic vacuole assembly;autophagy;catabolic process;cell communication;cellular catabolic process;cellular component assembly;cellular component assembly at cellular level;cellular component organization;cellular c
biological regulation;biosynthetic process;cellular biosynthetic process;cellular macromolecule biosynthetic process;cellular macromolecule metabolic process;cellular metabolic process;cellular nitrogen compound metabolic
response to chemical stimulus;response to organic substance;response to pheromone;response to stimulus
cellular process;establishment of localization;establishment of protein localization;ion transport;oligopeptide transport;peptide transport;protein transport;transmembrane transport;transport
cellular component organization;cellular component organization at cellular level;cellular component organization or biogenesis;cellular component organization or biogenesis at cellular level;cellular membrane fusion;cellular
biological regulation;cell cycle;cell cycle process;cellular component organization;cellular component organization at cellular level;cellular component organization or biogenesis;cellular component organization or biogenesis
biological regulation;cellular process;cellular response to stimulus;defense response;immune response;immune system process;innate immune response;intracellular signal transduction;regulation of biological process;regula
cellular macromolecule metabolic process;cellular metabolic process;cellular process;cellular protein metabolic process;establishment of localization;establishment of localization in cell;establishment of protein localization;es
biological regulation;biosynthetic process;cellular biosynthetic process;cellular macromolecule metabolic process;cellular metabolic process;cellular nitrogen compound metabolic process;cellular process;developmental proc
apoptosis;biological regulation;cell death;cell surface receptor linked signaling pathway;cellular macromolecule metabolic process;cellular metabolic process;cellular process;cellular protein metabolic process;cellular respons
cellular component assembly;cellular component organization;cellular component organization or biogenesis;macromolecular complex assembly;macromolecular complex subunit organization;metabolic process;protein com
cellular macromolecule metabolic process;cellular metabolic process;cellular process;cellular protein metabolic process;macromolecule metabolic process;metabolic process;primary metabolic process;protein folding;protein
cellular process;establishment of localization;establishment of localization in cell;establishment of protein localization;intracellular protein transport;intracellular transport;protein transport;transport;vesicle-mediated transport
metabolic process
acetyl-CoA catabolic process;acetyl-CoA metabolic process;biological regulation;catabolic process;cellular catabolic process;cellular metabolic process;cellular process;cellular response to chemical stimulus;cellular respons
biosynthetic process;carboxylic acid biosynthetic process;carboxylic acid metabolic process;cellular biosynthetic process;cellular ketone metabolic process;cellular lipid metabolic process;cellular macromolecule metabolic p
biological regulation;biosynthetic process;catabolic process;cell surface receptor linked signaling pathway;cellular biosynthetic process;cellular catabolic process;cellular macromolecule biosynthetic process;cellular macrom
autophagy;catabolic process;cellular catabolic process;cellular metabolic process;cellular process;metabolic process
biological regulation;cellular process;cellular response to stimulus;positive regulation of biological process;positive regulation of cell communication;positive regulation of cellular process;positive regulation of I-kappaB kinase
cell cycle;cell cycle process;cellular component movement;cellular component organization;cellular component organization at cellular level;cellular component organization or biogenesis;cellular component organization or b
anatomical structure morphogenesis;angiotensin maturation;anterior/posterior pattern specification;biological regulation;cellular macromolecule metabolic process;cellular metabolic process;cellular process;cellular protein m
cellular macromolecule metabolic process;cellular metabolic process;cellular nitrogen compound metabolic process;cellular process;macromolecule metabolic process;metabolic process;ncRNA metabolic process;ncRNA pr
cellular component organization;cellular component organization at cellular level;cellular component organization or biogenesis;cellular component organization or biogenesis at cellular level;cellular process;endocytic recyclin
anatomical structure morphogenesis;biological regulation;cellular component organization;cellular component organization at cellular level;cellular component organization or biogenesis;cellular component organization or bio
anatomical structure development;autophagy;binding of sperm to zona pellucida;catabolic process;cell recognition;cell-cell recognition;cellular catabolic process;cellular lipid metabolic process;cellular macromolecule metab
biological regulation;biosynthetic process;cell death;cellular biosynthetic process;cellular macromolecule biosynthetic process;cellular macromolecule metabolic process;cellular metabolic process;cellular nitrogen compound
biological regulation;cell differentiation;cellular developmental process;cellular macromolecule metabolic process;cellular metabolic process;cellular nitrogen compound metabolic process;cellular process;cellular response to
biological regulation;biosynthetic process;cellular biosynthetic process;cellular macromolecule biosynthetic process;cellular macromolecule metabolic process;cellular metabolic process;cellular nitrogen compound metabolic
biological regulation;biosynthetic process;cellular biosynthetic process;cellular macromolecule biosynthetic process;cellular macromolecule metabolic process;cellular metabolic process;cellular nitrogen compound metabolic
biological regulation;cellular component organization;cellular component organization or biogenesis;cellular process;cellular response to stimulus;establishment of localization;establishment of protein localization;intracellular
biological regulation;cellular process;cellular response to stimulus;endosome transport;establishment of localization;establishment of localization in cell;establishment of protein localization;intracellular protein transport;intrac
anion transport;biological regulation;cell volume homeostasis;cellular component assembly;cellular component assembly at cellular level;cellular component organization;cellular component organization at cellular level;cellula
biological regulation;biosynthetic process;cellular biosynthetic process;cellular macromolecule biosynthetic process;cellular macromolecule metabolic process;cellular metabolic process;cellular nitrogen compound metabolic
cellular macromolecule metabolic process;cellular metabolic process;cellular nitrogen compound metabolic process;cellular process;cellular response to stimulus;cellular response to stress;DNA metabolic process;DNA reco
catabolic process;cellular catabolic process;cellular macromolecule catabolic process;cellular macromolecule metabolic process;cellular metabolic process;cellular process;cellular protein metabolic process;macromolecule c
biological adhesion;biological regulation;blood coagulation;cell adhesion;cell migration;cell motility;cell surface receptor linked signaling pathway;cellular component movement;cellular component organization;cellular compo
biological regulation;cell surface receptor linked signaling pathway;cellular process;cellular response to chemical stimulus;cellular response to cytokine stimulus;cellular response to interferon-gamma;cellular response to orga
amine biosynthetic process;amine metabolic process;anatomical structure morphogenesis;biological regulation;biosynthetic process;carboxylic acid biosynthetic process;carboxylic acid metabolic process;cellular amine met
adult heart development;anatomical structure development;apoptosis;biological regulation;biosynthetic process;cell death;cell differentiation;cellular biosynthetic process;cellular developmental process;cellular macromolecu
biological regulation;cation homeostasis;cation transport;cellular cation homeostasis;cellular chemical homeostasis;cellular divalent inorganic cation homeostasis;cellular homeostasis;cellular ion homeostasis;cellular process
alcohol metabolic process;anatomical structure development;biological regulation;biosynthetic process;cellular process;cellular response to stimulus;cholesterol biosynthetic process;cholesterol metabolic process;developme
anatomical structure development;biological regulation;biosynthetic process;cell cycle arrest;cell cycle process;cellular biosynthetic process;cellular component organization;cellular component organization at cellular level;ce
biological regulation;cell proliferation;muscle cell proliferation;negative regulation of apoptosis;negative regulation of biological process;negative regulation of cell death;negative regulation of cellular process;negative regulati
cellular macromolecule metabolic process;cellular metabolic process;cellular nitrogen compound metabolic process;cellular process;establishment of localization;establishment of localization in cell;establishment of RNA loca
biological regulation;biosynthetic process;cellular biosynthetic process;cellular lipid metabolic process;cellular metabolic process;cellular process;glycerolipid biosynthetic process;glycerolipid metabolic process;glycerophos
cellular process;establishment of localization;transport;vesicle-mediated transport
biosynthetic process;carboxylic acid biosynthetic process;carboxylic acid metabolic process;cellular biosynthetic process;cellular ketone metabolic process;cellular lipid metabolic process;cellular metabolic process;cellular
cellular macromolecule metabolic process;cellular metabolic process;cellular process;cellular protein metabolic process;establishment of localization;establishment of localization in cell;establishment of protein localization;es
biological regulation;cellular macromolecule metabolic process;cellular metabolic process;cellular nitrogen compound metabolic process;cellular process;cellular protein metabolic process;cellular response to chemical stimu
cell differentiation;cellular component organization;cellular component organization at cellular level;cellular component organization or biogenesis;cellular component organization or biogenesis at cellular level;cellular develop
cellular macromolecule metabolic process;cellular metabolic process;cellular nitrogen compound metabolic process;cellular process;macromolecule metabolic process;metabolic process;mRNA metabolic process;mRNA pro
biological regulation;cation transport;cellular macromolecule metabolic process;cellular metabolic process;cellular process;cellular protein metabolic process;divalent inorganic cation transport;divalent metal ion transport;est
biological regulation;cell cycle cytokinesis;cell cycle phase;cell cycle process;cellular component organization;cellular component organization at cellular level;cellular component organization or biogenesis;cellular componen
biological regulation;cell differentiation;cellular component organization;cellular component organization or biogenesis;cellular developmental process;cellular membrane organization;cellular process;developmental process;e
metabolic process;response to chemical stimulus;response to external stimulus;response to extracellular stimulus;response to nutrient;response to nutrient levels;response to retinoic acid;response to stimulus;response to vit
biological regulation;cellular process;cellular response to stimulus;intracellular signal transduction;regulation of biological process;regulation of cellular process;regulation of response to stimulus;regulation of signal transducti
attachment of GPI anchor to protein;cellular macromolecule metabolic process;cellular metabolic process;cellular process;cellular protein metabolic process;C-terminal protein amino acid modification;C-terminal protein lipid
cellular macromolecule metabolic process;cellular metabolic process;cellular nitrogen compound metabolic process;cellular process;macromolecule metabolic process;metabolic process;ncRNA metabolic process;ncRNA pr
cation transport;cellular process;drug transmembrane transport;drug transport;establishment of localization;excretion;ion transport;multicellular organismal process;organic cation transport;secretion;system process;transme
cellular macromolecule metabolic process;cellular metabolic process;cellular nitrogen compound metabolic process;cellular process;macromolecule metabolic process;metabolic process;ncRNA metabolic process;ncRNA pr
biosynthetic process;cellular biosynthetic process;cellular macromolecule metabolic process;cellular metabolic process;cellular nitrogen compound metabolic process;cellular process;cellular protein metabolic process;gene
biosynthetic process;cellular biosynthetic process;cellular component disassembly;cellular component disassembly at cellular level;cellular component organization;cellular component organization at cellular level;cellular co
autophagy;biological adhesion;biological regulation;catabolic process;cell adhesion;cell junction assembly;cell junction organization;cell-cell adhesion;cellular catabolic process;cellular component assembly;cellular compone
biological regulation;biosynthetic process;cellular biosynthetic process;cellular component organization;cellular component organization or biogenesis;cellular macromolecule biosynthetic process;cellular macromolecule met
catabolic process;cellular macromolecule metabolic process;cellular metabolic process;cellular process;glycoprotein catabolic process;glycoprotein metabolic process;macromolecule catabolic process;macromolecule metal
biological regulation;cellular process;early endosome to late endosome transport;endosome transport;establishment of localization;establishment of localization in cell;establishment of protein localization;intracellular transpo
catabolic process;cellular catabolic process;cellular macromolecule catabolic process;cellular macromolecule metabolic process;cellular metabolic process;cellular nitrogen compound metabolic process;cellular process;cellu
activation of NF-kappaB-inducing kinase activity;activation of protein kinase activity;biological regulation;cellular macromolecule metabolic process;cellular metabolic process;cellular process;cellular protein metabolic proces
cell cycle phase;cell cycle process;cell division;cellular component organization;cellular component organization at cellular level;cellular component organization or biogenesis;cellular component organization or biogenesis at
biosynthetic process;cellular biosynthetic process;cellular component disassembly;cellular component disassembly at cellular level;cellular component organization;cellular component organization at cellular level;cellular co
biological regulation;cell differentiation;cellular developmental process;cellular process;developmental process;neuron differentiation;positive regulation of biological process;positive regulation of biosynthetic process;positive
multicellular organismal process;neurological system process;sensory perception;sensory perception of mechanical stimulus;sensory perception of sound;system process
biological regulation;biosynthetic process;cellular biosynthetic process;cellular component organization;cellular component organization at cellular level;cellular component organization or biogenesis;cellular component orga
adenosine metabolic process;catabolic process;cellular aromatic compound metabolic process;cellular catabolic process;cellular metabolic process;cellular nitrogen compound catabolic process;cellular nitrogen compound
cellular macromolecule metabolic process;cellular metabolic process;cellular process;cellular protein metabolic process;ER to Golgi vesicle-mediated transport;establishment of localization;establishment of localization in cel
cellular component organization;cellular component organization at cellular level;cellular component organization or biogenesis;cellular component organization or biogenesis at cellular level;cellular process;chromatin organiz
cellular macromolecule metabolic process;cellular metabolic process;cellular nitrogen compound metabolic process;cellular process;macromolecule metabolic process;macromolecule modification;metabolic process;ncRNA
biosynthetic process;cellular biosynthetic process;cellular component organization;cellular component organization at cellular level;cellular component organization or biogenesis;cellular component organization or biogenesis
biosynthetic process;cellular biosynthetic process;cellular component organization;cellular component organization at cellular level;cellular component organization or biogenesis;cellular component organization or biogenesis

cellular component assembly;cellular component assembly at cellular level;cellular component organization;cellular component organization at cellular level;cellular component organization or biogenesis;cellular component c
biological regulation;biosynthetic process;cellular biosynthetic process;cellular macromolecule biosynthetic process;cellular macromolecule metabolic process;cellular metabolic process;cellular nitrogen compound metabolic
biological regulation;biosynthetic process;cation transport;cellular biosynthetic process;cellular macromolecule biosynthetic process;cellular macromolecule metabolic process;cellular metabolic process;cellular nitrogen com
anatomical structure homeostasis;biological regulation;bone resorption;cell projection organization;cellular component organization;cellular component organization at cellular level;cellular component organization or biogenes
anatomical structure morphogenesis;biological regulation;biosynthetic process;cell differentiation;cellular biosynthetic process;cellular developmental process;cellular macromolecule biosynthetic process;cellular macromolec
gamete generation;male gamete generation;multicellular organismal process;multicellular organismal reproductive process;reproductive process;spermatogenesis
carbohydrate catabolic process;carbohydrate metabolic process;catabolic process;cell wall macromolecule catabolic process;cell wall macromolecule metabolic process;cell wall organization or biogenesis;cell wall polysacc
anatomical structure development;biological regulation;brain development;cellular process;cellular response to stimulus;detection of abiotic stimulus;detection of external stimulus;detection of light stimulus;detection of stimu
biological adhesion;biological regulation;catabolic process;cell adhesion;cell migration;cell motility;cell projection organization;cellular catabolic process;cellular component assembly;cellular component movement;cellular co
cellular component organization;cellular component organization at cellular level;cellular component organization or biogenesis;cellular component organization or biogenesis at cellular level;cellular membrane fusion;cellular
alcohol biosynthetic process;alcohol metabolic process;biological regulation;biosynthetic process;carbohydrate biosynthetic process;carbohydrate metabolic process;catabolic process;cellular biosynthetic process;cellular c
acyl-CoA metabolic process;bile acid biosynthetic process;bile acid metabolic process;biosynthetic process;carboxylic acid biosynthetic process;carboxylic acid catabolic process;carboxylic acid metabolic process;catabolic
biosynthetic process;cellular biosynthetic process;cellular component disassembly;cellular component disassembly at cellular level;cellular component organization;cellular component organization at cellular level;cellular co
biological regulation;cell surface receptor linked signaling pathway;cellular component organization;cellular component organization at cellular level;cellular component organization or biogenesis;cellular component organizat
biological regulation;biosynthetic process;catabolic process;cellular biosynthetic process;cellular catabolic process;cellular component assembly;cellular component assembly at cellular level;cellular component organization;
anion homeostasis;anion transport;bicarbonate transport;biological regulation;cellular metabolic process;cellular process;chemical homeostasis;chloride ion homeostasis;establishment of localization;homeostatic process;inc
biological regulation;cellular localization;cellular macromolecule localization;cellular macromolecule metabolic process;cellular metabolic process;cellular nitrogen compound metabolic process;cellular process;cellular protein
cell cycle phase;cell cycle process;cell division;cellular component organization;cellular component organization at cellular level;cellular component organization or biogenesis;cellular component organization or biogenesis at
cellular macromolecule metabolic process;cellular metabolic process;cellular nitrogen compound metabolic process;cellular process;macromolecule metabolic process;metabolic process;nitrogen compound metabolic proce
biological regulation;cell surface receptor linked signaling pathway;cellular process;cellular response to chemical stimulus;cellular response to cytokine stimulus;cellular response to organic substance;cellular response to stim
biological regulation;cellular component assembly;cellular component organization;cellular component organization at cellular level;cellular component organization or biogenesis;cellular component organization or biogenesis
biosynthetic process;cellular biosynthetic process;cellular macromolecule biosynthetic process;cellular macromolecule metabolic process;cellular metabolic process;cellular nitrogen compound metabolic process;cellular pro
cellular component assembly;cellular component assembly at cellular level;cellular component organization;cellular component organization at cellular level;cellular component organization or biogenesis;cellular component c
biological regulation;cation homeostasis;cation transport;cellular cation homeostasis;cellular chemical homeostasis;cellular component assembly;cellular component assembly at cellular level;cellular component organization;
biological regulation;regulation of biological process;regulation of cell proliferation;regulation of cellular process
biological regulation;cellular process;cellular response to chemical stimulus;cellular response to endogenous stimulus;cellular response to growth factor stimulus;cellular response to hormone stimulus;cellular response to org
cellular macromolecule metabolic process;cellular metabolic process;cellular nitrogen compound metabolic process;cellular process;macromolecule metabolic process;metabolic process;mRNA metabolic process;mRNA pro
biological regulation;cellular component assembly;cellular component organization;cellular component organization or biogenesis;macromolecular complex assembly;macromolecular complex subunit organization;positive re
acute inflammatory response;acute-phase response;anatomical structure development;biological regulation;cellular component organization;cellular component organization or biogenesis;cellular membrane organization;cellu
biological adhesion;cell adhesion;cellular process
activation of immune response;adaptive immune response;adaptive immune response based on somatic recombination of immune receptors built from immunoglobulin superfamily domains;biological regulation;cellular proce
butyrate catabolic process;butyrate metabolic process;carboxylic acid catabolic process;carboxylic acid metabolic process;catabolic process;cellular catabolic process;cellular component assembly;cellular component orga
biological regulation;biosynthetic process;cellular biosynthetic process;cellular macromolecule biosynthetic process;cellular macromolecule metabolic process;cellular metabolic process;cellular nitrogen compound metabolic
activation of immune response;activation of innate immune response;apoptosis;B cell homeostasis;B-1 B cell homeostasis;biological regulation;cell death;cellular component assembly;cellular component organization;cellula
anatomical structure development;biosynthetic process;cellular biosynthetic process;cellular macromolecule metabolic process;cellular metabolic process;cellular nitrogen compound metabolic process;cellular process;centr
adrenal gland development;aging;anatomical structure development;anatomical structure morphogenesis;anion transport;apoptosis;axon regeneration;axonogenesis;behavior;behavioral response to pain;biological regulation
biological regulation;biosynthetic process;catabolic process;cellular biosynthetic process;cellular catabolic process;cellular macromolecule biosynthetic process;cellular macromolecule catabolic process;cellular macromolec
biological regulation;dosage compensation;dosage compensation, by inactivation of X chromosome;posttranscriptional regulation of gene expression;regulation of biological process;regulation of biosynthetic process;regulat
actin cytoskeleton organization;actin filament bundle assembly;actin filament organization;actin filament-based process;biological regulation;biosynthetic process;blood coagulation;catabolic process;cell activation;cell surfac
anaphase-promoting complex-dependent proteasomal ubiquitin-dependent protein catabolic process;biological regulation;catabolic process;cell cycle;cell cycle checkpoint;cell cycle process;cell surface receptor linked sign
cellular macromolecule metabolic process;cellular metabolic process;cellular process;cellular protein metabolic process;macromolecule metabolic process;macromolecule modification;metabolic process;multicellular organis
aging;biological regulation;cell aging;cellular process;cellular response to chemical stimulus;cellular response to oxidative stress;cellular response to reactive oxygen species;cellular response to stimulus;cellular response to s
biological regulation;cell cycle;cell cycle process;cell division;cell proliferation;cellular process;G1/S transition of mitotic cell cycle;mitotic cell cycle;regulation of biological process;regulation of catalytic activity;regulation of c
biosynthetic process;cellular biosynthetic process;cellular component assembly;cellular component assembly at cellular level;cellular component organization;cellular component organization at cellular level;cellular compone
antibacterial humoral response;antimicrobial humoral response;biosynthetic process;catabolic process;cellular biosynthetic process;cellular catabolic process;cellular component disassembly;cellular component disassembly
biological regulation;cell communication;cell surface receptor linked signaling pathway;cell-cell signaling;cellular metabolic process;cellular process;cellular response to chemical stimulus;cellular response to endogenous sti
adenosine to inosine editing;base conversion or substitution editing;biological regulation;cellular macromolecule metabolic process;cellular metabolic process;cellular nitrogen compound metabolic process;cellular process;d
biosynthetic process;cellular biosynthetic process;cellular component disassembly;cellular component disassembly at cellular level;cellular component organization;cellular component organization at cellular level;cellular co
biological regulation;biosynthetic process;cellular biosynthetic process;cellular macromolecule biosynthetic process;cellular macromolecule metabolic process;cellular metabolic process;cellular nitrogen compound metabolic
amine catabolic process;amine metabolic process;branched chain family amino acid catabolic process;branched chain family amino acid metabolic process;brown fat cell differentiation;carboxylic acid catabolic process;carb
biological regulation;biosynthetic process;cellular biosynthetic process;cellular macromolecule biosynthetic process;cellular macromolecule metabolic process;cellular metabolic process;cellular nitrogen compound metabolic
biological regulation;biosynthetic process;cellular biosynthetic process;cellular component assembly;cellular component assembly at cellular level;cellular component organization;cellular component organization at cellular le
biological regulation;cell communication;cell-cell signaling;cellular component organization;cellular component organization or biogenesis;cellular membrane organization;cellular process;cellular response to stimulus;detectio
biological regulation;cell communication;cell-cell signaling;cellular process;cellular response to stimulus;establishment of localization;establishment of protein localization;protein transport;regulation of biological process;regu
anatomical structure development;anatomical structure formation involved in morphogenesis;biological regulation;biosynthetic process;carbohydrate metabolic process;catabolic process;cell development;cellular biosyntheti
activation of immune response;activation of innate immune response;apoptosis;biological regulation;biosynthetic process;cell death;cell surface receptor linked signaling pathway;cellular biosynthetic process;cellular macrom
alcohol metabolic process;biological regulation;carbohydrate homeostasis;carbohydrate metabolic process;carboxylic acid metabolic process;cellular carbohydrate metabolic process;cellular chemical homeostasis;cellular g
axon guidance;biological regulation;cell surface receptor linked signaling pathway;cellular component organization;cellular component organization or biogenesis;cellular membrane organization;cellular process;cellular respo
biological regulation;biosynthetic process;cellular biosynthetic process;cellular macromolecule biosynthetic process;cellular macromolecule metabolic process;cellular metabolic process;cellular nitrogen compound metabolic
apoptosis;biological regulation;cell death;cellular process;cellular response to chemical stimulus;cellular response to cytokine stimulus;cellular response to organic substance;cellular response to stimulus;cellular response to
biosynthetic process;cellular biosynthetic process;cellular component disassembly;cellular component disassembly at cellular level;cellular component organization;cellular component organization at cellular level;cellular co
biological regulation;cell cycle;cell cycle phase;cell cycle process;cell division;cellular component organization;cellular component organization at cellular level;cellular component organization or biogenesis;cellular componen
anatomical structure development;biological regulation;cell redox homeostasis;cellular homeostasis;cellular process;developmental process;hemopoiesis;hemopoietic or lymphoid organ development;homeostatic process;or
lipid metabolic process;metabolic process;primary metabolic process
cell cycle phase;cell cycle process;cell division;cellular component organization;cellular component organization at cellular level;cellular component organization or biogenesis;cellular component organization or biogenesis at
biological regulation;calcium ion homeostasis;calcium ion transport;cation homeostasis;cation transport;cellular calcium ion homeostasis;cellular cation homeostasis;cellular chemical homeostasis;cellular divalent inorganic c
biological regulation;cellular metabolic process;cellular process;electron transport chain;generation of precursor metabolites and energy;metabolic process;negative regulation of biological process;negative regulation of cellu
anatomical structure development;anatomical structure formation involved in morphogenesis;angiogenesis;biological regulation;blood vessel development;catabolic process;cellular process;cellular response to stimulus;deve
cation transport;cellular process;divalent inorganic cation transport;divalent metal ion transport;establishment of localization;ion transport;magnesium ion transport;metal ion transport;transmembrane transport;transport
activation of caspase activity;aging;apoptotic mitochondrial changes;autophagy;biological regulation;biosynthetic process;catabolic process;cell aging;cell cycle arrest;cell cycle process;cell division;cellular biosynthetic proc
biological regulation;cellular component assembly;cellular component assembly at cellular level;cellular component organization;cellular component organization at cellular level;cellular component organization or biogenesis;
3'-phosphoadenosine 5'-phosphosulfate biosynthetic process;3'-phosphoadenosine 5'-phosphosulfate metabolic process;3'-phosphoadenosine 5'-phosphosulfate transport;amine metabolic process;aminoglycan metabolic
biological regulation;biosynthetic process;cellular biosynthetic process;cellular component organization;cellular component organization at cellular level;cellular component organization or biogenesis;cellular component orga
anatomical structure development;biological regulation;biosynthetic process;cellular biosynthetic process;cellular macromolecule biosynthetic process;cellular macromolecule metabolic process;cellular metabolic process;cel
amine metabolic process;carboxylic acid metabolic process;cellular amine metabolic process;cellular amino acid metabolic process;cellular ketone metabolic process;cellular metabolic process;cellular nitrogen compound m
biological regulation;biosynthetic process;carbohydrate biosynthetic process;carbohydrate metabolic process;carbohydrate transport;cellular carbohydrate metabolic process;cellular component organization;cellular compon
biological regulation;posttranscriptional regulation of gene expression;protein destabilization;regulation of biological process;regulation of biological quality;regulation of gene expression;regulation of macromolecule metaboli
cellular process;establishment of localization;establishment of localization in cell;exocytosis;secretion;secretion by cell;transport;vesicle-mediated transport
cellular macromolecule metabolic process;cellular metabolic process;cellular nitrogen compound metabolic process;cellular process;macromolecule metabolic process;macromolecule methylation;macromolecule modificatio
actin cytoskeleton organization;actin filament organization;actin filament-based process;actin rod assembly;biological regulation;catabolic process;cellular catabolic process;cellular component assembly;cellular component a
activation of immune response;activation of innate immune response;antigen processing and presentation;antigen processing and presentation of exogenous antigen;antigen processing and presentation of exogenous peptic
acylglycerol catabolic process;acylglycerol metabolic process;arachidonic acid metabolic process;biological regulation;biosynthetic process;blood coagulation;carboxylic acid biosynthetic process;carboxylic acid metabolic p
anatomical structure development;biological regulation;biosynthetic process;cellular biosynthetic process;cellular macromolecule biosynthetic process;cellular macromolecule metabolic process;cellular metabolic process;cel
viral reproduction
biosynthetic process;cellular biosynthetic process;cellular macromolecule biosynthetic process;cellular macromolecule metabolic process;cellular metabolic process;cellular nitrogen compound metabolic process;cellular pro
biological regulation;catabolic process;cellular catabolic process;cellular macromolecule catabolic process;cellular macromolecule metabolic process;cellular metabolic process;cellular process;cellular protein metabolic proc
anatomical structure morphogenesis;developmental process;embryonic morphogenesis
amine metabolic process;aminoglycan catabolic process;aminoglycan metabolic process;carbohydrate catabolic process;carbohydrate metabolic process;catabolic process;chitin catabolic process;chitin metabolic process;
anatomical structure development;apoptosis;autophagic vacuole assembly;autophagy;biological regulation;blood vessel remodeling;cardiac cell development;cardiac muscle cell development;catabolic process;cell communi
biological regulation;biosynthetic process;cellular biosynthetic process;cellular component organization;cellular component organization at cellular level;cellular component organization or biogenesis;cellular component orga
biological regulation;negative regulation of apoptosis;negative regulation of biological process;negative regulation of cell death;negative regulation of cellular process;negative regulation of programmed cell death;positive reg
apoptosis;biological regulation;biosynthetic process;cell communication;cell death;cell growth;cellular biosynthetic process;cellular localization;cellular macromolecule biosynthetic process;cellular macromolecule localization
biological regulation;biosynthetic process;carbohydrate metabolic process;cellular biosynthetic process;cellular carbohydrate metabolic process;cellular component organization;cellular component organization at cellular lev
anatomical structure development;biological regulation;biosynthetic process;brain development;cation homeostasis;cellular biosynthetic process;cellular cation homeostasis;cellular chemical homeostasis;cellular homeostasi
biological regulation;cellular macromolecule metabolic process;cellular metabolic process;cellular nitrogen compound metabolic process;cellular process;cellular protein metabolic process;dephosphorylation;dephosphorylati
biological regulation;catabolic process;cellular catabolic process;cellular macromolecule metabolic process;cellular metabolic process;cellular nitrogen compound catabolic process;cellular nitrogen compound metabolic proc
biological regulation;biosynthetic process;cell differentiation;cellular biosynthetic process;cellular component assembly;cellular component assembly at cellular level;cellular component organization;cellular component organi
biological regulation;biosynthetic process;cell differentiation;cellular biosynthetic process;cellular component assembly;cellular component assembly at cellular level;cellular component organization;cellular component organi
box C/D snoRNP assembly;cell differentiation;cellular component assembly;cellular component assembly at cellular level;cellular component organization;cellular component organization at cellular level;cellular component o
biosynthetic process;cellular biosynthetic process;cellular component disassembly;cellular component disassembly at cellular level;cellular component organization;cellular component organization at cellular level;cellular co
biological regulation;cellular process;cellular response to abiotic stimulus;cellular response to chemical stimulus;cellular response to light stimulus;cellular response to oxidative stress;cellular response to radiation;cellular res
biological adhesion;biological regulation;cell adhesion;cell junction maintenance;cell junction organization;cell-cell adhesion;cell-cell junction maintenance;cell-cell junction organization;cellular component maintenance;cellula
catabolic process;cellular catabolic process;cellular macromolecule catabolic process;cellular macromolecule metabolic process;cellular metabolic process;cellular process;cellular protein metabolic process;macromolecule
biological regulation;biosynthetic process;cellular biosynthetic process;cellular macromolecule biosynthetic process;cellular macromolecule metabolic process;cellular metabolic process;cellular nitrogen compound metabolic
cellular metabolic process;cellular process;electron transport chain;generation of precursor metabolites and energy;metabolic process;multicellular organismal process;oxidation-reduction process;respiratory electron transpo
biological regulation;cellular process;cellular response to stimulus;negative regulation of biological process;negative regulation of cell communication;negative regulation of cellular process;negative regulation of Ras protein s
biological adhesion;cell adhesion;cell-cell adhesion;cellular component organization;cellular component organization or biogenesis;cellular membrane organization;cellular process;endocytosis;establishment of localization;m
biological regulation;cellular component assembly;cellular component assembly at cellular level;cellular component biogenesis;cellular component organization;cellular component organization at cellular level;cellular compo
biosynthetic process;cellular biosynthetic process;cellular metabolic process;cellular process;cofactor biosynthetic process;cofactor metabolic process;iron-sulfur cluster assembly;metabolic process;metallo-sulfur cluster as

cellular component biogenesis;cellular component biogenesis at cellular level;cellular component organization;cellular component organization at cellular level;cellular component organization or biogenesis;cellular componen
biological regulation;biosynthetic process;cellular biosynthetic process;cellular component organization;cellular component organization at cellular level;cellular component organization or biogenesis;cellular component orgar
establishment of localization;establishment of protein localization;establishment of RNA localization;mRNA transport;nucleic acid transport;nucleobase-containing compound transport;protein transport;RNA transport;transpo
cellular macromolecule metabolic process;cellular metabolic process;cellular nitrogen compound metabolic process;cellular process;macromolecule metabolic process;metabolic process;mRNA metabolic process;mRNA pro
anatomical structure development;anatomical structure homeostasis;biological regulation;bone resorption;catabolic process;cell differentiation;cellular catabolic process;cellular component organization;cellular component or
biological regulation;cellular metabolic process;cellular process;cellular response to stimulus;lipid metabolic process;metabolic process;oxidation-reduction process;primary metabolic process;regulation of biological process
axon ensheathment;biological regulation;cell cycle process;cellular component assembly;cellular component assembly at cellular level;cellular component organization;cellular component organization at cellular level;cellular
biological regulation;biosynthetic process;cellular biosynthetic process;cellular macromolecule biosynthetic process;cellular macromolecule metabolic process;cellular metabolic process;cellular nitrogen compound metabolic
biological regulation;biosynthetic process;cell surface receptor linked signaling pathway;cellular biosynthetic process;cellular macromolecule biosynthetic process;cellular macromolecule metabolic process;cellular metabolic
amide transport;antigen processing and presentation;antigen processing and presentation of endogenous antigen;antigen processing and presentation of endogenous peptide antigen;antigen processing and presentation of
biological regulation;regulation of biological process;regulation of cellular process;regulation of response to stimulus;regulation of signal transduction;regulation of signaling;regulation of TOR signaling cascade
anatomical structure development;biological regulation;biosynthetic process;cell cycle checkpoint;cellular biosynthetic process;cellular macromolecule biosynthetic process;cellular macromolecule metabolic process;cellular
acidic amino acid transport;amine transport;amino acid transport;anion transport;carboxylic acid transport;establishment of localization;establishment of protein localization;ion transport;L-glutamate transport;nitrogen compo
biological regulation;negative regulation of biological process;negative regulation of catalytic activity;negative regulation of cellular metabolic process;negative regulation of cellular process;negative regulation of cellular prote
biological regulation;calcium ion homeostasis;calcium ion transport;calcium ion transport into cytosol;cation homeostasis;cation transport;cellular calcium ion homeostasis;cellular cation homeostasis;cellular chemical homeo
apoptosis;cell death;cellular component assembly;cellular component assembly at cellular level;cellular component organization;cellular component organization at cellular level;cellular component organization or biogenesis;
cellular component organization;cellular component organization or biogenesis;cellular process;endosome transport;ER to Golgi vesicle-mediated transport;establishment of localization;establishment of localization in cell;est
assembly of spliceosomal tri-snRNP;biological regulation;cellular component assembly;cellular component assembly at cellular level;cellular component organization;cellular component organization at cellular level;cellular co
anion transport;biological regulation;cellular process;cellular response to stimulus;chloride transport;establishment of localization;inorganic anion transport;ion transport;regulation of biological process;regulation of cellular pr
actin cytoskeleton organization;actin filament branching;actin filament organization;actin filament-based process;activated T cell proliferation;activation of immune response;alpha-beta T cell activation;alpha-beta T cell differe
acylglycerol metabolic process;acylglycerol transport;biological regulation;blood coagulation, intrinsic pathway;cellular lipid metabolic process;cellular metabolic process;cellular process;establishment of localization;glycerol
biological regulation;negative regulation of binding;negative regulation of biological process;negative regulation of molecular function;negative regulation of protein binding;negative regulation of reproductive process;negative
biological regulation;biosynthetic process;catabolic process;cell surface receptor linked signaling pathway;cellular biosynthetic process;cellular catabolic process;cellular macromolecule biosynthetic process;cellular macrom
activation of MAPK activity;amine metabolic process;aminoglycan catabolic process;aminoglycan metabolic process;anatomical structure development;anatomical structure morphogenesis;biological regulation;biosynthetic p
apoptotic mitochondrial changes;biological regulation;cell surface receptor linked signaling pathway;cellular component organization;cellular component organization at cellular level;cellular component organization or biogen
biological regulation;biosynthetic process;carbohydrate biosynthetic process;carbohydrate metabolic process;cellular carbohydrate metabolic process;cellular lipid metabolic process;cellular macromolecule metabolic proces
acute inflammatory response;alcohol metabolic process;amine metabolic process;aminoglycan biosynthetic process;aminoglycan metabolic process;anatomical structure development;anatomical structure formation involved
activation of immune response;activation of innate immune response;biological regulation;cell surface receptor linked signaling pathway;cellular component assembly;cellular component assembly at cellular level;cellular com
adherens junction organization;anatomical structure morphogenesis;biological adhesion;biological regulation;blood vessel morphogenesis;calcium-dependent cell-cell adhesion;cell adhesion;cell adhesion mediated by integri
activation of immune response;activation of innate immune response;anaphase-promoting complex-dependent proteasomal ubiquitin-dependent protein catabolic process;antigen processing and presentation;antigen proces
ATP biosynthetic process;ATP metabolic process;ATP synthesis coupled proton transport;biosynthetic process;cation transport;cellular biosynthetic process;cellular metabolic process;cellular nitrogen compound biosynthetic
anaphase-promoting complex-dependent proteasomal ubiquitin-dependent protein catabolic process;biological regulation;catabolic process;cell cycle;cell cycle checkpoint;cell cycle process;cell proliferation;cellular catabol
apoptosis;biological regulation;cell death;cell surface receptor linked signaling pathway;cellular component disassembly;cellular component disassembly at cellular level;cellular component disassembly involved in apoptosis;
amine biosynthetic process;amine catabolic process;amine metabolic process;biological regulation;biosynthetic process;carbohydrate homeostasis;carboxylic acid biosynthetic process;carboxylic acid metabolic process;cat
carboxylic acid metabolic process;cellular aromatic compound metabolic process;cellular ketone metabolic process;cellular metabolic process;cellular process;coenzyme metabolic process;cofactor metabolic process;folic a
apoptosis;cell death;cellular metabolic process;cellular nitrogen compound metabolic process;cellular process;death;metabolic process;nitrogen compound metabolic process;nucleobase-containing compound metabolic pr
acylglycerol transport;calcium ion-dependent exocytosis;cell activation;cell activation involved in immune response;cellular component assembly;cellular component assembly at cellular level;cellular component organization;
biological regulation;biosynthetic process;cellular biosynthetic process;cellular macromolecule biosynthetic process;cellular macromolecule metabolic process;cellular metabolic process;cellular nitrogen compound metabolic
biological regulation;cell cycle;cell cycle phase;cell cycle process;cell division;cell surface receptor linked signaling pathway;cellular component organization;cellular component organization at cellular level;cellular componen
activation of MAPK activity;ameboidal cell migration;anatomical structure development;anatomical structure morphogenesis;apoptosis;axon guidance;biological regulation;brain development;branching morphogenesis of a tu
activation of immune response;activation of innate immune response;apoptosis;biological regulation;biosynthetic process;BMP signaling pathway;catabolic process;cell death;cell surface receptor linked signaling pathway;ce
cellular macromolecule metabolic process;cellular metabolic process;cellular process;cellular protein metabolic process;developmental process;macromolecule metabolic process;macromolecule modification;metabolic proc
anatomical structure development;biological regulation;biosynthetic process;cellular biosynthetic process;cellular component organization;cellular component organization at cellular level;cellular component organization or b
cellular component organization;cellular component organization at cellular level;cellular component organization or biogenesis;cellular component organization or biogenesis at cellular level;cellular macromolecule metabolic
biological regulation;cellular component assembly;cellular component assembly at cellular level;cellular component organization;cellular component organization at cellular level;cellular component organization or biogenesis;
antibacterial humoral response;antimicrobial humoral response;biosynthetic process;catabolic process;cellular biosynthetic process;cellular catabolic process;cellular component disassembly;cellular component disassembly
biological regulation;biosynthetic process;catabolic process;cell surface receptor linked signaling pathway;cellular biosynthetic process;cellular catabolic process;cellular macromolecule biosynthetic process;cellular macrom
biological regulation;biosynthetic process;cellular biosynthetic process;cellular component organization;cellular component organization at cellular level;cellular component organization or biogenesis;cellular component orgar
actin cytoskeleton organization;actin cytoskeleton reorganization;actin filament-based process;anatomical structure development;anatomical structure homeostasis;biological adhesion;biological regulation;blood coagulation;
cellular macromolecule metabolic process;cellular metabolic process;cellular nitrogen compound metabolic process;cellular process;macromolecule metabolic process;metabolic process;nitrogen compound metabolic proce
alcohol metabolic process;carbohydrate metabolic process;cellular carbohydrate metabolic process;cellular metabolic process;cellular process;galactose metabolic process;hexose metabolic process;metabolic process;mor
antigen processing and presentation;antigen processing and presentation of exogenous antigen;antigen processing and presentation of exogenous peptide antigen;antigen processing and presentation of exogenous peptide
anatomical structure development;anatomical structure morphogenesis;B cell activation;B cell differentiation;biological regulation;biosynthetic process;cell activation;cell differentiation;cellular biosynthetic process;cellular de
anatomical structure morphogenesis;anterior/posterior pattern specification;biological regulation;biosynthetic process;camera-type eye morphogenesis;cellular biosynthetic process;cellular component organization;cellular co
B cell activation;biological regulation;cell activation;cell communication;cell proliferation;cell surface receptor linked signaling pathway;cell-cell signaling;cellular process;cellular response to chemical stimulus;cellular respons
cellular macromolecule metabolic process;cellular metabolic process;cellular process;cellular protein metabolic process;dephosphorylation;macromolecule metabolic process;macromolecule modification;metabolic process;p
apoptosis;biological regulation;biosynthetic process;cell death;cellular biosynthetic process;cellular metabolic process;cellular nitrogen compound biosynthetic process;cellular nitrogen compound metabolic process;cellular
anatomical structure development;biological regulation;biosynthetic process;cap-independent translational initiation;cell cycle process;cell surface receptor linked signaling pathway;cellular biosynthetic process;cellular macr
anatomical structure maturation;anatomical structure morphogenesis;axon ensheathment;axon guidance;basement membrane organization;biological adhesion;biological regulation;branching involved in salivary gland morph
biological regulation;biosynthetic process;cellular biosynthetic process;cellular macromolecule biosynthetic process;cellular macromolecule metabolic process;cellular metabolic process;cellular nitrogen compound metabolic
biological regulation;biosynthetic process;cellular biosynthetic process;cellular component organization;cellular component organization at cellular level;cellular component organization or biogenesis;cellular component orgar
aerobic respiration;biological regulation;cellular component assembly;cellular component assembly at cellular level;cellular component biogenesis;cellular component organization;cellular component organization at cellular le
apoptosis;cell death;cellular component organization;cellular component organization or biogenesis;cellular membrane organization;cellular process;death;endocytosis;establishment of localization;establishment of protein lo
alcohol metabolic process;carbohydrate metabolic process;carbohydrate phosphorylation;cellular carbohydrate metabolic process;cellular metabolic process;cellular process;fructose 2,6-bisphosphate metabolic process;fru
biological regulation;cellular process;cellular response to stimulus;establishment of localization;establishment of localization in cell;establishment of protein localization;intracellular protein transport;intracellular signal transdu
cellular macromolecule metabolic process;cellular metabolic process;cellular nitrogen compound metabolic process;cellular process;cleavage in ITS2 between 5.8S rRNA and LSU-rRNA of tricistronic rRNA transcript (SSU-r
actin filament-based movement;actin filament-based process;anatomical structure formation involved in morphogenesis;autophagy;biological regulation;catabolic process;cell projection assembly;cell projection organization;
biological regulation;biosynthetic process;cellular biosynthetic process;cellular macromolecule biosynthetic process;cellular macromolecule metabolic process;cellular metabolic process;cellular nitrogen compound metabolic
response to abiotic stimulus;response to ionizing radiation;response to radiation;response to stimulus
autophagic vacuole assembly;autophagy;catabolic process;cellular catabolic process;cellular component assembly;cellular component assembly at cellular level;cellular component organization;cellular component organizati
ameboidal cell migration;biological regulation;cell cycle phase;cell cycle process;cell division;cell migration;cell motility;cellular component assembly;cellular component assembly at cellular level;cellular component movemer
cellular macromolecule metabolic process;cellular metabolic process;cellular nitrogen compound metabolic process;cellular process;macromolecule metabolic process;metabolic process;mRNA metabolic process;mRNA pro
apoptosis;biological regulation;biosynthetic process;cell death;cellular biosynthetic process;cellular component organization;cellular component organization at cellular level;cellular component organization or biogenesis;cellu
anatomical structure development;biological regulation;carbohydrate metabolic process;cardiovascular system development;cell surface receptor linked signaling pathway;cellular carbohydrate metabolic process;cellular ma
biological regulation;cell surface receptor linked signaling pathway;cellular process;cellular response to chemical stimulus;cellular response to fibroblast growth factor stimulus;cellular response to growth factor stimulus;cellul
biological regulation;biosynthetic process;carbohydrate metabolic process;carbohydrate transport;cell cycle;cell cycle phase;cell cycle process;cell division;cell surface receptor linked signaling pathway;cellular biosynthetic p
adhesion to host;adhesion to other organism involved in symbiotic interaction;alcohol metabolic process;androgen biosynthetic process;androgen metabolic process;biological adhesion;biological regulation;biosynthetic proc
amine metabolic process;amine transport;amino acid transmembrane transport;amino acid transport;biological regulation;blood coagulation;carboxylic acid metabolic process;carboxylic acid transport;cell migration;cell moti
biological regulation;biosynthetic process;catabolic process;cell surface receptor linked signaling pathway;cellular biosynthetic process;cellular catabolic process;cellular macromolecule biosynthetic process;cellular macrom
biological regulation;cell cycle checkpoint;mitotic cell cycle checkpoint;regulation of biological process;regulation of cell cycle;regulation of cell cycle arrest;regulation of cell cycle process;regulation of cellular process;regulat
catabolic process;cellular catabolic process;cellular macromolecule catabolic process;cellular macromolecule metabolic process;cellular metabolic process;cellular nitrogen compound metabolic process;cellular process;exo
cellular macromolecule metabolic process;cellular metabolic process;cellular nitrogen compound metabolic process;cellular process;cellular protein metabolic process;macromolecule metabolic process;macromolecule modi
alcohol metabolic process;carbohydrate metabolic process;cellular carbohydrate metabolic process;cellular metabolic process;cellular process;galactose metabolic process;glucose metabolic process;hexose metabolic proc
biological regulation;catabolic process;cellular catabolic process;cellular macromolecule catabolic process;cellular macromolecule metabolic process;cellular metabolic process;cellular nitrogen compound metabolic process
biosynthetic process;cellular biosynthetic process;cellular component disassembly;cellular component disassembly at cellular level;cellular component organization;cellular component organization at cellular level;cellular cor
biosynthetic process;cellular biosynthetic process;cellular component disassembly;cellular component disassembly at cellular level;cellular component organization;cellular component organization at cellular level;cellular cor
cellular component biogenesis;cellular component biogenesis at cellular level;cellular component organization or biogenesis;cellular component organization or biogenesis at cellular level;cellular macromolecule metabolic pro
abscission;aging;anaphase-promoting complex-dependent proteasomal ubiquitin-dependent protein catabolic process;attachment of spindle microtubules to chromosome;attachment of spindle microtubules to kinetochore;
amine biosynthetic process;amine metabolic process;amino acid salvage;apoptosis;aspartate family amino acid biosynthetic process;aspartate family amino acid metabolic process;biological regulation;biosynthetic process;
biological regulation;cellular macromolecule metabolic process;cellular metabolic process;cellular nitrogen compound metabolic process;cellular process;cellular response to stimulus;cellular response to stress;cytoplasmic s
abscission;biological regulation;cell cycle;cell division;cellular process;developmental process;negative regulation of biological process;negative regulation of cell division;negative regulation of cellular process;negative regula
biological regulation;biosynthetic process;cellular biosynthetic process;cellular macromolecule biosynthetic process;cellular macromolecule metabolic process;cellular metabolic process;cellular nitrogen compound metabolic
biological regulation;biosynthetic process;cellular biosynthetic process;cellular macromolecule biosynthetic process;cellular macromolecule metabolic process;cellular metabolic process;cellular nitrogen compound metabolic
cellular macromolecule metabolic process;cellular metabolic process;cellular nitrogen compound metabolic process;cellular process;macromolecule metabolic process;maturation of 5.8S rRNA;metabolic process;ncRNA met
behavior;biological regulation;body fluid secretion;carbohydrate homeostasis;cellular chemical homeostasis;cellular glucose homeostasis;cellular homeostasis;cellular ion homeostasis;cellular metabolic process;cellular nitrog
anatomical structure development;axon guidance;biological regulation;cellular process;cellular response to stimulus;chemotaxis;developmental process;locomotion;nervous system development;regulation of biological proce
cell cycle;cellular process
cell cycle phase;cell cycle process;cell division;cellular component assembly;cellular component assembly at cellular level;cellular component organization;cellular component organization at cellular level;cellular component
biological regulation;negative regulation of biological process;negative regulation of cellular metabolic process;negative regulation of cellular process;negative regulation of macromolecule metabolic process;negative regulati
cell cycle process;cellular component assembly;cellular component assembly at cellular level;cellular component organization;cellular component organization at cellular level;cellular component organization or biogenesis;ce
cellular macromolecule metabolic process;cellular metabolic process;cellular nitrogen compound metabolic process;cellular process;macromolecule metabolic process;metabolic process;ncRNA metabolic process;ncRNA pr
establishment of localization;lipid transport;organic substance transport;transport
biological regulation;cellular process;cellular response to abiotic stimulus;cellular response to gamma radiation;cellular response to ionizing radiation;cellular response to radiation;cellular response to stimulus;cellular respons
biosynthetic process;cellular biosynthetic process;cellular macromolecule biosynthetic process;cellular macromolecule metabolic process;cellular metabolic process;cellular nitrogen compound metabolic process;cellular pro
cellular macromolecule metabolic process;cellular metabolic process;cellular nitrogen compound metabolic process;cellular process;gene expression;macromolecule metabolic process;metabolic process;mRNA metabolic p
biosynthetic process;cellular biosynthetic process;cellular component disassembly;cellular component disassembly at cellular level;cellular component organization;cellular component organization at cellular level;cellular cor
biosynthetic process;cellular biosynthetic process;cellular component disassembly;cellular component disassembly at cellular level;cellular component organization;cellular component organization at cellular level;cellular cor

biological regulation;cell cycle;cell cycle phase;cell cycle process;cell division;cellular component organization;cellular component organization at cellular level;cellular component organization or biogenesis;cellular componen
cellular macromolecule metabolic process;cellular metabolic process;cellular process;cellular protein metabolic process;cullin deneddylation;macromolecule metabolic process;macromolecule modification;metabolic process
biological regulation;biosynthetic process;cellular biosynthetic process;cellular component organization;cellular component organization at cellular level;cellular component organization or biogenesis;cellular component orgar
biological regulation;biosynthetic process;cellular biosynthetic process;cellular component organization;cellular component organization at cellular level;cellular component organization or biogenesis;cellular component orgar
B cell activation;B cell activation involved in immune response;biological regulation;catabolic process;cell activation;cell activation involved in immune response;cellular catabolic process;cellular localization;cellular macromo
apoptosis;biological regulation;carbohydrate homeostasis;cell death;cellular component organization;cellular component organization or biogenesis;cellular membrane organization;cellular process;cellular response to biotic s
anatomical structure morphogenesis;asymmetric protein localization;biological regulation;cell communication;cell-cell signaling;cellular process;developmental process;establishment of localization;establishment of localizatio
cellular metabolic process;cellular process;electron transport chain;generation of precursor metabolites and energy;metabolic process;oxidation-reduction process;respiratory electron transport chain;small molecule metaboli
biological regulation;biosynthetic process;cellular biosynthetic process;cellular component assembly;cellular component assembly at cellular level;cellular component organization;cellular component organization at cellular le
cellular metabolic process;cellular process;electron transport chain;generation of precursor metabolites and energy;metabolic process;mitochondrial electron transport, ubiquinol to cytochrome c;oxidation-reduction process;
autophagy;catabolic process;cellular catabolic process;cellular metabolic process;cellular process;endosome transport;establishment of localization;establishment of localization in cell;establishment of protein localization;int
anatomical structure morphogenesis;apoptotic mitochondrial changes;autophagy;biological regulation;calcium ion homeostasis;calcium-mediated signaling;calcium-mediated signaling using intracellular calcium source;catab
cellular macromolecule metabolic process;cellular metabolic process;cellular process;cellular protein metabolic process;chaperone-mediated protein transport;establishment of localization;establishment of localization in cell;
cellular macromolecule metabolic process;cellular metabolic process;cellular process;cellular protein metabolic process;chaperone-mediated protein transport;establishment of localization;establishment of localization in cell;
anatomical structure morphogenesis;biological regulation;biosynthetic process;cell morphogenesis;cell part morphogenesis;cell proliferation;cellular biosynthetic process;cellular component morphogenesis;cellular componen
biosynthetic process;carboxylic acid biosynthetic process;carboxylic acid metabolic process;cellular biosynthetic process;cellular ketone metabolic process;cellular lipid metabolic process;cellular metabolic process;cellular p
cellular component organization;cellular component organization at cellular level;cellular component organization or biogenesis;cellular component organization or biogenesis at cellular level;cellular membrane organization;ce
biological regulation;biosynthetic process;cell cycle;cell surface receptor linked signaling pathway;cellular biosynthetic process;cellular component organization;cellular component organization at cellular level;cellular compor
catabolic process;cell differentiation;cellular catabolic process;cellular developmental process;cellular macromolecule catabolic process;cellular macromolecule metabolic process;cellular metabolic process;cellular nitrogen c
activation of anaphase-promoting complex activity;anaphase-promoting complex-dependent proteasomal ubiquitin-dependent protein catabolic process;biological regulation;catabolic process;cell cycle;cell cycle checkpoint
amine catabolic process;amine metabolic process;branched chain family amino acid catabolic process;branched chain family amino acid metabolic process;carboxylic acid catabolic process;carboxylic acid metabolic proces
biological regulation;cell communication;cell migration;cell motility;cell surface receptor linked signaling pathway;cell-cell signaling;cellular component movement;cellular process;cellular response to stimulus;endothelial cell r
cellular process;establishment of localization;establishment of localization in cell;establishment of protein localization;Golgi vesicle transport;intracellular transport;post-Golgi vesicle-mediated transport;protein transport;trans
biological regulation;cellular component organization;cellular component organization or biogenesis;cellular localization;cellular macromolecule localization;cellular membrane organization;cellular process;cellular protein local
metabolic process;oxidation-reduction process
cellular process;establishment of localization;transport;vesicle-mediated transport
cellular metabolic process;cellular process;electron transport chain;generation of precursor metabolites and energy;metabolic process;mitochondrial electron transport, NADH to ubiquinone;oxidation-reduction process;respi
establishment of localization;establishment of localization in cell;establishment of protein localization;intracellular protein transport;intracellular transport;protein targeting;protein transport;transport
anatomical structure development;cellular macromolecule metabolic process;cellular metabolic process;cellular process;cellular protein metabolic process;chaperone-mediated protein transport;developmental process;estab
alternative nuclear mRNA splicing, via spliceosome;biological regulation;biosynthetic process;cell projection organization;cellular biosynthetic process;cellular component organization;cellular component organization at cellul
cellular macromolecule metabolic process;cellular metabolic process;cellular nitrogen compound metabolic process;cellular process;macromolecule metabolic process;metabolic process;ncRNA metabolic process;ncRNA pr
ATP biosynthetic process;ATP metabolic process;ATP synthesis coupled proton transport;biosynthetic process;cation transport;cellular biosynthetic process;cellular metabolic process;cellular nitrogen compound biosynthetic
cellular component assembly;cellular component assembly at cellular level;cellular component organization;cellular component organization at cellular level;cellular component organization or biogenesis;cellular component c
cellular metabolic process;cellular process;electron transport chain;generation of precursor metabolites and energy;metabolic process;mitochondrial electron transport, NADH to ubiquinone;oxidation-reduction process;respi
cellular macromolecule metabolic process;cellular metabolic process;cellular nitrogen compound metabolic process;cellular process;macromolecule metabolic process;metabolic process;ncRNA metabolic process;ncRNA pr
biological regulation;cell cycle cytokinesis;cell cycle process;cellular macromolecule metabolic process;cellular metabolic process;cellular process;cellular protein metabolic process;cellular response to stimulus;cytokinesis;c
anatomical structure development;ATP-dependent chromatin remodeling;biological regulation;biosynthetic process;cellular biosynthetic process;cellular component organization;cellular component organization at cellular lev
biological regulation;cellular component assembly;cellular component organization;cellular component organization at cellular level;cellular component organization or biogenesis;cellular component organization or biogenesis
carboxylic acid metabolic process;cellular ketone metabolic process;cellular metabolic process;cellular process;L-ascorbic acid metabolic process;metabolic process;organic acid metabolic process;oxoacid metabolic proce
amine biosynthetic process;amine metabolic process;aromatic compound biosynthetic process;biological regulation;biosynthetic process;carboxylic acid biosynthetic process;carboxylic acid metabolic process;cell cycle;cell
alcohol biosynthetic process;alcohol metabolic process;anatomical structure development;anatomical structure formation involved in morphogenesis;anatomical structure homeostasis;anatomical structure morphogenesis;ap
adult behavior;adult locomotory behavior;behavior;biological regulation;locomotory behavior;multicellular organismal process;negative regulation of biological process;negative regulation of catalytic activity;negative regulatio
biological regulation;carbohydrate catabolic process;carbohydrate metabolic process;catabolic process;cellular carbohydrate catabolic process;cellular carbohydrate metabolic process;cellular catabolic process;cellular lipid
anatomical structure development;anatomical structure morphogenesis;biological regulation;biosynthetic process;cell differentiation;cell surface receptor linked signaling pathway;cellular biosynthetic process;cellular develop
activation of MAPK activity;adult behavior;anatomical structure development;anatomical structure morphogenesis;axon guidance;behavior;biological regulation;brain development;branching morphogenesis of a tube;carbohy
amine catabolic process;amine metabolic process;anatomical structure development;aromatic amino acid family catabolic process;aromatic amino acid family metabolic process;aromatic compound biosynthetic process;aro
biological regulation;carbohydrate metabolic process;cellular carbohydrate metabolic process;cellular macromolecule metabolic process;cellular metabolic process;cellular process;cellular protein metabolic process;cellular r
biosynthetic process;cellular biosynthetic process;cellular macromolecule metabolic process;cellular metabolic process;cellular nitrogen compound metabolic process;cellular process;electron transport chain;gene expressio
biological regulation;biosynthetic process;cellular biosynthetic process;cellular macromolecule biosynthetic process;cellular macromolecule metabolic process;cellular metabolic process;cellular nitrogen compound metabolic
apoptosis;biological regulation;biosynthetic process;catabolic process;cell death;cell differentiation;cellular biosynthetic process;cellular catabolic process;cellular developmental process;cellular macromolecule biosynthetic p
cell migration;cell motility;cellular component movement;cellular process;endothelial cell migration;locomotion;response to chemical stimulus;response to organic substance;response to stimulus
cellular macromolecule metabolic process;cellular metabolic process;cellular process;cellular protein metabolic process;macromolecule metabolic process;macromolecule modification;metabolic process;peptidyl-amino acid
biological regulation;biosynthetic process;catabolic process;cell surface receptor linked signaling pathway;cellular aromatic compound metabolic process;cellular biosynthetic process;cellular catabolic process;cellular compo
carboxylic acid catabolic process;carboxylic acid metabolic process;carboxylic acid transport;catabolic process;cellular catabolic process;cellular component organization;cellular component organization at cellular level;cellu
anatomical structure homeostasis;base-excision repair;biological regulation;biosynthetic process;catabolic process;cell cycle;cell cycle process;cellular biosynthetic process;cellular catabolic process;cellular component asse
anatomical structure development;anatomical structure morphogenesis;biological regulation;cell development;cell part morphogenesis;cell projection morphogenesis;cell projection organization;cell surface receptor linked sig
aging;amine metabolic process;arachidonic acid metabolic process;biological regulation;carboxylic acid metabolic process;catabolic process;cellular amine metabolic process;cellular amino acid metabolic process;cellular ca
aging;biological regulation;biosynthetic process;carbohydrate metabolic process;carbohydrate transport;cell aging;cell cycle;cell cycle process;cell death;cell surface receptor linked signaling pathway;cellular biosynthetic pro
biological regulation;cell cycle;cell cycle arrest;cell cycle process;cell differentiation;cellular developmental process;cellular process;developmental process;G1/S transition of mitotic cell cycle;glial cell differentiation;mitotic ce
acute inflammatory response;anatomical structure formation involved in morphogenesis;angiogenesis;angiogenesis involved in wound healing;biological regulation;blood coagulation;blood coagulation, intrinsic pathway;carb
cellular macromolecule metabolic process;cellular metabolic process;cellular process;cellular protein metabolic process;macromolecule metabolic process;macromolecule modification;metabolic process;peptidyl-amino acid
biological regulation;cell cycle checkpoint;cellular component organization;cellular component organization at cellular level;cellular component organization or biogenesis;cellular component organization or biogenesis at cellu
amine biosynthetic process;amine metabolic process;apoptotic mitochondrial changes;biological regulation;biosynthetic process;carboxylic acid biosynthetic process;carboxylic acid metabolic process;cellular amine metabo
biosynthetic process;cellular biosynthetic process;cellular lipid metabolic process;cellular metabolic process;cellular process;glycerolipid biosynthetic process;glycerolipid metabolic process;glycerophospholipid biosynthetic
anatomical structure development;anatomical structure morphogenesis;biological regulation;cell proliferation;cellular process;cellular response to abiotic stimulus;cellular response to antibiotic;cellular response to chemical st
androgen receptor signaling pathway;biological regulation;biosynthetic process;catabolic process;cell cycle;cell cycle arrest;cell cycle process;cell division;cell proliferation;cellular biosynthetic process;cellular catabolic proce
adult behavior;adult locomotory behavior;anatomical structure development;associative learning;behavior;biological regulation;brain development;catabolic process;cation homeostasis;cell development;cellular catabolic pro
activation of caspase activity;apoptosis;autophagy;biological regulation;catabolic process;cell communication;cell death;cellular catabolic process;cellular metabolic process;cellular process;cellular response to amino acid st
acetyl-CoA catabolic process;acetyl-CoA metabolic process;carbohydrate metabolic process;carboxylic acid metabolic process;catabolic process;cellular catabolic process;cellular ketone metabolic process;cellular metabol
biological regulation;catabolic process;cellular catabolic process;cellular macromolecule catabolic process;cellular macromolecule metabolic process;cellular metabolic process;cellular nitrogen compound metabolic process
activation of caspase activity;activation of caspase activity by cytochrome c;aging;anatomical structure formation involved in morphogenesis;apoptosis;biological regulation;cell death;cell differentiation;cell surface receptor li
ATP biosynthetic process;ATP metabolic process;ATP synthesis coupled proton transport;biosynthetic process;cation transport;cellular biosynthetic process;cellular metabolic process;cellular nitrogen compound biosynthetic
alcohol metabolic process;amine metabolic process;anatomical structure development;anatomical structure morphogenesis;benzene-containing compound metabolic process;biological regulation;catecholamine metabolic p
cellular component organization;cellular component organization at cellular level;cellular component organization or biogenesis;cellular component organization or biogenesis at cellular level;cellular process;COPI coating of G
establishment of localization;establishment of localization in cell;establishment of protein localization;intracellular protein transport;intracellular transport;nuclear export;nuclear transport;nucleocytoplasmic transport;protein e
catabolic process;cellular catabolic process;cellular macromolecule catabolic process;cellular macromolecule metabolic process;cellular metabolic process;cellular nitrogen compound metabolic process;cellular process;exo
catabolic process;cellular catabolic process;cellular macromolecule catabolic process;cellular macromolecule metabolic process;cellular metabolic process;cellular nitrogen compound metabolic process;cellular process;exo
90S preribosome assembly;anatomical structure development;anatomical structure morphogenesis;axial mesoderm development;biological regulation;biosynthetic process;catabolic process;cellular biosynthetic process;cell
adiponectin-mediated signaling pathway;anatomical structure morphogenesis;axon guidance;biological regulation;cell cycle;cell surface receptor linked signaling pathway;cellular component assembly;cellular component ass
anatomical structure formation involved in morphogenesis;anatomical structure morphogenesis;axon cargo transport;axon guidance;biological adhesion;biological regulation;cell adhesion;cell junction assembly;cell junction c
biological regulation;cellular component organization;cellular component organization or biogenesis;cellular process;cellular response to stimulus;intracellular signal transduction;positive regulation of actin filament polymeriza
B cell activation;biological regulation;cell activation;cellular macromolecule metabolic process;cellular metabolic process;cellular nitrogen compound metabolic process;cellular process;cellular response to stimulus;immune s
biological regulation;biosynthetic process;body fluid secretion;cellular aromatic compound metabolic process;cellular biosynthetic process;cellular metabolic process;cellular nitrogen compound biosynthetic process;cellular
anatomical structure formation involved in morphogenesis;angiogenesis;cell differentiation;cellular developmental process;cellular localization;cellular macromolecule localization;cellular process;cellular protein complex local
antigen processing and presentation;antigen processing and presentation of endogenous antigen;antigen processing and presentation of endogenous peptide antigen;antigen processing and presentation of endogenous pep
amine metabolic process;anatomical structure development;anatomical structure morphogenesis;brain development;carboxylic acid metabolic process;cellular amine metabolic process;cellular amino acid metabolic process
activation of immune response;apoptosis;biological regulation;biosynthetic process;blood coagulation;blood coagulation, intrinsic pathway;cell death;cellular biosynthetic process;cellular component assembly;cellular compo
biological regulation;biosynthetic process;cellular biosynthetic process;cellular macromolecule biosynthetic process;cellular macromolecule metabolic process;cellular metabolic process;cellular nitrogen compound metabolic
biological regulation;blood coagulation;cell activation;cellular component assembly;cellular component assembly at cellular level;cellular component organization;cellular component organization at cellular level;cellular compo
biological regulation;biosynthetic process;cellular biosynthetic process;cellular macromolecule biosynthetic process;cellular macromolecule metabolic process;cellular metabolic process;cellular nitrogen compound metabolic
anatomical structure development;biological regulation;biosynthetic process;cell development;cellular biosynthetic process;cellular developmental process;cellular macromolecule biosynthetic process;cellular macromolecule
biological regulation;cell cycle;cellular macromolecule metabolic process;cellular metabolic process;cellular nitrogen compound metabolic process;cellular process;macromolecule metabolic process;macromolecule methylat
anatomical structure homeostasis;base-excision repair;biological regulation;biosynthetic process;cell cycle;cellular biosynthetic process;cellular component organization;cellular component organization at cellular level;cellula
aromatic compound catabolic process;catabolic process;cellular aromatic compound metabolic process;cellular catabolic process;cellular metabolic process;cellular process;metabolic process;response to chemical stimulus
biological regulation;biosynthetic process;cellular biosynthetic process;cellular component organization;cellular component organization at cellular level;cellular component organization or biogenesis;cellular component orgar
biological regulation;cellular process;cellular response to stimulus;chemotaxis;establishment of localization;intracellular signal transduction;locomotion;metabolic process;positive regulation of catalytic activity;positive regulat
biological regulation;cell surface receptor linked signaling pathway;cellular process;cellular response to chemical stimulus;cellular response to cytokine stimulus;cellular response to interferon-gamma;cellular response to orga
anatomical structure development;axon ensheathment;biological regulation;biosynthetic process;cell differentiation;cell proliferation;cellular biosynthetic process;cellular developmental process;cellular macromolecule biosyn
biological regulation;cell differentiation;cell differentiation in hindbrain;cell projection organization;cellular component organization;cellular component organization at cellular level;cellular component organization or biogenesis
activin receptor signaling pathway;anatomical structure development;anatomical structure formation involved in morphogenesis;anatomical structure morphogenesis;anterior/posterior pattern specification;axis specification;b
acyl-CoA biosynthetic process;acyl-CoA metabolic process;acylglycerol biosynthetic process;acylglycerol metabolic process;biological regulation;biosynthetic process;carboxylic acid biosynthetic process;carboxylic acid me
biosynthetic process;cellular biosynthetic process;cellular component disassembly;cellular component disassembly at cellular level;cellular component organization;cellular component organization at cellular level;cellular cor
cell cycle;cell projection organization;cellular component organization;cellular component organization at cellular level;cellular component organization or biogenesis;cellular component organization or biogenesis at cellular le
biological regulation;cellular process;cellular response to chemical stimulus;cellular response to hypoxia;cellular response to oxygen levels;cellular response to redox state;cellular response to stimulus;cellular response to stre
acyl-CoA biosynthetic process;acyl-CoA metabolic process;acylglycerol biosynthetic process;acylglycerol metabolic process;biosynthetic process;carboxylic acid biosynthetic process;carboxylic acid metabolic process;cellu
alcohol metabolic process;amine metabolic process;biosynthetic process;cellular amine metabolic process;cellular biogenic amine metabolic process;cellular biosynthetic process;cellular component organization;cellular com
biological regulation;catabolic process;cellular catabolic process;cellular macromolecule catabolic process;cellular macromolecule metabolic process;cellular metabolic process;cellular nitrogen compound metabolic process
biological regulation;biosynthetic process;cellular biosynthetic process;cellular macromolecule biosynthetic process;cellular macromolecule metabolic process;cellular metabolic process;cellular nitrogen compound metabolic
cellular macromolecule metabolic process;cellular metabolic process;cellular nitrogen compound metabolic process;cellular process;macromolecule metabolic process;macromolecule modification;metabolic process;nitroger
biological regulation;biosynthetic process;cellular biosynthetic process;cellular macromolecule biosynthetic process;cellular macromolecule metabolic process;cellular metabolic process;cellular nitrogen compound metabolic
macromolecule metabolic process;membrane protein proteolysis;metabolic process;primary metabolic process;protein metabolic process;proteolysis
biological regulation;cell projection assembly;cell projection organization;cellular component assembly;cellular component assembly at cellular level;cellular component organization;cellular component organization at cellular
amyloid precursor protein catabolic process;amyloid precursor protein metabolic process;axon guidance;beta-amyloid metabolic process;biological regulation;catabolic process;cell activation;cell proliferation;cell surface rec
apoptosis;biological regulation;biosynthetic process;cell death;cellular biosynthetic process;cellular macromolecule biosynthetic process;cellular macromolecule metabolic process;cellular metabolic process;cellular nitrogen

biological regulation;biosynthetic process;cellular biosynthetic process;cellular macromolecule biosynthetic process;cellular macromolecule metabolic process;cellular metabolic process;cellular nitrogen compound metabolic
cellular component organization;cellular component organization or biogenesis;cellular membrane organization;cellular process;endocytosis;establishment of localization;membrane invagination;membrane organization;recep
cellular component organization;cellular component organization at cellular level;cellular component organization or biogenesis;cellular component organization or biogenesis at cellular level;cellular macromolecule metabolic
cellular component organization;cellular component organization or biogenesis;cellular membrane organization;cellular process;endocytosis;establishment of localization;membrane invagination;membrane organization;transp
adenosine metabolic process;cellular macromolecule metabolic process;cellular metabolic process;cellular nitrogen compound metabolic process;cellular process;cellular protein metabolic process;heterocycle metabolic pro
establishment of localization;lipid transport;organic substance transport;transport
biological regulation;cellular macromolecule metabolic process;cellular metabolic process;cellular process;cellular protein metabolic process;cellular response to chemical stimulus;cellular response to organic substance;cellu
anatomical structure morphogenesis;anterior/posterior axis specification;axis specification;biological regulation;biosynthetic process;cell cycle;cellular biosynthetic process;cellular component organization;cellular componen
carboxylic acid transport;cellular lipid metabolic process;cellular metabolic process;cellular process;establishment of localization;fatty acid transport;lipid localization;lipid metabolic process;lipid storage;lipid transport;localiz
biological regulation;biosynthetic process;cellular biosynthetic process;cellular macromolecule metabolic process;cellular metabolic process;cellular nitrogen compound metabolic process;cellular process;cellular response to
acylglycerol biosynthetic process;acylglycerol metabolic process;biological regulation;biosynthetic process;carboxylic acid biosynthetic process;carboxylic acid metabolic process;CDP-diacylglycerol biosynthetic process;CD
agmatine biosynthetic process;amine biosynthetic process;amine metabolic process;biosynthetic process;cellular amine metabolic process;cellular biogenic amine biosynthetic process;cellular biogenic amine metabolic proc
activation of immune response;activation of innate immune response;biological regulation;cellular process;cellular response to stimulus;defense response;immune response;immune response-activating signal transduction;im
alcohol metabolic process;biological regulation;carbohydrate catabolic process;carbohydrate metabolic process;catabolic process;cellular carbohydrate catabolic process;cellular carbohydrate metabolic process;cellular met
cellular component organization;cellular component organization at cellular level;cellular component organization or biogenesis;cellular component organization or biogenesis at cellular level;cellular macromolecule metabolic
cellular macromolecule metabolic process;cellular metabolic process;cellular nitrogen compound metabolic process;cellular process;macromolecule metabolic process;maturation of SSU-rRNA;maturation of SSU-rRNA from
biosynthetic process;cellular biosynthetic process;cellular component disassembly;cellular component disassembly at cellular level;cellular component organization;cellular component organization at cellular level;cellular cor
biological regulation;biosynthetic process;cellular biosynthetic process;cellular component assembly;cellular component organization;cellular component organization or biogenesis;cellular macromolecule biosynthetic proces
biological regulation;biosynthetic process;cellular biosynthetic process;cellular lipid metabolic process;cellular macromolecule biosynthetic process;cellular macromolecule metabolic process;cellular metabolic process;cellula
antigen processing and presentation;biological regulation;cellular component organization;cellular component organization at cellular level;cellular component organization or biogenesis;cellular component organization or bic
biological regulation;biosynthetic process;canonical Wnt receptor signaling pathway;catabolic process;cell surface receptor linked signaling pathway;cellular biosynthetic process;cellular catabolic process;cellular component
cellular macromolecule metabolic process;cellular metabolic process;cellular nitrogen compound metabolic process;cellular process;cellular protein metabolic process;macromolecule metabolic process;macromolecule modi
activation of immune response;anatomical structure formation involved in morphogenesis;anatomical structure morphogenesis;angiogenesis;antigen receptor-mediated signaling pathway;biological adhesion;biological regula
anatomical structure development;cranial nerve development;developmental process;nerve development;optic nerve development
biological regulation;cell redox homeostasis;cellular homeostasis;cellular macromolecule metabolic process;cellular metabolic process;cellular process;cellular protein metabolic process;cellular response to stimulus;cellular r
biological regulation;biosynthetic process;cellular biosynthetic process;cellular macromolecule biosynthetic process;cellular macromolecule metabolic process;cellular metabolic process;cellular nitrogen compound metabolic
anatomical structure development;appendage development;biological regulation;cell differentiation;cellular component organization;cellular component organization at cellular level;cellular component organization or biogene
cellular macromolecule metabolic process;cellular metabolic process;cellular nitrogen compound metabolic process;cellular process;macromolecule metabolic process;metabolic process;mRNA 3'-end processing;mRNA cle
biological regulation;cellular process;cellular response to stimulus;hippo signaling cascade;intracellular signal transduction;regulation of biological process;regulation of cellular process;response to stimulus;signal transductio
behavior;cellular component movement;cellular component organization;cellular component organization at cellular level;cellular component organization or biogenesis;cellular component organization or biogenesis at cellula
biological regulation;catabolic process;cellular catabolic process;cellular macromolecule catabolic process;cellular macromolecule metabolic process;cellular metabolic process;cellular nitrogen compound metabolic process
catabolic process;cellular catabolic process;cellular macromolecule catabolic process;cellular macromolecule metabolic process;cellular metabolic process;cellular nitrogen compound metabolic process;cellular process;defe
behavior;biological regulation;biosynthetic process;carbohydrate metabolic process;carbohydrate transport;cell cycle;cell cycle process;cell surface receptor linked signaling pathway;cellular biosynthetic process;cellular com
biosynthetic process;cellular biosynthetic process;cellular component disassembly;cellular component disassembly at cellular level;cellular component organization;cellular component organization at cellular level;cellular cor
cation transport;establishment of localization;ion transport;metal ion transport;transport
cellular macromolecule metabolic process;cellular metabolic process;cellular nitrogen compound metabolic process;cellular process;macromolecule metabolic process;macromolecule modification;metabolic process;ncRNA
cellular process;cellular response to chemical stimulus;cellular response to oxidative stress;cellular response to oxygen radical;cellular response to reactive oxygen species;cellular response to stimulus;cellular response to str
alcohol biosynthetic process;alcohol metabolic process;amine metabolic process;aminoglycan metabolic process;anatomical structure development;anatomical structure morphogenesis;biosynthetic process;carbohydrate bi
cation transport;cellular macromolecule metabolic process;cellular metabolic process;cellular nitrogen compound metabolic process;cellular process;establishment of localization;ion transport;macromolecule metabolic proce
biosynthetic process;cell cycle;cell cycle process;cell proliferation;cellular biosynthetic process;cellular macromolecule biosynthetic process;cellular macromolecule metabolic process;cellular metabolic process;cellular nitrog
biological regulation;cell surface receptor linked signaling pathway;cellular process;cellular response to stimulus;G-protein coupled receptor protein signaling pathway;multi-organism process;regulation of biological process;r
activation of JUN kinase activity;activation of MAPK activity;androgen receptor signaling pathway;apoptosis;biological regulation;biosynthetic process;cell cycle cytokinesis;cell cycle process;cell death;cellular biosynthetic p
biological regulation;cellular localization;cellular macromolecule localization;cellular process;cellular protein localization;localization;macromolecule localization;positive regulation of biological process;positive regulation of bio
biological regulation;regulation of autophagic vacuole assembly;regulation of autophagy;regulation of biological process;regulation of catabolic process;regulation of cell communication;regulation of cellular catabolic process
amine biosynthetic process;amine metabolic process;amino acid salvage;aspartate family amino acid biosynthetic process;aspartate family amino acid metabolic process;biosynthetic process;carboxylic acid biosynthetic pro
establishment of localization;establishment of localization in cell;establishment of protein localization;intracellular protein transport;intracellular transport;nuclear import;nuclear transport;nucleocytoplasmic transport;protein in
cellular macromolecule metabolic process;cellular metabolic process;cellular process;cellular protein metabolic process;macromolecule metabolic process;macromolecule methylation;macromolecule modification;metabolic
anatomical structure development;anatomical structure morphogenesis;biological regulation;developmental process;epidermis morphogenesis;epithelium development;lung alveolus development;lung epithelium developmen
biological regulation;cellular component organization;cellular component organization at cellular level;cellular component organization or biogenesis;cellular component organization or biogenesis at cellular level;cellular meml
biological regulation;biosynthetic process;blood coagulation;cell differentiation;cellular biosynthetic process;cellular developmental process;cellular macromolecule biosynthetic process;cellular macromolecule metabolic proc
antigen processing and presentation;biological regulation;blood coagulation;cell migration;cell motility;cellular component movement;cellular process;coagulation;hemostasis;immune response;immune system process;leuko
biological regulation;biosynthetic process;cell surface receptor linked signaling pathway;cellular biosynthetic process;cellular macromolecule biosynthetic process;cellular macromolecule metabolic process;cellular metabolic
biological regulation;biosynthetic process;cellular biosynthetic process;cellular macromolecule biosynthetic process;cellular macromolecule metabolic process;cellular metabolic process;cellular nitrogen compound metabolic
cellular process;establishment of localization;transport;vesicle-mediated transport
catabolic process;cellular catabolic process;cellular macromolecule catabolic process;cellular macromolecule metabolic process;cellular metabolic process;cellular nitrogen compound metabolic process;cellular process;exo
biological regulation;cellular macromolecule metabolic process;cellular metabolic process;cellular nitrogen compound metabolic process;cellular process;humoral immune response;immune response;immune system process
cellular component organization;cellular component organization at cellular level;cellular component organization or biogenesis;cellular component organization or biogenesis at cellular level;cellular macromolecule metabolic
5S class rRNA transcription from RNA polymerase III type 1 promoter;biosynthetic process;cell differentiation;cellular biosynthetic process;cellular developmental process;cellular macromolecule biosynthetic process;cellular
biological regulation;biosynthetic process;cellular biosynthetic process;cellular macromolecule biosynthetic process;cellular macromolecule metabolic process;cellular metabolic process;cellular nitrogen compound metabolic
activation of caspase activity;anatomical structure development;axon choice point recognition;axon guidance;axon midline choice point recognition;biological adhesion;biological regulation;blood vessel endothelial cell migra
amine transport;biological regulation;cellular component assembly;cellular component assembly at cellular level;cellular component organization;cellular component organization at cellular level;cellular component organizatio
biosynthetic process;cellular biosynthetic process;cellular component organization;cellular component organization at cellular level;cellular component organization or biogenesis;cellular component organization or biogenesis
biological regulation;cellular macromolecule metabolic process;cellular metabolic process;cellular nitrogen compound metabolic process;cellular process;macromolecule metabolic process;maturation of SSU-rRNA;metabolic
binding of sperm to zona pellucida;cell recognition;cell-cell recognition;cellular component assembly;cellular component organization;cellular component organization or biogenesis;cellular macromolecule metabolic process;
biological regulation;cell cycle;cell cycle phase;cell cycle process;cell division;cellular component organization;cellular component organization at cellular level;cellular component organization or biogenesis;cellular componen
biosynthetic process;cellular biosynthetic process;cellular macromolecule biosynthetic process;cellular macromolecule metabolic process;cellular metabolic process;cellular nitrogen compound metabolic process;cellular pro
establishment of localization;establishment of protein localization;protein transport;transport
alcohol metabolic process;biosynthetic process;carbohydrate biosynthetic process;carbohydrate metabolic process;cellular biosynthetic process;cellular carbohydrate metabolic process;cellular lipid metabolic process;cellul
biological regulation;cation homeostasis;cellular cation homeostasis;cellular chemical homeostasis;cellular copper ion homeostasis;cellular homeostasis;cellular ion homeostasis;cellular process;chemical homeostasis;coppe
anatomical structure morphogenesis;biological regulation;cellular macromolecule metabolic process;cellular metabolic process;cellular nitrogen compound metabolic process;cellular process;cellular protein metabolic proces
biological regulation;carboxylic acid metabolic process;catabolic process;cellular catabolic process;cellular ketone metabolic process;cellular lipid metabolic process;cellular macromolecule catabolic process;cellular macrom
activation of immune response;activation of innate immune response;anaphase-promoting complex-dependent proteasomal ubiquitin-dependent protein catabolic process;antigen processing and presentation;antigen proces
anatomical structure development;biological regulation;developmental process;lung alveolus development;negative regulation of biological process;negative regulation of biosynthetic process;negative regulation of cell comm
biological regulation;cellular macromolecule metabolic process;cellular metabolic process;cellular process;cellular protein metabolic process;cellular response to chemical stimulus;cellular response to organic substance;cellu
biological regulation;cellular process;establishment of localization;regulation of biological process;regulation of biosynthetic process;regulation of cellular biosynthetic process;regulation of cellular macromolecule biosynthetic
cellular metabolic process;cellular process;electron transport chain;generation of precursor metabolites and energy;metabolic process;mitochondrial electron transport, NADH to ubiquinone;oxidation-reduction process;respi
cell proliferation;cell proliferation in external granule layer;cell proliferation in hindbrain;cellular component assembly;cellular component assembly at cellular level;cellular component organization;cellular component organizat
biological regulation;cell redox homeostasis;cellular homeostasis;cellular process;homeostatic process;regulation of biological process;regulation of biological quality;regulation of cellular process
activation of adenylate cyclase activity;activation of immune response;activation of innate immune response;activation of MAPKK activity;activation of protein kinase activity;anatomical structure development;apoptosis;axon
anatomical structure development;biosynthetic process;cell proliferation;cellular aromatic compound metabolic process;cellular biosynthetic process;cellular component assembly;cellular component organization;cellular con
2'-deoxyribonucleotide biosynthetic process;2'-deoxyribonucleotide metabolic process;aging;anatomical structure development;anatomical structure morphogenesis;biological regulation;biosynthetic process;cartilage develo
biosynthetic process;cellular biosynthetic process;cellular macromolecule biosynthetic process;cellular macromolecule metabolic process;cellular metabolic process;cellular nitrogen compound metabolic process;cellular pro
biosynthetic process;cellular aromatic compound metabolic process;cellular biosynthetic process;cellular macromolecule metabolic process;cellular metabolic process;cellular nitrogen compound biosynthetic process;cellula
cellular component assembly;cellular component assembly at cellular level;cellular component organization;cellular component organization at cellular level;cellular component organization or biogenesis;cellular component c
3'-phosphoadenosine 5'-phosphosulfate metabolic process;alcohol metabolic process;amine metabolic process;benzene-containing compound metabolic process;biological regulation;catecholamine metabolic process;cate
biological regulation;cellular macromolecule metabolic process;cellular metabolic process;cellular process;cellular protein metabolic process;macromolecule metabolic process;macromolecule modification;metabolic process
biosynthetic process;cellular biosynthetic process;cellular macromolecule metabolic process;cellular metabolic process;cellular nitrogen compound metabolic process;cellular process;electron transport chain;gene expressio
biological regulation;blood coagulation;blood coagulation, extrinsic pathway;coagulation;hemostasis;macromolecule metabolic process;metabolic process;multicellular organismal process;negative regulation of catalytic activ
anatomical structure development;cellular component organization;cellular component organization at cellular level;cellular component organization or biogenesis;cellular component organization or biogenesis at cellular level
carbohydrate metabolic process;carbohydrate transmembrane transport;carbohydrate transport;carboxylic acid metabolic process;cation transport;cellular ketone metabolic process;cellular metabolic process;cellular proces
2'-deoxyribonucleotide metabolic process;base-excision repair;base-excision repair, AP site formation;biological regulation;catabolic process;cellular catabolic process;cellular macromolecule catabolic process;cellular macro
biological regulation;blood coagulation;cell activation;cellular process;coagulation;establishment of localization;establishment of localization in cell;exocytosis;hemostasis;multicellular organismal process;platelet activation;pla
biological regulation;blood coagulation;cell activation;cell surface receptor linked signaling pathway;cellular process;cellular response to stimulus;coagulation;defense response;hemostasis;immune response;immune system
acute inflammatory response;acute-phase response;alpha-beta T cell activation;anatomical structure development;biological regulation;biosynthetic process;brown fat cell differentiation;CD4-positive, alpha-beta T cell activa
B cell activation;B cell differentiation;biological regulation;carbohydrate homeostasis;cell activation;cell differentiation;cell surface receptor linked signaling pathway;cellular developmental process;cellular macromolecule meta
aging;anatomical structure development;anatomical structure morphogenesis;artery morphogenesis;biological adhesion;biological regulation;blood vessel morphogenesis;BMP signaling pathway;bone development;branching
apoptosis;biological regulation;cell death;cell differentiation;cell proliferation;cellular developmental process;cellular macromolecule metabolic process;cellular metabolic process;cellular process;cellular protein metabolic proc
biological regulation;biosynthetic process;cellular biosynthetic process;cellular macromolecule biosynthetic process;cellular macromolecule metabolic process;cellular metabolic process;cellular nitrogen compound metabolic
anatomical structure development;ATP biosynthetic process;ATP metabolic process;ATP synthesis coupled proton transport;biosynthetic process;cation transport;cellular biosynthetic process;cellular metabolic process;cellu
activation of MAPKK activity;activation of protein kinase activity;avoidance of defenses of other organism involved in symbiotic interaction;avoidance of host defenses;biological regulation;biosynthetic process;cellular biosyn
arachidonic acid metabolic process;biological regulation;biosynthetic process;carboxylic acid biosynthetic process;carboxylic acid metabolic process;cellular biosynthetic process;cellular ketone metabolic process;cellular lip
biological regulation;biosynthetic process;cellular biosynthetic process;cellular component assembly;cellular component assembly at cellular level;cellular component organization;cellular component organization at cellular le
ADP biosynthetic process;ADP metabolic process;AMP metabolic process;anatomical structure development;ATP metabolic process;biosynthetic process;brain development;cellular biosynthetic process;cellular metabolic pr
activation of immune response;activation of innate immune response;anaphase-promoting complex-dependent proteasomal ubiquitin-dependent protein catabolic process;antigen processing and presentation;antigen proces
anatomical structure development;blood vessel development;cellular component assembly;cellular component assembly at cellular level;cellular component organization;cellular component organization at cellular level;cellula
anatomical structure development;anatomical structure morphogenesis;apoptotic cell clearance;biological adhesion;biological regulation;blood vessel remodeling;cell activation;cell adhesion;cell communication;cell differenti
amine transport;amino acid transport;carboxylic acid transport;cellular process;establishment of localization;ion transport;nitrogen compound transport;organic acid transport;organic substance transport;transmembrane tran
actin cytoskeleton organization;actin filament-based process;biological regulation;cellular component organization;cellular component organization at cellular level;cellular component organization or biogenesis;cellular compo
acyl-CoA metabolic process;amine catabolic process;amine metabolic process;anatomical structure development;biosynthetic process;branched chain family amino acid catabolic process;branched chain family amino acid
activation of caspase activity;activation of caspase activity by cytochrome c;anatomical structure development;anatomical structure formation involved in morphogenesis;apoptosis;apoptotic mitochondrial changes;B cell ho
anatomical structure development;biological regulation;biosynthetic process;cell development;cellular biosynthetic process;cellular component assembly;cellular component assembly at cellular level;cellular component orga
anatomical structure homeostasis;biological regulation;biosynthetic process;cell cycle;cell cycle process;cellular biosynthetic process;cellular component organization;cellular component organization at cellular level;cellular c
antigen processing and presentation;biological regulation;blood coagulation;cell activation;cell activation involved in immune response;cell differentiation;cellular developmental process;cellular localization;cellular macromole
cellular metabolic process;cellular nitrogen compound metabolic process;cellular process;metabolic process;methylation;nitrogen compound metabolic process;nucleobase-containing compound metabolic process;one-carb
biological regulation;biosynthetic process;catabolic process;cell cycle;cell cycle process;cellular biosynthetic process;cellular catabolic process;cellular macromolecule biosynthetic process;cellular macromolecule catabolic p
actin cytoskeleton organization;actin filament-based process;biological regulation;cell projection assembly;cell projection organization;cellular component assembly;cellular component assembly at cellular level;cellular compo
abscission;biological regulation;cell division;cellular process;cytokinesis;developmental process;establishment of localization;establishment of protein localization;localization;macromolecule localization;positive regulation of
ATP biosynthetic process;ATP metabolic process;ATP synthesis coupled proton transport;biosynthetic process;cation transport;cellular biosynthetic process;cellular metabolic process;cellular nitrogen compound biosynthetic

cellular component assembly;cellular component assembly at cellular level;cellular component biogenesis;cellular component biogenesis at cellular level;cellular component organization;cellular component organization at ce
cellular component assembly;cellular component assembly at cellular level;cellular component organization;cellular component organization at cellular level;cellular component organization or biogenesis;cellular component c
antigen processing and presentation;antigen processing and presentation of exogenous antigen;antigen processing and presentation of exogenous peptide antigen;antigen processing and presentation of exogenous peptide
antigen processing and presentation;biological regulation;cellular component organization;cellular component organization at cellular level;cellular component organization or biogenesis;cellular component organization or bio
biological regulation;biosynthetic process;catabolic process;cellular biosynthetic process;cellular catabolic process;cellular component disassembly;cellular component disassembly at cellular level;cellular component organiz
B cell activation;biological regulation;cell activation;cellular process;cellular response to stimulus;immune system process;leukocyte activation;lymphocyte activation;negative regulation of biological process;negative regulatio
biological regulation;negative regulation of biological process;negative regulation of biosynthetic process;negative regulation of cellular biosynthetic process;negative regulation of cellular macromolecule biosynthetic process
actin cytoskeleton organization;actin filament-based process;adult behavior;anatomical structure development;anatomical structure formation involved in morphogenesis;anatomical structure morphogenesis;apoptosis;assoc [illegible]
anatomical structure development;apoptosis;biological regulation;cell death;cell development;cell division;cellular developmental process;cellular process;cellular process involved in reproduction;death;defense response;dev
amine metabolic process;amine transport;amino acid transmembrane transport;amino acid transport;anatomical structure development;aromatic amino acid transport;biological regulation;blood coagulation;branched-chain a
anatomical structure morphogenesis;biological regulation;biosynthetic process;cell surface receptor linked signaling pathway;cellular biosynthetic process;cellular macromolecule biosynthetic process;cellular macromolecule
catabolic process;cellular catabolic process;cellular component assembly;cellular component organization;cellular component organization or biogenesis;cellular metabolic process;cellular process;macromolecular complex a
biological regulation;biosynthetic process;cellular biosynthetic process;cellular lipid metabolic process;cellular metabolic process;cellular process;cellular response to stimulus;glycerolipid biosynthetic process;glycerolipid me
biological regulation;biosynthetic process;cellular biosynthetic process;cellular macromolecule biosynthetic process;cellular macromolecule metabolic process;cellular metabolic process;cellular nitrogen compound metabolic
biosynthetic process;cell cycle;cellular biosynthetic process;cellular component assembly;cellular component assembly at cellular level;cellular component organization;cellular component organization at cellular level;cellular
biological regulation;cellular component disassembly;cellular component disassembly at cellular level;cellular component organization;cellular component organization at cellular level;cellular component organization or bioge
anatomical structure development;biological regulation;biosynthetic process;cell activation;cell differentiation;cell maturation;cellular biosynthetic process;cellular developmental process;cellular macromolecule biosynthetic p
biosynthetic process;cellular biosynthetic process;cellular component disassembly;cellular component disassembly at cellular level;cellular component organization;cellular component organization at cellular level;cellular cor
anatomical structure development;biological regulation;cell surface receptor linked signaling pathway;cellular localization;cellular macromolecule localization;cellular macromolecule metabolic process;cellular metabolic proce
biological regulation;biosynthetic process;catabolic process;cellular biosynthetic process;cellular catabolic process;cellular macromolecule biosynthetic process;cellular macromolecule catabolic process;cellular macromolecu
anatomical structure development;anatomical structure morphogenesis;biological regulation;cargo loading into vesicle;cellular component assembly;cellular component assembly at cellular level;cellular component organizati
anatomical structure morphogenesis;biological regulation;catabolic process;cell surface receptor linked signaling pathway;cellular catabolic process;cellular macromolecule catabolic process;cellular macromolecule metaboli
2'-deoxyribonucleotide biosynthetic process;2'-deoxyribonucleotide metabolic process;alcohol metabolic process;ATP metabolic process;biosynthetic process;carbohydrate metabolic process;cellular biosynthetic process;c
cellular macromolecule metabolic process;cellular metabolic process;cellular process;cellular protein metabolic process;macromolecule metabolic process;metabolic process;primary metabolic process;protein folding;protein
anatomical structure development;biological regulation;cellular macromolecule metabolic process;cellular metabolic process;cellular nitrogen compound metabolic process;cellular process;developmental process;hemopoiet
biological regulation;cell differentiation;cellular developmental process;cellular macromolecule metabolic process;cellular metabolic process;cellular nitrogen compound metabolic process;cellular process;demethylation;deve
alcohol metabolic process;carbohydrate metabolic process;cellular carbohydrate metabolic process;cellular metabolic process;cellular process;inositol metabolic process;inositol phosphate metabolic process;metabolic proc
anatomical structure development;axis elongation;biological regulation;branch elongation of an epithelium;bud outgrowth involved in lung branching;cell development;cellular developmental process;cellular process;developm
biological regulation;biosynthetic process;cellular biosynthetic process;cellular component organization;cellular component organization at cellular level;cellular component organization or biogenesis;cellular component orga
biological regulation;biosynthetic process;cellular biosynthetic process;cellular macromolecule biosynthetic process;cellular macromolecule metabolic process;cellular metabolic process;cellular nitrogen compound metabolic
anatomical structure development;biological regulation;developmental process;liver development;organ development;regulation of biological process;regulation of lipid metabolic process;regulation of metabolic process;regu
anatomical structure development;cell differentiation;cellular developmental process;cellular process;developmental process;digestive tract development;ectodermal cell differentiation;embryonic digestive tract development;
cellular macromolecule metabolic process;cellular metabolic process;cellular process;cellular protein metabolic process;macromolecule metabolic process;macromolecule modification;metabolic process;primary metabolic p
developmental process;keratinization
biological regulation;catabolic process;cell communication;cellular catabolic process;cellular macromolecule catabolic process;cellular macromolecule metabolic process;cellular metabolic process;cellular process;cellular pro
biological regulation;biosynthetic process;carbohydrate metabolic process;carbohydrate transport;cell cycle;cell cycle process;cell surface receptor linked signaling pathway;cellular biosynthetic process;cellular component c
cell cycle;cell cycle phase;cell cycle process;cell division;cellular component disassembly;cellular component disassembly at cellular level;cellular component organization;cellular component organization at cellular level;cellu
cellular component organization;cellular component organization at cellular level;cellular component organization or biogenesis;cellular component organization or biogenesis at cellular level;cellular macromolecule metabolic
response to chemical stimulus;response to inorganic substance;response to oxidative stress;response to reactive oxygen species;response to stimulus;response to stress
alcohol metabolic process;amine metabolic process;amine transport;biosynthetic process;cation transport;cellular amine metabolic process;cellular biogenic amine metabolic process;cellular biosynthetic process;cellular lipi
anatomical structure development;anatomical structure morphogenesis;axon guidance;biological regulation;cell communication;cell-cell signaling;cellular component organization;cellular component organization or biogenesi
anatomical structure development;biosynthetic process;catabolic process;cellular biosynthetic process;cellular catabolic process;cellular macromolecule biosynthetic process;cellular macromolecule catabolic process;cellula
biological regulation;cell cycle checkpoint;cellular component assembly;cellular component assembly at cellular level;cellular component organization;cellular component organization at cellular level;cellular component organ
biological regulation;defense response;immune response;immune system process;innate immune response;regulation of biological process;regulation of cellular metabolic process;regulation of cellular process;regulation of m
alcohol biosynthetic process;alcohol metabolic process;amino sugar biosynthetic process;amino sugar metabolic process;anatomical structure development;biosynthetic process;carbohydrate biosynthetic process;carbohyd
apoptosis;biological regulation;biosynthetic process;cell death;cellular biosynthetic process;cellular macromolecule biosynthetic process;cellular macromolecule metabolic process;cellular metabolic process;cellular nitrogen
biological regulation;cellular process;cellular response to stimulus;cellular response to stress;positive regulation of molecular function;positive regulation of NF-kappaB transcription factor activity;positive regulation of sequen
biological regulation;metabolic process;positive regulation of biological process;positive regulation of biosynthetic process;positive regulation of cellular biosynthetic process;positive regulation of cellular metabolic process;p
apoptosis;biological regulation;calcium ion homeostasis;cation homeostasis;cell death;cell differentiation;cellular calcium ion homeostasis;cellular cation homeostasis;cellular chemical homeostasis;cellular developmental pro
actin cytoskeleton organization;actin filament organization;actin filament-based process;actin nucleation;Arp2/3 complex-mediated actin nucleation;biological regulation;cellular component organization;cellular component or
cellular macromolecule metabolic process;cellular metabolic process;cellular nitrogen compound metabolic process;cellular process;cellular protein metabolic process;cellular response to stimulus;cellular response to stress;
biological regulation;cell differentiation;cellular component assembly;cellular component assembly at cellular level;cellular component organization;cellular component organization at cellular level;cellular component organiza
defense response;detection of abiotic stimulus;detection of external stimulus;detection of stimulus;detection of stimulus involved in sensory perception;detection of temperature stimulus;detection of temperature stimulus inv
catabolic process;cellular catabolic process;cellular component organization;cellular component organization at cellular level;cellular component organization or biogenesis;cellular component organization or biogenesis at ce
cell cycle process;cellular component assembly;cellular component assembly at cellular level;cellular component organization;cellular component organization at cellular level;cellular component organization or biogenesis;ce
biosynthetic process;cellular biosynthetic process;cellular component disassembly;cellular component disassembly at cellular level;cellular component organization;cellular component organization at cellular level;cellular cor
behavior;biosynthetic process;cellular aromatic compound metabolic process;cellular biosynthetic process;cellular metabolic compound salvage;cellular metabolic process;cellular nitrogen compound biosynthetic process;ce
biological regulation;biosynthetic process;cellular biosynthetic process;cellular component organization;cellular component organization at cellular level;cellular component organization or biogenesis;cellular component orga
carbohydrate metabolic process;cation transport;cellular carbohydrate metabolic process;cellular macromolecule metabolic process;cellular metabolic process;cellular process;cellular protein metabolic process;cognition;div
biological regulation;biosynthetic process;cation homeostasis;cellular biosynthetic process;cellular cation homeostasis;cellular chemical homeostasis;cellular homeostasis;cellular ion homeostasis;cellular iron ion homeostasis
cellular component organization;cellular component organization at cellular level;cellular component organization or biogenesis;cellular component organization or biogenesis at cellular level;cellular localization;cellular proces
cellular process;establishment of localization;establishment of localization in cell;establishment of protein localization;intracellular protein transport;intracellular transport;protein transport;transport;vesicle-mediated transport
cellular macromolecule metabolic process;cellular metabolic process;cellular nitrogen compound metabolic process;cellular process;cellular protein metabolic process;macromolecule metabolic process;macromolecule modi
establishment of localization;establishment of localization in cell;establishment of protein localization;intracellular transport;protein transport;transport
cellular macromolecule metabolic process;cellular metabolic process;cellular nitrogen compound metabolic process;cellular process;macromolecule metabolic process;metabolic process;nitrogen compound metabolic proce
biological regulation;cellular macromolecule metabolic process;cellular metabolic process;cellular process;cellular protein metabolic process;cellular response to stimulus;cellular response to stress;macromolecule metabolic
chordate embryonic development;developmental process;embryo development;embryo development ending in birth or egg hatching;in utero embryonic development
biological regulation;catabolic process;cellular catabolic process;cellular macromolecule catabolic process;cellular macromolecule metabolic process;cellular metabolic process;cellular process;cellular response to chemical s
apoptosis;biosynthetic process;cell death;cellular biosynthetic process;cellular component disassembly;cellular component disassembly at cellular level;cellular component organization;cellular component organization at cel
aromatic compound biosynthetic process;biosynthetic process;cellular aromatic compound metabolic process;cellular biosynthetic process;cellular component organization;cellular component organization or biogenesis;cellu
biosynthetic process;carboxylic acid biosynthetic process;carboxylic acid metabolic process;cellular biosynthetic process;cellular ketone metabolic process;cellular lipid metabolic process;cellular metabolic process;cellular p
carbohydrate catabolic process;carbohydrate metabolic process;catabolic process;cellular carbohydrate catabolic process;cellular carbohydrate metabolic process;cellular metabolic process;cellular process;glucuronoside c
biological regulation;positive regulation of biological process;positive regulation of cytokine production;positive regulation of interleukin-6 production;positive regulation of multicellular organismal process;regulation of biologi
biological regulation;cellular process;cellular response to stimulus;cellular response to stress;negative regulation of biological process;negative regulation of cell growth;negative regulation of cellular process;negative regulatio
cellular component assembly;cellular component assembly at cellular level;cellular component biogenesis;cellular component organization;cellular component organization at cellular level;cellular component organization or b
amine biosynthetic process;amine catabolic process;amine metabolic process;aspartate family amino acid biosynthetic process;aspartate family amino acid metabolic process;biological regulation;biosynthetic process;carbo
cellular process;endosome transport;establishment of localization;establishment of localization in cell;establishment of protein localization;intracellular protein transport;intracellular transport;protein transport;retrograde trans
cellular macromolecule metabolic process;cellular metabolic process;cellular nitrogen compound metabolic process;cellular process;macromolecule metabolic process;macromolecule methylation;macromolecule modificatio
biological regulation;catabolic process;cellular catabolic process;cellular component assembly;cellular component assembly at cellular level;cellular component organization;cellular component organization at cellular level;ce
catabolic process;cellular catabolic process;cellular macromolecule catabolic process;cellular macromolecule metabolic process;cellular metabolic process;cellular nitrogen compound metabolic process;cellular process;exo
biological regulation;cellular process;cellular response to stimulus;establishment of localization;establishment of localization in cell;establishment of protein localization;intracellular protein transport;intracellular signal transdu
biosynthetic process;cellular biosynthetic process;cellular macromolecule biosynthetic process;cellular macromolecule metabolic process;cellular metabolic process;cellular process;cellular protein metabolic process;cotrans
biological regulation;regulation of biological process;regulation of catalytic activity;regulation of cellular metabolic process;regulation of cellular process;regulation of dephosphorylation;regulation of hydrolase activity;regulatio
biological regulation;cell surface receptor linked signaling pathway;cellular process;cellular response to chemical stimulus;cellular response to fibroblast growth factor stimulus;cellular response to growth factor stimulus;cellul
cellular component assembly;cellular component assembly at cellular level;cellular component organization;cellular component organization at cellular level;cellular component organization or biogenesis;cellular component c
biological regulation;biosynthetic process;cellular biosynthetic process;cellular macromolecule biosynthetic process;cellular macromolecule metabolic process;cellular metabolic process;cellular nitrogen compound metabolic
biological regulation;biosynthetic process;cellular biosynthetic process;cellular component organization;cellular component organization at cellular level;cellular component organization or biogenesis;cellular component orga
catabolic process;cellular catabolic process;cellular metabolic process;cellular nitrogen compound catabolic process;cellular nitrogen compound metabolic process;cellular process;deoxyribonucleoside catabolic process;de
cellular metabolic process;cellular nitrogen compound metabolic process;cellular process;metabolic process;nitrogen compound metabolic process;nucleic acid metabolic process;nucleic acid phosphodiester bond hydrolys
biosynthetic process;cellular biosynthetic process;cellular component disassembly;cellular component disassembly at cellular level;cellular component organization;cellular component organization at cellular level;cellular cor
aldehyde biosynthetic process;androgen receptor signaling pathway;biological regulation;biosynthetic process;cellular aldehyde metabolic process;cellular biosynthetic process;cellular component organization;cellular compo
actin filament-based movement;actin filament-based process;anatomical structure morphogenesis;axon ensheathment;biological regulation;biosynthetic process;carboxylic acid biosynthetic process;carboxylic acid metaboli
biological regulation;regulation of biological process;regulation of cellular process;regulation of response to stimulus;regulation of signal transduction;regulation of signaling;regulation of TOR signaling cascade
biological regulation;cation homeostasis;cation transport;cellular cation homeostasis;cellular chemical homeostasis;cellular copper ion homeostasis;cellular homeostasis;cellular ion homeostasis;cellular process;chemical hor
adaptive immune response;biological regulation;biosynthetic process;cell activation;cell chemotaxis;cell migration;cell motility;cell surface receptor linked signaling pathway;cellular biosynthetic process;cellular component as
cellular metabolic process;cellular process;electron transport chain;generation of precursor metabolites and energy;metabolic process;mitochondrial electron transport, NADH to ubiquinone;oxidation-reduction process;respi
anatomical structure development;anatomical structure formation involved in morphogenesis;biological adhesion;biological regulation;cell adhesion;cell development;cell migration;cell motility;cellular component movement;c
biological regulation;cation transport;cellular metabolic process;cellular process;cellular response to chemical stimulus;cellular response to oxidative stress;cellular response to oxygen radical;cellular response to reactive oxy
acute inflammatory response;arachidonic acid metabolic process;biological regulation;biosynthetic process;carboxylic acid biosynthetic process;carboxylic acid metabolic process;cellular biosynthetic process;cellular ketone
biological regulation;biosynthetic process;cellular biosynthetic process;cellular macromolecule biosynthetic process;cellular macromolecule metabolic process;cellular metabolic process;cellular nitrogen compound metabolic
biological regulation;biosynthetic process;carbon catabolite regulation of transcription;cell communication;cellular biosynthetic process;cellular component organization;cellular component organization at cellular level;cellular
apoptosis;biological regulation;biosynthetic process;cell death;cell differentiation;cellular biosynthetic process;cellular component organization;cellular component organization at cellular level;cellular component organization
biological regulation;negative regulation of biological process;negative regulation of cell communication;negative regulation of cellular process;negative regulation of G-protein coupled receptor protein signaling pathway;nega
attachment of spindle microtubules to chromosome;attachment of spindle microtubules to kinetochore;biological regulation;cell cycle checkpoint;cell cycle process;cell division;cellular component organization;cellular compo
viral reproduction
biosynthetic process;cellular biosynthetic process;cellular component assembly;cellular component organization;cellular component organization or biogenesis;cellular macromolecule biosynthetic process;cellular macromole
anatomical structure development;biological regulation;biosynthetic process;cation homeostasis;cation transport;cellular biosynthetic process;cellular cation homeostasis;cellular chemical homeostasis;cellular component as
anatomical structure development;biological regulation;biosynthetic process;blood coagulation;cell development;cell differentiation;cell proliferation;cellular biosynthetic process;cellular component organization;cellular comp [illegible]
biosynthetic process;cellular biosynthetic process;cellular macromolecule biosynthetic process;cellular macromolecule metabolic process;cellular metabolic process;cellular nitrogen compound metabolic process;cellular pro
actin cytoskeleton organization;actin filament organization;actin filament polymerization;actin filament-based process;actin polymerization or depolymerization;biological regulation;cellular component assembly;cellular compo
biosynthetic process;cellular biosynthetic process;cellular macromolecule biosynthetic process;cellular macromolecule metabolic process;cellular metabolic process;cellular nitrogen compound metabolic process;cellular pro
biological regulation;cellular component organization;cellular component organization at cellular level;cellular component organization or biogenesis;cellular component organization or biogenesis at cellular level;cellular macro
biological regulation;cellular component movement;cellular component organization;cellular component organization at cellular level;cellular component organization or biogenesis;cellular component organization or biogenes
biological regulation;biosynthetic process;cellular biosynthetic process;cellular metabolic process;cellular process;chromosome segregation;cofactor biosynthetic process;cofactor metabolic process;iron-sulfur cluster assem
anatomical structure development;anatomical structure formation involved in morphogenesis;anatomical structure morphogenesis;appendage morphogenesis;biological regulation;cell morphogenesis;cell morphogenesis invo

carboxylic acid metabolic process;cellular ketone metabolic process;cellular lipid metabolic process;cellular metabolic process;cellular process;fatty acid metabolic process;lipid metabolic process;metabolic process;monoca
antigen processing and presentation;antigen processing and presentation of peptide antigen;antigen processing and presentation of peptide antigen via MHC class I;biological regulation;immune response;immune system pr
biological adhesion;biological regulation;cell adhesion;cell adhesion mediated by integrin;cell differentiation;cell-matrix adhesion;cell-substrate adhesion;cellular component organization;cellular component organization at cel
alcohol metabolic process;amine metabolic process;aminoglycan catabolic process;aminoglycan metabolic process;carbohydrate catabolic process;carbohydrate metabolic process;catabolic process;cellular carbohydrate c
anatomical structure morphogenesis;biological regulation;biosynthetic process;cellular biosynthetic process;cellular macromolecule biosynthetic process;cellular macromolecule metabolic process;cellular metabolic process;
bile acid metabolic process;carboxylic acid catabolic process;carboxylic acid metabolic process;catabolic process;cellular catabolic process;cellular ketone metabolic process;cellular lipid catabolic process;cellular lipid meta
biosynthetic process;cellular biosynthetic process;cellular macromolecule biosynthetic process;cellular macromolecule metabolic process;cellular metabolic process;cellular process;cellular protein metabolic process;cotrans
biosynthetic process;cellular biosynthetic process;cellular macromolecule metabolic process;cellular metabolic process;cellular nitrogen compound metabolic process;cellular process;electron transport chain;gene expressio
biological regulation;cell surface receptor linked signaling pathway;cellular process;cellular response to chemical stimulus;cellular response to cytokine stimulus;cellular response to dsRNA;cellular response to exogenous ds
amine catabolic process;amine metabolic process;branched chain family amino acid catabolic process;branched chain family amino acid metabolic process;carboxylic acid catabolic process;carboxylic acid metabolic proces
adherens junction organization;biological adhesion;biological regulation;cell adhesion;cell junction assembly;cell junction organization;cell-cell junction organization;cellular component assembly;cellular component assembly
anatomical structure development;biological regulation;biosynthetic process;blood vessel development;cell differentiation;cell proliferation;cell surface receptor linked signaling pathway;cellular biosynthetic process;cellular d
anatomical structure homeostasis;anatomical structure morphogenesis;base-excision repair;biological regulation;biosynthetic process;cell cycle;cell division;cellular biosynthetic process;cellular component organization;cellu
biological regulation;blood coagulation;cellular component organization;cellular component organization or biogenesis;cellular macromolecule metabolic process;cellular membrane organization;cellular metabolic process;cel
biosynthetic process;cellular biosynthetic process;cellular macromolecule biosynthetic process;cellular macromolecule metabolic process;cellular metabolic process;cellular nitrogen compound metabolic process;cellular pro
apoptosis;biological regulation;cell cycle phase;cell cycle process;cell death;cell proliferation;cellular component organization;cellular component organization at cellular level;cellular component organization or biogenesis;cel
activation of immune response;activation of innate immune response;anaphase-promoting complex-dependent proteasomal ubiquitin-dependent protein catabolic process;antigen processing and presentation;antigen proces
activation of immune response;activation of innate immune response;anaphase-promoting complex-dependent proteasomal ubiquitin-dependent protein catabolic process;antigen processing and presentation;antigen proces
2'-deoxyribonucleotide metabolic process;base-excision repair;base-excision repair, AP site formation;catabolic process;cellular catabolic process;cellular macromolecule catabolic process;cellular macromolecule metabolic
alcohol catabolic process;alcohol metabolic process;amine catabolic process;amine metabolic process;branched chain family amino acid catabolic process;branched chain family amino acid metabolic process;carbohydrate
anatomical structure development;auditory receptor cell differentiation;biological regulation;cell differentiation;cellular developmental process;cellular process;developmental process;inner ear receptor cell differentiation;kidne
B cell activation;B cell activation involved in immune response;biological regulation;catabolic process;cell activation;cell activation involved in immune response;cell cycle process;cellular catabolic process;cellular componen
cap-independent translational initiation;cellular component assembly;cellular component assembly at cellular level;cellular component disassembly;cellular component disassembly at cellular level;cellular component organiz
biological regulation;biosynthetic process;cellular biosynthetic process;cellular component organization;cellular component organization at cellular level;cellular component organization or biogenesis;cellular component orga
actin cytoskeleton organization;actin filament organization;actin filament-based process;anatomical structure development;biological regulation;cell development;cell junction assembly;cell junction organization;cell migration
activation of immune response;activation of innate immune response;anaphase-promoting complex-dependent proteasomal ubiquitin-dependent protein catabolic process;antigen processing and presentation;antigen proces
anatomical structure development;anatomical structure morphogenesis;biological regulation;carbohydrate metabolic process;cardiac chamber morphogenesis;cardiac left ventricle morphogenesis;cardiac ventricle morphoge
acetate metabolic process;alcohol metabolic process;amine catabolic process;amine metabolic process;anatomical structure development;biological regulation;carbohydrate metabolic process;carboxylic acid catabolic proc
2-oxoglutarate metabolic process;acute inflammatory response;acute-phase response;allantoin metabolic process;amine metabolic process;anatomical structure development;biological regulation;biosynthetic process;body
anion transport;behavioral response to nutrient;biological regulation;blood coagulation;carbohydrate homeostasis;carboxylic acid metabolic process;carboxylic acid transport;cell migration;cell motility;cellular component mo
anatomical structure homeostasis;antigen processing and presentation;antigen processing and presentation of exogenous antigen;antigen processing and presentation of exogenous peptide antigen;antigen processing and p
biosynthetic process;catabolic process;cell differentiation;cellular biosynthetic process;cellular catabolic process;cellular component assembly;cellular component assembly at cellular level;cellular component biogenesis;cell
anatomical structure development;biological regulation;cell surface receptor linked signaling pathway;cellular component organization;cellular component organization at cellular level;cellular component organization or bioge
anion transport;bicarbonate transport;biological regulation;catabolic process;cellular catabolic process;cellular component assembly;cellular component organization;cellular component organization or biogenesis;cellular me
biological regulation;cellular process;cellular response to stimulus;establishment of localization;establishment of protein localization;intracellular signal transduction;metabolic process;protein transport;regulation of biological
antigen processing and presentation;antigen processing and presentation of endogenous antigen;antigen processing and presentation of endogenous peptide antigen;antigen processing and presentation of endogenous pep
biological regulation;biosynthetic process;catabolic process;cell proliferation;cellular biosynthetic process;cellular catabolic process;cellular macromolecule biosynthetic process;cellular macromolecule catabolic process;cellu
activation of MAPKK activity;activation of protein kinase activity;anatomical structure development;anatomical structure formation involved in morphogenesis;anatomical structure morphogenesis;apoptosis;biological regulati
cellular macromolecule metabolic process;cellular metabolic process;cellular process;cellular protein metabolic process;establishment of localization;establishment of localization in cell;establishment of protein localization;es
anatomical structure development;biological regulation;calcium ion transport;cation transport;cell communication;cell cycle process;cell differentiation;cell surface receptor linked signaling pathway;cell-cell signaling;cellular c
anatomical structure development;apoptosis;biological regulation;camera-type eye development;cell death;cell fate specification;cell surface receptor linked signaling pathway;cellular developmental process;cellular macrom
cellular process;cellular response to cold;cellular response to freezing;cellular response to stimulus;cellular response to stress;response to abiotic stimulus;response to cold;response to freezing;response to stimulus;response
cotranslational protein targeting to membrane;establishment of localization;establishment of localization in cell;establishment of protein localization;intracellular protein transport;intracellular transport;protein targeting;protein
biological regulation;developmental process;macromolecule metabolic process;metabolic process;multicellular organismal development;multicellular organismal process;positive regulation of catalytic activity;positive regulati
biological regulation;biosynthetic process;cell cycle arrest;cell cycle process;cell surface receptor linked signaling pathway;cellular biosynthetic process;cellular macromolecule metabolic process;cellular metabolic process;c
aerobic respiration;ATP biosynthetic process;ATP metabolic process;biosynthetic process;cellular biosynthetic process;cellular component assembly;cellular component assembly at cellular level;cellular component organizat
anatomical structure morphogenesis;biological adhesion;biological regulation;calcium ion-dependent exocytosis;cell adhesion;cell morphogenesis;cell morphogenesis involved in differentiation;cell-substrate adhesion;cellular
alcohol catabolic process;alcohol metabolic process;carbohydrate catabolic process;carbohydrate metabolic process;catabolic process;cellular carbohydrate catabolic process;cellular carbohydrate metabolic process;cellula
cellular process;establishment of localization;establishment of localization in cell;establishment of protein localization;Golgi to plasma membrane transport;Golgi vesicle transport;intracellular transport;post-Golgi vesicle-med
anatomical structure development;anatomical structure morphogenesis;apoptosis;autophagy;biological regulation;brain development;catabolic process;cell death;cell part morphogenesis;cell projection morphogenesis;cell p
apoptosis;cell death;cellular process;death;programmed cell death
cell differentiation;cellular developmental process;cellular process;developmental process;hemopoietic progenitor cell differentiation
anatomical structure formation involved in morphogenesis;blastocyst formation;cellular macromolecule metabolic process;cellular metabolic process;cellular nitrogen compound metabolic process;cellular process;developme
biological regulation;cell redox homeostasis;cellular homeostasis;cellular macromolecule metabolic process;cellular metabolic process;cellular process;cellular protein metabolic process;homeostatic process;macromolecule
adaptive immune response;adaptive immune response based on somatic recombination of immune receptors built from immunoglobulin superfamily domains;biological regulation;cell activation;cell activation involved in imm
biological regulation;directional locomotion;locomotion;negative regulation of actin nucleation;negative regulation of biological process;negative regulation of cell migration;negative regulation of cell motility;negative regulatio
catabolic process;cellular catabolic process;cellular component assembly;cellular component assembly at cellular level;cellular component organization;cellular component organization at cellular level;cellular component org
biological regulation;catabolic process;cellular catabolic process;cellular macromolecule catabolic process;cellular macromolecule metabolic process;cellular metabolic process;cellular process;cellular protein metabolic proc
adipose tissue development;anatomical structure development;biological regulation;carbohydrate homeostasis;carboxylic acid metabolic process;cell differentiation;cellular chemical homeostasis;cellular developmental proc
cellular macromolecule metabolic process;cellular metabolic process;cellular nitrogen compound metabolic process;cellular process;macromolecule metabolic process;metabolic process;nitrogen compound metabolic proce
amine metabolic process;AMP biosynthetic process;AMP metabolic process;aspartate family amino acid metabolic process;aspartate metabolic process;biosynthetic process;carboxylic acid metabolic process;cellular amine
biological regulation;cellular process;cellular response to stimulus;intracellular signal transduction;regulation of biological process;regulation of cellular process;regulation of response to stimulus;regulation of signal transducti
biological regulation;cell proliferation;cellular component assembly;cellular component assembly at cellular level;cellular component organization;cellular component organization at cellular level;cellular component organizatio
aging;autophagy;biological regulation;catabolic process;cellular catabolic process;cellular metabolic process;cellular process;developmental process;metabolic process;mitochondrion degradation;multicellular organismal ag
cellular process;establishment of localization;transmembrane transport;transport
cellular macromolecule metabolic process;cellular metabolic process;cellular process;cellular protein metabolic process;macromolecule metabolic process;macromolecule modification;metabolic process;post-translational pr
biological regulation;calcium ion homeostasis;calcium ion transport;calcium-mediated signaling;carbohydrate homeostasis;cation homeostasis;cation transport;cellular calcium ion homeostasis;cellular cation homeostasis;ce
biological regulation;positive regulation of catalytic activity;positive regulation of GTPase activity;positive regulation of hydrolase activity;positive regulation of molecular function;regulation of biological process;regulation of ca
biological regulation;cellular component assembly;cellular component assembly at cellular level;cellular component organization;cellular component organization at cellular level;cellular component organization or biogenesis;
amine transport;amino acid transmembrane transport;amino acid transport;carboxylic acid transport;cellular process;establishment of localization;glycine transport;ion transport;neutral amino acid transport;nitrogen compou
biological regulation;cellular process;cellular response to stimulus;multicellular organismal process;ossification;regulation of biological process;regulation of cellular process;response to stimulus;signal transduction
acyl-CoA biosynthetic process;acyl-CoA metabolic process;amine catabolic process;amine metabolic process;aromatic amino acid family metabolic process;aspartate family amino acid catabolic process;aspartate family am
alcohol catabolic process;alcohol metabolic process;carbohydrate catabolic process;carbohydrate metabolic process;catabolic process;cellular carbohydrate catabolic process;cellular carbohydrate metabolic process;cellula
cellular localization;cellular process;establishment of localization;establishment of localization in cell;establishment of organelle localization;establishment of protein localization;establishment of ribosome localization;intracellu
biosynthetic process;cellular biosynthetic process;cellular lipid metabolic process;cellular metabolic process;cellular process;ceramide biosynthetic process;ceramide metabolic process;lipid biosynthetic process;lipid metabo
actin cytoskeleton organization;actin filament bundle assembly;actin filament organization;actin filament-based process;apoptosis;biological regulation;biosynthetic process;cell death;cellular biosynthetic process;cellular con
apoptosis;cell death;cellular process;death;programmed cell death
biological regulation;cellular component organization;cellular component organization at cellular level;cellular component organization or biogenesis;cellular component organization or biogenesis at cellular level;cellular macr
biological regulation;cellular process;cellular response to stimulus;endosome to lysosome transport;endosome transport;establishment of cell polarity;establishment of localization;establishment of localization in cell;establish
anatomical structure development;anatomical structure formation involved in morphogenesis;biosynthetic process;cellular biosynthetic process;cellular lipid metabolic process;cellular metabolic process;cellular process;deve
cellular macromolecule metabolic process;cellular metabolic process;cellular nitrogen compound metabolic process;cellular process;macromolecule metabolic process;metabolic process;ncRNA metabolic process;ncRNA pr
3'-UTR-mediated mRNA destabilization;anatomical structure development;axon ensheathment;biological regulation;biosynthetic process;carboxylic acid biosynthetic process;carboxylic acid metabolic process;cell developm
biological regulation;biosynthetic process;cellular biosynthetic process;cellular macromolecule biosynthetic process;cellular macromolecule metabolic process;cellular metabolic process;cellular nitrogen compound metabolic
biological regulation;biosynthetic process;cellular biosynthetic process;cellular macromolecule biosynthetic process;cellular macromolecule metabolic process;cellular metabolic process;cellular nitrogen compound metabolic
carbohydrate metabolic process;cellular carbohydrate metabolic process;cellular macromolecule metabolic process;cellular metabolic process;cellular process;cellular protein metabolic process;glycosylation;macromolecule
cell cycle;cell cycle process;cellular component assembly;cellular component organization;cellular component organization at cellular level;cellular component organization or biogenesis;cellular component organization or bi
alanyl-tRNA aminoacylation;amine metabolic process;amino acid activation;carboxylic acid metabolic process;cellular amine metabolic process;cellular amino acid metabolic process;cellular ketone metabolic process;cellula
biosynthetic process;cellular biosynthetic process;cellular lipid metabolic process;cellular metabolic process;cellular process;glycerolipid biosynthetic process;glycerolipid metabolic process;glycerophospholipid biosynthetic
anatomical structure formation involved in morphogenesis;angiogenesis;apoptosis;biological regulation;cell death;cell differentiation;cellular developmental process;cellular process;death;developmental process;establishme
establishment of localization;lipid transport;organic substance transport;transport
biological regulation;biosynthetic process;catabolic process;cell surface receptor linked signaling pathway;cellular biosynthetic process;cellular catabolic process;cellular component organization;cellular component organizat
anatomical structure development;anatomical structure morphogenesis;biological regulation;biosynthetic process;cell development;cell motility;cell part morphogenesis;cellular biosynthetic process;cellular component morph
cell cycle process;cellular component organization;cellular component organization at cellular level;cellular component organization or biogenesis;cellular component organization or biogenesis at cellular level;cellular macrom
biological regulation;cellular macromolecule metabolic process;cellular metabolic process;cellular nitrogen compound metabolic process;cellular process;induction of apoptosis;induction of apoptosis by extracellular signals;
metabolic process
catabolic process;cellular catabolic process;cellular component assembly;cellular component organization;cellular component organization or biogenesis;cellular metabolic process;cellular nitrogen compound catabolic proce
cellular component assembly;cellular component assembly at cellular level;cellular component organization;cellular component organization at cellular level;cellular component organization or biogenesis;cellular component c
cell cycle;cellular component organization;cellular component organization at cellular level;cellular component organization or biogenesis;cellular component organization or biogenesis at cellular level;cellular process;mitotic
biological regulation;catabolic process;cell surface receptor linked signaling pathway;cellular catabolic process;cellular component assembly;cellular component assembly at cellular level;cellular component organization;cellu
biological regulation;cellular macromolecule metabolic process;cellular metabolic process;cellular nitrogen compound metabolic process;cellular process;macromolecule metabolic process;metabolic process;mRNA metaboli
anatomical structure development;ATP-dependent chromatin remodeling;biological regulation;biosynthetic process;brain development;canonical Wnt receptor signaling pathway;cell surface receptor linked signaling pathway
biosynthetic process;cellular biosynthetic process;cellular component disassembly;cellular component disassembly at cellular level;cellular component organization;cellular component organization at cellular level;cellular co
biological regulation;biosynthetic process;cellular biosynthetic process;cellular component organization;cellular component organization at cellular level;cellular component organization or biogenesis;cellular component orga
cellular component organization;cellular component organization at cellular level;cellular component organization or biogenesis;cellular component organization or biogenesis at cellular level;cellular process;establishment of l
biological regulation;regulation of biological process;regulation of catabolic process;regulation of cellular catabolic process;regulation of cellular metabolic process;regulation of cellular process;regulation of cellular protein me
biological regulation;cell cycle;cell division;cellular process;cellular response to stimulus;chromosome segregation;intracellular signal transduction;metabolic process;regulation of biological process;regulation of cellular proce
biological regulation;cell cycle phase;cell cycle process;cell division;cell motility;cellular component movement;cellular component organization;cellular component organization at cellular level;cellular component organization
biological regulation;biosynthetic process;cellular biosynthetic process;cellular metabolic process;cellular process;coenzyme biosynthetic process;coenzyme metabolic process;cofactor biosynthetic process;cofactor metabo
biosynthetic process;catabolic process;cellular biosynthetic process;cellular catabolic process;cellular macromolecule biosynthetic process;cellular macromolecule catabolic process;cellular macromolecule metabolic proces
biological regulation;cell differentiation;cell surface receptor linked signaling pathway;cellular component biogenesis;cellular component biogenesis at cellular level;cellular component organization or biogenesis;cellular comp
biological regulation;cell cycle checkpoint;cellular component organization;cellular component organization at cellular level;cellular component organization or biogenesis;cellular component organization or biogenesis at cellu
biological regulation;cellular macromolecule metabolic process;cellular metabolic process;cellular nitrogen compound metabolic process;cellular process;cellular protein metabolic process;macromolecule metabolic process;
anatomical structure formation involved in morphogenesis;angiogenesis;biological regulation;biosynthetic process;cellular biosynthetic process;cellular macromolecule biosynthetic process;cellular macromolecule metabolic
biological regulation;cell cycle process;cellular component organization;cellular component organization at cellular level;cellular component organization or biogenesis;cellular component organization or biogenesis at cellular
biological regulation;cell communication;cellular component organization;cellular component organization at cellular level;cellular component organization or biogenesis;cellular component organization or biogenesis at cellula
biosynthetic process;cellular aromatic compound metabolic process;cellular biosynthetic process;cellular metabolic compound salvage;cellular metabolic process;cellular nitrogen compound biosynthetic process;cellular nitr
biological regulation;biosynthetic process;cell proliferation;cellular biosynthetic process;cellular component organization;cellular component organization at cellular level;cellular component organization or biogenesis;cellular
biological regulation;catabolic process;cell surface receptor linked signaling pathway;cellular catabolic process;cellular macromolecule catabolic process;cellular macromolecule metabolic process;cellular metabolic process;

biological adhesion;biological regulation;cell adhesion;cellular process;developmental process;multicellular organismal development;multicellular organismal process;negative regulation of apoptosis;negative regulation of bio
activation of immune response;activation of innate immune response;biological regulation;cellular process;cellular response to stimulus;cytokine production;defense response;defense response to bacterium;defense respons
developmental process;multicellular organismal development;multicellular organismal process
antigen processing and presentation;antigen processing and presentation of exogenous antigen;antigen processing and presentation of exogenous peptide antigen;antigen processing and presentation of exogenous peptide
alcohol catabolic process;alcohol metabolic process;carbohydrate catabolic process;carbohydrate metabolic process;catabolic process;cellular carbohydrate catabolic process;cellular carbohydrate metabolic process;cellula
biological regulation;blood coagulation;cellular component organization;cellular component organization at cellular level;cellular component organization or biogenesis;cellular component organization or biogenesis at cellular
biological regulation;biosynthetic process;cellular biosynthetic process;cellular macromolecule metabolic process;cellular metabolic process;cellular nitrogen compound metabolic process;cellular process;gene expression;m
apoptosis;biological regulation;catabolic process;cell death;cellular catabolic process;cellular macromolecule catabolic process;cellular macromolecule metabolic process;cellular metabolic process;cellular process;cytoplasr
cellular macromolecule metabolic process;cellular metabolic process;cellular nitrogen compound metabolic process;cellular process;macromolecule metabolic process;metabolic process;ncRNA metabolic process;ncRNA pr
cellular macromolecule metabolic process;cellular metabolic process;cellular nitrogen compound metabolic process;cellular process;gene expression;macromolecule metabolic process;metabolic process;mRNA metabolic p
cellular macromolecule metabolic process;cellular metabolic process;cellular nitrogen compound metabolic process;cellular process;cellular protein metabolic process;macromolecule metabolic process;macromolecule modi
apoptosis;biological regulation;cell death;cellular process;death;metabolic process;negative regulation of anoikis;negative regulation of apoptosis;negative regulation of biological process;negative regulation of cell death;neg
biological regulation;BMP signaling pathway;catabolic process;cell surface receptor linked signaling pathway;cellular catabolic process;cellular component organization;cellular component organization at cellular level;cellular
biological regulation;catabolic process;cellular catabolic process;cellular macromolecule catabolic process;cellular macromolecule metabolic process;cellular metabolic process;cellular nitrogen compound metabolic process
biological regulation;biosynthetic process;catabolic process;cellular biosynthetic process;cellular catabolic process;cellular macromolecule biosynthetic process;cellular macromolecule catabolic process;cellular macromolec
biological adhesion;biological regulation;calcium ion homeostasis;cation homeostasis;cell adhesion;cell surface receptor linked signaling pathway;cell-cell adhesion;cellular calcium ion homeostasis;cellular cation homeostasi
activation of protein kinase activity;apoptosis;biological regulation;cell death;cellular component disassembly;cellular component disassembly at cellular level;cellular component disassembly involved in apoptosis;cellular cor
biological regulation;biosynthetic process;cellular biosynthetic process;cellular component organization;cellular component organization at cellular level;cellular component organization or biogenesis;cellular component orga
cell cycle process;cellular macromolecule metabolic process;cellular metabolic process;cellular process;cellular protein metabolic process;dephosphorylation;G1/S transition of mitotic cell cycle;macromolecule metabolic pro
bile acid biosynthetic process;bile acid metabolic process;biosynthetic process;carboxylic acid biosynthetic process;carboxylic acid catabolic process;carboxylic acid metabolic process;catabolic process;cellular biosyntheti
cellular component organization;cellular component organization or biogenesis;cellular membrane organization;cellular process;endocytosis;establishment of localization;establishment of localization in cell;establishment of p
biological regulation;cellular process;cellular response to chemical stimulus;cellular response to endogenous stimulus;cellular response to hormone stimulus;cellular response to organic substance;cellular response to steroid
amine metabolic process;amino acid activation;biological regulation;carboxylic acid metabolic process;cellular amine metabolic process;cellular amino acid metabolic process;cellular ketone metabolic process;cellular macro
biological regulation;catabolic process;cell differentiation;cell proliferation;cellular catabolic process;cellular developmental process;cellular metabolic process;cellular nitrogen compound catabolic process;cellular nitrogen co
biological regulation;cation homeostasis;cellular cation homeostasis;cellular chemical homeostasis;cellular homeostasis;cellular ion homeostasis;cellular iron ion homeostasis;cellular metal ion homeostasis;cellular process;ch
ATP biosynthetic process;ATP metabolic process;biological regulation;biosynthetic process;cellular biosynthetic process;cellular metabolic process;cellular nitrogen compound biosynthetic process;cellular nitrogen compoun
alpha-beta T cell activation;alpha-beta T cell differentiation;anatomical structure development;biological regulation;biosynthetic process;CD4-positive, alpha-beta T cell activation;CD4-positive, alpha-beta T cell differentiation
biological regulation;biosynthetic process;cellular biosynthetic process;cellular component assembly;cellular component assembly at cellular level;cellular component organization;cellular component organization at cellular le
apoptosis;cell death;cellular component organization;cellular component organization at cellular level;cellular component organization or biogenesis;cellular component organization or biogenesis at cellular level;cellular proce
alcohol biosynthetic process;alcohol metabolic process;biosynthetic process;cellular biosynthetic process;cellular lipid metabolic process;cellular metabolic process;cellular process;cholesterol biosynthetic process;choleste
cellular metabolic process;cellular process;electron transport chain;generation of precursor metabolites and energy;metabolic process;mitochondrial electron transport, NADH to ubiquinone;oxidation-reduction process;respi
biological regulation;cell differentiation;cellular developmental process;cellular process;developmental process;multicellular organismal development;multicellular organismal process;regulation of biological process;regulation
biosynthetic process;cellular biosynthetic process;cellular macromolecule metabolic process;cellular metabolic process;cellular nitrogen compound metabolic process;cellular process;electron transport chain;gene expressio
2'-deoxyribonucleotide metabolic process;adenosine catabolic process;adenosine metabolic process;aging;amine transport;anatomical structure development;B cell activation;B cell activation involved in immune response;E
anatomical structure homeostasis;anatomical structure morphogenesis;antibacterial humoral response;antifungal humoral response;antimicrobial humoral response;biological regulation;biosynthetic process;bone morphogen
amide biosynthetic process;amine biosynthetic process;amine catabolic process;amine metabolic process;arginine biosynthetic process;arginine biosynthetic process via ornithine;arginine catabolic process;arginine metaboli
biosynthetic process;cellular aromatic compound metabolic process;cellular biosynthetic process;cellular metabolic process;cellular nitrogen compound biosynthetic process;cellular nitrogen compound metabolic process;ce
acylglycerol metabolic process;aging;amine metabolic process;anatomical structure development;anatomical structure formation involved in morphogenesis;anatomical structure morphogenesis;angiogenesis;angiogenesis in
anatomical structure development;anatomical structure morphogenesis;axonogenesis;biological regulation;brain development;cell part morphogenesis;cell projection morphogenesis;cell projection organization;cell surface re
biological regulation;biosynthetic process;cellular biosynthetic process;cellular macromolecule biosynthetic process;cellular macromolecule metabolic process;cellular metabolic process;cellular nitrogen compound metabolic
aromatic compound biosynthetic process;biosynthetic process;cellular aromatic compound metabolic process;cellular biosynthetic process;cellular metabolic process;cellular nitrogen compound biosynthetic process;cellular
aging;biological regulation;biosynthetic process;carboxylic acid biosynthetic process;carboxylic acid metabolic process;cell aging;cell proliferation;cell surface receptor linked signaling pathway;cellular biosynthetic process;c
anatomical structure morphogenesis;developmental process
anaphase-promoting complex-dependent proteasomal ubiquitin-dependent protein catabolic process;anatomical structure development;anatomical structure morphogenesis;biological regulation;cardiac cell development;ca
anatomical structure development;biosynthetic process;cellular biosynthetic process;cellular macromolecule metabolic process;cellular metabolic process;cellular nitrogen compound metabolic process;cellular process;devel
amine metabolic process;aminoglycan catabolic process;aminoglycan metabolic process;carbohydrate catabolic process;carbohydrate metabolic process;catabolic process;cellular catabolic process;cellular metabolic proce
behavior;cognition;learning or memory;memory;multicellular organismal process;neurological system process;response to stimulus;system process
activation of immune response;activation of MAPKK activity;activation of phospholipase C activity;activation of protein kinase activity;antigen receptor-mediated signaling pathway;axon guidance;biological regulation;blood c
biosynthetic process;cellular biosynthetic process;cellular macromolecule metabolic process;cellular metabolic process;cellular nitrogen compound metabolic process;cellular process;electron transport chain;gene expressio
amine metabolic process;carboxylic acid catabolic process;carboxylic acid metabolic process;catabolic process;cellular amine metabolic process;cellular amino acid metabolic process;cellular catabolic process;cellular keto
alcohol metabolic process;biological regulation;biosynthetic process;cation homeostasis;cell differentiation;cellular aromatic compound metabolic process;cellular biosynthetic process;cellular component assembly;cellular c
cellular component organization;cellular component organization at cellular level;cellular component organization or biogenesis;cellular component organization or biogenesis at cellular level;cellular process;embryo implantat
cellular macromolecule metabolic process;cellular metabolic process;cellular process;cellular protein metabolic process;chaperone-mediated protein folding;macromolecule metabolic process;macromolecule modification;me
anatomical structure development;biological regulation;biosynthetic process;cardiac cell differentiation;cardiac muscle cell differentiation;cardiac muscle cell proliferation;cell communication;cell differentiation;cell proliferatio
alcohol biosynthetic process;alcohol metabolic process;biosynthetic process;carbohydrate biosynthetic process;carbohydrate metabolic process;cellular biosynthetic process;cellular carbohydrate biosynthetic process;cellul
amine metabolic process;aminoglycan biosynthetic process;aminoglycan catabolic process;aminoglycan metabolic process;anatomical structure formation involved in morphogenesis;axon guidance;biological regulation;bios
biological regulation;cell redox homeostasis;cellular chemical homeostasis;cellular homeostasis;cellular ion homeostasis;cellular macromolecule metabolic process;cellular metabolic process;cellular nitrogen compound meta
biological regulation;biosynthetic process;cell cycle arrest;cell cycle process;cell surface receptor linked signaling pathway;cellular biosynthetic process;cellular macromolecule biosynthetic process;cellular macromolecule me
activation of caspase activity;adult behavior;adult locomotory behavior;aging;alcohol biosynthetic process;alcohol metabolic process;amine biosynthetic process;amine metabolic process;amine transport;ATP synthesis coup
activation of adenylate cyclase activity;axon guidance;biological regulation;cellular component organization;cellular component organization at cellular level;cellular component organization or biogenesis;cellular component c
carboxylic acid catabolic process;carboxylic acid metabolic process;catabolic process;cellular catabolic process;cellular ketone metabolic process;cellular lipid catabolic process;cellular lipid metabolic process;cellular metab
acid secretion;alcohol metabolic process;amine metabolic process;arachidonic acid metabolic process;arachidonic acid secretion;biological regulation;biosynthetic process;blood coagulation;carboxylic acid biosynthetic pro
biological regulation;positive regulation of biological process;positive regulation of cell proliferation;positive regulation of cellular process;regulation of biological process;regulation of cell proliferation;regulation of cellular proc
3'-UTR-mediated mRNA stabilization;activation of immune response;activation of innate immune response;activation of MAPK activity;anatomical structure development;arachidonic acid metabolic process;biological regulati
anatomical structure homeostasis;biological regulation;biosynthetic process;cell cycle;cell cycle process;cellular biosynthetic process;cellular component organization;cellular component organization at cellular level;cellular c
cellular macromolecule metabolic process;cellular metabolic process;cellular process;cellular protein metabolic process;macromolecule metabolic process;macromolecule modification;metabolic process;primary metabolic p
biological regulation;biosynthetic process;catabolic process;cellular biosynthetic process;cellular catabolic process;cellular component assembly;cellular component assembly at cellular level;cellular component organization;
biological regulation;biosynthetic process;cation homeostasis;cell growth;cellular biosynthetic process;cellular cation homeostasis;cellular chemical homeostasis;cellular homeostasis;cellular ion homeostasis;cellular iron ion
cellular component biogenesis;cellular component biogenesis at cellular level;cellular component organization or biogenesis;cellular component organization or biogenesis at cellular level;cellular macromolecule metabolic pro
biological regulation;biosynthetic process;cellular biosynthetic process;cellular macromolecule biosynthetic process;cellular macromolecule metabolic process;cellular metabolic process;cellular process;cellular protein metab
apoptosis;apoptosis in response to endoplasmic reticulum stress;biological regulation;catabolic process;cell death;cellular catabolic process;cellular macromolecule catabolic process;cellular macromolecule metabolic proce
biological regulation;biosynthetic process;cellular biosynthetic process;cellular lipid metabolic process;cellular metabolic process;cellular process;cellular response to stimulus;establishment of localization;establishment of pr
biological regulation;cellular macromolecule metabolic process;cellular metabolic process;cellular process;cellular protein metabolic process;macromolecule metabolic process;macromolecule modification;metabolic process
actin cytoskeleton organization;actin filament organization;actin filament-based process;biological regulation;blood coagulation;cell activation;cellular component organization;cellular component organization at cellular level;c
anatomical structure formation involved in morphogenesis;angiogenesis;apoptosis;axon guidance;biological adhesion;biological regulation;blood coagulation;cell activation;cell adhesion;cell death;cellular process;cellular res
actin cytoskeleton organization;actin filament-based process;anatomical structure development;anatomical structure morphogenesis;antigen processing and presentation;antigen processing and presentation of exogenous a
actin cytoskeleton organization;actin filament-based process;axon guidance;biological regulation;blood coagulation;cell activation;cell chemotaxis;cell migration;cell motility;cellular component movement;cellular component
anatomical structure development;anatomical structure morphogenesis;biological regulation;cell differentiation;cellular developmental process;cellular process;developmental process;muscle organ development;muscle struc
biological regulation;biosynthetic process;cellular biosynthetic process;cellular macromolecule biosynthetic process;cellular macromolecule metabolic process;cellular metabolic process;cellular nitrogen compound metabolic
biosynthetic process;cellular biosynthetic process;cellular macromolecule biosynthetic process;cellular macromolecule metabolic process;cellular metabolic process;cellular nitrogen compound metabolic process;cellular pro
activation of immune response;activation of innate immune response;activation of phospholipase C activity;adaptive immune response;adaptive immune response based on somatic recombination of immune receptors built f
biological regulation;catabolic process;cellular catabolic process;cellular macromolecule catabolic process;cellular macromolecule metabolic process;cellular metabolic process;cellular nitrogen compound metabolic process
activation of caspase activity;activation of NF-kappaB-inducing kinase activity;activation of protein kinase activity;apoptosis;apoptosis in response to endoplasmic reticulum stress;biological regulation;catabolic process;cell c
cellular macromolecule metabolic process;cellular metabolic process;cellular nitrogen compound metabolic process;cellular process;macromolecule metabolic process;maturation of SSU-rRNA;maturation of SSU-rRNA from
biosynthetic process;cellular biosynthetic process;cellular lipid metabolic process;cellular metabolic process;cellular process;dephosphorylation;glycerolipid biosynthetic process;glycerolipid metabolic process;glycerophosph
actin cytoskeleton organization;actin filament-based process;anatomical structure development;cellular component organization;cellular component organization at cellular level;cellular component organization or biogenesis;
antigen processing and presentation;antigen processing and presentation of peptide antigen;antigen processing and presentation of peptide antigen via MHC class I;cellular macromolecule metabolic process;cellular metabo
axon guidance;biological regulation;cell differentiation;cell junction assembly;cell junction organization;cell projection assembly;cell projection organization;cell surface receptor linked signaling pathway;cell-substrate junction
behavior;behavioral response to pain;biological regulation;multicellular organismal process;multicellular organismal response to stress;regulation of biological process;regulation of cellular process;regulation of G-protein coup
cellular macromolecule metabolic process;cellular metabolic process;cellular nitrogen compound metabolic process;cellular process;macromolecule metabolic process;metabolic process;mRNA metabolic process;mRNA pro
activation of immune response;activation of innate immune response;anaphase-promoting complex-dependent proteasomal ubiquitin-dependent protein catabolic process;antigen processing and presentation;antigen proces
biological regulation;biosynthetic process;cellular biosynthetic process;cellular component assembly;cellular component assembly at cellular level;cellular component organization;cellular component organization at cellular le
alcohol metabolic process;anatomical structure development;anatomical structure morphogenesis;biological regulation;carbohydrate metabolic process;cellular carbohydrate metabolic process;cellular component organizatio
cellular component assembly;cellular component organization;cellular component organization or biogenesis;macromolecular complex assembly;macromolecular complex subunit organization;macromolecule metabolic proc
amine catabolic process;amine metabolic process;carboxylic acid catabolic process;carboxylic acid metabolic process;catabolic process;cell differentiation;cellular amine metabolic process;cellular amino acid catabolic proc
amine biosynthetic process;amine metabolic process;biosynthetic process;carboxylic acid biosynthetic process;carboxylic acid metabolic process;cellular amine metabolic process;cellular amino acid biosynthetic process;ce
biosynthetic process;catabolic process;cell communication;cellular aromatic compound metabolic process;cellular biosynthetic process;cellular catabolic process;cellular metabolic compound salvage;cellular metabolic proc
biological regulation;regulation of biological process;regulation of catalytic activity;regulation of cellular metabolic process;regulation of cellular process;regulation of dephosphorylation;regulation of hydrolase activity;regulatic
B cell activation;B cell activation involved in immune response;catabolic process;cell activation;cell activation involved in immune response;cellular catabolic process;cellular macromolecule catabolic process;cellular macrom
cell cycle;cell division;cellular process
cellular metabolic process;cellular process;dephosphorylation;metabolic process;phosphate-containing compound metabolic process;phosphorus metabolic process
biological regulation;cell chemotaxis;cell migration;cell motility;cell surface receptor linked signaling pathway;cellular component movement;cellular process;cellular response to chemical stimulus;cellular response to stimulus
cellular macromolecule metabolic process;cellular metabolic process;cellular nitrogen compound metabolic process;cellular process;macromolecule metabolic process;metabolic process;mRNA metabolic process;mRNA pro
biological regulation;cellular process;cellular response to stimulus;intracellular signal transduction;regulation of biological process;regulation of cellular process;regulation of response to stimulus;regulation of signal transducti
cellular macromolecule metabolic process;cellular metabolic process;cellular nitrogen compound metabolic process;cellular process;macromolecule metabolic process;metabolic process;mRNA metabolic process;mRNA pro
catabolic process;lipid catabolic process;lipid metabolic process;metabolic process;primary metabolic process
biological regulation;catabolic process;cellular component organization;cellular component organization or biogenesis;cellular macromolecule metabolic process;cellular membrane organization;cellular metabolic process;cell
alcohol metabolic process;carbohydrate metabolic process;cellular carbohydrate metabolic process;cellular component assembly;cellular component organization;cellular component organization or biogenesis;cellular metab
biological regulation;biosynthetic process;cellular biosynthetic process;cellular macromolecule biosynthetic process;cellular macromolecule metabolic process;cellular metabolic process;cellular nitrogen compound metabolic
biosynthetic process;catabolic process;cellular biosynthetic process;cellular lipid metabolic process;cellular metabolic process;cellular process;glycerolipid biosynthetic process;glycerolipid metabolic process;glycerophosph
cellular component assembly;cellular component assembly at cellular level;cellular component organization;cellular component organization at cellular level;cellular component organization or biogenesis;cellular component c
behavior;biological regulation;cellular component organization;cellular component organization at cellular level;cellular component organization or biogenesis;cellular component organization or biogenesis at cellular level;cellu
biological regulation;biosynthetic process;carbohydrate metabolic process;carbohydrate transport;cell cycle;cell cycle phase;cell cycle process;cell division;cell surface receptor linked signaling pathway;cellular biosynthetic p
biological regulation;biosynthetic process;cellular biosynthetic process;cellular macromolecule biosynthetic process;cellular macromolecule metabolic process;cellular metabolic process;cellular nitrogen compound metabolic
anatomical structure formation involved in morphogenesis;angiogenesis;apoptosis;biological regulation;cell death;cellular macromolecule metabolic process;cellular metabolic process;cellular process;cellular protein metabol
activation of caspase activity;apoptosis;biological regulation;cell death;cellular process;cellular response to chemical stimulus;cellular response to hypoxia;cellular response to oxygen levels;cellular response to stimulus;cellu
anatomical structure development;cell differentiation;cellular developmental process;cellular process;developmental process;establishment of localization;establishment of localization in cell;intracellular transport;mitochondri
catabolic process;cellular catabolic process;cellular macromolecule catabolic process;cellular macromolecule metabolic process;cellular metabolic process;cellular nitrogen compound metabolic process;cellular process;exo
amine catabolic process;amine metabolic process;aspartate family amino acid catabolic process;aspartate family amino acid metabolic process;carboxylic acid catabolic process;carboxylic acid metabolic process;catabolic

attachment of spindle microtubules to chromosome;attachment of spindle microtubules to kinetochore;attachment of spindle microtubules to kinetochore involved in mitotic sister chromatid segregation;biological regulation;c
activation of NF-kappaB-inducing kinase activity;activation of protein kinase activity;biological regulation;cellular macromolecule metabolic process;cellular metabolic process;cellular process;cellular protein metabolic proces
biological regulation;catabolic process;cell cycle;cell differentiation;cell proliferation;cellular catabolic process;cellular developmental process;cellular macromolecule catabolic process;cellular macromolecule metabolic proce
activation of immune response;activation of innate immune response;anaphase-promoting complex-dependent proteasomal ubiquitin-dependent protein catabolic process;antigen processing and presentation;antigen proces
anatomical structure development;biological regulation;cellular macromolecule metabolic process;cellular metabolic process;cellular process;cellular protein metabolic process;'de novo' posttranslational protein folding;'de n
biological regulation;cell cycle;cell cycle phase;cell cycle process;cell division;cell surface receptor linked signaling pathway;cellular component disassembly;cellular component disassembly at cellular level;cellular componen
biological regulation;calcium ion homeostasis;calcium ion import;calcium ion transmembrane transport;calcium ion transport;cation homeostasis;cation transport;cellular calcium ion homeostasis;cellular cation homeostasis;c
biosynthetic process;cellular biosynthetic process;cellular component disassembly;cellular component disassembly at cellular level;cellular component organization;cellular component organization at cellular level;cellular co
biological regulation;metabolic process;oxidation-reduction process;regulation of biological process;regulation of cell differentiation;regulation of cellular process;regulation of developmental process;regulation of immune sys
developmental process;multicellular organismal development;multicellular organismal process
biological regulation;cell cycle cytokinesis;cell cycle process;cellular component organization;cellular component organization at cellular level;cellular component organization or biogenesis;cellular component organization or
adipose tissue development;anatomical structure development;biological regulation;cellular macromolecule metabolic process;cellular metabolic process;cellular nitrogen compound metabolic process;cellular process;demet
biological regulation;catabolic process;cellular catabolic process;cellular macromolecule catabolic process;cellular macromolecule metabolic process;cellular metabolic process;cellular nitrogen compound metabolic process
amine transport;amino acid transmembrane transport;amino acid transport;biological regulation;carboxylic acid transport;cell communication;cell-cell signaling;cellular process;establishment of localization;ion transport;neur
biological regulation;biosynthetic process;cell cycle;cell differentiation;cell surface receptor linked signaling pathway;cellular biosynthetic process;cellular developmental process;cellular macromolecule biosynthetic process;c
cellular component organization;cellular component organization or biogenesis;cellular membrane organization;cellular process;endocytosis;establishment of localization;establishment of protein localization;membrane invagi
viral reproduction
anatomical structure development;biological regulation;biosynthetic process;cell migration;cell motility;cellular biosynthetic process;cellular component movement;cellular component organization;cellular component organiza
biological regulation;biosynthetic process;cellular biosynthetic process;cellular component biogenesis;cellular component biogenesis at cellular level;cellular component organization;cellular component organization at cellula
anatomical structure development;biological regulation;biosynthetic process;cell development;cellular biosynthetic process;cellular developmental process;cellular macromolecule metabolic process;cellular metabolic proces
cellular process;establishment of localization;establishment of localization in cell;establishment of protein localization;intracellular protein transport;intracellular transport;protein transport;transport;vesicle-mediated transport
apoptotic mitochondrial changes;autophagic vacuole assembly;autophagy;catabolic process;cellular catabolic process;cellular component assembly;cellular component assembly at cellular level;cellular component organiza
biosynthetic process;catabolic process;cellular biosynthetic process;cellular catabolic process;cellular lipid catabolic process;cellular lipid metabolic process;cellular metabolic process;cellular process;cellular response to ch
actin cytoskeleton organization;actin filament organization;actin filament-based process;actin polymerization or depolymerization;biological regulation;cell migration;cell motility;cellular component movement;cellular compon
biological regulation;regulation of biological process;regulation of cellular metabolic process;regulation of cellular process;regulation of cellular respiration;regulation of generation of precursor metabolites and energy;regulatio
activation of caspase activity;biological regulation;cellular component organization;cellular component organization or biogenesis;cellular membrane organization;cellular process;establishment of localization;membrane orga
biosynthetic process;cellular biosynthetic process;cellular component disassembly;cellular component disassembly at cellular level;cellular component organization;cellular component organization at cellular level;cellular co
biosynthetic process;cellular biosynthetic process;cellular macromolecule biosynthetic process;cellular macromolecule metabolic process;cellular metabolic process;cellular nitrogen compound metabolic process;cellular pro
biological regulation;cell surface receptor linked signaling pathway;cellular component organization;cellular component organization or biogenesis;cellular membrane organization;cellular process;cellular response to stimulus
anatomical structure development;biological regulation;cell communication;cell surface receptor linked signaling pathway;cell-cell signaling;cellular metabolic process;cellular process;cellular response to chemical stimulus;ce
apoptosis;cell death;cell differentiation;cellular developmental process;cellular localization;cellular macromolecule localization;cellular process;cellular protein localization;death;developmental process;ER to Golgi vesicle-med
carboxylic acid catabolic process;carboxylic acid metabolic process;catabolic process;cellular catabolic process;cellular component assembly;cellular component organization;cellular component organization or biogenesis;c
biosynthetic process;cellular biosynthetic process;cellular component disassembly;cellular component disassembly at cellular level;cellular component organization;cellular component organization at cellular level;cellular co
anterograde axon cargo transport;anterograde synaptic vesicle transport;axon cargo transport;cellular component movement;cellular process;cytoskeleton-dependent intracellular transport;establishment of localization;estab
biological regulation;metabolic process;negative regulation of catalytic activity;negative regulation of molecular function;negative regulation of monooxygenase activity;negative regulation of nitric-oxide synthase activity;nega
establishment of localization;establishment of protein localization;protein transport;transport
biosynthetic process;cellular biosynthetic process;cellular component disassembly;cellular component disassembly at cellular level;cellular component organization;cellular component organization at cellular level;cellular co
2'-deoxyribonucleotide metabolic process;biological regulation;catabolic process;cell surface receptor linked signaling pathway;cellular catabolic process;cellular component assembly;cellular component organization;cellula
anatomical structure morphogenesis;biological regulation;cell part morphogenesis;cell projection morphogenesis;cell projection organization;cellular component assembly;cellular component assembly at cellular level;cellular
biological regulation;catabolic process;cell surface receptor linked signaling pathway;cellular catabolic process;cellular macromolecule catabolic process;cellular macromolecule metabolic process;cellular metabolic process;
anatomical structure formation involved in morphogenesis;biological regulation;cation homeostasis;cation transport;cell projection assembly;cell projection organization;cell surface receptor linked signaling pathway;cellular c
activation of immune response;activation of innate immune response;activation of MAPK activity;activation of NF-kappaB-inducing kinase activity;activation of protein kinase activity;antigen receptor-mediated signaling pathw
cellular component assembly;cellular component assembly at cellular level;cellular component biogenesis;cellular component organization;cellular component organization at cellular level;cellular component organization or b
activation of immune response;activation of innate immune response;anaphase-promoting complex-dependent proteasomal ubiquitin-dependent protein catabolic process;antigen processing and presentation;antigen proces
alcohol metabolic process;carbohydrate metabolic process;cellular carbohydrate metabolic process;cellular macromolecule metabolic process;cellular metabolic process;cellular process;cellular protein metabolic process;he
biological regulation;negative regulation of binding;negative regulation of biological process;negative regulation of biosynthetic process;negative regulation of cellular biosynthetic process;negative regulation of cellular macro
apoptosis;biological regulation;carbohydrate metabolic process;cell communication;cell death;cellular carbohydrate metabolic process;cellular component organization;cellular component organization at cellular level;cellular
alcohol biosynthetic process;alcohol metabolic process;biosynthetic process;carbohydrate biosynthetic process;carbohydrate metabolic process;cellular biosynthetic process;cellular carbohydrate biosynthetic process;cellul
amine biosynthetic process;amine catabolic process;amine metabolic process;biological regulation;biosynthetic process;branched chain family amino acid biosynthetic process;branched chain family amino acid catabolic pr
cellular macromolecule metabolic process;cellular metabolic process;cellular nitrogen compound metabolic process;cellular process;gene expression;macromolecule metabolic process;metabolic process;mRNA metabolic p
biological regulation;cellular component assembly;cellular component organization;cellular component organization or biogenesis;cellular macromolecule metabolic process;cellular metabolic process;cellular nitrogen compo
cellular component organization;cellular component organization or biogenesis;cellular membrane organization;cellular process;endocytosis;endosome transport;establishment of localization;establishment of localization in c
biological regulation;biosynthetic process;catabolic process;cell differentiation;cellular biosynthetic process;cellular catabolic process;cellular developmental process;cellular macromolecule biosynthetic process;cellular macr
activation of caspase activity;anatomical structure formation involved in morphogenesis;angiogenesis;biological regulation;cellular process;cellular response to heat;cellular response to stimulus;cellular response to stress;dev
anatomical structure development;biological regulation;cell cycle;cell differentiation;cell division;cell proliferation;cellular component organization;cellular component organization at cellular level;cellular component organizatio
cellular macromolecule metabolic process;cellular metabolic process;cellular process;cellular protein metabolic process;establishment of localization;establishment of localization in cell;establishment of protein localization;es
acidic amino acid transport;amine transport;amino acid transport;anion transport;biological regulation;carboxylic acid transport;cellular chemical homeostasis;cellular homeostasis;cellular ion homeostasis;cellular process;che
amine catabolic process;amine metabolic process;arginine catabolic process;arginine metabolic process;biological regulation;carboxylic acid catabolic process;carboxylic acid metabolic process;catabolic process;cellular am
alcohol biosynthetic process;alcohol metabolic process;amine metabolic process;amino sugar biosynthetic process;amino sugar metabolic process;biosynthetic process;carbohydrate biosynthetic process;carbohydrate meta
cellular macromolecule metabolic process;cellular metabolic process;cellular process;cellular protein metabolic process;macromolecule metabolic process;macromolecule modification;metabolic process;primary metabolic p
anatomical structure formation involved in morphogenesis;anatomical structure morphogenesis;apoptosis;autophagy;biological regulation;blastocyst formation;blood coagulation;camera-type eye morphogenesis;catabolic pr
biological regulation;catabolic process;cellular catabolic process;cellular macromolecule catabolic process;cellular macromolecule metabolic process;cellular metabolic process;cellular process;cellular protein metabolic proc
adult behavior;adult locomotory behavior;adult walking behavior;amine metabolic process;anatomical structure development;anatomical structure morphogenesis;autophagy;axonogenesis;behavior;biological regulation;carbo
biological regulation;biosynthetic process;cellular biosynthetic process;cellular macromolecule biosynthetic process;cellular macromolecule metabolic process;cellular metabolic process;cellular nitrogen compound metabolic
activation of protein kinase activity;biological regulation;cellular hypotonic response;cellular macromolecule metabolic process;cellular metabolic process;cellular process;cellular protein metabolic process;cellular response to
cellular macromolecule metabolic process;cellular metabolic process;cellular process;cellular protein metabolic process;establishment of localization;establishment of localization in cell;establishment of protein localization;es
biological regulation;C21-steroid hormone metabolic process;cellular hormone metabolic process;cellular metabolic process;cellular process;cellular response to stimulus;establishment of localization;hormone metabolic proc
age-dependent response to oxidative stress;age-dependent response to reactive oxygen species;amine metabolic process;anatomical structure development;apoptotic mitochondrial changes;behavior;biological regulation;b
axon guidance;biological regulation;blood coagulation;carbohydrate transport;cell differentiation;cell migration;cell motility;cell surface receptor linked signaling pathway;cellular component movement;cellular developmental
biosynthetic process;cellular biosynthetic process;cellular component disassembly;cellular component disassembly at cellular level;cellular component organization;cellular component organization at cellular level;cellular co
amine metabolic process;benzene-containing compound metabolic process;carboxylic acid metabolic process;catabolic process;cellular amine metabolic process;cellular amino acid metabolic process;cellular aromatic com
biological regulation;cellular process;cellular response to chemical stimulus;cellular response to endogenous stimulus;cellular response to estradiol stimulus;cellular response to estrogen stimulus;cellular response to hormone
amine metabolic process;carboxylic acid metabolic process;cell differentiation;cellular amine metabolic process;cellular amino acid metabolic process;cellular component assembly;cellular component organization;cellular co
apoptosis;biological regulation;cell communication;cell death;cell surface receptor linked signaling pathway;cellular process;cellular response to chemical stimulus;cellular response to dsRNA;cellular response to exogenous
anatomical structure development;anatomical structure morphogenesis;biological regulation;cell cycle;cell cycle process;cell division;cellular component organization;cellular component organization at cellular level;cellular co
amine metabolic process;carboxylic acid metabolic process;cellular amine metabolic process;cellular amino acid metabolic process;cellular ketone metabolic process;cellular metabolic process;cellular nitrogen compound m
actin cytoskeleton organization;actin filament organization;actin filament-based process;activation of immune response;antigen receptor-mediated signaling pathway;axon guidance;biological regulation;cell activation;cell pro
activation of phospholipase D activity;anatomical structure development;biological regulation;brain development;cell migration;cell motility;cell surface receptor linked signaling pathway;cellular component movement;cellular
anatomical structure development;base-excision repair;cellular macromolecule metabolic process;cellular metabolic process;cellular nitrogen compound metabolic process;cellular process;cellular response to stimulus;cellula
biological regulation;biosynthetic process;cellular biosynthetic process;cellular macromolecule biosynthetic process;cellular macromolecule metabolic process;cellular metabolic process;cellular nitrogen compound metabolic
antigen processing and presentation;biological regulation;cellular component organization;cellular component organization or biogenesis;cellular process;cellular response to stimulus;establishment of localization;establishme
alcohol catabolic process;alcohol metabolic process;amine catabolic process;amine metabolic process;behavior;benzene-containing compound metabolic process;biological regulation;biosynthetic process;catabolic process
axon guidance;biological adhesion;biological regulation;cell adhesion;cell surface receptor linked signaling pathway;cell-cell adhesion;cellular process;cellular response to stimulus;chemotaxis;enzyme linked receptor protein
activation of immune response;activation of innate immune response;anaphase-promoting complex-dependent proteasomal ubiquitin-dependent protein catabolic process;antigen processing and presentation;antigen proces
biological regulation;cellular metabolic process;cellular process;energy derivation by oxidation of organic compounds;energy reserve metabolic process;generation of precursor metabolites and energy;metabolic process;oxic
biosynthetic process;cellular aromatic compound metabolic process;cellular biosynthetic process;cellular metabolic process;cellular nitrogen compound biosynthetic process;cellular nitrogen compound metabolic process;ce
amine biosynthetic process;amine catabolic process;amine metabolic process;betaine biosynthetic process;betaine metabolic process;biosynthetic process;carboxylic acid biosynthetic process;carboxylic acid catabolic proc
alkaloid metabolic process;anatomical structure development;anatomical structure formation involved in morphogenesis;angiogenesis;arachidonic acid metabolic process;behavior;biological regulation;biomineral tissue deve
actin cytoskeleton organization;actin filament bundle assembly;actin filament capping;actin filament organization;actin filament-based process;anatomical structure homeostasis;anatomical structure morphogenesis;apoptosi
ATP hydrolysis coupled proton transport;biological regulation;cation homeostasis;cation transport;cell surface receptor linked signaling pathway;cellular cation homeostasis;cellular chemical homeostasis;cellular component
biological regulation;biosynthetic process;catabolic process;cell cycle;cell proliferation;cellular biosynthetic process;cellular catabolic process;cellular component disassembly;cellular component disassembly at cellular level;
acidic amino acid transport;aging;amide biosynthetic process;amine metabolic process;amine transport;amino acid import;amino acid transport;anatomical structure development;anatomical structure formation involved in m
activation of immune response;activation of innate immune response;activation of MAPK activity;biological regulation;cardiac muscle contraction;cellular macromolecule metabolic process;cellular metabolic process;cellular
activation of immune response;activation of innate immune response;anaphase-promoting complex-dependent proteasomal ubiquitin-dependent protein catabolic process;antigen processing and presentation;antigen proces
anatomical structure homeostasis;base-excision repair;biological regulation;biosynthetic process;cell cycle;cellular biosynthetic process;cellular component organization;cellular component organization at cellular level;cellula
biological regulation;cell cycle;cell cycle process;cell surface receptor linked signaling pathway;cellular component organization;cellular component organization at cellular level;cellular component organization or biogenesis;
biological regulation;cellular component assembly;cellular component assembly at cellular level;cellular component organization;cellular component organization at cellular level;cellular component organization or biogenesis;
adherens junction organization;anatomical structure development;axon guidance;biological regulation;cell differentiation;cell division;cell junction organization;cell surface receptor linked signaling pathway;cell-cell junction or
anatomical structure development;biological regulation;cell cycle;cell cycle process;cell differentiation;cellular component disassembly;cellular component disassembly at cellular level;cellular component organization;cellular
biological regulation;biosynthetic process;cell proliferation;cell surface receptor linked signaling pathway;cellular biosynthetic process;cellular macromolecule biosynthetic process;cellular macromolecule metabolic process;c
apoptotic mitochondrial changes;biosynthetic process;cellular biosynthetic process;cellular component disassembly;cellular component disassembly at cellular level;cellular component organization;cellular component organ
biosynthetic process;cellular biosynthetic process;cellular macromolecule biosynthetic process;cellular macromolecule metabolic process;cellular metabolic process;cellular process;cellular protein metabolic process;cotrans
cellular metabolic process;cellular process;electron transport chain;generation of precursor metabolites and energy;metabolic process;mitochondrial electron transport, NADH to ubiquinone;oxidation-reduction process;respi
anatomical structure morphogenesis;developmental process;metabolic process;organ morphogenesis;oxidation-reduction process
activation of immune response;activation of innate immune response;antigen receptor-mediated signaling pathway;biological regulation;catabolic process;cell surface receptor linked signaling pathway;cellular catabolic proce
actin cytoskeleton organization;actin filament-based process;anatomical structure development;apoptosis;biological adhesion;biological regulation;cell adhesion;cell cycle;cell death;cell division;cell projection organization;ce
actin cytoskeleton organization;actin filament organization;actin filament-based process;biological regulation;cellular component organization;cellular component organization at cellular level;cellular component organization c
cellular macromolecule metabolic process;cellular metabolic process;cellular nitrogen compound metabolic process;cellular process;macromolecule metabolic process;metabolic process;ncRNA metabolic process;ncRNA pr
biological regulation;biosynthetic process;cell cycle;cell differentiation;cellular biosynthetic process;cellular developmental process;cellular metabolic process;cellular nitrogen compound biosynthetic process;cellular nitrogen
biosynthetic process;cellular biosynthetic process;cellular macromolecule biosynthetic process;cellular macromolecule metabolic process;cellular metabolic process;cellular nitrogen compound metabolic process;cellular pro
alcohol biosynthetic process;alcohol metabolic process;biosynthetic process;cellular biosynthetic process;cellular lipid metabolic process;cellular metabolic process;cellular process;cholesterol biosynthetic process;choleste
activation of adenylate cyclase activity;activation of adenylate cyclase activity by G-protein signaling pathway;activation of phospholipase D activity;anatomical structure morphogenesis;biological regulation;blood coagulatio
biological regulation;biosynthetic process;cellular biosynthetic process;cellular metabolic process;cellular nitrogen compound biosynthetic process;cellular nitrogen compound metabolic process;cellular process;metabolic pr

catabolic process;cellular catabolic process;cellular macromolecule catabolic process;cellular macromolecule metabolic process;cellular metabolic process;cellular nitrogen compound metabolic process;cellular process;exo
biological regulation;biosynthetic process;cellular biosynthetic process;cellular component organization;cellular component organization at cellular level;cellular component organization or biogenesis;cellular component orga
biological regulation;cell cycle process;cellular component organization;cellular component organization at cellular level;cellular component organization or biogenesis;cellular component organization or biogenesis at cellular
biological adhesion;biological regulation;cell adhesion;cell junction assembly;cell junction organization;cell migration;cell motility;cell surface receptor linked signaling pathway;cell-substrate junction assembly;cellular compon
biological regulation;biosynthetic process;cellular biosynthetic process;cellular macromolecule biosynthetic process;cellular macromolecule metabolic process;cellular metabolic process;cellular nitrogen compound metabolic
biological regulation;biosynthetic process;cellular biosynthetic process;cellular component assembly;cellular component organization;cellular component organization or biogenesis;cellular macromolecule biosynthetic proces
biological regulation;cellular process;chromosome segregation;positive regulation of biological process;positive regulation of cellular metabolic process;positive regulation of cellular process;positive regulation of cellular prote
amine transport;amino acid transport;carboxylic acid transport;cellular process;establishment of localization;extracellular amino acid transport;extracellular transport;glutamine transport;ion transport;neutral amino acid transp
activation of blood coagulation via clotting cascade;anatomical structure development;anion transport;biological regulation;biomineral tissue development;blood coagulation;bone mineralization;bone mineralization involved i
actin cytoskeleton organization;actin filament organization;actin filament uncapping;actin filament-based process;barbed-end actin filament uncapping;biological regulation;blood coagulation;cell migration;cell motility;cell pro
cellular macromolecule metabolic process;cellular metabolic process;cellular nitrogen compound metabolic process;cellular process;developmental process;macromolecule metabolic process;maturation of SSU-rRNA;matur
cellular macromolecule metabolic process;cellular metabolic process;cellular nitrogen compound metabolic process;cellular process;macromolecule metabolic process;metabolic process;mRNA metabolic process;mRNA pro
cellular component organization;cellular component organization at cellular level;cellular component organization or biogenesis;cellular component organization or biogenesis at cellular level;cellular macromolecule metabolic
biological regulation;cellular process;establishment of localization;establishment of localization in cell;establishment of protein localization;exocytosis;negative regulation of adiponectin secretion;negative regulation of biologic
biological regulation;biosynthetic process;cellular biosynthetic process;cellular macromolecule biosynthetic process;cellular macromolecule metabolic process;cellular metabolic process;cellular nitrogen compound metabolic
cellular macromolecule metabolic process;cellular metabolic process;cellular process;cellular protein metabolic process;cullin deneddylation;macromolecule metabolic process;macromolecule modification;metabolic process
cellular macromolecule metabolic process;cellular metabolic process;cellular nitrogen compound metabolic process;cellular process;macromolecule metabolic process;metabolic process;nitrogen compound metabolic proce
biological regulation;biosynthetic process;cell fate commitment;cell fate commitment involved in formation of primary germ layers;cell surface receptor linked signaling pathway;cellular biosynthetic process;cellular componen
biological regulation;cellular component organization;cellular component organization at cellular level;cellular component organization or biogenesis;cellular component organization or biogenesis at cellular level;cellular proce
alcohol metabolic process;amino sugar metabolic process;biosynthetic process;carbohydrate biosynthetic process;carbohydrate metabolic process;carboxylic acid metabolic process;cellular carbohydrate metabolic process
catabolic process;cellular catabolic process;cellular macromolecule catabolic process;cellular macromolecule metabolic process;cellular metabolic process;cellular process;ER-associated protein catabolic process;macromo
biological adhesion;biological regulation;cell adhesion;cellular macromolecule metabolic process;cellular metabolic process;cellular nitrogen compound metabolic process;cellular process;macromolecule metabolic process;r
biological regulation;induction of apoptosis;induction of apoptosis by intracellular signals;induction of apoptosis by oxidative stress;induction of programmed cell death;positive regulation of apoptosis;positive regulation of bi
activation of immune response;activation of innate immune response;anatomical structure formation involved in morphogenesis;angiogenesis;biological regulation;canonical Wnt receptor signaling pathway;cell surface recept
anatomical structure development;biological regulation;cell differentiation;cell proliferation;cellular developmental process;cellular process;developmental process;gamete generation;male gamete generation;maternal placent
cellular macromolecule metabolic process;cellular metabolic process;cellular nitrogen compound metabolic process;cellular process;establishment of localization;establishment of localization in cell;establishment of protein lo
catabolic process;cellular catabolic process;cellular lipid metabolic process;cellular macromolecule catabolic process;cellular macromolecule metabolic process;cellular metabolic process;cellular nitrogen compound metabo
biological regulation;biosynthetic process;cellular biosynthetic process;cellular macromolecule biosynthetic process;cellular macromolecule metabolic process;cellular metabolic process;cellular nitrogen compound metabolic
biological regulation;cell activation;cell activation involved in immune response;cellular process;cytokine secretion;establishment of localization;establishment of localization in cell;establishment of protein localization;exocyto
cellular component assembly;cellular component assembly at cellular level;cellular component organization;cellular component organization at cellular level;cellular component organization or biogenesis;cellular component o
acute inflammatory response;acute-phase response;alcohol metabolic process;cellular macromolecule metabolic process;cellular metabolic process;cellular process;cholesterol metabolic process;defense response;developr
alcohol catabolic process;alcohol metabolic process;carbohydrate catabolic process;carbohydrate metabolic process;catabolic process;cellular carbohydrate catabolic process;cellular carbohydrate metabolic process;cellula
ATP-dependent chromatin remodeling;cellular component organization;cellular component organization at cellular level;cellular component organization or biogenesis;cellular component organization or biogenesis at cellular
7-methylguanosine biosynthetic process;7-methylguanosine metabolic process;biosynthetic process;cellular biosynthetic process;cellular macromolecule metabolic process;cellular metabolic process;cellular nitrogen compo
biological regulation;blood coagulation;cell activation;cellular process;coagulation;establishment of localization;establishment of localization in cell;exocytosis;hemostasis;multicellular organismal process;platelet activation;pl
4-hydroxyproline metabolic process;amine metabolic process;anatomical structure development;anatomical structure formation involved in morphogenesis;anatomical structure morphogenesis;biological regulation;carboxylic
biosynthetic process;cellular biosynthetic process;cellular component disassembly;cellular component disassembly at cellular level;cellular component organization;cellular component organization at cellular level;cellular con
biosynthetic process;establishment of localization;lipid biosynthetic process;lipid metabolic process;metabolic process;primary metabolic process;steroid biosynthetic process;steroid metabolic process;transport
biological regulation;cellular component organization;cellular component organization or biogenesis;cellular process;cellular response to stimulus;intracellular signal transduction;positive regulation of actin filament polymeriza
acidic amino acid transport;amine transport;amino acid transport;anion transport;carboxylic acid transport;establishment of localization;ion transport;L-glutamate transport;nitrogen compound transport;organic acid transpor
biological regulation;cellular component organization;cellular component organization at cellular level;cellular component organization or biogenesis;cellular component organization or biogenesis at cellular level;cellular macro
amine metabolic process;biosynthetic process;carboxylic acid metabolic process;cellular amine metabolic process;cellular amino acid metabolic process;cellular biosynthetic process;cellular ketone metabolic process;cellula
anatomical structure development;anatomical structure morphogenesis;catabolic process;cellular catabolic process;cellular macromolecule catabolic process;cellular macromolecule metabolic process;cellular metabolic proc
ameboidal cell migration;cell junction assembly;cell junction organization;cell migration;cell motility;cell-substrate junction assembly;cellular component assembly;cellular component assembly at cellular level;cellular compon
biological regulation;biosynthetic process;carbohydrate homeostasis;cellular biosynthetic process;cellular macromolecule biosynthetic process;cellular macromolecule metabolic process;cellular metabolic process;cellular nit
anatomical structure development;biosynthetic process;blastocyst development;cellular biosynthetic process;cellular macromolecule biosynthetic process;cellular macromolecule metabolic process;cellular metabolic process
apoptosis;biological regulation;biosynthetic process;cell death;cell differentiation;cell proliferation;cellular biosynthetic process;cellular developmental process;cellular macromolecule biosynthetic process;cellular macromolec
ameboidal cell migration;anatomical structure development;associative learning;basement membrane organization;behavior;biological adhesion;biological regulation;cAMP-mediated signaling;cell adhesion;cell migration;cell
biosynthetic process;cellular biosynthetic process;cellular component disassembly;cellular component disassembly at cellular level;cellular component organization;cellular component organization at cellular level;cellular con
defense response;defense response to virus;immune effector process;immune system process;multi-organism process;response to biotic stimulus;response to other organism;response to stimulus;response to stress;respons
anatomical structure development;biological regulation;cell proliferation;developmental process;negative regulation of biological process;negative regulation of cell communication;negative regulation of cellular process;negat
biological regulation;cellular component organization;cellular component organization at cellular level;cellular component organization or biogenesis;cellular component organization or biogenesis at cellular level;cellular proce
cellular macromolecule metabolic process;cellular metabolic process;cellular nitrogen compound metabolic process;cellular process;gene expression;macromolecule metabolic process;metabolic process;mRNA metabolic p
activation of JUN kinase activity;activation of MAPK activity;biological regulation;biosynthetic process;carboxylic acid biosynthetic process;carboxylic acid metabolic process;cellular biosynthetic process;cellular ketone meta
biological regulation;cell cycle;cell differentiation;cellular developmental process;cellular macromolecule metabolic process;cellular metabolic process;cellular nitrogen compound metabolic process;cellular process;cellular re
alcohol metabolic process;anatomical structure development;biological regulation;biosynthetic process;blood vessel development;cell differentiation;cellular developmental process;cellular process;cholesterol biosynthetic pr
alcohol biosynthetic process;alcohol metabolic process;amine catabolic process;amine metabolic process;biosynthetic process;carbohydrate biosynthetic process;carbohydrate metabolic process;carboxylic acid catabolic p
biological regulation;cell differentiation;cellular developmental process;cellular process;cellular response to stimulus;developmental process;gamete generation;male gamete generation;multicellular organismal process;multic
biological regulation;cellular macromolecule metabolic process;cellular metabolic process;cellular process;cellular protein metabolic process;'de novo' posttranslational protein folding;'de novo' protein folding;macromolecule
ATP hydrolysis coupled proton transport;biological regulation;cation homeostasis;cation transport;cell surface receptor linked signaling pathway;cellular cation homeostasis;cellular chemical homeostasis;cellular component c
cellular macromolecule metabolic process;cellular metabolic process;cellular nitrogen compound metabolic process;cellular process;macromolecule metabolic process;metabolic process;mRNA metabolic process;mRNA pro
anatomical structure development;biological adhesion;cell adhesion;cell development;cell-cell adhesion;cellular developmental process;cellular process;cellular process involved in reproduction;developmental process;develo
biosynthetic process;cellular biosynthetic process;cellular component disassembly;cellular component disassembly at cellular level;cellular component organization;cellular component organization at cellular level;cellular con
cellular macromolecule metabolic process;cellular metabolic process;cellular nitrogen compound metabolic process;cellular process;macromolecule metabolic process;metabolic process;ncRNA metabolic process;ncRNA pr
biological regulation;blood vessel endothelial cell migration;blood vessel endothelial cell migration involved in intussusceptive angiogenesis;cell differentiation;cell migration;cell motility;cellular component movement;cellular c
biological regulation;cellular macromolecule metabolic process;cellular metabolic process;cellular nitrogen compound metabolic process;cellular process;cellular response to stimulus;intracellular signal transduction;macromo
cellular component biogenesis;cellular component biogenesis at cellular level;cellular component organization or biogenesis;cellular component organization or biogenesis at cellular level;cellular process;ribonucleoprotein co
cellular macromolecule metabolic process;cellular metabolic process;cellular process;cellular protein metabolic process;cellular response to stimulus;cellular response to stress;macromolecule metabolic process;macromolec
cellular metabolic process;cellular process;electron transport chain;generation of precursor metabolites and energy;metabolic process;mitochondrial electron transport, NADH to ubiquinone;multicellular organismal process;n
carbohydrate metabolic process;cellular macromolecule metabolic process;cellular metabolic process;cellular process;cellular protein metabolic process;dephosphorylation;macromolecule metabolic process;macromolecule
biological regulation;biosynthetic process;cellular biosynthetic process;cellular macromolecule biosynthetic process;cellular macromolecule metabolic process;cellular metabolic process;cellular nitrogen compound metabolic
biological regulation;cell surface receptor linked signaling pathway;cellular component assembly;cellular component organization;cellular component organization or biogenesis;cellular process;cellular response to chemical s
biological regulation;biosynthetic process;cell differentiation;cellular biosynthetic process;cellular developmental process;cellular macromolecule biosynthetic process;cellular macromolecule metabolic process;cellular metab
anatomical structure development;anatomical structure formation involved in morphogenesis;androgen receptor signaling pathway;ATP-dependent chromatin remodeling;biological regulation;biosynthetic process;blood vess
acid secretion;actin cytoskeleton organization;actin filament-based process;activation of adenylate cyclase activity;activation of adenylate cyclase activity by dopamine receptor signaling pathway;activation of adenylate cycla
biological regulation;cell surface receptor linked signaling pathway;cellular process;cellular response to chemical stimulus;cellular response to cytokine stimulus;cellular response to interferon-alpha;cellular response to organi
anatomical structure development;cell development;cellular developmental process;cellular metabolic process;cellular process;central nervous system neuron development;cerebellar Purkinje cell layer development;develop
cellular component organization;cellular component organization at cellular level;cellular component organization or biogenesis;cellular component organization or biogenesis at cellular level;cellular membrane fusion;cellular
carbohydrate metabolic process;cellular aldehyde metabolic process;cellular metabolic process;cellular process;metabolic process;primary metabolic process;small molecule metabolic process;xenobiotic metabolic process
biological regulation;cell cycle phase;cell cycle process;cellular component organization;cellular component organization at cellular level;cellular component organization or biogenesis;cellular component organization or bioge
anatomical structure development;biological regulation;biosynthetic process;blood coagulation;cellular biosynthetic process;cellular macromolecule biosynthetic process;cellular macromolecule metabolic process;cellular me
establishment of localization;establishment of localization in cell;establishment of RNA localization;intracellular transport;mRNA export from nucleus;mRNA transport;nuclear export;nuclear transport;nucleic acid transport;nuc
anatomical structure maturation;biological regulation;blood vessel maturation;defense response;defense response to bacterium;developmental maturation;developmental process;immune response;immune system process;i
biological regulation;cation homeostasis;cation transport;cell surface receptor linked signaling pathway;cellular cation homeostasis;cellular chemical homeostasis;cellular component organization;cellular component organizat
anatomical structure development;biological regulation;cellular metabolic process;cellular process;developmental process;electron transport chain;generation of precursor metabolites and energy;metabolic process;mitochon
cellular component biogenesis;cellular component biogenesis at cellular level;cellular component organization or biogenesis;cellular component organization or biogenesis at cellular level;cellular process;ribonucleoprotein co
CAAX-box protein processing;catabolic process;cellular catabolic process;cellular component organization;cellular component organization or biogenesis;cellular macromolecule catabolic process;cellular macromolecule me
antigen processing and presentation;antigen processing and presentation of exogenous antigen;antigen processing and presentation of exogenous peptide antigen;antigen processing and presentation of exogenous peptide
biological regulation;cell redox homeostasis;cellular homeostasis;cellular process;homeostatic process;negative regulation of biological process;negative regulation of cardiac muscle hypertrophy;negative regulation of multic
biological regulation;cellular macromolecule metabolic process;cellular metabolic process;cellular process;cellular protein metabolic process;macromolecule metabolic process;metabolic process;positive regulation of catalyt
anatomical structure development;biological regulation;biosynthetic process;cellular biosynthetic process;cellular macromolecule biosynthetic process;cellular macromolecule metabolic process;cellular metabolic process;ce
alcohol catabolic process;alcohol metabolic process;carbohydrate catabolic process;carbohydrate metabolic process;catabolic process;cellular carbohydrate catabolic process;cellular carbohydrate metabolic process;cellula
amine catabolic process;amine metabolic process;carboxylic acid catabolic process;carboxylic acid metabolic process;catabolic process;cellular amine metabolic process;cellular amino acid catabolic process;cellular amino
anatomical structure development;ATP-dependent chromatin remodeling;biological regulation;biosynthetic process;cellular biosynthetic process;cellular component organization;cellular component organization at cellular lev
activation of immune response;activation of innate immune response;activation of MAPKK activity;activation of protein kinase activity;axon guidance;behavior;behavioral interaction between organisms;biological regulation;bl
biological regulation;cellular process;cellular response to cadmium ion;cellular response to chemical stimulus;cellular response to inorganic substance;cellular response to metal ion;cellular response to stimulus;cellular respo
biosynthetic process;catabolic process;cellular biosynthetic process;cellular catabolic process;cellular component disassembly;cellular component disassembly at cellular level;cellular component organization;cellular compo
adenine biosynthetic process;adenine metabolic process;adenine salvage;AMP biosynthetic process;AMP metabolic process;AMP salvage;behavior;biological regulation;biosynthetic process;body fluid secretion;cellular aron
biological regulation;cellular macromolecule metabolic process;cellular metabolic process;cellular nitrogen compound metabolic process;cellular process;gene expression;macromolecule metabolic process;metabolic proces
biological regulation;biosynthetic process;carbohydrate biosynthetic process;carbohydrate metabolic process;carbohydrate transmembrane transport;carbohydrate transport;carboxylic acid metabolic process;cation transpo
actin cytoskeleton organization;actin cytoskeleton reorganization;actin filament-based process;biological regulation;cell surface receptor linked signaling pathway;cellular component organization;cellular component organizat
autophagic cell death;autophagy;biological regulation;catabolic process;cell death;cellular catabolic process;cellular metabolic process;cellular process;death;establishment of localization;establishment of localization in cell;
alcohol metabolic process;anatomical structure development;biosynthetic process;carbohydrate biosynthetic process;carbohydrate metabolic process;cellular biosynthetic process;cellular carbohydrate biosynthetic process
aerobic respiration;cellular metabolic process;cellular process;cellular respiration;electron transport chain;energy derivation by oxidation of organic compounds;generation of precursor metabolites and energy;metabolic proce
anatomical structure formation involved in morphogenesis;angiogenesis;biosynthetic process;catabolic process;cell differentiation;cellular aromatic compound metabolic process;cellular biosynthetic process;cellular catabolic
bile acid biosynthetic process;bile acid metabolic process;biological regulation;biosynthetic process;C21-steroid hormone biosynthetic process;C21-steroid hormone metabolic process;carboxylic acid biosynthetic process;c
anatomical structure development;biological regulation;cell proliferation;cellular macromolecule metabolic process;cellular metabolic process;cellular nitrogen compound metabolic process;cellular process;cellular protein me
anaphase-promoting complex-dependent proteasomal ubiquitin-dependent protein catabolic process;biological regulation;biosynthetic process;blood coagulation;catabolic process;cation transport;cell cycle;cell cycle check
amine catabolic process;amine metabolic process;branched chain family amino acid catabolic process;branched chain family amino acid metabolic process;carboxylic acid catabolic process;carboxylic acid metabolic proces
anatomical structure development;anatomical structure formation involved in morphogenesis;anatomical structure morphogenesis;angiogenesis;axon guidance;axonal fasciculation;axonogenesis;behavior;biological regulatio
anatomical structure development;anatomical structure morphogenesis;appendage morphogenesis;biological regulation;biosynthetic process;carboxylic acid metabolic process;cellular biosynthetic process;cellular hormone
biological regulation;biosynthetic process;catabolic process;cellular biosynthetic process;cellular catabolic process;cellular macromolecule metabolic process;cellular metabolic process;cellular nitrogen compound metabolic

biosynthetic process;cellular biosynthetic process;cellular metabolic process;cellular process;melanin biosynthetic process;melanin metabolic process;metabolic process;pigment biosynthetic process;pigment metabolic proc
amine biosynthetic process;amine catabolic process;amine metabolic process;biological regulation;biosynthetic process;carboxylic acid biosynthetic process;carboxylic acid catabolic process;carboxylic acid metabolic proc
alcohol metabolic process;biological regulation;cell differentiation;cellular component assembly;cellular component organization;cellular component organization or biogenesis;cellular developmental process;cellular lipid met
anatomical structure homeostasis;base-excision repair;biological regulation;biosynthetic process;cell cycle;cellular biosynthetic process;cellular component organization;cellular component organization at cellular level;cellula
activation of MAPKK activity;activation of protein kinase activity;biological regulation;blood coagulation;cell activation;cell surface receptor linked signaling pathway;cellular macromolecule metabolic process;cellular metaboli
alcohol metabolic process;amine metabolic process;biosynthetic process;CDP-choline pathway;cellular amine metabolic process;cellular biogenic amine metabolic process;cellular biosynthetic process;cellular lipid metaboli
actin cytoskeleton organization;actin filament-based process;biological regulation;cell chemotaxis;cell junction assembly;cell junction organization;cell migration;cell motility;cell projection organization;cell-cell junction assem
biological regulation;catabolic process;cellular catabolic process;cellular component organization;cellular component organization at cellular level;cellular component organization or biogenesis;cellular component organizatio
cellular component organization;cellular component organization at cellular level;cellular component organization or biogenesis;cellular component organization or biogenesis at cellular level;cellular process;extracellular matri
anatomical structure morphogenesis;B cell activation;B cell differentiation;cell activation;cell differentiation;cellular developmental process;cellular process;developmental process;establishment of localization;immune system
biological regulation;biosynthetic process;cellular biosynthetic process;cellular macromolecule biosynthetic process;cellular macromolecule metabolic process;cellular metabolic process;cellular process;macromolecule biosy
anatomical structure development;behavior;biological regulation;cellular macromolecule metabolic process;cellular metabolic process;cellular process;cellular protein metabolic process;cellular response to stimulus;cognition
activation of MAPKK activity;activation of protein kinase activity;anatomical structure development;biological regulation;blood coagulation;cell activation;cell development;cell surface receptor linked signaling pathway;cellular
activation of immune response;activation of innate immune response;anaphase-promoting complex-dependent proteasomal ubiquitin-dependent protein catabolic process;biological regulation;catabolic process;cell cycle;ce
anatomical structure development;biological regulation;brain development;catabolic process;developmental process;gamete generation;lipid catabolic process;lipid metabolic process;male gamete generation;metabolic proc
biosynthetic process;cellular biosynthetic process;cellular macromolecule biosynthetic process;cellular macromolecule metabolic process;cellular metabolic process;cellular nitrogen compound metabolic process;cellular pro
acetaldehyde metabolic process;activation of immune response;activation of innate immune response;aging;anatomical structure development;anatomical structure morphogenesis;antigen receptor-mediated signaling pathw
anatomical structure morphogenesis;biological regulation;biosynthetic process;cell part morphogenesis;cellular biosynthetic process;cellular component morphogenesis;cellular component organization;cellular component or
anatomical structure morphogenesis;anterior/posterior pattern specification;cellular macromolecule metabolic process;cellular metabolic process;cellular nitrogen compound metabolic process;cellular process;developmenta
establishment of localization;establishment of protein localization;protein transport;transport
behavior;biological regulation;biosynthetic process;catabolic process;cellular biosynthetic process;cellular catabolic process;cellular component organization;cellular component organization at cellular level;cellular componen
activation of immune response;activation of innate immune response;antigen receptor-mediated signaling pathway;biological regulation;cell differentiation;cell surface receptor linked signaling pathway;cellular developmental
biological regulation;cell cycle;cell surface receptor linked signaling pathway;cellular macromolecule metabolic process;cellular metabolic process;cellular process;cellular protein metabolic process;cellular response to chemi
biological regulation;catabolic process;cellular catabolic process;cellular localization;cellular macromolecule catabolic process;cellular macromolecule localization;cellular macromolecule metabolic process;cellular metabolic
biological regulation;biosynthetic process;cellular biosynthetic process;cellular component organization;cellular component organization at cellular level;cellular component organization or biogenesis;cellular component orga
biological regulation;cell redox homeostasis;cellular homeostasis;cellular macromolecule metabolic process;cellular metabolic process;cellular process;cellular protein metabolic process;cellular response to chemical stimulus
activation of immune response;activation of innate immune response;alcohol metabolic process;anatomical structure development;biological regulation;carbohydrate metabolic process;cell surface receptor linked signaling p
amine metabolic process;amino acid activation;carboxylic acid metabolic process;cellular amine metabolic process;cellular amino acid metabolic process;cellular ketone metabolic process;cellular macromolecule metabolic p
cellular component assembly;cellular component assembly at cellular level;cellular component organization;cellular component organization at cellular level;cellular component organization or biogenesis;cellular component c
actin cytoskeleton organization;actin filament-based process;anatomical structure morphogenesis;axonogenesis;biological regulation;cell part morphogenesis;cell projection morphogenesis;cell projection organization;cellula
biological regulation;cell junction assembly;cell junction organization;cellular component assembly;cellular component assembly at cellular level;cellular component organization;cellular component organization at cellular leve
anatomical structure development;biological adhesion;biological regulation;cell adhesion;cellular process;cellular response to chemical stimulus;cellular response to endogenous stimulus;cellular response to hormone stimulu
cation transport;cellular metabolic process;cellular process;electron transport chain;establishment of localization;generation of precursor metabolites and energy;ion transport;metabolic process;metal ion transport;mitochond
cellular component organization;cellular component organization at cellular level;cellular component organization or biogenesis;cellular component organization or biogenesis at cellular level;cellular process;endosome organi
biosynthetic process;cellular biosynthetic process;cellular metabolic process;cellular nitrogen compound biosynthetic process;cellular nitrogen compound metabolic process;cellular process;coenzyme biosynthetic process;c
amine biosynthetic process;amine metabolic process;biosynthetic process;carboxylic acid biosynthetic process;carboxylic acid metabolic process;cellular amine metabolic process;cellular amino acid biosynthetic process;ce
cellular macromolecule metabolic process;cellular metabolic process;cellular process;cellular protein metabolic process;macromolecule metabolic process;metabolic process;primary metabolic process;protein folding;protein
anatomical structure development;biological regulation;cell migration;cell motility;cellular component movement;cellular process;developmental process;locomotion;lung alveolus development;positive regulation of biological
amine catabolic process;amine metabolic process;branched chain family amino acid catabolic process;branched chain family amino acid metabolic process;carboxylic acid catabolic process;carboxylic acid metabolic proces
alkaloid metabolic process;biosynthetic process;cellular amide metabolic process;cellular biosynthetic process;cellular metabolic compound salvage;cellular metabolic process;cellular nitrogen compound biosynthetic proces
activation of protein kinase activity;autophagy;biological regulation;catabolic process;cell migration;cell motility;cell surface receptor linked signaling pathway;cellular catabolic process;cellular component movement;cellular l
biological regulation;cellular macromolecule metabolic process;cellular metabolic process;cellular nitrogen compound metabolic process;cellular process;macromolecule metabolic process;macromolecule methylation;macro
cell cycle process;cell division;cellular component assembly;cellular component assembly at cellular level;cellular component organization;cellular component organization at cellular level;cellular component organization or b
metabolic process;nitrogen compound metabolic process
cell projection organization;cellular component movement;cellular component organization;cellular component organization at cellular level;cellular component organization or biogenesis;cellular component organization or bio
anatomical structure development;biological regulation;calcium ion homeostasis;calcium ion transport;calcium ion transport into cytosol;cation homeostasis;cation transport;cellular calcium ion homeostasis;cellular cation ho
cellular macromolecule metabolic process;cellular metabolic process;cellular nitrogen compound metabolic process;cellular process;macromolecule metabolic process;macromolecule methylation;macromolecule modificatio
arachidonic acid metabolic process;biological regulation;biosynthetic process;blood coagulation;carboxylic acid metabolic process;catabolic process;cell activation;cell proliferation;cellular biosynthetic process;cellular keton
acylglycerol biosynthetic process;acylglycerol metabolic process;anatomical structure development;anatomical structure homeostasis;biological regulation;biosynthetic process;carboxylic acid biosynthetic process;carboxylic
biological regulation;cellular macromolecule metabolic process;cellular metabolic process;cellular nitrogen compound metabolic process;cellular process;macromolecule metabolic process;metabolic process;mRNA metaboli
biological regulation;cell surface receptor linked signaling pathway;cellular component organization;cellular component organization at cellular level;cellular component organization or biogenesis;cellular component organizat
biological regulation;cellular process;cellular response to stimulus;positive regulation of biological process;positive regulation of cell communication;positive regulation of cellular process;positive regulation of I-kappaB kinase
cellular component biogenesis;cellular component biogenesis at cellular level;cellular component organization or biogenesis;cellular component organization or biogenesis at cellular level;cellular macromolecule metabolic pro
acetyl-CoA catabolic process;acetyl-CoA metabolic process;alcohol catabolic process;alcohol metabolic process;carbohydrate catabolic process;carbohydrate metabolic process;catabolic process;cell differentiation;cellular
alcohol biosynthetic process;alcohol metabolic process;biosynthetic process;carbohydrate biosynthetic process;carbohydrate metabolic process;cellular biosynthetic process;cellular carbohydrate biosynthetic process;cellul
amine metabolic process;aromatic amino acid family metabolic process;carboxylic acid metabolic process;cellular amine metabolic process;cellular amino acid metabolic process;cellular aromatic compound metabolic proce
anatomical structure development;biological regulation;brain development;cell cycle checkpoint;cell cycle process;cell proliferation;cellular macromolecule metabolic process;cellular metabolic process;cellular process;cellula
biological regulation;cell redox homeostasis;cellular homeostasis;cellular macromolecule metabolic process;cellular metabolic process;cellular process;cellular protein metabolic process;cellular response to stimulus;cellular r
actin cytoskeleton organization;actin cytoskeleton reorganization;actin filament organization;actin filament reorganization involved in cell cycle;actin filament-based process;biological regulation;cell cycle process;cellular com
biological regulation;biosynthetic process;cellular biosynthetic process;cellular component organization;cellular component organization at cellular level;cellular component organization or biogenesis;cellular component orga
cellular macromolecule metabolic process;cellular metabolic process;cellular process;cellular protein metabolic process;macromolecule metabolic process;metabolic process;primary metabolic process;protein folding;protein
amine catabolic process;amine metabolic process;branched chain family amino acid catabolic process;branched chain family amino acid metabolic process;carboxylic acid catabolic process;carboxylic acid metabolic proces
establishment of localization;transport
biological regulation;cellular macromolecule metabolic process;cellular metabolic process;cellular process;cellular protein metabolic process;cytokine production;defense response;defense response to virus;immune effector
biological regulation;posttranscriptional regulation of gene expression;regulation of biological process;regulation of biosynthetic process;regulation of cellular biosynthetic process;regulation of cellular macromolecule biosynth
biological regulation;cellular component organization;cellular component organization at cellular level;cellular component organization or biogenesis;cellular component organization or biogenesis at cellular level;cellular proce
anatomical structure formation involved in morphogenesis;angiogenesis;biological regulation;cell migration;cell motility;cellular component assembly;cellular component assembly at cellular level;cellular component movemen
biological regulation;establishment of localization;establishment of localization in cell;establishment of RNA localization;intracellular transport;mRNA export from nucleus;mRNA transport;nuclear export;nuclear transport;nucle
biological regulation;cell differentiation;cellular developmental process;cellular localization;cellular macromolecule localization;cellular process;cellular protein localization;developmental process;establishment of localization;f
biological regulation;regulation of biological process;regulation of biosynthetic process;regulation of cellular biosynthetic process;regulation of cellular macromolecule biosynthetic process;regulation of cellular metabolic proc
cellular component biogenesis;cellular component biogenesis at cellular level;cellular component organization or biogenesis;cellular component organization or biogenesis at cellular level;cellular process;establishment of loc
biological regulation;cell redox homeostasis;cellular component organization;cellular component organization or biogenesis;cellular homeostasis;cellular process;homeostatic process;positive regulation of catalytic activity;po
biological regulation;negative regulation of catalytic activity;negative regulation of hydrolase activity;negative regulation of molecular function;negative regulation of phosphatase activity;regulation of biological process;regulat
behavior;exploration behavior;locomotory behavior;locomotory exploration behavior;pigmentation;response to stimulus
cellular component organization;cellular component organization or biogenesis;cellular process;endosome transport;establishment of localization;establishment of localization in cell;establishment of protein localization;intrac
adenosine metabolic process;cellular macromolecule metabolic process;cellular metabolic process;cellular nitrogen compound metabolic process;cellular process;heterocycle metabolic process;macromolecule metabolic pro
biological regulation;biosynthetic process;cellular biosynthetic process;cellular component organization;cellular component organization at cellular level;cellular component organization or biogenesis;cellular component orga
biological regulation;cellular macromolecule metabolic process;cellular metabolic process;cellular process;cellular protein metabolic process;dephosphorylation;macromolecule metabolic process;macromolecule modification
biological regulation;cell cycle;cellular macromolecule metabolic process;cellular metabolic process;cellular nitrogen compound metabolic process;cellular process;cellular response to stimulus;cellular response to stress;DNA
biological regulation;cellular macromolecule metabolic process;cellular metabolic process;cellular nitrogen compound metabolic process;cellular process;cellular response to chemical stimulus;cellular response to drug;cellula
anaphase-promoting complex-dependent proteasomal ubiquitin-dependent protein catabolic process;biological regulation;catabolic process;cell cycle;cell cycle checkpoint;cell cycle phase;cell cycle process;cell division;cel
biosynthetic process;cellular biosynthetic process;cellular macromolecule biosynthetic process;cellular macromolecule metabolic process;cellular metabolic process;cellular nitrogen compound metabolic process;cellular pro
cell proliferation
anatomical structure morphogenesis;cell part morphogenesis;cellular component morphogenesis;cellular component organization;cellular component organization at cellular level;cellular component organization or biogenesi
biological regulation;biosynthetic process;cellular biosynthetic process;cellular macromolecule biosynthetic process;cellular macromolecule metabolic process;cellular metabolic process;cellular process;cellular protein metab
biological regulation;cell cycle;cellular process;positive regulation of catalytic activity;positive regulation of GTPase activity;positive regulation of hydrolase activity;positive regulation of molecular function;regulation of biologic
biological regulation;biosynthetic process;cellular biosynthetic process;cellular macromolecule biosynthetic process;cellular macromolecule metabolic process;cellular metabolic process;cellular nitrogen compound metabolic
alcohol metabolic process;anatomical structure development;biological regulation;brain development;carbohydrate homeostasis;carbohydrate metabolic process;cell proliferation;cell surface receptor linked signaling pathway
cellular macromolecule metabolic process;cellular metabolic process;cellular nitrogen compound metabolic process;cellular process;macromolecule metabolic process;metabolic process;ncRNA metabolic process;ncRNA pr
establishment of localization;establishment of localization in cell;establishment of protein localization;intracellular protein transport;intracellular transport;nuclear import;nuclear transport;nucleocytoplasmic transport;protein in
biological regulation;biosynthetic process;cell surface receptor linked signaling pathway;cellular biosynthetic process;cellular component organization;cellular component organization at cellular level;cellular component orga
amine metabolic process;aromatic compound biosynthetic process;biosynthetic process;carboxylic acid metabolic process;cellular amine metabolic process;cellular amino acid metabolic process;cellular aromatic compound
biological regulation;cellular component organization;cellular component organization at cellular level;cellular component organization or biogenesis;cellular component organization or biogenesis at cellular level;cellular macro
biological regulation;cellular component organization;cellular component organization at cellular level;cellular component organization or biogenesis;cellular component organization or biogenesis at cellular level;cellular memb
biological regulation;cell cycle;cell cycle checkpoint;cell cycle phase;cell cycle process;cell division;cellular component organization;cellular component organization at cellular level;cellular component organization or biogene
carboxylic acid metabolic process;cellular ketone metabolic process;cellular lipid metabolic process;cellular metabolic process;cellular process;fatty acid metabolic process;lipid metabolic process;metabolic process;monoca
anatomical structure development;antigen processing and presentation;antigen processing and presentation of exogenous antigen;antigen processing and presentation of exogenous peptide antigen;antigen processing and p
biological regulation;cellular process;cellular response to stimulus;immune response;immune system process;metabolic process;positive regulation of biological process;positive regulation of catabolic process;positive regula
cellular process;establishment of localization;establishment of localization in cell;establishment of protein localization;Golgi vesicle transport;intracellular transport;post-Golgi vesicle-mediated transport;protein transport;respo
amine biosynthetic process;amine metabolic process;biosynthetic process;carboxylic acid biosynthetic process;carboxylic acid metabolic process;cellular amine metabolic process;cellular amino acid biosynthetic process;ce
biological adhesion;biological regulation;calcium ion import;calcium ion transmembrane transport;calcium ion transport;cation transport;cell adhesion;cell communication;cell-cell signaling;cellular component organization;cel
alcohol biosynthetic process;alcohol metabolic process;amine metabolic process;biosynthetic process;cellular biosynthetic process;cellular lipid metabolic process;cellular metabolic process;cellular nitrogen compound bios
biological regulation;cell cycle;cell cycle checkpoint;cell cycle phase;cell cycle process;cell division;cellular component assembly;cellular component organization;cellular component organization at cellular level;cellular comp
cap-independent translational initiation;cellular component assembly;cellular component assembly at cellular level;cellular component disassembly;cellular component disassembly at cellular level;cellular component organiza
anion transport;biological regulation;cellular localization;cellular macromolecule localization;cellular macromolecule metabolic process;cellular metabolic process;cellular process;cellular protein localization;cellular protein me
biological regulation;biosynthetic process;cellular biosynthetic process;cellular macromolecule biosynthetic process;cellular macromolecule metabolic process;cellular metabolic process;cellular nitrogen compound metabolic
apoptosis;biological regulation;cell cycle;cell cycle phase;cell cycle process;cell death;cell division;cellular component organization;cellular component organization at cellular level;cellular component organization or biogenes
apoptosis;biological regulation;cell death;cell differentiation;cellular developmental process;cellular process;death;developmental process;inner ear receptor cell differentiation;mechanoreceptor differentiation;multicellular org
apoptosis;cell death;cellular component organization;cellular component organization or biogenesis;cellular membrane organization;cellular process;clathrin-mediated endocytosis;death;endocytosis;establishment of localiza
assembly of spliceosomal tri-snRNP;biological regulation;cellular component assembly;cellular component assembly at cellular level;cellular component organization;cellular component organization at cellular level;cellular co
carboxylic acid catabolic process;carboxylic acid metabolic process;catabolic process;cellular catabolic process;cellular ketone metabolic process;cellular lipid catabolic process;cellular lipid metabolic process;cellular metab
cell cycle;cell cycle process;cellular component disassembly;cellular component disassembly at cellular level;cellular component organization;cellular component organization at cellular level;cellular component organization o
acidic amino acid transport;alcohol biosynthetic process;alcohol metabolic process;amine transport;amino acid transport;anion transport;aspartate transport;biosynthetic process;carbohydrate biosynthetic process;carbohyd
cellular macromolecule metabolic process;cellular metabolic process;cellular nitrogen compound metabolic process;cellular process;gene expression;macromolecule metabolic process;metabolic process;mRNA metabolic p
ameboidal cell migration;anatomical structure development;anatomical structure morphogenesis;androgen metabolic process;biological regulation;biosynthetic process;carboxylic acid metabolic process;catabolic process;ce
biological regulation;multicellular organismal process;negative regulation of biological process;negative regulation of cellular component organization;negative regulation of cellular process;negative regulation of cytoskeleton
adenine biosynthetic process;adenine metabolic process;adenine salvage;alcohol metabolic process;amine metabolic process;anatomical structure development;anatomical structure morphogenesis;behavior;benzene-conta
actin cytoskeleton organization;actin filament-based process;activation of immune response;activation of innate immune response;activation of MAPKK activity;activation of protein kinase activity;anatomical structure morpho
antigen processing and presentation;antigen processing and presentation of exogenous antigen;antigen processing and presentation of exogenous peptide antigen;antigen processing and presentation of exogenous peptide
apoptosis;catabolic process;cell death;cellular catabolic process;cellular component assembly;cellular component assembly at cellular level;cellular component disassembly;cellular component disassembly at cellular level;ce
aerobic respiration;cellular metabolic process;cellular process;cellular respiration;electron transport chain;energy derivation by oxidation of organic compounds;generation of precursor metabolites and energy;metabolic proce
cellular macromolecule metabolic process;cellular metabolic process;cellular nitrogen compound metabolic process;cellular process;gene expression;macromolecule metabolic process;metabolic process;mRNA metabolic p
cellular metabolic process;cellular process;dephosphorylation;metabolic process;phosphate-containing compound metabolic process;phosphorus metabolic process

biological regulation;biosynthetic process;cellular biosynthetic process;cellular component assembly;cellular component assembly at cellular level;cellular component organization;cellular component organization at cellular le
catabolic process;cellular catabolic process;cellular macromolecule catabolic process;cellular macromolecule metabolic process;cellular metabolic process;cellular process;cellular protein metabolic process;macromolecule
amine catabolic process;amine metabolic process;arginine catabolic process;arginine metabolic process;aromatic amino acid family catabolic process;aromatic amino acid family metabolic process;aromatic compound catab
antigen processing and presentation;biological regulation;cellular component movement;cellular localization;cellular macromolecule localization;cellular macromolecule metabolic process;cellular metabolic process;cellular pr
cell cycle;cell cycle process;cellular component organization;cellular component organization at cellular level;cellular component organization or biogenesis;cellular component organization or biogenesis at cellular level;cellula
activation of immune response;activation of innate immune response;anaphase-promoting complex-dependent proteasomal ubiquitin-dependent protein catabolic process;antigen processing and presentation;antigen proces
catabolic process;cellular catabolic process;cellular metabolic process;cellular nitrogen compound catabolic process;cellular nitrogen compound metabolic process;cellular process;cofactor catabolic process;cofactor metab
biological regulation;catabolic process;cation homeostasis;cellular catabolic process;cellular cation homeostasis;cellular chemical homeostasis;cellular homeostasis;cellular ion homeostasis;cellular iron ion homeostasis;cellu
anatomical structure development;biological regulation;calcium ion transport;cation transport;cellular chemical homeostasis;cellular homeostasis;cellular ion homeostasis;cellular process;cellular response to stimulus;chemica
anatomical structure homeostasis;base-excision repair;biological regulation;biosynthetic process;cell cycle;cellular biosynthetic process;cellular component organization;cellular component organization at cellular level;cellula
anatomical structure morphogenesis;biological regulation;biosynthetic process;cell fate commitment;cell proliferation;cell surface receptor linked signaling pathway;cellular biosynthetic process;cellular developmental process
aging;biological regulation;cell aging;cell cycle;cell cycle arrest;cell cycle process;cellular component assembly;cellular component assembly at cellular level;cellular component organization;cellular component organization a
acyl-CoA metabolic process;carboxylic acid metabolic process;cellular ketone metabolic process;cellular lipid metabolic process;cellular metabolic process;cellular process;coenzyme metabolic process;cofactor metabolic p
anatomical structure development;anatomical structure formation involved in morphogenesis;anatomical structure morphogenesis;axon guidance;biological regulation;canonical Wnt receptor signaling pathway;canonical Wnt
biological regulation;cellular process;cellular response to stimulus;establishment of localization;establishment of localization in cell;establishment of protein localization;intracellular protein transport;intracellular signal transdu
actin cytoskeleton organization;actin filament-based process;biological regulation;calcium ion homeostasis;cation homeostasis;cellular calcium ion homeostasis;cellular cation homeostasis;cellular chemical homeostasis;cellu
biological regulation;biosynthetic process;cellular biosynthetic process;cellular component disassembly;cellular component disassembly at cellular level;cellular component organization;cellular component organization at cell
anatomical structure development;biological regulation;developmental process;hemopoiesis;hemopoietic or lymphoid organ development;organ development;positive regulation of biological process;positive regulation of cell
anatomical structure development;apical protein localization;asymmetric protein localization;biological regulation;brain development;cell communication;cell differentiation;cell-cell signaling;cellular component disassembly;ce
biological regulation;cellular process;cellular response to stimulus;regulation of biological process;regulation of cellular process;response to stimulus;signal transduction
cellular macromolecule metabolic process;cellular metabolic process;cellular nitrogen compound metabolic process;cellular process;macromolecule metabolic process;metabolic process;ncRNA metabolic process;ncRNA pr
biological regulation;cellular metabolic process;cellular process;dephosphorylation;metabolic process;phosphate-containing compound metabolic process;phosphorus metabolic process;regulation of biological process;regu
activation of immune response;activation of innate immune response;biological regulation;cell surface receptor linked signaling pathway;cellular macromolecule metabolic process;cellular metabolic process;cellular process;c
anatomical structure formation involved in morphogenesis;anatomical structure morphogenesis;biological regulation;calcium ion transmembrane transport;calcium ion transport;cardiac muscle tissue morphogenesis;cation tr
aging;anatomical structure formation involved in morphogenesis;anatomical structure morphogenesis;axon guidance;biological regulation;blood coagulation;branching involved in embryonic placenta morphogenesis;cell acti
biological regulation;biosynthetic process;catabolic process;cell proliferation;cellular biosynthetic process;cellular catabolic process;cellular macromolecule biosynthetic process;cellular macromolecule catabolic process;cellu
biological regulation;cation homeostasis;cellular cation homeostasis;cellular chemical homeostasis;cellular divalent inorganic cation homeostasis;cellular homeostasis;cellular ion homeostasis;cellular process;cellular respons
biological regulation;biosynthetic process;cellular biosynthetic process;cellular macromolecule biosynthetic process;cellular macromolecule metabolic process;cellular metabolic process;cellular nitrogen compound metabolic
anatomical structure development;biological regulation;biosynthetic process;cell differentiation;cellular biosynthetic process;cellular developmental process;cellular macromolecule biosynthetic process;cellular macromolecule
anatomical structure development;biological adhesion;cell adhesion;cell differentiation;cellular component disassembly;cellular component disassembly at cellular level;cellular component organization;cellular component org
attachment of GPI anchor to protein;biological regulation;blood coagulation;cellular component movement;cellular macromolecule metabolic process;cellular metabolic process;cellular process;cellular protein metabolic proc
cellular macromolecule metabolic process;cellular metabolic process;cellular process;cellular protein metabolic process;'de novo' cotranslational protein folding;'de novo' protein folding;macromolecule metabolic process;me
activation of store-operated calcium channel activity;biological regulation;blood coagulation;calcium ion transport;cation transport;coagulation;detection of calcium ion;detection of chemical stimulus;detection of stimulus;div
carbohydrate metabolic process;cellular carbohydrate metabolic process;cellular macromolecule metabolic process;cellular metabolic process;cellular process;cellular protein metabolic process;glycosylation;macromolecule
biological regulation;positive regulation of biological process;positive regulation of cellular component organization;positive regulation of cellular process;positive regulation of establishment of protein localization in plasma m
activation of immune response;activation of innate immune response;antigen processing and presentation;antigen receptor-mediated signaling pathway;B cell receptor signaling pathway;biological regulation;cell surface rece
biological regulation;posttranscriptional regulation of gene expression;regulation of biological process;regulation of biosynthetic process;regulation of cellular biosynthetic process;regulation of cellular macromolecule biosynth
biological regulation;cell cycle phase;cell cycle process;cell division;cell proliferation;cellular component organization;cellular component organization at cellular level;cellular component organization or biogenesis;cellular com
cellular macromolecule metabolic process;cellular metabolic process;cellular nitrogen compound metabolic process;cellular process;DNA metabolic process;DNA recombination;gene expression;macromolecule metabolic pr
anatomical structure development;biosynthetic process;cell cycle phase;cell cycle process;cell development;cell maturation;cellular biosynthetic process;cellular component assembly;cellular component assembly at cellular
3'-UTR-mediated mRNA stabilization;activation of immune response;activation of innate immune response;activation of MAPK activity;aging;alcohol metabolic process;anatomical structure development;anatomical structure
cellular component assembly;cellular component organization;cellular component organization or biogenesis;cellular macromolecule metabolic process;cellular metabolic process;cellular nitrogen compound metabolic proce
biological regulation;biosynthetic process;carbohydrate metabolic process;carbohydrate transport;cell cycle;cell cycle process;cell surface receptor linked signaling pathway;cellular biosynthetic process;cellular component c
biological regulation;cell cycle;cellular process;cellular response to stimulus;cellular response to stress;developmental process;negative regulation of biological process;negative regulation of cell differentiation;negative regula
biological regulation;cell cycle checkpoint;cellular macromolecule metabolic process;cellular metabolic process;cellular nitrogen compound metabolic process;cellular process;cellular response to stimulus;cellular response t
biological regulation;biosynthetic process;cellular biosynthetic process;cellular component organization;cellular component organization at cellular level;cellular component organization or biogenesis;cellular component orga
anatomical structure development;anatomical structure formation involved in morphogenesis;biological regulation;blood coagulation;cell development;cell differentiation;cellular component organization;cellular component or
biological regulation;biosynthetic process;cellular biosynthetic process;cellular macromolecule biosynthetic process;cellular macromolecule metabolic process;cellular metabolic process;cellular nitrogen compound metabolic
biological regulation;biosynthetic process;carbohydrate metabolic process;carbohydrate transport;cell cycle;cell cycle process;cell surface receptor linked signaling pathway;cellular biosynthetic process;cellular component c
biological regulation;biosynthetic process;cellular biosynthetic process;cellular macromolecule biosynthetic process;cellular macromolecule metabolic process;cellular metabolic process;cellular nitrogen compound metabolic
biological regulation;cellular process;cellular response to stimulus;establishment of localization;establishment of protein localization;positive regulation of biological process;positive regulation of cell communication;positive re
cell differentiation;cell envelope organization;cellular component organization;cellular component organization or biogenesis;cellular developmental process;cellular process;developmental process;external encapsulating stru
apoptosis;apoptosis in response to endoplasmic reticulum stress;biological regulation;catabolic process;cell death;cell redox homeostasis;cellular catabolic process;cellular homeostasis;cellular macromolecule catabolic proc
biological regulation;cellular component movement;cellular homeostasis;cellular process;cellular response to stimulus;cytoskeleton-dependent intracellular transport;establishment of localization;establishment of localization
biological regulation;biosynthetic process;cellular biosynthetic process;cellular macromolecule biosynthetic process;cellular macromolecule metabolic process;cellular metabolic process;cellular nitrogen compound metabolic
acylglycerol biosynthetic process;acylglycerol metabolic process;alcohol catabolic process;alcohol metabolic process;alditol catabolic process;alditol metabolic process;biological regulation;biosynthetic process;carbohydrat
biological regulation;cellular component organization;cellular component organization at cellular level;cellular component organization or biogenesis;cellular component organization or biogenesis at cellular level;cellular proce
ATP-dependent chromatin remodeling;biological regulation;biosynthetic process;cellular biosynthetic process;cellular component disassembly;cellular component disassembly at cellular level;cellular component organization
apoptosis;attachment of GPI anchor to protein;cell death;cell differentiation;cellular developmental process;cellular macromolecule metabolic process;cellular metabolic process;cellular process;cellular protein metabolic proc
cellular process;ER to Golgi vesicle-mediated transport;establishment of localization;establishment of localization in cell;Golgi vesicle transport;intracellular transport;transport;vesicle-mediated transport
cellular macromolecule metabolic process;cellular metabolic process;cellular nitrogen compound metabolic process;cellular process;gene expression;macromolecule metabolic process;metabolic process;mRNA metabolic p
biosynthetic process;cellular biosynthetic process;cellular component disassembly;cellular component disassembly at cellular level;cellular component organization;cellular component organization at cellular level;cellular co
biological regulation;biosynthetic process;cellular biosynthetic process;cellular component disassembly;cellular component disassembly at cellular level;cellular component organization;cellular component organization at cell
alternative nuclear mRNA splicing, via spliceosome;cellular macromolecule metabolic process;cellular metabolic process;cellular nitrogen compound metabolic process;cellular process;macromolecule metabolic process;me
base-excision repair;biological regulation;catabolic process;cellular catabolic process;cellular macromolecule catabolic process;cellular macromolecule metabolic process;cellular metabolic process;cellular nitrogen compou
biological regulation;cell cycle phase;cell cycle process;cellular component organization;cellular component organization at cellular level;cellular component organization or biogenesis;cellular component organization or bioge
anatomical structure development;behavior;cell development;cellular developmental process;cellular process;cellular process involved in reproduction;developmental process;developmental process involved in reproduction;
cell differentiation;cellular developmental process;cellular process;developmental process;establishment of localization;establishment of protein localization;gamete generation;gene expression;macromolecule metabolic proc
catabolic process;cell cycle;cell division;cell proliferation;cellular catabolic process;cellular component organization;cellular component organization at cellular level;cellular component organization or biogenesis;cellular comp
amine biosynthetic process;amine metabolic process;amino acid salvage;aspartate family amino acid biosynthetic process;aspartate family amino acid metabolic process;biosynthetic process;carboxylic acid biosynthetic pro
biosynthetic process;cellular biosynthetic process;cellular component disassembly;cellular component disassembly at cellular level;cellular component organization;cellular component organization at cellular level;cellular co
biological regulation;biosynthetic process;cellular biosynthetic process;cellular component assembly;cellular component assembly at cellular level;cellular component organization;cellular component organization at cellular le
actin cytoskeleton organization;actin filament-based process;actomyosin structure organization;anion transport;antigen processing and presentation;antigen processing and presentation of exogenous antigen;antigen proces
asymmetric Golgi ribbon formation;biological adhesion;biological regulation;biosynthetic process;cell adhesion;cell adhesion molecule production;cell migration;cell motility;cell projection assembly;cell projection organization
cellular macromolecule metabolic process;cellular metabolic process;cellular nitrogen compound metabolic process;cellular process;establishment of localization;establishment of localization in cell;establishment of RNA loc
cellular macromolecule metabolic process;cellular metabolic process;cellular nitrogen compound metabolic process;cellular process;macromolecule metabolic process;metabolic process;nitrogen compound metabolic proce
anatomical structure development;cell differentiation;cellular developmental process;cellular process;developmental process;nervous system development;system development
anatomical structure formation involved in morphogenesis;anatomical structure morphogenesis;angiogenesis;apoptosis;axonal fasciculation;biological regulation;cardiac epithelial to mesenchymal transition;cell death;cell mig
biological regulation;cell activation;cellular component movement;cellular process;cellular response to stimulus;establishment of localization;establishment of localization in cell;establishment of protein localization;immune sy
anatomical structure development;biological regulation;biosynthetic process;cell development;cellular biosynthetic process;cellular developmental process;cellular macromolecule biosynthetic process;cellular macromolecule
actin cytoskeleton organization;actin filament-based process;cellular component biogenesis;cellular component biogenesis at cellular level;cellular component organization;cellular component organization at cellular level;cell
biological regulation;biosynthetic process;cellular biosynthetic process;cellular macromolecule biosynthetic process;cellular macromolecule metabolic process;cellular metabolic process;cellular nitrogen compound metabolic
cellular macromolecule metabolic process;cellular metabolic process;cellular nitrogen compound metabolic process;cellular process;macromolecule metabolic process;metabolic process;mRNA metabolic process;mRNA pro
biological regulation;cellular component organization;cellular component organization at cellular level;cellular component organization or biogenesis;cellular component organization or biogenesis at cellular level;cellular locali
aggresome assembly;autophagy;biological regulation;biosynthetic process;catabolic process;cell communication;cellular biosynthetic process;cellular catabolic process;cellular component assembly;cellular component asse
aging;anatomical structure development;biological regulation;cellular macromolecule metabolic process;cellular metabolic process;cellular process;cellular protein metabolic process;cellular response to chemical stimulus;ce
alcohol metabolic process;biosynthetic process;lipid biosynthetic process;lipid metabolic process;metabolic process;primary metabolic process;small molecule metabolic process;steroid biosynthetic process;steroid metabo
alcohol catabolic process;alcohol metabolic process;amino sugar catabolic process;amino sugar metabolic process;carbohydrate catabolic process;carbohydrate metabolic process;carbohydrate phosphorylation;carboxylic
developmental process
biological regulation;biosynthetic process;cap-independent translational initiation;cell cycle;cellular biosynthetic process;cellular component assembly;cellular component assembly at cellular level;cellular component disasse
cellular macromolecule metabolic process;cellular metabolic process;cellular nitrogen compound metabolic process;cellular process;macromolecule metabolic process;maturation of SSU-rRNA;maturation of SSU-rRNA from
alcohol biosynthetic process;alcohol metabolic process;amino sugar biosynthetic process;amino sugar metabolic process;biological adhesion;biosynthetic process;carbohydrate biosynthetic process;carbohydrate metabolic
biological regulation;cell cycle phase;cell cycle process;cellular component organization;cellular component organization at cellular level;cellular component organization or biogenesis;cellular component organization or bioge
biological regulation;biosynthetic process;cellular biosynthetic process;cellular macromolecule biosynthetic process;cellular macromolecule metabolic process;cellular metabolic process;cellular process;cellular protein metab
biological regulation;cell cycle cytokinesis;cell cycle phase;cell cycle process;cellular component assembly;cellular component assembly at cellular level;cellular component organization;cellular component organization at cel
cellular localization;cellular macromolecule localization;cellular process;cellular protein localization;localization;macromolecule localization;protein localization;protein localization in mitochondrion;protein localization to organe
biological regulation;biosynthetic process;cellular biosynthetic process;cellular component assembly;cellular component assembly at cellular level;cellular component organization;cellular component organization at cellular le
biological regulation;carboxylic acid metabolic process;cellular ketone metabolic process;cellular metabolic process;cellular process;metabolic process;monocarboxylic acid metabolic process;organic acid metabolic process
establishment of localization;transport
cellular component organization;cellular component organization at cellular level;cellular component organization or biogenesis;cellular component organization or biogenesis at cellular level;cellular macromolecule metabolic
activation of immune response;activation of innate immune response;anaphase-promoting complex-dependent proteasomal ubiquitin-dependent protein catabolic process;antigen processing and presentation;antigen proces
cellular component organization;cellular component organization at cellular level;cellular component organization or biogenesis;cellular component organization or biogenesis at cellular level;cellular process;COPI coating of
axon guidance;biological regulation;cell surface receptor linked signaling pathway;cellular process;cellular response to stimulus;chemotaxis;enzyme linked receptor protein signaling pathway;ephrin receptor signaling pathwa
cellular component assembly;cellular component assembly at cellular level;cellular component organization;cellular component organization at cellular level;cellular component organization or biogenesis;cellular component o
alcohol biosynthetic process;alcohol metabolic process;amine metabolic process;biosynthetic process;cellular biosynthetic process;cellular lipid metabolic process;cellular metabolic process;cellular nitrogen compound bios
anion transport;biological regulation;blood coagulation;carboxylic acid metabolic process;carboxylic acid transport;cell migration;cell motility;cellular component movement;cellular ketone metabolic process;cellular metaboli
biosynthetic process;cellular biosynthetic process;cellular macromolecule biosynthetic process;cellular macromolecule metabolic process;cellular metabolic process;cellular nitrogen compound metabolic process;cellular pro
biological regulation;cell redox homeostasis;cellular homeostasis;cellular macromolecule metabolic process;cellular metabolic process;cellular process;cellular protein metabolic process;cellular response to chemical stimulus
biological regulation;biosynthetic process;cation transport;cellular biosynthetic process;cellular component organization;cellular component organization at cellular level;cellular component organization or biogenesis;cellular
biological regulation;establishment of localization;establishment of localization in cell;establishment of protein localization;intracellular protein transport;intracellular transport;maternal process involved in female pregnancy;mu
biological regulation;biosynthetic process;cell cycle process;cell proliferation;cellular biosynthetic process;cellular component organization;cellular component organization at cellular level;cellular component organization or b
actin cytoskeleton organization;actin filament-based process;anatomical structure development;B cell activation;biological regulation;cell activation;cell cycle;cellular component organization;cellular component organization
amine biosynthetic process;amine metabolic process;apoptotic mitochondrial changes;biosynthetic process;carboxylic acid biosynthetic process;carboxylic acid metabolic process;cellular amine metabolic process;cellular a
biological regulation;cell cycle checkpoint;cellular process;cellular response to stimulus;cellular response to stress;DNA damage checkpoint;DNA integrity checkpoint;negative regulation of catalytic activity;negative regulation
anatomical structure morphogenesis;biological regulation;biosynthetic process;cell morphogenesis;cellular biosynthetic process;cellular component morphogenesis;cellular component organization;cellular component organi
biological regulation;cellular macromolecule metabolic process;cellular metabolic process;cellular nitrogen compound metabolic process;cellular process;macromolecule metabolic process;metabolic process;mRNA metabol
cellular macromolecule metabolic process;cellular metabolic process;cellular process;cellular protein metabolic process;chaperone-mediated protein folding;macromolecule metabolic process;macromolecule modification;me
biological regulation;cellular component organization;cellular component organization at cellular level;cellular component organization or biogenesis;cellular component organization or biogenesis at cellular level;cellular macro
antigen processing and presentation;antigen processing and presentation of exogenous antigen;antigen processing and presentation of exogenous peptide antigen;antigen processing and presentation of exogenous peptide

autophagic vacuole fusion;cellular component assembly;cellular component assembly at cellular level;cellular component organization;cellular component organization at cellular level;cellular component organization or bioge
establishment of localization;fat-soluble vitamin metabolic process;lipid metabolic process;metabolic process;primary metabolic process;small molecule metabolic process;steroid metabolic process;transport;vitamin D meta
acylglycerol catabolic process;acylglycerol metabolic process;biological regulation;carboxylic acid metabolic process;catabolic process;cellular catabolic process;cellular ketone metabolic process;cellular lipid catabolic proc
anatomical structure development;biological regulation;biosynthetic process;catabolic process;cell cycle process;cell surface receptor linked signaling pathway;cellular biosynthetic process;cellular catabolic process;cellular
amine metabolic process;aminoglycan catabolic process;aminoglycan metabolic process;carbohydrate catabolic process;carbohydrate metabolic process;catabolic process;cellular catabolic process;cellular lipid metabolic
activation of protein kinase activity;activation of protein kinase B activity;biological regulation;biosynthetic process;cell communication;cell proliferation;cell redox homeostasis;cell-cell signaling;cellular biosynthetic process;c
amine metabolic process;amine transport;amino acid transport;carboxylic acid metabolic process;carboxylic acid transport;cellular amine metabolic process;cellular amino acid metabolic process;cellular aromatic compound
aging;base-excision repair;biological regulation;biosynthetic process;cell aging;cellular biosynthetic process;cellular component assembly;cellular component assembly at cellular level;cellular component disassembly;cellular
apoptosis;axon guidance;biological regulation;biosynthetic process;cell cycle;cell cycle checkpoint;cell death;cell surface receptor linked signaling pathway;cellular biosynthetic process;cellular macromolecule biosynthetic p
anatomical structure morphogenesis;anterior/posterior pattern specification;biological regulation;camera-type eye morphogenesis;cell differentiation;cellular component organization;cellular component organization at cellular
biological regulation;cellular macromolecule metabolic process;cellular metabolic process;cellular process;cellular protein metabolic process;cellular response to heat;cellular response to stimulus;cellular response to stress;c
anatomical structure morphogenesis;biological regulation;cell morphogenesis;cell morphogenesis involved in differentiation;cellular component morphogenesis;cellular component organization;cellular component organization
activation-induced cell death of T cells;aging;alcohol metabolic process;amine metabolic process;aminoglycan metabolic process;anatomical structure development;apoptosis;apoptotic mitochondrial changes;biological regu
2'-deoxyribonucleotide biosynthetic process;2'-deoxyribonucleotide metabolic process;biosynthetic process;catabolic process;cellular aromatic compound metabolic process;cellular biosynthetic process;cellular catabolic p
alpha-beta T cell activation;alpha-beta T cell differentiation;biosynthetic process;catabolic process;cell activation;cell differentiation;cellular biosynthetic process;cellular catabolic process;cellular component disassembly;cell
biological regulation;cellular macromolecule metabolic process;cellular metabolic process;cellular nitrogen compound metabolic process;cellular process;macromolecule metabolic process;metabolic process;mRNA metaboli
biosynthetic process;catabolic process;cellular biosynthetic process;cellular catabolic process;cellular component disassembly;cellular component disassembly at cellular level;cellular component organization;cellular compo
anatomical structure formation involved in morphogenesis;anatomical structure morphogenesis;angiogenesis;angiogenesis involved in wound healing;biological adhesion;biological regulation;cell adhesion;cellular process;de
cellular metabolic process;cellular process;electron transport chain;generation of precursor metabolites and energy;metabolic process;oxidation-reduction process;respiratory electron transport chain;response to antibiotic;re
biosynthetic process;carbohydrate metabolic process;carboxylic acid metabolic process;cellular biosynthetic process;cellular component assembly;cellular component organization;cellular component organization or biogen
biological adhesion;cell activation;cell adhesion;cell junction assembly;cell junction organization;cell migration;cell motility;cell proliferation;cell-substrate junction assembly;cellular component assembly;cellular component as
biosynthetic process;cellular biosynthetic process;cellular component disassembly;cellular component disassembly at cellular level;cellular component organization;cellular component organization at cellular level;cellular cor
biological regulation;negative regulation of biological process;negative regulation of biosynthetic process;negative regulation of cellular biosynthetic process;negative regulation of cellular macromolecule biosynthetic process
activation of immune response;activation of innate immune response;anaphase-promoting complex-dependent proteasomal ubiquitin-dependent protein catabolic process;antigen processing and presentation;antigen proces
apoptosis;biological regulation;catabolic process;cell death;cell surface receptor linked signaling pathway;cellular catabolic process;cellular component disassembly;cellular component disassembly at cellular level;cellular co
activation of caspase activity;adaptive immune response;adaptive immune response based on somatic recombination of immune receptors built from immunoglobulin superfamily domains;aging;biological regulation;cell killin
biological regulation;cellular component assembly;cellular component assembly at cellular level;cellular component organization;cellular component organization at cellular level;cellular component organization or biogenesis;
biological regulation;cell cycle;cellular component organization;cellular component organization at cellular level;cellular component organization or biogenesis;cellular component organization or biogenesis at cellular level;cell
biological regulation;biosynthetic process;catabolic process;cellular biosynthetic process;cellular catabolic process;cellular macromolecule biosynthetic process;cellular macromolecule catabolic process;cellular macromolec
antigen processing and presentation;antigen processing and presentation of exogenous antigen;antigen processing and presentation of exogenous peptide antigen;antigen processing and presentation of exogenous peptide
biosynthetic process;cellular biosynthetic process;cellular component assembly;cellular component assembly at cellular level;cellular component organization;cellular component organization at cellular level;cellular compone
actin cytoskeleton organization;actin filament organization;actin filament polymerization;actin filament-based process;actin polymerization or depolymerization;anatomical structure arrangement;anatomical structure homeost
biological regulation;biosynthetic process;cellular biosynthetic process;cellular macromolecule biosynthetic process;cellular macromolecule metabolic process;cellular metabolic process;cellular process;cellular protein metab
biological regulation;biosynthetic process;carbohydrate metabolic process;carbohydrate transport;cell cycle;cell cycle process;cell surface receptor linked signaling pathway;cellular biosynthetic process;cellular component c
cellular process;establishment of localization;establishment of localization in cell;exocytosis;secretion;secretion by cell;transport;vesicle-mediated transport
actin cytoskeleton organization;actin filament organization;actin filament-based process;activation of protein kinase activity;adaptive immune response;adaptive immune response based on somatic recombination of immune
biosynthetic process;cellular biosynthetic process;cellular macromolecule biosynthetic process;cellular macromolecule metabolic process;cellular metabolic process;cellular nitrogen compound metabolic process;cellular pro
amine metabolic process;biosynthetic process;carboxylic acid metabolic process;cellular amine metabolic process;cellular amino acid metabolic process;cellular biosynthetic process;cellular ketone metabolic process;cellula
amine metabolic process;amino acid activation;apoptosis;biological regulation;carboxylic acid metabolic process;cell death;cell differentiation;cellular amine metabolic process;cellular amino acid metabolic process;cellular d
biological regulation;biosynthetic process;cellular biosynthetic process;cellular component assembly;cellular component assembly at cellular level;cellular component organization;cellular component organization at cellular le
biological regulation;cell differentiation;cellular component organization;cellular component organization at cellular level;cellular component organization or biogenesis;cellular component organization or biogenesis at cellular
activation of MAPKK activity;activation of protein kinase activity;biological adhesion;biological regulation;cell activation;cell adhesion;cell adhesion mediated by integrin;cell differentiation;cell surface receptor linked signaling
autophagy;biological regulation;catabolic process;cell communication;cell differentiation;cell surface receptor linked signaling pathway;cellular catabolic process;cellular component assembly;cellular component organization;
biological adhesion;cell adhesion;cell differentiation;cell proliferation;cellular developmental process;cellular macromolecule metabolic process;cellular metabolic process;cellular nitrogen compound metabolic process;cellula
anatomical structure development;biological adhesion;biological regulation;cell adhesion;cellular component assembly;cellular component assembly at cellular level;cellular component organization;cellular component organi
alcohol catabolic process;alcohol metabolic process;carbohydrate catabolic process;carbohydrate metabolic process;catabolic process;cellular carbohydrate catabolic process;cellular carbohydrate metabolic process;cellula
biological regulation;cellular component organization;cellular component organization or biogenesis;cellular process;establishment of localization;establishment of localization in cell;Golgi vesicle transport;intracellular transpo
alcohol metabolic process;amyloid precursor protein catabolic process;amyloid precursor protein metabolic process;anatomical structure development;apoptosis;biological regulation;biosynthetic process;catabolic process;c
cellular component assembly;cellular component assembly at cellular level;cellular component organization;cellular component organization at cellular level;cellular component organization or biogenesis;cellular component c
biological regulation;catabolic process;cell proliferation;cellular catabolic process;cellular macromolecule catabolic process;cellular macromolecule metabolic process;cellular metabolic process;cellular nitrogen compound m
ameboidal cell migration;apical junction assembly;apoptosis;biological adhesion;biological regulation;biosynthetic process;cell adhesion;cell cycle;cell death;cell division;cell junction assembly;cell junction organization;cell m
cell differentiation;cellular developmental process;cellular process;developmental process;multicellular organismal development;multicellular organismal process
aging;apoptosis;biological regulation;cell aging;cell death;cellular process;death;developmental process;negative regulation of biological process;negative regulation of biosynthetic process;negative regulation of catalytic act
biological adhesion;cell adhesion;cellular process
anatomical structure development;apoptosis;biological regulation;biosynthetic process;cell death;cellular biosynthetic process;cellular metabolic process;cellular process;cofactor biosynthetic process;cofactor metabolic proc
anatomical structure morphogenesis;biological regulation;catabolic process;cell part morphogenesis;cellular catabolic process;cellular component maintenance;cellular component maintenance at cellular level;cellular compo
biological regulation;carboxylic acid metabolic process;cellular ketone metabolic process;cellular metabolic process;cellular process;metabolic process;monocarboxylic acid metabolic process;organic acid metabolic process
cellular component assembly;cellular component organization;cellular component organization or biogenesis;cellular metabolic process;cellular nitrogen compound metabolic process;cellular process;coenzyme metabolic pro
cellular macromolecule metabolic process;cellular metabolic process;cellular nitrogen compound metabolic process;cellular process;establishment of localization;establishment of localization in cell;establishment of RNA loca
adaptation of rhodopsin mediated signaling;adaptation of signaling pathway;apocarotenoid metabolic process;biological regulation;cellular aldehyde metabolic process;cellular hormone metabolic process;cellular lipid metab
biological regulation;biosynthetic process;cellular biosynthetic process;cellular macromolecule biosynthetic process;cellular macromolecule metabolic process;cellular metabolic process;cellular nitrogen compound metabolic
behavior;behavioral interaction between organisms;multi-organism process;response to stimulus;social behavior
biological regulation;cell cycle;cellular component assembly;cellular component assembly at cellular level;cellular component organization;cellular component organization at cellular level;cellular component organization or bi
catabolic process;cellular catabolic process;cellular component organization;cellular component organization at cellular level;cellular component organization or biogenesis;cellular component organization or biogenesis at ce
anatomical structure development;ATP-dependent chromatin remodeling;biological regulation;cellular component disassembly;cellular component disassembly at cellular level;cellular component organization;cellular compor
cell cycle;cell cycle process;cellular component organization;cellular component organization at cellular level;cellular component organization or biogenesis;cellular component organization or biogenesis at cellular level;cellula
biological regulation;biosynthetic process;cell differentiation;cell proliferation;cellular biosynthetic process;cellular developmental process;cellular macromolecule biosynthetic process;cellular macromolecule metabolic proces
antigen processing and presentation;antigen processing and presentation of exogenous antigen;antigen processing and presentation of exogenous peptide antigen;antigen processing and presentation of exogenous peptide
biological regulation;cell surface receptor linked signaling pathway;cellular process;cellular response to chemical stimulus;cellular response to cytokine stimulus;cellular response to organic substance;cellular response to stim
biological regulation;cellular component organization;cellular component organization at cellular level;cellular component organization or biogenesis;cellular component organization or biogenesis at cellular level;cellular proce
biological regulation;cation homeostasis;chemical homeostasis;homeostatic process;ion homeostasis;iron ion homeostasis;regulation of biological quality
anatomical structure formation involved in morphogenesis;associative learning;behavior;cellular component maintenance;cellular component maintenance at cellular level;cellular component organization;cellular component c
biological regulation;biosynthetic process;cellular biosynthetic process;cellular macromolecule biosynthetic process;cellular macromolecule metabolic process;cellular metabolic process;cellular nitrogen compound metabolic
cellular metabolic process;cellular process;metabolic process;phosphate-containing compound metabolic process;phosphorus metabolic process;phosphorylation
amine catabolic process;amine metabolic process;biotin metabolic process;branched chain family amino acid catabolic process;branched chain family amino acid metabolic process;carboxylic acid catabolic process;carbox
biological regulation;cellular process;establishment of localization;establishment of localization in cell;establishment of protein localization;intracellular protein transport;intracellular transport;positive regulation of catalytic acti
amine metabolic process;amino acid activation;carboxylic acid metabolic process;cellular amine metabolic process;cellular amino acid metabolic process;cellular ketone metabolic process;cellular macromolecule metabolic p
biological regulation;cellular component assembly;cellular component assembly at cellular level;cellular component organization;cellular component organization at cellular level;cellular component organization or biogenesis;
biological regulation;biosynthetic process;cellular biosynthetic process;cellular macromolecule biosynthetic process;cellular macromolecule metabolic process;cellular metabolic process;cellular nitrogen compound metabolic
alternative nuclear mRNA splicing, via spliceosome;anatomical structure development;anatomical structure morphogenesis;appendage morphogenesis;biological regulation;cell differentiation;cellular component assembly;ce
actin cytoskeleton organization;actin cytoskeleton reorganization;actin filament-based process;actin-mediated cell contraction;anatomical structure formation involved in morphogenesis;anatomical structure morphogenesis;a
biosynthetic process;cellular biosynthetic process;cellular component disassembly;cellular component disassembly at cellular level;cellular component organization;cellular component organization at cellular level;cellular cor
anatomical structure development;cell projection organization;cellular component organization;cellular component organization at cellular level;cellular component organization or biogenesis;cellular component organization o
carboxylic acid metabolic process;cellular aldehyde metabolic process;cellular component assembly;cellular component organization;cellular component organization or biogenesis;cellular ketone metabolic process;cellular r
anatomical structure morphogenesis;angiotensin maturation;biological regulation;branching morphogenesis of a tube;cellular macromolecule metabolic process;cellular metabolic process;cellular process;cellular protein meta
establishment of localization;establishment of localization in cell;establishment of RNA localization;gene expression;intracellular transport;macromolecule metabolic process;metabolic process;mRNA export from nucleus;mR
ADP biosynthetic process;ADP metabolic process;AMP metabolic process;biological regulation;biosynthetic process;blood coagulation;cellular biosynthetic process;cellular metabolic process;cellular nitrogen compound bios
multicellular organismal process;neurological system process;sensory perception;sensory perception of light stimulus;system process;visual perception
biological regulation;cellular component organization;cellular component organization at cellular level;cellular component organization or biogenesis;cellular component organization or biogenesis at cellular level;cellular mem
anion transport;biological regulation;cation transport;cell volume homeostasis;cellular homeostasis;cellular process;chloride transport;establishment of localization;homeostatic process;inorganic anion transport;ion transport;
biological regulation;negative regulation of biological process;negative regulation of gene expression;negative regulation of macromolecule metabolic process;negative regulation of metabolic process;positive regulation of bio
cell differentiation;cellular developmental process;cellular macromolecule metabolic process;cellular metabolic process;cellular process;cellular protein metabolic process;developmental process;gamete generation;macromo
biological regulation;cell surface receptor linked signaling pathway;cellular metabolic process;cellular nitrogen compound metabolic process;cellular process;cellular response to chemical stimulus;cellular response to cytokin
biosynthetic process;cellular biosynthetic process;cellular macromolecule biosynthetic process;cellular macromolecule metabolic process;cellular metabolic process;cellular nitrogen compound metabolic process;cellular pro
adipose tissue development;anatomical structure development;biological regulation;bone development;cell differentiation;cellular component assembly;cellular component disassembly;cellular component disassembly at cell
anatomical structure development;biological adhesion;biological regulation;blood coagulation;brain development;carbohydrate transport;cell activation;cell activation involved in immune response;cell adhesion;cell-cell adhes
axon guidance;chemotaxis;locomotion;response to chemical stimulus;response to external stimulus;response to stimulus;taxis
biosynthetic process;cell proliferation;cellular biosynthetic process;cellular metabolic compound salvage;cellular metabolic process;cellular process;coenzyme biosynthetic process;coenzyme metabolic process;cofactor bios
actin cytoskeleton organization;actin filament organization;actin filament-based process;actin nucleation;Arp2/3 complex-mediated actin nucleation;axon guidance;biological regulation;cell surface receptor linked signaling pa
biological regulation;cell cycle process;cellular component assembly;cellular component assembly at cellular level;cellular component organization;cellular component organization at cellular level;cellular component organiza
catabolic process;cellular catabolic process;cellular macromolecule catabolic process;cellular macromolecule metabolic process;cellular metabolic process;cellular process;ER-associated protein catabolic process;macromo
ATP biosynthetic process;ATP metabolic process;ATP synthesis coupled proton transport;biosynthetic process;cation transport;cellular biosynthetic process;cellular metabolic process;cellular nitrogen compound biosynthetic
cellular component assembly;cellular component assembly at cellular level;cellular component organization;cellular component organization at cellular level;cellular component organization or biogenesis;cellular component c
anatomical structure morphogenesis;biological regulation;cell maturation;cellular developmental process;cellular macromolecule metabolic process;cellular metabolic process;cellular nitrogen compound metabolic process;ce
amine catabolic process;amine metabolic process;arginine catabolic process;arginine metabolic process;biological regulation;biosynthetic process;carboxylic acid catabolic process;carboxylic acid metabolic process;catabo
biological regulation;cation homeostasis;cell surface receptor linked signaling pathway;cellular cation homeostasis;cellular chemical homeostasis;cellular copper ion homeostasis;cellular homeostasis;cellular ion homeostasis
anatomical structure development;anatomical structure morphogenesis;behavior;biological regulation;blood coagulation;cellular component organization;cellular component organization at cellular level;cellular component or
cellular metabolic process;cellular process;electron transport chain;generation of precursor metabolites and energy;metabolic process;oxidation-reduction process;respiratory electron transport chain;response to chemical st
anatomical structure morphogenesis;axon guidance;biological regulation;cell communication;cell differentiation;cell-cell signaling;cellular developmental process;cellular process;cellular response to stimulus;chemotaxis;deve
antigen processing and presentation;antigen processing and presentation of exogenous antigen;antigen processing and presentation of exogenous peptide antigen;antigen processing and presentation of exogenous peptide
amine catabolic process;amine metabolic process;branched chain family amino acid catabolic process;branched chain family amino acid metabolic process;carboxylic acid catabolic process;carboxylic acid metabolic proces
alcohol metabolic process;aldehyde catabolic process;biosynthetic process;carbohydrate catabolic process;carbohydrate metabolic process;carboxylic acid biosynthetic process;carboxylic acid catabolic process;carboxylic
anatomical structure homeostasis;base-excision repair;biological regulation;biosynthetic process;catabolic process;cell cycle;cell cycle checkpoint;cell cycle process;cellular biosynthetic process;cellular catabolic process;ce
activation of immune response;activation of innate immune response;activation of phospholipase C activity;activation of protein kinase A activity;activation of protein kinase activity;acylglycerol catabolic process;acylglycerol
anatomical structure development;biological regulation;cell chemotaxis;cell differentiation;cell migration;cell motility;cellular component movement;cellular component organization;cellular component organization at cellular le
biological regulation;cell cycle phase;cell cycle process;cell division;cellular component assembly;cellular component assembly at cellular level;cellular component organization;cellular component organization at cellular level
biological regulation;cellular component organization;cellular component organization or biogenesis;cellular membrane organization;cellular process;cellular response to stimulus;endocytosis;endosome to lysosome transport
ATP hydrolysis coupled proton transport;ATP metabolic process;biological regulation;cation homeostasis;cation transport;cell surface receptor linked signaling pathway;cellular cation homeostasis;cellular chemical homeosta
cellular macromolecule metabolic process;cellular metabolic process;cellular process;cellular protein metabolic process;macromolecule metabolic process;macromolecule methylation;macromolecule modification;metabolic
alcohol metabolic process;biological regulation;biosynthetic process;C21-steroid hormone biosynthetic process;C21-steroid hormone metabolic process;cellular biosynthetic process;cellular hormone metabolic process;cellu
carboxylic acid metabolic process;cellular ketone metabolic process;cellular metabolic process;cellular process;dicarboxylic acid metabolic process;malate metabolic process;metabolic process;organic acid metabolic proce

activation of immune response;activation of innate immune response;anaphase-promoting complex-dependent proteasomal ubiquitin-dependent protein catabolic process;antigen processing and presentation;antigen proces
apoptosis;biological regulation;cell death;cell surface receptor linked signaling pathway;cellular macromolecule metabolic process;cellular metabolic process;cellular process;cellular protein metabolic process;cellular respons
amine biosynthetic process;amine metabolic process;biological regulation;biosynthetic process;carboxylic acid biosynthetic process;carboxylic acid metabolic process;cellular amine metabolic process;cellular amino acid bio
biosynthetic process;cellular biosynthetic process;cellular macromolecule biosynthetic process;cellular macromolecule metabolic process;cellular metabolic process;cellular nitrogen compound metabolic process;cellular pro
arachidonic acid metabolic process;carboxylic acid metabolic process;cellular alkene metabolic process;cellular ketone metabolic process;cellular lipid metabolic process;cellular metabolic process;cellular process;cobalami
cellular component organization;cellular component organization at cellular level;cellular component organization or biogenesis;cellular component organization or biogenesis at cellular level;cellular macromolecule metabolic
anatomical structure development;anatomical structure morphogenesis;biological regulation;bradykinin catabolic process;calcitonin catabolic process;catabolic process;cellular catabolic process;cellular hormone metabolic p
biological regulation;biosynthetic process;cellular biosynthetic process;cellular component organization;cellular component organization at cellular level;cellular component organization or biogenesis;cellular component orga
alcohol catabolic process;alcohol metabolic process;amino sugar catabolic process;amino sugar metabolic process;carbohydrate catabolic process;carbohydrate metabolic process;catabolic process;cellular carbohydrate ca
anatomical structure development;anatomical structure morphogenesis;biological regulation;biosynthetic process;cell differentiation;cell morphogenesis;cell proliferation;cellular biosynthetic process;cellular component morp
ATP biosynthetic process;ATP metabolic process;ATP synthesis coupled proton transport;biosynthetic process;cation transport;cellular biosynthetic process;cellular metabolic process;cellular nitrogen compound biosynthetic
biological regulation;cell cycle phase;cell cycle process;cell division;cell surface receptor linked signaling pathway;cellular component organization;cellular component organization at cellular level;cellular component organiza
biological regulation;biosynthetic process;cellular biosynthetic process;cellular macromolecule biosynthetic process;cellular macromolecule metabolic process;cellular metabolic process;cellular process;cellular protein metab
biological regulation;cell cycle;cell cycle checkpoint;cell cycle phase;cell cycle process;cell division;cellular component assembly;cellular component organization;cellular component organization at cellular level;cellular comp
biological adhesion;biological regulation;cell adhesion;cell-matrix adhesion;cell-substrate adhesion;cellular process;cellular response to stimulus;regulation of biological process;regulation of cellular process;response to stimu
acetyl-CoA catabolic process;acetyl-CoA metabolic process;carboxylic acid metabolic process;catabolic process;cellular catabolic process;cellular ketone metabolic process;cellular metabolic process;cellular process;coenz
biological regulation;blood coagulation;cation homeostasis;cation transport;cell migration;cell motility;cellular cation homeostasis;cellular chemical homeostasis;cellular component movement;cellular component organization
activation of immune response;activation of innate immune response;anaphase-promoting complex-dependent proteasomal ubiquitin-dependent protein catabolic process;antigen processing and presentation;antigen proces
antigen processing and presentation;antigen processing and presentation of exogenous antigen;antigen processing and presentation of exogenous peptide antigen;antigen processing and presentation of exogenous peptide
biosynthetic process;catabolic process;cellular biosynthetic process;cellular catabolic process;cellular component disassembly;cellular component disassembly at cellular level;cellular component organization;cellular compo
biological regulation;cell differentiation;cellular developmental process;cellular process;cellular response to stimulus;developmental process;intracellular signal transduction;metabolic process;osteoblast differentiation;positive
biological regulation;biosynthetic process;catabolic process;cell differentiation;cellular biosynthetic process;cellular catabolic process;cellular component assembly;cellular component assembly at cellular level;cellular compo
biosynthetic process;catabolic process;cellular biosynthetic process;cellular catabolic process;cellular component disassembly;cellular component disassembly at cellular level;cellular component organization;cellular compo
biosynthetic process;catabolic process;cell proliferation;cellular biosynthetic process;cellular catabolic process;cellular component disassembly;cellular component disassembly at cellular level;cellular component organizatio
activation of immune response;activation of innate immune response;activation of MAPK activity;alcohol metabolic process;anaphase-promoting complex-dependent proteasomal ubiquitin-dependent protein catabolic proce
amine biosynthetic process;amine metabolic process;biosynthetic process;carboxylic acid biosynthetic process;carboxylic acid metabolic process;cellular amine metabolic process;cellular amino acid biosynthetic process;ce
biosynthetic process;cellular biosynthetic process;cellular component disassembly;cellular component disassembly at cellular level;cellular component organization;cellular component organization at cellular level;cellular cor
biosynthetic process;cellular biosynthetic process;cellular component disassembly;cellular component disassembly at cellular level;cellular component organization;cellular component organization at cellular level;cellular cor
biosynthetic process;cellular biosynthetic process;cellular component disassembly;cellular component disassembly at cellular level;cellular component organization;cellular component organization at cellular level;cellular cor
amine metabolic process;aminoglycan metabolic process;biological regulation;carbohydrate metabolic process;glycosaminoglycan metabolic process;hyaluronan metabolic process;macromolecule metabolic process;metabo
cell differentiation;cellular developmental process;cellular process;developmental process
apoptosis;biological regulation;cell cycle checkpoint;cell death;cellular macromolecule metabolic process;cellular metabolic process;cellular process;cellular protein metabolic process;cellular response to stimulus;death;DNA
biological adhesion;cell adhesion;cellular process;establishment of localization;establishment of localization in cell;establishment of protein localization;establishment of protein localization to organelle;establishment of protei
cellular process;cytokinesis;viral reproduction
biological regulation;biosynthetic process;cellular biosynthetic process;cellular macromolecule biosynthetic process;cellular macromolecule metabolic process;cellular metabolic process;cellular nitrogen compound metabolic
biological regulation;carboxylic acid catabolic process;carboxylic acid metabolic process;catabolic process;cellular catabolic process;cellular component organization;cellular component organization at cellular level;cellular c
acetyl-CoA metabolic process;alcohol metabolic process;biosynthetic process;cellular biosynthetic process;cellular lipid metabolic process;cellular metabolic process;cellular process;cholesterol biosynthetic process;cholest
anatomical structure morphogenesis;anion transport;bicarbonate transport;cellular metabolic process;cellular process;cellular response to chemical stimulus;cellular response to hypoxia;cellular response to oxygen levels;cel
apoptosis;biological regulation;biosynthetic process;cell death;cellular biosynthetic process;cellular macromolecule biosynthetic process;cellular macromolecule metabolic process;cellular metabolic process;cellular nitrogen
biological regulation;biosynthetic process;cell fate commitment;cell fate commitment involved in formation of primary germ layers;cell surface receptor linked signaling pathway;cellular biosynthetic process;cellular componen
anatomical structure development;cellular macromolecule metabolic process;cellular metabolic process;cellular process;cellular protein metabolic process;chordate embryonic development;developmental process;embryo de
cellular macromolecule metabolic process;cellular metabolic process;cellular nitrogen compound metabolic process;cellular process;enzyme-directed rRNA 2'-O-methylation;macromolecule metabolic process;macromolecul
biosynthetic process;cellular biosynthetic process;cellular metabolic process;cellular nitrogen compound biosynthetic process;cellular nitrogen compound metabolic process;cellular process;coenzyme biosynthetic process;c
activation of immune response;activation of innate immune response;biological regulation;cell cycle;cell surface receptor linked signaling pathway;cellular macromolecule metabolic process;cellular metabolic process;cellular
biological regulation;cell surface receptor linked signaling pathway;cellular process;cellular response to chemical stimulus;cellular response to cytokine stimulus;cellular response to organic substance;cellular response to stim
biological regulation;biosynthetic process;cell fate commitment;cell fate commitment involved in formation of primary germ layers;cell surface receptor linked signaling pathway;cellular biosynthetic process;cellular componen
biosynthetic process;cellular biosynthetic process;cellular macromolecule biosynthetic process;cellular macromolecule metabolic process;cellular metabolic process;cellular nitrogen compound metabolic process;cellular pro
response to calcium ion;response to chemical stimulus;response to inorganic substance;response to metal ion;response to stimulus
biological regulation;cellular macromolecule metabolic process;cellular metabolic process;cellular nitrogen compound metabolic process;cellular process;cellular protein metabolic process;cellular response to stimulus;cellula
asymmetric protein localization;basal protein localization;biological adhesion;biological regulation;cell adhesion;cell cycle;cell growth;cell surface receptor linked signaling pathway;cellular component organization;cellular com
multicellular organismal process;neurological system process;sensory perception;sensory perception of pain;system process
cell cycle;cell cycle process;cell division;cellular component organization;cellular component organization at cellular level;cellular component organization or biogenesis;cellular component organization or biogenesis at cellula
adherens junction assembly;adherens junction organization;biological regulation;cell junction assembly;cell junction organization;cell-cell junction assembly;cell-cell junction organization;cellular component assembly;cellular
biosynthetic process;cellular biosynthetic process;cellular component disassembly;cellular component disassembly at cellular level;cellular component organization;cellular component organization at cellular level;cellular cor
biological regulation;cell proliferation;cell surface receptor linked signaling pathway;cellular component biogenesis;cellular component biogenesis at cellular level;cellular component organization or biogenesis;cellular compor
cellular macromolecule metabolic process;cellular metabolic process;cellular nitrogen compound metabolic process;cellular process;macromolecule metabolic process;metabolic process;mRNA metabolic process;mRNA pro
biological regulation;cell cycle process;cellular component assembly;cellular component organization;cellular component organization at cellular level;cellular component organization or biogenesis;cellular component organiz
alcohol metabolic process;anatomical structure development;anatomical structure formation involved in morphogenesis;angiogenesis;anterior/posterior pattern specification;biological regulation;carbohydrate metabolic proce
anatomical structure development;biological regulation;cell development;cellular developmental process;cellular macromolecule metabolic process;cellular metabolic process;cellular process;cellular protein metabolic proces
cation transport;establishment of localization;ion transport;transport
biological regulation;biosynthetic process;cell cycle;cellular biosynthetic process;cellular macromolecule biosynthetic process;cellular macromolecule metabolic process;cellular metabolic process;cellular nitrogen compound
biological regulation;cellular macromolecule metabolic process;cellular metabolic process;cellular process;cellular protein metabolic process;macromolecule metabolic process;metabolic process;negative regulation of cataly
amine biosynthetic process;amine metabolic process;biological regulation;biosynthetic process;carboxylic acid biosynthetic process;carboxylic acid metabolic process;cellular amine metabolic process;cellular amino acid bio
cell cycle;cell division;cellular process
anatomical structure development;axon ensheathment;biological regulation;biosynthetic process;cell development;cellular biosynthetic process;cellular developmental process;cellular macromolecule biosynthetic process;ce
activation of immune response;alpha-beta T cell activation;antigen receptor-mediated signaling pathway;ATP biosynthetic process;ATP metabolic process;ATP synthesis coupled proton transport;biological regulation;biosynth
biological regulation;biosynthetic process;carbohydrate metabolic process;carbohydrate transport;cell cycle;cell cycle process;cell surface receptor linked signaling pathway;cellular biosynthetic process;cellular component c
activation of protein kinase activity;apoptosis;biological regulation;cell cycle phase;cell cycle process;cell death;cell division;cell proliferation;cellular component organization;cellular component organization at cellular level;ce
actin cytoskeleton organization;actin filament-based process;actomyosin structure organization;cellular component organization;cellular component organization at cellular level;cellular component organization or biogenesis;
actin crosslink formation;actin cytoskeleton organization;actin filament bundle assembly;actin filament organization;actin filament-based process;anatomical structure development;anatomical structure morphogenesis;axono
amine metabolic process;amino acid activation;biosynthetic process;carboxylic acid metabolic process;cellular amine metabolic process;cellular amino acid metabolic process;cellular biosynthetic process;cellular ketone me
anatomical structure development;anatomical structure morphogenesis;biological regulation;biosynthetic process;catabolic process;cell surface receptor linked signaling pathway;cellular biosynthetic process;cellular catabol
actin cytoskeleton organization;actin filament-based movement;actin filament-based process;ameboidal cell migration;anatomical structure development;anatomical structure formation involved in morphogenesis;anatomical
axon guidance;biological regulation;chemotaxis;locomotion;regulation of biological process;regulation of cellular process;regulation of establishment of cell polarity;regulation of establishment or maintenance of cell polarity;re
adult behavior;adult locomotory behavior;adult walking behavior;alcohol metabolic process;autophagy;behavior;bile acid metabolic process;biological regulation;carbohydrate metabolic process;carboxylic acid metabolic pro
actin cytoskeleton organization;actin filament organization;actin filament-based process;actin nucleation;Arp2/3 complex-mediated actin nucleation;axon guidance;biological regulation;cell migration;cell motility;cell surface re
biological regulation;cell surface receptor linked signaling pathway;cellular process;cellular response to stimulus;G-protein coupled receptor protein signaling pathway;regulation of biological process;regulation of cellular proc
cellular macromolecule metabolic process;cellular metabolic process;cellular nitrogen compound metabolic process;cellular process;macromolecule metabolic process;metabolic process;mRNA metabolic process;mRNA pro
axon guidance;biological regulation;cell cycle;cell cycle phase;cell cycle process;cell division;cellular component organization;cellular component organization at cellular level;cellular component organization or biogenesis;ce
cellular metabolic process;cellular process;electron transport chain;generation of precursor metabolites and energy;metabolic process;mitochondrial electron transport, NADH to ubiquinone;oxidation-reduction process;respi
cellular process;endosome transport;establishment of localization;establishment of localization in cell;establishment of protein localization;intracellular protein transport;intracellular transport;protein transport;retrograde trans
adult behavior;adult locomotory behavior;aggressive behavior;behavior;behavioral interaction between organisms;biological regulation;cell communication;cell-cell signaling;cellular component organization;cellular componen
catabolic process;cellular catabolic process;cellular macromolecule catabolic process;cellular macromolecule metabolic process;cellular metabolic process;cellular process;ER-associated protein catabolic process;macromo
anterograde axon cargo transport;antigen processing and presentation;antigen processing and presentation of exogenous antigen;antigen processing and presentation of exogenous peptide antigen;antigen processing and p
alcohol metabolic process;biosynthetic process;blood circulation;carboxylic acid metabolic process;cellular ketone metabolic process;cellular metabolic process;cellular process;cholesterol biosynthetic process;cholesterol m
biological regulation;negative regulation of biological process;negative regulation of cell proliferation;negative regulation of cellular process;regulation of biological process;regulation of cell proliferation;regulation of cellular pr
anatomical structure morphogenesis;axonogenesis;biological regulation;cell part morphogenesis;cell projection morphogenesis;cell projection organization;cellular component morphogenesis;cellular component organization
anatomical structure homeostasis;biological regulation;biosynthetic process;cell cycle;cell cycle process;cell proliferation;cellular biosynthetic process;cellular component organization;cellular component organization at cellul
biosynthetic process;cellular biosynthetic process;cellular macromolecule metabolic process;cellular metabolic process;cellular nitrogen compound metabolic process;cellular process;electron transport chain;gene expressio
3'-UTR-mediated mRNA stabilization;anatomical structure development;biological regulation;cellular hyperosmotic response;cellular macromolecule metabolic process;cellular metabolic process;cellular nitrogen compound m
activation of immune response;activation of innate immune response;anaphase-promoting complex-dependent proteasomal ubiquitin-dependent protein catabolic process;antigen processing and presentation;antigen proces
biosynthetic process;catabolic process;cellular biosynthetic process;cellular catabolic process;cellular metabolic process;cellular nitrogen compound catabolic process;cellular nitrogen compound metabolic process;cellular p
carboxylic acid catabolic process;carboxylic acid metabolic process;catabolic process;cellular catabolic process;cellular component organization;cellular component organization at cellular level;cellular component organizati
alcohol biosynthetic process;alcohol metabolic process;biological regulation;biosynthetic process;carbohydrate biosynthetic process;carbohydrate metabolic process;catabolic process;cellular biosynthetic process;cellular ca
activation of MAPK activity;biological regulation;cellular macromolecule metabolic process;cellular metabolic process;cellular process;cellular protein metabolic process;establishment of localization;establishment of localizati
apoptotic mitochondrial changes;biological regulation;cell death;cellular component organization;cellular component organization at cellular level;cellular component organization or biogenesis;cellular component organization
anatomical structure development;axon guidance;biological adhesion;biological regulation;blood coagulation;cell adhesion;cell adhesion mediated by integrin;cell migration;cell motility;cell surface receptor linked signaling pa
biological regulation;biosynthetic process;cellular biosynthetic process;cellular macromolecule biosynthetic process;cellular macromolecule metabolic process;cellular metabolic process;cellular nitrogen compound metabolic
anatomical structure development;anatomical structure morphogenesis;biological regulation;blood coagulation;carboxylic acid metabolic process;cell migration;cell motility;cell surface receptor linked signaling pathway;cellul
acylglycerol catabolic process;acylglycerol metabolic process;biological regulation;carbohydrate metabolic process;catabolic process;cell cycle;cell differentiation;cell division;cell surface receptor linked signaling pathway;ce
biological regulation;blood coagulation;coagulation;hemostasis;multicellular organismal process;negative regulation of biological process;negative regulation of biosynthetic process;negative regulation of cellular biosynthetic
biosynthetic process;catabolic process;cellular biosynthetic process;cellular catabolic process;cellular component disassembly;cellular component disassembly at cellular level;cellular component organization;cellular compo
biosynthetic process;cellular biosynthetic process;cellular macromolecule biosynthetic process;cellular macromolecule metabolic process;cellular metabolic process;cellular process;cellular protein metabolic process;gene ex
anatomical structure development;axon ensheathment;biological regulation;biosynthetic process;cell development;cellular biosynthetic process;cellular developmental process;cellular macromolecule biosynthetic process;ce
adenosine metabolic process;catabolic process;cellular aromatic compound metabolic process;cellular catabolic process;cellular metabolic process;cellular nitrogen compound catabolic process;cellular nitrogen compound
2'-deoxyribonucleotide biosynthetic process;2'-deoxyribonucleotide metabolic process;adenosine metabolic process;AMP biosynthetic process;AMP metabolic process;AMP salvage;biological regulation;biosynthetic proces
anatomical structure development;astrocyte development;axon extension;axon extension involved in development;axon extension involved in regeneration;axon guidance;biological adhesion;cell adhesion;cell development;c
actin cytoskeleton organization;actin filament branching;actin filament bundle assembly;actin filament organization;actin filament-based process;adherens junction organization;anatomical structure development;anatomical s
cellular component assembly;cellular component assembly at cellular level;cellular component organization;cellular component organization at cellular level;cellular component organization or biogenesis;cellular component c
biosynthetic process;cellular biosynthetic process;cellular component disassembly;cellular component disassembly at cellular level;cellular component organization;cellular component organization at cellular level;cellular cor
biosynthetic process;cellular biosynthetic process;cellular macromolecule biosynthetic process;cellular macromolecule metabolic process;cellular metabolic process;cellular nitrogen compound metabolic process;cellular pro
biosynthetic process;cellular biosynthetic process;cellular macromolecule biosynthetic process;cellular macromolecule metabolic process;cellular metabolic process;cellular nitrogen compound metabolic process;cellular pro
antigen processing and presentation;antigen processing and presentation of exogenous antigen;antigen processing and presentation of exogenous peptide antigen;antigen processing and presentation of exogenous peptide
biological regulation;catabolic process;cellular catabolic process;cellular macromolecule catabolic process;cellular macromolecule metabolic process;cellular metabolic process;cellular process;macromolecule catabolic proc
amine metabolic process;biological regulation;carbohydrate metabolic process;carboxylic acid metabolic process;cell differentiation;cellular aldehyde metabolic process;cellular amine metabolic process;cellular amino acid m
cellular process;establishment of localization;establishment of localization in cell;Golgi vesicle transport;intracellular transport;intra-Golgi vesicle-mediated transport;transport;vesicle-mediated transport
anatomical structure development;astrocyte development;astrocyte differentiation;axon ensheathment;biological regulation;biosynthetic process;cell development;cell differentiation;cellular biosynthetic process;cellular devel
biological regulation;biosynthetic process;cellular biosynthetic process;cellular component organization;cellular component organization at cellular level;cellular component organization or biogenesis;cellular component orga
anatomical structure development;anatomical structure morphogenesis;axonogenesis;biological regulation;cargo loading into vesicle;cation homeostasis;cell part morphogenesis;cell projection morphogenesis;cell projection
anatomical structure morphogenesis;biological regulation;cell part morphogenesis;cell projection morphogenesis;cell projection organization;cellular component morphogenesis;cellular component organization;cellular compo
axon guidance;biological adhesion;biological regulation;cell adhesion;cellular process;cellular response to stimulus;chemotaxis;locomotion;motor axon guidance;regulation of biological process;regulation of cellular process;r
adaptive immune response;adaptive immune response based on somatic recombination of immune receptors built from immunoglobulin superfamily domains;anatomical structure formation involved in morphogenesis;angiog
biological regulation;cell cycle checkpoint;cellular macromolecule metabolic process;cellular metabolic process;cellular nitrogen compound metabolic process;cellular process;cellular protein metabolic process;cellular respo

cellular component assembly;cellular component assembly at cellular level;cellular component organization;cellular component organization at cellular level;cellular component organization or biogenesis;cellular component c
autophagy;biological regulation;biosynthetic process;catabolic process;cellular biosynthetic process;cellular catabolic process;cellular macromolecule biosynthetic process;cellular macromolecule catabolic process;cellular m
arachidonic acid metabolic process;carboxylic acid metabolic process;cellular alkene metabolic process;cellular ketone metabolic process;cellular lipid metabolic process;cellular metabolic process;cellular process;cyclooxyg
anatomical structure development;brain development;catabolic process;developmental process;gamete generation;lipid catabolic process;lipid metabolic process;male gamete generation;metabolic process;multicellular orga
anatomical structure development;camera-type eye development;chordate embryonic development;developmental process;embryo development;embryo development ending in birth or egg hatching;eye development;in uter
establishment of localization;establishment of localization in cell;establishment of protein localization;establishment of protein localization in mitochondrion;establishment of protein localization to organelle;intracellular protein
catabolic process;cell cycle;cell division;cellular catabolic process;cellular component assembly;cellular component assembly at cellular level;cellular component organization;cellular component organization at cellular level;c
alanyl-tRNA aminoacylation;amine metabolic process;amino acid activation;carboxylic acid metabolic process;cellular amine metabolic process;cellular amino acid metabolic process;cellular component assembly;cellular cor
cellular macromolecule metabolic process;cellular metabolic process;cellular nitrogen compound metabolic process;cellular process;macromolecule metabolic process;metabolic process;mRNA metabolic process;mRNA pro
biological regulation;biosynthetic process;cell cycle;cell fate commitment;cell fate commitment involved in formation of primary germ layers;cell surface receptor linked signaling pathway;cellular biosynthetic process;cellular
biological regulation;biosynthetic process;cellular biosynthetic process;cellular macromolecule biosynthetic process;cellular macromolecule metabolic process;cellular metabolic process;cellular nitrogen compound metabolic
cellular component organization;cellular component organization at cellular level;cellular component organization or biogenesis;cellular component organization or biogenesis at cellular level;cellular macromolecule metabolic
biological regulation;cellular component assembly;cellular component assembly at cellular level;cellular component organization;cellular component organization at cellular level;cellular component organization or biogenesis;
biological regulation;cell cycle process;cell division;cellular component organization;cellular component organization at cellular level;cellular component organization or biogenesis;cellular component organization or biogenes
anatomical structure morphogenesis;biological regulation;biosynthetic process;bone morphogenesis;cellular biosynthetic process;cellular component organization;cellular component organization at cellular level;cellular comp
biosynthetic process;cellular biosynthetic process;cellular macromolecule biosynthetic process;cellular macromolecule metabolic process;cellular metabolic process;cellular nitrogen compound metabolic process;cellular pro
actin cytoskeleton organization;actin filament organization;actin filament-based process;actin polymerization or depolymerization;anatomical structure development;anatomical structure formation involved in morphogenesis;a
cellular process;establishment of localization;establishment of protein localization;protein transport;transport;vesicle-mediated transport
catabolic process;lipid catabolic process;lipid metabolic process;metabolic process;primary metabolic process
anatomical structure homeostasis;biological regulation;cellular macromolecule metabolic process;cellular metabolic process;cellular nitrogen compound metabolic process;cellular process;homeostatic process;macromolecu
biological regulation;gamete generation;male gamete generation;multicellular organismal process;multicellular organismal reproductive process;positive regulation of biological process;positive regulation of cell proliferation;p
anatomical structure morphogenesis;biological regulation;cell morphogenesis;cellular component morphogenesis;cellular component organization;cellular component organization or biogenesis;cellular developmental proces
biological regulation;biosynthetic process;cellular biosynthetic process;cellular macromolecule biosynthetic process;cellular macromolecule metabolic process;cellular metabolic process;cellular nitrogen compound metabolic
apoptosis;biological regulation;cell death;cellular macromolecule metabolic process;cellular metabolic process;cellular nitrogen compound metabolic process;cellular process;death;gene expression;immune system process;
biological regulation;posttranscriptional regulation of gene expression;protein destabilization;regulation of biological process;regulation of biological quality;regulation of cellular component biogenesis;regulation of cellular cor
amine biosynthetic process;amine metabolic process;biosynthetic process;carboxylic acid biosynthetic process;carboxylic acid metabolic process;cellular amine metabolic process;cellular amino acid biosynthetic process;ce
biosynthetic process;cellular biosynthetic process;cellular component disassembly;cellular component disassembly at cellular level;cellular component organization;cellular component organization at cellular level;cellular cor
attachment of spindle microtubules to chromosome;attachment of spindle microtubules to kinetochore;attachment of spindle microtubules to kinetochore involved in mitotic sister chromatid segregation;biological regulation;
biological regulation;cell death;cellular metabolic process;cellular process;cellular response to stimulus;death;dephosphorylation;intracellular signal transduction;metabolic process;necroptosis;necrotic cell death;phosphate-
biological regulation;catabolic process;cellular catabolic process;cellular macromolecule catabolic process;cellular macromolecule metabolic process;cellular metabolic process;cellular nitrogen compound metabolic process
biosynthetic process;cell cycle phase;cell cycle process;cell division;cellular biosynthetic process;cellular component organization;cellular component organization at cellular level;cellular component organization or biogenesi
cellular component organization;cellular component organization or biogenesis;cellular membrane organization;cellular process;membrane organization;nuclear membrane organization
antigen processing and presentation;antigen processing and presentation of exogenous antigen;antigen processing and presentation of exogenous peptide antigen;antigen processing and presentation of exogenous peptide
cellular macromolecule metabolic process;cellular metabolic process;cellular nitrogen compound metabolic process;cellular process;macromolecule metabolic process;metabolic process;nitrogen compound metabolic proce
biological regulation;cellular macromolecule metabolic process;cellular metabolic process;cellular nitrogen compound metabolic process;cellular process;macromolecule metabolic process;maturation of SSU-rRNA;maturatic
biological regulation;biosynthetic process;carbon catabolite regulation of transcription;carbon catabolite repression of transcription;cell communication;cell cycle process;cell division;cellular biosynthetic process;cellular com
amine catabolic process;amine metabolic process;carboxylic acid catabolic process;carboxylic acid metabolic process;catabolic process;cellular amine metabolic process;cellular amino acid catabolic process;cellular amino
apoptosis;autophagic cell death;biological regulation;catabolic process;cell communication;cell death;cellular catabolic process;cellular component assembly;cellular component organization;cellular component organization
biological regulation;biosynthetic process;cellular biosynthetic process;cellular macromolecule biosynthetic process;cellular macromolecule metabolic process;cellular metabolic process;cellular process;cellular protein metab
cellular component organization;cellular component organization at cellular level;cellular component organization or biogenesis;cellular component organization or biogenesis at cellular level;cellular macromolecule metabolic
biological regulation;cell differentiation;cell proliferation;cellular component assembly;cellular component assembly at cellular level;cellular component organization;cellular component organization at cellular level;cellular com
biological regulation;cellular component assembly;cellular component assembly at cellular level;cellular component organization;cellular component organization at cellular level;cellular component organization or biogenesis;
3'-phosphoadenosine 5'-phosphosulfate biosynthetic process;3'-phosphoadenosine 5'-phosphosulfate metabolic process;amine metabolic process;aminoglycan metabolic process;anatomical structure development;biosynt
cellular macromolecule metabolic process;cellular metabolic process;cellular nitrogen compound metabolic process;cellular process;macromolecule metabolic process;metabolic process;ncRNA metabolic process;ncRNA pr
biological adhesion;biological regulation;cell adhesion;cellular process;developmental process;multicellular organismal development;multicellular organismal process;positive regulation of biological process;positive regulation
alcohol biosynthetic process;alcohol metabolic process;biological regulation;biosynthetic process;carbohydrate biosynthetic process;carbohydrate metabolic process;cell surface receptor linked signaling pathway;cellular bio
biological regulation;biosynthetic process;cell differentiation;cellular biosynthetic process;cellular developmental process;cellular macromolecule biosynthetic process;cellular macromolecule metabolic process;cellular metab
biological regulation;cellular macromolecule metabolic process;cellular metabolic process;cellular process;cellular protein metabolic process;macromolecule metabolic process;metabolic process;positive regulation of biologi
anatomical structure development;biological regulation;biosynthetic process;Cajal body organization;cell cycle process;cell proliferation;cellular biosynthetic process;cellular component organization;cellular component organ
biological regulation;cellular process;ER to Golgi vesicle-mediated transport;establishment of localization;establishment of localization in cell;establishment of protein localization;Golgi vesicle transport;intracellular transport;p
antigen processing and presentation;antigen processing and presentation of exogenous antigen;antigen processing and presentation of exogenous peptide antigen;antigen processing and presentation of exogenous peptide
activation of signaling protein activity involved in unfolded protein response;biological regulation;biosynthetic process;calcium ion homeostasis;cation homeostasis;cellular biosynthetic process;cellular calcium ion homeostas
cellular metabolic process;cellular process;electron transport chain;generation of precursor metabolites and energy;metabolic process;mitochondrial electron transport, NADH to ubiquinone;oxidation-reduction process;respi
amine metabolic process;biological regulation;biosynthetic process;carboxylic acid metabolic process;cell redox homeostasis;cellular amine metabolic process;cellular amino acid metabolic process;cellular biosynthetic proc
activation of MAPK activity;aging;amine metabolic process;anatomical structure development;anatomical structure homeostasis;anterograde axon cargo transport;auditory receptor cell stereocilium organization;axon cargo tr
alcohol catabolic process;alcohol metabolic process;biological regulation;biosynthetic process;carbohydrate metabolic process;catabolic process;cell communication;cell-cell signaling;cellular biosynthetic process;cellular m
apical junction assembly;axon guidance;biological regulation;cell junction assembly;cell junction organization;cell migration;cell motility;cell-cell junction assembly;cell-cell junction organization;cellular component assembly;c
amine metabolic process;anatomical structure development;biological regulation;carboxylic acid metabolic process;cell proliferation;cellular amine metabolic process;cellular amino acid metabolic process;cellular ketone met
antigen processing and presentation;antigen processing and presentation of exogenous antigen;antigen processing and presentation of exogenous peptide antigen;antigen processing and presentation of exogenous peptide
alcohol metabolic process;anatomical structure development;anatomical structure homeostasis;anatomical structure morphogenesis;behavior;biological regulation;carbohydrate catabolic process;carbohydrate metabolic pro
acetyl-CoA catabolic process;acetyl-CoA metabolic process;alcohol metabolic process;biological regulation;carbohydrate metabolic process;carboxylic acid metabolic process;catabolic process;cellular carbohydrate metabo
amine biosynthetic process;amine metabolic process;biosynthetic process;cellular amine metabolic process;cellular biogenic amine biosynthetic process;cellular biogenic amine metabolic process;cellular biosynthetic proces
amine metabolic process;aminoglycan metabolic process;biological regulation;carbohydrate metabolic process;glycosaminoglycan metabolic process;hyaluronan metabolic process;macromolecule metabolic process;metabo
biosynthetic process;cellular biosynthetic process;cellular macromolecule biosynthetic process;cellular macromolecule metabolic process;cellular metabolic process;cellular process;cellular protein metabolic process;gene ex
biological regulation;biosynthetic process;cellular biosynthetic process;cellular macromolecule biosynthetic process;cellular macromolecule metabolic process;cellular metabolic process;cellular nitrogen compound metabolic
cell migration;cell motility;cellular component movement;cellular process;endothelial cell migration;locomotion
apoptosis;apoptosis involved in morphogenesis;cell death;cellular developmental process;cellular process;death;developmental process;developmental programmed cell death;programmed cell death
biological regulation;biosynthetic process;cell cycle;cell cycle process;cellular biosynthetic process;cellular component assembly;cellular component organization;cellular component organization or biogenesis;cellular macror
biological regulation;cellular process;cellular response to stimulus;negative regulation of biological process;negative regulation of biosynthetic process;negative regulation of cell proliferation;negative regulation of cellular bios
anatomical structure homeostasis;base-excision repair;biological regulation;biosynthetic process;cell cycle;cellular biosynthetic process;cellular component organization;cellular component organization at cellular level;cellula
anatomical structure homeostasis;base-excision repair;biological regulation;biosynthetic process;cell cycle;cellular biosynthetic process;cellular component organization;cellular component organization at cellular level;cellula
anatomical structure homeostasis;base-excision repair;biological regulation;biosynthetic process;cell cycle;cellular biosynthetic process;cellular component organization;cellular component organization at cellular level;cellula
biological regulation;cellular process;cellular response to stimulus;establishment of localization;establishment of localization in cell;intracellular transport;regulation of biological process;regulation of catalytic activity;regulation
anatomical structure development;apoptosis;apoptosis in response to endoplasmic reticulum stress;biological regulation;brain development;catabolic process;cell cycle process;cell death;cell differentiation;cellular catabolic
biological regulation;cation homeostasis;cation transport;cellular cation homeostasis;cellular chemical homeostasis;cellular homeostasis;cellular ion homeostasis;cellular iron ion homeostasis;cellular metal ion homeostasis;ce
amine metabolic process;amino acid activation;biosynthetic process;carboxylic acid metabolic process;cellular amine metabolic process;cellular amino acid metabolic process;cellular biosynthetic process;cellular ketone me
activation of immune response;activation of innate immune response;anaphase-promoting complex-dependent proteasomal ubiquitin-dependent protein catabolic process;antigen processing and presentation;antigen proces
anatomical structure homeostasis;base-excision repair;biological regulation;biosynthetic process;cell cycle checkpoint;cell cycle process;cell proliferation;cellular biosynthetic process;cellular component organization;cellular
biological regulation;biosynthetic process;cell cycle phase;cell cycle process;cellular biosynthetic process;cellular component assembly;cellular component organization;cellular component organization at cellular level;cellula
biological regulation;biosynthetic process;cell differentiation;cell proliferation;cellular biosynthetic process;cellular developmental process;cellular macromolecule biosynthetic process;cellular macromolecule metabolic proces
biosynthetic process;catabolic process;cellular biosynthetic process;cellular catabolic process;cellular component disassembly;cellular component disassembly at cellular level;cellular component organization;cellular compo
biosynthetic process;cellular biosynthetic process;cellular macromolecule biosynthetic process;cellular macromolecule metabolic process;cellular metabolic process;cellular nitrogen compound metabolic process;cellular pro
apoptosis;biological regulation;cell death;cellular component assembly;cellular component organization;cellular component organization at cellular level;cellular component organization or biogenesis;cellular component orga
5S class rRNA transcription from RNA polymerase III type 1 promoter;biosynthetic process;cellular biosynthetic process;cellular macromolecule biosynthetic process;cellular macromolecule metabolic process;cellular metabo
4-hydroxyproline metabolic process;amine metabolic process;anatomical structure development;biological regulation;carboxylic acid metabolic process;cell redox homeostasis;cellular amine metabolic process;cellular amino
cellular macromolecule metabolic process;cellular metabolic process;cellular nitrogen compound metabolic process;cellular process;cellular protein metabolic process;macromolecule metabolic process;macromolecule modi
alcohol biosynthetic process;alcohol metabolic process;biological adhesion;biosynthetic process;carbohydrate biosynthetic process;carbohydrate metabolic process;cell adhesion;cell death;cell-cell adhesion;cellular biosynt
cellular component biogenesis;cellular component biogenesis at cellular level;cellular component organization or biogenesis;cellular component organization or biogenesis at cellular level;cellular process;metabolic process;ri
biological regulation;biosynthetic process;cellular biosynthetic process;cellular macromolecule biosynthetic process;cellular macromolecule metabolic process;cellular metabolic process;cellular nitrogen compound metabolic
biological regulation;biosynthetic process;cellular biosynthetic process;cellular macromolecule biosynthetic process;cellular macromolecule metabolic process;cellular metabolic process;cellular nitrogen compound metabolic
antigen processing and presentation;biological regulation;cell projection organization;cellular component organization;cellular component organization at cellular level;cellular component organization or biogenesis;cellular co
activation of JUN kinase activity;activation of MAPK activity;ameboidal cell migration;biological regulation;biosynthetic process;cell migration;cell motility;cellular biosynthetic process;cellular component movement;cellular co
cellular component assembly;cellular component assembly at cellular level;cellular component organization;cellular component organization at cellular level;cellular component organization or biogenesis;cellular component c
biological regulation;cellular component organization;cellular component organization or biogenesis;cellular macromolecule metabolic process;cellular membrane organization;cellular metabolic process;cellular process;cellula
cellular process;endosome transport;establishment of localization;establishment of localization in cell;establishment of protein localization;intracellular transport;protein transport;retrograde transport, endosome to Golgi;trans
response to abiotic stimulus;response to ionizing radiation;response to radiation;response to stimulus;response to X-ray
biological regulation;cell cycle;cell cycle phase;cell cycle process;cell division;cellular component organization;cellular component organization at cellular level;cellular component organization or biogenesis;cellular componen
biological regulation;cellular component organization;cellular component organization at cellular level;cellular component organization or biogenesis;cellular component organization or biogenesis at cellular level;cellular macro
biological regulation;cell differentiation;cellular developmental process;cellular process;developmental process;muscle cell differentiation;negative regulation of biological process;negative regulation of cell communication;ne
cell differentiation;cellular developmental process;cellular process;developmental process;fat cell differentiation
assembly of spliceosomal tri-snRNP;cellular component assembly;cellular component assembly at cellular level;cellular component organization;cellular component organization at cellular level;cellular component organizatio
adherens junction organization;antigen processing and presentation;biological regulation;cell junction organization;cell-cell junction organization;cellular component organization;cellular component organization at cellular leve
biological regulation;cell cycle arrest;cell cycle process;cellular metabolic process;cellular process;cellular respiration;energy derivation by oxidation of organic compounds;generation of precursor metabolites and energy;met
biological regulation;cellular component organization;cellular component organization at cellular level;cellular component organization or biogenesis;cellular component organization or biogenesis at cellular level;cellular proce
cellular macromolecule metabolic process;cellular metabolic process;cellular nitrogen compound metabolic process;cellular process;macromolecule metabolic process;metabolic process;nitrogen compound metabolic proce
cellular component assembly;cellular component assembly at cellular level;cellular component organization;cellular component organization at cellular level;cellular component organization or biogenesis;cellular component c
cellular macromolecule metabolic process;cellular metabolic process;cellular nitrogen compound metabolic process;cellular process;macromolecule metabolic process;macromolecule modification;metabolic process;ncRNA
anatomical structure morphogenesis;biological regulation;cell differentiation;cell differentiation in hindbrain;cell part morphogenesis;cell projection morphogenesis;cell projection organization;cellular component assembly;cell
amine biosynthetic process;amine metabolic process;anatomical structure development;aspartate family amino acid biosynthetic process;aspartate family amino acid metabolic process;biosynthetic process;carboxylic acid b
cellular component biogenesis;cellular component biogenesis at cellular level;cellular component organization or biogenesis;cellular component organization or biogenesis at cellular level;cellular process;ribonucleoprotein co
cellular component organization;cellular component organization at cellular level;cellular component organization or biogenesis;cellular component organization or biogenesis at cellular level;cellular macromolecule metabolic
biological regulation;cell proliferation;cellular component biogenesis;cellular component biogenesis at cellular level;cellular component organization or biogenesis;cellular component organization or biogenesis at cellular level
actin cytoskeleton organization;actin filament-based process;cell communication;cell-cell signaling;cellular component organization;cellular component organization at cellular level;cellular component organization or biogene
apoptosis;cell death;cellular macromolecule metabolic process;cellular metabolic process;cellular process;cellular protein metabolic process;death;macromolecule metabolic process;macromolecule modification;metabolic p
cellular macromolecule metabolic process;cellular metabolic process;cellular process;cellular protein metabolic process;establishment of localization;establishment of localization in cell;establishment of protein localization;es
cellular component assembly;cellular component organization;cellular component organization or biogenesis;macromolecular complex assembly;macromolecular complex subunit organization;metabolic process;protein com

activation of JUN kinase activity;activation of MAPK activity;activation of MAPKK activity;activation of protein kinase activity;biological regulation;cell cycle arrest;cell cycle checkpoint;cell cycle process;cell death;cell differen
anatomical structure formation involved in morphogenesis;angiogenesis;antigen processing and presentation;antigen processing and presentation of endogenous antigen;antigen processing and presentation of endogenous
cellular component biogenesis;cellular component biogenesis at cellular level;cellular component organization or biogenesis;cellular component organization or biogenesis at cellular level;cellular process;ribonucleoprotein co
anatomical structure homeostasis;biological regulation;cell cycle process;cellular component organization;cellular component organization at cellular level;cellular component organization or biogenesis;cellular component org
actin cytoskeleton organization;actin filament depolymerization;actin filament organization;actin filament-based process;actin polymerization or depolymerization;actomyosin structure organization;anatomical structure homeo
anatomical structure development;catabolic process;cellular aromatic compound metabolic process;cellular catabolic process;cellular metabolic process;cellular nitrogen compound catabolic process;cellular nitrogen compo
apoptosis;biological regulation;biosynthetic process;cell death;cellular biosynthetic process;cellular component assembly;cellular component assembly at cellular level;cellular component organization;cellular component orga
biological regulation;biosynthetic process;cell cycle checkpoint;cellular biosynthetic process;cellular component organization;cellular component organization at cellular level;cellular component organization or biogenesis;cell
cellular component organization;cellular component organization at cellular level;cellular component organization or biogenesis;cellular component organization or biogenesis at cellular level;cellular membrane organization;ce
biological regulation;cellular macromolecule metabolic process;cellular metabolic process;cellular nitrogen compound metabolic process;cellular process;macromolecule metabolic process;macromolecule modification;metab
acrosome assembly;anatomical structure formation involved in morphogenesis;cellular component assembly;cellular component assembly at cellular level;cellular component assembly involved in morphogenesis;cellular com
apoptosis;biological regulation;catabolic process;cell death;cellular catabolic process;cellular component disassembly;cellular component disassembly at cellular level;cellular component disassembly involved in apoptosis;c
biological regulation;cell surface receptor linked signaling pathway;cellular component organization;cellular component organization or biogenesis;cellular localization;cellular macromolecule localization;cellular membrane org
actin filament capping;adult behavior;anatomical structure morphogenesis;antigen processing and presentation;antigen processing and presentation of exogenous antigen;antigen processing and presentation of exogenous p
biological regulation;regulation of biological process;regulation of cell proliferation;regulation of cellular process
anaphase-promoting complex-dependent proteasomal ubiquitin-dependent protein catabolic process;attachment of spindle microtubules to chromosome;attachment of spindle microtubules to kinetochore;biological regulati
anatomical structure development;biological regulation;biosynthetic process;bone development;cellular biosynthetic process;cellular metabolic process;cellular nitrogen compound biosynthetic process;cellular nitrogen comp
activation of JUN kinase activity;activation of MAPK activity;biological regulation;cell differentiation;cellular component assembly;cellular component organization;cellular component organization or biogenesis;cellular develop
actin cytoskeleton organization;actin filament-based process;activation of protein kinase activity;apoptosis;biological regulation;cell cycle;cell death;cell growth;cell migration;cell motility;cell proliferation;cellular component m
cellular macromolecule metabolic process;cellular metabolic process;cellular nitrogen compound metabolic process;cellular process;gene expression;macromolecule metabolic process;metabolic process;mRNA metabolic p
actin cytoskeleton organization;actin filament-based process;activation of phospholipase C activity;activation of protein kinase A activity;activation of protein kinase activity;actomyosin structure organization;anatomical struc
acetyl-CoA biosynthetic process;acetyl-CoA biosynthetic process from pyruvate;acetyl-CoA catabolic process;acetyl-CoA metabolic process;alcohol metabolic process;biological regulation;biosynthetic process;carbohydrat
biological regulation;calcium ion homeostasis;calcium ion transport;cation homeostasis;cation transport;cellular calcium ion homeostasis;cellular cation homeostasis;cellular chemical homeostasis;cellular divalent inorganic c
biological regulation;cell cycle;cell cycle checkpoint;cellular component assembly;cellular component assembly at cellular level;cellular component organization;cellular component organization at cellular level;cellular compor
ATP hydrolysis coupled proton transport;biological regulation;cation homeostasis;cation transport;cell surface receptor linked signaling pathway;cellular cation homeostasis;cellular chemical homeostasis;cellular component c
box C/D snoRNA 3'-end processing;box C/D snoRNA metabolic process;box C/D snoRNA processing;cell differentiation;cellular developmental process;cellular macromolecule metabolic process;cellular metabolic process;c
biological regulation;catabolic process;cellular catabolic process;cellular component assembly;cellular component assembly at cellular level;cellular component organization;cellular component organization at cellular level;ce
biological regulation;blastocyst hatching;cellular process;cellular response to stimulus;developmental process;embryo implantation;hatching;organism emergence from protective structure;positive regulation of biological proc
carboxylic acid catabolic process;carboxylic acid metabolic process;catabolic process;cellular catabolic process;cellular ketone metabolic process;cellular lipid catabolic process;cellular lipid metabolic process;cellular metab
biological regulation;cell surface receptor linked signaling pathway;cellular process;cellular response to chemical stimulus;cellular response to cytokine stimulus;cellular response to interferon-gamma;cellular response to orga
amine biosynthetic process;amine catabolic process;amine metabolic process;biosynthetic process;carboxylic acid biosynthetic process;carboxylic acid catabolic process;carboxylic acid metabolic process;catabolic process
adaptive immune response;adaptive immune response based on somatic recombination of immune receptors built from immunoglobulin superfamily domains;biological regulation;cellular component biogenesis;cellular comp
actin filament capping;barbed-end actin filament capping;biological regulation;blood coagulation;cellular component assembly;cellular component movement;cellular component organization;cellular component organization
anatomical structure development;cargo loading into vesicle;cellular component assembly;cellular component assembly at cellular level;cellular component organization;cellular component organization at cellular level;cellula
biological regulation;biosynthetic process;catabolic process;cellular biosynthetic process;cellular catabolic process;cellular macromolecule biosynthetic process;cellular macromolecule metabolic process;cellular metabolic p
biological regulation;cell surface receptor linked signaling pathway;cellular macromolecule metabolic process;cellular metabolic process;cellular nitrogen compound metabolic process;cellular process;cellular response to che
anatomical structure formation involved in morphogenesis;angiogenesis;axon guidance;biological regulation;blood coagulation;cell activation;cell migration;cell motility;cell projection assembly;cell projection organization;cell
amine biosynthetic process;amine metabolic process;aspartate family amino acid metabolic process;biosynthetic process;carboxylic acid metabolic process;cellular amine metabolic process;cellular amino acid metabolic pro
biological regulation;cellular process;cellular response to stimulus;regulation of biological process;regulation of biosynthetic process;regulation of cellular biosynthetic process;regulation of cellular macromolecule biosynthetic
biological regulation;biosynthetic process;cell differentiation;cellular biosynthetic process;cellular developmental process;cellular macromolecule biosynthetic process;cellular macromolecule metabolic process;cellular metab
antigen processing and presentation;antigen processing and presentation of exogenous antigen;antigen processing and presentation of exogenous peptide antigen;antigen processing and presentation of exogenous peptide
activation of immune response;activation of innate immune response;anaphase-promoting complex-dependent proteasomal ubiquitin-dependent protein catabolic process;antigen processing and presentation;antigen proces
actin cytoskeleton organization;actin filament-based process;anatomical structure development;anatomical structure morphogenesis;apical junction assembly;apolipoprotein A-I-mediated signaling pathway;axon guidance;bi
biosynthetic process;catabolic process;cellular biosynthetic process;cellular catabolic process;cellular component disassembly;cellular component disassembly at cellular level;cellular component organization;cellular compo
acylglycerol catabolic process;acylglycerol metabolic process;anatomical structure development;anatomical structure morphogenesis;biological regulation;branching morphogenesis of a tube;carbohydrate metabolic process
acylglycerol catabolic process;acylglycerol metabolic process;biological regulation;carbohydrate metabolic process;catabolic process;cell cycle;cell cycle process;cell division;cell surface receptor linked signaling pathway;ce
biological regulation;biosynthetic process;catabolic process;cellular biosynthetic process;cellular catabolic process;cellular component disassembly;cellular component disassembly at cellular level;cellular component organiz
biological regulation;biosynthetic process;catabolic process;cellular biosynthetic process;cellular catabolic process;cellular component disassembly;cellular component disassembly at cellular level;cellular component organiz
biosynthetic process;catabolic process;cellular biosynthetic process;cellular catabolic process;cellular component biogenesis;cellular component biogenesis at cellular level;cellular component disassembly;cellular componen
biosynthetic process;catabolic process;cellular biosynthetic process;cellular catabolic process;cellular component biogenesis;cellular component biogenesis at cellular level;cellular component disassembly;cellular componen
anatomical structure development;anatomical structure morphogenesis;apoptosis;appendage morphogenesis;atrial septum morphogenesis;biological regulation;biosynthetic process;blood vessel development;bone remodel
amine catabolic process;amine metabolic process;anatomical structure development;anatomical structure morphogenesis;biological regulation;biosynthetic process;body fluid secretion;carboxylic acid catabolic process;carb
activation of immune response;activation of innate immune response;anaphase-promoting complex-dependent proteasomal ubiquitin-dependent protein catabolic process;antigen processing and presentation;antigen proces
anatomical structure development;biological regulation;central nervous system development;developmental process;establishment of localization;establishment of RNA localization;mRNA transport;negative regulation of biol
biological regulation;biosynthetic process;cell differentiation;cellular biosynthetic process;cellular developmental process;cellular macromolecule biosynthetic process;cellular macromolecule metabolic process;cellular metab
biosynthetic process;cellular biosynthetic process;cellular macromolecule biosynthetic process;cellular macromolecule metabolic process;cellular metabolic process;cellular nitrogen compound metabolic process;cellular pro
cellular process;endosome transport;establishment of localization;establishment of localization in cell;establishment of protein localization;intracellular transport;protein transport;transport;vesicle-mediated transport
biological regulation;cell surface receptor linked signaling pathway;cellular macromolecule metabolic process;cellular metabolic process;cellular process;cellular protein metabolic process;cellular response to stimulus;enzym
amine biosynthetic process;amine metabolic process;beta-alanine biosynthetic process;beta-alanine metabolic process;biosynthetic process;carboxylic acid biosynthetic process;carboxylic acid metabolic process;catabolic
biological regulation;biosynthetic process;cellular biosynthetic process;cellular macromolecule biosynthetic process;cellular macromolecule metabolic process;cellular metabolic process;cellular nitrogen compound metabolic
biological regulation;biosynthetic process;cellular biosynthetic process;cellular macromolecule biosynthetic process;cellular macromolecule metabolic process;cellular metabolic process;cellular nitrogen compound metabolic
amine biosynthetic process;amine metabolic process;amino acid salvage;aspartate family amino acid biosynthetic process;aspartate family amino acid metabolic process;biosynthetic process;carboxylic acid biosynthetic pro
cellular macromolecule metabolic process;cellular metabolic process;cellular nitrogen compound metabolic process;cellular process;macromolecule metabolic process;metabolic process;mRNA metabolic process;mRNA pro
anatomical structure development;anatomical structure morphogenesis;biological regulation;cell differentiation;cellular developmental process;cellular process;developmental process;muscle organ development;muscle struc
biological regulation;cellular component assembly;cellular component assembly at cellular level;cellular component organization;cellular component organization at cellular level;cellular component organization or biogenesis;
biological regulation;cell proliferation;cellular component biogenesis;cellular component biogenesis at cellular level;cellular component organization or biogenesis;cellular component organization or biogenesis at cellular level
cellular component assembly;cellular component assembly at cellular level;cellular component organization;cellular component organization at cellular level;cellular component organization or biogenesis;cellular component c
biological regulation;biosynthetic process;cellular biosynthetic process;cellular macromolecule biosynthetic process;cellular macromolecule metabolic process;cellular metabolic process;cellular process;cellular protein metab
anatomical structure homeostasis;arachidonic acid metabolic process;biological regulation;biosynthetic process;carboxylic acid biosynthetic process;carboxylic acid metabolic process;cellular biosynthetic process;cellular co
actin cytoskeleton organization;actin filament-based process;actomyosin structure organization;cell differentiation;cellular component organization;cellular component organization at cellular level;cellular component organiza
biological regulation;biosynthetic process;cell differentiation;cellular biosynthetic process;cellular component assembly;cellular component assembly at cellular level;cellular component organization;cellular component organi
actin cytoskeleton organization;actin filament-based process;biological regulation;cell communication;cellular component organization;cellular component organization at cellular level;cellular component organization or bioge
biological regulation;biosynthetic process;cell junction assembly;cell junction organization;cell-substrate junction assembly;cellular biosynthetic process;cellular component assembly;cellular component assembly at cellular l
biological regulation;biosynthetic process;cellular biosynthetic process;cellular macromolecule biosynthetic process;cellular macromolecule metabolic process;cellular metabolic process;cellular nitrogen compound metabolic
anatomical structure development;biological regulation;bone development;cell growth;cellular component organization;cellular component organization at cellular level;cellular component organization or biogenesis;cellular co
alcohol metabolic process;alditol metabolic process;biological regulation;carbohydrate metabolic process;carbohydrate phosphorylation;cellular carbohydrate metabolic process;cellular metabolic process;cellular process;de
apoptotic mitochondrial changes;cellular component organization;cellular component organization at cellular level;cellular component organization or biogenesis;cellular component organization or biogenesis at cellular level;c
biological regulation;cellular macromolecule metabolic process;cellular metabolic process;cellular nitrogen compound metabolic process;cellular process;macromolecule metabolic process;metabolic process;mRNA metaboli
carboxylic acid metabolic process;cellular ketone metabolic process;cellular metabolic process;cellular process;metabolic process;organic acid metabolic process;oxoacid metabolic process;small molecule metabolic proces
biological regulation;biosynthetic process;cellular biosynthetic process;cellular component organization;cellular component organization at cellular level;cellular component organization or biogenesis;cellular component orga
biological adhesion;cell adhesion;cellular process
biosynthetic process;cellular biosynthetic process;cellular component assembly;cellular component organization;cellular component organization or biogenesis;cellular macromolecule biosynthetic process;cellular macromole
anatomical structure development;anatomical structure morphogenesis;apoptosis;biological regulation;biosynthetic process;camera-type eye development;cardiac chamber morphogenesis;cardiac muscle contraction;cardia
ATP-dependent chromatin remodeling;biosynthetic process;cellular biosynthetic process;cellular component organization;cellular component organization at cellular level;cellular component organization or biogenesis;cellula
biosynthetic process;cellular biosynthetic process;cellular component disassembly;cellular component disassembly at cellular level;cellular component organization;cellular component organization at cellular level;cellular cor
anatomical structure development;anatomical structure formation involved in morphogenesis;anatomical structure morphogenesis;ATP-dependent chromatin remodeling;biological regulation;biosynthetic process;blood coagu
anatomical structure development;biological regulation;cell development;cellular component assembly;cellular component assembly at cellular level;cellular component organization;cellular component organization at cellular
anatomical structure development;biological regulation;cell proliferation;cellular component movement;cellular process;developmental process;fertilization;muscle tissue development;nervous system development;regulation
cellular component organization;cellular component organization at cellular level;cellular component organization or biogenesis;cellular component organization or biogenesis at cellular level;cellular macromolecule metabolic
cell cycle;cell division;cellular process
immune system process
biological regulation;cellular component organization;cellular component organization at cellular level;cellular component organization or biogenesis;cellular component organization or biogenesis at cellular level;cellular macro
anatomical structure development;cell differentiation;cellular developmental process;cellular macromolecule metabolic process;cellular metabolic process;cellular process;cellular protein metabolic process;'de novo' posttran
biological regulation;negative regulation of biological process;negative regulation of cellular component organization;negative regulation of cellular process;negative regulation of developmental process;negative regulation of
biological regulation;biosynthetic process;cellular biosynthetic process;cellular lipid metabolic process;cellular metabolic process;cellular process;cellular response to calcium ion;cellular response to chemical stimulus;cellula
anatomical structure development;calcium ion-dependent exocytosis;cell differentiation;cellular developmental process;cellular process;cerebellum development;developmental process;establishment of localization;establish
biological regulation;biosynthetic process;carbohydrate metabolic process;carbohydrate transport;cell chemotaxis;cell cycle;cell cycle process;cell migration;cell motility;cell projection assembly;cell projection organization;ce
biological regulation;cellular macromolecule metabolic process;cellular metabolic process;cellular process;cellular protein metabolic process;endosome transport;establishment of localization;establishment of localization in c
cellular process;cellular response to heat;cellular response to stimulus;cellular response to stress;response to abiotic stimulus;response to heat;response to stimulus;response to stress;response to temperature stimulus
biological regulation;cellular macromolecule metabolic process;cellular metabolic process;cellular process;cellular protein metabolic process;dephosphorylation;macromolecule metabolic process;macromolecule modificatior
biological regulation;biosynthetic process;carboxylic acid biosynthetic process;carboxylic acid metabolic process;cell redox homeostasis;cellular biosynthetic process;cellular homeostasis;cellular ketone metabolic process;c
amine metabolic process;asparagine metabolic process;aspartate family amino acid metabolic process;carboxylic acid metabolic process;cellular amine metabolic process;cellular amino acid metabolic process;cellular ketor
actin cytoskeleton organization;actin filament capping;actin filament organization;actin filament-based process;actomyosin structure organization;anatomical structure development;anatomical structure formation involved in
acyl-CoA biosynthetic process;acyl-CoA metabolic process;acylglycerol biosynthetic process;acylglycerol metabolic process;biosynthetic process;carboxylic acid biosynthetic process;carboxylic acid metabolic process;cellu
biological regulation;cellular macromolecule metabolic process;cellular metabolic process;cellular nitrogen compound metabolic process;cellular process;CRD-mediated mRNA stabilization;establishment of localization;estab
actin filament-based movement;actin filament-based process;biological regulation;cell cycle process;cellular component movement;cellular component organization;cellular component organization at cellular level;cellular co
actin crosslink formation;actin cytoskeleton organization;actin filament bundle assembly;actin filament organization;actin filament-based process;biological regulation;cell surface receptor linked signaling pathway;cellular con
biological regulation;cellular lipid metabolic process;cellular metabolic process;cellular process;cellular response to stimulus;defense response;inflammatory response;lipid metabolic process;metabolic process;organophosph
biosynthetic process;cellular biosynthetic process;cellular macromolecule biosynthetic process;cellular macromolecule metabolic process;cellular metabolic process;cellular nitrogen compound metabolic process;cellular pro
amine metabolic process;carboxylic acid metabolic process;cell differentiation;cellular amine metabolic process;cellular amino acid metabolic process;cellular developmental process;cellular ketone metabolic process;cellula
cell growth;cellular macromolecule metabolic process;cellular metabolic process;cellular nitrogen compound metabolic process;cellular process;establishment of localization;establishment of localization in cell;establishment
anatomical structure homeostasis;biological regulation;cell surface receptor linked signaling pathway;cellular component assembly;cellular component assembly at cellular level;cellular component organization;cellular compo
biological regulation;cell cycle phase;cell cycle process;cell division;cell projection assembly;cell projection organization;cellular component assembly;cellular component assembly at cellular level;cellular component organiza
cellular macromolecule metabolic process;cellular metabolic process;cellular nitrogen compound metabolic process;cellular process;macromolecule metabolic process;metabolic process;nitrogen compound metabolic proce
biological regulation;biosynthetic process;cellular biosynthetic process;cellular component assembly;cellular component assembly at cellular level;cellular component organization;cellular component organization at cellular le
cellular macromolecule metabolic process;cellular metabolic process;cellular process;cellular protein metabolic process;establishment of localization;establishment of localization in cell;establishment of protein localization;es
2-oxoglutarate metabolic process;acetyl-CoA catabolic process;acetyl-CoA metabolic process;carboxylic acid metabolic process;catabolic process;cellular catabolic process;cellular ketone metabolic process;cellular metabo
2'-deoxyribonucleotide metabolic process;aging;alcohol metabolic process;amino sugar metabolic process;biological regulation;carbohydrate metabolic process;catabolic process;cell death;cellular carbohydrate metabolic p
anatomical structure morphogenesis;cell part morphogenesis;cell projection morphogenesis;cell projection organization;cellular component assembly;cellular component morphogenesis;cellular component organization;cellu
catabolic process;cellular catabolic process;cellular component organization;cellular component organization at cellular level;cellular component organization or biogenesis;cellular component organization or biogenesis at ce
biological regulation;catabolic process;cation transport;cell cycle process;cellular catabolic process;cellular component disassembly;cellular component disassembly at cellular level;cellular component organization;cellular co
cellular macromolecule metabolic process;cellular metabolic process;cellular process;cellular protein metabolic process;establishment of localization;establishment of localization in cell;establishment of protein localization;es
biological regulation;biosynthetic process;cellular biosynthetic process;cellular component assembly;cellular component assembly at cellular level;cellular component organization;cellular component organization at cellular le
alcohol biosynthetic process;alcohol metabolic process;amino sugar biosynthetic process;amino sugar metabolic process;anatomical structure development;biosynthetic process;carbohydrate biosynthetic process;carbohyd
biological regulation;positive regulation of ATPase activity;positive regulation of catalytic activity;positive regulation of hydrolase activity;positive regulation of molecular function;regulation of ATPase activity;regulation of biolo

biological regulation;biosynthetic process;catabolic process;cellular aromatic compound metabolic process;cellular biosynthetic process;cellular catabolic process;cellular metabolic compound salvage;cellular metabolic proc
aging;biosynthetic process;carbohydrate metabolic process;cellular biosynthetic process;cellular carbohydrate metabolic process;cellular macromolecule biosynthetic process;cellular macromolecule metabolic process;cellul
activation of immune response;adaptive immune response;adaptive immune response based on somatic recombination of immune receptors built from immunoglobulin superfamily domains;biological regulation;calcium ion h
anatomical structure development;biological regulation;biosynthetic process;carbohydrate homeostasis;cellular biosynthetic process;cellular chemical homeostasis;cellular component organization;cellular component organiz
amine metabolic process;aminoglycan catabolic process;aminoglycan metabolic process;anatomical structure development;anatomical structure morphogenesis;biological adhesion;biological regulation;blood coagulation;br
biological adhesion;biological regulation;biosynthetic process;cell adhesion;cell surface receptor linked signaling pathway;cellular biosynthetic process;cellular macromolecule biosynthetic process;cellular macromolecule me
amine transport;amino acid transmembrane transport;amino acid transport;betaine transport;carboxylic acid catabolic process;carboxylic acid metabolic process;carboxylic acid transport;carnitine shuttle;carnitine transport;
2'-deoxyribonucleotide biosynthetic process;2'-deoxyribonucleotide metabolic process;biosynthetic process;cell cycle;cell differentiation;cell proliferation;cellular biosynthetic process;cellular developmental process;cellular r
biological regulation;cellular component assembly;cellular component organization;cellular component organization or biogenesis;macromolecular complex assembly;macromolecular complex subunit organization;positive re
activation of immune response;activation of innate immune response;activation of MAPK activity;activation of MAPKK activity;activation of protein kinase activity;anatomical structure development;anatomical structure morph
anatomical structure development;biological regulation;catabolic process;cell differentiation;cellular catabolic process;cellular chemical homeostasis;cellular component organization;cellular component organization at cellular
biosynthetic process;catabolic process;cellular biosynthetic process;cellular catabolic process;cellular component disassembly;cellular component disassembly at cellular level;cellular component organization;cellular compo
biological regulation;cellular component organization;cellular component organization or biogenesis;cellular macromolecule metabolic process;cellular membrane organization;cellular metabolic process;cellular process;cellul
biosynthetic process;cellular aromatic compound metabolic process;cellular biosynthetic process;cellular metabolic process;cellular nitrogen compound biosynthetic process;cellular nitrogen compound metabolic process;ce
biosynthetic process;carbohydrate metabolic process;cell activation;cellular biosynthetic process;cellular carbohydrate metabolic process;cellular macromolecule biosynthetic process;cellular macromolecule metabolic proce
biosynthetic process;catabolic process;cellular biosynthetic process;cellular catabolic process;cellular component disassembly;cellular component disassembly at cellular level;cellular component organization;cellular compo
anatomical structure homeostasis;antigen processing and presentation;antigen processing and presentation of exogenous antigen;antigen processing and presentation of exogenous peptide antigen;antigen processing and p
alcohol metabolic process;anatomical structure development;cellular aldehyde metabolic process;cellular lipid metabolic process;cellular metabolic process;cellular process;central nervous system development;development
antigen processing and presentation;antigen processing and presentation of exogenous antigen;antigen processing and presentation of exogenous peptide antigen;antigen processing and presentation of exogenous peptide
acyl-CoA biosynthetic process;acyl-CoA metabolic process;acylglycerol biosynthetic process;acylglycerol metabolic process;alcohol biosynthetic process;alcohol metabolic process;anion transport;biosynthetic process;carb
biological adhesion;biological regulation;cell adhesion;cell surface receptor linked signaling pathway;cell-cell adhesion;cellular macromolecule metabolic process;cellular metabolic process;cellular process;cellular protein me
biological regulation;biosynthetic process;cellular biosynthetic process;cellular component assembly;cellular component assembly at cellular level;cellular component organization;cellular component organization at cellular le
actin filament-based movement;actin filament-based process;actin-mediated cell contraction;actin-myosin filament sliding;anatomical structure development;axon guidance;biological regulation;cell surface receptor linked si
cellular macromolecule metabolic process;cellular metabolic process;cellular process;cellular protein metabolic process;'de novo' posttranslational protein folding;'de novo' protein folding;macromolecule metabolic process;r
biological regulation;biosynthetic process;cellular biosynthetic process;cellular component assembly;cellular component assembly at cellular level;cellular component organization;cellular component organization at cellular le
biological regulation;biosynthetic process;cellular aromatic compound metabolic process;cellular biosynthetic process;cellular metabolic compound salvage;cellular metabolic process;cellular nitrogen compound biosynthetic
biological regulation;biosynthetic process;cellular biosynthetic process;cellular macromolecule biosynthetic process;cellular macromolecule metabolic process;cellular metabolic process;cellular nitrogen compound metabolic
biological regulation;cell surface receptor linked signaling pathway;cellular macromolecule metabolic process;cellular metabolic process;cellular nitrogen compound metabolic process;cellular process;cellular response to stin
biological regulation;cellular component organization;cellular component organization or biogenesis;maintenance of location;maintenance of protein location;negative regulation of actin filament polymerization;negative regula
alcohol metabolic process;amine metabolic process;amino acid activation;anatomical structure formation involved in morphogenesis;angiogenesis;apoptosis;biological adhesion;biological regulation;carbohydrate metabolic p
3'-UTR-mediated mRNA stabilization;biological regulation;biosynthetic process;cellular biosynthetic process;cellular component organization;cellular component organization or biogenesis;cellular macromolecule biosynthetic
biological regulation;biosynthetic process;cellular biosynthetic process;cellular component assembly;cellular component assembly at cellular level;cellular component organization;cellular component organization at cellular le
biological regulation;cell proliferation;cellular process;cellular response to stimulus;regulation of biological process;regulation of cellular process;response to stimulus;signal transduction
anatomical structure development;anatomical structure formation involved in morphogenesis;anatomical structure morphogenesis;ATP-dependent chromatin remodeling;biological regulation;biosynthetic process;blood coagu
alkene biosynthetic process;biosynthetic process;carboxylic acid biosynthetic process;carboxylic acid metabolic process;cellular alkene metabolic process;cellular biosynthetic process;cellular ketone metabolic process;cellu
biological regulation;blood coagulation;cAMP catabolic process;cAMP metabolic process;cAMP-mediated signaling;catabolic process;cell maturation;cellular catabolic process;cellular developmental process;cellular lipid me
alcohol metabolic process;biosynthetic process;cholesterol biosynthetic process;cholesterol metabolic process;lipid biosynthetic process;lipid metabolic process;metabolic process;primary metabolic process;small molecule
anatomical structure formation involved in morphogenesis;biological regulation;cell cycle;cell cycle process;cell projection assembly;cell projection organization;cellular component assembly;cellular component assembly at c
adult behavior;adult locomotory behavior;anatomical structure morphogenesis;axonogenesis;behavior;cell part morphogenesis;cell projection morphogenesis;cell projection organization;cellular component morphogenesis;ce
actin cytoskeleton organization;actin cytoskeleton reorganization;actin filament-based process;biological regulation;cell surface receptor linked signaling pathway;cellular component organization;cellular component organizat
biological regulation;biosynthetic process;cellular biosynthetic process;cellular component organization;cellular component organization at cellular level;cellular component organization or biogenesis;cellular component orga
cellular component assembly;cellular component assembly at cellular level;cellular component organization;cellular component organization at cellular level;cellular component organization or biogenesis;cellular component c
biosynthetic process;cellular biosynthetic process;cellular macromolecule biosynthetic process;cellular macromolecule metabolic process;cellular metabolic process;cellular nitrogen compound metabolic process;cellular pro
activation of caspase activity;anatomical structure development;biological regulation;cellular component assembly;cellular component assembly at cellular level;cellular component organization;cellular component organizatio
cellular macromolecule metabolic process;cellular metabolic process;cellular process;cellular protein metabolic process;chaperone-mediated protein folding;macromolecule metabolic process;macromolecule modification;me
biological regulation;biosynthetic process;cellular biosynthetic process;cellular macromolecule biosynthetic process;cellular macromolecule metabolic process;cellular metabolic process;cellular nitrogen compound metabolic
activation of immune response;activation of innate immune response;aging;biological regulation;biosynthetic process;blood coagulation;carbohydrate homeostasis;cell differentiation;cellular biosynthetic process;cellular chen
biological regulation;biosynthetic process;carbohydrate metabolic process;carbohydrate transport;cell cycle;cell cycle process;cell surface receptor linked signaling pathway;cellular biosynthetic process;cellular component c
biological regulation;cell projection organization;cell surface receptor linked signaling pathway;cellular component organization;cellular component organization at cellular level;cellular component organization or biogenesis;c
anatomical structure development;biological regulation;cellular component organization;cellular component organization or biogenesis;cellular membrane organization;cellular process;cellular response to stimulus;central nerv
cellular macromolecule metabolic process;cellular metabolic process;cellular nitrogen compound metabolic process;cellular process;macromolecule metabolic process;metabolic process;nitrogen compound metabolic proce
cellular macromolecule metabolic process;cellular metabolic process;cellular process;cellular protein metabolic process;cullin deneddylation;macromolecule metabolic process;macromolecule modification;metabolic process
amine metabolic process;amino acid activation;carboxylic acid metabolic process;cellular amine metabolic process;cellular amino acid metabolic process;cellular ketone metabolic process;cellular macromolecule metabolic p
actin cytoskeleton organization;actin filament capping;actin filament-based process;actin modification;barbed-end actin filament capping;biological regulation;cell cycle phase;cell cycle process;cell division;cellular compone
anatomical structure morphogenesis;biological regulation;catabolic process;cell part morphogenesis;cell projection morphogenesis;cell projection organization;cellular catabolic process;cellular component morphogenesis;ce
cellular component assembly;cellular component assembly at cellular level;cellular component biogenesis;cellular component organization;cellular component organization at cellular level;cellular component organization or b
biosynthetic process;cellular biosynthetic process;cellular lipid metabolic process;cellular metabolic process;cellular process;dephosphorylation;glycerolipid biosynthetic process;glycerolipid metabolic process;glycerophosph
anatomical structure formation involved in morphogenesis;angiogenesis;biological regulation;biosynthetic process;cell differentiation;cellular biosynthetic process;cellular chemical homeostasis;cellular component assembly;c
acidic amino acid transport;alcohol biosynthetic process;alcohol metabolic process;amine transport;amino acid transport;anion transport;aspartate transport;ATP biosynthetic process;ATP metabolic process;biosynthetic pro
5S class rRNA transcription from RNA polymerase III type 1 promoter;biological regulation;biosynthetic process;cellular biosynthetic process;cellular component organization;cellular component organization at cellular level;c
biological regulation;cellular component organization;cellular component organization at cellular level;cellular component organization or biogenesis;cellular component organization or biogenesis at cellular level;cellular meml
biological regulation;cellular process;cellular response to stimulus;cytoplasmic sequestering of NF-kappaB;cytoplasmic sequestering of protein;cytoplasmic sequestering of transcription factor;establishment of localization;es
biological regulation;catabolic process;cell surface receptor linked signaling pathway;cellular catabolic process;cellular macromolecule catabolic process;cellular macromolecule metabolic process;cellular metabolic process;
biological regulation;catabolic process;cellular catabolic process;cellular macromolecule catabolic process;cellular macromolecule metabolic process;cellular metabolic process;cellular process;cellular protein metabolic proc
cellular macromolecule metabolic process;cellular metabolic process;cellular nitrogen compound metabolic process;cellular process;macromolecule metabolic process;metabolic process;mRNA metabolic process;mRNA pro
biosynthetic process;catabolic process;cellular biosynthetic process;cellular catabolic process;cellular component disassembly;cellular component disassembly at cellular level;cellular component organization;cellular compo
biological regulation;cellular macromolecule metabolic process;cellular metabolic process;cellular process;cellular protein metabolic process;demethylation;macromolecule metabolic process;macromolecule modification;met
biological regulation;cell cycle phase;cell cycle process;cell division;cellular component movement;cellular component organization;cellular component organization at cellular level;cellular component organization or biogenes
biological regulation;cellular macromolecule metabolic process;cellular metabolic process;cellular process;cellular protein metabolic process;cellular response to stimulus;establishment of localization;establishment of localiza
establishment of localization;establishment of localization in cell;establishment of protein localization;establishment of RNA localization;intracellular protein transport;intracellular transport;nuclear export;nuclear transport;nuc
anatomical structure homeostasis;biological regulation;biosynthetic process;box H/ACA snoRNA 3'-end processing;box H/ACA snoRNA metabolic process;box H/ACA snoRNA processing;cell proliferation;cellular biosynthet
cellular macromolecule metabolic process;cellular metabolic process;cellular nitrogen compound metabolic process;cellular process;gene expression;macromolecule metabolic process;metabolic process;mRNA metabolic p
biological regulation;cell differentiation;cellular developmental process;cellular macromolecule metabolic process;cellular metabolic process;cellular process;cellular protein metabolic process;cellular response to stimulus;cell
cellular macromolecule metabolic process;cellular metabolic process;cellular process;cellular protein metabolic process;cellular response to heat;cellular response to stimulus;cellular response to stress;macromolecule metab
biological adhesion;biological regulation;blood coagulation;calcium ion homeostasis;calcium-mediated signaling;cardiac muscle contraction;cation homeostasis;cation transport;cell adhesion;cell migration;cell motility;cellula
amine biosynthetic process;amine metabolic process;anatomical structure development;asparagine biosynthetic process;asparagine metabolic process;aspartate family amino acid biosynthetic process;aspartate family amin
apoptosis;B cell activation;B cell activation involved in immune response;B cell differentiation;biological regulation;carbohydrate homeostasis;cell activation;cell activation involved in immune response;cell death;cell differenti
anatomical structure development;anatomical structure morphogenesis;biological regulation;biosynthetic process;cell part morphogenesis;cell projection morphogenesis;cell projection organization;cell surface receptor linked
anatomical structure development;anatomical structure homeostasis;base-excision repair;biological regulation;biosynthetic process;cell cycle;cell cycle process;cell differentiation;cell proliferation;cellular biosynthetic process
biological regulation;biosynthetic process;cellular biosynthetic process;cellular component organization;cellular component organization at cellular level;cellular component organization or biogenesis;cellular component orga
activation of protein kinase activity;activation of protein kinase C activity by G-protein coupled receptor protein signaling pathway;biological regulation;blood coagulation;cell activation;cell surface receptor linked signaling pa
biosynthetic process;catabolic process;cellular biosynthetic process;cellular catabolic process;cellular component disassembly;cellular component disassembly at cellular level;cellular component organization;cellular compo
alcohol catabolic process;alcohol metabolic process;amine catabolic process;amine metabolic process;anatomical structure development;benzene-containing compound metabolic process;biological regulation;biosynthetic
2'-deoxyribonucleotide biosynthetic process;2'-deoxyribonucleotide metabolic process;benzene-containing compound metabolic process;biosynthetic process;carboxylic acid metabolic process;CDP biosynthetic process;C
acetyl-CoA catabolic process;acetyl-CoA metabolic process;amine catabolic process;amine metabolic process;aspartate family amino acid catabolic process;aspartate family amino acid metabolic process;carboxylic acid ca
biosynthetic process;cellular biosynthetic process;cellular macromolecule biosynthetic process;cellular macromolecule metabolic process;cellular metabolic process;cellular process;cellular protein metabolic process;cotrans
anion transport;biological regulation;establishment of localization;ion transport;negative regulation of biological process;negative regulation of cellular component organization;negative regulation of cellular process;negative r
2-oxoglutarate metabolic process;acetyl-CoA catabolic process;acetyl-CoA metabolic process;carbohydrate metabolic process;carboxylic acid metabolic process;catabolic process;cellular aldehyde metabolic process;cellul
cellular metabolic process;cellular process;electron transport chain;generation of precursor metabolites and energy;metabolic process;mitochondrial electron transport, NADH to ubiquinone;oxidation-reduction process;respi
biological regulation;cell surface receptor linked signaling pathway;cellular component movement;cellular process;cellular response to stimulus;enzyme linked receptor protein signaling pathway;intracellular signal transductio
actin cytoskeleton organization;actin filament organization;actin filament polymerization;actin filament-based process;actin nucleation;actin polymerization or depolymerization;Arp2/3 complex-mediated actin nucleation;axon
anatomical structure formation involved in morphogenesis;anatomical structure morphogenesis;axonogenesis;biological regulation;cell cycle;cell cycle process;cell part morphogenesis;cell projection assembly;cell projection
biosynthetic process;cellular biosynthetic process;cellular component assembly;cellular component assembly at cellular level;cellular component organization;cellular component organization at cellular level;cellular compone
anatomical structure morphogenesis;antigen processing and presentation;axonogenesis;biological regulation;cell differentiation;cell part morphogenesis;cell projection morphogenesis;cell projection organization;cellular comp
biological regulation;cell surface receptor linked signaling pathway;cellular component assembly;cellular component assembly at cellular level;cellular component organization;cellular component organization at cellular level;c
axon target recognition;biological adhesion;biological regulation;cell adhesion;cell communication;cell-cell adhesion;cell-cell signaling;cellular component organization;cellular component organization at cellular level;cellular c
alcohol metabolic process;biological regulation;biosynthetic process;cellular biosynthetic process;cellular macromolecule biosynthetic process;cellular macromolecule metabolic process;cellular metabolic process;cellular nitr
biosynthetic process;catabolic process;cellular biosynthetic process;cellular catabolic process;cellular component disassembly;cellular component disassembly at cellular level;cellular component organization;cellular compo
biological regulation;biosynthetic process;catabolic process;cellular biosynthetic process;cellular catabolic process;cellular component biogenesis;cellular component biogenesis at cellular level;cellular component disassem
biosynthetic process;catabolic process;cellular biosynthetic process;cellular catabolic process;cellular component disassembly;cellular component disassembly at cellular level;cellular component organization;cellular compo
biological regulation;catabolic process;cell cycle;cell cycle process;cellular catabolic process;cellular component organization;cellular component organization or biogenesis;cellular macromolecule catabolic process;cellular r
axon guidance;biological regulation;biosynthetic process;cell cycle;cell cycle checkpoint;cell surface receptor linked signaling pathway;cellular biosynthetic process;cellular macromolecule biosynthetic process;cellular macro
cellular macromolecule metabolic process;cellular metabolic process;cellular process;cellular protein metabolic process;chaperone-mediated protein folding;macromolecule metabolic process;macromolecule modification;me
amine metabolic process;carboxylic acid metabolic process;cellular amine metabolic process;cellular amino acid metabolic process;cellular ketone metabolic process;cellular metabolic process;cellular nitrogen compound m
activation of immune response;activation of innate immune response;anatomical structure development;biological regulation;calcineurin-NFAT signaling pathway;calcium ion transport;calcium-mediated signaling;carbohydrat
activation of immune response;adrenergic receptor signaling pathway;aging;anatomical structure development;antigen receptor-mediated signaling pathway;behavior;biological regulation;cAMP catabolic process;cAMP meta
activation of MAPK activity;alcohol metabolic process;autophagy;biological regulation;biosynthetic process;carbohydrate homeostasis;carbohydrate metabolic process;carboxylic acid biosynthetic process;carboxylic acid me
cellular macromolecule metabolic process;cellular metabolic process;cellular nitrogen compound metabolic process;cellular process;cellular protein metabolic process;macromolecule metabolic process;macromolecule modi
biological regulation;biosynthetic process;cell communication;cell surface receptor linked signaling pathway;cellular biosynthetic process;cellular macromolecule biosynthetic process;cellular macromolecule metabolic proces
activation of immune response;activation of innate immune response;anaphase-promoting complex-dependent proteasomal ubiquitin-dependent protein catabolic process;anatomical structure morphogenesis;biological regu
biological regulation;cellular macromolecule metabolic process;cellular metabolic process;cellular nitrogen compound metabolic process;cellular process;macromolecule metabolic process;metabolic process;ncRNA metabo
cell cycle process;cell differentiation;cellular component biogenesis;cellular component biogenesis at cellular level;cellular component organization or biogenesis;cellular component organization or biogenesis at cellular level;
biological regulation;biosynthetic process;cellular biosynthetic process;cellular macromolecule biosynthetic process;cellular macromolecule metabolic process;cellular metabolic process;cellular process;cellular protein metab
cell differentiation;cellular developmental process;cellular macromolecule metabolic process;cellular metabolic process;cellular process;cellular protein metabolic process;developmental process;gamete generation;macromo
actin filament capping;barbed-end actin filament capping;biological regulation;cell communication;cell projection organization;cellular component organization;cellular component organization at cellular level;cellular compone
biological regulation;biosynthetic process;cellular biosynthetic process;cellular macromolecule biosynthetic process;cellular macromolecule metabolic process;cellular metabolic process;cellular nitrogen compound metabolic
anatomical structure development;biological regulation;biosynthetic process;cell cycle phase;cell cycle process;cellular biosynthetic process;cellular component organization;cellular component organization at cellular level;c
biological regulation;biosynthetic process;cellular biosynthetic process;cellular macromolecule biosynthetic process;cellular macromolecule metabolic process;cellular metabolic process;cellular nitrogen compound metabolic
cell death;cellular component assembly;cellular component organization;cellular component organization or biogenesis;cellular process;death;macromolecular complex assembly;macromolecular complex subunit organizatio
ATP-dependent chromatin remodeling;biological regulation;biosynthetic process;cellular biosynthetic process;cellular component disassembly;cellular component disassembly at cellular level;cellular component organization
cellular component assembly;cellular component assembly at cellular level;cellular component organization;cellular component organization at cellular level;cellular component organization or biogenesis;cellular component c
biological regulation;positive regulation of biological process;positive regulation of biosynthetic process;positive regulation of cellular biosynthetic process;positive regulation of cellular metabolic process;positive regulation of
biosynthetic process;cellular biosynthetic process;cellular component organization;cellular component organization at cellular level;cellular component organization or biogenesis;cellular component organization or biogenesis

anatomical structure development;anatomical structure morphogenesis;biological regulation;blood vessel development;branching morphogenesis of a tube;cardiac septum morphogenesis;developmental process;hemopoieti
biological regulation;cap-independent translational initiation;cell differentiation;cellular developmental process;cellular macromolecule metabolic process;cellular metabolic process;cellular nitrogen compound metabolic proce
biological regulation;biosynthetic process;cellular biosynthetic process;cellular macromolecule biosynthetic process;cellular macromolecule metabolic process;cellular metabolic process;cellular nitrogen compound metabolic
biological regulation;cell cycle phase;cell cycle process;cellular component organization;cellular component organization at cellular level;cellular component organization or biogenesis;cellular component organization or bioge
biosynthetic process;metabolic process
biological regulation;cellular macromolecule metabolic process;cellular metabolic process;cellular nitrogen compound metabolic process;cellular process;cellular protein metabolic process;cellular response to chemical stimu
apoptosis;biological regulation;cell cycle;cell death;cell proliferation;cell surface receptor linked signaling pathway;cellular process;cellular response to chemical stimulus;cellular response to endogenous stimulus;cellular resp
activation of immune response;activation of innate immune response;anaphase-promoting complex-dependent proteasomal ubiquitin-dependent protein catabolic process;antigen processing and presentation;antigen proces
cellular macromolecule metabolic process;cellular metabolic process;cellular nitrogen compound metabolic process;cellular process;establishment of localization;establishment of localization in cell;establishment of protein lo
anatomical structure development;cell differentiation;cellular developmental process;cellular process;developmental process;nervous system development;system development
anterograde axon cargo transport;anterograde synaptic vesicle transport;antigen processing and presentation;antigen processing and presentation of exogenous antigen;antigen processing and presentation of lipid antigen v
biological regulation;biosynthetic process;cellular biosynthetic process;cellular macromolecule biosynthetic process;cellular macromolecule metabolic process;cellular metabolic process;cellular nitrogen compound metabolic
antigen processing and presentation;antigen processing and presentation of exogenous antigen;antigen processing and presentation of exogenous peptide antigen;antigen processing and presentation of exogenous peptide
biological regulation;cell migration;cell motility;cell proliferation;cellular component movement;cellular component organization;cellular component organization or biogenesis;cellular process;locomotion;macromolecule metab
biological regulation;cell differentiation;cellular component organization;cellular component organization at cellular level;cellular component organization or biogenesis;cellular component organization or biogenesis at cellular
cellular component assembly;cellular component assembly at cellular level;cellular component organization;cellular component organization at cellular level;cellular component organization or biogenesis;cellular component c
biological regulation;cellular component assembly;cellular component assembly at cellular level;cellular component organization;cellular component organization at cellular level;cellular component organization or biogenesis;
acetyl-CoA catabolic process;acetyl-CoA metabolic process;carbohydrate metabolic process;catabolic process;cellular carbohydrate metabolic process;cellular catabolic process;cellular metabolic process;cellular process;c
biological regulation;cellular component maintenance;cellular component maintenance at cellular level;cellular component organization;cellular component organization at cellular level;cellular component organization or bioge
biological regulation;biosynthetic process;cellular biosynthetic process;cellular component assembly;cellular component organization;cellular component organization at cellular level;cellular component organization or bioger
biological regulation;cellular component disassembly;cellular component disassembly at cellular level;cellular component organization;cellular component organization at cellular level;cellular component organization or bioge
anatomical structure homeostasis;biological adhesion;biological regulation;cell adhesion;cell surface receptor linked signaling pathway;cell-cell adhesion;cellular component movement;cellular process;cellular response to ch
biological regulation;blood coagulation;cell activation;cell differentiation;cellular developmental process;cellular lipid metabolic process;cellular metabolic process;cellular process;cellular process involved in reproduction;cellu
aging;alcohol biosynthetic process;alcohol metabolic process;anatomical structure development;anatomical structure morphogenesis;biosynthetic process;carbohydrate biosynthetic process;carbohydrate metabolic process
alcohol biosynthetic process;alcohol metabolic process;biosynthetic process;cellular biosynthetic process;cellular lipid metabolic process;cellular metabolic process;cellular process;cholesterol biosynthetic process;cholester
aging;biological regulation;biosynthetic process;cell communication;cell-cell signaling;cellular biosynthetic process;cellular metabolic process;cellular nitrogen compound biosynthetic process;cellular nitrogen compound met
activation of adenylate cyclase activity;activation of phospholipase C activity;anatomical structure formation involved in morphogenesis;angiogenesis;biological adhesion;biological regulation;blood coagulation;calcium ion ho
actin cytoskeleton organization;actin cytoskeleton reorganization;actin filament-based process;activation of signaling protein activity involved in unfolded protein response;biological regulation;blood coagulation;cell activation
biological regulation;cellular component organization;cellular component organization at cellular level;cellular component organization or biogenesis;cellular component organization or biogenesis at cellular level;cellular macro
anatomical structure development;anatomical structure morphogenesis;axon guidance;biological adhesion;biological regulation;biosynthetic process;blood vessel development;cell adhesion;cell migration;cell motility;cellular
anatomical structure development;ATP biosynthetic process;ATP metabolic process;ATP synthesis coupled proton transport;biosynthetic process;cation transport;cellular biosynthetic process;cellular metabolic process;cellu
activation of immune response;activation of innate immune response;anaphase-promoting complex-dependent proteasomal ubiquitin-dependent protein catabolic process;antigen processing and presentation;antigen proces
cell differentiation;cellular developmental process;cellular macromolecule metabolic process;cellular metabolic process;cellular nitrogen compound metabolic process;cellular process;developmental process;epithelial cell diff
actin filament capping;barbed-end actin filament capping;biological regulation;cell projection assembly;cell projection organization;cellular component assembly;cellular component assembly at cellular level;cellular compone
carbohydrate metabolic process;cellular carbohydrate metabolic process;cellular macromolecule metabolic process;cellular metabolic process;cellular process;cellular protein metabolic process;co-translational protein modif
alcohol metabolic process;biosynthetic process;cholesterol biosynthetic process;cholesterol metabolic process;lipid biosynthetic process;lipid metabolic process;metabolic process;primary metabolic process;small molecule
biosynthetic process;cellular biosynthetic process;cellular lipid metabolic process;cellular metabolic process;cellular process;chordate embryonic development;developmental process;embryo development;embryo developm
acylglycerol metabolic process;alcohol metabolic process;amine metabolic process;amine transport;amino acid transmembrane transport;amino acid transport;behavior;betaine metabolic process;betaine transport;biological
biological regulation;cell surface receptor linked signaling pathway;cellular macromolecule metabolic process;cellular metabolic process;cellular process;cellular protein metabolic process;cellular response to stimulus;detecti
alcohol catabolic process;alcohol metabolic process;alditol metabolic process;carbohydrate catabolic process;carbohydrate metabolic process;catabolic process;cellular carbohydrate catabolic process;cellular carbohydrate
biological regulation;biosynthetic process;carbohydrate metabolic process;carbohydrate transport;cell cycle;cell cycle process;cell surface receptor linked signaling pathway;cellular biosynthetic process;cellular component a
amine biosynthetic process;amine metabolic process;apoptosis;biological regulation;biosynthetic process;carboxylic acid biosynthetic process;carboxylic acid metabolic process;cell death;cellular amine metabolic process;c
biological regulation;biosynthetic process;cellular biosynthetic process;cellular macromolecule biosynthetic process;cellular macromolecule metabolic process;cellular metabolic process;cellular nitrogen compound metabolic
biological regulation;biosynthetic process;cellular biosynthetic process;cellular macromolecule biosynthetic process;cellular macromolecule metabolic process;cellular metabolic process;cellular nitrogen compound metabolic
alcohol biosynthetic process;alcohol metabolic process;alpha-ketoglutarate transport;anion transport;biosynthetic process;carbohydrate biosynthetic process;carbohydrate metabolic process;carboxylic acid transport;cellula
biological adhesion;biological regulation;cell adhesion;cellular component organization;cellular component organization or biogenesis;cellular defense response;cellular membrane organization;cellular process;cellular respons
cellular macromolecule metabolic process;cellular metabolic process;cellular process;cellular protein metabolic process;establishment of localization;establishment of localization in cell;establishment of protein localization;es
biological regulation;cellular process;establishment of localization;establishment of localization in cell;establishment of protein localization;Golgi vesicle transport;intracellular transport;positive regulation of biological process;
alternative nuclear mRNA splicing, via spliceosome;biological regulation;biosynthetic process;cellular biosynthetic process;cellular component assembly;cellular component assembly at cellular level;cellular component orgar
actin cytoskeleton organization;actin filament bundle assembly;actin filament organization;actin filament-based process;anatomical structure morphogenesis;biological regulation;catabolic process;cell cycle arrest;cell cycle p
carbohydrate metabolic process;cellular carbohydrate metabolic process;cellular macromolecule metabolic process;cellular metabolic process;cellular process;cellular protein metabolic process;glycosylation;macromolecule
biological regulation;cellular macromolecule metabolic process;cellular metabolic process;cellular nitrogen compound metabolic process;cellular process;cellular protein metabolic process;cellular response to stimulus;cellula
biological regulation;cell cycle;cell cycle phase;cell cycle process;cell division;cell proliferation;cellular component organization;cellular component organization at cellular level;cellular component organization or biogenesis;c
alcohol metabolic process;anatomical structure development;biological regulation;biosynthetic process;blood vessel development;cell surface receptor linked signaling pathway;cellular process;cellular response to stimulus;c
activation of immune response;activation of innate immune response;anaphase-promoting complex-dependent proteasomal ubiquitin-dependent protein catabolic process;antigen processing and presentation;antigen proces
alcohol catabolic process;alcohol metabolic process;biological regulation;carbohydrate catabolic process;carbohydrate homeostasis;carbohydrate metabolic process;carbohydrate phosphorylation;catabolic process;cellular
biological regulation;cellular component organization;cellular component organization or biogenesis;cellular localization;cellular macromolecule localization;cellular membrane organization;cellular process;cellular protein local
biological regulation;blood coagulation;cellular component assembly;cellular component assembly at cellular level;cellular component organization;cellular component organization at cellular level;cellular component organiza
axon guidance;biological regulation;cell cycle;cell cycle process;cell division;cellular component organization;cellular component organization at cellular level;cellular component organization or biogenesis;cellular component
biological regulation;biosynthetic process;cell differentiation;cellular biosynthetic process;cellular developmental process;cellular macromolecule biosynthetic process;cellular macromolecule metabolic process;cellular metab
cellular component assembly;cellular component assembly at cellular level;cellular component organization;cellular component organization at cellular level;cellular component organization or biogenesis;cellular component c
carbohydrate metabolic process;catabolic process;cellular carbohydrate metabolic process;cellular catabolic process;cellular macromolecule catabolic process;cellular macromolecule metabolic process;cellular metabolic pr
cellular component assembly;cellular component assembly at cellular level;cellular component organization;cellular component organization at cellular level;cellular component organization or biogenesis;cellular component c
catabolic process;cell cycle;cellular catabolic process;cellular macromolecule catabolic process;cellular macromolecule metabolic process;cellular metabolic process;cellular process;cellular protein metabolic process;macrom
cellular component assembly;cellular component assembly at cellular level;cellular component organization;cellular component organization at cellular level;cellular component organization or biogenesis;cellular component c
amine biosynthetic process;amine metabolic process;biosynthetic process;carboxylic acid biosynthetic process;carboxylic acid metabolic process;cellular amine metabolic process;cellular amino acid biosynthetic process;ce
apoptosis;biological regulation;cell death;cellular process;death;negative regulation of apoptosis;negative regulation of biological process;negative regulation of cell death;negative regulation of cellular process;negative regul
anatomical structure development;cellular metabolic process;cellular process;cellular response to chemical stimulus;cellular response to endogenous stimulus;cellular response to glucagon stimulus;cellular response to horm
cellular process;establishment of localization;establishment of localization in cell;establishment of protein localization;Golgi vesicle transport;intracellular protein transport;intracellular transport;intra-Golgi vesicle-mediated tra
biological regulation;cellular macromolecule metabolic process;cellular metabolic process;cellular process;cellular protein metabolic process;chaperone-mediated protein folding;macromolecule metabolic process;metabolic
biological regulation;catabolic process;cellular catabolic process;cellular macromolecule catabolic process;cellular macromolecule metabolic process;cellular metabolic process;cellular process;cellular protein metabolic proc
adenine transport;cellular process;establishment of localization;nitrogen compound transport;nucleobase transport;nucleobase-containing compound transport;purine base transport;purine-containing compound transmemb
activation of protein kinase activity;biological regulation;cell cycle arrest;cell cycle process;cell surface receptor linked signaling pathway;cellular component assembly;cellular component organization;cellular component orga
anatomical structure development;biological regulation;biomineral tissue development;bone marrow development;bone mineralization;cell chemotaxis;cell cycle process;cell migration;cell motility;cell proliferation;cellular com
anatomical structure formation involved in morphogenesis;anatomical structure morphogenesis;angiogenesis;anion transport;apoptosis;biological regulation;branching morphogenesis of a tube;cation homeostasis;cell death;
biological regulation;biosynthetic process;cell activation;cell proliferation;cellular biosynthetic process;cellular component assembly;cellular component assembly at cellular level;cellular component organization;cellular comp
biological regulation;cell cycle arrest;cell cycle process;cellular component assembly;cellular component assembly at cellular level;cellular component organization;cellular component organization at cellular level;cellular com
biological regulation;cell cycle;cell division;cell migration;cell motility;cellular component movement;cellular component organization;cellular component organization at cellular level;cellular component organization or biogene
anatomical structure morphogenesis;axonogenesis;biological regulation;cell part morphogenesis;cell projection morphogenesis;cell projection organization;cellular component assembly;cellular component assembly at cellula
amine biosynthetic process;amine catabolic process;amine metabolic process;behavior;behavioral interaction between organisms;biological regulation;biosynthetic process;carboxylic acid biosynthetic process;carboxylic aci
biological regulation;blood coagulation;blood coagulation, intrinsic pathway;cell activation;cell differentiation;cellular component disassembly;cellular component disassembly at cellular level;cellular component organization;c
cellular metabolic process;cellular process;dephosphorylation;metabolic process;phosphate-containing compound metabolic process;phosphorus metabolic process
acute inflammatory response;acute inflammatory response to antigenic stimulus;adhesion to host;adhesion to other organism involved in symbiotic interaction;aging;anatomical structure development;antigen processing and
cellular component organization;cellular component organization or biogenesis;cellular membrane organization;cellular process;clathrin-mediated endocytosis;endocytosis;establishment of localization;establishment of localiz
biological regulation;biosynthetic process;body fluid secretion;cellular aromatic compound metabolic process;cellular biosynthetic process;cellular metabolic process;cellular nitrogen compound biosynthetic process;cellular
activation of phospholipase C activity;activation of protein kinase A activity;activation of protein kinase activity;biological regulation;blood coagulation;cell surface receptor linked signaling pathway;cellular metabolic process;
acetyl-CoA catabolic process;acetyl-CoA metabolic process;aerobic respiration;carboxylic acid metabolic process;catabolic process;cellular catabolic process;cellular ketone metabolic process;cellular metabolic process;cel
androgen receptor signaling pathway;biological regulation;cell motility;cellular component movement;cellular localization;cellular macromolecule localization;cellular macromolecule metabolic process;cellular metabolic proce
cellular macromolecule metabolic process;cellular metabolic process;cellular nitrogen compound metabolic process;cellular process;gene expression;macromolecule metabolic process;metabolic process;mRNA metabolic pr
biological regulation;biosynthetic process;cellular biosynthetic process;cellular macromolecule biosynthetic process;cellular macromolecule metabolic process;cellular metabolic process;cellular nitrogen compound metabolic
activation of immune response;activation of innate immune response;anaphase-promoting complex-dependent proteasomal ubiquitin-dependent protein catabolic process;anatomical structure development;antigen processi
biological regulation;regulation of biological process;regulation of cellular component organization;regulation of cellular metabolic process;regulation of cellular process;regulation of chromosome organization;regulation of DN
biological regulation;biosynthetic process;cellular biosynthetic process;cellular macromolecule biosynthetic process;cellular macromolecule metabolic process;cellular metabolic process;cellular process;cellular protein metab
biological regulation;circulatory system process;multicellular organismal process;positive regulation of blood pressure;positive regulation of systemic arterial blood pressure;regulation of anatomical structure size;regulation of
activation of immune response;activation of innate immune response;activation of MAPK activity;alcohol metabolic process;anaphase-promoting complex-dependent proteasomal ubiquitin-dependent protein catabolic proce
anatomical structure development;anatomical structure morphogenesis;anterior/posterior pattern specification;biological regulation;cell differentiation;cell differentiation involved in kidney development;cell morphogenesis;cel
biological regulation;biosynthetic process;cell cycle arrest;cell cycle process;cell proliferation;cell surface receptor linked signaling pathway;cellular biosynthetic process;cellular macromolecule biosynthetic process;cellular m
biological regulation;cellular process;cellular response to stimulus;intracellular signal transduction;positive regulation of biological process;positive regulation of catalytic activity;positive regulation of cell communication;positi
biological regulation;biosynthetic process;carbohydrate metabolic process;carbohydrate transport;cell cycle;cell cycle process;cell surface receptor linked signaling pathway;cellular biosynthetic process;cellular component c
antigen processing and presentation;antigen processing and presentation of exogenous antigen;antigen processing and presentation of exogenous peptide antigen;antigen processing and presentation of exogenous peptide
activation of immune response;activation of innate immune response;activation of phospholipase C activity;apoptosis;apoptosis in response to endoplasmic reticulum stress;biological regulation;blood coagulation;calcium ion
cellular process;ER to Golgi vesicle-mediated transport;establishment of localization;establishment of localization in cell;establishment of protein localization;Golgi vesicle transport;intracellular protein transport;intracellular tra
biological regulation;cell activation;cell activation involved in immune response;cell killing;cellular process;establishment of localization;establishment of localization in cell;establishment of protein localization;exocytosis;immu
carboxylic acid catabolic process;carboxylic acid metabolic process;catabolic process;cellular catabolic process;cellular component assembly;cellular component organization;cellular component organization or biogenesis;c
actin cytoskeleton organization;actin filament-based process;biological regulation;cellular component organization;cellular component organization at cellular level;cellular component organization or biogenesis;cellular compo
anatomical structure development;biological regulation;cell development;cellular component organization;cellular component organization at cellular level;cellular component organization or biogenesis;cellular component org
cellular component assembly;cellular component organization;cellular component organization at cellular level;cellular component organization or biogenesis;cellular component organization or biogenesis at cellular level;cellu
anatomical structure development;anatomical structure morphogenesis;appendage morphogenesis;biological regulation;brain development;cell cycle;cell cycle process;cell differentiation;cellular component organization;cellu
biological regulation;cellular macromolecule metabolic process;cellular metabolic process;cellular nitrogen compound metabolic process;cellular process;macromolecule metabolic process;metabolic process;mRNA metaboli
adult behavior;adult locomotory behavior;anatomical structure homeostasis;behavior;biological regulation;cell cycle checkpoint;cell differentiation;cell differentiation in spinal cord;cell surface receptor linked signaling pathway
biological regulation;cell cycle;cell cycle phase;cell cycle process;cell division;cellular component organization;cellular component organization at cellular level;cellular component organization or biogenesis;cellular componer
biological adhesion;cell adhesion;cellular process
biosynthetic process;cellular biosynthetic process;cellular macromolecule biosynthetic process;cellular macromolecule metabolic process;cellular metabolic process;cellular process;cellular protein metabolic process;macron
apoptosis;biological regulation;cell communication;cell death;cell migration;cell motility;cell surface receptor linked signaling pathway;cell-cell signaling;cellular component movement;cellular component organization;cellular
biological regulation;cell redox homeostasis;cellular homeostasis;cellular macromolecule metabolic process;cellular metabolic process;cellular process;cellular protein metabolic process;cellular response to stimulus;cellular r
biological regulation;cell differentiation;cellular component biogenesis;cellular component biogenesis at cellular level;cellular component organization or biogenesis;cellular component organization or biogenesis at cellular lev
biological regulation;cellular process;cellular response to stimulus;cellular response to stress;negative regulation of biological process;negative regulation of cellular metabolic process;negative regulation of cellular process;ne
amine metabolic process;amino acid activation;carboxylic acid metabolic process;cellular amine metabolic process;cellular amino acid metabolic process;cellular ketone metabolic process;cellular macromolecule metabolic p
cellular component assembly;cellular component assembly at cellular level;cellular component organization;cellular component organization at cellular level;cellular component organization or biogenesis;cellular component c
biological regulation;cellular component organization;cellular component organization at cellular level;cellular component organization or biogenesis;cellular component organization or biogenesis at cellular level;cellular meml
anatomical structure formation involved in morphogenesis;cellular macromolecule metabolic process;cellular metabolic process;cellular process;cellular protein metabolic process;developmental process;macromolecule meta
aging;anatomical structure formation involved in morphogenesis;anatomical structure morphogenesis;angiogenesis;biological adhesion;biological regulation;biosynthetic process;cell adhesion;cell migration;cell morphogenes
biological regulation;cell communication;cellular process;cellular response to chemical stimulus;cellular response to external stimulus;cellular response to extracellular stimulus;cellular response to glucose starvation;cellular r
anatomical structure morphogenesis;biological regulation;developmental process;establishment of localization;establishment of RNA localization;gene expression;macromolecule metabolic process;metabolic process;mRNA
anatomical structure morphogenesis;biological regulation;biosynthetic process;cellular biosynthetic process;cellular macromolecule biosynthetic process;cellular macromolecule metabolic process;cellular metabolic process;c

biological regulation;cell cycle arrest;cell cycle process;cellular macromolecule metabolic process;cellular metabolic process;cellular process;cellular protein metabolic process;dephosphorylation;macromolecule metabolic pr
antigen processing and presentation;antigen processing and presentation of exogenous antigen;antigen processing and presentation of exogenous peptide antigen;antigen processing and presentation of exogenous peptide
assembly of spliceosomal tri-snRNP;cellular component assembly;cellular component assembly at cellular level;cellular component organization;cellular component organization at cellular level;cellular component organizatio
amine metabolic process;biological regulation;carboxylic acid metabolic process;cellular amine metabolic process;cellular amino acid metabolic process;cellular ketone metabolic process;cellular macromolecule metabolic pr
cellular component organization;cellular component organization at cellular level;cellular component organization or biogenesis;cellular component organization or biogenesis at cellular level;cellular membrane organization;ce
antigen processing and presentation;antigen processing and presentation of exogenous antigen;antigen processing and presentation of exogenous peptide antigen;antigen processing and presentation of exogenous peptide
activation of immune response;activation of innate immune response;activation of MAPK activity;activation of MAPKK activity;activation of protein kinase activity;aging;anaphase-promoting complex-dependent proteasomal
cellular metabolic process;cellular process;electron transport chain;generation of precursor metabolites and energy;metabolic process;oxidation-reduction process;respiratory electron transport chain;small molecule metaboli
biological regulation;establishment of localization;lipid transport;modulation of growth of symbiont involved in interaction with host;organic substance transport;positive regulation of biological process;positive regulation of gr
amine metabolic process;AMP biosynthetic process;AMP metabolic process;aspartate family amino acid metabolic process;aspartate metabolic process;biosynthetic process;carboxylic acid metabolic process;cellular amine
acrosome assembly;actin cytoskeleton organization;actin filament-based process;adult behavior;adult locomotory behavior;alcohol metabolic process;ameboidal cell migration;amine metabolic process;anatomical structure
biosynthetic process;catabolic process;cellular biosynthetic process;cellular catabolic process;cellular component disassembly;cellular component disassembly at cellular level;cellular component organization;cellular compo
amine biosynthetic process;amine metabolic process;apoptotic mitochondrial changes;biological regulation;biosynthetic process;carboxylic acid biosynthetic process;carboxylic acid metabolic process;cell redox homeostasi
biological regulation;blood coagulation;carbohydrate metabolic process;cellular carbohydrate metabolic process;cellular component organization;cellular component organization at cellular level;cellular component organizati
acetyl-CoA catabolic process;acetyl-CoA metabolic process;carbohydrate metabolic process;catabolic process;cellular catabolic process;cellular metabolic process;cellular process;coenzyme catabolic process;coenzyme m
catabolic process;cellular catabolic process;cellular metabolic process;cellular nitrogen compound catabolic process;cellular nitrogen compound metabolic process;cellular process;cofactor catabolic process;cofactor metab
antigen processing and presentation;antigen processing and presentation of exogenous antigen;antigen processing and presentation of exogenous peptide antigen;antigen processing and presentation of exogenous peptide
biosynthetic process;catabolic process;cellular biosynthetic process;cellular catabolic process;cellular component disassembly;cellular component disassembly at cellular level;cellular component organization;cellular compo
anatomical structure homeostasis;ATP-dependent chromatin remodeling;biological regulation;cell cycle;cellular component assembly;cellular component assembly at cellular level;cellular component organization;cellular com
biological regulation;calcium-mediated signaling;carboxylic acid metabolic process;catabolic process;cellular catabolic process;cellular ketone metabolic process;cellular metabolic process;cellular process;cellular response
biosynthetic process;catabolic process;cellular biosynthetic process;cellular catabolic process;cellular component disassembly;cellular component disassembly at cellular level;cellular component organization;cellular compo
activation of immune response;activation of innate immune response;adaptive immune response;adaptive immune response based on somatic recombination of immune receptors built from immunoglobulin superfamily dom
alcohol biosynthetic process;alcohol catabolic process;alcohol metabolic process;alditol catabolic process;alditol metabolic process;biosynthetic process;carbohydrate biosynthetic process;carbohydrate catabolic process;c
biosynthetic process;catabolic process;cellular biosynthetic process;cellular catabolic process;cellular component disassembly;cellular component disassembly at cellular level;cellular component organization;cellular compo
cell communication;cell-cell signaling;cellular process;immune response;immune system process;response to stimulus;signaling
biological regulation;cellular macromolecule metabolic process;cellular metabolic process;cellular process;cellular protein metabolic process;cellular response to chemical stimulus;cellular response to organic substance;cellu
biological regulation;cell cycle arrest;cell cycle process;cellular macromolecule metabolic process;cellular metabolic process;cellular process;cellular protein metabolic process;cellular response to chemical stimulus;cellular re
gamete generation;male gamete generation;multicellular organismal process;multicellular organismal reproductive process;reproductive process;spermatogenesis
biological regulation;catabolic process;cellular catabolic process;cellular macromolecule catabolic process;cellular macromolecule metabolic process;cellular metabolic process;cellular nitrogen compound metabolic process
biological regulation;cation transport;cellular macromolecule metabolic process;cellular metabolic process;cellular process;cellular response to abiotic stimulus;cellular response to acidity;cellular response to pH;cellular resp
biological regulation;biosynthetic process;cellular biosynthetic process;cellular macromolecule biosynthetic process;cellular macromolecule metabolic process;cellular metabolic process;cellular nitrogen compound metabolic
catabolic process;cellular catabolic process;cellular macromolecule catabolic process;cellular macromolecule metabolic process;cellular metabolic process;cellular nitrogen compound metabolic process;cellular process;exo
amine metabolic process;amino acid activation;asparaginyl-tRNA aminoacylation;carboxylic acid metabolic process;cellular amine metabolic process;cellular amino acid metabolic process;cellular ketone metabolic process;
biological regulation;cell junction assembly;cell junction organization;cell surface receptor linked signaling pathway;cell-cell junction assembly;cell-cell junction organization;cellular component assembly;cellular component as
actin cytoskeleton organization;actin filament organization;actin filament-based process;actin polymerization or depolymerization;activation of immune response;anatomical structure formation involved in morphogenesis;anti
cellular component organization;cellular component organization at cellular level;cellular component organization or biogenesis;cellular component organization or biogenesis at cellular level;cellular process;establishment of l
biological regulation;calcium ion homeostasis;cation homeostasis;cell differentiation;cellular developmental process;cellular process;cellular response to biotic stimulus;cellular response to stimulus;cellular response to stress
developmental process;multicellular organismal development;multicellular organismal process
cellular component assembly;cellular component assembly at cellular level;cellular component organization;cellular component organization at cellular level;cellular component organization or biogenesis;cellular component c
lipid metabolic process;metabolic process;primary metabolic process
biological regulation;cellular component assembly;cellular component organization;cellular component organization or biogenesis;cellular membrane organization;cellular process;cellular response to chemical stimulus;cellula
biological regulation;cation homeostasis;cation transport;cell differentiation;cellular developmental process;cellular process;chemical homeostasis;developmental process;erythrocyte differentiation;establishment of localizatio
biological regulation;biosynthetic process;cell surface receptor linked signaling pathway;cellular biosynthetic process;cellular macromolecule biosynthetic process;cellular macromolecule metabolic process;cellular metabolic
biosynthetic process;carbohydrate biosynthetic process;carbohydrate metabolic process;cellular biosynthetic process;cellular carbohydrate biosynthetic process;cellular carbohydrate metabolic process;cellular lipid metabol
cellular macromolecule metabolic process;cellular metabolic process;cellular nitrogen compound metabolic process;cellular process;macromolecule metabolic process;metabolic process;mRNA metabolic process;mRNA pro
apoptosis;biological regulation;biosynthetic process;cell death;cellular biosynthetic process;cellular macromolecule biosynthetic process;cellular macromolecule metabolic process;cellular metabolic process;cellular nitrogen
establishment of localization;establishment of localization in cell;establishment of protein localization;establishment of RNA localization;intracellular protein transport;intracellular transport;mRNA transport;nuclear export;nucle
activation of JUN kinase activity;activation of MAPK activity;anatomical structure morphogenesis;apoptosis;biological regulation;cell death;cell part morphogenesis;cell projection morphogenesis;cell projection organization;c
autophagic vacuole assembly;biological regulation;catabolic process;cellular catabolic process;cellular component assembly;cellular component assembly at cellular level;cellular component organization;cellular component
biological regulation;cellular component assembly;cellular component assembly at cellular level;cellular component organization;cellular component organization at cellular level;cellular component organization or biogenesis;
biological regulation;catabolic process;cellular catabolic process;cellular macromolecule catabolic process;cellular macromolecule metabolic process;cellular metabolic process;cellular nitrogen compound metabolic process
biological regulation;cellular component organization;cellular component organization at cellular level;cellular component organization or biogenesis;cellular component organization or biogenesis at cellular level;cellular proce
biosynthetic process;carboxylic acid biosynthetic process;carboxylic acid catabolic process;carboxylic acid metabolic process;catabolic process;cellular biosynthetic process;cellular catabolic process;cellular ketone metabo
anterograde axon cargo transport;anterograde synaptic vesicle transport;antigen processing and presentation;antigen processing and presentation of exogenous antigen;antigen processing and presentation of lipid antigen v
biological regulation;cellular process;cellular response to chemical stimulus;cellular response to endogenous stimulus;cellular response to growth factor stimulus;cellular response to organic substance;cellular response to sti
biological regulation;biosynthetic process;cellular biosynthetic process;cellular macromolecule biosynthetic process;cellular macromolecule metabolic process;cellular metabolic process;cellular nitrogen compound metabolic
biological regulation;biosynthetic process;cellular biosynthetic process;cellular component assembly;cellular component assembly at cellular level;cellular component organization;cellular component organization at cellular le
biological regulation;blood coagulation;cell activation;cellular process;coagulation;establishment of localization;establishment of localization in cell;exocytosis;hemostasis;multicellular organismal process;platelet activation;pla
biological regulation;catabolic process;cell proliferation;cellular catabolic process;cellular macromolecule catabolic process;cellular macromolecule metabolic process;cellular metabolic process;cellular nitrogen compound m
3'-phosphoadenosine 5'-phosphosulfate metabolic process;anatomical structure development;cellular lipid metabolic process;cellular metabolic process;cellular nitrogen compound metabolic process;cellular process;depho
biosynthetic process;catabolic process;cellular biosynthetic process;cellular catabolic process;cellular component biogenesis;cellular component biogenesis at cellular level;cellular component disassembly;cellular componen
aromatic compound catabolic process;catabolic process;cellular aromatic compound metabolic process;cellular catabolic process;cellular metabolic process;cellular process;metabolic process;response to chemical stimulus
activation of immune response;activation of innate immune response;anatomical structure development;biological regulation;cell differentiation;cellular component disassembly;cellular component disassembly at cellular leve
biological regulation;biosynthetic process;cellular biosynthetic process;cellular macromolecule biosynthetic process;cellular macromolecule metabolic process;cellular metabolic process;cellular process;cellular protein metab
biological regulation;biosynthetic process;catabolic process;cellular biosynthetic process;cellular catabolic process;cellular component assembly;cellular component assembly at cellular level;cellular component biogenesis;c
biological adhesion;biosynthetic process;catabolic process;cell adhesion;cellular biosynthetic process;cellular catabolic process;cellular component assembly;cellular component assembly at cellular level;cellular component
aldehyde catabolic process;catabolic process;cellular aldehyde metabolic process;cellular catabolic process;cellular metabolic process;cellular process;formaldehyde catabolic process;formaldehyde metabolic process;meta
amine biosynthetic process;amine metabolic process;betaine biosynthetic process;betaine metabolic process;biosynthetic process;carboxylic acid biosynthetic process;carboxylic acid catabolic process;carboxylic acid meta
beta-amyloid metabolic process;cellular macromolecule metabolic process;cellular metabolic process;cellular process;glycoprotein metabolic process;macromolecule metabolic process;metabolic process;primary metabolic
amine biosynthetic process;amine catabolic process;amine metabolic process;biological regulation;biosynthetic process;carboxylic acid biosynthetic process;carboxylic acid catabolic process;carboxylic acid metabolic proce
actin cytoskeleton organization;actin filament bundle assembly;actin filament organization;actin filament-based process;antigen processing and presentation;antigen processing and presentation of exogenous antigen;antiger
anatomical structure development;biosynthetic process;cell proliferation;cellular biosynthetic process;cellular macromolecule biosynthetic process;cellular macromolecule metabolic process;cellular metabolic process;cellular
amine metabolic process;anatomical structure development;benzene-containing compound metabolic process;carboxylic acid metabolic process;catabolic process;cell development;cellular amine metabolic process;cellular
activation of immune response;activation of innate immune response;anaphase-promoting complex-dependent proteasomal ubiquitin-dependent protein catabolic process;antigen processing and presentation;antigen proces
anatomical structure development;anatomical structure homeostasis;base-excision repair;biological regulation;biosynthetic process;catabolic process;cell cycle;cell cycle checkpoint;cell cycle phase;cell cycle process;cellula
biological regulation;biosynthetic process;catabolic process;cellular biosynthetic process;cellular catabolic process;cellular macromolecule metabolic process;cellular metabolic process;cellular nitrogen compound metabolic
biosynthetic process;catabolic process;cellular biosynthetic process;cellular catabolic process;cellular component disassembly;cellular component disassembly at cellular level;cellular component organization;cellular compo
biological regulation;cellular process;cellular response to abiotic stimulus;cellular response to osmotic stress;cellular response to stimulus;cellular response to stress;multicellular organismal process;negative regulation of cat
alcohol metabolic process;biosynthetic process;carbohydrate biosynthetic process;carbohydrate catabolic process;carbohydrate metabolic process;catabolic process;cellular biosynthetic process;cellular carbohydrate biosy
biological regulation;biosynthetic process;carbohydrate metabolic process;carbohydrate transport;cell cycle;cell cycle process;cell surface receptor linked signaling pathway;cellular biosynthetic process;cellular component c
cellular metabolic process;cellular process;electron transport chain;generation of precursor metabolites and energy;metabolic process;oxidation-reduction process;respiratory electron transport chain;small molecule metaboli
anatomical structure morphogenesis;cellular metabolic process;cellular process;developmental process;metabolic process;methylation;one-carbon metabolic process;organ morphogenesis;organ regeneration;regeneration;re
alcohol catabolic process;alcohol metabolic process;anatomical structure development;androgen biosynthetic process;androgen metabolic process;apocarotenoid metabolic process;arachidonic acid metabolic process;biolo
biosynthetic process;catabolic process;cellular biosynthetic process;cellular catabolic process;cellular component disassembly;cellular component disassembly at cellular level;cellular component organization;cellular compo
antigen processing and presentation;antigen processing and presentation of peptide antigen;antigen processing and presentation of peptide antigen via MHC class I;apoptosis;biological regulation;calcium ion homeostasis;c
biosynthetic process;catabolic process;cellular biosynthetic process;cellular catabolic process;cellular component assembly;cellular component assembly at cellular level;cellular component disassembly;cellular component c
activation of adenylate cyclase activity;activation of adenylate cyclase activity by adrenergic receptor signaling pathway;activation of adenylate cyclase activity by dopamine receptor signaling pathway;activation of adenylate
biological regulation;blood coagulation;cAMP-mediated signaling;cell activation;cell communication;cell cycle;cell division;cell surface receptor linked signaling pathway;cell-cell signaling;cellular process;cellular response to s
cellular macromolecule metabolic process;cellular metabolic process;cellular nitrogen compound metabolic process;cellular process;macromolecule metabolic process;metabolic process;mRNA metabolic process;mRNA pro
acylglycerol catabolic process;acylglycerol metabolic process;alcohol metabolic process;amine metabolic process;anatomical structure development;biosynthetic process;carbohydrate metabolic process;carbohydrate trans
biological regulation;catabolic process;cellular catabolic process;cellular localization;cellular macromolecule catabolic process;cellular macromolecule localization;cellular macromolecule metabolic process;cellular metabolic
biological regulation;catabolic process;cell communication;cell-cell signaling;cellular process;cellular response to stimulus;inositol phosphate metabolic process;intracellular signal transduction;lipid catabolic process;lipid me
cellular component assembly;cellular component assembly at cellular level;cellular component organization;cellular component organization at cellular level;cellular component organization or biogenesis;cellular component c
activation of immune response;activation of innate immune response;activation of MAPK activity;activation of MAPKK activity;activation of protein kinase activity;aging;anatomical structure development;axon guidance;biolog
abortive mitotic cell cycle;activation of MAPK activity;acylglycerol metabolic process;anatomical structure development;anatomical structure formation involved in morphogenesis;anatomical structure morphogenesis;axon gu
biological regulation;cell cycle;cellular macromolecule metabolic process;cellular metabolic process;cellular process;cellular protein metabolic process;cellular response to stimulus;cellular response to stress;cullin deneddyla
biological adhesion;biological regulation;cell adhesion;cell communication;cell-cell signaling;cell-matrix adhesion;cell-substrate adhesion;cellular process;cellular response to stimulus;establishment of localization;establishme
actin cytoskeleton organization;actin cytoskeleton reorganization;actin filament-based process;anatomical structure development;anatomical structure formation involved in morphogenesis;anatomical structure morphogenes
cell cycle;cell cycle process;cell division;cellular component organization;cellular component organization at cellular level;cellular component organization or biogenesis;cellular component organization or biogenesis at cellula
actin cytoskeleton organization;actin filament organization;actin filament-based process;biological regulation;cell communication;cell communication by chemical coupling;cell communication by electrical coupling;cell prolife
cellular macromolecule metabolic process;cellular metabolic process;cellular nitrogen compound metabolic process;cellular process;localization;macromolecule localization;macromolecule metabolic process;metabolic proce
biosynthetic process;catabolic process;cellular biosynthetic process;cellular catabolic process;cellular component organization;cellular component organization at cellular level;cellular component organization or biogenesis;c
actin cytoskeleton organization;actin filament organization;actin filament-based process;actin nucleation;Arp2/3 complex-mediated actin nucleation;axon guidance;biological regulation;cell surface receptor linked signaling pa
acetyl-CoA catabolic process;acetyl-CoA metabolic process;carboxylic acid metabolic process;catabolic process;cellular catabolic process;cellular ketone metabolic process;cellular metabolic process;cellular process;coenz
biological regulation;cell cycle;cell cycle process;cell division;cell proliferation;cellular component organization;cellular component organization at cellular level;cellular component organization or biogenesis;cellular componen
biological regulation;cell surface receptor linked signaling pathway;cellular process;cellular response to stimulus;G-protein coupled receptor protein signaling pathway;opioid receptor signaling pathway;regulation of biologica
acyl-CoA metabolic process;cellular metabolic process;cellular process;coenzyme metabolic process;cofactor metabolic process;metabolic process;thioester metabolic process
biological regulation;cell proliferation;cellular component biogenesis;cellular component biogenesis at cellular level;cellular component organization;cellular component organization at cellular level;cellular component organiza
cellular macromolecule metabolic process;cellular metabolic process;cellular nitrogen compound metabolic process;cellular process;macromolecule metabolic process;macromolecule modification;metabolic process;ncRNA
biological regulation;biosynthetic process;cellular biosynthetic process;cellular component assembly;cellular component assembly at cellular level;cellular component organization;cellular component organization at cellular le
antigen processing and presentation;antigen processing and presentation of exogenous antigen;antigen processing and presentation of exogenous peptide antigen;antigen processing and presentation of exogenous peptide
amine metabolic process;aminoglycan catabolic process;aminoglycan metabolic process;anatomical structure development;astrocyte cell migration;axon ensheathment;behavior;biological regulation;biosynthetic process;cal
actin cytoskeleton organization;actin filament-based process;anatomical structure formation involved in morphogenesis;biological regulation;blood coagulation;cell activation;cellular component organization;cellular compone
anatomical structure formation involved in morphogenesis;angiogenesis;axon guidance;behavior;biological adhesion;biological regulation;blood coagulation;cell adhesion;cell adhesion mediated by integrin;cell differentiation;
aging;alcohol catabolic process;alcohol metabolic process;aldehyde catabolic process;biological regulation;catabolic process;cellular aldehyde metabolic process;cellular catabolic process;cellular lipid metabolic process;cel
5-phosphoribose 1-diphosphate biosynthetic process;5-phosphoribose 1-diphosphate metabolic process;alcohol biosynthetic process;alcohol metabolic process;AMP biosynthetic process;AMP metabolic process;anatomic
biosynthetic process;catabolic process;cellular biosynthetic process;cellular catabolic process;cellular component biogenesis;cellular component biogenesis at cellular level;cellular component disassembly;cellular componen
aerobic respiration;cellular metabolic process;cellular process;cellular respiration;electron transport chain;energy derivation by oxidation of organic compounds;generation of precursor metabolites and energy;metabolic proce
aging;anatomical structure homeostasis;base-excision repair;biological regulation;biosynthetic process;cell cycle;cell redox homeostasis;cellular biosynthetic process;cellular component organization;cellular component orga
activation of immune response;activation of innate immune response;activation of MAPK activity;activation of MAPKK activity;activation of protein kinase activity;anatomical structure development;anatomical structure morph
aerobic respiration;AMP biosynthetic process;AMP metabolic process;biosynthetic process;cellular aromatic compound metabolic process;cellular biosynthetic process;cellular component assembly;cellular component orga
alcohol catabolic process;alcohol metabolic process;carbohydrate metabolic process;catabolic process;ethanol catabolic process;ethanol metabolic process;metabolic process;primary alcohol catabolic process;primary alco
cellular macromolecule metabolic process;cellular metabolic process;cellular nitrogen compound metabolic process;cellular process;macromolecule metabolic process;maturation of 5.8S rRNA;metabolic process;mRNA met
biological regulation;carboxylic acid catabolic process;carboxylic acid metabolic process;catabolic process;cell differentiation;cellular catabolic process;cellular developmental process;cellular ketone metabolic process;cellul
activation of immune response;activation of innate immune response;anatomical structure development;axon guidance;biological regulation;cell communication;cell cycle;cell surface receptor linked signaling pathway;cell-ce
2'-deoxyribonucleotide metabolic process;ADP biosynthetic process;ADP metabolic process;AMP metabolic process;anatomical structure development;biosynthetic process;brain development;cellular biosynthetic process;
actin cytoskeleton organization;actin filament depolymerization;actin filament organization;actin filament severing;actin filament-based process;actin polymerization or depolymerization;biological regulation;cellular componen

biosynthetic process;catabolic process;cellular biosynthetic process;cellular catabolic process;cellular component disassembly;cellular component disassembly at cellular level;cellular component organization;cellular compo
biosynthetic process;catabolic process;cellular biosynthetic process;cellular catabolic process;cellular component disassembly;cellular component disassembly at cellular level;cellular component organization;cellular compo
biosynthetic process;catabolic process;cellular biosynthetic process;cellular catabolic process;cellular component disassembly;cellular component disassembly at cellular level;cellular component organization;cellular compo
biological regulation;cellular macromolecule metabolic process;cellular metabolic process;cellular nitrogen compound metabolic process;cellular process;macromolecule metabolic process;metabolic process;mRNA metaboli
anion transport;cellular metabolic process;cellular process;establishment of localization;generation of precursor metabolites and energy;inorganic anion transport;ion transmembrane transport;ion transport;metabolic process
anatomical structure development;cell differentiation;cellular developmental process;cellular process;developmental process;epithelial cell differentiation;muscle organ development;muscle structure development;organ devel
anatomical structure development;anatomical structure morphogenesis;axonogenesis;cell cycle process;cell migration;cell motility;cell part morphogenesis;cell projection morphogenesis;cell projection organization;cellular co
activation of immune response;activation of innate immune response;anaphase-promoting complex-dependent proteasomal ubiquitin-dependent protein catabolic process;antigen processing and presentation;antigen proces
anatomical structure formation involved in morphogenesis;biological regulation;cell cycle phase;cell cycle process;cell division;cell projection assembly;cell projection organization;cell surface receptor linked signaling pathwa
anatomical structure development;anatomical structure morphogenesis;biological regulation;brain development;camera-type eye development;cellular chemical homeostasis;cellular component organization;cellular compone
cellular macromolecule metabolic process;cellular metabolic process;cellular nitrogen compound metabolic process;cellular process;gene expression;macromolecule metabolic process;metabolic process;mRNA metabolic p
3'-UTR-mediated mRNA stabilization;biological regulation;cellular macromolecule metabolic process;cellular metabolic process;cellular nitrogen compound metabolic process;cellular process;developmental process;gene ex
cellular component organization;cellular component organization at cellular level;cellular component organization or biogenesis;cellular component organization or biogenesis at cellular level;cellular process;cytoskeleton orga
adenine nucleotide transport;ATP transport;biological regulation;cellular process;cellular response to calcium ion;cellular response to chemical stimulus;cellular response to inorganic substance;cellular response to metal ion;c
aromatic compound biosynthetic process;biosynthetic process;carboxylic acid metabolic process;cellular aromatic compound metabolic process;cellular biosynthetic process;cellular ketone metabolic process;cellular metab
biological regulation;cellular macromolecule metabolic process;cellular metabolic process;cellular process;cellular protein metabolic process;developmental process;endosome transport;establishment of localization;establish
anatomical structure morphogenesis;biological regulation;catabolic process;cell cycle arrest;cell cycle process;cell part morphogenesis;cellular catabolic process;cellular component assembly;cellular component morphogen
anatomical structure development;biological regulation;biosynthetic process;carbohydrate metabolic process;carbohydrate transport;cell cycle;cell cycle process;cell surface receptor linked signaling pathway;cellular biosynt
actin cytoskeleton organization;actin filament-based process;actomyosin structure organization;anatomical structure development;biological regulation;cellular component organization;cellular component organization at cellu
anatomical structure morphogenesis;biological regulation;cell morphogenesis;cell morphogenesis involved in differentiation;cellular component morphogenesis;cellular component organization;cellular component organizatio
biological regulation;establishment of localization;establishment of localization in cell;establishment of protein localization;establishment of protein localization in endoplasmic reticulum membrane;establishment of protein loc
establishment of localization;establishment of localization in cell;establishment of protein localization;establishment of RNA localization;gene expression;intracellular protein transport;intracellular transport;macromolecule met
aging;biological regulation;cardiac cell differentiation;cardiac muscle cell differentiation;cell differentiation;cellular developmental process;cellular process;cellular response to calcium ion;cellular response to chemical stimulus
acetyl-CoA catabolic process;acetyl-CoA metabolic process;biosynthetic process;carboxylic acid metabolic process;catabolic process;cellular biosynthetic process;cellular catabolic process;cellular ketone metabolic proces
biosynthetic process;cellular biosynthetic process;cellular lipid metabolic process;cellular metabolic process;cellular process;ether lipid biosynthetic process;ether lipid metabolic process;glycerol ether biosynthetic process;g
biological regulation;regulation of biological process;regulation of biosynthetic process;regulation of cellular biosynthetic process;regulation of cellular macromolecule biosynthetic process;regulation of cellular metabolic proc
biological regulation;biosynthetic process;cell cycle;cellular biosynthetic process;cellular component organization;cellular component organization at cellular level;cellular component organization or biogenesis;cellular compo
actin cytoskeleton organization;actin filament organization;actin filament-based process;actin nucleation;Arp2/3 complex-mediated actin nucleation;axon guidance;biological regulation;cell surface receptor linked signaling pa
biosynthetic process;cellular biosynthetic process;cellular macromolecule biosynthetic process;cellular macromolecule metabolic process;cellular metabolic process;cellular process;cellular protein metabolic process;cotrans
biological regulation;blood coagulation;cAMP-mediated signaling;cell activation;cell communication;cell cycle;cell division;cell surface receptor linked signaling pathway;cell-cell signaling;cellular component organization;cellu
biological adhesion;cell adhesion;cell-cell adhesion;cellular process;homotypic cell-cell adhesion;platelet aggregation
anatomical structure development;behavior;biological adhesion;biological regulation;blood coagulation;cell adhesion;cell differentiation;cell migration;cell motility;cell surface receptor linked signaling pathway;cell-matrix adhe
biological regulation;cell cycle;cell cycle phase;cell cycle process;cellular component organization;cellular component organization at cellular level;cellular component organization or biogenesis;cellular component organizatio
cellular component assembly;cellular component assembly at cellular level;cellular component organization;cellular component organization at cellular level;cellular component organization or biogenesis;cellular component o
ATP biosynthetic process;ATP metabolic process;ATP synthesis coupled proton transport;biosynthetic process;cation transport;cellular biosynthetic process;cellular metabolic process;cellular nitrogen compound biosyntheti
cell differentiation;cellular developmental process;cellular process;developmental process;epithelial cell differentiation
ATP hydrolysis coupled proton transport;ATP metabolic process;biological regulation;cation homeostasis;cation transport;cell surface receptor linked signaling pathway;cellular cation homeostasis;cellular chemical homeosta
acute inflammatory response;acute-phase response;anatomical structure development;astrocyte differentiation;behavior;biological regulation;carbohydrate homeostasis;cell differentiation;cell proliferation;cell surface recepto
alcohol metabolic process;biosynthetic process;carboxylic acid metabolic process;cellular ketone metabolic process;cellular lipid metabolic process;cellular metabolic process;cellular process;cellular response to chemical st
biological regulation;cell cycle;cellular process;cellular response to stimulus;intracellular signal transduction;mitotic cell cycle;negative regulation of biological process;negative regulation of intracellular protein transport;negat
activation of MAPK activity;anatomical structure morphogenesis;biological adhesion;biological regulation;branching morphogenesis of a tube;cell adhesion;cell junction assembly;cell junction organization;cell morphogenesis
anatomical structure development;apoptosis;biological regulation;cell death;cell differentiation;cellular developmental process;cellular process;death;developmental process;muscle organ development;muscle structure devel
biological regulation;cellular macromolecule metabolic process;cellular metabolic process;cellular nitrogen compound metabolic process;cellular process;gene expression;macromolecule metabolic process;metabolic proces
actin filament capping;barbed-end actin filament capping;biological regulation;blood coagulation;cellular component assembly;cellular component movement;cellular component organization;cellular component organization
biological regulation;biosynthetic process;carbohydrate metabolic process;carbohydrate transport;cell cycle;cell cycle process;cell surface receptor linked signaling pathway;cellular biosynthetic process;cellular component a
biological regulation;catabolic process;cell communication;cell-cell signaling;cellular catabolic process;cellular macromolecule catabolic process;cellular macromolecule metabolic process;cellular metabolic process;cellular p
anatomical structure development;antibacterial humoral response;antimicrobial humoral response;defense response;defense response to bacterium;defense response to Gram-positive bacterium;developmental process;hum
cellular component assembly;cellular component assembly at cellular level;cellular component organization;cellular component organization at cellular level;cellular component organization or biogenesis;cellular component o
5-phosphoribose 1-diphosphate biosynthetic process;5-phosphoribose 1-diphosphate metabolic process;alcohol biosynthetic process;alcohol metabolic process;AMP biosynthetic process;AMP metabolic process;anatomic
anatomical structure morphogenesis;autophagic vacuole assembly;autophagy;biological adhesion;biological regulation;cargo loading into COPII-coated vesicle;cargo loading into vesicle;catabolic process;cell adhesion;cell c
biological regulation;cell communication;cell surface receptor linked signaling pathway;cell-cell signaling;cellular metabolic process;cellular process;cellular response to chemical stimulus;cellular response to endogenous stin
biological regulation;biosynthetic process;cell cycle;cellular biosynthetic process;cellular component assembly;cellular component assembly at cellular level;cellular component disassembly;cellular component disassembly a
axon guidance;biological regulation;cell surface receptor linked signaling pathway;cellular component organization;cellular component organization at cellular level;cellular component organization or biogenesis;cellular comp
anatomical structure development;anatomical structure formation involved in morphogenesis;anatomical structure morphogenesis;angiogenesis;apoptosis;axon guidance;axonogenesis;biological regulation;blood coagulation
carboxylic acid catabolic process;carboxylic acid metabolic process;catabolic process;cellular catabolic process;cellular ketone metabolic process;cellular lipid catabolic process;cellular lipid metabolic process;cellular metab
3'-UTR-mediated mRNA stabilization;biological regulation;cellular macromolecule metabolic process;cellular metabolic process;cellular nitrogen compound metabolic process;cellular process;defense response;gene express
actin cytoskeleton organization;actin filament-based process;activation of immune response;activation of innate immune response;activation of protein kinase activity;antigen receptor-mediated signaling pathway;apoptosis;a
biological regulation;biosynthetic process;cellular biosynthetic process;cellular component assembly;cellular component assembly at cellular level;cellular component organization;cellular component organization at cellular le
amine metabolic process;amino acid activation;carboxylic acid metabolic process;cellular amine metabolic process;cellular amino acid metabolic process;cellular ketone metabolic process;cellular macromolecule metabolic p
activation of immune response;activation of innate immune response;axon guidance;biological regulation;cell communication;cell cycle;cell proliferation;cell surface receptor linked signaling pathway;cell-cell signaling;cellular
actin cytoskeleton organization;actin filament bundle assembly;actin filament organization;actin filament-based process;biological adhesion;biological regulation;cell adhesion;cell communication;cell surface receptor linked s
acetyl-CoA metabolic process;amine catabolic process;amine metabolic process;anthranilate metabolic process;aromatic amino acid family catabolic process;aromatic amino acid family metabolic process;aromatic compou
biological regulation;carboxylic acid catabolic process;carboxylic acid metabolic process;catabolic process;cellular catabolic process;cellular ketone metabolic process;cellular lipid catabolic process;cellular lipid metabolic p
biological regulation;biosynthetic process;cellular biosynthetic process;cellular macromolecule biosynthetic process;cellular macromolecule metabolic process;cellular metabolic process;cellular nitrogen compound metabolic
cellular component assembly;cellular component assembly at cellular level;cellular component organization;cellular component organization at cellular level;cellular component organization or biogenesis;cellular component o
anatomical structure development;anatomical structure morphogenesis;artery development;blood vessel development;cardiac septum development;developmental process;embryonic morphogenesis;morphogenesis of an ep
apoptosis;biological regulation;biosynthetic process;cell cycle;cell death;cellular biosynthetic process;cellular macromolecule biosynthetic process;cellular macromolecule metabolic process;cellular metabolic process;cellular
biological regulation;cell activation;cellular component organization;cellular component organization or biogenesis;cellular membrane organization;cellular process;cellular response to chemical stimulus;cellular response to hy
anatomical structure morphogenesis;axonogenesis;biological regulation;cell differentiation;cell part morphogenesis;cell projection morphogenesis;cell projection organization;cellular component morphogenesis;cellular comp
establishment of localization;establishment of localization in cell;establishment of protein localization;intracellular protein transport;intracellular transport;nuclear import;nuclear transport;nucleocytoplasmic transport;protein in
activation of protein kinase activity;activation of protein kinase B activity;adult behavior;adult locomotory behavior;alcohol biosynthetic process;alcohol metabolic process;aldehyde catabolic process;amine transport;autopha
actin cytoskeleton organization;actin filament branching;actin filament bundle assembly;actin filament organization;actin filament-based process;biological regulation;cell chemotaxis;cell migration;cell motility;cell projection o
biological regulation;cellular component assembly;cellular component assembly at cellular level;cellular component organization;cellular component organization at cellular level;cellular component organization or biogenesis;
bradykinin catabolic process;catabolic process;cellular catabolic process;cellular metabolic process;cellular process;macromolecule metabolic process;metabolic process;peptide catabolic process;peptide metabolic proces
cell cycle;cell division;cellular component assembly;cellular component organization;cellular component organization or biogenesis;cellular process;macromolecular complex assembly;macromolecular complex subunit organ
macromolecule metabolic process;metabolic process;primary metabolic process;protein metabolic process;proteolysis
biological regulation;cellular macromolecule metabolic process;cellular metabolic process;cellular process;cellular protein metabolic process;macromolecule metabolic process;macromolecule modification;metabolic process
actin cytoskeleton organization;actin filament-based process;anatomical structure morphogenesis;biological regulation;caveola assembly;caveolin-mediated endocytosis;cell part morphogenesis;cell projection morphogenesi
biological regulation;cellular component organization;cellular component organization at cellular level;cellular component organization or biogenesis;cellular component organization or biogenesis at cellular level;cellular meml
activation of immune response;activation of innate immune response;anaphase-promoting complex-dependent proteasomal ubiquitin-dependent protein catabolic process;antigen processing and presentation;antigen proces
biological regulation;biosynthetic process;catabolic process;cellular biosynthetic process;cellular catabolic process;cellular macromolecule biosynthetic process;cellular macromolecule catabolic process;cellular macromolecu
anion transport;biological adhesion;biological regulation;cell adhesion;cell-cell adhesion;cellular chemical homeostasis;cellular homeostasis;cellular ion homeostasis;cellular process;cellular response to stimulus;chemical hor
activation of immune response;activation of innate immune response;anaphase-promoting complex-dependent proteasomal ubiquitin-dependent protein catabolic process;antigen processing and presentation;antigen proces
actin cytoskeleton organization;actin filament organization;actin filament-based process;alcohol metabolic process;biological adhesion;biological regulation;biosynthetic process;carbohydrate metabolic process;cell adhesion
biosynthetic process;cellular biosynthetic process;cellular lipid metabolic process;cellular macromolecule metabolic process;cellular metabolic process;cellular process;cellular protein metabolic process;cellular response to c
cellular macromolecule metabolic process;cellular metabolic process;cellular process;cellular protein metabolic process;establishment of localization;establishment of localization in cell;establishment of protein localization;es
3'-phosphoadenosine 5'-phosphosulfate biosynthetic process;3'-phosphoadenosine 5'-phosphosulfate metabolic process;amine metabolic process;aminoglycan metabolic process;anatomical structure development;biologic
activation of immune response;biological regulation;carboxylic acid metabolic process;cell surface receptor linked signaling pathway;cellular ketone metabolic process;cellular lipid metabolic process;cellular metabolic proces
adenine transport;apoptotic mitochondrial changes;biological regulation;cellular component organization;cellular component organization at cellular level;cellular component organization or biogenesis;cellular component orga
alcohol biosynthetic process;alcohol metabolic process;biological regulation;biosynthetic process;C21-steroid hormone biosynthetic process;C21-steroid hormone metabolic process;carbohydrate biosynthetic process;carbo
arachidonic acid metabolic process;carboxylic acid metabolic process;cell differentiation;cellular developmental process;cellular ketone metabolic process;cellular lipid metabolic process;cellular metabolic process;cellular pr
amine metabolic process;betaine metabolic process;biological regulation;carboxylic acid metabolic process;carnitine metabolic process;catabolic process;cellular amine metabolic process;cellular amino acid metabolic proce
biological regulation;negative regulation of catalytic activity;negative regulation of endopeptidase activity;negative regulation of hydrolase activity;negative regulation of molecular function;negative regulation of peptidase activ
biological regulation;biosynthetic process;cell differentiation;cellular biosynthetic process;cellular component organization;cellular component organization at cellular level;cellular component organization or biogenesis;cellula
biological regulation;biosynthetic process;cellular biosynthetic process;cellular component organization;cellular component organization at cellular level;cellular component organization or biogenesis;cellular component orga
B cell activation;cell activation;cell proliferation;cellular process;establishment of localization;establishment of localization in cell;exocytosis;immune system process;leukocyte activation;lymphocyte activation;secretion;secret
aging;biological regulation;cell aging;cell junction assembly;cell junction organization;cellular component assembly;cellular component assembly at cellular level;cellular component organization;cellular component organizatio
amine biosynthetic process;amine metabolic process;anatomical structure development;betaine biosynthetic process;betaine metabolic process;biological regulation;biosynthetic process;carboxylic acid biosynthetic process
biological regulation;biosynthetic process;catabolic process;cellular biosynthetic process;cellular catabolic process;cellular component assembly;cellular component assembly at cellular level;cellular component organization;
biological regulation;biosynthetic process;catabolic process;cellular biosynthetic process;cellular catabolic process;cellular component disassembly;cellular component disassembly at cellular level;cellular component organiz
activation of adenylate cyclase activity;activation of adenylate cyclase activity by dopamine receptor signaling pathway;activation of adenylate cyclase activity by G-protein signaling pathway;activation of phospholipase C ac
antigen processing and presentation;antigen processing and presentation of exogenous antigen;antigen processing and presentation of exogenous peptide antigen;antigen processing and presentation of exogenous peptide
cellular component organization;cellular component organization at cellular level;cellular component organization or biogenesis;cellular component organization or biogenesis at cellular level;cellular localization;cellular macro
cell cycle;cell cycle process;cell division;cellular component organization;cellular component organization at cellular level;cellular component organization or biogenesis;cellular component organization or biogenesis at cellula
catabolic process;cellular catabolic process;cellular macromolecule catabolic process;cellular macromolecule metabolic process;cellular metabolic process;cellular nitrogen compound metabolic process;cellular process;mac
cellular macromolecule metabolic process;cellular metabolic process;cellular process;cellular protein metabolic process;cellular response to heat;cellular response to stimulus;cellular response to stress;chaperone cofactor-d
autophagy;biological regulation;catabolic process;cellular catabolic process;cellular component assembly;cellular component assembly at cellular level;cellular component organization;cellular component organization at cellu
cellular macromolecule metabolic process;cellular metabolic process;cellular nitrogen compound metabolic process;cellular process;macromolecule metabolic process;metabolic process;nitrogen compound metabolic proce
establishment of localization;transport
cellular macromolecule metabolic process;cellular metabolic process;cellular nitrogen compound metabolic process;cellular process;macromolecule metabolic process;metabolic process;mRNA metabolic process;mRNA pro
amine biosynthetic process;amine metabolic process;biosynthetic process;carboxylic acid biosynthetic process;carboxylic acid metabolic process;cellular amine metabolic process;cellular amino acid biosynthetic process;ce
biological regulation;cellular macromolecule metabolic process;cellular metabolic process;cellular nitrogen compound metabolic process;cellular process;cellular response to stimulus;cellular response to stress;establishment
biological regulation;cell surface receptor linked signaling pathway;cellular process;cellular response to chemical stimulus;cellular response to cytokine stimulus;cellular response to organic substance;cellular response to stim
cellular macromolecule metabolic process;cellular metabolic process;cellular nitrogen compound metabolic process;cellular process;macromolecule metabolic process;metabolic process;mRNA metabolic process;mRNA pro
developmental process;keratinization
ADP biosynthetic process;ADP metabolic process;AMP metabolic process;ATP metabolic process;biological regulation;biosynthetic process;cell cycle arrest;cell cycle process;cellular biosynthetic process;cellular metabolic p
amine biosynthetic process;amine metabolic process;biosynthetic process;carboxylic acid biosynthetic process;carboxylic acid metabolic process;cellular amine metabolic process;cellular amino acid biosynthetic process;ce
beta-amyloid metabolic process;biological regulation;bradykinin catabolic process;catabolic process;cell surface receptor linked signaling pathway;cellular catabolic process;cellular component assembly;cellular component
biological regulation;cellular component assembly;cellular component assembly at cellular level;cellular component organization;cellular component organization at cellular level;cellular component organization or biogenesis;
2-oxoglutarate metabolic process;alcohol biosynthetic process;alcohol metabolic process;alditol biosynthetic process;alditol metabolic process;amine biosynthetic process;amine catabolic process;amine metabolic process;
biological regulation;negative regulation of apoptosis;negative regulation of biological process;negative regulation of catalytic activity;negative regulation of cell death;negative regulation of cellular process;negative regulation
actin cytoskeleton organization;actin filament depolymerization;actin filament organization;actin filament-based process;actin polymerization or depolymerization;ameboidal cell migration;anatomical structure morphogenesis;
anatomical structure development;ATP-dependent chromatin remodeling;biological regulation;biosynthetic process;brain development;cell differentiation;cellular biosynthetic process;cellular component organization;cellular c
biological regulation;cellular process;cellular response to stimulus;establishment of localization;establishment of protein localization;intracellular signal transduction;negative regulation of axonogenesis;negative regulation of b
activation of immune response;activation of innate immune response;activation of MAPK activity;activation of MAPKK activity;activation of protein kinase activity;axon guidance;biological regulation;cell surface receptor linke
biological regulation;cellular macromolecule metabolic process;cellular metabolic process;cellular nitrogen compound metabolic process;cellular process;macromolecule metabolic process;metabolic process;ncRNA metabo

biosynthetic process;catabolic process;cellular biosynthetic process;cellular catabolic process;cellular component disassembly;cellular component disassembly at cellular level;cellular component organization;cellular compo
adipose tissue development;anatomical structure development;biological regulation;brain development;catabolic process;cellular catabolic process;cellular ketone body metabolic process;cellular ketone metabolic process;c
activation of MAPK activity;axon guidance;biological adhesion;biological regulation;blood coagulation;cell adhesion;cell chemotaxis;cell migration;cell motility;cell surface receptor linked signaling pathway;cell-matrix adhesio
biosynthetic process;catabolic process;cellular biosynthetic process;cellular catabolic process;cellular component disassembly;cellular component disassembly at cellular level;cellular component organization;cellular compo
biological regulation;cell cycle;cellular process;mitotic cell cycle;negative regulation of binding;negative regulation of molecular function;negative regulation of protein binding;regulation of binding;regulation of molecular funct
ATP-dependent chromatin remodeling;biological regulation;biosynthetic process;cell cycle;cell cycle process;cellular biosynthetic process;cellular component assembly;cellular component assembly at cellular level;cellular co
cellular component assembly;cellular component assembly at cellular level;cellular component organization;cellular component organization at cellular level;cellular component organization or biogenesis;cellular component c
biological regulation;negative regulation of biological process;negative regulation of biosynthetic process;negative regulation of cellular biosynthetic process;negative regulation of cellular macromolecule biosynthetic process
biological regulation;cellular component organization;cellular component organization or biogenesis;cellular membrane organization;cellular process;cellular response to stimulus;endocytosis;establishment of localization;mem
alcohol biosynthetic process;alcohol metabolic process;biological regulation;biosynthetic process;carbohydrate biosynthetic process;carbohydrate homeostasis;carbohydrate metabolic process;carboxylic acid metabolic pro
biological regulation;biosynthetic process;cellular biosynthetic process;cellular macromolecule biosynthetic process;cellular macromolecule metabolic process;cellular metabolic process;cellular nitrogen compound metabolic
cell cycle phase;cell cycle process;cell division;cellular component organization;cellular component organization at cellular level;cellular component organization or biogenesis;cellular component organization or biogenesis at
biological regulation;catabolic process;cellular catabolic process;cellular component organization;cellular component organization at cellular level;cellular component organization or biogenesis;cellular component organizatio
anatomical structure morphogenesis;biological adhesion;biological regulation;cell adhesion;cell junction assembly;cell junction organization;cell morphogenesis;cell morphogenesis involved in differentiation;cell surface recep
biological regulation;biosynthetic process;cellular biosynthetic process;cellular component organization;cellular component organization at cellular level;cellular component organization or biogenesis;cellular component orga
actin cytoskeleton organization;actin filament-based process;biological regulation;cell differentiation;cellular component organization;cellular component organization at cellular level;cellular component organization or biogen
amine metabolic process;amino acid activation;biosynthetic process;carboxylic acid metabolic process;cellular amine metabolic process;cellular amino acid metabolic process;cellular biosynthetic process;cellular ketone me
B cell activation;B cell activation involved in immune response;cell activation;cell activation involved in immune response;cellular macromolecule metabolic process;cellular metabolic process;cellular nitrogen compound meta
apoptosis;biological regulation;biosynthetic process;cell death;cellular biosynthetic process;cellular macromolecule biosynthetic process;cellular macromolecule metabolic process;cellular metabolic process;cellular nitrogen
anatomical structure development;cellular macromolecule metabolic process;cellular metabolic process;cellular nitrogen compound metabolic process;cellular process;chordate embryonic development;developmental proce
activation of immune response;activation of innate immune response;anaphase-promoting complex-dependent proteasomal ubiquitin-dependent protein catabolic process;antigen processing and presentation;antigen proces
anatomical structure development;anatomical structure formation involved in morphogenesis;anatomical structure morphogenesis;angiogenesis;apoptosis;apoptosis involved in heart morphogenesis;apoptosis involved in mo
biological regulation;cell cycle;cell cycle phase;cell cycle process;cellular component organization;cellular component organization at cellular level;cellular component organization or biogenesis;cellular component organizatio
anatomical structure development;anatomical structure formation involved in morphogenesis;biological regulation;brain development;cell proliferation;cell surface receptor linked signaling pathway;cellular process;cellular res
actin cytoskeleton organization;actin filament organization;actin filament-based process;actin nucleation;Arp2/3 complex-mediated actin nucleation;biological regulation;cellular component movement;cellular component orga
biological regulation;cellular component organization;cellular component organization at cellular level;cellular component organization or biogenesis;cellular component organization or biogenesis at cellular level;cellular macr
2-oxoglutarate metabolic process;4-hydroxyproline catabolic process;4-hydroxyproline metabolic process;alcohol biosynthetic process;alcohol metabolic process;amine biosynthetic process;amine catabolic process;amine r
amine metabolic process;anatomical structure development;anion homeostasis;biological regulation;carboxylic acid metabolic process;cellular amine metabolic process;cellular amino acid metabolic process;cellular anion ho
biological regulation;carbohydrate metabolic process;cellular carbohydrate metabolic process;cellular macromolecule metabolic process;cellular metabolic process;cellular process;cellular protein metabolic process;cellular r
autophagy;behavior;behavioral interaction between organisms;biological regulation;calcium ion homeostasis;catabolic process;cation homeostasis;cell differentiation;cell proliferation;cell volume homeostasis;cellular calcium
anatomical structure development;biological regulation;bone development;cellular component organization;cellular component organization at cellular level;cellular component organization or biogenesis;cellular component o
aerobic respiration;cellular metabolic process;cellular process;cellular respiration;electron transport chain;energy derivation by oxidation of organic compounds;generation of precursor metabolites and energy;metabolic proce
acyl-CoA biosynthetic process;acyl-CoA metabolic process;acylglycerol biosynthetic process;acylglycerol metabolic process;adiponectin-mediated signaling pathway;biological regulation;biosynthetic process;carboxylic acid
acetyl-CoA catabolic process;acetyl-CoA metabolic process;alcohol biosynthetic process;alcohol metabolic process;biological regulation;biosynthetic process;carbohydrate biosynthetic process;carbohydrate metabolic proc
alcohol metabolic process;amine biosynthetic process;amine catabolic process;amine metabolic process;aspartate family amino acid catabolic process;aspartate family amino acid metabolic process;betaine biosynthetic pro
biological regulation;cellular component assembly;cellular component organization;cellular component organization or biogenesis;cellular macromolecule metabolic process;cellular metabolic process;cellular process;cellular
biological regulation;biosynthetic process;cell cycle;cellular biosynthetic process;cellular component organization;cellular component organization at cellular level;cellular component organization or biogenesis;cellular compo
biological regulation;cell proliferation;cellular macromolecule metabolic process;cellular metabolic process;cellular process;cellular protein metabolic process;cellular response to chemical stimulus;cellular response to organic
activation of immune response;activation of innate immune response;anaphase-promoting complex-dependent proteasomal ubiquitin-dependent protein catabolic process;antigen processing and presentation;antigen proces
activation of caspase activity;biological regulation;cell differentiation;cellular developmental process;cellular macromolecule metabolic process;cellular metabolic process;cellular process;cellular protein metabolic process;dev
biosynthetic process;catabolic process;cellular biosynthetic process;cellular catabolic process;cellular component biogenesis;cellular component biogenesis at cellular level;cellular component disassembly;cellular componen
activation of caspase activity;anatomical structure morphogenesis;biological regulation;carbohydrate homeostasis;cell cycle;cell differentiation;cellular chemical homeostasis;cellular developmental process;cellular glucose ho
anatomical structure development;apoptosis;biological regulation;catabolic process;cell cycle;cell cycle process;cell death;cell surface receptor linked signaling pathway;cellular catabolic process;cellular component assembl
biological regulation;biosynthetic process;catabolic process;cellular biosynthetic process;cellular catabolic process;cellular component assembly;cellular component assembly at cellular level;cellular component organization;
biological regulation;cellular macromolecule metabolic process;cellular metabolic process;cellular nitrogen compound metabolic process;cellular process;cellular response to stimulus;DNA conformation change;DNA duplex u
anatomical structure development;apoptosis;biological adhesion;cardiac Purkinje fiber development;cell adhesion;cell death;cell-cell adhesion;cellular component disassembly;cellular component disassembly at cellular level
biological regulation;biosynthetic process;catabolic process;cell surface receptor linked signaling pathway;cellular biosynthetic process;cellular catabolic process;cellular macromolecule biosynthetic process;cellular macrom
alcohol biosynthetic process;alcohol metabolic process;amino sugar biosynthetic process;amino sugar metabolic process;biosynthetic process;carbohydrate biosynthetic process;carbohydrate metabolic process;cellular bio
apoptotic cell clearance;biological regulation;cell redox homeostasis;cellular component organization;cellular component organization or biogenesis;cellular homeostasis;cellular macromolecule metabolic process;cellular me
cellular component assembly;cellular component assembly at cellular level;cellular component organization;cellular component organization at cellular level;cellular component organization or biogenesis;cellular component c
actin cytoskeleton organization;actin filament-based process;actomyosin structure organization;asymmetric Golgi ribbon formation;biological regulation;cell migration;cell motility;cellular component movement;cellular compo
anatomical structure development;anatomical structure morphogenesis;ATP-dependent chromatin remodeling;biological regulation;biosynthetic process;cell surface receptor linked signaling pathway;cellular biosynthetic proc
antigen processing and presentation;antigen processing and presentation of exogenous antigen;antigen processing and presentation of exogenous peptide antigen;antigen processing and presentation of exogenous peptide
biological regulation;biosynthetic process;cell cycle;cell proliferation;cellular biosynthetic process;cellular component organization;cellular component organization at cellular level;cellular component organization or biogenesi
biological regulation;biosynthetic process;cell death;cell projection organization;cellular biosynthetic process;cellular component organization;cellular component organization at cellular level;cellular component organization o
amine metabolic process;amino acid activation;carboxylic acid metabolic process;cellular amine metabolic process;cellular amino acid metabolic process;cellular ketone metabolic process;cellular macromolecule metabolic p
cellular component assembly;cellular component assembly at cellular level;cellular component organization;cellular component organization at cellular level;cellular component organization or biogenesis;cellular component c
biological adhesion;cell adhesion;cell junction assembly;cell junction organization;cell-cell adhesion;cell-cell junction assembly;cell-cell junction organization;cellular component assembly;cellular component assembly at cellu
anatomical structure development;anatomical structure morphogenesis;axon ensheathment;axonogenesis;biological regulation;cell development;cell part morphogenesis;cell projection morphogenesis;cell projection organiza
catabolic process;cellular catabolic process;cellular macromolecule catabolic process;cellular macromolecule metabolic process;cellular metabolic process;cellular process;cellular protein metabolic process;macromolecule c
cellular component organization;cellular component organization or biogenesis;cellular membrane organization;cellular process;endocytosis;establishment of localization;lipid transport;membrane invagination;membrane orga
biological regulation;biosynthetic process;cellular biosynthetic process;cellular macromolecule biosynthetic process;cellular macromolecule metabolic process;cellular metabolic process;cellular nitrogen compound metabolic
amine metabolic process;amino acid activation;asparaginyl-tRNA aminoacylation;carboxylic acid metabolic process;cellular amine metabolic process;cellular amino acid metabolic process;cellular ketone metabolic process;c
activation of MAPKK activity;activation of protein kinase activity;adenosine receptor signaling pathway;biological regulation;blood coagulation;cAMP-mediated signaling;cell activation;cell communication;cell cycle;cell divisio
adult behavior;adult locomotory behavior;aging;anatomical structure development;anatomical structure morphogenesis;axonogenesis;behavior;biological regulation;catabolic process;cell communication;cell differentiation;ce
catabolic process;cell cycle process;cellular catabolic process;cellular component disassembly;cellular component disassembly at cellular level;cellular component organization;cellular component organization at cellular leve
alcohol catabolic process;alcohol metabolic process;biological regulation;carbohydrate catabolic process;carbohydrate metabolic process;carbohydrate phosphorylation;catabolic process;cellular carbohydrate catabolic proc
biological regulation;biosynthetic process;cellular biosynthetic process;cellular macromolecule biosynthetic process;cellular macromolecule metabolic process;cellular metabolic process;cellular nitrogen compound metabolic
anatomical structure homeostasis;base-excision repair;base-excision repair, gap-filling;biological regulation;biosynthetic process;cell cycle;cellular biosynthetic process;cellular component organization;cellular component org
activation of caspase activity;aging;anatomical structure morphogenesis;apoptosis;apoptosis in response to endoplasmic reticulum stress;biological regulation;biosynthetic process;branching involved in mammary gland duc
amine biosynthetic process;amine metabolic process;biosynthetic process;carboxylic acid biosynthetic process;carboxylic acid metabolic process;cellular amine metabolic process;cellular amino acid biosynthetic process;ce
anatomical structure development;apoptosis;biological regulation;biosynthetic process;blood circulation;cell death;cell differentiation;cell differentiation involved in kidney development;cell differentiation involved in metaneph
ATP metabolic process;biological regulation;cation transport;cell communication;cell-cell signaling;cellular component organization;cellular component organization or biogenesis;cellular membrane fusion;cellular membrane
biosynthetic process;catabolic process;cellular biosynthetic process;cellular catabolic process;cellular component disassembly;cellular component disassembly at cellular level;cellular component organization;cellular compo
actin cytoskeleton organization;actin filament capping;actin filament-based process;barbed-end actin filament capping;biological regulation;blood coagulation;cellular component movement;cellular component organization;c
actin cytoskeleton organization;actin filament organization;actin filament-based process;actin polymerization or depolymerization;activation of immune response;anatomical structure formation involved in morphogenesis;anti
anatomical structure development;anatomical structure formation involved in morphogenesis;anatomical structure morphogenesis;aortic smooth muscle cell differentiation;appendage morphogenesis;ATP-dependent chroma
activation of immune response;activation of innate immune response;anaphase-promoting complex-dependent proteasomal ubiquitin-dependent protein catabolic process;antigen processing and presentation;antigen proces
actin cytoskeleton organization;actin filament-based process;anatomical structure development;androgen receptor signaling pathway;biological regulation;cell cycle phase;cell cycle process;cell development;cell division;cell
biosynthetic process;catabolic process;cellular biosynthetic process;cellular catabolic process;cellular component biogenesis;cellular component biogenesis at cellular level;cellular component disassembly;cellular componen
biological regulation;biosynthetic process;blood coagulation;cell activation;cell migration;cell motility;cellular biosynthetic process;cellular component assembly;cellular component assembly at cellular level;cellular componen
antigen processing and presentation;antigen processing and presentation of exogenous antigen;antigen processing and presentation of exogenous peptide antigen;antigen processing and presentation of exogenous peptide
biosynthetic process;carbohydrate metabolic process;cellular carbohydrate metabolic process;cellular macromolecule metabolic process;cellular metabolic process;cellular process;cellular protein metabolic process;glycosyl
biological regulation;biosynthetic process;blood coagulation;catabolic process;cellular biosynthetic process;cellular catabolic process;cellular macromolecule biosynthetic process;cellular macromolecule catabolic process;ce
biological regulation;biosynthetic process;cell proliferation;cellular biosynthetic process;cellular macromolecule biosynthetic process;cellular macromolecule metabolic process;cellular metabolic process;cellular nitrogen com
biological regulation;cell proliferation;cell surface receptor linked signaling pathway;cellular process;cellular response to stimulus;enzyme linked receptor protein signaling pathway;intracellular signal transduction;negative reg
alcohol catabolic process;alcohol metabolic process;biosynthetic process;carbohydrate biosynthetic process;carbohydrate catabolic process;carbohydrate metabolic process;catabolic process;cellular biosynthetic process;c
4-hydroxyproline metabolic process;amine metabolic process;apoptosis;apoptosis in response to endoplasmic reticulum stress;biological regulation;brown fat cell differentiation;calcium ion homeostasis;calcium ion transport
biosynthetic process;cellular biosynthetic process;cellular macromolecule biosynthetic process;cellular macromolecule metabolic process;cellular metabolic process;cellular nitrogen compound metabolic process;cellular pro
biological regulation;biosynthetic process;catabolic process;cell growth;cellular biosynthetic process;cellular catabolic process;cellular macromolecule catabolic process;cellular macromolecule metabolic process;cellular met
cellular component organization;cellular component organization at cellular level;cellular component organization or biogenesis;cellular component organization or biogenesis at cellular level;cellular process;lipid particle orga
assembly of spliceosomal tri-snRNP;biological regulation;biosynthetic process;cell proliferation;cellular component assembly;cellular component assembly at cellular level;cellular component organization;cellular component
antigen processing and presentation;antigen processing and presentation of exogenous antigen;antigen processing and presentation of exogenous peptide antigen;antigen processing and presentation of exogenous peptide
alcohol catabolic process;alcohol metabolic process;anatomical structure homeostasis;biological regulation;carbohydrate catabolic process;carbohydrate homeostasis;carbohydrate metabolic process;carbohydrate phospho
acetyl-CoA biosynthetic process;acetyl-CoA biosynthetic process from pyruvate;acetyl-CoA catabolic process;acetyl-CoA metabolic process;alcohol catabolic process;alcohol metabolic process;biological regulation;biosynth
alcohol biosynthetic process;alcohol metabolic process;biosynthetic process;carbohydrate biosynthetic process;carbohydrate metabolic process;cellular biosynthetic process;cellular carbohydrate biosynthetic process;cellul
biological regulation;biosynthetic process;catabolic process;cellular biosynthetic process;cellular catabolic process;cellular component disassembly;cellular component disassembly at cellular level;cellular component organiz
biological regulation;biosynthetic process;cell differentiation;cellular biosynthetic process;cellular developmental process;cellular macromolecule biosynthetic process;cellular macromolecule metabolic process;cellular metab
anatomical structure development;apoptosis;base-excision repair, DNA ligation;biological regulation;catabolic process;cell chemotaxis;cell death;cell migration;cell motility;cellular catabolic process;cellular component assem
antigen processing and presentation;antigen processing and presentation of peptide antigen;antigen processing and presentation of peptide antigen via MHC class I;cellular macromolecule metabolic process;cellular metabo
adherens junction assembly;adherens junction organization;anatomical structure development;anatomical structure formation involved in morphogenesis;anatomical structure homeostasis;anatomical structure morphogenesi
adaptive immune response;adaptive immune response based on somatic recombination of immune receptors built from immunoglobulin superfamily domains;anatomical structure development;B cell activation;B cell activati
biological regulation;cellular macromolecule metabolic process;cellular metabolic process;cellular nitrogen compound metabolic process;cellular process;macromolecule metabolic process;metabolic process;mRNA metaboli
cell cycle;cell division;cellular component organization;cellular component organization or biogenesis;cellular membrane organization;cellular process;endocytosis;establishment of localization;membrane invagination;membra
anatomical structure development;biological regulation;biosynthetic process;catabolic process;cell cycle arrest;cell cycle process;cellular biosynthetic process;cellular catabolic process;cellular macromolecule biosynthetic pr
binding of sperm to zona pellucida;cell recognition;cell-cell recognition;cellular process;cellular process involved in reproduction;reproductive process;sperm-egg recognition
anatomical structure development;anatomical structure formation involved in morphogenesis;androgen receptor signaling pathway;atrial cardiac muscle cell development;biological regulation;biosynthetic process;cardiac cel
biological regulation;biosynthetic process;cell surface receptor linked signaling pathway;cellular biosynthetic process;cellular macromolecule biosynthetic process;cellular macromolecule metabolic process;cellular metabolic
apoptotic mitochondrial changes;biological regulation;cell cycle;cellular component organization;cellular component organization at cellular level;cellular component organization or biogenesis;cellular component organization
biological regulation;cellular macromolecule metabolic process;cellular metabolic process;cellular nitrogen compound metabolic process;cellular process;macromolecule metabolic process;metabolic process;mRNA metaboli
activation of immune response;activation of innate immune response;anaphase-promoting complex-dependent proteasomal ubiquitin-dependent protein catabolic process;antigen processing and presentation;antigen proces
biological adhesion;biological regulation;biosynthetic process;cell adhesion;cellular biosynthetic process;cellular macromolecule biosynthetic process;cellular macromolecule metabolic process;cellular metabolic process;cellu
biosynthetic process;cellular biosynthetic process;cellular macromolecule biosynthetic process;cellular macromolecule metabolic process;cellular metabolic process;cellular process;cellular protein metabolic process;cotrans
activation of immune response;activation of innate immune response;anaphase-promoting complex-dependent proteasomal ubiquitin-dependent protein catabolic process;antigen processing and presentation;antigen proces
anatomical structure development;anatomical structure morphogenesis;basement membrane assembly;basement membrane organization;biological regulation;cell morphogenesis;cell morphogenesis involved in differentiatio
acylglycerol metabolic process;aerobic respiration;aging;alcohol metabolic process;anatomical structure development;biological regulation;catabolic process;cell differentiation;cellular aromatic compound metabolic process;
carbohydrate metabolic process;carboxylic acid metabolic process;cellular ketone metabolic process;cellular metabolic process;cellular nitrogen compound metabolic process;cellular process;coenzyme metabolic process;c
amine transport;amino acid import;amino acid transport;aromatic amino acid transport;biological regulation;blood coagulation;branched-chain aliphatic amino acid transport;calcium ion transport;carbohydrate metabolic proc
4-hydroxyproline metabolic process;amine metabolic process;carboxylic acid metabolic process;cellular amine metabolic process;cellular amino acid metabolic process;cellular component organization;cellular component or
adherens junction assembly;adherens junction organization;anatomical structure development;biological adhesion;biological regulation;cell adhesion;cell junction assembly;cell junction organization;cell migration;cell motility;
amine metabolic process;amino acid activation;anatomical structure formation involved in morphogenesis;angiogenesis;biological regulation;biosynthetic process;carboxylic acid metabolic process;cellular amine metabolic p
biological regulation;blood coagulation;calcium ion homeostasis;calcium ion transmembrane transport;calcium ion transport;cation homeostasis;cation transport;cellular calcium ion homeostasis;cellular cation homeostasis;c
adipose tissue development;amine catabolic process;amine metabolic process;anatomical structure development;biosynthetic process;brain development;branched chain family amino acid catabolic process;branched chain
biological regulation;biosynthetic process;cellular biosynthetic process;cellular macromolecule biosynthetic process;cellular macromolecule metabolic process;cellular metabolic process;cellular nitrogen compound metabolic

alcohol biosynthetic process;alcohol catabolic process;alcohol metabolic process;biosynthetic process;carbohydrate biosynthetic process;carbohydrate catabolic process;carbohydrate metabolic process;catabolic process;c
anatomical structure morphogenesis;biological regulation;catabolic process;cell surface receptor linked signaling pathway;cellular catabolic process;cellular macromolecule catabolic process;cellular macromolecule metaboli
adult behavior;adult locomotory behavior;anatomical structure maturation;behavior;cellular component organization;cellular component organization at cellular level;cellular component organization or biogenesis;cellular comp
amine metabolic process;amino acid activation;carboxylic acid metabolic process;cellular amine metabolic process;cellular amino acid metabolic process;cellular ketone metabolic process;cellular macromolecule metabolic p
cellular process;endosome transport;establishment of localization;establishment of localization in cell;intracellular transport;transport;vesicle-mediated transport
biological regulation;cellular metabolic process;cellular process;cellular response to stimulus;intracellular signal transduction;metabolic process;peptide metabolic process;regulation of biological process;regulation of cellular
biosynthetic process;catabolic process;cellular biosynthetic process;cellular catabolic process;cellular component biogenesis;cellular component biogenesis at cellular level;cellular component disassembly;cellular componen
biosynthetic process;catabolic process;cellular biosynthetic process;cellular catabolic process;cellular component disassembly;cellular component disassembly at cellular level;cellular component organization;cellular compo
activation of immune response;activation of innate immune response;anaphase-promoting complex-dependent proteasomal ubiquitin-dependent protein catabolic process;antigen processing and presentation;antigen proces
amine biosynthetic process;amine metabolic process;anatomical structure development;aspartate family amino acid biosynthetic process;aspartate family amino acid metabolic process;biosynthetic process;carboxylic acid b
cellular macromolecule metabolic process;cellular metabolic process;cellular process;cellular protein metabolic process;macromolecule metabolic process;macromolecule modification;metabolic process;primary metabolic p
biological regulation;cellular macromolecule metabolic process;cellular metabolic process;cellular process;cellular protein metabolic process;establishment of localization;establishment of localization in cell;establishment of p
biological regulation;biosynthetic process;cellular biosynthetic process;cellular macromolecule biosynthetic process;cellular macromolecule metabolic process;cellular metabolic process;cellular nitrogen compound metabolic
biological regulation;regulation of anatomical structure morphogenesis;regulation of biological process;regulation of cell development;regulation of cell differentiation;regulation of cell morphogenesis;regulation of cell morpho
actin cytoskeleton organization;actin filament-based process;biological regulation;blood coagulation;cellular component organization;cellular component organization at cellular level;cellular component organization or biogen
biological regulation;cell cycle;cell cycle phase;cell cycle process;cell division;cell proliferation;cellular component organization;cellular component organization at cellular level;cellular component organization or biogenesis;c
actin cytoskeleton organization;actin filament-based process;ameboidal cell migration;anatomical structure development;anatomical structure formation involved in morphogenesis;anatomical structure morphogenesis;anteri
actin cytoskeleton organization;actin cytoskeleton reorganization;actin filament-based process;actomyosin structure organization;biological regulation;cell migration;cell motility;cellular component movement;cellular compon
actin cytoskeleton organization;actin filament-based process;cellular component organization;cellular component organization at cellular level;cellular component organization or biogenesis;cellular component organization or
2-oxoglutarate metabolic process;acetyl-CoA catabolic process;acetyl-CoA metabolic process;amine metabolic process;anatomical structure development;carbohydrate metabolic process;carboxylic acid metabolic process
activation of protein kinase activity;anatomical structure morphogenesis;biological regulation;cell part morphogenesis;cell projection morphogenesis;cell projection organization;cellular component morphogenesis;cellular con
activation of phospholipase A2 activity;activation of phospholipase A2 activity by calcium-mediated signaling;activation of phospholipase C activity;alkanesulfonate metabolic process;anatomical structure development;anato
biological regulation;biosynthetic process;cellular biosynthetic process;cellular macromolecule biosynthetic process;cellular macromolecule metabolic process;cellular metabolic process;cellular process;cellular protein metab
acetyl-CoA catabolic process;acetyl-CoA metabolic process;amine catabolic process;amine metabolic process;anatomical structure morphogenesis;aspartate family amino acid catabolic process;aspartate family amino acid
cellular component assembly;cellular component assembly at cellular level;cellular component organization;cellular component organization at cellular level;cellular component organization or biogenesis;cellular component c
biological regulation;blood coagulation;cellular component organization;cellular component organization at cellular level;cellular component organization or biogenesis;cellular component organization or biogenesis at cellular
amine metabolic process;B cell activation;B cell proliferation;biosynthetic process;carboxylic acid metabolic process;cell activation;cell proliferation;cellular amine metabolic process;cellular amino acid metabolic process;cell
biosynthetic process;cellular biosynthetic process;cellular macromolecule biosynthetic process;cellular macromolecule metabolic process;cellular metabolic process;cellular nitrogen compound metabolic process;cellular pro
biological regulation;biosynthetic process;catabolic process;cellular biosynthetic process;cellular catabolic process;cellular component disassembly;cellular component disassembly at cellular level;cellular component organiz
macromolecule metabolic process;metabolic process;primary metabolic process;protein metabolic process;proteolysis
anatomical structure development;ATP-dependent chromatin remodeling;biosynthetic process;blastocyst development;cell cycle;cell proliferation;cellular biosynthetic process;cellular component assembly;cellular componen
biological regulation;biosynthetic process;carbohydrate metabolic process;carbohydrate transport;cell cycle;cell cycle process;cell surface receptor linked signaling pathway;cellular biosynthetic process;cellular component a
anatomical structure morphogenesis;antigen processing and presentation;antigen processing and presentation of exogenous antigen;antigen processing and presentation of exogenous peptide antigen;antigen processing an
biosynthetic process;catabolic process;cellular biosynthetic process;cellular catabolic process;cellular component biogenesis;cellular component biogenesis at cellular level;cellular component disassembly;cellular componen
alcohol biosynthetic process;alcohol catabolic process;alcohol metabolic process;aldonic acid metabolic process;biosynthetic process;carbohydrate biosynthetic process;carbohydrate catabolic process;carbohydrate metab
B cell activation;B cell activation involved in immune response;biological regulation;cell activation;cell activation involved in immune response;cell cycle process;cellular macromolecule metabolic process;cellular metabolic pr
cellular macromolecule metabolic process;cellular metabolic process;cellular nitrogen compound metabolic process;cellular process;gene expression;macromolecule metabolic process;metabolic process;mRNA metabolic p
activation of immune response;activation of innate immune response;anaphase-promoting complex-dependent proteasomal ubiquitin-dependent protein catabolic process;antigen processing and presentation;antigen proces
biosynthetic process;catabolic process;cellular biosynthetic process;cellular catabolic process;cellular component biogenesis;cellular component biogenesis at cellular level;cellular component disassembly;cellular componen
anatomical structure development;axon guidance;biological regulation;biosynthetic process;catabolic process;cell cycle checkpoint;cell cycle process;cellular biosynthetic process;cellular catabolic process;cellular compone
alcohol biosynthetic process;alcohol metabolic process;amine metabolic process;amino sugar biosynthetic process;amino sugar metabolic process;biological regulation;biosynthetic process;carbohydrate biosynthetic proce
actin cytoskeleton organization;actin filament severing;actin filament-based process;biological regulation;biosynthetic process;cellular biosynthetic process;cellular component organization;cellular component organization at
cellular macromolecule metabolic process;cellular metabolic process;cellular nitrogen compound metabolic process;cellular process;macromolecule metabolic process;maturation of 5.8S rRNA;maturation of 5.8S rRNA from
anatomical structure morphogenesis;assembly of spliceosomal tri-snRNP;biological regulation;cell morphogenesis;cell proliferation;cellular component assembly;cellular component assembly at cellular level;cellular compone
amine metabolic process;amino acid activation;biosynthetic process;bis(5'-nucleosidyl) oligophosphate biosynthetic process;bis(5'-nucleosidyl) oligophosphate metabolic process;carboxylic acid metabolic process;cellular a
apoptotic cell clearance;biological regulation;cell redox homeostasis;cellular component organization;cellular component organization or biogenesis;cellular homeostasis;cellular macromolecule metabolic process;cellular me
ATP-dependent chromatin remodeling;biological regulation;biosynthetic process;cell proliferation;cellular biosynthetic process;cellular component assembly;cellular component assembly at cellular level;cellular component or
cellular macromolecule metabolic process;cellular metabolic process;cellular process;cellular protein metabolic process;macromolecule metabolic process;macromolecule modification;metabolic process;primary metabolic p
cellular macromolecule metabolic process;cellular metabolic process;cellular nitrogen compound metabolic process;cellular process;macromolecule metabolic process;metabolic process;nitrogen compound metabolic proce
biological regulation;cellular macromolecule metabolic process;cellular metabolic process;cellular process;cellular protein metabolic process;macromolecule metabolic process;macromolecule modification;metabolic process
amine metabolic process;aminoglycan biosynthetic process;aminoglycan catabolic process;aminoglycan metabolic process;axon guidance;biological regulation;biosynthetic process;carbohydrate biosynthetic process;carbo
4-hydroxyproline metabolic process;amine metabolic process;carboxylic acid metabolic process;cellular amine metabolic process;cellular amino acid metabolic process;cellular ketone metabolic process;cellular macromolec
acyl-CoA biosynthetic process;acyl-CoA metabolic process;acylglycerol biosynthetic process;acylglycerol metabolic process;anatomical structure development;biological regulation;biosynthetic process;carboxylic acid meta
biological regulation;biosynthetic process;cellular biosynthetic process;cellular macromolecule biosynthetic process;cellular macromolecule metabolic process;cellular metabolic process;cellular process;cellular protein metab
activation of caspase activity;apoptosis;apoptosis in response to endoplasmic reticulum stress;biological regulation;catabolic process;cell death;cell differentiation;cell redox homeostasis;cellular catabolic process;cellular co
cellular component assembly;cellular component assembly at cellular level;cellular component organization;cellular component organization at cellular level;cellular component organization or biogenesis;cellular component c
biosynthetic process;catabolic process;cellular biosynthetic process;cellular catabolic process;cellular component disassembly;cellular component disassembly at cellular level;cellular component organization;cellular compo
activation of immune response;activation of innate immune response;anaphase-promoting complex-dependent proteasomal ubiquitin-dependent protein catabolic process;antigen processing and presentation;antigen proces
actin cytoskeleton organization;actin filament-based process;aging;antigen processing and presentation;antigen processing and presentation of exogenous antigen;antigen processing and presentation of exogenous peptide
catabolic process;cellular catabolic process;cellular lipid catabolic process;cellular lipid metabolic process;cellular metabolic process;cellular process;cellular response to chemical stimulus;cellular response to hydrogen pero
apoptosis;biological regulation;catabolic process;cell cycle;cell cycle process;cell death;cell surface receptor linked signaling pathway;cellular catabolic process;cellular component assembly;cellular component organization;
activation of immune response;activation of innate immune response;anaphase-promoting complex-dependent proteasomal ubiquitin-dependent protein catabolic process;antigen processing and presentation;antigen proces
biological regulation;biosynthetic process;catabolic process;cellular biosynthetic process;cellular catabolic process;cellular component disassembly;cellular component disassembly at cellular level;cellular component organiz
anatomical structure development;androgen metabolic process;bile acid biosynthetic process;bile acid metabolic process;biological regulation;biosynthetic process;carboxylic acid biosynthetic process;carboxylic acid catab
cellular component organization;cellular component organization at cellular level;cellular component organization or biogenesis;cellular component organization or biogenesis at cellular level;cellular process;COPI coating of
alcohol biosynthetic process;alcohol catabolic process;alcohol metabolic process;biosynthetic process;carbohydrate biosynthetic process;carbohydrate catabolic process;carbohydrate metabolic process;catabolic process;c
actin cytoskeleton organization;actin filament organization;actin filament-based process;actin nucleation;anatomical structure formation involved in morphogenesis;Arp2/3 complex-mediated actin nucleation;asymmetric cell
biosynthetic process;catabolic process;cellular biosynthetic process;cellular catabolic process;cellular component disassembly;cellular component disassembly at cellular level;cellular component organization;cellular compo
activation-induced cell death of T cells;anatomical structure development;anatomical structure morphogenesis;apoptosis;biological regulation;biosynthetic process;carbohydrate homeostasis;catabolic process;cell activation
anatomical structure morphogenesis;biosynthetic process;catabolic process;cellular biosynthetic process;cellular catabolic process;cellular component disassembly;cellular component disassembly at cellular level;cellular co
antigen processing and presentation;antigen processing and presentation of exogenous antigen;antigen processing and presentation of exogenous peptide antigen;antigen processing and presentation of exogenous peptide
anatomical structure development;apoptosis;biological regulation;biosynthetic process;C21-steroid hormone catabolic process;C21-steroid hormone metabolic process;catabolic process;cell death;cellular biosynthetic proce
anatomical structure development;anatomical structure homeostasis;biological regulation;biosynthetic process;catabolic process;cell killing;cell proliferation;cellular biosynthetic process;cellular catabolic process;cellular mac
biological regulation;biosynthetic process;cardiac muscle contraction;cellular biosynthetic process;cellular component assembly;cellular component assembly at cellular level;cellular component organization;cellular compone
antigen processing and presentation;antigen processing and presentation of exogenous antigen;antigen processing and presentation of exogenous peptide antigen;antigen processing and presentation of exogenous peptide
aging;ameboidal cell migration;anatomical structure development;anatomical structure formation involved in morphogenesis;anatomical structure morphogenesis;axon ensheathment;biological adhesion;biological regulation;
biological regulation;biosynthetic process;cellular biosynthetic process;cellular macromolecule biosynthetic process;cellular macromolecule metabolic process;cellular metabolic process;cellular nitrogen compound metabolic
anatomical structure development;anatomical structure formation involved in morphogenesis;anatomical structure morphogenesis;apoptosis;apoptosis involved in morphogenesis;astrocyte cell migration;asymmetric protein l
biological regulation;establishment of localization;ion transport;positive regulation of biological process;positive regulation of cell communication;positive regulation of cellular process;positive regulation of response to stimulu
cell cycle;cell cycle phase;cell cycle process;cellular component organization;cellular component organization at cellular level;cellular component organization or biogenesis;cellular component organization or biogenesis at ce
antigen processing and presentation;antigen processing and presentation of exogenous antigen;antigen processing and presentation of exogenous peptide antigen;antigen processing and presentation of exogenous peptide
anatomical structure development;axon guidance;biological regulation;cellular component organization;cellular component organization at cellular level;cellular component organization or biogenesis;cellular component organ
biological regulation;cellular component assembly;cellular component assembly at cellular level;cellular component organization;cellular component organization at cellular level;cellular component organization or biogenesis;
ATP-dependent chromatin remodeling;biological regulation;biosynthetic process;cell cycle phase;cell cycle process;cell division;cellular biosynthetic process;cellular component assembly;cellular component assembly at cell
cellular process;establishment of localization;transport;vesicle-mediated transport
cell cycle;cellular component organization;cellular component organization at cellular level;cellular component organization or biogenesis;cellular component organization or biogenesis at cellular level;cellular membrane fusio
biological regulation;biosynthetic process;carbohydrate metabolic process;carbohydrate transport;cell cycle;cell cycle process;cell surface receptor linked signaling pathway;cellular biosynthetic process;cellular component c
acyl-CoA biosynthetic process;acyl-CoA metabolic process;acylglycerol biosynthetic process;acylglycerol metabolic process;anatomical structure development;biological regulation;biosynthetic process;brain development;ca
actin filament-based movement;actin filament-based process;actin-mediated cell contraction;actin-myosin filament sliding;cellular component movement;cellular process;multicellular organismal process;muscle contraction;
acetyl-CoA catabolic process;acetyl-CoA metabolic process;anatomical structure homeostasis;biological regulation;carboxylic acid metabolic process;catabolic process;cellular catabolic process;cellular ketone metabolic pr
biological regulation;cellular macromolecule metabolic process;cellular metabolic process;cellular nitrogen compound metabolic process;cellular process;gene expression;macromolecule metabolic process;metabolic proces
biological regulation;cell differentiation;cellular developmental process;cellular process;cellular response to stimulus;developmental process;epithelial cell differentiation;negative regulation of apoptosis;negative regulation of b
anion transport;apoptosis;behavior;behavioral defense response;behavioral fear response;cell communication;cell death;cell differentiation;cell-cell signaling;cellular developmental process;cellular process;cognition;death;de
apoptosis;biological regulation;biosynthetic process;catabolic process;cell cycle phase;cell cycle process;cell death;cell division;cellular biosynthetic process;cellular catabolic process;cellular component disassembly;cellular
amine metabolic process;carboxylic acid metabolic process;cellular amine metabolic process;cellular amino acid metabolic process;cellular ketone metabolic process;cellular metabolic process;cellular modified amino acid m
biosynthetic process;catabolic process;cellular biosynthetic process;cellular catabolic process;cellular component disassembly;cellular component disassembly at cellular level;cellular component organization;cellular compo
apoptotic mitochondrial changes;ATP metabolic process;biological regulation;cellular chemical homeostasis;cellular component organization;cellular component organization at cellular level;cellular component organization o
anatomical structure development;anatomical structure formation involved in morphogenesis;anatomical structure morphogenesis;angiogenesis;axial mesoderm formation;axon guidance;biological adhesion;biological regula
biological regulation;regulation of biological process;regulation of biosynthetic process;regulation of cellular biosynthetic process;regulation of cellular macromolecule biosynthetic process;regulation of cellular metabolic proc
biological regulation;catabolic process;cellular catabolic process;cellular macromolecule catabolic process;cellular macromolecule metabolic process;cellular metabolic process;cellular nitrogen compound metabolic process
biosynthetic process;catabolic process;cellular biosynthetic process;cellular catabolic process;cellular component biogenesis;cellular component biogenesis at cellular level;cellular component disassembly;cellular componen
biosynthetic process;catabolic process;cellular biosynthetic process;cellular catabolic process;cellular component disassembly;cellular component disassembly at cellular level;cellular component organization;cellular compo
activation of immune response;activation of innate immune response;anaphase-promoting complex-dependent proteasomal ubiquitin-dependent protein catabolic process;antigen processing and presentation;antigen proces
acetyl-CoA catabolic process;acetyl-CoA metabolic process;biological regulation;catabolic process;cation transport;cell redox homeostasis;cellular catabolic process;cellular homeostasis;cellular metabolic process;cellular p
biological regulation;cellular component assembly;cellular component assembly at cellular level;cellular component organization;cellular component organization at cellular level;cellular component organization or biogenesis;
biological regulation;biosynthetic process;catabolic process;cellular biosynthetic process;cellular catabolic process;cellular macromolecule biosynthetic process;cellular macromolecule catabolic process;cellular macromolec
biological regulation;cellular component assembly;cellular component assembly at cellular level;cellular component organization;cellular component organization at cellular level;cellular component organization or biogenesis;
alcohol metabolic process;biosynthetic process;carbohydrate biosynthetic process;carbohydrate metabolic process;carboxylic acid biosynthetic process;carboxylic acid metabolic process;cellular biosynthetic process;cellula
anatomical structure formation involved in morphogenesis;biological regulation;biosynthetic process;cell proliferation;cell redox homeostasis;cellular biosynthetic process;cellular homeostasis;cellular lipid metabolic process;c
biological regulation;biosynthetic process;cellular biosynthetic process;cellular macromolecule biosynthetic process;cellular macromolecule metabolic process;cellular metabolic process;cellular nitrogen compound metabolic
biological regulation;biosynthetic process;cellular biosynthetic process;cellular macromolecule biosynthetic process;cellular macromolecule metabolic process;cellular metabolic process;cellular process;cellular protein metab
biological regulation;biosynthetic process;carbohydrate metabolic process;carbohydrate transport;cell cycle;cell cycle process;cell surface receptor linked signaling pathway;cellular biosynthetic process;cellular component a
biological regulation;biosynthetic process;cellular biosynthetic process;cellular macromolecule biosynthetic process;cellular macromolecule metabolic process;cellular metabolic process;cellular nitrogen compound metabolic
anatomical structure development;cell differentiation;cellular developmental process;cellular macromolecule metabolic process;cellular metabolic process;cellular process;cellular protein metabolic process;chaperone-mediat
biological regulation;cell cycle;cell cycle phase;cell cycle process;cell division;cell junction assembly;cell junction organization;cell surface receptor linked signaling pathway;cell-substrate junction assembly;cellular componen
actin cytoskeleton organization;actin filament-based process;ameboidal cell migration;biological regulation;cell migration;cell motility;cellular component movement;cellular component organization;cellular component organiz
amine catabolic process;amine metabolic process;carboxylic acid catabolic process;carboxylic acid metabolic process;catabolic process;cellular amine metabolic process;cellular amino acid catabolic process;cellular amino
aging;anatomical structure formation involved in morphogenesis;apoptosis;axon cargo transport;axon transport of mitochondrion;biological regulation;cell aging;cell death;cellular component movement;cellular component o
actin cytoskeleton organization;actin filament-based process;anatomical structure formation involved in morphogenesis;anatomical structure morphogenesis;axon guidance;biological regulation;cell part morphogenesis;cell p
biological regulation;cell differentiation;cellular developmental process;cellular process;developmental process;osteoblast differentiation;regulation of apoptosis;regulation of biological process;regulation of cell aging;regulatio
ATP-dependent chromatin remodeling;biological regulation;cellular component organization;cellular component organization at cellular level;cellular component organization or biogenesis;cellular component organization or b
anatomical structure formation involved in morphogenesis;anatomical structure morphogenesis;angiogenesis;antigen processing and presentation;antigen processing and presentation of exogenous antigen;antigen processi
biological regulation;catabolic process;cellular catabolic process;cellular macromolecule catabolic process;cellular macromolecule metabolic process;cellular metabolic process;cellular nitrogen compound metabolic process
anatomical structure development;biosynthetic process;cell cycle;cell proliferation;cell proliferation in forebrain;cellular aromatic compound metabolic process;cellular biosynthetic process;cellular component assembly;cellula
alternative nuclear mRNA splicing, via spliceosome;biological regulation;cellular macromolecule metabolic process;cellular metabolic process;cellular nitrogen compound metabolic process;cellular process;gene expression;
anatomical structure development;anatomical structure morphogenesis;aromatic compound biosynthetic process;biosynthetic process;brainstem development;cellular aromatic compound metabolic process;cellular biosynth

alcohol biosynthetic process;alcohol catabolic process;alcohol metabolic process;alditol catabolic process;alditol metabolic process;anatomical structure development;biosynthetic process;camera-type eye development;car
anatomical structure development;anatomical structure morphogenesis;biological regulation;brain development;camera-type eye development;cell cycle process;cell part morphogenesis;cell projection morphogenesis;cell pr
biosynthetic process;cellular biosynthetic process;cellular component organization;cellular component organization at cellular level;cellular component organization or biogenesis;cellular component organization or biogenesis
alcohol catabolic process;alcohol metabolic process;apoptotic mitochondrial changes;biological regulation;body fluid secretion;carbohydrate catabolic process;carbohydrate homeostasis;carbohydrate metabolic process;car
anatomical structure development;apoptosis;biological regulation;biosynthetic process;cell cycle;cell cycle process;cell death;cell migration;cell motility;cell surface receptor linked signaling pathway;cellular biosynthetic proc
biosynthetic process;catabolic process;cellular biosynthetic process;cellular catabolic process;cellular component disassembly;cellular component disassembly at cellular level;cellular component organization;cellular compo
biological regulation;biosynthetic process;cellular biosynthetic process;cellular component organization;cellular component organization at cellular level;cellular component organization or biogenesis;cellular component orga
actin filament-based movement;actin filament-based process;anatomical structure development;biological regulation;cell development;cell differentiation;cell surface receptor linked signaling pathway;cellular component mov
cellular component organization;cellular component organization at cellular level;cellular component organization or biogenesis;cellular component organization or biogenesis at cellular level;cellular process;Golgi organization
biological regulation;cell cycle;cell cycle process;cell division;cellular component organization;cellular component organization at cellular level;cellular component organization or biogenesis;cellular component organization or
biological regulation;cellular component assembly;cellular component assembly at cellular level;cellular component organization;cellular component organization at cellular level;cellular component organization or biogenesis;
cellular component organization;cellular component organization at cellular level;cellular component organization or biogenesis;cellular component organization or biogenesis at cellular level;cellular process;extracellular matri
biological regulation;biosynthetic process;carbohydrate metabolic process;carbohydrate transport;cell cycle;cell cycle process;cell surface receptor linked signaling pathway;cellular biosynthetic process;cellular component c
biological regulation;biosynthetic process;cellular biosynthetic process;cellular macromolecule biosynthetic process;cellular macromolecule metabolic process;cellular metabolic process;cellular nitrogen compound metabolic
cellular component assembly;cellular component assembly at cellular level;cellular component organization;cellular component organization at cellular level;cellular component organization or biogenesis;cellular component c
anatomical structure morphogenesis;biological regulation;biosynthetic process;cellular biosynthetic process;cellular component organization;cellular component organization at cellular level;cellular component organization o
biological regulation;cell cycle;cell surface receptor linked signaling pathway;cellular localization;cellular macromolecule localization;cellular process;cellular protein localization;cellular response to stimulus;enzyme linked rece
activation of immune response;activation of innate immune response;anaphase-promoting complex-dependent proteasomal ubiquitin-dependent protein catabolic process;antigen processing and presentation;antigen proces
alcohol metabolic process;amine metabolic process;aminoglycan biosynthetic process;aminoglycan metabolic process;anatomical structure morphogenesis;biosynthetic process;carbohydrate biosynthetic process;carbohyd
cellular macromolecule metabolic process;cellular metabolic process;cellular nitrogen compound metabolic process;cellular process;histone mRNA metabolic process;macromolecule metabolic process;macromolecule modi
alkene biosynthetic process;arachidonic acid metabolic process;biosynthetic process;carboxylic acid biosynthetic process;carboxylic acid metabolic process;catabolic process;cellular alkene metabolic process;cellular biosy
apoptotic chromosome condensation;biological regulation;cell cycle;cell cycle process;cell differentiation;cell division;cellular component organization;cellular component organization at cellular level;cellular component orgar
anatomical structure development;anatomical structure morphogenesis;axonogenesis;biological regulation;brain development;cell cycle process;cell part morphogenesis;cell projection morphogenesis;cell projection organiza
activation of immune response;activation of innate immune response;anaphase-promoting complex-dependent proteasomal ubiquitin-dependent protein catabolic process;anatomical structure development;antigen processi
anatomical structure development;apoptosis;biological regulation;biosynthetic process;cell death;cellular biosynthetic process;cellular component organization;cellular component organization or biogenesis;cellular macromo
anatomical structure development;apoptosis;apoptotic mitochondrial changes;biological regulation;biosynthetic process;cell death;cell development;cellular biosynthetic process;cellular component organization;cellular comp
biological regulation;biosynthetic process;catabolic process;cell chemotaxis;cell differentiation;cell migration;cell motility;cellular biosynthetic process;cellular catabolic process;cellular component assembly;cellular compone
anatomical structure homeostasis;base-excision repair;behavior;biological regulation;biosynthetic process;catabolic process;cell cycle;cellular biosynthetic process;cellular catabolic process;cellular component organization;c
amine metabolic process;amino acid activation;biosynthetic process;carboxylic acid metabolic process;cellular amine metabolic process;cellular amino acid metabolic process;cellular biosynthetic process;cellular ketone me
amine metabolic process;biosynthetic process;carboxylic acid metabolic process;cellular amine metabolic process;cellular amino acid metabolic process;cellular aromatic compound metabolic process;cellular biosynthetic p
anatomical structure development;biological regulation;biosynthetic process;cell development;cellular component organization;cellular component organization at cellular level;cellular component organization or biogenesis;c
cellular macromolecule metabolic process;cellular metabolic process;cellular nitrogen compound metabolic process;cellular process;gene expression;macromolecule metabolic process;metabolic process;mRNA metabolic p
amine biosynthetic process;amine metabolic process;biosynthetic process;carboxylic acid biosynthetic process;carboxylic acid metabolic process;cellular amine metabolic process;cellular amino acid biosynthetic process;ce
actin cytoskeleton organization;actin filament organization;actin filament-based process;actin nucleation;anatomical structure morphogenesis;Arp2/3 complex-mediated actin nucleation;asymmetric cell division;axon guidanc
anatomical structure formation involved in morphogenesis;apoptosis;biological regulation;blastocyst formation;carbohydrate homeostasis;cell death;cell junction assembly;cell junction organization;cell-cell junction assembly
anatomical structure morphogenesis;biological regulation;cell cycle phase;cell cycle process;cell part morphogenesis;cell projection morphogenesis;cell projection organization;cellular component assembly;cellular compone
actin cytoskeleton organization;actin filament organization;actin filament-based process;anatomical structure morphogenesis;biological regulation;cell cycle phase;cell cycle process;cell division;cell morphogenesis;cell surfac
cellular process;endosome transport;establishment of localization;establishment of localization in cell;establishment of protein localization;intracellular protein transport;intracellular transport;protein transport;retrograde trans
establishment of localization;lipid transport;organic substance transport;transport
actin cytoskeleton organization;actin filament-based process;actomyosin structure organization;cell differentiation;cell differentiation involved in embryonic placenta development;cellular component organization;cellular comp
apoptosis;base-excision repair, DNA ligation;biological regulation;catabolic process;cell activation;cell chemotaxis;cell death;cell migration;cell motility;cell projection organization;cellular catabolic process;cellular component
aging;alcohol biosynthetic process;alcohol metabolic process;anatomical structure morphogenesis;apoptosis;biosynthetic process;carbohydrate biosynthetic process;carbohydrate metabolic process;cell death;cell differentia
cell differentiation;cellular developmental process;cellular process;cellular response to calcium ion;cellular response to chemical stimulus;cellular response to inorganic substance;cellular response to metal ion;cellular respons
biological regulation;blood coagulation;cellular process;coagulation;establishment of localization;hemostasis;ion transmembrane transport;ion transport;multicellular organismal process;regulation of biological quality;regulatio
anatomical structure development;biological adhesion;biological regulation;blood coagulation;brown fat cell differentiation;cell adhesion;cell adhesion mediated by integrin;cell communication;cell differentiation;cell junction a
biosynthetic process;catabolic process;cellular biosynthetic process;cellular catabolic process;cellular component disassembly;cellular component disassembly at cellular level;cellular component organization;cellular compo
biological regulation;biosynthetic process;cellular biosynthetic process;cellular macromolecule biosynthetic process;cellular macromolecule metabolic process;cellular metabolic process;cellular nitrogen compound metabolic
activation of MAPKK activity;activation of protein kinase activity;apoptosis;axon guidance;biological regulation;biosynthetic process;cell death;cell surface receptor linked signaling pathway;cellular biosynthetic process;cellul
biosynthetic process;carboxylic acid catabolic process;carboxylic acid metabolic process;cardiolipin acyl-chain remodeling;cardiolipin metabolic process;catabolic process;cellular biosynthetic process;cellular catabolic proc
2-oxoglutarate metabolic process;acetyl-CoA catabolic process;acetyl-CoA metabolic process;alcohol catabolic process;alcohol metabolic process;amine catabolic process;amine metabolic process;anatomical structure dev
biological regulation;biosynthetic process;carbohydrate metabolic process;carbohydrate transport;cell cycle;cell cycle process;cell surface receptor linked signaling pathway;cellular biosynthetic process;cellular component a
cellular component organization;cellular component organization at cellular level;cellular component organization or biogenesis;cellular component organization or biogenesis at cellular level;cellular process;cytoskeleton orga
acetyl-CoA catabolic process;acetyl-CoA metabolic process;carboxylic acid metabolic process;catabolic process;cellular catabolic process;cellular ketone metabolic process;cellular metabolic process;cellular process;citrate
biological regulation;blood coagulation;cell projection organization;cellular component assembly;cellular component organization;cellular component organization at cellular level;cellular component organization or biogenesis
biological regulation;negative regulation of biological process;negative regulation of cell proliferation;negative regulation of cellular process;regulation of biological process;regulation of cell proliferation;regulation of cellular pr
cellular component movement;cellular component organization;cellular component organization at cellular level;cellular component organization or biogenesis;cellular component organization or biogenesis at cellular level;cel
biological regulation;cellular process;cellular response to stimulus;defense response;establishment of localization;establishment of localization in cell;establishment of protein localization;immune response;immune system pro
alcohol biosynthetic process;alcohol catabolic process;alcohol metabolic process;biological regulation;biosynthetic process;carbohydrate biosynthetic process;carbohydrate catabolic process;carbohydrate metabolic proces
aging;antigen processing and presentation;antigen processing and presentation of exogenous antigen;antigen processing and presentation of exogenous peptide antigen;antigen processing and presentation of exogenous pe
anatomical structure development;cellular component organization;cellular component organization at cellular level;cellular component organization or biogenesis;cellular component organization or biogenesis at cellular level
acetyl-CoA catabolic process;acetyl-CoA metabolic process;alcohol biosynthetic process;alcohol metabolic process;biosynthetic process;carbohydrate biosynthetic process;carbohydrate metabolic process;carboxylic acid r
biological regulation;catabolic process;cellular catabolic process;cellular macromolecule catabolic process;cellular macromolecule metabolic process;cellular metabolic process;cellular process;cellular protein metabolic proc
anatomical structure development;androgen receptor signaling pathway;biological regulation;cation transport;cellular component assembly;cellular component assembly at cellular level;cellular component organization;cellula
biological regulation;biosynthetic process;catabolic process;cellular biosynthetic process;cellular catabolic process;cellular component disassembly;cellular component disassembly at cellular level;cellular component organiz
alcohol metabolic process;biosynthetic process;carbohydrate biosynthetic process;carbohydrate metabolic process;cellular biosynthetic process;cellular carbohydrate biosynthetic process;cellular carbohydrate metabolic pr
activation of store-operated calcium channel activity;anatomical structure development;anatomical structure morphogenesis;appendage morphogenesis;biological regulation;calcium ion transmembrane transport;calcium ion
activation of immune response;activation of innate immune response;anatomical structure development;autophagy;biological regulation;catabolic process;cell communication;cell differentiation;cell proliferation;cellular catab
biological regulation;macromolecule metabolic process;metabolic process;positive regulation of catalytic activity;positive regulation of molecular function;primary metabolic process;protein metabolic process;proteolysis;regu
biological regulation;cell proliferation;cellular process;cellular response to stimulus;intracellular signal transduction;positive regulation of autophagy;positive regulation of biological process;positive regulation of biosynthetic p
biological regulation;defense response;defense response to virus;immune effector process;immune response;immune system process;innate immune response;multi-organism process;negative regulation of biological proces
biological regulation;cellular macromolecule metabolic process;cellular metabolic process;cellular process;cellular protein metabolic process;macromolecule metabolic process;macromolecule modification;metabolic process
biological regulation;biosynthetic process;catabolic process;cellular biosynthetic process;cellular catabolic process;cellular macromolecule biosynthetic process;cellular macromolecule catabolic process;cellular macromolec
alkene biosynthetic process;biosynthetic process;carboxylic acid biosynthetic process;carboxylic acid metabolic process;cellular alkene metabolic process;cellular biosynthetic process;cellular ketone metabolic process;cellu
biological regulation;cell cycle cytokinesis;cell cycle phase;cell cycle process;cell differentiation;cellular component assembly;cellular component assembly at cellular level;cellular component organization;cellular component
anatomical structure development;cellular component organization;cellular component organization at cellular level;cellular component organization or biogenesis;cellular component organization or biogenesis at cellular level
antigen processing and presentation;antigen processing and presentation of exogenous antigen;antigen processing and presentation of exogenous peptide antigen;antigen processing and presentation of exogenous peptide
anatomical structure development;bone development;developmental process
acetyl-CoA catabolic process;acetyl-CoA metabolic process;anatomical structure development;carboxylic acid metabolic process;catabolic process;cellular catabolic process;cellular ketone metabolic process;cellular metab
amine biosynthetic process;amine metabolic process;biological regulation;biosynthetic process;carboxylic acid biosynthetic process;carboxylic acid metabolic process;cellular amine metabolic process;cellular amino acid bio
biosynthetic process;cellular biosynthetic process;cellular macromolecule biosynthetic process;cellular macromolecule metabolic process;cellular metabolic process;cellular process;cellular protein metabolic process;establis
anatomical structure development;biological regulation;biosynthetic process;catabolic process;cell communication;cell cycle arrest;cell cycle process;cell development;cell growth;cell projection organization;cell surface rece
biological regulation;biosynthetic process;catabolic process;cellular biosynthetic process;cellular catabolic process;cellular component disassembly;cellular component disassembly at cellular level;cellular component organiz
amine transport;apoptosis;biological regulation;biosynthetic process;blood coagulation;cell activation;cell death;cellular biosynthetic process;cellular component assembly;cellular component assembly at cellular level;cellular
biological regulation;cell cycle arrest;cell cycle process;cell death;cell surface receptor linked signaling pathway;cellular process;cellular response to chemical stimulus;cellular response to cytokine stimulus;cellular response t
biological adhesion;biological regulation;cell adhesion;cell cycle arrest;cell cycle process;cell junction assembly;cell junction organization;cell motility;cell surface receptor linked signaling pathway;cell-substrate junction asse
cellular macromolecule metabolic process;cellular metabolic process;cellular nitrogen compound metabolic process;cellular process;gene expression;macromolecule metabolic process;metabolic process;mRNA metabolic p
biological regulation;biosynthetic process;cell cycle;cellular biosynthetic process;cellular component organization;cellular component organization at cellular level;cellular component organization or biogenesis;cellular compo
anatomical structure development;anatomical structure morphogenesis;biological regulation;biosynthetic process;branching involved in mammary gland duct morphogenesis;branching morphogenesis of a tube;cellular biosy
apoptosis;biological regulation;biosynthetic process;cell death;cellular biosynthetic process;cellular macromolecule biosynthetic process;cellular macromolecule metabolic process;cellular metabolic process;cellular nitrogen
biological regulation;biosynthetic process;cell cycle arrest;cell cycle process;cell proliferation;cellular biosynthetic process;cellular macromolecule biosynthetic process;cellular macromolecule metabolic process;cellular metal
androgen receptor signaling pathway;biological regulation;biosynthetic process;catabolic process;cellular biosynthetic process;cellular catabolic process;cellular macromolecule catabolic process;cellular macromolecule meta
amine biosynthetic process;amine metabolic process;anatomical structure development;aspartate family amino acid metabolic process;biological regulation;biosynthetic process;brain development;carboxylic acid biosynthet
cellular macromolecule metabolic process;cellular metabolic process;cellular nitrogen compound metabolic process;cellular process;gene expression;macromolecule metabolic process;metabolic process;mRNA metabolic p
cellular component organization;cellular component organization at cellular level;cellular component organization or biogenesis;cellular component organization or biogenesis at cellular level;cellular localization;cellular proces
anatomical structure development;biological regulation;catabolic process;cellular catabolic process;cellular macromolecule catabolic process;cellular macromolecule metabolic process;cellular metabolic process;cellular nitro
aging;ATP-dependent chromatin remodeling;biological regulation;cell aging;cell cycle process;cellular component assembly;cellular component assembly at cellular level;cellular component organization;cellular component o
biological regulation;catabolic process;cellular catabolic process;cellular component disassembly;cellular component disassembly at cellular level;cellular component organization;cellular component organization at cellular le
anatomical structure development;biosynthetic process;cell activation;cell proliferation;cellular aromatic compound metabolic process;cellular biosynthetic process;cellular component assembly;cellular component organizati
acetyl-CoA catabolic process;acetyl-CoA metabolic process;biological regulation;carboxylic acid metabolic process;catabolic process;cation homeostasis;cellular catabolic process;cellular cation homeostasis;cellular chemic
biosynthetic process;cellular aromatic compound metabolic process;cellular biosynthetic process;cellular metabolic process;cellular nitrogen compound biosynthetic process;cellular nitrogen compound metabolic process;ce
biosynthetic process;cellular biosynthetic process;cellular macromolecule biosynthetic process;cellular macromolecule metabolic process;cellular metabolic process;cellular process;cellular protein metabolic process;gene ex
biological regulation;cellular macromolecule metabolic process;cellular metabolic process;cellular nitrogen compound metabolic process;cellular process;gene expression;macromolecule metabolic process;metabolic proces
alcohol biosynthetic process;alcohol catabolic process;alcohol metabolic process;biosynthetic process;carbohydrate biosynthetic process;carbohydrate catabolic process;carbohydrate metabolic process;catabolic process;c
amine metabolic process;amino acid activation;arginyl-tRNA aminoacylation;carboxylic acid metabolic process;cellular amine metabolic process;cellular amino acid metabolic process;cellular ketone metabolic process;cellula
biosynthetic process;catabolic process;cellular biosynthetic process;cellular catabolic process;cellular component disassembly;cellular component disassembly at cellular level;cellular component organization;cellular compo
axon guidance;cell cycle phase;cell cycle process;cellular component assembly;cellular component assembly at cellular level;cellular component organization;cellular component organization at cellular level;cellular compone
cellular component assembly;cellular component assembly at cellular level;cellular component organization;cellular component organization at cellular level;cellular component organization or biogenesis;cellular component c
cellular component assembly;cellular component assembly at cellular level;cellular component organization;cellular component organization at cellular level;cellular component organization or biogenesis;cellular component c
anatomical structure formation involved in morphogenesis;angiogenesis;biological regulation;biosynthetic process;cell differentiation;cellular biosynthetic process;cellular developmental process;cellular macromolecule biosyn
anatomical structure formation involved in morphogenesis;angiogenesis;ATP biosynthetic process;ATP hydrolysis coupled proton transport;ATP metabolic process;ATP synthesis coupled proton transport;biological regulation
3'-UTR-mediated mRNA stabilization;ATP-dependent chromatin remodeling;biological regulation;cell differentiation;cellular component organization;cellular component organization at cellular level;cellular component organiz
actin cytoskeleton organization;actin filament-based movement;actin filament-based process;actin-mediated cell contraction;actin-myosin filament sliding;actomyosin structure organization;anatomical structure morphogene
amine metabolic process;amino acid activation;biosynthetic process;carboxylic acid metabolic process;cellular amine metabolic process;cellular amino acid metabolic process;cellular biosynthetic process;cellular componen
cellular macromolecule metabolic process;cellular metabolic process;cellular nitrogen compound metabolic process;cellular process;gene expression;macromolecule metabolic process;metabolic process;mRNA metabolic p
anatomical structure development;anatomical structure morphogenesis;axon guidance;biological adhesion;biological regulation;blood coagulation;cell adhesion;cell adhesion mediated by integrin;cell differentiation;cell migra
anatomical structure development;cell activation;cell migration;cell motility;cell proliferation;cellular component movement;cellular process;developmental process;epidermis development;keratinization;keratinocyte activation
binding of sperm to zona pellucida;cell recognition;cell-cell recognition;cellular macromolecule metabolic process;cellular metabolic process;cellular process;cellular process involved in reproduction;cellular protein metabolic
biological regulation;biosynthetic process;catabolic process;cellular biosynthetic process;cellular catabolic process;cellular component disassembly;cellular component disassembly at cellular level;cellular component organiz
biological regulation;cellular macromolecule metabolic process;cellular metabolic process;cellular nitrogen compound metabolic process;cellular process;cellular response to stimulus;gene expression;macromolecule metabo
apoptosis;biological regulation;biosynthetic process;cell cycle;cell cycle process;cell death;cellular biosynthetic process;cellular component organization;cellular component organization at cellular level;cellular component org
actin cytoskeleton organization;actin filament organization;actin filament-based movement;actin filament-based process;actin-mediated cell contraction;actin-myosin filament sliding;actomyosin structure organization;anatom
anatomical structure development;antigen processing and presentation;antigen processing and presentation of exogenous antigen;antigen processing and presentation of exogenous peptide antigen;antigen processing and
catabolic process;cellular catabolic process;cellular macromolecule catabolic process;cellular macromolecule metabolic process;cellular metabolic process;cellular process;ER-associated protein catabolic process;macromo
biological regulation;biosynthetic process;cellular biosynthetic process;cellular component assembly;cellular component assembly at cellular level;cellular component organization;cellular component organization at cellular le
biological regulation;biosynthetic process;cellular biosynthetic process;cellular macromolecule biosynthetic process;cellular macromolecule metabolic process;cellular metabolic process;cellular nitrogen compound metabolic

biological regulation;biosynthetic process;cell proliferation;cellular biosynthetic process;cellular macromolecule biosynthetic process;cellular macromolecule metabolic process;cellular metabolic process;cellular nitrogen com
anatomical structure morphogenesis;apoptosis;apoptotic mitochondrial changes;biological regulation;cell death;cell part morphogenesis;cellular component assembly;cellular component assembly at cellular level;cellular cor
amine metabolic process;biosynthetic process;carboxylic acid metabolic process;cellular amine metabolic process;cellular amino acid metabolic process;cellular aromatic compound metabolic process;cellular biosynthetic p
anatomical structure development;axon extension;biological regulation;cell growth;cell projection organization;cellular component movement;cellular component organization;cellular component organization at cellular level;c
amine metabolic process;amino acid activation;anatomical structure development;brain development;carboxylic acid metabolic process;cellular amine metabolic process;cellular amino acid metabolic process;cellular ketone
biosynthetic process;catabolic process;cellular biosynthetic process;cellular catabolic process;cellular component biogenesis;cellular component biogenesis at cellular level;cellular component disassembly;cellular componen
biological regulation;positive regulation of apoptosis;positive regulation of biological process;positive regulation of catalytic activity;positive regulation of cell death;positive regulation of cellular process;positive regulation of k
biological regulation;cellular macromolecule metabolic process;cellular metabolic process;cellular process;cellular protein metabolic process;chaperone-mediated protein folding;macromolecule metabolic process;metabolic
anatomical structure arrangement;anatomical structure development;axon extension;biological regulation;biosynthetic process;BMP signaling pathway;catabolic process;cell cycle phase;cell cycle process;cell division;cell gr
cellular component organization;cellular component organization at cellular level;cellular component organization or biogenesis;cellular component organization or biogenesis at cellular level;cellular macromolecule metabolic
biological regulation;cell differentiation;cellular developmental process;cellular macromolecule metabolic process;cellular metabolic process;cellular nitrogen compound metabolic process;cellular process;cellular response to
antigen processing and presentation;antigen processing and presentation of exogenous antigen;antigen processing and presentation of exogenous peptide antigen;antigen processing and presentation of exogenous peptide
adenine transport;biological regulation;cellular metabolic process;cellular process;chromosome segregation;energy derivation by oxidation of organic compounds;energy reserve metabolic process;establishment of localizatio
actin cytoskeleton organization;actin filament-based process;actomyosin structure organization;anatomical structure homeostasis;anatomical structure morphogenesis;associative learning;axon extension;axon guidance;B ce
anatomical structure development;anatomical structure formation involved in morphogenesis;biological regulation;blastocyst development;cellular process;cellular response to acid;cellular response to amine stimulus;cellular
biological regulation;biosynthetic process;cellular biosynthetic process;cellular macromolecule biosynthetic process;cellular macromolecule metabolic process;cellular metabolic process;cellular nitrogen compound metabolic
cellular macromolecule metabolic process;cellular metabolic process;cellular nitrogen compound metabolic process;cellular process;gene expression;macromolecule metabolic process;metabolic process;mRNA metabolic p
anatomical structure formation involved in morphogenesis;angiogenesis;biological adhesion;biological regulation;cell adhesion;cell differentiation;cell proliferation;cellular component organization;cellular component organizat
axon extension;biological regulation;cell growth;cell projection assembly;cell projection organization;cell surface receptor linked signaling pathway;cellular component assembly;cellular component assembly at cellular level;c
cell differentiation;cellular developmental process;cellular process;developmental process;hemopoietic progenitor cell differentiation
biological regulation;cellular process;cellular response to acid;cellular response to amine stimulus;cellular response to amino acid stimulus;cellular response to chemical stimulus;cellular response to endogenous stimulus;cell
ameboidal cell migration;anatomical structure development;anatomical structure formation involved in morphogenesis;anatomical structure morphogenesis;angiogenesis;biological adhesion;biological regulation;branching inv
anatomical structure morphogenesis;biological regulation;cell cycle;cell surface receptor linked signaling pathway;cellular component organization;cellular component organization at cellular level;cellular component organizat
anatomical structure development;anatomical structure morphogenesis;axon guidance;biological adhesion;biological regulation;cell adhesion;cell junction assembly;cell junction organization;cell migration;cell morphogenesis
active induction of host immune response by virus;adenine nucleotide transport;ADP transport;apoptosis;ATP transport;biological regulation;cell death;cellular macromolecule metabolic process;cellular metabolic process;cel
amine metabolic process;amino acid activation;aspartyl-tRNA aminoacylation;biosynthetic process;carboxylic acid metabolic process;cellular amine metabolic process;cellular amino acid metabolic process;cellular biosynthe
aging;anatomical structure development;anatomical structure morphogenesis;biological regulation;cell differentiation;cell part morphogenesis;cell projection morphogenesis;cell projection organization;cellular component mo
activation of protein kinase activity;apoptosis;biological regulation;cell death;cellular macromolecule metabolic process;cellular metabolic process;cellular process;cellular protein metabolic process;cellular response to stimul
biological regulation;cell growth;cellular process;growth;negative regulation of apoptosis;negative regulation of biological process;negative regulation of cell death;negative regulation of cellular process;negative regulation of
biological regulation;biosynthetic process;cellular biosynthetic process;cellular component organization;cellular component organization at cellular level;cellular component organization or biogenesis;cellular component orga
assembly of spliceosomal tri-snRNP;biological regulation;cellular component assembly;cellular component assembly at cellular level;cellular component organization;cellular component organization at cellular level;cellular co
actin cytoskeleton organization;actin filament capping;actin filament organization;actin filament polymerization;actin filament severing;actin filament-based process;actin nucleation;actin polymerization or depolymerization;ag
actin filament-based movement;actin filament-based process;actin-mediated cell contraction;actin-myosin filament sliding;biological regulation;cellular component movement;cellular process;multicellular organismal process
actin cytoskeleton organization;actin filament organization;actin filament-based process;adherens junction organization;aging;anatomical structure development;anatomical structure morphogenesis;apical junction assembly;
anatomical structure development;anatomical structure morphogenesis;biological regulation;cellular component disassembly;cellular component disassembly at cellular level;cellular component organization;cellular compone
amine metabolic process;amino acid activation;carboxylic acid metabolic process;cellular amine metabolic process;cellular amino acid metabolic process;cellular ketone metabolic process;cellular macromolecule metabolic
biological regulation;biosynthetic process;calcium ion homeostasis;cation homeostasis;cellular biosynthetic process;cellular calcium ion homeostasis;cellular cation homeostasis;cellular chemical homeostasis;cellular divalen
apoptosis;biological regulation;catabolic process;cell death;cell surface receptor linked signaling pathway;cellular catabolic process;cellular component disassembly;cellular component disassembly at cellular level;cellular co
biological regulation;cellular macromolecule metabolic process;cellular metabolic process;cellular nitrogen compound metabolic process;cellular process;macromolecule metabolic process;metabolic process;mRNA metaboli
biological regulation;biosynthetic process;cellular biosynthetic process;cellular macromolecule biosynthetic process;cellular macromolecule metabolic process;cellular metabolic process;cellular nitrogen compound metabolic
ATP-dependent chromatin remodeling;biological regulation;biosynthetic process;cellular biosynthetic process;cellular component assembly;cellular component assembly at cellular level;cellular component organization;cellula
cell cycle;cell cycle process;cell division;cellular component organization;cellular component organization at cellular level;cellular component organization or biogenesis;cellular component organization or biogenesis at cellula
aging;anatomical structure development;cell differentiation;cell junction assembly;cell junction organization;cell-substrate junction assembly;cellular component assembly;cellular component assembly at cellular level;cellular
amine metabolic process;anatomical structure development;aromatic compound biosynthetic process;biosynthetic process;brainstem development;carboxylic acid metabolic process;cellular amine metabolic process;cellula
aging;biological regulation;catabolic process;cellular catabolic process;cellular component assembly;cellular component assembly at cellular level;cellular component organization;cellular component organization at cellular le
biological regulation;biosynthetic process;cellular biosynthetic process;cellular component assembly;cellular component assembly at cellular level;cellular component organization;cellular component organization at cellular le
cellular component organization;cellular component organization at cellular level;cellular component organization or biogenesis;cellular component organization or biogenesis at cellular level;cellular membrane fusion;cellular
biological regulation;biosynthetic process;cellular biosynthetic process;cellular component organization;cellular component organization at cellular level;cellular component organization or biogenesis;cellular component orga
biological regulation;negative regulation of apoptosis;negative regulation of biological process;negative regulation of cell death;negative regulation of cellular process;negative regulation of programmed cell death;regulation o
alternative nuclear mRNA splicing, via spliceosome;cellular macromolecule metabolic process;cellular metabolic process;cellular nitrogen compound metabolic process;cellular process;establishment of localization;establish
biological regulation;biosynthetic process;catabolic process;cellular biosynthetic process;cellular catabolic process;cellular macromolecule biosynthetic process;cellular macromolecule catabolic process;cellular macromolec
actin cytoskeleton organization;actin filament-based process;activation of adenylate cyclase activity;ameboidal cell migration;axon guidance;biological regulation;blood coagulation;cell activation;cell migration;cell motility;cel
cellular component movement;cellular process;multicellular organismal process;muscle contraction;muscle system process;system process
acetyl-CoA metabolic process;acyl-CoA biosynthetic process;acyl-CoA metabolic process;acylglycerol biosynthetic process;acylglycerol metabolic process;amine transport;amino acid transmembrane transport;amino acid t
biological regulation;cellular component assembly;cellular component organization;cellular component organization or biogenesis;cellular macromolecule metabolic process;cellular metabolic process;cellular nitrogen compo
apoptosis;biological regulation;cell death;cellular component disassembly;cellular component disassembly at cellular level;cellular component disassembly involved in apoptosis;cellular component organization;cellular comp
biological regulation;cellular component organization;cellular component organization or biogenesis;cellular macromolecule metabolic process;cellular membrane organization;cellular metabolic process;cellular process;cellul
cellular macromolecule metabolic process;cellular metabolic process;cellular nitrogen compound metabolic process;cellular process;macromolecule metabolic process;metabolic process;mRNA metabolic process;mRNA pro
cellular macromolecule metabolic process;cellular metabolic process;cellular process;cellular protein metabolic process;macromolecule metabolic process;metabolic process;primary metabolic process;protein folding;protein
amine biosynthetic process;amine catabolic process;amine metabolic process;anatomical structure development;biological regulation;biosynthetic process;carboxylic acid biosynthetic process;carboxylic acid catabolic proc
biological regulation;cation homeostasis;cation transport;cell differentiation;cellular cation homeostasis;cellular chemical homeostasis;cellular developmental process;cellular homeostasis;cellular ion homeostasis;cellular iron
biological regulation;cell activation;cell activation involved in immune response;cellular component organization;cellular component organization or biogenesis;cellular membrane organization;cellular process;defense respons
biosynthetic process;catabolic process;cellular biosynthetic process;cellular catabolic process;cellular component biogenesis;cellular component biogenesis at cellular level;cellular component disassembly;cellular componen
biosynthetic process;cell cycle;cell cycle process;cellular biosynthetic process;cellular macromolecule biosynthetic process;cellular macromolecule metabolic process;cellular metabolic process;cellular nitrogen compound m
biosynthetic process;catabolic process;cellular biosynthetic process;cellular catabolic process;cellular component biogenesis;cellular component biogenesis at cellular level;cellular component disassembly;cellular componen
apoptosis;cell death;cell proliferation;cellular process;death;establishment of localization;establishment of localization in cell;establishment of protein localization;intracellular protein transport;intracellular transport;nuclear exp
adenosine to inosine editing;anatomical structure development;base conversion or substitution editing;biological regulation;cell differentiation;cell surface receptor linked signaling pathway;cellular component assembly;cellul
anatomical structure morphogenesis;biological regulation;biosynthetic process;catabolic process;cell surface receptor linked signaling pathway;cellular biosynthetic process;cellular catabolic process;cellular macromolecule
actin filament-based movement;actin filament-based process;actin-mediated cell contraction;actin-myosin filament sliding;cell differentiation;cellular component movement;cellular developmental process;cellular process;dev
cell cycle phase;cell cycle process;cell division;cellular component organization;cellular component organization at cellular level;cellular component organization or biogenesis;cellular component organization or biogenesis at
cellular macromolecule metabolic process;cellular metabolic process;cellular process;cellular protein metabolic process;chaperone-mediated protein folding;macromolecule metabolic process;macromolecule modification;me
carbohydrate metabolic process;cellular carbohydrate metabolic process;cellular macromolecule metabolic process;cellular metabolic process;cellular process;cellular protein metabolic process;'de novo' posttranslational pr
anatomical structure morphogenesis;axon guidance;biological adhesion;biological regulation;cell adhesion;cell differentiation;cell morphogenesis;cell morphogenesis involved in differentiation;cell projection organization;cell-
biological regulation;biosynthetic process;cell death;cell division;cellular biosynthetic process;cellular component organization;cellular component organization at cellular level;cellular component organization or biogenesis;ce
alcohol biosynthetic process;alcohol catabolic process;alcohol metabolic process;amine metabolic process;anatomical structure development;biological regulation;biosynthetic process;carbohydrate biosynthetic process;car
alcohol biosynthetic process;alcohol metabolic process;biological regulation;biosynthetic process;biotin metabolic process;carbohydrate biosynthetic process;carbohydrate metabolic process;carboxylic acid metabolic proce
anatomical structure formation involved in morphogenesis;angiogenesis;angiotensin maturation;biological regulation;cell differentiation;cellular developmental process;cellular macromolecule metabolic process;cellular metab
amine metabolic process;amino acid activation;biosynthetic process;bis(5'-nucleosidyl) oligophosphate biosynthetic process;bis(5'-nucleosidyl) oligophosphate metabolic process;carboxylic acid metabolic process;cellular a
binding of sperm to zona pellucida;cell recognition;cell-cell recognition;cellular component assembly;cellular component assembly at cellular level;cellular component organization;cellular component organization at cellular le
5-phosphoribose 1-diphosphate biosynthetic process;5-phosphoribose 1-diphosphate metabolic process;alcohol biosynthetic process;alcohol metabolic process;biological regulation;biosynthetic process;carbohydrate bios
binding of sperm to zona pellucida;biological regulation;cell recognition;cell-cell recognition;cellular component assembly;cellular component assembly at cellular level;cellular component organization;cellular component orga
biological regulation;posttranscriptional regulation of gene expression;regulation of biological process;regulation of gene expression;regulation of macromolecule metabolic process;regulation of metabolic process
biological regulation;blood coagulation;cell activation;cell cycle;cell cycle process;cellular component assembly;cellular component assembly at cellular level;cellular component organization;cellular component organization a
biological regulation;cell cycle;cell cycle process;cell killing;cellular component assembly;cellular component assembly at cellular level;cellular component movement;cellular component organization;cellular component orga
anatomical structure homeostasis;biological regulation;biosynthetic process;catabolic process;cellular biosynthetic process;cellular catabolic process;cellular component organization;cellular component organization at cellul
actin cytoskeleton organization;actin filament bundle assembly;actin filament organization;actin filament-based process;biological regulation;cell projection organization;cellular component assembly;cellular component asse
activation of immune response;anatomical structure homeostasis;biological regulation;complement activation;complement activation, lectin pathway;defense response;fibrinolysis;homeostatic process;humoral immune respo
biological regulation;cell cycle;cell cycle process;cell division;cell killing;cellular component assembly;cellular component assembly at cellular level;cellular component movement;cellular component organization;cellular comp
anatomical structure development;anatomical structure homeostasis;biological regulation;biosynthetic process;brain development;cellular biosynthetic process;cellular component organization;cellular component organizatio
anatomical structure development;anatomical structure homeostasis;biological regulation;biosynthetic process;brain development;cell differentiation;cell proliferation;cellular biosynthetic process;cellular component organizat
biological regulation;cellular macromolecule metabolic process;cellular metabolic process;cellular nitrogen compound metabolic process;cellular process;establishment of localization;establishment of RNA localization;gene e
biological regulation;biosynthetic process;cellular biosynthetic process;cellular component organization;cellular component organization at cellular level;cellular component organization or biogenesis;cellular component orga
alkaloid metabolic process;biological regulation;biosynthetic process;cell communication;cell-cell signaling;cellular amide metabolic process;cellular biosynthetic process;cellular metabolic process;cellular nitrogen compoun
biosynthetic process;cell cycle;cell cycle process;cellular biosynthetic process;cellular component assembly;cellular component assembly at cellular level;cellular component organization;cellular component organization at c
binding of sperm to zona pellucida;cell recognition;cell-cell recognition;cellular macromolecule metabolic process;cellular metabolic process;cellular process;cellular process involved in reproduction;cellular protein metabolic
alcohol metabolic process;carbohydrate metabolic process;carbohydrate phosphorylation;cellular carbohydrate metabolic process;cellular metabolic process;cellular process;fructose 6-phosphate metabolic process;fructos
biological regulation;cell surface receptor linked signaling pathway;cellular process;cellular response to stimulus;establishment of localization;establishment of localization in cell;establishment of protein localization;G-protein
biological regulation;biosynthetic process;catabolic process;cell surface receptor linked signaling pathway;cellular biosynthetic process;cellular catabolic process;cellular component organization;cellular component organizat
anatomical structure morphogenesis;biological regulation;blood coagulation;bone morphogenesis;cell migration;cell motility;cellular component movement;cellular process;coagulation;developmental process;hemostasis;imm
apoptosis;apoptotic chromosome condensation;biological regulation;cell death;cell differentiation;cellular component disassembly;cellular component disassembly at cellular level;cellular component disassembly involved in
adherens junction organization;anatomical structure development;biological adhesion;biological regulation;biosynthetic process;brain development;cell adhesion;cell junction assembly;cell junction organization;cell surface re
biological regulation;biosynthetic process;catabolic process;cell surface receptor linked signaling pathway;cellular biosynthetic process;cellular catabolic process;cellular macromolecule biosynthetic process;cellular macrom
alanyl-tRNA aminoacylation;amine metabolic process;amino acid activation;anatomical structure development;biological regulation;carboxylic acid metabolic process;cellular amine metabolic process;cellular amino acid met
antigen processing and presentation;antigen processing and presentation of peptide antigen;antigen processing and presentation of peptide antigen via MHC class I;cellular macromolecule metabolic process;cellular metabo
biological regulation;cell proliferation;establishment of localization;establishment of protein localization;establishment of RNA localization;macromolecule metabolic process;metabolic process;mRNA transport;negative regula
ATP-dependent chromatin remodeling;biological regulation;biosynthetic process;cellular biosynthetic process;cellular component assembly;cellular component assembly at cellular level;cellular component organization;cellula
anatomical structure homeostasis;biological regulation;biosynthetic process;cell cycle process;cellular biosynthetic process;cellular component organization;cellular component organization at cellular level;cellular componen
cell division;cellular component assembly;cellular component assembly at cellular level;cellular component organization;cellular component organization at cellular level;cellular component organization or biogenesis;cellular c
anatomical structure morphogenesis;biological regulation;cell differentiation;cellular developmental process;cellular process;developmental process;morphogenesis of an epithelium;positive regulation of biological process;po
viral reproduction
biological regulation;cellular process;cellular response to stimulus;establishment of localization;establishment of protein localization;intracellular signal transduction;protein transport;regulation of biological process;regulation
biosynthetic process;cell cycle;cell cycle process;cellular biosynthetic process;cellular macromolecule biosynthetic process;cellular macromolecule metabolic process;cellular metabolic process;cellular nitrogen compound m
biological regulation;cell differentiation;cellular component assembly;cellular component assembly at cellular level;cellular component organization;cellular component organization at cellular level;cellular component organizat
binding of sperm to zona pellucida;cell recognition;cell-cell recognition;cellular macromolecule metabolic process;cellular metabolic process;cellular process;cellular process involved in reproduction;cellular protein metabolic
cell cycle;cell cycle process;cell division;cellular component organization;cellular component organization at cellular level;cellular component organization or biogenesis;cellular component organization or biogenesis at cellula
biosynthetic process;carbohydrate metabolic process;cellular biosynthetic process;cellular carbohydrate metabolic process;cellular macromolecule biosynthetic process;cellular macromolecule metabolic process;cellular met
biological regulation;blood coagulation;cellular process;cellular response to stimulus;coagulation;hemostasis;multicellular organismal process;negative regulation of apoptosis;negative regulation of biological process;negative
biosynthetic process;cell cycle;cell cycle process;cellular biosynthetic process;cellular macromolecule biosynthetic process;cellular macromolecule metabolic process;cellular metabolic process;cellular nitrogen compound m
amine metabolic process;amino acid activation;apoptosis;biological regulation;carboxylic acid metabolic process;cell death;cellular amine metabolic process;cellular amino acid metabolic process;cellular ketone metabolic p
anatomical structure development;anatomical structure morphogenesis;biological regulation;cellular component organization;cellular component organization at cellular level;cellular component organization or biogenesis;cell
actin cytoskeleton organization;actin filament bundle assembly;actin filament organization;actin filament-based process;cell migration;cell motility;cell proliferation;cellular component assembly;cellular component assembly a
biological regulation;biosynthetic process;catabolic process;cellular biosynthetic process;cellular catabolic process;cellular macromolecule biosynthetic process;cellular macromolecule catabolic process;cellular macromolec
biosynthetic process;cell differentiation;cellular biosynthetic process;cellular developmental process;cellular macromolecule biosynthetic process;cellular macromolecule metabolic process;cellular metabolic process;cellular
amine metabolic process;amino acid activation;biosynthetic process;carboxylic acid metabolic process;cellular amine metabolic process;cellular amino acid metabolic process;cellular biosynthetic process;cellular componen
biological regulation;biosynthetic process;cellular biosynthetic process;cellular component disassembly;cellular component disassembly at cellular level;cellular component organization;cellular component organization at cell
biological regulation;biosynthetic process;cellular biosynthetic process;cellular component assembly;cellular component assembly at cellular level;cellular component organization;cellular component organization at cellular le
alcohol catabolic process;alcohol metabolic process;anatomical structure development;carbohydrate catabolic process;carbohydrate metabolic process;carboxylic acid metabolic process;catabolic process;cell communicati
acute inflammatory response;acute-phase response;aging;amide biosynthetic process;amine biosynthetic process;amine metabolic process;anatomical structure development;arginine biosynthetic process;arginine metabolic

anatomical structure development;biological adhesion;biological regulation;blood coagulation;calcium ion homeostasis;calcium ion transmembrane transport;calcium ion transport;calcium-mediated signaling;cation homeost
binding of sperm to zona pellucida;cell recognition;cell-cell recognition;cellular component assembly;cellular component assembly at cellular level;cellular component organization;cellular component organization at cellular le
apoptosis;biological regulation;cell cycle;cell death;cellular process;cytokinetic cell separation;cytokinetic process;death;establishment of localization;establishment of protein localization;programmed cell death;protein trans
biological regulation;biosynthetic process;cellular biosynthetic process;cellular component assembly;cellular component assembly at cellular level;cellular component organization;cellular component organization at cellular le
actin cytoskeleton organization;actin filament organization;actin filament polymerization;actin filament-based process;actin polymerization or depolymerization;biological regulation;cell projection organization;cellular compone
actin cytoskeleton organization;actin filament organization;actin filament-based process;alcohol biosynthetic process;alcohol catabolic process;alcohol metabolic process;anatomical structure homeostasis;ATP biosynthetic p
actin filament capping;anatomical structure development;apical protein localization;asymmetric protein localization;biological regulation;cell development;cell projection assembly;cell projection organization;cellular compone
cellular component organization;cellular component organization at cellular level;cellular component organization or biogenesis;cellular component organization or biogenesis at cellular level;cellular process;COPI coating of
binding of sperm to zona pellucida;cell recognition;cell-cell recognition;cellular macromolecule metabolic process;cellular metabolic process;cellular process;cellular process involved in reproduction;cellular protein metabolic
anatomical structure morphogenesis;biological regulation;biosynthetic process;cell morphogenesis;cell morphogenesis involved in differentiation;cellular biosynthetic process;cellular component assembly;cellular component
biological regulation;biosynthetic process;cell communication;cell differentiation;cellular biosynthetic process;cellular developmental process;cellular macromolecule biosynthetic process;cellular macromolecule metabolic pro
biosynthetic process;carboxylic acid catabolic process;carboxylic acid metabolic process;cardiolipin acyl-chain remodeling;cardiolipin metabolic process;catabolic process;cellular biosynthetic process;cellular catabolic proc
adherens junction organization;anatomical structure development;anatomical structure homeostasis;ATP-dependent chromatin remodeling;axon guidance;biological adhesion;biological regulation;blood coagulation;cell adhes
actin cytoskeleton organization;actin cytoskeleton reorganization;actin filament organization;actin filament polymerization;actin filament-based process;actin polymerization or depolymerization;anatomical structure morphoge
biological regulation;cellular component organization;cellular component organization or biogenesis;cellular macromolecule metabolic process;cellular membrane organization;cellular metabolic process;cellular process;cellul
biological regulation;cell cycle;cell cycle phase;cell cycle process;cell division;cellular component organization;cellular component organization at cellular level;cellular component organization or biogenesis;cellular componen
anatomical structure formation involved in morphogenesis;anatomical structure morphogenesis;apoptotic cell clearance;biological regulation;blood vessel remodeling;branching involved in salivary gland morphogenesis;calci
biological regulation;biosynthetic process;cell cycle;cell cycle process;cell proliferation;cellular biosynthetic process;cellular macromolecule biosynthetic process;cellular macromolecule metabolic process;cellular metabolic p
biological regulation;cell cycle;cell cycle phase;cell cycle process;cell division;cellular component organization;cellular component organization at cellular level;cellular component organization or biogenesis;cellular componen
cell cycle;cell division;cellular component assembly;cellular component organization;cellular component organization or biogenesis;cellular process;macromolecular complex assembly;macromolecular complex subunit organ
alcohol catabolic process;alcohol metabolic process;biological regulation;carbohydrate catabolic process;carbohydrate homeostasis;carbohydrate metabolic process;carbohydrate phosphorylation;carbohydrate transport;ca
biosynthetic process;cell cycle;cell cycle process;cellular biosynthetic process;cellular macromolecule biosynthetic process;cellular macromolecule metabolic process;cellular metabolic process;cellular nitrogen compound m
activation of immune response;activation of innate immune response;activation of phospholipase C activity;behavior;biological regulation;blood coagulation;calcium ion homeostasis;calcium ion transport;calcium ion transpo
activation of MAPK activity;amine metabolic process;aminoglycan biosynthetic process;aminoglycan catabolic process;aminoglycan metabolic process;anatomical structure formation involved in morphogenesis;angiogenesis
biological regulation;blood coagulation;cell activation;cellular process;coagulation;establishment of localization;establishment of localization in cell;exocytosis;hemostasis;multicellular organismal process;neurological system
acid secretion;alpha-beta T cell activation;alpha-beta T cell differentiation;anatomical structure development;arachidonic acid secretion;biological regulation;carboxylic acid transport;cell activation;cell differentiation;cell surfa
alcohol biosynthetic process;alcohol metabolic process;apoptosis;biological regulation;biosynthetic process;carbohydrate biosynthetic process;carbohydrate metabolic process;cell death;cellular biosynthetic process;cellula
biological regulation;cell redox homeostasis;cellular homeostasis;cellular macromolecule metabolic process;cellular metabolic process;cellular process;cellular protein metabolic process;cellular response to stimulus;cellular r
biological regulation;biosynthetic process;cell differentiation;cellular biosynthetic process;cellular component assembly;cellular component assembly at cellular level;cellular component organization;cellular component organi
anatomical structure development;biosynthetic process;cellular biosynthetic process;cellular macromolecule biosynthetic process;cellular macromolecule metabolic process;cellular metabolic process;cellular nitrogen compo
biological regulation;cell cycle;cell cycle checkpoint;cell cycle phase;cell cycle process;cell division;cellular component organization;cellular component organization at cellular level;cellular component organization or biogene
binding of sperm to zona pellucida;cell recognition;cell-cell recognition;cellular component assembly;cellular component assembly at cellular level;cellular component organization;cellular component organization at cellular le
catabolic process;cellular catabolic process;cellular macromolecule catabolic process;cellular macromolecule metabolic process;cellular metabolic process;cellular process;cellular protein metabolic process;cellular response
amine metabolic process;amino acid activation;biosynthetic process;carboxylic acid metabolic process;cellular amine metabolic process;cellular amino acid metabolic process;cellular biosynthetic process;cellular ketone me
biological regulation;biosynthetic process;catabolic process;cell differentiation;cellular biosynthetic process;cellular catabolic process;cellular component disassembly;cellular component disassembly at cellular level;cellular
actin cytoskeleton organization;actin filament-based process;actomyosin structure organization;biological regulation;cell differentiation;cell differentiation involved in embryonic placenta development;cell surface receptor link
cell cycle process;cell division;cellular component movement;cellular component organization;cellular component organization at cellular level;cellular component organization or biogenesis;cellular component organization o
biological regulation;biosynthetic process;cellular biosynthetic process;cellular macromolecule biosynthetic process;cellular macromolecule metabolic process;cellular metabolic process;cellular nitrogen compound metabolic
biological regulation;calcium ion homeostasis;cation homeostasis;cellular calcium ion homeostasis;cellular cation homeostasis;cellular chemical homeostasis;cellular divalent inorganic cation homeostasis;cellular homeostasi
biological regulation;biosynthetic process;cell growth;cellular biosynthetic process;cellular macromolecule biosynthetic process;cellular macromolecule metabolic process;cellular metabolic process;cellular nitrogen compoun
acyl-CoA biosynthetic process;acyl-CoA metabolic process;acylglycerol biosynthetic process;acylglycerol metabolic process;biological regulation;biosynthetic process;carbohydrate metabolic process;carboxylic acid metabo
cellular macromolecule metabolic process;cellular metabolic process;cellular process;cellular protein metabolic process;macromolecule metabolic process;macromolecule modification;metabolic process;primary metabolic p
actin cytoskeleton organization;actin filament bundle assembly;actin filament organization;actin filament-based movement;actin filament-based process;cellular component assembly;cellular component assembly at cellular le
biological regulation;cardiac muscle contraction;cation homeostasis;cation transport;cellular cation homeostasis;cellular chemical homeostasis;cellular homeostasis;cellular ion homeostasis;cellular metal ion homeostasis;cell
10-formyltetrahydrofolate biosynthetic process;10-formyltetrahydrofolate metabolic process;amine biosynthetic process;amine metabolic process;anatomical structure development;anatomical structure formation involved in
axon guidance;biological regulation;cellular chemical homeostasis;cellular component disassembly;cellular component disassembly at cellular level;cellular component movement;cellular component organization;cellular com
anatomical structure development;cell development;cell junction assembly;cell junction organization;cellular component assembly;cellular component assembly at cellular level;cellular component organization;cellular compo
ATP biosynthetic process;ATP hydrolysis coupled proton transport;ATP metabolic process;ATP synthesis coupled proton transport;biological regulation;biosynthetic process;cation transport;cellular biosynthetic process;cellu
biosynthetic process;catabolic process;cellular biosynthetic process;cellular catabolic process;cellular component disassembly;cellular component disassembly at cellular level;cellular component organization;cellular compo
carbohydrate metabolic process;cellular carbohydrate metabolic process;cellular macromolecule metabolic process;cellular metabolic process;cellular process;cellular protein metabolic process;glycosylation;macromolecule
biological regulation;biosynthetic process;cellular biosynthetic process;cellular macromolecule biosynthetic process;cellular macromolecule metabolic process;cellular metabolic process;cellular nitrogen compound metabolic
biological regulation;biosynthetic process;cellular biosynthetic process;cellular macromolecule biosynthetic process;cellular macromolecule metabolic process;cellular metabolic process;cellular nitrogen compound metabolic
amine metabolic process;amino acid activation;carboxylic acid metabolic process;cellular amine metabolic process;cellular amino acid metabolic process;cellular ketone metabolic process;cellular macromolecule metabolic p
cellular macromolecule metabolic process;cellular metabolic process;cellular nitrogen compound metabolic process;cellular process;macromolecule metabolic process;metabolic process;mRNA metabolic process;mRNA pro
alcohol biosynthetic process;alcohol catabolic process;alcohol metabolic process;aldehyde biosynthetic process;biological regulation;biosynthetic process;carbohydrate biosynthetic process;carbohydrate catabolic process;
antigen processing and presentation;antigen processing and presentation of exogenous antigen;antigen processing and presentation of exogenous peptide antigen;antigen processing and presentation of exogenous peptide
biological regulation;cellular component organization;cellular component organization or biogenesis;defense response;establishment of localization;establishment of localization in cell;establishment of protein localization;esta
amine metabolic process;aminoglycan biosynthetic process;aminoglycan catabolic process;aminoglycan metabolic process;anatomical structure development;anatomical structure formation involved in morphogenesis;anato
alcohol biosynthetic process;alcohol metabolic process;biosynthetic process;carbohydrate biosynthetic process;carbohydrate metabolic process;cell differentiation;cellular biosynthetic process;cellular carbohydrate biosynth
amine metabolic process;amino acid activation;carboxylic acid metabolic process;cellular amine metabolic process;cellular amino acid metabolic process;cellular ketone metabolic process;cellular macromolecule metabolic p
amide biosynthetic process;amine biosynthetic process;amine catabolic process;amine metabolic process;anatomical structure development;anatomical structure morphogenesis;arginine biosynthetic process;arginine metab
amine metabolic process;amino acid activation;carboxylic acid metabolic process;cell differentiation;cellular amine metabolic process;cellular amino acid metabolic process;cellular developmental process;cellular ketone met
anterior/posterior pattern specification;cellular macromolecule metabolic process;cellular metabolic process;cellular nitrogen compound metabolic process;cellular process;developmental process;gene expression;macromol
4-hydroxyproline metabolic process;amine metabolic process;biological regulation;carboxylic acid metabolic process;cell redox homeostasis;cellular amine metabolic process;cellular amino acid metabolic process;cellular co
actin cytoskeleton organization;actin cytoskeleton reorganization;actin filament bundle assembly;actin filament organization;actin filament-based process;anatomical structure development;axon guidance;biological adhesion
cell differentiation;cellular component assembly;cellular component assembly at cellular level;cellular component organization;cellular component organization at cellular level;cellular component organization or biogenesis;ce
alcohol biosynthetic process;alcohol metabolic process;aldehyde biosynthetic process;aldehyde catabolic process;anatomical structure formation involved in morphogenesis;angiogenesis;behavior;biological regulation;biosy
apoptosis;cell death;cellular component disassembly;cellular component disassembly at cellular level;cellular component disassembly involved in apoptosis;cellular component organization;cellular component organization a
biological regulation;cellular macromolecule metabolic process;cellular metabolic process;cellular process;cellular protein metabolic process;establishment of localization;establishment of localization in cell;establishment of p
biological regulation;cell differentiation;cellular developmental process;cellular macromolecule metabolic process;cellular metabolic process;cellular nitrogen compound metabolic process;cellular process;circadian regulation
alternative nuclear mRNA splicing, via spliceosome;biological regulation;biosynthetic process;cellular biosynthetic process;cellular component organization;cellular component organization at cellular level;cellular component
anatomical structure development;anatomical structure morphogenesis;biological regulation;cell surface receptor linked signaling pathway;cellular component organization;cellular component organization or biogenesis;cellul
activation of caspase activity;aggresome assembly;biological regulation;biosynthetic process;carbohydrate metabolic process;catabolic process;cellular biosynthetic process;cellular carbohydrate metabolic process;cellular
biological regulation;biosynthetic process;catabolic process;cell surface receptor linked signaling pathway;cellular biosynthetic process;cellular catabolic process;cellular macromolecule biosynthetic process;cellular macrom
anatomical structure formation involved in morphogenesis;angiogenesis;biological regulation;body fluid secretion;cellular component assembly;cellular component assembly at cellular level;cellular component organization;ce
axon guidance;biological regulation;biosynthetic process;cell communication;cell-cell signaling;cellular biosynthetic process;cellular component disassembly;cellular component disassembly at cellular level;cellular componen
anatomical structure formation involved in morphogenesis;angiogenesis;developmental process
cell cycle phase;cell cycle process;cell proliferation;cellular process;meiosis
activation of signaling protein activity involved in unfolded protein response;anatomical structure arrangement;anatomical structure development;biological regulation;blood coagulation;catabolic process;cell activation;cell co
alcohol metabolic process;carbohydrate catabolic process;carbohydrate metabolic process;catabolic process;cellular carbohydrate catabolic process;cellular carbohydrate metabolic process;cellular glucan metabolic proces
response to stimulus;response to stress
biological regulation;cell differentiation;cellular developmental process;cellular macromolecule metabolic process;cellular metabolic process;cellular nitrogen compound metabolic process;cellular process;cellular response to
biosynthetic process;catabolic process;cellular biosynthetic process;cellular catabolic process;cellular component disassembly;cellular component disassembly at cellular level;cellular component organization;cellular compo
biological regulation;biosynthetic process;cellular biosynthetic process;cellular component movement;cellular macromolecule biosynthetic process;cellular macromolecule metabolic process;cellular metabolic process;cellula
actin filament-based movement;actin filament-based process;ameboidal cell migration;biological regulation;cell migration;cell motility;cellular component movement;cellular component organization;cellular component organi
alcohol biosynthetic process;alcohol metabolic process;biological regulation;biosynthetic process;carbohydrate biosynthetic process;carbohydrate metabolic process;cellular biosynthetic process;cellular carbohydrate biosy
actin crosslink formation;actin cytoskeleton organization;actin filament bundle assembly;actin filament organization;actin filament-based process;biological regulation;blood coagulation;cell activation;cell junction assembly;ce
actin filament capping;apoptosis;axon guidance;biological regulation;cell death;cellular component disassembly;cellular component disassembly at cellular level;cellular component disassembly involved in apoptosis;cellular
biological regulation;biosynthetic process;cell differentiation;cellular biosynthetic process;cellular developmental process;cellular macromolecule biosynthetic process;cellular macromolecule metabolic process;cellular metab
biological regulation;catabolic process;cellular catabolic process;cellular component organization;cellular component organization at cellular level;cellular component organization or biogenesis;cellular component organizatio
activation of caspase activity;activation of immune response;activation of innate immune response;adaptive immune response;adaptive immune response based on somatic recombination of immune receptors built from imm
activation of protein kinase activity;activation of protein kinase C activity by G-protein coupled receptor protein signaling pathway;aging;alcohol metabolic process;anatomical structure morphogenesis;aorta morphogenesis;a
biological regulation;calcium ion transport;cation transport;cellular component assembly;cellular component organization;cellular component organization or biogenesis;cellular process;divalent inorganic cation transport;diva
adherens junction assembly;adherens junction organization;anatomical structure morphogenesis;apical junction assembly;axon extension;biological adhesion;biological regulation;blood coagulation;cell activation;cell adhesi
biological regulation;body fluid secretion;cellular component organization;cellular component organization at cellular level;cellular component organization or biogenesis;cellular component organization or biogenesis at cellula
assembly of spliceosomal tri-snRNP;cellular component assembly;cellular component assembly at cellular level;cellular component organization;cellular component organization at cellular level;cellular component organizatio
alcohol metabolic process;cholesterol metabolic process;establishment of localization;lipid metabolic process;lipid transport;metabolic process;organic substance transport;primary metabolic process;small molecule metabo
anatomical structure homeostasis;base-excision repair;biological regulation;biosynthetic process;cell differentiation;cell surface receptor linked signaling pathway;cellular biosynthetic process;cellular component organization
actin cytoskeleton organization;actin filament organization;actin filament-based process;actin rod assembly;activation of immune response;activation of innate immune response;biological regulation;catabolic process;cellular
biological regulation;cellular component movement;cellular process;cellular response to stimulus;intracellular signal transduction;microtubule-based movement;microtubule-based process;regulation of biological process;regu
anatomical structure development;biological adhesion;biological regulation;cell adhesion;cell development;cell migration;cell motility;cell-cell adhesion;cellular component movement;cellular developmental process;cellular pr
cellular component assembly;cellular component assembly at cellular level;cellular component organization;cellular component organization at cellular level;cellular component organization or biogenesis;cellular component
biological regulation;biosynthetic process;cell differentiation;cellular biosynthetic process;cellular developmental process;cellular macromolecule biosynthetic process;cellular macromolecule metabolic process;cellular metab
alcohol metabolic process;carbohydrate metabolic process;cell death;cellular carbohydrate metabolic process;cellular metabolic process;cellular process;death;glucose metabolic process;hexose metabolic process;metaboli
alternative nuclear mRNA splicing, via spliceosome;cellular macromolecule metabolic process;cellular metabolic process;cellular nitrogen compound metabolic process;cellular process;gene expression;macromolecule metab
anatomical structure development;autophagy;biological adhesion;biological regulation;catabolic process;cell adhesion;cell differentiation;cell junction assembly;cell junction organization;cell motility;cell projection assembly;c
apoptosis;axon guidance;biological regulation;cardiac muscle cell apoptosis;cell cycle;cell cycle process;cell death;cell migration;cell motility;cell surface receptor linked signaling pathway;cellular component assembly;cellul
biological regulation;biosynthetic process;carbohydrate metabolic process;carbohydrate transport;cell cycle;cell cycle process;cell surface receptor linked signaling pathway;cellular biosynthetic process;cellular component
actin filament-based movement;actin filament-based process;actin-mediated cell contraction;actin-myosin filament sliding;anatomical structure development;apoptosis;astrocyte development;astrocyte differentiation;Bergma
anatomical structure development;biological regulation;cell development;cell surface receptor linked signaling pathway;cellular developmental process;cellular metabolic process;cellular process;cellular response to calcium i
actin cytoskeleton organization;actin filament bundle assembly;actin filament organization;actin filament-based process;biological regulation;blood coagulation;cell activation;cell junction assembly;cell junction organization;c
actin filament capping;axon guidance;biological regulation;cell cycle cytokinesis;cell cycle process;cellular component assembly;cellular component assembly at cellular level;cellular component organization;cellular compone
anatomical structure development;apoptosis;biological regulation;cardiac cell development;cardiac muscle cell development;cell cycle;cell cycle process;cell death;cell development;cellular component disassembly;cellular c
anatomical structure development;axon guidance;biological regulation;cellular macromolecule metabolic process;cellular metabolic process;cellular process;cellular protein metabolic process;cellular response to chemical sti
biological regulation;biosynthetic process;cellular biosynthetic process;cellular component assembly;cellular component assembly at cellular level;cellular component organization;cellular component organization at cellular le
biological regulation;biosynthetic process;cellular biosynthetic process;cellular macromolecule biosynthetic process;cellular macromolecule metabolic process;cellular metabolic process;cellular process;cellular protein metab
base-excision repair;cell differentiation;cellular component organization;cellular component organization at cellular level;cellular component organization or biogenesis;cellular component organization or biogenesis at cellular
anatomical structure formation involved in morphogenesis;biological regulation;blood circulation;cellular component organization;cellular component organization or biogenesis;cellular membrane organization;cellular process
amine metabolic process;amino acid activation;biological regulation;carboxylic acid metabolic process;cellular amine metabolic process;cellular amino acid metabolic process;cellular component assembly;cellular componen
biosynthetic process;cell differentiation;cellular biosynthetic process;cellular developmental process;cellular macromolecule biosynthetic process;cellular macromolecule metabolic process;cellular metabolic process;cellular
actin cytoskeleton organization;actin filament-based movement;actin filament-based process;actomyosin structure organization;adult heart development;anatomical structure development;anatomical structure formation invo
cell cycle;cell cycle phase;cell cycle process;cell differentiation;cell division;cellular component organization;cellular component organization at cellular level;cellular component organization or biogenesis;cellular component
biological regulation;biosynthetic process;carbohydrate metabolic process;carbohydrate transport;cell cycle;cell cycle checkpoint;cell cycle phase;cell cycle process;cell division;cell surface receptor linked signaling pathway
antigen processing and presentation;antigen processing and presentation of exogenous antigen;antigen processing and presentation of exogenous peptide antigen;antigen processing and presentation of exogenous peptide

acylglycerol catabolic process;acylglycerol metabolic process;amide biosynthetic process;amine biosynthetic process;amine catabolic process;amine metabolic process;anatomical structure development;anion homeostasis;
actin crosslink formation;actin cytoskeleton organization;actin cytoskeleton reorganization;actin filament organization;actin filament-based process;anatomical structure formation involved in morphogenesis;anatomical struct
actin cytoskeleton organization;actin filament-based process;anatomical structure development;biological regulation;cell differentiation;cell surface receptor linked signaling pathway;cellular component organization;cellular c
acetyl-CoA metabolic process;acyl-CoA biosynthetic process;acyl-CoA metabolic process;acylglycerol biosynthetic process;acylglycerol metabolic process;amine metabolic process;biological regulation;biosynthetic process
actin cytoskeleton organization;actin filament-based process;axon guidance;biological adhesion;biological regulation;blood coagulation;cell activation;cell adhesion;cell junction assembly;cell junction organization;cell-cell ad
adherens junction organization;anatomical structure development;anatomical structure morphogenesis;apoptosis;cardiac muscle tissue morphogenesis;cell death;cell differentiation;cell junction organization;cell-cell junction
antigen processing and presentation;antigen processing and presentation of exogenous antigen;antigen processing and presentation of exogenous peptide antigen;antigen processing and presentation of exogenous peptide
anatomical structure development;anatomical structure formation involved in morphogenesis;anatomical structure homeostasis;anterior/posterior pattern specification;B cell lineage commitment;biological regulation;brain de
actin cytoskeleton organization;actin cytoskeleton reorganization;actin filament-based movement;actin filament-based process;actomyosin structure organization;anatomical structure formation involved in morphogenesis;an
biological regulation;cellular component assembly;cellular component organization;cellular component organization or biogenesis;macromolecular complex assembly;macromolecular complex subunit organization;protein co
apoptosis;cell death;cell junction assembly;cell junction organization;cell-substrate junction assembly;cellular component assembly;cellular component assembly at cellular level;cellular component disassembly;cellular comp

C: GOMF name
binding;cation binding;enzyme activator activity;enzyme regulator activity;GTPase activator activity;GTPase regulator activity;ion binding;metal ion binding;nucleoside-triphosphatase regulator activity;transition metal ion bind
binding;catalytic activity;cofactor binding;oxidoreductase activity;oxidoreductase activity, acting on CH-OH group of donors;oxidoreductase activity, acting on the CH-OH group of donors, disulfide as acceptor;quinone bindi
binding;G-protein coupled receptor activity;molecular transducer activity;odorant binding;olfactory receptor activity;receptor activity;signal transducer activity;signaling receptor activity;transmembrane signaling receptor activ
catalytic activity;hydrolase activity;peptidase activity
binding;nucleic acid binding;RNA binding;RNA cap binding
catalytic activity;methyltransferase activity;transferase activity;transferase activity, transferring one-carbon groups
active transmembrane transporter activity;macromolecule transmembrane transporter activity;P-P-bond-hydrolysis-driven protein transmembrane transporter activity;P-P-bond-hydrolysis-driven transmembrane transporter a
acetyltransferase activity;binding;C-acetyltransferase activity;C-acyltransferase activity;catalytic activity;cofactor binding;glycine C-acetyltransferase activity;pyridoxal phosphate binding;transferase activity;transferase activity
catalytic activity;NADH dehydrogenase (quinone) activity;NADH dehydrogenase (ubiquinone) activity;NADH dehydrogenase activity;oxidoreductase activity;oxidoreductase activity, acting on NADH or NADPH;oxidoreductase
binding;catalytic activity;Mo-molybdopterin synthase activity;nucleotide binding
catalytic activity;oxidoreductase activity;oxidoreductase activity, acting on a sulfur group of donors
amine binding;amino acid binding;binding;carboxylic acid binding;enzyme inhibitor activity;enzyme regulator activity
binding;catalytic activity;enzyme binding;hydrolase activity;hydrolase activity, acting on ester bonds;kinase binding;MAP kinase phosphatase activity;phosphatase activity;phosphoprotein phosphatase activity;phosphoric est
binding;catalytic activity;GTP binding;GTPase activity;guanyl nucleotide binding;guanyl ribonucleotide binding;hydrolase activity;hydrolase activity, acting on acid anhydrides;hydrolase activity, acting on acid anhydrides, in pl
binding;cation binding;copper chaperone activity;copper ion binding;ion binding;metal ion binding;metallochaperone activity;transition metal ion binding
binding;carbohydrate binding;cation binding;DNA binding;glycosaminoglycan binding;heparin binding;ion binding;metal ion binding;nucleic acid binding;nucleic acid binding transcription factor activity;pattern binding;polysac
binding;nucleotide binding
binding;DNA binding;hormone receptor binding;nuclear hormone receptor binding;nucleic acid binding;protein binding;receptor binding;thyroid hormone receptor binding;transcription factor binding
adenyl nucleotide binding;adenyl ribonucleotide binding;ATP binding;binding;nucleotide binding;purine nucleotide binding;purine ribonucleoside triphosphate binding;purine ribonucleotide binding;ribonucleotide binding
binding;lipid binding;phosphatidylinositol binding;phosphatidylinositol-3,5-bisphosphate binding;phosphatidylinositol-3-phosphate binding;phospholipid binding
binding;cation binding;DNA binding;ion binding;metal ion binding;nucleic acid binding
G-protein coupled receptor activity;molecular transducer activity;pheromone receptor activity;receptor activity;signal transducer activity;signaling receptor activity;transmembrane signaling receptor activity
active transmembrane transporter activity;amine transmembrane transporter activity;amino acid transmembrane transporter activity;basic amino acid transmembrane transporter activity;carboxylic acid transmembrane transp
binding;cation binding;ion binding;metal ion binding
protein binding transcription factor activity;RNA polymerase II transcription cofactor activity;RNA polymerase II transcription factor binding transcription factor activity;transcription cofactor activity;transcription factor binding
molecular transducer activity;receptor signaling protein activity;signal transducer activity
acyl-CoA hydrolase activity;catalytic activity;CoA hydrolase activity;hydrolase activity;hydrolase activity, acting on ester bonds;thiolester hydrolase activity
binding;catalytic activity;GTP binding;guanyl nucleotide binding;guanyl ribonucleotide binding;hydrolase activity;nucleotide binding;purine nucleotide binding;purine ribonucleoside triphosphate binding;purine ribonucleotide b
ADP-ribosylation factor binding;binding;enzyme binding;GTPase binding;protein binding;small GTPase binding
catalytic activity;hydrolase activity;hydrolase activity, acting on ester bonds;palmitoyl-(protein) hydrolase activity;thiolester hydrolase activity
binding;cation binding;ion binding;metal ion binding
binding;catalytic activity;cation binding;cofactor binding;electron carrier activity;heme binding;ion binding;iron ion binding;metal ion binding;oxidoreductase activity;oxidoreductase activity, acting on the CH-CH group of donc
acyl carrier activity;binding;calcium ion binding;carboxylic acid binding;catalytic activity;cation binding;fatty acid binding;ion binding;lipid binding;metal ion binding;monocarboxylic acid binding;NADH dehydrogenase (quinon
acid-amino acid ligase activity;catalytic activity;ISG15 ligase activity;ligase activity;ligase activity, forming carbon-nitrogen bonds;small conjugating protein ligase activity;ubiquitin-protein ligase activity
binding;cation binding;ion binding;metal ion binding;transition metal ion binding;zinc ion binding
molecular transducer activity;signal transducer activity
adenyl nucleotide binding;adenyl ribonucleotide binding;ATP binding;binding;catalytic activity;kinase activity;nucleotide binding;phosphotransferase activity, alcohol group as acceptor;purine nucleotide binding;purine ribonuc
aspartic-type endopeptidase activity;aspartic-type peptidase activity;catalytic activity;endopeptidase activity;hydrolase activity;peptidase activity;peptidase activity, acting on L-amino acid peptides;receptor activity
catalytic activity;endonuclease activity;endonuclease activity, active with either ribo- or deoxyribonucleic acids and producing 5'-phosphomonoesters;endoribonuclease activity;endoribonuclease activity, producing 5'-phosph
binding;epidermal growth factor receptor binding;growth factor receptor binding;insulin receptor binding;lipid binding;phosphatidylinositol binding;phospholipid binding;protein binding;protein complex binding;receptor bindir
binding;chromatin binding;DNA binding;nucleic acid binding
arylsulfatase activity;binding;calcium ion binding;catalytic activity;cation binding;cerebroside-sulfatase activity;hydrolase activity;hydrolase activity, acting on ester bonds;ion binding;metal ion binding;sulfuric ester hydrolase a
binding;DNA binding;double-stranded DNA binding;nucleic acid binding;nucleic acid binding transcription factor activity;regulatory region DNA binding;regulatory region nucleic acid binding;RNA polymerase II regulatory regi
binding;calcium ion binding;catalytic activity;cation binding;damaged DNA binding;DNA binding;DNA-methyltransferase activity;ion binding;metal ion binding;methylated-DNA-[protein]-cysteine S-methyltransferase activity;m
binding;cation binding;DNA binding;ion binding;metal ion binding;nucleic acid binding;sequence-specific DNA binding
binding;DNA binding;nucleic acid binding
binding;cation binding;enzyme regulator activity;GTPase regulator activity;guanyl-nucleotide exchange factor activity;ion binding;metal ion binding;nucleoside-triphosphatase regulator activity;transition metal ion binding;zinc
binding;catalytic activity;GDP binding;GTP binding;GTPase activity;guanyl nucleotide binding;guanyl ribonucleotide binding;hydrolase activity;hydrolase activity, acting on acid anhydrides;hydrolase activity, acting on acid anh
binding;protein binding;protein dimerization activity;protein heterodimerization activity
binding;cation binding;ion binding;metal ion binding
acid-amino acid ligase activity;adenyl nucleotide binding;adenyl ribonucleotide binding;ATP binding;binding;catalytic activity;enzyme binding;ligase activity;ligase activity, forming carbon-nitrogen bonds;nucleotide binding;pr
molecular transducer activity;receptor activity;signal transducer activity;signaling receptor activity;thrombospondin receptor activity
binding;cation binding;DNA binding;ion binding;metal ion binding;nucleic acid binding;transition metal ion binding;zinc ion binding
catalytic activity;guanidinoacetate N-methyltransferase activity;methyltransferase activity;S-adenosylmethionine-dependent methyltransferase activity;transferase activity;transferase activity, transferring one-carbon groups
activating transcription factor binding;binding;DNA binding;enzyme binding;histone deacetylase binding;nucleic acid binding;nucleic acid binding transcription factor activity;protein binding;regulatory region DNA binding;regu
cation transmembrane transporter activity;divalent inorganic cation transmembrane transporter activity;ferrous iron transmembrane transporter activity;inorganic cation transmembrane transporter activity;ion transmembrane
C-8 sterol isomerase activity;catalytic activity;cholestenol delta-isomerase activity;drug transmembrane transporter activity;intramolecular oxidoreductase activity;intramolecular oxidoreductase activity, transposing C=C bonc
binding;catalytic activity;chromatin binding;chromatin DNA binding;core promoter binding;DNA binding;histone methyltransferase activity;histone methyltransferase activity (H3-K27 specific);histone-lysine N-methyltransferas
binding;nucleic acid binding;RNA binding
1-acylglycerophosphocholine O-acyltransferase activity;catalytic activity;O-acyltransferase activity;transferase activity;transferase activity, transferring acyl groups;transferase activity, transferring acyl groups other than aminc
endopeptidase inhibitor activity;endopeptidase regulator activity;enzyme inhibitor activity;enzyme regulator activity;peptidase inhibitor activity;peptidase regulator activity;serine-type endopeptidase inhibitor activity
[acyl-carrier-protein] S-malonyltransferase activity;catalytic activity;fatty acid synthase activity;malonyltransferase activity;S-acyltransferase activity;S-malonyltransferase activity;transferase activity;transferase activity, transfe
catalytic activity;disulfide oxidoreductase activity;oxidoreductase activity;oxidoreductase activity, acting on a sulfur group of donors;protein disulfide oxidoreductase activity
binding;nucleic acid binding;RNA binding;translation factor activity, nucleic acid binding;translation initiation factor activity
cation transmembrane transporter activity;ion transmembrane transporter activity;substrate-specific transmembrane transporter activity;substrate-specific transporter activity;transmembrane transporter activity;transporter ac
binding;identical protein binding;lipid binding;phosphatidylinositol binding;phospholipid binding;protein binding
catalytic activity;hydrolase activity
binding;cation binding;enzyme activator activity;enzyme regulator activity;GTPase activator activity;GTPase regulator activity;ion binding;metal ion binding;nucleoside-triphosphatase regulator activity
catalytic activity;cysteine-type endopeptidase activity;cysteine-type peptidase activity;endopeptidase activity;GPI-anchor transamidase activity;hydrolase activity;intramolecular oxidoreductase activity;intramolecular oxidored
catalytic activity;endonuclease activity;endonuclease activity, active with either ribo- or deoxyribonucleic acids and producing 5'-phosphomonoesters;endoribonuclease activity;endoribonuclease activity, producing 5'-phosph
active transmembrane transporter activity;binding;drug transmembrane transporter activity;enzyme binding;protein binding;secondary active transmembrane transporter activity;symporter activity;transmembrane transporter
binding;catalytic activity;cation binding;hydrolase activity;ion binding;metal ion binding;transition metal ion binding;zinc ion binding
protein binding transcription factor activity;RNA polymerase II transcription cofactor activity;RNA polymerase II transcription factor binding transcription factor activity;transcription cofactor activity;transcription factor binding
binding;channel regulator activity;identical protein binding;protein binding;protein C-terminus binding;protein dimerization activity;protein homodimerization activity;protein N-terminus binding;transcription factor binding
binding;catalytic activity;cation binding;hydrolase activity;hydrolase activity, acting on carbon-nitrogen (but not peptide) bonds;hydrolase activity, acting on carbon-nitrogen (but not peptide) bonds, in linear amides;ion binding
binding;enzyme activator activity;enzyme binding;enzyme regulator activity;GTPase activator activity;GTPase binding;GTPase regulator activity;guanyl-nucleotide exchange factor activity;nucleoside-triphosphatase regulator
3'-flap endonuclease activity;binding;catalytic activity;cation binding;deoxyribonuclease activity;DNA binding;endodeoxyribonuclease activity;endodeoxyribonuclease activity, producing 3'-phosphomonoesters;endonuclease
binding;catalytic activity;GTP binding;GTPase activity;guanyl nucleotide binding;guanyl ribonucleotide binding;hydrolase activity;hydrolase activity, acting on acid anhydrides;hydrolase activity, acting on acid anhydrides, in pl
binding;nucleic acid binding;RNA binding;rRNA binding;structural constituent of ribosome;structural molecule activity
binding;identical protein binding;protein binding;transcription factor binding
protein binding transcription factor activity;transcription coactivator activity;transcription cofactor activity;transcription factor binding transcription factor activity
2'-phosphotransferase activity;5'-nucleotidase activity;binding;catalytic activity;cation binding;hydrolase activity;hydrolase activity, acting on ester bonds;ion binding;magnesium ion binding;metal ion binding;nucleotidase act
binding;carbohydrate binding;cation binding;ion binding;mannose binding;metal ion binding;monosaccharide binding;sugar binding
binding;catalytic activity;cation binding;histone methyltransferase activity;histone-lysine N-methyltransferase activity;ion binding;lysine N-methyltransferase activity;metal ion binding;methyltransferase activity;N-methyltransfe
binding;catalytic activity;GTP binding;GTPase activity;guanyl nucleotide binding;guanyl ribonucleotide binding;hydrolase activity;hydrolase activity, acting on acid anhydrides;hydrolase activity, acting on acid anhydrides, in pl
binding;nucleic acid binding;RNA binding;snoRNA binding
binding;catalytic activity;DNA binding;DNA polymerase activity;DNA-directed DNA polymerase activity;nucleic acid binding;nucleotidyltransferase activity;sequence-specific DNA binding;transferase activity;transferase activit
binding;catalytic activity;DNA binding;DNA polymerase activity;DNA-directed DNA polymerase activity;nucleic acid binding;nucleotidyltransferase activity;sequence-specific DNA binding;transferase activity;transferase activit

binding;lamin binding;protein binding
binding;DNA binding;nucleic acid binding;nucleic acid binding transcription factor activity;sequence-specific DNA binding;sequence-specific DNA binding RNA polymerase II transcription factor activity;sequence-specific DN
catalytic activity;endopeptidase activity;hydrolase activity;peptidase activity;peptidase activity, acting on L-amino acid peptides;serine hydrolase activity;serine-type endopeptidase activity;serine-type peptidase activity
beta-mannosidase activity;catalytic activity;hydrolase activity;hydrolase activity, acting on glycosyl bonds;hydrolase activity, hydrolyzing O-glycosyl compounds;mannosidase activity
binding;DNA binding;nucleic acid binding
binding;cation binding;ion binding;lipid transporter activity;metal ion binding;phosphatidylinositol transporter activity;phospholipid transporter activity;substrate-specific transporter activity;transporter activity
adenyl nucleotide binding;adenyl ribonucleotide binding;ATP binding;ATPase activity;binding;catalytic activity;cytoskeletal protein binding;hydrolase activity;hydrolase activity, acting on acid anhydrides;hydrolase activity, acti
binding;protein binding;SNAP receptor activity;SNARE binding;syntaxin binding
binding;catalytic activity;cation binding;hydrolase activity;hydrolase activity, acting on ester bonds;identical protein binding;inositol monophosphate phosphatase activity;inositol or phosphatidylinositol phosphatase activity;in
acyl-CoA hydrolase activity;acyl-CoA thioesterase activity;binding;carboxylic ester hydrolase activity;catalytic activity;choloyl-CoA hydrolase activity;CoA hydrolase activity;hydrolase activity;hydrolase activity, acting on ester
structural constituent of ribosome;structural molecule activity
binding;catalytic activity;cation binding;cytoskeletal protein binding;enzyme binding;identical protein binding;ion binding;ligase activity;metal ion binding;microtubule binding;phosphoprotein binding;protein binding;protein di
binding;catalytic activity;DNA-directed RNA polymerase activity;nucleotide binding;nucleotidyltransferase activity;protein binding;RNA polymerase activity;transferase activity;transferase activity, transferring phosphorus-cont
binding;carbonate dehydratase activity;carbon-oxygen lyase activity;catalytic activity;cation binding;hydro-lyase activity;ion binding;lyase activity;metal ion binding;transition metal ion binding;zinc ion binding
molecular transducer activity;protein binding transcription factor activity;signal transducer activity;transcription cofactor activity;transcription corepressor activity;transcription factor binding transcription factor activity
binding;cation binding;ion binding;metal ion binding
binding;enzyme binding;nucleic acid binding;protein binding;RNA binding;RNA cap binding;translation factor activity, nucleic acid binding;translation initiation factor activity;ubiquitin protein ligase binding
beta-tubulin binding;binding;cytoskeletal protein binding;microtubule binding;protein binding;protein binding transcription factor activity;protein N-terminus binding;receptor binding;transcription cofactor activity;transcription
binding;GTP binding;guanyl nucleotide binding;guanyl ribonucleotide binding;nucleic acid binding;nucleotide binding;purine nucleotide binding;purine ribonucleoside triphosphate binding;purine ribonucleotide binding;ribonu
binding;cation binding;copper ion binding;ion binding;metal ion binding;transition metal ion binding
catalytic activity;hydrolase activity
binding;chromatin binding;enzyme inhibitor activity;enzyme regulator activity;phosphatase inhibitor activity;phosphatase regulator activity;protein binding transcription factor activity;protein phosphatase inhibitor activity;prote
binding;nucleic acid binding;RNA binding;snRNA binding;U6 snRNA 3'-end binding;U6 snRNA binding
binding;cation binding;ion binding;metal ion binding
cysteine-type endopeptidase inhibitor activity;endopeptidase inhibitor activity;endopeptidase regulator activity;enzyme inhibitor activity;enzyme regulator activity;kinase inhibitor activity;kinase regulator activity;peptidase inhil
binding;cadherin binding;catalytic activity;cell adhesion molecule binding;endopeptidase activity;enzyme inhibitor activity;enzyme regulator activity;hydrolase activity;peptidase activity;peptidase activity, acting on L-amino ac
acyl-CoA dehydrogenase activity;binding;butyryl-CoA dehydrogenase activity;carboxylic acid binding;catalytic activity;coenzyme binding;cofactor binding;fatty acid binding;fatty-acyl-CoA binding;flavin adenine dinucleotide
binding;catalytic activity;DNA binding;DNA-directed RNA polymerase activity;nucleic acid binding;nucleotidyltransferase activity;RNA polymerase activity;transferase activity;transferase activity, transferring phosphorus-conta
acid-amino acid ligase activity;binding;catalytic activity;cation binding;cysteine-type peptidase activity;DNA binding;enzyme binding;hydrolase activity;ion binding;ligase activity;ligase activity, forming carbon-nitrogen bonds;
catalytic activity;cation transmembrane transporter activity;cytochrome-c oxidase activity;heme-copper terminal oxidase activity;hydrogen ion transmembrane transporter activity;inorganic cation transmembrane transporter a
androgen binding;benzodiazepine receptor activity;binding;cholesterol binding;hormone binding;lipid binding;molecular transducer activity;neurotransmitter binding;neurotransmitter receptor activity;receptor activity;signal tra
binding;nucleic acid binding;RNA binding;translation factor activity, nucleic acid binding;translation initiation factor activity
angiotensin receptor binding;binding;caspase inhibitor activity;caspase regulator activity;cysteine-type endopeptidase inhibitor activity;DNA binding;endopeptidase inhibitor activity;endopeptidase regulator activity;enzyme ac
acid-amino acid ligase activity;adenyl nucleotide binding;adenyl ribonucleotide binding;ATP binding;binding;catalytic activity;ISG15 ligase activity;ligase activity;ligase activity, forming carbon-nitrogen bonds;nucleotide bindin
binding;catalytic activity;cation binding;enzyme binding;GTPase binding;ion binding;metal ion binding;prenyltransferase activity;protein binding;protein geranylgeranyltransferase activity;protein prenyltransferase activity;Rab
cyclin-dependent protein kinase regulator activity;enzyme regulator activity;kinase regulator activity;protein kinase regulator activity
binding;nucleic acid binding;RNA binding
binding;nucleic acid binding;RNA binding;structural constituent of ribosome;structural molecule activity
binding;catalytic activity;GTPase activity;hydrolase activity;hydrolase activity, acting on acid anhydrides;hydrolase activity, acting on acid anhydrides, in phosphorus-containing anhydrides;molecular transducer activity;nucleo
adenosine deaminase activity;binding;catalytic activity;cation binding;deaminase activity;double-stranded RNA adenosine deaminase activity;double-stranded RNA binding;hydrolase activity;hydrolase activity, acting on carb
structural constituent of ribosome;structural molecule activity
binding;cation binding;core promoter binding;DNA binding;hormone receptor binding;ion binding;metal ion binding;nuclear hormone receptor binding;nucleic acid binding;protein binding;receptor binding;regulatory region DN
aldehyde dehydrogenase (NAD) activity;binding;carboxylic acid binding;catalytic activity;coenzyme binding;cofactor binding;fatty acid binding;fatty-acyl-CoA binding;lipid binding;malonate-semialdehyde dehydrogenase (ace
binding;core promoter proximal region DNA binding;core promoter proximal region sequence-specific DNA binding;DNA binding;nucleic acid binding;nucleic acid binding transcription factor activity;protein binding;regulatory
binding;nucleic acid binding;RNA binding
binding;lipid binding
binding;protein binding;receptor binding
catalytic activity;hydrolase activity;hydrolase activity, acting on ester bonds;inositol or phosphatidylinositol phosphatase activity;lipid phosphatase activity;phosphatase activity;phosphatidylinositol bisphosphate phosphatase
binding;DNA binding;identical protein binding;nucleic acid binding;nucleic acid binding transcription factor activity;protein binding;protein binding transcription factor activity;protein dimerization activity;protein homodimeriza
adenyl nucleotide binding;adenyl ribonucleotide binding;ATP binding;binding;catalytic activity;kinase activity;nucleotide binding;phosphotransferase activity, alcohol group as acceptor;protein kinase activity;protein serine/thre
binding;calcium ion binding;cation binding;enzyme activator activity;enzyme regulator activity;GTPase regulator activity;guanyl-nucleotide exchange factor activity;ion binding;kinase activator activity;kinase regulator activity;
binding;cation binding;DNA binding;ion binding;metal ion binding;nucleic acid binding;polyubiquitin binding;protein binding;small conjugating protein binding;transition metal ion binding;ubiquitin binding;zinc ion binding
structural constituent of ribosome;structural molecule activity
2 iron, 2 sulfur cluster binding;binding;catalytic activity;cation binding;disulfide oxidoreductase activity;electron carrier activity;ion binding;iron-sulfur cluster binding;metal cluster binding;metal ion binding;oxidoreductase acti
catalytic activity;hydrolase activity
acetyltransferase activity;catalytic activity;N-acetyltransferase activity;N-acyltransferase activity;transferase activity;transferase activity, transferring acyl groups;transferase activity, transferring acyl groups other than amino-a
binding;cation binding;enzyme binding;ion binding;kinase binding;metal ion binding;protein binding;protein kinase binding;transition metal ion binding;zinc ion binding
binding;calcium ion binding;cation binding;ion binding;metal ion binding;protein binding;protein dimerization activity;protein heterodimerization activity
catalytic activity;NADH dehydrogenase (quinone) activity;NADH dehydrogenase (ubiquinone) activity;NADH dehydrogenase activity;oxidoreductase activity;oxidoreductase activity, acting on NADH or NADPH;oxidoreductase
binding;catalytic activity;cation binding;hydrolase activity;hydrolase activity, acting on ester bonds;ion binding;lipase activity;metal ion binding;molecular transducer activity;phosphatidylinositol phospholipase C activity;phosp
cation transmembrane transporter activity;divalent inorganic cation transmembrane transporter activity;inorganic cation transmembrane transporter activity;ion transmembrane transporter activity;magnesium ion transmembra
binding;DNA binding;enzyme inhibitor activity;enzyme regulator activity;ligase inhibitor activity;ligase regulator activity;nucleic acid binding;p53 binding;protein binding;transcription factor binding;ubiquitin-protein ligase inhibi
binding;DNA binding;double-stranded DNA binding;double-stranded RNA binding;nucleic acid binding;RNA binding;single-stranded RNA binding;structure-specific DNA binding
acid-amino acid ligase activity;binding;catalytic activity;cation binding;ion binding;ligase activity;ligase activity, forming carbon-nitrogen bonds;metal ion binding;small conjugating protein ligase activity;transition metal ion bin
3'-phosphoadenosine 5'-phosphosulfate transmembrane transporter activity;molecular transducer activity;nucleobase-containing compound transmembrane transporter activity;nucleoside transmembrane transporter activity
binding;catalytic activity;histone methyltransferase activity;histone-lysine N-methyltransferase activity;lysine N-methyltransferase activity;methyltransferase activity;N-methyltransferase activity;p53 binding;protein binding;pro
binding;cation binding;ion binding;metal ion binding;transition metal ion binding;zinc ion binding
catalytic activity;exopeptidase activity;gamma-glutamyl-peptidase activity;hydrolase activity;omega peptidase activity;peptidase activity;peptidase activity, acting on L-amino acid peptides
lipid transporter activity;substrate-specific transporter activity;transporter activity
catalytic activity
catalytic activity;dipeptidase activity;exopeptidase activity;hydrolase activity;peptidase activity;peptidase activity, acting on L-amino acid peptides
catalytic activity;methyltransferase activity;RNA methyltransferase activity;S-adenosylmethionine-dependent methyltransferase activity;transferase activity;transferase activity, transferring one-carbon groups;tRNA (adenine) m
binding;catalytic activity;cation binding;heat shock protein binding;hydrolase activity;hydrolase activity, acting on ester bonds;ion binding;magnesium ion binding;metal ion binding;phosphatase activity;phosphoprotein phosp
catalytic activity;cysteine-type endopeptidase activity;cysteine-type peptidase activity;endopeptidase activity;hydrolase activity;peptidase activity;peptidase activity, acting on L-amino acid peptides
acylglycerol lipase activity;binding;carboxylic ester hydrolase activity;catalytic activity;hydrolase activity;hydrolase activity, acting on ester bonds;identical protein binding;lipase activity;lipid binding;lysophospholipase activity;
binding;DNA binding;nucleic acid binding;nucleic acid binding transcription factor activity;sequence-specific DNA binding transcription factor activity
binding;catalytic activity;cation binding;ion binding;ligase activity;metal ion binding;transition metal ion binding;zinc ion binding
binding;carbohydrate binding;catalytic activity;chitin binding;chitinase activity;hydrolase activity;hydrolase activity, acting on glycosyl bonds;hydrolase activity, hydrolyzing O-glycosyl compounds;oligosaccharide binding;patt
enzyme regulator activity;phosphatase regulator activity
binding;catalytic activity;cation binding;demethylase activity;histone demethylase activity;histone demethylase activity (H3-K36 specific);histone demethylase activity (H3-K4 specific);ion binding;iron ion binding;metal ion bind
binding;catalytic activity;cation binding;GDP binding;GTP binding;GTPase activity;guanyl nucleotide binding;guanyl ribonucleotide binding;hydrolase activity;hydrolase activity, acting on acid anhydrides;hydrolase activity, act
binding;DNA binding;nucleic acid binding
active transmembrane transporter activity;adenyl nucleotide binding;adenyl ribonucleotide binding;ATP binding;ATPase activity;ATPase activity, coupled;ATPase activity, coupled to movement of substances;ATPase activity, c
catalytic activity;CTD phosphatase activity;hydrolase activity;hydrolase activity, acting on ester bonds;phosphatase activity;phosphoprotein phosphatase activity;phosphoric ester hydrolase activity;protein serine/threonine ph
binding;catalytic activity;cation binding;hydrolase activity;hydrolase activity, acting on carbon-nitrogen (but not peptide) bonds;hydrolase activity, acting on carbon-nitrogen (but not peptide) bonds, in cyclic amidines;ion bindi
[myelin basic protein]-arginine N-methyltransferase activity;arginine N-methyltransferase activity;binding;catalytic activity;histone binding;histone methyltransferase activity;histone methyltransferase activity (H4-R3 specific);h
binding;nucleic acid binding;RNA binding;rRNA binding;structural constituent of ribosome;structural molecule activity
adenyl nucleotide binding;adenyl ribonucleotide binding;ATP binding;binding;nucleotide binding;purine nucleotide binding;purine ribonucleoside triphosphate binding;purine ribonucleotide binding;ribonucleotide binding
binding;cytoskeletal protein binding;microtubule binding;microtubule minus-end binding;protein binding;tubulin binding
acid-amino acid ligase activity;binding;catalytic activity;cation binding;copper ion binding;ion binding;ligase activity;ligase activity, forming carbon-nitrogen bonds;metal ion binding;NEDD8 ligase activity;small conjugating pro
binding;cation binding;enzyme binding;ion binding;metal ion binding;protein binding;transition metal ion binding;ubiquitin conjugating enzyme binding;zinc ion binding
catalytic activity;electron carrier activity;NADH dehydrogenase (quinone) activity;NADH dehydrogenase (ubiquinone) activity;NADH dehydrogenase activity;oxidoreductase activity;oxidoreductase activity, acting on NADH or N
binding;identical protein binding;protein binding
enzyme activator activity;enzyme regulator activity;GTPase activator activity;GTPase regulator activity;nucleoside-triphosphatase regulator activity
binding;integrin binding;protein binding;protein complex binding;receptor binding
catalytic activity;methyltransferase activity;N-methyltransferase activity;RNA methyltransferase activity;rRNA (adenine) methyltransferase activity;rRNA (adenine-N6,N6-)-dimethyltransferase activity;rRNA methyltransferase ac
4 iron, 4 sulfur cluster binding;adenyl nucleotide binding;adenyl ribonucleotide binding;ATP binding;binding;cation binding;ion binding;iron-sulfur cluster binding;metal cluster binding;metal ion binding;nucleotide binding;purin

binding;protein binding;protein C-terminus binding
binding;DNA binding;nucleic acid binding;protein binding transcription factor activity;transcription coactivator activity;transcription cofactor activity;transcription factor binding transcription factor activity
binding;nucleic acid binding;RNA binding
binding;catalytic activity;cation binding;endopeptidase activity;exopeptidase activity;hydrolase activity;ion binding;metal ion binding;peptidase activity;peptidase activity, acting on L-amino acid peptides;peptide binding;serin
antioxidant activity;catalytic activity;glutathione peroxidase activity;glutathione transferase activity;oxidoreductase activity;oxidoreductase activity, acting on peroxide as acceptor;peroxidase activity;transferase activity;transfe
binding;cytoskeletal protein binding;enzyme regulator activity;GTPase regulator activity;guanyl-nucleotide exchange factor activity;kinesin binding;nucleoside-triphosphatase regulator activity;protein binding;Ras guanyl-nucle
binding;catalytic activity;DNA binding;DNA-directed RNA polymerase activity;nucleic acid binding;nucleotidyltransferase activity;RNA polymerase activity;transferase activity;transferase activity, transferring phosphorus-conta
catalytic activity;DNA-directed RNA polymerase activity;nucleotidyltransferase activity;RNA polymerase activity;transferase activity;transferase activity, transferring phosphorus-containing groups
active transmembrane transporter activity;antigen binding;ATPase activity;ATPase activity, coupled;ATPase activity, coupled to movement of substances;ATPase activity, coupled to transmembrane movement of substances;b
3'-5' exonuclease activity;binding;catalytic activity;damaged DNA binding;deoxyribonuclease activity;DNA binding;exodeoxyribonuclease activity;exodeoxyribonuclease activity, producing 5'-phosphomonoesters;exodeoxyrib
binding;enzyme binding;enzyme inhibitor activity;enzyme regulator activity;phosphatase binding;phosphatase inhibitor activity;phosphatase regulator activity;protein binding;protein phosphatase 1 binding;protein phosphatas
binding;calcium ion binding;caspase inhibitor activity;caspase regulator activity;cation binding;cysteine-type endopeptidase inhibitor activity;endopeptidase inhibitor activity;endopeptidase regulator activity;enzyme inhibitor a
binding;enzyme binding;protein binding;small conjugating protein binding;transcription factor binding;ubiquitin binding;ubiquitin protein ligase binding
binding;catalytic activity;cation binding;endonuclease activity;hydrolase activity;hydrolase activity, acting on ester bonds;ion binding;metal ion binding;nuclease activity;nucleic acid binding
binding;protein binding;SNAP receptor activity
binding;ribonucleoprotein binding
binding;catalytic activity;cation binding;coenzyme binding;cofactor binding;ion binding;metal ion binding;NADP binding;NADPH:quinone reductase activity;nucleotide binding;oxidoreductase activity;oxidoreductase activity, a
anion channel activity;anion transmembrane transporter activity;channel activity;chloride channel activity;gated channel activity;ion channel activity;ion transmembrane transporter activity;passive transmembrane transporter a
catalytic activity;hydrolase activity
actin binding;actin monomer binding;adenyl nucleotide binding;adenyl ribonucleotide binding;adenylyltransferase activity;ATP binding;binding;catalytic activity;cation binding;cytoskeletal protein binding;DNA binding;enzyme
binding;carbohydrate binding;enzyme activator activity;enzyme regulator activity;glycoprotein binding;glycosaminoglycan binding;heparin binding;identical protein binding;lipase activator activity;lipid binding;lipoprotein lipas
binding;endopeptidase inhibitor activity;endopeptidase regulator activity;enzyme binding;enzyme inhibitor activity;enzyme regulator activity;peptidase inhibitor activity;peptidase regulator activity;protein binding;serine-type e
protein tag
binding;carbohydrate binding;chemoattractant activity;cytokine activity;fibroblast growth factor receptor binding;glycosaminoglycan binding;growth factor activity;growth factor receptor binding;heparin binding;ligand-depend
carboxypeptidase activity;catalytic activity;enzyme activator activity;enzyme regulator activity;exopeptidase activity;hydrolase activity;peptidase activity;peptidase activity, acting on L-amino acid peptides;serine hydrolase ac
alpha-tubulin binding;beta-N-acetylglucosaminylglycopeptide beta-1,4-galactosyltransferase activity;beta-tubulin binding;binding;catalytic activity;cation binding;cytoskeletal protein binding;galactosyltransferase activity;iden
binding;DNA binding;nucleic acid binding;nucleic acid binding transcription factor activity;regulatory region DNA binding;regulatory region nucleic acid binding;RNA polymerase II regulatory region DNA binding;RNA polymera
alpha-catenin binding;beta-catenin binding;binding;calcium ion binding;cation binding;gamma-catenin binding;ion binding;metal ion binding;protein binding
catalytic activity;endopeptidase activity;hydrolase activity;peptidase activity;peptidase activity, acting on L-amino acid peptides;threonine-type endopeptidase activity;threonine-type peptidase activity
active transmembrane transporter activity;ATPase activity;ATPase activity, coupled;ATPase activity, coupled to movement of substances;ATPase activity, coupled to transmembrane movement of ions;ATPase activity, coupled
binding;enzyme binding;phosphatase binding;protein binding;protein phosphatase binding
acetylcholine receptor regulator activity;alpha-tubulin binding;binding;CAAX-protein geranylgeranyltransferase activity;catalytic activity;cytoskeletal protein binding;microtubule binding;prenyltransferase activity;protein bindin
catalytic activity;deoxyhypusine synthase activity;transferase activity;transferase activity, transferring alkyl or aryl (other than methyl) groups
5-formyltetrahydrofolate cyclo-ligase activity;adenyl nucleotide binding;adenyl ribonucleotide binding;amine binding;amino acid binding;ATP binding;binding;carboxylic acid binding;catalytic activity;cation binding;cyclo-ligase
binding;bis(5'-nucleosyl)-tetraphosphatase (asymmetrical) activity;bis(5'-nucleosyl)-tetraphosphatase (symmetrical) activity;bis(5'-nucleosyl)-tetraphosphatase activity;catalytic activity;GTP binding;guanyl nucleotide binding;g
binding;protein binding;SNAP receptor activity;SNARE binding
binding;catalytic activity;DNA binding;DNA-directed RNA polymerase activity;nucleic acid binding;nucleotidyltransferase activity;RNA polymerase activity;transferase activity;transferase activity, transferring phosphorus-conta
binding;channel regulator activity;enzyme binding;enzyme inhibitor activity;enzyme regulator activity;phosphatase binding;phosphatase inhibitor activity;phosphatase regulator activity;potassium channel regulator activity;pro
actin binding;binding;chemokine binding;chemokine receptor activity;coreceptor activity;C-X-C chemokine binding;C-X-C chemokine receptor activity;cytokine binding;cytoskeletal protein binding;enzyme binding;G-protein c
acid-amino acid ligase activity;adenyl nucleotide binding;adenyl ribonucleotide binding;ATP binding;binding;catalytic activity;ligase activity;ligase activity, forming carbon-nitrogen bonds;nucleotide binding;purine nucleotide b
binding;cation binding;chaperone binding;identical protein binding;ion binding;metal ion binding;protein binding;protein dimerization activity;protein homodimerization activity;protein transporter activity;substrate-specific tran
binding;nucleic acid binding;RNA binding
binding;nucleic acid binding;RNA binding;structural constituent of ribosome;structural molecule activity
acid-amino acid ligase activity;binding;catalytic activity;cation binding;enzyme binding;ion binding;ligase activity;ligase activity, forming carbon-nitrogen bonds;metal ion binding;NEDD8 ligase activity;protein binding;small co
binding;cation binding;ion binding;metal ion binding;nucleic acid binding transcription factor activity;protein binding;protein dimerization activity;protein heterodimerization activity;sequence-specific DNA binding transcription
beta-catenin binding;binding;cell adhesion molecule binding;connexin binding;identical protein binding;integrin binding;PDZ domain binding;protein binding;protein complex binding;protein domain specific binding;receptor a
binding;DNA binding;nucleic acid binding;nucleotide binding;RNA binding
adenyl nucleotide binding;adenyl ribonucleotide binding;ATP binding;binding;carbohydrate kinase activity;catalytic activity;galactokinase activity;kinase activity;N-acetylgalactosamine kinase activity;nucleotide binding;phosp
adenyl nucleotide binding;adenyl ribonucleotide binding;ATP binding;ATPase activity;binding;catalytic activity;hydrolase activity;hydrolase activity, acting on acid anhydrides;hydrolase activity, acting on acid anhydrides, in ph
binding;cation binding;chromatin binding;core promoter binding;core promoter proximal region DNA binding;core promoter proximal region sequence-specific DNA binding;core promoter sequence-specific DNA binding;DNA
acid-amino acid ligase activity;binding;catalytic activity;cation binding;chromatin binding;ion binding;ligase activity;ligase activity, forming carbon-nitrogen bonds;metal ion binding;small conjugating protein ligase activity;trans
binding;endopeptidase inhibitor activity;endopeptidase regulator activity;enzyme inhibitor activity;enzyme regulator activity;identical protein binding;metalloendopeptidase inhibitor activity;metalloenzyme inhibitor activity;meta
catalytic activity;hydrolase activity;hydrolase activity, acting on ester bonds;phosphatase activity;phosphoprotein phosphatase activity;phosphoric ester hydrolase activity;prenylated protein tyrosine phosphatase activity;prote
adenyl nucleotide binding;adenyl ribonucleotide binding;ATP binding;binding;catalytic activity;cation binding;ion binding;kinase activity;metal ion binding;nucleobase-containing compound kinase activity;nucleoside diphosph
binding;eukaryotic initiation factor 4E binding;protein binding;translation initiation factor binding;translation regulator activity;translation repressor activity
actin binding;actinin binding;alpha-actinin binding;binding;calcium ion binding;cation binding;cytoskeletal protein binding;extracellular matrix binding;ion binding;laminin binding;laminin-1 binding;metal ion binding;protein bin
binding;protein binding;transcription factor binding
binding;identical protein binding;protein binding;protein dimerization activity;protein homodimerization activity
binding;enzyme activator activity;enzyme regulator activity;GTPase activator activity;GTPase regulator activity;identical protein binding;nucleoside-triphosphatase regulator activity;protein binding;protein dimerization activity;
protein binding transcription factor activity;RNA polymerase II transcription cofactor activity;RNA polymerase II transcription factor binding transcription factor activity;transcription cofactor activity;transcription factor binding
6-phosphofructo-2-kinase activity;adenyl nucleotide binding;adenyl ribonucleotide binding;ATP binding;binding;carbohydrate kinase activity;carbohydrate phosphatase activity;catalytic activity;fructose-2,6-bisphosphate 2-p
binding;catalytic activity;GTP binding;GTPase activity;guanyl nucleotide binding;guanyl ribonucleotide binding;hydrolase activity;hydrolase activity, acting on acid anhydrides;hydrolase activity, acting on acid anhydrides, in ph
catalytic activity;cation transmembrane transporter activity;cytochrome-c oxidase activity;heme-copper terminal oxidase activity;hydrogen ion transmembrane transporter activity;inorganic cation transmembrane transporter a
binding;catalytic activity;cation binding;ion binding;metal ion binding;oxidoreductase activity;transition metal ion binding;zinc ion binding
adenyl nucleotide binding;adenyl ribonucleotide binding;ATP binding;binding;catalytic activity;kinase activity;nucleic acid binding;nucleobase-containing compound kinase activity;nucleotide binding;phosphotransferase activ
binding;lipid binding
aspartic-type endopeptidase activity;aspartic-type peptidase activity;catalytic activity;endopeptidase activity;hydrolase activity;peptidase activity;peptidase activity, acting on L-amino acid peptides
binding;cation binding;DNA binding;ion binding;metal ion binding;nucleic acid binding
carbon-sulfur lyase activity;catalytic activity;lyase activity
binding;cation binding;DNA binding;ion binding;metal ion binding;nucleic acid binding;protein binding transcription factor activity;transcription cofactor activity;transcription corepressor activity;transcription factor binding tra
catalytic activity;glucosyltransferase activity;protein xylosyltransferase activity;transferase activity;transferase activity, transferring glycosyl groups;transferase activity, transferring hexosyl groups;transferase activity, transferrin
catalytic activity;oxidoreductase activity
1-phosphatidylinositol binding;apolipoprotein A-I binding;apolipoprotein binding;binding;cargo receptor activity;high-density lipoprotein particle binding;high-density lipoprotein particle receptor activity;lipid binding;lipopolysa
active transmembrane transporter activity;amine transmembrane transporter activity;amino acid transmembrane transporter activity;antiporter activity;carboxylic acid transmembrane transporter activity;L-amino acid transme
binding;identical protein binding;protein binding;protein dimerization activity;protein domain specific binding;protein homodimerization activity
AU-rich element binding;binding;nucleic acid binding;RNA binding
catalytic activity;cis-trans isomerase activity;isomerase activity;peptidyl-prolyl cis-trans isomerase activity
aldose 1-epimerase activity;binding;carbohydrate binding;catalytic activity;isomerase activity;racemase and epimerase activity;racemase and epimerase activity, acting on carbohydrates and derivatives
binding;catalytic activity;hydrolase activity;hydrolase activity, acting on acid anhydrides;hydrolase activity, acting on acid anhydrides, in phosphorus-containing anhydrides;m7G(5')pppN diphosphatase activity;nucleic acid bi
structural constituent of ribosome;structural molecule activity
adenyl nucleotide binding;adenyl ribonucleotide binding;ATP binding;binding;catalytic activity;cation binding;histone kinase activity;histone serine kinase activity;ion binding;kinase activity;metal ion binding;nucleotide binding
binding;carbon-oxygen lyase activity;catalytic activity;cation binding;hydro-lyase activity;identical protein binding;ion binding;lyase activity;metal ion binding;methylthioribulose 1-phosphate dehydratase activity;protein bindin
binding;histone binding;protein binding;protein binding transcription factor activity;transcription cofactor activity;transcription corepressor activity;transcription factor binding transcription factor activity
binding;cation binding;ion binding;lipid binding;metal ion binding;phosphatidylinositol binding;phosphatidylinositol-3-phosphate binding;phospholipid binding;transition metal ion binding;zinc ion binding
binding;cation binding;DNA binding;ion binding;metal ion binding;nucleic acid binding
protein binding transcription factor activity;transcription coactivator activity;transcription cofactor activity;transcription factor binding transcription factor activity
binding;nucleic acid binding;RNA binding
nucleobase-containing compound transmembrane transporter activity;nucleoside transmembrane transporter activity;substrate-specific transmembrane transporter activity;substrate-specific transporter activity;transmembra
catalytic activity;hydrolase activity;hydrolase activity, acting on carbon-nitrogen (but not peptide) bonds
binding;nucleotide binding
structural constituent of nuclear pore;structural molecule activity
catalytic activity;endonuclease activity;endonuclease activity, active with either ribo- or deoxyribonucleic acids and producing 5'-phosphomonoesters;endoribonuclease activity;endoribonuclease activity, producing 5'-phosph
binding;catalytic activity;DNA binding;DNA-directed RNA polymerase activity;nucleic acid binding;nucleotidyltransferase activity;RNA polymerase activity;transferase activity;transferase activity, transferring phosphorus-conta
structural constituent of ribosome;structural molecule activity
5S rRNA binding;binding;nucleic acid binding;RNA binding;rRNA binding;structural constituent of ribosome;structural molecule activity

acid-amino acid ligase activity;binding;catalytic activity;histone binding;ligase activity;ligase activity, forming carbon-nitrogen bonds;methylated histone residue binding;nucleic acid binding;protein binding;RNA binding;single
binding;chromatin binding;DNA binding;nucleic acid binding;nucleic acid binding transcription factor activity;sequence-specific DNA binding transcription factor activity
3'-5' exonuclease activity;3'-5'-exoribonuclease activity;binding;catalytic activity;exonuclease activity;exonuclease activity, active with either ribo- or deoxyribonucleic acids and producing 5'-phosphomonoesters;exoribonucl
binding;calcium ion binding;calcium-dependent protein binding;cation binding;ion binding;lipid transporter activity;metal ion binding;phospholipid scramblase activity;phospholipid transporter activity;protein binding;substrate
binding;cytoskeletal protein binding;L27 domain binding;protein binding;protein domain specific binding
binding;catalytic activity;cation binding;ion binding;ligase activity;metal ion binding;transition metal ion binding;zinc ion binding
binding;nucleic acid binding;ribonucleoprotein binding;ribosome binding;RNA binding;translation factor activity, nucleic acid binding;translation initiation factor activity
catalytic activity;cation transmembrane transporter activity;hydrogen ion transmembrane transporter activity;inorganic cation transmembrane transporter activity;ion transmembrane transporter activity;monovalent inorganic c
binding;protein binding;receptor binding
binding;cation binding;ion binding;metal ion binding;transition metal ion binding;zinc ion binding
binding;cation binding;ion binding;metal ion binding;transition metal ion binding;zinc ion binding
binding;catalytic activity;ceramide binding;ceramide transporter activity;kinase activity;lipid binding;lipid transporter activity;phosphatidylinositol binding;phosphatidylinositol-4-phosphate binding;phospholipid binding;phosph
binding;catalytic activity;cation binding;heme binding;ion binding;iron ion binding;metal ion binding;oxidoreductase activity;oxidoreductase activity, acting on paired donors, with incorporation or reduction of molecular oxyge
binding;identical protein binding;lipid binding;phosphatidylinositol binding;phospholipid binding;protein binding
binding;histone binding;methylated histone residue binding;protein binding
binding;catalytic activity;deoxyribonuclease activity;deoxyribonuclease II activity;DNA binding;endodeoxyribonuclease activity;endodeoxyribonuclease activity, producing 3'-phosphomonoesters;endonuclease activity;endonu
acid-amino acid ligase activity;adenyl nucleotide binding;adenyl ribonucleotide binding;ATP binding;binding;catalytic activity;enzyme binding;ligase activity;ligase activity, forming carbon-nitrogen bonds;nucleotide binding;pr
[3-methyl-2-oxobutanoate dehydrogenase (acetyl-transferring)] kinase activity;adenyl nucleotide binding;adenyl ribonucleotide binding;ATP binding;binding;catalytic activity;kinase activity;nucleotide binding;phosphotransfera
actin binding;binding;cytoskeletal protein binding;identical protein binding;myosin binding;protein binding;protein dimerization activity;protein homodimerization activity;receptor binding
binding;cation binding;heme binding;ion binding;iron ion binding;metal ion binding;tetrapyrrole binding;transition metal ion binding
catalytic activity;NADH dehydrogenase (quinone) activity;NADH dehydrogenase (ubiquinone) activity;NADH dehydrogenase activity;oxidoreductase activity;oxidoreductase activity, acting on NADH or NADPH;oxidoreductase
binding;cation binding;ion binding;metal ion binding
binding;DNA binding;nucleic acid binding;protein binding transcription factor activity;ribonucleoprotein binding;transcription coactivator activity;transcription cofactor activity;transcription factor binding transcription factor ac
catalytic activity;endonuclease activity;endonuclease activity, active with either ribo- or deoxyribonucleic acids and producing 5'-phosphomonoesters;endoribonuclease activity;endoribonuclease activity, producing 5'-phosph
cation transmembrane transporter activity;hydrogen ion transmembrane transporter activity;inorganic cation transmembrane transporter activity;ion transmembrane transporter activity;monovalent inorganic cation transmemb
binding;nucleic acid binding;protein transporter activity;RNA binding;RNA cap binding;substrate-specific transporter activity;transporter activity
catalytic activity;NADH dehydrogenase (quinone) activity;NADH dehydrogenase (ubiquinone) activity;NADH dehydrogenase activity;oxidoreductase activity;oxidoreductase activity, acting on NADH or NADPH;oxidoreductase
binding;catalytic activity;cation binding;cysteine-type peptidase activity;hydrolase activity;ion binding;metal ion binding;metallopeptidase activity;peptidase activity;peptidase activity, acting on L-amino acid peptides;protein b
binding;chromatin binding;DNA binding;methyl-CpG binding;nucleic acid binding;nucleotide binding;sequence-specific DNA binding
binding;identical protein binding;protein binding;protein dimerization activity;protein homodimerization activity
aldo-keto reductase (NADP) activity;binding;catalytic activity;cation binding;cation transmembrane transporter activity;cytochrome-c oxidase activity;enzyme binding;heme binding;heme-copper terminal oxidase activity;hydr
binding;catalytic activity;coenzyme binding;cofactor binding;dihydrofolate reductase activity;drug binding;mRNA binding;NADP binding;nucleic acid binding;nucleotide binding;oxidoreductase activity;oxidoreductase activity,
binding;isoprenoid binding;lipid binding;retinal binding;retinoid binding;retinol binding;retinol transporter activity;transmembrane transporter activity;transporter activity;vitamin binding;vitamin transporter activity
binding;cysteine-type endopeptidase inhibitor activity;endopeptidase inhibitor activity;endopeptidase regulator activity;enzyme binding;enzyme inhibitor activity;enzyme regulator activity;peptidase inhibitor activity;peptidase
alpha-galactosidase activity;binding;carbohydrate binding;catalytic activity;galactosidase activity;galactoside binding;hydrolase activity;hydrolase activity, acting on glycosyl bonds;hydrolase activity, hydrolyzing O-glycosyl co
bHLH transcription factor binding;binding;cation binding;core promoter binding;core promoter proximal region DNA binding;core promoter proximal region sequence-specific DNA binding;core promoter sequence-specific DN
adenyl nucleotide binding;adenyl ribonucleotide binding;ATP binding;binding;catalytic activity;enzyme binding;hepatocyte growth factor-activated receptor activity;kinase activity;molecular transducer activity;nucleotide bindi
6,7-dihydropteridine reductase activity;binding;catalytic activity;coenzyme binding;cofactor binding;electron carrier activity;NAD binding;NADH binding;NADP binding;NADPH binding;nucleotide binding;oxidoreductase activi
binding;core promoter proximal region DNA binding;core promoter proximal region sequence-specific DNA binding;DNA binding;nucleic acid binding;p53 binding;protein binding;protein binding transcription factor activity;reg
catalytic activity;cation transmembrane transporter activity;cytochrome-c oxidase activity;heme-copper terminal oxidase activity;hydrogen ion transmembrane transporter activity;inorganic cation transmembrane transporter a
binding;catalytic activity;DNA binding;DNA-directed RNA polymerase activity;nucleic acid binding;nucleotidyltransferase activity;RNA polymerase activity;transferase activity;transferase activity, transferring phosphorus-conta
3'-5' DNA helicase activity;adenyl deoxyribonucleotide binding;adenyl nucleotide binding;adenyl ribonucleotide binding;ATP binding;ATPase activity;ATPase activity, coupled;ATP-dependent DNA helicase activity;ATP-depend
binding;calcium ion binding;calcium-dependent protein binding;cation binding;ion binding;magnesium ion binding;metal ion binding;protein binding
binding;catalytic activity;cis-trans isomerase activity;cyclosporin A binding;drug binding;isomerase activity;peptide binding;peptidyl-prolyl cis-trans isomerase activity
binding;catalytic activity;cation binding;cytidine deaminase activity;deaminase activity;hydrolase activity;hydrolase activity, acting on carbon-nitrogen (but not peptide) bonds;hydrolase activity, acting on carbon-nitrogen (but
active transmembrane transporter activity;adenyl nucleotide binding;adenyl ribonucleotide binding;ATP binding;ATPase activity;ATPase activity, coupled;ATPase activity, coupled to movement of substances;ATPase activity, c
binding;damaged DNA binding;DNA binding;nucleic acid binding;single-stranded DNA binding;structure-specific DNA binding
binding;catalytic activity;cation binding;cytoskeletal protein binding;GDP binding;GTP binding;GTPase activity;guanyl nucleotide binding;guanyl ribonucleotide binding;hydrolase activity;hydrolase activity, acting on acid anhy
amine binding;amino acid binding;antioxidant activity;binding;carboxylic acid binding;catalytic activity;glutathione binding;glutathione peroxidase activity;modified amino acid binding;oxidoreductase activity;oxidoreductase a
binding;chromatin binding;hormone receptor binding;nuclear hormone receptor binding;protein binding;protein complex scaffold;protein domain specific binding;receptor binding;receptor signaling complex scaffold activity;S
binding;cyclin-dependent protein kinase inhibitor activity;cyclin-dependent protein kinase regulator activity;enzyme binding;enzyme inhibitor activity;enzyme regulator activity;kinase binding;kinase inhibitor activity;kinase regu
carboxypeptidase activity;catalytic activity;exopeptidase activity;hydrolase activity;peptidase activity;peptidase activity, acting on L-amino acid peptides;serine hydrolase activity;serine-type carboxypeptidase activity;serine-t
binding;catalytic activity;cis-trans isomerase activity;cyclosporin A binding;drug binding;isomerase activity;peptide binding;peptidyl-prolyl cis-trans isomerase activity
binding;catalytic activity;cation binding;cysteine-type peptidase activity;enzyme regulator activity;hydrolase activity;ion binding;metal ion binding;metallopeptidase activity;peptidase activity;peptidase activity, acting on L-ami
acid-amino acid ligase activity;binding;catalytic activity;enzyme binding;enzyme regulator activity;glutamate-cysteine ligase activity;glutamate-cysteine ligase catalytic subunit binding;ligase activity;ligase activity, forming carb
catalytic activity;transferase activity
AT DNA binding;binding;cation binding;DNA bending activity;DNA binding;ion binding;metal ion binding;nucleic acid binding;peptide binding;sequence-specific DNA binding;transition metal ion binding;zinc ion binding
adenyl nucleotide binding;adenyl ribonucleotide binding;androgen receptor binding;ATP binding;ATPase activity;ATPase activity, coupled;binding;catalytic activity;cyclin-dependent protein kinase activity;DNA-dependent ATP
acyl-CoA thioesterase activity;catalytic activity;CoA hydrolase activity;hydrolase activity;hydrolase activity, acting on ester bonds;palmitoyl-(protein) hydrolase activity;palmitoyl-CoA hydrolase activity;thiolester hydrolase activ
binding;death domain binding;protein binding;protein domain specific binding
adenyl nucleotide binding;adenyl ribonucleotide binding;ATP binding;binding;catalytic activity;cation binding;coenzyme binding;cofactor binding;ion binding;isocitrate dehydrogenase (NAD+) activity;isocitrate dehydrogenase
binding;damaged DNA binding;DNA binding;nucleic acid binding;polyubiquitin binding;protein binding;single-stranded DNA binding;small conjugating protein binding;structure-specific DNA binding;ubiquitin binding
binding;catalytic activity;cysteine-type endopeptidase activity;cysteine-type peptidase activity;endopeptidase activity;enzyme activator activity;enzyme binding;enzyme regulator activity;hydrolase activity;kinase binding;pepti
active transmembrane transporter activity;ATPase activity;ATPase activity, coupled;ATPase activity, coupled to movement of substances;ATPase activity, coupled to transmembrane movement of ions;ATPase activity, couplec
binding;enzyme binding;GTPase binding;protein binding;Ran GTPase binding;Ras GTPase binding;small GTPase binding;transporter activity
binding;nucleic acid binding;RNA binding;structural constituent of ribosome;structural molecule activity
binding;cation binding;chromatin binding;enzyme regulator activity;identical protein binding;ion binding;kinase regulator activity;metal ion binding;protein binding;protein domain specific binding;protein kinase regulator activit
binding;binding, bridging;protein binding;protein binding, bridging;protein domain specific binding;receptor activity;SH3 domain binding;SH3/SH2 adaptor activity;signaling adaptor activity
catalytic activity;hydrolase activity
binding;enzyme binding;GTPase binding;GTP-Rho binding;protein binding;Ras GTPase binding;Rho GTPase binding;small GTPase binding
binding;nucleotide binding;protein binding;protein kinase A binding
binding;catalytic activity;coenzyme binding;cofactor binding;dihydroorotate dehydrogenase activity;drug binding;FMN binding;nucleotide binding;oxidoreductase activity;oxidoreductase activity, acting on the CH-CH group o
binding;protein binding;SNARE binding
active transmembrane transporter activity;adenyl nucleotide binding;adenyl ribonucleotide binding;ADP binding;antigen binding;ATP binding;ATPase activity;ATPase activity, coupled;ATPase activity, coupled to movement of s
acid-amino acid ligase activity;adenyl nucleotide binding;adenyl ribonucleotide binding;ATP binding;binding;catalytic activity;ligase activity;ligase activity, forming carbon-nitrogen bonds;nucleotide binding;purine nucleotide b
adrenergic receptor binding;binding;carbohydrate binding;complement binding;complement component C1q binding;glycosaminoglycan binding;G-protein-coupled receptor binding;hyaluronic acid binding;kininogen binding
binding;DNA binding;nucleic acid binding;nucleic acid binding transcription factor activity;protein binding;protein C-terminus binding;sequence-specific DNA binding;sequence-specific DNA binding transcription factor activit
binding;enzyme activator activity;enzyme regulator activity;lipase activator activity;phospholipase activator activity;protein binding;SNAP receptor activity;SNARE binding;sphingomyelin phosphodiesterase activator activity
protein binding transcription factor activity;transcription cofactor activity;transcription factor binding transcription factor activity
binding;enzyme regulator activity;GDP binding;GTP binding;guanyl nucleotide binding;guanyl ribonucleotide binding;nucleic acid binding;nucleotide binding;purine nucleotide binding;purine ribonucleoside triphosphate bindi
catalytic activity;DNA polymerase activity;DNA-directed DNA polymerase activity;nucleotidyltransferase activity;transferase activity;transferase activity, transferring phosphorus-containing groups
arylesterase activity;binding;carboxylic ester hydrolase activity;catalytic activity;cation binding;hydrolase activity;hydrolase activity, acting on ester bonds;identical protein binding;ion binding;metal ion binding;protein binding
binding;DNA binding;histone binding;methylated histone residue binding;nucleic acid binding;protein binding;regulatory region DNA binding;regulatory region nucleic acid binding;transcription regulatory region DNA binding
ATPase activity;ATPase activity, coupled;ATPase activity, coupled to movement of substances;binding;catalytic activity;enzyme activator activity;enzyme binding;enzyme regulator activity;GTPase activator activity;GTPase bi
adenyl nucleotide binding;adenyl ribonucleotide binding;ATP binding;binding;catalytic activity;DNA binding;double-stranded RNA binding;hormone receptor binding;nuclear hormone receptor binding;nucleic acid binding;nuc
protein binding transcription factor activity;transcription cofactor activity;transcription corepressor activity;transcription factor binding transcription factor activity
acid-amino acid ligase activity;ARF guanyl-nucleotide exchange factor activity;catalytic activity;enzyme regulator activity;GTPase regulator activity;guanyl-nucleotide exchange factor activity;ligase activity;ligase activity, form
activating transcription factor binding;binding;cation binding;chromatin binding;core promoter proximal region DNA binding;core promoter proximal region sequence-specific DNA binding;co-SMAD binding;cytokine receptor
binding;carbohydrate binding;catalytic activity;estradiol 17-beta-dehydrogenase activity;glycosaminoglycan binding;heparin binding;oxidoreductase activity;oxidoreductase activity, acting on CH-OH group of donors;oxidored
structural constituent of ribosome;structural molecule activity
binding;cytoskeletal protein binding;kinesin binding;protein binding;structural molecule activity
binding;cytokine binding;growth factor binding;protein binding;transforming growth factor beta binding
binding;calcium ion binding;cation binding;ion binding;metal ion binding
3-hydroxyacyl-CoA dehydratase activity;binding;carbon-oxygen lyase activity;catalytic activity;enzyme binding;hydro-lyase activity;lyase activity;protein binding
1-acylglycerol-3-phosphate O-acyltransferase activity;1-acylglycerophosphocholine O-acyltransferase activity;1-alkylglycerophosphocholine O-acetyltransferase activity;acetyltransferase activity;acylglycerol O-acyltransferas
3'-5' exonuclease activity;3'-5'-exoribonuclease activity;binding;catalytic activity;cation binding;exonuclease activity;exonuclease activity, active with either ribo- or deoxyribonucleic acids and producing 5'-phosphomonoeste
binding;nucleotide binding
binding;nucleotide binding
catalytic activity;intramolecular transferase activity;isomerase activity;pseudouridine synthase activity
binding;cation binding;DNA binding;ion binding;metal ion binding;nucleic acid binding
aspartic endopeptidase activity, intramembrane cleaving;aspartic-type endopeptidase activity;aspartic-type peptidase activity;binding;catalytic activity;endopeptidase activity;enzyme binding;hydrolase activity;identical prote
binding;enzyme binding;enzyme regulator activity;GTPase binding;GTPase regulator activity;GTP-dependent protein binding;guanyl-nucleotide exchange factor activity;lipid binding;nucleoside-triphosphatase regulator activit
catalytic activity;endopeptidase activity;hydrolase activity;peptidase activity;peptidase activity, acting on L-amino acid peptides
binding;cation binding;core promoter proximal region DNA binding;core promoter proximal region sequence-specific DNA binding;DNA binding;ion binding;metal ion binding;nucleic acid binding;regulatory region DNA bindin

binding;calcium ion binding;cation binding;ion binding;metal ion binding;protein binding;protein domain specific binding;SH3 domain binding
binding;enzyme binding;GTPase binding;kinase binding;protein binding;protein kinase binding;Ral GTPase binding;Ras GTPase binding;small GTPase binding
binding;cation binding;DNA binding;ion binding;metal ion binding;nucleic acid binding;transition metal ion binding;zinc ion binding
binding;identical protein binding;lipid binding;protein binding
adenyl nucleotide binding;adenyl ribonucleotide binding;ATP binding;binding;catalytic activity;hydrolase activity;kinase activity;nucleotide binding;p53 binding;phosphotransferase activity, alcohol group as acceptor;protein bi
binding;lipid binding
protein transporter activity;receptor activity;substrate-specific transporter activity;transporter activity
acid-amino acid ligase activity;binding;catalytic activity;cation binding;chromatin binding;ion binding;ligase activity;ligase activity, forming carbon-nitrogen bonds;metal ion binding;protein binding;protein domain specific bind
binding;DNA binding;nucleic acid binding;nucleic acid binding transcription factor activity;protein binding;RNA polymerase II transcription factor binding;sequence-specific DNA binding transcription factor activity;transcriptio
1-acylglycerol-3-phosphate O-acyltransferase activity;acylglycerol O-acyltransferase activity;catalytic activity;O-acyltransferase activity;transferase activity;transferase activity, transferring acyl groups;transferase activity, tran
agmatinase activity;binding;catalytic activity;cation binding;hydrolase activity;hydrolase activity, acting on carbon-nitrogen (but not peptide) bonds;hydrolase activity, acting on carbon-nitrogen (but not peptide) bonds, in linea
alpha-L-fucosidase activity;binding;carbohydrate binding;catalytic activity;fucose binding;fucosidase activity;hydrolase activity;hydrolase activity, acting on glycosyl bonds;hydrolase activity, hydrolyzing O-glycosyl compound
catalytic activity;methyltransferase activity;N-terminal protein N-methyltransferase activity;protein methyltransferase activity;transferase activity;transferase activity, transferring one-carbon groups
structural constituent of ribosome;structural molecule activity
binding;nucleic acid binding;nucleotide binding;RNA binding
adenyl nucleotide binding;adenyl ribonucleotide binding;ATP binding;binding;catalytic activity;cation binding;DNA binding;helicase activity;hydrolase activity;hydrolase activity, acting on acid anhydrides;hydrolase activity, act
binding;catalytic activity;enzyme binding;GTP binding;GTPase activity;GTPase binding;guanyl nucleotide binding;guanyl ribonucleotide binding;hydrolase activity;hydrolase activity, acting on acid anhydrides;hydrolase activity
beta-catenin binding;binding;DNA binding;histone binding;nucleic acid binding;protein binding;protein binding transcription factor activity;protein N-terminus binding;regulatory region DNA binding;regulatory region nucleic ac
protein tag
adenyl nucleotide binding;adenyl ribonucleotide binding;ATP binding;binding;catalytic activity;cation binding;ion binding;kinase activity;metal ion binding;nucleotide binding;phosphotransferase activity, alcohol group as acce
catalytic activity;disulfide oxidoreductase activity;intramolecular oxidoreductase activity;intramolecular oxidoreductase activity, interconverting keto- and enol-groups;intramolecular oxidoreductase activity, transposing S-S bo
binding;chromatin binding
binding;calcium ion binding;cation binding;extracellular matrix binding;ion binding;metal ion binding
adenyl nucleotide binding;adenyl ribonucleotide binding;adenylyltransferase activity;ATP binding;binding;catalytic activity;cation binding;enzyme binding;ion binding;metal ion binding;mRNA 3'-UTR binding;mRNA binding;nu
binding;cation binding;ion binding;metal ion binding
catalytic activity;hydrolase activity;hydrolase activity, acting on acid anhydrides;hydrolase activity, acting on acid anhydrides, in phosphorus-containing anhydrides;microtubule motor activity;motor activity;nucleoside-triphosp
binding;carbohydrate binding
3'-5' exonuclease activity;3'-5'-exoribonuclease activity;AU-rich element binding;binding;catalytic activity;exonuclease activity;exonuclease activity, active with either ribo- or deoxyribonucleic acids and producing 5'-phospho
3'-5' exonuclease activity;3'-5'-exoribonuclease activity;binding;catalytic activity;exonuclease activity;exonuclease activity, active with either ribo- or deoxyribonucleic acids and producing 5'-phosphomonoesters;exoribonucl
structural constituent of ribosome;structural molecule activity
binding;cation binding;ion binding;metal ion binding
binding;nucleic acid binding;RNA binding;snoRNA binding
binding;catalytic activity;cation binding;hydrolase activity;hydrolase activity, acting on glycosyl bonds;hydrolase activity, hydrolyzing O-glycosyl compounds;ion binding;metal ion binding;poly(ADP-ribose) glycohydrolase activ
3'(2'),5'-bisphosphate nucleotidase activity;3'-nucleotidase activity;binding;catalytic activity;cation binding;hydrolase activity;hydrolase activity, acting on ester bonds;inositol monophosphate phosphatase activity;inositol or p
cation channel activity;cation transmembrane transporter activity;channel activity;ion channel activity;ion transmembrane transporter activity;passive transmembrane transporter activity;substrate-specific channel activity;sub
binding;DNA binding;DNA replication origin binding;nucleic acid binding;sequence-specific DNA binding
G-protein coupled receptor activity;molecular transducer activity;receptor activity;signal transducer activity;signaling receptor activity;transmembrane signaling receptor activity;viral receptor activity
androgen receptor binding;binding;enzyme activator activity;enzyme binding;enzyme regulator activity;heat shock protein binding;histone binding;hormone receptor binding;identical protein binding;kinase activator activity;ki
acireductone synthase activity;binding;catalytic activity;cation binding;hydrolase activity;hydrolase activity, acting on ester bonds;ion binding;magnesium ion binding;metal ion binding;phosphatase activity;phosphoric ester h
protein transporter activity;substrate-specific transporter activity;transporter activity
catalytic activity;methyltransferase activity;protein methyltransferase activity;transferase activity;transferase activity, transferring one-carbon groups
binding;enzyme activator activity;enzyme binding;enzyme regulator activity;GTPase activator activity;GTPase regulator activity;kinase binding;nucleoside-triphosphatase regulator activity;protein binding;protein kinase binding
binding;catalytic activity;GDP binding;GTP binding;GTPase activity;guanyl nucleotide binding;guanyl ribonucleotide binding;hydrolase activity;hydrolase activity, acting on acid anhydrides;hydrolase activity, acting on acid anh
binding;DNA binding;nucleic acid binding;nucleic acid binding transcription factor activity;sequence-specific DNA binding;sequence-specific DNA binding transcription factor activity
receptor activity
acid-amino acid ligase activity;binding;catalytic activity;cation binding;co-SMAD binding;DNA binding;ion binding;ligase activity;ligase activity, forming carbon-nitrogen bonds;metal ion binding;nucleic acid binding;protein bin
acetyltransferase activity;binding;catalytic activity;histone acetyltransferase activity;histone binding;lysine N-acetyltransferase activity;methylated histone residue binding;N-acetyltransferase activity;N-acyltransferase activity;
binding;nucleic acid binding;RNA binding
binding;cation binding;chaperone binding;identical protein binding;ion binding;metal ion binding;protein binding;protein dimerization activity;protein homodimerization activity;transition metal ion binding;transporter activity;zi
binding;DNA binding;nucleic acid binding
binding;DNA binding;nucleic acid binding;protein binding;sequence-specific DNA binding;transcription factor binding
axon guidance receptor activity;binding;identical protein binding;LRR domain binding;molecular transducer activity;protein binding;protein domain specific binding;receptor activity;signal transducer activity;signaling receptor
binding;protein binding;SNAP receptor activity;SNARE binding;syntaxin binding
binding;catalytic activity;DNA binding;DNA-directed RNA polymerase activity;nucleic acid binding;nucleotidyltransferase activity;RNA polymerase activity;transferase activity;transferase activity, transferring phosphorus-conta
binding;calcium ion binding;cation binding;ion binding;metal ion binding;protein binding;unfolded protein binding
binding;channel inhibitor activity;channel regulator activity;enzyme binding;enzyme inhibitor activity;enzyme regulator activity;ion channel inhibitor activity;phosphatase binding;phosphatase inhibitor activity;phosphatase regu
binding;catalytic activity;DNA binding;nucleic acid binding;transferase activity;transferase activity, transferring acyl groups
alcohol binding;binding;carbohydrate binding;catalytic activity;dolichyl-phosphate beta-D-mannosyltransferase activity;dolichyl-phosphate-mannose-protein mannosyltransferase activity;mannose binding;mannosyltransferas
binding;double-stranded RNA binding;enzyme activator activity;enzyme binding;enzyme regulator activity;identical protein binding;nucleic acid binding;protein binding;protein dimerization activity;protein homodimerization ac
carboxylic ester hydrolase activity;catalytic activity;hydrolase activity;hydrolase activity, acting on ester bonds;lipase activity;lysophospholipase activity;palmitoyl-(protein) hydrolase activity;phospholipase activity;thiolester hy
binding;protein binding;transcription factor binding
binding;cation binding;ion binding;metal ion binding;transition metal ion binding;zinc ion binding
binding;cofactor binding;pyridoxal phosphate binding;vitamin B6 binding;vitamin binding
binding;cation binding;DNA binding;ion binding;metal ion binding;nucleic acid binding;transition metal ion binding;zinc ion binding
catalytic activity;NADH dehydrogenase (quinone) activity;NADH dehydrogenase (ubiquinone) activity;NADH dehydrogenase activity;oxidoreductase activity;oxidoreductase activity, acting on NADH or NADPH;oxidoreductase
catalytic activity;oxidoreductase activity;oxidoreductase activity, acting on a sulfur group of donors;oxidoreductase activity, acting on a sulfur group of donors, disulfide as acceptor;protein-disulfide reductase (glutathione) ac
adenyl nucleotide binding;adenyl ribonucleotide binding;ATP binding;binding;catalytic activity;cation binding;identical protein binding;ion binding;kinase activity;MAP kinase kinase kinase activity;metal ion binding;molecular t
adenyl nucleotide binding;adenyl ribonucleotide binding;ATP binding;binding;catalytic activity;cation binding;deoxynucleoside kinase activity;identical protein binding;ion binding;kinase activity;metal ion binding;nucleobase-c
5,10-methylenetetrahydrofolate-dependent methyltransferase activity;amine binding;amino acid binding;binding;carboxylic acid binding;catalytic activity;cofactor binding;drug binding;folic acid binding;methyltransferase activ
catalytic activity;hydroxymethylbilane synthase activity;transferase activity;transferase activity, transferring alkyl or aryl (other than methyl) groups
binding;cation binding;identical protein binding;ion binding;metal ion binding;mRNA binding;nucleic acid binding;protein binding;protein dimerization activity;protein homodimerization activity;RNA binding;single-stranded RN
aryl sulfotransferase activity;catalytic activity;sulfotransferase activity;transferase activity;transferase activity, transferring sulfur-containing groups
adenyl nucleotide binding;adenyl ribonucleotide binding;ATP binding;binding;catalytic activity;cation binding;ion binding;kinase activity;metal ion binding;molecular transducer activity;nucleotide binding;phosphotransferase a
binding;catalytic activity;cation binding;cation transmembrane transporter activity;cytochrome-c oxidase activity;heme-copper terminal oxidase activity;hydrogen ion transmembrane transporter activity;inorganic cation transr
endopeptidase inhibitor activity;endopeptidase regulator activity;enzyme inhibitor activity;enzyme regulator activity;peptidase inhibitor activity;peptidase regulator activity;serine-type endopeptidase inhibitor activity
acid phosphatase activity;catalytic activity;hydrolase activity;hydrolase activity, acting on ester bonds;phosphatase activity;phosphoric ester hydrolase activity
active transmembrane transporter activity;carbohydrate transmembrane transporter activity;cation transmembrane transporter activity;cation:sugar symporter activity;glucose transmembrane transporter activity;hexose trans
catalytic activity;DNA N-glycosylase activity;hydrolase activity;hydrolase activity, acting on glycosyl bonds;hydrolase activity, hydrolyzing N-glycosyl compounds;uracil DNA N-glycosylase activity
binding;enzyme binding;protein binding
binding;complement binding;protein binding
binding;chromatin binding;core promoter proximal region DNA binding;core promoter proximal region sequence-specific DNA binding;DNA binding;identical protein binding;nucleic acid binding;nucleic acid binding transcripti
binding;catalytic activity;enzyme binding;hydrolase activity;hydrolase activity, acting on ester bonds;integrin binding;kinase binding;phosphatase activity;phosphoprotein phosphatase activity;phosphoric ester hydrolase activi
activin binding;binding;carbohydrate binding;catalytic activity;cytokine binding;cytokine receptor binding;galactose binding;glycosaminoglycan binding;growth factor binding;identical protein binding;kinase activity;molecular
binding;cation binding;enzyme activator activity;enzyme regulator activity;fibronectin binding;growth factor binding;insulin-like growth factor binding;insulin-like growth factor I binding;insulin-like growth factor II binding;ion bi
binding;nucleic acid binding;nucleotide binding;RNA binding
cation transmembrane transporter activity;hydrogen ion transmembrane transporter activity;inorganic cation transmembrane transporter activity;ion transmembrane transporter activity;monovalent inorganic cation transmemb
adenyl nucleotide binding;adenyl ribonucleotide binding;ATP binding;binding;catalytic activity;double-stranded RNA binding;enzyme regulator activity;eukaryotic translation initiation factor 2alpha kinase activity;kinase activity
antioxidant activity;binding;catalytic activity;cation binding;dioxygenase activity;heme binding;ion binding;iron ion binding;metal ion binding;oxidoreductase activity;oxidoreductase activity, acting on paired donors, with incorp
binding;chromatin binding;histone acetyl-lysine binding;histone binding;protein binding
adenyl nucleotide binding;adenyl ribonucleotide binding;adenylate kinase activity;ATP binding;binding;catalytic activity;GTP binding;guanyl nucleotide binding;guanyl ribonucleotide binding;kinase activity;nucleobase-contain
catalytic activity;endopeptidase activity;hydrolase activity;peptidase activity;peptidase activity, acting on L-amino acid peptides;threonine-type endopeptidase activity;threonine-type peptidase activity
actin binding;actin filament binding;binding;cytoskeletal protein binding;protein binding
binding;catalytic activity;cation binding;copper ion binding;ion binding;metal ion binding;oxidoreductase activity;oxidoreductase activity, acting on the CH-NH2 group of donors;oxidoreductase activity, acting on the CH-NH2
adenyl nucleotide binding;adenyl ribonucleotide binding;ATP binding;binding;catalytic activity;kinase activity;lipid binding;molecular transducer activity;nucleotide binding;phosphatidylserine binding;phospholipid binding;pho
active transmembrane transporter activity;amine transmembrane transporter activity;amino acid transmembrane transporter activity;antiporter activity;arginine transmembrane transporter activity;basic amino acid transmemb
actin binding;actin monomer binding;adenyl nucleotide binding;adenyl-nucleotide exchange factor activity;ATPase regulator activity;binding;cytoskeletal protein binding;enzyme regulator activity;lipid binding;nucleoside-triph
binding;carbon-carbon lyase activity;carboxylic acid binding;catalytic activity;cation binding;coenzyme binding;cofactor binding;fatty acid binding;fatty-acyl-CoA binding;hydroxymethylglutaryl-CoA lyase activity;identical pro
aspartic-type endopeptidase activity;aspartic-type peptidase activity;catalytic activity;cyclin-dependent protein kinase inhibitor activity;cyclin-dependent protein kinase regulator activity;cysteine-type endopeptidase activity;c
adenyl nucleotide binding;adenyl ribonucleotide binding;ATP binding;ATPase activity;ATPase activity, coupled;binding;catalytic activity;cation binding;chromatin binding;chromo shadow domain binding;DNA binding;DNA heli
binding;catalytic activity;cation binding;DNA primase activity;DNA-directed RNA polymerase activity;ion binding;metal ion binding;nucleotidyltransferase activity;RNA polymerase activity;transferase activity;transferase activity
binding;catalytic activity;GDP binding;GTP binding;GTPase activity;guanyl nucleotide binding;guanyl ribonucleotide binding;hydrolase activity;hydrolase activity, acting on acid anhydrides;hydrolase activity, acting on acid anh
catalytic activity;methyltransferase activity;S-adenosylmethionine-dependent methyltransferase activity;S-methyltransferase activity;thiopurine S-methyltransferase activity;transferase activity;transferase activity, transferring c
catalytic activity;cyclin-dependent protein kinase regulator activity;enzyme regulator activity;kinase activity;kinase regulator activity;protein kinase regulator activity;transferase activity;transferase activity, transferring phospho
binding;enzyme binding;GTP binding;GTPase binding;GTP-dependent protein binding;guanyl nucleotide binding;guanyl ribonucleotide binding;lipid binding;nucleotide binding;phosphatidylinositol binding;phosphatidylinosito
binding;protein binding;protein complex binding;protein domain specific binding
cation transmembrane transporter activity;hydrogen ion transmembrane transporter activity;inorganic cation transmembrane transporter activity;ion transmembrane transporter activity;monovalent inorganic cation transmemb

binding;nucleic acid binding;ribonucleoprotein binding;ribosomal large subunit binding;ribosome binding;RNA binding;translation factor activity, nucleic acid binding;translation initiation factor activity
structural constituent of nuclear pore;structural molecule activity
binding;epidermal growth factor binding;growth factor binding;hormone binding;protein binding
binding;catalytic activity;GDP binding;GTP binding;GTPase activity;GTP-dependent protein binding;guanyl nucleotide binding;guanyl ribonucleotide binding;hydrolase activity;hydrolase activity, acting on acid anhydrides;hyd
binding;mRNA binding;nucleic acid binding;RNA binding;structural constituent of ribosome;structural molecule activity
binding;enzyme binding;enzyme regulator activity;phosphatase binding;phosphatase regulator activity;protein binding;protein phosphatase 2A binding;protein phosphatase binding;protein phosphatase regulator activity;prote
binding;chromatin binding;enzyme binding;protein binding
acetylcholine receptor activator activity;acetylcholine receptor regulator activity;adenyl nucleotide binding;adenyl ribonucleotide binding;ATP binding;binding;catalytic activity;cyclin-dependent protein kinase activity;ErbB-2 cl
AU-rich element binding;binding;nucleic acid binding;nucleotide binding;RNA binding
active transmembrane transporter activity;amine transmembrane transporter activity;amino acid transmembrane transporter activity;antigen binding;antiporter activity;aromatic amino acid transmembrane transporter activity;
binding;catalytic activity;cation binding;coenzyme binding;cofactor binding;ion binding;metal ion binding;mRNA 3'-UTR binding;mRNA binding;NADP binding;NADPH binding;NADPH:quinone reductase activity;nucleic acid b
receptor activity
binding;catalytic activity;cation binding;DNA binding;ion binding;ligase activity;metal ion binding;nucleic acid binding;nucleic acid binding transcription factor activity;regulatory region DNA binding;regulatory region nucleic ac
binding;chromatin binding;chromo shadow domain binding;identical protein binding;protein binding;protein domain specific binding;unfolded protein binding
binding;protein binding;protein N-terminus binding;SNAP receptor activity;SNARE binding
binding;DNA binding;nucleic acid binding;nucleic acid binding transcription factor activity;protein binding transcription factor activity;sequence-specific DNA binding transcription factor activity;transcription coactivator activit
aminoacyl-tRNA hydrolase activity;binding;carboxylic ester hydrolase activity;catalytic activity;hydrolase activity;hydrolase activity, acting on ester bonds;nucleic acid binding;RNA binding;translation factor activity, nucleic aci
binding;calcium ion binding;cation binding;ion binding;metal ion binding
binding;mRNA 3'-UTR binding;mRNA binding;nucleic acid binding;nucleotide binding;RNA binding
binding;enzyme binding;protein binding;ubiquitin protein ligase binding
adenyl nucleotide binding;adenyl ribonucleotide binding;ATP binding;binding;catalytic activity;guanylate kinase activity;kinase activity;nucleobase-containing compound kinase activity;nucleotide binding;nucleotide kinase ac
adenyl nucleotide binding;adenyl ribonucleotide binding;ATP binding;binding;nucleotide binding;purine nucleotide binding;purine ribonucleoside triphosphate binding;purine ribonucleotide binding;ribonucleotide binding
catalytic activity;oxidoreductase activity
binding;catalytic activity;cation binding;demethylase activity;ion binding;metal ion binding;oxidative RNA demethylase activity;oxidoreductase activity;oxidoreductase activity, acting on paired donors, with incorporation or red
acid phosphatase activity;adenyl nucleotide binding;adenyl ribonucleotide binding;ATP binding;binding;catalytic activity;diphosphoinositol-pentakisphosphate kinase activity;hydrolase activity;hydrolase activity, acting on este
catalytic activity;histone methyltransferase activity;histone methyltransferase activity (H3-K36 specific);histone methyltransferase activity (H3-K4 specific);histone-lysine N-methyltransferase activity;lysine N-methyltransferase
binding;nucleic acid binding;nucleotide binding;RNA binding
binding;protein binding;SNARE binding;syntaxin binding
catalytic activity;cysteine-type peptidase activity;hydrolase activity;peptidase activity;peptidase activity, acting on L-amino acid peptides
binding;catalytic activity;cation binding;ion binding;ligase activity;metal ion binding;transition metal ion binding;zinc ion binding
acid-amino acid ligase activity;catalytic activity;ligase activity;ligase activity, forming carbon-nitrogen bonds;small conjugating protein ligase activity;ubiquitin-protein ligase activity
adenyl nucleotide binding;adenyl ribonucleotide binding;ATP binding;binding;catalytic activity;cation binding;enzyme binding;ion binding;kinase activity;kinase binding;metal ion binding;nucleotide binding;phosphotransferase
acid-amino acid ligase activity;binding;catalytic activity;cation binding;histone binding;ion binding;ligase activity;ligase activity, forming carbon-nitrogen bonds;metal ion binding;protein binding;small conjugating protein ligase
antioxidant activity;catalytic activity;glutathione peroxidase activity;oxidoreductase activity;oxidoreductase activity, acting on peroxide as acceptor;peroxidase activity
active transmembrane transporter activity;amine transmembrane transporter activity;choline transmembrane transporter activity;substrate-specific transmembrane transporter activity;substrate-specific transporter activity;tra
binding;catalytic activity;enzyme binding;glutamate receptor binding;guanylate kinase activity;ionotropic glutamate receptor binding;kinase activity;kinase binding;nucleobase-containing compound kinase activity;nucleotide k
catalytic activity;cysteine-type peptidase activity;hydrolase activity;peptidase activity;peptidase activity, acting on L-amino acid peptides;small conjugating protein-specific protease activity;ubiquitin-specific protease activity
enzyme inhibitor activity;enzyme regulator activity;phosphatase inhibitor activity;phosphatase regulator activity;protein phosphatase inhibitor activity;protein phosphatase regulator activity
acetyltransferase activity;binding;carbohydrate binding;catalytic activity;glucosamine 6-phosphate N-acetyltransferase activity;identical protein binding;monosaccharide binding;N-acetyltransferase activity;N-acyltransferase a
binding;DNA binding;nucleic acid binding;RNA binding
binding;cation binding;ion binding;metal ion binding;nucleic acid binding
catalytic activity;hydrolase activity
catalytic activity;deacetylase activity;hydrolase activity;hydrolase activity, acting on glycosyl bonds
ligand-dependent nuclear receptor transcription coactivator activity;protein binding transcription factor activity;transcription coactivator activity;transcription cofactor activity;transcription factor binding transcription factor act
binding;carbohydrate binding;endopeptidase inhibitor activity;endopeptidase regulator activity;enzyme inhibitor activity;enzyme regulator activity;glycosaminoglycan binding;heparin binding;metalloendopeptidase inhibitor ac
adenyl nucleotide binding;adenyl ribonucleotide binding;ATP binding;binding;catalytic activity;hydrolase activity;hydrolase activity, acting on acid anhydrides;hydrolase activity, acting on acid anhydrides, in phosphorus-conta
binding;catalytic activity;cation binding;dITP diphosphatase activity;hydrolase activity;hydrolase activity, acting on acid anhydrides;hydrolase activity, acting on acid anhydrides, in phosphorus-containing anhydrides;ion bindi
ATPase binding;binding;enzyme binding;identical protein binding;lipid binding;protein binding;protein dimerization activity;protein homodimerization activity
structural constituent of ribosome;structural molecule activity
adenyl nucleotide binding;adenyl ribonucleotide binding;ATP binding;binding;catalytic activity;kinase activity;nucleobase-containing compound kinase activity;nucleoside kinase activity;nucleotide binding;purine nucleotide bi
binding;identical protein binding;protein binding;protein dimerization activity;protein homodimerization activity
cation transmembrane transporter activity;divalent inorganic cation transmembrane transporter activity;inorganic cation transmembrane transporter activity;ion transmembrane transporter activity;magnesium ion transmembra
2 iron, 2 sulfur cluster binding;4 iron, 4 sulfur cluster binding;binding;cation binding;ferrous iron binding;ion binding;iron ion binding;iron-sulfur cluster binding;metal cluster binding;metal ion binding;protein binding;protein con
binding;cation binding;ion binding;metal ion binding;nucleotide binding;protein binding;SNARE binding;syntaxin binding;transition metal ion binding;zinc ion binding
catalytic activity;cis-trans isomerase activity;isomerase activity;peptidyl-prolyl cis-trans isomerase activity
catalytic activity;cysteine-type peptidase activity;hydrolase activity;peptidase activity;peptidase activity, acting on L-amino acid peptides
binding;enzyme binding;GTPase binding;protein binding;Ran GTPase binding;Ras GTPase binding;R-SMAD binding;SMAD binding;small GTPase binding
adenyl nucleotide binding;adenyl ribonucleotide binding;ATP binding;ATPase activity;ATPase activity, coupled;ATP-dependent helicase activity;ATP-dependent RNA helicase activity;binding;catalytic activity;helicase activity;h
binding;carboxyl-O-methyltransferase activity;catalytic activity;enzyme binding;methyltransferase activity;O-methyltransferase activity;protein binding;protein carboxyl O-methyltransferase activity;protein methyltransferase ac
structural constituent of ribosome;structural molecule activity
adenyl nucleotide binding;adenyl ribonucleotide binding;ATP binding;binding;cation binding;ion binding;metal ion binding;nucleotide binding;purine nucleotide binding;purine ribonucleoside triphosphate binding;purine ribonu
carbon-carbon lyase activity;carboxy-lyase activity;catalytic activity;lyase activity;methylmalonyl-CoA decarboxylase activity
2,4-dienoyl-CoA reductase (NADPH) activity;binding;catalytic activity;oxidoreductase activity;oxidoreductase activity, acting on the CH-CH group of donors;oxidoreductase activity, acting on the CH-CH group of donors, NAD
catalytic activity;hydrolase activity;hydrolase activity, acting on glycosyl bonds;hydrolase activity, hydrolyzing O-glycosyl compounds
binding;nucleic acid binding;p53 binding;protein binding;RNA binding
[methionine synthase] reductase activity;aquacobalamin reductase (NADPH) activity;binding;catalytic activity;cation binding;coenzyme binding;cofactor binding;FAD binding;flavin adenine dinucleotide binding;FMN binding;io
binding;cation binding;ion binding;metal ion binding;transition metal ion binding;zinc ion binding
binding;protein binding;protein complex binding;small conjugating protein binding;ubiquitin binding
binding;nucleic acid binding;RNA binding;snRNA binding;U6 snRNA binding
binding;catalytic activity;GDP binding;GTP binding;GTPase activity;guanyl nucleotide binding;guanyl ribonucleotide binding;hydrolase activity;hydrolase activity, acting on acid anhydrides;hydrolase activity, acting on acid anh
binding;enzyme binding;phosphatase binding;protein binding;protein phosphatase binding
binding;nucleotide binding
basal RNA polymerase II transcription machinery binding;basal transcription machinery binding;binding;enzyme binding;identical protein binding;nucleic acid binding;protein binding;RNA binding;RNA polymerase binding;RN
binding;catalytic activity;cation binding;demethylase activity;DNA binding;histone demethylase activity;histone demethylase activity (H3-K36 specific);ion binding;metal ion binding;nucleic acid binding;oxidoreductase activity
aldehyde-lyase activity;carbon-carbon lyase activity;catalytic activity;deoxyribose-phosphate aldolase activity;lyase activity
3'-5' exonuclease activity;3'-5'-exoribonuclease activity;binding;catalytic activity;exonuclease activity;exonuclease activity, active with either ribo- or deoxyribonucleic acids and producing 5'-phosphomonoesters;exoribonucl
structural constituent of ribosome;structural molecule activity
androgen receptor binding;binding;catalytic activity;cation binding;core promoter binding;dioxygenase activity;DNA binding;hormone receptor binding;ion binding;iron ion binding;metal ion binding;nuclear hormone receptor b
adenyl nucleotide binding;adenyl ribonucleotide binding;ATP binding;binding;calcium ion binding;catalytic activity;cation binding;enzyme binding;GTPase binding;hydrolase activity;hydrolase activity, acting on acid anhydride
binding;protein binding;protein complex binding
binding;cation binding;cation transmembrane transporter activity;copper chaperone activity;copper ion binding;copper ion transmembrane transporter activity;copper-dependent protein binding;inorganic cation transmembra
1-phosphatidylinositol-3-kinase activity;adenyl nucleotide binding;adenyl ribonucleotide binding;ATP binding;binding;catalytic activity;inositol or phosphatidylinositol kinase activity;kinase activity;lipid binding;lipid kinase activ
binding;catalytic activity;NADH dehydrogenase (quinone) activity;NADH dehydrogenase (ubiquinone) activity;NADH dehydrogenase activity;oxidoreductase activity;oxidoreductase activity, acting on NADH or NADPH;oxidored
antioxidant activity;binding;catalytic activity;cation binding;cation transmembrane transporter activity;copper ion binding;copper ion transmembrane transporter activity;disulfide oxidoreductase activity;inorganic cation transm
amine binding;amino acid binding;binding;carboxylic acid binding;catalytic activity;glutathione binding;intramolecular oxidoreductase activity;isomerase activity;modified amino acid binding;peptide binding;prostaglandin-E sy
binding;catalytic activity;cation binding;chromatin binding;DNA binding;DNA-directed RNA polymerase activity;ion binding;metal ion binding;nucleic acid binding;nucleoside binding;nucleotidyltransferase activity;ribonucleosi
binding;catalytic activity;histone binding;methylated histone residue binding;methyltransferase activity;protein binding;S-adenosylmethionine-dependent methyltransferase activity;transferase activity;transferase activity, trans
adenyl nucleotide binding;adenyl ribonucleotide binding;ATP binding;binding;calmodulin-dependent protein kinase activity;cAMP response element binding protein binding;catalytic activity;enzyme binding;GTPase binding;id
enzyme activator activity;enzyme regulator activity;GTPase activator activity;GTPase regulator activity;nucleoside-triphosphatase regulator activity
binding;carbohydrate binding;cation binding;cytoskeletal protein binding;DNA binding;glycosaminoglycan binding;heparin binding;ion binding;metal ion binding;microtubule binding;nucleic acid binding;nucleic acid binding tr
AU-rich element binding;binding;catalytic activity;enzyme binding;histone deacetylase binding;hydrolase activity;identical protein binding;mRNA binding;nucleic acid binding;protein binding;protein dimerization activity;protei
binding;cation binding;copper ion binding;ion binding;metal ion binding;transition metal ion binding
androgen receptor binding;bHLH transcription factor binding;binding;catalytic activity;chromatin binding;coenzyme binding;cofactor binding;demethylase activity;DNA binding;enzyme binding;flavin adenine dinucleotide bind
binding;protein binding;receptor binding
binding;catalytic activity;GTP binding;guanyl nucleotide binding;guanyl ribonucleotide binding;guanylyltransferase activity;hydrolase activity;hydrolase activity, acting on acid anhydrides;hydrolase activity, acting on acid anhy
binding;protein binding;protein complex binding
binding;growth factor activity;lipoprotein particle receptor binding;low-density lipoprotein particle receptor binding;molecular transducer activity;protein binding;receptor antagonist activity;receptor binding;receptor inhibitor a

catalytic activity;hydrolase activity
antigen binding;beta-2-microglobulin binding;binding;peptide antigen binding;peptide binding;protein binding
binding;carbohydrate binding;cargo receptor activity;extracellular matrix binding;glycosaminoglycan binding;heparin binding;integrin binding;pattern binding;polysaccharide binding;protein binding;protein complex binding;re
alpha-L-fucosidase activity;binding;carbohydrate binding;catalytic activity;fucose binding;fucosidase activity;hydrolase activity;hydrolase activity, acting on glycosyl bonds;hydrolase activity, hydrolyzing O-glycosyl compound
binding;core promoter proximal region DNA binding;core promoter proximal region sequence-specific DNA binding;DNA binding;double-stranded DNA binding;nucleic acid binding;nucleic acid binding transcription factor act
acetyl-CoA C-acyltransferase activity;acyl-CoA oxidase activity;C-acyltransferase activity;catalytic activity;oxidoreductase activity;oxidoreductase activity, acting on the CH-CH group of donors;oxidoreductase activity, acting
7S RNA binding;binding;nucleic acid binding;RNA binding
catalytic activity;cation transmembrane transporter activity;cytochrome-c oxidase activity;heme-copper terminal oxidase activity;hydrogen ion transmembrane transporter activity;inorganic cation transmembrane transporter a
binding;nucleic acid binding;RNA binding
binding;catalytic activity;dihydrolipoyllysine-residue (2-methylpropanoyl)transferase activity;enzyme binding;protein binding;transferase activity;transferase activity, transferring acyl groups;transferase activity, transferring acyl
binding;cell adhesion molecule binding;protein binding;receptor activity
binding;core promoter proximal region DNA binding;core promoter proximal region sequence-specific DNA binding;DNA binding;double-stranded DNA binding;nucleic acid binding;nucleic acid binding transcription factor act
adenyl nucleotide binding;adenyl ribonucleotide binding;ATP binding;binding;catalytic activity;cation binding;DNA binding;DNA ligase (ATP) activity;DNA ligase activity;ion binding;ligase activity;ligase activity, forming phospho
binding;catalytic activity;GDP binding;GTP binding;GTPase activity;guanyl nucleotide binding;guanyl ribonucleotide binding;hydrolase activity;hydrolase activity, acting on acid anhydrides;hydrolase activity, acting on acid anh
adenyl nucleotide binding;adenyl ribonucleotide binding;ATP binding;binding;catalytic activity;cyclin-dependent protein kinase activity;kinase activity;nucleotide binding;phosphotransferase activity, alcohol group as acceptor
acid phosphatase activity;catalytic activity;hydrolase activity;hydrolase activity, acting on ester bonds;non-membrane spanning protein tyrosine phosphatase activity;phosphatase activity;phosphoprotein phosphatase activity
binding;catalytic activity;endopeptidase activity;hydrolase activity;lipopolysaccharide binding;peptidase activity;peptidase activity, acting on L-amino acid peptides;threonine-type endopeptidase activity;threonine-type peptic
catalytic activity;endopeptidase activity;hydrolase activity;peptidase activity;peptidase activity, acting on L-amino acid peptides;threonine-type endopeptidase activity;threonine-type peptidase activity
alkylbase DNA N-glycosylase activity;binding;catalytic activity;damaged DNA binding;DNA binding;DNA N-glycosylase activity;DNA-3-methyladenine glycosylase I activity;DNA-7-methylguanine glycosylase activity;hydrolase
3-hydroxyisobutyrate dehydrogenase activity;binding;catalytic activity;coenzyme binding;cofactor binding;NAD binding;nucleotide binding;oxidoreductase activity;oxidoreductase activity, acting on CH-OH group of donors;ox
binding;chromatin binding;DNA binding;nucleic acid binding;regulatory region DNA binding;regulatory region nucleic acid binding;RNA polymerase II regulatory region DNA binding;RNA polymerase II regulatory region sequer
adenyl nucleotide binding;adenyl ribonucleotide binding;ATP binding;ATPase activity;binding;catalytic activity;DNA binding;double-stranded DNA binding;guanine/thymine mispair binding;hydrolase activity;hydrolase activity,
binding;nucleic acid binding;receptor activity;RNA binding;translation factor activity, nucleic acid binding;translation initiation factor activity
binding;catalytic activity;cation binding;demethylase activity;DNA binding;histone demethylase activity;histone demethylase activity (H3-K4 specific);ion binding;metal ion binding;nucleic acid binding;oxidoreductase activity;c
adenyl nucleotide binding;adenyl ribonucleotide binding;ATP binding;binding;catalytic activity;cation binding;ion binding;kinase activity;lipid binding;metal ion binding;nucleotide binding;phospholipid binding;phosphotransfer
binding;nucleotide binding
catalytic activity;endopeptidase activity;hydrolase activity;peptidase activity;peptidase activity, acting on L-amino acid peptides;threonine-type endopeptidase activity;threonine-type peptidase activity
adenyl nucleotide binding;adenyl ribonucleotide binding;ATP binding;binding;catalytic activity;enzyme binding;kinase activity;kinase binding;nucleotide binding;phosphotransferase activity, alcohol group as acceptor;protein b
aldehyde dehydrogenase (NAD) activity;binding;catalytic activity;identical protein binding;oxidoreductase activity;oxidoreductase activity, acting on the aldehyde or oxo group of donors;oxidoreductase activity, acting on the a
binding;chromatin binding;core promoter binding;core promoter sequence-specific DNA binding;DNA binding;double-stranded DNA binding;glucocorticoid receptor binding;hormone receptor binding;molecular transducer ac
active transmembrane transporter activity;carboxylic acid transmembrane transporter activity;lactate transmembrane transporter activity;mevalonate transmembrane transporter activity;monocarboxylic acid transmembrane t
binding;identical protein binding;protein binding
binding;DNA binding;nucleic acid binding;structural constituent of ribosome;structural molecule activity
acid-amino acid ligase activity;binding;catalytic activity;enzyme binding;ligase activity;ligase activity, forming carbon-nitrogen bonds;protein binding;small conjugating protein ligase activity;SUMO ligase activity;transcription f
binding;cation binding;heme binding;ion binding;iron ion binding;metal ion binding;oxygen binding;oxygen transporter activity;substrate-specific transporter activity;tetrapyrrole binding;transition metal ion binding;transporter
binding;catalytic activity;GTP binding;GTPase activity;guanyl nucleotide binding;guanyl ribonucleotide binding;hydrolase activity;hydrolase activity, acting on acid anhydrides;hydrolase activity, acting on acid anhydrides, in ph
active transmembrane transporter activity;adenyl nucleotide binding;adenyl ribonucleotide binding;ATP binding;ATPase activity;ATPase activity, coupled;ATPase activity, coupled to movement of substances;ATPase activity, c
binding;catalytic activity;DNA binding;endonuclease activity;enzyme inhibitor activity;enzyme regulator activity;hydrolase activity;hydrolase activity, acting on ester bonds;nuclease activity;nucleic acid binding;phosphatase inh
adenyl nucleotide binding;adenyl ribonucleotide binding;ATP binding;binding;catalytic activity;cation binding;enzyme activator activity;enzyme regulator activity;identical protein binding;ion binding;kinase activator activity;kin
adenyl nucleotide binding;adenyl ribonucleotide binding;ATP binding;binding;calcium-dependent protein serine/threonine phosphatase activity;calmodulin binding;calmodulin-dependent protein kinase activity;catalytic activity
binding;catalytic activity;cation binding;cis-trans isomerase activity;drug binding;FK506 binding;identical protein binding;ion binding;isomerase activity;macrolide binding;metal ion binding;peptidyl-prolyl cis-trans isomerase a
binding;polyubiquitin binding;protein binding;small conjugating protein binding;ubiquitin binding
binding;carbohydrate binding;collagen binding;enzyme activator activity;enzyme regulator activity;glycosaminoglycan binding;heparin binding;pattern binding;peptidase activator activity;peptidase regulator activity;polysacch
binding;catalytic activity;cation binding;GDP binding;GTP binding;GTPase activity;guanyl nucleotide binding;guanyl ribonucleotide binding;hydrolase activity;hydrolase activity, acting on acid anhydrides;hydrolase activity, act
catalytic activity;cation transmembrane transporter activity;cytochrome-c oxidase activity;heme-copper terminal oxidase activity;hydrogen ion transmembrane transporter activity;inorganic cation transmembrane transporter a
binding;protein binding;SNAP receptor activity;SNARE binding
binding;catalytic activity;coenzyme binding;cofactor binding;DNA binding;histone binding;methylated histone residue binding;NAD binding;nucleic acid binding;nucleotide binding;oxidoreductase activity;oxidoreductase activ
binding;calcium ion binding;cation binding;enzyme activator activity;enzyme regulator activity;GTPase activator activity;GTPase regulator activity;ion binding;metal ion binding;nucleoside-triphosphatase regulator activity
actin binding;actin filament binding;beta-tubulin binding;binding;cytoskeletal protein binding;DNA binding;microtubule binding;nucleic acid binding;protein binding;tubulin binding
catalytic activity;flavin-linked sulfhydryl oxidase activity;intramolecular oxidoreductase activity;intramolecular oxidoreductase activity, interconverting keto- and enol-groups;intramolecular oxidoreductase activity, transposing
binding;enzyme binding;GTPase binding;protein binding;Rab GTPase binding;Ras GTPase binding;small GTPase binding
binding;nucleic acid binding;poly(G) RNA binding;poly(U) RNA binding;poly-purine tract binding;poly-pyrimidine tract binding;RNA binding;single-stranded RNA binding
catalytic activity;cysteine-type endopeptidase activity;cysteine-type peptidase activity;endopeptidase activity;hydrolase activity;peptidase activity;peptidase activity, acting on L-amino acid peptides;small conjugating protein-
acetoacetate-CoA ligase activity;acid-thiol ligase activity;adenyl nucleotide binding;adenyl ribonucleotide binding;ATP binding;binding;butyrate-CoA ligase activity;catalytic activity;ligase activity;ligase activity, forming carbon
binding;catalytic activity;methyltransferase activity;nucleotide binding;RNA methyltransferase activity;transferase activity;transferase activity, transferring one-carbon groups
adenylosuccinate synthase activity;anion binding;binding;catalytic activity;cation binding;GTP binding;GTPase activity;guanyl nucleotide binding;guanyl ribonucleotide binding;hydrolase activity;hydrolase activity, acting on ac
enzyme activator activity;enzyme regulator activity;GTPase activator activity;GTPase regulator activity;nucleoside-triphosphatase regulator activity
binding;carboxylic acid binding;catalytic activity;cation binding;ion binding;iron ion binding;L-ascorbic acid binding;metal ion binding;oxidoreductase activity;oxidoreductase activity, acting on paired donors, with incorporatio
2 iron, 2 sulfur cluster binding;binding;cation binding;identical protein binding;ion binding;iron-sulfur cluster binding;metal cluster binding;metal ion binding;protein binding;protein dimerization activity;protein homodimerizatio
binding;cation binding;ion binding;metal ion binding
active transmembrane transporter activity;calcium channel activity;cation channel activity;cation transmembrane transporter activity;channel activity;ion channel activity;ion transmembrane transporter activity;passive transm
catalytic activity;hydrolase activity
enzyme activator activity;enzyme regulator activity;GTPase activator activity;GTPase regulator activity;nucleoside-triphosphatase regulator activity
adenyl nucleotide binding;adenyl ribonucleotide binding;ATP binding;binding;catalytic activity;cation binding;ion binding;kinase activity;metal ion binding;nucleotide binding;phosphotransferase activity, alcohol group as acce
active transmembrane transporter activity;amine transmembrane transporter activity;amino acid transmembrane transporter activity;carboxylic acid transmembrane transporter activity;glycine transmembrane transporter acti
acyl-CoA dehydrogenase activity;binding;carboxylic acid binding;catalytic activity;coenzyme binding;cofactor binding;fatty acid binding;fatty-acyl-CoA binding;flavin adenine dinucleotide binding;glutaryl-CoA dehydrogenase
binding;catalytic activity;cation binding;identical protein binding;ion binding;isomerase activity;metal ion binding;protein binding;protein dimerization activity;protein homodimerization activity;racemase and epimerase activity;
binding;binding, bridging;protein binding;protein binding, bridging;ribonucleoprotein binding;ribosomal large subunit binding
binding;catalytic activity;DNA binding;N-acyltransferase activity;nucleic acid binding;sphingosine N-acyltransferase activity;transferase activity;transferase activity, transferring acyl groups;transferase activity, transferring acyl
actin binding;binding;cytoskeletal protein binding;enzyme binding;leucine zipper domain binding;LRR domain binding;protein binding;protein binding transcription factor activity;protein domain specific binding;transcription c
binding;C2H2 zinc finger domain binding;catalytic activity;cation binding;histone methyltransferase activity;histone methyltransferase activity (H3-K27 specific);histone methyltransferase activity (H3-K9 specific);histone-lysine
binding;lipid binding;phosphatidylinositol binding;phosphatidylinositol-3-phosphate binding;phospholipid binding
binding;cation binding;ion binding;metal ion binding;transition metal ion binding;zinc ion binding
catalytic activity;lysophospholipid acyltransferase activity;transferase activity;transferase activity, transferring acyl groups;transferase activity, transferring acyl groups other than amino-acyl groups
catalytic activity;methyltransferase activity;transferase activity;transferase activity, transferring one-carbon groups
binding;mRNA binding;nucleic acid binding;RNA binding
adenyl nucleotide binding;adenyl ribonucleotide binding;ATP binding;ATPase activity;binding;catalytic activity;cation binding;DNA binding;hydrolase activity;hydrolase activity, acting on acid anhydrides;hydrolase activity, acti
binding;binding, bridging;estrogen receptor binding;hormone receptor binding;nuclear hormone receptor binding;protein binding;protein binding transcription factor activity;protein binding, bridging;protein complex scaffold;r
catalytic activity;dolichyl-phosphate-mannose-protein mannosyltransferase activity;mannosyltransferase activity;transferase activity;transferase activity, transferring glycosyl groups;transferase activity, transferring hexosyl gro
adenyl nucleotide binding;adenyl ribonucleotide binding;alanine-tRNA ligase activity;aminoacyl-tRNA ligase activity;ATP binding;binding;catalytic activity;cation binding;ion binding;ligase activity;ligase activity, forming aminoa
1-phosphatidylinositol 4-kinase activity;adenyl nucleotide binding;adenyl ribonucleotide binding;AP-3 adaptor complex binding;ATP binding;binding;catalytic activity;cation binding;inositol or phosphatidylinositol kinase activi
catalytic activity;oxidoreductase activity;protein binding transcription factor activity;transcription coactivator activity;transcription cofactor activity;transcription factor binding transcription factor activity
binding;cholesterol binding;lipid binding;steroid binding;sterol binding
binding;nucleotide binding
acetyltransferase activity;catalytic activity;H4 histone acetyltransferase activity;histone acetyltransferase activity;lysine N-acetyltransferase activity;N-acetyltransferase activity;N-acyltransferase activity;peptide alpha-N-acetyl
adenyl nucleotide binding;adenyl ribonucleotide binding;ATP binding;ATPase activity;ATPase activity, coupled;ATP-dependent helicase activity;ATP-dependent RNA helicase activity;binding;catalytic activity;helicase activity;h
binding;catalytic activity;cation binding;hydrolase activity;hydrolase activity, acting on ester bonds;ion binding;metal ion binding;transition metal ion binding;zinc ion binding
binding;catalytic activity;cation binding;dCTP diphosphatase activity;deoxyribonucleotide binding;hydrolase activity;hydrolase activity, acting on acid anhydrides;hydrolase activity, acting on acid anhydrides, in phosphorus-c
binding;nucleic acid binding;RNA binding;toxin binding
binding;cation binding;ion binding;metal ion binding
binding;double-stranded RNA binding;miRNA binding;nucleic acid binding;pre-miRNA binding;RNA binding;single-stranded RNA binding
binding;nucleic acid binding;RNA binding
adenyl nucleotide binding;adenyl ribonucleotide binding;ATP binding;ATPase activity;ATPase activity, coupled;beta-catenin binding;binding;catalytic activity;chromatin binding;DNA binding;DNA helicase activity;DNA-depend
structural constituent of ribosome;structural molecule activity
ligand-dependent nuclear receptor transcription coactivator activity;protein binding transcription factor activity;transcription coactivator activity;transcription cofactor activity;transcription factor binding transcription factor act
binding;identical protein binding;protein binding;protein complex binding
binding;identical protein binding;polyubiquitin binding;protein binding;small conjugating protein binding;ubiquitin binding
alpha-tubulin binding;beta-tubulin binding;binding;catalytic activity;cytoskeletal protein binding;GDP binding;GTP binding;GTPase activity;guanyl nucleotide binding;guanyl ribonucleotide binding;hydrolase activity;hydrolase
binding;catalytic activity;coenzyme binding;cofactor binding;FMN binding;nucleotide binding;oxidoreductase activity;oxidoreductase activity, acting on the CH-NH2 group of donors;oxidoreductase activity, acting on the CH-
adenyl nucleotide binding;adenyl ribonucleotide binding;adenylyltransferase activity;ATP binding;binding;catalytic activity;cation binding;identical protein binding;ion binding;magnesium ion binding;manganese ion binding;me
adenyl nucleotide binding;adenyl ribonucleotide binding;ATP binding;binding;catalytic activity;cyclin binding;cyclin-dependent protein kinase activity;kinase activity;nucleotide binding;phosphotransferase activity, alcohol grou
binding;DNA binding;nucleic acid binding;nucleic acid binding transcription factor activity;protein binding transcription factor activity;sequence-specific DNA binding;sequence-specific DNA binding transcription factor activit
adenyl nucleotide binding;adenyl ribonucleotide binding;ATP binding;binding;catalytic activity;NADH dehydrogenase (quinone) activity;NADH dehydrogenase (ubiquinone) activity;NADH dehydrogenase activity;nucleotide bin
binding;catalytic activity;cation binding;GMP reductase activity;ion binding;metal ion binding;oxidoreductase activity;oxidoreductase activity, acting on NADH or NADPH;oxidoreductase activity, acting on NADH or NADPH, ni
binding;chromatin binding;protein binding;protein N-terminus binding

adenyl nucleotide binding;adenyl ribonucleotide binding;ATP binding;binding;catalytic activity;kinase activity;nucleic acid binding;nucleotide binding;phosphoprotein binding;phosphotransferase activity, alcohol group as acce
binding;protein binding;protein N-terminus binding
ADP-ribose diphosphatase activity;ADP-sugar diphosphatase activity;binding;catalytic activity;cation binding;hydrolase activity;hydrolase activity, acting on acid anhydrides;hydrolase activity, acting on acid anhydrides, in pho
binding;chromatin binding;DNA binding;nucleic acid binding;nucleic acid binding transcription factor activity;regulatory region DNA binding;regulatory region nucleic acid binding;RNA polymerase II core promoter proximal reg
protein binding transcription factor activity;transcription coactivator activity;transcription cofactor activity;transcription factor binding transcription factor activity
binding;enzyme binding;enzyme regulator activity;heat shock protein binding;kinase binding;kinase regulator activity;NF-kappaB binding;protein binding;protein kinase binding;protein kinase regulator activity;small conjugatin
bent DNA binding;binding;catalytic activity;cis-trans isomerase activity;DNA binding;double-stranded DNA binding;isomerase activity;nucleic acid binding;peptidyl-prolyl cis-trans isomerase activity;structure-specific DNA bin
binding;nucleotide binding
catalytic activity;cis-trans isomerase activity;isomerase activity;peptidyl-prolyl cis-trans isomerase activity
aminoacyl-tRNA hydrolase activity;carboxylic ester hydrolase activity;catalytic activity;hydrolase activity;hydrolase activity, acting on ester bonds
binding;catalytic activity;cysteine-type endopeptidase activity;cysteine-type peptidase activity;cytokine receptor binding;endopeptidase activity;hydrolase activity;identical protein binding;peptidase activity;peptidase activity,
binding;mRNA binding;nucleic acid binding;nucleotide binding;RNA binding
binding;catalytic activity;cell adhesion molecule binding;fibroblast growth factor receptor binding;growth factor receptor binding;kinase activity;molecular transducer activity;phosphotransferase activity, alcohol group as acce
adenyl nucleotide binding;adenyl ribonucleotide binding;ATP binding;binding;catalytic activity;cation binding;ion binding;kinase activity;metal ion binding;nucleotide binding;phosphotransferase activity, alcohol group as accep
protein binding transcription factor activity;transcription cofactor activity;transcription corepressor activity;transcription factor binding transcription factor activity
binding;catalytic activity;cation binding;hydrolase activity;hydrolase activity, acting on ester bonds;ion binding;metal ion binding;phosphatase activity;phosphoprotein phosphatase activity;phosphoric ester hydrolase activity;p
acid-thiol ligase activity;adenyl nucleotide binding;adenyl ribonucleotide binding;ATP binding;binding;catalytic activity;enzyme binding;fatty acid ligase activity;fatty acid transporter activity;ligase activity;ligase activity, forming
binding;cation binding;heme binding;ion binding;iron ion binding;ligand-dependent nuclear receptor activity;lipid binding;metal ion binding;molecular transducer activity;receptor activity;signal transducer activity;signaling rec
catalytic activity;deoxyribonucleoside 5'-monophosphate N-glycosidase activity;hydrolase activity;hydrolase activity, acting on glycosyl bonds;hydrolase activity, hydrolyzing N-glycosyl compounds;nucleoside deoxyribosyltra
active transmembrane transporter activity;adenyl nucleotide binding;adenyl ribonucleotide binding;ATP binding;ATPase activity;ATPase activity, coupled;ATPase activity, coupled to movement of substances;ATPase activity, c
activating transcription factor binding;binding;chromatin binding;DNA binding;enzyme binding;enzyme regulator activity;histone deacetylase binding;histone deacetylase regulator activity;hormone receptor binding;nuclear ho
binding;nucleic acid binding;RNA binding;translation factor activity, nucleic acid binding;translation initiation factor activity
binding;nucleotide binding
binding;catalytic activity;cation binding;dimethylallyltranstransferase activity;farnesyltranstransferase activity;geranyltranstransferase activity;ion binding;metal ion binding;prenyltransferase activity;transferase activity;transfera
catalytic activity;NADH dehydrogenase (quinone) activity;NADH dehydrogenase (ubiquinone) activity;NADH dehydrogenase activity;oxidoreductase activity;oxidoreductase activity, acting on NADH or NADPH;oxidoreductase
binding;catalytic activity;cation binding;cation transmembrane transporter activity;copper ion binding;cytochrome-c oxidase activity;heme-copper terminal oxidase activity;hydrogen ion transmembrane transporter activity;inc
adenosine deaminase activity;binding;catalytic activity;cation binding;deaminase activity;hydrolase activity;hydrolase activity, acting on carbon-nitrogen (but not peptide) bonds;hydrolase activity, acting on carbon-nitrogen (b
binding;carbohydrate binding;catalytic activity;cation binding;DNA binding;endopeptidase activity;enzyme activator activity;enzyme regulator activity;glycosaminoglycan binding;heparin binding;hydrolase activity;ion binding;i
amidine-lyase activity;argininosuccinate lyase activity;carbon-nitrogen lyase activity;catalytic activity;lyase activity
binding;DNA binding;nucleic acid binding
carbon-carbon lyase activity;carboxy-lyase activity;catalytic activity;lyase activity;uroporphyrinogen decarboxylase activity
amine binding;amino acid binding;antioxidant activity;binding;carboxylic acid binding;catalytic activity;glutathione binding;glutathione peroxidase activity;modified amino acid binding;oxidoreductase activity;oxidoreductase a
adenyl nucleotide binding;adenyl ribonucleotide binding;ATP binding;binding;catalytic activity;enzyme binding;growth factor binding;hormone binding;identical protein binding;insulin binding;insulin receptor binding;insulin rec
adenyl nucleotide binding;adenyl ribonucleotide binding;ATP binding;binding;catalytic activity;DNA binding;helicase activity;hydrolase activity;hydrolase activity, acting on acid anhydrides;hydrolase activity, acting on acid anh
anion binding;binding;catalytic activity;cation binding;cyclohydrolase activity;hydrolase activity;hydrolase activity, acting on carbon-nitrogen (but not peptide) bonds;hydrolase activity, acting on carbon-nitrogen (but not peptic
binding;catalytic activity;chemoattractant activity;cytokine activity;cytokine receptor binding;dopachrome isomerase activity;intramolecular oxidoreductase activity;intramolecular oxidoreductase activity, interconverting keto-
binding;carboxypeptidase activity;catalytic activity;cation binding;exopeptidase activity;hydrolase activity;ion binding;metal ion binding;metallocarboxypeptidase activity;metalloexopeptidase activity;metallopeptidase activity;
binding;catalytic activity;enzyme binding;histone kinase activity;kinase activity;kinase binding;patched binding;phosphotransferase activity, alcohol group as acceptor;protein binding;protein kinase activity;protein kinase bind
catalytic activity;cation transmembrane transporter activity;cytochrome-c oxidase activity;heme-copper terminal oxidase activity;hydrogen ion transmembrane transporter activity;inorganic cation transmembrane transporter a
binding;catalytic activity;cation binding;hydrolase activity;hydrolase activity, acting on ester bonds;ion binding;metal ion binding;N-acetylglucosamine-6-sulfatase activity;sulfuric ester hydrolase activity
binding;catalytic activity;cation binding;dihydronicotinamide riboside quinone reductase activity;electron carrier activity;ion binding;metal ion binding;NADPH dehydrogenase (quinone) activity;NADPH dehydrogenase activity;
binding;calcium ion binding;catalytic activity;cation binding;cytokine receptor binding;enzyme binding;hydrolase activity;hydrolase activity, acting on ester bonds;ion binding;kinase binding;lipase activity;metal ion binding;mo
binding;catalytic activity;cation binding;cation transmembrane transporter activity;cytochrome-c oxidase activity;electron carrier activity;heme-copper terminal oxidase activity;hydrogen ion transmembrane transporter activity
amine binding;amino acid binding;binding;carboxylic acid binding;catalytic activity;cation binding;cobalamin binding;intramolecular transferase activity;ion binding;isomerase activity;metal ion binding;methylmalonyl-CoA mut
2 iron, 2 sulfur cluster binding;binding;catalytic activity;cation binding;ferrochelatase activity;ferrous iron binding;heme binding;ion binding;iron ion binding;iron-responsive element binding;iron-sulfur cluster binding;lyase activ
binding;calcium ion binding;cation binding;enzyme activator activity;enzyme regulator activity;extracellular matrix structural constituent;ion binding;metal ion binding;peptidase activator activity;peptidase regulator activity;stru
binding;catalytic activity;cis-trans isomerase activity;drug binding;FK506 binding;isomerase activity;macrolide binding;peptidyl-prolyl cis-trans isomerase activity
binding;cation binding;DNA binding;enhancer binding;enhancer sequence-specific DNA binding;ion binding;ligand-dependent nuclear receptor activity;metal ion binding;molecular transducer activity;nucleic acid binding;nucl
binding;catalytic activity;cation binding;intramolecular oxidoreductase activity;intramolecular oxidoreductase activity, interconverting aldoses and ketoses;ion binding;isomerase activity;mannose-6-phosphate isomerase activ
binding;cation binding;copper ion binding;extracellular matrix binding;fibroblast growth factor binding;growth factor binding;ion binding;laminin binding;metal ion binding;protein binding;transition metal ion binding
binding;catalytic activity;disulfide oxidoreductase activity;electron carrier activity;glutathione disulfide oxidoreductase activity;oxidoreductase activity;oxidoreductase activity, acting on a sulfur group of donors;peptide disulfid
binding;calcium-dependent protein serine/threonine phosphatase activity;calmodulin-dependent protein phosphatase activity;catalytic activity;cation binding;hydrolase activity;hydrolase activity, acting on ester bonds;ion bin
alpha-tubulin binding;binding;calcium ion binding;caspase inhibitor activity;caspase regulator activity;catalytic activity;cation binding;copper ion binding;cysteine-type endopeptidase inhibitor activity;cytoskeletal protein bind
catalytic activity;dodecenoyl-CoA delta-isomerase activity;intramolecular oxidoreductase activity;intramolecular oxidoreductase activity, transposing C=C bonds;isomerase activity
binding;calcium ion binding;calcium-dependent phospholipase A2 activity;calcium-dependent phospholipid binding;carboxylic ester hydrolase activity;catalytic activity;cation binding;hydrolase activity;hydrolase activity, actin
adenyl nucleotide binding;adenyl ribonucleotide binding;ATP binding;binding;calcium-dependent protein kinase activity;calcium-dependent protein serine/threonine kinase activity;calmodulin binding;calmodulin-dependent pr
4 iron, 4 sulfur cluster binding;binding;catalytic activity;cation binding;DNA binding;DNA primase activity;DNA-directed RNA polymerase activity;ion binding;iron-sulfur cluster binding;metal cluster binding;metal ion binding;nu
adenyl nucleotide binding;adenyl ribonucleotide binding;ATP binding;binding;catalytic activity;GTP binding;guanyl nucleotide binding;guanyl ribonucleotide binding;identical protein binding;kinase activity;nucleotide binding;p
binding;nucleic acid binding;nucleotide binding;RNA 7-methylguanosine cap binding;RNA binding;RNA cap binding
4 iron, 4 sulfur cluster binding;adenyl nucleotide binding;adenyl ribonucleotide binding;ATP binding;binding;cation binding;ion binding;iron-sulfur cluster binding;metal cluster binding;metal ion binding;nucleotide binding;purin
binding;box C/D snoRNA binding;nucleic acid binding;RNA binding;snoRNA binding;snRNA binding;U3 snoRNA binding;U4 snRNA binding
catalytic activity;hydrolase activity;peptidase activity
acid-amino acid ligase activity;adenyl nucleotide binding;adenyl ribonucleotide binding;ATP binding;binding;catalytic activity;enzyme binding;ligase activity;ligase activity, forming carbon-nitrogen bonds;nucleotide binding;pro
binding;catalytic activity;GTP binding;GTPase activity;guanyl nucleotide binding;guanyl ribonucleotide binding;hydrolase activity;hydrolase activity, acting on acid anhydrides;hydrolase activity, acting on acid anhydrides, in ph
acid-amino acid ligase activity;binding;catalytic activity;enzyme binding;ligase activity;ligase activity, forming carbon-nitrogen bonds;protein binding;small conjugating protein ligase activity;SUMO ligase activity;ubiquitin prot
binding;catalytic activity;GDP binding;GTP binding;GTPase activity;guanyl nucleotide binding;guanyl ribonucleotide binding;hydrolase activity;hydrolase activity, acting on acid anhydrides;hydrolase activity, acting on acid anh
catalytic activity;enzyme regulator activity;hydrolase activity;hydrolase activity, acting on acid anhydrides;hydrolase activity, acting on acid anhydrides, in phosphorus-containing anhydrides;motor activity;nitric-oxide synthase
binding;catalytic activity;GTP binding;GTPase activity;guanyl nucleotide binding;guanyl ribonucleotide binding;hydrolase activity;hydrolase activity, acting on acid anhydrides;hydrolase activity, acting on acid anhydrides, in ph
binding;DNA binding;nucleic acid binding;nucleic acid binding transcription factor activity;regulatory region DNA binding;regulatory region nucleic acid binding;RNA polymerase II regulatory region DNA binding;RNA polymera
binding;cation binding;identical protein binding;ion binding;metal ion binding;nucleic acid binding;nucleotide binding;protein binding;RNA binding;transition metal ion binding;zinc ion binding
binding;nucleotide binding
adenyl nucleotide binding;adenyl ribonucleotide binding;ATP binding;binding;calcium-independent protein kinase C activity;catalytic activity;cation binding;enzyme activator activity;enzyme binding;enzyme regulator activity;i
3'-5' exonuclease activity;3'-5'-exoribonuclease activity;AU-rich element binding;binding;catalytic activity;exonuclease activity;exonuclease activity, active with either ribo- or deoxyribonucleic acids and producing 5'-phosph
acid-amino acid ligase activity;binding;catalytic activity;cation binding;CD40 receptor binding;cytokine receptor binding;enzyme binding;identical protein binding;ion binding;kinase binding;ligase activity;ligase activity, forming
catalytic activity;hydrolase activity;hydrolase activity, acting on ester bonds;inositol or phosphatidylinositol phosphatase activity;lipid phosphatase activity;phosphatase activity;phosphatidylinositol bisphosphate phosphatase
binding;cation binding;ion binding;metal ion binding;transition metal ion binding;zinc ion binding
aminopeptidase activity;carboxypeptidase activity;catalytic activity;cysteine-type endopeptidase activity;cysteine-type peptidase activity;endopeptidase activity;exopeptidase activity;hydrolase activity;peptidase activity;pept
binding;enzyme binding;enzyme regulator activity;GTPase regulator activity;guanyl-nucleotide exchange factor activity;kinase binding;nucleoside-triphosphatase regulator activity;protein binding;protein kinase binding;Ras gu
binding;cation binding;enzyme activator activity;enzyme regulator activity;GTPase activator activity;GTPase regulator activity;ion binding;metal ion binding;nucleoside-triphosphatase regulator activity;transition metal ion bind
adenyl nucleotide binding;adenyl ribonucleotide binding;ATP binding;ATPase activity;ATPase activity, coupled;ATP-dependent helicase activity;ATP-dependent RNA helicase activity;binding;catalytic activity;helicase activity;h
binding;enzyme activator activity;enzyme regulator activity;histone acetyl-lysine binding;histone binding;peptidase activator activity;peptidase regulator activity;protein binding
binding;endopeptidase activator activity;endopeptidase regulator activity;enzyme activator activity;enzyme binding;enzyme regulator activity;peptidase activator activity;peptidase regulator activity;protease binding;proteaso
binding;carbohydrate binding;catalytic activity;cation binding;hydrolase activity;hydrolase activity, acting on glycosyl bonds;hydrolase activity, hydrolyzing N-glycosyl compounds;hydrolase activity, hydrolyzing O-glycosyl co
catalytic activity;endopeptidase activity;hydrolase activity;peptidase activity;peptidase activity, acting on L-amino acid peptides;serine hydrolase activity;serine-type endopeptidase activity;serine-type peptidase activity
5S rRNA binding;binding;catalytic activity;nucleic acid binding;RNA binding;rRNA binding;sulfurtransferase activity;thiosulfate sulfurtransferase activity;transferase activity;transferase activity, transferring sulfur-containing gro
binding;catalytic activity;cation binding;hydrolase activity;hydrolase activity, acting on ester bonds;hydroxyacylglutathione hydrolase activity;ion binding;metal ion binding;thiolester hydrolase activity;transition metal ion bindin
catalytic activity;transferase activity;transferase activity, transferring glycosyl groups;transferase activity, transferring pentosyl groups;uridine phosphorylase activity
binding;catalytic activity;cation binding;hydrolase activity;ion binding;metal ion binding
binding;enzyme binding;phosphatase binding;protein binding;protein phosphatase binding
catalytic activity;methyltransferase activity;transferase activity;transferase activity, transferring one-carbon groups
5'-nucleotidase activity;binding;catalytic activity;cation binding;hydrolase activity;hydrolase activity, acting on ester bonds;ion binding;metal ion binding;nucleotidase activity;phosphatase activity;phosphoric ester hydrolase a
binding;enzyme binding;enzyme regulator activity;GTPase binding;GTPase regulator activity;guanyl-nucleotide exchange factor activity;nucleoside-triphosphatase regulator activity;PDZ domain binding;protein binding;protein
enzyme activator activity;enzyme regulator activity;GTPase activator activity;GTPase regulator activity;nucleoside-triphosphatase regulator activity
binding;cation binding;ion binding;metal ion binding;transition metal ion binding;zinc ion binding
carboxylic ester hydrolase activity;catalytic activity;hydrolase activity;hydrolase activity, acting on ester bonds
acid-amino acid ligase activity;binding;catalytic activity;cation binding;ion binding;ligase activity;ligase activity, forming carbon-nitrogen bonds;metal ion binding;small conjugating protein ligase activity;transition metal ion bin
catalytic activity;L-xylulose reductase (NADP+) activity;oxidoreductase activity;oxidoreductase activity, acting on CH-OH group of donors;oxidoreductase activity, acting on NADH or NADPH;oxidoreductase activity, acting on
binding;catalytic activity;cation binding;ion binding;ligase activity;metal ion binding
catalytic activity;hydrolase activity;hydrolase activity, acting on ester bonds;lipase activity;NAPE-specific phospholipase D activity;phospholipase activity;phospholipase D activity;phosphoric diester hydrolase activity;phosph
binding;cytoskeletal protein binding;microtubule binding;protein binding;structural molecule activity;tubulin binding
catalytic activity;methyltransferase activity;transferase activity;transferase activity, transferring one-carbon groups
adenyl nucleotide binding;adenyl ribonucleotide binding;ATP binding;ATPase activity;ATPase activity, coupled;binding;catalytic activity;hydrolase activity;hydrolase activity, acting on acid anhydrides;hydrolase activity, acting
binding;GTP binding;guanyl nucleotide binding;guanyl ribonucleotide binding;nucleotide binding;purine nucleotide binding;purine ribonucleoside triphosphate binding;purine ribonucleotide binding;ribonucleotide binding
binding;catalytic activity;cation binding;cysteine-type peptidase activity;hydrolase activity;ion binding;metal ion binding;metallopeptidase activity;nucleic acid binding;peptidase activity;peptidase activity, acting on L-amino ac
binding;cytoskeletal protein binding;kinesin binding;protein binding
binding;identical protein binding;nucleic acid binding;protein binding;RNA binding
ammonia-lyase activity;binding;carbon-nitrogen lyase activity;catalytic activity;cofactor binding;L-serine ammonia-lyase activity;L-threonine ammonia-lyase activity;lyase activity;pyridoxal phosphate binding;vitamin B6 bindin

binding;cation binding;ion binding;metal ion binding;nucleic acid binding
acid-amino acid ligase activity;binding;catalytic activity;cation binding;ion binding;ligase activity;ligase activity, forming carbon-nitrogen bonds;metal ion binding;nucleic acid binding;small conjugating protein ligase activity;ub
acid-amino acid ligase activity;binding;catalytic activity;cation binding;DNA binding;histone binding;ion binding;ligase activity;ligase activity, forming carbon-nitrogen bonds;metal ion binding;nucleic acid binding;protein bindi
catalytic activity;endopeptidase activity;hydrolase activity;peptidase activity;peptidase activity, acting on L-amino acid peptides;threonine-type endopeptidase activity;threonine-type peptidase activity
protein binding transcription factor activity;transcription cofactor activity;transcription corepressor activity;transcription factor binding transcription factor activity
adenyl nucleotide binding;adenyl ribonucleotide binding;ATP binding;binding;catalytic activity;chromatin binding;enzyme binding;histone binding;histone kinase activity;histone kinase activity (H3-S10 specific);histone kinase
binding;calcium ion binding;cation binding;identical protein binding;ion binding;metal ion binding;protein binding;protein dimerization activity;protein heterodimerization activity
antioxidant activity
binding;DNA binding;nucleic acid binding
binding;catalytic activity;cation binding;demethylase activity;DNA demethylase activity;DNA-N1-methyladenine dioxygenase activity;ferrous iron binding;ion binding;iron ion binding;metal ion binding;oxidative DNA demethyla
binding;nucleic acid binding;RNA binding;translation activator activity;translation regulator activity;translation regulator activity, nucleic acid binding
active transmembrane transporter activity;amine transmembrane transporter activity;amino acid transmembrane transporter activity;carboxylic acid transmembrane transporter activity;cation transmembrane transporter activ
binding;enzyme binding;enzyme inhibitor activity;enzyme regulator activity;protein binding;ubiquitin protein ligase binding
enzyme activator activity;enzyme regulator activity;GTPase activator activity;GTPase regulator activity;nucleoside-triphosphatase regulator activity
acetyltransferase activity;binding;catalytic activity;cation binding;enzyme regulator activity;H3 histone acetyltransferase activity;H4 histone acetyltransferase activity;histone acetyltransferase activity;ion binding;iron-sulfur clus
binding;catalytic activity;cation binding;DNA binding;DNA-directed RNA polymerase activity;ion binding;metal ion binding;nucleic acid binding;nucleoside binding;nucleotidyltransferase activity;ribonucleoside binding;RNA po
carbohydrate phosphatase activity;catalytic activity;fructose-2,6-bisphosphate 2-phosphatase activity;hydrolase activity;hydrolase activity, acting on ester bonds;phosphatase activity;phosphoric ester hydrolase activity;suga
binding;GTP binding;guanyl nucleotide binding;guanyl ribonucleotide binding;nucleotide binding;purine nucleotide binding;purine ribonucleoside triphosphate binding;purine ribonucleotide binding;ribonucleotide binding
acid-amino acid ligase activity;Atg12 ligase activity;Atg8 ligase activity;binding;catalytic activity;enzyme binding;ligase activity;ligase activity, forming carbon-nitrogen bonds;protein binding;small conjugating protein ligase act
enzyme activator activity;enzyme regulator activity;GTPase activator activity;GTPase regulator activity;nucleoside-triphosphatase regulator activity
binding;catalytic activity;cation binding;hydrolase activity;hydrolase activity, acting on ester bonds;ion binding;lipase activity;metal ion binding;phospholipase activity;phosphoric diester hydrolase activity;phosphoric ester hyd
binding;binding, bridging;cytoskeletal adaptor activity;cytoskeletal protein binding;DNA binding;enzyme binding;kinase binding;nucleic acid binding;proline-rich region binding;protein binding;protein binding, bridging;protein
2 iron, 2 sulfur cluster binding;binding;cation binding;ion binding;iron-sulfur cluster binding;metal cluster binding;metal ion binding
structural constituent of ribosome;structural molecule activity
binding;nucleic acid binding;RNA binding
binding;calcium ion binding;cation binding;ion binding;metal ion binding
anion binding;binding;ion binding;molecular transducer activity;PDZ domain binding;phosphate ion binding;protein binding;protein domain specific binding;signal transducer activity
binding;carbon-carbon lyase activity;catalytic activity;cation binding;coenzyme binding;cofactor binding;identical protein binding;ion binding;lyase activity;magnesium ion binding;metal ion binding;protein binding;receptor bi
binding;cation binding;ion binding;metal ion binding
binding;nucleic acid binding;RNA binding;structural constituent of ribosome;structural molecule activity
binding;enzyme binding;GTPase binding;protein binding;Rab GTPase binding;Ras GTPase binding;small GTPase binding
structural constituent of ribosome;structural molecule activity
binding;catalytic activity;cation binding;deoxyribonucleotide binding;dGTP binding;dGTPase activity;guanyl deoxyribonucleotide binding;guanyl nucleotide binding;hydrolase activity;hydrolase activity, acting on ester bonds;i
enzyme regulator activity;GTPase regulator activity;guanyl-nucleotide exchange factor activity;nucleoside-triphosphatase regulator activity;Rac guanyl-nucleotide exchange factor activity;Ras guanyl-nucleotide exchange fact
acid-amino acid ligase activity;binding;catalytic activity;cation binding;enzyme binding;ion binding;ligase activity;ligase activity, forming carbon-nitrogen bonds;metal ion binding;protein binding;small conjugating protein ligas
active transmembrane transporter activity;ATPase activity;ATPase activity, coupled;ATPase activity, coupled to movement of substances;ATPase activity, coupled to transmembrane movement of substances;catalytic activity;
binding;cation binding;enzyme binding;identical protein binding;ion binding;K63-linked polyubiquitin binding;metal ion binding;molecular transducer activity;polyubiquitin binding;protein binding;protein dimerization activity;pr
4 iron, 4 sulfur cluster binding;binding;catalytic activity;cation binding;ion binding;iron-sulfur cluster binding;metal cluster binding;metal ion binding;NADH dehydrogenase (quinone) activity;NADH dehydrogenase (ubiquinone)
bHLH transcription factor binding;binding;protein binding;protein binding transcription factor activity;transcription coactivator activity;transcription cofactor activity;transcription factor binding;transcription factor binding trans
alpha-mannosidase activity;binding;carbohydrate binding;catalytic activity;cation binding;hydrolase activity;hydrolase activity, acting on glycosyl bonds;hydrolase activity, hydrolyzing O-glycosyl compounds;ion binding;mann
adenyl nucleotide binding;adenyl ribonucleotide binding;ATP binding;binding;catalytic activity;DNA binding;helicase activity;hydrolase activity;hydrolase activity, acting on acid anhydrides;hydrolase activity, acting on acid anh
acetylglucosaminyltransferase activity;binding;catalytic activity;enzyme activator activity;enzyme regulator activity;lipid binding;phosphatidylinositol binding;phosphatidylinositol-3,4,5-trisphosphate binding;phospholipid bindi
catalytic activity;intramolecular transferase activity;intramolecular transferase activity, phosphotransferases;isomerase activity;phosphomannomutase activity
branched-chain-amino-acid transaminase activity;catalytic activity;L-isoleucine transaminase activity;L-leucine transaminase activity;L-valine transaminase activity;transaminase activity;transferase activity;transferase activity
binding;nucleic acid binding;RNA binding;snRNA binding;U4 snRNA binding;U6 snRNA binding
binding;catalytic activity;cis-trans isomerase activity;cyclosporin A binding;drug binding;isomerase activity;peptide binding;peptidyl-prolyl cis-trans isomerase activity;ribonucleoprotein binding
binding;clathrin binding;protein binding
binding;binding, bridging;calcium ion binding;calcium-dependent cysteine-type endopeptidase activity;calcium-dependent protein binding;catalytic activity;cation binding;cysteine-type endopeptidase activity;cysteine-type p
amine binding;amino acid binding;binding;carboxylic acid binding;catalytic activity;cation binding;dimethylargininase activity;hydrolase activity;hydrolase activity, acting on carbon-nitrogen (but not peptide) bonds;hydrolase a
binding;carbohydrate binding;catalytic activity;glutamine-fructose-6-phosphate transaminase (isomerizing) activity;transaminase activity;transferase activity;transferase activity, transferring nitrogenous groups
acid-amino acid ligase activity;binding;catalytic activity;cation binding;ion binding;ligase activity;ligase activity, forming carbon-nitrogen bonds;metal ion binding;small conjugating protein ligase activity;transition metal ion bin
binding;catalytic activity;enzyme binding;GTP binding;GTPase activity;guanyl nucleotide binding;guanyl ribonucleotide binding;hydrolase activity;hydrolase activity, acting on acid anhydrides;hydrolase activity, acting on acid
acid-amino acid ligase activity;binding;catalytic activity;enzyme binding;ligase activity;ligase activity, forming carbon-nitrogen bonds;protein binding;small conjugating protein ligase activity;ubiquitin-protein ligase activity;ubic
APG12 activating enzyme activity;APG8 activating enzyme activity;binding;catalytic activity;identical protein binding;protein binding;protein dimerization activity;protein homodimerization activity;small protein activating enzym
binding;catalytic activity;cation binding;DNA binding;DNA-directed RNA polymerase activity;ion binding;metal ion binding;nucleic acid binding;nucleotidyltransferase activity;RNA polymerase activity;transferase activity;transf
catalytic activity;methyltransferase activity;O-methyltransferase activity;transferase activity;transferase activity, transferring one-carbon groups
adenyl nucleotide binding;adenyl ribonucleotide binding;ATP binding;binding;catalytic activity;cation binding;ion binding;kinase activity;magnesium ion binding;metal ion binding;molecular transducer activity;nucleotide bindir
channel activity;macromolecule transmembrane transporter activity;passive transmembrane transporter activity;porin activity;protein transmembrane transporter activity;protein transporter activity;substrate-specific transmem
binding;cation binding;ion binding;metal ion binding
antioxidant activity;binding;catalytic activity;cation binding;DNA binding;identical protein binding;ion binding;manganese ion binding;metal ion binding;nucleic acid binding;oxidoreductase activity;oxidoreductase activity, actir
adenyl nucleotide binding;adenyl ribonucleotide binding;ATP binding;binding;catalytic activity;enzyme binding;ion channel binding;kinase activity;non-membrane spanning protein tyrosine kinase activity;nucleotide binding;ph
binding;nucleic acid binding;RNA binding;structural constituent of ribosome;structural molecule activity
amine binding;amino acid binding;binding;carboxylic acid binding;catalytic activity;enzyme binding;glutathione binding;glutathione transferase activity;identical protein binding;modified amino acid binding;peptide binding;pro
binding;chromatin binding;chromatin DNA binding;core promoter binding;core promoter proximal region DNA binding;core promoter proximal region sequence-specific DNA binding;core promoter sequence-specific DNA bin
amine binding;amino acid binding;antioxidant activity;binding;carboxylic acid binding;catalytic activity;glutathione binding;glutathione peroxidase activity;glutathione transferase activity;modified amino acid binding;oxidoredu
ATPase binding;binding;catalytic activity;enzyme binding;GDP binding;GTP binding;GTPase activity;guanyl nucleotide binding;guanyl ribonucleotide binding;hydrolase activity;hydrolase activity, acting on acid anhydrides;hyd
adenyl nucleotide binding;adenyl ribonucleotide binding;ATP binding;binding;catalytic activity;cyclin binding;cyclin-dependent protein kinase activity;cyclin-dependent protein kinase regulator activity;enzyme regulator activity
aminopeptidase activity;binding;carboxypeptidase activity;catalytic activity;cation binding;dipeptidase activity;exopeptidase activity;hydrolase activity;ion binding;manganese ion binding;metal ion binding;metallocarboxypept
binding;binding, bridging;cytoskeletal adaptor activity;cytoskeletal protein binding;enzyme inhibitor activity;enzyme regulator activity;eukaryotic initiation factor eIF2 binding;kinase inhibitor activity;kinase regulator activity;pro
binding;catalytic activity;epidermal growth factor receptor binding;growth factor receptor binding;GTP binding;GTPase activity;guanyl nucleotide binding;guanyl ribonucleotide binding;hydrolase activity;hydrolase activity, acti
binding;damaged DNA binding;DNA binding;enzyme binding;nucleic acid binding;protein binding
binding;catalytic activity;GDP binding;GTP binding;GTPase activity;guanyl nucleotide binding;guanyl ribonucleotide binding;hydrolase activity;hydrolase activity, acting on acid anhydrides;hydrolase activity, acting on acid anh
binding;catalytic activity;catechol O-methyltransferase activity;cation binding;ion binding;magnesium ion binding;metal ion binding;methyltransferase activity;O-methyltransferase activity;S-adenosylmethionine-dependent me
binding;calcium ion binding;cation binding;ion binding;metal ion binding;structural constituent of muscle;structural molecule activity
catalytic activity;endopeptidase activity;hydrolase activity;peptidase activity;peptidase activity, acting on L-amino acid peptides;threonine-type endopeptidase activity;threonine-type peptidase activity
actin binding;actin filament binding;binding;calmodulin binding;cytoskeletal protein binding;protein binding
binding;catalytic activity;cation binding;dCMP deaminase activity;deaminase activity;hydrolase activity;hydrolase activity, acting on carbon-nitrogen (but not peptide) bonds;hydrolase activity, acting on carbon-nitrogen (but n
aldehyde-lyase activity;amine binding;amino acid binding;binding;carbon-carbon lyase activity;carboxylic acid binding;catalytic activity;cofactor binding;glycine hydroxymethyltransferase activity;hydroxymethyl-, formyl- and
antioxidant activity;arachidonate 15-lipoxygenase activity;binding;catalytic activity;cation binding;dioxygenase activity;enzyme binding;heme binding;ion binding;iron ion binding;lipid binding;metal ion binding;oxidoreductase
actin binding;actin filament binding;binding;cytoskeletal protein binding;identical protein binding;protein binding;protein dimerization activity;protein heterodimerization activity;protein homodimerization activity;spectrin bindin
active transmembrane transporter activity;ATPase activity;ATPase activity, coupled;ATPase activity, coupled to movement of substances;ATPase activity, coupled to transmembrane movement of ions;ATPase activity, couplec
binding;cation binding;DNA binding;ion binding;metal ion binding;nucleic acid binding;structural constituent of ribosome;structural molecule activity;transition metal ion binding;zinc ion binding
beta-tubulin binding;binding;cytoskeletal protein binding;diazepam binding;drug binding;dynactin binding;dynein binding;dynein intermediate chain binding;identical protein binding;ion channel binding;p53 binding;profilin bin
adenyl nucleotide binding;adenyl ribonucleotide binding;ATP binding;binding;catalytic activity;enzyme binding;kinase activity;kinase binding;MAP kinase kinase activity;nucleotide binding;phosphotransferase activity, alcohol
binding;catalytic activity;DNA binding;DNA polymerase activity;DNA-directed DNA polymerase activity;nucleic acid binding;nucleotidyltransferase activity;transferase activity;transferase activity, transferring phosphorus-conta
adenyl nucleotide binding;adenyl ribonucleotide binding;ATP binding;binding;catalytic activity;kinase activity;nucleotide binding;phosphotransferase activity, alcohol group as acceptor;protein kinase activity;protein serine/thre
binding;cation binding;chromatin binding;chromatin DNA binding;chromatin insulator sequence binding;core promoter proximal region DNA binding;core promoter proximal region sequence-specific DNA binding;DNA binding
actin binding;beta-tubulin binding;binding;cytoskeletal protein binding;protein binding;tubulin binding
adenyl nucleotide binding;adenyl ribonucleotide binding;ATP binding;binding;catalytic activity;chromatin binding;cyclin-dependent protein kinase activity;DNA binding;kinase activity;nucleic acid binding;nucleotide binding;ph
binding;catalytic activity;NADH dehydrogenase (quinone) activity;NADH dehydrogenase (ubiquinone) activity;NADH dehydrogenase activity;oxidoreductase activity;oxidoreductase activity, acting on NADH or NADPH;oxidored
binding;carbon-sulfur lyase activity;catalytic activity;cation binding;holocytochrome-c synthase activity;ion binding;lyase activity;metal ion binding
acid-amino acid ligase activity;adenyl nucleotide binding;adenyl ribonucleotide binding;ATP binding;binding;catalytic activity;enzyme binding;ligase activity;ligase activity, forming carbon-nitrogen bonds;nucleotide binding;pro
binding;catalytic activity;enzyme binding;GTP binding;GTPase activity;guanyl nucleotide binding;guanyl ribonucleotide binding;hydrolase activity;hydrolase activity, acting on acid anhydrides;hydrolase activity, acting on acid
catalytic activity;endonuclease activity;endonuclease activity, active with either ribo- or deoxyribonucleic acids and producing 5'-phosphomonoesters;endoribonuclease activity;endoribonuclease activity, producing 5'-phosph
binding;catalytic activity;cation binding;hydrolase activity;intramolecular oxidoreductase activity;intramolecular oxidoreductase activity, transposing C=C bonds;ion binding;isomerase activity;isopentenyl-diphosphate delta-iso
angiotensin receptor binding;binding;catalytic activity;cation binding;D5 dopamine receptor binding;dopamine receptor binding;G-protein beta/gamma-subunit complex binding;G-protein-coupled receptor binding;GTP bindi
binding;catalytic activity;cation binding;diphosphotransferase activity;enzyme inhibitor activity;enzyme regulator activity;ion binding;magnesium ion binding;metal ion binding;ribose phosphate diphosphokinase activity;transfe

3'-5' exonuclease activity;3'-5'-exoribonuclease activity;binding;catalytic activity;exonuclease activity;exonuclease activity, active with either ribo- or deoxyribonucleic acids and producing 5'-phosphomonoesters;exoribonucl
binding;chromatin binding;histone acetyl-lysine binding;histone binding;protein binding
adenyl nucleotide binding;adenyl ribonucleotide binding;ATP binding;binding;catalytic activity;cis-trans isomerase activity;enzyme activator activity;enzyme binding;enzyme regulator activity;identical protein binding;isomeras
beta-catenin binding;binding;catalytic activity;enzyme binding;gamma-catenin binding;hydrolase activity;hydrolase activity, acting on ester bonds;kinase binding;molecular transducer activity;phosphatase activity;phosphopro
binding;enzyme binding;protein binding;ubiquitin protein ligase binding
enzyme regulator activity;phosphatase regulator activity;protein phosphatase regulator activity;protein phosphatase type 1 regulator activity
active transmembrane transporter activity;amine transmembrane transporter activity;amino acid transmembrane transporter activity;anion transmembrane transporter activity;anion:cation symporter activity;carboxylic acid tra
catalytic activity;nucleotidyltransferase activity;transferase activity;transferase activity, transferring phosphorus-containing groups;uridylyltransferase activity
anion channel activity;anion transmembrane transporter activity;calcium activated cation channel activity;cation channel activity;cation transmembrane transporter activity;channel activity;chloride channel activity;gated chann
binding;protein binding;protein complex binding
binding;nucleic acid binding;RNA binding;rRNA binding;snoRNA binding;U3 snoRNA binding
binding;chromatin binding
binding;enzyme binding;GTPase binding;protein binding;Rab GTPase binding;Ras GTPase binding;small GTPase binding
binding;cation binding;DNA binding;ion binding;metal ion binding;nucleic acid binding;nucleic acid binding transcription factor activity;regulatory region DNA binding;regulatory region nucleic acid binding;RNA polymerase II c
adenyl nucleotide binding;adenyl ribonucleotide binding;ATP binding;ATPase activity;ATPase activity, coupled;ATP-dependent helicase activity;ATP-dependent RNA helicase activity;binding;catalytic activity;helicase activity;h
basal RNA polymerase II transcription machinery binding;basal transcription machinery binding;binding;chromatin binding;enzyme binding;protein binding;RNA polymerase binding;RNA polymerase core enzyme binding;RNA
catalytic activity;cytidylyltransferase activity;N-acylneuraminate cytidylyltransferase activity;nucleotidyltransferase activity;transferase activity;transferase activity, transferring phosphorus-containing groups
binding;cation binding;ion binding;metal ion binding;transition metal ion binding;zinc ion binding
catalytic activity;cysteine-type peptidase activity;hydrolase activity;peptidase activity;peptidase activity, acting on L-amino acid peptides;small conjugating protein-specific protease activity;ubiquitin-specific protease activity
binding;nucleic acid binding;nucleotide binding;RNA binding;RNA stem-loop binding
adenyl nucleotide binding;adenyl ribonucleotide binding;adenylyltransferase activity;ATP binding;binding;catalytic activity;nucleic acid binding;nucleotide binding;nucleotidyltransferase activity;purine nucleotide binding;purine
adenyl nucleotide binding;adenyl ribonucleotide binding;ATP binding;binding;catalytic activity;cation binding;ion binding;kinase activity;metal ion binding;nucleotide binding;phosphotransferase activity, alcohol group as acce
binding;DNA binding;nucleic acid binding
binding;calcium ion binding;cation binding;copper ion binding;fibroblast growth factor binding;growth factor binding;identical protein binding;ion binding;lipid binding;metal ion binding;protein binding;protein dimerization acti
binding;cation binding;ion binding;metal ion binding;transition metal ion binding;zinc ion binding
binding;high-density lipoprotein particle binding;lipid binding;lipoprotein particle binding;protein binding;protein-lipid complex binding;receptor binding
ADP-specific glucokinase activity;binding;catalytic activity;cation binding;ion binding;kinase activity;metal ion binding;phosphotransferase activity, alcohol group as acceptor;transferase activity;transferase activity, transferring
binding;enzyme inhibitor activity;enzyme regulator activity;histone binding;phosphatase inhibitor activity;phosphatase regulator activity;protein binding
binding;catalytic activity;cation binding;ion binding;metal ion binding;queuine tRNA-ribosyltransferase activity;transferase activity;transferase activity, transferring glycosyl groups;transferase activity, transferring pentosyl grou
adenyl nucleotide binding;adenyl ribonucleotide binding;ATP binding;binding;nucleotide binding;purine nucleotide binding;purine ribonucleoside triphosphate binding;purine ribonucleotide binding;ribonucleotide binding
binding;carboxylic acid binding;catalytic activity;cation binding;enzyme binding;ion binding;iron ion binding;L-ascorbic acid binding;metal ion binding;oxidoreductase activity;oxidoreductase activity, acting on paired donors, v
catalytic activity;hydrolase activity
carboxypeptidase activity;catalytic activity;exopeptidase activity;hydrolase activity;peptidase activity;peptidase activity, acting on L-amino acid peptides;serine hydrolase activity;serine-type carboxypeptidase activity;serine-t
binding;carboxylic acid binding;coenzyme binding;cofactor binding;fatty acid binding;fatty-acyl-CoA binding;lipid binding;monocarboxylic acid binding
binding;enzyme binding;GTPase binding;GTP-Rho binding;protein binding;Ras GTPase binding;Rho GTPase binding;small GTPase binding
acidic amino acid transmembrane transporter activity;active transmembrane transporter activity;amine transmembrane transporter activity;amino acid transmembrane transporter activity;carboxylic acid transmembrane trans
binding;catalytic activity;cation binding;histone methyltransferase activity;histone methyltransferase activity (H3-K27 specific);histone methyltransferase activity (H3-K9 specific);histone-lysine N-methyltransferase activity;ion
acid-amino acid ligase activity;catalytic activity;ligase activity;ligase activity, forming carbon-nitrogen bonds;phosphopantothenate--cysteine ligase activity
binding;nucleic acid binding;RNA binding
binding;chromatin binding
ligand-dependent nuclear receptor transcription coactivator activity;protein binding transcription factor activity;transcription coactivator activity;transcription cofactor activity;transcription factor binding transcription factor act
binding;enzyme activator activity;enzyme regulator activity;G-protein alpha-subunit binding;GTPase activator activity;GTPase regulator activity;guanyl-nucleotide exchange factor activity;nucleoside-triphosphatase regulator a
binding;protein binding;protein domain specific binding;structural constituent of ribosome;structural molecule activity
catalytic activity;hydrolase activity;hydrolase activity, acting on acid anhydrides;hydrolase activity, acting on acid anhydrides, in phosphorus-containing anhydrides;microtubule motor activity;motor activity;nucleoside-triphosp
binding;nucleotide binding
adenyl nucleotide binding;adenyl ribonucleotide binding;ATP binding;ATPase activity;binding;catalytic activity;hydrolase activity;hydrolase activity, acting on acid anhydrides;hydrolase activity, acting on acid anhydrides, in ph
binding;cytoskeletal protein binding;protein binding
binding;nucleic acid binding;RNA binding
3-hydroxyacyl-CoA dehydratase activity;binding;carbon-oxygen lyase activity;catalytic activity;enzyme activator activity;enzyme binding;enzyme regulator activity;GTPase activator activity;GTPase regulator activity;hydro-lyas
enzyme regulator activity;kinase regulator activity
7-dehydrocholesterol reductase activity;catalytic activity;oxidoreductase activity;oxidoreductase activity, acting on the CH-CH group of donors;oxidoreductase activity, acting on the CH-CH group of donors, NAD or NADP as
carboxylic acid transmembrane transporter activity;dicarboxylic acid transmembrane transporter activity;organic acid transmembrane transporter activity;substrate-specific transmembrane transporter activity;substrate-speci
binding;protein binding;protein binding involved in protein folding;unfolded protein binding
active transmembrane transporter activity;ATPase activity;ATPase activity, coupled;ATPase activity, coupled to movement of substances;ATPase activity, coupled to transmembrane movement of ions;ATPase activity, coupled
binding;catalytic activity;cation binding;endonuclease activity;endonuclease activity, active with either ribo- or deoxyribonucleic acids and producing 5'-phosphomonoesters;endoribonuclease activity;endoribonuclease activit
adenyl nucleotide binding;adenyl ribonucleotide binding;ATP binding;ATPase activity;ATPase activity, coupled;ATP-dependent helicase activity;ATP-dependent RNA helicase activity;binding;catalytic activity;helicase activity;h
binding;DNA binding;nucleic acid binding;nucleotide binding
binding;DNA binding;enzyme binding;mRNA 3'-UTR binding;mRNA binding;nucleic acid binding;phosphatase binding;protein binding;RNA binding
acid-amino acid ligase activity;catalytic activity;ligase activity;ligase activity, forming carbon-nitrogen bonds;small conjugating protein ligase activity;UFM1 conjugating enzyme activity
catalytic activity;NADH dehydrogenase (quinone) activity;NADH dehydrogenase (ubiquinone) activity;NADH dehydrogenase activity;oxidoreductase activity;oxidoreductase activity, acting on NADH or NADPH;oxidoreductase
binding;catalytic activity;cation binding;hydrolase activity;hydrolase activity, acting on ester bonds;ion binding;magnesium ion binding;metal ion binding;phosphatase activity;phosphoglycolate phosphatase activity;phosphop
binding;DNA binding;nucleic acid binding
binding;nuclear localization sequence binding;peptide binding;protein transporter activity;signal sequence binding;substrate-specific transporter activity;transporter activity
binding;catalytic activity;cation binding;dioxygenase activity;ion binding;metal ion binding;oxidoreductase activity;oxidoreductase activity, acting on single donors with incorporation of molecular oxygen;oxidoreductase activi
binding;DNA binding;ligand-dependent nuclear receptor binding;nucleic acid binding;protein binding;protein binding transcription factor activity;receptor binding;transcription coactivator activity;transcription cofactor activity;
adrenergic receptor binding;beta-2 adrenergic receptor binding;beta-catenin binding;binding;channel regulator activity;chloride channel regulator activity;enzyme binding;G-protein-coupled receptor binding;growth factor rece
binding;identical protein binding;protein binding
catalytic activity;cation transmembrane transporter activity;hydrogen ion transmembrane transporter activity;inorganic cation transmembrane transporter activity;ion transmembrane transporter activity;monovalent inorganic c
adenyl nucleotide binding;adenyl ribonucleotide binding;ATP binding;binding;nucleotide binding;purine nucleotide binding;purine ribonucleoside triphosphate binding;purine ribonucleotide binding;ribonucleotide binding
binding;catalytic activity;palmitoyltransferase activity;protein binding;protein-cysteine S-acyltransferase activity;protein-cysteine S-palmitoleyltransferase activity;S-acyltransferase activity;SNAP receptor activity;SNARE bindi
alcohol dehydrogenase (NADP+) activity;alditol:NADP+ 1-oxidoreductase activity;aldo-keto reductase (NADP) activity;catalytic activity;electron carrier activity;epoxide hydrolase activity;ether hydrolase activity;hydrolase activ
binding;cation binding;DNA binding;double-stranded DNA binding;ion binding;metal ion binding;nucleic acid binding;structure-specific DNA binding;transition metal ion binding;zinc ion binding
binding;DNA binding;nucleic acid binding;nucleic acid binding transcription factor activity;regulatory region DNA binding;regulatory region nucleic acid binding;sequence-specific DNA binding;sequence-specific DNA binding
binding;cation binding;ion binding;metal ion binding
binding;chromatin binding
active transmembrane transporter activity;ATPase activity;ATPase activity, coupled;ATPase activity, coupled to movement of substances;ATPase activity, coupled to transmembrane movement of ions;ATPase activity, coupled
catalytic activity;electron carrier activity;NADH dehydrogenase (quinone) activity;NADH dehydrogenase (ubiquinone) activity;NADH dehydrogenase activity;oxidoreductase activity;oxidoreductase activity, acting on NADH or N
binding;DNA binding;nucleic acid binding;RNA binding
binding;catalytic activity;cation binding;endopeptidase activity;exopeptidase activity;hydrolase activity;ion binding;metal ion binding;metalloendopeptidase activity;metalloexopeptidase activity;metallopeptidase activity;peptic
structural molecule activity
binding;catalytic activity;cation binding;disulfide oxidoreductase activity;electron carrier activity;ion binding;iron-sulfur cluster binding;metal cluster binding;metal ion binding;oxidoreductase activity;oxidoreductase activity, ac
adenyl nucleotide binding;adenyl ribonucleotide binding;ATP binding;ATPase activity;binding;catalytic activity;cation binding;enzyme activator activity;enzyme regulator activity;hydrolase activity;hydrolase activity, acting on a
binding;cyclin-dependent protein kinase inhibitor activity;cyclin-dependent protein kinase regulator activity;enzyme inhibitor activity;enzyme regulator activity;kinase inhibitor activity;kinase regulator activity;nucleic acid bindir
6-phosphogluconolactonase activity;binding;carbohydrate binding;carboxylic ester hydrolase activity;catalytic activity;hydrolase activity;hydrolase activity, acting on ester bonds;monosaccharide binding;sugar binding
binding;catalytic activity;cation binding;dioxygenase activity;ion binding;iron ion binding;metal ion binding;oxidoreductase activity;oxidoreductase activity, acting on single donors with incorporation of molecular oxygen;oxido
binding;chromatin binding;protein binding transcription factor activity;transcription coactivator activity;transcription cofactor activity;transcription factor binding transcription factor activity
binding;GDP binding;GMP binding;GTP binding;guanyl nucleotide binding;guanyl ribonucleotide binding;nucleotide binding;protein binding;protein complex binding;purine nucleotide binding;purine ribonucleoside triphospha
binding;cation binding;ion binding;metal ion binding;transition metal ion binding;zinc ion binding
binding;nucleic acid binding;RNA binding;structural constituent of ribosome;structural molecule activity
adenine binding;adenine phosphoribosyltransferase activity;adenyl nucleotide binding;adenyl ribonucleotide binding;AMP binding;binding;catalytic activity;nucleobase binding;nucleotide binding;purine base binding;purine nu
binding;nucleic acid binding;nucleotide binding;RNA binding;snRNA binding
active transmembrane transporter activity;binding;carbohydrate transmembrane transporter activity;cation transmembrane transporter activity;cation:sugar symporter activity;dehydroascorbic acid transporter activity;D-gluco
ATPase binding;binding;catalytic activity;cytoskeletal protein binding;Edg-2 lysophosphatidic acid receptor binding;endothelial differentiation G-protein coupled receptor binding;enzyme binding;GDP binding;G-protein-coupl
binding;enzyme binding;protein binding
binding;carbohydrate binding;catalytic activity;enzyme binding;glucose binding;glucosyltransferase activity;glycogen (starch) synthase activity;kinase binding;monosaccharide binding;protein binding;protein kinase binding;su
binding;catalytic activity;growth factor receptor binding;phosphorylase activity;platelet-derived growth factor receptor binding;protein binding;pyrimidine-nucleoside phosphorylase activity;receptor binding;thymidine phospho
binding;C-acyltransferase activity;carboxylic acid binding;catalytic activity;cholesterol binding;coenzyme binding;cofactor binding;fatty acid binding;fatty-acyl-CoA binding;lipid binding;lipid transporter activity;monocarboxylic
binding;growth factor binding;insulin-like growth factor binding;insulin-like growth factor I binding;insulin-like growth factor II binding;protein binding
adenyl nucleotide binding;adenyl ribonucleotide binding;ATP binding;binding;catalytic activity;cation binding;cyclin binding;cyclin-dependent protein kinase activity;ion binding;kinase activity;metal ion binding;nucleotide bind
acyl-CoA dehydrogenase activity;binding;catalytic activity;coenzyme binding;cofactor binding;flavin adenine dinucleotide binding;isovaleryl-CoA dehydrogenase activity;oxidoreductase activity;oxidoreductase activity, acting
adenyl nucleotide binding;adenyl ribonucleotide binding;ATP binding;axon guidance receptor activity;binding;catalytic activity;ephrin receptor activity;kinase activity;molecular transducer activity;nucleotide binding;phosphotr
binding;carboxylic acid binding;isoprenoid binding;lipid binding;monocarboxylic acid binding;retinal binding;retinoic acid binding;retinoid binding;retinol binding;transporter activity;vitamin binding
antioxidant activity;binding;caspase inhibitor activity;caspase regulator activity;catalytic activity;cysteine-type endopeptidase inhibitor activity;DNA binding;endopeptidase inhibitor activity;endopeptidase regulator activity;enz

carbon-carbon lyase activity;carboxy-lyase activity;catalytic activity;D-dopachrome decarboxylase activity;dopachrome isomerase activity;intramolecular oxidoreductase activity;intramolecular oxidoreductase activity, transpo
binding;carbon-sulfur lyase activity;catalytic activity;cofactor binding;cystathionine gamma-lyase activity;homocysteine desulfhydrase activity;L-cysteine desulfhydrase activity;lyase activity;pyridoxal phosphate binding;vitam
binding;carboxylic acid binding;catalytic activity;cholesterol binding;cholesterol O-acyltransferase activity;coenzyme binding;cofactor binding;fatty acid binding;fatty-acyl-CoA binding;lipid binding;monocarboxylic acid bindin
adenyl nucleotide binding;adenyl ribonucleotide binding;ATP binding;binding;DNA binding;enzyme binding;nucleic acid binding;nucleotide binding;protein binding;purine nucleotide binding;purine ribonucleoside triphosphate
binding;ephrin receptor binding;protein binding;protein domain specific binding;receptor binding;SH2 domain binding
binding;catalytic activity;choline-phosphate cytidylyltransferase activity;cytidylyltransferase activity;lipid binding;nucleotidyltransferase activity;transferase activity;transferase activity, transferring phosphorus-containing group
binding;catalytic activity;GTP binding;GTPase activity;guanyl nucleotide binding;guanyl ribonucleotide binding;hydrolase activity;hydrolase activity, acting on acid anhydrides;hydrolase activity, acting on acid anhydrides, in ph
binding;damaged DNA binding;DNA binding;nucleic acid binding;polyubiquitin binding;protein binding;single-stranded DNA binding;small conjugating protein binding;structure-specific DNA binding;ubiquitin binding
binding;calcium ion binding;cation binding;identical protein binding;ion binding;metal ion binding;protein binding;protein dimerization activity;protein heterodimerization activity;protein homodimerization activity
binding;catalytic activity;GTP binding;GTPase activity;guanyl nucleotide binding;guanyl ribonucleotide binding;hydrolase activity;hydrolase activity, acting on acid anhydrides;hydrolase activity, acting on acid anhydrides, in ph
enzyme activator activity;enzyme inhibitor activity;enzyme regulator activity;kinase inhibitor activity;kinase regulator activity;molecular transducer activity;protein kinase inhibitor activity;protein kinase regulator activity;signal t
binding;catalytic activity;enzyme regulator activity;GTP binding;GTPase activity;GTPase regulator activity;guanyl nucleotide binding;guanyl ribonucleotide binding;guanyl-nucleotide exchange factor activity;hydrolase activity;
acid-amino acid ligase activity;catalytic activity;ligase activity;ligase activity, forming carbon-nitrogen bonds;small conjugating protein ligase activity;ubiquitin-protein ligase activity
1-alkyl-2-acetylglycerophosphocholine esterase activity;acetyltransferase activity;carboxylic ester hydrolase activity;catalytic activity;hydrolase activity;hydrolase activity, acting on ester bonds;platelet-activating factor acetylt
binding;cation binding;ion binding;metal ion binding;nucleotide binding
activating transcription factor binding;anion binding;binding;chromatin binding;core promoter proximal region DNA binding;core promoter proximal region sequence-specific DNA binding;DNA binding;enhancer binding;enhan
binding;chromatin binding;DNA binding;nucleic acid binding;single-stranded DNA binding;structure-specific DNA binding
binding;mRNA binding;nucleic acid binding;nucleotide binding;RNA binding
binding;selenium binding
binding;cation binding;chromatin binding;core promoter binding;core promoter sequence-specific DNA binding;DNA binding;ion binding;metal ion binding;nucleic acid binding;nucleic acid binding transcription factor activity;
acid-amino acid ligase activity;binding;catalytic activity;enzyme binding;ligase activity;ligase activity, forming carbon-nitrogen bonds;protein binding;small conjugating protein ligase activity;ubiquitin protein ligase binding
amine binding;amino acid binding;binding;carboxylic acid binding;catalytic activity;cis-trans isomerase activity;enzyme binding;GTPase activating protein binding;isomerase activity;kinase binding;mitogen-activated protein k
3'-5' exonuclease activity;3'-5'-exoribonuclease activity;7S RNA binding;binding;catalytic activity;exonuclease activity;exonuclease activity, active with either ribo- or deoxyribonucleic acids and producing 5'-phosphomonoes
adenyl nucleotide binding;adenyl ribonucleotide binding;ATP binding;ATPase activity;binding;catalytic activity;cation binding;DNA binding;helicase activity;hydrolase activity;hydrolase activity, acting on acid anhydrides;hydrol
catalytic activity;intramolecular oxidoreductase activity;intramolecular oxidoreductase activity, interconverting keto- and enol-groups;intramolecular oxidoreductase activity, transposing S-S bonds;isomerase activity;oxidored
catalytic activity;enzyme regulator activity;hydrolase activity;hydrolase activity, acting on ester bonds;phosphatase activity;phosphatase regulator activity;phosphoprotein phosphatase activity;phosphoric ester hydrolase activ
adenyl nucleotide binding;adenyl ribonucleotide binding;aminoacyl-tRNA editing activity;aminoacyl-tRNA ligase activity;ATP binding;binding;carboxylic ester hydrolase activity;catalytic activity;hydrolase activity;hydrolase acti
binding;nucleic acid binding;RNA binding;snoRNA binding
binding;enzyme activator activity;enzyme binding;enzyme regulator activity;GTPase activator activity;GTPase regulator activity;kinase binding;nucleoside-triphosphatase regulator activity;protein binding;protein kinase binding
binding;catalytic activity;coenzyme binding;cofactor binding;NADH dehydrogenase (quinone) activity;NADH dehydrogenase (ubiquinone) activity;NADH dehydrogenase activity;oxidoreductase activity;oxidoreductase activity,
adenyl nucleotide binding;adenyl ribonucleotide binding;ATP binding;binding;catalytic activity;identical protein binding;kinase activity;NAD+ kinase activity;nucleotide binding;phosphotransferase activity, alcohol group as acc
catalytic activity;oxidoreductase activity;oxidoreductase activity, acting on the CH-NH group of donors;oxidoreductase activity, acting on the CH-NH group of donors, NAD or NADP as acceptor;pyrroline-5-carboxylate reduct
binding;cation binding;ion binding;metal ion binding;transition metal ion binding;zinc ion binding
binding;cation binding;ion binding;metal ion binding
3-hydroxyisobutyryl-CoA hydrolase activity;catalytic activity;CoA hydrolase activity;hydrolase activity;hydrolase activity, acting on ester bonds;thiolester hydrolase activity
catalytic activity;ligase activity;nicotinate phosphoribosyltransferase activity;nicotinate-nucleotide diphosphorylase (carboxylating) activity;transferase activity;transferase activity, transferring glycosyl groups;transferase activit
adenyl nucleotide binding;adenyl ribonucleotide binding;ATP binding;binding;catalytic activity;cation binding;enzyme activator activity;enzyme regulator activity;ion binding;kinase activator activity;kinase activity;kinase regula
catalytic activity;methyltransferase activity;RNA methyltransferase activity;S-adenosylmethionine-dependent methyltransferase activity;transferase activity;transferase activity, transferring one-carbon groups
binding;cation binding;DNA binding;ion binding;metal ion binding;nucleic acid binding
catalytic activity;hydrolase activity;hydrolase activity, acting on carbon-nitrogen (but not peptide) bonds;hydrolase activity, acting on carbon-nitrogen (but not peptide) bonds, in nitriles;nitrilase activity
binding;dynein binding;dynein intermediate chain binding;protein binding
binding;ion channel binding;protein binding
binding;catalytic activity;methyltransferase activity;mRNA (nucleoside-2'-O-)-methyltransferase activity;mRNA methyltransferase activity;nucleic acid binding;O-methyltransferase activity;RNA methyltransferase activity;S-ade
binding;catalytic activity;cation binding;hydrolase activity;hydrolase activity, acting on ester bonds;ion binding;lipase activity;metal ion binding
1-acylglycerophosphocholine O-acyltransferase activity;1-alkenylglycerophosphocholine O-acyltransferase activity;1-alkylglycerophosphocholine O-acetyltransferase activity;1-alkylglycerophosphocholine O-acyltransferase a
binding;mRNA binding;nucleic acid binding;nucleotide binding;RNA binding
binding;histone acetyl-lysine binding;histone binding;insulin receptor binding;protein binding;protein complex binding;receptor binding
molecular transducer activity;signal transducer activity
binding;catalytic activity;methyltransferase activity;nucleic acid binding;RNA binding;RNA methyltransferase activity;rRNA (pseudouridine) methyltransferase activity;rRNA binding;rRNA methyltransferase activity;S-adenosylm
binding;catalytic activity;coenzyme binding;cofactor binding;oxidoreductase activity;oxidoreductase activity, acting on the aldehyde or oxo group of donors;oxidoreductase activity, acting on the aldehyde or oxo group of don
catalytic activity;nucleotidyltransferase activity;transferase activity;transferase activity, transferring phosphorus-containing groups
4-hydroxyphenylpyruvate dioxygenase activity;binding;catalytic activity;cation binding;dioxygenase activity;ion binding;metal ion binding;oxidoreductase activity;oxidoreductase activity, acting on single donors with incorpora
binding;cyclin binding;enzyme binding;kinase binding;NF-kappaB binding;protein binding;protein kinase binding;transcription factor binding
catalytic activity;intramolecular oxidoreductase activity;intramolecular oxidoreductase activity, interconverting keto- and enol-groups;intramolecular oxidoreductase activity, transposing S-S bonds;isomerase activity;protein d
binding;cation binding;enzyme activator activity;enzyme regulator activity;GTPase activator activity;GTPase regulator activity;ion binding;lipid binding;metal ion binding;nucleoside-triphosphatase regulator activity;phosphatid
binding;chromatin binding;DNA binding;heat shock protein binding;histone binding;Hsp70 protein binding;nucleic acid binding;protein binding;small conjugating protein binding;ubiquitin binding
binding;protein binding;unfolded protein binding
3-hydroxy-2-methylbutyryl-CoA dehydrogenase activity;3-hydroxyacyl-CoA dehydrogenase activity;catalytic activity;cholate 7-alpha-dehydrogenase activity;oxidoreductase activity;oxidoreductase activity, acting on CH-OH g
acid-amino acid ligase activity;binding;catalytic activity;cation binding;ion binding;ligase activity;ligase activity, forming carbon-nitrogen bonds;metal ion binding;small conjugating protein ligase activity;transition metal ion bin
binding;cation binding;chromatin binding;DNA binding;ion binding;metal ion binding;nucleic acid binding;nucleic acid binding transcription factor activity;sequence-specific DNA binding;sequence-specific DNA binding transc
binding;enzyme binding;identical protein binding;kinase binding;protein binding;protein dimerization activity;protein homodimerization activity;protein kinase binding;protein N-terminus binding
binding;nucleotide binding
binding;cholesterol binding;lipid binding;steroid binding;sterol binding
catalytic activity;exonuclease activity;hydrolase activity;hydrolase activity, acting on ester bonds;nuclease activity;nucleic acid binding transcription factor activity;sequence-specific DNA binding transcription factor activity
binding;catalytic activity;GTP binding;GTPase activity;guanyl nucleotide binding;guanyl ribonucleotide binding;hydrolase activity;hydrolase activity, acting on acid anhydrides;hydrolase activity, acting on acid anhydrides, in ph
binding;catalytic activity;disulfide oxidoreductase activity;electron carrier activity;enzyme activator activity;enzyme regulator activity;GTPase activator activity;GTPase regulator activity;nucleoside-triphosphatase regulator acti
binding;enzyme binding;phosphatase binding;protein binding
binding;protein binding;protein C-terminus binding
binding;catalytic activity;cation binding;ion binding;metal ion binding;transferase activity;transferase activity, transferring acyl groups
binding;cation binding;ion binding;metal ion binding;transition metal ion binding;zinc ion binding
binding;calcium channel inhibitor activity;calcium channel regulator activity;catalytic activity;channel inhibitor activity;channel regulator activity;hydrolase activity;hydrolase activity, acting on ester bonds;ion channel binding;io
binding;DNA binding;DNA polymerase binding;enzyme binding;nucleic acid binding;protein binding
binding;calcium-dependent protein binding;cation binding;ion binding;metal ion binding;nucleic acid binding;nucleocytoplasmic transporter activity;nucleotide binding;pre-mRNA binding;protein binding;RNA binding;snRNA b
catalytic activity;dipeptidyl-peptidase activity;exopeptidase activity;hydrolase activity;peptidase activity;peptidase activity, acting on L-amino acid peptides;serine hydrolase activity;serine-type peptidase activity
binding;enzyme binding;phosphatase binding;protein binding;protein phosphatase binding
5'-3' exonuclease activity;binding;catalytic activity;cation binding;endonuclease activity;endoribonuclease activity;exonuclease activity;hydrolase activity;hydrolase activity, acting on ester bonds;ion binding;metal ion binding;
binding;DNA binding;nucleic acid binding
structural constituent of ribosome;structural molecule activity
binding;cytoskeletal protein binding;DNA binding;enzyme activator activity;enzyme binding;enzyme regulator activity;GTPase activator activity;GTPase binding;GTPase regulator activity;nucleic acid binding;nucleoside-tripho
binding;enzyme binding;insulin receptor binding;kinase binding;molecular transducer activity;phosphatidylinositol 3-kinase binding;protein binding;protein complex binding;receptor binding;signal transducer activity
receptor activity
binding;chromatin binding;DNA binding;enzyme binding;histone deacetylase binding;Notch binding;nucleic acid binding;protein binding;protein binding transcription factor activity;protein N-terminus binding;receptor binding;
binding;catalytic activity;cation binding;cofactor binding;cysteine desulfurase activity;identical protein binding;ion binding;iron-sulfur cluster binding;metal cluster binding;metal ion binding;protein binding;protein dimerization
ARF guanyl-nucleotide exchange factor activity;binding;enzyme regulator activity;GABA receptor binding;GTPase regulator activity;guanyl-nucleotide exchange factor activity;nucleoside-triphosphatase regulator activity;prote
ARF guanyl-nucleotide exchange factor activity;binding;cytoskeletal protein binding;enzyme regulator activity;GTPase regulator activity;guanyl-nucleotide exchange factor activity;myosin binding;nucleoside-triphosphatase re
binding;cation binding;ion binding;metal ion binding;transition metal ion binding;zinc ion binding
adenyl nucleotide binding;adenyl ribonucleotide binding;ATP binding;ATPase activity;binding;catalytic activity;hydrolase activity;hydrolase activity, acting on acid anhydrides;hydrolase activity, acting on acid anhydrides, in ph
binding;catalytic activity;GTP binding;GTPase activity;guanyl nucleotide binding;guanyl ribonucleotide binding;hydrolase activity;hydrolase activity, acting on acid anhydrides;hydrolase activity, acting on acid anhydrides, in ph
binding;enzyme binding;protein binding;ubiquitin protein ligase binding
5-oxoprolinase (ATP-hydrolyzing) activity;adenyl nucleotide binding;adenyl ribonucleotide binding;ATP binding;binding;catalytic activity;hydrolase activity;hydrolase activity, acting on carbon-nitrogen (but not peptide) bonds;h
adenyl nucleotide binding;adenyl ribonucleotide binding;ATP binding;binding;catalytic activity;guanylate kinase activity;kinase activity;nucleobase-containing compound kinase activity;nucleotide binding;nucleotide kinase ac
binding;C-acyltransferase activity;catalytic activity;cofactor binding;C-palmitoyltransferase activity;palmitoyltransferase activity;pyridoxal phosphate binding;serine C-palmitoyltransferase activity;transferase activity;transferas
binding;centromeric DNA binding;DNA binding;nucleic acid binding;sequence-specific DNA binding
binding;nucleic acid binding;RNA binding;translation factor activity, nucleic acid binding;translation initiation factor activity
adenyl nucleotide binding;adenyl ribonucleotide binding;anion transmembrane transporter activity;arsenite transmembrane transporter activity;ATP binding;ATPase activity;binding;catalytic activity;cation binding;hydrolase ac
binding;nucleotide binding
binding;lipid binding;phosphatidylinositol binding;phospholipid binding
binding;nucleic acid binding;nucleotide binding;protein binding;protein domain specific binding;RNA binding;RS domain binding;unfolded protein binding
binding;carboxylic acid binding;catalytic activity;coenzyme binding;cofactor binding;dodecenoyl-CoA delta-isomerase activity;fatty acid binding;fatty-acyl-CoA binding;intramolecular oxidoreductase activity;intramolecular ox
binding;DNA binding;nucleic acid binding
acidic amino acid transmembrane transporter activity;active transmembrane transporter activity;amine transmembrane transporter activity;amino acid transmembrane transporter activity;binding;calcium ion binding;carboxyli
aldehyde-lyase activity;binding;carbon-carbon lyase activity;carboxy-lyase activity;catalytic activity;cofactor binding;lyase activity;pyridoxal phosphate binding;sphinganine-1-phosphate aldolase activity;vitamin B6 binding;vi
binding;cytoskeletal protein binding;microtubule binding;protein binding;tubulin binding
binding;catalytic activity;cation binding;guanine phosphoribosyltransferase activity;hypoxanthine phosphoribosyltransferase activity;identical protein binding;ion binding;magnesium ion binding;metal ion binding;nucleotide bi
binding;GTP binding;guanyl nucleotide binding;guanyl ribonucleotide binding;nucleotide binding;protein binding;protein complex binding;purine nucleotide binding;purine ribonucleoside triphosphate binding;purine ribonucle
antigen binding;binding;peptide antigen binding;peptide binding;protein binding;receptor binding
binding;chromatin binding;chromatin DNA binding;DNA binding;nucleic acid binding;structure-specific DNA binding
catalytic activity;cation transmembrane transporter activity;hydrogen ion transmembrane transporter activity;inorganic cation transmembrane transporter activity;ion transmembrane transporter activity;monovalent inorganic c
binding;nucleic acid binding;nucleotide binding;RNA binding;snRNA binding
alkaline phosphatase activity;binding;catalytic activity;cation binding;enzyme binding;hydrolase activity;hydrolase activity, acting on ester bonds;ion binding;magnesium ion binding;metal ion binding;phosphatase activity;pho

binding;histone pre-mRNA DCP binding;nucleic acid binding;RNA binding
binding;catalytic activity;cysteine-type peptidase activity;hydrolase activity;peptidase activity;peptidase activity, acting on L-amino acid peptides;protein binding;small conjugating protein binding;small conjugating protein-sp
binding;catalytic activity;cation binding;fumarylacetoacetase activity;hydrolase activity;hydrolase activity, acting on acid carbon-carbon bonds;hydrolase activity, acting on acid carbon-carbon bonds, in ketonic substances;io
binding;catalytic activity;cytoskeletal protein binding;GTP binding;GTPase activity;guanyl nucleotide binding;guanyl ribonucleotide binding;hydrolase activity;hydrolase activity, acting on acid anhydrides;hydrolase activity, act
binding;catalytic activity;GTP binding;GTPase activity;guanyl nucleotide binding;guanyl ribonucleotide binding;hydrolase activity;hydrolase activity, acting on acid anhydrides;hydrolase activity, acting on acid anhydrides, in p
catalytic activity;endopeptidase activity;hydrolase activity;peptidase activity;peptidase activity, acting on L-amino acid peptides;threonine-type endopeptidase activity;threonine-type peptidase activity
biliverdin reductase activity;catalytic activity;flavin reductase activity;oxidoreductase activity;oxidoreductase activity, acting on the CH-CH group of donors;oxidoreductase activity, acting on the CH-CH group of donors, NAD
binding;catalytic activity;cation binding;heme oxygenase (decyclizing) activity;ion binding;metal ion binding;oxidoreductase activity;oxidoreductase activity, acting on paired donors, with incorporation or reduction of molecula
binding;calcium channel regulator activity;calcium ion binding;calcium-dependent cysteine-type endopeptidase activity;catalytic activity;cation binding;channel regulator activity;cysteine-type endopeptidase activity;cysteine-
adenyl nucleotide binding;adenyl ribonucleotide binding;ATP binding;binding;DNA binding;nucleic acid binding;nucleotide binding;purine nucleotide binding;purine ribonucleoside triphosphate binding;purine ribonucleotide bi
binding;core promoter binding;core promoter sequence-specific DNA binding;DNA binding;enzyme binding;identical protein binding;molecular transducer activity;nucleic acid binding;nucleic acid binding transcription factor
binding;cyclin-dependent protein kinase inhibitor activity;cyclin-dependent protein kinase regulator activity;enzyme binding;enzyme inhibitor activity;enzyme regulator activity;kinase binding;kinase inhibitor activity;kinase regu
acyl-CoA hydrolase activity;acyl-CoA thioesterase activity;binding;carboxylic ester hydrolase activity;catalytic activity;CoA hydrolase activity;hydrolase activity;hydrolase activity, acting on ester bonds;palmitoyl-CoA hydrolas
adenyl nucleotide binding;adenyl ribonucleotide binding;ATP binding;beta-catenin binding;binding;catalytic activity;enzyme binding;kinase activity;kinase binding;NF-kappaB binding;nucleotide binding;p53 binding;phosphot
binding;catalytic activity;GDP binding;GTP binding;GTPase activity;guanyl nucleotide binding;guanyl ribonucleotide binding;hydrolase activity;hydrolase activity, acting on acid anhydrides;hydrolase activity, acting on acid an
enzyme activator activity;enzyme regulator activity;GTPase activator activity;GTPase regulator activity;nucleoside-triphosphatase regulator activity
binding;nucleic acid binding;RNA binding;structural constituent of ribosome;structural molecule activity
binding;cation binding;ion binding;metal ion binding;transition metal ion binding;zinc ion binding
binding;protein binding;SNARE binding;soluble NSF attachment protein activity;syntaxin binding
binding;GTP binding;guanyl nucleotide binding;guanyl ribonucleotide binding;nucleotide binding;purine nucleotide binding;purine ribonucleoside triphosphate binding;purine ribonucleotide binding;ribonucleotide binding
binding;catalytic activity;cation binding;hydrolase activity;hydrolase activity, acting on ester bonds;ion binding;kinase activity;metal ion binding;molecular transducer activity;NF-kappaB-inducing kinase activity;phosphatase
acid-amino acid ligase activity;adenyl nucleotide binding;adenyl ribonucleotide binding;ATP binding;binding;catalytic activity;ligase activity;ligase activity, forming carbon-nitrogen bonds;NEDD8 ligase activity;nucleotide bind
activin binding;binding;catalytic activity;cis-trans isomerase activity;cytokine receptor binding;drug binding;FK506 binding;ion channel binding;isomerase activity;macrolide binding;molecular transducer activity;peptidyl-proly
binding;binding, bridging;cytokine receptor binding;enzyme binding;ephrin receptor binding;epidermal growth factor receptor binding;growth factor receptor binding;identical protein binding;insulin receptor substrate binding;
acid-amino acid ligase activity;adenyl nucleotide binding;adenyl ribonucleotide binding;ATP binding;binding;catalytic activity;enzyme binding;enzyme regulator activity;ligase activity;ligase activity, forming carbon-nitrogen bo
binding;cation binding;ion binding;metal ion binding;transition metal ion binding;zinc ion binding
binding;nucleic acid binding;nucleotide binding;ribonucleoprotein binding;ribosomal large subunit binding;RNA binding
binding;DNA binding;double-stranded DNA binding;double-stranded telomeric DNA binding;nucleic acid binding;nucleic acid binding transcription factor activity;protein binding;purine-rich negative regulatory element binding
endopeptidase inhibitor activity;endopeptidase regulator activity;enzyme inhibitor activity;enzyme regulator activity;peptidase inhibitor activity;peptidase regulator activity;serine-type endopeptidase inhibitor activity
binding;enzyme binding;molecular transducer activity;protein binding;protein domain specific binding;receptor activity;receptor binding;signal transducer activity;signaling receptor activity;U-plasminogen activator receptor a
adenyl nucleotide binding;adenyl ribonucleotide binding;ATP binding;binding;nucleotide binding;purine nucleotide binding;purine ribonucleoside triphosphate binding;purine ribonucleotide binding;ribonucleotide binding
binding;calcium channel regulator activity;calcium ion binding;cation binding;channel regulator activity;cytoskeletal protein binding;identical protein binding;ion binding;metal ion binding;microtubule binding;microtubule plus-
binding;carbohydrate binding;enzyme binding;protein binding
actin binding;actin filament binding;binding;calcium ion binding;cation binding;cytoskeletal protein binding;ion binding;metal ion binding;protein binding;structural constituent of cytoskeleton;structural molecule activity
binding;double-stranded RNA binding;nucleic acid binding;RNA binding
binding;DNA binding;mRNA binding;nucleic acid binding;RNA binding
binding;catalytic activity;DNA binding;endonuclease activity;endoribonuclease activity;hydrolase activity;hydrolase activity, acting on ester bonds;mRNA binding;nuclease activity;nucleic acid binding;ribonuclease activity;RN
adenyl nucleotide binding;adenyl ribonucleotide binding;ATP binding;binding;identical protein binding;nucleotide binding;protein binding;protein binding transcription factor activity;purine nucleotide binding;purine ribonucleo
adenyl nucleotide binding;adenyl ribonucleotide binding;ATP binding;binding;catalytic activity;kinase activity;MAP kinase activity;MAP kinase kinase activity;molecular transducer activity;NFAT protein binding;nucleotide bind
binding;mRNA binding;nucleic acid binding;nucleotide binding;RNA binding
binding;DNA binding;nucleic acid binding
binding;lipid binding;phospholipid binding
binding;DNA binding;nucleic acid binding;nucleic acid binding transcription factor activity;sequence-specific DNA binding transcription factor activity
nucleocytoplasmic transporter activity;transporter activity
binding;cation binding;ion binding;metal ion binding
molecular transducer activity;signal transducer activity
binding;calcium ion binding;cation binding;ion binding;metal ion binding
ATPase activator activity;ATPase binding;ATPase regulator activity;binding;catalytic activity;chaperone binding;disulfide oxidoreductase activity;enzyme activator activity;enzyme binding;enzyme regulator activity;heat shock
binding;calcium ion binding;catalytic activity;cation binding;GTP binding;GTPase activity;guanyl nucleotide binding;guanyl ribonucleotide binding;hydrolase activity;hydrolase activity, acting on acid anhydrides;hydrolase activ
binding;chromatin binding;protein binding;transcription factor binding
binding;catalytic activity;channel regulator activity;coenzyme binding;cofactor binding;glycerol-3-phosphate dehydrogenase [NAD+] activity;ion channel binding;NAD binding;nucleotide binding;oxidoreductase activity;oxidor
ATPase activator activity;ATPase binding;ATPase regulator activity;binding;enzyme activator activity;enzyme binding;enzyme regulator activity;nucleoside-triphosphatase regulator activity;protein binding
protein binding transcription factor activity;transcription coactivator activity;transcription cofactor activity;transcription factor binding transcription factor activity
catalytic activity;GPI-anchor transamidase activity;hydrolase activity
binding;nucleic acid binding;ribonucleoprotein binding;ribosomal small subunit binding;RNA binding;rRNA binding
binding;nucleic acid binding;nucleotide binding;RNA binding
binding;catalytic activity;cysteine-type endopeptidase activity;cysteine-type peptidase activity;endopeptidase activity;hydrolase activity;peptidase activity;peptidase activity, acting on L-amino acid peptides;protein binding;p
adenyl nucleotide binding;adenyl ribonucleotide binding;ATP binding;binding;catalytic activity;kinase activity;nucleotide binding;phosphotransferase activity, alcohol group as acceptor;protein kinase activity;protein serine/thr
binding;DNA binding;double-stranded RNA binding;nucleic acid binding;RNA binding;single-stranded RNA binding
binding;catalytic activity;cation binding;DNA binding;endonuclease activity;endoribonuclease activity;hydrolase activity;hydrolase activity, acting on ester bonds;ion binding;metal ion binding;nuclease activity;nucleic acid bin
binding;catalytic activity;cation binding;endonuclease activity;hydrolase activity;hydrolase activity, acting on ester bonds;ion binding;metal ion binding;nuclease activity
binding;catalytic activity;identical protein binding;intramolecular oxidoreductase activity;intramolecular oxidoreductase activity, interconverting aldoses and ketoses;isomerase activity;protein binding;S-methyl-5-thioribose-1-
binding;catalytic activity;methyltransferase activity;nucleic acid binding;RNA binding;transferase activity;transferase activity, transferring one-carbon groups
structural constituent of ribosome;structural molecule activity
binding;catalytic activity;DNA binding;DNA-directed RNA polymerase activity;nucleic acid binding;nucleotidyltransferase activity;RNA polymerase activity;transferase activity;transferase activity, transferring phosphorus-cont
alpha-tubulin binding;beta-tubulin binding;binding;cation binding;cytoskeletal protein binding;enzyme activator activity;enzyme binding;enzyme regulator activity;gamma-tubulin binding;GTPase activator activity;GTPase regu
binding;enzyme binding;lipid binding;phosphatidylinositol binding;phosphatidylinositol-4-phosphate binding;phospholipid binding;protein binding
adenyl nucleotide binding;adenyl ribonucleotide binding;ATP binding;ATPase activity;ATPase activity, coupled;ATP-dependent helicase activity;ATP-dependent RNA helicase activity;binding;catalytic activity;enzyme binding;
receptor activity
14-3-3 protein binding;adenyl nucleotide binding;adenyl ribonucleotide binding;ATP binding;binding;catalytic activity;enzyme binding;hydrolase activity;hydrolase activity, acting on acid anhydrides;hydrolase activity, acting o
binding;catalytic activity;enzyme regulator activity;GTPase regulator activity;guanyl-nucleotide exchange factor activity;nucleic acid binding;nucleoside-triphosphatase regulator activity;nucleotidyltransferase activity;RNA bin
binding;cation binding;ion binding;metal ion binding;nucleotide binding
binding;cation binding;enzyme binding;GTPase binding;ion binding;metal ion binding;protein binding;Rab GTPase binding;Ras GTPase binding;small GTPase binding
alpha-tubulin binding;beta-catenin binding;binding;catalytic activity;cation binding;core promoter binding;cytoskeletal protein binding;deacetylase activity;DNA binding;dynein complex binding;enzyme binding;heat shock pro
protein transporter activity;receptor activity;substrate-specific transporter activity;transporter activity
catalytic activity;cytochrome-b5 reductase activity;oxidoreductase activity;oxidoreductase activity, acting on NADH or NADPH;oxidoreductase activity, acting on NADH or NADPH, heme protein as acceptor
adenyl nucleotide binding;adenyl ribonucleotide binding;ATP binding;binding;carbohydrate kinase activity;catalytic activity;kinase activity;N-acetylglucosamine kinase activity;N-acylmannosamine kinase activity;nucleotide bi
binding;nucleic acid binding;RNA binding;translation factor activity, nucleic acid binding;translation initiation factor activity
binding;nucleic acid binding;RNA binding;snoRNA binding
adenyl nucleotide binding;adenyl ribonucleotide binding;ATP binding;binding;carbohydrate kinase activity;catalytic activity;cation binding;hydrolase activity;hydrolase activity, acting on glycosyl bonds;hydrolase activity, hydr
binding;GTP binding;guanyl nucleotide binding;guanyl ribonucleotide binding;nucleotide binding;purine nucleotide binding;purine ribonucleoside triphosphate binding;purine ribonucleotide binding;ribonucleotide binding
1-phosphatidylinositol binding;Arp2/3 complex binding;binding;enzyme binding;identical protein binding;lipid binding;phosphatidylinositol binding;phospholipid binding;protein binding;protein complex binding;protein dimeri
binding;catalytic activity;cysteine-type peptidase activity;hydrolase activity;nucleic acid binding;peptidase activity;peptidase activity, acting on L-amino acid peptides;RNA binding;small conjugating protein-specific protease
catalytic activity;transferase activity;transferase activity, transferring acyl groups
binding;enzyme binding;GTPase binding;protein binding;Ral GTPase binding;Ras GTPase binding;small GTPase binding
binding;catalytic activity;cation binding;endopeptidase activator activity;endopeptidase regulator activity;enzyme activator activity;enzyme regulator activity;hydrolase activity;ion binding;metal ion binding;metallopeptidase a
structural molecule activity
binding;calcium ion binding;cation binding;ion binding;metal ion binding
binding;chaperone binding;protein binding;unfolded protein binding
binding;C-acyltransferase activity;catalytic activity;cofactor binding;C-palmitoyltransferase activity;palmitoyltransferase activity;pyridoxal phosphate binding;serine C-palmitoyltransferase activity;transferase activity;transfera
active transmembrane transporter activity;carboxylic acid transmembrane transporter activity;lactate transmembrane transporter activity;monocarboxylic acid transmembrane transporter activity;organic acid transmembrane
binding;catalytic activity;methyltransferase activity;mRNA (guanine-N7-)-methyltransferase activity;mRNA methyltransferase activity;N-methyltransferase activity;nucleic acid binding;RNA binding;RNA methyltransferase activ
catalytic activity;disulfide oxidoreductase activity;oxidoreductase activity;oxidoreductase activity, acting on a sulfur group of donors;oxidoreductase activity, acting on a sulfur group of donors, disulfide as acceptor;protein dis
binding;enzyme binding;lipid binding;phosphatase binding;phosphatidylinositol binding;phosphatidylinositol-3,5-bisphosphate binding;phosphatidylinositol-3-phosphate binding;phosphatidylinositol-4-phosphate binding;pho
protein transporter activity;substrate-specific transporter activity;transporter activity
binding;chromatin binding;DNA binding;histone acetyl-lysine binding;histone binding;nucleic acid binding;p53 binding;protein binding
binding;nucleic acid binding transcription factor activity;protein binding;protein binding involved in protein folding;sequence-specific DNA binding transcription factor activity
binding;catalytic activity;gamma-glutamylcyclotransferase activity;identical protein binding;protein binding;protein dimerization activity;protein homodimerization activity;transferase activity;transferase activity, transferring acy
adenyl nucleotide binding;adenyl ribonucleotide binding;ATP binding;binding;catalytic activity;enzyme activator activity;enzyme regulator activity;GTP binding;GTPase activator activity;GTPase regulator activity;guanyl nucleo
binding;cation binding;ion binding;metal ion binding;nucleic acid binding;nucleic acid binding transcription factor activity;RNA binding;sequence-specific DNA binding transcription factor activity;transition metal ion binding;zi
binding;calcium ion binding;catalytic activity;cation binding;cis-trans isomerase activity;drug binding;FK506 binding;ion binding;isomerase activity;macrolide binding;metal ion binding;peptidyl-prolyl cis-trans isomerase activi
binding;cation binding;cytokine binding;DNA binding;growth factor binding;interleukin-1 binding;ion binding;metal ion binding;NACHT domain binding;nucleic acid binding;protein binding;protein domain specific binding;trans
binding;cation binding;ion binding;metal ion binding;transition metal ion binding;zinc ion binding

binding;protein binding;SNAP receptor activity;SNARE binding;syntaxin binding
actin binding;binding;cytoskeletal protein binding;lipid binding;protein binding;steroid binding;transmembrane transporter activity;transporter activity;vitamin binding;vitamin D binding;vitamin transporter activity
binding;carboxylic acid binding;cytoskeletal protein binding;fatty acid binding;fatty acid transporter activity;icosanoid binding;icosatetraenoic acid binding;lipid binding;lipid transporter activity;long-chain fatty acid transporter
binding;nucleic acid binding;RNA binding;RNA cap binding;translation factor activity, nucleic acid binding;translation initiation factor activity
beta-N-acetylhexosaminidase activity;binding;catalytic activity;hexosaminidase activity;hydrolase activity;hydrolase activity, acting on glycosyl bonds;hydrolase activity, hydrolyzing O-glycosyl compounds;protein binding;pro
catalytic activity;disulfide oxidoreductase activity;oxidoreductase activity;oxidoreductase activity, acting on a sulfur group of donors;oxidoreductase activity, acting on a sulfur group of donors, disulfide as acceptor;peptide di
amine binding;amino acid binding;binding;carboxylic acid binding;cofactor transporter activity;folic acid binding;folic acid transporter activity;receptor activity;transmembrane transporter activity;transporter activity;vitamin bin
5'-deoxyribose-5-phosphate lyase activity;AT DNA binding;binding;carbon-oxygen lyase activity;catalytic activity;DNA binding;DNA-(apurinic or apyrimidinic site) lyase activity;enzyme binding;hormone receptor binding;ligand
adenyl nucleotide binding;adenyl ribonucleotide binding;ATP binding;binding;catalytic activity;kinase activity;nucleotide binding;phosphotransferase activity, alcohol group as acceptor;protein binding;protein kinase activity;pr
binding;cation binding;DNA binding;DNA secondary structure binding;enhancer binding;enhancer sequence-specific DNA binding;four-way junction DNA binding;ion binding;metal ion binding;nucleic acid binding;nucleic acid
ATPase activator activity;ATPase binding;ATPase regulator activity;binding;chaperone binding;enzyme activator activity;enzyme binding;enzyme regulator activity;heat shock protein binding;Hsp70 protein binding;nucleoside-
binding;calcium ion binding;cation binding;ion binding;metal ion binding;protein binding;RAGE receptor binding;receptor binding
14-3-3 protein binding;adenyl nucleotide binding;adenyl ribonucleotide binding;ATP binding;binding;catalytic activity;enzyme binding;enzyme regulator activity;identical protein binding;kinase activity;lipid binding;nitric-oxide
binding;catalytic activity;cation binding;dUTP diphosphatase activity;hydrolase activity;hydrolase activity, acting on acid anhydrides;hydrolase activity, acting on acid anhydrides, in phosphorus-containing anhydrides;ion bind
binding;carbohydrate binding;glycosaminoglycan binding;heparin binding;nucleic acid binding;pattern binding;polysaccharide binding;RNA binding;structural constituent of ribosome;structural molecule activity
nucleic acid binding transcription factor activity;sequence-specific DNA binding transcription factor activity
binding;mRNA binding;nucleic acid binding;RNA binding;structural constituent of ribosome;structural molecule activity
2 iron, 2 sulfur cluster binding;binding;catalytic activity;cation binding;cation transmembrane transporter activity;hydrogen ion transmembrane transporter activity;inorganic cation transmembrane transporter activity;ion bindin
adenyl nucleotide binding;adenyl ribonucleotide binding;ADP binding;binding;carbon-carbon lyase activity;carboxy-lyase activity;catalytic activity;cation binding;coenzyme binding;cofactor binding;electron carrier activity;ion
binding;integrin binding;protein binding;protein complex binding;receptor binding
structural constituent of ribosome;structural molecule activity
binding;identical protein binding;mRNA binding;nucleic acid binding;protein binding;RNA binding
binding;identical protein binding;protein binding;protein dimerization activity;protein homodimerization activity
binding;nuclear localization sequence binding;peptide binding;protein transporter activity;signal sequence binding;substrate-specific transporter activity;transporter activity
anion binding;apoptotic protease activator activity;binding;caspase activator activity;caspase regulator activity;catalytic activity;chaperone binding;chloride ion binding;cysteine-type endopeptidase activity;cysteine-type pept
binding;calcium ion binding;cation binding;identical protein binding;ion binding;ion channel binding;metal ion binding;protein binding;protein dimerization activity;protein homodimerization activity
binding;catalytic activity;GDP binding;GTP binding;GTPase activity;guanyl nucleotide binding;guanyl ribonucleotide binding;hydrolase activity;hydrolase activity, acting on acid anhydrides;hydrolase activity, acting on acid anh
binding;ribonucleoprotein binding;ribosome binding
binding;enzyme binding;histone pre-mRNA DCP binding;nucleic acid binding;protein binding;RNA binding
binding;catalytic activity;enzyme binding;GDP-dissociation inhibitor binding;GTP binding;GTPase activity;guanyl nucleotide binding;guanyl ribonucleotide binding;hydrolase activity;hydrolase activity, acting on acid anhydride
catalytic activity;hydrolase activity;peptidase activity;peptidase activity, acting on L-amino acid peptides;serine hydrolase activity;serine-type peptidase activity
binding;cytoskeletal protein binding;microtubule binding;nucleic acid binding;protein binding;RNA binding;tubulin binding
catalytic activity;dipeptidase activity;exopeptidase activity;hydrolase activity;peptidase activity;peptidase activity, acting on L-amino acid peptides
binding;catalytic activity;channel regulator activity;cytoskeletal protein binding;enzyme binding;glutamate receptor binding;guanylate kinase activity;hydrolase activity;hydrolase activity, acting on ester bonds;ion channel bind
binding;mRNA binding;nucleic acid binding;RNA binding
adenyl nucleotide binding;adenyl ribonucleotide binding;adenylyltransferase activity;ATP binding;binding;catalytic activity;dephospho-CoA kinase activity;kinase activity;nucleotide binding;nucleotidyltransferase activity;pante
binding;nucleotide binding
binding;catalytic activity;coenzyme binding;cofactor binding;NAD binding;nucleic acid binding transcription factor activity;nucleotide binding;oxidoreductase activity;oxidoreductase activity, acting on CH-OH group of donors
binding;catalytic activity;cation binding;collagen binding;endopeptidase activity;enzyme binding;extracellular matrix binding;hydrolase activity;integrin binding;ion binding;kinase binding;laminin binding;metal ion binding;meta
binding;catalytic activity;cation binding;enzyme binding;identical protein binding;ion binding;kinase activity;kinase binding;metal ion binding;phosphotransferase activity, alcohol group as acceptor;protein binding;protein dom
binding;catalytic activity;coenzyme binding;cofactor binding;identical protein binding;isomerase activity;protein binding;protein dimerization activity;protein homodimerization activity;racemase and epimerase activity;racemas
antigen binding;binding;catalytic activity;coenzyme binding;cofactor binding;delta24(24-1) sterol reductase activity;delta24-sterol reductase activity;enzyme binding;flavin adenine dinucleotide binding;oxidoreductase activity;
binding;cation binding;ion binding;metal ion binding;transition metal ion binding;zinc ion binding
acid-amino acid ligase activity;binding;catalytic activity;enzyme binding;ligase activity;ligase activity, forming carbon-nitrogen bonds;protein binding;small conjugating protein ligase activity;ubiquitin protein ligase binding
adenyl nucleotide binding;adenyl ribonucleotide binding;ATP binding;binding;catalytic activity;enzyme binding;histone deacetylase binding;kinase activity;nucleotide binding;phosphotransferase activity, alcohol group as acce
binding;cation binding;ion binding;metal ion binding;transition metal ion binding;zinc ion binding
binding;nucleic acid binding;RNA binding
2 iron, 2 sulfur cluster binding;binding;cation binding;electron carrier activity;ion binding;iron-sulfur cluster binding;metal cluster binding;metal ion binding
3'-5' RNA helicase activity;adenyl nucleotide binding;adenyl ribonucleotide binding;ATP binding;ATPase activity;ATPase activity, coupled;ATP-dependent helicase activity;ATP-dependent RNA helicase activity;binding;catalyti
catalytic activity;oxidoreductase activity
binding;cation binding;ion binding;metal ion binding;nucleotide binding
binding;nucleic acid binding;RNA binding
alcohol dehydrogenase (NADP+) activity;aldo-keto reductase (NADP) activity;catalytic activity;NADP-retinol dehydrogenase activity;oxidoreductase activity;oxidoreductase activity, acting on CH-OH group of donors;oxidoredu
adenyl nucleotide binding;adenyl ribonucleotide binding;ATP binding;ATPase activity;ATPase activity, coupled;ATP-dependent helicase activity;ATP-dependent RNA helicase activity;binding;catalytic activity;estrogen receptor
binding;DNA binding;nucleic acid binding;nucleic acid binding transcription factor activity;sequence-specific DNA binding transcription factor activity
binding;damaged DNA binding;DNA binding;nucleic acid binding
acetyltransferase activity;binding;catalytic activity;chromatin binding;DNA binding;ligand-dependent nuclear receptor binding;N-acetyltransferase activity;N-acyltransferase activity;nucleic acid binding;protein binding;protein
binding;cytoskeletal protein binding;gamma-tubulin binding;protein binding;structural constituent of cytoskeleton;structural molecule activity;tubulin binding
binding;DNA binding;double-stranded DNA binding;mRNA binding;nucleic acid binding;nucleic acid binding transcription factor activity;protein binding;RNA binding;sequence-specific DNA binding transcription factor activity
adenyl nucleotide binding;adenyl ribonucleotide binding;ATP binding;ATPase activity;binding;catalytic activity;centromeric DNA binding;cytoskeletal protein binding;DNA binding;hydrolase activity;hydrolase activity, acting on
antioxidant activity;catalytic activity;electron carrier activity;oxidoreductase activity;oxidoreductase activity, acting on a sulfur group of donors;oxidoreductase activity, acting on a sulfur group of donors, NAD or NADP as acce
binding;protein binding;SNARE binding;syntaxin binding
ion transmembrane transporter activity;substrate-specific transmembrane transporter activity;substrate-specific transporter activity;transmembrane transporter activity;transporter activity
binding;cation binding;chromatin binding;DNA binding;ion binding;metal ion binding;nucleic acid binding
catalytic activity;kinase activity;transferase activity;transferase activity, transferring phosphorus-containing groups
adenyl nucleotide binding;adenyl ribonucleotide binding;ATP binding;binding;catalytic activity;CoA carboxylase activity;ligase activity;ligase activity, forming carbon-carbon bonds;methylcrotonoyl-CoA carboxylase activity;nu
binding;cation binding;enzyme activator activity;enzyme regulator activity;GTPase activator activity;GTPase regulator activity;ion binding;metal ion binding;nucleoside-triphosphatase regulator activity;protein transporter activ
adenyl nucleotide binding;adenyl ribonucleotide binding;aminoacyl-tRNA ligase activity;ATP binding;binding;catalytic activity;ligase activity;ligase activity, forming aminoacyl-tRNA and related compounds;ligase activity, formi
binding;identical protein binding;mRNA binding;nucleic acid binding;protein binding;RNA binding
basal RNA polymerase II transcription machinery binding;basal transcription machinery binding;binding;enzyme binding;protein binding;RNA polymerase binding;RNA polymerase core enzyme binding;RNA polymerase II core
binding;cation binding;double-stranded RNA binding;ion binding;metal ion binding;nucleic acid binding;RNA binding
binding;nucleotide binding
catalytic activity
binding;carboxylic acid binding;catalytic activity;coenzyme binding;cofactor binding;glycerate dehydrogenase activity;glyoxylate reductase (NADP) activity;hydroxypyruvate reductase activity;identical protein binding;NAD bin
catalytic activity;cysteine-type endopeptidase activity;cysteine-type peptidase activity;endopeptidase activity;hydrolase activity;peptidase activity;peptidase activity, acting on L-amino acid peptides
binding;mRNA binding;nucleic acid binding;nucleocytoplasmic transporter activity;nucleotide binding;RNA binding;transporter activity
adenyl nucleotide binding;adenyl ribonucleotide binding;ATP binding;binding;catalytic activity;GTP binding;guanyl nucleotide binding;guanyl ribonucleotide binding;kinase activity;nucleobase-containing compound kinase act
binding;cation binding;ion binding;metal ion binding
binding;cation binding;DEAD/H-box RNA helicase binding;DNA binding;enzyme binding;ion binding;metal ion binding;nucleic acid binding;protein binding
binding;enzyme binding;lipid binding;phosphatidylinositol binding;phospholipid binding;protein binding
active transmembrane transporter activity;anion transmembrane transporter activity;anion:cation symporter activity;binding;cation transmembrane transporter activity;cation:chloride symporter activity;chloride transmembran
binding;catalytic activity;cation binding;ion binding;ligase activity;metal ion binding;transition metal ion binding;zinc ion binding
2'-5'-oligoadenylate synthetase activity;adenyl nucleotide binding;adenyl ribonucleotide binding;adenylyltransferase activity;ATP binding;binding;catalytic activity;cation binding;double-stranded RNA binding;ion binding;meta
binding;lipid binding;phosphatidylinositol binding;phosphatidylinositol-3,5-bisphosphate binding;phosphatidylinositol-3-phosphate binding;phosphatidylinositol-4-phosphate binding;phosphatidylinositol-5-phosphate binding
binding;protein binding;SNARE binding;syntaxin binding
binding;cation binding;heme binding;ion binding;iron ion binding;lipid binding;metal ion binding;steroid binding;tetrapyrrole binding;transition metal ion binding
adenyl nucleotide binding;adenyl ribonucleotide binding;alkali metal ion binding;ATP binding;binding;catalytic activity;cation binding;cofactor binding;identical protein binding;ion binding;kinase activity;lithium ion binding;mag
structural constituent of cytoskeleton;structural molecule activity
alcohol binding;amine binding;binding;cation binding;ion binding;lipid binding;phosphatidylcholine binding;phospholipid binding;protein binding;protein domain specific binding;quaternary ammonium group binding
cation transmembrane transporter activity;hydrogen ion transmembrane transporter activity;inorganic cation transmembrane transporter activity;ion transmembrane transporter activity;monovalent inorganic cation transmemb
binding;DNA binding;mRNA binding;nucleic acid binding;RNA binding
binding;nucleic acid binding;nucleotide binding;RNA binding
amine binding;amino acid binding;binding;carboxylic acid binding;catalytic activity;dimethylargininase activity;hydrolase activity;hydrolase activity, acting on carbon-nitrogen (but not peptide) bonds;hydrolase activity, acting o
binding;cation binding;drug binding;ion binding;metal ion binding;transition metal ion binding;zinc ion binding
binding;carbohydrate binding;endopeptidase inhibitor activity;endopeptidase regulator activity;enzyme inhibitor activity;enzyme regulator activity;glycosaminoglycan binding;heparin binding;pattern binding;peptidase inhibitor
binding;cation binding;electron carrier activity;electron transporter, transferring electrons from CoQH2-cytochrome c reductase complex and cytochrome c oxidase complex activity;heme binding;ion binding;iron ion binding;
binding;catalytic activity;GDP binding;GTP binding;GTPase activity;guanyl nucleotide binding;guanyl ribonucleotide binding;hydrolase activity;hydrolase activity, acting on acid anhydrides;hydrolase activity, acting on acid anh
antigen binding;binding;peptide antigen binding;peptide binding
3-methyl-2-oxobutanoate dehydrogenase (2-methylpropanoyl-transferring) activity;alpha-ketoacid dehydrogenase activity;binding;carbon-carbon lyase activity;carboxy-lyase activity;catalytic activity;cation binding;ion binding
alcohol dehydrogenase (NADP+) activity;alditol:NADP+ 1-oxidoreductase activity;aldo-keto reductase (NADP) activity;catalytic activity;electron carrier activity;L-glucuronate reductase activity;oxidoreductase activity;oxidored
binding;damaged DNA binding;DNA binding;enzyme binding;nucleic acid binding;phosphatase binding;protein binding;protein phosphatase binding;single-stranded DNA binding;structure-specific DNA binding;ubiquitin prot
adenyl nucleotide binding;adenyl ribonucleotide binding;ATP binding;binding;cAMP-dependent protein kinase activity;catalytic activity;cyclic nucleotide-dependent protein kinase activity;enzyme binding;kinase activity;kinase
binding;carbohydrate binding;chemoattractant activity;extracellular matrix binding;IgE binding;immunoglobulin binding;laminin binding;protein binding;protein complex binding
binding;chromatin binding;chromatin DNA binding;DNA binding;enzyme regulator activity;GTPase regulator activity;guanyl-nucleotide exchange factor activity;histone binding;nucleic acid binding;nucleoside-triphosphatase r
binding;carbohydrate binding;carbohydrate transmembrane transporter activity;hexose transmembrane transporter activity;mannose binding;mannose transmembrane transporter activity;molecular transducer activity;monos
active transmembrane transporter activity;adenyl nucleotide binding;adenyl ribonucleotide binding;ATP binding;ATPase activity;ATPase activity, coupled;ATPase activity, coupled to movement of substances;ATPase activity, c
carboxyl-O-methyltransferase activity;catalytic activity;methyltransferase activity;O-methyltransferase activity;protein carboxyl O-methyltransferase activity;protein methyltransferase activity;protein-L-isoaspartate (D-aspartat
catalytic activity;ferredoxin-NADP+ reductase activity;NADPH-adrenodoxin reductase activity;oxidoreductase activity;oxidoreductase activity, acting on iron-sulfur proteins as donors;oxidoreductase activity, acting on iron-sul
binding;carbon-carbon lyase activity;carboxy-lyase activity;catalytic activity;cation binding;coenzyme binding;cofactor binding;electron carrier activity;ion binding;lyase activity;malate dehydrogenase (decarboxylating) activity

catalytic activity;endopeptidase activity;hydrolase activity;peptidase activity;peptidase activity, acting on L-amino acid peptides;threonine-type endopeptidase activity;threonine-type peptidase activity
catalytic activity;glycylpeptide N-tetradecanoyltransferase activity;myristoyltransferase activity;N-acyltransferase activity;transferase activity;transferase activity, transferring acyl groups;transferase activity, transferring acyl gr
binding;catalytic activity;identical protein binding;oxidoreductase activity;oxidoreductase activity, acting on the CH-NH group of donors;oxidoreductase activity, acting on the CH-NH group of donors, NAD or NADP as accept
binding;nucleic acid binding;nucleotide binding;RNA binding
active transmembrane transporter activity;adenyl nucleotide binding;adenyl ribonucleotide binding;ATP binding;ATPase activity;ATPase activity, coupled;ATPase activity, coupled to movement of substances;ATPase activity, c
acetyltransferase activity;catalytic activity;N-acetyltransferase activity;N-acyltransferase activity;transferase activity;transferase activity, transferring acyl groups;transferase activity, transferring acyl groups other than amino-ac
binding;catalytic activity;cation binding;endopeptidase activity;hormone binding;hydrolase activity;identical protein binding;ion binding;metal ion binding;metalloendopeptidase activity;metallopeptidase activity;peptidase acti
binding;nucleic acid binding;RNA binding;translation elongation factor activity;translation factor activity, nucleic acid binding
catalytic activity;deaminase activity;glucosamine-6-phosphate deaminase activity;hydrolase activity;intramolecular oxidoreductase activity;intramolecular oxidoreductase activity, interconverting aldoses and ketoses;isomeras
binding;chromatin binding;core promoter binding;core promoter sequence-specific DNA binding;DNA binding;nucleic acid binding;protein binding transcription factor activity;regulatory region DNA binding;regulatory region n
binding;cation transmembrane transporter activity;drug binding;hydrogen ion transmembrane transporter activity;hydrogen ion transporting ATP synthase activity, rotational mechanism;inorganic cation transmembrane transp
adenyl nucleotide binding;adenyl ribonucleotide binding;ATP binding;binding;catalytic activity;kinase activity;nucleotide binding;phosphotransferase activity, alcohol group as acceptor;protein kinase activity;protein serine/thre
7S RNA binding;binding;nucleic acid binding;ribonucleoprotein binding;RNA binding;signal recognition particle binding
binding;extracellular matrix binding;laminin binding;laminin receptor activity;molecular transducer activity;protein binding;protein C-terminus binding;receptor activity;signal transducer activity;signaling receptor activity;transm
acid-thiol ligase activity;ATP citrate synthase activity;binding;catalytic activity;CoA-ligase activity;cofactor binding;GDP binding;GTP binding;guanyl nucleotide binding;guanyl ribonucleotide binding;ligase activity;ligase activit
active transmembrane transporter activity;ATPase activator activity;ATPase activity;ATPase activity, coupled;ATPase activity, coupled to movement of substances;ATPase activity, coupled to transmembrane movement of ions
binding;identical protein binding;protein binding
binding;cation binding;ion binding;metal ion binding;structural constituent of ribosome;structural molecule activity
binding;catalytic activity;GTP binding;GTPase activity;guanyl nucleotide binding;guanyl ribonucleotide binding;hydrolase activity;hydrolase activity, acting on acid anhydrides;hydrolase activity, acting on acid anhydrides, in pl
binding;mRNA 5'-UTR binding;mRNA binding;nucleic acid binding;RNA binding;structural constituent of ribosome;structural molecule activity;translation regulator activity
binding;cation binding;ion binding;metal ion binding;structural constituent of ribosome;structural molecule activity;transition metal ion binding;zinc ion binding
binding;nucleic acid binding;nucleotide binding;RNA binding;rRNA binding;structural constituent of ribosome;structural molecule activity
structural constituent of ribosome;structural molecule activity
binding;calcium ion binding;catalytic activity;cation binding;hydrolase activity;hydrolase activity, acting on ester bonds;identical protein binding;ion binding;magnesium ion binding;metal ion binding;phosphatase activity;phos
structural constituent of ribosome;structural molecule activity
structural constituent of ribosome;structural molecule activity
endopeptidase inhibitor activity;endopeptidase regulator activity;enzyme inhibitor activity;enzyme regulator activity;peptidase inhibitor activity;peptidase regulator activity;serine-type endopeptidase inhibitor activity
adenyl nucleotide binding;adenyl ribonucleotide binding;ATP binding;binding;nucleotide binding;purine nucleotide binding;purine ribonucleoside triphosphate binding;purine ribonucleotide binding;ribonucleotide binding
binding;catalytic activity;enzyme binding;NEDD8 activating enzyme activity;protein binding;protein dimerization activity;protein heterodimerization activity;small protein activating enzyme activity;ubiquitin protein ligase binding
binding;enzyme binding;protein binding;receptor activity
binding;GTP binding;guanyl nucleotide binding;guanyl ribonucleotide binding;nucleotide binding;purine nucleotide binding;purine ribonucleoside triphosphate binding;purine ribonucleotide binding;ribonucleotide binding
binding;DNA binding;nucleic acid binding
acyl-CoA dehydrogenase activity;acyl-CoA oxidase activity;binding;carboxylic acid binding;catalytic activity;coenzyme binding;cofactor binding;FAD binding;fatty acid binding;flavin adenine dinucleotide binding;lipid binding;
adenyl nucleotide binding;adenyl ribonucleotide binding;ATP binding;binding;catalytic activity;kinase activity;nucleotide binding;phosphomevalonate kinase activity;phosphotransferase activity, phosphate group as acceptor;p
binding;carbonate dehydratase activity;carbon-oxygen lyase activity;catalytic activity;cation binding;hydro-lyase activity;ion binding;lyase activity;metal ion binding;transition metal ion binding;zinc ion binding
basal RNA polymerase II transcription machinery binding;basal transcription machinery binding;binding;enzyme binding;protein binding;RNA polymerase binding;RNA polymerase core enzyme binding;RNA polymerase II core
catalytic activity;oxidoreductase activity
binding;catalytic activity;cation binding;hydrolase activity;ion binding;metal ion binding;metallopeptidase activity;peptidase activity;peptidase activity, acting on L-amino acid peptides
acid-amino acid ligase activity;binding;catalytic activity;cation binding;ion binding;ligase activity;ligase activity, forming carbon-nitrogen bonds;metal ion binding;small conjugating protein ligase activity;transition metal ion bin
catalytic activity;methyltransferase activity;RNA methyltransferase activity;rRNA methyltransferase activity;S-adenosylmethionine-dependent methyltransferase activity;transferase activity;transferase activity, transferring one-
adenyl nucleotide binding;adenyl ribonucleotide binding;adenylyltransferase activity;ATP binding;binding;catalytic activity;FMN adenylyltransferase activity;nucleotide binding;nucleotidyltransferase activity;purine nucleotide b
acid-amino acid ligase activity;adenyl nucleotide binding;adenyl ribonucleotide binding;ATP binding;binding;catalytic activity;ligase activity;ligase activity, forming carbon-nitrogen bonds;NEDD8 activating enzyme activity;nuc
binding;cation binding;ion binding;metal ion binding
adenyl nucleotide binding;adenyl ribonucleotide binding;ATP binding;ATPase activity;ATPase activity, coupled;ATP-dependent helicase activity;ATP-dependent RNA helicase activity;binding;catalytic activity;helicase activity;h
catalytic activity;hydrolase activity
binding;calcium ion binding;cation binding;identical protein binding;ion binding;metal ion binding;protein binding;protein dimerization activity;protein homodimerization activity
binding;catalytic activity;cysteine-type peptidase activity;enzyme binding;hydrolase activity;NEDD8-specific protease activity;peptidase activity;peptidase activity, acting on L-amino acid peptides;protein binding;small conjug
binding;ErbB-2 class receptor binding;integrin binding;protein binding;protein complex binding;receptor binding;structural constituent of cytoskeleton;structural molecule activity
binding;neurotransmitter binding
beta-tubulin binding;binding;chaperone binding;cytoskeletal protein binding;enzyme activator activity;enzyme regulator activity;GTPase activator activity;GTPase regulator activity;nucleoside-triphosphatase regulator activity;
binding;ribonucleoprotein binding
binding;nucleotide binding
binding;identical protein binding;protein binding;protein dimerization activity;protein homodimerization activity
catalytic activity;fucosyltransferase activity;peptide-O-fucosyltransferase activity;transferase activity;transferase activity, transferring glycosyl groups;transferase activity, transferring hexosyl groups
adenyl nucleotide binding;adenyl ribonucleotide binding;ATP binding;binding;catalytic activity;channel inhibitor activity;channel regulator activity;chloride channel inhibitor activity;chloride channel regulator activity;enzyme bin
adenyl nucleotide binding;adenyl ribonucleotide binding;ATP binding;ATPase activity;binding;catalytic activity;cation binding;hydrolase activity;hydrolase activity, acting on acid anhydrides;hydrolase activity, acting on acid an
binding;protein binding;protein dimerization activity;protein heterodimerization activity
enzyme inhibitor activity;enzyme regulator activity
binding;enzyme binding;enzyme regulator activity;methionine adenosyltransferase regulator activity;protein binding
binding;GTP binding;guanyl nucleotide binding;guanyl ribonucleotide binding;nucleotide binding;purine nucleotide binding;purine ribonucleoside triphosphate binding;purine ribonucleotide binding;ribonucleotide binding
binding;nucleic acid binding;protein binding;RNA binding;translation factor activity, nucleic acid binding;translation initiation factor activity;translation initiation factor binding
binding;enzyme binding;GTPase binding;protein binding;receptor binding
adenyl nucleotide binding;adenyl ribonucleotide binding;ATP binding;binding;enzyme binding;GTP binding;guanyl nucleotide binding;guanyl ribonucleotide binding;kinase binding;nucleotide binding;protein binding;protein kin
binding;cation binding;ion binding;metal ion binding;transition metal ion binding;zinc ion binding
binding;binding, bridging;cytoskeletal adaptor activity;cytoskeletal protein binding;identical protein binding;proline-rich region binding;protein binding;protein binding, bridging;protein C-terminus binding
adenyl nucleotide binding;adenyl ribonucleotide binding;amine binding;amino acid binding;aminoacyl-tRNA ligase activity;ATP binding;binding;carboxylic acid binding;catalytic activity;identical protein binding;ligase activity;li
binding;catalytic activity;cysteine-type peptidase activity;endopeptidase inhibitor activity;endopeptidase regulator activity;enzyme inhibitor activity;enzyme regulator activity;exopeptidase activity;hydrolase activity;omega pep
actin binding;binding;cytoskeletal protein binding;protein binding;protein complex binding
binding;cholesterol binding;hedgehog receptor activity;lipid binding;lipid transporter activity;molecular transducer activity;receptor activity;signal transducer activity;signaling receptor activity;steroid binding;sterol binding;ster
structural constituent of cytoskeleton;structural molecule activity
amine binding;amino acid binding;binding;carboxylic acid binding;catalytic activity;cation binding;glutathione binding;G-protein coupled receptor activity;ion binding;lipoprotein particle receptor binding;low-density lipoprotein
alpha(1,2)-fucosyltransferase activity;binding;catalytic activity;cytoskeletal protein binding;fucosyltransferase activity;galactoside 2-alpha-L-fucosyltransferase activity;microtubule binding;microtubule plus-end binding;protein
4 iron, 4 sulfur cluster binding;binding;catalytic activity;cation binding;coenzyme binding;cofactor binding;electron carrier activity;enzyme binding;ion binding;iron-sulfur cluster binding;metal cluster binding;metal ion binding;
protein transporter activity;substrate-specific transporter activity;transporter activity
adenyl nucleotide binding;adenyl ribonucleotide binding;ATP binding;ATPase activity;binding;catalytic activity;DNA binding;hydrolase activity;hydrolase activity, acting on acid anhydrides;hydrolase activity, acting on acid anhy
adenyl nucleotide binding;adenyl ribonucleotide binding;ADP binding;AMP binding;binding;catalytic activity;coenzyme binding;cofactor binding;cytochrome-b5 reductase activity;FAD binding;flavin adenine dinucleotide bindi
binding;DNA binding;nucleic acid binding
binding;calcium ion binding;calcium-dependent protein binding;cation binding;cytoskeletal protein binding;identical protein binding;ion binding;ion transmembrane transporter activity;metal ion binding;protein binding;protein
3'-5' exonuclease activity;4 iron, 4 sulfur cluster binding;binding;catalytic activity;cation binding;chromatin binding;DNA binding;DNA polymerase activity;DNA-directed DNA polymerase activity;enzyme binding;exonuclease a
binding;DNA binding;enzyme binding;nucleic acid binding;protein binding
catalytic activity;cation transmembrane transporter activity;cytochrome-c oxidase activity;heme-copper terminal oxidase activity;hydrogen ion transmembrane transporter activity;inorganic cation transmembrane transporter a
binding;DNA binding;double-stranded DNA binding;enzyme binding;GTPase binding;mRNA 3'-UTR binding;mRNA binding;nucleic acid binding;nucleic acid binding transcription factor activity;protein binding;protein binding
catalytic activity;endopeptidase activity;hydrolase activity;peptidase activity;peptidase activity, acting on L-amino acid peptides;threonine-type endopeptidase activity;threonine-type peptidase activity
3-mercaptopyruvate sulfurtransferase activity;catalytic activity;sulfurtransferase activity;thiosulfate sulfurtransferase activity;transferase activity;transferase activity, transferring sulfur-containing groups
active transmembrane transporter activity;adenyl nucleotide binding;adenyl ribonucleotide binding;ATP binding;ATPase activity;ATPase activity, coupled;ATPase activity, coupled to movement of substances;ATPase activity, c
alkali metal ion binding;binding;catalytic activity;cation binding;hydrolase activity;hydrolase activity, acting on ester bonds;identical protein binding;inositol monophosphate phosphatase activity;inositol or phosphatidylinositol
binding;chaperone binding;identical protein binding;protein binding;protein dimerization activity;protein homodimerization activity
binding;catalytic activity;cis-trans isomerase activity;cyclosporin A binding;drug binding;isomerase activity;peptide binding;peptidyl-prolyl cis-trans isomerase activity
binding;carbohydrate binding;monosaccharide binding;sialic acid binding;sugar binding
binding;catalytic activity;DNA binding;enzyme activator activity;enzyme regulator activity;nucleic acid binding;phosphatase activator activity;phosphatase regulator activity;protein binding;protein binding transcription factor a
binding;carbohydrate binding;mannose binding;monosaccharide binding;sugar binding
binding;catalytic activity;cation binding;enzyme binding;hydrolase activity;hydrolase activity, acting on ester bonds;ion binding;kinase binding;metal ion binding;phosphatase activity;phosphoprotein phosphatase activity;phos
binding;binding, bridging;chromatin binding;histone binding;methylated histone residue binding;protein binding;protein binding, bridging;repressing transcription factor binding;transcription factor binding
binding;nucleic acid binding;RNA binding;structural constituent of ribosome;structural molecule activity
binding;nucleic acid binding;RNA binding;translation factor activity, nucleic acid binding;translation initiation factor activity
adenyl nucleotide binding;adenyl ribonucleotide binding;ATP binding;binding;GTP binding;guanyl nucleotide binding;guanyl ribonucleotide binding;nucleic acid binding;nucleotide binding;purine nucleotide binding;purine ribo
5'-nucleotidase activity;binding;catalytic activity;cation binding;hydrolase activity;hydrolase activity, acting on ester bonds;ion binding;kinase activity;metal ion binding;nucleobase-containing compound kinase activity;nucleo
adenosine kinase activity;adenyl nucleotide binding;adenyl ribonucleotide binding;ATP binding;binding;catalytic activity;cation binding;ion binding;kinase activity;metal ion binding;nucleobase-containing compound kinase ac
structural molecule activity
apolipoprotein A-I receptor binding;apolipoprotein receptor binding;binding;catalytic activity;enzyme binding;GTP binding;GTPase activity;guanyl nucleotide binding;guanyl ribonucleotide binding;hydrolase activity;hydrolase
binding;calcium ion binding;cation binding;DNA binding;ion binding;metal ion binding;nucleic acid binding
structural constituent of ribosome;structural molecule activity
binding;nucleic acid binding;nucleotide binding;RNA binding
binding;nucleotide binding;protein binding transcription factor activity;transcription cofactor activity;transcription corepressor activity;transcription factor binding transcription factor activity
adenyl nucleotide binding;adenyl ribonucleotide binding;ATP binding;ATPase activity;binding;catalytic activity;cytoskeletal protein binding;hydrolase activity;hydrolase activity, acting on acid anhydrides;hydrolase activity, acti
ATPase binding;binding;enzyme binding;K6-linked polyubiquitin binding;polyubiquitin binding;protein binding;small conjugating protein binding;ubiquitin binding;ubiquitin protein ligase binding
binding;carbon-sulfur lyase activity;catalytic activity;cation binding;ion binding;lactoylglutathione lyase activity;lyase activity;metal ion binding;transition metal ion binding;zinc ion binding
transporter activity
binding;enzyme regulator activity;GTPase regulator activity;guanyl-nucleotide exchange factor activity;nucleic acid binding;nucleoside-triphosphatase regulator activity;protein binding;RNA binding;translation factor activity, n
binding;enzyme binding;identical protein binding;protein binding;protein domain specific binding
1-phosphatidylinositol binding;binding;binding, bridging;clathrin adaptor activity;clathrin binding;clathrin heavy chain binding;lipid binding;phosphatidylinositol binding;phospholipid binding;protein binding;protein binding, bri
binding;epidermal growth factor receptor binding;growth factor receptor binding;identical protein binding;insulin receptor binding;lipid binding;phosphatidylinositol binding;phospholipid binding;protein binding;protein comple
binding;protein binding;receptor binding
binding;cytokine activity;protein binding;receptor binding
binding;FHA domain binding;protein binding;protein C-terminus binding;protein domain specific binding

adenyl nucleotide binding;adenyl ribonucleotide binding;ATP binding;binding;nucleotide binding;purine nucleotide binding;purine ribonucleoside triphosphate binding;purine ribonucleotide binding;ribonucleotide binding
binding;catalytic activity;cysteine-type endopeptidase activity;cysteine-type peptidase activity;endopeptidase activity;hydrolase activity;ion channel binding;p53 binding;peptidase activity;peptidase activity, acting on L-amino
15-oxoprostaglandin 13-oxidase activity;2-alkenal reductase [NAD(P)] activity;binding;catalytic activity;cation binding;ion binding;metal ion binding;oxidoreductase activity;oxidoreductase activity, acting on the CH-CH group
1-alkyl-2-acetylglycerophosphocholine esterase activity;acetyltransferase activity;binding;carboxylic ester hydrolase activity;catalytic activity;hydrolase activity;hydrolase activity, acting on ester bonds;identical protein binding
binding;calcium ion binding;cation binding;ion binding;metal ion binding
binding;heat shock protein binding;protein binding
binding;cation binding;ion binding;metal ion binding;transition metal ion binding;zinc ion binding
adenyl nucleotide binding;adenyl ribonucleotide binding;alanine-tRNA ligase activity;aminoacyl-tRNA ligase activity;ATP binding;binding;catalytic activity;cation binding;ion binding;ligase activity;ligase activity, forming amino
3'-5' exonuclease activity;3'-5'-exoribonuclease activity;binding;catalytic activity;cation binding;exonuclease activity;exonuclease activity, active with either ribo- or deoxyribonucleic acids and producing 5'-phosphomonoeste
basal RNA polymerase II transcription machinery binding;basal transcription machinery binding;binding;enzyme binding;protein binding;RNA polymerase binding;RNA polymerase core enzyme binding;RNA polymerase II core
binding;core promoter proximal region DNA binding;core promoter proximal region sequence-specific DNA binding;DNA binding;molecular transducer activity;nucleic acid binding;nucleic acid binding transcription factor activ
catalytic activity;methyltransferase activity;transferase activity;transferase activity, transferring one-carbon groups
binding;nucleic acid binding;RNA binding;translation factor activity, nucleic acid binding;translation initiation factor activity
binding;protein binding;protein domain specific binding
binding;nucleic acid binding;RNA binding
binding;cation binding;ion binding;metal ion binding
arginine N-methyltransferase activity;beta-catenin binding;binding;catalytic activity;DNA binding;histone acetyl-lysine binding;histone binding;histone methyltransferase activity;histone methyltransferase activity (H3-R17 spec
binding;DNA binding;nucleic acid binding
binding;cytoskeletal protein binding;enzyme activator activity;enzyme regulator activity;kinase activator activity;kinase regulator activity;protein binding;protein complex binding;protein kinase activator activity;protein kinase r
binding;cation binding;enzyme activator activity;enzyme regulator activity;GTPase activator activity;GTPase regulator activity;ion binding;metal ion binding;nucleoside-triphosphatase regulator activity;transition metal ion bind
catalytic activity;hydrolase activity
binding;protein binding;SNARE binding;syntaxin binding
binding;enzyme binding;protein binding
catalytic activity;oxidoreductase activity;oxidoreductase activity, acting on the CH-NH group of donors;oxidoreductase activity, acting on the CH-NH group of donors, NAD or NADP as acceptor;pyrroline-5-carboxylate reduct
binding;catalytic activity;enzyme activator activity;enzyme regulator activity;GTPase activator activity;GTPase regulator activity;hydrolase activity;hydrolase activity, acting on ester bonds;molecular transducer activity;nucleos
binding;ribonucleoprotein binding;ribosome binding
binding;chromatin binding
adenyl nucleotide binding;adenyl ribonucleotide binding;ATP binding;ATPase activity;ATPase activity, coupled;ATP-dependent helicase activity;ATP-dependent RNA helicase activity;binding;catalytic activity;helicase activity;h
binding;nucleic acid binding;RNA binding;snoRNA binding
binding;catalytic activity;cation binding;hydrolase activity;identical protein binding;ion binding;metal ion binding;metallopeptidase activity;peptidase activity;peptidase activity, acting on L-amino acid peptides;protein binding;
binding;enzyme binding;protein binding
active transmembrane transporter activity;anion transmembrane transporter activity;ATPase activity;ATPase activity, coupled;ATPase activity, coupled to movement of substances;ATPase activity, coupled to transmembrane m
binding;identical protein binding;lipid binding;protein binding;protein dimerization activity;protein homodimerization activity
binding;catalytic activity;GTP binding;GTPase activity;guanyl nucleotide binding;guanyl ribonucleotide binding;hydrolase activity;hydrolase activity, acting on acid anhydrides;hydrolase activity, acting on acid anhydrides, in ph
binding;cytoskeletal protein binding;enzyme binding;identical protein binding;protein binding;RNA polymerase binding;tau protein binding
acetyltransferase activity;catalytic activity;H4 histone acetyltransferase activity;histone acetyltransferase activity;lysine N-acetyltransferase activity;N-acetyltransferase activity;N-acyltransferase activity;transferase activity;tran
adenyl nucleotide binding;adenyl ribonucleotide binding;adenylylsulfate kinase activity;adenylyltransferase activity;ATP binding;binding;catalytic activity;kinase activity;nucleotide binding;nucleotidyltransferase activity;phosph
binding;nucleic acid binding;RNA binding;snoRNA binding
binding;calcium ion binding;cation binding;integrin binding;ion binding;metal ion binding;protein binding;protein complex binding;receptor binding
binding;carbon-oxygen lyase activity;catalytic activity;coenzyme binding;cofactor binding;GDP-mannose 4,6-dehydratase activity;hydro-lyase activity;lyase activity;NADP binding;NADP+ binding;nucleotide binding
binding;DNA binding;nucleic acid binding;protein binding transcription factor activity;sequence-specific DNA binding;transcription coactivator activity;transcription cofactor activity;transcription factor binding transcription fac
adenyl nucleotide binding;adenyl ribonucleotide binding;ATP binding;binding;cation binding;chaperone binding;ion binding;metal ion binding;nucleotide binding;protein binding;purine nucleotide binding;purine ribonucleoside
binding;cation binding;ion binding;metal ion binding;protein binding;transition metal ion binding;translation initiation factor binding;zinc ion binding
binding;cation binding;ion binding;metal ion binding;transition metal ion binding;zinc ion binding
beta-tubulin binding;binding;cytoskeletal protein binding;enzyme binding;FFAT motif binding;identical protein binding;microtubule binding;protein binding;protein dimerization activity;protein domain specific binding;protein he
adenyl nucleotide binding;adenyl ribonucleotide binding;ATP binding;binding;catalytic activity;kinase activity;NADH dehydrogenase (quinone) activity;NADH dehydrogenase (ubiquinone) activity;NADH dehydrogenase activity;
antioxidant activity;binding;catalytic activity;coenzyme binding;cofactor binding;disulfide oxidoreductase activity;electron carrier activity;flavin adenine dinucleotide binding;glutathione disulfide oxidoreductase activity;glutathi
antioxidant activity;binding;catalytic activity;cation binding;chaperone binding;copper ion binding;enzyme binding;GTPase binding;identical protein binding;ion binding;metal ion binding;oxidoreductase activity;oxidoreductas
aldehyde dehydrogenase (NAD) activity;aldehyde dehydrogenase [NAD(P)+] activity;catalytic activity;electron carrier activity;oxidoreductase activity;oxidoreductase activity, acting on the aldehyde or oxo group of donors;oxid
binding;GTP binding;guanyl nucleotide binding;guanyl ribonucleotide binding;molecular transducer activity;nucleotide binding;purine nucleotide binding;purine ribonucleoside triphosphate binding;purine ribonucleotide bindin
binding;catalytic activity;dinitrosyl-iron complex binding;enzyme binding;enzyme regulator activity;glutathione transferase activity;JUN kinase binding;kinase binding;kinase regulator activity;nitric oxide binding;protein binding
binding;clathrin binding;clathrin heavy chain binding;peptide binding;protein binding;structural molecule activity
alpha-glucosidase activity;binding;carbohydrate binding;catalytic activity;glucosidase activity;hydrolase activity;hydrolase activity, acting on glycosyl bonds;hydrolase activity, hydrolyzing O-glycosyl compounds;maltose alph
acetyltransferase activity;catalytic activity;dihydrolipoamide S-acyltransferase activity;dihydrolipoyllysine-residue acetyltransferase activity;S-acetyltransferase activity;S-acyltransferase activity;transferase activity;transferase
binding;catalytic activity;identical protein binding;protein binding;protein dimerization activity;protein homodimerization activity;spermidine synthase activity;transferase activity;transferase activity, transferring alkyl or aryl (oth
endopeptidase inhibitor activity;endopeptidase regulator activity;enzyme inhibitor activity;enzyme regulator activity;peptidase inhibitor activity;peptidase regulator activity;serine-type endopeptidase inhibitor activity
binding;nucleic acid binding;RNA binding;translation elongation factor activity;translation factor activity, nucleic acid binding
binding;enzyme binding;nucleotide binding;protein binding
binding;calcium ion binding;cation binding;identical protein binding;ion binding;metal ion binding;protein binding
adenyl nucleotide binding;adenyl ribonucleotide binding;ATP binding;binding;endopeptidase inhibitor activity;endopeptidase regulator activity;enzyme binding;enzyme inhibitor activity;enzyme regulator activity;kinase binding
binding;catalytic activity;cation binding;ion binding;metal ion binding;oxidoreductase activity;oxidoreductase activity, acting on CH or CH2 groups;oxidoreductase activity, acting on CH or CH2 groups, disulfide as acceptor;ri
binding;calcium ion binding;calcium-dependent protein binding;cation binding;identical protein binding;ion binding;metal ion binding;protein binding;protein dimerization activity;protein homodimerization activity
adenyl nucleotide binding;adenyl ribonucleotide binding;ATP binding;binding;DNA binding;nucleic acid binding;nucleotide binding;purine nucleotide binding;purine ribonucleoside triphosphate binding;purine ribonucleotide bi
adenyl nucleotide binding;adenyl ribonucleotide binding;ATP binding;ATPase activity;ATPase activity, coupled;binding;catalytic activity;DNA binding;DNA clamp loader activity;DNA-dependent ATPase activity;double-strande
ATPase activity;ATPase activity, coupled;binding;catalytic activity;DNA binding;DNA clamp loader activity;DNA-dependent ATPase activity;hydrolase activity;hydrolase activity, acting on acid anhydrides;hydrolase activity, acti
binding;enzyme activator activity;enzyme binding;enzyme regulator activity;GDP-dissociation inhibitor activity;GTPase activator activity;GTPase binding;GTPase regulator activity;nucleoside-triphosphatase regulator activity;
binding;enzyme binding;polyubiquitin binding;proteasome binding;protein binding;protein complex binding;ribonucleoprotein binding;ribosome binding;small conjugating protein binding;ubiquitin binding;ubiquitin protein ligas
4 iron, 4 sulfur cluster binding;binding;cation binding;ion binding;iron-responsive element binding;iron-sulfur cluster binding;metal cluster binding;metal ion binding;mRNA binding;nucleic acid binding;RNA binding;translation
adenyl nucleotide binding;adenyl ribonucleotide binding;aminoacyl-tRNA ligase activity;ATP binding;binding;catalytic activity;histidine-tRNA ligase activity;ligase activity;ligase activity, forming aminoacyl-tRNA and related co
catalytic activity;endopeptidase activity;hydrolase activity;peptidase activity;peptidase activity, acting on L-amino acid peptides;threonine-type endopeptidase activity;threonine-type peptidase activity
3'-5' exonuclease activity;binding;catalytic activity;cation binding;deoxyribonuclease activity;DNA binding;double-stranded DNA binding;endodeoxyribonuclease activity;endonuclease activity;exonuclease activity;hydrolase a
binding;catalytic activity;cation binding;hydrolase activity;hydrolase activity, acting on ester bonds;identical protein binding;ion binding;lipid binding;metal ion binding;molecular transducer activity;nucleic acid binding;phosph
molecular transducer activity;protein binding transcription factor activity;signal transducer activity;transcription cofactor activity;transcription corepressor activity;transcription factor binding transcription factor activity
structural constituent of ribosome;structural molecule activity
binding;nucleic acid binding;RNA binding
binding;catalytic activity;cis-trans isomerase activity;cyclosporin A binding;drug binding;estrogen receptor binding;heat shock protein binding;hormone receptor binding;Hsp70 protein binding;Hsp90 protein binding;isomeras
binding;DNA binding;nucleic acid binding
antioxidant activity;catalytic activity;oxidoreductase activity;oxidoreductase activity, acting on paired donors, with incorporation or reduction of molecular oxygen;oxidoreductase activity, acting on peroxide as acceptor;perox
binding;catalytic activity;cis-trans isomerase activity;cyclosporin A binding;drug binding;isomerase activity;peptide binding;peptidyl-prolyl cis-trans isomerase activity
binding;catalytic activity;coenzyme binding;cofactor binding;electron carrier activity;GDP-4-dehydro-D-rhamnose reductase activity;GDP-L-fucose synthase activity;isomerase activity;oxidoreductase activity;oxidoreductase a
binding;catalytic activity;GTP binding;GTPase activity;guanyl nucleotide binding;guanyl ribonucleotide binding;hydrolase activity;hydrolase activity, acting on acid anhydrides;hydrolase activity, acting on acid anhydrides, in ph
binding;nucleotide binding
binding;cation binding;DNA binding;double-stranded DNA binding;ion binding;metal ion binding;nucleic acid binding;nucleotide binding;RNA binding;structure-specific DNA binding;transition metal ion binding;zinc ion bindin
binding;catalytic activity;GDP binding;GTP binding;GTPase activity;guanyl nucleotide binding;guanyl ribonucleotide binding;hydrolase activity;hydrolase activity, acting on acid anhydrides;hydrolase activity, acting on acid anh
structural constituent of cytoskeleton;structural molecule activity
adenyl nucleotide binding;adenyl ribonucleotide binding;androgen receptor binding;ATP binding;binding;catalytic activity;chromatin binding;enzyme binding;GTPase binding;GTP-Rho binding;histone binding;histone deacetyl
binding;lipid binding
binding;enzyme binding;enzyme regulator activity;phosphatase binding;phosphatase regulator activity;protein binding;protein phosphatase 2A binding;protein phosphatase binding;protein phosphatase regulator activity;prote
binding;cation binding;enzyme activator activity;enzyme regulator activity;GTPase activator activity;GTPase regulator activity;ion binding;metal ion binding;nucleoside-triphosphatase regulator activity;transition metal ion bind
binding;chromatin binding
binding;ribonucleoprotein binding;snRNP binding
binding;catalytic activity;GDP binding;GTP binding;GTPase activity;guanyl nucleotide binding;guanyl ribonucleotide binding;hydrolase activity;hydrolase activity, acting on acid anhydrides;hydrolase activity, acting on acid anh
catalytic activity;kinase activity;phosphotransferase activity, alcohol group as acceptor;protein kinase activity;transferase activity;transferase activity, transferring phosphorus-containing groups
binding;enzyme binding;enzyme inhibitor activity;enzyme regulator activity;lipase binding;lipase inhibitor activity;protein binding;small conjugating protein binding;ubiquitin binding;ubiquitin protein ligase binding
binding;catalytic activity;cation binding;coenzyme binding;cofactor binding;flavin adenine dinucleotide binding;ion binding;metal ion binding;oxidoreductase activity;oxidoreductase activity, acting on the CH-CH group of dono
adenyl nucleotide binding;adenyl ribonucleotide binding;ATP binding;ATPase activity;ATPase activity, coupled;ATP-dependent helicase activity;ATP-dependent RNA helicase activity;binding;catalytic activity;helicase activity;h
binding;nucleic acid binding;RNA binding
binding;catalytic activity;enzyme binding;intramolecular transferase activity;isomerase activity;protein binding;pseudouridine synthase activity
binding;epidermal growth factor receptor binding;growth factor receptor binding;nucleic acid binding;protein binding;protein C-terminus binding;receptor binding;RNA binding
5-methyltetrahydrofolate-dependent methyltransferase activity;binding;catalytic activity;cation binding;cobalamin binding;homocysteine S-methyltransferase activity;ion binding;metal ion binding;methionine synthase activity;
3'-tRNA processing endoribonuclease activity;binding;catalytic activity;cation binding;endonuclease activity;endonuclease activity, active with either ribo- or deoxyribonucleic acids and producing 5'-phosphomonoesters;end
binding;catalytic activity;GTP binding;GTPase activity;guanyl nucleotide binding;guanyl ribonucleotide binding;hydrolase activity;hydrolase activity, acting on acid anhydrides;hydrolase activity, acting on acid anhydrides, in ph
structural molecule activity
acid-amino acid ligase activity;adenyl nucleotide binding;adenyl ribonucleotide binding;ATP binding;binding;catalytic activity;enzyme binding;ligase activity;ligase activity, forming carbon-nitrogen bonds;nucleotide binding;pro
adenyl nucleotide binding;adenyl-nucleotide exchange factor activity;ATPase regulator activity;binding;enzyme regulator activity;nucleoside-triphosphatase regulator activity;nucleotide binding;protein binding;purine nucleotic
adenyl nucleotide binding;adenyl ribonucleotide binding;ATP binding;ATPase activity;binding;catalytic activity;hydrolase activity;hydrolase activity, acting on acid anhydrides;hydrolase activity, acting on acid anhydrides, in ph
binding;catalytic activity;cation binding;ion binding;metal ion binding;methyltransferase activity;N-methyltransferase activity;nucleic acid binding;RNA binding;RNA methyltransferase activity;S-adenosylmethionine-dependent

adenyl nucleotide binding;adenyl ribonucleotide binding;ATP binding;binding;catalytic activity;cation binding;ion binding;kinase activity;magnesium ion binding;MAP kinase kinase kinase activity;metal ion binding;molecular tr
aminopeptidase activity;binding;catalytic activity;cation binding;cytokine receptor binding;exopeptidase activity;growth factor receptor binding;hydrolase activity;interleukin-1 receptor binding;interleukin-1, Type II receptor bir
binding;protein binding;protein domain specific binding
binding;catalytic activity;cation binding;deaminase activity;guanine deaminase activity;hydrolase activity;hydrolase activity, acting on carbon-nitrogen (but not peptide) bonds;hydrolase activity, acting on carbon-nitrogen (but
binding;chromatin binding;histone binding;nucleosome binding;protein binding;protein binding transcription factor activity;repressing transcription factor binding;transcription cofactor activity;transcription corepressor activity
catalytic activity;kinase activity;phosphotransferase activity, alcohol group as acceptor;protein binding transcription factor activity;transcription coactivator activity;transcription cofactor activity;transcription factor binding trar
binding;cytoskeletal protein binding;dynactin binding;lipid binding;phosphatidylinositol binding;phospholipid binding;protein binding
carbon-oxygen lyase activity;catalytic activity;hydro-lyase activity;intramolecular transferase activity;isomerase activity;ligand-dependent nuclear receptor transcription coactivator activity;lyase activity;protein binding transcri
binding;cation binding;ion binding;metal ion binding
binding;cation binding;ion binding;metal ion binding;protein binding;protein domain specific binding
actin binding;binding;cytoskeletal protein binding;lipid binding;phospholipid binding;protein binding;structural constituent of cytoskeleton;structural molecule activity
binding;identical protein binding;protein binding;protein dimerization activity;protein heterodimerization activity;protein homodimerization activity
binding;catalytic activity;cation binding;diphosphotransferase activity;enzyme inhibitor activity;enzyme regulator activity;ion binding;magnesium ion binding;metal ion binding;ribose phosphate diphosphokinase activity;transfe
binding;binding, bridging;cytoskeletal protein binding;enzyme binding;JUN kinase binding;kinase binding;kinesin binding;MAP-kinase scaffold activity;protein binding;protein complex scaffold;protein kinase binding;receptor s
adenyl nucleotide binding;adenyl ribonucleotide binding;ATP binding;binding;catalytic activity;enzyme binding;GTPase binding;kinase activity;nucleotide binding;phosphotransferase activity, alcohol group as acceptor;protein
adenyl nucleotide binding;adenyl ribonucleotide binding;AMP binding;binding;cAMP binding;cAMP-dependent protein kinase inhibitor activity;cAMP-dependent protein kinase regulator activity;cyclic nucleotide binding;enzyr
catalytic activity;oxidoreductase activity;oxidoreductase activity, acting on the aldehyde or oxo group of donors;oxidoreductase activity, acting on the aldehyde or oxo group of donors, disulfide as acceptor;pyruvate dehydro
binding;calcium ion binding;cation binding;ion binding;metal ion binding
binding;damaged DNA binding;DNA binding;enzyme binding;histone binding;nucleic acid binding;protein binding
active transmembrane transporter activity;ATPase activity;ATPase activity, coupled;ATPase activity, coupled to movement of substances;ATPase activity, coupled to transmembrane movement of ions;ATPase activity, couplec
binding;catalytic activity;methyltransferase activity;nucleic acid binding;RNA binding;RNA methyltransferase activity;rRNA methyltransferase activity;S-adenosylmethionine-dependent methyltransferase activity;transferase ac
adenyl nucleotide binding;adenyl ribonucleotide binding;ATP binding;ATPase activity;ATPase activity, coupled;ATP-dependent helicase activity;ATP-dependent RNA helicase activity;binding;catalytic activity;helicase activity;h
binding;growth factor activity;protein binding;receptor binding
carbon-oxygen lyase activity;catalytic activity;enoyl-CoA hydratase activity;hydro-lyase activity;lyase activity
actin binding;binding;catalytic activity;cytokine binding;cytoskeletal protein binding;enzyme binding;GTP binding;GTPase activity;guanyl nucleotide binding;guanyl ribonucleotide binding;heat shock protein binding;Hsp90 pro
amine binding;amino acid binding;binding;carbon monoxide binding;carbon-oxygen lyase activity;carboxylic acid binding;catalytic activity;cation binding;cofactor binding;cystathionine beta-synthase activity;enzyme binding;
binding;catalytic activity;GTP binding;GTPase activity;guanyl nucleotide binding;guanyl ribonucleotide binding;hydrolase activity;hydrolase activity, acting on acid anhydrides;hydrolase activity, acting on acid anhydrides, in ph
binding;protein binding;SNARE binding;syntaxin binding
binding;catalytic activity;enzyme binding;hydrolase activity;kinase binding;nucleotide binding;protein binding;protein kinase binding;protein kinase C binding
binding;enzyme binding;histone deacetylase binding;nuclear localization sequence binding;peptide binding;protein binding;protein transporter activity;signal sequence binding;substrate-specific transporter activity;transporte
binding;cation binding;enzyme regulator activity;GTPase regulator activity;guanyl-nucleotide exchange factor activity;ion binding;metal ion binding;nucleoside-triphosphatase regulator activity;Ras guanyl-nucleotide exchange
catalytic activity;spermine synthase activity;transferase activity;transferase activity, transferring alkyl or aryl (other than methyl) groups
binding;DNA binding;nucleic acid binding;protein binding transcription factor activity;single-stranded DNA binding;structure-specific DNA binding;transcription coactivator activity;transcription cofactor activity;transcription fa
binding;catalytic activity;chromatin binding;coenzyme binding;cofactor binding;NAD binding;nucleotide binding;oxidoreductase activity;oxidoreductase activity, acting on CH-OH group of donors;oxidoreductase activity, actin
adenyl nucleotide binding;adenyl ribonucleotide binding;ATP binding;binding;nucleotide binding;purine nucleotide binding;purine ribonucleoside triphosphate binding;purine ribonucleotide binding;ribonucleotide binding
binding;endopeptidase activator activity;endopeptidase regulator activity;enzyme activator activity;enzyme regulator activity;identical protein binding;p53 binding;peptidase activator activity;peptidase regulator activity;protein
binding;catalytic activity;cytoskeletal protein binding;GTP binding;GTPase activity;guanyl nucleotide binding;guanyl ribonucleotide binding;hydrolase activity;hydrolase activity, acting on acid anhydrides;hydrolase activity, act
binding;cation binding;ion binding;metal ion binding;nucleic acid binding;RNA binding;rRNA binding;structural constituent of ribosome;structural molecule activity
binding;catalytic activity;cation binding;hydrolase activity;hydrolase activity, acting on ester bonds;ion binding;metal ion binding;phosphatase activity;phosphoprotein phosphatase activity;phosphoric ester hydrolase activity;p
binding;catalytic activity;cation binding;enzyme binding;hydrolase activity;hydrolase activity, acting on ester bonds;ion binding;kinase binding;metal ion binding;myosin phosphatase activity;myosin-light-chain-phosphatase a
binding;nucleic acid binding;RNA binding;structural constituent of ribosome;structural molecule activity
binding;nucleic acid binding;ribonucleoprotein binding;ribosome binding;RNA binding;translation factor activity, nucleic acid binding;translation release factor activity;translation release factor activity, codon specific;translatio
structural constituent of ribosome;structural molecule activity
binding;protein binding;protein N-terminus binding;structural constituent of ribosome;structural molecule activity
binding;calcium ion binding;cation binding;ion binding;metal ion binding;receptor activity
4 iron, 4 sulfur cluster binding;amidophosphoribosyltransferase activity;binding;catalytic activity;cation binding;ion binding;iron-sulfur cluster binding;metal cluster binding;metal ion binding;transferase activity;transferase acti
endopeptidase activator activity;endopeptidase regulator activity;enzyme activator activity;enzyme regulator activity;peptidase activator activity;peptidase regulator activity
binding;mRNA binding;nucleic acid binding;RNA binding
binding;nucleotide binding
binding;mRNA 3'-UTR binding;mRNA binding;nucleic acid binding;RNA binding
binding;cAMP-dependent protein kinase activity;catalytic activity;cation binding;cyclic nucleotide-dependent protein kinase activity;enzyme regulator activity;GTPase regulator activity;guanyl-nucleotide exchange factor activ
4 iron, 4 sulfur cluster binding;binding;catalytic activity;cation binding;coenzyme binding;cofactor binding;dihydroorotate dehydrogenase activity;dihydropyrimidine dehydrogenase (NADP+) activity;flavin adenine dinucleotide
activating transcription factor binding;binding;damaged DNA binding;DNA binding;histone binding;methylated histone residue binding;nucleic acid binding;p53 binding;protein binding;protein binding transcription factor activ
adenyl nucleotide binding;adenyl ribonucleotide binding;ATP binding;binding;catalytic activity;DNA binding;double-stranded RNA binding;nucleic acid binding;nucleotide binding;purine nucleotide binding;purine ribonucleosic
catalytic activity;phosphorylase activity;purine-nucleoside phosphorylase activity;S-methyl-5-thioadenosine phosphorylase activity;transferase activity;transferase activity, transferring glycosyl groups;transferase activity, trans
binding;nucleotide binding
binding;cation binding;ion binding;ion channel binding;metal ion binding;protein binding;transition metal ion binding;zinc ion binding
binding;mRNA 3'-UTR binding;mRNA binding;nucleic acid binding;nucleotide binding;RNA binding;rRNA binding;SSU rRNA binding;translation regulator activity;translation repressor activity
binding;ribonucleoprotein binding
binding;clathrin binding;lipid binding;protein binding
catalytic activity;hydrolase activity;peptidase activity
binding;catalytic activity;DNA polymerase activity;intramolecular oxidoreductase activity;isomerase activity;nucleotidyltransferase activity;prostaglandin-E synthase activity;protein binding;RNA-directed DNA polymerase activ
binding;cation binding;ion binding;metal ion binding;nucleic acid binding;protein binding transcription factor activity;RNA binding;transcription cofactor activity;transcription corepressor activity;transcription factor binding trar
binding;identical protein binding;lipid binding;protein binding
binding;cation binding;cytokine receptor binding;enzyme binding;growth factor receptor binding;hormone receptor binding;interleukin-1 receptor binding;ion binding;kinase binding;metal ion binding;nuclear hormone recepto
binding;cation binding;ion binding;metal ion binding;nucleotide binding;transition metal ion binding;zinc ion binding
binding;carboxylic acid binding;catalytic activity;cation binding;ion binding;iron ion binding;L-ascorbic acid binding;metal ion binding;oxidoreductase activity;oxidoreductase activity, acting on paired donors, with incorporatio
adenyl nucleotide binding;adenyl ribonucleotide binding;ATP binding;binding;catalytic activity;cation binding;cyclase activity;FAD-AMP lyase (cyclizing) activity;glycerone kinase activity;ion binding;kinase activity;lyase activity
binding;catalytic activity;cytokine receptor binding;growth factor receptor binding;hydrolase activity;hydrolase activity, acting on ester bonds;interleukin-2 receptor binding;nucleic acid binding;phosphatase activity;phosphop
binding;cation binding;ion binding;metal ion binding;nucleotide binding
binding;carbon-carbon lyase activity;carboxy-lyase activity;catalytic activity;cofactor binding;lyase activity;pyridoxal phosphate binding;vitamin B6 binding;vitamin binding
binding;catalytic activity;cation binding;dioxygenase activity;ion binding;metal ion binding;oxidoreductase activity
binding;nucleotide binding
binding;identical protein binding;protein binding;protein binding transcription factor activity;transcription cofactor activity;transcription corepressor activity;transcription factor binding;transcription factor binding transcription
binding;cation binding;DNA binding;ion binding;metal ion binding;nucleic acid binding;nucleic acid binding transcription factor activity;sequence-specific DNA binding;sequence-specific DNA binding transcription factor activ
binding;catalytic activity;chromatin binding;chromatin DNA binding;core promoter binding;deacetylase activity;DNA binding;enzyme binding;histone deacetylase activity;histone deacetylase activity (H3-K14 specific);histone d
binding;BRE binding;mRNA binding;nucleic acid binding;nucleotide binding;RNA binding;translation regulator activity;translation regulator activity, nucleic acid binding;translation repressor activity;translation repressor activity
binding;enzyme binding;GTPase binding;protein binding;protein N-terminus binding;Ral GTPase binding;Ras GTPase binding;small GTPase binding
adenyl nucleotide binding;adenyl ribonucleotide binding;ATP binding;binding;catalytic activity;cation binding;ion binding;kinase activity;magnesium ion binding;metal ion binding;nucleotide binding;phosphotransferase activity
binding;catalytic activity;cation binding;ion binding;metal ion binding;oxidoreductase activity;transition metal ion binding;zinc ion binding
binding;calcium ion binding;calcium-dependent phospholipid binding;catalytic activity;cation binding;endopeptidase activity;hydrolase activity;identical protein binding;ion binding;lipid binding;metal ion binding;NF-kappaB b
binding;calcium ion binding;cation binding;identical protein binding;ion binding;metal ion binding;protein binding
acid-amino acid ligase activity;adenyl nucleotide binding;adenyl ribonucleotide binding;ATP binding;binding;catalytic activity;enzyme binding;ligase activity;ligase activity, forming carbon-nitrogen bonds;nucleotide binding;pro
adenyl nucleotide binding;adenyl ribonucleotide binding;ATP binding;binding;nucleotide binding;purine nucleotide binding;purine ribonucleoside triphosphate binding;purine ribonucleotide binding;ribonucleotide binding
binding;catalytic activity;cation binding;hydrolase activity;hydrolase activity, acting on ester bonds;ion binding;metal ion binding;phosphatase activity;phosphoprotein phosphatase activity;phosphoric ester hydrolase activity;p
amine binding;amino acid binding;binding;carboxylic acid binding;catalytic activity;cation binding;disulfide oxidoreductase activity;DNA binding;electron carrier activity;glutathione binding;heme binding;intramolecular oxidore
catalytic activity;hydrolase activity;hydrolase activity, acting on carbon-nitrogen (but not peptide) bonds;hydrolase activity, acting on carbon-nitrogen (but not peptide) bonds, in linear amides;omega-amidase activity
binding;cytoskeletal protein binding;protein binding;tropomyosin binding
catalytic activity;oxidoreductase activity;oxidoreductase activity, acting on the CH-CH group of donors
binding;mRNA 3'-UTR binding;mRNA 5'-UTR binding;mRNA binding;nucleic acid binding;nucleotide binding;RNA binding;translation regulator activity
binding;cytoskeletal protein binding;identical protein binding;lamin binding;microtubule binding;protein binding;tubulin binding
binding;binding, bridging;cytoskeletal adaptor activity;cytoskeletal protein binding;proline-rich region binding;protein binding;protein binding, bridging
enzyme activator activity;enzyme regulator activity;lipase activator activity;phospholipase A2 activator activity;phospholipase activator activity
binding;GTP binding;guanyl nucleotide binding;guanyl ribonucleotide binding;identical protein binding;nucleotide binding;protein binding;purine nucleotide binding;purine ribonucleoside triphosphate binding;purine ribonucle
antioxidant activity;binding;catalytic activity;disulfide oxidoreductase activity;glutathione peroxidase activity;glutathione transferase activity;oxidoreductase activity;oxidoreductase activity, acting on a sulfur group of donors;o
binding;nucleic acid binding;RNA binding;snoRNA binding
binding;protein binding;protein domain specific binding;SH3 domain binding;structural constituent of cytoskeleton;structural molecule activity
binding;catalytic activity;cation binding;coenzyme binding;cofactor binding;ion binding;magnesium ion binding;metal ion binding;thiamine pyrophosphate binding;transferase activity;vitamin binding
binding;nucleotide binding
binding;nucleic acid binding;RNA binding;translation factor activity, nucleic acid binding;translation initiation factor activity
active transmembrane transporter activity;adenyl nucleotide binding;adenyl ribonucleotide binding;ATP binding;binding;chaperone binding;macromolecule transmembrane transporter activity;nucleotide binding;P-P-bond-hy
binding;catalytic activity;cation binding;coenzyme binding;cofactor binding;electron carrier activity;ion binding;isocitrate dehydrogenase (NAD+) activity;isocitrate dehydrogenase activity;magnesium ion binding;metal ion bind
acetyltransferase activity;beta-N-acetylglucosaminidase activity;beta-N-acetylhexosaminidase activity;catalytic activity;hexosaminidase activity;histone acetyltransferase activity;hyaluronoglucosaminidase activity;hydrolase
binding;epidermal growth factor receptor binding;growth factor receptor binding;insulin receptor binding;lipid binding;phosphatidylinositol binding;phospholipid binding;protein binding;protein complex binding;receptor bindir
acid-amino acid ligase activity;binding;catalytic activity;cation binding;enzyme binding;identical protein binding;ion binding;ligase activity;ligase activity, forming carbon-nitrogen bonds;metal ion binding;mRNA 3'-UTR binding
adenyl nucleotide binding;adenyl ribonucleotide binding;ATP binding;ATPase activity;ATPase activity, coupled;binding;catalytic activity;hydrolase activity;hydrolase activity, acting on acid anhydrides;hydrolase activity, acting
binding;catalytic activity;cation binding;endopeptidase activity;hydrolase activity;ion binding;metal ion binding;metalloendopeptidase activity;metallopeptidase activity;peptidase activity;peptidase activity, acting on L-amino a
binding;nucleic acid binding;nucleotide binding;RNA binding;translation factor activity, nucleic acid binding;translation initiation factor activity
binding;catalytic activity;cation binding;intramolecular transferase activity;intramolecular transferase activity, phosphotransferases;ion binding;isomerase activity;magnesium ion binding;metal ion binding;phosphoacetylgluco
ATPase activator activity;ATPase regulator activity;binding;chaperone binding;enzyme activator activity;enzyme regulator activity;nucleoside-triphosphatase regulator activity;protein binding

anion binding;binding;catalytic activity;drug binding;ion binding;nucleobase binding;nucleoside binding;phosphate ion binding;purine base binding;purine-nucleoside phosphorylase activity;transferase activity;transferase act
binding;catalytic activity;ribonucleoprotein binding;ribosome binding;transferase activity;transferase activity, transferring glycosyl groups
binding;lipid binding;receptor activity;viral receptor activity
adenyl nucleotide binding;adenyl ribonucleotide binding;ATP binding;binding;catalytic activity;cation binding;deoxyribonuclease activity;DNA binding;GTP binding;guanyl nucleotide binding;guanyl ribonucleotide binding;hydr
binding;carbohydrate binding;catalytic activity;collagen binding;glycosaminoglycan binding;hexosaminidase activity;hyaluronic acid binding;hyaluronoglucosaminidase activity;hydrolase activity;hydrolase activity, acting on g
adenyl nucleotide binding;adenyl ribonucleotide binding;ATP binding;binding;catalytic activity;cation binding;DNA binding;ion binding;kinase activity;metal ion binding;nucleic acid binding;nucleic acid binding transcription fac
carnitine O-acyltransferase activity;carnitine O-palmitoyltransferase activity;catalytic activity;O-acyltransferase activity;O-palmitoyltransferase activity;palmitoyltransferase activity;transferase activity;transferase activity, transf
adenyl nucleotide binding;adenyl ribonucleotide binding;ATP binding;binding;catalytic activity;kinase activity;nucleobase-containing compound kinase activity;nucleoside phosphate kinase activity;nucleotide binding;nucleoti
binding;enzyme binding;protein binding;RNA polymerase binding
adenyl nucleotide binding;adenyl ribonucleotide binding;ATP binding;binding;catalytic activity;enzyme binding;kinase activity;MAP kinase activity;molecular transducer activity;nucleotide binding;phosphatase binding;phosph
alkyl hydroperoxide reductase activity;antioxidant activity;binding;caspase inhibitor activity;caspase regulator activity;catalytic activity;cysteine-type endopeptidase inhibitor activity;endopeptidase inhibitor activity;endopeptic
structural constituent of ribosome;structural molecule activity
asialoglycoprotein receptor activity;binding;carbohydrate binding;cargo receptor activity;glycosaminoglycan binding;heparin binding;lipoprotein particle receptor binding;low-density lipoprotein particle receptor binding;patter
binding;catalytic activity;coproporphyrinogen oxidase activity;identical protein binding;oxidoreductase activity;oxidoreductase activity, acting on the CH-CH group of donors;oxidoreductase activity, acting on the CH-CH grou
catalytic activity;dolichyl-diphosphooligosaccharide-protein glycotransferase activity;oligosaccharyl transferase activity;transferase activity;transferase activity, transferring glycosyl groups;transferase activity, transferring hex
binding;carbohydrate binding;glycosaminoglycan binding;heparin binding;nucleic acid binding;pattern binding;polysaccharide binding;RNA binding;structural constituent of ribosome;structural molecule activity
binding;catalytic activity;GDP binding;GTP binding;GTPase activity;guanyl nucleotide binding;guanyl ribonucleotide binding;hydrolase activity;hydrolase activity, acting on acid anhydrides;hydrolase activity, acting on acid anh
3-chloroallyl aldehyde dehydrogenase activity;aldehyde dehydrogenase (NAD) activity;aldehyde dehydrogenase [NAD(P)+] activity;catalytic activity;long-chain-alcohol oxidase activity;long-chain-aldehyde dehydrogenase acti
adenyl nucleotide binding;adenyl ribonucleotide binding;ATP binding;ATPase activity;binding;catalytic activity;enzyme binding;hydrolase activity;hydrolase activity, acting on acid anhydrides;hydrolase activity, acting on acid a
carboxylic acid transmembrane transporter activity;citrate transmembrane transporter activity;organic acid transmembrane transporter activity;substrate-specific transmembrane transporter activity;substrate-specific transpo
aminopeptidase activity;binding;catalytic activity;cation binding;exopeptidase activity;hydrolase activity;ion binding;metal ion binding;metalloaminopeptidase activity;metalloexopeptidase activity;metallopeptidase activity;pep
actin-dependent ATPase activity;ATPase activity;ATPase activity, coupled;binding;calcium ion binding;catalytic activity;cation binding;hydrolase activity;hydrolase activity, acting on acid anhydrides;hydrolase activity, acting o
binding;chromatin binding;core promoter proximal region DNA binding;core promoter proximal region sequence-specific DNA binding;DNA bending activity;DNA binding;mitochondrial light strand promoter sense binding;nuc
AMP deaminase activity;binding;catalytic activity;cation binding;deaminase activity;hydrolase activity;hydrolase activity, acting on carbon-nitrogen (but not peptide) bonds;hydrolase activity, acting on carbon-nitrogen (but not
binding;core promoter proximal region DNA binding;core promoter proximal region sequence-specific DNA binding;DNA binding;nucleic acid binding;nucleic acid binding transcription factor activity;regulatory region DNA bin
binding;molecular transducer activity;nucleic acid binding;receptor signaling protein activity;RNA binding;signal transducer activity;snoRNA binding
actin binding;actin monomer binding;adenyl nucleotide binding;adenyl ribonucleotide binding;ATP binding;binding;cytoskeletal protein binding;lipid binding;nucleotide binding;phosphatidylinositol binding;phosphatidylinositol
binding;cytokine activity;enzyme binding;GTPase binding;identical protein binding;nucleic acid binding;protein binding;protein dimerization activity;protein homodimerization activity;receptor binding;RNA binding;tRNA bindin
binding;DNA binding;double-stranded DNA binding;identical protein binding;mRNA 3'-UTR binding;mRNA binding;nucleic acid binding;nucleic acid binding transcription factor activity;nucleotide binding;protein binding;RNA
binding;nucleotide binding
activating transcription factor binding;binding;catalytic activity;core promoter binding;deacetylase activity;DNA binding;enzyme binding;histone deacetylase activity;histone deacetylase activity (H3-K14 specific);histone deac
actin binding;binding;cytoskeletal protein binding;enzyme binding;protein binding
3',5'-cyclic-AMP phosphodiesterase activity;3',5'-cyclic-nucleotide phosphodiesterase activity;adenyl nucleotide binding;adenyl ribonucleotide binding;AMP binding;binding;cAMP binding;catalytic activity;cation binding;cGM
binding;catalytic activity;chromo shadow domain binding;DNA binding;lamin binding;nucleic acid binding;oxidoreductase activity;oxidoreductase activity, acting on the CH-CH group of donors;oxidoreductase activity, acting
binding;identical protein binding;protein binding
binding;chaperone binding;protein binding
binding;enzyme binding;kinase binding;protein binding;protein kinase binding;ribonucleoprotein binding;ribosome binding
binding;DNA binding;histone binding;nucleic acid binding;nucleic acid binding transcription factor activity;protein binding;sequence-specific DNA binding transcription factor activity
binding;catalytic activity;GTP binding;GTPase activity;guanyl nucleotide binding;guanyl ribonucleotide binding;hydrolase activity;hydrolase activity, acting on acid anhydrides;hydrolase activity, acting on acid anhydrides, in ph
binding;cation binding;ion binding;metal ion binding;transition metal ion binding;zinc ion binding
binding;GTP binding;guanyl nucleotide binding;guanyl ribonucleotide binding;nucleotide binding;purine nucleotide binding;purine ribonucleoside triphosphate binding;purine ribonucleotide binding;ribonucleotide binding
binding;enzyme binding;histone binding;protein binding;RNA polymerase binding
binding;calcium ion binding;catalytic activity;cation binding;cis-trans isomerase activity;drug binding;FK506 binding;ion binding;isomerase activity;macrolide binding;metal ion binding;peptidyl-prolyl cis-trans isomerase activi
adenyl nucleotide binding;adenyl ribonucleotide binding;ATP binding;binding;catalytic activity;DNA binding;kinase activity;nucleic acid binding;nucleotide binding;phosphotransferase activity, alcohol group as acceptor;protei
activating transcription factor binding;binding;catalytic activity;chromatin binding;deacetylase activity;DNA binding;hydrolase activity;nucleic acid binding;nucleic acid binding transcription factor activity;protein binding;protei
transporter activity
arginine N-methyltransferase activity;binding;catalytic activity;histone methyltransferase activity;histone methyltransferase activity (H4-R3 specific);histone-arginine N-methyltransferase activity;identical protein binding;methyl
binding;identical protein binding;lipid binding;protein binding
adenyl nucleotide binding;adenyl ribonucleotide binding;ATP binding;ATPase activity;ATPase activity, coupled;ATP-dependent helicase activity;ATP-dependent RNA helicase activity;binding;catalytic activity;helicase activity;h
adenyl nucleotide binding;adenyl ribonucleotide binding;aminoacyl-tRNA ligase activity;ATP binding;binding;catalytic activity;ligase activity;ligase activity, forming aminoacyl-tRNA and related compounds;ligase activity, formi
actin binding;actin filament binding;binding;cytoskeletal protein binding;enzyme binding;GTPase binding;GTP-Rho binding;myosin binding;myosin II binding;protein binding;Ras GTPase binding;Rho GTPase binding;small GT
binding;catalytic activity;enzyme binding;hydrolase activity;hydrolase activity, acting on ester bonds;kinase binding;phosphatase activity;phosphoprotein phosphatase activity;phosphoric ester hydrolase activity;protein bindin
acyl-CoA dehydrogenase activity;binding;catalytic activity;coenzyme binding;cofactor binding;flavin adenine dinucleotide binding;oxidoreductase activity;oxidoreductase activity, acting on the CH-CH group of donors
catalytic activity;hydrolase activity;hydrolase activity, acting on ester bonds;inositol or phosphatidylinositol phosphatase activity;lipid phosphatase activity;phosphatase activity;phosphatidylinositol bisphosphate phosphatase
angiostatin binding;ATPase binding;ATPase inhibitor activity;ATPase regulator activity;binding;calmodulin binding;enzyme binding;enzyme inhibitor activity;enzyme regulator activity;identical protein binding;nucleoside-triphos
acidic amino acid transmembrane transporter activity;active transmembrane transporter activity;amine transmembrane transporter activity;amino acid transmembrane transporter activity;binding;calcium ion binding;carboxyli
acetyltransferase activity;binding;catalytic activity;DNA binding;enzyme activator activity;enzyme regulator activity;histone acetyltransferase activity;lysine N-acetyltransferase activity;N-acetyltransferase activity;N-acyltransfe
binding;calcium channel inhibitor activity;calcium channel regulator activity;channel inhibitor activity;channel regulator activity;cytoskeletal protein binding;ion channel inhibitor activity;lipid binding;phospholipid binding;protein
binding;nucleotide binding;protein binding;protein complex scaffold;receptor signaling complex scaffold activity;structural molecule activity
acid-amino acid ligase activity;binding;binding, bridging;catalytic activity;enzyme binding;G-protein-coupled receptor binding;heat shock protein binding;Hsp70 protein binding;Hsp90 protein binding;identical protein binding;
binding;DNA binding;enzyme regulator activity;nucleic acid binding;phosphatase regulator activity;protein binding;protein domain specific binding;protein phosphatase regulator activity;protein phosphatase type 1 regulator a
structural constituent of ribosome;structural molecule activity
binding;carboxylic ester hydrolase activity;catalytic activity;enzyme binding;enzyme inhibitor activity;enzyme regulator activity;hydrolase activity;hydrolase activity, acting on ester bonds;phosphatase binding;phosphatase inh
adenyl nucleotide binding;adenyl ribonucleotide binding;ATP binding;binding;catalytic activity;hydrolase activity;hydrolase activity, acting on acid anhydrides;hydrolase activity, acting on acid anhydrides, in phosphorus-conta
binding;GAF domain binding;molecular transducer activity;protein binding;protein binding transcription factor activity;protein domain specific binding;signal transducer activity;transcription coactivator activity;transcription co
binding;nucleic acid binding;RNA binding;tRNA binding
binding;catalytic activity;DNA polymerase activity;intramolecular transferase activity;isomerase activity;nucleic acid binding;nucleotidyltransferase activity;pseudouridine synthase activity;RNA binding;RNA-directed DNA poly
acid-amino acid ligase activity;catalytic activity;ligase activity;ligase activity, forming carbon-nitrogen bonds;small conjugating protein ligase activity;UFM1 conjugating enzyme activity
binding;identical protein binding;protein binding
active transmembrane transporter activity;antigen binding;ATPase activator activity;ATPase activity;ATPase activity, coupled;ATPase activity, coupled to movement of substances;ATPase activity, coupled to transmembrane m
adenyl nucleotide binding;adenyl ribonucleotide binding;asparagine synthase (glutamine-hydrolyzing) activity;ATP binding;binding;carbon-nitrogen ligase activity, with glutamine as amido-N-donor;catalytic activity;cofactor bi
binding;carbohydrate binding;disaccharide binding;galactoside binding;lactose binding;molecular transducer activity;monosaccharide binding;signal transducer activity;sugar binding
binding;cation binding;ion binding;metal ion binding;nucleic acid binding;RNA binding;snRNA binding;U2 snRNA binding
binding;catalytic activity;chromatin binding;damaged DNA binding;dinucleotide insertion or deletion binding;DNA binding;DNA insertion or deletion binding;DNA N-glycosylase activity;DNA polymerase binding;DNA polymera
binding;chromatin binding;DNA binding;nucleic acid binding
adenyl nucleotide binding;adenyl ribonucleotide binding;ATP binding;binding;calcium ion binding;catalytic activity;cation binding;diacylglycerol kinase activity;ion binding;kinase activity;lipid binding;metal ion binding;NAD+ ki
structural constituent of ribosome;structural molecule activity
binding;catalytic activity;coenzyme binding;cofactor binding;electron carrier activity;flavin adenine dinucleotide binding;oxidoreductase activity;oxidoreductase activity, acting on the CH-NH2 group of donors;oxidoreductase
adenyl nucleotide binding;adenyl ribonucleotide binding;ATP binding;binding;catalytic activity;cytidylate kinase activity;kinase activity;nucleobase-containing compound kinase activity;nucleoside diphosphate kinase activity;n
catalytic activity;dihydrolipoyllysine-residue succinyltransferase activity;S-acyltransferase activity;S-succinyltransferase activity;succinyltransferase activity;transferase activity;transferase activity, transferring acyl groups;trans
7S RNA binding;binding;endoplasmic reticulum signal peptide binding;nucleic acid binding;peptide binding;RNA binding;signal sequence binding
anion channel activity;anion transmembrane transporter activity;binding;channel activity;gated channel activity;ion channel activity;ion transmembrane transporter activity;nucleotide binding;passive transmembrane transporte
binding;catalytic activity;cation binding;coenzyme binding;cofactor binding;ion binding;isocitrate dehydrogenase (NADP+) activity;isocitrate dehydrogenase activity;magnesium ion binding;metal ion binding;NAD binding;nucle
4 iron, 4 sulfur cluster binding;binding;catalytic activity;cation binding;coenzyme binding;cofactor binding;FMN binding;ion binding;iron-sulfur cluster binding;metal cluster binding;metal ion binding;NAD binding;NADH dehyd
enzyme activator activity;enzyme regulator activity;GDP-dissociation inhibitor activity;GTPase activator activity;GTPase regulator activity;nucleoside-triphosphatase regulator activity;Rho GDP-dissociation inhibitor activity;sm
binding;enzyme binding;protein binding;structural constituent of cytoskeleton;structural molecule activity
binding;catalytic activity;cytoskeletal protein binding;enzyme binding;GDP binding;GTP binding;GTPase activity;GTPase binding;guanyl nucleotide binding;guanyl ribonucleotide binding;hydrolase activity;hydrolase activity, a
7S RNA binding;binding;catalytic activity;drug binding;endoplasmic reticulum signal peptide binding;GDP binding;GTP binding;GTPase activity;guanyl nucleotide binding;guanyl ribonucleotide binding;hydrolase activity;hydro
binding;catalytic activity;cytoskeletal protein binding;GDP binding;GTP binding;GTPase activity;guanyl nucleotide binding;guanyl ribonucleotide binding;hydrolase activity;hydrolase activity, acting on acid anhydrides;hydrolas
binding;catalytic activity;cytoskeletal protein binding;GDP binding;GTP binding;GTPase activity;guanyl nucleotide binding;guanyl ribonucleotide binding;hydrolase activity;hydrolase activity, acting on acid anhydrides;hydrolas
binding;identical protein binding;protein binding;SNARE binding;syntaxin binding;syntaxin-1 binding
binding;cation binding;DNA binding;ion binding;metal ion binding;nucleic acid binding;nucleic acid binding transcription factor activity;RNA binding;sequence-specific DNA binding transcription factor activity;single-stranded
structural constituent of ribosome;structural molecule activity
binding;nucleotide binding;protein binding;structural constituent of ribosome;structural molecule activity;translation initiation factor binding
binding;nucleic acid binding;RNA binding;structural constituent of ribosome;structural molecule activity
catalytic activity;enzyme regulator activity;hydrolase activity;hydrolase activity, acting on ester bonds;phosphatase activity;phosphatase regulator activity;phosphoprotein phosphatase activity;phosphoric ester hydrolase activ
adenyl nucleotide binding;adenyl ribonucleotide binding;ATP binding;binding;catalytic activity;heat shock protein binding;Hsp90 protein binding;kinase activity;nucleotide binding;phosphotransferase activity, alcohol group as
binding;catalytic activity;cis-trans isomerase activity;drug binding;FK506 binding;isomerase activity;macrolide binding;peptidyl-prolyl cis-trans isomerase activity;receptor activity
aminoacylase activity;binding;catalytic activity;cation binding;hydrolase activity;hydrolase activity, acting on carbon-nitrogen (but not peptide) bonds;hydrolase activity, acting on carbon-nitrogen (but not peptide) bonds, in lin
binding;calcium ion binding;calcium-dependent protein serine/threonine phosphatase activity;calmodulin binding;calmodulin-dependent protein phosphatase activity;catalytic activity;cation binding;drug binding;enzyme bind
3',5'-cyclic-AMP phosphodiesterase activity;3',5'-cyclic-nucleotide phosphodiesterase activity;adenyl nucleotide binding;adenyl ribonucleotide binding;adrenergic receptor binding;AMP binding;ATPase binding;beta-2 adrene
[acetyl-CoA carboxylase] kinase activity;[hydroxymethylglutaryl-CoA reductase (NADPH)] kinase activity;adenyl nucleotide binding;adenyl ribonucleotide binding;AMP-activated protein kinase activity;ATP binding;binding;cAM
adenyl nucleotide binding;adenyl ribonucleotide binding;ATP binding;binding;catalytic activity;kinase activity;nucleotide binding;phosphotransferase activity, alcohol group as acceptor;protein kinase activity;protein serine/thre
binding;hormone receptor binding;Notch binding;nuclear hormone receptor binding;protein binding;protein binding transcription factor activity;receptor binding;retinoic acid receptor binding;SMAD binding;transcription coact
binding;nucleic acid binding;nucleotide binding;RNA binding;translation factor activity, nucleic acid binding;translation initiation factor activity
acid-amino acid ligase activity;catalytic activity;ligase activity;ligase activity, forming carbon-nitrogen bonds;small conjugating protein ligase activity;ubiquitin-protein ligase activity
actin binding;actin monomer binding;adenyl nucleotide binding;adenyl ribonucleotide binding;ATP binding;binding;cytoskeletal protein binding;enzyme binding;kinase binding;lipid binding;nucleotide binding;phosphatidylinos
binding;chromatin binding;DNA binding;nucleic acid binding
binding;chromatin binding;DNA binding;nucleic acid binding
binding;cation binding;cytoskeletal protein binding;DNA binding;enzyme binding;histone binding;histone deacetylase binding;ion binding;metal ion binding;nucleic acid binding;phosphoprotein binding;protein binding;ribonuc
aldehyde dehydrogenase (NAD) activity;catalytic activity;oxidoreductase activity;oxidoreductase activity, acting on the aldehyde or oxo group of donors;oxidoreductase activity, acting on the aldehyde or oxo group of donors,
adenyl nucleotide binding;adenyl ribonucleotide binding;ATP binding;binding;catalytic activity;kinase activity;nucleotide binding;phosphotransferase activity, alcohol group as acceptor;protein binding;protein complex binding
binding;chromatin binding;DNA binding;nucleic acid binding;protein binding transcription factor activity;transcription coactivator activity;transcription cofactor activity;transcription factor binding transcription factor activity
binding;cation binding;DNA binding;ion binding;metal ion binding;nucleic acid binding;nucleotide binding;transition metal ion binding;zinc ion binding
binding;nucleic acid binding
binding;calcium ion binding;cation binding;ion binding;metal ion binding
binding;catalytic activity;GTP binding;GTPase activity;guanyl nucleotide binding;guanyl ribonucleotide binding;hydrolase activity;hydrolase activity, acting on acid anhydrides;hydrolase activity, acting on acid anhydrides, in ph

binding;nucleotide binding
binding;cation binding;ion binding;metal ion binding;miRNA binding;mRNA 3'-UTR binding;mRNA binding;nucleic acid binding;nucleotide binding;RNA binding;transition metal ion binding;zinc ion binding
binding;catalytic activity;cation binding;endopeptidase activity;hydrolase activity;ion binding;metal ion binding;metalloendopeptidase activity;metallopeptidase activity;peptidase activity;peptidase activity, acting on L-amino a
adenyl nucleotide binding;adenyl ribonucleotide binding;ATP binding;ATPase activity;ATPase activity, coupled;ATP-dependent helicase activity;ATP-dependent RNA helicase activity;binding;catalytic activity;core promoter bin
amine-lyase activity;arylesterase activity;carbon-nitrogen lyase activity;carboxylic ester hydrolase activity;catalytic activity;hydrolase activity;hydrolase activity, acting on ester bonds;lyase activity;strictosidine synthase activity
binding;enzyme binding;identical protein binding;kinase binding;protein binding;protein kinase B binding;protein kinase binding
adenyl nucleotide binding;adenyl ribonucleotide binding;ATP binding;ATPase activity;ATPase activity, coupled;ATP-dependent helicase activity;ATP-dependent RNA helicase activity;binding;catalytic activity;helicase activity;h
transporter activity
binding;DNA binding;nucleic acid binding
binding;cytoskeletal protein binding;enzyme binding;GTPase binding;GTP-dependent protein binding;kinesin binding;protein binding;protein transporter activity;Rab GTPase binding;Ras GTPase binding;small GTPase bindin
binding;catalytic activity;cation binding;endopeptidase activity;epidermal growth factor binding;growth factor binding;hormone binding;hydrolase activity;ion binding;metal ion binding;metalloendopeptidase activity;metallope
binding;chaperone binding;protein binding
binding;chromatin binding;chromatin DNA binding;core promoter binding;core promoter sequence-specific DNA binding;DNA binding;double-stranded DNA binding;double-stranded methylated DNA binding;enzyme binding;
catalytic activity;citrate (Si)-synthase activity;transferase activity;transferase activity, transferring acyl groups;transferase activity, transferring acyl groups, acyl groups converted into alkyl on transfer
binding;chromatin binding;DNA binding;nucleic acid binding;RNA binding
enzyme regulator activity;kinase regulator activity;molecular transducer activity;phosphorylase kinase regulator activity;protein kinase regulator activity;signal transducer activity
binding;calcium ion binding;calcium-dependent cysteine-type endopeptidase activity;catalytic activity;cation binding;cysteine-type endopeptidase activity;cysteine-type peptidase activity;endopeptidase activity;hydrolase ac
binding;enzyme binding;enzyme inhibitor activity;enzyme regulator activity;identical protein binding;kinase binding;kinase inhibitor activity;kinase regulator activity;protein binding;protein kinase binding;protein kinase C bindi
binding;enzyme activator activity;enzyme regulator activity;lipid binding
binding;carbon-oxygen lyase activity;catalytic activity;cation binding;hydro-lyase activity;ion binding;lyase activity;magnesium ion binding;metal ion binding;phosphopyruvate hydratase activity
binding;catalytic activity;cation binding;dimethylallyltranstransferase activity;geranyltranstransferase activity;ion binding;metal ion binding;prenyltransferase activity;transferase activity;transferase activity, transferring alkyl or a
antioxidant activity;binding;catalytic activity;cytochrome-b5 reductase activity;identical protein binding;NAD(P)H dehydrogenase (quinone) activity;oxidoreductase activity;oxidoreductase activity, acting on NADH or NADPH;o
adenyl nucleotide binding;adenyl ribonucleotide binding;ATP binding;binding;calcium-dependent protein kinase activity;calcium-dependent protein kinase C activity;calcium-dependent protein serine/threonine kinase activity;
binding;catalytic activity;cation binding;enzyme binding;ephrin receptor binding;hydrolase activity;hydrolase activity, acting on ester bonds;ion binding;kinase binding;metal ion binding;phosphatase activity;phosphoprotein ph
binding;DNA binding;nucleic acid binding;RNA binding
binding;carbohydrate binding;cation binding;extracellular matrix structural constituent;glycoprotein binding;glycosaminoglycan binding;growth factor binding;heparin binding;integrin binding;ion binding;metal ion binding;patt
cation transmembrane transporter activity;hydrogen ion transmembrane transporter activity;inorganic cation transmembrane transporter activity;ion transmembrane transporter activity;monovalent inorganic cation transmemb
catalytic activity;endopeptidase activity;hydrolase activity;peptidase activity;peptidase activity, acting on L-amino acid peptides;threonine-type endopeptidase activity;threonine-type peptidase activity
binding;nucleic acid binding;nucleotide binding;RNA binding
binding;protein binding;protein complex binding;protein domain specific binding
catalytic activity;dolichyl-diphosphooligosaccharide-protein glycotransferase activity;oligosaccharyl transferase activity;transferase activity;transferase activity, transferring glycosyl groups;transferase activity, transferring hex
catalytic activity;intramolecular transferase activity;isomerase activity;lanosterol synthase activity;oxidosqualene cyclase activity
binding;lipid binding
carnitine O-acyltransferase activity;carnitine O-palmitoyltransferase activity;catalytic activity;O-acyltransferase activity;O-palmitoyltransferase activity;palmitoyltransferase activity;transferase activity;transferase activity, transf
aminopeptidase activity;binding;catalytic activity;cation binding;exopeptidase activity;hydrolase activity;ion binding;metal ion binding;metalloaminopeptidase activity;metalloexopeptidase activity;metallopeptidase activity;pep
adenyl nucleotide binding;adenyl ribonucleotide binding;ATP binding;binding;carbohydrate binding;carbohydrate kinase activity;catalytic activity;galactokinase activity;galactose binding;kinase activity;monosaccharide bindin
nucleocytoplasmic transporter activity;structural constituent of nuclear pore;structural molecule activity;transporter activity
binding;nucleic acid binding;protein binding;protein N-terminus binding;ribonucleoprotein binding;ribosome binding;RNA binding;snRNA binding;translation elongation factor activity;translation factor activity, nucleic acid binc
binding;core promoter proximal region DNA binding;core promoter proximal region sequence-specific DNA binding;DNA binding;double-stranded DNA binding;enzyme binding;GTPase binding;nucleic acid binding;nucleic ac
binding;DNA binding;nucleic acid binding
active transmembrane transporter activity;anion transmembrane transporter activity;anion:anion antiporter activity;antiporter activity;C4-dicarboxylate transmembrane transporter activity;carboxylic acid transmembrane trans
cargo receptor activity;receptor activity;scavenger receptor activity
binding;catalytic activity;cation binding;endopeptidase activity;hydrolase activity;ion binding;metal ion binding;metalloendopeptidase activity;metallopeptidase activity;peptidase activity;peptidase activity, acting on L-amino a
binding;carbohydrate binding;cation binding;glycoprotein binding;heat shock protein binding;ion binding;mannose binding;metal ion binding;monosaccharide binding;protein binding;sugar binding
binding;nucleic acid binding;nucleotide binding;pre-mRNA binding;RNA binding
binding;POZ domain binding;protein binding;protein domain specific binding
catalytic activity;glucosidase activity;hydrolase activity;hydrolase activity, acting on glycosyl bonds;hydrolase activity, hydrolyzing O-glycosyl compounds;mannosyl-oligosaccharide glucosidase activity
acid-amino acid ligase activity;binding;catalytic activity;hormone receptor binding;ligase activity;ligase activity, forming carbon-nitrogen bonds;nuclear hormone receptor binding;protein binding;receptor binding;small conjuga
binding;cytoskeletal protein binding;microtubule binding;microtubule plus-end binding;protein binding;protein C-terminus binding;tubulin binding
3-beta-hydroxy-delta5-steroid dehydrogenase activity;catalytic activity;oxidoreductase activity;oxidoreductase activity, acting on CH-OH group of donors;oxidoreductase activity, acting on the CH-OH group of donors, NAD c
adenyl nucleotide binding;adenyl ribonucleotide binding;ATP binding;binding;carbohydrate kinase activity;catalytic activity;fructokinase activity;glucokinase activity;hexokinase activity;kinase activity;mannokinase activity;nuc
ATPase activator activity;ATPase binding;ATPase regulator activity;binding;cytoskeletal protein binding;enzyme activator activity;enzyme binding;enzyme regulator activity;nucleoside-triphosphatase regulator activity;protein b
binding;DNA binding;histone binding;nucleic acid binding;protein binding
binding;cytoskeletal protein binding;kinetochore binding;microtubule binding;microtubule plus-end binding;protein binding;tubulin binding
binding;nucleotide binding
binding;DNA binding;nucleic acid binding
catalytic activity;dolichyl-diphosphooligosaccharide-protein glycotransferase activity;oligosaccharyl transferase activity;transferase activity;transferase activity, transferring glycosyl groups;transferase activity, transferring hex
binding;DNA binding;nucleic acid binding
binding;carboxypeptidase activity;catalytic activity;cation binding;dipeptidase activity;exopeptidase activity;hydrolase activity;ion binding;metal ion binding;metallopeptidase activity;peptidase activity;peptidase activity, acting
binding;fibroblast growth factor binding;growth factor binding;protein binding
binding;molecular transducer activity;protein binding;protein complex binding;signal transducer activity
structural molecule activity
binding;cation binding;heat shock protein binding;Hsp90 protein binding;ion binding;metal ion binding;protein binding;transition metal ion binding;zinc ion binding
catalytic activity;cysteine-type endopeptidase activity;cysteine-type peptidase activity;endopeptidase activity;hydrolase activity;peptidase activity;peptidase activity, acting on L-amino acid peptides;small conjugating protein-
anion channel activity;anion transmembrane transporter activity;binding;channel activity;gated channel activity;ion channel activity;ion transmembrane transporter activity;nucleotide binding;passive transmembrane transporte
binding;catalytic activity;enzyme activator activity;enzyme binding;enzyme regulator activity;kinase activator activity;kinase activity;kinase binding;kinase regulator activity;phosphotransferase activity, alcohol group as accept
binding;cytoskeletal protein binding;microtubule binding;nucleic acid binding;protein binding;ribonucleoprotein binding;ribosome binding;RNA binding;rRNA binding;tubulin binding
anion channel activity;anion transmembrane transporter activity;channel activity;chloride channel activity;gated channel activity;ion channel activity;ion transmembrane transporter activity;passive transmembrane transporter
arginine N-methyltransferase activity;binding;catalytic activity;chromatin binding;core promoter binding;core promoter sequence-specific DNA binding;DNA binding;histone methyltransferase activity;histone-arginine N-methy
binding;enzyme binding;protease binding;protein binding
catalytic activity;glutaminase activity;hydrolase activity;hydrolase activity, acting on carbon-nitrogen (but not peptide) bonds;hydrolase activity, acting on carbon-nitrogen (but not peptide) bonds, in linear amides
binding;calcium-dependent protein binding;chemokine binding;C-X-C chemokine binding;cytokine binding;endopeptidase inhibitor activity;endopeptidase regulator activity;enzyme binding;enzyme inhibitor activity;enzyme re
alkaline phosphatase activity;binding;catalytic activity;cation binding;hydrolase activity;hydrolase activity, acting on ester bonds;ion binding;magnesium ion binding;metal ion binding;phosphatase activity;phosphoric ester hy
binding;integrin binding;molecular transducer activity;protein binding;protein complex binding;receptor activity;receptor binding;signal transducer activity;signaling receptor activity;transmembrane signaling receptor activity;v
binding;clathrin binding;clathrin heavy chain binding;peptide binding;protein binding;structural molecule activity
carbon-carbon lyase activity;carboxy-lyase activity;catalytic activity;lyase activity;orotate phosphoribosyltransferase activity;orotidine-5'-phosphate decarboxylase activity;transferase activity;transferase activity, transferring g
adenyl nucleotide binding;adenyl ribonucleotide binding;AMP binding;binding;cAMP binding;cAMP-dependent protein kinase inhibitor activity;cAMP-dependent protein kinase regulator activity;cyclic nucleotide binding;enzy
2 iron, 2 sulfur cluster binding;3 iron, 4 sulfur cluster binding;4 iron, 4 sulfur cluster binding;binding;catalytic activity;cation binding;cofactor binding;electron carrier activity;ion binding;iron-sulfur cluster binding;metal cluster b
adenyl nucleotide binding;adenyl ribonucleotide binding;ATP binding;binding;C3HC4-type RING finger domain binding;cation binding;chaperone binding;enzyme binding;G-protein-coupled receptor binding;heat shock protei
binding;cation binding;DNA binding;identical protein binding;ion binding;metal ion binding;nucleic acid binding;nucleotide binding;protein binding;protein binding transcription factor activity;RNA binding;transcription coactiva
adenyl nucleotide binding;adenyl ribonucleotide binding;ATP binding;ATPase activity;binding;catalytic activity;hydrolase activity;hydrolase activity, acting on acid anhydrides;hydrolase activity, acting on acid anhydrides, in ph
nucleic acid binding transcription factor activity;RNA polymerase II core promoter proximal region sequence-specific DNA binding transcription factor activity;RNA polymerase II core promoter proximal region sequence-spec
binding;catalytic activity;GTP binding;GTPase activity;guanyl nucleotide binding;guanyl ribonucleotide binding;hydrolase activity;hydrolase activity, acting on acid anhydrides;hydrolase activity, acting on acid anhydrides, in pl
binding;cation binding;ion binding;metal ion binding;structural constituent of ribosome;structural molecule activity
binding;DNA binding;nucleic acid binding;protein binding;protein binding transcription factor activity;protein domain specific binding;regulatory region DNA binding;regulatory region nucleic acid binding;transcription cofactor
binding;binding, bridging;DNA binding;identical protein binding;mRNA binding;nucleic acid binding;poly(A) RNA binding;poly(U) RNA binding;poly-purine tract binding;poly-pyrimidine tract binding;protein binding;protein bind
binding;binding, bridging;enzyme activator activity;enzyme regulator activity;GTPase activator activity;GTPase regulator activity;nucleoside-triphosphatase regulator activity;protein binding;protein binding, bridging;SH3/SH2
nucleocytoplasmic transporter activity;transporter activity
catalytic activity;hydrolase activity;hydrolase activity, acting on acid anhydrides;hydrolase activity, acting on acid anhydrides, in phosphorus-containing anhydrides;microtubule motor activity;motor activity;nucleoside-triphos
binding;calcium channel activity;calcium channel inhibitor activity;calcium channel regulator activity;calcium ion transmembrane transporter activity;calcium-release channel activity;cation channel activity;cation transmembra
binding;cation binding;ion binding;metal ion binding;transition metal ion binding;zinc ion binding
binding;protein binding;SNARE binding;syntaxin binding;syntaxin-3 binding
2,4-dienoyl-CoA reductase (NADPH) activity;binding;catalytic activity;coenzyme binding;cofactor binding;NADP binding;NADPH binding;nucleotide binding;oxidoreductase activity;oxidoreductase activity, acting on NADH or
binding;catalytic activity;GTP binding;GTPase activity;guanyl nucleotide binding;guanyl ribonucleotide binding;hydrolase activity;hydrolase activity, acting on acid anhydrides;hydrolase activity, acting on acid anhydrides, in pl
binding;chromatin binding;chromo shadow domain binding;enzyme binding;histone deacetylase binding;protein binding;protein C-terminus binding;protein domain specific binding;protein N-terminus binding
binding;cation binding;ion binding;metal ion binding;mRNA binding;nucleic acid binding;poly(A) RNA binding;poly-purine tract binding;RNA binding;single-stranded RNA binding
binding;chromatin binding
binding;cation binding;ion binding;metal ion binding;transition metal ion binding;zinc ion binding
structural constituent of ribosome;structural molecule activity
catalytic activity;intramolecular oxidoreductase activity;intramolecular oxidoreductase activity, interconverting keto- and enol-groups;intramolecular oxidoreductase activity, transposing S-S bonds;isomerase activity;protein d
binding;catalytic activity;GTP binding;GTPase activity;guanyl nucleotide binding;guanyl ribonucleotide binding;hydrolase activity;hydrolase activity, acting on acid anhydrides;hydrolase activity, acting on acid anhydrides, in pl
binding;catalytic activity;cation binding;hydrolase activity;hydrolase activity, acting on acid anhydrides;hydrolase activity, acting on acid anhydrides, in phosphorus-containing anhydrides;hydrolase activity, acting on ester bor
binding;nucleic acid binding;RNA binding;rRNA binding
binding;enzyme binding;phosphatase binding;protein binding;protein complex scaffold;structural molecule activity
acid-amino acid ligase activity;binding;catalytic activity;cation binding;ion binding;ligase activity;ligase activity, forming carbon-nitrogen bonds;metal ion binding;small conjugating protein ligase activity;ubiquitin-protein ligase
binding;carbohydrate binding;cargo receptor activity;catalytic activity;cation binding;chromatin binding;copper ion binding;electron carrier activity;histone binding;ion binding;metal ion binding;methylated histone residue binc
binding;enzyme binding;kinase binding;phosphoprotein binding;protein binding;protein kinase binding;translation initiation factor binding
binding;mRNA 3'-UTR binding;mRNA 5'-UTR binding;mRNA binding;nucleic acid binding;nucleotide binding;RNA binding;translation regulator activity
binding;mRNA 3'-UTR binding;mRNA 5'-UTR binding;mRNA binding;nucleic acid binding;nucleotide binding;RNA binding;translation regulator activity

binding;catalytic activity;cation binding;hydrolase activity;hydrolase activity, acting on ester bonds;ion binding;metal ion binding;phosphatase activity;phosphoprotein phosphatase activity;phosphoric ester hydrolase activity;p
adenyl nucleotide binding;adenyl ribonucleotide binding;ATP binding;binding;catalytic activity;hydrolase activity;hydrolase activity, acting on acid anhydrides;hydrolase activity, acting on acid anhydrides, in phosphorus-conta
binding;identical protein binding;protein binding
catalytic activity;hydrolase activity
binding;calcium ion binding;cation binding;ion binding;metal ion binding
antigen binding;beta-2-microglobulin binding;binding;peptide antigen binding;peptide binding;protein binding;TAP binding
adenyl nucleotide binding;adenyl ribonucleotide binding;ATP binding;binding;catalytic activity;cyclin-dependent protein kinase activity;histone kinase activity;kinase activity;nucleotide binding;phosphotransferase activity, alco
binding;catalytic activity;coenzyme binding;cofactor binding;electron carrier activity;flavin adenine dinucleotide binding;oxidoreductase activity
binding;lipid binding;oxysterol binding;protein binding;protein domain specific binding;steroid binding;sterol binding
adenylosuccinate synthase activity;anion binding;binding;catalytic activity;cation binding;GTP binding;guanyl nucleotide binding;guanyl ribonucleotide binding;ion binding;ligase activity;ligase activity, forming carbon-nitrogen
binding;carbohydrate binding;cytoskeletal protein binding;dynactin binding;dynein binding;enzyme binding;glycosaminoglycan binding;heparin binding;identical protein binding;microtubule binding;pattern binding;phospholip
acid-amino acid ligase activity;adenyl nucleotide binding;adenyl ribonucleotide binding;ADP binding;amine binding;amino acid binding;ATP binding;binding;carboxylic acid binding;catalytic activity;cation binding;coenzyme b
binding;carbohydrate binding;cation binding;ion binding;mannose binding;metal ion binding;monosaccharide binding;protein binding;sugar binding;unfolded protein binding
binding;catalytic activity;cation binding;coenzyme binding;cofactor binding;ion binding;isocitrate dehydrogenase (NAD+) activity;isocitrate dehydrogenase activity;magnesium ion binding;metal ion binding;NAD binding;nucleo
biliverdin reductase activity;binding;catalytic activity;cation binding;ion binding;metal ion binding;oxidoreductase activity;oxidoreductase activity, acting on the CH-CH group of donors;oxidoreductase activity, acting on the Cl
binding;cation binding;ion binding;metal ion binding;transition metal ion binding;zinc ion binding
structural constituent of ribosome;structural molecule activity
binding;catalytic activity;demethylase activity;DNA binding;histone binding;histone demethylase activity;histone demethylase activity (H4-K20 specific);nucleic acid binding;protein binding
antioxidant activity;catalytic activity;disulfide oxidoreductase activity;glutathione dehydrogenase (ascorbate) activity;glutathione disulfide oxidoreductase activity;glutathione transferase activity;methylarsonate reductase activ
structural constituent of ribosome;structural molecule activity
binding;chromatin binding;core promoter proximal region DNA binding;core promoter proximal region sequence-specific DNA binding;DNA binding;nucleic acid binding;nucleic acid binding transcription factor activity;protein
binding;carbohydrate binding;catalytic activity;cation binding;coenzyme binding;cofactor binding;ion binding;L-iditol 2-dehydrogenase activity;metal ion binding;NAD binding;nucleotide binding;oxidoreductase activity;oxidor
binding;nucleic acid binding;RNA binding;structural constituent of ribosome;structural molecule activity
binding;identical protein binding;protein binding
enzyme inhibitor activity;enzyme regulator activity;kinase inhibitor activity;kinase regulator activity;protein kinase inhibitor activity;protein kinase regulator activity
binding;enzyme binding;protein binding;ubiquitin protein ligase binding
binding;DNA binding;enzyme binding;nucleic acid binding;protein binding;RNA binding;ubiquitin protein ligase binding
binding;catalytic activity;cytoskeletal protein binding;GDP binding;GTP binding;GTPase activity;guanyl nucleotide binding;guanyl ribonucleotide binding;hydrolase activity;hydrolase activity, acting on acid anhydrides;hydrolas
basal RNA polymerase II transcription machinery binding;basal transcription machinery binding;binding;cation binding;DNA binding;enzyme binding;ion binding;metal ion binding;nucleic acid binding;protein binding;RNA poly
adenyl nucleotide binding;adenyl ribonucleotide binding;aminoacyl-tRNA ligase activity;aspartate-tRNA ligase activity;aspartate-tRNA(Asn) ligase activity;ATP binding;binding;catalytic activity;identical protein binding;ligase ac
binding;double-stranded RNA binding;NF-kappaB binding;nucleic acid binding;protein binding;protein binding transcription factor activity;RNA binding;RNA polymerase II transcription factor binding;transcription coactivator
binding;protein binding;protein domain specific binding;WW domain binding
binding;nuclear localization sequence binding;peptide binding;signal sequence binding
binding;calcium ion binding;cation binding;ion binding;metal ion binding
binding;cation binding;DNA binding;ion binding;metal ion binding;nucleic acid binding;transition metal ion binding;zinc ion binding
binding;protein binding;unfolded protein binding
acetyl-CoA C-acetyltransferase activity;acetyl-CoA C-acyltransferase activity;acetyltransferase activity;C-acetyltransferase activity;C-acyltransferase activity;catalytic activity;transferase activity;transferase activity, transferring
adenyl nucleotide binding;adenyl ribonucleotide binding;ATP binding;binding;calcium ion binding;cation binding;GTP binding;guanyl nucleotide binding;guanyl ribonucleotide binding;ion binding;metal ion binding;nucleic acid
ion transmembrane transporter activity;substrate-specific transmembrane transporter activity;substrate-specific transporter activity;transmembrane transporter activity;transporter activity
adenyl nucleotide binding;adenyl ribonucleotide binding;ATP binding;binding;catalytic activity;helicase activity;hydrolase activity;hydrolase activity, acting on acid anhydrides;hydrolase activity, acting on acid anhydrides, in ph
catalytic activity;cytidylyltransferase activity;N-acetylneuraminate synthase activity;N-acylneuraminate cytidylyltransferase activity;N-acylneuraminate-9-phosphate synthase activity;nucleotidyltransferase activity;transferase a
adenyl nucleotide binding;adenyl ribonucleotide binding;ATP binding;ATPase activity;binding;catalytic activity;cation binding;GTP binding;guanyl nucleotide binding;guanyl ribonucleotide binding;hydrolase activity;hydrolase a
binding;nucleotide binding
binding;nucleotide binding
binding;nuclear export signal receptor activity;protein binding
actin binding;actin filament binding;binding;cytoskeletal protein binding;enzyme activator activity;enzyme regulator activity;protein binding
binding;enzyme regulator activity;lipid binding;phosphatase regulator activity;protein binding;protein phosphatase regulator activity;protein phosphatase type 1 regulator activity;small conjugating protein binding;ubiquitin binc
binding;nucleic acid binding;RNA binding;translation factor activity, nucleic acid binding;translation initiation factor activity
3'-5' exonuclease activity;3'-5'-exoribonuclease activity;binding;catalytic activity;endonuclease activity;enzyme regulator activity;exonuclease activity;exonuclease activity, active with either ribo- or deoxyribonucleic acids anc
adenyl nucleotide binding;adenyl ribonucleotide binding;ATP binding;binding;nucleotide binding;purine nucleotide binding;purine ribonucleoside triphosphate binding;purine ribonucleotide binding;ribonucleotide binding
acyl-CoA thioesterase activity;binding;carboxylic acid binding;carboxylic ester hydrolase activity;catalytic activity;CoA hydrolase activity;coenzyme binding;cofactor binding;fatty acid binding;fatty-acyl-CoA binding;hydrolase
binding;enzyme binding;GTP-dependent protein binding;phosphatase binding;protein binding;protein phosphatase binding
acetyltransferase activator activity;beta-tubulin binding;binding;carbohydrate binding;cytoskeletal protein binding;DNA binding;enzyme activator activity;enzyme regulator activity;glycosaminoglycan binding;heparin binding;n
binding;DNA binding;double-stranded DNA binding;mRNA binding;nucleic acid binding;nucleotide binding;poly(A) RNA binding;poly(G) RNA binding;poly-purine tract binding;RNA binding;single-stranded DNA binding;single-
binding;nucleic acid binding;RNA binding;translation factor activity, nucleic acid binding;translation initiation factor activity
binding;calcium ion binding;cation binding;ion binding;metal ion binding
acid-amino acid ligase activity;binding;catalytic activity;cation binding;ion binding;ligase activity;ligase activity, forming carbon-nitrogen bonds;metal ion binding;nucleic acid binding;RNA binding;small conjugating protein liga
3'(2'),5'-bisphosphate nucleotidase activity;binding;catalytic activity;cation binding;hydrolase activity;hydrolase activity, acting on ester bonds;inositol bisphosphate phosphatase activity;inositol or phosphatidylinositol phosph
binding;nucleic acid binding;RNA binding;structural constituent of ribosome;structural molecule activity
catalytic activity;cis-stilbene-oxide hydrolase activity;epoxide hydrolase activity;ether hydrolase activity;hydrolase activity;hydrolase activity, acting on ether bonds
binding;catalytic activity;collagen binding;cysteine-type endopeptidase activity;cysteine-type peptidase activity;endopeptidase activity;glycoprotein binding;hydrolase activity;peptidase activity;peptidase activity, acting on L-a
binding;catalytic activity;GTP binding;GTPase activity;guanyl nucleotide binding;guanyl ribonucleotide binding;hydrolase activity;hydrolase activity, acting on acid anhydrides;hydrolase activity, acting on acid anhydrides, in ph
structural constituent of ribosome;structural molecule activity
binding;laminin receptor activity;receptor activity;ribonucleoprotein binding;ribosome binding;structural constituent of ribosome;structural molecule activity
carboxylic ester hydrolase activity;catalytic activity;deacetylase activity;hydrolase activity;hydrolase activity, acting on ester bonds;methylumbelliferyl-acetate deacetylase activity;S-formylglutathione hydrolase activity;thiolest
acyl-CoA dehydrogenase activity;binding;catalytic activity;coenzyme binding;cofactor binding;flavin adenine dinucleotide binding;identical protein binding;medium-chain-acyl-CoA dehydrogenase activity;oxidoreductase acti
catalytic activity;endopeptidase activity;exopeptidase activity;hydrolase activity;omega peptidase activity;peptidase activity;peptidase activity, acting on L-amino acid peptides;serine hydrolase activity;serine-type endopeptic
acid-ammonia (or amide) ligase activity;adenyl nucleotide binding;adenyl ribonucleotide binding;amine binding;amino acid binding;ammonia ligase activity;ATP binding;binding;carbon-carbon lyase activity;carboxylic acid bin
receptor activity
binding;cation binding;ion binding;metal ion binding;nucleic acid binding;RNA binding;translation factor activity, nucleic acid binding;translation initiation factor activity
amine binding;amino acid binding;binding;carboxylic acid binding;catalytic activity;enzyme binding;glutathione binding;glutathione transferase activity;identical protein binding;modified amino acid binding;peptide binding;pro
catalytic activity;endopeptidase activity;hydrolase activity;peptidase activity;peptidase activity, acting on L-amino acid peptides;threonine-type endopeptidase activity;threonine-type peptidase activity
binding;cation binding;chromatin binding;damaged DNA binding;DNA binding;ion binding;metal ion binding;nucleic acid binding;single-stranded DNA binding;structure-specific DNA binding
antioxidant activity;catalytic activity;oxidoreductase activity;oxidoreductase activity, acting on paired donors, with incorporation or reduction of molecular oxygen;oxidoreductase activity, acting on peroxide as acceptor;perox
binding;nucleic acid binding;RNA binding;rRNA binding;structural constituent of ribosome;structural molecule activity
binding;endopeptidase inhibitor activity;endopeptidase regulator activity;enzyme binding;enzyme inhibitor activity;enzyme regulator activity;peptidase inhibitor activity;peptidase regulator activity;protease binding;protein binc
4-alpha-glucanotransferase activity;alpha-glucosidase activity;amylo-alpha-1,6-glucosidase activity;binding;carbohydrate binding;catalytic activity;glucosidase activity;glycogen debranching enzyme activity;hydrolase activity
nucleocytoplasmic transporter activity;transporter activity
electron carrier activity
catalytic activity;methyltransferase activity;nicotinamide N-methyltransferase activity;N-methyltransferase activity;S-adenosylmethionine-dependent methyltransferase activity;transferase activity;transferase activity, transferrin
15-hydroxyprostaglandin-D dehydrogenase (NADP+) activity;alcohol dehydrogenase (NADP+) activity;alditol:NADP+ 1-oxidoreductase activity;aldo-keto reductase (NADP) activity;androsterone dehydrogenase activity;catalyt
binding;nucleic acid binding;RNA binding;structural constituent of ribosome;structural molecule activity
binding;nucleic acid binding;RNA binding;structural constituent of ribosome;structural molecule activity
adenylate cyclase activity;binding;catalytic activity;cation binding;cyclase activity;GTP binding;GTPase activity;guanyl nucleotide binding;guanyl ribonucleotide binding;hydrolase activity;hydrolase activity, acting on acid anhy
binding;catalytic activity;cation binding;G-protein beta/gamma-subunit complex binding;G-protein-coupled receptor binding;GTP binding;GTPase activity;guanyl nucleotide binding;guanyl ribonucleotide binding;hydrolase ac
binding;cation binding;identical protein binding;ion binding;metal ion binding;nucleotide binding;protein binding;protein complex binding;transition metal ion binding;zinc ion binding
binding;carboxylic acid binding;fatty acid binding;lipid binding;monocarboxylic acid binding;transporter activity
3'-5' exonuclease activity;binding;catalytic activity;exonuclease activity;exoribonuclease activity;hydrolase activity;hydrolase activity, acting on ester bonds;nuclease activity;nucleotide binding;ribonuclease activity
binding;calcium ion binding;calmodulin binding;catalytic activity;cation binding;hydrolase activity;hydrolase activity, acting on ester bonds;ion binding;lipase activity;metal ion binding;molecular transducer activity;phosphatidy
binding;chromatin binding;chromatin DNA binding;DNA binding;nucleic acid binding;structure-specific DNA binding
adenyl nucleotide binding;adenyl ribonucleotide binding;ATP binding;binding;catalytic activity;enzyme activator activity;enzyme regulator activity;hydrolase activity;hydrolase activity, acting on ester bonds;kinase activator act
binding;binding, bridging;catalytic activity;hydrolase activity;hydrolase activity, acting on ester bonds;insulin receptor binding;non-membrane spanning protein tyrosine phosphatase activity;phosphatase activity;phosphoprote
enzyme inhibitor activity;enzyme regulator activity;GTPase inhibitor activity;GTPase regulator activity;nucleoside-triphosphatase regulator activity
molecular transducer activity;signal transducer activity
adenyl nucleotide binding;adenyl ribonucleotide binding;ATP binding;binding;catalytic activity;kinase activity;nucleotide binding;phosphotransferase activity, alcohol group as acceptor;protein kinase activity;protein tyrosine ki
actin binding;binding;cytoskeletal protein binding;profilin binding;protein binding
adenyl nucleotide binding;adenyl ribonucleotide binding;ATP binding;ATPase activity;ATPase activity, coupled;ATP-dependent helicase activity;ATP-dependent RNA helicase activity;binding;catalytic activity;helicase activity;h
binding;cation binding;enzyme binding;ion binding;metal ion binding;protein binding;small conjugating protein binding;transition metal ion binding;ubiquitin binding;ubiquitin protein ligase binding;zinc ion binding
actin binding;actin filament binding;binding;cytoskeletal protein binding;protein binding
acid-thiol ligase activity;adenyl nucleotide binding;adenyl ribonucleotide binding;ATP binding;binding;catalytic activity;CoA-ligase activity;GDP binding;GTP binding;guanyl nucleotide binding;guanyl ribonucleotide binding;liga
adenyl nucleotide binding;adenyl ribonucleotide binding;ATP binding;binding;DNA binding;nucleic acid binding;nucleotide binding;purine nucleotide binding;purine ribonucleoside triphosphate binding;purine ribonucleotide bi
binding;G-protein coupled receptor activity;molecular transducer activity;opioid receptor activity;peptide binding;peptide receptor activity;peptide receptor activity, G-protein coupled;receptor activity;signal transducer activity
acetyl-CoA hydrolase activity;carboxylic ester hydrolase activity;catalytic activity;CoA hydrolase activity;hydrolase activity;hydrolase activity, acting on ester bonds;thiolester hydrolase activity
binding;ribonucleoprotein binding
binding;nucleic acid binding;RNA binding;snoRNA binding
activating transcription factor binding;binding;chromatin binding;DNA binding;nucleic acid binding;protein binding;protein binding transcription factor activity;RNA polymerase II transcription coactivator activity;RNA polymera
aspartic-type endopeptidase activity;aspartic-type peptidase activity;catalytic activity;endopeptidase activity;hydrolase activity;peptidase activity;peptidase activity, acting on L-amino acid peptides
beta-N-acetylhexosaminidase activity;binding;catalytic activity;hexosaminidase activity;hydrolase activity;hydrolase activity, acting on glycosyl bonds;hydrolase activity, hydrolyzing O-glycosyl compounds;identical protein bir
actin binding;actin monomer binding;adenyl nucleotide binding;adenyl-nucleotide exchange factor activity;ATPase regulator activity;binding;cytoskeletal protein binding;enzyme regulator activity;lipid binding;nucleoside-triph
binding;cation binding;cytokine receptor binding;growth factor receptor binding;ion binding;metal ion binding;platelet-derived growth factor receptor binding;protein binding;receptor binding;vascular endothelial growth factor
alcohol dehydrogenase (NAD) activity;binding;carboxylic acid binding;catalytic activity;cation binding;electron carrier activity;fatty acid binding;formaldehyde dehydrogenase activity;ion binding;lipid binding;metal ion binding;
adenyl nucleotide binding;adenyl ribonucleotide binding;ADP binding;AMP binding;ATP binding;binding;carbohydrate binding;catalytic activity;cation binding;diphosphotransferase activity;GDP binding;guanyl nucleotide binc
binding;nucleic acid binding;RNA binding;structural constituent of ribosome;structural molecule activity;tRNA binding
binding;catalytic activity;cation binding;endopeptidase activity;hydrolase activity;ion binding;metal ion binding;metalloendopeptidase activity;metallopeptidase activity;peptidase activity;peptidase activity, acting on L-amino a
3'-5' exonuclease activity;3'-5'-exodeoxyribonuclease activity;binding;carbon-oxygen lyase activity;catalytic activity;cation binding;chromatin binding;chromatin DNA binding;damaged DNA binding;deoxyribonuclease activity
adenyl nucleotide binding;adenyl ribonucleotide binding;ATP binding;binding;catalytic activity;DNA binding;enzyme binding;kinase activity;MAP kinase activity;molecular transducer activity;nucleic acid binding;nucleotide binc
(S)-2-(5-amino-1-(5-phospho-D-ribosyl)imidazole-4-carboxamido)succinate AMP-lyase (fumarate-forming) activity;amidine-lyase activity;carbon-nitrogen lyase activity;catalytic activity;lyase activity;N6-(1,2-dicarboxyethyl)AM
aldehyde dehydrogenase (NAD) activity;catalytic activity;oxidoreductase activity;oxidoreductase activity, acting on the aldehyde or oxo group of donors;oxidoreductase activity, acting on the aldehyde or oxo group of donors,
adenyl nucleotide binding;adenyl ribonucleotide binding;ATP binding;binding;catalytic activity;helicase activity;hydrolase activity;hydrolase activity, acting on acid anhydrides;hydrolase activity, acting on acid anhydrides, in ph
acyl-CoA dehydrogenase activity;binding;catalytic activity;coenzyme binding;cofactor binding;flavin adenine dinucleotide binding;long-chain-acyl-CoA dehydrogenase activity;oxidoreductase activity;oxidoreductase activity, a
adenyl nucleotide binding;adenyl ribonucleotide binding;ATP binding;binding;caspase inhibitor activity;caspase regulator activity;catalytic activity;cation binding;cysteine-type endopeptidase inhibitor activity;endopeptidase ir
adenyl nucleotide binding;adenyl ribonucleotide binding;adenylate kinase activity;ATP binding;binding;catalytic activity;kinase activity;nucleobase-containing compound kinase activity;nucleotide binding;nucleotide kinase act

structural constituent of ribosome;structural molecule activity
binding;nucleic acid binding;RNA binding;structural constituent of ribosome;structural molecule activity
structural constituent of ribosome;structural molecule activity
binding;mRNA binding;nucleic acid binding;nucleotide binding;RNA binding
active transmembrane transporter activity;anion transmembrane transporter activity;binding;inorganic anion transmembrane transporter activity;ion transmembrane transporter activity;phosphate ion carrier activity;phosphate
adenyl nucleotide binding;adenyl ribonucleotide binding;ATP binding;ATPase activity;ATPase activity, coupled;binding;catalytic activity;cation binding;chromatin binding;DNA bending activity;DNA binding;DNA topoisomerase
ATPase activity;catalytic activity;hydrolase activity;hydrolase activity, acting on acid anhydrides;hydrolase activity, acting on acid anhydrides, in phosphorus-containing anhydrides;nucleoside-triphosphatase activity;pyrophos
binding;enzyme regulator activity;GTP binding;guanyl nucleotide binding;guanyl ribonucleotide binding;nucleotide binding;purine nucleotide binding;purine ribonucleoside triphosphate binding;purine ribonucleotide binding;ri
binding;enzyme activator activity;enzyme binding;enzyme regulator activity;GTPase activator activity;GTPase binding;GTPase regulator activity;nucleoside-triphosphatase regulator activity;protein binding;Rab GTPase bindin
binding;DNA binding;nucleic acid binding;RNA binding;single-stranded DNA binding;structure-specific DNA binding
AU-rich element binding;binding;double-stranded RNA binding;enzyme binding;kinase binding;mRNA 3'-UTR AU-rich region binding;mRNA 3'-UTR binding;mRNA binding;nucleic acid binding;nucleotide binding;protein bind
structural molecule activity
adenine nucleotide transmembrane transporter activity;ATP transmembrane transporter activity;binding;calcium ion binding;cation binding;ion binding;metal ion binding;nucleobase-containing compound transmembrane tran
adenyl nucleotide binding;adenyl ribonucleotide binding;ATP binding;binding;catalytic activity;formate-tetrahydrofolate ligase activity;identical protein binding;ligase activity;ligase activity, forming carbon-nitrogen bonds;nucle
binding;enzyme binding;GTPase binding;leucine zipper domain binding;LRR domain binding;PDZ domain binding;protein binding;protein domain specific binding;Rab GTPase binding;Ras GTPase binding;small GTPase bind
3'-5' exonuclease activity;3'-5'-exoribonuclease activity;binding;catalytic activity;exonuclease activity;exonuclease activity, active with either ribo- or deoxyribonucleic acids and producing 5'-phosphomonoesters;exoribonucl
nucleocytoplasmic transporter activity;transporter activity
actin binding;binding;cytoskeletal protein binding;protein binding
binding;mRNA binding;nucleic acid binding;nucleotide binding;RNA binding
binding;enzyme activator activity;enzyme binding;enzyme regulator activity;GTPase activator activity;GTPase binding;GTPase regulator activity;nucleoside-triphosphatase regulator activity;protein binding;protein dimerization
binding;enzyme binding;GTPase binding;mRNA binding;nucleic acid binding;pre-miRNA binding;protein binding;protein transporter activity;Ran GTPase binding;Ras GTPase binding;RNA binding;small GTPase binding;subst
binding;identical protein binding;protein binding;protein dimerization activity;protein homodimerization activity
acid-thiol ligase activity;adenyl nucleotide binding;adenyl ribonucleotide binding;ATP binding;binding;catalytic activity;cation binding;CoA-ligase activity;ion binding;ligase activity;ligase activity, forming carbon-sulfur bonds;m
alkylglycerone-phosphate synthase activity;binding;catalytic activity;coenzyme binding;cofactor binding;FAD binding;flavin adenine dinucleotide binding;oxidoreductase activity;oxidoreductase activity, acting on CH-OH grou
binding;cation binding;ion binding;metal ion binding;protein binding transcription factor activity;transcription coactivator activity;transcription cofactor activity;transcription factor binding transcription factor activity;transition r
binding;chromatin binding;enzyme binding;protein binding;protein dimerization activity;protein heterodimerization activity
structural constituent of cytoskeleton;structural molecule activity
7S RNA binding;binding;nucleic acid binding;ribonucleoprotein binding;RNA binding;signal recognition particle binding
binding;catalytic activity;cation binding;G-protein beta/gamma-subunit complex binding;G-protein-coupled receptor binding;GTP binding;GTPase activity;guanyl nucleotide binding;guanyl ribonucleotide binding;hydrolase ac
binding;cation binding;ion binding;metal ion binding;transition metal ion binding;zinc ion binding
binding;cation binding;enzyme binding;glycoprotein binding;ion binding;metal ion binding;protease binding;protein binding;protein dimerization activity;protein heterodimerization activity
binding;cation binding;cytoskeletal protein binding;identical protein binding;ion binding;metal ion binding;microtubule binding;microtubule plus-end binding;nucleic acid binding;protein binding;protein dimerization activity;pro
binding;DNA binding;nucleic acid binding
active transmembrane transporter activity;ATPase activity;ATPase activity, coupled;ATPase activity, coupled to movement of substances;ATPase activity, coupled to transmembrane movement of ions;ATPase activity, couplec
active transmembrane transporter activity;adenyl nucleotide binding;adenyl ribonucleotide binding;ATP binding;ATPase activity;ATPase activity, coupled;ATPase activity, coupled to movement of substances;ATPase activity, c
binding;core promoter proximal region DNA binding;core promoter proximal region sequence-specific DNA binding;DNA binding;enzyme binding;kinase binding;ligand-dependent nuclear receptor activity;molecular transduce
acetyl-CoA C-acyltransferase activity;C-acyltransferase activity;catalytic activity;transferase activity;transferase activity, transferring acyl groups;transferase activity, transferring acyl groups other than amino-acyl groups
enzyme activator activity;enzyme regulator activity;GTPase activator activity;GTPase regulator activity;nucleoside-triphosphatase regulator activity
beta-catenin binding;binding;cation binding;cytoskeletal protein binding;ion binding;metal ion binding;protein binding;transition metal ion binding;vinculin binding;zinc ion binding
binding;G-quadruplex RNA binding;mRNA 3'-UTR binding;mRNA binding;nucleic acid binding;RNA binding
binding;nucleic acid binding;nucleotide binding;RNA binding;single-stranded RNA binding
actin binding;binding;cytoskeletal protein binding;protein binding
binding;peptide binding;structural constituent of nuclear pore;structural molecule activity;transporter activity
binding;catalytic activity;cysteine-type endopeptidase activity;cysteine-type peptidase activity;endopeptidase activity;endopeptidase inhibitor activity;endopeptidase regulator activity;enzyme inhibitor activity;enzyme regulato
binding;DNA binding;nucleic acid binding
binding;DNA binding;nucleic acid binding
adenyl nucleotide binding;adenyl ribonucleotide binding;ADP binding;AMP binding;ATP binding;binding;carbohydrate binding;catalytic activity;cation binding;diphosphotransferase activity;GDP binding;guanyl nucleotide binc
binding;catalytic activity;GTP binding;GTPase activity;guanyl nucleotide binding;guanyl ribonucleotide binding;hydrolase activity;hydrolase activity, acting on acid anhydrides;hydrolase activity, acting on acid anhydrides, in pl
binding;calcium channel regulator activity;catalytic activity;channel regulator activity;enzyme binding;GTPase activity;GTPase binding;hydrolase activity;hydrolase activity, acting on acid anhydrides;hydrolase activity, acting o
binding;carbohydrate binding
binding;DNA binding;enzyme inhibitor activity;enzyme regulator activity;histone binding;nucleic acid binding;phosphatase inhibitor activity;phosphatase regulator activity;protein binding;protein phosphatase inhibitor activity;p
binding;catalytic activity;enzyme binding;GTP binding;GTPase activity;guanyl nucleotide binding;guanyl ribonucleotide binding;hydrolase activity;hydrolase activity, acting on acid anhydrides;hydrolase activity, acting on acid
actin binding;adenyl nucleotide binding;adenyl ribonucleotide binding;ATP binding;binding;catalytic activity;cytoskeletal protein binding;enzyme binding;JUN kinase binding;kinase activity;kinase binding;molecular transducer
binding;catalytic activity;isomerase activity;protein binding;receptor binding
AU-rich element binding;binding;enzyme binding;kinase binding;nucleic acid binding;nucleotide binding;protein binding;protein kinase binding;RNA binding
adenyl nucleotide binding;adenyl ribonucleotide binding;ATP binding;binding;catalytic activity;enzyme activator activity;enzyme binding;enzyme regulator activity;GTPase binding;identical protein binding;kinase activator activ
binding;nucleic acid binding;RNA binding;translation factor activity, nucleic acid binding;translation initiation factor activity
binding;catalytic activity;cation binding;hydrolase activity;hydrolase activity, acting on acid anhydrides;hydrolase activity, acting on acid anhydrides, in phosphorus-containing anhydrides;inorganic diphosphatase activity;ion b
adenyl nucleotide binding;adenyl ribonucleotide binding;ATP binding;binding;caspase inhibitor activity;caspase regulator activity;catalytic activity;cation binding;cysteine-type endopeptidase inhibitor activity;endopeptidase ir
binding;cation binding;ion binding;metal ion binding;transition metal ion binding;zinc ion binding
binding;catalytic activity;cofactor binding;hydrolase activity;hydrolase activity, acting on acid carbon-carbon bonds;hydrolase activity, acting on acid carbon-carbon bonds, in ketonic substances;identical protein binding;kynu
3-hydroxyacyl-CoA dehydrogenase activity;binding;catalytic activity;coenzyme binding;cofactor binding;NAD binding;NAD+ binding;nucleotide binding;oxidoreductase activity;oxidoreductase activity, acting on CH-OH group
binding;DNA binding;double-stranded RNA binding;identical protein binding;nucleic acid binding;protein binding;protein dimerization activity;protein homodimerization activity;RNA binding
binding;DNA binding;nucleic acid binding
ligand-dependent nuclear receptor transcription coactivator activity;protein binding transcription factor activity;transcription coactivator activity;transcription cofactor activity;transcription factor binding transcription factor act
binding;cadherin binding;cell adhesion molecule binding;cytoskeletal protein binding;enzyme binding;gamma-tubulin binding;GTPase binding;microtubule binding;protein binding;Rab GTPase binding;Ras GTPase binding;sm
binding;enzyme binding;enzyme regulator activity;GTPase binding;GTPase regulator activity;guanyl-nucleotide exchange factor activity;nucleoside-triphosphatase regulator activity;protein binding;Rac GTPase binding;Ras G
binding;histone binding;protein binding;protein transporter activity;substrate-specific transporter activity;transporter activity
androgen receptor binding;binding;catalytic activity;cation binding;copper chaperone activity;copper ion binding;cytokine binding;enzyme binding;glyoxalase III activity;hormone receptor binding;hydrolase activity;identical pr
actin binding;actin filament binding;Arp2/3 complex binding;binding;cytoskeletal protein binding;identical protein binding;protein binding;protein complex binding
binding;nucleic acid binding;ribonucleoprotein binding;ribosome binding;RNA binding;translation factor activity, nucleic acid binding;translation initiation factor activity;tRNA binding
aminopeptidase activity;binding;catalytic activity;cation binding;exopeptidase activity;hydrolase activity;identical protein binding;ion binding;manganese ion binding;metal ion binding;metalloaminopeptidase activity;metalloex
binding;GTP binding;guanyl nucleotide binding;guanyl ribonucleotide binding;nucleotide binding;purine nucleotide binding;purine ribonucleoside triphosphate binding;purine ribonucleotide binding;ribonucleotide binding
binding;catalytic activity;cation binding;dipeptidyl-peptidase activity;exopeptidase activity;hydrolase activity;ion binding;metal ion binding;metallopeptidase activity;peptidase activity;peptidase activity, acting on L-amino acid
ATP-dependent protein binding;binding;catalytic activity;enzyme activator activity;enzyme regulator activity;protein binding;protein C-terminus binding;protein dimerization activity;protein heterodimerization activity;small prot
binding;cytoskeletal protein binding;identical protein binding;lipid binding;phosphatidic acid binding;phospholipid binding;protein binding;transporter activity
binding;cytoskeletal protein binding;dynactin binding;identical protein binding;lipid binding;phosphatidylinositol binding;phospholipid binding;protein binding;protein dimerization activity;protein homodimerization activity
armadillo repeat domain binding;binding;estrogen receptor binding;hormone receptor binding;nuclear hormone receptor binding;protein binding;protein complex scaffold;protein domain specific binding;receptor binding;retin
anion channel activity;anion transmembrane transporter activity;channel activity;chloride channel activity;gated channel activity;ion channel activity;ion transmembrane transporter activity;passive transmembrane transporter
binding;catalytic activity;endopeptidase activity;hydrolase activity;identical protein binding;peptidase activity;peptidase activity, acting on L-amino acid peptides;protein binding;threonine-type endopeptidase activity;threonin
binding;catalytic activity;hydrolase activity;protein binding;protein domain specific binding;SH2 domain binding
binding;calcium-dependent phospholipid binding;catalytic activity;kinase activity;lipid binding;phospholipid binding;phosphotransferase activity, alcohol group as acceptor;protein kinase activity;protein serine/threonine kinas
macromolecule transmembrane transporter activity;protein transmembrane transporter activity;protein transporter activity;substrate-specific transmembrane transporter activity;substrate-specific transporter activity;transmer
adenyl nucleotide binding;adenyl ribonucleotide binding;adenylylsulfate kinase activity;adenylyltransferase activity;ATP binding;binding;catalytic activity;kinase activity;nucleotide binding;nucleotidyltransferase activity;phosph
binding;C5a anaphylatoxin chemotactic receptor binding;C5L2 anaphylatoxin chemotactic receptor binding;endopeptidase inhibitor activity;endopeptidase regulator activity;enzyme inhibitor activity;enzyme regulator activity;
adenine transmembrane transporter activity;nucleobase transmembrane transporter activity;nucleobase-containing compound transmembrane transporter activity;purine base transmembrane transporter activity;substrate-sp
alcohol dehydrogenase (NADP+) activity;alditol:NADP+ 1-oxidoreductase activity;aldo-keto reductase (NADP) activity;catalytic activity;electron carrier activity;glyceraldehyde oxidoreductase activity;oxidoreductase activity;ox
15-hydroxyprostaglandin dehydrogenase (NADP+) activity;carbonyl reductase (NADPH) activity;catalytic activity;oxidoreductase activity;oxidoreductase activity, acting on CH-OH group of donors;oxidoreductase activity, actii
binding;catalytic activity;cation binding;coenzyme binding;cofactor binding;cytochrome-b5 reductase activity;dioxygenase activity;electron carrier activity;flavin adenine dinucleotide binding;FMN binding;hydrolase activity;ior
endopeptidase inhibitor activity;endopeptidase regulator activity;enzyme inhibitor activity;enzyme regulator activity;peptidase inhibitor activity;peptidase regulator activity;serine-type endopeptidase inhibitor activity
binding;complement binding;complement component C3a binding;complement component C3b binding;DNA binding;enzyme binding;G-protein-coupled receptor binding;histone deacetylase binding;nucleic acid binding;nu
binding;DNA binding;histone binding;nucleic acid binding;protein binding
binding;cytokine activity;growth factor receptor binding;high molecular weight B cell growth factor receptor binding;protein binding;receptor binding
binding;cation binding;enzyme binding;ion binding;kinase binding;metal ion binding;protein binding;protein kinase binding;transition metal ion binding;zinc ion binding
1-pyrroline dehydrogenase activity;3-chloroallyl aldehyde dehydrogenase activity;4-trimethylammoniobutyraldehyde dehydrogenase activity;aldehyde dehydrogenase (NAD) activity;amine binding;aminobutyraldehyde dehydro
binding;nucleic acid binding;protein binding;protein N-terminus binding;RNA binding;translation factor activity, nucleic acid binding;translation initiation factor activity
binding;mRNA binding;nucleic acid binding;RNA binding;rRNA binding;SSU rRNA binding;structural constituent of ribosome;structural molecule activity
binding;catalytic activity;enzyme binding;GTPase activity;GTPase binding;hydrolase activity;hydrolase activity, acting on acid anhydrides;hydrolase activity, acting on acid anhydrides, in phosphorus-containing anhydrides;mo
binding;calcium ion binding;cation binding;DNA binding;ion binding;metal ion binding;nucleic acid binding
binding;catalytic activity;cytoskeletal protein binding;hydrolase activity;hydrolase activity, acting on acid anhydrides;hydrolase activity, acting on acid anhydrides, in phosphorus-containing anhydrides;motor activity;nucleosid
binding;protein binding;receptor binding;structural molecule activity
binding;histone binding;protein binding
catalytic activity;endonuclease activity;endonuclease activity, active with either ribo- or deoxyribonucleic acids and producing 5'-phosphomonoesters;endoribonuclease activity;endoribonuclease activity, producing 5'-phosph
binding;heat shock protein binding;protein binding
binding;catalytic activity;GTP binding;GTPase activity;guanyl nucleotide binding;guanyl ribonucleotide binding;hydrolase activity;hydrolase activity, acting on acid anhydrides;hydrolase activity, acting on acid anhydrides, in pl
adenyl nucleotide binding;adenyl ribonucleotide binding;ATP binding;ATPase activity;ATPase activity, coupled;ATP-dependent helicase activity;ATP-dependent RNA helicase activity;binding;catalytic activity;helicase activity;h
adenyl nucleotide binding;adenyl ribonucleotide binding;ATP binding;ATPase activity;binding;catalytic activity;hydrolase activity;hydrolase activity, acting on acid anhydrides;hydrolase activity, acting on acid anhydrides, in ph
binding;nucleic acid binding;nucleotide binding;RNA binding
binding;catalytic activity;cofactor binding;O-phospho-L-serine:2-oxoglutarate aminotransferase activity;pyridoxal phosphate binding;transaminase activity;transferase activity;transferase activity, transferring nitrogenous grou
adenyl nucleotide binding;adenyl ribonucleotide binding;ATP binding;ATPase activity;ATPase activity, coupled;ATP-dependent helicase activity;ATP-dependent RNA helicase activity;binding;catalytic activity;helicase activity;h
binding;nucleic acid binding;RNA binding;RNA cap binding;translation factor activity, nucleic acid binding;translation initiation factor activity
adenyl nucleotide binding;adenyl ribonucleotide binding;ATP binding;ATPase activity;ATPase activity, coupled;ATP-dependent helicase activity;ATP-dependent RNA helicase activity;binding;catalytic activity;helicase activity;h
structural constituent of cytoskeleton;structural molecule activity
adenyl nucleotide binding;adenyl ribonucleotide binding;adenylate kinase activity;ATP binding;binding;catalytic activity;kinase activity;nucleobase-containing compound kinase activity;nucleoside diphosphate kinase activity;r
binding;catalytic activity;cofactor binding;ornithine-oxo-acid transaminase activity;pyridoxal phosphate binding;transaminase activity;transferase activity;transferase activity, transferring nitrogenous groups;vitamin B6 binding
adenyl nucleotide binding;adenyl ribonucleotide binding;ATP binding;ATPase activity;beta-amyloid binding;beta-endorphin binding;binding;catalytic activity;cation binding;endopeptidase activity;glycoprotein binding;hormone
binding;chromatin binding;chromatin DNA binding;DNA binding;enzyme binding;histone deacetylase binding;nucleic acid binding;protein binding;structure-specific DNA binding
binding;carbon-carbon lyase activity;carboxylic acid binding;carboxy-lyase activity;catalytic activity;cofactor binding;L-aspartate:2-oxoglutarate aminotransferase activity;L-cysteine:2-oxoglutarate aminotransferase activity;L
cysteine-type endopeptidase inhibitor activity;endopeptidase inhibitor activity;endopeptidase regulator activity;enzyme inhibitor activity;enzyme regulator activity;peptidase inhibitor activity;peptidase regulator activity
adenyl nucleotide binding;adenyl ribonucleotide binding;ATP binding;ATPase activity;binding;catalytic activity;chromatin binding;DNA binding;helicase activity;hydrolase activity;hydrolase activity, acting on acid anhydrides;hy
enzyme activator activity;enzyme regulator activity;GDP-dissociation inhibitor activity;GTPase activator activity;GTPase regulator activity;nucleoside-triphosphatase regulator activity;Rab GDP-dissociation inhibitor activity;sm
adenyl nucleotide binding;adenyl ribonucleotide binding;ATP binding;binding;catalytic activity;enzyme activator activity;enzyme regulator activity;kinase activator activity;kinase activity;kinase regulator activity;MAP kinase kin
catalytic activity;methyltransferase activity;S-adenosylmethionine-dependent methyltransferase activity;transferase activity;transferase activity, transferring one-carbon groups

binding;nucleic acid binding;RNA binding;structural constituent of ribosome;structural molecule activity
3-oxoacid CoA-transferase activity;binding;catalytic activity;CoA-transferase activity;identical protein binding;protein binding;protein dimerization activity;protein homodimerization activity;transferase activity;transferase activ
binding;cation binding;collagen binding;enzyme binding;ion binding;metal ion binding;phosphatase binding;protein binding;protein phosphatase binding
structural constituent of ribosome;structural molecule activity
binding;enzyme binding;kinase binding;protein binding;protein kinase binding;SNARE binding;syntaxin binding
binding;enzyme binding;histone binding;histone deacetylase binding;protein binding
binding;cation binding;ion binding;metal ion binding;transition metal ion binding;zinc ion binding
binding;nucleic acid binding;RNA binding
binding;enzyme activator activity;enzyme regulator activity;GTPase activating protein binding;GTPase activator activity;GTPase regulator activity;guanyl-nucleotide exchange factor activity;nucleoside-triphosphatase regulato
binding;carbon-carbon lyase activity;carboxy-lyase activity;catalytic activity;cation binding;GTP binding;guanyl nucleotide binding;guanyl ribonucleotide binding;ion binding;lyase activity;metal ion binding;nucleotide binding;p
binding;catalytic activity;cysteine-type endopeptidase activity;cysteine-type peptidase activity;endopeptidase activity;enzyme binding;hydrolase activity;p53 binding;peptidase activity;peptidase activity, acting on L-amino aci
binding;lipid binding;phosphatidylinositol binding;phosphatidylinositol-3,4,5-trisphosphate binding;phospholipid binding
binding;binding, bridging;ligand-dependent nuclear receptor transcription coactivator activity;nucleic acid binding;nucleotide binding;protein binding;protein binding transcription factor activity;protein binding, bridging;RNA b
binding;cation binding;ion binding;metal ion binding;transition metal ion binding;zinc ion binding
adenyl nucleotide binding;adenyl ribonucleotide binding;aminoacyl-tRNA ligase activity;ATP binding;binding;catalytic activity;cation binding;ion binding;ligase activity;ligase activity, forming aminoacyl-tRNA and related compo
adenyl nucleotide binding;adenyl ribonucleotide binding;ATP binding;binding;calcium ion binding;cation binding;DNA binding;ion binding;metal ion binding;nucleic acid binding;nucleotide binding;purine nucleotide binding;pu
binding;DNA binding;identical protein binding;nucleic acid binding;nucleotide binding;protein binding
adenyl nucleotide binding;adenyl ribonucleotide binding;ATP binding;binding;catalytic activity;cation binding;cytoskeletal protein binding;ion binding;ligase activity;ligase activity, forming phosphoric ester bonds;metal ion bin
binding;carbohydrate binding;extracellular matrix binding;glycosaminoglycan binding;heparin binding;integrin binding;pattern binding;polysaccharide binding;protein binding;protein complex binding;receptor binding
14-3-3 protein binding;binding;enzyme binding;enzyme inhibitor activity;enzyme regulator activity;kinase binding;molecular transducer activity;phosphatase regulator activity;protein binding;protein kinase binding;signal trans
molecular transducer activity;receptor activity;semaphorin receptor activity;signal transducer activity;signaling receptor activity;transmembrane signaling receptor activity
structural constituent of cytoskeleton;structural molecule activity
binding;chromatin binding;DNA bending activity;DNA binding;double-stranded DNA binding;nucleic acid binding;structure-specific DNA binding
binding;catalytic activity;cofactor binding;kynurenine-oxoglutarate transaminase activity;L-aspartate:2-oxoglutarate aminotransferase activity;L-phenylalanine aminotransferase activity;L-phenylalanine:2-oxoglutarate aminotra
adenyl nucleotide binding;adenyl ribonucleotide binding;ATP binding;binding;catalytic activity;creatine kinase activity;enzyme binding;kinase activity;nucleotide binding;phosphotransferase activity, nitrogenous group as acce
binding;calcium ion binding;cation binding;enzyme binding;ion binding;ion channel binding;kinase binding;metal ion binding;phosphoprotein binding;protein binding;protein kinase binding;protein kinase C binding
binding;calcium ion binding;calcium-dependent phospholipid binding;calcium-dependent protein binding;cation binding;integrin binding;ion binding;lipid binding;metal ion binding;phospholipid binding;protein binding;protein
binding;catalytic activity;cis-trans isomerase activity;enzyme binding;isomerase activity;peptide binding;peptidyl-prolyl cis-trans isomerase activity;protein binding;protein complex binding;RNA polymerase binding;unfolded p
binding;catalytic activity;cation binding;cation transmembrane transporter activity;enzyme binding;hydrogen ion transmembrane transporter activity;inorganic cation transmembrane transporter activity;ion binding;ion transme
adenyl nucleotide binding;adenyl ribonucleotide binding;ATP binding;binding;catalytic activity;fatty acid ligase activity;ligase activity;ligase activity, forming carbon-sulfur bonds;long-chain fatty acid-CoA ligase activity;nucleot
binding;catalytic activity;coenzyme binding;cofactor binding;diiodophenylpyruvate reductase activity;L-malate dehydrogenase activity;malate dehydrogenase activity;malic enzyme activity;NAD binding;nucleotide binding;oxi
aldehyde dehydrogenase (NAD) activity;betaine-aldehyde dehydrogenase activity;catalytic activity;L-aminoadipate-semialdehyde dehydrogenase activity;oxidoreductase activity;oxidoreductase activity, acting on the aldehyd
adenyl deoxyribonucleotide binding;adenyl nucleotide binding;binding;binding, bridging;dATP binding;deoxyribonucleotide binding;nucleotide binding;protein binding;protein binding, bridging;purine deoxyribonucleotide bind
binding;chromatin binding;identical protein binding;nucleic acid binding transcription factor activity;protein binding;protein binding transcription factor activity;sequence-specific DNA binding transcription factor activity;transc
binding;carbohydrate binding;DNA binding;glycosaminoglycan binding;heparin binding;nucleic acid binding;nucleotide binding;pattern binding;polysaccharide binding;protein binding transcription factor activity;RNA polymer
binding;catalytic activity;endopeptidase activity;hydrolase activity;NF-kappaB binding;nucleic acid binding;nucleotide binding;peptidase activity;peptidase activity, acting on L-amino acid peptides;protein binding;purine nucle
adenyl nucleotide binding;adenyl ribonucleotide binding;ATP binding;binding;chaperone binding;nucleotide binding;protein binding;purine nucleotide binding;purine ribonucleoside triphosphate binding;purine ribonucleotide b
binding;nucleic acid binding;RNA binding;structural constituent of ribosome;structural molecule activity
apoptotic protease activator activity;binding;binding, bridging;caspase activator activity;caspase regulator activity;channel inhibitor activity;channel regulator activity;enzyme activator activity;enzyme binding;enzyme inhibitor
binding;catalytic activity;cation binding;hydrolase activity;hydrolase activity, acting on ester bonds;ion binding;metal ion binding;phosphatase activity;phosphoprotein phosphatase activity;phosphoric ester hydrolase activity;p
binding;nucleic acid binding;RNA binding;RNA cap binding
adenyl nucleotide binding;adenyl ribonucleotide binding;ATP binding;ATPase activity;ATPase activity, coupled;ATP-dependent DNA helicase activity;ATP-dependent helicase activity;ATP-dependent RNA helicase activity;bind
binding;calcium ion binding;cation binding;cell adhesion molecule binding;ion binding;metal ion binding;protein binding
adenyl nucleotide binding;adenyl ribonucleotide binding;ATP binding;ATPase activity;ATPase activity, coupled;ATP-dependent helicase activity;ATP-dependent RNA helicase activity;binding;catalytic activity;helicase activity;h
binding;carbohydrate binding;catalytic activity;identical protein binding;nucleotidyltransferase activity;protein binding;transferase activity;transferase activity, transferring phosphorus-containing groups;UDP-N-acetylglucosam
catalytic activity;intramolecular oxidoreductase activity;intramolecular oxidoreductase activity, interconverting keto- and enol-groups;intramolecular oxidoreductase activity, transposing S-S bonds;isomerase activity;protein d
binding;nucleic acid binding;RNA binding;snRNA binding
actin binding;actin filament binding;adenyl nucleotide binding;adenyl ribonucleotide binding;ADP binding;ATP binding;binding;catalytic activity;cytoskeletal protein binding;DNA binding;hydrolase activity;hydrolase activity, ac
binding;chromatin binding;DNA binding;nucleic acid binding;protein binding;protein binding transcription factor activity;protein N-terminus binding;transcription coactivator activity;transcription cofactor activity;transcription fa
binding;lipid binding;lipoprotein particle receptor binding;low-density lipoprotein particle receptor binding;peptide binding;protein binding;receptor binding;signal sequence binding;transporter activity
acid-amino acid ligase activity;binding;catalytic activity;cation binding;chromatin binding;core promoter proximal region DNA binding;core promoter proximal region sequence-specific DNA binding;DNA binding;histone bindin
binding;cytoskeletal protein binding;FFAT motif binding;microtubule binding;molecular transducer activity;protein binding;protein dimerization activity;protein domain specific binding;protein heterodimerization activity;signal t
adenyl nucleotide binding;adenyl ribonucleotide binding;aminoacyl-tRNA ligase activity;ATP binding;binding;catalytic activity;ligase activity;ligase activity, forming aminoacyl-tRNA and related compounds;ligase activity, formi
binding;enzyme binding;mRNA binding;nucleic acid binding;protein binding;RNA binding
alpha-catenin binding;binding;cell adhesion molecule binding;protein binding
adenyl nucleotide binding;adenyl ribonucleotide binding;ATP binding;binding;catalytic activity;cation binding;endopeptidase activity;hydrolase activity;ion binding;metal ion binding;metalloendopeptidase activity;metallopeptic
adenyl nucleotide binding;adenyl ribonucleotide binding;ATP binding;binding;catalytic activity;FAT10 activating enzyme activity;nucleotide binding;purine nucleotide binding;purine ribonucleoside triphosphate binding;purine r
alcohol binding;amine binding;binding;calcium ion binding;calcium-dependent phospholipid binding;cation binding;identical protein binding;ion binding;lipid binding;metal ion binding;phosphatidylcholine binding;phosphatidy
binding;protein binding;protein binding transcription factor activity;repressing transcription factor binding;RNA polymerase II repressing transcription factor binding;RNA polymerase II transcription cofactor activity;RNA polym
adenyl nucleotide binding;adenyl ribonucleotide binding;aminoacyl-tRNA ligase activity;asparagine-tRNA ligase activity;ATP binding;binding;catalytic activity;ligase activity;ligase activity, forming aminoacyl-tRNA and related c
binding;catalytic activity;cation binding;G-protein beta/gamma-subunit complex binding;G-protein-coupled receptor binding;GTP binding;GTPase activity;guanyl nucleotide binding;guanyl ribonucleotide binding;hydrolase ac
2',3'-cyclic-nucleotide 3'-phosphodiesterase activity;binding;catalytic activity;cyclic nucleotide binding;cyclic-nucleotide phosphodiesterase activity;hydrolase activity;hydrolase activity, acting on ester bonds;nucleic acid bin
binding;catalytic activity;GTP binding;GTPase activity;guanyl nucleotide binding;guanyl ribonucleotide binding;hydrolase activity;hydrolase activity, acting on acid anhydrides;hydrolase activity, acting on acid anhydrides, in ph
6-phosphofructokinase activity;adenyl nucleotide binding;adenyl ribonucleotide binding;ATP binding;binding;carbohydrate binding;carbohydrate kinase activity;catalytic activity;cation binding;enzyme binding;fructose binding
binding;catalytic activity;cation binding;DNA binding;DNA-directed RNA polymerase activity;enzyme binding;ion binding;metal ion binding;nucleic acid binding;nucleotidyltransferase activity;protein binding;RNA polymerase a
3'-5' exonuclease activity;3'-5'-exodeoxyribonuclease activity;4 iron, 4 sulfur cluster binding;binding;catalytic activity;cation binding;chromatin binding;damaged DNA binding;deoxyribonuclease activity;DNA binding;DNA pol
binding;cation binding;cobalt ion binding;DNA binding;enzyme binding;identical protein binding;ion binding;metal ion binding;nucleic acid binding;protein binding;protein binding transcription factor activity;protein dimerizatio
adenyl nucleotide binding;adenyl ribonucleotide binding;ATP binding;binding;catalytic activity;cation binding;ion binding;metal ion binding;methionine adenosyltransferase activity;nucleotide binding;purine nucleotide binding;
binding;core promoter binding;core promoter proximal region DNA binding;core promoter proximal region sequence-specific DNA binding;core promoter sequence-specific DNA binding;cytokine receptor binding;DNA binding
adenyl nucleotide binding;adenyl ribonucleotide binding;ATP binding;ATPase activity;ATPase activity, coupled;binding;catalytic activity;cation binding;enzyme binding;GTPase binding;hydrolase activity;hydrolase activity, actin
binding;nucleic acid binding;RNA binding;structural constituent of ribosome;structural molecule activity
actin binding;actin filament binding;binding;cytoskeletal protein binding;protein binding
binding;profilin binding;protein binding
adenyl nucleotide binding;adenyl ribonucleotide binding;androgen receptor binding;ATP binding;ATPase activity;ATPase activity, coupled;binding;catalytic activity;chromatin binding;DNA binding;DNA polymerase binding;DN
adenyl nucleotide binding;adenyl ribonucleotide binding;ATP binding;ATPase activity;binding;catalytic activity;hydrolase activity;hydrolase activity, acting on acid anhydrides;hydrolase activity, acting on acid anhydrides, in ph
androgen receptor binding;binding;catalytic activity;chromatin binding;GDP binding;GTP binding;GTPase activity;guanyl nucleotide binding;guanyl ribonucleotide binding;hormone receptor binding;hydrolase activity;hydrolas
binding;nucleic acid binding;RNA binding;rRNA binding;structural constituent of ribosome;structural molecule activity
binding;catalytic activity;cis-trans isomerase activity;isomerase activity;peptide binding;peptidyl-prolyl cis-trans isomerase activity;protein binding;unfolded protein binding;virion binding
catalytic activity;hydrolase activity;hydrolase activity, acting on acid anhydrides;hydrolase activity, acting on acid anhydrides, in phosphorus-containing anhydrides;microtubule motor activity;motor activity;nucleoside-triphos
acetylgalactosaminyltransferase activity;binding;carbohydrate binding;catalytic activity;cation binding;ion binding;manganese ion binding;metal ion binding;polypeptide N-acetylgalactosaminyltransferase activity;transferase a
binding;mRNA binding;nucleic acid binding;nucleotide binding;poly(A) RNA binding;poly(U) RNA binding;poly-purine tract binding;poly-pyrimidine tract binding;RNA binding;single-stranded RNA binding
3'-5' DNA helicase activity;adenyl nucleotide binding;adenyl ribonucleotide binding;ATP binding;ATPase activity;ATPase activity, coupled;ATP-dependent 3'-5' DNA helicase activity;ATP-dependent DNA helicase activity;ATP-
enzyme activator activity;enzyme regulator activity;GTPase activator activity;GTPase regulator activity;guanyl-nucleotide exchange factor activity;nucleoside-triphosphatase regulator activity;Ras guanyl-nucleotide exchange
binding;catalytic activity;cation binding;intramolecular transferase activity;intramolecular transferase activity, phosphotransferases;ion binding;isomerase activity;magnesium ion binding;metal ion binding;phosphoglucomutas
catalytic activity;disulfide oxidoreductase activity;intramolecular oxidoreductase activity;intramolecular oxidoreductase activity, interconverting keto- and enol-groups;intramolecular oxidoreductase activity, transposing S-S bo
binding;cation binding;ion binding;metal ion binding;transition metal ion binding;zinc ion binding
5'-3' exonuclease activity;5'-3' exoribonuclease activity;binding;catalytic activity;cation binding;DNA binding;exonuclease activity;exonuclease activity, active with either ribo- or deoxyribonucleic acids and producing 5'-phos
acid-amino acid ligase activity;binding;catalytic activity;identical protein binding;ligase activity;ligase activity, forming carbon-nitrogen bonds;protein binding;small conjugating protein ligase activity;ubiquitin-protein ligase acti
binding;enzyme binding;kinase binding;lipid binding;protein binding;protein kinase binding;protein transporter activity;substrate-specific transporter activity;transporter activity
6-phosphofructokinase activity;adenyl nucleotide binding;adenyl ribonucleotide binding;ATP binding;binding;carbohydrate binding;carbohydrate kinase activity;catalytic activity;cation binding;enzyme binding;fructose binding
catalytic activity;oxidoreductase activity;oxidoreductase activity, acting on the aldehyde or oxo group of donors;oxidoreductase activity, acting on the aldehyde or oxo group of donors, disulfide as acceptor;pyruvate dehydro
binding;carbon-oxygen lyase activity;catalytic activity;cation binding;hydro-lyase activity;ion binding;lyase activity;magnesium ion binding;metal ion binding;phosphopyruvate hydratase activity
binding;enzyme binding;fibroblast growth factor binding;growth factor binding;mRNA binding;nucleic acid binding;protein binding;RNA binding;structural constituent of ribosome;structural molecule activity
binding;cation binding;DNA binding;ion binding;metal ion binding;nucleic acid binding;transition metal ion binding;zinc ion binding
binding;chemoattractant activity;chromatin binding;damaged DNA binding;DNA bending activity;DNA binding;double-stranded DNA binding;nucleic acid binding;nucleic acid binding transcription factor activity;protein binding
aminopeptidase activity;binding;catalytic activity;endopeptidase activity;exopeptidase activity;hydrolase activity;peptidase activity;peptidase activity, acting on L-amino acid peptides;peptide binding;serine hydrolase activity;
activating transcription factor binding;alpha-catenin binding;androgen receptor binding;binding;cadherin binding;cell adhesion molecule binding;DNA binding;double-stranded DNA binding;enzyme binding;estrogen receptor
binding;DNA binding;lamin binding;nucleic acid binding;protein binding
adenyl nucleotide binding;adenyl ribonucleotide binding;ATP binding;ATPase activity;ATPase activity, coupled;binding;catalytic activity;centromeric DNA binding;damaged DNA binding;DNA binding;DNA insertion or deletion
binding;mRNA binding;nucleic acid binding;nucleotide binding;RNA binding
antigen binding;binding;calcium ion binding;calcium-dependent phospholipid binding;calcium-dependent protein binding;cation binding;ion binding;lipid binding;metal ion binding;MHC class II protein complex binding;MHC
androgen receptor binding;binding;cation binding;hormone receptor binding;identical protein binding;ion binding;metal ion binding;nuclear hormone receptor binding;protein binding;protein binding transcription factor activity
acid-amino acid ligase activity;binding;catalytic activity;cation binding;ion binding;ligase activity;ligase activity, forming carbon-nitrogen bonds;metal ion binding;nucleic acid binding transcription factor activity;sequence-spec
basal RNA polymerase II transcription machinery binding;basal transcription machinery binding;binding;enzyme binding;enzyme inhibitor activity;enzyme regulator activity;protein binding;RNA polymerase binding;RNA polym
binding;mRNA 3'-UTR binding;mRNA binding;nucleic acid binding;RNA binding
enzyme regulator activity
binding;DNA binding;nucleic acid binding;structural molecule activity
binding;cation binding;ion binding;metal ion binding
7S RNA binding;binding;endoplasmic reticulum signal peptide binding;nucleic acid binding;peptide binding;ribonucleoprotein binding;RNA binding;signal recognition particle binding;signal sequence binding
binding;carboxylic acid binding;catalytic activity;cation binding;galactosyltransferase activity;glucosyltransferase activity;ion binding;iron ion binding;L-ascorbic acid binding;metal ion binding;oxidoreductase activity;oxidored
aminoacylase activity;antioxidant activity;binding;catalase activity;catalytic activity;cation binding;coenzyme binding;cofactor binding;enzyme binding;heme binding;hydrolase activity;hydrolase activity, acting on carbon-nitro
binding;catalytic activity;coenzyme binding;cofactor binding;lactate dehydrogenase activity;L-lactate dehydrogenase activity;NAD binding;nucleotide binding;oxidoreductase activity;oxidoreductase activity, acting on CH-OH
active transmembrane transporter activity;amine transmembrane transporter activity;amino acid transmembrane transporter activity;antiporter activity;binding;calcium ion transmembrane transporter activity;calcium:cation an
binding;carboxylic acid binding;catalytic activity;cation binding;dioxygenase activity;ion binding;iron ion binding;L-ascorbic acid binding;metal ion binding;oxidoreductase activity;oxidoreductase activity, acting on paired don
alpha-catenin binding;binding;cadherin binding;cell adhesion molecule binding;enzyme binding;identical protein binding;molecular transducer activity;phosphatase binding;protein binding;protein binding transcription factor a
adenyl nucleotide binding;adenyl ribonucleotide binding;aminoacyl-tRNA ligase activity;ATP binding;binding;catalytic activity;ligase activity;ligase activity, forming aminoacyl-tRNA and related compounds;ligase activity, formi
active transmembrane transporter activity;adenyl nucleotide binding;adenyl ribonucleotide binding;ATP binding;ATPase activity;ATPase activity, coupled;ATPase activity, coupled to movement of substances;ATPase activity, c
acetyl-CoA C-acetyltransferase activity;acetyl-CoA C-acyltransferase activity;acetyltransferase activity;binding;C-acetyltransferase activity;C-acyltransferase activity;catalytic activity;cation binding;coenzyme binding;cofactor
activating transcription factor binding;binding;DNA binding;heat shock protein binding;molecular transducer activity;nucleic acid binding;protein binding;RNA binding;signal transducer activity;transcription factor binding;trans

catalytic activity;sedoheptulose-7-phosphate:D-glyceraldehyde-3-phosphate glyceronetransferase activity;transferase activity;transferase activity, transferring aldehyde or ketonic groups
adenyl nucleotide binding;adenyl ribonucleotide binding;ATP binding;ATPase activity;ATPase activity, coupled;ATP-dependent helicase activity;ATP-dependent RNA helicase activity;binding;catalytic activity;helicase activity;h
adenyl nucleotide binding;adenyl ribonucleotide binding;aminoacyl-tRNA ligase activity;ATP binding;binding;catalytic activity;cation binding;cysteine-tRNA ligase activity;identical protein binding;ion binding;ligase activity;liga
binding;lipid binding;phospholipid binding
binding;catalytic activity;cation binding;endopeptidase activity;hydrolase activity;ion binding;metal ion binding;metalloendopeptidase activity;metallopeptidase activity;peptidase activity;peptidase activity, acting on L-amino a
binding;nucleic acid binding;RNA binding;structural constituent of ribosome;structural molecule activity
binding;nucleic acid binding;RNA binding;structural constituent of ribosome;structural molecule activity
adenyl nucleotide binding;adenyl ribonucleotide binding;ATP binding;ATPase activity;binding;binding, bridging;catalytic activity;hydrolase activity;hydrolase activity, acting on acid anhydrides;hydrolase activity, acting on acid
binding;carboxylic acid binding;catalytic activity;cation binding;identical protein binding;ion binding;iron ion binding;L-ascorbic acid binding;metal ion binding;oxidoreductase activity;oxidoreductase activity, acting on paired
binding;chaperone binding;enzyme binding;heat shock protein binding;kinase binding;protein binding;unfolded protein binding
binding;core promoter binding;DNA binding;nucleic acid binding;nucleotide binding;regulatory region DNA binding;regulatory region nucleic acid binding;transcription regulatory region DNA binding
actin binding;actinin binding;binding;cation binding;cytoskeletal protein binding;enzyme binding;ion binding;kinase binding;metal ion binding;protein binding;protein kinase binding;protein kinase C binding;transition metal ion
adenyl nucleotide binding;adenyl ribonucleotide binding;ATP binding;binding;calcium ion binding;catalytic activity;cation binding;GTP binding;guanyl nucleotide binding;guanyl ribonucleotide binding;hydrolase activity;ion bin
binding;protein binding;protein complex binding
adenyl nucleotide binding;adenyl ribonucleotide binding;ATP binding;binding;catalytic activity;cation binding;ion binding;kinase activity;magnesium ion binding;metal ion binding;nucleotide binding;phosphotransferase activity
structural molecule activity
binding;catalytic activity;cation binding;coenzyme binding;cofactor binding;identical protein binding;ion binding;isocitrate dehydrogenase (NADP+) activity;isocitrate dehydrogenase activity;magnesium ion binding;metal ion b
adenyl nucleotide binding;adenyl ribonucleotide binding;ATP binding;binding;catalytic activity;creatine kinase activity;kinase activity;nucleotide binding;phosphotransferase activity, alcohol group as acceptor;phosphotransfer
actin binding;actin filament binding;adenyl nucleotide binding;adenyl ribonucleotide binding;ATP binding;binding;catalytic activity;chromatin binding;cytoskeletal protein binding;DNA binding;double-stranded DNA binding;en
binding;nucleic acid binding;ribonucleoprotein binding;ribosome binding;RNA binding;translation factor activity, nucleic acid binding;translation initiation factor activity
binding;catalytic activity;coenzyme binding;cofactor binding;dihydrolipoyl dehydrogenase activity;flavin adenine dinucleotide binding;oxidoreductase activity;oxidoreductase activity, acting on a sulfur group of donors;oxidore
binding;chromatin binding;chromatin DNA binding;DNA binding;nucleic acid binding;structure-specific DNA binding
actin binding;actin filament binding;binding;calcium ion binding;calcium-dependent phospholipid binding;cation binding;cytoskeletal protein binding;ion binding;lipid binding;metal ion binding;phosphatidylinositol binding;pho
adenyl nucleotide binding;adenyl ribonucleotide binding;ATP binding;binding;catalytic activity;CTP synthase activity;ligase activity;ligase activity, forming carbon-nitrogen bonds;nucleotide binding;purine nucleotide binding;p
3'-5' DNA helicase activity;adenyl nucleotide binding;adenyl ribonucleotide binding;ATP binding;ATPase activity;ATPase activity, coupled;ATP-dependent 3'-5' DNA helicase activity;ATP-dependent DNA helicase activity;ATP-
binding;mRNA binding;nucleic acid binding;RNA binding;structural constituent of ribosome;structural molecule activity
catalytic activity;endopeptidase activity;exopeptidase activity;hydrolase activity;peptidase activity;peptidase activity, acting on L-amino acid peptides;serine hydrolase activity;serine-type endopeptidase activity;serine-type ex
binding;heat shock protein binding;Hsp90 protein binding;protein binding;protein complex binding
binding;cation binding;DNA binding;identical protein binding;ion binding;metal ion binding;nucleic acid binding;nucleocytoplasmic transporter activity;protein anchor;protein binding;structural constituent of nuclear pore;struc
binding;catalytic activity;cytoskeletal protein binding;enzyme binding;GTP binding;GTPase activity;guanyl nucleotide binding;guanyl ribonucleotide binding;hydrolase activity;hydrolase activity, acting on acid anhydrides;hydro
binding;nucleic acid binding;RNA binding;structural constituent of ribosome;structural molecule activity
binding;catalytic activity;coenzyme binding;cofactor binding;NADP binding;nucleotide binding;oxidoreductase activity;oxidoreductase activity, acting on CH-OH group of donors;oxidoreductase activity, acting on the CH-OH
adenyl nucleotide binding;adenyl ribonucleotide binding;ATP binding;ATPase activity;ATPase activity, coupled;binding;catalytic activity;chromatin binding;damaged DNA binding;DNA binding;DNA-dependent ATPase activity;
binding;nucleic acid binding;nucleotide binding;RNA binding
adenyl nucleotide binding;adenyl ribonucleotide binding;ATP binding;ATPase activity;binding;catalytic activity;G-protein-coupled receptor binding;hormone receptor binding;hydrolase activity;hydrolase activity, acting on acid
binding;nucleic acid binding;RNA binding;rRNA binding;structural constituent of ribosome;structural molecule activity
binding;nucleic acid binding;RNA binding;structural constituent of ribosome;structural molecule activity
amine binding;amino acid binding;binding;carbohydrate binding;carboxylic acid binding;catalytic activity;glutamine-fructose-6-phosphate transaminase (isomerizing) activity;transaminase activity;transferase activity;transfera
actin binding;binding;cytoskeletal protein binding;protein binding
binding;protein binding;transcription factor binding
binding;histone binding;nucleic acid binding;nucleotide binding;protein binding;RNA binding;snRNA binding;U4 snRNA binding;U6 snRNA binding;U6atac snRNA binding
adenyl nucleotide binding;adenyl ribonucleotide binding;amine binding;amino acid binding;aminoacyl-tRNA ligase activity;ATP binding;binding;carboxylic acid binding;catalytic activity;cation binding;ion binding;ligase activity
catalytic activity;intramolecular oxidoreductase activity;intramolecular oxidoreductase activity, interconverting keto- and enol-groups;intramolecular oxidoreductase activity, transposing S-S bonds;isomerase activity;protein d
binding;nucleic acid binding;RNA binding
acid-amino acid ligase activity;ATPase activity;binding;catalytic activity;cation binding;hydrolase activity;hydrolase activity, acting on acid anhydrides;hydrolase activity, acting on acid anhydrides, in phosphorus-containing an
adenyl nucleotide binding;adenyl ribonucleotide binding;ATP binding;ATPase activity;ATPase activity, coupled;ATP-dependent helicase activity;ATP-dependent RNA helicase activity;binding;catalytic activity;chromatin binding
acetyltransferase activity;adenyl nucleotide binding;adenyl ribonucleotide binding;ATP binding;binding;catalytic activity;N-acetyltransferase activity;N-acyltransferase activity;nucleotide binding;purine nucleotide binding;purin
adenyl nucleotide binding;adenyl ribonucleotide binding;ATP binding;binding;catalytic activity;cation binding;enzyme activator activity;enzyme regulator activity;ion binding;metal ion binding;nucleotide binding;protein binding
acetylcholine receptor regulator activity;binding;calcium ion binding;carbohydrate binding;cation binding;chondroitin sulfate binding;dystroglycan binding;extracellular matrix binding;glycoprotein binding;glycosaminoglycan b
binding;carboxylic acid binding;catalytic activity;cation binding;dioxygenase activity;electron carrier activity;ion binding;iron ion binding;L-ascorbic acid binding;metal ion binding;oxidoreductase activity;oxidoreductase activit
acid-thiol ligase activity;adenyl nucleotide binding;adenyl ribonucleotide binding;arachidonate-CoA ligase activity;ATP binding;binding;catalytic activity;fatty acid ligase activity;ligase activity;ligase activity, forming carbon-sulf
binding;catalytic activity;GTP binding;GTPase activity;guanyl nucleotide binding;guanyl ribonucleotide binding;hydrolase activity;hydrolase activity, acting on acid anhydrides;hydrolase activity, acting on acid anhydrides, in ph
binding;catalytic activity;coenzyme binding;cofactor binding;DNA binding;electron carrier activity;FAD binding;flavin adenine dinucleotide binding;NAD(P)H oxidase activity;nucleic acid binding;oxidoreductase activity;oxidore
binding;chromatin binding;chromatin DNA binding;DNA binding;nucleic acid binding;structure-specific DNA binding
structural constituent of ribosome;structural molecule activity
binding;catalytic activity;endopeptidase activity;hydrolase activity;nucleic acid binding;peptidase activity;peptidase activity, acting on L-amino acid peptides;RNA binding;threonine-type endopeptidase activity;threonine-type
androgen receptor binding;binding;calcium ion binding;carbohydrate binding;cation binding;chaperone binding;complement binding;complement component C1q binding;DNA binding;enzyme binding;glycoprotein binding;h
antioxidant activity;binding;carboxylic ester hydrolase activity;catalytic activity;enzyme binding;glutathione peroxidase activity;hydrolase activity;hydrolase activity, acting on ester bonds;lipase activity;oxidoreductase activity;
antigen binding;binding;catalytic activity;enzyme regulator activity;hydrolase activity;hydrolase activity, acting on ester bonds;phosphatase activity;phosphatase regulator activity;phosphoprotein phosphatase activity;phospho
adenyl nucleotide binding;adenyl ribonucleotide binding;ATP binding;ATPase activity;binding;catalytic activity;hydrolase activity;hydrolase activity, acting on acid anhydrides;hydrolase activity, acting on acid anhydrides, in ph
structural constituent of ribosome;structural molecule activity
3alpha,7alpha,12alpha-trihydroxy-5beta-cholest-24-enoyl-CoA hydratase activity;3-hydroxyacyl-CoA dehydrogenase activity;binding;carbon-oxygen lyase activity;catalytic activity;hydro-lyase activity;identical protein binding
structural molecule activity
binding;catalytic activity;enzyme binding;intramolecular oxidoreductase activity;intramolecular oxidoreductase activity, interconverting aldoses and ketoses;isomerase activity;protein binding;triose-phosphate isomerase activ
adenyl nucleotide binding;adenyl ribonucleotide binding;ATP binding;binding;nucleotide binding;purine nucleotide binding;purine ribonucleoside triphosphate binding;purine ribonucleotide binding;ribonucleotide binding;struc
binding;nucleic acid binding;RNA binding;rRNA binding;structural constituent of ribosome;structural molecule activity
binding;enzyme binding;kinase binding;protein binding;protein kinase binding;structural constituent of ribosome;structural molecule activity
structural constituent of ribosome;structural molecule activity
binding;clathrin binding;peptide binding;protein binding;protein transporter activity;signal sequence binding;substrate-specific transporter activity;transporter activity
binding;channel regulator activity;enzyme binding;glucocorticoid receptor binding;hormone receptor binding;insulin-like growth factor receptor binding;ion channel binding;nuclear hormone receptor binding;protein binding;pr
antioxidant activity;binding;catalytic activity;cation binding;heme binding;ion binding;iron ion binding;metal ion binding;oxidoreductase activity;oxidoreductase activity, acting on paired donors, with incorporation or reduction
binding;mRNA binding;nucleic acid binding;nucleotide binding;RNA binding
binding;enzyme binding;kinase binding;protein binding;protein kinase binding;protein transporter activity;substrate-specific transporter activity;transporter activity
adenyl nucleotide binding;adenyl ribonucleotide binding;ATP binding;binding;catalytic activity;enzyme binding;kinase activity;kinase binding;molecular transducer activity;nucleotide binding;phosphotransferase activity, alcoh
binding;DNA binding;identical protein binding;nucleic acid binding;nucleotide binding;protein binding
binding;binding, bridging;cation binding;ion binding;ion transmembrane transporter activity;metal ion binding;protein binding;protein binding, bridging;SH3/SH2 adaptor activity;signaling adaptor activity;substrate-specific tra
adenyl nucleotide binding;adenyl ribonucleotide binding;ATP binding;binding;GTP binding;guanyl nucleotide binding;guanyl ribonucleotide binding;nucleotide binding;purine nucleotide binding;purine ribonucleoside triphosph
binding;cation binding;ion binding;metal ion binding;transition metal ion binding;zinc ion binding
catalytic activity;dihydropyrimidinase activity;hydrolase activity;hydrolase activity, acting on carbon-nitrogen (but not peptide) bonds;hydrolase activity, acting on carbon-nitrogen (but not peptide) bonds, in cyclic amides
adenyl nucleotide binding;adenyl ribonucleotide binding;ATP binding;binding;catalytic activity;DNA helicase activity;helicase activity;hydrolase activity;hydrolase activity, acting on acid anhydrides;hydrolase activity, acting on
protein transporter activity;substrate-specific transporter activity;transporter activity
structural constituent of nuclear pore;structural molecule activity;transporter activity
adenyl nucleotide binding;adenyl ribonucleotide binding;ATP binding;binding;catalytic activity;enzyme binding;fatty acid ligase activity;kinase binding;ligase activity;ligase activity, forming carbon-sulfur bonds;long-chain fatty
carbon-oxygen lyase activity;catalytic activity;fumarate hydratase activity;hydro-lyase activity;lyase activity
binding;nucleic acid binding;nucleotide binding;RNA binding
binding;calcium ion binding;calcium-dependent phospholipid binding;calcium-dependent protein binding;cation binding;enzyme inhibitor activity;enzyme regulator activity;identical protein binding;ion binding;lipase inhibitor a
anion channel activity;anion transmembrane transporter activity;binding;channel activity;enzyme binding;gated channel activity;ion channel activity;ion transmembrane transporter activity;kinase binding;passive transmembra
binding;carbon-oxygen lyase activity;catalytic activity;damaged DNA binding;DNA binding;DNA N-glycosylase activity;DNA-(apurinic or apyrimidinic site) lyase activity;enzyme binding;hydrolase activity;hydrolase activity, acti
adenosylhomocysteinase activity;catalytic activity;hydrolase activity;hydrolase activity, acting on ether bonds;trialkylsulfonium hydrolase activity
structural constituent of ribosome;structural molecule activity
2 iron, 2 sulfur cluster binding;4 iron, 4 sulfur cluster binding;binding;catalytic activity;cation binding;electron carrier activity;ion binding;iron-sulfur cluster binding;metal cluster binding;metal ion binding;NADH dehydrogenase
adenyl nucleotide binding;adenyl ribonucleotide binding;ATP binding;binding;catalytic activity;ephrin receptor activity;kinase activity;molecular transducer activity;nucleotide binding;phosphotransferase activity, alcohol group
binding;DNA binding;lamin binding;nucleic acid binding;protein binding
adenyl nucleotide binding;adenyl ribonucleotide binding;ATP binding;ATPase activity;binding;catalytic activity;enzyme inhibitor activity;enzyme regulator activity;hydrolase activity;hydrolase activity, acting on acid anhydrides;
binding;nucleic acid binding;RNA binding;structural constituent of ribosome;structural molecule activity
binding;nucleic acid binding;RNA binding;structural constituent of ribosome;structural molecule activity
enzyme regulator activity
binding;catalytic activity;cation transmembrane transporter activity;coenzyme binding;cofactor binding;hydrogen ion transmembrane transporter activity;inorganic cation transmembrane transporter activity;ion transmembran
adenyl nucleotide binding;adenyl ribonucleotide binding;ATP binding;ATPase activity;ATPase activity, coupled;ATP-dependent helicase activity;ATP-dependent protein binding;ATP-dependent RNA helicase activity;binding;ca
binding;DNA binding;nucleic acid binding;nucleotide binding;RNA binding;sequence-specific DNA binding;telomeric DNA binding
binding;nucleic acid binding;RNA binding
binding;carbohydrate binding;catalytic activity;cation binding;glucose binding;identical protein binding;ion binding;metal ion binding;monosaccharide binding;nucleotide binding;nucleotidyltransferase activity;protein binding;
antioxidant activity;binding;catalytic activity;coenzyme binding;cofactor binding;disulfide oxidoreductase activity;electron carrier activity;flavin adenine dinucleotide binding;oxidoreductase activity;oxidoreductase activity, acti
binding;nucleic acid binding;RNA binding;rRNA binding;rRNA primary transcript binding
adenyl nucleotide binding;adenyl ribonucleotide binding;ATP binding;ATPase activity;binding;catalytic activity;hydrolase activity;hydrolase activity, acting on acid anhydrides;hydrolase activity, acting on acid anhydrides, in ph
structural constituent of nuclear pore;structural molecule activity
binding;enzyme binding;nucleic acid binding;protein binding;RNA binding
binding;cytoskeletal protein binding;enzyme binding;GTPase binding;kinase binding;microtubule binding;protein binding;protein domain specific binding;protein kinase binding;Rac GTPase binding;Ras GTPase binding;Rho
actin binding;actin filament binding;binding;cytoskeletal protein binding;protein binding
binding;catalytic activity;cofactor binding;oxidoreductase activity;oxidoreductase activity, acting on a sulfur group of donors;oxidoreductase activity, acting on a sulfur group of donors, quinone or similar compound as accept
binding;catalytic activity;cation binding;GTP binding;GTPase activity;guanyl nucleotide binding;guanyl ribonucleotide binding;hydrolase activity;hydrolase activity, acting on acid anhydrides;hydrolase activity, acting on acid an
adenyl nucleotide binding;adenyl ribonucleotide binding;ATP binding;binding;catalytic activity;cation binding;ion binding;kinase activity;metal ion binding;nucleotide binding;phosphotransferase activity, alcohol group as acce
binding;catalytic activity;cation binding;chromatin binding;deacetylase activity;DNA binding;histone deacetylase activity;hydrolase activity;hydrolase activity, acting on carbon-nitrogen (but not peptide) bonds;hydrolase activi
binding;calcium channel activity;cation binding;cation channel activity;cation transmembrane transporter activity;channel activity;cytokine binding;enzyme binding;extracellular matrix binding;fibronectin binding;gated channe
enzyme inhibitor activity;enzyme regulator activity;ribonuclease inhibitor activity
adenyl nucleotide binding;adenyl ribonucleotide binding;ATP binding;binding;catalytic activity;nucleotide binding;oxidoreductase activity;oxidoreductase activity, acting on CH or CH2 groups;oxidoreductase activity, acting on
binding;nucleic acid binding;nucleotide binding;poly-pyrimidine tract binding;pre-mRNA binding;RNA binding;single-stranded RNA binding
binding;catalytic activity;cyclohydrolase activity;hydrolase activity;hydrolase activity, acting on carbon-nitrogen (but not peptide) bonds;hydrolase activity, acting on carbon-nitrogen (but not peptide) bonds, in cyclic amidines;

binding;calcium ion binding;catalytic activity;cation binding;glycerol-3-phosphate dehydrogenase activity;ion binding;metal ion binding;oxidoreductase activity;oxidoreductase activity, acting on CH-OH group of donors;oxido
binding;intermediate filament binding;protein binding;protein complex binding;structural molecule activity
binding;catalytic activity;GTP binding;GTPase activity;guanyl nucleotide binding;guanyl ribonucleotide binding;hydrolase activity;hydrolase activity, acting on acid anhydrides;hydrolase activity, acting on acid anhydrides, in pl
adenyl nucleotide binding;adenyl ribonucleotide binding;ATP binding;binding;carbohydrate binding;carbohydrate kinase activity;catalytic activity;fructokinase activity;glucokinase activity;glucose binding;hexokinase activity;ki
amine binding;amino acid binding;antigen binding;binding;carboxylic acid binding;channel regulator activity;enzyme binding;histone deacetylase binding;ion channel binding;MHC class II protein complex binding;MHC protei
binding;nucleic acid binding;RNA binding;structural constituent of ribosome;structural molecule activity
binding;chromatin binding;DNA binding;nucleic acid binding
actin binding;actin filament binding;adenyl nucleotide binding;adenyl ribonucleotide binding;ATP binding;ATPase activity;ATPase activity, coupled;binding;calmodulin binding;catalytic activity;cytoskeletal protein binding;hydro
binding;chromatin binding;DNA binding;nucleic acid binding;nucleosome binding
catalytic activity;galactosyltransferase activity;procollagen galactosyltransferase activity;transferase activity;transferase activity, transferring glycosyl groups;transferase activity, transferring hexosyl groups;UDP-galactosyltran
binding;chromatin binding;DNA binding;nucleic acid binding;protein binding;protein domain specific binding;WD40-repeat domain binding
binding;catalytic activity;GTP binding;GTPase activity;guanyl nucleotide binding;guanyl ribonucleotide binding;hydrolase activity;hydrolase activity, acting on acid anhydrides;hydrolase activity, acting on acid anhydrides, in pl
acetylcholine receptor regulator activity;adenyl nucleotide binding;adenyl ribonucleotide binding;ATP binding;binding;catalytic activity;cation binding;chromatin binding;histone acetyl-lysine binding;histone binding;histone kin
binding;nucleic acid binding;nucleocytoplasmic transporter activity;protein transporter activity;RNA binding;substrate-specific transporter activity;transporter activity
enzyme regulator activity
binding;catalytic activity;coenzyme binding;cofactor binding;electron carrier activity;NAD binding;nucleotide binding;oxidoreductase activity;oxidoreductase activity, acting on CH-OH group of donors;oxidoreductase activity,
binding;mRNA binding;nucleic acid binding;nucleotide binding;RNA binding;tRNA binding
aminopeptidase activity;binding;catalytic activity;cation binding;epoxide hydrolase activity;ether hydrolase activity;exopeptidase activity;hydrolase activity;hydrolase activity, acting on ether bonds;ion binding;leukotriene-A4 h
adenyl nucleotide binding;adenyl ribonucleotide binding;ATP binding;ATPase activity;ATPase activity, coupled;binding;catalytic activity;cation binding;chromatin binding;DNA bending activity;DNA binding;DNA topoisomerase
binding;cytoskeletal protein binding;molecular transducer activity;protein binding;signal transducer activity;tubulin binding
adenyl nucleotide binding;adenyl ribonucleotide binding;ATP binding;ATPase activity;binding;catalytic activity;hydrolase activity;hydrolase activity, acting on acid anhydrides;hydrolase activity, acting on acid anhydrides, in ph
binding;protein binding;protein N-terminus binding
enzyme inhibitor activity;enzyme regulator activity;kinase inhibitor activity;kinase regulator activity;protein kinase C inhibitor activity;protein kinase inhibitor activity;protein kinase regulator activity;protein serine/threonine kinas
binding;enzyme binding;fibroblast growth factor binding;growth factor binding;identical protein binding;kinase binding;protein binding;protein dimerization activity;protein homodimerization activity;protein kinase binding;struc
5'-3' exonuclease activity;5'-flap endonuclease activity;binding;catalytic activity;cation binding;damaged DNA binding;deoxyribonuclease activity;DNA binding;double-stranded DNA binding;double-stranded DNA specific exo
adenyl nucleotide binding;adenyl ribonucleotide binding;aminoacyl-tRNA ligase activity;ATP binding;binding;catalytic activity;ligase activity;ligase activity, forming aminoacyl-tRNA and related compounds;ligase activity, formi
adenyl nucleotide binding;adenyl ribonucleotide binding;ATP binding;binding;carbon-nitrogen ligase activity, with glutamine as amido-N-donor;catalytic activity;GMP synthase (glutamine-hydrolyzing) activity;GMP synthase ac
binding;collagen binding;endopeptidase inhibitor activity;endopeptidase regulator activity;enzyme inhibitor activity;enzyme regulator activity;peptidase inhibitor activity;peptidase regulator activity;protein binding;serine-type e
binding;nucleic acid binding;nucleotide binding;RNA binding
adenyl nucleotide binding;adenyl ribonucleotide binding;amino acid kinase activity;ATP binding;binding;catalytic activity;coenzyme binding;cofactor binding;glutamate 5-kinase activity;glutamate-5-semialdehyde dehydrogen
adenyl nucleotide binding;adenyl ribonucleotide binding;ATP binding;binding;nucleotide binding;purine nucleotide binding;purine ribonucleoside triphosphate binding;purine ribonucleotide binding;ribonucleotide binding;struc
binding;GTP binding;guanyl nucleotide binding;guanyl ribonucleotide binding;identical protein binding;nucleotide binding;protein binding;purine nucleotide binding;purine ribonucleoside triphosphate binding;purine ribonucle
adenyl nucleotide binding;adenyl ribonucleotide binding;ATP binding;binding;nucleotide binding;purine nucleotide binding;purine ribonucleoside triphosphate binding;purine ribonucleotide binding;ribonucleotide binding
binding;cation binding;cytoskeletal protein binding;enzyme binding;enzyme regulator activity;GTPase binding;GTPase regulator activity;guanyl-nucleotide exchange factor activity;ion binding;metal ion binding;microtubule bin
protein transporter activity;substrate-specific transporter activity;transporter activity
binding;cation binding;ion binding;lipid binding;metal ion binding;phospholipid binding
binding;cation binding;ion binding;metal ion binding;transition metal ion binding;zinc ion binding
structural constituent of cytoskeleton;structural constituent of muscle;structural molecule activity
binding;chemoattractant activity;chromatin binding;cytokine activity;damaged DNA binding;DNA bending activity;DNA binding;double-stranded DNA binding;nucleic acid binding;nucleic acid binding transcription factor activi
aldehyde-lyase activity;binding;carbon-carbon lyase activity;catalytic activity;cytoskeletal protein binding;fructose-bisphosphate aldolase activity;lyase activity;protein binding
structural constituent of epidermis;structural molecule activity
active transmembrane transporter activity;adenyl nucleotide binding;adenyl ribonucleotide binding;ATP binding;ATPase activity;ATPase activity, coupled;ATPase activity, coupled to movement of substances;ATPase activity, c
binding;cation binding;ion binding;metal ion binding
binding;nucleic acid binding;RNA binding;structural constituent of ribosome;structural molecule activity
binding;catalytic activity;cation binding;chromatin binding;DNA binding;DNA-directed RNA polymerase activity;ion binding;metal ion binding;nucleic acid binding;nucleoside binding;nucleotidyltransferase activity;ribonucleosi
amine binding;amino acid binding;binding;carboxylic acid binding;enzyme binding;histone deacetylase binding;phosphoprotein binding;phosphoserine binding;protein binding;protein binding transcription factor activity;prote
3-hydroxyacyl-CoA dehydrogenase activity;acetyl-CoA C-acyltransferase activity;binding;C-acyltransferase activity;carbon-oxygen lyase activity;carboxylic acid binding;catalytic activity;coenzyme binding;cofactor binding;en
binding;catalytic activity;cation binding;coenzyme binding;cofactor binding;ion binding;metal ion binding;oxidoreductase activity;oxidoreductase activity, acting on the aldehyde or oxo group of donors;oxidoreductase activity
structural constituent of nuclear pore;structural molecule activity
actinin binding;alpha-actinin binding;binding;cytoskeletal protein binding;muscle alpha-actinin binding;protein binding
3 iron, 4 sulfur cluster binding;4 iron, 4 sulfur cluster binding;aconitate hydratase activity;binding;carbon-oxygen lyase activity;catalytic activity;cation binding;hydro-lyase activity;ion binding;iron ion binding;iron-sulfur cluster
adenyl nucleotide binding;adenyl ribonucleotide binding;ATP binding;binding;calcium ion binding;cation binding;enzyme binding;GTP binding;GTPase binding;guanyl nucleotide binding;guanyl ribonucleotide binding;ion bindi
binding;cation binding;ion binding;metal ion binding;transition metal ion binding;zinc ion binding
structural molecule activity
binding;enzyme binding;enzyme regulator activity;GTPase binding;GTPase regulator activity;nucleoside-triphosphatase regulator activity;protein binding;Ran GTPase binding;Ras GTPase binding;small GTPase binding;transp
binding;bisphosphoglycerate 2-phosphatase activity;bisphosphoglycerate mutase activity;bisphosphoglycerate phosphatase activity;catalytic activity;enzyme binding;hydrolase activity;hydrolase activity, acting on ester bond
binding;calcium ion binding;carbohydrate binding;cation binding;ion binding;metal ion binding
structural constituent of cytoskeleton;structural molecule activity
catalytic activity;L-malate dehydrogenase activity;malate dehydrogenase (NADP+) activity;malate dehydrogenase activity;oxidoreductase activity;oxidoreductase activity, acting on CH-OH group of donors;oxidoreductase act
binding;catalytic activity;cation binding;cysteine-type endopeptidase activity;cysteine-type peptidase activity;endopeptidase activity;exopeptidase activity;hydrolase activity;ion binding;metal ion binding;omega peptidase act
adenyl nucleotide binding;adenyl ribonucleotide binding;ATP binding;binding;binding, bridging;catalytic activity;cis-trans isomerase activity;drug binding;FK506 binding;GTP binding;guanyl nucleotide binding;guanyl ribonucle
binding;DNA binding;nucleic acid binding;RNA binding;structural constituent of ribosome;structural molecule activity
1,4-alpha-glucan branching enzyme activity;binding;catalytic activity;cation binding;hydrolase activity;hydrolase activity, acting on glycosyl bonds;hydrolase activity, hydrolyzing O-glycosyl compounds;ion binding;transferase
binding;calcium ion binding;catalytic activity;cation binding;electron carrier activity;ion binding;ion channel binding;metal ion binding;oxidoreductase activity;oxidoreductase activity, acting on paired donors, with incorporation
binding;core promoter binding;core promoter proximal region DNA binding;core promoter proximal region sequence-specific DNA binding;DNA binding;double-stranded DNA binding;identical protein binding;nucleic acid binc
binding;catalytic activity;cation binding;endopeptidase activity;enzyme activator activity;enzyme regulator activity;hydrolase activity;ion binding;metal ion binding;metalloendopeptidase activity;metallopeptidase activity;peptic
binding;eukaryotic initiation factor 4E binding;mRNA 3'-UTR binding;mRNA 5'-UTR binding;mRNA binding;nucleic acid binding;protein binding;RNA binding;RNA cap binding;translation activator activity;translation initiation f
binding;catalytic activity;cation binding;ion binding;metal ion binding;NAD+ ADP-ribosyltransferase activity;transferase activity;transferase activity, transferring glycosyl groups;transferase activity, transferring pentosyl groups
acid-amino acid ligase activity;binding;catalytic activity;cation binding;ion binding;ligase activity;ligase activity, forming carbon-nitrogen bonds;metal ion binding;small conjugating protein ligase activity;transition metal ion bin
adenyl nucleotide binding;adenyl ribonucleotide binding;ATP binding;ATPase activity;ATPase activity, coupled;ATP-dependent helicase activity;ATP-dependent RNA helicase activity;binding;catalytic activity;cation binding;chr
aminopeptidase activity;binding;catalytic activity;cation binding;epoxide hydrolase activity;ether hydrolase activity;exopeptidase activity;hydrolase activity;hydrolase activity, acting on ether bonds;ion binding;metal ion binding
actin binding;binding;cytoskeletal protein binding;protein binding
binding;enzyme binding;kinase binding;protein binding;protein kinase binding;protein transporter activity;substrate-specific transporter activity;transporter activity
binding;calcium ion binding;cation binding;ion binding;metal ion binding
binding;catalytic activity;coenzyme binding;cofactor binding;flavin adenine dinucleotide binding;oxidoreductase activity;oxidoreductase activity, acting on the CH-CH group of donors;oxidoreductase activity, acting on the CH
aldehyde-lyase activity;amine binding;amino acid binding;binding;carbon-carbon lyase activity;carboxylic acid binding;catalytic activity;chromatin binding;cofactor binding;glycine hydroxymethyltransferase activity;hydroxyme
binding;catalytic activity;GTP binding;GTPase activity;guanyl nucleotide binding;guanyl ribonucleotide binding;hydrolase activity;hydrolase activity, acting on acid anhydrides;hydrolase activity, acting on acid anhydrides, in pl
5S rDNA binding;adenyl nucleotide binding;adenyl ribonucleotide binding;ATP binding;binding;catalytic activity;DNA binding;drug binding;kinase activity;nucleic acid binding;nucleotide binding;phosphoprotein binding;phosp
binding;nucleic acid binding;RNA binding;rRNA binding;structural constituent of ribosome;structural molecule activity
binding;enzyme binding;identical protein binding;kinase binding;protein binding;protein kinase binding;transcription factor binding;ubiquitin protein ligase binding
binding;nucleic acid binding;RNA binding;translation factor activity, nucleic acid binding;translation initiation factor activity
binding;calcium ion binding;cation binding;cytoskeletal protein binding;identical protein binding;integrin binding;ion binding;metal ion binding;microtubule binding;microtubule plus-end binding;protein binding;protein complex
binding;estrogen receptor binding;hormone receptor binding;nuclear hormone receptor binding;protein binding;protein N-terminus binding;receptor binding;steroid hormone receptor binding
binding;DNA binding;nucleic acid binding
binding;DNA binding;enzyme binding;nucleic acid binding;nucleic acid binding transcription factor activity;protein binding;sequence-specific DNA binding transcription factor activity;ubiquitin protein ligase binding
adenyl nucleotide binding;adenyl ribonucleotide binding;ATP binding;binding;core promoter binding;core promoter sequence-specific DNA binding;DNA binding;hormone receptor binding;ligand-dependent nuclear receptor t
binding;catalytic activity;coenzyme binding;cofactor binding;electron carrier activity;NAD binding;nucleotide binding;oxidoreductase activity;oxidoreductase activity, acting on CH-OH group of donors;oxidoreductase activity,
binding;nucleic acid binding;nucleotide binding;RNA binding
binding;mRNA binding;nucleic acid binding;RNA binding
binding;DNA binding;nucleic acid binding
binding;enzyme binding;enzyme inhibitor activity;enzyme regulator activity;histone binding;identical protein binding;kinase binding;kinase inhibitor activity;kinase regulator activity;NF-kappaB binding;nucleic acid binding;prot
binding;calcium ion binding;calcium-dependent cysteine-type endopeptidase activity;catalytic activity;cation binding;cysteine-type endopeptidase activity;cysteine-type peptidase activity;endopeptidase activity;hydrolase ac
binding;catalytic activity;cation binding;DNA binding;IMP dehydrogenase activity;ion binding;metal ion binding;nucleic acid binding;nucleotide binding;oxidoreductase activity;oxidoreductase activity, acting on CH-OH group
4 iron, 4 sulfur cluster binding;aconitate hydratase activity;binding;carbon-oxygen lyase activity;catalytic activity;cation binding;hydro-lyase activity;ion binding;iron-responsive element binding;iron-sulfur cluster binding;lyase
acid-amino acid ligase activity;adenyl nucleotide binding;adenyl ribonucleotide binding;ATP binding;binding;carbon-carbon lyase activity;carboxy-lyase activity;catalytic activity;identical protein binding;ligase activity;ligase ac
binding;nucleic acid binding;RNA binding;translation elongation factor activity;translation factor activity, nucleic acid binding
binding;nucleic acid binding;nucleotide binding;poly(U) RNA binding;poly-pyrimidine tract binding;RNA binding;single-stranded RNA binding
binding;catalytic activity;cation binding;intramolecular transferase activity;intramolecular transferase activity, phosphotransferases;ion binding;isomerase activity;magnesium ion binding;metal ion binding;phosphoglucomutas
adenyl nucleotide binding;adenyl ribonucleotide binding;amine binding;amino acid binding;aminoacyl-tRNA ligase activity;arginine binding;arginine-tRNA ligase activity;ATP binding;binding;carboxylic acid binding;catalytic ac
binding;nucleic acid binding;RNA binding;rRNA binding;structural constituent of ribosome;structural molecule activity
binding;catalytic activity;GTP binding;GTPase activity;guanyl nucleotide binding;guanyl ribonucleotide binding;hydrolase activity;hydrolase activity, acting on acid anhydrides;hydrolase activity, acting on acid anhydrides, in pl
binding;catalytic activity;GTP binding;GTPase activity;guanyl nucleotide binding;guanyl ribonucleotide binding;hydrolase activity;hydrolase activity, acting on acid anhydrides;hydrolase activity, acting on acid anhydrides, in pl
adenyl nucleotide binding;adenyl ribonucleotide binding;ATP binding;ATPase activity;ATPase activity, coupled;ATP-dependent helicase activity;ATP-dependent RNA helicase activity;binding;catalytic activity;helicase activity;h
acetyltransferase activity;binding;catalytic activity;N-acetyltransferase activity;N-acyltransferase activity;ribonucleoprotein binding;ribosome binding;transferase activity;transferase activity, transferring acyl groups;transferase
active transmembrane transporter activity;adenyl nucleotide binding;adenyl ribonucleotide binding;ATP binding;ATPase activity;ATPase activity, coupled;ATPase activity, coupled to movement of substances;ATPase activity, c
binding;chromatin binding;chromatin DNA binding;core promoter proximal region DNA binding;core promoter proximal region sequence-specific DNA binding;DNA binding;enhancer binding;enhancer sequence-specific DNA
actin binding;binding;cytoskeletal protein binding;protein binding;structural constituent of cytoskeleton;structural constituent of muscle;structural molecule activity
adenyl nucleotide binding;adenyl ribonucleotide binding;aminoacyl-tRNA ligase activity;ATP binding;binding;catalytic activity;histidine-tRNA ligase activity;ligase activity;ligase activity, forming aminoacyl-tRNA and related cor
binding;DNA binding;nucleic acid binding;nucleotide binding;regulatory region DNA binding;regulatory region nucleic acid binding;RNA binding;transcription regulatory region DNA binding
binding;cation binding;collagen binding;ion binding;metal ion binding;protein binding;receptor activity;viral receptor activity
structural constituent of cytoskeleton;structural molecule activity
adenyl nucleotide binding;adenyl ribonucleotide binding;ATP binding;binding;nucleotide binding;protein binding;purine nucleotide binding;purine ribonucleoside triphosphate binding;purine ribonucleotide binding;ribonucleoti
binding;nucleic acid binding;RNA binding;rRNA binding;structural constituent of ribosome;structural molecule activity;translation regulator activity
binding;core promoter proximal region DNA binding;core promoter proximal region sequence-specific DNA binding;DNA binding;nucleic acid binding;nucleic acid binding transcription factor activity;regulatory region DNA bin
binding;enzyme binding;enzyme inhibitor activity;enzyme regulator activity;insulin-like growth factor receptor binding;kinase binding;kinase inhibitor activity;kinase regulator activity;protein binding;protein kinase binding;prote
adenyl nucleotide binding;adenyl ribonucleotide binding;ATP binding;ATPase activity;binding;catalytic activity;cytoskeletal protein binding;hydrolase activity;hydrolase activity, acting on acid anhydrides;hydrolase activity, acti
catalytic activity;hydrolase activity;hydrolase activity, acting on acid anhydrides;hydrolase activity, acting on acid anhydrides, in phosphorus-containing anhydrides;motor activity;nucleoside-triphosphatase activity;pyrophosp
adenyl nucleotide binding;adenyl ribonucleotide binding;ATP binding;ATPase activity;ATPase activity, coupled;ATP-dependent helicase activity;ATP-dependent RNA helicase activity;binding;catalytic activity;chromatin binding
binding;core promoter proximal region DNA binding;core promoter proximal region sequence-specific DNA binding;DNA binding;nucleic acid binding;nucleic acid binding transcription factor activity;regulatory region DNA bin

binding;DNA binding;nucleic acid binding
binding;catalytic activity;enzyme binding;GTP binding;GTPase activity;guanyl nucleotide binding;guanyl ribonucleotide binding;hydrolase activity;hydrolase activity, acting on acid anhydrides;hydrolase activity, acting on acid
adenyl nucleotide binding;adenyl ribonucleotide binding;ATP binding;binding;carbon-nitrogen ligase activity, with glutamine as amido-N-donor;catalytic activity;cation binding;ion binding;ligase activity;ligase activity, forming
structural molecule activity
adenyl nucleotide binding;adenyl ribonucleotide binding;aminoacyl-tRNA ligase activity;ATP binding;binding;catalytic activity;glutamine-tRNA ligase activity;ligase activity;ligase activity, forming aminoacyl-tRNA and related c
binding;nucleic acid binding;RNA binding;structural constituent of ribosome;structural molecule activity
binding;catalytic activity;enzyme binding;GTP binding;GTPase activity;guanyl nucleotide binding;guanyl ribonucleotide binding;hydrolase activity;hydrolase activity, acting on acid anhydrides;hydrolase activity, acting on acid
adenyl nucleotide binding;adenyl ribonucleotide binding;ATP binding;binding;cytokine receptor binding;enzyme binding;kinase binding;nucleotide binding;protein binding;protein kinase binding;purine nucleotide binding;purin
binding;catalytic activity;co-SMAD binding;cysteine-type endopeptidase activity;cysteine-type peptidase activity;endopeptidase activity;hydrolase activity;peptidase activity;peptidase activity, acting on L-amino acid peptides
binding;carboxylic acid binding;catalytic activity;cation binding;ion binding;iron ion binding;L-ascorbic acid binding;metal ion binding;oxidoreductase activity;oxidoreductase activity, acting on paired donors, with incorporatio
binding;mRNA binding;nucleic acid binding;nucleotide binding;poly(A) RNA binding;poly-purine tract binding;RNA binding;single-stranded RNA binding
binding;calcium-dependent protein binding;protein binding
adenine transmembrane transporter activity;binding;enzyme binding;nucleobase transmembrane transporter activity;nucleobase-containing compound transmembrane transporter activity;protein binding;purine base transme
actin binding;binding;cation binding;cell adhesion molecule binding;cytoskeletal protein binding;enzyme binding;ion binding;metal ion binding;peptide binding;protease binding;protein binding;protein dimerization activity;pro
binding;calcium ion binding;calcium-dependent cysteine-type endopeptidase activity;catalytic activity;cation binding;cysteine-type endopeptidase activity;cysteine-type peptidase activity;cytoskeletal protein binding;endope
binding;DNA binding;double-stranded RNA binding;nucleic acid binding;RNA binding
binding;catalytic activity;GTP binding;GTPase activity;guanyl nucleotide binding;guanyl ribonucleotide binding;hydrolase activity;hydrolase activity, acting on acid anhydrides;hydrolase activity, acting on acid anhydrides, in p
binding;collagen binding;extracellular matrix binding;integrin binding;protein binding;protein complex binding;receptor binding
actin binding;actin filament binding;binding;cytoskeletal protein binding;enzyme binding;GTPase binding;protein binding;protein complex binding;Rac GTPase binding;Ras GTPase binding;Rho GTPase binding;small GTPase
binding;protein binding;protein C-terminus binding
binding;enzyme binding;enzyme inhibitor activity;enzyme regulator activity;GTPase binding;GTPase inhibitor activity;GTPase regulator activity;nucleoside-triphosphatase regulator activity;protein binding;protein transporter a
binding;integrin binding;protein binding;protein complex binding;receptor binding;structural molecule activity
binding;protein binding;scaffold protein binding;structural molecule activity
binding;extracellular matrix structural constituent;glycolipid binding;glycosphingolipid binding;lipid binding;sphingolipid binding;structural molecule activity
active transmembrane transporter activity;antiporter activity;ATP:ADP antiporter activity;secondary active transmembrane transporter activity;solute:solute antiporter activity;transmembrane transporter activity;transporter act
adenyl nucleotide binding;adenyl ribonucleotide binding;aminoacylase activity;aminoacyl-tRNA ligase activity;aspartate-tRNA ligase activity;ATP binding;binding;catalytic activity;hydrolase activity;hydrolase activity, acting on
actin binding;binding;cation binding;cytoskeletal protein binding;enzyme binding;integrin binding;ion binding;kinase binding;metal ion binding;protein binding;protein complex binding;protein kinase binding;receptor binding;t
adenyl nucleotide binding;adenyl ribonucleotide binding;ATP binding;binding;catalytic activity;identical protein binding;kinase activity;nucleotide binding;phosphotransferase activity, alcohol group as acceptor;protein binding
adenyl nucleotide binding;adenyl ribonucleotide binding;ATP binding;binding;nucleotide binding;purine nucleotide binding;purine ribonucleoside triphosphate binding;purine ribonucleotide binding;ribonucleotide binding
5'-3' DNA helicase activity;adenyl nucleotide binding;adenyl ribonucleotide binding;ATP binding;ATPase activity;ATPase activity, coupled;ATP-dependent 5'-3' DNA helicase activity;ATP-dependent DNA helicase activity;ATP-
androgen receptor binding;binding;hormone receptor binding;nuclear hormone receptor binding;nucleic acid binding;protein binding;protein binding transcription factor activity;receptor binding;ribonucleoprotein binding;RNA
actin binding;binding;calcium ion binding;cation binding;cytoskeletal protein binding;ion binding;metal ion binding;myosin binding;myosin II binding;protein binding;protein domain specific binding
actin binding;binding;cytoskeletal protein binding;protein binding;structural constituent of muscle;structural molecule activity
beta-catenin binding;binding;cadherin binding;cell adhesion molecule binding;cytoskeletal protein binding;gamma-catenin binding;protein binding;structural molecule activity;vinculin binding
binding;calcium ion binding;cation binding;extracellular matrix structural constituent;ion binding;metal ion binding;structural molecule activity
adenyl nucleotide binding;adenyl ribonucleotide binding;aminoacyl-tRNA ligase activity;ATP binding;binding;catalytic activity;ligase activity;ligase activity, forming aminoacyl-tRNA and related compounds;ligase activity, formi
binding;DNA binding;nucleic acid binding;nucleic acid binding transcription factor activity;sequence-specific DNA binding transcription factor activity
binding;cation binding;enzyme binding;ion binding;metal ion binding;nuclear localization sequence binding;peptide binding;protein binding;protein domain specific binding;protein transporter activity;signal sequence binding;
adenyl nucleotide binding;adenyl ribonucleotide binding;ATP binding;ATPase activity;ATPase activity, coupled;ATP-dependent helicase activity;ATP-dependent RNA helicase activity;binding;catalytic activity;helicase activity;h
binding;DNA binding;nucleic acid binding;nucleic acid binding transcription factor activity;sequence-specific DNA binding transcription factor activity;single-stranded DNA binding;structure-specific DNA binding
adenyl nucleotide binding;adenyl ribonucleotide binding;ATP binding;ATPase activity;binding;catalytic activity;chromatin binding;DNA binding;helicase activity;hydrolase activity;hydrolase activity, acting on acid anhydrides;hy
adenyl nucleotide binding;adenyl ribonucleotide binding;ATP binding;binding;nucleotide binding;protein binding;protein dimerization activity;protein heterodimerization activity;purine nucleotide binding;purine ribonucleoside
structural constituent of cytoskeleton;structural molecule activity
adenyl nucleotide binding;adenyl ribonucleotide binding;ATP binding;binding;catalytic activity;cation binding;cyclo-ligase activity;hydroxymethyl-, formyl- and related transferase activity;ion binding;ligase activity;ligase activit
adenyl nucleotide binding;adenyl ribonucleotide binding;ADP binding;ATP binding;ATPase activity;ATPase activity, coupled;ATP-dependent peptidase activity;binding;catalytic activity;DNA binding;DNA polymerase binding;e
binding;nucleic acid binding;nucleotide binding;protein binding;protein complex scaffold;RNA binding;structural molecule activity;translation factor activity, nucleic acid binding;translation initiation factor activity
1-phosphatidylinositol binding;binding;calmodulin binding;cation binding;GTP-dependent protein binding;identical protein binding;ion binding;lipid binding;metal ion binding;phosphatidylinositol binding;phospholipid binding;
binding;chromatin binding;core promoter binding;DNA binding;double-stranded DNA binding;nucleic acid binding;nucleotide binding;regulatory region DNA binding;regulatory region nucleic acid binding;structure-specific DN
binding;DNA binding;nucleic acid binding;nucleotide binding;RNA binding;single-stranded DNA binding;single-stranded RNA binding;structure-specific DNA binding
binding;mRNA binding;nucleic acid binding;nucleotide binding;poly(A) RNA binding;poly(U) RNA binding;poly-purine tract binding;poly-pyrimidine tract binding;protein binding;protein C-terminus binding;RNA binding;single-s
actin binding;binding;calmodulin binding;cytoskeletal protein binding;protein binding;tropomyosin binding
acetyl-CoA carboxylase activity;adenyl nucleotide binding;adenyl ribonucleotide binding;ATP binding;binding;biotin carboxylase activity;catalytic activity;cation binding;CoA carboxylase activity;ion binding;ligase activity;ligas
binding;nucleic acid binding;RNA binding
binding;binding, bridging;catalytic activity;guanylate kinase activity;kinase activity;nucleobase-containing compound kinase activity;nucleotide kinase activity;phosphotransferase activity, phosphate group as acceptor;protein
adenyl nucleotide binding;adenyl ribonucleotide binding;ATP binding;binding;chaperone binding;nucleotide binding;protein binding;purine nucleotide binding;purine ribonucleoside triphosphate binding;purine ribonucleotide
adenyl nucleotide binding;adenyl ribonucleotide binding;ATP binding;ATPase activity;ATPase activity, coupled;ATP-dependent helicase activity;ATP-dependent RNA helicase activity;binding;catalytic activity;double-stranded
adenyl nucleotide binding;adenyl ribonucleotide binding;ATP binding;binding;nucleotide binding;purine nucleotide binding;purine ribonucleoside triphosphate binding;purine ribonucleotide binding;ribonucleotide binding
adenyl nucleotide binding;adenyl ribonucleotide binding;ADP binding;amine binding;amino acid binding;ATP binding;binding;carboxylic acid binding;catalytic activity;coenzyme binding;cofactor binding;glutamate dehydroge
binding;cargo receptor activity;double-stranded RNA binding;identical protein binding;nucleic acid binding;protein binding;receptor activity;RNA binding;transferrin receptor activity
binding;calcium ion binding;calcium-dependent phospholipid binding;calcium-dependent protein binding;cation binding;enzyme inhibitor activity;enzyme regulator activity;ion binding;lipase inhibitor activity;lipid binding;meta
binding;DNA binding;identical protein binding;mRNA binding;nucleic acid binding;protein binding;protein dimerization activity;protein homodimerization activity;RNA binding;structural constituent of ribosome;structural molec
adenyl nucleotide binding;adenyl ribonucleotide binding;ATP binding;binding;catalytic activity;DNA binding;DNA helicase activity;helicase activity;hydrolase activity;hydrolase activity, acting on acid anhydrides;hydrolase activ
5S rRNA binding;binding;nucleic acid binding;RNA binding;rRNA binding;structural constituent of ribosome;structural molecule activity
binding;importin-alpha export receptor activity;nuclear export signal receptor activity;protein binding;protein transporter activity;substrate-specific transporter activity;transporter activity
adenosine deaminase activity;binding;catalytic activity;cation binding;deaminase activity;DNA binding;double-stranded RNA adenosine deaminase activity;hydrolase activity;hydrolase activity, acting on carbon-nitrogen (but
adenyl nucleotide binding;adenyl ribonucleotide binding;ATP binding;ATPase activity;ATPase activity, coupled;ATP-dependent helicase activity;ATP-dependent RNA helicase activity;binding;catalytic activity;double-stranded
binding;calcium ion binding;cation binding;ion binding;metal ion binding;structural constituent of muscle;structural molecule activity
binding;catalytic activity;methyltransferase activity;nucleic acid binding;RNA binding;RNA methyltransferase activity;S-adenosylmethionine-dependent methyltransferase activity;transferase activity;transferase activity, transfe
binding;catalytic activity;cis-trans isomerase activity;drug binding;FK506 binding;heat shock protein binding;isomerase activity;macrolide binding;peptidyl-prolyl cis-trans isomerase activity;protein binding
binding;catalytic activity;glucosyltransferase activity;protein binding;transferase activity;transferase activity, transferring glycosyl groups;transferase activity, transferring hexosyl groups;UDP-glucose:glycoprotein glucosyltrans
binding;extracellular matrix structural constituent;glycolipid binding;glycosphingolipid binding;lipid binding;sphingolipid binding;structural molecule activity
ATPase activity;ATPase activity, coupled;binding;catalytic activity;chromatin binding;core promoter binding;core promoter sequence-specific DNA binding;DNA binding;DNA topoisomerase (ATP-hydrolyzing) activity;DNA topo
binding;carbohydrate binding;catalytic activity;coenzyme binding;cofactor binding;glucose binding;glucose-6-phosphate dehydrogenase activity;identical protein binding;monosaccharide binding;NADP binding;nucleotide bi
adenyl nucleotide binding;adenyl ribonucleotide binding;ATP binding;binding;biotin binding;biotin carboxylase activity;carboxylic acid binding;catalytic activity;cation binding;DNA binding;ion binding;ligase activity;ligase activ
aminopeptidase activity;binding;catalytic activity;cation binding;exopeptidase activity;hydrolase activity;ion binding;metal ion binding;metallopeptidase activity;peptidase activity;peptidase activity, acting on L-amino acid pep
adenyl nucleotide binding;adenyl ribonucleotide binding;aminoacyl-tRNA ligase activity;ATP binding;binding;catalytic activity;glycine-tRNA ligase activity;ligase activity;ligase activity, forming aminoacyl-tRNA and related com
adenyl nucleotide binding;adenyl ribonucleotide binding;ATP binding;binding;nucleotide binding;protein binding;purine nucleotide binding;purine ribonucleoside triphosphate binding;purine ribonucleotide binding;ribonucleoti
adenyl nucleotide binding;adenyl ribonucleotide binding;AMP binding;ATP binding;bile acid binding;binding;carbohydrate binding;carboxylic acid binding;catalytic activity;cofactor binding;drug binding;glucose binding;glycog
adenyl nucleotide binding;adenyl ribonucleotide binding;ATP binding;binding;enzyme binding;nucleotide binding;protein binding;purine nucleotide binding;purine ribonucleoside triphosphate binding;purine ribonucleotide bin
binding;cation binding;ion binding;metal ion binding;nucleotide binding;structural molecule activity;transition metal ion binding;zinc ion binding
binding;catalytic activity;GTP binding;GTPase activity;guanyl nucleotide binding;guanyl ribonucleotide binding;hydrolase activity;hydrolase activity, acting on acid anhydrides;hydrolase activity, acting on acid anhydrides, in p
binding;catalytic activity;double-stranded RNA binding;GTP binding;GTPase activity;guanyl nucleotide binding;guanyl ribonucleotide binding;hydrolase activity;hydrolase activity, acting on acid anhydrides;hydrolase activity,
ankyrin binding;binding;cytoskeletal protein binding;enzyme binding;protein binding
actin binding;actin filament binding;actin monomer binding;binding;cation binding;cytoskeletal protein binding;ion binding;metal ion binding;protein binding;transition metal ion binding;zinc ion binding
binding;carbohydrate binding;receptor activity;structural molecule activity
binding;catalytic activity;enzyme binding;GTP binding;GTPase activity;guanyl nucleotide binding;guanyl ribonucleotide binding;hydrolase activity;hydrolase activity, acting on acid anhydrides;hydrolase activity, acting on acid
5'-deoxyribose-5-phosphate lyase activity;adenyl nucleotide binding;adenyl ribonucleotide binding;ATP binding;ATPase activity;ATPase activity, coupled;ATP-dependent DNA helicase activity;ATP-dependent helicase activity;
adenyl nucleotide binding;adenyl ribonucleotide binding;ATP binding;ATPase activity;ATPase activity, coupled;ATP-dependent DNA helicase activity;ATP-dependent helicase activity;binding;catalytic activity;damaged DNA bi
binding;DNA binding;nucleic acid binding;nucleotide binding;RNA binding;sequence-specific DNA binding;single-stranded DNA binding;single-stranded telomeric DNA binding;structure-specific DNA binding;telomeric DNA b
binding;catalytic activity;cation binding;chromatin binding;DNA (cytosine-5-)-methyltransferase activity;DNA binding;DNA-methyltransferase activity;ion binding;metal ion binding;methyl-CpG binding;methyltransferase activit
binding;catalytic activity;cytokine activity;drug binding;nicotinamide phosphoribosyltransferase activity;nicotinate-nucleotide diphosphorylase (carboxylating) activity;protein binding;receptor binding;transferase activity;transf
adenyl nucleotide binding;adenyl ribonucleotide binding;ATP binding;binding;catalytic activity;cation binding;DNA binding;DNA helicase activity;DNA replication origin binding;helicase activity;hydrolase activity;hydrolase acti
adenyl nucleotide binding;adenyl ribonucleotide binding;ATP binding;binding;nucleotide binding;protein binding;purine nucleotide binding;purine ribonucleoside triphosphate binding;purine ribonucleotide binding;ribonucleoti
6-phosphofructokinase activity;adenyl nucleotide binding;adenyl ribonucleotide binding;ATP binding;binding;carbohydrate kinase activity;catalytic activity;cation binding;ion binding;kinase activity;metal ion binding;nucleotide
adenylate cyclase binding;binding;enzyme binding;protein binding;protein kinase A binding
binding;damaged DNA binding;DNA binding;nucleic acid binding
binding;protein binding;receptor binding
ATPase activity;binding;catalytic activity;enzyme binding;hydrolase activity;hydrolase activity, acting on acid anhydrides;hydrolase activity, acting on acid anhydrides, in phosphorus-containing anhydrides;nucleic acid binding
binding;cadherin binding;cell adhesion molecule binding;protein binding;receptor binding
binding;nucleic acid binding;nucleotide binding;RNA binding;translation factor activity, nucleic acid binding;translation initiation factor activity
adenyl nucleotide binding;adenyl ribonucleotide binding;alanine-tRNA ligase activity;amine binding;amino acid binding;aminoacyl-tRNA editing activity;aminoacyl-tRNA ligase activity;ATP binding;binding;carboxylic acid bindi
aminopeptidase activity;binding;catalytic activity;cation binding;exopeptidase activity;hydrolase activity;ion binding;metal ion binding;metallopeptidase activity;peptidase activity;peptidase activity, acting on L-amino acid pep
binding;enzyme binding;kinase binding;phosphatase binding;protein binding;protein kinase binding;protein phosphatase binding
adenyl nucleotide binding;adenyl ribonucleotide binding;ATP binding;ATPase activity;ATPase activity, coupled;ATP-dependent DNA helicase activity;ATP-dependent helicase activity;binding;catalytic activity;cation binding;DN
adenyl nucleotide binding;adenyl ribonucleotide binding;ATP binding;ATPase activity;binding;binding, bridging;catalytic activity;cation binding;DNA binding;hydrolase activity;hydrolase activity, acting on acid anhydrides;hydr
binding;catalytic activity;GTP binding;GTPase activity;guanyl nucleotide binding;guanyl ribonucleotide binding;hydrolase activity;hydrolase activity, acting on acid anhydrides;hydrolase activity, acting on acid anhydrides, in p
structural constituent of cytoskeleton;structural molecule activity
structural molecule activity
enzyme activator activity;enzyme regulator activity;GDP-dissociation inhibitor activity;GTPase activator activity;GTPase regulator activity;nucleoside-triphosphatase regulator activity;Rab GDP-dissociation inhibitor activity;sm
adenyl nucleotide binding;adenyl ribonucleotide binding;ATP binding;binding;catalytic activity;DNA binding;DNA helicase activity;helicase activity;hydrolase activity;hydrolase activity, acting on acid anhydrides;hydrolase activ
adenyl nucleotide binding;adenyl ribonucleotide binding;ATP binding;binding;nucleotide binding;protein binding;purine nucleotide binding;purine ribonucleoside triphosphate binding;purine ribonucleotide binding;ribonucleoti
adenyl nucleotide binding;adenyl ribonucleotide binding;ATP binding;binding;nucleotide binding;protein binding;protein dimerization activity;protein heterodimerization activity;purine nucleotide binding;purine ribonucleoside
catalytic activity;dolichyl-diphosphooligosaccharide-protein glycotransferase activity;oligosaccharyl transferase activity;transferase activity;transferase activity, transferring glycosyl groups;transferase activity, transferring hex
binding;calcium ion binding;calcium-dependent phospholipid binding;cation binding;enzyme inhibitor activity;enzyme regulator activity;ion binding;lipase inhibitor activity;lipid binding;metal ion binding;phospholipase inhibito
adenyl nucleotide binding;adenyl ribonucleotide binding;ATP binding;binding;catalytic activity;chromatin binding;DNA binding;DNA helicase activity;DNA replication origin binding;helicase activity;hydrolase activity;hydrolase
adenyl nucleotide binding;adenyl ribonucleotide binding;aminoacyl-tRNA ligase activity;ATP binding;binding;catalytic activity;chemokine receptor binding;CXCR chemokine receptor binding;cytokine receptor binding;G-protei
binding;MHC class II protein binding;MHC class II receptor activity;MHC protein binding;molecular transducer activity;protein binding;receptor activity;receptor binding;signal transducer activity;signaling receptor activity;stru
actin binding;actin filament binding;binding;cytoskeletal protein binding;drug binding;protein binding
binding;DNA binding;mRNA binding;nucleic acid binding;RNA binding
adenyl nucleotide binding;adenyl ribonucleotide binding;ATP binding;ATPase activity;ATPase activity, coupled;ATP-dependent helicase activity;ATP-dependent RNA helicase activity;binding;catalytic activity;double-stranded
adenyl nucleotide binding;adenyl ribonucleotide binding;aminoacyl-tRNA editing activity;aminoacyl-tRNA ligase activity;ATP binding;binding;carboxylic ester hydrolase activity;catalytic activity;hydrolase activity;hydrolase act
adenyl nucleotide binding;adenyl ribonucleotide binding;ATP binding;ATPase activity;ATPase activity, coupled;ATP-dependent DNA helicase activity;ATP-dependent helicase activity;ATP-dependent RNA helicase activity;bind
catalytic activity;lactate dehydrogenase activity;L-lactate dehydrogenase activity;oxidoreductase activity;oxidoreductase activity, acting on CH-OH group of donors;oxidoreductase activity, acting on the CH-OH group of don
adenyl nucleotide binding;adenyl ribonucleotide binding;amine binding;amino acid binding;argininosuccinate synthase activity;ATP binding;binding;carboxylic acid binding;catalytic activity;identical protein binding;ligase activ

active transmembrane transporter activity;adenyl nucleotide binding;adenyl ribonucleotide binding;ATP binding;ATPase activity;ATPase activity, coupled;ATPase activity, coupled to movement of substances;ATPase activity, c
adenyl nucleotide binding;adenyl ribonucleotide binding;ATP binding;binding;enzyme binding;nucleotide binding;protein binding;purine nucleotide binding;purine ribonucleoside triphosphate binding;purine ribonucleotide bin
structural molecule activity
binding;calcium-dependent protein binding;G-protein-coupled receptor binding;identical protein binding;protein binding;protein dimerization activity;protein homodimerization activity;proteinase activated receptor binding;rec
binding;nucleic acid binding;RNA binding;translation factor activity, nucleic acid binding;translation initiation factor activity
binding;ion channel binding;protein binding;receptor binding
actin binding;aldehyde-lyase activity;binding;carbohydrate binding;carbon-carbon lyase activity;catalytic activity;cytoskeletal protein binding;fructose binding;fructose-bisphosphate aldolase activity;identical protein binding;l
structural molecule activity
adenyl nucleotide binding;adenyl ribonucleotide binding;ATP binding;beta-tubulin binding;binding;cytoskeletal protein binding;G-protein beta-subunit binding;nucleotide binding;protein binding;purine nucleotide binding;purin
acid-amino acid ligase activity;binding;catalytic activity;cation binding;chromo shadow domain binding;DNA binding;enzyme binding;ion binding;kinase activity;Krueppel-associated box domain binding;ligase activity;ligase a
binding;catalytic activity;core promoter binding;DNA binding;DNA polymerase activity;DNA-directed DNA polymerase activity;nucleic acid binding;nucleotidyltransferase activity;protein binding;protein binding transcription fa
3-hydroxyacyl-CoA dehydrogenase activity;acetyl-CoA C-acetyltransferase activity;acetyl-CoA C-acyltransferase activity;acetyltransferase activity;binding;C-acetyltransferase activity;C-acyltransferase activity;carbon-oxyger
adenyl nucleotide binding;adenyl ribonucleotide binding;ATP binding;binding;cytoskeletal protein binding;enzyme binding;identical protein binding;kinesin binding;nitric-oxide synthase binding;nucleotide binding;protein bindi
binding;profilin binding;protein binding
adenyl nucleotide binding;adenyl ribonucleotide binding;ATP binding;binding;nucleotide binding;purine nucleotide binding;purine ribonucleoside triphosphate binding;purine ribonucleotide binding;ribonucleotide binding
adenyl nucleotide binding;adenyl ribonucleotide binding;ATP binding;binding;catalytic activity;chromatin binding;dynein binding;hydrolase activity;hydrolase activity, acting on acid anhydrides;hydrolase activity, acting on acid
binding;catalytic activity;cation binding;GTP binding;guanyl nucleotide binding;guanyl ribonucleotide binding;ion binding;metal ion binding;nucleotide binding;protein-glutamine gamma-glutamyltransferase activity;purine nucl
adenyl nucleotide binding;adenyl ribonucleotide binding;ATP binding;binding;catalytic activity;DNA binding;DNA helicase activity;helicase activity;hydrolase activity;hydrolase activity, acting on acid anhydrides;hydrolase activ
binding;catalytic activity;GTP binding;GTPase activity;guanyl nucleotide binding;guanyl ribonucleotide binding;hydrolase activity;hydrolase activity, acting on acid anhydrides;hydrolase activity, acting on acid anhydrides, in pl
adenyl nucleotide binding;adenyl ribonucleotide binding;ATP binding;binding;carbohydrate kinase activity;catalytic activity;fructokinase activity;glucokinase activity;hexokinase activity;kinase activity;mannokinase activity;nuc
adenyl nucleotide binding;adenyl ribonucleotide binding;ATP binding;binding;catalytic activity;DNA binding;DNA helicase activity;helicase activity;hydrolase activity;hydrolase activity, acting on acid anhydrides;hydrolase activ
alcohol binding;binding;calcium channel activity;calcium-release channel activity;cation channel activity;cation transmembrane transporter activity;channel activity;gated channel activity;inositol 1,3,4,5 tetrakisphosphate bind
binding;enzyme binding;kinase binding;molecular transducer activity;protein binding;protein kinase binding;signal transducer activity
ATPase activity;ATPase activity, coupled;binding;binding, bridging;calcium ion binding;calcium-dependent phospholipid binding;calcium-dependent protein binding;catalytic activity;cation binding;DNA binding;DNA-depende
binding;catalytic activity;coenzyme binding;cofactor binding;cytoskeletal protein binding;glyceraldehyde-3-phosphate dehydrogenase (NAD+) (phosphorylating) activity;identical protein binding;microtubule binding;NAD bindi
catalytic activity;intramolecular oxidoreductase activity;intramolecular oxidoreductase activity, interconverting keto- and enol-groups;intramolecular oxidoreductase activity, transposing S-S bonds;isomerase activity;protein d
binding;chromatin binding;core promoter binding;DNA binding;mRNA binding;nucleic acid binding;nucleotide binding;regulatory region DNA binding;regulatory region nucleic acid binding;RNA binding;single-stranded RNA bi
transporter activity
adenyl nucleotide binding;adenyl ribonucleotide binding;ATP binding;binding;catalytic activity;chromatin binding;hydrolase activity;hydrolase activity, acting on acid anhydrides;hydrolase activity, acting on acid anhydrides, in
adenyl nucleotide binding;adenyl ribonucleotide binding;ATP binding;ATPase activity;ATPase activity, coupled;binding;catalytic activity;hydrolase activity;hydrolase activity, acting on acid anhydrides;hydrolase activity, acting
acid-amino acid ligase activity;adenyl nucleotide binding;adenyl ribonucleotide binding;ATP binding;binding;catalytic activity;ligase activity;ligase activity, forming carbon-nitrogen bonds;nucleotide binding;purine nucleotide b
adenyl nucleotide binding;adenyl ribonucleotide binding;aminoacyl-tRNA ligase activity;ATP binding;binding;catalytic activity;identical protein binding;ligase activity;ligase activity, forming aminoacyl-tRNA and related compou
binding;nucleic acid binding;RNA binding;structural constituent of ribosome;structural molecule activity
binding;protein binding;scaffold protein binding;structural molecule activity
structural molecule activity
binding;catalytic activity;enzyme binding;GTP binding;GTPase activity;guanyl nucleotide binding;guanyl ribonucleotide binding;hydrolase activity;hydrolase activity, acting on acid anhydrides;hydrolase activity, acting on acid
adenyl nucleotide binding;adenyl ribonucleotide binding;androgen receptor binding;ATP binding;ATPase activity;ATPase activity, coupled;ATP-dependent helicase activity;ATP-dependent RNA helicase activity;binding;catalyti
acid-thiol ligase activity;adenyl nucleotide binding;adenyl ribonucleotide binding;ATP binding;ATP citrate synthase activity;binding;catalytic activity;cation binding;CoA-ligase activity;cofactor binding;ion binding;ligase activity
acid-amino acid ligase activity;binding;catalytic activity;cation binding;ion binding;ligase activity;ligase activity, forming carbon-nitrogen bonds;metal ion binding;small conjugating protein ligase activity;transition metal ion bin
actin binding;actin filament binding;adenyl nucleotide binding;adenyl ribonucleotide binding;ATP binding;binding;catalytic activity;cytoskeletal protein binding;hydrolase activity;hydrolase activity, acting on acid anhydrides;hy
active transmembrane transporter activity;adenyl nucleotide binding;adenyl ribonucleotide binding;ADP binding;alkali metal ion binding;ATP binding;ATPase activity;ATPase activity, coupled;ATPase activity, coupled to movem
adenyl nucleotide binding;adenyl ribonucleotide binding;ATP binding;binding;catalytic activity;cyclohydrolase activity;formate-tetrahydrofolate ligase activity;hydrolase activity;hydrolase activity, acting on carbon-nitrogen (but
adenyl nucleotide binding;adenyl ribonucleotide binding;ATP binding;ATPase activity;binding;catalytic activity;cytoskeletal protein binding;hydrolase activity;hydrolase activity, acting on acid anhydrides;hydrolase activity, acti
ankyrin binding;binding;cytoskeletal protein binding;protein binding
active transmembrane transporter activity;adenyl nucleotide binding;adenyl ribonucleotide binding;ATP binding;ATPase activity;ATPase activity, coupled;ATPase activity, coupled to movement of substances;ATPase activity, c
binding;nucleic acid binding;RNA binding;structural constituent of ribosome;structural molecule activity
alpha-glucosidase activity;binding;carbohydrate binding;catalytic activity;glucan 1,3-alpha-glucosidase activity;glucosidase activity;hydrolase activity;hydrolase activity, acting on glycosyl bonds;hydrolase activity, hydrolyzing
binding;core promoter binding;DNA binding;identical protein binding;nucleic acid binding;nucleotide binding;protein binding;regulatory region DNA binding;regulatory region nucleic acid binding;transcription regulatory region
adenyl nucleotide binding;adenyl ribonucleotide binding;ATP binding;ATPase activity;ATPase activity, coupled;ATP-dependent helicase activity;ATP-dependent RNA helicase activity;binding;catalytic activity;estrogen receptor
adenyl nucleotide binding;adenyl ribonucleotide binding;aminoacyl-tRNA editing activity;aminoacyl-tRNA ligase activity;ATP binding;binding;carboxylic ester hydrolase activity;catalytic activity;hydrolase activity;hydrolase acti
binding;C2H2 zinc finger domain binding;protein binding;protein domain specific binding;protein N-terminus binding
binding;catalytic activity;cation binding;cofactor binding;identical protein binding;ion binding;metal ion binding;protein binding;protein dimerization activity;protein homodimerization activity;transferase activity;transferase acti
catalytic activity;cysteine-type endopeptidase activity;cysteine-type peptidase activity;disulfide oxidoreductase activity;endopeptidase activity;hydrolase activity;hydrolase activity, acting on ester bonds;intramolecular oxidore
actin-dependent ATPase activity;adenyl nucleotide binding;adenyl ribonucleotide binding;ATP binding;ATPase activity;ATPase activity, coupled;binding;catalytic activity;enzyme binding;GTPase binding;hydrolase activity;hydr
binding;cation binding;ion binding;metal ion binding;protein binding;protein C-terminus binding
adenyl nucleotide binding;adenyl ribonucleotide binding;ATP binding;binding;catalytic activity;kinase activity;nucleotide binding;phosphoglycerate kinase activity;phosphotransferase activity, carboxyl group as acceptor;purin
adenyl nucleotide binding;adenyl ribonucleotide binding;aminoacyl-tRNA editing activity;aminoacyl-tRNA ligase activity;ATP binding;binding;carboxylic ester hydrolase activity;catalytic activity;hydrolase activity;hydrolase acti
adenyl nucleotide binding;adenyl ribonucleotide binding;amine binding;amino acid binding;aspartate binding;aspartate carbamoyltransferase activity;ATP binding;binding;carbamoyl-phosphate synthase (ammonia) activity;ca
adenyl nucleotide binding;adenyl ribonucleotide binding;aminoacyl-tRNA editing activity;aminoacyl-tRNA ligase activity;ATP binding;binding;carboxylic ester hydrolase activity;catalytic activity;enzyme binding;GTPase binding
binding;chromatin binding
catalytic activity;endopeptidase activity;hydrolase activity;intramolecular oxidoreductase activity;intramolecular oxidoreductase activity, interconverting keto- and enol-groups;intramolecular oxidoreductase activity, transposin
actin binding;actin filament binding;binding;cell adhesion molecule binding;cytoskeletal protein binding;protein binding
adenyl nucleotide binding;adenyl ribonucleotide binding;ATP binding;ATPase activity;ATPase activity, coupled;ATP-dependent helicase activity;ATP-dependent RNA helicase activity;binding;catalytic activity;helicase activity;h
binding;carbohydrate binding;catalytic activity;glucose-6-phosphate isomerase activity;intramolecular oxidoreductase activity;intramolecular oxidoreductase activity, interconverting aldoses and ketoses;intramolecular transfe
structural molecule activity
adenyl nucleotide binding;adenyl ribonucleotide binding;ATP binding;binding;enzyme binding;nucleotide binding;protein binding;purine nucleotide binding;purine ribonucleoside triphosphate binding;purine ribonucleotide bin
adenyl nucleotide binding;adenyl ribonucleotide binding;ATP binding;binding;core promoter binding;DNA binding;nucleic acid binding;nucleotide binding;purine nucleotide binding;purine ribonucleoside triphosphate binding;p
binding;core promoter binding;DNA binding;enzyme binding;histone deacetylase binding;nucleic acid binding;nucleotide binding;protein binding;regulatory region DNA binding;regulatory region nucleic acid binding;sequence
binding;carbohydrate binding;carboxylic acid binding;catalytic activity;glycoprotein binding;G-protein coupled receptor activity;identical protein binding;insulin-like growth factor-activated receptor activity;isoprenoid binding;l
adenyl nucleotide binding;adenyl ribonucleotide binding;ADP binding;ATP binding;ATPase activity;binding;catalytic activity;enzyme activator activity;enzyme binding;enzyme regulator activity;hydrolase activity;hydrolase activ
binding;nucleic acid binding;RNA binding;translation factor activity, nucleic acid binding;translation initiation factor activity
binding;calcium ion binding;calcium-dependent phospholipid binding;calcium-dependent protein binding;cation binding;enzyme inhibitor activity;enzyme regulator activity;ion binding;lipase inhibitor activity;lipid binding;meta
adenyl nucleotide binding;adenyl ribonucleotide binding;antigen binding;ATP binding;ATPase activity;ATPase activity, coupled;binding;C3HC4-type RING finger domain binding;catalytic activity;enzyme binding;G-protein-cou
binding;DNA binding;identical protein binding;nucleic acid binding;nucleotide binding;protein binding;protein C-terminus binding;RNA binding;sequence-specific DNA binding;telomeric DNA binding
adenyl nucleotide binding;adenyl ribonucleotide binding;ATP binding;binding;nucleotide binding;protein binding;protein C-terminus binding;purine nucleotide binding;purine ribonucleoside triphosphate binding;purine ribonuc
adenyl nucleotide binding;adenyl ribonucleotide binding;ATP binding;ATPase activity;binding;calcium ion binding;catalytic activity;cation binding;chaperone binding;enzyme binding;glycoprotein binding;hydrolase activity;hyd
binding;catalytic activity;cofactor binding;glycogen phosphorylase activity;phosphorylase activity;pyridoxal phosphate binding;transferase activity;transferase activity, transferring glycosyl groups;transferase activity, transferri
adenyl nucleotide binding;adenyl ribonucleotide binding;ATP binding;ATPase activity;ATPase activity, coupled;ATP-dependent DNA helicase activity;ATP-dependent helicase activity;ATP-dependent RNA helicase activity;bind
binding;nucleic acid binding;RNA binding;structural constituent of ribosome;structural molecule activity
beta-tubulin binding;binding;cytoskeletal protein binding;DNA binding;enzyme binding;microtubule binding;nucleic acid binding;protein binding;RNA binding;single-stranded DNA binding;structure-specific DNA binding;tubuli
actin binding;binding;cytoskeletal protein binding;protein binding
binding;carbon-oxygen lyase activity;catalytic activity;cation binding;DNA binding;enzyme binding;GTPase binding;hydro-lyase activity;ion binding;lyase activity;magnesium ion binding;metal ion binding;nucleic acid binding;r
binding;calcium ion binding;cation binding;cytoskeletal protein binding;double-stranded RNA binding;integrin binding;ion binding;ion channel binding;ligand-dependent nuclear receptor transcription coactivator activity;metal
actin binding;binding;calcium ion binding;cation binding;cytoskeletal protein binding;ion binding;metal ion binding;protein binding;structural constituent of cytoskeleton;structural molecule activity
catalytic activity;hydrolase activity;hydrolase activity, acting on ester bonds;nuclease activity;protein binding transcription factor activity;transcription cofactor activity;transcription factor binding transcription factor activity
antioxidant activity;binding;catalytic activity;cation binding;cytokine receptor binding;extracellular matrix structural constituent;heme binding;interleukin-1 receptor antagonist activity;ion binding;iron ion binding;metal ion binc
adenyl nucleotide binding;adenyl ribonucleotide binding;ATP binding;ATPase activity;binding;catalytic activity;chaperone binding;DNA binding;DNA replication origin binding;double-stranded RNA binding;enzyme binding;hyc
apolipoprotein binding;binding;calcium ion binding;cation binding;ion binding;lipoprotein particle receptor binding;lipoprotein transporter activity;metal ion binding;protein binding;protein complex binding;protein transporter a
binding;calcium ion binding;calcium-dependent phospholipid binding;calcium-dependent protein binding;cation binding;channel activity;cholesterol binding;gated channel activity;GTP binding;guanyl nucleotide binding;guan
actin binding;alpha-catenin binding;beta-catenin binding;binding;cadherin binding;cell adhesion molecule binding;cytoskeletal protein binding;dystroglycan binding;enzyme binding;glycoprotein binding;protein binding;structu
structural molecule activity
binding;catalytic activity;K63-linked polyubiquitin binding;nucleic acid binding;polyubiquitin binding;protein binding;RNA binding;second spliceosomal transesterification activity;small conjugating protein binding;snRNA bindi
binding;lipid binding
binding;catalytic activity;cation binding;coenzyme binding;cofactor binding;DNA binding;enzyme binding;identical protein binding;ion binding;kinase binding;metal ion binding;NAD binding;NAD+ ADP-ribosyltransferase activi
adenyl nucleotide binding;adenyl ribonucleotide binding;ATP binding;binding;calcium ion binding;cation binding;enzyme binding;ion binding;lipoprotein particle receptor binding;low-density lipoprotein particle receptor bindin
binding;cell adhesion molecule binding;double-stranded RNA binding;enzyme binding;kinase binding;nucleic acid binding;protein binding;protein kinase binding;receptor binding;RNA binding;structural constituent of cytoske
adenyl nucleotide binding;adenyl ribonucleotide binding;ATP binding;binding;nucleotide binding;purine nucleotide binding;purine ribonucleoside triphosphate binding;purine ribonucleotide binding;ribonucleotide binding
binding;catalytic activity;enzyme binding;GTP binding;GTPase activity;guanyl nucleotide binding;guanyl ribonucleotide binding;hydrolase activity;hydrolase activity, acting on acid anhydrides;hydrolase activity, acting on acid
adenyl nucleotide binding;adenyl ribonucleotide binding;alkali metal ion binding;antigen binding;ATP binding;binding;catalytic activity;cation binding;ion binding;kinase activity;magnesium ion binding;metal ion binding;MHC c
binding;nucleic acid binding;nucleotide binding;protein binding;protein domain specific binding;RNA binding
binding;G-protein-coupled receptor binding;protein binding;receptor activity;receptor binding
adenyl deoxyribonucleotide binding;adenyl nucleotide binding;adenyl ribonucleotide binding;antigen binding;ATP binding;ATPase activity;binding;catalytic activity;CTP binding;dATP binding;deoxyribonucleotide binding;enzy
binding;catalytic activity;cation binding;cis-trans isomerase activity;enzyme binding;GTPase binding;ion binding;isomerase activity;ligase activity;metal ion binding;nucleic acid binding;peptidyl-prolyl cis-trans isomerase activ
binding;double-stranded RNA binding;glycoprotein binding;identical protein binding;nucleic acid binding;protein binding;protein C-terminus binding;RNA binding;scaffold protein binding;structural constituent of cytoskeleton;
binding;calcium ion binding;calmodulin binding;cation binding;enzyme activator activity;enzyme binding;enzyme inhibitor activity;enzyme regulator activity;GTPase activator activity;GTPase inhibitor activity;GTPase regulator
actin binding;actin filament binding;binding;calcium ion binding;cation binding;cytoskeletal protein binding;hormone receptor binding;identical protein binding;integrin binding;ion binding;ligand-dependent nuclear receptor tra
actin binding;ankyrin binding;binding;cytoskeletal protein binding;enzyme binding;GTPase binding;lipid binding;phospholipid binding;protein binding;structural constituent of cytoskeleton;structural molecule activity
structural molecule activity
adenyl nucleotide binding;adenyl ribonucleotide binding;antigen binding;ATP binding;binding;double-stranded RNA binding;enzyme binding;enzyme regulator activity;kinase binding;MHC class II protein complex binding;MH
binding;nucleic acid binding;RNA binding;structural molecule activity;translation factor activity, nucleic acid binding;translation initiation factor activity
binding;nucleic acid binding;RNA binding;translation factor activity, nucleic acid binding
acid-amino acid ligase activity;binding;catalytic activity;DNA binding;ligase activity;ligase activity, forming carbon-nitrogen bonds;nucleic acid binding;small conjugating protein ligase activity;ubiquitin-protein ligase activity
binding;lipid binding;phospholipid binding
adenyl nucleotide binding;adenyl ribonucleotide binding;aminoacyl-tRNA ligase activity;ATP binding;binding;catalytic activity;enzyme binding;glutamate-tRNA ligase activity;GTPase binding;ligase activity;ligase activity, formi
receptor activity
actin binding;actin filament binding;actin-dependent ATPase activity;adenyl nucleotide binding;adenyl ribonucleotide binding;ADP binding;ATP binding;ATPase activity;ATPase activity, coupled;binding;catalytic activity;cytosk
structural molecule activity
binding;chromatin binding;cytoskeletal protein binding;dynein complex binding;enzyme binding;heat shock protein binding;identical protein binding;kinase binding;mitogen-activated protein kinase binding;mRNA binding;nuc
binding;clathrin binding;clathrin light chain binding;double-stranded RNA binding;enzyme binding;kinase binding;nucleic acid binding;protein binding;protein kinase binding;RNA binding;structural molecule activity

adenyl nucleotide binding;adenyl ribonucleotide binding;amine binding;amino acid binding;ATP binding;binding;calcium ion binding;carbamoyl-phosphate synthase (ammonia) activity;carbamoyl-phosphate synthase (glutami
actin binding;actin filament binding;binding;cytoskeletal protein binding;enzyme binding;Fc-gamma receptor I complex binding;glycoprotein binding;GTPase binding;identical protein binding;immunoglobulin receptor binding;
actin binding;binding;cytoskeletal protein binding;identical protein binding;protein binding
[acyl-carrier-protein] S-acetyltransferase activity;[acyl-carrier-protein] S-malonyltransferase activity;3-hydroxyacyl-[acyl-carrier-protein] dehydratase activity;3-hydroxyoctanoyl-[acyl-carrier-protein] dehydratase activity;3-hydro
binding;cytoskeletal protein binding;integrin binding;LIM domain binding;protein binding;protein complex binding;protein domain specific binding;receptor binding;structural constituent of cytoskeleton;structural molecule act
binding;binding, bridging;enzyme binding;kinase binding;protein binding;protein binding, bridging;protein kinase binding;protein kinase C binding;scaffold protein binding;structural constituent of cytoskeleton;structural molec
adenyl nucleotide binding;adenyl ribonucleotide binding;ATP binding;ATPase activity;binding;catalytic activity;hydrolase activity;hydrolase activity, acting on acid anhydrides;hydrolase activity, acting on acid anhydrides, in ph
adenyl nucleotide binding;adenyl ribonucleotide binding;ATP binding;binding;catalytic activity;DNA binding;DNA-dependent protein kinase activity;kinase activity;nucleic acid binding;nucleotide binding;phosphotransferase ac
actin binding;actin filament binding;actin-dependent ATPase activity;adenyl nucleotide binding;adenyl ribonucleotide binding;ADP binding;ATP binding;ATPase activity;ATPase activity, coupled;binding;catalytic activity;cytosk
ankyrin binding;binding;cytoskeletal protein binding;protein binding;structural constituent of muscle;structural molecule activity

C: GOCC name	C: KEGG name	C: Organism	N: Protein Probability	N: Combined Total Peptides
cell part;integral to membrane;integral to plasma membrane;intrinsic to membrane;intrinsic to plasma membrane;membrane part;plasma membrane part		Homo sapiens	0.9999	1
cell part;cytoplasmic part;endosomal part;endosome membrane;intracellular organelle part;intracellular part;membrane;organelle membrane;organelle part;recycling endosome membrane	Endocytosis	Homo sapiens	0.9999	1
cell part;cytoplasmic part;endoplasmic reticulum membrane;endoplasmic reticulum part;integral to membrane;intracellular organelle part;intracellular part;intrinsic to membrane;membrane;membrane part;organelle membrane;organelle part		Homo sapiens	0.9601	1
cell part;integral to membrane;integral to plasma membrane;intrinsic to membrane;intrinsic to plasma membrane;membrane;membrane part;plasma membrane;plasma membrane part	Olfactory transduction	Homo sapiens	0.9165	1
cell part;cytoplasmic part;endoplasmic reticulum membrane;endoplasmic reticulum part;integral to endoplasmic reticulum membrane;integral to membrane;integral to organelle membrane;intracellular organelle part;intracellu	Protein export	Homo sapiens	0.9999	1
cell part;cytoplasm;cytoplasmic mRNA processing body;cytoplasmic part;cytosol;intracellular membrane-bounded organelle;intracellular non-membrane-bounded organelle;intracellular organelle;intracellular part;macromolec	RNA degradation	Homo sapiens	0.9999	2
cell part;cytoplasm;intracellular non-membrane-bounded organelle;intracellular organelle;intracellular organelle part;intracellular part;non-membrane-bounded organelle;nuclear part;nucleolus;organelle;organelle part	Histidine metabolism;Polycyclic aromatic hydrocarbon degradation;Tyrosine metabolism	Homo sapiens	0.9996	2
cell part;cytoplasmic part;integral to membrane;integral to mitochondrial inner membrane;integral to mitochondrial membrane;integral to organelle membrane;intracellular organelle part;intracellular part;intrinsic to membrane;intrinsic to mitochondrial inner membrane;intrinsic to organelle membrane;macromolecular complex;membrane;mem		Homo sapiens	0.9998	2
cell part;cytoplasmic part;intracellular membrane-bounded organelle;intracellular organelle;intracellular organelle part;intracellular part;membrane-bounded organelle;mitochondrion;nuclear part;nucleoplasm;organelle;organel	Glycine, serine and threonine metabolism	Homo sapiens	0.9999	2
cell part;cytoplasmic part;integral to membrane;intracellular membrane-bounded organelle;intracellular organelle;intracellular organelle part;intracellular part;intrinsic to membrane;macromolecular complex;membrane;membra	Alzheimer's disease;Huntington's disease;Oxidative phosphorylation;Parkinson's disease	Homo sapiens	0.9999	2
cell part;integral to membrane;integral to plasma membrane;intrinsic to membrane;intrinsic to plasma membrane;membrane;membrane part;plasma membrane part		Homo sapiens	0.9999	2
cell part;cytoplasmic part;cytosol;extracellular membrane-bounded organelle;extracellular organelle;extracellular region part;extracellular vesicular exosome;intracellular part;macromolecular complex;membrane-bounded org	Folate biosynthesis;Sulfur relay system	Homo sapiens	0.9997	2
cell junction;cell part;cytoplasm;cytoplasmic part;extracellular region;intracellular membrane-bounded organelle;intracellular organelle;intracellular organelle lumen;intracellular organelle part;intracellular part;lysosomal lumen;	Antigen processing and presentation	Homo sapiens	0.9999	2
cell part;cell projection;cytoplasm;cytoplasmic membrane-bounded vesicle;cytoplasmic part;cytoplasmic vesicle;cytosol;dendrite;intracellular membrane-bounded organelle;intracellular organelle;intracellular organelle part;intracellular part;macromolecular complex;melanosome;membrane;membrane-bounded organelle;membrane-bounde		Homo sapiens	0.9999	2
cell part;cytoplasmic part;endoplasmic reticulum;endoplasmic reticulum membrane;endoplasmic reticulum part;integral to membrane;intracellular membrane-bounded organelle;intracellular organelle;intracellular organelle pa	Protein processing in endoplasmic reticulum	Homo sapiens	0.9999	2
cell part;cytoplasm;cytoplasmic part;cytosol;extracellular membrane-bounded organelle;extracellular organelle;extracellular region part;extracellular vesicular exosome;immunological synapse;intracellular membrane-bounded	MAPK signaling pathway	Homo sapiens	0.9998	2
cell part;integral to membrane;intrinsic to membrane;membrane part		Homo sapiens	1	2
cell part;cytoplasmic part;Golgi apparatus;Golgi apparatus part;intracellular membrane-bounded organelle;intracellular organelle;intracellular organelle part;intracellular part;membrane;membrane-bounded organelle;organelle;organelle part;trans-Golgi network		Homo sapiens	0.9999	2
cell part;cytoplasm;cytoplasmic part;intracellular membrane-bounded organelle;intracellular organelle;intracellular organelle part;intracellular part;membrane-bounded organelle;membrane-enclosed lumen;mitochondrial inter	Oxidative phosphorylation	Homo sapiens	1	2
cell part;cytoplasm;intracellular membrane-bounded organelle;intracellular non-membrane-bounded organelle;intracellular organelle;intracellular organelle part;intracellular part;membrane-bounded organelle;non-membrane-bounded organelle;nuclear part;nucleolus;nucleus;organelle;organelle part		Homo sapiens	0.9988	2
cell part;intracellular organelle part;intracellular part;macromolecular complex;nuclear part;nucleoplasm;organelle part;ribonucleoprotein complex;spliceosomal complex;U12-type spliceosomal complex	Spliceosome	Homo sapiens	0.9999	2
cell part;chromatin;chromosomal part;cytoplasm;intracellular organelle part;intracellular part;nuclear part;nucleoplasm;organelle part		Homo sapiens	1	2
cell part;cytoplasmic part;endoplasmic reticulum lumen;endoplasmic reticulum part;intracellular organelle lumen;intracellular organelle part;intracellular part;membrane-enclosed lumen;organelle lumen;organelle part		Homo sapiens	0.9999	2
cell part;cytoplasmic part;cytosol;extrinsic to membrane;intracellular part;membrane;membrane part;pre-autophagosomal structure membrane		Homo sapiens	0.9999	2
cell part;cytoplasmic part;Golgi apparatus;integral to membrane;intracellular membrane-bounded organelle;intracellular organelle;intracellular part;intrinsic to membrane;membrane part;membrane-bounded organelle;organelle		Homo sapiens	0.9999	2
cell part;intracellular membrane-bounded organelle;intracellular organelle;intracellular part;membrane-bounded organelle;nucleus;organelle		Homo sapiens	0.9999	2
cell part;integral to membrane;intrinsic to membrane;membrane;membrane part;plasma membrane		Homo sapiens	0.9865	2
		Homo sapiens	0.9999	2
cell part;intracellular organelle part;intracellular part;nuclear part;nucleoplasm;organelle part		Homo sapiens	0.9998	2
cell part;cytoplasmic part;integral to membrane;intracellular organelle part;intracellular part;intrinsic to membrane;lysosomal membrane;membrane;membrane part;organelle membrane;organelle part;vacuolar membrane;vacuolar part		Homo sapiens	0.9989	2
cell part;cytoplasm;cytoplasmic part;integral to membrane;integral to mitochondrial membrane;integral to mitochondrial outer membrane;integral to organelle membrane;intracellular membrane-bounded organelle;intracellular organelle;intracellular organelle part;intracellular part;intrinsic to membrane;intrinsic to mitochondrial outer membrar		Homo sapiens	0.9992	2
cell part;cell projection;centrosome;cilium;cytoplasm;cytoplasmic part;cytoskeletal part;cytosol;intracellular membrane-bounded organelle;intracellular non-membrane-bounded organelle;intracellular organelle;intracellular organelle part;intracellular part;membrane-bounded organelle;microtubule organizing center;non-membrane-bounded c		Homo sapiens	0.9781	2
cell part;cytoplasmic part;cytoskeleton;cytosol;intracellular non-membrane-bounded organelle;intracellular organelle;intracellular part;non-membrane-bounded organelle;organelle		Homo sapiens	0.9999	2
cell part;cytoplasmic part;intracellular membrane-bounded organelle;intracellular organelle;intracellular organelle part;intracellular part;macromolecular complex;membrane part;membrane-bounded organelle;mitochondrial membrane part;mitochondrial outer membrane translocase complex;mitochondrial part;mitochondrion;organelle;organ		Homo sapiens	0.9999	2
		Homo sapiens	0.9999	2
cell part;cytoplasm;intracellular membrane-bounded organelle;intracellular organelle;intracellular organelle part;intracellular part;macromolecular complex;mediator complex;membrane;membrane-bounded organelle;nuclear part;nucleoplasm;nucleoplasm part;nucleus;organelle;organelle part;protein complex		Homo sapiens	1	2
cell part;cytoplasm;cytoplasmic part;cytosol;intracellular membrane-bounded organelle;intracellular organelle;intracellular part;membrane-bounded organelle;nucleus;organelle	Protein processing in endoplasmic reticulum	Homo sapiens	0.9999	2
cell part;centrosome;cytoplasm;cytoplasmic part;cytoskeletal part;intracellular non-membrane-bounded organelle;intracellular organelle;intracellular organelle part;intracellular part;microtubule organizing center;midbody;non-membrane-bounded organelle;organelle;organelle part;spindle pole		Homo sapiens	0.9999	2
cell part;cytoplasmic part;cytoskeletal part;cytosol;extracellular membrane-bounded organelle;extracellular organelle;extracellular region part;extracellular vesicular exosome;intracellular membrane-bounded organelle;intracellular non-membrane-bounded organelle;intracellular organelle;intracellular organelle part;intracellular part;membrane		Homo sapiens	0.9999	2
cell part;ER membrane protein complex;integral to membrane;intrinsic to membrane;macromolecular complex;membrane part;protein complex		Homo sapiens	0.9999	2
macromolecular complex;protein complex		Homo sapiens	0.9984	2
AP-type membrane coat adaptor complex;cell part;clathrin adaptor complex;clathrin-coated vesicle;coated vesicle;cytoplasmic membrane-bounded vesicle;cytoplasmic part;cytoplasmic vesicle;endosomal part;endosome m	Lysosome	Homo sapiens	0.9999	2
cell part;cytoplasmic part;extracellular membrane-bounded organelle;extracellular organelle;extracellular region part;extracellular vesicular exosome;intracellular membrane-bounded organelle;intracellular organelle;intracellul	Fatty acid elongation in mitochondria;Lysosome	Homo sapiens	0.9999	2
cell part;cytoplasmic part;extracellular region;intracellular membrane-bounded organelle;intracellular organelle;intracellular part;membrane-bounded organelle;mitochondrion;organelle		Homo sapiens	0.9999	2
		Homo sapiens	1	2
cell part;cytoplasmic part;envelope;fumarate reductase complex;integral to membrane;intracellular membrane-bounded organelle;intracellular organelle;intracellular organelle part;intracellular part;intrinsic to membrane;macro	Alzheimer's disease;Citrate cycle (TCA cycle);Huntington's disease;Oxidative phosphorylation;Parkinson's disease	Homo sapiens	1	3
cell part;cytoplasmic part;intracellular organelle lumen;intracellular organelle part;intracellular part;macromolecular complex;membrane;membrane part;membrane-enclosed lumen;mitochondrial inner membrane;mitochondria	Alzheimer's disease;Huntington's disease;Oxidative phosphorylation;Parkinson's disease	Homo sapiens	1	3
		Homo sapiens	0.9999	3
cell part;cytoplasmic part;cytosol;intracellular organelle part;intracellular part;nuclear part;nucleoplasm;organelle part	Parkinson's disease;Ubiquitin mediated proteolysis	Homo sapiens	1	3
autophagic vacuole membrane;cell part;cytoplasmic part;cytoplasmic vesicle;integral to membrane;intracellular organelle;intracellular organelle part;intracellular part;intrinsic to membrane;lysosomal membrane;membrane;membrane part;organelle;organelle membrane;organelle part;vacuolar membrane;vacuolar part;vesicle		Homo sapiens	1	3
		Homo sapiens	1	3
cell part;extracellular membrane-bounded organelle;extracellular organelle;extracellular region part;extracellular vesicular exosome;integral to membrane;intrinsic to membrane;membrane part;membrane-bounded organelle;membrane-bounded vesicle;organelle;vesicle		Homo sapiens	1	3
cell part;centrosome;cytoplasmic part;cytoskeletal part;cytosol;intracellular membrane-bounded organelle;intracellular non-membrane-bounded organelle;intracellular organelle;intracellular organelle part;intracellular part;membrane-bounded organelle;microtubule organizing center;non-membrane-bounded organelle;nucleus;organelle;orgar		Homo sapiens	1	3
cell part;cytoplasmic part;Golgi apparatus;Golgi apparatus part;Golgi membrane;integral to membrane;intracellular membrane-bounded organelle;intracellular organelle;intracellular organelle part;intracellular part;intrinsic to membrane;membrane;membrane part;membrane-bounded organelle;nuclear part;nucleoplasm;organelle;organelle me		Homo sapiens	1	3
cell body;cell part;cell projection;external side of plasma membrane;extracellular membrane-bounded organelle;extracellular organelle;extracellular region part;extracellular vesicular exosome;integral to membrane;intrinsic to membrane;membrane;membrane part;membrane-bounded organelle;membrane-bounded vesicle;neuron projection		Homo sapiens	1	3
cell part;intracellular membrane-bounded organelle;intracellular organelle;intracellular organelle part;intracellular part;macromolecular complex;membrane-bounded organelle;multimeric ribonuclease P complex;nuclear part;n	Ribosome biogenesis in eukaryotes;RNA transport	Homo sapiens	1	3
cell part;cytoplasm;cytoplasmic dynein complex;cytoplasmic part;cytoskeletal part;dynein complex;early endosome membrane;endosomal part;endosome membrane;extrinsic to membrane;intracellular organelle part;intracellular part;macromolecular complex;membrane;membrane part;microtubule associated complex;organelle membrane		Homo sapiens	1	3
cell part;chromatin;chromosomal part;cytoplasm;intracellular membrane-bounded organelle;intracellular organelle;intracellular organelle part;intracellular part;membrane-bounded organelle;nucleus;organelle;organelle part		Homo sapiens	1	3
acrosomal vesicle;cell part;cytoplasmic membrane-bounded vesicle;cytoplasmic part;cytoplasmic vesicle;endoplasmic reticulum lumen;endoplasmic reticulum part;endosome;extracellular membrane-bounded organelle;extra	Lysosome;Sphingolipid metabolism	Homo sapiens	1	3
cell part;intracellular membrane-bounded organelle;intracellular organelle;intracellular organelle part;intracellular part;membrane-bounded organelle;nuclear part;nucleoplasm;nucleus;organelle;organelle part	Osteoclast differentiation	Homo sapiens	1	3
cell part;intracellular membrane-bounded organelle;intracellular organelle;intracellular organelle part;intracellular part;membrane;membrane-bounded organelle;nuclear part;nucleoplasm;nucleus;organelle;organelle part		Homo sapiens	1	3
cell part;cytoskeleton;intermediate filament cytoskeleton;intracellular non-membrane-bounded organelle;intracellular organelle;intracellular organelle part;intracellular part;non-membrane-bounded organelle;nuclear part;nucle	Basal transcription factors	Homo sapiens	1	3
cell part;cytoplasm;intracellular membrane-bounded organelle;intracellular organelle;intracellular organelle part;intracellular part;macromolecular complex;membrane-bounded organelle;nuclear part;nucleoplasm;nucleoplasm	Basal transcription factors	Homo sapiens	1	3
		Homo sapiens	1	3
cell part;cytoplasmic membrane-bounded vesicle;cytoplasmic part;cytoplasmic vesicle;cytoplasmic vesicle membrane;cytoplasmic vesicle part;endocytic vesicle;endocytic vesicle membrane;endoplasmic reticulum membrar	Measles	Homo sapiens	1	3
cell part;cytoplasm;cytoplasmic part;cytoskeleton;cytosol;intracellular membrane-bounded organelle;intracellular non-membrane-bounded organelle;intracellular organelle;intracellular organelle part;intracellular part;macromc	RNA transport	Homo sapiens	1	3
cell part;intracellular membrane-bounded organelle;intracellular organelle;intracellular part;membrane-bounded organelle;nucleus;organelle		Homo sapiens	1	3
cell part;intracellular part;macromolecular complex;proteasome complex;protein complex	Homologous recombination;Proteasome	Homo sapiens	1	3
cell part;cytoplasm;intracellular part	Ubiquitin mediated proteolysis	Homo sapiens	1	3
cell part;extracellular membrane-bounded organelle;extracellular organelle;extracellular region part;extracellular vesicular exosome;integral to membrane;integral to plasma membrane;intrinsic to membrane;intrinsic to plasma	ECM-receptor interaction	Homo sapiens	1	3
cell part;cytoplasmic part;cytosol;intracellular part		Homo sapiens	1	3
cell part;cytoplasmic part;cytosol;extracellular membrane-bounded organelle;extracellular organelle;extracellular region part;extracellular vesicular exosome;intracellular part;membrane-bounded organelle;membrane-bounde	Arginine and proline metabolism;Glycine, serine and threonine metabolism	Homo sapiens	1	3
cell part;intracellular membrane-bounded organelle;intracellular organelle;intracellular organelle part;intracellular part;membrane-bounded organelle;nuclear part;nucleoplasm;nucleus;organelle;organelle part		Homo sapiens	0.9997	3
cell part;cell projection;cytoplasmic part;endoplasmic reticulum;Golgi apparatus;integral to membrane;integral to plasma membrane;intracellular membrane-bounded organelle;intracellular organelle;intracellular part;intrinsic to membrane;intrinsic to plasma membrane;lamellipodium;membrane;membrane part;membrane-bounded organelle;		Homo sapiens	1	3
cell part;cytoplasmic part;endoplasmic reticulum;endoplasmic reticulum membrane;endoplasmic reticulum part;integral to membrane;integral to plasma membrane;intracellular membrane-bounded organelle;intracellular orga	Steroid biosynthesis	Homo sapiens	1	3
cell part;chromatin;chromosomal part;cytoplasm;ESC/E(Z) complex;histone methyltransferase complex;intracellular membrane-bounded organelle;intracellular organelle;intracellular organelle part;intracellular part;macromolecular complex;membrane-bounded organelle;methyltransferase complex;nuclear chromatin;nuclear chromosome pa		Homo sapiens	1	3
cell part;cytoplasm;cytoplasmic part;intracellular part;macromolecular complex;NatC complex;N-terminal protein acetyltransferase complex;protein acetyltransferase complex;protein complex		Homo sapiens	1	3
cell part;cytoplasm;intracellular membrane-bounded organelle;intracellular organelle;intracellular organelle part;intracellular part;macromolecular complex;membrane-bounded organelle;nuclear body;nuclear part;nuclear spec	RNA transport	Homo sapiens	1	3
cell part;cytoplasmic part;endoplasmic reticulum membrane;endoplasmic reticulum part;integral to membrane;intracellular organelle part;intracellular part;intrinsic to membrane;membrane;membrane part;organelle membran	Glycerophospholipid metabolism	Homo sapiens	1	3
extracellular region		Homo sapiens	1	3
		Homo sapiens	1	3
cell part;cytoplasmic part;endoplasmic reticulum;Golgi apparatus;Golgi apparatus part;intracellular membrane-bounded organelle;intracellular organelle;intracellular organelle part;intracellular part;macromolecular complex;membrane-bounded organelle;organelle;organelle part;protein complex;TRAPP complex		Homo sapiens	1	3
cell part;cytoplasmic part;intracellular membrane-bounded organelle;intracellular organelle;intracellular part;membrane-bounded organelle;mitochondrion;organelle	Fatty acid biosynthesis	Homo sapiens	1	3
cell part;intracellular membrane-bounded organelle;intracellular organelle;intracellular part;membrane-bounded organelle;nucleus;organelle		Homo sapiens	1	3
cell part;cytoplasmic part;integral to membrane;intracellular membrane-bounded organelle;intracellular organelle;intracellular organelle part;intracellular part;intrinsic to membrane;macromolecular complex;membrane part;membrane-bounded organelle;mitochondrial membrane part;mitochondrial outer membrane translocase complex;mitoc		Homo sapiens	1	3
cell part;cytoplasmic part;intracellular membrane-bounded organelle;intracellular organelle;intracellular organelle part;intracellular part;membrane-bounded organelle;membrane-enclosed lumen;mitochondrial intermembrane space;mitochondrial part;mitochondrion;organelle;organelle envelope lumen;organelle part		Homo sapiens	1	3
cell part;cytoplasmic part;endoplasmic reticulum;endoplasmic reticulum membrane;endoplasmic reticulum part;integral to membrane;intracellular membrane-bounded organelle;intracellular organelle;intracellular organelle part;intracellular part;intrinsic to membrane;membrane;membrane part;membrane-bounded organelle;organelle;organel		Homo sapiens	1	3
cell part;cytoskeleton;intermediate filament cytoskeleton;intracellular membrane-bounded organelle;intracellular non-membrane-bounded organelle;intracellular organelle;intracellular organelle part;intracellular part;membrane-bounded organelle;non-membrane-bounded organelle;nuclear part;nucleoplasm;organelle;organelle part		Homo sapiens	1	3
cell part;intracellular membrane-bounded organelle;intracellular organelle;intracellular organelle part;intracellular part;macromolecular complex;membrane;membrane-bounded organelle;nuclear membrane;nuclear part;nucleu	Spliceosome	Homo sapiens	1	3
cell part;cytoplasm;cytoplasmic membrane-bounded vesicle;cytoplasmic part;cytoplasmic vesicle;extracellular membrane-bounded organelle;extracellular organelle;extracellular region part;extracellular vesicular exosome;Golgi apparatus;integral to membrane;intracellular membrane-bounded organelle;intracellular organelle;intracellular par		Homo sapiens	1	3
	Spliceosome	Homo sapiens	1	3
cell part;cytoplasmic membrane-bounded vesicle;cytoplasmic part;cytoplasmic vesicle;cytoplasmic vesicle membrane;cytoplasmic vesicle part;cytosol;endosome;extrinsic to membrane;intracellular membrane-bounded organelle;intracellular organelle;intracellular organelle part;intracellular part;membrane;membrane part;membrane-bound		Homo sapiens	0.9993	3
AP-type membrane coat adaptor complex;cell junction;cell part;cell projection;clathrin adaptor complex;clathrin-coated vesicle;coated vesicle;cytoplasm;cytoplasmic membrane-bounded vesicle;cytoplasmic part;cytoplasmic vesicle;intracellular membrane-bounded organelle;intracellular non-membrane-bounded organelle;intracellular orga		Homo sapiens	1	3
cell part;cytoplasm;intracellular membrane-bounded organelle;intracellular organelle;intracellular part;membrane-bounded organelle;nucleus;organelle		Homo sapiens	1	3
cell part;cytoplasmic part;cytosol;intracellular part;membrane		Homo sapiens	1	3
cell part;cytoplasmic part;endoplasmic reticulum membrane;endoplasmic reticulum part;GPI-anchor transamidase complex;integral to endoplasmic reticulum membrane;integral to membrane;integral to organelle membrane;	Glycosylphosphatidylinositol(GPI)-anchor biosynthesis	Homo sapiens	1	3
cell part;intracellular organelle part;intracellular part;macromolecular complex;multimeric ribonuclease P complex;nuclear part;nucleolar part;nucleolar ribonuclease P complex;organelle part;ribonuclease P complex;ribonucle	Ribosome biogenesis in eukaryotes;RNA transport	Homo sapiens	1	3
		Homo sapiens	1	3
apical plasma membrane;cell part;cytoplasm;envelope;integral to membrane;intracellular organelle part;intracellular part;intrinsic to membrane;membrane;membrane part;nuclear envelope;nuclear part;organelle envelope;organelle part;plasma membrane;plasma membrane part		Homo sapiens	0.9995	3
		Homo sapiens	0.9998	3
cell part;intracellular organelle part;intracellular part;macromolecular complex;mediator complex;nuclear part;nucleoplasm;nucleoplasm part;organelle part;protein complex		Homo sapiens	0.998	3
cell part;cytoplasmic part;intracellular non-membrane-bounded organelle;intracellular organelle;intracellular organelle part;intracellular part;macromolecular complex;membrane;mitochondrial inner membrane;mitochondrial membrane;mitochondrial part;mitochondrial ribosome;non-membrane-bounded organelle;organellar ribosome;organel		Homo sapiens	1	3
autophagic vacuole membrane;cell part;cytoplasmic part;endoplasmic reticulum;endoplasmic reticulum-Golgi intermediate compartment membrane;integral to membrane;intracellular membrane-bounded organelle;intracellular organelle;intracellular organelle part;intracellular part;intrinsic to membrane;membrane;membrane part;membrane		Homo sapiens	1	3
cell part;cytoplasm;cytoplasmic part;cytosol;endosomal part;endosome;endosome membrane;ESCRT II complex;extracellular membrane-bounded organelle;extracellular organelle;extracellular region part;extracellular vesicu	Endocytosis	Homo sapiens	1	3
cell part;cytoplasm;intracellular part	Protein processing in endoplasmic reticulum	Homo sapiens	1	3
cell part;cytoplasm;cytoplasmic membrane-bounded vesicle;cytoplasmic part;cytoplasmic vesicle;cytoplasmic vesicle membrane;cytoplasmic vesicle part;early endosome;endosome;intracellular membrane-bounded organelle;intracellular organelle;intracellular organelle part;intracellular part;late endosome;lysosome;lytic vacuole;melanosor		Homo sapiens	1	3
cell junction;cell part;cytoplasm;cytoskeletal part;intracellular organelle part;intracellular part;membrane;organelle part;postsynaptic density;postsynaptic membrane;synapse part;synaptic membrane		Homo sapiens	0.9941	3
cell part;intracellular membrane-bounded organelle;intracellular non-membrane-bounded organelle;intracellular organelle;intracellular organelle part;intracellular part;membrane-bounded organelle;non-membrane-bounded or	Homologous recombination	Homo sapiens	1	3
		Homo sapiens	1	3
cell part;integral to membrane;intrinsic to membrane;membrane part		Homo sapiens	1	3
cell part;cytoplasm;cytoplasmic part;extracellular membrane-bounded organelle;extracellular organelle;extracellular region part;extracellular vesicular exosome;intracellular membrane-bounded organelle;intracellular organelle;intracellular organelle part;intracellular part;macromolecular complex;membrane-bounded organelle;membrane-bou		Homo sapiens	1	3
		Homo sapiens	1	3
cell division site part;cell part;cleavage furrow;cytoplasm;cytoplasmic part;cytoskeletal part;intracellular membrane-bounded organelle;intracellular organelle;intracellular organelle part;intracellular part;macromolecular complex;membrane-bounded organelle;microtubule;microtubule organizing center;nucleus;organelle;organelle part;protein		Homo sapiens	1	3
cell part;intracellular membrane-bounded organelle;intracellular organelle;intracellular part;membrane-bounded organelle;organelle		Homo sapiens	1	3
cell part;cytoplasmic part;intracellular non-membrane-bounded organelle;intracellular organelle;intracellular organelle part;intracellular part;macromolecular complex;membrane;mitochondrial inner membrane;mitochondrial membrane;mitochondrial part;mitochondrial ribosome;non-membrane-bounded organelle;organellar ribosome;organel		Homo sapiens	1	3
cell part;cytoplasm;cytoplasmic part;intracellular membrane-bounded organelle;intracellular organelle;intracellular part;membrane-bounded organelle;mitochondrion;nucleus;organelle		Homo sapiens	1	3
		Homo sapiens	1	3
cell part;histone acetyltransferase complex;intracellular organelle part;intracellular part;macromolecular complex;nuclear part;nucleoplasm;nucleoplasm part;organelle part;protein complex;SAGA-type complex;STAGA complex		Homo sapiens	1	3
cell part;cytoplasm;cytoplasmic part;cytosol;endoplasmic reticulum;intracellular membrane-bounded organelle;intracellular organelle;intracellular part;membrane-bounded organelle;mitochondrion;organelle	Nicotinate and nicotinamide metabolism;Purine metabolism;Pyrimidine metabolism	Homo sapiens	1	3
cell part;coated vesicle;cytoplasmic membrane-bounded vesicle;cytoplasmic part;cytoplasmic vesicle;endoplasmic reticulum membrane;endoplasmic reticulum part;ER to Golgi transport vesicle;Golgi apparatus;Golgi apparatus part;Golgi membrane;integral to membrane;intracellular membrane-bounded organelle;intracellular organelle;intr		Homo sapiens	1	3
cell part;integral to membrane;intrinsic to membrane;membrane part		Homo sapiens	1	3
cell part;cytoplasm;intracellular organelle part;intracellular part;nuclear part;nucleoplasm;organelle part		Homo sapiens	1	3
cell part;cytoplasm;intracellular membrane-bounded organelle;intracellular organelle;intracellular part;membrane-bounded organelle;nucleus;organelle		Homo sapiens	1	3
box H/ACA RNP complex;Cajal body;cell part;intracellular non-membrane-bounded organelle;intracellular organelle;intracellular organelle part;intracellular part;macromolecular complex;non-membrane-bounded organelle;nu	Ribosome biogenesis in eukaryotes	Homo sapiens	1	3
Ada2/Gcn5/Ada3 transcription activator complex;cell part;chromosomal part;DNA polymerase complex;epsilon DNA polymerase complex;histone acetyltransferase complex;intracellular membrane-bounded organelle;intrace	Base excision repair;DNA replication;Nucleotide excision repair;Purine metabolism;Pyrimidine metabolism	Homo sapiens	1	3
cell part;chromatin accessibility complex;chromatin remodeling complex;chromosomal part;DNA polymerase complex;epsilon DNA polymerase complex;intracellular organelle part;intracellular part;macromolecular complex;nuclear chromosome part;nuclear part;organelle part;protein complex		Homo sapiens	1	3

cell part;cytoplasm;cytoplasmic part;cytosol;intracellular membrane-bounded organelle;intracellular organelle;intracellular organelle part;intracellular part;macromolecular complex;membrane-bounded organelle;nuclear part;r	RNA transport	Homo sapiens	1	3
cell part;intracellular organelle part;intracellular part;macromolecular complex;mediator complex;nuclear part;nucleoplasm;nucleoplasm part;organelle part;protein complex		Homo sapiens	1	3
cell part;cytoplasmic membrane-bounded vesicle;cytoplasmic part;cytoplasmic vesicle;intracellular membrane-bounded organelle;intracellular organelle;intracellular part;membrane-bounded organelle;membrane-bounded vesicle;nucleus;organelle;vesicle		Homo sapiens	1	3
cell body;cell part;cell projection;cytoplasmic part;cytosol;dendrite;intracellular part;membrane;neuron projection;neuronal cell body		Homo sapiens	1	3
cell part;cytoskeletal part;intermediate filament;intracellular membrane-bounded organelle;intracellular non-membrane-bounded organelle;intracellular organelle;intracellular organelle lumen;intracellular organelle part;intracellular part;lamin filament;macromolecular complex;membrane-bounded organelle;membrane-enclosed lumen;non-mem		Homo sapiens	1	3
cell part;intracellular membrane-bounded organelle;intracellular organelle;intracellular part;membrane-bounded organelle;nucleus;organelle		Homo sapiens	1	3
anchored to membrane;cell part;cytoplasm;intracellular part;intrinsic to membrane;membrane;membrane part;plasma membrane		Homo sapiens	1	3
cell part;cytoplasmic part;intracellular membrane-bounded organelle;intracellular organelle;intracellular part;lysosome;lytic vacuole;membrane-bounded organelle;organelle;vacuole	Lysosome;Other glycan degradation	Homo sapiens	1	4
cell part;chromatin;chromosomal part;intracellular membrane-bounded organelle;intracellular organelle;intracellular organelle part;intracellular part;membrane-bounded organelle;nucleus;organelle;organelle part		Homo sapiens	0.9999	4
cell division site part;cell part;cleavage furrow;cytoplasm;cytoplasmic part;endoplasmic reticulum membrane;endoplasmic reticulum part;Golgi apparatus part;Golgi cisterna membrane;Golgi membrane;intracellular organelle part;intracellular part;lipid particle;membrane;membrane part;midbody;organelle membrane;organelle part		Homo sapiens	1	4
cell junction;cell part;cell projection part;clathrin-coated vesicle;coated vesicle;cytoplasmic membrane-bounded vesicle;cytoplasmic part;cytoplasmic vesicle;cytoplasmic vesicle membrane;cytoplasmic vesicle part;cytoskeleton;endoplasmic reticulum;endoplasmic reticulum lumen;endoplasmic reticulum part;envelope;extracellular membra		Homo sapiens	1	4
adherens junction;anchoring junction;cell junction;cell part;cell-substrate adherens junction;cell-substrate junction;cytoplasm;cytoplasmic part;cytosol;endomembrane system;focal adhesion;Golgi apparatus;Golgi apparatus	SNARE interactions in vesicular transport	Homo sapiens	1	4
cell part;cytoplasm;cytoplasmic part;cytosol;intracellular part	Inositol phosphate metabolism;Phosphatidylinositol signaling system;Streptomycin biosynthesis	Homo sapiens	1	4
cell part;cytoplasmic part;intracellular membrane-bounded organelle;intracellular organelle;intracellular organelle lumen;intracellular organelle part;intracellular part;membrane-bounded organelle;membrane-enclosed lumen;m	Peroxisome	Homo sapiens	1	4
cell part;cytoplasmic part;intracellular non-membrane-bounded organelle;intracellular organelle;intracellular organelle part;intracellular part;macromolecular complex;membrane;mitochondrial inner membrane;mitochondrial membrane;mitochondrial part;mitochondrial ribosome;non-membrane-bounded organelle;organellar ribosome;organel		Homo sapiens	1	4
cell part;cytoplasmic part;cytoskeletal part;cytosol;intracellular non-membrane-bounded organelle;intracellular organelle;intracellular organelle part;intracellular part;macromolecular complex;microtubule;microtubule associat	Ubiquitin mediated proteolysis	Homo sapiens	1	4
cell part;cytoplasmic mRNA processing body;cytoplasmic part;DNA-directed RNA polymerase complex;DNA-directed RNA polymerase II, core complex;intracellular membrane-bounded organelle;intracellular non-membrane	Huntington's disease;Purine metabolism;Pyrimidine metabolism;RNA polymerase	Homo sapiens	1	4
cell part;integral to membrane;intrinsic to membrane;membrane;membrane part;plasma membrane	Nitrogen metabolism	Homo sapiens	1	4
catalytic step 2 spliceosome;cell part;intracellular membrane-bounded organelle;intracellular organelle;intracellular organelle part;intracellular part;macromolecular complex;membrane-bounded organelle;nuclear body;nuclea	Spliceosome	Homo sapiens	1	4
		Homo sapiens	1	4
cell part;extracellular region part;extracellular space;intracellular		Homo sapiens	1	4
cell part;cytoplasmic part;cytosol;intracellular part;macromolecular complex;mRNA cap binding complex;protein complex;RNA cap binding complex	Insulin signaling pathway;mTOR signaling pathway;RNA transport	Homo sapiens	1	4
cell part;cytoplasmic part;integral to membrane;intracellular;intracellular membrane-bounded organelle;intracellular non-membrane-bounded organelle;intracellular organelle;intracellular organelle part;intracellular part;intrinsic	Peroxisome	Homo sapiens	1	4
cell part;intracellular non-membrane-bounded organelle;intracellular organelle;intracellular organelle part;intracellular part;non-membrane-bounded organelle;nuclear part;nucleolus;organelle;organelle part		Homo sapiens	1	4
actin cytoskeleton;cell part;cytoplasm;cytoplasmic part;cytoskeleton;intermediate filament cytoskeleton;intracellular membrane-bounded organelle;intracellular non-membrane-bounded organelle;intracellular organelle;intracellular organelle lumen;intracellular organelle part;intracellular part;membrane;membrane-bounded organelle;membra		Homo sapiens	1	4
cell part;contractile fiber;cytoplasmic part;intracellular membrane-bounded organelle;intracellular non-membrane-bounded organelle;intracellular organelle;intracellular organelle part;intracellular part;membrane;membrane-bounded organelle;mitochondrial inner membrane;mitochondrial membrane;mitochondrial part;mitochondrion;myofibril		Homo sapiens	1	4
cell part;cytoplasm;extracellular membrane-bounded organelle;extracellular organelle;extracellular region part;extracellular vesicular exosome;intracellular membrane-bounded organelle;intracellular non-membrane-bounded organelle;intracellular organelle;intracellular organelle part;intracellular part;membrane-bounded organelle;membrane		Homo sapiens	1	4
cell part;cell projection;cytoplasm;cytoplasmic part;dendrite;DNA-directed RNA polymerase complex;DNA-directed RNA polymerase II, core complex;intracellular membrane-bounded organelle;intracellular organelle;intracellular organelle part;intracellular part;macromolecular complex;membrane-bounded organelle;mitochondrion;neuron pr		Homo sapiens	1	4
cell part;intracellular organelle part;intracellular part;macromolecular complex;nuclear part;organelle part;ribonucleoprotein complex;small nuclear ribonucleoprotein complex;spliceosomal complex;U4/U6 x U5 tri-snRNP com	RNA degradation;Spliceosome	Homo sapiens	1	4
cell part;extracellular membrane-bounded organelle;extracellular organelle;extracellular region part;extracellular vesicular exosome;intracellular membrane-bounded organelle;intracellular organelle;intracellular part;membrane-bounded organelle;membrane-bounded vesicle;nucleus;organelle;vesicle		Homo sapiens	1	4
blood microparticle;extracellular membrane-bounded organelle;extracellular organelle;extracellular region;extracellular region part;extracellular space;extracellular vesicular exosome;membrane-bounded organelle;membrane-bounded vesicle;organelle;vesicle		Homo sapiens	1	4
adherens junction;anchoring junction;cell junction;cell part;cell-substrate adherens junction;cell-substrate junction;cytoplasm;focal adhesion;integral to membrane;integral to plasma membrane;intracellular part;intrinsic to me	Cell adhesion molecules (CAMs);Leukocyte transendothelial migration	Homo sapiens	1	4
adherens junction;anchoring junction;basolateral plasma membrane;cell junction;cell part;cell surface;cell-substrate adherens junction;cell-substrate junction;cytoplasmic part;cytoplasmic vesicle part;extracellular membrane-	Complement and coagulation cascades;Measles	Homo sapiens	1	4
cell part;cytoplasmic part;intracellular membrane-bounded organelle;intracellular organelle;intracellular organelle lumen;intracellular organelle part;intracellular part;membrane;membrane-bounded organelle;membrane-enclos	Butanoate metabolism;Fatty acid metabolism;Valine, leucine and isoleucine degradation	Homo sapiens	1	4
cell part;cytoplasm;cytoskeleton;DNA-directed RNA polymerase complex;DNA-directed RNA polymerase II, core complex;intracellular membrane-bounded organelle;intracellular non-membrane-bounded organelle;intracellula	Huntington's disease;Purine metabolism;Pyrimidine metabolism;RNA polymerase	Homo sapiens	1	4
cell part;centrosome;cytoplasm;cytoplasmic part;cytoskeletal part;cytosol;extracellular membrane-bounded organelle;extracellular organelle;extracellular region part;extracellular vesicular exosome;intracellular membrane-bo	Measles;NOD-like receptor signaling pathway	Homo sapiens	1	4
cell part;cytoplasmic part;integral to membrane;intracellular organelle part;intracellular part;intrinsic to membrane;membrane;membrane part;mitochondrial inner membrane;mitochondrial membrane;mitochondrial membrane	Alzheimer's disease;Cardiac muscle contraction;Huntington's disease;Oxidative phosphorylation;Parkinson's diseas	Homo sapiens	1	4
cell part;cytoplasm;cytoplasmic part;extracellular membrane-bounded organelle;extracellular organelle;extracellular region part;extracellular vesicular exosome;integral to membrane;intracellular membrane-bounded organelle	Neuroactive ligand-receptor interaction	Homo sapiens	1	4
carboxy-terminal domain protein kinase complex;cell part;core TFIIH complex;holo TFIIH complex;intracellular organelle part;intracellular part;macromolecular complex;nuclear part;nucleoplasm;nucleoplasm part;organelle pa	Basal transcription factors;Nucleotide excision repair	Homo sapiens	1	4
cell part;cytoplasm;intracellular membrane-bounded organelle;intracellular organelle;intracellular part;membrane-bounded organelle;nucleus;organelle	RNA transport	Homo sapiens	1	4
cell part;cell projection;chromatin;chromosomal part;coated pit;cytoplasm;cytoplasmic part;cytoplasmic vesicle;cytoplasmic vesicle membrane;cytoplasmic vesicle part;cytosol;Golgi apparatus part;Golgi membrane;intracellu	Chemokine signaling pathway;Endocytosis;MAPK signaling pathway;Phototransduction	Homo sapiens	1	4
cell part;cytoplasmic part;cytosol;intracellular membrane-bounded organelle;intracellular organelle;intracellular organelle part;intracellular part;macromolecular complex;membrane-bounded organelle;nuclear part;nucleoplasm	Protein processing in endoplasmic reticulum;Ubiquitin mediated proteolysis	Homo sapiens	0.9999	4
cell part;cytoplasmic part;intracellular part;macromolecular complex;protein complex;Rab-protein geranylgeranyltransferase complex		Homo sapiens	1	4
cell part;cytoplasmic part;integral to membrane;intracellular membrane-bounded organelle;intracellular organelle;intracellular organelle part;intracellular part;intrinsic to membrane;membrane;membrane part;membrane-bounded organelle;mitochondrial inner membrane;mitochondrial membrane;mitochondrial part;mitochondrion;organelle;or		Homo sapiens	1	4
cell part;intracellular organelle part;intracellular part;nuclear part;nucleoplasm;organelle part	Cell cycle - yeast;Pathways in cancer;Small cell lung cancer	Homo sapiens	1	4
catalytic step 2 spliceosome;cell part;cytoplasmic part;cytosol;extracellular membrane-bounded organelle;extracellular organelle;extracellular region part;extracellular vesicular exosome;intracellular membrane-bounded orga	Spliceosome	Homo sapiens	1	4
cell part;cytoplasmic part;cytosol;cytosolic large ribosomal subunit;extracellular region part;extracellular space;intracellular organelle part;intracellular part;large ribosomal subunit;macromolecular complex;organelle part;ribor	Ribosome	Homo sapiens	1	4
cell part;extracellular membrane-bounded organelle;extracellular organelle;extracellular region part;extracellular vesicular exosome;extrinsic to internal side of plasma membrane;extrinsic to membrane;extrinsic to plasma me	Chemokine signaling pathway	Homo sapiens	1	4
cell part;cytoplasm;intracellular membrane-bounded organelle;intracellular non-membrane-bounded organelle;intracellular organelle;intracellular organelle part;intracellular part;membrane-bounded organelle;non-membrane-bounded organelle;nuclear part;nucleolus;nucleoplasm;nucleus;organelle;organelle part		Homo sapiens	1	4
cell part;cytoplasmic part;intracellular membrane-bounded organelle;intracellular organelle;intracellular organelle part;intracellular part;macromolecular complex;membrane;membrane-bounded organelle;mitochondrial inner membrane;mitochondrial membrane;mitochondrial part;mitochondrial small ribosomal subunit;mitochondrion;organel		Homo sapiens	1	4
cell part;intracellular membrane-bounded organelle;intracellular non-membrane-bounded organelle;intracellular organelle;intracellular organelle part;intracellular part;membrane-bounded organelle;non-membrane-bounded or	Basal transcription factors	Homo sapiens	1	4
cell part;cytoplasmic part;extracellular membrane-bounded organelle;extracellular organelle;extracellular region part;extracellular vesicular exosome;intracellular membrane-bounded organelle;intracellular organelle;intracellula	Inositol phosphate metabolism;Propanoate metabolism;Valine, leucine and isoleucine degradation	Homo sapiens	1	4
cell junction;cell part;intracellular membrane-bounded organelle;intracellular organelle;intracellular organelle part;intracellular part;membrane-bounded organelle;nuclear part;nucleoplasm;nucleus;organelle;organelle part		Homo sapiens	1	4
cell part;intracellular membrane-bounded organelle;intracellular organelle;intracellular part;membrane-bounded organelle;nucleus;organelle		Homo sapiens	1	4
cell part;centrosome;cytoplasmic part;cytoskeletal part;cytosol;extracellular region part;intercellular bridge;intracellular non-membrane-bounded organelle;intracellular organelle;intracellular organelle part;intracellular part;microtubule organizing center;non-membrane-bounded organelle;organelle;organelle part;spindle midzone;spindle pole		Homo sapiens	1	4
cell part;cytoplasmic part;endoplasmic reticulum;endoplasmic reticulum membrane;endoplasmic reticulum part;endoplasmic reticulum-Golgi intermediate compartment;endoplasmic reticulum-Golgi intermediate compartment membrane;Golgi apparatus;integral to membrane;intracellular membrane-bounded organelle;intracellular organelle		Homo sapiens	1	4
axon;cell part;cell projection;cell projection part;clathrin-coated vesicle;coated vesicle;cytoplasm;cytoplasmic membrane-bounded vesicle;cytoplasmic part;cytoplasmic vesicle;cytosol;dendrite;dendritic spine;early endosom	Fructose and mannose metabolism	Homo sapiens	1	4
cell part;cytoplasm;cytoplasmic part;cytosol;intracellular membrane-bounded organelle;intracellular organelle;intracellular organelle part;intracellular part;membrane-bounded organelle;nuclear part;nucleoplasm;nucleus;orgar	Cytosolic DNA-sensing pathway;Hepatitis C;Measles;RIG-I-like receptor signaling pathway;Toll-like receptor signalii	Homo sapiens	1	4
cell part;cytoplasmic part;intracellular membrane-bounded organelle;intracellular organelle;intracellular organelle lumen;intracellular organelle part;intracellular part;membrane-bounded organelle;membrane-enclosed lumen;mitochondrial matrix;mitochondrial part;mitochondrion;organelle;organelle lumen;organelle part		Homo sapiens	1	4
cell junction;cell part;cell projection;coated pit;cytoplasmic membrane-bounded vesicle;cytoplasmic part;cytoplasmic vesicle;cytosol;endocytic vesicle;intracellular membrane-bounded organelle;intracellular organelle;intracellular part;lamellipodium;membrane;membrane part;membrane-bounded organelle;membrane-bounded vesicle;neurc		Homo sapiens	1	4
cell part;cytoplasm;intracellular membrane-bounded organelle;intracellular organelle;intracellular part;membrane-bounded organelle;nucleus;organelle		Homo sapiens	1	4
cell part;cytoplasmic part;integral to membrane;intracellular membrane-bounded organelle;intracellular organelle;intracellular organelle part;intracellular part;intrinsic to membrane;membrane;membrane part;membrane-bounded organelle;mitochondrial inner membrane;mitochondrial membrane;mitochondrial part;mitochondrion;organelle;or		Homo sapiens	1	4
cell part;cytoplasm;intracellular part		Homo sapiens	1	4
cell part;cytoplasmic part;intracellular non-membrane-bounded organelle;intracellular organelle;intracellular organelle part;intracellular part;macromolecular complex;membrane;mitochondrial inner membrane;mitochondrial membrane;mitochondrial part;non-membrane-bounded organelle;organelle;organelle inner membrane;organelle memb		Homo sapiens	1	4
cell part;extracellular membrane-bounded organelle;extracellular organelle;extracellular region part;extracellular vesicular exosome;integral to membrane;intrinsic to membrane;membrane part;membrane-bounded organelle;membrane-bounded vesicle;organelle;vesicle		Homo sapiens	1	4
cell part;chromatin;chromosomal part;chromosome;chromosome, centromeric region;cohesin complex;cytoplasm;cytoplasmic part;cytoskeleton;cytosol;intracellular membrane-bounded organelle;intracellular non-membrane-bounded organelle;intracellular organelle;intracellular organelle part;intracellular part;macromolecular complex;mem		Homo sapiens	1	4
cell part;cytoplasmic part;intracellular membrane-bounded organelle;intracellular organelle;intracellular part;membrane-bounded organelle;mitochondrion;nucleus;organelle		Homo sapiens	1	4
cell part;cytoplasmic part;endosomal part;endosome membrane;integral to membrane;intracellular organelle part;intracellular part;intrinsic to membrane;late endosome membrane;lysosomal membrane;membrane;membrane part;organelle membrane;organelle part;vacuolar membrane;vacuolar part		Homo sapiens	1	4
		Homo sapiens	1	4
cell part;intracellular membrane-bounded organelle;intracellular organelle;intracellular organelle part;intracellular part;membrane;membrane-bounded organelle;nuclear membrane;nuclear part;nucleus;organelle;organelle membrane;organelle part		Homo sapiens	1	4
cell part;membrane;plasma membrane		Homo sapiens	1	4
calcium channel complex;cation channel complex;cell part;cytoplasmic part;intracellular membrane-bounded organelle;intracellular organelle;intracellular organelle part;intracellular part;ion channel complex;macromolecular complex;membrane part;membrane-bounded organelle;membrane-enclosed lumen;mitochondrial intermembrane sp		Homo sapiens	1	4
cell part;cytoplasmic part;intracellular membrane-bounded organelle;intracellular organelle;intracellular part;membrane-bounded organelle;mitochondrion;organelle		Homo sapiens	1	4
cell division site part;cell part;cleavage furrow;cytoplasmic part;cytosol;intracellular part;membrane;plasma membrane	Calcium signaling pathway;Inositol phosphate metabolism;Phosphatidylinositol signaling system	Homo sapiens	1	4
cell part;cytoplasmic part;early endosome;early endosome membrane;endosomal part;endosome;endosome membrane;ER membrane protein complex;Golgi apparatus;Golgi apparatus part;Golgi membrane;integral to membrane;intracellular membrane-bounded organelle;intracellular organelle;intracellular organelle part;intracellular part;in		Homo sapiens	1	4
cell part;cytoplasmic part;intracellular membrane-bounded organelle;intracellular non-membrane-bounded organelle;intracellular organelle;intracellular organelle part;intracellular part;macromolecular complex;membrane-bou	Bladder cancer;Cell cycle;Chronic myeloid leukemia;Glioma;Melanoma;Non-small cell lung cancer;p53 signaling pa	Homo sapiens	1	4
cell part;cytoplasm;cytoplasmic mRNA processing body;cytoplasmic part;intracellular membrane-bounded organelle;intracellular non-membrane-bounded organelle;intracellular organelle;intracellular part;macromolecular complex;membrane-bounded organelle;non-membrane-bounded organelle;organelle;ribonucleoprotein complex;RNA g		Homo sapiens	1	4
cell part;cytoplasm;cytoplasmic part;intracellular membrane-bounded organelle;intracellular organelle;intracellular part;membrane-bounded organelle;mitochondrion;organelle;perinuclear region of cytoplasm		Homo sapiens	1	4
		Homo sapiens	1	4
cell part;cytoplasmic part;Golgi apparatus;Golgi apparatus part;Golgi membrane;integral to membrane;intracellular membrane-bounded organelle;intracellular organelle;intracellular organelle part;intracellular part;intrinsic to membrane;membrane;membrane part;membrane-bounded organelle;organelle;organelle membrane;organelle part		Homo sapiens	1	4
cell part;chromosome;intracellular non-membrane-bounded organelle;intracellular organelle;intracellular organelle part;intracellular part;non-membrane-bounded organelle;nuclear part;nucleolus;nucleoplasm;organelle;organe	Lysine degradation	Homo sapiens	1	4
cell part;chromatin remodeling complex;intracellular organelle part;intracellular part;macromolecular complex;npBAF complex;nuclear part;organelle part;protein complex;SWI/SNF-type complex		Homo sapiens	1	4
cell part;cytoplasmic membrane-bounded vesicle;cytoplasmic part;cytoplasmic vesicle;cytosol;extracellular membrane-bounded organelle;extracellular organelle;extracellular region part;extracellular space;extracellular vesic	Folate biosynthesis	Homo sapiens	1	4
cell part;cytoplasmic part;endoplasmic reticulum membrane;endoplasmic reticulum part;integral to membrane;intracellular organelle part;intracellular part;intrinsic to membrane;membrane;membrane part;organelle membrane	N-Glycan biosynthesis	Homo sapiens	1	4
cell part;cytoplasm;cytoplasmic part;intracellular membrane-bounded organelle;intracellular organelle;intracellular part;membrane-bounded organelle;mitochondrion;nucleus;organelle		Homo sapiens	1	4
		Homo sapiens	1	4
		Homo sapiens	1	4
extracellular membrane-bounded organelle;extracellular organelle;extracellular region part;extracellular vesicular exosome;membrane-bounded organelle;membrane-bounded vesicle;organelle;vesicle		Homo sapiens	1	4
cell part;cytoplasmic part;intracellular membrane-bounded organelle;intracellular organelle;intracellular part;macromolecular complex;membrane-bounded organelle;methyltransferase complex;nucleus;organelle;protein complex;tRNA (m1A) methyltransferase complex;tRNA methyltransferase complex		Homo sapiens	1	4
cell part;cytoplasm;intracellular organelle part;intracellular part;nuclear part;nucleoplasm;organelle part		Homo sapiens	1	4
cell part;cell projection membrane;cell projection part;cytoplasmic part;cytoskeleton;cytosol;extracellular membrane-bounded organelle;extracellular organelle;extracellular region part;extracellular vesicular exosome;intracellu	Vitamin B6 metabolism	Homo sapiens	1	4
apical part of cell;cell part;cytoplasmic part;endosome;extracellular membrane-bounded organelle;extracellular organelle;extracellular region part;extracellular vesicular exosome;intracellular membrane-bounded organelle;intr	Antigen processing and presentation;Lysosome	Homo sapiens	1	4
axon part;cell part;cell projection part;cytoplasmic part;cytosol;endoplasmic reticulum membrane;endoplasmic reticulum part;extrinsic to membrane;intracellular organelle part;intracellular part;membrane;membrane part;nuc	Glycerolipid metabolism	Homo sapiens	1	4
cell part;intracellular membrane-bounded organelle;intracellular organelle;intracellular part;membrane-bounded organelle;nucleus;organelle		Homo sapiens	1	4
cell part;intracellular part;macromolecular complex;proteasome complex;protein complex		Homo sapiens	1	4
cell part;intracellular organelle part;intracellular part;nuclear part;nucleoplasm;organelle part		Homo sapiens	1	4
		Homo sapiens	1	4
cell part;cytoplasm;intracellular membrane-bounded organelle;intracellular organelle;intracellular part;membrane-bounded organelle;nucleus;organelle		Homo sapiens	1	4
cell part;intracellular part;macromolecular complex;protein complex;tRNA-splicing ligase complex		Homo sapiens	1	4
cell part;cytoplasmic part;endosome;extracellular membrane-bounded organelle;extracellular organelle;extracellular region part;extracellular space;extracellular vesicular exosome;Golgi apparatus part;intracellular membrane-bounded organelle;intracellular organelle;intracellular organelle part;intracellular part;late endosome;lysosome;lytic v		Homo sapiens	1	4
cell part;cytoplasm;intracellular part		Homo sapiens	1	4
cell part;intracellular membrane-bounded organelle;intracellular organelle;intracellular part;membrane-bounded organelle;nucleus;organelle		Homo sapiens	1	4
cell part;intracellular membrane-bounded organelle;intracellular organelle;intracellular part;membrane-bounded organelle;nucleus;organelle		Homo sapiens	1	4
Atg12-Atg5-Atg16 complex;autophagic vacuole;axoneme;cell part;cell projection part;cytoplasm;cytoplasmic part;endoplasmic reticulum part;ER-mitochondrion membrane contact site;intracellular membrane-bounded orgar	Regulation of autophagy;RIG-I-like receptor signaling pathway;Shigellosis	Homo sapiens	1	4
cell part;intracellular membrane-bounded organelle;intracellular non-membrane-bounded organelle;intracellular organelle;intracellular organelle part;intracellular part;membrane-bounded organelle;non-membrane-bounded organelle;nuclear part;nucleolus;nucleoplasm;nucleus;organelle;organelle part		Homo sapiens	1	4
cell part;cytoplasm;intracellular part		Homo sapiens	1	4
cell part;cytoplasm;cytoplasmic part;EGO complex;intracellular membrane-bounded organelle;intracellular organelle;intracellular organelle part;intracellular part;lysosome;lytic vacuole;macromolecular complex;membrane part;membrane-bounded organelle;nuclear part;nucleoplasm;nucleus;organelle;organelle part;protein complex;vacuolar		Homo sapiens	1	4
cell part;cytoplasmic part;endoplasmic reticulum membrane;endoplasmic reticulum part;Golgi apparatus part;Golgi membrane;integral to membrane;intracellular membrane-bounded organelle;intracellular organelle;intracellu	Protein processing in endoplasmic reticulum	Homo sapiens	1	4
ATP-binding cassette (ABC) transporter complex;cell part;cytoplasmic part;endoplasmic reticulum;endosome;envelope;extracellular membrane-bounded organelle;extracellular organelle;extracellular region part;extracellular v	ABC transporters	Homo sapiens	1	4
cell part;cytoplasm;intracellular membrane-bounded organelle;intracellular organelle;intracellular part;membrane-bounded organelle;nucleus;organelle		Homo sapiens	1	4
cell part;cytoplasm;intracellular membrane-bounded organelle;intracellular organelle;intracellular part;membrane-bounded organelle;nucleus;organelle	Atrazine degradation	Homo sapiens	1	4
		Homo sapiens	1	4
cell part;cytoplasmic part;cytosol;intracellular membrane-bounded organelle;intracellular non-membrane-bounded organelle;intracellular organelle;intracellular organelle part;intracellular part;membrane;membrane-bounded organelle;non-membrane-bounded organelle;nuclear part;nucleolus;nucleoplasm;nucleus;organelle;organelle part;plas		Homo sapiens	1	4
cell part;chromatin;chromosomal part;intracellular organelle part;intracellular part;macromolecular complex;nuclear chromatin;nuclear chromosome part;nuclear part;nucleoplasm;organelle part;protein complex		Homo sapiens	1	4
cell part;intracellular part;macromolecular complex;pre-snoRNP complex;ribonucleoprotein complex		Homo sapiens	1	4
cell part;cytoplasmic part;intracellular organelle part;intracellular part;large ribosomal subunit;macromolecular complex;membrane;mitochondrial inner membrane;mitochondrial large ribosomal subunit;mitochondrial membrane;mitochondrial part;organellar large ribosomal subunit;organelle inner membrane;organelle membrane;organelle pa		Homo sapiens	1	4
cell part;cytoplasm;intracellular membrane-bounded organelle;intracellular organelle;intracellular part;membrane-bounded organelle;nucleus;organelle		Homo sapiens	1	4
cell part;integral to membrane;intrinsic to membrane;membrane part		Homo sapiens	0.9999	4
cell part;integral to membrane;intrinsic to membrane;membrane part		Homo sapiens	1	4
adherens junction;anchoring junction;cell junction;cell part;cell-cell adherens junction;cell-cell junction;cytoplasm;intracellular part;zonula adherens		Homo sapiens	1	4
cell part;Cul2-RING ubiquitin ligase complex;Cul3-RING ubiquitin ligase complex;CUL4 RING ubiquitin ligase complex;Cul5-RING ubiquitin ligase complex;Cul7-RING ubiquitin ligase complex;cullin-RING ubiquitin ligase com	Ubiquitin mediated proteolysis	Homo sapiens	1	4
cell part;cytoplasmic part;cytosol;intracellular organelle part;intracellular part;nuclear body;nuclear part;nucleoplasm part;organelle part;PML body		Homo sapiens	1	4
cell part;cytoplasm;cytoplasmic part;intracellular membrane-bounded organelle;intracellular organelle;intracellular organelle part;intracellular part;macromolecular complex;membrane;membrane part;membrane-bounded orga	Alzheimer's disease;Huntington's disease;Oxidative phosphorylation;Parkinson's disease	Homo sapiens	1	4
cell junction;cell part;clathrin-coated vesicle;coated vesicle;cytoplasmic membrane-bounded vesicle;cytoplasmic part;cytoplasmic vesicle;Golgi apparatus;integral to membrane;intracellular membrane-bounded organelle;intracellular organelle;intracellular part;intrinsic to membrane;membrane;membrane part;membrane-bounded organelle;		Homo sapiens	1	4
cell part;cytoplasm;intracellular part;intrinsic to internal side of plasma membrane;intrinsic to membrane;intrinsic to plasma membrane;membrane part;plasma membrane part		Homo sapiens	1	4
cell part;integral to membrane;integral to plasma membrane;intrinsic to membrane;intrinsic to plasma membrane;membrane;membrane part;plasma membrane;plasma membrane part		Homo sapiens	1	4
cell part;cytoplasm;intracellular membrane-bounded organelle;intracellular non-membrane-bounded organelle;intracellular organelle;intracellular organelle part;intracellular part;membrane-bounded organelle;non-membrane-bounded organelle;nuclear part;nucleolus;nucleus;organelle;organelle part		Homo sapiens	1	4
cell part;cytoplasmic part;integral to membrane;integral to mitochondrial inner membrane;integral to mitochondrial membrane;integral to organelle membrane;intracellular membrane-bounded organelle;intracellular organelle;intracellular organelle part;intracellular part;intrinsic to membrane;intrinsic to mitochondrial inner membrane;intrinsic t		Homo sapiens	1	4
cell part;centrosome;cytoplasm;cytoplasmic part;cytoskeletal part;intracellular membrane-bounded organelle;intracellular non-membrane-bounded organelle;intracellular organelle;intracellular organelle part;intracellular part;membrane-bounded organelle;microtubule organizing center;non-membrane-bounded organelle;nucleus;organelle;or		Homo sapiens	1	4

cell part;cytoplasmic part;endoplasmic reticulum;endoplasmic reticulum membrane;endoplasmic reticulum part;integral to membrane;integral to organelle membrane;integral to peroxisomal membrane;intracellular membrane	Peroxisome	Homo sapiens	1	4
cell part;chromatin remodeling complex;extracellular membrane-bounded organelle;extracellular organelle;extracellular region part;extracellular vesicular exosome;histone acetyltransferase complex;histone deacetylase comp	Basal transcription factors	Homo sapiens	0.9998	4
		Homo sapiens	1	5
cell part;cytoplasmic part;endoplasmic reticulum membrane;endoplasmic reticulum part;intracellular organelle part;intracellular part;macromolecular complex;membrane;membrane part;nuclear membrane;nuclear part;nuclear pore;organelle membrane;organelle part;pore complex;protein complex		Homo sapiens	1	5
cell part;intracellular organelle part;intracellular part;macromolecular complex;nuclear part;organelle part;ribonucleoprotein complex;small nucleolar ribonucleoprotein complex;spliceosomal complex		Homo sapiens	1	5
		Homo sapiens	1	5
		Homo sapiens	1	5
cell part;cytoplasmic membrane-bounded vesicle;cytoplasmic part;cytoplasmic vesicle;extracellular membrane-bounded organelle;extracellular organelle;extracellular region part;extracellular vesicular exosome;intracellular m	Lysosome	Homo sapiens	1	5
cell part;cytoplasmic part;endoplasmic reticulum membrane;endoplasmic reticulum part;envelope;extracellular membrane-bounded organelle;extracellular organelle;extracellular region part;extracellular vesicular exosome;int	Drug metabolism - cytochrome P450;Glutathione metabolism;Metabolism of xenobiotics by cytochrome P450	Homo sapiens	1	5
cell part;centrosome;cytoplasmic part;cytoskeletal part;cytosol;intracellular non-membrane-bounded organelle;intracellular organelle;intracellular organelle part;intracellular part;microtubule organizing center;non-membrane-bounded organelle;organelle;organelle part		Homo sapiens	1	5
cell part;cytoplasmic part;cytosol;DNA-directed RNA polymerase complex;DNA-directed RNA polymerase I complex;DNA-directed RNA polymerase III complex;intracellular organelle part;intracellular part;macromolecular co	Cytosolic DNA-sensing pathway;Purine metabolism;Pyrimidine metabolism;RNA polymerase	Homo sapiens	1	5
cell part;chromosome;cytoplasm;cytoplasmic part;DNA-directed RNA polymerase complex;DNA-directed RNA polymerase I complex;intracellular membrane-bounded organelle;intracellular non-membrane-bounded organelle;intracellular organelle;intracellular organelle part;intracellular part;macromolecular complex;membrane-bounded or		Homo sapiens	1	5
cell part;cytoplasmic part;endoplasmic reticulum;endoplasmic reticulum membrane;endoplasmic reticulum part;Golgi apparatus part;Golgi membrane;integral to endoplasmic reticulum membrane;integral to lumenal side of e	Antigen processing and presentation	Homo sapiens	1	5
cell part;cytoplasm;intracellular part;macromolecular complex;protein complex;TORC1 complex;TORC2 complex		Homo sapiens	1	5
cell part;checkpoint clamp complex;chromosomal part;chromosome;intracellular membrane-bounded organelle;intracellular non-membrane-bounded organelle;intracellular organelle;intracellular organelle part;intracellular pa	Cell cycle - yeast;Meiosis - yeast	Homo sapiens	1	5
cell part;cytoplasmic part;endosomal part;endosome membrane;integral to membrane;intracellular organelle part;intracellular part;intrinsic to membrane;membrane;membrane part;organelle membrane;organelle part		Homo sapiens	1	5
cell part;cytoplasm;intracellular membrane-bounded organelle;intracellular organelle;intracellular part;membrane-bounded organelle;nucleus;organelle		Homo sapiens	1	5
cell part;cytoplasm;cytoplasmic part;cytosol;endoplasmic reticulum;intracellular membrane-bounded organelle;intracellular non-membrane-bounded organelle;intracellular organelle;intracellular organelle part;intracellular part;membrane-bounded organelle;mitochondrion;non-membrane-bounded organelle;nuclear part;nucleolus;organelle;o		Homo sapiens	1	5
Cajal body;cell part;cytoplasm;cytoskeleton;intermediate filament cytoskeleton;intracellular membrane-bounded organelle;intracellular non-membrane-bounded organelle;intracellular organelle;intracellular organelle part;intrac	Spliceosome	Homo sapiens	1	5
Cdc48p-Npl4p-Ufd1p AAA ATPase complex;cell part;cytoplasmic part;endoplasmic reticulum part;intracellular organelle part;intracellular part;macromolecular complex;membrane part;nuclear part;nucleoplasm;organelle part;protein complex		Homo sapiens	1	5
cell part;extracellular membrane-bounded organelle;extracellular organelle;extracellular region part;extracellular vesicular exosome;membrane;membrane-bounded organelle;membrane-bounded vesicle;organelle;vesicle	Apoptosis	Homo sapiens	1	5
cell part;cis-Golgi network;cytoplasmic part;Golgi apparatus;Golgi apparatus part;Golgi membrane;integral to membrane;intracellular membrane-bounded organelle;intracellular organelle;intracellular organelle part;intracellula	SNARE interactions in vesicular transport	Homo sapiens	1	5
cell part;cytoplasm;intracellular membrane-bounded organelle;intracellular organelle;intracellular organelle part;intracellular part;macromolecular complex;membrane-bounded organelle;nuclear part;nucleoplasm;nucleus;organelle;organelle part;ribonucleoprotein complex;small nuclear ribonucleoprotein complex;U5 snRNP		Homo sapiens	1	5
cell part;cytoplasmic part;cytosol;intracellular part		Homo sapiens	1	5
cell part;chloride channel complex;cytoplasm;extracellular membrane-bounded organelle;extracellular organelle;extracellular region part;extracellular vesicular exosome;intracellular membrane-bounded organelle;intracellular organelle;intracellular part;ion channel complex;macromolecular complex;membrane part;membrane-bounded orga		Homo sapiens	1	5
cell part;integral to membrane;intrinsic to membrane;membrane part		Homo sapiens	1	5
actin cytoskeleton;cell leading edge;cell part;cytoplasm;cytoplasmic part;cytoskeleton;cytosol;extrinsic to internal side of plasma membrane;extrinsic to membrane;extrinsic to plasma membrane;intracellular membrane-bour	Axon guidance;Cell cycle;Chronic myeloid leukemia;ErbB signaling pathway;Neurotrophin signaling pathway;Pathog	Homo sapiens	1	5
cell part;cell surface;chylomicron;extracellular membrane-bounded organelle;extracellular organelle;extracellular region part;extracellular space;extracellular vesicular exosome;high-density lipoprotein particle;macromolecular complex;membrane-bounded organelle;membrane-bounded vesicle;organelle;plasma lipoprotein particle;protein-li		Homo sapiens	1	5
extracellular membrane-bounded organelle;extracellular organelle;extracellular region part;extracellular vesicular exosome;membrane-bounded organelle;membrane-bounded vesicle;organelle;vesicle		Homo sapiens	1	5
cell part;cytoplasmic part;cytosol;extracellular region;intracellular organelle part;intracellular part;nuclear part;nucleoplasm;organelle part	RIG-I-like receptor signaling pathway	Homo sapiens	1	5
cell part;extracellular region;extracellular region part;extracellular space;intracellular membrane-bounded organelle;intracellular organelle;intracellular part;membrane-bounded organelle;nucleus;organelle	MAPK signaling pathway;Melanoma;Pathways in cancer;Regulation of actin cytoskeleton	Homo sapiens	1	5
cell part;cytoplasm;cytoplasmic part;cytosol;endoplasmic reticulum;intracellular membrane-bounded organelle;intracellular organelle;intracellular part;membrane-bounded organelle;organelle		Homo sapiens	1	5
cell part;cytoplasmic part;endoplasmic reticulum;extracellular membrane-bounded organelle;extracellular organelle;extracellular region part;extracellular vesicular exosome;intracellular membrane-bounded organelle;intracellu	Lysosome;Renin-angiotensin system	Homo sapiens	1	5
anchoring junction;basolateral plasma membrane;brush border membrane;cell junction;cell part;cell projection;cell projection membrane;cell projection part;cell-cell junction;cytoplasmic part;desmosome;external encapsulati	Galactose metabolism;Glycosaminoglycan biosynthesis - keratan sulfate;Glycosphingolipid biosynthesis - lacto anc	Homo sapiens	1	5
cell part;intracellular membrane-bounded organelle;intracellular organelle;intracellular organelle part;intracellular part;membrane-bounded organelle;nuclear part;nucleoplasm;nucleus;organelle;organelle part		Homo sapiens	0.9999	5
adherens junction;anchoring junction;apical plasma membrane;catenin complex;cell junction;cell part;cell projection;cell-cell adherens junction;cell-cell contact zone;cell-cell junction;cell-substrate adherens junction;cell-subs	Arrhythmogenic right ventricular cardiomyopathy (ARVC);Cell adhesion molecules (CAMs)	Homo sapiens	1	5
cell part;cytoplasm;cytoplasmic part;cytosol;extracellular membrane-bounded organelle;extracellular organelle;extracellular region part;extracellular vesicular exosome;intracellular membrane-bounded organelle;intracellular c	Proteasome	Homo sapiens	1	5
cell part;cytoplasmic part;intracellular membrane-bounded organelle;intracellular organelle;intracellular organelle lumen;intracellular organelle part;intracellular part;macromolecular complex;membrane;membrane part;membr	Alzheimer's disease;Huntington's disease;Oxidative phosphorylation;Parkinson's disease	Homo sapiens	1	5
anaphase-promoting complex;cell part;centrosome;cullin-RING ubiquitin ligase complex;cytoplasm;cytoplasmic part;cytoskeletal part;cytosol;intracellular membrane-bounded organelle;intracellular non-membrane-bounded	Cell cycle;Cell cycle - yeast;Meiosis - yeast;Oocyte meiosis;Progesterone-mediated oocyte maturation;Ubiquitin me	Homo sapiens	1	5
CAAX-protein geranylgeranyltransferase complex;cell part;cytoplasm;cytoplasmic part;cytoskeletal part;cytosol;intracellular organelle part;intracellular part;macromolecular complex;microtubule associated complex;organelle part;protein complex;protein farnesyltransferase complex		Homo sapiens	1	5
cell part;cytoplasmic part;cytosol;intracellular part		Homo sapiens	1	5
cell part;cytoplasm;intracellular part	One carbon pool by folate	Homo sapiens	1	5
cell part;cytoplasmic part;intracellular membrane-bounded organelle;intracellular organelle;intracellular part;membrane-bounded organelle;mitochondrion;organelle	Purine metabolism;Pyrimidine metabolism	Homo sapiens	1	5
apical part of cell;azurophil granule membrane;cell junction;cell part;cell projection;cell surface;cytoplasm;cytoplasmic membrane-bounded vesicle;cytoplasmic part;cytoplasmic vesicle;cytoplasmic vesicle membrane;cytopla	SNARE interactions in vesicular transport	Homo sapiens	1	5
cell part;cytoplasmic part;cytosol;DNA-directed RNA polymerase complex;DNA-directed RNA polymerase I complex;DNA-directed RNA polymerase II, core complex;DNA-directed RNA polymerase III complex;intracellular m	Cytosolic DNA-sensing pathway;Huntington's disease;Purine metabolism;Pyrimidine metabolism;RNA polymerase	Homo sapiens	1	5
cell part;cytoplasm;intracellular organelle part;intracellular part;nuclear part;nucleoplasm;organelle part		Homo sapiens	1	5
cell part;cytoplasmic part;integral to membrane;intracellular membrane-bounded organelle;intracellular organelle;intracellular organelle part;intracellular part;intrinsic to membrane;membrane;membrane part;membrane-bounded organelle;mitochondrial membrane;mitochondrial outer membrane;mitochondrial part;mitochondrion;organelle;or		Homo sapiens	1	5
cell junction;cell leading edge;cell part;cell projection part;cell surface;cell-cell junction;cytoplasm;cytoplasmic membrane-bounded vesicle;cytoplasmic part;cytoplasmic vesicle;early endosome;endosome;external side of pla	Axon guidance;Chemokine signaling pathway;Cytokine-cytokine receptor interaction;Endocytosis;Intestinal immune	Homo sapiens	1	5
cell part;cytoplasmic part;cytosol;endosomal part;endosome membrane;extracellular membrane-bounded organelle;extracellular organelle;extracellular region part;extracellular vesicular exosome;intracellular organelle part;in	Protein processing in endoplasmic reticulum;Ubiquitin mediated proteolysis	Homo sapiens	1	5
cell part;CUL4 RING ubiquitin ligase complex;cullin-RING ubiquitin ligase complex;cytoplasm;intracellular organelle part;intracellular part;macromolecular complex;nuclear matrix;nuclear part;nucleoplasm;organelle part;protein complex;ubiquitin ligase complex		Homo sapiens	1	5
Ada2/Gcn5/Ada3 transcription activator complex;cell part;chromatin remodeling complex;histone acetyltransferase complex;histone methyltransferase complex;intracellular membrane-bounded organelle;intracellular organelle;intracellular organelle part;intracellular part;macromolecular complex;membrane-bounded organelle;methyltransfer		Homo sapiens	1	5
cell part;cytoplasmic part;intracellular membrane-bounded organelle;intracellular organelle;intracellular organelle part;intracellular part;macromolecular complex;membrane;membrane part;membrane-bounded organelle;membrane-enclosed lumen;mitochondrial inner membrane;mitochondrial inner membrane presequence translocase comp		Homo sapiens	1	5
catalytic step 2 spliceosome;cell part;cytoplasmic part;cytosol;intracellular membrane-bounded organelle;intracellular organelle;intracellular organelle part;intracellular part;macromolecular complex;membrane-bounded orgar	Spliceosome;Systemic lupus erythematosus	Homo sapiens	1	5
cell part;cytoplasmic part;cytosol;cytosolic small ribosomal subunit;extracellular region part;extracellular space;intracellular organelle part;intracellular part;macromolecular complex;organelle part;ribonucleoprotein complex;small ribosomal subunit		Homo sapiens	1	5
cell part;Cul2-RING ubiquitin ligase complex;Cul3-RING ubiquitin ligase complex;CUL4 RING ubiquitin ligase complex;Cul4A-RING ubiquitin ligase complex;Cul4B-RING ubiquitin ligase complex;Cul5-RING ubiquitin ligase c	Cell cycle;Cell cycle - yeast;Circadian rhythm - mammal;Nucleotide excision repair;Oocyte meiosis;Pathways in can	Homo sapiens	1	5
cell part;DSIF complex;intracellular membrane-bounded organelle;intracellular organelle;intracellular organelle part;intracellular part;macromolecular complex;membrane-bounded organelle;nuclear part;nucleoplasm;nucleoplasm part;nucleus;organelle;organelle part;protein complex;transcription elongation factor complex		Homo sapiens	1	5
acrosomal vesicle;adherens junction;anchoring junction;apicolateral plasma membrane;basolateral plasma membrane;cell body;cell junction;cell part;cell projection;cell projection part;cell-cell contact zone;cell-cell junction;c	Viral myocarditis	Homo sapiens	1	5
cell part;intracellular membrane-bounded organelle;intracellular organelle;intracellular part;membrane-bounded organelle;nucleus;organelle		Homo sapiens	1	5
cell part;cytoplasm;intracellular part	Amino sugar and nucleotide sugar metabolism;Galactose metabolism	Homo sapiens	1	5
cell part;chromosomal part;chromosome;chromosome, centromeric region;condensed chromosome kinetochore;condensed chromosome outer kinetochore;condensed chromosome, centromeric region;condensed nuclear chromosome kinetochore;cytoplasm;cytoplasmic part;cytoskeletal part;cytoskeleton;cytosol;intracellular membrane-b		Homo sapiens	0.9999	5
cell part;cytoplasm;cytoplasmic part;Golgi apparatus;intracellular membrane-bounded organelle;intracellular organelle;intracellular organelle part;intracellular part;macromolecular complex;membrane;membrane-bounded organelle;nuclear body;nuclear part;nucleoplasm;nucleoplasm part;nucleus;organelle;organelle part;plasma membrane;		Homo sapiens	1	5
cell part;chromatin;chromosomal part;cytoplasm;heterochromatin;intracellular membrane-bounded organelle;intracellular organelle;intracellular organelle part;intracellular part;macromolecular complex;membrane-bounded organelle;nuclear body;nuclear chromatin;nuclear chromosome part;nuclear heterochromatin;nuclear part;nuclear spec		Homo sapiens	0.9999	5
anchored to membrane;apical plasma membrane;cell part;cell surface;cytoplasm;cytoplasmic part;endosome;extracellular membrane-bounded organelle;extracellular organelle;extracellular region part;extracellular vesicular exosome;Golgi apparatus;integral to membrane;integral to plasma membrane;intracellular membrane-bounded orgar		Homo sapiens	1	5
cell part;cytoplasm;cytoplasmic part;early endosome;endosome;extracellular membrane-bounded organelle;extracellular organelle;extracellular region part;extracellular vesicular exosome;intracellular membrane-bounded organelle;intracellular organelle;intracellular part;membrane;membrane-bounded organelle;membrane-bounded vesicle;		Homo sapiens	1	5
cell part;cytoplasmic part;cytosol;extracellular membrane-bounded organelle;extracellular organelle;extracellular region part;extracellular vesicular exosome;intracellular membrane-bounded organelle;intracellular organelle;int	Purine metabolism;Pyrimidine metabolism	Homo sapiens	1	5
cell part;cytoplasm;cytoplasmic part;cytosol;intracellular organelle part;intracellular part;macromolecular complex;nuclear part;nucleoplasm;organelle part;protein complex	Acute myeloid leukemia;ErbB signaling pathway;Insulin signaling pathway;mTOR signaling pathway;RNA transport	Homo sapiens	1	5
adherens junction;anchoring junction;axon part;basement membrane;basolateral plasma membrane;cell junction;cell outer membrane;cell part;cell projection;cell projection part;cell-cell adherens junction;cell-cell junction;cel	Arrhythmogenic right ventricular cardiomyopathy (ARVC);Dilated cardiomyopathy;ECM-receptor interaction;Hypertr	Homo sapiens	1	5
cell part;Cul3-RING ubiquitin ligase complex;cullin-RING ubiquitin ligase complex;cytoplasm;cytoplasmic part;cytoskeletal part;cytosol;endoplasmic reticulum;intracellular membrane-bounded organelle;intracellular organelle	Ubiquitin mediated proteolysis	Homo sapiens	1	5
cell part;intracellular non-membrane-bounded organelle;intracellular organelle;intracellular organelle part;intracellular part;non-membrane-bounded organelle;nuclear part;nucleolus;nucleoplasm;organelle;organelle part		Homo sapiens	1	5
cell part;cytoplasmic part;integral to membrane;integral to mitochondrial membrane;integral to organelle membrane;intracellular membrane-bounded organelle;intracellular organelle;intracellular organelle part;intracellular part	Bacterial secretion system;Protein export	Homo sapiens	1	5
cell part;cytoplasmic membrane-bounded vesicle;cytoplasmic part;cytoplasmic vesicle;early endosome;endocytic vesicle;endosome;intracellular membrane-bounded organelle;intracellular organelle;intracellular part;membra	Endocytosis	Homo sapiens	1	5
cell part;cytoplasm;intracellular organelle part;intracellular part;macromolecular complex;mediator complex;nuclear part;nucleoplasm part;organelle part;protein complex		Homo sapiens	1	5
cell part;cytoplasmic part;cytosol;intracellular part	Fructose and mannose metabolism	Homo sapiens	1	5
cell part;cytoplasmic part;intracellular membrane-bounded organelle;intracellular organelle;intracellular part;membrane-bounded organelle;mitochondrion;organelle		Homo sapiens	1	5
cell part;endomembrane system		Homo sapiens	1	5
cell part;cytoplasmic part;intracellular membrane-bounded organelle;intracellular organelle;intracellular part;membrane-bounded organelle;mitochondrion;organelle		Homo sapiens	1	5
cell part;integral to membrane;intrinsic to membrane;membrane part		Homo sapiens	1	5
cell part;intracellular non-membrane-bounded organelle;intracellular organelle;intracellular organelle part;intracellular part;membrane;non-membrane-bounded organelle;nuclear part;nucleolus;organelle;organelle part		Homo sapiens	1	5
cell cortex;cell part;cytoplasm;cytoplasmic membrane-bounded vesicle;cytoplasmic part;cytoplasmic vesicle;cytoskeleton;extracellular membrane-bounded organelle;extracellular organelle;extracellular region part;extracellular vesicular exosome;intracellular membrane-bounded organelle;intracellular non-membrane-bounded organelle;intra		Homo sapiens	1	5
		Homo sapiens	1	5
cell part;cytoplasm;intracellular organelle part;intracellular part;nuclear part;nucleoplasm;organelle part		Homo sapiens	1	5
cell part;intracellular membrane-bounded organelle;intracellular organelle;intracellular part;membrane-bounded organelle;nucleus;organelle		Homo sapiens	1	5
cell part;intracellular membrane-bounded organelle;intracellular organelle;intracellular part;membrane;membrane-bounded organelle;nucleus;organelle		Homo sapiens	1	5
autophagic vacuole;cell part;cytoplasmic part;cytoplasmic vesicle;endoplasmic reticulum membrane;endoplasmic reticulum part;endosomal part;endosome;endosome membrane;Golgi apparatus part;integral to membrane;intracellular membrane-bounded organelle;intracellular organelle;intracellular organelle part;intracellular part;intrinsic t		Homo sapiens	1	5
cell part;chromosomal part;condensed chromosome kinetochore;cytoplasm;cytoskeletal part;cytoskeleton;intracellular membrane-bounded organelle;intracellular non-membrane-bounded organelle;intracellular organelle;intracellular organelle part;intracellular part;kinetochore;macromolecular complex;membrane-bounded organelle;microtu		Homo sapiens	1	5
extracellular membrane-bounded organelle;extracellular organelle;extracellular region part;extracellular vesicular exosome;membrane-bounded organelle;membrane-bounded vesicle;organelle;vesicle		Homo sapiens	1	5
cell part;intracellular organelle part;intracellular part;nuclear part;nucleoplasm;organelle part		Homo sapiens	1	5
cell part;integral to membrane;intrinsic to membrane;membrane;membrane part		Homo sapiens	1	5
cell part;cytoplasm;intracellular organelle part;intracellular part;nuclear part;nucleoplasm;organelle part		Homo sapiens	1	5
		Homo sapiens	1	5
cell part;cytoplasmic part;endoplasmic reticulum lumen;endoplasmic reticulum part;extracellular membrane-bounded organelle;extracellular organelle;extracellular region part;extracellular vesicular exosome;intracellular orgar	Other types of O-glycan biosynthesis	Homo sapiens	1	5
cell part;cytoplasmic part;intracellular membrane-bounded organelle;intracellular organelle;intracellular part;lipid particle;membrane;membrane-bounded organelle;midbody;mitochondrion;nucleus;organelle		Homo sapiens	1	5
		Homo sapiens	1	5
cell part;chromosomal part;condensed chromosome kinetochore;cytoplasmic part;cytosol;envelope;intracellular non-membrane-bounded organelle;intracellular organelle;intracellular organelle part;intracellular part;kinetochor	RNA transport	Homo sapiens	1	5
cell part;cytoplasm;intracellular membrane-bounded organelle;intracellular organelle;intracellular organelle part;intracellular part;membrane-bounded organelle;nuclear body;nuclear part;nuclear speck;nucleoplasm;nucleoplasm part;nucleus;organelle;organelle part		Homo sapiens	1	5
caveola;cell part;cell projection membrane;cell projection part;cell surface;cytoplasmic part;cytoplasmic vesicle membrane;cytoplasmic vesicle part;endocytic vesicle membrane;extracellular membrane-bounded organelle;ex	Bile secretion;Fat digestion and absorption;Hepatitis C;Phagosome;Vitamin digestion and absorption	Homo sapiens	1	5
cell part;cytoplasmic part;intracellular membrane-bounded organelle;intracellular organelle;intracellular part;membrane-bounded organelle;mitochondrion;organelle		Homo sapiens	1	5
basolateral plasma membrane;cell part;integral to membrane;integral to plasma membrane;intrinsic to membrane;intrinsic to plasma membrane;membrane;membrane part;plasma membrane;plasma membrane part		Homo sapiens	1	5
CCR4-NOT complex;cell part;cytoplasmic mRNA processing body;cytoplasmic part;cytosol;intracellular membrane-bounded organelle;intracellular non-membrane-bounded organelle;intracellular organelle;intracellular organe	RNA degradation	Homo sapiens	1	5
cell part;intracellular membrane-bounded organelle;intracellular organelle;intracellular organelle part;intracellular part;membrane-bounded organelle;nuclear part;nucleoplasm;nucleus;organelle;organelle part		Homo sapiens	1	5
cell part;cytoplasm;cytoplasmic part;cytosol;exosome (RNase complex);intracellular membrane-bounded organelle;intracellular non-membrane-bounded organelle;intracellular organelle;intracellular organelle part;intracellular	RNA degradation	Homo sapiens	1	5
catalytic step 2 spliceosome;cell part;intracellular membrane-bounded organelle;intracellular organelle;intracellular organelle part;intracellular part;macromolecular complex;membrane-bounded organelle;nuclear part;nucleoplasm;nucleus;organelle;organelle part;ribonucleoprotein complex;spliceosomal complex		Homo sapiens	1	5
cell part;cytoplasm;extracellular membrane-bounded organelle;extracellular organelle;extracellular region part;extracellular vesicular exosome;intracellular part;membrane-bounded organelle;membrane-bounded vesicle;orga	Glycolysis / Gluconeogenesis	Homo sapiens	1	5
cell part;cytoplasm;cytoplasmic part;cytosol;intracellular membrane-bounded organelle;intracellular organelle;intracellular organelle part;intracellular part;membrane-bounded organelle;mitochondrion;nuclear part;nucleoplasr	RNA degradation	Homo sapiens	1	5
cell part;cytoplasmic part;intracellular organelle part;intracellular part;large ribosomal subunit;macromolecular complex;membrane;mitochondrial inner membrane;mitochondrial large ribosomal subunit;mitochondrial membrane;mitochondrial part;mitochondrial small ribosomal subunit;organellar large ribosomal subunit;organellar small riboso		Homo sapiens	1	5
cell part;cytoplasmic part;intracellular non-membrane-bounded organelle;intracellular organelle;intracellular organelle part;intracellular part;macromolecular complex;membrane;mitochondrial inner membrane;mitochondrial membrane;mitochondrial part;non-membrane-bounded organelle;organelle;organelle inner membrane;organelle memb		Homo sapiens	1	5
90S preribosome;cell part;intracellular membrane-bounded organelle;intracellular non-membrane-bounded organelle;intracellular organelle;intracellular organelle part;intracellular part;macromolecular complex;membrane-bounded organelle;non-membrane-bounded organelle;nuclear part;nucleolus;nucleus;organelle;organelle part;preriboso		Homo sapiens	1	5
cell part;centrosome;chromocenter;chromosomal part;chromosome passenger complex;chromosome, centromeric region;condensed chromosome, centromeric region;condensed nuclear chromosome, centromeric region;cytoplasmic part;cytoskeletal part;cytosol;intracellular membrane-bounded organelle;intracellular non-membrane-bou		Homo sapiens	1	5
cell part;cytoplasm;intracellular part	Cysteine and methionine metabolism	Homo sapiens	1	5
cell part;chromosomal part;cytoplasm;intracellular organelle part;intracellular part;nuclear chromosome part;nuclear part;nuclear replication fork;nucleoplasm;organelle part;replication fork		Homo sapiens	1	5
cell division site part;cell part;centrosome;cleavage furrow;cytoplasm;cytoplasmic part;cytoskeletal part;intracellular non-membrane-bounded organelle;intracellular organelle;intracellular organelle part;intracellular part;microtubule organizing center;midbody;non-membrane-bounded organelle;organelle;organelle part		Homo sapiens	1	5
cell part;intracellular membrane-bounded organelle;intracellular organelle;intracellular part;membrane-bounded organelle;nucleus;organelle		Homo sapiens	1	5
		Homo sapiens	1	5
cell part;centrosome;cytoplasm;cytoplasmic part;cytoskeletal part;intracellular membrane-bounded organelle;intracellular non-membrane-bounded organelle;intracellular organelle;intracellular organelle part;intracellular part;membrane-bounded organelle;microtubule organizing center;mitochondrion;non-membrane-bounded organelle;nucle		Homo sapiens	1	5
cell part;cytoplasm;exosome (RNase complex);intracellular membrane-bounded organelle;intracellular non-membrane-bounded organelle;intracellular organelle;intracellular organelle part;intracellular part;macromolecular con	RNA degradation	Homo sapiens	1	5
apical plasma membrane;basolateral plasma membrane;cell part;integral to membrane;integral to plasma membrane;intrinsic to membrane;intrinsic to plasma membrane;membrane;membrane part;plasma membrane;plasma membrane part		Homo sapiens	1	5
cell body;cell part;cell projection;cytoplasmic part;cytosol;dendrite;intracellular part;neuron projection;neuronal cell body	Axon guidance	Homo sapiens	0.9999	5
		Homo sapiens	1	5
cell part;cytoplasm;intracellular part;membrane		Homo sapiens	1	5
cell part;centrosome;cytoplasm;cytoplasmic part;cytoskeletal part;HAUS complex;intracellular non-membrane-bounded organelle;intracellular organelle;intracellular organelle part;intracellular part;macromolecular complex;microtubule;microtubule associated complex;microtubule organizing center;non-membrane-bounded organelle;organe		Homo sapiens	1	5
cell part;cytoplasm;intracellular membrane-bounded organelle;intracellular organelle;intracellular part;membrane-bounded organelle;nucleus;organelle		Homo sapiens	1	5
cell part;cytoplasm;intracellular organelle part;intracellular part;macromolecular complex;membrane;membrane part;nuclear membrane;nuclear part;nuclear pore;organelle membrane;organelle part;pore complex;protein com	RNA transport	Homo sapiens	1	5
cell part;cytoplasmic part;cytoskeletal part;intracellular organelle part;intracellular part;microtubule organizing center;nuclear part;nucleoplasm;organelle part	Ribosome biogenesis in eukaryotes;RNA transport	Homo sapiens	1	5
cell part;cytoplasmic part;extracellular region part;extracellular space;high-density lipoprotein particle;integral to membrane;intracellular membrane-bounded organelle;intracellular organelle;intracellular part;intrinsic to membrane;low-density lipoprotein particle;macromolecular complex;membrane part;membrane-bounded organelle;mitocho		Homo sapiens	1	5
cell part;cytoplasmic part;endoplasmic reticulum membrane;endoplasmic reticulum part;extracellular membrane-bounded organelle;extracellular organelle;extracellular region part;extracellular vesicular exosome;integral to membrane;intracellular organelle part;intracellular part;intrinsic to membrane;membrane;membrane part;membrane-bo		Homo sapiens	1	5
cell part;intracellular membrane-bounded organelle;intracellular organelle;intracellular part;membrane-bounded organelle;nucleus;organelle		Homo sapiens	1	5
cell part;intracellular membrane-bounded organelle;intracellular organelle;intracellular organelle part;intracellular part;macromolecular complex;membrane-bounded organelle;nuclear part;nucleoplasm;nucleus;organelle;organ	Spliceosome	Homo sapiens	1	5
cell part;cytoplasmic part;intracellular non-membrane-bounded organelle;intracellular organelle;intracellular organelle part;intracellular part;large ribosomal subunit;macromolecular complex;membrane;mitochondrial inner membrane;mitochondrial membrane;mitochondrial part;mitochondrial ribosome;non-membrane-bounded organelle;orga		Homo sapiens	1	5
cell part;cytoplasmic part;extracellular region part;extracellular space;intracellular membrane-bounded organelle;intracellular non-membrane-bounded organelle;intracellular organelle;intracellular organelle part;intracellular part;macromolecular complex;membrane;membrane-bounded organelle;mitochondrial inner membrane;mitochondrial		Homo sapiens	1	5

cell part;cytoplasm;intracellular organelle part;intracellular part;nuclear part;nucleoplasm;organelle part		Homo sapiens	1	5
cell part;chromosomal part;cytoplasm;cytoplasmic part;cytosol;intracellular membrane-bounded organelle;intracellular organelle;intracellular organelle part;intracellular part;macromolecular complex;membrane-bounded organelle;MIS12/MIND type complex;nucleus;organelle;organelle part;protein complex		Homo sapiens	1	5
cell part;cytoplasm;intracellular organelle part;intracellular part;macromolecular complex;nuclear part;nucleoplasm;organelle part;protein complex;signalosome		Homo sapiens	1	5
cell part;cytoplasm;cytoplasmic part;intracellular membrane-bounded organelle;intracellular organelle;intracellular part;membrane-bounded organelle;nucleus;organelle;perinuclear region of cytoplasm		Homo sapiens	1	5
cell part;chromatin;chromosomal part;heterochromatin;intracellular membrane-bounded organelle;intracellular organelle;intracellular organelle part;intracellular part;macromolecular complex;membrane-bounded organelle;nuclear chromatin;nuclear chromosome part;nuclear part;nucleoplasm;nucleus;organelle;organelle part;PcG protein cor		Homo sapiens	1	5
cell part;intracellular membrane-bounded organelle;intracellular organelle;intracellular part;membrane-bounded organelle;nucleus;organelle		Homo sapiens	1	5
cell part;chromatin;chromosomal part;cytoplasm;cytoplasmic exosome (RNase complex);cytoplasmic part;cytosol;exosome (RNase complex);intracellular membrane-bounded organelle;intracellular non-membrane-bounded	RNA degradation	Homo sapiens	1	5
cell part;cytoplasmic part;integral to membrane;intracellular organelle part;intracellular part;intrinsic to membrane;membrane;membrane part;mitochondrial membrane;mitochondrial part;organelle membrane;organelle part;plasma membrane		Homo sapiens	1	5
basolateral plasma membrane;cell junction;cell part;cell projection;cell-cell junction;cytoplasm;cytoskeletal part;extracellular membrane-bounded organelle;extracellular organelle;extracellular region part;extracellular vesicular exosome;intracellular organelle part;intracellular part;macromolecular complex;membrane;membrane part;membrar		Homo sapiens	1	5
cell part;cytoplasmic part;integral to membrane;intracellular membrane-bounded organelle;intracellular organelle;intracellular organelle part;intracellular part;intrinsic to membrane;macromolecular complex;membrane;membra	Alzheimer's disease;Huntington's disease;Oxidative phosphorylation;Parkinson's disease	Homo sapiens	1	5
		Homo sapiens	1	5
cell part;cytoplasm;cytoplasmic part;cytosol;eukaryotic 43S preinitiation complex;eukaryotic 48S preinitiation complex;eukaryotic translation initiation factor 3 complex;extracellular membrane-bounded organelle;extracellular organelle;extracellular region part;extracellular vesicular exosome;intracellular membrane-bounded organelle;intrace		Homo sapiens	1	5
cell part;cytoplasmic part;extracellular membrane-bounded organelle;extracellular organelle;extracellular region part;extracellular vesicular exosome;intracellular organelle part;intracellular part;macromolecular complex;memb	Alzheimer's disease;Cardiac muscle contraction;Huntington's disease;Oxidative phosphorylation;Parkinson's diseas	Homo sapiens	1	5
cell part;cytoplasmic part;cytoplasmic vesicle part;GARP complex;Golgi apparatus;Golgi apparatus part;integral to membrane;intracellular membrane-bounded organelle;intracellular organelle;intracellular organelle part;intracellular part;intrinsic to membrane;macromolecular complex;membrane part;membrane-bounded organelle;organelle;		Homo sapiens	1	5
		Homo sapiens	1	5
cell part;cytoplasmic part;integral to membrane;integral to mitochondrial membrane;integral to mitochondrial outer membrane;integral to organelle membrane;integral to peroxisomal membrane;intracellular membrane-bounded organelle;intracellular organelle;intracellular organelle part;intracellular part;intrinsic to membrane;intrinsic to mitoc		Homo sapiens	1	5
cell part;cytoplasmic part;extracellular region part;extracellular space;intracellular organelle part;intracellular part;macromolecular complex;membrane;mitochondrial inner membrane;mitochondrial intermembrane space protein transporter complex;mitochondrial membrane;mitochondrial part;organelle inner membrane;organelle membrane;c		Homo sapiens	1	5
cell part;cytoplasmic part;intracellular membrane-bounded organelle;intracellular non-membrane-bounded organelle;intracellular organelle;intracellular organelle part;intracellular part;macromolecular complex;membrane;membrane-bounded organelle;mitochondrial inner membrane;mitochondrial intermembrane space protein transporter cc		Homo sapiens	1	5
cell part;cytoplasmic part;cytosol;endoplasmic reticulum membrane;endoplasmic reticulum part;Golgi apparatus;intracellular membrane-bounded organelle;intracellular organelle;intracellular organelle part;intracellular part;membrane;membrane part;membrane-bounded organelle;mitochondrion;nuclear part;nucleoplasm;organelle;organelle		Homo sapiens	1	5
cell part;cytoplasmic part;endoplasmic reticulum membrane;endoplasmic reticulum part;integral to membrane;intracellular organelle part;intracellular part;intrinsic to membrane;membrane;membrane part;organelle membrane;organelle part		Homo sapiens	1	5
cell part;cytoplasmic part;early endosome membrane;endosomal part;endosome membrane;extrinsic to membrane;intracellular membrane-bounded organelle;intracellular organelle;intracellular organelle part;intracellular part;membrane;membrane part;membrane-bounded organelle;organelle;organelle membrane;organelle part		Homo sapiens	1	5
cell part;cytoskeletal part;intracellular membrane-bounded organelle;intracellular non-membrane-bounded organelle;intracellular organelle;intracellular organelle part;intracellular part;membrane-bounded organelle;non-membrane-bounded organelle;nuclear part;nucleolus;nucleus;organelle;organelle part;spindle		Homo sapiens	1	5
cell part;cytoplasmic part;extracellular membrane-bounded organelle;extracellular organelle;extracellular region part;extracellular vesicular exosome;intracellular;intracellular membrane-bounded organelle;intracellular organel	Lysosome	Homo sapiens	1	6
anaphase-promoting complex;cell part;cullin-RING ubiquitin ligase complex;cytoplasm;cytoplasmic part;cytosol;intracellular organelle part;intracellular part;macromolecular complex;nuclear part;nuclear ubiquitin ligase comp	Ubiquitin mediated proteolysis	Homo sapiens	1	6
cell part;cytoplasmic part;dihydrolipoyl dehydrogenase complex;intracellular membrane-bounded organelle;intracellular organelle;intracellular organelle part;intracellular part;macromolecular complex;membrane-bounded organelle;mitochondrial alpha-ketoglutarate dehydrogenase complex;mitochondrial part;mitochondrial tricarboxylic acid		Homo sapiens	1	6
brush border;cell cortex;cell part;cell projection;cell projection part;clathrin-coated vesicle;coated vesicle;cytoplasm;cytoplasmic membrane-bounded vesicle;cytoplasmic part;cytoplasmic vesicle;cytosol;dendritic shaft;dendritic spine;endocytic vesicle;extracellular membrane-bounded organelle;extracellular organelle;extracellular region pa		Homo sapiens	1	6
cell part;cytoplasmic membrane-bounded vesicle;cytoplasmic part;cytoplasmic vesicle;endosomal part;endosome membrane;extracellular membrane-bounded organelle;extracellular organelle;extracellular region part;extracellular vesicular exosome;Golgi apparatus;Golgi apparatus part;Golgi membrane;integral to membrane;intracellular m		Homo sapiens	1	6
cell part;cell surface;cytoplasmic part;endoplasmic reticulum-Golgi intermediate compartment;Golgi apparatus;Golgi apparatus part;integral to Golgi membrane;integral to membrane;integral to organelle membrane;intracellular membrane-bounded organelle;intracellular organelle;intracellular organelle part;intracellular part;intrinsic to Golgi r		Homo sapiens	1	6
cell part;cytoplasmic part;integral to membrane;intracellular organelle part;intracellular part;intrinsic to membrane;membrane;membrane part;mitochondrial membrane;mitochondrial outer membrane;mitochondrial part;organelle membrane;organelle outer membrane;organelle part;outer membrane		Homo sapiens	1	6
cell part;cytoplasmic part;endoplasmic reticulum;Golgi apparatus;intracellular membrane-bounded organelle;intracellular organelle;intracellular part;membrane-bounded organelle;organelle		Homo sapiens	1	6
cell part;cytoplasmic part;integral to membrane;intracellular membrane-bounded organelle;intracellular organelle;intracellular organelle part;intracellular part;intrinsic to membrane;macromolecular complex;membrane;membra	Alzheimer's disease;Huntington's disease;Oxidative phosphorylation;Parkinson's disease	Homo sapiens	1	6
cell part;clathrin coated vesicle membrane;coated vesicle membrane;cytoplasmic part;cytoplasmic vesicle membrane;cytoplasmic vesicle part;extracellular membrane-bounded organelle;extracellular organelle;extracellular region part;extracellular vesicular exosome;integral to membrane;intracellular organelle part;intracellular part;intrinsic t		Homo sapiens	1	6
cell part;cytoplasmic part;intracellular membrane-bounded organelle;intracellular organelle;intracellular organelle part;intracellular part;membrane;membrane-bounded organelle;membrane-enclosed lumen;mitochondrial inner membrane;mitochondrial intermembrane space;mitochondrial membrane;mitochondrial part;mitochondrion;organel		Homo sapiens	1	6
cell part;cell projection part;cytoplasm;cytoplasmic part;intracellular membrane-bounded organelle;intracellular non-membrane-bounded organelle;intracellular organelle;intracellular organelle part;intracellular part;macromole	Spliceosome	Homo sapiens	1	6
cell part;cytoplasmic part;intracellular membrane-bounded organelle;intracellular organelle;intracellular organelle part;intracellular part;macromolecular complex;membrane-bounded organelle;mitochondrial part;mitochondria	Ribosome biogenesis in eukaryotes;RNA transport	Homo sapiens	1	6
cell part;cytoplasmic part;extracellular membrane-bounded organelle;extracellular organelle;extracellular region part;extracellular vesicular exosome;intracellular membrane-bounded organelle;intracellular organelle;intracellula	Oxidative phosphorylation	Homo sapiens	1	6
cell part;cytoplasmic part;cytosol;extracellular membrane-bounded organelle;extracellular organelle;extracellular region part;extracellular vesicular exosome;intracellular organelle part;intracellular part;macromolecular comple	RNA transport	Homo sapiens	1	6
cell part;cytoplasmic part;endoplasmic reticulum;integral to membrane;intracellular membrane-bounded organelle;intracellular organelle;intracellular organelle part;intracellular part;intrinsic to membrane;macromolecular com	Alzheimer's disease;Huntington's disease;Oxidative phosphorylation;Parkinson's disease	Homo sapiens	1	6
cell part;intracellular membrane-bounded organelle;intracellular non-membrane-bounded organelle;intracellular organelle;intracellular organelle part;intracellular part;macromolecular complex;membrane-bounded organelle;non-membrane-bounded organelle;nuclear part;nucleolus;nucleus;organelle;organelle part;ribonucleoprotein complex		Homo sapiens	1	6
cell division site part;cell part;cleavage furrow;cytoplasm;cytoplasmic part;early endosome;endosome;extracellular membrane-bounded organelle;extracellular organelle;extracellular region part;extracellular vesicular exosome	Endocytosis	Homo sapiens	1	6
		Homo sapiens	1	6
cell part;chromatin;chromatin remodeling complex;chromosomal part;cytoplasm;heterochromatin;histone deacetylase complex;intracellular organelle part;intracellular part;macromolecular complex;nuclear chromatin;nuclear chromosome part;nuclear part;nucleoplasm;nucleoplasm part;NuRD complex;organelle part;protein complex;transcr		Homo sapiens	1	6
cell part;cytoplasmic part;extracellular membrane-bounded organelle;extracellular organelle;extracellular region part;extracellular vesicular exosome;integral to membrane;integral to organelle membrane;integral to peroxisom	Peroxisome	Homo sapiens	1	6
cell part;cytoplasmic part;endoplasmic reticulum membrane;endoplasmic reticulum part;extracellular membrane-bounded organelle;extracellular organelle;extracellular region part;extracellular vesicular exosome;integral to membrane;intracellular organelle part;intracellular part;intrinsic to membrane;membrane;membrane part;membrane-bo		Homo sapiens	1	6
cell part;cytoplasmic part;cytosol;intracellular organelle part;intracellular part;nuclear part;nucleoplasm;organelle part	Folate biosynthesis;One carbon pool by folate	Homo sapiens	1	6
cell part;cytoplasmic part;cytosol;extracellular membrane-bounded organelle;extracellular organelle;extracellular region;extracellular region part;extracellular space;extracellular vesicular exosome;intracellular part;macromolecular complex;membrane-bounded organelle;membrane-bounded vesicle;organelle;protein complex;vesicle		Homo sapiens	1	6
cell part;cytoplasm;extracellular membrane-bounded organelle;extracellular organelle;extracellular region part;extracellular space;extracellular vesicular exosome;intracellular non-membrane-bounded organelle;intracellular organelle;intracellular organelle part;intracellular part;membrane-bounded organelle;membrane-bounded vesicle;non-n		Homo sapiens	1	6
cell part;cytoplasm;cytoplasmic part;extracellular membrane-bounded organelle;extracellular organelle;extracellular region;extracellular region part;extracellular vesicular exosome;Golgi apparatus;intracellular membrane-bou	Galactose metabolism;Glycerolipid metabolism;Glycosphingolipid biosynthesis - globo series;Lysosome;Sphingolip	Homo sapiens	1	6
cell part;cytoplasm;intracellular membrane-bounded organelle;intracellular organelle;intracellular organelle part;intracellular part;macromolecular complex;membrane-bounded organelle;nuclear part;nucleoplasm;nucleus;orga	Huntington's disease;TGF-beta signaling pathway	Homo sapiens	1	6
basal plasma membrane;cell part;cell surface;extracellular region;integral to membrane;integral to plasma membrane;intrinsic to membrane;intrinsic to plasma membrane;membrane;membrane part;plasma membrane;plasma	Adherens junction;Axon guidance;Bacterial invasion of epithelial cells;Cytokine-cytokine receptor interaction;Endoc	Homo sapiens	1	6
cell part;cell projection;cytoplasm;cytoplasmic part;cytosol;extracellular membrane-bounded organelle;extracellular organelle;extracellular region part;extracellular vesicular exosome;intracellular membrane-bounded organell	Folate biosynthesis	Homo sapiens	1	6
apical plasma membrane;cell part;chromatin;chromosomal part;cytoplasmic part;extracellular membrane-bounded organelle;extracellular organelle;extracellular region part;extracellular space;extracellular vesicular exosome;Golgi apparatus part;Golgi lumen;integral to membrane;integral to plasma membrane;intracellular organelle lumen;in		Homo sapiens	1	6
cell part;cytoplasmic part;integral to membrane;intracellular membrane-bounded organelle;intracellular organelle;intracellular organelle part;intracellular part;intrinsic to membrane;membrane;membrane part;membrane-bounc	Alzheimer's disease;Cardiac muscle contraction;Huntington's disease;Oxidative phosphorylation;Parkinson's diseas	Homo sapiens	1	6
cell part;cytoplasmic part;cytosol;DNA-directed RNA polymerase complex;DNA-directed RNA polymerase I complex;DNA-directed RNA polymerase II, core complex;DNA-directed RNA polymerase III complex;intracellular m	Cytosolic DNA-sensing pathway;Huntington's disease;Purine metabolism;Pyrimidine metabolism;RNA polymerase	Homo sapiens	1	6
carboxy-terminal domain protein kinase complex;cell part;core TFIIH complex;holo TFIIH complex;intracellular membrane-bounded organelle;intracellular organelle;intracellular organelle part;intracellular part;macromolecular	Basal transcription factors;Nucleotide excision repair	Homo sapiens	1	6
cell part;cell projection membrane;cell projection part;cytoplasm;extracellular membrane-bounded organelle;extracellular organelle;extracellular region part;extracellular vesicular exosome;intracellular membrane-bounded organelle;intracellular organelle;intracellular part;membrane part;membrane-bounded organelle;membrane-bounded ve		Homo sapiens	1	6
cell part;membrane		Homo sapiens	1	6
cell part;cytoplasmic part;cytosol;extracellular region;intracellular part	Drug metabolism - other enzymes;Pyrimidine metabolism	Homo sapiens	1	6
cell part;cytoplasm;cytoplasmic part;cytosol;integral to membrane;integral to organelle membrane;integral to peroxisomal membrane;intracellular membrane-bounded organelle;intracellular organelle;intracellular organelle par	ABC transporters;Peroxisome	Homo sapiens	1	6
cell part;chromosomal part;DNA replication factor A complex;intracellular organelle part;intracellular part;macromolecular complex;nuclear chromosome part;nuclear part;nucleoplasm;organelle part;protein complex	DNA replication;Homologous recombination;Mismatch repair;Nucleotide excision repair	Homo sapiens	1	6
cell part;cell projection;centrosome;cilium;cytoplasmic microtubule;cytoplasmic part;cytoskeletal part;extracellular membrane-bounded organelle;extracellular organelle;extracellular region part;extracellular vesicular exosome;Golgi apparatus;Golgi apparatus part;Golgi membrane;intracellular membrane-bounded organelle;intracellular non-r		Homo sapiens	1	6
cell part;cytoplasmic part;cytosol;extracellular membrane-bounded organelle;extracellular organelle;extracellular region part;extracellular vesicular exosome;intracellular membrane-bounded organelle;intracellular organelle;int	Glutathione metabolism	Homo sapiens	1	6
cell part;cytoplasm;cytoskeletal part;envelope;intracellular membrane-bounded organelle;intracellular organelle;intracellular organelle part;intracellular part;macromolecular complex;membrane;membrane part;membrane-bou	RNA transport	Homo sapiens	1	6
cell part;cytoplasm;cytoplasmic part;cytosol;intracellular membrane-bounded organelle;intracellular organelle;intracellular part;membrane-bounded organelle;nucleus;organelle	Cell cycle	Homo sapiens	1	6
basal part of cell;cell part;cytoplasmic part;extracellular membrane-bounded organelle;extracellular organelle;extracellular region part;extracellular vesicular exosome;intracellular membrane-bounded organelle;intracellular org	Protein digestion and absorption	Homo sapiens	1	6
cell part;cytoplasm;extracellular membrane-bounded organelle;extracellular organelle;extracellular region part;extracellular vesicular exosome;intracellular part;membrane-bounded organelle;membrane-bounded vesicle;organelle;vesicle		Homo sapiens	1	6
BRCA1-A complex;BRISC complex;cell part;intracellular membrane-bounded organelle;intracellular organelle;intracellular organelle part;intracellular part;macromolecular complex;membrane-bounded organelle;nuclear part;nuclear ubiquitin ligase complex;nucleoplasm;nucleus;organelle;organelle part;protein complex;ubiquitin ligase compl		Homo sapiens	1	6
cell part;cytoplasmic part;cytosol;glutamate-cysteine ligase complex;intracellular part;macromolecular complex;protein complex	Glutathione metabolism	Homo sapiens	1	6
cell part;cytoplasmic part;endoplasmic reticulum membrane;endoplasmic reticulum part;integral to membrane;intracellular organelle part;intracellular part;intrinsic to membrane;membrane;membrane part;organelle membrane	Glycerophospholipid metabolism	Homo sapiens	1	6
cell part;cytoplasm;intracellular part		Homo sapiens	1	6
cell part;cytoplasm;cytoplasmic part;holo TFIIH complex;intracellular membrane-bounded organelle;intracellular organelle;intracellular organelle part;intracellular part;macromolecular complex;membrane-bounded organelle;n	Basal transcription factors;Cell cycle;Nucleotide excision repair	Homo sapiens	1	6
axon;cell body;cell part;cell projection;clathrin-coated vesicle;coated vesicle;cytoplasmic membrane-bounded vesicle;cytoplasmic part;cytoplasmic vesicle;cytosol;dendrite;extracellular membrane-bounded organelle;extrace	Fatty acid elongation in mitochondria;Lysosome	Homo sapiens	1	6
		Homo sapiens	1	6
cell part;cytoplasmic part;intracellular membrane-bounded organelle;intracellular non-membrane-bounded organelle;intracellular organelle;intracellular organelle lumen;intracellular organelle part;intracellular part;membrane-b	Citrate cycle (TCA cycle)	Homo sapiens	1	6
cell part;cytoplasm;intracellular organelle part;intracellular part;macromolecular complex;nuclear part;nucleoplasm;organelle part;proteasome complex;protein complex	Nucleotide excision repair;Protein processing in endoplasmic reticulum	Homo sapiens	1	6
apoptosome;cell part;cytoplasmic part;cytosol;cytosolic part;intracellular membrane-bounded organelle;intracellular organelle;intracellular part;macromolecular complex;membrane-bounded organelle;mitochondrion;nucleus;	Alzheimer's disease;Amyotrophic lateral sclerosis (ALS);Apoptosis;Colorectal cancer;Endometrial cancer;Huntingtor	Homo sapiens	1	6
cell part;cytoplasmic part;intracellular organelle lumen;intracellular organelle part;intracellular part;macromolecular complex;membrane part;membrane-enclosed lumen;mitochondrial matrix;mitochondrial membrane part;mitc	Alzheimer's disease;Huntington's disease;Oxidative phosphorylation;Parkinson's disease	Homo sapiens	1	6
axon;cell part;cell projection;cytoplasmic part;cytoskeletal part;cytosol;extracellular membrane-bounded organelle;extracellular organelle;extracellular region part;extracellular vesicular exosome;F-actin capping protein complex;intracellular membrane-bounded organelle;intracellular organelle;intracellular organelle part;intracellular part;macı		Homo sapiens	1	6
cell part;COPI vesicle coat;cytoplasmic part;cytoplasmic vesicle part;cytosol;Golgi apparatus part;intracellular organelle part;intracellular part;macromolecular complex;membrane coat;membrane part;organelle part;protein complex;vesicle coat		Homo sapiens	1	6
cell part;cytoplasmic part;cytosol;extracellular membrane-bounded organelle;extracellular organelle;extracellular region part;extracellular vesicular exosome;intracellular organelle part;intracellular part;macromolecular complex;membrane part;membrane-bounded organelle;membrane-bounded vesicle;nuclear part;nuclear pore;organelle;org		Homo sapiens	1	6
catalytic step 2 spliceosome;cell part;cytoplasmic part;cytosol;intracellular membrane-bounded organelle;intracellular organelle;intracellular organelle part;intracellular part;macromolecular complex;membrane-bounded orgar	RNA degradation;Spliceosome	Homo sapiens	1	6
cell part;cytoplasmic mRNA processing body;cytoplasmic part;cytosol;extracellular membrane-bounded organelle;extracellular organelle;extracellular region part;extracellular vesicular exosome;intracellular non-membrane-b	RNA degradation;Spliceosome	Homo sapiens	1	6
adherens junction;anchoring junction;cell junction;cell part;cell-substrate adherens junction;cell-substrate junction;cytoplasmic part;cytosol;cytosolic large ribosomal subunit;eukaryotic 80S initiation complex;focal adhesion;ir	Ribosome	Homo sapiens	1	6
cell part;cytoplasm;cytoplasmic part;cytosol;extracellular membrane-bounded organelle;extracellular organelle;extracellular region part;extracellular vesicular exosome;intracellular membrane-bounded organelle;intracellular c	Adherens junction;Circadian rhythm - plant;Measles;Ribosome biogenesis in eukaryotes;Tight junction;Wnt signaling	Homo sapiens	1	6
axon part;cation channel complex;cell part;cell projection part;integral to membrane;integral to plasma membrane;intrinsic to membrane;intrinsic to plasma membrane;ion channel complex;macromolecular complex;membrar	Cell adhesion molecules (CAMs)	Homo sapiens	1	6
cell part;cytoplasm;cytoplasmic part;intracellular membrane-bounded organelle;intracellular organelle;intracellular part;membrane-bounded organelle;mitochondrion;organelle		Homo sapiens	1	6
actin cytoskeleton;adherens junction;anchoring junction;cell junction;cell part;cell-substrate adherens junction;cell-substrate junction;cytoplasm;cytoplasmic part;cytoskeleton;focal adhesion;Golgi apparatus;intracellular membrane-bounded organelle;intracellular non-membrane-bounded organelle;intracellular organelle;intracellular part;mei		Homo sapiens	1	6
cell part;cytoplasm;intracellular membrane-bounded organelle;intracellular organelle;intracellular organelle part;intracellular part;macromolecular complex;membrane-bounded organelle;nuclear body;nuclear part;nuclear speck;nucleoplasm;nucleoplasm part;nucleus;organelle;organelle part;ribonucleoprotein complex;spliceosomal complex		Homo sapiens	1	6
cell body;cell part;cytoplasmic part;integral to membrane;intracellular membrane-bounded organelle;intracellular organelle;intracellular organelle part;intracellular part;intrinsic to membrane;intrinsic to mitochondrial inner men	Pyrimidine metabolism	Homo sapiens	1	6
cell cortex part;cell part;cytoplasmic part;exocyst;extracellular region part;extracellular space;intracellular part;macromolecular complex;protein complex		Homo sapiens	1	6
cell part;cytoplasmic part;endoplasmic reticulum membrane;endoplasmic reticulum part;integral to endoplasmic reticulum membrane;integral to membrane;integral to organelle membrane;intracellular membrane-bounded or	ABC transporters;Antigen processing and presentation;Phagosome;Primary immunodeficiency	Homo sapiens	1	6
cell part;cytoplasm;cytoplasmic part;cytosol;intracellular membrane-bounded organelle;intracellular organelle;intracellular organelle lumen;intracellular organelle part;intracellular part;membrane;membrane-bounded organelle	Folate biosynthesis	Homo sapiens	1	6
cell part;cell surface;cytoplasm;cytoplasmic part;cytosol;extracellular region part;extracellular space;intracellular membrane-bounded organelle;intracellular non-membrane-bounded organelle;intracellular organelle;intracellular organelle lumen;intracellular organelle part;intracellular part;membrane;membrane-bounded organelle;membrane-e		Homo sapiens	1	6
cell part;intracellular membrane-bounded organelle;intracellular organelle;intracellular part;membrane-bounded organelle;nucleus;organelle		Homo sapiens	1	6
basolateral plasma membrane;cell part;cell projection;cell projection part;cell surface;clathrin-coated vesicle;coated vesicle;cytoplasmic membrane-bounded vesicle;cytoplasmic part;cytoplasmic vesicle;cytosol;dendritic spi	SNARE interactions in vesicular transport;Vasopressin-regulated water reabsorption	Homo sapiens	1	6
cell part;cytoplasm;intracellular organelle part;intracellular part;nuclear part;nucleoplasm;organelle part		Homo sapiens	1	6
cell part;cytoplasm;cytoplasmic part;cytosol;eukaryotic translation initiation factor 2B complex;intracellular part;macromolecular complex;membrane;plasma membrane;protein complex	RNA transport	Homo sapiens	1	6
cell part;intracellular membrane-bounded organelle;intracellular organelle;intracellular organelle part;intracellular part;membrane;membrane-bounded organelle;nuclear body;nuclear membrane;nuclear part;nuclear speck;nucleoplasm;nucleoplasm part;nucleus;organelle;organelle membrane;organelle part		Homo sapiens	1	6
cell part;chromosomal part;cytoplasmic part;delta DNA polymerase complex;DNA polymerase complex;intracellular membrane-bounded organelle;intracellular organelle;intracellular organelle part;intracellular part;macromole	Base excision repair;DNA replication;Homologous recombination;Mismatch repair;Nucleotide excision repair;Purine	Homo sapiens	1	6
cell part;cytoplasmic part;extracellular region;intracellular membrane-bounded organelle;intracellular organelle;intracellular part;lysosome;lytic vacuole;membrane;membrane-bounded organelle;mitochondrion;nucleus;organe	Aminobenzoate degradation;Bisphenol degradation	Homo sapiens	1	6
cell part;chromatin remodeling complex;histone methyltransferase complex;intracellular membrane-bounded organelle;intracellular non-membrane-bounded organelle;intracellular organelle;intracellular organelle part;intracellular part;macromolecular complex;membrane-bounded organelle;methyltransferase complex;MLL1 complex;non-me		Homo sapiens	1	6
cell part;cytoplasmic part;cytosol;intracellular part;membrane	Pancreatic cancer;Pathways in cancer	Homo sapiens	1	6
cell part;cytoplasm;cytoplasmic part;cytosol;intracellular non-membrane-bounded organelle;intracellular organelle;intracellular organelle part;intracellular part;membrane;non-membrane-bounded organelle;nuclear part;nucleolus;organelle;organelle part		Homo sapiens	1	6
cell part;intracellular membrane-bounded organelle;intracellular organelle;intracellular part;membrane-bounded organelle;nucleus;organelle		Homo sapiens	1	6
cell part;cytoplasm;cytoplasmic part;cytosol;Golgi apparatus;intracellular membrane-bounded organelle;intracellular organelle;intracellular part;membrane;membrane-bounded organelle;organelle	Ubiquitin mediated proteolysis	Homo sapiens	1	6
activin responsive factor complex;cell part;chromatin;chromosomal part;cytoplasm;cytoplasmic part;cytosol;intracellular membrane-bounded organelle;intracellular organelle;intracellular organelle part;intracellular part;macro	Adherens junction;Cell cycle;Chagas disease (American trypanosomiasis);Colorectal cancer;Endocytosis;Pancreatic	Homo sapiens	1	6
cell part;intracellular non-membrane-bounded organelle;intracellular organelle;intracellular organelle part;intracellular part;non-membrane-bounded organelle;nuclear part;nucleolus;organelle;organelle part		Homo sapiens	1	6
cell part;cytoplasmic part;endoplasmic reticulum membrane;endoplasmic reticulum part;extracellular matrix;extracellular region part;integral to membrane;intracellular organelle part;intracellular part;intrinsic to membrane;me	Biosynthesis of unsaturated fatty acids;Steroid hormone biosynthesis	Homo sapiens	1	6
cell part;cytoplasmic part;intracellular non-membrane-bounded organelle;intracellular organelle;intracellular organelle part;intracellular part;macromolecular complex;membrane;mitochondrial inner membrane;mitochondrial membrane;mitochondrial part;non-membrane-bounded organelle;organelle;organelle inner membrane;organelle memb		Homo sapiens	1	6
actin cytoskeleton;cell part;cell projection part;centriole;centrosome;ciliary rootlet;cilium part;cytoplasm;cytoplasmic part;cytoskeletal part;cytoskeleton;extracellular membrane-bounded organelle;extracellular organelle;extracellular region part;extracellular vesicular exosome;intracellular non-membrane-bounded organelle;intracellular organ		Homo sapiens	1	6
adherens junction;anchoring junction;cell junction;cell part;cell-substrate adherens junction;cell-substrate junction;focal adhesion;integral to membrane;intrinsic to membrane;membrane part		Homo sapiens	1	6
cell part;cell surface;cytoplasmic part;extracellular membrane-bounded organelle;extracellular organelle;extracellular region part;extracellular space;extracellular vesicular exosome;integral to membrane;intracellular membrane-bounded organelle;intracellular organelle;intracellular organelle part;intracellular part;intrinsic to membrane;lysosor		Homo sapiens	1	6
cell part;cytoplasmic part;endoplasmic reticulum;extracellular region part;extracellular space;Golgi apparatus;intracellular membrane-bounded organelle;intracellular organelle;intracellular part;membrane-bounded organelle;organelle		Homo sapiens	1	6
cell part;cytoplasmic part;integral to membrane;intracellular organelle part;intracellular part;intrinsic to membrane;membrane;membrane part;mitochondrial inner membrane;mitochondrial membrane;mitochondrial part;organelle inner membrane;organelle membrane;organelle part		Homo sapiens	1	6
cell part;cytoplasmic part;endoplasmic reticulum;endoplasmic reticulum membrane;endoplasmic reticulum part;integral to membrane;intracellular membrane-bounded organelle;intracellular organelle;intracellular organelle pa	Biosynthesis of unsaturated fatty acids	Homo sapiens	1	6
cell part;cytoplasmic part;endoplasmic reticulum;endoplasmic reticulum membrane;endoplasmic reticulum part;Golgi apparatus part;Golgi membrane;Golgi stack;integral to membrane;intracellular membrane-bounded organ	Ether lipid metabolism;Glycerophospholipid metabolism	Homo sapiens	1	6
cell part;cytoplasmic part;endoplasmic reticulum lumen;endoplasmic reticulum part;intracellular organelle lumen;intracellular organelle part;intracellular part;membrane-enclosed lumen;organelle lumen;organelle part		Homo sapiens	1	6
		Homo sapiens	1	6
		Homo sapiens	1	6
cell part;cytoplasm;histone pre-mRNA 3'end processing complex;intracellular membrane-bounded organelle;intracellular non-membrane-bounded organelle;intracellular organelle;intracellular organelle part;intracellular part;macromolecular complex;membrane-bounded organelle;non-membrane-bounded organelle;nuclear part;nucleolus;nu		Homo sapiens	1	6
cell part;intracellular organelle part;intracellular part;macromolecular complex;membrane;NELF complex;nuclear part;nucleoplasm;nucleoplasm part;organelle part;protein complex;transcription elongation factor complex		Homo sapiens	1	6
		Homo sapiens	1	6
cell part;cytoplasm;intracellular membrane-bounded organelle;intracellular organelle;intracellular part;membrane-bounded organelle;nucleus;organelle		Homo sapiens	1	6
		Homo sapiens	1	6
cell part;intracellular membrane-bounded organelle;intracellular non-membrane-bounded organelle;intracellular organelle;intracellular organelle part;intracellular part;membrane-bounded organelle;non-membrane-bounded organelle;nuclear part;nucleolus;nucleus;organelle;organelle part		Homo sapiens	1	6
cell part;cell surface;cytoplasmic part;endoplasmic reticulum;endoplasmic reticulum part;integral to cytosolic side of endoplasmic reticulum membrane;integral to endoplasmic reticulum membrane;integral to lumenal side of endoplasmic reticulum membrane;integral to membrane;integral to organelle membrane;intracellular membrane-bou		Homo sapiens	1	6
apical plasma membrane;cell part;cytoplasmic membrane-bounded vesicle;cytoplasmic part;cytoplasmic vesicle;cytosol;endocytic vesicle;intracellular membrane-bounded organelle;intracellular organelle;intracellular part;membrane;membrane part;membrane-bounded organelle;membrane-bounded vesicle;organelle;plasma membrane;pla		Homo sapiens	1	6
adherens junction;anchoring junction;cell junction;cell part;cell-substrate adherens junction;cell-substrate junction;cytoplasmic membrane-bounded vesicle;cytoplasmic part;cytoplasmic vesicle;endoplasmic reticulum;extrac	Alzheimer's disease;Notch signaling pathway	Homo sapiens	1	6
cell part;centrosome;chromatin;chromosomal part;cytoplasm;cytoplasmic part;cytoskeletal part;intracellular membrane-bounded organelle;intracellular non-membrane-bounded organelle;intracellular organelle;intracellular organelle part;intracellular part;membrane-bounded organelle;microtubule organizing center;non-membrane-bounded (		Homo sapiens	1	6

cell part;intracellular non-membrane-bounded organelle;intracellular organelle;intracellular organelle part;intracellular part;macromolecular complex;non-membrane-bounded organelle;nuclear part;nucleolar part;nucleolus;org	Ribosome biogenesis in eukaryotes	Homo sapiens	1	6
cell part;coated pit;membrane;membrane part;plasma membrane		Homo sapiens	1	6
cell part;cytoplasmic part;integral to membrane;intracellular membrane-bounded organelle;intracellular organelle;intracellular organelle part;intracellular part;intrinsic to membrane;membrane part;membrane-bounded organelle;mitochondrion;nuclear part;nucleoplasm;organelle;organelle part		Homo sapiens	1	6
		Homo sapiens	1	6
cell cortex part;cell part;cytoplasmic part;cytosol;exocyst;intracellular membrane-bounded organelle;intracellular organelle;intracellular part;macromolecular complex;membrane;membrane-bounded organelle;organelle;plasma membrane;protein complex		Homo sapiens	1	6
cell part;intracellular membrane-bounded organelle;intracellular organelle;intracellular part;membrane-bounded organelle;nucleus;organelle	Spliceosome	Homo sapiens	1	6
cell cortex;cell part;coated pit;cytoplasmic membrane-bounded vesicle;cytoplasmic part;cytoplasmic vesicle;cytoskeleton;intracellular membrane-bounded organelle;intracellular non-membrane-bounded organelle;intracellular organelle;intracellular part;lysosome;lytic vacuole;membrane;membrane part;membrane-bounded organelle;memb		Homo sapiens	1	6
cell part;cytoplasmic part;cytosol;EKC/KEOPS complex;intracellular membrane-bounded organelle;intracellular organelle;intracellular part;macromolecular complex;membrane;membrane-bounded organelle;nucleus;organelle;protein complex		Homo sapiens	1	6
cell part;cytoplasm;cytoplasmic part;endosomal part;endosome membrane;Golgi apparatus;intracellular membrane-bounded organelle;intracellular organelle;intracellular organelle part;intracellular part;late endosome membrane;membrane;membrane-bounded organelle;organelle;organelle membrane;organelle part		Homo sapiens	1	6
		Homo sapiens	1	6
aggresome;cell part;cytoplasmic part;cytoskeleton;cytosol;endoplasmic reticulum;endoplasmic reticulum membrane;endoplasmic reticulum part;inclusion body;integral to membrane;intermediate filament cytoskeleton;intrac	Protein export;Protein processing in endoplasmic reticulum	Homo sapiens	1	6
cell part;chromatin;chromosomal part;euchromatin;heterochromatin;histone methyltransferase complex;intracellular membrane-bounded organelle;intracellular organelle;intracellular organelle part;intracellular part;macromolecular complex;membrane-bounded organelle;methyltransferase complex;MLL1 complex;nuclear body;nuclear chrom		Homo sapiens	1	6
cell part;cytoplasmic part;cytosol;endoplasmic reticulum;extracellular region;intracellular membrane-bounded organelle;intracellular organelle;intracellular part;lipid particle;membrane;membrane-bounded organelle;nucleus;organelle;plasma membrane		Homo sapiens	1	6
cell part;intracellular organelle part;intracellular part;macromolecular complex;nuclear part;nucleoplasm;nucleoplasm part;organelle part;protein complex;transcription factor complex		Homo sapiens	0.9999	6
cell part;cytoplasmic part;endoplasmic reticulum;endoplasmic reticulum membrane;endoplasmic reticulum part;integral to membrane;intracellular membrane-bounded organelle;intracellular organelle;intracellular organelle pa	Fat digestion and absorption;Glycerolipid metabolism;Glycerophospholipid metabolism	Homo sapiens	1	6
		Homo sapiens	1	6
cell part;cytoplasmic part;extracellular membrane-bounded organelle;extracellular organelle;extracellular region part;extracellular vesicular exosome;intracellular membrane-bounded organelle;intracellular organelle;intracellula	Arginine and proline metabolism	Homo sapiens	1	6
cell part;cytoplasmic part;endoplasmic reticulum lumen;endoplasmic reticulum part;intracellular organelle lumen;intracellular organelle part;intracellular part;membrane-enclosed lumen;organelle lumen;organelle part		Homo sapiens	1	6
cell part;cytoplasmic part;extracellular membrane-bounded organelle;extracellular organelle;extracellular region part;extracellular space;extracellular vesicular exosome;intracellular membrane-bounded organelle;intracellular (	Other glycan degradation	Homo sapiens	1	6
		Homo sapiens	1	6
cell part;cytoplasm;intracellular membrane-bounded organelle;intracellular organelle;intracellular organelle part;intracellular part;membrane-bounded organelle;nuclear part;nucleoplasm;nucleus;organelle;organelle part		Homo sapiens	1	6
cell part;cytoplasmic part;integral to membrane;intracellular membrane-bounded organelle;intracellular non-membrane-bounded organelle;intracellular organelle;intracellular organelle part;intracellular part;intrinsic to membrane;macromolecular complex;membrane;membrane part;membrane-bounded organelle;Noc complex;Noc4p-Nop14p		Homo sapiens	1	6
cell part;cytoplasmic part;cytosolic part;cytosolic ribosome;intracellular non-membrane-bounded organelle;intracellular organelle;intracellular organelle part;intracellular part;macromolecular complex;membrane;mitochondrial inner membrane;mitochondrial membrane;mitochondrial part;non-membrane-bounded organelle;organelle;organelle		Homo sapiens	1	6
cell part;chromosome;condensed chromosome;condensed nuclear chromosome;cytoplasm;intracellular membrane-bounded organelle;intracellular non-membrane-bounded organelle;intracellular organelle;intracellular organelle part;intracellular part;membrane-bounded organelle;non-membrane-bounded organelle;nuclear chromosome;nu		Homo sapiens	1	6
cell part;intracellular organelle part;intracellular part;membrane;nuclear part;nucleoplasm;organelle part		Homo sapiens	1	6
cell part;cytoplasmic membrane-bounded vesicle;cytoplasmic part;cytoplasmic vesicle;cytoplasmic vesicle membrane;cytoplasmic vesicle part;endocytic vesicle;endocytic vesicle membrane;extracellular membrane-bounded organelle;extracellular organelle;extracellular region part;extracellular vesicular exosome;Golgi apparatus;Golgi app		Homo sapiens	1	6
cell part;cytoskeletal part;intracellular membrane-bounded organelle;intracellular organelle;intracellular organelle part;intracellular part;macromolecular complex;membrane-bounded organelle;microtubule;nuclear part;nucleop	Wnt signaling pathway	Homo sapiens	1	6
cell part;cytoplasm;intracellular membrane-bounded organelle;intracellular organelle;intracellular part;membrane-bounded organelle;nucleus;organelle		Homo sapiens	1	6
cell part;cytoplasm;cytoplasmic part;Golgi apparatus;intracellular membrane-bounded organelle;intracellular organelle;intracellular organelle part;intracellular part;membrane;membrane-bounded organelle;nuclear part;nucleoplasm;nucleus;organelle;organelle part;plasma membrane		Homo sapiens	1	6
cell part;cytoplasmic part;integral to membrane;intracellular membrane-bounded organelle;intracellular organelle;intracellular organelle part;intracellular part;intrinsic to membrane;membrane;membrane part;membrane-bounded organelle;mitochondrial inner membrane;mitochondrial membrane;mitochondrial part;mitochondrion;organelle;or		Homo sapiens	1	6
cell part;cytoplasmic part;endoplasmic reticulum;endoplasmic reticulum membrane;endoplasmic reticulum part;integral to membrane;intracellular membrane-bounded organelle;intracellular non-membrane-bounded organelle;intracellular organelle;intracellular organelle part;intracellular part;intrinsic to membrane;membrane;membrane part;		Homo sapiens	1	6
cell part;cytoplasm;intracellular organelle part;intracellular part;macromolecular complex;NELF complex;nuclear part;nucleoplasm;nucleoplasm part;organelle part;protein complex;transcription elongation factor complex		Homo sapiens	1	6
basement membrane;extracellular matrix part;extracellular region part		Homo sapiens	1	6
cell part;extracellular region part;intercellular bridge;intracellular non-membrane-bounded organelle;intracellular organelle;intracellular organelle part;intracellular part;non-membrane-bounded organelle;nuclear body;nuclear part;nuclear speck;nucleolus;nucleoplasm;nucleoplasm part;organelle;organelle part		Homo sapiens	1	6
cell part;cytoplasmic part;cytosol;extracellular membrane-bounded organelle;extracellular organelle;extracellular region part;extracellular vesicular exosome;intracellular part;membrane-bounded organelle;membrane-bounde	Cell cycle - yeast	Homo sapiens	0.9999	6
cell part;cell projection;centrosome;cilium;cytoplasm;cytoplasmic dynein complex;cytoplasmic part;cytoskeletal part;dynein complex;intracellular non-membrane-bounded organelle;intracellular organelle;intracellular organelle part;intracellular part;macromolecular complex;membrane;microtubule;microtubule associated complex;microtubul		Homo sapiens	1	6
cell part;ER membrane protein complex;integral to membrane;intrinsic to membrane;macromolecular complex;membrane part;protein complex		Homo sapiens	1	6
cell part;chromatin;chromosomal part;cytoplasm;cytoplasmic exosome (RNase complex);cytoplasmic part;cytosol;exosome (RNase complex);intracellular membrane-bounded organelle;intracellular non-membrane-bounded	RNA degradation	Homo sapiens	1	6
cell part;chromatin;chromosomal part;cytoplasm;cytoplasmic exosome (RNase complex);cytoplasmic part;cytosol;exosome (RNase complex);intracellular membrane-bounded organelle;intracellular non-membrane-bounded	RNA degradation	Homo sapiens	1	6
cell part;centrosome;cytoplasm;cytoplasmic part;cytoskeletal part;envelope;intracellular membrane-bounded organelle;intracellular non-membrane-bounded organelle;intracellular organelle;intracellular organelle part;intracell	RNA transport	Homo sapiens	1	6
cell part;cytoplasmic part;intracellular organelle part;intracellular part;macromolecular complex;membrane;mitochondrial inner membrane;mitochondrial membrane;mitochondrial part;mitochondrial small ribosomal subunit;organellar small ribosomal subunit;organelle inner membrane;organelle membrane;organelle part;ribonucleoprotein con		Homo sapiens	1	6
cell part;cytoplasmic part;intracellular membrane-bounded organelle;intracellular organelle;intracellular part;membrane-bounded organelle;mitochondrion;organelle		Homo sapiens	1	6
Cajal body;cell part;cytoplasm;intracellular non-membrane-bounded organelle;intracellular organelle;intracellular organelle part;intracellular part;macromolecular complex;non-membrane-bounded organelle;nuclear body;nucl	Ribosome biogenesis in eukaryotes	Homo sapiens	1	6
cell part;cytoplasmic part;intracellular organelle lumen;intracellular organelle part;intracellular part;membrane-enclosed lumen;mitochondrial matrix;mitochondrial part;nuclear part;nucleoplasm;organelle lumen;organelle part		Homo sapiens	1	6
cell part;cytoplasmic part;Golgi apparatus;integral to membrane;intracellular membrane-bounded organelle;intracellular organelle;intracellular part;intrinsic to membrane;membrane;membrane part;membrane-bounded organelle;organelle		Homo sapiens	1	6
box H/ACA RNP complex;box H/ACA snoRNP complex;Cajal body;cell part;intracellular membrane-bounded organelle;intracellular non-membrane-bounded organelle;intracellular organelle;intracellular organelle part;intracell	Ribosome biogenesis in eukaryotes	Homo sapiens	1	6
cell part;cytoplasmic part;intracellular membrane-bounded organelle;intracellular organelle;intracellular part;membrane-bounded organelle;mitochondrion;organelle		Homo sapiens	1	6
cell part;chromosomal part;intracellular organelle part;intracellular part;macromolecular complex;nuclear chromosome part;nuclear origin of replication recognition complex;nuclear part;nucleoplasm;organelle part;origin recog	Cell cycle;Cell cycle - yeast;Meiosis - yeast	Homo sapiens	1	6
cell part;cytoplasmic part;Golgi apparatus;integral to membrane;integral to plasma membrane;intracellular membrane-bounded organelle;intracellular organelle;intracellular part;intrinsic to membrane;intrinsic to plasma membrane;membrane;membrane part;membrane-bounded organelle;organelle;plasma membrane;plasma membrane part		Homo sapiens	1	6
cell part;chromatin;chromatin remodeling complex;chromosomal part;chromosome, centromeric region;cytoplasm;cytoplasmic part;cytosol;heterochromatin;intracellular membrane-bounded organelle;intracellular non-membr	Amyotrophic lateral sclerosis (ALS);MAPK signaling pathway	Homo sapiens	1	6
cell part;cytoplasmic part;integral to membrane;integral to mitochondrial membrane;integral to mitochondrial outer membrane;integral to organelle membrane;intracellular organelle part;intracellular part;intrinsic to membrane;intrinsic to mitochondrial outer membrane;intrinsic to organelle membrane;membrane part;mitochondrial membrane		Homo sapiens	1	6
cell part;cytoplasm;intracellular membrane-bounded organelle;intracellular organelle;intracellular part;membrane;membrane-bounded organelle;nucleus;organelle;plasma membrane	Protein processing in endoplasmic reticulum	Homo sapiens	1	6
cell part;cytoplasmic part;cytosol;extracellular membrane-bounded organelle;extracellular organelle;extracellular region part;extracellular vesicular exosome;intracellular membrane-bounded organelle;intracellular organelle;in	Cysteine and methionine metabolism	Homo sapiens	1	6
cell part;cytoplasm;intracellular membrane-bounded organelle;intracellular organelle;intracellular part;membrane-bounded organelle;nucleus;organelle		Homo sapiens	1	6
extracellular membrane-bounded organelle;extracellular organelle;extracellular region part;extracellular vesicular exosome;macromolecular complex;membrane-bounded organelle;membrane-bounded vesicle;organelle;protein complex;vesicle		Homo sapiens	1	6
cell part;cytoplasm;extrinsic to internal side of plasma membrane;extrinsic to membrane;extrinsic to plasma membrane;intracellular membrane-bounded organelle;intracellular organelle;intracellular part;membrane part;membrane-bounded organelle;nucleus;organelle;plasma membrane part		Homo sapiens	1	6
actin cytoskeleton;cell part;cell projection;cytoplasmic membrane-bounded vesicle;cytoplasmic part;cytoplasmic vesicle;cytoplasmic vesicle membrane;cytoplasmic vesicle part;cytoskeleton;early endosome;endocytic vesicl	Endocytosis	Homo sapiens	1	6
cell part;intracellular organelle part;intracellular part;nuclear part;nucleoplasm;organelle part		Homo sapiens	0.9999	6
adherens junction;anchoring junction;cell junction;cell part;cell surface;cell-substrate adherens junction;cell-substrate junction;centrosome;cytoplasmic part;cytoskeletal part;extracellular membrane-bounded organelle;extracellular organelle;extracellular region;extracellular region part;extracellular vesicular exosome;focal adhesion;integral t		Homo sapiens	1	6
cell part;intracellular membrane-bounded organelle;intracellular organelle;intracellular organelle part;intracellular part;membrane-bounded organelle;nuclear part;nucleoplasm;nucleus;organelle;organelle part		Homo sapiens	1	6
cell part;intracellular membrane-bounded organelle;intracellular organelle;intracellular organelle part;intracellular part;membrane-bounded organelle;nuclear part;nucleoplasm;nucleus;organelle;organelle part		Homo sapiens	1	6
cell part;cytoplasmic part;endoplasmic reticulum membrane;endoplasmic reticulum part;endoplasmic reticulum-Golgi intermediate compartment membrane;Golgi apparatus;integral to membrane;intracellular membrane-bounded organelle;intracellular organelle;intracellular organelle part;intracellular part;intrinsic to membrane;membrane;me		Homo sapiens	1	6
cell part;cytoplasm;cytoplasmic part;cytosol;exosome (RNase complex);intracellular membrane-bounded organelle;intracellular non-membrane-bounded organelle;intracellular organelle;intracellular organelle part;intracellular	RNA degradation	Homo sapiens	1	6
cell part;cytoplasmic mRNA processing body;cytoplasmic part;intracellular non-membrane-bounded organelle;intracellular organelle;intracellular part;macromolecular complex;non-membrane-bounded organelle;organelle;ribonucleoprotein complex;RNA granule		Homo sapiens	1	6
cell part;cytoplasmic part;intracellular membrane-bounded organelle;intracellular organelle;intracellular organelle part;intracellular part;macromolecular complex;membrane;membrane-bounded organelle;membrane-enclosed lumen;mitochondrial inner membrane;mitochondrial intermembrane space;mitochondrial intermembrane space prot		Homo sapiens	1	6
cell part;intracellular organelle part;intracellular part;macromolecular complex;nuclear part;nucleoplasm;nucleoplasm part;organelle part;protein complex;transcription factor complex;transcription factor TFIIIC complex		Homo sapiens	1	6
cell part;cytoplasmic part;intracellular membrane-bounded organelle;intracellular organelle;intracellular part;membrane-bounded organelle;mitochondrion;nucleus;organelle		Homo sapiens	1	6
axolemma;axon part;cell part;cell projection membrane;cell projection part;cell surface;cytoplasm;integral to membrane;integral to plasma membrane;intracellular part;intrinsic to membrane;intrinsic to plasma membrane;leac	Axon guidance	Homo sapiens	1	6
adherens junction;anchoring junction;azurophil granule;cell junction;cell part;cell projection;cell-substrate adherens junction;cell-substrate junction;cytoplasm;cytoplasmic membrane-bounded vesicle;cytoplasmic part;cytopla	SNARE interactions in vesicular transport	Homo sapiens	1	7
cell part;cytoplasmic part;intracellular membrane-bounded organelle;intracellular non-membrane-bounded organelle;intracellular organelle;intracellular organelle lumen;intracellular organelle part;intracellular part;membrane-bounded organelle;membrane-enclosed lumen;mitochondrial matrix;mitochondrial nucleoid;mitochondrial part;mitoch		Homo sapiens	1	7
cell part;chromosome;intracellular non-membrane-bounded organelle;intracellular organelle;intracellular organelle part;intracellular part;macromolecular complex;Mpp10 complex;non-membrane-bounded organelle;nuclear p	Ribosome biogenesis in eukaryotes	Homo sapiens	1	7
cell part;cytoplasmic part;endoplasmic reticulum;endoplasmic reticulum membrane;endoplasmic reticulum part;envelope;integral to membrane;intracellular membrane-bounded organelle;intracellular organelle;intracellular organelle part;intracellular part;intrinsic to membrane;membrane;membrane part;membrane-bounded organelle;nuclear		Homo sapiens	1	7
cell part;cytoplasm;cytoplasmic part;ER membrane protein complex;intracellular membrane-bounded organelle;intracellular organelle;intracellular part;macromolecular complex;membrane;membrane-bounded organelle;mitochondrion;nucleus;organelle;protein complex		Homo sapiens	1	7
cell part;cytoplasm;intracellular organelle part;intracellular part;nuclear part;nucleoplasm;organelle part		Homo sapiens	1	7
cell part;cytoplasm;cytoplasmic part;cytosol;intracellular membrane-bounded organelle;intracellular organelle;intracellular organelle part;intracellular part;macromolecular complex;membrane;membrane part;membrane-bounded organelle;nuclear membrane;nuclear part;nuclear pore;nucleoplasm;nucleus;organelle;organelle membrane;orga		Homo sapiens	1	7
cell part;cytoplasm;intracellular membrane-bounded organelle;intracellular organelle;intracellular part;membrane-bounded organelle;nucleus;organelle		Homo sapiens	1	7
cell part;cytoplasmic part;dolichol-phosphate-mannose synthase complex;endoplasmic reticulum;endoplasmic reticulum membrane;endoplasmic reticulum part;intracellular membrane-bounded organelle;intracellular organel	N-Glycan biosynthesis	Homo sapiens	1	7
cell part;cytoplasmic part;endosome;intracellular membrane-bounded organelle;intracellular organelle;intracellular part;membrane-bounded organelle;organelle		Homo sapiens	1	7
cell part;cytoplasm;cytoplasmic part;cytosol;intracellular organelle part;intracellular part;membrane;nuclear part;nucleoplasm;organelle part;perinuclear region of cytoplasm		Homo sapiens	1	7
cell part;cytoplasmic part;cytosol;extracellular membrane-bounded organelle;extracellular organelle;extracellular region part;extracellular vesicular exosome;intracellular membrane-bounded organelle;intracellular organelle;in	Glycerophospholipid metabolism	Homo sapiens	1	7
cell part;cytoplasm;cytoplasmic part;cytoskeleton;cytosol;intermediate filament cytoskeleton;intracellular membrane-bounded organelle;intracellular non-membrane-bounded organelle;intracellular organelle;intracellular organelle part;intracellular part;macromolecular complex;membrane-bounded organelle;non-membrane-bounded organell		Homo sapiens	1	7
cell part;intracellular membrane-bounded organelle;intracellular organelle;intracellular part;membrane-bounded organelle;nucleus;organelle		Homo sapiens	1	7
cell part;cytoplasm;cytoplasmic part;extracellular membrane-bounded organelle;extracellular organelle;extracellular region part;extracellular vesicular exosome;intracellular;intracellular membrane-bounded organelle;intracellular organelle;intracellular part;membrane-bounded organelle;membrane-bounded vesicle;mitochondrion;organelle;ve		Homo sapiens	1	7
cell part;cytoplasmic part;cytoskeletal part;endoplasmic reticulum membrane;endoplasmic reticulum part;endoplasmic reticulum-Golgi intermediate compartment;Golgi apparatus;Golgi apparatus part;Golgi membrane;integral to membrane;intracellular membrane-bounded organelle;intracellular organelle;intracellular organelle part;intracellu		Homo sapiens	1	7
cell part;cytoplasmic part;Golgi apparatus;integral to membrane;intracellular membrane-bounded organelle;intracellular organelle;intracellular part;intrinsic to membrane;membrane part;membrane-bounded organelle;nucleus;organelle		Homo sapiens	1	7
cell part;cytoplasmic part;extracellular membrane-bounded organelle;extracellular organelle;extracellular region part;extracellular vesicular exosome;integral to membrane;intracellular membrane-bounded organelle;intracellula	Alzheimer's disease;Huntington's disease;Oxidative phosphorylation;Parkinson's disease	Homo sapiens	1	7
cell part;cytoplasm;cytoplasmic part;endoplasmic reticulum;intracellular membrane-bounded organelle;intracellular organelle;intracellular part;membrane-bounded organelle;nucleus;organelle		Homo sapiens	1	7
cell part;cytoplasmic part;endoplasmic reticulum lumen;endoplasmic reticulum part;intracellular organelle lumen;intracellular organelle part;intracellular part;membrane-enclosed lumen;organelle lumen;organelle part	Glutathione metabolism	Homo sapiens	1	7
cell part;cell projection;cytoplasm;cytoplasmic part;cytosol;Golgi apparatus;intracellular membrane-bounded organelle;intracellular organelle;intracellular organelle part;intracellular part;membrane;membrane-bounded organe	Acute myeloid leukemia;B cell receptor signaling pathway;Bladder cancer;Chemokine signaling pathway;Chronic my	Homo sapiens	1	7
cell part;cytoplasmic part;cytosol;intracellular part	Drug metabolism - other enzymes;Pyrimidine metabolism	Homo sapiens	1	7
cell part;cytoplasm;cytoplasmic part;cytosol;intracellular membrane-bounded organelle;intracellular non-membrane-bounded organelle;intracellular organelle;intracellular organelle lumen;intracellular organelle part;intracellula	One carbon pool by folate;Pyrimidine metabolism	Homo sapiens	1	7
cell part;cytoplasm;extracellular membrane-bounded organelle;extracellular organelle;extracellular region part;extracellular vesicular exosome;intracellular membrane-bounded organelle;intracellular organelle;intracellular organelle part;intracellular part;membrane-bounded organelle;membrane-bounded vesicle;nuclear part;nucleoplasm;nuc		Homo sapiens	1	7
cell part;cytoplasmic part;cytosol;intracellular part	Porphyrin and chlorophyll metabolism	Homo sapiens	1	7
Cajal body;cell part;commitment complex;intracellular organelle part;intracellular part;macromolecular complex;nuclear body;nuclear part;nucleoplasm;nucleoplasm part;organelle part;prespliceosome;ribonucleoprotein comp	Spliceosome	Homo sapiens	1	7
cell part;cytoplasmic part;cytosol;intracellular part	Sulfur metabolism	Homo sapiens	0.9981	7
	Acute myeloid leukemia;Bladder cancer;Chronic myeloid leukemia;Colorectal cancer;Endometrial cancer;ErbB signa	Homo sapiens	1	7
cell part;cytoplasmic part;extracellular membrane-bounded organelle;extracellular organelle;extracellular region part;extracellular vesicular exosome;intracellular membrane-bounded organelle;intracellular organelle;intracellula	Alzheimer's disease;Cardiac muscle contraction;Huntington's disease;Oxidative phosphorylation;Parkinson's diseas	Homo sapiens	1	7
anchored to membrane;cell part;cell surface;cytoplasmic part;endoplasmic reticulum;extracellular region;extracellular region part;extracellular space;intracellular membrane-bounded organelle;intracellular organelle;intracellula	Complement and coagulation cascades	Homo sapiens	1	7
cell part;cytoplasmic part;extracellular membrane-bounded organelle;extracellular organelle;extracellular region part;extracellular vesicular exosome;integral to membrane;intracellular membrane-bounded organelle;intracellula	Riboflavin metabolism	Homo sapiens	1	7
cell part;extracellular membrane-bounded organelle;extracellular organelle;extracellular region part;extracellular vesicular exosome;integral to membrane;integral to plasma membrane;intrinsic to membrane;intrinsic to plasma membrane;membrane;membrane part;membrane-bounded organelle;membrane-bounded vesicle;organelle;plasma		Homo sapiens	1	7
cell part;cytoplasmic part;intracellular membrane-bounded organelle;intracellular organelle;intracellular organelle part;intracellular part;membrane-bounded organelle;mitochondrion;nuclear part;nucleoplasm;nucleus;organelle	Base excision repair;Primary immunodeficiency	Homo sapiens	1	7
cell part;cytoplasmic part;cytoplasmic vesicle membrane;cytoplasmic vesicle part;endocytic vesicle membrane;endosomal part;endosome;endosome membrane;extracellular membrane-bounded organelle;extracellular orgar	ko05152;Lysosome;Phagosome	Homo sapiens	1	7
adherens junction;anchored to external side of plasma membrane;anchored to membrane;anchored to plasma membrane;anchoring junction;cell junction;cell part;cell surface;cell-substrate adherens junction;cell-substrate ju	Complement and coagulation cascades;Hematopoietic cell lineage	Homo sapiens	1	7
cell part;chromatin;chromosomal part;chromosome, centromeric region;condensed chromosome, centromeric region;cytoplasm;intracellular membrane-bounded organelle;intracellular organelle;intracellular organelle part;intr	ko05152	Homo sapiens	1	7
cell part;cytoplasmic part;endoplasmic reticulum;endoplasmic reticulum-Golgi intermediate compartment;intracellular membrane-bounded organelle;intracellular organelle;intracellular organelle part;intracellular part;membrane;membrane-bounded organelle;nuclear part;nucleoplasm;nucleus;organelle;organelle part;plasma membrane		Homo sapiens	1	7
adherens junction;anchoring junction;blood microparticle;cell junction;cell part;cell surface;cell-substrate adherens junction;cell-substrate junction;cytoplasm;endothelial microparticle;external side of plasma membrane;extracellular region part;extracellular space;focal adhesion;intracellular organelle part;intracellular part;macromolecular cor		Homo sapiens	1	7
cell part;extracellular membrane-bounded organelle;extracellular organelle;extracellular region;extracellular region part;extracellular space;extracellular vesicular exosome;insulin-like growth factor binding protein complex;intr	p53 signaling pathway	Homo sapiens	1	7
cell part;cytoplasmic part;intracellular membrane-bounded organelle;intracellular organelle;intracellular organelle part;intracellular part;macromolecular complex;membrane-bounded organelle;mitochondrion;NELF complex;nuclear part;nucleoplasm;nucleoplasm part;nucleus;organelle;organelle part;protein complex;transcription elongation f		Homo sapiens	1	7
cell part;cytoplasmic part;intracellular membrane-bounded organelle;intracellular organelle;intracellular organelle part;intracellular part;macromolecular complex;membrane;membrane part;membrane-bounded organelle;mitoc	Alzheimer's disease;Huntington's disease;Oxidative phosphorylation;Parkinson's disease	Homo sapiens	1	7
cell part;cytoplasm;cytoplasmic part;cytosol;intracellular membrane-bounded organelle;intracellular non-membrane-bounded organelle;intracellular organelle;intracellular part;macromolecular complex;membrane;membrane-	Hepatitis C;Measles;Protein processing in endoplasmic reticulum	Homo sapiens	1	7
cell part;cell projection;cilium;cytoplasm;cytoplasmic part;endoplasmic reticulum membrane;endoplasmic reticulum part;extracellular membrane-bounded organelle;extracellular organelle;extracellular region part;extracellular	Arachidonic acid metabolism	Homo sapiens	1	7
cell part;cytoplasm;intracellular membrane-bounded organelle;intracellular organelle;intracellular part;membrane-bounded organelle;nucleus;organelle		Homo sapiens	1	7
cell part;cytoplasmic part;extracellular membrane-bounded organelle;extracellular organelle;extracellular region part;extracellular vesicular exosome;intracellular membrane-bounded organelle;intracellular organelle;intracellula	Purine metabolism	Homo sapiens	1	7
cell part;cytoplasm;cytoplasmic part;cytosol;extracellular membrane-bounded organelle;extracellular organelle;extracellular region part;extracellular vesicular exosome;intracellular membrane-bounded organelle;intracellular c	Proteasome	Homo sapiens	1	7
cell part;cytoplasm;intracellular membrane-bounded organelle;intracellular organelle;intracellular organelle part;intracellular part;membrane;membrane-bounded organelle;nuclear part;nucleoplasm;nucleus;organelle;organelle part;plasma membrane		Homo sapiens	1	7
cell part;collagen;extracellular matrix;extracellular matrix part;extracellular region;extracellular region part;extracellular space;intracellular membrane-bounded organelle;intracellular organelle;intracellular part;membrane-bounded organelle;nucleus;organelle;proteinaceous extracellular matrix		Homo sapiens	1	7
cell part;cell surface;extracellular membrane-bounded organelle;extracellular organelle;extracellular region part;extracellular space;extracellular vesicular exosome;host cell part;host cell surface;integral to membrane;integral to plasma membrane;intrinsic to membrane;intrinsic to plasma membrane;membrane;membrane part;membrane-bo		Homo sapiens	1	7
cell part;integral to membrane;integral to plasma membrane;intrinsic to membrane;intrinsic to plasma membrane;membrane;membrane part;plasma membrane;plasma membrane part		Homo sapiens	1	7
axon part;cell part;cell projection part;cytoplasm;cytoskeleton;extracellular membrane-bounded organelle;extracellular organelle;extracellular region part;extracellular vesicular exosome;intracellular non-membrane-bounded (	Regulation of actin cytoskeleton;Shigellosis	Homo sapiens	1	7
cell part;cytoplasmic part;intracellular membrane-bounded organelle;intracellular organelle;intracellular organelle lumen;intracellular organelle part;intracellular part;membrane;membrane-bounded organelle;membrane-enclos	Butanoate metabolism;Geraniol degradation;Peroxisome;Synthesis and degradation of ketone bodies;Valine, leucin	Homo sapiens	1	7
cell part;cytoplasmic part;cytosol;intracellular membrane-bounded organelle;intracellular organelle;intracellular organelle part;intracellular part;membrane;membrane-bounded organelle;nuclear part;nucleoplasm;nucleus;orga	Alzheimer's disease;Amoebiasis;Amyotrophic lateral sclerosis (ALS);Apoptosis;Colorectal cancer;Epithelial cell signa	Homo sapiens	1	7
cell part;chromatin;chromatin remodeling complex;chromosomal part;heterochromatin;intracellular membrane-bounded organelle;intracellular organelle;intracellular organelle part;intracellular part;macromolecular complex;membrane-bounded organelle;nuclear body;nuclear chromatin;nuclear chromosome part;nuclear heterochromatin;nucl		Homo sapiens	1	7
cell part;intracellular organelle part;intracellular part;membrane;nuclear part;nucleoplasm;organelle part	DNA replication;Purine metabolism;Pyrimidine metabolism	Homo sapiens	1	7
apical plasma membrane;cell part;cell projection;cilium;cytoplasmic membrane-bounded vesicle;cytoplasmic part;cytoplasmic vesicle;cytoplasmic vesicle membrane;cytoplasmic vesicle part;dendrite;endosomal part;endosome;endosome membrane;exocytic vesicle;extracellular membrane-bounded organelle;extracellular organelle;extrace		Homo sapiens	1	7
cell part;cytoplasmic part;cytosol;extracellular membrane-bounded organelle;extracellular organelle;extracellular region part;extracellular vesicular exosome;intracellular part;membrane-bounded organelle;membrane-bounde	Drug metabolism - other enzymes	Homo sapiens	1	7
cell part;cyclin-dependent protein kinase activating kinase holoenzyme complex;holo TFIIH complex;intracellular membrane-bounded organelle;intracellular organelle;intracellular organelle part;intracellular part;macromolecul	Basal transcription factors;Cell cycle;Nucleotide excision repair	Homo sapiens	1	7
cell cortex;cell part;cell projection;cytoplasm;cytoplasmic part;Golgi apparatus part;Golgi membrane;intracellular organelle part;intracellular part;membrane;organelle membrane;organelle part;plasma membrane;ruffle;trans-Golgi network membrane		Homo sapiens	1	7
cell part;centrosome;cytoplasmic membrane-bounded vesicle;cytoplasmic part;cytoplasmic vesicle;cytoskeletal part;cytosol;endoplasmic reticulum-Golgi intermediate compartment;extracellular membrane-bounded organelle;extracellular organelle;extracellular region part;extracellular vesicular exosome;intracellular membrane-bounded or		Homo sapiens	1	7
cell part;cytoplasmic part;intracellular membrane-bounded organelle;intracellular organelle;intracellular organelle part;intracellular part;macromolecular complex;membrane;membrane part;membrane-bounded organelle;mitoc	Oxidative phosphorylation	Homo sapiens	1	7

cell part;cytoplasm;cytoskeletal part;extracellular membrane-bounded organelle;extracellular organelle;extracellular region part;extracellular vesicular exosome;intermediate filament;intracellular membrane-bounded organelle	Ribosome biogenesis in eukaryotes	Homo sapiens	1	7
cell part;cytoplasmic membrane-bounded vesicle;cytoplasmic part;cytoplasmic vesicle;endoplasmic reticulum;extracellular membrane-bounded organelle;extracellular organelle;extracellular region part;extracellular vesicular exosome;intracellular membrane-bounded organelle;intracellular organelle;intracellular organelle part;intracellular par		Homo sapiens	1	7
cell part;cytoplasmic part;cytosol;endoplasmic reticulum;endoplasmic reticulum membrane;endoplasmic reticulum part;endoplasmic reticulum Sec complex;integral to membrane;intracellular membrane-bounded organelle;ir	Phagosome;Protein export;Protein processing in endoplasmic reticulum;Vibrio cholerae infection	Homo sapiens	1	7
cell part;cytoplasmic membrane-bounded vesicle;cytoplasmic part;cytoplasmic vesicle;early endosome;early endosome membrane;endocytic vesicle;endosomal part;endosome;endosome membrane;extracellular membrane	Amoebiasis;Endocytosis;ko05152;Phagosome;Vasopressin-regulated water reabsorption	Homo sapiens	1	7
cell part;cytoplasm;cytoplasmic part;cytosol;cytosolic small ribosomal subunit;extracellular membrane-bounded organelle;extracellular organelle;extracellular region part;extracellular vesicular exosome;intracellular non-meml	Ribosome	Homo sapiens	1	7
cell part;cytoplasm;cytoplasmic part;cytoskeletal part;cytosol;intracellular organelle part;intracellular part;macromolecular complex;microtubule;organelle part;protein complex		Homo sapiens	1	7
cell part;centromeric heterochromatin;chromatin;chromocenter;chromosomal part;chromosome, centromeric region;cytoskeletal part;female pronucleus;heterochromatin;intracellular membrane-bounded organelle;intracellular non-membrane-bounded organelle;intracellular organelle;intracellular organelle part;intracellular part;male pronucle		Homo sapiens	1	7
axon;cell body;cell junction;cell part;cell projection;cell projection part;cyclin-dependent protein kinase 5 holoenzyme complex;cyclin-dependent protein kinase holoenzyme complex;cytoplasm;cytoplasmic part;cytoskeletal p	Alzheimer's disease;Axon guidance	Homo sapiens	1	7
cell part;cytoplasm;cytoplasmic part;extracellular membrane-bounded organelle;extracellular organelle;extracellular region part;extracellular vesicular exosome;intracellular membrane-bounded organelle;intracellular non-membrane-bounded organelle;intracellular organelle;intracellular part;lysosome;lytic vacuole;macromolecular complex;m		Homo sapiens	1	7
apical plasma membrane;cell part;cytoplasm;cytoplasmic part;cytosol;extracellular membrane-bounded organelle;extracellular organelle;extracellular region part;extracellular vesicular exosome;integral to membrane;integral to plasma membrane;intracellular membrane-bounded organelle;intracellular non-membrane-bounded organelle;intra		Homo sapiens	1	7
cell part;intracellular membrane-bounded organelle;intracellular organelle;intracellular organelle part;intracellular part;membrane-bounded organelle;nuclear part;nucleoplasm;nucleus;organelle;organelle part		Homo sapiens	1	7
cell part;cytoplasm;cytoplasmic part;cytosol;extracellular membrane-bounded organelle;extracellular organelle;extracellular region part;extracellular vesicular exosome;Golgi apparatus;intracellular membrane-bounded organelle;intracellular organelle;intracellular part;membrane-bounded organelle;membrane-bounded vesicle;organelle;vesic		Homo sapiens	1	7
cell part;cytoplasmic part;early endosome membrane;endoplasmic reticulum;endosomal part;endosome membrane;Golgi apparatus part;Golgi membrane;intracellular membrane-bounded organelle;intracellular organelle;intracellular organelle part;intracellular part;late endosome membrane;macromolecular complex;membrane;membrane p		Homo sapiens	1	7
cell part;cytoplasm;intracellular membrane-bounded organelle;intracellular organelle;intracellular organelle part;intracellular part;membrane;membrane-bounded organelle;nuclear part;nucleoplasm;nucleus;organelle;organelle part;plasma membrane		Homo sapiens	1	7
CAF-1 complex;cell part;chromatin;chromatin assembly complex;chromosomal part;intracellular organelle part;intracellular part;macromolecular complex;nuclear chromatin;nuclear chromosome part;nuclear part;organelle part;protein complex		Homo sapiens	1	7
cell part;cytoplasmic part;endoplasmic reticulum;endoplasmic reticulum-Golgi intermediate compartment membrane;Golgi apparatus;Golgi apparatus part;Golgi membrane;integral to membrane;intracellular membrane-boun	SNARE interactions in vesicular transport	Homo sapiens	1	7
cell part;intracellular membrane-bounded organelle;intracellular organelle;intracellular part;membrane;membrane-bounded organelle;nucleus;organelle		Homo sapiens	1	7
cell part;cytoplasmic part;intracellular membrane-bounded organelle;intracellular organelle;intracellular organelle lumen;intracellular organelle part;intracellular part;large ribosomal subunit;macromolecular complex;membrane;membrane-bounded organelle;membrane-enclosed lumen;mitochondrial inner membrane;mitochondrial large ribos		Homo sapiens	1	7
cell part;cytoplasmic part;endoplasmic reticulum;endoplasmic reticulum lumen;endoplasmic reticulum part;intracellular membrane-bounded organelle;intracellular non-membrane-bounded organelle;intracellular organelle;intracellular organelle lumen;intracellular organelle part;intracellular part;membrane-bounded organelle;membrane-enclo		Homo sapiens	1	7
cell part;cytoplasmic part;endoplasmic reticulum;intracellular membrane-bounded organelle;intracellular organelle;intracellular part;membrane;membrane-bounded organelle;organelle;plasma membrane		Homo sapiens	1	7
cell part;cytoplasm;cytoplasmic part;cytosol;intracellular membrane-bounded organelle;intracellular organelle;intracellular organelle part;intracellular part;membrane-bounded organelle;nuclear body;nuclear part;nuclear spec	mRNA surveillance pathway;RNA transport	Homo sapiens	1	7
cell part;coated vesicle;coated vesicle membrane;COPI coated vesicle membrane;COPI-coated vesicle;cytoplasmic membrane-bounded vesicle;cytoplasmic part;cytoplasmic vesicle;cytoplasmic vesicle membrane;cytoplasmic vesicle part;endoplasmic reticulum;endoplasmic reticulum membrane;endoplasmic reticulum part;endoplasmic re		Homo sapiens	1	7
cell part;cytoplasmic part;cytosol;extracellular membrane-bounded organelle;extracellular organelle;extracellular region part;extracellular vesicular exosome;intracellular membrane-bounded organelle;intracellular organelle;intracellular part;membrane-bounded organelle;membrane-bounded vesicle;nucleus;organelle;vesicle		Homo sapiens	1	7
cell part;cytoplasmic part;cytosol;intracellular part	Purine metabolism	Homo sapiens	1	7
cell part;intracellular organelle part;intracellular part;nuclear part;nucleoplasm;organelle part		Homo sapiens	1	7
cell part;cytoplasm;intracellular part		Homo sapiens	1	7
cell part;cytoplasmic part;cytosol;endosomal part;endosome membrane;Golgi apparatus;intracellular membrane-bounded organelle;intracellular organelle;intracellular organelle part;intracellular part;membrane;membrane-bounded organelle;organelle;organelle membrane;organelle part;perinuclear region of cytoplasm		Homo sapiens	1	7
		Homo sapiens	1	7
cell part;cytoplasmic part;endoplasmic reticulum membrane;endoplasmic reticulum part;integral to membrane;intracellular organelle part;intracellular part;intrinsic to membrane;membrane;membrane part;organelle membrane;organelle part		Homo sapiens	1	7
cell part;intracellular membrane-bounded organelle;intracellular organelle;intracellular organelle part;intracellular part;membrane-bounded organelle;nuclear body;nuclear part;nuclear speck;nucleoplasm part;nucleus;organelle;organelle part		Homo sapiens	1	7
cell part;cytoplasm;cytoplasmic part;cytosol;intracellular organelle part;intracellular part;membrane;nuclear part;nucleoplasm;organelle part;plasma membrane		Homo sapiens	1	7
cell part;intracellular non-membrane-bounded organelle;intracellular organelle;intracellular organelle part;intracellular part;non-membrane-bounded organelle;nuclear part;nucleolus;organelle;organelle part		Homo sapiens	1	7
cell part;integral to membrane;intrinsic to membrane;membrane part		Homo sapiens	1	7
cell part;chromatin;chromosomal part;intracellular organelle part;intracellular part;nuclear chromatin;nuclear chromosome part;nuclear part;organelle part		Homo sapiens	1	7
cell part;cytoplasm;intracellular membrane-bounded organelle;intracellular organelle;intracellular organelle part;intracellular part;macromolecular complex;membrane-bounded organelle;nuclear part;nucleoplasm;nucleus;orga	mRNA surveillance pathway	Homo sapiens	1	7
cell part;chylomicron;extracellular membrane-bounded organelle;extracellular organelle;extracellular region part;extracellular vesicular exosome;integral to membrane;intrinsic to membrane;low-density lipoprotein particle;macromolecular complex;membrane;membrane part;membrane-bounded organelle;membrane-bounded vesicle;organe		Homo sapiens	1	7
cell part;cytoplasmic part;endosomal part;endosome membrane;Golgi apparatus;intracellular membrane-bounded organelle;intracellular organelle;intracellular organelle part;intracellular part;membrane;membrane-bounded organelle;organelle;organelle membrane;organelle part;perinuclear region of cytoplasm		Homo sapiens	1	7
		Homo sapiens	1	7
cell part;cytoplasm;intracellular part		Homo sapiens	1	7
cell part;cytoplasm;cytoplasmic part;Golgi apparatus;intracellular membrane-bounded organelle;intracellular organelle;intracellular organelle part;intracellular part;membrane-bounded organelle;nuclear part;nucleoplasm;organelle;organelle part		Homo sapiens	1	7
cell part;contractile fiber part;cytoplasmic part;intracellular membrane-bounded organelle;intracellular organelle;intracellular part;membrane-bounded organelle;nucleus;organelle;organelle part;Z disc		Homo sapiens	1	7
cell part;cytoskeleton;envelope;intermediate filament cytoskeleton;intracellular membrane-bounded organelle;intracellular non-membrane-bounded organelle;intracellular organelle;intracellular organelle part;intracellular part;r	RNA transport	Homo sapiens	1	7
cell part;cytoplasmic part;cytosol;intracellular membrane-bounded organelle;intracellular organelle;intracellular part;membrane-bounded organelle;nucleus;organelle		Homo sapiens	1	7
cell part;cytoplasm;intracellular membrane-bounded organelle;intracellular organelle;intracellular organelle part;intracellular part;membrane-bounded organelle;nuclear part;nucleoplasm;nucleus;organelle;organelle part	Notch signaling pathway	Homo sapiens	1	7
cell part;cytoplasmic part;endoplasmic reticulum lumen;endoplasmic reticulum part;integral to membrane;intracellular organelle lumen;intracellular organelle part;intracellular part;intrinsic to membrane;membrane part;membrane-enclosed lumen;organelle lumen;organelle part		Homo sapiens	1	7
cell part;cytoplasmic part;extracellular membrane-bounded organelle;extracellular organelle;extracellular region part;extracellular vesicular exosome;integral to membrane;intracellular organelle part;intracellular part;intrinsic to membrane;membrane;membrane part;membrane-bounded organelle;membrane-bounded vesicle;mitochondrial me		Homo sapiens	1	7
alpha-amino-3-hydroxy-5-methyl-4-isoxazolepropionic acid selective glutamate receptor complex;basolateral plasma membrane;cell body;cell junction;cell part;cell projection part;cell-cell junction;cytoplasm;cytoskeletal part;dendritic shaft;extracellular region part;extracellular space;growth cone;intracellular organelle part;intracellular part;		Homo sapiens	1	7
cell part;cytoplasmic part;cytosol;intracellular membrane-bounded organelle;intracellular organelle;intracellular organelle part;intracellular part;membrane-bounded organelle;nuclear part;nucleoplasm;nucleus;organelle;organe	Protein processing in endoplasmic reticulum	Homo sapiens	1	7
cell part;intracellular membrane-bounded organelle;intracellular organelle;intracellular part;membrane-bounded organelle;nucleus;organelle		Homo sapiens	1	7
cell part;cytoplasm;intracellular part		Homo sapiens	1	7
cell part;cytoplasmic part;cytosol;endoplasmic reticulum-Golgi intermediate compartment;endosomal part;endosome;endosome membrane;Golgi apparatus part;Golgi membrane;intracellular membrane-bounded organelle;ir	Amino sugar and nucleotide sugar metabolism	Homo sapiens	1	7
cell part;cytoplasm;extracellular region part;intercellular bridge;intracellular membrane-bounded organelle;intracellular organelle;intracellular organelle part;intracellular part;macromolecular complex;membrane-bounded orgar	RNA transport;Spliceosome	Homo sapiens	1	7
cell part;intracellular membrane-bounded organelle;intracellular non-membrane-bounded organelle;intracellular organelle;intracellular organelle part;intracellular part;membrane-bounded organelle;non-membrane-bounded organelle;nuclear body;nuclear part;nuclear speck;nucleolus;nucleoplasm;nucleoplasm part;nucleus;organelle;organell		Homo sapiens	1	7
cell part;cytoplasmic part;endoplasmic reticulum;endoplasmic reticulum membrane;endoplasmic reticulum part;intracellular membrane-bounded organelle;intracellular organelle;intracellular organelle part;intracellular part;membrane;membrane part;membrane-bounded organelle;organelle;organelle membrane;organelle part		Homo sapiens	1	7
cell part;cytoplasm;cytoplasmic part;cytosol;extracellular membrane-bounded organelle;extracellular organelle;extracellular region part;extracellular vesicular exosome;intracellular membrane-bounded organelle;intracellular non-membrane-bounded organelle;intracellular organelle;intracellular organelle part;intracellular part;membrane-boun		Homo sapiens	1	7
cell part;cytoplasmic part;cytoskeletal part;integral to membrane;intracellular membrane-bounded organelle;intracellular organelle;intracellular organelle part;intracellular part;intrinsic to membrane;macromolecular complex;membrane;membrane part;membrane-bounded organelle;microtubule;mitochondrial membrane;mitochondrial outer m		Homo sapiens	1	7
adherens junction;anchoring junction;Arp2/3 protein complex;cell junction;cell part;cell-substrate adherens junction;cell-substrate junction;cytoplasm;cytoskeletal part;extracellular membrane-bounded organelle;extracellular	Bacterial invasion of epithelial cells;Fc gamma R-mediated phagocytosis;Pathogenic Escherichia coli infection;Regu	Homo sapiens	1	7
cell part;cytoplasm;intracellular membrane-bounded organelle;intracellular non-membrane-bounded organelle;intracellular organelle;intracellular organelle part;intracellular part;membrane-bounded organelle;non-membrane-bounded organelle;nuclear part;nucleolus;nucleus;organelle;organelle part		Homo sapiens	1	7
cell part;cytoplasmic part;intracellular membrane-bounded organelle;intracellular organelle;intracellular part;membrane-bounded organelle;mitochondrion;nucleus;organelle		Homo sapiens	1	7
cell part;cytoplasm;cytoplasmic part;cytosol;Golgi apparatus;intracellular membrane-bounded organelle;intracellular organelle;intracellular organelle part;intracellular part;macromolecular complex;membrane-bounded organelle;methylosome;methyltransferase complex;nuclear part;nucleoplasm;nucleus;organelle;organelle part;protein comp		Homo sapiens	1	7
cell part;cytoplasm;extracellular membrane-bounded organelle;extracellular organelle;extracellular region part;extracellular vesicular exosome;intracellular part;membrane-bounded organelle;membrane-bounded vesicle;organelle;vesicle		Homo sapiens	1	7
cell part;extracellular membrane-bounded organelle;extracellular organelle;extracellular region part;extracellular vesicular exosome;membrane;membrane-bounded organelle;membrane-bounded vesicle;organelle;vesicle	Purine metabolism;Thiamine metabolism	Homo sapiens	1	7
cell part;cytoplasmic part;endoplasmic reticulum membrane;endoplasmic reticulum part;integral to membrane;intracellular organelle part;intracellular part;intrinsic to membrane;membrane;membrane part;organelle membrane;organelle part		Homo sapiens	1	7
cell part;cytoplasm;cytoplasmic part;cytosol;intracellular part	Drug metabolism - other enzymes;Purine metabolism;Pyrimidine metabolism	Homo sapiens	1	7
cell part;cytoplasm;cytoplasmic part;cytoplasmic vesicle membrane;cytoplasmic vesicle part;cytosol;endosomal part;endosome membrane;ESCRT III complex;extracellular membrane-bounded organelle;extracellular organel	Endocytosis	Homo sapiens	1	7
cell part;cytoplasmic part;intracellular non-membrane-bounded organelle;intracellular organelle;intracellular organelle part;intracellular part;large ribosomal subunit;macromolecular complex;membrane;mitochondrial inner me	Ribosome	Homo sapiens	1	7
cell part;cytoplasmic part;cytosol;intracellular membrane-bounded organelle;intracellular organelle;intracellular part;membrane-bounded organelle;organelle	Drug metabolism - other enzymes;Pyrimidine metabolism	Homo sapiens	1	7
cell part;chromatin remodeling complex;cytoplasmic part;Golgi apparatus;Golgi apparatus part;histone methyltransferase complex;intracellular membrane-bounded organelle;intracellular organelle;intracellular organelle part;intracellular part;macromolecular complex;membrane-bounded organelle;methyltransferase complex;nuclear part;nuc		Homo sapiens	1	7
cell part;cytoplasmic part;endoplasmic reticulum part;integral to membrane;integral to plasma membrane;intracellular organelle part;intracellular part;intrinsic to membrane;intrinsic to plasma membrane;macromolecular complex;membrane;membrane part;oligosaccharyltransferase complex;organelle part;plasma membrane;plasma membra		Homo sapiens	1	7
cell part;cytoplasm;cytoplasmic part;cytosol;intracellular membrane-bounded organelle;intracellular organelle;intracellular organelle lumen;intracellular organelle part;intracellular part;membrane-bounded organelle;membrane-enclosed lumen;mitochondrial matrix;mitochondrial part;mitochondrion;nucleus;organelle;organelle lumen;organelle		Homo sapiens	1	7
cell part;cytoplasm;cytoplasmic membrane-bounded vesicle;cytoplasmic part;cytoplasmic vesicle;endosomal part;endosome;endosome membrane;intracellular membrane-bounded organelle;intracellular organelle;intracellular organelle part;intracellular part;late endosome;late endosome membrane;lysosomal membrane;lysosome;lytic vac		Homo sapiens	1	7
cell part;cytoplasmic membrane-bounded vesicle;cytoplasmic part;cytoplasmic vesicle;endocytic vesicle;endosomal part;endosome;endosome membrane;HOPS complex;intracellular membrane-bounded organelle;intracellular organelle;intracellular organelle part;intracellular part;late endosome;late endosome membrane;lysosomal membr		Homo sapiens	1	7
catalytic step 2 spliceosome;cell part;intracellular organelle part;intracellular part;macromolecular complex;nuclear part;organelle part;ribonucleoprotein complex;spliceosomal complex		Homo sapiens	1	7
cell part;cytoplasm;histone methyltransferase complex;intracellular membrane-bounded organelle;intracellular non-membrane-bounded organelle;intracellular organelle;intracellular organelle part;intracellular part;macromolecular complex;membrane-bounded organelle;methyltransferase complex;MLL1 complex;non-membrane-bounded org		Homo sapiens	1	7
cell part;cytoplasm;intracellular membrane-bounded organelle;intracellular organelle;intracellular organelle part;intracellular part;membrane-bounded organelle;nuclear part;nucleoplasm;nucleus;organelle;organelle part		Homo sapiens	1	7
cell part;cytoplasmic part;Golgi apparatus;intracellular membrane-bounded organelle;intracellular non-membrane-bounded organelle;intracellular organelle;intracellular organelle part;intracellular part;membrane-bounded organelle;non-membrane-bounded organelle;nuclear part;nucleolus;organelle;organelle part		Homo sapiens	1	7
		Homo sapiens	1	7
		Homo sapiens	1	7
cell part;cytoplasmic part;endoplasmic reticulum lumen;endoplasmic reticulum part;intracellular organelle lumen;intracellular organelle part;intracellular part;membrane;membrane-enclosed lumen;organelle lumen;organelle part		Homo sapiens	1	7
cell part;integral to membrane;intrinsic to membrane;membrane part		Homo sapiens	1	7
cell part;cytoplasmic part;intracellular non-membrane-bounded organelle;intracellular organelle;intracellular organelle part;intracellular part;macromolecular complex;membrane;mitochondrial inner membrane;mitochondrial membrane;mitochondrial part;non-membrane-bounded organelle;organelle;organelle inner membrane;organelle memb		Homo sapiens	1	7
cell junction;cell part;cytoplasm;cytoskeleton;inhibitory synapse;intracellular non-membrane-bounded organelle;intracellular organelle;intracellular part;membrane;non-membrane-bounded organelle;organelle;plasma membrane;postsynaptic membrane;synapse;synapse part;synaptic membrane		Homo sapiens	1	7
cell part;cytoplasmic part;cytosol;extracellular membrane-bounded organelle;extracellular organelle;extracellular region part;extracellular vesicular exosome;intracellular part;membrane-bounded organelle;membrane-bounded vesicle;organelle;vesicle		Homo sapiens	1	7
cell part;cytoplasmic part;intracellular membrane-bounded organelle;intracellular organelle;intracellular organelle part;intracellular part;membrane;membrane-bounded organelle;microbody;microbody membrane;microbody p	Peroxisome	Homo sapiens	1	7
cell part;cytoplasmic part;cytosol;intracellular membrane-bounded organelle;intracellular organelle;intracellular part;membrane-bounded organelle;mitochondrion;organelle		Homo sapiens	1	7
		Homo sapiens	1	7
cell part;cytoplasm;granular component;intracellular membrane-bounded organelle;intracellular non-membrane-bounded organelle;intracellular organelle;intracellular organelle part;intracellular part;membrane-bounded organelle;non-membrane-bounded organelle;nuclear part;nucleolar part;nucleolus;nucleoplasm;nucleus;organelle;organell		Homo sapiens	1	7
cell part;cytoplasmic part;intracellular membrane-bounded organelle;intracellular organelle;intracellular organelle part;intracellular part;membrane;membrane-bounded organelle;mitochondrial membrane;mitochondrial part;mitochondrion;organelle;organelle membrane;organelle part		Homo sapiens	1	7
cell part;cytoplasm;cytoplasmic part;cytoskeleton;cytosol;intermediate filament cytoskeleton;intracellular non-membrane-bounded organelle;intracellular organelle;intracellular organelle part;intracellular part;non-membrane-bounded organelle;nuclear part;nucleoplasm;organelle;organelle part		Homo sapiens	1	7
cell part;cytoplasm;cytoplasmic part;cytosol;early endosome;endosomal part;endosome;endosome membrane;extracellular membrane-bounded organelle;extracellular organelle;extracellular region part;extracellular vesicular exosome;intracellular membrane-bounded organelle;intracellular organelle;intracellular organelle part;intracellular pa		Homo sapiens	1	7
cell part;cytoplasmic part;intracellular membrane-bounded organelle;intracellular organelle;intracellular part;macromolecular complex;membrane-bounded organelle;methyltransferase complex;nucleus;organelle;protein complex;tRNA (m1A) methyltransferase complex;tRNA methyltransferase complex		Homo sapiens	1	7
cell part;cytoplasm;cytoplasmic part;cytosol;early endosome;endosomal part;endosome;endosome membrane;ESCRT I complex;extracellular membrane-bounded organelle;extracellular organelle;extracellular region part;ext	Endocytosis	Homo sapiens	1	7
cell part;cytoplasmic part;cytosol;intracellular organelle part;intracellular part;macromolecular complex;nuclear part;organelle part;ribonucleoprotein complex;small nucleolar ribonucleoprotein complex;spliceosomal complex	RNA degradation;Spliceosome	Homo sapiens	1	7
adherens junction;anchoring junction;cell division site part;cell junction;cell part;cell-substrate adherens junction;cell-substrate junction;cleavage furrow;cytoplasmic part;cytoplasmic vesicle membrane;cytoplasmic vesicle part;endoplasmic reticulum membrane;endoplasmic reticulum part;endosome;extracellular membrane-bounded organe		Homo sapiens	1	7
cell part;cytoplasmic part;endoplasmic reticulum part;integral to endoplasmic reticulum membrane;integral to membrane;integral to organelle membrane;intracellular organelle part;intracellular part;intrinsic to endoplasmic ret	Protein processing in endoplasmic reticulum	Homo sapiens	1	7
		Homo sapiens	1	7
cell part;cytoplasm;intracellular part		Homo sapiens	1	7
cell part;cytoplasmic mRNA processing body;cytoplasmic part;cytosol;intracellular non-membrane-bounded organelle;intracellular organelle;intracellular part;macromolecular complex;non-membrane-bounded organelle;organelle;ribonucleoprotein complex;RNA granule		Homo sapiens	1	7
cell part;intracellular non-membrane-bounded organelle;intracellular organelle;intracellular organelle part;intracellular part;non-membrane-bounded organelle;nuclear part;nucleolus;organelle;organelle part		Homo sapiens	1	7
cell part;cytoplasm;cytoplasmic part;cytoskeletal part;cytosol;intracellular membrane-bounded organelle;intracellular organelle;intracellular organelle part;intracellular part;macromolecular complex;membrane-bounded organelle;microtubule organizing center;nuclear part;nucleoplasm;nucleus;organelle;organelle part;perinuclear region of cy		Homo sapiens	1	7
cell part;chromatin;chromosomal part;intracellular membrane-bounded organelle;intracellular non-membrane-bounded organelle;intracellular organelle;intracellular organelle part;intracellular part;membrane-bounded organelle;non-membrane-bounded organelle;nuclear chromatin;nuclear chromosome part;nuclear part;nucleolus;nucleoplasi		Homo sapiens	1	7
cell part;cytoplasm;extracellular membrane-bounded organelle;extracellular organelle;extracellular region part;extracellular vesicular exosome;intracellular membrane-bounded organelle;intracellular organelle;intracellular part	Pentose phosphate pathway	Homo sapiens	1	7
adherens junction;anchoring junction;cell junction;cell part;cell-substrate adherens junction;cell-substrate junction;cytoplasmic part;focal adhesion;intracellular membrane-bounded organelle;intracellular non-membrane-boun	Ribosome biogenesis in eukaryotes	Homo sapiens	1	7
cell part;cytoplasmic part;cytoskeleton;intermediate filament cytoskeleton;intracellular membrane-bounded organelle;intracellular non-membrane-bounded organelle;intracellular organelle;intracellular organelle part;intracellular part;macromolecular complex;membrane;membrane-bounded organelle;mitochondrial inner membrane;mitochonc		Homo sapiens	1	7
cell part;cytoplasm;intracellular membrane-bounded organelle;intracellular organelle;intracellular organelle part;intracellular part;membrane;membrane-bounded organelle;nuclear part;nucleoplasm;nucleus;organelle;organelle part		Homo sapiens	1	7
actomyosin;cell body;cell part;cell projection;cell projection part;cilium;cytoplasm;cytoplasmic membrane-bounded vesicle;cytoplasmic part;cytoplasmic vesicle;cytoskeletal part;cytosol;extracellular membrane-bounded organelle;extracellular organelle;extracellular region part;extracellular vesicular exosome;filopodium tip;Golgi apparatus;(		Homo sapiens	1	7
cell part;chromosomal part;chromosome, telomeric region;cytoplasm;intracellular;intracellular membrane-bounded organelle;intracellular organelle;intracellular organelle part;intracellular part;macromolecular complex;membrane;membrane-bounded organelle;nucleus;organelle;organelle part;protein complex;TORC1 complex;TORC2 comple		Homo sapiens	1	7
cell part;cytoplasmic part;cytosol;intracellular part	Mineral absorption	Homo sapiens	1	8
cell part;clathrin-coated vesicle;coated vesicle;cytoplasm;cytoplasmic membrane-bounded vesicle;cytoplasmic part;cytoplasmic vesicle;cytosol;cytosolic part;extracellular membrane-bounded organelle;extracellular organell	Inositol phosphate metabolism;Phosphatidylinositol signaling system	Homo sapiens	1	8
cell part;cytoplasmic part;extracellular membrane-bounded organelle;extracellular organelle;extracellular region part;extracellular vesicular exosome;intracellular membrane-bounded organelle;intracellular organelle;intracellula	Alzheimer's disease;Huntington's disease;Oxidative phosphorylation;Parkinson's disease	Homo sapiens	1	8
apical plasma membrane;cell body;cell part;cell projection;cell projection membrane;cell projection part;cytoplasm;extracellular membrane-bounded organelle;extracellular organelle;extracellular region part;extracellular space;extracellular vesicular exosome;filopodium;integral to membrane;integral to plasma membrane;intracellular part;intr		Homo sapiens	1	8
cell part;cytoplasm;cytoplasmic part;cytosol;intracellular membrane-bounded organelle;intracellular organelle;intracellular part;membrane-bounded organelle;nucleus;organelle	Amyotrophic lateral sclerosis (ALS)	Homo sapiens	1	8
cell part;cytoplasmic part;endoplasmic reticulum membrane;endoplasmic reticulum part;integral to membrane;intracellular organelle part;intracellular part;intrinsic to membrane;membrane;membrane part;membrane-enclose	Arachidonic acid metabolism	Homo sapiens	1	8
cell part;cytoplasmic part;cytosol;DNA-directed RNA polymerase complex;DNA-directed RNA polymerase III complex;intracellular organelle part;intracellular part;macromolecular complex;membrane;nuclear DNA-directed RI	Cytosolic DNA-sensing pathway;Purine metabolism;Pyrimidine metabolism;RNA polymerase	Homo sapiens	1	8
cell part;chromatin;chromatin remodeling complex;chromatin silencing complex;chromosomal part;cytoplasm;heterochromatin;intracellular membrane-bounded organelle;intracellular non-membrane-bounded organelle;intracellular organelle;intracellular organelle part;intracellular part;macromolecular complex;membrane;membrane-bounde		Homo sapiens	1	8
cell part;cytoplasm;intracellular membrane-bounded organelle;intracellular organelle;intracellular organelle part;intracellular part;membrane part;membrane raft;membrane-bounded organelle;nuclear body;nuclear part;nucleo	Bladder cancer;Pathways in cancer	Homo sapiens	1	8
axon part;axon terminus;cell body;cell part;cell projection;cell projection part;cytoplasm;dendritic spine;intracellular membrane-bounded organelle;intracellular organelle;intracellular part;membrane;membrane-bounded organelle;neuron projection;neuron projection terminus;neuron spine;neuronal cell body;nucleus;organelle;plasma membra		Homo sapiens	1	8
cell part;chromosomal part;condensed chromosome kinetochore;cytoskeletal part;intracellular membrane-bounded organelle;intracellular non-membrane-bounded organelle;intracellular organelle;intracellular organelle part;intracellular part;kinetochore;macromolecular complex;membrane-bounded organelle;microtubule;non-membrane-bou		Homo sapiens	1	8
cell part;cytoplasm;intracellular part		Homo sapiens	1	8
cell part;centrosome;cytoplasmic part;cytoskeletal part;intracellular membrane-bounded organelle;intracellular non-membrane-bounded organelle;intracellular organelle;intracellular organelle part;intracellular part;macromolec	mRNA surveillance pathway	Homo sapiens	1	8
cell part;contractile fiber;cytoplasmic part;intracellular membrane-bounded organelle;intracellular non-membrane-bounded organelle;intracellular organelle;intracellular organelle lumen;intracellular organelle part;intracellular part;membrane;membrane-bounded organelle;membrane-enclosed lumen;mitochondrial inner membrane;mitochondr		Homo sapiens	1	8
cell part;chromatin;chromosomal part;intracellular membrane-bounded organelle;intracellular organelle;intracellular organelle part;intracellular part;macromolecular complex;membrane-bounded organelle;nuclear chromatin;nuclear chromosome part;nuclear part;nucleoplasm;nucleoplasm part;nucleus;organelle;organelle part;protein comple		Homo sapiens	1	8
catalytic step 2 spliceosome;cell part;intracellular organelle part;intracellular part;macromolecular complex;nuclear part;nucleoplasm;organelle part;ribonucleoprotein complex;spliceosomal complex	Spliceosome	Homo sapiens	1	8
cell part;cytoplasmic part;cytosol;early endosome;endosome;Golgi apparatus;intracellular membrane-bounded organelle;intracellular non-membrane-bounded organelle;intracellular organelle;intracellular organelle part;intrac	Regulation of actin cytoskeleton	Homo sapiens	1	8
cell part;intracellular membrane-bounded organelle;intracellular organelle;intracellular organelle part;intracellular part;membrane-bounded organelle;nuclear part;nucleoplasm;nucleus;organelle;organelle part	mRNA surveillance pathway	Homo sapiens	1	8
cell part;cytoplasmic part;endoplasmic reticulum;endoplasmic reticulum lumen;endoplasmic reticulum part;extracellular matrix;extracellular region part;extracellular space;intracellular membrane-bounded organelle;intracellular organelle;intracellular organelle lumen;intracellular organelle part;intracellular part;macromolecular complex;memb		Homo sapiens	1	8
apicolateral plasma membrane;axon;basolateral plasma membrane;cell part;cell projection;cell projection membrane;cell projection part;cytoplasmic membrane-bounded vesicle;cytoplasmic part;cytoplasmic vesicle;dendrite membrane;dendritic spine membrane;filopodium membrane;integral to membrane;integral to plasma membrane;intu		Homo sapiens	1	8
cell part;cytoskeletal part;intracellular organelle part;intracellular part;macromolecular complex;MMXD complex;organelle part;protein complex		Homo sapiens	1	8
cell part;cytoplasmic part;early endosome membrane;endosomal part;endosome membrane;extracellular region;extracellular region part;extracellular space;intracellular organelle part;intracellular part;membrane;organelle me	Wnt signaling pathway	Homo sapiens	1	8

cell part;cytoplasm;extracellular membrane-bounded organelle;extracellular organelle;extracellular region part;extracellular vesicular exosome;intracellular part;membrane-bounded organelle;membrane-bounded vesicle;orga	Glycerophospholipid metabolism	Homo sapiens	1	8
cell part;cell surface;integral to membrane;integral to plasma membrane;intrinsic to membrane;intrinsic to plasma membrane;macromolecular complex;membrane part;MHC class I protein complex;MHC protein complex;plasma membrane part;protein complex		Homo sapiens	0.9949	8
alphav-beta3 integrin-vitronectin complex;blood microparticle;cell part;extracellular matrix;extracellular membrane-bounded organelle;extracellular organelle;extracellular region;extracellular region part;extracellular space;ext	ECM-receptor interaction;Focal adhesion	Homo sapiens	1	8
cell part;cytoplasm;cytoplasmic part;extracellular membrane-bounded organelle;extracellular organelle;extracellular region part;extracellular vesicular exosome;intracellular membrane-bounded organelle;intracellular organelle	Other glycan degradation	Homo sapiens	1	8
cell part;intracellular membrane-bounded organelle;intracellular non-membrane-bounded organelle;intracellular organelle;intracellular organelle part;intracellular part;membrane-bounded organelle;non-membrane-bounded organelle;nuclear part;nucleolus;nucleoplasm;nucleus;organelle;organelle part		Homo sapiens	1	8
cell part;cytoplasmic part;intracellular membrane-bounded organelle;intracellular organelle;intracellular organelle lumen;intracellular organelle part;intracellular part;membrane;membrane-bounded organelle;membrane-enclos	Benzoate degradation;Biosynthesis of unsaturated fatty acids;Fatty acid metabolism;Peroxisome;PPAR signaling pa	Homo sapiens	1	8
cell part;cytoplasm;cytoplasmic part;cytosol;intracellular membrane-bounded organelle;intracellular non-membrane-bounded organelle;intracellular organelle;intracellular organelle part;intracellular part;macromolecular comp	Protein export	Homo sapiens	1	8
cell part;cytoplasmic part;integral to membrane;intracellular membrane-bounded organelle;intracellular organelle;intracellular organelle part;intracellular part;intrinsic to membrane;membrane;membrane part;membrane-bounc	Alzheimer's disease;Cardiac muscle contraction;Huntington's disease;Oxidative phosphorylation;Parkinson's diseas	Homo sapiens	1	8
cell part;cytoplasm;cytoplasmic part;cytosol;intracellular part	Hepatitis C	Homo sapiens	1	8
cell part;cytoplasmic part;dihydrolipoyl dehydrogenase complex;intracellular membrane-bounded organelle;intracellular non-membrane-bounded organelle;intracellular organelle;intracellular organelle lumen;intracellular orgar	Valine, leucine and isoleucine degradation	Homo sapiens	1	8
adherens junction;anchoring junction;cell junction;cell part;cell surface;cell-substrate adherens junction;cell-substrate junction;cytoplasm;extracellular membrane-bounded organelle;extracellular organelle;extracellular region	Cell adhesion molecules (CAMs)	Homo sapiens	1	8
cell part;chromatin;chromosomal part;intracellular organelle part;intracellular part;macromolecular complex;nuclear chromatin;nuclear chromosome part;nuclear part;nucleoplasm;nucleoplasm part;organelle part;protein comp	Osteoclast differentiation	Homo sapiens	1	8
cell part;cytoplasmic part;Golgi apparatus;intracellular membrane-bounded organelle;intracellular organelle;intracellular organelle part;intracellular part;membrane-bounded organelle;mitochondrion;nuclear part;nucleoplasm;r	Base excision repair;DNA replication;Mismatch repair;Nucleotide excision repair	Homo sapiens	1	8
actin cytoskeleton;axon;axon part;axon terminus;cell body;cell part;cell projection;cell projection part;clathrin-coated vesicle;coated vesicle;cytoplasm;cytoplasmic membrane-bounded vesicle;cytoplasmic part;cytoplasmic v	Amoebiasis;Amyotrophic lateral sclerosis (ALS);Endocytosis;ko05152;Phagosome;Vasopressin-regulated water reat	Homo sapiens	1	8
cell part;intracellular membrane-bounded organelle;intracellular organelle;intracellular part;membrane-bounded organelle;nucleus;organelle		Homo sapiens	1	8
cell part;cytoplasm;intracellular membrane-bounded organelle;intracellular organelle;intracellular part;membrane-bounded organelle;nucleus;organelle		Homo sapiens	1	8
cell part;cytoplasm;extracellular membrane-bounded organelle;extracellular organelle;extracellular region part;extracellular vesicular exosome;internal side of plasma membrane;intracellular part;membrane part;membrane-bo	Riboflavin metabolism	Homo sapiens	1	8
cell part;cytoplasm;cytoplasmic part;cytosol;extracellular membrane-bounded organelle;extracellular organelle;extracellular region part;extracellular vesicular exosome;intracellular membrane-bounded organelle;intracellular o	Proteasome	Homo sapiens	1	8
cell part;cytoplasm;cytoplasmic part;cytosol;extracellular membrane-bounded organelle;extracellular organelle;extracellular region part;extracellular vesicular exosome;Golgi apparatus;intracellular membrane-bounded organe	Proteasome	Homo sapiens	1	8
cell part;cytoplasmic part;intracellular non-membrane-bounded organelle;intracellular organelle;intracellular organelle part;intracellular part;mitochondrial nucleoid;mitochondrial part;non-membrane-bounded organelle;nuclea	Base excision repair	Homo sapiens	1	8
cell part;cytoplasmic part;intracellular organelle lumen;intracellular organelle part;intracellular part;membrane-enclosed lumen;mitochondrial matrix;mitochondrial part;organelle lumen;organelle part	Valine, leucine and isoleucine degradation	Homo sapiens	1	8
cell part;cytoplasmic part;cytosol;intracellular membrane-bounded organelle;intracellular organelle;intracellular part;membrane-bounded organelle;nucleus;organelle		Homo sapiens	1	8
cell part;chiasma;chromosomal part;germ cell nucleus;intracellular membrane-bounded organelle;intracellular organelle;intracellular organelle part;intracellular part;macromolecular complex;male germ cell nucleus;membrane	Colorectal cancer;Endometrial cancer;Mismatch repair;Pathways in cancer	Homo sapiens	1	8
cell part;cytoplasm;intracellular membrane-bounded organelle;intracellular organelle;intracellular organelle part;intracellular part;membrane-bounded organelle;nuclear part;nucleoplasm;nucleus;organelle;organelle part		Homo sapiens	1	8
cell part;intracellular membrane-bounded organelle;intracellular organelle;intracellular organelle part;intracellular part;membrane-bounded organelle;nuclear part;nucleoplasm;nucleus;organelle;organelle part		Homo sapiens	1	8
apical plasma membrane;cell junction;cell leading edge;cell part;cell-cell junction;cytoplasm;cytoplasmic part;cytoskeleton;cytosol;endosome;extracellular membrane-bounded organelle;extracellular organelle;extracellular re	Endocytosis;Insulin signaling pathway;Tight junction	Homo sapiens	1	8
cell part;intracellular membrane-bounded organelle;intracellular non-membrane-bounded organelle;intracellular organelle;intracellular organelle part;intracellular part;membrane-bounded organelle;non-membrane-bounded organelle;nuclear part;nucleolus;nucleus;organelle;organelle part		Homo sapiens	1	8
actin cytoskeleton;cell part;cytoplasm;cytoplasmic part;cytoskeleton;cytosol;extracellular membrane-bounded organelle;extracellular organelle;extracellular region part;extracellular vesicular exosome;Golgi apparatus;intracel	Proteasome	Homo sapiens	1	8
beta-catenin destruction complex;cell part;cytoplasmic part;cytosol;intracellular part;macromolecular complex;protein complex	Chemokine signaling pathway	Homo sapiens	1	8
cell part;cytoplasmic part;intracellular membrane-bounded organelle;intracellular organelle;intracellular organelle lumen;intracellular organelle part;intracellular part;membrane-bounded organelle;membrane-enclosed lumen;m	Alanine, aspartate and glutamate metabolism;Butanoate metabolism	Homo sapiens	1	8
cell part;cytoplasm;cytoplasmic part;cytosol;intracellular membrane-bounded organelle;intracellular organelle;intracellular organelle part;intracellular part;membrane-bounded organelle;nuclear part;nucleoplasm;nucleus;orga	Acute myeloid leukemia;Chemokine signaling pathway;Chronic myeloid leukemia;ErbB signaling pathway;Jak-STAT	Homo sapiens	1	8
cell part;centrosome;cytoplasmic part;cytoskeletal part;extracellular membrane-bounded organelle;extracellular organelle;extracellular region part;extracellular vesicular exosome;integral to membrane;integral to plasma membrane;intracellular membrane-bounded organelle;intracellular non-membrane-bounded organelle;intracellular organe		Homo sapiens	1	8
adherens junction;anchoring junction;cell junction;cell part;cell-substrate adherens junction;cell-substrate junction;coated vesicle membrane;cytoplasm;cytoplasmic part;cytoplasmic vesicle membrane;cytoplasmic vesicle pa	Antigen processing and presentation	Homo sapiens	1	8
adherens junction;anchoring junction;cell junction;cell part;cell-substrate adherens junction;cell-substrate junction;cytoplasm;cytoplasmic part;cytosol;cytosolic small ribosomal subunit;focal adhesion;intracellular membrane-	Ribosome	Homo sapiens	1	8
cell part;cell projection;cytoplasm;dendrite;fibrillar center;intracellular membrane-bounded organelle;intracellular non-membrane-bounded organelle;intracellular organelle;intracellular organelle part;intracellular part;macromol	RNA transport	Homo sapiens	1	8
blood microparticle;cell part;cytoplasmic membrane-bounded vesicle lumen;cytoplasmic part;cytoplasmic vesicle part;cytosol;cytosolic part;cytosolic small ribosomal subunit;endocytic vesicle lumen;extracellular membrane-	African trypanosomiasis;Malaria	Homo sapiens	1	8
cell part;cytoplasmic part;extracellular membrane-bounded organelle;extracellular organelle;extracellular region part;extracellular vesicular exosome;Golgi apparatus;intracellular membrane-bounded organelle;intracellular organelle;intracellular part;membrane;membrane-bounded organelle;membrane-bounded vesicle;organelle;perinuclear r		Homo sapiens	1	8
cell part;cytoplasmic part;endoplasmic reticulum membrane;endoplasmic reticulum part;integral to endoplasmic reticulum membrane;integral to membrane;integral to organelle membrane;intracellular organelle part;intracellu	ABC transporters;Antigen processing and presentation;Phagosome;Primary immunodeficiency	Homo sapiens	1	8
cell part;cytoplasm;intracellular membrane-bounded organelle;intracellular organelle;intracellular organelle part;intracellular part;macromolecular complex;membrane-bounded organelle;nuclear body;nuclear part;nuclear speck;nucleoplasm;nucleoplasm part;nucleus;organelle;organelle part;ribonucleoprotein complex;spliceosomal complex		Homo sapiens	1	8
cell part;cytoplasm;cytoplasmic part;cytosol;intracellular membrane-bounded organelle;intracellular organelle;intracellular organelle part;intracellular part;membrane-bounded organelle;nuclear part;nucleoplasm;nucleus;orgar	MAPK signaling pathway;Non-small cell lung cancer;Pathways in cancer	Homo sapiens	1	8
cell part;cytoplasmic part;integral to membrane;intracellular organelle part;intracellular part;intrinsic to membrane;membrane;membrane part;mitochondrial membrane;mitochondrial outer membrane;mitochondrial part;organelle membrane;organelle outer membrane;organelle part;outer membrane		Homo sapiens	1	8
cell part;cytoplasmic part;cytoplasmic vesicle membrane;cytoplasmic vesicle part;cytosol;endocytic vesicle membrane;endoplasmic reticulum membrane;endoplasmic reticulum part;intracellular organelle part;intracellular pa	Calcium signaling pathway;ErbB signaling pathway;Gastric acid secretion;Glioma;GnRH signaling pathway;ko05152	Homo sapiens	1	8
cell part;cytoplasmic part;endoplasmic reticulum membrane;endoplasmic reticulum part;integral to endoplasmic reticulum membrane;integral to membrane;integral to organelle membrane;intracellular organelle part;intracellular part;intrinsic to endoplasmic reticulum membrane;intrinsic to membrane;intrinsic to organelle membrane;membrai		Homo sapiens	1	8
BRISC complex;cell part;cytoplasm;intracellular organelle part;intracellular part;macromolecular complex;nuclear part;organelle part;protein complex		Homo sapiens	1	8
cell part;cytoplasmic part;cytosol;endoplasmic reticulum part;integral to membrane;intracellular organelle part;intracellular part;intrinsic to membrane;macromolecular complex;membrane;membrane part;organelle part;protein complex;Sec61 translocon complex;translocon complex		Homo sapiens	1	8
extracellular membrane-bounded organelle;extracellular organelle;extracellular region part;extracellular space;extracellular vesicular exosome;membrane-bounded organelle;membrane-bounded vesicle;organelle;vesicle		Homo sapiens	1	8
cell body;cell part;cell projection;cytoplasmic part;cytosol;dendrite;endoplasmic reticulum membrane;endoplasmic reticulum part;extracellular membrane-bounded organelle;extracellular organelle;extracellular region part;ext	Insulin signaling pathway;mTOR signaling pathway	Homo sapiens	1	8
cell part;cytoplasmic part;integral to membrane;intracellular organelle part;intracellular part;intrinsic to membrane;membrane part;mitochondrial membrane part;mitochondrial part;mitochondrial respiratory chain;organelle part;respiratory chain		Homo sapiens	1	8
apical plasma membrane;cell junction;cell part;cell projection;cell surface;clathrin coated vesicle membrane;clathrin-coated vesicle;coated vesicle;coated vesicle membrane;cytoplasmic membrane-bounded vesicle;cytoplasr	Phagosome;SNARE interactions in vesicular transport	Homo sapiens	1	8
cell part;cytoplasm;cytoplasmic part;Golgi apparatus;intracellular membrane-bounded organelle;intracellular organelle;intracellular organelle part;intracellular part;membrane-bounded organelle;nuclear part;nucleoplasm;organelle;organelle part		Homo sapiens	1	8
cell part;cytoplasmic part;early endosome;endosome;intracellular membrane-bounded organelle;intracellular organelle;intracellular part;membrane-bounded organelle;organelle		Homo sapiens	1	8
extracellular membrane-bounded organelle;extracellular organelle;extracellular region part;extracellular vesicular exosome;membrane-bounded organelle;membrane-bounded vesicle;organelle;vesicle		Homo sapiens	1	8
cell part;integral to membrane;intrinsic to membrane;membrane part		Homo sapiens	1	8
cell body;cell junction;cell part;cell projection;cytoplasm;cytoplasmic part;cytoskeletal part;cytosol;dendrite;intracellular membrane-bounded organelle;intracellular non-membrane-bounded organelle;intracellular organelle;intracellular organelle part;intracellular part;macromolecular complex;membrane-bounded organelle;microtubule;neuror		Homo sapiens	1	8
cell part;cytoplasm;cytoplasmic microtubule;cytoplasmic part;cytoskeletal part;endoplasmic reticulum;endoplasmic reticulum membrane;endoplasmic reticulum part;Golgi apparatus;integral to membrane;intracellular membrane-bounded organelle;intracellular organelle;intracellular organelle part;intracellular part;intrinsic to membrane;macr		Homo sapiens	1	8
		Homo sapiens	1	8
cell part;intracellular membrane-bounded organelle;intracellular non-membrane-bounded organelle;intracellular organelle;intracellular organelle part;intracellular part;membrane-bounded organelle;non-membrane-bounded organelle;nuclear part;nucleolus;nucleus;organelle;organelle part		Homo sapiens	1	8
cell part;cytoplasmic part;extracellular region part;extracellular space;Golgi apparatus part;integral to Golgi membrane;integral to membrane;integral to organelle membrane;intracellular organelle part;intracellular part;intrinsic to Golgi membrane;intrinsic to membrane;intrinsic to organelle membrane;membrane;membrane part;nuclear memb		Homo sapiens	1	8
cell part;clathrin-coated vesicle;coated vesicle;cytoplasmic membrane-bounded vesicle;cytoplasmic part;cytoplasmic vesicle;endosome;exocytic vesicle;intracellular membrane-bounded organelle;intracellular organelle;intracellular part;late endosome;lysosome;lytic vacuole;membrane;membrane-bounded organelle;membrane-bounded ve		Homo sapiens	1	8
cell part;cell projection;lamellipodium		Homo sapiens	1	8
cell part;cytoplasmic mRNA processing body;cytoplasmic part;cytosol;intracellular membrane-bounded organelle;intracellular non-membrane-bounded organelle;intracellular organelle;intracellular organelle part;intracellular p	RNA degradation	Homo sapiens	1	8
cell part;cytoplasm;cytoplasmic part;intracellular membrane-bounded organelle;intracellular organelle;intracellular organelle part;intracellular part;membrane-bounded organelle;mitochondrion;nuclear part;nucleoplasm;organelle;organelle part		Homo sapiens	1	8
cell part;cytoplasmic part;cytosol;intracellular part	Butanoate metabolism	Homo sapiens	1	8
		Homo sapiens	1	8
cell part;cytoplasm;cytoplasmic part;cytosol;intracellular part	Alanine, aspartate and glutamate metabolism;Purine metabolism	Homo sapiens	1	8
cell part;cytoplasmic part;cytosol;intracellular part		Homo sapiens	1	8
cell part;cytoplasm;cytoplasmic part;intracellular membrane-bounded organelle;intracellular non-membrane-bounded organelle;intracellular organelle;intracellular part;macromolecular complex;membrane-bounded organelle;non-membrane-bounded organelle;nucleus;organelle;ribonucleoprotein complex;RNA granule;stress granule		Homo sapiens	1	8
cell part;cytoplasmic part;endoplasmic reticulum;endoplasmic reticulum membrane;endoplasmic reticulum part;integral to membrane;intracellular membrane-bounded organelle;intracellular organelle;intracellular organelle part;intracellular part;intrinsic to membrane;macromolecular complex;membrane;membrane part;membrane-bounded o		Homo sapiens	1	8
cell part;integral to membrane;intrinsic to membrane;membrane part		Homo sapiens	1	8
cell part;cytoplasmic part;endoplasmic reticulum lumen;endoplasmic reticulum part;intracellular organelle lumen;intracellular organelle part;intracellular part;membrane-enclosed lumen;organelle lumen;organelle part		Homo sapiens	1	8
calcium channel complex;cation channel complex;cell part;cytoplasmic part;integral to membrane;integral to mitochondrial inner membrane;integral to mitochondrial membrane;integral to organelle membrane;intracellular membrane-bounded organelle;intracellular organelle;intracellular organelle part;intracellular part;intrinsic to membrane;i		Homo sapiens	1	8
cell part;cytoplasmic part;intracellular membrane-bounded organelle;intracellular organelle;intracellular part;membrane-bounded organelle;mitochondrion;organelle		Homo sapiens	1	8
cell part;cytoplasm;cytoplasmic part;extracellular membrane-bounded organelle;extracellular organelle;extracellular region part;extracellular vesicular exosome;intracellular membrane-bounded organelle;intracellular organelle;intracellular part;membrane-bounded organelle;membrane-bounded vesicle;mitochondrion;organelle;vesicle		Homo sapiens	1	8
cell part;cytoplasm;cytoplasmic part;cytoskeletal part;intracellular organelle part;intracellular part;macromolecular complex;microtubule;microtubule organizing center;nuclear part;nucleoplasm;organelle part;protein complex;spindle pole		Homo sapiens	1	8
cell part;integral to membrane;integral to plasma membrane;intrinsic to membrane;intrinsic to plasma membrane;membrane;membrane part;plasma membrane;plasma membrane part		Homo sapiens	1	8
cell part;chromosomal part;condensed chromosome kinetochore;cytoplasm;cytoplasmic part;cytoskeletal part;cytoskeleton;extracellular membrane-bounded organelle;extracellular organelle;extracellular region part;extracellular vesicular exosome;intracellular;intracellular non-membrane-bounded organelle;intracellular organelle;intracellular		Homo sapiens	1	8
cell part;integral to membrane;intrinsic to membrane;membrane part		Homo sapiens	1	8
cell part;cytoplasm;extracellular membrane-bounded organelle;extracellular organelle;extracellular region part;extracellular vesicular exosome;intracellular;intracellular part;membrane-bounded organelle;membrane-bounded vesicle;organelle;vesicle		Homo sapiens	1	8
cell part;cytoplasmic part;intracellular membrane-bounded organelle;intracellular organelle;intracellular organelle lumen;intracellular organelle part;intracellular part;membrane;membrane-bounded organelle;membrane-enclos	Benzoate degradation;Fatty acid metabolism;Lysine degradation;Tryptophan metabolism	Homo sapiens	1	8
cell part;cytoplasmic part;cytosol;extracellular membrane-bounded organelle;extracellular organelle;extracellular region part;extracellular vesicular exosome;intracellular part;membrane-bounded organelle;membrane-bounde	Carbon fixation in photosynthetic organisms;Pentose and glucuronate interconversions;Pentose phosphate pathwa	Homo sapiens	1	8
cell part;cytoplasm;intracellular membrane-bounded organelle;intracellular non-membrane-bounded organelle;intracellular organelle;intracellular organelle part;intracellular part;membrane;membrane-bounded organelle;non-r	Ribosome biogenesis in eukaryotes;RNA transport	Homo sapiens	1	8
cell part;cytoplasmic part;endoplasmic reticulum;endoplasmic reticulum membrane;endoplasmic reticulum part;integral to membrane;intracellular membrane-bounded organelle;intracellular organelle;intracellular organelle part;intracellular part;intrinsic to membrane;membrane;membrane part;membrane-bounded organelle;nuclear membrar		Homo sapiens	1	8
actin cytoskeleton;cell part;cytoplasm;cytoskeleton;intracellular membrane-bounded organelle;intracellular non-membrane-bounded organelle;intracellular organelle;intracellular part;membrane;membrane-bounded organelle;non-membrane-bounded organelle;nucleus;organelle;plasma membrane		Homo sapiens	1	8
cell part;integral to membrane;intrinsic to membrane;membrane;membrane part		Homo sapiens	1	8
cell part;chromatin;chromosomal part;cytoskeleton;intermediate filament cytoskeleton;intracellular membrane-bounded organelle;intracellular non-membrane-bounded organelle;intracellular organelle;intracellular organelle pa	Lysine degradation	Homo sapiens	1	8
cell part;cytoplasm;cytoplasmic part;cytosol;early endosome;early endosome membrane;endosomal part;endosome;endosome membrane;immunological synapse;intracellular membrane-bounded organelle;intracellular organelle;intracellular organelle part;intracellular part;membrane;membrane part;membrane-bounded organelle;nuclear p		Homo sapiens	1	8
cell part;cytoplasm;intracellular membrane-bounded organelle;intracellular organelle;intracellular part;membrane-bounded organelle;nucleus;organelle		Homo sapiens	1	8
cell part;cytoplasmic part;Golgi apparatus;intracellular membrane-bounded organelle;intracellular organelle;intracellular part;membrane-bounded organelle;organelle		Homo sapiens	1	8
cell part;cytoplasmic part;endoplasmic reticulum membrane;endoplasmic reticulum part;integral to membrane;intracellular organelle part;intracellular part;intrinsic to membrane;membrane;membrane part;organelle membrane	Glycerophospholipid metabolism	Homo sapiens	1	8
cell part;intracellular membrane-bounded organelle;intracellular non-membrane-bounded organelle;intracellular organelle;intracellular organelle part;intracellular part;membrane-bounded organelle;non-membrane-bounded organelle;nuclear part;nucleolus;nucleus;organelle;organelle part		Homo sapiens	1	8
cell part;cytoplasm;intracellular membrane-bounded organelle;intracellular organelle;intracellular part;membrane-bounded organelle;nucleus;organelle		Homo sapiens	1	8
cell part;cytoplasmic part;intracellular membrane-bounded organelle;intracellular organelle;intracellular part;membrane;membrane-bounded organelle;nucleus;organelle;perinuclear region of cytoplasm		Homo sapiens	1	8
cell part;cytoplasm;cytoskeletal part;holo TFIIH complex;intracellular membrane-bounded organelle;intracellular non-membrane-bounded organelle;intracellular organelle;intracellular organelle part;intracellular part;macromolecular complex;membrane;membrane-bounded organelle;MMXD complex;non-membrane-bounded organelle;nuclea		Homo sapiens	1	8
cell part;extracellular region part;extracellular space;membrane		Homo sapiens	1	8
		Homo sapiens	1	8
cell part;centrosome;cytoplasmic microtubule;cytoplasmic part;cytoskeletal part;cytosol;intracellular non-membrane-bounded organelle;intracellular organelle;intracellular organelle part;intracellular part;macromolecular complex;membrane;microtubule;microtubule organizing center;non-membrane-bounded organelle;nuclear part;nucleopla		Homo sapiens	1	8
cell part;cytoplasm;intracellular membrane-bounded organelle;intracellular organelle;intracellular part;membrane-bounded organelle;nucleus;organelle		Homo sapiens	1	8
cell body;cell junction;cell part;cell pole;cell projection;cell tip;cytoplasmic membrane-bounded vesicle;cytoplasmic part;cytoplasmic vesicle;cytosol;dendrite;endosome;extracellular region part;Golgi apparatus;growing cell ti	Inositol phosphate metabolism;Phosphatidylinositol signaling system	Homo sapiens	1	8
cell part;cytoplasm;envelope;intracellular membrane-bounded organelle;intracellular organelle;intracellular organelle part;intracellular part;membrane;membrane-bounded organelle;nuclear envelope;nuclear part;nucleus;organelle;organelle envelope;organelle part		Homo sapiens	1	8
		Homo sapiens	1	8
Cdc73/Paf1 complex;cell part;chromatin;chromosomal part;cytoplasm;cytoplasmic part;intracellular membrane-bounded organelle;intracellular organelle;intracellular organelle part;intracellular part;macromolecular complex;r	RNA degradation	Homo sapiens	1	8
acrosomal vesicle;cell part;cytoplasmic membrane-bounded vesicle;cytoplasmic part;cytoplasmic vesicle;intracellular membrane-bounded organelle;intracellular organelle;intracellular part;macromolecular complex;membrane-bounded organelle;membrane-bounded vesicle;mitochondrion;nucleus;organelle;perinuclear region of cytoplasm;r		Homo sapiens	1	8
cell part;cytoplasm;extracellular membrane-bounded organelle;extracellular organelle;extracellular region part;extracellular vesicular exosome;intracellular part;membrane-bounded organelle;membrane-bounded vesicle;organelle;vesicle		Homo sapiens	1	8
cell part;cytoplasm;intracellular non-membrane-bounded organelle;intracellular organelle;intracellular organelle part;intracellular part;membrane;non-membrane-bounded organelle;nuclear part;nucleolus;organelle;organelle part		Homo sapiens	1	8
cell part;extracellular membrane-bounded organelle;extracellular organelle;extracellular region part;extracellular vesicular exosome;intracellular membrane-bounded organelle;intracellular organelle;intracellular part;membrane-bounded organelle;membrane-bounded vesicle;nucleus;organelle;vesicle		Homo sapiens	1	8
cell part;cytoplasmic part;cytosol;intracellular part		Homo sapiens	1	8
Cajal body;cell body;cell part;cytoplasmic part;cytosol;intracellular membrane-bounded organelle;intracellular non-membrane-bounded organelle;intracellular organelle;intracellular organelle part;intracellular part;membrane-b	RNA transport	Homo sapiens	1	8
cell part;membrane		Homo sapiens	1	8
cell part;cytoplasmic part;Golgi apparatus;Golgi apparatus part;Golgi membrane;intracellular membrane-bounded organelle;intracellular organelle;intracellular organelle part;intracellular part;membrane;membrane-bounded organelle;organelle;organelle membrane;organelle part		Homo sapiens	1	8
cell part;cytoplasm;cytoplasmic mRNA processing body;cytoplasmic part;cytosol;intracellular non-membrane-bounded organelle;intracellular organelle;intracellular part;macromolecular complex;membrane;micro-ribonucleoprotein complex;non-membrane-bounded organelle;organelle;ribonucleoprotein complex;RISC-loading complex;RNA		Homo sapiens	1	8
catalytic step 2 spliceosome;cell part;cytoplasm;cytoplasmic part;Golgi apparatus;intracellular membrane-bounded organelle;intracellular organelle;intracellular organelle part;intracellular part;macromolecular complex;membrane-bounded organelle;nuclear body;nuclear part;nuclear speck;nucleoplasm;nucleoplasm part;organelle;organelle		Homo sapiens	1	8
cell part;histone methyltransferase complex;intracellular membrane-bounded organelle;intracellular organelle;intracellular organelle part;intracellular part;macromolecular complex;membrane-bounded organelle;methyltransfe	Wnt signaling pathway	Homo sapiens	1	8
cell part;cytoplasmic part;intracellular membrane-bounded organelle;intracellular organelle;intracellular organelle part;intracellular part;large ribosomal subunit;macromolecular complex;membrane;membrane-bounded organelle;mitochondrial inner membrane;mitochondrial large ribosomal subunit;mitochondrial membrane;mitochondrial part		Homo sapiens	1	8
cell part;DUBm complex;histone acetyltransferase complex;intracellular organelle part;intracellular part;macromolecular complex;membrane part;nuclear part;nuclear pore;nucleoplasm part;organelle part;pore complex;protein complex;SAGA complex;SAGA-type complex;transcription export complex 2		Homo sapiens	1	8
BBSome;cell part;cytoplasmic part;cytosol;intracellular part;macromolecular complex;protein complex		Homo sapiens	1	8
cell part;cytoplasm;cytoplasmic part;cytosol;endoplasmic reticulum membrane;endoplasmic reticulum part;intracellular membrane-bounded organelle;intracellular organelle;intracellular organelle part;intracellular part;membra	Protein processing in endoplasmic reticulum	Homo sapiens	1	8
cell part;cytoplasm;cytoplasmic part;cytoskeletal part;endosomal part;endosome membrane;extracellular membrane-bounded organelle;extracellular organelle;extracellular region part;extracellular vesicular exosome;intracellular organelle part;intracellular part;late endosome membrane;lysosomal membrane;membrane;membrane-bounded		Homo sapiens	1	8
cell part;cytoplasm;intracellular membrane-bounded organelle;intracellular organelle;intracellular part;membrane-bounded organelle;nucleus;organelle		Homo sapiens	1	8
cell part;cytoplasm;cytoplasmic part;cytosol;extracellular membrane-bounded organelle;extracellular organelle;extracellular region part;extracellular vesicular exosome;intracellular organelle part;intracellular part;membrane-b	Vitamin B6 metabolism	Homo sapiens	1	8
cell part;cytoplasmic part;intracellular membrane-bounded organelle;intracellular organelle;intracellular part;membrane-bounded organelle;mitochondrion;organelle		Homo sapiens	1	8
cell part;intracellular membrane-bounded organelle;intracellular non-membrane-bounded organelle;intracellular organelle;intracellular organelle part;intracellular part;membrane-bounded organelle;non-membrane-bounded organelle;nuclear part;nucleolus;nucleus;organelle;organelle part		Homo sapiens	1	8
BRCA1-A complex;BRISC complex;cell part;cytoplasm;intracellular membrane-bounded organelle;intracellular organelle;intracellular organelle part;intracellular part;macromolecular complex;membrane-bounded organelle;nuclear part;nucleoplasm;nucleus;organelle;organelle part;protein complex		Homo sapiens	1	8
cell part;cyclin-dependent protein kinase holoenzyme complex;intracellular membrane-bounded organelle;intracellular non-membrane-bounded organelle;intracellular organelle;intracellular organelle part;intracellular part;macromolecular complex;membrane-bounded organelle;non-membrane-bounded organelle;nuclear body;nuclear cyclin-		Homo sapiens	1	8
cell part;cytoplasm;intracellular membrane-bounded organelle;intracellular organelle;intracellular organelle part;intracellular part;membrane-bounded organelle;nuclear part;nucleoplasm;nucleus;organelle;organelle part		Homo sapiens	1	8
cell part;cytoplasmic part;cytosol;endosomal part;endosome membrane;extracellular membrane-bounded organelle;extracellular organelle;extracellular region part;extracellular vesicular exosome;intracellular membrane-bour	Endocytosis	Homo sapiens	1	8
cell part;cytoplasm;cytoplasmic part;extracellular membrane-bounded organelle;extracellular organelle;extracellular region part;extracellular vesicular exosome;integral to membrane;intracellular membrane-bounded organelle	Alzheimer's disease;Huntington's disease;Oxidative phosphorylation;Parkinson's disease	Homo sapiens	1	8
cell part;cytoplasmic part;cytosol;intracellular part	Purine metabolism	Homo sapiens	1	8
cell part;chromatin remodeling complex;chromosomal part;H4/H2A histone acetyltransferase complex;histone acetyltransferase complex;histone deacetylase complex;intracellular organelle part;intracellular part;macromolecular complex;NuA4 histone acetyltransferase complex;nuclear chromosome part;nuclear part;nucleoplasm;nucleopla		Homo sapiens	1	8
cell part;cytoplasmic part;cytoplasmic ubiquitin ligase complex;endoplasmic reticulum membrane;endoplasmic reticulum part;ER ubiquitin ligase complex;Hrd1p ubiquitin ligase complex;integral to membrane;intracellular org	Protein processing in endoplasmic reticulum	Homo sapiens	1	8

cell part;chromosomal part;condensed chromosome kinetochore;intracellular non-membrane-bounded organelle;intracellular organelle;intracellular organelle part;intracellular part;kinetochore;macromolecular complex;non-membrane-bounded organelle;organelle;organelle part;protein complex		Homo sapiens	1	8
cell part;cytoplasm;cytoplasmic part;cytosol;endosomal part;endosome membrane;intracellular organelle part;intracellular part;membrane;organelle membrane;organelle part	Cytosolic DNA-sensing pathway;Hepatitis C;Measles;RIG-I-like receptor signaling pathway;Toll-like receptor signali	Homo sapiens	1	8
cell part;extracellular membrane-bounded organelle;extracellular organelle;extracellular region part;extracellular vesicular exosome;integral to membrane;intrinsic to membrane;membrane part;membrane-bounded organelle;membrane-bounded vesicle;organelle;vesicle		Homo sapiens	1	8
cell part;centrosome;cytoplasm;cytoplasmic dynein complex;cytoplasmic part;cytoskeletal part;cytosol;dynactin complex;dynein complex;intracellular membrane-bounded organelle;intracellular non-membrane-bounded orga	Huntington's disease;Vasopressin-regulated water reabsorption	Homo sapiens	1	8
cell part;cytoplasmic part;cytosol;extracellular membrane-bounded organelle;extracellular organelle;extracellular region part;extracellular vesicular exosome;intracellular;intracellular membrane-bounded organelle;intracellular	Purine metabolism	Homo sapiens	1	8
cell part;intracellular membrane-bounded organelle;intracellular organelle;intracellular organelle part;intracellular part;macromolecular complex;membrane-bounded organelle;nuclear part;nucleoplasm;nucleoplasm part;nucle	Huntington's disease	Homo sapiens	1	8
cell part;intracellular organelle part;intracellular part;macromolecular complex;mediator complex;nuclear part;nucleoplasm;nucleoplasm part;organelle part;protein complex;transcription factor complex		Homo sapiens	1	8
CD95 death-inducing signaling complex;Cdc48p-Npl4p-Ufd1p AAA ATPase complex;cell part;cytoplasmic part;cytosol;death-inducing signaling complex;endoplasmic reticulum part;envelope;intracellular membrane-bounded organelle;intracellular organelle;intracellular organelle part;intracellular part;macromolecular complex;membrane pa		Homo sapiens	1	8
cell part;cytoplasm;cytoplasmic part;cytoskeletal part;extracellular membrane-bounded organelle;extracellular organelle;extracellular region part;extracellular vesicular exosome;intracellular membrane-bounded organelle;intracellular non-membrane-bounded organelle;intracellular organelle;intracellular organelle lumen;intracellular organelle		Homo sapiens	1	8
cell part;intracellular organelle part;intracellular part;macromolecular complex;nuclear part;nucleoplasm;organelle part;ribonucleoprotein complex;spliceosomal complex;U12-type spliceosomal complex	Spliceosome	Homo sapiens	1	8
catalytic step 2 spliceosome;cell part;extracellular membrane-bounded organelle;extracellular organelle;extracellular region part;extracellular vesicular exosome;intracellular organelle part;intracellular part;macromolecular cor	Spliceosome	Homo sapiens	1	8
cell part;cytoplasmic part;cytosol;intracellular membrane-bounded organelle;intracellular organelle;intracellular part;membrane;membrane-bounded organelle;mitochondrion;organelle		Homo sapiens	1	8
cell part;cytoplasm;intracellular membrane-bounded organelle;intracellular organelle;intracellular part;membrane-bounded organelle;nucleus;organelle		Homo sapiens	1	8
cell part;cytoplasmic part;cytosol;intracellular organelle part;intracellular part;macromolecular complex;nuclear part;organelle part;ribonucleoprotein complex;small nuclear ribonucleoprotein complex;spliceosomal complex;U	RNA degradation;Spliceosome	Homo sapiens	1	8
catalytic step 2 spliceosome;cell part;cytoplasm;cytoplasmic part;cytosol;exon-exon junction complex;intracellular membrane-bounded organelle;intracellular organelle;intracellular organelle part;intracellular part;macromolec	mRNA surveillance pathway;RNA transport;Spliceosome	Homo sapiens	1	8
cell part;cell surface;integral to membrane;intrinsic to membrane;membrane;membrane part;presynaptic membrane;synapse part;synaptic membrane		Homo sapiens	1	8
cell part;cytoplasm;cytoplasmic part;cytosol;extracellular membrane-bounded organelle;extracellular organelle;extracellular region part;extracellular vesicular exosome;intracellular membrane-bounded organelle;intracellular non-membrane-bounded organelle;intracellular organelle;intracellular organelle part;intracellular part;membrane;mem		Homo sapiens	1	8
cell part;chromatin remodeling complex;cytoplasm;histone deacetylase complex;intracellular organelle part;intracellular part;macromolecular complex;nuclear body;nuclear part;nuclear speck;nucleoplasm;nucleoplasm part;c	mRNA surveillance pathway;RNA transport	Homo sapiens	1	9
cell part;cytoplasmic part;cytosol;intracellular part		Homo sapiens	1	9
cell part;cytoplasmic part;endoplasmic reticulum lumen;endoplasmic reticulum membrane;endoplasmic reticulum part;extracellular membrane-bounded organelle;extracellular organelle;extracellular region part;extracellular ve	Peroxisome;PPAR signaling pathway	Homo sapiens	1	9
cell part;clathrin coated vesicle membrane;clathrin-coated vesicle;coated vesicle;coated vesicle membrane;cytoplasmic membrane-bounded vesicle;cytoplasmic part;cytoplasmic vesicle;cytoplasmic vesicle membrane;cytoplasmic vesicle part;endosomal part;endosome membrane;Golgi apparatus part;integral to membrane;intracellular me		Homo sapiens	1	9
cell part;endomembrane system;integral to membrane;intrinsic to membrane;membrane;membrane part		Homo sapiens	1	9
cell part;cytoplasm;cytoplasmic part;cytosol;extracellular membrane-bounded organelle;extracellular organelle;extracellular region part;extracellular vesicular exosome;intracellular membrane-bounded organelle;intracellular organelle;intracellular part;membrane-bounded organelle;membrane-bounded vesicle;nucleus;organelle;vesicle		Homo sapiens	1	9
cell part;cytoplasm;extracellular membrane-bounded organelle;extracellular organelle;extracellular region part;extracellular vesicular exosome;intracellular membrane-bounded organelle;intracellular organelle;intracellular organelle part;intracellular part;membrane-bounded organelle;membrane-bounded vesicle;nuclear part;nucleoplasm;nuc		Homo sapiens	1	9
cell part;cytoplasmic part;integral to membrane;intracellular membrane-bounded organelle;intracellular organelle;intracellular organelle part;intracellular part;intrinsic to membrane;membrane;membrane part;membrane-bounc	ABC transporters	Homo sapiens	1	9
cell part;cytoplasmic part;integral to membrane;integral to plasma membrane;intracellular membrane-bounded organelle;intracellular organelle;intracellular part;intrinsic to membrane;intrinsic to plasma membrane;membrane;membrane part;membrane-bounded organelle;mitochondrion;organelle;plasma membrane part		Homo sapiens	1	9
cell part;chromatin;chromosomal part;cytoplasm;cytoskeletal part;intracellular membrane-bounded organelle;intracellular organelle;intracellular organelle part;intracellular part;macromolecular complex;membrane;membrane-bounded organelle;microtubule;nuclear chromatin;nuclear chromosome part;nuclear part;nucleoplasm;nucleoplasm		Homo sapiens	1	9
cell part;cytoplasmic part;cytosol;eukaryotic 43S preinitiation complex;eukaryotic 48S preinitiation complex;eukaryotic translation initiation factor 3 complex;intracellular part;macromolecular complex;protein complex;ribonuc	RNA transport	Homo sapiens	1	9
cell part;intracellular organelle part;intracellular part;nuclear part;nucleoplasm;organelle part		Homo sapiens	1	9
cell part;cytoplasmic part;endoplasmic reticulum;endoplasmic reticulum membrane;endoplasmic reticulum part;extracellular membrane-bounded organelle;extracellular organelle;extracellular region part;extracellular space;extracellular vesicular exosome;Golgi apparatus part;Golgi membrane;integral to membrane;intracellular membrane-b		Homo sapiens	1	9
cell part;cytoplasmic part;cytosol;intracellular part	Terpenoid backbone biosynthesis	Homo sapiens	1	9
cell part;cytoplasmic part;extracellular membrane-bounded organelle;extracellular organelle;extracellular region part;extracellular vesicular exosome;intracellular organelle part;intracellular part;macromolecular complex;memb	Alzheimer's disease;Huntington's disease;Oxidative phosphorylation;Parkinson's disease	Homo sapiens	1	9
cell part;external side of plasma membrane;integral to membrane;integral to plasma membrane;intrinsic to membrane;intrinsic to plasma membrane;membrane;membrane part;plasma membrane part		Homo sapiens	1	9
cell part;cytoplasmic part;extracellular membrane-bounded organelle;extracellular organelle;extracellular region part;extracellular vesicular exosome;integral to membrane;intracellular membrane-bounded organelle;intracellul	Alzheimer's disease;Cardiac muscle contraction;Huntington's disease;Oxidative phosphorylation;Parkinson's diseas	Homo sapiens	1	9
cell body;cell junction;cell part;cell projection cytoplasm;cell projection part;cell surface;cytoplasm;cytoplasmic membrane-bounded vesicle lumen;cytoplasmic part;cytoplasmic vesicle part;cytosol;dendrite cytoplasm;extern	Primary immunodeficiency;Purine metabolism	Homo sapiens	1	9
cell part;cytoplasm;cytoplasmic membrane-bounded vesicle;cytoplasmic membrane-bounded vesicle lumen;cytoplasmic part;cytoplasmic vesicle;cytoplasmic vesicle part;endocytic vesicle lumen;extracellular membrane-bounded organelle;extracellular organelle;extracellular region;extracellular region part;extracellular space;extracellular v		Homo sapiens	1	9
cell part;cytoplasm;cytoplasmic part;cytosol;extracellular membrane-bounded organelle;extracellular organelle;extracellular region part;extracellular vesicular exosome;intracellular part;membrane-bounded organelle;membra	Alanine, aspartate and glutamate metabolism;Arginine and proline metabolism	Homo sapiens	1	9
cell part;chromatin;chromosomal part;cytoplasm;intracellular membrane-bounded organelle;intracellular organelle;intracellular organelle part;intracellular part;membrane-bounded organelle;nucleus;organelle;organelle part		Homo sapiens	1	9
cell part;cytoplasm;cytoplasmic part;cytosol;intracellular organelle part;intracellular part;nuclear part;nucleoplasm;organelle part	Porphyrin and chlorophyll metabolism	Homo sapiens	1	9
cell part;cytoplasm;cytoplasmic part;cytosol;extracellular membrane-bounded organelle;extracellular organelle;extracellular region part;extracellular vesicular exosome;intracellular membrane-bounded organelle;intracellular o	Arachidonic acid metabolism;Glutathione metabolism	Homo sapiens	1	9
caveola;cell part;cell projection;integral to membrane;integral to plasma membrane;intracellular membrane-bounded organelle;intracellular organelle;intracellular part;intrinsic to membrane;intrinsic to plasma membrane;macr	Adherens junction;Endocytosis;Focal adhesion;Glioma;Long-term depression;Melanoma;Oocyte meiosis;Pathways	Homo sapiens	1	9
cell part;cytoskeleton;intracellular membrane-bounded organelle;intracellular non-membrane-bounded organelle;intracellular organelle;intracellular organelle part;intracellular part;macromolecular complex;membrane-bounde	Basal transcription factors	Homo sapiens	1	9
cell part;cytoplasmic part;extracellular region part;extracellular space;intracellular membrane-bounded organelle;intracellular organelle;intracellular part;membrane-bounded organelle;mitochondrion;organelle	One carbon pool by folate	Homo sapiens	1	9
cell part;cell surface;cytoplasm;extracellular membrane-bounded organelle;extracellular organelle;extracellular region;extracellular region part;extracellular space;extracellular vesicular exosome;intracellular organelle part;intra	Phenylalanine metabolism;Tyrosine metabolism	Homo sapiens	1	9
anchored to membrane;cell part;cell surface;extracellular membrane-bounded organelle;extracellular organelle;extracellular region part;extracellular vesicular exosome;intrinsic to membrane;membrane;membrane part;membrane-bounded organelle;membrane-bounded vesicle;organelle;plasma membrane;vesicle		Homo sapiens	1	9
cell part;centrosome;chromosomal part;condensed chromosome outer kinetochore;condensed nuclear chromosome outer kinetochore;cytoplasm;cytoplasmic part;cytoskeletal part;cytosol;intracellular membrane-bounded c	Cell cycle;p53 signaling pathway;Progesterone-mediated oocyte maturation	Homo sapiens	1	9
cell part;cytoplasmic part;intracellular membrane-bounded organelle;intracellular organelle;intracellular organelle part;intracellular part;membrane;membrane-bounded organelle;membrane-enclosed lumen;mitochondrial inne	Alzheimer's disease;Cardiac muscle contraction;Huntington's disease;Oxidative phosphorylation;Parkinson's diseas	Homo sapiens	1	9
cell part;cytoplasmic part;extracellular membrane-bounded organelle;extracellular organelle;extracellular region part;extracellular vesicular exosome;intracellular organelle lumen;intracellular organelle part;intracellular part;lys	Glycosaminoglycan degradation;Lysosome	Homo sapiens	1	9
cell part;cytoplasm;extracellular membrane-bounded organelle;extracellular organelle;extracellular region part;extracellular vesicular exosome;intracellular organelle part;intracellular part;membrane-bounded organelle;membrane-bounded vesicle;nuclear part;nucleoplasm;organelle;organelle part;vesicle		Homo sapiens	1	9
cell junction;cell part;cell projection;cell-cell junction;cytoplasm;cytoplasmic part;cytosol;intracellular organelle part;intracellular part;lamellipodium;macromolecular complex;membrane;nuclear part;organelle part;plasma mem	Calcium signaling pathway;Epithelial cell signaling in Helicobacter pylori infection;ErbB signaling pathway;Fc epsilor	Homo sapiens	1	9
cell part;cytoplasmic part;extracellular membrane-bounded organelle;extracellular organelle;extracellular region part;extracellular vesicular exosome;intracellular organelle part;intracellular part;membrane;membrane-boundec	Alzheimer's disease;Cardiac muscle contraction;Huntington's disease;Oxidative phosphorylation;Parkinson's diseas	Homo sapiens	1	9
cell part;cytoplasmic part;intracellular membrane-bounded organelle;intracellular organelle;intracellular organelle lumen;intracellular organelle part;intracellular part;membrane-bounded organelle;membrane-enclosed lumen;m	Carbon fixation pathways in prokaryotes;Glyoxylate and dicarboxylate metabolism;Propanoate metabolism;Valine, l	Homo sapiens	1	9
cell part;cytoplasmic part;intracellular membrane-bounded organelle;intracellular organelle;intracellular organelle lumen;intracellular organelle part;intracellular part;membrane;membrane-bounded organelle;membrane-enclos	Porphyrin and chlorophyll metabolism	Homo sapiens	1	9
basement membrane;extracellular matrix;extracellular matrix part;extracellular membrane-bounded organelle;extracellular organelle;extracellular region;extracellular region part;extracellular space;extracellular vesicular exosome;membrane-bounded organelle;membrane-bounded vesicle;organelle;proteinaceous extracellular matrix;vesicle		Homo sapiens	1	9
cell part;cytoplasmic part;endoplasmic reticulum;endoplasmic reticulum membrane;endoplasmic reticulum part;extracellular membrane-bounded organelle;extracellular organelle;extracellular region part;extracellular vesicular exosome;intracellular membrane-bounded organelle;intracellular organelle;intracellular organelle part;intracellular pa		Homo sapiens	1	9
cell part;intracellular membrane-bounded organelle;intracellular organelle;intracellular organelle part;intracellular part;membrane-bounded organelle;nuclear part;nucleoplasm;nucleus;organelle;organelle part	Adipocytokine signaling pathway;Non-small cell lung cancer;Pathways in cancer;PPAR signaling pathway;Small cell	Homo sapiens	1	9
cell part;cytoplasmic part;cytosol;extracellular membrane-bounded organelle;extracellular organelle;extracellular region part;extracellular vesicular exosome;intracellular part;membrane-bounded organelle;membrane-bounde	Amino sugar and nucleotide sugar metabolism;Fructose and mannose metabolism	Homo sapiens	1	9
anchored to membrane;cell part;cytoplasmic part;endosome;extracellular matrix;extracellular membrane-bounded organelle;extracellular organelle;extracellular region part;extracellular space;extracellular vesicular exosome;Golgi apparatus part;Golgi lumen;integral to membrane;integral to plasma membrane;intracellular membrane-bounde		Homo sapiens	1	9
cell part;cytoplasmic part;cytosol;extracellular membrane-bounded organelle;extracellular organelle;extracellular region part;extracellular vesicular exosome;intracellular membrane-bounded organelle;intracellular organelle;intracellular part;membrane-bounded organelle;membrane-bounded vesicle;mitochondrion;nucleus;organelle;vesicle		Homo sapiens	1	9
calcium channel complex;cation channel complex;cell part;cell projection;cytoplasmic part;cytosol;intracellular membrane-bounded organelle;intracellular organelle;intracellular organelle part;intracellular part;ion channel com	MAPK signaling pathway	Homo sapiens	1	9
actin cytoskeleton;axon;axon part;cell cortex;cell junction;cell part;cell projection;cell projection part;clathrin-coated vesicle;coated vesicle;cytoplasm;cytoplasmic membrane-bounded vesicle;cytoplasmic part;cytoplasmic ve	Alzheimer's disease;Parkinson's disease	Homo sapiens	1	9
cell part;membrane;plasma membrane		Homo sapiens	1	9
cell part;cytoplasmic part;extracellular membrane-bounded organelle;extracellular organelle;extracellular region part;extracellular vesicular exosome;intracellular membrane-bounded organelle;intracellular organelle;intracellul	Fatty acid metabolism	Homo sapiens	1	9
cell part;cytoplasm;cytoplasmic membrane-bounded vesicle;cytoplasmic part;cytoplasmic vesicle;cytosol;endoplasmic reticulum membrane;endoplasmic reticulum part;Golgi apparatus;intracellular membrane-bounded orga	alpha-Linolenic acid metabolism;Arachidonic acid metabolism;Ether lipid metabolism;Fat digestion and absorption;	Homo sapiens	1	9
cell part;cytoplasm;extracellular membrane-bounded organelle;extracellular organelle;extracellular region part;extracellular vesicular exosome;intracellular part;membrane;membrane-bounded organelle;membrane-bounded v	Fc gamma R-mediated phagocytosis;Leishmaniasis	Homo sapiens	1	9
cell part;centrosome;cytoplasm;cytoplasmic part;cytoskeletal part;cytosol;extracellular membrane-bounded organelle;extracellular organelle;extracellular region part;extracellular vesicular exosome;intracellular membrane-bo	MAPK signaling pathway;Neurotrophin signaling pathway;VEGF signaling pathway	Homo sapiens	1	9
cell part;intracellular organelle part;intracellular part;nuclear part;nucleoplasm;organelle part	DNA replication;Purine metabolism;Pyrimidine metabolism	Homo sapiens	1	9
cell part;cytoplasm;intracellular organelle part;intracellular part;membrane;nuclear membrane;nuclear part;organelle membrane;organelle part;plasma membrane	Selenocompound metabolism	Homo sapiens	1	9
cell part;cytoplasm;cytoplasmic part;cytosol;intracellular membrane-bounded organelle;intracellular organelle;intracellular organelle part;intracellular part;macromolecular complex;membrane-bounded organelle;mRNA cap bi	mRNA surveillance pathway;RNA transport;Spliceosome	Homo sapiens	1	9
cell part;cytoplasm;cytoplasmic part;cytosol;extracellular membrane-bounded organelle;extracellular organelle;extracellular region part;extracellular vesicular exosome;intracellular part;membrane;membrane-bounded organelle;membrane-bounded vesicle;organelle;plasma membrane;vesicle		Homo sapiens	1	9
box C/D snoRNP complex;cell part;intracellular membrane-bounded organelle;intracellular non-membrane-bounded organelle;intracellular organelle;intracellular organelle part;intracellular part;macromolecular complex;memt	Ribosome biogenesis in eukaryotes;Spliceosome	Homo sapiens	1	9
cell part;cytoplasmic part;endoplasmic reticulum;endoplasmic reticulum membrane;endoplasmic reticulum part;integral to membrane;intracellular membrane-bounded organelle;intracellular organelle;intracellular organelle pa	Protein export	Homo sapiens	1	9
actin cytoskeleton;cell part;cytoplasm;cytoskeleton;extracellular membrane-bounded organelle;extracellular organelle;extracellular region part;extracellular vesicular exosome;intermediate filament cytoskeleton;intracellular m	Ubiquitin mediated proteolysis	Homo sapiens	1	9
cell part;cytoplasmic part;extracellular membrane-bounded organelle;extracellular organelle;extracellular region part;extracellular vesicular exosome;Golgi apparatus part;Golgi membrane;intracellular organelle part;intracellular part;membrane;membrane-bounded organelle;membrane-bounded vesicle;organelle;organelle membrane;organell		Homo sapiens	1	9
cell part;intracellular membrane-bounded organelle;intracellular organelle;intracellular organelle part;intracellular part;membrane-bounded organelle;nuclear body;nuclear part;nucleoplasm;nucleoplasm part;nucleus;organelle;	RNA transport	Homo sapiens	1	9
cell part;cytoplasmic membrane-bounded vesicle lumen;cytoplasmic part;cytoplasmic vesicle part;cytoskeleton;cytosol;extracellular region;intracellular membrane-bounded organelle;intracellular non-membrane-bounded or	Regulation of actin cytoskeleton	Homo sapiens	1	9
adherens junction;anchoring junction;cell division site part;cell junction;cell part;cell-substrate adherens junction;cell-substrate junction;cleavage furrow;cytoplasmic part;cytosol;early endosome;endosomal part;endosome;endosome membrane;extracellular membrane-bounded organelle;extracellular organelle;extracellular region part;extra		Homo sapiens	1	9
cell part;cell projection;centrosome;chromosomal part;cilium;cytoplasm;cytoplasmic dynein complex;cytoplasmic part;cytoskeletal part;cytosol;dynein complex;extracellular membrane-bounded organelle;extracellular organe	Vasopressin-regulated water reabsorption	Homo sapiens	1	9
adherens junction;anchoring junction;cell junction;cell part;cell-substrate adherens junction;cell-substrate junction;cytoplasmic part;cytosol;endoplasmic reticulum membrane;endoplasmic reticulum part;extracellular membra	Bacterial invasion of epithelial cells;Shigellosis	Homo sapiens	1	9
cell part;cytoplasmic part;cytosol;intracellular membrane-bounded organelle;intracellular organelle;intracellular part;membrane-bounded organelle;nucleus;organelle		Homo sapiens	1	9
cell part;intracellular membrane-bounded organelle;intracellular non-membrane-bounded organelle;intracellular organelle;intracellular organelle part;intracellular part;membrane-bounded organelle;non-membrane-bounded organelle;nuclear part;nucleolus;nucleoplasm;nucleus;organelle;organelle part		Homo sapiens	1	9
cell part;cytoplasm;intracellular membrane-bounded organelle;intracellular non-membrane-bounded organelle;intracellular organelle;intracellular organelle part;intracellular part;membrane;membrane-bounded organelle;non-membrane-bounded organelle;nuclear part;nucleolus;nucleoplasm;nucleus;organelle;organelle part;plasma membran		Homo sapiens	1	9
cell part;intracellular membrane-bounded organelle;intracellular organelle;intracellular organelle part;intracellular part;membrane-bounded organelle;nuclear part;nucleoplasm;nucleus;organelle;organelle part		Homo sapiens	1	9
cell junction;cell part;cell-cell junction;cytoplasm;cytoplasmic part;cytosol;endoplasmic reticulum;extracellular membrane-bounded organelle;extracellular organelle;extracellular region part;extracellular vesicular exosome;intr	Tight junction;Vascular smooth muscle contraction	Homo sapiens	1	9
cell part;chromosome;cytoplasm;cytoplasmic part;cytoskeleton;cytosol;exosome (RNase complex);extracellular membrane-bounded organelle;extracellular organelle;extracellular region part;extracellular vesicular exosome;ir	RNA degradation	Homo sapiens	1	9
CD40 receptor complex;cell cortex;cell part;cytoplasm;cytoplasmic part;cytosol;internal side of plasma membrane;intracellular part;macromolecular complex;membrane;membrane part;membrane raft;organelle membrane;or	Adipocytokine signaling pathway;Apoptosis;Hepatitis C;MAPK signaling pathway;Osteoclast differentiation;Pathway	Homo sapiens	1	9
cell part;cytoplasm;extracellular region part;intercellular bridge;intracellular membrane-bounded organelle;intracellular non-membrane-bounded organelle;intracellular organelle;intracellular organelle part;intracellular part;macromolecular complex;membrane;membrane-bounded organelle;non-membrane-bounded organelle;nuclear part;nucl		Homo sapiens	1	9
cell part;cytoplasm;cytoplasmic part;cytosol;intracellular part;membrane;plasma membrane	Fructose and mannose metabolism	Homo sapiens	1	9
actin filament bundle;actomyosin;adherens junction;anchoring junction;cell junction;cell part;cell-substrate adherens junction;cell-substrate junction;contractile fiber part;cytoplasmic part;cytoskeletal part;focal adhesion;intracellular membrane-bounded organelle;intracellular organelle;intracellular organelle part;intracellular part;membrane-b		Homo sapiens	1	9
cell part;cytoplasm;cytoplasmic part;cytosol;extracellular membrane-bounded organelle;extracellular organelle;extracellular region part;extracellular vesicular exosome;intracellular membrane-bounded organelle;intracellular organelle;intracellular organelle part;intracellular part;membrane-bounded organelle;membrane-bounded vesicle;nucle		Homo sapiens	1	9
adherens junction;anchoring junction;cell body;cell cortex;cell junction;cell part;cell projection;cell-substrate adherens junction;cell-substrate junction;cytoplasmic part;cytosol;focal adhesion;intracellular part;lamellipodium;m	Regulation of actin cytoskeleton	Homo sapiens	1	9
adherens junction;anchoring junction;cell junction;cell part;cell-substrate adherens junction;cell-substrate junction;focal adhesion;intracellular organelle part;intracellular part;nuclear part;nucleoplasm;organelle part	Endocytosis	Homo sapiens	1	9
catalytic step 2 spliceosome;cell part;cytoplasm;intracellular membrane-bounded organelle;intracellular organelle;intracellular organelle part;intracellular part;macromolecular complex;membrane-bounded organelle;nuclear p	Spliceosome	Homo sapiens	1	9
cell part;cytoplasmic part;cytosol;intracellular membrane-bounded organelle;intracellular organelle;intracellular organelle part;intracellular part;membrane-bounded organelle;nuclear body;nuclear part;nuclear speck;nucleopla	Proteasome	Homo sapiens	1	9
cell part;cytoplasm;cytoplasmic part;endoplasmic reticulum;ER membrane protein complex;intracellular membrane-bounded organelle;intracellular non-membrane-bounded organelle;intracellular organelle;intracellular organelle part;intracellular part;macromolecular complex;membrane-bounded organelle;mitochondrion;non-membrane-bou		Homo sapiens	1	9
cell part;cytoplasm;integral to membrane;integral to plasma membrane;intracellular organelle part;intracellular part;intrinsic to membrane;intrinsic to plasma membrane;macromolecular complex;membrane;membrane part;nuclear part;nucleoplasm;organelle part;plasma membrane;plasma membrane part;proteasome complex;protein compl		Homo sapiens	1	9
cell part;cis-Golgi network;cytoplasmic part;extracellular membrane-bounded organelle;extracellular organelle;extracellular region part;extracellular vesicular exosome;Golgi apparatus part;Golgi membrane;integral to membra	N-Glycan biosynthesis;Various types of N-glycan biosynthesis	Homo sapiens	1	9
cell part;cytoplasmic part;endopeptidase Clp complex;intracellular membrane-bounded organelle;intracellular organelle;intracellular organelle lumen;intracellular organelle part;intracellular part;macromolecular complex;memb	Cell cycle - Caulobacter	Homo sapiens	1	9
cell part;cytoplasmic part;extracellular membrane-bounded organelle;extracellular organelle;extracellular region part;extracellular space;extracellular vesicular exosome;intracellular membrane-bounded organelle;intracellular c	Cysteine and methionine metabolism;Sulfur relay system	Homo sapiens	1	9
cell part;cytoplasm;cytoplasmic part;extracellular membrane-bounded organelle;extracellular organelle;extracellular region part;extracellular vesicular exosome;intracellular organelle lumen;intracellular organelle part;intracellu	Pyruvate metabolism	Homo sapiens	1	9
cell part;cytoplasmic part;cytosol;intracellular part	Drug metabolism - other enzymes;Pyrimidine metabolism	Homo sapiens	1	9
cell part;cytoplasmic part;intracellular membrane-bounded organelle;intracellular organelle;intracellular part;membrane-bounded organelle;mitochondrion;organelle		Homo sapiens	1	9
cell part;cytoplasm;intracellular organelle part;intracellular part;membrane;nuclear part;nucleoplasm;organelle part;plasma membrane		Homo sapiens	1	9
cell part;cytoplasmic exosome (RNase complex);cytoplasmic part;cytosol;exosome (RNase complex);intracellular non-membrane-bounded organelle;intracellular organelle;intracellular organelle part;intracellular part;macromo	RNA degradation	Homo sapiens	1	9
cell part;chromosomal part;condensed chromosome kinetochore;intracellular non-membrane-bounded organelle;intracellular organelle;intracellular organelle part;intracellular part;kinetochore;macromolecular complex;non-membrane-bounded organelle;organelle;organelle part;protein complex		Homo sapiens	1	9
		Homo sapiens	1	9
		Homo sapiens	1	9
cell part;cytoplasmic part;cytosol;intracellular part		Homo sapiens	1	9
cell part;intracellular membrane-bounded organelle;intracellular non-membrane-bounded organelle;intracellular organelle;intracellular organelle part;intracellular part;membrane-bounded organelle;non-membrane-bounded organelle;nuclear body;nuclear part;nuclear speck;nucleolus;nucleoplasm part;nucleus;organelle;organelle part		Homo sapiens	1	9
cell junction;cell part;cell-cell junction;cytoplasmic part;cytosol;intracellular part;membrane;occluding junction;plasma membrane;tight junction		Homo sapiens	1	9
catalytic step 2 spliceosome;cell part;intracellular membrane-bounded organelle;intracellular organelle;intracellular organelle part;intracellular part;macromolecular complex;membrane-bounded organelle;nuclear part;nucleoplasm;nucleus;organelle;organelle part;ribonucleoprotein complex;spliceosomal complex		Homo sapiens	1	9
cell part;cytoplasmic part;endoplasmic reticulum;integral to membrane;intracellular membrane-bounded organelle;intracellular organelle;intracellular part;intrinsic to membrane;membrane;membrane part;membrane-bounded	Bile secretion	Homo sapiens	1	9
cell part;cytoplasm;extracellular region;intracellular part;membrane		Homo sapiens	1	9
brush border;cell part;cell projection;cytoplasmic microtubule;cytoplasmic part;cytoskeletal part;extracellular membrane-bounded organelle;extracellular organelle;extracellular region part;extracellular vesicular exosome;intra	Pentose and glucuronate interconversions	Homo sapiens	1	9
aggresome;cell part;inclusion body;intracellular membrane-bounded organelle;intracellular organelle;intracellular organelle part;intracellular part;membrane-bounded organelle;nuclear part;nucleoplasm;organelle;organelle part		Homo sapiens	1	9
cell part;cytoplasmic part;endoplasmic reticulum membrane;endoplasmic reticulum part;extracellular membrane-bounded organelle;extracellular organelle;extracellular region part;extracellular vesicular exosome;integral to membrane;intracellular organelle part;intracellular part;intrinsic to membrane;membrane;membrane part;membrane-bo		Homo sapiens	1	9
actin cytoskeleton;cell part;centrosome;cytoplasm;cytoplasmic part;cytoskeletal part;cytoskeleton;intracellular membrane-bounded organelle;intracellular non-membrane-bounded organelle;intracellular organelle;intracellular organelle part;intracellular part;macromolecular complex;membrane;membrane-bounded organelle;microtubule;micr		Homo sapiens	1	9
		Homo sapiens	1	9
cell junction;cell part;cytoplasmic part;intracellular membrane-bounded organelle;intracellular organelle;intracellular organelle part;intracellular part;membrane;membrane-bounded organelle;microbody;microbody membrane;microbody part;mitochondrion;nucleus;organelle;organelle membrane;organelle part;peroxisomal membrane;peroxis		Homo sapiens	1	9
cell part;chromosomal part;condensed chromosome kinetochore;cytoplasmic part;cytosol;envelope;intracellular membrane-bounded organelle;intracellular non-membrane-bounded organelle;intracellular organelle;intracellula	RNA transport	Homo sapiens	1	9
		Homo sapiens	1	9
cell junction;cell part;clathrin-coated vesicle;coated vesicle;cytoplasm;cytoplasmic membrane-bounded vesicle;cytoplasmic part;cytoplasmic vesicle;eukaryotic translation initiation factor 3 complex;intracellular membrane-bounded organelle;intracellular organelle;intracellular organelle part;intracellular part;macromolecular complex;membra		Homo sapiens	1	9
cell part;cytoplasmic part;endoplasmic reticulum lumen;endoplasmic reticulum part;endoplasmic reticulum-Golgi intermediate compartment;extracellular membrane-bounded organelle;extracellular organelle;extracellular region part;extracellular space;extracellular vesicular exosome;intracellular membrane-bounded organelle;intracellular or		Homo sapiens	1	9
cell part;cytoplasm;cytoplasmic part;extracellular membrane-bounded organelle;extracellular organelle;extracellular region part;extracellular vesicular exosome;integral to membrane;intracellular membrane-bounded organelle;intracellular organelle;intracellular part;intrinsic to membrane;membrane part;membrane-bounded organelle;membr		Homo sapiens	1	9
cell part;cytoplasmic part;intracellular organelle part;intracellular part;membrane-enclosed lumen;mitochondrial intermembrane space;mitochondrial part;organelle envelope lumen;organelle part		Homo sapiens	1	9
cell part;cytoplasm;cytoplasmic part;intracellular membrane-bounded organelle;intracellular organelle;intracellular part;membrane-bounded organelle;mitochondrion;organelle		Homo sapiens	1	9
cell part;cytoplasmic part;cytoskeleton;intracellular membrane-bounded organelle;intracellular non-membrane-bounded organelle;intracellular organelle;intracellular part;membrane-bounded organelle;mitochondrion;non-membrane-bounded organelle;organelle		Homo sapiens	1	9
cell part;cytoplasmic mRNA processing body;cytoplasmic part;cytosol;intracellular non-membrane-bounded organelle;intracellular organelle;intracellular part;macromolecular complex;membrane;non-membrane-bounded org	RNA degradation	Homo sapiens	1	9
cell part;cytoplasm;cytoplasmic part;extracellular membrane-bounded organelle;extracellular organelle;extracellular region part;extracellular vesicular exosome;intracellular membrane-bounded organelle;intracellular organelle;intracellular part;membrane-bounded organelle;membrane-bounded vesicle;mitochondrion;organelle;vesicle		Homo sapiens	1	9

cell part;chromosomal part;chromosome;condensed chromosome;condensed chromosome kinetochore;cytoplasm;cytoskeletal part;intracellular membrane-bounded organelle;intracellular non-membrane-bounded organelle;intracellular organelle;intracellular organelle part;intracellular part;kinetochore;macromolecular complex;membrane-b		Homo sapiens	1	9
cell part;intracellular membrane-bounded organelle;intracellular organelle;intracellular part;membrane-bounded organelle;nucleus;organelle		Homo sapiens	1	9
cell part;chromatin;chromosomal part;heterochromatin;intracellular membrane-bounded organelle;intracellular organelle;intracellular organelle part;intracellular part;membrane-bounded organelle;nuclear chromatin;nuclear chromosome part;nuclear heterochromatin;nuclear part;nucleoplasm;nucleus;organelle;organelle part		Homo sapiens	1	9
cell part;cytoplasm;cytoplasmic part;cytosol;extracellular membrane-bounded organelle;extracellular organelle;extracellular region part;extracellular vesicular exosome;intracellular membrane-bounded organelle;intracellular c	Proteasome	Homo sapiens	1	9
cell part;cytoplasm;cytoplasmic part;cytosolic part;intracellular membrane-bounded organelle;intracellular organelle;intracellular part;macromolecular complex;membrane-bounded organelle;nucleus;organelle;prefoldin complex;protein complex		Homo sapiens	1	9
cell part;cytoplasm;cytoplasmic part;cytoskeletal part;cytosol;Golgi apparatus part;Golgi stack;intracellular membrane-bounded organelle;intracellular non-membrane-bounded organelle;intracellular organelle;intracellular organelle part;intracellular part;membrane-bounded organelle;non-membrane-bounded organelle;nuclear part;nucleolus		Homo sapiens	1	9
calcium channel complex;cation channel complex;cell part;cytoplasmic part;integral to membrane;integral to mitochondrial membrane;integral to organelle membrane;intracellular;intracellular membrane-bounded organelle;intracellular organelle;intracellular organelle part;intracellular part;intrinsic to membrane;intrinsic to organelle membran		Homo sapiens	1	9
cell part;cytoplasmic part;intracellular membrane-bounded organelle;intracellular non-membrane-bounded organelle;intracellular organelle;intracellular organelle part;intracellular part;macromolecular complex;membrane;membrane-bounded organelle;mitochondrial inner membrane;mitochondrial membrane;mitochondrial part;mitochondrio		Homo sapiens	1	9
cell part;cytoplasm;cytoplasmic part;extracellular membrane-bounded organelle;extracellular organelle;extracellular region part;extracellular vesicular exosome;intracellular membrane-bounded organelle;intracellular organelle;intracellular part;membrane-bounded organelle;membrane-bounded vesicle;mitochondrion;organelle;vesicle		Homo sapiens	1	9
cell part;cytoplasm;intracellular organelle part;intracellular part;nuclear part;nucleoplasm;organelle part		Homo sapiens	1	9
cell part;cytoplasm;intracellular membrane-bounded organelle;intracellular organelle;intracellular organelle part;intracellular part;membrane;membrane-bounded organelle;nuclear part;nucleoplasm;nucleus;organelle;organelle part;plasma membrane		Homo sapiens	1	9
cell part;chromosome;cytoplasm;cytoskeletal part;intracellular membrane-bounded organelle;intracellular non-membrane-bounded organelle;intracellular organelle;intracellular organelle part;intracellular part;macromolecular complex;membrane-bounded organelle;microtubule;non-membrane-bounded organelle;nuclear part;nucleolus;nucle		Homo sapiens	1	9
cell part;intracellular membrane-bounded organelle;intracellular non-membrane-bounded organelle;intracellular organelle;intracellular organelle part;intracellular part;membrane-bounded organelle;non-membrane-bounded organelle;nuclear part;nucleolus;organelle;organelle part		Homo sapiens	1	9
		Homo sapiens	1	9
cell part;intracellular membrane-bounded organelle;intracellular organelle;intracellular organelle part;intracellular part;membrane-bounded organelle;nuclear body;nuclear part;nuclear speck;nucleoplasm part;nucleus;organelle;organelle part		Homo sapiens	1	9
cell part;intracellular membrane-bounded organelle;intracellular organelle;intracellular part;membrane-bounded organelle;nucleus;organelle		Homo sapiens	1	9
cell part;cytoplasm;cytoplasmic part;cytosol;intracellular part	RNA transport	Homo sapiens	1	9
cell part;extracellular membrane-bounded organelle;extracellular organelle;extracellular region part;extracellular vesicular exosome;integral to membrane;integral to plasma membrane;intrinsic to membrane;intrinsic to plasma membrane;membrane;membrane part;membrane-bounded organelle;membrane-bounded vesicle;organelle;plasma		Homo sapiens	1	9
cell part;cytoplasm;cytoplasmic part;cytosol;intracellular membrane-bounded organelle;intracellular organelle;intracellular organelle part;intracellular part;membrane-bounded organelle;membrane-enclosed lumen;mitochondrial intermembrane space;mitochondrial part;nucleus;organelle;organelle envelope lumen;organelle part		Homo sapiens	1	9
cell part;cytoplasm;cytoplasmic part;early endosome;endosome;intracellular membrane-bounded organelle;intracellular organelle;intracellular part;membrane-bounded organelle;organelle		Homo sapiens	1	9
cell part;intracellular membrane-bounded organelle;intracellular non-membrane-bounded organelle;intracellular organelle;intracellular organelle part;intracellular part;membrane-bounded organelle;non-membrane-bounded organelle;nuclear part;nucleolus;nucleoplasm;nucleus;organelle;organelle part		Homo sapiens	1	9
		Homo sapiens	1	9
		Homo sapiens	1	9
		Homo sapiens	1	9
cell part;membrane		Homo sapiens	1	9
cell part;cytoplasm;Elongator holoenzyme complex;histone acetyltransferase complex;intracellular non-membrane-bounded organelle;intracellular organelle;intracellular organelle part;intracellular part;macromolecular complex;non-membrane-bounded organelle;nuclear part;nucleolus;nucleoplasm part;organelle;organelle part;protein compl		Homo sapiens	1	9
cell part;cytoplasm;DNA-directed RNA polymerase complex;DNA-directed RNA polymerase I complex;intracellular non-membrane-bounded organelle;intracellular organelle;intracellular organelle part;intracellular part;macron	Purine metabolism;Pyrimidine metabolism;RNA polymerase	Homo sapiens	1	9
cell part;cytoplasmic part;cytosol;intracellular;intracellular part	Fructose and mannose metabolism	Homo sapiens	1	9
cell part;cytoplasmic part;endoplasmic reticulum;extracellular membrane-bounded organelle;extracellular organelle;extracellular region part;extracellular vesicular exosome;Golgi apparatus;intracellular membrane-bounded o	Protein processing in endoplasmic reticulum	Homo sapiens	1	9
cell part;intracellular membrane-bounded organelle;intracellular non-membrane-bounded organelle;intracellular organelle;intracellular organelle part;intracellular part;membrane-bounded organelle;non-membrane-bounded organelle;nuclear part;nucleolus;nucleus;organelle;organelle part		Homo sapiens	1	9
cell part;cytoplasmic part;cytoplasmic ubiquitin ligase complex;cytosol;intracellular part;macromolecular complex;protein complex;ubiquitin ligase complex	Regulation of autophagy	Homo sapiens	1	9
		Homo sapiens	1	9
extracellular membrane-bounded organelle;extracellular organelle;extracellular region part;extracellular vesicular exosome;membrane-bounded organelle;membrane-bounded vesicle;organelle;vesicle		Homo sapiens	1	9
cell part;cytoplasmic part;endoplasmic reticulum;endoplasmic reticulum membrane;endoplasmic reticulum part;Golgi apparatus;Golgi apparatus part;Golgi membrane;integral to membrane;intracellular membrane-bounded o	Sphingolipid metabolism	Homo sapiens	1	9
cell part;cytoplasm;histone methyltransferase complex;integral to membrane;intracellular non-membrane-bounded organelle;intracellular organelle;intracellular organelle part;intracellular part;intrinsic to membrane;macromolecular complex;membrane;membrane part;methyltransferase complex;MLL1 complex;non-membrane-bounded orga		Homo sapiens	1	9
cell part;cell projection;cytoplasm;cytoplasmic part;cytoskeleton;cytosol;filopodium;intracellular non-membrane-bounded organelle;intracellular organelle;intracellular part;lamellipodium;macromolecular complex;non-membra	Regulation of actin cytoskeleton	Homo sapiens	1	9
cell part;cytoplasmic part;extracellular membrane-bounded organelle;extracellular organelle;extracellular region part;extracellular vesicular exosome;integral to membrane;intracellular membrane-bounded organelle;intracellular organelle;intracellular organelle part;intracellular part;intrinsic to membrane;membrane;membrane part;membrane-		Homo sapiens	1	9
cell part;cytoplasmic part;integral to membrane;intracellular;intracellular membrane-bounded organelle;intracellular organelle;intracellular organelle part;intracellular part;intrinsic to membrane;membrane;membrane part;membrane-bounded organelle;mitochondrial inner membrane;mitochondrial membrane;mitochondrial part;mitochondrion;		Homo sapiens	1	9
cell part;cytoplasmic part;intracellular membrane-bounded organelle;intracellular organelle;intracellular organelle part;intracellular part;large ribosomal subunit;macromolecular complex;membrane;membrane-bounded organelle;mitochondrial inner membrane;mitochondrial large ribosomal subunit;mitochondrial membrane;mitochondrial part		Homo sapiens	1	9
		Homo sapiens	1	9
cell part;intracellular organelle part;intracellular part;macromolecular complex;membrane;mRNA cleavage and polyadenylation specificity factor complex;mRNA cleavage factor complex;nuclear part;nucleoplasm;nucleoplasn	mRNA surveillance pathway	Homo sapiens	1	9
cell part;clathrin coat;clathrin coat of coated pit;cytoplasmic part;intracellular membrane-bounded organelle;intracellular organelle;intracellular part;macromolecular complex;membrane;membrane coat;membrane part;membrane-bounded organelle;nucleus;organelle;plasma membrane;plasma membrane part;protein complex		Homo sapiens	1	9
actin filament;cell part;cytoskeletal part;extracellular membrane-bounded organelle;extracellular organelle;extracellular region part;extracellular vesicular exosome;extrinsic to internal side of plasma membrane;extrinsic to me	Chemokine signaling pathway;MAPK signaling pathway;Regulation of actin cytoskeleton	Homo sapiens	1	9
cell part;cytoplasmic part;endoplasmic reticulum membrane;endoplasmic reticulum part;integral to membrane;intracellular organelle part;intracellular part;intrinsic to membrane;membrane;membrane part;organelle membrane;organelle part		Homo sapiens	1	9
cell part;cytoplasmic part;intracellular membrane-bounded organelle;intracellular organelle;intracellular organelle lumen;intracellular organelle part;intracellular part;membrane-bounded organelle;membrane-enclosed lumen;m	Peroxisome	Homo sapiens	1	9
cell part;cytoplasmic part;endoplasmic reticulum;endoplasmic reticulum membrane;endoplasmic reticulum part;Golgi apparatus part;Golgi membrane;integral to membrane;intracellular membrane-bounded organelle;intracellular organelle;intracellular organelle part;intracellular part;intrinsic to membrane;membrane;membrane part;membran		Homo sapiens	1	9
		Homo sapiens	1	9
cell part;cytoplasmic part;cytosolic small ribosomal subunit;intracellular organelle part;intracellular part;macromolecular complex;membrane;mitochondrial inner membrane;mitochondrial membrane;mitochondrial part;organelle inner membrane;organelle membrane;organelle part;ribonucleoprotein complex;small ribosomal subunit		Homo sapiens	1	9
AP-type membrane coat adaptor complex;cell part;clathrin adaptor complex;cytoplasmic part;cytoplasmic vesicle membrane;cytoplasmic vesicle part;Golgi apparatus;intracellular membrane-bounded organelle;intracellular c	Lysosome	Homo sapiens	1	9
cell part;cytoplasm;cytoplasmic part;cytosol;Golgi apparatus part;Golgi membrane;intracellular membrane-bounded organelle;intracellular organelle;intracellular organelle part;intracellular part;membrane;membrane-bounded organelle;nuclear part;nucleoplasm;nucleus;organelle;organelle membrane;organelle part		Homo sapiens	1	9
cell part;COPI vesicle coat;COPII vesicle coat;cytoplasmic part;cytoplasmic vesicle part;endoplasmic reticulum;endoplasmic reticulum membrane;endoplasmic reticulum part;endoplasmic reticulum-Golgi intermediate compartment;endoplasmic reticulum-Golgi intermediate compartment membrane;extracellular membrane-bounded organe		Homo sapiens	1	9
cell part;cytoplasmic part;intracellular non-membrane-bounded organelle;intracellular organelle;intracellular organelle part;intracellular part;macromolecular complex;membrane;mitochondrial inner membrane;mitochondrial membrane;mitochondrial part;mitochondrial ribosome;non-membrane-bounded organelle;organellar ribosome;organel		Homo sapiens	1	9
cell part;intracellular;intracellular membrane-bounded organelle;intracellular organelle;intracellular organelle part;intracellular part;membrane;membrane-bounded organelle;nuclear part;nucleoplasm;nucleus;organelle;organelle part;plasma membrane		Homo sapiens	1	9
cell junction;cell part;cell projection;cell projection part;cytoplasm;cytoplasmic part;cytoskeleton;cytosol;dendrite;dendritic spine;extrinsic to internal side of plasma membrane;extrinsic to membrane;extrinsic to plasma membrane;filopodium;intracellular non-membrane-bounded organelle;intracellular organelle;intracellular part;membrane pa		Homo sapiens	1	9
cell part;cytoplasm;cytoplasmic part;cytosol;intracellular part;macromolecular complex;protein complex;ubiquitin ligase complex		Homo sapiens	1	9
cell part;cytoplasmic part;cytosol;extracellular membrane-bounded organelle;extracellular organelle;extracellular region part;extracellular vesicular exosome;intracellular organelle part;intracellular part;lysosomal membrane;m	Collecting duct acid secretion;Epithelial cell signaling in Helicobacter pylori infection;Oxidative phosphorylation;Pha	Homo sapiens	1	9
cell part;cytoplasm;cytoplasmic part;cytoskeletal part;cytosol;cytosolic part;IkappaB kinase complex;intracellular;intracellular membrane-bounded organelle;intracellular non-membrane-bounded organelle;intracellular organe	Acute myeloid leukemia;Adipocytokine signaling pathway;Apoptosis;B cell receptor signaling pathway;Chagas dise	Homo sapiens	1	9
cell part;cytoplasmic part;intracellular membrane-bounded organelle;intracellular organelle;intracellular organelle part;intracellular part;macromolecular complex;membrane;membrane part;membrane-bounded organelle;mitoc	Alzheimer's disease;Huntington's disease;Oxidative phosphorylation;Parkinson's disease	Homo sapiens	1	10
cell part;cytoplasm;cytoplasmic part;cytosol;intracellular membrane-bounded organelle;intracellular organelle;intracellular organelle part;intracellular part;macromolecular complex;membrane-bounded organelle;nuclear part;nucleoplasm;nucleus;organelle;organelle part;proteasome regulatory particle;protein complex		Homo sapiens	1	10
cell part;cytoplasmic part;extracellular membrane-bounded organelle;extracellular organelle;extracellular region part;extracellular vesicular exosome;intracellular membrane-bounded organelle;intracellular organelle;intracellula	Lysosome;Other glycan degradation	Homo sapiens	1	10
cell part;intracellular membrane-bounded organelle;intracellular organelle;intracellular part;membrane-bounded organelle;nucleus;organelle		Homo sapiens	1	10
cell part;cytoplasm;cytoplasmic part;cytoskeletal part;cytosol;histone acetyltransferase complex;histone methyltransferase complex;intracellular membrane-bounded organelle;intracellular organelle;intracellular organelle part	Other types of O-glycan biosynthesis	Homo sapiens	1	10
cell body;cell part;cytoplasmic part;cytosol;extracellular membrane-bounded organelle;extracellular organelle;extracellular region part;extracellular vesicular exosome;intracellular part;membrane-bounded organelle;membran	Amino sugar and nucleotide sugar metabolism;Fructose and mannose metabolism	Homo sapiens	1	10
cell part;cytoplasmic part;intracellular membrane-bounded organelle;intracellular organelle;intracellular organelle lumen;intracellular organelle part;intracellular part;membrane-bounded organelle;membrane-enclosed lumen;m	Pantothenate and CoA biosynthesis;Valine, leucine and isoleucine biosynthesis;Valine, leucine and isoleucine degra	Homo sapiens	1	10
Cajal body;cell part;intracellular membrane-bounded organelle;intracellular organelle;intracellular organelle part;intracellular part;macromolecular complex;membrane-bounded organelle;nuclear body;nuclear part;nuclear spec	Spliceosome	Homo sapiens	1	10
cell part;cytoplasm;intracellular organelle part;intracellular part;macromolecular complex;nuclear body;nuclear part;nuclear speck;nucleoplasm part;organelle part;ribonucleoprotein complex;small nuclear ribonucleoprotein cc	Spliceosome	Homo sapiens	1	10
cell part;cytoplasm;cytoplasmic part;cytosol;early endosome;endosome;extracellular membrane-bounded organelle;extracellular organelle;extracellular region part;extracellular vesicular exosome;intracellular membrane-bounded organelle;intracellular organelle;intracellular part;membrane;membrane-bounded organelle;membrane-bounded		Homo sapiens	1	10
CCR4-NOT complex;cell part;cytoplasmic mRNA processing body;cytoplasmic part;cytosol;intracellular membrane-bounded organelle;intracellular non-membrane-bounded organelle;intracellular organelle;intracellular organe	RNA degradation	Homo sapiens	1	10
cell part;cytoplasm;cytoplasmic part;cytoplasmic vesicle;endoplasmic reticulum;endoplasmic reticulum exit site;endoplasmic reticulum membrane;endoplasmic reticulum part;endosome;extracellular membrane-bounded organelle;extracellular organelle;extracellular region part;extracellular vesicular exosome;intracellular membrane-bounde		Homo sapiens	1	10
cell part;cytoplasm;cytoplasmic part;cytoskeletal part;cytoskeleton;intermediate filament cytoskeleton;intracellular membrane-bounded organelle;intracellular non-membrane-bounded organelle;intracellular organelle;intracellular organelle part;intracellular part;membrane-bounded organelle;microtubule cytoskeleton;microtubule organizing c		Homo sapiens	1	10
cell part;cytoplasmic part;intracellular organelle part;intracellular part;membrane;mitochondrial membrane;mitochondrial outer membrane;mitochondrial part;organelle membrane;organelle outer membrane;organelle part;outer membrane		Homo sapiens	1	10
cell part;cytoplasmic part;endoplasmic reticulum;endoplasmic reticulum membrane;endoplasmic reticulum part;extracellular membrane-bounded organelle;extracellular organelle;extracellular region part;extracellular vesicular exosome;integral to membrane;intracellular membrane-bounded organelle;intracellular organelle;intracellular organe		Homo sapiens	1	10
cell part;cytoplasmic part;extracellular membrane-bounded organelle;extracellular organelle;extracellular region part;extracellular vesicular exosome;intracellular membrane-bounded organelle;intracellular organelle;intracellular part;membrane-bounded organelle;membrane-bounded vesicle;mitochondrion;organelle;vesicle		Homo sapiens	1	10
cell part;cytoplasmic part;cytosol;intracellular part	Alanine, aspartate and glutamate metabolism;Amino sugar and nucleotide sugar metabolism	Homo sapiens	1	10
		Homo sapiens	1	10
cell part;cytoplasmic part;cytoskeleton;cytosol;integral to membrane;intracellular membrane-bounded organelle;intracellular non-membrane-bounded organelle;intracellular organelle;intracellular organelle part;intracellular part;intrinsic to membrane;intrinsic to mitochondrial outer membrane;intrinsic to organelle membrane;membrane;memb		Homo sapiens	1	10
cell part;cytoplasm;intracellular membrane-bounded organelle;intracellular organelle;intracellular part;macromolecular complex;membrane-bounded organelle;nucleus;organelle;protein complex;ubiquitin ligase complex	Protein processing in endoplasmic reticulum;Ubiquitin mediated proteolysis	Homo sapiens	1	10
axoneme;cell part;cell projection part;cytoplasm;cytoplasmic part;cytosol;intracellular part;organelle part;pre-autophagosomal structure	Regulation of autophagy	Homo sapiens	1	10
cell part;cytoplasm;DNA-directed RNA polymerase complex;DNA-directed RNA polymerase I complex;intracellular membrane-bounded organelle;intracellular non-membrane-bounded organelle;intracellular organelle;intracell	Purine metabolism;Pyrimidine metabolism;RNA polymerase	Homo sapiens	1	10
cell part;cytoplasm;intracellular part		Homo sapiens	1	10
cell part;cytoplasm;extracellular membrane-bounded organelle;extracellular organelle;extracellular region part;extracellular vesicular exosome;intracellular part;membrane-bounded organelle;membrane-bounded vesicle;organelle;vesicle		Homo sapiens	1	10
cell part;cytoplasm;cytoplasmic part;extracellular membrane-bounded organelle;extracellular organelle;extracellular region part;extracellular vesicular exosome;integral to membrane;integral to mitochondrial membrane;integr	Amyotrophic lateral sclerosis (ALS)	Homo sapiens	1	10
cell part;cytoplasm;extracellular region part;extracellular space;intracellular part		Homo sapiens	1	10
cell part;cytoplasmic part;extracellular membrane-bounded organelle;extracellular organelle;extracellular region part;extracellular vesicular exosome;intracellular membrane-bounded organelle;intracellular non-membrane-bou	Huntington's disease;Peroxisome	Homo sapiens	1	10
actin filament;adherens junction;anchoring junction;cell junction;cell part;cell-substrate adherens junction;cell-substrate junction;cytoplasm;cytoplasmic part;cytoskeletal part;cytosol;extracellular membrane-bounded organel	Adherens junction;Tight junction	Homo sapiens	1	10
cell part;cytoplasmic part;intracellular organelle part;intracellular part;large ribosomal subunit;macromolecular complex;membrane;mitochondrial inner membrane;mitochondrial large ribosomal subunit;mitochondrial membrane;mitochondrial part;organellar large ribosomal subunit;organelle inner membrane;organelle membrane;organelle pa		Homo sapiens	1	10
cell part;cytoplasm;cytoplasmic part;cytosol;intracellular part	Drug metabolism - cytochrome P450;Glutathione metabolism;Metabolism of xenobiotics by cytochrome P450	Homo sapiens	1	10
cell part;chromatin;chromosomal part;euchromatin;extracellular membrane-bounded organelle;extracellular organelle;extracellular region part;extracellular vesicular exosome;heterochromatin;intracellular membrane-bounded	Systemic lupus erythematosus	Homo sapiens	1	10
apical part of cell;cell part;cytoplasmic part;endoplasmic reticulum;endoplasmic reticulum membrane;endoplasmic reticulum part;integral to membrane;intracellular membrane-bounded organelle;intracellular organelle;intrace	Drug metabolism - cytochrome P450;Glutathione metabolism;Metabolism of xenobiotics by cytochrome P450	Homo sapiens	1	10
cell part;extracellular membrane-bounded organelle;extracellular organelle;extracellular region part;extracellular vesicular exosome;membrane;membrane-bounded organelle;membrane-bounded vesicle;midbody;organelle;pl	Pancreatic cancer;Pathways in cancer	Homo sapiens	1	10
cell junction;cell part;cell-cell junction;chromatin;chromosomal part;cyclin-dependent protein kinase holoenzyme complex;cytoplasmic part;cytosol;intracellular membrane-bounded organelle;intracellular non-membrane-bour	Bladder cancer;Cell cycle;Chronic myeloid leukemia;Glioma;Measles;Melanoma;Non-small cell lung cancer;p53 sig	Homo sapiens	1	10
extracellular membrane-bounded organelle;extracellular organelle;extracellular region part;extracellular vesicular exosome;membrane-bounded organelle;membrane-bounded vesicle;organelle;vesicle		Homo sapiens	1	10
cell junction;cell part;cell-cell junction;cytoplasm;cytoplasmic part;cytosol;endoplasmic reticulum;intracellular membrane-bounded organelle;intracellular non-membrane-bounded organelle;intracellular organelle;intracellular p	ErbB signaling pathway;Pathogenic Escherichia coli infection;T cell receptor signaling pathway	Homo sapiens	1	10
cell part;cell projection membrane;cell projection part;cytoplasmic part;cytosol;extracellular membrane-bounded organelle;extracellular organelle;extracellular region part;extracellular vesicular exosome;Golgi apparatus;intracellular membrane-bounded organelle;intracellular organelle;intracellular part;leading edge membrane;membrane;mer		Homo sapiens	1	10
cell part;intracellular membrane-bounded organelle;intracellular organelle;intracellular organelle part;intracellular part;membrane-bounded organelle;nuclear part;nucleoplasm;nucleus;organelle;organelle part	Base excision repair	Homo sapiens	1	10
cell part;cytoplasm;intracellular membrane-bounded organelle;intracellular organelle;intracellular part;membrane-bounded organelle;nucleus;organelle		Homo sapiens	1	10
cell part;cytoplasmic membrane-bounded vesicle;cytoplasmic part;cytoplasmic vesicle;endosome;extracellular membrane-bounded organelle;extracellular organelle;extracellular region part;extracellular vesicular exosome;in	Endocytosis	Homo sapiens	1	10
axon;cell body;cell part;cell projection;cell projection part;cytoplasmic part;cytosol;dendritic spine;extracellular membrane-bounded organelle;extracellular organelle;extracellular region part;extracellular vesicular exosome;int	Betalain biosynthesis;Steroid hormone biosynthesis;Tyrosine metabolism	Homo sapiens	1	10
actin filament bundle;actomyosin;cell part;contractile fiber part;cytoplasmic part;cytoskeletal part;cytosol;intracellular organelle part;intracellular part;macromolecular complex;muscle myosin complex;myosin complex;myosir	Focal adhesion;Leukocyte transendothelial migration;Regulation of actin cytoskeleton;Tight junction;Vascular smoot	Homo sapiens	1	10
cell part;centrosome;cytoplasm;cytoplasmic part;cytoskeletal part;cytosol;extracellular membrane-bounded organelle;extracellular organelle;extracellular region part;extracellular vesicular exosome;intracellular membrane-bo	Proteasome	Homo sapiens	1	10
actin cytoskeleton;adherens junction;anchoring junction;cell cortex;cell junction;cell part;cell-substrate adherens junction;cell-substrate junction;centrosome;cytoplasmic part;cytoskeletal part;cytoskeleton;extracellular memb	Fc gamma R-mediated phagocytosis	Homo sapiens	1	10
cell part;cytoplasmic part;cytosol;extracellular membrane-bounded organelle;extracellular organelle;extracellular region part;extracellular vesicular exosome;intracellular part;membrane-bounded organelle;membrane-bounde	Pyrimidine metabolism	Homo sapiens	1	10
cell part;cytoplasm;cytoplasmic part;cytosol;extracellular membrane-bounded organelle;extracellular organelle;extracellular region part;extracellular vesicular exosome;intracellular membrane-bounded organelle;intracellular c	Cyanoamino acid metabolism;Glycine, serine and threonine metabolism;Methane metabolism;One carbon pool by f	Homo sapiens	1	10
caveola;cell part;cell projection;cytoplasm;cytoplasmic part;endoplasmic reticulum membrane;endoplasmic reticulum part;intracellular membrane-bounded organelle;intracellular organelle;intracellular organelle part;intracellul	Arachidonic acid metabolism;Leishmaniasis;Pathways in cancer;Small cell lung cancer;VEGF signaling pathway	Homo sapiens	1	10
adherens junction;anchoring junction;cell junction;cell part;cell projection;cell projection part;cell-substrate adherens junction;cell-substrate junction;cytoplasmic part;cytoskeletal part;cytoskeleton;cytosol;dendritic spine;F-actin capping protein complex;focal adhesion;intermediate filament cytoskeleton;intracellular membrane-bounded orga		Homo sapiens	1	10
apical plasma membrane;cell part;cell projection;cytoplasmic part;cytosol;endosome;extracellular membrane-bounded organelle;extracellular organelle;extracellular region part;extracellular vesicular exosome;intracellular me	Collecting duct acid secretion;Epithelial cell signaling in Helicobacter pylori infection;Oxidative phosphorylation;Pha	Homo sapiens	1	10
cell part;cytoplasmic part;cytosol;cytosolic small ribosomal subunit;intracellular membrane-bounded organelle;intracellular non-membrane-bounded organelle;intracellular organelle;intracellular organelle part;intracellular part	Ribosome	Homo sapiens	1	10
autophagic vacuole;axon;cell part;cell projection;cytoplasm;cytoplasmic part;cytoplasmic vesicle membrane;cytoplasmic vesicle part;cytosol;dendrite;endoplasmic reticulum;endosome;Golgi apparatus;inclusion body;intrace	Huntington's disease	Homo sapiens	1	10
cell part;cytoplasm;cytoplasmic part;cytosol;intracellular organelle part;intracellular part;membrane;nuclear part;nucleoplasm;organelle part	Amyotrophic lateral sclerosis (ALS);Fc epsilon RI signaling pathway;GnRH signaling pathway;MAPK signaling pathw	Homo sapiens	1	10
cell part;cytoplasmic part;cytosol;extracellular membrane-bounded organelle;extracellular organelle;extracellular region part;extracellular vesicular exosome;intracellular membrane-bounded organelle;intracellular organelle;int	Proteasome	Homo sapiens	1	10
cell part;intracellular membrane-bounded organelle;intracellular organelle;intracellular organelle part;intracellular part;membrane-bounded organelle;nuclear part;nucleoplasm;nucleus;organelle;organelle part	Base excision repair;DNA replication;Homologous recombination;Mismatch repair;Nucleotide excision repair;Purine	Homo sapiens	1	10
cell part;cytoplasm;cytoplasmic part;cytosol;intracellular membrane-bounded organelle;intracellular organelle;intracellular part;macromolecular complex;membrane-bounded organelle;nucleus;organelle;ribonucleoprotein con	Circadian rhythm - fly;Circadian rhythm - mammal;Hedgehog signaling pathway;Wnt signaling pathway	Homo sapiens	1	10
cell part;chromosomal part;chromosome;chromosome, centromeric region;condensed chromosome;intracellular membrane-bounded organelle;intracellular non-membrane-bounded organelle;intracellular organelle;intracellular organelle part;intracellular part;membrane-bounded organelle;non-membrane-bounded organelle;nuclear part;nuc		Homo sapiens	1	10
adherens junction;anchoring junction;apical part of cell;basolateral plasma membrane;cell junction;cell part;cell-substrate adherens junction;cell-substrate junction;clathrin-coated vesicle;coated vesicle;cytoplasmic membrar	Notch signaling pathway	Homo sapiens	1	10
cell part;cytoplasmic part;cytoskeletal part;endoplasmic reticulum;envelope;integral to membrane;intracellular membrane-bounded organelle;intracellular organelle;intracellular organelle part;intracellular part;intrinsic to memb	Arrhythmogenic right ventricular cardiomyopathy (ARVC);Dilated cardiomyopathy;Hypertrophic cardiomyopathy (HC	Homo sapiens	1	10
carboxy-terminal domain protein kinase complex;cell part;cytoplasm;intracellular membrane-bounded organelle;intracellular organelle;intracellular organelle part;intracellular part;macromolecular complex;membrane;membrane-bounded organelle;nuclear body;nuclear part;nucleoplasm;nucleoplasm part;organelle;organelle part;PML body;p		Homo sapiens	1	10
cell part;cytoplasmic part;intracellular membrane-bounded organelle;intracellular non-membrane-bounded organelle;intracellular organelle;intracellular organelle part;intracellular part;macromolecular complex;membrane;membrane-bounded organelle;mitochondrial inner membrane;mitochondrial membrane;mitochondrial part;mitochondrial		Homo sapiens	1	10
cell part;cytoplasmic part;endoplasmic reticulum part;extracellular membrane-bounded organelle;extracellular organelle;extracellular region part;extracellular vesicular exosome;integral to membrane;intracellular organelle par	Protein processing in endoplasmic reticulum	Homo sapiens	1	10
cell part;cytoplasmic part;intracellular membrane-bounded organelle;intracellular organelle;intracellular organelle part;intracellular part;macromolecular complex;membrane;membrane part;membrane-bounded organelle;mem	Alzheimer's disease;Huntington's disease;Oxidative phosphorylation;Parkinson's disease	Homo sapiens	1	10
cell part;cytoplasmic part;intracellular membrane-bounded organelle;intracellular organelle;intracellular organelle part;intracellular part;membrane;membrane-bounded organelle;mitochondrial inner membrane;mitochondrial m	Porphyrin and chlorophyll metabolism	Homo sapiens	1	10
cell part;cytoplasm;cytoplasmic part;cytosol;extracellular membrane-bounded organelle;extracellular organelle;extracellular region part;extracellular vesicular exosome;intracellular membrane-bounded organelle;intracellular c	Ubiquitin mediated proteolysis	Homo sapiens	1	10
adherens junction;anchoring junction;cell cortex;cell division site part;cell junction;cell part;cell projection;cell projection membrane;cell projection part;cell-substrate adherens junction;cell-substrate junction;cleavage furrow;c	Endocytosis;Fc gamma R-mediated phagocytosis	Homo sapiens	1	10
cell part;cytoplasm;cytoskeleton;intracellular non-membrane-bounded organelle;intracellular organelle;intracellular part;non-membrane-bounded organelle;organelle		Homo sapiens	1	10
cell part;intracellular membrane-bounded organelle;intracellular organelle;intracellular organelle part;intracellular part;macromolecular complex;membrane-bounded organelle;multimeric ribonuclease P complex;nuclear part;n	Ribosome biogenesis in eukaryotes;RNA transport	Homo sapiens	1	10
cell part;membrane;midbody		Homo sapiens	1	10
cell part;cytoplasmic part;Golgi apparatus;intracellular membrane-bounded organelle;intracellular non-membrane-bounded organelle;intracellular organelle;intracellular organelle part;intracellular part;membrane-bounded organelle;non-membrane-bounded organelle;nuclear part;nucleolus;nucleus;organelle;organelle part		Homo sapiens	1	10
cell part;cytoplasmic part;cytosol;intracellular membrane-bounded organelle;intracellular organelle;intracellular part;membrane-bounded organelle;microbody;mitochondrion;organelle;peroxisome	Terpenoid backbone biosynthesis	Homo sapiens	1	10
adherens junction;anchoring junction;brush border membrane;cell junction;cell part;cell projection membrane;cell projection part;cell-substrate adherens junction;cell-substrate junction;cytoplasmic membrane-bounded vesic	Long-term depression;Regulation of actin cytoskeleton;Vascular smooth muscle contraction	Homo sapiens	1	10
macromolecular complex;protein complex;ribose phosphate diphosphokinase complex		Homo sapiens	1	10

cell part;cytoplasmic part;cytosol;exosome (RNase complex);intracellular membrane-bounded organelle;intracellular non-membrane-bounded organelle;intracellular organelle;intracellular organelle part;intracellular part;macro	RNA degradation	Homo sapiens	1	10
cell part;intracellular membrane-bounded organelle;intracellular organelle;intracellular part;membrane-bounded organelle;nucleus;organelle		Homo sapiens	1	10
calcium channel complex;cation channel complex;cell part;cytoplasm;extracellular membrane-bounded organelle;extracellular organelle;extracellular region part;extracellular vesicular exosome;intracellular membrane-bounded organelle;intracellular organelle;intracellular organelle part;intracellular part;ion channel complex;macromolecular c		Homo sapiens	1	10
adherens junction;anchoring junction;axon;cell body;cell junction;cell part;cell projection;cell surface;cell-cell junction;cilium;dendrite;integral to membrane;integral to plasma membrane;intrinsic to membrane;intrinsic to plasma membrane;leading edge membrane;membrane part;neuron projection;neuronal cell body;nonmotile primary cilium		Homo sapiens	1	10
cell part;cytoplasmic part;cytosol;elongin complex;intracellular organelle part;intracellular part;macromolecular complex;nuclear part;nucleoplasm;nucleoplasm part;organelle part;protein complex;transcription elongation fact	Pathways in cancer;Renal cell carcinoma;Ubiquitin mediated proteolysis	Homo sapiens	1	10
cell part;Cul2-RING ubiquitin ligase complex;Cul5-RING ubiquitin ligase complex;cullin-RING ubiquitin ligase complex;cytoplasmic part;cytoplasmic ubiquitin ligase complex;cytosol;elongin complex;extracellular membrane-b	Pathways in cancer;Renal cell carcinoma;Ubiquitin mediated proteolysis	Homo sapiens	1	10
cell part;chromosome;cytoplasm;extracellular membrane-bounded organelle;extracellular organelle;extracellular region part;extracellular vesicular exosome;intracellular membrane-bounded organelle;intracellular non-membrane-bounded organelle;intracellular organelle;intracellular part;membrane-bounded organelle;membrane-bounded ve		Homo sapiens	1	10
cell part;cytoplasmic membrane-bounded vesicle;cytoplasmic part;cytoplasmic vesicle;extracellular membrane-bounded organelle;extracellular organelle;extracellular region part;extracellular vesicular exosome;Golgi apparat	Protein digestion and absorption	Homo sapiens	1	10
	Amino sugar and nucleotide sugar metabolism	Homo sapiens	1	10
cell part;cell surface;chloride channel complex;extracellular membrane-bounded organelle;extracellular organelle;extracellular region part;extracellular vesicular exosome;intracellular;ion channel complex;macromolecular complex;membrane;membrane part;membrane-bounded organelle;membrane-bounded vesicle;organelle;plasma memb		Homo sapiens	1	10
cell part;extracellular membrane-bounded organelle;extracellular organelle;extracellular region part;extracellular vesicular exosome;membrane;membrane-bounded organelle;membrane-bounded vesicle;organelle;vesicle		Homo sapiens	1	10
cell part;cell projection;cytoplasm;cytoplasmic part;cytoskeletal part;cytosol;extracellular membrane-bounded organelle;extracellular organelle;extracellular region part;extracellular vesicular exosome;filamentous actin;intracellular membrane-bounded organelle;intracellular organelle;intracellular organelle part;intracellular part;lamellipodium;		Homo sapiens	1	10
cell part;intracellular non-membrane-bounded organelle;intracellular organelle;intracellular organelle part;intracellular part;macromolecular complex;non-membrane-bounded organelle;nuclear part;nucleolus;organelle;organelle part;preribosome;ribonucleoprotein complex;small-subunit processome		Homo sapiens	1	10
		Homo sapiens	1	10
cell part;intracellular organelle part;intracellular part;macromolecular complex;mRNA cleavage and polyadenylation specificity factor complex;mRNA cleavage factor complex;nuclear part;nucleoplasm;nucleoplasm part;orgar	mRNA surveillance pathway	Homo sapiens	1	10
cell part;chromatin;chromatin remodeling complex;chromosomal part;histone methyltransferase complex;intracellular organelle part;intracellular part;macromolecular complex;methyltransferase complex;nuclear part;nucleoplasm part;organelle part;protein complex;protein serine/threonine phosphatase complex;PTW/PP1 phosphatase com		Homo sapiens	1	10
cell part;cytoplasm;cytoplasmic part;cytoplasmic vesicle membrane;cytoplasmic vesicle part;endocytic vesicle membrane;endosome;intracellular membrane-bounded organelle;intracellular organelle;intracellular organelle pa	Endocytosis	Homo sapiens	1	10
cell part;integral to membrane;intracellular membrane-bounded organelle;intracellular organelle;intracellular part;intrinsic to membrane;membrane;membrane part;membrane-bounded organelle;nucleus;organelle		Homo sapiens	1	10
cell part;cytoplasm;extracellular membrane-bounded organelle;extracellular organelle;extracellular region part;extracellular vesicular exosome;intracellular organelle part;intracellular part;macromolecular complex;membrane-bounded organelle;membrane-bounded vesicle;nuclear part;nucleoplasm;organelle;organelle part;protein complex;si		Homo sapiens	1	10
cell part;cytoplasmic part;eukaryotic 43S preinitiation complex;intracellular membrane-bounded organelle;intracellular organelle;intracellular part;macromolecular complex;membrane-bounded organelle;mitochondrion;nucleus;organelle;ribonucleoprotein complex;translation preinitiation complex		Homo sapiens	1	10
		Homo sapiens	1	10
cell part;cytoplasm;intracellular part		Homo sapiens	1	10
Cdc73/Paf1 complex;cell part;cytoplasm;intracellular organelle part;intracellular part;macromolecular complex;membrane;nuclear part;nucleoplasm part;organelle part;protein complex;transcription elongation factor complex		Homo sapiens	1	10
cell part;cytoplasmic part;extracellular membrane-bounded organelle;extracellular organelle;extracellular region part;extracellular space;extracellular vesicular exosome;Golgi apparatus;integral to membrane;integral to plasma membrane;intracellular membrane-bounded organelle;intracellular organelle;intracellular part;intrinsic to membrane		Homo sapiens	1	10
cell part;intracellular membrane-bounded organelle;intracellular organelle;intracellular organelle part;intracellular part;membrane;membrane-bounded organelle;nuclear part;nucleoplasm;nucleus;organelle;organelle part	Amino sugar and nucleotide sugar metabolism	Homo sapiens	1	10
extracellular region		Homo sapiens	1	10
cell part;cytoplasm;cytoplasmic part;endoplasmic reticulum;endoplasmic reticulum membrane;endoplasmic reticulum part;envelope;intracellular membrane-bounded organelle;intracellular organelle;intracellular organelle part;intracellular part;membrane;membrane part;membrane-bounded organelle;nuclear envelope;nuclear part;organelle;		Homo sapiens	1	10
cell junction;cell part;cell-cell junction;cytoplasm;cytoskeleton;intracellular non-membrane-bounded organelle;intracellular organelle;intracellular organelle part;intracellular part;membrane;non-membrane-bounded organelle;n	mRNA surveillance pathway;Tight junction	Homo sapiens	1	10
cell part;cytoplasm;cytoplasmic part;Golgi apparatus;intracellular membrane-bounded organelle;intracellular non-membrane-bounded organelle;intracellular organelle;intracellular organelle part;intracellular part;membrane-bounded organelle;non-membrane-bounded organelle;nuclear part;nucleolus;organelle;organelle part		Homo sapiens	1	10
cell part;cytoplasm;intracellular part;LUBAC complex;macromolecular complex;protein complex;ubiquitin ligase complex		Homo sapiens	1	10
		Homo sapiens	1	10
cell part;cytoplasm;intracellular organelle part;intracellular part;nuclear part;nucleoplasm;organelle part	mRNA surveillance pathway	Homo sapiens	1	10
cell part;cytoplasmic part;intracellular;intracellular membrane-bounded organelle;intracellular organelle;intracellular part;membrane-bounded organelle;mitochondrion;organelle	RNA transport	Homo sapiens	1	10
cell part;cytoplasm;cytoplasmic part;cytosol;intracellular membrane-bounded organelle;intracellular organelle;intracellular part;membrane-bounded organelle;nucleus;organelle	mRNA surveillance pathway	Homo sapiens	1	10
cell part;cytoplasm;intracellular membrane-bounded organelle;intracellular organelle;intracellular organelle part;intracellular part;membrane-bounded organelle;nuclear part;nucleoplasm;nucleus;organelle;organelle part		Homo sapiens	1	10
cell part;cytoplasm;cytoplasmic part;cytosol;extracellular membrane-bounded organelle;extracellular organelle;extracellular region part;extracellular space;extracellular vesicular exosome;intracellular membrane-bounded organelle;intracellular organelle;intracellular part;lysosome;lytic vacuole;mast cell granule;membrane-bounded organelle		Homo sapiens	1	10
cell part;intracellular membrane-bounded organelle;intracellular non-membrane-bounded organelle;intracellular organelle;intracellular organelle part;intracellular part;membrane-bounded organelle;non-membrane-bounded organelle;nuclear part;nucleolus;nucleoplasm;nucleus;organelle;organelle part		Homo sapiens	1	10
cell part;cytoplasmic part;endoplasmic reticulum membrane;endoplasmic reticulum part;extracellular region;intracellular organelle part;intracellular part;membrane;membrane part;organelle membrane;organelle part		Homo sapiens	1	10
cell part;cytoplasmic part;endoplasmic reticulum;extracellular region;intracellular membrane-bounded organelle;intracellular organelle;intracellular part;membrane;membrane-bounded organelle;organelle	Glycolysis / Gluconeogenesis	Homo sapiens	1	10
cell part;chromatin remodeling complex;chromosomal part;cytoplasmic membrane-bounded vesicle;cytoplasmic part;cytoplasmic vesicle;intracellular membrane-bounded organelle;intracellular organelle;intracellular organelle part;intracellular part;macromolecular complex;membrane-bounded organelle;membrane-bounded vesicle;nuclear		Homo sapiens	1	10
		Homo sapiens	1	10
cell part;cytoplasmic part;intracellular membrane-bounded organelle;intracellular organelle;intracellular part;membrane-bounded organelle;mitochondrion;nucleus;organelle		Homo sapiens	1	10
cell part;cytoplasmic part;cytoskeleton;cytosol;extracellular membrane-bounded organelle;extracellular organelle;extracellular region;extracellular region part;extracellular vesicular exosome;intracellular non-membrane-bounded organelle;intracellular organelle;intracellular part;membrane-bounded organelle;membrane-bounded vesicle;non-		Homo sapiens	1	10
cell part;cytoplasm;cytoplasmic part;cytosol;intracellular membrane-bounded organelle;intracellular organelle;intracellular part;membrane-bounded organelle;nucleus;organelle	Pathways in cancer;Renal cell carcinoma	Homo sapiens	1	10
cell junction;cell part;cytoplasmic part;intracellular membrane-bounded organelle;intracellular non-membrane-bounded organelle;intracellular organelle;intracellular organelle part;intracellular part;macromolecular complex;membrane;membrane-bounded organelle;mitochondrial inner membrane;mitochondrial membrane;mitochondrial part;n		Homo sapiens	1	10
extracellular membrane-bounded organelle;extracellular organelle;extracellular region part;extracellular vesicular exosome;membrane-bounded organelle;membrane-bounded vesicle;organelle;vesicle		Homo sapiens	1	10
extracellular membrane-bounded organelle;extracellular organelle;extracellular region part;extracellular vesicular exosome;membrane-bounded organelle;membrane-bounded vesicle;organelle;vesicle		Homo sapiens	1	10
cell part;cytoplasmic part;Golgi apparatus;Golgi apparatus part;Golgi membrane;integral to membrane;intracellular membrane-bounded organelle;intracellular organelle;intracellular organelle part;intracellular part;intrinsic to membrane;membrane;membrane part;membrane-bounded organelle;mitochondrion;organelle;organelle membrane;o		Homo sapiens	1	10
actin cytoskeleton;cell part;cytoplasm;cytoskeleton;endomembrane system;intracellular non-membrane-bounded organelle;intracellular organelle;intracellular part;membrane;microtubule cytoskeleton;non-membrane-bounded organelle;organelle;plasma membrane		Homo sapiens	1	10
cell part;cytoplasmic part;integral to membrane;intracellular membrane-bounded organelle;intracellular organelle;intracellular organelle part;intracellular part;intrinsic to membrane;membrane;membrane part;membrane-bounded organelle;mitochondrial inner membrane;mitochondrial membrane;mitochondrial part;mitochondrion;nuclear part		Homo sapiens	1	10
cell part;chromosome;cytoplasm;intracellular membrane-bounded organelle;intracellular non-membrane-bounded organelle;intracellular organelle;intracellular organelle part;intracellular part;membrane;membrane-bounded o	Lysine degradation	Homo sapiens	1	10
cell part;cytoplasmic part;cytosol;extracellular membrane-bounded organelle;extracellular organelle;extracellular region part;extracellular vesicular exosome;intracellular part;membrane-bounded organelle;membrane-bounde	Pantothenate and CoA biosynthesis	Homo sapiens	1	10
cell part;cytoplasm;cytoplasmic part;cytosol;exon-exon junction complex;intracellular membrane-bounded organelle;intracellular organelle;intracellular organelle part;intracellular part;macromolecular complex;membrane-bou	mRNA surveillance pathway;RNA transport	Homo sapiens	1	10
adherens junction;anchoring junction;cell junction;cell part;cell surface;cell-substrate adherens junction;cell-substrate junction;cytoplasm;cytoskeleton;focal adhesion;intracellular non-membrane-bounded organelle;intracellular organelle;intracellular part;non-membrane-bounded organelle;organelle		Homo sapiens	1	10
actin cytoskeleton;cell part;cytoskeleton;extracellular region part;extracellular space;intracellular membrane-bounded organelle;intracellular non-membrane-bounded organelle;intracellular organelle;intracellular organelle part;intracellular part;membrane-bounded organelle;non-membrane-bounded organelle;nuclear part;nucleoplasm;nucleu		Homo sapiens	1	10
catalytic step 2 spliceosome;cell part;intracellular membrane-bounded organelle;intracellular organelle;intracellular organelle part;intracellular part;macromolecular complex;membrane;membrane-bounded organelle;nuclear p	Spliceosome	Homo sapiens	1	10
cell part;cytoplasm;cytoskeleton;extracellular region part;intercellular bridge;intracellular membrane-bounded organelle;intracellular non-membrane-bounded organelle;intracellular organelle;intracellular organelle part;intracellular part;macromolecular complex;membrane;membrane-bounded organelle;microtubule cytoskeleton;non-membra		Homo sapiens	1	10
cell part;cytoplasm;intracellular part;membrane;plasma membrane		Homo sapiens	1	10
cell part;cytoplasmic part;intracellular organelle part;intracellular part;large ribosomal subunit;macromolecular complex;membrane;mitochondrial inner membrane;mitochondrial large ribosomal subunit;mitochondrial membrane;mitochondrial part;organellar large ribosomal subunit;organelle inner membrane;organelle membrane;organelle pa		Homo sapiens	1	10
cell part;cytoplasm;cytoskeletal part;intracellular organelle part;intracellular part;kinesin complex;macromolecular complex;microtubule;microtubule associated complex;organelle part;protein complex		Homo sapiens	1	10
cell part;intracellular organelle part;intracellular part;nuclear part;nucleoplasm;organelle part		Homo sapiens	1	10
cell part;membrane		Homo sapiens	1	10
cell part;intracellular non-membrane-bounded organelle;intracellular organelle;intracellular organelle part;intracellular part;membrane;non-membrane-bounded organelle;nuclear part;nucleolus;nucleoplasm;organelle;organelle part;plasma membrane		Homo sapiens	1	10
cell part;cytoplasmic part;cytoskeletal part;cytosol;intermediate filament;intracellular membrane-bounded organelle;intracellular organelle;intracellular organelle part;intracellular part;macromolecular complex;membrane-bounded organelle;nucleus;organelle;organelle part;protein complex		Homo sapiens	1	10
catalytic step 2 spliceosome;cell part;intracellular membrane-bounded organelle;intracellular organelle;intracellular organelle part;intracellular part;macromolecular complex;membrane-bounded organelle;nuclear part;nucleop	Spliceosome	Homo sapiens	1	10
cell part;cytoplasm;intracellular membrane-bounded organelle;intracellular non-membrane-bounded organelle;intracellular organelle;intracellular organelle part;intracellular part;membrane-bounded organelle;non-membrane-bounded organelle;nuclear part;nucleolus;nucleus;organelle;organelle part		Homo sapiens	1	10
adherens junction;anchoring junction;cell junction;cell part;cell-substrate adherens junction;cell-substrate junction;cytoplasmic part;endoplasmic reticulum;endoplasmic reticulum membrane;endoplasmic reticulum part;focal adhesion;integral to membrane;intracellular membrane-bounded organelle;intracellular organelle;intracellular organell		Homo sapiens	1	10
cell part;cyclin-dependent protein kinase holoenzyme complex;intracellular membrane-bounded organelle;intracellular organelle;intracellular organelle part;intracellular part;macromolecular complex;membrane-bounded organelle;nuclear cyclin-dependent protein kinase holoenzyme complex;nuclear part;nucleus;organelle;organelle part;pro		Homo sapiens	1	10
cell part;cytoplasmic part;endoplasmic reticulum;endoplasmic reticulum membrane;endoplasmic reticulum part;integral to membrane;intracellular membrane-bounded organelle;intracellular organelle;intracellular organelle pa	Steroid biosynthesis	Homo sapiens	1	10
cell part;cytoplasmic part;integral to membrane;intracellular membrane-bounded organelle;intracellular organelle;intracellular organelle part;intracellular part;intrinsic to membrane;membrane;membrane part;membrane-bounc	Proximal tubule bicarbonate reclamation	Homo sapiens	1	10
cell part;cytoplasm;extracellular membrane-bounded organelle;extracellular organelle;extracellular region part;extracellular vesicular exosome;intracellular part;membrane-bounded organelle;membrane-bounded vesicle;organelle;vesicle		Homo sapiens	1	10
cell part;cytoplasm;cytoplasmic part;cytosolic part;extracellular membrane-bounded organelle;extracellular organelle;extracellular region part;extracellular vesicular exosome;intracellular membrane-bounded organelle;intracellular non-membrane-bounded organelle;intracellular organelle;intracellular organelle part;intracellular part;macromol		Homo sapiens	1	10
cell part;cytoplasmic part;cytosol;extracellular membrane-bounded organelle;extracellular organelle;extracellular region part;extracellular vesicular exosome;intracellular organelle part;intracellular part;lysosomal membrane;m	Epithelial cell signaling in Helicobacter pylori infection;ko05152;Lysosome;Oxidative phosphorylation;Phagosome;R	Homo sapiens	1	10
cell part;intracellular membrane-bounded organelle;intracellular organelle;intracellular part;membrane-bounded organelle;nucleus;organelle		Homo sapiens	1	10
acrosomal vesicle;cell part;cytoplasmic membrane-bounded vesicle;cytoplasmic part;cytoplasmic vesicle;cytosol;Golgi apparatus;Golgi apparatus part;Golgi membrane;integral to membrane;intracellular membrane-bounded organelle;intracellular organelle;intracellular organelle part;intracellular part;intrinsic to membrane;membrane;membr		Homo sapiens	1	10
cell part;cytoplasmic part;intracellular membrane-bounded organelle;intracellular organelle;intracellular organelle part;intracellular part;macromolecular complex;membrane;membrane-bounded organelle;mitochondrial inner membrane;mitochondrial membrane;mitochondrial part;mitochondrial small ribosomal subunit;mitochondrion;organell		Homo sapiens	1	10
cell part;cytoplasm;intracellular membrane-bounded organelle;intracellular non-membrane-bounded organelle;intracellular organelle;intracellular organelle part;intracellular part;membrane;membrane-bounded organelle;non-membrane-bounded organelle;nuclear part;nucleolus;nucleus;organelle;organelle part		Homo sapiens	1	10
cell part;integral to membrane;integral to nuclear inner membrane;integral to organelle membrane;intracellular organelle part;intracellular part;intrinsic to membrane;intrinsic to nuclear inner membrane;intrinsic to organelle membrane;membrane;membrane part;nuclear inner membrane;nuclear membrane;nuclear membrane part;nuclear part;		Homo sapiens	1	10
cell part;cytoplasmic mRNA processing body;cytoplasmic part;cytosol;extracellular membrane-bounded organelle;extracellular organelle;extracellular region part;extracellular vesicular exosome;intracellular non-membrane-bounded organelle;intracellular organelle;intracellular part;macromolecular complex;membrane-bounded organelle;me		Homo sapiens	1	10
cell part;cytoplasmic part;intracellular membrane-bounded organelle;intracellular non-membrane-bounded organelle;intracellular organelle;intracellular organelle part;intracellular part;membrane-bounded organelle;mitochondrion;non-membrane-bounded organelle;nuclear part;nucleolus;nucleus;organelle;organelle part		Homo sapiens	1	10
cell part;intracellular membrane-bounded organelle;intracellular non-membrane-bounded organelle;intracellular organelle;intracellular organelle part;intracellular part;membrane-bounded organelle;non-membrane-bounded organelle;nuclear part;nucleolus;nucleus;organelle;organelle part		Homo sapiens	1	10
extracellular membrane-bounded organelle;extracellular organelle;extracellular region part;extracellular vesicular exosome;membrane-bounded organelle;membrane-bounded vesicle;organelle;vesicle		Homo sapiens	1	10
cell part;cytoplasmic part;extracellular membrane-bounded organelle;extracellular organelle;extracellular region part;extracellular vesicular exosome;intracellular membrane-bounded organelle;intracellular organelle;intracellul	Alzheimer's disease;Huntington's disease;Oxidative phosphorylation;Parkinson's disease	Homo sapiens	1	10
	Glyoxylate and dicarboxylate metabolism	Homo sapiens	1	11
cell part;cytoplasm;intracellular membrane-bounded organelle;intracellular organelle;intracellular part;membrane-bounded organelle;nucleus;organelle		Homo sapiens	1	11
		Homo sapiens	1	11
cell part;cytoplasmic part;cytosol;intracellular organelle part;intracellular part;macromolecular complex;membrane part;nuclear part;nuclear pore;nucleoplasm;organelle part;pore complex;protein complex		Homo sapiens	1	11
cell part;cytoplasm;intracellular membrane-bounded organelle;intracellular organelle;intracellular part;membrane-bounded organelle;nucleus;organelle		Homo sapiens	1	11
cell part;chromatin;chromatin remodeling complex;chromosomal part;intracellular membrane-bounded organelle;intracellular organelle;intracellular organelle part;intracellular part;macromolecular complex;membrane-bounded organelle;nBAF complex;npBAF complex;nuclear chromatin;nuclear chromosome part;nuclear part;nucleoplasm;nu		Homo sapiens	1	11
actin cytoskeleton;apical plasma membrane;brush border membrane;cell part;cell periphery;cell projection;cell projection membrane;cell projection part;centrosome;cytoplasm;cytoplasmic part;cytoskeletal part;cytoskeleton;endomembrane system;extracellular membrane-bounded organelle;extracellular organelle;extracellular region part;e		Homo sapiens	1	11
cell part;cytoplasm;cytoplasmic part;cytosol;intracellular membrane-bounded organelle;intracellular organelle;intracellular part;membrane-bounded organelle;mitochondrion;organelle		Homo sapiens	1	11
cell part;cytoplasmic part;intracellular membrane-bounded organelle;intracellular organelle;intracellular organelle part;intracellular part;membrane;membrane part;membrane-bounded organelle;mitochondrial inner membrane	Alzheimer's disease;Cardiac muscle contraction;Huntington's disease;Oxidative phosphorylation;Parkinson's diseas	Homo sapiens	1	11
cell part;cytoplasm;cytoplasmic part;intracellular membrane-bounded organelle;intracellular non-membrane-bounded organelle;intracellular organelle;intracellular organelle part;intracellular part;membrane;membrane-bounde	Ribosome biogenesis in eukaryotes	Homo sapiens	1	11
cell part;cytoplasm;cytoplasmic part;cytoplasmic vesicle membrane;cytoplasmic vesicle part;cytosol;endoplasmic reticulum;endosome;extracellular membrane-bounded organelle;extracellular organelle;extracellular region pa	SNARE interactions in vesicular transport	Homo sapiens	1	11
cell part;cytoplasmic part;cytosol;extracellular membrane-bounded organelle;extracellular organelle;extracellular region part;extracellular vesicular exosome;Golgi apparatus;intracellular membrane-bounded organelle;intracellular organelle;intracellular part;membrane-bounded organelle;membrane-bounded vesicle;mitochondrion;organelle;v		Homo sapiens	1	11
cell part;chromosome;condensed chromosome;cytoplasmic part;female pronucleus;Golgi apparatus;intracellular membrane-bounded organelle;intracellular non-membrane-bounded organelle;intracellular organelle;intracellular organelle part;intracellular part;membrane;membrane-bounded organelle;mitochondrion;non-membrane-bounded		Homo sapiens	1	11
cell part;intracellular organelle part;intracellular part;nuclear part;nucleoplasm;organelle part		Homo sapiens	1	11
		Homo sapiens	1	11
cell part;chromatin;chromosomal part;cytoplasm;intracellular membrane-bounded organelle;intracellular organelle;intracellular organelle part;intracellular part;membrane;membrane-bounded organelle;nucleus;organelle;organelle part		Homo sapiens	1	11
cell part;cytoplasmic part;cytosol;extracellular membrane-bounded organelle;extracellular organelle;extracellular region part;extracellular vesicular exosome;intracellular organelle part;intracellular part;lysosomal membrane;m	Collecting duct acid secretion;Epithelial cell signaling in Helicobacter pylori infection;Oxidative phosphorylation;Pha	Homo sapiens	1	11
cell part;cytoplasmic part;intracellular membrane-bounded organelle;intracellular organelle;intracellular organelle part;intracellular part;macromolecular complex;membrane;membrane part;membrane-bounded organelle;mitoc	Alzheimer's disease;Huntington's disease;Oxidative phosphorylation;Parkinson's disease	Homo sapiens	1	11
cell part;granular component;intracellular non-membrane-bounded organelle;intracellular organelle;intracellular organelle part;intracellular part;non-membrane-bounded organelle;nuclear part;nucleolar part;nucleolus;nucleoplasm;organelle;organelle part		Homo sapiens	1	11
cell part;cytoplasmic part;endoplasmic reticulum membrane;endoplasmic reticulum part;extracellular membrane-bounded organelle;extracellular organelle;extracellular region part;extracellular vesicular exosome;integral to membrane;intracellular organelle part;intracellular part;intrinsic to membrane;membrane;membrane part;membrane-bo		Homo sapiens	1	11
cell division site part;cell part;centrosome;chromosomal part;cleavage furrow;condensed chromosome kinetochore;cytoplasmic part;cytoskeletal part;cytosol;dynactin complex;intracellular non-membrane-bounded organelle;intracellular organelle;intracellular organelle part;intracellular part;kinetochore;macromolecular complex;microtubule		Homo sapiens	1	11
cell cortex;cell part;contractile fiber part;cytoplasmic part;extracellular membrane-bounded organelle;extracellular organelle;extracellular region part;extracellular vesicular exosome;intracellular part;membrane-bounded organelle;membrane-bounded vesicle;organelle;organelle part;vesicle;Z disc		Homo sapiens	1	11
cell part;cytoplasm;cytoplasmic part;endopeptidase Clp complex;intracellular membrane-bounded organelle;intracellular non-membrane-bounded organelle;intracellular organelle;intracellular organelle lumen;intracellular orga	Cell cycle - Caulobacter	Homo sapiens	1	11
cell part;cytoplasm;intracellular membrane-bounded organelle;intracellular organelle;intracellular organelle part;intracellular part;membrane-bounded organelle;nuclear part;nucleoplasm;nucleus;organelle;organelle part		Homo sapiens	1	11
cell part;cytoplasmic part;cytosol;extracellular membrane-bounded organelle;extracellular organelle;extracellular region part;extracellular vesicular exosome;intracellular part;membrane-bounded organelle;membrane-bounde	Pentose phosphate pathway	Homo sapiens	1	11
cell part;cytoplasm;cytoplasmic part;intracellular membrane-bounded organelle;intracellular organelle;intracellular organelle lumen;intracellular organelle part;intracellular part;membrane-bounded organelle;membrane-enclosed lumen;mitochondrial matrix;mitochondrial part;mitochondrion;nuclear part;nucleoplasm;organelle;organelle lumen		Homo sapiens	1	11
cell part;chromatin;chromatin remodeling complex;chromosomal part;DNA helicase complex;H4/H2A histone acetyltransferase complex;histone acetyltransferase complex;Ino80 complex;intracellular membrane-bounded organelle;intracellular organelle;intracellular organelle part;intracellular part;macromolecular complex;membrane;membr		Homo sapiens	1	11
adherens junction;anchoring junction;cell junction;cell part;cell-substrate adherens junction;cell-substrate junction;cytoplasm;cytoplasmic part;cytosol;extrinsic to internal side of plasma membrane;extrinsic to membrane;ext	Acute myeloid leukemia;Aldosterone-regulated sodium reabsorption;Axon guidance;B cell receptor signaling pathw	Homo sapiens	1	11
cell part;cytoplasm;cytoplasmic part;intracellular membrane-bounded organelle;intracellular organelle;intracellular part;membrane-bounded organelle;nucleus;organelle;perinuclear region of cytoplasm	Mineral absorption	Homo sapiens	1	11
adherens junction;anchoring junction;cell junction;cell part;cell-substrate adherens junction;cell-substrate junction;cytoplasmic part;cytosol;cytosolic large ribosomal subunit;extracellular membrane-bounded organelle;extrac	Ribosome	Homo sapiens	1	11
cell part;cytoplasm;cytoplasmic part;cytosol;extracellular membrane-bounded organelle;extracellular organelle;extracellular region part;extracellular vesicular exosome;intracellular organelle part;intracellular part;membrane-b	Purine metabolism	Homo sapiens	1	11
catalytic step 2 spliceosome;cell part;intracellular non-membrane-bounded organelle;intracellular organelle;intracellular organelle part;intracellular part;macromolecular complex;non-membrane-bounded organelle;nuclear par	Spliceosome	Homo sapiens	1	11
		Homo sapiens	1	11
basolateral plasma membrane;blood microparticle;caveola;cell cortex part;cell junction;cell part;cell-cell junction;cortical actin cytoskeleton;cortical cytoskeleton;cytoplasmic membrane-bounded vesicle;cytoplasmic part;cyto	Adipocytokine signaling pathway;Bile secretion;Pathways in cancer;Renal cell carcinoma	Homo sapiens	1	11
adherens junction;anchoring junction;cell division site part;cell junction;cell part;cell surface;cell-substrate adherens junction;cell-substrate junction;cleavage furrow;cytoplasmic part;cytoplasmic vesicle membrane;cytoplasm	Pancreatic cancer;Pathways in cancer	Homo sapiens	1	11
cell body;cell part;cell projection;cytolytic granule;cytoplasm;cytoplasmic membrane-bounded vesicle;cytoplasmic part;cytoplasmic vesicle;dendrite;endosomal part;endosome;endosome membrane;external side of plasma r	ko05152;Lysosome;Phagosome	Homo sapiens	1	11
cell part;cytoplasm;cytoplasmic part;cytosol;inclusion body;intracellular part;membrane	Insulin signaling pathway;Starch and sucrose metabolism	Homo sapiens	1	11
cell part;cytoplasmic part;intracellular organelle part;intracellular part;macromolecular complex;membrane;membrane part;mitochondrial inner membrane;mitochondrial membrane;mitochondrial membrane part;mitochondrial	Alzheimer's disease;Cardiac muscle contraction;Huntington's disease;Oxidative phosphorylation;Parkinson's diseas	Homo sapiens	1	11
cell part;cytoplasmic part;cytosol;intracellular part	Bladder cancer;Drug metabolism - other enzymes;Pyrimidine metabolism	Homo sapiens	1	11
cell part;cytoplasm;cytoplasmic part;extracellular membrane-bounded organelle;extracellular organelle;extracellular region part;extracellular vesicular exosome;intracellular membrane-bounded organelle;intracellular organelle	Peroxisome;PPAR signaling pathway;Primary bile acid biosynthesis	Homo sapiens	1	11
extracellular region;extracellular region part;extracellular space		Homo sapiens	1	11
Cajal body;cell part;centrosome;chromosomal part;chromosome;chromosome, telomeric region;condensed chromosome;cyclin-dependent protein kinase holoenzyme complex;cytoplasm;cytoplasmic part;cytoskeletal part;c	Cell cycle;Measles;Oocyte meiosis;p53 signaling pathway;Pathways in cancer;Progesterone-mediated oocyte matu	Homo sapiens	1	11
cell part;cytoplasmic part;intracellular membrane-bounded organelle;intracellular organelle;intracellular organelle lumen;intracellular organelle part;intracellular part;membrane-bounded organelle;membrane-enclosed lumen;m	Valine, leucine and isoleucine degradation	Homo sapiens	1	11
axon;cell body;cell part;cell projection;cytoplasmic part;cytosol;dendrite;extracellular region;integral to membrane;integral to plasma membrane;intracellular part;intrinsic to membrane;intrinsic to plasma membrane;membrane	Axon guidance	Homo sapiens	1	11
cell part;cytoplasm;cytoplasmic part;cytosol;endoplasmic reticulum;extracellular membrane-bounded organelle;extracellular organelle;extracellular region part;extracellular vesicular exosome;intracellular membrane-bounded organelle;intracellular organelle;intracellular organelle part;intracellular part;membrane-bounded organelle;membrane		Homo sapiens	1	11
cell part;cytoplasm;cytoplasmic part;cytoplasmic vesicle;cytosol;extracellular membrane-bounded organelle;extracellular organelle;extracellular region part;extracellular space;extracellular vesicular exosome;intracellular men	Peroxisome	Homo sapiens	1	11

cell part;cytoplasm;extracellular membrane-bounded organelle;extracellular organelle;extracellular region part;extracellular vesicular exosome;intracellular part;membrane-bounded organelle;membrane-bounded vesicle;organelle;vesicle		Homo sapiens	1	11
cell part;cytoplasm;cytoplasmic part;cytosol;extracellular membrane-bounded organelle;extracellular organelle;extracellular region part;extracellular vesicular exosome;intracellular membrane-bounded organelle;intracellular o	Cysteine and methionine metabolism;Glycine, serine and threonine metabolism;Nitrogen metabolism;Selenocompo	Homo sapiens	1	11
cell part;cytoplasmic part;endoplasmic reticulum;endoplasmic reticulum membrane;endoplasmic reticulum part;integral to membrane;intracellular membrane-bounded organelle;intracellular organelle;intracellular organelle pa	Steroid biosynthesis	Homo sapiens	1	11
cell part;chromosomal part;DNA replication factor C complex;intracellular membrane-bounded organelle;intracellular organelle;intracellular organelle part;intracellular part;macromolecular complex;membrane-bounded organe	DNA replication;Mismatch repair;Nucleotide excision repair	Homo sapiens	1	11
actin cytoskeleton;cell part;cytoplasm;cytoplasmic part;cytoskeleton;cytosol;extracellular membrane-bounded organelle;extracellular organelle;extracellular region part;extracellular vesicular exosome;intracellular membrane-	Bacterial invasion of epithelial cells;Chemokine signaling pathway;Chronic myeloid leukemia;ErbB signaling pathway	Homo sapiens	1	11
cell part;cytoplasmic part;cytosol;endoplasmic reticulum membrane;endoplasmic reticulum part;glycogen granule;intracellular organelle part;intracellular part;membrane;membrane part;organelle membrane;organelle part	Glycerophospholipid metabolism;Phosphonate and phosphinate metabolism	Homo sapiens	1	11
cell junction;cell part;cell projection;cell-cell junction;cytoplasm;cytoplasmic membrane-bounded vesicle;cytoplasmic part;cytoplasmic vesicle;cytoplasmic vesicle membrane;cytoplasmic vesicle part;endocytic vesicle;endos	Tight junction	Homo sapiens	1	11
cell part;cytoplasm;intracellular membrane-bounded organelle;intracellular organelle;intracellular organelle part;intracellular part;macromolecular complex;membrane-bounded organelle;nuclear part;nucleoplasm;nucleotide-e	Nucleotide excision repair;Protein processing in endoplasmic reticulum	Homo sapiens	1	11
extracellular matrix part;extracellular region;extracellular region part;fibril;microfibril		Homo sapiens	1	11
cell part;cytoplasm;cytoplasmic part;endoplasmic reticulum;intracellular membrane-bounded organelle;intracellular organelle;intracellular part;membrane-bounded organelle;organelle;perinuclear region of cytoplasm		Homo sapiens	1	11
cell part;cytoplasmic part;intracellular membrane-bounded organelle;intracellular organelle;intracellular part;macromolecular complex;membrane-bounded organelle;mitochondrion;nucleus;organelle;ribonucleoprotein complex		Homo sapiens	1	11
cell part;intracellular		Homo sapiens	1	11
cell junction;cell part;cell projection;cytoplasm;cytoplasmic part;cytosol;early endosome;endosome;extracellular membrane-bounded organelle;extracellular organelle;extracellular region part;extracellular vesicular exosome;g	Chemokine signaling pathway;Focal adhesion;Leukocyte transendothelial migration;Long-term potentiation;MAPK s	Homo sapiens	1	11
cell part;Cul7-RING ubiquitin ligase complex;cullin-RING ubiquitin ligase complex;cytoplasmic part;cytosol;extracellular membrane-bounded organelle;extracellular organelle;extracellular region part;extracellular vesicular exc	Cell cycle;Cell cycle - yeast;Circadian rhythm - mammal;Oocyte meiosis;Protein processing in endoplasmic reticulu	Homo sapiens	1	11
cell part;cytoplasm;cytoplasmic part;cytosol;extracellular membrane-bounded organelle;extracellular organelle;extracellular region part;extracellular vesicular exosome;intracellular non-membrane-bounded organelle;intracellu	Ether lipid metabolism	Homo sapiens	1	11
Cajal body;catalytic step 2 spliceosome;cell part;intracellular organelle part;intracellular part;macromolecular complex;nuclear body;nuclear part;nuclear speck;nucleoplasm;nucleoplasm part;organelle part;ribonucleoprotein	Shigellosis;Spliceosome	Homo sapiens	1	11
cell part;cytoplasm;cytoplasmic part;cytosol;I-kappaB/NF-kappaB complex;intracellular membrane-bounded organelle;intracellular organelle;intracellular organelle part;intracellular part;macromolecular complex;membrane-b	Acute myeloid leukemia;Adipocytokine signaling pathway;Amoebiasis;Apoptosis;B cell receptor signaling pathway;	Homo sapiens	1	11
cell part;cytoplasmic part;extracellular membrane-bounded organelle;extracellular organelle;extracellular region part;extracellular vesicular exosome;intracellular membrane-bounded organelle;intracellular non-membrane-bou	DNA replication;Homologous recombination;Mismatch repair	Homo sapiens	1	11
cell part;cytoplasm;cytoplasmic part;intracellular membrane-bounded organelle;intracellular non-membrane-bounded organelle;intracellular organelle;intracellular organelle part;intracellular part;membrane-bounded organelle;mitochondrial nucleoid;mitochondrial part;mitochondrion;non-membrane-bounded organelle;nucleoid;organelle;orga		Homo sapiens	1	11
cell part;cytoplasmic part;cytosol;extracellular membrane-bounded organelle;extracellular organelle;extracellular region part;extracellular space;extracellular vesicular exosome;intracellular non-membrane-bounded organelle;intracellular organelle;intracellular organelle part;intracellular part;membrane;membrane-bounded organelle;membrar		Homo sapiens	1	11
cell part;chromatin remodeling complex;cytoplasm;cytoskeletal part;envelope;histone deacetylase complex;intracellular membrane-bounded organelle;intracellular organelle;intracellular organelle part;intracellular part;macromolecular complex;membrane-bounded organelle;microtubule;nuclear envelope;nuclear part;nucleoplasm;nucleoplas		Homo sapiens	1	11
cell part;cytoplasm;cytoplasmic part;cytosol;extracellular membrane-bounded organelle;extracellular organelle;extracellular region part;extracellular vesicular exosome;intracellular membrane-bounded organelle;intracellular organelle;intracellular part;macromolecular complex;membrane-bounded organelle;membrane-bounded vesicle;nucle		Homo sapiens	1	11
cell part;cytoplasm;intracellular membrane-bounded organelle;intracellular organelle;intracellular organelle part;intracellular part;membrane-bounded organelle;midbody;nuclear body;nuclear part;nuclear speck;nucleoplasm;r	RIG-I-like receptor signaling pathway	Homo sapiens	1	11
cell part;cytoplasm;cytoplasmic exosome (RNase complex);cytoplasmic part;cytosol;exosome (RNase complex);intracellular membrane-bounded organelle;intracellular non-membrane-bounded organelle;intracellular organell	RNA degradation	Homo sapiens	1	11
cell part;cytoplasm;intracellular membrane-bounded organelle;intracellular non-membrane-bounded organelle;intracellular organelle;intracellular organelle part;intracellular part;membrane;membrane-bounded organelle;non-membrane-bounded organelle;nuclear part;nucleolus;nucleoplasm;nucleus;organelle;organelle part		Homo sapiens	1	11
cell part;cytoplasmic part;endoplasmic reticulum;endoplasmic reticulum lumen;endoplasmic reticulum membrane;endoplasmic reticulum part;intracellular membrane-bounded organelle;intracellular organelle;intracellular organelle lumen;intracellular organelle part;intracellular part;membrane;membrane part;membrane-bounded organelle;me		Homo sapiens	1	11
cell part;cytoplasmic part;cytosol;intracellular membrane-bounded organelle;intracellular organelle;intracellular organelle part;intracellular part;macromolecular complex;membrane-bounded organelle;nuclear part;nucleoplasn	Meiosis - yeast;mRNA surveillance pathway;Oocyte meiosis;Wnt signaling pathway	Homo sapiens	1	11
cell part;cytoplasmic part;intracellular membrane-bounded organelle;intracellular organelle;intracellular organelle lumen;intracellular organelle part;intracellular part;membrane-bounded organelle;membrane-enclosed lumen;m	Aminoacyl-tRNA biosynthesis;Valine, leucine and isoleucine biosynthesis	Homo sapiens	1	11
cell part;cytoplasm;intracellular non-membrane-bounded organelle;intracellular organelle;intracellular organelle part;intracellular part;macromolecular complex;non-membrane-bounded organelle;nuclear part;nucleolar part;nu	Ribosome biogenesis in eukaryotes	Homo sapiens	1	11
axon;cell cortex part;cell part;cell projection;cortical actin cytoskeleton;cortical cytoskeleton;cytoplasm;cytoplasmic part;cytoskeletal part;cytoskeleton;early endosome membrane;endosomal part;endosome membrane;Golg	Tight junction	Homo sapiens	1	11
adherens junction;anchoring junction;cell junction;cell part;cell-substrate adherens junction;cell-substrate junction;cytoplasmic part;cytosol;extracellular membrane-bounded organelle;extracellular organelle;extracellular region part;extracellular vesicular exosome;focal adhesion;intracellular part;membrane-bounded organelle;membrane-bou		Homo sapiens	1	11
extracellular membrane-bounded organelle;extracellular organelle;extracellular region;extracellular region part;extracellular space;extracellular vesicular exosome;membrane-bounded organelle;membrane-bounded vesicle;organelle;vesicle		Homo sapiens	1	11
cell part;cytoplasmic part;intracellular membrane-bounded organelle;intracellular organelle;intracellular organelle lumen;intracellular organelle part;intracellular part;macromolecular complex;membrane;membrane part;membr	Alzheimer's disease;Huntington's disease;Oxidative phosphorylation;Parkinson's disease	Homo sapiens	1	11
cell part;cytoplasmic part;early endosome;endosome;intracellular membrane-bounded organelle;intracellular organelle;intracellular organelle part;intracellular part;macromolecular complex;membrane-bounded organelle;nuclear part;nucleoplasm;organelle;organelle part;protein complex;WASH complex		Homo sapiens	1	11
cell part;cytoplasmic part;intracellular membrane-bounded organelle;intracellular organelle;intracellular part;membrane-bounded organelle;mitochondrion;organelle		Homo sapiens	1	11
	Arginine and proline metabolism	Homo sapiens	1	11
cell part;cytoplasmic part;intracellular non-membrane-bounded organelle;intracellular organelle;intracellular part;macromolecular complex;non-membrane-bounded organelle;organelle;ribonucleoprotein complex;ribosome		Homo sapiens	1	11
		Homo sapiens	1	11
adherens junction;anchoring junction;cell junction;cell part;cell-substrate adherens junction;cell-substrate junction;cytoplasm;focal adhesion;intracellular part		Homo sapiens	1	11
cell part;cytoplasmic part;extracellular membrane-bounded organelle;extracellular organelle;extracellular region part;extracellular vesicular exosome;intracellular organelle lumen;intracellular organelle part;intracellular part;me	beta-Alanine metabolism;Propanoate metabolism;Valine, leucine and isoleucine degradation	Homo sapiens	1	11
cell part;cytoplasm;cytoplasmic part;cytosol;extracellular membrane-bounded organelle;extracellular organelle;extracellular region part;extracellular vesicular exosome;Golgi apparatus;intracellular membrane-bounded organe	Nicotinate and nicotinamide metabolism	Homo sapiens	1	11
actin filament;basal cortex;cell cortex part;cell part;cytoplasmic part;cytoskeletal part;intracellular membrane-bounded organelle;intracellular organelle;intracellular organelle part;intracellular part;lateral plasma membrane;macromolecular complex;membrane;membrane part;membrane-bounded organelle;mitochondrion;nucleus;organelle;or		Homo sapiens	1	11
		Homo sapiens	1	11
cell part;chromatin;chromosomal part;cytoplasm;intracellular membrane-bounded organelle;intracellular organelle;intracellular organelle part;intracellular part;membrane-bounded organelle;nuclear chromatin;nuclear chromosome part;nuclear part;nucleoplasm;nucleus;organelle;organelle part		Homo sapiens	1	11
cell part;cytoplasmic part;extracellular membrane-bounded organelle;extracellular organelle;extracellular region part;extracellular vesicular exosome;intracellular membrane-bounded organelle;intracellular organelle;intracellular part;membrane-bounded organelle;membrane-bounded vesicle;mitochondrion;nucleus;organelle;vesicle		Homo sapiens	1	11
cell part;cytoplasm;intracellular part		Homo sapiens	1	11
cell part;cytoplasm;cytoplasmic part;endoplasmic reticulum membrane;endoplasmic reticulum part;intracellular organelle part;intracellular part;membrane;membrane part;organelle membrane;organelle part;perinuclear regior	Spliceosome	Homo sapiens	1	11
cell part;cytoplasm;intracellular membrane-bounded organelle;intracellular organelle;intracellular organelle part;intracellular part;membrane-bounded organelle;nuclear part;nucleoplasm;nucleus;organelle;organelle part		Homo sapiens	1	11
cell part;cytoplasmic part;integral to membrane;intracellular organelle part;intracellular part;intrinsic to membrane;lysosomal membrane;membrane;membrane part;organelle membrane;organelle part;plasma membrane;vacuolar membrane;vacuolar part		Homo sapiens	1	11
cell part;cytoplasmic part;endoplasmic reticulum;endoplasmic reticulum membrane;endoplasmic reticulum part;Golgi apparatus;Golgi apparatus part;Golgi membrane;integral to membrane;intracellular membrane-bounded c	Ether lipid metabolism;Glycerophospholipid metabolism	Homo sapiens	1	11
cell part;intracellular membrane-bounded organelle;intracellular organelle;intracellular part;macromolecular complex;membrane;membrane-bounded organelle;nucleus;organelle;ribonucleoprotein complex		Homo sapiens	1	11
cell part;extracellular membrane-bounded organelle;extracellular organelle;extracellular region part;extracellular vesicular exosome;intracellular membrane-bounded organelle;intracellular organelle;intracellular part;membrane-bounded organelle;membrane-bounded vesicle;nucleus;organelle;vesicle		Homo sapiens	1	11
cell part;cytoplasm;extracellular membrane-bounded organelle;extracellular organelle;extracellular region part;extracellular vesicular exosome;intracellular part;membrane-bounded organelle;membrane-bounded vesicle;orga	Pathways in cancer;Thyroid cancer	Homo sapiens	1	11
cell part;cytoplasm;intracellular membrane-bounded organelle;intracellular non-membrane-bounded organelle;intracellular organelle;intracellular organelle part;intracellular part;membrane-bounded organelle;non-membrane-l	Ribosome biogenesis in eukaryotes	Homo sapiens	1	11
cell part;cytoplasmic part;intracellular membrane-bounded organelle;intracellular organelle;intracellular part;macromolecular complex;membrane-bounded organelle;mitochondrion;organelle;oxoglutarate dehydrogenase complex;protein complex		Homo sapiens	1	11
cell part;cytoplasm;extracellular membrane-bounded organelle;extracellular organelle;extracellular region part;extracellular vesicular exosome;intracellular part;membrane-bounded organelle;membrane-bounded vesicle;orga	Amino sugar and nucleotide sugar metabolism;Fructose and mannose metabolism	Homo sapiens	1	11
		Homo sapiens	1	11
cell part;centrosome;cytoplasm;cytoplasmic part;cytoskeletal part;endomembrane system;intracellular membrane-bounded organelle;intracellular non-membrane-bounded organelle;intracellular organelle;intracellular organelle part;intracellular part;macromolecular complex;membrane;membrane-bounded organelle;microtubule organizing c		Homo sapiens	1	11
cell part;cell surface;cytoplasmic part;endoplasmic reticulum;endoplasmic reticulum membrane;endoplasmic reticulum part;integral to membrane;intracellular membrane-bounded organelle;intracellular organelle;intracellular organelle part;intracellular part;intrinsic to membrane;membrane;membrane part;membrane-bounded organelle;orga		Homo sapiens	1	11
cell part;cytoplasmic part;cytoplasmic vesicle;cytosol;Golgi apparatus;Golgi apparatus part;Golgi cisterna membrane;Golgi membrane;intracellular membrane-bounded organelle;intracellular organelle;intracellular organelle p	Endocytosis	Homo sapiens	1	11
cell part;cytoplasm;cytoplasmic part;cytosol;intracellular membrane-bounded organelle;intracellular organelle;intracellular organelle part;intracellular part;membrane;membrane-bounded organelle;nuclear membrane;nuclear part;nucleoplasm;nucleus;organelle;organelle membrane;organelle part		Homo sapiens	1	11
cell part;cytoplasm;cytoplasmic part;intracellular membrane-bounded organelle;intracellular organelle;intracellular organelle part;intracellular part;membrane;membrane-bounded organelle;microbody;microbody membrane;microbody part;organelle;organelle membrane;organelle part;peroxisomal membrane;peroxisomal part		Homo sapiens	1	11
cell part;cytoplasm;cytoplasmic part;endoplasmic reticulum;intracellular membrane-bounded organelle;intracellular organelle;intracellular organelle lumen;intracellular organelle part;intracellular part;membrane;membrane-bou	Alzheimer's disease;Valine, leucine and isoleucine degradation	Homo sapiens	1	11
cell part;cytoplasmic part;endosomal part;endosome;endosome membrane;extracellular membrane-bounded organelle;extracellular organelle;extracellular region part;extracellular vesicular exosome;integral to membrane;integral to plasma membrane;intracellular membrane-bounded organelle;intracellular organelle;intracellular organelle pa		Homo sapiens	1	11
cell part;cytoplasm;cytoplasmic part;cytosol;intracellular part		Homo sapiens	1	11
cell part;cytoplasmic part;intracellular membrane-bounded organelle;intracellular organelle;intracellular organelle part;intracellular part;membrane-bounded organelle;mitochondrion;nuclear part;nucleoplasm;nucleus;organelle;organelle part		Homo sapiens	1	11
cell part;intracellular membrane-bounded organelle;intracellular organelle;intracellular organelle part;intracellular part;membrane-bounded organelle;nuclear part;nucleoplasm;nucleus;organelle;organelle part		Homo sapiens	1	11
cell part;cytoplasm;cytoplasmic part;cytosol;extracellular membrane-bounded organelle;extracellular organelle;extracellular region part;extracellular vesicular exosome;Golgi apparatus;Golgi apparatus part;Golgi membrane;intracellular membrane-bounded organelle;intracellular organelle;intracellular organelle part;intracellular part;membrar		Homo sapiens	1	11
		Homo sapiens	1	11
cell part;cytoplasm;intracellular membrane-bounded organelle;intracellular organelle;intracellular organelle part;intracellular part;membrane-bounded organelle;nuclear body;nuclear part;nuclear speck;nucleoplasm;nucleoplasm part;organelle;organelle part		Homo sapiens	1	11
cell part;intracellular organelle part;intracellular part;membrane;nuclear membrane;nuclear part;organelle membrane;organelle part		Homo sapiens	1	11
cell part;intracellular membrane-bounded organelle;intracellular non-membrane-bounded organelle;intracellular organelle;intracellular organelle part;intracellular part;membrane-bounded organelle;non-membrane-bounded organelle;nuclear part;nucleolus;nucleus;organelle;organelle part		Homo sapiens	1	11
Cajal body;cell part;cytoplasm;cytoplasmic part;cytosol;endoplasmic reticulum;intracellular membrane-bounded organelle;intracellular organelle;intracellular organelle part;intracellular part;membrane;membrane-bounded org	Ribosome biogenesis in eukaryotes	Homo sapiens	1	11
cell part;cell projection;cytoplasm;extracellular membrane-bounded organelle;extracellular organelle;extracellular region part;extracellular vesicular exosome;intracellular membrane-bounded organelle;intracellular organelle;intracellular part;lamellipodium;membrane-bounded organelle;membrane-bounded vesicle;nucleus;organelle;vesicle		Homo sapiens	1	11
cell part;cytoplasm;intracellular part		Homo sapiens	1	11
cell part;cytoplasmic part;extracellular membrane-bounded organelle;extracellular organelle;extracellular region part;extracellular vesicular exosome;intracellular membrane-bounded organelle;intracellular organelle;intracellular organelle part;intracellular part;membrane-bounded organelle;membrane-bounded vesicle;mitochondrion;nuclear		Homo sapiens	1	11
cell part;cytoplasmic part;extracellular membrane-bounded organelle;extracellular organelle;extracellular region part;extracellular vesicular exosome;intracellular membrane-bounded organelle;intracellular organelle;intracellular part;membrane-bounded organelle;membrane-bounded vesicle;mitochondrion;organelle;vesicle		Homo sapiens	1	11
cell part;cytoplasmic part;cytosol;endosomal part;endosome membrane;extracellular membrane-bounded organelle;extracellular organelle;extracellular region part;extracellular vesicular exosome;intracellular organelle part;in	Endocytosis	Homo sapiens	1	11
cell part;cytoplasm;EKC/KEOPS complex;intracellular membrane-bounded organelle;intracellular organelle;intracellular part;macromolecular complex;membrane-bounded organelle;nucleus;organelle;protein complex		Homo sapiens	1	11
cell part;intracellular membrane-bounded organelle;intracellular non-membrane-bounded organelle;intracellular organelle;intracellular organelle part;intracellular part;membrane-bounded organelle;non-membrane-bounded organelle;nuclear part;nucleolus;nucleus;organelle;organelle part		Homo sapiens	1	11
ACF complex;cell part;chromatin accessibility complex;chromatin assembly complex;chromatin remodeling complex;chromosome;intracellular non-membrane-bounded organelle;intracellular organelle;intracellular organelle part;intracellular part;macromolecular complex;non-membrane-bounded organelle;nuclear chromosome;nuclear part;		Homo sapiens	1	11
cell part;cytoplasmic part;cytosol;extracellular membrane-bounded organelle;extracellular organelle;extracellular region part;extracellular vesicular exosome;intracellular part;membrane-bounded organelle;membrane-bounde	Fructose and mannose metabolism	Homo sapiens	1	11
cell part;cytoplasm;intracellular membrane-bounded organelle;intracellular organelle;intracellular organelle part;intracellular part;membrane;membrane-bounded organelle;nuclear part;nucleoplasm;nucleus;organelle;organelle part;plasma membrane		Homo sapiens	1	11
catalytic step 2 spliceosome;cell part;cytoplasm;intracellular membrane-bounded organelle;intracellular organelle;intracellular organelle part;intracellular part;macromolecular complex;membrane-bounded organelle;nuclear p	Spliceosome	Homo sapiens	1	11
		Homo sapiens	1	11
cell part;cytoplasmic membrane-bounded vesicle;cytoplasmic part;cytoplasmic vesicle;cytosol;extracellular membrane-bounded organelle;extracellular organelle;extracellular region part;extracellular vesicular exosome;Golgi apparatus;intracellular membrane-bounded organelle;intracellular organelle;intracellular part;lysosome;lytic vacuole;		Homo sapiens	1	11
anaphase-promoting complex;cell part;cullin-RING ubiquitin ligase complex;cytoplasmic part;cytosol;intracellular membrane-bounded organelle;intracellular organelle;intracellular organelle part;intracellular part;macromolecu	Cell cycle;Cell cycle - yeast;Meiosis - yeast;Oocyte meiosis;Progesterone-mediated oocyte maturation;Ubiquitin me	Homo sapiens	1	11
cell part;intracellular organelle part;intracellular part;macromolecular complex;mRNA cleavage and polyadenylation specificity factor complex;mRNA cleavage factor complex;nuclear part;nucleoplasm;nucleoplasm part;orgar	mRNA surveillance pathway	Homo sapiens	1	11
cell part;cytoplasm;intracellular non-membrane-bounded organelle;intracellular organelle;intracellular organelle part;intracellular part;membrane;non-membrane-bounded organelle;nuclear part;nucleolus;organelle;organelle part;plasma membrane		Homo sapiens	1	11
cell part;cytoplasmic part;intracellular membrane-bounded organelle;intracellular non-membrane-bounded organelle;intracellular organelle;intracellular organelle part;intracellular part;membrane-bounded organelle;mitochondrial nucleoid;mitochondrial part;mitochondrion;non-membrane-bounded organelle;nucleoid;nucleus;organelle;organe		Homo sapiens	1	11
cell part;cytoplasmic part;intracellular membrane-bounded organelle;intracellular organelle;intracellular organelle part;intracellular part;macromolecular complex;membrane-bounded organelle;mitochondrial part;mitochondrial small ribosomal subunit;mitochondrion;organellar small ribosomal subunit;organelle;organelle part;ribonucleoprotei		Homo sapiens	1	11
cell part;centrosome;cytoplasmic part;cytoskeletal part;cytosol;intracellular non-membrane-bounded organelle;intracellular organelle;intracellular organelle part;intracellular part;macromolecular complex;microtubule associated complex;microtubule organizing center;non-membrane-bounded organelle;organelle;organelle part;protein compl		Homo sapiens	1	11
cell part;intracellular membrane-bounded organelle;intracellular non-membrane-bounded organelle;intracellular organelle;intracellular organelle part;intracellular part;macromolecular complex;membrane-bounded organelle;non-membrane-bounded organelle;nuclear body;nuclear part;nuclear speck;nucleolus;nucleoplasm;nucleoplasm part;		Homo sapiens	1	11
cell part;cytoplasmic part;cytosol;intracellular part;membrane;plasma membrane	Adipocytokine signaling pathway;Aldosterone-regulated sodium reabsorption;Insulin signaling pathway;Neurotrophi	Homo sapiens	1	11
cell part;cytoplasm;cytoplasmic part;cytoskeletal part;histone methyltransferase complex;intracellular non-membrane-bounded organelle;intracellular organelle;intracellular organelle part;intracellular part;macromolecular complex;membrane;methyltransferase complex;microtubule organizing center;MLL1 complex;non-membrane-bounded		Homo sapiens	1	11
cell part;cytoplasm;intracellular membrane-bounded organelle;intracellular organelle;intracellular part;membrane-bounded organelle;nucleus;organelle		Homo sapiens	1	11
cell part;intracellular membrane-bounded organelle;intracellular organelle;intracellular organelle part;intracellular part;macromolecular complex;membrane;membrane-bounded organelle;nuclear body;nuclear matrix;nuclear pa	Notch signaling pathway	Homo sapiens	1	11
cell part;cytoplasm;cytoplasmic part;cytosol;intracellular membrane-bounded organelle;intracellular non-membrane-bounded organelle;intracellular organelle;intracellular organelle lumen;intracellular organelle part;intracellula	Sulfur relay system;Thiamine metabolism	Homo sapiens	1	11
asymmetric synapse;axonemal microtubule;axoneme part;cell junction;cell part;cell projection;cell projection part;cytoplasmic membrane-bounded vesicle;cytoplasmic part;cytoplasmic vesicle;cytoskeletal part;cytosol;dendritic spine;endosome;Golgi apparatus part;Golgi membrane;intracellular membrane-bounded organelle;intracellular or		Homo sapiens	1	11
cell part;cytoplasm;cytoplasmic part;cytosol;Golgi apparatus;Golgi apparatus part;Golgi membrane;intracellular membrane-bounded organelle;intracellular non-membrane-bounded organelle;intracellular organelle;intracellular organelle part;intracellular part;macromolecular complex;membrane;membrane-bounded organelle;non-membrane		Homo sapiens	1	11
actin cytoskeleton;cell part;centrosome;chromosomal part;condensed chromosome kinetochore;cytoplasm;cytoplasmic part;cytoskeletal part;cytoskeleton;cytosol;intracellular membrane-bounded organelle;intracellular non-	Cell cycle;Progesterone-mediated oocyte maturation	Homo sapiens	1	11
cell part;cytoplasm;cytoplasmic part;cytosol;intracellular membrane-bounded organelle;intracellular organelle;intracellular organelle part;intracellular part;membrane-bounded organelle;nuclear part;nucleoplasm;nucleus;organelle;organelle part		Homo sapiens	1	11
cell part;centrosome;cytoplasmic part;cytoskeletal part;cytosol;intracellular non-membrane-bounded organelle;intracellular organelle;intracellular organelle part;intracellular part;kinesin complex;macromolecular complex;membrane;microtubule;microtubule associated complex;microtubule organizing center;non-membrane-bounded organel		Homo sapiens	1	12
cell part;cytoplasmic exosome (RNase complex);cytoplasmic part;cytosol;exosome (RNase complex);intracellular part;macromolecular complex;membrane;protein complex		Homo sapiens	1	12
cell part;cytoplasmic part;extracellular membrane-bounded organelle;extracellular organelle;extracellular region part;extracellular vesicular exosome;Golgi apparatus part;Golgi membrane;integral to membrane;intracellular organelle part;intracellular part;intrinsic to membrane;membrane;membrane part;membrane-bounded organelle;membr		Homo sapiens	1	12
cell part;cytoplasmic part;cytosol;intracellular part	Glutathione metabolism	Homo sapiens	1	12
actin cytoskeleton;adherens junction;anchoring junction;basement membrane;basolateral plasma membrane;cell junction;cell part;cell projection membrane;cell projection part;cell-cell junction;cell-substrate adherens junctio	Tight junction	Homo sapiens	1	12
cell part;cytoplasmic part;endoplasmic reticulum membrane;endoplasmic reticulum palmitoyltransferase complex;endoplasmic reticulum part;integral to membrane;intracellular membrane-bounded organelle;intracellular orga	Sphingolipid metabolism	Homo sapiens	1	12
cell part;chromosomal part;condensed chromosome kinetochore;cytoplasm;cytoplasmic part;cytoskeletal part;cytosol;endoplasmic reticulum;endoplasmic reticulum membrane;endoplasmic reticulum part;intracellular membrane-bounded organelle;intracellular non-membrane-bounded organelle;intracellular organelle;intracellular organelle		Homo sapiens	1	12
		Homo sapiens	1	12
cell part;cytoplasm;cytoplasmic part;endoplasmic reticulum membrane;endoplasmic reticulum part;extracellular membrane-bounded organelle;extracellular organelle;extracellular region part;extracellular vesicular exosome;intracellular membrane-bounded organelle;intracellular non-membrane-bounded organelle;intracellular organelle;intrac		Homo sapiens	1	12
cell part;intracellular membrane-bounded organelle;intracellular organelle;intracellular organelle part;intracellular part;membrane-bounded organelle;nuclear part;nucleoplasm;nucleus;organelle;organelle part		Homo sapiens	1	12
cell part;chromatin;chromosomal part;chromosome;chromosome, centromeric region;cohesin complex;cytoplasmic part;cytosol;intracellular non-membrane-bounded organelle;intracellular organelle;intracellular organelle par	Cell cycle;Cell cycle - yeast	Homo sapiens	1	12
cell part;cytoplasm;intracellular part		Homo sapiens	1	12
cell part;clathrin coated vesicle membrane;clathrin-coated vesicle;coated pit;coated vesicle;coated vesicle membrane;cytoplasm;cytoplasmic membrane-bounded vesicle;cytoplasmic part;cytoplasmic vesicle;cytoplasmic vesicle membrane;cytoplasmic vesicle part;cytoskeleton;intracellular membrane-bounded organelle;intracellular non-m		Homo sapiens	1	12
cell part;cytoplasm;intracellular membrane-bounded organelle;intracellular organelle;intracellular organelle part;intracellular part;membrane-bounded organelle;nuclear body;nuclear part;nuclear speck;nucleoplasm;nucleoplas	Spliceosome	Homo sapiens	1	12
cell part;cytoplasmic part;intracellular membrane-bounded organelle;intracellular organelle;intracellular organelle lumen;intracellular organelle part;intracellular part;membrane;membrane-bounded organelle;membrane-enclos	Fatty acid metabolism;Peroxisome	Homo sapiens	1	12
cell part;chromosome;cytoplasm;cytoplasmic part;cytosol;extracellular membrane-bounded organelle;extracellular organelle;extracellular region part;extracellular vesicular exosome;intracellular membrane-bounded organelle;intracellular non-membrane-bounded organelle;intracellular organelle;intracellular organelle part;intracellular part;me		Homo sapiens	1	12
cell part;cytoplasmic part;integral to membrane;intracellular membrane-bounded organelle;intracellular organelle;intracellular organelle part;intracellular part;intrinsic to membrane;membrane;membrane part;membrane-bounded organelle;mitochondrial inner membrane;mitochondrial membrane;mitochondrial part;mitochondrion;organelle;or		Homo sapiens	1	12
cell junction;cell part;intracellular membrane-bounded organelle;intracellular non-membrane-bounded organelle;intracellular organelle;intracellular organelle part;intracellular part;macromolecular complex;membrane-boundec	Spliceosome	Homo sapiens	1	12
cell part;cytoplasmic part;endoplasmic reticulum;endoplasmic reticulum membrane;endoplasmic reticulum part;integral to endoplasmic reticulum membrane;integral to membrane;integral to organelle membrane;intracellular	Sphingolipid metabolism	Homo sapiens	1	12
cell part;cytoplasm;cytoskeletal part;intracellular non-membrane-bounded organelle;intracellular organelle;intracellular organelle part;intracellular part;macromolecular complex;microtubule;microtubule associated complex;non-membrane-bounded organelle;organelle;organelle part;protein complex;spindle		Homo sapiens	1	12
cell part;cytoplasm;cytoplasmic part;cytosol;extracellular membrane-bounded organelle;extracellular organelle;extracellular region part;extracellular vesicular exosome;intracellular part;membrane-bounded organelle;membra	Drug metabolism - other enzymes;Purine metabolism	Homo sapiens	1	12
cell part;cytoplasmic part;extracellular membrane-bounded organelle;extracellular organelle;extracellular region part;extracellular vesicular exosome;Golgi apparatus;Golgi apparatus part;Golgi membrane;intracellular membra	Acute myeloid leukemia;Axon guidance;B cell receptor signaling pathway;Bladder cancer;Chemokine signaling path	Homo sapiens	1	12
cell part;cell surface;coated vesicle membrane;cytoplasmic part;cytoplasmic vesicle membrane;cytoplasmic vesicle part;early endosome membrane;endocytic vesicle membrane;endoplasmic reticulum;endoplasmic reticulun	Allograft rejection;Antigen processing and presentation;Autoimmune thyroid disease;Cell adhesion molecules (CAM	Homo sapiens	1	12
actin cytoskeleton;cell part;chromatin;chromosomal part;cytoplasmic part;cytoskeleton;euchromatin;Golgi apparatus;intracellular membrane-bounded organelle;intracellular non-membrane-bounded organelle;intracellular organelle;intracellular organelle part;intracellular part;macromolecular complex;membrane-bounded organelle;non-mem		Homo sapiens	1	12
cell part;cytoplasmic part;intracellular membrane-bounded organelle;intracellular organelle;intracellular organelle part;intracellular part;membrane;membrane part;membrane-bounded organelle;mitochondrial inner membrane	Alzheimer's disease;Cardiac muscle contraction;Huntington's disease;Oxidative phosphorylation;Parkinson's diseas	Homo sapiens	1	12
cell part;cytoplasm;intracellular non-membrane-bounded organelle;intracellular organelle;intracellular organelle part;intracellular part;macromolecular complex;non-membrane-bounded organelle;nuclear part;nucleolus;nucleo	Spliceosome	Homo sapiens	1	12
anchored to membrane;cell part;integral to membrane;intrinsic to membrane;membrane;membrane part;plasma membrane	Aminobenzoate degradation;Folate biosynthesis;Two-component system	Homo sapiens	1	12

catalytic step 2 spliceosome;cell part;cytoplasmic part;cytosol;extracellular membrane-bounded organelle;extracellular organelle;extracellular region part;extracellular vesicular exosome;histone pre-mRNA 3'end processing c	Spliceosome;Systemic lupus erythematosus	Homo sapiens	1	12
cell part;cytoplasm;extracellular membrane-bounded organelle;extracellular organelle;extracellular region part;extracellular vesicular exosome;intracellular membrane-bounded organelle;intracellular organelle;intracellular organelle part;intracellular part;membrane-bounded organelle;membrane-bounded vesicle;nuclear part;nucleoplasm;nuc		Homo sapiens	1	12
cell part;cytoplasmic part;cytosol;extracellular membrane-bounded organelle;extracellular organelle;extracellular region part;extracellular vesicular exosome;intracellular part;membrane-bounded organelle;membrane-bounde	Styrene degradation;Tyrosine metabolism	Homo sapiens	1	12
cell part;cytoplasmic part;cytoplasmic vesicle;cytosol;extracellular membrane-bounded organelle;extracellular organelle;extracellular region part;extracellular vesicular exosome;Golgi apparatus;Golgi apparatus part;Golgi membrane;intracellular membrane-bounded organelle;intracellular organelle;intracellular organelle part;intracellular part;		Homo sapiens	1	12
cell part;centrosome;chromosome;condensed chromosome;condensed nuclear chromosome;cytoplasm;cytoplasmic part;cytoskeletal part;cytosol;endosome;gamma-tubulin complex;intracellular membrane-bounded organelle;intracellular non-membrane-bounded organelle;intracellular organelle;intracellular organelle part;intracellular part;		Homo sapiens	1	12
cell part;cytoplasm;cytoplasmic mRNA processing body;cytoplasmic part;cytosol;extracellular membrane-bounded organelle;extracellular organelle;extracellular region part;extracellular vesicular exosome;intracellular membr	Proteasome	Homo sapiens	1	12
cell part;cytoplasm;cytoplasmic part;cytosol;extracellular membrane-bounded organelle;extracellular organelle;extracellular region part;extracellular vesicular exosome;intracellular organelle part;intracellular part;membrane;m	Porphyrin and chlorophyll metabolism;Riboflavin metabolism	Homo sapiens	1	12
cell part;cytoplasmic part;endoplasmic reticulum membrane;endoplasmic reticulum part;intracellular organelle part;intracellular part;membrane;membrane part;organelle membrane;organelle part;plasma membrane	Mineral absorption;Porphyrin and chlorophyll metabolism	Homo sapiens	1	12
axon part;axon terminus;cell part;cell projection part;contractile fiber part;cytoplasm;cytoplasmic part;cytosol;dendritic spine neck;endoplasmic reticulum;endoplasmic reticulum membrane;endoplasmic reticulum part;extracellular membrane-bounded organelle;extracellular organelle;extracellular region part;extracellular vesicular exosome;i		Homo sapiens	1	12
cell part;chromosomal part;cytoplasmic part;DNA replication factor C complex;Golgi apparatus;intracellular membrane-bounded organelle;intracellular organelle;intracellular organelle part;intracellular part;macromolecular co	DNA replication;Mismatch repair;Nucleotide excision repair	Homo sapiens	1	12
cell part;chromatin;chromosomal part;cytoplasm;cytoplasmic part;cytosol;intracellular organelle part;intracellular part;membrane part;membrane raft;nuclear chromatin;nuclear chromosome part;nuclear part;nucleoplasm;org	Jak-STAT signaling pathway;ko05152	Homo sapiens	1	12
cell part;chromatin;chromosomal part;cytoplasm;cytoplasmic part;cytosol;heterochromatin;intracellular membrane-bounded organelle;intracellular organelle;intracellular organelle part;intracellular part;membrane-bounded org	Bladder cancer;Cell cycle;Chronic myeloid leukemia;Glioma;Melanoma;Non-small cell lung cancer;p53 signaling pa	Homo sapiens	1	12
cell part;cytoplasmic part;extracellular membrane-bounded organelle;extracellular organelle;extracellular region part;extracellular vesicular exosome;intracellular membrane-bounded organelle;intracellular organelle;intracellula	Biosynthesis of unsaturated fatty acids	Homo sapiens	1	12
beta-catenin destruction complex;cell body;cell part;cell projection;cell projection part;centrosome;cytoplasm;cytoplasmic part;cytoskeletal part;cytosol;dendritic shaft;dendritic spine;growth cone;intracellular membrane-bou	Alzheimer's disease;Axon guidance;B cell receptor signaling pathway;Basal cell carcinoma;Cell cycle;Chemokine si	Homo sapiens	1	12
cell part;cytoplasmic membrane-bounded vesicle;cytoplasmic part;cytoplasmic vesicle;early endosome;early endosome membrane;endocytic vesicle;endosomal part;endosome;endosome membrane;extracellular membrane	Amoebiasis;Endocytosis;ko05152;Phagosome;Vasopressin-regulated water reabsorption	Homo sapiens	1	12
cell part;cytoplasmic part;cytosol;endoplasmic reticulum;extracellular membrane-bounded organelle;extracellular organelle;extracellular region part;extracellular vesicular exosome;intracellular membrane-bounded organelle;intracellular organelle;intracellular part;membrane-bounded organelle;membrane-bounded vesicle;mitochondrion;orga		Homo sapiens	1	12
cell part;cytoplasmic part;intracellular membrane-bounded organelle;intracellular organelle;intracellular organelle part;intracellular part;large ribosomal subunit;macromolecular complex;membrane;membrane-bounded organelle;mitochondrial inner membrane;mitochondrial large ribosomal subunit;mitochondrial membrane;mitochondrial part		Homo sapiens	1	12
cell cortex;cell part;cytoplasmic part;intracellular part		Homo sapiens	1	12
cell part;cytoplasmic part;cytosol;extracellular membrane-bounded organelle;extracellular organelle;extracellular region part;extracellular vesicular exosome;intracellular organelle part;intracellular part;macromolecular complex;membrane;membrane part;membrane-bounded organelle;membrane-bounded vesicle;organelle;organelle membra		Homo sapiens	1	12
cell part;cytoplasm;cytoplasmic part;intracellular membrane-bounded organelle;intracellular organelle;intracellular part;membrane;membrane-bounded organelle;mitochondrion;organelle		Homo sapiens	1	12
cell part;intracellular membrane-bounded organelle;intracellular non-membrane-bounded organelle;intracellular organelle;intracellular organelle part;intracellular part;macromolecular complex;membrane-bounded organelle;non-membrane-bounded organelle;nuclear part;nucleolus;nucleus;organelle;organelle part;preribosome;preribosome,		Homo sapiens	1	12
cell part;cytoplasm;cytoplasmic part;cytoskeletal part;intracellular membrane-bounded organelle;intracellular organelle;intracellular organelle part;intracellular part;macromolecular complex;membrane;membrane-bounded organelle;microtubule organizing center;nuclear part;nucleoplasm;nucleus;organelle;organelle part;plasma membrane;p		Homo sapiens	1	12
cell part;cytoplasm;cytoplasmic part;cytosol;extracellular membrane-bounded organelle;extracellular organelle;extracellular region part;extracellular vesicular exosome;intracellular part;membrane-bounded organelle;membra	Ubiquitin mediated proteolysis	Homo sapiens	1	12
cell part;contractile fiber part;cytoplasm;cytoplasmic part;cytosol;endoplasmic reticulum membrane;endoplasmic reticulum part;extracellular membrane-bounded organelle;extracellular organelle;extracellular region part;extracellular vesicular exosome;intracellular organelle part;intracellular part;membrane;membrane part;membrane-bounde		Homo sapiens	1	12
cell junction;cell part;cell-cell junction;cytoplasm;cytoplasmic part;cytosol;endosome;extracellular membrane-bounded organelle;extracellular organelle;extracellular region part;extracellular vesicular exosome;Golgi apparatus	Acute myeloid leukemia;B cell receptor signaling pathway;Chemokine signaling pathway;Chronic myeloid leukemia;	Homo sapiens	1	12
cell part;cytoplasm;extracellular membrane-bounded organelle;extracellular organelle;extracellular region part;extracellular vesicular exosome;intracellular membrane-bounded organelle;intracellular organelle;intracellular part	Parkinson's disease;Ubiquitin mediated proteolysis	Homo sapiens	1	12
cell part;cytoplasm;cytoplasmic part;intracellular membrane-bounded organelle;intracellular organelle;intracellular part;membrane-bounded organelle;nucleus;organelle;perinuclear region of cytoplasm	Mineral absorption	Homo sapiens	1	12
cell part;cell projection;cytoplasm;dendrite;intracellular membrane-bounded organelle;intracellular organelle;intracellular part;membrane-bounded organelle;neuron projection;nucleus;organelle		Homo sapiens	1	12
cell body;cell part;cell projection;chromosomal part;chromosome, telomeric region;cytoplasm;dendrite;DNA replication factor A complex;intracellular membrane-bounded organelle;intracellular organelle;intracellular organelle part;intracellular part;macromolecular complex;membrane-bounded organelle;neuron projection;neuronal cell body;r		Homo sapiens	1	12
cell part;extracellular membrane-bounded organelle;extracellular organelle;extracellular region part;extracellular vesicular exosome;integral to membrane;intrinsic to membrane;membrane part;membrane-bounded organelle;membrane-bounded vesicle;organelle;vesicle		Homo sapiens	1	12
anchoring collagen;basement membrane;cell part;collagen;collagen type VII;cytoplasmic part;endoplasmic reticulum lumen;endoplasmic reticulum part;extracellular matrix part;extracellular region;extracellular region part;extracellular space;intracellular organelle lumen;intracellular organelle part;intracellular part;membrane-enclosed lumen;o		Homo sapiens	1	12
adherens junction;anchored to membrane;anchoring junction;cell junction;cell part;cell projection membrane;cell projection part;cell-substrate adherens junction;cell-substrate junction;cytoplasmic part;endoplasmic reticulum	Complement and coagulation cascades	Homo sapiens	1	12
cell part;cytoplasmic part;cytosol;intracellular part;membrane		Homo sapiens	1	12
adherens junction;anchoring junction;cell junction;cell part;cell-substrate adherens junction;cell-substrate junction;cytoplasm;cytoplasmic part;cytoplasmic vesicle membrane;cytoplasmic vesicle part;cytoskeletal part;dystrophin-associated glycoprotein complex;extracellular membrane-bounded organelle;extracellular organelle;extracellular		Homo sapiens	1	12
cell cortex part;cell part;cortical endoplasmic reticulum;cytoplasmic part;cytoskeletal part;endoplasmic reticulum;endoplasmic reticulum membrane;endoplasmic reticulum part;integral to endoplasmic reticulum membrane;integral to membrane;integral to organelle membrane;integral to plasma membrane;intracellular membrane-bounded o		Homo sapiens	1	12
cell part;cytoplasmic part;endoplasmic reticulum;endoplasmic reticulum membrane;endoplasmic reticulum part;integral to membrane;intracellular membrane-bounded organelle;intracellular organelle;intracellular organelle part;intracellular part;intrinsic to membrane;membrane;membrane part;membrane-bounded organelle;organelle;organel		Homo sapiens	1	12
brush border;cell part;cell projection;cytoplasm;extracellular membrane-bounded organelle;extracellular organelle;extracellular region part;extracellular vesicular exosome;intracellular part;membrane-bounded organelle;membrane-bounded vesicle;organelle;vesicle		Homo sapiens	1	12
cell part;cytoplasm;cytoplasmic part;endosome;extracellular membrane-bounded organelle;extracellular organelle;extracellular region part;extracellular vesicular exosome;intracellular membrane-bounded organelle;intracellular organelle;intracellular part;macromolecular complex;membrane;membrane part;membrane raft;membrane-bounde		Homo sapiens	1	12
cell part;cytoplasmic part;endoplasmic reticulum;intracellular membrane-bounded organelle;intracellular non-membrane-bounded organelle;intracellular organelle;intracellular organelle part;intracellular part;membrane-bounded organelle;non-membrane-bounded organelle;nuclear part;nucleolus;organelle;organelle part		Homo sapiens	1	12
cell part;cytoplasm;cytoskeletal part;cytoskeleton;intracellular non-membrane-bounded organelle;intracellular organelle;intracellular organelle part;intracellular part;microtubule cytoskeleton;microtubule plus end;non-membrane-bounded organelle;organelle;organelle part		Homo sapiens	1	12
cell part;cytoplasm;cytoplasmic part;cytosol;intracellular membrane-bounded organelle;intracellular organelle;intracellular part;membrane-bounded organelle;nucleus;organelle		Homo sapiens	1	12
cell part;germ cell nucleus;intracellular membrane-bounded organelle;intracellular organelle;intracellular part;male germ cell nucleus;membrane-bounded organelle;nucleus;organelle		Homo sapiens	1	12
cell part;cytoplasm;cytoplasmic part;cytoskeletal part;cytosol;extracellular membrane-bounded organelle;extracellular organelle;extracellular region part;extracellular vesicular exosome;intracellular membrane-bounded organ	Amyotrophic lateral sclerosis (ALS);Chagas disease (American trypanosomiasis);Epithelial cell signaling in Helicobac	Homo sapiens	1	12
cell part;intracellular membrane-bounded organelle;intracellular organelle;intracellular organelle part;intracellular part;macromolecular complex;membrane;membrane-bounded organelle;mRNA cleavage factor complex;nucle	mRNA surveillance pathway	Homo sapiens	1	12
cell part;envelope;intracellular organelle part;intracellular part;macromolecular complex;membrane;membrane part;nuclear envelope;nuclear part;nuclear pore;organelle envelope;organelle part;pore complex;protein complex	RNA transport	Homo sapiens	1	12
cell part;chromosomal part;intracellular membrane-bounded organelle;intracellular organelle;intracellular organelle part;intracellular part;macromolecular complex;membrane-bounded organelle;nucleosome;nucleus;organelle;organelle part;protein-DNA complex		Homo sapiens	1	12
cell part;chromosomal part;chromosome, telomeric region;cytoplasm;cytoskeletal part;female pronucleus;intracellular membrane-bounded organelle;intracellular non-membrane-bounded organelle;intracellular organelle;intracellular organelle part;intracellular part;male pronucleus;membrane;membrane-bounded organelle;non-membrane-bo		Homo sapiens	1	12
		Homo sapiens	1	12
cell part;integrator complex;intracellular membrane-bounded organelle;intracellular organelle;intracellular organelle part;intracellular part;macromolecular complex;membrane-bounded organelle;nuclear part;nucleoplasm part;nucleus;organelle;organelle part;protein complex;SOSS complex		Homo sapiens	1	12
cell part;cytoplasm;Elongator holoenzyme complex;histone acetyltransferase complex;intracellular organelle part;intracellular part;macromolecular complex;nuclear part;nucleoplasm part;organelle part;protein complex;transcription elongation factor complex		Homo sapiens	1	12
cell part;cytoplasmic part;endoplasmic reticulum;extrinsic to membrane;intracellular membrane-bounded organelle;intracellular organelle;intracellular part;membrane;membrane part;membrane-bounded organelle;organelle		Homo sapiens	1	12
cell part;intracellular organelle part;intracellular part;macromolecular complex;nuclear body;nuclear matrix;nuclear part;nuclear speck;nucleoplasm;nucleoplasm part;organelle part;ribonucleoprotein complex;small nuclear ribo	Spliceosome	Homo sapiens	1	12
cell part;cytoplasm;envelope;intracellular organelle part;intracellular part;macromolecular complex;membrane;membrane part;nuclear envelope;nuclear membrane;nuclear part;nuclear pore;nucleoplasm;organelle envelope;or	RNA transport	Homo sapiens	1	12
cell part;cytoplasm;intracellular membrane-bounded organelle;intracellular organelle;intracellular organelle part;intracellular part;membrane-bounded organelle;nuclear part;nucleoplasm;nucleus;organelle;organelle part		Homo sapiens	1	12
cell part;cytoplasmic part;endoplasmic reticulum membrane;endoplasmic reticulum part;extracellular membrane-bounded organelle;extracellular organelle;extracellular region part;extracellular vesicular exosome;integral to membrane;intracellular organelle part;intracellular part;intrinsic to membrane;membrane;membrane part;membrane-bo		Homo sapiens	1	12
cell envelope;cell part;cornified envelope;cytoplasm;cytoplasmic part;cytoskeleton;envelope;extracellular membrane-bounded organelle;extracellular organelle;extracellular region part;extracellular vesicular exosome;intracellular membrane-bounded organelle;intracellular non-membrane-bounded organelle;intracellular organelle;intracellular		Homo sapiens	1	12
cell part;cytoplasmic part;endoplasmic reticulum;endoplasmic reticulum chaperone complex;endoplasmic reticulum lumen;endoplasmic reticulum part;intracellular membrane-bounded organelle;intracellular organelle;intracel	Protein processing in endoplasmic reticulum	Homo sapiens	1	12
cell part;cytoplasmic part;cytosol;extracellular membrane-bounded organelle;extracellular organelle;extracellular region part;extracellular vesicular exosome;integral to membrane;integral to mitochondrial membrane;integral to mitochondrial outer membrane;integral to organelle membrane;intracellular organelle part;intracellular part;intrinsic		Homo sapiens	1	12
cell part;chromatin;chromosomal part;cytoplasm;histone methyltransferase complex;intracellular membrane-bounded organelle;intracellular non-membrane-bounded organelle;intracellular organelle;intracellular organelle part;intracellular part;macromolecular complex;membrane;membrane-bounded organelle;methyltransferase complex;ML		Homo sapiens	1	12
cell part;cytoplasmic part;cytosol;extracellular membrane-bounded organelle;extracellular organelle;extracellular region part;extracellular vesicular exosome;glycerol-3-phosphate dehydrogenase complex;intracellular part;ma	Glycerophospholipid metabolism	Homo sapiens	1	12
cell part;membrane		Homo sapiens	1	12
cell part;cytoplasm;cytoplasmic part;endoplasmic reticulum;endoplasmic reticulum membrane;endoplasmic reticulum part;integral to membrane;intracellular membrane-bounded organelle;intracellular organelle;intracellular organelle part;intracellular part;intrinsic to membrane;membrane;membrane part;membrane-bounded organelle;nuclea		Homo sapiens	1	12
cell part;chromatin;chromatin remodeling complex;chromosomal part;intracellular membrane-bounded organelle;intracellular organelle;intracellular organelle part;intracellular part;macromolecular complex;membrane-bounded organelle;nuclear chromatin;nuclear chromosome part;nuclear part;nucleus;organelle;organelle part;protein comple		Homo sapiens	1	12
cell part;cytoplasmic membrane-bounded vesicle;cytoplasmic part;cytoplasmic vesicle;endoplasmic reticulum membrane;endoplasmic reticulum part;GPI-anchor transamidase complex;integral to endoplasmic reticulum mer	Glycosylphosphatidylinositol(GPI)-anchor biosynthesis	Homo sapiens	1	12
cell part;cytoplasmic part;endoplasmic reticulum membrane;endoplasmic reticulum part;endoplasmic reticulum-Golgi intermediate compartment;endoplasmic reticulum-Golgi intermediate compartment membrane;Golgi apparatus part;Golgi membrane;integral to membrane;intracellular membrane-bounded organelle;intracellular organelle;i		Homo sapiens	1	12
cell part;cytoplasm;cytoplasmic part;extracellular membrane-bounded organelle;extracellular organelle;extracellular region part;extracellular vesicular exosome;Golgi apparatus;intracellular membrane-bounded organelle;intracellular organelle;intracellular organelle part;intracellular part;membrane-bounded organelle;membrane-bounded ves		Homo sapiens	1	12
catalytic step 2 spliceosome;cell part;cytoplasm;intracellular organelle part;intracellular part;macromolecular complex;nuclear part;nucleoplasm;organelle part;ribonucleoprotein complex;small nuclear ribonucleoprotein comp	Spliceosome	Homo sapiens	1	12
cell part;cytoplasmic part;intracellular organelle lumen;intracellular organelle part;intracellular part;membrane-enclosed lumen;mitochondrial matrix;mitochondrial part;organelle lumen;organelle part		Homo sapiens	1	12
cell part;cytoplasmic part;intracellular membrane-bounded organelle;intracellular organelle;intracellular organelle part;intracellular part;membrane;membrane-bounded organelle;mitochondrial inner membrane;mitochondrial membrane;mitochondrial part;mitochondrion;organelle;organelle inner membrane;organelle membrane;organelle part		Homo sapiens	1	12
cell part;intracellular organelle part;intracellular part;macromolecular complex;nuclear part;nucleoplasm;organelle part;ribonucleoprotein complex;spliceosomal complex	Spliceosome	Homo sapiens	1	12
cell part;cullin-RING ubiquitin ligase complex;cytoplasm;intracellular membrane-bounded organelle;intracellular organelle;intracellular part;macromolecular complex;membrane-bounded organelle;nucleus;organelle;protein complex;SCF ubiquitin ligase complex;ubiquitin ligase complex		Homo sapiens	1	12
		Homo sapiens	1	12
cell part;cytoplasm;cytoskeleton;intracellular membrane-bounded organelle;intracellular non-membrane-bounded organelle;intracellular organelle;intracellular part;membrane-bounded organelle;microtubule cytoskeleton;non-membrane-bounded organelle;nucleus;organelle		Homo sapiens	1	12
cell part;cytoplasm;cytoplasmic part;cytosol;Golgi apparatus;intracellular membrane-bounded organelle;intracellular organelle;intracellular part;membrane-bounded organelle;nucleus;organelle;perinuclear region of cytoplasm		Homo sapiens	1	12
cell part;cytoplasm;intracellular membrane-bounded organelle;intracellular organelle;intracellular part;membrane-bounded organelle;nucleus;organelle	mRNA surveillance pathway	Homo sapiens	1	12
cell part;cell projection;cytoplasm;cytoplasmic part;cytosol;intracellular non-membrane-bounded organelle;intracellular organelle;intracellular organelle part;intracellular part;non-membrane-bounded organelle;nuclear part;nu	Cysteine and methionine metabolism	Homo sapiens	1	12
cell part;cytoplasm;intracellular non-membrane-bounded organelle;intracellular organelle;intracellular organelle part;intracellular part;non-membrane-bounded organelle;nuclear part;nucleolus;organelle;organelle part		Homo sapiens	1	12
cell part;cytoplasmic part;intracellular membrane-bounded organelle;intracellular non-membrane-bounded organelle;intracellular organelle;intracellular organelle part;intracellular part;macromolecular complex;membrane;membrane-bounded organelle;mitochondrial inner membrane;mitochondrial membrane;mitochondrial part;mitochondrial		Homo sapiens	1	12
cell part;DNA-directed RNA polymerase complex;DNA-directed RNA polymerase I complex;intracellular membrane-bounded organelle;intracellular non-membrane-bounded organelle;intracellular organelle;intracellular organe	Purine metabolism;Pyrimidine metabolism;RNA polymerase	Homo sapiens	1	12
acrosomal vesicle;cell division site part;cell part;centralspindlin complex;cleavage furrow;cytoplasmic membrane-bounded vesicle;cytoplasmic part;cytoplasmic vesicle;cytoskeletal part;cytosol;extracellular membrane-bounded organelle;extracellular organelle;extracellular region part;extracellular vesicular exosome;extrinsic to internal side		Homo sapiens	1	12
cell part;cytoplasmic part;cytosol;endosome;Golgi apparatus;Golgi apparatus part;Golgi cisterna;Golgi cisterna membrane;Golgi membrane;intracellular membrane-bounded organelle;intracellular organelle;intracellular organelle part;intracellular part;membrane;membrane-bounded organelle;membrane-enclosed lumen;mitochondrial interm		Homo sapiens	1	12
cell part;chromosome;condensed chromosome;condensed nuclear chromosome;CURI complex;cytoplasmic part;intracellular membrane-bounded organelle;intracellular non-membrane-bounded organelle;intracellular organe	Ribosome biogenesis in eukaryotes	Homo sapiens	1	12
cell part;cytoplasmic part;endoplasmic reticulum;intracellular membrane-bounded organelle;intracellular organelle;intracellular part;membrane-bounded organelle;nucleus;organelle		Homo sapiens	1	12
cell part;integral to membrane;intrinsic to membrane;membrane part		Homo sapiens	1	12
cell body;cell part;cell projection;cytoplasmic part;endoplasmic reticulum;endoplasmic reticulum part;envelope;extracellular membrane-bounded organelle;extracellular organelle;extracellular region part;extracellular vesicular exosome;integral to endoplasmic reticulum membrane;integral to membrane;integral to organelle membrane;intrace		Homo sapiens	1	12
axon;cell part;cell projection;cytoplasm;cytoskeletal part;intracellular organelle part;intracellular part;kinesin complex;macromolecular complex;microtubule;microtubule associated complex;neuron projection;organelle part;protein complex		Homo sapiens	1	12
cell part;cytoplasm;cytoplasmic part;cytosol;eukaryotic translation initiation factor 2B complex;intracellular part;macromolecular complex;protein complex	RNA transport	Homo sapiens	1	12
cell part;cytoplasmic part;extracellular membrane-bounded organelle;extracellular organelle;extracellular region part;extracellular vesicular exosome;intracellular organelle part;intracellular part;membrane;membrane-bounded organelle;membrane-bounded vesicle;mitochondrial inner membrane;mitochondrial membrane;mitochondrial part;o		Homo sapiens	1	12
cell part;intracellular membrane-bounded organelle;intracellular non-membrane-bounded organelle;intracellular organelle;intracellular organelle part;intracellular part;membrane-bounded organelle;non-membrane-bounded organelle;nuclear part;nucleolus;nucleus;organelle;organelle part		Homo sapiens	1	12
cell part;cytoplasmic part;intracellular membrane-bounded organelle;intracellular organelle;intracellular organelle part;intracellular part;membrane-bounded organelle;mitochondrion;nuclear part;nucleoplasm;organelle;organelle part		Homo sapiens	1	12
cell part;cytoplasmic part;endosome;intracellular membrane-bounded organelle;intracellular organelle;intracellular part;membrane;membrane-bounded organelle;mitochondrion;organelle		Homo sapiens	1	12
cell part;cytoplasm;intracellular organelle part;intracellular part;nuclear body;nuclear part;nuclear speck;nucleoplasm;nucleoplasm part;organelle part		Homo sapiens	1	12
cell part;cytoplasmic part;early endosome;endosomal part;endosome;endosome membrane;extracellular membrane-bounded organelle;extracellular organelle;extracellular region part;extracellular vesicular exosome;intracellular membrane-bounded organelle;intracellular organelle;intracellular organelle part;intracellular part;lysosomal memb		Homo sapiens	1	12
aggresome;axon;caveola;cell leading edge;cell part;cell projection;chromatin remodeling complex;cytoplasm;cytoplasmic microtubule;cytoplasmic part;cytoskeletal part;cytosol;dendrite;histone deacetylase complex;inclusion body;intracellular membrane-bounded organelle;intracellular organelle;intracellular organelle part;intracellular part;n		Homo sapiens	1	12
cell part;cytoplasmic part;cytosol;endoplasmic reticulum;endoplasmic reticulum membrane;endoplasmic reticulum part;integral to membrane;intracellular membrane-bounded organelle;intracellular organelle;intracellular orga	Protein export;Protein processing in endoplasmic reticulum	Homo sapiens	1	12
cell part;cytoplasmic part;extracellular membrane-bounded organelle;extracellular organelle;extracellular region part;extracellular vesicular exosome;integral to membrane;intracellular membrane-bounded organelle;intracellul	Amino sugar and nucleotide sugar metabolism	Homo sapiens	1	12
extracellular membrane-bounded organelle;extracellular organelle;extracellular region part;extracellular vesicular exosome;membrane-bounded organelle;membrane-bounded vesicle;organelle;vesicle	Amino sugar and nucleotide sugar metabolism	Homo sapiens	1	12
cell part;intracellular membrane-bounded organelle;intracellular organelle;intracellular part;membrane-bounded organelle;nucleus;organelle		Homo sapiens	1	12
cell part;cytoplasm;intracellular part;membrane;plasma membrane		Homo sapiens	1	12
cell part;intracellular membrane-bounded organelle;intracellular non-membrane-bounded organelle;intracellular organelle;intracellular organelle part;intracellular part;macromolecular complex;membrane;membrane-bounded	Ribosome biogenesis in eukaryotes	Homo sapiens	1	12
cell part;cytoplasm;cytoplasmic part;cytosol;intracellular part	Amino sugar and nucleotide sugar metabolism	Homo sapiens	1	12
cell part;chromosomal part;cytoplasm;cytoplasmic part;cytosol;intracellular membrane-bounded organelle;intracellular non-membrane-bounded organelle;intracellular organelle;intracellular organelle part;intracellular part;kine	NOD-like receptor signaling pathway;Plant-pathogen interaction	Homo sapiens	1	12
cell part;cytoplasm;cytoplasmic microtubule;cytoplasmic part;cytoskeletal part;endoplasmic reticulum membrane;endoplasmic reticulum part;integral to membrane;intracellular organelle part;intracellular part;intrinsic to mem	Protein export	Homo sapiens	1	12
cell part;cell projection;clathrin-coated vesicle;coated vesicle;cytoplasm;cytoplasmic membrane-bounded vesicle;cytoplasmic part;cytoplasmic vesicle;cytoplasmic vesicle membrane;cytoplasmic vesicle part;endosome;extracellular membrane-bounded organelle;extracellular organelle;extracellular region part;extracellular vesicular exosom		Homo sapiens	1	12
cell part;cytoplasmic part;extracellular membrane-bounded organelle;extracellular organelle;extracellular region part;extracellular vesicular exosome;integral to membrane;intracellular membrane-bounded organelle;intracellular organelle;intracellular organelle part;intracellular part;intrinsic to membrane;membrane;membrane part;membrane-		Homo sapiens	1	12
cell part;cytoplasmic part;cytosol;eukaryotic 43S preinitiation complex;eukaryotic 48S preinitiation complex;eukaryotic translation initiation factor 3 complex;intracellular part;macromolecular complex;membrane;protein comp	RNA transport	Homo sapiens	1	13
cell part;cytoplasmic part;intracellular organelle lumen;intracellular organelle part;intracellular part;membrane-enclosed lumen;mitochondrial matrix;mitochondrial part;organelle lumen;organelle part	Citrate cycle (TCA cycle);Glycolysis / Gluconeogenesis;Pyruvate metabolism	Homo sapiens	1	13
cell part;cis-Golgi network;cytoplasmic membrane-bounded vesicle;cytoplasmic part;cytoplasmic vesicle;endocytic vesicle;endosomal part;endosome membrane;Golgi apparatus;Golgi apparatus part;Golgi cisterna membrane;Golgi lumen;Golgi membrane;integral to membrane;intracellular membrane-bounded organelle;intracellular organe		Homo sapiens	1	13
cell part;cytoplasm;cytoplasmic part;cytosol;intracellular part		Homo sapiens	1	13
cell part;cytoplasmic part;cytosol;extracellular membrane-bounded organelle;extracellular organelle;extracellular region part;extracellular vesicular exosome;intracellular membrane-bounded organelle;intracellular organelle;int	Proteasome	Homo sapiens	1	13
cell part;COPI vesicle coat;cytoplasmic part;cytoplasmic vesicle part;cytosol;Golgi apparatus;Golgi apparatus part;intracellular membrane-bounded organelle;intracellular organelle;intracellular organelle part;intracellular part;macromolecular complex;membrane coat;membrane part;membrane-bounded organelle;nuclear part;nucleoplasm;o		Homo sapiens	1	13
actin filament bundle;actomyosin;apical part of cell;cell part;contractile fiber part;cytoplasmic part;cytoskeletal part;cytosol;extracellular membrane-bounded organelle;extracellular organelle;extracellular region part;extracellu	Focal adhesion;Leukocyte transendothelial migration;Regulation of actin cytoskeleton;Tight junction	Homo sapiens	1	13
cell part;cytoplasm;cytoplasmic part;cytosolic part;intracellular part;macromolecular complex;prefoldin complex;protein complex		Homo sapiens	1	13
cell part;cytoplasmic part;endoplasmic reticulum membrane;endoplasmic reticulum palmitoyltransferase complex;endoplasmic reticulum part;integral to membrane;intracellular organelle part;intracellular part;intrinsic to mem	Sphingolipid metabolism	Homo sapiens	1	13
actin cytoskeleton;cell part;cytoskeleton;integral to membrane;integral to plasma membrane;intracellular non-membrane-bounded organelle;intracellular organelle;intracellular organelle part;intracellular part;intrinsic to membrane;intrinsic to plasma membrane;membrane;membrane part;non-membrane-bounded organelle;nuclear membrane		Homo sapiens	1	13
cell part;intracellular membrane-bounded organelle;intracellular non-membrane-bounded organelle;intracellular organelle;intracellular organelle part;intracellular part;macromolecular complex;membrane-bounded organelle;m	mRNA surveillance pathway	Homo sapiens	1	13
cell part;cytoplasm;extracellular membrane-bounded organelle;extracellular organelle;extracellular region part;extracellular vesicular exosome;intracellular membrane-bounded organelle;intracellular organelle;intracellular part;macromolecular complex;membrane-bounded organelle;membrane-bounded vesicle;nucleus;organelle;proteasome		Homo sapiens	1	13
cell part;intracellular non-membrane-bounded organelle;intracellular organelle;intracellular organelle part;intracellular part;non-membrane-bounded organelle;nuclear part;nucleolus;organelle;organelle part		Homo sapiens	1	13
cell part;clathrin-coated vesicle;coated vesicle;cytoplasm;cytoplasmic membrane-bounded vesicle;cytoplasmic part;cytoplasmic vesicle;cytosol;early endosome;early endosome membrane;early phagosome;endocytic vesicle;endosomal part;endosome;endosome membrane;extracellular membrane-bounded organelle;extracellular organell		Homo sapiens	1	13
cell part;cytoplasm;intracellular membrane-bounded organelle;intracellular organelle;intracellular part;membrane;membrane-bounded organelle;nucleus;organelle		Homo sapiens	1	13
carboxy-terminal domain protein kinase complex;cell part;chromatin;chromosomal part;chromosome;condensed chromosome;condensed nuclear chromosome;cytoplasm;intracellular membrane-bounded organelle;intracellular non-membrane-bounded organelle;intracellular organelle;intracellular organelle part;intracellular part;macromolec		Homo sapiens	1	13
cell part;cytoplasmic part;cytosolic part;intracellular part;macromolecular complex;prefoldin complex;protein complex		Homo sapiens	1	13
cell part;cytoplasmic part;cytosol;extracellular membrane-bounded organelle;extracellular organelle;extracellular region part;extracellular vesicular exosome;intracellular part;membrane-bounded organelle;membrane-bounde	Glutathione metabolism	Homo sapiens	1	13
cell part;cytoplasm;intracellular membrane-bounded organelle;intracellular organelle;intracellular part;membrane-bounded organelle;organelle		Homo sapiens	1	13
cell part;cell projection;centriole;cilium;cytoplasm;cytoplasmic part;cytoplasmic vesicle;cytoskeletal part;extracellular membrane-bounded organelle;extracellular organelle;extracellular region part;extracellular vesicular exosome;Golgi apparatus;intracellular membrane-bounded organelle;intracellular non-membrane-bounded organelle;intrac		Homo sapiens	1	13
cell part;intracellular membrane-bounded organelle;intracellular organelle;intracellular organelle part;intracellular part;membrane-bounded organelle;nuclear part;nucleoplasm;nucleus;organelle;organelle part		Homo sapiens	1	13
cell part;cytoplasmic part;endoplasmic reticulum;endoplasmic reticulum membrane;endoplasmic reticulum part;intracellular membrane-bounded organelle;intracellular organelle;intracellular organelle part;intracellular part;membrane;membrane part;membrane-bounded organelle;organelle;organelle membrane;organelle part		Homo sapiens	1	13
cell part;cytoplasm;intracellular organelle part;intracellular part;nuclear body;nuclear part;nucleoplasm part;organelle part;PML body		Homo sapiens	1	13
cell part;coated vesicle membrane;COPII vesicle coat;cytoplasmic part;cytoplasmic vesicle membrane;cytoplasmic vesicle part;cytosol;endoplasmic reticulum membrane;endoplasmic reticulum part;ER to Golgi transport ves	Protein processing in endoplasmic reticulum	Homo sapiens	1	13

cell junction;cell part;cell projection;centrosome;cytoplasm;cytoplasmic part;cytoskeletal part;intracellular non-membrane-bounded organelle;intracellular organelle;intracellular organelle part;intracellular part;macromolecular	SNARE interactions in vesicular transport	Homo sapiens	1	13
blood microparticle;cell part;cytoplasmic part;cytosol;extracellular membrane-bounded organelle;extracellular organelle;extracellular region;extracellular region part;extracellular space;extracellular vesicular exosome;intracellular organelle lumen;intracellular organelle part;intracellular part;lysosomal lumen;membrane-bounded organelle;mem		Homo sapiens	1	13
cell part;cytoplasm;cytoplasmic part;cytosol;extracellular membrane-bounded organelle;extracellular organelle;extracellular region part;extracellular space;extracellular vesicular exosome;intracellular part;membrane-boundec	PPAR signaling pathway	Homo sapiens	1	13
cell part;chromatoid body;cytoplasm;cytoplasmic mRNA processing body;cytoplasmic part;cytosol;eukaryotic translation initiation factor 4F complex;extracellular membrane-bounded organelle;extracellular organelle;extrace	Insulin signaling pathway;mTOR signaling pathway;RNA transport	Homo sapiens	1	13
cell part;cytoplasmic part;extracellular membrane-bounded organelle;extracellular organelle;extracellular region part;extracellular vesicular exosome;intracellular organelle lumen;intracellular organelle part;intracellular part;lys	Amino sugar and nucleotide sugar metabolism;Glycosaminoglycan degradation;Glycosphingolipid biosynthesis - ga	Homo sapiens	1	13
cell part;cytoplasm;cytoplasmic part;cytosol;extracellular membrane-bounded organelle;extracellular organelle;extracellular region part;extracellular vesicular exosome;intracellular membrane-bounded organelle;intracellular organelle;intracellular organelle part;intracellular part;membrane-bounded organelle;membrane-bounded vesicle;mito		Homo sapiens	1	13
anchored to external side of plasma membrane;anchored to membrane;anchored to plasma membrane;apical plasma membrane;brush border membrane;cell part;cell projection membrane;cell projection part;cell surface;cla	Endocytosis	Homo sapiens	1	13
adherens junction;anchoring junction;cell junction;cell part;cell-substrate adherens junction;cell-substrate junction;chromatin;chromosomal part;cytoplasmic part;cytosol;focal adhesion;heterochromatin;intracellular membrane-bounded organelle;intracellular organelle;intracellular organelle part;intracellular part;macromolecular complex;men		Homo sapiens	1	13
cell part;cytoplasmic part;cytosol;intracellular membrane-bounded organelle;intracellular organelle;intracellular part;membrane-bounded organelle;nucleus;organelle	Adherens junction;Circadian rhythm - plant;Measles;Ribosome biogenesis in eukaryotes;Tight junction;Wnt signaling	Homo sapiens	1	13
cell part;chromatin remodeling complex;chromosomal part;DNA helicase complex;Ino80 complex;intracellular membrane-bounded organelle;intracellular organelle;intracellular organelle part;intracellular part;macromolecular complex;membrane-bounded organelle;nuclear chromosome part;nuclear matrix;nuclear part;nucleoplasm part;nucle		Homo sapiens	1	13
cell part;cytoplasmic part;cytosol;extracellular membrane-bounded organelle;extracellular organelle;extracellular region part;extracellular vesicular exosome;intracellular membrane-bounded organelle;intracellular non-membr	Protein processing in endoplasmic reticulum	Homo sapiens	1	13
cell part;cell projection;cytoplasmic part;extracellular membrane-bounded organelle;extracellular organelle;extracellular region part;extracellular space;extracellular vesicular exosome;intracellular membrane-bounded organelle;intracellular organelle;intracellular part;membrane-bounded organelle;membrane-bounded vesicle;neuron projectio		Homo sapiens	1	13
cell junction;cell part;cell-cell junction;cytoplasm;cytoplasmic part;cytoskeletal part;cytoskeleton;cytosol;intracellular membrane-bounded organelle;intracellular non-membrane-bounded organelle;intracellular organelle;intrac	Acute myeloid leukemia;Adipocytokine signaling pathway;Apoptosis;B cell receptor signaling pathway;Carbohydrat	Homo sapiens	1	13
cell part;cytoplasmic part;extracellular membrane-bounded organelle;extracellular organelle;extracellular region part;extracellular vesicular exosome;intracellular membrane-bounded organelle;intracellular organelle;intracellula	Pyrimidine metabolism	Homo sapiens	1	13
adherens junction;anchoring junction;cell junction;cell part;cell-substrate adherens junction;cell-substrate junction;cytoplasm;cytoplasmic part;cytosol;cytosolic large ribosomal subunit;extracellular membrane-bounded orga	Ribosome	Homo sapiens	1	13
cell part;intracellular membrane-bounded organelle;intracellular organelle;intracellular organelle part;intracellular part;macromolecular complex;membrane-bounded organelle;nuclear part;nucleus;organelle;organelle part;ribo	Spliceosome	Homo sapiens	1	13
cell part;cytoplasm;cytoplasmic part;cytosol;cytosolic large ribosomal subunit;intracellular non-membrane-bounded organelle;intracellular organelle;intracellular organelle part;intracellular part;large ribosomal subunit;macrom	Ribosome	Homo sapiens	1	13
adherens junction;anchoring junction;cell junction;cell part;cell-substrate adherens junction;cell-substrate junction;external side of plasma membrane;extracellular region part;extracellular space;focal adhesion;integral to membrane;intracellular membrane-bounded organelle;intracellular organelle;intracellular part;intrinsic to membrane;mem		Homo sapiens	1	13
cell part;cytoplasmic part;intracellular membrane-bounded organelle;intracellular organelle;intracellular organelle part;intracellular part;macromolecular complex;membrane;membrane part;membrane-bounded organelle;mito	Alzheimer's disease;Cardiac muscle contraction;Huntington's disease;Oxidative phosphorylation;Parkinson's diseas	Homo sapiens	1	13
cell part;cytoplasmic part;cytosol;intracellular membrane-bounded organelle;intracellular organelle;intracellular part;membrane-bounded organelle;mitochondrion;organelle	Carbon fixation in photosynthetic organisms;Pyruvate metabolism	Homo sapiens	1	13
adherens junction;anchoring junction;basement membrane;cell junction;cell part;cell-substrate adherens junction;cell-substrate junction;cytoplasmic part;cytosol;extracellular matrix part;extracellular region part;focal adhesion;integral to membrane;integral to plasma membrane;intracellular part;intrinsic to membrane;intrinsic to plasma mem		Homo sapiens	1	13
cell part;cytoplasmic part;intracellular membrane-bounded organelle;intracellular non-membrane-bounded organelle;intracellular organelle;intracellular organelle part;intracellular part;macromolecular complex;membrane;membrane-bounded organelle;mitochondrial inner membrane;mitochondrial membrane;mitochondrial part;non-membrar		Homo sapiens	1	13
cell part;cytoplasm;cytosolic large ribosomal subunit;extracellular membrane-bounded organelle;extracellular organelle;extracellular region part;extracellular vesicular exosome;intracellular membrane-bounded organelle;intracellular organelle;intracellular organelle part;intracellular part;large ribosomal subunit;macromolecular complex;memb		Homo sapiens	1	13
cell part;cytoplasmic part;cytosol;extracellular membrane-bounded organelle;extracellular organelle;extracellular region part;extracellular vesicular exosome;intracellular membrane-bounded organelle;intracellular organelle;int	Proteasome	Homo sapiens	1	13
cell part;cell projection;cytoplasm;cytoplasmic part;cytosol;dendrite;intracellular membrane-bounded organelle;intracellular organelle;intracellular organelle part;intracellular part;macromolecular complex;membrane part;membrane-bounded organelle;neuron projection;nuclear part;nuclear pore;nucleoplasm;nucleus;organelle;organelle part;p		Homo sapiens	1	13
cell part;cytoplasmic part;endoplasmic reticulum;extracellular membrane-bounded organelle;extracellular organelle;extracellular region part;extracellular space;extracellular vesicular exosome;Golgi apparatus;intracellular me	Lysosome	Homo sapiens	1	13
cell part;extracellular membrane-bounded organelle;extracellular organelle;extracellular region part;extracellular vesicular exosome;extrinsic to membrane;extrinsic to plasma membrane;membrane part;membrane-bounded organelle;membrane-bounded vesicle;organelle;plasma membrane part;vesicle		Homo sapiens	1	13
cell part;cytoplasmic membrane-bounded vesicle;cytoplasmic part;cytoplasmic vesicle;endoplasmic reticulum membrane;endoplasmic reticulum part;endoplasmic reticulum-Golgi intermediate compartment membrane;extracellular membrane-bounded organelle;extracellular organelle;extracellular region part;extracellular vesicular exosome		Homo sapiens	1	13
catalytic step 2 spliceosome;cell part;cytoplasmic part;cytosol;exon-exon junction complex;intracellular membrane-bounded organelle;intracellular organelle;intracellular organelle part;intracellular part;macromolecular compl	mRNA surveillance pathway;RNA transport;Spliceosome	Homo sapiens	1	13
cell part;cytoplasmic part;cytosol;endoplasmic reticulum;endoplasmic reticulum part;integral to endoplasmic reticulum membrane;integral to membrane;integral to organelle membrane;intracellular membrane-bounded organ	Phagosome;Protein export;Protein processing in endoplasmic reticulum;Vibrio cholerae infection	Homo sapiens	1	13
catalytic step 2 spliceosome;cell part;cytoplasm;cytoplasmic part;cytosol;extracellular membrane-bounded organelle;extracellular organelle;extracellular region part;extracellular vesicular exosome;intracellular organelle part;i	Spliceosome;Systemic lupus erythematosus	Homo sapiens	1	13
adherens junction;anchoring junction;cell junction;cell part;cell projection;cell projection membrane;cell projection part;cell-substrate adherens junction;cell-substrate junction;cytoplasm;cytoplasmic membrane-bounded vesi	Adherens junction;Amyotrophic lateral sclerosis (ALS);Axon guidance;B cell receptor signaling pathway;Bacterial inv	Homo sapiens	1	13
cell part;cytoplasmic part;endoplasmic reticulum membrane;endoplasmic reticulum part;extracellular membrane-bounded organelle;extracellular organelle;extracellular region part;extracellular vesicular exosome;integral to m	Protein export	Homo sapiens	1	13
cell part;cytoplasm;cytoskeleton;envelope;intracellular membrane-bounded organelle;intracellular non-membrane-bounded organelle;intracellular organelle;intracellular organelle part;intracellular part;macromolecular comple	RNA transport	Homo sapiens	1	13
cell part;cytoplasm;intracellular membrane-bounded organelle;intracellular organelle;intracellular organelle part;intracellular part;membrane;membrane-bounded organelle;nuclear membrane;nuclear part;nucleus;organelle;organelle membrane;organelle part		Homo sapiens	1	13
axon part;basal lamina;basolateral plasma membrane;cell junction;cell part;cell projection membrane;cell projection part;cell-cell contact zone;cell-cell junction;cytoplasm;cytoplasmic part;cytoskeletal part;cytosol;endoplasm	T cell receptor signaling pathway	Homo sapiens	1	13
cell part;intracellular membrane-bounded organelle;intracellular organelle;intracellular organelle part;intracellular part;membrane-bounded organelle;nuclear part;nucleoplasm;nucleus;organelle;organelle part	mRNA surveillance pathway	Homo sapiens	1	13
cell part;cytoplasm;cytoplasmic part;extracellular membrane-bounded organelle;extracellular organelle;extracellular region part;extracellular vesicular exosome;intracellular organelle lumen;intracellular organelle part;intracellu	Pantothenate and CoA biosynthesis	Homo sapiens	1	13
cell part;cytoplasmic part;cytosol;intracellular membrane-bounded organelle;intracellular organelle;intracellular part;membrane;membrane-bounded organelle;nucleus;organelle		Homo sapiens	1	13
cell part;cytoplasm;intracellular membrane-bounded organelle;intracellular non-membrane-bounded organelle;intracellular organelle;intracellular organelle part;intracellular part;membrane-bounded organelle;non-membrane-b	Spliceosome	Homo sapiens	1	13
cell part;cytoplasmic part;cytosol;intracellular membrane-bounded organelle;intracellular organelle;intracellular organelle part;intracellular part;macromolecular complex;membrane-bounded organelle;nuclear part;nucleoplasm	Chronic myeloid leukemia;Notch signaling pathway;Pathways in cancer;Wnt signaling pathway	Homo sapiens	1	13
adherens junction;anchoring junction;basolateral plasma membrane;cell junction;cell part;cell surface;cell-substrate adherens junction;cell-substrate junction;cytoplasm;extracellular membrane-bounded organelle;extracellular organelle;extracellular region part;extracellular space;extracellular vesicular exosome;focal adhesion;integral to mem		Homo sapiens	1	13
aggresome;autophagic vacuole;cell part;cytoplasm;cytoplasmic mRNA processing body;cytoplasmic part;cytoplasmic vesicle;cytosol;endoplasmic reticulum;endosome;extracellular membrane-bounded organelle;extracellula	Osteoclast differentiation	Homo sapiens	1	13
apical part of cell;cell part;cytoplasm;intracellular membrane-bounded organelle;intracellular non-membrane-bounded organelle;intracellular organelle;intracellular organelle part;intracellular part;membrane;membrane-bounded organelle;non-membrane-bounded organelle;nuclear part;nucleolus;nucleus;organelle;organelle part		Homo sapiens	1	13
acrosomal membrane;adherens junction;anchoring junction;caveola;cell junction;cell part;cell-substrate adherens junction;cell-substrate junction;cytoplasmic membrane-bounded vesicle;cytoplasmic part;cytoplasmic vesicle	Insulin signaling pathway	Homo sapiens	1	13
cell part;cytoplasmic part;cytosol;extracellular membrane-bounded organelle;extracellular organelle;extracellular region part;extracellular vesicular exosome;intracellular part;membrane-bounded organelle;membrane-bounde	Amino sugar and nucleotide sugar metabolism;Galactose metabolism	Homo sapiens	1	13
cell part;cytoplasmic part;cytosol;intracellular part		Homo sapiens	1	13
cell part;cytoplasmic part;cytoskeleton;cytosol;endoplasmic reticulum;endoplasmic reticulum membrane;endoplasmic reticulum part;Golgi apparatus part;Golgi membrane;integral to membrane;intracellular membrane-bounc	Steroid biosynthesis	Homo sapiens	1	13
catalytic step 2 spliceosome;cell part;intracellular organelle part;intracellular part;macromolecular complex;nuclear part;nucleoplasm;organelle part;ribonucleoprotein complex;small nuclear ribonucleoprotein complex;spliceo	Spliceosome	Homo sapiens	1	13
cell part;cytoplasm;extracellular membrane-bounded organelle;extracellular organelle;extracellular region part;extracellular vesicular exosome;intracellular membrane-bounded organelle;intracellular organelle;intracellular part;macromolecular complex;membrane-bounded organelle;membrane-bounded vesicle;nucleus;organelle;protein com		Homo sapiens	1	13
apical junction complex;cell division site part;cell junction;cell part;cell projection;cell-cell junction;centrosome;cleavage furrow;cytoplasm;cytoplasmic part;cytoskeletal part;cytoskeleton;cytosol;intermediate filament cytoskeleton;intracellular membrane-bounded organelle;intracellular non-membrane-bounded organelle;intracellular organell		Homo sapiens	1	13
adherens junction;anchoring junction;cell junction;cell part;cell-substrate adherens junction;cell-substrate junction;focal adhesion;intracellular membrane-bounded organelle;intracellular organelle;intracellular part;membrane-bounded organelle;nucleus;organelle		Homo sapiens	1	13
cell part;cytoplasm;intracellular membrane-bounded organelle;intracellular organelle;intracellular part;macromolecular complex;membrane-bounded organelle;nucleus;organelle;protein complex;tRNA-splicing ligase complex		Homo sapiens	1	13
cell part;cytoplasm;cytoplasmic part;cytosol;intracellular membrane-bounded organelle;intracellular organelle;intracellular organelle part;intracellular part;membrane-bounded organelle;nuclear part;nucleoplasm;nucleus;organelle;organelle part		Homo sapiens	1	13
cell part;cytoplasm;intracellular membrane-bounded organelle;intracellular organelle;intracellular part;membrane-bounded organelle;organelle		Homo sapiens	1	13
cell part;intracellular membrane-bounded organelle;intracellular organelle;intracellular part;membrane-bounded organelle;nucleus;organelle		Homo sapiens	1	13
cell part;cytoplasm;cytoplasmic part;intracellular non-membrane-bounded organelle;intracellular organelle;intracellular organelle part;intracellular part;membrane-enclosed lumen;mitochondrial intermembrane space;mitochondrial part;non-membrane-bounded organelle;nuclear part;nucleolus;nucleoplasm;organelle;organelle envelope lumer		Homo sapiens	1	13
cell part;chromatin;chromosomal part;cytoplasm;cytoplasmic part;intracellular membrane-bounded organelle;intracellular organelle;intracellular organelle part;intracellular part;macromolecular complex;membrane-bounded o	RNA degradation	Homo sapiens	1	13
cell part;cytoplasmic exosome (RNase complex);cytoplasmic part;exosome (RNase complex);intracellular membrane-bounded organelle;intracellular non-membrane-bounded organelle;intracellular organelle;intracellular organelle lumen;intracellular organelle part;intracellular part;macromolecular complex;membrane-bounded organelle;mem		Homo sapiens	1	13
cell part;cytoplasm;cytoplasmic part;intracellular organelle lumen;intracellular organelle part;intracellular part;membrane-enclosed lumen;mitochondrial matrix;mitochondrial part;organelle lumen;organelle part		Homo sapiens	1	13
cell body;cell part;cell projection;cilium;cytoplasm;cytoplasmic part;extracellular membrane-bounded organelle;extracellular organelle;extracellular region;extracellular region part;extracellular space;extracellular vesicular exosome;intracellular membrane-bounded organelle;intracellular organelle;intracellular organelle part;intracellular part;m		Homo sapiens	1	13
cell part;intracellular organelle part;intracellular part;macromolecular complex;nuclear body;nuclear part;nuclear speck;nucleoplasm;nucleoplasm part;organelle part;protein complex;THO complex;THO complex part of transc	RNA transport;Spliceosome	Homo sapiens	1	13
cell part;cytoplasmic part;endoplasmic reticulum membrane;endoplasmic reticulum part;integral to membrane;intracellular;intracellular organelle part;intracellular part;intrinsic to membrane;membrane;membrane part;organell	Retinol metabolism	Homo sapiens	1	13
cell part;cytoplasm;intracellular membrane-bounded organelle;intracellular non-membrane-bounded organelle;intracellular organelle;intracellular organelle part;intracellular part;membrane;membrane-bounded organelle;non-membrane-bounded organelle;nuclear part;nucleolus;nucleus;organelle;organelle part		Homo sapiens	1	13
cell part;intracellular membrane-bounded organelle;intracellular organelle;intracellular part;membrane-bounded organelle;nucleus;organelle		Homo sapiens	1	13
cell part;chromosomal part;condensed chromosome kinetochore;cytoplasmic part;cytosol;extracellular membrane-bounded organelle;extracellular organelle;extracellular region part;extracellular vesicular exosome;intracellular membrane-bounded organelle;intracellular non-membrane-bounded organelle;intracellular organelle;intracellular or		Homo sapiens	1	13
cell junction;cell part;CUL4 RING ubiquitin ligase complex;Cul4B-RING ubiquitin ligase complex;cullin-RING ubiquitin ligase complex;intracellular organelle part;intracellular part;macromolecular complex;nuclear part;nucleop	Nucleotide excision repair;p53 signaling pathway;Ubiquitin mediated proteolysis	Homo sapiens	1	13
cell part;chromatin;chromatin remodeling complex;chromosomal part;chromosome;intracellular membrane-bounded organelle;intracellular non-membrane-bounded organelle;intracellular organelle;intracellular organelle part;intracellular part;macromolecular complex;membrane-bounded organelle;nBAF complex;non-membrane-bounded or		Homo sapiens	1	13
cell part;centriole;centrosome;cytoplasm;cytoplasmic part;cytoskeletal part;cytosol;intracellular non-membrane-bounded organelle;intracellular organelle;intracellular organelle part;intracellular part;macromolecular complex;membrane;microtubule;microtubule organizing center;microtubule organizing center part;non-membrane-bounded or		Homo sapiens	1	13
cell part;chromosomal part;DNA replication factor A complex;intracellular membrane-bounded organelle;intracellular organelle;intracellular organelle part;intracellular part;macromolecular complex;membrane-bounded organelle;nuclear chromosome part;nuclear part;nucleoplasm;nucleus;organelle;organelle part;protein complex		Homo sapiens	1	13
cell part;chromosomal part;chromosome, centromeric region;condensed chromosome kinetochore;cytoplasmic part;cytoskeletal part;cytoskeleton;cytosol;intracellular membrane-bounded organelle;intracellular non-membrane-bounded organelle;intracellular organelle;intracellular organelle part;intracellular part;kinesin complex;kinetochore		Homo sapiens	1	13
[truncated: 930,565 more chars]
