# Supplementary material for: Extracellular Vesicles Bearing Vimentin Drive Epithelial–Mesenchymal Transition
Source: Mol Cell Proteomics. 2025 Jul 4;24(12):101028. doi: 10.1016/j.mcpro.2025.101028 (PMC12719745; doi:10.1016/j.mcpro.2025.101028)
Supplement: Supplemental Data 6 [file mmc9.pdf]

sheet 6.MCF7 imputed data

MCF7_EV_KO_1 MaxLFQ Intensity	MCF7_EV_KO_2 MaxLFQ Intensity	MCF7_EV_KO_3 MaxLFQ Intensity	MCF7_EV_KO_4 MaxLFQ Intensity	MCF7_EV_WT_1 MaxLFQ Intensity	MCF7_EV_WT_2 MaxLFQ Intensity	MCF7_EV_WT_3 MaxLFQ Intensity	MCF7_EV_WT_4 MaxLFQ Intensity	C: Protein Existence
27.4001	27.3898	27.4871	27.4208	27.3386	27.3699	27.3318	27.3817	1:Experimental evidence at protein level
26.8367	27.0004	27.1125	27.0692	26.7284	26.5669	26.7446	26.8368	1:Experimental evidence at protein level
24.7692	26.0287	25.7484	24.509	26.6084	26.9061	26.5109	27.4261	1:Experimental evidence at protein level
27.7389	27.6447	27.6299	27.8962	27.9132	27.9754	28.2058	28.1613	1:Experimental evidence at protein level
27.2104	27.1673	27.2314	27.2783	27.4507	27.3046	27.2944	27.461	1:Experimental evidence at protein level
26.8245	26.8268	26.7645	26.9266	26.5497	26.4451	26.4346	26.6017	1:Experimental evidence at protein level
26.1304	26.1354	25.4516	25.4138	26.4558	26.8624	26.4768	26.7394	1:Experimental evidence at protein level
26.9024	27.0711	27.0873	27.1185	27.3256	27.3443	27.32	27.4177	4:Protein predicted
27.6948	27.6721	27.7367	27.78	28.2332	28.2217	28.124	28.1246	1:Experimental evidence at protein level
25.6749	25.9552	25.9439	26.149	26.2076	26.1781	26.2475	26.4018	1:Experimental evidence at protein level
27.3717	27.3272	27.4912	27.4806	27.7557	27.49	27.6902	27.8523	1:Experimental evidence at protein level
26.9968	26.9734	27.163	27.2672	27.2891	27.299	27.4102	27.4863	1:Experimental evidence at protein level
27.362	27.2245	27.2626	27.3262	27.7878	27.7499	27.8276	28.2142	1:Experimental evidence at protein level
26.2254	26.3166	26.448	26.4624	26.8603	26.6138	26.8389	26.5732	1:Experimental evidence at protein level
28.5695	28.6125	28.7098	28.952	28.349	28.3752	28.3658	28.0044	1:Experimental evidence at protein level
28.3927	28.3763	28.4139	28.3646	28.6605	28.6386	28.6021	28.6308	1:Experimental evidence at protein level
28.8467	28.8604	28.9381	28.976	29.2356	29.2691	29.2574	29.1995	1:Experimental evidence at protein level
24.789	24.8863	24.9749	25.1764	25.4522	25.7467	25.3905	25.9744	1:Experimental evidence at protein level
28.1325	28.2196	28.1498	28.0577	27.9852	28.0279	27.9991	28.072	1:Experimental evidence at protein level
26.5519	26.586	26.6766	26.9102	27.0125	27.0494	27.1227	27.1758	1:Experimental evidence at protein level
27.4703	27.4717	27.4403	27.417	26.5449	26.69	26.5496	26.8987	1:Experimental evidence at protein level
30.0871	30.1587	30.2812	30.2684	28.0811	28.0728	28.134	28.3624	1:Experimental evidence at protein level
26.5954	26.5491	26.4288	26.6064	28.3375	28.1702	28.2356	27.5126	1:Experimental evidence at protein level
26.5025	26.5596	26.6313	26.6307	27.0214	27.0974	27.3434	27.024	1:Experimental evidence at protein level
26.3017	26.3432	26.1117	26.2091	26.6208	26.5099	26.6555	26.8404	1:Experimental evidence at protein level
27.3897	27.3377	27.3152	27.4829	28.3707	28.3659	28.2333	27.669	1:Experimental evidence at protein level
27.4644	27.4663	27.4392	27.3568	27.2457	27.2761	27.104	27.2957	1:Experimental evidence at protein level
27.2344	27.6443	27.6303	27.474	27.9597	28.1798	28.0732	27.9032	1:Experimental evidence at protein level
25.6452	25.4998	25.5301	25.1397	26.2827	26.4584	25.9655	25.7017	1:Experimental evidence at protein level
26.8723	26.8513	26.8101	26.9271	27.0645	27.1204	27.1527	27.0122	1:Experimental evidence at protein level
28.0699	28.2528	28.261	28.446	27.3442	27.2842	27.3995	27.9875	1:Experimental evidence at protein level
28.7863	28.8501	28.7115	28.9983	29.0121	29.1348	29.1254	29.2404	1:Experimental evidence at protein level
27.4194	27.3837	27.5452	27.4387	27.6118	27.5828	27.6117	27.8006	1:Experimental evidence at protein level
26.4058	26.8805	27.0377	26.7732	26.0112	26.056	26.261	26.1233	1:Experimental evidence at protein level
29.4444	29.5123	29.6118	29.5439	29.3403	29.3932	29.3661	29.4071	1:Experimental evidence at protein level
27.9632	28.2457	27.869	27.9295	26.9102	26.931	26.8458	27.4349	1:Experimental evidence at protein level
25.0535	25.1524	24.7693	23.8965	25.9392	25.9252	26.1407	25.6378	1:Experimental evidence at protein level
24.044	26.0376	24.1921	26.057	27.335	27.4949	27.3127	27.501	1:Experimental evidence at protein level
28.1305	28.3124	28.3846	28.3282	28.7278	28.5484	28.4939	28.5601	1:Experimental evidence at protein level
27.7685	28.0055	27.737	27.7563	28.0688	28.276	28.2012	28.4198	1:Experimental evidence at protein level
27.5642	27.7257	27.5446	27.6169	27.895	27.959	27.9346	28.2665	1:Experimental evidence at protein level
25.1431	25.2495	25.2112	25.2712	25.7321	25.6503	25.58	25.9538	1:Experimental evidence at protein level
26.9454	26.8512	26.5996	26.7008	25.7682	25.8802	25.8194	26.2074	1:Experimental evidence at protein level
26.3364	26.0837	26.1174	26.0878	26.5701	26.4672	26.4535	27.1013	1:Experimental evidence at protein level
26.0158	26.2352	26.1615	26.2767	26.5593	26.6801	26.4412	26.7454	1:Experimental evidence at protein level
27.8156	27.8281	27.7441	27.9634	28.1373	28.0301	28.2135	28.3414	1:Experimental evidence at protein level
26.9301	26.9379	26.9743	27.0354	27.2388	27.1484	27.2359	27.4571	1:Experimental evidence at protein level
28.9476	29.0043	28.9191	29.0409	29.2769	29.1996	29.0859	29.3267	1:Experimental evidence at protein level
28.2499	28.2784	28.2937	28.2741	28.334	28.3783	28.4192	28.338	1:Experimental evidence at protein level
24.3448	25.1918	25.2012	25.5912	25.9554	25.7779	25.8248	26.8277	1:Experimental evidence at protein level
27.9927	27.8621	27.7239	27.7343	27.5664	27.3104	27.3783	27.5501	1:Experimental evidence at protein level
30.1607	29.9783	29.9342	29.9408	29.3714	29.36	29.0293	28.9054	1:Experimental evidence at protein level
28.6415	28.8244	28.9897	28.7528	27.0376	27.0004	27.0549	27.2647	1:Experimental evidence at protein level
27.2318	27.2581	27.3568	27.0208	27.0498	26.978	26.969	26.8277	1:Experimental evidence at protein level
30.1782	30.1621	30.1425	30.3804	31.1405	31.0689	31.0743	31.0293	1:Experimental evidence at protein level
26.5604	26.4308	26.4181	26.6469	26.8722	26.807	26.8551	27.1736	1:Experimental evidence at protein level
26.7012	26.827	26.8407	26.8083	26.9322	27.0059	26.8509	27.0485	1:Experimental evidence at protein level
27.6095	27.5211	27.4053	27.4122	27.1485	27.2145	27.2221	27.3105	1:Experimental evidence at protein level
28.5757	28.5926	28.4646	28.5367	28.7995	28.9004	28.8806	28.5716	1:Experimental evidence at protein level
26.3617	26.3145	26.2785	26.7958	24.5179	23.4765	23.7739	25.0147	1:Experimental evidence at protein level
26.53	26.7147	26.8586	26.8596	27.2516	27.2935	27.4408	27.5328	1:Experimental evidence at protein level
26.1401	25.9389	26.1332	26.1971	26.6319	26.2513	26.5274	27.1626	1:Experimental evidence at protein level
27.4032	27.3947	27.5395	27.3869	27.747	27.609	27.739	27.7449	1:Experimental evidence at protein level
26.9782	26.7893	26.933	26.9529	27.1131	27.0059	27.1174	27.3167	1:Experimental evidence at protein level
28.1733	28.2698	28.2656	28.2964	28.57	28.5145	28.5899	28.7101	1:Experimental evidence at protein level
28.4095	28.4944	28.8709	28.7415	28.2287	28.2173	28.382	28.1832	1:Experimental evidence at protein level
26.3127	26.3878	26.4521	26.3653	26.7647	26.9863	26.7758	26.8308	1:Experimental evidence at protein level
25.7612	25.219	24.9103	25.3185	26.2818	26.0773	25.8895	25.8999	1:Experimental evidence at protein level
30.0155	30.0983	30.2821	30.0742	29.7936	29.8606	29.7616	29.2447	1:Experimental evidence at protein level
26.4843	26.5273	26.588	26.5431	26.6855	26.6179	26.7624	26.9506	1:Experimental evidence at protein level
26.9949	26.7796	26.9419	26.556	26.4157	26.2551	26.1615	25.6489	1:Experimental evidence at protein level
26.127	26.4939	25.9408	26.1871	25.613	25.6638	25.9095	25.3016	1:Experimental evidence at protein level
26.6529	26.7693	26.9441	26.9975	27.2112	27.205	27.2362	27.0903	1:Experimental evidence at protein level
26.2331	26.1151	26.2757	26.4078	25.7836	25.6836	25.6802	26.1605	1:Experimental evidence at protein level
26.9758	26.9162	27.0801	27.2097	26.6273	26.9873	26.4668	26.7596	1:Experimental evidence at protein level
28.509	28.4357	28.4327	28.5598	28.0159	27.8041	27.9621	28.1789	1:Experimental evidence at protein level
27.2694	27.2749	27.398	27.2138	27.4532	27.6659	27.4508	27.4578	1:Experimental evidence at protein level
29.4326	29.336	29.6892	29.7568	29.1192	28.9434	29.1862	28.7761	1:Experimental evidence at protein level
24.5669	24.6804	24.76	24.8744	25.0773	25.0888	25.0422	25.1438	1:Experimental evidence at protein level
26.6896	26.6473	26.6234	26.8967	25.542	25.3373	25.0969	25.8826	1:Experimental evidence at protein level
29.1373	29.1002	29.3033	29.2366	29.0491	28.9608	28.9489	29.0348	1:Experimental evidence at protein level
27.5037	27.443	27.6865	27.6064	27.2667	27.3419	27.2439	27.4961	1:Experimental evidence at protein level
27.2304	27.1906	27.2883	27.1733	27.0024	27.0047	27.0306	26.6409	1:Experimental evidence at protein level
28.5635	28.5959	28.5127	28.5104	28.8485	28.7965	28.7985	28.5704	1:Experimental evidence at protein level
29.0822	29.203	29.2019	29.2166	29.4425	29.3904	29.4406	29.5311	1:Experimental evidence at protein level
26.0366	26.0884	26.3061	26.1527	24.5363	24.5296	25.2437	24.6738	1:Experimental evidence at protein level
28.5472	28.3389	28.5756	28.8011	27.7787	27.5151	27.6287	28.0307	1:Experimental evidence at protein level
27.6471	27.885	27.3104	27.582	26.7321	26.5967	26.5696	27.1593	1:Experimental evidence at protein level
25.4054	25.6038	25.8551	25.7602	24.9369	24.4555	24.9132	24.9826	1:Experimental evidence at protein level
28.5906	28.6149	28.5951	28.5701	28.9637	28.9387	28.9171	28.9317	1:Experimental evidence at protein level
26.4502	26.2538	26.52	26.5414	26.6266	26.6254	26.659	26.8462	1:Experimental evidence at protein level
25.2156	25.4483	25.1344	25.6571	25.9817	26.3086	26.1124	26.3351	1:Experimental evidence at protein level
25.7832	25.9766	25.0847	24.8803	27.0912	27.0574	26.8923	27.1106	1:Experimental evidence at protein level
27.5876	27.7411	27.4945	27.5276	27.9218	28.0656	27.8395	27.8243	1:Experimental evidence at protein level
27.4492	27.5207	27.256	27.3674	27.6841	27.6734	27.6224	28.0389	1:Experimental evidence at protein level
29.9128	29.4482	27.6045	27.3576	26.6653	27.3202	26.0578	26.2996	1:Experimental evidence at protein level
26.3993	26.3126	26.2494	26.7465	24.4769	24.8549	25.6811	24.7637	1:Experimental evidence at protein level
28.6971	28.6945	28.8658	28.9914	29.0986	29.1657	29.181	29.5088	1:Experimental evidence at protein level
24.5883	24.3668	24.5475	24.4374	25.5069	25.3967	25.4645	25.9365	1:Experimental evidence at protein level
28.7273	28.805	28.5809	28.4617	28.3135	28.3488	28.368	28.4737	1:Experimental evidence at protein level
27.3881	27.5263	27.4855	27.5311	26.9546	26.8669	27.1005	27.0173	1:Experimental evidence at protein level
27.2616	27.1118	26.8101	26.5293	28.2252	27.9768	27.4547	27.4959	1:Experimental evidence at protein level
27.9593	27.9748	28.049	27.6693	27.6741	27.7266	27.5227	27.2435	1:Experimental evidence at protein level
26.8257	26.8188	26.9159	26.7571	27.1907	27.2602	27.2145	26.8432	1:Experimental evidence at protein level
24.3486	23.9389	23.9457	24.5065	26.3159	26.0869	26.1716	26.9014	1:Experimental evidence at protein level
24.3459	24.4834	24.9487	24.2095	25.5795	25.5845	24.8656	25.5539	1:Experimental evidence at protein level
26.0314	26.1765	26.1975	26.2271	26.4185	26.2387	26.6356	26.8745	1:Experimental evidence at protein level
28.8349	28.8525	28.9277	28.9387	29.1605	29.1394	29.2686	28.9436	1:Experimental evidence at protein level
26.6491	26.5064	26.4901	26.8301	27.0901	27.2004	27.0581	27.2704	1:Experimental evidence at protein level
26.7165	26.7253	26.8866	26.6571	26.5479	26.6496	26.5746	26.4811	1:Experimental evidence at protein level
27.0947	27.2538	27.3796	27.3978	27.6271	28.1779	28.0254	28.2338	1:Experimental evidence at protein level
29.3677	28.9743	29.1309	29.1919	29.0487	28.6441	28.7537	28.7429	1:Experimental evidence at protein level
26.6047	26.7808	26.9491	26.7574	26.2979	26.5155	26.4884	25.9394	1:Experimental evidence at protein level
27.2803	27.2693	27.1274	27.3383	26.8136	26.8848	26.6865	27.1791	1:Experimental evidence at protein level
25.7272	26.0157	25.7615	26.0082	25.1183	25.0963	25.0215	25.4401	1:Experimental evidence at protein level
26.6338	26.735	26.6161	26.5313	27.2889	27.2823	27.395	27.4423	1:Experimental evidence at protein level
26.8705	26.7097	26.6272	26.6646	27.2241	27.3993	27.4652	27.6538	1:Experimental evidence at protein level
26.3985	26.3709	26.4979	26.5933	25.9809	25.8251	25.8391	26.4048	1:Experimental evidence at protein level
26.0003	25.4061	25.6337	24.6526	26.3468	26.3981	25.8514	26.6332	1:Experimental evidence at protein level
24.2193	23.4156	26.2866	26.3761	28.4317	28.0964	26.8095	27.5679	1:Experimental evidence at protein level
26.4349	26.4728	26.2264	26.3461	26.8424	26.6948	26.6656	27.3351	1:Experimental evidence at protein level
26.8742	26.9501	27.0873	27.0503	27.0825	27.119	27.222	27.1314	1:Experimental evidence at protein level
25.4214	25.4614	25.2666	25.4841	26.0048	25.8806	25.9217	26.2911	1:Experimental evidence at protein level
25.9689	26.0901	26.1668	26.5214	26.7114	26.7084	26.5479	27.2737	1:Experimental evidence at protein level
27.5138	27.7884	27.7488	27.7201	27.2284	27.2266	27.4783	27.2976	1:Experimental evidence at protein level
26.9041	26.7627	26.8162	27.0073	27.0768	27.1076	27.1187	27.1991	1:Experimental evidence at protein level

27.0156	24.5318	24.3983	24.3297	27.5991	27.1914	27.6091	28.4518	1:Experimental evidence at protein level
28.1339	28.133	28.2721	28.1894	28.5952	28.4955	28.3666	28.3555	1:Experimental evidence at protein level
26.0017	26.0175	26.1154	26.3605	26.4138	26.4061	26.3931	26.779	1:Experimental evidence at protein level
25.6069	25.2644	24.5896	24.5153	26.6416	26.7997	26.5386	26.3649	1:Experimental evidence at protein level
26.6585	26.5828	26.599	26.7931	27.1432	27.0311	27.046	27.4471	1:Experimental evidence at protein level
27.946	27.9625	28.0785	28.25	27.1268	27.2719	27.3076	27.7208	1:Experimental evidence at protein level
27.3196	27.3419	27.3144	27.5106	27.0537	27.2519	27.1451	27.0684	1:Experimental evidence at protein level
25.3463	25.5461	25.1966	25.5996	25.8487	25.6868	25.717	26.06	1:Experimental evidence at protein level
25.1216	25.4848	24.441	25.1846	25.549	25.9152	25.5494	25.8712	1:Experimental evidence at protein level
28.3093	28.1522	28.1944	28.0885	28.5016	28.5704	28.4753	28.4575	1:Experimental evidence at protein level
26.9455	27.0556	27.2794	27.1387	26.2906	25.9659	26.1499	26.9033	1:Experimental evidence at protein level
25.5742	25.7254	25.8311	25.6066	24.1833	23.7036	23.6099	25.2432	2:Experimental evidence at transcript lev
26.7035	26.5222	26.5602	26.7522	26.9292	26.9327	27.0957	27.2659	1:Experimental evidence at protein level
28.6589	28.6964	28.7754	28.6958	28.0118	27.8863	28.0124	27.9259	1:Experimental evidence at protein level
26.2063	26.0835	26.2851	26.2196	26.6401	26.4063	26.4002	26.8298	1:Experimental evidence at protein level
26.562	26.4825	26.1251	26.7082	27.0522	27.174	26.995	27.4691	1:Experimental evidence at protein level
28.0592	28.4616	28.0361	27.9937	28.5203	28.7206	28.7025	28.6258	1:Experimental evidence at protein level
25.7273	25.7319	25.7367	25.6058	25.0388	25.4698	25.1418	24.6464	1:Experimental evidence at protein level
34.0682	34.0308	34.0958	34.282	33.2234	33.1815	33.1912	33.0956	1:Experimental evidence at protein level
24.6745	25.0746	25.0727	25.8611	26.2662	25.8992	26.2571	25.7768	1:Experimental evidence at protein level
33.729	33.8905	34.1741	34.0271	32.401	32.5009	32.3976	32.1748	1:Experimental evidence at protein level
31.7316	31.7122	31.8999	31.9178	32.1399	31.9868	32.0659	32.0454	1:Experimental evidence at protein level
27.7129	27.1259	27.9449	28.4834	29.3628	29.2638	29.1165	29.173	1:Experimental evidence at protein level
30.1549	30.2329	29.9652	30.1431	29.7054	29.5893	29.8983	30.007	5:Protein uncertain
27.6491	27.536	27.6063	27.5682	28.1576	28.2435	28.2065	28.0992	1:Experimental evidence at protein level
28.092	28.2083	28.2677	28.3465	25.7158	25.5763	25.7852	25.6808	1:Experimental evidence at protein level
26.5051	26.4039	26.5411	26.5852	25.4662	24.8896	25.5248	25.3434	1:Experimental evidence at protein level
29.228	29.3335	29.407	29.2944	28.8045	28.7874	28.9028	28.907	1:Experimental evidence at protein level
25.5928	26.4037	26.5447	26.2747	25.4315	25.1964	25.5801	25.8651	1:Experimental evidence at protein level
33.5424	33.5928	33.7981	33.8207	32.4184	32.3599	32.5342	32.3325	1:Experimental evidence at protein level
29.6211	29.4764	29.2696	28.7707	30.8342	30.9071	30.8247	30.6414	1:Experimental evidence at protein level
35.1085	35.1752	35.3052	35.3064	34.4628	34.3716	34.4709	34.2045	1:Experimental evidence at protein level
25.8458	25.9015	25.5432	26.0915	25.5553	25.5792	25.4212	25.5051	1:Experimental evidence at protein level
31.963	32.0689	31.9878	32.2097	32.7587	32.6152	32.7431	32.4115	1:Experimental evidence at protein level
25.6217	26.0433	25.5902	26.1979	26.508	26.4367	26.6069	27.3133	1:Experimental evidence at protein level
29.6781	29.5919	29.7896	29.7907	29.4234	29.3722	29.3979	29.4341	1:Experimental evidence at protein level
28.264	28.1821	28.2515	28.3201	27.9617	27.7861	27.8622	28.1449	1:Experimental evidence at protein level
33.6773	33.3432	31.8467	31.7294	30.8798	31.2281	30.5846	30.7873	1:Experimental evidence at protein level
28.7818	28.7553	28.7466	29.0304	29.2738	29.296	29.2814	29.621	1:Experimental evidence at protein level
31.7215	31.6981	31.762	31.8587	32.0066	32.0569	32.0607	32.334	1:Experimental evidence at protein level
28.8906	28.9907	29.0651	29.1397	29.6113	29.744	29.6795	29.3695	1:Experimental evidence at protein level
27.9821	27.836	27.2661	27.2045	28.9919	29.6315	28.2976	28.0181	1:Experimental evidence at protein level
27.7411	27.7424	27.7773	27.983	26.9548	26.8884	27.0692	27.4047	1:Experimental evidence at protein level
31.0729	31.07	31.0317	31.0092	32.2139	32.2495	32.1569	31.2844	1:Experimental evidence at protein level
31.0331	30.8228	30.7045	30.7037	32.1822	32.1796	31.8692	31.0612	1:Experimental evidence at protein level
29.2763	29.1467	29.3375	29.3296	29.5702	29.4809	29.4169	29.5853	1:Experimental evidence at protein level
34.5297	34.579	34.6215	34.7506	34.1286	33.9838	33.9693	34.0277	1:Experimental evidence at protein level
28.2024	28.0641	28.1616	28.0519	27.5335	27.5242	27.439	27.7007	1:Experimental evidence at protein level
32.7648	32.8164	33.0372	33.1295	31.3643	31.3843	31.3931	31.5729	1:Experimental evidence at protein level
33.6353	33.5424	33.5054	33.5262	33.3295	33.1806	33.3998	32.9798	1:Experimental evidence at protein level
26.3833	26.5117	26.548	26.478	26.8591	26.9773	26.9437	26.8137	1:Experimental evidence at protein level
26.3299	26.0391	25.8769	26.0282	24.1602	25.6552	25.2122	25.2098	1:Experimental evidence at protein level
27.1953	27.4306	27.8026	27.2167	25.0691	25.9628	26.465	26.72	1:Experimental evidence at protein level
30.8729	30.8948	31.0788	30.9088	30.3384	30.2582	30.3312	29.882	1:Experimental evidence at protein level
28.7621	28.852	29.1616	28.7581	28.5872	28.6306	28.4144	28.6396	1:Experimental evidence at protein level
29.4755	29.0175	27.3395	27.0924	31.859	32.0062	31.8336	31.9113	1:Experimental evidence at protein level
27.7154	27.7769	28.0441	27.9305	27.3424	27.2217	27.3822	27.7628	1:Experimental evidence at protein level
29.9381	29.8769	29.8494	29.8436	30.0771	29.9856	29.9618	30.0918	1:Experimental evidence at protein level
29.4781	29.6412	29.8195	29.6696	28.9339	28.9336	29.1508	28.8794	1:Experimental evidence at protein level
26.6467	26.5076	26.5286	26.6887	27.0198	26.9413	26.861	27.276	1:Experimental evidence at protein level
26.0882	25.9647	24.0489	24.4893	28.547	28.5116	28.3887	28.1524	1:Experimental evidence at protein level
29.6701	29.6892	29.5172	29.1069	28.2534	28.4159	28.2692	28.1084	1:Experimental evidence at protein level
30.6995	30.7818	30.9842	30.8062	31.1841	31.0848	31.1499	30.9349	1:Experimental evidence at protein level
26.4905	26.7201	27.0099	26.7682	27.9589	27.8968	28.1177	28.2634	1:Experimental evidence at protein level
28.3684	28.3755	28.3746	28.2913	28.5418	28.5769	28.6329	28.6731	1:Experimental evidence at protein level
29.4459	29.3845	29.5027	29.5621	28.603	28.4378	28.5263	28.5833	1:Experimental evidence at protein level
26.6976	26.675	26.3705	26.5468	27.2221	26.7658	26.7931	27.1923	1:Experimental evidence at protein level
31.914	31.7198	31.4169	31.3983	32.321	32.5078	32.0918	31.7686	1:Experimental evidence at protein level
30.3938	30.3127	30.0445	29.7304	30.9294	30.9641	30.9169	31.176	1:Experimental evidence at protein level
30.2497	30.1212	30.0277	30.1333	31.1214	31.2192	30.7421	30.2429	1:Experimental evidence at protein level
28.0609	27.978	28.0857	27.8603	30.3085	30.2585	30.4201	30.5917	1:Experimental evidence at protein level
26.2399	26.2117	25.9982	26.1064	26.7126	26.7235	26.6398	26.8455	1:Experimental evidence at protein level
25.007	25.0171	24.8362	23.3247	25.8177	25.9756	26.3216	26.0095	1:Experimental evidence at protein level
29.8314	29.955	30.2265	30.3382	28.1306	27.7585	28.1506	28.7293	1:Experimental evidence at protein level
31.7608	31.8768	32.0274	31.8488	31.5898	31.4724	31.4973	31.2603	1:Experimental evidence at protein level
27.4183	27.6028	27.5851	27.6368	29.0557	29.0499	29.203	29.1548	1:Experimental evidence at protein level
24.6728	24.6672	23.6471	23.659	28.366	28.2846	28.2468	28.5457	1:Experimental evidence at protein level
27.3732	27.3526	27.3894	27.4786	27.5148	27.647	27.5351	27.6426	1:Experimental evidence at protein level
29.662	29.6582	29.5643	29.5404	29.7358	29.7117	29.7451	29.6871	1:Experimental evidence at protein level
26.0952	26.1177	26.3236	25.8154	26.8088	26.6203	26.2453	26.3957	1:Experimental evidence at protein level
23.7514	23.6012	23.8933	24.2198	25.3625	25.206	24.958	25.4274	1:Experimental evidence at protein level
24.5575	24.5513	24.4055	24.5213	25.6092	25.5765	25.4434	26.1879	1:Experimental evidence at protein level
27.9612	27.9293	27.644	27.422	27.3075	27.4815	26.7806	27.0142	1:Experimental evidence at protein level
32.1562	32.1695	32.2958	32.2345	29.9293	29.8116	30.0211	30.4229	1:Experimental evidence at protein level
31.1299	31.1052	31.3213	31.2193	30.9074	30.7461	30.9252	30.6793	1:Experimental evidence at protein level
25.7488	25.8319	26.0915	26.1354	26.3979	26.2941	26.292	26.2097	1:Experimental evidence at protein level
28.2046	28.1283	28.1803	28.2393	27.2951	27.1276	27.3424	27.7671	1:Experimental evidence at protein level
25.2288	25.4353	25.1494	25.4611	25.9746	26.278	25.9619	26.4712	1:Experimental evidence at protein level
27.2492	27.0788	26.9332	27.3193	28.2107	28.3398	27.9255	28.3447	1:Experimental evidence at protein level
31.7225	31.699	31.893	31.7941	31.2574	31.1854	31.2659	30.9837	1:Experimental evidence at protein level
27.9316	27.9539	27.9736	27.8703	27.5548	27.4368	27.5174	27.5087	1:Experimental evidence at protein level
30.2542	30.134	30.1028	30.3534	28.8214	28.6719	28.6811	29.2069	1:Experimental evidence at protein level
26.6607	26.922	27.0196	26.8449	25.7726	25.7904	24.7853	25.6453	1:Experimental evidence at protein level
27.5662	27.4657	27.6164	27.6566	27.8218	27.929	27.8661	27.8018	1:Experimental evidence at protein level
28.4422	28.4492	28.5272	28.4643	28.3017	28.328	28.3974	28.1162	1:Experimental evidence at protein level
29.027	29.0103	29.1594	29.1816	29.5832	29.4479	29.4372	29.4543	1:Experimental evidence at protein level
27.1247	27.193	27.2254	27.3151	27.4166	27.3633	27.4933	27.6323	1:Experimental evidence at protein level
29.9641	29.9872	29.9911	30.1359	30.3901	30.3087	30.381	30.5604	1:Experimental evidence at protein level
27.4318	27.5675	27.6617	27.5231	27.8719	27.8277	27.9316	27.8222	1:Experimental evidence at protein level
28.5233	28.4112	28.4932	28.5598	28.789	28.8505	28.7627	29.0859	1:Experimental evidence at protein level
29.275	29.29	29.4007	29.3282	29.5067	29.4809	29.5	29.5167	1:Experimental evidence at protein level
29.1115	29.1363	29.1725	29.0803	28.7246	28.8377	28.8625	28.7125	1:Experimental evidence at protein level
30.4701	30.5968	30.8429	30.7776	30.1796	30.1797	30.3859	30.4735	1:Experimental evidence at protein level
28.0196	28.0134	28.0829	27.9266	29.3054	29.23	29.1474	29.272	1:Experimental evidence at protein level
27.0597	27.07	27.1319	27.232	26.7744	26.4951	26.7486	27.1022	1:Experimental evidence at protein level
25.7556	26.0429	26.0802	25.9596	26.3483	26.6276	26.5212	26.2013	1:Experimental evidence at protein level
30.8276	30.7646	30.7971	30.7581	30.7597	30.67	30.6432	30.7225	1:Experimental evidence at protein level
29.7943	29.7712	29.8533	29.7396	29.6427	29.5319	29.5939	29.5457	1:Experimental evidence at protein level
26.5868	26.5038	25.814	25.8027	27.0329	26.6481	27.2885	26.8071	1:Experimental evidence at protein level
28.7453	28.8647	28.9382	28.8563	28.6463	28.3977	28.6329	28.5522	1:Experimental evidence at protein level
31.6663	31.6031	31.7267	31.7406	31.9391	31.8738	31.9385	31.9048	1:Experimental evidence at protein level
29.8273	29.9089	30.047	30.0444	29.2375	29.2389	29.3915	29.2838	1:Experimental evidence at protein level
28.7386	28.8326	28.9487	28.7935	26.6398	26.4885	26.6548	26.4362	1:Experimental evidence at protein level
29.5816	29.4699	29.5322	29.6845	30.0992	29.9519	29.8975	29.7845	1:Experimental evidence at protein level
26.8369	26.6356	26.2804	26.1917	25.7901	25.791	25.3591	26.0881	1:Experimental evidence at protein level
27.5115	27.3915	27.3672	27.4479	28.0359	27.8774	27.7669	28.0486	1:Experimental evidence at protein level
26.4542	26.2907	26.4557	26.6609	27.6616	27.5991	27.3617	27.8415	1:Experimental evidence at protein level
26.5518	26.3305	26.205	26.38	26.412	26.9639	27.1233	27.4873	1:Experimental evidence at protein level
26.3925	26.6376	26.2983	26.7815	27.103	27.1453	27.1602	27.5762	1:Experimental evidence at protein level
30.383	30.4315	30.633	30.6377	30.2212	30.0437	30.1982	30.1313	1:Experimental evidence at protein level
27.3166	27.0897	27.0817	26.8855	25.7319	25.5168	25.0153	25.9799	1:Experimental evidence at protein level
34.408	34.3497	34.3412	34.5906	34.2052	34.0417	34.058	34.2342	1:Experimental evidence at protein level
32.0462	32.0187	32.2286	32.2877	31.1247	31.0396	31.2066	31.1898	1:Experimental evidence at protein level
25.9529	25.9559	26.0926	25.9565	26.4131	26.6167	26.3774	26.272	1:Experimental evidence at protein level
29.1272	29.1102	29.1243	28.9596	28.6653	28.8476	28.5642	28.4821	1:Experimental evidence at protein level
24.1375	23.5194	24.3897	24.9925	25.1551	25.8281	24.925	26.0191	1:Experimental evidence at protein level
25.7938	26.0951	26.1621	25.8798	26.8766	27.0451	26.827	26.3521	1:Experimental evidence at protein level
26.3997	26.6841	26.6007	26.2434	24.2096	25.6753	24.774	25.3933	1:Experimental evidence at protein level
26.7738	26.7918	26.9921	26.8915	25.7191	26.4551	25.7967	25.6531	1:Experimental evidence at protein level

29.2784	29.3001	29.5576	29.5665	29.7873	29.7782	29.7394	29.9132	1:Experimental evidence at protein level
28.6546	28.7792	28.8491	28.8049	28.6282	28.2592	28.4428	28.5396	1:Experimental evidence at protein level
31.1474	31.082	30.9386	30.9648	31.3079	31.169	31.2579	31.4247	1:Experimental evidence at protein level
26.4993	26.6598	26.5601	26.7364	27.0323	27.2901	26.7804	27.3887	1:Experimental evidence at protein level
27.6468	27.5936	27.6999	27.5444	27.3272	26.9073	27.1799	27.0105	1:Experimental evidence at protein level
27.3351	26.8939	26.5519	26.5194	25.8159	25.643	25.7841	26.1848	1:Experimental evidence at protein level
26.701	26.8493	27.3283	27.5785	26.3017	26.7528	25.9856	26.4852	1:Experimental evidence at protein level
29.7558	29.7589	30.0835	30.0256	29.6519	29.6137	29.64	29.7483	1:Experimental evidence at protein level
29.3501	29.2493	29.2921	29.3428	29.0054	29.0218	29.037	29.0624	1:Experimental evidence at protein level
27.3568	27.3232	27.7477	27.6252	26.3013	26.139	26.2055	26.5192	1:Experimental evidence at protein level
26.5602	26.6513	26.7451	26.5446	26.8431	26.9284	26.8458	26.8102	1:Experimental evidence at protein level
28.5201	28.3699	28.3629	28.5713	28.2128	28.1867	28.1215	28.1966	1:Experimental evidence at protein level
26.688	26.8547	26.8	26.9023	25.782	25.1194	25.5019	26.2832	1:Experimental evidence at protein level
27.7832	27.692	27.7844	27.586	28.438	28.3909	28.2743	28.2718	1:Experimental evidence at protein level
25.2054	24.8744	24.6643	25.0513	25.9875	25.7682	25.9381	26.2786	1:Experimental evidence at protein level
28.3432	28.3005	28.392	28.4294	28.828	28.8277	28.7239	28.7655	1:Experimental evidence at protein level
27.9881	28.0965	28.1944	28.1743	27.6936	27.6666	27.362	27.5852	1:Experimental evidence at protein level
28.9399	29.0012	29.1959	29.4086	24.4173	24.2873	24.1055	23.6335	1:Experimental evidence at protein level
30.6505	30.3971	29.9378	29.9281	30.9695	31.1189	30.6862	30.5265	1:Experimental evidence at protein level
31.2228	31.0937	30.9108	30.9854	31.4655	31.521	31.3593	31.5443	1:Experimental evidence at protein level
28.8527	28.7912	28.9072	29.0051	29.4222	29.425	29.3773	28.8703	1:Experimental evidence at protein level
30.4932	30.3335	29.9574	30.123	30.5689	30.604	30.5582	30.5042	1:Experimental evidence at protein level
32.2983	32.4775	32.6339	32.4842	31.9122	32.0407	31.8644	31.801	1:Experimental evidence at protein level
25.0964	25.1344	25.1276	25.3316	24.7335	24.0331	25.0873	24.1493	1:Experimental evidence at protein level
26.3256	26.4309	26.3214	26.3605	26.6993	26.6475	26.7737	26.957	1:Experimental evidence at protein level
31.9488	31.8861	31.8084	31.9047	31.6271	31.6532	31.7549	31.3199	1:Experimental evidence at protein level
26.884	27.0513	27.4134	27.2579	27.9014	27.8008	27.8424	27.9294	1:Experimental evidence at protein level
28.4841	28.3808	28.2969	28.5437	28.7402	28.7742	28.6258	28.9447	1:Experimental evidence at protein level
26.1664	26.1642	25.6926	25.7592	28.2892	28.2162	28.3458	28.1568	1:Experimental evidence at protein level
29.5292	29.4959	29.5823	29.6374	30.0339	29.9338	29.8976	30.0519	1:Experimental evidence at protein level
25.0437	25.093	24.1991	25.0028	25.9348	26.0658	25.583	25.3414	1:Experimental evidence at protein level
27.004	27.0279	27.1759	27.0673	26.754	26.8506	26.8385	26.7113	1:Experimental evidence at protein level
29.5679	29.5813	29.8024	29.6846	29.4573	29.4271	29.2889	29.5696	1:Experimental evidence at protein level
28.407	28.3607	28.3906	28.4004	28.1725	28.1789	28.2527	28.3718	1:Experimental evidence at protein level
28.0599	28.0624	28.1482	28.0103	25.7378	26.133	25.9859	26.5741	1:Experimental evidence at protein level
27.7816	27.7173	27.7704	27.7092	27.9329	28.0822	27.8422	28.0135	1:Experimental evidence at protein level
29.4557	29.4685	29.5565	29.68	29.9625	29.8468	29.9106	30.0588	1:Experimental evidence at protein level
30.9154	30.7861	30.7002	30.7532	31.3969	31.423	31.286	30.8466	1:Experimental evidence at protein level
27.766	27.8436	27.9221	27.9275	27.401	27.5171	27.485	27.3795	1:Experimental evidence at protein level
28.4216	28.5099	28.5878	28.4997	28.1744	28.1123	28.1562	28.3207	1:Experimental evidence at protein level
25.2016	25.6434	25.6185	25.3938	23.2852	23.3422	23.6909	24.7262	1:Experimental evidence at protein level
26.1239	26.3075	25.8123	26.3195	26.8378	26.6755	26.8924	27.3944	1:Experimental evidence at protein level
27.9519	27.8658	27.982	27.9893	28.3264	28.2288	28.345	28.4381	1:Experimental evidence at protein level
26.1603	26.3252	26.1204	26.4098	25.1535	24.7714	24.9015	25.7448	1:Experimental evidence at protein level
31.3553	31.4623	31.5734	31.4141	31.0904	31.1993	31.1472	31.0839	1:Experimental evidence at protein level
30.2519	30.2337	30.4312	30.36	30.9394	30.8365	30.8372	31.0331	1:Experimental evidence at protein level
29.5456	29.6344	29.7988	29.6683	29.9383	29.9206	29.8996	29.7036	1:Experimental evidence at protein level
29.4792	29.6329	29.6472	29.4968	30.0276	30.0029	30.1151	29.7155	1:Experimental evidence at protein level
28.7424	28.7243	28.6008	28.8615	28.2543	28.4702	28.412	28.6583	1:Experimental evidence at protein level
28.553	28.4981	28.6846	28.4607	28.4695	28.35	28.3857	28.415	1:Experimental evidence at protein level
24.7634	24.2413	24.921	26.5713	27.1419	27.131	27.2705	26.9325	1:Experimental evidence at protein level
29.9822	29.9528	29.7799	29.8324	30.1114	30.0941	30.06	29.9875	1:Experimental evidence at protein level
26.8654	27.1294	27.1879	27.0627	27.4716	27.5038	27.4753	27.4045	1:Experimental evidence at protein level
29.8458	29.9268	30.0236	29.8678	29.625	29.6959	29.593	29.694	1:Experimental evidence at protein level
29.4185	29.6436	29.837	29.6561	29.2301	29.2941	29.4393	29.4259	1:Experimental evidence at protein level
27.3207	27.3268	27.3238	27.3639	27.7318	27.7744	27.7909	27.9855	1:Experimental evidence at protein level
31.4514	31.3588	30.9963	31.2232	31.8144	31.8661	31.6471	31.8452	1:Experimental evidence at protein level
30.12	30.4472	30.741	29.8918	30.8551	30.9019	31.2672	30.8441	1:Experimental evidence at protein level
29.7362	29.849	29.7704	29.8138	30.0868	29.963	30.1163	29.9293	1:Experimental evidence at protein level
28.3274	28.1454	27.698	27.7603	28.5884	28.7162	28.5658	28.7029	1:Experimental evidence at protein level
26.7507	27.0529	27.1711	27.059	26.4102	26.548	26.4928	26.4681	1:Experimental evidence at protein level
31.1636	31.0367	30.7006	30.6445	31.3129	31.3405	31.2927	31.6344	1:Experimental evidence at protein level
28.5261	28.6567	28.7212	28.6527	28.3243	28.3474	28.3219	28.0762	1:Experimental evidence at protein level
28.2184	28.2733	28.2801	28.2406	28.6397	28.6231	28.6154	28.4757	1:Experimental evidence at protein level
28.817	28.9145	28.8892	28.8507	29.081	29.12	29.1701	29.2944	1:Experimental evidence at protein level
30.8117	30.8327	30.8664	30.8327	30.5312	30.3279	30.5202	30.3778	1:Experimental evidence at protein level
29.2301	29.2654	29.5557	29.3042	29.0431	29.0013	29.1095	28.6099	1:Experimental evidence at protein level
27.1387	27.1533	27.1144	27.1193	27.3696	27.4531	27.3387	27.6247	1:Experimental evidence at protein level
27.0865	27.2377	27.2235	27.0605	25.1858	24.5636	24.6763	25.6709	1:Experimental evidence at protein level
25.6728	25.5851	25.6479	24.8564	24.3873	24.3971	23.8879	24.1542	1:Experimental evidence at protein level
24.0314	24.5418	24.4097	24.5079	25.2485	24.5747	25.4546	25.5671	1:Experimental evidence at protein level
27.6961	27.743	27.7465	27.9049	27.2021	27.1407	27.0763	27.6271	1:Experimental evidence at protein level
27.8109	27.9084	28.1449	27.6464	26.3759	26.4899	26.3962	26.4401	1:Experimental evidence at protein level
30.9908	30.9533	31.0764	31.0524	31.2174	31.1188	31.2684	31.164	1:Experimental evidence at protein level
31.0254	30.9744	31.1465	30.9969	30.7842	30.6898	30.7969	30.5438	1:Experimental evidence at protein level
31.983	32.0138	32.1663	32.2112	31.4353	31.1788	31.4037	31.5806	1:Experimental evidence at protein level
27.0809	27.1832	27.1682	27.3824	27.5187	27.4078	27.5026	28.0018	1:Experimental evidence at protein level
27.4228	27.5311	27.5147	27.4956	26.8296	26.7551	26.8444	26.5503	1:Experimental evidence at protein level
27.9594	27.9651	27.9416	28.1484	28.4419	28.2902	28.2496	28.5622	1:Experimental evidence at protein level
30.6403	30.6725	30.7991	30.7731	29.4714	29.4428	29.3556	29.3361	1:Experimental evidence at protein level
26.6399	26.6911	27.0398	26.8273	27.2735	27.3251	27.4405	27.4867	1:Experimental evidence at protein level
26.5113	26.3827	26.4493	26.8166	26.789	26.8559	26.7749	27.1845	1:Experimental evidence at protein level
29.4728	29.5933	29.7764	29.6429	28.9537	28.9027	28.9755	29.0192	1:Experimental evidence at protein level
31.9648	31.9062	32.0518	32.1581	31.7844	31.7029	31.657	31.7741	1:Experimental evidence at protein level
25.7205	25.5287	25.5061	25.1775	24.6352	24.8725	24.9531	24.7368	1:Experimental evidence at protein level
31.4953	31.4883	31.7739	31.7591	30.7283	30.4523	30.8445	30.572	1:Experimental evidence at protein level
27.9326	28.0971	27.9157	28.1731	28.3598	28.4754	28.2563	28.668	1:Experimental evidence at protein level
25.8471	25.8366	25.8806	26.1854	26.4999	26.4127	26.567	26.9761	1:Experimental evidence at protein level
26.6627	26.8252	26.7373	26.9281	27.2101	27.3553	27.0725	27.9248	1:Experimental evidence at protein level
29.9289	29.6508	29.4633	29.5609	30.021	29.9447	29.8513	30.1676	1:Experimental evidence at protein level
28.9368	28.9381	29.1697	29.2801	29.5514	29.4885	29.5474	29.578	1:Experimental evidence at protein level
29.825	29.7801	29.6259	29.644	29.8701	29.8934	29.833	29.9301	1:Experimental evidence at protein level
27.9039	28.1154	27.8881	28.0602	28.0543	28.2287	28.2826	28.4053	1:Experimental evidence at protein level
29.5229	29.5287	29.6146	29.5768	30.0023	30.0263	29.9696	29.8161	1:Experimental evidence at protein level
28.3855	28.336	28.4321	28.4262	28.6565	28.6928	28.6151	28.5205	1:Experimental evidence at protein level
28.94	28.9713	29.2389	29.084	28.5637	28.5814	28.531	28.2989	1:Experimental evidence at protein level
28.2544	28.3685	28.5821	28.4337	27.9877	27.7711	27.9943	28.0569	1:Experimental evidence at protein level
28.4227	28.4285	28.424	28.6442	24.4229	24.9832	24.0998	24.7763	1:Experimental evidence at protein level
26.9493	26.8665	27.0291	27.1238	27.9268	28.094	28.0681	28.2524	1:Experimental evidence at protein level
27.7015	27.6023	27.7991	27.7683	27.5173	27.5551	27.4887	27.6499	1:Experimental evidence at protein level
31.285	31.2713	31.4891	31.2574	30.7564	30.6872	30.5455	30.2522	1:Experimental evidence at protein level
27.0081	27.2647	27.7579	27.4547	24.8241	24.1151	26.1951	24.7623	1:Experimental evidence at protein level
27.064	27.1761	27.273	27.1404	27.512	27.5572	27.5643	27.2173	1:Experimental evidence at protein level
26.0916	25.8778	26.0951	25.6703	26.362	26.5713	26.0676	26.5116	1:Experimental evidence at protein level
25.743	26.0332	25.963	25.7043	24.9063	25.3845	25.306	25.6838	1:Experimental evidence at protein level
31.1885	31.1111	30.7707	30.8478	31.4062	31.3708	31.3109	31.5412	1:Experimental evidence at protein level
28.8228	28.8851	29.0504	28.8982	28.5071	28.6189	28.4793	28.0806	1:Experimental evidence at protein level
26.5463	26.552	26.5742	26.3757	25.9442	26.1024	25.8238	25.8828	1:Experimental evidence at protein level
29.0186	29.0963	29.0857	29.0506	28.8997	28.9466	28.9971	28.7427	1:Experimental evidence at protein level
26.7571	27.0722	27.4369	27.2667	26.2118	26.205	26.2083	26.0524	1:Experimental evidence at protein level
31.2708	31.1895	30.8662	31.0304	31.4818	31.4783	31.4752	31.6064	1:Experimental evidence at protein level
26.1461	26.1386	26.1227	26.2138	26.7129	26.7827	26.9833	26.6248	1:Experimental evidence at protein level
29.9286	29.8259	29.5568	29.7158	30.1767	30.0279	29.9868	30.2706	1:Experimental evidence at protein level
29.222	29.2531	29.3009	29.302	29.4532	29.4946	29.4264	29.5725	1:Experimental evidence at protein level
27.514	27.4026	27.0617	27.2549	27.8652	27.9143	27.9179	27.9554	1:Experimental evidence at protein level
26.4326	26.2539	26.445	26.5201	23.727	25.3454	23.7515	25.0799	1:Experimental evidence at protein level
28.2962	28.4087	28.5327	28.4228	29.0608	29.0065	29.0853	28.8421	1:Experimental evidence at protein level
29.2116	29.1712	29.2699	29.2345	29.3315	29.3677	29.3139	29.3652	1:Experimental evidence at protein level
26.1598	26.2391	26.0218	26.1824	26.8258	26.7637	26.995	27.2126	1:Experimental evidence at protein level
27.2434	27.3634	27.4975	27.6625	27.8414	27.7222	27.6723	27.6882	1:Experimental evidence at protein level
29.5082	29.6101	29.6731	29.4998	29.3793	29.2951	29.4287	29.1418	1:Experimental evidence at protein level
27.0328	26.8989	27.1174	26.9811	27.565	27.5403	27.4685	27.5671	1:Experimental evidence at protein level
27.008	27.3462	27.292	27.1947	26.2913	26.6669	26.5736	26.5075	1:Experimental evidence at protein level
25.5005	25.5111	25.5447	25.5449	25.705	25.5874	25.7198	25.9785	1:Experimental evidence at protein level
31.498	31.4804	31.1169	31.2417	31.7381	31.8703	31.8301	31.7804	1:Experimental evidence at protein level
30.6009	30.3279	29.9907	30.1025	30.8663	30.8021	30.7339	30.9755	1:Experimental evidence at protein level
27.8261	27.9771	28.1375	27.971	28.6198	28.3069	28.5886	28.9087	1:Experimental evidence at protein level
25.8329	25.6664	25.9908	25.8749	26.2426	26.0106	26.0585	26.2359	1:Experimental evidence at protein level
25.9718	26.0165	26.0559	26.372	26.8712	26.3768	26.7955	27.0977	1:Experimental evidence at protein level

30.2861	30.285	30.3383	30.2824	30.1019	30.0488	30.1453	30.1933	1:Experimental evidence at protein level
30.2261	30.1939	30.2948	30.2391	29.2188	29.1468	29.1899	29.1119	1:Experimental evidence at protein level
28.3657	28.5251	28.5685	28.5595	28.3655	28.3003	28.3791	28.4084	1:Experimental evidence at protein level
27.0284	26.9504	26.9655	26.992	26.7501	26.6776	26.6734	26.9321	1:Experimental evidence at protein level
27.5489	27.6092	27.6537	27.6523	27.8318	27.8872	27.8022	27.8315	1:Experimental evidence at protein level
27.548	27.5139	27.5908	27.5428	27.2699	27.2789	27.2136	27.1965	1:Experimental evidence at protein level
25.1079	25.0217	24.8688	25.1134	24.2868	24.5957	24.791	24.3909	1:Experimental evidence at protein level
31.2049	31.0506	30.5336	30.8181	31.6641	31.6427	31.4646	31.7314	1:Experimental evidence at protein level
31.0945	31.0415	30.8358	30.809	31.2686	31.2992	31.1346	31.3039	1:Experimental evidence at protein level
30.5619	30.4789	30.0794	30.2741	30.8383	30.7771	30.7088	31.0341	1:Experimental evidence at protein level
31.2687	31.0724	30.6251	30.8311	31.5385	31.6543	31.4158	31.676	1:Experimental evidence at protein level
31.3191	31.2341	31.0194	31.0794	31.4042	31.3406	31.3286	31.3446	1:Experimental evidence at protein level
30.1093	30.0039	29.7677	29.9585	30.2584	30.2456	30.2394	30.3117	1:Experimental evidence at protein level
31.0804	31.0521	30.7568	30.7201	31.1076	31.2687	31.1637	31.2485	1:Experimental evidence at protein level
26.6914	26.4412	26.3139	26.2418	27.1759	27.3844	27.1083	27.4039	1:Experimental evidence at protein level
25.842	25.9626	26.0025	23.801	27.0545	26.9322	26.9339	27.3536	1:Experimental evidence at protein level
26.4409	26.5612	26.6342	26.0991	25.6721	25.8195	25.6914	24.2915	1:Experimental evidence at protein level
28.4351	28.3079	28.1389	28.3188	28.7629	28.6591	28.6601	29.0923	1:Experimental evidence at protein level
28.0948	28.1371	28.0883	28.2089	28.2817	28.2093	28.2339	28.1943	1:Experimental evidence at protein level
26.1573	26.3192	26.2896	26.3397	26.7264	26.6476	26.5882	27.009	1:Experimental evidence at protein level
25.6618	25.7253	26.0301	25.7066	26.3012	26.2177	26.0477	26.4133	1:Experimental evidence at protein level
26.2031	26.1849	26.0511	26.431	26.5016	26.4962	26.3614	26.7935	1:Experimental evidence at protein level
26.9773	26.9573	26.8993	27.0084	27.2411	27.1855	27.1356	27.149	1:Experimental evidence at protein level
28.0073	27.8589	27.8572	28.0644	28.4316	28.6681	28.6312	28.5444	1:Experimental evidence at protein level
27.5026	27.6064	27.5309	27.6231	27.7741	27.8871	27.7122	28.0877	1:Experimental evidence at protein level
26.701	26.8381	26.8543	26.6753	26.6074	26.5652	26.4796	26.4466	1:Experimental evidence at protein level
26.4524	26.5661	26.5503	26.4332	26.8719	26.8962	26.9624	26.9894	1:Experimental evidence at protein level
25.6778	25.916	25.4772	25.9021	25.9325	26.2426	26.2346	26.4699	1:Experimental evidence at protein level
31.2657	31.1482	30.7274	31.0579	31.399	31.3979	31.4975	31.6769	1:Experimental evidence at protein level
28.4612	28.4364	28.681	28.7008	27.7873	27.8023	27.8613	28.0002	1:Experimental evidence at protein level
29.8481	29.7732	29.8906	29.948	30.2126	30.1333	30.173	30.2869	1:Experimental evidence at protein level
26.2214	26.4648	26.5842	26.5233	26.0561	25.8877	26.0526	26.3255	1:Experimental evidence at protein level
30.3601	30.2786	30.3486	30.391	30.6285	30.584	30.5402	30.4077	1:Experimental evidence at protein level
26.3104	26.4172	26.0727	26.1082	27.0087	26.9725	26.9707	26.9212	1:Experimental evidence at protein level
29.3074	29.4103	29.5297	29.5361	29.6588	29.6508	29.7372	29.7179	1:Experimental evidence at protein level
29.2147	29.1707	29.1783	29.2394	29.013	28.953	29.0418	28.7677	1:Experimental evidence at protein level
29.1534	29.352	29.5075	29.2582	28.9198	29.0984	28.9838	28.6608	1:Experimental evidence at protein level
28.9968	29.0323	29.2493	29.1191	29.8415	29.7442	29.8961	29.4673	1:Experimental evidence at protein level
27.9463	27.9365	28.0173	28.0733	28.5497	28.5292	28.5505	28.8835	1:Experimental evidence at protein level
26.1716	26.1168	26.0489	26.0764	26.5699	26.6047	26.1418	26.5407	1:Experimental evidence at protein level
28.1313	28.1518	28.2789	28.2958	28.6436	28.6338	28.5911	28.7225	1:Experimental evidence at protein level
29.1457	29.1332	29.2583	29.2376	29.9542	29.6422	29.7482	29.6426	1:Experimental evidence at protein level
26.7288	26.6425	26.8381	27.0397	27.3797	27.0098	27.1979	27.4177	1:Experimental evidence at protein level
26.5335	26.5976	26.6764	26.7213	26.8318	26.785	26.904	27.2507	1:Experimental evidence at protein level
27.8602	28.0246	27.8998	28.0718	28.1997	28.1009	28.1752	28.4123	1:Experimental evidence at protein level
29.3314	29.4392	29.4823	29.5308	29.1352	29.0587	29.1765	29.2554	1:Experimental evidence at protein level
27.5678	27.6786	27.6272	27.6173	28.1903	28.096	28.1213	28.4082	1:Experimental evidence at protein level
29.3616	29.4284	29.5551	29.3855	29.9225	29.7838	29.7304	29.993	1:Experimental evidence at protein level
26.7261	26.6946	26.6272	26.9003	27.0167	26.9727	27.1702	27.5238	1:Experimental evidence at protein level
30.8846	30.6944	30.6359	30.7061	31.0877	30.9326	30.8841	31.3144	1:Experimental evidence at protein level
23.832	24.0999	24.009	24.2509	27.3885	27.2335	27.0997	27.4077	1:Experimental evidence at protein level
25.2817	25.3234	25.5714	25.2714	25.0676	25.0607	25.0368	24.931	1:Experimental evidence at protein level
28.1542	28.2387	28.1362	28.4147	28.5267	28.3858	28.6004	28.4673	1:Experimental evidence at protein level
26.6468	26.7372	26.6979	26.7647	26.8259	27.1038	26.9659	27.2878	1:Experimental evidence at protein level
29.4472	29.6364	29.7141	29.4323	29.113	29.1801	29.1701	28.8364	1:Experimental evidence at protein level
28.5209	28.4872	28.5705	28.4768	28.3446	28.4123	28.3152	28.0922	1:Experimental evidence at protein level
27.308	27.2699	27.267	27.1286	27.4294	27.5095	27.4147	27.3421	1:Experimental evidence at protein level
30.0086	30.1336	30.1977	30.0827	29.9707	29.7863	29.8839	29.561	1:Experimental evidence at protein level
27.2371	27.244	27.3092	27.4176	28.2448	28.2593	28.3081	28.4908	1:Experimental evidence at protein level
26.9222	27.0067	26.9379	27.0906	27.3408	27.3928	27.346	27.4906	1:Experimental evidence at protein level
26.784	26.7806	26.794	26.5617	26.328	26.1625	26.4675	26.2494	1:Experimental evidence at protein level
25.2846	25.4701	25.767	25.4639	25.8293	26.002	25.9067	25.7934	1:Experimental evidence at protein level
26.4629	26.2944	26.18	25.8316	25.7933	24.1011	24.196	24.9873	1:Experimental evidence at protein level
26.8975	27.0373	27.139	27.0824	27.254	27.359	27.2946	27.346	1:Experimental evidence at protein level
28.6372	28.6739	29.0352	28.8978	28.5843	28.4568	28.6199	28.4087	1:Experimental evidence at protein level
28.7935	28.7691	28.61	28.6773	26.2061	26.436	26.1988	27.157	1:Experimental evidence at protein level
28.9951	29.0163	29.1383	29.2004	29.3073	29.2722	29.2838	29.4675	1:Experimental evidence at protein level
28.604	28.8201	29.1219	28.6259	28.3953	28.3737	28.3658	28.1477	1:Experimental evidence at protein level
26.7743	26.9247	26.9188	27.0134	27.1145	27.0302	27.2683	27.558	1:Experimental evidence at protein level
27.9702	27.9658	28.0817	28.1188	27.9457	27.8868	27.9034	27.8209	1:Experimental evidence at protein level
29.865	29.9288	29.8331	29.9637	30.3539	30.2614	30.0669	30.0804	1:Experimental evidence at protein level
29.3475	29.3017	29.4463	29.3496	30.1144	30.0927	30.135	29.7332	1:Experimental evidence at protein level
27.6289	27.5931	27.5726	27.5799	28.39	28.2639	28.3115	28.1128	1:Experimental evidence at protein level
29.9909	30.085	30.3662	30.2337	29.9264	29.9651	29.9223	29.9291	1:Experimental evidence at protein level
28.3781	28.3558	28.1388	28.1881	28.016	28.0158	28.0113	28.1456	1:Experimental evidence at protein level
25.917	26.2222	26.5567	25.9356	26.6363	26.6976	26.479	26.6327	1:Experimental evidence at protein level
25.1215	25.224	25.2058	25.0469	24.8156	23.8486	24.5684	23.7436	1:Experimental evidence at protein level
28.5018	28.4811	28.9511	28.8135	28.1797	28.0134	28.3973	28.4748	1:Experimental evidence at protein level
29.2007	29.0891	29.1058	29.187	29.4468	29.4007	29.3782	29.507	1:Experimental evidence at protein level
26.7809	26.9564	26.8766	26.9054	27.3765	27.5837	27.5348	27.5214	1:Experimental evidence at protein level
27.2982	27.3432	27.4088	27.0895	27.5062	27.608	27.4501	27.5175	1:Experimental evidence at protein level
27.2425	27.1915	26.9866	27.0078	28.3203	28.3547	28.2522	28.3902	1:Experimental evidence at protein level
27.4293	27.3893	26.4495	27.2645	27.8134	27.7614	27.6227	28.1022	1:Experimental evidence at protein level
30.9602	31.9409	31.9309	31.8883	30.2987	30.2527	30.6121	30.1451	1:Experimental evidence at protein level
28.4719	28.4375	28.3946	28.2244	28.5814	28.7176	28.5814	28.5167	1:Experimental evidence at protein level
31.9452	31.9517	32.0792	32.1088	32.3596	32.3529	32.3466	32.3042	1:Experimental evidence at protein level
31.6542	31.6618	31.418	31.4652	31.7649	31.865	31.7801	31.8005	1:Experimental evidence at protein level
29.1764	29.1993	29.3785	29.1797	29.002	28.9352	29.0385	28.9976	1:Experimental evidence at protein level
29.7834	29.728	29.6723	29.8429	30.1463	30.1353	30.1686	29.8857	1:Experimental evidence at protein level
27.9986	28.256	28.3918	28.275	28.624	28.5702	28.8272	28.7706	1:Experimental evidence at protein level
27.8969	27.9812	27.5685	27.9866	27.2635	27.4952	27.2749	27.5043	1:Experimental evidence at protein level
29.0763	29.0515	28.986	29.2637	29.3771	29.4874	29.2371	29.3813	1:Experimental evidence at protein level
31.2872	31.2179	31.1061	31.0458	31.3667	31.3757	31.2586	31.418	1:Experimental evidence at protein level
31.0346	31.0189	30.697	30.8209	31.3421	31.4062	31.3755	31.5994	1:Experimental evidence at protein level
31.2566	31.1689	30.8145	30.8869	31.5979	31.5692	31.5435	31.6816	1:Experimental evidence at protein level
31.0154	31.0788	30.7865	30.8166	31.3036	31.3751	31.3245	31.5142	1:Experimental evidence at protein level
29.4373	29.2684	28.3945	28.9643	29.7798	29.9144	29.7001	30.1586	1:Experimental evidence at protein level
31.3997	31.3733	31.3881	31.1695	31.1524	31.1367	31.1161	30.8045	1:Experimental evidence at protein level
32.4349	32.574	32.7368	32.7924	32.2762	32.2065	32.3592	32.2468	1:Experimental evidence at protein level
26.5097	26.0234	26.1302	26.6199	23.9873	23.7331	26.1664	24.8186	1:Experimental evidence at protein level
30.5061	30.4452	30.2869	30.3474	30.5316	30.5178	30.5756	30.5486	1:Experimental evidence at protein level
24.2187	24.2588	24.8083	24.1529	25.8356	25.9478	26.013	26.5047	1:Experimental evidence at protein level
31.1595	31.0653	30.9141	31.0942	31.2481	31.1583	31.2418	31.4584	1:Experimental evidence at protein level
30.2505	30.2881	29.8801	30.045	30.4796	30.4265	30.4786	30.7226	1:Experimental evidence at protein level
31.1012	31.0062	30.836	30.6582	31.1449	31.1286	31.1859	31.2354	1:Experimental evidence at protein level
31.1497	31.0752	30.3705	30.3153	31.2639	31.623	31.265	31.4788	1:Experimental evidence at protein level
30.8408	30.783	30.5598	30.5407	30.9519	30.98	30.9632	31.065	1:Experimental evidence at protein level
27.3386	27.7213	27.6069	27.5024	26.9783	27.0383	27.0384	26.4584	1:Experimental evidence at protein level
27.0326	27.2904	27.811	27.4904	26.5901	26.9733	26.9638	26.8789	1:Experimental evidence at protein level
28.1407	28.1097	28.3444	28.2507	27.8826	27.8338	28.0051	28.1454	1:Experimental evidence at protein level
31.3444	31.2136	30.8984	31.0486	31.57	31.5722	31.4744	31.7247	1:Experimental evidence at protein level
29.4241	29.6328	29.7269	29.6513	29.8916	29.8451	29.9379	30.0752	1:Experimental evidence at protein level
27.5719	27.5123	27.6663	27.8512	26.3683	26.2079	26.3272	26.6826	1:Experimental evidence at protein level
30.1812	30.0143	29.6973	29.8791	30.411	30.4298	30.2589	30.2746	1:Experimental evidence at protein level
29.5076	29.4095	29.2011	29.2255	29.5429	29.6389	29.5235	29.9439	1:Experimental evidence at protein level
31.7191	31.7168	31.2686	31.0691	31.9483	32.0218	31.9095	32.0445	1:Experimental evidence at protein level
29.6302	29.6298	29.184	29.3448	30.1217	30.1508	30.052	30.2137	1:Experimental evidence at protein level
30.4846	30.5041	30.5158	30.3503	30.2198	30.255	30.3912	30.2536	1:Experimental evidence at protein level
28.8517	28.5572	28.5985	28.5875	28.9254	29.4601	28.9774	28.8615	1:Experimental evidence at protein level
30.5614	30.2112	29.7339	30.2651	30.6781	30.8104	30.5614	30.8457	1:Experimental evidence at protein level
31.4607	31.2006	30.796	30.983	31.6075	31.5917	31.5568	31.7382	1:Experimental evidence at protein level
30.6159	30.5761	30.1195	30.3232	30.9088	31.0697	30.8454	30.966	1:Experimental evidence at protein level
34.0981	34.3868	34.4763	34.3654	34.0456	33.8793	34.0969	34.0233	1:Experimental evidence at protein level
26.6639	26.9451	26.9979	26.9537	26.2773	26.2038	26.3348	26.4386	1:Experimental evidence at protein level
34.0528	34.0636	34.0706	34.1644	34.5251	34.4189	34.5229	34.172	1:Experimental evidence at protein level
32.3995	32.3154	32.5913	32.5332	32.7973	32.6206	32.6285	32.6559	1:Experimental evidence at protein level
27.7031	27.7702	27.8065	27.678	27.5922	27.5745	27.5257	27.4419	1:Experimental evidence at protein level
26.9288	27.2936	27.3381	27.3357	28.2794	28.3325	28.3142	28.3164	1:Experimental evidence at protein level
27.5144	27.448	27.5228	27.5955	27.6565	27.6875	27.644	27.7609	1:Experimental evidence at protein level

26.9237	27.0776	27.4928	27.081	27.6904	27.7041	27.3785	27.8509	1:Experimental evidence at protein level
25.2429	25.4354	24.9217	25.0667	24.325	24.3881	24.3294	25.1014	1:Experimental evidence at protein level
26.8571	26.974	27.1204	26.8313	26.4119	26.3696	26.3896	26.4052	1:Experimental evidence at protein level
26.4491	26.4525	26.4029	26.4934	26.7571	26.7182	26.7118	27.3009	1:Experimental evidence at protein level
26.7062	26.5871	26.6649	26.7047	27.0952	27.0284	27.0977	27.4862	1:Experimental evidence at protein level
25.928	25.9171	25.8249	25.6251	26.4655	26.379	26.2512	26.1593	1:Experimental evidence at protein level
30.2189	29.9538	29.7098	29.8404	30.4472	30.4667	30.2059	30.4607	1:Experimental evidence at protein level
30.7705	30.6195	30.2381	30.4301	31.0115	31.0939	30.8807	31.0577	1:Experimental evidence at protein level
27.4487	27.3948	27.2023	27.6669	27.8682	27.7026	27.8344	28.2696	1:Experimental evidence at protein level
30.2136	30.1758	29.8455	29.9462	30.4908	30.585	30.5193	30.9416	1:Experimental evidence at protein level
27.0628	26.9969	26.7399	26.9151	26.2797	26.3215	26.1834	26.6542	1:Experimental evidence at protein level
27.7123	27.7216	27.7031	27.7486	27.8964	27.8979	27.8332	28.0052	1:Experimental evidence at protein level
26.5012	26.8252	26.6483	26.6423	27.1558	27.2528	27.1446	27.8733	1:Experimental evidence at protein level
27.0666	26.9292	27.1085	27.1255	27.2033	27.3847	27.31	27.624	1:Experimental evidence at protein level
26.1084	26.0984	26.2326	26.1057	26.6501	26.6581	26.6175	26.4873	1:Experimental evidence at protein level
31.6825	31.677	31.72	31.6996	31.5766	31.4997	31.6039	31.5205	1:Experimental evidence at protein level
29.6639	29.7221	29.8577	29.7173	29.414	29.5373	29.6246	29.2562	1:Experimental evidence at protein level
27.4223	27.3437	27.2445	27.6116	26.6512	26.7333	26.6971	27.3031	1:Experimental evidence at protein level
29.4492	29.5953	29.7861	29.605	29.3779	29.2965	29.4251	29.2564	1:Experimental evidence at protein level
28.8781	28.948	28.9947	28.9316	29.2801	29.2429	29.1615	28.9367	1:Experimental evidence at protein level
30.7179	30.6954	30.618	30.8016	31.3078	31.2909	31.1905	31.0424	1:Experimental evidence at protein level
29.7029	29.6996	29.785	29.8214	29.2912	29.2681	29.2347	29.3968	1:Experimental evidence at protein level
25.6978	25.7074	26.2072	26.2828	27.5722	27.3966	27.5147	27.6108	1:Experimental evidence at protein level
26.1497	26.2276	26.4009	26.5026	25.8684	25.5963	25.9093	26.2388	1:Experimental evidence at protein level
29.7113	29.6376	29.2847	29.6122	29.9627	30.0158	29.8953	30.2092	1:Experimental evidence at protein level
28.1926	28.2684	28.4052	28.3536	27.9415	27.9868	27.9627	27.9067	1:Experimental evidence at protein level
29.1245	29.1255	29.1991	29.1452	27.4721	27.5929	27.5272	27.4967	1:Experimental evidence at protein level
26.7558	26.9771	27.0916	26.9726	27.2759	27.0439	27.2492	27.3892	1:Experimental evidence at protein level
32.1333	32.0536	31.6959	31.8669	32.366	32.3784	32.2715	32.313	1:Experimental evidence at protein level
27.7431	27.9061	28.0656	28.074	27.1565	26.6383	26.9726	27.4026	1:Experimental evidence at protein level
28.5243	28.4241	27.9929	28.3926	27.4172	27.5079	27.2174	27.7671	1:Experimental evidence at protein level
26.9857	27.0126	27.0172	27.3258	27.4127	27.5552	27.3996	27.7347	1:Experimental evidence at protein level
27.7245	27.7974	28.0274	28.0804	27.2233	27.0538	27.316	27.1933	1:Experimental evidence at protein level
27.9175	28.1671	28.1706	28.3286	28.4987	28.3278	28.7312	28.7886	1:Experimental evidence at protein level
28.9187	29.0004	29.112	29.0663	27.6254	27.6033	27.6929	27.7532	1:Experimental evidence at protein level
29.726	29.702	29.7594	29.8651	29.9438	29.9333	29.9209	30.0681	1:Experimental evidence at protein level
25.5868	25.5791	25.5769	25.6246	26.3194	26.1829	26.6086	27.0243	1:Experimental evidence at protein level
26.1846	26.1745	26.3815	25.6069	25.4662	25.1389	25.5706	25.2267	1:Experimental evidence at protein level
29.945	29.8554	30.0061	30.2358	30.3059	30.4176	30.3926	30.3646	1:Experimental evidence at protein level
31.4912	31.4321	31.3573	31.6298	32.0163	31.8615	31.891	31.9777	1:Experimental evidence at protein level
27.9248	27.9557	28.0078	28.159	28.8337	28.7865	28.7775	28.715	1:Experimental evidence at protein level
25.7082	26.1553	26.0589	26.4424	26.6589	26.482	26.9164	26.9302	1:Experimental evidence at protein level
27.1413	27.0065	27.1013	27.179	27.5316	27.397	27.385	27.5458	1:Experimental evidence at protein level
30.817	30.7721	30.3529	30.4606	31.0798	31.0086	31.0211	31.2494	1:Experimental evidence at protein level
29.7199	29.6834	29.7723	29.7729	29.1919	29.028	29.1326	28.8505	1:Experimental evidence at protein level
26.947	26.7634	26.2826	26.9477	27.4276	27.2966	27.5781	28.0264	1:Experimental evidence at protein level
28.4941	28.5067	28.5343	28.549	27.2938	27.3446	27.2434	27.6431	1:Experimental evidence at protein level
27.7771	27.9662	27.8369	27.6891	27.1484	27.1621	27.2824	27.5246	1:Experimental evidence at protein level
24.0864	23.7011	24.6324	25.1555	25.0096	25.2035	25.4157	25.7268	1:Experimental evidence at protein level
29.0875	29.1745	29.1349	28.9775	29.4351	29.3072	29.2813	29.5658	1:Experimental evidence at protein level
29.2695	28.9305	27.9218	27.9914	30.0411	30.1053	29.8272	29.4289	1:Experimental evidence at protein level
29.4199	29.4004	29.2908	29.6066	29.1166	28.9791	29.0877	29.3625	1:Experimental evidence at protein level
27.1559	27.1657	27.2075	27.0249	27.2474	27.455	27.3434	27.2272	1:Experimental evidence at protein level
26.687	26.7648	26.5371	26.213	24.4459	25.3925	25.5005	24.9126	1:Experimental evidence at protein level
27.1585	27.1413	27.4179	27.3621	27.4634	27.4625	27.4561	27.5333	1:Experimental evidence at protein level
28.5271	28.5095	28.4525	28.6123	27.9716	28.0058	28.0314	28.3886	1:Experimental evidence at protein level
26.5314	26.5993	26.544	26.7042	27.1139	26.9197	27.0576	27.2859	1:Experimental evidence at protein level
26.0757	26.5425	26.1381	26.3577	24.9754	24.2791	25.3663	24.8841	1:Experimental evidence at protein level
24.222	24.2942	24.8252	25.5234	26.3177	25.974	26.1721	27.4198	1:Experimental evidence at protein level
29.7034	29.6801	29.8201	29.6185	29.245	29.2119	29.0946	28.7214	1:Experimental evidence at protein level
25.7924	25.7291	25.8438	25.7817	26.5203	26.5578	26.3223	25.7469	1:Experimental evidence at protein level
29.3012	29.3654	29.5455	29.5263	29.2602	29.2155	29.2523	29.2541	1:Experimental evidence at protein level
25.7352	26.0584	26.1064	26.3016	23.844	23.7346	23.765	24.7218	1:Experimental evidence at protein level
26.5599	26.6494	26.6327	26.7068	27.135	27.1164	27.0861	27.5543	1:Experimental evidence at protein level
28.3186	28.4748	28.3751	28.573	28.1665	28.2438	28.2151	28.3099	1:Experimental evidence at protein level
28.4683	28.361	28.3924	28.5457	28.607	28.6221	28.5587	28.614	1:Experimental evidence at protein level
29.3192	29.4054	29.5395	29.55	29.0143	29.0527	29.0328	28.9275	1:Experimental evidence at protein level
29.6845	29.5819	29.4621	29.3997	29.2277	29.2201	29.3156	29.2301	1:Experimental evidence at protein level
27.9667	28.0806	28.2874	28.1227	27.6697	27.7945	27.6677	27.5807	1:Experimental evidence at protein level
27.641	27.721	27.7104	27.1257	27.0238	27.2012	27.2107	26.6743	1:Experimental evidence at protein level
30.2942	30.3243	30.3373	30.3614	30.3828	30.4181	30.4102	30.5523	1:Experimental evidence at protein level
29.0188	29.1095	29.2446	29.0357	29.4594	29.3247	29.3628	29.356	1:Experimental evidence at protein level
29.0418	29.0891	29.0301	29.0658	29.3069	29.1207	29.275	29.4909	1:Experimental evidence at protein level
26.7964	26.8607	26.8846	26.8528	27.4206	27.3215	27.3995	27.3397	1:Experimental evidence at protein level
29.1254	29.0557	28.9371	28.8973	28.8258	28.8099	28.8004	28.6717	1:Experimental evidence at protein level
25.7822	25.8089	25.83	25.9358	26.4146	26.16	26.4731	26.7641	1:Experimental evidence at protein level
26.4004	26.3796	26.2885	26.3533	26.247	25.9571	26.0036	26.1378	1:Experimental evidence at protein level
26.7714	26.8883	26.9604	26.9037	28.2748	28.1073	28.26	28.3477	1:Experimental evidence at protein level
27.0477	26.8531	26.5717	26.6404	27.5332	27.7559	27.2753	27.0717	1:Experimental evidence at protein level
25.0978	25.0226	25.0321	23.9582	25.5224	25.7632	25.7846	26.482	1:Experimental evidence at protein level
24.3677	24.5948	24.9191	24.382	24.2693	23.7661	24.1399	23.9783	1:Experimental evidence at protein level
25.1362	25.1659	24.621	25.4599	25.7926	25.531	25.7574	26.2815	1:Experimental evidence at protein level
27.6985	27.7495	27.6244	27.6399	27.4851	27.6133	27.5326	27.5161	1:Experimental evidence at protein level
26.0846	26.3856	26.3023	26.0446	27.0719	27.1469	27.159	26.5451	1:Experimental evidence at protein level
26.1655	26.4901	26.2065	26.5486	26.8345	26.6268	27.0736	26.7153	1:Experimental evidence at protein level
23.8711	24.5513	24.7522	23.8657	25.1878	24.9276	24.9778	25.374	1:Experimental evidence at protein level
25.0684	25.3687	25.0452	24.9769	25.4616	25.4491	25.3272	25.9345	1:Experimental evidence at protein level
27.7018	28.0718	27.799	28.0084	27.3453	27.2384	27.6187	27.782	1:Experimental evidence at protein level
26.2496	26.2515	25.7872	26.0557	26.5577	26.4058	26.623	26.88	1:Experimental evidence at protein level
30.4823	30.3528	30.4556	30.3365	30.7752	30.7143	30.6831	30.4689	1:Experimental evidence at protein level
26.3158	25.9407	26.1126	26.3822	27.1351	27.107	27.2926	27.8391	1:Experimental evidence at protein level
26.3776	26.0431	25.5974	26.0916	26.5244	26.5003	26.4336	27.0092	1:Experimental evidence at protein level
31.2812	31.1483	30.6244	30.7865	29.9312	30.3462	30.0133	29.8485	1:Experimental evidence at protein level
28.108	28.0935	28.1797	28.2334	28.0042	27.9107	27.9353	27.7559	1:Experimental evidence at protein level
27.0582	26.9853	27.2235	26.9905	26.6158	26.7505	26.2491	26.4322	1:Experimental evidence at protein level
27.96	27.9295	27.9892	28.0481	27.7767	27.6961	27.768	27.7905	1:Experimental evidence at protein level
27.4291	27.5111	27.6252	27.4515	28.3749	28.3188	28.4198	28.7222	1:Experimental evidence at protein level
26.3034	26.2829	26.3113	26.5094	26.488	26.566	26.6715	26.8103	1:Experimental evidence at protein level
29.2591	29.222	29.2383	29.1759	29.3644	29.3452	29.2489	29.3915	1:Experimental evidence at protein level
26.9691	27.2131	27.0623	27.3546	27.9345	28.0818	28.053	28.2473	1:Experimental evidence at protein level
27.3952	27.463	27.4633	27.429	27.6509	27.6059	27.543	27.8584	1:Experimental evidence at protein level
26.3525	26.4075	26.5437	26.4747	26.674	26.8567	26.587	26.6119	1:Experimental evidence at protein level
27.4184	27.6722	27.8862	27.6798	28.0393	27.7519	28.1034	28.2594	1:Experimental evidence at protein level
27.437	27.4406	27.5878	27.6063	26.9163	26.8456	26.7003	26.9486	1:Experimental evidence at protein level
26.149	26.1536	26.371	26.2791	25.5527	25.3246	25.5397	25.9207	1:Experimental evidence at protein level
25.182	24.9289	24.5746	24.7016	25.3995	25.7115	25.475	25.9623	1:Experimental evidence at protein level
26.347	26.1908	26.0481	26.4281	25.5574	25.5794	25.3871	26.2168	1:Experimental evidence at protein level
25.8561	25.8664	25.8785	25.9452	26.3461	26.4311	26.363	26.3725	1:Experimental evidence at protein level
28.0426	27.9663	27.9289	27.8463	27.646	27.765	27.6494	27.7307	1:Experimental evidence at protein level
27.3112	27.3786	27.6057	27.328	27.1516	26.9727	27.0939	26.9	1:Experimental evidence at protein level
30.5906	30.5705	30.7767	30.7236	30.368	30.262	30.3075	30.1373	1:Experimental evidence at protein level
27.198	27.1388	26.9913	27.0957	25.774	25.986	25.9099	26.2436	1:Experimental evidence at protein level
31.0086	31.0368	31.1557	31.1124	31.2556	31.19	31.1958	31.2014	1:Experimental evidence at protein level
29.2352	29.2608	29.2519	29.1406	28.8573	28.9205	28.9036	28.7564	1:Experimental evidence at protein level
25.5938	25.0962	25.2287	25.5825	25.6946	25.8878	25.7374	26.0339	1:Experimental evidence at protein level
27.0966	26.9788	27.0223	27.2055	27.3906	27.4694	27.4278	27.8981	1:Experimental evidence at protein level
26.5743	26.5709	26.5023	26.5428	26.1342	26.0032	26.1116	26.4051	1:Experimental evidence at protein level
26.0513	26.6468	26.4254	26.6359	25.7768	25.5995	26.0564	26.1087	1:Experimental evidence at protein level
28.5134	28.4906	28.4685	28.4969	28.3826	28.3331	28.3233	28.2901	1:Experimental evidence at protein level
26.299	26.4019	26.328	26.4045	26.9905	26.7749	26.7824	27.0468	1:Experimental evidence at protein level
26.2906	26.4468	26.41	26.1108	26.9177	26.6994	26.9386	27.2567	1:Experimental evidence at protein level
26.7396	26.6426	26.9606	27.0656	27.3952	27.27	27.1243	27.8463	1:Experimental evidence at protein level
26.1519	26.0759	26.1891	26.2588	26.4856	26.5328	26.4232	26.5552	1:Experimental evidence at protein level
24.5622	24.6425	24.5649	24.5979	25.3942	25.3456	25.4987	25.405	1:Experimental evidence at protein level
25.6407	25.6361	25.7905	25.701	25.3981	25.6288	25.3091	25.0797	1:Experimental evidence at protein level
26.0665	25.5288	25.6217	25.8274	25.1091	25.2914	25.167	24.8482	1:Experimental evidence at protein level
29.9817	30.1001	30.2646	30.3082	29.4745	29.2721	29.6314	29.6736	1:Experimental evidence at protein level
25.52	25.3914	25.5727	25.3748	24.9397	25.1148	25.0514	25.229	1:Experimental evidence at protein level
27.241	27.1075	27.2761	27.1956	23.3693	24.9368	24.7772	24.5816	1:Experimental evidence at protein level

28.7824	28.6395	28.3997	28.4841	29.4662	29.5525	29.472	29.4459	1:Experimental evidence at protein level
26.2139	26.3391	26.4451	26.1565	25.8313	26.1482	25.6799	25.8884	1:Experimental evidence at protein level
29.7432	29.8079	29.9052	29.8492	29.6805	29.7243	29.7387	29.7174	1:Experimental evidence at protein level
24.7927	24.3706	24.3678	24.3966	25.2207	25.3192	25.4386	25.5868	1:Experimental evidence at protein level
31.1488	31.1354	31.1917	31.457	30.9109	30.7948	30.9613	31.1513	1:Experimental evidence at protein level
26.3005	26.1729	26.2753	26.2596	26.7701	26.688	26.8922	26.4605	1:Experimental evidence at protein level
26.8239	26.5946	26.8844	26.9495	27.4696	27.5161	27.3872	27.1763	1:Experimental evidence at protein level
26.0335	25.9254	25.5898	25.4932	26.3885	26.673	26.2749	26.8713	1:Experimental evidence at protein level
27.7757	27.9166	28.0473	27.9189	28.2633	28.1771	28.3288	28.7127	1:Experimental evidence at protein level
28.2769	28.2472	28.3022	28.7027	29.4237	29.53	29.4658	29.6037	1:Experimental evidence at protein level
26.5168	25.6935	26.6392	26.5764	25.5545	24.4142	24.7637	24.8303	1:Experimental evidence at protein level
29.5087	29.4974	29.6166	29.6784	29.7188	29.6459	29.7432	29.7878	1:Experimental evidence at protein level
28.3565	28.5084	28.6422	28.6424	29.113	29.0042	29.1903	29.0833	1:Experimental evidence at protein level
25.2815	25.2796	25.5615	25.4392	26.4849	26.5405	26.5736	26.7949	1:Experimental evidence at protein level
24.8032	24.4572	24.4374	24.774	25.2714	25.1044	25.0835	25.5473	1:Experimental evidence at protein level
27.5337	27.5576	27.8219	27.8383	26.9604	26.8472	27.0672	27.1986	1:Experimental evidence at protein level
28.5245	28.4792	28.5456	28.3705	28.2693	28.3679	28.1874	28.1234	1:Experimental evidence at protein level
27.7041	27.8222	27.9032	27.867	28.0562	28.1551	28.12	28.0943	1:Experimental evidence at protein level
30.0321	30.1082	30.2537	30.2516	30.4082	30.3486	30.3681	30.3554	1:Experimental evidence at protein level
27.8078	27.6943	27.9005	28.0001	27.6071	27.6645	27.524	27.7137	1:Experimental evidence at protein level
25.3821	25.3329	25.3903	25.1225	25.9177	25.7565	25.7816	26.298	1:Experimental evidence at protein level
29.7355	29.7547	30.1134	29.9052	29.3578	29.1137	29.2181	29.4611	1:Experimental evidence at protein level
27.3314	27.4252	27.2641	27.5124	27.5683	27.5535	27.7678	27.9788	1:Experimental evidence at protein level
26.4005	26.5406	26.7196	26.5746	25.8485	25.9599	26.0596	25.8887	1:Experimental evidence at protein level
30.1943	30.0847	29.9798	30.1884	26.3292	28.3643	27.9579	27.7634	1:Experimental evidence at protein level
29.1187	29.0359	29.0884	29.3478	30.4284	30.2941	30.3714	30.6679	1:Experimental evidence at protein level
28.3441	28.3923	28.6585	28.6044	28.19	28.1346	28.2708	28.2938	1:Experimental evidence at protein level
29.6087	29.4609	29.4506	29.4959	29.1333	29.0363	29.0455	29.3958	1:Experimental evidence at protein level
26.3542	26.4781	26.5908	26.5784	26.925	26.7989	26.85	26.8561	1:Experimental evidence at protein level
26.1687	26.26	26.1855	25.7991	25.5306	25.5142	25.7321	25.3014	1:Experimental evidence at protein level
25.7596	25.811	25.6337	25.7378	26.7496	26.7111	26.7608	27.1806	1:Experimental evidence at protein level
27.4522	27.5152	27.476	27.5375	27.0727	27.0651	27.1074	27.0564	1:Experimental evidence at protein level
24.9967	24.8096	24.5233	24.6086	25.416	25.4486	25.3914	25.7483	1:Experimental evidence at protein level
26.4043	26.3613	26.7805	26.8242	27.1865	27.1769	26.8852	27.5571	1:Experimental evidence at protein level
27.774	27.82	27.8195	27.9792	27.4483	27.4564	27.4616	27.4352	1:Experimental evidence at protein level
26.9241	27.1261	27.081	27.0384	27.418	27.4771	27.5609	27.4309	1:Experimental evidence at protein level
25.2708	25.2082	25.3141	25.2964	25.4626	25.7105	25.532	25.8116	1:Experimental evidence at protein level
26.7245	26.6391	26.7397	26.8241	26.9564	27.0232	26.8762	27.1629	1:Experimental evidence at protein level
25.454	25.3434	25.4733	25.5744	25.6684	25.7912	25.6661	25.723	1:Experimental evidence at protein level
27.2661	27.3276	27.246	27.4817	27.6011	27.7166	27.7313	28.052	1:Experimental evidence at protein level
24.1069	24.7257	24.7302	24.7583	25.8505	25.9895	25.9825	25.8952	1:Experimental evidence at protein level
25.564	25.4634	25.7506	25.7031	25.9458	25.8318	26	26.0479	1:Experimental evidence at protein level
25.6055	25.7236	25.6997	25.8327	25.177	25.3102	25.4277	25.5965	1:Experimental evidence at protein level
28.5288	28.5391	28.6317	28.717	28.3614	28.4775	28.2365	28.0921	1:Experimental evidence at protein level
25.45	25.6113	26.0369	26.2524	24.4657	24.0967	24.4506	25.2665	1:Experimental evidence at protein level
26.3325	26.5256	26.4751	26.6031	25.5143	25.4035	25.6165	26.2406	1:Experimental evidence at protein level
26.3065	26.0487	26.0118	26.35	26.6163	26.767	26.5599	26.8949	1:Experimental evidence at protein level
26.4232	26.3763	26.3287	26.5748	26.8021	26.6793	26.5653	27.0195	1:Experimental evidence at protein level
24.7999	24.7962	24.8244	24.494	25.3455	24.8797	25.0176	25.0624	1:Experimental evidence at protein level
26.6363	26.7614	26.7206	26.5258	26.9207	27.0212	27.2822	27.4817	1:Experimental evidence at protein level
24.8444	25.0003	25.1628	24.9785	24.4929	24.8355	24.7566	24.1962	1:Experimental evidence at protein level
24.8593	24.9462	24.8542	24.7671	25.5902	25.5921	25.7	25.7779	1:Experimental evidence at protein level
25.2147	24.9224	25.085	25.4317	25.7322	25.5793	25.6687	25.6795	1:Experimental evidence at protein level
26.6855	26.7373	24.7664	26.9805	27.8712	27.7946	28.1449	28.2251	1:Experimental evidence at protein level
27.3805	27.3146	27.3483	27.3878	27.5472	27.5704	27.4139	27.6684	1:Experimental evidence at protein level
25.5711	25.5979	25.5765	25.5801	25.2593	25.3709	25.5646	25.1695	1:Experimental evidence at protein level
25.269	25.542	25.7945	25.7208	25.8429	25.8529	25.9684	26.111	1:Experimental evidence at protein level
25.8593	25.9607	25.7073	26.0028	26.3806	26.2593	26.2292	26.9657	1:Experimental evidence at protein level
26.8906	27.1431	27.2451	27.1133	27.3631	27.2433	27.4912	27.48	1:Experimental evidence at protein level
26.9081	26.9206	26.8124	26.9839	26.7061	26.8182	26.8507	26.7154	1:Experimental evidence at protein level
26.782	26.6295	26.8434	26.8881	27.47	27.2924	27.2138	27.8751	1:Experimental evidence at protein level
24.3036	24.4308	24.4153	25.0443	25.4531	26.0804	25.7538	26.2815	1:Experimental evidence at protein level
24.9686	25.1813	24.8807	25.1606	25.4746	25.9067	25.732	25.9946	1:Experimental evidence at protein level
26.6608	26.662	26.6609	26.7422	26.8363	26.8392	26.8751	27.113	1:Experimental evidence at protein level
25.7217	25.3253	25.3079	25.5483	25.5979	25.9403	25.8809	26.265	1:Experimental evidence at protein level
26.21	25.1086	24.2762	26.3256	26.9304	27.5896	27.0679	27.6482	1:Experimental evidence at protein level
24.6911	25.1675	24.8413	25.0787	24.5278	24.2536	24.4155	24.8337	1:Experimental evidence at protein level
24.9113	24.7093	24.5704	24.7007	26.0088	25.9863	25.8456	26.7515	1:Experimental evidence at protein level
23.6467	23.7199	23.563	23.8953	25.3539	25.5188	24.422	23.9771	1:Experimental evidence at protein level
25.705	25.6153	25.4032	25.7492	26.1635	25.7811	26.1239	26.5719	1:Experimental evidence at protein level
26.8084	27.0283	26.9457	27.1245	26.3389	26.5223	26.4701	26.8879	1:Experimental evidence at protein level
29.62	29.5205	29.4572	29.512	29.6601	29.6008	29.619	29.7753	1:Experimental evidence at protein level
29.9856	30.1461	30.3729	30.1782	29.0411	29.1803	29.0785	28.8752	1:Experimental evidence at protein level
26.8709	26.5702	26.5382	26.7586	27.3038	27.288	27.2006	27.4644	1:Experimental evidence at protein level
26.5398	26.4359	27.1142	26.8848	23.8014	23.7587	25.9885	25.1681	1:Experimental evidence at protein level
27.4662	27.5101	27.5334	27.5892	27.6168	27.6598	27.7285	27.8593	1:Experimental evidence at protein level
23.9405	24.9412	23.9838	24.5238	25.1448	24.9517	24.8019	25.4941	1:Experimental evidence at protein level
26.2467	26.2632	26.5422	26.4897	25.8909	25.8282	25.935	26.3428	1:Experimental evidence at protein level
27.8905	27.9411	27.9636	28.0658	27.555	27.5872	27.6271	27.8776	1:Experimental evidence at protein level
27.3488	27.4448	27.4229	27.5286	27.6311	27.5797	27.6001	27.9178	1:Experimental evidence at protein level
25.7071	25.9247	25.8734	25.9351	25.1117	25.1274	25.0307	25.7994	1:Experimental evidence at protein level
26.2517	26.205	26.3553	26.3261	26.4468	26.6303	26.6588	26.4704	1:Experimental evidence at protein level
25.6282	25.6924	25.4814	25.5872	25.8577	26.0947	25.8377	25.9716	1:Experimental evidence at protein level
25.8536	25.9597	25.9343	25.9734	25.5868	25.7615	25.615	25.5074	1:Experimental evidence at protein level
30.3383	30.2891	30.2883	29.6767	28.9761	29.0233	28.7685	27.1791	2:Experimental evidence at transcript lev
26.975	27.0483	27.294	27.2937	26.6462	26.2384	26.4361	26.8599	1:Experimental evidence at protein level
28.1836	28.3008	28.3689	28.2971	27.6265	27.39	27.8051	27.7632	1:Experimental evidence at protein level
27.9186	27.8137	27.6568	27.7522	27.4731	27.3577	27.3701	27.5496	1:Experimental evidence at protein level
25.4547	25.8508	25.6861	26.0638	26.2062	26.3513	26.1015	26.7262	1:Experimental evidence at protein level
28.3005	28.0745	28.4846	28.2217	28.1137	27.9877	28.0254	27.8532	1:Experimental evidence at protein level
28.1058	28.0794	28.0849	28.0854	27.6778	27.6785	27.5976	27.7738	1:Experimental evidence at protein level
25.981	25.6546	25.7279	25.5959	26.2414	26.4844	26.1717	26.3691	1:Experimental evidence at protein level
26.6261	26.6453	26.6493	26.74	26.8871	26.889	27.0376	27.1546	1:Experimental evidence at protein level
25.5078	25.3851	25.4656	25.3892	25.8289	25.7115	26.0931	26.3753	1:Experimental evidence at protein level
26.0594	26.1868	26.4044	26.054	25.5941	25.931	25.8427	25.3443	1:Experimental evidence at protein level
24.447	25.3851	25.4225	25.2873	26.0581	25.9321	25.8176	26.1083	1:Experimental evidence at protein level
25.4308	25.3809	25.4473	25.4342	25.8581	25.991	26.1427	26.6234	1:Experimental evidence at protein level
26.0355	25.7914	26.2652	26.0828	24.7336	24.1105	25.0628	24.1367	1:Experimental evidence at protein level
24.554	24.3106	23.9628	24.4265	24.9803	24.9982	25.1681	24.9228	1:Experimental evidence at protein level
26.5107	26.4176	26.2497	26.3721	27.4396	27.3245	27.3841	27.6644	1:Experimental evidence at protein level
23.7156	23.5014	23.6787	23.9728	25.1272	24.8288	24.7528	25.9309	1:Experimental evidence at protein level
27.514	27.5796	27.6313	27.485	27.373	27.3088	27.2386	27.3701	1:Experimental evidence at protein level
26.7972	26.8142	26.7678	26.9169	24.991	25.0539	24.6949	25.7351	1:Experimental evidence at protein level
23.9295	23.9606	24.2279	24.0783	24.7264	24.5757	24.7762	24.8915	1:Experimental evidence at protein level
25.8817	25.8977	25.9149	26.0241	25.3536	24.8156	25.3355	25.2891	1:Experimental evidence at protein level
26.4355	26.3889	26.3088	26.2918	26.0749	26.3499	26.0838	26.1132	1:Experimental evidence at protein level
26.3838	26.5368	26.42	26.5659	26.7853	27.3688	26.9617	27.8236	1:Experimental evidence at protein level
26.4952	26.5345	26.7554	26.574	27.057	26.8632	26.9808	27.137	1:Experimental evidence at protein level
24.5977	24.4172	24.3597	23.6732	26.5792	26.6696	26.6179	26.8082	1:Experimental evidence at protein level
26.8807	26.853	26.9542	26.928	27.2541	27.1554	27.3856	27.1662	1:Experimental evidence at protein level
25.71	25.2359	25.397	25.4493	24.3198	24.4263	23.6722	25.2254	1:Experimental evidence at protein level
25.6779	25.9514	25.5652	25.9617	26.3412	26.2036	26.1457	26.6063	1:Experimental evidence at protein level
28.4613	28.4431	28.4094	28.3767	28.0838	27.9972	27.8491	28.185	1:Experimental evidence at protein level
25.7452	25.9238	25.9318	25.8769	26.2518	26.0427	26.3566	26.23	1:Experimental evidence at protein level
27.8996	28.0728	28.1225	28.2264	27.6103	27.4583	27.4421	27.8412	1:Experimental evidence at protein level
24.5455	24.5431	24.1924	24.5818	25.6325	25.9598	25.4778	24.745	1:Experimental evidence at protein level
25.4019	25.4317	25.36	25.3325	26.0251	26.3386	25.9841	26.4218	1:Experimental evidence at protein level
26.4909	23.8649	26.1276	25.1968	27.2833	27.3198	27.2838	26.9684	1:Experimental evidence at protein level
26.3655	26.3	25.9663	26.1165	26.7672	26.8062	27.0405	26.9376	1:Experimental evidence at protein level
24.8347	24.7087	24.3743	24.9623	25.1462	25.097	25.0733	25.3117	1:Experimental evidence at protein level
26.3446	26.4613	26.5443	26.4984	27.0396	26.6295	26.8993	26.9632	1:Experimental evidence at protein level
26.4319	26.5339	26.2266	26.3039	26.7541	26.7753	26.6212	26.6546	1:Experimental evidence at protein level
27.1513	27.2037	27.0139	27.3277	27.7258	27.6865	27.7913	28.1192	1:Experimental evidence at protein level
26.3907	26.5071	26.9739	26.9049	28.4821	28.5475	28.5561	28.3631	1:Experimental evidence at protein level
26.3002	26.4501	26.5127	26.1173	26.1325	24.1807	24.836	24.7309	1:Experimental evidence at protein level
26.8541	26.9457	26.9253	27.0736	26.162	26.4192	26.103	26.7286	1:Experimental evidence at protein level
27.626	27.7346	27.6219	27.6274	27.845	27.9444	27.8641	28.0803	1:Experimental evidence at protein level
26.7939	26.7943	26.624	26.6848	26.2991	26.4987	26.3498	26.1627	1:Experimental evidence at protein level
27.3348	26.9362	27.0393	27.0445	26.6455	26.541	26.4697	26.3361	1:Experimental evidence at protein level

25.3157	25.3279	25.6551	25.2857	26.0183	25.9368	26.0751	26.2226	1:Experimental evidence at protein level
25.5653	25.4893	25.5614	25.8231	26.138	26.0501	26.2453	26.5792	1:Experimental evidence at protein level
24.9433	25.114	25.12	25.3497	24.5853	24.6539	24.6204	24.9873	1:Experimental evidence at protein level
32.2751	32.3476	32.4772	32.5717	26.9714	26.8871	26.9349	27.3459	1:Experimental evidence at protein level
27.3335	27.4362	27.5422	27.6671	27.8965	27.7256	27.787	28.0604	1:Experimental evidence at protein level
26.5898	26.498	26.5895	26.7383	25.7487	26.0496	26.0021	26.1568	1:Experimental evidence at protein level
25.2036	25.36	24.9702	25.3604	24.2494	23.8428	24.7361	24.7315	1:Experimental evidence at protein level
25.1579	25.1751	24.9907	25.1033	25.9052	25.957	26.066	26.3162	1:Experimental evidence at protein level
27.9014	27.8718	27.9153	27.9741	27.6095	27.879	27.6473	27.6673	1:Experimental evidence at protein level
27.2367	27.2683	27.9766	27.7585	26.5758	26.7345	26.7372	26.9113	1:Experimental evidence at protein level
30.2779	30.2652	30.3615	30.2929	30.1899	30.1234	30.1464	30.1431	1:Experimental evidence at protein level
26.7316	26.7356	26.7484	26.8779	26.3179	26.2525	26.4233	26.6683	1:Experimental evidence at protein level
27.8316	27.8025	27.9781	27.9893	27.5045	27.6826	27.6649	27.3776	1:Experimental evidence at protein level
27.1662	27.4134	27.2556	27.3317	27.4918	27.4764	27.5737	27.644	1:Experimental evidence at protein level
27.5314	27.5634	27.5987	27.5671	26.834	26.77	26.9115	26.9618	1:Experimental evidence at protein level
28.3486	28.3419	28.3236	28.3091	28.4291	28.445	28.5393	28.6577	1:Experimental evidence at protein level
26.2901	26.2502	26.5088	26.5387	25.7831	25.8436	25.8633	25.8659	1:Experimental evidence at protein level
29.982	30.089	30.2298	30.176	29.834	29.8199	29.9105	29.7106	1:Experimental evidence at protein level
28.8973	28.99	29.0936	29.0531	29.2869	29.1661	29.2402	29.1829	1:Experimental evidence at protein level
27.3155	27.1025	26.9596	27.3558	28.1437	28.1975	27.9781	28.574	1:Experimental evidence at protein level
27.0185	27.0345	27.0938	27.0735	26.8711	26.9557	26.8225	26.8076	1:Experimental evidence at protein level
23.1021	23.5538	23.0428	23.1449	23.9432	23.7333	25.0594	24.4005	1:Experimental evidence at protein level
28.4651	28.6934	29.0155	28.7871	28.274	28.2168	28.4386	28.2093	1:Experimental evidence at protein level
26.3276	26.1773	26.3173	26.4023	24.825	24.9149	24.9919	24.7418	1:Experimental evidence at protein level
28.3395	27.9433	27.5919	27.8371	28.4305	28.5307	28.701	28.4379	1:Experimental evidence at protein level
26.3004	26.2698	26.0687	26.2805	26.7408	26.7878	26.8186	26.8429	1:Experimental evidence at protein level
27.5074	27.4869	27.5447	27.517	27.2825	27.2888	27.1975	27.4134	1:Experimental evidence at protein level
25.7688	26.0736	25.3022	24.9723	26.4032	26.5062	26.4472	26.984	1:Experimental evidence at protein level
26.5204	26.6582	26.8301	26.7833	27.0452	27.0804	27.1531	27.1536	1:Experimental evidence at protein level
29.0606	29.0412	29.2147	29.1728	27.9506	27.8531	27.7844	27.8357	1:Experimental evidence at protein level
25.0729	25.0189	24.7905	24.7708	25.421	25.5012	25.2597	26.0012	1:Experimental evidence at protein level
27.3089	27.4757	27.5174	27.4774	27.8258	27.8998	27.9113	28.1048	1:Experimental evidence at protein level
29.5034	29.4878	29.6687	29.6491	29.4489	29.3124	29.3724	29.125	1:Experimental evidence at protein level
26.9086	26.971	26.8456	26.9442	27.0741	27.1442	27.1875	27.562	1:Experimental evidence at protein level
29.1973	29.2632	29.2492	29.3103	28.5351	28.6163	28.6228	28.9211	1:Experimental evidence at protein level
26.9698	26.9762	26.4078	26.4945	27.106	27.1927	27.1015	27.178	1:Experimental evidence at protein level
24.1088	24.2634	23.6226	24.1264	25.0456	24.9175	24.965	25.6245	1:Experimental evidence at protein level
26.4608	26.1721	25.9966	26.1689	25.6147	25.1206	25.6599	25.975	1:Experimental evidence at protein level
26.4438	26.3839	25.6488	25.6133	26.7967	26.9172	26.7629	26.8493	1:Experimental evidence at protein level
26.5332	26.836	26.9551	26.8133	27.0709	26.9684	27.1741	27.3889	1:Experimental evidence at protein level
26.8798	27.218	27.2633	27.1606	27.3232	27.3975	27.3932	27.3608	1:Experimental evidence at protein level
28.0343	28.0899	28.1763	28.0821	27.6853	27.8903	27.7822	27.387	1:Experimental evidence at protein level
25.5866	25.6885	25.9225	25.8535	25.5741	25.0601	25.3362	25.2156	1:Experimental evidence at protein level
29.1146	29.1418	29.1418	29.2185	29.0185	28.92	28.8132	28.9626	1:Experimental evidence at protein level
27.5464	27.6893	27.7717	27.4628	27.5034	27.0771	27.3782	27.197	1:Experimental evidence at protein level
27.4468	27.4813	27.624	27.5842	27.6665	27.7146	27.7599	27.9674	1:Experimental evidence at protein level
28.3248	28.2002	28.1794	28.4215	27.8866	27.7273	27.7984	28.1626	1:Experimental evidence at protein level
25.85	25.907	26.0896	26.0548	26.2624	26.3156	26.2869	26.4848	1:Experimental evidence at protein level
26.3274	26.4004	26.1518	26.4419	26.9411	27.195	27.0195	27.2684	1:Experimental evidence at protein level
24.3951	25.4523	24.8592	24.3319	26.0927	25.895	25.9487	26.0943	1:Experimental evidence at protein level
24.4941	26.521	26.4715	26.2533	27.3212	27.3209	27.1005	27.4485	1:Experimental evidence at protein level
26.5804	26.8018	26.5834	26.7683	26.9319	26.8455	26.9443	27.2305	1:Experimental evidence at protein level
24.0959	24.2329	24.2255	25.6869	26.5217	26.8061	26.7434	26.907	1:Experimental evidence at protein level
25.1993	25.5164	25.5378	25.4511	25.6874	26.0715	25.6198	25.8148	1:Experimental evidence at protein level
24.5271	24.2811	24.5879	24.1764	25.0277	24.8123	24.8938	25.2315	1:Experimental evidence at protein level
29.1512	29.2074	29.4115	29.2157	26.8637	26.9388	26.8328	26.766	1:Experimental evidence at protein level
25.5557	24.9665	24.8621	25.3371	24.1928	23.7606	24.7243	24.7952	1:Experimental evidence at protein level
29.0471	29.0648	29.1987	29.0606	28.9322	28.8365	28.9346	28.6926	1:Experimental evidence at protein level
25.6374	25.9104	25.7645	25.8495	25.9492	26.1413	26.0317	26.2281	1:Experimental evidence at protein level
27.1449	27.2863	27.2074	27.5023	28.2636	28.1241	28.207	28.0709	1:Experimental evidence at protein level
25.8488	26.1385	26.4533	26.2209	25.4961	25.7059	26.0234	25.5803	1:Experimental evidence at protein level
27.3375	27.3603	27.4503	27.3877	27.2693	26.8675	26.8547	26.9875	1:Experimental evidence at protein level
27.1491	27.2471	27.4679	27.4556	26.6067	26.5483	26.9811	27.0757	1:Experimental evidence at protein level
26.9404	27.0178	26.9699	27.0426	25.0689	25.3546	25.0203	24.1659	1:Experimental evidence at protein level
24.2081	24.2596	23.8466	24.0947	24.7161	24.7192	24.3816	25.3208	1:Experimental evidence at protein level
25.8313	26.0172	25.8255	26.0195	26.3145	26.1794	26.3101	26.907	1:Experimental evidence at protein level
25.7117	25.5835	25.9739	26.0037	25.4837	24.3586	24.9441	24.9062	1:Experimental evidence at protein level
26.3588	26.5752	26.7004	26.7328	25.5854	25.7449	24.6548	25.5776	1:Experimental evidence at protein level
26.9717	26.7752	26.6807	26.7587	26.2231	26.2194	26.1683	25.7324	1:Experimental evidence at protein level
26.5485	26.3737	26.0128	26.1563	26.6373	26.7894	26.6084	26.548	1:Experimental evidence at protein level
25.0241	25.0878	24.9294	24.9996	25.3584	25.5898	25.5009	26.184	1:Experimental evidence at protein level
26.4608	26.4525	26.5745	26.378	26.1824	26.2809	26.2649	26.3841	1:Experimental evidence at protein level
27.1343	27.0752	27.0286	27.2159	27.6047	27.3778	27.4907	27.9211	1:Experimental evidence at protein level
29.1695	29.1967	29.3782	29.3981	28.532	28.5006	28.5322	28.4936	1:Experimental evidence at protein level
28.4637	28.4064	28.4948	28.5831	28.9914	29.0707	28.9535	28.7026	1:Experimental evidence at protein level
26.6419	26.7373	26.9607	26.8791	24.3714	24.4276	24.6419	25.1368	1:Experimental evidence at protein level
25.9647	26.0526	26.052	26.0086	25.5516	25.5922	25.6222	25.8345	1:Experimental evidence at protein level
26.2255	26.2909	25.9057	26.0464	26.4119	26.443	26.3339	26.8363	1:Experimental evidence at protein level
26.7559	26.5935	26.6317	26.8184	27.1565	26.8827	27.0111	27.4893	1:Experimental evidence at protein level
27.6889	27.6078	27.6285	27.7623	27.8361	27.9231	27.8628	28.0441	1:Experimental evidence at protein level
27.0921	27.3722	27.2174	27.3095	27.5204	27.5405	27.5249	27.7362	1:Experimental evidence at protein level
26.7201	27.0205	27.1409	27.0139	27.3218	27.1476	27.3992	27.4906	1:Experimental evidence at protein level
26.3091	26.4039	26.187	26.3927	26.7462	26.7939	26.7264	27.223	1:Experimental evidence at protein level
29.0588	28.8452	29.1025	29.1354	29.3253	29.3944	29.2237	29.3482	1:Experimental evidence at protein level
25.2208	25.6949	25.6921	25.3102	26.183	26.2573	26.2615	26.2862	1:Experimental evidence at protein level
26.2326	25.9535	25.8759	26.2922	26.7046	26.6686	26.394	26.5879	1:Experimental evidence at protein level
26.3886	26.5486	26.4456	26.3982	26.1721	26.1441	26.2392	26.2873	1:Experimental evidence at protein level
25.891	26.1395	26.1179	26.0353	26.4085	26.4569	26.4928	26.772	1:Experimental evidence at protein level
27.9351	27.5578	27.8817	27.9476	27.3656	27.3112	27.2707	27.518	1:Experimental evidence at protein level
25.7101	23.6751	25.7184	24.2658	25.8466	27.0223	26.8072	27.2479	1:Experimental evidence at protein level
28.1251	28.2566	28.4856	28.5643	27.7123	27.7128	27.8733	27.5108	1:Experimental evidence at protein level
27.7984	27.7431	27.4225	27.4413	27.9804	27.951	27.8697	28.034	1:Experimental evidence at protein level
26.3068	26.1341	25.9041	26.2356	26.7546	26.6263	26.8905	27.1847	1:Experimental evidence at protein level
26.2824	26.1988	26.3168	26.2417	27.1172	27.1526	26.7183	26.6136	1:Experimental evidence at protein level
24.3962	25.7638	25.2781	25.4107	27.1161	26.8737	26.2795	26.8177	1:Experimental evidence at protein level
24.9112	27.1531	24.2501	26.446	27.9646	28.2541	27.4307	28.284	1:Experimental evidence at protein level
28.2379	28.3212	28.4282	28.3347	27.7815	27.5936	27.7462	28.2845	1:Experimental evidence at protein level
24.6843	24.7003	24.7435	24.6285	25.2163	24.9667	24.8316	25.5819	1:Experimental evidence at protein level
26.5304	26.6466	26.6673	26.8624	26.8876	27.0189	26.8839	27.3388	1:Experimental evidence at protein level
25.5563	25.5688	25.6739	25.3521	25.1602	25.3005	25.0705	24.8943	1:Experimental evidence at protein level
26.1996	26.1516	25.9232	26.27	26.8105	26.7134	26.6068	27.1874	1:Experimental evidence at protein level
27.6568	27.9352	27.7133	27.7273	27.2052	27.4437	27.5859	27.157	1:Experimental evidence at protein level
28.1389	28.0791	28.1442	28.2081	28.3523	28.2558	28.3003	28.4421	1:Experimental evidence at protein level
26.1276	26.342	25.0679	24.8042	26.8452	27.2512	26.8781	27.2631	1:Experimental evidence at protein level
27.5285	27.5392	27.4893	27.5123	27.2415	27.2152	27.2867	27.4932	1:Experimental evidence at protein level
26.8424	26.965	26.8464	27.0256	27.2895	27.3673	27.2518	27.6387	1:Experimental evidence at protein level
29.3848	29.334	29.3622	29.4008	29.2828	29.1397	29.3188	29.2842	1:Experimental evidence at protein level
28.032	28.0827	28.2151	28.0987	28.5172	28.6704	28.5819	28.595	1:Experimental evidence at protein level
28.6956	28.8233	28.7547	28.7301	28.309	28.32	28.4011	28.3341	1:Experimental evidence at protein level
26.4388	26.5458	26.4848	26.7113	25.5886	25.7483	25.8416	26.0249	1:Experimental evidence at protein level
25.2782	25.2561	25.3971	24.9213	24.2664	24.6649	24.3781	24.7227	1:Experimental evidence at protein level
25.5912	25.6206	25.7024	25.81	26.4653	25.8321	26.099	26.0585	1:Experimental evidence at protein level
23.8279	23.8199	23.8276	24.2488	25.5454	25.8767	25.9123	26.1057	1:Experimental evidence at protein level
25.3189	25.3441	25.1805	25.1854	25.9765	26.0024	26.0102	26.0763	1:Experimental evidence at protein level
27.1725	26.6843	26.5558	26.9375	27.6109	27.4068	27.0749	27.9612	1:Experimental evidence at protein level
27.2649	27.4342	27.211	27.2653	27.6084	27.6854	27.5396	27.7072	1:Experimental evidence at protein level
27.6203	27.4342	27.5339	27.4897	27.7922	27.6147	27.656	27.6344	1:Experimental evidence at protein level
25.4081	25.2071	25.4156	25.5948	25.7879	25.9498	26.1677	26.5555	1:Experimental evidence at protein level
25.1096	24.873	25.0691	24.1616	25.7507	25.7799	25.533	25.942	1:Experimental evidence at protein level
25.3664	25.5439	25.1995	24.9164	25.9666	26.1688	25.6223	26.0066	1:Experimental evidence at protein level
25.3468	25.3515	25.2692	25.0646	25.788	25.5548	25.5521	26.1346	1:Experimental evidence at protein level
25.695	25.7846	25.7861	25.9533	25.9542	26.1186	26.0904	26.1821	1:Experimental evidence at protein level
27.6327	27.6386	27.7656	27.4456	25.1924	25.9057	25.446	25.8224	5:Protein uncertain
27.1351	27.0884	26.8561	26.9941	27.3036	27.4381	27.1127	27.5544	1:Experimental evidence at protein level
25.3143	25.2108	25.2985	25.4502	25.6275	25.6271	25.4663	25.5817	1:Experimental evidence at protein level
26.7474	26.607	26.6475	26.8535	27.7483	27.6732	27.7457	27.9813	1:Experimental evidence at protein level
28.728	28.7516	28.9542	28.8642	28.5222	28.5411	28.5867	28.4048	1:Experimental evidence at protein level
25.857	26.281	26.5045	26.4161	25.3579	25.4789	25.8556	24.2721	1:Experimental evidence at protein level
25.309	25.3882	25.1763	25.5414	26.0774	26.2216	26.1436	26.4583	1:Experimental evidence at protein level

27.4402	27.6591	27.2956	27.5955	27.7102	27.8963	27.8191	27.9137	1:Experimental evidence at protein level
26.9538	27.0171	26.8139	27.1256	27.3994	27.2802	27.3532	27.8227	1:Experimental evidence at protein level
27.104	27.0041	26.8151	26.9648	27.3171	27.221	27.1332	27.5256	1:Experimental evidence at protein level
27.3676	27.553	27.6068	27.3846	27.0757	27.2105	27.1977	26.6411	1:Experimental evidence at protein level
27.0908	27.0903	27.3416	27.1701	25.047	25.1522	25.1531	25.2644	1:Experimental evidence at protein level
28.2279	28.4184	28.1413	28.4819	28.7088	28.6333	28.8041	29.1088	1:Experimental evidence at protein level
26.4398	26.6475	26.7814	26.8237	26.9893	27.1654	26.9188	26.8146	1:Experimental evidence at protein level
24.6184	24.9532	25.0166	24.6361	25.244	25.5636	25.3169	25.0932	1:Experimental evidence at protein level
28.0253	27.9946	27.8405	27.9562	27.5998	27.4418	27.39	27.8308	1:Experimental evidence at protein level
25.801	25.9355	25.7685	25.8183	26.3558	26.5779	26.4177	26.2983	1:Experimental evidence at protein level
25.5022	25.5631	25.3217	25.4939	24.8221	24.0774	24.6832	24.7268	1:Experimental evidence at protein level
28.9259	29.108	29.2624	29.1282	28.6269	28.8007	28.6385	29.01	1:Experimental evidence at protein level
25.959	26.056	25.7925	25.8589	28.0027	28.0473	28.0181	28.0562	1:Experimental evidence at protein level
25.9549	25.9805	25.7944	26.0743	26.3239	26.5162	26.4173	26.803	1:Experimental evidence at protein level
27.9558	27.9185	28.0481	27.9435	27.5382	27.7262	27.568	27.6988	1:Experimental evidence at protein level
26.6853	26.6835	26.3651	26.6061	24.5675	23.9225	22.3668	24.9993	1:Experimental evidence at protein level
29.1828	29.072	29.2031	29.3399	28.8844	28.8407	28.8612	29.1587	1:Experimental evidence at protein level
26.9229	26.9439	27.1919	27.1728	27.2466	27.3747	27.3448	27.467	1:Experimental evidence at protein level
26.4546	26.532	26.6005	26.6477	26.8633	26.8926	26.8621	27.0686	1:Experimental evidence at protein level
24.7618	24.6061	24.8105	25.4621	25.9742	25.5315	25.8666	26.1678	1:Experimental evidence at protein level
27.2508	27.3329	27.0095	27.2752	28.0542	28.0137	28.0262	28.0391	1:Experimental evidence at protein level
27.0467	26.8201	26.9382	26.8643	27.3278	27.1803	27.2295	27.126	1:Experimental evidence at protein level
25.8676	25.857	25.904	25.7642	24.8329	24.8408	24.3397	24.37	1:Experimental evidence at protein level
28.2643	28.3625	28.3231	28.3389	28.7793	28.5624	28.8507	29.195	1:Experimental evidence at protein level
25.9507	25.9831	26.5877	26.0075	26.6314	26.5713	26.664	26.5561	1:Experimental evidence at protein level
25.5746	25.6002	25.7424	25.5946	26.2223	26.2417	26.0526	26.1	1:Experimental evidence at protein level
26.188	26.116	26.4095	26.2578	25.091	25.3039	25.146	25.6863	1:Experimental evidence at protein level
25.1705	25.2864	24.8918	25.646	26.5333	26.6281	26.5684	27.1963	1:Experimental evidence at protein level
23.62	25.3472	24.494	23.9886	25.8472	25.8896	25.9092	26.2428	1:Experimental evidence at protein level
27.4795	27.4893	27.6368	27.4754	26.8103	26.7115	26.8563	26.3216	1:Experimental evidence at protein level
25.4624	25.1465	25.082	25.2379	25.9276	26.1194	26.0454	25.8806	1:Experimental evidence at protein level
26.8563	26.71	26.6098	27.0805	25.9054	26.038	25.8953	24.3394	1:Experimental evidence at protein level
25.9342	25.9557	25.7483	25.4848	24.8254	25.1092	24.6583	24.6923	1:Experimental evidence at protein level
26.0614	26.2467	26.3561	26.1721	26.3292	26.5956	26.5819	26.5165	1:Experimental evidence at protein level
26.0044	25.2559	25.8024	25.8592	26.2596	26.2083	26.1305	26.7368	1:Experimental evidence at protein level
25.1882	25.2972	25.6317	25.7123	25.809	25.9343	25.8294	25.7378	1:Experimental evidence at protein level
27.3608	27.5742	27.6456	27.6454	24.9352	25.2832	25.4463	25.5725	1:Experimental evidence at protein level
25.6547	25.5138	25.4452	25.6296	26.1041	26.2459	25.8677	26.0797	1:Experimental evidence at protein level
28.0669	28.1686	28.1486	28.2553	26.4987	26.6081	26.623	27.033	1:Experimental evidence at protein level
26.8141	26.9816	27.054	27.0944	26.5598	26.4465	26.4738	26.7785	1:Experimental evidence at protein level
25.2562	25.3832	24.7471	24.619	25.5704	25.6617	25.5894	26.2101	1:Experimental evidence at protein level
27.3095	27.2583	27.1034	27.2769	28.4509	28.3703	28.3241	28.6823	1:Experimental evidence at protein level
28.8275	28.6217	28.3476	28.2993	29.2926	29.3124	29.1005	29.0428	1:Experimental evidence at protein level
27.155	27.0523	27.1283	26.8649	26.9076	26.828	26.9028	26.7462	1:Experimental evidence at protein level
27.5628	27.4978	27.5515	27.5612	27.8732	27.8526	27.8991	27.9132	1:Experimental evidence at protein level
24.5238	24.7181	24.7741	24.5603	25.3373	25.0462	25.4969	26.1454	1:Experimental evidence at protein level
26.4423	26.6311	26.8988	26.5651	27.0926	27.2392	27.2388	27.2952	1:Experimental evidence at protein level
26.5386	26.5168	26.5396	26.414	26.914	27.1363	27.093	26.5289	1:Experimental evidence at protein level
29.234	29.3228	29.0135	29.1234	29.7495	29.8513	29.7691	30.0116	1:Experimental evidence at protein level
23.9652	25.2665	24.367	24.8862	25.4342	25.3598	25.6569	25.5015	1:Experimental evidence at protein level
26.7695	26.8739	26.8955	26.9859	26.5331	26.5566	26.7863	26.4552	1:Experimental evidence at protein level
27.2502	27.4384	27.4322	27.2177	27.2262	27.0264	27.0592	27.0113	1:Experimental evidence at protein level
27.7104	27.5958	27.4774	27.5434	27.6869	28.013	28.0574	28.3338	1:Experimental evidence at protein level
23.9906	23.8428	23.7083	24.4155	24.8183	24.4116	24.58	25.1596	1:Experimental evidence at protein level
26.3211	26.473	26.4033	26.6447	26.7945	26.87	26.8158	26.9376	1:Experimental evidence at protein level
28.3088	28.5051	28.5848	28.7256	28.182	27.6616	28.0294	28.3751	1:Experimental evidence at protein level
28.6184	28.6818	28.8371	28.8723	28.0979	28.1137	28.1821	28.2751	1:Experimental evidence at protein level
27.6884	27.7786	27.8604	27.7436	27.4341	27.3982	27.3053	27.2606	1:Experimental evidence at protein level
27.1164	27.3105	26.8721	27.3216	27.7814	28.0932	27.8126	27.7871	1:Experimental evidence at protein level
27.8422	27.7892	27.7762	27.8494	28.207	28.1614	28.194	28.6033	1:Experimental evidence at protein level
26.0594	26.1669	26.4071	26.3833	25.3599	24.9963	25.41	26.1601	1:Experimental evidence at protein level
25.3737	25.5369	25.523	25.8204	24.4232	24.8981	24.53	25.2002	3:Protein inferred from homology
26.5034	26.5928	26.5671	26.3757	25.8664	25.8428	25.9307	26.2389	1:Experimental evidence at protein level
26.4594	26.3196	25.967	26.276	26.8429	26.883	26.6027	27.5026	1:Experimental evidence at protein level
26.0948	25.7615	25.8068	26.0395	26.6412	26.5518	26.2813	26.8817	1:Experimental evidence at protein level
29.1439	29.1316	29.2257	29.3504	29.6078	29.5098	29.553	29.7074	1:Experimental evidence at protein level
24.598	24.6534	24.6577	24.8827	25.2814	25.4006	25.0191	25.0855	1:Experimental evidence at protein level
27.635	27.7254	27.5966	27.6204	26.9831	27.007	27.2805	27.3176	1:Experimental evidence at protein level
28.4333	28.3368	28.3326	28.2622	28.5077	28.5344	28.4009	28.5207	1:Experimental evidence at protein level
25.9444	26.08	26.0034	25.9157	26.3705	26.3864	26.1467	26.6985	1:Experimental evidence at protein level
26.0699	26.1453	26.1968	26.2452	26.3669	26.4227	26.2444	26.683	1:Experimental evidence at protein level
26.141	26.2045	26.3756	26.3336	26.375	26.4823	26.5943	26.679	1:Experimental evidence at protein level
27.6346	27.1064	27.3381	27.6347	28.0718	27.9513	27.7799	27.8195	1:Experimental evidence at protein level
28.4118	28.3967	28.5833	28.6104	28.7465	28.7821	28.6313	28.6973	1:Experimental evidence at protein level
28.8208	28.9961	28.8931	28.9032	28.5102	28.4773	28.5833	28.6081	1:Experimental evidence at protein level
25.1007	25.1881	25.1294	25.2477	24.5763	24.7094	24.8819	25.1231	1:Experimental evidence at protein level
25.6818	26.3276	25.4347	25.8788	27.0186	26.6386	26.3289	27.0905	1:Experimental evidence at protein level
24.8754	24.9062	24.9492	24.8766	24.3446	24.5641	24.7831	24.7063	1:Experimental evidence at protein level
28.0643	28.0467	27.8801	27.8995	28.7259	28.7302	28.6518	29.0337	1:Experimental evidence at protein level
26.2511	26.4773	26.5212	25.8747	26.9073	26.8725	26.7802	27.2907	1:Experimental evidence at protein level
27.596	27.6914	27.7045	27.6212	28.4174	28.4884	28.2769	28.5134	1:Experimental evidence at protein level
27.2725	27.2682	27.0689	27.3285	28.0416	28.0121	27.6849	27.7045	1:Experimental evidence at protein level
27.0668	27.03	27.1946	26.9934	26.5722	26.3961	26.3764	26.0908	1:Experimental evidence at protein level
27.9435	28.0571	28.2181	28.3135	27.2641	27.2571	27.4876	27.6635	1:Experimental evidence at protein level
24.1893	24.394	24.1501	23.4828	24.5841	24.8717	24.7088	25.3754	1:Experimental evidence at protein level
26.177	26.3671	26.1248	26.3413	24.8564	24.2046	24.1025	24.4178	1:Experimental evidence at protein level
24.1644	24.9777	24.8567	25.0337	25.9588	26.048	26.1948	26.3687	1:Experimental evidence at protein level
26.4362	26.4972	26.4706	26.6007	26.6169	26.6903	26.6498	26.862	1:Experimental evidence at protein level
25.1083	25.457	24.8407	25.1294	25.9921	26.0133	26.1317	26.6948	1:Experimental evidence at protein level
26.8659	26.7743	26.8046	26.6786	27.202	27.0491	27.1141	27.4705	1:Experimental evidence at protein level
25.8423	25.9781	25.9323	26.05	26.3438	26.3346	26.4707	26.0477	1:Experimental evidence at protein level
25.6256	25.8105	25.7572	25.7461	25.4997	25.2718	25.4491	25.5769	1:Experimental evidence at protein level
23.6383	23.3586	23.4986	23.5436	23.941	23.8587	24.1917	24.8073	1:Experimental evidence at protein level
26.5542	26.9996	27.168	27.3735	24.4259	24.1729	23.7891	24.9309	1:Experimental evidence at protein level
25.9776	26.3097	25.9915	26.1471	26.3436	26.6407	26.4332	26.6301	1:Experimental evidence at protein level
28.2985	28.3512	28.1256	28.424	28.63	28.4574	28.6717	28.7677	1:Experimental evidence at protein level
28.2544	28.3936	28.2372	28.3037	28.5379	28.4517	28.5572	28.7714	1:Experimental evidence at protein level
26.5087	26.5193	26.6655	26.4471	26.2416	26.3129	26.3067	26.2827	1:Experimental evidence at protein level
29.1168	28.9957	29.1184	29.1124	29.6202	29.4159	29.4108	29.2697	1:Experimental evidence at protein level
26.5596	26.5577	26.5053	26.5914	27.1968	27.3044	27.249	27.5258	1:Experimental evidence at protein level
26.684	26.8782	26.8352	26.925	25.7779	25.6588	25.8575	25.9782	1:Experimental evidence at protein level
24.9676	24.9841	24.9206	24.4276	23.5198	24.619	23.78	24.0228	1:Experimental evidence at protein level
27.6959	27.8504	27.969	27.7046	28.1129	28.0819	28.0166	27.9205	1:Experimental evidence at protein level
28.037	28.0102	28.1497	28.0275	27.8329	27.9232	27.9161	27.8391	1:Experimental evidence at protein level
26.0351	25.7309	25.9725	25.8489	26.3477	26.1977	26.0188	26.1965	1:Experimental evidence at protein level
27.0075	27.0729	26.843	27.1029	27.2252	27.3637	27.1571	27.5918	1:Experimental evidence at protein level
27.9068	27.836	27.6728	27.8287	27.4365	27.5665	27.5485	27.4784	1:Experimental evidence at protein level
27.679	27.6402	27.7094	27.6306	27.9323	27.7303	27.7845	27.8256	1:Experimental evidence at protein level
27.2875	27.3897	27.4611	27.3837	27.7303	27.6124	27.7159	27.4548	1:Experimental evidence at protein level
26.2727	26.0439	26.2367	26.4118	25.8761	25.8414	26.0356	25.7899	1:Experimental evidence at protein level
25.9832	26.1576	26.3229	26.2842	24.7443	24.7326	24.8705	25.4128	1:Experimental evidence at protein level
27.1805	27.1671	27.169	27.2628	27.3426	27.3818	27.2393	27.4315	1:Experimental evidence at protein level
28.7357	28.6147	28.7316	28.6704	29.0352	29.0678	28.839	28.7421	1:Experimental evidence at protein level
26.4763	26.8601	25.8243	26.366	27.27	27.8581	27.6152	27.4358	1:Experimental evidence at protein level
24.7891	23.6573	24.1756	24.6644	26.684	26.8991	26.418	25.9841	1:Experimental evidence at protein level
24.776	23.782	24.9807	24.4085	26.0802	25.9507	25.9644	25.9502	1:Experimental evidence at protein level
27.2105	27.2599	27.465	27.4727	27.52	27.6466	27.5818	27.4963	1:Experimental evidence at protein level
26.4386	26.3355	26.4505	26.2632	26.6099	26.8667	26.8854	27.1203	1:Experimental evidence at protein level
24.7627	24.2512	24.0035	24.7914	24.9256	25.2309	25.5252	25.3179	1:Experimental evidence at protein level
28.7185	28.7839	28.8387	28.8074	29.0057	28.9648	28.9437	28.801	1:Experimental evidence at protein level
27.4988	27.4128	27.3609	27.4767	27.7314	27.8132	27.8131	27.8142	1:Experimental evidence at protein level
28.2122	28.2187	28.3082	28.2609	28.5423	28.4971	28.4792	28.7148	1:Experimental evidence at protein level
26.8863	27.0064	26.9052	26.8649	26.319	26.6749	26.4037	26.322	1:Experimental evidence at protein level
26.9671	27.0545	26.6115	27.0469	27.6131	27.5171	27.365	27.7514	1:Experimental evidence at protein level
26.3157	26.4408	26.2243	26.5753	27.0896	27.2958	27.1397	27.7766	1:Experimental evidence at protein level
27.1811	27.2076	27.3519	27.41	27.7184	27.7458	27.7053	27.9086	1:Experimental evidence at protein level
25.1868	25.1517	25.267	25.1284	25.9309	25.993	25.9706	26.2611	1:Experimental evidence at protein level
30.2907	30.1706	29.9174	30.1127	30.603	30.8185	30.5164	30.7767	1:Experimental evidence at protein level
25.4337	25.1793	25.2331	25.2854	26.3875	26.2148	26.3184	26.7959	1:Experimental evidence at protein level

26.9966	27.0437	26.8686	27.1325	26.4503	26.557	26.5703	26.9996	1:Experimental evidence at protein level
26.3465	26.3018	26.4941	26.5316	26.8597	26.9693	26.8572	27.1077	1:Experimental evidence at protein level
26.2105	26.0924	26.3513	26.3911	25.9228	26.0583	25.8393	26.0274	1:Experimental evidence at protein level
28.1348	27.8899	27.9489	27.9116	25.872	25.6621	25.7525	25.7513	1:Experimental evidence at protein level
28.2773	28.3398	28.2238	28.0604	27.9454	28.0432	28.0622	27.8929	1:Experimental evidence at protein level
26.6599	26.7132	26.632	26.7705	27.2233	27.1483	27.0389	27.4779	1:Experimental evidence at protein level
28.3176	28.3256	28.5137	28.5638	28.0147	27.9954	28.136	28.3106	1:Experimental evidence at protein level
26.0956	26.3086	26.4012	26.3012	26.6663	26.5908	26.8631	26.6492	1:Experimental evidence at protein level
25.9503	26.0267	25.976	26.0569	26.581	26.5935	26.421	26.7963	1:Experimental evidence at protein level
26.6405	26.7832	26.7874	26.9474	27.0952	26.967	27.2119	27.5113	1:Experimental evidence at protein level
25.5715	25.7602	25.8166	26.0713	26.3509	26.195	26.1059	27.0244	1:Experimental evidence at protein level
25.9767	25.9757	25.9645	25.9809	26.4394	26.3754	26.3939	26.6508	1:Experimental evidence at protein level
28.6405	28.9937	29.0948	28.8169	29.8253	29.7936	29.7847	29.1507	1:Experimental evidence at protein level
26.8355	26.6599	26.2819	26.2497	26.9841	26.989	26.8232	27.4649	1:Experimental evidence at protein level
28.1652	28.1182	28.2562	28.2101	28.2802	28.361	28.33	28.3906	1:Experimental evidence at protein level
26.3181	26.2777	26.0733	25.9536	26.5163	26.6184	26.9063	27.1237	1:Experimental evidence at protein level
29.073	29.1	29.0115	28.8223	29.2424	29.2961	29.2697	29.1279	1:Experimental evidence at protein level
27.0109	27.0982	27.2127	26.9906	27.2686	27.3422	27.3615	27.3266	1:Experimental evidence at protein level
29.2108	29.0753	29.3232	29.2104	28.3701	28.9015	28.5519	28.6129	1:Experimental evidence at protein level
26.6726	26.8718	26.6856	27.0034	24.1825	25.4558	23.8714	24.453	1:Experimental evidence at protein level
25.6966	25.7166	25.7101	25.935	26.2959	26.4716	26.0481	26.4414	1:Experimental evidence at protein level
25.44	25.5377	25.5036	25.3841	25.7894	25.6563	26.0556	26.4265	1:Experimental evidence at protein level

C: GOBP name
catabolic process;cellular catabolic process;cellular macromolecule catabolic process;cellular macromolecule metabolic process;cellular metabolic process;cellular process;cellular protein metabolic process;macromolecule
cellular component organization;cellular component organization or biogenesis;cellular membrane organization;cellular process;endocytosis;establishment of localization;lipid transport;membrane invagination;membrane orga
adipose tissue development;anatomical structure development;biological regulation;bone development;cell differentiation;cellular component assembly;cellular component disassembly;cellular component disassembly at cell
carbohydrate metabolic process;cellular macromolecule metabolic process;cellular metabolic process;cellular process;cellular protein metabolic process;dephosphorylation;macromolecule metabolic process;macromolecule
biological regulation;cellular component organization;cellular component organization at cellular level;cellular component organization or biogenesis;cellular component organization or biogenesis at cellular level;cellular proce
establishment of localization;establishment of protein localization;establishment of RNA localization;mRNA transport;nucleic acid transport;nucleobase-containing compound transport;protein transport;RNA transport;transpo
biosynthetic process;cellular biosynthetic process;cellular lipid metabolic process;cellular metabolic process;cellular process;ether lipid biosynthetic process;ether lipid metabolic process;glycerol ether biosynthetic process;g
anatomical structure development;antigen processing and presentation;antigen processing and presentation of exogenous antigen;antigen processing and presentation of exogenous peptide antigen;antigen processing and
biological regulation;regulation of biological process;regulation of biosynthetic process;regulation of cellular biosynthetic process;regulation of cellular macromolecule biosynthetic process;regulation of cellular metabolic proc
biosynthetic process;carboxylic acid biosynthetic process;carboxylic acid catabolic process;carboxylic acid metabolic process;catabolic process;cellular biosynthetic process;cellular catabolic process;cellular ketone metabo
biological regulation;cellular component organization;cellular component organization or biogenesis;defense response;establishment of localization;establishment of localization in cell;establishment of protein localization;esta
cellular component assembly;cellular component assembly at cellular level;cellular component biogenesis;cellular component organization;cellular component organization at cellular level;cellular component organization or b
axon guidance;chemotaxis;locomotion;response to chemical stimulus;response to external stimulus;response to stimulus;taxis
biological regulation;biosynthetic process;cellular biosynthetic process;cellular component assembly;cellular component assembly at cellular level;cellular component organization;cellular component organization at cellular le
biological regulation;cellular process;cellular response to acid;cellular response to amine stimulus;cellular response to amino acid stimulus;cellular response to chemical stimulus;cellular response to endogenous stimulus;cell
biosynthetic process;cellular biosynthetic process;cellular component organization;cellular component organization at cellular level;cellular component organization or biogenesis;cellular component organization or biogenesis
anatomical structure morphogenesis;biological regulation;biosynthetic process;cellular biosynthetic process;cellular macromolecule biosynthetic process;cellular macromolecule metabolic process;cellular metabolic process;
establishment of localization;transport
amine metabolic process;aminoglycan biosynthetic process;aminoglycan catabolic process;aminoglycan metabolic process;axon guidance;biological regulation;biosynthetic process;carbohydrate biosynthetic process;carbo
cellular component organization;cellular component organization at cellular level;cellular component organization or biogenesis;cellular component organization or biogenesis at cellular level;cellular macromolecule metabolic
cellular metabolic process;cellular process;electron transport chain;generation of precursor metabolites and energy;metabolic process;mitochondrial electron transport, NADH to ubiquinone;oxidation-reduction process;respi
biological regulation;cell differentiation;cell proliferation;cellular component assembly;cellular component assembly at cellular level;cellular component organization;cellular component organization at cellular level;cellular com
biological regulation;cell surface receptor linked signaling pathway;cellular component assembly;cellular component organization;cellular component organization or biogenesis;cellular process;cellular response to chemical s
anatomical structure development;anatomical structure formation involved in morphogenesis;biological adhesion;biological regulation;cell adhesion;cell development;cell migration;cell motility;cellular component movement;
alcohol metabolic process;carbohydrate metabolic process;cellular carbohydrate metabolic process;cellular macromolecule metabolic process;cellular metabolic process;cellular process;cellular protein metabolic process;he
biosynthetic process;cell proliferation;cellular biosynthetic process;cellular metabolic compound salvage;cellular metabolic process;cellular process;coenzyme biosynthetic process;coenzyme metabolic process;cofactor bios
anterograde axon cargo transport;anterograde synaptic vesicle transport;antigen processing and presentation;antigen processing and presentation of exogenous antigen;antigen processing and presentation of lipid antigen v
biological regulation;cation transport;cellular metabolic process;cellular process;cellular response to chemical stimulus;cellular response to oxidative stress;cellular response to oxygen radical;cellular response to reactive oxy
acute inflammatory response;arachidonic acid metabolic process;biological regulation;biosynthetic process;carboxylic acid biosynthetic process;carboxylic acid metabolic process;cellular biosynthetic process;cellular ketone
biological regulation;biosynthetic process;cell activation;cell proliferation;cellular biosynthetic process;cellular component assembly;cellular component assembly at cellular level;cellular component organization;cellular comp
acid secretion;actin cytoskeleton organization;actin filament-based process;activation of adenylate cyclase activity;activation of adenylate cyclase activity by dopamine receptor signaling pathway;activation of adenylate cycla
activation of immune response;activation of innate immune response;anaphase-promoting complex-dependent proteasomal ubiquitin-dependent protein catabolic process;antigen processing and presentation;antigen proces
amine catabolic process;amine metabolic process;branched chain family amino acid catabolic process;branched chain family amino acid metabolic process;carboxylic acid catabolic process;carboxylic acid metabolic proces
biological regulation;cell surface receptor linked signaling pathway;cellular process;cellular response to chemical stimulus;cellular response to cytokine stimulus;cellular response to interferon-alpha;cellular response to organi
biological regulation;cell communication;cell migration;cell motility;cell surface receptor linked signaling pathway;cell-cell signaling;cellular component movement;cellular process;cellular response to stimulus;endothelial cell
biological regulation;cellular component assembly;cellular component assembly at cellular level;cellular component organization;cellular component organization at cellular level;cellular component organization or biogenesis;
anatomical structure development;cell development;cellular developmental process;cellular metabolic process;cellular process;central nervous system neuron development;cerebellar Purkinje cell layer development;develop
biological regulation;cell cycle;cell cycle phase;cell cycle process;cellular component organization;cellular component organization at cellular level;cellular component organization or biogenesis;cellular component organizatio
axon ensheathment;biological regulation;cell cycle process;cellular component assembly;cellular component assembly at cellular level;cellular component organization;cellular component organization at cellular level;cellular
ameboidal cell migration;anatomical structure development;anatomical structure formation involved in morphogenesis;anatomical structure morphogenesis;angiogenesis;biological adhesion;biological regulation;branching inv
biosynthetic process;cellular biosynthetic process;cellular component disassembly;cellular component disassembly at cellular level;cellular component organization;cellular component organization at cellular level;cellular co
alcohol biosynthetic process;alcohol metabolic process;amine metabolic process;biosynthetic process;cellular biosynthetic process;cellular lipid metabolic process;cellular metabolic process;cellular nitrogen compound bios
biological regulation;cell cycle arrest;cell cycle process;cellular macromolecule metabolic process;cellular metabolic process;cellular process;cellular protein metabolic process;dephosphorylation;macromolecule metabolic pr
actin cytoskeleton organization;actin filament organization;actin filament-based process;alcohol metabolic process;biological adhesion;biological regulation;biosynthetic process;carbohydrate metabolic process;cell adhesion
biological regulation;biosynthetic process;cellular biosynthetic process;cellular component assembly;cellular component assembly at cellular level;cellular component organization;cellular component organization at cellular le
biological regulation;biosynthetic process;cellular biosynthetic process;cellular component assembly;cellular component assembly at cellular level;cellular component organization;cellular component organization at cellular le
amine biosynthetic process;amine catabolic process;amine metabolic process;biological regulation;biosynthetic process;branched chain family amino acid biosynthetic process;branched chain family amino acid catabolic pr
anion transport;biological regulation;blood coagulation;carboxylic acid metabolic process;carboxylic acid transport;cell migration;cell motility;cellular component movement;cellular ketone metabolic process;cellular metaboli
4-hydroxyproline metabolic process;amine metabolic process;carboxylic acid metabolic process;cellular amine metabolic process;cellular amino acid metabolic process;cellular ketone metabolic process;cellular macromolec
cellular macromolecule metabolic process;cellular metabolic process;cellular nitrogen compound metabolic process;cellular process;gene expression;macromolecule metabolic process;metabolic process;mRNA metabolic p
amine biosynthetic process;amine metabolic process;anatomical structure development;aspartate family amino acid metabolic process;biological regulation;biosynthetic process;brain development;carboxylic acid biosynthet
antigen processing and presentation;antigen processing and presentation of exogenous antigen;antigen processing and presentation of exogenous peptide antigen;antigen processing and presentation of exogenous peptide
3'-phosphoadenosine 5'-phosphosulfate biosynthetic process;3'-phosphoadenosine 5'-phosphosulfate metabolic process;amine metabolic process;aminoglycan metabolic process;anatomical structure development;biosynt
biological regulation;cell cycle arrest;cell cycle process;cellular component assembly;cellular component assembly at cellular level;cellular component organization;cellular component organization at cellular level;cellular com
amine metabolic process;amino acid activation;biological regulation;carboxylic acid metabolic process;cellular amine metabolic process;cellular amino acid metabolic process;cellular ketone metabolic process;cellular macro
anion homeostasis;anion transport;bicarbonate transport;biological regulation;cellular metabolic process;cellular process;chemical homeostasis;chloride ion homeostasis;establishment of localization;homeostatic process;inc
cellular macromolecule metabolic process;cellular metabolic process;cellular process;cellular protein metabolic process;establishment of localization;establishment of localization in cell;establishment of protein localization;es
cellular metabolic process;cellular process;electron transport chain;generation of precursor metabolites and energy;metabolic process;mitochondrial electron transport, NADH to ubiquinone;oxidation-reduction process;respi
anion transport;biological regulation;cellular localization;cellular macromolecule localization;cellular macromolecule metabolic process;cellular metabolic process;cellular process;cellular protein localization;cellular protein me
biological regulation;biosynthetic process;cellular biosynthetic process;cellular macromolecule biosynthetic process;cellular macromolecule metabolic process;cellular metabolic process;cellular nitrogen compound metaboli
amine metabolic process;amino acid activation;asparaginyl-tRNA aminoacylation;carboxylic acid metabolic process;cellular amine metabolic process;cellular amino acid metabolic process;cellular ketone metabolic process;
biological regulation;cell surface receptor linked signaling pathway;cellular process;cellular response to stimulus;G-protein coupled receptor protein signaling pathway;regulation of biological process;regulation of cellular proc
2-oxoglutarate metabolic process;acetyl-CoA catabolic process;acetyl-CoA metabolic process;carboxylic acid metabolic process;catabolic process;cellular catabolic process;cellular ketone metabolic process;cellular metabo
biological regulation;cell migration;cell motility;cell proliferation;cellular component movement;cellular component organization;cellular component organization or biogenesis;cellular process;locomotion;macromolecule metab
biological regulation;blood coagulation;cell activation;cellular process;coagulation;establishment of localization;establishment of localization in cell;exocytosis;hemostasis;multicellular organismal process;platelet activation;pla
aging;anatomical structure formation involved in morphogenesis;apoptosis;axon cargo transport;axon transport of mitochondrion;biological regulation;cell aging;cell death;cellular component movement;cellular component o
anatomical structure development;biological regulation;biosynthetic process;blood coagulation;cell development;cell differentiation;cell proliferation;cellular biosynthetic process;cellular component organization;cellular comp
developmental process;keratinization
acyl-CoA biosynthetic process;acyl-CoA metabolic process;acylglycerol biosynthetic process;acylglycerol metabolic process;anatomical structure development;biological regulation;biosynthetic process;carboxylic acid meta
2'-deoxyribonucleotide metabolic process;aging;alcohol metabolic process;amino sugar metabolic process;biological regulation;carbohydrate metabolic process;catabolic process;cell death;cellular carbohydrate metabolic p
alcohol biosynthetic process;alcohol metabolic process;biological regulation;biosynthetic process;carbohydrate biosynthetic process;carbohydrate metabolic process;cell surface receptor linked signaling pathway;cellular bio
anatomical structure development;anatomical structure morphogenesis;basement membrane assembly;basement membrane organization;biological regulation;cell morphogenesis;cell morphogenesis involved in differentiatio
actin cytoskeleton organization;actin filament organization;actin filament polymerization;actin filament-based process;actin polymerization or depolymerization;biological regulation;cell projection organization;cellular compone
cellular process;establishment of localization;transport;vesicle-mediated transport
anatomical structure development;biological regulation;biosynthetic process;cell cycle checkpoint;cellular biosynthetic process;cellular macromolecule biosynthetic process;cellular macromolecule metabolic process;cellular
anatomical structure development;biological regulation;biosynthetic process;blood coagulation;cellular biosynthetic process;cellular macromolecule biosynthetic process;cellular macromolecule metabolic process;cellular me
alcohol metabolic process;amine metabolic process;aminoglycan biosynthetic process;aminoglycan metabolic process;anatomical structure morphogenesis;biosynthetic process;carbohydrate biosynthetic process;carbohyd
alcohol metabolic process;biosynthetic process;carbohydrate biosynthetic process;carbohydrate metabolic process;cellular biosynthetic process;cellular carbohydrate metabolic process;cellular lipid metabolic process;cellul
cell cycle;cellular component organization;cellular component organization at cellular level;cellular component organization or biogenesis;cellular component organization or biogenesis at cellular level;cellular membrane fusio
alternative nuclear mRNA splicing, via spliceosome;biological regulation;biosynthetic process;cell projection organization;cellular biosynthetic process;cellular component organization;cellular component organization at cellul
biological regulation;biosynthetic process;cell differentiation;cellular biosynthetic process;cellular developmental process;cellular macromolecule biosynthetic process;cellular macromolecule metabolic process;cellular metab
actin cytoskeleton organization;actin filament organization;actin filament polymerization;actin filament-based process;actin polymerization or depolymerization;biological regulation;cellular component assembly;cellular compo
biological regulation;calcium ion homeostasis;calcium ion transport;calcium ion transport into cytosol;cation homeostasis;cation transport;cellular calcium ion homeostasis;cellular cation homeostasis;cellular chemical homeo
biosynthetic process;cellular biosynthetic process;cellular macromolecule biosynthetic process;cellular macromolecule metabolic process;cellular metabolic process;cellular nitrogen compound metabolic process;cellular pro
biological regulation;blood coagulation;cell activation;cellular process;coagulation;establishment of localization;establishment of localization in cell;exocytosis;hemostasis;multicellular organismal process;neurological system
actin cytoskeleton organization;actin filament-based process;anatomical structure formation involved in morphogenesis;anatomical structure morphogenesis;axon guidance;biological regulation;cell part morphogenesis;cell p
axon guidance;biological regulation;cell cycle;cell cycle phase;cell cycle process;cell division;cellular component organization;cellular component organization at cellular level;cellular component organization or biogenesis;ce
biosynthetic process;cellular biosynthetic process;cellular lipid metabolic process;cellular macromolecule metabolic process;cellular metabolic process;cellular process;cellular protein metabolic process;cellular response to c
establishment of localization;establishment of localization in cell;establishment of RNA localization;intracellular transport;mRNA export from nucleus;mRNA transport;nuclear export;nuclear transport;nucleic acid transport;nuc
biological regulation;cation homeostasis;cation transport;cell surface receptor linked signaling pathway;cellular cation homeostasis;cellular chemical homeostasis;cellular component organization;cellular component organizat
biological regulation;cellular component assembly;cellular component assembly at cellular level;cellular component organization;cellular component organization at cellular level;cellular component organization or biogenesis;
alpha-beta T cell activation;alpha-beta T cell differentiation;anatomical structure development;biological regulation;biosynthetic process;CD4-positive, alpha-beta T cell activation;CD4-positive, alpha-beta T cell differentiation
acetyl-CoA catabolic process;acetyl-CoA metabolic process;carbohydrate metabolic process;catabolic process;cellular carbohydrate metabolic process;cellular catabolic process;cellular metabolic process;cellular process;c
anatomical structure development;biological regulation;cell cycle;cell differentiation;cell division;cell proliferation;cellular component organization;cellular component organization at cellular level;cellular component organizatio
biological regulation;biosynthetic process;cellular biosynthetic process;cellular component assembly;cellular component assembly at cellular level;cellular component organization;cellular component organization at cellular le
catabolic process;cellular catabolic process;cellular macromolecule catabolic process;cellular macromolecule metabolic process;cellular metabolic process;cellular process;ER-associated protein catabolic process;macromo
anatomical structure development;biological regulation;cellular metabolic process;cellular process;developmental process;electron transport chain;generation of precursor metabolites and energy;metabolic process;mitochon
assembly of spliceosomal tri-snRNP;biological regulation;cellular component assembly;cellular component assembly at cellular level;cellular component organization;cellular component organization at cellular level;cellular co
anatomical structure development;biological regulation;catabolic process;cellular catabolic process;cellular macromolecule catabolic process;cellular macromolecule metabolic process;cellular metabolic process;cellular nitro
cellular component assembly;cellular component assembly at cellular level;cellular component organization;cellular component organization at cellular level;cellular component organization or biogenesis;cellular component c
acidic amino acid transport;alcohol biosynthetic process;alcohol metabolic process;amine transport;amino acid transport;anion transport;aspartate transport;biosynthetic process;carbohydrate biosynthetic process;carbohyc
cellular macromolecule metabolic process;cellular metabolic process;cellular nitrogen compound metabolic process;cellular process;macromolecule metabolic process;metabolic process;ncRNA metabolic process;ncRNA pr
biological regulation;biosynthetic process;cellular biosynthetic process;cellular component assembly;cellular component assembly at cellular level;cellular component organization;cellular component organization at cellular le
biological regulation;biosynthetic process;cellular biosynthetic process;cellular metabolic process;cellular process;chromosome segregation;cofactor biosynthetic process;cofactor metabolic process;iron-sulfur cluster assem
biosynthetic process;cellular biosynthetic process;cellular macromolecule biosynthetic process;cellular macromolecule metabolic process;cellular metabolic process;cellular process;cellular protein metabolic process;cotrans
amine catabolic process;amine metabolic process;arginine catabolic process;arginine metabolic process;biological regulation;carboxylic acid catabolic process;carboxylic acid metabolic process;catabolic process;cellular am
alcohol biosynthetic process;alcohol metabolic process;amine metabolic process;amino sugar biosynthetic process;amino sugar metabolic process;biosynthetic process;carbohydrate biosynthetic process;carbohydrate meta
catabolic process;cellular catabolic process;cellular macromolecule catabolic process;cellular macromolecule metabolic process;cellular metabolic process;cellular process;ER-associated protein catabolic process;macromo
anatomical structure development;anatomical structure formation involved in morphogenesis;anatomical structure morphogenesis;appendage morphogenesis;biological regulation;cell morphogenesis;cell morphogenesis invo
amine biosynthetic process;amine catabolic process;amine metabolic process;behavior;behavioral interaction between organisms;biological regulation;biosynthetic process;carboxylic acid biosynthetic process;carboxylic aci
biological regulation;cellular macromolecule metabolic process;cellular metabolic process;cellular process;cellular protein metabolic process;cellular response to chemical stimulus;cellular response to organic substance;cellu
biological regulation;catabolic process;cell proliferation;cellular catabolic process;cellular macromolecule catabolic process;cellular macromolecule metabolic process;cellular metabolic process;cellular nitrogen compound m
cellular metabolic process;cellular process;electron transport chain;generation of precursor metabolites and energy;metabolic process;mitochondrial electron transport, NADH to ubiquinone;oxidation-reduction process;respi
cellular metabolic process;cellular process;electron transport chain;generation of precursor metabolites and energy;metabolic process;mitochondrial electron transport, NADH to ubiquinone;oxidation-reduction process;respi
cellular metabolic process;cellular process;electron transport chain;generation of precursor metabolites and energy;metabolic process;mitochondrial electron transport, NADH to ubiquinone;oxidation-reduction process;respi
cellular component assembly;cellular component assembly at cellular level;cellular component organization;cellular component organization at cellular level;cellular component organization or biogenesis;cellular component c
anterograde axon cargo transport;antigen processing and presentation;antigen processing and presentation of exogenous antigen;antigen processing and presentation of exogenous peptide antigen;antigen processing and p
cellular metabolic process;cellular process;electron transport chain;generation of precursor metabolites and energy;metabolic process;mitochondrial electron transport, NADH to ubiquinone;oxidation-reduction process;respi
cellular macromolecule metabolic process;cellular metabolic process;cellular process;cellular protein metabolic process;chaperone-mediated protein folding;macromolecule metabolic process;macromolecule modification;me
cell cycle;cell cycle process;cell division;cellular component organization;cellular component organization at cellular level;cellular component organization or biogenesis;cellular component organization or biogenesis at cellula

biological regulation;cellular component organization;cellular component organization at cellular level;cellular component organization or biogenesis;cellular component organization or biogenesis at cellular level;cellular macr
biological regulation;cellular process;cellular response to stimulus;defense response;establishment of localization;establishment of localization in cell;establishment of protein localization;immune response;immune system pr
ameboidal cell migration;anatomical structure development;anatomical structure morphogenesis;androgen metabolic process;biological regulation;biosynthetic process;carboxylic acid metabolic process;catabolic process;ce
cellular macromolecule metabolic process;cellular metabolic process;cellular nitrogen compound metabolic process;cellular process;macromolecule metabolic process;metabolic process;ncRNA metabolic process;ncRNA pr
acyl-CoA biosynthetic process;acyl-CoA metabolic process;acylglycerol biosynthetic process;acylglycerol metabolic process;anatomical structure development;biological regulation;biosynthetic process;brain development;c
activation of protein kinase activity;biological regulation;cellular hypotonic response;cellular macromolecule metabolic process;cellular metabolic process;cellular process;cellular protein metabolic process;cellular response t
alcohol biosynthetic process;alcohol metabolic process;biosynthetic process;cellular biosynthetic process;cellular lipid metabolic process;cellular metabolic process;cellular process;cholesterol biosynthetic process;cholester
biological regulation;cellular component assembly;cellular component organization;cellular component organization or biogenesis;macromolecular complex assembly;macromolecular complex subunit organization;positive re
cellular macromolecule metabolic process;cellular metabolic process;cellular process;cellular protein metabolic process;cellular response to heat;cellular response to stimulus;cellular response to stress;macromolecule metab
biological regulation;multicellular organismal process;negative regulation of biological process;negative regulation of cellular component organization;negative regulation of cellular process;negative regulation of cytoskeleton
amine catabolic process;amine metabolic process;arginine catabolic process;arginine metabolic process;biological regulation;biosynthetic process;carboxylic acid catabolic process;carboxylic acid metabolic process;catabo
biological regulation;cell redox homeostasis;cellular homeostasis;cellular process;homeostatic process;regulation of biological process;regulation of biological quality;regulation of cellular process
cellular metabolic process;cellular process;electron transport chain;generation of precursor metabolites and energy;metabolic process;mitochondrial electron transport, NADH to ubiquinone;oxidation-reduction process;respi
biological regulation;cell differentiation;cellular developmental process;cellular process;developmental process;multicellular organismal development;multicellular organismal process;regulation of biological process;regulation
cellular macromolecule metabolic process;cellular metabolic process;cellular process;cellular protein metabolic process;establishment of localization;establishment of localization in cell;establishment of protein localization;es
biological regulation;cellular component assembly;cellular component organization;cellular component organization at cellular level;cellular component organization or biogenesis;cellular component organization or biogenesi
alcohol catabolic process;alcohol metabolic process;anatomical structure development;carbohydrate catabolic process;carbohydrate metabolic process;carboxylic acid metabolic process;catabolic process;cell communicati
amine metabolic process;biological regulation;biosynthetic process;carboxylic acid metabolic process;cell redox homeostasis;cellular amine metabolic process;cellular amino acid metabolic process;cellular biosynthetic proc
alcohol biosynthetic process;alcohol metabolic process;biosynthetic process;carbohydrate biosynthetic process;carbohydrate metabolic process;cell differentiation;cellular biosynthetic process;cellular carbohydrate biosynth
acute inflammatory response;acute-phase response;aging;amide biosynthetic process;amine biosynthetic process;amine metabolic process;anatomical structure development;arginine biosynthetic process;arginine metaboli
biological regulation;blood coagulation;blood coagulation, intrinsic pathway;cell activation;cell differentiation;cellular component disassembly;cellular component disassembly at cellular level;cellular component organization;c
antigen processing and presentation;antigen processing and presentation of peptide antigen;antigen processing and presentation of peptide antigen via MHC class I;biological regulation;immune response;immune system pr
biological regulation;cation homeostasis;cation transport;cell differentiation;cellular cation homeostasis;cellular chemical homeostasis;cellular developmental process;cellular homeostasis;cellular ion homeostasis;cellular iron
biological regulation;negative regulation of binding;negative regulation of biological process;negative regulation of molecular function;negative regulation of protein binding;negative regulation of reproductive process;negative
biological adhesion;biological regulation;cell adhesion;cell adhesion mediated by integrin;cell differentiation;cell-matrix adhesion;cell-substrate adhesion;cellular component organization;cellular component organization at cel
acylglycerol metabolic process;aerobic respiration;aging;alcohol metabolic process;anatomical structure development;biological regulation;catabolic process;cell differentiation;cellular aromatic compound metabolic process;
alcohol metabolic process;amine metabolic process;aminoglycan catabolic process;aminoglycan metabolic process;carbohydrate catabolic process;carbohydrate metabolic process;catabolic process;cellular carbohydrate c
actin cytoskeleton organization;actin filament organization;actin filament-based process;alcohol biosynthetic process;alcohol catabolic process;alcohol metabolic process;anatomical structure homeostasis;ATP biosynthetic p
acid secretion;alpha-beta T cell activation;alpha-beta T cell differentiation;anatomical structure development;arachidonic acid secretion;biological regulation;carboxylic acid transport;cell activation;cell differentiation;cell surfa
alcohol biosynthetic process;alcohol metabolic process;apoptosis;biological regulation;biosynthetic process;carbohydrate biosynthetic process;carbohydrate metabolic process;cell death;cellular biosynthetic process;cellula
amide biosynthetic process;amine biosynthetic process;amine catabolic process;amine metabolic process;arginine biosynthetic process;arginine biosynthetic process via ornithine;arginine catabolic process;arginine metaboli
anatomical structure homeostasis;biological adhesion;biological regulation;cell adhesion;cell surface receptor linked signaling pathway;cell-cell adhesion;cellular component movement;cellular process;cellular response to ch
2'-deoxyribonucleotide biosynthetic process;2'-deoxyribonucleotide metabolic process;aging;anatomical structure development;anatomical structure morphogenesis;biological regulation;biosynthetic process;cartilage devel
biosynthetic process;carbohydrate metabolic process;cellular biosynthetic process;cellular carbohydrate metabolic process;cellular macromolecule biosynthetic process;cellular macromolecule metabolic process;cellular me
activation of MAPKK activity;activation of protein kinase activity;adenosine receptor signaling pathway;biological regulation;blood coagulation;cAMP-mediated signaling;cell activation;cell communication;cell cycle;cell divisio
biological regulation;negative regulation of biological process;negative regulation of cell proliferation;negative regulation of cellular process;regulation of biological process;regulation of cell proliferation;regulation of cellular pr
biological regulation;cardiac muscle contraction;cation homeostasis;cation transport;cellular cation homeostasis;cellular chemical homeostasis;cellular homeostasis;cellular ion homeostasis;cellular metal ion homeostasis;cell
adenine transport;biological regulation;cellular metabolic process;cellular process;chromosome segregation;energy derivation by oxidation of organic compounds;energy reserve metabolic process;establishment of localizati
biological regulation;biosynthetic process;catabolic process;cell surface receptor linked signaling pathway;cellular biosynthetic process;cellular catabolic process;cellular macromolecule biosynthetic process;cellular macrom
cellular metabolic process;cellular process;dephosphorylation;metabolic process;phosphate-containing compound metabolic process;phosphorus metabolic process
acute inflammatory response;acute inflammatory response to antigenic stimulus;adhesion to host;adhesion to other organism involved in symbiotic interaction;aging;anatomical structure development;antigen processing and
anatomical structure morphogenesis;biological regulation;cell cycle;cell surface receptor linked signaling pathway;cellular component organization;cellular component organization at cellular level;cellular component organiza
actin cytoskeleton organization;actin filament-based process;actomyosin structure organization;biological regulation;cell differentiation;cell differentiation involved in embryonic placenta development;cell surface receptor link
activation of immune response;activation of innate immune response;activation of MAPK activity;activation of MAPKK activity;activation of protein kinase activity;aging;anaphase-promoting complex-dependent proteasomal
alcohol biosynthetic process;alcohol metabolic process;biological regulation;biosynthetic process;carbohydrate biosynthetic process;carbohydrate metabolic process;cellular biosynthetic process;cellular carbohydrate biosy
5-phosphoribose 1-diphosphate biosynthetic process;5-phosphoribose 1-diphosphate metabolic process;alcohol biosynthetic process;alcohol metabolic process;biological regulation;biosynthetic process;carbohydrate bios
alcohol biosynthetic process;alcohol metabolic process;aldehyde biosynthetic process;aldehyde catabolic process;anatomical structure formation involved in morphogenesis;angiogenesis;behavior;biological regulation;biosy
aging;ATP-dependent chromatin remodeling;biological regulation;cell aging;cell cycle process;cellular component assembly;cellular component assembly at cellular level;cellular component organization;cellular component o
anatomical structure formation involved in morphogenesis;anatomical structure morphogenesis;angiogenesis;antigen processing and presentation;antigen processing and presentation of exogenous antigen;antigen processi
anatomical structure development;anatomical structure morphogenesis;behavior;biological regulation;blood coagulation;cellular component organization;cellular component organization at cellular level;cellular component or
acylglycerol metabolic process;aging;amine metabolic process;anatomical structure development;anatomical structure formation involved in morphogenesis;anatomical structure morphogenesis;angiogenesis;angiogenesis in
4-hydroxyproline metabolic process;amine metabolic process;biological regulation;carboxylic acid metabolic process;cell redox homeostasis;cellular amine metabolic process;cellular amino acid metabolic process;cellular c
antigen processing and presentation;antigen processing and presentation of exogenous antigen;antigen processing and presentation of exogenous peptide antigen;antigen processing and presentation of exogenous peptide
anatomical structure formation involved in morphogenesis;angiogenesis;biological regulation;body fluid secretion;cellular component assembly;cellular component assembly at cellular level;cellular component organization;ce
amine metabolic process;aminoglycan catabolic process;aminoglycan metabolic process;anatomical structure development;astrocyte cell migration;axon ensheathment;behavior;biological regulation;biosynthetic process;ca
amine metabolic process;amino acid activation;biological regulation;carboxylic acid metabolic process;cellular amine metabolic process;cellular amino acid metabolic process;cellular component assembly;cellular componen
activation of immune response;activation of innate immune response;anatomical structure development;biological regulation;cell differentiation;cellular component disassembly;cellular component disassembly at cellular leve
anatomical structure morphogenesis;axon guidance;biological adhesion;biological regulation;cell adhesion;cell differentiation;cell morphogenesis;cell morphogenesis involved in differentiation;cell projection organization;cell-
biological regulation;calcium ion transport;cation transport;cellular component assembly;cellular component organization;cellular component organization or biogenesis;cellular process;divalent inorganic cation transport;diva
activation of immune response;adaptive immune response;adaptive immune response based on somatic recombination of immune receptors built from immunoglobulin superfamily domains;biological regulation;calcium ion
amine transport;amino acid import;amino acid transport;aromatic amino acid transport;biological regulation;blood coagulation;branched-chain aliphatic amino acid transport;calcium ion transport;carbohydrate metabolic pro
amine biosynthetic process;amine metabolic process;anatomical structure development;asparagine biosynthetic process;asparagine metabolic process;aspartate family amino acid biosynthetic process;aspartate family amin
acetyl-CoA biosynthetic process;acetyl-CoA biosynthetic process from pyruvate;acetyl-CoA catabolic process;acetyl-CoA metabolic process;alcohol catabolic process;alcohol metabolic process;biological regulation;biosynt
anatomical structure formation involved in morphogenesis;angiogenesis;axon guidance;behavior;biological adhesion;biological regulation;blood coagulation;cell adhesion;cell adhesion mediated by integrin;cell differentiation;
anatomical structure morphogenesis;biological regulation;biosynthetic process;cellular biosynthetic process;cellular macromolecule biosynthetic process;cellular macromolecule metabolic process;cellular metabolic process;
actin filament-based movement;actin filament-based process;actin-mediated cell contraction;actin-myosin filament sliding;anatomical structure development;apoptosis;astrocyte development;astrocyte differentiation;Bergma
biological regulation;biosynthetic process;catabolic process;cellular biosynthetic process;cellular catabolic process;cellular component assembly;cellular component assembly at cellular level;cellular component biogenesis;c
viral reproduction
biological regulation;blood coagulation;cellular process;cellular response to stimulus;coagulation;hemostasis;multicellular organismal process;negative regulation of apoptosis;negative regulation of biological process;negative
biosynthetic process;cellular biosynthetic process;cellular component disassembly;cellular component disassembly at cellular level;cellular component organization;cellular component organization at cellular level;cellular co
activation of MAPK activity;amine metabolic process;aminoglycan catabolic process;aminoglycan metabolic process;anatomical structure development;anatomical structure morphogenesis;biological regulation;biosynthetic
alcohol biosynthetic process;alcohol metabolic process;biosynthetic process;carbohydrate biosynthetic process;carbohydrate metabolic process;cellular biosynthetic process;cellular carbohydrate biosynthetic process;cellul
apoptosis;B cell activation;B cell activation involved in immune response;B cell differentiation;biological regulation;carbohydrate homeostasis;cell activation;cell activation involved in immune response;cell death;cell different
actin cytoskeleton organization;actin filament-based movement;actin filament-based process;actin-mediated cell contraction;actin-myosin filament sliding;actomyosin structure organization;anatomical structure morphogene
biological regulation;cell differentiation;cellular developmental process;cellular process;cellular response to stimulus;developmental process;epithelial cell differentiation;negative regulation of apoptosis;negative regulation of
adult behavior;adult locomotory behavior;aging;anatomical structure development;anatomical structure morphogenesis;axonogenesis;behavior;biological regulation;catabolic process;cell communication;cell differentiation;ce
anatomical structure homeostasis;base-excision repair;biological regulation;biosynthetic process;cell differentiation;cell surface receptor linked signaling pathway;cellular biosynthetic process;cellular component organization
anatomical structure homeostasis;biological regulation;biosynthetic process;cell cycle;cell cycle process;cell proliferation;cellular biosynthetic process;cellular component organization;cellular component organization at cellu
apoptotic mitochondrial changes;biological regulation;cell surface receptor linked signaling pathway;cellular component organization;cellular component organization at cellular level;cellular component organization or biogen
biological regulation;cell surface receptor linked signaling pathway;cellular process;cellular response to chemical stimulus;cellular response to cytokine stimulus;cellular response to dsRNA;cellular response to exogenous ds
cellular metabolic process;cellular process;dephosphorylation;metabolic process;phosphate-containing compound metabolic process;phosphorus metabolic process
aging;alcohol biosynthetic process;alcohol metabolic process;anatomical structure morphogenesis;apoptosis;biosynthetic process;carbohydrate biosynthetic process;carbohydrate metabolic process;cell death;cell different
anatomical structure development;anatomical structure morphogenesis;biological regulation;biosynthetic process;cell part morphogenesis;cell projection morphogenesis;cell projection organization;cell surface receptor linke
alcohol metabolic process;anatomical structure development;anatomical structure homeostasis;anatomical structure morphogenesis;behavior;biological regulation;carbohydrate catabolic process;carbohydrate metabolic pro
biological regulation;cellular macromolecule metabolic process;cellular metabolic process;cellular process;cellular protein metabolic process;macromolecule metabolic process;macromolecule modification;metabolic process
actin cytoskeleton organization;actin filament-based process;activation of phospholipase C activity;activation of protein kinase A activity;activation of protein kinase activity;actomyosin structure organization;anatomical struc
activation of signaling protein activity involved in unfolded protein response;anatomical structure arrangement;anatomical structure development;biological regulation;blood coagulation;catabolic process;cell activation;cell c
anatomical structure development;anatomical structure morphogenesis;axon guidance;biological adhesion;biological regulation;cell adhesion;cell junction assembly;cell junction organization;cell migration;cell morphogenesis
biological regulation;biosynthetic process;carbohydrate biosynthetic process;carbohydrate metabolic process;carbohydrate transmembrane transport;carbohydrate transport;carboxylic acid metabolic process;cation transpo
carbohydrate metabolic process;carbohydrate transmembrane transport;carbohydrate transport;carboxylic acid metabolic process;cation transport;cellular ketone metabolic process;cellular metabolic process;cellular proces
biological regulation;biosynthetic process;body fluid secretion;cellular aromatic compound metabolic process;cellular biosynthetic process;cellular metabolic process;cellular nitrogen compound biosynthetic process;cellular
actin cytoskeleton organization;actin cytoskeleton reorganization;actin filament-based process;biological regulation;cell surface receptor linked signaling pathway;cellular component organization;cellular component organiza
alcohol biosynthetic process;alcohol catabolic process;alcohol metabolic process;amine metabolic process;anatomical structure development;biological regulation;biosynthetic process;carbohydrate biosynthetic process;ca
alcohol biosynthetic process;alcohol metabolic process;biological regulation;biosynthetic process;biotin metabolic process;carbohydrate biosynthetic process;carbohydrate metabolic process;carboxylic acid metabolic proc
10-formyltetrahydrofolate biosynthetic process;10-formyltetrahydrofolate metabolic process;amine biosynthetic process;amine metabolic process;anatomical structure development;anatomical structure formation involved in
anatomical structure development;anatomical structure morphogenesis;biological regulation;cell cycle;cell cycle process;cell division;cellular component organization;cellular component organization at cellular level;cellular c
active induction of host immune response by virus;adenine nucleotide transport;ADP transport;apoptosis;ATP transport;biological regulation;cell death;cellular macromolecule metabolic process;cellular metabolic process;ce
anatomical structure development;biosynthetic process;cell activation;cell proliferation;cellular aromatic compound metabolic process;cellular biosynthetic process;cellular component assembly;cellular component organizat
biological regulation;biosynthetic process;carbohydrate metabolic process;carbohydrate transport;cell cycle;cell cycle checkpoint;cell cycle phase;cell cycle process;cell division;cell surface receptor linked signaling pathway
amine metabolic process;anatomical structure development;anion homeostasis;biological regulation;carboxylic acid metabolic process;cellular amine metabolic process;cellular amino acid metabolic process;cellular anion h
biological regulation;cell activation;cell activation involved in immune response;cellular component organization;cellular component organization or biogenesis;cellular membrane organization;cellular process;defense respons
amine catabolic process;amine metabolic process;branched chain family amino acid catabolic process;branched chain family amino acid metabolic process;carboxylic acid catabolic process;carboxylic acid metabolic proce
amine metabolic process;carboxylic acid metabolic process;cellular amine metabolic process;cellular amino acid metabolic process;cellular ketone metabolic process;cellular metabolic process;cellular nitrogen compound m
anatomical structure development;anatomical structure homeostasis;biological regulation;biosynthetic process;brain development;cellular biosynthetic process;cellular component organization;cellular component organizatio
anatomical structure development;anatomical structure homeostasis;biological regulation;biosynthetic process;brain development;cell differentiation;cell proliferation;cellular biosynthetic process;cellular component organiza
2'-deoxyribonucleotide metabolic process;base-excision repair;base-excision repair, AP site formation;biological regulation;catabolic process;cellular catabolic process;cellular macromolecule catabolic process;cellular macr
biological regulation;catabolic process;cellular catabolic process;cellular macromolecule catabolic process;cellular macromolecule metabolic process;cellular metabolic process;cellular nitrogen compound metabolic process
biological regulation;biosynthetic process;cell differentiation;cellular biosynthetic process;cellular developmental process;cellular macromolecule biosynthetic process;cellular macromolecule metabolic process;cellular metab
biological regulation;cell redox homeostasis;cellular homeostasis;cellular macromolecule metabolic process;cellular metabolic process;cellular process;cellular protein metabolic process;cellular response to stimulus;cellular
4-hydroxyproline metabolic process;amine metabolic process;carboxylic acid metabolic process;cellular amine metabolic process;cellular amino acid metabolic process;cellular component organization;cellular component or
anatomical structure development;bone development;developmental process
alcohol metabolic process;anatomical structure development;biosynthetic process;carbohydrate biosynthetic process;carbohydrate metabolic process;cellular biosynthetic process;cellular carbohydrate biosynthetic process;
biological regulation;blood coagulation;cellular component organization;cellular component organization at cellular level;cellular component organization or biogenesis;cellular component organization or biogenesis at cellular
aging;alcohol biosynthetic process;alcohol metabolic process;anatomical structure development;anatomical structure morphogenesis;biosynthetic process;carbohydrate biosynthetic process;carbohydrate metabolic process
aromatic compound biosynthetic process;biosynthetic process;cellular aromatic compound metabolic process;cellular biosynthetic process;cellular metabolic process;cellular nitrogen compound biosynthetic process;cellular
biological adhesion;cell adhesion;cellular process
biological regulation;carbohydrate metabolic process;cellular carbohydrate metabolic process;cellular macromolecule metabolic process;cellular metabolic process;cellular process;cellular protein metabolic process;cellular
anatomical structure morphogenesis;developmental process
alcohol metabolic process;carbohydrate metabolic process;cell death;cellular carbohydrate metabolic process;cellular metabolic process;cellular process;death;glucose metabolic process;hexose metabolic process;metaboli
actin cytoskeleton organization;actin filament organization;actin filament-based process;actin rod assembly;activation of immune response;activation of innate immune response;biological regulation;catabolic process;cellular
beta-amyloid metabolic process;biological regulation;bradykinin catabolic process;catabolic process;cell surface receptor linked signaling pathway;cellular catabolic process;cellular component assembly;cellular component
amine metabolic process;amino acid activation;aspartyl-tRNA aminoacylation;biosynthetic process;carboxylic acid metabolic process;cellular amine metabolic process;cellular amino acid metabolic process;cellular biosynthe
amine biosynthetic process;amine catabolic process;amine metabolic process;biological regulation;biosynthetic process;carboxylic acid biosynthetic process;carboxylic acid catabolic process;carboxylic acid metabolic proc
acute inflammatory response;alcohol metabolic process;amine metabolic process;aminoglycan biosynthetic process;aminoglycan metabolic process;anatomical structure development;anatomical structure formation involved
biological regulation;biosynthetic process;cell death;cellular biosynthetic process;cellular macromolecule biosynthetic process;cellular macromolecule metabolic process;cellular metabolic process;cellular nitrogen compound
activation of immune response;adaptive immune response;adaptive immune response based on somatic recombination of immune receptors built from immunoglobulin superfamily domains;biological regulation;cellular proce

aging;biological regulation;biosynthetic process;cell communication;cell-cell signaling;cellular biosynthetic process;cellular metabolic process;cellular nitrogen compound biosynthetic process;cellular nitrogen compound met
amine metabolic process;aminoglycan catabolic process;aminoglycan metabolic process;carbohydrate catabolic process;carbohydrate metabolic process;catabolic process;cellular catabolic process;cellular metabolic proce
biological regulation;biosynthetic process;catabolic process;cellular biosynthetic process;cellular catabolic process;cellular component disassembly;cellular component disassembly at cellular level;cellular component organiz
adherens junction organization;anatomical structure development;anatomical structure morphogenesis;apoptosis;cardiac muscle tissue morphogenesis;cell death;cell differentiation;cell junction organization;cell-cell junction
anatomical structure homeostasis;base-excision repair;biological regulation;biosynthetic process;catabolic process;cell cycle;cell cycle checkpoint;cell cycle process;cellular biosynthetic process;cellular catabolic process;ce
biological regulation;carbohydrate metabolic process;cellular carbohydrate metabolic process;cellular macromolecule metabolic process;cellular metabolic process;cellular process;cellular protein metabolic process;cellular r
behavior;cognition;learning or memory;memory;multicellular organismal process;neurological system process;response to stimulus;system process
arachidonic acid metabolic process;carboxylic acid metabolic process;cell differentiation;cellular developmental process;cellular ketone metabolic process;cellular lipid metabolic process;cellular metabolic process;cellular pr
anatomical structure development;biological adhesion;biological regulation;blood coagulation;calcium ion homeostasis;calcium ion transmembrane transport;calcium ion transport;calcium-mediated signaling;cation homeost
anatomical structure development;anatomical structure morphogenesis;axon guidance;biological adhesion;biological regulation;blood coagulation;cell adhesion;cell adhesion mediated by integrin;cell differentiation;cell migra
activation of immune response;activation of innate immune response;activation of phospholipase C activity;activation of protein kinase A activity;activation of protein kinase activity;acylglycerol catabolic process;acylglycerol
anatomical structure development;anatomical structure formation involved in morphogenesis;biological regulation;blastocyst development;cellular process;cellular response to acid;cellular response to amine stimulus;cellular
acute inflammatory response;acute-phase response;alpha-beta T cell activation;anatomical structure development;biological regulation;biosynthetic process;brown fat cell differentiation;CD4-positive, alpha-beta T cell activa
amine metabolic process;B cell activation;B cell proliferation;biosynthetic process;carboxylic acid metabolic process;cell activation;cell proliferation;cellular amine metabolic process;cellular amino acid metabolic process;cell
aging;anatomical structure development;anatomical structure morphogenesis;artery morphogenesis;biological adhesion;biological regulation;blood vessel morphogenesis;BMP signaling pathway;bone development;branching
biological regulation;biosynthetic process;cell growth;cellular biosynthetic process;cellular macromolecule biosynthetic process;cellular macromolecule metabolic process;cellular metabolic process;cellular nitrogen compoun
alcohol catabolic process;alcohol metabolic process;biological regulation;carbohydrate catabolic process;carbohydrate metabolic process;carbohydrate phosphorylation;catabolic process;cellular carbohydrate catabolic pro
apoptosis;biological regulation;cell death;cell differentiation;cell proliferation;cellular developmental process;cellular macromolecule metabolic process;cellular metabolic process;cellular process;cellular protein metabolic pro
biosynthetic process;catabolic process;cellular biosynthetic process;cellular catabolic process;cellular component biogenesis;cellular component biogenesis at cellular level;cellular component disassembly;cellular componen
biosynthetic process;catabolic process;cellular biosynthetic process;cellular catabolic process;cellular component biogenesis;cellular component biogenesis at cellular level;cellular component disassembly;cellular componen
adherens junction assembly;adherens junction organization;anatomical structure morphogenesis;apical junction assembly;axon extension;biological adhesion;biological regulation;blood coagulation;cell activation;cell adhesio
biosynthetic process;catabolic process;cellular biosynthetic process;cellular catabolic process;cellular component disassembly;cellular component disassembly at cellular level;cellular component organization;cellular compo
alcohol biosynthetic process;alcohol catabolic process;alcohol metabolic process;biological regulation;biosynthetic process;carbohydrate biosynthetic process;carbohydrate catabolic process;carbohydrate metabolic proces
anatomical structure homeostasis;anatomical structure morphogenesis;base-excision repair;biological regulation;biosynthetic process;cell cycle;cell division;cellular biosynthetic process;cellular component organization;cellu
anatomical structure development;base-excision repair;cellular macromolecule metabolic process;cellular metabolic process;cellular nitrogen compound metabolic process;cellular process;cellular response to stimulus;cellula
anatomical structure formation involved in morphogenesis;angiogenesis;developmental process
amine metabolic process;aminoglycan metabolic process;biological regulation;carbohydrate metabolic process;glycosaminoglycan metabolic process;hyaluronan metabolic process;macromolecule metabolic process;metab
anatomical structure development;biosynthetic process;cell proliferation;cellular biosynthetic process;cellular macromolecule biosynthetic process;cellular macromolecule metabolic process;cellular metabolic process;cellular
autophagy;behavior;behavioral interaction between organisms;biological regulation;calcium ion homeostasis;catabolic process;cation homeostasis;cell differentiation;cell proliferation;cell volume homeostasis;cellular calcium
biological regulation;biosynthetic process;cellular biosynthetic process;cellular macromolecule biosynthetic process;cellular macromolecule metabolic process;cellular metabolic process;cellular nitrogen compound metabolic
antigen processing and presentation;biological regulation;cellular component organization;cellular component organization or biogenesis;cellular process;cellular response to stimulus;establishment of localization;establishme
antigen processing and presentation;biological regulation;cellular component movement;cellular localization;cellular macromolecule localization;cellular macromolecule metabolic process;cellular metabolic process;cellular pr
activation of immune response;activation of innate immune response;anaphase-promoting complex-dependent proteasomal ubiquitin-dependent protein catabolic process;antigen processing and presentation;antigen proces
biological regulation;negative regulation of apoptosis;negative regulation of biological process;negative regulation of catalytic activity;negative regulation of cell death;negative regulation of cellular process;negative regulation
anatomical structure development;anatomical structure morphogenesis;axon guidance;biological adhesion;biological regulation;biosynthetic process;blood vessel development;cell adhesion;cell migration;cell motility;cellular
ATP hydrolysis coupled proton transport;ATP metabolic process;biological regulation;cation homeostasis;cation transport;cell surface receptor linked signaling pathway;cellular cation homeostasis;cellular chemical homeosta
biological adhesion;cell adhesion;cell-cell adhesion;cellular process;homotypic cell-cell adhesion;platelet aggregation
actin crosslink formation;actin cytoskeleton organization;actin cytoskeleton reorganization;actin filament organization;actin filament-based process;anatomical structure formation involved in morphogenesis;anatomical structu
acetyl-CoA catabolic process;acetyl-CoA metabolic process;biological regulation;carboxylic acid metabolic process;catabolic process;cation homeostasis;cellular catabolic process;cellular cation homeostasis;cellular chemic
acetyl-CoA catabolic process;acetyl-CoA metabolic process;aerobic respiration;carboxylic acid metabolic process;catabolic process;cellular catabolic process;cellular ketone metabolic process;cellular metabolic process;cel
anatomical structure development;biological regulation;cell proliferation;cellular macromolecule metabolic process;cellular metabolic process;cellular nitrogen compound metabolic process;cellular process;cellular protein me
cellular component organization;cellular component organization at cellular level;cellular component organization or biogenesis;cellular component organization or biogenesis at cellular level;cellular process;embryo implantat
biological regulation;biosynthetic process;cell differentiation;cellular biosynthetic process;cellular developmental process;cellular macromolecule biosynthetic process;cellular macromolecule metabolic process;cellular metab
arachidonic acid metabolic process;biological regulation;biosynthetic process;carboxylic acid biosynthetic process;carboxylic acid metabolic process;cellular biosynthetic process;cellular ketone metabolic process;cellular lip
anatomical structure development;biological regulation;bone development;cellular component organization;cellular component organization at cellular level;cellular component organization or biogenesis;cellular component o
amine metabolic process;amino acid activation;anatomical structure formation involved in morphogenesis;angiogenesis;biological regulation;biosynthetic process;carboxylic acid metabolic process;cellular amine metabolic p
amine metabolic process;carboxylic acid metabolic process;cellular amine metabolic process;cellular amino acid metabolic process;cellular ketone metabolic process;cellular metabolic process;cellular modified amino acid m
biological regulation;biosynthetic process;catabolic process;cell surface receptor linked signaling pathway;cellular biosynthetic process;cellular catabolic process;cellular macromolecule biosynthetic process;cellular macrom
biological regulation;blood coagulation;calcium ion homeostasis;calcium ion transmembrane transport;calcium ion transport;cation homeostasis;cation transport;cellular calcium ion homeostasis;cellular cation homeostasis;c
anatomical structure development;biosynthetic process;cell cycle;cell proliferation;cell proliferation in forebrain;cellular aromatic compound metabolic process;cellular biosynthetic process;cellular component assembly;cellula
anatomical structure development;biosynthetic process;cellular biosynthetic process;cellular macromolecule metabolic process;cellular metabolic process;cellular nitrogen compound metabolic process;cellular process;centr
biosynthetic process;cell cycle;cell cycle process;cellular biosynthetic process;cellular macromolecule biosynthetic process;cellular macromolecule metabolic process;cellular metabolic process;cellular nitrogen compound m
biosynthetic process;catabolic process;cellular biosynthetic process;cellular catabolic process;cellular metabolic process;cellular nitrogen compound catabolic process;cellular nitrogen compound metabolic process;cellular
activation of immune response;activation of innate immune response;anaphase-promoting complex-dependent proteasomal ubiquitin-dependent protein catabolic process;antigen processing and presentation;antigen proces
activation of immune response;activation of innate immune response;anaphase-promoting complex-dependent proteasomal ubiquitin-dependent protein catabolic process;antigen processing and presentation;antigen proces
biological regulation;biosynthetic process;cellular biosynthetic process;cellular component organization;cellular component organization at cellular level;cellular component organization or biogenesis;cellular component orga
biosynthetic process;catabolic process;cellular biosynthetic process;cellular catabolic process;cellular component disassembly;cellular component disassembly at cellular level;cellular component organization;cellular compo
anatomical structure morphogenesis;biological regulation;cell morphogenesis;cell morphogenesis involved in differentiation;cellular component morphogenesis;cellular component organization;cellular component organizatio
amine metabolic process;amino acid activation;biosynthetic process;carboxylic acid metabolic process;cellular amine metabolic process;cellular amino acid metabolic process;cellular biosynthetic process;cellular ketone me
biological regulation;cellular component assembly;cellular component organization;cellular component organization or biogenesis;macromolecular complex assembly;macromolecular complex subunit organization;positive re
ADP biosynthetic process;ADP metabolic process;AMP metabolic process;anatomical structure development;ATP metabolic process;biosynthetic process;brain development;cellular biosynthetic process;cellular metabolic pr
biosynthetic process;catabolic process;cellular biosynthetic process;cellular catabolic process;cellular component disassembly;cellular component disassembly at cellular level;cellular component organization;cellular compo
anatomical structure development;anatomical structure homeostasis;base-excision repair;biological regulation;biosynthetic process;catabolic process;cell cycle;cell cycle checkpoint;cell cycle phase;cell cycle process;cellula
amide biosynthetic process;amine biosynthetic process;amine catabolic process;amine metabolic process;anatomical structure development;anatomical structure morphogenesis;arginine biosynthetic process;arginine metab
cell cycle process;cell division;cellular component movement;cellular component organization;cellular component organization at cellular level;cellular component organization or biogenesis;cellular component organization or
aging;antigen processing and presentation;antigen processing and presentation of exogenous antigen;antigen processing and presentation of exogenous peptide antigen;antigen processing and presentation of exogenous pe
activation of immune response;activation of innate immune response;anaphase-promoting complex-dependent proteasomal ubiquitin-dependent protein catabolic process;antigen processing and presentation;antigen proces
carboxylic acid catabolic process;carboxylic acid metabolic process;catabolic process;cellular catabolic process;cellular component organization;cellular component organization at cellular level;cellular component organizati
anatomical structure development;blood vessel development;cellular component assembly;cellular component assembly at cellular level;cellular component organization;cellular component organization at cellular level;cellula
biological regulation;biosynthetic process;cellular biosynthetic process;cellular macromolecule biosynthetic process;cellular macromolecule metabolic process;cellular metabolic process;cellular nitrogen compound metabolic
anatomical structure development;anatomical structure formation involved in morphogenesis;anatomical structure morphogenesis;angiogenesis;axon guidance;axonal fasciculation;axonogenesis;behavior;biological regulatio
2'-deoxyribonucleotide metabolic process;base-excision repair;base-excision repair, AP site formation;catabolic process;cellular catabolic process;cellular macromolecule catabolic process;cellular macromolecule metabolic
anatomical structure development;anatomical structure morphogenesis;appendage morphogenesis;biological regulation;biosynthetic process;carboxylic acid metabolic process;cellular biosynthetic process;cellular hormone
biological regulation;biosynthetic process;cellular biosynthetic process;cellular macromolecule biosynthetic process;cellular macromolecule metabolic process;cellular metabolic process;cellular nitrogen compound metabolic
biological regulation;biosynthetic process;catabolic process;cellular biosynthetic process;cellular catabolic process;cellular macromolecule metabolic process;cellular metabolic process;cellular nitrogen compound metabolic
antigen processing and presentation;antigen processing and presentation of exogenous antigen;antigen processing and presentation of exogenous peptide antigen;antigen processing and presentation of exogenous peptide
amine metabolic process;AMP biosynthetic process;AMP metabolic process;aspartate family amino acid metabolic process;aspartate metabolic process;biosynthetic process;carboxylic acid metabolic process;cellular amine
biological regulation;cellular component organization;cellular component organization or biogenesis;cellular macromolecule metabolic process;cellular membrane organization;cellular metabolic process;cellular process;cellula
aerobic respiration;AMP biosynthetic process;AMP metabolic process;biosynthetic process;cellular aromatic compound metabolic process;cellular biosynthetic process;cellular component assembly;cellular component orga
biological regulation;negative regulation of catalytic activity;negative regulation of endopeptidase activity;negative regulation of hydrolase activity;negative regulation of molecular function;negative regulation of peptidase acti
amine transport;amino acid transport;carboxylic acid transport;cellular process;establishment of localization;ion transport;nitrogen compound transport;organic acid transport;organic substance transport;transmembrane tran
alcohol catabolic process;alcohol metabolic process;carbohydrate metabolic process;catabolic process;ethanol catabolic process;ethanol metabolic process;metabolic process;primary alcohol catabolic process;primary alco
acetyl-CoA catabolic process;acetyl-CoA metabolic process;anatomical structure development;carboxylic acid metabolic process;catabolic process;cellular catabolic process;cellular ketone metabolic process;cellular metab
acylglycerol catabolic process;acylglycerol metabolic process;amide biosynthetic process;amine biosynthetic process;amine catabolic process;amine metabolic process;anatomical structure development;anion homeostasis;
activation-induced cell death of T cells;aging;alcohol metabolic process;amine metabolic process;aminoglycan metabolic process;anatomical structure development;apoptosis;apoptotic mitochondrial changes;biological regu
biological regulation;biosynthetic process;catabolic process;cellular biosynthetic process;cellular catabolic process;cellular macromolecule metabolic process;cellular metabolic process;cellular nitrogen compound metabolic
amine biosynthetic process;amine metabolic process;biological regulation;biosynthetic process;carboxylic acid biosynthetic process;carboxylic acid metabolic process;cellular amine metabolic process;cellular amino acid bio
biological regulation;cell surface receptor linked signaling pathway;cellular process;cellular response to chemical stimulus;cellular response to cytokine stimulus;cellular response to interferon-gamma;cellular response to orga
amine biosynthetic process;amine catabolic process;amine metabolic process;biological regulation;biosynthetic process;carboxylic acid biosynthetic process;carboxylic acid catabolic process;carboxylic acid metabolic proc
biosynthetic process;catabolic process;cellular biosynthetic process;cellular catabolic process;cellular component disassembly;cellular component disassembly at cellular level;cellular component organization;cellular compo
2'-deoxyribonucleotide biosynthetic process;2'-deoxyribonucleotide metabolic process;biosynthetic process;catabolic process;cellular aromatic compound metabolic process;cellular biosynthetic process;cellular catabolic p
biosynthetic process;cell cycle;cell cycle process;cellular biosynthetic process;cellular macromolecule biosynthetic process;cellular macromolecule metabolic process;cellular metabolic process;cellular nitrogen compound m
amine biosynthetic process;amine catabolic process;amine metabolic process;betaine biosynthetic process;betaine metabolic process;biosynthetic process;carboxylic acid biosynthetic process;carboxylic acid catabolic proc
amine biosynthetic process;amine metabolic process;biological regulation;biosynthetic process;carboxylic acid biosynthetic process;carboxylic acid metabolic process;cellular amine metabolic process;cellular amino acid bio
actin cytoskeleton organization;actin filament organization;actin filament-based process;adherens junction organization;aging;anatomical structure development;anatomical structure morphogenesis;apical junction assembly;a
biological regulation;cellular process;cellular response to abiotic stimulus;cellular response to osmotic stress;cellular response to stimulus;cellular response to stress;multicellular organismal process;negative regulation of cata
anatomical structure homeostasis;base-excision repair;biological regulation;biosynthetic process;catabolic process;cell cycle;cell cycle process;cellular biosynthetic process;cellular catabolic process;cellular component asse
alkaloid metabolic process;anatomical structure development;anatomical structure formation involved in morphogenesis;angiogenesis;arachidonic acid metabolic process;behavior;biological regulation;biomineral tissue deve
amine biosynthetic process;amine catabolic process;amine metabolic process;biosynthetic process;carboxylic acid biosynthetic process;carboxylic acid catabolic process;carboxylic acid metabolic process;catabolic process
actin cytoskeleton organization;actin filament-based movement;actin filament-based process;actomyosin structure organization;adult heart development;anatomical structure development;anatomical structure formation invo
anatomical structure development;anatomical structure morphogenesis;biological regulation;blood coagulation;carboxylic acid metabolic process;cell migration;cell motility;cell surface receptor linked signaling pathway;cellul
biological regulation;cell redox homeostasis;cellular chemical homeostasis;cellular homeostasis;cellular ion homeostasis;cellular macromolecule metabolic process;cellular metabolic process;cellular nitrogen compound meta
activation of immune response;activation of innate immune response;activation of MAPK activity;activation of MAPKK activity;activation of protein kinase activity;axon guidance;biological regulation;cell surface receptor linke
ATP hydrolysis coupled proton transport;biological regulation;cation homeostasis;cation transport;cell surface receptor linked signaling pathway;cellular cation homeostasis;cellular chemical homeostasis;cellular component
biosynthetic process;cellular aromatic compound metabolic process;cellular biosynthetic process;cellular metabolic process;cellular nitrogen compound biosynthetic process;cellular nitrogen compound metabolic process;ce
biosynthetic process;catabolic process;cellular biosynthetic process;cellular catabolic process;cellular component disassembly;cellular component disassembly at cellular level;cellular component organization;cellular compo
alcohol biosynthetic process;alcohol catabolic process;alcohol metabolic process;biosynthetic process;carbohydrate biosynthetic process;carbohydrate catabolic process;carbohydrate metabolic process;catabolic process;
aging;amine metabolic process;arachidonic acid metabolic process;biological regulation;carboxylic acid metabolic process;catabolic process;cellular amine metabolic process;cellular amino acid metabolic process;cellular ca
biosynthetic process;cellular biosynthetic process;cellular macromolecule biosynthetic process;cellular macromolecule metabolic process;cellular metabolic process;cellular process;cellular protein metabolic process;cotrans
activation of caspase activity;adult behavior;adult locomotory behavior;aging;alcohol biosynthetic process;alcohol metabolic process;amine biosynthetic process;amine metabolic process;amine transport;ATP synthesis coup
biosynthetic process;catabolic process;cellular biosynthetic process;cellular catabolic process;cellular component disassembly;cellular component disassembly at cellular level;cellular component organization;cellular compo
activation of adenylate cyclase activity;axon guidance;biological regulation;cellular component organization;cellular component organization at cellular level;cellular component organization or biogenesis;cellular component
biological regulation;biosynthetic process;catabolic process;cellular biosynthetic process;cellular catabolic process;cellular component disassembly;cellular component disassembly at cellular level;cellular component organiz
biosynthetic process;cellular biosynthetic process;cellular macromolecule biosynthetic process;cellular macromolecule metabolic process;cellular metabolic process;cellular process;cellular protein metabolic process;establis
biological regulation;cellular macromolecule metabolic process;cellular metabolic process;cellular nitrogen compound metabolic process;cellular process;macromolecule metabolic process;metabolic process;mRNA metaboli
biological regulation;biosynthetic process;cellular biosynthetic process;cellular component organization;cellular component organization at cellular level;cellular component organization or biogenesis;cellular component orga
amine metabolic process;amino acid activation;biosynthetic process;bis(5'-nucleosidyl) oligophosphate biosynthetic process;bis(5'-nucleosidyl) oligophosphate metabolic process;carboxylic acid metabolic process;cellular a
amine metabolic process;amino acid activation;carboxylic acid metabolic process;cell differentiation;cellular amine metabolic process;cellular amino acid metabolic process;cellular developmental process;cellular ketone met
actin cytoskeleton organization;actin filament organization;actin filament-based process;anatomical structure development;biological regulation;cell development;cell junction assembly;cell junction organization;cell migration
carboxylic acid catabolic process;carboxylic acid metabolic process;catabolic process;cellular catabolic process;cellular ketone metabolic process;cellular lipid catabolic process;cellular lipid metabolic process;cellular metab
anatomical structure development;apoptosis;biological regulation;biosynthetic process;blood circulation;cell death;cell differentiation;cell differentiation involved in kidney development;cell differentiation involved in metaneph
alcohol catabolic process;alcohol metabolic process;anatomical structure development;androgen biosynthetic process;androgen metabolic process;apocarotenoid metabolic process;arachidonic acid metabolic process;biolo
anatomical structure development;biological regulation;biosynthetic process;catabolic process;cell communication;cell cycle arrest;cell cycle process;cell development;cell growth;cell projection organization;cell surface rece
biological regulation;biosynthetic process;catabolic process;cell cycle;cell proliferation;cellular biosynthetic process;cellular catabolic process;cellular component disassembly;cellular component disassembly at cellular level;
biosynthetic process;catabolic process;cellular biosynthetic process;cellular catabolic process;cellular component disassembly;cellular component disassembly at cellular level;cellular component organization;cellular compo
aging;biological regulation;cell aging;cell cycle;cell cycle arrest;cell cycle process;cellular component assembly;cellular component assembly at cellular level;cellular component organization;cellular component organization a
anatomical structure development;anatomical structure morphogenesis;biological regulation;bradykinin catabolic process;calcitonin catabolic process;catabolic process;cellular catabolic process;cellular hormone metabolic
anatomical structure formation involved in morphogenesis;anatomical structure morphogenesis;angiogenesis;angiogenesis involved in wound healing;biological adhesion;biological regulation;cell adhesion;cellular process;de

biological regulation;posttranscriptional regulation of gene expression;regulation of biological process;regulation of gene expression;regulation of macromolecule metabolic process;regulation of metabolic process
alkaloid metabolic process;biological regulation;biosynthetic process;cell communication;cell-cell signaling;cellular amide metabolic process;cellular biosynthetic process;cellular metabolic process;cellular nitrogen compoun
biological regulation;catabolic process;cellular catabolic process;cellular macromolecule catabolic process;cellular macromolecule metabolic process;cellular metabolic process;cellular process;cellular protein metabolic proc
cell cycle phase;cell cycle process;cell proliferation;cellular process;meiosis
biological regulation;cell cycle;cellular process;cellular response to stimulus;intracellular signal transduction;mitotic cell cycle;negative regulation of biological process;negative regulation of intracellular protein transport;negat
biosynthetic process;cellular biosynthetic process;cellular macromolecule biosynthetic process;cellular macromolecule metabolic process;cellular metabolic process;cellular nitrogen compound metabolic process;cellular pro
anatomical structure development;biological regulation;biosynthetic process;cell development;cellular biosynthetic process;cellular component assembly;cellular component assembly at cellular level;cellular component orga
biosynthetic process;catabolic process;cellular biosynthetic process;cellular catabolic process;cellular component disassembly;cellular component disassembly at cellular level;cellular component organization;cellular compo
biosynthetic process;catabolic process;cellular biosynthetic process;cellular catabolic process;cellular component biogenesis;cellular component biogenesis at cellular level;cellular component disassembly;cellular componen
biosynthetic process;catabolic process;cellular biosynthetic process;cellular catabolic process;cellular component disassembly;cellular component disassembly at cellular level;cellular component organization;cellular compo
biosynthetic process;catabolic process;cellular biosynthetic process;cellular catabolic process;cellular component disassembly;cellular component disassembly at cellular level;cellular component organization;cellular compo
biological regulation;biosynthetic process;catabolic process;cellular biosynthetic process;cellular catabolic process;cellular component disassembly;cellular component disassembly at cellular level;cellular component organiz
biological regulation;biosynthetic process;catabolic process;cellular biosynthetic process;cellular catabolic process;cellular component disassembly;cellular component disassembly at cellular level;cellular component organiz
biosynthetic process;catabolic process;cellular biosynthetic process;cellular catabolic process;cellular component disassembly;cellular component disassembly at cellular level;cellular component organization;cellular compo
anatomical structure development;axon extension;biological regulation;cell growth;cell projection organization;cellular component movement;cellular component organization;cellular component organization at cellular level;c
biological regulation;cellular component organization;cellular component organization or biogenesis;cellular process;cellular response to stimulus;establishment of localization;establishment of protein localization;intracellular
acid secretion;alcohol metabolic process;amine metabolic process;arachidonic acid metabolic process;arachidonic acid secretion;biological regulation;biosynthetic process;blood coagulation;carboxylic acid biosynthetic pro
amine metabolic process;amino acid activation;anatomical structure development;brain development;carboxylic acid metabolic process;cellular amine metabolic process;cellular amino acid metabolic process;cellular ketone
ATP biosynthetic process;ATP metabolic process;ATP synthesis coupled proton transport;biosynthetic process;cation transport;cellular biosynthetic process;cellular metabolic process;cellular nitrogen compound biosynthetic
aging;biological regulation;cell aging;cell junction assembly;cell junction organization;cellular component assembly;cellular component assembly at cellular level;cellular component organization;cellular component organizatio
biosynthetic process;carbohydrate metabolic process;carboxylic acid metabolic process;cellular biosynthetic process;cellular component assembly;cellular component organization;cellular component organization or biogen
alcohol metabolic process;biosynthetic process;cholesterol biosynthetic process;cholesterol metabolic process;lipid biosynthetic process;lipid metabolic process;metabolic process;primary metabolic process;small molecule
amine biosynthetic process;amine metabolic process;apoptotic mitochondrial changes;biological regulation;biosynthetic process;carboxylic acid biosynthetic process;carboxylic acid metabolic process;cellular amine metabo
biological adhesion;cell activation;cell adhesion;cell junction assembly;cell junction organization;cell migration;cell motility;cell proliferation;cell-substrate junction assembly;cellular component assembly;cellular component as
biosynthetic process;cellular biosynthetic process;cellular lipid metabolic process;cellular metabolic process;cellular process;chordate embryonic development;developmental process;embryo development;embryo developm
anatomical structure homeostasis;base-excision repair;biological regulation;biosynthetic process;cell cycle;cellular biosynthetic process;cellular component organization;cellular component organization at cellular level;cellula
activation of MAPK activity;anatomical structure morphogenesis;biological adhesion;biological regulation;branching morphogenesis of a tube;cell adhesion;cell junction assembly;cell junction organization;cell morphogenesis
3'-UTR-mediated mRNA stabilization;activation of immune response;activation of innate immune response;activation of MAPK activity;anatomical structure development;arachidonic acid metabolic process;biological regulati
biosynthetic process;catabolic process;cellular biosynthetic process;cellular catabolic process;cellular component disassembly;cellular component disassembly at cellular level;cellular component organization;cellular compo
biological regulation;blood coagulation;carbohydrate metabolic process;cellular carbohydrate metabolic process;cellular component organization;cellular component organization at cellular level;cellular component organizati
acetyl-CoA metabolic process;acyl-CoA biosynthetic process;acyl-CoA metabolic process;acylglycerol biosynthetic process;acylglycerol metabolic process;amine metabolic process;biological regulation;biosynthetic process
amine biosynthetic process;amine catabolic process;amine metabolic process;biological regulation;biosynthetic process;carbohydrate homeostasis;carboxylic acid biosynthetic process;carboxylic acid metabolic process;cat
binding of sperm to zona pellucida;cell recognition;cell-cell recognition;cellular component assembly;cellular component assembly at cellular level;cellular component organization;cellular component organization at cellular le
biosynthetic process;cellular biosynthetic process;cellular component disassembly;cellular component disassembly at cellular level;cellular component organization;cellular component organization at cellular level;cellular co
biosynthetic process;cellular biosynthetic process;cellular component organization;cellular component organization at cellular level;cellular component organization or biogenesis;cellular component organization or biogenesis
alcohol metabolic process;amine biosynthetic process;amine catabolic process;amine metabolic process;aspartate family amino acid catabolic process;aspartate family amino acid metabolic process;betaine biosynthetic pro
biological regulation;biosynthetic process;cellular biosynthetic process;cellular macromolecule biosynthetic process;cellular macromolecule metabolic process;cellular metabolic process;cellular process;cellular protein metab
alanyl-tRNA aminoacylation;amine metabolic process;amino acid activation;anatomical structure development;biological regulation;carboxylic acid metabolic process;cellular amine metabolic process;cellular amino acid meta
amine metabolic process;amino acid activation;carboxylic acid metabolic process;cellular amine metabolic process;cellular amino acid metabolic process;cellular ketone metabolic process;cellular macromolecule metabolic
amine metabolic process;amino acid activation;biosynthetic process;carboxylic acid metabolic process;cellular amine metabolic process;cellular amino acid metabolic process;cellular biosynthetic process;cellular ketone me
amine metabolic process;amino acid activation;biosynthetic process;carboxylic acid metabolic process;cellular amine metabolic process;cellular amino acid metabolic process;cellular biosynthetic process;cellular ketone me
acyl-CoA metabolic process;carboxylic acid metabolic process;cellular ketone metabolic process;cellular lipid metabolic process;cellular metabolic process;cellular process;coenzyme metabolic process;cofactor metabolic p
anatomical structure development;anatomical structure morphogenesis;biological regulation;carbohydrate metabolic process;cardiac chamber morphogenesis;cardiac left ventricle morphogenesis;cardiac ventricle morphoge
anatomical structure development;anatomical structure formation involved in morphogenesis;anatomical structure morphogenesis;axon guidance;biological regulation;canonical Wnt receptor signaling pathway;canonical Wnt
acetyl-CoA catabolic process;acetyl-CoA metabolic process;carbohydrate metabolic process;catabolic process;cellular catabolic process;cellular metabolic process;cellular process;coenzyme catabolic process;coenzyme m
anatomical structure development;biological regulation;cell cycle;cell cycle process;cell differentiation;cellular component disassembly;cellular component disassembly at cellular level;cellular component organization;cellular
acylglycerol metabolic process;alcohol metabolic process;amine metabolic process;amine transport;amino acid transmembrane transport;amino acid transport;behavior;betaine metabolic process;betaine transport;biological
biological regulation;cellular component assembly;cellular component organization;cellular component organization or biogenesis;cellular macromolecule metabolic process;cellular metabolic process;cellular process;cellular
biological adhesion;biological regulation;cell adhesion;cell-matrix adhesion;cell-substrate adhesion;cellular process;cellular response to stimulus;regulation of biological process;regulation of cellular process;response to stimu
biosynthetic process;catabolic process;cellular biosynthetic process;cellular catabolic process;cellular component biogenesis;cellular component biogenesis at cellular level;cellular component disassembly;cellular componen
cell cycle;cell division;cellular component organization;cellular component organization or biogenesis;cellular membrane organization;cellular process;endocytosis;establishment of localization;membrane invagination;membra
antigen processing and presentation;biological regulation;blood coagulation;cell activation;cell activation involved in immune response;cell differentiation;cellular developmental process;cellular localization;cellular macromole
activation of caspase activity;apoptosis;autophagy;biological regulation;catabolic process;cell communication;cell death;cellular catabolic process;cellular metabolic process;cellular process;cellular response to amino acid st
acetyl-CoA catabolic process;acetyl-CoA metabolic process;carbohydrate metabolic process;carboxylic acid metabolic process;catabolic process;cellular catabolic process;cellular ketone metabolic process;cellular metabol
antigen processing and presentation;antigen processing and presentation of peptide antigen;antigen processing and presentation of peptide antigen via MHC class I;apoptosis;biological regulation;calcium ion homeostasis;c
anatomical structure development;androgen metabolic process;bile acid biosynthetic process;bile acid metabolic process;biological regulation;biosynthetic process;carboxylic acid biosynthetic process;carboxylic acid catab
activation of immune response;activation of innate immune response;anatomical structure development;axon guidance;biological regulation;cell communication;cell cycle;cell surface receptor linked signaling pathway;cell-ce
biological regulation;cell proliferation;cellular macromolecule metabolic process;cellular metabolic process;cellular process;cellular protein metabolic process;cellular response to chemical stimulus;cellular response to organic
biological regulation;cell surface receptor linked signaling pathway;cellular macromolecule metabolic process;cellular metabolic process;cellular nitrogen compound metabolic process;cellular process;cellular response to che
apoptosis;biological regulation;catabolic process;cell death;cell surface receptor linked signaling pathway;cellular catabolic process;cellular component disassembly;cellular component disassembly at cellular level;cellular co
biological regulation;biosynthetic process;catabolic process;cellular biosynthetic process;cellular catabolic process;cellular component assembly;cellular component assembly at cellular level;cellular component organization;
actin cytoskeleton organization;actin filament-based process;biological regulation;calcium ion homeostasis;cation homeostasis;cellular calcium ion homeostasis;cellular cation homeostasis;cellular chemical homeostasis;cellu
biological regulation;biosynthetic process;cellular biosynthetic process;cellular macromolecule biosynthetic process;cellular macromolecule metabolic process;cellular metabolic process;cellular nitrogen compound metabolic
anatomical structure formation involved in morphogenesis;angiogenesis;axon guidance;biological regulation;blood coagulation;cell activation;cell migration;cell motility;cell projection assembly;cell projection organization;cell
amine biosynthetic process;amine metabolic process;aspartate family amino acid metabolic process;biosynthetic process;carboxylic acid metabolic process;cellular amine metabolic process;cellular amino acid metabolic pro
alcohol catabolic process;alcohol metabolic process;apoptotic mitochondrial changes;biological regulation;body fluid secretion;carbohydrate catabolic process;carbohydrate homeostasis;carbohydrate metabolic process;car
acyl-CoA biosynthetic process;acyl-CoA metabolic process;acylglycerol biosynthetic process;acylglycerol metabolic process;biological regulation;biosynthetic process;carbohydrate metabolic process;carboxylic acid metabo
activation of caspase activity;adaptive immune response;adaptive immune response based on somatic recombination of immune receptors built from immunoglobulin superfamily domains;aging;biological regulation;cell killin
anatomical structure morphogenesis;developmental process;metabolic process;organ morphogenesis;oxidation-reduction process
anion transport;behavioral response to nutrient;biological regulation;blood coagulation;carbohydrate homeostasis;carboxylic acid metabolic process;carboxylic acid transport;cell migration;cell motility;cellular component mo
biological regulation;cellular process;cellular response to stimulus;regulation of biological process;regulation of biosynthetic process;regulation of cellular biosynthetic process;regulation of cellular macromolecule biosynthetic
amine metabolic process;amino acid activation;apoptosis;biological regulation;carboxylic acid metabolic process;cell death;cellular amine metabolic process;cellular amino acid metabolic process;cellular ketone metabolic pr
biological regulation;blood coagulation;cation homeostasis;cation transport;cell migration;cell motility;cellular cation homeostasis;cellular chemical homeostasis;cellular component movement;cellular component organization
2'-deoxyribonucleotide metabolic process;ADP biosynthetic process;ADP metabolic process;AMP metabolic process;anatomical structure development;biosynthetic process;brain development;cellular biosynthetic process;c
amine biosynthetic process;amine metabolic process;biosynthetic process;carboxylic acid biosynthetic process;carboxylic acid metabolic process;cellular amine metabolic process;cellular amino acid biosynthetic process;ce
anatomical structure development;apical protein localization;asymmetric protein localization;biological regulation;brain development;cell communication;cell differentiation;cell-cell signaling;cellular component disassembly;ce
anatomical structure development;astrocyte development;axon extension;axon extension involved in development;axon extension involved in regeneration;axon guidance;biological adhesion;cell adhesion;cell development;c
anatomical structure morphogenesis;B cell activation;B cell differentiation;cell activation;cell differentiation;cellular developmental process;cellular process;developmental process;establishment of localization;immune system
amine metabolic process;amino acid activation;carboxylic acid metabolic process;cellular amine metabolic process;cellular amino acid metabolic process;cellular ketone metabolic process;cellular macromolecule metabolic
activation of MAPK activity;axon guidance;biological adhesion;biological regulation;blood coagulation;cell adhesion;cell chemotaxis;cell migration;cell motility;cell surface receptor linked signaling pathway;cell-matrix adhesio
cellular component assembly;cellular component assembly at cellular level;cellular component organization;cellular component organization at cellular level;cellular component organization or biogenesis;cellular component c
biological regulation;biosynthetic process;cellular biosynthetic process;cellular macromolecule biosynthetic process;cellular macromolecule metabolic process;cellular metabolic process;cellular nitrogen compound metabolic
alcohol biosynthetic process;alcohol catabolic process;alcohol metabolic process;biosynthetic process;carbohydrate biosynthetic process;carbohydrate catabolic process;carbohydrate metabolic process;catabolic process;c
biological regulation;biosynthetic process;catabolic process;cellular biosynthetic process;cellular catabolic process;cellular component assembly;cellular component assembly at cellular level;cellular component organization;
anatomical structure morphogenesis;biological regulation;biosynthetic process;catabolic process;cell surface receptor linked signaling pathway;cellular biosynthetic process;cellular catabolic process;cellular macromolecule b
biosynthetic process;catabolic process;cellular biosynthetic process;cellular catabolic process;cellular component disassembly;cellular component disassembly at cellular level;cellular component organization;cellular compo
activation of immune response;activation of innate immune response;anaphase-promoting complex-dependent proteasomal ubiquitin-dependent protein catabolic process;antigen processing and presentation;antigen proces
actin cytoskeleton organization;actin filament branching;actin filament bundle assembly;actin filament organization;actin filament-based process;adherens junction organization;anatomical structure development;anatomical s
actin cytoskeleton organization;actin filament depolymerization;actin filament organization;actin filament severing;actin filament-based process;actin polymerization or depolymerization;biological regulation;cellular componen
activation of MAPK activity;ameboidal cell migration;anatomical structure development;anatomical structure morphogenesis;apoptosis;axon guidance;biological regulation;brain development;branching morphogenesis of a tu
activation of immune response;activation of innate immune response;biological regulation;cell surface receptor linked signaling pathway;cellular macromolecule metabolic process;cellular metabolic process;cellular process;c
biological regulation;biosynthetic process;catabolic process;cell differentiation;cellular biosynthetic process;cellular catabolic process;cellular component disassembly;cellular component disassembly at cellular level;cellular c
biosynthetic process;catabolic process;cellular biosynthetic process;cellular catabolic process;cellular component biogenesis;cellular component biogenesis at cellular level;cellular component disassembly;cellular componen
biosynthetic process;catabolic process;cellular biosynthetic process;cellular catabolic process;cellular component disassembly;cellular component disassembly at cellular level;cellular component organization;cellular compo
biosynthetic process;catabolic process;cellular biosynthetic process;cellular catabolic process;cellular component disassembly;cellular component disassembly at cellular level;cellular component organization;cellular compo
biosynthetic process;catabolic process;cellular biosynthetic process;cellular catabolic process;cellular component disassembly;cellular component disassembly at cellular level;cellular component organization;cellular compo
biological regulation;cellular macromolecule metabolic process;cellular metabolic process;cellular nitrogen compound metabolic process;cellular process;cellular response to stimulus;gene expression;macromolecule metabo
apoptosis;biological regulation;biosynthetic process;cell cycle;cell cycle process;cell death;cellular biosynthetic process;cellular component organization;cellular component organization at cellular level;cellular component org
biological regulation;cell differentiation;cellular developmental process;cellular process;cellular response to stimulus;developmental process;intracellular signal transduction;metabolic process;osteoblast differentiation;positive
biosynthetic process;catabolic process;cellular biosynthetic process;cellular catabolic process;cellular component biogenesis;cellular component biogenesis at cellular level;cellular component disassembly;cellular componen
catabolic process;cellular catabolic process;cellular macromolecule catabolic process;cellular macromolecule metabolic process;cellular metabolic process;cellular process;cellular protein metabolic process;macromolecule c
biological regulation;biosynthetic process;catabolic process;cell differentiation;cellular biosynthetic process;cellular catabolic process;cellular component assembly;cellular component assembly at cellular level;cellular compo
biosynthetic process;catabolic process;cellular biosynthetic process;cellular catabolic process;cellular component disassembly;cellular component disassembly at cellular level;cellular component organization;cellular compo
biosynthetic process;catabolic process;cellular biosynthetic process;cellular catabolic process;cellular component biogenesis;cellular component biogenesis at cellular level;cellular component disassembly;cellular componen
biosynthetic process;catabolic process;cellular biosynthetic process;cellular catabolic process;cellular component disassembly;cellular component disassembly at cellular level;cellular component organization;cellular compo
biosynthetic process;catabolic process;cellular biosynthetic process;cellular catabolic process;cellular component disassembly;cellular component disassembly at cellular level;cellular component organization;cellular compo
catabolic process;cellular catabolic process;cellular macromolecule catabolic process;cellular macromolecule metabolic process;cellular metabolic process;cellular nitrogen compound metabolic process;cellular process;exo
actin cytoskeleton organization;actin filament-based process;anatomical structure development;apoptosis;biological adhesion;biological regulation;cell adhesion;cell cycle;cell death;cell division;cell projection organization;ce
activation of immune response;activation of innate immune response;anaphase-promoting complex-dependent proteasomal ubiquitin-dependent protein catabolic process;antigen processing and presentation;antigen proces
biosynthetic process;catabolic process;cellular biosynthetic process;cellular catabolic process;cellular component biogenesis;cellular component biogenesis at cellular level;cellular component disassembly;cellular componen
alcohol metabolic process;biological regulation;biosynthetic process;cellular biosynthetic process;cellular macromolecule biosynthetic process;cellular macromolecule metabolic process;cellular metabolic process;cellular nit
anatomical structure formation involved in morphogenesis;angiogenesis;apoptosis;axon guidance;biological adhesion;biological regulation;blood coagulation;cell activation;cell adhesion;cell death;cellular process;cellular res
biosynthetic process;catabolic process;cellular biosynthetic process;cellular catabolic process;cellular component disassembly;cellular component disassembly at cellular level;cellular component organization;cellular compo
biological regulation;biosynthetic process;catabolic process;cellular biosynthetic process;cellular catabolic process;cellular component biogenesis;cellular component biogenesis at cellular level;cellular component disassemb
biosynthetic process;catabolic process;cellular biosynthetic process;cellular catabolic process;cellular component assembly;cellular component assembly at cellular level;cellular component disassembly;cellular component c
biological regulation;biosynthetic process;catabolic process;cellular biosynthetic process;cellular catabolic process;cellular component disassembly;cellular component disassembly at cellular level;cellular component organiz
biosynthetic process;catabolic process;cellular biosynthetic process;cellular catabolic process;cellular component biogenesis;cellular component biogenesis at cellular level;cellular component disassembly;cellular componen
antibacterial humoral response;antimicrobial humoral response;biosynthetic process;catabolic process;cellular biosynthetic process;cellular catabolic process;cellular component disassembly;cellular component disassembly
biosynthetic process;catabolic process;cellular biosynthetic process;cellular catabolic process;cellular component disassembly;cellular component disassembly at cellular level;cellular component organization;cellular compo
biosynthetic process;catabolic process;cellular biosynthetic process;cellular catabolic process;cellular component biogenesis;cellular component biogenesis at cellular level;cellular component disassembly;cellular componen
biosynthetic process;catabolic process;cellular biosynthetic process;cellular catabolic process;cellular component disassembly;cellular component disassembly at cellular level;cellular component organization;cellular compo
biological regulation;biosynthetic process;blood coagulation;cell activation;cell migration;cell motility;cellular biosynthetic process;cellular component assembly;cellular component assembly at cellular level;cellular componen
biological regulation;blood coagulation;cAMP-mediated signaling;cell activation;cell communication;cell cycle;cell division;cell surface receptor linked signaling pathway;cell-cell signaling;cellular process;cellular response to
biological regulation;biosynthetic process;cellular biosynthetic process;cellular macromolecule biosynthetic process;cellular macromolecule metabolic process;cellular metabolic process;cellular nitrogen compound metabolic
biological regulation;blood coagulation;cell activation;cell cycle;cell cycle process;cellular component assembly;cellular component assembly at cellular level;cellular component organization;cellular component organization a
amine biosynthetic process;amine metabolic process;biosynthetic process;carboxylic acid biosynthetic process;carboxylic acid metabolic process;cellular amine metabolic process;cellular amino acid biosynthetic process;ce
biological regulation;cell cycle arrest;cell cycle process;cell death;cell surface receptor linked signaling pathway;cellular process;cellular response to chemical stimulus;cellular response to cytokine stimulus;cellular response t

cellular macromolecule metabolic process;cellular metabolic process;cellular nitrogen compound metabolic process;cellular process;macromolecule metabolic process;metabolic process;ncRNA metabolic process;ncRNA pr
adenosine to inosine editing;base conversion or substitution editing;biological regulation;cellular macromolecule metabolic process;cellular metabolic process;cellular nitrogen compound metabolic process;cellular process;d
biosynthetic process;cellular biosynthetic process;cellular component disassembly;cellular component disassembly at cellular level;cellular component organization;cellular component organization at cellular level;cellular cor
biosynthetic process;cellular biosynthetic process;cellular component disassembly;cellular component disassembly at cellular level;cellular component organization;cellular component organization at cellular level;cellular cor
biosynthetic process;cellular biosynthetic process;cellular component disassembly;cellular component disassembly at cellular level;cellular component organization;cellular component organization at cellular level;cellular cor
anatomical structure development;axon guidance;biological regulation;biosynthetic process;catabolic process;cell cycle checkpoint;cell cycle process;cellular biosynthetic process;cellular catabolic process;cellular compone
biosynthetic process;catabolic process;cellular biosynthetic process;cellular catabolic process;cellular component disassembly;cellular component disassembly at cellular level;cellular component organization;cellular compo
actin cytoskeleton organization;actin filament-based process;axon guidance;biological regulation;blood coagulation;cell activation;cell chemotaxis;cell migration;cell motility;cellular component movement;cellular component
biosynthetic process;catabolic process;cellular biosynthetic process;cellular catabolic process;cellular component disassembly;cellular component disassembly at cellular level;cellular component organization;cellular compo
amine metabolic process;aminoglycan biosynthetic process;aminoglycan catabolic process;aminoglycan metabolic process;anatomical structure development;anatomical structure formation involved in morphogenesis;anato
alcohol metabolic process;cholesterol metabolic process;establishment of localization;lipid metabolic process;lipid transport;metabolic process;organic substance transport;primary metabolic process;small molecule metabo
actin cytoskeleton organization;actin filament-based process;adult behavior;anatomical structure development;anatomical structure formation involved in morphogenesis;anatomical structure morphogenesis;apoptosis;assoc
anatomical structure development;biological regulation;biosynthetic process;cell differentiation;cellular biosynthetic process;cellular developmental process;cellular macromolecule biosynthetic process;cellular macromolecule
biological regulation;cellular component organization;cellular component organization or biogenesis;cellular process;cellular response to stimulus;intracellular signal transduction;positive regulation of actin filament polymeriza
biological regulation;cell differentiation;cellular developmental process;cellular macromolecule metabolic process;cellular metabolic process;cellular nitrogen compound metabolic process;cellular process;circadian regulation
biosynthetic process;cellular biosynthetic process;cellular macromolecule biosynthetic process;cellular macromolecule metabolic process;cellular metabolic process;cellular nitrogen compound metabolic process;cellular pro
biological regulation;biosynthetic process;cellular aromatic compound metabolic process;cellular biosynthetic process;cellular metabolic compound salvage;cellular metabolic process;cellular nitrogen compound biosynthetic
acylglycerol catabolic process;acylglycerol metabolic process;alcohol metabolic process;amine metabolic process;anatomical structure development;biosynthetic process;carbohydrate metabolic process;carbohydrate trans
actin cytoskeleton organization;actin filament-based process;activation of adenylate cyclase activity;ameboidal cell migration;axon guidance;biological regulation;blood coagulation;cell activation;cell migration;cell motility;cel
amine metabolic process;amine transport;amino acid transmembrane transport;amino acid transport;anatomical structure development;aromatic amino acid transport;biological regulation;blood coagulation;branched-chain a
alcohol metabolic process;carbohydrate metabolic process;carbohydrate phosphorylation;cellular carbohydrate metabolic process;cellular metabolic process;cellular process;fructose 6-phosphate metabolic process;fructose
anatomical structure development;cell differentiation;cellular developmental process;cellular process;developmental process;epithelial cell differentiation;muscle organ development;muscle structure development;organ devel
anatomical structure development;biological adhesion;cell adhesion;cell differentiation;cellular component disassembly;cellular component disassembly at cellular level;cellular component organization;cellular component org
biosynthetic process;catabolic process;cellular biosynthetic process;cellular catabolic process;cellular component disassembly;cellular component disassembly at cellular level;cellular component organization;cellular compo
activation of immune response;activation of innate immune response;activation of MAPK activity;activation of MAPKK activity;activation of protein kinase activity;aging;anatomical structure development;axon guidance;biolog
amine biosynthetic process;amine metabolic process;anatomical structure development;aspartate family amino acid biosynthetic process;aspartate family amino acid metabolic process;biosynthetic process;carboxylic acid b
biological regulation;biosynthetic process;catabolic process;cellular biosynthetic process;cellular catabolic process;cellular component disassembly;cellular component disassembly at cellular level;cellular component organiz
amine metabolic process;carboxylic acid metabolic process;cellular amine metabolic process;cellular amino acid metabolic process;cellular ketone metabolic process;cellular metabolic process;cellular nitrogen compound m
attachment of GPI anchor to protein;biological regulation;blood coagulation;cellular component movement;cellular macromolecule metabolic process;cellular metabolic process;cellular process;cellular protein metabolic proc
antigen processing and presentation;antigen processing and presentation of endogenous antigen;antigen processing and presentation of endogenous peptide antigen;antigen processing and presentation of endogenous pep
acetaldehyde metabolic process;activation of immune response;activation of innate immune response;aging;anatomical structure development;anatomical structure morphogenesis;antigen receptor-mediated signaling pathw
biological regulation;catabolic process;cellular catabolic process;cellular macromolecule catabolic process;cellular macromolecule metabolic process;cellular metabolic process;cellular process;macromolecule catabolic proc
alcohol metabolic process;biosynthetic process;carbohydrate biosynthetic process;carbohydrate metabolic process;cellular biosynthetic process;cellular carbohydrate biosynthetic process;cellular carbohydrate metabolic pr
biological regulation;biosynthetic process;catabolic process;cell surface receptor linked signaling pathway;cellular biosynthetic process;cellular catabolic process;cellular macromolecule biosynthetic process;cellular macrom
anatomical structure development;anatomical structure morphogenesis;apoptosis;appendage morphogenesis;atrial septum morphogenesis;biological regulation;biosynthetic process;blood vessel development;bone remodel
anatomical structure morphogenesis;biological regulation;biosynthetic process;cell surface receptor linked signaling pathway;cellular biosynthetic process;cellular macromolecule biosynthetic process;cellular macromolecule
anatomical structure development;apoptosis;biological regulation;biosynthetic process;C21-steroid hormone catabolic process;C21-steroid hormone metabolic process;catabolic process;cell death;cellular biosynthetic proce
biological regulation;positive regulation of apoptosis;positive regulation of biological process;positive regulation of catalytic activity;positive regulation of cell death;positive regulation of cellular process;positive regulation of k
cellular component movement;cellular process;multicellular organismal process;muscle contraction;muscle system process;system process
amine metabolic process;aminoglycan metabolic process;biological regulation;carbohydrate metabolic process;glycosaminoglycan metabolic process;hyaluronan metabolic process;macromolecule metabolic process;metabo
alcohol biosynthetic process;alcohol metabolic process;amine metabolic process;amino sugar biosynthetic process;amino sugar metabolic process;biological regulation;biosynthetic process;carbohydrate biosynthetic proces
biosynthetic process;catabolic process;cellular biosynthetic process;cellular catabolic process;cellular component disassembly;cellular component disassembly at cellular level;cellular component organization;cellular compo
anatomical structure formation involved in morphogenesis;apoptosis;biological regulation;blastocyst formation;carbohydrate homeostasis;cell death;cell junction assembly;cell junction organization;cell-cell junction assembly
activation of protein kinase activity;activation of protein kinase C activity by G-protein coupled receptor protein signaling pathway;aging;alcohol metabolic process;anatomical structure morphogenesis;aorta morphogenesis;a
activation of immune response;adrenergic receptor signaling pathway;aging;anatomical structure development;antigen receptor-mediated signaling pathway;behavior;biological regulation;cAMP catabolic process;cAMP meta
cell differentiation;cellular developmental process;cellular process;developmental process
ATP-dependent chromatin remodeling;biological regulation;biosynthetic process;cell cycle;cell cycle process;cellular biosynthetic process;cellular component assembly;cellular component assembly at cellular level;cellular co
biological regulation;cellular component assembly;cellular component organization;cellular component organization or biogenesis;macromolecular complex assembly;macromolecular complex subunit organization;protein co
biosynthetic process;carbohydrate metabolic process;cellular carbohydrate metabolic process;cellular macromolecule metabolic process;cellular metabolic process;cellular process;cellular protein metabolic process;glycosyl
antigen processing and presentation;antigen processing and presentation of exogenous antigen;antigen processing and presentation of exogenous peptide antigen;antigen processing and presentation of exogenous peptide
B cell activation;biological regulation;cell activation;cell communication;cell proliferation;cell surface receptor linked signaling pathway;cell-cell signaling;cellular process;cellular response to chemical stimulus;cellular respons
cellular macromolecule metabolic process;cellular metabolic process;cellular process;cellular protein metabolic process;establishment of localization;establishment of localization in cell;establishment of protein localization;es
activation of store-operated calcium channel activity;anatomical structure development;anatomical structure morphogenesis;appendage morphogenesis;biological regulation;calcium ion transmembrane transport;calcium ion
anatomical structure morphogenesis;anterior/posterior pattern specification;cellular macromolecule metabolic process;cellular metabolic process;cellular nitrogen compound metabolic process;cellular process;developmenta
amine biosynthetic process;amine metabolic process;beta-alanine biosynthetic process;beta-alanine metabolic process;biosynthetic process;carboxylic acid biosynthetic process;carboxylic acid metabolic process;catabolic
biological regulation;cell surface receptor linked signaling pathway;cellular process;cellular response to chemical stimulus;cellular response to cytokine stimulus;cellular response to interferon-gamma;cellular response to orga
biological regulation;cellular process;establishment of localization;establishment of localization in cell;establishment of protein localization;Golgi vesicle transport;intracellular transport;positive regulation of biological process;
cellular macromolecule metabolic process;cellular metabolic process;cellular process;cellular protein metabolic process;dephosphorylation;macromolecule metabolic process;macromolecule modification;metabolic process;p
carboxylic acid catabolic process;carboxylic acid metabolic process;catabolic process;cellular catabolic process;cellular ketone metabolic process;cellular lipid catabolic process;cellular lipid metabolic process;cellular metab
activation of MAPKK activity;activation of protein kinase activity;anatomical structure development;anatomical structure formation involved in morphogenesis;anatomical structure morphogenesis;apoptosis;biological regulati
acetyl-CoA metabolic process;acyl-CoA biosynthetic process;acyl-CoA metabolic process;acylglycerol biosynthetic process;acylglycerol metabolic process;amine transport;amino acid transmembrane transport;amino acid t
3'-UTR-mediated mRNA stabilization;biological regulation;biosynthetic process;cellular biosynthetic process;cellular component organization;cellular component organization or biogenesis;cellular macromolecule biosynthetic
amine metabolic process;amino acid activation;apoptosis;biological regulation;carboxylic acid metabolic process;cell death;cell differentiation;cellular amine metabolic process;cellular amino acid metabolic process;cellular d
4-hydroxyproline metabolic process;amine metabolic process;anatomical structure development;biological regulation;carboxylic acid metabolic process;cell redox homeostasis;cellular amine metabolic process;cellular amino
biological regulation;biosynthetic process;cellular biosynthetic process;cellular component organization;cellular component organization at cellular level;cellular component organization or biogenesis;cellular component orga
establishment of localization;establishment of protein localization;protein transport;transport
biological regulation;biosynthetic process;cellular biosynthetic process;cellular component assembly;cellular component assembly at cellular level;cellular component organization;cellular component organization at cellular le
anatomical structure morphogenesis;biological regulation;biosynthetic process;cell morphogenesis;cell morphogenesis involved in differentiation;cellular biosynthetic process;cellular component assembly;cellular component
biological regulation;cellular macromolecule metabolic process;cellular metabolic process;cellular nitrogen compound metabolic process;cellular process;cellular response to stimulus;DNA conformation change;DNA duplex u
biological regulation;biosynthetic process;cellular biosynthetic process;cellular component assembly;cellular component assembly at cellular level;cellular component organization;cellular component organization at cellular le
aging;ameboidal cell migration;anatomical structure development;anatomical structure formation involved in morphogenesis;anatomical structure morphogenesis;axon ensheathment;biological adhesion;biological regulation;
cellular macromolecule metabolic process;cellular metabolic process;cellular nitrogen compound metabolic process;cellular process;gene expression;macromolecule metabolic process;metabolic process;mRNA metabolic p
activation of MAPKK activity;activation of protein kinase activity;biological adhesion;biological regulation;cell activation;cell adhesion;cell adhesion mediated by integrin;cell differentiation;cell surface receptor linked signaling
biological regulation;cell communication;cell-cell signaling;cellular process;cellular response to stimulus;establishment of localization;establishment of protein localization;protein transport;regulation of biological process;regu
autophagy;biological regulation;catabolic process;cell communication;cell differentiation;cell surface receptor linked signaling pathway;cellular catabolic process;cellular component assembly;cellular component organization;
cellular macromolecule metabolic process;cellular metabolic process;cellular process;cellular protein metabolic process;establishment of localization;establishment of localization in cell;establishment of protein localization;es
anatomical structure development;biological regulation;calcium ion transport;cation transport;cell communication;cell cycle process;cell differentiation;cell surface receptor linked signaling pathway;cell-cell signaling;cellular c
biosynthetic process;cellular biosynthetic process;cellular lipid metabolic process;cellular metabolic process;cellular process;dephosphorylation;glycerolipid biosynthetic process;glycerolipid metabolic process;glycerophosph
anatomical structure development;anatomical structure formation involved in morphogenesis;biological regulation;biosynthetic process;carbohydrate metabolic process;catabolic process;cell development;cellular biosyntheti
biological regulation;biosynthetic process;cell cycle;cellular biosynthetic process;cellular component organization;cellular component organization at cellular level;cellular component organization or biogenesis;cellular compo
actin cytoskeleton organization;actin filament-based process;anatomical structure development;cellular component organization;cellular component organization at cellular level;cellular component organization or biogenesis;
axon guidance;biological adhesion;biological regulation;cell adhesion;cellular process;cellular response to stimulus;chemotaxis;locomotion;motor axon guidance;regulation of biological process;regulation of cellular process;r
anatomical structure morphogenesis;biological regulation;cellular localization;cellular macromolecule localization;cellular process;cellular protein localization;cellular response to stimulus;developmental process;embryonic mo
cellular component biogenesis;cellular component biogenesis at cellular level;cellular component organization or biogenesis;cellular component organization or biogenesis at cellular level;cellular process;metabolic process;ri
antigen processing and presentation;antigen processing and presentation of peptide antigen;antigen processing and presentation of peptide antigen via MHC class I;cellular macromolecule metabolic process;cellular metabo
biological adhesion;cell adhesion;cell differentiation;cell proliferation;cellular developmental process;cellular macromolecule metabolic process;cellular metabolic process;cellular nitrogen compound metabolic process;cellula
alkene biosynthetic process;biosynthetic process;carboxylic acid biosynthetic process;carboxylic acid metabolic process;cellular alkene metabolic process;cellular biosynthetic process;cellular ketone metabolic process;cellu
adaptive immune response;adaptive immune response based on somatic recombination of immune receptors built from immunoglobulin superfamily domains;anatomical structure formation involved in morphogenesis;angiog
biological regulation;cell proliferation;cellular component biogenesis;cellular component biogenesis at cellular level;cellular component organization or biogenesis;cellular component organization or biogenesis at cellular level
biological regulation;biosynthetic process;cellular biosynthetic process;cellular macromolecule biosynthetic process;cellular macromolecule metabolic process;cellular metabolic process;cellular nitrogen compound metabolic
binding of sperm to zona pellucida;cell recognition;cell-cell recognition;cellular process;cellular process involved in reproduction;reproductive process;sperm-egg recognition
carbohydrate metabolic process;cellular carbohydrate metabolic process;cellular macromolecule metabolic process;cellular metabolic process;cellular process;cellular protein metabolic process;glycosylation;macromolecule
cellular macromolecule metabolic process;cellular metabolic process;cellular process;cellular protein metabolic process;macromolecule metabolic process;macromolecule modification;metabolic process;primary metabolic p
anatomical structure development;anatomical structure formation involved in morphogenesis;androgen receptor signaling pathway;atrial cardiac muscle cell development;biological regulation;biosynthetic process;cardiac cel
anatomical structure development;antigen processing and presentation;antigen processing and presentation of exogenous antigen;antigen processing and presentation of exogenous peptide antigen;antigen processing and p
antigen processing and presentation;antigen processing and presentation of exogenous antigen;antigen processing and presentation of exogenous peptide antigen;antigen processing and presentation of exogenous peptide
cellular component organization;cellular component organization at cellular level;cellular component organization or biogenesis;cellular component organization or biogenesis at cellular level;cellular localization;cellular macro
biological regulation;biosynthetic process;cell surface receptor linked signaling pathway;cellular biosynthetic process;cellular macromolecule biosynthetic process;cellular macromolecule metabolic process;cellular metabolic
anatomical structure development;cell development;cell junction assembly;cell junction organization;cellular component assembly;cellular component assembly at cellular level;cellular component organization;cellular compo
gamete generation;male gamete generation;multicellular organismal process;multicellular organismal reproductive process;reproductive process;spermatogenesis
alcohol catabolic process;alcohol metabolic process;carbohydrate catabolic process;carbohydrate metabolic process;catabolic process;cellular carbohydrate catabolic process;cellular carbohydrate metabolic process;cellula
biological regulation;cell redox homeostasis;cellular homeostasis;cellular macromolecule metabolic process;cellular metabolic process;cellular process;cellular protein metabolic process;cellular response to chemical stimulus
cellular macromolecule metabolic process;cellular metabolic process;cellular nitrogen compound metabolic process;cellular process;macromolecule metabolic process;metabolic process;mRNA metabolic process;mRNA pro
activation of immune response;activation of innate immune response;activation of phospholipase C activity;apoptosis;apoptosis in response to endoplasmic reticulum stress;biological regulation;blood coagulation;calcium ion
biological regulation;positive regulation of biological process;positive regulation of cellular component organization;positive regulation of cellular process;positive regulation of establishment of protein localization in plasma m
biological regulation;cell cycle;cell cycle checkpoint;cell cycle phase;cell cycle process;cell division;cellular component organization;cellular component organization at cellular level;cellular component organization or biogene
anatomical structure development;biological regulation;cell surface receptor linked signaling pathway;cellular localization;cellular macromolecule localization;cellular macromolecule metabolic process;cellular metabolic proce
carbohydrate metabolic process;cellular carbohydrate metabolic process;cellular macromolecule metabolic process;cellular metabolic process;cellular process;cellular protein metabolic process;glycosylation;macromolecule
biological regulation;cell proliferation;establishment of localization;establishment of protein localization;establishment of RNA localization;macromolecule metabolic process;metabolic process;mRNA transport;negative regula
apoptosis;biological regulation;catabolic process;cell death;cell surface receptor linked signaling pathway;cellular catabolic process;cellular component disassembly;cellular component disassembly at cellular level;cellular co
cell cycle;cell cycle phase;cell cycle process;cell differentiation;cell division;cellular component organization;cellular component organization at cellular level;cellular component organization or biogenesis;cellular component o
activation of immune response;activation of innate immune response;anaphase-promoting complex-dependent proteasomal ubiquitin-dependent protein catabolic process;antigen processing and presentation;antigen proces
cell cycle;cell cycle process;cell division;cellular component organization;cellular component organization at cellular level;cellular component organization or biogenesis;cellular component organization or biogenesis at cellula
biological regulation;cell cycle;cellular macromolecule metabolic process;cellular metabolic process;cellular nitrogen compound metabolic process;cellular process;macromolecule metabolic process;macromolecule methylat
biological regulation;biosynthetic process;cellular biosynthetic process;cellular component organization;cellular component organization at cellular level;cellular component organization or biogenesis;cellular component orga
anatomical structure formation involved in morphogenesis;biological regulation;cell cycle phase;cell cycle process;cell division;cell projection assembly;cell projection organization;cell surface receptor linked signaling pathwa
cell cycle;cell cycle process;cell division;cellular component organization;cellular component organization at cellular level;cellular component organization or biogenesis;cellular component organization or biogenesis at cellula
amine metabolic process;amino acid activation;carboxylic acid metabolic process;cellular amine metabolic process;cellular amino acid metabolic process;cellular ketone metabolic process;cellular macromolecule metabolic p
cotranslational protein targeting to membrane;establishment of localization;establishment of localization in cell;establishment of protein localization;intracellular protein transport;intracellular transport;protein targeting;protein
anatomical structure development;anatomical structure morphogenesis;biological regulation;brain development;camera-type eye development;cellular chemical homeostasis;cellular component organization;cellular compone
biological regulation;cation homeostasis;cation transport;cellular cation homeostasis;cellular chemical homeostasis;cellular divalent inorganic cation homeostasis;cellular homeostasis;cellular ion homeostasis;cellular process
anatomical structure homeostasis;base-excision repair;biological regulation;biosynthetic process;cell cycle;cellular biosynthetic process;cellular component organization;cellular component organization at cellular level;cellula
apoptotic cell clearance;biological regulation;cell redox homeostasis;cellular component organization;cellular component organization or biogenesis;cellular homeostasis;cellular macromolecule metabolic process;cellular me
biological regulation;developmental process;macromolecule metabolic process;metabolic process;multicellular organismal development;multicellular organismal process;positive regulation of catalytic activity;positive regulati
alcohol metabolic process;biological regulation;carbohydrate homeostasis;carbohydrate metabolic process;carboxylic acid metabolic process;cellular carbohydrate metabolic process;cellular chemical homeostasis;cellular g

apoptosis;cell death;cell junction assembly;cell junction organization;cell-substrate junction assembly;cellular component assembly;cellular component assembly at cellular level;cellular component disassembly;cellular comp
aromatic compound catabolic process;catabolic process;cellular aromatic compound metabolic process;cellular catabolic process;cellular metabolic process;cellular process;metabolic process;response to chemical stimulus
biological regulation;biosynthetic process;cellular biosynthetic process;cellular macromolecule biosynthetic process;cellular macromolecule metabolic process;cellular metabolic process;cellular nitrogen compound metabolic
biological adhesion;biological regulation;cell adhesion;cell junction assembly;cell junction organization;cell migration;cell motility;cell surface receptor linked signaling pathway;cell-substrate junction assembly;cellular compor
cellular macromolecule metabolic process;cellular metabolic process;cellular nitrogen compound metabolic process;cellular process;gene expression;macromolecule metabolic process;metabolic process;mRNA metabolic p
biological regulation;cell junction assembly;cell junction organization;cellular component assembly;cellular component assembly at cellular level;cellular component organization;cellular component organization at cellular leve
cellular macromolecule metabolic process;cellular metabolic process;cellular nitrogen compound metabolic process;cellular process;DNA metabolic process;DNA recombination;gene expression;macromolecule metabolic pr
biological regulation;cell surface receptor linked signaling pathway;cellular process;cellular response to chemical stimulus;cellular response to cytokine stimulus;cellular response to interferon-gamma;cellular response to orga
biological regulation;biosynthetic process;cell junction assembly;cell junction organization;cell-substrate junction assembly;cellular biosynthetic process;cellular component assembly;cellular component assembly at cellular l
amine transport;amino acid transport;carboxylic acid transport;cellular process;establishment of localization;extracellular amino acid transport;extracellular transport;glutamine transport;ion transport;neutral amino acid transp
activin receptor signaling pathway;anatomical structure development;anatomical structure formation involved in morphogenesis;anatomical structure morphogenesis;anterior/posterior pattern specification;axis specification;b
biological regulation;cation transport;cellular macromolecule metabolic process;cellular metabolic process;cellular process;cellular response to abiotic stimulus;cellular response to acidity;cellular response to pH;cellular respo
actin cytoskeleton organization;actin filament bundle assembly;actin filament organization;actin filament-based process;biological adhesion;biological regulation;cell adhesion;cell communication;cell surface receptor linked s
anatomical structure development;biological adhesion;biological regulation;cell adhesion;cellular process;cellular response to chemical stimulus;cellular response to endogenous stimulus;cellular response to hormone stimulu
activation of JUN kinase activity;activation of MAPK activity;ameboidal cell migration;biological regulation;biosynthetic process;cell migration;cell motility;cellular biosynthetic process;cellular component movement;cellular co
cell differentiation;cellular developmental process;cellular process;developmental process;multicellular organismal development;multicellular organismal process
biological regulation;biosynthetic process;catabolic process;cell surface receptor linked signaling pathway;cellular biosynthetic process;cellular catabolic process;cellular component organization;cellular component organizat
3'-UTR-mediated mRNA stabilization;activation of immune response;activation of innate immune response;activation of MAPK activity;aging;alcohol metabolic process;anatomical structure development;anatomical structure
actin cytoskeleton organization;actin filament bundle assembly;actin filament organization;actin filament-based process;cell migration;cell motility;cell proliferation;cellular component assembly;cellular component assembly a
carboxylic acid catabolic process;carboxylic acid metabolic process;catabolic process;cellular catabolic process;cellular component assembly;cellular component organization;cellular component organization or biogenesis;c
alcohol metabolic process;anatomical structure development;anatomical structure morphogenesis;biological regulation;carbohydrate metabolic process;cellular carbohydrate metabolic process;cellular component organizatic
acetyl-CoA metabolic process;amine catabolic process;amine metabolic process;anthranilate metabolic process;aromatic amino acid family catabolic process;aromatic amino acid family metabolic process;aromatic compou
cellular component assembly;cellular component organization;cellular component organization or biogenesis;macromolecular complex assembly;macromolecular complex subunit organization;macromolecule metabolic proc
2'-deoxyribonucleotide biosynthetic process;2'-deoxyribonucleotide metabolic process;alcohol metabolic process;ATP metabolic process;biosynthetic process;carbohydrate metabolic process;cellular biosynthetic process;c
anatomical structure morphogenesis;anion transport;bicarbonate transport;cellular metabolic process;cellular process;cellular response to chemical stimulus;cellular response to hypoxia;cellular response to oxygen levels;cel
alcohol biosynthetic process;alcohol metabolic process;biological regulation;biosynthetic process;carbohydrate biosynthetic process;carbohydrate homeostasis;carbohydrate metabolic process;carboxylic acid metabolic pro
alcohol metabolic process;biosynthetic process;carbohydrate biosynthetic process;carbohydrate metabolic process;carboxylic acid biosynthetic process;carboxylic acid metabolic process;cellular biosynthetic process;cellula
actin cytoskeleton organization;actin filament-based process;biological regulation;cellular component organization;cellular component organization at cellular level;cellular component organization or biogenesis;cellular compo
alcohol catabolic process;alcohol metabolic process;biological regulation;carbohydrate catabolic process;carbohydrate homeostasis;carbohydrate metabolic process;carbohydrate phosphorylation;catabolic process;cellular
anatomical structure development;biological regulation;bone development;cell growth;cellular component organization;cellular component organization at cellular level;cellular component organization or biogenesis;cellular co
cellular component organization;cellular component organization at cellular level;cellular component organization or biogenesis;cellular component organization or biogenesis at cellular level;cellular process;cytoskeleton orga
alcohol metabolic process;alditol metabolic process;biological regulation;carbohydrate metabolic process;carbohydrate phosphorylation;cellular carbohydrate metabolic process;cellular metabolic process;cellular process;de
apoptotic mitochondrial changes;cellular component organization;cellular component organization at cellular level;cellular component organization or biogenesis;cellular component organization or biogenesis at cellular level;c
activation of blood coagulation via clotting cascade;anatomical structure development;anion transport;biological regulation;biomineral tissue development;blood coagulation;bone mineralization;bone mineralization involved i
aging;apoptosis;biological regulation;cell aging;cell death;cellular process;death;developmental process;negative regulation of biological process;negative regulation of biosynthetic process;negative regulation of catalytic act
amine biosynthetic process;amine metabolic process;biosynthetic process;carboxylic acid biosynthetic process;carboxylic acid metabolic process;cellular amine metabolic process;cellular amino acid biosynthetic process;ce
cellular macromolecule metabolic process;cellular metabolic process;cellular process;cellular protein metabolic process;macromolecule metabolic process;metabolic process;primary metabolic process;protein folding;protein
biological regulation;cellular process;cellular response to stimulus;establishment of localization;establishment of localization in cell;establishment of protein localization;intracellular protein transport;intracellular signal transduc
biological regulation;cellular component organization;cellular component organization or biogenesis;cellular localization;cellular macromolecule localization;cellular membrane organization;cellular process;cellular protein local
autophagic vacuole assembly;autophagy;catabolic process;cell communication;cellular catabolic process;cellular component assembly;cellular component assembly at cellular level;cellular component organization;cellular co
cellular macromolecule metabolic process;cellular metabolic process;cellular process;cellular protein metabolic process;macromolecule metabolic process;macromolecule modification;metabolic process;primary metabolic p
actin cytoskeleton organization;actin filament organization;actin filament uncapping;actin filament-based process;barbed-end actin filament uncapping;biological regulation;blood coagulation;cell migration;cell motility;cell pro
anatomical structure development;biological regulation;cell migration;cell motility;cellular component movement;cellular process;developmental process;locomotion;lung alveolus development;positive regulation of biological
biological regulation;cellular process;cellular response to stimulus;intracellular signal transduction;regulation of biological process;regulation of cellular process;regulation of response to stimulus;regulation of signal transducti
biological regulation;cellular process;cellular response to chemical stimulus;cellular response to hypoxia;cellular response to oxygen levels;cellular response to redox state;cellular response to stimulus;cellular response to stre
actin filament capping;barbed-end actin filament capping;biological regulation;cell communication;cell projection organization;cellular component organization;cellular component organization at cellular level;cellular compone
anatomical structure development;anatomical structure morphogenesis;appendage morphogenesis;biological regulation;brain development;cell cycle;cell cycle process;cell differentiation;cellular component organization;cellu
cell differentiation;cellular developmental process;cellular process;developmental process;hemopoietic progenitor cell differentiation
biological regulation;biosynthetic process;cellular biosynthetic process;cellular macromolecule biosynthetic process;cellular macromolecule metabolic process;cellular metabolic process;cellular nitrogen compound metabolic
carboxylic acid metabolic process;cellular ketone metabolic process;cellular metabolic process;cellular process;metabolic process;organic acid metabolic process;oxoacid metabolic process;small molecule metabolic proces
biological regulation;biosynthetic process;cell fate commitment;cell fate commitment involved in formation of primary germ layers;cell surface receptor linked signaling pathway;cellular biosynthetic process;cellular componen
amine metabolic process;amino acid activation;asparaginyl-tRNA aminoacylation;carboxylic acid metabolic process;cellular amine metabolic process;cellular amino acid metabolic process;cellular ketone metabolic process;c
response to abiotic stimulus;response to ionizing radiation;response to radiation;response to stimulus
aromatic compound biosynthetic process;biosynthetic process;carboxylic acid metabolic process;cellular aromatic compound metabolic process;cellular biosynthetic process;cellular ketone metabolic process;cellular metab
acyl-CoA biosynthetic process;acyl-CoA metabolic process;acylglycerol biosynthetic process;acylglycerol metabolic process;biosynthetic process;carboxylic acid biosynthetic process;carboxylic acid metabolic process;cellu
biological regulation;biosynthetic process;cellular biosynthetic process;cellular macromolecule biosynthetic process;cellular macromolecule metabolic process;cellular metabolic process;cellular nitrogen compound metabolic
biological regulation;biosynthetic process;cellular biosynthetic process;cellular macromolecule biosynthetic process;cellular macromolecule metabolic process;cellular metabolic process;cellular nitrogen compound metabolic
anatomical structure development;anatomical structure formation involved in morphogenesis;biological regulation;blood coagulation;cell development;cell differentiation;cellular component organization;cellular component or
adaptive immune response;adaptive immune response based on somatic recombination of immune receptors built from immunoglobulin superfamily domains;biological regulation;cell activation;cell activation involved in imm
response to abiotic stimulus;response to ionizing radiation;response to radiation;response to stimulus;response to X-ray
biological regulation;biosynthetic process;cell differentiation;cellular biosynthetic process;cellular developmental process;cellular macromolecule biosynthetic process;cellular macromolecule metabolic process;cellular metab
biological regulation;biosynthetic process;cellular biosynthetic process;cellular macromolecule biosynthetic process;cellular macromolecule metabolic process;cellular metabolic process;cellular nitrogen compound metabolic
cellular macromolecule metabolic process;cellular metabolic process;cellular nitrogen compound metabolic process;cellular process;macromolecule metabolic process;metabolic process;nitrogen compound metabolic proce
axon extension;biological regulation;cell growth;cell projection assembly;cell projection organization;cell surface receptor linked signaling pathway;cellular component assembly;cellular component assembly at cellular level;c
alcohol metabolic process;amine metabolic process;biosynthetic process;cellular amine metabolic process;cellular biogenic amine metabolic process;cellular biosynthetic process;cellular component organization;cellular com
biological regulation;biosynthetic process;cellular biosynthetic process;cellular component organization;cellular component organization at cellular level;cellular component organization or biogenesis;cellular component orga
biological regulation;cell cycle process;cell division;cellular component organization;cellular component organization at cellular level;cellular component organization or biogenesis;cellular component organization or biogenes
biological regulation;defense response;defense response to virus;immune effector process;immune response;immune system process;innate immune response;multi-organism process;negative regulation of biological proces
biological regulation;biosynthetic process;carbohydrate metabolic process;carbohydrate transport;cell cycle;cell cycle process;cell surface receptor linked signaling pathway;cellular biosynthetic process;cellular component c
axon guidance;biological regulation;cell cycle;cell cycle process;cell division;cellular component organization;cellular component organization at cellular level;cellular component organization or biogenesis;cellular component
response to chemical stimulus;response to organic substance;response to pheromone;response to stimulus
biological regulation;biosynthetic process;cellular biosynthetic process;cellular macromolecule biosynthetic process;cellular macromolecule metabolic process;cellular metabolic process;cellular nitrogen compound metabolic
biological regulation;cellular process;cellular response to stimulus;establishment of localization;establishment of protein localization;positive regulation of biological process;positive regulation of cell communication;positive r
ameboidal cell migration;biological regulation;cell cycle phase;cell cycle process;cell division;cell migration;cell motility;cellular component assembly;cellular component assembly at cellular level;cellular component movemer
biological regulation;biosynthetic process;cellular biosynthetic process;cellular component organization;cellular component organization at cellular level;cellular component organization or biogenesis;cellular component orga
biological regulation;biosynthetic process;cellular biosynthetic process;cellular macromolecule biosynthetic process;cellular macromolecule metabolic process;cellular metabolic process;cellular nitrogen compound metabolic
biological regulation;cell junction assembly;cell junction organization;cell surface receptor linked signaling pathway;cell-cell junction assembly;cell-cell junction organization;cellular component assembly;cellular component as
lipid metabolic process;metabolic process;primary metabolic process
adipose tissue development;anatomical structure development;biological regulation;carbohydrate homeostasis;carboxylic acid metabolic process;cell differentiation;cellular chemical homeostasis;cellular developmental proce
anatomical structure development;anatomical structure morphogenesis;artery development;blood vessel development;cardiac septum development;developmental process;embryonic morphogenesis;morphogenesis of an ep
anatomical structure development;biological regulation;developmental process;liver development;organ development;regulation of biological process;regulation of lipid metabolic process;regulation of metabolic process;regu
biological regulation;cell cycle;cell cycle phase;cell cycle process;cell division;cellular component organization;cellular component organization at cellular level;cellular component organization or biogenesis;cellular componen
biological regulation;biosynthetic process;cellular biosynthetic process;cellular macromolecule biosynthetic process;cellular macromolecule metabolic process;cellular metabolic process;cellular nitrogen compound metabolic
cellular process;establishment of localization;transport;vesicle-mediated transport
cell cycle phase;cell cycle process;cell division;cellular component organization;cellular component organization at cellular level;cellular component organization or biogenesis;cellular component organization or biogenesis at
amine metabolic process;AMP biosynthetic process;AMP metabolic process;aspartate family amino acid metabolic process;aspartate metabolic process;biosynthetic process;carboxylic acid metabolic process;cellular amine
biological regulation;cellular process;cellular response to stimulus;intracellular signal transduction;regulation of biological process;regulation of cellular process;regulation of response to stimulus;regulation of signal transducti
biological regulation;cell cycle;cell cycle phase;cell cycle process;cell division;cellular component organization;cellular component organization at cellular level;cellular component organization or biogenesis;cellular componen
cell differentiation;cellular component organization;cellular component organization at cellular level;cellular component organization or biogenesis;cellular component organization or biogenesis at cellular level;cellular develop
biological regulation;cellular component organization;cellular component organization at cellular level;cellular component organization or biogenesis;cellular component organization or biogenesis at cellular level;cellular macro
activation of caspase activity;aging;apoptotic mitochondrial changes;autophagy;biological regulation;biosynthetic process;catabolic process;cell aging;cell cycle arrest;cell cycle process;cell division;cellular biosynthetic proc
actin cytoskeleton organization;actin filament organization;actin filament-based process;actin polymerization or depolymerization;activation of immune response;anatomical structure formation involved in morphogenesis;anti
cell death;cellular component assembly;cellular component organization;cellular component organization or biogenesis;cellular process;death;macromolecular complex assembly;macromolecular complex subunit organizatio
cellular component organization;cellular component organization at cellular level;cellular component organization or biogenesis;cellular component organization or biogenesis at cellular level;cellular process;extracellular matri
biological regulation;cell differentiation;cellular developmental process;cellular process;developmental process;muscle cell differentiation;negative regulation of biological process;negative regulation of cell communication;neg
acylglycerol biosynthetic process;acylglycerol metabolic process;anatomical structure development;anatomical structure homeostasis;biological regulation;biosynthetic process;carboxylic acid biosynthetic process;carboxylic
cellular component organization;cellular component organization at cellular level;cellular component organization or biogenesis;cellular component organization or biogenesis at cellular level;cellular macromolecule metabolic
biological regulation;biosynthetic process;cellular biosynthetic process;cellular macromolecule biosynthetic process;cellular macromolecule metabolic process;cellular metabolic process;cellular nitrogen compound metabolic
response to chemical stimulus;response to inorganic substance;response to oxidative stress;response to reactive oxygen species;response to stimulus;response to stress
biological regulation;cellular process;cellular response to stimulus;defense response;immune response;immune system process;innate immune response;intracellular signal transduction;regulation of biological process;regula
biological regulation;biosynthetic process;cellular biosynthetic process;cellular component organization;cellular component organization at cellular level;cellular component organization or biogenesis;cellular component orga
anatomical structure development;anatomical structure morphogenesis;apoptosis;biological regulation;biosynthetic process;camera-type eye development;cardiac chamber morphogenesis;cardiac muscle contraction;cardia
amine transport;amino acid transmembrane transport;amino acid transport;carboxylic acid transport;cellular process;establishment of localization;glycine transport;ion transport;neutral amino acid transport;nitrogen compou
biological regulation;cell cycle cytokinesis;cell cycle phase;cell cycle process;cellular component organization;cellular component organization at cellular level;cellular component organization or biogenesis;cellular componen
biological regulation;cellular macromolecule metabolic process;cellular metabolic process;cellular process;cellular protein metabolic process;macromolecule metabolic process;macromolecule modification;metabolic process
biological regulation;cellular component assembly;cellular component assembly at cellular level;cellular component organization;cellular component organization at cellular level;cellular component organization or biogenesis;
assembly of spliceosomal tri-snRNP;cellular component assembly;cellular component assembly at cellular level;cellular component organization;cellular component organization at cellular level;cellular component organizatic
actin filament-based movement;actin filament-based process;ameboidal cell migration;biological regulation;cell migration;cell motility;cellular component movement;cellular component organization;cellular component organi

metabolic process;response to chemical stimulus;response to external stimulus;response to extracellular stimulus;response to nutrient;response to nutrient levels;response to retinoic acid;response to stimulus;response to vit
biosynthetic process;cellular biosynthetic process;cellular macromolecule biosynthetic process;cellular macromolecule metabolic process;cellular metabolic process;cellular nitrogen compound metabolic process;cellular pro
biological regulation;cell activation;cellular component organization;cellular component organization or biogenesis;cellular membrane organization;cellular process;cellular response to chemical stimulus;cellular response to hy
biological regulation;cellular component organization;cellular component organization or biogenesis;cellular macromolecule metabolic process;cellular membrane organization;cellular metabolic process;cellular process;cellul
biological regulation;cellular process;cellular response to stimulus;intracellular signal transduction;regulation of biological process;regulation of cellular process;regulation of response to stimulus;regulation of signal transducti
biological regulation;catabolic process;cellular catabolic process;cellular component organization;cellular component organization at cellular level;cellular component organization or biogenesis;cellular component organizatio
actin cytoskeleton organization;actin filament organization;actin filament-based process;actin nucleation;Arp2/3 complex-mediated actin nucleation;axon guidance;biological regulation;cell surface receptor linked signaling pa
amine metabolic process;carboxylic acid metabolic process;cellular amine metabolic process;cellular amino acid metabolic process;cellular ketone metabolic process;cellular metabolic process;cellular nitrogen compound m
biological regulation;biosynthetic process;cellular biosynthetic process;cellular macromolecule biosynthetic process;cellular macromolecule metabolic process;cellular metabolic process;cellular nitrogen compound metabolic
anatomical structure homeostasis;biological regulation;biosynthetic process;cell cycle process;cellular biosynthetic process;cellular component organization;cellular component organization at cellular level;cellular componen
anatomical structure development;biological regulation;cell development;cellular component assembly;cellular component assembly at cellular level;cellular component organization;cellular component organization at cellular
biological regulation;cell proliferation;cell surface receptor linked signaling pathway;cellular process;cellular response to stimulus;enzyme linked receptor protein signaling pathway;intracellular signal transduction;negative reg
anatomical structure morphogenesis;biological regulation;blood coagulation;bone morphogenesis;cell migration;cell motility;cellular component movement;cellular process;coagulation;developmental process;hemostasis;imn
biological regulation;biosynthetic process;catabolic process;cellular biosynthetic process;cellular catabolic process;cellular macromolecule biosynthetic process;cellular macromolecule catabolic process;cellular macromolecu
adherens junction organization;antigen processing and presentation;biological regulation;cell junction organization;cell-cell junction organization;cellular component organization;cellular component organization at cellular leve
biological regulation;biosynthetic process;catabolic process;cellular biosynthetic process;cellular catabolic process;cellular macromolecule biosynthetic process;cellular macromolecule catabolic process;cellular macromolecu
cellular component organization;cellular component organization at cellular level;cellular component organization or biogenesis;cellular component organization or biogenesis at cellular level;cellular process;establishment of l
actin cytoskeleton organization;actin filament organization;actin filament-based process;anatomical structure morphogenesis;biological regulation;cell cycle phase;cell cycle process;cell division;cell morphogenesis;cell surfac
biological regulation;catabolic process;cellular catabolic process;cellular component organization;cellular component organization at cellular level;cellular component organization or biogenesis;cellular component organizatio
cellular macromolecule metabolic process;cellular metabolic process;cellular nitrogen compound metabolic process;cellular process;macromolecule metabolic process;metabolic process;ncRNA metabolic process;ncRNA pr
anatomical structure formation involved in morphogenesis;angiogenesis;apoptosis;biological regulation;cell death;cellular macromolecule metabolic process;cellular metabolic process;cellular process;cellular protein metabol
anatomical structure development;biological regulation;cell proliferation;cellular component movement;cellular process;developmental process;fertilization;muscle tissue development;nervous system development;regulation
biosynthetic process;cellular biosynthetic process;cellular macromolecule biosynthetic process;cellular macromolecule metabolic process;cellular metabolic process;cellular process;cellular protein metabolic process;macron
biological regulation;induction of apoptosis;induction of apoptosis by intracellular signals;induction of apoptosis by oxidative stress;induction of programmed cell death;positive regulation of apoptosis;positive regulation of bi
cellular process;ER to Golgi vesicle-mediated transport;establishment of localization;establishment of localization in cell;Golgi vesicle transport;intracellular transport;transport;vesicle-mediated transport
biological regulation;biosynthetic process;cellular biosynthetic process;cellular macromolecule biosynthetic process;cellular macromolecule metabolic process;cellular metabolic process;cellular nitrogen compound metabolic
biological regulation;cell cycle arrest;cell cycle process;cellular metabolic process;cellular process;cellular respiration;energy derivation by oxidation of organic compounds;generation of precursor metabolites and energy;met
activation of caspase activity;apoptosis;biological regulation;cell death;cellular process;cellular response to chemical stimulus;cellular response to hypoxia;cellular response to oxygen levels;cellular response to stimulus;cellul
cellular component organization;cellular component organization at cellular level;cellular component organization or biogenesis;cellular component organization or biogenesis at cellular level;cellular macromolecule metabolic
anatomical structure morphogenesis;biological adhesion;biological regulation;cell adhesion;cell junction assembly;cell junction organization;cell morphogenesis;cell morphogenesis involved in differentiation;cell surface recep
biological regulation;biosynthetic process;cellular biosynthetic process;cellular macromolecule biosynthetic process;cellular macromolecule metabolic process;cellular metabolic process;cellular nitrogen compound metabolic
alcohol catabolic process;alcohol metabolic process;carbohydrate catabolic process;carbohydrate metabolic process;catabolic process;cellular carbohydrate catabolic process;cellular carbohydrate metabolic process;cellula
cellular macromolecule metabolic process;cellular metabolic process;cellular process;cellular protein metabolic process;chaperone-mediated protein folding;macromolecule metabolic process;macromolecule modification;me
cellular macromolecule metabolic process;cellular metabolic process;cellular process;cellular protein metabolic process;establishment of localization;establishment of localization in cell;establishment of protein localization;es
cation transport;cellular process;drug transmembrane transport;drug transport;establishment of localization;excretion;ion transport;multicellular organismal process;organic cation transport;secretion;system process;transmer
cellular macromolecule metabolic process;cellular metabolic process;cellular nitrogen compound metabolic process;cellular process;cellular protein metabolic process;macromolecule metabolic process;macromolecule modi
alcohol metabolic process;carbohydrate metabolic process;cellular carbohydrate metabolic process;cellular metabolic process;cellular process;galactose metabolic process;glucose metabolic process;hexose metabolic proc
amine biosynthetic process;amine metabolic process;biosynthetic process;carboxylic acid biosynthetic process;carboxylic acid metabolic process;cellular amine metabolic process;cellular amino acid biosynthetic process;ce
biological regulation;catabolic process;cellular catabolic process;cellular macromolecule catabolic process;cellular macromolecule metabolic process;cellular metabolic process;cellular nitrogen compound metabolic process
biological regulation;cellular component organization;cellular component organization at cellular level;cellular component organization or biogenesis;cellular component organization or biogenesis at cellular level;cellular proce
cell cycle;cell division;cellular process
cellular localization;cellular process;establishment of localization;establishment of localization in cell;establishment of organelle localization;establishment of protein localization;establishment of ribosome localization;intracellu
biosynthetic process;cellular biosynthetic process;cellular component disassembly;cellular component disassembly at cellular level;cellular component organization;cellular component organization at cellular level;cellular con
cellular component biogenesis;cellular component biogenesis at cellular level;cellular component organization or biogenesis;cellular component organization or biogenesis at cellular level;cellular macromolecule metabolic pro
cellular macromolecule metabolic process;cellular metabolic process;cellular nitrogen compound metabolic process;cellular process;macromolecule metabolic process;metabolic process;ncRNA metabolic process;ncRNA pr
biosynthetic process;cellular biosynthetic process;cellular component disassembly;cellular component disassembly at cellular level;cellular component organization;cellular component organization at cellular level;cellular con
biological regulation;biosynthetic process;cellular biosynthetic process;cellular component organization;cellular component organization or biogenesis;cellular macromolecule biosynthetic process;cellular macromolecule met
4-hydroxyproline metabolic process;amine metabolic process;apoptosis;apoptosis in response to endoplasmic reticulum stress;biological regulation;brown fat cell differentiation;calcium ion homeostasis;calcium ion transport
biological regulation;cellular process;cellular response to stimulus;cellular response to stress;positive regulation of molecular function;positive regulation of NF-kappaB transcription factor activity;positive regulation of sequen
biological regulation;biosynthetic process;cellular biosynthetic process;cellular macromolecule biosynthetic process;cellular macromolecule metabolic process;cellular metabolic process;cellular nitrogen compound metabolic
alcohol biosynthetic process;alcohol metabolic process;biosynthetic process;carbohydrate biosynthetic process;carbohydrate metabolic process;cellular biosynthetic process;cellular carbohydrate biosynthetic process;cellul
actin cytoskeleton organization;actin filament bundle assembly;actin filament organization;actin filament-based process;apoptosis;biological regulation;biosynthetic process;cell death;cellular biosynthetic process;cellular con
anatomical structure development;biological regulation;brain development;cell cycle checkpoint;cell cycle process;cell proliferation;cellular macromolecule metabolic process;cellular metabolic process;cellular process;cellula
base-excision repair;biological regulation;catabolic process;cellular catabolic process;cellular macromolecule catabolic process;cellular macromolecule metabolic process;cellular metabolic process;cellular nitrogen compour
amine biosynthetic process;amine metabolic process;biosynthetic process;carboxylic acid biosynthetic process;carboxylic acid metabolic process;cellular amine metabolic process;cellular amino acid biosynthetic process;ce
catabolic process;cellular catabolic process;cellular macromolecule catabolic process;cellular macromolecule metabolic process;cellular metabolic process;cellular nitrogen compound metabolic process;cellular process;cellu
actin cytoskeleton organization;actin cytoskeleton reorganization;actin filament organization;actin filament reorganization involved in cell cycle;actin filament-based process;biological regulation;cell cycle process;cellular com
cellular component organization;cellular component organization or biogenesis;cellular membrane organization;cellular process;endocytosis;establishment of localization;membrane invagination;membrane organization;transp
biological regulation;biosynthetic process;cellular biosynthetic process;cellular macromolecule biosynthetic process;cellular macromolecule metabolic process;cellular metabolic process;cellular nitrogen compound metabolic
biological regulation;cellular component organization;cellular component organization at cellular level;cellular component organization or biogenesis;cellular component organization or biogenesis at cellular level;cellular macro
establishment of localization;lipid transport;organic substance transport;transport
anatomical structure development;anatomical structure morphogenesis;biological regulation;blood vessel development;branching morphogenesis of a tube;cardiac septum morphogenesis;developmental process;hemopoieti
biological regulation;biosynthetic process;cell cycle;cell proliferation;cellular biosynthetic process;cellular component organization;cellular component organization at cellular level;cellular component organization or biogenesi
biological regulation;negative regulation of apoptosis;negative regulation of biological process;negative regulation of cell death;negative regulation of cellular process;negative regulation of programmed cell death;regulation o
actin cytoskeleton organization;actin filament-based process;actomyosin structure organization;anatomical structure development;biological regulation;cellular component organization;cellular component organization at cellu
carboxylic acid transport;cellular lipid metabolic process;cellular metabolic process;cellular process;establishment of localization;fatty acid transport;lipid localization;lipid metabolic process;lipid storage;lipid transport;localiza
cellular component assembly;cellular component assembly at cellular level;cellular component organization;cellular component organization at cellular level;cellular component organization or biogenesis;cellular component o
biological regulation;biosynthetic process;cellular biosynthetic process;cellular macromolecule metabolic process;cellular metabolic process;cellular nitrogen compound metabolic process;cellular process;cellular response to
cell differentiation;cellular developmental process;cellular process;developmental process;establishment of localization;establishment of protein localization;gamete generation;gene expression;macromolecule metabolic proc
biological regulation;biosynthetic process;cellular biosynthetic process;cellular component assembly;cellular component assembly at cellular level;cellular component organization;cellular component organization at cellular le
cellular macromolecule metabolic process;cellular metabolic process;cellular process;cellular protein metabolic process;macromolecule metabolic process;metabolic process;primary metabolic process;protein folding;protein
cellular macromolecule metabolic process;cellular metabolic process;cellular process;cellular protein metabolic process;cellular response to heat;cellular response to stimulus;cellular response to stress;chaperone cofactor-d
anatomical structure morphogenesis;biological regulation;cell differentiation;cell differentiation in hindbrain;cell part morphogenesis;cell projection morphogenesis;cell projection organization;cellular component assembly;cell
amine catabolic process;amine metabolic process;branched chain family amino acid catabolic process;branched chain family amino acid metabolic process;carboxylic acid catabolic process;carboxylic acid metabolic proces
behavior;biological regulation;body fluid secretion;carbohydrate homeostasis;cellular chemical homeostasis;cellular glucose homeostasis;cellular homeostasis;cellular ion homeostasis;cellular metabolic process;cellular nitrog
cell cycle;cell cycle process;cell division;cellular component organization;cellular component organization at cellular level;cellular component organization or biogenesis;cellular component organization or biogenesis at cellula
cellular macromolecule metabolic process;cellular metabolic process;cellular nitrogen compound metabolic process;cellular process;macromolecule metabolic process;metabolic process;nitrogen compound metabolic proce
cellular component organization;cellular component organization at cellular level;cellular component organization or biogenesis;cellular component organization or biogenesis at cellular level;cellular macromolecule metabolic
cellular macromolecule metabolic process;cellular metabolic process;cellular nitrogen compound metabolic process;cellular process;cellular protein metabolic process;cellular response to stimulus;cellular response to stress;
anatomical structure development;biological regulation;biosynthetic process;cellular biosynthetic process;cellular macromolecule biosynthetic process;cellular macromolecule metabolic process;cellular metabolic process;ce
acute inflammatory response;acute-phase response;alcohol metabolic process;cellular macromolecule metabolic process;cellular metabolic process;cellular process;cholesterol metabolic process;defense response;develop
actin cytoskeleton organization;actin filament branching;actin filament bundle assembly;actin filament organization;actin filament-based process;biological regulation;cell chemotaxis;cell migration;cell motility;cell projection o
biosynthetic process;cellular biosynthetic process;cellular component disassembly;cellular component disassembly at cellular level;cellular component organization;cellular component organization at cellular level;cellular con
cell cycle;cellular process
biological regulation;cell redox homeostasis;cellular homeostasis;cellular macromolecule metabolic process;cellular metabolic process;cellular process;cellular protein metabolic process;cellular response to stimulus;cellular r
cell cycle phase;cell cycle process;cell division;cellular component organization;cellular component organization at cellular level;cellular component organization or biogenesis;cellular component organization or biogenesis at
adherens junction assembly;adherens junction organization;biological regulation;cell junction assembly;cell junction organization;cell-cell junction assembly;cell-cell junction organization;cellular component assembly;cellular
alcohol metabolic process;biological regulation;carbohydrate catabolic process;carbohydrate metabolic process;catabolic process;cellular carbohydrate catabolic process;cellular carbohydrate metabolic process;cellular met
cellular component assembly;cellular component assembly at cellular level;cellular component organization;cellular component organization at cellular level;cellular component organization or biogenesis;cellular component o
cellular macromolecule metabolic process;cellular metabolic process;cellular nitrogen compound metabolic process;cellular process;macromolecule metabolic process;metabolic process;ncRNA metabolic process;ncRNA pr
cellular component assembly;cellular component assembly at cellular level;cellular component organization;cellular component organization at cellular level;cellular component organization or biogenesis;cellular component o
biological regulation;cellular process;cellular response to abiotic stimulus;cellular response to gamma radiation;cellular response to ionizing radiation;cellular response to radiation;cellular response to stimulus;cellular respons
lipid metabolic process;metabolic process;primary metabolic process
biological regulation;cap-independent translational initiation;cell differentiation;cellular developmental process;cellular macromolecule metabolic process;cellular metabolic process;cellular nitrogen compound metabolic proce
biological regulation;cation homeostasis;chemical homeostasis;homeostatic process;ion homeostasis;iron ion homeostasis;regulation of biological quality
amine metabolic process;aminoglycan catabolic process;aminoglycan metabolic process;carbohydrate catabolic process;carbohydrate metabolic process;catabolic process;chitin catabolic process;chitin metabolic process;
establishment of localization;lipid transport;organic substance transport;transport
biological regulation;cellular component assembly;cellular component assembly at cellular level;cellular component organization;cellular component organization at cellular level;cellular component organization or biogenesis;
biosynthetic process;cellular biosynthetic process;cellular component disassembly;cellular component disassembly at cellular level;cellular component organization;cellular component organization at cellular level;cellular con
biosynthetic process;cellular biosynthetic process;cellular component disassembly;cellular component disassembly at cellular level;cellular component organization;cellular component organization at cellular level;cellular con
biosynthetic process;cellular biosynthetic process;cellular component disassembly;cellular component disassembly at cellular level;cellular component organization;cellular component organization at cellular level;cellular con
biological regulation;biosynthetic process;cellular biosynthetic process;cellular lipid metabolic process;cellular macromolecule biosynthetic process;cellular macromolecule metabolic process;cellular metabolic process;cellula
biosynthetic process;cellular biosynthetic process;cellular component disassembly;cellular component disassembly at cellular level;cellular component organization;cellular component organization at cellular level;cellular con
biological regulation;blood coagulation;cell activation;cellular process;coagulation;establishment of localization;establishment of localization in cell;exocytosis;hemostasis;multicellular organismal process;platelet activation;pla
biological regulation;cell differentiation;cellular component biogenesis;cellular component biogenesis at cellular level;cellular component organization or biogenesis;cellular component organization or biogenesis at cellular lev
antigen processing and presentation;biological regulation;cellular component organization;cellular component organization at cellular level;cellular component organization or biogenesis;cellular component organization or bio
biological regulation;cellular process;cellular response to stimulus;cellular response to stress;negative regulation of biological process;negative regulation of cellular metabolic process;negative regulation of cellular process;ne
apoptosis;biological regulation;cell death;cellular process;death;negative regulation of apoptosis;negative regulation of biological process;negative regulation of cell death;negative regulation of cellular process;negative regula
adipose tissue development;anatomical structure development;biological regulation;cellular macromolecule metabolic process;cellular metabolic process;cellular nitrogen compound metabolic process;cellular process;demet
anatomical structure formation involved in morphogenesis;associative learning;behavior;cellular component maintenance;cellular component maintenance at cellular level;cellular component organization;cellular component o

biological regulation;cell proliferation;cell surface receptor linked signaling pathway;cellular component biogenesis;cellular component biogenesis at cellular level;cellular component organization or biogenesis;cellular compor
biological regulation;regulation of biological process;regulation of biosynthetic process;regulation of cellular biosynthetic process;regulation of cellular macromolecule biosynthetic process;regulation of cellular metabolic proc
biological regulation;biosynthetic process;cellular biosynthetic process;cellular component assembly;cellular component assembly at cellular level;cellular component organization;cellular component organization at cellular le
biological regulation;biosynthetic process;catabolic process;cell surface receptor linked signaling pathway;cellular biosynthetic process;cellular catabolic process;cellular component organization;cellular component organizat
4-hydroxyproline metabolic process;amine metabolic process;anatomical structure development;anatomical structure formation involved in morphogenesis;anatomical structure morphogenesis;biological regulation;carboxylic
cell cycle process;cellular component organization;cellular component organization at cellular level;cellular component organization or biogenesis;cellular component organization or biogenesis at cellular level;cellular macrom
cellular process;cellular response to heat;cellular response to stimulus;cellular response to stress;response to abiotic stimulus;response to heat;response to stimulus;response to stress;response to temperature stimulus
cellular component biogenesis;cellular component biogenesis at cellular level;cellular component organization or biogenesis;cellular component organization or biogenesis at cellular level;cellular process;establishment of loca
biosynthetic process;cellular biosynthetic process;cellular component disassembly;cellular component disassembly at cellular level;cellular component organization;cellular component organization at cellular level;cellular cor
cellular process;establishment of localization;establishment of localization in cell;establishment of protein localization;intracellular protein transport;intracellular transport;protein transport;transport;vesicle-mediated transport
cellular macromolecule metabolic process;cellular metabolic process;cellular nitrogen compound metabolic process;cellular process;cellular protein metabolic process;macromolecule metabolic process;macromolecule modi
amine transport;amino acid transmembrane transport;amino acid transport;biological regulation;carboxylic acid transport;cell communication;cell-cell signaling;cellular process;establishment of localization;ion transport;neuro
biological regulation;biosynthetic process;cellular biosynthetic process;cellular macromolecule biosynthetic process;cellular macromolecule metabolic process;cellular metabolic process;cellular nitrogen compound metabolic
biological regulation;biosynthetic process;cell cycle;cell differentiation;cell surface receptor linked signaling pathway;cellular biosynthetic process;cellular developmental process;cellular macromolecule biosynthetic process;c
biological regulation;cell redox homeostasis;cellular homeostasis;cellular macromolecule metabolic process;cellular metabolic process;cellular process;cellular protein metabolic process;cellular response to stimulus;cellular r
biological regulation;biosynthetic process;cellular biosynthetic process;cellular macromolecule biosynthetic process;cellular macromolecule metabolic process;cellular metabolic process;cellular nitrogen compound metabolic
anatomical structure development;cell differentiation;cellular developmental process;cellular macromolecule metabolic process;cellular metabolic process;cellular process;cellular protein metabolic process;chaperone-mediat
anatomical structure development;appendage development;biological regulation;cell differentiation;cellular component organization;cellular component organization at cellular level;cellular component organization or biogene
actin cytoskeleton organization;actin filament-based process;cell communication;cell-cell signaling;cellular component organization;cellular component organization at cellular level;cellular component organization or biogene
biological regulation;blood coagulation;cell projection organization;cellular component assembly;cellular component organization;cellular component organization at cellular level;cellular component organization or biogenesis
catabolic process;cellular catabolic process;cellular component assembly;cellular component organization;cellular component organization or biogenesis;cellular metabolic process;cellular nitrogen compound catabolic proce
biological regulation;negative regulation of catalytic activity;negative regulation of hydrolase activity;negative regulation of molecular function;negative regulation of phosphatase activity;regulation of biological process;regulat
cellular macromolecule metabolic process;cellular metabolic process;cellular nitrogen compound metabolic process;cellular process;macromolecule metabolic process;metabolic process;nitrogen compound metabolic proce
biological regulation;cellular component organization;cellular component organization at cellular level;cellular component organization or biogenesis;cellular component organization or biogenesis at cellular level;cellular macro
cellular macromolecule metabolic process;cellular metabolic process;cellular process;cellular protein metabolic process;establishment of localization;establishment of localization in cell;establishment of protein localization;es
amine catabolic process;amine metabolic process;biotin metabolic process;branched chain family amino acid catabolic process;branched chain family amino acid metabolic process;carboxylic acid catabolic process;carbox
anatomical structure development;biological regulation;biosynthetic process;brain development;cation homeostasis;cellular biosynthetic process;cellular cation homeostasis;cellular chemical homeostasis;cellular homeostasi
biological regulation;biosynthetic process;cellular biosynthetic process;cellular component organization;cellular component organization at cellular level;cellular component organization or biogenesis;cellular component orga
amine metabolic process;amino acid activation;carboxylic acid metabolic process;cellular amine metabolic process;cellular amino acid metabolic process;cellular ketone metabolic process;cellular macromolecule metabolic
apoptosis;biosynthetic process;cell death;cellular biosynthetic process;cellular component disassembly;cellular component disassembly at cellular level;cellular component organization;cellular component organization at cel
ameboidal cell migration;anatomical structure development;associative learning;basement membrane organization;behavior;biological adhesion;biological regulation;cAMP-mediated signaling;cell adhesion;cell migration;cell
anatomical structure development;cell differentiation;cellular developmental process;cellular process;developmental process;nervous system development;system development
B cell activation;B cell activation involved in immune response;biological regulation;catabolic process;cell activation;cell activation involved in immune response;cellular catabolic process;cellular localization;cellular macromo
biological regulation;cell cycle cytokinesis;cell cycle phase;cell cycle process;cell differentiation;cellular component assembly;cellular component assembly at cellular level;cellular component organization;cellular component
bradykinin catabolic process;catabolic process;cellular catabolic process;cellular metabolic process;cellular process;macromolecule metabolic process;metabolic process;peptide catabolic process;peptide metabolic proces
aromatic compound biosynthetic process;biosynthetic process;cellular aromatic compound metabolic process;cellular biosynthetic process;cellular component organization;cellular component organization or biogenesis;cell
actin cytoskeleton organization;actin filament-based process;biological regulation;cell differentiation;cellular component organization;cellular component organization at cellular level;cellular component organization or biogen
biosynthetic process;cell differentiation;cellular biosynthetic process;cellular developmental process;cellular macromolecule biosynthetic process;cellular macromolecule metabolic process;cellular metabolic process;cellular
biological regulation;cell cycle;cell cycle process;cell division;cell proliferation;cellular component organization;cellular component organization at cellular level;cellular component organization or biogenesis;cellular componen
cell cycle;cell cycle process;cell division;cellular component organization;cellular component organization at cellular level;cellular component organization or biogenesis;cellular component organization or biogenesis at cellula
actin cytoskeleton organization;actin cytoskeleton reorganization;actin filament-based process;actin-mediated cell contraction;anatomical structure formation involved in morphogenesis;anatomical structure morphogenesis;a
biological regulation;cell growth;cellular process;growth;negative regulation of apoptosis;negative regulation of biological process;negative regulation of cell death;negative regulation of cellular process;negative regulation of
actin cytoskeleton organization;actin filament-based process;cellular component biogenesis;cellular component biogenesis at cellular level;cellular component organization;cellular component organization at cellular level;cell
biological regulation;biosynthetic process;cellular biosynthetic process;cellular macromolecule biosynthetic process;cellular macromolecule metabolic process;cellular metabolic process;cellular nitrogen compound metabolic
actin cytoskeleton organization;actin filament capping;actin filament organization;actin filament-based process;actomyosin structure organization;anatomical structure development;anatomical structure formation involved in
carbohydrate metabolic process;cellular carbohydrate metabolic process;cellular macromolecule metabolic process;cellular metabolic process;cellular process;cellular protein metabolic process;'de novo' posttranslational pr
activation of caspase activity;biological regulation;cellular component organization;cellular component organization or biogenesis;cellular membrane organization;cellular process;establishment of localization;membrane orga
anatomical structure formation involved in morphogenesis;biological regulation;blood circulation;cellular component organization;cellular component organization or biogenesis;cellular membrane organization;cellular process
actin cytoskeleton organization;actin filament-based process;biological regulation;blood coagulation;cellular component organization;cellular component organization at cellular level;cellular component organization or biogen
biological regulation;cell surface receptor linked signaling pathway;cellular process;cellular response to stimulus;detection of chemical stimulus;detection of chemical stimulus involved in sensory perception;detection of chem
biological regulation;cellular component organization;cellular component organization at cellular level;cellular component organization or biogenesis;cellular component organization or biogenesis at cellular level;cellular proce
biosynthetic process;cellular biosynthetic process;cellular component disassembly;cellular component disassembly at cellular level;cellular component organization;cellular component organization at cellular level;cellular cor
biological regulation;cell communication;cellular component organization;cellular component organization at cellular level;cellular component organization or biogenesis;cellular component organization or biogenesis at cellula
biological regulation;cell cycle;cell cycle phase;cell cycle process;cell division;cell junction assembly;cell junction organization;cell surface receptor linked signaling pathway;cell-substrate junction assembly;cellular componen
biosynthetic process;cell differentiation;cellular biosynthetic process;cellular developmental process;cellular macromolecule biosynthetic process;cellular macromolecule metabolic process;cellular metabolic process;cellular
amine metabolic process;amino acid activation;carboxylic acid metabolic process;cellular amine metabolic process;cellular amino acid metabolic process;cellular ketone metabolic process;cellular macromolecule metabolic
cellular macromolecule metabolic process;cellular metabolic process;cellular nitrogen compound metabolic process;cellular process;macromolecule metabolic process;metabolic process;mRNA metabolic process;mRNA pro
biological regulation;cellular component organization;cellular component organization at cellular level;cellular component organization or biogenesis;cellular component organization or biogenesis at cellular level;cellular locali
acetyl-CoA catabolic process;acetyl-CoA metabolic process;biosynthetic process;carboxylic acid metabolic process;catabolic process;cellular biosynthetic process;cellular catabolic process;cellular ketone metabolic proces
biological regulation;biosynthetic process;cellular biosynthetic process;cellular component assembly;cellular component assembly at cellular level;cellular component organization;cellular component organization at cellular le
carboxylic acid metabolic process;cellular aldehyde metabolic process;cellular component assembly;cellular component organization;cellular component organization or biogenesis;cellular ketone metabolic process;cellular r
anatomical structure morphogenesis;angiotensin maturation;biological regulation;branching morphogenesis of a tube;cellular macromolecule metabolic process;cellular metabolic process;cellular process;cellular protein meta
biological regulation;biosynthetic process;cellular biosynthetic process;cellular macromolecule biosynthetic process;cellular macromolecule metabolic process;cellular metabolic process;cellular nitrogen compound metabolic
apoptosis;biological regulation;cell death;cellular component disassembly;cellular component disassembly at cellular level;cellular component disassembly involved in apoptosis;cellular component organization;cellular comp
activation of JUN kinase activity;activation of MAPK activity;androgen receptor signaling pathway;apoptosis;biological regulation;biosynthetic process;cell cycle cytokinesis;cell cycle process;cell death;cellular biosynthetic pr
establishment of localization;transport
biological regulation;cellular localization;cellular macromolecule localization;cellular process;cellular protein localization;localization;macromolecule localization;positive regulation of biological process;positive regulation of bio
actin cytoskeleton organization;actin filament bundle assembly;actin filament organization;actin filament-based process;biological regulation;cell projection organization;cellular component assembly;cellular component assem
biological regulation;cellular macromolecule metabolic process;cellular metabolic process;cellular process;cellular protein metabolic process;chaperone-mediated protein folding;macromolecule metabolic process;metabolic
amine catabolic process;amine metabolic process;carboxylic acid catabolic process;carboxylic acid metabolic process;catabolic process;cellular amine metabolic process;cellular amino acid catabolic process;cellular amino
developmental process;multicellular organismal development;multicellular organismal process
actin crosslink formation;actin cytoskeleton organization;actin filament bundle assembly;actin filament organization;actin filament-based process;biological regulation;cell surface receptor linked signaling pathway;cellular con
ATP hydrolysis coupled proton transport;biological regulation;cation homeostasis;cation transport;cell surface receptor linked signaling pathway;cellular cation homeostasis;cellular chemical homeostasis;cellular component
anatomical structure morphogenesis;biological regulation;biosynthetic process;cellular biosynthetic process;cellular component organization;cellular component organization at cellular level;cellular component organization or
alcohol catabolic process;alcohol metabolic process;amino sugar catabolic process;amino sugar metabolic process;carbohydrate catabolic process;carbohydrate metabolic process;carbohydrate phosphorylation;carboxylic
carboxylic acid catabolic process;carboxylic acid metabolic process;catabolic process;cellular catabolic process;cellular component assembly;cellular component organization;cellular component organization or biogenesis;c
biological regulation;cellular process;cellular response to stimulus;negative regulation of biological process;negative regulation of cell communication;negative regulation of cellular process;negative regulation of Ras protein s
anatomical structure development;anatomical structure morphogenesis;biological regulation;developmental process;epidermis morphogenesis;epithelium development;lung alveolus development;lung epithelium developmen
antigen processing and presentation;antigen processing and presentation of exogenous antigen;antigen processing and presentation of exogenous peptide antigen;antigen processing and presentation of exogenous peptide
developmental process
biological regulation;cellular component organization;cellular component organization at cellular level;cellular component organization or biogenesis;cellular component organization or biogenesis at cellular level;cellular meml
biological regulation;biosynthetic process;carbohydrate metabolic process;carbohydrate transport;cell cycle;cell cycle process;cell surface receptor linked signaling pathway;cellular biosynthetic process;cellular component c
cell proliferation
multicellular organismal process;neurological system process;sensory perception;sensory perception of light stimulus;system process;visual perception
biological regulation;biosynthetic process;blood coagulation;cell differentiation;cellular biosynthetic process;cellular developmental process;cellular macromolecule biosynthetic process;cellular macromolecule metabolic proc
biological adhesion;cell adhesion;cell-cell adhesion;cellular component organization;cellular component organization or biogenesis;cellular membrane organization;cellular process;endocytosis;establishment of localization;m
cellular macromolecule metabolic process;cellular metabolic process;cellular nitrogen compound metabolic process;cellular process;establishment of localization;establishment of localization in cell;establishment of protein lo
actin cytoskeleton organization;actin filament-based process;anatomical structure morphogenesis;biological regulation;caveola assembly;caveolin-mediated endocytosis;cell part morphogenesis;cell projection morphogenesi
antigen processing and presentation;biological regulation;blood coagulation;cell migration;cell motility;cellular component movement;cellular process;coagulation;hemostasis;immune response;immune system process;leuko
biological regulation;cell cycle;cell cycle phase;cell cycle process;cell division;cellular component organization;cellular component organization at cellular level;cellular component organization or biogenesis;cellular componen
biological regulation;cellular component assembly;cellular component assembly at cellular level;cellular component organization;cellular component organization at cellular level;cellular component organization or biogenesis;
actin cytoskeleton organization;actin filament depolymerization;actin filament organization;actin filament-based process;actin polymerization or depolymerization;actomyosin structure organization;anatomical structure homeo
cellular process;establishment of localization;transport;vesicle-mediated transport
anatomical structure development;biological adhesion;cell adhesion;cell development;cell-cell adhesion;cellular developmental process;cellular process;cellular process involved in reproduction;developmental process;develo
biological regulation;catabolic process;cellular catabolic process;cellular macromolecule catabolic process;cellular macromolecule metabolic process;cellular metabolic process;cellular nitrogen compound metabolic process
amine metabolic process;carboxylic acid metabolic process;cell differentiation;cellular amine metabolic process;cellular amino acid metabolic process;cellular developmental process;cellular ketone metabolic process;cellular
cellular macromolecule metabolic process;cellular metabolic process;cellular nitrogen compound metabolic process;cellular process;macromolecule metabolic process;metabolic process;ncRNA metabolic process;ncRNA pr
biosynthetic process;cellular biosynthetic process;cellular component disassembly;cellular component disassembly at cellular level;cellular component organization;cellular component organization at cellular level;cellular cor
amine metabolic process;amino acid activation;biosynthetic process;carboxylic acid metabolic process;cellular amine metabolic process;cellular amino acid metabolic process;cellular biosynthetic process;cellular ketone me
acyl-CoA metabolic process;cellular metabolic process;cellular process;coenzyme metabolic process;cofactor metabolic process;metabolic process;thioester metabolic process
biological regulation;metabolic process;negative regulation of catalytic activity;negative regulation of molecular function;negative regulation of monooxygenase activity;negative regulation of nitric-oxide synthase activity;nega
catabolic process;cellular catabolic process;cellular metabolic process;cellular nitrogen compound catabolic process;cellular nitrogen compound metabolic process;cellular process;deoxyribonucleoside catabolic process;de
cellular component assembly;cellular component assembly at cellular level;cellular component organization;cellular component organization at cellular level;cellular component organization or biogenesis;cellular component c
biological regulation;biosynthetic process;cellular biosynthetic process;cellular macromolecule biosynthetic process;cellular macromolecule metabolic process;cellular metabolic process;cellular process;cellular protein metab
anatomical structure development;biological regulation;biomineral tissue development;bone marrow development;bone mineralization;cell chemotaxis;cell cycle process;cell migration;cell motility;cell proliferation;cellular com
establishment of localization;establishment of protein localization;protein transport;transport
biosynthetic process;cellular biosynthetic process;cellular component disassembly;cellular component disassembly at cellular level;cellular component organization;cellular component organization at cellular level;cellular cor
cellular metabolic process;cellular nitrogen compound metabolic process;cellular process;metabolic process;nitrogen compound metabolic process;nucleic acid metabolic process;nucleic acid phosphodiester bond hydrolys
cellular macromolecule metabolic process;cellular metabolic process;cellular process;cellular protein metabolic process;cellular response to stimulus;cellular response to stress;macromolecule metabolic process;macromolec
biological regulation;cell cycle;cellular process;positive regulation of catalytic activity;positive regulation of GTPase activity;positive regulation of hydrolase activity;positive regulation of molecular function;regulation of biologic
biosynthetic process;catabolic process;cellular biosynthetic process;cellular catabolic process;cellular component disassembly;cellular component disassembly at cellular level;cellular component organization;cellular compo
2'-deoxyribonucleotide metabolic process;biological regulation;catabolic process;cell surface receptor linked signaling pathway;cellular catabolic process;cellular component assembly;cellular component organization;cellula

biological adhesion;cell adhesion;cell junction assembly;cell junction organization;cell-cell adhesion;cell-cell junction assembly;cell-cell junction organization;cellular component assembly;cellular component assembly at cellu
biological regulation;BMP signaling pathway;catabolic process;cell surface receptor linked signaling pathway;cellular catabolic process;cellular component organization;cellular component organization at cellular level;cellular
anatomical structure morphogenesis;biological regulation;cell part morphogenesis;cell projection morphogenesis;cell projection organization;cellular component assembly;cellular component assembly at cellular level;cellular
aging;anatomical structure formation involved in morphogenesis;anatomical structure morphogenesis;angiogenesis;biological adhesion;biological regulation;biosynthetic process;cell adhesion;cell migration;cell morphogenes
biological regulation;catabolic process;cellular catabolic process;cellular macromolecule catabolic process;cellular macromolecule metabolic process;cellular metabolic process;cellular nitrogen compound metabolic process
cell differentiation;cellular developmental process;cellular process;developmental process;hemopoietic progenitor cell differentiation
biological regulation;cellular macromolecule metabolic process;cellular metabolic process;cellular process;cellular protein metabolic process;demethylation;macromolecule metabolic process;macromolecule modification;met
cellular macromolecule metabolic process;cellular metabolic process;cellular process;cellular protein metabolic process;chaperone-mediated protein transport;establishment of localization;establishment of localization in cell;
anatomical structure formation involved in morphogenesis;biological regulation;cation homeostasis;cation transport;cell projection assembly;cell projection organization;cell surface receptor linked signaling pathway;cellular c
biosynthetic process;cellular biosynthetic process;cellular metabolic process;cellular process;cofactor biosynthetic process;cofactor metabolic process;iron-sulfur cluster assembly;metabolic process;metallo-sulfur cluster as
cellular component biogenesis;cellular component biogenesis at cellular level;cellular component organization;cellular component organization at cellular level;cellular component organization or biogenesis;cellular componen
biological regulation;cellular macromolecule metabolic process;cellular metabolic process;cellular nitrogen compound metabolic process;cellular process;macromolecule metabolic process;macromolecule modification;metab
amine biosynthetic process;amine metabolic process;biosynthetic process;carboxylic acid biosynthetic process;carboxylic acid metabolic process;cellular amine metabolic process;cellular amino acid biosynthetic process;ce
biological regulation;biosynthetic process;cell cycle;cell surface receptor linked signaling pathway;cellular biosynthetic process;cellular component organization;cellular component organization at cellular level;cellular compor
cellular component movement;cellular component organization;cellular component organization at cellular level;cellular component organization or biogenesis;cellular component organization or biogenesis at cellular level;cel
amine metabolic process;aromatic compound biosynthetic process;biosynthetic process;carboxylic acid metabolic process;cellular amine metabolic process;cellular amino acid metabolic process;cellular aromatic compound
cellular localization;cellular macromolecule localization;cellular process;cellular protein localization;localization;macromolecule localization;protein localization;protein localization in mitochondrion;protein localization to organe
biological regulation;cell cycle phase;cell cycle process;cell division;cellular component movement;cellular component organization;cellular component organization at cellular level;cellular component organization or biogene
biological regulation;biosynthetic process;cellular biosynthetic process;cellular macromolecule biosynthetic process;cellular macromolecule metabolic process;cellular metabolic process;cellular nitrogen compound metabolic
biological regulation;biosynthetic process;cellular biosynthetic process;cellular component organization;cellular component organization at cellular level;cellular component organization or biogenesis;cellular component orga
biological regulation;cell surface receptor linked signaling pathway;cellular metabolic process;cellular nitrogen compound metabolic process;cellular process;cellular response to chemical stimulus;cellular response to cytokin
gamete generation;male gamete generation;multicellular organismal process;multicellular organismal reproductive process;reproductive process;spermatogenesis

C: GOMF name
adenyl nucleotide binding;adenyl ribonucleotide binding;ATP binding;binding;catalytic activity;FAT10 activating enzyme activity;nucleotide binding;purine nucleotide binding;purine ribonucleoside triphosphate binding;purine r
alcohol binding;amine binding;binding;calcium ion binding;calcium-dependent phospholipid binding;cation binding;identical protein binding;ion binding;lipid binding;metal ion binding;phosphatidylcholine binding;phosphatidy
binding;lipid binding;phosphatidylinositol binding;phosphatidylinositol-3,5-bisphosphate binding;phosphatidylinositol-3-phosphate binding;phosphatidylinositol-4-phosphate binding;phosphatidylinositol-5-phosphate binding
binding;catalytic activity;cation binding;hydrolase activity;hydrolase activity, acting on ester bonds;ion binding;magnesium ion binding;metal ion binding;phosphatase activity;phosphoglycolate phosphatase activity;phosphop
adenyl nucleotide binding;adenyl ribonucleotide binding;ATP binding;binding;nucleotide binding;purine nucleotide binding;purine ribonucleoside triphosphate binding;purine ribonucleotide binding;ribonucleotide binding
alkylglycerone-phosphate synthase activity;binding;catalytic activity;coenzyme binding;cofactor binding;FAD binding;flavin adenine dinucleotide binding;oxidoreductase activity;oxidoreductase activity, acting on CH-OH grou
adenyl nucleotide binding;adenyl ribonucleotide binding;ATP binding;ATPase activity;binding;catalytic activity;hydrolase activity;hydrolase activity, acting on acid anhydrides;hydrolase activity, acting on acid anhydrides, in ph
binding;cation binding;ion binding;metal ion binding;protein binding transcription factor activity;transcription coactivator activity;transcription cofactor activity;transcription factor binding transcription factor activity;transition r
acyl-CoA thioesterase activity;binding;carboxylic acid binding;carboxylic ester hydrolase activity;catalytic activity;CoA hydrolase activity;coenzyme binding;cofactor binding;fatty acid binding;fatty-acyl-CoA binding;hydrolase
actin-dependent ATPase activity;adenyl nucleotide binding;adenyl ribonucleotide binding;ATP binding;ATPase activity;ATPase activity, coupled;binding;catalytic activity;enzyme binding;GTPase binding;hydrolase activity;hydr
4 iron, 4 sulfur cluster binding;binding;catalytic activity;cation binding;ion binding;iron-sulfur cluster binding;metal cluster binding;metal ion binding;NADH dehydrogenase (quinone) activity;NADH dehydrogenase (ubiquinone)
binding;cation binding;heme binding;ion binding;iron ion binding;lipid binding;metal ion binding;steroid binding;tetrapyrrole binding;transition metal ion binding
binding;catalytic activity;cysteine-type peptidase activity;hydrolase activity;nucleic acid binding;peptidase activity;peptidase activity, acting on L-amino acid peptides;RNA binding;small conjugating protein-specific protease a
binding;enzyme binding;enzyme inhibitor activity;enzyme regulator activity;GTPase binding;GTPase inhibitor activity;GTPase regulator activity;nucleoside-triphosphatase regulator activity;protein binding;protein transporter ac
binding;catalytic activity;DNA binding;DNA-directed RNA polymerase activity;nucleic acid binding;nucleotidyltransferase activity;RNA polymerase activity;transferase activity;transferase activity, transferring phosphorus-conta
binding;mRNA 3'-UTR binding;mRNA 5'-UTR binding;mRNA binding;nucleic acid binding;nucleotide binding;RNA binding;translation regulator activity
acetylcholine receptor regulator activity;binding;calcium ion binding;carbohydrate binding;cation binding;chondroitin sulfate binding;dystroglycan binding;extracellular matrix binding;glycoprotein binding;glycosaminoglycan b
binding;carboxylic acid binding;catalytic activity;cation binding;ion binding;iron ion binding;L-ascorbic acid binding;metal ion binding;oxidoreductase activity;oxidoreductase activity, acting on paired donors, with incorporatio
binding;catalytic activity;NADH dehydrogenase (quinone) activity;NADH dehydrogenase (ubiquinone) activity;NADH dehydrogenase activity;oxidoreductase activity;oxidoreductase activity, acting on NADH or NADPH;oxidored
binding;cytoskeletal protein binding;enzyme binding;identical protein binding;protein binding;RNA polymerase binding;tau protein binding
binding;nuclear localization sequence binding;peptide binding;protein transporter activity;signal sequence binding;substrate-specific transporter activity;transporter activity
alpha-mannosidase activity;binding;carbohydrate binding;catalytic activity;cation binding;hydrolase activity;hydrolase activity, acting on glycosyl bonds;hydrolase activity, hydrolyzing O-glycosyl compounds;ion binding;mann
adenyl nucleotide binding;adenyl ribonucleotide binding;alkali metal ion binding;ATP binding;binding;catalytic activity;cation binding;cofactor binding;identical protein binding;ion binding;kinase activity;lithium ion binding;mag
transporter activity
antioxidant activity;binding;catalytic activity;cation binding;cation transmembrane transporter activity;copper ion binding;copper ion transmembrane transporter activity;disulfide oxidoreductase activity;inorganic cation transm
amine binding;amino acid binding;binding;carboxylic acid binding;catalytic activity;glutathione binding;intramolecular oxidoreductase activity;isomerase activity;modified amino acid binding;peptide binding;prostaglandin-E sy
arginine N-methyltransferase activity;binding;catalytic activity;chromatin binding;core promoter binding;core promoter sequence-specific DNA binding;DNA binding;histone methyltransferase activity;histone-arginine N-methy
adrenergic receptor binding;beta-2 adrenergic receptor binding;beta-catenin binding;binding;channel regulator activity;chloride channel regulator activity;enzyme binding;G-protein-coupled receptor binding;growth factor rece
binding;catalytic activity;endopeptidase activity;hydrolase activity;identical protein binding;peptidase activity;peptidase activity, acting on L-amino acid peptides;protein binding;threonine-type endopeptidase activity;threonin
[3-methyl-2-oxobutanoate dehydrogenase (acetyl-transferring)] kinase activity;adenyl nucleotide binding;adenyl ribonucleotide binding;ATP binding;binding;catalytic activity;kinase activity;nucleotide binding;phosphotransfera
binding;identical protein binding;protein binding
actin binding;binding;cytoskeletal protein binding;identical protein binding;myosin binding;protein binding;protein dimerization activity;protein homodimerization activity;receptor binding
acetyltransferase activity;catalytic activity;H4 histone acetyltransferase activity;histone acetyltransferase activity;lysine N-acetyltransferase activity;N-acetyltransferase activity;N-acyltransferase activity;transferase activity;tran
catalytic activity;cation transmembrane transporter activity;hydrogen ion transmembrane transporter activity;inorganic cation transmembrane transporter activity;ion transmembrane transporter activity;monovalent inorganic c
14-3-3 protein binding;binding;enzyme binding;enzyme inhibitor activity;enzyme regulator activity;kinase binding;molecular transducer activity;phosphatase regulator activity;protein binding;protein kinase binding;signal trans
binding;cytoskeletal protein binding;enzyme regulator activity;GTPase regulator activity;guanyl-nucleotide exchange factor activity;kinesin binding;nucleoside-triphosphatase regulator activity;protein binding;Ras guanyl-nucle
binding;integrin binding;protein binding;protein complex binding;receptor binding;structural molecule activity
structural constituent of ribosome;structural molecule activity
binding;C-acyltransferase activity;catalytic activity;cofactor binding;C-palmitoyltransferase activity;palmitoyltransferase activity;pyridoxal phosphate binding;serine C-palmitoyltransferase activity;transferase activity;transferas
binding;catalytic activity;cation binding;hydrolase activity;hydrolase activity, acting on ester bonds;ion binding;metal ion binding;phosphatase activity;phosphoprotein phosphatase activity;phosphoric ester hydrolase activity;p
binding;catalytic activity;hydrolase activity;protein binding;protein domain specific binding;SH2 domain binding
binding;nucleic acid binding;RNA binding;translation factor activity, nucleic acid binding;translation initiation factor activity
binding;nucleic acid binding;RNA binding;translation factor activity, nucleic acid binding;translation initiation factor activity
adenyl nucleotide binding;adenyl ribonucleotide binding;ATP binding;binding;nucleotide binding;purine nucleotide binding;purine ribonucleoside triphosphate binding;purine ribonucleotide binding;ribonucleotide binding
branched-chain-amino-acid transaminase activity;catalytic activity;L-isoleucine transaminase activity;L-leucine transaminase activity;L-valine transaminase activity;transaminase activity;transferase activity;transferase activity
active transmembrane transporter activity;carboxylic acid transmembrane transporter activity;lactate transmembrane transporter activity;monocarboxylic acid transmembrane transporter activity;organic acid transmembrane
binding;carboxylic acid binding;catalytic activity;cation binding;dioxygenase activity;electron carrier activity;ion binding;iron ion binding;L-ascorbic acid binding;metal ion binding;oxidoreductase activity;oxidoreductase activit
binding;nucleic acid binding;RNA binding;snRNA binding;U4 snRNA binding;U6 snRNA binding
binding;catalytic activity;coenzyme binding;cofactor binding;electron carrier activity;NAD binding;nucleotide binding;oxidoreductase activity;oxidoreductase activity, acting on CH-OH group of donors;oxidoreductase activity,
adenyl nucleotide binding;adenyl ribonucleotide binding;ATP binding;binding;catalytic activity;hydrolase activity;hydrolase activity, acting on acid anhydrides;hydrolase activity, acting on acid anhydrides, in phosphorus-conta
adenyl nucleotide binding;adenyl ribonucleotide binding;adenylylsulfate kinase activity;adenylyltransferase activity;ATP binding;binding;catalytic activity;kinase activity;nucleotide binding;nucleotidyltransferase activity;phosph
binding;carbonate dehydratase activity;carbon-oxygen lyase activity;catalytic activity;cation binding;hydro-lyase activity;ion binding;lyase activity;metal ion binding;transition metal ion binding;zinc ion binding
active transmembrane transporter activity;adenyl nucleotide binding;adenyl ribonucleotide binding;ATP binding;binding;chaperone binding;macromolecule transmembrane transporter activity;nucleotide binding;P-P-bond-hy
catalytic activity;NADH dehydrogenase (quinone) activity;NADH dehydrogenase (ubiquinone) activity;NADH dehydrogenase activity;oxidoreductase activity;oxidoreductase activity, acting on NADH or NADPH;oxidoreductase
adenyl nucleotide binding;adenyl ribonucleotide binding;anion transmembrane transporter activity;arsenite transmembrane transporter activity;ATP binding;ATPase activity;binding;catalytic activity;cation binding;hydrolase ac
binding;nucleotide binding
adenyl nucleotide binding;adenyl ribonucleotide binding;aminoacyl-tRNA ligase activity;asparagine-tRNA ligase activity;ATP binding;binding;catalytic activity;ligase activity;ligase activity, forming aminoacyl-tRNA and related c
amine binding;amino acid binding;binding;carboxylic acid binding;catalytic activity;cation binding;glutathione binding;G-protein coupled receptor activity;ion binding;lipoprotein particle receptor binding;low-density lipoprotei
binding;catalytic activity;cation binding;coenzyme binding;cofactor binding;electron carrier activity;ion binding;isocitrate dehydrogenase (NAD+) activity;isocitrate dehydrogenase activity;magnesium ion binding;metal ion binc
binding;catalytic activity;cation binding;endopeptidase activity;epidermal growth factor binding;growth factor binding;hormone binding;hydrolase activity;ion binding;metal ion binding;metalloendopeptidase activity;metallope
binding;calcium ion binding;cation binding;ion binding;metal ion binding
binding;catalytic activity;cation binding;GTP binding;GTPase activity;guanyl nucleotide binding;guanyl ribonucleotide binding;hydrolase activity;hydrolase activity, acting on acid anhydrides;hydrolase activity, acting on acid a
androgen receptor binding;bHLH transcription factor binding;binding;catalytic activity;chromatin binding;coenzyme binding;cofactor binding;demethylase activity;DNA binding;enzyme binding;flavin adenine dinucleotide bind
structural constituent of cytoskeleton;structural molecule activity
acid-thiol ligase activity;adenyl nucleotide binding;adenyl ribonucleotide binding;arachidonate-CoA ligase activity;ATP binding;binding;catalytic activity;fatty acid ligase activity;ligase activity;ligase activity, forming carbon-sulf
acetyltransferase activity;beta-N-acetylglucosaminidase activity;beta-N-acetylhexosaminidase activity;catalytic activity;hexosaminidase activity;histone acetyltransferase activity;hyaluronoglucosaminidase activity;hydrolase
binding;carbon-oxygen lyase activity;catalytic activity;coenzyme binding;cofactor binding;GDP-mannose 4,6-dehydratase activity;hydro-lyase activity;lyase activity;NADP binding;NADP+ binding;nucleotide binding
binding;carboxylic acid binding;catalytic activity;cation binding;galactosyltransferase activity;glucosyltransferase activity;ion binding;iron ion binding;L-ascorbic acid binding;metal ion binding;oxidoreductase activity;oxidored
binding;ion channel binding;protein binding;receptor binding
3'-5' exonuclease activity;binding;catalytic activity;damaged DNA binding;deoxyribonuclease activity;DNA binding;exodeoxyribonuclease activity;exodeoxyribonuclease activity, producing 5'-phosphomonoesters;exodeoxyrib
binding;DNA binding;nucleic acid binding;nucleic acid binding transcription factor activity;regulatory region DNA binding;regulatory region nucleic acid binding;sequence-specific DNA binding;sequence-specific DNA binding
binding;catalytic activity;coenzyme binding;cofactor binding;electron carrier activity;NAD binding;nucleotide binding;oxidoreductase activity;oxidoreductase activity, acting on CH-OH group of donors;oxidoreductase activity,
alcohol binding;binding;carbohydrate binding;catalytic activity;dolichyl-phosphate beta-D-mannosyltransferase activity;dolichyl-phosphate-mannose-protein mannosyltransferase activity;mannose binding;mannosyltransferas
protein transporter activity;substrate-specific transporter activity;transporter activity
binding;DNA binding;nucleic acid binding;protein binding transcription factor activity;ribonucleoprotein binding;transcription coactivator activity;transcription cofactor activity;transcription factor binding transcription factor ac
binding;DNA binding;nucleic acid binding;protein binding transcription factor activity;sequence-specific DNA binding;transcription coactivator activity;transcription cofactor activity;transcription factor binding transcription fac
binding;protein binding;receptor binding
binding;calcium ion binding;caspase inhibitor activity;caspase regulator activity;cation binding;cysteine-type endopeptidase inhibitor activity;endopeptidase inhibitor activity;endopeptidase regulator activity;enzyme inhibitor a
binding;catalytic activity;GTP binding;guanyl nucleotide binding;guanyl ribonucleotide binding;guanylyltransferase activity;hydrolase activity;hydrolase activity, acting on acid anhydrides;hydrolase activity, acting on acid anhy
adenyl nucleotide binding;adenyl ribonucleotide binding;ATP binding;binding;catalytic activity;kinase activity;nucleotide binding;phosphotransferase activity, alcohol group as acceptor;purine nucleotide binding;purine ribonuc
adenyl nucleotide binding;adenyl ribonucleotide binding;ATP binding;binding;catalytic activity;cation binding;ion binding;kinase activity;metal ion binding;nucleotide binding;phosphotransferase activity, alcohol group as accep
alpha(1,2)-fucosyltransferase activity;binding;catalytic activity;cytoskeletal protein binding;fucosyltransferase activity;galactoside 2-alpha-L-fucosyltransferase activity;microtubule binding;microtubule plus-end binding;protein
binding;calcium-dependent phospholipid binding;catalytic activity;kinase activity;lipid binding;phospholipid binding;phosphotransferase activity, alcohol group as acceptor;protein kinase activity;protein serine/threonine kinas
binding;cation binding;ion binding;metal ion binding
active transmembrane transporter activity;ATPase activity;ATPase activity, coupled;ATPase activity, coupled to movement of substances;ATPase activity, coupled to transmembrane movement of ions;ATPase activity, couplec
binding;chromatin binding;chromatin DNA binding;core promoter binding;core promoter sequence-specific DNA binding;DNA binding;double-stranded DNA binding;double-stranded methylated DNA binding;enzyme binding;
activating transcription factor binding;binding;chromatin binding;DNA binding;enzyme binding;enzyme regulator activity;histone deacetylase binding;histone deacetylase regulator activity;hormone receptor binding;nuclear ho
catalytic activity;citrate (Si)-synthase activity;transferase activity;transferase activity, transferring acyl groups;transferase activity, transferring acyl groups, acyl groups converted into alkyl on transfer
activating transcription factor binding;binding;chromatin binding;DNA binding;nucleic acid binding;protein binding;protein binding transcription factor activity;RNA polymerase II transcription coactivator activity;RNA polymera
catalytic activity;electron carrier activity;NADH dehydrogenase (quinone) activity;NADH dehydrogenase (ubiquinone) activity;NADH dehydrogenase activity;oxidoreductase activity;oxidoreductase activity, acting on NADH or N
binding;nucleic acid binding;nucleotide binding;protein binding;protein domain specific binding;RNA binding;RS domain binding;unfolded protein binding
binding;DNA binding;nucleic acid binding
binding;GTP binding;guanyl nucleotide binding;guanyl ribonucleotide binding;nucleic acid binding;nucleotide binding;purine nucleotide binding;purine ribonucleoside triphosphate binding;purine ribonucleotide binding;ribonu
acidic amino acid transmembrane transporter activity;active transmembrane transporter activity;amine transmembrane transporter activity;amino acid transmembrane transporter activity;binding;calcium ion binding;carboxyli
catalytic activity;endonuclease activity;endonuclease activity, active with either ribo- or deoxyribonucleic acids and producing 5'-phosphomonoesters;endoribonuclease activity;endoribonuclease activity, producing 5'-phosph
binding;nucleic acid binding;nucleotide binding;RNA binding;translation factor activity, nucleic acid binding;translation initiation factor activity
7S RNA binding;binding;nucleic acid binding;ribonucleoprotein binding;RNA binding;signal recognition particle binding
amine binding;amino acid binding;binding;carboxylic acid binding;catalytic activity;cation binding;dimethylargininase activity;hydrolase activity;hydrolase activity, acting on carbon-nitrogen (but not peptide) bonds;hydrolase a
binding;carbohydrate binding;catalytic activity;glutamine-fructose-6-phosphate transaminase (isomerizing) activity;transaminase activity;transferase activity;transferase activity, transferring nitrogenous groups
binding;enzyme binding;protein binding;small conjugating protein binding;transcription factor binding;ubiquitin binding;ubiquitin protein ligase binding
binding;growth factor activity;lipoprotein particle receptor binding;low-density lipoprotein particle receptor binding;molecular transducer activity;protein binding;receptor antagonist activity;receptor binding;receptor inhibitor a
catalytic activity;glutaminase activity;hydrolase activity;hydrolase activity, acting on carbon-nitrogen (but not peptide) bonds;hydrolase activity, acting on carbon-nitrogen (but not peptide) bonds, in linear amides
acid-amino acid ligase activity;binding;catalytic activity;cation binding;ion binding;ligase activity;ligase activity, forming carbon-nitrogen bonds;metal ion binding;nucleic acid binding;RNA binding;small conjugating protein liga
catalytic activity;NADH dehydrogenase (quinone) activity;NADH dehydrogenase (ubiquinone) activity;NADH dehydrogenase activity;oxidoreductase activity;oxidoreductase activity, acting on NADH or NADPH;oxidoreductase
catalytic activity;NADH dehydrogenase (quinone) activity;NADH dehydrogenase (ubiquinone) activity;NADH dehydrogenase activity;oxidoreductase activity;oxidoreductase activity, acting on NADH or NADPH;oxidoreductase
catalytic activity;NADH dehydrogenase (quinone) activity;NADH dehydrogenase (ubiquinone) activity;NADH dehydrogenase activity;oxidoreductase activity;oxidoreductase activity, acting on NADH or NADPH;oxidoreductase
binding;DNA binding;mRNA binding;nucleic acid binding;RNA binding
adenyl nucleotide binding;adenyl ribonucleotide binding;ATP binding;ATPase activity;binding;catalytic activity;DNA binding;hydrolase activity;hydrolase activity, acting on acid anhydrides;hydrolase activity, acting on acid anhy
adenyl nucleotide binding;adenyl ribonucleotide binding;ATP binding;binding;catalytic activity;kinase activity;NADH dehydrogenase (quinone) activity;NADH dehydrogenase (ubiquinone) activity;NADH dehydrogenase activity;
binding;calcium ion binding;catalytic activity;cation binding;cis-trans isomerase activity;drug binding;FK506 binding;ion binding;isomerase activity;macrolide binding;metal ion binding;peptidyl-prolyl cis-trans isomerase activi
adenyl nucleotide binding;adenyl ribonucleotide binding;ATP binding;binding;nucleotide binding;protein binding;protein dimerization activity;protein heterodimerization activity;purine nucleotide binding;purine ribonucleoside t

binding;cation binding;cytokine binding;DNA binding;growth factor binding;interleukin-1 binding;ion binding;metal ion binding;NACHT domain binding;nucleic acid binding;protein binding;protein domain specific binding;trans
binding;enzyme binding;enzyme regulator activity;GTPase binding;GTPase regulator activity;nucleoside-triphosphatase regulator activity;protein binding;Ran GTPase binding;Ras GTPase binding;small GTPase binding;transp
aldehyde-lyase activity;binding;carbon-carbon lyase activity;carboxy-lyase activity;catalytic activity;cofactor binding;lyase activity;pyridoxal phosphate binding;sphinganine-1-phosphate aldolase activity;vitamin B6 binding;vi
adenyl nucleotide binding;adenyl ribonucleotide binding;ATP binding;binding;catalytic activity;enzyme binding;fatty acid ligase activity;kinase binding;ligase activity;ligase activity, forming carbon-sulfur bonds;long-chain fatty
catalytic activity;methyltransferase activity;O-methyltransferase activity;transferase activity;transferase activity, transferring one-carbon groups
adenyl nucleotide binding;adenyl ribonucleotide binding;ATP binding;binding;catalytic activity;cation binding;ion binding;kinase activity;magnesium ion binding;metal ion binding;molecular transducer activity;nucleotide bindir
binding;catalytic activity;cation binding;dimethylallyltranstransferase activity;farnesyltranstransferase activity;geranyltranstransferase activity;ion binding;metal ion binding;prenyltransferase activity;transferase activity;transfera
binding;cation binding;ion binding;metal ion binding
binding;identical protein binding;protein binding
binding;cytoskeletal protein binding;microtubule binding;protein binding;tubulin binding
amine binding;amino acid binding;binding;carboxylic acid binding;catalytic activity;dimethylargininase activity;hydrolase activity;hydrolase activity, acting on carbon-nitrogen (but not peptide) bonds;hydrolase activity, acting c
catalytic activity;oxidoreductase activity;oxidoreductase activity, acting on a sulfur group of donors;oxidoreductase activity, acting on a sulfur group of donors, disulfide as acceptor;protein-disulfide reductase (glutathione) act
catalytic activity;NADH dehydrogenase (quinone) activity;NADH dehydrogenase (ubiquinone) activity;NADH dehydrogenase activity;oxidoreductase activity;oxidoreductase activity, acting on NADH or NADPH;oxidoreductase
channel activity;macromolecule transmembrane transporter activity;passive transmembrane transporter activity;porin activity;protein transmembrane transporter activity;protein transporter activity;substrate-specific transmen
binding;identical protein binding;protein binding;protein dimerization activity;protein homodimerization activity
catalytic activity;lactate dehydrogenase activity;L-lactate dehydrogenase activity;oxidoreductase activity;oxidoreductase activity, acting on CH-OH group of donors;oxidoreductase activity, acting on the CH-OH group of donc
antioxidant activity;binding;catalytic activity;coenzyme binding;cofactor binding;disulfide oxidoreductase activity;electron carrier activity;flavin adenine dinucleotide binding;glutathione disulfide oxidoreductase activity;glutathi
adenyl nucleotide binding;adenyl ribonucleotide binding;ATP binding;binding;catalytic activity;kinase activity;nucleotide binding;phosphoglycerate kinase activity;phosphotransferase activity, carboxyl group as acceptor;purin
adenyl nucleotide binding;adenyl ribonucleotide binding;amine binding;amino acid binding;argininosuccinate synthase activity;ATP binding;binding;carboxylic acid binding;catalytic activity;identical protein binding;ligase activ
binding;calcium-dependent protein binding;chemokine binding;C-X-C chemokine binding;cytokine binding;endopeptidase inhibitor activity;endopeptidase regulator activity;enzyme binding;enzyme inhibitor activity;enzyme re
antigen binding;beta-2-microglobulin binding;binding;peptide antigen binding;peptide binding;protein binding
binding;cargo receptor activity;double-stranded RNA binding;identical protein binding;nucleic acid binding;protein binding;receptor activity;RNA binding;transferrin receptor activity
binding;endopeptidase inhibitor activity;endopeptidase regulator activity;enzyme binding;enzyme inhibitor activity;enzyme regulator activity;peptidase inhibitor activity;peptidase regulator activity;protein binding;serine-type e
binding;carbohydrate binding;cargo receptor activity;extracellular matrix binding;glycosaminoglycan binding;heparin binding;integrin binding;pattern binding;polysaccharide binding;protein binding;protein complex binding;re
aminoacylase activity;antioxidant activity;binding;catalase activity;catalytic activity;cation binding;coenzyme binding;cofactor binding;enzyme binding;heme binding;hydrolase activity;hydrolase activity, acting on carbon-nitro
alpha-L-fucosidase activity;binding;carbohydrate binding;catalytic activity;fucose binding;fucosidase activity;hydrolase activity;hydrolase activity, acting on glycosyl bonds;hydrolase activity, hydrolyzing O-glycosyl compounc
actin binding;aldehyde-lyase activity;binding;carbohydrate binding;carbon-carbon lyase activity;catalytic activity;cytoskeletal protein binding;fructose binding;fructose-bisphosphate aldolase activity;identical protein binding;l
ATPase activity;ATPase activity, coupled;binding;binding, bridging;calcium ion binding;calcium-dependent phospholipid binding;calcium-dependent protein binding;catalytic activity;cation binding;DNA binding;DNA-depende
binding;catalytic activity;coenzyme binding;cofactor binding;cytoskeletal protein binding;glyceraldehyde-3-phosphate dehydrogenase (NAD+) (phosphorylating) activity;identical protein binding;microtubule binding;NAD bindi
amidine-lyase activity;argininosuccinate lyase activity;carbon-nitrogen lyase activity;catalytic activity;lyase activity
binding;enzyme binding;enzyme inhibitor activity;enzyme regulator activity;identical protein binding;kinase binding;kinase inhibitor activity;kinase regulator activity;protein binding;protein kinase binding;protein kinase C bindir
5,10-methylenetetrahydrofolate-dependent methyltransferase activity;amine binding;amino acid binding;binding;carboxylic acid binding;catalytic activity;cofactor binding;drug binding;folic acid binding;methyltransferase activ
catalytic activity;dolichyl-diphosphooligosaccharide-protein glycotransferase activity;oligosaccharyl transferase activity;transferase activity;transferase activity, transferring glycosyl groups;transferase activity, transferring hex
binding;catalytic activity;cation binding;G-protein beta/gamma-subunit complex binding;G-protein-coupled receptor binding;GTP binding;GTPase activity;guanyl nucleotide binding;guanyl ribonucleotide binding;hydrolase ac
binding;DNA binding;nucleic acid binding
active transmembrane transporter activity;adenyl nucleotide binding;adenyl ribonucleotide binding;ADP binding;alkali metal ion binding;ATP binding;ATPase activity;ATPase activity, coupled;ATPase activity, coupled to moven
adenine transmembrane transporter activity;binding;enzyme binding;nucleobase transmembrane transporter activity;nucleobase-containing compound transmembrane transporter activity;protein binding;purine base transme
protein tag
alkaline phosphatase activity;binding;catalytic activity;cation binding;hydrolase activity;hydrolase activity, acting on ester bonds;ion binding;magnesium ion binding;metal ion binding;phosphatase activity;phosphoric ester hy
binding;integrin binding;molecular transducer activity;protein binding;protein complex binding;receptor activity;receptor binding;signal transducer activity;signaling receptor activity;transmembrane signaling receptor activity;v
binding;protein binding;scaffold protein binding;structural molecule activity
binding;protein binding;scaffold protein binding;structural molecule activity
adenyl nucleotide binding;adenyl ribonucleotide binding;ATP binding;binding;catalytic activity;cyclin-dependent protein kinase activity;histone kinase activity;kinase activity;nucleotide binding;phosphotransferase activity, alco
binding;carbon-oxygen lyase activity;catalytic activity;cation binding;DNA binding;enzyme binding;GTPase binding;hydro-lyase activity;ion binding;lyase activity;magnesium ion binding;metal ion binding;nucleic acid binding;
adenyl nucleotide binding;adenyl ribonucleotide binding;AMP binding;ATP binding;bile acid binding;binding;carbohydrate binding;carboxylic acid binding;catalytic activity;cofactor binding;drug binding;glucose binding;glycog
binding;carbohydrate binding;catalytic activity;glucose-6-phosphate isomerase activity;intramolecular oxidoreductase activity;intramolecular oxidoreductase activity, interconverting aldoses and ketoses;intramolecular transfe
binding;enzyme binding;enzyme inhibitor activity;enzyme regulator activity;histone binding;identical protein binding;kinase binding;kinase inhibitor activity;kinase regulator activity;NF-kappaB binding;nucleic acid binding;prot
binding;calcium channel activity;cation binding;cation channel activity;cation transmembrane transporter activity;channel activity;cytokine binding;enzyme binding;extracellular matrix binding;fibronectin binding;gated channe
binding;carbohydrate binding;endopeptidase inhibitor activity;endopeptidase regulator activity;enzyme inhibitor activity;enzyme regulator activity;glycosaminoglycan binding;heparin binding;pattern binding;peptidase inhibito
amine binding;amino acid binding;antioxidant activity;binding;carboxylic acid binding;catalytic activity;glutathione binding;glutathione peroxidase activity;modified amino acid binding;oxidoreductase activity;oxidoreductase a
catalytic activity;endopeptidase activity;hydrolase activity;intramolecular oxidoreductase activity;intramolecular oxidoreductase activity, interconverting keto- and enol-groups;intramolecular oxidoreductase activity, transposin
aspartic-type endopeptidase activity;aspartic-type peptidase activity;catalytic activity;endopeptidase activity;hydrolase activity;peptidase activity;peptidase activity, acting on L-amino acid peptides
binding;calcium ion binding;calcium-dependent phospholipid binding;calcium-dependent protein binding;cation binding;enzyme inhibitor activity;enzyme regulator activity;ion binding;lipase inhibitor activity;lipid binding;meta
beta-N-acetylhexosaminidase activity;binding;catalytic activity;hexosaminidase activity;hydrolase activity;hydrolase activity, acting on glycosyl bonds;hydrolase activity, hydrolyzing O-glycosyl compounds;identical protein bir
adenyl nucleotide binding;adenyl ribonucleotide binding;aminoacyl-tRNA ligase activity;ATP binding;binding;catalytic activity;enzyme binding;glutamate-tRNA ligase activity;GTPase binding;ligase activity;ligase activity, formir
binding;catalytic activity;collagen binding;cysteine-type endopeptidase activity;cysteine-type peptidase activity;endopeptidase activity;glycoprotein binding;hydrolase activity;peptidase activity;peptidase activity, acting on L-
binding;extracellular matrix structural constituent;glycolipid binding;glycosphingolipid binding;lipid binding;sphingolipid binding;structural molecule activity
binding;calcium ion binding;calcium-dependent phospholipid binding;calcium-dependent protein binding;cation binding;channel activity;cholesterol binding;gated channel activity;GTP binding;guanyl nucleotide binding;guan
binding;lipid binding;receptor activity;viral receptor activity
active transmembrane transporter activity;amine transmembrane transporter activity;amino acid transmembrane transporter activity;antiporter activity;binding;calcium ion transmembrane transporter activity;calcium:cation ar
adenyl nucleotide binding;adenyl ribonucleotide binding;asparagine synthase (glutamine-hydrolyzing) activity;ATP binding;binding;carbon-nitrogen ligase activity, with glutamine as amido-N-donor;catalytic activity;cofactor bi
catalytic activity;oxidoreductase activity;oxidoreductase activity, acting on the aldehyde or oxo group of donors;oxidoreductase activity, acting on the aldehyde or oxo group of donors, disulfide as acceptor;pyruvate dehydro
binding;cation binding;cytokine receptor binding;growth factor receptor binding;ion binding;metal ion binding;platelet-derived growth factor receptor binding;protein binding;receptor binding;vascular endothelial growth factor
binding;core promoter proximal region DNA binding;core promoter proximal region sequence-specific DNA binding;DNA binding;double-stranded DNA binding;nucleic acid binding;nucleic acid binding transcription factor act
binding;double-stranded RNA binding;glycoprotein binding;identical protein binding;nucleic acid binding;protein binding;protein C-terminus binding;RNA binding;scaffold protein binding;structural constituent of cytoskeleton;
structural constituent of ribosome;structural molecule activity
structural molecule activity
binding;calcium ion binding;calcium-dependent phospholipid binding;cation binding;enzyme inhibitor activity;enzyme regulator activity;ion binding;lipase inhibitor activity;lipid binding;metal ion binding;phospholipase inhibito
binding;nucleic acid binding;RNA binding;structural constituent of ribosome;structural molecule activity
binding;carbohydrate binding;chemoattractant activity;cytokine activity;fibroblast growth factor receptor binding;glycosaminoglycan binding;growth factor activity;growth factor receptor binding;heparin binding;ligand-depenc
binding;carbon-oxygen lyase activity;catalytic activity;cation binding;hydro-lyase activity;ion binding;lyase activity;magnesium ion binding;metal ion binding;phosphopyruvate hydratase activity
binding;carbohydrate binding;disaccharide binding;galactoside binding;lactose binding;molecular transducer activity;monosaccharide binding;signal transducer activity;sugar binding
actin binding;binding;cytoskeletal protein binding;protein binding;structural constituent of cytoskeleton;structural constituent of muscle;structural molecule activity
binding;calcium ion binding;calcium-dependent phospholipid binding;calcium-dependent protein binding;cation binding;enzyme inhibitor activity;enzyme regulator activity;identical protein binding;ion binding;lipase inhibitor a
2',3'-cyclic-nucleotide 3'-phosphodiesterase activity;binding;catalytic activity;cyclic nucleotide binding;cyclic-nucleotide phosphodiesterase activity;hydrolase activity;hydrolase activity, acting on ester bonds;nucleic acid bin
binding;catalytic activity;cation binding;coenzyme binding;cofactor binding;DNA binding;enzyme binding;identical protein binding;ion binding;kinase binding;metal ion binding;NAD binding;NAD+ ADP-ribosyltransferase activi
3'-5' exonuclease activity;4 iron, 4 sulfur cluster binding;binding;catalytic activity;cation binding;chromatin binding;DNA binding;DNA polymerase activity;DNA-directed DNA polymerase activity;enzyme binding;exonuclease a
binding;nucleic acid binding;RNA binding
alkaline phosphatase activity;binding;catalytic activity;cation binding;enzyme binding;hydrolase activity;hydrolase activity, acting on ester bonds;ion binding;magnesium ion binding;metal ion binding;phosphatase activity;pho
aldehyde-lyase activity;binding;carbon-carbon lyase activity;catalytic activity;cytoskeletal protein binding;fructose-bisphosphate aldolase activity;lyase activity;protein binding
binding;cation binding;ion binding;metal ion binding;nucleic acid binding;RNA binding;snRNA binding;U2 snRNA binding
alpha-glucosidase activity;binding;carbohydrate binding;catalytic activity;glucosidase activity;hydrolase activity;hydrolase activity, acting on glycosyl bonds;hydrolase activity, hydrolyzing O-glycosyl compounds;maltose alph
adenyl nucleotide binding;adenyl ribonucleotide binding;ATP binding;binding;catalytic activity;cation binding;ion binding;kinase activity;metal ion binding;molecular transducer activity;nucleotide binding;phosphotransferase a
adenyl nucleotide binding;adenyl ribonucleotide binding;AMP binding;binding;cAMP binding;cAMP-dependent protein kinase inhibitor activity;cAMP-dependent protein kinase regulator activity;cyclic nucleotide binding;enzyr
adenyl nucleotide binding;adenyl ribonucleotide binding;ATP binding;ATPase activity;binding;calcium ion binding;catalytic activity;cation binding;chaperone binding;enzyme binding;glycoprotein binding;hydrolase activity;hyd
binding;extracellular matrix structural constituent;glycolipid binding;glycosphingolipid binding;lipid binding;sphingolipid binding;structural molecule activity
active transmembrane transporter activity;binding;carbohydrate transmembrane transporter activity;cation transmembrane transporter activity;cation:sugar symporter activity;dehydroascorbic acid transporter activity;D-gluco
active transmembrane transporter activity;carbohydrate transmembrane transporter activity;cation transmembrane transporter activity;cation:sugar symporter activity;glucose transmembrane transporter activity;hexose trans
carbon-carbon lyase activity;carboxy-lyase activity;catalytic activity;lyase activity;orotate phosphoribosyltransferase activity;orotidine-5'-phosphate decarboxylase activity;transferase activity;transferase activity, transferring g
ATPase binding;binding;catalytic activity;cytoskeletal protein binding;Edg-2 lysophosphatidic acid receptor binding;endothelial differentiation G-protein coupled receptor binding;enzyme binding;GDP binding;G-protein-coupl
binding;carbohydrate binding;catalytic activity;coenzyme binding;cofactor binding;glucose binding;glucose-6-phosphate dehydrogenase activity;identical protein binding;monosaccharide binding;NADP binding;nucleotide bi
adenyl nucleotide binding;adenyl ribonucleotide binding;ATP binding;binding;biotin binding;biotin carboxylase activity;carboxylic acid binding;catalytic activity;cation binding;DNA binding;ion binding;ligase activity;ligase activ
adenyl nucleotide binding;adenyl ribonucleotide binding;ATP binding;binding;catalytic activity;cyclohydrolase activity;formate-tetrahydrofolate ligase activity;hydrolase activity;hydrolase activity, acting on carbon-nitrogen (but
adenyl nucleotide binding;adenyl ribonucleotide binding;ATP binding;binding;catalytic activity;cyclin binding;cyclin-dependent protein kinase activity;cyclin-dependent protein kinase regulator activity;enzyme regulator activity
active transmembrane transporter activity;antiporter activity;ATP:ADP antiporter activity;secondary active transmembrane transporter activity;solute:solute antiporter activity;transmembrane transporter activity;transporter act
binding;catalytic activity;cation binding;DNA binding;IMP dehydrogenase activity;ion binding;metal ion binding;nucleic acid binding;nucleotide binding;oxidoreductase activity;oxidoreductase activity, acting on CH-OH group
binding;chromatin binding;cytoskeletal protein binding;dynein complex binding;enzyme binding;heat shock protein binding;identical protein binding;kinase binding;mitogen-activated protein kinase binding;mRNA binding;nuc
adenyl nucleotide binding;adenyl ribonucleotide binding;ATP binding;binding;catalytic activity;creatine kinase activity;enzyme binding;kinase activity;nucleotide binding;phosphotransferase activity, nitrogenous group as acce
binding;calcium ion binding;calcium-dependent phospholipid binding;calcium-dependent protein binding;cation binding;enzyme inhibitor activity;enzyme regulator activity;ion binding;lipase inhibitor activity;lipid binding;meta
3-methyl-2-oxobutanoate dehydrogenase (2-methylpropanoyl-transferring) activity;alpha-ketoacid dehydrogenase activity;binding;carbon-carbon lyase activity;carboxy-lyase activity;catalytic activity;cation binding;ion bindin
aminopeptidase activity;binding;carboxypeptidase activity;catalytic activity;cation binding;dipeptidase activity;exopeptidase activity;hydrolase activity;ion binding;manganese ion binding;metal ion binding;metallocarboxypept
5'-deoxyribose-5-phosphate lyase activity;adenyl nucleotide binding;adenyl ribonucleotide binding;ATP binding;ATPase activity;ATPase activity, coupled;ATP-dependent DNA helicase activity;ATP-dependent helicase activity;
adenyl nucleotide binding;adenyl ribonucleotide binding;ATP binding;ATPase activity;ATPase activity, coupled;ATP-dependent DNA helicase activity;ATP-dependent helicase activity;binding;catalytic activity;damaged DNA bi
catalytic activity;DNA N-glycosylase activity;hydrolase activity;hydrolase activity, acting on glycosyl bonds;hydrolase activity, hydrolyzing N-glycosyl compounds;uracil DNA N-glycosylase activity
enzyme inhibitor activity;enzyme regulator activity;ribonuclease inhibitor activity
binding;catalytic activity;enzyme binding;GTP binding;GTPase activity;guanyl nucleotide binding;guanyl ribonucleotide binding;hydrolase activity;hydrolase activity, acting on acid anhydrides;hydrolase activity, acting on acid
catalytic activity;intramolecular oxidoreductase activity;intramolecular oxidoreductase activity, interconverting keto- and enol-groups;intramolecular oxidoreductase activity, transposing S-S bonds;isomerase activity;protein d
binding;carboxylic acid binding;catalytic activity;cation binding;dioxygenase activity;ion binding;iron ion binding;L-ascorbic acid binding;metal ion binding;oxidoreductase activity;oxidoreductase activity, acting on paired donc
binding;calcium ion binding;cation binding;ion binding;metal ion binding
binding;carbohydrate binding;catalytic activity;enzyme binding;glucose binding;glucosyltransferase activity;glycogen (starch) synthase activity;kinase binding;monosaccharide binding;protein binding;protein kinase binding;su
actin binding;actin filament binding;binding;calcium ion binding;calcium-dependent phospholipid binding;cation binding;cytoskeletal protein binding;ion binding;lipid binding;metal ion binding;phosphatidylinositol binding;pho
binding;carbon-oxygen lyase activity;catalytic activity;cation binding;hydro-lyase activity;ion binding;lyase activity;magnesium ion binding;metal ion binding;phosphopyruvate hydratase activity
anion binding;binding;catalytic activity;cation binding;cyclohydrolase activity;hydrolase activity;hydrolase activity, acting on carbon-nitrogen (but not peptide) bonds;hydrolase activity, acting on carbon-nitrogen (but not pepti
binding;calcium ion binding;cation binding;enzyme binding;ion binding;ion channel binding;kinase binding;metal ion binding;phosphoprotein binding;protein binding;protein kinase binding;protein kinase C binding
binding;carboxypeptidase activity;catalytic activity;cation binding;exopeptidase activity;hydrolase activity;ion binding;metal ion binding;metallocarboxypeptidase activity;metalloexopeptidase activity;metallopeptidase activity;
adenyl nucleotide binding;adenyl ribonucleotide binding;alkali metal ion binding;antigen binding;ATP binding;binding;catalytic activity;cation binding;ion binding;kinase activity;magnesium ion binding;metal ion binding;MHC c
adenyl nucleotide binding;adenyl ribonucleotide binding;ATP binding;binding;calcium ion binding;cation binding;enzyme binding;ion binding;lipoprotein particle receptor binding;low-density lipoprotein particle receptor bindin
adenyl nucleotide binding;adenyl ribonucleotide binding;ATP binding;ATPase activity;beta-amyloid binding;beta-endorphin binding;binding;catalytic activity;cation binding;endopeptidase activity;glycoprotein binding;hormone
adenyl nucleotide binding;adenyl ribonucleotide binding;aminoacylase activity;aminoacyl-tRNA ligase activity;aspartate-tRNA ligase activity;ATP binding;binding;catalytic activity;hydrolase activity;hydrolase activity, acting on
acid-ammonia (or amide) ligase activity;adenyl nucleotide binding;adenyl ribonucleotide binding;amine binding;amino acid binding;ammonia ligase activity;ATP binding;binding;carbon-carbon lyase activity;carboxylic acid bin
alpha-tubulin binding;beta-N-acetylglucosaminylglycopeptide beta-1,4-galactosyltransferase activity;beta-tubulin binding;binding;catalytic activity;cation binding;cytoskeletal protein binding;galactosyltransferase activity;iden
binding;DNA binding;double-stranded DNA binding;nucleic acid binding;nucleic acid binding transcription factor activity;regulatory region DNA binding;regulatory region nucleic acid binding;RNA polymerase II regulatory regi
binding;cadherin binding;catalytic activity;cell adhesion molecule binding;endopeptidase activity;enzyme inhibitor activity;enzyme regulator activity;hydrolase activity;peptidase activity;peptidase activity, acting on L-amino ac

antioxidant activity;binding;catalytic activity;cytochrome-b5 reductase activity;identical protein binding;NAD(P)H dehydrogenase (quinone) activity;oxidoreductase activity;oxidoreductase activity, acting on NADH or NADPH;o
binding;catalytic activity;cation binding;hydrolase activity;hydrolase activity, acting on ester bonds;ion binding;metal ion binding;N-acetylglucosamine-6-sulfatase activity;sulfuric ester hydrolase activity
binding;enzyme binding;fibroblast growth factor binding;growth factor binding;mRNA binding;nucleic acid binding;protein binding;RNA binding;structural constituent of ribosome;structural molecule activity
binding;binding, bridging;enzyme binding;kinase binding;protein binding;protein binding, bridging;protein kinase binding;protein kinase C binding;scaffold protein binding;structural constituent of cytoskeleton;structural molec
binding;damaged DNA binding;DNA binding;enzyme binding;nucleic acid binding;phosphatase binding;protein binding;protein phosphatase binding;single-stranded DNA binding;structure-specific DNA binding;ubiquitin prote
binding;core promoter proximal region DNA binding;core promoter proximal region sequence-specific DNA binding;DNA binding;nucleic acid binding;p53 binding;protein binding;protein binding transcription factor activity;reg
binding;catalytic activity;cation binding;dihydronicotinamide riboside quinone reductase activity;electron carrier activity;ion binding;metal ion binding;NADPH dehydrogenase (quinone) activity;NADPH dehydrogenase activity;
15-hydroxyprostaglandin dehydrogenase (NADP+) activity;carbonyl reductase (NADPH) activity;catalytic activity;oxidoreductase activity;oxidoreductase activity, acting on CH-OH group of donors;oxidoreductase activity, acti
active transmembrane transporter activity;adenyl nucleotide binding;adenyl ribonucleotide binding;ATP binding;ATPase activity;ATPase activity, coupled;ATPase activity, coupled to movement of substances;ATPase activity, c
binding;cation binding;collagen binding;ion binding;metal ion binding;protein binding;receptor activity;viral receptor activity
adenyl nucleotide binding;adenyl ribonucleotide binding;ATP binding;binding;cAMP-dependent protein kinase activity;catalytic activity;cyclic nucleotide-dependent protein kinase activity;enzyme binding;kinase activity;kinase
binding;calcium ion binding;calcium-dependent cysteine-type endopeptidase activity;catalytic activity;cation binding;cysteine-type endopeptidase activity;cysteine-type peptidase activity;cytoskeletal protein binding;endopep
binding;chromatin binding;core promoter proximal region DNA binding;core promoter proximal region sequence-specific DNA binding;DNA binding;identical protein binding;nucleic acid binding;nucleic acid binding transcripti
adenyl nucleotide binding;adenyl ribonucleotide binding;ATP binding;binding;catalytic activity;CTP synthase activity;ligase activity;ligase activity, forming carbon-nitrogen bonds;nucleotide binding;purine nucleotide binding;p
activin binding;binding;carbohydrate binding;catalytic activity;cytokine binding;cytokine receptor binding;galactose binding;glycosaminoglycan binding;growth factor binding;identical protein binding;kinase activity;molecular
adenyl nucleotide binding;adenyl ribonucleotide binding;androgen receptor binding;ATP binding;ATPase activity;ATPase activity, coupled;ATP-dependent helicase activity;ATP-dependent RNA helicase activity;binding;catalyti
6-phosphofructokinase activity;adenyl nucleotide binding;adenyl ribonucleotide binding;ATP binding;binding;carbohydrate binding;carbohydrate kinase activity;catalytic activity;cation binding;enzyme binding;fructose binding
binding;cation binding;enzyme activator activity;enzyme regulator activity;fibronectin binding;growth factor binding;insulin-like growth factor binding;insulin-like growth factor I binding;insulin-like growth factor II binding;ion bi
binding;nucleic acid binding;RNA binding;structural constituent of ribosome;structural molecule activity;tRNA binding
binding;DNA binding;identical protein binding;mRNA binding;nucleic acid binding;protein binding;protein dimerization activity;protein homodimerization activity;RNA binding;structural constituent of ribosome;structural molec
actin binding;alpha-catenin binding;beta-catenin binding;binding;cadherin binding;cell adhesion molecule binding;cytoskeletal protein binding;dystroglycan binding;enzyme binding;glycoprotein binding;protein binding;structu
structural constituent of ribosome;structural molecule activity
binding;bisphosphoglycerate 2-phosphatase activity;bisphosphoglycerate mutase activity;bisphosphoglycerate phosphatase activity;catalytic activity;enzyme binding;hydrolase activity;hydrolase activity, acting on ester bonc
adenyl nucleotide binding;adenyl ribonucleotide binding;ATP binding;binding;catalytic activity;cation binding;DNA binding;DNA ligase (ATP) activity;DNA ligase activity;ion binding;ligase activity;ligase activity, forming phospho
binding;damaged DNA binding;DNA binding;enzyme binding;nucleic acid binding;protein binding
binding;DNA binding;identical protein binding;nucleic acid binding;nucleotide binding;protein binding;protein C-terminus binding;RNA binding;sequence-specific DNA binding;telomeric DNA binding
endopeptidase inhibitor activity;endopeptidase regulator activity;enzyme inhibitor activity;enzyme regulator activity;peptidase inhibitor activity;peptidase regulator activity;serine-type endopeptidase inhibitor activity
binding;cation binding;ion binding;metal ion binding;nucleic acid binding;RNA binding;translation factor activity, nucleic acid binding;translation initiation factor activity
binding;calcium ion binding;calcium-dependent phospholipid binding;calcium-dependent protein binding;cation binding;integrin binding;ion binding;lipid binding;metal ion binding;phospholipid binding;protein binding;protein
binding;catalytic activity;GDP binding;GTP binding;GTPase activity;guanyl nucleotide binding;guanyl ribonucleotide binding;hydrolase activity;hydrolase activity, acting on acid anhydrides;hydrolase activity, acting on acid anh
binding;catalytic activity;cytoskeletal protein binding;GTP binding;GTPase activity;guanyl nucleotide binding;guanyl ribonucleotide binding;hydrolase activity;hydrolase activity, acting on acid anhydrides;hydrolase activity, act
catalytic activity;endopeptidase activity;hydrolase activity;peptidase activity;peptidase activity, acting on L-amino acid peptides;threonine-type endopeptidase activity;threonine-type peptidase activity
cysteine-type endopeptidase inhibitor activity;endopeptidase inhibitor activity;endopeptidase regulator activity;enzyme inhibitor activity;enzyme regulator activity;peptidase inhibitor activity;peptidase regulator activity
binding;carbohydrate binding;cation binding;extracellular matrix structural constituent;glycoprotein binding;glycosaminoglycan binding;growth factor binding;heparin binding;integrin binding;ion binding;metal ion binding;patt
active transmembrane transporter activity;adenyl nucleotide binding;adenyl ribonucleotide binding;ATP binding;ATPase activity;ATPase activity, coupled;ATPase activity, coupled to movement of substances;ATPase activity, c
binding;cation binding;ion binding;metal ion binding;transition metal ion binding;zinc ion binding
actin binding;actin filament binding;binding;cytoskeletal protein binding;enzyme binding;Fc-gamma receptor I complex binding;glycoprotein binding;GTPase binding;identical protein binding;immunoglobulin receptor binding;
4 iron, 4 sulfur cluster binding;aconitate hydratase activity;binding;carbon-oxygen lyase activity;catalytic activity;cation binding;hydro-lyase activity;ion binding;iron-responsive element binding;iron-sulfur cluster binding;lyase
2 iron, 2 sulfur cluster binding;3 iron, 4 sulfur cluster binding;4 iron, 4 sulfur cluster binding;binding;catalytic activity;cation binding;cofactor binding;electron carrier activity;ion binding;iron-sulfur cluster binding;metal cluster b
binding;growth factor binding;insulin-like growth factor binding;insulin-like growth factor I binding;insulin-like growth factor II binding;protein binding
binding;calcium ion binding;cation binding;enzyme activator activity;enzyme regulator activity;extracellular matrix structural constituent;ion binding;metal ion binding;peptidase activator activity;peptidase regulator activity;stru
binding;cation binding;DNA binding;ion binding;metal ion binding;nucleic acid binding;transition metal ion binding;zinc ion binding
antioxidant activity;binding;catalytic activity;cation binding;dioxygenase activity;heme binding;ion binding;iron ion binding;metal ion binding;oxidoreductase activity;oxidoreductase activity, acting on paired donors, with incorp
binding;catalytic activity;cis-trans isomerase activity;enzyme binding;isomerase activity;peptide binding;peptidyl-prolyl cis-trans isomerase activity;protein binding;protein complex binding;RNA polymerase binding;unfolded p
adenyl nucleotide binding;adenyl ribonucleotide binding;aminoacyl-tRNA ligase activity;ATP binding;binding;catalytic activity;ligase activity;ligase activity, forming aminoacyl-tRNA and related compounds;ligase activity, formi
adenosylhomocysteinase activity;catalytic activity;hydrolase activity;hydrolase activity, acting on ether bonds;trialkylsulfonium hydrolase activity
binding;nucleic acid binding;nucleotide binding;RNA binding;translation factor activity, nucleic acid binding;translation initiation factor activity
active transmembrane transporter activity;adenyl nucleotide binding;adenyl ribonucleotide binding;ATP binding;ATPase activity;ATPase activity, coupled;ATPase activity, coupled to movement of substances;ATPase activity, c
adenyl nucleotide binding;adenyl ribonucleotide binding;ATP binding;binding;catalytic activity;nucleotide binding;oxidoreductase activity;oxidoreductase activity, acting on CH or CH2 groups;oxidoreductase activity, acting or
catalytic activity;cation transmembrane transporter activity;cytochrome-c oxidase activity;heme-copper terminal oxidase activity;hydrogen ion transmembrane transporter activity;inorganic cation transmembrane transporter a
adenyl nucleotide binding;adenyl ribonucleotide binding;ATP binding;binding;catalytic activity;DNA binding;DNA helicase activity;helicase activity;hydrolase activity;hydrolase activity, acting on acid anhydrides;hydrolase activ
3-mercaptopyruvate sulfurtransferase activity;catalytic activity;sulfurtransferase activity;thiosulfate sulfurtransferase activity;transferase activity;transferase activity, transferring sulfur-containing groups
binding;catalytic activity;endopeptidase activity;hydrolase activity;nucleic acid binding;peptidase activity;peptidase activity, acting on L-amino acid peptides;RNA binding;threonine-type endopeptidase activity;threonine-type
catalytic activity;endopeptidase activity;hydrolase activity;peptidase activity;peptidase activity, acting on L-amino acid peptides;threonine-type endopeptidase activity;threonine-type peptidase activity
binding;catalytic activity;cation binding;chromatin binding;DNA (cytosine-5-)-methyltransferase activity;DNA binding;DNA-methyltransferase activity;ion binding;metal ion binding;methyl-CpG binding;methyltransferase activity
binding;nucleic acid binding;RNA binding;structural constituent of ribosome;structural molecule activity
binding;calcium ion binding;cation binding;ion binding;metal ion binding;protein binding;RAGE receptor binding;receptor binding
adenyl nucleotide binding;adenyl ribonucleotide binding;aminoacyl-tRNA ligase activity;ATP binding;binding;catalytic activity;identical protein binding;ligase activity;ligase activity, forming aminoacyl-tRNA and related compou
binding;enzyme binding;protein binding;RNA polymerase binding
adenyl nucleotide binding;adenyl ribonucleotide binding;adenylate kinase activity;ATP binding;binding;catalytic activity;GTP binding;guanyl nucleotide binding;guanyl ribonucleotide binding;kinase activity;nucleobase-contain
structural constituent of ribosome;structural molecule activity
binding;cation binding;chromatin binding;damaged DNA binding;DNA binding;ion binding;metal ion binding;nucleic acid binding;single-stranded DNA binding;structure-specific DNA binding
adenyl nucleotide binding;adenyl ribonucleotide binding;amine binding;amino acid binding;aspartate binding;aspartate carbamoyltransferase activity;ATP binding;binding;carbamoyl-phosphate synthase (ammonia) activity;ca
structural molecule activity
binding;calcium ion binding;carbohydrate binding;cation binding;ion binding;metal ion binding
catalytic activity;endopeptidase activity;hydrolase activity;peptidase activity;peptidase activity, acting on L-amino acid peptides;threonine-type endopeptidase activity;threonine-type peptidase activity
active transmembrane transporter activity;adenyl nucleotide binding;adenyl ribonucleotide binding;ATP binding;ATPase activity;ATPase activity, coupled;ATPase activity, coupled to movement of substances;ATPase activity, c
binding;catalytic activity;cation binding;copper ion binding;ion binding;metal ion binding;oxidoreductase activity;oxidoreductase activity, acting on the CH-NH2 group of donors;oxidoreductase activity, acting on the CH-NH2
binding;cation binding;DNA binding;ion binding;metal ion binding;nucleic acid binding;sequence-specific DNA binding
adenyl nucleotide binding;adenyl ribonucleotide binding;ATP binding;axon guidance receptor activity;binding;catalytic activity;ephrin receptor activity;kinase activity;molecular transducer activity;nucleotide binding;phosphotr
alkylbase DNA N-glycosylase activity;binding;catalytic activity;damaged DNA binding;DNA binding;DNA N-glycosylase activity;DNA-3-methyladenine glycosylase I activity;DNA-7-methylguanine glycosylase activity;hydrolase
binding;carboxylic acid binding;isoprenoid binding;lipid binding;monocarboxylic acid binding;retinal binding;retinoic acid binding;retinoid binding;retinol binding;transporter activity;vitamin binding
activating transcription factor binding;binding;DNA binding;heat shock protein binding;molecular transducer activity;nucleic acid binding;protein binding;RNA binding;signal transducer activity;transcription factor binding;trans
antioxidant activity;binding;caspase inhibitor activity;caspase regulator activity;catalytic activity;cysteine-type endopeptidase inhibitor activity;DNA binding;endopeptidase inhibitor activity;endopeptidase regulator activity;enz
catalytic activity;cysteine-type endopeptidase activity;cysteine-type peptidase activity;disulfide oxidoreductase activity;endopeptidase activity;hydrolase activity;hydrolase activity, acting on ester bonds;intramolecular oxidore
adenylosuccinate synthase activity;anion binding;binding;catalytic activity;cation binding;GTP binding;guanyl nucleotide binding;guanyl ribonucleotide binding;ion binding;ligase activity;ligase activity, forming carbon-nitrogen
asialoglycoprotein receptor activity;binding;carbohydrate binding;cargo receptor activity;glycosaminoglycan binding;heparin binding;lipoprotein particle receptor binding;low-density lipoprotein particle receptor binding;patter
(S)-2-(5-amino-1-(5-phospho-D-ribosyl)imidazole-4-carboxamido)succinate AMP-lyase (fumarate-forming) activity;amidine-lyase activity;carbon-nitrogen lyase activity;catalytic activity;lyase activity;N6-(1,2-dicarboxyethyl)AM
endopeptidase inhibitor activity;endopeptidase regulator activity;enzyme inhibitor activity;enzyme regulator activity;peptidase inhibitor activity;peptidase regulator activity;serine-type endopeptidase inhibitor activity
active transmembrane transporter activity;amine transmembrane transporter activity;amino acid transmembrane transporter activity;antiporter activity;arginine transmembrane transporter activity;basic amino acid transmemb
aldehyde dehydrogenase (NAD) activity;catalytic activity;oxidoreductase activity;oxidoreductase activity, acting on the aldehyde or oxo group of donors;oxidoreductase activity, acting on the aldehyde or oxo group of donors,
binding;catalytic activity;coenzyme binding;cofactor binding;flavin adenine dinucleotide binding;oxidoreductase activity;oxidoreductase activity, acting on the CH-CH group of donors;oxidoreductase activity, acting on the CH
adenyl nucleotide binding;adenyl ribonucleotide binding;amine binding;amino acid binding;ATP binding;binding;calcium ion binding;carbamoyl-phosphate synthase (ammonia) activity;carbamoyl-phosphate synthase (glutami
14-3-3 protein binding;adenyl nucleotide binding;adenyl ribonucleotide binding;ATP binding;binding;catalytic activity;enzyme binding;enzyme regulator activity;identical protein binding;kinase activity;lipid binding;nitric-oxide
antioxidant activity;catalytic activity;oxidoreductase activity;oxidoreductase activity, acting on paired donors, with incorporation or reduction of molecular oxygen;oxidoreductase activity, acting on peroxide as acceptor;perox
binding;catalytic activity;identical protein binding;oxidoreductase activity;oxidoreductase activity, acting on the CH-NH group of donors;oxidoreductase activity, acting on the CH-NH group of donors, NAD or NADP as accept
actin binding;binding;catalytic activity;cytokine binding;cytoskeletal protein binding;enzyme binding;GTP binding;GTPase activity;guanyl nucleotide binding;guanyl ribonucleotide binding;heat shock protein binding;Hsp90 pro
binding;carbon-sulfur lyase activity;catalytic activity;cofactor binding;cystathionine gamma-lyase activity;homocysteine desulfhydrase activity;L-cysteine desulfhydrase activity;lyase activity;pyridoxal phosphate binding;vitam
binding;nucleic acid binding;RNA binding;rRNA binding;structural constituent of ribosome;structural molecule activity
binding;catalytic activity;cation binding;dUTP diphosphatase activity;hydrolase activity;hydrolase activity, acting on acid anhydrides;hydrolase activity, acting on acid anhydrides, in phosphorus-containing anhydrides;ion bind
adenyl nucleotide binding;adenyl ribonucleotide binding;ATP binding;binding;catalytic activity;DNA binding;DNA helicase activity;helicase activity;hydrolase activity;hydrolase activity, acting on acid anhydrides;hydrolase activ
aldehyde-lyase activity;amine binding;amino acid binding;binding;carbon-carbon lyase activity;carboxylic acid binding;catalytic activity;cofactor binding;glycine hydroxymethyltransferase activity;hydroxymethyl-, formyl- and
aldehyde-lyase activity;amine binding;amino acid binding;binding;carbon-carbon lyase activity;carboxylic acid binding;catalytic activity;chromatin binding;cofactor binding;glycine hydroxymethyltransferase activity;hydroxyme
beta-catenin binding;binding;cadherin binding;cell adhesion molecule binding;cytoskeletal protein binding;gamma-catenin binding;protein binding;structural molecule activity;vinculin binding
binding;endopeptidase inhibitor activity;endopeptidase regulator activity;enzyme binding;enzyme inhibitor activity;enzyme regulator activity;peptidase inhibitor activity;peptidase regulator activity;protease binding;protein binc
binding;damaged DNA binding;DNA binding;nucleic acid binding;single-stranded DNA binding;structure-specific DNA binding
antioxidant activity;arachidonate 15-lipoxygenase activity;binding;catalytic activity;cation binding;dioxygenase activity;enzyme binding;heme binding;ion binding;iron ion binding;lipid binding;metal ion binding;oxidoreductase
amine binding;amino acid binding;binding;carbon monoxide binding;carbon-oxygen lyase activity;carboxylic acid binding;catalytic activity;cation binding;cofactor binding;cystathionine beta-synthase activity;enzyme binding;
actin binding;actin filament binding;actin-dependent ATPase activity;adenyl nucleotide binding;adenyl ribonucleotide binding;ADP binding;ATP binding;ATPase activity;ATPase activity, coupled;binding;catalytic activity;cytosk
binding;carbohydrate binding;mannose binding;monosaccharide binding;sugar binding
binding;catalytic activity;disulfide oxidoreductase activity;electron carrier activity;glutathione disulfide oxidoreductase activity;oxidoreductase activity;oxidoreductase activity, acting on a sulfur group of donors;peptide disulfid
adenyl nucleotide binding;adenyl ribonucleotide binding;ATP binding;binding;catalytic activity;enzyme activator activity;enzyme regulator activity;kinase activator activity;kinase activity;kinase regulator activity;MAP kinase kin
active transmembrane transporter activity;ATPase activity;ATPase activity, coupled;ATPase activity, coupled to movement of substances;ATPase activity, coupled to transmembrane movement of ions;ATPase activity, couplec
binding;catalytic activity;coproporphyrinogen oxidase activity;identical protein binding;oxidoreductase activity;oxidoreductase activity, acting on the CH-CH group of donors;oxidoreductase activity, acting on the CH-CH grou
binding;nucleic acid binding;RNA binding;structural constituent of ribosome;structural molecule activity
binding;catalytic activity;cation binding;intramolecular transferase activity;intramolecular transferase activity, phosphotransferases;ion binding;isomerase activity;magnesium ion binding;metal ion binding;phosphoglucomutas
amine binding;amino acid binding;antioxidant activity;binding;carboxylic acid binding;catalytic activity;glutathione binding;glutathione peroxidase activity;modified amino acid binding;oxidoreductase activity;oxidoreductase a
7S RNA binding;binding;endoplasmic reticulum signal peptide binding;nucleic acid binding;peptide binding;RNA binding;signal sequence binding
alpha-tubulin binding;binding;calcium ion binding;caspase inhibitor activity;caspase regulator activity;catalytic activity;cation binding;copper ion binding;cysteine-type endopeptidase inhibitor activity;cytoskeletal protein bind
binding;nucleic acid binding;RNA binding;structural constituent of ribosome;structural molecule activity
structural constituent of ribosome;structural molecule activity
binding;catalytic activity;GTP binding;GTPase activity;guanyl nucleotide binding;guanyl ribonucleotide binding;hydrolase activity;hydrolase activity, acting on acid anhydrides;hydrolase activity, acting on acid anhydrides, in pl
nucleic acid binding transcription factor activity;sequence-specific DNA binding transcription factor activity
binding;catalytic activity;cation binding;demethylase activity;DNA binding;histone demethylase activity;histone demethylase activity (H3-K4 specific);ion binding;metal ion binding;nucleic acid binding;oxidoreductase activity;o
adenyl nucleotide binding;adenyl ribonucleotide binding;aminoacyl-tRNA ligase activity;ATP binding;binding;catalytic activity;glycine-tRNA ligase activity;ligase activity;ligase activity, forming aminoacyl-tRNA and related com
adenyl nucleotide binding;adenyl ribonucleotide binding;aminoacyl-tRNA editing activity;aminoacyl-tRNA ligase activity;ATP binding;binding;carboxylic ester hydrolase activity;catalytic activity;enzyme binding;GTPase binding
adenyl nucleotide binding;adenyl ribonucleotide binding;ATP binding;binding;catalytic activity;cation binding;ion binding;kinase activity;lipid binding;metal ion binding;nucleotide binding;phospholipid binding;phosphotransfer
catalytic activity;dodecenoyl-CoA delta-isomerase activity;intramolecular oxidoreductase activity;intramolecular oxidoreductase activity, transposing C=C bonds;isomerase activity
binding;DNA binding;lamin binding;nucleic acid binding;protein binding
binding;core promoter binding;core promoter proximal region DNA binding;core promoter proximal region sequence-specific DNA binding;core promoter sequence-specific DNA binding;cytokine receptor binding;DNA bindin
15-hydroxyprostaglandin-D dehydrogenase (NADP+) activity;alcohol dehydrogenase (NADP+) activity;alditol:NADP+ 1-oxidoreductase activity;aldo-keto reductase (NADP) activity;androsterone dehydrogenase activity;catalyt
5S rDNA binding;adenyl nucleotide binding;adenyl ribonucleotide binding;ATP binding;binding;catalytic activity;DNA binding;drug binding;kinase activity;nucleic acid binding;nucleotide binding;phosphoprotein binding;phosp
binding;cation binding;DNA binding;ion binding;metal ion binding;nucleic acid binding;structural constituent of ribosome;structural molecule activity;transition metal ion binding;zinc ion binding
binding;mRNA binding;nucleic acid binding;RNA binding;structural constituent of ribosome;structural molecule activity
binding;cyclin-dependent protein kinase inhibitor activity;cyclin-dependent protein kinase regulator activity;enzyme binding;enzyme inhibitor activity;enzyme regulator activity;kinase binding;kinase inhibitor activity;kinase regu
binding;catalytic activity;cation binding;endopeptidase activity;hormone binding;hydrolase activity;identical protein binding;ion binding;metal ion binding;metalloendopeptidase activity;metallopeptidase activity;peptidase acti

binding;cation binding;ion binding;metal ion binding;nucleotide binding;structural molecule activity;transition metal ion binding;zinc ion binding
binding;catalytic activity;cytokine activity;drug binding;nicotinamide phosphoribosyltransferase activity;nicotinate-nucleotide diphosphorylase (carboxylating) activity;protein binding;receptor binding;transferase activity;transf
binding;catalytic activity;cation binding;cysteine-type endopeptidase activity;cysteine-type peptidase activity;endopeptidase activity;exopeptidase activity;hydrolase activity;ion binding;metal ion binding;omega peptidase act
adenyl nucleotide binding;adenyl ribonucleotide binding;ATP binding;binding;nucleotide binding;protein binding;protein C-terminus binding;purine nucleotide binding;purine ribonucleoside triphosphate binding;purine ribonuc
enzyme activator activity;enzyme regulator activity;GTPase activator activity;GTPase regulator activity;nucleoside-triphosphatase regulator activity
3'-5' DNA helicase activity;adenyl nucleotide binding;adenyl ribonucleotide binding;ATP binding;ATPase activity;ATPase activity, coupled;ATP-dependent 3'-5' DNA helicase activity;ATP-dependent DNA helicase activity;ATP-
adenyl nucleotide binding;adenyl ribonucleotide binding;ATP binding;ATPase activity;ATPase activity, coupled;binding;catalytic activity;cation binding;chromatin binding;chromo shadow domain binding;DNA binding;DNA heli
binding;nucleic acid binding;RNA binding;structural constituent of ribosome;structural molecule activity
5S rRNA binding;binding;nucleic acid binding;RNA binding;rRNA binding;structural constituent of ribosome;structural molecule activity
binding;nucleic acid binding;RNA binding;structural constituent of ribosome;structural molecule activity
binding;nucleic acid binding;RNA binding;structural constituent of ribosome;structural molecule activity
binding;nucleic acid binding;RNA binding;rRNA binding;structural constituent of ribosome;structural molecule activity;translation regulator activity
binding;mRNA binding;nucleic acid binding;RNA binding;structural constituent of ribosome;structural molecule activity
structural molecule activity
binding;cation binding;enzyme regulator activity;GTPase regulator activity;guanyl-nucleotide exchange factor activity;ion binding;metal ion binding;nucleoside-triphosphatase regulator activity;transition metal ion binding;zinc
binding;calcium ion binding;calcium-dependent phospholipase A2 activity;calcium-dependent phospholipid binding;carboxylic ester hydrolase activity;catalytic activity;cation binding;hydrolase activity;hydrolase activity, actin
adenyl nucleotide binding;adenyl ribonucleotide binding;aminoacyl-tRNA ligase activity;ATP binding;binding;catalytic activity;glutamine-tRNA ligase activity;ligase activity;ligase activity, forming aminoacyl-tRNA and related co
binding;cation transmembrane transporter activity;drug binding;hydrogen ion transmembrane transporter activity;hydrogen ion transporting ATP synthase activity, rotational mechanism;inorganic cation transmembrane transp
binding;cation binding;enzyme binding;ion binding;kinase binding;metal ion binding;protein binding;protein kinase binding;transition metal ion binding;zinc ion binding
adenyl nucleotide binding;adenyl ribonucleotide binding;ADP binding;binding;carbon-carbon lyase activity;carboxy-lyase activity;catalytic activity;cation binding;coenzyme binding;cofactor binding;electron carrier activity;ion
catalytic activity;intramolecular transferase activity;isomerase activity;lanosterol synthase activity;oxidosqualene cyclase activity
acid-amino acid ligase activity;binding;catalytic activity;enzyme binding;enzyme regulator activity;glutamate-cysteine ligase activity;glutamate-cysteine ligase catalytic subunit binding;ligase activity;ligase activity, forming carl
binding;integrin binding;protein binding;protein complex binding;receptor binding
binding;lipid binding
binding;catalytic activity;DNA binding;DNA polymerase activity;DNA-directed DNA polymerase activity;nucleic acid binding;nucleotidyltransferase activity;transferase activity;transferase activity, transferring phosphorus-conta
beta-catenin binding;binding;cation binding;cytoskeletal protein binding;ion binding;metal ion binding;protein binding;transition metal ion binding;vinculin binding;zinc ion binding
adenyl nucleotide binding;adenyl ribonucleotide binding;ATP binding;binding;calcium-dependent protein kinase activity;calcium-dependent protein serine/threonine kinase activity;calmodulin binding;calmodulin-dependent p
binding;nucleic acid binding;RNA binding;structural constituent of ribosome;structural molecule activity
binding;carbohydrate binding;cation binding;ion binding;mannose binding;metal ion binding;monosaccharide binding;protein binding;sugar binding;unfolded protein binding
[acyl-carrier-protein] S-acetyltransferase activity;[acyl-carrier-protein] S-malonyltransferase activity;3-hydroxyacyl-[acyl-carrier-protein] dehydratase activity;3-hydroxyoctanoyl-[acyl-carrier-protein] dehydratase activity;3-hydro
catalytic activity;deoxyhypusine synthase activity;transferase activity;transferase activity, transferring alkyl or aryl (other than methyl) groups
adenyl nucleotide binding;adenyl ribonucleotide binding;ATP binding;binding;nucleotide binding;protein binding;purine nucleotide binding;purine ribonucleoside triphosphate binding;purine ribonucleotide binding;ribonucleoti
structural constituent of ribosome;structural molecule activity
binding;catalytic activity;GTP binding;GTPase activity;guanyl nucleotide binding;guanyl ribonucleotide binding;hydrolase activity;hydrolase activity, acting on acid anhydrides;hydrolase activity, acting on acid anhydrides, in pl
aldehyde dehydrogenase (NAD) activity;betaine-aldehyde dehydrogenase activity;catalytic activity;L-aminoadipate-semialdehyde dehydrogenase activity;oxidoreductase activity;oxidoreductase activity, acting on the aldehyd
7S RNA binding;binding;nucleic acid binding;ribonucleoprotein binding;RNA binding;signal recognition particle binding
adenyl nucleotide binding;adenyl ribonucleotide binding;alanine-tRNA ligase activity;amine binding;amino acid binding;aminoacyl-tRNA editing activity;aminoacyl-tRNA ligase activity;ATP binding;binding;carboxylic acid bindi
adenyl nucleotide binding;adenyl ribonucleotide binding;aminoacyl-tRNA ligase activity;ATP binding;binding;catalytic activity;cation binding;cysteine-tRNA ligase activity;identical protein binding;ion binding;ligase activity;ligas
adenyl nucleotide binding;adenyl ribonucleotide binding;aminoacyl-tRNA ligase activity;ATP binding;binding;catalytic activity;histidine-tRNA ligase activity;ligase activity;ligase activity, forming aminoacyl-tRNA and related cor
adenyl nucleotide binding;adenyl ribonucleotide binding;aminoacyl-tRNA ligase activity;ATP binding;binding;catalytic activity;ligase activity;ligase activity, forming aminoacyl-tRNA and related compounds;ligase activity, formi
acyl-CoA hydrolase activity;acyl-CoA thioesterase activity;binding;carboxylic ester hydrolase activity;catalytic activity;CoA hydrolase activity;hydrolase activity;hydrolase activity, acting on ester bonds;palmitoyl-CoA hydrolase
adenyl nucleotide binding;adenyl ribonucleotide binding;ATP binding;binding;catalytic activity;enzyme binding;kinase activity;kinase binding;nucleotide binding;phosphotransferase activity, alcohol group as acceptor;protein b
adenyl nucleotide binding;adenyl ribonucleotide binding;ATP binding;beta-catenin binding;binding;catalytic activity;enzyme binding;kinase activity;kinase binding;NF-kappaB binding;nucleotide binding;p53 binding;phosphotr
binding;catalytic activity;cation binding;coenzyme binding;cofactor binding;ion binding;isocitrate dehydrogenase (NAD+) activity;isocitrate dehydrogenase activity;magnesium ion binding;metal ion binding;NAD binding;nucleo
actin binding;beta-tubulin binding;binding;cytoskeletal protein binding;protein binding;tubulin binding
carnitine O-acyltransferase activity;carnitine O-palmitoyltransferase activity;catalytic activity;O-acyltransferase activity;O-palmitoyltransferase activity;palmitoyltransferase activity;transferase activity;transferase activity, transfe
adenyl deoxyribonucleotide binding;adenyl nucleotide binding;binding;binding, bridging;dATP binding;deoxyribonucleotide binding;nucleotide binding;protein binding;protein binding, bridging;purine deoxyribonucleotide bind
binding;extracellular matrix binding;laminin binding;laminin receptor activity;molecular transducer activity;protein binding;protein C-terminus binding;receptor activity;signal transducer activity;signaling receptor activity;transm
binding;nucleic acid binding;RNA binding;structural constituent of ribosome;structural molecule activity
antigen binding;binding;calcium ion binding;calcium-dependent phospholipid binding;calcium-dependent protein binding;cation binding;ion binding;lipid binding;metal ion binding;MHC class II protein complex binding;MHC
binding;catalytic activity;GDP binding;GTP binding;GTPase activity;guanyl nucleotide binding;guanyl ribonucleotide binding;hydrolase activity;hydrolase activity, acting on acid anhydrides;hydrolase activity, acting on acid anh
binding;death domain binding;protein binding;protein domain specific binding
adenyl nucleotide binding;adenyl ribonucleotide binding;ATP binding;binding;catalytic activity;cation binding;coenzyme binding;cofactor binding;ion binding;isocitrate dehydrogenase (NAD+) activity;isocitrate dehydrogenase
3alpha,7alpha,12alpha-trihydroxy-5beta-cholest-24-enoyl-CoA hydratase activity;3-hydroxyacyl-CoA dehydrogenase activity;binding;carbon-oxygen lyase activity;catalytic activity;hydro-lyase activity;identical protein binding
adenyl nucleotide binding;adenyl ribonucleotide binding;ATP binding;binding;caspase inhibitor activity;caspase regulator activity;catalytic activity;cation binding;cysteine-type endopeptidase inhibitor activity;endopeptidase ir
binding;carbohydrate binding;DNA binding;glycosaminoglycan binding;heparin binding;nucleic acid binding;nucleotide binding;pattern binding;polysaccharide binding;protein binding transcription factor activity;RNA polymer
binding;enzyme binding;histone deacetylase binding;nuclear localization sequence binding;peptide binding;protein binding;protein transporter activity;signal sequence binding;substrate-specific transporter activity;transporte
binding;nuclear localization sequence binding;peptide binding;protein transporter activity;signal sequence binding;substrate-specific transporter activity;transporter activity
binding;nucleic acid binding;nucleotide binding;RNA 7-methylguanosine cap binding;RNA binding;RNA cap binding
enzyme activator activity;enzyme regulator activity;GTPase activator activity;GTPase regulator activity;nucleoside-triphosphatase regulator activity
binding;catalytic activity;DNA binding;DNA-directed RNA polymerase activity;nucleic acid binding;nucleotidyltransferase activity;RNA polymerase activity;transferase activity;transferase activity, transferring phosphorus-conta
binding;cation binding;enzyme regulator activity;GTPase regulator activity;guanyl-nucleotide exchange factor activity;ion binding;metal ion binding;nucleoside-triphosphatase regulator activity;Ras guanyl-nucleotide exchang
catalytic activity;spermine synthase activity;transferase activity;transferase activity, transferring alkyl or aryl (other than methyl) groups
adenyl nucleotide binding;adenyl ribonucleotide binding;ATP binding;binding;carbohydrate binding;carbohydrate kinase activity;catalytic activity;fructokinase activity;glucokinase activity;glucose binding;hexokinase activity;ki
acid-thiol ligase activity;adenyl nucleotide binding;adenyl ribonucleotide binding;ATP binding;ATP citrate synthase activity;binding;catalytic activity;cation binding;CoA-ligase activity;cofactor binding;ion binding;ligase activity
anion binding;apoptotic protease activator activity;binding;caspase activator activity;caspase regulator activity;catalytic activity;chaperone binding;chloride ion binding;cysteine-type endopeptidase activity;cysteine-type pept
binding;carbon-sulfur lyase activity;catalytic activity;cation binding;holocytochrome-c synthase activity;ion binding;lyase activity;metal ion binding
active transmembrane transporter activity;carboxylic acid transmembrane transporter activity;lactate transmembrane transporter activity;mevalonate transmembrane transporter activity;monocarboxylic acid transmembrane t
binding;DNA binding;nucleic acid binding;protein binding transcription factor activity;single-stranded DNA binding;structure-specific DNA binding;transcription coactivator activity;transcription cofactor activity;transcription fa
adenyl nucleotide binding;adenyl ribonucleotide binding;aminoacyl-tRNA ligase activity;ATP binding;binding;catalytic activity;chemokine receptor binding;CXCR chemokine receptor binding;cytokine receptor binding;G-protei
active transmembrane transporter activity;ATPase activator activity;ATPase activity;ATPase activity, coupled;ATPase activity, coupled to movement of substances;ATPase activity, coupled to transmembrane movement of ions
adenyl nucleotide binding;adenyl ribonucleotide binding;adenylate kinase activity;ATP binding;binding;catalytic activity;kinase activity;nucleobase-containing compound kinase activity;nucleotide binding;nucleotide kinase ac
adenyl nucleotide binding;adenyl ribonucleotide binding;amino acid kinase activity;ATP binding;binding;catalytic activity;coenzyme binding;cofactor binding;glutamate 5-kinase activity;glutamate-5-semialdehyde dehydrogen
binding;protein binding;SNARE binding;soluble NSF attachment protein activity;syntaxin binding
structural molecule activity
binding;calcium ion binding;cation binding;identical protein binding;ion binding;metal ion binding;protein binding;protein dimerization activity;protein heterodimerization activity;protein homodimerization activity
adenyl nucleotide binding;adenyl ribonucleotide binding;aminoacyl-tRNA ligase activity;ATP binding;binding;catalytic activity;ligase activity;ligase activity, forming aminoacyl-tRNA and related compounds;ligase activity, formi
binding;cation binding;collagen binding;enzyme binding;ion binding;metal ion binding;phosphatase binding;protein binding;protein phosphatase binding
structural constituent of nuclear pore;structural molecule activity
binding;cation binding;ion binding;metal ion binding
binding;catalytic activity;enzyme binding;intramolecular oxidoreductase activity;intramolecular oxidoreductase activity, interconverting aldoses and ketoses;isomerase activity;protein binding;triose-phosphate isomerase activ
binding;nucleic acid binding;protein binding;protein N-terminus binding;RNA binding;translation factor activity, nucleic acid binding;translation initiation factor activity
adenyl nucleotide binding;adenyl ribonucleotide binding;ATP binding;ATPase activity;ATPase activity, coupled;ATP-dependent helicase activity;ATP-dependent RNA helicase activity;binding;catalytic activity;double-stranded F
structural constituent of ribosome;structural molecule activity
binding;catalytic activity;endopeptidase activity;hydrolase activity;NF-kappaB binding;nucleic acid binding;nucleotide binding;peptidase activity;peptidase activity, acting on L-amino acid peptides;protein binding;purine nucle
apolipoprotein A-I receptor binding;apolipoprotein receptor binding;binding;catalytic activity;enzyme binding;GTP binding;GTPase activity;guanyl nucleotide binding;guanyl ribonucleotide binding;hydrolase activity;hydrolase
actin binding;binding;chemokine binding;chemokine receptor activity;coreceptor activity;C-X-C chemokine binding;C-X-C chemokine receptor activity;cytokine binding;cytoskeletal protein binding;enzyme binding;G-protein c
acid-amino acid ligase activity;adenyl nucleotide binding;adenyl ribonucleotide binding;ATP binding;binding;catalytic activity;ligase activity;ligase activity, forming carbon-nitrogen bonds;NEDD8 ligase activity;nucleotide bindi
binding;nucleic acid binding;RNA binding;structural constituent of ribosome;structural molecule activity
binding;nucleic acid binding;RNA binding;structural constituent of ribosome;structural molecule activity
binding;nucleic acid binding;RNA binding;structural constituent of ribosome;structural molecule activity
binding;cation binding;ion binding;metal ion binding;structural constituent of ribosome;structural molecule activity
binding;cation binding;ion binding;metal ion binding;nucleic acid binding;RNA binding;rRNA binding;structural constituent of ribosome;structural molecule activity
binding;core promoter proximal region DNA binding;core promoter proximal region sequence-specific DNA binding;DNA binding;nucleic acid binding;nucleic acid binding transcription factor activity;regulatory region DNA bin
binding;enzyme binding;enzyme inhibitor activity;enzyme regulator activity;insulin-like growth factor receptor binding;kinase binding;kinase inhibitor activity;kinase regulator activity;protein binding;protein kinase binding;prote
binding;catalytic activity;GTP binding;GTPase activity;guanyl nucleotide binding;guanyl ribonucleotide binding;hydrolase activity;hydrolase activity, acting on acid anhydrides;hydrolase activity, acting on acid anhydrides, in pl
binding;nucleic acid binding;RNA binding;structural constituent of ribosome;structural molecule activity
acid-amino acid ligase activity;adenyl nucleotide binding;adenyl ribonucleotide binding;ATP binding;binding;catalytic activity;enzyme binding;ligase activity;ligase activity, forming carbon-nitrogen bonds;nucleotide binding;pr
binding;mRNA 5'-UTR binding;mRNA binding;nucleic acid binding;RNA binding;structural constituent of ribosome;structural molecule activity;translation regulator activity
structural constituent of ribosome;structural molecule activity
binding;nucleic acid binding;RNA binding;rRNA binding;structural constituent of ribosome;structural molecule activity
binding;cation binding;ion binding;metal ion binding;structural constituent of ribosome;structural molecule activity;transition metal ion binding;zinc ion binding
binding;nucleic acid binding;RNA binding;rRNA binding;structural constituent of ribosome;structural molecule activity
binding;catalytic activity;enzyme binding;GTP binding;GTPase activity;guanyl nucleotide binding;guanyl ribonucleotide binding;hydrolase activity;hydrolase activity, acting on acid anhydrides;hydrolase activity, acting on acid
adenyl nucleotide binding;adenyl ribonucleotide binding;ATP binding;ATPase activity;binding;binding, bridging;catalytic activity;hydrolase activity;hydrolase activity, acting on acid anhydrides;hydrolase activity, acting on acid
binding;nucleic acid binding;RNA binding;structural constituent of ribosome;structural molecule activity
binding;cation binding;DNA binding;ion binding;metal ion binding;nucleic acid binding;nucleic acid binding transcription factor activity;RNA binding;sequence-specific DNA binding transcription factor activity;single-stranded
binding;catalytic activity;GDP binding;GTP binding;GTPase activity;guanyl nucleotide binding;guanyl ribonucleotide binding;hydrolase activity;hydrolase activity, acting on acid anhydrides;hydrolase activity, acting on acid anh
structural constituent of ribosome;structural molecule activity
binding;nucleotide binding;protein binding;structural constituent of ribosome;structural molecule activity;translation initiation factor binding
binding;nucleic acid binding;RNA binding;structural constituent of ribosome;structural molecule activity
binding;mRNA binding;nucleic acid binding;RNA binding;structural constituent of ribosome;structural molecule activity
structural constituent of ribosome;structural molecule activity
binding;nucleic acid binding;RNA binding;structural constituent of ribosome;structural molecule activity
binding;nucleic acid binding;RNA binding;structural constituent of ribosome;structural molecule activity
binding;nucleic acid binding;RNA binding;rRNA binding;structural constituent of ribosome;structural molecule activity
binding;nucleic acid binding;RNA binding;rRNA binding;structural constituent of ribosome;structural molecule activity
binding;catalytic activity;cis-trans isomerase activity;isomerase activity;peptide binding;peptidyl-prolyl cis-trans isomerase activity;protein binding;unfolded protein binding;virion binding
binding;catalytic activity;cation binding;G-protein beta/gamma-subunit complex binding;G-protein-coupled receptor binding;GTP binding;GTPase activity;guanyl nucleotide binding;guanyl ribonucleotide binding;hydrolase ac
binding;catalytic activity;enzyme binding;GTP binding;GTPase activity;guanyl nucleotide binding;guanyl ribonucleotide binding;hydrolase activity;hydrolase activity, acting on acid anhydrides;hydrolase activity, acting on acid
binding;catalytic activity;GTP binding;GTPase activity;guanyl nucleotide binding;guanyl ribonucleotide binding;hydrolase activity;hydrolase activity, acting on acid anhydrides;hydrolase activity, acting on acid anhydrides, in pl
binding;carbohydrate binding
binding;calcium ion binding;catalytic activity;cation binding;hydrolase activity;hydrolase activity, acting on ester bonds;identical protein binding;ion binding;magnesium ion binding;metal ion binding;phosphatase activity;phos
binding;nucleic acid binding;RNA binding;translation factor activity, nucleic acid binding;translation initiation factor activity

catalytic activity;endonuclease activity;endonuclease activity, active with either ribo- or deoxyribonucleic acids and producing 5'-phosphomonoesters;endoribonuclease activity;endoribonuclease activity, producing 5'-phosph
adenosine deaminase activity;binding;catalytic activity;cation binding;deaminase activity;double-stranded RNA adenosine deaminase activity;double-stranded RNA binding;hydrolase activity;hydrolase activity, acting on carb
binding;calcium ion binding;cation binding;DNA binding;ion binding;metal ion binding;nucleic acid binding
structural constituent of ribosome;structural molecule activity
structural constituent of ribosome;structural molecule activity
structural constituent of ribosome;structural molecule activity
binding;nucleic acid binding;RNA binding;structural constituent of ribosome;structural molecule activity
structural constituent of ribosome;structural molecule activity
binding;catalytic activity;GTP binding;GTPase activity;guanyl nucleotide binding;guanyl ribonucleotide binding;hydrolase activity;hydrolase activity, acting on acid anhydrides;hydrolase activity, acting on acid anhydrides, in ph
binding;nucleic acid binding;RNA binding;structural constituent of ribosome;structural molecule activity
binding;cation binding;ion binding;metal ion binding;protein binding;protein C-terminus binding
binding;lipid binding
acetylcholine receptor activator activity;acetylcholine receptor regulator activity;adenyl nucleotide binding;adenyl ribonucleotide binding;ATP binding;binding;catalytic activity;cyclin-dependent protein kinase activity;ErbB-2 cl
binding;DNA binding;double-stranded DNA binding;double-stranded telomeric DNA binding;nucleic acid binding;nucleic acid binding transcription factor activity;protein binding;purine-rich negative regulatory element binding
binding;enzyme binding;GTPase binding;GTP-Rho binding;protein binding;Ras GTPase binding;Rho GTPase binding;small GTPase binding
adenyl nucleotide binding;adenyl ribonucleotide binding;ATP binding;binding;core promoter binding;DNA binding;nucleic acid binding;nucleotide binding;purine nucleotide binding;purine ribonucleoside triphosphate binding;
binding;nucleotide binding;protein binding transcription factor activity;transcription cofactor activity;transcription corepressor activity;transcription factor binding transcription factor activity
AMP deaminase activity;binding;catalytic activity;cation binding;deaminase activity;hydrolase activity;hydrolase activity, acting on carbon-nitrogen (but not peptide) bonds;hydrolase activity, acting on carbon-nitrogen (but not
binding;carboxylic acid binding;fatty acid binding;lipid binding;monocarboxylic acid binding;transporter activity
active transmembrane transporter activity;amine transmembrane transporter activity;amino acid transmembrane transporter activity;antigen binding;antiporter activity;aromatic amino acid transmembrane transporter activity;
6-phosphofructokinase activity;adenyl nucleotide binding;adenyl ribonucleotide binding;ATP binding;binding;carbohydrate kinase activity;catalytic activity;cation binding;ion binding;kinase activity;metal ion binding;nucleotide
endopeptidase inhibitor activity;endopeptidase regulator activity;enzyme inhibitor activity;enzyme regulator activity;peptidase inhibitor activity;peptidase regulator activity;serine-type endopeptidase inhibitor activity
binding;nucleic acid binding;RNA binding;structural constituent of ribosome;structural molecule activity
adenyl nucleotide binding;adenyl ribonucleotide binding;ATP binding;binding;catalytic activity;enzyme activator activity;enzyme regulator activity;hydrolase activity;hydrolase activity, acting on ester bonds;kinase activator act
binding;carboxylic acid binding;catalytic activity;cation binding;identical protein binding;ion binding;iron ion binding;L-ascorbic acid binding;metal ion binding;oxidoreductase activity;oxidoreductase activity, acting on paired
binding;calcium ion binding;cation binding;DNA binding;ion binding;metal ion binding;nucleic acid binding
binding;DNA binding;nucleic acid binding;RNA binding;structural constituent of ribosome;structural molecule activity
aminoacylase activity;binding;catalytic activity;cation binding;hydrolase activity;hydrolase activity, acting on carbon-nitrogen (but not peptide) bonds;hydrolase activity, acting on carbon-nitrogen (but not peptide) bonds, in lin
binding;enzyme binding;molecular transducer activity;protein binding;protein domain specific binding;receptor activity;receptor binding;signal transducer activity;signaling receptor activity;U-plasminogen activator receptor ac
active transmembrane transporter activity;adenyl nucleotide binding;adenyl ribonucleotide binding;ADP binding;antigen binding;ATP binding;ATPase activity;ATPase activity, coupled;ATPase activity, coupled to movement of
activating transcription factor binding;anion binding;binding;chromatin binding;core promoter proximal region DNA binding;core promoter proximal region sequence-specific DNA binding;DNA binding;enhancer binding;enhan
ATPase binding;binding;enzyme binding;K6-linked polyubiquitin binding;polyubiquitin binding;protein binding;small conjugating protein binding;ubiquitin binding;ubiquitin protein ligase binding
1,4-alpha-glucan branching enzyme activity;binding;catalytic activity;cation binding;hydrolase activity;hydrolase activity, acting on glycosyl bonds;hydrolase activity, hydrolyzing O-glycosyl compounds;ion binding;transferase
binding;nucleic acid binding;RNA binding;translation factor activity, nucleic acid binding;translation initiation factor activity
binding;calcium ion binding;cation binding;ion binding;metal ion binding;receptor activity
binding;channel regulator activity;enzyme binding;glucocorticoid receptor binding;hormone receptor binding;insulin-like growth factor receptor binding;ion channel binding;nuclear hormone receptor binding;protein binding;p
binding;catalytic activity;enzyme binding;GTP binding;GTPase activity;guanyl nucleotide binding;guanyl ribonucleotide binding;hydrolase activity;hydrolase activity, acting on acid anhydrides;hydrolase activity, acting on acid
actin binding;binding;calmodulin binding;cytoskeletal protein binding;protein binding;tropomyosin binding
endopeptidase inhibitor activity;endopeptidase regulator activity;enzyme inhibitor activity;enzyme regulator activity;peptidase inhibitor activity;peptidase regulator activity;serine-type endopeptidase inhibitor activity
amine binding;amino acid binding;binding;carbohydrate binding;carboxylic acid binding;catalytic activity;glutamine-fructose-6-phosphate transaminase (isomerizing) activity;transaminase activity;transferase activity;transfera
binding;nucleic acid binding;RNA binding;structural constituent of ribosome;structural molecule activity
apolipoprotein binding;binding;calcium ion binding;cation binding;ion binding;lipoprotein particle receptor binding;lipoprotein transporter activity;metal ion binding;protein binding;protein complex binding;protein transporter a
3',5'-cyclic-AMP phosphodiesterase activity;3',5'-cyclic-nucleotide phosphodiesterase activity;adenyl nucleotide binding;adenyl ribonucleotide binding;adrenergic receptor binding;AMP binding;ATPase binding;beta-2 adrene
adenyl nucleotide binding;adenyl ribonucleotide binding;ATP binding;binding;nucleotide binding;purine nucleotide binding;purine ribonucleoside triphosphate binding;purine ribonucleotide binding;ribonucleotide binding
binding;enzyme binding;histone binding;histone deacetylase binding;protein binding
acetylgalactosaminyltransferase activity;binding;carbohydrate binding;catalytic activity;cation binding;ion binding;manganese ion binding;metal ion binding;polypeptide N-acetylgalactosaminyltransferase activity;transferase a
binding;enzyme binding;kinase binding;protein binding;protein kinase binding;protein transporter activity;substrate-specific transporter activity;transporter activity
binding;endopeptidase inhibitor activity;endopeptidase regulator activity;enzyme inhibitor activity;enzyme regulator activity;identical protein binding;metalloendopeptidase inhibitor activity;metalloenzyme inhibitor activity;meta
binding;catalytic activity;cation binding;endopeptidase activity;hydrolase activity;ion binding;metal ion binding;metalloendopeptidase activity;metallopeptidase activity;peptidase activity;peptidase activity, acting on L-amino a
binding;calcium ion binding;catalytic activity;cation binding;electron carrier activity;ion binding;ion channel binding;metal ion binding;oxidoreductase activity;oxidoreductase activity, acting on paired donors, with incorporation
binding;mRNA binding;nucleic acid binding;nucleotide binding;RNA binding
4 iron, 4 sulfur cluster binding;binding;catalytic activity;cation binding;coenzyme binding;cofactor binding;dihydroorotate dehydrogenase activity;dihydropyrimidine dehydrogenase (NADP+) activity;flavin adenine dinucleotide
binding;cation binding;DNA binding;ion binding;metal ion binding;nucleic acid binding;transition metal ion binding;zinc ion binding
binding;carbohydrate binding;cation binding;glycoprotein binding;heat shock protein binding;ion binding;mannose binding;metal ion binding;monosaccharide binding;protein binding;sugar binding
catalytic activity;hydrolase activity;hydrolase activity, acting on ester bonds;phosphatase activity;phosphoprotein phosphatase activity;phosphoric ester hydrolase activity;prenylated protein tyrosine phosphatase activity;prot
binding;catalytic activity;isomerase activity;protein binding;receptor binding
adenyl nucleotide binding;adenyl ribonucleotide binding;ATP binding;binding;catalytic activity;cation binding;enzyme activator activity;enzyme regulator activity;identical protein binding;ion binding;kinase activator activity;kin
acetyl-CoA carboxylase activity;adenyl nucleotide binding;adenyl ribonucleotide binding;ATP binding;binding;biotin carboxylase activity;catalytic activity;cation binding;CoA carboxylase activity;ion binding;ligase activity;ligas
binding;DNA binding;double-stranded DNA binding;identical protein binding;mRNA 3'-UTR binding;mRNA binding;nucleic acid binding;nucleic acid binding transcription factor activity;nucleotide binding;protein binding;RNA
antioxidant activity;catalytic activity;oxidoreductase activity;oxidoreductase activity, acting on paired donors, with incorporation or reduction of molecular oxygen;oxidoreductase activity, acting on peroxide as acceptor;perox
binding;enzyme binding;identical protein binding;protein binding;protein domain specific binding
binding;selenium binding
binding;nucleotide binding
acid-amino acid ligase activity;binding;catalytic activity;cation binding;chromo shadow domain binding;DNA binding;enzyme binding;ion binding;kinase activity;Krueppel-associated box domain binding;ligase activity;ligase a
adenyl nucleotide binding;adenyl ribonucleotide binding;ATP binding;ATPase activity;ATPase activity, coupled;ATP-dependent DNA helicase activity;ATP-dependent helicase activity;ATP-dependent RNA helicase activity;bind
binding;nucleic acid binding;RNA binding;translation factor activity, nucleic acid binding;translation initiation factor activity
adenyl nucleotide binding;adenyl ribonucleotide binding;ATP binding;binding;catalytic activity;enzyme binding;kinase activity;kinase binding;molecular transducer activity;nucleotide binding;phosphotransferase activity, alcoh
binding;catalytic activity;cation binding;collagen binding;endopeptidase activity;enzyme binding;extracellular matrix binding;hydrolase activity;integrin binding;ion binding;kinase binding;laminin binding;metal ion binding;meta
binding;protein binding;receptor binding
binding;catalytic activity;cation binding;enzyme binding;identical protein binding;ion binding;kinase activity;kinase binding;metal ion binding;phosphotransferase activity, alcohol group as acceptor;protein binding;protein dom
adenyl nucleotide binding;adenyl ribonucleotide binding;ATP binding;binding;calcium-dependent protein serine/threonine phosphatase activity;calmodulin binding;calmodulin-dependent protein kinase activity;catalytic activit
catalytic activity;hydrolase activity;hydrolase activity, acting on ester bonds;inositol or phosphatidylinositol phosphatase activity;lipid phosphatase activity;phosphatase activity;phosphatidylinositol bisphosphate phosphatase
catalytic activity;hydrolase activity;hydrolase activity, acting on ester bonds;inositol or phosphatidylinositol phosphatase activity;lipid phosphatase activity;phosphatase activity;phosphatidylinositol bisphosphate phosphatase
binding;cation binding;ion binding;metal ion binding;transition metal ion binding;zinc ion binding
binding;protein binding;receptor binding
binding;catalytic activity;GTP binding;GTPase activity;guanyl nucleotide binding;guanyl ribonucleotide binding;hydrolase activity;hydrolase activity, acting on acid anhydrides;hydrolase activity, acting on acid anhydrides, in ph
binding;catalytic activity;GTP binding;GTPase activity;guanyl nucleotide binding;guanyl ribonucleotide binding;hydrolase activity;hydrolase activity, acting on acid anhydrides;hydrolase activity, acting on acid anhydrides, in ph
aminopeptidase activity;carboxypeptidase activity;catalytic activity;cysteine-type endopeptidase activity;cysteine-type peptidase activity;endopeptidase activity;exopeptidase activity;hydrolase activity;peptidase activity;pept
actin binding;binding;cytoskeletal protein binding;enzyme binding;protein binding
binding;cytokine activity;protein binding;receptor binding
binding;ribonucleoprotein binding
binding;protein binding;transcription factor binding
binding;carbohydrate binding;enzyme binding;protein binding
androgen receptor binding;binding;cation binding;hormone receptor binding;identical protein binding;ion binding;metal ion binding;nuclear hormone receptor binding;protein binding;protein binding transcription factor activity
catalytic activity;hydrolase activity;hydrolase activity, acting on acid anhydrides;hydrolase activity, acting on acid anhydrides, in phosphorus-containing anhydrides;motor activity;nucleoside-triphosphatase activity;pyrophosp
adenyl nucleotide binding;adenyl ribonucleotide binding;ATP binding;ATPase activity;binding;catalytic activity;hydrolase activity;hydrolase activity, acting on acid anhydrides;hydrolase activity, acting on acid anhydrides, in ph
binding;protein binding;receptor binding;structural molecule activity
acid-amino acid ligase activity;binding;catalytic activity;cation binding;ion binding;ligase activity;ligase activity, forming carbon-nitrogen bonds;metal ion binding;nucleic acid binding transcription factor activity;sequence-spec
ankyrin binding;binding;cytoskeletal protein binding;protein binding
binding;catalytic activity;coenzyme binding;cofactor binding;identical protein binding;isomerase activity;protein binding;protein dimerization activity;protein homodimerization activity;racemase and epimerase activity;racemas
catalytic activity;intramolecular oxidoreductase activity;intramolecular oxidoreductase activity, interconverting keto- and enol-groups;intramolecular oxidoreductase activity, transposing S-S bonds;isomerase activity;oxidored
adenyl nucleotide binding;adenyl ribonucleotide binding;ATP binding;ATPase activity;ATPase activity, coupled;ATP-dependent helicase activity;ATP-dependent RNA helicase activity;binding;catalytic activity;helicase activity;h
binding;calcium channel activity;calcium channel inhibitor activity;calcium channel regulator activity;calcium ion transmembrane transporter activity;calcium-release channel activity;cation channel activity;cation transmembra
actin binding;actin filament binding;binding;calcium ion binding;cation binding;cytoskeletal protein binding;ion binding;metal ion binding;protein binding;structural constituent of cytoskeleton;structural molecule activity
adenyl nucleotide binding;adenyl ribonucleotide binding;ATP binding;binding;catalytic activity;chromatin binding;hydrolase activity;hydrolase activity, acting on acid anhydrides;hydrolase activity, acting on acid anhydrides, in
alpha-glucosidase activity;binding;carbohydrate binding;catalytic activity;glucan 1,3-alpha-glucosidase activity;glucosidase activity;hydrolase activity;hydrolase activity, acting on glycosyl bonds;hydrolase activity, hydrolyzing
binding;enzyme binding;kinase binding;phosphatase binding;protein binding;protein kinase binding;protein phosphatase binding
binding;cation binding;enzyme binding;ion binding;metal ion binding;nuclear localization sequence binding;peptide binding;protein binding;protein domain specific binding;protein transporter activity;signal sequence binding;
structural molecule activity
binding;enzyme activator activity;enzyme regulator activity;histone acetyl-lysine binding;histone binding;peptidase activator activity;peptidase regulator activity;protein binding
binding;enzyme regulator activity;GTP binding;guanyl nucleotide binding;guanyl ribonucleotide binding;nucleotide binding;purine nucleotide binding;purine ribonucleoside triphosphate binding;purine ribonucleotide binding;ri
binding;histone binding;protein binding
adenyl nucleotide binding;adenyl ribonucleotide binding;aminoacyl-tRNA editing activity;aminoacyl-tRNA ligase activity;ATP binding;binding;carboxylic ester hydrolase activity;catalytic activity;hydrolase activity;hydrolase act
binding;enzyme activator activity;enzyme binding;enzyme regulator activity;GTPase activator activity;GTPase binding;GTPase regulator activity;nucleoside-triphosphatase regulator activity;protein binding;Rab GTPase bindin
cation transmembrane transporter activity;divalent inorganic cation transmembrane transporter activity;ferrous iron transmembrane transporter activity;inorganic cation transmembrane transporter activity;ion transmembrane
catalytic activity;DNA polymerase activity;DNA-directed DNA polymerase activity;nucleotidyltransferase activity;transferase activity;transferase activity, transferring phosphorus-containing groups
catalytic activity;intramolecular oxidoreductase activity;intramolecular oxidoreductase activity, interconverting keto- and enol-groups;intramolecular oxidoreductase activity, transposing S-S bonds;isomerase activity;protein d
binding;carbohydrate binding;collagen binding;enzyme activator activity;enzyme regulator activity;glycosaminoglycan binding;heparin binding;pattern binding;peptidase activator activity;peptidase regulator activity;polysacch
adenyl nucleotide binding;adenyl ribonucleotide binding;ATP binding;binding;catalytic activity;kinase activity;nucleotide binding;phosphotransferase activity, alcohol group as acceptor;protein kinase activity;protein serine/thre

ankyrin binding;binding;cytoskeletal protein binding;protein binding;structural constituent of muscle;structural molecule activity
arylesterase activity;binding;carboxylic ester hydrolase activity;catalytic activity;cation binding;hydrolase activity;hydrolase activity, acting on ester bonds;identical protein binding;ion binding;metal ion binding;protein binding
binding;core promoter binding;DNA binding;identical protein binding;nucleic acid binding;nucleotide binding;protein binding;regulatory region DNA binding;regulatory region nucleic acid binding;transcription regulatory region
beta-catenin binding;binding;catalytic activity;enzyme binding;gamma-catenin binding;hydrolase activity;hydrolase activity, acting on ester bonds;kinase binding;molecular transducer activity;phosphatase activity;phosphopr
binding;DNA binding;nucleic acid binding;RNA binding;single-stranded DNA binding;structure-specific DNA binding
binding;catalytic activity;DNA binding;endonuclease activity;endoribonuclease activity;hydrolase activity;hydrolase activity, acting on ester bonds;mRNA binding;nuclease activity;nucleic acid binding;ribonuclease activity;RN
adenyl nucleotide binding;adenyl ribonucleotide binding;ATP binding;binding;catalytic activity;DNA binding;double-stranded RNA binding;hormone receptor binding;nuclear hormone receptor binding;nucleic acid binding;nuc
binding;cation binding;cytokine receptor binding;enzyme binding;growth factor receptor binding;hormone receptor binding;interleukin-1 receptor binding;ion binding;kinase binding;metal ion binding;nuclear hormone recepto
active transmembrane transporter activity;amine transmembrane transporter activity;amino acid transmembrane transporter activity;anion transmembrane transporter activity;anion:cation symporter activity;carboxylic acid tr
activating transcription factor binding;binding;cation binding;chromatin binding;core promoter proximal region DNA binding;core promoter proximal region sequence-specific DNA binding;co-SMAD binding;cytokine receptor
binding;catalytic activity;cytoskeletal protein binding;GDP binding;GTP binding;GTPase activity;guanyl nucleotide binding;guanyl ribonucleotide binding;hydrolase activity;hydrolase activity, acting on acid anhydrides;hydrolas
binding;cation binding;ion binding;metal ion binding;transition metal ion binding;zinc ion binding
adenyl nucleotide binding;adenyl ribonucleotide binding;androgen receptor binding;ATP binding;binding;catalytic activity;chromatin binding;enzyme binding;GTPase binding;GTP-Rho binding;histone binding;histone deacety
binding;cation binding;ion binding;metal ion binding;transition metal ion binding;zinc ion binding
binding;damaged DNA binding;DNA binding;nucleic acid binding
adenyl nucleotide binding;adenyl ribonucleotide binding;ATP binding;binding;catalytic activity;kinase activity;MAP kinase activity;MAP kinase kinase activity;molecular transducer activity;NFAT protein binding;nucleotide bind
actin binding;actin filament binding;binding;cytoskeletal protein binding;drug binding;protein binding
2,4-dienoyl-CoA reductase (NADPH) activity;binding;catalytic activity;coenzyme binding;cofactor binding;NADP binding;NADPH binding;nucleotide binding;oxidoreductase activity;oxidoreductase activity, acting on NADH or
binding;carbohydrate binding;catalytic activity;cation binding;hydrolase activity;hydrolase activity, acting on glycosyl bonds;hydrolase activity, hydrolyzing N-glycosyl compounds;hydrolase activity, hydrolyzing O-glycosyl co
binding;catalytic activity;cofactor binding;hydrolase activity;hydrolase activity, acting on acid carbon-carbon bonds;hydrolase activity, acting on acid carbon-carbon bonds, in ketonic substances;identical protein binding;kyn
catalytic activity;endopeptidase activity;hydrolase activity;peptidase activity;peptidase activity, acting on L-amino acid peptides;serine hydrolase activity;serine-type endopeptidase activity;serine-type peptidase activity
adenyl nucleotide binding;adenyl ribonucleotide binding;ATP binding;binding;catalytic activity;guanylate kinase activity;kinase activity;nucleobase-containing compound kinase activity;nucleotide binding;nucleotide kinase ac
binding;carbonate dehydratase activity;carbon-oxygen lyase activity;catalytic activity;cation binding;hydro-lyase activity;ion binding;lyase activity;metal ion binding;transition metal ion binding;zinc ion binding
binding;carbon-carbon lyase activity;carboxy-lyase activity;catalytic activity;cation binding;GTP binding;guanyl nucleotide binding;guanyl ribonucleotide binding;ion binding;lyase activity;metal ion binding;nucleotide binding;
binding;carbohydrate binding;catalytic activity;cation binding;glucose binding;identical protein binding;ion binding;metal ion binding;monosaccharide binding;nucleotide binding;nucleotidyltransferase activity;protein binding;
adenyl nucleotide binding;adenyl ribonucleotide binding;ATP binding;binding;carbohydrate kinase activity;catalytic activity;fructokinase activity;glucokinase activity;hexokinase activity;kinase activity;mannokinase activity;nuc
binding;carboxylic acid binding;catalytic activity;cation binding;ion binding;iron ion binding;L-ascorbic acid binding;metal ion binding;oxidoreductase activity;oxidoreductase activity, acting on paired donors, with incorporatio
structural molecule activity
catalytic activity;nucleotidyltransferase activity;transferase activity;transferase activity, transferring phosphorus-containing groups;uridylyltransferase activity
adenyl nucleotide binding;adenyl ribonucleotide binding;ATP binding;binding;catalytic activity;cation binding;cyclase activity;FAD-AMP lyase (cyclizing) activity;glycerone kinase activity;ion binding;kinase activity;lyase activity
binding;catalytic activity;cytokine receptor binding;growth factor receptor binding;hydrolase activity;hydrolase activity, acting on ester bonds;interleukin-2 receptor binding;nucleic acid binding;phosphatase activity;phospho
anion channel activity;anion transmembrane transporter activity;calcium activated cation channel activity;cation channel activity;cation transmembrane transporter activity;channel activity;chloride channel activity;gated chan
binding;nucleic acid binding;RNA binding
catalytic activity;oxidoreductase activity;oxidoreductase activity, acting on the CH-NH group of donors;oxidoreductase activity, acting on the CH-NH group of donors, NAD or NADP as acceptor;pyrroline-5-carboxylate reduc
binding;cation binding;ion binding;metal ion binding;transition metal ion binding;zinc ion binding
binding;catalytic activity;GTP binding;GTPase activity;guanyl nucleotide binding;guanyl ribonucleotide binding;hydrolase activity;hydrolase activity, acting on acid anhydrides;hydrolase activity, acting on acid anhydrides, in p
ATPase activator activity;ATPase binding;ATPase regulator activity;binding;cytoskeletal protein binding;enzyme activator activity;enzyme binding;enzyme regulator activity;nucleoside-triphosphatase regulator activity;protein b
binding;lipid binding;phosphatidylinositol binding;phosphatidylinositol-3,5-bisphosphate binding;phosphatidylinositol-3-phosphate binding;phospholipid binding
acid-amino acid ligase activity;binding;catalytic activity;cation binding;ion binding;ligase activity;ligase activity, forming carbon-nitrogen bonds;metal ion binding;small conjugating protein ligase activity;transition metal ion bin
binding;protein binding;protein complex binding
enzyme activator activity;enzyme regulator activity;GTPase activator activity;GTPase regulator activity;nucleoside-triphosphatase regulator activity
binding;cytokine binding;growth factor binding;protein binding;transforming growth factor beta binding
actin binding;actin monomer binding;adenyl nucleotide binding;adenyl ribonucleotide binding;ATP binding;binding;cytoskeletal protein binding;enzyme binding;kinase binding;lipid binding;nucleotide binding;phosphatidylinos
binding;chromatin binding;chromo shadow domain binding;enzyme binding;histone deacetylase binding;protein binding;protein C-terminus binding;protein domain specific binding;protein N-terminus binding
binding;core promoter proximal region DNA binding;core promoter proximal region sequence-specific DNA binding;DNA binding;molecular transducer activity;nucleic acid binding;nucleic acid binding transcription factor acti
binding;carbon-carbon lyase activity;carboxy-lyase activity;catalytic activity;cofactor binding;lyase activity;pyridoxal phosphate binding;vitamin B6 binding;vitamin binding
basal RNA polymerase II transcription machinery binding;basal transcription machinery binding;binding;enzyme binding;protein binding;RNA polymerase binding;RNA polymerase core enzyme binding;RNA polymerase II cor
adenyl nucleotide binding;adenyl ribonucleotide binding;aminoacyl-tRNA ligase activity;aspartate-tRNA ligase activity;aspartate-tRNA(Asn) ligase activity;ATP binding;binding;catalytic activity;identical protein binding;ligase a
adenyl nucleotide binding;adenyl ribonucleotide binding;ATP binding;binding;catalytic activity;formate-tetrahydrofolate ligase activity;identical protein binding;ligase activity;ligase activity, forming carbon-nitrogen bonds;nucle
3-hydroxyacyl-CoA dehydratase activity;binding;carbon-oxygen lyase activity;catalytic activity;enzyme binding;hydro-lyase activity;lyase activity;protein binding
binding;cation binding;DNA binding;ion binding;metal ion binding;nucleic acid binding
binding;cation binding;DNA binding;ion binding;metal ion binding;nucleic acid binding;nucleic acid binding transcription factor activity;regulatory region DNA binding;regulatory region nucleic acid binding;RNA polymerase II
binding;lipid binding;phospholipid binding
binding;enzyme binding;GTPase binding;protein binding;Rab GTPase binding;Ras GTPase binding;small GTPase binding
catalytic activity;hydrolase activity;hydrolase activity, acting on ester bonds;nuclease activity;protein binding transcription factor activity;transcription cofactor activity;transcription factor binding transcription factor activity
adenyl nucleotide binding;adenyl ribonucleotide binding;ATP binding;ATPase activity;ATPase activity, coupled;ATP-dependent helicase activity;ATP-dependent RNA helicase activity;binding;catalytic activity;chromatin binding
actin binding;actin filament binding;binding;cytoskeletal protein binding;enzyme binding;GTPase binding;protein binding;protein complex binding;Rac GTPase binding;Ras GTPase binding;Rho GTPase binding;small GTPase
1-acylglycerol-3-phosphate O-acyltransferase activity;1-acylglycerophosphocholine O-acyltransferase activity;1-alkylglycerophosphocholine O-acetyltransferase activity;acetyltransferase activity;acylglycerol O-acyltransfera
binding;catalytic activity;cation binding;dioxygenase activity;ion binding;metal ion binding;oxidoreductase activity
binding;protein binding;protein domain specific binding
binding;catalytic activity;cation binding;ion binding;metal ion binding;NAD+ ADP-ribosyltransferase activity;transferase activity;transferase activity, transferring glycosyl groups;transferase activity, transferring pentosyl groups
nucleocytoplasmic transporter activity;transporter activity
binding;nucleic acid binding;RNA binding
binding;cytoskeletal protein binding;kinetochore binding;microtubule binding;microtubule plus-end binding;protein binding;tubulin binding
G-protein coupled receptor activity;molecular transducer activity;pheromone receptor activity;receptor activity;signal transducer activity;signaling receptor activity;transmembrane signaling receptor activity
binding;cation binding;ion binding;metal ion binding
molecular transducer activity;signal transducer activity
carbon-sulfur lyase activity;catalytic activity;lyase activity
catalytic activity;histone methyltransferase activity;histone methyltransferase activity (H3-K36 specific);histone methyltransferase activity (H3-K4 specific);histone-lysine N-methyltransferase activity;lysine N-methyltransferase
binding;nucleic acid binding;nucleotide binding;RNA binding
binding;double-stranded RNA binding;NF-kappaB binding;nucleic acid binding;protein binding;protein binding transcription factor activity;RNA binding;RNA polymerase II transcription factor binding;transcription coactivator
acetyltransferase activity;catalytic activity;N-acetyltransferase activity;N-acyltransferase activity;transferase activity;transferase activity, transferring acyl groups;transferase activity, transferring acyl groups other than amino-a
acetoacetate-CoA ligase activity;acid-thiol ligase activity;adenyl nucleotide binding;adenyl ribonucleotide binding;ATP binding;binding;butyrate-CoA ligase activity;catalytic activity;ligase activity;ligase activity, forming carbon
binding;enzyme binding;enzyme regulator activity;phosphatase binding;phosphatase regulator activity;protein binding;protein phosphatase 2A binding;protein phosphatase binding;protein phosphatase regulator activity;prote
binding;catalytic activity;cation binding;ion binding;ligase activity;metal ion binding
binding;nucleotide binding
adenylosuccinate synthase activity;anion binding;binding;catalytic activity;cation binding;GTP binding;GTPase activity;guanyl nucleotide binding;guanyl ribonucleotide binding;hydrolase activity;hydrolase activity, acting on a
enzyme activator activity;enzyme regulator activity;GTPase activator activity;GTPase regulator activity;nucleoside-triphosphatase regulator activity
binding;chromatin binding
binding;cation binding;enzyme activator activity;enzyme regulator activity;GTPase activator activity;GTPase regulator activity;ion binding;metal ion binding;nucleoside-triphosphatase regulator activity;transition metal ion bind
binding;DNA binding;enzyme inhibitor activity;enzyme regulator activity;ligase inhibitor activity;ligase regulator activity;nucleic acid binding;p53 binding;protein binding;transcription factor binding;ubiquitin-protein ligase inhib
binding;protein binding;protein domain specific binding;WW domain binding
binding;nucleic acid binding;RNA binding;translation factor activity, nucleic acid binding;translation initiation factor activity
adenyl nucleotide binding;adenyl ribonucleotide binding;ATP binding;binding;catalytic activity;kinase activity;nucleotide binding;phosphotransferase activity, alcohol group as acceptor;protein binding;protein complex binding
catalytic activity;galactosyltransferase activity;procollagen galactosyltransferase activity;transferase activity;transferase activity, transferring glycosyl groups;transferase activity, transferring hexosyl groups;UDP-galactosyltran
1-acylglycerophosphocholine O-acyltransferase activity;1-alkenylglycerophosphocholine O-acyltransferase activity;1-alkylglycerophosphocholine O-acetyltransferase activity;1-alkylglycerophosphocholine O-acyltransferase
acid-amino acid ligase activity;binding;catalytic activity;cation binding;ion binding;ligase activity;ligase activity, forming carbon-nitrogen bonds;metal ion binding;small conjugating protein ligase activity;transition metal ion bin
acid-amino acid ligase activity;binding;catalytic activity;cation binding;histone binding;ion binding;ligase activity;ligase activity, forming carbon-nitrogen bonds;metal ion binding;protein binding;small conjugating protein ligase
adenyl nucleotide binding;adenyl ribonucleotide binding;ATP binding;ATPase activity;ATPase activity, coupled;ATP-dependent helicase activity;ATP-dependent RNA helicase activity;binding;catalytic activity;estrogen receptor
antioxidant activity;catalytic activity;glutathione peroxidase activity;oxidoreductase activity;oxidoreductase activity, acting on peroxide as acceptor;peroxidase activity
binding;catalytic activity;histone methyltransferase activity;histone-lysine N-methyltransferase activity;lysine N-methyltransferase activity;methyltransferase activity;N-methyltransferase activity;p53 binding;protein binding;pro
binding;identical protein binding;protein binding;protein binding transcription factor activity;transcription cofactor activity;transcription corepressor activity;transcription factor binding;transcription factor binding transcription
active transmembrane transporter activity;amine transmembrane transporter activity;amino acid transmembrane transporter activity;carboxylic acid transmembrane transporter activity;glycine transmembrane transporter acti
binding;identical protein binding;lipid binding;phosphatidylinositol binding;phospholipid binding;protein binding
acid-amino acid ligase activity;binding;catalytic activity;cation binding;ion binding;ligase activity;ligase activity, forming carbon-nitrogen bonds;metal ion binding;small conjugating protein ligase activity;transition metal ion bin
binding;ribonucleoprotein binding;snRNP binding
actin binding;binding;cytoskeletal protein binding;protein binding

catalytic activity;hydrolase activity
binding;GTP binding;guanyl nucleotide binding;guanyl ribonucleotide binding;nucleotide binding;purine nucleotide binding;purine ribonucleoside triphosphate binding;purine ribonucleotide binding;ribonucleotide binding
binding;cation binding;ion binding;metal ion binding;transition metal ion binding;zinc ion binding
binding;cadherin binding;cell adhesion molecule binding;cytoskeletal protein binding;enzyme binding;gamma-tubulin binding;GTPase binding;microtubule binding;protein binding;Rab GTPase binding;Ras GTPase binding;sm
adenyl nucleotide binding;adenyl ribonucleotide binding;ATP binding;binding;nucleotide binding;purine nucleotide binding;purine ribonucleoside triphosphate binding;purine ribonucleotide binding;ribonucleotide binding
binding;GTP binding;guanyl nucleotide binding;guanyl ribonucleotide binding;nucleotide binding;purine nucleotide binding;purine ribonucleoside triphosphate binding;purine ribonucleotide binding;ribonucleotide binding
binding;cation binding;enzyme activator activity;enzyme regulator activity;GTPase activator activity;GTPase regulator activity;ion binding;metal ion binding;nucleoside-triphosphatase regulator activity
antioxidant activity;binding;catalytic activity;cation binding;cytokine receptor binding;extracellular matrix structural constituent;heme binding;interleukin-1 receptor antagonist activity;ion binding;iron ion binding;metal ion binc
actin binding;actin filament binding;binding;cytoskeletal protein binding;protein binding
catalytic activity;exopeptidase activity;gamma-glutamyl-peptidase activity;hydrolase activity;omega peptidase activity;peptidase activity;peptidase activity, acting on L-amino acid peptides
adenyl nucleotide binding;adenyl ribonucleotide binding;ATP binding;ATPase activity;ATPase activity, coupled;ATP-dependent helicase activity;ATP-dependent RNA helicase activity;binding;catalytic activity;estrogen receptor
adenyl nucleotide binding;adenyl ribonucleotide binding;ATP binding;ATPase activity;binding;binding, bridging;catalytic activity;cation binding;DNA binding;hydrolase activity;hydrolase activity, acting on acid anhydrides;hydro
binding;BRE binding;mRNA binding;nucleic acid binding;nucleotide binding;RNA binding;translation regulator activity;translation regulator activity, nucleic acid binding;translation repressor activity;translation repressor activity
enzyme activator activity;enzyme regulator activity;GTPase activator activity;GTPase regulator activity;guanyl-nucleotide exchange factor activity;nucleoside-triphosphatase regulator activity;Ras guanyl-nucleotide exchange
binding;protein binding;receptor binding
adenyl nucleotide binding;adenyl ribonucleotide binding;ATP binding;ATPase activity;ATPase activity, coupled;ATP-dependent helicase activity;ATP-dependent RNA helicase activity;binding;catalytic activity;cation binding;chr
binding;catalytic activity;GDP binding;GTP binding;GTPase activity;guanyl nucleotide binding;guanyl ribonucleotide binding;hydrolase activity;hydrolase activity, acting on acid anhydrides;hydrolase activity, acting on acid anh
binding;DNA binding;mRNA binding;nucleic acid binding;RNA binding
binding;nuclear localization sequence binding;peptide binding;signal sequence binding
binding;cation binding;cytoskeletal protein binding;enzyme binding;enzyme regulator activity;GTPase binding;GTPase regulator activity;guanyl-nucleotide exchange factor activity;ion binding;metal ion binding;microtubule bin
binding;catalytic activity;cysteine-type endopeptidase activity;cysteine-type peptidase activity;endopeptidase activity;enzyme binding;hydrolase activity;p53 binding;peptidase activity;peptidase activity, acting on L-amino aci
catalytic activity;endonuclease activity;endonuclease activity, active with either ribo- or deoxyribonucleic acids and producing 5'-phosphomonoesters;endoribonuclease activity;endoribonuclease activity, producing 5'-phosph
structural constituent of ribosome;structural molecule activity
binding;cation binding;ion binding;metal ion binding;transition metal ion binding;zinc ion binding
catalytic activity;kinase activity;phosphotransferase activity, alcohol group as acceptor;protein kinase activity;transferase activity;transferase activity, transferring phosphorus-containing groups
binding;enzyme binding;GTPase binding;protein binding;protein N-terminus binding;Ral GTPase binding;Ras GTPase binding;small GTPase binding
binding;lipid binding;phosphatidylinositol binding;phosphatidylinositol-3,4,5-trisphosphate binding;phospholipid binding
binding;DNA binding;nucleic acid binding;nucleic acid binding transcription factor activity;sequence-specific DNA binding transcription factor activity;single-stranded DNA binding;structure-specific DNA binding
binding;catalytic activity;cation binding;identical protein binding;ion binding;isomerase activity;metal ion binding;protein binding;protein dimerization activity;protein homodimerization activity;racemase and epimerase activity;
binding;calcium ion binding;catalytic activity;cation binding;cis-trans isomerase activity;drug binding;FK506 binding;ion binding;isomerase activity;macrolide binding;metal ion binding;peptidyl-prolyl cis-trans isomerase activi
binding;cation binding;ion binding;metal ion binding
active transmembrane transporter activity;binding;drug transmembrane transporter activity;enzyme binding;protein binding;secondary active transmembrane transporter activity;symporter activity;transmembrane transporter
catalytic activity;cis-trans isomerase activity;isomerase activity;peptidyl-prolyl cis-trans isomerase activity
binding;calcium ion binding;cation binding;ion binding;metal ion binding
aldose 1-epimerase activity;binding;carbohydrate binding;catalytic activity;isomerase activity;racemase and epimerase activity;racemase and epimerase activity, acting on carbohydrates and derivatives
catalytic activity;oxidoreductase activity;oxidoreductase activity, acting on the CH-NH group of donors;oxidoreductase activity, acting on the CH-NH group of donors, NAD or NADP as acceptor;pyrroline-5-carboxylate reduc
binding;catalytic activity;hydrolase activity;hydrolase activity, acting on acid anhydrides;hydrolase activity, acting on acid anhydrides, in phosphorus-containing anhydrides;m7G(5')pppN diphosphatase activity;nucleic acid bi
binding;enzyme binding;enzyme inhibitor activity;enzyme regulator activity;lipase binding;lipase inhibitor activity;protein binding;small conjugating protein binding;ubiquitin binding;ubiquitin protein ligase binding
binding;binding, bridging;protein binding;protein binding, bridging;ribonucleoprotein binding;ribosomal large subunit binding
binding;catalytic activity;cation binding;hydrolase activity;ion binding;metal ion binding;transition metal ion binding;zinc ion binding
binding;catalytic activity;cation binding;coenzyme binding;cofactor binding;flavin adenine dinucleotide binding;ion binding;metal ion binding;oxidoreductase activity;oxidoreductase activity, acting on the CH-CH group of don
binding;channel regulator activity;identical protein binding;protein binding;protein C-terminus binding;protein dimerization activity;protein homodimerization activity;protein N-terminus binding;transcription factor binding
catalytic activity;disulfide oxidoreductase activity;intramolecular oxidoreductase activity;intramolecular oxidoreductase activity, interconverting keto- and enol-groups;intramolecular oxidoreductase activity, transposing S-S bo
binding;core promoter proximal region DNA binding;core promoter proximal region sequence-specific DNA binding;DNA binding;nucleic acid binding;nucleic acid binding transcription factor activity;regulatory region DNA bin
catalytic activity;nucleotidyltransferase activity;transferase activity;transferase activity, transferring phosphorus-containing groups
actin binding;binding;cytoskeletal protein binding;enzyme binding;leucine zipper domain binding;LRR domain binding;protein binding;protein binding transcription factor activity;protein domain specific binding;transcription c
binding;cyclin binding;enzyme binding;kinase binding;NF-kappaB binding;protein binding;protein kinase binding;transcription factor binding
binding;catalytic activity;cysteine-type endopeptidase activity;cysteine-type peptidase activity;endopeptidase activity;hydrolase activity;peptidase activity;peptidase activity, acting on L-amino acid peptides;protein binding;pr
binding;carboxypeptidase activity;catalytic activity;cation binding;dipeptidase activity;exopeptidase activity;hydrolase activity;ion binding;metal ion binding;metallopeptidase activity;peptidase activity;peptidase activity, acting
3'-flap endonuclease activity;binding;catalytic activity;cation binding;deoxyribonuclease activity;DNA binding;endodeoxyribonuclease activity;endodeoxyribonuclease activity, producing 3'-phosphomonoesters;endonuclease
binding;cation binding;enzyme activator activity;enzyme regulator activity;GTPase activator activity;GTPase regulator activity;ion binding;lipid binding;metal ion binding;nucleoside-triphosphatase regulator activity;phosphatid
binding;identical protein binding;lipid binding;protein binding
adenyl nucleotide binding;adenyl ribonucleotide binding;ATP binding;ATPase activity;binding;catalytic activity;cation binding;DNA binding;hydrolase activity;hydrolase activity, acting on acid anhydrides;hydrolase activity, acti
adenyl nucleotide binding;adenyl ribonucleotide binding;ATP binding;binding;catalytic activity;cation binding;ion binding;kinase activity;magnesium ion binding;metal ion binding;nucleotide binding;phosphotransferase activity
binding;lipid binding
binding;nucleotide binding
acid-amino acid ligase activity;binding;catalytic activity;cation binding;chromatin binding;core promoter proximal region DNA binding;core promoter proximal region sequence-specific DNA binding;DNA binding;histone bindi
actin binding;binding;cytoskeletal protein binding;protein binding
binding;cation binding;ion binding;metal ion binding;transition metal ion binding;zinc ion binding
binding;DNA binding;nucleic acid binding;nucleic acid binding transcription factor activity;protein binding;RNA polymerase II transcription factor binding;sequence-specific DNA binding transcription factor activity;transcriptio
binding;catalytic activity;cation binding;DNA binding;endonuclease activity;endoribonuclease activity;hydrolase activity;hydrolase activity, acting on ester bonds;ion binding;metal ion binding;nuclease activity;nucleic acid binc
binding;nucleic acid binding;RNA binding;translation factor activity, nucleic acid binding;translation initiation factor activity
binding;protein binding;unfolded protein binding
binding;heat shock protein binding;protein binding
binding;epidermal growth factor receptor binding;growth factor receptor binding;nucleic acid binding;protein binding;protein C-terminus binding;receptor binding;RNA binding
3-hydroxy-2-methylbutyryl-CoA dehydrogenase activity;3-hydroxyacyl-CoA dehydrogenase activity;catalytic activity;cholate 7-alpha-dehydrogenase activity;oxidoreductase activity;oxidoreductase activity, acting on CH-OH g
nucleobase-containing compound transmembrane transporter activity;nucleoside transmembrane transporter activity;substrate-specific transmembrane transporter activity;substrate-specific transporter activity;transmembra
adenyl nucleotide binding;adenyl ribonucleotide binding;ATP binding;ATPase activity;ATPase activity, coupled;ATP-dependent helicase activity;ATP-dependent RNA helicase activity;binding;catalytic activity;helicase activity;h
3'-tRNA processing endoribonuclease activity;binding;catalytic activity;cation binding;endonuclease activity;endonuclease activity, active with either ribo- or deoxyribonucleic acids and producing 5'-phosphomonoesters;end
catalytic activity;deacetylase activity;hydrolase activity;hydrolase activity, acting on glycosyl bonds
binding;DNA binding;nucleic acid binding;nucleic acid binding transcription factor activity;sequence-specific DNA binding transcription factor activity
binding;high-density lipoprotein particle binding;lipid binding;lipoprotein particle binding;protein binding;protein-lipid complex binding;receptor binding
actin binding;actin filament binding;Arp2/3 complex binding;binding;cytoskeletal protein binding;identical protein binding;protein binding;protein complex binding
catalytic activity;intramolecular oxidoreductase activity;intramolecular oxidoreductase activity, interconverting keto- and enol-groups;intramolecular oxidoreductase activity, transposing S-S bonds;isomerase activity;protein d
beta-tubulin binding;binding;chaperone binding;cytoskeletal protein binding;enzyme activator activity;enzyme regulator activity;GTPase activator activity;GTPase regulator activity;nucleoside-triphosphatase regulator activity;
alpha-L-fucosidase activity;binding;carbohydrate binding;catalytic activity;fucose binding;fucosidase activity;hydrolase activity;hydrolase activity, acting on glycosyl bonds;hydrolase activity, hydrolyzing O-glycosyl compound
binding;catalytic activity;GTP binding;GTPase activity;guanyl nucleotide binding;guanyl ribonucleotide binding;hydrolase activity;hydrolase activity, acting on acid anhydrides;hydrolase activity, acting on acid anhydrides, in pl
catalytic activity;endonuclease activity;endonuclease activity, active with either ribo- or deoxyribonucleic acids and producing 5'-phosphomonoesters;endoribonuclease activity;endoribonuclease activity, producing 5'-phosph
adenyl nucleotide binding;adenyl ribonucleotide binding;ATP binding;ATPase activity;ATPase activity, coupled;ATP-dependent helicase activity;ATP-dependent RNA helicase activity;binding;catalytic activity;helicase activity;h
binding;catalytic activity;methyltransferase activity;nucleic acid binding;RNA binding;transferase activity;transferase activity, transferring one-carbon groups
acetyl-CoA C-acetyltransferase activity;acetyl-CoA C-acyltransferase activity;acetyltransferase activity;C-acetyltransferase activity;C-acyltransferase activity;catalytic activity;transferase activity;transferase activity, transferrin
binding;cation binding;ion binding;metal ion binding;miRNA binding;mRNA 3'-UTR binding;mRNA binding;nucleic acid binding;nucleotide binding;RNA binding;transition metal ion binding;zinc ion binding
ion transmembrane transporter activity;substrate-specific transmembrane transporter activity;substrate-specific transporter activity;transmembrane transporter activity;transporter activity
binding;carbohydrate binding;catalytic activity;chitin binding;chitinase activity;hydrolase activity;hydrolase activity, acting on glycosyl bonds;hydrolase activity, hydrolyzing O-glycosyl compounds;oligosaccharide binding;patt
binding;catalytic activity;GTP binding;GTPase activity;guanyl nucleotide binding;guanyl ribonucleotide binding;hydrolase activity;hydrolase activity, acting on acid anhydrides;hydrolase activity, acting on acid anhydrides, in pl
binding;cholesterol binding;lipid binding;steroid binding;sterol binding
binding;nucleic acid binding;ribonucleoprotein binding;ribosome binding;RNA binding;translation factor activity, nucleic acid binding;translation initiation factor activity;tRNA binding
structural constituent of ribosome;structural molecule activity
structural constituent of ribosome;structural molecule activity
structural constituent of ribosome;structural molecule activity
adenyl nucleotide binding;adenyl ribonucleotide binding;ATP binding;binding;catalytic activity;cation binding;DNA binding;helicase activity;hydrolase activity;hydrolase activity, acting on acid anhydrides;hydrolase activity, act
adenyl nucleotide binding;adenyl ribonucleotide binding;ATP binding;binding;nucleotide binding;purine nucleotide binding;purine ribonucleoside triphosphate binding;purine ribonucleotide binding;ribonucleotide binding
binding;catalytic activity;GTP binding;GTPase activity;guanyl nucleotide binding;guanyl ribonucleotide binding;hydrolase activity;hydrolase activity, acting on acid anhydrides;hydrolase activity, acting on acid anhydrides, in pl
binding;catalytic activity;enzyme binding;GTP binding;GTPase activity;GTPase binding;guanyl nucleotide binding;guanyl ribonucleotide binding;hydrolase activity;hydrolase activity, acting on acid anhydrides;hydrolase activity
binding;fibroblast growth factor binding;growth factor binding;protein binding
binding;catalytic activity;cation binding;demethylase activity;DNA demethylase activity;DNA-N1-methyladenine dioxygenase activity;ferrous iron binding;ion binding;iron ion binding;metal ion binding;oxidative DNA demethyla

binding;ribonucleoprotein binding
catalytic activity;exonuclease activity;hydrolase activity;hydrolase activity, acting on ester bonds;nuclease activity;nucleic acid binding transcription factor activity;sequence-specific DNA binding transcription factor activity
binding;catalytic activity;DNA binding;DNA-directed RNA polymerase activity;nucleic acid binding;nucleotidyltransferase activity;RNA polymerase activity;transferase activity;transferase activity, transferring phosphorus-conta
binding;carboxylic acid binding;catalytic activity;cation binding;enzyme binding;ion binding;iron ion binding;L-ascorbic acid binding;metal ion binding;oxidoreductase activity;oxidoreductase activity, acting on paired donors,
acetyltransferase activity;catalytic activity;H4 histone acetyltransferase activity;histone acetyltransferase activity;lysine N-acetyltransferase activity;N-acetyltransferase activity;N-acyltransferase activity;peptide alpha-N-acetyl
adenyl nucleotide binding;adenyl ribonucleotide binding;ATP binding;binding;nucleotide binding;purine nucleotide binding;purine ribonucleoside triphosphate binding;purine ribonucleotide binding;ribonucleotide binding
binding;catalytic activity;GTP binding;GTPase activity;guanyl nucleotide binding;guanyl ribonucleotide binding;hydrolase activity;hydrolase activity, acting on acid anhydrides;hydrolase activity, acting on acid anhydrides, in p
acetyltransferase activity;adenyl nucleotide binding;adenyl ribonucleotide binding;ATP binding;binding;catalytic activity;N-acetyltransferase activity;N-acyltransferase activity;nucleotide binding;purine nucleotide binding;purin
5S rRNA binding;binding;nucleic acid binding;RNA binding;rRNA binding;structural constituent of ribosome;structural molecule activity
binding;cation binding;ion binding;metal ion binding;nucleotide binding;protein binding;SNARE binding;syntaxin binding;transition metal ion binding;zinc ion binding
catalytic activity;cis-trans isomerase activity;isomerase activity;peptidyl-prolyl cis-trans isomerase activity
active transmembrane transporter activity;amine transmembrane transporter activity;amino acid transmembrane transporter activity;carboxylic acid transmembrane transporter activity;cation transmembrane transporter activ
adenyl nucleotide binding;adenyl ribonucleotide binding;ATP binding;ATPase activity;ATPase activity, coupled;ATP-dependent helicase activity;ATP-dependent RNA helicase activity;binding;catalytic activity;core promoter bin
carboxypeptidase activity;catalytic activity;exopeptidase activity;hydrolase activity;peptidase activity;peptidase activity, acting on L-amino acid peptides;serine hydrolase activity;serine-type carboxypeptidase activity;serine-t
binding;enzyme binding;enzyme inhibitor activity;enzyme regulator activity;protein binding;ubiquitin protein ligase binding
catalytic activity;disulfide oxidoreductase activity;intramolecular oxidoreductase activity;intramolecular oxidoreductase activity, interconverting keto- and enol-groups;intramolecular oxidoreductase activity, transposing S-S b
binding;chromatin binding
binding;calcium ion binding;cation binding;extracellular matrix binding;ion binding;metal ion binding
structural molecule activity
adenyl nucleotide binding;adenyl ribonucleotide binding;ATP binding;binding;calcium ion binding;cation binding;enzyme binding;GTP binding;GTPase binding;guanyl nucleotide binding;guanyl ribonucleotide binding;ion bindi
binding;catalytic activity;cation binding;dCTP diphosphatase activity;deoxyribonucleotide binding;hydrolase activity;hydrolase activity, acting on acid anhydrides;hydrolase activity, acting on acid anhydrides, in phosphorus-c
binding;enzyme binding;phosphatase binding;protein binding
adenyl nucleotide binding;adenyl ribonucleotide binding;ATP binding;ATPase activity;ATPase activity, coupled;ATP-dependent helicase activity;ATP-dependent RNA helicase activity;binding;catalytic activity;helicase activity;h
binding;cation binding;ion binding;metal ion binding
binding;catalytic activity;cation binding;histone methyltransferase activity;histone methyltransferase activity (H3-K27 specific);histone methyltransferase activity (H3-K9 specific);histone-lysine N-methyltransferase activity;ion
adenyl nucleotide binding;adenyl-nucleotide exchange factor activity;ATPase regulator activity;binding;enzyme regulator activity;nucleoside-triphosphatase regulator activity;nucleotide binding;protein binding;purine nucleotic
adenyl nucleotide binding;adenyl ribonucleotide binding;ATP binding;binding;catalytic activity;CoA carboxylase activity;ligase activity;ligase activity, forming carbon-carbon bonds;methylcrotonoyl-CoA carboxylase activity;nu
active transmembrane transporter activity;adenyl nucleotide binding;adenyl ribonucleotide binding;ATP binding;ATPase activity;ATPase activity, coupled;ATPase activity, coupled to movement of substances;ATPase activity, c
binding;chromatin binding;DNA binding;nucleic acid binding;nucleic acid binding transcription factor activity;sequence-specific DNA binding transcription factor activity
adenyl nucleotide binding;adenyl ribonucleotide binding;aminoacyl-tRNA ligase activity;ATP binding;binding;catalytic activity;ligase activity;ligase activity, forming aminoacyl-tRNA and related compounds;ligase activity, formi
structural constituent of ribosome;structural molecule activity
binding;enzyme activator activity;enzyme regulator activity;G-protein alpha-subunit binding;GTPase activator activity;GTPase regulator activity;guanyl-nucleotide exchange factor activity;nucleoside-triphosphatase regulator a
receptor activity
3'-5' exonuclease activity;3'-5'-exoribonuclease activity;binding;catalytic activity;exonuclease activity;exonuclease activity, active with either ribo- or deoxyribonucleic acids and producing 5'-phosphomonoesters;exoribonucl
actin binding;binding;cytoskeletal protein binding;protein binding
aminopeptidase activity;binding;catalytic activity;cation binding;exopeptidase activity;hydrolase activity;identical protein binding;ion binding;manganese ion binding;metal ion binding;metalloaminopeptidase activity;metalloex
adenyl nucleotide binding;adenyl ribonucleotide binding;ATP binding;binding;cation binding;ion binding;metal ion binding;nucleotide binding;purine nucleotide binding;purine ribonucleoside triphosphate binding;purine ribonu
binding;cation binding;ion binding;metal ion binding;transition metal ion binding;zinc ion binding
adenyl nucleotide binding;adenyl ribonucleotide binding;ATP binding;ATPase activity;ATPase activity, coupled;ATP-dependent helicase activity;ATP-dependent RNA helicase activity;binding;catalytic activity;double-stranded
adenyl nucleotide binding;adenyl ribonucleotide binding;ATP binding;binding;DNA binding;nucleic acid binding;nucleotide binding;purine nucleotide binding;purine ribonucleoside triphosphate binding;purine ribonucleotide bi
adenyl nucleotide binding;adenyl ribonucleotide binding;ATP binding;binding;nucleotide binding;protein binding;protein dimerization activity;protein heterodimerization activity;purine nucleotide binding;purine ribonucleoside t
enzyme activator activity;enzyme regulator activity;GTPase activator activity;GTPase regulator activity;nucleoside-triphosphatase regulator activity
adenyl nucleotide binding;adenyl ribonucleotide binding;ATP binding;binding;nucleotide binding;purine nucleotide binding;purine ribonucleoside triphosphate binding;purine ribonucleotide binding;ribonucleotide binding
adenyl nucleotide binding;adenyl ribonucleotide binding;ATP binding;binding;nucleotide binding;purine nucleotide binding;purine ribonucleoside triphosphate binding;purine ribonucleotide binding;ribonucleotide binding
binding;catalytic activity;cation binding;ion binding;metal ion binding;methyltransferase activity;N-methyltransferase activity;nucleic acid binding;RNA binding;RNA methyltransferase activity;S-adenosylmethionine-dependent
binding;cytoskeletal protein binding;protein binding;tropomyosin binding
binding;catalytic activity;glucosyltransferase activity;protein binding;transferase activity;transferase activity, transferring glycosyl groups;transferase activity, transferring hexosyl groups;UDP-glucose:glycoprotein glucosyltrans
binding;lipid binding;phospholipid binding
adenyl nucleotide binding;adenyl ribonucleotide binding;ATP binding;binding;calcium ion binding;catalytic activity;cation binding;GTP binding;guanyl nucleotide binding;guanyl ribonucleotide binding;hydrolase activity;ion bin
binding;G-protein coupled receptor activity;molecular transducer activity;odorant binding;olfactory receptor activity;receptor activity;signal transducer activity;signaling receptor activity;transmembrane signaling receptor acti
binding;cytoskeletal protein binding;protein binding
structural constituent of ribosome;structural molecule activity
adenyl nucleotide binding;adenyl ribonucleotide binding;ATP binding;binding;catalytic activity;NADH dehydrogenase (quinone) activity;NADH dehydrogenase (ubiquinone) activity;NADH dehydrogenase activity;nucleotide bin
binding;cytoskeletal protein binding;enzyme binding;GTPase binding;kinase binding;microtubule binding;protein binding;protein domain specific binding;protein kinase binding;Rac GTPase binding;Ras GTPase binding;Rho
catalytic activity
receptor activity
adenyl nucleotide binding;adenyl ribonucleotide binding;aminoacyl-tRNA editing activity;aminoacyl-tRNA ligase activity;ATP binding;binding;carboxylic ester hydrolase activity;catalytic activity;hydrolase activity;hydrolase act
binding;cation binding;ion binding;metal ion binding;nucleotide binding
binding;cation binding;enzyme binding;GTPase binding;ion binding;metal ion binding;protein binding;Rab GTPase binding;Ras GTPase binding;small GTPase binding
acid-thiol ligase activity;adenyl nucleotide binding;adenyl ribonucleotide binding;ATP binding;binding;catalytic activity;cation binding;CoA-ligase activity;ion binding;ligase activity;ligase activity, forming carbon-sulfur bonds;m
binding;nucleic acid binding;ribonucleoprotein binding;ribosome binding;RNA binding;translation factor activity, nucleic acid binding;translation initiation factor activity
binding;carboxylic acid binding;catalytic activity;coenzyme binding;cofactor binding;glycerate dehydrogenase activity;glyoxylate reductase (NADP) activity;hydroxypyruvate reductase activity;identical protein binding;NAD bin
catalytic activity;cysteine-type endopeptidase activity;cysteine-type peptidase activity;endopeptidase activity;hydrolase activity;peptidase activity;peptidase activity, acting on L-amino acid peptides
binding;cation binding;enzyme binding;ion binding;metal ion binding;protein binding;transition metal ion binding;ubiquitin conjugating enzyme binding;zinc ion binding
binding;binding, bridging;catalytic activity;guanylate kinase activity;kinase activity;nucleobase-containing compound kinase activity;nucleotide kinase activity;phosphotransferase activity, phosphate group as acceptor;protein
androgen receptor binding;binding;enzyme activator activity;enzyme binding;enzyme regulator activity;heat shock protein binding;histone binding;hormone receptor binding;identical protein binding;kinase activator activity;ki
adenyl nucleotide binding;adenyl ribonucleotide binding;ATP binding;ATPase activity;binding;catalytic activity;hydrolase activity;hydrolase activity, acting on acid anhydrides;hydrolase activity, acting on acid anhydrides, in ph
actin binding;actin filament binding;actin monomer binding;binding;cation binding;cytoskeletal protein binding;ion binding;metal ion binding;protein binding;transition metal ion binding;zinc ion binding
binding;cation binding;heat shock protein binding;Hsp90 protein binding;ion binding;metal ion binding;protein binding;transition metal ion binding;zinc ion binding
active transmembrane transporter activity;anion transmembrane transporter activity;ATPase activity;ATPase activity, coupled;ATPase activity, coupled to movement of substances;ATPase activity, coupled to transmembrane
catalytic activity;dipeptidyl-peptidase activity;exopeptidase activity;hydrolase activity;peptidase activity;peptidase activity, acting on L-amino acid peptides;serine hydrolase activity;serine-type peptidase activity
binding;lamin binding;protein binding
binding;binding, bridging;cytoskeletal adaptor activity;cytoskeletal protein binding;proline-rich region binding;protein binding;protein binding, bridging
active transmembrane transporter activity;ATPase activity;ATPase activity, coupled;ATPase activity, coupled to movement of substances;ATPase activity, coupled to transmembrane movement of ions;ATPase activity, couple
binding;identical protein binding;protein binding
acetylcholine receptor regulator activity;adenyl nucleotide binding;adenyl ribonucleotide binding;ATP binding;binding;catalytic activity;cation binding;chromatin binding;histone acetyl-lysine binding;histone binding;histone kin
adenyl nucleotide binding;adenyl ribonucleotide binding;ATP binding;binding;carbohydrate kinase activity;catalytic activity;kinase activity;N-acetylglucosamine kinase activity;N-acylmannosamine kinase activity;nucleotide bi
binding;carbon-carbon lyase activity;catalytic activity;cation binding;coenzyme binding;cofactor binding;identical protein binding;ion binding;lyase activity;magnesium ion binding;metal ion binding;protein binding;receptor bi
enzyme activator activity;enzyme regulator activity;GTPase activator activity;GTPase regulator activity;nucleoside-triphosphatase regulator activity
binding;enzyme activator activity;enzyme binding;enzyme regulator activity;GTPase activator activity;GTPase regulator activity;kinase binding;nucleoside-triphosphatase regulator activity;protein binding;protein kinase binding
binding;protein binding;protein N-terminus binding
binding;calcium channel inhibitor activity;calcium channel regulator activity;channel inhibitor activity;channel regulator activity;cytoskeletal protein binding;ion channel inhibitor activity;lipid binding;phospholipid binding;protein
binding;cation binding;ion binding;metal ion binding
binding;DNA binding;nucleic acid binding;nucleic acid binding transcription factor activity;sequence-specific DNA binding;sequence-specific DNA binding transcription factor activity
binding;integrin binding;protein binding;protein complex binding;receptor binding
adenyl nucleotide binding;adenyl ribonucleotide binding;ATP binding;ATPase activity;ATPase activity, coupled;ATP-dependent helicase activity;ATP-dependent RNA helicase activity;binding;catalytic activity;helicase activity;h
binding;cytoskeletal protein binding;identical protein binding;lipid binding;phosphatidic acid binding;phospholipid binding;protein binding;transporter activity
receptor activity
adenyl nucleotide binding;adenyl ribonucleotide binding;ATP binding;binding;catalytic activity;chromatin binding;dynein binding;hydrolase activity;hydrolase activity, acting on acid anhydrides;hydrolase activity, acting on acid
binding;nucleic acid binding;RNA binding;translation factor activity, nucleic acid binding;translation initiation factor activity
3'-5' exonuclease activity;3'-5'-exoribonuclease activity;binding;catalytic activity;endonuclease activity;enzyme regulator activity;exonuclease activity;exonuclease activity, active with either ribo- or deoxyribonucleic acids an
antioxidant activity;binding;catalytic activity;disulfide oxidoreductase activity;glutathione peroxidase activity;glutathione transferase activity;oxidoreductase activity;oxidoreductase activity, acting on a sulfur group of donors;o
adenyl nucleotide binding;adenyl ribonucleotide binding;ATP binding;ATPase activity;ATPase activity, coupled;ATP-dependent helicase activity;ATP-dependent RNA helicase activity;binding;catalytic activity;helicase activity;h
binding;nucleic acid binding;RNA binding;structural constituent of ribosome;structural molecule activity
adenyl nucleotide binding;adenyl ribonucleotide binding;amine binding;amino acid binding;aminoacyl-tRNA ligase activity;ATP binding;binding;carboxylic acid binding;catalytic activity;identical protein binding;ligase activity;li
acetyl-CoA hydrolase activity;carboxylic ester hydrolase activity;catalytic activity;CoA hydrolase activity;hydrolase activity;hydrolase activity, acting on ester bonds;thiolester hydrolase activity
aldehyde-lyase activity;carbon-carbon lyase activity;catalytic activity;deoxyribose-phosphate aldolase activity;lyase activity
binding;enzyme binding;mRNA binding;nucleic acid binding;protein binding;RNA binding
structural constituent of ribosome;structural molecule activity
binding;cytoskeletal protein binding;microtubule binding;nucleic acid binding;protein binding;ribonucleoprotein binding;ribosome binding;RNA binding;rRNA binding;tubulin binding
structural constituent of ribosome;structural molecule activity
3'-5' exonuclease activity;3'-5'-exoribonuclease activity;binding;catalytic activity;exonuclease activity;exonuclease activity, active with either ribo- or deoxyribonucleic acids and producing 5'-phosphomonoesters;exoribonucl
acid-amino acid ligase activity;catalytic activity;ligase activity;ligase activity, forming carbon-nitrogen bonds;small conjugating protein ligase activity;UFM1 conjugating enzyme activity
binding;cytoskeletal protein binding;DNA binding;enzyme activator activity;enzyme binding;enzyme regulator activity;GTPase activator activity;GTPase binding;GTPase regulator activity;nucleic acid binding;nucleoside-tripho
structural constituent of ribosome;structural molecule activity
binding;catalytic activity;cation binding;deoxyribonucleotide binding;dGTP binding;dGTPase activity;guanyl deoxyribonucleotide binding;guanyl nucleotide binding;hydrolase activity;hydrolase activity, acting on ester bonds;i

alpha-catenin binding;binding;cell adhesion molecule binding;protein binding
binding;catalytic activity;cysteine-type endopeptidase activity;cysteine-type peptidase activity;cytokine receptor binding;endopeptidase activity;hydrolase activity;identical protein binding;peptidase activity;peptidase activity,
enzyme regulator activity;GTPase regulator activity;guanyl-nucleotide exchange factor activity;nucleoside-triphosphatase regulator activity;Rac guanyl-nucleotide exchange factor activity;Ras guanyl-nucleotide exchange fact
binding;carbohydrate binding;cargo receptor activity;catalytic activity;cation binding;chromatin binding;copper ion binding;electron carrier activity;histone binding;ion binding;metal ion binding;methylated histone residue binc
binding;protein binding;protein C-terminus binding
binding;carboxylic ester hydrolase activity;catalytic activity;enzyme binding;enzyme inhibitor activity;enzyme regulator activity;hydrolase activity;hydrolase activity, acting on ester bonds;phosphatase binding;phosphatase inh
binding;cation binding;ion binding;metal ion binding;transition metal ion binding;zinc ion binding
active transmembrane transporter activity;ATPase activity;ATPase activity, coupled;ATPase activity, coupled to movement of substances;ATPase activity, coupled to transmembrane movement of substances;catalytic activity;
4 iron, 4 sulfur cluster binding;adenyl nucleotide binding;adenyl ribonucleotide binding;ATP binding;binding;cation binding;ion binding;iron-sulfur cluster binding;metal cluster binding;metal ion binding;nucleotide binding;purir
binding;protein binding;protein C-terminus binding
carbon-oxygen lyase activity;catalytic activity;hydro-lyase activity;intramolecular transferase activity;isomerase activity;ligand-dependent nuclear receptor transcription coactivator activity;lyase activity;protein binding transcri
binding;catalytic activity;cofactor binding;O-phospho-L-serine:2-oxoglutarate aminotransferase activity;pyridoxal phosphate binding;transaminase activity;transferase activity;transferase activity, transferring nitrogenous grou
binding;histone binding;methylated histone residue binding;protein binding
structural molecule activity
binding;catalytic activity;cation binding;cofactor binding;cysteine desulfurase activity;identical protein binding;ion binding;iron-sulfur cluster binding;metal cluster binding;metal ion binding;protein binding;protein dimerization
adenyl nucleotide binding;adenyl ribonucleotide binding;ATP binding;binding;catalytic activity;hydrolase activity;hydrolase activity, acting on acid anhydrides;hydrolase activity, acting on acid anhydrides, in phosphorus-conta
binding;DNA binding;nucleic acid binding;protein binding;sequence-specific DNA binding;transcription factor binding
binding;DNA binding;nucleic acid binding;protein binding transcription factor activity;transcription coactivator activity;transcription cofactor activity;transcription factor binding transcription factor activity
2'-5'-oligoadenylate synthetase activity;adenyl nucleotide binding;adenyl ribonucleotide binding;adenylyltransferase activity;ATP binding;binding;catalytic activity;cation binding;double-stranded RNA binding;ion binding;meta
catalytic activity;endopeptidase activity;hydrolase activity;peptidase activity;peptidase activity, acting on L-amino acid peptides;serine hydrolase activity;serine-type endopeptidase activity;serine-type peptidase activity

C: GOCC name	C: KEGG name	C: Organism
cell part;cytoplasm;cytoplasmic part;cytosol;intracellular part	Ubiquitin mediated proteolysis	Homo sapiens
cell part;cytoplasmic part;endoplasmic reticulum part;extrinsic to internal side of plasma membrane;extrinsic to membrane;extrinsic to plasma membrane;integral to membrane;integral to plasma membrane;intracellular organelle part;intracellular part;intrinsic to endoplasmic reticulum membrane;intrinsic to membrane;intrinsic to organelle membrar		Homo sapiens
cell junction;cell part;cell projection;cytoplasm;cytoskeletal part;intracellular non-membrane-bounded organelle;intracellular organelle;intracellular organelle part;intracellular part;macromolecular complex;non-membrane-bounded organelle;organelle;organelle part;podosome;protein complex		Homo sapiens
	Glyoxylate and dicarboxylate metabolism	Homo sapiens
		Homo sapiens
Barr body;cell part;chromosome;intracellular non-membrane-bounded organelle;intracellular organelle;intracellular organelle part;intracellular part;non-membrane-bounded organelle;nuclear chromosome;nuclear part;organelle;organelle part;sex chromosome;X chromosome		Homo sapiens
cell part;cytoplasmic part;endoplasmic reticulum membrane;endoplasmic reticulum part;intracellular organelle part;intracellular part;macromolecular complex;membrane;membrane part;nuclear membrane;nuclear part;nuclear pore;organelle membrane;organelle part;pore complex;protein complex		Homo sapiens
		Homo sapiens
cell part;cytoplasmic part;intracellular membrane-bounded organelle;intracellular non-membrane-bounded organelle;intracellular organelle;intracellular organelle lumen;intracellular organelle part;intracellular part;membrane;m	Ether lipid metabolism;Peroxisome	Homo sapiens
cell part;centrosome;cytoplasmic part;cytoskeletal part;cytosol;intracellular non-membrane-bounded organelle;intracellular organelle;intracellular organelle part;intracellular part;kinesin complex;macromolecular complex;membrane;microtubule;microtubule associated complex;microtubule organizing center;non-membrane-bounded organelle;orga		Homo sapiens
adherens junction;anchoring junction;cell junction;cell part;cell-substrate adherens junction;cell-substrate junction;cytoplasm;cytoskeleton;focal adhesion;intracellular non-membrane-bounded organelle;intracellular organelle;intracellular organelle part;intracellular part;macromolecular complex;non-membrane-bounded organelle;nuclear part;nucle		Homo sapiens
cell body;cell part;cell projection;cytoplasm;cytoplasmic part;cytosol;extracellular membrane-bounded organelle;extracellular organelle;extracellular region part;extracellular vesicular exosome;intracellular membrane-bounde	Biosynthesis of unsaturated fatty acids	Homo sapiens
basal plasma membrane;brush border;cell part;cell projection;cell projection membrane;cell projection part;cytoplasm;cytoplasmic membrane-bounded vesicle;cytoplasmic part;cytoplasmic vesicle;cytoskeletal part;cytosol;extracellular membrane-bounded organelle;extracellular organelle;extracellular region part;extracellular vesicular exosome;fila		Homo sapiens
cell part;cytoplasmic part;intracellular membrane-bounded organelle;intracellular organelle;intracellular organelle part;intracellular part;macromolecular complex;membrane;membrane part;membrane-bounded organelle;mito	Alzheimer's disease;Huntington's disease;Oxidative phosphorylation;Parkinson's disease	Homo sapiens
cell part;cytoplasmic part;endoplasmic reticulum;endoplasmic reticulum membrane;endoplasmic reticulum part;extracellular membrane-bounded organelle;extracellular organelle;extracellular region part;extracellular vesicular exosome;integral to membrane;intracellular membrane-bounded organelle;intracellular non-membrane-bounded organelle;		Homo sapiens
cell part;cytoplasmic part;cytosol;eukaryotic 43S preinitiation complex;eukaryotic 48S preinitiation complex;eukaryotic translation initiation factor 3 complex;intracellular part;macromolecular complex;membrane;protein comp	RNA transport	Homo sapiens
cell part;cytoplasm;cytoplasmic part;Golgi apparatus;intracellular membrane-bounded organelle;intracellular non-membrane-bounded organelle;intracellular organelle;intracellular organelle part;intracellular part;macromolecular complex;membrane;membrane part;membrane-bounded organelle;non-membrane-bounded organelle;nuclear membran		Homo sapiens
cell part;cytoplasmic part;intracellular membrane-bounded organelle;intracellular non-membrane-bounded organelle;intracellular organelle;intracellular organelle lumen;intracellular organelle part;intracellular part;membrane-bounded organelle;membrane-enclosed lumen;mitochondrial matrix;mitochondrial nucleoid;mitochondrial part;mitochondrion		Homo sapiens
cell part;cytoplasm;cytoplasmic part;cytosol;intracellular membrane-bounded organelle;intracellular organelle;intracellular part;membrane-bounded organelle;nucleus;organelle		Homo sapiens
cell part;cis-Golgi network;cytoplasmic membrane-bounded vesicle;cytoplasmic part;cytoplasmic vesicle;endocytic vesicle;endosomal part;endosome membrane;Golgi apparatus;Golgi apparatus part;Golgi cisterna membrane;Golgi lumen;Golgi membrane;integral to membrane;intracellular membrane-bounded organelle;intracellular organelle;intra		Homo sapiens
basal lamina;cell junction;cell part;cell surface;cytoplasm;cytoplasmic part;extracellular matrix;extracellular matrix part;extracellular membrane-bounded organelle;extracellular organelle;extracellular region;extracellular region	ECM-receptor interaction	Homo sapiens
cell part;cytoplasmic part;endoplasmic reticulum;endoplasmic reticulum membrane;endoplasmic reticulum part;extracellular membrane-bounded organelle;extracellular organelle;extracellular region part;extracellular vesicula	Lysine degradation	Homo sapiens
cell part;cytoplasmic part;extracellular membrane-bounded organelle;extracellular organelle;extracellular region part;extracellular vesicular exosome;intracellular membrane-bounded organelle;intracellular organelle;intracellula	Alzheimer's disease;Huntington's disease;Oxidative phosphorylation;Parkinson's disease	Homo sapiens
actin cytoskeleton;axon;axon part;axon terminus;cell part;cell projection;cell projection part;cerebellar mossy fiber;clathrin-coated vesicle;coated vesicle;contractile fiber part;cytoplasm;cytoplasmic membrane-bounded vesicle;cytoplasmic part;cytoplasmic vesicle;cytoskeleton;I band;initial segment;intracellular membrane-bounded organelle;intra		Homo sapiens
cell part;cytoplasmic part;cytosol;intracellular organelle part;intracellular part;macromolecular complex;membrane part;nuclear part;nuclear pore;nucleoplasm;organelle part;pore complex;protein complex		Homo sapiens
apical plasma membrane;cell body;cell part;cell projection;cell projection membrane;cell projection part;cytoplasm;extracellular membrane-bounded organelle;extracellular organelle;extracellular region part;extracellular space;extracellular vesicular exosome;filopodium;integral to membrane;integral to plasma membrane;intracellular part;intrinsic to		Homo sapiens
cell part;cytoplasmic part;extracellular membrane-bounded organelle;extracellular organelle;extracellular region part;extracellular vesicular exosome;intracellular membrane-bounded organelle;intracellular organelle;intracellula	Lysosome;Other glycan degradation	Homo sapiens
cell part;cytoplasm;cytoplasmic part;cytosol;extracellular membrane-bounded organelle;extracellular organelle;extracellular region part;extracellular vesicular exosome;intracellular membrane-bounded organelle;intracellular c	Vitamin B6 metabolism	Homo sapiens
		Homo sapiens
axon part;cell part;cell projection part;cytoplasmic part;endosomal part;endosome membrane;Golgi apparatus;Golgi apparatus part;Golgi membrane;intracellular membrane-bounded organelle;intracellular organelle;intracellu	Lysosome	Homo sapiens
cell part;cytoplasm;cytoplasmic part;cytosol;intracellular membrane-bounded organelle;intracellular organelle;intracellular part;membrane-bounded organelle;nucleus;organelle	Amyotrophic lateral sclerosis (ALS)	Homo sapiens
cell part;cytoplasmic part;endoplasmic reticulum membrane;endoplasmic reticulum part;integral to membrane;intracellular organelle part;intracellular part;intrinsic to membrane;membrane;membrane part;membrane-enclose	Arachidonic acid metabolism	Homo sapiens
cell part;cytoplasm;cytoplasmic part;cytosol;intracellular membrane-bounded organelle;intracellular organelle;intracellular organelle part;intracellular part;macromolecular complex;membrane-bounded organelle;methylosome	Cell cycle - yeast;RNA transport	Homo sapiens
actin cytoskeleton;apical plasma membrane;brush border membrane;cell part;cell periphery;cell projection;cell projection membrane;cell projection part;centrosome;cytoplasm;cytoplasmic part;cytoskeletal part;cytoskeleton;endomembrane system;extracellular membrane-bounded organelle;extracellular organelle;extracellular region part;extracell		Homo sapiens
cell part;cytoplasm;cytoplasmic part;cytosol;extracellular membrane-bounded organelle;extracellular organelle;extracellular region part;extracellular vesicular exosome;intracellular membrane-bounded organelle;intracellular c	Proteasome	Homo sapiens
cell part;cytoplasmic part;dihydrolipoyl dehydrogenase complex;intracellular membrane-bounded organelle;intracellular organelle;intracellular organelle part;intracellular part;macromolecular complex;membrane-bounded organelle;mitochondrial alpha-ketoglutarate dehydrogenase complex;mitochondrial part;mitochondrial tricarboxylic acid cycle e		Homo sapiens
cell part;cytoplasm;cytoplasmic part;cytosol;intracellular membrane-bounded organelle;intracellular organelle;intracellular part;membrane-bounded organelle;mitochondrion;organelle		Homo sapiens
brush border;cell cortex;cell part;cell projection;cell projection part;clathrin-coated vesicle;coated vesicle;cytoplasm;cytoplasmic membrane-bounded vesicle;cytoplasmic part;cytoplasmic vesicle;cytosol;dendritic shaft;dendritic spine;endocytic vesicle;extracellular membrane-bounded organelle;extracellular organelle;extracellular region part;extra		Homo sapiens
cell part;chromatin;chromosomal part;cytoplasm;intracellular membrane-bounded organelle;intracellular organelle;intracellular organelle part;intracellular part;macromolecular complex;membrane-bounded organelle;nuclear chromatin;nuclear chromosome part;nuclear matrix;nuclear part;nucleoplasm;nucleus;organelle;organelle part;protein comple		Homo sapiens
cell part;cytoplasmic part;intracellular membrane-bounded organelle;intracellular organelle;intracellular organelle part;intracellular part;membrane;membrane part;membrane-bounded organelle;mitochondrial inner membrane	Alzheimer's disease;Cardiac muscle contraction;Huntington's disease;Oxidative phosphorylation;Parkinson's disease	Homo sapiens
actin cytoskeleton;adherens junction;anchoring junction;cell junction;cell part;cell-substrate adherens junction;cell-substrate junction;centrosome;chromosomal part;contractile fiber;cytoplasm;cytoplasmic part;cytoskeletal pa	Focal adhesion;Long-term potentiation;Regulation of actin cytoskeleton;Vascular smooth muscle contraction	Homo sapiens
cell part;centrosome;cytoplasmic part;cytoskeletal part;cytosol;intracellular non-membrane-bounded organelle;intracellular organelle;intracellular organelle part;intracellular part;microtubule organizing center;non-membrane-bounded organelle;organelle;organelle part		Homo sapiens
basal lamina;basement membrane;cell part;extracellular matrix;extracellular matrix part;extracellular membrane-bounded organelle;extracellular organelle;extracellular region;extracellular region part;extracellular space;extrac	Amoebiasis;ECM-receptor interaction;Focal adhesion;Pathways in cancer;Small cell lung cancer;Toxoplasmosis	Homo sapiens
cell part;cytoplasmic part;intracellular non-membrane-bounded organelle;intracellular organelle;intracellular organelle part;intracellular part;macromolecular complex;membrane;mitochondrial inner membrane;mitochondrial membrane;mitochondrial part;mitochondrial ribosome;non-membrane-bounded organelle;organellar ribosome;organelle;orga		Homo sapiens
cell part;cytoplasmic part;endoplasmic reticulum membrane;endoplasmic reticulum palmitoyltransferase complex;endoplasmic reticulum part;integral to membrane;intracellular organelle part;intracellular part;intrinsic to mem	Sphingolipid metabolism	Homo sapiens
cell part;cytoplasm;intracellular membrane-bounded organelle;intracellular organelle;intracellular organelle part;intracellular part;membrane-bounded organelle;nuclear part;nucleoplasm;nucleus;organelle;organelle part		Homo sapiens
cell part;cell projection;cytoplasm;cytoplasmic part;cytoskeleton;cytosol;filopodium;Golgi apparatus;intracellular membrane-bounded organelle;intracellular non-membrane-bounded organelle;intracellular organelle;intracellula	Inositol phosphate metabolism;Phosphatidylinositol signaling system	Homo sapiens
cell part;cytoplasmic part;cytosol;eukaryotic 43S preinitiation complex;eukaryotic 48S preinitiation complex;eukaryotic translation initiation factor 3 complex;eukaryotic translation initiation factor 3 complex, eIF3m;intracellula	RNA transport	Homo sapiens
cell part;cytoplasmic part;cytosol;eukaryotic 43S preinitiation complex;eukaryotic 48S preinitiation complex;eukaryotic translation initiation factor 3 complex;eukaryotic translation initiation factor 3 complex, eIF3m;extracellula	Measles;RNA transport	Homo sapiens
cell part;cytoplasm;cytoplasmic part;intracellular membrane-bounded organelle;intracellular non-membrane-bounded organelle;intracellular organelle;intracellular organelle part;intracellular part;membrane;membrane-bounde	Ribosome biogenesis in eukaryotes	Homo sapiens
cell part;cytoplasmic part;intracellular membrane-bounded organelle;intracellular organelle;intracellular organelle lumen;intracellular organelle part;intracellular part;membrane-bounded organelle;membrane-enclosed lumen;m	Pantothenate and CoA biosynthesis;Valine, leucine and isoleucine biosynthesis;Valine, leucine and isoleucine degradation	Homo sapiens
actin cytoskeleton;cell part;cytoskeleton;integral to membrane;integral to plasma membrane;intracellular non-membrane-bounded organelle;intracellular organelle;intracellular organelle part;intracellular part;intrinsic to membrane;intrinsic to plasma membrane;membrane;membrane part;non-membrane-bounded organelle;nuclear membrane;nuclea		Homo sapiens
cell part;cytoplasm;cytoplasmic part;endoplasmic reticulum;endoplasmic reticulum lumen;endoplasmic reticulum part;intracellular membrane-bounded organelle;intracellular organelle;intracellular organelle lumen;intracellular	Arginine and proline metabolism	Homo sapiens
Cajal body;cell part;intracellular membrane-bounded organelle;intracellular organelle;intracellular organelle part;intracellular part;macromolecular complex;membrane-bounded organelle;nuclear body;nuclear part;nuclear spe	Spliceosome	Homo sapiens
cell part;cytoplasmic part;cytosol;extracellular membrane-bounded organelle;extracellular organelle;extracellular region part;extracellular vesicular exosome;intracellular part;membrane-bounded organelle;membrane-bounde	Glycine, serine and threonine metabolism;Methane metabolism	Homo sapiens
cell part;centrosome;cytoplasmic dynein complex;cytoplasmic part;cytoskeletal part;cytosol;dynein complex;intracellular non-membrane-bounded organelle;intracellular organelle;intracellular organelle part;intracellular part;m	Phagosome;Vasopressin-regulated water reabsorption	Homo sapiens
cell part;cytoplasmic part;cytosol;intracellular part	Purine metabolism;Selenocompound metabolism;Sulfur metabolism	Homo sapiens
Cajal body;catalytic step 2 spliceosome;cell part;cytoplasm;cytoplasmic part;cytosol;Golgi apparatus;intracellular membrane-bounded organelle;intracellular organelle;intracellular organelle part;intracellular part;macromolecu	Spliceosome	Homo sapiens
cell part;cytoplasm;cytoplasmic part;cytosol;extracellular membrane-bounded organelle;extracellular organelle;extracellular region part;extracellular vesicular exosome;intracellular membrane-bounded organelle;intracellular organelle;intracellular part;membrane-bounded organelle;membrane-bounded vesicle;nucleus;organelle;vesicle		Homo sapiens
cell part;integral to membrane;intrinsic to membrane;membrane;membrane part;plasma membrane	Nitrogen metabolism	Homo sapiens
cell part;cytoplasmic part;intracellular membrane-bounded organelle;intracellular organelle;intracellular organelle lumen;intracellular organelle part;intracellular part;membrane;membrane-bounded organelle;membrane-enclosed lumen;mitochondrial inner membrane;mitochondrial matrix;mitochondrial membrane;mitochondrial part;mitochondrion;o		Homo sapiens
cell part;cytoplasmic part;integral to membrane;intracellular membrane-bounded organelle;intracellular organelle;intracellular organelle part;intracellular part;intrinsic to membrane;macromolecular complex;membrane;membra	Alzheimer's disease;Huntington's disease;Oxidative phosphorylation;Parkinson's disease	Homo sapiens
cell part;cytoplasm;cytoplasmic part;endoplasmic reticulum membrane;endoplasmic reticulum part;extracellular membrane-bounded organelle;extracellular organelle;extracellular region part;extracellular vesicular exosome;intracellular membrane-bounded organelle;intracellular non-membrane-bounded organelle;intracellular organelle;intracellular		Homo sapiens
cell part;intracellular membrane-bounded organelle;intracellular organelle;intracellular organelle part;intracellular part;membrane-bounded organelle;nuclear part;nucleoplasm;nucleus;organelle;organelle part		Homo sapiens
cell part;cytoplasm;cytoplasmic part;cytosol;extracellular membrane-bounded organelle;extracellular organelle;extracellular region part;extracellular vesicular exosome;intracellular membrane-bounded organelle;intracellular c	Aminoacyl-tRNA biosynthesis	Homo sapiens
cell part;cytoplasm;extracellular membrane-bounded organelle;extracellular organelle;extracellular region part;extracellular vesicular exosome;integral to membrane;integral to plasma membrane;intracellular membrane-bounded organelle;intracellular organelle;intracellular part;intrinsic to membrane;intrinsic to plasma membrane;membrane part;me		Homo sapiens
cell part;cytoplasmic part;intracellular membrane-bounded organelle;intracellular organelle;intracellular organelle lumen;intracellular organelle part;intracellular part;membrane-bounded organelle;membrane-enclosed lumen;m	Citrate cycle (TCA cycle)	Homo sapiens
cell part;cell surface;cytoplasmic part;cytosol;intracellular part		Homo sapiens
cell part;cytoplasmic membrane-bounded vesicle;cytoplasmic part;cytoplasmic vesicle;endoplasmic reticulum;endoplasmic reticulum lumen;endoplasmic reticulum part;extracellular region;Golgi apparatus;intracellular membrane-bounded organelle;intracellular organelle;intracellular organelle lumen;intracellular organelle part;intracellular part;mela		Homo sapiens
cell part;cell projection;cytoplasm;cytoplasmic part;dendrite;integral to membrane;intracellular membrane-bounded organelle;intracellular organelle;intracellular organelle part;intracellular part;intrinsic to membrane;membrane;membrane part;membrane-bounded organelle;membrane-enclosed lumen;mitochondrial crista;mitochondrial inner membr		Homo sapiens
cell part;chromatin;chromosomal part;intracellular membrane-bounded organelle;intracellular organelle;intracellular organelle part;intracellular part;macromolecular complex;membrane-bounded organelle;nuclear chromatin;nuclear chromosome part;nuclear part;nucleoplasm;nucleoplasm part;nucleus;organelle;organelle part;protein complex;trans		Homo sapiens
anchoring junction;cell junction;cell part;cell-cell junction;cytoplasmic part;cytoskeleton;desmosome;extracellular membrane-bounded organelle;extracellular organelle;extracellular region part;extracellular vesicular exosome;intracellular membrane-bounded organelle;intracellular non-membrane-bounded organelle;intracellular organelle;intracellula		Homo sapiens
cell body;cell part;cytoplasm;cytoplasmic part;endoplasmic reticulum membrane;endoplasmic reticulum part;ER-mitochondrion membrane contact site;extracellular membrane-bounded organelle;extracellular organelle;extra	Adipocytokine signaling pathway;Fatty acid metabolism;Peroxisome;PPAR signaling pathway	Homo sapiens
cell part;cytoplasmic part;cytosol;intracellular membrane-bounded organelle;intracellular organelle;intracellular part;membrane;membrane-bounded organelle;nucleus;organelle		Homo sapiens
cell part;cytoplasm;extracellular membrane-bounded organelle;extracellular organelle;extracellular region part;extracellular vesicular exosome;intracellular part;membrane-bounded organelle;membrane-bounded vesicle;orga	Amino sugar and nucleotide sugar metabolism;Fructose and mannose metabolism	Homo sapiens
cell part;cytoplasmic part;endoplasmic reticulum;endoplasmic reticulum membrane;endoplasmic reticulum part;extracellular membrane-bounded organelle;extracellular organelle;extracellular region part;extracellular vesicula	Lysine degradation;Other types of O-glycan biosynthesis	Homo sapiens
cell part;cell projection;cell projection membrane;cell projection part;cytoplasmic part;cytoskeletal part;cytosol;intracellular non-membrane-bounded organelle;intracellular organelle;intracellular organelle part;intracellular part;	Focal adhesion;Regulation of actin cytoskeleton;Shigellosis	Homo sapiens
cell part;cytoplasm;cytoplasmic part;endosomal part;endosome;endosome membrane;Golgi apparatus;intracellular membrane-bounded organelle;intracellular organelle;intracellular organelle part;intracellular part;lipid particle;membrane;membrane-bounded organelle;organelle;organelle membrane;organelle part		Homo sapiens
cell part;checkpoint clamp complex;chromosomal part;chromosome;intracellular membrane-bounded organelle;intracellular non-membrane-bounded organelle;intracellular organelle;intracellular organelle part;intracellular pa	Cell cycle - yeast;Meiosis - yeast	Homo sapiens
cell part;intracellular organelle part;intracellular part;nuclear part;nucleoplasm;organelle part		Homo sapiens
cell part;cytoplasmic part;cytosol;extracellular membrane-bounded organelle;extracellular organelle;extracellular region part;extracellular vesicular exosome;intracellular organelle part;intracellular part;membrane-bounded org	Amino sugar and nucleotide sugar metabolism;Ascorbate and aldarate metabolism;Pentose and glucuronate interconversio	Homo sapiens
cell part;cytoplasmic part;dolichol-phosphate-mannose synthase complex;endoplasmic reticulum;endoplasmic reticulum membrane;endoplasmic reticulum part;intracellular membrane-bounded organelle;intracellular organel	N-Glycan biosynthesis	Homo sapiens
cell part;coated vesicle membrane;cytoplasmic part;cytoplasmic vesicle membrane;cytoplasmic vesicle part;cytosol;endoplasmic reticulum;ER to Golgi transport vesicle membrane;Golgi apparatus;Golgi apparatus part;Golgi membrane;Golgi stack;intracellular membrane-bounded organelle;intracellular non-membrane-bounded organelle;intracellu		Homo sapiens
cell part;cell projection part;cytoplasm;cytoplasmic part;intracellular membrane-bounded organelle;intracellular non-membrane-bounded organelle;intracellular organelle;intracellular organelle part;intracellular part;macromole	Spliceosome	Homo sapiens
cell part;cytoplasm;extracellular membrane-bounded organelle;extracellular organelle;extracellular region part;extracellular vesicular exosome;intracellular;intracellular membrane-bounded organelle;intracellular non-membrane-bounded organelle;intracellular organelle;intracellular organelle part;intracellular part;membrane-bounded organelle;memb		Homo sapiens
cell part;cytoplasmic part;cytosol;early endosome;endosome;Golgi apparatus;intracellular membrane-bounded organelle;intracellular non-membrane-bounded organelle;intracellular organelle;intracellular organelle part;intrac	Regulation of actin cytoskeleton	Homo sapiens
cell part;cytoplasm;cytoplasmic part;cytosol;endoplasmic reticulum;intracellular membrane-bounded organelle;intracellular non-membrane-bounded organelle;intracellular organelle;intracellular organelle part;intracellular part;membrane-bounded organelle;mitochondrion;non-membrane-bounded organelle;nuclear part;nucleolus;organelle;organelle		Homo sapiens
cell part;intracellular membrane-bounded organelle;intracellular organelle;intracellular organelle part;intracellular part;membrane-bounded organelle;nuclear part;nucleoplasm;nucleus;organelle;organelle part	mRNA surveillance pathway	Homo sapiens
cell part;cytoplasmic part;Golgi apparatus;Golgi apparatus part;Golgi membrane;integral to membrane;intracellular membrane-bounded organelle;intracellular organelle;intracellular organelle part;intracellular part;intrinsic to membrane;membrane;membrane part;membrane-bounded organelle;nuclear part;nucleoplasm;organelle;organelle membran		Homo sapiens
actin filament;cell junction;cell part;cell projection;cytoplasm;cytoplasmic part;cytoskeletal part;cytosol;extracellular membrane-bounded organelle;extracellular organelle;extracellular region;extracellular region part;extracellular vesicular exosome;intracellular non-membrane-bounded organelle;intracellular organelle;intracellular organelle part;intrac		Homo sapiens
cell part;centrosome;cytoplasmic part;cytoskeletal part;cytosol;intracellular membrane-bounded organelle;intracellular non-membrane-bounded organelle;intracellular organelle;intracellular organelle part;intracellular part;mer	Axon guidance;Chemokine signaling pathway;Focal adhesion;Leukocyte transendothelial migration;Pathogenic Escherichia	Homo sapiens
cell cortex;cell part;cell projection membrane;cell projection part;chromosomal part;condensed chromosome kinetochore;cytoplasm;cytoplasmic microtubule;cytoplasmic part;cytoskeletal part;cytosol;Golgi apparatus;Golgi apparatus part;intracellular membrane-bounded organelle;intracellular non-membrane-bounded organelle;intracellular organ		Homo sapiens
cell part;cytoplasm;cytoplasmic part;cytosol;extracellular membrane-bounded organelle;extracellular organelle;extracellular region part;extracellular vesicular exosome;intracellular membrane-bounded organelle;intracellular organelle;intracellular part;membrane;membrane-bounded organelle;membrane-bounded vesicle;nucleus;organelle;plasma r		Homo sapiens
		Homo sapiens
cell part;cytoplasmic part;cytosol;extracellular membrane-bounded organelle;extracellular organelle;extracellular region part;extracellular vesicular exosome;intracellular organelle part;intracellular part;lysosomal membrane;m	Collecting duct acid secretion;Epithelial cell signaling in Helicobacter pylori infection;Oxidative phosphorylation;Phagosome	Homo sapiens
Barr body;cell part;centromeric heterochromatin;chromatin;chromosomal part;chromosome;condensed chromosome;extracellular membrane-bounded organelle;extracellular organelle;extracellular region part;extracellular ve	Systemic lupus erythematosus	Homo sapiens
cell part;chromatin;chromosomal part;cytoplasm;cytoskeletal part;intracellular membrane-bounded organelle;intracellular organelle;intracellular organelle part;intracellular part;macromolecular complex;membrane;membrane-bounded organelle;microtubule;nuclear chromatin;nuclear chromosome part;nuclear part;nucleoplasm;nucleoplasm part;nu		Homo sapiens
cell part;cytoplasmic part;extracellular membrane-bounded organelle;extracellular organelle;extracellular region part;extracellular vesicular exosome;intracellular membrane-bounded organelle;intracellular organelle;intracellula	Citrate cycle (TCA cycle);Glyoxylate and dicarboxylate metabolism	Homo sapiens
cell part;cytoplasm;cytoplasmic part;cytoskeletal part;cytoskeleton;intermediate filament cytoskeleton;intracellular membrane-bounded organelle;intracellular non-membrane-bounded organelle;intracellular organelle;intracellular organelle part;intracellular part;membrane-bounded organelle;microtubule cytoskeleton;microtubule organizing center;n		Homo sapiens
cell part;chromatin;chromosomal part;cytoplasmic part;cytosol;heterochromatin;intracellular membrane-bounded organelle;intracellular organelle;intracellular organelle part;intracellular part;membrane-bounded organelle;nuclear chromatin;nuclear chromosome part;nuclear heterochromatin;nuclear part;nuclear periphery;nucleoplasm;nucleus;orgar		Homo sapiens
cell part;cytoplasmic part;endoplasmic reticulum;endoplasmic reticulum membrane;endoplasmic reticulum part;integral to membrane;intracellular membrane-bounded organelle;intracellular organelle;intracellular organelle part;intracellular part;intrinsic to membrane;macromolecular complex;membrane;membrane part;membrane-bounded organell		Homo sapiens
cell part;cytoplasmic part;intracellular membrane-bounded organelle;intracellular organelle;intracellular organelle part;intracellular part;macromolecular complex;membrane;membrane part;membrane-bounded organelle;mito	Alzheimer's disease;Huntington's disease;Oxidative phosphorylation;Parkinson's disease	Homo sapiens
cell part;cytoplasm;intracellular membrane-bounded organelle;intracellular organelle;intracellular organelle part;intracellular part;membrane-bounded organelle;nuclear body;nuclear part;nuclear speck;nucleoplasm;nucleoplas	Spliceosome	Homo sapiens
cell part;CRD-mediated mRNA stability complex;cytoplasm;cytoplasmic part;Golgi apparatus;intracellular membrane-bounded organelle;intracellular organelle;intracellular organelle part;intracellular part;macromolecular complex;membrane;membrane-bounded organelle;mitochondrial inner membrane;mitochondrial membrane;mitochondrial part;c		Homo sapiens
actin cytoskeleton;cell part;cytoplasm;cytoplasmic part;cytoskeleton;intermediate filament cytoskeleton;intracellular membrane-bounded organelle;intracellular non-membrane-bounded organelle;intracellular organelle;intracellular organelle lumen;intracellular organelle part;intracellular part;membrane;membrane-bounded organelle;membrane-encl		Homo sapiens
cell part;cytoplasmic part;integral to membrane;intracellular membrane-bounded organelle;intracellular organelle;intracellular organelle part;intracellular part;intrinsic to membrane;membrane;membrane part;membrane-bounded organelle;mitochondrial inner membrane;mitochondrial membrane;mitochondrial part;mitochondrion;organelle;organelle		Homo sapiens
cell part;intracellular membrane-bounded organelle;intracellular organelle;intracellular organelle part;intracellular part;macromolecular complex;membrane-bounded organelle;multimeric ribonuclease P complex;nuclear part;n	Ribosome biogenesis in eukaryotes;RNA transport	Homo sapiens
cell part;cytoplasm;cytoplasmic part;cytosol;eukaryotic 43S preinitiation complex;eukaryotic 48S preinitiation complex;eukaryotic translation initiation factor 3 complex;intracellular part;macromolecular complex;perinuclear re	RNA transport	Homo sapiens
cell part;cytoskeletal part;intracellular organelle part;intracellular part;macromolecular complex;MMXD complex;organelle part;protein complex		Homo sapiens
cell part;cytoplasm;cytoplasmic part;cytosol;endoplasmic reticulum;intracellular membrane-bounded organelle;intracellular non-membrane-bounded organelle;intracellular organelle;intracellular organelle part;intracellular part	Protein export	Homo sapiens
cell part;cytoplasmic part;extracellular membrane-bounded organelle;extracellular organelle;extracellular region part;extracellular vesicular exosome;intracellular membrane-bounded organelle;intracellular organelle;intracellular part;membrane-bounded organelle;membrane-bounded vesicle;mitochondrion;organelle;vesicle		Homo sapiens
cell part;cytoplasmic part;cytosol;intracellular part	Alanine, aspartate and glutamate metabolism;Amino sugar and nucleotide sugar metabolism	Homo sapiens
Cdc48p-Npl4p-Ufd1p AAA ATPase complex;cell part;cytoplasmic part;endoplasmic reticulum part;intracellular organelle part;intracellular part;macromolecular complex;membrane part;nuclear part;nucleoplasm;organelle part;protein complex		Homo sapiens
cell part;cytoplasm;cytoplasmic part;endoplasmic reticulum;endoplasmic reticulum membrane;endoplasmic reticulum part;extracellular membrane-bounded organelle;extracellular organelle;extracellular region part;extracellular vesicular exosome;integral to membrane;intracellular membrane-bounded organelle;intracellular organelle;intracellular org		Homo sapiens
cell part;cytoplasmic part;early endosome membrane;endosomal part;endosome membrane;extracellular region;extracellular region part;extracellular space;intracellular organelle part;intracellular part;membrane;organelle me	Wnt signaling pathway	Homo sapiens
cell part;cytoplasmic part;cytosol;intracellular membrane-bounded organelle;intracellular organelle;intracellular organelle lumen;intracellular organelle part;intracellular part;membrane-bounded organelle;membrane-enclosed l	Alanine, aspartate and glutamate metabolism;Arginine and proline metabolism;D-Glutamine and D-glutamate metabolism;N	Homo sapiens
cell part;cytoplasmic part;cytoskeletal part;endoplasmic reticulum membrane;endoplasmic reticulum part;endoplasmic reticulum-Golgi intermediate compartment;Golgi apparatus;Golgi apparatus part;Golgi membrane;integral to membrane;intracellular membrane-bounded organelle;intracellular organelle;intracellular organelle part;intracellular part		Homo sapiens
cell part;cytoplasm;intracellular membrane-bounded organelle;intracellular organelle;intracellular organelle part;intracellular part;membrane;membrane-bounded organelle;nuclear part;nucleoplasm;nucleus;organelle;organelle	Ubiquitin mediated proteolysis	Homo sapiens
cell part;cytoplasmic part;integral to membrane;intracellular membrane-bounded organelle;intracellular organelle;intracellular organelle part;intracellular part;intrinsic to membrane;macromolecular complex;membrane;membra	Alzheimer's disease;Huntington's disease;Oxidative phosphorylation;Parkinson's disease	Homo sapiens
cell part;cytoplasmic part;extracellular membrane-bounded organelle;extracellular organelle;extracellular region part;extracellular vesicular exosome;integral to membrane;intracellular membrane-bounded organelle;intracellula	Alzheimer's disease;Huntington's disease;Oxidative phosphorylation;Parkinson's disease	Homo sapiens
cell part;cytoplasmic part;endoplasmic reticulum;integral to membrane;intracellular membrane-bounded organelle;intracellular organelle;intracellular organelle part;intracellular part;intrinsic to membrane;macromolecular comp	Alzheimer's disease;Huntington's disease;Oxidative phosphorylation;Parkinson's disease	Homo sapiens
cell part;intracellular membrane-bounded organelle;intracellular organelle;intracellular organelle part;intracellular part;macromolecular complex;membrane-bounded organelle;nuclear body;nuclear part;nuclear speck;nucleoplasm;nucleoplasm part;nucleus;organelle;organelle part;ribonucleoprotein complex;small nuclear ribonucleoprotein complex;		Homo sapiens
cell part;chromosome;cytoplasm;cytoplasmic part;cytoskeletal part;cytosol;intracellular non-membrane-bounded organelle;intracellular organelle;intracellular organelle part;intracellular part;kinesin complex;macromolecular complex;membrane;microtubule;microtubule associated complex;midbody;non-membrane-bounded organelle;nuclear matri		Homo sapiens
cell part;cytoplasmic part;intracellular organelle lumen;intracellular organelle part;intracellular part;macromolecular complex;membrane;membrane part;membrane-enclosed lumen;mitochondrial inner membrane;mitochondria	Alzheimer's disease;Huntington's disease;Oxidative phosphorylation;Parkinson's disease	Homo sapiens
cell part;cytoplasmic part;endoplasmic reticulum;endoplasmic reticulum membrane;endoplasmic reticulum part;intracellular membrane-bounded organelle;intracellular organelle;intracellular organelle part;intracellular part;membrane;membrane part;membrane-bounded organelle;organelle;organelle membrane;organelle part		Homo sapiens
cell part;chromosomal part;chromosome;condensed chromosome;condensin complex;cytoplasm;cytoplasmic part;cytosol;extracellular membrane-bounded organelle;extracellular organelle;extracellular region part;extracellu	Cell cycle - yeast	Homo sapiens

cell part;cytoplasm;intracellular organelle part;intracellular part;nuclear body;nuclear part;nucleoplasm part;organelle part;PML body		Homo sapiens
cell part;cytoplasm;intracellular organelle part;intracellular part;macromolecular complex;membrane;membrane part;nuclear part;nuclear pore;nucleoplasm;organelle part;pore complex;protein complex		Homo sapiens
cell part;cytoplasmic part;endoplasmic reticulum;endoplasmic reticulum membrane;endoplasmic reticulum part;integral to endoplasmic reticulum membrane;integral to membrane;integral to organelle membrane;intracellular	Sphingolipid metabolism	Homo sapiens
cell part;intracellular membrane-bounded organelle;intracellular non-membrane-bounded organelle;intracellular organelle;intracellular organelle part;intracellular part;macromolecular complex;membrane-bounded organelle;non-membrane-bounded organelle;nuclear part;nucleolus;nucleus;organelle;organelle part;ribonucleoprotein complex		Homo sapiens
cell part;cytoplasmic part;endoplasmic reticulum;endoplasmic reticulum membrane;endoplasmic reticulum part;Golgi apparatus;integral to membrane;intracellular membrane-bounded organelle;intracellular organelle;intracell	Adipocytokine signaling pathway;Fatty acid metabolism;Peroxisome;PPAR signaling pathway	Homo sapiens
cell part;cytoplasm;intracellular part		Homo sapiens
cell part;cytoplasm;extracellular membrane-bounded organelle;extracellular organelle;extracellular region part;extracellular vesicular exosome;intracellular part;membrane-bounded organelle;membrane-bounded vesicle;organelle;vesicle		Homo sapiens
cell part;cytoplasmic part;cytosol;intracellular part	Terpenoid backbone biosynthesis	Homo sapiens
cell part;extracellular membrane-bounded organelle;extracellular organelle;extracellular region part;extracellular vesicular exosome;intracellular membrane-bounded organelle;intracellular organelle;intracellular part;membrane-bounded organelle;membrane-bounded vesicle;nucleus;organelle;vesicle		Homo sapiens
cell part;cytoplasmic part;cytosol;intracellular part	Protein processing in endoplasmic reticulum	Homo sapiens
cell part;cytoplasm;cytoskeletal part;intracellular non-membrane-bounded organelle;intracellular organelle;intracellular organelle part;intracellular part;macromolecular complex;microtubule;microtubule associated complex;non-membrane-bounded organelle;organelle;organelle part;protein complex;spindle		Homo sapiens
cell part;integral to membrane;integral to plasma membrane;intrinsic to membrane;intrinsic to plasma membrane;membrane;membrane part;plasma membrane part		Homo sapiens
cell part;centrosome;cytoplasm;cytoplasmic part;cytoskeletal part;extracellular membrane-bounded organelle;extracellular organelle;extracellular region part;extracellular vesicular exosome;intracellular membrane-bounded organelle;intracellular non-membrane-bounded organelle;intracellular organelle;intracellular organelle part;intracellular part;m		Homo sapiens
cell part;cytoplasmic part;endoplasmic reticulum lumen;endoplasmic reticulum part;intracellular organelle lumen;intracellular organelle part;intracellular part;membrane-enclosed lumen;organelle lumen;organelle part	Glutathione metabolism	Homo sapiens
cell part;cytoplasmic part;extracellular membrane-bounded organelle;extracellular organelle;extracellular region part;extracellular vesicular exosome;intracellular organelle part;intracellular part;macromolecular complex;memb	Alzheimer's disease;Huntington's disease;Oxidative phosphorylation;Parkinson's disease	Homo sapiens
cell part;external side of plasma membrane;integral to membrane;integral to plasma membrane;intrinsic to membrane;intrinsic to plasma membrane;membrane;membrane part;plasma membrane part		Homo sapiens
cell part;cytoplasm;cytoplasmic part;extracellular membrane-bounded organelle;extracellular organelle;extracellular region part;extracellular vesicular exosome;integral to membrane;integral to mitochondrial membrane;integr	Amyotrophic lateral sclerosis (ALS)	Homo sapiens
cell part;cytoplasmic part;extracellular membrane-bounded organelle;extracellular organelle;extracellular region part;extracellular vesicular exosome;integral to membrane;integral to organelle membrane;integral to peroxisom	Peroxisome	Homo sapiens
cell part;cell projection part;cytoplasmic part;cytosol;extracellular membrane-bounded organelle;extracellular organelle;extracellular region part;extracellular vesicular exosome;flagellar fibrous sheath;flagellum part;intracellula	Cysteine and methionine metabolism;Glycolysis / Gluconeogenesis;Propanoate metabolism;Pyruvate metabolism	Homo sapiens
cell part;cytoplasmic part;cytosol;external side of plasma membrane;extracellular membrane-bounded organelle;extracellular organelle;extracellular region part;extracellular vesicular exosome;intracellular organelle lumen;intr	Glutathione metabolism	Homo sapiens
cell part;cytoplasmic part;cytosol;extracellular membrane-bounded organelle;extracellular organelle;extracellular region part;extracellular vesicular exosome;intracellular part;membrane;membrane part;membrane raft;membra	Carbon fixation in photosynthetic organisms;Glycolysis / Gluconeogenesis	Homo sapiens
cell body fiber;cell part;cell projection;cytoplasm;cytoplasmic part;cytosol;endoplasmic reticulum;extracellular membrane-bounded organelle;extracellular organelle;extracellular region part;extracellular vesicular exosome;intr	Alanine, aspartate and glutamate metabolism;Arginine and proline metabolism	Homo sapiens
blood microparticle;cell part;cytoplasmic membrane-bounded vesicle lumen;cytoplasmic part;cytoplasmic vesicle part;cytosol;extracellular membrane-bounded organelle;extracellular organelle;extracellular region;extracellula	Complement and coagulation cascades	Homo sapiens
cell part;cell surface;integral to membrane;integral to plasma membrane;intrinsic to membrane;intrinsic to plasma membrane;macromolecular complex;membrane part;MHC class I protein complex;MHC protein complex;plasma membrane part;protein complex		Homo sapiens
blood microparticle;cell part;cell surface;coated pit;cytoplasmic membrane-bounded vesicle;cytoplasmic part;cytoplasmic vesicle;endosome;external side of plasma membrane;extracellular membrane-bounded organelle;ext	Endocytosis;Hematopoietic cell lineage;Phagosome	Homo sapiens
extracellular membrane-bounded organelle;extracellular organelle;extracellular region part;extracellular vesicular exosome;membrane-bounded organelle;membrane-bounded vesicle;organelle;vesicle		Homo sapiens
alphav-beta3 integrin-vitronectin complex;blood microparticle;cell part;extracellular matrix;extracellular membrane-bounded organelle;extracellular organelle;extracellular region;extracellular region part;extracellular space;ext	ECM-receptor interaction;Focal adhesion	Homo sapiens
adherens junction;anchoring junction;cell junction;cell part;cell-substrate adherens junction;cell-substrate junction;cytoplasmic part;cytosol;endoplasmic reticulum;extracellular membrane-bounded organelle;extracellular orga	Amyotrophic lateral sclerosis (ALS);Methane metabolism;Peroxisome;Tryptophan metabolism	Homo sapiens
cell part;cytoplasm;cytoplasmic part;extracellular membrane-bounded organelle;extracellular organelle;extracellular region part;extracellular vesicular exosome;intracellular membrane-bounded organelle;intracellular organelle	Other glycan degradation	Homo sapiens
actin cytoskeleton;cell part;contractile fiber part;cytoplasmic membrane-bounded vesicle lumen;cytoplasmic part;cytoplasmic vesicle part;cytoskeleton;cytosol;extracellular membrane-bounded organelle;extracellular organe	Carbon fixation in photosynthetic organisms;Fructose and mannose metabolism;Glycolysis / Gluconeogenesis;Pentose ph	Homo sapiens
adherens junction;anchoring junction;basolateral plasma membrane;cell junction;cell part;cell projection;cell surface;cell-substrate adherens junction;cell-substrate junction;cilium;cornified envelope;cytoplasm;cytoplasmic part;cytoskeleton;endosome;extracellular membrane-bounded organelle;extracellular organelle;extracellular region;extracellula		Homo sapiens
cell part;cytoplasm;cytoplasmic part;cytoskeleton;cytosol;extracellular membrane-bounded organelle;extracellular organelle;extracellular region part;extracellular vesicular exosome;intracellular membrane-bounded organelle	Alzheimer's disease;Glycolysis / Gluconeogenesis	Homo sapiens
cell part;cytoplasm;cytoplasmic part;cytosol;extracellular membrane-bounded organelle;extracellular organelle;extracellular region part;extracellular vesicular exosome;intracellular part;membrane-bounded organelle;membra	Alanine, aspartate and glutamate metabolism;Arginine and proline metabolism	Homo sapiens
adherens junction;anchoring junction;cell junction;cell part;cell-substrate adherens junction;cell-substrate junction;contractile fiber part;cytoplasm;cytoplasmic part;cytoskeletal part;cytoskeleton;cytosol;extracellular membra	Amoebiasis;MAPK signaling pathway;VEGF signaling pathway	Homo sapiens
cell part;cytoplasm;cytoplasmic part;cytosol;intracellular membrane-bounded organelle;intracellular non-membrane-bounded organelle;intracellular organelle;intracellular organelle lumen;intracellular organelle part;intracellula	One carbon pool by folate;Pyrimidine metabolism	Homo sapiens
cell part;cytoplasmic membrane-bounded vesicle;cytoplasmic part;cytoplasmic vesicle;endoplasmic reticulum;endoplasmic reticulum membrane;endoplasmic reticulum part;integral to membrane;intracellular membrane-bou	N-Glycan biosynthesis;Protein processing in endoplasmic reticulum;Various types of N-glycan biosynthesis	Homo sapiens
cell part;centrosome;cytoplasm;cytoplasmic part;cytoskeletal part;cytosol;extracellular membrane-bounded organelle;extracellular organelle;extracellular region part;extracellular vesicular exosome;extrinsic to internal side of	Axon guidance;Chagas disease (American trypanosomiasis);Chemokine signaling pathway;Gap junction;Gastric acid secre	Homo sapiens
cell part;chromosomal part;extracellular membrane-bounded organelle;extracellular organelle;extracellular region part;extracellular vesicular exosome;intracellular membrane-bounded organelle;intracellular organelle;intracellu	Systemic lupus erythematosus	Homo sapiens
apical plasma membrane;basolateral plasma membrane;caveola;cell part;cytoplasmic membrane-bounded vesicle;cytoplasmic part;cytoplasmic vesicle;endoplasmic reticulum;endosome;extracellular membrane-bounded or	Aldosterone-regulated sodium reabsorption;Bile secretion;Carbohydrate digestion and absorption;Cardiac muscle contract	Homo sapiens
cell part;cytoplasmic part;cytoskeletal part;extracellular membrane-bounded organelle;extracellular organelle;extracellular region part;extracellular vesicular exosome;integral to membrane;integral to plasma membrane;intrac	Calcium signaling pathway;Huntington's disease;Parkinson's disease	Homo sapiens
cell part;cytoplasmic part;cytosol;extracellular region;intracellular organelle part;intracellular part;nuclear part;nucleoplasm;organelle part	RIG-I-like receptor signaling pathway	Homo sapiens
anchored to membrane;cell part;cell surface;integral to membrane;intrinsic to membrane;membrane;membrane part;plasma membrane	Aminobenzoate degradation;Folate biosynthesis;Two-component system	Homo sapiens
adherens junction;anchoring junction;cell junction;cell part;cell surface;cell-substrate adherens junction;cell-substrate junction;external side of plasma membrane;extracellular membrane-bounded organelle;extracellular orgar	African trypanosomiasis;Cell adhesion molecules (CAMs);Leukocyte transendothelial migration;Malaria;Natural killer cell me	Homo sapiens
cell part;cell periphery;centriolar satellite;cytoplasm;cytoplasmic part;cytoskeletal part;extracellular membrane-bounded organelle;extracellular organelle;extracellular region part;extracellular vesicular exosome;intermediate filament;intracellular non-membrane-bounded organelle;intracellular organelle;intracellular organelle part;intracellular part;kera		Homo sapiens
cell junction;cell part;cell-cell junction;contractile fiber part;costamere;cytoplasm;cytoplasmic part;cytoskeletal part;cytoskeleton;dystrophin-associated glycoprotein complex;extracellular membrane-bounded organelle;extracellular organelle;extracellular region part;extracellular vesicular exosome;intermediate filament;intermediate filament cytosk		Homo sapiens
cell part;centrosome;cytoplasm;cytoplasmic part;cytoskeletal part;cytosol;extracellular membrane-bounded organelle;extracellular organelle;extracellular region part;extracellular vesicular exosome;intracellular membrane-bo	Cell cycle;Gap junction;Oocyte meiosis;p53 signaling pathway;Progesterone-mediated oocyte maturation	Homo sapiens
cell part;contractile fiber part;cytoplasm;cytoplasmic part;cytosol;cytosolic part;extracellular membrane-bounded organelle;extracellular organelle;extracellular region part;extracellular space;extracellular vesicular exosome;in	Glycolysis / Gluconeogenesis;Methane metabolism;RNA degradation	Homo sapiens
cell part;cytoplasm;cytoplasmic part;cytosol;extracellular membrane-bounded organelle;extracellular organelle;extracellular region part;extracellular vesicular exosome;intracellular part;membrane;membrane-bounded organe	Insulin signaling pathway;Starch and sucrose metabolism	Homo sapiens
cell part;cell projection;cytoplasm;cytoplasmic part;cytosol;extracellular membrane-bounded organelle;extracellular organelle;extracellular region part;extracellular space;extracellular vesicular exosome;intracellular organelle	Amino sugar and nucleotide sugar metabolism;Glycolysis / Gluconeogenesis;Pentose phosphate pathway;Starch and sucr	Homo sapiens
adherens junction;anchoring junction;cell junction;cell part;cell-substrate adherens junction;cell-substrate junction;centrosome;cytoplasm;cytoplasmic part;cytoskeletal part;cytosol;focal adhesion;intracellular membrane-bounded organelle;intracellular non-membrane-bounded organelle;intracellular organelle;intracellular organelle part;intracellular		Homo sapiens
adherens junction;alphav-beta3 integrin complex;alphav-beta3 integrin-IGF-1-IGF1R complex;alphav-beta5 integrin complex;alphav-beta8 integrin complex;anchoring junction;cell junction;cell part;cell projection membrane;c	Arrhythmogenic right ventricular cardiomyopathy (ARVC);Cell adhesion molecules (CAMs);Dilated cardiomyopathy;ECM-re	Homo sapiens
cell part;cytoplasmic membrane-bounded vesicle;cytoplasmic part;cytoplasmic vesicle;cytosol;extracellular matrix;extracellular region;extracellular region part;extracellular space;extrinsic to external side of plasma membrane;extrinsic to membrane;extrinsic to plasma membrane;intracellular membrane-bounded organelle;intracellular organelle;intr		Homo sapiens
cell part;cytoplasm;cytoplasmic part;cytosol;extracellular membrane-bounded organelle;extracellular organelle;extracellular region part;extracellular vesicular exosome;intracellular membrane-bounded organelle;intracellular c	Arachidonic acid metabolism;Glutathione metabolism	Homo sapiens
adherens junction;anchoring junction;cell junction;cell part;cell-substrate adherens junction;cell-substrate junction;cytoplasmic membrane-bounded vesicle;cytoplasmic part;cytoplasmic vesicle;endoplasmic reticulum;endopl	Protein processing in endoplasmic reticulum	Homo sapiens
cell part;cytoplasmic membrane-bounded vesicle;cytoplasmic part;cytoplasmic vesicle;extracellular membrane-bounded organelle;extracellular organelle;extracellular region;extracellular region part;extracellular space;extrac	ko05152;Lysosome	Homo sapiens
basement membrane;basolateral plasma membrane;cell cortex;cell part;cell projection;cell surface;cytoplasmic membrane-bounded vesicle;cytoplasmic part;cytoplasmic vesicle;early endosome;endosomal part;endosome;endosome membrane;extracellular matrix part;extracellular membrane-bounded organelle;extracellular organelle;extracellula		Homo sapiens
acrosomal vesicle;cell part;cytoplasmic membrane-bounded vesicle;cytoplasmic part;cytoplasmic vesicle;extracellular membrane-bounded organelle;extracellular organelle;extracellular region part;extracellular vesicular exos	Amino sugar and nucleotide sugar metabolism;Glycosaminoglycan degradation;Glycosphingolipid biosynthesis - ganglio s	Homo sapiens
aminoacyl-tRNA synthetase multienzyme complex;cell part;cytoplasm;cytoplasmic part;cytosol;intracellular part;macromolecular complex;membrane;protein complex;ribonucleoprotein complex	Aminoacyl-tRNA biosynthesis;Porphyrin and chlorophyll metabolism	Homo sapiens
cell part;cytoplasmic membrane-bounded vesicle;cytoplasmic part;cytoplasmic vesicle;extracellular membrane-bounded organelle;extracellular organelle;extracellular region;extracellular region part;extracellular space;extrac	Antigen processing and presentation;Lysosome	Homo sapiens
basement membrane;cell part;cytoplasmic part;extracellular matrix;extracellular matrix part;extracellular membrane-bounded organelle;extracellular organelle;extracellular region;extracellular region part;extracellular space;ex	Amoebiasis;ECM-receptor interaction;Focal adhesion;Pathways in cancer;Small cell lung cancer;Toxoplasmosis	Homo sapiens
adherens junction;anchoring junction;cell junction;cell part;cell-substrate adherens junction;cell-substrate junction;cytoplasmic membrane-bounded vesicle;cytoplasmic part;cytoplasmic vesicle;endosomal part;endosome membrane;extracellular membrane-bounded organelle;extracellular organelle;extracellular region part;extracellular vesicular ex		Homo sapiens
anchored to membrane;cell part;cell surface;extracellular membrane-bounded organelle;extracellular organelle;extracellular region;extracellular region part;extracellular vesicular exosome;integral to membrane;integral to plas	Complement and coagulation cascades;Hematopoietic cell lineage;Viral myocarditis	Homo sapiens
apical plasma membrane;cell part;cell surface;cytoplasm;cytoplasmic membrane-bounded vesicle;cytoplasmic part;cytoplasmic vesicle;extracellular membrane-bounded organelle;extracellular organelle;extracellular region p	Protein digestion and absorption	Homo sapiens
cell part;cytoplasmic part;cytosol;intracellular part	Alanine, aspartate and glutamate metabolism;Nitrogen metabolism	Homo sapiens
cell part;cytoplasmic part;intracellular membrane-bounded organelle;intracellular organelle;intracellular organelle lumen;intracellular organelle part;intracellular part;macromolecular complex;membrane-bounded organelle;mer	Butanoate metabolism;Citrate cycle (TCA cycle);Glycolysis / Gluconeogenesis;Pyruvate metabolism;Valine, leucine and iso	Homo sapiens
adherens junction;alphav-beta3 integrin-vitronectin complex;anchoring junction;cell junction;cell part;cell projection;cell surface;cell-substrate adherens junction;cell-substrate junction;cytoplasm;external side of plasma mem	Arrhythmogenic right ventricular cardiomyopathy (ARVC);Bacterial invasion of epithelial cells;Dilated cardiomyopathy;ECM-	Homo sapiens
cell part;intracellular membrane-bounded organelle;intracellular non-membrane-bounded organelle;intracellular organelle;intracellular organelle part;intracellular part;membrane-bounded organelle;non-membrane-bounded organelle;nuclear part;nucleolus;nucleoplasm;nucleus;organelle;organelle part		Homo sapiens
adherens junction;anchoring junction;cell junction;cell leading edge;cell part;cell projection;cell-substrate adherens junction;cell-substrate junction;cytoplasm;cytoplasmic part;cytoskeletal part;cytoskeleton;cytosol;extracellular membrane-bounded organelle;extracellular organelle;extracellular region part;extracellular vesicular exosome;focal adhes		Homo sapiens
adherens junction;anchoring junction;cell junction;cell part;cell-substrate adherens junction;cell-substrate junction;cytoplasmic part;cytosol;cytosolic small ribosomal subunit;extracellular membrane-bounded organelle;extrac	Ribosome	Homo sapiens
cell part;cytoplasm;cytoskeletal part;extracellular membrane-bounded organelle;extracellular organelle;extracellular region part;extracellular vesicular exosome;intermediate filament;intracellular membrane-bounded organelle;intracellular organelle;intracellular organelle part;intracellular part;keratin filament;macromolecular complex;membrane-bou		Homo sapiens
adherens junction;anchoring junction;blood microparticle;cell junction;cell part;cell-substrate adherens junction;cell-substrate junction;cytoplasm;endothelial microparticle;external side of plasma membrane;extracellular membrane-bounded organelle;extracellular organelle;extracellular region part;extracellular vesicular exosome;focal adhesion;intr		Homo sapiens
cell part;cytoplasmic part;intracellular organelle part;intracellular part;large ribosomal subunit;macromolecular complex;membrane;mitochondrial inner membrane;mitochondrial large ribosomal subunit;mitochondrial membrane;mitochondrial part;organellar large ribosomal subunit;organelle inner membrane;organelle membrane;organelle part;ribonu		Homo sapiens
cell part;extracellular region;extracellular region part;extracellular space;intracellular membrane-bounded organelle;intracellular organelle;intracellular part;membrane-bounded organelle;nucleus;organelle	MAPK signaling pathway;Melanoma;Pathways in cancer;Regulation of actin cytoskeleton	Homo sapiens
cell part;cytoplasmic part;cytosol;cytosolic part;extracellular membrane-bounded organelle;extracellular organelle;extracellular region part;extracellular space;extracellular vesicular exosome;intracellular part;macromolecular	Glycolysis / Gluconeogenesis;Methane metabolism;RNA degradation	Homo sapiens
cell part;cell surface;cytoplasm;extracellular matrix;extracellular membrane-bounded organelle;extracellular organelle;extracellular region part;extracellular space;extracellular vesicular exosome;intracellular;intracellular membrane-bounded organelle;intracellular organelle;intracellular part;membrane-bounded organelle;membrane-bounded vesicle;		Homo sapiens
actin filament bundle;actomyosin;bleb;cell part;cell projection;cell projection membrane;cell projection part;contractile fiber part;cytoplasmic part;cytoskeletal part;cytoskeleton;cytosol;filamentous actin;intracellular non-mem	Cardiac muscle contraction;Dilated cardiomyopathy;Hypertrophic cardiomyopathy (HCM)	Homo sapiens
cell part;cell surface;cytoplasm;cytoplasmic part;extracellular membrane-bounded organelle;extracellular organelle;extracellular region part;extracellular vesicular exosome;intracellular membrane-bounded organelle;intracellular organelle;intracellular organelle part;intracellular part;membrane;membrane-bounded organelle;membrane-bounded vesi		Homo sapiens
cell part;cell projection;cytoplasm;cytoplasmic membrane-bounded vesicle;cytoplasmic part;cytoplasmic vesicle;cytoskeletal part;extracellular membrane-bounded organelle;extracellular organelle;extracellular region part;extracellular space;extracellular vesicular exosome;intracellular membrane-bounded organelle;intracellular organelle;intracellul		Homo sapiens
cell part;envelope;intracellular membrane-bounded organelle;intracellular non-membrane-bounded organelle;intracellular organelle;intracellular organelle part;intracellular part;macromolecular complex;membrane;membrane-	Base excision repair	Homo sapiens
alpha DNA polymerase:primase complex;cell part;chromosomal part;cytoplasm;envelope;intracellular membrane-bounded organelle;intracellular non-membrane-bounded organelle;intracellular organelle;intracellular organelle	DNA replication;Purine metabolism;Pyrimidine metabolism	Homo sapiens
cell part;cytoplasm;cytoplasmic part;cytosol;endoplasmic reticulum;intracellular membrane-bounded organelle;intracellular organelle;intracellular part;membrane-bounded organelle;organelle		Homo sapiens
cell part;cytoplasm;cytoplasmic part;cytosol;intracellular part	Hepatitis C	Homo sapiens
anchored to membrane;cell part;integral to membrane;intrinsic to membrane;membrane;membrane part;plasma membrane	Aminobenzoate degradation;Folate biosynthesis;Two-component system	Homo sapiens
axon;cell part;cell projection;cytoplasmic part;cytoskeleton;cytosol;extracellular membrane-bounded organelle;extracellular organelle;extracellular region part;extracellular vesicular exosome;intracellular membrane-bounded	Carbon fixation in photosynthetic organisms;Fructose and mannose metabolism;Glycolysis / Gluconeogenesis;Pentose ph	Homo sapiens
		Homo sapiens
cell part;cytoplasm;intracellular organelle part;intracellular part;macromolecular complex;nuclear part;nucleoplasm;organelle part;ribonucleoprotein complex	Systemic lupus erythematosus	Homo sapiens
cell part;cytoplasmic part;extracellular membrane-bounded organelle;extracellular organelle;extracellular region part;extracellular vesicular exosome;intracellular membrane-bounded organelle;intracellular organelle;intracellula	Galactose metabolism;Lysosome;Starch and sucrose metabolism	Homo sapiens
	Acute myeloid leukemia;Bladder cancer;Chronic myeloid leukemia;Colorectal cancer;Endometrial cancer;ErbB signaling pa	Homo sapiens
AMP-activated protein kinase complex;cAMP-dependent protein kinase complex;cell part;cytoplasmic part;cytosol;intracellular part;macromolecular complex;membrane;neuromuscular junction;plasma membrane;protein co	Apoptosis;Insulin signaling pathway	Homo sapiens
adherens junction;anchoring junction;cell junction;cell part;cell surface;cell-substrate adherens junction;cell-substrate junction;cytoplasmic membrane-bounded vesicle;cytoplasmic part;cytoplasmic vesicle;endoplasmic retic	Prion diseases;Protein export;Protein processing in endoplasmic reticulum	Homo sapiens
basement membrane;extracellular matrix;extracellular matrix part;extracellular membrane-bounded organelle;extracellular organelle;extracellular region;extracellular region part;extracellular space;extracellular vesicular exoso	Amoebiasis;ECM-receptor interaction;Focal adhesion;Pathways in cancer;Prion diseases;Small cell lung cancer;Toxoplasm	Homo sapiens
basolateral plasma membrane;blood microparticle;caveola;cell cortex part;cell junction;cell part;cell-cell junction;cortical actin cytoskeleton;cortical cytoskeleton;cytoplasmic membrane-bounded vesicle;cytoplasmic part;cyto	Adipocytokine signaling pathway;Bile secretion;Pathways in cancer;Renal cell carcinoma	Homo sapiens
cell part;extracellular membrane-bounded organelle;extracellular organelle;extracellular region part;extracellular vesicular exosome;integral to membrane;integral to plasma membrane;intrinsic to membrane;intrinsic to plasma membrane;membrane;membrane part;membrane-bounded organelle;membrane-bounded vesicle;organelle;plasma memb		Homo sapiens
cell part;cytoplasm;cytoplasmic part;cytosol;intracellular membrane-bounded organelle;intracellular organelle;intracellular part;membrane-bounded organelle;nucleus;organelle	Drug metabolism - other enzymes;Pyrimidine metabolism	Homo sapiens
adherens junction;anchoring junction;cell division site part;cell junction;cell part;cell surface;cell-substrate adherens junction;cell-substrate junction;cleavage furrow;cytoplasmic part;cytoplasmic vesicle membrane;cytoplasm	Pancreatic cancer;Pathways in cancer	Homo sapiens
cell part;centrosome;cytoplasm;cytoplasmic part;cytoskeletal part;cytosol;extracellular membrane-bounded organelle;extracellular organelle;extracellular region part;extracellular vesicular exosome;internal side of plasma me	Glutathione metabolism;Pentose phosphate pathway	Homo sapiens
cell part;cytoplasm;cytoplasmic part;cytosol;intracellular membrane-bounded organelle;intracellular organelle;intracellular organelle lumen;intracellular organelle part;intracellular part;membrane;membrane-bounded organelle	Citrate cycle (TCA cycle);Pyruvate metabolism	Homo sapiens
cell part;cytoplasmic part;cytosol;extracellular membrane-bounded organelle;extracellular organelle;extracellular region part;extracellular vesicular exosome;intracellular membrane-bounded organelle;intracellular organelle;int	One carbon pool by folate	Homo sapiens
cell junction;cell part;cell-cell junction;chromatin;chromosomal part;cyclin-dependent protein kinase holoenzyme complex;cytoplasmic part;cytosol;intracellular membrane-bounded organelle;intracellular non-membrane-bour	Bladder cancer;Cell cycle;Chronic myeloid leukemia;Glioma;Measles;Melanoma;Non-small cell lung cancer;p53 signaling p	Homo sapiens
cell part;cytoplasmic part;integral to membrane;intracellular membrane-bounded organelle;intracellular organelle;intracellular organelle part;intracellular part;intrinsic to membrane;macromolecular complex;membrane;membra	Calcium signaling pathway;Huntington's disease;Parkinson's disease	Homo sapiens
cell part;cytoplasm;cytoplasmic part;cytosol;extracellular membrane-bounded organelle;extracellular organelle;extracellular region part;extracellular vesicular exosome;intracellular membrane-bounded organelle;intracellular c	Drug metabolism - other enzymes;Purine metabolism	Homo sapiens
cell part;chromosomal part;cytoplasm;cytoplasmic dynein complex;cytoplasmic part;cytoskeletal part;dynein complex;envelope;extrinsic to membrane;inclusion body;intracellular membrane-bounded organelle;intracellular no	MAPK signaling pathway - yeast;Pathways in cancer;RNA transport;Thyroid cancer	Homo sapiens
cell part;cytoplasmic part;cytosol;extracellular membrane-bounded organelle;extracellular organelle;extracellular region part;extracellular vesicular exosome;intracellular membrane-bounded organelle;intracellular organelle;int	Arginine and proline metabolism	Homo sapiens
cell part;cytoplasm;cytoplasmic membrane-bounded vesicle;cytoplasmic part;cytoplasmic vesicle;cytoplasmic vesicle membrane;cytoplasmic vesicle part;endocytic vesicle membrane;extracellular membrane-bounded organelle;extracellular organelle;extracellular region part;extracellular vesicular exosome;intracellular membrane-bounded organe		Homo sapiens
cell part;cytoplasmic part;dihydrolipoyl dehydrogenase complex;intracellular membrane-bounded organelle;intracellular organelle;intracellular organelle lumen;intracellular organelle part;intracellular part;macromolecular comp	Valine, leucine and isoleucine degradation	Homo sapiens
extracellular membrane-bounded organelle;extracellular organelle;extracellular region part;extracellular vesicular exosome;membrane-bounded organelle;membrane-bounded vesicle;organelle;vesicle		Homo sapiens
cell part;chromosomal part;cytoplasmic part;cytosol;intracellular membrane-bounded organelle;intracellular non-membrane-bounded organelle;intracellular organelle;intracellular organelle part;intracellular part;Ku70:Ku80 cor	Non-homologous end-joining	Homo sapiens
cell part;chromosomal part;chromosome, telomeric region;cytoplasmic part;cytosol;intracellular membrane-bounded organelle;intracellular non-membrane-bounded organelle;intracellular organelle;intracellular organelle part;i	Non-homologous end-joining	Homo sapiens
cell part;cytoplasmic part;intracellular membrane-bounded organelle;intracellular organelle;intracellular organelle part;intracellular part;membrane-bounded organelle;mitochondrion;nuclear part;nucleoplasm;nucleus;organelle	Base excision repair;Primary immunodeficiency	Homo sapiens
angiogenin-PRI complex;cell part;cytoplasm;extracellular membrane-bounded organelle;extracellular organelle;extracellular region part;extracellular vesicular exosome;intracellular part;macromolecular complex;membrane-bounded organelle;membrane-bounded vesicle;organelle;protein complex;vesicle		Homo sapiens
aggresome;cell part;cytoplasm;cytoplasmic part;cytosol;extracellular membrane-bounded organelle;extracellular organelle;extracellular region part;extracellular vesicular exosome;inclusion body;intracellular membrane-bounded organelle;intracellular organelle;intracellular part;macromolecular complex;membrane;membrane-bounded organelle;me		Homo sapiens
cell part;cell surface;cytoplasmic membrane-bounded vesicle;cytoplasmic part;cytoplasmic vesicle;endoplasmic reticulum;endoplasmic reticulum lumen;endoplasmic reticulum part;intracellular membrane-bounded organelle	Protein processing in endoplasmic reticulum;Vibrio cholerae infection	Homo sapiens
cell part;cytoplasmic part;endoplasmic reticulum;endoplasmic reticulum lumen;endoplasmic reticulum part;intracellular membrane-bounded organelle;intracellular organelle;intracellular organelle lumen;intracellular organelle p	Arginine and proline metabolism	Homo sapiens
cell part;cytoplasm;intracellular part		Homo sapiens
cell part;cytoplasm;cytoplasmic part;cytosol;inclusion body;intracellular part;membrane	Insulin signaling pathway;Starch and sucrose metabolism	Homo sapiens
cell part;cytoplasmic part;cytosol;endosomal part;endosome membrane;intracellular organelle part;intracellular part;late endosome membrane;membrane;organelle membrane;organelle part;plasma membrane		Homo sapiens
cell part;cytoplasm;cytoplasmic part;cytosol;cytosolic part;extracellular membrane-bounded organelle;extracellular organelle;extracellular region part;extracellular space;extracellular vesicular exosome;intracellular part;macro	Glycolysis / Gluconeogenesis;Methane metabolism;RNA degradation	Homo sapiens
cell part;cytoplasmic part;extracellular region part;extracellular space;intracellular membrane-bounded organelle;intracellular organelle;intracellular part;membrane-bounded organelle;mitochondrion;organelle	One carbon pool by folate	Homo sapiens
adherens junction;anchoring junction;cell junction;cell part;cell-substrate adherens junction;cell-substrate junction;cytoplasm;focal adhesion;integral to membrane;integral to plasma membrane;intracellular part;intrinsic to me	Cell adhesion molecules (CAMs);Leukocyte transendothelial migration	Homo sapiens
cell part;cytoplasmic part;endoplasmic reticulum;endoplasmic reticulum lumen;endoplasmic reticulum part;intracellular;intracellular membrane-bounded organelle;intracellular organelle;intracellular organelle lumen;intracellula	Protein processing in endoplasmic reticulum	Homo sapiens
anchored to membrane;cell part;cell surface;extracellular membrane-bounded organelle;extracellular organelle;extracellular region part;extracellular vesicular exosome;intrinsic to membrane;membrane;membrane part;membrane-bounded organelle;membrane-bounded vesicle;organelle;plasma membrane;vesicle		Homo sapiens
cell part;cell projection;cilium;cytoplasm;cytoplasmic part;cytosol;extracellular matrix;extracellular membrane-bounded organelle;extracellular organelle;extracellular region part;extracellular vesicular exosome;intracellular me	Carbon fixation in photosynthetic organisms;Glycolysis / Gluconeogenesis;Purine metabolism;Pyruvate metabolism;Type II	Homo sapiens
adherens junction;anchoring junction;cell junction;cell part;cell-substrate adherens junction;cell-substrate junction;cytoplasmic membrane-bounded vesicle;cytoplasmic membrane-bounded vesicle lumen;cytoplasmic part;cy	NOD-like receptor signaling pathway;Pathways in cancer;Plant-pathogen interaction;Prostate cancer;Protein processing in	Homo sapiens
cell part;cell surface;cytoplasm;cytoplasmic part;cytosol;cytosolic part;cytosolic proteasome complex;extracellular region part;extracellular space;intracellular membrane-bounded organelle;intracellular organelle;intracellular	Alzheimer's disease	Homo sapiens
cell part;cytoplasm;cytoplasmic part;cytosol;extracellular membrane-bounded organelle;extracellular organelle;extracellular region part;extracellular vesicular exosome;intracellular part;membrane;membrane-bounded organe	Aminoacyl-tRNA biosynthesis	Homo sapiens
axon part;axon terminus;cell part;cell projection part;cytoplasm;cytoplasmic part;cytosol;endoplasmic reticulum;extracellular membrane-bounded organelle;extracellular organelle;extracellular region part;extracellular vesicula	Alanine, aspartate and glutamate metabolism;Arginine and proline metabolism;Nitrogen metabolism;Two-component syste	Homo sapiens
anchoring junction;basolateral plasma membrane;brush border membrane;cell junction;cell part;cell projection;cell projection membrane;cell projection part;cell-cell junction;cytoplasmic part;desmosome;external encapsulati	Galactose metabolism;Glycosaminoglycan biosynthesis - keratan sulfate;Glycosphingolipid biosynthesis - lacto and neolac	Homo sapiens
cell part;intracellular membrane-bounded organelle;intracellular organelle;intracellular organelle part;intracellular part;membrane-bounded organelle;nuclear part;nucleoplasm;nucleus;organelle;organelle part	Osteoclast differentiation	Homo sapiens
adherens junction;anchoring junction;basolateral plasma membrane;cell junction;cell part;cell surface;cell-substrate adherens junction;cell-substrate junction;cytoplasmic part;cytoplasmic vesicle part;extracellular membrane-	Complement and coagulation cascades;Measles	Homo sapiens

cell body;cell part;cytoplasm;cytoplasmic part;cytosol;extracellular membrane-bounded organelle;extracellular organelle;extracellular region part;extracellular vesicular exosome;intracellular part;membrane-bounded organelle;membrane-bounded vesicle;neuronal cell body;organelle;vesicle		Homo sapiens
cell part;cytoplasmic part;extracellular membrane-bounded organelle;extracellular organelle;extracellular region part;extracellular vesicular exosome;intracellular organelle lumen;intracellular organelle part;intracellular part;lyso	Glycosaminoglycan degradation;Lysosome	Homo sapiens
adherens junction;anchoring junction;cell junction;cell part;cell-substrate adherens junction;cell-substrate junction;cytoplasm;cytoplasmic part;cytosol;cytosolic small ribosomal subunit;extracellular membrane-bounded orga	Ribosome	Homo sapiens
adherens junction;anchoring junction;basolateral plasma membrane;cell junction;cell part;cell-cell adherens junction;cell-cell contact zone;cell-cell junction;cornified envelope;cytoplasmic part;cytoskeletal part;cytoskeleton;d	Arrhythmogenic right ventricular cardiomyopathy (ARVC)	Homo sapiens
cell part;chromatin;chromosomal part;DNA replication factor A complex;intracellular membrane-bounded organelle;intracellular organelle;intracellular organelle part;intracellular part;macromolecular complex;membrane-boun	DNA replication;Homologous recombination;Mismatch repair;Nucleotide excision repair	Homo sapiens
apical plasma membrane;cell part;chromatin;chromosomal part;cytoplasmic part;extracellular membrane-bounded organelle;extracellular organelle;extracellular region part;extracellular space;extracellular vesicular exosome;Golgi apparatus part;Golgi lumen;integral to membrane;integral to plasma membrane;intracellular organelle lumen;intracellul		Homo sapiens
cell part;cytoplasm;extracellular membrane-bounded organelle;extracellular organelle;extracellular region part;extracellular vesicular exosome;intracellular organelle part;intracellular part;membrane-bounded organelle;membrane-bounded vesicle;nuclear part;nucleoplasm;organelle;organelle part;vesicle		Homo sapiens
cell part;cytoplasmic part;cytosol;extracellular membrane-bounded organelle;extracellular organelle;extracellular region part;extracellular vesicular exosome;intracellular part;membrane-bounded organelle;membrane-bounde	Arachidonic acid metabolism	Homo sapiens
cell part;cytoplasmic part;endoplasmic reticulum;endoplasmic reticulum membrane;endoplasmic reticulum part;integral to membrane;integral to plasma membrane;intracellular membrane-bounded organelle;intracellular orga	Alzheimer's disease;Calcium signaling pathway;Pancreatic secretion	Homo sapiens
adherens junction;alpha2-beta1 integrin complex;anchoring junction;axon part;axon terminus;basal part of cell;cell junction;cell outer membrane;cell part;cell projection part;cell surface;cell-substrate adherens junction;cell-su	Arrhythmogenic right ventricular cardiomyopathy (ARVC);Dilated cardiomyopathy;ECM-receptor interaction;Focal adhesion	Homo sapiens
AMP-activated protein kinase complex;calcium channel complex;cAMP-dependent protein kinase complex;cation channel complex;cell part;cell projection;centrosome;cilium;cytoplasmic part;cytoskeletal part;cytosol;extrac	Amoebiasis;Apoptosis;Bile secretion;Calcium signaling pathway;Chemokine signaling pathway;Dilated cardiomyopathy;En	Homo sapiens
adherens junction;anchoring junction;cell cortex part;cell junction;cell part;cell projection;cell-substrate adherens junction;cell-substrate junction;chromatin;chromosomal part;cortical actin cytoskeleton;cortical cytoskeleton;c	Alzheimer's disease;Apoptosis;Focal adhesion;Protein processing in endoplasmic reticulum	Homo sapiens
cell part;chromatin;chromosomal part;chromosome, centromeric region;condensed chromosome, centromeric region;cytoplasm;intracellular membrane-bounded organelle;intracellular organelle;intracellular organelle part;intr	ko05152	Homo sapiens
cell part;cytoplasmic part;cytosol;intracellular part;membrane	Pyrimidine metabolism	Homo sapiens
adherens junction;anchoring junction;blood microparticle;cell junction;cell part;cell surface;cell-substrate adherens junction;cell-substrate junction;cytoplasm;endothelial microparticle;external side of plasma membrane;extracellular region part;extracellular space;focal adhesion;intracellular organelle part;intracellular part;macromolecular complex;r		Homo sapiens
catalytic step 2 spliceosome;cell part;cytoplasm;extracellular membrane-bounded organelle;extracellular organelle;extracellular region part;extracellular vesicular exosome;intracellular membrane-bounded organelle;intracellu	Spliceosome	Homo sapiens
6-phosphofructokinase complex;cell part;cytoplasmic part;cytosol;cytosolic part;extracellular membrane-bounded organelle;extracellular organelle;extracellular region part;extracellular vesicular exosome;intracellular part;ma	Fructose and mannose metabolism;Galactose metabolism;Glycolysis / Gluconeogenesis;Methane metabolism;Pentose ph	Homo sapiens
cell part;extracellular membrane-bounded organelle;extracellular organelle;extracellular region;extracellular region part;extracellular space;extracellular vesicular exosome;insulin-like growth factor binding protein complex;intr	p53 signaling pathway	Homo sapiens
cell part;cytoplasmic part;cytosol;cytosolic large ribosomal subunit;extracellular membrane-bounded organelle;extracellular organelle;extracellular region part;extracellular vesicular exosome;intracellular membrane-bounded	Ribosome	Homo sapiens
adherens junction;anchoring junction;cell junction;cell part;cell-substrate adherens junction;cell-substrate junction;cytoplasm;cytoplasmic part;cytosol;cytosolic large ribosomal subunit;extracellular membrane-bounded orga	Ribosome	Homo sapiens
actin cytoskeleton;adherens junction;anchoring junction;cell junction;cell part;cell-cell adherens junction;cell-cell junction;cell-substrate adherens junction;cell-substrate junction;contractile fiber part;costamere;cytoplasmic pa	Adherens junction;Amoebiasis;Bacterial invasion of epithelial cells;Focal adhesion;Leukocyte transendothelial migration;Re	Homo sapiens
cell part;cytoplasmic part;cytosol;cytosolic large ribosomal subunit;intracellular membrane-bounded organelle;intracellular organelle;intracellular organelle part;intracellular part;large ribosomal subunit;macromolecular comple	Ribosome	Homo sapiens
cell part;cytoplasm;cytoplasmic part;cytosol;extracellular membrane-bounded organelle;extracellular organelle;extracellular region part;extracellular vesicular exosome;intracellular part;membrane;membrane-bounded organe	Glycolysis / Gluconeogenesis;Methane metabolism	Homo sapiens
cell part;cytoplasmic part;Golgi apparatus;intracellular membrane-bounded organelle;intracellular organelle;intracellular organelle part;intracellular part;membrane-bounded organelle;mitochondrion;nuclear part;nucleoplasm;r	Base excision repair;DNA replication;Mismatch repair;Nucleotide excision repair	Homo sapiens
cell part;intracellular membrane-bounded organelle;intracellular organelle;intracellular organelle part;intracellular part;membrane-bounded organelle;nuclear part;nucleoplasm;nucleus;organelle;organelle part	Base excision repair	Homo sapiens
cell cortex;cell part;cytoplasmic part;extracellular membrane-bounded organelle;extracellular organelle;extracellular region part;extracellular vesicular exosome;intracellular membrane-bounded organelle;intracellular non-mem	Pathogenic Escherichia coli infection	Homo sapiens
blood microparticle;extracellular membrane-bounded organelle;extracellular organelle;extracellular region;extracellular region part;extracellular vesicular exosome;membrane-bounded organelle;membrane-bounded vesicle;organelle;vesicle		Homo sapiens
cell part;cytoplasm;cytoplasmic part;cytosol;eukaryotic translation initiation factor 2 complex;intracellular part;macromolecular complex;protein complex	RNA transport	Homo sapiens
cell part;cytoplasmic part;cytosol;endoplasmic reticulum membrane;endoplasmic reticulum part;envelope;extracellular membrane-bounded organelle;extracellular organelle;extracellular region part;extracellular vesicular exosome;intracellular membrane-bounded organelle;intracellular organelle;intracellular organelle part;intracellular part;membrane		Homo sapiens
cell part;cytoplasm;intracellular membrane-bounded organelle;intracellular organelle;intracellular part;membrane-bounded organelle;nucleus;organelle		Homo sapiens
cell part;cytoplasmic membrane-bounded vesicle;cytoplasmic part;cytoplasmic vesicle;endosome;extracellular membrane-bounded organelle;extracellular organelle;extracellular region part;extracellular vesicular exosome;in	Endocytosis	Homo sapiens
cell part;cytoplasmic part;cytoplasmic vesicle;cytosol;extracellular membrane-bounded organelle;extracellular organelle;extracellular region part;extracellular vesicular exosome;Golgi apparatus;Golgi apparatus part;Golgi membrane;intracellular membrane-bounded organelle;intracellular organelle;intracellular organelle part;intracellular part;membr		Homo sapiens
cell part;cytoplasm;cytoplasmic part;cytosol;extracellular membrane-bounded organelle;extracellular organelle;extracellular region part;extracellular vesicular exosome;intracellular membrane-bounded organelle;intracellular c	Proteasome	Homo sapiens
cell part;cytoplasm;cytoplasmic part;cytosol;intracellular part;membrane		Homo sapiens
basement membrane;cell part;collagen;collagen type V;cytoplasmic part;endoplasmic reticulum lumen;endoplasmic reticulum part;extracellular matrix;extracellular matrix part;extracellular membrane-bounded organelle;extra	Amoebiasis;ECM-receptor interaction;Focal adhesion;Protein digestion and absorption	Homo sapiens
cell part;cell projection;cytoplasmic membrane-bounded vesicle;cytoplasmic part;cytoplasmic vesicle;cytosol;endomembrane system;extracellular membrane-bounded organelle;extracellular organelle;extracellular region par	Collecting duct acid secretion;Epithelial cell signaling in Helicobacter pylori infection;Oxidative phosphorylation;Phagosom	Homo sapiens
adherens junction;anchoring junction;cell junction;cell part;cell-substrate adherens junction;cell-substrate junction;extracellular membrane-bounded organelle;extracellular organelle;extracellular region part;extracellular vesicular exosome;focal adhesion;intracellular membrane-bounded organelle;intracellular organelle;intracellular part;membrane-b		Homo sapiens
actin cytoskeleton;actin filament;adherens junction;anchoring junction;cell body;cell cortex part;cell junction;cell part;cell projection part;cell-substrate adherens junction;cell-substrate junction;chromosomal part;cortical cyto	Focal adhesion;MAPK signaling pathway	Homo sapiens
cell part;cytoplasm;cytoplasmic part;cytosol;endoplasmic reticulum;extracellular membrane-bounded organelle;extracellular organelle;extracellular region part;extracellular vesicular exosome;Golgi apparatus;intracellular mem	Carbon fixation pathways in prokaryotes;Citrate cycle (TCA cycle);Glyoxylate and dicarboxylate metabolism	Homo sapiens
cell part;cytoplasmic part;extracellular membrane-bounded organelle;extracellular organelle;extracellular region part;extracellular vesicular exosome;fumarate reductase complex;intracellular membrane-bounded organelle;int	Alzheimer's disease;Citrate cycle (TCA cycle);Huntington's disease;Oxidative phosphorylation;Parkinson's disease	Homo sapiens
extracellular region;extracellular region part;extracellular space		Homo sapiens
basement membrane;extracellular matrix;extracellular matrix part;extracellular membrane-bounded organelle;extracellular organelle;extracellular region;extracellular region part;extracellular space;extracellular vesicular exosome;membrane-bounded organelle;membrane-bounded vesicle;organelle;proteinaceous extracellular matrix;vesicle		Homo sapiens
cell part;intracellular membrane-bounded organelle;intracellular non-membrane-bounded organelle;intracellular organelle;intracellular organelle part;intracellular part;membrane-bounded organelle;non-membrane-bounded organelle;nuclear part;nucleolus;nucleoplasm;nucleus;organelle;organelle part		Homo sapiens
cell part;cell projection;cilium;cytoplasm;cytoplasmic part;endoplasmic reticulum membrane;endoplasmic reticulum part;extracellular membrane-bounded organelle;extracellular organelle;extracellular region part;extracellular	Arachidonic acid metabolism	Homo sapiens
adherens junction;anchoring junction;cell junction;cell part;cell-substrate adherens junction;cell-substrate junction;cytoplasmic membrane-bounded vesicle;cytoplasmic part;cytoplasmic vesicle;endoplasmic reticulum;endoplasmic reticulum lumen;endoplasmic reticulum part;extracellular membrane-bounded organelle;extracellular organelle;extrac		Homo sapiens
cell part;cytoplasm;cytoplasmic part;cytosol;extracellular membrane-bounded organelle;extracellular organelle;extracellular region part;extracellular vesicular exosome;intracellular membrane-bounded organelle;intracellular c	Aminoacyl-tRNA biosynthesis;Tryptophan metabolism	Homo sapiens
cell part;cytoplasm;cytoplasmic membrane-bounded vesicle;cytoplasmic part;cytoplasmic vesicle;cytosol;extracellular membrane-bounded organelle;extracellular organelle;extracellular region part;extracellular vesicular exos	Cysteine and methionine metabolism	Homo sapiens
cell part;cytoplasmic part;cytosol;eukaryotic translation initiation factor 4F complex;intracellular part;macromolecular complex;protein complex	mTOR signaling pathway;RNA transport	Homo sapiens
caveola;cell part;contractile fiber part;cytoplasmic part;integral to membrane;integral to plasma membrane;intracellular part;intrinsic to membrane;intrinsic to plasma membrane;macromolecular complex;membrane;membran	Calcium signaling pathway;Pancreatic secretion;Salivary secretion	Homo sapiens
cell body;cell part;cell projection;cytoplasm;cytoplasmic part;cytosol;envelope;extracellular membrane-bounded organelle;extracellular organelle;extracellular region part;extracellular vesicular exosome;intracellular organelle	Glutathione metabolism;Purine metabolism;Pyrimidine metabolism	Homo sapiens
cell part;cytoplasmic part;integral to membrane;intracellular organelle part;intracellular part;intrinsic to membrane;membrane;membrane part;mitochondrial inner membrane;mitochondrial membrane;mitochondrial membrane	Alzheimer's disease;Cardiac muscle contraction;Huntington's disease;Oxidative phosphorylation;Parkinson's disease	Homo sapiens
alpha DNA polymerase:primase complex;cell part;centrosome;chromosomal part;cytoplasmic part;cytoskeletal part;intracellular membrane-bounded organelle;intracellular non-membrane-bounded organelle;intracellular orga	Cell cycle;Cell cycle - yeast;DNA replication;Meiosis - yeast	Homo sapiens
cell junction;cell part;cell projection;cytoplasmic part;extracellular membrane-bounded organelle;extracellular organelle;extracellular region part;extracellular vesicular exosome;intracellular organelle part;intracellular part;mem	Cysteine and methionine metabolism;Sulfur relay system	Homo sapiens
cell part;centrosome;cytoplasm;cytoplasmic part;cytoskeletal part;cytosol;extracellular membrane-bounded organelle;extracellular organelle;extracellular region part;extracellular vesicular exosome;intracellular membrane-bo	Proteasome	Homo sapiens
cell part;cytoplasm;cytoplasmic part;cytosol;extracellular membrane-bounded organelle;extracellular organelle;extracellular region part;extracellular vesicular exosome;intracellular membrane-bounded organelle;intracellular c	Proteasome	Homo sapiens
cell part;centromeric heterochromatin;chromatin;chromosomal part;heterochromatin;intracellular membrane-bounded organelle;intracellular organelle;intracellular organelle part;intracellular part;membrane-bounded organelle	Cysteine and methionine metabolism	Homo sapiens
cell part;cytoplasm;cytoplasmic part;cytosol;cytosolic large ribosomal subunit;cytosolic part;cytosolic ribosome;intracellular membrane-bounded organelle;intracellular non-membrane-bounded organelle;intracellular organell	Ribosome	Homo sapiens
cell part;cell projection;cytoplasmic part;extracellular membrane-bounded organelle;extracellular organelle;extracellular region part;extracellular space;extracellular vesicular exosome;intracellular membrane-bounded organelle;intracellular organelle;intracellular part;membrane-bounded organelle;membrane-bounded vesicle;neuron projection;nucle		Homo sapiens
actin cytoskeleton;cell part;cytoplasm;cytoplasmic part;cytoskeleton;cytosol;extracellular membrane-bounded organelle;extracellular organelle;extracellular region part;extracellular vesicular exosome;intracellular non-membr	Aminoacyl-tRNA biosynthesis	Homo sapiens
blood microparticle;cell part;cytoplasm;cytoplasmic membrane-bounded vesicle;cytoplasmic part;cytoplasmic vesicle;cytoskeleton;endoplasmic reticulum;extracellular membrane-bounded organelle;extracellular organelle;extracellular region part;extracellular space;extracellular vesicular exosome;integral to membrane;integral to plasma membrar		Homo sapiens
cell part;cytoplasmic part;extracellular membrane-bounded organelle;extracellular organelle;extracellular region part;extracellular vesicular exosome;intracellular membrane-bounded organelle;intracellular organelle;intracellula	Purine metabolism	Homo sapiens
cell part;cytoplasmic part;cytosol;cytosolic large ribosomal subunit;endoplasmic reticulum;intracellular membrane-bounded organelle;intracellular organelle;intracellular organelle part;intracellular part;large ribosomal subunit;r	Ribosome	Homo sapiens
cell part;chromosomal part;DNA replication factor A complex;germ cell nucleus;intracellular membrane-bounded organelle;intracellular organelle;intracellular organelle part;intracellular part;lateral element;macromolecular cor	DNA replication;Homologous recombination;Mismatch repair;Nucleotide excision repair	Homo sapiens
axon part;cell body;cell part;cell projection;cell projection part;cytoplasmic part;cytosol;extracellular membrane-bounded organelle;extracellular organelle;extracellular region part;extracellular vesicular exosome;intracellular m	Alanine, aspartate and glutamate metabolism;Pyrimidine metabolism	Homo sapiens
cell part;cytoplasm;cytoskeletal part;cytoskeleton;extracellular membrane-bounded organelle;extracellular organelle;extracellular region part;extracellular vesicular exosome;intracellular non-membrane-bounded organelle;intracellular organelle;intracellular organelle part;intracellular part;macromolecular complex;membrane;membrane-bounded org		Homo sapiens
axon;cell body;cell part;cell projection;cell projection cytoplasm;cell projection part;cytoplasmic membrane-bounded vesicle;cytoplasmic part;cytoplasmic vesicle;dendrite cytoplasm;dendritic spine;endoplasmic reticulum;en	Antigen processing and presentation;Phagosome;Protein processing in endoplasmic reticulum	Homo sapiens
cell part;cytoplasm;cytoplasmic part;cytosol;extracellular membrane-bounded organelle;extracellular organelle;extracellular region part;extracellular vesicular exosome;Golgi apparatus;intracellular membrane-bounded organe	Proteasome	Homo sapiens
cell part;cytoplasmic part;cytosol;integral to membrane;intracellular membrane-bounded organelle;intracellular organelle;intracellular organelle lumen;intracellular organelle part;intracellular part;intrinsic to membrane;membra	ABC transporters;Peroxisome	Homo sapiens
cell part;collagen;extracellular matrix;extracellular matrix part;extracellular region;extracellular region part;extracellular space;intracellular membrane-bounded organelle;intracellular organelle;intracellular part;membrane-bounded organelle;nucleus;organelle;proteinaceous extracellular matrix		Homo sapiens
cell part;cytoskeleton;intermediate filament cytoskeleton;intracellular non-membrane-bounded organelle;intracellular organelle;intracellular organelle part;intracellular part;non-membrane-bounded organelle;nuclear part;nucle	Basal transcription factors	Homo sapiens
axon;cell body;cell part;cell projection;cytoplasmic part;cytosol;dendrite;extracellular region;integral to membrane;integral to plasma membrane;intracellular part;intrinsic to membrane;intrinsic to plasma membrane;membran	Axon guidance	Homo sapiens
cell part;cytoplasmic part;intracellular non-membrane-bounded organelle;intracellular organelle;intracellular organelle part;intracellular part;mitochondrial nucleoid;mitochondrial part;non-membrane-bounded organelle;nuclea	Base excision repair	Homo sapiens
cell part;cytoplasm;cytoplasmic part;cytosol;endoplasmic reticulum;extracellular membrane-bounded organelle;extracellular organelle;extracellular region part;extracellular vesicular exosome;intracellular membrane-bounded organelle;intracellular organelle;intracellular organelle part;intracellular part;membrane-bounded organelle;membrane-bounc		Homo sapiens
cell part;cytoplasm;cytoplasmic part;cytosol;eukaryotic translation elongation factor 1 complex;intracellular membrane-bounded organelle;intracellular non-membrane-bounded organelle;intracellular organelle;intracellular organelle part;intracellular part;macromolecular complex;membrane-bounded organelle;non-membrane-bounded organelle;nu		Homo sapiens
cell part;cytoplasm;cytoplasmic part;cytoplasmic vesicle;cytosol;extracellular membrane-bounded organelle;extracellular organelle;extracellular region part;extracellular space;extracellular vesicular exosome;intracellular mem	Peroxisome	Homo sapiens
adherens junction;anchoring junction;cell junction;cell part;cell surface;cell-substrate adherens junction;cell-substrate junction;cytoplasmic membrane-bounded vesicle;cytoplasmic part;cytoplasmic vesicle;endoplasmic retic	Antigen processing and presentation;Protein processing in endoplasmic reticulum	Homo sapiens
cell part;cytoplasm;cytoplasmic part;cytosol;extracellular membrane-bounded organelle;extracellular organelle;extracellular region part;extracellular vesicular exosome;intracellular part;membrane;membrane-bounded organe	Alanine, aspartate and glutamate metabolism;Purine metabolism	Homo sapiens
cell part;cell surface;cytoplasmic part;endoplasmic reticulum;endoplasmic reticulum lumen;endoplasmic reticulum part;extracellular region;integral to membrane;intracellular membrane-bounded organelle;intracellular organelle;intracellular organelle lumen;intracellular organelle part;intracellular part;intrinsic to membrane;membrane;membrane part		Homo sapiens
cell part;cytoplasmic part;cytosol;intracellular membrane-bounded organelle;intracellular organelle;intracellular part;membrane-bounded organelle;mitochondrion;organelle	Alanine, aspartate and glutamate metabolism;Purine metabolism	Homo sapiens
cell part;cytoplasm;extracellular membrane-bounded organelle;extracellular organelle;extracellular region part;extracellular space;extracellular vesicular exosome;intracellular part;membrane;membrane-bounded organelle;me	Amoebiasis	Homo sapiens
cell part;integral to membrane;integral to plasma membrane;intrinsic to membrane;intrinsic to plasma membrane;membrane;membrane part;plasma membrane;plasma membrane part		Homo sapiens
cell part;cytoplasmic part;intracellular membrane-bounded organelle;intracellular organelle;intracellular organelle lumen;intracellular organelle part;intracellular part;membrane-bounded organelle;membrane-enclosed lumen;m	Arginine and proline metabolism;Ascorbate and aldarate metabolism;beta-Alanine metabolism;Chloroalkane and chloroalke	Homo sapiens
cell part;cytoplasmic part;fumarate reductase complex;intracellular membrane-bounded organelle;intracellular organelle;intracellular organelle part;intracellular part;macromolecular complex;membrane;membrane part;memb	Alzheimer's disease;Citrate cycle (TCA cycle);Huntington's disease;Oxidative phosphorylation;Parkinson's disease	Homo sapiens
cell part;cytoplasmic part;intracellular non-membrane-bounded organelle;intracellular organelle;intracellular organelle lumen;intracellular organelle part;intracellular part;macromolecular complex;membrane;membrane-enclose	Alanine, aspartate and glutamate metabolism;Arginine and proline metabolism;Nitrogen metabolism	Homo sapiens
cell junction;cell part;cell-cell junction;cytoplasm;cytoplasmic part;cytoskeletal part;cytoskeleton;cytosol;intracellular membrane-bounded organelle;intracellular non-membrane-bounded organelle;intracellular organelle;intrac	Acute myeloid leukemia;Adipocytokine signaling pathway;Apoptosis;B cell receptor signaling pathway;Carbohydrate diges	Homo sapiens
cell part;cytoplasm;cytoplasmic part;cytosol;extracellular membrane-bounded organelle;extracellular organelle;extracellular region part;extracellular vesicular exosome;intracellular part;membrane-bounded organelle;membrane-bounded vesicle;organelle;vesicle		Homo sapiens
cell part;cytoplasmic part;intracellular membrane-bounded organelle;intracellular organelle;intracellular organelle lumen;intracellular organelle part;intracellular part;membrane-bounded organelle;membrane-enclosed lumen;m	Arginine and proline metabolism	Homo sapiens
cell part;cytoplasmic part;cytosol;extracellular region;Golgi apparatus part;Golgi membrane;intracellular organelle part;intracellular part;membrane;organelle membrane;organelle part		Homo sapiens
cell part;cytoplasm;cytoplasmic part;cytosol;extracellular membrane-bounded organelle;extracellular organelle;extracellular region part;extracellular vesicular exosome;intracellular membrane-bounded organelle;intracellular c	Cysteine and methionine metabolism;Glycine, serine and threonine metabolism;Nitrogen metabolism;Selenocompound me	Homo sapiens
adherens junction;anchoring junction;cell junction;cell part;cell-substrate adherens junction;cell-substrate junction;cytoplasm;cytoplasmic part;cytosol;cytosolic large ribosomal subunit;focal adhesion;intracellular membrane-	Ribosome	Homo sapiens
cell part;cytoplasmic part;extracellular membrane-bounded organelle;extracellular organelle;extracellular region part;extracellular vesicular exosome;intracellular membrane-bounded organelle;intracellular organelle;intracellula	Pyrimidine metabolism	Homo sapiens
cell part;chromosomal part;intracellular membrane-bounded organelle;intracellular organelle;intracellular organelle part;intracellular part;macromolecular complex;MCM complex;membrane;membrane-bounded organelle;nuc	Cell cycle;Cell cycle - yeast;DNA replication;Meiosis - yeast	Homo sapiens
cell part;cytoplasm;cytoplasmic part;cytosol;extracellular membrane-bounded organelle;extracellular organelle;extracellular region part;extracellular vesicular exosome;intracellular membrane-bounded organelle;intracellular c	Cyanoamino acid metabolism;Glycine, serine and threonine metabolism;Methane metabolism;One carbon pool by folate	Homo sapiens
cell part;cytoplasmic part;cytoskeleton;extracellular membrane-bounded organelle;extracellular organelle;extracellular region part;extracellular vesicular exosome;intracellular membrane-bounded organelle;intracellular non-m	Cyanoamino acid metabolism;Glycine, serine and threonine metabolism;Methane metabolism;One carbon pool by folate	Homo sapiens
acrosomal vesicle;actin cytoskeleton;adherens junction;anchoring junction;catenin complex;cell junction;cell part;cell projection;cell-cell adherens junction;cell-cell contact zone;cell-cell junction;cell-substrate adherens juncti	Adherens junction;Arrhythmogenic right ventricular cardiomyopathy (ARVC);Bacterial invasion of epithelial cells;Endometria	Homo sapiens
cell part;cytoplasm;cytoplasmic part;cytosol;extracellular membrane-bounded organelle;extracellular organelle;extracellular region part;extracellular space;extracellular vesicular exosome;intracellular part;macromolecular cor	Amoebiasis	Homo sapiens
cell part;chromosomal part;DNA replication factor A complex;intracellular organelle part;intracellular part;macromolecular complex;nuclear chromosome part;nuclear part;nucleoplasm;organelle part;protein complex	DNA replication;Homologous recombination;Mismatch repair;Nucleotide excision repair	Homo sapiens
caveola;cell part;cell projection;cytoplasm;cytoplasmic part;endoplasmic reticulum membrane;endoplasmic reticulum part;intracellular membrane-bounded organelle;intracellular organelle;intracellular organelle part;intracellul	Arachidonic acid metabolism;Leishmaniasis;Pathways in cancer;Small cell lung cancer;VEGF signaling pathway	Homo sapiens
cell part;cytoplasmic part;cytosol;intracellular membrane-bounded organelle;intracellular non-membrane-bounded organelle;intracellular organelle;intracellular organelle part;intracellular part;membrane-bounded organelle;no	Cysteine and methionine metabolism;Glycine, serine and threonine metabolism	Homo sapiens
actin cytoskeleton;actin filament bundle;actomyosin;axon;cell body;cell cortex;cell division site part;cell part;cell projection;cell projection part;cleavage furrow;cytoplasm;cytoplasmic part;cytoskeletal part;cytoskeleton;cytos	Tight junction;Viral myocarditis	Homo sapiens
acrosomal membrane;adherens junction;anchoring junction;cell junction;cell part;cell-substrate adherens junction;cell-substrate junction;cytoplasmic membrane-bounded vesicle;cytoplasmic part;cytoplasmic vesicle;cytoplasmic vesicle membrane;cytoplasmic vesicle part;extracellular membrane-bounded organelle;extracellular organelle;extracell		Homo sapiens
cell part;cytoplasmic part;cytosol;extracellular membrane-bounded organelle;extracellular organelle;extracellular region part;extracellular vesicular exosome;intracellular membrane-bounded organelle;intracellular organelle;intracellular part;membrane-bounded organelle;membrane-bounded vesicle;mitochondrion;nucleus;organelle;vesicle		Homo sapiens
adherens junction;anchoring junction;cell cortex;cell junction;cell part;cell-cell junction;cell-substrate adherens junction;cell-substrate junction;cytoplasm;cytoplasmic part;cytoskeletal part;cytosol;early endosome;endoplasm	Acute myeloid leukemia;B cell receptor signaling pathway;Bladder cancer;Chronic myeloid leukemia;Endometrial cancer;Er	Homo sapiens
apical plasma membrane;cell part;cell projection;cytoplasmic part;cytosol;endosome;extracellular membrane-bounded organelle;extracellular organelle;extracellular region part;extracellular vesicular exosome;intracellular me	Collecting duct acid secretion;Epithelial cell signaling in Helicobacter pylori infection;Oxidative phosphorylation;Phagosom	Homo sapiens
cell part;cytoplasm;cytoplasmic part;intracellular membrane-bounded organelle;intracellular organelle;intracellular organelle part;intracellular part;membrane;membrane-bounded organelle;membrane-enclosed lumen;mitoch	Porphyrin and chlorophyll metabolism	Homo sapiens
adherens junction;anchoring junction;cell junction;cell part;cell-substrate adherens junction;cell-substrate junction;cytoplasm;cytoplasmic part;cytosol;cytosolic large ribosomal subunit;extracellular membrane-bounded orga	Ribosome	Homo sapiens
actin cytoskeleton;cell part;cytoplasm;cytoplasmic part;cytoskeleton;cytosol;extracellular membrane-bounded organelle;extracellular organelle;extracellular region part;extracellular vesicular exosome;intracellular non-membr	Amino sugar and nucleotide sugar metabolism;Galactose metabolism;Glycolysis / Gluconeogenesis;Pentose phosphate pa	Homo sapiens
cell part;cytoplasmic part;cytosol;extracellular membrane-bounded organelle;extracellular organelle;extracellular region part;extracellular vesicular exosome;intracellular membrane-bounded organelle;intracellular organelle;int	Glutathione metabolism	Homo sapiens
cell part;cytoplasm;cytoplasmic part;cytosol;extracellular membrane-bounded organelle;extracellular organelle;extracellular region part;extracellular vesicular exosome;intercellular bridge;intracellular membrane-bounded orga	Protein export	Homo sapiens
actin cytoskeleton;axon;axon part;cell cortex;cell junction;cell part;cell projection;cell projection part;clathrin-coated vesicle;coated vesicle;cytoplasm;cytoplasmic membrane-bounded vesicle;cytoplasmic part;cytoplasmic ve	Alzheimer's disease;Parkinson's disease	Homo sapiens
adherens junction;anchoring junction;cell junction;cell part;cell-substrate adherens junction;cell-substrate junction;cytoplasm;cytoplasmic part;cytosol;cytosolic large ribosomal subunit;extracellular membrane-bounded orga	Ribosome	Homo sapiens
cell part;membrane;plasma membrane		Homo sapiens
adherens junction;anchoring junction;cell junction;cell part;cell-substrate adherens junction;cell-substrate junction;cytoplasm;cytoplasmic part;cytosol;cytosolic large ribosomal subunit;focal adhesion;intracellular membrane-	Ribosome	Homo sapiens
cell part;cytoplasm;cytoplasmic part;cytosol;extracellular membrane-bounded organelle;extracellular organelle;extracellular region part;extracellular vesicular exosome;intracellular part;membrane-bounded organelle;membra	RNA transport	Homo sapiens
cell part;intracellular membrane-bounded organelle;intracellular organelle;intracellular organelle part;intracellular part;macromolecular complex;membrane-bounded organelle;nuclear part;nucleus;organelle;organelle part;ribor	Spliceosome	Homo sapiens
cell part;intracellular membrane-bounded organelle;intracellular organelle;intracellular organelle part;intracellular part;membrane-bounded organelle;nuclear part;nucleoplasm;nucleus;organelle;organelle part		Homo sapiens
axon;cell part;cell projection;cytoplasm;cytoplasmic membrane-bounded vesicle;cytoplasmic part;cytoplasmic vesicle;cytosol;extracellular membrane-bounded organelle;extracellular organelle;extracellular region part;extrac	Aminoacyl-tRNA biosynthesis	Homo sapiens
cell part;cytoplasm;cytoplasmic part;cytosol;extracellular membrane-bounded organelle;extracellular organelle;extracellular region part;extracellular vesicular exosome;intracellular organelle part;intracellular part;membrane;m	Aminoacyl-tRNA biosynthesis;Valine, leucine and isoleucine biosynthesis	Homo sapiens
apical plasma membrane;cell junction;cell leading edge;cell part;cell-cell junction;cytoplasm;cytoplasmic part;cytoskeleton;cytosol;endosome;extracellular membrane-bounded organelle;extracellular organelle;extracellular re	Endocytosis;Insulin signaling pathway;Tight junction	Homo sapiens
cell part;cytoplasmic part;extracellular membrane-bounded organelle;extracellular organelle;extracellular region part;extracellular vesicular exosome;intracellular membrane-bounded organelle;intracellular organelle;intracellula	Fatty acid metabolism	Homo sapiens
cell part;cytoplasm;envelope;integral to membrane;intracellular membrane-bounded organelle;intracellular organelle;intracellular organelle part;intracellular part;intrinsic to membrane;membrane;membrane part;membrane-bounded organelle;nuclear envelope;nuclear inner membrane;nuclear membrane;nuclear part;nucleus;organelle;organelle enve		Homo sapiens
axon;cell part;cell projection;chromatin;chromosomal part;cytoplasm;cytoplasmic part;cytosol;dendrite;intracellular membrane-bounded organelle;intracellular non-membrane-bounded organelle;intracellular organelle;intracel	Chemokine signaling pathway;Hepatitis C;Jak-STAT signaling pathway;ko05152;Leishmaniasis;Measles;Osteoclast differer	Homo sapiens
cell part;cytoplasm;cytoplasmic part;cytosol;extracellular membrane-bounded organelle;extracellular organelle;extracellular region part;extracellular vesicular exosome;intracellular;intracellular membrane-bounded organelle;ii	Arachidonic acid metabolism;Metabolism of xenobiotics by cytochrome P450;Steroid hormone biosynthesis	Homo sapiens
cell body;cell part;cell projection;cytoplasm;cytoplasmic part;cytosol;cytosolic part;dendrite;endomembrane system;endoplasmic reticulum membrane;endoplasmic reticulum part;Golgi apparatus part;Golgi membrane;intrac	Acute myeloid leukemia;Adipocytokine signaling pathway;ErbB signaling pathway;Glioma;Insulin signaling pathway;mTOR	Homo sapiens
cell part;cytoplasmic part;cytosol;cytosolic small ribosomal subunit;intracellular membrane-bounded organelle;intracellular non-membrane-bounded organelle;intracellular organelle;intracellular organelle part;intracellular part	Ribosome	Homo sapiens
cell part;cytoplasm;cytoplasmic part;cytosol;cytosolic large ribosomal subunit;intracellular non-membrane-bounded organelle;intracellular organelle;intracellular organelle part;intracellular part;large ribosomal subunit;macrom	Ribosome	Homo sapiens
cell part;chromatin;chromosomal part;cytoplasm;cytoplasmic part;cytosol;heterochromatin;intracellular membrane-bounded organelle;intracellular organelle;intracellular organelle part;intracellular part;membrane-bounded org	Bladder cancer;Cell cycle;Chronic myeloid leukemia;Glioma;Melanoma;Non-small cell lung cancer;p53 signaling pathway;F	Homo sapiens
cell part;clathrin-coated vesicle;coated vesicle;cytoplasmic membrane-bounded vesicle;cytoplasmic part;cytoplasmic vesicle;early endosome;endosomal part;endosome;external side of plasma membrane;extracellular membrane-bounded organelle;extracellular organelle;extracellular region part;extracellular vesicular exosome;integral to membra		Homo sapiens
adherens junction;anchoring junction;cell junction;cell part;cell-substrate adherens junction;cell-substrate junction;external side of plasma membrane;extracellular region part;extracellular space;focal adhesion;integral to membrane;intracellular membrane-bounded organelle;intracellular organelle;intracellular part;intrinsic to membrane;membrane;r		Homo sapiens

cell part;intracellular organelle part;intracellular part;membrane;nuclear inner membrane;nuclear matrix;nuclear membrane;nuclear part;nucleoplasm;organelle inner membrane;organelle membrane;organelle part		Homo sapiens
cell part;cytoplasmic part;cytosol;extracellular membrane-bounded organelle;extracellular organelle;extracellular region part;extracellular space;extracellular vesicular exosome;intracellular membrane-bounded organelle;intra	Nicotinate and nicotinamide metabolism	Homo sapiens
cell part;cytoplasmic part;intracellular membrane-bounded organelle;intracellular organelle;intracellular part;lysosome;lytic vacuole;membrane-bounded organelle;organelle;vacuole		Homo sapiens
cell part;chromosomal part;chromosome;chromosome, centromeric region;condensed chromosome;cytoplasm;intracellular membrane-bounded organelle;intracellular non-membrane-bounded organelle;intracellular organelle;intracellular organelle part;intracellular part;membrane;membrane-bounded organelle;non-membrane-bounded organelle;n		Homo sapiens
cell part;chromosomal part;condensed chromosome kinetochore;cytoplasm;cytoplasmic part;cytoskeletal part;cytosol;intracellular non-membrane-bounded organelle;intracellular organelle;intracellular organelle part;intracellu	RNA transport	Homo sapiens
cell part;intracellular membrane-bounded organelle;intracellular organelle;intracellular organelle part;intracellular part;membrane;membrane-bounded organelle;nuclear part;nucleoplasm;nucleus;organelle;organelle part		Homo sapiens
cell part;chromatin;chromatin remodeling complex;chromosomal part;heterochromatin;intracellular membrane-bounded organelle;intracellular organelle;intracellular organelle part;intracellular part;macromolecular complex;membrane-bounded organelle;nuclear body;nuclear chromatin;nuclear chromosome part;nuclear heterochromatin;nuclear par		Homo sapiens
cell part;cytoplasmic part;cytosol;cytosolic large ribosomal subunit;intracellular organelle part;intracellular part;large ribosomal subunit;macromolecular complex;membrane;organelle part;ribonucleoprotein complex	Ribosome	Homo sapiens
adherens junction;anchoring junction;cell junction;cell part;cell-substrate adherens junction;cell-substrate junction;cytoplasm;cytoplasmic part;cytosol;cytosolic large ribosomal subunit;extracellular membrane-bounded orga	Ribosome	Homo sapiens
cell part;cytoplasm;cytoplasmic part;cytosol;cytosolic large ribosomal subunit;intracellular non-membrane-bounded organelle;intracellular organelle;intracellular organelle part;intracellular part;large ribosomal subunit;macrom	Ribosome	Homo sapiens
cell body;cell part;cell projection;cytoplasm;cytoplasmic part;cytosol;cytosolic large ribosomal subunit;dendrite;extracellular membrane-bounded organelle;extracellular organelle;extracellular region part;extracellular vesicula	Ribosome	Homo sapiens
adherens junction;anchoring junction;cell junction;cell part;cell-substrate adherens junction;cell-substrate junction;cytoplasm;cytoplasmic part;cytosol;cytosolic small ribosomal subunit;extracellular membrane-bounded orga	Ribosome	Homo sapiens
adherens junction;anchoring junction;cell junction;cell part;cell-substrate adherens junction;cell-substrate junction;cytoplasmic part;cytosol;cytosolic small ribosomal subunit;extracellular membrane-bounded organelle;extrac	Ribosome	Homo sapiens
adherens junction;anchoring junction;cell junction;cell part;cell-substrate adherens junction;cell-substrate junction;cytoplasm;cytoplasmic part;cytosol;cytosolic small ribosomal subunit;extracellular membrane-bounded orga	Ribosome	Homo sapiens
cell junction;cell part;cell projection;cell projection part;cytoplasm;cytoplasmic part;cytoskeletal part;cytosol;dendritic spine;intracellular organelle part;intracellular part;macromolecular complex;membrane;microtubule;microtubule associated complex;neuron projection;neuron spine;organelle part;plasma membrane;postsynaptic density;protein co		Homo sapiens
		Homo sapiens
cell part;cytoplasm;cytoplasmic membrane-bounded vesicle;cytoplasmic part;cytoplasmic vesicle;cytosol;endoplasmic reticulum membrane;endoplasmic reticulum part;Golgi apparatus;intracellular membrane-bounded orga	alpha-Linolenic acid metabolism;Arachidonic acid metabolism;Ether lipid metabolism;Fat digestion and absorption;Fc epsi	Homo sapiens
cell part;cytoplasm;cytoplasmic part;cytosol;intracellular organelle lumen;intracellular organelle part;intracellular part;membrane-enclosed lumen;mitochondrial matrix;mitochondrial part;organelle lumen;organelle part	Aminoacyl-tRNA biosynthesis	Homo sapiens
cell part;cytoplasmic part;extracellular membrane-bounded organelle;extracellular organelle;extracellular region part;extracellular vesicular exosome;intracellular membrane-bounded organelle;intracellular organelle;intracellul	Alzheimer's disease;Huntington's disease;Oxidative phosphorylation;Parkinson's disease	Homo sapiens
adherens junction;anchoring junction;cell junction;cell part;cell-cell junction;cell-substrate adherens junction;cell-substrate junction;cytoplasmic part;cytosol;focal adhesion;intracellular part;membrane;perinuclear region of cytoplasm;plasma membrane		Homo sapiens
cell part;cytoplasmic part;cytosol;intracellular membrane-bounded organelle;intracellular organelle;intracellular part;membrane-bounded organelle;mitochondrion;organelle	Carbon fixation in photosynthetic organisms;Pyruvate metabolism	Homo sapiens
cell part;cytoplasmic part;endoplasmic reticulum membrane;endoplasmic reticulum part;intracellular organelle part;intracellular part;lipid particle;membrane;membrane part;organelle membrane;organelle part	Steroid biosynthesis	Homo sapiens
cell part;cytoplasmic part;cytosol;glutamate-cysteine ligase complex;intracellular part;macromolecular complex;protein complex	Glutathione metabolism	Homo sapiens
adherens junction;anchoring junction;basement membrane;cell junction;cell part;cell-substrate adherens junction;cell-substrate junction;cytoplasmic part;cytosol;extracellular matrix part;extracellular region part;focal adhesion;integral to membrane;integral to plasma membrane;intracellular part;intrinsic to membrane;intrinsic to plasma membrane;		Homo sapiens
cell part;cytoplasmic part;endoplasmic reticulum membrane;endoplasmic reticulum part;extracellular membrane-bounded organelle;extracellular organelle;extracellular region part;extracellular vesicular exosome;Golgi apparatus part;Golgi membrane;intracellular organelle part;intracellular part;membrane;membrane part;membrane-bounded organ		Homo sapiens
cell part;intracellular membrane-bounded organelle;intracellular organelle;intracellular organelle part;intracellular part;membrane-bounded organelle;nuclear part;nucleoplasm;nucleus;organelle;organelle part	Base excision repair;DNA replication;Homologous recombination;Mismatch repair;Nucleotide excision repair;Purine metab	Homo sapiens
actin filament bundle;actomyosin;adherens junction;anchoring junction;cell cortex;cell junction;cell part;cell projection;cell-substrate adherens junction;cell-substrate junction;cytoplasm;cytoplasmic part;cytoskeletal part;cyto	Bacterial invasion of epithelial cells;Chemokine signaling pathway;Focal adhesion;Leukocyte transendothelial migration;Re	Homo sapiens
cell part;centrosome;cytoplasm;cytoplasmic part;cytoskeletal part;cytosol;extracellular membrane-bounded organelle;extracellular organelle;extracellular region part;extracellular vesicular exosome;intracellular membrane-bo	MAPK signaling pathway;Neurotrophin signaling pathway;VEGF signaling pathway	Homo sapiens
cell part;cytoplasm;cytoplasmic part;cytosol;cytosolic large ribosomal subunit;extracellular membrane-bounded organelle;extracellular organelle;extracellular region part;extracellular vesicular exosome;intracellular non-memb	Ribosome	Homo sapiens
cell part;coated vesicle membrane;contractile fiber part;cytoplasmic part;cytoplasmic vesicle membrane;cytoplasmic vesicle part;endoplasmic reticulum membrane;endoplasmic reticulum part;endoplasmic reticulum-Golgi in	Protein processing in endoplasmic reticulum	Homo sapiens
cell part;cytoplasm;cytoplasmic membrane-bounded vesicle;cytoplasmic part;cytoplasmic vesicle;cytosol;extracellular membrane-bounded organelle;extracellular organelle;extracellular region part;extracellular vesicular exos	Fatty acid biosynthesis;Insulin signaling pathway	Homo sapiens
cell part;cytoplasmic part;cytosol;intracellular part		Homo sapiens
cell body;cell part;chaperonin-containing T-complex;cytoplasm;cytoplasmic part;cytoskeletal part;cytoskeleton;cytosol;cytosolic part;extracellular membrane-bounded organelle;extracellular organelle;extracellular region part;extracellular vesicular exosome;intracellular non-membrane-bounded organelle;intracellular organelle;intracellular organelle		Homo sapiens
cell part;cytoplasmic part;intracellular membrane-bounded organelle;intracellular non-membrane-bounded organelle;intracellular organelle;intracellular organelle part;intracellular part;macromolecular complex;membrane;membrane-bounded organelle;mitochondrial inner membrane;mitochondrial membrane;mitochondrial part;non-membrane-bour		Homo sapiens
cell part;cytoplasmic part;extracellular membrane-bounded organelle;extracellular organelle;extracellular region part;extracellular vesicular exosome;intracellular membrane-bounded organelle;intracellular non-membrane-bou	Plant-pathogen interaction	Homo sapiens
cell part;cytoplasm;cytoplasmic part;cytosol;extracellular membrane-bounded organelle;extracellular organelle;extracellular region part;extracellular vesicular exosome;intracellular membrane-bounded organelle;intracellular o	Arginine and proline metabolism;Ascorbate and aldarate metabolism;beta-Alanine metabolism;Fatty acid metabolism;Glyc	Homo sapiens
cell part;cytoplasmic part;cytosol;endoplasmic reticulum part;extracellular membrane-bounded organelle;extracellular organelle;extracellular region part;extracellular vesicular exosome;intracellular organelle part;intracellular p	Protein export	Homo sapiens
cell part;cytoplasm;cytoplasmic part;cytosol;extracellular membrane-bounded organelle;extracellular organelle;extracellular region part;extracellular vesicular exosome;intracellular part;membrane;membrane-bounded organe	Aminoacyl-tRNA biosynthesis	Homo sapiens
cell part;cytoplasm;cytoplasmic part;cytosol;intracellular part	Aminoacyl-tRNA biosynthesis	Homo sapiens
cell part;cytoplasmic part;intracellular membrane-bounded organelle;intracellular organelle;intracellular organelle lumen;intracellular organelle part;intracellular part;membrane-bounded organelle;membrane-enclosed lumen;m	Aminoacyl-tRNA biosynthesis	Homo sapiens
cell part;cytoplasm;cytoplasmic part;cytosol;extracellular membrane-bounded organelle;extracellular organelle;extracellular region part;extracellular vesicular exosome;intracellular membrane-bounded organelle;intracellular o	Aminoacyl-tRNA biosynthesis	Homo sapiens
cell part;cytoplasmic part;extracellular membrane-bounded organelle;extracellular organelle;extracellular region part;extracellular vesicular exosome;intracellular membrane-bounded organelle;intracellular organelle;intracellul	Biosynthesis of unsaturated fatty acids	Homo sapiens
beta-catenin destruction complex;cell part;cytoplasmic part;cytosol;intracellular part;macromolecular complex;protein complex	Chemokine signaling pathway	Homo sapiens
beta-catenin destruction complex;cell body;cell part;cell projection;cell projection part;centrosome;cytoplasm;cytoplasmic part;cytoskeletal part;cytosol;dendritic shaft;dendritic spine;growth cone;intracellular membrane-bou	Alzheimer's disease;Axon guidance;B cell receptor signaling pathway;Basal cell carcinoma;Cell cycle;Chemokine signaling	Homo sapiens
cell part;cytoplasmic part;intracellular membrane-bounded organelle;intracellular organelle;intracellular organelle lumen;intracellular organelle part;intracellular part;membrane-bounded organelle;membrane-enclosed lumen;m	Citrate cycle (TCA cycle)	Homo sapiens
cell part;cytoplasmic part;cytoskeletal part;endoplasmic reticulum;envelope;integral to membrane;intracellular membrane-bounded organelle;intracellular organelle;intracellular organelle part;intracellular part;intrinsic to memb	Arrhythmogenic right ventricular cardiomyopathy (ARVC);Dilated cardiomyopathy;Hypertrophic cardiomyopathy (HCM)	Homo sapiens
cell part;cytoplasmic part;integral to membrane;integral to mitochondrial membrane;integral to mitochondrial outer membrane;integral to organelle membrane;intracellular membrane-bounded organelle;intracellular organelle;	Adipocytokine signaling pathway;Fatty acid metabolism;PPAR signaling pathway	Homo sapiens
cell part;cytoplasmic part;cytosol;extracellular membrane-bounded organelle;extracellular organelle;extracellular region part;extracellular vesicular exosome;intracellular part;macromolecular complex;membrane-bounded organelle;membrane-bounded vesicle;organelle;protein complex;vesicle		Homo sapiens
cell part;external side of plasma membrane;extracellular membrane-bounded organelle;extracellular organelle;extracellular region part;extracellular vesicular exosome;integral to membrane;integral to plasma membrane;intrinsic to membrane;intrinsic to plasma membrane;membrane;membrane part;membrane-bounded organelle;membrane-bound		Homo sapiens
cell part;cytoplasm;cytoplasmic part;cytosol;cytosolic large ribosomal subunit;extracellular membrane-bounded organelle;extracellular organelle;extracellular region part;extracellular vesicular exosome;intracellular organelle p	Ribosome	Homo sapiens
azurophil granule;cell part;cytoplasm;cytoplasmic membrane-bounded vesicle;cytoplasmic part;cytoplasmic vesicle;cytoskeletal part;endocytic vesicle;envelope;extracellular membrane-bounded organelle;extracellular organelle;extracellular region part;extracellular vesicular exosome;intracellular membrane-bounded organelle;intracellular non-me		Homo sapiens
apical plasma membrane;cell part;cell projection;cilium;cytoplasmic membrane-bounded vesicle;cytoplasmic part;cytoplasmic vesicle;cytoplasmic vesicle membrane;cytoplasmic vesicle part;dendrite;endosomal part;endosome;endosome membrane;exocytic vesicle;extracellular membrane-bounded organelle;extracellular organelle;extracellular re		Homo sapiens
		Homo sapiens
cell part;cytoplasmic part;intracellular membrane-bounded organelle;intracellular non-membrane-bounded organelle;intracellular organelle;intracellular organelle lumen;intracellular organelle part;intracellular part;membrane-b	Citrate cycle (TCA cycle)	Homo sapiens
cell part;clathrin-coated vesicle;coated vesicle;cytoplasmic membrane-bounded vesicle;cytoplasmic part;cytoplasmic vesicle;cytosol;endoplasmic reticulum;endoplasmic reticulum membrane;endoplasmic reticulum part;end	Protein processing in endoplasmic reticulum	Homo sapiens
cell part;cytoplasmic part;intracellular membrane-bounded organelle;intracellular organelle;intracellular organelle lumen;intracellular organelle part;intracellular part;membrane;membrane-bounded organelle;membrane-enclos	Peroxisome;Primary bile acid biosynthesis	Homo sapiens
cell part;cytoplasm;cytoplasmic part;cytosol;intracellular membrane-bounded organelle;intracellular organelle;intracellular organelle part;intracellular part;membrane-bounded organelle;nuclear part;nucleoplasm;nucleus;orga	Long-term potentiation;MAPK signaling pathway;mTOR signaling pathway;Neurotrophin signaling pathway;Oocyte meiosis	Homo sapiens
cell part;cytoplasm;extracellular region part;extracellular space;intracellular organelle part;intracellular part;macromolecular complex;nuclear part;nucleoplasm;nucleoplasm part;organelle part;protein complex;transcriptional repressor complex		Homo sapiens
cell part;cytoplasm;cytoplasmic part;cytosol;intracellular organelle part;intracellular part;membrane;nuclear part;nucleoplasm;organelle part		Homo sapiens
cell part;cell projection;cytoplasm;cytoplasmic part;cytosol;dendrite;intracellular membrane-bounded organelle;intracellular organelle;intracellular organelle part;intracellular part;macromolecular complex;membrane part;membrane-bounded organelle;neuron projection;nuclear part;nuclear pore;nucleoplasm;nucleus;organelle;organelle part;pore co		Homo sapiens
cell part;cytoplasm;cytoplasmic part;cytosol;intracellular membrane-bounded organelle;intracellular organelle;intracellular organelle part;intracellular part;macromolecular complex;membrane-bounded organelle;mRNA cap bi	mRNA surveillance pathway;RNA transport;Spliceosome	Homo sapiens
cell part;cytoplasmic part;cytosol;endoplasmic reticulum;extracellular membrane-bounded organelle;extracellular organelle;extracellular region part;extracellular vesicular exosome;intracellular membrane-bounded organelle;intracellular organelle;intracellular part;membrane-bounded organelle;membrane-bounded vesicle;mitochondrion;organelle;v		Homo sapiens
cell part;cytoplasmic part;cytosol;DNA-directed RNA polymerase complex;DNA-directed RNA polymerase I complex;DNA-directed RNA polymerase II, core complex;DNA-directed RNA polymerase III complex;intracellular m	Cytosolic DNA-sensing pathway;Huntington's disease;Purine metabolism;Pyrimidine metabolism;RNA polymerase	Homo sapiens
cell part;cytoplasmic part;cytosol;intracellular part;membrane;plasma membrane	B cell receptor signaling pathway;Chemokine signaling pathway;Fc epsilon RI signaling pathway;Fc gamma R-mediated ph	Homo sapiens
cell part;cytoplasmic part;cytosol;extracellular membrane-bounded organelle;extracellular organelle;extracellular region part;extracellular vesicular exosome;intracellular part;membrane-bounded organelle;membrane-bounde	Arginine and proline metabolism;beta-Alanine metabolism;Cysteine and methionine metabolism;Glutathione metabolism	Homo sapiens
cell part;cytoplasmic part;cytosol;intracellular organelle part;intracellular part;membrane;mitochondrial membrane;mitochondrial outer membrane;mitochondrial part;organelle membrane;organelle outer membrane;organelle p	Amino sugar and nucleotide sugar metabolism;Butirosin and neomycin biosynthesis;Carbohydrate digestion and absorptio	Homo sapiens
cell part;citrate lyase complex;cytoplasm;cytoplasmic part;cytosol;extracellular membrane-bounded organelle;extracellular organelle;extracellular region part;extracellular vesicular exosome;intracellular membrane-bounded o	Carbon fixation pathways in prokaryotes;Citrate cycle (TCA cycle)	Homo sapiens
cell part;cytoplasmic part;endoplasmic reticulum;extracellular membrane-bounded organelle;extracellular organelle;extracellular region part;extracellular space;extracellular vesicular exosome;Golgi apparatus;intracellular me	Lysosome	Homo sapiens
cell part;cytoplasmic part;intracellular membrane-bounded organelle;intracellular organelle;intracellular organelle part;intracellular part;membrane;membrane-bounded organelle;mitochondrial inner membrane;mitochondrial m	Porphyrin and chlorophyll metabolism	Homo sapiens
cell part;centrosome;cytoplasmic part;cytoskeletal part;extracellular membrane-bounded organelle;extracellular organelle;extracellular region part;extracellular vesicular exosome;integral to membrane;integral to plasma membrane;intracellular membrane-bounded organelle;intracellular non-membrane-bounded organelle;intracellular organelle;intra		Homo sapiens
cell part;extracellular membrane-bounded organelle;extracellular organelle;extracellular region part;extracellular vesicular exosome;intracellular membrane-bounded organelle;intracellular non-membrane-bounded organelle;intracellular organelle;intracellular organelle part;intracellular part;macromolecular complex;membrane-bounded organelle;me		Homo sapiens
cell part;cytoplasm;cytoplasmic part;cytosol;extracellular region part;extracellular space;intracellular membrane-bounded organelle;intracellular organelle;intracellular part;membrane-bounded organelle;nucleus;organelle	Aminoacyl-tRNA biosynthesis	Homo sapiens
caveola;cell part;cytoplasmic membrane-bounded vesicle;cytoplasmic part;cytoplasmic vesicle;extracellular membrane-bounded organelle;extracellular organelle;extracellular region part;extracellular vesicular exosome;intrac	Aldosterone-regulated sodium reabsorption;Bile secretion;Carbohydrate digestion and absorption;Cardiac muscle contract	Homo sapiens
cell part;cytoplasmic part;cytosol;extracellular membrane-bounded organelle;extracellular organelle;extracellular region part;extracellular vesicular exosome;intracellular organelle part;intracellular part;membrane;membrane-b	Purine metabolism	Homo sapiens
cell part;cytoplasm;cytoplasmic part;intracellular membrane-bounded organelle;intracellular organelle;intracellular organelle part;intracellular part;membrane;membrane-bounded organelle;mitochondrial inner membrane;mito	Arginine and proline metabolism	Homo sapiens
cell part;cytoplasmic part;cytosol;extracellular membrane-bounded organelle;extracellular organelle;extracellular region part;extracellular vesicular exosome;intracellular organelle part;intracellular part;macromolecular complex;membrane;membrane part;membrane-bounded organelle;membrane-bounded vesicle;organelle;organelle membrane;org		Homo sapiens
basal lamina;basement membrane;extracellular matrix part;extracellular membrane-bounded organelle;extracellular organelle;extracellular region;extracellular region part;extracellular vesicular exosome;laminin complex;lamir	Amoebiasis;ECM-receptor interaction;Focal adhesion;Pathways in cancer;Small cell lung cancer;Toxoplasmosis	Homo sapiens
cell part;cytoplasm;cytoplasmic part;endoplasmic reticulum;intracellular membrane-bounded organelle;intracellular organelle;intracellular part;membrane-bounded organelle;organelle;perinuclear region of cytoplasm		Homo sapiens
cell part;cytoplasm;cytoplasmic part;cytosol;extracellular membrane-bounded organelle;extracellular organelle;extracellular region part;extracellular vesicular exosome;intracellular membrane-bounded organelle;intracellular o	Aminoacyl-tRNA biosynthesis;Selenocompound metabolism	Homo sapiens
acrosomal vesicle;adherens junction;alpha1-beta1 integrin complex;anchoring junction;basal part of cell;cell junction;cell part;cell projection;cell surface;cell-substrate adherens junction;cell-substrate junction;cytoplasmic me	Arrhythmogenic right ventricular cardiomyopathy (ARVC);Dilated cardiomyopathy;ECM-receptor interaction;Focal adhesion	Homo sapiens
cell part;cytoplasmic membrane-bounded vesicle;cytoplasmic part;cytoplasmic vesicle;endoplasmic reticulum;extracellular membrane-bounded organelle;extracellular organelle;extracellular region part;extracellular vesicular exosome;intracellular membrane-bounded organelle;intracellular organelle;intracellular organelle part;intracellular part;macr		Homo sapiens
cell part;cytoplasm;cytoskeleton;intracellular non-membrane-bounded organelle;intracellular organelle;intracellular part;non-membrane-bounded organelle;organelle		Homo sapiens
cell part;intracellular membrane-bounded organelle;intracellular organelle;intracellular part;membrane-bounded organelle;nucleus;organelle		Homo sapiens
cell part;cytoplasmic part;cytosol;extracellular membrane-bounded organelle;extracellular organelle;extracellular region part;extracellular space;extracellular vesicular exosome;intracellular membrane-bounded organelle;intra	Carbon fixation in photosynthetic organisms;Fructose and mannose metabolism;Glycolysis / Gluconeogenesis;Inositol pho	Homo sapiens
cell part;cytoplasm;cytoplasmic part;cytosol;eukaryotic 43S preinitiation complex;eukaryotic 48S preinitiation complex;eukaryotic translation initiation factor 3 complex;extracellular membrane-bounded organelle;extracellular	Hepatitis C;RNA transport	Homo sapiens
cell part;cytoplasm;cytoplasmic part;cytosol;eukaryotic translation initiation factor 4F complex;extracellular membrane-bounded organelle;extracellular organelle;extracellular region part;extracellular vesicular exosome;intrace	RNA transport	Homo sapiens
cell part;cytoplasm;cytoplasmic part;cytosol;cytosolic small ribosomal subunit;extracellular membrane-bounded organelle;extracellular organelle;extracellular region part;extracellular vesicular exosome;intracellular organelle	Ribosome	Homo sapiens
cell part;contractile fiber;contractile fiber part;cytoplasm;cytoplasmic mRNA processing body;cytoplasmic part;cytosol;extracellular membrane-bounded organelle;extracellular organelle;extracellular region part;extracellular v	Proteasome	Homo sapiens
adherens junction;anchoring junction;apical part of cell;cell body;cell junction;cell part;cell projection;cell-cell junction;cell-substrate adherens junction;cell-substrate junction;cytoplasm;cytoplasmic membrane-bounded vesic	Adherens junction;Axon guidance;Bacterial invasion of epithelial cells;Chemokine signaling pathway;Endocytosis;Epithelial	Homo sapiens
actin cytoskeleton;cell cortex part;cell part;cortical actin cytoskeleton;cortical cytoskeleton;cytoplasmic part;cytoskeletal part;cytoskeleton;extracellular membrane-bounded organelle;extracellular organelle;extracellular region part;extracellular vesicular exosome;intracellular non-membrane-bounded organelle;intracellular organelle;intracellular org		Homo sapiens
cell junction;cell leading edge;cell part;cell projection part;cell surface;cell-cell junction;cytoplasm;cytoplasmic membrane-bounded vesicle;cytoplasmic part;cytoplasmic vesicle;early endosome;endosome;external side of pla	Axon guidance;Chemokine signaling pathway;Cytokine-cytokine receptor interaction;Endocytosis;Intestinal immune netwo	Homo sapiens
cell part;cytoplasm;cytoplasmic part;cytosol;extracellular membrane-bounded organelle;extracellular organelle;extracellular region part;extracellular vesicular exosome;intracellular part;membrane-bounded organelle;membra	Ubiquitin mediated proteolysis	Homo sapiens
adherens junction;anchoring junction;cell junction;cell part;cell-substrate adherens junction;cell-substrate junction;cytoplasm;cytoplasmic part;cytosol;cytosolic small ribosomal subunit;extracellular membrane-bounded orga	Ribosome	Homo sapiens
cell part;cytoplasmic part;cytosol;cytosolic large ribosomal subunit;extracellular membrane-bounded organelle;extracellular organelle;extracellular region part;extracellular vesicular exosome;intracellular organelle part;intrace	Ribosome	Homo sapiens
cell part;cytoplasmic part;cytosol;cytosolic large ribosomal subunit;extracellular membrane-bounded organelle;extracellular organelle;extracellular region part;extracellular vesicular exosome;intracellular membrane-bounded	Ribosome	Homo sapiens
adherens junction;anchoring junction;cell junction;cell part;cell-substrate adherens junction;cell-substrate junction;cytoplasmic part;cytosol;cytosolic large ribosomal subunit;extracellular membrane-bounded organelle;extrac	Ribosome	Homo sapiens
cell part;cytoplasmic part;cytosol;cytosolic large ribosomal subunit;intracellular organelle part;intracellular part;large ribosomal subunit;macromolecular complex;organelle part;ribonucleoprotein complex	Ribosome	Homo sapiens
adherens junction;anchoring junction;catalytic step 2 spliceosome;cell junction;cell part;cell projection;cell-substrate adherens junction;cell-substrate junction;chromatin;chromosomal part;cytoplasm;cytoskeletal part;extrace	Spliceosome	Homo sapiens
adherens junction;anchoring junction;cell junction;cell part;cell-substrate adherens junction;cell-substrate junction;cytoplasmic part;cytoplasmic vesicle membrane;cytoplasmic vesicle part;cytosol;extracellular membrane-bo	Cell cycle;Neurotrophin signaling pathway;Oocyte meiosis	Homo sapiens
adherens junction;anchoring junction;cell junction;cell part;cell-substrate adherens junction;cell-substrate junction;cytoplasmic part;endoplasmic reticulum;extracellular membrane-bounded organelle;extracellular organelle;ex	MAPK signaling pathway;Regulation of actin cytoskeleton;Tight junction	Homo sapiens
90S preribosome;adherens junction;anchoring junction;cell junction;cell part;cell-substrate adherens junction;cell-substrate junction;cytoplasm;cytoplasmic part;cytoskeletal part;cytosol;cytosolic small ribosomal subunit;extr	Ribosome	Homo sapiens
cell part;cytoplasm;intracellular part	Ubiquitin mediated proteolysis	Homo sapiens
adherens junction;anchoring junction;cell junction;cell part;cell-substrate adherens junction;cell-substrate junction;cytoplasm;cytoplasmic part;cytosol;cytosolic small ribosomal subunit;extracellular membrane-bounded orga	Ribosome	Homo sapiens
cell part;cytoplasm;cytoplasmic part;cytosol;cytosolic small ribosomal subunit;intracellular non-membrane-bounded organelle;intracellular organelle;intracellular organelle part;intracellular part;macromolecular complex;memb	Ribosome	Homo sapiens
adherens junction;anchoring junction;cell junction;cell part;cell-substrate adherens junction;cell-substrate junction;cytoplasm;cytoplasmic part;cytosol;cytosolic small ribosomal subunit;extracellular membrane-bounded orga	Ribosome	Homo sapiens
adherens junction;anchoring junction;cell junction;cell part;cell-substrate adherens junction;cell-substrate junction;cytoplasm;cytoplasmic part;cytosol;cytosolic small ribosomal subunit;extracellular membrane-bounded orga	Ribosome	Homo sapiens
adherens junction;anchoring junction;cell junction;cell part;cell-substrate adherens junction;cell-substrate junction;cytoplasm;cytoplasmic part;cytosol;cytosolic small ribosomal subunit;extracellular membrane-bounded orga	Ribosome	Homo sapiens
cell part;cytoplasmic mRNA processing body;cytoplasmic part;cytosol;extracellular membrane-bounded organelle;extracellular organelle;extracellular region part;extracellular vesicular exosome;intracellular non-membrane-b	RNA degradation;Spliceosome	Homo sapiens
adherens junction;anchoring junction;cell cortex;cell division site part;cell junction;cell part;cell projection;cell projection membrane;cell projection part;cell-substrate adherens junction;cell-substrate junction;cleavage furrow;	Endocytosis;Fc gamma R-mediated phagocytosis	Homo sapiens
cell part;cytoplasmic part;cytosol;cytosolic part;cytosolic proteasome complex;extracellular membrane-bounded organelle;extracellular organelle;extracellular region part;extracellular vesicular exosome;intracellular membran	Proteasome	Homo sapiens
adherens junction;anchoring junction;cell junction;cell part;cell-substrate adherens junction;cell-substrate junction;cytoplasm;cytoplasmic part;cytosol;cytosolic large ribosomal subunit;extracellular membrane-bounded orga	Ribosome	Homo sapiens
cell part;cytoplasmic part;cytosol;endoplasmic reticulum;intracellular membrane-bounded organelle;intracellular organelle;intracellular part;membrane-bounded organelle;nucleus;organelle		Homo sapiens
adherens junction;anchoring junction;cell division site part;cell junction;cell part;cell-substrate adherens junction;cell-substrate junction;cleavage furrow;cytoplasmic part;cytosol;early endosome;endosomal part;endosome;endosome membrane;extracellular membrane-bounded organelle;extracellular organelle;extracellular region part;extracellular		Homo sapiens
adherens junction;anchoring junction;cell junction;cell part;cell-substrate adherens junction;cell-substrate junction;cytoplasm;cytoplasmic part;cytosol;cytosolic large ribosomal subunit;extracellular membrane-bounded orga	Ribosome	Homo sapiens
cell part;cytoplasm;cytoplasmic part;cytosol;cytosolic small ribosomal subunit;intracellular membrane-bounded organelle;intracellular non-membrane-bounded organelle;intracellular organelle;intracellular organelle part;intrac	Ribosome	Homo sapiens
cell part;cytoplasm;cytoplasmic part;cytosol;cytosolic small ribosomal subunit;extracellular membrane-bounded organelle;extracellular organelle;extracellular region part;extracellular vesicular exosome;intracellular membrane	Ribosome	Homo sapiens
cell part;cytoplasm;cytoplasmic part;cytosol;cytosolic small ribosomal subunit;extracellular membrane-bounded organelle;extracellular organelle;extracellular region part;extracellular vesicular exosome;intracellular non-meml	Ribosome	Homo sapiens
cell part;cytoplasm;cytoplasmic part;cytosol;cytosolic small ribosomal subunit;extracellular membrane-bounded organelle;extracellular organelle;extracellular region part;extracellular vesicular exosome;intracellular organelle	Ribosome	Homo sapiens
cell part;cytoplasmic part;cytosol;cytosolic large ribosomal subunit;extracellular region part;extracellular space;intracellular organelle part;intracellular part;large ribosomal subunit;macromolecular complex;organelle part;ribor	Ribosome	Homo sapiens
adherens junction;anchoring junction;cell junction;cell part;cell-substrate adherens junction;cell-substrate junction;cytoplasmic part;cytosol;cytosolic large ribosomal subunit;extracellular membrane-bounded organelle;extrac	Ribosome	Homo sapiens
cell part;cytoplasm;cytoplasmic part;cytosol;cytosolic large ribosomal subunit;extracellular membrane-bounded organelle;extracellular organelle;extracellular region part;extracellular vesicular exosome;intracellular non-memb	Ribosome	Homo sapiens
adherens junction;anchoring junction;cell junction;cell part;cell-substrate adherens junction;cell-substrate junction;cytoplasm;cytoplasmic part;cytosol;cytosolic large ribosomal subunit;focal adhesion;intracellular non-membr	Ribosome	Homo sapiens
adherens junction;anchoring junction;cell junction;cell part;cell-substrate adherens junction;cell-substrate junction;cytoplasmic part;cytosol;extracellular membrane-bounded organelle;extracellular organelle;extracellular region;extracellular region part;extracellular space;extracellular vesicular exosome;focal adhesion;intracellular membrane-bound		Homo sapiens
cell part;centrosome;cytoplasm;cytoplasmic part;cytoskeletal part;extracellular membrane-bounded organelle;extracellular organelle;extracellular region part;extracellular vesicular exosome;extrinsic to internal side of plasma	Axon guidance;Chagas disease (American trypanosomiasis);Chemokine signaling pathway;Gap junction;Gastric acid secre	Homo sapiens
cell cortex part;cell part;cell projection membrane;cell projection part;cortical actin cytoskeleton;cortical cytoskeleton;cytoplasm;cytoplasmic part;cytoskeletal part;cytoskeleton;cytosol;eukaryotic translation elongation factor	RNA transport	Homo sapiens
cell part;cytoplasmic part;cytoskeletal part;cytosol;extracellular membrane-bounded organelle;extracellular organelle;extracellular region;extracellular region part;extracellular vesicular exosome;intracellular organelle part;intra	Gap junction;Pathogenic Escherichia coli infection;Phagosome	Homo sapiens
cell part;integral to membrane;intrinsic to membrane;membrane part		Homo sapiens
cell part;cell projection;cytoplasm;cytoplasmic part;cytosol;intracellular part;neuron projection	Glycine, serine and threonine metabolism;Methane metabolism	Homo sapiens
cell part;cytoplasmic part;cytosol;eukaryotic translation initiation factor 4F complex;intracellular part;macromolecular complex;membrane;protein complex	RNA transport;Viral myocarditis	Homo sapiens

cell part;intracellular membrane-bounded organelle;intracellular organelle;intracellular organelle part;intracellular part;macromolecular complex;membrane-bounded organelle;multimeric ribonuclease P complex;nuclear part;n	Ribosome biogenesis in eukaryotes;RNA transport	Homo sapiens
cell part;cytoplasm;intracellular membrane-bounded organelle;intracellular non-membrane-bounded organelle;intracellular organelle;intracellular organelle part;intracellular part;membrane-bounded organelle;non-membrane-bounded organelle;nuclear part;nucleolus;nucleoplasm;nucleus;organelle;organelle part		Homo sapiens
cell part;cytoplasmic part;cytosol;endoplasmic reticulum;endoplasmic reticulum-Golgi intermediate compartment;envelope;extracellular membrane-bounded organelle;extracellular organelle;extracellular region part;extracellular space;extracellular vesicular exosome;Golgi apparatus;intracellular membrane-bounded organelle;intracellular organelle;		Homo sapiens
cell part;cytoplasmic part;intracellular membrane-bounded organelle;intracellular non-membrane-bounded organelle;intracellular organelle;intracellular organelle part;intracellular part;macromolecular complex;membrane;membrane-bounded organelle;mitochondrial inner membrane;mitochondrial membrane;mitochondrial part;mitochondrial riboso		Homo sapiens
cell part;cytoplasmic part;intracellular membrane-bounded organelle;intracellular organelle;intracellular organelle part;intracellular part;macromolecular complex;membrane;membrane-bounded organelle;mitochondrial inner membrane;mitochondrial membrane;mitochondrial part;mitochondrial small ribosomal subunit;mitochondrion;organellar sma		Homo sapiens
cell part;cytoplasmic part;intracellular membrane-bounded organelle;intracellular non-membrane-bounded organelle;intracellular organelle;intracellular organelle part;intracellular part;macromolecular complex;membrane;membrane-bounded organelle;mitochondrial inner membrane;mitochondrial membrane;mitochondrial part;mitochondrial small r		Homo sapiens
cell part;cytoplasmic part;cytosol;cytosolic large ribosomal subunit;extracellular membrane-bounded organelle;extracellular organelle;extracellular region part;extracellular vesicular exosome;intracellular organelle part;intrace	Ribosome	Homo sapiens
cell part;cytoplasmic part;cytosol;cytosolic large ribosomal subunit;intracellular non-membrane-bounded organelle;intracellular organelle;intracellular organelle part;intracellular part;large ribosomal subunit;macromolecular co	Ribosome	Homo sapiens
adherens junction;anchoring junction;cell junction;cell part;cell-substrate adherens junction;cell-substrate junction;cytoplasmic part;cytosol;endoplasmic reticulum membrane;endoplasmic reticulum part;extracellular membra	Bacterial invasion of epithelial cells;Shigellosis	Homo sapiens
adherens junction;anchoring junction;cell junction;cell part;cell-substrate adherens junction;cell-substrate junction;cytoplasm;cytoplasmic part;cytosol;cytosolic large ribosomal subunit;focal adhesion;intracellular non-membr	Ribosome	Homo sapiens
adherens junction;anchoring junction;basal lamina;cell junction;cell part;cell-substrate adherens junction;cell-substrate junction;cytoplasmic part;extracellular matrix;extracellular matrix part;extracellular membrane-bounded c	ECM-receptor interaction	Homo sapiens
cell part;cytoplasm;extracellular region part;high-density lipoprotein particle;intracellular membrane-bounded organelle;intracellular organelle;intracellular part;macromolecular complex;membrane;membrane-bounded organelle;nucleus;organelle;plasma lipoprotein particle;plasma membrane;protein-lipid complex		Homo sapiens
axon;cell body;cell junction;cell part;cell projection;cell projection part;cyclin-dependent protein kinase 5 holoenzyme complex;cyclin-dependent protein kinase holoenzyme complex;cytoplasm;cytoplasmic part;cytoskeletal p	Alzheimer's disease;Axon guidance	Homo sapiens
cell body;cell part;cell projection;chromosomal part;chromosome, telomeric region;cytoplasm;dendrite;DNA replication factor A complex;intracellular membrane-bounded organelle;intracellular organelle;intracellular organelle part;intracellular part;macromolecular complex;membrane-bounded organelle;neuron projection;neuronal cell body;nuclear		Homo sapiens
actin cytoskeleton;adherens junction;anchoring junction;cell junction;cell part;cell-substrate adherens junction;cell-substrate junction;cytoplasm;cytoplasmic part;cytoskeleton;focal adhesion;Golgi apparatus;intracellular membrane-bounded organelle;intracellular non-membrane-bounded organelle;intracellular organelle;intracellular part;membrane		Homo sapiens
catalytic step 2 spliceosome;cell part;cell surface;CRD-mediated mRNA stability complex;cytoplasmic part;intracellular membrane-bounded organelle;intracellular organelle;intracellular organelle part;intracellular part;macrom	Spliceosome	Homo sapiens
cell part;cytoplasm;extracellular membrane-bounded organelle;extracellular organelle;extracellular region part;extracellular vesicular exosome;intracellular membrane-bounded organelle;intracellular organelle;intracellular orga	Spliceosome	Homo sapiens
cell part;cytoplasmic part;cytosol;intracellular part	Purine metabolism	Homo sapiens
cell part;cytoplasm;cytoplasmic part;cytosol;extracellular membrane-bounded organelle;extracellular organelle;extracellular region part;extracellular vesicular exosome;intracellular organelle part;intracellular part;membrane-b	PPAR signaling pathway	Homo sapiens
adherens junction;anchoring junction;cell cortex part;cell junction;cell part;cell-substrate adherens junction;cell-substrate junction;cortical actin cytoskeleton;cortical cytoskeleton;cytoplasmic part;cytoskeletal part;cytoskeleton;extracellular membrane-bounded organelle;extracellular organelle;extracellular region part;extracellular vesicular exosome		Homo sapiens
apical plasma membrane;cell part;cytoplasm;cytoplasmic part;cytosol;extracellular membrane-bounded organelle;extracellular organelle;extracellular region part;extracellular vesicular exosome;integral to membrane;integral to plasma membrane;intracellular membrane-bounded organelle;intracellular non-membrane-bounded organelle;intracellula		Homo sapiens
cell part;cytoplasmic part;cytosol;extracellular membrane-bounded organelle;extracellular organelle;extracellular region part;extracellular vesicular exosome;intracellular membrane-bounded organelle;intracellular organelle;int	Fructose and mannose metabolism;Galactose metabolism;Glycolysis / Gluconeogenesis;Methane metabolism;Pentose ph	Homo sapiens
cell part;cytoplasm;intracellular part		Homo sapiens
anchoring collagen;basement membrane;cell part;collagen;collagen type VII;cytoplasmic part;endoplasmic reticulum lumen;endoplasmic reticulum part;extracellular matrix part;extracellular region;extracellular region part;extracellular space;intracellular organelle lumen;intracellular organelle part;intracellular part;membrane-enclosed lumen;organell		Homo sapiens
cell part;cytoplasmic part;cytosol;cytosolic large ribosomal subunit;intracellular organelle part;intracellular part;large ribosomal subunit;macromolecular complex;membrane;organelle part;ribonucleoprotein complex	Ribosome	Homo sapiens
adherens junction;anchoring junction;axon;cell cortex;cell junction;cell part;cell projection;cell projection cytoplasm;cell projection part;cell-substrate adherens junction;cell-substrate junction;cytoplasm;cytoplasmic part;cyto	Acute myeloid leukemia;B cell receptor signaling pathway;Bladder cancer;Chemokine signaling pathway;Chronic myeloid l	Homo sapiens
cell part;cytoplasmic part;endoplasmic reticulum membrane;endoplasmic reticulum part;extracellular membrane-bounded organelle;extracellular organelle;extracellular region part;extracellular vesicular exosome;intracellular	Lysine degradation	Homo sapiens
cell part;cytoplasmic part;cytoskeleton;endoplasmic reticulum-Golgi intermediate compartment;extracellular membrane-bounded organelle;extracellular organelle;extracellular region part;extracellular space;extracellular vesicular exosome;Golgi apparatus;intracellular membrane-bounded organelle;intracellular non-membrane-bounded organelle;in		Homo sapiens
adherens junction;anchoring junction;cell junction;cell part;cell-substrate adherens junction;cell-substrate junction;cytoplasmic part;cytosol;cytosolic large ribosomal subunit;focal adhesion;intracellular membrane-bounded o	Ribosome	Homo sapiens
cell part;cytoplasmic part;cytosol;extracellular membrane-bounded organelle;extracellular organelle;extracellular region part;extracellular vesicular exosome;intracellular part;membrane-bounded organelle;membrane-bounde	Arginine and proline metabolism	Homo sapiens
adherens junction;anchored to membrane;anchoring junction;cell junction;cell part;cell projection membrane;cell projection part;cell-substrate adherens junction;cell-substrate junction;cytoplasmic part;endoplasmic reticulum	Complement and coagulation cascades	Homo sapiens
cell part;cytoplasmic part;endoplasmic reticulum membrane;endoplasmic reticulum part;integral to endoplasmic reticulum membrane;integral to membrane;integral to organelle membrane;intracellular membrane-bounded or	ABC transporters;Antigen processing and presentation;Phagosome;Primary immunodeficiency	Homo sapiens
cell part;cytoplasm;cytoplasmic part;cytosol;I-kappaB/NF-kappaB complex;intracellular membrane-bounded organelle;intracellular organelle;intracellular organelle part;intracellular part;macromolecular complex;membrane-b	Acute myeloid leukemia;Adipocytokine signaling pathway;Amoebiasis;Apoptosis;B cell receptor signaling pathway;Chagas	Homo sapiens
Cdc48p-Npl4p-Ufd1p AAA ATPase complex;cell body;cell part;cell projection;cytoplasm;cytoplasmic part;dendrite;endoplasmic reticulum part;intracellular membrane-bounded organelle;intracellular organelle;intracellular organelle part;intracellular part;macromolecular complex;membrane part;membrane-bounded organelle;neuron projection;neur		Homo sapiens
cell part;cytoplasmic part;cytosol;extracellular membrane-bounded organelle;extracellular organelle;extracellular region part;extracellular vesicular exosome;intracellular part;membrane-bounded organelle;membrane-bounde	Starch and sucrose metabolism	Homo sapiens
cell part;cytoplasmic part;cytosol;eukaryotic translation initiation factor 4F complex;intracellular part;macromolecular complex;membrane;protein complex	RNA transport;Viral myocarditis	Homo sapiens
cell part;cell projection;cell surface;cilium;cytoplasmic part;endoplasmic reticulum membrane;endoplasmic reticulum part;extracellular region;Golgi apparatus part;Golgi membrane;integral to membrane;integral to plasma me	Dorso-ventral axis formation;Notch signaling pathway	Homo sapiens
cell part;intracellular membrane-bounded organelle;intracellular organelle;intracellular organelle part;intracellular part;membrane-bounded organelle;nuclear part;nucleoplasm;nucleus;organelle;organelle part		Homo sapiens
cell junction;cell part;cell-cell contact zone;cell-cell junction;cytoplasm;cytoplasmic part;cytoplasmic vesicle membrane;cytoplasmic vesicle part;cytosol;extracellular membrane-bounded organelle;extracellular organelle;extra	Cell cycle;Neurotrophin signaling pathway;Oocyte meiosis	Homo sapiens
cell body;cell part;cytoplasm;cytoplasmic part;eukaryotic translation elongation factor 1 complex;intracellular part;macromolecular complex;neuronal cell body;protein complex	RNA transport	Homo sapiens
actin cap;actin cytoskeleton;cell cortex part;cell part;contractile fiber;cytoplasmic part;cytoskeletal part;cytoskeleton;cytosol;intracellular non-membrane-bounded organelle;intracellular organelle;intracellular organelle part;int	Vascular smooth muscle contraction	Homo sapiens
extracellular membrane-bounded organelle;extracellular organelle;extracellular region;extracellular region part;extracellular vesicular exosome;membrane-bounded organelle;membrane-bounded vesicle;organelle;vesicle		Homo sapiens
cell part;cytoplasmic part;cytosol;extracellular membrane-bounded organelle;extracellular organelle;extracellular region part;extracellular vesicular exosome;intracellular part;membrane-bounded organelle;membrane-bounde	Alanine, aspartate and glutamate metabolism;Amino sugar and nucleotide sugar metabolism	Homo sapiens
adherens junction;anchoring junction;cell junction;cell part;cell-substrate adherens junction;cell-substrate junction;cytoplasm;cytoplasmic part;cytosol;cytosolic large ribosomal subunit;focal adhesion;intracellular membrane-	Ribosome	Homo sapiens
cell part;cytoplasmic part;cytoskeleton;endoplasmic reticulum;endoplasmic reticulum membrane;endoplasmic reticulum part;extracellular membrane-bounded organelle;extracellular organelle;extracellular region part;extrace	Protein processing in endoplasmic reticulum	Homo sapiens
adherens junction;anchoring junction;apical junction complex;apical part of cell;apical plasma membrane;apicolateral plasma membrane;basolateral plasma membrane;cell junction;cell part;cell-cell adherens junction;cell-cel	Adherens junction;Epithelial cell signaling in Helicobacter pylori infection;Gap junction;Tight junction;Vibrio cholerae infecti	Homo sapiens
adherens junction;anchoring junction;cell body;cell junction;cell part;cell projection;cell-substrate adherens junction;cell-substrate junction;clathrin-coated vesicle;coated pit;coated vesicle;cytoplasm;cytoplasmic membrane-	Alzheimer's disease;Malaria	Homo sapiens
apical plasma membrane;calcium channel complex;cation channel complex;cell part;centrosome;contractile fiber;cytoplasmic part;cytoskeletal part;cytosol;Golgi apparatus;intracellular membrane-bounded organelle;intracel	Purine metabolism	Homo sapiens
cell part;intracellular membrane-bounded organelle;intracellular organelle;intracellular part;membrane-bounded organelle;nucleus;organelle		Homo sapiens
CAF-1 complex;cell part;chromatin;chromatin assembly complex;chromatin remodeling complex;chromosomal part;ESC/E(Z) complex;histone deacetylase complex;histone methyltransferase complex;intracellular membrane-bounded organelle;intracellular organelle;intracellular organelle part;intracellular part;ISWI complex;macromolecular comple		Homo sapiens
actin cytoskeleton;adherens junction;anchoring junction;cell junction;cell part;cell-cell contact zone;cell-cell junction;cell-substrate adherens junction;cell-substrate junction;contractile fiber part;costamere;cytoplasm;cytoplasmic part;cytoskeleton;cytosol;extracellular membrane-bounded organelle;extracellular organelle;extracellular region part;ext		Homo sapiens
cell part;cytoplasmic part;extracellular membrane-bounded organelle;extracellular organelle;extracellular region part;extracellular vesicular exosome;Golgi apparatus;Golgi apparatus part;Golgi cisterna membrane;Golgi mem	Mucin type O-Glycan biosynthesis	Homo sapiens
AP-type membrane coat adaptor complex;cell part;clathrin adaptor complex;clathrin coated vesicle membrane;clathrin-coated endocytic vesicle membrane;coated vesicle membrane;cytoplasmic part;cytoplasmic vesicle me	Lysosome	Homo sapiens
anchored to membrane;apical plasma membrane;cell part;cell surface;cytoplasm;cytoplasmic part;endosome;extracellular membrane-bounded organelle;extracellular organelle;extracellular region part;extracellular vesicular exosome;Golgi apparatus;integral to membrane;integral to plasma membrane;intracellular membrane-bounded organelle;int		Homo sapiens
cell part;cytoplasmic part;extracellular region part;extracellular space;intracellular membrane-bounded organelle;intracellular organelle;intracellular organelle lumen;intracellular organelle part;intracellular part;membrane;membrane-bounded organelle;membrane-enclosed lumen;mitochondrial inner membrane;mitochondrial matrix;mitochondrial me		Homo sapiens
calcium channel complex;cation channel complex;cell cortex part;cell part;cortical endoplasmic reticulum;cytoplasmic part;endoplasmic reticulum;endoplasmic reticulum lumen;endoplasmic reticulum membrane;endoplasmic reticulum part;integral to endoplasmic reticulum membrane;integral to membrane;integral to organelle membrane;intracellu		Homo sapiens
cell part;cytoplasm;cytoplasmic part;intracellular membrane-bounded organelle;intracellular non-membrane-bounded organelle;intracellular organelle;intracellular organelle part;intracellular part;membrane-bounded organelle;mitochondrial nucleoid;mitochondrial part;mitochondrion;non-membrane-bounded organelle;nucleoid;organelle;organelle p		Homo sapiens
cell part;cytoplasm;cytoplasmic part;cytosol;intracellular part	beta-Alanine metabolism;Drug metabolism - other enzymes;Pantothenate and CoA biosynthesis;Pyrimidine metabolism	Homo sapiens
cell part;cytoplasmic part;cytosol;intracellular part		Homo sapiens
cell part;cell surface;cytoplasmic part;endoplasmic reticulum membrane;endoplasmic reticulum part;endoplasmic reticulum-Golgi intermediate compartment;endoplasmic reticulum-Golgi intermediate compartment membran	Protein processing in endoplasmic reticulum	Homo sapiens
cell part;cytoplasm;cytoplasmic part;early endosome;endosome;extracellular membrane-bounded organelle;extracellular organelle;extracellular region part;extracellular vesicular exosome;intracellular membrane-bounded organelle;intracellular organelle;intracellular part;membrane;membrane-bounded organelle;membrane-bounded vesicle;organe		Homo sapiens
cell part;cytoplasmic part;extracellular membrane-bounded organelle;extracellular organelle;extracellular region part;extracellular vesicular exosome;intracellular membrane-bounded organelle;intracellular organelle;intracellula	Peroxisome	Homo sapiens
cell part;cytoplasm;cytoplasmic part;cytosol;intracellular membrane-bounded organelle;intracellular organelle;intracellular organelle part;intracellular part;membrane-bounded organelle;nuclear part;nucleoplasm;nucleus;orga	MAPK signaling pathway;Non-small cell lung cancer;Pathways in cancer	Homo sapiens
actin cytoskeleton;cell part;cytoplasm;cytoplasmic part;cytoskeleton;cytosol;extracellular membrane-bounded organelle;extracellular organelle;extracellular region part;extracellular vesicular exosome;intracellular membrane-	Fatty acid biosynthesis;Insulin signaling pathway;Propanoate metabolism;Pyruvate metabolism	Homo sapiens
cell part;intracellular membrane-bounded organelle;intracellular organelle;intracellular organelle part;intracellular part;membrane-bounded organelle;nuclear part;nucleoplasm;nucleus;organelle;organelle part		Homo sapiens
cell part;cytoplasmic part;cytosol;intracellular membrane-bounded organelle;intracellular organelle;intracellular part;membrane;membrane-bounded organelle;nucleus;organelle		Homo sapiens
cell part;cytoplasmic part;cytosol;endoplasmic reticulum;extracellular membrane-bounded organelle;extracellular organelle;extracellular region part;extracellular space;extracellular vesicular exosome;intracellular membrane-bounded organelle;intracellular organelle;intracellular part;membrane-bounded organelle;membrane-bounded vesicle;mitoch		Homo sapiens
cell part;centromeric heterochromatin;chromatin;chromosomal part;chromosome, centromeric region;condensed chromosome, centromeric region;cytoskeletal part;envelope;euchromatin;heterochromatin;intracellular membrane-bounded organelle;intracellular non-membrane-bounded organelle;intracellular organelle;intracellular organelle part;intra		Homo sapiens
cell part;cytoplasmic part;cytosol;extracellular membrane-bounded organelle;extracellular organelle;extracellular region part;extracellular space;extracellular vesicular exosome;intracellular non-membrane-bounded organelle;intracellular organelle;intracellular organelle part;intracellular part;membrane;membrane-bounded organelle;membrane-bour		Homo sapiens
cell part;intracellular non-membrane-bounded organelle;intracellular organelle;intracellular organelle part;intracellular part;non-membrane-bounded organelle;nuclear part;nucleolus;nucleoplasm;organelle;organelle part	Spliceosome	Homo sapiens
cell part;chromatin;chromosomal part;euchromatin;heterochromatin;intracellular membrane-bounded organelle;intracellular organelle;intracellular organelle part;intracellular part;membrane-bounded organelle;nuclear chromatin;nuclear chromosome part;nuclear euchromatin;nuclear heterochromatin;nuclear part;nucleoplasm;nucleus;organelle;orga		Homo sapiens
adherens junction;anchoring junction;cell junction;cell part;cell-substrate adherens junction;cell-substrate junction;cytoplasm;cytoplasmic part;cytosol;focal adhesion;intracellular membrane-bounded organelle;intracellular non-membrane-bounded organelle;intracellular organelle;intracellular part;macromolecular complex;membrane;membrane-bo		Homo sapiens
cell part;cytoplasmic part;cytosol;eukaryotic 43S preinitiation complex;eukaryotic 48S preinitiation complex;eukaryotic translation initiation factor 3 complex;eukaryotic translation initiation factor 3 complex, eIF3m;extracellula	RNA transport	Homo sapiens
actin filament bundle;actomyosin;adherens junction;anchoring junction;axon part;cell body;cell junction;cell part;cell projection;cell projection part;cell-cell junction;cell-substrate adherens junction;cell-substrate junction;cont	Bacterial invasion of epithelial cells;Endometrial cancer;Focal adhesion;PPAR signaling pathway	Homo sapiens
catalytic step 2 spliceosome;cell part;intracellular organelle part;intracellular part;macromolecular complex;nuclear part;nucleoplasm;organelle part;ribonucleoprotein complex;spliceosomal complex;U12-type spliceosomal cc	Spliceosome	Homo sapiens
adherens junction;anchoring junction;basolateral plasma membrane;cell junction;cell part;cell surface;cell-substrate adherens junction;cell-substrate junction;cytoplasm;extracellular membrane-bounded organelle;extracellular organelle;extracellular region part;extracellular space;extracellular vesicular exosome;focal adhesion;integral to membrane		Homo sapiens
cell part;cytoplasmic part;endoplasmic reticulum;endoplasmic reticulum membrane;endoplasmic reticulum part;endoplasmic reticulum-Golgi intermediate compartment;endoplasmic reticulum-Golgi intermediate compartment membrane;Golgi apparatus;integral to membrane;intracellular membrane-bounded organelle;intracellular organelle;intrace		Homo sapiens
aggresome;autophagic vacuole;cell part;cytoplasm;cytoplasmic mRNA processing body;cytoplasmic part;cytoplasmic vesicle;cytosol;endoplasmic reticulum;endosome;extracellular membrane-bounded organelle;extracellula	Osteoclast differentiation	Homo sapiens
cell part;cytoplasmic part;integral to membrane;intracellular organelle part;intracellular part;intrinsic to membrane;membrane;membrane part;mitochondrial membrane;mitochondrial outer membrane;mitochondrial part;organelle membrane;organelle outer membrane;organelle part;outer membrane		Homo sapiens
cell part;cytoplasmic part;cytoplasmic vesicle membrane;cytoplasmic vesicle part;cytosol;endocytic vesicle membrane;endoplasmic reticulum membrane;endoplasmic reticulum part;intracellular organelle part;intracellular pa	Calcium signaling pathway;ErbB signaling pathway;Gastric acid secretion;Glioma;GnRH signaling pathway;ko05152;Long-	Homo sapiens
cell part;cytoplasm;cytoplasmic part;cytosol;intracellular part;membrane;plasma membrane	Fructose and mannose metabolism	Homo sapiens
axon;cell part;cell projection;cell projection part;clathrin-coated vesicle;coated vesicle;cytoplasm;cytoplasmic membrane-bounded vesicle;cytoplasmic part;cytoplasmic vesicle;cytosol;dendrite;dendritic spine;early endosom	Fructose and mannose metabolism	Homo sapiens
cell part;CUL4 RING ubiquitin ligase complex;Cul4B-RING ubiquitin ligase complex;cullin-RING ubiquitin ligase complex;cytoplasm;extracellular membrane-bounded organelle;extracellular organelle;extracellular region part;e	Nucleotide excision repair;Ubiquitin mediated proteolysis	Homo sapiens
actin filament bundle;actomyosin;adherens junction;anchoring junction;cell junction;cell part;cell-substrate adherens junction;cell-substrate junction;contractile fiber part;cytoplasmic part;cytoskeletal part;focal adhesion;intracellular membrane-bounded organelle;intracellular organelle;intracellular organelle part;intracellular part;membrane-boundec		Homo sapiens
adherens junction;anchoring junction;axon;cell body;cell junction;cell part;cell projection;cell-substrate adherens junction;cell-substrate junction;external side of plasma membrane;extracellular membrane-bounded organelle;	Cell adhesion molecules (CAMs)	Homo sapiens
cell part;cytoplasmic part;Golgi apparatus;Golgi apparatus part;intracellular membrane-bounded organelle;intracellular organelle;intracellular organelle part;intracellular part;membrane;membrane-bounded organelle;organelle;organelle part;trans-Golgi network		Homo sapiens
cell part;intracellular membrane-bounded organelle;intracellular non-membrane-bounded organelle;intracellular organelle;intracellular organelle part;intracellular part;membrane;membrane-bounded organelle;non-membrane-	Ribosome biogenesis in eukaryotes	Homo sapiens
cell part;cytoplasm;cytoplasmic part;cytosol;extracellular membrane-bounded organelle;extracellular organelle;extracellular region part;extracellular vesicular exosome;intracellular membrane-bounded organelle;intracellular organelle;intracellular organelle part;intracellular part;membrane-bounded organelle;membrane-bounded vesicle;nuclear part		Homo sapiens
apical part of cell;cell part;cytoplasm;intracellular membrane-bounded organelle;intracellular non-membrane-bounded organelle;intracellular organelle;intracellular organelle part;intracellular part;membrane;membrane-bounded organelle;non-membrane-bounded organelle;nuclear part;nucleolus;nucleus;organelle;organelle part		Homo sapiens
cell part;cytoplasm;cytoskeleton;extracellular membrane-bounded organelle;extracellular organelle;extracellular region part;extracellular vesicular exosome;intracellular non-membrane-bounded organelle;intracellular organelle;intracellular organelle part;intracellular part;membrane;membrane-bounded organelle;membrane-bounded vesicle;non-me		Homo sapiens
cell part;cytoplasmic part;cytosol;extracellular membrane-bounded organelle;extracellular organelle;extracellular region;extracellular region part;extracellular space;extracellular vesicular exosome;intracellular part;membrane-	African trypanosomiasis;Cytokine-cytokine receptor interaction;Cytosolic DNA-sensing pathway;ko05152;Malaria;NOD-like	Homo sapiens
cell part;intracellular organelle part;intracellular part;macromolecular complex;nuclear part;nucleolar part;nucleoplasm;organelle part;PeBoW complex;preribosome;preribosome, large subunit precursor;protein complex;ribonucleoprotein complex		Homo sapiens
cell part;Cul3-RING ubiquitin ligase complex;cullin-RING ubiquitin ligase complex;cytoplasm;cytoplasmic part;cytoskeletal part;cytosol;endoplasmic reticulum;intracellular membrane-bounded organelle;intracellular organelle	Ubiquitin mediated proteolysis	Homo sapiens
cell part;intracellular organelle part;intracellular part;macromolecular complex;nuclear part;organelle part;PcG protein complex;protein complex		Homo sapiens
cell part;cytoplasmic part;endoplasmic reticulum;endoplasmic reticulum membrane;endoplasmic reticulum part;integral to membrane;intracellular membrane-bounded organelle;intracellular organelle;intracellular organelle part;intracellular part;intrinsic to membrane;membrane;membrane part;membrane-bounded organelle;organelle;organelle mem		Homo sapiens
		Homo sapiens
actin cytoskeleton;adherens junction;anchoring junction;cell junction;cell part;cell-substrate adherens junction;cell-substrate junction;contractile fiber part;cytoplasmic part;cytoskeleton;focal adhesion;intracellular membrane-	Osteoclast differentiation	Homo sapiens
cell leading edge;cell part;centrosome;chromosomal part;cytoplasm;cytoplasmic part;cytoskeletal part;cytosol;dynactin complex;dynein complex;intracellular non-membrane-bounded organelle;intracellular organelle;intracell	Huntington's disease;Vasopressin-regulated water reabsorption	Homo sapiens
cell part;cell projection;centrosome;cytoplasmic dynein complex;cytoplasmic part;cytoskeletal part;cytosol;dynein complex;extracellular membrane-bounded organelle;extracellular organelle;extracellular region part;extracellu	Phagosome;Vasopressin-regulated water reabsorption	Homo sapiens
basolateral plasma membrane;cell part;cytoplasm;cytoplasmic part;cytoskeletal part;cytoskeleton;intracellular non-membrane-bounded organelle;intracellular organelle;intracellular organelle part;intracellular part;macromolecular complex;membrane part;microtubule;microtubule associated complex;microtubule cytoskeleton;non-membrane-bounc		Homo sapiens
cell part;cytoplasm;cytoplasmic part;cytosol;intracellular organelle part;intracellular part;nuclear part;nucleoplasm;organelle part	RIG-I-like receptor signaling pathway	Homo sapiens
adherens junction;anchoring junction;cell junction;cell part;cell-substrate adherens junction;cell-substrate junction;contractile fiber part;costamere;cytoplasm;cytoplasmic part;cytoskeleton;cytosol;focal adhesion;intracellular	Focal adhesion;MAPK signaling pathway	Homo sapiens
cell part;intracellular membrane-bounded organelle;intracellular organelle;intracellular organelle part;intracellular part;membrane-bounded organelle;nuclear part;nucleoplasm;nucleus;organelle;organelle part		Homo sapiens
cell part;cytoplasmic part;cytosol;extracellular membrane-bounded organelle;extracellular organelle;extracellular region part;extracellular vesicular exosome;intracellular part;membrane-bounded organelle;membrane-bounde	Amino sugar and nucleotide sugar metabolism;Galactose metabolism	Homo sapiens
cell part;cytoplasmic part;endoplasmic reticulum;endoplasmic reticulum lumen;endoplasmic reticulum membrane;endoplasmic reticulum part;intracellular membrane-bounded organelle;intracellular organelle;intracellular organelle lumen;intracellular organelle part;intracellular part;membrane;membrane part;membrane-bounded organelle;membran		Homo sapiens
catalytic step 2 spliceosome;cell part;cytoplasm;intracellular membrane-bounded organelle;intracellular organelle;intracellular organelle part;intracellular part;macromolecular complex;membrane-bounded organelle;nuclear p	Spliceosome	Homo sapiens
calcineurin complex;cell part;cytoplasmic part;cytoplasmic vesicle membrane;cytoplasmic vesicle part;cytoskeletal part;endoplasmic reticulum;endoplasmic reticulum membrane;endoplasmic reticulum part;integral to memb	Alzheimer's disease;Calcium signaling pathway;Gap junction;Gastric acid secretion;GnRH signaling pathway;Huntington's	Homo sapiens
brush border;cell part;cell projection;cytoplasm;extracellular membrane-bounded organelle;extracellular organelle;extracellular region part;extracellular vesicular exosome;intracellular part;membrane-bounded organelle;membrane-bounded vesicle;organelle;vesicle		Homo sapiens
cell part;chromosomal part;chromosome;chromosome, centromeric region;cohesin complex;cohesin core heterodimer;condensed chromosome;condensed chromosome kinetochore;condensed nuclear chromosome;cytopla	Cell cycle;Cell cycle - yeast;Meiosis - yeast;Oocyte meiosis	Homo sapiens
cell part;cytoplasmic part;endoplasmic reticulum;intracellular membrane-bounded organelle;intracellular organelle;intracellular part;membrane;membrane-bounded organelle;organelle;plasma membrane		Homo sapiens
cell part;cytoplasmic membrane-bounded vesicle;cytoplasmic part;cytoplasmic vesicle;endoplasmic reticulum lumen;endoplasmic reticulum part;extracellular membrane-bounded organelle;extracellular organelle;extracellula	N-Glycan biosynthesis;Protein processing in endoplasmic reticulum	Homo sapiens
cell part;cytoplasm;cytoplasmic part;cytoskeleton;extracellular membrane-bounded organelle;extracellular organelle;extracellular region part;extracellular vesicular exosome;intracellular membrane-bounded organelle;intracellular non-membrane-bounded organelle;intracellular organelle;intracellular organelle part;intracellular part;macromolecular c		Homo sapiens
cell part;cytoplasm;cytoplasmic part;cytosol;endoplasmic reticulum part;endoplasmic reticulum tubular network;envelope;extracellular membrane-bounded organelle;extracellular organelle;extracellular region part;extracellula	RNA transport	Homo sapiens
apical part of cell;cell body;cell part;cell projection;chromosome;cytoplasm;cytoplasmic part;cytoskeletal part;cytosol;dendrite;extracellular membrane-bounded organelle;extracellular organelle;extracellular region part;extracellular vesicular exosome;Golgi apparatus part;Golgi membrane;intracellular membrane-bounded organelle;intracellular non		Homo sapiens
cell part;cytoplasmic part;cytosol;intracellular membrane-bounded organelle;intracellular organelle;intracellular organelle part;intracellular part;membrane-bounded organelle;nuclear body;nuclear part;nuclear speck;nucleopla	Proteasome	Homo sapiens
cell part;chromosomal part;condensin complex;cytoplasm;cytoplasmic part;cytosol;intracellular membrane-bounded organelle;intracellular organelle;intracellular organelle part;intracellular part;macromolecular complex;mem	Cell cycle - yeast	Homo sapiens
cell part;intracellular membrane-bounded organelle;intracellular organelle;intracellular organelle part;intracellular part;membrane;membrane-bounded organelle;nuclear body;nuclear membrane;nuclear part;nuclear speck;nucleoplasm;nucleoplasm part;nucleus;organelle;organelle membrane;organelle part		Homo sapiens
cell part;intracellular non-membrane-bounded organelle;intracellular organelle;intracellular organelle part;intracellular part;non-membrane-bounded organelle;nuclear part;nucleolus;nucleoplasm;organelle;organelle part		Homo sapiens
actin cytoskeleton;cell cortex part;cell division site part;cell part;cell projection membrane;cell projection part;chromosomal part;ciliary transition zone;cilium membrane;cilium part;cleavage furrow;condensed chromosome kinetochore;cytoplasm;cytoplasmic part;cytoskeletal part;cytoskeleton;exocyst;extracellular membrane-bounded organelle;ex		Homo sapiens
cell part;chromosomal part;chromosome;condensed chromosome;condensin complex;condensin core heterodimer;cytoplasm;cytoplasmic part;cytosol;intracellular membrane-bounded organelle;intracellular non-membrane-	Cell cycle - yeast	Homo sapiens
cell part;cytoplasmic part;intracellular membrane-bounded organelle;intracellular organelle;intracellular organelle lumen;intracellular organelle part;intracellular part;membrane-bounded organelle;membrane-enclosed lumen;m	Aminoacyl-tRNA biosynthesis;Valine, leucine and isoleucine biosynthesis	Homo sapiens
cell part;cytoplasmic part;cytosol;endoplasmic reticulum part;integral to membrane;intracellular organelle part;intracellular part;intrinsic to membrane;macromolecular complex;membrane;membrane part;organelle part;protein complex;Sec61 translocon complex;translocon complex		Homo sapiens
cell part;cytoplasm;cytoplasmic part;endoplasmic reticulum part;endoplasmic reticulum tubular network;extracellular membrane-bounded organelle;extracellular organelle;extracellular region part;extracellular vesicular exosome;intracellular organelle part;intracellular part;macromolecular complex;membrane-bounded organelle;membrane-boundec		Homo sapiens
cell part;cell projection;cytoplasmic part;endoplasmic reticulum;Golgi apparatus;integral to membrane;integral to plasma membrane;intracellular membrane-bounded organelle;intracellular organelle;intracellular part;intrinsic to membrane;intrinsic to plasma membrane;lamellipodium;membrane;membrane part;membrane-bounded organelle;organel		Homo sapiens
cell part;chromosomal part;cytoplasmic part;delta DNA polymerase complex;DNA polymerase complex;intracellular membrane-bounded organelle;intracellular organelle;intracellular organelle part;intracellular part;macromole	Base excision repair;DNA replication;Homologous recombination;Mismatch repair;Nucleotide excision repair;Purine metab	Homo sapiens
cell part;intracellular non-membrane-bounded organelle;intracellular organelle;intracellular organelle part;intracellular part;non-membrane-bounded organelle;nuclear part;nucleolus;organelle;organelle part	Ribosome biogenesis in eukaryotes	Homo sapiens
cell part;cytoplasmic membrane-bounded vesicle;cytoplasmic part;cytoplasmic vesicle;endoplasmic reticulum;endoplasmic reticulum chaperone complex;endoplasmic reticulum lumen;endoplasmic reticulum membrane;end	Protein processing in endoplasmic reticulum	Homo sapiens
extracellular membrane-bounded organelle;extracellular organelle;extracellular region part;extracellular space;extracellular vesicular exosome;membrane-bounded organelle;membrane-bounded vesicle;organelle;vesicle		Homo sapiens
cell part;cytoplasmic part;intracellular membrane-bounded organelle;intracellular organelle;intracellular organelle lumen;intracellular organelle part;intracellular part;membrane-bounded organelle;membrane-enclosed lumen;mitochondrial matrix;mitochondrial part;mitochondrion;organelle;organelle lumen;organelle part		Homo sapiens

adherens junction;anchoring junction;cell junction;cell part;cell-substrate adherens junction;cell-substrate junction;contractile fiber part;costamere;cytoplasm;cytoplasmic part;cytoskeleton;cytosol;extracellular membrane-bounded organelle;extracellular organelle;extracellular region part;extracellular vesicular exosome;focal adhesion;hemidesmos		Homo sapiens
cell part;cytoplasmic part;extracellular region;intracellular membrane-bounded organelle;intracellular organelle;intracellular part;lysosome;lytic vacuole;membrane;membrane-bounded organelle;mitochondrion;nucleus;organe	Aminobenzoate degradation;Bisphenol degradation	Homo sapiens
cell part;intracellular membrane-bounded organelle;intracellular non-membrane-bounded organelle;intracellular organelle;intracellular organelle part;intracellular part;membrane;membrane-bounded organelle;non-membrane-bounded organelle;nuclear body;nuclear matrix;nuclear part;nuclear speck;nucleolus;nucleoplasm;nucleoplasm part;nucleus		Homo sapiens
adherens junction;anchoring junction;axon;cell body;cell junction;cell part;cell projection;cell surface;cell-cell junction;cilium;dendrite;integral to membrane;integral to plasma membrane;intrinsic to membrane;intrinsic to plasma membrane;leading edge membrane;membrane part;neuron projection;neuronal cell body;nonmotile primary cilium;photo		Homo sapiens
cell part;cytoplasm;extracellular membrane-bounded organelle;extracellular organelle;extracellular region part;extracellular vesicular exosome;intracellular membrane-bounded organelle;intracellular organelle;intracellular orga	Spliceosome	Homo sapiens
adherens junction;anchoring junction;cell junction;cell part;cell-substrate adherens junction;cell-substrate junction;cytoplasmic part;cytosol;extracellular membrane-bounded organelle;extracellular organelle;extracellular region part;extracellular vesicular exosome;focal adhesion;intracellular part;membrane-bounded organelle;membrane-bounded v		Homo sapiens
cell part;cytoplasm;cytoplasmic part;cytosol;intracellular membrane-bounded organelle;intracellular organelle;intracellular part;membrane-bounded organelle;nucleus;organelle		Homo sapiens
cell part;cytoplasm;cytoplasmic part;cytosol;intracellular non-membrane-bounded organelle;intracellular organelle;intracellular organelle part;intracellular part;membrane;non-membrane-bounded organelle;nuclear part;nucleolus;organelle;organelle part		Homo sapiens
adherens junction;anchoring junction;cell junction;cell part;cell-substrate adherens junction;cell-substrate junction;cytoplasm;cytoskeleton;focal adhesion;intracellular membrane-bounded organelle;intracellular non-membran	NOD-like receptor signaling pathway	Homo sapiens
cell part;cytoplasmic membrane-bounded vesicle;cytoplasmic part;cytoplasmic vesicle;extracellular membrane-bounded organelle;extracellular organelle;extracellular region part;extracellular vesicular exosome;Golgi apparat	Protein digestion and absorption	Homo sapiens
activin responsive factor complex;cell part;chromatin;chromosomal part;cytoplasm;cytoplasmic part;cytosol;intracellular membrane-bounded organelle;intracellular organelle;intracellular organelle part;intracellular part;macro	Adherens junction;Cell cycle;Chagas disease (American trypanosomiasis);Colorectal cancer;Endocytosis;Pancreatic cance	Homo sapiens
cell junction;cell part;clathrin coated vesicle membrane;clathrin-coated vesicle;coated vesicle;coated vesicle membrane;cytoplasmic membrane-bounded vesicle;cytoplasmic part;cytoplasmic vesicle;cytoplasmic vesicle mer	Endocytosis;Vasopressin-regulated water reabsorption	Homo sapiens
actin filament bundle;actomyosin;adherens junction;anchoring junction;cell junction;cell part;cell-cell adherens junction;cell-cell junction;cell-substrate adherens junction;cell-substrate junction;cytoplasm;cytoskeletal part;foc	Focal adhesion	Homo sapiens
extracellular membrane-bounded organelle;extracellular organelle;extracellular region;extracellular region part;extracellular space;extracellular vesicular exosome;membrane-bounded organelle;membrane-bounded vesicle;organelle;vesicle		Homo sapiens
cell division site part;cell part;cleavage furrow;cytoplasm;cytoplasmic membrane-bounded vesicle;cytoplasmic part;cytoplasmic vesicle;cytosol;endosome;intracellular membrane-bounded organelle;intracellular organelle;intracellular organelle part;intracellular part;membrane;membrane-bounded organelle;membrane-bounded vesicle;midbody;nuc		Homo sapiens
adherens junction;anchoring junction;cell junction;cell part;cell-substrate adherens junction;cell-substrate junction;focal adhesion;intracellular membrane-bounded organelle;intracellular organelle;intracellular part;membrane-bounded organelle;nucleus;organelle		Homo sapiens
cell part;CUL4 RING ubiquitin ligase complex;Cul4A-RING ubiquitin ligase complex;Cul4B-RING ubiquitin ligase complex;cullin-RING ubiquitin ligase complex;cytoplasm;extracellular membrane-bounded organelle;extracellu	Nucleotide excision repair;Ubiquitin mediated proteolysis	Homo sapiens
cell part;cytoplasm;cytoplasmic part;cytoskeletal part;cytosol;extracellular membrane-bounded organelle;extracellular organelle;extracellular region part;extracellular vesicular exosome;intracellular membrane-bounded organ	Amyotrophic lateral sclerosis (ALS);Chagas disease (American trypanosomiasis);Epithelial cell signaling in Helicobacter pylo	Homo sapiens
actin cytoskeleton;actin filament bundle;actomyosin;cell junction;cell part;cell projection;cytoplasm;cytoplasmic part;cytoskeletal part;cytoskeleton;cytosol;extracellular membrane-bounded organelle;extracellular organelle;extracellular region part;extracellular vesicular exosome;filopodium;intracellular non-membrane-bounded organelle;intracellula		Homo sapiens
cell part;cytoplasm;cytoplasmic part;extracellular membrane-bounded organelle;extracellular organelle;extracellular region part;extracellular vesicular exosome;intracellular membrane-bounded organelle;intracellular organelle;intracellular organelle lumen;intracellular organelle part;intracellular part;membrane-bounded organelle;membrane-boundec		Homo sapiens
cell part;cis-Golgi network;cytoplasmic part;extracellular membrane-bounded organelle;extracellular organelle;extracellular region part;extracellular vesicular exosome;Golgi apparatus part;Golgi membrane;integral to membra	N-Glycan biosynthesis;Various types of N-glycan biosynthesis	Homo sapiens
cell part;cytoplasm;cytoplasmic part;cytosol;intracellular membrane-bounded organelle;intracellular organelle;intracellular organelle part;intracellular part;membrane-bounded organelle;mitochondrion;nuclear part;nucleoplasr	Tryptophan metabolism	Homo sapiens
cell part;cytoplasmic part;endopeptidase Clp complex;intracellular membrane-bounded organelle;intracellular organelle;intracellular organelle lumen;intracellular organelle part;intracellular part;macromolecular complex;memb	Cell cycle - Caulobacter	Homo sapiens
cell part;cytoplasmic part;cytosol;intracellular part	Purine metabolism	Homo sapiens
basolateral plasma membrane;cell part;cell projection membrane;cell projection part;integral to membrane;intracellular non-membrane-bounded organelle;intracellular organelle;intracellular organelle part;intracellular part;intri	Nitrogen metabolism	Homo sapiens
cell part;cytoplasmic part;extracellular membrane-bounded organelle;extracellular organelle;extracellular region part;extracellular vesicular exosome;intracellular membrane-bounded organelle;intracellular organelle;intracellula	Adipocytokine signaling pathway;Citrate cycle (TCA cycle);Glycolysis / Gluconeogenesis;Insulin signaling pathway;PPAR si	Homo sapiens
cell part;cytoplasmic part;cytosol;extracellular membrane-bounded organelle;extracellular organelle;extracellular region part;extracellular vesicular exosome;intracellular membrane-bounded organelle;intracellular organelle;int	Amino sugar and nucleotide sugar metabolism;Galactose metabolism;Pentose and glucuronate interconversions;Starch an	Homo sapiens
cell part;intracellular membrane-bounded organelle;intracellular organelle;intracellular part;membrane;membrane-bounded organelle;nucleus;organelle		Homo sapiens
cell part;cytoplasm;cytoplasmic part;endoplasmic reticulum;intracellular membrane-bounded organelle;intracellular organelle;intracellular part;membrane-bounded organelle;nucleus;organelle;perinuclear region of cytoplasm		Homo sapiens
cell part;intracellular organelle part;intracellular part;nuclear part;nucleoplasm;organelle part		Homo sapiens
cell part;cytoplasmic part;cytosol;intracellular part	Amino sugar and nucleotide sugar metabolism;Butirosin and neomycin biosynthesis;Carbohydrate digestion and absorptio	Homo sapiens
cell part;cytoplasmic part;endoplasmic reticulum;endoplasmic reticulum lumen;endoplasmic reticulum part;extracellular matrix;extracellular membrane-bounded organelle;extracellular organelle;extracellular region part;extracellular vesicular exosome;intracellular membrane-bounded organelle;intracellular organelle;intracellular organelle lumen;intra		Homo sapiens
cell part;cytoplasm;cytoskeletal part;intracellular non-membrane-bounded organelle;intracellular organelle;intracellular organelle part;intracellular part;macromolecular complex;microtubule;non-membrane-bounded organelle;organelle;organelle part;protein complex;spindle		Homo sapiens
	Amino sugar and nucleotide sugar metabolism	Homo sapiens
cell part;cytoplasmic part;cytosol;extracellular membrane-bounded organelle;extracellular organelle;extracellular region part;extracellular vesicular exosome;intracellular membrane-bounded organelle;intracellular organelle;int	Glycerolipid metabolism;Methane metabolism;RIG-I-like receptor signaling pathway	Homo sapiens
cell part;cytoplasmic part;integral to membrane;intracellular membrane-bounded organelle;intracellular organelle;intracellular organelle part;intracellular part;intrinsic to membrane;macromolecular complex;membrane;membrane part;membrane-bounded organelle;mitochondrial inner membrane;mitochondrial inner membrane presequence transloc		Homo sapiens
cell part;cell surface;chloride channel complex;extracellular membrane-bounded organelle;extracellular organelle;extracellular region part;extracellular vesicular exosome;intracellular;ion channel complex;macromolecular complex;membrane;membrane part;membrane-bounded organelle;membrane-bounded vesicle;organelle;plasma membrane;pr		Homo sapiens
cell part;cytoplasm;cytoplasmic part;cytosol;intracellular membrane-bounded organelle;intracellular organelle;intracellular organelle part;intracellular part;membrane-bounded organelle;nuclear part;nucleoplasm;nucleus;organelle;organelle part		Homo sapiens
cell part;integral to membrane;intrinsic to membrane;membrane part		Homo sapiens
	Arginine and proline metabolism	Homo sapiens
cell part;cytoplasmic part;endosome;intracellular membrane-bounded organelle;intracellular organelle;intracellular organelle part;intracellular part;membrane;membrane-bounded organelle;mitochondrial inner membrane;mitochondrial membrane;mitochondrial part;organelle;organelle inner membrane;organelle membrane;organelle part		Homo sapiens
cell part;cytoplasmic part;intracellular non-membrane-bounded organelle;intracellular organelle;intracellular part;macromolecular complex;non-membrane-bounded organelle;organelle;ribonucleoprotein complex;ribosome		Homo sapiens
cell part;endomembrane system		Homo sapiens
cell part;integral to membrane;intracellular membrane-bounded organelle;intracellular organelle;intracellular organelle part;intracellular part;intrinsic to membrane;membrane;membrane part;membrane-bounded organelle;nuclear inner membrane;nuclear membrane;nuclear part;nucleus;organelle;organelle inner membrane;organelle membrane;orga		Homo sapiens
cell part;cytoplasmic part;cytosol;extrinsic to membrane;intracellular part;membrane;membrane part;pre-autophagosomal structure membrane		Homo sapiens
extracellular membrane-bounded organelle;extracellular organelle;extracellular region part;extracellular vesicular exosome;membrane-bounded organelle;membrane-bounded vesicle;organelle;vesicle		Homo sapiens
cell part;centriole;centrosome;cytoplasm;cytoplasmic part;cytoskeletal part;intracellular non-membrane-bounded organelle;intracellular organelle;intracellular organelle part;intracellular part;macromolecular complex;membrane;microtubule;microtubule organizing center;microtubule organizing center part;non-membrane-bounded organelle;nuclear		Homo sapiens
cell part;centrosome;cytoplasm;cytoplasmic part;cytoskeletal part;integral to membrane;intracellular non-membrane-bounded organelle;intracellular organelle;intracellular organelle part;intracellular part;intrinsic to membrane;membrane;membrane part;microtubule organizing center;non-membrane-bounded organelle;nuclear part;nucleoplasm;orga		Homo sapiens
adherens junction;anchoring junction;cell junction;cell part;cell-substrate adherens junction;cell-substrate junction;focal adhesion;integral to membrane;intrinsic to membrane;membrane part		Homo sapiens
cell part;cell projection;cytoplasm;cytoplasmic part;cytoskeletal part;cytosol;extracellular membrane-bounded organelle;extracellular organelle;extracellular region part;extracellular vesicular exosome;filamentous actin;intracellular membrane-bounded organelle;intracellular organelle;intracellular organelle part;intracellular part;lamellipodium;macron		Homo sapiens
		Homo sapiens
adherens junction;anchoring junction;cell junction;cell part;cell-substrate adherens junction;cell-substrate junction;cytoplasm;focal adhesion;intracellular part		Homo sapiens
cell junction;cell part;cell-cell junction;cytoplasmic part;cytosol;intracellular part;membrane;occluding junction;plasma membrane;tight junction		Homo sapiens
cell part;cell surface;cytoplasmic part;extracellular membrane-bounded organelle;extracellular organelle;extracellular region part;extracellular space;extracellular vesicular exosome;integral to membrane;intracellular membrane-bounded organelle;intracellular organelle;intracellular organelle part;intracellular part;intrinsic to membrane;lysosomal mer		Homo sapiens
cell part;cell projection;cell projection part;contractile fiber;cytoplasm;cytoplasmic part;cytoskeleton;extracellular membrane-bounded organelle;extracellular organelle;extracellular region part;extracellular vesicular exosome;filopodium;growth cone;intracellular non-membrane-bounded organelle;intracellular organelle;intracellular part;lamellipodium		Homo sapiens
cell part;chromatin;chromosomal part;extracellular membrane-bounded organelle;extracellular organelle;extracellular region part;extracellular vesicular exosome;intracellular membrane-bounded organelle;intracellular organel	Cell cycle - yeast	Homo sapiens
		Homo sapiens
cell part;cytoplasm;cytoplasmic part;cytoskeletal part;extracellular membrane-bounded organelle;extracellular organelle;extracellular region part;extracellular vesicular exosome;intracellular membrane-bounded organelle;intracellular organelle;intracellular organelle part;intracellular part;membrane;membrane-bounded organelle;membrane-boundec		Homo sapiens
cell part;cytoplasmic part;Golgi apparatus;intracellular membrane-bounded organelle;intracellular organelle;intracellular part;membrane-bounded organelle;organelle		Homo sapiens
Cdc73/Paf1 complex;cell part;chromatin;chromosomal part;intracellular organelle part;intracellular part;macromolecular complex;nuclear body;nuclear part;nuclear speck;nucleoplasm part;organelle part;protein complex;transcription elongation factor complex;transcriptionally active chromatin		Homo sapiens
cell part;cytoplasmic part;intracellular membrane-bounded organelle;intracellular organelle;intracellular organelle lumen;intracellular organelle part;intracellular part;membrane-bounded organelle;membrane-enclosed lumen;m	Aminoacyl-tRNA biosynthesis	Homo sapiens
cell part;intracellular membrane-bounded organelle;intracellular organelle;intracellular part;membrane;membrane-bounded organelle;nucleus;organelle		Homo sapiens
cell part;intracellular non-membrane-bounded organelle;intracellular organelle;intracellular organelle part;intracellular part;non-membrane-bounded organelle;nuclear part;nucleolus;organelle;organelle part		Homo sapiens
cell part;cytoplasmic part;intracellular membrane-bounded organelle;intracellular organelle;intracellular part;membrane;membrane-bounded organelle;mitochondrion;organelle	One carbon pool by folate	Homo sapiens
actin cytoskeleton;adherens junction;anchoring junction;cell junction;cell part;cell-substrate adherens junction;cell-substrate junction;cytoplasm;cytoskeleton;focal adhesion;intracellular non-membrane-bounded organelle;intracellular organelle;intracellular part;non-membrane-bounded organelle;organelle		Homo sapiens
cell part;cytoplasmic part;endoplasmic reticulum;endoplasmic reticulum membrane;endoplasmic reticulum part;integral to membrane;intracellular membrane-bounded organelle;intracellular organelle;intracellular organelle pa	Biosynthesis of unsaturated fatty acids	Homo sapiens
cell part;intracellular membrane-bounded organelle;intracellular organelle;intracellular part;membrane-bounded organelle;nucleus;organelle		Homo sapiens
cell part;integral to membrane;intracellular membrane-bounded organelle;intracellular organelle;intracellular part;intrinsic to membrane;membrane;membrane part;membrane-bounded organelle;nucleus;organelle		Homo sapiens
cell part;cytoplasmic part;endoplasmic reticulum;extrinsic to membrane;intracellular membrane-bounded organelle;intracellular organelle;intracellular part;membrane;membrane part;membrane-bounded organelle;organelle		Homo sapiens
cell part;clathrin-coated vesicle;coated vesicle;cytoplasmic membrane-bounded vesicle;cytoplasmic part;cytoplasmic vesicle;endosome;exocytic vesicle;intracellular membrane-bounded organelle;intracellular organelle;intracellular part;late endosome;lysosome;lytic vacuole;membrane;membrane-bounded organelle;membrane-bounded vesicle;or		Homo sapiens
cell part;cytoplasmic part;endoplasmic reticulum;endoplasmic reticulum membrane;endoplasmic reticulum part;integral to membrane;intracellular membrane-bounded organelle;intracellular organelle;intracellular organelle part;intracellular part;intrinsic to membrane;membrane;membrane part;membrane-bounded organelle;organelle;organelle mem		Homo sapiens
cell part;cytoplasm;cytoplasmic membrane-bounded vesicle;cytoplasmic part;cytoplasmic vesicle;extracellular membrane-bounded organelle;extracellular organelle;extracellular region part;extracellular vesicular exosome;intracellular membrane-bounded organelle;intracellular organelle;intracellular part;macromolecular complex;melanosome;mem		Homo sapiens
cell part;cytoplasm;intracellular part;membrane		Homo sapiens
cell part;cytoplasm;cytoplasmic part;intracellular membrane-bounded organelle;intracellular non-membrane-bounded organelle;intracellular organelle;intracellular organelle part;intracellular part;membrane-bounded organelle;mitochondrial nucleoid;mitochondrial part;mitochondrion;non-membrane-bounded organelle;nucleoid;nucleus;organelle;or		Homo sapiens
		Homo sapiens
adherens junction;anchoring junction;cell junction;cell part;cell projection;cell-substrate adherens junction;cell-substrate junction;cytoplasmic part;cytosol;extracellular membrane-bounded organelle;extracellular organelle;ext	Regulation of actin cytoskeleton	Homo sapiens
cell part;cytoplasmic part;endoplasmic reticulum;endoplasmic reticulum membrane;endoplasmic reticulum part;Golgi apparatus part;Golgi membrane;Golgi stack;integral to membrane;intracellular membrane-bounded organ	Ether lipid metabolism;Glycerophospholipid metabolism	Homo sapiens
cell part;intracellular organelle part;intracellular part;nuclear part;nucleoplasm;organelle part		Homo sapiens
cell part;cytoplasmic part;cytosol;endosomal part;endosome membrane;ESCRT III complex;extracellular membrane-bounded organelle;extracellular organelle;extracellular region part;extracellular vesicular exosome;intracellu	Endocytosis	Homo sapiens
cell part;cytoplasm;cytoplasmic part;Golgi apparatus;intracellular membrane-bounded organelle;intracellular organelle;intracellular part;membrane-bounded organelle;nucleus;organelle		Homo sapiens
cell part;cytoplasm;envelope;intracellular organelle part;intracellular part;macromolecular complex;membrane;membrane part;nuclear envelope;nuclear membrane;nuclear part;nuclear pore;nucleoplasm;organelle envelope;or	RNA transport	Homo sapiens
cell part;cytoplasm;cytoplasmic part;intracellular membrane-bounded organelle;intracellular non-membrane-bounded organelle;intracellular organelle;intracellular part;macromolecular complex;membrane;membrane-bounded organelle;non-membrane-bounded organelle;nucleus;organelle;polysomal ribosome;ribonucleoprotein complex;ribosome		Homo sapiens
cell cortex;cell cortex part;cell part;centrosomal corona;centrosome;chromosomal part;condensed chromosome kinetochore;cortical cytoskeleton;cortical microtubule cytoskeleton;cytoplasmic microtubule;cytoplasmic part;cytoskeletal part;cytoskeleton;cytosol;extracellular membrane-bounded organelle;extracellular organelle;extracellular region		Homo sapiens
cell part;cytoplasmic part;endoplasmic reticulum lumen;endoplasmic reticulum part;intracellular organelle lumen;intracellular organelle part;intracellular part;membrane-enclosed lumen;organelle lumen;organelle part		Homo sapiens
cell part;integral to membrane;intrinsic to membrane;membrane;membrane part;plasma membrane		Homo sapiens
cell part;cytoplasm;intracellular membrane-bounded organelle;intracellular organelle;intracellular organelle part;intracellular part;membrane-bounded organelle;nuclear part;nucleoplasm;nucleus;organelle;organelle part		Homo sapiens
cell part;cytoplasmic part;endoplasmic reticulum membrane;endoplasmic reticulum part;extracellular membrane-bounded organelle;extracellular organelle;extracellular region part;extracellular vesicular exosome;integral to membrane;intracellular organelle part;intracellular part;intrinsic to membrane;membrane;membrane part;membrane-bounded		Homo sapiens
cell part;chromosomal part;condensed chromosome kinetochore;cytoplasm;cytoskeletal part;cytoskeleton;intracellular membrane-bounded organelle;intracellular non-membrane-bounded organelle;intracellular organelle;intracellular organelle part;intracellular part;kinetochore;macromolecular complex;membrane-bounded organelle;microtubule cy		Homo sapiens
cell part;chromatin;chromosomal part;intracellular organelle part;intracellular part;nuclear chromatin;nuclear chromosome part;nuclear part;organelle part		Homo sapiens
cell part;cytoplasm;intracellular membrane-bounded organelle;intracellular organelle;intracellular organelle part;intracellular part;macromolecular complex;membrane-bounded organelle;nuclear part;nucleoplasm;nucleus;orga	mRNA surveillance pathway	Homo sapiens
apical plasma membrane;cell junction;cell part;cell-cell junction;cytoplasm;cytoplasmic part;endoplasmic reticulum;endoplasmic reticulum membrane;endoplasmic reticulum part;integral to membrane;intercellular canaliculus;intracellular membrane-bounded organelle;intracellular non-membrane-bounded organelle;intracellular organelle;intracellula		Homo sapiens
		Homo sapiens
cell part;cytoplasmic part;cytosol;intracellular part	Butanoate metabolism	Homo sapiens
cell part;extracellular membrane-bounded organelle;extracellular organelle;extracellular region part;extracellular vesicular exosome;intracellular membrane-bounded organelle;intracellular organelle;intracellular part;membrane;membrane-bounded organelle;membrane-bounded vesicle;nucleus;organelle;vesicle		Homo sapiens
extracellular membrane-bounded organelle;extracellular organelle;extracellular region part;extracellular vesicular exosome;membrane-bounded organelle;membrane-bounded vesicle;organelle;vesicle		Homo sapiens
cell part;chylomicron;extracellular membrane-bounded organelle;extracellular organelle;extracellular region part;extracellular vesicular exosome;integral to membrane;intrinsic to membrane;low-density lipoprotein particle;macromolecular complex;membrane;membrane part;membrane-bounded organelle;membrane-bounded vesicle;organelle;plas		Homo sapiens
cell part;cytoplasmic part;endoplasmic reticulum membrane;endoplasmic reticulum part;integral to endoplasmic reticulum membrane;integral to membrane;integral to organelle membrane;intracellular organelle part;intracellular part;intrinsic to endoplasmic reticulum membrane;intrinsic to membrane;intrinsic to organelle membrane;membrane;mem		Homo sapiens
aggresome;cell part;inclusion body;intracellular membrane-bounded organelle;intracellular organelle;intracellular organelle part;intracellular part;membrane-bounded organelle;nuclear part;nucleoplasm;organelle;organelle part		Homo sapiens
cell part;cytoplasmic part;endoplasmic reticulum;Golgi apparatus;Golgi apparatus part;intracellular membrane-bounded organelle;intracellular organelle;intracellular organelle part;intracellular part;macromolecular complex;membrane-bounded organelle;organelle;organelle part;protein complex;TRAPP complex		Homo sapiens
adherens junction;anchoring junction;cell cortex;cell junction;cell part;cell-substrate adherens junction;cell-substrate junction;cytoplasmic part;cytoskeleton;focal adhesion;intracellular membrane-bounded organelle;intracellular non-membrane-bounded organelle;intracellular organelle;intracellular part;membrane;membrane-bounded organelle;non		Homo sapiens
cell part;membrane;plasma membrane		Homo sapiens
		Homo sapiens
cell part;cytoplasm;cytoplasmic part;cytosol;intracellular part	Alanine, aspartate and glutamate metabolism;Purine metabolism	Homo sapiens
cell part;cytoplasmic part;cytosol;intracellular part		Homo sapiens
cell part;membrane		Homo sapiens
actin cytoskeleton;cell part;chromatin;chromosomal part;chromosome;chromosome, centromeric region;cytoplasmic part;cytoskeleton;cytosol;intermediate filament cytoskeleton;intracellular membrane-bounded organelle;in	Cell cycle;Cell cycle - yeast;Meiosis - yeast	Homo sapiens
cell part;cytoplasmic part;endoplasmic reticulum;endoplasmic reticulum membrane;endoplasmic reticulum part;integral to membrane;intracellular membrane-bounded organelle;intracellular organelle;intracellular organelle part;intracellular part;intrinsic to membrane;membrane;membrane part;membrane-bounded organelle;organelle;organelle mem		Homo sapiens
cell part;cytoplasmic part;cytoplasmic vesicle membrane;cytoplasmic vesicle part;cytosol;Golgi apparatus part;Golgi membrane;Golgi-associated vesicle membrane;intracellular organelle part;intracellular part;membrane;orga	Endocytosis	Homo sapiens
cell part;cytoplasmic part;intracellular membrane-bounded organelle;intracellular non-membrane-bounded organelle;intracellular organelle;intracellular organelle part;intracellular part;macromolecular complex;membrane-bou	Bladder cancer;Cell cycle;Chronic myeloid leukemia;Glioma;Melanoma;Non-small cell lung cancer;p53 signaling pathway;F	Homo sapiens
actin filament bundle;actomyosin;adherens junction;anchoring junction;cell junction;cell part;cell projection;cell-substrate adherens junction;cell-substrate junction;cytoplasm;cytoplasmic part;cytoskeletal part;cytosol;filopodi	Regulation of actin cytoskeleton	Homo sapiens
cell part;cytoskeleton;intermediate filament cytoskeleton;intracellular membrane-bounded organelle;intracellular non-membrane-bounded organelle;intracellular organelle;intracellular organelle part;intracellular part;membrane-bounded organelle;non-membrane-bounded organelle;nuclear part;nucleoplasm;organelle;organelle part		Homo sapiens
cell part;cytoplasm;cytoplasmic part;cytosol;intracellular part;membrane;plasma membrane		Homo sapiens
cell part;cytoplasmic part;endoplasmic reticulum lumen;endoplasmic reticulum part;intracellular organelle lumen;intracellular organelle part;intracellular part;membrane;membrane-enclosed lumen;organelle lumen;organelle pa	Lysine degradation;Other types of O-glycan biosynthesis	Homo sapiens
cell part;integral to membrane;integral to nuclear inner membrane;integral to organelle membrane;intracellular organelle part;intracellular part;intrinsic to membrane;intrinsic to nuclear inner membrane;intrinsic to organelle membrane;membrane;membrane part;nuclear membrane;nuclear membrane part;nuclear part;organelle membrane;organelle p		Homo sapiens
cell part;cytoplasmic part;endoplasmic reticulum;endoplasmic reticulum membrane;endoplasmic reticulum part;Golgi apparatus;Golgi apparatus part;Golgi membrane;integral to membrane;intracellular membrane-bounded o	Ether lipid metabolism;Glycerophospholipid metabolism	Homo sapiens
		Homo sapiens
cell part;cytoplasm;intracellular membrane-bounded organelle;intracellular organelle;intracellular organelle part;intracellular part;membrane-bounded organelle;nuclear part;nucleoplasm;nucleus;organelle;organelle part	Notch signaling pathway	Homo sapiens
cell part;cytoplasm;intracellular membrane-bounded organelle;intracellular non-membrane-bounded organelle;intracellular organelle;intracellular organelle part;intracellular part;membrane;membrane-bounded organelle;non-membrane-bounded organelle;nuclear part;nucleolus;nucleus;organelle;organelle part		Homo sapiens
cell part;cytoplasmic part;endoplasmic reticulum lumen;endoplasmic reticulum part;integral to membrane;intracellular organelle lumen;intracellular organelle part;intracellular part;intrinsic to membrane;membrane part;membrane-enclosed lumen;organelle lumen;organelle part		Homo sapiens
cell part;cytoplasmic part;cytoskeleton;cytosol;intracellular non-membrane-bounded organelle;intracellular organelle;intracellular part;non-membrane-bounded organelle;organelle		Homo sapiens
cell part;chromosome;intracellular non-membrane-bounded organelle;intracellular organelle;intracellular organelle part;intracellular part;non-membrane-bounded organelle;nuclear part;nucleolus;nucleoplasm;organelle;organe	Lysine degradation	Homo sapiens
	Spliceosome	Homo sapiens
cell junction;cell part;cytoplasm;extracellular region part;intercellular bridge;intracellular membrane-bounded organelle;intracellular organelle;intracellular part;membrane-bounded organelle;nucleus;organelle		Homo sapiens
cell part;integral to membrane;integral to plasma membrane;intrinsic to membrane;intrinsic to plasma membrane;membrane;membrane part;plasma membrane;plasma membrane part		Homo sapiens
cell part;cytoplasmic membrane-bounded vesicle;cytoplasmic part;cytoplasmic vesicle;cytoplasmic vesicle membrane;cytoplasmic vesicle part;cytosol;endosome;extrinsic to membrane;intracellular membrane-bounded organelle;intracellular organelle;intracellular organelle part;intracellular part;membrane;membrane part;membrane-bounded orga		Homo sapiens
adherens junction;anchoring junction;cell junction;cell part;cell-substrate adherens junction;cell-substrate junction;cytoplasm;focal adhesion;intracellular membrane-bounded organelle;intracellular organelle;intracellular part;r	Adherens junction	Homo sapiens
cell part;cytoplasm;cytoplasmic part;intracellular non-membrane-bounded organelle;intracellular organelle;intracellular organelle part;intracellular part;macromolecular complex;membrane;non-membrane-bounded organelle;nuclear body;nuclear part;nuclear speck;nucleoplasm part;organelle;organelle part;ribonucleoprotein complex;RNA granule;s		Homo sapiens
Cajal body;cell part;histone methyltransferase complex;intracellular membrane-bounded organelle;intracellular organelle;intracellular organelle part;intracellular part;macromolecular complex;membrane-bounded organelle;me	Spliceosome	Homo sapiens
adherens junction;aggresome;anchoring junction;cell junction;cell part;cell projection membrane;cell projection part;cell-substrate adherens junction;cell-substrate junction;contractile fiber part;cytoplasm;cytoplasmic part;cytoskeleton;endoplasmic reticulum;endoplasmic reticulum membrane;endoplasmic reticulum part;envelope;extracellular mem		Homo sapiens

cell part;cytoplasm;intracellular membrane-bounded organelle;intracellular organelle;intracellular part;membrane-bounded organelle;nucleus;organelle		Homo sapiens
		Homo sapiens
cell part;intracellular membrane-bounded organelle;intracellular organelle;intracellular part;membrane-bounded organelle;nucleus;organelle		Homo sapiens
adherens junction;anchoring junction;cell junction;cell part;cell-cell adherens junction;cell-cell junction;centrosome;cytoplasm;cytoplasmic part;cytoskeletal part;cytoskeleton;cytosol;endosomal part;endosome membrane;extracellular membrane-bounded organelle;extracellular organelle;extracellular region part;extracellular vesicular exosome;intra		Homo sapiens
cell part;cytoplasm;cytoplasmic membrane-bounded vesicle lumen;cytoplasmic part;cytoplasmic vesicle part;cytoskeletal part;cytosol;endocytic vesicle lumen;extracellular membrane-bounded organelle;extracellular organel	Protein processing in endoplasmic reticulum	Homo sapiens
cell part;cytoplasm;cytoskeleton;intracellular non-membrane-bounded organelle;intracellular organelle;intracellular part;non-membrane-bounded organelle;organelle		Homo sapiens
cell part;cytoplasmic part;cytosol;intracellular part;membrane		Homo sapiens
cell part;cytoplasmic part;endoplasmic reticulum;extracellular matrix;extracellular membrane-bounded organelle;extracellular organelle;extracellular region part;extracellular space;extracellular vesicular exosome;intracellular membrane-bounded organelle;intracellular organelle;intracellular part;membrane-bounded organelle;membrane-bounded ve		Homo sapiens
actin cytoskeleton;Arp2/3 protein complex;cell part;cytoplasmic part;cytoskeletal part;cytoskeleton;cytosol;extracellular membrane-bounded organelle;extracellular organelle;extracellular region part;extracellular vesicular exc	Bacterial invasion of epithelial cells;Fc gamma R-mediated phagocytosis;Pathogenic Escherichia coli infection;Regulation c	Homo sapiens
cell part;cytoplasmic membrane-bounded vesicle;cytoplasmic part;cytoplasmic vesicle;cytosol;extracellular membrane-bounded organelle;extracellular organelle;extracellular region part;extracellular space;extracellular vesic	Folate biosynthesis	Homo sapiens
cell part;cytoplasm;intracellular membrane-bounded organelle;intracellular non-membrane-bounded organelle;intracellular organelle;intracellular organelle part;intracellular part;membrane;membrane-bounded organelle;non-membrane-bounded organelle;nuclear part;nucleolus;nucleoplasm;nucleus;organelle;organelle part		Homo sapiens
cell part;chromosomal part;chromosome, telomeric region;intracellular membrane-bounded organelle;intracellular organelle;intracellular organelle part;intracellular part;macromolecular complex;membrane;membrane-bounde	Homologous recombination;Non-homologous end-joining	Homo sapiens
cell part;cytoplasm;intracellular membrane-bounded organelle;intracellular organelle;intracellular organelle part;intracellular part;macromolecular complex;membrane;membrane-bounded organelle;nuclear part;nucleoplasm;nucleus;organelle;organelle part;ribonucleoprotein complex		Homo sapiens
cell part;cytoplasm;cytoplasmic part;cytosol;intracellular part;membrane;plasma membrane	ko05152;Regulation of actin cytoskeleton;Vascular smooth muscle contraction	Homo sapiens
cell part;cytoplasmic part;extracellular matrix;extracellular membrane-bounded organelle;extracellular organelle;extracellular region part;extracellular vesicular exosome;Golgi apparatus;Golgi apparatus part;Golgi membrane;	Cell adhesion molecules (CAMs)	Homo sapiens
cell part;chromatin;chromosomal part;cytoplasm;cytoplasmic mRNA processing body;cytoplasmic part;cytosol;exon-exon junction complex;intracellular membrane-bounded organelle;intracellular non-membrane-bounded or	mRNA surveillance pathway;RNA transport	Homo sapiens
cell part;cell pole;cell tip;clathrin-coated vesicle;coated vesicle;cytoplasmic membrane-bounded vesicle;cytoplasmic part;cytoplasmic vesicle;cytoplasmic vesicle membrane;cytoplasmic vesicle part;endocytic vesicle;endocytic vesicle membrane;endosome;extracellular membrane-bounded organelle;extracellular organelle;extracellular region part;		Homo sapiens
cell part;cytoplasmic part;cytosol;intracellular non-membrane-bounded organelle;intracellular organelle;intracellular organelle part;intracellular part;macromolecular complex;membrane;non-membrane-bounded organelle;nuclear part;nucleoplasm;organelle;organelle part;ribonucleoprotein complex;RNA granule;stress granule		Homo sapiens
cell part;cell projection;cilium;cytoplasm;cytoplasmic part;cytosol;extracellular membrane-bounded organelle;extracellular organelle;extracellular region part;extracellular vesicular exosome;intracellular membrane-bounded organelle;intracellular organelle;intracellular part;membrane-bounded organelle;membrane-bounded vesicle;nucleus;organelle		Homo sapiens
adherens junction;anchoring junction;cell body;cell junction;cell part;cell projection membrane;cell projection part;cell-cell junction;cell-substrate adherens junction;cell-substrate junction;cytoplasm;cytoplasmic membrane-bo	Pathogenic Escherichia coli infection	Homo sapiens
cell part;cytoplasmic part;cytosol;intracellular membrane-bounded organelle;intracellular organelle;intracellular organelle part;intracellular part;membrane-bounded organelle;nuclear body;nuclear part;nucleoplasm part;nucleus;organelle;organelle part;PML body		Homo sapiens
cell part;intracellular organelle part;intracellular part;macromolecular complex;multimeric ribonuclease P complex;nuclear part;nucleolar part;nucleolar ribonuclease P complex;organelle part;ribonuclease P complex;ribonucle	Ribosome biogenesis in eukaryotes;RNA transport	Homo sapiens
cell part;cytoplasmic part;endoplasmic reticulum lumen;endoplasmic reticulum part;endoplasmic reticulum-Golgi intermediate compartment;extracellular membrane-bounded organelle;extracellular organelle;extracellular region part;extracellular space;extracellular vesicular exosome;intracellular membrane-bounded organelle;intracellular organelle		Homo sapiens
axon;cell part;cell projection;extracellular membrane-bounded organelle;extracellular organelle;extracellular region part;extracellular vesicular exosome;integral to membrane;intrinsic to membrane;membrane;membrane part;membrane-bounded organelle;membrane-bounded vesicle;neuron projection;organelle;plasma membrane;synapse;vesicle		Homo sapiens
cell part;cytosolic large ribosomal subunit;intracellular organelle part;intracellular part;large ribosomal subunit;macromolecular complex;organelle part;ribonucleoprotein complex	Ribosome	Homo sapiens
cell part;cytoplasm;cytoplasmic part;Golgi apparatus;intracellular membrane-bounded organelle;intracellular non-membrane-bounded organelle;intracellular organelle;intracellular organelle part;intracellular part;membrane-bounded organelle;non-membrane-bounded organelle;nuclear part;nucleolus;organelle;organelle part		Homo sapiens
cell part;cytoplasmic part;endoplasmic reticulum membrane;endoplasmic reticulum part;endoplasmic reticulum-Golgi intermediate compartment;endoplasmic reticulum-Golgi intermediate compartment membrane;Golgi apparatus part;Golgi membrane;integral to membrane;intracellular membrane-bounded organelle;intracellular organelle;intracellu		Homo sapiens
cell part;intracellular non-membrane-bounded organelle;intracellular organelle;intracellular organelle part;intracellular part;macromolecular complex;non-membrane-bounded organelle;nuclear part;nucleolar part;nucleolus;org	Ribosome biogenesis in eukaryotes	Homo sapiens
cell part;cytoplasmic part;intracellular membrane-bounded organelle;intracellular organelle;intracellular part;membrane-bounded organelle;mitochondrion;organelle		Homo sapiens
cell part;cytoplasm;cytoplasmic part;extracellular membrane-bounded organelle;extracellular organelle;extracellular region part;extracellular vesicular exosome;integral to membrane;intracellular membrane-bounded organelle;intracellular organelle;intracellular part;intrinsic to membrane;membrane part;membrane-bounded organelle;membrane-bo		Homo sapiens
cell cortex part;cell part;cell projection part;cytoplasm;cytoplasmic part;cytosol;exocyst;growth cone membrane;intracellular part;macromolecular complex;membrane;membrane part;myelin sheath abaxonal region;plasma m	Tight junction	Homo sapiens
actin filament bundle;actomyosin;adherens junction;anchoring junction;cell cortex;cell junction;cell part;cell projection membrane;cell projection part;cell surface;cell-substrate adherens junction;cell-substrate junction;contractile fiber part;cytoplasm;cytoplasmic part;cytoskeletal part;cytosol;extrinsic to internal side of plasma membrane;extrinsic to		Homo sapiens
cell part;intracellular membrane-bounded organelle;intracellular organelle;intracellular organelle part;intracellular part;membrane-bounded organelle;nuclear part;nucleoplasm;nucleus;organelle;organelle part		Homo sapiens
cell part;cytoplasmic part;cytosol;extracellular membrane-bounded organelle;extracellular organelle;extracellular region part;extracellular vesicular exosome;intracellular part;membrane-bounded organelle;membrane-bounde	Carbon fixation in photosynthetic organisms;Pentose and glucuronate interconversions;Pentose phosphate pathway	Homo sapiens
cell part;cytoplasmic part;endoplasmic reticulum lumen;endoplasmic reticulum membrane;endoplasmic reticulum part;intracellular organelle lumen;intracellular organelle part;intracellular part;membrane;membrane part;membrane-enclosed lumen;organelle lumen;organelle membrane;organelle part		Homo sapiens
cell part;cytoplasmic part;intracellular membrane-bounded organelle;intracellular organelle;intracellular organelle part;intracellular part;macromolecular complex;membrane part;membrane-bounded organelle;mitochondrial membrane part;mitochondrial outer membrane translocase complex;mitochondrial part;mitochondrion;organelle;organelle par		Homo sapiens
		Homo sapiens
apical plasma membrane;cell part;cytoplasm;envelope;integral to membrane;intracellular organelle part;intracellular part;intrinsic to membrane;membrane;membrane part;nuclear envelope;nuclear part;organelle envelope;organelle part;plasma membrane;plasma membrane part		Homo sapiens
catalytic step 2 spliceosome;cell part;intracellular membrane-bounded organelle;intracellular organelle;intracellular organelle part;intracellular part;macromolecular complex;membrane-bounded organelle;nuclear part;nucleoplasm;nucleus;organelle;organelle part;ribonucleoprotein complex;spliceosomal complex		Homo sapiens
cell part;cytoplasmic part;intracellular organelle part;intracellular part;membrane-enclosed lumen;mitochondrial intermembrane space;mitochondrial part;organelle envelope lumen;organelle part		Homo sapiens
cell part;cytoplasm;cytoplasmic part;intracellular membrane-bounded organelle;intracellular organelle;intracellular part;membrane-bounded organelle;mitochondrion;organelle		Homo sapiens
cell part;membrane part;membrane raft		Homo sapiens
cell part;cytoplasm;extracellular membrane-bounded organelle;extracellular organelle;extracellular region part;extracellular vesicular exosome;intracellular part;membrane-bounded organelle;membrane-bounded vesicle;orga	Glycolysis / Gluconeogenesis	Homo sapiens
cell part;cytoplasm;intracellular part	Arginine and proline metabolism	Homo sapiens
cell part;cytoplasm;cytoplasmic part;cytosol;intracellular membrane-bounded organelle;intracellular organelle;intracellular organelle part;intracellular part;membrane-bounded organelle;mitochondrion;nuclear part;nucleoplasr	RNA degradation	Homo sapiens
Cdc48p-Npl4p-Ufd1p AAA ATPase complex;cell part;cytoplasmic part;endoplasmic reticulum;endoplasmic reticulum part;intracellular membrane-bounded organelle;intracellular organelle;intracellular organelle part;intracellular part;lipid particle;macromolecular complex;membrane part;membrane-bounded organelle;organelle;organelle part;protein		Homo sapiens
cell part;cytoplasm;cytoplasmic part;cytoskeletal part;intracellular organelle part;intracellular part;membrane;microtubule organizing center;midbody;organelle part;plasma membrane		Homo sapiens
cell part;cytoplasm;intracellular membrane-bounded organelle;intracellular non-membrane-bounded organelle;intracellular organelle;intracellular organelle part;intracellular part;membrane;membrane-bounded organelle;non-r	Ribosome biogenesis in eukaryotes;RNA transport	Homo sapiens
cell part;cytoplasmic part;intracellular non-membrane-bounded organelle;intracellular organelle;intracellular organelle part;intracellular part;macromolecular complex;membrane;mitochondrial inner membrane;mitochondrial membrane;mitochondrial part;non-membrane-bounded organelle;organelle;organelle inner membrane;organelle membrane;or		Homo sapiens
90S preribosome;cell part;intracellular membrane-bounded organelle;intracellular non-membrane-bounded organelle;intracellular organelle;intracellular organelle part;intracellular part;macromolecular complex;membrane-bounded organelle;non-membrane-bounded organelle;nuclear part;nucleolus;nucleus;organelle;organelle part;preribosome;ribo		Homo sapiens
		Homo sapiens
		Homo sapiens
cell part;cytoplasm;intracellular organelle part;intracellular part;nuclear part;nucleoplasm;organelle part		Homo sapiens
cell part;cytoplasmic part;intracellular non-membrane-bounded organelle;intracellular organelle;intracellular organelle part;intracellular part;macromolecular complex;membrane;mitochondrial inner membrane;mitochondrial membrane;mitochondrial part;mitochondrial ribosome;non-membrane-bounded organelle;organellar ribosome;organelle;orga		Homo sapiens
cell part;cytoplasm;cytoplasmic part;cytosol;endosomal part;endosome;endosome membrane;ESCRT II complex;extracellular membrane-bounded organelle;extracellular organelle;extracellular region part;extracellular vesicu	Endocytosis	Homo sapiens
cell part;cytoplasmic part;endoplasmic reticulum;endoplasmic reticulum lumen;endoplasmic reticulum membrane;endoplasmic reticulum part;intracellular membrane-bounded organelle;intracellular organelle;intracellular orga	Protein processing in endoplasmic reticulum;Vibrio cholerae infection	Homo sapiens
cell part;cytoplasmic part;endoplasmic reticulum;endoplasmic reticulum membrane;endoplasmic reticulum part;intracellular membrane-bounded organelle;intracellular organelle;intracellular organelle part;intracellular part;membrane;membrane part;membrane-bounded organelle;organelle;organelle membrane;organelle part		Homo sapiens
cell part;cytoplasm;intracellular membrane-bounded organelle;intracellular organelle;intracellular part;membrane;membrane-bounded organelle;nucleus;organelle		Homo sapiens
cell part;cytoplasm;extracellular membrane-bounded organelle;extracellular organelle;extracellular region part;extracellular vesicular exosome;intracellular part;membrane-bounded organelle;membrane-bounded vesicle;orga	Amino sugar and nucleotide sugar metabolism;Fructose and mannose metabolism	Homo sapiens
actin cytoskeleton;cell part;cytoplasm;cytoskeleton;intracellular membrane-bounded organelle;intracellular non-membrane-bounded organelle;intracellular organelle;intracellular part;membrane;membrane-bounded organelle;non-membrane-bounded organelle;nucleus;organelle;plasma membrane		Homo sapiens
cell part;centrosome;cytoplasm;cytoplasmic part;cytoskeletal part;endomembrane system;intracellular membrane-bounded organelle;intracellular non-membrane-bounded organelle;intracellular organelle;intracellular organelle part;intracellular part;macromolecular complex;membrane;membrane-bounded organelle;microtubule organizing center;n		Homo sapiens
cell part;cullin-RING ubiquitin ligase complex;cytoplasm;intracellular membrane-bounded organelle;intracellular organelle;intracellular part;macromolecular complex;membrane-bounded organelle;nucleus;organelle;protein complex;SCF ubiquitin ligase complex;ubiquitin ligase complex		Homo sapiens
cell part;cytoplasmic part;cytosol;extracellular membrane-bounded organelle;extracellular organelle;extracellular region part;extracellular vesicular exosome;intracellular part;membrane-bounded organelle;membrane-bounded vesicle;organelle;vesicle		Homo sapiens
cell junction;cell part;cytoplasm;cytoskeletal part;intracellular organelle part;intracellular part;membrane;organelle part;postsynaptic density;postsynaptic membrane;synapse part;synaptic membrane		Homo sapiens
cell part;intracellular membrane-bounded organelle;intracellular non-membrane-bounded organelle;intracellular organelle;intracellular organelle part;intracellular part;membrane-bounded organelle;non-membrane-bounded or	Homologous recombination	Homo sapiens
cell part;cytoplasmic part;cytoplasmic vesicle;cytosol;Golgi apparatus;Golgi apparatus part;Golgi cisterna membrane;Golgi membrane;intracellular membrane-bounded organelle;intracellular organelle;intracellular organelle p	Endocytosis	Homo sapiens
cell cortex;cell part;coated pit;cytoplasmic membrane-bounded vesicle;cytoplasmic part;cytoplasmic vesicle;cytoskeleton;intracellular membrane-bounded organelle;intracellular non-membrane-bounded organelle;intracellular organelle;intracellular part;lysosome;lytic vacuole;membrane;membrane part;membrane-bounded organelle;membrane-b		Homo sapiens
		Homo sapiens
cell part;cytoplasmic part;intracellular membrane-bounded organelle;intracellular organelle;intracellular part;membrane;membrane-bounded organelle;nucleus;organelle;perinuclear region of cytoplasm		Homo sapiens
cell part;cytoplasm;cytoplasmic part;endoplasmic reticulum;intracellular membrane-bounded organelle;intracellular organelle;intracellular organelle part;intracellular part;membrane;membrane-bounded organelle;nuclear matrix;nuclear part;nucleoplasm;nucleus;organelle;organelle part;plasma membrane		Homo sapiens
cell part;cytoplasm;cytoplasmic part;endosomal part;endosome membrane;Golgi apparatus;intracellular membrane-bounded organelle;intracellular organelle;intracellular organelle part;intracellular part;late endosome membrane;membrane;membrane-bounded organelle;organelle;organelle membrane;organelle part		Homo sapiens
cell part;intracellular organelle part;intracellular part;membrane;nuclear membrane;nuclear part;nucleoplasm;organelle membrane;organelle part		Homo sapiens
cell part;chromatin;chromosomal part;euchromatin;heterochromatin;intracellular membrane-bounded organelle;intracellular organelle;intracellular organelle part;intracellular part;membrane-bounded organelle;nuclear chromatin;nuclear chromosome part;nuclear heterochromatin;nuclear matrix;nuclear part;nucleus;organelle;organelle part;replicatio		Homo sapiens
actin cytoskeleton;adherens junction;anchoring junction;cell junction;cell part;cytoplasm;cytoplasmic part;cytoskeleton;cytosol;extracellular membrane-bounded organelle;extracellular organelle;extracellular region part;extracellular vesicular exosome;intracellular membrane-bounded organelle;intracellular non-membrane-bounded organelle;intrace		Homo sapiens
actin filament bundle;actomyosin;adherens junction;anchoring junction;cell junction;cell part;cell-cell junction;cell-substrate adherens junction;cell-substrate junction;cytoskeletal part;cytoskeleton;extracellular membrane-bounded organelle;extracellular organelle;extracellular region part;extracellular vesicular exosome;focal adhesion;intracellular n		Homo sapiens
cell part;cytoplasmic part;cytosol;endoplasmic reticulum;extracellular region;intracellular membrane-bounded organelle;intracellular organelle;intracellular part;lipid particle;membrane;membrane-bounded organelle;nucleus;organelle;plasma membrane		Homo sapiens
cell part;intracellular membrane-bounded organelle;intracellular non-membrane-bounded organelle;intracellular organelle;intracellular organelle part;intracellular part;membrane-bounded organelle;non-membrane-bounded organelle;nuclear part;nucleolus;nucleoplasm;nucleus;organelle;organelle part		Homo sapiens
cell part;intracellular organelle part;intracellular part;macromolecular complex;nuclear part;nucleoplasm;nucleoplasm part;organelle part;protein complex;transcription factor complex		Homo sapiens
cell part;cytoplasm;cytoplasmic part;cytosol;Golgi apparatus;intracellular membrane-bounded organelle;intracellular organelle;intracellular part;membrane-bounded organelle;nucleus;organelle;perinuclear region of cytoplasm		Homo sapiens
cell part;cytoplasmic part;cytosol;eukaryotic 43S preinitiation complex;eukaryotic 48S preinitiation complex;eukaryotic translation initiation factor 3 complex;intracellular part;macromolecular complex;protein complex;ribonuc	RNA transport	Homo sapiens
cell part;cytoplasm;cytoplasmic part;intracellular membrane-bounded organelle;intracellular organelle;intracellular organelle part;intracellular part;membrane;membrane-bounded organelle;microbody;microbody membrane;microbody part;organelle;organelle membrane;organelle part;peroxisomal membrane;peroxisomal part		Homo sapiens
cell part;cytoplasm;cytoplasmic part;cytoskeleton;cytosol;extracellular membrane-bounded organelle;extracellular organelle;extracellular region part;extracellular vesicular exosome;intracellular non-membrane-bounded organelle;intracellular organelle;intracellular organelle part;intracellular part;membrane;membrane-bounded organelle;membrane-		Homo sapiens
cell part;cytoplasm;cytoplasmic part;Golgi apparatus;Golgi apparatus part;intracellular membrane-bounded organelle;intracellular non-membrane-bounded organelle;intracellular organelle;intracellular organelle part;intracellular part;macromolecular complex;membrane;membrane-bounded organelle;non-membrane-bounded organelle;nuclear part		Homo sapiens
cell part;cytoplasm;cytoplasmic part;endoplasmic reticulum;intracellular membrane-bounded organelle;intracellular organelle;intracellular organelle lumen;intracellular organelle part;intracellular part;membrane;membrane-bou	Alzheimer's disease;Valine, leucine and isoleucine degradation	Homo sapiens
apical plasma membrane;basolateral plasma membrane;cell part;integral to membrane;integral to plasma membrane;intrinsic to membrane;intrinsic to plasma membrane;membrane;membrane part;plasma membrane;plasma membrane part		Homo sapiens
actin cytoskeleton;cell part;centrosome;chromosomal part;condensin complex;cytoplasm;cytoplasmic part;cytoskeletal part;cytoskeleton;cytosol;intracellular membrane-bounded organelle;intracellular non-membrane-bounc	Cell cycle - yeast	Homo sapiens
cell part;cytoplasm;intracellular non-membrane-bounded organelle;intracellular organelle;intracellular organelle part;intracellular part;membrane;non-membrane-bounded organelle;nuclear part;nucleolus;organelle;organelle part;plasma membrane		Homo sapiens
cell part;cytoplasmic part;intracellular membrane-bounded organelle;intracellular organelle;intracellular organelle part;intracellular part;membrane-bounded organelle;mitochondrion;nuclear part;nucleoplasm;nucleus;organelle	RNA transport	Homo sapiens
cell part;cytoplasmic part;intracellular membrane-bounded organelle;intracellular organelle;intracellular part;membrane-bounded organelle;mitochondrion;nucleus;organelle		Homo sapiens
cell part;intracellular membrane-bounded organelle;intracellular organelle;intracellular part;membrane-bounded organelle;nucleus;organelle		Homo sapiens
cell part;cytoplasmic part;endoplasmic reticulum membrane;endoplasmic reticulum part;extracellular region;intracellular organelle part;intracellular part;membrane;membrane part;organelle membrane;organelle part		Homo sapiens
actin cytoskeleton;actin filament;actin filament bundle;actomyosin;adherens junction;anchoring junction;cell junction;cell leading edge;cell part;cell periphery;cell projection;cell-substrate adherens junction;cell-substrate junction;cytoplasm;cytoplasmic part;cytoskeletal part;cytoskeleton;cytosol;extracellular membrane-bounded organelle;extracellu		Homo sapiens
cell part;cytoplasmic part;intracellular membrane-bounded organelle;intracellular non-membrane-bounded organelle;intracellular organelle;intracellular organelle part;intracellular part;macromolecular complex;membrane;membrane-bounded organelle;mitochondrial inner membrane;mitochondrial membrane;mitochondrial part;mitochondrion;non-n		Homo sapiens
		Homo sapiens
cell part;cytoplasm;intracellular part;membrane		Homo sapiens
		Homo sapiens
cell part;cell surface;cytoplasmic part;endoplasmic reticulum lumen;endoplasmic reticulum membrane;endoplasmic reticulum part;endoplasmic reticulum-Golgi intermediate compartment;extracellular membrane-bounded organelle;extracellular organelle;extracellular region part;extracellular vesicular exosome;intracellular membrane-bounded orga		Homo sapiens
cell division site part;cell part;cleavage furrow;cytoplasm;cytoplasmic part;cytoskeletal part;intracellular membrane-bounded organelle;intracellular organelle;intracellular organelle part;intracellular part;macromolecular complex;membrane-bounded organelle;microtubule;microtubule organizing center;nucleus;organelle;organelle part;protein comple		Homo sapiens
adherens junction;anchoring junction;cell junction;cell part;cell-cell junction;cytoplasm;cytoskeletal part;intracellular organelle part;intracellular part;lateral plasma membrane;macromolecular complex;membrane part;microtubule;occluding junction;organelle part;plasma membrane part;protein complex;tight junction		Homo sapiens
cell part;cytoplasmic part;extracellular membrane-bounded organelle;extracellular organelle;extracellular region part;extracellular space;extracellular vesicular exosome;intracellular membrane-bounded organelle;intracellular	Other glycan degradation	Homo sapiens
		Homo sapiens
		Homo sapiens
cell part;cytoplasm;cytoskeletal part;extracellular membrane-bounded organelle;extracellular organelle;extracellular region part;extracellular vesicular exosome;intracellular membrane-bounded organelle;intracellular organelle	Gap junction;Pathogenic Escherichia coli infection;Phagosome	Homo sapiens
cell part;cytoplasmic part;cytoskeletal part;intracellular organelle part;intracellular part;microtubule organizing center;nuclear part;nucleoplasm;organelle part	Ribosome biogenesis in eukaryotes;RNA transport	Homo sapiens
catalytic step 2 spliceosome;cell part;cytoplasmic part;extracellular membrane-bounded organelle;extracellular organelle;extracellular region part;extracellular vesicular exosome;intracellular membrane-bounded organelle;int	Spliceosome	Homo sapiens
cell part;cytoplasm;intracellular non-membrane-bounded organelle;intracellular organelle;intracellular organelle part;intracellular part;non-membrane-bounded organelle;nuclear part;nucleolus;organelle;organelle part		Homo sapiens
cell part;cytoplasmic part;endoplasmic reticulum membrane;endoplasmic reticulum part;extracellular membrane-bounded organelle;extracellular organelle;extracellular region part;extracellular vesicular exosome;integral to membrane;intracellular organelle part;intracellular part;intrinsic to membrane;membrane;membrane part;membrane-bounded		Homo sapiens
cell part;cytoplasm;extracellular membrane-bounded organelle;extracellular organelle;extracellular region part;extracellular vesicular exosome;intracellular membrane-bounded organelle;intracellular non-membrane-bounded	Benzoate degradation;Butanoate metabolism;Carbon fixation pathways in prokaryotes;Fatty acid metabolism;Glyoxylate ar	Homo sapiens
cell part;cytoplasm;cytoplasmic part;intracellular membrane-bounded organelle;intracellular non-membrane-bounded organelle;intracellular organelle;intracellular organelle part;intracellular part;macromolecular complex;membrane-bounded organelle;non-membrane-bounded organelle;nuclear body;nuclear part;nuclear speck;nucleolus;nucleoplas		Homo sapiens
cell part;cytoplasmic part;integral to membrane;intracellular membrane-bounded organelle;intracellular organelle;intracellular organelle part;intracellular part;intrinsic to membrane;membrane;membrane part;membrane-bounded organelle;mitochondrial membrane;mitochondrial part;mitochondrion;organelle;organelle membrane;organelle part		Homo sapiens
cell part;cytoplasmic part;endosome;extracellular membrane-bounded organelle;extracellular organelle;extracellular region part;extracellular space;extracellular vesicular exosome;Golgi apparatus part;intracellular membrane-bounded organelle;intracellular organelle;intracellular organelle part;intracellular part;late endosome;lysosome;lytic vacuole;		Homo sapiens
cell part;cytoplasm;intracellular membrane-bounded organelle;intracellular organelle;intracellular organelle part;intracellular part;membrane;membrane-bounded organelle;nuclear part;nucleoplasm;nucleus;organelle;organelle part;plasma membrane		Homo sapiens
cell part;intracellular membrane-bounded organelle;intracellular organelle;intracellular part;membrane-bounded organelle;organelle		Homo sapiens
		Homo sapiens
cell part;intracellular membrane-bounded organelle;intracellular non-membrane-bounded organelle;intracellular organelle;intracellular organelle part;intracellular part;membrane-bounded organelle;non-membrane-bounded organelle;nuclear part;nucleolus;organelle;organelle part		Homo sapiens
		Homo sapiens
blood microparticle;cell part;cytoplasm;cytoplasmic part;eukaryotic translation initiation factor 2 complex;extracellular region part;extracellular space;intracellular part;macromolecular complex;protein complex		Homo sapiens
cell part;cytoplasmic part;intracellular non-membrane-bounded organelle;intracellular organelle;intracellular organelle part;intracellular part;large ribosomal subunit;macromolecular complex;membrane;mitochondrial inner membrane;mitochondrial membrane;mitochondrial part;mitochondrial ribosome;non-membrane-bounded organelle;organellar r		Homo sapiens
cell part;cytoplasmic part;intracellular non-membrane-bounded organelle;intracellular organelle;intracellular organelle part;intracellular part;large ribosomal subunit;macromolecular complex;membrane;mitochondrial inner me	Ribosome	Homo sapiens
cell part;cytoplasmic part;cytosolic part;cytosolic ribosome;intracellular non-membrane-bounded organelle;intracellular organelle;intracellular organelle part;intracellular part;macromolecular complex;membrane;mitochondrial inner membrane;mitochondrial membrane;mitochondrial part;non-membrane-bounded organelle;organelle;organelle inner r		Homo sapiens
cell part;intracellular organelle part;intracellular part;membrane;nuclear part;nucleoplasm;organelle part		Homo sapiens
cell part;cytoplasmic part;intracellular membrane-bounded organelle;intracellular organelle;intracellular organelle part;intracellular part;macromolecular complex;membrane;membrane-bounded organelle;mitochondrial inner membrane;mitochondrial membrane;mitochondrial part;mitochondrial small ribosomal subunit;mitochondrion;nuclear part;nu		Homo sapiens
cell part;cytoplasmic part;cytoskeleton;cytosol;extracellular membrane-bounded organelle;extracellular organelle;extracellular region;extracellular region part;extracellular vesicular exosome;intracellular non-membrane-bounded organelle;intracellular organelle;intracellular part;membrane-bounded organelle;membrane-bounded vesicle;non-membr		Homo sapiens
cell part;cytoplasm;cytoplasmic part;Golgi apparatus;intracellular membrane-bounded organelle;intracellular non-membrane-bounded organelle;intracellular organelle;intracellular organelle part;intracellular part;membrane;me	Ribosome biogenesis in eukaryotes	Homo sapiens
cell part;cytoplasmic membrane-bounded vesicle;cytoplasmic part;cytoplasmic vesicle;cytoplasmic vesicle membrane;cytoplasmic vesicle part;endocytic vesicle;endocytic vesicle membrane;extracellular membrane-bounded organelle;extracellular organelle;extracellular region part;extracellular vesicular exosome;Golgi apparatus;Golgi apparatus		Homo sapiens
cell part;cytoplasm;extracellular membrane-bounded organelle;extracellular organelle;extracellular region part;extracellular vesicular exosome;intracellular part;membrane;membrane-bounded organelle;membrane-bounded vesicle;organelle;plasma membrane;vesicle		Homo sapiens
cell part;cytoplasm;intracellular membrane-bounded organelle;intracellular organelle;intracellular organelle part;intracellular part;macromolecular complex;membrane;membrane-bounded organelle;nuclear part;nucleoplasm;nucleus;organelle;organelle part;ribonucleoprotein complex;spliceosomal complex		Homo sapiens
cell part;intracellular membrane-bounded organelle;intracellular organelle;intracellular organelle part;intracellular part;membrane-bounded organelle;nuclear body;nuclear part;nuclear speck;nucleoplasm part;nucleus;organelle;organelle part		Homo sapiens
axon part;axon terminus;cell body;cell junction;cell part;cell projection;cell projection part;cytoskeletal part;dendrite;intracellular organelle part;intracellular part;membrane;neuron projection;neuron projection terminus;neuronal cell body;organelle part;postsynaptic density;postsynaptic membrane;synapse part;synaptic membrane		Homo sapiens

cell part;intracellular non-membrane-bounded organelle;intracellular organelle;intracellular organelle part;intracellular part;macromolecular complex;non-membrane-bounded organelle;nuclear part;nucleolar part;nucleolus;nucleoplasm;organelle;organelle part;PeBoW complex;preribosome;preribosome, large subunit precursor;protein complex;ribo		Homo sapiens
cell part;intracellular membrane-bounded organelle;intracellular non-membrane-bounded organelle;intracellular organelle;intracellular organelle part;intracellular part;membrane-bounded organelle;non-membrane-bounded organelle;nuclear part;nucleolus;nucleus;organelle;organelle part		Homo sapiens
cell part;DNA-directed RNA polymerase complex;DNA-directed RNA polymerase I complex;intracellular membrane-bounded organelle;intracellular non-membrane-bounded organelle;intracellular organelle;intracellular organe	Purine metabolism;Pyrimidine metabolism;RNA polymerase	Homo sapiens
Cdc73/Paf1 complex;cell part;chromatin;chromosomal part;cytoplasm;cytoplasmic part;intracellular membrane-bounded organelle;intracellular organelle;intracellular organelle part;intracellular part;macromolecular complex;r	RNA degradation	Homo sapiens
cell part;cytoplasm;cytoplasmic part;cytosol;intracellular membrane-bounded organelle;intracellular organelle;intracellular part;membrane-bounded organelle;nucleus;organelle	Pathways in cancer;Renal cell carcinoma	Homo sapiens
cell part;cytoplasm;extracellular membrane-bounded organelle;extracellular organelle;extracellular region part;extracellular vesicular exosome;intracellular part;membrane-bounded organelle;membrane-bounded vesicle;organelle;vesicle		Homo sapiens
cell part;cytoplasmic part;intracellular membrane-bounded organelle;intracellular organelle;intracellular part;membrane-bounded organelle;mitochondrion;organelle		Homo sapiens
Cajal body;cell part;cytoplasm;cytoplasmic part;cytosol;endoplasmic reticulum;intracellular membrane-bounded organelle;intracellular organelle;intracellular organelle part;intracellular part;membrane;membrane-bounded org	Ribosome biogenesis in eukaryotes	Homo sapiens
cell part;intracellular membrane-bounded organelle;intracellular non-membrane-bounded organelle;intracellular organelle;intracellular organelle part;intracellular part;membrane;membrane-bounded organelle;non-membrane-	Ribosome biogenesis in eukaryotes	Homo sapiens
cell part;cytoplasmic part;extracellular region part;extracellular space;intracellular membrane-bounded organelle;intracellular non-membrane-bounded organelle;intracellular organelle;intracellular organelle part;intracellular part;macromolecular complex;membrane;membrane-bounded organelle;mitochondrial inner membrane;mitochondrial membra		Homo sapiens
cell part;cytoplasmic membrane-bounded vesicle;cytoplasmic part;cytoplasmic vesicle;endocytic vesicle;endosomal part;endosome;endosome membrane;HOPS complex;intracellular membrane-bounded organelle;intracellular organelle;intracellular organelle part;intracellular part;late endosome;late endosome membrane;lysosomal membrane;lys		Homo sapiens
catalytic step 2 spliceosome;cell part;intracellular organelle part;intracellular part;macromolecular complex;nuclear part;organelle part;ribonucleoprotein complex;spliceosomal complex		Homo sapiens
cell part;extracellular membrane-bounded organelle;extracellular organelle;extracellular region part;extracellular vesicular exosome;integral to membrane;integral to plasma membrane;intrinsic to membrane;intrinsic to plasma membrane;membrane;membrane part;membrane-bounded organelle;membrane-bounded vesicle;organelle;plasma memb		Homo sapiens
cell part;chromosomal part;chromosome, telomeric region;cytoplasm;cytoplasmic part;cytosol;extracellular membrane-bounded organelle;extracellular organelle;extracellular region part;extracellular vesicular exosome;intrace	RNA degradation	Homo sapiens
extracellular membrane-bounded organelle;extracellular organelle;extracellular region part;extracellular vesicular exosome;membrane-bounded organelle;membrane-bounded vesicle;organelle;vesicle		Homo sapiens
cell part;cytoplasm;cytoplasmic part;cytosol;intracellular membrane-bounded organelle;intracellular organelle;intracellular organelle part;intracellular part;membrane-bounded organelle;membrane-enclosed lumen;mitochondrial intermembrane space;mitochondrial part;nucleus;organelle;organelle envelope lumen;organelle part		Homo sapiens
cell part;cytoplasmic part;endoplasmic reticulum;endoplasmic reticulum membrane;endoplasmic reticulum part;integral to membrane;intracellular membrane-bounded organelle;intracellular non-membrane-bounded organelle;intracellular organelle;intracellular organelle part;intracellular part;intrinsic to membrane;membrane;membrane part;membra		Homo sapiens
cell part;cytoplasm;intracellular organelle part;intracellular part;macromolecular complex;NELF complex;nuclear part;nucleoplasm;nucleoplasm part;organelle part;protein complex;transcription elongation factor complex		Homo sapiens
cell part;cytoplasm;cytoplasmic part;Golgi apparatus;intracellular membrane-bounded organelle;intracellular organelle;intracellular organelle part;intracellular part;membrane-bounded organelle;nuclear part;nucleoplasm;organelle;organelle part;perinuclear region of cytoplasm		Homo sapiens
basement membrane;extracellular matrix part;extracellular region part		Homo sapiens
cell part;cytoplasmic part;cytoskeleton;cytosol;extrinsic to membrane;intracellular non-membrane-bounded organelle;intracellular organelle;intracellular part;membrane;membrane part;non-membrane-bounded organelle;orga	Tight junction	Homo sapiens
cell part;cytoplasmic part;early endosome membrane;endosomal part;endosome membrane;extracellular membrane-bounded organelle;extracellular organelle;extracellular region part;extracellular vesicular exosome;intracell	Endocytosis	Homo sapiens
cell part;extracellular region;integral to membrane;intrinsic to membrane;membrane;membrane part;plasma membrane		Homo sapiens
cell part;cytoplasmic part;cytosol;intracellular part		Homo sapiens
cell part;cytoplasm;intracellular part		Homo sapiens
cell part;cytoplasmic part;Golgi apparatus;intracellular membrane-bounded organelle;intracellular non-membrane-bounded organelle;intracellular organelle;intracellular organelle part;intracellular part;membrane-bounded organelle;non-membrane-bounded organelle;nuclear part;nucleolus;organelle;organelle part		Homo sapiens
cell part;membrane		Homo sapiens
		Homo sapiens
cell part;chromosome;cytoplasm;intracellular membrane-bounded organelle;intracellular non-membrane-bounded organelle;intracellular organelle;intracellular organelle part;intracellular part;membrane;membrane-bounded o	Lysine degradation	Homo sapiens
cell part;cytoplasmic part;intracellular membrane-bounded organelle;intracellular organelle;intracellular organelle lumen;intracellular organelle part;intracellular part;membrane-bounded organelle;membrane-enclosed lumen;mitochondrial matrix;mitochondrial part;mitochondrion;organelle;organelle lumen;organelle part		Homo sapiens
cell part;cytoplasmic part;cytosol;intracellular membrane-bounded organelle;intracellular organelle;intracellular organelle lumen;intracellular organelle part;intracellular part;membrane-bounded organelle;membrane-enclosed l	Valine, leucine and isoleucine degradation	Homo sapiens
ATP-binding cassette (ABC) transporter complex;cell part;cytoplasmic part;endoplasmic reticulum;endosome;envelope;extracellular membrane-bounded organelle;extracellular organelle;extracellular region part;extracellular v	ABC transporters	Homo sapiens
cell part;intracellular membrane-bounded organelle;intracellular organelle;intracellular part;membrane-bounded organelle;nucleus;organelle		Homo sapiens
cell part;cytoplasmic part;intracellular membrane-bounded organelle;intracellular organelle;intracellular organelle lumen;intracellular organelle part;intracellular part;membrane-bounded organelle;membrane-enclosed lumen;m	Aminoacyl-tRNA biosynthesis	Homo sapiens
cell part;cytoplasmic part;intracellular non-membrane-bounded organelle;intracellular organelle;intracellular organelle part;intracellular part;macromolecular complex;membrane;mitochondrial inner membrane;mitochondrial membrane;mitochondrial part;non-membrane-bounded organelle;organelle;organelle inner membrane;organelle membrane;or		Homo sapiens
cell part;cytoplasm;intracellular part;membrane;plasma membrane		Homo sapiens
cell part;integral to membrane;intrinsic to membrane;membrane part		Homo sapiens
cell part;chromatin;chromosomal part;cytoplasm;cytoplasmic exosome (RNase complex);cytoplasmic part;cytosol;exosome (RNase complex);intracellular membrane-bounded organelle;intracellular non-membrane-bounded	RNA degradation	Homo sapiens
actin cytoskeleton;actomyosin contractile ring;cell cortex part;cell division site part;cell part;contractile ring;cytoplasmic part;cytoskeletal part;cytoskeleton;intracellular membrane-bounded organelle;intracellular non-membrane-bounded organelle;intracellular organelle;intracellular organelle part;intracellular part;membrane-bounded organelle;non-		Homo sapiens
cell part;cytoplasm;cytoplasmic part;cytosol;extracellular membrane-bounded organelle;extracellular organelle;extracellular region part;extracellular vesicular exosome;intracellular part;membrane-bounded organelle;membrane-bounded vesicle;organelle;vesicle		Homo sapiens
cell junction;cell part;cytoplasm;cytoskeleton;inhibitory synapse;intracellular non-membrane-bounded organelle;intracellular organelle;intracellular part;membrane;non-membrane-bounded organelle;organelle;plasma membrane;postsynaptic membrane;synapse;synapse part;synaptic membrane		Homo sapiens
actin cytoskeleton;actin filament bundle;actomyosin;adherens junction;anchoring junction;cell junction;cell part;cell projection;cell-substrate adherens junction;cell-substrate junction;cytoplasm;cytoskeletal part;cytoskeleton;focal adhesion;intracellular membrane-bounded organelle;intracellular non-membrane-bounded organelle;intracellular organ		Homo sapiens
cell part;cytoplasm;intracellular membrane-bounded organelle;intracellular non-membrane-bounded organelle;intracellular organelle;intracellular organelle part;intracellular part;membrane;membrane-bounded organelle;non-membrane-bounded organelle;nuclear part;nucleolus;nucleoplasm;nucleus;organelle;organelle part		Homo sapiens
cell part;chromatin;chromosomal part;chromosome;chromosome, centromeric region;cytoplasmic part;cytosol;intracellular membrane-bounded organelle;intracellular non-membrane-bounded organelle;intracellular organelle;intracellular organelle part;intracellular part;membrane-bounded organelle;non-membrane-bounded organelle;nuclear part;r		Homo sapiens
cell part;chromosomal part;condensin complex;cytoplasm;cytoplasmic part;cytosol;intracellular membrane-bounded organelle;intracellular organelle;intracellular organelle part;intracellular part;macromolecular complex;mem	Cell cycle - yeast	Homo sapiens
cell part;cytoplasm;intracellular membrane-bounded organelle;intracellular non-membrane-bounded organelle;intracellular organelle;intracellular organelle part;intracellular part;macromolecular complex;membrane-bounded organelle;non-membrane-bounded organelle;nuclear part;nucleolus;nucleus;organelle;organelle part;protein complex		Homo sapiens
actin cytoskeleton;adherens junction;anchoring junction;cell junction;cell part;cell-substrate adherens junction;cell-substrate junction;contractile fiber part;cytoplasm;cytoplasmic part;cytoskeleton;cytosol;focal adhesion;intra	Focal adhesion	Homo sapiens
		Homo sapiens
cell part;cytoplasmic part;integral to membrane;intracellular membrane-bounded organelle;intracellular non-membrane-bounded organelle;intracellular organelle;intracellular organelle part;intracellular part;intrinsic to membrane;membrane;membrane part;membrane-bounded organelle;mitochondrial inner membrane;mitochondrial membrane;mitoc		Homo sapiens
cell part;intracellular membrane-bounded organelle;intracellular non-membrane-bounded organelle;intracellular organelle;intracellular organelle part;intracellular part;membrane-bounded organelle;non-membrane-bounded organelle;nuclear part;nucleolus;nucleus;organelle;organelle part		Homo sapiens
cell part;cytoplasmic part;intracellular membrane-bounded organelle;intracellular organelle;intracellular organelle part;intracellular part;membrane-bounded organelle;mitochondrion;nuclear part;nucleoplasm;organelle;organelle part		Homo sapiens
extracellular membrane-bounded organelle;extracellular organelle;extracellular region part;extracellular vesicular exosome;membrane-bounded organelle;membrane-bounded vesicle;organelle;vesicle		Homo sapiens
cell part;cytoplasmic part;endosome;intracellular membrane-bounded organelle;intracellular organelle;intracellular part;membrane;membrane-bounded organelle;mitochondrion;organelle		Homo sapiens
cell part;integral to membrane;intrinsic to membrane;membrane part		Homo sapiens
cell part;cytoplasmic part;intracellular membrane-bounded organelle;intracellular organelle;intracellular part;membrane-bounded organelle;mitochondrion;nucleus;organelle		Homo sapiens
cell part;cytoplasmic part;intracellular membrane-bounded organelle;intracellular organelle;intracellular part;membrane-bounded organelle;mitochondrion;organelle		Homo sapiens
cell part;contractile fiber part;cytoplasmic part;cytoskeletal part;intracellular organelle part;intracellular part;organelle part;striated muscle thin filament		Homo sapiens
cell part;cytoplasmic part;endoplasmic reticulum;endoplasmic reticulum lumen;endoplasmic reticulum part;endoplasmic reticulum-Golgi intermediate compartment;extracellular membrane-bounded organelle;extracellular org	Protein processing in endoplasmic reticulum	Homo sapiens
cell part;cytoplasmic part;integral to membrane;intracellular;intracellular membrane-bounded organelle;intracellular organelle;intracellular organelle part;intracellular part;intrinsic to membrane;membrane;membrane part;membrane-bounded organelle;mitochondrial inner membrane;mitochondrial membrane;mitochondrial part;mitochondrion;organe		Homo sapiens
caveola;cell part;cytoplasmic part;cytoplasmic vesicle;cytoplasmic vesicle membrane;cytoplasmic vesicle part;envelope;extracellular membrane-bounded organelle;extracellular organelle;extracellular region part;extracellular vesicular exosome;integral to membrane;intracellular membrane-bounded organelle;intracellular organelle;intracellular orga		Homo sapiens
caveola;cell part;cytoplasmic part;endosomal part;endosome membrane;extracellular membrane-bounded organelle;extracellular organelle;extracellular region part;extracellular vesicular exosome;intracellular membrane-bou	Endocytosis	Homo sapiens
cell part;integral to membrane;integral to plasma membrane;intrinsic to membrane;intrinsic to plasma membrane;membrane;membrane part;plasma membrane;plasma membrane part	Olfactory transduction	Homo sapiens
cell part;cytoplasmic part;cytoskeletal part;cytosol;intermediate filament;intracellular membrane-bounded organelle;intracellular organelle;intracellular organelle part;intracellular part;macromolecular complex;membrane-bounded organelle;nucleus;organelle;organelle part;protein complex		Homo sapiens
cell part;cytoplasmic part;intracellular membrane-bounded organelle;intracellular organelle;intracellular organelle part;intracellular part;large ribosomal subunit;macromolecular complex;membrane;membrane-bounded organelle;mitochondrial inner membrane;mitochondrial large ribosomal subunit;mitochondrial membrane;mitochondrial part;mitoch		Homo sapiens
cell part;cytoplasm;cytoplasmic part;extracellular membrane-bounded organelle;extracellular organelle;extracellular region part;extracellular vesicular exosome;integral to membrane;intracellular membrane-bounded organelle	Alzheimer's disease;Huntington's disease;Oxidative phosphorylation;Parkinson's disease	Homo sapiens
cell part;chromosomal part;chromosome, centromeric core region;cytoplasmic part;cytoskeletal part;cytosol;intracellular non-membrane-bounded organelle;intracellular organelle;intracellular organelle part;intracellular part;macromolecular complex;microtubule;midbody;non-membrane-bounded organelle;nuclear part;nucleolus;organelle;organelle		Homo sapiens
cell part;cytoplasm;extracellular membrane-bounded organelle;extracellular organelle;extracellular region part;extracellular vesicular exosome;intracellular membrane-bounded organelle;intracellular organelle;intracellular part;membrane;membrane-bounded organelle;membrane-bounded vesicle;nucleus;organelle;vesicle		Homo sapiens
cell part;cytoplasmic part;endoplasmic reticulum;endoplasmic reticulum part;integral to endoplasmic reticulum membrane;integral to membrane;integral to organelle membrane;intracellular membrane-bounded organelle;intra	Protein processing in endoplasmic reticulum	Homo sapiens
cell part;cytoplasm;cytoplasmic part;cytosol;intracellular membrane-bounded organelle;intracellular organelle;intracellular part;membrane-bounded organelle;nucleus;organelle	Aminoacyl-tRNA biosynthesis;Valine, leucine and isoleucine biosynthesis	Homo sapiens
cell part;cytoplasm;intracellular organelle part;intracellular part;nuclear body;nuclear part;nuclear speck;nucleoplasm;nucleoplasm part;organelle part		Homo sapiens
cell part;cytoplasmic part;early endosome;endosomal part;endosome;endosome membrane;extracellular membrane-bounded organelle;extracellular organelle;extracellular region part;extracellular vesicular exosome;intracellular membrane-bounded organelle;intracellular organelle;intracellular organelle part;intracellular part;lysosomal membrane;m		Homo sapiens
cell part;cytoplasmic part;extracellular membrane-bounded organelle;extracellular organelle;extracellular region part;extracellular vesicular exosome;intracellular membrane-bounded organelle;intracellular organelle;intracellula	Citrate cycle (TCA cycle);Propanoate metabolism	Homo sapiens
cell part;cytoplasm;cytoplasmic part;cytosol;eukaryotic 43S preinitiation complex;eukaryotic 48S preinitiation complex;eukaryotic translation initiation factor 3 complex;extracellular membrane-bounded organelle;extracellular organelle;extracellular region part;extracellular vesicular exosome;intracellular membrane-bounded organelle;intracellular or		Homo sapiens
cell part;cytoplasm;cytoplasmic part;cytosol;extracellular membrane-bounded organelle;extracellular organelle;extracellular region part;extracellular vesicular exosome;intracellular organelle lumen;intracellular organelle part;i	Glyoxylate and dicarboxylate metabolism;Pyruvate metabolism	Homo sapiens
cell part;cytoplasmic part;endoplasmic reticulum;extracellular membrane-bounded organelle;extracellular organelle;extracellular region part;extracellular space;extracellular vesicular exosome;intracellular membrane-boundec	Lysosome	Homo sapiens
cell part;cytoplasmic part;cytosol;intracellular organelle part;intracellular part;nuclear body;nuclear part;nucleoplasm part;organelle part;PML body		Homo sapiens
adherens junction;anchoring junction;cell junction;cell part;cell-cell junction;cytoplasm;cytoplasmic part;cytosol;intracellular organelle part;intracellular part;membrane;nuclear part;nucleoplasm;occluding junction;organelle pa	Tight junction;Vibrio cholerae infection	Homo sapiens
cell part;chromatin;chromatin remodeling complex;chromosomal part;chromosome, centromeric region;cytoplasm;cytoplasmic part;cytosol;heterochromatin;intracellular membrane-bounded organelle;intracellular non-memb	Amyotrophic lateral sclerosis (ALS);MAPK signaling pathway	Homo sapiens
ATP-binding cassette (ABC) transporter complex;cell part;cytoplasmic part;envelope;intracellular organelle part;intracellular part;macromolecular complex;membrane;membrane part;mitochondrial envelope;mitochondrial part;organelle envelope;organelle part;protein complex		Homo sapiens
cell part;cytoplasmic part;integral to membrane;integral to mitochondrial membrane;integral to mitochondrial outer membrane;integral to organelle membrane;intracellular organelle part;intracellular part;intrinsic to membrane;intrinsic to mitochondrial outer membrane;intrinsic to organelle membrane;membrane part;mitochondrial membrane part;mi		Homo sapiens
actin cytoskeleton;actin filament bundle;actomyosin;adherens junction;anchoring junction;cell division site part;cell junction;cell part;cell-substrate adherens junction;cell-substrate junction;cleavage furrow;cytoplasm;cytoskeletal part;cytoskeleton;focal adhesion;intracellular non-membrane-bounded organelle;intracellular organelle;intracellular orga		Homo sapiens
		Homo sapiens
cell part;cytoplasmic part;extracellular membrane-bounded organelle;extracellular organelle;extracellular region part;extracellular vesicular exosome;intracellular membrane-bounded organelle;intracellular organelle;intracellular organelle part;intracellular part;lysosome;lytic vacuole;macromolecular complex;membrane;membrane-bounded organelle		Homo sapiens
cell part;cytoplasmic membrane-bounded vesicle;cytoplasmic part;cytoplasmic vesicle;cytosol;extracellular membrane-bounded organelle;extracellular organelle;extracellular region part;extracellular vesicular exosome;Golgi apparatus;intracellular membrane-bounded organelle;intracellular organelle;intracellular part;lysosome;lytic vacuole;membra		Homo sapiens
cell part;extracellular membrane-bounded organelle;extracellular organelle;extracellular region part;extracellular vesicular exosome;integral to membrane;intrinsic to membrane;membrane part;membrane-bounded organelle;membrane-bounded vesicle;organelle;vesicle		Homo sapiens
cell part;cytoskeletal part;intermediate filament;intracellular membrane-bounded organelle;intracellular non-membrane-bounded organelle;intracellular organelle;intracellular organelle lumen;intracellular organelle part;intracellular part;lamin filament;macromolecular complex;membrane-bounded organelle;membrane-enclosed lumen;non-membrane		Homo sapiens
cell part;cytoplasm;cytoplasmic part;cytoskeleton;cytosol;extracellular membrane-bounded organelle;extracellular organelle;extracellular region part;extracellular vesicular exosome;intracellular non-membrane-bounded organelle;intracellular organelle;intracellular organelle part;intracellular part;membrane;membrane-bounded organelle;membrane-		Homo sapiens
cell part;cytoplasmic part;cytosol;extracellular membrane-bounded organelle;extracellular organelle;extracellular region part;extracellular vesicular exosome;intracellular organelle part;intracellular part;lysosomal membrane;m	Epithelial cell signaling in Helicobacter pylori infection;ko05152;Lysosome;Oxidative phosphorylation;Phagosome;Rheumat	Homo sapiens
cell junction;cell part;clathrin-coated vesicle;coated vesicle;cytoplasmic membrane-bounded vesicle;cytoplasmic part;cytoplasmic vesicle;Golgi apparatus;integral to membrane;intracellular membrane-bounded organelle;intracellular organelle;intracellular part;intrinsic to membrane;membrane;membrane part;membrane-bounded organelle;membra		Homo sapiens
cell part;centromeric heterochromatin;chromatin;chromosomal part;chromosome;condensed chromosome;heterochromatin;intracellular membrane-bounded organelle;intracellular non-membrane-bounded organelle;intracellular organelle;intracellular organelle part;intracellular part;membrane-bounded organelle;non-membrane-bounded organelle;		Homo sapiens
extracellular membrane-bounded organelle;extracellular organelle;extracellular region part;extracellular vesicular exosome;membrane-bounded organelle;membrane-bounded vesicle;organelle;vesicle	Amino sugar and nucleotide sugar metabolism	Homo sapiens
cell part;cytoplasmic part;intracellular membrane-bounded organelle;intracellular organelle;intracellular organelle lumen;intracellular organelle part;intracellular part;membrane-bounded organelle;membrane-enclosed lumen;m	Peroxisome	Homo sapiens
cell part;cytoplasm;intracellular part;intrinsic to internal side of plasma membrane;intrinsic to membrane;intrinsic to plasma membrane;membrane part;plasma membrane part		Homo sapiens
cell part;cytoplasm;extrinsic to internal side of plasma membrane;extrinsic to membrane;extrinsic to plasma membrane;intracellular membrane-bounded organelle;intracellular organelle;intracellular part;membrane part;membrane-bounded organelle;nucleus;organelle;plasma membrane part		Homo sapiens
cell part;centrosome;cytoplasm;cytoplasmic dynein complex;cytoplasmic part;cytoskeletal part;cytosol;dynactin complex;dynein complex;intracellular membrane-bounded organelle;intracellular non-membrane-bounded orga	Huntington's disease;Vasopressin-regulated water reabsorption	Homo sapiens
cell part;intracellular membrane-bounded organelle;intracellular organelle;intracellular part;membrane-bounded organelle;nucleus;organelle		Homo sapiens
cell part;cytoplasm;cytoplasmic part;endosome;extracellular membrane-bounded organelle;extracellular organelle;extracellular region part;extracellular vesicular exosome;intracellular membrane-bounded organelle;intracellular organelle;intracellular organelle part;intracellular part;membrane;membrane-bounded organelle;membrane-bounded vesic		Homo sapiens
cell part;cytoplasm;intracellular organelle part;intracellular part;macromolecular complex;membrane;membrane part;nuclear membrane;nuclear part;nuclear pore;nucleoplasm;organelle membrane;organelle part;pore complex	RNA transport	Homo sapiens
cell part;cytoplasm;intracellular non-membrane-bounded organelle;intracellular organelle;intracellular organelle part;intracellular part;membrane;non-membrane-bounded organelle;nuclear part;nucleolus;organelle;organelle part;plasma membrane		Homo sapiens
cell part;intracellular membrane-bounded organelle;intracellular organelle;intracellular part;membrane-bounded organelle;nucleus;organelle	Ribosome biogenesis in eukaryotes	Homo sapiens
cell part;intracellular organelle part;intracellular part;nuclear part;nucleoplasm;organelle part		Homo sapiens
cell part;integral to membrane;integral to plasma membrane;intrinsic to membrane;intrinsic to plasma membrane;membrane;membrane part;plasma membrane;plasma membrane part		Homo sapiens
cell part;cytoplasm;envelope;extracellular membrane-bounded organelle;extracellular organelle;extracellular region part;extracellular vesicular exosome;intracellular non-membrane-bounded organelle;intracellular organelle;intracellular organelle part;intracellular part;macromolecular complex;membrane;membrane part;membrane-bounded organell		Homo sapiens
adherens junction;anchoring junction;caveola;cell junction;cell part;cell projection membrane;cell projection part;cell-cell junction;cell-substrate adherens junction;cell-substrate junction;cytoplasm;cytoplasmic part;cytoplasmic vesicle membrane;cytoplasmic vesicle part;cytoskeleton;cytosol;early endosome;endosomal part;endosome;endosome r		Homo sapiens
adherens junction;anchoring junction;cell junction;cell part;cell surface;cell-substrate adherens junction;cell-substrate junction;centrosome;cytoplasmic part;cytoskeletal part;extracellular membrane-bounded organelle;extracellular organelle;extracellular region;extracellular region part;extracellular vesicular exosome;focal adhesion;integral to memb		Homo sapiens
		Homo sapiens
basement membrane;cell part;chromatin;chromosomal part;chromosome;chromosome, centromeric region;cohesin complex;cohesin core heterodimer;cytoplasm;cytoplasmic part;cytoskeletal part;cytosol;extracellular matrix	Cell cycle;Cell cycle - yeast;Meiosis - yeast;Oocyte meiosis	Homo sapiens
cell part;cytoplasmic part;eukaryotic 43S preinitiation complex;eukaryotic 48S preinitiation complex;eukaryotic translation initiation factor 3 complex;fibrillar center;intracellular organelle part;intracellular part;macromolecular complex;membrane;nuclear part;nucleolar part;nucleoplasm;organelle part;protein complex;ribonucleoprotein complex;trans		Homo sapiens
actin cytoskeleton;cell part;contractile fiber part;cytoplasmic part;cytoskeleton;extracellular membrane-bounded organelle;extracellular organelle;extracellular region part;extracellular space;extracellular vesicular exosome;I b	Axon guidance;Fc gamma R-mediated phagocytosis;Regulation of actin cytoskeleton	Homo sapiens
cell part;cytoplasmic part;endoplasmic reticulum membrane;endoplasmic reticulum part;endoplasmic reticulum-Golgi intermediate compartment membrane;Golgi apparatus;integral to membrane;intracellular membrane-bounded organelle;intracellular organelle;intracellular organelle part;intracellular part;intrinsic to membrane;membrane;membran		Homo sapiens
acrosomal vesicle;cell part;cytoplasmic membrane-bounded vesicle;cytoplasmic part;cytoplasmic vesicle;cytosol;Golgi apparatus;Golgi apparatus part;Golgi membrane;integral to membrane;intracellular membrane-bounded organelle;intracellular organelle;intracellular organelle part;intracellular part;intrinsic to membrane;membrane;membrane pa		Homo sapiens
cell part;cytoplasmic exosome (RNase complex);cytoplasmic part;cytosol;exosome (RNase complex);intracellular organelle part;intracellular part;macromolecular complex;membrane;nuclear exosome (RNase complex);nuclea	RNA degradation	Homo sapiens
cell part;cytoplasmic part;extracellular membrane-bounded organelle;extracellular organelle;extracellular region part;extracellular vesicular exosome;intracellular;intracellular membrane-bounded organelle;intracellular organel	Drug metabolism - cytochrome P450;Glutathione metabolism;Metabolism of xenobiotics by cytochrome P450;Peroxisome	Homo sapiens
cell part;cytoplasm;intracellular membrane-bounded organelle;intracellular non-membrane-bounded organelle;intracellular organelle;intracellular organelle part;intracellular part;membrane;membrane-bounded organelle;non-membrane-bounded organelle;nuclear part;nucleolus;nucleus;organelle;organelle part		Homo sapiens
cell part;cytoplasmic part;cytosolic small ribosomal subunit;intracellular organelle part;intracellular part;macromolecular complex;membrane;mitochondrial inner membrane;mitochondrial membrane;mitochondrial part;organelle inner membrane;organelle membrane;organelle part;ribonucleoprotein complex;small ribosomal subunit		Homo sapiens
cell part;cytoplasmic part;cytosol;intracellular membrane-bounded organelle;intracellular organelle;intracellular organelle lumen;intracellular organelle part;intracellular part;membrane-bounded organelle;membrane-enclosed l	Aminoacyl-tRNA biosynthesis	Homo sapiens
cell part;cytoplasmic part;intracellular membrane-bounded organelle;intracellular organelle;intracellular part;membrane-bounded organelle;mitochondrion;organelle		Homo sapiens
cell part;cytoplasm;cytoplasmic part;cytosol;Golgi apparatus part;Golgi membrane;intracellular membrane-bounded organelle;intracellular organelle;intracellular organelle part;intracellular part;membrane;membrane-bounded organelle;nuclear part;nucleoplasm;nucleus;organelle;organelle membrane;organelle part		Homo sapiens
cell part;cytoplasm;extracellular membrane-bounded organelle;extracellular organelle;extracellular region part;extracellular vesicular exosome;intracellular membrane-bounded organelle;intracellular organelle;intracellular part	Pentose phosphate pathway	Homo sapiens
cell part;intracellular organelle part;intracellular part;macromolecular complex;nuclear body;nuclear part;nuclear speck;nucleoplasm part;organelle part;ribonucleoprotein complex;small nuclear ribonucleoprotein complex;U1 snRNP		Homo sapiens
cell part;cytoplasmic part;intracellular membrane-bounded organelle;intracellular organelle;intracellular organelle part;intracellular part;macromolecular complex;membrane-bounded organelle;mitochondrial part;mitochondrial small ribosomal subunit;mitochondrion;organellar small ribosomal subunit;organelle;organelle part;ribonucleoprotein compl		Homo sapiens
cell part;cytoplasm;cytoskeletal part;intracellular membrane-bounded organelle;intracellular non-membrane-bounded organelle;intracellular organelle;intracellular organelle part;intracellular part;membrane-bounded organelle	Ribosome biogenesis in eukaryotes	Homo sapiens
cell part;COPI vesicle coat;COPII vesicle coat;cytoplasmic part;cytoplasmic vesicle part;endoplasmic reticulum;endoplasmic reticulum membrane;endoplasmic reticulum part;endoplasmic reticulum-Golgi intermediate compartment;endoplasmic reticulum-Golgi intermediate compartment membrane;extracellular membrane-bounded organelle;extra		Homo sapiens
cell part;cytoplasmic part;intracellular non-membrane-bounded organelle;intracellular organelle;intracellular organelle part;intracellular part;macromolecular complex;membrane;mitochondrial inner membrane;mitochondrial membrane;mitochondrial part;mitochondrial ribosome;non-membrane-bounded organelle;organellar ribosome;organelle;orga		Homo sapiens
adherens junction;anchoring junction;cell junction;cell part;cell-substrate adherens junction;cell-substrate junction;cytoplasmic part;focal adhesion;intracellular membrane-bounded organelle;intracellular non-membrane-boun	Ribosome biogenesis in eukaryotes	Homo sapiens
extracellular membrane-bounded organelle;extracellular organelle;extracellular region part;extracellular vesicular exosome;membrane-bounded organelle;membrane-bounded vesicle;organelle;vesicle		Homo sapiens
cell part;centrosome;cytoplasmic part;cytoskeletal part;cytosol;intracellular non-membrane-bounded organelle;intracellular organelle;intracellular organelle part;intracellular part;macromolecular complex;microtubule associated complex;microtubule organizing center;non-membrane-bounded organelle;organelle;organelle part;protein complex		Homo sapiens
cell part;cytoplasm;cytoplasmic part;cytosol;cytosolic large ribosomal subunit;intracellular non-membrane-bounded organelle;intracellular organelle;intracellular organelle part;intracellular part;large ribosomal subunit;macrom	Ribosome	Homo sapiens
cell part;intracellular;intracellular membrane-bounded organelle;intracellular organelle;intracellular organelle part;intracellular part;membrane;membrane-bounded organelle;nuclear part;nucleoplasm;nucleus;organelle;organelle part;plasma membrane		Homo sapiens

anchoring junction;cell junction;cell part;cell-cell junction;desmosome;intracellular membrane-bounded organelle;intracellular organelle;intracellular part;membrane;membrane-bounded organelle;nucleus;organelle;plasma membrane		Homo sapiens
cell part;cytoplasm;intracellular membrane-bounded organelle;intracellular organelle;intracellular part;membrane-bounded organelle;nucleus;organelle		Homo sapiens
cell junction;cell part;cell projection;cell projection part;cytoplasm;cytoplasmic part;cytoskeleton;cytosol;dendrite;dendritic spine;extrinsic to internal side of plasma membrane;extrinsic to membrane;extrinsic to plasma membrane;filopodium;intracellular non-membrane-bounded organelle;intracellular organelle;intracellular part;membrane part;neur		Homo sapiens
basement membrane;cell part;chromosome;extracellular matrix part;extracellular region part;extracellular space;intracellular membrane-bounded organelle;intracellular non-membrane-bounded organelle;intracellular organelle;intracellular organelle part;intracellular part;membrane;membrane-bounded organelle;non-membrane-bounded organelle;		Homo sapiens
cell part;cytoplasmic part;cytosol;intracellular organelle part;intracellular part;macromolecular complex;nuclear part;organelle part;ribonucleoprotein complex;small nuclear ribonucleoprotein complex;spliceosomal complex;U	RNA degradation;Spliceosome	Homo sapiens
cell part;membrane		Homo sapiens
		Homo sapiens
cell part;cytoplasmic part;extracellular region part;extracellular space;intracellular organelle part;intracellular part;macromolecular complex;membrane;mitochondrial inner membrane;mitochondrial intermembrane space protein transporter complex;mitochondrial membrane;mitochondrial part;organelle inner membrane;organelle membrane;organell		Homo sapiens
cell part;cytoplasmic part;cytosol;extracellular membrane-bounded organelle;extracellular organelle;extracellular region part;extracellular vesicular exosome;intracellular organelle part;intracellular part;lysosomal membrane;m	Collecting duct acid secretion;Epithelial cell signaling in Helicobacter pylori infection;Oxidative phosphorylation;Phagosom	Homo sapiens
cell part;centrosome;cytoplasm;cytoplasmic part;cytoskeletal part;intracellular membrane-bounded organelle;intracellular non-membrane-bounded organelle;intracellular organelle;intracellular organelle part;intracellular part;membrane-bounded organelle;microtubule organizing center;non-membrane-bounded organelle;nucleus;organelle;organelle		Homo sapiens
cell part;cytoplasmic part;endoplasmic reticulum;endoplasmic reticulum membrane;endoplasmic reticulum part;integral to membrane;integral to organelle membrane;integral to peroxisomal membrane;intracellular membrane	Peroxisome	Homo sapiens
cell part;chromatin;chromosomal part;cytoplasmic part;intracellular membrane-bounded organelle;intracellular non-membrane-bounded organelle;intracellular organelle;intracellular organelle part;intracellular part;macromolecular complex;membrane-bounded organelle;mitochondrion;non-membrane-bounded organelle;nuclear chromatin;nuclear c		Homo sapiens
cell part;cytoplasm;cytoplasmic part;cytosol;extracellular membrane-bounded organelle;extracellular organelle;extracellular region part;extracellular vesicular exosome;intracellular part;membrane-bounded organelle;membra	Glycine, serine and threonine metabolism;Methane metabolism;Vitamin B6 metabolism	Homo sapiens
cell part;cytoskeletal part;intracellular membrane-bounded organelle;intracellular non-membrane-bounded organelle;intracellular organelle;intracellular organelle part;intracellular part;membrane-bounded organelle;non-membrane-bounded organelle;nuclear part;nucleolus;nucleus;organelle;organelle part;spindle		Homo sapiens
cell part;COPI vesicle coat;cytoplasmic part;cytoplasmic vesicle part;cytosol;Golgi apparatus part;Golgi membrane;intracellular organelle part;intracellular part;macromolecular complex;membrane;membrane coat;membrane part;organelle membrane;organelle part;protein complex;vesicle coat		Homo sapiens
cell part;cytoplasm;cytoplasmic part;cytosol;intracellular membrane-bounded organelle;intracellular non-membrane-bounded organelle;intracellular organelle;intracellular organelle lumen;intracellular organelle part;intracellula	Sulfur relay system;Thiamine metabolism	Homo sapiens
cell part;cytoplasmic part;extracellular membrane-bounded organelle;extracellular organelle;extracellular region part;extracellular vesicular exosome;integral to membrane;intracellular membrane-bounded organelle;intracellular organelle;intracellular organelle part;intracellular part;intrinsic to membrane;membrane;membrane part;membrane-bound		Homo sapiens
cell part;centrosome;chromosomal part;condensed chromosome kinetochore;cytoplasm;cytoplasmic dynein complex;cytoplasmic part;cytoskeletal part;dynein complex;intracellular non-membrane-bounded organelle;intrace	Phagosome;Vasopressin-regulated water reabsorption	Homo sapiens
cell part;cytoplasmic part;intracellular membrane-bounded organelle;intracellular organelle;intracellular part;membrane-bounded organelle;mitochondrion;nucleus;organelle		Homo sapiens
cell part;chromatin remodeling complex;extracellular membrane-bounded organelle;extracellular organelle;extracellular region part;extracellular vesicular exosome;histone acetyltransferase complex;histone deacetylase comp	Basal transcription factors	Homo sapiens
cell part;cytoplasm;cytoplasmic part;cytosol;extracellular region part;extracellular space;intracellular membrane-bounded organelle;intracellular organelle;intracellular organelle part;intracellular part;membrane;membrane-bou	Hepatitis C;Measles	Homo sapiens
anchored to membrane;cell part;cytoplasm;intracellular part;intrinsic to membrane;membrane;membrane part;plasma membrane		Homo sapiens

C: Student's T-test Significant MCF7 + WT EVs_MCF7 + Vim -/- EVs	C: Student's T-test significant	N: Protein Probability	N: Combined Total Peptides	N: Combined Spectral Count	N: Combined Unique Spectral Count	N: Combined Total Spectral Count	N: Top Peptide Probability	N: Protein Length	N: -Log Student's T-test p-value MCF7 + WT EVs_MCF7 + Vim -/- EVs
+	MCF7 + WT EVs_MCF7 + Vim -/- EVs	1	29	791	791	791	0.999	1052	1.48504
+	MCF7 + WT EVs_MCF7 + Vim -/- EVs	1	29	400	400	400	0.999	921	1.87017
+	MCF7 + WT EVs_MCF7 + Vim -/- EVs	1	14	139	125	139	0.999	911	2.03965
+	MCF7 + WT EVs_MCF7 + Vim -/- EVs	1	11	292	292	292	0.999	321	1.9418
+	MCF7 + WT EVs_MCF7 + Vim -/- EVs	1	5	60	60	60	0.999	294	1.65842
+	MCF7 + WT EVs_MCF7 + Vim -/- EVs	1	23	145	145	145	0.999	2005	3.0957
+	MCF7 + WT EVs_MCF7 + Vim -/- EVs	1	5	120	4	120	0.999	1229	2.03279
+	MCF7 + WT EVs_MCF7 + Vim -/- EVs	1	5	107	107	107	0.999	123	2.91685
+	MCF7 + WT EVs_MCF7 + Vim -/- EVs	1	25	448	448	448	0.999	658	4.67263
+	MCF7 + WT EVs_MCF7 + Vim -/- EVs	1	12	86	86	158	0.999	706	1.61904
+	MCF7 + WT EVs_MCF7 + Vim -/- EVs	1	25	748	748	748	0.999	329	1.74326
+	MCF7 + WT EVs_MCF7 + Vim -/- EVs	1	23	456	368	456	0.999	380	1.73829
+	MCF7 + WT EVs_MCF7 + Vim -/- EVs	1	68	1212	1180	1242	0.999	1063	2.76557
+	MCF7 + WT EVs_MCF7 + Vim -/- EVs	1	10	136	136	136	0.999	210	2.06509
+	MCF7 + WT EVs_MCF7 + Vim -/- EVs	1	14	322	322	322	0.999	195	1.90493
+	MCF7 + WT EVs_MCF7 + Vim -/- EVs	1	13	519	519	519	0.999	357	5.30062
+	MCF7 + WT EVs_MCF7 + Vim -/- EVs	1	44	1371	1317	1371	0.999	1097	4.16044
+	MCF7 + WT EVs_MCF7 + Vim -/- EVs	1	7	37	37	37	0.999	1230	2.29803
+	MCF7 + WT EVs_MCF7 + Vim -/- EVs	1	22	606	457	606	0.999	579	1.67491
+	MCF7 + WT EVs_MCF7 + Vim -/- EVs	1	13	210	210	210	0.999	696	2.43789
+	MCF7 + WT EVs_MCF7 + Vim -/- EVs	1	33	347	347	347	0.999	2068	4.04774
+	MCF7 + WT EVs_MCF7 + Vim -/- EVs	1	43	942	942	942	0.999	737	6.54375
+	MCF7 + WT EVs_MCF7 + Vim -/- EVs	1	8	259	259	259	0.999	81	3.67305
+	MCF7 + WT EVs_MCF7 + Vim -/- EVs	1	16	314	263	314	0.999	593	3.22823
+	MCF7 + WT EVs_MCF7 + Vim -/- EVs	1	11	277	162	277	0.999	521	2.53882
+	MCF7 + WT EVs_MCF7 + Vim -/- EVs	1	8	104	104	104	0.999	558	2.41349
+	MCF7 + WT EVs_MCF7 + Vim -/- EVs	1	10	133	133	133	0.999	1011	2.14425
+	MCF7 + WT EVs_MCF7 + Vim -/- EVs	1	14	341	341	341	0.999	312	2.47924
+	MCF7 + WT EVs_MCF7 + Vim -/- EVs	0.9999	3	86	86	86	0.999	309	1.75
+	MCF7 + WT EVs_MCF7 + Vim -/- EVs	1	20	251	251	251	0.999	1153	2.87548
+	MCF7 + WT EVs_MCF7 + Vim -/- EVs	1	8	291	291	291	0.999	274	2.23754
+	MCF7 + WT EVs_MCF7 + Vim -/- EVs	1	8	259	259	259	0.999	152	2.04827
+	MCF7 + WT EVs_MCF7 + Vim -/- EVs	1	21	580	580	580	0.999	637	1.81878
+	MCF7 + WT EVs_MCF7 + Vim -/- EVs	1	11	257	257	257	0.999	358	2.41592
+	MCF7 + WT EVs_MCF7 + Vim -/- EVs	1	26	786	517	786	0.999	248	2.15093
+	MCF7 + WT EVs_MCF7 + Vim -/- EVs	1	6	46	46	46	0.999	412	3.04758
+	MCF7 + WT EVs_MCF7 + Vim -/- EVs	1	11	53	51	53	0.999	490	2.11126
+	MCF7 + WT EVs_MCF7 + Vim -/- EVs	1	6	124	124	124	0.999	333	2.22455
+	MCF7 + WT EVs_MCF7 + Vim -/- EVs	1	16	224	224	224	0.999	419	2.11318
+	MCF7 + WT EVs_MCF7 + Vim -/- EVs	1	11	358	358	358	0.999	82	2.33439
+	MCF7 + WT EVs_MCF7 + Vim -/- EVs	1	28	455	454	455	0.999	1030	2.26719
+	MCF7 + WT EVs_MCF7 + Vim -/- EVs	1	5	95	95	95	0.999	1369	2.99464
+	MCF7 + WT EVs_MCF7 + Vim -/- EVs	1	44	319	319	320	0.999	3695	3.31397
+	MCF7 + WT EVs_MCF7 + Vim -/- EVs	1	4	125	125	125	0.999	138	1.61028
+	MCF7 + WT EVs_MCF7 + Vim -/- EVs	1	13	281	281	281	0.999	473	2.57277
+	MCF7 + WT EVs_MCF7 + Vim -/- EVs	1	22	710	710	710	0.999	546	2.28857
+	MCF7 + WT EVs_MCF7 + Vim -/- EVs	1	26	274	243	274	0.999	1258	2.29009
+	MCF7 + WT EVs_MCF7 + Vim -/- EVs	1	23	962	962	962	0.999	548	2.20991
+	MCF7 + WT EVs_MCF7 + Vim -/- EVs	1	18	404	404	404	0.999	352	2.27246
+	MCF7 + WT EVs_MCF7 + Vim -/- EVs	1	11	86	86	86	0.999	856	1.51428
+	MCF7 + WT EVs_MCF7 + Vim -/- EVs	1	10	205	205	205	0.999	392	2.25095
+	MCF7 + WT EVs_MCF7 + Vim -/- EVs	1	13	572	572	572	0.999	465	3.19066
+	MCF7 + WT EVs_MCF7 + Vim -/- EVs	1	33	545	545	545	0.999	535	5.74951
+	MCF7 + WT EVs_MCF7 + Vim -/- EVs	1	10	298	298	298	0.999	522	1.66614
+	MCF7 + WT EVs_MCF7 + Vim -/- EVs	1	40	1487	1487	1487	0.999	533	5.15085
+	MCF7 + WT EVs_MCF7 + Vim -/- EVs	1	22	324	323	324	0.999	492	2.21681
+	MCF7 + WT EVs_MCF7 + Vim -/- EVs	1	16	356	331	356	0.999	624	1.65877
+	MCF7 + WT EVs_MCF7 + Vim -/- EVs	1	21	451	451	451	0.999	800	2.36962
+	MCF7 + WT EVs_MCF7 + Vim -/- EVs	1	9	354	354	354	0.999	174	1.64673
+	MCF7 + WT EVs_MCF7 + Vim -/- EVs	1	4	91	91	91	0.999	354	3.03706
+	MCF7 + WT EVs_MCF7 + Vim -/- EVs	1	18	325	325	325	0.999	452	3.12119
+	MCF7 + WT EVs_MCF7 + Vim -/- EVs	1	6	169	169	169	0.999	189	1.45985
+	MCF7 + WT EVs_MCF7 + Vim -/- EVs	1	12	397	397	397	0.999	348	2.87225
+	MCF7 + WT EVs_MCF7 + Vim -/- EVs	1	12	208	208	208	0.999	755	1.56566
+	MCF7 + WT EVs_MCF7 + Vim -/- EVs	1	29	955	955	955	0.999	548	3.37661
+	MCF7 + WT EVs_MCF7 + Vim -/- EVs	1	15	334	334	334	0.999	399	1.75764
+	MCF7 + WT EVs_MCF7 + Vim -/- EVs	1	18	351	351	351	0.999	385	3.64347
+	MCF7 + WT EVs_MCF7 + Vim -/- EVs	1	20	274	274	274	0.999	1151	1.99599
+	MCF7 + WT EVs_MCF7 + Vim -/- EVs	1	23	648	648	648	0.999	315	1.59667
+	MCF7 + WT EVs_MCF7 + Vim -/- EVs	1	35	661	661	661	0.999	960	1.57076
+	MCF7 + WT EVs_MCF7 + Vim -/- EVs	1	8	131	131	131	0.999	852	1.9546
+	MCF7 + WT EVs_MCF7 + Vim -/- EVs	1	27	263	263	263	0.999	1756	1.80168
+	MCF7 + WT EVs_MCF7 + Vim -/- EVs	1	33	590	590	680	0.999	711	2.15693
+	MCF7 + WT EVs_MCF7 + Vim -/- EVs	1	18	210	210	210	0.999	916	1.81003
+	MCF7 + WT EVs_MCF7 + Vim -/- EVs	1	16	255	255	255	0.999	372	1.40767
+	MCF7 + WT EVs_MCF7 + Vim -/- EVs	1	31	602	602	602	0.999	738	2.99258
+	MCF7 + WT EVs_MCF7 + Vim -/- EVs	1	56	1030	1015	1030	0.999	1272	1.79275
+	MCF7 + WT EVs_MCF7 + Vim -/- EVs	1	34	1224	1224	1224	0.999	434	2.1524
+	MCF7 + WT EVs_MCF7 + Vim -/- EVs	1	5	44	44	44	0.999	282	2.77593
+	MCF7 + WT EVs_MCF7 + Vim -/- EVs	1	11	37	35	58	0.999	156	3.38427
+	MCF7 + WT EVs_MCF7 + Vim -/- EVs	1	36	767	767	767	0.999	494	1.99973
+	MCF7 + WT EVs_MCF7 + Vim -/- EVs	1	7	184	184	184	0.999	260	1.52772
+	MCF7 + WT EVs_MCF7 + Vim -/- EVs	1	34	792	792	792	0.999	962	1.68472
+	MCF7 + WT EVs_MCF7 + Vim -/- EVs	1	6	166	166	166	0.999	265	1.71385
+	MCF7 + WT EVs_MCF7 + Vim -/- EVs	1	16	374	374	374	0.999	148	3.16896
+	MCF7 + WT EVs_MCF7 + Vim -/- EVs	1	8	106	106	106	0.999	1101	3.63687
+	MCF7 + WT EVs_MCF7 + Vim -/- EVs	1	5	101	101	101	0.999	208	2.88447
+	MCF7 + WT EVs_MCF7 + Vim -/- EVs	1	8	74	74	74	0.999	597	2.46249
+	MCF7 + WT EVs_MCF7 + Vim -/- EVs	1	3	54	54	54	0.999	409	2.73576
+	MCF7 + WT EVs_MCF7 + Vim -/- EVs	1	60	2457	2457	2457	0.999	606	6.64678
+	MCF7 + WT EVs_MCF7 + Vim -/- EVs	1	35	319	257	319	0.999	1388	1.58947
+	MCF7 + WT EVs_MCF7 + Vim -/- EVs	1	15	92	92	123	0.999	1294	2.88398
+	MCF7 + WT EVs_MCF7 + Vim -/- EVs	1	26	580	551	580	0.999	537	2.99706
+	MCF7 + WT EVs_MCF7 + Vim -/- EVs	1	11	131	25	131	0.999	810	2.23741
+	MCF7 + WT EVs_MCF7 + Vim -/- EVs	1	11	296	249	296	0.999	118	1.73176
+	MCF7 + WT EVs_MCF7 + Vim -/- EVs	1	20	435	386	435	0.999	369	1.53462
+	MCF7 + WT EVs_MCF7 + Vim -/- EVs	1	9	41	41	41	0.999	2440	2.7257
+	MCF7 + WT EVs_MCF7 + Vim -/- EVs	1	20	529	529	529	0.999	466	1.97275
+	MCF7 + WT EVs_MCF7 + Vim -/- EVs	1	10	102	102	103	0.999	805	3.76455
+	MCF7 + WT EVs_MCF7 + Vim -/- EVs	1	24	394	394	411	0.999	530	1.72741
+	MCF7 + WT EVs_MCF7 + Vim -/- EVs	1	14	405	142	405	0.999	348	3.80044
+	MCF7 + WT EVs_MCF7 + Vim -/- EVs	1	11	213	213	213	0.999	264	1.87093
+	MCF7 + WT EVs_MCF7 + Vim -/- EVs	1	12	268	242	268	0.999	262	1.45645
+	MCF7 + WT EVs_MCF7 + Vim -/- EVs	1	40	839	839	839	0.999	798	1.58758
+	MCF7 + WT EVs_MCF7 + Vim -/- EVs	1	4	49	49	49	0.999	437	4.07501
+	MCF7 + WT EVs_MCF7 + Vim -/- EVs	1	12	122	122	180	0.999	678	2.02812
+	MCF7 + WT EVs_MCF7 + Vim -/- EVs	1	3	83	83	83	0.999	363	1.42701
+	MCF7 + WT EVs_MCF7 + Vim -/- EVs	1	18	622	622	622	0.999	320	1.78596
+	MCF7 + WT EVs_MCF7 + Vim -/- EVs	1	8	142	142	142	0.999	339	2.92435
+	MCF7 + WT EVs_MCF7 + Vim -/- EVs	1	25	300	300	300	0.999	671	1.64168
+	MCF7 + WT EVs_MCF7 + Vim -/- EVs	1	10	102	102	127	0.999	285	2.51246
+	MCF7 + WT EVs_MCF7 + Vim -/- EVs	1	10	102	102	199	0.999	682	1.67057
+	MCF7 + WT EVs_MCF7 + Vim -/- EVs	1	5	124	124	124	0.999	489	1.66322
+	MCF7 + WT EVs_MCF7 + Vim -/- EVs	1	15	129	129	392	0.999	339	1.72658
+	MCF7 + WT EVs_MCF7 + Vim -/- EVs	1	8	39	32	39	0.999	266	2.96457
+	MCF7 + WT EVs_MCF7 + Vim -/- EVs	1	21	356	356	356	0.999	669	4.80645
+	MCF7 + WT EVs_MCF7 + Vim -/- EVs	1	7	168	168	168	0.999	293	3.34646
+	MCF7 + WT EVs_MCF7 + Vim -/- EVs	1	23	220	220	220	0.999	2799	1.69211
+	MCF7 + WT EVs_MCF7 + Vim -/- EVs	0.9999	2	54	54	54	0.999	84	1.44382
+	MCF7 + WT EVs_MCF7 + Vim -/- EVs	1	7	182	182	182	0.999	129	1.74068
+	MCF7 + WT EVs_MCF7 + Vim -/- EVs	1	6	202	202	202	0.999	186	1.69039
+	MCF7 + WT EVs_MCF7 + Vim -/- EVs	1	14	170	170	172	0.999	432	1.39827
+	MCF7 + WT EVs_MCF7 + Vim -/- EVs	1	15	108	48	108	0.999	1232	2.97153
+	MCF7 + WT EVs_MCF7 + Vim -/- EVs	1	16	287	287	287	0.999	355	1.69823
+	MCF7 + WT EVs_MCF7 + Vim -/- EVs	1	13	214	164	216	0.999	570	2.39546
+	MCF7 + WT EVs_MCF7 + Vim -/- EVs	1	46	711	711	711	0.999	1197	2.26882

+	MCF7 + WT EVs_MCF7 + Vim -/- EVs	1	13	193	137	193	0.999	564	2.02981
+	MCF7 + WT EVs_MCF7 + Vim -/- EVs	1	38	754	754	754	0.999	1038	2.20772
+	MCF7 + WT EVs_MCF7 + Vim -/- EVs	1	12	217	217	217	0.999	568	1.615
+	MCF7 + WT EVs_MCF7 + Vim -/- EVs	1	6	62	62	62	0.999	260	2.89349
+	MCF7 + WT EVs_MCF7 + Vim -/- EVs	1	34	779	689	779	0.999	720	2.48501
+	MCF7 + WT EVs_MCF7 + Vim -/- EVs	1	10	165	165	165	0.999	621	2.53544
+	MCF7 + WT EVs_MCF7 + Vim -/- EVs	1	10	182	161	182	0.999	527	2.00327
+	MCF7 + WT EVs_MCF7 + Vim -/- EVs	1	9	118	118	118	0.999	300	1.74469
+	MCF7 + WT EVs_MCF7 + Vim -/- EVs	1	4	16	16	16	0.999	1651	1.47288
+	MCF7 + WT EVs_MCF7 + Vim -/- EVs	1	19	355	355	355	0.999	211	3.00837
+	MCF7 + WT EVs_MCF7 + Vim -/- EVs	1	12	258	258	258	0.999	649	1.95257
+	MCF7 + WT EVs_MCF7 + Vim -/- EVs	0.9999	2	37	28	37	0.999	204	2.12576
+	MCF7 + WT EVs_MCF7 + Vim -/- EVs	1	14	147	122	147	0.999	285	2.30682
+	MCF7 + WT EVs_MCF7 + Vim -/- EVs	1	7	348	348	348	0.999	172	5.81619
+	MCF7 + WT EVs_MCF7 + Vim -/- EVs	1	9	236	236	236	0.999	172	1.79685
+	MCF7 + WT EVs_MCF7 + Vim -/- EVs	1	9	159	159	159	0.999	669	2.30134
+	MCF7 + WT EVs_MCF7 + Vim -/- EVs	1	10	219	219	219	0.999	361	2.28219
+	MCF7 + WT EVs_MCF7 + Vim -/- EVs	1	6	53	53	53	0.999	259	1.9595
+	MCF7 + WT EVs_MCF7 + Vim -/- EVs	1	55	3524	3017	3524	0.999	332	5.29377
+	MCF7 + WT EVs_MCF7 + Vim -/- EVs	1	16	356	356	356	0.999	522	1.70861
+	MCF7 + WT EVs_MCF7 + Vim -/- EVs	1	69	4297	3892	4297	0.999	417	4.99241
+	MCF7 + WT EVs_MCF7 + Vim -/- EVs	1	55	1451	1414	1451	0.999	412	2.09175
+	MCF7 + WT EVs_MCF7 + Vim -/- EVs	1	21	510	368	510	0.999	1474	2.58092
+	MCF7 + WT EVs_MCF7 + Vim -/- EVs	0.9949	8	168	2	298	0.999	362	1.59476
+	MCF7 + WT EVs_MCF7 + Vim -/- EVs	1	48	1264	1181	1264	0.999	760	5.22196
+	MCF7 + WT EVs_MCF7 + Vim -/- EVs	1	5	104	104	104	0.999	132	7.56815
+	MCF7 + WT EVs_MCF7 + Vim -/- EVs	1	8	102	102	102	0.999	478	3.71409
+	MCF7 + WT EVs_MCF7 + Vim -/- EVs	1	31	710	710	710	0.999	527	4.1097
+	MCF7 + WT EVs_MCF7 + Vim -/- EVs	1	8	76	76	76	0.999	466	1.45314
+	MCF7 + WT EVs_MCF7 + Vim -/- EVs	1	56	3663	479	3663	0.999	364	5.30207
+	MCF7 + WT EVs_MCF7 + Vim -/- EVs	1	60	2701	2680	2701	0.999	346	3.63426
+	MCF7 + WT EVs_MCF7 + Vim -/- EVs	1	60	7618	7289	7618	0.999	335	4.40495
+	MCF7 + WT EVs_MCF7 + Vim -/- EVs	1	9	96	96	96	0.999	464	1.49345
+	MCF7 + WT EVs_MCF7 + Vim -/- EVs	1	20	1178	1178	1178	0.999	205	2.97238
+	MCF7 + WT EVs_MCF7 + Vim -/- EVs	1	7	102	102	102	0.999	313	1.82442
+	MCF7 + WT EVs_MCF7 + Vim -/- EVs	1	54	2010	2010	2010	0.999	607	3.0548
+	MCF7 + WT EVs_MCF7 + Vim -/- EVs	1	29	936	533	936	0.999	355	2.05904
+	MCF7 + WT EVs_MCF7 + Vim -/- EVs	1	15	330	0	877	0.999	130	1.85013
+	MCF7 + WT EVs_MCF7 + Vim -/- EVs	1	65	2279	1542	2279	0.999	1023	2.60415
+	MCF7 + WT EVs_MCF7 + Vim -/- EVs	1	43	2018	525	2018	0.999	298	2.29926
+	MCF7 + WT EVs_MCF7 + Vim -/- EVs	1	5	180	180	180	0.999	165	2.99234
+	MCF7 + WT EVs_MCF7 + Vim -/- EVs	1	21	232	85	232	0.999	535	1.51703
+	MCF7 + WT EVs_MCF7 + Vim -/- EVs	1	21	296	296	296	0.999	532	2.89676
+	MCF7 + WT EVs_MCF7 + Vim -/- EVs	1	44	1221	1184	1288	0.999	430	2.15316
+	MCF7 + WT EVs_MCF7 + Vim -/- EVs	1	63	1553	1267	1698	0.999	483	1.97288
+	MCF7 + WT EVs_MCF7 + Vim -/- EVs	1	22	629	491	629	0.999	297	2.17997
+	MCF7 + WT EVs_MCF7 + Vim -/- EVs	1	79	6854	6470	6854	0.999	434	4.23041
+	MCF7 + WT EVs_MCF7 + Vim -/- EVs	1	50	996	996	1261	0.999	847	3.88026
+	MCF7 + WT EVs_MCF7 + Vim -/- EVs	1	71	2970	2911	2970	0.999	558	5.27705
+	MCF7 + WT EVs_MCF7 + Vim -/- EVs	1	40	2810	2755	2810	0.999	294	1.83536
+	MCF7 + WT EVs_MCF7 + Vim -/- EVs	1	35	598	598	598	0.999	1048	3.72174
+	MCF7 + WT EVs_MCF7 + Vim -/- EVs	1	14	97	97	97	0.999	398	1.64548
+	MCF7 + WT EVs_MCF7 + Vim -/- EVs	1	9	296	296	296	0.999	203	1.87931
+	MCF7 + WT EVs_MCF7 + Vim -/- EVs	1	70	3344	3344	3344	0.999	508	3.10183
+	MCF7 + WT EVs_MCF7 + Vim -/- EVs	1	24	558	558	558	0.999	412	1.56478
+	MCF7 + WT EVs_MCF7 + Vim -/- EVs	1	75	4421	1025	4421	0.999	339	3.07221
+	MCF7 + WT EVs_MCF7 + Vim -/- EVs	1	24	489	449	489	0.999	556	1.71416
+	MCF7 + WT EVs_MCF7 + Vim -/- EVs	1	117	4147	4147	4147	0.999	1512	2.09618
+	MCF7 + WT EVs_MCF7 + Vim -/- EVs	1	23	760	760	760	0.999	339	3.48665
+	MCF7 + WT EVs_MCF7 + Vim -/- EVs	1	49	467	467	467	0.999	1786	2.29689
+	MCF7 + WT EVs_MCF7 + Vim -/- EVs	1	81	1328	1328	1328	0.999	673	3.09545
+	MCF7 + WT EVs_MCF7 + Vim -/- EVs	1	18	290	290	290	0.999	381	3.77329
+	MCF7 + WT EVs_MCF7 + Vim -/- EVs	1	31	1451	1451	1451	0.999	630	1.79627
+	MCF7 + WT EVs_MCF7 + Vim -/- EVs	1	19	292	292	292	0.999	561	4.17261
+	MCF7 + WT EVs_MCF7 + Vim -/- EVs	1	30	791	637	791	0.999	390	3.41588
+	MCF7 + WT EVs_MCF7 + Vim -/- EVs	1	24	567	567	567	0.999	1049	5.67033
+	MCF7 + WT EVs_MCF7 + Vim -/- EVs	1	8	96	96	121	0.999	508	1.56751
+	MCF7 + WT EVs_MCF7 + Vim -/- EVs	1	99	4493	3941	4493	0.999	466	1.48902
+	MCF7 + WT EVs_MCF7 + Vim -/- EVs	1	23	852	852	852	0.999	135	2.78691
+	MCF7 + WT EVs_MCF7 + Vim -/- EVs	1	53	1309	1195	1632	0.999	469	1.66901
+	MCF7 + WT EVs_MCF7 + Vim -/- EVs	1	54	1659	1659	1659	0.999	320	6.74468
+	MCF7 + WT EVs_MCF7 + Vim -/- EVs	1	10	252	252	252	0.999	348	3.83588
+	MCF7 + WT EVs_MCF7 + Vim -/- EVs	1	5	49	49	49	0.999	288	1.89888
+	MCF7 + WT EVs_MCF7 + Vim -/- EVs	1	30	349	330	597	0.999	434	3.73955
+	MCF7 + WT EVs_MCF7 + Vim -/- EVs	1	19	1481	1481	1481	0.999	135	2.50402
+	MCF7 + WT EVs_MCF7 + Vim -/- EVs	1	41	383	383	1679	0.999	284	6.59533
+	MCF7 + WT EVs_MCF7 + Vim -/- EVs	1	34	738	738	738	0.999	319	5.0728
+	MCF7 + WT EVs_MCF7 + Vim -/- EVs	1	29	610	610	610	0.999	421	2.2375
+	MCF7 + WT EVs_MCF7 + Vim -/- EVs	1	86	3037	3037	3037	0.999	1014	1.80597
+	MCF7 + WT EVs_MCF7 + Vim -/- EVs	1	15	157	157	157	0.999	1462	1.42042
+	MCF7 + WT EVs_MCF7 + Vim -/- EVs	1	5	32	32	34	0.999	472	3.73671
+	MCF7 + WT EVs_MCF7 + Vim -/- EVs	1	8	86	86	86	0.999	478	3.39892
+	MCF7 + WT EVs_MCF7 + Vim -/- EVs	1	12	40	40	104	0.999	528	1.59244
+	MCF7 + WT EVs_MCF7 + Vim -/- EVs	1	37	493	485	1132	0.999	364	5.40262
+	MCF7 + WT EVs_MCF7 + Vim -/- EVs	1	66	2671	0	3190	0.999	641	2.55592
+	MCF7 + WT EVs_MCF7 + Vim -/- EVs	1	19	342	342	342	0.999	538	1.82441
+	MCF7 + WT EVs_MCF7 + Vim -/- EVs	1	16	202	202	202	0.999	952	2.95092
+	MCF7 + WT EVs_MCF7 + Vim -/- EVs	1	7	22	22	36	0.999	606	2.96035
+	MCF7 + WT EVs_MCF7 + Vim -/- EVs	1	17	299	229	299	0.999	381	3.71704
+	MCF7 + WT EVs_MCF7 + Vim -/- EVs	1	77	3373	3312	3538	0.999	654	3.58859
+	MCF7 + WT EVs_MCF7 + Vim -/- EVs	1	44	693	693	693	0.999	1609	4.86307
+	MCF7 + WT EVs_MCF7 + Vim -/- EVs	1	11	386	386	386	0.999	492	4.21362
+	MCF7 + WT EVs_MCF7 + Vim -/- EVs	1	7	100	17	100	0.999	496	2.78647
+	MCF7 + WT EVs_MCF7 + Vim -/- EVs	1	21	479	479	479	0.999	480	2.84999
+	MCF7 + WT EVs_MCF7 + Vim -/- EVs	1	11	452	291	452	0.999	206	1.5805
+	MCF7 + WT EVs_MCF7 + Vim -/- EVs	1	49	1413	1413	1413	0.999	515	3.33734
+	MCF7 + WT EVs_MCF7 + Vim -/- EVs	1	49	800	800	800	0.999	1178	2.00245
+	MCF7 + WT EVs_MCF7 + Vim -/- EVs	1	65	2687	2589	2687	0.999	935	2.97679
+	MCF7 + WT EVs_MCF7 + Vim -/- EVs	1	10	158	158	296	0.999	303	2.96479
+	MCF7 + WT EVs_MCF7 + Vim -/- EVs	1	44	515	377	2006	0.999	298	2.46949
+	MCF7 + WT EVs_MCF7 + Vim -/- EVs	1	40	1289	1169	1289	0.999	514	3.05639
+	MCF7 + WT EVs_MCF7 + Vim -/- EVs	1	133	2847	2806	2847	0.999	2363	3.6662
+	MCF7 + WT EVs_MCF7 + Vim -/- EVs	1	28	582	565	582	0.999	381	1.75786
+	MCF7 + WT EVs_MCF7 + Vim -/- EVs	1	48	972	972	972	0.999	323	6.69223
+	MCF7 + WT EVs_MCF7 + Vim -/- EVs	1	14	223	223	223	0.999	445	1.40723
+	MCF7 + WT EVs_MCF7 + Vim -/- EVs	1	10	158	158	158	0.999	493	2.10534
+	MCF7 + WT EVs_MCF7 + Vim -/- EVs	1	51	2006	2006	2006	0.999	609	1.54688
+	MCF7 + WT EVs_MCF7 + Vim -/- EVs	1	51	1795	1795	1795	0.999	732	3.03952
+	MCF7 + WT EVs_MCF7 + Vim -/- EVs	1	7	27	27	27	0.999	313	1.6256
+	MCF7 + WT EVs_MCF7 + Vim -/- EVs	1	35	1127	1127	1127	0.999	461	2.25565
+	MCF7 + WT EVs_MCF7 + Vim -/- EVs	1	89	5887	5625	5887	0.999	858	3.21521
+	MCF7 + WT EVs_MCF7 + Vim -/- EVs	1	60	2131	2131	2131	0.999	645	4.31189
+	MCF7 + WT EVs_MCF7 + Vim -/- EVs	1	31	521	521	521	0.999	534	7.2328
+	MCF7 + WT EVs_MCF7 + Vim -/- EVs	1	39	1126	1007	1126	0.999	630	2.43415
+	MCF7 + WT EVs_MCF7 + Vim -/- EVs	1	11	154	153	154	0.999	737	1.85088
+	MCF7 + WT EVs_MCF7 + Vim -/- EVs	1	32	474	10	474	0.999	327	3.27931
+	MCF7 + WT EVs_MCF7 + Vim -/- EVs	1	20	77	77	480	0.999	434	4.0372
+	MCF7 + WT EVs_MCF7 + Vim -/- EVs	1	9	244	244	244	0.999	350	1.43864
+	MCF7 + WT EVs_MCF7 + Vim -/- EVs	1	4	156	156	156	0.999	185	2.42782
+	MCF7 + WT EVs_MCF7 + Vim -/- EVs	1	28	1052	1052	1052	0.999	528	2.52385
+	MCF7 + WT EVs_MCF7 + Vim -/- EVs	1	9	81	81	81	0.999	443	3.32809
+	MCF7 + WT EVs_MCF7 + Vim -/- EVs	1	95	6447	6231	6447	0.999	531	2.03353
+	MCF7 + WT EVs_MCF7 + Vim -/- EVs	1	86	4247	3596	4500	0.999	803	4.9198
+	MCF7 + WT EVs_MCF7 + Vim -/- EVs	1	27	375	375	375	0.999	1019	2.77255
+	MCF7 + WT EVs_MCF7 + Vim -/- EVs	1	44	1354	1354	1354	0.999	501	2.60046
+	MCF7 + WT EVs_MCF7 + Vim -/- EVs	1	23	262	262	262	0.999	373	1.64067
+	MCF7 + WT EVs_MCF7 + Vim -/- EVs	1	5	91	62	91	0.999	398	2.43367
+	MCF7 + WT EVs_MCF7 + Vim -/- EVs	1	3	61	61	61	0.999	326	2.28998
+	MCF7 + WT EVs_MCF7 + Vim -/- EVs	1	4	20	20	20	0.999	392	2.60097

+	MCF7 + WT EVs_MCF7 + Vim -/- EVs	1	20	901	901	901	0.999	274		2.30888
+	MCF7 + WT EVs_MCF7 + Vim -/- EVs	1	9	204	204	204	0.999	552		1.84239
+	MCF7 + WT EVs_MCF7 + Vim -/- EVs	1	30	1110	1110	1110	0.999	293		1.91188
+	MCF7 + WT EVs_MCF7 + Vim -/- EVs	1	209	2648	2648	2648	0.999	2871		1.87942
+	MCF7 + WT EVs_MCF7 + Vim -/- EVs	1	14	261	261	261	0.999	270		2.70684
+	MCF7 + WT EVs_MCF7 + Vim -/- EVs	1	6	64	64	64	0.999	1255		2.31876
+	MCF7 + WT EVs_MCF7 + Vim -/- EVs	1	9	96	96	96	0.999	231		1.51437
+	MCF7 + WT EVs_MCF7 + Vim -/- EVs	1	26	1020	752	1020	0.999	277		1.42077
+	MCF7 + WT EVs_MCF7 + Vim -/- EVs	1	55	1852	1517	1852	0.999	1042		4.34377
+	MCF7 + WT EVs_MCF7 + Vim -/- EVs	1	41	809	809	809	0.999	1181		4.03761
+	MCF7 + WT EVs_MCF7 + Vim -/- EVs	1	14	250	76	250	0.999	351		2.3353
+	MCF7 + WT EVs_MCF7 + Vim -/- EVs	1	43	1441	1440	1441	0.999	700		2.56533
+	MCF7 + WT EVs_MCF7 + Vim -/- EVs	1	7	86	86	86	0.999	345		2.42007
+	MCF7 + WT EVs_MCF7 + Vim -/- EVs	1	32	673	670	673	0.999	591		4.24452
+	MCF7 + WT EVs_MCF7 + Vim -/- EVs	1	7	102	102	102	0.999	658		3.24487
+	MCF7 + WT EVs_MCF7 + Vim -/- EVs	1	64	1211	1211	1922	0.999	614		4.48907
+	MCF7 + WT EVs_MCF7 + Vim -/- EVs	1	29	556	551	771	0.999	780		3.03688
+	MCF7 + WT EVs_MCF7 + Vim -/- EVs	1	7	35	35	35	0.999	291		6.56236
+	MCF7 + WT EVs_MCF7 + Vim -/- EVs	1	24	845	845	845	0.999	110		1.43517
+	MCF7 + WT EVs_MCF7 + Vim -/- EVs	1	48	1822	1822	1822	0.999	248		2.72986
+	MCF7 + WT EVs_MCF7 + Vim -/- EVs	1	82	2699	2699	2699	0.999	1134		1.45032
+	MCF7 + WT EVs_MCF7 + Vim -/- EVs	1	33	1379	1330	1379	0.999	184		1.49641
+	MCF7 + WT EVs_MCF7 + Vim -/- EVs	1	38	2306	916	2306	0.999	254		3.25709
+	MCF7 + WT EVs_MCF7 + Vim -/- EVs	1	8	59	59	59	0.999	919		1.41594
+	MCF7 + WT EVs_MCF7 + Vim -/- EVs	1	10	140	140	140	0.999	633		2.88789
+	MCF7 + WT EVs_MCF7 + Vim -/- EVs	1	75	2793	2793	2793	0.999	710		1.63931
+	MCF7 + WT EVs_MCF7 + Vim -/- EVs	1	16	245	245	245	0.999	946		3.01376
+	MCF7 + WT EVs_MCF7 + Vim -/- EVs	1	23	591	591	591	0.999	333		2.15931
+	MCF7 + WT EVs_MCF7 + Vim -/- EVs	1	28	786	785	786	0.999	488		5.60647
+	MCF7 + WT EVs_MCF7 + Vim -/- EVs	1	10	405	405	405	0.999	206		3.85898
+	MCF7 + WT EVs_MCF7 + Vim -/- EVs	1	10	109	109	214	0.999	218		1.80117
+	MCF7 + WT EVs_MCF7 + Vim -/- EVs	1	12	122	10	227	0.999	208		2.82984
+	MCF7 + WT EVs_MCF7 + Vim -/- EVs	1	15	554	554	554	0.999	241		1.51414
+	MCF7 + WT EVs_MCF7 + Vim -/- EVs	1	27	812	812	812	0.999	708		1.65908
+	MCF7 + WT EVs_MCF7 + Vim -/- EVs	1	20	186	171	187	0.999	1838		4.48181
+	MCF7 + WT EVs_MCF7 + Vim -/- EVs	1	14	351	146	351	0.999	511		2.17768
+	MCF7 + WT EVs_MCF7 + Vim -/- EVs	1	25	809	807	809	0.999	193		2.9822
+	MCF7 + WT EVs_MCF7 + Vim -/- EVs	1	156	5366	4781	5366	0.999	2647		1.71793
+	MCF7 + WT EVs_MCF7 + Vim -/- EVs	1	40	895	888	895	0.999	889		3.78758
+	MCF7 + WT EVs_MCF7 + Vim -/- EVs	1	21	523	519	523	0.999	280		2.84
+	MCF7 + WT EVs_MCF7 + Vim -/- EVs	1	11	53	53	53	0.999	258		2.55346
+	MCF7 + WT EVs_MCF7 + Vim -/- EVs	1	9	94	94	94	0.999	703		2.22005
+	MCF7 + WT EVs_MCF7 + Vim -/- EVs	1	30	639	581	639	0.999	301		3.5476
+	MCF7 + WT EVs_MCF7 + Vim -/- EVs	1	7	51	51	51	0.999	599		2.57107
+	MCF7 + WT EVs_MCF7 + Vim -/- EVs	1	28	1412	1412	1412	0.999	216		3.01304
+	MCF7 + WT EVs_MCF7 + Vim -/- EVs	1	31	924	924	924	0.999	471		3.95964
+	MCF7 + WT EVs_MCF7 + Vim -/- EVs	1	34	1977	1908	1977	0.999	432		1.44404
+	MCF7 + WT EVs_MCF7 + Vim -/- EVs	1	52	1248	1248	1248	0.999	611		2.20915
+	MCF7 + WT EVs_MCF7 + Vim -/- EVs	1	31	588	331	588	0.999	1241		1.54267
+	MCF7 + WT EVs_MCF7 + Vim -/- EVs	1	35	912	912	912	0.999	792		1.39868
+	MCF7 + WT EVs_MCF7 + Vim -/- EVs	1	4	43	43	43	0.9985	80		2.10899
+	MCF7 + WT EVs_MCF7 + Vim -/- EVs	1	48	1089	1089	1089	0.999	808		1.72147
+	MCF7 + WT EVs_MCF7 + Vim -/- EVs	1	15	415	415	415	0.999	297		2.8182
+	MCF7 + WT EVs_MCF7 + Vim -/- EVs	1	33	1453	1453	1453	0.999	263		2.85253
+	MCF7 + WT EVs_MCF7 + Vim -/- EVs	1	23	1040	1040	1040	0.999	255		1.57595
+	MCF7 + WT EVs_MCF7 + Vim -/- EVs	1	51	735	735	735	0.999	1616		3.83945
+	MCF7 + WT EVs_MCF7 + Vim -/- EVs	1	37	1518	1518	1518	0.999	211		2.54022
+	MCF7 + WT EVs_MCF7 + Vim -/- EVs	1	13	500	500	500	0.999	101		1.70461
+	MCF7 + WT EVs_MCF7 + Vim -/- EVs	1	62	1874	1649	1874	0.999	723		2.36405
+	MCF7 + WT EVs_MCF7 + Vim -/- EVs	1	18	570	521	570	0.999	288		2.25587
+	MCF7 + WT EVs_MCF7 + Vim -/- EVs	1	7	127	127	127	0.999	223		2.85691
+	MCF7 + WT EVs_MCF7 + Vim -/- EVs	1	34	1639	1034	1639	0.999	214		1.83438
+	MCF7 + WT EVs_MCF7 + Vim -/- EVs	1	23	425	425	425	0.999	616		2.56477
+	MCF7 + WT EVs_MCF7 + Vim -/- EVs	1	69	1510	1510	1611	0.999	2225		3.77046
+	MCF7 + WT EVs_MCF7 + Vim -/- EVs	1	63	1676	1675	1676	0.999	1152		2.95518
+	MCF7 + WT EVs_MCF7 + Vim -/- EVs	1	38	1519	1491	1519	0.999	592		3.56737
+	MCF7 + WT EVs_MCF7 + Vim -/- EVs	1	8	392	392	392	0.999	239		1.59464
+	MCF7 + WT EVs_MCF7 + Vim -/- EVs	1	15	370	370	370	0.999	659		2.55242
+	MCF7 + WT EVs_MCF7 + Vim -/- EVs	1	7	47	47	47	0.999	417		3.75921
+	MCF7 + WT EVs_MCF7 + Vim -/- EVs	1	3	56	56	56	0.999	439		2.76888
+	MCF7 + WT EVs_MCF7 + Vim -/- EVs	1	11	51	50	106	0.999	1055		1.80553
+	MCF7 + WT EVs_MCF7 + Vim -/- EVs	1	8	103	103	103	0.999	298		2.07478
+	MCF7 + WT EVs_MCF7 + Vim -/- EVs	1	11	135	98	135	0.999	138		4.99796
+	MCF7 + WT EVs_MCF7 + Vim -/- EVs	1	31	1399	1322	1399	0.999	281		2.17197
+	MCF7 + WT EVs_MCF7 + Vim -/- EVs	1	11	443	443	443	0.999	214		2.50187
+	MCF7 + WT EVs_MCF7 + Vim -/- EVs	1	67	2947	2947	2947	0.999	505		3.34761
+	MCF7 + WT EVs_MCF7 + Vim -/- EVs	1	22	502	463	502	0.999	456		1.46596
+	MCF7 + WT EVs_MCF7 + Vim -/- EVs	1	18	395	395	395	0.999	357		4.33243
+	MCF7 + WT EVs_MCF7 + Vim -/- EVs	1	24	603	603	603	0.999	484		2.34489
+	MCF7 + WT EVs_MCF7 + Vim -/- EVs	1	26	741	704	741	0.999	379		6.68191
+	MCF7 + WT EVs_MCF7 + Vim -/- EVs	1	7	170	170	170	0.999	629		2.89886
+	MCF7 + WT EVs_MCF7 + Vim -/- EVs	1	24	452	349	452	0.999	517		1.42556
+	MCF7 + WT EVs_MCF7 + Vim -/- EVs	1	39	1372	1372	1372	0.999	664		4.18206
+	MCF7 + WT EVs_MCF7 + Vim -/- EVs	1	138	2718	2617	2718	0.999	1500		2.45161
+	MCF7 + WT EVs_MCF7 + Vim -/- EVs	1	13	175	88	175	0.999	480		2.67134
+	MCF7 + WT EVs_MCF7 + Vim -/- EVs	1	23	994	994	1090	0.999	198		3.79866
+	MCF7 + WT EVs_MCF7 + Vim -/- EVs	1	14	228	218	228	0.999	319		2.03962
+	MCF7 + WT EVs_MCF7 + Vim -/- EVs	1	17	243	156	243	0.999	592		2.39192
+	MCF7 + WT EVs_MCF7 + Vim -/- EVs	1	11	94	94	94	0.999	405		1.66287
+	MCF7 + WT EVs_MCF7 + Vim -/- EVs	1	23	1002	1002	1002	0.999	192		1.54322
+	MCF7 + WT EVs_MCF7 + Vim -/- EVs	1	13	452	452	452	0.999	252		2.70694
+	MCF7 + WT EVs_MCF7 + Vim -/- EVs	1	59	1335	1280	1335	0.999	863		1.64783
+	MCF7 + WT EVs_MCF7 + Vim -/- EVs	1	10	182	182	183	0.999	483		1.45965
+	MCF7 + WT EVs_MCF7 + Vim -/- EVs	1	39	1625	1624	1625	0.999	504		3.55468
+	MCF7 + WT EVs_MCF7 + Vim -/- EVs	1	45	1238	940	1238	0.999	906		2.7079
+	MCF7 + WT EVs_MCF7 + Vim -/- EVs	1	23	695	645	695	0.999	376		3.01087
+	MCF7 + WT EVs_MCF7 + Vim -/- EVs	1	6	124	124	124	0.999	121		2.58317
+	MCF7 + WT EVs_MCF7 + Vim -/- EVs	1	10	58	58	58	0.999	604		5.90301
+	MCF7 + WT EVs_MCF7 + Vim -/- EVs	1	17	220	220	220	0.999	551		4.82353
+	MCF7 + WT EVs_MCF7 + Vim -/- EVs	1	129	1734	1652	3323	0.999	1976		1.59142
+	MCF7 + WT EVs_MCF7 + Vim -/- EVs	1	15	637	637	637	0.999	385		3.07322
+	MCF7 + WT EVs_MCF7 + Vim -/- EVs	1	9	125	125	125	0.999	106		2.67645
+	MCF7 + WT EVs_MCF7 + Vim -/- EVs	1	27	387	387	639	0.999	400		1.73502
+	MCF7 + WT EVs_MCF7 + Vim -/- EVs	1	10	178	114	178	0.999	226		1.58263
+	MCF7 + WT EVs_MCF7 + Vim -/- EVs	1	18	274	274	274	0.999	454		1.62698
+	MCF7 + WT EVs_MCF7 + Vim -/- EVs	1	66	2785	2785	2785	0.999	427		2.05724
+	MCF7 + WT EVs_MCF7 + Vim -/- EVs	1	40	939	939	939	0.999	562		2.08584
+	MCF7 + WT EVs_MCF7 + Vim -/- EVs	1	6	117	117	117	0.999	197		3.56852
+	MCF7 + WT EVs_MCF7 + Vim -/- EVs	1	19	782	782	782	0.999	136		1.55118
+	MCF7 + WT EVs_MCF7 + Vim -/- EVs	1	9	122	116	122	0.999	140		3.1584
+	MCF7 + WT EVs_MCF7 + Vim -/- EVs	1	78	3229	2902	3229	0.999	403		2.35385
+	MCF7 + WT EVs_MCF7 + Vim -/- EVs	1	9	94	94	154	0.999	477		3.65106
+	MCF7 + WT EVs_MCF7 + Vim -/- EVs	1	33	1135	1000	1135	0.999	203		1.87768
+	MCF7 + WT EVs_MCF7 + Vim -/- EVs	1	39	1717	320	1717	0.999	472		2.94413
+	MCF7 + WT EVs_MCF7 + Vim -/- EVs	1	13	203	203	203	0.999	144		3.04426
+	MCF7 + WT EVs_MCF7 + Vim -/- EVs	1	8	65	18	65	0.999	1560		2.37684
+	MCF7 + WT EVs_MCF7 + Vim -/- EVs	1	49	1709	1709	1709	0.999	739		3.68871
+	MCF7 + WT EVs_MCF7 + Vim -/- EVs	1	69	1635	1635	1635	0.999	1262		2.61495
+	MCF7 + WT EVs_MCF7 + Vim -/- EVs	1	8	115	81	115	0.999	596		3.4473
+	MCF7 + WT EVs_MCF7 + Vim -/- EVs	1	9	221	221	221	0.999	302		1.59554
+	MCF7 + WT EVs_MCF7 + Vim -/- EVs	1	30	1122	605	1122	0.999	454		1.87401
+	MCF7 + WT EVs_MCF7 + Vim -/- EVs	1	29	485	485	485	0.999	750		4.30663
+	MCF7 + WT EVs_MCF7 + Vim -/- EVs	1	23	522	116	522	0.999	323		3.17086
+	MCF7 + WT EVs_MCF7 + Vim -/- EVs	1	39	471	471	471	0.999	2549		1.4327
+	MCF7 + WT EVs_MCF7 + Vim -/- EVs	1	10	303	121	303	0.999	84		2.53506
+	MCF7 + WT EVs_MCF7 + Vim -/- EVs	1	13	520	520	520	0.999	123		2.18846
+	MCF7 + WT EVs_MCF7 + Vim -/- EVs	1	12	142	93	142	0.999	156		2.40308
+	MCF7 + WT EVs_MCF7 + Vim -/- EVs	1	14	100	100	100	0.999	770		1.77671
+	MCF7 + WT EVs_MCF7 + Vim -/- EVs	1	13	63	63	63	0.999	646		2.0843

+	MCF7 + WT EVs_MCF7 + Vim -/- EVs	1	50	1476	1476	1476	0.999	847	2.70573
+	MCF7 + WT EVs_MCF7 + Vim -/- EVs	1	51	1200	1200	1200	0.999	491	7.36171
+	MCF7 + WT EVs_MCF7 + Vim -/- EVs	1	38	786	786	786	0.999	858	1.44481
+	MCF7 + WT EVs_MCF7 + Vim -/- EVs	1	76	852	852	852	0.999	3256	1.93802
+	MCF7 + WT EVs_MCF7 + Vim -/- EVs	1	25	696	696	696	0.999	587	3.47585
+	MCF7 + WT EVs_MCF7 + Vim -/- EVs	1	32	881	831	881	0.999	649	4.68406
+	MCF7 + WT EVs_MCF7 + Vim -/- EVs	1	7	68	68	68	0.999	2492	2.18512
+	MCF7 + WT EVs_MCF7 + Vim -/- EVs	1	15	556	556	556	0.999	148	2.43865
+	MCF7 + WT EVs_MCF7 + Vim -/- EVs	1	48	2210	2210	2210	0.999	297	2.01027
+	MCF7 + WT EVs_MCF7 + Vim -/- EVs	1	29	1388	1388	1388	0.999	160	2.0522
+	MCF7 + WT EVs_MCF7 + Vim -/- EVs	1	27	1462	1462	1462	0.999	137	2.18472
+	MCF7 + WT EVs_MCF7 + Vim -/- EVs	1	41	1726	1726	1726	0.999	194	1.44747
+	MCF7 + WT EVs_MCF7 + Vim -/- EVs	1	32	1141	1141	1141	0.999	204	2.21992
+	MCF7 + WT EVs_MCF7 + Vim -/- EVs	1	22	1248	1036	1249	0.999	165	1.55338
+	MCF7 + WT EVs_MCF7 + Vim -/- EVs	1	42	361	346	361	0.999	2468	3.32187
+	MCF7 + WT EVs_MCF7 + Vim -/- EVs	1	3	52	52	52	0.999	123	1.65549
+	MCF7 + WT EVs_MCF7 + Vim -/- EVs	1	9	72	72	72	0.999	749	1.51024
+	MCF7 + WT EVs_MCF7 + Vim -/- EVs	1	42	1189	1189	1189	0.999	775	2.21474
+	MCF7 + WT EVs_MCF7 + Vim -/- EVs	1	14	492	492	492	0.999	213	1.56049
+	MCF7 + WT EVs_MCF7 + Vim -/- EVs	1	26	316	192	316	0.999	325	2.42461
+	MCF7 + WT EVs_MCF7 + Vim -/- EVs	1	13	185	185	185	0.999	572	2.18163
+	MCF7 + WT EVs_MCF7 + Vim -/- EVs	1	20	356	356	356	0.999	732	1.42684
+	MCF7 + WT EVs_MCF7 + Vim -/- EVs	1	6	230	230	230	0.999	274	3.23517
+	MCF7 + WT EVs_MCF7 + Vim -/- EVs	1	13	304	268	304	0.999	253	3.7982
+	MCF7 + WT EVs_MCF7 + Vim -/- EVs	1	20	495	446	495	0.999	271	1.85152
+	MCF7 + WT EVs_MCF7 + Vim -/- EVs	1	10	70	70	70	0.999	469	2.19476
+	MCF7 + WT EVs_MCF7 + Vim -/- EVs	1	25	764	764	764	0.999	591	4.2052
+	MCF7 + WT EVs_MCF7 + Vim -/- EVs	1	9	79	53	79	0.999	400	1.69899
+	MCF7 + WT EVs_MCF7 + Vim -/- EVs	1	23	750	750	750	0.999	117	1.80251
+	MCF7 + WT EVs_MCF7 + Vim -/- EVs	1	22	817	817	817	0.999	510	3.77653
+	MCF7 + WT EVs_MCF7 + Vim -/- EVs	1	178	5910	5857	5910	0.999	2511	3.31524
+	MCF7 + WT EVs_MCF7 + Vim -/- EVs	1	5	106	106	106	0.999	369	1.64878
+	MCF7 + WT EVs_MCF7 + Vim -/- EVs	1	49	2126	2116	2126	0.999	545	1.9813
+	MCF7 + WT EVs_MCF7 + Vim -/- EVs	1	13	207	207	207	0.999	292	3.92543
+	MCF7 + WT EVs_MCF7 + Vim -/- EVs	1	35	1401	1401	1401	0.999	455	2.24054
+	MCF7 + WT EVs_MCF7 + Vim -/- EVs	1	28	836	836	836	0.999	539	2.16638
+	MCF7 + WT EVs_MCF7 + Vim -/- EVs	1	14	423	423	423	0.999	86	1.82298
+	MCF7 + WT EVs_MCF7 + Vim -/- EVs	1	52	1625	1625	1625	0.999	968	2.9251
+	MCF7 + WT EVs_MCF7 + Vim -/- EVs	1	31	704	704	704	0.999	748	3.36269
+	MCF7 + WT EVs_MCF7 + Vim -/- EVs	1	16	120	120	367	0.999	506	1.75023
+	MCF7 + WT EVs_MCF7 + Vim -/- EVs	1	36	691	691	691	0.999	514	3.85989
+	MCF7 + WT EVs_MCF7 + Vim -/- EVs	1	12	200	5	200	0.999	483	3.34269
+	MCF7 + WT EVs_MCF7 + Vim -/- EVs	1	8	89	89	270	0.999	483	1.86903
+	MCF7 + WT EVs_MCF7 + Vim -/- EVs	1	12	317	136	317	0.999	420	1.47148
+	MCF7 + WT EVs_MCF7 + Vim -/- EVs	1	22	603	603	603	0.999	366	1.66837
+	MCF7 + WT EVs_MCF7 + Vim -/- EVs	1	10	311	311	311	0.999	254	2.56695
+	MCF7 + WT EVs_MCF7 + Vim -/- EVs	1	20	470	470	470	0.999	773	3.63183
+	MCF7 + WT EVs_MCF7 + Vim -/- EVs	1	28	699	116	699	0.999	369	2.90234
+	MCF7 + WT EVs_MCF7 + Vim -/- EVs	1	14	151	151	151	0.999	628	1.70044
+	MCF7 + WT EVs_MCF7 + Vim -/- EVs	1	32	1177	1177	1177	0.999	215	1.58019
+	MCF7 + WT EVs_MCF7 + Vim -/- EVs	1	30	600	575	601	0.999	505	6.90665
+	MCF7 + WT EVs_MCF7 + Vim -/- EVs	1	7	118	80	118	0.999	221	2.32223
+	MCF7 + WT EVs_MCF7 + Vim -/- EVs	1	6	109	109	109	0.999	102	1.79202
+	MCF7 + WT EVs_MCF7 + Vim -/- EVs	1	6	144	144	144	0.999	393	1.77438
+	MCF7 + WT EVs_MCF7 + Vim -/- EVs	1	23	646	646	646	0.999	246	2.39369
+	MCF7 + WT EVs_MCF7 + Vim -/- EVs	1	33	682	682	682	0.999	736	1.6612
+	MCF7 + WT EVs_MCF7 + Vim -/- EVs	1	24	257	256	362	0.999	740	1.86911
+	MCF7 + WT EVs_MCF7 + Vim -/- EVs	1	28	883	865	883	0.999	240	1.70286
+	MCF7 + WT EVs_MCF7 + Vim -/- EVs	1	17	350	350	350	0.999	529	5.17756
+	MCF7 + WT EVs_MCF7 + Vim -/- EVs	1	13	313	212	313	0.999	538	3.62673
+	MCF7 + WT EVs_MCF7 + Vim -/- EVs	1	9	178	136	178	0.999	156	2.60816
+	MCF7 + WT EVs_MCF7 + Vim -/- EVs	1	12	256	200	256	0.999	607	1.89729
+	MCF7 + WT EVs_MCF7 + Vim -/- EVs	1	5	81	81	81	0.999	150	1.84576
+	MCF7 + WT EVs_MCF7 + Vim -/- EVs	1	17	221	221	221	0.999	878	2.53042
+	MCF7 + WT EVs_MCF7 + Vim -/- EVs	1	17	779	779	779	0.999	366	1.4747
+	MCF7 + WT EVs_MCF7 + Vim -/- EVs	1	35	408	408	570	0.999	917	4.14271
+	MCF7 + WT EVs_MCF7 + Vim -/- EVs	1	64	2742	2742	2742	0.999	1101	1.97609
+	MCF7 + WT EVs_MCF7 + Vim -/- EVs	1	13	431	431	431	0.999	463	1.9165
+	MCF7 + WT EVs_MCF7 + Vim -/- EVs	1	10	257	257	257	0.999	268	1.4229
+	MCF7 + WT EVs_MCF7 + Vim -/- EVs	1	8	280	280	280	0.999	500	1.67387
+	MCF7 + WT EVs_MCF7 + Vim -/- EVs	1	17	512	512	512	0.999	127	2.0702
+	MCF7 + WT EVs_MCF7 + Vim -/- EVs	1	54	1564	1564	1564	0.999	528	3.21924
+	MCF7 + WT EVs_MCF7 + Vim -/- EVs	1	14	239	239	239	0.999	279	4.54728
+	MCF7 + WT EVs_MCF7 + Vim -/- EVs	1	24	714	714	714	0.999	239	1.50865
+	MCF7 + WT EVs_MCF7 + Vim -/- EVs	1	36	1380	1380	1380	0.999	795	1.72917
+	MCF7 + WT EVs_MCF7 + Vim -/- EVs	1	12	249	226	249	0.999	295	1.55454
+	MCF7 + WT EVs_MCF7 + Vim -/- EVs	1	15	87	87	87	0.999	1798	1.83065
+	MCF7 + WT EVs_MCF7 + Vim -/- EVs	1	11	263	263	263	0.999	224	1.4399
+	MCF7 + WT EVs_MCF7 + Vim -/- EVs	1	45	1466	1466	1466	0.999	900	3.43361
+	MCF7 + WT EVs_MCF7 + Vim -/- EVs	1	27	292	292	292	0.999	1179	4.42576
+	MCF7 + WT EVs_MCF7 + Vim -/- EVs	1	7	185	185	185	0.999	247	1.66729
+	MCF7 + WT EVs_MCF7 + Vim -/- EVs	1	88	874	874	1179	0.999	5088	5.6145
+	MCF7 + WT EVs_MCF7 + Vim -/- EVs	1	3	59	59	59	0.999	83	1.47707
+	MCF7 + WT EVs_MCF7 + Vim -/- EVs	1	33	1812	1812	1812	0.999	249	2.69451
+	MCF7 + WT EVs_MCF7 + Vim -/- EVs	1	26	687	687	687	0.999	445	1.69396
+	MCF7 + WT EVs_MCF7 + Vim -/- EVs	1	48	2018	914	2018	0.999	406	3.44653
+	MCF7 + WT EVs_MCF7 + Vim -/- EVs	1	22	921	921	921	0.999	119	2.03563
+	MCF7 + WT EVs_MCF7 + Vim -/- EVs	1	28	1050	1050	1050	0.999	246	2.39612
+	MCF7 + WT EVs_MCF7 + Vim -/- EVs	1	15	665	529	665	0.999	191	2.29992
+	MCF7 + WT EVs_MCF7 + Vim -/- EVs	1	24	795	795	1200	0.999	165	2.4146
+	MCF7 + WT EVs_MCF7 + Vim -/- EVs	1	5	83	83	83	0.999	352	2.13637
+	MCF7 + WT EVs_MCF7 + Vim -/- EVs	1	12	256	256	256	0.999	183	1.89982
+	MCF7 + WT EVs_MCF7 + Vim -/- EVs	1	62	2997	2997	2997	0.999	264	1.60537
+	MCF7 + WT EVs_MCF7 + Vim -/- EVs	1	31	1503	451	1503	0.999	145	2.7753
+	MCF7 + WT EVs_MCF7 + Vim -/- EVs	1	31	1303	1303	1303	0.999	204	2.65076
+	MCF7 + WT EVs_MCF7 + Vim -/- EVs	1	14	532	414	532	0.999	92	2.72195
+	MCF7 + WT EVs_MCF7 + Vim -/- EVs	1	17	329	329	329	0.999	97	1.88452
+	MCF7 + WT EVs_MCF7 + Vim -/- EVs	1	41	2255	2255	2255	0.999	463	1.51783
+	MCF7 + WT EVs_MCF7 + Vim -/- EVs	1	41	1530	1210	1929	0.999	247	2.22123
+	MCF7 + WT EVs_MCF7 + Vim -/- EVs	1	14	174	156	174	0.999	204	1.56411
+	MCF7 + WT EVs_MCF7 + Vim -/- EVs	1	34	1243	1243	1243	0.999	194	1.56787
+	MCF7 + WT EVs_MCF7 + Vim -/- EVs	1	3	76	76	76	0.999	183	3.72653
+	MCF7 + WT EVs_MCF7 + Vim -/- EVs	1	14	634	634	634	0.999	151	1.42033
+	MCF7 + WT EVs_MCF7 + Vim -/- EVs	1	16	668	668	668	0.999	143	1.91502
+	MCF7 + WT EVs_MCF7 + Vim -/- EVs	1	32	1388	1388	1388	0.999	152	1.46055
+	MCF7 + WT EVs_MCF7 + Vim -/- EVs	1	14	338	338	338	0.999	56	1.52807
+	MCF7 + WT EVs_MCF7 + Vim -/- EVs	1	33	1614	1614	1614	0.999	158	2.06067
+	MCF7 + WT EVs_MCF7 + Vim -/- EVs	1	6	153	153	153	0.999	80	2.18961
+	MCF7 + WT EVs_MCF7 + Vim -/- EVs	1	10	190	190	190	0.999	175	1.59868
+	MCF7 + WT EVs_MCF7 + Vim -/- EVs	1	31	1003	1003	1003	0.999	389	1.49715
+	MCF7 + WT EVs_MCF7 + Vim -/- EVs	1	42	1988	1988	1988	0.999	266	2.23199
+	MCF7 + WT EVs_MCF7 + Vim -/- EVs	1	19	805	805	805	0.999	177	2.15981
+	MCF7 + WT EVs_MCF7 + Vim -/- EVs	1	9	55	55	130	0.999	196	4.23618
+	MCF7 + WT EVs_MCF7 + Vim -/- EVs	1	19	785	785	785	0.999	140	1.93434
+	MCF7 + WT EVs_MCF7 + Vim -/- EVs	1	19	552	552	552	0.999	133	1.43464
+	MCF7 + WT EVs_MCF7 + Vim -/- EVs	1	23	819	819	819	0.999	125	1.74466
+	MCF7 + WT EVs_MCF7 + Vim -/- EVs	1	7	126	49	126	0.999	115	2.99674
+	MCF7 + WT EVs_MCF7 + Vim -/- EVs	1	17	764	764	764	0.999	69	1.84216
+	MCF7 + WT EVs_MCF7 + Vim -/- EVs	1	4	79	1	79	0.999	51	1.4292
+	MCF7 + WT EVs_MCF7 + Vim -/- EVs	1	24	744	744	744	0.999	125	1.56207
+	MCF7 + WT EVs_MCF7 + Vim -/- EVs	1	29	1364	1364	1364	0.999	178	1.86879
+	MCF7 + WT EVs_MCF7 + Vim -/- EVs	1	40	1769	1769	1769	0.999	257	2.28927
+	MCF7 + WT EVs_MCF7 + Vim -/- EVs	1	29	3233	2945	3233	0.999	165	1.84589
+	MCF7 + WT EVs_MCF7 + Vim -/- EVs	1	23	81	81	484	0.999	354	3.14121
+	MCF7 + WT EVs_MCF7 + Vim -/- EVs	1	63	6701	75	6701	0.999	462	1.99768
+	MCF7 + WT EVs_MCF7 + Vim -/- EVs	1	50	1964	481	4198	0.999	448	1.54855
+	MCF7 + WT EVs_MCF7 + Vim -/- EVs	1	25	583	0	583	0.999	1222	2.42948
+	MCF7 + WT EVs_MCF7 + Vim -/- EVs	1	14	239	239	239	0.999	225	4.45433
+	MCF7 + WT EVs_MCF7 + Vim -/- EVs	1	39	898	898	898	0.999	907	2.23548

+	MCF7 + WT EVs_MCF7 + Vim -/- EVs	1	10	144	144	144	0.999	268	1.7605
+	MCF7 + WT EVs_MCF7 + Vim -/- EVs	1	4	28	28	28	0.999	741	1.55021
+	MCF7 + WT EVs_MCF7 + Vim -/- EVs	1	15	244	244	264	0.999	420	3.77284
+	MCF7 + WT EVs_MCF7 + Vim -/- EVs	1	14	142	142	142	0.999	360	1.57688
+	MCF7 + WT EVs_MCF7 + Vim -/- EVs	1	14	262	262	262	0.999	430	2.49223
+	MCF7 + WT EVs_MCF7 + Vim -/- EVs	1	15	317	317	317	0.999	396	2.6211
+	MCF7 + WT EVs_MCF7 + Vim -/- EVs	1	32	1095	1095	1095	0.999	157	1.99903
+	MCF7 + WT EVs_MCF7 + Vim -/- EVs	1	22	900	160	900	0.999	106	2.14051
+	MCF7 + WT EVs_MCF7 + Vim -/- EVs	1	9	161	161	297	0.999	191	1.70877
+	MCF7 + WT EVs_MCF7 + Vim -/- EVs	1	35	1238	1238	1238	0.999	196	2.29003
+	MCF7 + WT EVs_MCF7 + Vim -/- EVs	1	68	414	414	414	0.999	4391	2.42971
+	MCF7 + WT EVs_MCF7 + Vim -/- EVs	1	85	1314	1314	1314	0.999	1268	2.63031
+	MCF7 + WT EVs_MCF7 + Vim -/- EVs	1	7	146	146	284	0.999	292	2.03367
+	MCF7 + WT EVs_MCF7 + Vim -/- EVs	1	12	238	238	246	0.999	322	1.7513
+	MCF7 + WT EVs_MCF7 + Vim -/- EVs	1	6	124	124	124	0.999	391	4.01706
+	MCF7 + WT EVs_MCF7 + Vim -/- EVs	1	72	4195	4195	4195	0.999	825	2.84085
+	MCF7 + WT EVs_MCF7 + Vim -/- EVs	1	15	741	594	741	0.999	221	1.69515
+	MCF7 + WT EVs_MCF7 + Vim -/- EVs	1	18	153	152	153	0.999	879	1.76073
+	MCF7 + WT EVs_MCF7 + Vim -/- EVs	1	23	1098	930	1098	0.999	135	1.85001
+	MCF7 + WT EVs_MCF7 + Vim -/- EVs	1	47	1595	1535	1595	0.999	475	1.44561
+	MCF7 + WT EVs_MCF7 + Vim -/- EVs	1	7	399	301	399	0.999	507	3.36283
+	MCF7 + WT EVs_MCF7 + Vim -/- EVs	1	51	2042	1813	2042	0.999	784	4.18896
+	MCF7 + WT EVs_MCF7 + Vim -/- EVs	1	24	420	402	420	0.999	201	4.09528
+	MCF7 + WT EVs_MCF7 + Vim -/- EVs	1	12	66	65	66	0.999	2944	1.45085
+	MCF7 + WT EVs_MCF7 + Vim -/- EVs	1	34	1370	1370	1370	0.999	176	2.12407
+	MCF7 + WT EVs_MCF7 + Vim -/- EVs	1	23	776	524	776	0.999	393	3.41611
+	MCF7 + WT EVs_MCF7 + Vim -/- EVs	1	31	789	761	789	0.999	727	8.45495
+	MCF7 + WT EVs_MCF7 + Vim -/- EVs	1	26	440	420	440	0.999	461	1.55757
+	MCF7 + WT EVs_MCF7 + Vim -/- EVs	1	38	2121	2121	2121	0.999	288	2.10154
+	MCF7 + WT EVs_MCF7 + Vim -/- EVs	1	19	382	382	382	0.999	408	2.63401
+	MCF7 + WT EVs_MCF7 + Vim -/- EVs	1	12	213	213	213	0.999	335	2.713
+	MCF7 + WT EVs_MCF7 + Vim -/- EVs	1	6	69	69	69	0.999	748	2.11027
+	MCF7 + WT EVs_MCF7 + Vim -/- EVs	1	11	280	280	280	0.999	551	3.35891
+	MCF7 + WT EVs_MCF7 + Vim -/- EVs	1	15	399	399	399	0.999	297	1.74815
+	MCF7 + WT EVs_MCF7 + Vim -/- EVs	1	38	1046	1046	1046	0.999	702	6.577
+	MCF7 + WT EVs_MCF7 + Vim -/- EVs	1	74	2303	1846	2303	0.999	1599	2.19601
+	MCF7 + WT EVs_MCF7 + Vim -/- EVs	1	17	136	131	136	0.999	2471	2.63379
+	MCF7 + WT EVs_MCF7 + Vim -/- EVs	1	7	76	24	76	0.999	772	2.03493
+	MCF7 + WT EVs_MCF7 + Vim -/- EVs	1	33	958	958	1486	0.999	246	2.26743
+	MCF7 + WT EVs_MCF7 + Vim -/- EVs	1	42	287	287	5096	0.999	463	3.28451
+	MCF7 + WT EVs_MCF7 + Vim -/- EVs	1	47	1232	1232	1232	0.999	793	4.96204
+	MCF7 + WT EVs_MCF7 + Vim -/- EVs	1	14	153	153	153	0.999	890	1.90453
+	MCF7 + WT EVs_MCF7 + Vim -/- EVs	1	32	765	668	765	0.999	699	3.13477
+	MCF7 + WT EVs_MCF7 + Vim -/- EVs	1	22	794	794	794	0.999	188	2.07027
+	MCF7 + WT EVs_MCF7 + Vim -/- EVs	1	65	2249	2212	2249	0.999	602	3.92425
+	MCF7 + WT EVs_MCF7 + Vim -/- EVs	1	36	453	453	503	0.999	1748	2.04376
+	MCF7 + WT EVs_MCF7 + Vim -/- EVs	1	80	820	816	820	0.999	4544	4.81234
+	MCF7 + WT EVs_MCF7 + Vim -/- EVs	1	19	239	227	239	0.999	809	2.66864
+	MCF7 + WT EVs_MCF7 + Vim -/- EVs	1	14	168	168	168	0.999	891	1.4361
+	MCF7 + WT EVs_MCF7 + Vim -/- EVs	1	27	455	455	998	0.999	425	2.09292
+	MCF7 + WT EVs_MCF7 + Vim -/- EVs	1	324	7199	7188	7199	0.999	5890	1.92841
+	MCF7 + WT EVs_MCF7 + Vim -/- EVs	1	29	740	740	740	0.999	571	1.51355
+	MCF7 + WT EVs_MCF7 + Vim -/- EVs	1	33	379	379	876	0.999	949	1.47663
+	MCF7 + WT EVs_MCF7 + Vim -/- EVs	1	5	54	54	54	0.999	180	2.81432
+	MCF7 + WT EVs_MCF7 + Vim -/- EVs	1	20	686	686	686	0.999	525	1.54894
+	MCF7 + WT EVs_MCF7 + Vim -/- EVs	1	38	870	870	870	0.999	758	2.2209
+	MCF7 + WT EVs_MCF7 + Vim -/- EVs	1	11	157	157	157	0.999	480	2.95803
+	MCF7 + WT EVs_MCF7 + Vim -/- EVs	1	17	110	110	110	0.999	1025	2.87332
+	MCF7 + WT EVs_MCF7 + Vim -/- EVs	1	3	44	44	44	0.999	539	2.13204
+	MCF7 + WT EVs_MCF7 + Vim -/- EVs	1	20	548	548	548	0.999	356	2.61424
+	MCF7 + WT EVs_MCF7 + Vim -/- EVs	1	5	85	15	85	0.999	167	1.4199
+	MCF7 + WT EVs_MCF7 + Vim -/- EVs	1	25	714	714	714	0.999	328	1.67741
+	MCF7 + WT EVs_MCF7 + Vim -/- EVs	1	8	80	70	80	0.999	487	3.60217
+	MCF7 + WT EVs_MCF7 + Vim -/- EVs	1	47	746	676	746	0.999	2346	2.65131
+	MCF7 + WT EVs_MCF7 + Vim -/- EVs	1	18	514	514	514	0.999	414	1.71292
+	MCF7 + WT EVs_MCF7 + Vim -/- EVs	1	13	495	495	495	0.999	320	1.96005
+	MCF7 + WT EVs_MCF7 + Vim -/- EVs	1	16	537	537	851	0.999	271	3.43993
+	MCF7 + WT EVs_MCF7 + Vim -/- EVs	1	15	759	555	759	0.999	183	2.25454
+	MCF7 + WT EVs_MCF7 + Vim -/- EVs	1	11	118	118	118	0.999	472	2.80935
+	MCF7 + WT EVs_MCF7 + Vim -/- EVs	1	18	344	344	364	0.999	221	1.47895
+	MCF7 + WT EVs_MCF7 + Vim -/- EVs	1	56	2086	2086	2086	0.999	835	1.48362
+	MCF7 + WT EVs_MCF7 + Vim -/- EVs	1	28	1401	1262	1401	0.999	466	2.44708
+	MCF7 + WT EVs_MCF7 + Vim -/- EVs	1	25	873	873	873	0.999	325	1.6949
+	MCF7 + WT EVs_MCF7 + Vim -/- EVs	1	33	621	621	621	0.999	452	5.62476
+	MCF7 + WT EVs_MCF7 + Vim -/- EVs	1	39	1416	1416	1416	0.999	895	1.93076
+	MCF7 + WT EVs_MCF7 + Vim -/- EVs	1	13	206	206	206	0.999	819	2.51266
+	MCF7 + WT EVs_MCF7 + Vim -/- EVs	1	4	59	59	59	0.999	227	2.06432
+	MCF7 + WT EVs_MCF7 + Vim -/- EVs	1	13	465	465	465	0.999	440	6.15336
+	MCF7 + WT EVs_MCF7 + Vim -/- EVs	1	8	141	141	141	0.999	466	1.85169
+	MCF7 + WT EVs_MCF7 + Vim -/- EVs	1	8	32	6	48	0.999	558	1.75103
+	MCF7 + WT EVs_MCF7 + Vim -/- EVs	1	9	88	88	88	0.999	665	1.69384
+	MCF7 + WT EVs_MCF7 + Vim -/- EVs	1	4	34	32	34	0.999	643	1.71201
+	MCF7 + WT EVs_MCF7 + Vim -/- EVs	1	39	654	478	654	0.999	913	1.92738
+	MCF7 + WT EVs_MCF7 + Vim -/- EVs	1	9	207	207	207	0.999	280	2.43903
+	MCF7 + WT EVs_MCF7 + Vim -/- EVs	1	15	287	287	287	0.999	583	1.8116
+	MCF7 + WT EVs_MCF7 + Vim -/- EVs	0.9999	2	27	27	27	0.999	201	1.84084
+	MCF7 + WT EVs_MCF7 + Vim -/- EVs	1	16	168	168	168	0.999	731	1.43899
+	MCF7 + WT EVs_MCF7 + Vim -/- EVs	1	9	122	122	122	0.999	455	1.40775
+	MCF7 + WT EVs_MCF7 + Vim -/- EVs	1	13	195	195	195	0.999	437	1.94076
+	MCF7 + WT EVs_MCF7 + Vim -/- EVs	1	18	736	736	736	0.999	142	1.80629
+	MCF7 + WT EVs_MCF7 + Vim -/- EVs	1	15	310	310	310	0.999	193	2.95761
+	MCF7 + WT EVs_MCF7 + Vim -/- EVs	1	17	296	296	296	0.999	746	1.52207
+	MCF7 + WT EVs_MCF7 + Vim -/- EVs	1	5	120	120	120	0.999	624	2.57632
+	MCF7 + WT EVs_MCF7 + Vim -/- EVs	1	30	973	973	973	0.999	1087	2.1898
+	MCF7 + WT EVs_MCF7 + Vim -/- EVs	1	12	176	176	176	0.999	292	2.39083
+	MCF7 + WT EVs_MCF7 + Vim -/- EVs	1	31	651	651	651	0.999	644	3.30701
+	MCF7 + WT EVs_MCF7 + Vim -/- EVs	1	30	657	657	657	0.999	279	4.11593
+	MCF7 + WT EVs_MCF7 + Vim -/- EVs	1	41	656	656	656	0.999	1278	1.74291
+	MCF7 + WT EVs_MCF7 + Vim -/- EVs	1	267	7478	7478	7478	0.999	4646	1.72146
+	MCF7 + WT EVs_MCF7 + Vim -/- EVs	1	26	239	236	239	0.999	749	3.90174
+	MCF7 + WT EVs_MCF7 + Vim -/- EVs	1	30	907	907	907	0.999	630	1.74825
+	MCF7 + WT EVs_MCF7 + Vim -/- EVs	1	65	476	476	1006	0.999	2725	1.74568
+	MCF7 + WT EVs_MCF7 + Vim -/- EVs	1	22	354	306	354	0.999	339	1.40198
+	MCF7 + WT EVs_MCF7 + Vim -/- EVs	1	13	267	267	267	0.999	348	4.05164
+	MCF7 + WT EVs_MCF7 + Vim -/- EVs	1	11	226	226	226	0.999	519	2.54505
+	MCF7 + WT EVs_MCF7 + Vim -/- EVs	1	9	42	42	96	0.999	1220	2.28576
+	MCF7 + WT EVs_MCF7 + Vim -/- EVs	1	21	60	52	120	0.999	2758	1.5227
+	MCF7 + WT EVs_MCF7 + Vim -/- EVs	1	12	143	143	209	0.999	629	5.72634
+	MCF7 + WT EVs_MCF7 + Vim -/- EVs	1	60	942	923	942	0.999	1233	2.5744
+	MCF7 + WT EVs_MCF7 + Vim -/- EVs	1	7	318	318	318	0.999	234	2.26178
+	MCF7 + WT EVs_MCF7 + Vim -/- EVs	1	66	2591	2591	2591	0.999	944	2.88095
+	MCF7 + WT EVs_MCF7 + Vim -/- EVs	1	52	1272	1272	1272	0.999	893	4.34727
+	MCF7 + WT EVs_MCF7 + Vim -/- EVs	1	45	1751	1751	1751	0.999	876	1.92874
+	MCF7 + WT EVs_MCF7 + Vim -/- EVs	1	132	2602	2600	2602	0.999	2115	3.65179
+	MCF7 + WT EVs_MCF7 + Vim -/- EVs	1	9	119	119	119	0.999	1843	1.69553
+	MCF7 + WT EVs_MCF7 + Vim -/- EVs	1	23	457	419	457	0.999	741	1.98006
+	MCF7 + WT EVs_MCF7 + Vim -/- EVs	1	6	167	167	167	0.999	396	2.34525
+	MCF7 + WT EVs_MCF7 + Vim -/- EVs	1	5	81	81	81	0.999	288	1.62984
+	MCF7 + WT EVs_MCF7 + Vim -/- EVs	1	24	790	790	790	0.999	361	3.54563
+	MCF7 + WT EVs_MCF7 + Vim -/- EVs	1	26	388	387	388	0.999	1401	3.43799
+	MCF7 + WT EVs_MCF7 + Vim -/- EVs	1	11	145	132	145	0.999	903	2.45343
+	MCF7 + WT EVs_MCF7 + Vim -/- EVs	1	8	197	197	197	0.999	203	1.63559
+	MCF7 + WT EVs_MCF7 + Vim -/- EVs	1	24	416	416	416	0.999	981	3.33151
+	MCF7 + WT EVs_MCF7 + Vim -/- EVs	1	3	22	22	22	0.999	492	6.25043
+	MCF7 + WT EVs_MCF7 + Vim -/- EVs	1	6	41	41	41	0.999	466	1.5289
+	MCF7 + WT EVs_MCF7 + Vim -/- EVs	1	15	153	153	153	0.999	677	2.3103
+	MCF7 + WT EVs_MCF7 + Vim -/- EVs	1	32	1410	1410	1410	0.999	440	2.8228
+	MCF7 + WT EVs_MCF7 + Vim -/- EVs	1	8	64	64	64	0.999	449	2.57503
+	MCF7 + WT EVs_MCF7 + Vim -/- EVs	1	4	26	26	26	0.999	406	3.62543

+	MCF7 + WT EVs_MCF7 + Vim -/- EVs	1	512	10698	10363	10698	0.999	4684	4.31643
+	MCF7 + WT EVs_MCF7 + Vim -/- EVs	1	6	111	111	111	0.999	354	1.85639
+	MCF7 + WT EVs_MCF7 + Vim -/- EVs	1	66	2139	2136	2283	0.999	471	1.65338
+	MCF7 + WT EVs_MCF7 + Vim -/- EVs	1	10	112	111	112	0.999	1439	3.36443
+	MCF7 + WT EVs_MCF7 + Vim -/- EVs	1	24	1027	655	1027	0.999	356	1.40839
+	MCF7 + WT EVs_MCF7 + Vim -/- EVs	1	11	262	262	262	0.999	277	2.49506
+	MCF7 + WT EVs_MCF7 + Vim -/- EVs	1	12	358	358	358	0.999	228	2.74859
+	MCF7 + WT EVs_MCF7 + Vim -/- EVs	1	6	28	28	28	0.999	514	2.25396
+	MCF7 + WT EVs_MCF7 + Vim -/- EVs	1	17	534	534	534	0.999	476	1.88671
+	MCF7 + WT EVs_MCF7 + Vim -/- EVs	1	10	414	414	414	0.999	541	4.19368
+	MCF7 + WT EVs_MCF7 + Vim -/- EVs	1	6	35	19	35	0.999	467	2.38037
+	MCF7 + WT EVs_MCF7 + Vim -/- EVs	1	22	739	9	819	0.999	218	1.51629
+	MCF7 + WT EVs_MCF7 + Vim -/- EVs	1	25	774	774	774	0.999	572	3.42901
+	MCF7 + WT EVs_MCF7 + Vim -/- EVs	1	11	156	149	156	0.999	282	4.80446
+	MCF7 + WT EVs_MCF7 + Vim -/- EVs	1	16	86	86	90	0.999	942	2.31584
+	MCF7 + WT EVs_MCF7 + Vim -/- EVs	1	13	329	329	331	0.999	193	3.01998
+	MCF7 + WT EVs_MCF7 + Vim -/- EVs	1	51	1495	1495	1495	0.999	1140	1.99494
+	MCF7 + WT EVs_MCF7 + Vim -/- EVs	1	12	255	204	255	0.999	360	2.9676
+	MCF7 + WT EVs_MCF7 + Vim -/- EVs	1	54	2371	2371	2371	0.999	493	1.9924
+	MCF7 + WT EVs_MCF7 + Vim -/- EVs	1	21	544	544	544	0.999	335	1.56524
+	MCF7 + WT EVs_MCF7 + Vim -/- EVs	1	9	114	114	114	0.999	1144	2.3937
+	MCF7 + WT EVs_MCF7 + Vim -/- EVs	1	25	611	611	611	0.999	465	2.64375
+	MCF7 + WT EVs_MCF7 + Vim -/- EVs	1	9	105	105	105	0.999	277	1.58277
+	MCF7 + WT EVs_MCF7 + Vim -/- EVs	1	7	211	211	211	0.999	197	3.60782
+	MCF7 + WT EVs_MCF7 + Vim -/- EVs	1	14	144	144	144	0.999	459	2.87034
+	MCF7 + WT EVs_MCF7 + Vim -/- EVs	1	27	384	384	384	0.999	640	4.73193
+	MCF7 + WT EVs_MCF7 + Vim -/- EVs	1	34	788	788	788	0.999	508	1.75118
+	MCF7 + WT EVs_MCF7 + Vim -/- EVs	1	44	1372	1372	1372	0.999	747	2.06934
+	MCF7 + WT EVs_MCF7 + Vim -/- EVs	1	21	358	358	358	0.999	1249	2.97144
+	MCF7 + WT EVs_MCF7 + Vim -/- EVs	1	7	97	97	97	0.999	776	2.29342
+	MCF7 + WT EVs_MCF7 + Vim -/- EVs	1	20	117	117	240	0.999	917	4.12584
+	MCF7 + WT EVs_MCF7 + Vim -/- EVs	1	17	264	264	264	0.999	736	5.85172
+	MCF7 + WT EVs_MCF7 + Vim -/- EVs	1	24	189	189	192	0.999	841	2.89457
+	MCF7 + WT EVs_MCF7 + Vim -/- EVs	1	10	70	70	117	0.999	507	1.79203
+	MCF7 + WT EVs_MCF7 + Vim -/- EVs	1	17	309	309	309	0.999	575	3.91516
+	MCF7 + WT EVs_MCF7 + Vim -/- EVs	1	17	366	366	366	0.999	353	3.67525
+	MCF7 + WT EVs_MCF7 + Vim -/- EVs	1	10	127	127	127	0.999	910	2.28192
+	MCF7 + WT EVs_MCF7 + Vim -/- EVs	1	13	273	273	273	0.999	469	2.05403
+	MCF7 + WT EVs_MCF7 + Vim -/- EVs	1	14	175	175	175	0.999	1204	2.38684
+	MCF7 + WT EVs_MCF7 + Vim -/- EVs	1	11	124	124	124	0.999	274	2.16126
+	MCF7 + WT EVs_MCF7 + Vim -/- EVs	1	17	335	335	335	0.999	154	3.79509
+	MCF7 + WT EVs_MCF7 + Vim -/- EVs	1	11	137	137	137	0.999	531	2.23635
+	MCF7 + WT EVs_MCF7 + Vim -/- EVs	1	5	134	134	134	0.999	236	1.81567
+	MCF7 + WT EVs_MCF7 + Vim -/- EVs	1	20	567	567	567	0.999	583	1.80024
+	MCF7 + WT EVs_MCF7 + Vim -/- EVs	0.9999	2	70	70	70	0.999	344	2.19657
+	MCF7 + WT EVs_MCF7 + Vim -/- EVs	1	8	84	84	84	0.999	469	2.16511
+	MCF7 + WT EVs_MCF7 + Vim -/- EVs	1	35	517	446	517	0.999	1584	2.43506
+	MCF7 + WT EVs_MCF7 + Vim -/- EVs	1	64	971	971	971	0.999	5183	1.66137
+	MCF7 + WT EVs_MCF7 + Vim -/- EVs	1	6	62	62	62	0.999	1801	1.49114
+	MCF7 + WT EVs_MCF7 + Vim -/- EVs	1	10	87	87	87	0.999	1371	2.02294
+	MCF7 + WT EVs_MCF7 + Vim -/- EVs	1	12	118	118	118	0.999	838	1.44081
+	MCF7 + WT EVs_MCF7 + Vim -/- EVs	1	11	107	107	107	0.999	1445	5.05303
+	MCF7 + WT EVs_MCF7 + Vim -/- EVs	1	9	84	60	84	0.999	881	2.37369
+	MCF7 + WT EVs_MCF7 + Vim -/- EVs	1	6	53	53	53	0.999	673	1.77457
+	MCF7 + WT EVs_MCF7 + Vim -/- EVs	1	19	392	392	504	0.999	349	1.88745
+	MCF7 + WT EVs_MCF7 + Vim -/- EVs	1	21	92	92	92	0.999	2804	1.51584
+	MCF7 + WT EVs_MCF7 + Vim -/- EVs	1	8	127	127	127	0.999	501	1.46726
+	MCF7 + WT EVs_MCF7 + Vim -/- EVs	1	15	137	137	137	0.999	951	1.69024
+	MCF7 + WT EVs_MCF7 + Vim -/- EVs	1	17	274	267	274	0.999	788	1.69364
+	MCF7 + WT EVs_MCF7 + Vim -/- EVs	1	14	188	188	188	0.999	1173	1.40989
+	MCF7 + WT EVs_MCF7 + Vim -/- EVs	1	22	523	523	523	0.999	645	2.28693
+	MCF7 + WT EVs_MCF7 + Vim -/- EVs	1	5	65	65	65	0.999	821	2.78893
+	MCF7 + WT EVs_MCF7 + Vim -/- EVs	1	7	54	54	54	0.999	289	2.76108
+	MCF7 + WT EVs_MCF7 + Vim -/- EVs	1	24	408	408	506	0.999	978	1.8293
+	MCF7 + WT EVs_MCF7 + Vim -/- EVs	1	24	223	223	223	0.999	1025	1.41412
+	MCF7 + WT EVs_MCF7 + Vim -/- EVs	1	6	106	106	106	0.999	254	1.90514
+	MCF7 + WT EVs_MCF7 + Vim -/- EVs	0.9999	2	6	6	6	0.999	896	1.42949
+	MCF7 + WT EVs_MCF7 + Vim -/- EVs	1	10	85	85	85	0.999	911	3.23291
+	MCF7 + WT EVs_MCF7 + Vim -/- EVs	1	12	37	37	37	0.999	2754	1.5925
+	MCF7 + WT EVs_MCF7 + Vim -/- EVs	1	8	47	47	47	0.999	1090	1.63292
+	MCF7 + WT EVs_MCF7 + Vim -/- EVs	1	16	224	224	224	0.999	350	1.68585
+	MCF7 + WT EVs_MCF7 + Vim -/- EVs	1	79	3313	3313	3313	0.999	910	1.40953
+	MCF7 + WT EVs_MCF7 + Vim -/- EVs	1	27	884	827	884	0.999	419	4.49315
+	MCF7 + WT EVs_MCF7 + Vim -/- EVs	1	32	413	413	413	0.999	1194	3.21732
+	MCF7 + WT EVs_MCF7 + Vim -/- EVs	1	3	53	53	53	0.999	434	1.96406
+	MCF7 + WT EVs_MCF7 + Vim -/- EVs	1	43	868	423	868	0.999	1253	1.75861
+	MCF7 + WT EVs_MCF7 + Vim -/- EVs	1	6	115	115	115	0.999	544	1.43079
+	MCF7 + WT EVs_MCF7 + Vim -/- EVs	1	17	199	197	199	0.999	1761	1.49089
+	MCF7 + WT EVs_MCF7 + Vim -/- EVs	1	15	326	326	326	0.999	199	1.99283
+	MCF7 + WT EVs_MCF7 + Vim -/- EVs	1	38	595	595	595	0.999	902	1.5132
+	MCF7 + WT EVs_MCF7 + Vim -/- EVs	1	12	104	104	104	0.999	507	1.72116
+	MCF7 + WT EVs_MCF7 + Vim -/- EVs	1	15	142	142	142	0.999	695	2.22695
+	MCF7 + WT EVs_MCF7 + Vim -/- EVs	1	20	265	234	265	0.999	1538	2.44978
+	MCF7 + WT EVs_MCF7 + Vim -/- EVs	1	6	56	56	56	0.999	507	2.71967
+	MCF7 + WT EVs_MCF7 + Vim -/- EVs	0.9865	2	111	111	111	0.9957	357	1.92307
+	MCF7 + WT EVs_MCF7 + Vim -/- EVs	1	12	102	100	102	0.999	587	2.07704
+	MCF7 + WT EVs_MCF7 + Vim -/- EVs	1	12	208	208	265	0.999	227	3.14363
+	MCF7 + WT EVs_MCF7 + Vim -/- EVs	1	5	91	91	91	0.999	275	2.55794
+	MCF7 + WT EVs_MCF7 + Vim -/- EVs	1	7	115	115	115	0.999	594	1.67652
+	MCF7 + WT EVs_MCF7 + Vim -/- EVs	1	7	334	245	334	0.999	306	1.4619
+	MCF7 + WT EVs_MCF7 + Vim -/- EVs	1	22	754	754	754	0.999	582	4.5093
+	MCF7 + WT EVs_MCF7 + Vim -/- EVs	1	4	117	117	117	0.999	237	2.72439
+	MCF7 + WT EVs_MCF7 + Vim -/- EVs	1	8	156	156	156	0.999	672	2.48076
+	MCF7 + WT EVs_MCF7 + Vim -/- EVs	1	25	236	236	236	0.999	1076	2.02137
+	MCF7 + WT EVs_MCF7 + Vim -/- EVs	1	5	30	30	30	0.999	208	1.73193
+	MCF7 + WT EVs_MCF7 + Vim -/- EVs	1	7	63	63	63	0.999	649	1.89716
+	MCF7 + WT EVs_MCF7 + Vim -/- EVs	1	16	110	110	110	0.999	938	2.3205
+	MCF7 + WT EVs_MCF7 + Vim -/- EVs	1	9	49	49	50	0.999	584	3.03666
+	MCF7 + WT EVs_MCF7 + Vim -/- EVs	1	3	82	82	82	0.999	188	2.66062
+	MCF7 + WT EVs_MCF7 + Vim -/- EVs	1	27	391	391	391	0.999	679	4.60484
+	MCF7 + WT EVs_MCF7 + Vim -/- EVs	1	4	47	47	47	0.999	547	2.6267
+	MCF7 + WT EVs_MCF7 + Vim -/- EVs	1	6	109	109	109	0.999	1001	2.62467
+	MCF7 + WT EVs_MCF7 + Vim -/- EVs	1	8	45	45	84	0.999	457	3.59123
+	MCF7 + WT EVs_MCF7 + Vim -/- EVs	1	8	99	99	99	0.999	663	3.49353
+	MCF7 + WT EVs_MCF7 + Vim -/- EVs	1	12	110	109	110	0.999	1523	2.82998
+	MCF7 + WT EVs_MCF7 + Vim -/- EVs	1	21	270	268	270	0.999	1231	1.46548
+	MCF7 + WT EVs_MCF7 + Vim -/- EVs	1	3	55	55	55	0.999	183	1.74226
+	MCF7 + WT EVs_MCF7 + Vim -/- EVs	1	16	246	246	246	0.999	406	2.66489
+	MCF7 + WT EVs_MCF7 + Vim -/- EVs	1	4	37	37	37	0.999	132	4.59152
+	MCF7 + WT EVs_MCF7 + Vim -/- EVs	1	22	604	596	604	0.999	591	2.9417
+	MCF7 + WT EVs_MCF7 + Vim -/- EVs	1	3	36	36	36	0.999	165	1.68143
+	MCF7 + WT EVs_MCF7 + Vim -/- EVs	1	19	161	161	161	0.999	471	2.01985
+	MCF7 + WT EVs_MCF7 + Vim -/- EVs	1	35	789	759	789	0.999	622	2.76057
+	MCF7 + WT EVs_MCF7 + Vim -/- EVs	1	16	209	209	209	0.999	503	2.37871
+	MCF7 + WT EVs_MCF7 + Vim -/- EVs	1	11	171	171	171	0.999	534	2.2846
+	MCF7 + WT EVs_MCF7 + Vim -/- EVs	1	4	62	62	62	0.999	745	1.9575
+	MCF7 + WT EVs_MCF7 + Vim -/- EVs	1	7	97	97	97	0.999	740	3.44781
+	MCF7 + WT EVs_MCF7 + Vim -/- EVs	1	13	181	181	181	0.999	881	1.6382
+	MCF7 + WT EVs_MCF7 + Vim -/- EVs	1	7	46	46	46	0.999	209	3.15236
+	MCF7 + WT EVs_MCF7 + Vim -/- EVs	0.9999	2	25	25	25	0.999	440	1.72137
+	MCF7 + WT EVs_MCF7 + Vim -/- EVs	1	4	96	96	96	0.999	366	2.27127
+	MCF7 + WT EVs_MCF7 + Vim -/- EVs	1	3	20	20	20	0.999	166	2.25079
+	MCF7 + WT EVs_MCF7 + Vim -/- EVs	1	17	301	301	301	0.999	828	2.84431
+	MCF7 + WT EVs_MCF7 + Vim -/- EVs	1	8	98	98	98	0.999	472	4.66644
+	MCF7 + WT EVs_MCF7 + Vim -/- EVs	0.9993	3	18	18	18	0.999	574	1.76007
+	MCF7 + WT EVs_MCF7 + Vim -/- EVs	1	38	325	325	325	0.999	1683	2.13748
+	MCF7 + WT EVs_MCF7 + Vim -/- EVs	1	33	777	739	777	0.999	1075	2.46739
+	MCF7 + WT EVs_MCF7 + Vim -/- EVs	1	16	284	284	284	0.999	499	2.55982
+	MCF7 + WT EVs_MCF7 + Vim -/- EVs	1	78	576	525	576	0.999	6885	2.8161

+	MCF7 + WT EVs_MCF7 + Vim -/- EVs	1	3	75	75	75	0.999	227	3.13287
+	MCF7 + WT EVs_MCF7 + Vim -/- EVs	1	9	128	128	128	0.999	623	2.47646
+	MCF7 + WT EVs_MCF7 + Vim -/- EVs	1	18	167	167	167	0.999	2039	1.81934
+	MCF7 + WT EVs_MCF7 + Vim -/- EVs	1	25	1262	1262	1262	0.999	394	7.99582
+	MCF7 + WT EVs_MCF7 + Vim -/- EVs	1	57	1380	1245	1520	0.999	858	1.96285
+	MCF7 + WT EVs_MCF7 + Vim -/- EVs	1	18	212	212	366	0.999	483	3.07596
+	MCF7 + WT EVs_MCF7 + Vim -/- EVs	1	3	25	25	25	0.999	1136	1.92426
+	MCF7 + WT EVs_MCF7 + Vim -/- EVs	1	79	416	416	416	0.999	1479	4.11139
+	MCF7 + WT EVs_MCF7 + Vim -/- EVs	1	23	578	578	583	0.999	370	1.80702
+	MCF7 + WT EVs_MCF7 + Vim -/- EVs	1	4	171	171	171	0.999	318	2.24268
+	MCF7 + WT EVs_MCF7 + Vim -/- EVs	1	66	2238	1527	2238	0.999	729	2.9374
+	MCF7 + WT EVs_MCF7 + Vim -/- EVs	1	52	873	873	873	0.999	1312	1.97522
+	MCF7 + WT EVs_MCF7 + Vim -/- EVs	1	17	515	406	515	0.999	486	2.12099
+	MCF7 + WT EVs_MCF7 + Vim -/- EVs	1	29	560	560	560	0.999	912	2.09003
+	MCF7 + WT EVs_MCF7 + Vim -/- EVs	1	51	944	944	944	0.999	1179	5.3659
+	MCF7 + WT EVs_MCF7 + Vim -/- EVs	1	38	700	631	700	0.999	1129	1.89447
+	MCF7 + WT EVs_MCF7 + Vim -/- EVs	1	16	68	68	554	0.999	207	3.47368
+	MCF7 + WT EVs_MCF7 + Vim -/- EVs	1	54	1709	1496	1709	0.999	711	2.34782
+	MCF7 + WT EVs_MCF7 + Vim -/- EVs	1	22	736	465	736	0.999	898	2.21473
+	MCF7 + WT EVs_MCF7 + Vim -/- EVs	1	36	517	517	517	0.999	986	3.25138
+	MCF7 + WT EVs_MCF7 + Vim -/- EVs	1	27	561	561	561	0.999	1102	2.6427
+	MCF7 + WT EVs_MCF7 + Vim -/- EVs	1	3	21	21	21	0.999	163	1.83817
+	MCF7 + WT EVs_MCF7 + Vim -/- EVs	1	9	384	384	384	0.999	173	1.95539
+	MCF7 + WT EVs_MCF7 + Vim -/- EVs	1	17	101	101	101	0.999	613	5.99914
+	MCF7 + WT EVs_MCF7 + Vim -/- EVs	1	21	126	126	866	0.999	106	1.92143
+	MCF7 + WT EVs_MCF7 + Vim -/- EVs	1	10	163	163	163	0.999	477	4.16655
+	MCF7 + WT EVs_MCF7 + Vim -/- EVs	1	12	309	309	309	0.999	290	2.49789
+	MCF7 + WT EVs_MCF7 + Vim -/- EVs	1	6	63	63	63	0.999	686	2.04071
+	MCF7 + WT EVs_MCF7 + Vim -/- EVs	1	16	233	233	233	0.999	631	2.8203
+	MCF7 + WT EVs_MCF7 + Vim -/- EVs	1	9	339	339	339	0.999	154	6.36845
+	MCF7 + WT EVs_MCF7 + Vim -/- EVs	1	17	168	168	168	0.999	974	1.92353
+	MCF7 + WT EVs_MCF7 + Vim -/- EVs	1	27	365	359	365	0.999	680	3.2003
+	MCF7 + WT EVs_MCF7 + Vim -/- EVs	1	45	1177	1034	1390	0.999	644	1.69356
+	MCF7 + WT EVs_MCF7 + Vim -/- EVs	1	8	247	245	247	0.999	228	1.5546
+	MCF7 + WT EVs_MCF7 + Vim -/- EVs	1	18	317	312	317	0.999	582	3.23852
+	MCF7 + WT EVs_MCF7 + Vim -/- EVs	0.9999	2	73	73	73	0.999	74	1.5159
+	MCF7 + WT EVs_MCF7 + Vim -/- EVs	0.9999	2	17	17	17	0.999	188	2.66609
+	MCF7 + WT EVs_MCF7 + Vim -/- EVs	0.9995	3	30	30	30	0.9989	424	1.63036
+	MCF7 + WT EVs_MCF7 + Vim -/- EVs	1	5	80	80	80	0.999	646	1.90997
+	MCF7 + WT EVs_MCF7 + Vim -/- EVs	1	9	186	186	186	0.999	231	1.55763
+	MCF7 + WT EVs_MCF7 + Vim -/- EVs	1	9	273	273	273	0.999	138	1.45554
+	MCF7 + WT EVs_MCF7 + Vim -/- EVs	1	19	488	457	488	0.999	240	1.97146
+	MCF7 + WT EVs_MCF7 + Vim -/- EVs	1	5	64	64	64	0.999	342	1.89897
+	MCF7 + WT EVs_MCF7 + Vim -/- EVs	1	15	228	228	238	0.999	320	2.44123
+	MCF7 + WT EVs_MCF7 + Vim -/- EVs	1	5	184	184	184	0.999	337	1.50671
+	MCF7 + WT EVs_MCF7 + Vim -/- EVs	1	16	389	389	389	0.999	445	1.67646
+	MCF7 + WT EVs_MCF7 + Vim -/- EVs	1	17	381	381	381	0.999	223	1.88981
+	MCF7 + WT EVs_MCF7 + Vim -/- EVs	1	8	178	178	178	0.999	503	2.49423
+	MCF7 + WT EVs_MCF7 + Vim -/- EVs	1	5	116	116	116	0.999	112	3.6353
+	MCF7 + WT EVs_MCF7 + Vim -/- EVs	1	5	18	18	18	0.999	259	2.49017
+	MCF7 + WT EVs_MCF7 + Vim -/- EVs	0.9998	3	46	46	46	0.999	351	1.49827
+	MCF7 + WT EVs_MCF7 + Vim -/- EVs	1	16	146	146	146	0.999	650	1.60441
+	MCF7 + WT EVs_MCF7 + Vim -/- EVs	1	4	49	49	49	0.999	475	2.88568
+	MCF7 + WT EVs_MCF7 + Vim -/- EVs	1	3	66	66	66	0.999	212	1.58835
+	MCF7 + WT EVs_MCF7 + Vim -/- EVs	1	3	65	65	65	0.999	258	2.36783
+	MCF7 + WT EVs_MCF7 + Vim -/- EVs	1	29	667	594	667	0.999	468	7.48816
+	MCF7 + WT EVs_MCF7 + Vim -/- EVs	1	7	91	91	92	0.999	314	1.49681
+	MCF7 + WT EVs_MCF7 + Vim -/- EVs	1	41	958	958	1239	0.999	572	1.96263
+	MCF7 + WT EVs_MCF7 + Vim -/- EVs	1	11	131	131	131	0.999	420	1.88725
+	MCF7 + WT EVs_MCF7 + Vim -/- EVs	1	8	132	132	132	0.999	340	4.2147
+	MCF7 + WT EVs_MCF7 + Vim -/- EVs	1	11	156	156	156	0.999	506	1.4642
+	MCF7 + WT EVs_MCF7 + Vim -/- EVs	1	12	159	159	159	0.999	1375	2.1071
+	MCF7 + WT EVs_MCF7 + Vim -/- EVs	1	20	516	516	516	0.999	475	1.85267
+	MCF7 + WT EVs_MCF7 + Vim -/- EVs	0.9941	3	32	32	34	0.9948	1537	3.72783
+	MCF7 + WT EVs_MCF7 + Vim -/- EVs	1	3	7	7	7	0.9987	551	1.70711
+	MCF7 + WT EVs_MCF7 + Vim -/- EVs	1	11	115	115	115	0.999	1450	1.58358
+	MCF7 + WT EVs_MCF7 + Vim -/- EVs	1	6	35	35	35	0.999	617	1.92315
+	MCF7 + WT EVs_MCF7 + Vim -/- EVs	1	3	25	25	25	0.999	204	2.42267
+	MCF7 + WT EVs_MCF7 + Vim -/- EVs	1	8	49	49	49	0.999	665	2.74483
+	MCF7 + WT EVs_MCF7 + Vim -/- EVs	1	17	262	200	262	0.999	655	1.5608
+	MCF7 + WT EVs_MCF7 + Vim -/- EVs	1	6	82	82	138	0.999	736	1.89821
+	MCF7 + WT EVs_MCF7 + Vim -/- EVs	1	19	296	245	296	0.999	977	1.75778
+	MCF7 + WT EVs_MCF7 + Vim -/- EVs	1	28	472	418	472	0.999	793	2.10632
+	MCF7 + WT EVs_MCF7 + Vim -/- EVs	1	46	1406	1406	1406	0.999	746	4.84602
+	MCF7 + WT EVs_MCF7 + Vim -/- EVs	1	24	729	693	729	0.999	309	2.63211
+	MCF7 + WT EVs_MCF7 + Vim -/- EVs	1	6	68	68	68	0.999	437	4.58135
+	MCF7 + WT EVs_MCF7 + Vim -/- EVs	1	10	198	198	198	0.999	1463	2.84055
+	MCF7 + WT EVs_MCF7 + Vim -/- EVs	0.9999	6	50	3	50	0.999	435	1.46821
+	MCF7 + WT EVs_MCF7 + Vim -/- EVs	1	12	286	286	286	0.999	290	1.6687
+	MCF7 + WT EVs_MCF7 + Vim -/- EVs	1	55	1805	152	1805	0.999	913	2.25975
+	MCF7 + WT EVs_MCF7 + Vim -/- EVs	1	11	266	266	266	0.999	292	2.22286
+	MCF7 + WT EVs_MCF7 + Vim -/- EVs	1	26	424	424	424	0.999	494	1.7159
+	MCF7 + WT EVs_MCF7 + Vim -/- EVs	1	16	203	203	241	0.999	1313	2.28953
+	MCF7 + WT EVs_MCF7 + Vim -/- EVs	1	11	396	396	396	0.999	261	2.07333
+	MCF7 + WT EVs_MCF7 + Vim -/- EVs	1	5	96	96	96	0.999	456	3.0348
+	MCF7 + WT EVs_MCF7 + Vim -/- EVs	1	14	202	202	202	0.999	1015	2.17042
+	MCF7 + WT EVs_MCF7 + Vim -/- EVs	1	18	146	146	210	0.999	737	2.52066
+	MCF7 + WT EVs_MCF7 + Vim -/- EVs	1	16	297	297	297	0.999	826	2.56953
+	MCF7 + WT EVs_MCF7 + Vim -/- EVs	1	7	135	135	135	0.999	325	2.31516
+	MCF7 + WT EVs_MCF7 + Vim -/- EVs	1	4	89	89	89	0.999	676	1.69471
+	MCF7 + WT EVs_MCF7 + Vim -/- EVs	1	10	173	173	173	0.999	337	2.70273
+	MCF7 + WT EVs_MCF7 + Vim -/- EVs	1	25	620	529	626	0.999	489	1.8527
+	MCF7 + WT EVs_MCF7 + Vim -/- EVs	1	9	140	140	140	0.999	306	2.54125
+	MCF7 + WT EVs_MCF7 + Vim -/- EVs	1	5	94	94	94	0.999	194	2.42745
+	MCF7 + WT EVs_MCF7 + Vim -/- EVs	1	5	63	63	63	0.999	358	2.42667
+	MCF7 + WT EVs_MCF7 + Vim -/- EVs	1	3	72	72	72	0.999	129	1.77474
+	MCF7 + WT EVs_MCF7 + Vim -/- EVs	1	21	708	571	708	0.999	406	1.67033
+	MCF7 + WT EVs_MCF7 + Vim -/- EVs	1	3	33	33	33	0.999	496	1.48483
+	MCF7 + WT EVs_MCF7 + Vim -/- EVs	1	14	284	284	284	0.999	1192	1.5047
+	MCF7 + WT EVs_MCF7 + Vim -/- EVs	1	6	37	37	37	0.9989	467	2.13654
+	MCF7 + WT EVs_MCF7 + Vim -/- EVs	1	10	75	75	75	0.999	390	2.48665
+	MCF7 + WT EVs_MCF7 + Vim -/- EVs	1	6	97	97	97	0.999	263	1.87874
+	MCF7 + WT EVs_MCF7 + Vim -/- EVs	1	35	1201	876	2571	0.999	446	2.18211
+	MCF7 + WT EVs_MCF7 + Vim -/- EVs	1	5	21	21	21	0.999	199	1.99761
+	MCF7 + WT EVs_MCF7 + Vim -/- EVs	1	40	788	788	788	0.999	820	1.75844
+	MCF7 + WT EVs_MCF7 + Vim -/- EVs	1	12	97	97	97	0.999	507	2.4994
+	MCF7 + WT EVs_MCF7 + Vim -/- EVs	1	5	179	179	179	0.999	243	1.44616
+	MCF7 + WT EVs_MCF7 + Vim -/- EVs	1	22	422	422	422	0.999	397	4.1644
+	MCF7 + WT EVs_MCF7 + Vim -/- EVs	1	19	321	131	321	0.999	364	4.7042
+	MCF7 + WT EVs_MCF7 + Vim -/- EVs	1	13	172	172	250	0.999	321	3.31781
+	MCF7 + WT EVs_MCF7 + Vim -/- EVs	1	4	63	63	63	0.999	393	2.47449
+	MCF7 + WT EVs_MCF7 + Vim -/- EVs	1	9	97	97	97	0.999	796	1.67096
+	MCF7 + WT EVs_MCF7 + Vim -/- EVs	1	3	16	16	16	0.999	602	4.74632
+	MCF7 + WT EVs_MCF7 + Vim -/- EVs	1	8	175	116	175	0.999	764	5.38487
+	MCF7 + WT EVs_MCF7 + Vim -/- EVs	1	9	119	119	119	0.999	300	1.58002
+	MCF7 + WT EVs_MCF7 + Vim -/- EVs	1	11	115	115	115	0.999	306	2.82989
+	MCF7 + WT EVs_MCF7 + Vim -/- EVs	1	25	629	629	629	0.999	585	1.47967
+	MCF7 + WT EVs_MCF7 + Vim -/- EVs	1	5	93	93	93	0.999	188	2.07354
+	MCF7 + WT EVs_MCF7 + Vim -/- EVs	1	7	189	189	189	0.999	178	2.15969
+	MCF7 + WT EVs_MCF7 + Vim -/- EVs	1	6	91	91	91	0.999	311	2.09172
+	MCF7 + WT EVs_MCF7 + Vim -/- EVs	1	6	11	11	11	0.999	2649	1.76639
+	MCF7 + WT EVs_MCF7 + Vim -/- EVs	1	14	321	321	321	0.999	205	2.09794
+	MCF7 + WT EVs_MCF7 + Vim -/- EVs	1	10	53	45	516	0.999	375	4.54899
+	MCF7 + WT EVs_MCF7 + Vim -/- EVs	1	21	458	458	458	0.999	634	1.59244
+	MCF7 + WT EVs_MCF7 + Vim -/- EVs	1	6	117	117	117	0.999	259	2.20805
+	MCF7 + WT EVs_MCF7 + Vim -/- EVs	1	21	231	231	231	0.999	928	4.76875
+	MCF7 + WT EVs_MCF7 + Vim -/- EVs	1	20	420	420	420	0.999	524	2.50753
+	MCF7 + WT EVs_MCF7 + Vim -/- EVs	1	9	85	85	85	0.999	505	1.49144
+	MCF7 + WT EVs_MCF7 + Vim -/- EVs	1	13	88	88	88	0.999	1861	3.60756

+	MCF7 + WT EVs_MCF7 + Vim -/- EVs	1	14	211	211	211	0.999	423	1.94184
+	MCF7 + WT EVs_MCF7 + Vim -/- EVs	1	11	110	110	110	0.999	422	1.89968
+	MCF7 + WT EVs_MCF7 + Vim -/- EVs	1	12	152	152	152	0.999	419	1.71066
+	MCF7 + WT EVs_MCF7 + Vim -/- EVs	1	8	161	161	161	0.999	305	1.64876
+	MCF7 + WT EVs_MCF7 + Vim -/- EVs	1	10	108	108	108	0.999	426	6.79667
+	MCF7 + WT EVs_MCF7 + Vim -/- EVs	1	8	218	218	218	0.999	169	2.03688
+	MCF7 + WT EVs_MCF7 + Vim -/- EVs	1	17	314	314	314	0.999	707	1.40943
+	MCF7 + WT EVs_MCF7 + Vim -/- EVs	1	11	43	43	43	0.999	658	1.883
+	MCF7 + WT EVs_MCF7 + Vim -/- EVs	1	32	480	480	480	0.999	1025	1.96151
+	MCF7 + WT EVs_MCF7 + Vim -/- EVs	1	5	114	114	114	0.999	180	3.76795
+	MCF7 + WT EVs_MCF7 + Vim -/- EVs	1	7	65	65	65	0.999	941	2.63027
+	MCF7 + WT EVs_MCF7 + Vim -/- EVs	1	7	77	77	77	0.999	161	1.60774
+	MCF7 + WT EVs_MCF7 + Vim -/- EVs	1	9	125	125	125	0.999	487	7.50063
+	MCF7 + WT EVs_MCF7 + Vim -/- EVs	1	19	256	228	256	0.999	1008	2.49804
+	MCF7 + WT EVs_MCF7 + Vim -/- EVs	1	10	178	178	178	0.999	476	3.05573
+	MCF7 + WT EVs_MCF7 + Vim -/- EVs	1	9	64	64	64	0.999	391	2.39025
+	MCF7 + WT EVs_MCF7 + Vim -/- EVs	1	6	118	118	118	0.999	280	1.52832
+	MCF7 + WT EVs_MCF7 + Vim -/- EVs	1	6	106	106	106	0.999	528	1.90521
+	MCF7 + WT EVs_MCF7 + Vim -/- EVs	1	34	572	572	572	0.999	944	2.8553
+	MCF7 + WT EVs_MCF7 + Vim -/- EVs	1	6	18	18	18	0.999	434	2.25187
+	MCF7 + WT EVs_MCF7 + Vim -/- EVs	1	16	123	122	142	0.999	881	4.55571
+	MCF7 + WT EVs_MCF7 + Vim -/- EVs	1	37	736	522	736	0.999	534	2.41359
+	MCF7 + WT EVs_MCF7 + Vim -/- EVs	1	19	213	213	213	0.999	836	3.92079
+	MCF7 + WT EVs_MCF7 + Vim -/- EVs	1	8	193	193	193	0.999	170	2.12165
+	MCF7 + WT EVs_MCF7 + Vim -/- EVs	1	11	136	119	136	0.999	454	1.65712
+	MCF7 + WT EVs_MCF7 + Vim -/- EVs	1	7	69	69	69	0.999	851	3.90903
+	MCF7 + WT EVs_MCF7 + Vim -/- EVs	1	8	59	59	59	0.999	726	3.13358
+	MCF7 + WT EVs_MCF7 + Vim -/- EVs	1	9	59	59	59	0.999	626	3.27518
+	MCF7 + WT EVs_MCF7 + Vim -/- EVs	1	10	34	34	34	0.999	1298	2.23668
+	MCF7 + WT EVs_MCF7 + Vim -/- EVs	1	16	479	479	479	0.999	217	3.24299
+	MCF7 + WT EVs_MCF7 + Vim -/- EVs	1	13	155	155	155	0.999	563	3.58641
+	MCF7 + WT EVs_MCF7 + Vim -/- EVs	1	4	50	50	50	0.999	842	1.6496
+	MCF7 + WT EVs_MCF7 + Vim -/- EVs	1	5	47	47	47	0.999	347	3.16722
+	MCF7 + WT EVs_MCF7 + Vim -/- EVs	1	13	180	180	180	0.999	518	1.83924
+	MCF7 + WT EVs_MCF7 + Vim -/- EVs	1	7	91	91	96	0.999	439	1.52121
+	MCF7 + WT EVs_MCF7 + Vim -/- EVs	1	10	107	106	107	0.999	531	1.49344
+	MCF7 + WT EVs_MCF7 + Vim -/- EVs	1	12	157	157	157	0.999	837	5.19356
+	MCF7 + WT EVs_MCF7 + Vim -/- EVs	1	5	23	23	23	0.999	275	2.84584
+	MCF7 + WT EVs_MCF7 + Vim -/- EVs	1	38	374	374	374	0.999	1124	4.6682
+	MCF7 + WT EVs_MCF7 + Vim -/- EVs	1	25	686	634	686	0.999	623	2.30464
+	MCF7 + WT EVs_MCF7 + Vim -/- EVs	1	7	45	45	45	0.999	736	1.69455
+	MCF7 + WT EVs_MCF7 + Vim -/- EVs	1	27	787	787	787	0.999	457	4.95001
+	MCF7 + WT EVs_MCF7 + Vim -/- EVs	1	54	1284	1220	1284	0.999	783	2.47985
+	MCF7 + WT EVs_MCF7 + Vim -/- EVs	1	23	365	365	388	0.999	1447	1.44609
+	MCF7 + WT EVs_MCF7 + Vim -/- EVs	1	53	979	979	979	0.999	1288	5.52841
+	MCF7 + WT EVs_MCF7 + Vim -/- EVs	1	17	197	197	197	0.999	558	1.93878
+	MCF7 + WT EVs_MCF7 + Vim -/- EVs	1	13	291	211	291	0.999	372	2.82156
+	MCF7 + WT EVs_MCF7 + Vim -/- EVs	1	9	124	124	124	0.999	400	1.58442
+	MCF7 + WT EVs_MCF7 + Vim -/- EVs	1	44	1208	292	1208	0.999	634	3.53146
+	MCF7 + WT EVs_MCF7 + Vim -/- EVs	1	12	60	60	60	0.999	687	1.59684
+	MCF7 + WT EVs_MCF7 + Vim -/- EVs	1	12	166	166	166	0.999	273	1.92056
+	MCF7 + WT EVs_MCF7 + Vim -/- EVs	1	9	222	222	222	0.999	160	1.79133
+	MCF7 + WT EVs_MCF7 + Vim -/- EVs	1	12	282	282	282	0.999	245	1.68565
+	MCF7 + WT EVs_MCF7 + Vim -/- EVs	1	4	26	26	26	0.999	423	1.82498
+	MCF7 + WT EVs_MCF7 + Vim -/- EVs	1	16	250	250	250	0.999	659	2.69353
+	MCF7 + WT EVs_MCF7 + Vim -/- EVs	1	6	118	118	118	0.999	87	1.44343
+	MCF7 + WT EVs_MCF7 + Vim -/- EVs	1	17	602	558	602	0.999	352	3.69504
+	MCF7 + WT EVs_MCF7 + Vim -/- EVs	1	48	750	727	750	0.999	1555	3.61399
+	MCF7 + WT EVs_MCF7 + Vim -/- EVs	1	9	171	171	171	0.999	389	2.82328
+	MCF7 + WT EVs_MCF7 + Vim -/- EVs	1	115	2023	1990	2023	0.999	2061	2.38706
+	MCF7 + WT EVs_MCF7 + Vim -/- EVs	1	31	500	480	562	0.999	543	1.61387
+	MCF7 + WT EVs_MCF7 + Vim -/- EVs	0.9165	1	49	49	49	0.9977	309	2.14196
+	MCF7 + WT EVs_MCF7 + Vim -/- EVs	1	10	103	103	103	0.999	722	2.69997
+	MCF7 + WT EVs_MCF7 + Vim -/- EVs	1	9	81	81	81	0.999	296	1.74119
+	MCF7 + WT EVs_MCF7 + Vim -/- EVs	1	8	165	165	165	0.999	144	2.36174
+	MCF7 + WT EVs_MCF7 + Vim -/- EVs	1	34	891	891	891	0.999	522	2.93199
+	MCF7 + WT EVs_MCF7 + Vim -/- EVs	1	13	133	132	133	0.999	1576	2.43729
+	MCF7 + WT EVs_MCF7 + Vim -/- EVs	1	118	2496	2456	2496	0.999	1410	2.7668
+	MCF7 + WT EVs_MCF7 + Vim -/- EVs	1	66	1496	1496	1496	0.999	1176	1.73985
+	MCF7 + WT EVs_MCF7 + Vim -/- EVs	1	12	146	146	146	0.999	1060	1.88279
+	MCF7 + WT EVs_MCF7 + Vim -/- EVs	1	12	163	163	163	0.999	1169	1.42347
+	MCF7 + WT EVs_MCF7 + Vim -/- EVs	1	24	343	343	343	0.999	463	1.69277
+	MCF7 + WT EVs_MCF7 + Vim -/- EVs	1	5	158	158	158	0.999	218	1.78878
+	MCF7 + WT EVs_MCF7 + Vim -/- EVs	1	13	436	436	436	0.999	328	1.78415
+	MCF7 + WT EVs_MCF7 + Vim -/- EVs	1	13	415	415	415	0.999	303	3.56587
+	MCF7 + WT EVs_MCF7 + Vim -/- EVs	1	4	21	21	21	0.999	1377	1.5105
+	MCF7 + WT EVs_MCF7 + Vim -/- EVs	1	47	583	533	583	0.999	1190	1.95702
+	MCF7 + WT EVs_MCF7 + Vim -/- EVs	1	6	57	57	57	0.999	740	1.6691
+	MCF7 + WT EVs_MCF7 + Vim -/- EVs	1	26	439	439	439	0.999	623	3.79314
+	MCF7 + WT EVs_MCF7 + Vim -/- EVs	1	6	55	55	55	0.999	379	1.97986
+	MCF7 + WT EVs_MCF7 + Vim -/- EVs	1	50	756	715	756	0.999	759	4.89377
+	MCF7 + WT EVs_MCF7 + Vim -/- EVs	1	20	358	358	358	0.999	332	2.86156
+	MCF7 + WT EVs_MCF7 + Vim -/- EVs	1	15	183	183	183	0.999	505	3.21666
+	MCF7 + WT EVs_MCF7 + Vim -/- EVs	1	11	187	187	187	0.999	492	2.85658
+	MCF7 + WT EVs_MCF7 + Vim -/- EVs	1	8	34	34	34	0.999	1383	1.70649
+	MCF7 + WT EVs_MCF7 + Vim -/- EVs	1	3	19	19	19	0.999	456	4.34901
+	MCF7 + WT EVs_MCF7 + Vim -/- EVs	1	17	132	132	132	0.999	511	3.12059
+	MCF7 + WT EVs_MCF7 + Vim -/- EVs	1	10	238	238	238	0.999	483	1.69293
+	MCF7 + WT EVs_MCF7 + Vim -/- EVs	1	4	56	56	56	0.999	185	2.68236
+	MCF7 + WT EVs_MCF7 + Vim -/- EVs	1	35	345	345	345	0.999	1483	2.27306
+	MCF7 + WT EVs_MCF7 + Vim -/- EVs	1	12	152	152	152	0.999	344	1.8946
+	MCF7 + WT EVs_MCF7 + Vim -/- EVs	1	9	101	101	101	0.999	578	2.03481
+	MCF7 + WT EVs_MCF7 + Vim -/- EVs	1	4	84	84	84	0.999	1139	1.67717
+	MCF7 + WT EVs_MCF7 + Vim -/- EVs	1	6	28	28	28	0.999	462	4.00484
+	MCF7 + WT EVs_MCF7 + Vim -/- EVs	1	8	173	173	173	0.999	460	2.03828
+	MCF7 + WT EVs_MCF7 + Vim -/- EVs	1	12	355	355	355	0.999	154	1.97297
+	MCF7 + WT EVs_MCF7 + Vim -/- EVs	1	18	365	365	365	0.999	424	1.99227
+	MCF7 + WT EVs_MCF7 + Vim -/- EVs	1	14	281	281	281	0.999	468	2.64598
+	MCF7 + WT EVs_MCF7 + Vim -/- EVs	1	11	317	317	317	0.999	258	2.33712
+	MCF7 + WT EVs_MCF7 + Vim -/- EVs	1	13	297	297	297	0.999	412	4.30386
+	MCF7 + WT EVs_MCF7 + Vim -/- EVs	0.9999	6	75	54	75	0.999	164	4.67097
+	MCF7 + WT EVs_MCF7 + Vim -/- EVs	1	4	19	19	19	0.999	924	1.68087
+	MCF7 + WT EVs_MCF7 + Vim -/- EVs	1	19	399	43	399	0.999	479	1.58055
+	MCF7 + WT EVs_MCF7 + Vim -/- EVs	1	25	500	500	500	0.999	486	2.37194
+	MCF7 + WT EVs_MCF7 + Vim -/- EVs	1	6	117	117	117	0.999	238	1.6631
+	MCF7 + WT EVs_MCF7 + Vim -/- EVs	1	7	120	120	120	0.999	99	1.57689
+	MCF7 + WT EVs_MCF7 + Vim -/- EVs	1	57	833	833	833	0.999	1217	2.71257
+	MCF7 + WT EVs_MCF7 + Vim -/- EVs	1	22	709	709	709	0.999	564	1.78524
+	MCF7 + WT EVs_MCF7 + Vim -/- EVs	1	16	197	197	924	0.999	166	1.84054
+	MCF7 + WT EVs_MCF7 + Vim -/- EVs	1	6	114	114	114	0.999	383	2.06871
+	MCF7 + WT EVs_MCF7 + Vim -/- EVs	1	10	50	50	50	0.999	1198	3.37554
+	MCF7 + WT EVs_MCF7 + Vim -/- EVs	1	22	361	361	361	0.999	958	1.78135
+	MCF7 + WT EVs_MCF7 + Vim -/- EVs	1	17	482	482	482	0.999	226	1.5048
+	MCF7 + WT EVs_MCF7 + Vim -/- EVs	1	10	89	89	89	0.999	599	2.47181
+	MCF7 + WT EVs_MCF7 + Vim -/- EVs	1	9	79	79	79	0.999	242	3.27016
+	MCF7 + WT EVs_MCF7 + Vim -/- EVs	1	14	124	124	124	0.999	477	2.88374
+	MCF7 + WT EVs_MCF7 + Vim -/- EVs	1	23	527	527	527	0.999	439	1.47738
+	MCF7 + WT EVs_MCF7 + Vim -/- EVs	1	9	231	231	231	0.999	301	2.33889
+	MCF7 + WT EVs_MCF7 + Vim -/- EVs	1	7	44	44	44	0.999	318	1.86882
+	MCF7 + WT EVs_MCF7 + Vim -/- EVs	1	28	1037	718	1037	0.999	392	1.48496
+	MCF7 + WT EVs_MCF7 + Vim -/- EVs	1	11	303	303	303	0.999	296	4.10956
+	MCF7 + WT EVs_MCF7 + Vim -/- EVs	1	20	403	403	403	0.999	250	2.73639
+	MCF7 + WT EVs_MCF7 + Vim -/- EVs	1	9	155	155	155	0.999	224	2.78691
+	MCF7 + WT EVs_MCF7 + Vim -/- EVs	1	9	163	163	163	0.999	192	2.54045
+	MCF7 + WT EVs_MCF7 + Vim -/- EVs	1	7	141	141	141	0.999	237	2.76791
+	MCF7 + WT EVs_MCF7 + Vim -/- EVs	1	10	260	260	260	0.999	167	3.24394
+	MCF7 + WT EVs_MCF7 + Vim -/- EVs	1	11	119	101	119	0.999	1069	4.37118
+	MCF7 + WT EVs_MCF7 + Vim -/- EVs	1	18	849	849	849	0.999	105	2.72086
+	MCF7 + WT EVs_MCF7 + Vim -/- EVs	1	9	98	98	98	0.999	626	3.77246

+	MCF7 + WT EVs_MCF7 + Vim -/- EVs	1	28	383	383	383	0.999	797	1.47515
+	MCF7 + WT EVs_MCF7 + Vim -/- EVs	1	8	152	103	152	0.999	981	3.20677
+	MCF7 + WT EVs_MCF7 + Vim -/- EVs	1	9	127	127	127	0.999	1045	1.90877
+	MCF7 + WT EVs_MCF7 + Vim -/- EVs	1	21	168	168	168	0.999	774	7.15568
+	MCF7 + WT EVs_MCF7 + Vim -/- EVs	1	8	322	322	322	0.999	139	1.79563
+	MCF7 + WT EVs_MCF7 + Vim -/- EVs	1	43	635	579	635	0.999	2896	2.77088
+	MCF7 + WT EVs_MCF7 + Vim -/- EVs	1	18	438	438	438	0.999	386	1.77336
+	MCF7 + WT EVs_MCF7 + Vim -/- EVs	1	5	96	96	96	0.999	83	2.50035
+	MCF7 + WT EVs_MCF7 + Vim -/- EVs	1	9	132	132	132	0.999	247	3.5013
+	MCF7 + WT EVs_MCF7 + Vim -/- EVs	1	4	130	130	130	0.999	271	1.6633
+	MCF7 + WT EVs_MCF7 + Vim -/- EVs	1	4	42	42	42	0.999	336	1.41689
+	MCF7 + WT EVs_MCF7 + Vim -/- EVs	1	16	253	253	253	0.999	427	3.60593
+	MCF7 + WT EVs_MCF7 + Vim -/- EVs	1	26	859	859	859	0.999	370	2.11483
+	MCF7 + WT EVs_MCF7 + Vim -/- EVs	1	5	66	66	66	0.999	262	1.50425
+	MCF7 + WT EVs_MCF7 + Vim -/- EVs	1	37	929	876	929	0.999	874	2.168
+	MCF7 + WT EVs_MCF7 + Vim -/- EVs	1	11	135	135	135	0.999	457	2.0993
+	MCF7 + WT EVs_MCF7 + Vim -/- EVs	1	12	515	515	515	0.999	303	1.72786
+	MCF7 + WT EVs_MCF7 + Vim -/- EVs	1	18	248	248	249	0.999	523	2.40268
+	MCF7 + WT EVs_MCF7 + Vim -/- EVs	1	6	158	42	158	0.999	151	2.57089
+	MCF7 + WT EVs_MCF7 + Vim -/- EVs	0.9998	4	32	32	32	0.999	622	3.22978
+	MCF7 + WT EVs_MCF7 + Vim -/- EVs	1	13	68	68	68	0.999	1087	2.5667
+	MCF7 + WT EVs_MCF7 + Vim -/- EVs	1	3	47	47	47	0.999	314	1.60661

N: Student's T-test q-value MCF7 + WT EVs_MCF7 + Vim -/- EVs	N: Student's T-test Difference MCF7 + WT EVs_MCF7 + Vim -/- EVs	N: Student's T-test Test statistic MCF7 + WT EVs_MCF7 + Vim -/- EVs	T: Protein	T: Gene	T: Protein ID	T: Description	T: Entry Name	T: Indistinguishable Proteins
0.0418171	-0.0689526	-2.76284	sp|A0AVT1|UBA6_HUMAN	UBA6	A0AVT1	Ubiquitin-like modifier-activating enzyme 6	UBA6_HUMAN	
0.0200111	-0.285539	-3.45897	sp|A0FGR8|ESYT2_HUMAN	ESYT2	A0FGR8	Extended synaptotagmin-2	ESYT2_HUMAN	
0.0150877	1.59906	3.78493	sp|A1X283|SPD2B_HUMAN	SH3PXD2B	A1X283	SH3 and PX domain-containing protein 2B	SPD2B_HUMAN	
0.017203	0.336491	3.59508	sp|A6NDG6|PGP_HUMAN	PGP	A6NDG6	Glycerol-3-phosphate phosphatase	PGP_HUMAN	
0.0296777	0.155846	3.06945	sp|A6NDU8|RIMC1_HUMAN	RIMOC1	A6NDU8	RAB7A-interacting MON1-CCZ1 complex subunit 1	RIMC1_HUMAN	
0.00305306	-0.327852	-6.2135	sp|A6NHR9|SMHD1_HUMAN	SMCHD1	A6NHR9	Structural maintenance of chromosomes flexible hinge domain-containing protein 1	SMHD1_HUMAN	
0.0150629	0.850836	3.77146	sp|A8CG34|P121C_HUMAN	POM121C	A8CG34	Nuclear envelope pore membrane protein POM 121C	P121C_HUMAN	
0.0047474	0.307056	5.74395	sp|E9PRG8|CK098_HUMAN	C11orf98	E9PRG8	Uncharacterized protein C11orf98	CK098_HUMAN	
0	0.454993	11.9062	sp|O00116|ADAS_HUMAN	AGPS	O00116	Alkyldihydroxyacetonephosphate synthase, peroxisomal	ADAS_HUMAN	
0.0320894	0.328001	2.99893	sp|O00139|KIF2A_HUMAN	KIF2A	O00139	Kinesin-like protein KIF2A	KIF2A_HUMAN	
0.0251414	0.279388	3.22334	sp|O00151|PDLI1_HUMAN	PDLIM1	O00151	PDZ and LIM domain protein 1	PDLI1_HUMAN	
0.0255851	0.27104	3.21425	sp|O00154|BACH_HUMAN	ACOT7	O00154	Cytosolic acyl coenzyme A thioester hydrolase	BACH_HUMAN	
0.00505325	0.601075	5.36764	sp|O00159|MYO1C_HUMAN	MYO1C	O00159	Unconventional myosin-Ic	MYO1C_HUMAN	
0.014295	0.358459	3.83508	sp|O00217|NDUS8_HUMAN	NDUFS8	O00217	NADH dehydrogenase [ubiquinone] iron-sulfur protein 8, mitochondrial	NDUS8_HUMAN	
0.0185194	-0.437352	-3.52471	sp|O00264|PGRC1_HUMAN	PGRMC1	O00264	Membrane-associated progesterone receptor component 1	PGRC1_HUMAN	
0	0.246129	15.2575	sp|O00303|EIF3F_HUMAN	EIF3F	O00303	Eukaryotic translation initiation factor 3 subunit F	EIF3F_HUMAN	
0	0.335068	9.69446	sp|O00410|IPO5_HUMAN	IPO5	O00410	Importin-5	IPO5_HUMAN	
0.00928827	0.684307	4.31049	sp|O00411|RPOM_HUMAN	POLRMT	O00411	DNA-directed RNA polymerase, mitochondrial	RPOM_HUMAN	
0.0291963	-0.118869	-3.09916	sp|O00425|IF2B3_HUMAN	IGF2BP3	O00425	Insulin-like growth factor 2 mRNA-binding protein 3	IF2B3_HUMAN	
0.00764126	0.408914	4.6113	sp|O00461|GOLI4_HUMAN	GOLIM4	O00461	Golgi integral membrane protein 4	GOLI4_HUMAN	
0	-0.779042	-9.26077	sp|O00468|AGRIN_HUMAN	AGRN	O00468	Agrin	AGRIN_HUMAN	
0	-2.03625	-24.7547	sp|O00469|PLOD2_HUMAN	PLOD2	O00469	Procollagen-lysine,2-oxoglutarate 5-dioxygenase 2	PLOD2_HUMAN	
0.000789474	1.51904	7.93893	sp|O00483|NDUA4_HUMAN	NDUFA4	O00483	Cytochrome c oxidase subunit NDUFA4	NDUA4_HUMAN	
0.00223214	0.540508	6.57994	sp|O00499|BIN1_HUMAN	BIN1	O00499	Myc box-dependent-interacting protein 1	BIN1_HUMAN	
0.00708068	0.415222	4.83623	sp|O00505|IMA4_HUMAN	KPNA3	O00505	Importin subunit alpha-4	IMA4_HUMAN	
0.0078961	0.778334	4.55794	sp|O00592|PODXL_HUMAN	PODXL	O00592	Podocalyxin	PODXL_HUMAN	
0.0123655	-0.201287	-3.99326	sp|O00754|MA2B1_HUMAN	MAN2B1	O00754	Lysosomal alpha-mannosidase	MA2B1_HUMAN	
0.0072963	0.533238	4.70264	sp|O00764|PDXK_HUMAN	PDXK	O00764	Pyridoxal kinase	PDXK_HUMAN	
0.0249685	0.648369	3.2357	sp|O14562|UBFD1_HUMAN	UBFD1	O14562	Ubiquitin domain-containing protein UBFD1	UBFD1_HUMAN	
0.00502326	0.222236	5.6392	sp|O14617|AP3D1_HUMAN	AP3D1	O14617	AP-3 complex subunit delta-1	AP3D1_HUMAN	
0.0102772	-0.753584	-4.18409	sp|O14618|CCS_HUMAN	CCS	O14618	Copper chaperone for superoxide dismutase	CCS_HUMAN	
0.0147756	0.291655	3.80189	sp|O14684|PTGES_HUMAN	PTGES	O14684	Prostaglandin E synthase	PTGES_HUMAN	
0.0226152	0.204988	3.36273	sp|O14744|ANM5_HUMAN	PRMT5	O14744	Protein arginine N-methyltransferase 5	ANM5_HUMAN	
0.00794771	-0.661407	-4.56323	sp|O14745|NHRF1_HUMAN	NHERF1	O14745	Na(+)/H(+) exchange regulatory cofactor NHE-RF1	NHRF1_HUMAN	
0.0120622	-0.151424	-4.00677	sp|O14818|PSA7_HUMAN	PSMA7	O14818	Proteasome subunit alpha type-7	PSA7_HUMAN	
0.00366798	-0.971364	-6.08442	sp|O14874|BCKD_HUMAN	BCKDK	O14874	[3-methyl-2-oxobutanoate dehydrogenase [lipoamide]] kinase, mitochondrial	BCKD_HUMAN	
0.0133626	1.19279	3.92694	sp|O14879|IFIT3_HUMAN	IFIT3	O14879	Interferon-induced protein with tetratricopeptide repeats 3	IFIT3_HUMAN	
0.010214	2.32823	4.15723	sp|O14908|GIPC1_HUMAN	GIPC1	O14908	PDZ domain-containing protein GIPC1	GIPC1_HUMAN	
0.0133851	0.29365	3.93079	sp|O14929|HAT1_HUMAN	HAT1	O14929	Histone acetyltransferase type B catalytic subunit	HAT1_HUMAN	
0.00891429	0.424608	4.38753	sp|O14949|QCR8_HUMAN	UQCRQ	O14949	Cytochrome b-c1 complex subunit 8	QCR8_HUMAN	
0.00983142	0.400932	4.24579	sp|O14974|MYPT1_HUMAN	PPP1R12A	O14974	Protein phosphatase 1 regulatory subunit 12A	MYPT1_HUMAN	
0.00370037	0.5103	5.94479	sp|O15013|ARHGA_HUMAN	ARHGEF10	O15013	Rho guanine nucleotide exchange factor 10	ARHGA_HUMAN	
0.00189474	-0.855468	-6.82593	sp|O15230|LAMA5_HUMAN	LAMA5	O15230	Laminin subunit alpha-5	LAMA5_HUMAN	
0.0326192	0.49171	2.98332	sp|O15235|RT12_HUMAN	MRPS12	O15235	Small ribosomal subunit protein uS12m	RT12_HUMAN	
0.00644898	0.4342	4.91344	sp|O15269|SPTC1_HUMAN	SPTLC1	O15269	Serine palmitoyltransferase 1	SPTC1_HUMAN	
0.00945597	0.342775	4.29059	sp|O15355|PPM1G_HUMAN	PPM1G	O15355	Protein phosphatase 1G	PPM1G_HUMAN	
0.00954941	0.30065	4.29378	sp|O15357|SHIP2_HUMAN	INPPL1	O15357	Phosphatidylinositol 3,4,5-trisphosphate 5-phosphatase 2	SHIP2_HUMAN	
0.0102505	0.244299	4.12707	sp|O15371|EIF3D_HUMAN	EIF3D	O15371	Eukaryotic translation initiation factor 3 subunit D	EIF3D_HUMAN	
0.00979923	0.0933456	4.25681	sp|O15372|EIF3H_HUMAN	EIF3H	O15372	Eukaryotic translation initiation factor 3 subunit H	EIF3H_HUMAN	
0.0395722	1.0142	2.81386	sp|O15381|NVL_HUMAN	NVL	O15381	Nuclear valosin-containing protein-like	NVL_HUMAN	
0.0101132	-0.376944	-4.21192	sp|O15382|BCAT2_HUMAN	BCAT2	O15382	Branched-chain-amino-acid aminotransferase, mitochondrial	BCAT2_HUMAN	
0.00240693	-0.836963	-6.4744	sp|O15427|MOT4_HUMAN	SLC16A3	O15427	Monocarboxylate transporter 4	MOT4_HUMAN	
0	-1.7127	-18.1852	sp|O15460|P4HA2_HUMAN	P4HA2	O15460	Prolyl 4-hydroxylase subunit alpha-2	P4HA2_HUMAN	
0.0294781	-0.260775	-3.08336	sp|O43172|PRP4_HUMAN	PRPF4	O43172	U4/U6 small nuclear ribonucleoprotein Prp4	PRP4_HUMAN	
0	0.862421	14.3855	sp|O43175|SERA_HUMAN	PHGDH	O43175	D-3-phosphoglycerate dehydrogenase	SERA_HUMAN	
0.0102044	0.41295	4.14127	sp|O43237|DC1L2_HUMAN	DYNC1LI2	O43237	Cytoplasmic dynein 1 light intermediate chain 2	DC1L2_HUMAN	
0.0297117	0.165067	3.07009	sp|O43252|PAPS1_HUMAN	PAPSS1	O43252	Bifunctional 3'-phosphoadenosine 5'-phosphosulfate synthase 1	PAPS1_HUMAN	
0.00890188	-0.263153	-4.46294	sp|O43290|SNUT1_HUMAN	SART1	O43290	U4/U6.U5 tri-snRNP-associated protein 1	SNUT1_HUMAN	
0.0308448	0.245613	3.04847	sp|O43324|MCA3_HUMAN	EEF1E1	O43324	Eukaryotic translation elongation factor 1 epsilon-1	MCA3_HUMAN	
0.00385938	-2.24186	-6.05649	sp|O43570|CAH12_HUMAN	CA12	O43570	Carbonic anhydrase 12	CAH12_HUMAN	
0.00280992	0.638974	6.28272	sp|O43615|TIM44_HUMAN	TIMM44	O43615	Mitochondrial import inner membrane translocase subunit TIM44	TIM44_HUMAN	
0.0441394	0.540968	2.71907	sp|O43674|NDUB5_HUMAN	NDUFB5	O43674	NADH dehydrogenase [ubiquinone] 1 beta subcomplex subunit 5, mitochondrial	NDUB5_HUMAN	
0.0049901	0.278899	5.63109	sp|O43681|GET3_HUMAN	GET3	O43681	ATPase GET3	GET3_HUMAN	
0.0355484	0.224908	2.90419	sp|O43719|HTSF1_HUMAN	HTATSF1	O43719	HIV Tat-specific factor 1	HTSF1_HUMAN	
0.0014433	0.34487	7.01024	sp|O43776|SYNC_HUMAN	NARS1	O43776	Asparagine--tRNA ligase, cytoplasmic	SYNC_HUMAN	
0.0250558	-0.376235	-3.2497	sp|O43813|LANC1_HUMAN	LANCL1	O43813	Glutathione S-transferase LANCL1	LANC1_HUMAN	
0.000769231	0.459916	7.84187	sp|O43837|IDH3B_HUMAN	IDH3B	O43837	Isocitrate dehydrogenase [NAD] subunit beta, mitochondrial	IDH3B_HUMAN	
0.0155562	0.73492	3.69963	sp|O43847|NRDC_HUMAN	NRDC	O43847	Nardilysin	NRDC_HUMAN	
0.0339713	-0.452406	-2.9591	sp|O43852|CALU_HUMAN	CALU	O43852	Calumenin	CALU_HUMAN	
0.0353355	0.218406	2.91319	sp|O60313|OPA1_HUMAN	OPA1	O60313	Dynamin-like 120 kDa protein, mitochondrial	OPA1_HUMAN	
0.0165714	-0.697836	-3.61965	sp|O60341|KDM1A_HUMAN	KDM1A	O60341	Lysine-specific histone demethylase 1A	KDM1A_HUMAN	
0.0234158	-0.56525	-3.33097	sp|O60437|PEPL_HUMAN	PPL	O60437	Periplakin	PEPL_HUMAN	
0.0119167	0.344744	4.01892	sp|O60488|ACSL4_HUMAN	ACSL4	O60488	Long-chain-fatty-acid--CoA ligase 4	ACSL4_HUMAN	
0.0231141	-0.430958	-3.34646	sp|O60502|OGA_HUMAN	OGA	O60502	Protein O-GlcNAcase	OGA_HUMAN	
0.0490162	-0.33517	-2.62897	sp|O60547|GMDS_HUMAN	GMDS	O60547	GDP-mannose 4,6 dehydratase	GMDS_HUMAN	
0.00368657	-0.494032	-5.93941	sp|O60568|PLOD3_HUMAN	PLOD3	O60568	Multifunctional procollagen lysine hydroxylase and glycosyltransferase LH3	PLOD3_HUMAN	
0.0238956	0.217899	3.31443	sp|O60610|DIAP1_HUMAN	DIAPH1	O60610	Protein diaphanous homolog 1	DIAP1_HUMAN	
0.0119654	-0.547478	-4.00975	sp|O60664|PLIN3_HUMAN	PLIN3	O60664	Perilipin-3	PLIN3_HUMAN	
0.00516012	0.367579	5.39283	sp|O60671|RAD1_HUMAN	RAD1	O60671	Cell cycle checkpoint protein RAD1	RAD1_HUMAN	
0.00145078	-1.24957	-7.03305	sp|O60675|MAFK_HUMAN	MAFK	O60675	Transcription factor MafK	MAFK_HUMAN	
0.0155907	-0.195949	-3.7069	sp|O60701|UGDH_HUMAN	UGDH	O60701	UDP-glucose 6-dehydrogenase	UGDH_HUMAN	
0.0381784	-0.222764	-2.83741	sp|O60762|DPM1_HUMAN	DPM1	O60762	Dolichol-phosphate mannosyltransferase subunit 1	DPM1_HUMAN	
0.0286125	-0.300992	-3.11687	sp|O60763|USO1_HUMAN	USO1	O60763	General vesicular transport factor p115	USO1_HUMAN	
0.0272732	0.207828	3.16968	sp|O60828|PQBP1_HUMAN	PQBP1	O60828	Polyglutamine-binding protein 1	PQBP1_HUMAN	
0.00238627	0.275227	6.41405	sp|O60869|EDF1_HUMAN	EDF1	O60869	Endothelial differentiation-related factor 1	EDF1_HUMAN	
0.000764331	-1.4001	-7.82035	sp|O60879|DIAP2_HUMAN	DIAPH2	O60879	Protein diaphanous homolog 2	DIAP2_HUMAN	
0.00509091	-0.82738	-5.66185	sp|O60936|NOL3_HUMAN	NOL3	O60936	Nucleolar protein 3	NOL3_HUMAN	
0.00731507	-0.841705	-4.6655	sp|O60942|MCE1_HUMAN	RNGTT	O60942	mRNA-capping enzyme	MCE1_HUMAN	
0.00546512	-0.83402	-5.29559	sp|O75063|XYLK_HUMAN	FAM20B	O75063	Glycosaminoglycan xylosylkinase	XYLK_HUMAN	
0	0.345129	25.7615	sp|O75083|WDR1_HUMAN	WDR1	O75083	WD repeat-containing protein 1	WDR1_HUMAN	
0.0339759	0.247956	2.94633	sp|O75116|ROCK2_HUMAN	ROCK2	O75116	Rho-associated protein kinase 2	ROCK2_HUMAN	
0.00507383	0.820587	5.66061	sp|O75122|CLAP2_HUMAN	CLASP2	O75122	CLIP-associating protein 2	CLAP2_HUMAN	
0.0037283	1.60666	5.95113	sp|O75131|CPNE3_HUMAN	CPNE3	O75131	Copine-3	CPNE3_HUMAN	
0.0102388	0.325112	4.18383	sp|O75152|ZC11A_HUMAN	ZC3H11A	O75152	Zinc finger CCCH domain-containing protein 11A	ZC11A_HUMAN	
0.0259059	0.356371	3.20233	sp|O75348|VATG1_HUMAN	ATP6V1G1	O75348	V-type proton ATPase subunit G 1	VATG1_HUMAN	
0.0376312	-1.99504	-2.84952	sp|O75367|H2AY_HUMAN	MACROH2A	O75367	Core histone macro-H2A.1	H2AY_HUMAN	
0.0056185	-1.48281	-5.27143	sp|O75376|NCOR1_HUMAN	NCOR1	O75376	Nuclear receptor corepressor 1	NCOR1_HUMAN	
0.0162965	0.426341	3.65462	sp|O75390|CISY_HUMAN	CS	O75390	Citrate synthase, mitochondrial	CISY_HUMAN	
0.000371429	1.09115	8.24564	sp|O75410|TACC1_HUMAN	TACC1	O75410	Transforming acidic coiled-coil-containing protein 1	TACC1_HUMAN	
0.0265499	-0.267772	-3.19438	sp|O75475|PSIP1_HUMAN	PSIP1	O75475	PC4 and SFRS1-interacting protein	PSIP1_HUMAN	
0	-0.497901	-8.36868	sp|O75477|ERLN1_HUMAN	ERLIN1	O75477	Erlin-1	ERLN1_HUMAN	
0.0199667	0.859972	3.46039	sp|O75489|NDUS3_HUMAN	NDUFS3	O75489	NADH dehydrogenase [ubiquinone] iron-sulfur protein 3, mitochondrial	NDUS3_HUMAN	
0.0444612	-0.371375	-2.71318	sp|O75494|SRS10_HUMAN	SRSF10	O75494	Serine/arginine-rich splicing factor 10	SRS10_HUMAN	
0.0340109	0.297786	2.94297	sp|O75534|CSDE1_HUMAN	CSDE1	O75534	Cold shock domain-containing protein E1	CSDE1_HUMAN	
0	2.18404	9.36412	sp|O75616|ERAL1_HUMAN	ERAL1	O75616	GTPase Era, mitochondrial	ERAL1_HUMAN	
0.0150909	0.898993	3.76231	sp|O75746|S2512_HUMAN	SLC25A12	O75746	Electrogenic aspartate/glutamate antiporter SLC25A12, mitochondrial	S2512_HUMAN	
0.0468816	0.383687	2.66227	sp|O75818|RPP40_HUMAN	RPP40	O75818	Ribonuclease P protein subunit p40	RPP40_HUMAN	
0.0239274	0.239586	3.30187	sp|O75821|EIF3G_HUMAN	EIF3G	O75821	Eukaryotic translation initiation factor 3 subunit G	EIF3G_HUMAN	
0.00476389	0.535824	5.76311	sp|O76071|CIAO1_HUMAN	CIAO1	O76071	Probable cytosolic iron-sulfur protein assembly protein CIAO1	CIAO1_HUMAN	
0.0309198	-0.183049	-3.03941	sp|O76094|SRP72_HUMAN	SRP72	O76094	Signal recognition particle subunit SRP72	SRP72_HUMAN	
0.00722115	0.734569	4.77682	sp|O94760|DDAH1_HUMAN	DDAH1	O94760	N(G),N(G)-dimethylarginine dimethylaminohydrolase 1	DDAH1_HUMAN	
0.0295274	-0.36887	-3.09133	sp|O94808|GFPT2_HUMAN	GFPT2	O94808	Glutamine--fructose-6-phosphate aminotransferase [isomerizing] 2	GFPT2_HUMAN	
0.0296221	-0.462708	-3.07809	sp|O94888|UBXN7_HUMAN	UBXN7	O94888	UBX domain-containing protein 7	UBXN7_HUMAN	
0.0265172	-0.362782	-3.19288	sp|O94905|ERLN2_HUMAN	ERLIN2	O94905	Erlin-2	ERLN2_HUMAN	
0.00450542	-0.709096	-5.86655	sp|O94907|DKK1_HUMAN	DKK 1.00	O94907	Dickkopf-related protein 1	DKK1_HUMAN	
0	0.723054	12.5562	sp|O94925|GLSK_HUMAN	GLS	O94925	Glutaminase kidney isoform, mitochondrial	GLSK_HUMAN	
0.00197015	0.717595	6.92104	sp|O95070|YIF1A_HUMAN	YIF1A	O95070	Protein YIF1A	YIF1A_HUMAN	
0.0281517	-0.452689	-3.13025	sp|O95071|UBR5_HUMAN	UBR5	O95071	E3 ubiquitin-protein ligase UBR5	UBR5_HUMAN	
0.045353	0.884215	2.69131	sp|O95167|NDUA3_HUMAN	NDUFA3	O95167	NADH dehydrogenase [ubiquinone] 1 alpha subcomplex subunit 3	NDUA3_HUMAN	
0.0253699	2.65197	3.21863	sp|O95168|NDUB4_HUMAN	NDUFB4	O95168	NADH dehydrogenase [ubiquinone] 1 beta subcomplex subunit 4	NDUB4_HUMAN	
0.0283124	0.514423	3.12712	sp|O95169|NDUB8_HUMAN	NDUFB8	O95169	NADH dehydrogenase [ubiquinone] 1 beta subcomplex subunit 8, mitochondrial	NDUB8_HUMAN	
0.0497735	0.148265	2.61281	sp|O95232|LC7L3_HUMAN	LUC7L3	O95232	Luc7-like protein 3	LC7L3_HUMAN	
0.00435165	0.616179	5.88459	sp|O95239|KIF4A_HUMAN	KIF4A	O95239	Chromosome-associated kinesin KIF4A	KIF4A_HUMAN	
0.0279615	0.623561	3.14132	sp|O95299|NDUAA_HUMAN	NDUFA10	O95299	NADH dehydrogenase [ubiquinone] 1 alpha subcomplex subunit 10, mitochondrial	NDUAA_HUMAN	
0.00827468	-0.385042	-4.51875	sp|O95302|FKBP9_HUMAN	FKBP9	O95302	Peptidyl-prolyl cis-trans isomerase FKBP9	FKBP9_HUMAN	
0.00976154	0.253001	4.24919	sp|O95347|SMC2_HUMAN	SMC2	O95347	Structural maintenance of chromosomes protein 2	SMC2_HUMAN	

0.0151146	2.64401	3.76562	sp|O95361|TRI16_HUMAN	TRIM16	O95361	Tripartite motif-containing protein 16	TRI16_HUMAN	
0.010426	0.271093	4.12256	sp|O95373|IPO7_HUMAN	IPO7	O95373	Importin-7	IPO7_HUMAN	
0.0324643	0.374233	2.99172	sp|O95470|SGPL1_HUMAN	SGPL1	O95470	Sphingosine-1-phosphate lyase 1	SGPL1_HUMAN	
0.00485714	1.59218	5.68463	sp|O95478|NSA2_HUMAN	NSA2	O95478	Ribosome biogenesis protein NSA2 homolog	NSA2_HUMAN	
0.00734732	0.508506	4.71547	sp|O95573|ACSL3_HUMAN	ACSL3	O95573	Fatty acid CoA ligase Acsl3	ACSL3_HUMAN	
0.00706341	-0.702435	-4.82858	sp|O95671|ASML_HUMAN	ASMTL	O95671	Probable bifunctional dTTP/UTP pyrophosphatase/methyltransferase protein	ASML_HUMAN	
0.0156392	-0.241872	-3.7138	sp|O95747|OXSR1_HUMAN	OXSR1	O95747	Serine/threonine-protein kinase OSR1	OXSR1_HUMAN	
0.0251343	0.405968	3.22596	sp|O95749|GGPPS_HUMAN	GGPS1	O95749	Geranylgeranyl pyrophosphate synthase	GGPPS_HUMAN	
0.0431196	0.663206	2.74169	sp|O95785|WIZ_HUMAN	WIZ	O95785	Protein Wiz	WIZ_HUMAN	
0.00374242	0.315057	5.98077	sp|O95816|BAG2_HUMAN	BAG2	O95816	BAG family molecular chaperone regulator 2	BAG2_HUMAN	
0.01663	-0.777361	-3.61574	sp|O95834|EMAL2_HUMAN	EML2	O95834	Echinoderm microtubule-associated protein-like 2	EMAL2_HUMAN	
0.0130153	-1.49929	-3.95602	sp|O95857|TSN13_HUMAN	TSPAN13	O95857	Tetraspanin-13	TSN13_HUMAN	
0.00921285	0.421363	4.32905	sp|O95865|DDAH2_HUMAN	DDAH2	O95865	N(G),N(G)-dimethylarginine dimethylaminohydrolase 2	DDAH2_HUMAN	
0	-0.74754	-18.664	sp|O95881|TXD12_HUMAN	TXNDC12	O95881	Thioredoxin domain-containing protein 12	TXD12_HUMAN	
0.0237746	0.37047	3.32202	sp|O96000|NDUBA_HUMAN	NDUFB10	O96000	NADH dehydrogenase [ubiquinone] 1 beta subcomplex subunit 10	NDUBA_HUMAN	
0.009344	0.703117	4.31749	sp|O96005|CLPT1_HUMAN	CLPTM1	O96005	Putative lipid scramblase CLPTM1	CLPT1_HUMAN	
0.00963107	0.504674	4.27718	sp|O96008|TOM40_HUMAN	TOMM40	O96008	Mitochondrial import receptor subunit TOM40 homolog	TOM40_HUMAN	
0.0165868	-0.62623	-3.62907	sp|O96011|PX11B_HUMAN	PEX11B	O96011	Peroxisomal membrane protein 11B	PX11B_HUMAN	
0	-0.946283	-15.2166	sp|P00338|LDHA_HUMAN	LDHA	P00338	L-lactate dehydrogenase A chain	LDHA_HUMAN	
0.0275103	0.879133	3.16016	sp|P00390|GSHR_HUMAN	GSR	P00390	Glutathione reductase, mitochondrial	GSHR_HUMAN	
0	-1.58661	-13.5148	sp|P00558|PGK1_HUMAN	PGK 1.00	P00558	Phosphoglycerate kinase 1	PGK1_HUMAN	
0.0137815	0.244102	3.88799	sp|P00966|ASSY_HUMAN	ASS1	P00966	Argininosuccinate synthase	ASSY_HUMAN	
0.00651546	1.41222	4.9321	sp|P01023|A2MG_HUMAN	A2M	P01023	Alpha-2-macroglobulin	A2MG_HUMAN	
0.0338964	-0.324036	-2.95571	sp|P01893|HLAH_HUMAN	HLA-H	P01893	Putative HLA class I histocompatibility antigen, alpha chain H	HLAH_HUMAN	
0	0.586799	14.7935	sp|P02786|TFR1_HUMAN	TFRC	P02786	Transferrin receptor protein 1	TFR1_HUMAN	
0	-2.53909	-36.7622	sp|P03973|SLPI_HUMAN	SLPI	P03973	Antileukoproteinase	SLPI_HUMAN	
0.000351351	-1.20285	-8.07525	sp|P04004|VTNC_HUMAN	VTN	P04004	Vitronectin	VTNC_HUMAN	
0	-0.465362	-9.49701	sp|P04040|CATA_HUMAN	CAT	P04040	Catalase	CATA_HUMAN	
0.0446905	-0.685685	-2.70743	sp|P04066|FUCO_HUMAN	FUCA1	P04066	Tissue alpha-L-fucosidase	FUCO_HUMAN	
0	-1.27729	-15.2662	sp|P04075|ALDOA_HUMAN	ALDOA	P04075	Fructose-bisphosphate aldolase A	ALDOA_HUMAN	
0.000754717	1.51738	7.81186	sp|P04083|ANXA1_HUMAN	ANXA1	P04083	Annexin A1	ANXA1_HUMAN	
0	-0.846343	-10.6986	sp|P04406|G3P_HUMAN	GAPDH	P04406	Glyceraldehyde-3-phosphate dehydrogenase	G3P_HUMAN	
0.0408875	-0.330312	-2.77748	sp|P04424|ARLY_HUMAN	ASL	P04424	Argininosuccinate lyase	ARLY_HUMAN	
0.00436765	0.574747	5.88678	sp|P04792|HSPB1_HUMAN	HSPB1	P04792	Heat shock protein beta-1	HSPB1_HUMAN	
0.0222704	0.852983	3.37325	sp|P04818|TYSY_HUMAN	TYMS	P04818	Thymidylate synthase	TYSY_HUMAN	
0.00368254	-0.305662	-6.10368	sp|P04843|RPN1_HUMAN	RPN1	P04843	Dolichyl-diphosphooligosaccharide--protein glycosyltransferase subunit 1	RPN1_HUMAN	
0.0145161	-0.315675	-3.82311	sp|P04899|GNAI2_HUMAN	GNAI2	P04899	Guanine nucleotide-binding protein G(i) subunit alpha-2	GNAI2_HUMAN	
0.0209235	-1.77921	-3.4213	sp|P04908|H2A1B_HUMAN	H2AC4	P04908	Histone H2A type 1-B/E	H2A1B_HUMAN	sp|Q7L7L0|H2A3_HUMAN
0.00658333	0.539524	4.98552	sp|P05023|AT1A1_HUMAN	ATP1A1	P05023	Sodium/potassium-transporting ATPase subunit alpha-1	AT1A1_HUMAN	
0.00930677	0.354487	4.31309	sp|P05141|ADT2_HUMAN	SLC25A5	P05141	ADP/ATP translocase 2	ADT2_HUMAN	
0.00367286	0.579527	5.93877	sp|P05161|ISG15_HUMAN	ISG15	P05161	Ubiquitin-like protein ISG15	ISG15_HUMAN	
0.0392735	1.16261	2.81869	sp|P05187|PPB1_HUMAN	ALPP	P05187	Alkaline phosphatase, placental type	PPB1_HUMAN	
0.00489041	-0.731694	-5.69293	sp|P05362|ICAM1_HUMAN	ICAM1	P05362	Intercellular adhesion molecule 1	ICAM1_HUMAN	
0.0119861	0.930218	4.01129	sp|P05783|K1C18_HUMAN	KRT18	P05783	Keratin, type I cytoskeletal 18	K1C18_HUMAN	
0.0163212	1.00699	3.65487	sp|P05787|K2C8_HUMAN	KRT8	P05787	Keratin, type II cytoskeletal 8	K2C8_HUMAN	
0.010846	0.240793	4.06576	sp|P06493|CDK1_HUMAN	CDK1	P06493	Cyclin-dependent kinase 1	CDK1_HUMAN	
0	-0.592852	-9.97274	sp|P06733|ENOA_HUMAN	ENO1	P06733	Alpha-enolase	ENOA_HUMAN	
0	-0.570673	-8.64802	sp|P06737|PYGL_HUMAN	PYGL	P06737	Glycogen phosphorylase, liver form	PYGL_HUMAN	
0	-1.5083	-15.117	sp|P06744|G6PI_HUMAN	GPI	P06744	Glucose-6-phosphate isomerase	G6PI_HUMAN	
0.0214124	-0.329906	-3.39365	sp|P06748|NPM_HUMAN	NPM1	P06748	Nucleophosmin	NPM_HUMAN	
0.000356164	0.418222	8.10089	sp|P06756|ITAV_HUMAN	ITGAV	P06756	Integrin alpha-V	ITAV_HUMAN	
0.0308597	-1.00918	-3.04621	sp|P07093|GDN_HUMAN	SERPINE2	P07093	Glia-derived nexin	GDN_HUMAN	
0.0197877	-1.35707	-3.4762	sp|P07203|GPX1_HUMAN	GPX1	P07203	Glutathione peroxidase 1	GPX1_HUMAN	
0.00306557	-0.736371	-6.23009	sp|P07237|PDIA1_HUMAN	P4HB	P07237	Protein disulfide-isomerase	PDIA1_HUMAN	
0.0354721	-0.31549	-2.90263	sp|P07339|CATD_HUMAN	CTSD	P07339	Cathepsin D	CATD_HUMAN	
0.00300402	3.67129	6.15024	sp|P07355|ANXA2_HUMAN	ANXA2	P07355	Annexin A2	ANXA2_HUMAN	
0.0272381	-0.439432	-3.17026	sp|P07686|HEXB_HUMAN	HEXB	P07686	Beta-hexosaminidase subunit beta	HEXB_HUMAN	
0.0138272	0.152089	3.89681	sp|P07814|SYEP_HUMAN	EPRS1	P07814	Bifunctional glutamate/proline--tRNA ligase	SYEP_HUMAN	
0.00124444	-0.677678	-7.34384	sp|P07858|CATB_HUMAN	CTSB	P07858	Cathepsin B	CATB_HUMAN	
0.00926984	0.431617	4.30811	sp|P07942|LAMB1_HUMAN	LAMB1	P07942	Laminin subunit beta-1	LAMB1_HUMAN	
0.00304065	3.25214	6.21284	sp|P08133|ANXA6_HUMAN	ANXA6	P08133	Annexin A6	ANXA6_HUMAN	
0.000385185	-1.2341	-8.27543	sp|P08174|DAF_HUMAN	CD55	P08174	Complement decay-accelerating factor	DAF_HUMAN	
0.0237435	0.270542	3.32094	sp|P08195|4F2_HUMAN	SLC3A2	P08195	4F2 cell-surface antigen heavy chain	4F2_HUMAN	
0	1.31206	9.74233	sp|P08243|ASNS_HUMAN	ASNS	P08243	Asparagine synthetase [glutamine-hydrolyzing]	ASNS_HUMAN	
0.00117277	0.253719	7.12785	sp|P08559|ODPA_HUMAN	PDHA1	P08559	Pyruvate dehydrogenase E1 component subunit alpha, somatic form, mitochondrial	ODPA_HUMAN	
0	-0.936162	-17.6322	sp|P08648|ITA5_HUMAN	ITGA5	P08648	Integrin alpha-5	ITA5_HUMAN	
0.0355178	0.420842	2.90746	sp|P08651|NFIC_HUMAN	NFIC	P08651	Nuclear factor 1 C-type	NFIC_HUMAN	
0.0413835	0.560062	2.76976	sp|P08670|VIME_HUMAN	VIM	P08670	Vimentin	VIME_HUMAN	
0.00520732	0.876266	5.4196	sp|P08708|RS17_HUMAN	RPS17	P08708	Small ribosomal subunit protein eS17	RS17_HUMAN	
0.0294803	0.698425	3.08852	sp|P08729|K2C7_HUMAN	KRT7	P08729	Keratin, type II cytoskeletal 7	K2C7_HUMAN	
0	2.39848	26.7553	sp|P08758|ANXA5_HUMAN	ANXA5	P08758	Annexin A5	ANXA5_HUMAN	
0	0.591284	8.49172	sp|P09001|RM03_HUMAN	MRPL3	P09001	Large ribosomal subunit protein uL3m	RM03_HUMAN	
0.0187635	1.48488	3.51323	sp|P09038|FGF2_HUMAN	FGF2	P09038	Fibroblast growth factor 2	FGF2_HUMAN	
0.000366197	-1.89557	-8.16083	sp|P09104|ENOG_HUMAN	ENO2	P09104	Gamma-enolase	ENOG_HUMAN	
0.00732057	-0.423487	-4.7579	sp|P09382|LEG1_HUMAN	LGALS1	P09382	Galectin-1	LEG1_HUMAN	
0	1.55512	25.2538	sp|P09493|TPM1_HUMAN	TPM1	P09493	Tropomyosin alpha-1 chain	TPM1_HUMAN	
0	4.19924	13.9501	sp|P09525|ANXA4_HUMAN	ANXA4	P09525	Annexin A4	ANXA4_HUMAN	
0.0102579	0.186415	4.18401	sp|P09543|CN37_HUMAN	CNP	P09543	2',3'-cyclic-nucleotide 3'-phosphodiesterase	CN37_HUMAN	
0.0233712	0.113711	3.33893	sp|P09874|PARP1_HUMAN	PARP1	P09874	Poly [ADP-ribose] polymerase 1	PARP1_HUMAN	
0.0474286	0.429561	2.65092	sp|P09884|DPOLA_HUMAN	POLA1	P09884	DNA polymerase alpha catalytic subunit	DPOLA_HUMAN	
0.000363636	1.37202	8.15125	sp|P09913|IFIT2_HUMAN	IFIT2	P09913	Interferon-induced protein with tetratricopeptide repeats 2	IFIT2_HUMAN	
0.00116667	1.19532	7.07685	sp|P09914|IFIT1_HUMAN	IFIT1	P09914	Interferon-induced protein with tetratricopeptide repeats 1	IFIT1_HUMAN	
0.033809	-0.593151	-2.9516	sp|P09923|PPBI_HUMAN	ALPI	P09923	Intestinal-type alkaline phosphatase	PPBI_HUMAN	
0	-2.16777	-15.88	sp|P09972|ALDOC_HUMAN	ALDOC	P09972	Fructose-bisphosphate aldolase C	ALDOC_HUMAN	
0.00656716	-0.379417	-4.87502	sp|P0DMV8|HS71A_HUMAN	HSPA1A	P0DMV8	Heat shock 70 kDa protein 1A	HS71A_HUMAN	sp|P0DMV9|HS71B_HUMAN
0.0222406	0.346569	3.37323	sp|P10155|RO60_HUMAN	RO60	P10155	RNA-binding protein RO60	RO60_HUMAN	
0.00466667	-0.805076	-5.83128	sp|P10253|LYAG_HUMAN	GAA	P10253	Lysosomal alpha-glucosidase	LYAG_HUMAN	
0.00448921	0.852745	5.85564	sp|P10398|ARAF_HUMAN	ARAF	P10398	Serine/threonine-protein kinase A-Raf	ARAF_HUMAN	
0.000353741	1.06009	8.08512	sp|P10644|KAP0_HUMAN	PRKAR1A	P10644	cAMP-dependent protein kinase type I-alpha regulatory subunit	KAP0_HUMAN	
0.000710059	-0.604076	-7.66446	sp|P11021|BIP_HUMAN	HSPA5	P11021	Endoplasmic reticulum chaperone BiP	BIP_HUMAN	
0	-0.427901	-12.8411	sp|P11047|LAMC1_HUMAN	LAMC1	P11047	Laminin subunit gamma-1	LAMC1_HUMAN	
0	-1.36575	-9.9053	sp|P11166|GTR1_HUMAN	SLC2A1	P11166	Solute carrier family 2, facilitated glucose transporter member 1	GTR1_HUMAN	
0.00517576	-1.36341	-5.41853	sp|P11169|GTR3_HUMAN	SLC2A3	P11169	Solute carrier family 2, facilitated glucose transporter member 3	GTR3_HUMAN	
0.0052129	0.278409	5.57537	sp|P11172|UMPS_HUMAN	UMPS	P11172	Uridine 5'-monophosphate synthase	UMPS_HUMAN	
0.0343366	-0.184879	-2.93044	sp|P11233|RALA_HUMAN	RALA	P11233	Ras-related protein Ral-A	RALA_HUMAN	
0.00195074	0.386068	6.89423	sp|P11413|G6PD_HUMAN	G6PD	P11413	Glucose-6-phosphate 1-dehydrogenase	G6PD_HUMAN	
0.0156149	0.261835	3.71218	sp|P11498|PYC_HUMAN	PC	P11498	Pyruvate carboxylase, mitochondrial	PYC_HUMAN	
0.00413284	0.390445	5.89824	sp|P11586|C1TC_HUMAN	MTHFD1	P11586	C-1-tetrahydrofolate synthase, cytoplasmic	C1TC_HUMAN	
0.00452174	0.317302	5.86712	sp|P11802|CDK4_HUMAN	CDK4	P11802	Cyclin-dependent kinase 4	CDK4_HUMAN	
0.00734862	0.37516	4.68099	sp|P12236|ADT3_HUMAN	SLC25A6	P12236	ADP/ATP translocase 3	ADT3_HUMAN	
0.003712	0.177603	6.10791	sp|P12268|IMDH2_HUMAN	IMPDH2	P12268	Inosine-5'-monophosphate dehydrogenase 2	IMDH2_HUMAN	
0.000784314	-0.340808	-7.91634	sp|P12270|TPR_HUMAN	TPR	P12270	Nucleoprotein TPR	TPR_HUMAN	
0.0251196	-0.367167	-3.25011	sp|P12277|KCRB_HUMAN	CKB	P12277	Creatine kinase B-type	KCRB_HUMAN	
0	1.22805	26.2183	sp|P12429|ANXA3_HUMAN	ANXA3	P12429	Annexin A3	ANXA3_HUMAN	
0.0489695	-0.343292	-2.62822	sp|P12694|ODBA_HUMAN	BCKDHA	P12694	2-oxoisovalerate dehydrogenase subunit alpha, mitochondrial	ODBA_HUMAN	
0.0134515	0.465031	3.9151	sp|P12955|PEPD_HUMAN	PEPD	P12955	Xaa-Pro dipeptidase	PEPD_HUMAN	
0.0369049	-0.0880241	-2.87108	sp|P12956|XRCC6_HUMAN	XRCC6	P12956	X-ray repair cross-complementing protein 6	XRCC6_HUMAN	
0.00387451	-0.211019	-6.06301	sp|P13010|XRCC5_HUMAN	XRCC5	P13010	X-ray repair cross-complementing protein 5	XRCC5_HUMAN	
0.0319463	0.767311	3.01064	sp|P13051|UNG_HUMAN	UNG	P13051	Uracil-DNA glycosylase	UNG_HUMAN	
0.0100989	-0.293854	-4.22172	sp|P13489|RINI_HUMAN	RNH1	P13489	Ribonuclease inhibitor	RINI_HUMAN	
0.00219298	0.229866	6.54321	sp|P13639|EF2_HUMAN	EEF2	P13639	Elongation factor 2	EF2_HUMAN	
0	-0.668984	-10.3059	sp|P13667|PDIA4_HUMAN	PDIA4	P13667	Protein disulfide-isomerase A4	PDIA4_HUMAN	
0	-2.27353	-32.3044	sp|P13674|P4HA1_HUMAN	P4HA1	P13674	Prolyl 4-hydroxylase subunit alpha-1	P4HA1_HUMAN	
0.00774165	0.366213	4.6031	sp|P13797|PLST_HUMAN	PLS3	P13797	Plastin-3	PLST_HUMAN	
0.0209521	-0.729093	-3.42271	sp|P13807|GYS1_HUMAN	GYS1	P13807	Glycogen [starch] synthase, muscle	GYS1_HUMAN	
0.00235849	0.502632	6.72563	sp|P13928|ANXA8_HUMAN	ANXA8	P13928	Annexin A8	ANXA8_HUMAN	
0	1.15059	9.22112	sp|P13929|ENOB_HUMAN	ENO3	P13929	Beta-enolase	ENOB_HUMAN	
0.0454912	0.6298	2.68235	sp|P13995|MTDC_HUMAN	MTHFD2	P13995	Bifunctional methylenetetrahydrofolate dehydrogenase/cyclohydrolase, mitochondria	MTDC_HUMAN	
0.00767329	0.718741	4.58924	sp|P14209|CD99_HUMAN	CD99	P14209	CD99 antigen	CD99_HUMAN	
0.00727361	-0.372695	-4.80242	sp|P14314|GLU2B_HUMAN	PRKCSH	P14314	Glucosidase 2 subunit beta	GLU2B_HUMAN	
0.00193171	-1.5324	-6.86712	sp|P14384|CBPM_HUMAN	CPM	P14384	Carboxypeptidase M	CBPM_HUMAN	
0.0149858	-0.287615	-3.77291	sp|P14618|KPYM_HUMAN	PKM	P14618	Pyruvate kinase PKM	KPYM_HUMAN	
0	-1.00509	-13.1326	sp|P14625|ENPL_HUMAN	HSP90B1	P14625	Endoplasmin	ENPL_HUMAN	
0.00512913	0.43032	5.3846	sp|P14735|IDE_HUMAN	IDE	P14735	Insulin-degrading enzyme	IDE_HUMAN	
0.00654922	-0.440514	-4.97701	sp|P14868|SYDC_HUMAN	DARS1	P14868	Aspartate--tRNA ligase, cytoplasmic	SYDC_HUMAN	
0.0310023	1.22205	3.0376	sp|P15104|GLNA_HUMAN	GLUL	P15104	Glutamine synthetase	GLNA_HUMAN	
0.00772444	0.79251	4.60205	sp|P15291|B4GT1_HUMAN	B4GALT1	P15291	Beta-1,4-galactosyltransferase 1	B4GT1_HUMAN	
0.00951181	-1.46888	-4.29356	sp|P15408|FOSL2_HUMAN	FOSL2	P15408	Fos-related antigen 2	FOSL2_HUMAN	
0.00656623	-0.956281	-4.97817	sp|P15529|MCP_HUMAN	CD46	P15529	Membrane cofactor protein	MCP_HUMAN	

0.00923139	0.37888	4.3334	sp|P15559|NQO1_HUMAN	NQO1	P15559	NAD(P)H dehydrogenase [quinone] 1	NQO1_HUMAN	
0.0210217	-0.304504	-3.40681	sp|P15586|GNS_HUMAN	GNS	P15586	N-acetylglucosamine-6-sulfatase	GNS_HUMAN	
0.0181098	0.256687	3.53794	sp|P15880|RS2_HUMAN	RPS2	P15880	Small ribosomal subunit protein uS5	RS2_HUMAN	
0.0197427	0.508983	3.4764	sp|P15924|DESP_HUMAN	DSP	P15924	Desmoplakin	DESP_HUMAN	
0.00547606	-0.514975	-5.22635	sp|P15927|RFA2_HUMAN	RPA2	P15927	Replication protein A 32 kDa subunit	RFA2_HUMAN	
0.00908722	-0.968147	-4.35432	sp|P15941|MUC1_HUMAN	MUC1	P15941	Mucin-1	MUC1_HUMAN	
0.0395509	-0.732936	-2.81403	sp|P16083|NQO2_HUMAN	NQO2	P16083	Ribosyldihydronicotinamide dehydrogenase [quinone]	NQO2_HUMAN	
0.0474744	-0.242497	-2.65153	sp|P16152|CBR1_HUMAN	CBR1	P16152	Carbonyl reductase [NADPH] 1	CBR1_HUMAN	
0	-0.27692	-10.4389	sp|P16615|AT2A2_HUMAN	ATP2A2	P16615	Sarcoplasmic/endoplasmic reticulum calcium ATPase 2	AT2A2_HUMAN	
0	-1.22195	-9.22268	sp|P17301|ITA2_HUMAN	ITGA2	P17301	Integrin alpha-2	ITA2_HUMAN	
0.00893252	0.231548	4.38947	sp|P17612|KAPCA_HUMAN	PRKACA	P17612	cAMP-dependent protein kinase catalytic subunit alpha	KAPCA_HUMAN	
0.00635176	-0.276623	-4.89644	sp|P17655|CAN2_HUMAN	CAPN2	P17655	Calpain-2 catalytic subunit	CAN2_HUMAN	
0.00770306	-1.13962	-4.57229	sp|P17676|CEBPB_HUMAN	CEBPB	P17676	CCAAT/enhancer-binding protein beta	CEBPB_HUMAN	
0	0.632345	10.0297	sp|P17812|PYRG1_HUMAN	CTPS1	P17812	CTP synthase 1	PYRG1_HUMAN	
0.00230415	1.04421	6.62714	sp|P17813|EGLN_HUMAN	ENG	P17813	Endoglin	EGLN_HUMAN	
0	0.420022	11.0654	sp|P17844|DDX5_HUMAN	DDX5	P17844	Probable ATP-dependent RNA helicase DDX5	DDX5_HUMAN	
0.00384436	-0.536496	-6.05602	sp|P17858|PFKAL_HUMAN	PFKL	P17858	ATP-dependent 6-phosphofructokinase, liver type	PFKAL_HUMAN	
0	-5.02549	-24.9336	sp|P17936|IBP3_HUMAN	IGFBP3	P17936	Insulin-like growth factor-binding protein 3	IBP3_HUMAN	
0.0460312	0.596896	2.67636	sp|P18077|RL35A_HUMAN	RPL35A	P18077	Large ribosomal subunit protein eL33	RL35A_HUMAN	
0.00563478	0.419378	5.28141	sp|P18124|RL7_HUMAN	RPL7	P18124	Large ribosomal subunit protein uL30	RL7_HUMAN	
0.0448079	0.384635	2.70256	sp|P18206|VINC_HUMAN	VCL	P18206	Vinculin	VINC_HUMAN	
0.040696	0.332041	2.78266	sp|P18621|RL17_HUMAN	RPL17	P18621	Large ribosomal subunit protein uL22	RL17_HUMAN	
0.00232558	-0.568868	-6.66195	sp|P18669|PGAM1_HUMAN	PGAM1	P18669	Phosphoglycerate mutase 1	PGAM1_HUMAN	
0.0477615	-0.671693	-2.6432	sp|P18858|DNLI1_HUMAN	LIG1	P18858	DNA ligase 1	DNLI1_HUMAN	
0.00512542	0.409784	5.67048	sp|P18887|XRCC1_HUMAN	XRCC1	P18887	DNA repair protein XRCC1	XRCC1_HUMAN	
0.0309673	-0.298216	-3.03516	sp|P19338|NUCL_HUMAN	NCL	P19338	Nucleolin	NUCL_HUMAN	
0.00378544	0.716826	5.99493	sp|P19823|ITIH2_HUMAN	ITIH2	P19823	Inter-alpha-trypsin inhibitor heavy chain H2	ITIH2_HUMAN	
0.0115478	0.344884	4.02375	sp|P20042|IF2B_HUMAN	EIF2S2	P20042	Eukaryotic translation initiation factor 2 subunit 2	IF2B_HUMAN	
0	2.3064	17.198	sp|P20073|ANXA7_HUMAN	ANXA7	P20073	Annexin A7	ANXA7_HUMAN	
0	0.418114	8.57279	sp|P20290|BTF3_HUMAN	BTF3	P20290	Transcription factor BTF3	BTF3_HUMAN	
0.0235375	0.896598	3.33002	sp|P20338|RAB4A_HUMAN	RAB4A	P20338	Ras-related protein Rab-4A	RAB4A_HUMAN	
0.00508176	-0.28019	-5.52528	sp|P20340|RAB6A_HUMAN	RAB6A	P20340	Ras-related protein Rab-6A	RAB6A_HUMAN	
0.0395311	-0.223272	-2.81363	sp|P20618|PSB1_HUMAN	PSMB1	P20618	Proteasome subunit beta type-1	PSB1_HUMAN	
0.0296816	-0.145703	-3.07065	sp|P20810|ICAL_HUMAN	CAST	P20810	Calpastatin	ICAL_HUMAN	
0	-1.96252	-11.0333	sp|P20908|CO5A1_HUMAN	COL5A1	P20908	Collagen alpha-1(V) chain	CO5A1_HUMAN	
0.0109187	0.223074	4.06108	sp|P21281|VATB2_HUMAN	ATP6V1B2	P21281	V-type proton ATPase subunit B, brain isoform	VATB2_HUMAN	
0.00414815	0.404518	5.91233	sp|P21291|CSRP1_HUMAN	CSRP1	P21291	Cysteine and glycine-rich protein 1	CSRP1_HUMAN	
0.0269621	0.449378	3.17712	sp|P21333|FLNA_HUMAN	FLNA	P21333	Filamin-A	FLNA_HUMAN	
0	-0.419185	-8.32441	sp|P21399|ACOHC_HUMAN	ACO1	P21399	Cytoplasmic aconitate hydratase	ACOHC_HUMAN	
0.00513016	-0.313847	-5.55049	sp|P21912|SDHB_HUMAN	SDHB	P21912	Succinate dehydrogenase [ubiquinone] iron-sulfur subunit, mitochondrial	SDHB_HUMAN	
0.00655087	-1.7032	-4.86942	sp|P22692|IBP4_HUMAN	IGFBP4	P22692	Insulin-like growth factor-binding protein 4	IBP4_HUMAN	
0.0102418	0.80918	4.14795	sp|P23142|FBLN1_HUMAN	FBLN1	P23142	Fibulin-1	FBLN1_HUMAN	
0.000685714	0.38731	7.53416	sp|P23193|TCEA1_HUMAN	TCEA1	P23193	Transcription elongation factor A protein 1	TCEA1_HUMAN	
0.00643257	-1.11111	-4.90956	sp|P23219|PGH1_HUMAN	PTGS1	P23219	Prostaglandin G/H synthase 1	PGH1_HUMAN	
0.00377099	-0.321076	-5.99305	sp|P23284|PPIB_HUMAN	PPIB	P23284	Peptidyl-prolyl cis-trans isomerase B	PPIB_HUMAN	
0	0.592375	8.93388	sp|P23381|SYWC_HUMAN	WARS1	P23381	Tryptophan--tRNA ligase, cytoplasmic	SYWC_HUMAN	
0.0453976	0.203787	2.69169	sp|P23526|SAHH_HUMAN	AHCY	P23526	Adenosylhomocysteinase	SAHH_HUMAN	
0.0102319	0.401248	4.1255	sp|P23588|IF4B_HUMAN	EIF4B	P23588	Eukaryotic translation initiation factor 4B	IF4B_HUMAN	
0.0371139	-0.283571	-2.86367	sp|P23634|AT2B4_HUMAN	ATP2B4	P23634	Plasma membrane calcium-transporting ATPase 4	AT2B4_HUMAN	
0.0497676	-0.144047	-2.61352	sp|P23921|RIR1_HUMAN	RRM1	P23921	Ribonucleoside-diphosphate reductase large subunit	RIR1_HUMAN	
0.0134252	1.99476	3.92239	sp|P24311|COX7B_HUMAN	COX7B	P24311	Cytochrome c oxidase subunit 7B, mitochondrial	COX7B_HUMAN	
0.026829	0.176473	3.18355	sp|P25205|MCM3_HUMAN	MCM3	P25205	DNA replication licensing factor MCM3	MCM3_HUMAN	
0.00516409	0.402454	5.49648	sp|P25325|THTM_HUMAN	MPST	P25325	3-mercaptopyruvate sulfurtransferase	THTM_HUMAN	
0.00522977	-0.26399	-5.58172	sp|P25786|PSA1_HUMAN	PSMA1	P25786	Proteasome subunit alpha type-1	PSA1_HUMAN	
0.0344104	-0.291459	-2.92238	sp|P25788|PSA3_HUMAN	PSMA3	P25788	Proteasome subunit alpha type-3	PSA3_HUMAN	
0	0.486854	8.50421	sp|P26358|DNMT1_HUMAN	DNMT1	P26358	DNA (cytosine-5)-methyltransferase 1	DNMT1_HUMAN	
0.00709804	0.535747	4.83939	sp|P26373|RL13_HUMAN	RPL13	P26373	Large ribosomal subunit protein eL13	RL13_HUMAN	
0.0276039	0.667053	3.15291	sp|P26447|S10A4_HUMAN	S100A4	P26447	Protein S100-A4	S10A4_HUMAN	
0.00886486	0.231483	4.45096	sp|P26639|SYTC_HUMAN	TARS1	P26639	Threonine--tRNA ligase 1, cytoplasmic	SYTC_HUMAN	
0.0100038	0.660502	4.22218	sp|P27105|STOM_HUMAN	STOM	P27105	Stomatin	STOM_HUMAN	
0.00511111	-0.528626	-5.59265	sp|P27144|KAD4_HUMAN	AK4	P27144	Adenylate kinase 4, mitochondrial	KAD4_HUMAN	
0.0214751	0.508765	3.39182	sp|P27635|RL10_HUMAN	RPL10	P27635	Large ribosomal subunit protein uL16	RL10_HUMAN	
0.00647619	-0.371707	-4.89518	sp|P27694|RFA1_HUMAN	RPA1	P27694	Replication protein A 70 kDa DNA-binding subunit	RFA1_HUMAN	
0.000376812	0.335415	8.26579	sp|P27708|PYR1_HUMAN	CAD	P27708	CAD protein	PYR1_HUMAN	
0.00468327	0.298543	5.84228	sp|P27816|MAP4_HUMAN	MAP4	P27816	Microtubule-associated protein 4	MAP4_HUMAN	
0.000697674	-0.396612	-7.59677	sp|P27824|CALX_HUMAN	CANX	P27824	Calnexin	CALX_HUMAN	
0.033859	-0.397931	-2.95549	sp|P28072|PSB6_HUMAN	PSMB6	P28072	Proteasome subunit beta type-6	PSB6_HUMAN	
0.00653465	0.315106	4.86706	sp|P28288|ABCD3_HUMAN	ABCD3	P28288	ATP-binding cassette sub-family D member 3	ABCD3_HUMAN	
0.000368794	-2.12791	-8.22745	sp|P28300|LYOX_HUMAN	LOX	P28300	Protein-lysine 6-oxidase	LYOX_HUMAN	
0.00509851	-1.23395	-5.37567	sp|P29083|T2EA_HUMAN	GTF2E1	P29083	General transcription factor IIE subunit 1	T2EA_HUMAN	
0.0234142	0.838517	3.33812	sp|P29323|EPHB2_HUMAN	EPHB2	P29323	Ephrin type-B receptor 2	EPHB2_HUMAN	
0.0140787	-0.511042	-3.85426	sp|P29372|3MG_HUMAN	MPG	P29372	DNA-3-methyladenine glycosylase	3MG_HUMAN	
0	-1.45214	-13.5444	sp|P29373|RABP2_HUMAN	CRABP2	P29373	Cellular retinoic acid-binding protein 2	RABP2_HUMAN	
0.0109912	0.173895	4.04947	sp|P29692|EF1D_HUMAN	EEF1D	P29692	Elongation factor 1-delta	EF1D_HUMAN	
0.0073031	-0.332093	-4.75309	sp|P30044|PRDX5_HUMAN	PRDX5	P30044	Peroxiredoxin-5, mitochondrial	PRDX5_HUMAN	
0.00198	-0.694008	-6.92442	sp|P30101|PDIA3_HUMAN	PDIA3	P30101	Protein disulfide-isomerase A3	PDIA3_HUMAN	
0.0435435	0.404068	2.72968	sp|P30520|PURA2_HUMAN	ADSS2	P30520	Adenylosuccinate synthetase isozyme 2	PURA2_HUMAN	
0	-0.746192	-10.3914	sp|P30533|AMRP_HUMAN	LRPAP1	P30533	Alpha-2-macroglobulin receptor-associated protein	AMRP_HUMAN	
0.00877366	0.382354	4.40993	sp|P30566|PUR8_HUMAN	ADSL	P30566	Adenylosuccinate lyase	PUR8_HUMAN	
0	-1.31979	-26.1139	sp|P30740|ILEU_HUMAN	SERPINB1	P30740	Leukocyte elastase inhibitor	ILEU_HUMAN	
0.00490722	0.581921	5.69825	sp|P30825|CTR1_HUMAN	SLC7A1	P30825	High affinity cationic amino acid transporter 1	CTR1_HUMAN	
0.0468566	0.361091	2.65977	sp|P30837|AL1B1_HUMAN	ALDH1B1	P30837	Aldehyde dehydrogenase X, mitochondrial	AL1B1_HUMAN	
0	-0.658578	-9.77969	sp|P31040|SDHA_HUMAN	SDHA	P31040	Succinate dehydrogenase [ubiquinone] flavoprotein subunit, mitochondrial	SDHA_HUMAN	
0.00743636	-0.290602	-4.64147	sp|P31327|CPSM_HUMAN	CPS1	P31327	Carbamoyl-phosphate synthase [ammonia], mitochondrial	CPSM_HUMAN	
0.00587328	-0.68377	-5.14222	sp|P31749|AKT1_HUMAN	AKT1	P31749	RAC-alpha serine/threonine-protein kinase	AKT1_HUMAN	
0	-0.979853	-8.36252	sp|P32119|PRDX2_HUMAN	PRDX2	P32119	Peroxiredoxin-2	PRDX2_HUMAN	
0.0150637	0.410217	3.78488	sp|P32322|P5CR1_HUMAN	PYCR1	P32322	Pyrroline-5-carboxylate reductase 1, mitochondrial	P5CR1_HUMAN	
0.00822175	0.676493	4.51109	sp|P32455|GBP1_HUMAN	GBP1	P32455	Guanylate-binding protein 1	GBP1_HUMAN	
0.029554	0.602321	3.07746	sp|P32929|CGL_HUMAN	CTH	P32929	Cystathionine gamma-lyase	CGL_HUMAN	
0.0371531	0.345175	2.86463	sp|P32969|RL9_HUMAN	RPL9	P32969	Large ribosomal subunit protein uL6	RL9_HUMAN	
0.00549153	0.460123	5.22658	sp|P33316|DUT_HUMAN	DUT	P33316	Deoxyuridine 5'-triphosphate nucleotidohydrolase, mitochondrial	DUT_HUMAN	
0.0308798	0.16292	3.05044	sp|P33991|MCM4_HUMAN	MCM4	P33991	DNA replication licensing factor MCM4	MCM4_HUMAN	
0.0440955	0.250803	2.71872	sp|P34896|GLYC_HUMAN	SHMT1	P34896	Serine hydroxymethyltransferase, cytosolic	GLYC_HUMAN	
0.000689655	0.392815	7.55653	sp|P34897|GLYM_HUMAN	SHMT2	P34897	Serine hydroxymethyltransferase, mitochondrial	GLYM_HUMAN	
0.00550708	0.226284	5.22888	sp|P35221|CTNA1_HUMAN	CTNNA1	P35221	Catenin alpha-1	CTNA1_HUMAN	
0.00375665	-0.564823	-5.98735	sp|P35237|SPB6_HUMAN	SERPINB6	P35237	Serpin B6	SPB6_HUMAN	
0.0065323	-0.45715	-4.93726	sp|P35244|RFA3_HUMAN	RPA3	P35244	Replication protein A 14 kDa subunit	RFA3_HUMAN	
0	-3.90929	-19.3056	sp|P35354|PGH2_HUMAN	PTGS2	P35354	Prostaglandin G/H synthase 2	PGH2_HUMAN	
0	1.09317	12.6415	sp|P35520|CBS_HUMAN	CBS	P35520	Cystathionine beta-synthase	CBS_HUMAN	
0.0338202	-0.165042	-2.94978	sp|P35580|MYH10_HUMAN	MYH10	P35580	Myosin-10	MYH10_HUMAN	
0.00301613	-0.765395	-6.15295	sp|P35613|BASI_HUMAN	BSG	P35613	Basigin	BASI_HUMAN	
0.00570166	-2.39721	-5.15428	sp|P35754|GLRX1_HUMAN	GLRX	P35754	Glutaredoxin-1	GLRX1_HUMAN	
0.0257618	0.299357	3.20828	sp|P36507|MP2K2_HUMAN	MAP2K2	P36507	Dual specificity mitogen-activated protein kinase kinase 2	MP2K2_HUMAN	
0.0343504	0.444431	2.9342	sp|P36543|VATE1_HUMAN	ATP6V1E1	P36543	V-type proton ATPase subunit E 1	VATE1_HUMAN	
0.0318477	-0.540679	-3.0131	sp|P36551|HEM6_HUMAN	CPOX	P36551	Oxygen-dependent coproporphyrinogen-III oxidase, mitochondrial	HEM6_HUMAN	
0.0146602	0.427741	3.81957	sp|P36578|RL4_HUMAN	RPL4	P36578	Large ribosomal subunit protein uL4	RL4_HUMAN	
0.013799	-0.492632	-3.87623	sp|P36871|PGM1_HUMAN	PGM1	P36871	Phosphoglucomutase-1	PGM1_HUMAN	
0.000701754	-0.573756	-7.60041	sp|P36969|GPX4_HUMAN	GPX4	P36969	Phospholipid hydroperoxide glutathione peroxidase	GPX4_HUMAN	
0.0363822	-0.166256	-2.87865	sp|P37108|SRP14_HUMAN	SRP14	P37108	Signal recognition particle 14 kDa protein	SRP14_HUMAN	
0.00236596	-0.963874	-6.38485	sp|P37840|SYUA_HUMAN	SNCA	P37840	Alpha-synuclein	SYUA_HUMAN	
0.00882816	0.421234	4.4291	sp|P39023|RL3_HUMAN	RPL3	P39023	Large ribosomal subunit protein uL3	RL3_HUMAN	
0.000774194	0.620637	7.86665	sp|P40123|CAP2_HUMAN	CAP2	P40123	Adenylyl cyclase-associated protein 2	CAP2_HUMAN	
0.0198273	0.358738	3.47313	sp|P40429|RL13A_HUMAN	RPL13A	P40429	Large ribosomal subunit protein uL13	RL13A_HUMAN	
0.00484806	0.217204	5.81382	sp|P41091|IF2G_HUMAN	EIF2S3	P41091	Eukaryotic translation initiation factor 2 subunit 3	IF2G_HUMAN	
0.00388976	0.604909	6.07561	sp|P41223|BUD31_HUMAN	BUD31	P41223	Protein BUD31 homolog	BUD31_HUMAN	
0.00855462	-1.93694	-4.47849	sp|P41229|KDM5C_HUMAN	KDM5C	P41229	Lysine-specific demethylase 5C	KDM5C_HUMAN	
0.0008	0.583549	7.9907	sp|P41250|GARS_HUMAN	GARS1	P41250	Glycine--tRNA ligase	GARS_HUMAN	
0.00630971	0.122776	5.01049	sp|P41252|SYIC_HUMAN	IARS1	P41252	Isoleucine--tRNA ligase, cytoplasmic	SYIC_HUMAN	
0.00121739	0.798529	7.22308	sp|P41743|KPCI_HUMAN	PRKCI	P41743	Protein kinase C iota type	KPCI_HUMAN	
0.0339338	0.28935	2.9571	sp|P42126|ECI1_HUMAN	ECI1	P42126	Enoyl-CoA delta isomerase 1, mitochondrial	ECI1_HUMAN	
0.0198776	-0.261576	-3.46619	sp|P42167|LAP2B_HUMAN	TMPO	P42167	Lamina-associated polypeptide 2, isoforms beta/gamma	LAP2B_HUMAN	
0	0.527706	10.284	sp|P42224|STAT1_HUMAN	STAT1	P42224	Signal transducer and activator of transcription 1-alpha/beta	STAT1_HUMAN	
0.00239655	-0.700403	-6.41933	sp|P42330|AK1C3_HUMAN	AKR1C3	P42330	Aldo-keto reductase family 1 member C3	AK1C3_HUMAN	
0.0463431	0.222377	2.6721	sp|P42345|MTOR_HUMAN	MTOR	P42345	Serine/threonine-protein kinase mTOR	MTOR_HUMAN	
0.00704623	0.470466	4.82772	sp|P42677|RS27_HUMAN	RPS27	P42677	Small ribosomal subunit protein eS27	RS27_HUMAN	
0.01085	0.588971	4.0831	sp|P42766|RL35_HUMAN	RPL35	P42766	Large ribosomal subunit protein uL29	RL35_HUMAN	
0.00808639	0.628107	4.5353	sp|P42771|CDN2A_HUMAN	CDKN2A	P42771	Cyclin-dependent kinase inhibitor 2A	CDN2A_HUMAN	
0.0245316	0.295642	3.28479	sp|P42892|ECE1_HUMAN	ECE1	P42892	Endothelin-converting enzyme 1	ECE1_HUMAN	
0.0138816	0.68124	3.87316	sp|P43121|MUC18_HUMAN	MCAM	P43121	Cell surface glycoprotein MUC18	MUC18_HUMAN	

0.00546067	-0.175633	-5.22371	sp|P43243|MATR3_HUMAN	MATR3	P43243	Matrin-3	MATR3_HUMAN	
0	-1.07165	-33.9507	sp|P43490|NAMPT_HUMAN	NAMPT	P43490	Nicotinamide phosphoribosyltransferase	NAMPT_HUMAN	
0.0453754	-0.141356	-2.69302	sp|P45974|UBP5_HUMAN	USP5	P45974	Ubiquitin carboxyl-terminal hydrolase 5	UBP5_HUMAN	
0.0171268	-0.225737	-3.58783	sp|P46013|KI67_HUMAN	MKI67	P46013	Proliferation marker protein Ki-67	KI67_HUMAN	
0.00123757	0.222135	7.31052	sp|P46060|RAGP1_HUMAN	RANGAP1	P46060	Ran GTPase-activating protein 1	RAGP1_HUMAN	
0	-0.309163	-11.9605	sp|P46063|RECQ1_HUMAN	RECQL	P46063	ATP-dependent DNA helicase Q1	RECQ1_HUMAN	
0.0109234	-0.511846	-4.07627	sp|P46100|ATRX_HUMAN	ATRX	P46100	Transcriptional regulator ATRX	ATRX_HUMAN	
0.00765843	0.723888	4.61298	sp|P46776|RL27A_HUMAN	RPL27A	P46776	Large ribosomal subunit protein uL15	RL27A_HUMAN	
0.0154206	0.306376	3.72742	sp|P46777|RL5_HUMAN	RPL5	P46777	Large ribosomal subunit protein uL18	RL5_HUMAN	
0.0147994	0.491005	3.80962	sp|P46778|RL21_HUMAN	RPL21	P46778	Large ribosomal subunit protein eL21	RL21_HUMAN	
0.0109039	0.621797	4.07545	sp|P46779|RL28_HUMAN	RPL28	P46779	Large ribosomal subunit protein eL28	RL28_HUMAN	
0.0451276	0.191498	2.69762	sp|P46781|RS9_HUMAN	RPS9	P46781	Small ribosomal subunit protein uS4	RS9_HUMAN	
0.010223	0.303966	4.14768	sp|P46782|RS5_HUMAN	RPS5	P46782	Small ribosomal subunit protein uS7	RS5_HUMAN	
0.0361615	0.294753	2.88252	sp|P46783|RS10_HUMAN	RPS10	P46783	Small ribosomal subunit protein eS10	RS10_HUMAN	
0.00192233	0.846072	6.84896	sp|P46821|MAP1B_HUMAN	MAP1B	P46821	Microtubule-associated protein 1B	MAP1B_HUMAN	
0.030057	1.66654	3.06418	sp|P47224|MSS4_HUMAN	RABIF	P47224	Guanine nucleotide exchange factor MSS4	MSS4_HUMAN	
0.0396281	-1.06523	-2.8068	sp|P47712|PA24A_HUMAN	PLA2G4A	P47712	Cytosolic phospholipase A2	PA24A_HUMAN	
0.0102878	0.493424	4.13701	sp|P47897|SYQ_HUMAN	QARS1	P47897	Glutamine--tRNA ligase	SYQ_HUMAN	
0.0356453	0.097528	2.89507	sp|P48047|ATPO_HUMAN	ATP5PO	P48047	ATP synthase subunit O, mitochondrial	ATPO_HUMAN	
0.00762281	0.466327	4.58221	sp|P48059|LIMS1_HUMAN	LIMS1	P48059	LIM and senescent cell antigen-like-containing domain protein 1	LIMS1_HUMAN	
0.0108652	0.464038	4.06915	sp|P48163|MAOX_HUMAN	ME1	P48163	NADP-dependent malic enzyme	MAOX_HUMAN	
0.0468361	0.320649	2.66198	sp|P48449|LSS_HUMAN	LSS	P48449	Lanosterol synthase	LSS_HUMAN	
0.00226244	0.217234	6.59959	sp|P48507|GSH0_HUMAN	GCLM	P48507	Glutamate--cysteine ligase regulatory subunit	GSH0_HUMAN	
0	0.621871	8.36094	sp|P48509|CD151_HUMAN	CD151	P48509	CD151 antigen	CD151_HUMAN	
0.0209808	0.299518	3.42391	sp|P48739|PIPNB_HUMAN	PITPNB	P48739	Phosphatidylinositol transfer protein beta isoform	PIPNB_HUMAN	
0.0104919	-0.242446	-4.09597	sp|P49005|DPOD2_HUMAN	POLD2	P49005	DNA polymerase delta subunit 2	DPOD2_HUMAN	
0	0.429479	9.87164	sp|P49023|PAXI_HUMAN	PXN	P49023	Paxillin	PAXI_HUMAN	
0.0278171	0.476594	3.1427	sp|P49137|MAPK2_HUMAN	MAPKAPK2	P49137	MAP kinase-activated protein kinase 2	MAPK2_HUMAN	
0.0234466	0.443005	3.33251	sp|P49207|RL34_HUMAN	RPL34	P49207	Large ribosomal subunit protein eL34	RL34_HUMAN	
0	-0.707084	-8.28651	sp|P49257|LMAN1_HUMAN	LMAN1	P49257	Protein ERGIC-53	LMAN1_HUMAN	
0.00190385	0.336471	6.82962	sp|P49327|FAS_HUMAN	FASN	P49327	Fatty acid synthase	FAS_HUMAN	
0.0309	-0.367974	-3.05215	sp|P49366|DHYS_HUMAN	DHPS	P49366	Deoxyhypusine synthase	DHYS_HUMAN	
0.0161407	0.195524	3.67115	sp|P49368|TCPG_HUMAN	CCT3	P49368	T-complex protein 1 subunit gamma	TCPG_HUMAN	
0	0.741134	8.80969	sp|P49406|RM19_HUMAN	MRPL19	P49406	Large ribosomal subunit protein bL19m	RM19_HUMAN	
0.0100563	0.24531	4.19032	sp|P49411|EFTU_HUMAN	TUFM	P49411	Elongation factor Tu, mitochondrial	EFTU_HUMAN	
0.0113614	-0.256925	-4.0381	sp|P49419|AL7A1_HUMAN	ALDH7A1	P49419	Alpha-aminoadipic semialdehyde dehydrogenase	AL7A1_HUMAN	
0.022275	-0.402088	-3.37056	sp|P49458|SRP09_HUMAN	SRP9	P49458	Signal recognition particle 9 kDa protein	SRP09_HUMAN	
0.00478049	0.637895	5.76503	sp|P49588|SYAC_HUMAN	AARS1	P49588	Alanine--tRNA ligase, cytoplasmic	SYAC_HUMAN	
0.00173737	0.634896	6.96896	sp|P49589|SYCC_HUMAN	CARS1	P49589	Cysteine--tRNA ligase, cytoplasmic	SYCC_HUMAN	
0.025	0.360855	3.23612	sp|P49590|SYHM_HUMAN	HARS2	P49590	Histidine--tRNA ligase, mitochondrial	SYHM_HUMAN	
0	0.433286	8.57598	sp|P49591|SYSC_HUMAN	SARS1	P49591	Serine--tRNA ligase, cytoplasmic	SYSC_HUMAN	
0.0019604	0.55311	6.90994	sp|P49753|ACOT2_HUMAN	ACOT2	P49753	Acyl-coenzyme A thioesterase 2, mitochondrial	ACOT2_HUMAN	
0.0199557	0.438958	3.45682	sp|P49840|GSK3A_HUMAN	GSK3A	P49840	Glycogen synthase kinase-3 alpha	GSK3A_HUMAN	
0.0432369	0.310699	2.73926	sp|P49841|GSK3B_HUMAN	GSK3B	P49841	Glycogen synthase kinase-3 beta	GSK3B_HUMAN	
0.029412	0.257907	3.08736	sp|P50213|IDH3A_HUMAN	IDH3A	P50213	Isocitrate dehydrogenase [NAD] subunit alpha, mitochondrial	IDH3A_HUMAN	
0.00638384	-0.289461	-4.90014	sp|P50402|EMD_HUMAN	EMD	P50402	Emerin	EMD_HUMAN	
0.00075	0.581186	7.80396	sp|P50416|CPT1A_HUMAN	CPT1A	P50416	Carnitine O-palmitoyltransferase 1, liver isoform	CPT1A_HUMAN	
0.00473103	0.424762	5.70706	sp|P50502|F10A1_HUMAN	ST13	P50502	Hsc70-interacting protein	F10A1_HUMAN	
0.0277735	0.433777	3.14533	sp|P50895|BCAM_HUMAN	BCAM	P50895	Basal cell adhesion molecule	BCAM_HUMAN	
0.0342993	0.324437	2.92988	sp|P50914|RL14_HUMAN	RPL14	P50914	Large ribosomal subunit protein eL14	RL14_HUMAN	
0	3.23439	28.4834	sp|P50995|ANX11_HUMAN	ANXA11	P50995	Annexin A11	ANX11_HUMAN	
0.00912424	-0.337959	-4.36168	sp|P51159|RB27A_HUMAN	RAB27A	P51159	Ras-related protein Rab-27A	RB27A_HUMAN	
0.0238333	0.259101	3.31308	sp|P51397|DAP1_HUMAN	DAP	P51397	Death-associated protein 1	DAP1_HUMAN	
0.0245193	0.334191	3.28049	sp|P51553|IDH3G_HUMAN	IDH3G	P51553	Isocitrate dehydrogenase [NAD] subunit gamma, mitochondrial	IDH3G_HUMAN	
0.00823932	-0.482565	-4.5149	sp|P51572|BAP31_HUMAN	BCAP31	P51572	B-cell receptor-associated protein 31	BAP31_HUMAN	
0.0294862	-0.222793	-3.07445	sp|P51659|DHB4_HUMAN	HSD17B4	P51659	Peroxisomal multifunctional enzyme type 2	DHB4_HUMAN	
0.0199834	0.180569	3.45697	sp|P51812|KS6A3_HUMAN	RPS6KA3	P51812	Ribosomal protein S6 kinase alpha-3	KS6A3_HUMAN	
0.0277298	-0.305203	-3.14972	sp|P51858|HDGF_HUMAN	HDGF	P51858	Hepatoma-derived growth factor	HDGF_HUMAN	
0	1.02378	14.5375	sp|P52292|IMA1_HUMAN	KPNA2	P52292	Importin subunit alpha-1	IMA1_HUMAN	
0.000745342	0.403207	7.78738	sp|P52294|IMA5_HUMAN	KPNA1	P52294	Importin subunit alpha-5	IMA5_HUMAN	
0.00645431	-0.428197	-4.99478	sp|P52298|NCBP2_HUMAN	NCBP2	P52298	Nuclear cap-binding protein subunit 2	NCBP2_HUMAN	
0.0188068	0.386467	3.51021	sp|P52306|GDS1_HUMAN	RAP1GDS1	P52306	Rap1 GTPase-GDP dissociation stimulator 1	GDS1_HUMAN	
0.0208381	-1.42278	-3.41312	sp|P52434|RPAB3_HUMAN	POLR2H	P52434	DNA-directed RNA polymerases I, II, and III subunit RPABC3	RPAB3_HUMAN	
0.00702913	0.274379	4.81724	sp|P52735|VAV2_HUMAN	VAV2	P52735	Guanine nucleotide exchange factor VAV2	VAV2_HUMAN	
0.0430423	-0.293604	-2.74485	sp|P52788|SPSY_HUMAN	SMS	P52788	Spermine synthase	SPSY_HUMAN	
0	-2.21297	-9.62503	sp|P52789|HXK2_HUMAN	HK2	P52789	Hexokinase-2	HXK2_HUMAN	
0.0163957	0.245163	3.66107	sp|P53396|ACLY_HUMAN	ACLY	P53396	ATP-citrate synthase	ACLY_HUMAN	
0.0180928	-0.472373	-3.54672	sp|P53634|CATC_HUMAN	CTSC	P53634	Dipeptidyl peptidase 1	CATC_HUMAN	
0.047354	0.334982	2.65519	sp|P53701|CCHL_HUMAN	HCCS	P53701	Holocytochrome c-type synthase	CCHL_HUMAN	
0.0292415	-0.144926	-3.09728	sp|P53985|MOT1_HUMAN	SLC16A1	P53985	Monocarboxylate transporter 1	MOT1_HUMAN	
0.0141889	0.293036	3.84518	sp|P53999|TCP4_HUMAN	SUB1	P53999	Activated RNA polymerase II transcriptional coactivator p15	TCP4_HUMAN	
0.00222222	0.657515	6.55458	sp|P54577|SYYC_HUMAN	YARS1	P54577	Tyrosine--tRNA ligase, cytoplasmic	SYYC_HUMAN	
0	0.675882	11.3259	sp|P54709|AT1B3_HUMAN	ATP1B3	P54709	Sodium/potassium-transporting ATPase subunit beta-3	AT1B3_HUMAN	
0.0398679	-0.233225	-2.80403	sp|P54819|KAD2_HUMAN	AK2	P54819	Adenylate kinase 2, mitochondrial	KAD2_HUMAN	
0.0261656	-0.218037	-3.1976	sp|P54886|P5CS_HUMAN	ALDH18A1	P54886	Delta-1-pyrroline-5-carboxylate synthase	P5CS_HUMAN	
0.036	0.453519	2.88457	sp|P54920|SNAA_HUMAN	NAPA	P54920	Alpha-soluble NSF attachment protein	SNAA_HUMAN	
0.0217634	-0.90551	-3.38487	sp|P55268|LAMB2_HUMAN	LAMB2	P55268	Laminin subunit beta-2	LAMB2_HUMAN	
0.0455294	-0.420567	-2.68453	sp|P55327|TPD52_HUMAN	TPD52	P55327	Tumor protein D52	TPD52_HUMAN	
0.00119149	0.287521	7.18144	sp|P56192|SYMC_HUMAN	MARS1	P56192	Methionine--tRNA ligase, cytoplasmic	SYMC_HUMAN	
0	0.624265	10.7883	sp|P56199|ITA1_HUMAN	ITGA1	P56199	Integrin alpha-1	ITA1_HUMAN	
0.0295121	0.235506	3.08543	sp|P57088|TMM33_HUMAN	TMEM33	P57088	Transmembrane protein 33	TMM33_HUMAN	
0	1.22225	17.2521	sp|P58107|EPIPL_HUMAN	EPPK1	P58107	Epiplakin	EPIPL_HUMAN	
0.0426034	0.691752	2.74897	sp|P60002|ELOF1_HUMAN	ELOF1	P60002	Transcription elongation factor 1 homolog	ELOF1_HUMAN	
0.0057493	-1.35294	-5.19703	sp|P60174|TPIS_HUMAN	TPI1	P60174	Triosephosphate isomerase	TPIS_HUMAN	
0.02821	0.217145	3.13359	sp|P60228|EIF3E_HUMAN	EIF3E	P60228	Eukaryotic translation initiation factor 3 subunit E	EIF3E_HUMAN	
0.00121081	0.319603	7.22074	sp|P60842|IF4A1_HUMAN	EIF4A1	P60842	Eukaryotic initiation factor 4A-I	IF4A1_HUMAN	
0.0149921	0.252848	3.77704	sp|P60866|RS20_HUMAN	RPS20	P60866	Small ribosomal subunit protein uS10	RS20_HUMAN	
0.00829247	-0.240108	-4.52017	sp|P60900|PSA6_HUMAN	PSMA6	P60900	Proteasome subunit alpha type-6	PSA6_HUMAN	
0.00932535	0.32734	4.31448	sp|P60953|CDC42_HUMAN	CDC42	P60953	Cell division control protein 42 homolog	CDC42_HUMAN	
0.00793043	0.467677	4.56036	sp|P60981|DEST_HUMAN	DSTN	P60981	Destrin	DEST_HUMAN	
0.0126564	-0.473863	-3.97737	sp|P61073|CXCR4_HUMAN	CXCR4	P61073	C-X-C chemokine receptor type 4	CXCR4_HUMAN	
0.0187124	0.276371	3.51502	sp|P61081|UBC12_HUMAN	UBE2M	P61081	NEDD8-conjugating enzyme Ubc12	UBC12_HUMAN	
0.0330255	0.190499	2.97458	sp|P61247|RS3A_HUMAN	RPS3A	P61247	Small ribosomal subunit protein eS1	RS3A_HUMAN	
0.00514458	0.537926	5.39129	sp|P61254|RL26_HUMAN	RPL26	P61254	Large ribosomal subunit protein uL24	RL26_HUMAN	
0.00607046	0.566299	5.09387	sp|P61313|RL15_HUMAN	RPL15	P61313	Large ribosomal subunit protein eL15	RL15_HUMAN	
0.00558621	0.454996	5.26246	sp|P61513|RL37A_HUMAN	RPL37A	P61513	Large ribosomal subunit protein eL43	RL37A_HUMAN	
0.0193146	0.872097	3.48605	sp|P61927|RL37_HUMAN	RPL37	P61927	Large ribosomal subunit protein eL37	RL37_HUMAN	
0.0393145	-0.280193	-2.82008	sp|P61978|HNRPK_HUMAN	HNRNPK	P61978	Heterogeneous nuclear ribonucleoprotein K	HNRPK_HUMAN	
0.0101765	-0.36235	-4.15038	sp|P61981|1433G_HUMAN	YWHAG	P61981	14-3-3 protein gamma	1433G_HUMAN	
0.0354341	-1.64446	-2.90145	sp|P62070|RRAS2_HUMAN	RRAS2	P62070	Ras-related protein R-Ras2	RRAS2_HUMAN	
0.0354957	0.147003	2.90809	sp|P62081|RS7_HUMAN	RPS7	P62081	Small ribosomal subunit protein eS7	RS7_HUMAN	
0.000358621	1.71562	8.11698	sp|P62256|UBE2H_HUMAN	UBE2H	P62256	Ubiquitin-conjugating enzyme E2 H	UBE2H_HUMAN	
0.0473828	0.218338	2.65076	sp|P62263|RS14_HUMAN	RPS14	P62263	Small ribosomal subunit protein uS11	RS14_HUMAN	
0.0180666	0.410911	3.5439	sp|P62266|RS23_HUMAN	RPS23	P62266	Small ribosomal subunit protein uS12	RS23_HUMAN	
0.0440638	0.273313	2.72029	sp|P62269|RS18_HUMAN	RPS18	P62269	Small ribosomal subunit protein uS13	RS18_HUMAN	
0.0382185	0.680023	2.83804	sp|P62273|RS29_HUMAN	RPS29	P62273	Small ribosomal subunit protein uS14	RS29_HUMAN	
0.0143393	0.308909	3.82633	sp|P62280|RS11_HUMAN	RPS11	P62280	Small ribosomal subunit protein uS17	RS11_HUMAN	
0.0108694	-0.663939	-4.08545	sp|P62312|LSM6_HUMAN	LSM 6.00	P62312	U6 snRNA-associated Sm-like protein LSm6	LSM6_HUMAN	
0.0340465	-0.554583	-2.96269	sp|P62330|ARF6_HUMAN	ARF6	P62330	ADP-ribosylation factor 6	ARF6_HUMAN	
0.0406646	-0.244666	-2.78394	sp|P62333|PRS10_HUMAN	PSMC6	P62333	26S proteasome regulatory subunit 10B	PRS10_HUMAN	
0.010163	0.45912	4.17261	sp|P62424|RL7A_HUMAN	RPL7A	P62424	Large ribosomal subunit protein eL8	RL7A_HUMAN	
0.0114904	0.328683	4.02476	sp|P62633|CNBP_HUMAN	CNBP	P62633	CCHC-type zinc finger nucleic acid binding protein	CNBP_HUMAN	
0	-1.25389	-9.99598	sp|P62745|RHOB_HUMAN	RHOB	P62745	Rho-related GTP-binding protein RhoB	RHOB_HUMAN	
0.0172607	0.400613	3.58078	sp|P62829|RL23_HUMAN	RPL23	P62829	Large ribosomal subunit protein uL14	RL23_HUMAN	
0.0459863	0.326376	2.67544	sp|P62847|RS24_HUMAN	RPS24	P62847	Small ribosomal subunit protein eS24	RS24_HUMAN	
0.0251028	0.537629	3.2259	sp|P62851|RS25_HUMAN	RPS25	P62851	Small ribosomal subunit protein eS25	RS25_HUMAN	
0.00371429	0.687343	5.95028	sp|P62854|RS26_HUMAN	RPS26	P62854	Small ribosomal subunit protein eS26	RS26_HUMAN	
0.0209932	-0.183801	-3.40637	sp|P62857|RS28_HUMAN	RPS28	P62857	Small ribosomal subunit protein eS28	RS28_HUMAN	
0.046655	0.407371	2.66605	sp|P62891|RL39_HUMAN	RPL39	P62891	Large ribosomal subunit protein eL39	RL39_HUMAN	
0.0356617	0.530989	2.89785	sp|P62899|RL31_HUMAN	RPL31	P62899	Large ribosomal subunit protein eL31	RL31_HUMAN	
0.019989	0.513447	3.45636	sp|P62913|RL11_HUMAN	RPL11	P62913	Large ribosomal subunit protein uL5	RL11_HUMAN	
0.00947451	0.538782	4.29206	sp|P62917|RL8_HUMAN	RPL8	P62917	Large ribosomal subunit protein uL2	RL8_HUMAN	
0.0208665	-0.320357	-3.41336	sp|P62937|PPIA_HUMAN	PPIA	P62937	Peptidyl-prolyl cis-trans isomerase A	PPIA_HUMAN	
0.00257143	-0.576529	-6.33752	sp|P63096|GNAI1_HUMAN	GNAI1	P63096	Guanine nucleotide-binding protein G(i) subunit alpha-1	GNAI1_HUMAN	
0.0156043	0.321901	3.70291	sp|P68104|EF1A1_HUMAN	EEF1A1	P68104	Elongation factor 1-alpha 1	EF1A1_HUMAN	
0.0367577	0.215742	2.87402	sp|P68366|TBA4A_HUMAN	TUBA4A	P68366	Tubulin alpha-4A chain	TBA4A_HUMAN	
0.00769027	-0.205897	-4.59287	sp|P69849|NOMO3_HUMAN	NOMO3	P69849	BOS complex subunit NOMO3	NOMO3_HUMAN	sp|Q5JPE7|NOMO2_HUMAN
0	1.08655	10.9126	sp|P78330|SERB_HUMAN	PSPH	P78330	Phosphoserine phosphatase	SERB_HUMAN	
0.0101818	0.16707	4.17983	sp|P78344|IF4G2_HUMAN	EIF4G2	P78344	Eukaryotic translation initiation factor 4 gamma 2	IF4G2_HUMAN	

0.0251765	0.512177	3.25496	sp|P78346|RPP30_HUMAN	RPP30	P78346	Ribonuclease P protein subunit p30	RPP30_HUMAN
0.0365302	-0.630726	-2.87694	sp|P78563|RED1_HUMAN	ADARB1	P78563	Double-stranded RNA-specific editase 1	RED1_HUMAN
0.000382353	-0.551599	-8.27391	sp|P80303|NUCB2_HUMAN	NUCB2	P80303	Nucleobindin-2	NUCB2_HUMAN
0.034387	0.422559	2.92402	sp|P82650|RT22_HUMAN	MRPS22	P82650	Small ribosomal subunit protein mS22	RT22_HUMAN
0.00729577	0.511158	4.73156	sp|P82675|RT05_HUMAN	MRPS5	P82675	Small ribosomal subunit protein uS5m	RT05_HUMAN
0.00622105	0.489997	5.02474	sp|P82933|RT09_HUMAN	MRPS9	P82933	Small ribosomal subunit protein uS9m	RT09_HUMAN
0.0155666	0.46441	3.70555	sp|P83731|RL24_HUMAN	RPL24	P83731	Large ribosomal subunit protein eL24	RL24_HUMAN
0.0125086	0.496408	3.98572	sp|P83881|RL36A_HUMAN	RPL36A	P83881	Large ribosomal subunit protein eL42	RL36A_HUMAN
0.0275437	0.490532	3.16046	sp|P84095|RHOG_HUMAN	RHOG	P84095	Rho-related GTP-binding protein RhoG	RHOG_HUMAN
0.00953057	0.588903	4.29366	sp|P84098|RL19_HUMAN	RPL19	P84098	Large ribosomal subunit protein eL19	RL19_HUMAN
0.00770732	-0.568981	-4.59338	sp|P98160|PGBM_HUMAN	HSPG2	P98160	Basement membrane-specific heparan sulfate proteoglycan core protein	PGBM_HUMAN
0.00628723	0.186776	5.04613	sp|Q00341|VIGLN_HUMAN	HDLBP	Q00341	Vigilin	VIGLN_HUMAN
0.0150095	0.702382	3.77318	sp|Q00535|CDK5_HUMAN	CDK5	Q00535	Cyclin-dependent kinase 5	CDK5_HUMAN
0.0250951	0.323049	3.23808	sp|Q00577|PURA_HUMAN	PURA	Q00577	Transcriptional activator protein Pur-alpha	PURA_HUMAN
0	0.466949	9.14576	sp|Q00587|BORG5_HUMAN	CDC42EP1	Q00587	Cdc42 effector protein 1	BORG5_HUMAN
0.00516294	-0.144623	-5.55259	sp|Q00839|HNRPU_HUMAN	HNRNPU	Q00839	Heterogeneous nuclear ribonucleoprotein U	HNRPU_HUMAN
0.0281343	-0.282209	-3.13575	sp|Q01130|SRSF2_HUMAN	SRSF2	Q01130	Serine/arginine-rich splicing factor 2	SRSF2_HUMAN
0.0252087	-0.559369	-3.25539	sp|Q01433|AMPD2_HUMAN	AMPD2	Q01433	AMP deaminase 2	AMPD2_HUMAN
0.020895	-0.269904	-3.42107	sp|Q01469|FABP5_HUMAN	FABP5	Q01469	Fatty acid-binding protein 5	FABP5_HUMAN
0.0452505	0.217226	2.69441	sp|Q01518|CAP1_HUMAN	CAP1	Q01518	Adenylyl cyclase-associated protein 1	CAP1_HUMAN
0.00174619	0.499683	6.96937	sp|Q01650|LAT1_HUMAN	SLC7A5	Q01650	Large neutral amino acids transporter small subunit 1	LAT1_HUMAN
0	-0.454493	-9.80702	sp|Q01813|PFKAP_HUMAN	PFKP	Q01813	ATP-dependent 6-phosphofructokinase, platelet type	PFKAP_HUMAN
0	1.54977	9.44156	sp|Q01995|TAGL_HUMAN	TAGLN	Q01995	Transgelin	TAGL_HUMAN
0.0448523	-0.416994	-2.70348	sp|Q02388|CO7A1_HUMAN	COL7A1	Q02388	Collagen alpha-1(VII) chain	CO7A1_HUMAN
0.0129932	0.45933	3.95262	sp|Q02543|RL18A_HUMAN	RPL18A	Q02543	Large ribosomal subunit protein eL20	RL18A_HUMAN
0.00117895	-0.355518	-7.12853	sp|Q02750|MP2K1_HUMAN	MAP2K1	Q02750	Dual specificity mitogen-activated protein kinase kinase 1	MP2K1_HUMAN
0	-1.62635	-51.715	sp|Q02809|PLOD1_HUMAN	PLOD1	Q02809	Procollagen-lysine,2-oxoglutarate 5-dioxygenase 1	PLOD1_HUMAN
0.0359147	0.290299	2.88991	sp|Q02818|NUCB1_HUMAN	NUCB1	Q02818	Nucleobindin-1	NUCB1_HUMAN
0.0136027	0.394764	3.9075	sp|Q02878|RL6_HUMAN	RPL6	Q02878	Large ribosomal subunit protein eL6	RL6_HUMAN
0.0063378	-0.904679	-5.05476	sp|Q03154|ACY1_HUMAN	ACY1	Q03154	Aminoacylase-1	ACY1_HUMAN
0.00553846	-0.856058	-5.24105	sp|Q03405|UPAR_HUMAN	PLAUR	Q03405	Urokinase plasminogen activator surface receptor	UPAR_HUMAN
0.0133401	0.440178	3.92495	sp|Q03518|TAP1_HUMAN	TAP1	Q03518	Antigen peptide transporter 1	TAP1_HUMAN
0.00198995	-0.710838	-6.95777	sp|Q04206|TF65_HUMAN	RELA	Q04206	Transcription factor p65	TF65_HUMAN
0.0249711	0.440647	3.2323	sp|Q04323|UBXN1_HUMAN	UBXN1	Q04323	UBX domain-containing protein 1	UBXN1_HUMAN
0	-1.35566	-25.0753	sp|Q04446|GLGB_HUMAN	GBE1	Q04446	1,4-alpha-glucan-branching enzyme	GLGB_HUMAN
0.0105108	0.203388	4.09853	sp|Q04637|IF4G1_HUMAN	EIF4G1	Q04637	Eukaryotic translation initiation factor 4 gamma 1	IF4G1_HUMAN
0.00632086	0.941966	5.05423	sp|Q04721|NOTC2_HUMAN	NOTCH2	Q04721	Neurogenic locus notch homolog protein 2	NOTC2_HUMAN
0.0149684	-0.73629	-3.77566	sp|Q04726|TLE3_HUMAN	TLE3	Q04726	Transducin-like enhancer protein 3	TLE3_HUMAN
0.00985029	0.35956	4.24629	sp|Q04917|1433F_HUMAN	YWHAH	Q04917	14-3-3 protein eta	1433F_HUMAN
0.00236967	0.459019	6.74059	sp|Q05639|EF1A2_HUMAN	EEF1A2	Q05639	Elongation factor 1-alpha 2	EF1A2_HUMAN
0	0.766355	13.3537	sp|Q05682|CALD1_HUMAN	CALD1	Q05682	Caldesmon	CALD1_HUMAN
0.0185788	0.655674	3.52395	sp|Q06033|ITIH3_HUMAN	ITIH3	Q06033	Inter-alpha-trypsin inhibitor heavy chain H3	ITIH3_HUMAN
0.00256067	0.357835	6.31983	sp|Q06210|GFPT1_HUMAN	GFPT1	Q06210	Glutamine--fructose-6-phosphate aminotransferase [isomerizing] 1	GFPT1_HUMAN
0.0142121	0.489097	3.84532	sp|Q07020|RL18_HUMAN	RPL18	Q07020	Large ribosomal subunit protein eL18	RL18_HUMAN
0	-0.686388	-8.80542	sp|Q07065|CKAP4_HUMAN	CKAP4	Q07065	Cytoskeleton-associated protein 4	CKAP4_HUMAN
0.0149376	0.846994	3.79301	sp|Q07157|ZO1_HUMAN	TJP1	Q07157	Tight junction protein ZO-1	ZO1_HUMAN
0	-1.1398	-12.5856	sp|Q07954|LRP1_HUMAN	LRP1	Q07954	Prolow-density lipoprotein receptor-related protein 1	LRP1_HUMAN
0.006	-0.537969	-5.13585	sp|Q08499|PDE4D_HUMAN	PDE4D	Q08499	cAMP-specific 3',5'-cyclic phosphodiesterase 4D	PDE4D_HUMAN
0.0458377	0.945086	2.67796	sp|Q08AF3|SLFN5_HUMAN	SLFN5	Q08AF3	Schlafen family member 5	SLFN5_HUMAN
0.0138043	0.303766	3.89032	sp|Q09028|RBBP4_HUMAN	RBBP4	Q09028	Histone-binding protein RBBP4	RBBP4_HUMAN
0.0174487	1.32231	3.56944	sp|Q09666|AHNK_HUMAN	AHNAK	Q09666	Neuroblast differentiation-associated protein AHNAK	AHNK_HUMAN
0.0395482	-0.29295	-2.81259	sp|Q10471|GALT2_HUMAN	GALNT2	Q10471	Polypeptide N-acetylgalactosaminyltransferase 2	GALT2_HUMAN
0.0425605	0.17977	2.7482	sp|Q10567|AP1B1_HUMAN	AP1B1	Q10567	AP-1 complex subunit beta-1	AP1B1_HUMAN
0.00513231	-1.48759	-5.48691	sp|Q10589|BST2_HUMAN	BST2	Q10589	Bone marrow stromal antigen 2	BST2_HUMAN
0.0367373	0.208839	2.87471	sp|Q10713|MPPA_HUMAN	PMPCA	Q10713	Mitochondrial-processing peptidase subunit alpha	MPPA_HUMAN
0.0102606	-0.425974	-4.14969	sp|Q12797|ASPH_HUMAN	ASPH	Q12797	Aspartyl/asparaginyl beta-hydroxylase	ASPH_HUMAN
0.00447312	0.499578	5.84964	sp|Q12849|GRSF1_HUMAN	GRSF1	Q12849	G-rich sequence factor 1	GRSF1_HUMAN
0.00500662	-1.40222	-5.63379	sp|Q12882|DPYD_HUMAN	DPYD	Q12882	Dihydropyrimidine dehydrogenase [NADP(+)]	DPYD_HUMAN
0.0126348	1.75467	3.96864	sp|Q12899|TRI26_HUMAN	TRIM26	Q12899	Tripartite motif-containing protein 26	TRI26_HUMAN
0.0064712	-0.637306	-5.00884	sp|Q12907|LMAN2_HUMAN	LMAN2	Q12907	Vesicular integral-membrane protein VIP36	LMAN2_HUMAN
0.0474952	0.500074	2.65002	sp|Q12974|TP4A2_HUMAN	PTP4A2	Q12974	Protein tyrosine phosphatase type IVA 2	TP4A2_HUMAN
0.0291221	-0.189092	-3.10367	sp|Q13011|ECH1_HUMAN	ECH1	Q13011	Delta(3,5)-Delta(2,4)-dienoyl-CoA isomerase, mitochondrial	ECH1_HUMAN
0.000718563	-2.03405	-7.70803	sp|Q13043|STK4_HUMAN	STK4	Q13043	Serine/threonine-protein kinase 4	STK4_HUMAN
0.00608696	0.585743	5.09516	sp|Q13085|ACACA_HUMAN	ACACA	Q13085	Acetyl-CoA carboxylase 1	ACACA_HUMAN
0.0273423	-0.201496	-3.16801	sp|Q13148|TADBP_HUMAN	TARDBP	Q13148	TAR DNA-binding protein 43	TADBP_HUMAN
0.0166117	0.158599	3.63013	sp|Q13155|AIMP2_HUMAN	AIMP2	Q13155	Aminoacyl tRNA synthase complex-interacting multifunctional protein 2	AIMP2_HUMAN
0.0012043	-0.446659	-7.20064	sp|Q13162|PRDX4_HUMAN	PRDX4	Q13162	Peroxiredoxin-4	PRDX4_HUMAN
0.0100797	-0.283658	-4.21941	sp|Q13185|CBX3_HUMAN	CBX3	Q13185	Chromobox protein homolog 3	CBX3_HUMAN
0.00511656	-0.436185	-5.47465	sp|Q13228|SBP1_HUMAN	SELENBP1	Q13228	Methanethiol oxidase	SBP1_HUMAN
0.0424469	-0.522052	-2.75223	sp|Q13242|SRSF9_HUMAN	SRSF9	Q13242	Serine/arginine-rich splicing factor 9	SRSF9_HUMAN
0.0418845	0.111544	2.76036	sp|Q13263|TIF1B_HUMAN	TRIM28	Q13263	Transcription intermediary factor 1-beta	TIF1B_HUMAN
0.00740271	0.273552	4.63151	sp|Q13283|G3BP1_HUMAN	G3BP1	Q13283	Ras GTPase-activating protein-binding protein 1	G3BP1_HUMAN
0.0282299	0.241678	3.13529	sp|Q13347|EIF3I_HUMAN	EIF3I	Q13347	Eukaryotic translation initiation factor 3 subunit I	EIF3I_HUMAN
0	0.521698	17.3213	sp|Q13418|ILK_HUMAN	ILK	Q13418	Integrin-linked protein kinase	ILK_HUMAN
0.0174	-0.226918	-3.57393	sp|Q13435|SF3B2_HUMAN	SF3B2	Q13435	Splicing factor 3B subunit 2	SF3B2_HUMAN
0.00723855	0.613777	4.77726	sp|Q13443|ADAM9_HUMAN	ADAM9	Q13443	Disintegrin and metalloproteinase domain-containing protein 9	ADAM9_HUMAN
0.0142718	-0.269067	-3.83355	sp|Q13445|TMED1_HUMAN	TMED1	Q13445	Transmembrane emp24 domain-containing protein 1	TMED1_HUMAN
0	1.36651	21.2786	sp|Q13501|SQSTM_HUMAN	SQSTM1	Q13501	Sequestosome-1	SQSTM_HUMAN
0.0210096	0.630797	3.42422	sp|Q13505|MTX1_HUMAN	MTX1	Q13505	Metaxin-1	MTX1_HUMAN
0.0250316	1.11036	3.23757	sp|Q13555|KCC2G_HUMAN	CAMK2G	Q13555	Calcium/calmodulin-dependent protein kinase type II subunit gamma	KCC2G_HUMAN
0.0281764	-0.527467	-3.13337	sp|Q13613|MTMR1_HUMAN	MTMR1	Q13613	Myotubularin-related protein 1	MTMR1_HUMAN
0.027309	0.744862	3.16634	sp|Q13614|MTMR2_HUMAN	MTMR2	Q13614	Myotubularin-related protein 2	MTMR2_HUMAN
0.0174231	-0.141308	-3.56748	sp|Q13620|CUL4B_HUMAN	CUL4B	Q13620	Cullin-4B	CUL4B_HUMAN
0.00767568	0.776458	4.6138	sp|Q13643|FHL3_HUMAN	FHL3	Q13643	Four and a half LIM domains protein 3	FHL3_HUMAN
0.0229748	0.459908	3.34938	sp|Q13740|CD166_HUMAN	ALCAM	Q13740	CD166 antigen	CD166_HUMAN
0.0210461	0.856723	3.40389	sp|Q13795|ARFRP_HUMAN	ARFRP1	Q13795	ADP-ribosylation factor-related protein 1	ARFRP_HUMAN
0.0455357	0.428274	2.68296	sp|Q13823|NOG2_HUMAN	GNL2	Q13823	Nucleolar GTP-binding protein 2	NOG2_HUMAN
0.0490631	-0.399137	-2.6291	sp|Q13867|BLMH_HUMAN	BLMH	Q13867	Bleomycin hydrolase	BLMH_HUMAN
0.0171775	0.530603	3.59307	sp|Q13895|BYST_HUMAN	BYSL	Q13895	Bystin	BYST_HUMAN
0.0234021	0.253588	3.33951	sp|Q14019|COTL1_HUMAN	COTL1	Q14019	Coactosin-like protein	COTL1_HUMAN
0.00445714	1.15563	5.84855	sp|Q14116|IL18_HUMAN	IL18	Q14116	Interleukin-18	IL18_HUMAN
0.0387435	0.589486	2.82752	sp|Q14137|BOP1_HUMAN	BOP 1.00	Q14137	Ribosome biogenesis protein BOP1	BOP1_HUMAN
0.00649871	-0.92531	-4.92156	sp|Q14145|KEAP1_HUMAN	KEAP1	Q14145	Kelch-like ECH-associated protein 1	KEAP1_HUMAN
0.0108889	-0.252117	-4.08582	sp|Q14157|UBP2L_HUMAN	UBAP2L	Q14157	Ubiquitin-associated protein 2-like	UBP2L_HUMAN
0.00830638	-0.552471	-4.50873	sp|Q14165|MLEC_HUMAN	MLEC	Q14165	Malectin	MLEC_HUMAN
0.00213333	-0.223905	-6.80569	sp|Q14166|TTL12_HUMAN	TTLL12	Q14166	Tubulin--tyrosine ligase-like protein 12	TTL12_HUMAN
0	0.954701	9.52104	sp|Q14192|FHL2_HUMAN	FHL2	Q14192	Four and a half LIM domains protein 2	FHL2_HUMAN
0.02511	0.282177	3.22271	sp|Q14203|DCTN1_HUMAN	DCTN1	Q14203	Dynactin subunit 1	DCTN1_HUMAN
0.0267961	0.113704	3.18354	sp|Q14204|DYHC1_HUMAN	DYNC1H1	Q14204	Cytoplasmic dynein 1 heavy chain 1	DYHC1_HUMAN
0	0.929367	8.72458	sp|Q14244|MAP7_HUMAN	MAP7	Q14244	Ensconsin	MAP7_HUMAN
0.0250025	0.226933	3.23248	sp|Q14258|TRI25_HUMAN	TRIM25	Q14258	E3 ubiquitin/ISG15 ligase TRIM25	TRI25_HUMAN
0.0250905	0.237766	3.22777	sp|Q14315|FLNC_HUMAN	FLNC	Q14315	Filamin-C	FLNC_HUMAN
0.0493232	0.374359	2.61918	sp|Q14320|FA50A_HUMAN	FAM50A	Q14320	Protein FAM50A	FA50A_HUMAN
0	-0.665258	-9.27552	sp|Q14376|GALE_HUMAN	GALE	Q14376	UDP-glucose 4-epimerase	GALE_HUMAN
0.00679506	-0.653776	-4.85034	sp|Q14554|PDIA5_HUMAN	PDIA5	Q14554	Protein disulfide-isomerase A5	PDIA5_HUMAN
0.0094191	0.790316	4.28468	sp|Q14562|DHX8_HUMAN	DHX8	Q14562	ATP-dependent RNA helicase DHX8	DHX8_HUMAN
0.0387841	-0.568304	-2.82862	sp|Q14643|ITPR1_HUMAN	ITPR1	Q14643	Inositol 1,4,5-trisphosphate receptor type 1	ITPR1_HUMAN
0	0.491654	18.0216	sp|Q14651|PLSI_HUMAN	PLS1	Q14651	Plastin-1	PLSI_HUMAN
0.00646547	-0.248247	-4.91716	sp|Q14683|SMC1A_HUMAN	SMC1A	Q14683	Structural maintenance of chromosomes protein 1A	SMC1A_HUMAN
0.00993499	-0.376278	-4.23448	sp|Q14696|MESD_HUMAN	MESD	Q14696	LRP chaperone MESD	MESD_HUMAN
0.00504	-0.39662	-5.65297	sp|Q14697|GANAB_HUMAN	GANAB	Q14697	Neutral alpha-glucosidase AB	GANAB_HUMAN
0	-1.12759	-10.4536	sp|Q14764|MVP_HUMAN	MVP	Q14764	Major vault protein	MVP_HUMAN
0.0174743	0.132314	3.57007	sp|Q14974|IMB1_HUMAN	KPNB1	Q14974	Importin subunit beta-1	IMB1_HUMAN
0.000779221	-0.362704	-7.86906	sp|Q14980|NUMA1_HUMAN	NUMA1	Q14980	Nuclear mitotic apparatus protein 1	NUMA1_HUMAN
0.0281681	0.463101	3.13643	sp|Q14997|PSME4_HUMAN	PSME4	Q14997	Proteasome activator complex subunit 4	PSME4_HUMAN
0.0162443	0.470671	3.66874	sp|Q15003|CND2_HUMAN	NCAPH	Q15003	Condensin complex subunit 2	CND2_HUMAN
0.00879175	-0.384063	-4.4107	sp|Q15007|FL2D_HUMAN	WTAP	Q15007	Pre-mRNA-splicing regulator WTAP	FL2D_HUMAN
0.031565	-0.554519	-3.01822	sp|Q15014|MO4L2_HUMAN	MORF4L2	Q15014	Mortality factor 4-like protein 2	MO4L2_HUMAN
0.000681818	-0.160067	-7.52794	sp|Q15019|SEPT2_HUMAN	SEPTIN2	Q15019	Septin-2	SEPT2_HUMAN
0.00119786	0.540303	7.19476	sp|Q15021|CND1_HUMAN	NCAPD2	Q15021	Condensin complex subunit 1	CND1_HUMAN
0.0074533	0.638532	4.64549	sp|Q15031|SYLM_HUMAN	LARS2	Q15031	Leucine--tRNA ligase, mitochondrial	SYLM_HUMAN
0.0312531	0.556831	3.0285	sp|Q15041|AR6P1_HUMAN	ARL6IP1	Q15041	ADP-ribosylation factor-like protein 6-interacting protein 1	AR6P1_HUMAN
0.00194118	0.330258	6.87713	sp|Q15042|RB3GP_HUMAN	RAB3GAP1	Q15042	Rab3 GTPase-activating protein catalytic subunit	RB3GP_HUMAN
0	0.818999	22.0954	sp|Q15043|S39AE_HUMAN	SLC39A14	Q15043	Metal cation symporter ZIP14	S39AE_HUMAN
0.0381853	-0.338182	-2.83948	sp|Q15054|DPOD3_HUMAN	POLD3	Q15054	DNA polymerase delta subunit 3	DPOD3_HUMAN
0.00914516	-0.657197	-4.3364	sp|Q15061|WDR43_HUMAN	WDR43	Q15061	WD repeat-containing protein 43	WDR43_HUMAN
0.0052125	-0.650768	-5.50785	sp|Q15084|PDIA6_HUMAN	PDIA6	Q15084	Protein disulfide-isomerase A6	PDIA6_HUMAN
0.00648205	-0.381022	-4.91861	sp|Q15113|PCOC1_HUMAN	PCOLCE	Q15113	Procollagen C-endopeptidase enhancer 1	PCOC1_HUMAN
0.000740741	-2.78885	-7.78316	sp|Q15120|PDK3_HUMAN	PDK3	Q15120	[Pyruvate dehydrogenase (acetyl-transferring)] kinase isozyme 3, mitochondrial	PDK3_HUMAN

<table>
<tr><td>0</td><td>0.907706</td><td>10.3247</td><td>sp|Q15149|PLEC_HUMAN</td><td>PLEC</td><td>Q15149</td><td>Plectin</td><td>PLEC_HUMAN</td><td></td></tr>
<tr><td>0.0205179</td><td>-0.40169</td><td>-3.43306</td><td>sp|Q15165|PON2_HUMAN</td><td>PON2</td><td>Q15165</td><td>Serum paraoxonase/arylesterase 2</td><td>PON2_HUMAN</td><td></td></tr>
<tr><td>0.0302551</td><td>-0.111142</td><td>-3.06039</td><td>sp|Q15233|NONO_HUMAN</td><td>NONO</td><td>Q15233</td><td>Non-POU domain-containing octamer-binding protein</td><td>NONO_HUMAN</td><td></td></tr>
<tr><td>0.0017551</td><td>0.909428</td><td>6.97409</td><td>sp|Q15262|PTPRK_HUMAN</td><td>PTPRK</td><td>Q15262</td><td>Receptor-type tyrosine-protein phosphatase kappa</td><td>PTPRK_HUMAN</td><td></td></tr>
<tr><td>0.0488995</td><td>-0.278644</td><td>-2.6302</td><td>sp|Q15365|PCBP1_HUMAN</td><td>PCBP1</td><td>Q15365</td><td>Poly(rC)-binding protein 1</td><td>PCBP1_HUMAN</td><td></td></tr>
<tr><td>0.00733019</td><td>0.450603</td><td>4.73786</td><td>sp|Q15404|RSU1_HUMAN</td><td>RSU1</td><td>Q15404</td><td>Ras suppressor protein 1</td><td>RSU1_HUMAN</td><td></td></tr>
<tr><td>0.00532551</td><td>0.57422</td><td>5.32651</td><td>sp|Q15631|TSN_HUMAN</td><td>TSN</td><td>Q15631</td><td>Translin</td><td>TSN_HUMAN</td><td></td></tr>
<tr><td>0.0100606</td><td>0.791455</td><td>4.2182</td><td>sp|Q15646|OASL_HUMAN</td><td>OASL</td><td>Q15646</td><td>2'-5'-oligoadenylate synthase-like protein</td><td>OASL_HUMAN</td><td></td></tr>
<tr><td>0.0191899</td><td>0.455816</td><td>3.49018</td><td>sp|Q15654|TRIP6_HUMAN</td><td>TRIP6</td><td>Q15654</td><td>Thyroid receptor-interacting protein 6</td><td>TRIP6_HUMAN</td><td></td></tr>
<tr><td>0</td><td>1.12356</td><td>9.82575</td><td>sp|Q15758|AAAT_HUMAN</td><td>SLC1A5</td><td>Q15758</td><td>Neutral amino acid transporter B(0)</td><td>AAAT_HUMAN</td><td></td></tr>
<tr><td>0.00847257</td><td>-1.46577</td><td>-4.4861</td><td>sp|Q15796|SMAD2_HUMAN</td><td>SMAD2</td><td>Q15796</td><td>Mothers against decapentaplegic homolog 2</td><td>SMAD2_HUMAN</td><td></td></tr>
<tr><td>0.0393702</td><td>0.148632</td><td>2.81739</td><td>sp|Q15907|RB11B_HUMAN</td><td>RAB11B</td><td>Q15907</td><td>Ras-related protein Rab-11B</td><td>RB11B_HUMAN</td><td></td></tr>
<tr><td>0.00118519</td><td>0.560327</td><td>7.16751</td><td>sp|Q15942|ZYX_HUMAN</td><td>ZYX</td><td>Q15942</td><td>Zyxin</td><td>ZYX_HUMAN</td><td></td></tr>
<tr><td>0</td><td>1.20801</td><td>12.5463</td><td>sp|Q16270|IBP7_HUMAN</td><td>IGFBP7</td><td>Q16270</td><td>Insulin-like growth factor-binding protein 7</td><td>IBP7_HUMAN</td><td></td></tr>
<tr><td>0.00906883</td><td>0.633653</td><td>4.34813</td><td>sp|Q16512|PKN1_HUMAN</td><td>PKN1</td><td>Q16512</td><td>Serine/threonine-protein kinase N1</td><td>PKN1_HUMAN</td><td></td></tr>
<tr><td>0.0038</td><td>-0.669519</td><td>-6.01132</td><td>sp|Q16527|CSRP2_HUMAN</td><td>CSRP2</td><td>Q16527</td><td>Cysteine and glycine-rich protein 2</td><td>CSRP2_HUMAN</td><td></td></tr>
<tr><td>0.0155323</td><td>-0.242967</td><td>-3.69759</td><td>sp|Q16531|DDB1_HUMAN</td><td>DDB1</td><td>Q16531</td><td>DNA damage-binding protein 1</td><td>DDB1_HUMAN</td><td></td></tr>
<tr><td>0.00453818</td><td>0.282284</td><td>5.87441</td><td>sp|Q16539|MK14_HUMAN</td><td>MAPK14</td><td>Q16539</td><td>Mitogen-activated protein kinase 14</td><td>MK14_HUMAN</td><td></td></tr>
<tr><td>0.0156687</td><td>0.208699</td><td>3.69266</td><td>sp|Q16658|FSCN1_HUMAN</td><td>FSCN1</td><td>Q16658</td><td>Fascin</td><td>FSCN1_HUMAN</td><td></td></tr>
<tr><td>0.0355102</td><td>-0.22336</td><td>-2.90344</td><td>sp|Q16698|DECR_HUMAN</td><td>DECR1</td><td>Q16698</td><td>2,4-dienoyl-CoA reductase [(3E)-enoyl-CoA-producing], mitochondrial</td><td>DECR_HUMAN</td><td></td></tr>
<tr><td>0.00825696</td><td>0.631495</td><td>4.51494</td><td>sp|Q16706|MA2A1_HUMAN</td><td>MAN2A1</td><td>Q16706</td><td>Alpha-mannosidase 2</td><td>MA2A1_HUMAN</td><td></td></tr>
<tr><td>0.00603774</td><td>-0.589549</td><td>-5.07748</td><td>sp|Q16719|KYNU_HUMAN</td><td>KYNU</td><td>Q16719</td><td>Kynureninase</td><td>KYNU_HUMAN</td><td></td></tr>
<tr><td>0.0343878</td><td>0.333813</td><td>2.93445</td><td>sp|Q16740|CLPP_HUMAN</td><td>CLPP</td><td>Q16740</td><td>ATP-dependent Clp protease proteolytic subunit, mitochondrial</td><td>CLPP_HUMAN</td><td></td></tr>
<tr><td>0.000731707</td><td>-0.619651</td><td>-7.72623</td><td>sp|Q16774|KGUA_HUMAN</td><td>GUK1</td><td>Q16774</td><td>Guanylate kinase</td><td>KGUA_HUMAN</td><td></td></tr>
<tr><td>0.00497368</td><td>-2.5081</td><td>-5.6263</td><td>sp|Q16790|CAH9_HUMAN</td><td>CA9</td><td>Q16790</td><td>Carbonic anhydrase 9</td><td>CAH9_HUMAN</td><td></td></tr>
<tr><td>0</td><td>1.29273</td><td>12.1903</td><td>sp|Q16822|PCKGM_HUMAN</td><td>PCK2</td><td>Q16822</td><td>Phosphoenolpyruvate carboxykinase [GTP], mitochondrial</td><td>PCKGM_HUMAN</td><td></td></tr>
<tr><td>0.0250633</td><td>-0.277496</td><td>-3.23787</td><td>sp|Q16851|UGPA_HUMAN</td><td>UGP2</td><td>Q16851</td><td>UTP--glucose-1-phosphate uridylyltransferase</td><td>UGPA_HUMAN</td><td></td></tr>
<tr><td>0.0143415</td><td>-0.351333</td><td>-3.84347</td><td>sp|Q1KMD3|HNRL2_HUMAN</td><td>HNRNPUL2</td><td>Q1KMD3</td><td>Heterogeneous nuclear ribonucleoprotein U-like protein 2</td><td>HNRL2_HUMAN</td><td></td></tr>
<tr><td>0.00433577</td><td>0.357122</td><td>5.88437</td><td>sp|Q27J81|INF2_HUMAN</td><td>INF2</td><td>Q27J81</td><td>Inverted formin-2</td><td>INF2_HUMAN</td><td></td></tr>
<tr><td>0.00956832</td><td>-0.583776</td><td>-4.30079</td><td>sp|Q2TAL8|QRIC1_HUMAN</td><td>QRICH1</td><td>Q2TAL8</td><td>Transcriptional regulator QRICH1</td><td>QRIC1_HUMAN</td><td></td></tr>
<tr><td>0</td><td>1.11502</td><td>9.5594</td><td>sp|Q2TB90|HKDC1_HUMAN</td><td>HKDC1</td><td>Q2TB90</td><td>Hexokinase HKDC1</td><td>HKDC1_HUMAN</td><td></td></tr>
<tr><td>0</td><td>-0.41984</td><td>-18.924</td><td>sp|Q32P28|P3H1_HUMAN</td><td>P3H1</td><td>Q32P28</td><td>Prolyl 3-hydroxylase 1</td><td>P3H1_HUMAN</td><td></td></tr>
<tr><td>0.00487372</td><td>0.766517</td><td>5.68738</td><td>sp|Q3KQU3|MA7D1_HUMAN</td><td>MAP7D1</td><td>Q3KQU3</td><td>MAP7 domain-containing protein 1</td><td>MA7D1_HUMAN</td><td></td></tr>
<tr><td>0.0238644</td><td>0.608866</td><td>3.31309</td><td>sp|Q3KQV9|UAP1L_HUMAN</td><td>UAP1L1</td><td>Q3KQV9</td><td>UDP-N-acetylhexosamine pyrophosphorylase-like protein 1</td><td>UAP1L_HUMAN</td><td></td></tr>
<tr><td>0</td><td>-0.397799</td><td>-8.7727</td><td>sp|Q3LXA3|TKFC_HUMAN</td><td>TKFC</td><td>Q3LXA3</td><td>Triokinase/FMN cyclase</td><td>TKFC_HUMAN</td><td></td></tr>
<tr><td>0.000794702</td><td>0.429347</td><td>7.94619</td><td>sp|Q3ZCQ8|TIM50_HUMAN</td><td>TIMM50</td><td>Q3ZCQ8</td><td>Mitochondrial import inner membrane translocase subunit TIM50</td><td>TIM50_HUMAN</td><td></td></tr>
<tr><td>0.0096124</td><td>0.356797</td><td>4.27663</td><td>sp|Q4KMQ2|ANO6_HUMAN</td><td>ANO6</td><td>Q4KMQ2</td><td>Anoctamin-6</td><td>ANO6_HUMAN</td><td></td></tr>
<tr><td>0.0148232</td><td>0.272834</td><td>3.81322</td><td>sp|Q53EL6|PDCD4_HUMAN</td><td>PDCD4</td><td>Q53EL6</td><td>Programmed cell death protein 4</td><td>PDCD4_HUMAN</td><td></td></tr>
<tr><td>0.0082537</td><td>0.250916</td><td>4.50009</td><td>sp|Q53EP0|FND3B_HUMAN</td><td>FNDC3B</td><td>Q53EP0</td><td>Fibronectin type III domain-containing protein 3B</td><td>FND3B_HUMAN</td><td></td></tr>
<tr><td>0.0114126</td><td>0.444898</td><td>4.0277</td><td>sp|Q53H96|P5CR3_HUMAN</td><td>PYCR3</td><td>Q53H96</td><td>Pyrroline-5-carboxylate reductase 3</td><td>P5CR3_HUMAN</td><td></td></tr>
<tr><td>0</td><td>1.34915</td><td>8.35023</td><td>sp|Q56VL3|OCAD2_HUMAN</td><td>OCIAD2</td><td>Q56VL3</td><td>OCIA domain-containing protein 2</td><td>OCAD2_HUMAN</td><td></td></tr>
<tr><td>0.0102007</td><td>0.336127</td><td>4.18162</td><td>sp|Q5F1R6|DJC21_HUMAN</td><td>DNAJC21</td><td>Q5F1R6</td><td>DnaJ homolog subfamily C member 21</td><td>DJC21_HUMAN</td><td></td></tr>
<tr><td>0.0227872</td><td>-0.33754</td><td>-3.35696</td><td>sp|Q5HYI8|RABL3_HUMAN</td><td>RABL3</td><td>Q5HYI8</td><td>Rab-like protein 3</td><td>RABL3_HUMAN</td><td></td></tr>
<tr><td>0.0235066</td><td>-0.312299</td><td>-3.32831</td><td>sp|Q5JTV8|TOIP1_HUMAN</td><td>TOR1AIP1</td><td>Q5JTV8</td><td>Torsin-1A-interacting protein 1</td><td>TOIP1_HUMAN</td><td></td></tr>
<tr><td>0.0105297</td><td>-1.26777</td><td>-4.0997</td><td>sp|Q5MNZ6|WIPI3_HUMAN</td><td>WDR45B</td><td>Q5MNZ6</td><td>WD repeat domain phosphoinositide-interacting protein 3</td><td>WIPI3_HUMAN</td><td></td></tr>
<tr><td>0.0113415</td><td>-0.79038</td><td>-4.03552</td><td>sp|Q5R3I4|TTC38_HUMAN</td><td>TTC38</td><td>Q5R3I4</td><td>Tetratricopeptide repeat protein 38</td><td>TTC38_HUMAN</td><td></td></tr>
<tr><td>0.00775893</td><td>0.530312</td><td>4.60509</td><td>sp|Q5SW79|CE170_HUMAN</td><td>CEP170</td><td>Q5SW79</td><td>Centrosomal protein of 170 kDa</td><td>CE170_HUMAN</td><td></td></tr>
<tr><td>0.0295201</td><td>0.340815</td><td>3.07476</td><td>sp|Q5T4S7|UBR4_HUMAN</td><td>UBR4</td><td>Q5T4S7</td><td>E3 ubiquitin-protein ligase UBR4</td><td>UBR4_HUMAN</td><td></td></tr>
<tr><td>0.0413496</td><td>0.34771</td><td>2.77345</td><td>sp|Q5VW36|FOCAD_HUMAN</td><td>FOCAD</td><td>Q5VW36</td><td>Focadhesin</td><td>FOCAD_HUMAN</td><td></td></tr>
<tr><td>0.0151487</td><td>0.515441</td><td>3.75217</td><td>sp|Q5VZK9|CARL1_HUMAN</td><td>CARMIL1</td><td>Q5VZK9</td><td>F-actin-uncapping protein LRRC16A</td><td>CARL1_HUMAN</td><td></td></tr>
<tr><td>0.0455741</td><td>-0.426193</td><td>-2.68611</td><td>sp|Q658Y4|F91A1_HUMAN</td><td>FAM91A1</td><td>Q658Y4</td><td>Protein FAM91A1</td><td>F91A1_HUMAN</td><td></td></tr>
<tr><td>0</td><td>0.808354</td><td>13.8419</td><td>sp|Q68CZ2|TENS3_HUMAN</td><td>TNS3</td><td>Q68CZ2</td><td>Tensin-3</td><td>TENS3_HUMAN</td><td></td></tr>
<tr><td>0.00865409</td><td>0.501473</td><td>4.4717</td><td>sp|Q68EM7|RHG17_HUMAN</td><td>ARHGAP17</td><td>Q68EM7</td><td>Rho GTPase-activating protein 17</td><td>RHG17_HUMAN</td><td></td></tr>
<tr><td>0.0245508</td><td>1.71651</td><td>3.28084</td><td>sp|Q6EMK4|VASN_HUMAN</td><td>VASN</td><td>Q6EMK4</td><td>Vasorin</td><td>VASN_HUMAN</td><td></td></tr>
<tr><td>0.019244</td><td>0.192176</td><td>3.49158</td><td>sp|Q6IBS0|TWF2_HUMAN</td><td>TWF2</td><td>Q6IBS0</td><td>Twinfilin-2</td><td>TWF2_HUMAN</td><td></td></tr>
<tr><td>0.0395463</td><td>-0.240322</td><td>-2.81661</td><td>sp|Q6KC79|NIPBL_HUMAN</td><td>NIPBL</td><td>Q6KC79</td><td>Nipped-B-like protein</td><td>NIPBL_HUMAN</td><td></td></tr>
<tr><td>0.0435872</td><td>0.362239</td><td>2.73192</td><td>sp|Q6NXE6|ARMC6_HUMAN</td><td>ARMC6</td><td>Q6NXE6</td><td>Armadillo repeat-containing protein 6</td><td>ARMC6_HUMAN</td><td></td></tr>
<tr><td>0.028279</td><td>0.576158</td><td>3.12686</td><td>sp|Q6P1N0|C2D1A_HUMAN</td><td>CC2D1A</td><td>Q6P1N0</td><td>Coiled-coil and C2 domain-containing protein 1A</td><td>C2D1A_HUMAN</td><td></td></tr>
<tr><td>0.0282143</td><td>0.296392</td><td>3.133</td><td>sp|Q6P996|PDXD1_HUMAN</td><td>PDXDC1</td><td>Q6P996</td><td>Pyridoxal-dependent decarboxylase domain-containing protein 1</td><td>PDXD1_HUMAN</td><td></td></tr>
<tr><td>0.0486603</td><td>-0.133651</td><td>-2.63279</td><td>sp|Q6PD62|CTR9_HUMAN</td><td>CTR9</td><td>Q6PD62</td><td>RNA polymerase-associated protein CTR9 homolog</td><td>CTR9_HUMAN</td><td></td></tr>
<tr><td>0.0094375</td><td>0.677069</td><td>4.28714</td><td>sp|Q6PI48|SYDM_HUMAN</td><td>DARS2</td><td>Q6PI48</td><td>Aspartate--tRNA ligase, mitochondrial</td><td>SYDM_HUMAN</td><td></td></tr>
<tr><td>0.00522324</td><td>1.34371</td><td>5.42454</td><td>sp|Q6PJG6|BRAT1_HUMAN</td><td>BRAT1</td><td>Q6PJG6</td><td>BRCA1-associated ATM activator 1</td><td>BRAT1_HUMAN</td><td></td></tr>
<tr><td>0.00520354</td><td>0.72917</td><td>5.35674</td><td>sp|Q6PK04|CC137_HUMAN</td><td>CCDC137</td><td>Q6PK04</td><td>Coiled-coil domain-containing protein 137</td><td>CC137_HUMAN</td><td></td></tr>
<tr><td>0.0218792</td><td>0.234432</td><td>3.38235</td><td>sp|Q6UB35|C1TM_HUMAN</td><td>MTHFD1L</td><td>Q6UB35</td><td>Monofunctional C1-tetrahydrofolate synthase, mitochondrial</td><td>C1TM_HUMAN</td><td></td></tr>
<tr><td>0.0481575</td><td>0.44523</td><td>2.64006</td><td>sp|Q6WCQ1|MPRIP_HUMAN</td><td>MPRIP</td><td>Q6WCQ1</td><td>Myosin phosphatase Rho-interacting protein</td><td>MPRIP_HUMAN</td><td></td></tr>
<tr><td>0.018546</td><td>1.82891</td><td>3.52512</td><td>sp|Q6Y1H2|HACD2_HUMAN</td><td>HACD2</td><td>Q6Y1H2</td><td>Very-long-chain (3R)-3-hydroxyacyl-CoA dehydratase 2</td><td>HACD2_HUMAN</td><td></td></tr>
<tr><td>0.0465992</td><td>-0.437025</td><td>-2.66656</td><td>sp|Q6ZN55|ZN574_HUMAN</td><td>ZNF574</td><td>Q6ZN55</td><td>Zinc finger protein 574</td><td>ZN574_HUMAN</td><td></td></tr>
<tr><td>0.00225225</td><td>1.42511</td><td>6.59319</td><td>sp|Q6ZNB6|NFXL1_HUMAN</td><td>NFXL1</td><td>Q6ZNB6</td><td>NF-X1-type zinc finger protein NFXL1</td><td>NFXL1_HUMAN</td><td></td></tr>
<tr><td>0.0338834</td><td>1.11174</td><td>2.9517</td><td>sp|Q6ZNJ1|NBEL2_HUMAN</td><td>NBEAL2</td><td>Q6ZNJ1</td><td>Neurobeachin-like protein 2</td><td>NBEL2_HUMAN</td><td></td></tr>
<tr><td>0.0312809</td><td>0.541915</td><td>3.02372</td><td>sp|Q70J99|UN13D_HUMAN</td><td>UNC13D</td><td>Q70J99</td><td>Protein unc-13 homolog D</td><td>UN13D_HUMAN</td><td></td></tr>
<tr><td>0.02868</td><td>-0.421918</td><td>-3.11891</td><td>sp|Q70UQ0|IKIP_HUMAN</td><td>IKBIP</td><td>Q70UQ0</td><td>Inhibitor of nuclear factor kappa-B kinase-interacting protein</td><td>IKIP_HUMAN</td><td></td></tr>
<tr><td>0.0487939</td><td>0.136372</td><td>2.63216</td><td>sp|Q7KZF4|SND1_HUMAN</td><td>SND1</td><td>Q7KZF4</td><td>Staphylococcal nuclease domain-containing protein 1</td><td>SND1_HUMAN</td><td></td></tr>
<tr><td>0</td><td>-1.12694</td><td>-11.0835</td><td>sp|Q7L1Q6|5MP2_HUMAN</td><td>BZW1</td><td>Q7L1Q6</td><td>eIF5-mimic protein 2</td><td>5MP2_HUMAN</td><td></td></tr>
<tr><td>0.00221239</td><td>0.629725</td><td>6.54915</td><td>sp|Q7L2E3|DHX30_HUMAN</td><td>DHX30</td><td>Q7L2E3</td><td>ATP-dependent RNA helicase DHX30</td><td>DHX30_HUMAN</td><td></td></tr>
<tr><td>0.0167119</td><td>-2.06447</td><td>-3.63785</td><td>sp|Q7L4I2|RSRC2_HUMAN</td><td>RSRC2</td><td>Q7L4I2</td><td>Arginine/serine-rich coiled-coil protein 2</td><td>RSRC2_HUMAN</td><td></td></tr>
<tr><td>0.0251837</td><td>0.191355</td><td>3.25149</td><td>sp|Q7L576|CYFP1_HUMAN</td><td>CYFIP1</td><td>Q7L576</td><td>Cytoplasmic FMR1-interacting protein 1</td><td>CYFP1_HUMAN</td><td></td></tr>
<tr><td>0.0465355</td><td>0.750801</td><td>2.6688</td><td>sp|Q7L5N7|PCAT2_HUMAN</td><td>LPCAT2</td><td>Q7L5N7</td><td>Lysophosphatidylcholine acyltransferase 2</td><td>PCAT2_HUMAN</td><td></td></tr>
<tr><td>0.0413075</td><td>-0.386246</td><td>-2.77302</td><td>sp|Q7LBC6|KDM3B_HUMAN</td><td>KDM3B</td><td>Q7LBC6</td><td>Lysine-specific demethylase 3B</td><td>KDM3B_HUMAN</td><td></td></tr>
<tr><td>0.0155883</td><td>-0.303527</td><td>-3.6935</td><td>sp|Q7LBR1|CHM1B_HUMAN</td><td>CHMP1B</td><td>Q7LBR1</td><td>Charged multivesicular body protein 1b</td><td>CHM1B_HUMAN</td><td></td></tr>
<tr><td>0.0395072</td><td>0.245907</td><td>2.81198</td><td>sp|Q7Z2W4|ZCCHV_HUMAN</td><td>ZC3HAV1</td><td>Q7Z2W4</td><td>Zinc finger CCCH-type antiviral protein 1</td><td>ZCCHV_HUMAN</td><td></td></tr>
<tr><td>0.0267892</td><td>-0.592817</td><td>-3.18299</td><td>sp|Q7Z3B4|NUP54_HUMAN</td><td>NUP54</td><td>Q7Z3B4</td><td>Nucleoporin p54</td><td>NUP54_HUMAN</td><td></td></tr>
<tr><td>0.0101442</td><td>0.267029</td><td>4.16219</td><td>sp|Q7Z417|NUFP2_HUMAN</td><td>NUFIP2</td><td>Q7Z417</td><td>FMR1-interacting protein NUFIP2</td><td>NUFP2_HUMAN</td><td></td></tr>
<tr><td>0.0074195</td><td>0.343142</td><td>4.63744</td><td>sp|Q7Z460|CLAP1_HUMAN</td><td>CLASP1</td><td>Q7Z460</td><td>CLIP-associating protein 1</td><td>CLAP1_HUMAN</td><td></td></tr>
<tr><td>0.00555429</td><td>-0.312572</td><td>-5.25699</td><td>sp|Q7Z4H8|PLGT3_HUMAN</td><td>POGLUT3</td><td>Q7Z4H8</td><td>Protein O-glucosyltransferase 3</td><td>PLGT3_HUMAN</td><td></td></tr>
<tr><td>0.0176769</td><td>-1.66138</td><td>-3.55925</td><td>sp|Q7Z5H4|VN1R5_HUMAN</td><td>VN1R5</td><td>Q7Z5H4</td><td>Vomeronasal type-1 receptor 5</td><td>VN1R5_HUMAN</td><td></td></tr>
<tr><td>0.0140361</td><td>-0.607635</td><td>-3.85875</td><td>sp|Q7Z5L9|I2BP2_HUMAN</td><td>IRF2BP2</td><td>Q7Z5L9</td><td>Interferon regulatory factor 2-binding protein 2</td><td>I2BP2_HUMAN</td><td></td></tr>
<tr><td>0.00234599</td><td>-0.641417</td><td>-6.34416</td><td>sp|Q7Z7H5|TMED4_HUMAN</td><td>TMED4</td><td>Q7Z7H5</td><td>Transmembrane emp24 domain-containing protein 4</td><td>TMED4_HUMAN</td><td></td></tr>
<tr><td>0.00658354</td><td>-0.347707</td><td>-4.8796</td><td>sp|Q7Z7K6|CENPV_HUMAN</td><td>CENPV</td><td>Q7Z7K6</td><td>Centromere protein V</td><td>CENPV_HUMAN</td><td></td></tr>
<tr><td>0.0291288</td><td>0.58246</td><td>3.10206</td><td>sp|Q86TU7|SETD3_HUMAN</td><td>SETD3</td><td>Q86TU7</td><td>Actin-histidine N-methyltransferase</td><td>SETD3_HUMAN</td><td></td></tr>
<tr><td>0.0437725</td><td>-0.275342</td><td>-2.72263</td><td>sp|Q86U42|PABP2_HUMAN</td><td>PABPN1</td><td>Q86U42</td><td>Polyadenylate-binding protein 2</td><td>PABP2_HUMAN</td><td></td></tr>
<tr><td>0</td><td>-0.406959</td><td>-11.1553</td><td>sp|Q86UE4|LYRIC_HUMAN</td><td>MTDH</td><td>Q86UE4</td><td>Protein LYRIC</td><td>LYRIC_HUMAN</td><td></td></tr>
<tr><td>0.00560231</td><td>0.576793</td><td>5.2683</td><td>sp|Q86UY6|NAA40_HUMAN</td><td>NAA40</td><td>Q86UY6</td><td>N-alpha-acetyltransferase 40</td><td>NAA40_HUMAN</td><td></td></tr>
<tr><td>0.00733023</td><td>0.326895</td><td>4.70602</td><td>sp|Q86V21|AACS_HUMAN</td><td>AACS</td><td>Q86V21</td><td>Acetoacetyl-CoA synthetase</td><td>AACS_HUMAN</td><td></td></tr>
<tr><td>0.015125</td><td>0.565292</td><td>3.7491</td><td>sp|Q86V48|LUZP1_HUMAN</td><td>LUZP1</td><td>Q86V48</td><td>Leucine zipper protein 1</td><td>LUZP1_HUMAN</td><td></td></tr>
<tr><td>0.025938</td><td>-0.498155</td><td>-3.20264</td><td>sp|Q86WR0|CCD25_HUMAN</td><td>CCDC25</td><td>Q86WR0</td><td>Coiled-coil domain-containing protein 25</td><td>CCD25_HUMAN</td><td></td></tr>
<tr><td>0.0187801</td><td>0.843523</td><td>3.50998</td><td>sp|Q86X29|LSR_HUMAN</td><td>LSR</td><td>Q86X29</td><td>Lipolysis-stimulated lipoprotein receptor</td><td>LSR_HUMAN</td><td></td></tr>
<tr><td>0.00910569</td><td>0.730483</td><td>4.35801</td><td>sp|Q86XL3|ANKL2_HUMAN</td><td>ANKLE2</td><td>Q86XL3</td><td>Ankyrin repeat and LEM domain-containing protein 2</td><td>ANKL2_HUMAN</td><td></td></tr>
<tr><td>0.00382946</td><td>-1.53281</td><td>-6.05542</td><td>sp|Q8IU81|I2BP1_HUMAN</td><td>IRF2BP1</td><td>Q8IU81</td><td>Interferon regulatory factor 2-binding protein 1</td><td>I2BP1_HUMAN</td><td></td></tr>
<tr><td>0.00595095</td><td>0.703865</td><td>5.11699</td><td>sp|Q8IUR0|TPPC5_HUMAN</td><td>TRAPPC5</td><td>Q8IUR0</td><td>Trafficking protein particle complex subunit 5</td><td>TPPC5_HUMAN</td><td></td></tr>
<tr><td>0</td><td>1.06564</td><td>11.5891</td><td>sp|Q8IVT2|MISP_HUMAN</td><td>MISP</td><td>Q8IVT2</td><td>Mitotic interactor and substrate of PLK1</td><td>MISP_HUMAN</td><td></td></tr>
<tr><td>0.00625397</td><td>1.4428</td><td>5.03776</td><td>sp|Q8IWB1|IPRI_HUMAN</td><td>ITPRIP</td><td>Q8IWB1</td><td>Inositol 1,4,5-trisphosphate receptor-interacting protein</td><td>IPRI_HUMAN</td><td></td></tr>
<tr><td>0.00623747</td><td>-0.229828</td><td>-5.03303</td><td>sp|Q8IXT5|RB12B_HUMAN</td><td>RBM12B</td><td>Q8IXT5</td><td>RNA-binding protein 12B</td><td>RB12B_HUMAN</td><td></td></tr>
<tr><td>0.000714286</td><td>-1.70531</td><td>-7.6729</td><td>sp|Q8N142|PURA1_HUMAN</td><td>ADSS1</td><td>Q8N142</td><td>Adenylosuccinate synthetase isozyme 1</td><td>PURA1_HUMAN</td><td></td></tr>
<tr><td>0.0012514</td><td>0.6934</td><td>7.36511</td><td>sp|Q8N392|RHG18_HUMAN</td><td>ARHGAP18</td><td>Q8N392</td><td>Rho GTPase-activating protein 18</td><td>RHG18_HUMAN</td><td></td></tr>
<tr><td>0.00511392</td><td>-0.731139</td><td>-5.52562</td><td>sp|Q8N3D4|EH1L1_HUMAN</td><td>EHBP1L1</td><td>Q8N3D4</td><td>EH domain-binding protein 1-like protein 1</td><td>EH1L1_HUMAN</td><td></td></tr>
<tr><td>0.0435</td><td>-0.200802</td><td>-2.72884</td><td>sp|Q8N3U4|STAG2_HUMAN</td><td>STAG2</td><td>Q8N3U4</td><td>Cohesin subunit SA-2</td><td>STAG2_HUMAN</td><td></td></tr>
<tr><td>0.0250787</td><td>0.758227</td><td>3.22151</td><td>sp|Q8N5M9|JAGN1_HUMAN</td><td>JAGN1</td><td>Q8N5M9</td><td>Protein jagunal homolog 1</td><td>JAGN1_HUMAN</td><td></td></tr>
<tr><td>0.00596721</td><td>0.419745</td><td>5.12705</td><td>sp|Q8N6T3|ARFG1_HUMAN</td><td>ARFGAP1</td><td>Q8N6T3</td><td>ADP-ribosylation factor GTPase-activating protein 1</td><td>ARFG1_HUMAN</td><td></td></tr>
<tr><td>0</td><td>2.40675</td><td>11.5277</td><td>sp|Q8N726|ARF_HUMAN</td><td>CDKN2A</td><td>Q8N726</td><td>Tumor suppressor ARF</td><td>ARF_HUMAN</td><td></td></tr>
<tr><td>0.00483099</td><td>0.336374</td><td>5.80757</td><td>sp|Q8N8S7|ENAH_HUMAN</td><td>ENAH</td><td>Q8N8S7</td><td>Protein enabled homolog</td><td>ENAH_HUMAN</td><td></td></tr>
<tr><td>0.0289129</td><td>-1.03714</td><td>-3.11092</td><td>sp|Q8N9N8|EIF1A_HUMAN</td><td>EIF1AD</td><td>Q8N9N8</td><td>Probable RNA-binding protein EIF1AD</td><td>EIF1A_HUMAN</td><td></td></tr>
<tr><td>0.0151014</td><td>0.535157</td><td>3.74613</td><td>sp|Q8NB16|MLKL_HUMAN</td><td>MLKL</td><td>Q8NB16</td><td>Mixed lineage kinase domain-like protein</td><td>MLKL_HUMAN</td><td></td></tr>
<tr><td>0.00518824</td><td>-0.39383</td><td>-5.35549</td><td>sp|Q8NBJ5|GT251_HUMAN</td><td>COLGALT1</td><td>Q8NBJ5</td><td>Procollagen galactosyltransferase 1</td><td>GT251_HUMAN</td><td></td></tr>
<tr><td>0.00857263</td><td>0.35087</td><td>4.48253</td><td>sp|Q8NC56|LEMD2_HUMAN</td><td>LEMD2</td><td>Q8NC56</td><td>LEM domain-containing protein 2</td><td>LEMD2_HUMAN</td><td></td></tr>
<tr><td>0.00951751</td><td>-0.492336</td><td>-4.28226</td><td>sp|Q8NF37|PCAT1_HUMAN</td><td>LPCAT1</td><td>Q8NF37</td><td>Lysophosphatidylcholine acyltransferase 1</td><td>PCAT1_HUMAN</td><td></td></tr>
<tr><td>0.0166457</td><td>0.988064</td><td>3.62521</td><td>sp|Q8TB52|FBX30_HUMAN</td><td>FBXO30</td><td>Q8TB52</td><td>F-box only protein 30</td><td>FBX30_HUMAN</td><td></td></tr>
<tr><td>0.00122404</td><td>0.810846</td><td>7.22464</td><td>sp|Q8TDB6|DTX3L_HUMAN</td><td>DTX3L</td><td>Q8TDB6</td><td>E3 ubiquitin-protein ligase DTX3L</td><td>DTX3L_HUMAN</td><td></td></tr>
<tr><td>0.0309324</td><td>1.79378</td><td>3.03317</td><td>sp|Q8TDD1|DDX54_HUMAN</td><td>DDX54</td><td>Q8TDD1</td><td>ATP-dependent RNA helicase DDX54</td><td>DDX54_HUMAN</td><td></td></tr>
<tr><td>0.00235593</td><td>0.700825</td><td>6.36816</td><td>sp|Q8TED1|GPX8_HUMAN</td><td>GPX8</td><td>Q8TED1</td><td>Probable glutathione peroxidase 8</td><td>GPX8_HUMAN</td><td></td></tr>
<tr><td>0.0267632</td><td>0.43706</td><td>3.18338</td><td>sp|Q8TF74|WIPF2_HUMAN</td><td>WIPF2</td><td>Q8TF74</td><td>WAS/WASL-interacting protein family member 2</td><td>WIPF2_HUMAN</td><td></td></tr>
<tr><td>0.00978035</td><td>0.420759</td><td>4.25432</td><td>sp|Q8WTS6|SETD7_HUMAN</td><td>SETD7</td><td>Q8WTS6</td><td>Histone-lysine N-methyltransferase SETD7</td><td>SETD7_HUMAN</td><td></td></tr>
<tr><td>0.0100942</td><td>0.327209</td><td>4.2116</td><td>sp|Q8WUD4|CCD12_HUMAN</td><td>CCDC12</td><td>Q8WUD4</td><td>Coiled-coil domain-containing protein 12</td><td>CCD12_HUMAN</td><td></td></tr>
<tr><td>0.00517949</td><td>0.656539</td><td>5.56121</td><td>sp|Q8WUF5|IASPP_HUMAN</td><td>PPP1R13L</td><td>Q8WUF5</td><td>RelA-associated inhibitor</td><td>IASPP_HUMAN</td><td></td></tr>
<tr><td>0</td><td>1.79306</td><td>11.877</td><td>sp|Q8WUX1|S38A5_HUMAN</td><td>SLC38A5</td><td>Q8WUX1</td><td>Sodium-coupled neutral amino acid transporter 5</td><td>S38A5_HUMAN</td><td></td></tr>
<tr><td>0.0251443</td><td>-1.37503</td><td>-3.25417</td><td>sp|Q8WV41|SNX33_HUMAN</td><td>SNX33</td><td>Q8WV41</td><td>Sorting nexin-33</td><td>SNX33_HUMAN</td><td></td></tr>
<tr><td>0.0125901</td><td>-0.596477</td><td>-3.97961</td><td>sp|Q8WWI1|LMO7_HUMAN</td><td>LMO7</td><td>Q8WWI1</td><td>LIM domain only protein 7</td><td>LMO7_HUMAN</td><td></td></tr>
<tr><td>0.00733181</td><td>0.280991</td><td>4.67635</td><td>sp|Q8WWM7|ATX2L_HUMAN</td><td>ATXN2L</td><td>Q8WWM7</td><td>Ataxin-2-like protein</td><td>ATX2L_HUMAN</td><td></td></tr>
<tr><td>0.0066</td><td>-0.396676</td><td>-4.88388</td><td>sp|Q8WWY3|PRP31_HUMAN</td><td>PRPF31</td><td>Q8WWY3</td><td>U4/U6 small nuclear ribonucleoprotein Prp31</td><td>PRP31_HUMAN</td><td></td></tr>
<tr><td>0.00514815</td><td>-0.590643</td><td>-5.4913</td><td>sp|Q8WXH0|SYNE2_HUMAN</td><td>SYNE2</td><td>Q8WXH0</td><td>Nesprin-2</td><td>SYNE2_HUMAN</td><td></td></tr>
</table>

0.00253942	0.6671	6.31463	sp|Q8WZ82|OVCA2_HUMAN	OVCA2	Q8WZ82	Esterase OVCA2	OVCA2_HUMAN
0.00739954	0.643351	4.69645	sp|Q8WZA9|IRGQ_HUMAN	IRGQ	Q8WZA9	Immunity-related GTPase family Q protein	IRGQ_HUMAN
0.0226453	-0.420021	-3.36378	sp|Q92576|PHF3_HUMAN	PHF3	Q92576	PHD finger protein 3	PHF3_HUMAN
0	-5.38306	-43.3426	sp|Q92597|NDRG1_HUMAN	NDRG1	Q92597	Protein NDRG1	NDRG1_HUMAN
0.0166867	0.372613	3.63553	sp|Q92598|HS105_HUMAN	HSPH1	Q92598	Heat shock protein 105 kDa	HS105_HUMAN
0.00302834	-0.614565	-6.1603	sp|Q92599|SEPT8_HUMAN	SEPTIN8	Q92599	Septin-8	SEPT8_HUMAN
0.0176784	-0.833635	-3.56152	sp|Q92619|HMHA1_HUMAN	ARHGAP45	Q92619	Rho GTPase-activating protein 45	HMHA1_HUMAN
0	0.954339	9.50352	sp|Q92626|PXDN_HUMAN	PXDN	Q92626	Peroxidasin homolog	PXDN_HUMAN
0.0233483	-0.214859	-3.34088	sp|Q92747|ARC1A_HUMAN	ARPC1A	Q92747	Actin-related protein 2/3 complex subunit 1A	ARC1A_HUMAN
0.0100752	-0.820354	-4.19474	sp|Q92820|GGH_HUMAN	GGH	Q92820	Gamma-glutamyl hydrolase	GGH_HUMAN
0.00481404	-0.14868	-5.79651	sp|Q92841|DDX17_HUMAN	DDX17	Q92841	Probable ATP-dependent RNA helicase DDX17	DDX17_HUMAN
0.0163708	-0.357872	-3.65939	sp|Q92878|RAD50_HUMAN	RAD50	Q92878	DNA repair protein RAD50	RAD50_HUMAN
0.0130441	-0.342974	-3.94644	sp|Q92879|CELF1_HUMAN	CELF1	Q92879	CUGBP Elav-like family member 1	CELF1_HUMAN
0.013736	0.254742	3.88455	sp|Q92888|ARHG1_HUMAN	ARHGEF1	Q92888	Rho guanine nucleotide exchange factor 1	ARHG1_HUMAN
0	-0.695835	-15.6532	sp|Q92896|GSLG1_HUMAN	GLG1	Q92896	Golgi apparatus protein 1	GSLG1_HUMAN
0.0189703	0.18701	3.50487	sp|Q92900|RENT1_HUMAN	UPF1	Q92900	Regulator of nonsense transcripts 1	RENT1_HUMAN
0.00123077	-0.557926	-7.30383	sp|Q92930|RAB8B_HUMAN	RAB8B	Q92930	Ras-related protein Rab-8B	RAB8B_HUMAN
0.00880992	-0.300442	-4.41619	sp|Q92945|FUBP2_HUMAN	KHSRP	Q92945	Far upstream element-binding protein 2	FUBP2_HUMAN
0.0102691	0.210548	4.13697	sp|Q92973|TNPO1_HUMAN	TNPO1	Q92973	Transportin-1	TNPO1_HUMAN
0.00231481	1.04002	6.64566	sp|Q92974|ARHG2_HUMAN	ARHGEF2	Q92974	Rho guanine nucleotide exchange factor 2	ARHG2_HUMAN
0.00602151	-0.190821	-5.07503	sp|Q93009|UBP7_HUMAN	USP7	Q93009	Ubiquitin carboxyl-terminal hydrolase 7	UBP7_HUMAN
0.0212092	1.07317	3.39891	sp|Q969H6|POP5_HUMAN	POP5	Q969H6	Ribonuclease P/MRP protein subunit POP5	POP5_HUMAN
0.0165961	-0.455586	-3.62116	sp|Q969H8|MYDGF_HUMAN	MYDGF	Q969H8	Myeloid-derived growth factor	MYDGF_HUMAN
0	-1.43769	-20.0412	sp|Q969P0|IGSF8_HUMAN	IGSF8	Q969P0	Immunoglobulin superfamily member 8	IGSF8_HUMAN
0.0178023	0.597115	3.55611	sp|Q969Q0|RL36L_HUMAN	RPL36AL	Q969Q0	Ribosomal protein eL42-like	RL36L_HUMAN
0	0.567675	9.71846	sp|Q969S3|ZN622_HUMAN	ZNF622	Q969S3	Cytoplasmic 60S subunit biogenesis factor ZNF622	ZN622_HUMAN
0.00734752	-0.218453	-4.74419	sp|Q969X5|ERGI1_HUMAN	ERGIC1	Q969X5	Endoplasmic reticulum-Golgi intermediate compartment protein 1	ERGI1_HUMAN
0.0151118	1.05593	3.78701	sp|Q969X6|UTP4_HUMAN	UTP4	Q969X6	U3 small nucleolar RNA-associated protein 4 homolog	UTP4_HUMAN
0.00518012	0.410071	5.50168	sp|Q969Z0|FAKD4_HUMAN	TBRG4	Q969Z0	FAST kinase domain-containing protein 4	FAKD4_HUMAN
0	-1.2664	-23.1299	sp|Q96A26|F162A_HUMAN	FAM162A	Q96A26	Protein FAM162A	F162A_HUMAN
0.0176526	0.632518	3.56012	sp|Q96A65|EXOC4_HUMAN	EXOC4	Q96A65	Exocyst complex component 4	EXOC4_HUMAN
0.00241739	0.490536	6.50135	sp|Q96AC1|FERM2_HUMAN	FERMT2	Q96AC1	Fermitin family homolog 2	FERM2_HUMAN
0.0281807	-0.262572	-3.13287	sp|Q96AE4|FUBP1_HUMAN	FUBP1	Q96AE4	Far upstream element-binding protein 1	FUBP1_HUMAN
0.0360383	0.324586	2.88467	sp|Q96AT9|RPE_HUMAN	RPE	Q96AT9	Ribulose-phosphate 3-epimerase	RPE_HUMAN
0.00227273	-0.581177	-6.6091	sp|Q96AY3|FKB10_HUMAN	FKBP10	Q96AY3	Peptidyl-prolyl cis-trans isomerase FKBP10	FKB10_HUMAN
0.0395875	0.432495	2.8167	sp|Q96B49|TOM6_HUMAN	TOMM6	Q96B49	Mitochondrial import receptor subunit TOM6 homolog	TOM6_HUMAN
0.00598356	1.10786	5.12986	sp|Q96B54|ZN428_HUMAN	ZNF428	Q96B54	Zinc finger protein 428	ZN428_HUMAN
0.0315421	-0.607034	-3.01914	sp|Q96BI1|S22AI_HUMAN	SLC22A18	Q96BI1	Solute carrier family 22 member 18	S22AI_HUMAN
0.018228	0.809076	3.5343	sp|Q96BP3|PPWD1_HUMAN	PPWD1	Q96BP3	Peptidylprolyl isomerase domain and WD repeat-containing protein 1	PPWD1_HUMAN
0.035953	0.366171	2.89001	sp|Q96BR5|COA7_HUMAN	COA7	Q96BR5	Cytochrome c oxidase assembly factor 7	COA7_HUMAN
0.044572	0.238216	2.7116	sp|Q96C01|F136A_HUMAN	FAM136A	Q96C01	Protein FAM136A	F136A_HUMAN
0.0162719	-0.409463	-3.65212	sp|Q96C19|EFHD2_HUMAN	EFHD2	Q96C19	EF-hand domain-containing protein D2	EFHD2_HUMAN
0.0187903	-0.466249	-3.51341	sp|Q96C23|GALM_HUMAN	GALM	Q96C23	Galactose mutarotase	GALM_HUMAN
0.00750339	-0.225591	-4.61864	sp|Q96C36|P5CR2_HUMAN	PYCR2	Q96C36	Pyrroline-5-carboxylate reductase 2	P5CR2_HUMAN
0.039934	-0.328607	-2.80064	sp|Q96C86|DCPS_HUMAN	DCPS	Q96C86	m7GpppX diphosphatase	DCPS_HUMAN
0.0290947	0.243	3.10195	sp|Q96CS3|FAF2_HUMAN	FAF2	Q96CS3	FAS-associated factor 2	FAF2_HUMAN
0.0191921	-0.387768	-3.49606	sp|Q96CT7|CC124_HUMAN	CCDC124	Q96CT7	Coiled-coil domain-containing protein 124	CC124_HUMAN
0.00731294	0.362062	4.73601	sp|Q96D46|NMD3_HUMAN	NMD3	Q96D46	60S ribosomal export protein NMD3	NMD3_HUMAN
0.000759494	0.775661	7.81524	sp|Q96EL3|RM53_HUMAN	MRPL53	Q96EL3	Large ribosomal subunit protein mL53	RM53_HUMAN
0.00738173	1.24803	4.72695	sp|Q96EU6|RRP36_HUMAN	RRP36	Q96EU6	Ribosomal RNA processing protein 36 homolog	RRP36_HUMAN
0.0406612	1.36281	2.78589	sp|Q96EY9|ADAT3_HUMAN	ADAT3	Q96EY9	Probable inactive tRNA-specific adenosine deaminase-like protein 3	ADAT3_HUMAN
0.0329889	0.304595	2.97287	sp|Q96G46|DUS3L_HUMAN	DUS3L	Q96G46	tRNA-dihydrouridine(47) synthase [NAD(P)(+)]-like	DUS3L_HUMAN
0.00510811	2.18426	5.66492	sp|Q96GA3|LTV1_HUMAN	LTV1	Q96GA3	Protein LTV1 homolog	LTV1_HUMAN
0.0339387	0.372218	2.94433	sp|Q96GC5|RM48_HUMAN	MRPL48	Q96GC5	Large ribosomal subunit protein mL48	RM48_HUMAN
0.00888333	0.598195	4.45909	sp|Q96H20|SNF8_HUMAN	SNF8	Q96H20	Vacuolar-sorting protein SNF8	SNF8_HUMAN
0	-2.39614	-35.6464	sp|Q96HE7|ERO1A_HUMAN	ERO1A	Q96HE7	ERO1-like protein alpha	ERO1A_HUMAN
0.0406803	-0.812107	-2.78336	sp|Q96HY6|DDRGK_HUMAN	DDRGK1	Q96HY6	DDRGK domain-containing protein 1	DDRGK_HUMAN
0.0166617	-0.243828	-3.63509	sp|Q96I24|FUBP3_HUMAN	FUBP3	Q96I24	Far upstream element-binding protein 3	FUBP3_HUMAN
0.0192169	0.297103	3.4912	sp|Q96IJ6|GMPPA_HUMAN	GMPPA	Q96IJ6	Mannose-1-phosphate guanyltransferase alpha	GMPPA_HUMAN
0	0.881174	9.90962	sp|Q96IZ0|PAWR_HUMAN	PAWR	Q96IZ0	PRKC apoptosis WT1 regulator protein	PAWR_HUMAN
0.0435405	-0.46394	-2.72661	sp|Q96JB5|CK5P3_HUMAN	CDK5RAP3	Q96JB5	CDK5 regulatory subunit-associated protein 3	CK5P3_HUMAN
0.0134027	-0.389201	-3.91862	sp|Q96K76|UBP47_HUMAN	USP47	Q96K76	Ubiquitin carboxyl-terminal hydrolase 47	UBP47_HUMAN
0.0210385	-0.526949	-3.42607	sp|Q96KP4|CNDP2_HUMAN	CNDP2	Q96KP4	Cytosolic non-specific dipeptidase	CNDP2_HUMAN
0.000361111	-2.09025	-8.12134	sp|Q96NW7|LRRC7_HUMAN	LRRC7	Q96NW7	Leucine-rich repeat-containing protein 7	LRRC7_HUMAN
0.0276174	0.682197	3.15743	sp|Q96NY9|MUS81_HUMAN	MUS81	Q96NY9	Crossover junction endonuclease MUS81	MUS81_HUMAN
0.034181	0.504355	2.93588	sp|Q96P48|ARAP1_HUMAN	ARAP1	Q96P48	Arf-GAP with Rho-GAP domain, ANK repeat and PH domain-containing protein 1	ARAP1_HUMAN
0.0177026	-0.895047	-3.5594	sp|Q96RU3|FNBP1_HUMAN	FNBP1	Q96RU3	Formin-binding protein 1	FNBP1_HUMAN
0.00760613	-1.20114	-4.57797	sp|Q96S19|MTL26_HUMAN	METTL26	Q96S19	Methyltransferase-like 26	MTL26_HUMAN
0.00549708	-0.710736	-5.31744	sp|Q96S55|WRIP1_HUMAN	WRNIP1	Q96S55	ATPase WRNIP1	WRIP1_HUMAN
0.0356834	0.372936	2.8956	sp|Q96SB4|SRPK1_HUMAN	SRPK1	Q96SB4	SRSF protein kinase 1	SRPK1_HUMAN
0.0187368	0.648029	3.51196	sp|Q96SU4|OSBL9_HUMAN	OSBPL9	Q96SU4	Oxysterol-binding protein-related protein 9	OSBL9_HUMAN
0.0250877	-0.188364	-3.24997	sp|Q96T37|RBM15_HUMAN	RBM15	Q96T37	RNA-binding protein 15	RBM15_HUMAN
0.013474	0.485071	3.91705	sp|Q96T88|UHRF1_HUMAN	UHRF1	Q96T88	E3 ubiquitin-protein ligase UHRF1	UHRF1_HUMAN
0	-0.771009	-12.7547	sp|Q96TA1|NIBA2_HUMAN	NIBAN2	Q96TA1	Protein Niban 2	NIBA2_HUMAN
0.006304	0.442595	5.05032	sp|Q99439|CNN2_HUMAN	CNN2	Q99439	Calponin-2	CNN2_HUMAN
0	-2.16033	-11.481	sp|Q99541|PLIN2_HUMAN	PLIN2	Q99541	Perilipin-2	PLIN2_HUMAN
0.0051465	-0.369318	-5.55186	sp|Q99590|SCAFB_HUMAN	SCAF11	Q99590	Protein SCAF11	SCAFB_HUMAN
0.0434142	0.389138	2.73357	sp|Q99594|TEAD3_HUMAN	TEAD3	Q99594	Transcriptional enhancer factor TEF-5	TEAD3_HUMAN
0.0294461	0.435045	3.08795	sp|Q99598|TSNAX_HUMAN	TSNAX	Q99598	Translin-associated protein X	TSNAX_HUMAN
0.0100229	0.244631	4.23027	sp|Q99613|EIF3C_HUMAN	EIF3C	Q99613	Eukaryotic translation initiation factor 3 subunit C	EIF3C_HUMAN
0.0101952	0.332739	4.15374	sp|Q99614|TTC1_HUMAN	TTC1	Q99614	Tetratricopeptide repeat protein 1	TTC1_HUMAN
0.027066	0.365979	3.17342	sp|Q99615|DNJC7_HUMAN	DNAJC7	Q99615	DnaJ homolog subfamily C member 7	DNJC7_HUMAN
0.00949312	0.549171	4.29261	sp|Q99700|ATX2_HUMAN	ATXN2	Q99700	Ataxin-2	ATX2_HUMAN
0.0141438	0.287412	3.85138	sp|Q99714|HCD2_HUMAN	HSD17B10	Q99714	3-hydroxyacyl-CoA dehydrogenase type-2	HCD2_HUMAN
0.00381467	0.767505	6.05051	sp|Q99808|S29A1_HUMAN	SLC29A1	Q99808	Equilibrative nucleoside transporter 1	S29A1_HUMAN
0.0110634	0.50023	4.04631	sp|Q9BPX3|CND3_HUMAN	NCAPG	Q9BPX3	Condensin complex subunit 3	CND3_HUMAN
0.00725604	-0.234551	-4.79525	sp|Q9BQ39|DDX50_HUMAN	DDX50	Q9BQ39	ATP-dependent RNA helicase DDX50	DDX50_HUMAN
0.0064	0.486648	4.90603	sp|Q9BQ52|RNZ2_HUMAN	ELAC2	Q9BQ52	Zinc phosphodiesterase ELAC protein 2	RNZ2_HUMAN
0.00905051	-0.46416	-4.3467	sp|Q9BQ69|MACD1_HUMAN	MACROD1	Q9BQ69	ADP-ribose glycohydrolase MACROD1	MACD1_HUMAN
0.0281962	1.88865	3.13494	sp|Q9BQ70|TCF25_HUMAN	TCF25	Q9BQ70	Ribosome quality control complex subunit TCF25	TCF25_HUMAN
0.00559104	-0.655576	-5.21657	sp|Q9BQE5|APOL2_HUMAN	APOL2	Q9BQE5	Apolipoprotein L2	APOL2_HUMAN
0.0210674	0.357436	3.42613	sp|Q9BR76|COR1B_HUMAN	CORO1B	Q9BR76	Coronin-1B	COR1B_HUMAN
0.007133	0.718866	4.84173	sp|Q9BRJ2|RM45_HUMAN	MRPL45	Q9BRJ2	Large ribosomal subunit protein mL45	RM45_HUMAN
0.00765639	0.640486	4.58841	sp|Q9BRJ6|CG050_HUMAN	C7orf50	Q9BRJ6	Uncharacterized protein C7orf50	CG050_HUMAN
0.00763956	1.55952	4.58673	sp|Q9BRP1|PDD2L_HUMAN	PDCD2L	Q9BRP1	Programmed cell death protein 2-like	PDD2L_HUMAN
0.0245825	2.29327	3.28117	sp|Q9BRT6|LLPH_HUMAN	LLPH	Q9BRT6	Protein LLP homolog	LLPH_HUMAN
0.029493	-0.479036	-3.0909	sp|Q9BS26|ERP44_HUMAN	ERP44	Q9BS26	Endoplasmic reticulum resident protein 44	ERP44_HUMAN
0.0417323	0.459967	2.76247	sp|Q9BTV5|FSD1_HUMAN	FSD1	Q9BTV5	Fibronectin type III and SPRY domain-containing protein 1	FSD1_HUMAN
0.0399753	0.355662	2.79712	sp|Q9BTW9|TBCD_HUMAN	TBCD	Q9BTW9	Tubulin-specific chaperone D	TBCD_HUMAN
0.0126781	-0.431429	-3.97771	sp|Q9BTY2|FUCO2_HUMAN	FUCA2	Q9BTY2	Plasma alpha-L-fucosidase	FUCO2_HUMAN
0.00736449	0.693417	4.71912	sp|Q9BTY7|HGH1_HUMAN	HGH1	Q9BTY7	Protein HGH1 homolog	HGH1_HUMAN
0.0197601	-0.410203	-3.47513	sp|Q9BU76|MMTA2_HUMAN	MMTAG2	Q9BU76	Multiple myeloma tumor-associated protein 2	MMTA2_HUMAN
0.0108845	0.195065	4.07013	sp|Q9BUF5|TBB6_HUMAN	TUBB6	Q9BUF5	Tubulin beta-6 chain	TBB6_HUMAN
0.0155802	1.47398	3.70279	sp|Q9BUL9|RPP25_HUMAN	RPP25	Q9BUL9	Ribonuclease P protein subunit p25	RPP25_HUMAN
0.0251516	-0.208179	-3.25119	sp|Q9BUQ8|DDX23_HUMAN	DDX23	Q9BUQ8	Probable ATP-dependent RNA helicase DDX23	DDX23_HUMAN
0.00726841	0.466998	4.74757	sp|Q9BV44|THUM3_HUMAN	THUMPD3	Q9BV44	tRNA (guanine(6)-N2)-methyltransferase THUMP3	THUM3_HUMAN
0.0453399	-0.114086	-2.69536	sp|Q9BVC6|TM109_HUMAN	TMEM109	Q9BVC6	Transmembrane protein 109	TM109_HUMAN
0	0.484005	9.71	sp|Q9BWD1|THIC_HUMAN	ACAT2	Q9BWD1	Acetyl-CoA acetyltransferase, cytosolic	THIC_HUMAN
0	-0.409873	-12.0567	sp|Q9BWF3|RBM4_HUMAN	RBM4	Q9BWF3	RNA-binding protein 4	RBM4_HUMAN
0.00191304	-0.744339	-6.8371	sp|Q9BWM7|SFXN3_HUMAN	SFXN3	Q9BWM7	Sideroflexin-3	SFXN3_HUMAN
0.00738249	-0.705155	-4.69208	sp|Q9BWS9|CHID1_HUMAN	CHID1	Q9BWS9	Chitinase domain-containing protein 1	CHID1_HUMAN
0.0295618	0.432679	3.09203	sp|Q9BWU0|NADAP_HUMAN	SLC4A1AP	Q9BWU0	Kanadaptin	NADAP_HUMAN
0	1.92892	12.2602	sp|Q9BX10|GTPB2_HUMAN	GTPBP2	Q9BX10	GTP-binding protein 2	GTPB2_HUMAN
0	0.75914	15.77	sp|Q9BXB5|OSB10_HUMAN	OSBPL10	Q9BXB5	Oxysterol-binding protein-related protein 10	OSB10_HUMAN
0.0342622	0.675896	2.92959	sp|Q9BXY0|MAK16_HUMAN	MAK16	Q9BXY0	Protein MAK16 homolog	MAK16_HUMAN
0.00509779	0.341311	5.52541	sp|Q9BY42|RTF2_HUMAN	RTF2	Q9BY42	Replication termination factor 2	RTF2_HUMAN
0.0424899	0.15477	2.75349	sp|Q9BY44|EIF2A_HUMAN	EIF2A	Q9BY44	Eukaryotic translation initiation factor 2A	EIF2A_HUMAN
0.0141669	0.70883	3.85179	sp|Q9BYC8|RM32_HUMAN	MRPL32	Q9BYC8	Large ribosomal subunit protein bL32m	RM32_HUMAN
0.0115679	0.948044	4.02451	sp|Q9BYD1|RM13_HUMAN	MRPL13	Q9BYD1	Large ribosomal subunit protein uL13m	RM13_HUMAN
0.0137587	0.68455	3.88793	sp|Q9BYD3|RM04_HUMAN	MRPL4	Q9BYD3	Large ribosomal subunit protein uL4m	RM04_HUMAN
0.0246872	0.499351	3.2658	sp|Q9BYK8|HELZ2_HUMAN	HELZ2	Q9BYK8	Helicase with zinc finger domain 2	HELZ2_HUMAN
0.0137571	0.281603	3.90031	sp|Q9BYN8|RT26_HUMAN	MRPS26	Q9BYN8	Small ribosomal subunit protein mS26	RT26_HUMAN
0	-2.02898	-11.3337	sp|Q9BYX7|ACTBM_HUMAN	POTEKP	Q9BYX7	Putative beta-actin-like protein 3	ACTBM_HUMAN
0.0338462	0.333787	2.9516	sp|Q9BZE4|GTPB4_HUMAN	GTPBP4	Q9BZE4	GTP-binding protein 4	GTPB4_HUMAN
0.0104448	0.257211	4.12324	sp|Q9BZG1|RAB34_HUMAN	RAB34	Q9BZG1	Ras-related protein Rab-34	RAB34_HUMAN
0	1.07328	12.3699	sp|Q9BZQ8|NIBA1_HUMAN	NIBAN1	Q9BZQ8	Protein Niban 1	NIBA1_HUMAN
0.00720384	-0.310811	-4.76575	sp|Q9BZZ5|API5_HUMAN	API5	Q9BZZ5	Apoptosis inhibitor 5	API5_HUMAN
0.0413265	-1.02355	-2.77399	sp|Q9C0B1|FTO_HUMAN	FTO	Q9C0B1	Alpha-ketoglutarate-dependent dioxygenase FTO	FTO_HUMAN
0.000727273	0.871481	7.72541	sp|Q9C0D5|TANC1_HUMAN	TANC1	Q9C0D5	Protein TANC1	TANC1_HUMAN

0.0172285	0.3372	3.59514	sp|Q9GZL7|WDR12_HUMAN	WDR12	Q9GZL7	Ribosome biogenesis protein WDR12	WDR12_HUMAN
0.0187543	0.486255	3.51474	sp|Q9GZR2|REXO4_HUMAN	REXO4	Q9GZR2	RNA exonuclease 4	REXO4_HUMAN
0.0273439	0.327223	3.16388	sp|Q9GZS1|RPA49_HUMAN	POLR1E	Q9GZS1	DNA-directed RNA polymerase I subunit RPA49	RPA49_HUMAN
0.0308649	-0.446778	-3.05211	sp|Q9GZS3|SKI8_HUMAN	SKIC8	Q9GZS3	Superkiller complex protein 8	SKI8_HUMAN
0	-2.01904	-27.2985	sp|Q9GZT9|EGLN1_HUMAN	EGLN1	Q9GZT9	Egl nine homolog 1	EGLN1_HUMAN
0.0150159	0.496385	3.77948	sp|Q9GZZ1|NAA50_HUMAN	NAA50	Q9GZZ1	N-alpha-acetyltransferase 50	NAA50_HUMAN
0.0487471	0.298926	2.63199	sp|Q9H078|CLPB_HUMAN	CLPB	Q9H078	Mitochondrial disaggregase	CLPB_HUMAN
0.0194502	0.498336	3.48317	sp|Q9H089|LSG1_HUMAN	LSG1	Q9H089	Large subunit GTPase 1 homolog	LSG1_HUMAN
0.0166366	-0.388556	-3.63293	sp|Q9H0A0|NAT10_HUMAN	NAT10	Q9H0A0	RNA cytidine acetyltransferase	NAT10_HUMAN
0.000374101	0.581608	8.2572	sp|Q9H0U6|RM18_HUMAN	MRPL18	Q9H0U6	Large ribosomal subunit protein uL18m	RM18_HUMAN
0.00627056	-0.89288	-5.04606	sp|Q9H270|VPS11_HUMAN	VPS11	Q9H270	Vacuolar protein sorting-associated protein 11 homolog	VPS11_HUMAN
0.0328098	-0.33708	-2.9788	sp|Q9H2H8|PPIL3_HUMAN	PPIL3	Q9H2H8	Peptidyl-prolyl cis-trans isomerase-like 3	PPIL3_HUMAN
0	2.11449	35.8181	sp|Q9H2H9|S38A1_HUMAN	SLC38A1	Q9H2H9	Sodium-coupled neutral amino acid symporter 1	S38A1_HUMAN
0.00736493	0.564084	4.74452	sp|Q9H2U1|DHX36_HUMAN	DHX36	Q9H2U1	ATP-dependent DNA/RNA helicase DHX36	DHX36_HUMAN
0.00369721	-0.33367	-6.10615	sp|Q9H3G5|CPVL_HUMAN	CPVL	Q9H3G5	Probable serine carboxypeptidase CPVL	CPVL_HUMAN
0.00828875	-2.62098	-4.50746	sp|Q9H3M7|TXNIP_HUMAN	TXNIP	Q9H3M7	Thioredoxin-interacting protein	TXNIP_HUMAN
0.0382587	-0.263206	-2.83847	sp|Q9H3N1|TMX1_HUMAN	TMX1	Q9H3N1	Thioredoxin-related transmembrane protein 1	TMX1_HUMAN
0.0185727	0.300389	3.52525	sp|Q9H3P2|NELFA_HUMAN	NELFA	Q9H3P2	Negative elongation factor A	NELFA_HUMAN
0.00507792	0.362932	5.58862	sp|Q9H3U1|UN45A_HUMAN	UNC45A	Q9H3U1	Protein unc-45 homolog A	UN45A_HUMAN
0.0101323	0.974889	4.21384	sp|Q9H4F8|SMOC1_HUMAN	SMOC1	Q9H4F8	SPARC-related modular calcium-binding protein 1	SMOC1_HUMAN
0	0.816214	11.3641	sp|Q9H4G0|E41L1_HUMAN	EPB41L1	Q9H4G0	Band 4.1-like protein 1	E41L1_HUMAN
0.00791323	0.29856	4.55815	sp|Q9H4M9|EHD1_HUMAN	EHD1	Q9H4M9	EH domain-containing protein 1	EHD1_HUMAN
0	-1.25237	-8.79294	sp|Q9H5V8|CDCP1_HUMAN	CDCP1	Q9H5V8	CUB domain-containing protein 1	CDCP1_HUMAN
0.0130662	0.524653	3.94776	sp|Q9H773|DCTP1_HUMAN	DCTPP1	Q9H773	dCTP pyrophosphatase 1	DCTP1_HUMAN
0.0297032	0.473445	3.06713	sp|Q9H788|SH24A_HUMAN	SH2D4A	Q9H788	SH2 domain-containing protein 4A	SH24A_HUMAN
0	0.526227	8.7507	sp|Q9H8H2|DDX31_HUMAN	DDX31	Q9H8H2	Probable ATP-dependent RNA helicase DDX31	DDX31_HUMAN
0.00255	-0.936031	-6.31658	sp|Q9H8Y5|ANKZ1_HUMAN	ANKZF1	Q9H8Y5	tRNA endonuclease ANKZF1	ANKZ1_HUMAN
0.00234742	1.48281	6.71376	sp|Q9H967|WDR76_HUMAN	WDR76	Q9H967	WD repeat-containing protein 76	WDR76_HUMAN
0.0102197	1.60975	4.18231	sp|Q9H9B1|EHMT1_HUMAN	EHMT1	Q9H9B1	Histone-lysine N-methyltransferase EHMT1	EHMT1_HUMAN
0.00228311	-0.845363	-6.62179	sp|Q9HAV7|GRPE1_HUMAN	GRPEL1	Q9HAV7	GrpE protein homolog 1, mitochondrial	GRPE1_HUMAN
0.000705882	0.761101	7.65749	sp|Q9HCC0|MCCB_HUMAN	MCCC2	Q9HCC0	Methylcrotonoyl-CoA carboxylase beta chain, mitochondrial	MCCB_HUMAN
0.0309352	-1.26959	-3.05361	sp|Q9NP58|ABCB6_HUMAN	ABCB6	Q9NP58	ATP-binding cassette sub-family B member 6	ABCB6_HUMAN
0.00237607	-0.959434	-6.40922	sp|Q9NP66|HM20A_HUMAN	HMG20A	Q9NP66	High mobility group protein 20A	HM20A_HUMAN
0.0210595	0.29674	3.40091	sp|Q9NP81|SYSM_HUMAN	SARS2	Q9NP81	Serine--tRNA ligase, mitochondrial	SYSM_HUMAN
0.0388954	0.603344	2.826	sp|Q9NP92|RT30_HUMAN	MRPS30	Q9NP92	Large ribosomal subunit protein mL65	RT30_HUMAN
0.0408458	0.370296	2.77748	sp|Q9NPQ8|RIC8A_HUMAN	RIC8A	Q9NPQ8	Synembryn-A	RIC8A_HUMAN
0	-2.24717	-14.6293	sp|Q9NPR2|SEM4B_HUMAN	SEMA4B	Q9NPR2	Semaphorin-4B	SEM4B_HUMAN
0.00519614	0.513506	5.56502	sp|Q9NQT5|EXOS3_HUMAN	EXOSC3	Q9NQT5	Exosome complex component RRP40	EXOS3_HUMAN
0	-1.46917	-11.8853	sp|Q9NQW6|ANLN_HUMAN	ANLN	Q9NQW6	Anillin	ANLN_HUMAN
0.00919439	-0.421359	-4.32444	sp|Q9NQW7|XPP1_HUMAN	XPNPEP1	Q9NQW7	Xaa-Pro aminopeptidase 1	XPP1_HUMAN
0.0281625	0.756533	3.13465	sp|Q9NQX3|GEPH_HUMAN	GPHN	Q9NQX3	Gephyrin	GEPH_HUMAN
0	1.21985	13.2904	sp|Q9NR12|PDLI7_HUMAN	PDLIM7	Q9NR12	PDZ and LIM domain protein 7	PDLI7_HUMAN
0.00731323	0.663067	4.70398	sp|Q9NR30|DDX21_HUMAN	DDX21	Q9NR30	Nucleolar RNA helicase 2	DDX21_HUMAN
0.0452952	-0.203958	-2.69523	sp|Q9NTI5|PDS5B_HUMAN	PDS5B	Q9NTI5	Sister chromatid cohesion protein PDS5 homolog B	PDS5B_HUMAN
0	0.34119	16.6813	sp|Q9NTJ3|SMC4_HUMAN	SMC4	Q9NTJ3	Structural maintenance of chromosomes protein 4	SMC4_HUMAN
0.0171521	0.862347	3.58927	sp|Q9NUQ6|SPS2L_HUMAN	SPATS2L	Q9NUQ6	SPATS2-like protein	SPS2L_HUMAN
0.00519626	0.582102	5.50478	sp|Q9NVD7|PARVA_HUMAN	PARVA	Q9NVD7	Alpha-parvin	PARVA_HUMAN
0.0340961	0.415768	2.93737	sp|Q9NVG8|TBC13_HUMAN	TBC1D13	Q9NVG8	TBC1 domain family member 13	TBC13_HUMAN
0.000677966	0.671964	7.48336	sp|Q9NVI7|ATD3A_HUMAN	ATAD3A	Q9NVI7	ATPase family AAA domain-containing protein 3A	ATD3A_HUMAN
0.0340088	0.866863	2.95941	sp|Q9NVU7|SDA1_HUMAN	SDAD1	Q9NVU7	Protein SDA1 homolog	SDA1_HUMAN
0.0179448	-0.298358	-3.55446	sp|Q9NWB6|ARGL1_HUMAN	ARGLU1	Q9NWB6	Arginine and glutamate-rich protein 1	ARGL1_HUMAN
0.0238023	-0.253835	-3.3118	sp|Q9NWV4|CZIB_HUMAN	CZIB	Q9NWV4	CXXC motif containing zinc binding protein	CZIB_HUMAN
0.0286462	0.440989	3.11854	sp|Q9NX40|OCAD1_HUMAN	OCIAD1	Q9NX40	OCIA domain-containing protein 1	OCAD1_HUMAN
0.0222145	0.75308	3.37428	sp|Q9NXH8|TOR4A_HUMAN	TOR4A	Q9NXH8	Torsin-4A	TOR4A_HUMAN
0.00573333	0.393966	5.19471	sp|Q9NXH9|TRM1_HUMAN	TRMT1	Q9NXH9	tRNA (guanine(26)-N(2))-dimethyltransferase	TRM1_HUMAN
0.0453595	-0.469052	-2.69064	sp|Q9NYJ1|COA4_HUMAN	COA4	Q9NYJ1	Cytochrome c oxidase assembly factor 4 homolog, mitochondrial	COA4_HUMAN
0.000805369	-0.585226	-8.01174	sp|Q9NYL9|TMOD3_HUMAN	TMOD3	Q9NYL9	Tropomodulin-3	TMOD3_HUMAN
0.000736196	-0.418179	-7.74613	sp|Q9NYU2|UGGG1_HUMAN	UGGT1	Q9NYU2	UDP-glucose:glycoprotein glucosyltransferase 1	UGGG1_HUMAN
0.00522884	0.713413	5.50903	sp|Q9NZJ7|MTCH1_HUMAN	MTCH1	Q9NZJ7	Mitochondrial carrier homolog 1	MTCH1_HUMAN
0.00827119	0.477175	4.50055	sp|Q9NZM1|MYOF_HUMAN	MYOF	Q9NZM1	Myoferlin	MYOF_HUMAN
0.0324816	-0.772615	-2.98971	sp|Q9NZN4|EHD2_HUMAN	EHD2	Q9NZN4	EH domain-containing protein 2	EHD2_HUMAN
0.0124131	-0.800643	-3.98864	sp|Q9NZP5|O5AC2_HUMAN	OR5AC2	Q9NZP5	Olfactory receptor 5AC2	O5AC2_HUMAN
0.00557542	-0.540057	-5.20999	sp|Q9NZQ3|SPN90_HUMAN	NCKIPSD	Q9NZQ3	NCK-interacting protein with SH3 domain	SPN90_HUMAN
0.0253167	0.702327	3.21957	sp|Q9P015|RM15_HUMAN	MRPL15	Q9P015	Large ribosomal subunit protein uL15m	RM15_HUMAN
0.00884647	0.663351	4.446	sp|Q9P0J0|NDUAD_HUMAN	NDUFA13	Q9P0J0	NADH dehydrogenase [ubiquinone] 1 alpha subcomplex subunit 13	NDUAD_HUMAN
0.0047972	0.381596	5.78264	sp|Q9P258|RCC2_HUMAN	RCC2	Q9P258	Protein RCC2	RCC2_HUMAN
0.00777629	0.498691	4.60999	sp|Q9P265|DIP2B_HUMAN	DIP2B	Q9P265	Disco-interacting protein 2 homolog B	DIP2B_HUMAN
0.00506825	-0.497283	-5.37061	sp|Q9P2E9|RRBP1_HUMAN	RRBP1	Q9P2E9	Ribosome-binding protein 1	RRBP1_HUMAN
0.0254776	0.149702	3.2171	sp|Q9P2J5|SYLC_HUMAN	LARS1	Q9P2J5	Leucine--tRNA ligase, cytoplasmic	SYLC_HUMAN
0.0194902	0.414645	3.48277	sp|Q9P2N5|RBM27_HUMAN	RBM27	Q9P2N5	RNA-binding protein 27	RBM27_HUMAN
0.0472798	0.264935	2.65617	sp|Q9P2R3|ANFY1_HUMAN	ANKFY1	Q9P2R3	Rabankyrin-5	ANFY1_HUMAN
0.0281851	0.268985	3.13143	sp|Q9P2R7|SUCB1_HUMAN	SUCLA2	Q9P2R7	Succinate--CoA ligase [ADP-forming] subunit beta, mitochondrial	SUCB1_HUMAN
0.0238234	0.477196	3.30708	sp|Q9UBQ5|EIF3K_HUMAN	EIF3K	Q9UBQ5	Eukaryotic translation initiation factor 3 subunit K	EIF3K_HUMAN
0.0241501	0.213729	3.29852	sp|Q9UBQ7|GRHPR_HUMAN	GRHPR	Q9UBQ7	Glyoxylate reductase/hydroxypyruvate reductase	GRHPR_HUMAN
0.000693642	-0.358591	-7.592	sp|Q9UBR2|CATZ_HUMAN	CTSZ	Q9UBR2	Cathepsin Z	CATZ_HUMAN
0.0396691	-0.343849	-2.80725	sp|Q9UBW7|ZMYM2_HUMAN	ZMYM2	Q9UBW7	Zinc finger MYM-type protein 2	ZMYM2_HUMAN
0.0166209	0.938432	3.62429	sp|Q9UDY2|ZO2_HUMAN	TJP2	Q9UDY2	Tight junction protein ZO-2	ZO2_HUMAN
0.0295145	-0.302328	-3.08868	sp|Q9UER7|DAXX_HUMAN	DAXX	Q9UER7	Death domain-associated protein 6	DAXX_HUMAN
0	0.812774	8.34353	sp|Q9UG63|ABCF2_HUMAN	ABCF2	Q9UG63	ATP-binding cassette sub-family F member 2	ABCF2_HUMAN
0.0162195	0.681609	3.66837	sp|Q9UH62|ARMX3_HUMAN	ARMCX3	Q9UH62	Armadillo repeat-containing X-linked protein 3	ARMX3_HUMAN
0	0.770792	12.9981	sp|Q9UHB6|LIMA1_HUMAN	LIMA1	Q9UHB6	LIM domain and actin-binding protein 1	LIMA1_HUMAN
0.00495738	0.626243	5.60429	sp|Q9UHD1|CHRD1_HUMAN	CHORDC1	Q9UHD1	Cysteine and histidine-rich domain-containing protein 1	CHRD1_HUMAN
0.00220264	-0.712331	-6.54731	sp|Q9UHG3|PCYOX_HUMAN	PCYOX1	Q9UHG3	Prenylcysteine oxidase 1	PCYOX_HUMAN
0.00509446	-0.71498	-5.59183	sp|Q9UHL4|DPP2_HUMAN	DPP7	Q9UHL4	Dipeptidyl peptidase 2	DPP2_HUMAN
0.027584	0.830945	3.15632	sp|Q9UHN6|CEIP2_HUMAN	CEMIP2	Q9UHN6	Cell surface hyaluronidase	CEIP2_HUMAN
0	-1.85723	-10.4609	sp|Q9UHQ1|NARF_HUMAN	NARF	Q9UHQ1	Nuclear prelamin A recognition factor	NARF_HUMAN
0.00279835	1.38445	6.28109	sp|Q9UHR4|BI2L1_HUMAN	BAIAP2L1	Q9UHR4	Brain-specific angiogenesis inhibitor 1-associated protein 2-like protein 1	BI2L1_HUMAN
0.0282185	0.203596	3.13172	sp|Q9UI12|VATH_HUMAN	ATP6V1H	Q9UI12	V-type proton ATPase subunit H	VATH_HUMAN
0.00571745	1.07417	5.16824	sp|Q9UI14|PRAF1_HUMAN	RABAC1	Q9UI14	Prenylated Rab acceptor protein 1	PRAF1_HUMAN
0.00981818	0.428066	4.25806	sp|Q9UIG0|BAZ1B_HUMAN	BAZ1B	Q9UIG0	Tyrosine-protein kinase BAZ1B	BAZ1B_HUMAN
0.0189065	0.348496	3.50513	sp|Q9UJ70|NAGK_HUMAN	NAGK	Q9UJ70	N-acetyl-D-glucosamine kinase	NAGK_HUMAN
0.0149447	-0.285499	-3.77543	sp|Q9UJ83|HACL1_HUMAN	HACL1	Q9UJ83	2-hydroxyacyl-CoA lyase 1	HACL1_HUMAN
0.0290879	0.689874	3.10324	sp|Q9UJF2|NGAP_HUMAN	RASAL2	Q9UJF2	Ras GTPase-activating protein nGAP	NGAP_HUMAN
0	-2.69411	-9.10031	sp|Q9UJM3|ERRFI_HUMAN	ERRFI1	Q9UJM3	ERBB receptor feedback inhibitor 1	ERRFI_HUMAN
0.0150397	0.405395	3.78224	sp|Q9UJW0|DCTN4_HUMAN	DCTN4	Q9UJW0	Dynactin subunit 4	DCTN4_HUMAN
0.016346	0.331846	3.65505	sp|Q9UK76|JUPI1_HUMAN	JPT1	Q9UK76	Jupiter microtubule associated homolog 1	JUPI1_HUMAN
0.0156447	0.282327	3.69241	sp|Q9UKS6|PACN3_HUMAN	PACSIN3	Q9UKS6	Protein kinase C and casein kinase substrate in neurons protein 3	PACN3_HUMAN
0.00605405	-0.249183	-5.08269	sp|Q9UKX7|NUP50_HUMAN	NUP50	Q9UKX7	Nuclear pore complex protein Nup50	NUP50_HUMAN
0.00895082	0.343347	4.39335	sp|Q9UKY7|CDV3_HUMAN	CDV3	Q9UKY7	Protein CDV3 homolog	CDV3_HUMAN
0	0.765496	10.2726	sp|Q9ULX3|NOB1_HUMAN	NOB1	Q9ULX3	RNA-binding protein NOB1	NOB1_HUMAN
0	-1.01249	-11.8984	sp|Q9ULX9|MAFF_HUMAN	MAFF	Q9ULX9	Transcription factor MafF	MAFF_HUMAN
0.028879	-0.839554	-3.10991	sp|Q9UMF0|ICAM5_HUMAN	ICAM5	Q9UMF0	Intercellular adhesion molecule 5	ICAM5_HUMAN
0.0343739	0.228019	2.93052	sp|Q9UMR2|DD19B_HUMAN	DDX19B	Q9UMR2	ATP-dependent RNA helicase DDX19B	DD19B_HUMAN
0.00881172	-0.178277	-4.46793	sp|Q9UNF0|PACN2_HUMAN	PACSIN2	Q9UNF0	Protein kinase C and casein kinase substrate in neurons protein 2	PACN2_HUMAN
0.029588	0.293293	3.07788	sp|Q9UNN8|EPCR_HUMAN	PROCR	Q9UNN8	Endothelial protein C receptor	EPCR_HUMAN
0.0344242	0.327867	2.92403	sp|Q9UNZ5|L10K_HUMAN	C19orf53	Q9UNZ5	Leydig cell tumor 10 kDa protein homolog	L10K_HUMAN
0.00552273	-0.303593	-5.24003	sp|Q9UQE7|SMC3_HUMAN	SMC3	Q9UQE7	Structural maintenance of chromosomes protein 3	SMC3_HUMAN
0.0238964	0.153368	3.30054	sp|Q9Y262|EIF3L_HUMAN	EIF3L	Q9Y262	Eukaryotic translation initiation factor 3 subunit L	EIF3L_HUMAN
0.021088	0.24788	3.40334	sp|Q9Y281|COF2_HUMAN	CFL2	Q9Y281	Cofilin-2	COF2_HUMAN
0.0143182	-0.355566	-3.84224	sp|Q9Y282|ERGI3_HUMAN	ERGIC3	Q9Y282	Endoplasmic reticulum-Golgi intermediate compartment protein 3	ERGI3_HUMAN
0.0014359	-1.24693	-7.00705	sp|Q9Y2H6|FND3A_HUMAN	FNDC3A	Q9Y2H6	Fibronectin type-III domain-containing protein 3A	FND3A_HUMAN
0.024186	0.153962	3.29336	sp|Q9Y2L1|RRP44_HUMAN	DIS3	Q9Y2L1	Exosome complex exonuclease RRP44	RRP44_HUMAN
0.0400165	0.23292	2.79729	sp|Q9Y2Q3|GSTK1_HUMAN	GSTK1	Q9Y2Q3	Glutathione S-transferase kappa 1	GSTK1_HUMAN
0.00736552	1.16306	4.68613	sp|Q9Y2R4|DDX52_HUMAN	DDX52	Q9Y2R4	Probable ATP-dependent RNA helicase DDX52	DDX52_HUMAN
0.00233645	2.17474	6.69935	sp|Q9Y2R9|RT07_HUMAN	MRPS7	Q9Y2R9	Small ribosomal subunit protein uS7m	RT07_HUMAN
0.00505686	1.49961	5.66002	sp|Q9Y2Z4|SYYM_HUMAN	YARS2	Q9Y2Z4	Tyrosine--tRNA ligase, mitochondrial	SYYM_HUMAN
0.0425616	0.209156	2.74951	sp|Q9Y305|ACOT9_HUMAN	ACOT9	Q9Y305	Acyl-coenzyme A thioesterase 9, mitochondrial	ACOT9_HUMAN
0.0089692	0.498649	4.39713	sp|Q9Y314|NOSIP_HUMAN	NOSIP	Q9Y314	Nitric oxide synthase-interacting protein	NOSIP_HUMAN
0.0200166	0.797711	3.45642	sp|Q9Y315|DEOC_HUMAN	DERA	Q9Y315	Deoxyribose-phosphate aldolase	DEOC_HUMAN
0.0417746	0.141692	2.76269	sp|Q9Y383|LC7L2_HUMAN	LUC7L2	Q9Y383	Putative RNA-binding protein Luc7-like 2	LC7L2_HUMAN
0	0.355689	9.49646	sp|Q9Y399|RT02_HUMAN	MRPS2	Q9Y399	Small ribosomal subunit protein uS2m	RT02_HUMAN
0.00548105	0.308341	5.2971	sp|Q9Y3A5|SBDS_HUMAN	SBDS	Q9Y3A5	Ribosome maturation protein SBDS	SBDS_HUMAN
0.00519149	-0.485795	-5.4196	sp|Q9Y3B3|TMED7_HUMAN	TMED7	Q9Y3B3	Transmembrane emp24 domain-containing protein 7	TMED7_HUMAN
0.00711548	0.641599	4.83992	sp|Q9Y3B7|RM11_HUMAN	MRPL11	Q9Y3B7	Large ribosomal subunit protein uL11m	RM11_HUMAN
0.00508333	0.936349	5.37331	sp|Q9Y3B8|ORN_HUMAN	REXO2	Q9Y3B8	Oligoribonuclease, mitochondrial	ORN_HUMAN
0.00229358	0.481867	6.62448	sp|Q9Y3C8|UFC1_HUMAN	UFC1	Q9Y3C8	Ubiquitin-fold modifier-conjugating enzyme 1	UFC1_HUMAN
0	0.855451	10.5546	sp|Q9Y3P9|RBGP1_HUMAN	RABGAP1	Q9Y3P9	Rab GTPase-activating protein 1	RBGP1_HUMAN
0.0055702	0.555779	5.25984	sp|Q9Y3U8|RL36_HUMAN	RPL36	Q9Y3U8	Large ribosomal subunit protein eL36	RL36_HUMAN
0.000379562	1.14627	8.27261	sp|Q9Y3Z3|SAMH1_HUMAN	SAMHD1	Q9Y3Z3	Deoxynucleoside triphosphate triphosphohydrolase SAMHD1	SAMH1_HUMAN

0.0429849	-0.366079	-2.74563	sp|Q9Y446|PKP3_HUMAN	PKP3	Q9Y446	Plakophilin-3	PKP3_HUMAN	
0.00218341	0.529959	6.51948	sp|Q9Y4E8|UBP15_HUMAN	USP15	Q9Y4E8	Ubiquitin carboxyl-terminal hydrolase 15	UBP15_HUMAN	
0.0182017	-0.299355	-3.53202	sp|Q9Y4F1|FARP1_HUMAN	FARP1	Q9Y4F1	FERM, ARHGEF and pleckstrin domain-containing protein 1	FARP1_HUMAN	
0	-2.21183	-31.3575	sp|Q9Y4K0|LOXL2_HUMAN	LOXL2	Q9Y4K0	Lysyl oxidase homolog 2	LOXL2_HUMAN	
0.0237699	-0.239382	-3.31976	sp|Q9Y4Z0|LSM4_HUMAN	LSM 4.00	Q9Y4Z0	U6 snRNA-associated Sm-like protein LSm4	LSM4_HUMAN	
0.00511377	0.52825	5.38053	sp|Q9Y520|PRC2C_HUMAN	PRRC2C	Q9Y520	Protein PRRC2C	PRC2C_HUMAN	
0.0244878	-0.316022	-3.27863	sp|Q9Y570|PPME1_HUMAN	PPME1	Q9Y570	Protein phosphatase methylesterase 1	PPME1_HUMAN	
0.00728571	0.415694	4.74969	sp|Q9Y5J9|TIM8B_HUMAN	TIMM8B	Q9Y5J9	Mitochondrial import inner membrane translocase subunit Tim8 B	TIM8B_HUMAN	
0.000966292	0.595457	7.38922	sp|Q9Y5K8|VATD_HUMAN	ATP6V1D	Q9Y5K8	V-type proton ATPase subunit D	VATD_HUMAN	
0.0296563	0.406728	3.07824	sp|Q9Y5Y2|NUBP2_HUMAN	NUBP2	Q9Y5Y2	Cytosolic Fe-S cluster assembly factor NUBP2	NUBP2_HUMAN	
0.0477421	0.614131	2.64483	sp|Q9Y5Y5|PEX16_HUMAN	PEX16	Q9Y5Y5	Peroxisomal membrane protein PEX16	PEX16_HUMAN	
0.000722892	0.490453	7.72015	sp|Q9Y606|PUS1_HUMAN	PUS1	Q9Y606	Pseudouridylate synthase 1 homolog	PUS1_HUMAN	
0.0134078	0.75206	3.93408	sp|Q9Y617|SERC_HUMAN	PSAT1	Q9Y617	Phosphoserine aminotransferase	SERC_HUMAN	
0.0400206	0.558574	2.79634	sp|Q9Y657|SPIN1_HUMAN	SPIN1	Q9Y657	Spindlin-1	SPIN1_HUMAN	
0.0113814	0.153008	4.04139	sp|Q9Y678|COPG1_HUMAN	COPG1	Q9Y678	Coatomer subunit gamma-1	COPG1_HUMAN	
0.0136867	0.635534	3.90304	sp|Q9Y697|NFS1_HUMAN	NFS1	Q9Y697	Cysteine desulfurase	NFS1_HUMAN	
0.0263704	0.232371	3.1952	sp|Q9Y6C9|MTCH2_HUMAN	MTCH2	Q9Y6C9	Mitochondrial carrier homolog 2	MTCH2_HUMAN	
0.00806897	0.246642	4.53443	sp|Q9Y6G9|DC1L1_HUMAN	DYNC1LI1	Q9Y6G9	Cytoplasmic dynein 1 light intermediate chain 1	DC1L1_HUMAN	
0.00641624	-0.595788	-4.90914	sp|Q9Y6H1|CHCH2_HUMAN	CHCHD2	Q9Y6H1	Coiled-coil-helix-coiled-coil-helix domain-containing protein 2	CHCH2_HUMAN	
0.00224215	-2.31765	-6.58433	sp|Q9Y6J9|TAF6L_HUMAN	TAF6L	Q9Y6J9	TAF6-like RNA polymerase II p300/CBP-associated factor-associated factor 65 kDa s	TAF6L_HUMAN	
0.00636776	0.549677	4.89957	sp|Q9Y6K5|OAS3_HUMAN	OAS3	Q9Y6K5	2'-5'-oligoadenylate synthase 3	OAS3_HUMAN	
0.0328356	0.515633	2.97678	sp|Q9Y6M0|TEST_HUMAN	PRSS21	Q9Y6M0	Testisin	TEST_HUMAN	

T: Gene name
UBA6
ESYT2
SH3PXD2B
PGP
C5orf51
SMCHD1
POM121C
C11orf98
AGPS
KIF2A
PDLIM1
ACOT7
MYO1C
NDUFS8
PGRMC1
EIF3F
IPO5
POLRMT
IGF2BP3
GOLIM4
AGRN
PLOD2
NDUFA4
BIN1
KPNA3
PODXL
MAN2B1
PDXK
UBFD1
AP3D1
CCS
PTGES
PRMT5
SLC9A3R1
PSMA7
BCKDK
IFIT3
GIPC1
HAT1
UQCRQ
PPP1R12A
ARHGEF10
LAMA5
MRPS12
SPTLC1
PPM1G
INPPL1
EIF3D
EIF3H
NVL
BCAT2
SLC16A3
P4HA2
PRPF4
PHGDH
DYNC1LI2
PAPSS1
SART1
EEF1E1
CA12
TIMM44
NDUFB5
ASNA1
HTATSF1
NARS
LANCL1
IDH3B
NRD1
CALU
OPA1
KDM1A
PPL
ACSL4
MGEA5
GMDS
PLOD3
DIAPH1
PLIN3
RAD1
MAFK
UGDH
DPM1
USO1
PQBP1
EDF1
DIAPH2
NOL3
RNGTT
FAM20B
WDR1
ROCK2
CLASP2
CPNE3
ZC3H11A
ATP6V1G1
H2AFY
NCOR1
CS
TACC1
PSIP1
ERLIN1
NDUFS3
SRSF10
CSDE1
ERAL1
SLC25A12
RPP40
EIF3G
CIAO1
SRP72
DDAH1
GFPT2
UBXN7
ERLIN2
DKK 1.00
GLS
YIF1A
UBR5
NDUFA3
NDUFB4
NDUFB8
LUC7L3
KIF4A
NDUFA10
FKBP9
SMC2

TRIM16
IPO7
SGPL1
NSA2
ACSL3
ASMTL
OXSR1
GGPS1
WIZ
BAG2
EML2
TSPAN13
DDAH2
TXNDC12
NDUFB10
CLPTM1
TOMM40
PEX11B
LDHA
GSR
PGK 1.00
ASS1
A2M
HLA-H
TFRC
SLPI
VTN
CAT
FUCA1
ALDOA
ANXA1
GAPDH
ASL
HSPB1
TYMS
RPN1
GNAI2
HIST1H2AB
ATP1A1
SLC25A5
ISG15
ALPP
ICAM1
KRT18
KRT8
CDK1
ENO1
PYGL
GPI
NPM1
ITGAV
SERPINE2
GPX1
P4HB
CTSD
ANXA2
HEXB
EPRS
CTSB
LAMB1
ANXA6
CD55
SLC3A2
ASNS
PDHA1
ITGA5
NFIC
VIM
RPS17
KRT7
ANXA5
MRPL3
FGF2
ENO2
LGALS1
TPM1
ANXA4
CNP
PARP1
POLA1
IFIT2
IFIT1
ALPI
ALDOC
HSPA1A
TROVE2
GAA
ARAF
PRKAR1A
HSPA5
LAMC1
SLC2A1
SLC2A3
UMPS
RALA
G6PD
PC
MTHFD1
CDK4
SLC25A6
IMPDH2
TPR
CKB
ANXA3
BCKDHA
PEPD
XRCC6
XRCC5
UNG
RNH1
EEF2
PDIA4
P4HA1
PLS3
GYS1
ANXA8
ENO3
MTHFD2
CD99
PRKCSH
CPM
PKM
HSP90B1
IDE
DARS
GLUL
B4GALT1
FOSL2
CD46

NQO1
GNS
RPS2
DSP
RPA2
MUC1
NQO2
CBR1
ATP2A2
ITGA2
PRKACA
CAPN2
CEBPB
CTPS1
ENG
DDX5
PFKL
IGFBP3
RPL35A
RPL7
VCL
RPL17
PGAM1
LIG1
XRCC1
NCL
ITIH2
EIF2S2
ANXA7
BTF3
RAB4A
RAB6A
PSMB1
CAST
COL5A1
ATP6V1B2
CSRP1
FLNA
ACO1
SDHB
IGFBP4
FBLN1
TCEA1
PTGS1
PPIB
WARS
AHCY
EIF4B
ATP2B4
RRM1
COX7B
MCM3
MPST
PSMA1
PSMA3
DNMT1
RPL13
S100A4
TARS
STOM
AK4
RPL10
RPA1
CAD
MAP4
CANX
PSMB6
ABCD3
LOX
GTF2E1
EPHB2
MPG
CRABP2
EEF1D
PRDX5
PDIA3
ADSS
LRPAP1
ADSL
SERPINB1
SLC7A1
ALDH1B1
SDHA
CPS1
AKT1
PRDX2
PYCR1
GBP1
CTH
RPL9
DUT
MCM4
SHMT1
SHMT2
CTNNA1
SERPINB6
RPA3
PTGS2
CBS
MYH10
BSG
GLRX
MAP2K2
ATP6V1E1
CPOX
RPL4
PGM1
GPX4
SRP14
SNCA
RPL3
CAP2
RPL13A
EIF2S3
BUD31
KDM5C
GARS
IARS
PRKCI
ECI1
TMPO
STAT1
AKR1C3
MTOR
RPS27
RPL35
CDKN2A
ECE1
MCAM

MATR3
NAMPT
USP5
MKI67
RANGAP1
RECQL
ATRX
RPL27A
RPL5
RPL21
RPL28
RPS9
RPS5
RPS10
MAP1B
RABIF
PLA2G4A
QARS
ATP5O
LIMS1
ME1
LSS
GCLM
CD151
PITPNB
POLD2
PXN
MAPKAPK2
RPL34
LMAN1
FASN
DHPS
CCT3
MRPL19
TUFM
ALDH7A1
SRP9
AARS
CARS
HARS2
SARS
ACOT2
GSK3A
GSK3B
IDH3A
EMD
CPT1A
ST13
BCAM
RPL14
ANXA11
RAB27A
DAP
IDH3G
BCAP31
HSD17B4
RPS6KA3
HDGF
KPNA2
KPNA1
NCBP2
RAP1GDS1
POLR2H
VAV2
SMS
HK2
ACLY
CTSC
HCCS
SLC16A1
SUB1
YARS
ATP1B3
AK2
ALDH18A1
NAPA
LAMB2
TPD52
MARS
ITGA1
TMEM33
EPPK1
ELOF1
TPI1
EIF3E
EIF4A1
RPS20
PSMA6
CDC42
DSTN
CXCR4
UBE2M
RPS3A
RPL26
RPL15
RPL37A
RPL37
HNRNPK
YWHAG
RRAS2
RPS7
UBE2H
RPS14
RPS23
RPS18
RPS29
RPS11
LSM 6.00
ARF6
PSMC6
RPL7A
CNBP
RHOB
RPL23
RPS24
RPS25
RPS26
RPS28
RPL39
RPL31
RPL11
RPL8
PPIA
GNAI1
EEF1A1
TUBA4A
NOMO3
PSPH
EIF4G2

RPP30
ADARB1
NUCB2
MRPS22
MRPS5
MRPS9
RPL24
RPL36A
RHOG
RPL19
HSPG2
HDLBP
CDK5
PURA
CDC42EP1
HNRNPU
SRSF2
AMPD2
FABP5
CAP1
SLC7A5
PFKP
TAGLN
COL7A1
RPL18A
MAP2K1
PLOD1
NUCB1
RPL6
ACY1
PLAUR
TAP1
RELA
UBXN1
GBE1
EIF4G1
NOTCH2
TLE3
YWHAH
EEF1A2
CALD1
ITIH3
GFPT1
RPL18
CKAP4
TJP1
LRP1
PDE4D
SLFN5
RBBP4
AHNAK
GALNT2
AP1B1
BST2
PMPCA
ASPH
GRSF1
DPYD
TRIM26
LMAN2
PTP4A2
ECH1
STK4
ACACA
TARDBP
AIMP2
PRDX4
CBX3
SELENBP1
SRSF9
TRIM28
G3BP1
EIF3I
ILK
SF3B2
ADAM9
TMED1
SQSTM1
MTX1
CAMK2G
MTMR1
MTMR2
CUL4B
FHL3
ALCAM
ARFRP1
GNL2
BLMH
BYSL
COTL1
IL18
BOP 1.00
KEAP1
UBAP2L
MLEC
TTLL12
FHL2
DCTN1
DYNC1H1
MAP7
TRIM25
FLNC
FAM50A
GALE
PDIA5
DHX8
ITPR1
PLS1
SMC1A
MESDC2
GANAB
MVP
KPNB1
NUMA1
PSME4
NCAPH
WTAP
MORF4L2
SEPT2
NCAPD2
LARS2
ARL6IP1
RAB3GAP1
SLC39A14
POLD3
WDR43
PDIA6
PCOLCE
PDK3

PLEC
PON2
NONO
PTPRK
PCBP1
RSU1
TSN
OASL
TRIP6
SLC1A5
SMAD2
RAB11B
ZYX
IGFBP7
PKN1
CSRP2
DDB1
MAPK14
FSCN1
DECR1
MAN2A1
KYNU
CLPP
GUK1
CA9
PCK2
UGP2
HNRNPUL2
INF2
QRICH1
HKDC1
LEPRE1
MAP7D1
UAP1L1
DAK
TIMM50
ANO6
PDCD4
FNDC3B
PYCRL
OCIAD2
DNAJC21
RABL3
TOR1AIP1
WDR45B
TTC38
CEP170
UBR4
FOCAD
LRRC16A
FAM91A1
TNS3
ARHGAP17
VASN
TWF2
NIPBL
ARMC6
CC2D1A
PDXDC1
CTR9
DARS2
BRAT1
CCDC137
MTHFD1L
MPRIP
HACD2
ZNF574
NFXL1
NBEAL2
UNC13D
IKBIP
SND1
BZW1
DHX30
RSRC2
CYFIP1
LPCAT2
KDM3B
CHMP1B
ZC3HAV1
NUP54
NUFIP2
CLASP1
KDELC2
VN1R5
IRF2BP2
TMED4
CENPV
SETD3
PABPN1
MTDH
NAA40
AACS
LUZP1
CCDC25
LSR
ANKLE2
IRF2BP1
TRAPPC5
MISP
ITPRIP
RBM12B
ADSSL1
ARHGAP18
EHBP1L1
STAG2
JAGN1
ARFGAP1
CDKN2A
ENAH
EIF1AD
MLKL
COLGALT1
LEMD2
LPCAT1
FBXO30
DTX3L
DDX54
GPX8
WIPF2
SETD7
CCDC12
PPP1R13L
SLC38A5
SNX33
LMO7
ATXN2L
PRPF31
SYNE2

OVCA2
IRGQ
PHF3
NDRG1
HSPH1
SEPT8
HMHA1
PXDN
ARPC1A
GGH
DDX17
RAD50
CELF1
ARHGEF1
GLG1
UPF1
RAB8B
KHSRP
TNPO1
ARHGEF2
USP7
POP5
MYDGF
IGSF8
RPL36AL
ZNF622
ERGIC1
CIRH1A
TBRG4
FAM162A
EXOC4
FERMT2
FUBP1
RPE
FKBP10
TOMM6
ZNF428
SLC22A18
PPWD1
COA7
FAM136A
EFHD2
GALM
PYCR2
DCPS
FAF2
CCDC124
NMD3
MRPL53
RRP36
ADAT3
DUS3L
LTV1
MRPL48
SNF8
ERO1L
DDRGK1
FUBP3
GMPPA
PAWR
CDK5RAP3
USP47
CNDP2
LRRC7
MUS81
ARAP1
FNBP1
C16orf13
WRNIP1
SRPK1
OSBPL9
RBM15
UHRF1
FAM129B
CNN2
PLIN2
SCAF11
TEAD3
TSNAX
EIF3C
TTC1
DNAJC7
ATXN2
HSD17B10
SLC29A1
NCAPG
DDX50
ELAC2
MACROD1
TCF25
APOL2
CORO1B
MRPL45
C7orf50
PDCD2L
LLPH
ERP44
FSD1
TBCD
FUCA2
HGH1
MMTAG2
TUBB6
RPP25
DDX23
THUMPD3
TMEM109
ACAT2
RBM4
SFXN3
CHID1
SLC4A1AP
GTPBP2
OSBPL10
MAK16
RTFDC1
EIF2A
MRPL32
MRPL13
MRPL4
HELZ2
MRPS26
POTEKP
GTPBP4
RAB34
FAM129A
API5
FTO
TANC1

WDR12
REXO4
POLR1E
WDR61
EGLN1
NAA50
CLPB
LSG1
NAT10
MRPL18
VPS11
PPIL3
SLC38A1
DHX36
CPVL
TXNIP
TMX1
NELFA
UNC45A
SMOC1
EPB41L1
EHD1
CDCP1
DCTPP1
SH2D4A
DDX31
ANKZF1
WDR76
EHMT1
GRPEL1
MCCC2
ABCB6
HMG20A
SARS2
MRPS30
RIC8A
SEMA4B
EXOSC3
ANLN
XPNPEP1
GPHN
PDLIM7
DDX21
PDS5B
SMC4
SPATS2L
PARVA
TBC1D13
ATAD3A
SDAD1
ARGLU1
C1orf123
OCIAD1
TOR4A
TRMT1
COA4
TMOD3
UGGT1
MTCH1
MYOF
EHD2
OR5AC2
NCKIPSD
MRPL15
NDUFA13
RCC2
DIP2B
RRBP1
LARS
RBM27
ANKFY1
SUCLA2
EIF3K
GRHPR
CTSZ
ZMYM2
TJP2
DAXX
ABCF2
ARMCX3
LIMA1
CHORDC1
PCYOX1
DPP7
TMEM2
NARF
BAIAP2L1
ATP6V1H
RABAC1
BAZ1B
NAGK
HACL1
RASAL2
ERRFI1
DCTN4
HN1
PACSIN3
NUP50
CDV3
NOB1
MAFF
ICAM5
DDX19B
PACSIN2
PROCR
C19orf53
SMC3
EIF3L
CFL2
ERGIC3
FNDC3A
DIS3
GSTK1
DDX52
MRPS7
YARS2
ACOT9
NOSIP
DERA
LUC7L2
MRPS2
SBDS
TMED7
MRPL11
REXO2
UFC1
RABGAP1
RPL36
SAMHD1

PKP3
USP15
FARP1
LOXL2
LSM 4.00
PRRC2C
PPME1
TIMM8B
ATP6V1D
NUBP2
PEX16
PUS1
PSAT1
SPIN1
COPG1
NFS1
MTCH2
DYNC1LI1
CHCHD2
TAF6L
OAS3
PRSS21
