# Supplementary material for: Extracellular Vesicles Bearing Vimentin Drive Epithelial–Mesenchymal Transition
Source: Mol Cell Proteomics. 2025 Jul 4;24(12):101028. doi: 10.1016/j.mcpro.2025.101028 (PMC12719745; doi:10.1016/j.mcpro.2025.101028)
Supplement: Supplemental Data 8 [file mmc11.pdf]

Sheet 8.MCF10A_clean_data

MCF10A_EV_KO_2 MaxLFQ Intensity	MCF10A_EV_KO_3 MaxLFQ Intensity	MCF10A_EV_KO_4 MaxLFQ Intensity	MCF10A_EV_WT_1 MaxLFQ Intensity	MCF10A_EV_WT_2 MaxLFQ Intensity	MCF10A_EV_WT_3 MaxLFQ Intensity	C: Protein Existence
30.0075	29.6249	29.5582	29.6095	29.8047	29.6278	1:Experimental evidence at protein level
26.8971	27.185	27.2529	27.6901	27.4101	27.3159	1:Experimental evidence at protein level
25.1461	25.2178	25.1403	24.8905	24.9723	24.9017	1:Experimental evidence at protein level
24.8452	25.3878	25.4266	25.0149	24.6122	25.0604	1:Experimental evidence at protein level
26.0912	26.8652	26.8025	26.4806	26.3354	26.4817	1:Experimental evidence at protein level
26.0776	26.4645	26.163	25.9933	25.5608	25.9316	1:Experimental evidence at protein level
25.7996	25.1774	25.068	25.1336	25.0791	24.7472	1:Experimental evidence at protein level
28.2785	28.0961	27.3501	27.7818	27.828	27.6641	1:Experimental evidence at protein level
28.3796	29.3066	29.588	29.0729	28.7704	29.382	1:Experimental evidence at protein level
28.3984	28.0566	27.7788	27.4839	27.5461	27.2775	1:Experimental evidence at protein level
28.1382	28.4216	28.6016	28.8807	29.0544	28.5857	1:Experimental evidence at protein level
#NUM!	25.6263	26.1909	26.0813	26.3178	25.8289	1:Experimental evidence at protein level
27.3006	27.8974	28.1585	27.7333	27.2927	27.7224	1:Experimental evidence at protein level
27.2768	27.29	27.0028	27.354	27.8576	27.0211	1:Experimental evidence at protein level
25.3087	25.0854	25.2955	25.1986	25.6494	25.4517	1:Experimental evidence at protein level
28.6005	28.5024	28.7356	29.3328	30.0203	28.7509	2:Experimental evidence at transcript level
25.9795	26.0146	26.0489	25.4615	25.6778	25.3795	1:Experimental evidence at protein level
26.8601	26.6325	26.7385	26.3137	26.8292	26.2223	1:Experimental evidence at protein level
25.5697	25.3995	25.255	25.2105	25.4162	25.3528	1:Experimental evidence at protein level
27.5837	27.6031	27.5349	27.484	27.4925	27.2175	1:Experimental evidence at protein level
24.5206	25.301	25.0724	25.2952	25.1902	25.3466	1:Experimental evidence at protein level
25.0042	#NUM!	25.3554	25.6494	25.2912	25.6982	1:Experimental evidence at protein level
26.9802	26.819	26.9012	27.0223	27.3305	#NUM!	1:Experimental evidence at protein level
26.6009	25.6556	25.3862	25.1831	25.2602	25.4774	1:Experimental evidence at protein level
24.9899	25.3695	25.0932	25.1906	25.4365	25.0874	1:Experimental evidence at protein level
26.2759	25.9261	25.6382	25.6902	25.7227	26.2334	1:Experimental evidence at protein level
25.7272	25.6829	25.7461	25.5611	25.5615	25.7294	1:Experimental evidence at protein level
25.8522	25.0388	24.8743	24.9447	25.4623	24.8438	1:Experimental evidence at protein level
27.5092	27.2351	26.9838	27.2707	27.4548	27.2055	1:Experimental evidence at protein level
25.2782	#NUM!	25.4046	25.2769	25.1272	25.1735	1:Experimental evidence at protein level
#NUM!	26.2842	26.0944	27.046	26.9129	26.2296	1:Experimental evidence at protein level
27.546	#NUM!	27.7705	28.0164	27.458	27.8624	1:Experimental evidence at protein level
27.6094	27.3268	27.4144	25.7902	25.8777	25.4512	1:Experimental evidence at protein level
#NUM!	27.0742	27.0934	26.8244	26.9953	26.7209	1:Experimental evidence at protein level
25.1409	25.0649	24.8862	25.0395	25.1529	24.7174	1:Experimental evidence at protein level
26.0292	26.1182	26.4144	26.1202	25.7604	26.0897	1:Experimental evidence at protein level
29.0503	29.3018	29.4734	29.2704	28.9665	29.1937	1:Experimental evidence at protein level
26.498	27.4462	27.5067	27.0602	26.7021	27.1968	1:Experimental evidence at protein level
24.7665	25.1783	25.2986	25.2483	25.4409	24.8643	1:Experimental evidence at protein level
30.1825	30.1894	30.1711	30.3071	30.4759	30.3306	1:Experimental evidence at protein level
25.8015	25.7463	25.7406	25.7702	25.4282	25.3593	1:Experimental evidence at protein level
25.2248	#NUM!	25.507	25.5983	25.4016	25.6868	2:Experimental evidence at transcript level
27.0807	27.2085	26.9752	26.9906	26.0979	27.1294	1:Experimental evidence at protein level
26.8916	26.8079	#NUM!	26.5384	26.2176	26.2146	1:Experimental evidence at protein level
27.3534	26.0375	25.9401	25.838	26.9278	26.0805	1:Experimental evidence at protein level
25.86	25.5054	25.872	25.9139	26.0418	25.8063	1:Experimental evidence at protein level
24.2358	#NUM!	#NUM!	23.7234	23.9286	23.6057	1:Experimental evidence at protein level
28.5936	29.5887	29.6778	29.7717	29.199	29.9843	1:Experimental evidence at protein level
26.2289	25.8768	25.8906	25.7339	26.0993	25.6875	1:Experimental evidence at protein level
25.7933	26.2444	26.3971	26.3756	26.143	26.4872	1:Experimental evidence at protein level
26.6433	26.6086	26.6371	26.3969	26.1842	26.4196	1:Experimental evidence at protein level
25.8793	25.8341	25.8188	26.459	26.5412	26.3621	1:Experimental evidence at protein level
27.1118	27.2794	27.5327	27.1535	26.9067	27.3323	1:Experimental evidence at protein level
25.0871	25.8862	25.3796	25.7432	25.5296	25.581	1:Experimental evidence at protein level
27.4087	27.0736	26.914	26.7417	26.9252	26.7602	1:Experimental evidence at protein level
26.418	26.4879	26.5475	26.2567	26.3421	26.2809	1:Experimental evidence at protein level
25.0436	25.3756	25.3413	25.469	25.295	25.5668	1:Experimental evidence at protein level
#NUM!	25.8542	25.2911	26.8623	26.8609	26.689	1:Experimental evidence at protein level
25.7173	26.218	25.8447	26.2219	26.3818	26.4001	1:Experimental evidence at protein level
26.404	26.7509	26.595	25.8667	25.6011	25.9973	1:Experimental evidence at protein level
25.8936	25.9448	26.018	25.6599	25.3097	25.4835	1:Experimental evidence at protein level
#NUM!	24.3965	24.3141	24.0836	24.3384	23.8761	1:Experimental evidence at protein level
26.3204	26.0987	25.9545	25.9229	25.9398	26.2518	1:Experimental evidence at protein level
26.4945	26.3791	26.5557	26.1398	25.5548	26.1982	1:Experimental evidence at protein level
26.5758	26.236	26.1797	25.8858	26.2223	25.9709	1:Experimental evidence at protein level
26.4891	26.1549	26.272	26.46	26.4823	26.565	1:Experimental evidence at protein level
26.7422	26.8003	26.7823	26.6262	26.9021	26.5085	1:Experimental evidence at protein level
26.1044	25.6849	25.8418	26.3022	26.0923	26.0781	1:Experimental evidence at protein level
26.5749	27.0115	27.047	26.7705	26.5644	27.0108	1:Experimental evidence at protein level
26.6732	26.5999	26.2945	26.0474	26.2144	26.0403	1:Experimental evidence at protein level
26.165	25.9869	25.6181	27.1927	27.2433	27.1514	1:Experimental evidence at protein level
27.8808	27.0905	26.9614	27.0048	25.9997	26.7612	1:Experimental evidence at protein level
27.227	27.3476	27.6305	26.9596	26.9561	27.0187	1:Experimental evidence at protein level
26.2246	26.0311	26.1255	26.3525	26.4876	25.8388	1:Experimental evidence at protein level
27.0498	27.1621	27.0697	27.0388	26.9487	26.951	1:Experimental evidence at protein level
25.3528	24.9519	24.987	24.8739	25.0274	25.0924	1:Experimental evidence at protein level
26.3018	25.5283	25.9512	25.285	25.8706	25.4123	1:Experimental evidence at protein level
29.583	29.1817	29.3131	29.0525	29.0589	29.1509	1:Experimental evidence at protein level
25.6719	26.0833	25.8064	25.6737	25.2641	25.4399	1:Experimental evidence at protein level
26.8652	27.6634	27.6055	27.4542	27.492	27.457	1:Experimental evidence at protein level
27.8285	28.443	28.3086	28.0953	28.1789	27.721	1:Experimental evidence at protein level
27.3286	28.1716	28.257	27.9376	27.2452	27.8749	1:Experimental evidence at protein level
27.5459	27.2333	27.4037	27.1781	27.1833	26.9144	1:Experimental evidence at protein level
30.4832	30.3954	30.1268	30.1212	30.502	30.0568	1:Experimental evidence at protein level
28.1402	28.0851	27.9586	28.7808	28.7078	28.862	1:Experimental evidence at protein level
27.6212	27.6587	27.5707	27.4287	27.2571	27.4547	1:Experimental evidence at protein level
26.0049	25.5063	25.3187	25.9239	26.3527	25.741	1:Experimental evidence at protein level
27.6492	26.9639	26.7733	26.6755	27.6078	26.8218	1:Experimental evidence at protein level
28.0937	28.3769	28.334	28.3687	28.4546	28.2729	1:Experimental evidence at protein level
26.0909	26.3412	26.493	26.3821	26.7854	26.3929	1:Experimental evidence at protein level
27.3951	26.9607	27.292	26.9956	26.213	26.9726	1:Experimental evidence at protein level
26.1611	26.2348	25.9767	26.1057	26.3288	26.2863	1:Experimental evidence at protein level
27.6266	27.9538	27.5464	27.7831	27.6079	28.0614	1:Experimental evidence at protein level
26.8528	26.9941	27.0237	27.0402	26.7081	26.8715	1:Experimental evidence at protein level
26.7165	27.2803	27.3555	27.4196	#NUM!	27.2566	1:Experimental evidence at protein level
29.0396	28.5493	29.2935	28.3062	29.0617	28.1481	1:Experimental evidence at protein level
27.5495	26.9537	26.5848	26.738	27.2858	26.7281	1:Experimental evidence at protein level
25.9473	25.651	#NUM!	25.5867	25.7047	25.7892	1:Experimental evidence at protein level
27.5577	27.0805	27.175	26.7403	27.0537	26.7367	1:Experimental evidence at protein level
27.4505	26.5578	26.78	26.23	27.4304	26.4836	1:Experimental evidence at protein level
26.6304	26.8237	26.5749	26.5896	26.8619	26.6314	1:Experimental evidence at protein level
26.1228	26.1112	26.2048	26.1003	26.0305	26.2116	1:Experimental evidence at protein level
26.4983	26.0204	25.7947	25.7762	26.0054	25.7832	1:Experimental evidence at protein level
25.7653	26.358	26.4396	26.1882	25.8188	25.8166	1:Experimental evidence at protein level
#NUM!	25.3648	#NUM!	25.9423	25.743	25.6667	1:Experimental evidence at protein level
26.2772	26.4025	26.1833	26.2955	26.5003	26.2895	1:Experimental evidence at protein level
24.5653	24.75	24.863	24.992	25.0679	24.8465	1:Experimental evidence at protein level
25.6564	25.6266	25.6798	25.4685	25.6784	25.2453	1:Experimental evidence at protein level
26.283	26.3124	26.3622	26.2979	26.2991	26.0267	1:Experimental evidence at protein level
#NUM!	#NUM!	25.1993	25.6339	25.7942	25.18	1:Experimental evidence at protein level
26.748	27.057	27.0182	26.9526	26.7701	26.8521	1:Experimental evidence at protein level
26.0532	26.5382	26.5077	26.3334	26.321	26.3313	1:Experimental evidence at protein level
#NUM!	24.7168	24.6967	24.6867	24.5568	23.9728	1:Experimental evidence at protein level
27.2757	27.099	27.2019	27.4657	27.1599	27.2106	1:Experimental evidence at protein level
26.6275	27.219	27.145	27.1587	26.6278	26.7234	1:Experimental evidence at protein level
24.2855	24.7325	24.5074	25.6532	24.859	25.2495	1:Experimental evidence at protein level
25.0115	25.0103	25.1528	24.8068	#NUM!	24.8322	1:Experimental evidence at protein level
#NUM!	25.1819	25.1156	25.1883	25.4013	25.0493	1:Experimental evidence at protein level
27.3526	27.584	27.5745	27.3302	27.027	27.1649	1:Experimental evidence at protein level
27.4266	27.5001	27.7256	27.5448	27.3711	27.5974	1:Experimental evidence at protein level
#NUM!	26.1426	26.4132	25.9184	25.5842	26.0455	1:Experimental evidence at protein level
25.7351	25.8659	25.9387	25.5523	25.1746	25.4667	1:Experimental evidence at protein level
25.0112	24.4342	24.494	24.3774	25.2752	24.4806	1:Experimental evidence at protein level
25.215	25.891	26.0756	26.1319	25.6154	26.1908	1:Experimental evidence at protein level
28.8432	28.1671	28.1222	28.1634	28.4452	28.0613	1:Experimental evidence at protein level
27.0654	26.9616	26.8804	26.9571	26.2554	26.8112	1:Experimental evidence at protein level

26.2434	26.2248	26.2494	26.1835	25.4754	26.0856	1:Experimental evidence at protein level
27.8427	28.04	28.2016	27.6947	27.4051	27.8228	1:Experimental evidence at protein level
27.7741	27.6359	27.5632	27.4451	#NUM!	27.4437	1:Experimental evidence at protein level
26.611	27.0286	26.9057	26.9401	26.5809	26.9961	1:Experimental evidence at protein level
26.8457	#NUM!	27.3926	26.8682	26.7983	27.0869	1:Experimental evidence at protein level
25.8243	25.4027	25.4439	25.1529	25.4095	25.1148	1:Experimental evidence at protein level
26.6648	27.0788	26.8671	26.8879	26.6368	26.8454	1:Experimental evidence at protein level
25.7495	26.6311	26.289	26.2856	25.8506	26.0575	1:Experimental evidence at protein level
27.2833	#NUM!	27.5562	27.9386	27.7393	27.9015	1:Experimental evidence at protein level
24.391	24.752	24.596	24.5589	23.9994	24.8571	1:Experimental evidence at protein level
26.7989	26.443	26.4087	26.4177	26.7041	26.6144	1:Experimental evidence at protein level
25.718	24.7597	24.9289	24.548	24.471	24.949	1:Experimental evidence at protein level
28.8992	#NUM!	27.8866	28.401	28.7358	28.4286	1:Experimental evidence at protein level
26.3777	25.8066	25.67	25.7384	26.0029	26.2227	1:Experimental evidence at protein level
25.96	25.6101	25.679	25.8393	25.6426	25.5413	1:Experimental evidence at protein level
27.2595	27.7403	27.6803	27.3838	26.9841	27.2542	1:Experimental evidence at protein level
25.1201	25.3003	25.2173	25.2478	25.1134	24.9527	1:Experimental evidence at protein level
27.7718	28.2346	28.2202	27.946	27.6224	28.0213	1:Experimental evidence at protein level
25.6729	#NUM!	25.6766	25.629	25.3555	25.4785	1:Experimental evidence at protein level
26.2427	25.2251	25.4497	25.332	25.7885	25.4599	1:Experimental evidence at protein level
26.7424	27.3832	27.1988	27.5338	26.7816	27.2139	1:Experimental evidence at protein level
26.5343	26.5491	26.6187	26.3587	26.7257	26.5285	1:Experimental evidence at protein level
23.3967	23.8357	23.3662	23.5097	23.5231	23.4036	1:Experimental evidence at protein level
26.4785	26.0046	25.7326	25.8895	26.2898	25.9799	1:Experimental evidence at protein level
28.2713	27.651	27.6074	26.9328	27.5949	27.4218	1:Experimental evidence at protein level
27.2122	26.908	26.707	26.5015	26.9966	26.305	1:Experimental evidence at protein level
25.4424	25.3765	25.182	25.3904	25.299	25.3123	1:Experimental evidence at protein level
27.0849	26.6149	26.4474	26.5624	26.9589	26.3843	1:Experimental evidence at protein level
26.1083	26.4263	26.3594	26.218	26.1864	26.0695	1:Experimental evidence at protein level
27.1108	27.2135	27.0616	27.5678	27.2792	27.5707	1:Experimental evidence at protein level
26.1842	25.6421	25.6107	25.417	25.9633	25.4097	1:Experimental evidence at protein level
28.9549	28.8916	28.6275	28.1611	28.503	28.2808	5:Protein uncertain
27.3923	26.8484	27.0263	26.8072	27.048	26.9542	4:Protein predicted
25.0096	24.8861	24.7396	24.7862	24.2752	24.7443	3:Protein inferred from homology
26.2258	25.4754	25.3099	25.1449	25.4101	#NUM!	1:Experimental evidence at protein level
27.5758	28.0356	27.8961	27.8606	28.0841	28.0041	1:Experimental evidence at protein level
25.1057	25.0987	25.3657	25.3433	25.3054	25.4009	1:Experimental evidence at protein level
27.5635	27.7459	27.5367	27.4102	27.4851	27.5798	1:Experimental evidence at protein level
26.5368	26.7189	26.9832	26.9245	26.5416	26.7349	1:Experimental evidence at protein level
27.5689	28.036	27.8421	27.7363	27.6312	28.0027	1:Experimental evidence at protein level
28.0152	27.695	27.7865	27.515	27.7768	27.6127	1:Experimental evidence at protein level
26.2303	26.5417	26.4813	26.4112	26.2775	26.6808	1:Experimental evidence at protein level
24.9048	24.7365	24.5844	25.3024	24.9766	24.9129	1:Experimental evidence at protein level
28.4232	28.3769	28.3909	28.115	28.5169	28.5089	1:Experimental evidence at protein level
27.0841	27.0293	27.1369	26.7352	26.5935	26.6997	1:Experimental evidence at protein level
25.5692	25.7258	25.6047	25.733	25.5606	25.674	1:Experimental evidence at protein level
29.2086	28.8547	29.0661	28.7248	28.8682	28.5834	2:Experimental evidence at transcript level
25.8292	26.5178	26.5368	26.3402	25.4159	26.7229	1:Experimental evidence at protein level
26.1887	26.1604	25.8129	26.1208	26.2358	25.9282	1:Experimental evidence at protein level
#NUM!	26.3749	26.4342	26.1661	27.0918	26.3577	1:Experimental evidence at protein level
30.2671	30.6218	30.6878	30.579	30.8628	30.4751	1:Experimental evidence at protein level
25.895	26.4001	26.1015	26.7077	26.1769	26.1142	1:Experimental evidence at protein level
27.2202	27.4886	27.5615	27.0599	26.924	27.0908	1:Experimental evidence at protein level
#NUM!	26.019	25.9466	25.8924	25.5666	25.7867	1:Experimental evidence at protein level
26.8164	26.5648	26.5577	26.239	26.6931	26.4154	1:Experimental evidence at protein level
26.2207	26.0878	26.3469	26.6545	26.4544	26.5713	1:Experimental evidence at protein level
27.4426	27.0328	26.9981	26.9518	27.5507	27.1044	1:Experimental evidence at protein level
25.9612	26.1883	25.959	26.0923	25.715	25.7139	1:Experimental evidence at protein level
30.1982	29.6185	29.6888	29.6672	30.1817	29.6809	1:Experimental evidence at protein level
27.5335	27.2642	27.2258	27.0491	26.8016	27.3012	1:Experimental evidence at protein level
28.1549	28.4817	28.6479	27.7696	28.0828	27.9685	1:Experimental evidence at protein level
30.5466	29.9682	30.0997	29.8369	30.3702	30.0629	1:Experimental evidence at protein level
27.5232	26.4615	26.4758	26.8809	27.431	26.6257	1:Experimental evidence at protein level
25.5993	25.9355	25.8223	25.7306	25.6977	25.5592	1:Experimental evidence at protein level
#NUM!	29.3304	29.1325	29.3847	29.474	29.3189	1:Experimental evidence at protein level
26.2283	25.5319	25.3241	25.3978	25.902	25.3488	1:Experimental evidence at protein level
27.997	27.5767	27.6589	27.399	27.8595	27.5843	1:Experimental evidence at protein level
25.0166	25.3224	25.216	25.1045	25.2219	25.3154	1:Experimental evidence at protein level
29.2023	28.9517	28.9156	28.9596	29.3048	29.089	1:Experimental evidence at protein level
25.4844	26.1393	26.1998	25.9569	25.3066	26.0018	1:Experimental evidence at protein level
28.9538	29.0944	28.7804	29.1079	29.4977	29.3269	1:Experimental evidence at protein level
27.5013	27.1915	27.2637	27.3047	27.1531	26.9843	1:Experimental evidence at protein level
25.0479	25.371	25.3476	24.8036	25.0501	25.0581	1:Experimental evidence at protein level
25.2993	25.536	25.6129	25.4571	25.68	25.5324	1:Experimental evidence at protein level
26.9475	27.5837	27.4852	27.4022	26.7994	27.1439	1:Experimental evidence at protein level
25.7632	25.5439	25.466	25.3905	25.6572	25.061	1:Experimental evidence at protein level
28.0216	28.193	28.1031	27.7662	27.6977	27.5927	1:Experimental evidence at protein level
25.6622	25.9746	25.9128	25.7782	25.2491	25.9256	1:Experimental evidence at protein level
#NUM!	26.2966	26.1198	26.5669	25.3312	26.5382	1:Experimental evidence at protein level
27.4414	27.0711	26.9974	27.0715	26.804	26.9284	1:Experimental evidence at protein level
27.3177	27.3424	27.1357	27.1567	27.0971	27.1113	1:Experimental evidence at protein level
26.1048	26.3592	26.5973	26.1859	27.1385	26.5168	1:Experimental evidence at protein level
24.4637	#NUM!	24.7383	24.7473	24.8337	24.8604	1:Experimental evidence at protein level
25.6281	25.4276	25.5789	25.6152	25.7903	25.6348	1:Experimental evidence at protein level
26.6687	25.7983	25.3662	25.771	26.2084	26.6267	1:Experimental evidence at protein level
26.0187	26.4771	26.3943	26.4988	25.9954	26.3865	1:Experimental evidence at protein level
#NUM!	26.6284	26.5931	26.9768	26.5809	26.7804	1:Experimental evidence at protein level
26.6288	26.5292	26.3285	25.6428	25.5402	25.7082	1:Experimental evidence at protein level
25.3234	25.4503	25.2436	25.3112	25.0366	24.5754	1:Experimental evidence at protein level
25.9591	25.1698	25.2543	25.2119	25.7489	25.453	1:Experimental evidence at protein level
26.9842	26.299	26.2929	26.5459	26.2427	26.5092	1:Experimental evidence at protein level
25.5948	25.0378	25.379	24.9304	25.0423	25.0097	1:Experimental evidence at protein level
27.2079	27.038	26.8375	27.0684	26.8621	26.5696	1:Experimental evidence at protein level
26.206	26.0088	26.219	26.185	26.0854	26.3279	1:Experimental evidence at protein level
27.5211	26.259	26.0655	26.0691	26.0205	25.837	1:Experimental evidence at protein level
26.3386	26.2893	26.1696	26.012	26.0003	25.3721	1:Experimental evidence at protein level
26.2023	26.1111	25.8008	25.7506	25.825	25.5633	1:Experimental evidence at protein level
27.7513	27.7484	27.7128	27.6859	27.7239	27.5698	1:Experimental evidence at protein level
27.0023	26.8367	26.8756	26.923	26.8364	26.9996	1:Experimental evidence at protein level
27.3537	27.1216	27.3407	27.0968	27.0211	27.1093	1:Experimental evidence at protein level
27.6642	27.083	26.5727	26.4411	27.1318	26.5498	1:Experimental evidence at protein level
#NUM!	26.0108	25.9909	25.9737	26.2724	25.8962	1:Experimental evidence at protein level
26.9456	26.4857	26.5561	26.4693	26.7677	26.621	1:Experimental evidence at protein level
27.3697	27.7858	27.7659	27.5396	27.3792	27.3495	1:Experimental evidence at protein level
26.1769	25.0486	25.1552	25.2898	25.9142	25.9605	1:Experimental evidence at protein level
25.1961	25.686	25.6832	27.1422	26.6725	27.1345	1:Experimental evidence at protein level
27.3608	26.799	26.6265	26.6656	27.0651	26.533	1:Experimental evidence at protein level
26.1729	26.2467	26.083	26.1523	25.5904	25.9009	1:Experimental evidence at protein level
25.3915	26.0257	26.1713	#NUM!	25.2231	25.3107	1:Experimental evidence at protein level
26.9012	27.0271	27.1695	26.9818	27.2139	26.8494	1:Experimental evidence at protein level
29.0178	29.1061	28.8881	29.0106	29.2208	29.138	1:Experimental evidence at protein level
26.4634	26.5755	26.4467	26.3764	26.2671	26.1596	1:Experimental evidence at protein level
25.5197	25.6503	25.5898	25.6671	25.6861	25.5416	1:Experimental evidence at protein level
27.8281	27.9173	27.8606	27.6414	27.4593	27.791	1:Experimental evidence at protein level
26.9109	26.534	26.4489	26.3023	26.565	26.422	1:Experimental evidence at protein level
26.469	26.7052	26.6297	26.434	26.5002	26.483	1:Experimental evidence at protein level
26.8409	26.6539	26.7684	26.7658	26.9255	26.3616	1:Experimental evidence at protein level
24.5601	24.3847	25.028	24.6546	24.2224	24.6375	1:Experimental evidence at protein level
26.4998	26.328	26.3873	26.3691	26.4704	26.2939	1:Experimental evidence at protein level
24.997	25.2331	25.0512	25.679	25.6733	25.5794	1:Experimental evidence at protein level
26.1202	25.9628	26.0796	26.1604	26.1412	25.9696	1:Experimental evidence at protein level
26.9234	27.4381	27.4648	27.5779	27.1976	27.553	1:Experimental evidence at protein level
26.054	26.357	26.3447	26.0583	25.7211	26.121	1:Experimental evidence at protein level
26.7572	26.7263	26.6161	26.63	26.7253	26.4073	1:Experimental evidence at protein level
29.4316	28.8628	28.9437	28.5812	29.2101	28.6905	1:Experimental evidence at protein level
26.1353	25.8549	25.6288	25.7037	25.7375	26.0387	1:Experimental evidence at protein level
27.7001	27.5225	27.5521	27.6773	27.9864	27.5954	1:Experimental evidence at protein level
27.6428	28.0901	28.1459	28.0771	28.1488	27.9242	1:Experimental evidence at protein level
24.1173	#NUM!	24.1372	24.2054	24.2594	24.1706	1:Experimental evidence at protein level

25.0646	25.5149	25.3596	25.2451	24.8607	25.4066	1:Experimental evidence at protein level
27.934	27.8706	27.6688	27.6312	28.0001	27.752	1:Experimental evidence at protein level
27.19	27.7755	27.762	27.5478	26.6146	27.7618	1:Experimental evidence at protein level
26.4399	26.5987	26.5301	25.3758	25.3484	25.3559	1:Experimental evidence at protein level
26.472	27.1128	26.861	26.5205	26.2817	26.2262	1:Experimental evidence at protein level
25.8577	25.3957	25.6181	25.6131	25.5685	25.8245	1:Experimental evidence at protein level
26.0844	26.189	25.7865	26.1718	26.0435	26.0403	1:Experimental evidence at protein level
25.4369	25.6404	25.7839	25.6845	25.8005	25.5056	1:Experimental evidence at protein level
27.207	26.1017	26.5547	26.4387	26.5227	26.4056	1:Experimental evidence at protein level
25.8229	25.5777	25.0846	25.094	25.2591	25.2937	1:Experimental evidence at protein level
27.3269	27.3343	27.1421	27.5266	27.7662	27.4848	1:Experimental evidence at protein level
#NUM!	23.8278	24.5647	24.1903	24.9661	25.3014	1:Experimental evidence at protein level
27.6955	27.6637	27.7885	27.8234	27.8443	27.7833	1:Experimental evidence at protein level
27.0699	26.9921	27.2855	27.1439	27.0118	27.1774	1:Experimental evidence at protein level
28.0633	27.7905	27.924	27.5799	27.2526	27.7071	1:Experimental evidence at protein level
27.21	27.1373	27.1686	27.272	27.1737	27.2507	1:Experimental evidence at protein level
27.4226	27.3896	27.3382	27.2943	27.4824	27.3441	1:Experimental evidence at protein level
29.9495	29.7403	29.5854	29.8072	29.82	29.7546	1:Experimental evidence at protein level
28.5134	28.4971	28.4822	28.4072	28.1473	28.5368	1:Experimental evidence at protein level
26.5486	26.5306	26.4987	25.4547	25.8218	25.7809	1:Experimental evidence at protein level
26.7891	26.4999	26.6468	26.6315	26.8262	26.5199	1:Experimental evidence at protein level
26.3511	26.2143	26.1524	25.595	25.8213	25.6655	1:Experimental evidence at protein level
27.9868	27.7282	27.8809	27.1516	27.5405	27.3346	1:Experimental evidence at protein level
28.0654	28.1763	28.1385	28.1713	28.4054	27.8197	1:Experimental evidence at protein level
27.5117	26.6562	27.0662	27.1765	28.0018	27.5976	1:Experimental evidence at protein level
25.3974	26.3436	26.1083	26.0584	25.8081	26.0977	1:Experimental evidence at protein level
24.6907	25.1541	#NUM!	25.6015	25.8498	25.6006	1:Experimental evidence at protein level
26.1399	25.8284	26.2619	26.7983	26.6706	26.4093	1:Experimental evidence at protein level
26.8947	26.9296	26.9363	26.6253	26.4972	26.342	1:Experimental evidence at protein level
27.3736	28.1583	28.6319	27.8707	27.2864	28.0041	1:Experimental evidence at protein level
25.8291	26.2879	26.2853	26.3162	26.0996	26.2424	1:Experimental evidence at protein level
26.5945	26.639	26.2743	25.9403	25.8734	25.977	1:Experimental evidence at protein level
27.4053	27.9117	27.9308	27.6848	27.5403	27.7443	1:Experimental evidence at protein level
25.5249	25.5879	25.4868	25.5097	25.0222	25.291	1:Experimental evidence at protein level
24.9716	24.6201	24.7928	24.9764	24.5935	25.0964	1:Experimental evidence at protein level
27.0699	27.2136	27.1063	26.9991	26.6174	26.8213	1:Experimental evidence at protein level
26.7845	26.0066	25.8736	26.1101	27.0596	26.1717	1:Experimental evidence at protein level
27.2347	27.1293	27.0071	27.1637	27.0557	26.9214	1:Experimental evidence at protein level
26.6466	26.8647	26.6753	26.9135	26.6692	26.4083	1:Experimental evidence at protein level
26.4283	27.1285	27.1798	27.2811	26.637	27.2489	1:Experimental evidence at protein level
#NUM!	24.5616	24.8077	24.6594	24.667	24.6098	1:Experimental evidence at protein level
25.1132	25.2814	25.0024	25.2681	24.5515	25.2076	1:Experimental evidence at protein level
25.8458	25.5422	25.4213	25.6315	25.4353	25.507	1:Experimental evidence at protein level
25.4583	25.1406	25.1788	25.4456	26.0334	25.6303	1:Experimental evidence at protein level
26.1023	25.853	25.3747	25.5935	26.1805	25.749	1:Experimental evidence at protein level
26.8526	26.6102	26.5637	26.9111	27.3293	26.9131	1:Experimental evidence at protein level
26.0718	25.619	25.7718	25.6534	25.7222	25.7771	1:Experimental evidence at protein level
25.7152	25.3915	25.5373	25.0809	25.0878	25.316	1:Experimental evidence at protein level
28.5152	27.8303	27.9023	27.8843	27.8824	27.7975	1:Experimental evidence at protein level
27.2317	26.6738	26.5167	26.5065	26.6899	26.9134	1:Experimental evidence at protein level
28.5653	28.8721	28.8585	28.6531	28.5445	28.6861	1:Experimental evidence at protein level
28.1802	28.4116	28.5101	27.9142	27.6156	27.8236	1:Experimental evidence at protein level
27.0121	28.3315	28.3893	28.6999	27.3116	28.5582	1:Experimental evidence at protein level
27.5536	27.6902	27.7154	28.0029	27.5922	28.0248	1:Experimental evidence at protein level
28.9982	28.707	28.4599	28.5283	29.2979	28.1517	1:Experimental evidence at protein level
27.5973	26.925	26.8372	27.3359	27.4965	26.8301	1:Experimental evidence at protein level
27.6846	28.0353	28.1066	27.8192	27.5631	27.8236	1:Experimental evidence at protein level
30.7951	31.1884	31.3402	31.0581	30.6907	31.1298	1:Experimental evidence at protein level
26.9825	26.9665	27.1025	27.0319	26.7428	26.995	1:Experimental evidence at protein level
26.9874	26.8636	26.6633	26.5119	26.5846	26.5937	1:Experimental evidence at protein level
26.4358	26.4675	26.7315	26.4458	#NUM!	26.274	1:Experimental evidence at protein level
26.2837	26.3411	26.4027	26.0075	25.6216	25.6446	1:Experimental evidence at protein level
25.9837	24.6008	24.9824	24.6996	#NUM!	24.5906	1:Experimental evidence at protein level
#NUM!	#NUM!	#NUM!	24.0776	23.7666	23.8931	1:Experimental evidence at protein level
25.3325	25.5689	25.4053	25.1175	24.8547	24.9286	1:Experimental evidence at protein level
28.8434	28.9567	28.8825	28.7646	28.7117	28.4996	1:Experimental evidence at protein level
27.1044	26.6637	26.941	26.9759	27.0533	27.1576	1:Experimental evidence at protein level
25.5632	25.5015	25.4969	25.3648	25.554	25.3317	1:Experimental evidence at protein level
26.5156	26.0778	26.106	25.6093	25.602	25.298	1:Experimental evidence at protein level
27.0968	27.1384	27.1707	27.1001	26.5582	26.9686	1:Experimental evidence at protein level
#NUM!	24.3403	24.2855	24.3965	24.1979	24.3143	1:Experimental evidence at protein level
26.3978	26.2582	26.1638	26.1323	26.0016	26.1572	1:Experimental evidence at protein level
25.978	26.443	26.5697	26.6265	26.209	26.5413	1:Experimental evidence at protein level
25.9561	25.8812	25.8068	25.5442	25.8983	25.5406	1:Experimental evidence at protein level
25.4823	25.2067	25.077	25.2634	25.338	25.3069	1:Experimental evidence at protein level
30.7423	30.7141	30.4579	30.0408	29.9163	30.1859	1:Experimental evidence at protein level
24.6979	24.5868	24.6051	24.2477	24.3018	24.4418	1:Experimental evidence at protein level
27.2907	27.4139	27.5051	27.3906	27.4196	26.9901	1:Experimental evidence at protein level
26.1459	25.6048	25.4276	25.1972	25.7565	25.1241	1:Experimental evidence at protein level
24.2877	24.7382	24.8688	24.9783	24.1513	24.8002	1:Experimental evidence at protein level
27.2677	27.2895	27.1803	27.0807	27.3923	27.1078	1:Experimental evidence at protein level
24.6684	24.3046	24.4501	24.6506	24.6116	24.6937	1:Experimental evidence at protein level
26.286	26.2029	26.2093	26.0782	26.2068	26.2733	1:Experimental evidence at protein level
27.7372	27.5865	27.6276	27.2682	27.7286	27.4953	1:Experimental evidence at protein level
25.8075	25.7221	25.6333	26.2082	26.6687	25.9896	1:Experimental evidence at protein level
#NUM!	25.5982	25.46	25.3138	24.9311	25.4199	1:Experimental evidence at protein level
24.598	24.5985	24.8565	24.8328	24.8676	24.9136	1:Experimental evidence at protein level
25.9394	25.615	25.6004	25.7724	25.7103	25.5023	1:Experimental evidence at protein level
26.1884	26.2522	26.3432	26.2095	26.0361	26.1386	1:Experimental evidence at protein level
24.9821	25.4302	25.3359	24.7695	24.4648	25.0681	1:Experimental evidence at protein level
27.3004	26.7115	26.6134	26.5263	26.4425	26.5505	1:Experimental evidence at protein level
25.1674	25.474	25.4802	25.0643	24.8206	25.2781	1:Experimental evidence at protein level
26.0935	26.0003	25.9505	25.7703	25.9534	25.7783	1:Experimental evidence at protein level
24.8681	24.7033	24.9872	25.4912	24.7106	25.3529	1:Experimental evidence at protein level
26.8816	27.303	27.4267	27.5138	27.6715	27.6401	1:Experimental evidence at protein level
24.5337	25.0183	25.0396	24.9654	25.2938	24.7496	1:Experimental evidence at protein level
#NUM!	25.9864	26.0026	25.8873	26.9646	25.9743	1:Experimental evidence at protein level
26.3548	26.185	26.0704	26.2849	26.5519	25.763	1:Experimental evidence at protein level
27.0467	26.5188	26.0533	25.9591	26.5828	26.146	1:Experimental evidence at protein level
26.5868	26.4681	26.4722	26.4389	26.281	26.5152	1:Experimental evidence at protein level
26.0564	25.8199	25.6724	25.9084	25.8404	25.7891	1:Experimental evidence at protein level
26.6523	26.7238	26.8358	26.4019	26.1629	26.0456	1:Experimental evidence at protein level
26.645	27.008	27.0166	26.7856	26.5066	26.9833	1:Experimental evidence at protein level
26.1632	26.0543	26.2738	26.2868	26.0743	26.1734	1:Experimental evidence at protein level
28.4196	28.1502	27.8725	27.7104	28.0508	27.6853	1:Experimental evidence at protein level
24.5246	24.3965	24.8315	24.6615	#NUM!	24.7419	1:Experimental evidence at protein level
25.5186	25.9278	25.5974	25.2358	25.3078	25.3153	1:Experimental evidence at protein level
#NUM!	24.5451	24.9797	24.4799	24.6901	24.5874	1:Experimental evidence at protein level
25.0475	24.8348	24.7504	24.8708	25.213	24.8555	1:Experimental evidence at protein level
26.1803	25.5356	25.6781	25.4273	25.9116	25.6345	1:Experimental evidence at protein level
25.9383	25.2056	25.6822	25.7026	26.4306	25.825	1:Experimental evidence at protein level
26.0762	26.1596	26.0415	25.9351	26.2016	25.928	1:Experimental evidence at protein level
25.5062	26.1196	26.0899	26.4439	26.2027	26.4826	1:Experimental evidence at protein level
25.4153	25.5357	25.7851	24.8356	24.6676	24.5247	1:Experimental evidence at protein level
27.2666	27.1973	27.0188	27.3106	27.4681	27.1431	1:Experimental evidence at protein level
27.036	27.4575	27.5578	27.7652	27.3537	27.6321	1:Experimental evidence at protein level
26.1251	25.3741	25.2764	24.9205	25.1316	25.6726	1:Experimental evidence at protein level
26.2937	26.5824	26.8638	26.0556	25.8458	26.0483	1:Experimental evidence at protein level
25.8481	25.7183	25.6443	25.8739	26.6404	25.5896	1:Experimental evidence at protein level
25.1349	26.9014	25.6046	25.991	25.7036	25.8475	1:Experimental evidence at protein level
25.6336	26.0998	25.9171	25.7687	25.399	25.8778	1:Experimental evidence at protein level
25.1675	25.7103	25.515	25.7861	25.5982	25.6285	1:Experimental evidence at protein level
26.1146	27.0539	26.9193	26.8819	25.5507	27.0265	1:Experimental evidence at protein level
26.12	25.7187	25.8246	25.8244	25.9996	25.8003	1:Experimental evidence at protein level
26.122	26.486	26.4789	26.446	26.8478	26.6964	1:Experimental evidence at protein level
28.5901	28.2917	28.2667	28.4291	28.7324	28.3136	1:Experimental evidence at protein level
27.0426	26.2691	26.3802	26.1434	26.4742	26.0265	1:Experimental evidence at protein level
26.8235	26.6047	26.4405	26.3915	26.6054	26.222	1:Experimental evidence at protein level
26.597	26.839	26.6445	26.5029	26.155	26.7238	1:Experimental evidence at protein level

27.9053	28.3161	28.1882	28.4356	28.4983	28.2221	1:Experimental evidence at protein level
27.9734	27.6347	27.5604	27.591	27.8797	27.9991	1:Experimental evidence at protein level
25.9137	26.2038	26.1944	25.837	26.1971	25.654	1:Experimental evidence at protein level
27.0135	26.9838	26.7037	26.6352	26.5136	26.8165	1:Experimental evidence at protein level
26.0393	26.4663	26.4591	26.4379	26.2625	26.6607	1:Experimental evidence at protein level
25.8511	25.4888	25.5933	25.5339	25.6718	25.4305	1:Experimental evidence at protein level
27.7663	26.7175	26.7693	26.7751	27.4044	26.7768	1:Experimental evidence at protein level
26.3225	26.456	26.5301	26.5579	26.5466	26.5955	1:Experimental evidence at protein level
26.0753	25.8821	26.0769	26.0485	25.5556	26.0827	1:Experimental evidence at protein level
26.9724	26.7956	26.888	26.6767	26.7138	26.7444	1:Experimental evidence at protein level
27.0716	27.0179	27.0571	26.912	27.0372	27.2214	1:Experimental evidence at protein level
24.5153	24.8269	24.7568	24.6167	24.3562	24.6459	1:Experimental evidence at protein level
27.8054	28.4159	28.3508	27.9545	27.935	28.1095	1:Experimental evidence at protein level
26.0909	26.2134	26.2854	26.9749	26.4718	26.9642	1:Experimental evidence at protein level
26.5903	26.2336	26.1496	26.2661	26.2587	26.1364	1:Experimental evidence at protein level
26.9895	26.8448	26.6914	27.7889	27.822	27.5142	1:Experimental evidence at protein level
25.6778	25.979	26.1409	26.2599	26.8884	26.1147	1:Experimental evidence at protein level
25.4931	26.005	26.0361	26.4742	26.0698	26.0802	1:Experimental evidence at protein level
26.0835	26.2297	26.0593	26.2824	26.2141	26.1576	1:Experimental evidence at protein level
26.1853	26.4817	26.2476	25.7285	25.9285	25.8473	1:Experimental evidence at protein level
25.7911	25.2161	25.3001	25.2242	24.8787	25.0662	1:Experimental evidence at protein level
#NUM!	26.5445	26.3044	26.2487	26.4219	26.3554	1:Experimental evidence at protein level
26.9869	28.0938	27.8663	27.9088	27.0731	27.4442	1:Experimental evidence at protein level
26.2227	25.9973	26.1903	26.3321	25.978	25.9635	1:Experimental evidence at protein level
28.9031	28.8026	28.438	27.9905	28.7311	28.4586	1:Experimental evidence at protein level
25.7313	25.4679	25.7201	26.0428	25.7761	26.1758	1:Experimental evidence at protein level
27.7598	27.4169	27.5436	27.3369	27.6589	27.5078	1:Experimental evidence at protein level
24.8002	25.4986	25.4304	#NUM!	25.0378	25.5484	1:Experimental evidence at protein level
25.8696	26.3662	26.2068	26.299	26.0653	26.3545	1:Experimental evidence at protein level
24.4746	24.8105	25.007	24.8877	25.1997	25.1705	1:Experimental evidence at protein level
27.6675	27.448	27.2806	27.4031	27.5122	26.9871	1:Experimental evidence at protein level
28.8891	28.8041	28.8763	28.7767	28.6938	28.6147	1:Experimental evidence at protein level
26.2993	26.6535	26.5323	26.2879	25.9343	26.0029	1:Experimental evidence at protein level
26.3385	26.251	26.3745	26.2467	26.2137	26.067	1:Experimental evidence at protein level
26.0022	25.5814	25.5429	25.4106	26.0634	25.5132	1:Experimental evidence at protein level
27.8891	27.0748	27.085	27.3257	27.4462	27.0813	1:Experimental evidence at protein level
28.8776	28.953	28.9682	28.8464	28.85	28.7651	1:Experimental evidence at protein level
25.5544	25.0783	25.1658	25.385	25.2911	25.3298	1:Experimental evidence at protein level
25.298	25.9436	25.923	26.559	26.1721	26.1951	1:Experimental evidence at protein level
26.5572	26.732	26.8235	26.2633	26.286	26.0208	1:Experimental evidence at protein level
25.9783	25.6342	25.8449	25.7009	25.5997	25.8518	1:Experimental evidence at protein level
28.3138	28.2402	28.3143	28.1333	28.2244	28.2404	1:Experimental evidence at protein level
27.7607	27.9031	27.9454	27.7606	27.4698	27.7115	1:Experimental evidence at protein level
25.9556	26.4318	26.1201	26.035	25.9134	25.9748	1:Experimental evidence at protein level
28.4172	28.4665	28.484	28.5761	28.5903	28.3967	1:Experimental evidence at protein level
26.2847	26.0264	25.813	25.7558	#NUM!	26.1851	1:Experimental evidence at protein level
26.3525	26.1416	26.2063	26.0132	26.2322	25.9988	1:Experimental evidence at protein level
25.5265	25.4183	25.4172	24.6517	25.092	24.5323	1:Experimental evidence at protein level
31.1384	31.0163	30.7992	30.8281	31.6261	30.6896	1:Experimental evidence at protein level
#NUM!	24.382	24.6327	25.5423	25.355	25.7778	1:Experimental evidence at protein level
25.983	26.1558	26.2204	26.0382	25.7643	26.0146	1:Experimental evidence at protein level
28.4152	28.2082	28.0698	28.0224	28.3982	28.1593	1:Experimental evidence at protein level
26.5518	26.6453	26.674	26.3066	26.4447	25.8991	1:Experimental evidence at protein level
27.5218	27.4324	27.4156	27.3228	27.6504	27.2905	1:Experimental evidence at protein level
30.8562	30.5265	30.4019	30.2962	30.5978	30.2016	1:Experimental evidence at protein level
26.5875	26.2877	26.4471	27.2926	27.2411	27.3325	1:Experimental evidence at protein level
26.631	26.9727	26.8417	27.032	27.2574	26.9131	1:Experimental evidence at protein level
30.3867	29.7748	29.928	30.1638	30.22	30.2553	1:Experimental evidence at protein level
26.6195	26.6093	26.572	26.8429	26.6395	26.8188	1:Experimental evidence at protein level
27.2157	#NUM!	24.9443	24.6025	26.6724	24.5496	1:Experimental evidence at protein level
26.3956	26.4456	26.1483	26.2574	26.0345	26.1761	1:Experimental evidence at protein level
28.9448	29.7639	29.9785	29.7114	28.915	29.653	1:Experimental evidence at protein level
26.3999	26.0786	26.3027	26.1654	26.055	26.278	1:Experimental evidence at protein level
29.8419	29.8116	29.6691	29.4921	29.6831	29.9305	1:Experimental evidence at protein level
27.5295	27.6453	27.5451	27.4437	27.1907	27.3925	1:Experimental evidence at protein level
26.4904	26.3938	26.3482	25.9279	26.2722	26.197	1:Experimental evidence at protein level
29.3093	29.0452	29.0507	28.9278	28.9832	28.7184	1:Experimental evidence at protein level
27.0932	26.7585	26.8307	26.8907	26.8074	27.0286	1:Experimental evidence at protein level
25.7839	25.6507	25.4693	25.526	25.3112	25.2898	1:Experimental evidence at protein level
24.9638	25.2091	25.0497	25.0797	25.2127	25.1489	1:Experimental evidence at protein level
24.8993	25.3582	25.6003	25.539	25.6306	25.4075	1:Experimental evidence at protein level
25.6084	25.5237	25.2447	25.3451	24.9468	25.2701	1:Experimental evidence at protein level
25.7962	25.7065	25.0778	25.2938	25.9565	25.8115	1:Experimental evidence at protein level
26.4351	26.4948	26.6814	26.9045	27.1254	26.6279	1:Experimental evidence at protein level
27.1482	27.1991	27.001	27.0549	26.9349	26.9742	1:Experimental evidence at protein level
29.7357	29.0621	29.2544	29.1543	29.8332	29.2313	1:Experimental evidence at protein level
29.6276	29.5585	29.6814	29.5954	29.2029	29.5172	1:Experimental evidence at protein level
27.6511	28.0039	28.2474	27.7979	27.4776	27.7985	1:Experimental evidence at protein level
29.7714	29.3637	29.1942	29.7149	30.3921	29.9652	1:Experimental evidence at protein level
27.4444	27.0793	26.8915	27.5046	27.7478	27.5018	1:Experimental evidence at protein level
#NUM!	25.2514	25.4371	25.4461	25.7715	25.3878	1:Experimental evidence at protein level
25.9377	26.3903	26.3755	26.5188	25.9383	26.4763	1:Experimental evidence at protein level
25.7933	25.6138	25.5011	25.4784	25.7087	25.55	1:Experimental evidence at protein level
27.0713	27.2151	27.3043	27.399	26.8379	27.2756	1:Experimental evidence at protein level
30.2761	29.9903	29.97	29.9466	30.1915	29.9278	1:Experimental evidence at protein level
24.8915	25.1194	25.4127	25.282	24.7757	25.4198	1:Experimental evidence at protein level
24.7045	24.4135	24.5963	24.4667	25.1007	24.6553	1:Experimental evidence at protein level
26.5977	26.7694	26.9786	26.8948	26.6867	26.8247	1:Experimental evidence at protein level
26.3227	26.3242	26.4952	26.4432	26.2632	26.3982	1:Experimental evidence at protein level
24.9087	24.5166	24.6033	24.813	24.8067	25.0429	1:Experimental evidence at protein level
26.3274	25.7927	25.5748	25.4557	26.3541	25.8246	1:Experimental evidence at protein level
27.2641	26.812	26.9462	27.0529	26.9797	27.0307	1:Experimental evidence at protein level
27.0209	27.3061	27.297	27.1899	27.0837	27.0873	1:Experimental evidence at protein level
27.3823	27.716	27.6571	27.6518	27.3456	27.6509	1:Experimental evidence at protein level
24.5286	24.376	24.3463	25.0326	24.8549	25.111	1:Experimental evidence at protein level
26.8511	26.774	26.8146	27.0116	26.8559	26.8331	1:Experimental evidence at protein level
29.3163	29.4154	29.4312	29.3309	29.1921	29.6419	1:Experimental evidence at protein level
28.1999	28.5342	28.3355	28.5576	29.1419	28.5333	1:Experimental evidence at protein level
25.9341	25.2669	25.3686	25.418	25.8472	25.3755	1:Experimental evidence at protein level
27.6715	28.8277	28.5895	28.5328	27.867	28.5052	1:Experimental evidence at protein level
27.4903	27.2474	27.1706	26.9227	26.6332	27.0965	1:Experimental evidence at protein level
27.2783	27.2598	27.4355	27.3895	27.2465	27.1192	1:Experimental evidence at protein level
25.8992	26.2251	26.1178	26.0092	25.8964	26.0504	1:Experimental evidence at protein level
26.0316	25.9864	26.3463	26.0304	25.9928	25.782	1:Experimental evidence at protein level
25.7119	25.7485	25.7121	25.9096	25.9458	25.7721	1:Experimental evidence at protein level
26.1573	25.7674	25.4481	25.1448	25.3926	25.5871	1:Experimental evidence at protein level
#NUM!	26.1069	25.9607	25.8445	26.0561	25.8312	1:Experimental evidence at protein level
#NUM!	#NUM!	25.3366	25.516	25.1204	25.5171	1:Experimental evidence at protein level
25.6788	25.5978	25.4789	25.3754	25.3769	25.011	1:Experimental evidence at protein level
#NUM!	23.9368	23.8802	23.8616	23.6095	24.1414	1:Experimental evidence at protein level
25.0579	25.1717	25.3135	25.7778	25.5914	25.7918	1:Experimental evidence at protein level
25.2299	25.3452	25.3643	25.3765	25.2124	25.2647	1:Experimental evidence at protein level
26.9409	26.8553	27.052	27.3751	27.0515	27.3815	1:Experimental evidence at protein level
25.6431	25.4736	25.4495	25.4845	25.6628	25.3566	1:Experimental evidence at protein level
26.1969	25.8267	25.7419	25.6241	26.1401	25.7373	1:Experimental evidence at protein level
25.5049	24.5964	24.5814	24.0694	24.9253	23.9781	1:Experimental evidence at protein level
25.5621	24.9046	24.7034	24.8251	26.0184	24.7746	1:Experimental evidence at protein level
27.1848	27.2624	27.0697	27.2032	26.8435	26.7939	1:Experimental evidence at protein level
26.3463	25.9229	25.8488	25.8968	26.015	26.0393	1:Experimental evidence at protein level
27.4228	28.1666	28.0645	28.0963	27.3069	28.0611	1:Experimental evidence at protein level
25.7023	25.7076	25.2426	25.5749	25.9394	25.5508	1:Experimental evidence at protein level
25.9527	26.1603	26.3129	26.0528	26.1048	25.8246	1:Experimental evidence at protein level
26.5255	26.7765	26.9656	26.3386	26.4837	26.242	1:Experimental evidence at protein level
27.2537	27.1452	27.2026	26.8025	26.7291	26.8354	1:Experimental evidence at protein level
26.2788	26.1197	26.1029	25.6842	25.7833	25.8853	1:Experimental evidence at protein level
25.1636	24.7222	25.2902	25.0598	25.1576	25.104	1:Experimental evidence at protein level
27.7768	26.7715	27.1855	27.1397	27.499	27.2817	1:Experimental evidence at protein level
25.8847	25.9286	25.8442	25.7735	25.3289	25.5725	1:Experimental evidence at protein level
27.2457	26.1683	25.8628	26.0182	25.5883	26.152	1:Experimental evidence at protein level

25.1669	25.4789	25.197	24.8387	24.6999	25.0282	1:Experimental evidence at protein level
27.0098	27.6723	27.7256	27.5755	27.0947	27.3819	1:Experimental evidence at protein level
26.2498	26.0414	26.1257	26.1957	26.6606	26.0851	1:Experimental evidence at protein level
26.1509	25.5761	25.7169	25.8076	26.2095	25.566	1:Experimental evidence at protein level
27.0374	26.1722	26.2447	26.396	26.7988	26.1752	1:Experimental evidence at protein level
26.555	26.4299	26.6368	26.4152	26.5462	26.1583	1:Experimental evidence at protein level
28.0354	29.0262	28.7604	28.6361	28.3199	28.4954	1:Experimental evidence at protein level
29.309	29.0493	29.1539	29.1244	29.3936	29.2837	1:Experimental evidence at protein level
26.4724	26.25	25.8564	26.1514	25.9462	26.0839	1:Experimental evidence at protein level
26.1833	26.0003	26.5336	25.9766	25.7718	26.0301	1:Experimental evidence at protein level
25.9213	26.1781	26.0896	25.9537	25.6722	25.8485	1:Experimental evidence at protein level
25.2101	26.1399	25.5947	26.0094	25.8902	25.6153	1:Experimental evidence at protein level
27.1443	27.5592	27.4568	27.1778	27.1237	27.3032	1:Experimental evidence at protein level
26.809	27.018	26.9716	26.6112	26.5513	26.6003	1:Experimental evidence at protein level
26.209	#NUM!	26.4436	26.3506	25.9252	26.0095	1:Experimental evidence at protein level
27.0439	27.1366	27.2721	27.2212	26.7299	27.0225	1:Experimental evidence at protein level
26.0309	26.1205	25.915	25.5859	25.6116	25.3606	1:Experimental evidence at protein level
27.3416	28.1046	28.1928	27.8543	27.063	27.8058	1:Experimental evidence at protein level
28.055	27.2149	27.3863	27.2382	27.4736	27.295	1:Experimental evidence at protein level
25.4893	26.1894	26.2013	26.2362	26.3119	26.57	1:Experimental evidence at protein level
28.874	28.5901	28.613	28.3003	28.9352	28.173	1:Experimental evidence at protein level
28.1035	27.86	27.9725	27.6961	27.8147	27.5222	1:Experimental evidence at protein level
26.3616	26.792	26.6475	26.4349	26.4635	26.49	1:Experimental evidence at protein level
27.3927	28.1017	27.801	27.8131	27.4473	27.9342	1:Experimental evidence at protein level
#NUM!	24.7072	24.8045	24.6488	24.0011	24.8794	1:Experimental evidence at protein level
26.6456	26.8109	26.9374	27.0535	26.7199	27.0069	1:Experimental evidence at protein level
27.1405	27.4483	27.5722	27.2575	27.03	27.4769	1:Experimental evidence at protein level
26.2828	25.6622	25.9365	25.9158	26.1994	25.5834	1:Experimental evidence at protein level
25.9262	25.4557	25.5281	25.2898	25.6587	25.4334	1:Experimental evidence at protein level
28.9452	28.4925	28.4946	28.3124	28.5333	28.4929	1:Experimental evidence at protein level
25.1139	25.376	25.1962	25.0285	24.871	25.2354	1:Experimental evidence at protein level
26.081	26.9781	27.3998	27.2545	26.9321	27.1688	1:Experimental evidence at protein level
25.8566	24.3705	25.0125	24.5219	24.962	25.0264	1:Experimental evidence at protein level
28.4858	28.0881	27.9556	27.9281	27.7945	27.2241	1:Experimental evidence at protein level
27.2663	26.9895	26.614	27.1655	27.3031	26.7424	1:Experimental evidence at protein level
27.7202	28.1468	28.243	28.1931	27.8807	28.2487	1:Experimental evidence at protein level
27.1752	26.6542	26.7567	26.7662	26.7986	26.8112	1:Experimental evidence at protein level
27.5496	27.3983	27.3471	27.213	27.2433	27.2051	1:Experimental evidence at protein level
26.7403	26.4626	26.1869	25.8418	26.5988	26.0917	1:Experimental evidence at protein level
26.6165	27.0765	27.1356	26.7613	26.6458	26.5555	1:Experimental evidence at protein level
26.5754	26.2847	26.1834	26.8536	26.8771	26.7782	1:Experimental evidence at protein level
25.7855	25.9455	26.0083	25.5264	25.5468	26.0263	1:Experimental evidence at protein level
26.0635	26.3143	26.1777	26.9033	26.6834	27.0246	1:Experimental evidence at protein level
26.7417	26.8298	26.6591	26.5583	26.2807	26.6252	1:Experimental evidence at protein level
27.2062	27.4847	27.3174	27.4471	27.1881	27.2594	1:Experimental evidence at protein level
27.0899	26.9221	26.8032	26.7599	26.86	26.7292	1:Experimental evidence at protein level
27.4024	27.0531	27.0944	27.3108	27.1218	27.4605	1:Experimental evidence at protein level
27.8286	27.8063	27.9917	27.5935	27.2635	27.738	1:Experimental evidence at protein level
27.5166	27.731	27.647	27.7344	27.5817	27.3882	1:Experimental evidence at protein level
25.747	26.0662	25.6643	26.241	26.3376	26.1783	1:Experimental evidence at protein level
26.413	26.3017	26.3325	26.3637	26.0403	26.2922	1:Experimental evidence at protein level
26.473	26.778	26.7655	26.7108	26.5278	26.7168	1:Experimental evidence at protein level
27.2571	27.5534	27.3864	27.3051	26.9133	27.2501	1:Experimental evidence at protein level
27.702	28.3028	28.2849	28.2818	27.2234	28.4356	1:Experimental evidence at protein level
25.9244	25.3955	25.4183	25.4193	25.764	25.4182	1:Experimental evidence at protein level
25.3491	25.7463	25.7673	26.1685	25.548	25.7971	1:Experimental evidence at protein level
27.7298	26.8538	26.7587	26.8916	27.7577	26.6838	1:Experimental evidence at protein level
27.1467	26.5153	26.6763	26.3815	26.8841	26.2655	1:Experimental evidence at protein level
27.9863	27.7645	27.7812	27.4688	27.598	27.5036	1:Experimental evidence at protein level
26.3123	26.4699	26.7203	26.4724	26.4464	26.5232	1:Experimental evidence at protein level
29.0268	28.8193	28.7851	28.4521	28.5309	28.2807	1:Experimental evidence at protein level
24.8906	25.0749	25.0478	25.1845	24.7687	24.9027	1:Experimental evidence at protein level
29.1055	29.35	29.1369	29.0339	29.1155	29.0781	1:Experimental evidence at protein level
25.8965	25.5543	25.6296	25.4161	25.5789	25.565	1:Experimental evidence at protein level
24.6124	24.6207	24.551	24.8457	24.8085	24.8453	1:Experimental evidence at protein level
31.436	30.7893	30.6194	30.4669	30.8745	30.5066	1:Experimental evidence at protein level
24.8646	25.2219	25.1096	25.3779	25.293	25.1278	1:Experimental evidence at protein level
26.7398	26.1815	26.0014	25.9799	#NUM!	26.025	1:Experimental evidence at protein level
27.7036	27.906	28.0005	27.8831	27.4823	27.8064	1:Experimental evidence at protein level
27.0606	27.4873	27.4439	27.368	27.077	27.3219	1:Experimental evidence at protein level
27.4309	27.7756	27.7939	27.7019	27.674	27.6154	1:Experimental evidence at protein level
27.2188	26.7813	26.7399	26.4668	26.7861	26.5909	1:Experimental evidence at protein level
25.3811	26.0302	26.0036	26.4063	26.4075	26.2686	1:Experimental evidence at protein level
24.817	25.0562	25.471	25.1863	25.0515	25.32	1:Experimental evidence at protein level
26.4092	26.3617	26.3138	26.3564	26.6045	26.0857	1:Experimental evidence at protein level
28.8911	28.8553	28.8394	28.8923	28.7974	28.9851	1:Experimental evidence at protein level
25.828	25.8659	25.4338	25.6917	25.6917	25.7451	1:Experimental evidence at protein level
26.5414	25.755	25.7158	25.6671	26.1413	25.9375	1:Experimental evidence at protein level
26.5264	25.9993	25.7525	25.9034	26.3043	25.8436	1:Experimental evidence at protein level
27.7464	27.2538	27.1878	26.9235	27.3316	26.9119	1:Experimental evidence at protein level
27.1187	26.5884	26.6311	26.7114	26.6872	26.8976	1:Experimental evidence at protein level
30.219	29.8576	30.0215	29.6815	29.9153	29.9523	1:Experimental evidence at protein level
25.4847	25.8434	25.536	25.535	25.4009	25.5049	1:Experimental evidence at protein level
25.5755	25.259	25.3893	25.6174	25.6046	24.9657	1:Experimental evidence at protein level
27.6784	27.787	27.7286	27.2181	27.5388	27.6221	1:Experimental evidence at protein level
27.086	26.6613	26.7961	26.9556	27.104	26.9519	1:Experimental evidence at protein level
30.1145	30.2395	29.9902	30.0838	29.662	30.1081	1:Experimental evidence at protein level
25.6091	25.3656	25.3513	25.4296	25.3977	25.3606	1:Experimental evidence at protein level
27.2655	26.8437	26.9519	26.8044	27.1453	26.6462	1:Experimental evidence at protein level
25.8626	25.8257	25.8858	25.9698	26.2828	25.84	1:Experimental evidence at protein level
28.9048	28.528	28.7354	28.0859	28.8267	28.0981	1:Experimental evidence at protein level
29.5507	29.7508	29.6721	29.7743	29.3662	29.6074	1:Experimental evidence at protein level
27.0367	26.5275	26.3554	26.7042	26.9377	26.6545	1:Experimental evidence at protein level
26.6183	26.6697	26.7085	26.617	26.8567	26.4661	1:Experimental evidence at protein level
25.5716	26.0263	25.908	26.0904	25.4611	25.6571	1:Experimental evidence at protein level
25.4759	25.1655	25.5092	25.5304	25.6897	25.6166	1:Experimental evidence at protein level
25.9318	25.8445	25.7248	25.2357	25.6179	25.3885	1:Experimental evidence at protein level
28.1549	28.0195	27.7489	27.8033	28.0647	27.7033	1:Experimental evidence at protein level
27.0411	26.8123	26.8395	26.8157	27.1837	26.865	1:Experimental evidence at protein level
25.8425	26.0586	26.2397	25.7553	25.2948	25.9679	1:Experimental evidence at protein level
28.3117	28.4246	28.4327	28.4863	28.3582	28.1486	1:Experimental evidence at protein level
27.7676	27.8149	27.8148	28.0895	27.9969	27.8293	1:Experimental evidence at protein level
25.2264	24.7969	24.97	25.282	25.4367	24.7833	1:Experimental evidence at protein level
28.1455	28.1419	28.0057	27.3055	27.6037	27.3208	1:Experimental evidence at protein level
29.377	31.0134	30.6194	30.3677	29.961	30.3321	1:Experimental evidence at protein level
29.0456	28.5535	28.5657	28.2709	28.5186	28.311	1:Experimental evidence at protein level
26.0893	25.6579	26.058	25.8808	25.7696	25.753	1:Experimental evidence at protein level
25.8232	26.5602	26.6338	26.3878	26.2635	26.1237	1:Experimental evidence at protein level
26.8764	26.8442	26.8339	26.0181	26.0481	26.3609	1:Experimental evidence at protein level
27.0169	27.3589	27.474	27.4079	27.24	27.3523	1:Experimental evidence at protein level
25.0284	26.0509	25.846	26.2448	25.896	25.8752	1:Experimental evidence at protein level
26.7613	26.3849	26.4821	26.4344	26.6398	26.5179	1:Experimental evidence at protein level
25.4913	25.3326	25.2729	25.4624	25.7531	25.4414	1:Experimental evidence at protein level
27.2403	27.2575	27.5536	27.4294	27.4381	27.3959	1:Experimental evidence at protein level
26.8643	26.7386	26.814	26.7528	26.6523	26.5812	1:Experimental evidence at protein level
28.5962	28.529	28.626	28.7441	28.888	28.862	1:Experimental evidence at protein level
25.6963	25.0242	25.1275	24.9765	25.5769	25.0968	1:Experimental evidence at protein level
25.4071	25.2906	25.5635	25.6566	25.459	25.583	1:Experimental evidence at protein level
25.8342	25.7471	25.5942	25.8787	25.9005	25.8646	1:Experimental evidence at protein level
25.4974	#NUM!	25.3176	24.7625	24.2171	24.8323	1:Experimental evidence at protein level
26.2245	25.5763	25.4954	25.921	26.0304	25.6061	1:Experimental evidence at protein level
25.6423	25.6686	25.8723	25.4378	24.7468	25.4293	1:Experimental evidence at protein level
26.911	26.8597	26.6618	26.7844	26.8694	26.7004	1:Experimental evidence at protein level
26.6076	25.3393	25.3998	25.5774	25.5062	25.8886	1:Experimental evidence at protein level
25.884	25.7359	25.6064	24.8436	25.011	#NUM!	1:Experimental evidence at protein level
25.7594	25.134	25.3033	25.1708	25.0639	24.6224	1:Experimental evidence at protein level
25.9732	25.4731	25.7305	25.4352	25.5751	25.4444	1:Experimental evidence at protein level
25.8977	26.2085	26.0042	26.2437	26.138	26.1422	1:Experimental evidence at protein level
25.3077	25.6399	25.5256	25.6287	24.8934	25.5764	1:Experimental evidence at protein level

27.0178	26.5251	26.4842	26.7416	27.3087	26.9388	1:Experimental evidence at protein level
27.245	27.4452	27.5153	26.7505	26.4227	26.489	1:Experimental evidence at protein level
#NUM!	25.7197	25.564	25.6733	25.6257	25.6244	1:Experimental evidence at protein level
25.6177	25.2125	24.9273	24.8444	25.2843	25.0472	1:Experimental evidence at protein level
25.524	25.2715	25.3201	25.381	25.4143	25.5944	1:Experimental evidence at protein level
26.7582	26.5068	26.4345	26.648	26.3348	26.439	1:Experimental evidence at protein level
26.9778	26.7651	27.1193	26.7845	26.7855	26.8939	1:Experimental evidence at protein level
26.4122	26.7105	26.7944	26.73	26.3141	26.7738	1:Experimental evidence at protein level
26.1099	25.6962	25.6902	25.7869	25.8513	25.7732	1:Experimental evidence at protein level
27.6782	26.8864	27.091	27.1066	27.8791	27.0414	1:Experimental evidence at protein level
25.7426	26.2288	26.2976	26.0995	26.1437	26.3186	1:Experimental evidence at protein level
27.5558	27.2902	27.03	26.8312	27.4557	26.8129	1:Experimental evidence at protein level
25.8154	25.9807	25.9099	25.7651	25.8233	25.6768	1:Experimental evidence at protein level
28.17	27.7687	27.9796	27.9802	28.2954	27.9338	1:Experimental evidence at protein level
27.3512	27.2447	27.1631	27.3981	27.7112	27.2736	1:Experimental evidence at protein level
24.0601	24.44	24.5459	24.4644	26.4389	24.2537	1:Experimental evidence at protein level
26.3174	26.5132	26.4858	26.6078	26.3298	26.5992	1:Experimental evidence at protein level
25.7427	25.0254	25.5208	25.8418	25.5865	26.0398	1:Experimental evidence at protein level
27.5999	27.9285	27.7296	27.7349	27.5705	27.4433	1:Experimental evidence at protein level
27.0593	26.8591	26.7987	26.8643	26.885	26.9401	1:Experimental evidence at protein level
27.402	27.3206	27.2262	27.3727	27.2964	27.4017	1:Experimental evidence at protein level
26.1573	26.1877	26.3718	26.1092	26.2947	26.019	1:Experimental evidence at protein level
25.5666	25.6965	25.5742	26.6295	26.2697	25.7082	1:Experimental evidence at protein level
27.4863	27.4204	27.1099	26.9358	27.4026	27.0283	1:Experimental evidence at protein level
25.6137	25.9751	26.0746	25.0827	24.9461	25.2972	1:Experimental evidence at protein level
25.8923	25.6454	25.8979	25.8803	25.4902	25.8972	1:Experimental evidence at protein level
25.6072	25.3111	25.4555	24.851	24.8321	25.4209	1:Experimental evidence at protein level
25.8763	26.0262	26.0535	26.4702	25.8989	26.5188	1:Experimental evidence at protein level
26.054	#NUM!	26.3461	26.092	25.3108	26.0243	1:Experimental evidence at protein level
25.6891	25.2748	25.5775	25.7267	25.9309	25.6623	1:Experimental evidence at protein level
26.5178	27.273	26.8072	26.8594	26.6364	26.8261	1:Experimental evidence at protein level
26.473	27.04	27.2138	26.7969	26.5361	26.5793	1:Experimental evidence at protein level
28.2223	28.5317	28.4403	28.02	28.2245	27.6891	1:Experimental evidence at protein level
29.1937	28.7615	28.6577	28.5551	28.8459	28.557	1:Experimental evidence at protein level
26.5272	26.7153	26.7706	26.2402	25.7539	26.2219	1:Experimental evidence at protein level
29.1479	28.6253	28.5982	28.4294	28.8358	28.43	1:Experimental evidence at protein level
26.7975	27.0996	27.3633	27.0142	26.5467	26.8309	1:Experimental evidence at protein level
24.7974	24.9811	25.0032	24.4335	24.266	24.8614	1:Experimental evidence at protein level
27.5635	27.5434	27.6955	27.5907	27.3195	27.6635	1:Experimental evidence at protein level
27.1365	27.3888	27.2345	27.0976	27.1027	26.9719	1:Experimental evidence at protein level
27.7449	26.2207	27.0206	26.1853	27.1592	26.4	1:Experimental evidence at protein level
26.1863	26.0752	26.0146	26.3131	26.916	26.272	1:Experimental evidence at protein level
25.2949	25.1658	25.1118	24.9798	24.9152	24.954	1:Experimental evidence at protein level
27.3988	27.4634	27.083	26.9442	26.3774	26.6218	1:Experimental evidence at protein level
26.0089	25.9966	25.9211	25.5053	25.6936	25.4147	1:Experimental evidence at protein level
26.8559	26.8709	26.9686	26.8414	26.1256	26.662	1:Experimental evidence at protein level
28.0526	28.1569	28.0674	28.2277	28.2274	28.074	1:Experimental evidence at protein level
26.4859	26.6109	26.6779	26.5467	25.8647	26.6167	1:Experimental evidence at protein level
25.9944	26.712	25.6685	26.0279	26.4265	25.7105	1:Experimental evidence at protein level
28.0229	28.1571	28.1538	27.9532	27.7329	27.9492	1:Experimental evidence at protein level
27.2886	27.0622	26.9877	27.0429	26.8977	26.7455	1:Experimental evidence at protein level
25.9979	25.9552	25.9216	25.9278	26.0936	25.842	1:Experimental evidence at protein level
26.9218	26.7844	26.8322	26.6195	26.9536	26.7694	1:Experimental evidence at protein level
29.3864	29.1183	29.163	29.1957	29.3103	28.9309	1:Experimental evidence at protein level
26.5843	26.4313	26.6208	26.038	25.5427	26.3515	1:Experimental evidence at protein level
26.8668	27.3647	27.0819	27.3605	27.0422	26.9852	1:Experimental evidence at protein level
26.4556	#NUM!	#NUM!	27.7425	27.4301	27.7539	1:Experimental evidence at protein level
28.0613	28.0385	27.9902	28.0852	28.0109	27.9541	1:Experimental evidence at protein level
28.7706	28.5467	28.4635	28.3617	28.401	28.1951	1:Experimental evidence at protein level
27.4216	27.1669	27.0826	27.0934	26.979	27.1672	1:Experimental evidence at protein level
26.8291	26.545	26.6675	26.7826	26.7972	26.769	1:Experimental evidence at protein level
26.8578	25.741	26.0396	25.9881	26.6233	26.0832	1:Experimental evidence at protein level
28.3689	28.6058	28.313	28.3502	28.3387	28.2619	1:Experimental evidence at protein level
29.1662	28.6551	28.5808	28.5294	28.6053	29.0581	1:Experimental evidence at protein level
#NUM!	25.3069	25.3825	26.2563	26.0264	25.9943	1:Experimental evidence at protein level
26.6793	26.5994	26.523	26.8248	26.834	26.8286	1:Experimental evidence at protein level
25.7113	26.2018	26.4396	26.563	25.7082	26.6987	1:Experimental evidence at protein level
27.6239	27.6136	27.2809	27.3839	27.618	27.4947	1:Experimental evidence at protein level
27.2059	27.078	27.1082	27.1908	27.1182	26.981	1:Experimental evidence at protein level
26.4404	26.7034	26.7087	26.8592	26.6437	26.9918	1:Experimental evidence at protein level
26.1361	26.0264	26.3676	26.365	26.3393	26.2935	1:Experimental evidence at protein level
28.5566	28.8462	28.5654	28.4795	28.5079	28.3892	1:Experimental evidence at protein level
28.3713	28.4268	28.4374	28.1923	28.258	28.0237	1:Experimental evidence at protein level
25.5446	25.3651	25.272	25.4542	25.557	25.3548	1:Experimental evidence at protein level
27.2137	27.2326	26.9847	27.0102	27.2102	27.1108	1:Experimental evidence at protein level
26.2047	26.2053	26.207	26.1462	25.9202	26.0928	1:Experimental evidence at protein level
26.1998	26.2262	26.163	25.9377	25.7487	25.9778	1:Experimental evidence at protein level
27.8745	27.3895	27.6674	27.7691	27.8455	27.4759	1:Experimental evidence at protein level
26.0825	25.902	25.705	26.0192	26.3613	25.8953	1:Experimental evidence at protein level
27.595	27.7398	27.7299	27.2727	27.1814	27.6744	1:Experimental evidence at protein level
26.951	26.3996	26.3931	26.454	27.1739	26.4552	1:Experimental evidence at protein level
29.47	29.8715	29.8908	29.7516	29.4783	29.6418	1:Experimental evidence at protein level
26.712	26.7595	26.8574	26.6056	26.8114	26.9377	1:Experimental evidence at protein level
25.7391	25.0886	25.0521	25.0681	26.1114	25.3607	1:Experimental evidence at protein level
26.7075	27.1474	26.92	26.9239	27.0467	26.7367	1:Experimental evidence at protein level
27.6263	27.3669	27.3841	27.2988	27.5566	27.2404	1:Experimental evidence at protein level
28.7239	29.5235	29.4602	29.5777	29.1414	29.1829	1:Experimental evidence at protein level
27.7168	27.3894	27.2766	27.4228	27.5108	27.2023	1:Experimental evidence at protein level
27.1931	27.1765	27.2108	27.1917	27.2251	27.0081	1:Experimental evidence at protein level
27.5477	27.4348	27.5333	26.7186	26.4015	26.6068	1:Experimental evidence at protein level
32.2765	31.769	31.4317	32.33	32.6809	30.9652	1:Experimental evidence at protein level
30.2631	29.8646	29.91	29.5094	29.8051	29.3995	1:Experimental evidence at protein level
27.8319	28.1173	28.0953	27.8882	27.9501	28.359	1:Experimental evidence at protein level
26.1209	25.4865	25.3544	25.7132	25.9305	25.8038	1:Experimental evidence at protein level
30.2367	30.0541	30.1531	29.8815	29.8695	30.1982	1:Experimental evidence at protein level
25.881	26.1181	25.9144	25.9176	25.3707	25.7724	1:Experimental evidence at protein level
27.7904	27.137	27.215	27.1644	27.482	27.1114	1:Experimental evidence at protein level
27.2464	27.2787	26.7142	27.4212	27.4457	27.2444	1:Experimental evidence at protein level
27.5418	27.1816	27.361	27.0376	26.3572	27.5462	1:Experimental evidence at protein level
26.3734	26.2709	26.3362	26.4127	25.9988	26.4068	1:Experimental evidence at protein level
26.0978	26.3669	26.3127	26.3843	26.3687	26.3324	1:Experimental evidence at protein level
27.4687	27.3899	27.932	27.5826	27.3981	27.4871	1:Experimental evidence at protein level
25.7947	26.0168	26.2473	25.9757	25.7039	25.65	1:Experimental evidence at protein level
25.3762	25.3162	25.3094	25.4202	25.759	25.3714	1:Experimental evidence at protein level
27.0452	27.1627	27.0766	27.2366	27.1261	27.1559	1:Experimental evidence at protein level
25.9282	26.2378	26.3749	25.8904	25.6753	26.2429	1:Experimental evidence at protein level
#NUM!	26.2108	26.2861	26.4403	26.5422	26.3778	1:Experimental evidence at protein level
26.7377	26.7721	26.7469	26.7162	26.8706	26.6797	1:Experimental evidence at protein level
27.6971	27.4248	26.9578	26.811	27.1917	26.8407	1:Experimental evidence at protein level
27.6591	27.7974	27.8377	27.8719	27.5944	27.6873	1:Experimental evidence at protein level
27.8739	27.9114	27.9601	27.9214	27.7488	27.9104	1:Experimental evidence at protein level
29.7354	30.7462	30.7728	30.9666	30.2465	30.7593	1:Experimental evidence at protein level
28.2937	28.5657	28.4195	28.4165	28.7622	28.4286	1:Experimental evidence at protein level
32.528	32.2637	32.2437	33.0208	33.7104	32.8579	1:Experimental evidence at protein level
30.0834	30.1649	30.6863	30.4327	30.0522	30.5716	1:Experimental evidence at protein level
26.5215	26.3411	26.4378	26.3168	26.2611	26.3668	1:Experimental evidence at protein level
27.3975	27.011	26.8308	27.3931	27.7461	27.0541	1:Experimental evidence at protein level
26.6159	26.5217	26.6306	26.5698	26.6101	26.4695	1:Experimental evidence at protein level
27.586	27.8538	28.0899	27.8545	28.1605	28.0252	1:Experimental evidence at protein level
28.0405	28.2136	28.2375	28.1718	28.0958	28.1648	1:Experimental evidence at protein level
26.1968	26.41	26.4815	26.2968	26.2348	26.1709	1:Experimental evidence at protein level
26.7953	26.6001	26.6247	26.6329	27.1196	26.4852	1:Experimental evidence at protein level
26.9374	26.3786	26.2697	26.2538	26.4057	26.1883	1:Experimental evidence at protein level
24.6477	25.2037	25.1541	25.0553	24.6112	24.7146	1:Experimental evidence at protein level
25.3361	25.7361	25.7699	25.8257	25.7677	25.8439	1:Experimental evidence at protein level
27.2822	26.9025	27.351	27.024	27.2893	26.9357	1:Experimental evidence at protein level
25.9813	26.223	26.0929	25.9972	26.4428	25.9835	1:Experimental evidence at protein level
26.507	26.0671	26.1096	25.8631	25.8473	25.8887	1:Experimental evidence at protein level
27.2723	27.2106	27.1247	26.6452	26.5733	26.6063	1:Experimental evidence at protein level

26.4875	26.3025	26.3622	26.4736	26.5565	26.3509	1:Experimental evidence at protein level
28.3057	28.1812	28.1817	28.2754	28.3299	28.1806	1:Experimental evidence at protein level
25.9114	25.0378	25.1198	24.889	25.4207	24.8486	1:Experimental evidence at protein level
27.6613	28.0896	27.7759	27.9179	27.6797	27.8821	1:Experimental evidence at protein level
27.8019	27.6838	27.6837	27.6691	27.9631	27.8712	1:Experimental evidence at protein level
27.7094	27.6348	27.5798	27.6661	27.554	27.436	1:Experimental evidence at protein level
26.9531	27.0889	27.2218	26.997	26.8379	26.4481	1:Experimental evidence at protein level
28.1408	27.9288	28.0426	28.1804	28.2637	28.0732	1:Experimental evidence at protein level
27.2217	26.8878	26.842	26.8078	27.0296	26.8635	1:Experimental evidence at protein level
26.7817	27.2094	27.0707	26.6281	26.5607	27.0453	1:Experimental evidence at protein level
27.7092	27.5965	27.5209	27.2618	27.3105	27.1012	1:Experimental evidence at protein level
26.6906	26.7299	26.6397	26.6187	26.6139	26.4991	1:Experimental evidence at protein level
26.5861	26.011	25.8964	25.5453	25.9994	25.4314	1:Experimental evidence at protein level
26.4922	25.5586	25.569	25.8959	26.3613	26.1885	1:Experimental evidence at protein level
24.6436	24.6141	24.0453	24.8254	24.9078	24.6611	1:Experimental evidence at protein level
27.6827	28.0205	28.1736	27.7538	27.4096	27.5146	1:Experimental evidence at protein level
27.0624	26.7704	26.8321	27.0647	27.1202	27.0412	1:Experimental evidence at protein level
26.1669	25.9911	25.8742	25.7721	25.8687	25.5849	1:Experimental evidence at protein level
23.2179	#NUM!	23.5239	25.1568	25.4054	25.1871	1:Experimental evidence at protein level
25.7032	25.5808	25.5752	25.5246	25.6813	25.4933	1:Experimental evidence at protein level
26.3652	26.4401	26.4397	26.5107	26.2866	26.3515	1:Experimental evidence at protein level
27.126	26.9214	26.8238	26.7468	26.9708	26.8858	1:Experimental evidence at protein level
27.0651	27.1488	27.2934	27.1462	27.1094	27.0488	1:Experimental evidence at protein level
28.3807	28.465	28.5451	28.828	28.5347	28.5569	1:Experimental evidence at protein level
28.0945	28.1563	28.1529	27.8629	27.9154	27.8858	1:Experimental evidence at protein level
26.8655	26.994	26.9569	26.6657	26.5648	26.3356	1:Experimental evidence at protein level
27.692	27.3583	27.2639	27.3427	27.3884	27.3881	1:Experimental evidence at protein level
27.2879	26.7995	26.9539	26.6215	26.8269	26.556	1:Experimental evidence at protein level
26.504	26.2789	26.4771	26.0836	25.6964	26.1016	1:Experimental evidence at protein level
25.698	#NUM!	26.0516	26.0074	25.5516	25.6211	1:Experimental evidence at protein level
25.1724	#NUM!	25.0585	25.2236	25.4078	25.25	1:Experimental evidence at protein level
26.5012	26.3943	26.2278	26.4119	26.3468	26.3361	1:Experimental evidence at protein level
#NUM!	25.6505	25.4464	25.7163	25.5578	25.7399	1:Experimental evidence at protein level
25.3035	25.5019	25.4779	25.385	25.3223	25.4736	1:Experimental evidence at protein level
25.9103	25.3496	25.5212	25.4704	26.0888	25.4518	1:Experimental evidence at protein level
29.5329	29.8685	29.6731	29.6373	29.1273	29.6885	1:Experimental evidence at protein level
27.6461	28.3392	28.3493	28.2283	27.646	28.1552	1:Experimental evidence at protein level
26.4154	26.3833	26.2841	26.3073	26.1576	26.2565	1:Experimental evidence at protein level
25.0624	24.2251	24.6847	24.4099	24.477	24.512	1:Experimental evidence at protein level
27.3686	27.3017	27.1946	27.0527	27.431	27.1386	1:Experimental evidence at protein level
25.5422	25.7415	26.0648	25.927	26.2476	25.9208	1:Experimental evidence at protein level
26.7697	26.9896	27.0683	27.0821	26.7013	26.9794	1:Experimental evidence at protein level
27.739	27.1148	27.0205	26.6868	27.6017	26.5995	1:Experimental evidence at protein level
25.3563	26.0399	25.7053	25.4367	25.1136	25.2753	1:Experimental evidence at protein level
26.6445	25.8916	26.1481	26.1656	26.3637	26.0148	1:Experimental evidence at protein level
27.0152	26.8542	26.8426	26.453	26.6952	26.5075	1:Experimental evidence at protein level
25.8989	25.7706	26.1435	25.6025	25.1489	25.4541	1:Experimental evidence at protein level
27.2454	27.4443	27.4847	26.9971	26.916	27.2103	1:Experimental evidence at protein level
25.5956	26.0654	26.1731	25.7995	25.5536	25.8165	1:Experimental evidence at protein level
26.0497	25.493	25.4687	24.6388	#NUM!	24.9609	1:Experimental evidence at protein level
25.3546	24.2552	24.4737	24.9668	25.0917	24.9985	1:Experimental evidence at protein level
25.9686	26.1081	25.8031	25.9154	25.8213	26.0906	1:Experimental evidence at protein level
25.8901	25.8788	26.1209	25.8677	25.8093	26.2702	1:Experimental evidence at protein level
28.2286	28.3173	28.3127	28.5554	28.1236	28.185	1:Experimental evidence at protein level
27.8226	28.0022	28.0204	28.0169	27.3591	27.9078	1:Experimental evidence at protein level
25.9837	26.2735	26.3206	25.9558	25.9926	25.9551	1:Experimental evidence at protein level
26.4781	25.9029	25.8559	25.8998	26.4858	25.9746	1:Experimental evidence at protein level
26.852	26.9866	26.8764	26.8091	26.9095	26.8688	1:Experimental evidence at protein level
24.6201	25.364	25.5765	25.5693	24.6568	25.4314	1:Experimental evidence at protein level
25.7393	26.0071	26.1703	26.0408	25.4577	25.8499	1:Experimental evidence at protein level
27.8647	27.6521	27.8692	#NUM!	28.0575	27.8157	1:Experimental evidence at protein level
27.4031	27.1269	27.2216	27.2283	27.4282	27.2368	1:Experimental evidence at protein level
27.2987	26.0129	26.0164	25.7571	27.2517	25.8092	1:Experimental evidence at protein level
26.1637	25.9425	25.7619	25.9775	26.1846	26.1875	1:Experimental evidence at protein level
26.7301	26.0308	25.9883	26.1447	26.137	25.9674	1:Experimental evidence at protein level
25.1789	25.608	25.7416	25.6657	25.8057	25.9255	1:Experimental evidence at protein level
28.6068	28.5948	28.513	28.6756	28.4402	28.4933	1:Experimental evidence at protein level
25.9211	25.8607	25.9165	26.4703	26.5085	26.3715	1:Experimental evidence at protein level
28.2796	28.0284	28.0896	28.0157	28.0243	27.8325	1:Experimental evidence at protein level
25.646	26.0625	25.8834	25.9839	25.7633	25.9393	1:Experimental evidence at protein level
26.7417	26.7745	27.0218	26.8271	26.7461	26.795	1:Experimental evidence at protein level
26.1432	26.5632	26.494	26.8601	26.6891	26.7537	1:Experimental evidence at protein level
26.3071	26.4753	26.4109	26.436	26.1326	26.1497	1:Experimental evidence at protein level
27.2083	26.8186	26.8521	26.9069	27.2077	26.9302	1:Experimental evidence at protein level
27.1252	27.013	26.9527	27.1722	27.0544	27.3772	1:Experimental evidence at protein level
28.1883	27.7664	27.6224	27.4701	27.882	27.5418	1:Experimental evidence at protein level
25.2818	25.5286	24.9118	26.1518	25.6849	26.2294	1:Experimental evidence at protein level
26.7038	26.2362	26.389	26.2834	26.5692	26.7548	1:Experimental evidence at protein level
26.8249	26.767	26.6617	26.6731	26.789	26.8828	1:Experimental evidence at protein level
25.6881	25.5936	25.293	25.3868	25.7555	25.5725	1:Experimental evidence at protein level
27.9704	28.0912	27.9717	27.8401	27.6579	27.8244	1:Experimental evidence at protein level
27.0512	27.3066	27.4018	27.461	27.2254	27.3607	1:Experimental evidence at protein level
26.2797	26.0581	26.0865	25.8098	25.4948	25.7892	1:Experimental evidence at protein level
27.9391	27.6119	27.7556	27.5572	27.6322	27.4228	1:Experimental evidence at protein level
26.8773	27.2118	27.1819	26.9188	26.5683	27.1745	1:Experimental evidence at protein level
27.6792	27.7211	27.6278	27.6224	27.6301	27.3042	1:Experimental evidence at protein level
25.2794	25.3539	25.472	26.3704	26.6559	26.3867	1:Experimental evidence at protein level
26.325	26.1591	25.8848	25.9455	26.167	25.9452	1:Experimental evidence at protein level
26.9852	27.6783	27.6801	27.6206	27.2407	27.6066	1:Experimental evidence at protein level
26.9949	27.2802	27.2193	27.1512	26.9931	27.1771	1:Experimental evidence at protein level
27.7464	27.7057	27.8182	28.1975	28.2638	28.3248	1:Experimental evidence at protein level
27.8512	27.2945	27.2756	26.9812	27.309	27.144	1:Experimental evidence at protein level
27.8242	28.7431	28.6013	29.1387	28.8022	29.0299	1:Experimental evidence at protein level
24.7672	24.9178	24.8568	25.4952	25.4273	25.8138	1:Experimental evidence at protein level
25.8855	25.6945	25.8085	25.7312	25.5651	25.6936	1:Experimental evidence at protein level
#NUM!	#NUM!	25.6629	25.6062	25.5338	25.7038	1:Experimental evidence at protein level
26.9562	26.7677	26.8212	26.851	27.3813	26.9829	1:Experimental evidence at protein level
28.7198	28.8621	28.8703	28.5037	28.599	28.5757	1:Experimental evidence at protein level
29.5269	29.7092	29.7398	29.5768	29.6224	29.0712	1:Experimental evidence at protein level
24.8937	24.8214	24.7153	24.4263	24.325	24.4444	1:Experimental evidence at protein level
27.2362	27.4257	27.5304	27.4926	27.1232	27.1443	1:Experimental evidence at protein level
27.1001	27.1875	27.3996	27.0528	27.082	27.1102	1:Experimental evidence at protein level
28.7698	29.1624	29.4694	28.5182	28.5353	28.5654	1:Experimental evidence at protein level
29.147	28.8832	29.1526	29.1623	29.1432	28.946	1:Experimental evidence at protein level
25.2464	25.3915	25.2776	25.1878	25.6051	25.3354	1:Experimental evidence at protein level
26.3875	26.9991	26.9885	27.2946	27.022	27.3334	1:Experimental evidence at protein level
28.2344	28.643	29.045	28.8324	28.9765	28.7599	1:Experimental evidence at protein level
25.763	25.8297	25.8577	25.605	25.5978	25.6454	1:Experimental evidence at protein level
30.1595	30.2543	30.2229	30.0702	30.1198	29.9364	1:Experimental evidence at protein level
25.9113	26.254	26.339	26.3355	26.2295	26.5268	1:Experimental evidence at protein level
29.1879	29.343	29.3579	29.3313	29.236	29.1992	1:Experimental evidence at protein level
27.7987	27.158	27.1633	27.7876	28.4154	27.7018	1:Experimental evidence at protein level
28.3105	28.5684	28.4022	28.3694	28.3255	28.2196	1:Experimental evidence at protein level
27.6318	27.8625	27.5958	27.9495	28.2887	27.8089	1:Experimental evidence at protein level
26.6695	26.6188	26.7132	26.6768	27.0707	26.6061	1:Experimental evidence at protein level
26.5833	25.7318	25.9026	25.3883	25.9647	25.3582	1:Experimental evidence at protein level
26.0941	25.8086	25.7502	25.6941	25.5329	26.2758	1:Experimental evidence at protein level
26.5928	26.8149	26.7019	26.6404	26.6618	26.5445	1:Experimental evidence at protein level
31.1452	31.0146	31.0895	31.0365	31.2461	31.0405	1:Experimental evidence at protein level
25.5895	25.3556	25.2265	25.1975	25.4856	25.2993	1:Experimental evidence at protein level
27.0766	26.8354	26.8098	26.583	26.7753	26.4859	1:Experimental evidence at protein level
28.0711	28.2952	28.3608	28.4481	28.2265	28.4779	1:Experimental evidence at protein level
25.9046	25.9369	25.8253	26.1812	26.1872	26.0871	1:Experimental evidence at protein level
28.4655	28.3715	28.4001	28.3967	28.5954	28.5792	1:Experimental evidence at protein level
25.5699	25.5041	25.4348	25.2909	24.9808	25.0486	1:Experimental evidence at protein level
27.402	27.3626	27.4152	27.3031	27.218	27.1462	1:Experimental evidence at protein level
28.8858	28.8751	28.7828	28.9991	28.7762	28.9158	1:Experimental evidence at protein level
27.2595	27.7202	27.5959	27.4037	26.8768	26.9216	1:Experimental evidence at protein level
26.3765	25.8905	25.7332	25.6111	25.7527	25.5402	1:Experimental evidence at protein level

25.1166	25.3961	25.1077	25.3872	25.0824	25.1919	1:Experimental evidence at protein level
29.4483	28.7335	28.7748	28.9166	29.7182	28.7441	1:Experimental evidence at protein level
26.6953	28.0888	28.0674	28.1846	26.8768	27.9599	1:Experimental evidence at protein level
33.6183	34.3204	34.244	34.5801	34.4944	34.2641	1:Experimental evidence at protein level
27.1944	27.2605	27.2013	26.9008	27.0297	27.0084	1:Experimental evidence at protein level
31.0137	30.603	30.8113	30.9105	30.9152	30.7984	1:Experimental evidence at protein level
24.8102	25.5261	25.5451	25.4224	24.9725	25.3401	1:Experimental evidence at protein level
26.4294	26.5292	26.4679	26.19	26.3666	26.0694	1:Experimental evidence at protein level
28.5882	28.3403	28.262	28.2544	28.7411	27.8376	1:Experimental evidence at protein level
26.0365	26.1994	26.0877	26.006	26.3525	25.9074	1:Experimental evidence at protein level
26.5384	26.8666	#NUM!	26.7418	26.3623	26.5701	1:Experimental evidence at protein level
25.5053	26.1769	26.252	26.6566	27.0536	26.5776	1:Experimental evidence at protein level
27.4515	27.5652	27.6946	27.9254	27.8322	27.7054	1:Experimental evidence at protein level
25.1448	25.2362	25.0123	26.1327	26.0326	26.2539	1:Experimental evidence at protein level
28.5023	28.9042	28.9142	29.0873	28.5241	28.7468	1:Experimental evidence at protein level
28.0738	27.6982	27.8486	27.7609	28.1303	27.7932	1:Experimental evidence at protein level
26.2384	26.1825	26.435	26.4951	26.4299	26.011	1:Experimental evidence at protein level
29.0046	28.5499	28.3308	28.279	28.4188	28.3553	1:Experimental evidence at protein level
27.9652	28.1764	28.0823	28.1477	28.0346	28.1483	1:Experimental evidence at protein level
26.4644	26.6242	26.705	26.1151	26.1502	25.9745	1:Experimental evidence at protein level
25.7721	25.7828	25.9975	25.7001	25.1399	25.6618	1:Experimental evidence at protein level
26.8208	26.2303	26.1541	26.5657	26.7736	26.5451	1:Experimental evidence at protein level
26.7067	26.6748	26.5848	26.6145	26.5878	26.7719	1:Experimental evidence at protein level
26.1919	25.8834	25.9351	25.9131	25.9094	26.0038	1:Experimental evidence at protein level
26.467	25.6624	25.7545	24.852	25.2319	25.0144	1:Experimental evidence at protein level
26.7865	26.8849	26.8472	26.9252	26.4356	26.8872	1:Experimental evidence at protein level
25.3106	25.3119	25.0581	25.3665	25.679	25.7066	1:Experimental evidence at protein level
24.038	23.6436	23.8105	23.7738	24.1085	23.7999	1:Experimental evidence at protein level
26.4434	26.5941	26.6879	26.7574	26.7598	26.8333	1:Experimental evidence at protein level
25.0183	24.9153	24.9712	25.2727	24.3365	24.6377	1:Experimental evidence at protein level
27.0561	26.8123	26.857	26.5699	26.6895	26.2427	1:Experimental evidence at protein level
27.532	26.6705	26.886	27.1127	27.9588	27.2257	1:Experimental evidence at protein level
28.0353	27.8927	27.6495	27.7207	28.1888	27.8733	1:Experimental evidence at protein level
27.0045	#NUM!	26.6369	26.896	27.0591	26.9526	1:Experimental evidence at protein level
25.5156	25.4057	25.7214	25.624	25.8858	25.6557	1:Experimental evidence at protein level
25.5764	26.0251	26.1121	26.1812	25.7176	25.9359	1:Experimental evidence at protein level
25.9846	25.5561	25.5959	25.5134	25.8496	25.5225	1:Experimental evidence at protein level
25.7532	25.7949	25.5376	25.2856	25.7616	25.5806	1:Experimental evidence at protein level
26.152	26.2743	26.5073	26.4763	26.0942	26.3495	1:Experimental evidence at protein level
28.3309	28.0924	27.9378	27.9586	28.1084	28.0484	1:Experimental evidence at protein level
25.9089	25.7184	25.7995	25.5439	25.8833	25.5016	1:Experimental evidence at protein level
26.1599	26.4711	26.4276	26.2386	25.9301	26.2718	1:Experimental evidence at protein level
28.5655	29.1571	29.0207	29.3603	29.1664	29.4341	1:Experimental evidence at protein level
25.3699	25.4899	25.4377	25.2926	25.2067	25.1852	1:Experimental evidence at protein level
25.3853	25.1702	24.9737	25.4317	25.8946	25.4515	1:Experimental evidence at protein level
26.5467	26.3849	26.5412	26.4923	26.5643	26.4221	1:Experimental evidence at protein level
28.379	27.7371	27.3765	27.6496	28.0439	27.3291	1:Experimental evidence at protein level
24.5512	24.8227	24.8462	24.9461	24.4948	24.7618	1:Experimental evidence at protein level
26.3499	26.5837	26.6673	26.5689	26.0936	26.4157	1:Experimental evidence at protein level
#NUM!	24.4678	24.5559	24.8997	25.0388	25.246	1:Experimental evidence at protein level
27.1197	26.7269	26.932	26.74	26.6678	26.8962	1:Experimental evidence at protein level
26.7335	26.9273	26.8455	26.33	26.2569	26.3141	1:Experimental evidence at protein level
29.4483	29.1607	29.2125	29.1177	29.074	29.0504	1:Experimental evidence at protein level
26.6931	26.8195	26.8646	27.1376	26.8865	27.1065	1:Experimental evidence at protein level
25.9881	25.9026	25.7609	25.6897	25.7839	25.6876	1:Experimental evidence at protein level
25.6487	25.5303	25.6104	25.6255	25.4839	25.5304	1:Experimental evidence at protein level
27.0842	27.3542	27.451	27.2599	26.9449	27.0634	1:Experimental evidence at protein level
27.278	27.0025	27.0372	26.7	27.2348	26.6442	1:Experimental evidence at protein level
26.3116	26.4145	26.6024	26.4218	26.2432	26.257	1:Experimental evidence at protein level
26.8426	26.3381	26.5413	26.2691	26.5354	26.2444	1:Experimental evidence at protein level
24.9821	24.7236	24.7739	24.8783	24.5053	24.7867	1:Experimental evidence at protein level
25.8525	25.6413	25.5733	25.7542	25.7015	25.7904	1:Experimental evidence at protein level
24.5554	24.9678	25.019	24.2583	23.6206	24.4685	1:Experimental evidence at protein level
26.1242	25.5854	25.4734	25.5019	26.1851	25.2409	1:Experimental evidence at protein level
#NUM!	25.1774	25.9285	25.8024	25.561	25.6117	1:Experimental evidence at protein level
27.0418	26.8937	26.933	26.474	26.652	26.3465	1:Experimental evidence at protein level
25.7614	26.7422	26.7817	26.7162	26.5663	26.8998	1:Experimental evidence at protein level
24.797	24.9281	25.0033	24.7022	24.669	24.7577	1:Experimental evidence at protein level
26.3878	26.4978	26.6094	26.2479	26.279	26.396	1:Experimental evidence at protein level
24.5018	25.6146	25.4648	25.2313	24.5741	25.5478	1:Experimental evidence at protein level
26.2702	26.096	26.1449	26.0606	26.102	25.6596	1:Experimental evidence at protein level
26.1253	25.834	25.4025	25.3929	26.0258	25.231	1:Experimental evidence at protein level
25.7289	25.9271	26.0157	26.4055	26.3396	26.2296	1:Experimental evidence at protein level
27.6088	28.1554	27.8097	27.5689	27.2812	27.5636	1:Experimental evidence at protein level
28.1199	27.7781	27.8657	27.9001	27.5654	28.3744	1:Experimental evidence at protein level
26.8233	27.0383	27.1301	26.7048	26.5803	26.7382	1:Experimental evidence at protein level
26.9022	26.6316	26.5601	26.6543	26.6878	26.5765	1:Experimental evidence at protein level
29.1103	29.2478	29.135	29.0491	28.5565	29.0967	1:Experimental evidence at protein level
27.4532	27.1733	27.1198	27.041	27.0951	27.0689	1:Experimental evidence at protein level
25.9362	26.1167	26.2302	26.2372	25.9541	25.9027	1:Experimental evidence at protein level
#NUM!	24.4513	24.7803	24.8573	24.6804	24.9747	1:Experimental evidence at protein level
27.5666	27.3015	27.5137	27.286	27.408	27.2724	1:Experimental evidence at protein level
26.7659	26.3406	27.192	26.3378	26.6827	26.6277	1:Experimental evidence at protein level
25.8803	25.4785	25.161	25.3791	25.666	25.6753	1:Experimental evidence at protein level
27.3171	27.2212	26.9187	27.4062	27.9497	27.4863	1:Experimental evidence at protein level
27.1064	26.6808	26.582	26.1114	26.4862	26.1269	1:Experimental evidence at protein level
27.7343	28.0695	27.9677	27.5046	26.9961	27.4489	1:Experimental evidence at protein level
27.5672	27.445	27.3617	27.5516	27.6297	27.3355	1:Experimental evidence at protein level
28.1177	28.7276	28.6236	28.2727	27.9069	28.2512	1:Experimental evidence at protein level
24.9028	25.2526	25.4251	25.0692	26.2383	25.1617	1:Experimental evidence at protein level
27.6807	27.1803	27.2415	27.3429	27.4816	27.5268	1:Experimental evidence at protein level
29.581	30.1717	30.2709	30.2205	30.1142	29.9987	1:Experimental evidence at protein level
28.9205	29.557	29.4326	29.5656	29.1121	29.5537	1:Experimental evidence at protein level
27.0372	26.8898	26.9117	26.8275	27.1164	26.9976	1:Experimental evidence at protein level
27.2677	27.7247	27.4834	27.3883	27.52	27.1298	1:Experimental evidence at protein level
26.4431	26.9806	26.9469	27.0244	26.518	27.137	1:Experimental evidence at protein level
29.2293	28.8349	28.7928	28.6501	28.8076	28.7534	1:Experimental evidence at protein level
25.6538	25.6755	25.7436	25.6086	25.4849	25.4641	1:Experimental evidence at protein level
25.8432	25.5588	25.0304	24.706	25.4466	24.6891	1:Experimental evidence at protein level
24.9012	25.7492	26.0169	26.1172	25.4967	25.9229	1:Experimental evidence at protein level
27.4795	27.0188	26.8194	26.8801	27.0786	26.845	1:Experimental evidence at protein level
26.5249	26.4335	26.5253	26.3535	26.1933	26.4518	1:Experimental evidence at protein level
27.4507	27.0471	27.0273	26.9808	27.6751	26.9212	1:Experimental evidence at protein level
24.5866	25.2297	25.3815	24.9741	24.575	25.1042	1:Experimental evidence at protein level
28.0812	28.0445	27.7153	27.6837	28.0523	27.6764	1:Experimental evidence at protein level
27.822	28.4797	28.1187	28.1149	28.2864	28.1893	1:Experimental evidence at protein level
25.389	25.0145	24.821	24.5649	24.9525	24.5781	1:Experimental evidence at protein level
26.3975	26.239	25.7966	25.7132	25.8986	25.5087	1:Experimental evidence at protein level
25.8883	25.1833	25.507	25.025	25.2656	24.8841	1:Experimental evidence at protein level
28.0963	28.0581	28.082	28.273	28.2812	28.1361	1:Experimental evidence at protein level
26.4274	26.2814	26.1629	26.2704	26.3373	26.0834	1:Experimental evidence at protein level
28.3467	28.3791	28.3677	28.463	28.2959	28.5026	1:Experimental evidence at protein level
26.0859	26.2834	26.1805	25.937	25.6516	25.981	1:Experimental evidence at protein level
25.6855	25.9054	25.8352	25.7754	25.4336	25.8623	1:Experimental evidence at protein level
27.6121	27.8018	27.8957	27.8389	27.7749	27.701	1:Experimental evidence at protein level
27.7933	27.6465	27.6941	27.6211	27.7786	27.5987	1:Experimental evidence at protein level
27.6217	27.1181	27.0226	27.0612	26.9877	26.9898	1:Experimental evidence at protein level
#NUM!	26.0975	26.1526	26.0563	26.613	26.0231	1:Experimental evidence at protein level
27.3782	27.4233	27.444	27.4947	27.6811	27.4611	1:Experimental evidence at protein level
32.0432	28.215	29.1628	29.2236	31.7627	29.1018	1:Experimental evidence at protein level
29.5064	30.4152	30.4066	30.4125	30.0187	30.4043	1:Experimental evidence at protein level
29.4072	29.4219	29.4491	29.2922	29.3647	29.1287	1:Experimental evidence at protein level
28.4838	28.4554	28.3414	28.2782	28.4638	28.4588	1:Experimental evidence at protein level
26.2197	26.4971	26.4994	26.3414	26.0714	26.3417	1:Experimental evidence at protein level
31.3678	31.1408	31.2755	31.4315	31.9356	31.1542	1:Experimental evidence at protein level
27.1048	26.6618	26.6373	26.8991	27.1254	27.0415	1:Experimental evidence at protein level
28.1334	27.7738	27.7886	28.0374	28.115	28.0207	1:Experimental evidence at protein level
28.597	28.6165	28.9241	29.1849	28.9563	29.0339	1:Experimental evidence at protein level
26.4862	26.2512	26.415	26.1681	26.1599	26.3448	1:Experimental evidence at protein level

28.7626	28.873	28.6997	28.7311	28.5993	28.7066	1:Experimental evidence at protein level
28.1404	27.3739	27.4644	27.6824	27.7394	27.6407	1:Experimental evidence at protein level
27.8503	28.0467	28.0373	26.9167	26.8261	26.8226	1:Experimental evidence at protein level
25.5853	25.3747	25.182	25.7282	26.2981	25.8426	1:Experimental evidence at protein level
28.2323	28.2465	28.3598	27.8731	27.8232	27.7597	1:Experimental evidence at protein level
24.825	25.0601	24.8059	25.0745	25.6453	24.7791	1:Experimental evidence at protein level
27.0902	26.9766	26.987	26.5764	26.5745	26.5737	1:Experimental evidence at protein level
30.3261	29.9589	30.0207	30.1658	30.314	29.9582	1:Experimental evidence at protein level
30.125	30.0695	30.1892	30.0822	30.5716	30.0863	1:Experimental evidence at protein level
26.9159	26.4784	26.5578	26.3801	27.1482	26.2533	1:Experimental evidence at protein level
26.0372	26.2739	26.496	26.6023	26.4681	26.5286	1:Experimental evidence at protein level
26.9567	27.2686	27.1474	27.181	27.599	27.4982	1:Experimental evidence at protein level
25.8393	26.2665	26.3361	25.9943	25.6192	26.0112	1:Experimental evidence at protein level
26.7773	26.7635	26.7721	26.9131	26.9916	27.0175	1:Experimental evidence at protein level
27.4729	27.3903	27.4518	27.5061	27.5914	27.1265	1:Experimental evidence at protein level
26.8986	26.6146	26.4681	26.8727	26.6569	26.8578	1:Experimental evidence at protein level
26.699	26.9515	26.9245	26.9327	27.1635	26.8837	1:Experimental evidence at protein level
28.7544	28.2012	28.4087	28.2388	29.3743	28.1707	1:Experimental evidence at protein level
26.2548	26.6515	26.6081	26.3857	26.3099	26.4882	1:Experimental evidence at protein level
27.8378	27.776	27.7623	27.6768	27.3319	27.5944	1:Experimental evidence at protein level
26.597	26.2636	26.515	26.4148	26.3831	26.2551	1:Experimental evidence at protein level
27.2135	27.6388	27.6315	27.6131	27.0573	27.2383	1:Experimental evidence at protein level
28.7352	28.3876	28.2316	28.2256	28.435	28.4368	1:Experimental evidence at protein level
29.0798	29.163	29.1862	28.9688	28.7038	29.0982	1:Experimental evidence at protein level
28.8618	29.8959	29.7432	30.0496	29.3526	29.6818	1:Experimental evidence at protein level
28.335	28.5144	28.3717	28.3981	28.4308	28.3622	1:Experimental evidence at protein level
26.8157	26.7199	26.7522	26.6388	26.451	26.5688	1:Experimental evidence at protein level
27.3515	27.6177	27.5865	26.8219	26.6482	26.663	1:Experimental evidence at protein level
24.5556	24.3944	24.968	24.019	24.3349	24.2039	1:Experimental evidence at protein level
26.833	25.974	25.7367	26.1417	26.6412	25.5217	1:Experimental evidence at protein level
28.0108	27.7104	27.6968	27.5535	27.6886	27.4477	1:Experimental evidence at protein level
26.8731	27.0188	26.851	26.8469	26.2781	26.6542	1:Experimental evidence at protein level
25.3636	25.3891	25.386	24.6818	24.5414	24.5747	1:Experimental evidence at protein level
26.0733	26.1299	26.1539	26.5517	26.298	26.4265	1:Experimental evidence at protein level
26.8557	26.7039	26.8166	26.6068	26.1978	26.6161	1:Experimental evidence at protein level
25.9474	25.9122	26.0755	25.6378	24.9089	25.8141	1:Experimental evidence at protein level
26.7161	26.6886	26.509	26.7437	26.7594	26.5734	1:Experimental evidence at protein level
25.4086	25.0238	24.8548	26.2657	26.4522	26.3219	1:Experimental evidence at protein level
26.5611	27.073	27.1727	26.9766	26.5635	26.9336	1:Experimental evidence at protein level
26.2159	26.1807	26.2781	25.5784	25.2484	25.7198	1:Experimental evidence at protein level
25.946	26.0121	25.929	26.0503	25.7994	25.8295	1:Experimental evidence at protein level
26.391	26.2548	26.2522	26.3217	25.7736	26.1452	1:Experimental evidence at protein level
26.8319	26.7606	26.0202	26.0438	26.685	26.1878	1:Experimental evidence at protein level
26.4469	26.358	26.1503	26.2803	26.2663	26.0211	1:Experimental evidence at protein level
28.4698	28.4968	28.4612	28.2678	28.3336	28.3793	1:Experimental evidence at protein level
26.846	26.4463	26.4483	26.2556	26.8053	26.1801	1:Experimental evidence at protein level
25.9571	25.5139	25.688	25.8311	25.9951	25.7716	1:Experimental evidence at protein level
26.0046	26.1765	26.0258	25.8124	25.7403	25.7486	1:Experimental evidence at protein level
26.1133	25.9186	25.8681	25.9585	26.1711	25.6718	1:Experimental evidence at protein level
25.716	25.6829	25.3378	24.9609	25.077	25.0147	1:Experimental evidence at protein level
26.0728	26.0572	26.2269	25.9986	25.9067	26.0775	1:Experimental evidence at protein level
25.2795	25.162	25.2421	25.1676	25.3136	25.1547	1:Experimental evidence at protein level
26.6497	26.2782	26.2557	26.4742	26.6059	26.3163	1:Experimental evidence at protein level
26.3437	27.0648	27.0041	26.8912	26.5566	26.9881	1:Experimental evidence at protein level
26.8877	26.423	26.4692	26.476	27.7343	26.6506	1:Experimental evidence at protein level
26.1261	26.957	26.8136	26.3876	25.9883	26.1161	1:Experimental evidence at protein level
24.8024	25.0164	24.8708	25.1673	24.7715	25.172	1:Experimental evidence at protein level
25.8686	26.3332	26.103	25.9432	25.6811	25.5807	1:Experimental evidence at protein level
26.4822	26.7893	26.7102	26.665	26.474	26.5672	1:Experimental evidence at protein level
25.95	25.1498	25.2934	25.5601	25.5028	25.7288	1:Experimental evidence at protein level
25.5014	26.4144	26.5494	26.5619	25.4902	26.7694	1:Experimental evidence at protein level
26.9063	26.6906	26.8639	26.9525	27.1326	26.8141	1:Experimental evidence at protein level
25.4802	25.5137	25.5566	25.4025	24.9237	25.3711	1:Experimental evidence at protein level
26.804	26.7472	26.7029	26.6435	26.5868	26.5093	1:Experimental evidence at protein level
27.7152	27.4046	27.5055	27.5431	27.6652	27.4657	1:Experimental evidence at protein level
28.8145	28.7937	28.8432	28.6126	28.6063	28.8453	1:Experimental evidence at protein level
26.6231	26.9507	27.0944	27.3443	27.3173	27.3498	1:Experimental evidence at protein level
27.0872	26.6752	26.72	26.6987	26.9556	26.3533	1:Experimental evidence at protein level
26.6223	26.3379	26.3725	26.4904	26.5181	26.2606	1:Experimental evidence at protein level
26.8166	26.59	26.5677	26.4169	26.3378	26.1294	1:Experimental evidence at protein level
25.1287	25.1273	25.2416	24.8523	24.8167	24.6655	1:Experimental evidence at protein level
27.5623	27.6025	27.5747	27.759	27.1749	27.8834	1:Experimental evidence at protein level
26.5285	27.1306	27.1833	27.0527	26.5336	26.8937	1:Experimental evidence at protein level
26.2998	26.5253	26.5871	26.1586	26.3971	26.5905	1:Experimental evidence at protein level
26.1472	26.2344	26.5159	26.4222	26.0984	26.2677	1:Experimental evidence at protein level
26.1436	25.7009	25.7494	25.313	25.6525	25.2015	1:Experimental evidence at protein level
30.7175	30.1315	30.109	30.627	30.7049	30.5068	1:Experimental evidence at protein level
25.5526	25.149	24.9639	25.4333	25.3089	25.6118	1:Experimental evidence at protein level
27.2933	26.9683	26.9613	26.9602	27.0589	26.7647	1:Experimental evidence at protein level
28.4723	28.6134	28.5143	28.55	28.484	28.4249	1:Experimental evidence at protein level
27.8363	27.5981	27.8698	27.7623	27.567	27.7963	1:Experimental evidence at protein level
27.3623	26.9247	26.7123	26.394	26.6715	26.3568	2:Experimental evidence at transcript level
25.315	24.7235	24.5661	24.3554	24.0481	23.8695	1:Experimental evidence at protein level
28.883	29.2964	28.9594	29.112	28.6911	29.0624	1:Experimental evidence at protein level
26.556	26.471	26.3728	26.1136	26.3318	26.418	1:Experimental evidence at protein level
27.0167	27.0321	27.1713	27.111	27.0529	27.0717	1:Experimental evidence at protein level
25.9742	25.9528	25.9423	25.245	25.3815	25.6479	1:Experimental evidence at protein level
26.1991	26.0174	26.1235	25.764	25.7052	25.7177	1:Experimental evidence at protein level
25.8794	26.1942	25.9383	26.0274	25.7885	25.8719	1:Experimental evidence at protein level
29.0165	28.8858	28.8977	28.9799	29.1814	28.7723	1:Experimental evidence at protein level
27.308	26.7132	26.7846	26.5443	26.603	26.2651	1:Experimental evidence at protein level
27.2923	26.9818	27.0628	26.8401	26.5056	26.8319	1:Experimental evidence at protein level
28.6541	28.1389	28.2179	28.0098	28.1582	28.0522	1:Experimental evidence at protein level
28.7571	28.6428	28.6781	28.3641	28.5623	28.7103	1:Experimental evidence at protein level
24.0489	#NUM!	23.7299	23.6462	24.722	24.2541	1:Experimental evidence at protein level
25.1068	25.4931	25.4486	25.2893	25.6403	25.319	1:Experimental evidence at protein level
26.6103	26.0743	26.0444	25.9434	26.1779	25.7371	1:Experimental evidence at protein level
26.9984	26.8048	26.7839	26.6665	26.6806	26.6079	1:Experimental evidence at protein level
24.9102	24.9242	24.989	25.039	24.9721	25.1651	1:Experimental evidence at protein level
24.769	25.1368	25.1506	25.2586	25.13	25.0554	1:Experimental evidence at protein level
25.9491	25.4227	25.5986	25.4091	25.7229	25.4354	1:Experimental evidence at protein level
25.5888	25.4078	25.4836	25.138	25.2984	25.2994	1:Experimental evidence at protein level
26.0785	26.0895	25.8654	25.9056	26.1314	26.0393	1:Experimental evidence at protein level
27.1214	26.4232	26.3546	26.5761	27.1722	26.2523	1:Experimental evidence at protein level
25.1873	25.174	25.0836	25.2779	25.2982	25.3816	1:Experimental evidence at protein level
27.7754	27.5633	27.4246	27.5683	27.6444	27.5608	1:Experimental evidence at protein level
26.0561	25.5902	25.7444	25.9369	25.7034	25.9517	1:Experimental evidence at protein level
25.2288	25.1699	25.3013	25.6601	25.2996	25.4501	1:Experimental evidence at protein level
27.1086	26.3376	26.355	26.2457	26.9847	26.3174	1:Experimental evidence at protein level
27.3655	27.3398	27.2756	27.4564	27.9606	27.3026	1:Experimental evidence at protein level
27.453	27.7392	27.756	27.7638	27.5819	27.7862	1:Experimental evidence at protein level
26.5621	26.2898	25.8227	25.8821	26.1507	25.7958	1:Experimental evidence at protein level
26.3477	26.5668	26.696	27.0231	26.5721	26.8423	1:Experimental evidence at protein level
25.4351	24.9453	24.9199	25.3499	25.5815	25.4844	1:Experimental evidence at protein level
27.5064	28.3978	28.2273	28.1778	27.3083	28.1738	1:Experimental evidence at protein level
27.3936	26.8543	26.8376	26.6953	27.0092	26.6591	1:Experimental evidence at protein level
28.0861	29.0007	28.9119	28.9294	28.5646	28.6858	1:Experimental evidence at protein level
25.3711	25.5675	25.5764	25.7016	25.1781	25.6742	1:Experimental evidence at protein level
26.731	26.5434	26.4944	26.1891	26.164	26.527	1:Experimental evidence at protein level
26.093	26.0334	25.9886	26.3568	26.406	26.5304	1:Experimental evidence at protein level
26.8624	26.9882	26.8578	26.7163	26.6018	27.0068	1:Experimental evidence at protein level
27.6434	27.9743	28.0792	27.9422	27.9756	27.8756	1:Experimental evidence at protein level
26.5767	26.8493	26.7027	26.3453	26.2635	26.1884	1:Experimental evidence at protein level
27.0554	26.3543	26.6135	26.4104	26.4822	26.6303	1:Experimental evidence at protein level
26.867	26.9327	26.6271	27.0293	27.2237	27.2769	1:Experimental evidence at protein level
29.3632	29.5047	29.5611	29.3707	29.3645	29.3181	1:Experimental evidence at protein level
28.4574	28.1022	28.1961	27.9563	28.6881	28.1242	1:Experimental evidence at protein level
28.5765	28.6697	28.6939	27.9663	28.27	27.782	1:Experimental evidence at protein level
27.8295	27.701	27.6212	27.5472	27.8016	27.5257	1:Experimental evidence at protein level

27.3446	27.3672	27.1926	27.0322	27.2819	26.8977	1:Experimental evidence at protein level
26.4598	26.3	26.23	26.339	26.324	26.2833	1:Experimental evidence at protein level
27.6833	27.5487	27.4162	27.482	27.5479	27.2613	1:Experimental evidence at protein level
29.8906	30.0539	29.6678	29.6523	29.5706	29.3156	1:Experimental evidence at protein level
28.8373	28.6047	28.63	28.5686	28.7042	28.2993	1:Experimental evidence at protein level
27.7194	27.9196	27.8671	27.7676	27.9478	27.7378	1:Experimental evidence at protein level
28.7246	28.8009	28.8033	28.8713	28.5343	28.7035	1:Experimental evidence at protein level
29.1201	29.1858	29.1461	29.3562	29.2311	29.1334	1:Experimental evidence at protein level
26.8352	26.5972	26.8157	26.9067	26.9414	27.0014	1:Experimental evidence at protein level
29.2431	29.2229	29.5739	29.3978	29.4071	29.4171	1:Experimental evidence at protein level
26.6798	26.976	27.1376	27.0172	26.7581	26.8854	1:Experimental evidence at protein level
27.9067	28.1956	28.1906	28.0391	27.491	28.1898	1:Experimental evidence at protein level
26.5653	26.8336	26.839	27.6725	27.6703	27.5955	1:Experimental evidence at protein level
26.3144	26.7785	26.8989	26.969	26.652	26.7857	1:Experimental evidence at protein level
26.7614	26.4062	26.5019	26.3693	26.3465	26.3992	1:Experimental evidence at protein level
27.2149	27.1378	26.8579	26.815	26.8913	26.7213	1:Experimental evidence at protein level
27.6879	27.92	27.8495	28.0572	27.9224	27.6877	1:Experimental evidence at protein level
28.9138	28.5945	28.5925	28.5747	28.867	28.2776	1:Experimental evidence at protein level
27.4648	26.5103	26.2625	27.0157	27.8597	26.6136	1:Experimental evidence at protein level
29.1557	29.6909	29.6326	29.5201	29.1809	29.0249	1:Experimental evidence at protein level
25.8538	26.0611	25.9656	25.8514	26.0634	25.7988	1:Experimental evidence at protein level
27.5719	27.3213	27.3866	27.7247	28.012	27.6841	1:Experimental evidence at protein level
30.9328	30.0646	29.8967	30.32	31.1333	30.4321	1:Experimental evidence at protein level
28.1616	28.388	28.3884	27.7201	27.4535	27.562	1:Experimental evidence at protein level
27.5275	27.3549	27.2767	27.4553	27.6458	27.5745	1:Experimental evidence at protein level
26.9608	27.1387	27.1749	27.2771	27.335	27.3766	1:Experimental evidence at protein level
28.3982	28.4821	28.5662	28.3766	28.6414	28.5752	1:Experimental evidence at protein level
28.1259	28.1808	28.2303	28.1033	27.9472	28.0223	1:Experimental evidence at protein level
26.0129	26.0543	26.0329	26.041	25.7738	26.0373	1:Experimental evidence at protein level
25.2274	25.1663	25.4	25.1881	25.3803	25.4622	1:Experimental evidence at protein level
26.5439	26.9419	26.9524	27.2133	27.0653	27.0278	1:Experimental evidence at protein level
26.6631	26.2512	26.5603	26.3425	26.0743	26.5374	1:Experimental evidence at protein level
26.1431	26.429	26.3739	25.9203	25.666	25.9339	1:Experimental evidence at protein level
27.1239	27.1764	27.5396	27.2283	27.3858	27.1945	1:Experimental evidence at protein level
26.0935	26.5546	26.9135	26.4931	26.3124	26.5941	1:Experimental evidence at protein level
27.8547	27.734	27.7951	27.8587	27.6952	27.8106	1:Experimental evidence at protein level
26.5209	27.2264	27.1102	27.2913	26.9125	27.1723	1:Experimental evidence at protein level
32.2089	31.5318	31.8858	31.2077	31.7369	30.9921	1:Experimental evidence at protein level
25.3948	25.5608	25.8625	25.6941	24.9806	25.3941	1:Experimental evidence at protein level
25.8116	25.6952	25.5784	25.4577	25.2219	25.3832	1:Experimental evidence at protein level
26.8022	26.855	27.0632	26.6465	26.5711	26.8284	1:Experimental evidence at protein level
27.584	27.5839	27.456	27.2474	27.029	27.2934	1:Experimental evidence at protein level
25.8811	25.6243	25.6434	25.5985	25.002	25.8459	1:Experimental evidence at protein level
27.0663	27.3999	27.5497	27.2288	27.0587	27.0609	1:Experimental evidence at protein level
25.5846	25.3392	25.4502	25.7673	25.228	25.6054	1:Experimental evidence at protein level
26.5087	26.3281	26.3016	26.605	26.3468	26.4085	1:Experimental evidence at protein level
26.4409	26.4352	26.4069	26.4951	26.2665	26.4179	1:Experimental evidence at protein level
26.4526	26.5204	26.401	26.4117	26.2767	26.54	1:Experimental evidence at protein level
27.6943	27.6482	27.7951	27.2124	27.4501	27.1662	1:Experimental evidence at protein level
24.9224	24.6009	24.5469	24.7479	25.1562	25.4157	1:Experimental evidence at protein level
25.9054	26.0868	26.3688	26.406	26.2713	26.4973	1:Experimental evidence at protein level
25.8392	25.6887	25.6766	25.7641	26.1011	25.5972	1:Experimental evidence at protein level
23.6917	23.9402	24.0259	25.164	25.0507	24.9462	1:Experimental evidence at protein level
26.9366	26.6163	26.599	26.579	26.4382	26.4926	1:Experimental evidence at protein level
26.9996	26.764	26.8159	25.6978	25.5807	25.7701	1:Experimental evidence at protein level
25.8022	25.6595	25.8751	25.7471	25.67	25.495	1:Experimental evidence at protein level
25.358	25.0713	25.7289	25.2846	25.4174	25.2351	1:Experimental evidence at protein level
27.756	28.0161	28.13	28.205	27.8059	28.2524	1:Experimental evidence at protein level
27.2218	27.1387	27.2104	27.0538	27.1639	27.3712	1:Experimental evidence at protein level
27.4572	27.6723	27.1832	27.1847	27.111	27.1777	1:Experimental evidence at protein level
27.0325	27.3286	27.4275	27.2578	27.3465	27.193	1:Experimental evidence at protein level
27.1649	27.2856	27.4644	27.1484	26.8223	27.2618	1:Experimental evidence at protein level
26.2016	25.8183	25.6758	25.7149	25.7252	25.8401	1:Experimental evidence at protein level
26.4108	25.9804	25.8039	26.2382	27.3835	26.0055	1:Experimental evidence at protein level
25.7214	25.5058	25.573	25.4877	25.595	25.4409	1:Experimental evidence at protein level
26.8109	26.5774	26.6195	26.6717	26.6809	26.5364	1:Experimental evidence at protein level
26.2299	26.0546	25.9719	25.9464	25.9833	25.9163	1:Experimental evidence at protein level
26.7049	26.9313	26.9612	26.9384	26.7082	26.8413	1:Experimental evidence at protein level
28.0587	28.08	28.0209	27.8406	28.2738	27.955	1:Experimental evidence at protein level
27.0769	26.6203	26.555	26.5413	26.7502	26.3544	1:Experimental evidence at protein level
26.9286	#NUM!	26.4785	25.9912	26.2267	25.9538	1:Experimental evidence at protein level
25.0947	25.6269	25.5377	25.6498	25.6946	24.9121	1:Experimental evidence at protein level
26.1708	26.0791	26.3031	25.9398	25.8741	25.9871	1:Experimental evidence at protein level
25.6094	25.6873	25.4591	25.3327	25.6402	25.4912	1:Experimental evidence at protein level
31.6491	32.2447	32.2666	32.3199	31.9199	32.3167	1:Experimental evidence at protein level
26.647	26.8937	26.8178	27.0297	26.2626	26.7316	1:Experimental evidence at protein level
26.6489	26.1475	26.1909	26.6734	27.1487	26.6988	1:Experimental evidence at protein level
29.3131	29.5731	29.7218	30.0117	29.8808	29.9511	1:Experimental evidence at protein level
24.8779	24.4135	24.3072	24.4341	24.9386	24.5019	1:Experimental evidence at protein level
28.3627	28.4933	28.6334	28.3644	28.3772	28.3916	1:Experimental evidence at protein level
#NUM!	25.8011	25.4796	25.9067	25.3681	25.5198	1:Experimental evidence at protein level
#NUM!	#NUM!	24.579	25.0346	25.4391	25.8129	1:Experimental evidence at protein level
25.8241	26.2156	26.3218	26.0637	26.0013	26.1427	1:Experimental evidence at protein level
25.934	25.5953	25.4965	25.2121	25.5796	24.9149	1:Experimental evidence at protein level
25.913	26.5192	26.1872	26.3541	26.4241	26.5076	1:Experimental evidence at protein level
28.8545	28.5839	28.6418	28.2828	28.3657	28.3955	1:Experimental evidence at protein level
26.0309	25.4693	25.3783	25.465	25.8965	25.5673	1:Experimental evidence at protein level
25.6069	25.3997	25.4451	25.5154	25.3656	25.6643	1:Experimental evidence at protein level
26.9826	26.9499	26.9328	27.1495	27.1262	27.0261	1:Experimental evidence at protein level
27.4752	27.7799	27.7456	27.7904	27.5483	27.7547	1:Experimental evidence at protein level
28.3741	28.1121	28.0434	27.7396	28.2652	28.013	1:Experimental evidence at protein level
28.0196	27.952	27.8602	27.9328	28.144	27.6815	1:Experimental evidence at protein level
26.1184	26.5829	26.5053	26.2418	25.9522	26.3224	1:Experimental evidence at protein level
27.7074	27.812	27.8681	27.7111	27.6015	27.6384	1:Experimental evidence at protein level
27.6245	27.8279	27.8257	27.8339	27.175	27.846	1:Experimental evidence at protein level
26.8009	26.9913	27.0904	27.149	27.2451	27.1069	1:Experimental evidence at protein level
26.7917	26.7701	26.567	26.6293	26.4544	26.5618	1:Experimental evidence at protein level
28.0877	28.2542	28.3403	28.1709	28.1009	28.224	1:Experimental evidence at protein level
28.6501	28.8682	28.793	28.6326	28.6164	28.7604	1:Experimental evidence at protein level
26.8592	26.7	26.6156	26.2173	26.3601	26.51	1:Experimental evidence at protein level
25.5439	25.922	25.8127	25.4986	25.4629	25.5341	1:Experimental evidence at protein level
28.8728	28.8793	28.9885	28.8886	28.7088	28.8797	1:Experimental evidence at protein level
27.5344	26.5265	26.5756	26.7594	27.3711	26.21	1:Experimental evidence at protein level
28.3453	28.0473	28.0952	28.2658	28.135	28.3883	1:Experimental evidence at protein level
29.4136	28.9812	29.1071	29.3103	30.4112	29.2106	1:Experimental evidence at protein level
27.5917	27.8992	27.9582	27.7105	27.6983	27.7479	1:Experimental evidence at protein level
26.3705	26.1629	26.281	26.5443	26.5299	26.5938	1:Experimental evidence at protein level
29.4208	29.0912	29.2526	29.3632	29.903	29.2825	1:Experimental evidence at protein level
25.2322	25.4245	25.275	25.857	25.3603	25.2318	1:Experimental evidence at protein level
27.0298	26.73	26.7111	27.2351	27.2018	27.2647	1:Experimental evidence at protein level
29.1769	29.2495	29.3256	29.116	29.1268	29.1281	1:Experimental evidence at protein level
26.7445	27.2406	27.2804	27.1421	26.7466	27.2289	1:Experimental evidence at protein level
27.5435	27.477	27.4867	27.4937	27.389	27.5034	1:Experimental evidence at protein level
26.0771	25.797	26.0073	26.0608	25.9105	26.0119	1:Experimental evidence at protein level
28.9165	28.5787	28.5698	28.574	28.6287	28.4909	1:Experimental evidence at protein level
28.9296	28.8302	28.9445	28.8892	28.4781	28.8616	1:Experimental evidence at protein level
28.1015	27.9518	27.8588	27.8689	27.6475	27.8066	1:Experimental evidence at protein level
#NUM!	24.9056	24.9189	24.8861	25.152	24.9077	1:Experimental evidence at protein level
26.7632	26.2695	26.3029	26.2225	26.5133	26.1779	1:Experimental evidence at protein level
26.7648	26.5672	26.7026	27.0233	26.5792	26.9929	1:Experimental evidence at protein level
28.0362	27.8133	27.9568	28.3512	28.5616	28.3322	1:Experimental evidence at protein level
27.0471	26.437	26.3141	26.7593	27.1109	26.8978	1:Experimental evidence at protein level
25.3266	25.924	25.9251	25.9478	25.3824	25.7459	1:Experimental evidence at protein level
31.3449	31.5194	31.4891	29.4987	29.2096	29.5677	1:Experimental evidence at protein level
28.3943	28.8543	28.7079	28.7601	28.2533	28.4569	1:Experimental evidence at protein level
31.1895	32.0397	32.0633	32.2463	31.7652	31.8124	1:Experimental evidence at protein level
28.707	28.0412	28.2163	28.5615	28.8425	28.6719	1:Experimental evidence at protein level
27.6283	27.5585	27.6201	27.6735	27.6927	27.7104	1:Experimental evidence at protein level
26.9128	26.6048	26.5461	26.6152	26.8847	26.5689	1:Experimental evidence at protein level

27.9625	27.6914	27.4617	27.5659	27.7961	27.361	1:Experimental evidence at protein level
29.5098	30.2128	30.0595	29.9524	29.5593	29.973	1:Experimental evidence at protein level
25.1395	25.8421	25.8552	25.9049	25.5042	25.8554	1:Experimental evidence at protein level
28.3764	28.0903	27.9664	27.6477	27.9735	27.3534	1:Experimental evidence at protein level
30.6674	30.4386	30.5943	30.3918	30.2295	30.479	1:Experimental evidence at protein level
27.1574	26.7567	26.481	26.3478	26.5067	26.5351	1:Experimental evidence at protein level
30.5322	30.3178	30.5396	30.4597	30.7561	30.43	1:Experimental evidence at protein level
28.8647	29.052	29.1833	28.9156	28.7192	28.9763	1:Experimental evidence at protein level
26.4784	27.0076	27.0222	26.9531	26.6258	26.8157	1:Experimental evidence at protein level
29.0407	28.978	28.9048	29.0937	29.4884	28.9801	1:Experimental evidence at protein level
27.3782	27.4244	27.5164	27.3742	27.558	27.3701	1:Experimental evidence at protein level
26.0183	26.5542	26.6761	26.4437	26.103	26.5861	1:Experimental evidence at protein level
28.2344	28.4183	28.5607	28.5072	28.262	28.5565	1:Experimental evidence at protein level
27.5985	27.3429	27.3367	27.2872	27.4189	27.3495	1:Experimental evidence at protein level
27.7923	28.2221	28.192	28.5549	28.1924	28.3665	1:Experimental evidence at protein level
30.6108	31.6962	31.6618	32.3379	31.795	32.4369	1:Experimental evidence at protein level
27.5143	28.1022	28.276	28.218	27.8736	28.2005	1:Experimental evidence at protein level
29.241	29.1862	29.1999	29.0353	29.0642	29.243	1:Experimental evidence at protein level
29.0697	29.15	29.0307	29.3237	29.2995	29.1115	1:Experimental evidence at protein level
28.9218	29.1228	29.2552	28.3281	28.5941	28.2835	1:Experimental evidence at protein level
29.5055	29.1442	29.1837	29.3768	29.5251	29.275	1:Experimental evidence at protein level
27.7388	27.9043	27.8019	28.1701	28.2244	28.0837	1:Experimental evidence at protein level
28.078	28.1485	27.8558	27.5924	27.8952	27.826	1:Experimental evidence at protein level
26.6586	26.7909	26.4877	26.7667	26.7486	26.5818	1:Experimental evidence at protein level
26.263	25.8553	25.4876	#NUM!	25.9263	25.7249	1:Experimental evidence at protein level
26.2369	26.2394	26.0575	26.0422	26.2643	26.0412	1:Experimental evidence at protein level
26.7868	26.6312	26.7221	26.482	26.3314	26.4393	1:Experimental evidence at protein level
28.4938	28.5212	28.4439	28.3997	28.448	28.2851	1:Experimental evidence at protein level
27.0371	27.3021	27.1871	27.0039	27.2641	27.1501	1:Experimental evidence at protein level
27.0531	26.5844	26.8643	26.7871	26.9015	26.7076	1:Experimental evidence at protein level
25.7907	25.512	25.676	25.5372	25.6722	25.7889	1:Experimental evidence at protein level
28.3536	28.4277	28.4135	27.9506	27.8128	27.9341	1:Experimental evidence at protein level
26.7613	26.8073	26.8167	26.4961	26.0546	26.5277	1:Experimental evidence at protein level
25.9806	26.3268	26.1944	25.476	25.2274	25.5716	1:Experimental evidence at protein level
26.7243	27.2217	27.1699	27.1858	27.2353	26.8009	1:Experimental evidence at protein level
26.238	26.2199	26.269	26.1813	26.0285	26.1875	1:Experimental evidence at protein level
27.6699	27.7484	27.9005	27.5928	27.6516	27.6834	1:Experimental evidence at protein level
28.0819	28.4193	28.0694	28.1338	27.9798	28.13	1:Experimental evidence at protein level
29.4621	29.3511	29.5075	29.5249	29.7464	29.4122	1:Experimental evidence at protein level
26.9074	27.175	27.3469	27.432	27.4269	27.548	1:Experimental evidence at protein level
25.6214	25.7817	25.5133	26.0195	25.9725	26.0726	1:Experimental evidence at protein level
27.5934	27.5348	27.566	27.5402	27.448	27.5656	1:Experimental evidence at protein level
28.4421	28.3062	28.264	28.0291	28.1057	28.0119	1:Experimental evidence at protein level
27.1029	26.9551	26.8278	27.3028	27.4468	27.2956	1:Experimental evidence at protein level
26.5113	26.9008	26.9636	27.0089	26.7087	26.7429	1:Experimental evidence at protein level
28.1688	27.9623	28.04	27.9986	28.1362	28.1746	1:Experimental evidence at protein level
25.8579	25.5781	25.1302	25.413	26.0014	25.3469	1:Experimental evidence at protein level
26.7049	26.5338	26.1919	26.1366	26.919	26.3287	1:Experimental evidence at protein level
25.4805	25.5879	25.3541	26.0758	26.2974	26.0208	1:Experimental evidence at protein level
26.0576	26.3949	26.3543	26.3198	25.7974	26.0592	1:Experimental evidence at protein level
26.0789	25.9296	26.0006	26.0856	26.0116	25.862	1:Experimental evidence at protein level
29.0348	29.0388	28.9555	28.9392	28.8636	28.7396	1:Experimental evidence at protein level
26.0876	26.0783	26.1331	25.8717	25.6455	26.1839	1:Experimental evidence at protein level
25.5916	25.4123	25.5539	25.796	25.9548	25.6978	1:Experimental evidence at protein level
25.5541	26.2151	26.3652	26.5146	26.3218	26.5367	1:Experimental evidence at protein level
27.4686	27.613	27.3084	27.2462	27.4794	27.251	1:Experimental evidence at protein level
27.3482	27.275	27.3168	27.1829	26.8227	27.0957	1:Experimental evidence at protein level
25.6549	25.6544	25.4969	25.4126	25.2428	25.6926	1:Experimental evidence at protein level
26.6217	26.7704	26.6422	26.3203	26.5939	26.3112	1:Experimental evidence at protein level
25.9867	26.1612	26.1865	25.9305	26.3065	26.2271	1:Experimental evidence at protein level
26.6524	26.3008	26.3792	26.0783	26.2544	26.2078	1:Experimental evidence at protein level
26.3908	26.0221	25.7023	25.9622	26.1896	26.0062	1:Experimental evidence at protein level
27.2584	27.1523	27.2818	27.0891	27.0985	27.3632	1:Experimental evidence at protein level
29.2314	29.2359	29.3546	29.7252	29.588	29.595	1:Experimental evidence at protein level
29.4639	29.1595	29.3447	29.5266	29.4958	29.5612	1:Experimental evidence at protein level
26.0669	26.1728	26.0922	26.2481	26.3965	26.1687	1:Experimental evidence at protein level
26.6634	26.3642	26.39	26.4808	26.5462	26.2898	1:Experimental evidence at protein level
27.929	27.5787	27.4765	27.5387	27.7602	27.4794	1:Experimental evidence at protein level
25.3151	24.884	24.5758	24.4977	25.2689	24.1175	1:Experimental evidence at protein level
25.6235	25.2037	25.2959	25.7089	26.1011	25.6969	1:Experimental evidence at protein level
25.1039	25.2594	25.0342	24.9644	25.2326	25.1089	1:Experimental evidence at protein level
25.7675	26.0126	25.8265	25.6538	25.2565	25.6399	1:Experimental evidence at protein level
26.117	25.9173	25.7255	25.8428	26.0145	25.9097	1:Experimental evidence at protein level
26.8233	26.9854	26.55	26.5379	26.7133	26.624	1:Experimental evidence at protein level
26.8663	27.0574	26.9233	26.6458	26.7967	26.4888	1:Experimental evidence at protein level
26.0462	26.4144	26.322	26.4898	26.6189	26.7489	1:Experimental evidence at protein level
25.684	26.0753	26.0193	25.8068	25.6674	25.9538	1:Experimental evidence at protein level
27.4192	26.7678	27.275	27.0426	27.3147	27.3426	1:Experimental evidence at protein level
26.1455	25.7705	25.9132	25.9806	25.9642	25.8798	1:Experimental evidence at protein level
27.6527	27.6481	27.5576	27.6276	27.359	27.3328	1:Experimental evidence at protein level
26.4807	26.568	26.6445	26.9817	26.9918	26.8927	1:Experimental evidence at protein level
26.931	26.3287	26.1262	26.1101	26.3757	25.8761	1:Experimental evidence at protein level
24.003	24.5304	24.6215	24.9742	24.7095	24.7593	1:Experimental evidence at protein level
26.2898	26.7238	26.9319	26.4843	26.2896	26.5845	1:Experimental evidence at protein level
29.0938	29.0297	28.9463	28.7999	29.0335	28.5701	1:Experimental evidence at protein level
28.8384	28.7182	28.7042	28.8338	28.9953	28.6898	1:Experimental evidence at protein level
26.2019	25.9966	25.8	25.7799	25.9339	25.6988	1:Experimental evidence at protein level
28.1323	27.6702	27.7714	27.5576	27.8163	27.4772	1:Experimental evidence at protein level
27.0903	26.6789	26.6376	26.0524	26.5145	26.1275	1:Experimental evidence at protein level
26.6162	26.0211	26.4353	26.2182	26.3223	25.9736	1:Experimental evidence at protein level
26.7372	26.9419	26.8753	26.8578	26.6322	26.7701	1:Experimental evidence at protein level
26.0757	25.814	25.9201	25.9483	25.5074	26.4359	1:Experimental evidence at protein level
28.2317	27.9742	27.9352	28.0282	28.3132	28.0057	1:Experimental evidence at protein level
25.9981	26.0781	26.0506	26.0189	25.8282	25.9226	1:Experimental evidence at protein level
27.0067	26.224	26.4789	26.1942	26.9255	26.3591	1:Experimental evidence at protein level
26.862	26.8344	26.9387	27.0531	27.0792	26.9682	1:Experimental evidence at protein level
27.7652	27.6585	27.6486	27.6452	27.7431	27.8564	1:Experimental evidence at protein level
27.4743	27.514	27.4033	27.6172	27.4567	27.4854	1:Experimental evidence at protein level
28.4449	28.5572	28.5414	28.7511	28.7497	28.7653	1:Experimental evidence at protein level
28.9122	28.8195	28.7898	28.9049	29.1828	28.9304	1:Experimental evidence at protein level
27.103	27.0452	26.9015	26.9137	27.1176	26.969	1:Experimental evidence at protein level
25.3668	25.5012	25.4302	25.4561	24.7332	25.5015	1:Experimental evidence at protein level
27.4368	27.5848	27.5714	27.4572	26.9433	27.5298	1:Experimental evidence at protein level
29.5742	29.833	29.8014	29.6789	29.5772	29.5882	1:Experimental evidence at protein level
26.5702	26.8767	26.7615	26.9461	26.7791	27.2094	1:Experimental evidence at protein level
26.6704	26.4542	26.4685	26.6579	26.4022	26.601	1:Experimental evidence at protein level
34.9509	34.1772	33.1527	32.8281	35.3215	33.4416	1:Experimental evidence at protein level
29.6542	29.7664	29.7743	29.9342	30.0777	29.9046	1:Experimental evidence at protein level
#NUM!	25.0655	25.6199	26.2488	26.3522	26.3142	1:Experimental evidence at protein level
27.0291	26.9905	27.1022	26.7585	26.6066	26.6108	1:Experimental evidence at protein level
27.3623	27.521	27.5566	27.3159	26.9132	27.2201	1:Experimental evidence at protein level
26.0577	26.6228	26.6615	26.4229	25.8455	26.5825	1:Experimental evidence at protein level
25.5344	25.9726	25.7526	25.8603	25.7311	26.1365	1:Experimental evidence at protein level
28.4214	28.08	28.2409	28.4788	28.514	28.6021	1:Experimental evidence at protein level
28.9984	29.3102	29.3417	29.1381	29.1079	28.9004	1:Experimental evidence at protein level
27.3414	27.3282	27.3927	27.3138	27.4697	27.3549	1:Experimental evidence at protein level
26.2366	26.2392	26.0601	25.7179	25.4965	25.8098	1:Experimental evidence at protein level
28.6704	28.2513	28.2786	28.1626	28.2735	28.0523	1:Experimental evidence at protein level
29.6404	29.671	29.5798	29.5928	29.5559	29.4148	1:Experimental evidence at protein level
26.7623	26.633	26.666	26.7051	26.6514	26.4303	1:Experimental evidence at protein level
27.8086	27.7498	27.7522	27.6901	27.7699	27.699	1:Experimental evidence at protein level
30.5015	30.0309	30.0138	29.9804	30.231	29.7876	1:Experimental evidence at protein level
26.8694	27.1889	27.0672	27.2287	26.9894	27.0484	1:Experimental evidence at protein level
26.7818	26.5708	26.4461	25.7145	26.0089	25.8231	1:Experimental evidence at protein level
26.963	26.8678	26.9176	27.041	26.9127	27.0432	1:Experimental evidence at protein level
25.9074	26.122	26.0516	26.0368	25.8971	25.934	1:Experimental evidence at protein level
28.0771	28.2757	28.1473	27.964	27.7434	27.6753	1:Experimental evidence at protein level
28.1062	28.1295	28.2951	28.1815	28.0686	28.0452	1:Experimental evidence at protein level
27.2348	27.8443	27.7909	27.369	27.4459	26.9679	1:Experimental evidence at protein level

27.5223	27.1221	27.233	27.2113	27.7207	27.1213	1:Experimental evidence at protein level
29.5249	29.3877	29.3097	29.2654	29.4434	29.1592	1:Experimental evidence at protein level
27.9504	27.6539	27.7794	27.6532	27.8432	27.7678	1:Experimental evidence at protein level
28.3357	28.6113	28.5619	28.4476	28.4507	28.3481	1:Experimental evidence at protein level
28.2421	28.2036	28.0055	27.9269	27.8832	27.9319	1:Experimental evidence at protein level
29.0767	29.3512	29.4376	28.7427	28.9305	28.6978	1:Experimental evidence at protein level
28.8534	29.1583	29.1117	29.0027	28.7392	29.1163	1:Experimental evidence at protein level
27.9812	28.4454	28.4557	28.6599	28.2595	28.505	1:Experimental evidence at protein level
31.4116	31.0931	31.0884	30.8344	31.3812	30.9848	1:Experimental evidence at protein level
27.591	27.4793	27.4362	27.5011	27.4228	27.3492	1:Experimental evidence at protein level
31.3997	31.0547	30.9535	30.8998	31.0823	30.9491	1:Experimental evidence at protein level
31.1415	30.9405	30.7993	30.6115	31.0078	30.5022	1:Experimental evidence at protein level
29.8823	30.1537	30.1187	29.9347	29.8439	29.9994	1:Experimental evidence at protein level
29.6739	27.5076	27.7961	27.6338	29.2886	27.9999	1:Experimental evidence at protein level
27.4469	27.7498	27.8892	27.5871	27.4432	27.1853	1:Experimental evidence at protein level
27.4509	27.1743	27.1144	26.8984	26.9863	27.0179	1:Experimental evidence at protein level
27.5855	27.6224	27.5247	27.6115	27.6786	27.5303	1:Experimental evidence at protein level
26.8645	26.7496	26.8699	26.5478	26.6536	26.3703	1:Experimental evidence at protein level
26.6788	27.2863	27.3668	27.299	26.3753	27.6398	1:Experimental evidence at protein level
25.819	25.2866	25.1471	25.2027	25.476	25.1213	1:Experimental evidence at protein level
26.4818	26.7389	26.5045	26.7981	26.6628	26.5635	1:Experimental evidence at protein level
27.2543	27.5443	27.5644	27.4433	26.953	27.317	1:Experimental evidence at protein level
27.2943	27.7196	27.7578	27.9529	27.5025	27.9698	1:Experimental evidence at protein level
25.4641	25.1995	25.1891	24.9864	24.6297	25.131	1:Experimental evidence at protein level
27.5039	27.3154	27.254	27.1208	27.4185	26.9879	1:Experimental evidence at protein level
25.3768	#NUM!	25.1078	25.8517	25.8554	25.9002	1:Experimental evidence at protein level
26.0819	26.2488	26.2991	25.7248	25.1339	26.1153	1:Experimental evidence at protein level
27.2216	26.9567	26.7375	26.8649	27.0812	26.6148	1:Experimental evidence at protein level
26.146	26.2687	26.2932	26.1897	26.2417	26.2333	1:Experimental evidence at protein level
#NUM!	25.8853	25.2382	25.2522	25.5804	24.8349	1:Experimental evidence at protein level
25.4346	25.1209	25.0575	25.6212	25.5388	25.5405	1:Experimental evidence at protein level
26.2055	26.2645	26.23	26.1537	26.2595	26.3111	1:Experimental evidence at protein level
25.0966	24.6816	24.2189	24.9673	24.7221	24.5499	1:Experimental evidence at protein level
26.0324	25.9932	25.9358	25.875	25.8343	25.766	1:Experimental evidence at protein level
27.6818	27.8314	27.8842	27.7834	27.6152	27.7431	1:Experimental evidence at protein level
25.046	25.0032	25.1877	24.7199	24.4689	24.703	1:Experimental evidence at protein level
25.7528	25.4714	25.5184	25.777	25.9394	25.8084	1:Experimental evidence at protein level
26.8998	26.8948	27.027	26.8409	26.7169	26.7425	1:Experimental evidence at protein level
25.9349	26.2146	25.7571	25.6741	25.3737	25.735	1:Experimental evidence at protein level
27.409	26.7868	26.6442	26.6297	27.3868	26.4437	1:Experimental evidence at protein level
28.0459	28.0643	27.9857	27.9788	27.9113	27.8778	1:Experimental evidence at protein level
29.9563	30.1191	30.243	30.2694	29.9547	30.0983	1:Experimental evidence at protein level
27.6205	27.7418	27.7529	28.0205	27.9326	27.6651	1:Experimental evidence at protein level
25.1757	24.9473	24.675	24.7112	24.9268	24.8348	1:Experimental evidence at protein level
28.2497	28.4501	28.2351	28.3081	27.9377	28.3006	1:Experimental evidence at protein level
25.5878	25.5936	25.8647	25.5543	25.8072	25.6126	1:Experimental evidence at protein level
26.351	26.0819	26.1443	26.028	26.4909	26.0448	1:Experimental evidence at protein level
26.911	27.2459	27.1072	27.0215	26.6301	27.0057	1:Experimental evidence at protein level
28.1112	27.8735	27.6875	27.2677	27.4508	27.3151	1:Experimental evidence at protein level
26.6436	26.2091	26.0884	25.8813	26.039	25.9601	1:Experimental evidence at protein level
27.7396	27.7758	27.697	27.9116	27.8435	27.8288	1:Experimental evidence at protein level
26.7322	27.0602	27.1839	26.8797	26.783	26.7303	1:Experimental evidence at protein level
25.6025	25.4686	25.5469	25.4857	25.395	25.6108	1:Experimental evidence at protein level
26.1252	25.8696	26.3342	26.0046	25.6003	26.1503	1:Experimental evidence at protein level
25.4112	24.7977	24.3667	24.6913	24.4893	24.1628	1:Experimental evidence at protein level
26.9151	27.0646	27.0716	26.8975	26.5724	26.7195	1:Experimental evidence at protein level
27.5691	27.5959	27.5314	27.394	27.5306	27.2479	1:Experimental evidence at protein level
27.1833	26.7101	26.6116	27.0928	27.1791	27.2596	1:Experimental evidence at protein level
26.4254	26.467	26.579	26.4674	26.3033	26.5558	1:Experimental evidence at protein level
28.4672	28.6899	28.686	28.4039	28.2982	28.4729	1:Experimental evidence at protein level
25.9608	26.3751	26.4616	26.1067	25.6407	26.0714	1:Experimental evidence at protein level
25.9124	26.0226	25.5181	25.8063	25.2746	25.2848	1:Experimental evidence at protein level
26.5079	26.678	26.722	26.6061	26.5594	26.6317	1:Experimental evidence at protein level
27.6661	27.6345	27.7604	27.4442	27.4401	27.4108	1:Experimental evidence at protein level
26.1625	25.6388	25.4101	25.4676	25.8615	25.7135	1:Experimental evidence at protein level
26.3913	25.9952	26.2264	26.3402	26.6135	26.3975	1:Experimental evidence at protein level
25.629	26.0009	26.2345	26.4613	25.8591	26.2504	1:Experimental evidence at protein level
29.5133	29.4874	29.4839	29.4676	29.7591	29.598	1:Experimental evidence at protein level
27.0085	27.2643	27.4415	27.4715	26.9848	27.4602	1:Experimental evidence at protein level
25.5621	25.0732	24.9684	24.8708	25.2345	24.8579	1:Experimental evidence at protein level
28.3506	28.8095	28.8524	28.5245	28.0966	28.6574	1:Experimental evidence at protein level
27.5095	27.8984	27.9017	28.0642	28.0347	27.9036	1:Experimental evidence at protein level
27.8017	28.2711	28.2748	28.3646	27.8056	28.4446	1:Experimental evidence at protein level
26.9538	27.0781	26.885	26.8265	26.2596	26.9683	1:Experimental evidence at protein level
28.0621	28.155	28.2504	28.5471	28.3175	28.2894	1:Experimental evidence at protein level
31.0054	31.0081	31.0853	30.4225	30.2186	30.6815	1:Experimental evidence at protein level
33.1606	33.3505	33.4602	33.5505	33.855	33.6352	1:Experimental evidence at protein level
32.9655	33.3108	33.3573	32.9329	32.9794	32.9421	1:Experimental evidence at protein level
29.9854	30.3778	30.4101	30.3015	30.0332	30.3413	1:Experimental evidence at protein level
28.1267	27.5295	27.7162	27.3451	28.5116	27.3636	1:Experimental evidence at protein level
27.0204	26.8099	26.9608	26.8299	26.589	26.6875	1:Experimental evidence at protein level
29.4771	29.6956	29.5775	29.6337	29.4115	29.5081	1:Experimental evidence at protein level
27.8804	27.8664	27.7867	27.661	27.779	27.5327	1:Experimental evidence at protein level
26.793	26.6583	26.6184	26.4234	26.4218	26.2745	1:Experimental evidence at protein level
25.867	24.9564	24.7238	24.5308	24.8687	23.9708	1:Experimental evidence at protein level
26.99	26.9994	26.9301	27.1138	27.2396	26.6787	1:Experimental evidence at protein level
26.8607	26.6702	26.7154	26.7098	26.8617	26.4843	1:Experimental evidence at protein level
27.8959	28.5589	28.4365	28.5282	28.1168	28.3783	1:Experimental evidence at protein level
28.3109	27.7333	28.4477	27.4296	28.3114	27.7787	1:Experimental evidence at protein level
26.8181	26.8953	26.8556	26.4344	26.4142	26.2517	1:Experimental evidence at protein level
27.562	27.1181	27.2211	26.8406	27.308	26.7752	1:Experimental evidence at protein level
30.4725	31.0095	31.1802	31.1466	30.7255	31.0042	1:Experimental evidence at protein level
27.3758	26.8636	26.8543	26.4532	26.5932	26.8173	1:Experimental evidence at protein level
31.4365	30.3539	30.5489	30.3088	31.381	30.1437	1:Experimental evidence at protein level
28.5688	28.7605	28.8179	28.5682	28.6051	28.6105	1:Experimental evidence at protein level
26.3594	26.0147	26.189	26.1022	26.05	26.1159	1:Experimental evidence at protein level
26.3758	26.3948	26.2719	26.3841	26.4814	26.2298	1:Experimental evidence at protein level
26.5308	26.5352	26.6204	26.8384	26.5332	26.6941	1:Experimental evidence at protein level
26.521	26.3352	26.3411	26.775	26.6588	26.542	1:Experimental evidence at protein level
28.3639	28.4407	28.4167	28.3646	28.2196	28.2515	1:Experimental evidence at protein level
30.1332	29.9748	29.9227	30.0928	30.2289	30.0025	1:Experimental evidence at protein level
28.7098	28.5861	28.5975	27.934	28.2306	28.047	1:Experimental evidence at protein level
25.5021	25.8669	25.6507	25.8804	25.77	25.9036	1:Experimental evidence at protein level
26.6936	26.8908	26.7974	26.737	26.7088	26.7639	1:Experimental evidence at protein level
29.9361	30.1585	30.1724	30.0854	29.7808	30.0407	1:Experimental evidence at protein level
29.0931	29.7122	29.454	29.651	29.7252	29.8568	1:Experimental evidence at protein level
27.7253	27.2325	27.089	27.2679	27.5607	27.2819	1:Experimental evidence at protein level
28.315	28.6807	28.8526	28.4843	28.5314	28.3322	1:Experimental evidence at protein level
25.3448	25.2849	25.1957	24.9542	24.7346	25.6047	1:Experimental evidence at protein level
26.4243	26.1143	26.2528	26.0432	26.0405	26.1988	1:Experimental evidence at protein level
28.784	29.1219	29.0504	29.0312	28.6797	28.9206	1:Experimental evidence at protein level
26.3278	26.295	26.1415	26.5609	26.9423	26.6293	1:Experimental evidence at protein level
26.7134	26.3374	26.5521	26.8191	26.4198	26.7169	1:Experimental evidence at protein level
27.0921	26.9083	27.0107	27.5882	27.6313	27.8005	1:Experimental evidence at protein level
28.7693	29.1813	29.1504	29.3574	28.9353	29.0674	1:Experimental evidence at protein level
27.0648	26.6388	26.6231	26.2521	26.1988	26.4954	1:Experimental evidence at protein level
26.6492	26.7905	26.743	26.422	26.2146	26.4754	1:Experimental evidence at protein level
27.3628	27.2387	27.212	27.1719	27.5057	27.2364	1:Experimental evidence at protein level
26.5743	26.9464	26.9983	26.7342	26.5119	26.732	1:Experimental evidence at protein level
27.8916	27.9846	28.0388	28.0234	28.0167	28.0222	1:Experimental evidence at protein level
28.0423	28.2084	28.1441	28.1694	28.307	28.0913	1:Experimental evidence at protein level
26.4425	26.4075	26.3418	26.3902	26.3005	26.3287	1:Experimental evidence at protein level
27.2264	27.4454	27.4896	27.2136	27.1564	27.2752	1:Experimental evidence at protein level
26.774	26.727	26.8324	26.9322	26.7571	26.9704	1:Experimental evidence at protein level
27.1853	27.159	26.9877	26.9486	26.8673	26.9957	1:Experimental evidence at protein level
26.6487	26.7072	26.7614	26.6227	26.7298	26.6192	1:Experimental evidence at protein level
27.7936	27.8504	27.7553	27.6133	27.531	27.472	1:Experimental evidence at protein level
26.5905	26.7705	26.7496	26.8885	26.729	26.641	1:Experimental evidence at protein level
26.3056	25.9954	26.0529	25.8624	25.5241	25.6132	1:Experimental evidence at protein level

26.0921	25.7689	25.8119	25.738	25.7492	26.0974	1:Experimental evidence at protein level
29.0672	29.4362	29.5245	29.4173	29.2628	29.2323	1:Experimental evidence at protein level
27.1802	27.7238	27.7549	27.8168	27.5011	27.7655	1:Experimental evidence at protein level
27.8045	27.3965	27.2092	27.0531	27.357	27.0792	1:Experimental evidence at protein level
25.9877	26.3683	26.3632	26.2684	26.0726	26.1528	1:Experimental evidence at protein level
26.3014	26.2422	26.2965	25.9434	26.1428	26.1453	1:Experimental evidence at protein level
27.0285	27.0913	27.2377	27.2647	26.9352	27.1115	1:Experimental evidence at protein level
27.7014	28.008	27.9722	27.8956	27.9744	27.858	1:Experimental evidence at protein level
26.8476	25.7173	25.6676	26.1006	26.4555	26.1614	1:Experimental evidence at protein level
26.0721	26.2995	26.399	26.3451	26.2124	26.0433	1:Experimental evidence at protein level
25.9566	25.8864	25.8561	25.3467	25.6432	25.7445	1:Experimental evidence at protein level
25.7634	25.8625	25.6729	25.6645	24.7568	25.7206	1:Experimental evidence at protein level
28.73	28.2689	28.3565	28.2424	28.3779	28.5355	1:Experimental evidence at protein level
26.4871	26.5765	26.5346	26.5825	25.8575	26.6688	1:Experimental evidence at protein level
26.3946	26.1985	26.2429	26.1939	26.3824	26.2471	1:Experimental evidence at protein level
27.6075	27.4503	27.6058	27.4358	27.5694	27.4783	1:Experimental evidence at protein level
29.2275	28.976	28.8626	28.424	28.5806	28.2414	1:Experimental evidence at protein level
24.6414	25.2041	24.9436	25.5658	25.2972	25.7562	1:Experimental evidence at protein level
27.0693	27.3435	27.3736	27.1114	26.9192	26.9941	1:Experimental evidence at protein level
26.5869	26.4799	26.5585	26.5485	26.4559	26.5574	1:Experimental evidence at protein level
28.1595	28.3198	28.1683	27.9423	27.867	27.9401	1:Experimental evidence at protein level
28.63	28.2983	28.1248	28.2279	28.3842	28.1265	1:Experimental evidence at protein level
27.7656	27.444	27.5803	26.8969	27.3031	26.8271	1:Experimental evidence at protein level
27.3715	27.2232	27.6294	27.4544	27.8823	27.4031	1:Experimental evidence at protein level
26.0796	26.4508	26.5647	25.992	26.0259	26.3484	1:Experimental evidence at protein level
27.051	26.9868	27.073	27.2212	27.2573	27.23	1:Experimental evidence at protein level
25.8426	26.1957	26.171	25.926	25.447	26.2798	1:Experimental evidence at protein level
26.3219	26.2645	26.3561	25.795	25.5834	26.0481	1:Experimental evidence at protein level
27.1247	26.4229	26.2572	26.5657	26.8243	26.4553	1:Experimental evidence at protein level
26.5875	27.033	27.2197	27.1995	26.9827	27.1863	1:Experimental evidence at protein level
27.5644	27.6591	27.7541	27.7729	27.586	27.8841	1:Experimental evidence at protein level
26.6225	26.4852	26.5907	26.5383	26.462	26.6704	1:Experimental evidence at protein level
27.4787	27.586	27.6824	27.434	27.5623	27.6561	1:Experimental evidence at protein level
27.8019	27.4402	27.4875	27.0758	27.6112	27.2929	5:Protein uncertain
26.0501	26.1407	26.0969	26.2377	26.0434	26.0924	1:Experimental evidence at protein level
26.6879	26.9176	27.2235	27.1591	26.6139	26.5544	1:Experimental evidence at protein level
24.7804	24.8492	24.9592	24.7109	#NUM!	24.747	1:Experimental evidence at protein level
26.7726	26.3915	26.3551	26.4031	26.3572	26.3762	1:Experimental evidence at protein level
26.3455	26.5445	26.6116	26.2103	25.9374	26.1843	1:Experimental evidence at protein level
26.5402	26.579	26.6644	26.4204	26.3512	26.4227	1:Experimental evidence at protein level
27.7959	27.8493	27.8681	27.9497	28.0951	27.8767	1:Experimental evidence at protein level
26.8788	27.3181	27.2187	27.256	26.8527	27.0064	1:Experimental evidence at protein level
26.2672	25.989	26.1033	26.291	26.352	26.5699	1:Experimental evidence at protein level
26.1267	26.0978	25.8165	25.9374	26.0166	25.8217	1:Experimental evidence at protein level
27.0637	27.0631	27.1382	27.292	27.1144	27.1309	1:Experimental evidence at protein level
25.536	25.3955	25.8849	26.0999	26.0389	26.0766	1:Experimental evidence at protein level
27.7922	27.9512	27.952	28.0116	27.6193	28.0433	1:Experimental evidence at protein level
28.3349	28.1536	28.1939	28.0426	28.076	28.2401	1:Experimental evidence at protein level
27.084	27.3186	27.0582	24.8594	25.6395	25.856	1:Experimental evidence at protein level
31.0033	31.6537	31.5782	31.7242	31.2327	31.6642	1:Experimental evidence at protein level
32.8008	33.2669	33.241	31.0937	30.7494	31.0421	1:Experimental evidence at protein level
28.6352	28.1841	28.1742	28.4275	28.6144	28.4413	1:Experimental evidence at protein level
32.9682	33.1515	33.0998	33.4693	33.261	33.0887	1:Experimental evidence at protein level
27.5853	27.5905	27.5697	27.3968	27.2502	27.4794	1:Experimental evidence at protein level
28.4124	28.5324	28.672	28.1911	28.4055	28.4782	1:Experimental evidence at protein level
28.9911	29.0153	29.0869	29.0864	28.8548	28.8302	1:Experimental evidence at protein level
30.705	30.444	30.5159	30.409	30.4979	30.5014	1:Experimental evidence at protein level
29.4501	29.2031	29.1845	29.1293	29.2893	29.3085	1:Experimental evidence at protein level
33.4606	34.2472	34.168	34.6173	34.6043	34.6051	1:Experimental evidence at protein level
28.4963	28.8436	28.9285	28.7407	28.732	28.3614	1:Experimental evidence at protein level
26.6815	26.4271	26.4586	26.0838	26.031	25.9692	1:Experimental evidence at protein level
32.2767	32.471	32.3464	32.9083	32.7375	32.657	1:Experimental evidence at protein level
25.1057	#NUM!	24.9221	26.0077	25.6442	25.6515	1:Experimental evidence at protein level
26.4978	26.4099	26.3078	26.0063	26.0864	25.8091	1:Experimental evidence at protein level
25.4032	25.2186	25.1915	24.8372	23.7804	24.735	1:Experimental evidence at protein level
26.069	25.9325	26.0021	25.9136	25.7871	25.8079	1:Experimental evidence at protein level
30.9578	31.2463	31.1124	31.1201	31.0716	31.0345	1:Experimental evidence at protein level
27.4102	27.077	27.1837	26.8428	26.9496	26.823	1:Experimental evidence at protein level
26.8853	26.7846	26.9098	26.8794	26.7972	26.7784	1:Experimental evidence at protein level
28.9164	28.9843	28.9915	28.8701	28.9182	28.9747	1:Experimental evidence at protein level
28.087	27.6321	27.599	27.3904	27.6737	27.5459	1:Experimental evidence at protein level
27.6222	27.8258	27.6751	27.5915	27.6502	27.5341	1:Experimental evidence at protein level
27.5512	27.7276	27.9095	27.8954	27.5916	27.8685	1:Experimental evidence at protein level
30.4822	30.0515	30.0127	29.8691	30.3144	30.0665	1:Experimental evidence at protein level
26.9973	26.6159	26.7563	26.3215	26.5276	26.5026	1:Experimental evidence at protein level
28.1823	28.823	28.7986	28.3283	28.1849	28.2931	1:Experimental evidence at protein level
28.7818	28.6264	28.7145	28.6615	28.5413	28.6766	1:Experimental evidence at protein level
26.4224	26.8244	26.5981	26.4129	26.2473	26.5074	1:Experimental evidence at protein level
28.125	27.9466	27.7555	27.8872	28.1502	27.7125	1:Experimental evidence at protein level
26.1544	25.719	25.8754	25.2159	25.2791	25.3226	1:Experimental evidence at protein level
28.106	28.2291	27.9822	28.1209	28.3455	27.9961	1:Experimental evidence at protein level
29.5715	29.0163	29.0617	29.4004	29.7173	29.3298	1:Experimental evidence at protein level
28.0328	27.7712	27.8888	27.8092	28.0298	27.9484	1:Experimental evidence at protein level
26.6795	26.7111	26.7462	26.8139	26.5152	26.786	1:Experimental evidence at protein level
27.7222	27.6086	27.5246	27.1193	27.4711	27.2109	1:Experimental evidence at protein level
25.7172	26.6471	26.3014	26.7795	25.9565	26.9927	1:Experimental evidence at protein level
27.6963	27.4272	27.5187	27.5806	28.2282	27.4516	1:Experimental evidence at protein level
26.4117	26.4748	26.1386	26.3883	26.176	26.0592	1:Experimental evidence at protein level
26.1257	26.3245	26.2638	26.337	26.0438	26.2922	1:Experimental evidence at protein level
26.5734	26.543	26.5093	26.8397	26.9576	26.7915	1:Experimental evidence at protein level
25.6122	25.2646	25.4905	25.9776	25.9942	25.7572	1:Experimental evidence at protein level
25.1986	25.7275	25.963	25.7858	25.2803	26.0103	1:Experimental evidence at protein level
26.1914	26.4794	26.255	26.5706	26.5002	26.7479	1:Experimental evidence at protein level
25.7452	25.9766	25.9922	25.8226	25.5936	26.0614	1:Experimental evidence at protein level
26.3199	26.6125	26.4618	26.4668	26.2041	26.3609	1:Experimental evidence at protein level
27.0406	27.0266	27.037	26.5176	26.2726	26.3609	1:Experimental evidence at protein level
27.779	27.7787	27.7625	28.0013	27.9377	27.7625	1:Experimental evidence at protein level
26.0673	25.9685	25.9651	25.6105	25.681	25.6426	1:Experimental evidence at protein level
26.4959	26.6145	26.7401	26.4092	25.9914	26.566	1:Experimental evidence at protein level
26.3627	25.9436	25.8148	25.6476	26.1737	25.9012	1:Experimental evidence at protein level
25.9886	25.3045	25.5473	25.4265	25.4392	25.3412	1:Experimental evidence at protein level
26.251	25.6447	25.832	25.9959	26.1893	25.8616	1:Experimental evidence at protein level
24.6506	24.3979	24.2957	23.4875	24.0552	23.4127	1:Experimental evidence at protein level
27.7834	27.5662	27.5016	27.255	27.354	27.4676	1:Experimental evidence at protein level
26.2282	26.4707	26.232	25.7758	25.6584	25.4938	1:Experimental evidence at protein level
28.1594	28.2193	28.2054	27.7572	27.4512	28.0074	1:Experimental evidence at protein level
30.4385	30.3989	30.2114	30.3069	30.5193	30.2593	1:Experimental evidence at protein level
27.2136	27.0344	27.2337	27.1935	27.4298	27.0654	1:Experimental evidence at protein level
28.3021	28.7178	28.7349	28.5763	28.4193	28.5967	1:Experimental evidence at protein level
25.2812	25.0201	24.9054	24.682	25.1278	24.2703	1:Experimental evidence at protein level
26.23	25.9257	26.0984	25.6243	25.9876	26.0037	1:Experimental evidence at protein level
26.8512	26.3757	26.3699	26.3351	26.5131	26.2748	1:Experimental evidence at protein level
25.9725	25.7742	25.826	26.0259	26.4102	25.9618	1:Experimental evidence at protein level
26.0978	25.825	25.7302	26.1012	25.4547	26.2062	1:Experimental evidence at protein level
28.5652	28.581	28.6452	28.1934	28.2817	28.2887	1:Experimental evidence at protein level
26.5666	26.4635	26.127	26.4931	26.8743	26.5883	1:Experimental evidence at protein level
25.5281	26.2972	26.1875	26.4551	26.3166	26.0745	1:Experimental evidence at protein level
26.7463	26.6347	26.7276	26.478	26.7814	26.5768	1:Experimental evidence at protein level
27.2204	27.2062	27.27	27.023	26.7674	26.9315	1:Experimental evidence at protein level
26.4723	26.2241	26.0674	26.0573	26.3486	25.9282	1:Experimental evidence at protein level
27.7098	27.5851	27.682	27.7072	27.608	27.7823	1:Experimental evidence at protein level
26.3212	26.0708	26.1352	25.8589	25.8816	25.9626	1:Experimental evidence at protein level
26.4289	26.9317	27.0009	26.5928	26.3569	26.5493	1:Experimental evidence at protein level
26.9981	26.6292	26.799	26.8131	27.1469	26.917	1:Experimental evidence at protein level
29.0039	28.4416	28.5296	28.5908	28.4839	28.5015	1:Experimental evidence at protein level
28.3141	28.1913	28.235	28.0408	27.9361	27.926	1:Experimental evidence at protein level
27.0608	26.7546	26.8665	26.6751	26.5333	26.7977	1:Experimental evidence at protein level
26.5367	26.2243	26.292	26.246	26.3861	26.3654	1:Experimental evidence at protein level
25.9728	25.9064	26.1233	26.1468	26.0727	26.0542	1:Experimental evidence at protein level

25.8143	25.5163	25.7435	25.8265	26.0799	25.6672	1:Experimental evidence at protein level
27.031	27.5238	27.6588	27.3278	27.0384	27.4967	1:Experimental evidence at protein level
27.1775	26.9533	26.8528	26.6327	26.8378	26.4437	1:Experimental evidence at protein level
30.7009	30.73	30.6816	30.7002	30.6556	30.6925	1:Experimental evidence at protein level
30.3644	30.3865	30.2945	30.4455	30.1825	30.3524	1:Experimental evidence at protein level
26.9886	26.7876	26.9426	26.7494	26.8011	26.7013	1:Experimental evidence at protein level
28.4675	28.3929	28.4598	28.3804	28.5321	28.6762	1:Experimental evidence at protein level
27.641	27.7466	27.9399	27.91	27.6616	27.9135	1:Experimental evidence at protein level
26.8801	27.0992	26.4701	26.5759	26.973	26.3846	1:Experimental evidence at protein level
29.1735	29.1242	29.2029	29.2098	29.1181	29.0554	1:Experimental evidence at protein level
26.3526	25.9122	26.0052	26.36	26.4355	26.2375	1:Experimental evidence at protein level
26.9192	26.5047	26.3212	26.3673	26.6415	26.4532	1:Experimental evidence at protein level
28.858	28.953	28.8897	29.1471	28.9458	29.1533	1:Experimental evidence at protein level
28.8187	28.8674	28.8437	29.1527	28.8892	29.0597	1:Experimental evidence at protein level
31.5627	30.9959	31.0458	30.9966	31.433	30.6479	1:Experimental evidence at protein level
26.5502	26.5728	26.5005	24.8147	24.7806	24.5649	1:Experimental evidence at protein level
25.7825	25.6817	25.867	26.2574	26.1777	26.1237	1:Experimental evidence at protein level
27.8841	28.1446	28.0905	28.2297	28.1491	28.3364	1:Experimental evidence at protein level
28.947	28.3465	28.468	28.5996	29.1029	28.3308	1:Experimental evidence at protein level
27.7285	27.7049	27.7324	27.6346	27.5865	27.6984	1:Experimental evidence at protein level
28.4685	28.369	28.5043	28.6255	28.6266	28.7057	1:Experimental evidence at protein level
27.6768	27.8083	27.7118	27.5038	27.3609	27.3846	1:Experimental evidence at protein level
30.7033	30.5802	30.5734	30.7468	30.8055	30.7432	1:Experimental evidence at protein level
29.2478	28.992	28.4738	28.8943	29.5432	28.265	1:Experimental evidence at protein level
28.8941	28.9266	28.8463	28.8474	28.7484	28.9562	1:Experimental evidence at protein level
28.7146	28.6499	28.7419	28.5973	28.6798	28.6752	1:Experimental evidence at protein level
30.6091	30.9111	30.9594	30.8824	30.4844	30.9448	1:Experimental evidence at protein level
28.7045	28.937	29.0374	29.0462	28.9788	28.8502	1:Experimental evidence at protein level
30.6744	30.6227	30.6394	30.2832	30.1403	30.1928	1:Experimental evidence at protein level
31.1079	30.9391	30.9611	30.5447	30.7769	30.4389	1:Experimental evidence at protein level
27.324	27.2222	27.2271	26.8941	26.7513	26.8251	1:Experimental evidence at protein level
28.6408	28.777	28.8252	28.9513	28.8982	28.7768	1:Experimental evidence at protein level
26.4238	26.3774	26.1846	26.3701	26.3708	26.3742	1:Experimental evidence at protein level
27.941	27.2033	27.2722	27.4642	27.9128	27.5492	1:Experimental evidence at protein level
25.4457	25.6485	25.6655	25.5818	25.3523	25.6432	1:Experimental evidence at protein level
25.6813	25.7643	25.793	25.7154	25.365	25.8411	1:Experimental evidence at protein level
26.3616	26.0663	25.8539	26.1154	26.3172	26.2666	1:Experimental evidence at protein level
25.8704	25.5428	25.6317	25.7607	26.2866	25.7776	1:Experimental evidence at protein level
26.2565	26.4851	26.9176	26.4122	26.6936	27.0233	1:Experimental evidence at protein level
29.984	30.3878	30.4664	30.5052	30.2595	30.5218	1:Experimental evidence at protein level
24.4908	#NUM!	24.5436	25.4608	24.8745	24.9432	1:Experimental evidence at protein level
25.3975	25.3098	25.7105	25.5255	25.2563	25.5318	1:Experimental evidence at protein level
26.3834	26.3804	26.3358	26.2631	26.2486	26.3155	1:Experimental evidence at protein level
27.3549	27.3715	27.152	26.9918	27.1182	26.7776	1:Experimental evidence at protein level
26.8934	26.7025	26.8156	26.3985	26.4699	26.4352	1:Experimental evidence at protein level
27.5524	27.2543	27.3532	27.2758	27.1719	27.5415	1:Experimental evidence at protein level
26.1664	26.2692	26.2257	26.2467	26.2121	26.2953	1:Experimental evidence at protein level
28.0705	27.818	28.0167	28.1232	28.4008	28.0528	1:Experimental evidence at protein level
30.8317	30.8666	30.7995	30.7488	30.7219	30.4875	1:Experimental evidence at protein level
28.1216	28.4686	28.5712	28.1177	27.7653	28.0339	1:Experimental evidence at protein level
28.4529	28.4892	28.3359	28.4043	28.4324	28.2817	1:Experimental evidence at protein level
26.3464	26.3047	26.2012	26.4221	26.3413	26.3919	1:Experimental evidence at protein level
28.1035	28.0786	28.1619	27.8457	27.7668	27.6516	1:Experimental evidence at protein level
28.7074	29.0347	29.2837	28.9701	28.9821	29.2101	1:Experimental evidence at protein level
25.909	26.0356	25.9638	26.0742	26.1385	26.0774	1:Experimental evidence at protein level
26.5948	26.202	26.1912	26.1098	26.1165	26.0041	1:Experimental evidence at protein level
27.0364	27.0323	26.929	26.7828	26.9178	26.7906	1:Experimental evidence at protein level
27.6584	27.6469	27.4635	27.6474	27.6545	27.5885	1:Experimental evidence at protein level
27.1333	26.8724	26.695	26.5397	26.8796	26.7289	1:Experimental evidence at protein level
26.2616	26.3659	26.4974	26.2534	26.0143	26.326	1:Experimental evidence at protein level
24.2856	24.84	24.8228	24.8265	24.7352	24.9082	1:Experimental evidence at protein level
26.2654	26.2533	26.2353	26.16	25.8199	26.3585	1:Experimental evidence at protein level
25.202	25.3804	25.5295	25.8943	25.9277	25.7436	1:Experimental evidence at protein level
27.5457	27.4597	27.505	27.4385	27.4741	27.3054	1:Experimental evidence at protein level
27.0691	26.5168	26.6206	27.4255	27.6421	27.4367	1:Experimental evidence at protein level
25.8198	26.2788	26.1964	25.9159	25.9195	26.0336	1:Experimental evidence at protein level
27.2616	27.4417	27.3533	27.2592	26.7354	27.2708	1:Experimental evidence at protein level
26.5797	26.2836	26.4709	26.4062	26.3086	26.1109	1:Experimental evidence at protein level
26.8402	27.0781	27.0305	26.6571	26.7517	26.8563	1:Experimental evidence at protein level
27.3101	27.3644	27.3969	27.3855	27.3298	27.3766	1:Experimental evidence at protein level
25.3486	24.8773	24.8638	25.6323	25.9847	25.8304	1:Experimental evidence at protein level
25.649	25.8207	25.5134	25.3859	25.6056	25.2933	1:Experimental evidence at protein level
27.8725	27.6888	27.5285	27.8247	27.9774	27.8046	1:Experimental evidence at protein level
27.1654	27.6568	27.6827	27.6551	27.2634	27.3791	1:Experimental evidence at protein level
26.5307	26.4124	26.4404	26.2078	26.3813	26.2204	1:Experimental evidence at protein level
27.3372	27.7557	27.4901	27.557	27.034	27.5497	1:Experimental evidence at protein level
28.038	28.3299	28.2498	28.6053	28.4643	28.5364	1:Experimental evidence at protein level
29.3	29.1876	29.225	29.2628	29.2649	29.0785	1:Experimental evidence at protein level
26.0572	26.4068	26.4843	26.2377	26.0812	26.4099	1:Experimental evidence at protein level
26.1159	25.7712	25.7934	25.7017	25.9686	25.6199	1:Experimental evidence at protein level
26.8658	27.2168	27.257	27.1111	26.772	27.0825	1:Experimental evidence at protein level
26.4244	26.0549	26.1594	26.1566	26.0473	26.2057	1:Experimental evidence at protein level
26.8302	27.1734	27.125	27.075	27.0425	26.9157	1:Experimental evidence at protein level
28.2161	28.0568	28.1178	28.0408	27.8508	27.839	1:Experimental evidence at protein level
26.7782	26.925	26.8428	26.7461	26.5914	26.7749	1:Experimental evidence at protein level
25.9727	25.6006	25.4883	25.3958	25.8472	25.4128	1:Experimental evidence at protein level
28.6708	28.6826	28.5649	28.6061	28.5307	28.654	1:Experimental evidence at protein level
27.7775	28.1705	28.3726	28.2819	28.2268	28.1656	1:Experimental evidence at protein level
27.437	27.2787	27.1384	27.7823	27.8345	27.9332	1:Experimental evidence at protein level
25.736	25.2398	25.4655	25.8429	25.9383	25.9341	1:Experimental evidence at protein level
26.5225	26.7009	26.6622	26.7315	26.618	26.7539	1:Experimental evidence at protein level
27.6336	27.4551	27.6448	27.3617	27.3513	27.2966	1:Experimental evidence at protein level
28.5556	28.485	28.3229	28.3933	28.4155	28.2902	1:Experimental evidence at protein level
27.8981	27.6938	27.6555	27.636	27.6266	27.9798	1:Experimental evidence at protein level
27.9609	28.4669	28.4592	28.3523	27.8322	28.217	1:Experimental evidence at protein level
26.9062	26.5826	26.6236	26.7597	27.024	26.7021	1:Experimental evidence at protein level
26.1565	26.1707	26.0395	26.1887	26.3049	25.9592	1:Experimental evidence at protein level
26.7734	27.0385	26.9623	26.8073	26.6193	26.908	1:Experimental evidence at protein level
28.8096	28.6231	28.4797	28.393	28.5389	28.5723	1:Experimental evidence at protein level
27.2969	27.3835	27.2957	27.2946	27.2484	27.2749	1:Experimental evidence at protein level
28.1291	28.139	28.2896	28.1223	27.9385	28.041	1:Experimental evidence at protein level
28.1854	27.879	27.7842	27.8451	27.9955	27.6643	1:Experimental evidence at protein level
25.9657	25.5424	25.4921	25.4457	25.6287	25.2997	1:Experimental evidence at protein level
27.1213	27.0104	27.074	27.159	27.2454	26.9739	1:Experimental evidence at protein level
28.1443	28.1361	28.2089	28.017	27.8351	27.8005	1:Experimental evidence at protein level
29.1016	28.7958	28.8711	28.7805	28.9552	28.951	1:Experimental evidence at protein level
27.1432	27.204	27.1623	27.6741	27.5036	27.3483	1:Experimental evidence at protein level
27.8936	28.0769	28.0682	27.8557	27.675	27.7495	1:Experimental evidence at protein level
27.0127	26.923	27.1222	26.8612	26.9148	26.8839	1:Experimental evidence at protein level
29.2746	28.7515	28.6081	28.2977	28.4868	28.3882	1:Experimental evidence at protein level
27.768	28.2569	28.4286	28.4228	28.5138	28.1254	1:Experimental evidence at protein level
28.4953	28.6569	28.7327	29.0219	29.052	28.9383	1:Experimental evidence at protein level
29.0794	29.2083	29.223	28.8769	29.3695	28.6957	1:Experimental evidence at protein level
29.084	29.2082	29.2779	29.5158	29.4248	29.7396	1:Experimental evidence at protein level
33.5896	33.6577	33.5891	33.578	33.6769	33.3607	1:Experimental evidence at protein level
26.9914	27.1467	27.1422	26.9625	27.0317	27.0587	1:Experimental evidence at protein level
26.9716	26.6957	26.6859	26.5723	26.7421	26.5629	1:Experimental evidence at protein level
27.9905	27.7869	27.79	27.9113	27.9931	27.823	1:Experimental evidence at protein level
29.0258	29.3393	29.425	29.4761	29.2513	29.5708	1:Experimental evidence at protein level
30.7016	30.5964	30.5125	30.4923	30.5914	30.5985	1:Experimental evidence at protein level
26.6404	26.9744	26.7309	26.6477	26.7066	26.5012	1:Experimental evidence at protein level
26.6904	26.8533	26.9313	26.5673	26.679	26.4002	1:Experimental evidence at protein level
29.0574	29.2629	29.418	29.5923	29.3962	29.5306	1:Experimental evidence at protein level
29.9574	29.6485	28.4731	29.227	29.9697	29.0262	1:Experimental evidence at protein level
28.4323	28.5137	28.61	28.4029	28.6368	28.4612	1:Experimental evidence at protein level
28.1122	28.2656	28.2761	27.9623	27.992	27.9407	1:Experimental evidence at protein level
28.6858	29.3285	29.2312	29.1049	28.9795	29.0707	1:Experimental evidence at protein level
28.6936	28.5389	28.5088	28.4234	28.4336	28.4586	1:Experimental evidence at protein level
28.112	27.9795	27.9031	27.8339	27.8102	27.6084	1:Experimental evidence at protein level

29.2943	29.4676	29.3716	29.5042	29.1482	29.4069	1:Experimental evidence at protein level
30.5963	30.7022	30.7289	30.9688	30.7739	30.9415	1:Experimental evidence at protein level
28.4214	28.066	27.706	27.7821	28.1203	27.8909	1:Experimental evidence at protein level
28.2994	28.6048	28.6052	28.5209	28.1778	28.3099	1:Experimental evidence at protein level
26.9004	26.148	26.405	26.1043	26.4208	26.202	1:Experimental evidence at protein level
27.6464	27.0767	27.1148	26.9974	27.1248	26.9557	1:Experimental evidence at protein level
26.4382	25.8284	25.7884	25.9685	26.3621	25.8087	1:Experimental evidence at protein level
26.2471	26.3709	26.4661	25.898	25.6383	26.0343	1:Experimental evidence at protein level
28.3088	27.9647	27.8862	28.0481	28.3106	28.0823	1:Experimental evidence at protein level
29.2073	28.8856	28.908	28.8599	29.2146	28.8982	1:Experimental evidence at protein level
28.0726	28.0875	27.8946	28.0129	28.1498	27.9729	1:Experimental evidence at protein level
26.8567	27.8685	27.7345	27.5488	26.6771	27.5205	1:Experimental evidence at protein level
28.278	27.996	27.9541	27.9489	27.8325	28.1141	1:Experimental evidence at protein level
27.972	28.1981	28.3952	27.9992	27.975	27.9848	1:Experimental evidence at protein level
31.009	30.8372	30.7492	30.774	31.0808	30.6191	1:Experimental evidence at protein level
25.9868	26.1762	26.2	26.814	26.7034	26.9887	1:Experimental evidence at protein level
28.9679	28.9299	28.9511	28.8368	28.7373	28.7206	1:Experimental evidence at protein level
25.7936	25.6061	25.4166	24.8651	24.8758	24.7945	1:Experimental evidence at protein level
26.6917	26.6574	26.6469	26.513	26.5293	26.6646	1:Experimental evidence at protein level
25.2105	25.5381	25.2996	25.5293	25.1764	25.6344	1:Experimental evidence at protein level
26.9602	27.1114	26.9884	27.3158	26.9881	27.4469	1:Experimental evidence at protein level
25.3735	25.2785	25.5149	24.9874	24.9223	25.09	1:Experimental evidence at protein level
26.2298	25.9306	26.1623	26.0486	26.2981	26.1419	1:Experimental evidence at protein level
26.9773	26.7063	26.8535	26.8148	26.6818	26.8182	1:Experimental evidence at protein level
27.4051	26.8814	26.8783	27.2853	27.4772	27.1587	1:Experimental evidence at protein level
24.6328	24.4995	24.1389	24.19	24.2215	23.93	1:Experimental evidence at protein level
27.7656	27.7333	27.7298	27.6422	27.8144	27.9731	1:Experimental evidence at protein level
29.2684	29.5435	29.3272	29.0581	29.2069	29.1367	1:Experimental evidence at protein level
26.6179	26.5652	26.528	26.3372	26.2586	26.1724	1:Experimental evidence at protein level
26.6528	26.79	26.7288	26.7852	27.0585	26.6996	1:Experimental evidence at protein level
26.9995	27.3104	27.1568	27.3024	26.8028	27.3743	1:Experimental evidence at protein level
28.8764	29.347	29.5093	29.2372	28.8801	29.2486	1:Experimental evidence at protein level
27.3981	27.2308	27.4508	27.3265	27.5882	27.3515	1:Experimental evidence at protein level
26.0273	26.0487	26.128	25.5231	25.4967	25.9152	1:Experimental evidence at protein level
27.3885	27.7825	27.6466	27.7688	27.7127	27.821	1:Experimental evidence at protein level
26.3489	26.2201	26.2713	26.0642	26.0982	26.0918	1:Experimental evidence at protein level
25.1427	25.4537	25.4354	25.7153	25.2661	25.6409	1:Experimental evidence at protein level
25.5972	25.6177	25.6747	25.8155	25.5795	25.7912	1:Experimental evidence at protein level
27.752	27.7127	27.9122	27.5672	27.5556	27.7067	1:Experimental evidence at protein level
27.132	26.9145	27.03	27.0502	27.0785	27.1172	1:Experimental evidence at protein level
28.9052	28.7695	28.7392	28.837	28.88	28.8102	1:Experimental evidence at protein level
28.7326	28.7272	28.5611	28.5501	28.1214	28.5055	1:Experimental evidence at protein level
27.4512	27.6312	27.6502	27.592	27.2504	27.5175	1:Experimental evidence at protein level
26.0589	26.2201	26.2663	26.4431	25.8737	26.3621	1:Experimental evidence at protein level
25.141	25.4265	25.2986	25.0995	24.9299	25.2006	1:Experimental evidence at protein level
27.4871	26.946	26.9736	26.8071	27.3056	26.7051	1:Experimental evidence at protein level
27.8953	27.8658	27.7776	27.6485	27.3678	27.6132	1:Experimental evidence at protein level
27.061	26.9926	26.9008	26.8399	26.6803	26.6088	1:Experimental evidence at protein level
27.7416	27.9593	27.7736	27.8389	27.6595	27.808	1:Experimental evidence at protein level
29.3779	29.1414	29.0141	29.0388	28.9446	29.2528	1:Experimental evidence at protein level
30.7015	30.299	30.2723	30.1596	30.5201	30.2459	1:Experimental evidence at protein level
27.4147	27.1338	27.1439	27.331	27.3552	27.2966	1:Experimental evidence at protein level
27.4889	27.8302	27.8244	27.6866	27.4979	27.7033	1:Experimental evidence at protein level
27.1363	26.9295	26.9167	26.7561	26.9814	26.573	1:Experimental evidence at protein level
26.4423	26.7391	26.8798	26.8405	26.7357	26.87	1:Experimental evidence at protein level
26.9679	26.5824	26.7599	26.3983	26.5978	26.686	1:Experimental evidence at protein level
26.6143	26.7411	26.8027	26.6831	26.3071	26.788	1:Experimental evidence at protein level
26.4926	26.1288	26.2209	26.2372	26.2495	26.4322	1:Experimental evidence at protein level
27.1798	26.9914	27.181	27.1991	26.6841	26.5818	1:Experimental evidence at protein level
26.0068	26.5024	26.3224	26.4291	26.421	26.3192	1:Experimental evidence at protein level
26.3673	26.4639	26.376	26.2972	26.5143	26.2984	1:Experimental evidence at protein level
26.708	26.9497	26.8718	27.3104	26.8776	27.2752	1:Experimental evidence at protein level
32.7267	32.5555	32.5334	32.653	32.2878	32.5586	1:Experimental evidence at protein level
26.4804	26.7225	26.8812	27.3928	27.1448	27.3475	1:Experimental evidence at protein level
29.9361	29.7873	29.9085	29.1621	29.1999	29.0815	1:Experimental evidence at protein level
27.0386	26.9027	26.9164	26.6699	26.711	26.6522	1:Experimental evidence at protein level
28.5426	28.9177	28.9197	25.9453	26.7712	26.0478	1:Experimental evidence at protein level
28.331	28.4464	28.5978	28.4259	28.3881	28.3133	1:Experimental evidence at protein level
26.7546	26.595	26.5772	26.8837	26.9046	26.8351	1:Experimental evidence at protein level
30.175	30.9304	30.979	30.85	30.0502	30.8719	1:Experimental evidence at protein level
25.9869	25.6121	25.4631	25.754	25.604	25.6047	1:Experimental evidence at protein level
28.8434	29.053	28.9902	29.1604	28.8301	28.9293	1:Experimental evidence at protein level
27.6943	28.1596	28.4984	28.1548	28.1535	28.2275	1:Experimental evidence at protein level
28.4077	28.3691	28.3037	28.2476	28.342	28.2453	1:Experimental evidence at protein level
31.2043	31.1392	31.15	30.8862	30.5671	31.142	1:Experimental evidence at protein level
26.3075	26.0642	26.482	26.2255	26.404	26.2795	1:Experimental evidence at protein level
28.3294	28.8036	28.6806	28.412	27.731	28.4129	1:Experimental evidence at protein level
29.2533	29.759	29.7118	29.7695	29.3326	29.6397	1:Experimental evidence at protein level
28.1512	28.2043	28.0911	28.4561	28.5517	28.5993	1:Experimental evidence at protein level
27.032	27.0602	27.0368	27.2431	27.2571	26.8945	1:Experimental evidence at protein level
27.5967	27.5344	27.4306	27.4698	27.4845	27.4933	1:Experimental evidence at protein level
28.4154	28.7051	28.8649	28.897	28.7746	28.8015	1:Experimental evidence at protein level
28.8456	28.9323	29.0457	29.0826	28.827	29.0738	1:Experimental evidence at protein level
29.1617	29.2844	29.1978	28.7651	28.8442	28.6813	1:Experimental evidence at protein level
30.3733	30.2501	30.2177	30.2084	30.5216	30.2295	1:Experimental evidence at protein level
29.6988	29.1099	29.3022	29.0215	29.3401	29.1684	1:Experimental evidence at protein level
31.5633	31.324	31.2871	31.2893	31.3945	31.1816	1:Experimental evidence at protein level
27.8281	28.2287	28.1553	27.9625	27.6587	27.7376	1:Experimental evidence at protein level
28.7119	28.8758	28.8259	28.6083	28.5667	28.6907	1:Experimental evidence at protein level
27.7977	28.2679	28.3627	28.4261	28.0019	28.3936	1:Experimental evidence at protein level
27.6172	27.5346	27.4881	27.0948	27.2373	27.1705	1:Experimental evidence at protein level
25.8206	26.0052	26.0457	26.4025	26.127	26.5007	1:Experimental evidence at protein level
26.7324	26.7489	26.5561	26.6909	26.5444	26.6845	1:Experimental evidence at protein level
25.3966	25.5503	25.5377	25.72	25.8745	26.0004	1:Experimental evidence at protein level
26.8437	26.3063	26.4511	26.1782	25.9916	26.2916	1:Experimental evidence at protein level
26.0496	25.4715	25.572	25.63	25.7024	25.4712	1:Experimental evidence at protein level
26.9361	26.913	27.0703	26.9017	26.7083	27.1188	1:Experimental evidence at protein level
27.5862	27.4348	27.3977	27.2389	27.1603	27.434	1:Experimental evidence at protein level
27.6437	27.5992	27.6935	27.5562	27.6815	27.5193	1:Experimental evidence at protein level
27.1309	26.7225	26.814	26.8152	26.7086	26.7156	1:Experimental evidence at protein level
27.0166	27.014	27.1217	26.7186	26.7523	26.7668	1:Experimental evidence at protein level
27.4093	27.4291	27.3076	27.1765	27.2325	27.2578	1:Experimental evidence at protein level
29.7862	29.6445	29.5346	29.5948	29.8057	29.3887	1:Experimental evidence at protein level
26.8806	27.191	27.2619	26.9808	26.7805	26.9892	1:Experimental evidence at protein level
27.715	27.6289	27.649	27.9707	27.9029	27.865	1:Experimental evidence at protein level
27.1274	27.0036	26.8415	26.9743	27.089	26.912	1:Experimental evidence at protein level
26.3319	26.3113	26.4756	26.7201	26.5874	26.5738	1:Experimental evidence at protein level
25.9399	25.5249	25.484	25.5813	25.5144	25.7723	1:Experimental evidence at protein level
27.2589	27.0843	27.0519	26.9214	26.923	27.0114	1:Experimental evidence at protein level
26.4519	26.1698	26.286	26.3021	26.5277	26.3282	1:Experimental evidence at protein level
26.4938	26.4202	26.7204	26.2428	26.458	26.0976	1:Experimental evidence at protein level
26.6795	26.5664	26.4556	26.3997	26.2507	26.6498	1:Experimental evidence at protein level
29.2628	29.3421	29.3606	29.1219	29.1222	29.2286	1:Experimental evidence at protein level
27.8514	28.1096	28.0125	28.1881	28.2172	28.0639	1:Experimental evidence at protein level
26.0296	25.8073	25.6402	25.4992	25.9724	25.3882	1:Experimental evidence at protein level
28.1114	28.1819	28.0812	28.2253	28.5816	28.2246	1:Experimental evidence at protein level
27.8822	27.7542	27.7122	27.5908	27.3229	27.5579	1:Experimental evidence at protein level
26.2022	26.3538	26.3498	26.2165	25.8986	26.2224	1:Experimental evidence at protein level
27.0231	26.9051	26.9343	26.6544	26.5509	26.7231	1:Experimental evidence at protein level
28.1408	28.2301	28.1014	28.0864	27.8424	27.9874	1:Experimental evidence at protein level
26.7248	26.8128	26.9443	26.7779	26.5191	26.6802	1:Experimental evidence at protein level
26.0446	25.5385	25.5686	25.3433	25.6396	25.4023	1:Experimental evidence at protein level
25.8689	26.0127	26.1467	26.451	26.4143	26.0703	1:Experimental evidence at protein level
28.9627	28.2044	28.2212	28.6612	29.0254	28.6826	1:Experimental evidence at protein level
#NUM!	25.5304	25.4123	25.8212	26.1104	26.4977	1:Experimental evidence at protein level
27.9409	28.1407	28.2583	28.1142	27.9949	28.0302	1:Experimental evidence at protein level
27.4645	27.6223	27.5078	27.7651	28.1057	27.9016	1:Experimental evidence at protein level
27.2952	27.218	27.2195	27.3333	27.2943	27.0968	1:Experimental evidence at protein level
30.3798	30.4608	30.4702	30.4501	30.3171	30.2876	1:Experimental evidence at protein level

27.2397	27.4587	27.579	27.5609	27.2817	27.3823	1:Experimental evidence at protein level
26.7905	26.3444	26.3004	26.6706	26.9994	26.8027	1:Experimental evidence at protein level
27.7147	27.7093	27.6866	27.5157	27.5664	27.3259	1:Experimental evidence at protein level
27.0462	27.3081	27.4003	27.3773	27.0167	27.5642	1:Experimental evidence at protein level
25.7965	25.8428	25.7885	25.4583	25.1718	25.3053	1:Experimental evidence at protein level
27.5146	27.4243	27.3315	27.4264	27.4453	27.3832	1:Experimental evidence at protein level
25.4396	25.7246	25.9593	25.9097	25.8592	25.6935	1:Experimental evidence at protein level
25.6949	25.5448	25.6129	25.5307	25.4774	25.648	1:Experimental evidence at protein level
29.7955	29.9994	29.8926	29.9663	29.8481	29.8781	1:Experimental evidence at protein level
28.007	27.8562	27.9496	27.2126	27.1211	27.2592	1:Experimental evidence at protein level
29.797	29.738	29.7407	29.6218	29.832	29.5796	1:Experimental evidence at protein level
26.9389	27.069	27.0621	26.7816	26.6859	26.6547	1:Experimental evidence at protein level
26.6691	26.9354	26.956	26.2875	25.8689	26.2047	1:Experimental evidence at protein level
29.1854	29.1985	29.2733	29.4052	28.8693	29.3268	1:Experimental evidence at protein level
31.1657	31.1602	30.9062	32.363	32.5246	32.2197	1:Experimental evidence at protein level
29.127	28.992	29.0157	29.1743	29.3624	28.971	1:Experimental evidence at protein level
29.8716	29.8689	29.9377	29.9764	29.4126	29.9459	1:Experimental evidence at protein level
29.3685	29.4196	29.3459	29.5089	29.3832	29.482	1:Experimental evidence at protein level
27.6741	27.6466	27.7794	27.9315	27.8099	27.6663	1:Experimental evidence at protein level
25.7434	25.0014	25.3614	27.0377	27.5722	27.3182	1:Experimental evidence at protein level
27.7293	27.4245	27.4954	27.2852	27.5033	27.3381	1:Experimental evidence at protein level
29.6344	29.5272	29.6083	29.663	29.5312	29.8131	1:Experimental evidence at protein level
29.6273	30.0294	30.0115	29.8906	29.8493	29.6723	1:Experimental evidence at protein level
28.3511	28.8306	28.6737	28.5349	28.1212	28.5788	1:Experimental evidence at protein level
27.59	27.4007	27.286	27.4617	27.2131	27.4022	1:Experimental evidence at protein level
29.733	29.8514	29.7393	30.2176	30.0508	29.985	1:Experimental evidence at protein level
27.3607	27.698	27.6288	27.6949	27.5415	27.6617	1:Experimental evidence at protein level
27.6546	27.4062	27.517	26.6559	26.4296	26.2166	1:Experimental evidence at protein level
27.4985	27.4997	27.673	27.5568	27.5726	27.713	1:Experimental evidence at protein level
27.7557	27.7251	27.7494	27.7349	27.8398	27.5632	1:Experimental evidence at protein level
27.5402	27.4358	27.5133	27.5441	27.7134	27.6366	1:Experimental evidence at protein level
27.5913	27.7047	27.6498	27.5724	27.3807	27.4328	1:Experimental evidence at protein level
26.8208	26.856	26.9804	26.7219	26.6634	27.1218	1:Experimental evidence at protein level
33.1258	33.2855	33.3382	33.2312	33.2296	33.0882	1:Experimental evidence at protein level
29.9649	30.3365	30.308	30.0046	30.0212	30.0367	1:Experimental evidence at protein level
29.2276	28.8049	28.8702	28.6998	28.7891	28.8808	1:Experimental evidence at protein level
27.5787	27.791	27.8207	27.8896	27.8075	27.7863	1:Experimental evidence at protein level
26.8569	26.8201	26.9802	26.4089	26.4462	26.6786	1:Experimental evidence at protein level
27.9805	28.1303	28.0916	27.9128	27.7618	27.8694	1:Experimental evidence at protein level
28.9465	29.1693	29.2772	28.9372	28.983	28.8839	1:Experimental evidence at protein level
30.1827	29.5784	29.5822	29.4895	29.9166	29.712	1:Experimental evidence at protein level
24.4803	25.1687	25.082	25.1684	24.6292	25.204	1:Experimental evidence at protein level
27.8498	27.6772	27.7137	27.5483	27.5987	27.5871	1:Experimental evidence at protein level
27.4095	27.3992	27.4911	27.5169	27.3459	27.4869	1:Experimental evidence at protein level
25.3169	25.1066	25.2976	25.3288	25.735	25.2689	1:Experimental evidence at protein level
28.2254	28.4402	28.4793	28.5	28.1979	28.4959	1:Experimental evidence at protein level
27.1647	26.756	26.8209	27.0262	27.1793	27.0343	1:Experimental evidence at protein level
27.5737	27.5881	27.6709	27.7959	27.6142	27.6266	1:Experimental evidence at protein level
28.4973	28.4501	28.4565	28.6003	28.8092	28.6266	1:Experimental evidence at protein level
29.789	29.1526	29.4798	29.4005	29.7616	28.6391	1:Experimental evidence at protein level
25.4102	25.5941	25.5765	25.4113	25.7023	25.2272	1:Experimental evidence at protein level
29.4077	29.4096	29.2266	29.2485	29.3094	29.318	1:Experimental evidence at protein level
25.746	26.0355	26.2393	26.9367	26.3872	26.8132	1:Experimental evidence at protein level
26.2611	26.0964	26.2245	26.3134	26.0905	26.1753	1:Experimental evidence at protein level
26.1446	26.5351	26.3046	26.8067	26.7264	26.8992	1:Experimental evidence at protein level
27.9285	27.9365	28.0336	27.971	#NUM!	27.8788	1:Experimental evidence at protein level
28.3747	27.6566	27.5205	27.6926	28.0116	27.7656	1:Experimental evidence at protein level
27.1983	26.7445	27.0034	27.0142	27.0121	27.5383	1:Experimental evidence at protein level
28.2813	28.4041	28.2727	28.3761	28.3247	28.1137	1:Experimental evidence at protein level
27.2239	27.2189	27.3542	27.6193	27.4923	27.6399	1:Experimental evidence at protein level
28.6508	28.3354	28.4892	28.3659	28.1737	28.6471	1:Experimental evidence at protein level
27.0047	26.7502	26.6163	26.7278	27.0592	26.5188	1:Experimental evidence at protein level
26.0278	25.7222	26.2959	26.1145	26.0528	26.0996	1:Experimental evidence at protein level
27.4676	27.0373	27.0692	27.1225	27.2096	26.9991	1:Experimental evidence at protein level
25.8635	25.5255	25.7792	25.6726	25.6218	25.3806	1:Experimental evidence at protein level
29.067	29.322	29.454	29.1172	28.652	29.1688	1:Experimental evidence at protein level
27.2319	27.5688	27.4811	27.8882	27.4105	27.8225	1:Experimental evidence at protein level
28.0876	28.2608	28.348	28.4301	28.3673	28.1956	1:Experimental evidence at protein level
29.3602	28.8858	28.7832	29.1265	29.3108	29.0088	1:Experimental evidence at protein level
28.0772	28.0858	28.1951	28.0262	27.8805	28.016	1:Experimental evidence at protein level
27.0706	26.9305	26.8797	26.8094	26.83	26.9688	1:Experimental evidence at protein level
26.3448	26.9633	27.0033	26.9705	26.6319	27.0468	1:Experimental evidence at protein level
25.1969	25.4348	24.7036	25.0802	24.9818	24.8581	1:Experimental evidence at protein level
27.9316	28.3593	28.3922	28.3929	27.9189	28.3009	1:Experimental evidence at protein level
28.0187	27.9602	27.9653	28.0079	28.2856	27.419	1:Experimental evidence at protein level
26.6184	26.6394	26.7243	26.9343	26.7769	27.0427	1:Experimental evidence at protein level
29.0902	29.138	29.0227	29.2666	29.2546	29.2693	1:Experimental evidence at protein level
27.9539	27.6319	27.7021	27.5282	27.5406	27.5951	1:Experimental evidence at protein level
27.3305	27.4218	27.5396	27.3988	27.3636	27.3818	1:Experimental evidence at protein level
28.9625	28.9098	28.8796	28.9325	28.7589	28.8253	1:Experimental evidence at protein level
28.7768	28.9001	28.7862	28.5972	28.5623	28.5303	1:Experimental evidence at protein level
28.6608	28.58	28.3999	28.2575	28.3358	28.2955	1:Experimental evidence at protein level
29.9847	29.7793	29.8213	29.811	30.1694	29.7903	1:Experimental evidence at protein level
29.5126	29.6567	29.7568	29.5643	29.471	29.5535	1:Experimental evidence at protein level
28.5075	28.7057	28.5324	28.5429	28.4351	28.6698	1:Experimental evidence at protein level
25.7156	25.9858	25.9816	25.9838	25.8896	25.9071	1:Experimental evidence at protein level
28.2012	27.9597	28.079	28.2031	28.3802	28.4446	1:Experimental evidence at protein level
28.2093	28.2571	28.2934	28.8562	28.4901	28.594	1:Experimental evidence at protein level
29.0709	28.3576	28.4936	28.2657	28.6558	28.4869	1:Experimental evidence at protein level
28.5488	28.9059	28.6611	29.02	29.251	28.9711	1:Experimental evidence at protein level
29.566	29.4837	29.2993	29.4261	29.8313	29.5717	1:Experimental evidence at protein level
27.707	27.6989	27.7909	27.9183	27.7337	27.8041	1:Experimental evidence at protein level
#NUM!	25.8783	26.2665	26.0006	25.6204	26.1794	1:Experimental evidence at protein level
28.5311	28.6838	28.6545	28.8019	28.8383	28.6784	1:Experimental evidence at protein level
27.0691	27.0793	26.9579	27.0141	26.9339	27.0243	1:Experimental evidence at protein level
27.8766	27.7167	28.0188	27.9314	28.0428	27.8638	1:Experimental evidence at protein level
29.4437	29.2673	29.1885	29.357	29.3257	29.3853	1:Experimental evidence at protein level
26.7457	26.9047	26.9579	26.9691	26.7288	26.8774	1:Experimental evidence at protein level
27.3111	27.4306	27.2933	27.2685	27.7802	27.2868	1:Experimental evidence at protein level
27.1841	27.2977	27.41	27.5175	27.5896	27.6092	1:Experimental evidence at protein level
25.4257	25.295	25.3884	25.5743	25.6275	25.3652	1:Experimental evidence at protein level
26.5702	26.7376	26.6477	26.4026	26.334	26.57	1:Experimental evidence at protein level
26.6369	26.5425	26.3934	26.3461	26.2943	26.5378	1:Experimental evidence at protein level
25.7082	25.7483	25.6717	25.3163	25.3973	25.649	1:Experimental evidence at protein level
25.8837	25.9424	26.1759	26.3326	25.9985	26.3389	1:Experimental evidence at protein level
26.8667	26.8077	26.8263	26.8648	26.7965	26.8562	1:Experimental evidence at protein level
28.3993	28.2859	28.3193	28.3689	28.8828	28.613	1:Experimental evidence at protein level
26.8739	27.1353	27.1268	28.3582	28.1349	28.1666	1:Experimental evidence at protein level
27.7857	27.6538	27.7463	27.825	27.9039	27.7115	1:Experimental evidence at protein level
27.9217	27.9718	27.8306	27.5145	27.4588	27.6113	1:Experimental evidence at protein level
28.3078	28.7647	28.7875	28.716	28.5044	28.6888	1:Experimental evidence at protein level
27.5839	27.4773	27.5902	27.4214	27.379	27.4625	1:Experimental evidence at protein level
29.376	29.2601	28.9263	28.826	28.7129	28.9546	1:Experimental evidence at protein level
26.6087	26.2328	26.2535	26.3752	26.3869	26.3557	1:Experimental evidence at protein level
27.5133	27.5695	27.5425	27.6325	27.3993	27.5696	1:Experimental evidence at protein level
26.1594	25.8179	25.9148	26.1703	25.9388	26.1385	1:Experimental evidence at protein level
28.1537	28.0047	27.8862	27.8539	27.9084	27.8708	1:Experimental evidence at protein level
26.9277	26.6488	26.5153	26.7629	27.2158	26.7589	1:Experimental evidence at protein level
27.5223	27.366	27.2648	27.2107	27.317	27.3935	1:Experimental evidence at protein level
29.0913	28.8588	28.9322	28.7133	28.727	28.8043	1:Experimental evidence at protein level
26.3662	26.1073	26.2074	26.2894	26.3117	26.184	1:Experimental evidence at protein level
27.53	27.7813	27.8207	27.8521	27.5852	27.9129	1:Experimental evidence at protein level
26.2218	26.4895	26.937	25.8647	26.4371	26.0446	1:Experimental evidence at protein level
28.9246	28.7115	28.6558	27.6553	27.6969	27.6271	1:Experimental evidence at protein level
28.8124	29.0621	29.0878	29.0286	28.9186	29.0177	1:Experimental evidence at protein level
27.1043	27.0121	26.9469	27.065	26.9079	27.1334	1:Experimental evidence at protein level
28.2834	27.8276	27.8868	27.8816	28.2363	27.7625	1:Experimental evidence at protein level
28.0663	28.4702	28.45	28.3558	28.1904	28.3415	1:Experimental evidence at protein level
31.0759	31.0128	31.0509	30.9796	30.9762	30.9859	1:Experimental evidence at protein level

28.3707	28.0736	28.1715	27.9491	28.0764	27.964	1:Experimental evidence at protein level
25.9622	26.4063	26.267	26.2661	25.9054	26.2429	1:Experimental evidence at protein level
28.593	28.4762	28.4711	28.768	28.7097	28.8074	1:Experimental evidence at protein level
29.0077	29.0117	29.1124	29.0177	28.8363	29.03	1:Experimental evidence at protein level
27.7042	27.9464	28.0207	28.1174	27.7946	27.9064	1:Experimental evidence at protein level
28.2361	28.4517	28.5606	28.522	28.5082	28.638	1:Experimental evidence at protein level
31.7064	31.4046	31.3991	31.3848	31.588	31.3924	1:Experimental evidence at protein level
30.9696	30.9967	31.5508	30.938	31.0047	30.7265	1:Experimental evidence at protein level
29.2484	29.5643	29.4537	29.8273	29.6587	29.6784	1:Experimental evidence at protein level
30.8947	30.2096	30.2227	30.1039	30.737	30.1789	1:Experimental evidence at protein level
25.9245	25.8914	25.8388	25.9641	25.931	25.9618	1:Experimental evidence at protein level
28.5898	28.4931	28.3735	28.2653	28.6005	28.1394	1:Experimental evidence at protein level
30.9944	30.6309	30.6269	30.6103	30.8137	30.7068	1:Experimental evidence at protein level
28.6623	28.7266	28.7052	28.6321	28.5027	28.6566	1:Experimental evidence at protein level
28.4627	28.0084	27.9742	28.5298	28.601	28.4384	1:Experimental evidence at protein level
26.1494	26.0457	26.1189	26.1793	26.1353	25.9891	1:Experimental evidence at protein level
26.6737	25.9675	26.1857	26.2957	26.2253	25.545	1:Experimental evidence at protein level
29.4771	29.6413	29.6506	29.7109	29.7482	29.7204	1:Experimental evidence at protein level
29.7572	29.4149	29.4513	29.3543	29.3965	29.3152	1:Experimental evidence at protein level
26.2674	26.2483	26.0401	25.9263	25.9661	26.0212	1:Experimental evidence at protein level
27.2654	27.1803	27.0496	27.0833	27.0276	27.0411	1:Experimental evidence at protein level
27.8574	27.8788	27.9145	27.9696	27.9314	27.9203	1:Experimental evidence at protein level
28.5624	28.5272	28.6678	28.6953	28.5102	28.7046	1:Experimental evidence at protein level
27.4385	27.8629	27.8099	27.965	27.6264	27.851	1:Experimental evidence at protein level
28.2753	28.2917	28.4739	28.4056	28.2741	28.42	1:Experimental evidence at protein level
28.3718	28.1403	28.2237	28.2555	28.3725	28.1023	1:Experimental evidence at protein level
26.1202	25.8672	25.9559	25.6814	25.6785	25.8246	1:Experimental evidence at protein level
26.4493	26.4491	26.6663	26.818	26.9256	26.8055	1:Experimental evidence at protein level
29.0466	29.3496	29.2817	29.3332	29.0872	29.3336	1:Experimental evidence at protein level
29.2482	29.3088	29.1912	29.1787	29.308	29.0826	1:Experimental evidence at protein level
26.1294	25.9929	25.6067	25.8933	25.5035	25.6138	1:Experimental evidence at protein level
28.0405	27.8378	27.9465	27.8827	27.8325	27.8389	1:Experimental evidence at protein level
29.5805	29.2716	29.4147	29.3535	29.3909	29.371	1:Experimental evidence at protein level
26.7078	26.7502	26.5953	26.5498	26.7413	26.6655	1:Experimental evidence at protein level
28.7137	28.8158	28.7562	28.8159	28.5812	28.658	1:Experimental evidence at protein level
29.4845	29.6227	29.4647	29.7101	29.7267	29.5179	1:Experimental evidence at protein level
26.6029	26.705	26.5403	26.4066	26.5698	26.4273	1:Experimental evidence at protein level
26.6814	26.1206	25.9764	26.2314	26.2157	26.0738	1:Experimental evidence at protein level
26.9319	27.206	27.3784	27.1584	26.9029	27.1379	1:Experimental evidence at protein level
28.1413	27.9553	27.848	28.1641	28.0343	28.1221	1:Experimental evidence at protein level
27.7337	28.3285	28.1783	28.3394	27.961	28.4023	1:Experimental evidence at protein level
28.0597	28.2209	28.2932	28.2184	28.016	28.2069	1:Experimental evidence at protein level
27.6739	27.5342	27.5651	27.4709	27.5267	27.3565	1:Experimental evidence at protein level
26.5467	26.507	26.614	25.9951	26.0673	26.2649	1:Experimental evidence at protein level
28.6354	28.8614	28.7868	28.8836	28.7062	28.6688	1:Experimental evidence at protein level
27.5048	27.1798	27.2825	27.3692	27.3647	27.2633	1:Experimental evidence at protein level
27.6607	27.5171	27.5462	26.8544	26.7038	26.7738	1:Experimental evidence at protein level
28.4597	28.816	28.6471	28.6107	28.3562	28.48	1:Experimental evidence at protein level
30.0808	30.2375	30.1446	29.9966	29.6343	30.1391	1:Experimental evidence at protein level
29.4883	29.3087	29.2925	29.2241	29.3355	29.1829	1:Experimental evidence at protein level
28.7105	29.2293	29.1607	29.4858	29.3985	29.3217	1:Experimental evidence at protein level
26.3188	26.1898	26.2225	25.914	25.8603	26.2151	1:Experimental evidence at protein level
29.5771	29.2405	29.2594	29.2541	29.3867	29.1772	1:Experimental evidence at protein level
31.0969	31.2329	31.1441	31.1484	31.2755	31.1694	1:Experimental evidence at protein level
28.0947	28.3892	28.4901	28.4274	28.123	28.3104	1:Experimental evidence at protein level
29.9845	30.3692	30.2417	31.0597	30.6723	30.9563	1:Experimental evidence at protein level
27.1659	27.0976	27.1335	27.3678	27.2087	27.4468	1:Experimental evidence at protein level
31.0461	30.4186	30.321	30.2367	30.7623	30.4493	1:Experimental evidence at protein level
32.1431	32.3383	32.2488	32.1862	32.0518	32.1187	1:Experimental evidence at protein level
29.7145	29.3561	29.3836	29.2621	29.5001	28.9559	1:Experimental evidence at protein level
28.2812	28.2064	28.1194	28.111	28.0576	27.9815	1:Experimental evidence at protein level
28.0679	27.944	28.0162	27.7351	27.9562	27.7807	1:Experimental evidence at protein level
29.4015	29.097	29.0622	27.6891	28.1569	27.6706	1:Experimental evidence at protein level
26.3107	26.1723	26.0945	26.6906	26.3752	26.7556	1:Experimental evidence at protein level
28.4087	28.3557	28.4287	28.4305	28.3676	28.2909	1:Experimental evidence at protein level
29.6069	29.7577	29.7068	29.5797	29.2676	29.5172	1:Experimental evidence at protein level
27.2857	27.3561	27.4261	26.9766	26.8228	26.9446	1:Experimental evidence at protein level
30.6851	30.6668	30.5564	30.6687	30.5123	30.5241	1:Experimental evidence at protein level
30.0541	29.9613	30.1251	29.9322	29.9606	30.1963	1:Experimental evidence at protein level
27.8686	28.1575	28.3166	28.396	28.1041	28.2564	1:Experimental evidence at protein level
26.0144	26.1358	26.1078	26.1325	25.9925	26.1838	1:Experimental evidence at protein level
26.5939	26.2828	26.2565	26.2266	26.4427	26.5508	1:Experimental evidence at protein level
29.137	29.2478	29.2602	29.1614	29.0588	29.214	1:Experimental evidence at protein level
31.2449	31.2234	31.2969	30.9005	30.8051	30.9295	1:Experimental evidence at protein level
29.6707	29.7159	29.7734	30.0189	29.8766	29.8605	1:Experimental evidence at protein level
31.2885	31.0326	31.1048	30.9313	31.2635	31.0628	1:Experimental evidence at protein level
28.9942	29.6568	29.6403	29.7734	29.2991	29.7132	1:Experimental evidence at protein level
31.8406	31.3701	31.4043	31.2708	31.955	31.345	1:Experimental evidence at protein level
26.2847	25.9153	25.6369	25.8526	26.1491	25.9735	1:Experimental evidence at protein level
26.2916	26.3988	26.3106	26.3955	26.2035	26.1725	1:Experimental evidence at protein level
26.6157	26.2759	25.9264	26.2054	26.2081	25.9984	1:Experimental evidence at protein level
29.8289	30.0706	30.0773	30.0606	29.9204	30.0252	1:Experimental evidence at protein level
27.4279	27.3917	27.3353	27.0115	26.7878	27.0092	1:Experimental evidence at protein level
27.067	26.9119	27.0183	27.2245	27.2935	27.1663	1:Experimental evidence at protein level
30.9459	30.6645	30.5779	30.6635	30.9335	30.6632	1:Experimental evidence at protein level
28.3962	28.4117	28.5215	28.6023	28.4294	28.5864	1:Experimental evidence at protein level
27.4107	27.1821	27.2314	27.3026	27.2136	27.1697	1:Experimental evidence at protein level
27.2994	27.3033	27.2914	27.2873	27.1498	27.1679	1:Experimental evidence at protein level
25.4676	25.6102	25.678	25.572	25.1939	25.3511	1:Experimental evidence at protein level
26.6374	26.8957	26.9122	26.9989	27.2355	27.0623	1:Experimental evidence at protein level
27.0198	26.9473	26.8166	26.6593	26.8108	26.7927	1:Experimental evidence at protein level
#NUM!	#NUM!	#NUM!	26.524	26.7385	27.0746	1:Experimental evidence at protein level
26.156	26.3908	26.2968	26.4764	26.3979	26.6646	1:Experimental evidence at protein level
27.2403	27.2625	27.2429	27.039	27.0395	27.2028	1:Experimental evidence at protein level
26.8934	26.4972	26.2826	26.4193	26.715	26.279	1:Experimental evidence at protein level
27.8452	27.8382	27.78	27.8589	28.0345	27.7797	1:Experimental evidence at protein level
28.3036	28.8052	28.8009	28.6749	28.2623	28.7397	1:Experimental evidence at protein level
26.2932	26.8023	26.8426	26.7283	26.2098	26.8337	1:Experimental evidence at protein level
26.5008	25.9541	26.2554	26.2051	26.2717	25.8749	1:Experimental evidence at protein level
27.3901	27.5219	27.4388	27.5704	27.4123	27.5508	1:Experimental evidence at protein level
27.3918	27.4954	27.4721	27.0026	26.9733	27.0461	1:Experimental evidence at protein level
27.4379	27.5215	27.5197	27.5575	27.3779	27.7716	1:Experimental evidence at protein level
27.8363	27.7997	27.665	27.4736	27.0969	27.4961	1:Experimental evidence at protein level
29.5865	29.8534	29.8246	29.57	29.3199	29.489	1:Experimental evidence at protein level
26.9552	27.2495	27.3854	27.2056	26.8333	27.2499	1:Experimental evidence at protein level
32.6462	32.6052	32.5092	32.7117	32.6986	32.3818	1:Experimental evidence at protein level
27.3857	27.5103	27.5794	28.7163	28.5577	28.8675	1:Experimental evidence at protein level
29.0136	28.9513	28.9555	28.9535	29.0716	28.7939	1:Experimental evidence at protein level
26.6907	26.4537	26.6678	26.344	26.8459	26.2828	1:Experimental evidence at protein level
30.4666	30.0034	30.3051	30.1699	30.6255	30.051	1:Experimental evidence at protein level
29.7915	29.9563	29.8812	29.8051	29.6891	29.8909	1:Experimental evidence at protein level
29.3334	29.3004	29.2647	29.117	29.1737	29.107	1:Experimental evidence at protein level
28.5413	28.3542	28.4331	28.503	28.5225	28.4261	1:Experimental evidence at protein level
28.2859	28.1489	27.9797	28.0651	28.2257	27.9338	1:Experimental evidence at protein level
28.0828	28.097	28.1174	27.7985	27.7733	27.6882	1:Experimental evidence at protein level
26.7938	26.9235	26.9796	26.3066	26.3456	26.3999	1:Experimental evidence at protein level
28.0795	26.7862	27.9304	27.0078	27.8297	27.1713	1:Experimental evidence at protein level
26.2194	26.0925	26.1259	26.355	26.0441	26.353	1:Experimental evidence at protein level
29.9046	29.819	29.7004	29.4337	29.542	29.1316	1:Experimental evidence at protein level
29.608	29.3856	29.3017	29.377	29.4725	29.1823	1:Experimental evidence at protein level
31.1827	31.0805	31.1288	30.9643	30.9832	30.8818	1:Experimental evidence at protein level
30.6954	30.4841	30.3325	30.236	30.7475	30.2672	1:Experimental evidence at protein level
30.8209	30.1888	30.2614	30.0862	30.3071	30.3441	1:Experimental evidence at protein level
29.1031	29.5839	29.5083	29.3685	29.393	29.5218	1:Experimental evidence at protein level
30.3454	30.4122	30.5633	30.396	30.3294	30.437	1:Experimental evidence at protein level
25.33	25.4693	25.2604	25.6518	25.6088	25.3293	1:Experimental evidence at protein level
25.686	26.0394	26.1475	25.7121	25.6152	25.9679	1:Experimental evidence at protein level
27.5193	27.4133	27.4536	27.5247	27.2741	27.8385	1:Experimental evidence at protein level
28.6132	28.7564	28.665	28.8508	28.6523	28.7917	1:Experimental evidence at protein level

26.8101	26.8313	26.804	26.7532	26.5651	26.6997	1:Experimental evidence at protein level
31.1931	30.9599	31.0132	31.096	30.9501	31.0717	1:Experimental evidence at protein level
28.1591	28.8745	28.7363	28.5973	28.5076	28.6459	1:Experimental evidence at protein level
25.4853	25.5192	25.4605	25.7756	25.5151	25.8001	1:Experimental evidence at protein level
27.2581	27.2059	27.2194	27.3494	27.3578	27.2593	1:Experimental evidence at protein level
26.5486	26.5595	26.5441	26.4639	26.3555	26.6172	1:Experimental evidence at protein level
25.8897	25.6242	25.639	26.0027	25.6183	25.6175	1:Experimental evidence at protein level
26.0982	26.2768	26.2067	26.3812	25.9419	26.5032	1:Experimental evidence at protein level
27.7782	27.6135	27.7041	27.3679	27.2853	27.3493	1:Experimental evidence at protein level
26.6273	26.5247	26.6287	26.0316	26.2655	26.6914	1:Experimental evidence at protein level
28.798	28.8162	28.8113	29.0158	29.1884	28.9409	1:Experimental evidence at protein level
29.78	29.5759	29.3442	29.4317	29.2474	29.3766	1:Experimental evidence at protein level
26.9833	27.0704	27.1974	27.1079	26.8943	27.0407	1:Experimental evidence at protein level
26.3329	26.4597	26.4712	25.8496	25.5242	25.9509	1:Experimental evidence at protein level
29.0291	28.6535	28.7943	28.6465	29.2976	28.7283	1:Experimental evidence at protein level
28.0537	28.1061	28.1729	27.9953	27.7224	27.9615	1:Experimental evidence at protein level
27.0449	27.0231	27.024	26.9661	27.001	26.99	1:Experimental evidence at protein level
28.4305	28.5346	28.4434	28.7087	28.4394	28.5913	1:Experimental evidence at protein level
26.3078	26.1744	26.1761	25.8514	26.1221	25.9783	1:Experimental evidence at protein level
28.5275	28.9886	29.1396	29.1661	28.8992	29.093	1:Experimental evidence at protein level
27.1355	27.6023	27.5784	27.3533	26.9881	27.2862	1:Experimental evidence at protein level
#NUM!	#NUM!	#NUM!	25.1894	24.6049	25.0065	1:Experimental evidence at protein level
26.8857	27.3168	27.2457	27.4387	27.1299	27.6199	1:Experimental evidence at protein level
29.4017	29.1222	29.1949	29.27	29.5653	29.1453	1:Experimental evidence at protein level
28.4621	28.273	28.3452	27.9039	27.7936	27.7522	1:Experimental evidence at protein level
25.885	25.8381	25.6664	25.6902	25.8024	25.6964	1:Experimental evidence at protein level
31.031	29.6959	29.6916	29.2377	30.6771	29.3689	1:Experimental evidence at protein level
29.4849	29.8214	29.6546	29.7811	29.5618	29.7352	1:Experimental evidence at protein level
32.1379	31.9501	31.9126	32.4713	32.99	32.5234	1:Experimental evidence at protein level
27.9235	28.2158	28.2363	28.1594	28.0795	28.1934	1:Experimental evidence at protein level
27.7551	27.9959	28.1456	27.5883	27.4449	27.483	1:Experimental evidence at protein level
28.7906	28.7471	28.695	28.7711	28.7594	28.5702	1:Experimental evidence at protein level
27.5107	27.6691	27.6635	27.5231	27.3499	27.5957	1:Experimental evidence at protein level
28.0048	28.1601	28.1286	28.4798	28.4222	28.2726	1:Experimental evidence at protein level
27.9537	27.915	28.0309	27.9195	27.6725	27.9905	1:Experimental evidence at protein level
28.6003	29.0877	29.1565	29.0332	28.8455	29.1303	1:Experimental evidence at protein level
30.4849	30.4946	30.5657	30.6406	30.3786	30.611	1:Experimental evidence at protein level
26.9849	27.2012	27.0885	27.0878	26.8239	27.2435	1:Experimental evidence at protein level
28.6105	28.4665	28.4755	28.463	28.6306	28.4262	1:Experimental evidence at protein level
33.3583	32.3781	32.303	32.0061	32.7288	32.0646	1:Experimental evidence at protein level
27.8096	28.1069	28.1541	28.1809	27.8606	28.001	1:Experimental evidence at protein level
29.1718	28.7794	28.7064	28.9415	29.3846	29.1554	1:Experimental evidence at protein level
29.5627	29.2956	29.326	29.4398	29.6789	29.3568	1:Experimental evidence at protein level
27.3692	27.6701	27.5695	27.6136	27.6	27.5741	1:Experimental evidence at protein level
30.3313	30.9687	30.9036	30.8513	30.4623	30.8624	1:Experimental evidence at protein level
28.1095	27.8915	28.2243	28.408	28.3303	28.2855	1:Experimental evidence at protein level
28.9658	28.7986	28.8036	28.8232	28.7641	29.0479	1:Experimental evidence at protein level
29.1223	29.0925	29.0306	28.8592	28.9664	28.9201	1:Experimental evidence at protein level
28.1815	28.271	28.4097	28.4257	28.2298	28.4796	1:Experimental evidence at protein level
29.2758	29.0044	29.1003	28.9375	29.1067	28.8374	1:Experimental evidence at protein level
30.7384	30.9856	31.0465	30.9924	30.8952	30.7434	1:Experimental evidence at protein level
27.5452	27.5376	27.5418	27.6146	27.4485	27.5741	1:Experimental evidence at protein level
28.8717	28.7784	28.7341	29.1004	29.1301	29.0285	1:Experimental evidence at protein level
28.9399	29.3041	29.3803	29.2417	28.9316	29.1968	1:Experimental evidence at protein level
28.7666	29.0866	28.9638	28.922	28.5618	28.7404	1:Experimental evidence at protein level
28.411	28.326	28.2966	28.39	28.3235	28.6033	1:Experimental evidence at protein level
25.9562	25.7246	25.8772	26.07	25.992	25.7894	1:Experimental evidence at protein level
27.1609	27.2651	27.3279	27.4124	27.2822	27.5858	1:Experimental evidence at protein level
29.2741	29.0962	29.1423	30.022	30.0321	29.9929	1:Experimental evidence at protein level
25.3917	25.1693	25.1944	25.5373	25.3947	25.6	1:Experimental evidence at protein level
27.6049	27.8972	28.0673	28.0791	28.0023	27.8892	1:Experimental evidence at protein level
30.8326	31.0337	31.0291	31.0611	31.1826	30.8959	1:Experimental evidence at protein level
26.9704	26.5713	26.6902	26.7704	27.1231	26.7086	1:Experimental evidence at protein level
28.0956	28.3246	28.3897	28.3397	28.1345	28.3138	1:Experimental evidence at protein level
27.0296	27.048	26.9702	26.9628	27.1548	26.9142	1:Experimental evidence at protein level
27.954	28.4889	28.3393	28.4141	28.1928	28.4369	1:Experimental evidence at protein level
27.8684	27.7202	27.7308	27.9912	27.9359	27.8383	1:Experimental evidence at protein level
28.1482	28.2249	28.1904	28.1009	27.8601	28.0206	1:Experimental evidence at protein level
27.2269	27.3689	27.4115	27.4505	27.3791	27.5333	1:Experimental evidence at protein level
27.5462	27.6278	27.6662	27.7334	27.4165	27.6063	1:Experimental evidence at protein level
28.7958	28.8762	29.0677	28.9669	28.7023	28.9999	1:Experimental evidence at protein level
27.1015	27.0149	27.0981	27.0019	27.1806	27.0285	1:Experimental evidence at protein level
30.2754	30.2329	30.3449	30.6801	30.5448	30.4206	1:Experimental evidence at protein level
29.2681	29.2864	29.223	29.0589	29.0068	29.1621	1:Experimental evidence at protein level
28.1268	28.037	28.0821	28.0352	28.1117	27.9765	1:Experimental evidence at protein level
26.9842	27.2804	27.2532	26.8401	26.405	27.1695	1:Experimental evidence at protein level
26.2825	26.1933	26.17	26.5174	26.4705	26.4342	1:Experimental evidence at protein level
28.2865	28.496	28.5574	28.3088	28.1732	28.3353	1:Experimental evidence at protein level
26.6481	26.6349	26.3746	26.2037	26.6094	26.2114	1:Experimental evidence at protein level
30.4652	30.3916	30.5433	30.4213	30.9871	30.0354	1:Experimental evidence at protein level
26.5304	26.8955	27.0813	26.9417	26.5474	26.7326	1:Experimental evidence at protein level
31.1258	31.1027	31.1427	31.6505	31.6751	31.4563	1:Experimental evidence at protein level
29.4899	29.4173	29.3999	29.3081	29.2887	29.2406	1:Experimental evidence at protein level
27.2537	27.1082	27.5187	27.2566	27.1803	27.0629	1:Experimental evidence at protein level
28.7826	28.9263	28.9546	29.0397	28.8866	28.7003	1:Experimental evidence at protein level
29.8514	29.8077	29.7408	29.643	29.7187	29.5565	1:Experimental evidence at protein level
28.795	28.9114	28.9391	28.8879	28.7259	28.9154	1:Experimental evidence at protein level
27.5621	27.444	27.5453	27.335	27.2411	27.3766	1:Experimental evidence at protein level
26.6146	26.2629	26.2416	26.7797	27.0666	26.6157	1:Experimental evidence at protein level
28.5262	28.5906	28.5893	28.6184	28.4933	28.4078	1:Experimental evidence at protein level
28.696	28.8999	28.9071	28.7915	28.7131	28.7857	1:Experimental evidence at protein level
30.5594	30.3907	30.4219	30.3347	30.2569	30.4583	1:Experimental evidence at protein level
29.3675	29.7162	29.7797	29.8932	29.7907	29.7711	1:Experimental evidence at protein level
26.9996	27.087	27.1029	27.1798	26.8971	27.2643	1:Experimental evidence at protein level
29.2448	29.7041	29.7129	29.8181	29.3863	29.8453	1:Experimental evidence at protein level
26.1141	25.8338	25.8003	25.9809	25.3759	25.5623	1:Experimental evidence at protein level
26.242	26.3213	26.3738	26.0817	26.0105	25.964	1:Experimental evidence at protein level
27.8288	27.7513	27.5946	27.5413	27.5359	27.4579	1:Experimental evidence at protein level
27.105	27.4095	27.338	27.1869	27.0792	26.9395	1:Experimental evidence at protein level
29.8155	29.7342	29.8376	29.9512	29.7658	29.9146	1:Experimental evidence at protein level
28.1443	27.9383	27.8536	27.5105	27.6305	27.7813	1:Experimental evidence at protein level
27.9932	27.5581	27.6082	27.4524	27.7122	27.2085	1:Experimental evidence at protein level
28.0735	27.7398	27.9696	27.6101	27.5799	27.9444	1:Experimental evidence at protein level
28.8854	29.17	29.1149	28.7961	28.7231	28.7064	1:Experimental evidence at protein level
28.2248	28.1982	28.1889	28.0241	27.8742	28.3831	1:Experimental evidence at protein level
25.7484	25.5688	25.8004	25.4752	25.8126	25.7152	1:Experimental evidence at protein level
25.7043	25.6138	25.7278	25.5734	25.2	25.488	1:Experimental evidence at protein level
27.1519	27.2452	27.1511	26.0211	25.99	26.1883	1:Experimental evidence at protein level
28.9341	28.9558	28.7918	29.2072	29.1338	28.7975	1:Experimental evidence at protein level
29.7613	30.0114	29.8453	29.8175	29.4072	29.7954	1:Experimental evidence at protein level
26.9902	27.3997	27.546	27.3683	26.9792	27.3454	1:Experimental evidence at protein level
32.8004	32.3615	32.3524	32.4458	32.5963	32.6891	1:Experimental evidence at protein level
29.2116	29.179	29.094	29.0613	29.1854	29.0905	1:Experimental evidence at protein level
28.4591	28.3525	28.4033	28.4012	28.5658	28.3512	1:Experimental evidence at protein level
31.2408	31.5592	31.3159	31.7075	31.4636	31.2655	1:Experimental evidence at protein level
26.7967	26.3906	26.3785	26.9576	26.7055	26.8727	1:Experimental evidence at protein level
26.6902	26.8777	26.9588	26.9505	26.8433	26.8466	1:Experimental evidence at protein level
27.3542	27.421	27.4156	27.1181	27.0771	27.2999	1:Experimental evidence at protein level
31.3479	30.6886	30.6362	30.5907	31.4444	30.7912	1:Experimental evidence at protein level
26.3475	26.3902	26.3027	26.5485	26.6216	26.4988	1:Experimental evidence at protein level
27.6401	27.3206	27.2764	27.1242	27.2712	27.1159	1:Experimental evidence at protein level
31.753	31.4569	31.5449	31.3891	31.7063	31.4631	1:Experimental evidence at protein level
25.8041	25.6332	25.6073	25.7707	25.8653	25.6148	1:Experimental evidence at protein level
28.3864	28.6603	28.5587	28.3014	28.3199	28.2666	1:Experimental evidence at protein level
27.8099	28.2812	28.3269	28.2335	27.8558	28.2707	1:Experimental evidence at protein level
27.6652	27.9051	27.8446	27.5986	27.4343	27.7207	1:Experimental evidence at protein level
27.4549	27.1387	27.1749	27.1639	27.1303	27.0408	1:Experimental evidence at protein level
29.058	28.7129	28.611	28.559	28.9138	28.5693	1:Experimental evidence at protein level
29.1058	29.3488	29.3885	29.4085	29.228	29.3505	1:Experimental evidence at protein level

25.2527	24.9603	24.8558	25.3869	25.064	25.2079	1:Experimental evidence at protein level
27.0617	27.0879	27.0765	26.9789	26.9844	26.9447	1:Experimental evidence at protein level
25.6379	25.7608	25.7202	26.1317	25.9378	26.1933	1:Experimental evidence at protein level
28.7653	28.5415	28.6051	28.4867	28.5142	28.345	1:Experimental evidence at protein level
27.4455	27.0235	27.0984	28.0987	28.0834	28.0085	1:Experimental evidence at protein level
28.226	28.1822	28.2858	28.2751	28.1944	28.0621	1:Experimental evidence at protein level
27.7107	27.4569	27.5427	27.4478	27.5003	27.4277	1:Experimental evidence at protein level
26.0112	26.2926	26.3538	26.5814	26.1778	26.433	1:Experimental evidence at protein level
29.0397	29.076	29.2253	29.0758	28.8405	29.0295	1:Experimental evidence at protein level
29.0616	28.8885	28.8939	28.8294	28.9892	28.6806	1:Experimental evidence at protein level
28.5332	28.6485	28.701	28.6594	28.3515	28.7257	1:Experimental evidence at protein level
27.6771	27.4027	27.3207	27.5345	27.7516	27.5502	1:Experimental evidence at protein level
26.3024	26.0185	25.8847	26.3286	26.3802	26.4685	1:Experimental evidence at protein level
28.574	28.9159	28.8859	29.112	28.8536	29.1323	1:Experimental evidence at protein level
28.6458	28.8361	28.7712	28.8985	28.4196	28.8469	1:Experimental evidence at protein level
27.5849	27.8115	27.8081	28.325	28.1323	28.2193	1:Experimental evidence at protein level
27.3193	27.3437	29.5536	27.9085	27.8953	27.6089	1:Experimental evidence at protein level
29.5709	29.3271	29.3195	29.4302	29.5881	29.503	1:Experimental evidence at protein level
28.3058	28.3299	28.4117	28.5143	28.4272	28.4713	1:Experimental evidence at protein level
31.0742	31.0007	30.8237	31.011	30.81	31.0097	1:Experimental evidence at protein level
30.7497	30.7244	30.7476	30.6437	30.673	30.6355	1:Experimental evidence at protein level
29.6286	29.761	29.869	29.3242	29.2799	29.1301	1:Experimental evidence at protein level
30.6538	30.3124	30.2833	30.3101	30.6186	30.2323	1:Experimental evidence at protein level
29.5854	29.659	29.6163	29.5246	29.6805	29.5216	1:Experimental evidence at protein level
29.6659	29.5255	29.6112	29.5147	29.4553	29.5925	1:Experimental evidence at protein level
26.6231	26.6788	26.7191	26.6291	26.7574	26.8947	1:Experimental evidence at protein level
30.5679	30.4997	30.5653	30.6096	30.6081	30.5569	1:Experimental evidence at protein level
29.1689	29.228	29.1437	29.0453	28.9015	28.9222	1:Experimental evidence at protein level
31.1329	31.5349	31.5479	31.4433	31.0355	31.2443	1:Experimental evidence at protein level
31.384	31.4162	31.5543	31.3214	31.3407	31.3372	1:Experimental evidence at protein level
30.8645	31.2637	31.2554	31.0349	30.7853	31.0639	1:Experimental evidence at protein level
29.0711	28.9852	28.9622	29.0155	28.9397	29.0637	1:Experimental evidence at protein level
27	27.1537	27.2123	27.0276	26.9105	27.1306	1:Experimental evidence at protein level
29.0446	29.3066	29.2607	28.9919	28.7688	28.8565	1:Experimental evidence at protein level
25.2774	25.6559	25.7767	25.7431	25.4287	25.956	1:Experimental evidence at protein level
27.5036	27.3936	27.3871	26.9728	26.9038	27.2015	1:Experimental evidence at protein level
27.3254	27.6445	27.6963	27.8861	27.5562	27.8055	1:Experimental evidence at protein level
27.8632	27.8799	28.0135	27.9977	27.7485	27.9872	1:Experimental evidence at protein level
26.6092	26.6418	26.6633	26.2273	26.2977	26.0842	1:Experimental evidence at protein level
29.1332	29.058	29.1137	29.2279	28.9713	29.2947	1:Experimental evidence at protein level
26.9542	27.0249	27.1348	26.6264	26.3516	26.5789	1:Experimental evidence at protein level
25.9831	25.9139	25.9297	25.6799	25.5017	25.7965	1:Experimental evidence at protein level
26.3776	26.3048	26.4425	26.1115	26.1873	26.1573	1:Experimental evidence at protein level
27.7352	27.9957	28.117	28.2378	27.849	28.1303	1:Experimental evidence at protein level
25.1898	24.8762	24.6465	23.1075	24.1464	23.517	1:Experimental evidence at protein level
25.6692	25.197	24.9276	26.0789	25.81	26.1224	1:Experimental evidence at protein level
28.7052	29.2233	29.1747	29.3165	29.1184	29.3444	1:Experimental evidence at protein level
26.3113	27.2641	26.8653	26.5312	26.5506	26.7782	1:Experimental evidence at protein level
27.5435	27.5251	27.4754	27.4063	27.1526	27.2839	1:Experimental evidence at protein level
28.4255	28.6868	28.6399	28.6233	28.5517	28.5294	1:Experimental evidence at protein level
28.2872	27.5411	27.5294	27.5719	28.0032	27.7863	1:Experimental evidence at protein level
27.8496	27.8914	27.8517	27.7223	27.774	27.67	1:Experimental evidence at protein level
27.5645	27.4449	27.4403	27.6498	27.5663	27.4835	1:Experimental evidence at protein level
26.2884	26.4408	26.4708	26.628	26.4707	26.7171	1:Experimental evidence at protein level
27.6994	27.6875	27.752	27.5801	27.5405	27.4864	1:Experimental evidence at protein level
28.7458	28.9115	29.0353	28.9599	28.8113	28.7236	1:Experimental evidence at protein level
26.1355	26.3301	26.1115	25.8596	26.1141	26.224	1:Experimental evidence at protein level
28.2755	28.2993	28.3113	28.623	28.3758	28.4397	1:Experimental evidence at protein level
27.3315	27.3959	27.4061	27.2825	27.3695	27.3179	1:Experimental evidence at protein level
28.8741	29.0149	28.985	28.9738	28.8391	28.8379	1:Experimental evidence at protein level
27.348	27.3147	27.2724	27.2587	27.0991	27.3108	1:Experimental evidence at protein level
27.2954	26.8568	26.9503	26.8404	27.0409	27.0027	1:Experimental evidence at protein level
28.9389	29.0541	28.9824	28.8262	28.7641	28.6982	1:Experimental evidence at protein level
26.5416	26.9097	26.7491	26.6157	26.5359	26.4411	1:Experimental evidence at protein level
26.5396	26.5703	26.4782	26.699	26.6959	26.5947	1:Experimental evidence at protein level
30.6544	30.0647	30.3041	30.1873	30.6607	30.0761	1:Experimental evidence at protein level
29.593	29.7443	29.7941	29.9058	29.785	29.8702	1:Experimental evidence at protein level
28.4017	28.3454	28.3667	28.5786	28.4518	28.634	1:Experimental evidence at protein level
26.4969	26.4912	26.4074	26.4023	26.1773	26.3091	1:Experimental evidence at protein level
29.0431	29.1103	28.9447	29.1303	29.0511	29.1417	1:Experimental evidence at protein level
32.2262	32.4563	32.3104	32.4583	32.3798	32.2251	1:Experimental evidence at protein level
31.484	31.0724	31.0179	30.9834	31.4129	31.0444	1:Experimental evidence at protein level
34.3166	34.1132	34.056	33.9858	34.2386	33.7132	1:Experimental evidence at protein level
27.2626	27.3014	27.2497	27.2374	27.2653	27.2821	1:Experimental evidence at protein level
27.813	27.3003	27.208	27.7049	28.0077	27.5458	1:Experimental evidence at protein level
#NUM!	23.02	#NUM!	26.0532	25.3882	26.097	1:Experimental evidence at protein level
26.9185	27.618	27.7535	27.6685	27.462	27.6068	1:Experimental evidence at protein level
26.2159	26.1498	26.2253	26.0783	26.05	26.1015	1:Experimental evidence at protein level
27.171	26.891	26.9833	27.0221	27.1598	27.1388	1:Experimental evidence at protein level
29.2896	29.4154	29.2169	29.5727	29.5828	29.3787	1:Experimental evidence at protein level
28.4529	28.5528	28.5737	28.4321	28.5079	28.3403	1:Experimental evidence at protein level
27.286	27.7537	28.0119	28.0263	27.8908	28.0221	1:Experimental evidence at protein level
26.0266	25.9147	25.8199	26.0482	25.3487	25.6898	1:Experimental evidence at protein level
28.0683	28.0432	28.1161	27.8917	28.0314	28.0387	1:Experimental evidence at protein level
28.0384	27.8513	27.9106	28.0811	27.9464	28.0005	1:Experimental evidence at protein level
28.862	28.5781	28.5696	28.3909	28.353	28.4751	1:Experimental evidence at protein level
27.4708	27.3427	27.4324	27.5851	27.733	27.6633	1:Experimental evidence at protein level
27.028	26.6754	26.243	26.4201	26.519	26.2547	1:Experimental evidence at protein level
27.4297	27.4647	27.5285	27.3967	27.3553	27.348	1:Experimental evidence at protein level
29.0563	28.7977	28.8413	28.6586	28.6005	28.7493	1:Experimental evidence at protein level
31.1394	31.1997	31.1591	30.9523	31.0085	31.1663	1:Experimental evidence at protein level
27.4905	27.1607	27.1965	27.0994	27.2668	27.0604	1:Experimental evidence at protein level
29.0072	29.2545	29.1529	29.0159	28.7296	28.9115	1:Experimental evidence at protein level
26.6682	26.8995	26.9662	26.7775	26.6388	26.8383	1:Experimental evidence at protein level
28.4129	28.1762	28.2798	28.0317	28.3972	28.1744	1:Experimental evidence at protein level
28.5568	29.1241	29.0739	29.0609	28.6849	29.0957	1:Experimental evidence at protein level
26.5869	26.7395	26.9147	26.4813	26.1014	26.364	1:Experimental evidence at protein level
26.8143	26.874	26.949	27.0398	26.8848	27.1204	1:Experimental evidence at protein level
27.2274	27.4043	27.4869	27.3177	27.2284	27.1537	1:Experimental evidence at protein level
25.2381	25.6674	25.7829	25.6567	25.3055	25.6502	1:Experimental evidence at protein level
27.9696	27.8222	27.8255	27.8495	27.9614	27.892	1:Experimental evidence at protein level
27.7162	27.7184	27.6231	28.0088	27.9713	27.8779	1:Experimental evidence at protein level
27.8241	27.7791	27.792	27.8378	27.7658	27.8279	1:Experimental evidence at protein level
28.25	28.3818	28.4004	28.5072	28.4183	28.3354	1:Experimental evidence at protein level
30.7536	30.6723	30.5271	30.5024	30.6883	30.4162	1:Experimental evidence at protein level
28.4492	28.6884	28.7656	28.7712	28.7517	28.7521	1:Experimental evidence at protein level
27.5164	27.182	27.0574	27.0179	27.4356	27.2639	1:Experimental evidence at protein level
28.6156	28.392	28.3424	28.1362	28.2029	28.1959	1:Experimental evidence at protein level
27.3123	27.0383	27.1042	27.1112	27.2018	27.1987	1:Experimental evidence at protein level
28.6147	28.6606	28.7768	28.8912	28.5546	28.8294	1:Experimental evidence at protein level
27.9349	27.7808	27.8121	27.8901	27.7329	27.9676	1:Experimental evidence at protein level
27.352	27.1462	27.3534	27.1806	27.2571	27.1998	1:Experimental evidence at protein level
32.7358	32.4764	32.5177	32.4442	32.6233	32.4448	1:Experimental evidence at protein level
30.4839	30.5971	30.7186	30.4951	30.3206	30.2357	1:Experimental evidence at protein level
26.1778	26.5907	26.7431	27.1363	26.6842	27.0304	1:Experimental evidence at protein level
28.5553	28.4461	28.5024	28.2634	28.2318	28.5297	1:Experimental evidence at protein level
29.1349	29.2992	29.2359	29.181	29.0404	29.0933	1:Experimental evidence at protein level
28.2196	27.632	27.7045	28.2197	28.496	28.2985	1:Experimental evidence at protein level
29.6559	29.7031	29.7754	29.5446	29.4525	29.5864	1:Experimental evidence at protein level
30.4732	30.6823	30.6136	30.6903	30.6301	30.7067	1:Experimental evidence at protein level
29.6294	29.4606	29.6044	29.5453	29.6134	29.3973	1:Experimental evidence at protein level
28.8182	29.0545	29.183	28.8109	28.5575	28.9285	1:Experimental evidence at protein level
28.8731	28.8264	28.7895	28.9782	28.7553	28.9123	1:Experimental evidence at protein level
28.5381	28.2818	28.2893	28.4276	28.364	28.575	1:Experimental evidence at protein level
26.5864	26.7192	26.7091	26.6471	26.5845	26.6904	1:Experimental evidence at protein level
28.3805	28.2208	28.0927	28.1875	28.284	28.0862	1:Experimental evidence at protein level
29.7579	29.5923	29.7924	30.8847	30.8152	30.6692	1:Experimental evidence at protein level
31.3598	31.0723	31.0556	30.954	31.1538	31.0483	1:Experimental evidence at protein level
31.4426	31.1308	31.2099	31.0791	31.4718	31.0871	1:Experimental evidence at protein level

28.381	28.6368	28.6173	28.6907	28.1094	28.586	1:Experimental evidence at protein level
26.8939	26.9971	27.1479	27.6021	27.5551	27.7184	1:Experimental evidence at protein level
28.2578	28.1717	28.2726	28.1944	27.9925	28.0574	1:Experimental evidence at protein level
27.7682	28.1621	28.1468	28.0815	27.7675	28.0861	1:Experimental evidence at protein level
27.5775	27.5404	27.4441	27.3996	27.3257	27.2408	1:Experimental evidence at protein level
27.6306	27.7339	27.6742	27.5379	27.114	27.6487	1:Experimental evidence at protein level
27.8028	27.8532	27.9106	28.0501	28.1575	28.0049	1:Experimental evidence at protein level
27.9549	27.918	28.0216	28.2515	27.9518	28.2416	1:Experimental evidence at protein level
28.8251	28.9741	29.0288	28.656	28.7924	28.6441	1:Experimental evidence at protein level
27.1985	27.1242	27.2591	27.4203	27.2732	27.462	1:Experimental evidence at protein level
27.1538	27.1493	27.0858	27.0181	26.9815	26.9204	1:Experimental evidence at protein level
27.2787	26.8302	27.0143	27.3441	27.2805	27.4741	1:Experimental evidence at protein level
29.0262	29.1552	29.1006	29.1749	29.0397	29.0304	1:Experimental evidence at protein level
26.812	26.6574	26.6672	26.9976	27.05	26.9296	1:Experimental evidence at protein level
26.1654	26.1931	26.2205	27.0044	27.0405	26.8987	1:Experimental evidence at protein level
28.8561	28.8572	29.0278	28.8159	28.6099	28.8261	1:Experimental evidence at protein level
28.772	28.6056	28.5708	28.5551	28.6359	28.6455	1:Experimental evidence at protein level
32.2257	31.6024	31.8185	31.4847	31.8564	31.6302	1:Experimental evidence at protein level
28.8095	29.2422	29.2026	29.7021	29.2805	29.4991	1:Experimental evidence at protein level
27.8054	27.8813	28.0514	27.9467	27.8561	27.8143	1:Experimental evidence at protein level
26.1703	26.4974	26.5753	27.135	26.8542	27.2096	1:Experimental evidence at protein level
27.4314	27.7407	27.7366	27.8606	27.577	27.9004	1:Experimental evidence at protein level
30.1624	29.9232	29.8686	29.907	30.0138	29.784	1:Experimental evidence at protein level
27.67	27.9313	27.8293	27.8977	27.839	27.7525	1:Experimental evidence at protein level
28.7914	28.5357	28.6515	28.4903	28.4457	28.4645	1:Experimental evidence at protein level
26.1787	26.2263	26.0493	26.2242	25.8475	26.3808	1:Experimental evidence at protein level
29.6854	29.8028	29.8872	29.9993	29.6903	29.9484	1:Experimental evidence at protein level
31.1599	30.8393	30.8526	30.934	30.9907	30.8266	1:Experimental evidence at protein level
30.0962	30.0795	30.1687	30.1701	30.0559	30.0399	1:Experimental evidence at protein level
26.0022	26.1215	26.0894	25.9478	25.6977	25.6938	1:Experimental evidence at protein level
27.3413	27.3575	27.3888	27.4576	27.0397	27.5572	1:Experimental evidence at protein level
28.3631	28.2439	28.2698	28.3451	28.1069	28.3104	1:Experimental evidence at protein level
31.334	31.2049	31.0633	31.2135	31.0794	31.2349	1:Experimental evidence at protein level
30.2232	29.9016	29.9404	29.7309	30.186	29.6986	1:Experimental evidence at protein level
27.8438	27.686	27.9055	27.8751	27.7816	27.9472	1:Experimental evidence at protein level
27.064	26.9255	27.119	27.1041	27.2061	27.2312	1:Experimental evidence at protein level
27.5676	27.2378	27.1727	26.9027	26.8918	27.0376	1:Experimental evidence at protein level
27.5064	27.4403	27.4707	27.3252	27.2736	27.3314	1:Experimental evidence at protein level
28.8091	28.8333	28.8546	28.734	28.5013	28.7551	1:Experimental evidence at protein level
29.9487	29.9225	29.9368	30.2782	30.0726	30.3052	1:Experimental evidence at protein level
28.5263	28.7762	28.76	28.4875	28.5768	28.4836	1:Experimental evidence at protein level
24.9952	24.4774	24.4753	24.5539	24.8806	24.741	1:Experimental evidence at protein level
27.2011	26.8846	27.0527	26.6339	26.6556	26.6589	1:Experimental evidence at protein level
27.8164	27.6885	27.7433	27.4162	27.1179	27.5586	1:Experimental evidence at protein level
28.1614	28.1354	28.0421	28.0147	28.0657	27.9074	1:Experimental evidence at protein level
24.9454	#NUM!	#NUM!	25.4169	25.2021	25.39	1:Experimental evidence at protein level
26.9201	27.0913	26.8219	27.8787	27.7793	27.8729	1:Experimental evidence at protein level
28.1274	28.3864	28.4901	27.8478	27.6523	27.7386	1:Experimental evidence at protein level
28.8876	28.6747	28.8068	28.6295	28.6378	28.6689	1:Experimental evidence at protein level
27.9526	27.996	27.9111	27.8638	28.0526	27.9228	1:Experimental evidence at protein level
30.5295	30.0777	30.2166	30.1513	30.5488	30.0923	1:Experimental evidence at protein level
29.7477	29.756	29.611	29.3663	29.5522	29.2373	1:Experimental evidence at protein level
30.7588	31.0917	31.0792	31.6605	31.5074	31.5271	1:Experimental evidence at protein level
30.9352	31.5882	31.6669	31.5419	31.3943	31.4373	1:Experimental evidence at protein level
29.572	29.3455	29.4604	29.5118	29.6415	29.5056	1:Experimental evidence at protein level
28.7434	28.8245	28.778	28.8603	28.7365	28.8906	1:Experimental evidence at protein level
30.0712	30.0139	29.9369	30.0138	30.0843	29.9227	1:Experimental evidence at protein level
28.1829	28.3162	28.3809	28.1775	28.1206	28.0982	1:Experimental evidence at protein level
28.3108	28.5174	28.5776	28.6021	28.528	28.7134	1:Experimental evidence at protein level
32.0069	32.4491	32.1178	32.3874	32.1386	32.1993	1:Experimental evidence at protein level
28.8776	28.5989	28.6664	28.9446	28.8583	28.7366	1:Experimental evidence at protein level
30.9301	30.4567	30.6449	30.3155	30.7166	30.4638	1:Experimental evidence at protein level
31.8044	31.5643	31.6752	31.4128	31.4461	31.4385	1:Experimental evidence at protein level
31.3276	31.2365	31.3278	31.2847	31.1985	31.1955	1:Experimental evidence at protein level
28.2843	28.3837	28.3106	28.4237	28.5912	28.4296	1:Experimental evidence at protein level
30.1311	30.2669	30.1248	30.5246	30.4694	30.437	1:Experimental evidence at protein level
33.3228	33.1663	33.1918	33.2209	33.1891	33.0746	1:Experimental evidence at protein level
29.3697	29.8418	29.8496	29.7799	29.5816	29.832	1:Experimental evidence at protein level
27.2165	27.3333	27.5326	27.3619	27.3518	27.3199	1:Experimental evidence at protein level
27.0225	27.074	27.1814	27.3449	27.4463	27.4366	1:Experimental evidence at protein level
26.5493	26.6952	26.6687	26.5769	26.6143	26.6211	1:Experimental evidence at protein level
27.2534	26.9762	26.8016	26.7022	26.8654	26.8695	1:Experimental evidence at protein level
28.696	28.8949	28.8237	28.9976	28.9646	28.9087	1:Experimental evidence at protein level
27.6927	27.5927	27.464	26.8674	26.9745	27.1994	1:Experimental evidence at protein level
28.1687	28.4222	28.5706	28.7335	28.6262	28.6302	1:Experimental evidence at protein level
29.368	29.158	29.1445	29.4215	29.5783	29.3899	1:Experimental evidence at protein level
27.3527	27.1234	27.1615	27.0262	27.2999	27.0694	1:Experimental evidence at protein level
29.6256	29.6098	29.5955	29.4565	29.3656	29.5185	1:Experimental evidence at protein level
29.5585	29.9239	29.8204	30.2707	29.9235	30.2624	1:Experimental evidence at protein level
27.434	27.7428	27.8458	27.8773	27.6558	27.8814	1:Experimental evidence at protein level
26.7656	26.8085	26.9083	26.7398	26.7099	26.9789	1:Experimental evidence at protein level
28.3712	28.2941	28.3533	28.3971	28.2856	28.3534	1:Experimental evidence at protein level
30.3014	30.1679	30.2854	30.3691	30.8065	30.3835	1:Experimental evidence at protein level
31.1697	31.2274	31.1462	31.0733	31.3083	31.1065	1:Experimental evidence at protein level
28.4612	28.533	28.3399	28.3987	28.435	28.4285	1:Experimental evidence at protein level
28.9338	28.9191	28.8825	28.9347	29.0668	28.8292	1:Experimental evidence at protein level
31.2312	31.4582	31.4011	30.9644	30.8673	31.1113	1:Experimental evidence at protein level
30.924	30.9802	31.0235	30.9255	30.8556	30.897	1:Experimental evidence at protein level
29.9879	30.0724	30.2011	29.9932	29.8371	29.9014	1:Experimental evidence at protein level
31.3388	31.0236	31.16	30.9956	31.3156	30.9926	1:Experimental evidence at protein level
28.4322	28.258	28.2489	28.1303	27.932	28.1251	1:Experimental evidence at protein level
28.5872	28.5066	28.5065	28.2526	27.989	28.1156	1:Experimental evidence at protein level
28.6538	28.4965	28.5755	28.3892	28.1783	28.2338	1:Experimental evidence at protein level
30.6978	30.5335	30.5333	30.336	30.327	30.4196	1:Experimental evidence at protein level
29.8255	29.389	29.512	29.4699	29.7012	29.5322	1:Experimental evidence at protein level
28.6675	28.7818	28.8579	28.9408	28.7919	28.9442	1:Experimental evidence at protein level
27.0964	27.2682	27.28	27.1485	27.1583	27.4144	1:Experimental evidence at protein level
30.1823	30.2652	30.3858	30.1933	30.116	30.3594	1:Experimental evidence at protein level
30.4716	30.8893	30.8408	30.9221	30.7104	30.7715	1:Experimental evidence at protein level
28.2501	28.4754	28.4113	28.3234	28.1619	28.3713	1:Experimental evidence at protein level
28.7242	28.6824	28.5103	28.9207	28.7877	28.8554	1:Experimental evidence at protein level
27.1248	27.0366	27.4984	26.9981	26.9006	26.6898	1:Experimental evidence at protein level
29.5366	29.519	29.4467	29.4834	29.7742	29.6226	1:Experimental evidence at protein level
27.5019	27.9483	28.0256	27.9482	27.4703	27.9194	1:Experimental evidence at protein level
26.5828	26.9007	27.0688	26.6873	26.6808	26.9998	1:Experimental evidence at protein level
28.2995	27.9443	27.8414	27.4755	27.6708	27.4638	1:Experimental evidence at protein level
27.7123	27.3678	27.5901	27.7192	27.9921	27.4766	1:Experimental evidence at protein level
26.8373	26.7644	26.7109	26.714	26.6047	26.5655	1:Experimental evidence at protein level
28.5732	28.6368	28.53	28.478	28.5229	28.3561	1:Experimental evidence at protein level
28.5244	28.4403	28.4977	28.4143	28.6004	28.3515	1:Experimental evidence at protein level
29.3334	29.2633	29.3692	29.3375	29.2221	29.2845	1:Experimental evidence at protein level
28.7198	28.5555	28.5351	28.4422	28.5311	28.7151	1:Experimental evidence at protein level
27.2523	27.3792	27.2735	27.2512	27.1636	27.2649	1:Experimental evidence at protein level
25.7429	26.235	26.1997	26.2678	26.0346	26.3113	1:Experimental evidence at protein level
28.8907	28.6746	28.6922	28.3454	28.4631	28.7371	1:Experimental evidence at protein level
27.7033	27.3998	27.3797	26.9453	27.1056	27.1612	1:Experimental evidence at protein level
26.7544	27.0361	27.0758	28.6026	28.4097	28.7148	1:Experimental evidence at protein level
29.9777	30.0924	30.1904	30.1391	30.0465	30.2068	1:Experimental evidence at protein level
26.3049	26.5722	26.7085	26.3036	26.0605	26.0654	1:Experimental evidence at protein level
30.0808	30.1346	30.1285	30.1929	30.1436	30.2581	1:Experimental evidence at protein level
29.9626	29.8006	29.8075	29.602	29.7228	29.4176	1:Experimental evidence at protein level
28.5731	28.4677	28.5032	28.433	28.1867	28.4467	1:Experimental evidence at protein level
30.262	30.289	30.3363	30.2623	30.169	30.1789	1:Experimental evidence at protein level
26.8616	26.6214	26.7628	26.0643	#NUM!	26.1725	1:Experimental evidence at protein level
30.9504	31.001	31.005	30.8668	31.1465	30.8983	1:Experimental evidence at protein level
30.4278	29.8826	30.0172	29.7912	30.2337	29.8728	1:Experimental evidence at protein level
27.709	28.0658	28.1754	27.8835	27.5139	27.8427	1:Experimental evidence at protein level
27.3557	27.2935	27.3198	27.423	27.5357	27.5659	1:Experimental evidence at protein level
25.5974	25.9413	26.0034	26.0199	26.0139	26.0625	1:Experimental evidence at protein level

26.7061	26.7426	26.8746	26.5623	26.4452	26.602	1:Experimental evidence at protein level
28.2945	27.9536	28.0226	28.0045	28.1983	28.1374	1:Experimental evidence at protein level
26.6576	26.3385	26.2198	26.7239	26.947	26.7309	1:Experimental evidence at protein level
27.958	27.6956	27.8102	28.0924	28.0771	28.0405	1:Experimental evidence at protein level
26.0438	26.5274	26.3923	25.975	25.7346	26.3821	1:Experimental evidence at protein level
26.6215	26.6925	26.7257	26.4889	26.6038	26.6719	1:Experimental evidence at protein level
29.6743	29.4419	29.4611	29.5224	29.9068	29.5723	1:Experimental evidence at protein level
25.6011	25.8879	25.9101	25.3718	24.967	25.482	1:Experimental evidence at protein level
28.2811	28.3488	28.4664	28.4155	28.3423	28.3517	1:Experimental evidence at protein level
28.5226	28.4876	28.4933	28.426	28.3911	28.4289	1:Experimental evidence at protein level
27.6072	27.4618	27.3591	27.5936	27.3772	27.6125	1:Experimental evidence at protein level
28.7331	29.0951	29.2242	29.1362	29.3323	29.1934	1:Experimental evidence at protein level
29.6718	29.8879	29.9801	29.9499	29.882	29.7539	1:Experimental evidence at protein level
25.9038	26.1598	26.2471	24.8834	25.8401	25.3043	1:Experimental evidence at protein level
29.5609	29.6471	29.585	29.4703	29.5373	29.4931	1:Experimental evidence at protein level
29.899	30.3901	30.3042	30.1913	29.7759	30.1628	1:Experimental evidence at protein level
29.3903	29.1266	29.2318	29.2053	29.3537	29.1952	1:Experimental evidence at protein level
31.3798	31.1493	31.3146	31.4091	31.57	31.3611	1:Experimental evidence at protein level
32.5647	32.3078	32.2224	32.4367	32.8087	32.4146	1:Experimental evidence at protein level
30.5156	30.7381	30.8428	31.8647	31.6618	31.5439	1:Experimental evidence at protein level
32.4611	32.001	32.1417	32.2424	32.5104	32.0921	1:Experimental evidence at protein level
28.1463	28.0883	28.1476	27.9477	28.1123	28.0514	1:Experimental evidence at protein level
28.0647	28.1095	28.1253	28.1796	28.1295	28.0736	1:Experimental evidence at protein level
28.7661	28.6805	28.6172	28.5878	28.4162	28.5051	1:Experimental evidence at protein level
29.657	29.474	29.4242	29.6439	29.4998	29.72	1:Experimental evidence at protein level
30.0372	30.2367	30.1479	30.2016	30.025	30.3319	1:Experimental evidence at protein level
28.2653	28.2746	28.2829	27.9694	28.0096	28.0119	1:Experimental evidence at protein level
29.1105	29.2469	29.2986	29.4351	29.2536	29.3391	1:Experimental evidence at protein level
27.2664	26.8787	26.9233	27.2729	27.3409	27.1702	1:Experimental evidence at protein level
28.2808	28.3441	28.2218	28.5064	28.2148	28.4204	1:Experimental evidence at protein level
27.7544	27.8475	27.9692	27.639	27.5849	27.7656	1:Experimental evidence at protein level
27.6707	27.841	27.7409	27.8741	27.3203	28.0489	1:Experimental evidence at protein level
27.8413	27.3863	27.3731	27.6146	27.9568	27.5557	1:Experimental evidence at protein level
28.1102	28.2132	28.2766	28.3416	28.0787	28.2155	1:Experimental evidence at protein level
27.9471	27.9088	28.0891	28.312	28.1152	28.2379	1:Experimental evidence at protein level
25.1511	#NUM!	24.1561	25.6689	25.6516	25.6478	1:Experimental evidence at protein level
30.6172	30.7915	30.7213	30.9705	30.6853	30.826	1:Experimental evidence at protein level
29.3845	28.987	29.2642	29.0439	29.1701	29.0233	1:Experimental evidence at protein level
28.4519	28.724	27.1259	27.6131	27.5121	26.8848	1:Experimental evidence at protein level
27.218	26.1991	26.416	26.5669	26.3496	26.4535	1:Experimental evidence at protein level
28.5062	28.5609	28.5815	28.4322	28.2335	28.2461	1:Experimental evidence at protein level
31.7074	31.1869	31.2538	31.0623	31.5434	31.1651	1:Experimental evidence at protein level
26.6024	26.5219	26.5195	26.0114	26.1589	26.0791	1:Experimental evidence at protein level
30.2838	30.1759	30.1503	30.1886	30.2871	30.2996	1:Experimental evidence at protein level
30.1052	30.1914	30.2484	30.218	30.1962	30.1462	1:Experimental evidence at protein level
29.6177	29.6926	29.5995	29.5323	29.4515	29.5745	1:Experimental evidence at protein level
26.6847	27.183	27.1903	26.9825	26.738	27.3016	1:Experimental evidence at protein level
27.4501	27.3374	27.5672	28.2185	28.0948	28.0095	1:Experimental evidence at protein level
28.9987	29.1683	29.195	29.2018	29.031	29.0524	1:Experimental evidence at protein level
27.0695	27.371	27.4153	27.8633	27.7149	27.804	1:Experimental evidence at protein level
28.4043	28.2563	28.2157	28.7756	28.698	28.6669	1:Experimental evidence at protein level
27.2481	27.2233	27.0304	27.5907	27.6508	27.5429	1:Experimental evidence at protein level
28.5689	28.4855	28.5842	28.6766	28.6092	28.7354	1:Experimental evidence at protein level
27.6474	27.8683	28.0152	28.001	27.8064	27.98	1:Experimental evidence at protein level
32.2277	32.1682	32.0589	32.2696	32.4075	32.1419	1:Experimental evidence at protein level
30.6911	30.8206	30.7867	31.0436	31.105	30.9749	1:Experimental evidence at protein level
26.9651	27.638	25.807	27.0972	26.2762	26.7286	1:Experimental evidence at protein level
31.7691	31.7202	31.7228	31.6978	31.5624	31.5483	1:Experimental evidence at protein level
28.4753	28.4001	28.2734	28.3571	28.3779	28.3199	1:Experimental evidence at protein level
29.8499	30.0835	30.0889	29.901	29.94	29.8257	1:Experimental evidence at protein level
32.2932	31.8844	31.9021	31.6839	32.2385	31.8005	1:Experimental evidence at protein level
27.7346	27.8151	27.8753	28.1219	27.8471	27.9917	1:Experimental evidence at protein level
28.6225	28.4316	28.3227	28.5386	28.728	28.5121	1:Experimental evidence at protein level
29.3832	29.3883	29.3263	29.1256	29.0652	29.0377	1:Experimental evidence at protein level
27.8099	27.6706	27.6089	27.7679	27.7394	27.7745	1:Experimental evidence at protein level
27.9515	27.8772	27.8338	27.6067	27.5599	27.6556	1:Experimental evidence at protein level
26.8124	26.6057	26.5892	26.6183	26.8181	26.6623	1:Experimental evidence at protein level
25.2013	24.6979	24.7752	24.7499	25.0109	24.7566	1:Experimental evidence at protein level
28.52	28.439	28.6131	28.3945	28.2626	28.3386	1:Experimental evidence at protein level
29.9004	30.126	30.1308	30.2404	30.1496	30.1342	1:Experimental evidence at protein level
27.0257	26.6284	26.5679	26.3806	26.9197	26.5418	1:Experimental evidence at protein level
27.8467	28.0648	28.1084	28.2248	28.0999	28.2033	1:Experimental evidence at protein level
28.8379	28.7934	28.849	29.3524	29.5742	29.4082	1:Experimental evidence at protein level
28.8219	28.8627	28.8374	28.7693	28.7827	28.8288	1:Experimental evidence at protein level
30.2829	30.1399	30.052	30.0042	30.1802	30.0846	1:Experimental evidence at protein level
29.1061	28.8871	28.9401	28.9009	28.905	28.8602	1:Experimental evidence at protein level
25.4924	25.4447	25.3124	25.306	25.4233	25.1807	1:Experimental evidence at protein level
31.112	31.1491	31.1561	31.0763	31.0667	31.1218	1:Experimental evidence at protein level
33.5573	33.4768	33.4434	33.7696	33.8651	33.676	1:Experimental evidence at protein level
27.6107	27.4243	27.4328	27.5385	27.6665	27.5961	1:Experimental evidence at protein level
27.6681	27.4929	27.4968	27.1619	27.3837	27.1742	1:Experimental evidence at protein level
28.5876	28.7995	28.7471	28.6571	28.3648	28.8993	1:Experimental evidence at protein level
27.6802	27.8433	27.8394	28.3668	28.2406	28.3551	1:Experimental evidence at protein level
30.647	30.3395	30.3722	30.3116	30.3528	30.3975	1:Experimental evidence at protein level
28.1904	28.0156	28.0579	28.0655	28.0321	28.1677	1:Experimental evidence at protein level
30.6371	30.9102	30.7977	30.6759	30.5825	30.6426	1:Experimental evidence at protein level
28.6264	28.6213	28.5882	28.6279	28.5351	28.7249	1:Experimental evidence at protein level
29.6018	29.202	29.3349	29.2198	29.4109	29.11	1:Experimental evidence at protein level
29.4792	29.4943	29.4416	29.4837	29.4841	29.7116	1:Experimental evidence at protein level
26.9964	26.9769	27.0151	26.6669	26.6617	26.4782	1:Experimental evidence at protein level
26.7861	27.0559	27.2086	26.9671	26.9823	26.9949	1:Experimental evidence at protein level
34.1018	34.1722	34.1022	34.0978	33.9762	34.0112	1:Experimental evidence at protein level
28.7283	28.5526	28.6549	28.874	28.8487	28.6383	1:Experimental evidence at protein level
29.2035	29.1034	29.1218	28.8975	28.785	28.7863	1:Experimental evidence at protein level
27.3001	27.3647	27.3883	27.5475	27.4397	27.4979	1:Experimental evidence at protein level
29.4852	29.3391	29.4859	29.1781	29.3389	29.2925	1:Experimental evidence at protein level
30.068	30.4131	30.3528	30.411	30.2777	30.3895	1:Experimental evidence at protein level
29.4085	29.7974	29.8575	29.7878	29.6803	29.926	1:Experimental evidence at protein level
28.3582	28.8397	28.9309	29.0142	28.7646	28.9437	1:Experimental evidence at protein level
28.5011	28.6985	28.7572	28.7	28.4305	28.69	1:Experimental evidence at protein level
30.8354	30.3489	30.26	30.1955	30.633	30.3444	1:Experimental evidence at protein level
28.5765	28.2923	28.105	28.629	28.8552	28.4589	1:Experimental evidence at protein level
27.9096	27.0622	27.2594	27.6455	28.1826	27.557	1:Experimental evidence at protein level
27.087	26.993	26.9278	26.8275	26.7902	26.9445	1:Experimental evidence at protein level
28.0032	28.0319	28.0376	27.8045	27.7311	27.777	1:Experimental evidence at protein level
26.5745	26.4374	26.2903	26.3753	26.2229	26.445	1:Experimental evidence at protein level
32.6511	32.5797	32.6749	32.6836	32.5937	32.6909	1:Experimental evidence at protein level
31.3804	31.6281	31.6185	31.2982	31.1478	31.658	1:Experimental evidence at protein level
27.9441	28.0449	27.8636	28.3613	28.0601	28.6651	1:Experimental evidence at protein level
27.9644	28.1779	28.1308	28.1446	27.8896	28.0741	1:Experimental evidence at protein level
30.0298	30.0624	30.0117	29.9239	29.8894	30.0237	1:Experimental evidence at protein level
27.9656	28.173	28.3677	28.5343	28.3171	28.5022	1:Experimental evidence at protein level
27.5821	27.7405	26.7449	26.8986	26.7081	26.7545	1:Experimental evidence at protein level
30.7412	30.4158	30.5691	30.2881	30.3123	30.3453	1:Experimental evidence at protein level
31.3791	31.2237	31.2803	31.2177	31.1798	31.1913	1:Experimental evidence at protein level
30.9943	31.1147	31.1912	31.0647	30.9361	31.064	1:Experimental evidence at protein level
32.4714	32.4765	32.4219	32.5836	32.4715	32.4855	1:Experimental evidence at protein level
32.815	32.8813	33.095	33.3451	32.9392	33.2287	1:Experimental evidence at protein level
27.0326	26.8163	26.7947	26.9999	26.9007	26.9568	1:Experimental evidence at protein level
27.4099	27.6012	27.7263	27.6056	27.3302	27.614	1:Experimental evidence at protein level
28.6268	28.3167	28.3035	28.2414	28.3927	28.1585	1:Experimental evidence at protein level
28.2102	28.4235	28.5279	28.4494	28.1404	28.3969	1:Experimental evidence at protein level
27.9556	27.9402	28.0127	28.0135	27.9737	27.9896	1:Experimental evidence at protein level
27.484	27.5535	27.5741	27.6711	27.4224	27.7767	1:Experimental evidence at protein level
26.9588	26.8407	26.7972	26.8627	27.069	26.7778	1:Experimental evidence at protein level
28.1931	27.718	27.862	27.8829	28.0171	27.831	1:Experimental evidence at protein level
31.4623	31.5162	31.4453	31.5149	31.457	31.4548	1:Experimental evidence at protein level
29.0777	29.3645	29.4763	29.1128	28.777	28.9861	1:Experimental evidence at protein level
28.2634	28.3495	28.363	28.0292	27.8556	28.0068	1:Experimental evidence at protein level

26.9085	26.9127	26.9075	27.0164	26.9645	27.0809	1:Experimental evidence at protein level
27.9074	27.8424	27.9489	28.8353	28.699	28.8595	1:Experimental evidence at protein level
30.2622	30.4602	30.5828	30.3601	30.3889	30.5131	1:Experimental evidence at protein level
28.0189	28.0293	28.1332	28.2623	28.1671	28.2976	1:Experimental evidence at protein level
31.9901	31.8393	31.8437	31.7998	31.9784	31.7633	1:Experimental evidence at protein level
29.9658	30.3415	30.441	30.3655	30.1163	30.3692	1:Experimental evidence at protein level
29.592	29.5899	29.6204	29.8142	29.6709	29.7449	1:Experimental evidence at protein level
29.4573	29.4675	29.5421	29.3434	29.3377	29.5839	1:Experimental evidence at protein level
27.9817	28.0149	28.0208	27.7978	27.6004	27.9912	1:Experimental evidence at protein level
25.4467	25.5742	25.4297	26.7352	26.3611	26.721	1:Experimental evidence at protein level
27.9836	27.9579	28.0048	28.1062	28.1143	27.9887	1:Experimental evidence at protein level
26.7285	26.5657	26.6839	26.4328	26.6289	26.4079	1:Experimental evidence at protein level
28.5717	28.7525	28.8204	28.8159	28.7058	28.6798	1:Experimental evidence at protein level
24.722	24.3397	24.5385	24.7097	25.8304	24.9587	1:Experimental evidence at protein level
30.2235	29.8825	29.9742	29.8668	30.1444	29.9794	1:Experimental evidence at protein level
26.141	26.2962	26.2435	26.3235	25.9687	26.3164	1:Experimental evidence at protein level
28.7835	28.6502	28.5682	28.7026	28.5543	28.4807	1:Experimental evidence at protein level
28.6862	28.9898	29.0238	28.9389	28.5952	28.9209	1:Experimental evidence at protein level
26.3411	26.163	26.1096	26.1804	25.9755	26.1811	1:Experimental evidence at protein level
28.956	28.7284	28.7481	28.7701	28.7746	28.7289	1:Experimental evidence at protein level
27.0036	26.8639	27.1099	27.4471	27.0732	27.4566	1:Experimental evidence at protein level
29.724	29.2263	29.2444	29.1728	29.3186	29.2754	1:Experimental evidence at protein level
28.9354	29.0654	29.0864	28.8613	28.6953	28.8466	1:Experimental evidence at protein level
27.1938	26.9705	26.9318	26.8397	27.0277	26.9683	1:Experimental evidence at protein level
27.5092	27.6351	27.6133	28.6135	28.6555	28.6053	1:Experimental evidence at protein level
29.0769	29.3172	29.3084	29.3639	29.0079	29.4039	1:Experimental evidence at protein level
30.3072	30.1005	30.1271	29.6345	29.5465	29.5762	1:Experimental evidence at protein level
29.4795	29.6915	29.7178	29.7601	29.5184	29.8684	1:Experimental evidence at protein level
29.2367	29.231	29.3123	29.166	29.1006	29.248	1:Experimental evidence at protein level
27.43	27.4625	27.6073	27.279	27.2319	27.4108	1:Experimental evidence at protein level
30.7388	30.7926	30.9009	30.7787	30.6778	30.7363	1:Experimental evidence at protein level
28.2505	28.1382	28.1748	28.1587	28.0021	28.1446	1:Experimental evidence at protein level
29.1291	29.3645	29.3975	29.2609	29.1945	29.3189	1:Experimental evidence at protein level
26.9607	27.1591	27.2848	26.9695	26.7849	27.0466	1:Experimental evidence at protein level
26.6606	26.9101	26.8292	26.7064	26.3816	26.5968	1:Experimental evidence at protein level
28.9401	29.2198	28.7984	29.1421	29.1285	29.0854	1:Experimental evidence at protein level
30.3056	30.256	30.3791	30.027	29.8643	29.923	1:Experimental evidence at protein level
28.7146	28.7563	28.7702	28.705	28.5362	28.6336	1:Experimental evidence at protein level
29.5102	29.7544	29.8548	29.7217	29.4433	29.6653	1:Experimental evidence at protein level
26.1092	26.3236	26.1981	26.5661	26.448	26.5542	1:Experimental evidence at protein level
28.6217	28.5384	28.4953	28.5279	28.4469	28.523	1:Experimental evidence at protein level
28.6861	28.7698	28.7542	29.0427	28.8244	29.018	1:Experimental evidence at protein level
30.9636	31.1966	31.0919	31.0918	31.5066	31.2412	1:Experimental evidence at protein level
28.9039	29.149	29.3016	29.2037	29.1158	29.169	1:Experimental evidence at protein level
29.4227	29.5761	29.5657	29.7119	29.7453	29.7486	1:Experimental evidence at protein level
28.488	28.5176	28.4376	29.027	28.9543	29.0255	1:Experimental evidence at protein level
27.7274	27.6566	27.7767	27.1228	27.0479	27.1408	1:Experimental evidence at protein level
28.2648	28.3034	28.2782	28.2193	28.0421	28.3672	1:Experimental evidence at protein level
#NUM!	25.4129	25.539	25.2091	23.9525	25.4814	1:Experimental evidence at protein level
27.5255	27.498	27.3958	27.4581	27.4023	27.4919	1:Experimental evidence at protein level
28.3339	28.3321	28.3736	28.7438	28.8153	28.7242	1:Experimental evidence at protein level
28.5656	28.6326	28.6803	28.5195	28.3995	28.5403	1:Experimental evidence at protein level
27.7714	27.8563	27.8077	27.5411	27.3788	27.5442	1:Experimental evidence at protein level
30.3051	30.2758	30.3041	29.9984	30.014	29.8768	1:Experimental evidence at protein level
27.2129	27.5974	27.5238	27.1774	27.1352	26.9327	1:Experimental evidence at protein level
30.116	29.9534	29.9658	30.0211	30.2825	29.9089	1:Experimental evidence at protein level
31.3688	31.226	31.2465	31.2027	31.3209	31.2342	1:Experimental evidence at protein level
28.3023	28.1234	28.184	27.4982	27.7118	27.6175	1:Experimental evidence at protein level
31.2586	31.1965	31.063	31.1102	31.0523	31.1536	1:Experimental evidence at protein level
29.0061	29.1031	29.1929	28.9306	28.8282	28.9488	1:Experimental evidence at protein level
28.4251	28.3522	28.328	28.2543	28.2993	28.2161	1:Experimental evidence at protein level
31.9788	31.8988	32.0112	31.7736	31.7914	31.7584	1:Experimental evidence at protein level
31.4668	31.6368	31.5697	31.8744	31.672	31.9465	1:Experimental evidence at protein level
28.2013	28.1104	28.0831	27.9044	27.9036	27.8094	1:Experimental evidence at protein level
30.0209	29.9728	29.9818	29.6846	29.7676	29.6066	1:Experimental evidence at protein level
26.9067	27.1849	27.3746	27.4283	27.3066	27.2921	1:Experimental evidence at protein level
26.0444	26.2722	26.2342	26.4447	26.1722	26.3594	1:Experimental evidence at protein level
28.1501	28.227	28.3343	28.1	27.9496	28.2679	1:Experimental evidence at protein level
29.2209	29.2855	29.3722	29.3512	29.2132	29.264	1:Experimental evidence at protein level
28.3372	28.1648	28.189	27.8963	27.994	27.977	1:Experimental evidence at protein level
29.4042	29.4767	29.6007	29.8541	29.7227	29.958	1:Experimental evidence at protein level
28.8363	29.091	29.0838	29.0656	28.8761	29.0182	1:Experimental evidence at protein level
30.8741	30.6228	30.5795	30.4673	30.7741	30.5324	1:Experimental evidence at protein level
27.5576	27.5632	27.6095	27.8546	27.5899	27.7948	1:Experimental evidence at protein level
30.3356	30.4796	30.5289	30.3998	30.1242	30.4028	1:Experimental evidence at protein level
29.8842	29.95	29.9629	29.946	29.7749	30.0522	1:Experimental evidence at protein level
32.1706	32.4526	32.4675	32.7722	32.558	32.7038	1:Experimental evidence at protein level
31.7946	31.6219	31.7245	31.8709	31.9764	31.7931	1:Experimental evidence at protein level
28.2605	28.4946	28.3202	28.0972	28.1249	28.0943	1:Experimental evidence at protein level
27.3782	27.5121	27.3769	27.8563	27.6814	27.8503	1:Experimental evidence at protein level
28.3478	28.117	28.2402	28.2912	28.3271	28.1363	1:Experimental evidence at protein level
28.6775	29.0201	27.0309	28.0995	27.6233	27.6245	1:Experimental evidence at protein level
33.8372	33.6665	33.7211	33.8899	33.9186	33.8562	1:Experimental evidence at protein level
30.7869	30.7345	30.8199	30.8005	30.752	30.7317	1:Experimental evidence at protein level
29.6391	29.766	29.9356	29.7742	29.6338	29.7281	1:Experimental evidence at protein level
31.5814	31.4933	31.4369	31.4066	31.6694	31.3233	1:Experimental evidence at protein level
26.1032	26.2973	25.9962	25.6332	25.6443	25.5047	1:Experimental evidence at protein level
31.1016	31.2875	31.3303	30.6116	30.6453	30.5991	1:Experimental evidence at protein level
27.4287	27.7533	27.692	27.1257	26.9366	27.1263	1:Experimental evidence at protein level
30.554	30.3086	30.2723	30.1761	30.3251	30.1688	1:Experimental evidence at protein level
29.7435	29.7811	29.8087	30.0864	29.9454	30.051	1:Experimental evidence at protein level
28.1392	28.5273	28.5673	28.346	28.0144	28.3032	1:Experimental evidence at protein level
27.2323	27.2911	27.2181	27.582	27.3934	27.5403	1:Experimental evidence at protein level
27.9416	27.7426	27.7926	27.7589	27.7896	27.9692	1:Experimental evidence at protein level
28.6212	28.5548	28.5723	28.5811	28.6648	28.5412	1:Experimental evidence at protein level
29.1019	29.2809	29.1558	29.127	29.1345	29.2049	1:Experimental evidence at protein level
28.68	28.7621	28.8228	28.8272	28.7515	28.7969	1:Experimental evidence at protein level
28.115	28.0034	28.0549	28.1933	28.1138	28.1243	1:Experimental evidence at protein level
28.4666	28.3258	28.3654	28.3552	28.2797	28.4721	1:Experimental evidence at protein level
28.1061	27.9416	28.0641	27.7196	27.686	27.7534	1:Experimental evidence at protein level
27.1547	27.085	27.1449	26.8097	26.6369	26.9832	1:Experimental evidence at protein level
34.2793	34.2325	34.26	34.4674	34.4662	34.3177	1:Experimental evidence at protein level
30.5328	30.2468	30.3234	30.3461	30.7656	30.4268	1:Experimental evidence at protein level
26.517	26.3635	26.0052	26.0589	26.5549	26.2024	1:Experimental evidence at protein level
30.514	30.5247	30.4976	30.1399	30.4273	30.0667	1:Experimental evidence at protein level
29.9976	30.1238	30.1962	30.3217	30.1746	30.1955	1:Experimental evidence at protein level
27.6365	27.7133	27.7358	27.216	27.0332	27.2715	1:Experimental evidence at protein level
29.1915	29.2492	29.3386	29.4231	29.4468	29.3435	1:Experimental evidence at protein level
30.2328	30.2329	30.2358	30.2359	30.1463	30.1776	1:Experimental evidence at protein level
27.3928	27.3822	27.3475	27.0342	27.1774	27.0872	1:Experimental evidence at protein level
29.2026	29.2582	29.2324	29.4674	29.2963	29.503	1:Experimental evidence at protein level
30.5409	30.2272	30.2885	30.874	30.8484	30.892	1:Experimental evidence at protein level
28.1981	28.3343	28.335	27.7318	27.5433	27.8798	1:Experimental evidence at protein level
29.328	29.4428	29.5096	29.3892	29.4375	29.2742	1:Experimental evidence at protein level
30.4332	29.9077	29.9289	29.7135	30.3169	29.6747	1:Experimental evidence at protein level
30.5914	30.6286	30.5179	30.7498	30.8956	30.4638	1:Experimental evidence at protein level
29.5294	29.6243	29.6027	29.6151	29.3242	29.6202	1:Experimental evidence at protein level
29.0387	29.0625	29.1424	28.3823	28.2864	28.5023	1:Experimental evidence at protein level
29.6951	29.8898	30.0132	29.9808	29.6651	29.9219	1:Experimental evidence at protein level
28.9579	29.0345	29.0473	28.7669	28.6414	28.6463	1:Experimental evidence at protein level
28.5634	28.5873	28.6374	28.5296	28.4017	28.4821	1:Experimental evidence at protein level
33.7541	33.5281	33.4619	33.6587	33.8546	33.5822	1:Experimental evidence at protein level
29.2336	28.9295	28.9331	28.8604	28.9801	28.7492	1:Experimental evidence at protein level
29.3326	29.251	29.2552	29.4478	29.4979	29.4475	1:Experimental evidence at protein level
30.4839	30.5906	30.4892	30.4615	30.1906	30.4432	1:Experimental evidence at protein level
28.437	29.033	29.0322	28.7896	28.282	29.3046	1:Experimental evidence at protein level
28.2638	28.3558	28.4725	28.682	28.4394	28.6108	1:Experimental evidence at protein level
28.339	28.1456	28.2305	28.0064	27.9916	28.1502	1:Experimental evidence at protein level
28.1332	28.5109	28.5132	28.4911	28.2535	28.5752	1:Experimental evidence at protein level

33.0974	33.1937	33.0932	33.2199	33.0722	33.034	1:Experimental evidence at protein level
27.6552	27.8352	27.6985	28.0196	27.865	28.0522	1:Experimental evidence at protein level
28.4084	28.6248	28.7439	28.8377	28.7112	28.7715	1:Experimental evidence at protein level
30.2075	30.4243	30.4845	30.3397	30.0808	30.2667	1:Experimental evidence at protein level
29.8121	29.6374	29.713	29.5167	29.589	29.5633	1:Experimental evidence at protein level
28.314	28.4956	28.4921	28.125	27.9913	28.1044	1:Experimental evidence at protein level
29.8708	29.8469	29.8321	29.8627	29.755	29.9348	1:Experimental evidence at protein level
35.3425	35.4767	35.3626	35.6583	35.4999	35.553	1:Experimental evidence at protein level
29.1935	29.4801	29.3469	29.4666	29.143	29.4578	1:Experimental evidence at protein level
28.8639	28.9559	28.9986	28.9581	28.6772	28.9204	1:Experimental evidence at protein level
27.2006	27.3002	27.2363	26.9908	26.9106	27.0728	1:Experimental evidence at protein level
25.1926	25.0012	25.0148	29.7718	29.6656	29.564	1:Experimental evidence at protein level
27.551	27.4822	27.578	26.9086	26.9353	27.2145	1:Experimental evidence at protein level
27.3855	27.2618	27.4008	27.1954	27.1191	27.2636	1:Experimental evidence at protein level
29.3218	28.9825	28.9741	29.1053	29.1582	29.2248	1:Experimental evidence at protein level
29.1402	29.3348	29.3664	29.2795	29.0457	29.4467	1:Experimental evidence at protein level
28.1083	27.9086	27.9983	27.4149	27.3897	27.5926	1:Experimental evidence at protein level
28.4956	28.4015	28.6263	28.9979	28.6999	28.9816	1:Experimental evidence at protein level
29.706	29.6696	29.6968	30.0012	29.9128	29.879	1:Experimental evidence at protein level
31.8568	31.9535	31.9782	32.0658	31.9133	32.0273	1:Experimental evidence at protein level
34.4499	34.7167	34.6494	34.6693	34.5422	34.469	1:Experimental evidence at protein level
29.407	29.4363	29.4305	29.8469	29.7559	29.7008	1:Experimental evidence at protein level
28.7009	28.9684	28.9354	28.8736	28.5899	29.0693	1:Experimental evidence at protein level
28.551	28.4488	28.3181	28.3583	28.2995	28.5217	1:Experimental evidence at protein level
27.0103	27.4127	27.365	27.2004	26.9543	27.29	1:Experimental evidence at protein level
30.6184	30.459	30.4537	30.3646	30.3749	30.3743	1:Experimental evidence at protein level
29.6371	29.5284	29.495	29.8286	29.8009	29.7612	1:Experimental evidence at protein level
28.8184	28.7578	28.8716	28.9097	28.9641	28.8183	1:Experimental evidence at protein level
31.313	31.3961	31.33	31.2138	31.1133	31.3282	1:Experimental evidence at protein level
28.5436	28.6271	28.4449	28.5773	28.7279	28.8351	1:Experimental evidence at protein level
28.7164	28.7203	28.7966	28.9116	28.9376	28.9317	1:Experimental evidence at protein level
34.2382	34.2979	34.2676	34.4075	34.3726	34.2816	1:Experimental evidence at protein level
28.0175	28.4804	28.4713	28.2238	27.7252	28.3779	1:Experimental evidence at protein level
29.0043	29.1114	29.1254	28.7671	28.7102	28.7309	1:Experimental evidence at protein level
29.5762	29.4718	29.5262	29.2986	29.318	29.2894	1:Experimental evidence at protein level
26.744	26.6295	26.6867	26.8048	26.8745	26.7313	1:Experimental evidence at protein level
29.3892	29.4994	29.555	29.441	29.3718	29.3853	1:Experimental evidence at protein level
30.3889	30.1288	30.1111	29.8248	30.0062	29.8177	1:Experimental evidence at protein level
29.8482	29.5966	29.5868	29.4714	29.6707	29.4307	1:Experimental evidence at protein level
27.6312	27.794	27.8132	27.9307	27.6818	27.8585	1:Experimental evidence at protein level
29.2882	29.7264	29.6162	29.8476	29.5419	29.8126	1:Experimental evidence at protein level
29.9955	30.4295	30.3936	30.2643	30.0591	30.2873	1:Experimental evidence at protein level
32.3658	32.649	32.6735	32.7421	32.4449	32.6079	1:Experimental evidence at protein level
31.4666	31.1913	31.2041	31.1381	31.3637	31.0639	1:Experimental evidence at protein level
29.6616	29.6339	29.6606	29.8218	29.6739	29.8242	1:Experimental evidence at protein level
29.0146	29.4357	29.4184	29.3201	28.7431	29.4191	1:Experimental evidence at protein level
29.5134	29.3695	29.3696	29.3482	29.3466	29.3717	1:Experimental evidence at protein level
28.6186	28.6243	28.6077	28.636	28.4796	28.5409	1:Experimental evidence at protein level
28.8739	28.5964	28.602	28.6208	28.5609	28.678	1:Experimental evidence at protein level
30.6817	31.306	30.8412	31.3613	31.5697	31.1928	1:Experimental evidence at protein level
31.1824	31.1096	31.0648	31.4764	31.4824	31.3826	1:Experimental evidence at protein level
27.8402	27.7857	27.837	27.9365	28.036	27.9325	1:Experimental evidence at protein level
32.3334	32.5644	32.3801	32.5411	32.5095	32.3944	1:Experimental evidence at protein level
28.6023	28.5556	28.6748	28.5483	28.5474	28.5118	1:Experimental evidence at protein level
28.5394	28.6278	28.686	28.545	28.3682	28.531	1:Experimental evidence at protein level
29.7221	29.8589	30.0215	29.7616	29.4874	29.8502	1:Experimental evidence at protein level
28.433	28.4796	28.504	28.3781	28.4188	28.5013	1:Experimental evidence at protein level
29.8632	30.18	30.196	30.4698	30.3327	30.4342	1:Experimental evidence at protein level
29.4072	29.3983	29.3045	29.316	29.3682	29.3215	1:Experimental evidence at protein level
28.0316	28.0785	28.1213	27.9118	27.6279	28.0628	1:Experimental evidence at protein level
31.0262	31.0054	30.9378	31.085	30.979	31.0021	1:Experimental evidence at protein level
29.7676	30.0091	30.0049	29.8249	29.544	30.289	1:Experimental evidence at protein level
32.0378	31.7778	31.7598	31.5531	31.886	31.545	1:Experimental evidence at protein level
31.399	31.3751	31.4364	31.4547	31.3353	31.4519	1:Experimental evidence at protein level
30.5428	30.6243	30.6568	30.5123	30.3274	30.7181	1:Experimental evidence at protein level
26.0723	25.8224	26.1121	26.5551	26.5266	26.5866	1:Experimental evidence at protein level
30.0372	30.0243	30.0123	30.1957	30.2823	30.1624	1:Experimental evidence at protein level
29.8608	29.7998	29.781	29.6807	29.6989	29.7491	1:Experimental evidence at protein level
27.9315	28.1937	28.174	28.6492	28.3676	28.81	1:Experimental evidence at protein level
33.1995	33.0988	33.197	33.5874	33.5212	33.6328	1:Experimental evidence at protein level
32.6272	32.3042	32.2611	32.1983	32.463	32.1711	1:Experimental evidence at protein level
32.0969	32.2614	32.2044	32.0923	31.9975	32.1078	1:Experimental evidence at protein level
26.1761	26.2554	26.2332	24.9359	24.0896	25.2001	1:Experimental evidence at protein level
30.8408	30.8388	30.8395	31.5163	31.3221	31.5052	1:Experimental evidence at protein level
29.8207	29.7967	29.8497	29.9077	29.8464	29.915	1:Experimental evidence at protein level
29.6249	29.8232	29.7905	29.8065	29.705	29.7748	1:Experimental evidence at protein level
30.0227	30.0326	30.0332	29.9995	30.0869	30.0389	1:Experimental evidence at protein level
31.4937	31.1915	31.1772	31.0363	31.3436	31.0885	1:Experimental evidence at protein level
28.9737	29.3074	29.3525	28.9845	28.6588	28.9474	1:Experimental evidence at protein level
27.3851	27.4142	27.5271	27.6359	27.3308	27.6817	1:Experimental evidence at protein level
34.261	33.9325	33.8437	33.9282	34.3533	33.7594	1:Experimental evidence at protein level
29.7227	29.7604	30.1139	30.1108	30.5245	30.2178	1:Experimental evidence at protein level
27.5365	27.8846	27.9212	28.1969	27.9714	28.4304	1:Experimental evidence at protein level
29.9624	29.852	29.8713	30.0092	29.9606	29.9991	1:Experimental evidence at protein level
32.5677	32.6475	32.6438	32.7408	32.4152	32.6973	1:Experimental evidence at protein level
26.6668	27.1523	27.1635	27.682	27.4019	27.6951	1:Experimental evidence at protein level
25.1427	24.6895	25.0743	25.6613	25.8793	25.6021	1:Experimental evidence at protein level
28.5813	28.5501	28.5522	28.7944	28.8327	28.7868	1:Experimental evidence at protein level
28.8339	28.7621	28.8358	28.9404	28.9797	28.8139	1:Experimental evidence at protein level
27.4457	27.475	27.6045	27.2928	27.1932	27.4671	1:Experimental evidence at protein level
27.7944	27.7271	27.769	27.9993	27.9487	28.0303	1:Experimental evidence at protein level
30.0473	30.0963	30.109	29.9028	29.7837	29.9556	1:Experimental evidence at protein level
31.2833	31.2899	31.3567	31.7603	31.6096	31.7102	1:Experimental evidence at protein level
28.6482	28.8725	28.8487	29.0488	28.7211	29.1135	1:Experimental evidence at protein level
31.0893	31.561	31.4663	31.7051	31.502	31.7448	1:Experimental evidence at protein level
29.9146	30.0294	29.9275	29.9403	29.8341	29.8914	1:Experimental evidence at protein level
27.5317	27.6405	27.6315	27.1983	27.3122	27.1239	1:Experimental evidence at protein level
31.6983	31.6353	31.711	31.7647	31.7231	31.7012	1:Experimental evidence at protein level
34.3093	33.9588	33.8558	34.2383	34.4125	34.018	1:Experimental evidence at protein level
31.2335	30.9312	30.8697	31.0181	31.2139	31.0194	1:Experimental evidence at protein level
29.0386	28.7357	28.7853	28.7767	28.9568	28.8169	1:Experimental evidence at protein level
32.1821	32.2005	32.1615	32.162	31.9831	32.0015	1:Experimental evidence at protein level
27.765	27.7572	27.7933	27.7666	27.6057	28.0035	1:Experimental evidence at protein level
31.548	31.6011	31.5816	31.9601	31.8395	32.1241	1:Experimental evidence at protein level
28.9501	29.0227	29.0231	29.276	29.2106	29.2232	1:Experimental evidence at protein level
29.2157	29.2486	29.5376	29.5127	29.7456	29.4841	1:Experimental evidence at protein level
27.6681	27.5658	27.5508	28.9777	28.8295	28.9577	1:Experimental evidence at protein level
27.4567	27.5974	27.6857	27.5698	27.3846	27.6111	1:Experimental evidence at protein level
27.1101	27.0819	27.1138	29.4461	29.2685	29.4412	1:Experimental evidence at protein level
27.7065	27.7872	27.7986	28.199	27.9595	28.3775	1:Experimental evidence at protein level
30.7816	30.8166	30.7872	30.8106	31.026	31.1998	1:Experimental evidence at protein level
32.5334	32.5654	32.6163	32.4137	32.4152	32.3766	1:Experimental evidence at protein level
30.0678	29.9912	29.9236	29.8588	29.7392	29.8456	1:Experimental evidence at protein level
27.1186	26.8466	26.7985	30.0004	29.9191	30.0192	1:Experimental evidence at protein level
28.1092	28.2068	28.288	28.1676	27.9658	28.2033	1:Experimental evidence at protein level
27.2376	27.3331	27.2748	27.3818	27.3564	27.2856	1:Experimental evidence at protein level
28.3799	28.3218	28.4071	28.2156	28.074	28.0845	1:Experimental evidence at protein level
30.3377	30.4493	30.4569	30.3071	30.1727	30.2929	1:Experimental evidence at protein level
27.8138	27.9904	27.9338	28.3455	28.0614	28.4156	1:Experimental evidence at protein level
27.964	28.2239	28.2077	28.105	27.6453	28.3495	1:Experimental evidence at protein level
27.8639	28.1334	28.1548	27.9055	27.6671	28.0677	1:Experimental evidence at protein level
28.2551	28.7053	28.6845	28.4549	28.0579	28.8441	1:Experimental evidence at protein level
29.9725	30.1069	30.1532	30.2507	30.0892	30.2782	1:Experimental evidence at protein level
26.2331	25.6459	25.4181	25.9502	25.9964	26.0513	1:Experimental evidence at protein level
30.0976	29.9958	30.0178	30.2811	30.417	30.3523	1:Experimental evidence at protein level
29.6454	29.862	29.9803	30.3747	30.0932	30.5903	1:Experimental evidence at protein level
31.5725	31.5918	31.6417	31.4356	31.3584	31.3918	1:Experimental evidence at protein level
29.0558	29.0346	29.0306	29.2839	29.3015	29.3106	1:Experimental evidence at protein level
28.5419	28.6291	28.6348	28.0828	27.9275	28.1141	1:Experimental evidence at protein level

29.4243	29.5125	29.5667	29.6539	29.5068	29.6398	1:Experimental evidence at protein level
29.8026	29.9646	30.0154	29.877	29.7049	29.8174	1:Experimental evidence at protein level
30.6453	30.8214	30.8093	31.0735	30.8235	31.1726	1:Experimental evidence at protein level
30.6333	30.837	30.9703	31.2635	31.1641	31.5191	1:Experimental evidence at protein level
29.9732	30.1047	30.1383	30.5848	30.3683	30.6649	1:Experimental evidence at protein level

C: GOBP name
establishment of localization;establishment of protein localization;protein transport;transport
biological regulation;biosynthetic process;cellular biosynthetic process;cellular macromolecule biosynthetic process;cellular macromolecule metabolic process;cellular metabolic process;cellular process;cellular protein metab
cellular macromolecule metabolic process;cellular metabolic process;cellular nitrogen compound metabolic process;cellular process;macromolecule metabolic process;macromolecule methylation;macromolecule modificatio
catabolic process;cellular catabolic process;cellular macromolecule catabolic process;cellular macromolecule metabolic process;cellular metabolic process;cellular nitrogen compound metabolic process;cellular process;dea
biological regulation;cellular component organization;cellular component organization or biogenesis;cellular membrane organization;cellular process;cellular response to abiotic stimulus;cellular response to light stimulus;cellu
amine catabolic process;amine metabolic process;aspartate family amino acid catabolic process;aspartate family amino acid metabolic process;biosynthetic process;carboxylic acid catabolic process;carboxylic acid metabo
cellular metabolic process;cellular process;electron transport chain;generation of precursor metabolites and energy;metabolic process;mitochondrial electron transport, NADH to ubiquinone;oxidation-reduction process;respi
ATP biosynthetic process;ATP metabolic process;ATP synthesis coupled proton transport;biosynthetic process;cation transport;cellular biosynthetic process;cellular metabolic process;cellular nitrogen compound biosynthetic
biological regulation;cell communication;cell proliferation;cell-cell signaling;cellular process;cellular response to stimulus;immune response;immune system process;regulation of biological process;regulation of cellular proces
biological regulation;biosynthetic process;cellular biosynthetic process;cellular macromolecule biosynthetic process;cellular macromolecule metabolic process;cellular metabolic process;cellular process;cellular protein metab
biological regulation;biosynthetic process;cellular biosynthetic process;cellular component disassembly;cellular component disassembly at cellular level;cellular component organization;cellular component organization at cell
anatomical structure development;brain development;cation transport;cellular metabolic process;cellular process;copper ion transport;developmental process;establishment of localization;generation of precursor metabolites
biological regulation;cellular macromolecule metabolic process;cellular metabolic process;cellular nitrogen compound metabolic process;cellular process;gene expression;macromolecule metabolic process;metabolic proces
response to chemical stimulus;response to organic substance;response to pheromone;response to stimulus
cellular macromolecule metabolic process;cellular metabolic process;cellular process;cellular protein metabolic process;establishment of localization;establishment of localization in cell;establishment of protein localization;es
biosynthetic process;cellular biosynthetic process;cellular macromolecule metabolic process;cellular metabolic process;cellular process;cellular protein metabolic process;macromolecule metabolic process;macromolecule m
cellular component assembly;cellular component organization;cellular component organization or biogenesis;macromolecular complex assembly;macromolecular complex subunit organization;metabolic process;protein com
amine transport;amino acid transport;biological regulation;biosynthetic process;carboxylic acid transport;cellular biosynthetic process;cellular lipid metabolic process;cellular metabolic process;cellular process;establishment
cellular macromolecule metabolic process;cellular metabolic process;cellular process;cellular protein metabolic process;macromolecule metabolic process;metabolic process;primary metabolic process;protein folding;protei
cellular component organization;cellular component organization at cellular level;cellular component organization or biogenesis;cellular component organization or biogenesis at cellular level;cellular process;establishment of l
acylglycerol metabolic process;biosynthetic process;CDP-diacylglycerol metabolic process;cellular biosynthetic process;cellular lipid metabolic process;cellular metabolic process;cellular process;diacylglycerol metabolic pro
biological regulation;cell differentiation;cellular component organization;cellular component organization at cellular level;cellular component organization or biogenesis;cellular component organization or biogenesis at cellular
cellular metabolic process;cellular process;electron transport chain;generation of precursor metabolites and energy;metabolic process;mitochondrial electron transport, NADH to ubiquinone;oxidation-reduction process;respi
cellular component organization;cellular component organization at cellular level;cellular component organization or biogenesis;cellular component organization or biogenesis at cellular level;cellular process;endocytic recycli
amine metabolic process;amino acid activation;carboxylic acid metabolic process;cellular amine metabolic process;cellular amino acid metabolic process;cellular ketone metabolic process;cellular macromolecule metabolic
aging;biological regulation;blood coagulation;cell activation;cellular component disassembly;cellular component disassembly at cellular level;cellular component organization;cellular component organization at cellular level;ce
anatomical structure morphogenesis;biological regulation;cellular component organization;cellular component organization at cellular level;cellular component organization or biogenesis;cellular component organization or bio
activation of MAPK activity;acute inflammatory response;acute-phase response;biological regulation;calcium ion homeostasis;cation homeostasis;cell activation;cell chemotaxis;cell migration;cell motility;cellular calcium ion h
amine metabolic process;aminoglycan biosynthetic process;aminoglycan catabolic process;aminoglycan metabolic process;anatomical structure development;anatomical structure morphogenesis;biological regulation;biosy
catabolic process;lipid catabolic process;lipid metabolic process;metabolic process;primary metabolic process
biological regulation;cellular process;cellular response to stimulus;endosome transport;establishment of localization;establishment of localization in cell;establishment of protein localization;intracellular protein transport;intrac
ATP biosynthetic process;ATP metabolic process;ATP synthesis coupled proton transport;biosynthetic process;cation transport;cellular biosynthetic process;cellular metabolic process;cellular nitrogen compound biosynthetic
cellular metabolic process;cellular process;electron transport chain;generation of precursor metabolites and energy;metabolic process;mitochondrial electron transport, NADH to ubiquinone;oxidation-reduction process;respi
antibiotic transport;apoptosis;cell death;cellular process;death;drug transport;establishment of localization;organic alcohol transport;organic substance transport;programmed cell death;tetracycline transport;transmembrane
alcohol metabolic process;anatomical structure development;biological regulation;biosynthetic process;cellular process;cellular response to stimulus;cholesterol biosynthetic process;cholesterol metabolic process;developme
biological regulation;cell proliferation;muscle cell proliferation;negative regulation of apoptosis;negative regulation of biological process;negative regulation of cell death;negative regulation of cellular process;negative regulatio
biosynthetic process;cellular biosynthetic process;cellular macromolecule metabolic process;cellular metabolic process;cellular nitrogen compound metabolic process;cellular process;gene expression;macromolecule metabo
biological regulation;cellular macromolecule metabolic process;cellular metabolic process;cellular nitrogen compound metabolic process;cellular process;cellular protein metabolic process;cellular response to chemical stimu
biological regulation;cation transport;cellular macromolecule metabolic process;cellular metabolic process;cellular process;cellular protein metabolic process;divalent inorganic cation transport;divalent metal ion transport;est
catabolic process;cellular catabolic process;cellular component assembly;cellular component assembly at cellular level;cellular component organization;cellular component organization at cellular level;cellular component org
biological regulation;cell differentiation;cellular component organization;cellular component organization or biogenesis;cellular developmental process;cellular membrane organization;cellular process;developmental process;e
metabolic process;response to chemical stimulus;response to external stimulus;response to extracellular stimulus;response to nutrient;response to nutrient levels;response to retinoic acid;response to stimulus;response to vit
biosynthetic process;cellular biosynthetic process;cellular metabolic process;cellular nitrogen compound biosynthetic process;cellular nitrogen compound metabolic process;cellular process;cobalamin biosynthetic process;c
biosynthetic process;cellular biosynthetic process;cellular component disassembly;cellular component disassembly at cellular level;cellular component organization;cellular component organization at cellular level;cellular cor
activation of NF-kappaB-inducing kinase activity;activation of protein kinase activity;biological regulation;cellular macromolecule metabolic process;cellular metabolic process;cellular process;cellular protein metabolic proces
biosynthetic process;cellular biosynthetic process;cellular component disassembly;cellular component disassembly at cellular level;cellular component organization;cellular component organization at cellular level;cellular cor
biological regulation;cell differentiation;cellular developmental process;cellular process;developmental process;neuron differentiation;positive regulation of biological process;positive regulation of biosynthetic process;positive
adenosine metabolic process;catabolic process;cellular aromatic compound metabolic process;cellular catabolic process;cellular metabolic process;cellular nitrogen compound catabolic process;cellular nitrogen compound
anatomical structure development;biological regulation;cellular process;cellular response to stimulus;developmental process;establishment of localization;establishment of localization in cell;intracellular signal transduction;int
biological regulation;biosynthetic process;cellular biosynthetic process;cellular component organization;cellular component organization at cellular level;cellular component organization or biogenesis;cellular component orga
cellular component organization;cellular component organization at cellular level;cellular component organization or biogenesis;cellular component organization or biogenesis at cellular level;cellular process;chromatin organiz
cellular macromolecule metabolic process;cellular metabolic process;cellular nitrogen compound metabolic process;cellular process;macromolecule metabolic process;macromolecule modification;metabolic process;ncRNA
biosynthetic process;cellular biosynthetic process;cellular component organization;cellular component organization at cellular level;cellular component organization or biogenesis;cellular component organization or biogenesis
cellular component organization;cellular component organization at cellular level;cellular component organization or biogenesis;cellular component organization or biogenesis at cellular level;cellular macromolecule metabolic
biosynthetic process;cellular biosynthetic process;cellular macromolecule metabolic process;cellular metabolic process;cellular nitrogen compound metabolic process;cellular process;electron transport chain;gene expressio
biosynthetic process;carboxylic acid biosynthetic process;carboxylic acid metabolic process;cellular biosynthetic process;cellular ketone metabolic process;cellular lipid metabolic process;cellular metabolic process;cellular p
biosynthetic process;cellular biosynthetic process;cellular component disassembly;cellular component disassembly at cellular level;cellular component organization;cellular component organization at cellular level;cellular cor
anatomical structure development;biological regulation;brain development;cAMP-mediated signaling;cellular component assembly;cellular component assembly at cellular level;cellular component biogenesis;cellular compor
anion homeostasis;anion transport;bicarbonate transport;biological regulation;cellular metabolic process;cellular process;chemical homeostasis;chloride ion homeostasis;establishment of localization;homeostatic process;inc
cell cycle phase;cell cycle process;cell division;cellular component organization;cellular component organization at cellular level;cellular component organization or biogenesis;cellular component organization or biogenesis at
biological regulation;cell surface receptor linked signaling pathway;cellular process;cellular response to chemical stimulus;cellular response to cytokine stimulus;cellular response to organic substance;cellular response to stim
cellular component assembly;cellular component assembly at cellular level;cellular component biogenesis;cellular component organization;cellular component organization at cellular level;cellular component organization or b
biological regulation;cellular component assembly;cellular component organization;cellular component organization at cellular level;cellular component organization or biogenesis;cellular component organization or biogenesis
cellular component assembly;cellular component assembly at cellular level;cellular component organization;cellular component organization at cellular level;cellular component organization or biogenesis;cellular component c
biological regulation;cation homeostasis;cation transport;cellular cation homeostasis;cellular chemical homeostasis;cellular component assembly;cellular component assembly at cellular level;cellular component organization;
biological regulation;cellular process;cellular response to chemical stimulus;cellular response to endogenous stimulus;cellular response to growth factor stimulus;cellular response to hormone stimulus;cellular response to org
cellular macromolecule metabolic process;cellular metabolic process;cellular nitrogen compound metabolic process;cellular process;macromolecule metabolic process;metabolic process;mRNA metabolic process;mRNA pro
acute inflammatory response;acute-phase response;anatomical structure development;biological regulation;cellular component organization;cellular component organization or biogenesis;cellular membrane organization;cellu
anatomical structure homeostasis;biological regulation;blood coagulation;cation homeostasis;cation transport;cell activation;cellular cation homeostasis;cellular chemical homeostasis;cellular homeostasis;cellular ion homeos
biosynthetic process;cellular biosynthetic process;cellular macromolecule metabolic process;cellular metabolic process;cellular nitrogen compound metabolic process;cellular process;electron transport chain;gene expressio
biological adhesion;cell adhesion;cellular process
cellular metabolic process;cellular process;electron transport chain;generation of precursor metabolites and energy;metabolic process;mitochondrial electron transport, NADH to ubiquinone;oxidation-reduction process;respi
amine metabolic process;aminoglycan catabolic process;aminoglycan metabolic process;biological regulation;calcium-mediated signaling;carbohydrate catabolic process;carbohydrate metabolic process;cardiac cell differen
anatomical structure development;biosynthetic process;cellular biosynthetic process;cellular macromolecule metabolic process;cellular metabolic process;cellular nitrogen compound metabolic process;cellular process;centr
adrenal gland development;aging;anatomical structure development;anatomical structure morphogenesis;anion transport;apoptosis;axon regeneration;axonogenesis;behavior;behavioral response to pain;biological regulation
biological regulation;carbohydrate metabolic process;cellular carbohydrate metabolic process;cellular glucan metabolic process;cellular macromolecule metabolic process;cellular metabolic process;cellular polysaccharide m
biological regulation;dosage compensation;dosage compensation, by inactivation of X chromosome;posttranscriptional regulation of gene expression;regulation of biological process;regulation of biosynthetic process;regulat
alcohol metabolic process;carbohydrate catabolic process;carbohydrate metabolic process;catabolic process;cellular carbohydrate catabolic process;cellular carbohydrate metabolic process;cellular glucan metabolic proces
aging;anatomical structure morphogenesis;base-excision repair;biological regulation;cell aging;cell communication;cell cycle checkpoint;cell cycle phase;cell cycle process;cell differentiation;cell division;cell morphogenesis;c
cellular macromolecule metabolic process;cellular metabolic process;cellular process;cellular protein metabolic process;macromolecule metabolic process;macromolecule modification;metabolic process;multicellular organis
antigen processing and presentation;antigen processing and presentation of exogenous antigen;antigen processing and presentation of exogenous peptide antigen;antigen processing and presentation of exogenous peptide
cellular metabolic process;cellular process;electron transport chain;generation of precursor metabolites and energy;metabolic process;mitochondrial electron transport, NADH to ubiquinone;oxidation-reduction process;respi
alcohol metabolic process;biological regulation;biosynthetic process;cellular biosynthetic process;cellular hormone metabolic process;cellular metabolic process;cellular process;cholesterol biosynthetic process;cholesterol m
aging;biological regulation;cell aging;cellular process;cellular response to chemical stimulus;cellular response to oxidative stress;cellular response to reactive oxygen species;cellular response to stimulus;cellular response to s
biosynthetic process;cellular biosynthetic process;cellular component assembly;cellular component assembly at cellular level;cellular component organization;cellular component organization at cellular level;cellular compone
biological regulation;cell communication;cell surface receptor linked signaling pathway;cell-cell signaling;cellular metabolic process;cellular process;cellular response to chemical stimulus;cellular response to endogenous sti
biosynthetic process;cellular biosynthetic process;cellular component disassembly;cellular component disassembly at cellular level;cellular component organization;cellular component organization at cellular level;cellular cor
adipose tissue development;anatomical structure development;biosynthetic process;brain development;catabolic process;cellular biosynthetic process;cellular catabolic process;cellular ketone body metabolic process;cellula
biological regulation;biosynthetic process;cellular biosynthetic process;cellular macromolecule biosynthetic process;cellular macromolecule metabolic process;cellular metabolic process;cellular nitrogen compound metaboli
biological regulation;cell communication;cell-cell signaling;cellular process;cellular response to stimulus;establishment of localization;establishment of protein localization;protein transport;regulation of biological process;regu
activation of immune response;activation of innate immune response;apoptosis;biological regulation;biosynthetic process;cell death;cell surface receptor linked signaling pathway;cellular biosynthetic process;cellular macrom
biological regulation;cellular component organization;cellular component organization or biogenesis;cellular macromolecule metabolic process;cellular membrane organization;cellular metabolic process;cellular process;cellul
biosynthetic process;cellular biosynthetic process;cellular ketone metabolic process;cellular metabolic process;cellular process;coenzyme biosynthetic process;coenzyme metabolic process;cofactor biosynthetic process;co
biological regulation;cellular component organization;cellular component organization at cellular level;cellular component organization or biogenesis;cellular component organization or biogenesis at cellular level;cellular proce
biological regulation;catabolic process;cellular catabolic process;cellular macromolecule catabolic process;cellular macromolecule metabolic process;cellular metabolic process;cellular process;cellular protein metabolic proc
biosynthetic process;cellular biosynthetic process;cellular component disassembly;cellular component disassembly at cellular level;cellular component organization;cellular component organization at cellular level;cellular cor
cellular process;establishment of localization;transport;vesicle-mediated transport
anatomical structure development;biological regulation;cell redox homeostasis;cellular homeostasis;cellular process;developmental process;hemopoiesis;hemopoietic or lymphoid organ development;homeostatic process;or
biological regulation;cellular component movement;cellular homeostasis;cellular process;cellular response to stimulus;cytoskeleton-dependent intracellular transport;establishment of localization;establishment of localization
biological regulation;calcium ion homeostasis;calcium ion transport;cation homeostasis;cation transport;cellular calcium ion homeostasis;cellular cation homeostasis;cellular chemical homeostasis;cellular divalent inorganic c
biological regulation;cellular metabolic process;cellular process;electron transport chain;generation of precursor metabolites and energy;metabolic process;negative regulation of biological process;negative regulation of cellu
cation transport;cellular process;divalent inorganic cation transport;divalent metal ion transport;establishment of localization;ion transport;magnesium ion transport;metal ion transport;transmembrane transport;transport
biosynthetic process;cellular biosynthetic process;cellular component disassembly;cellular component disassembly at cellular level;cellular component organization;cellular component organization at cellular level;cellular cor
anatomical structure development;anatomical structure formation involved in morphogenesis;cell development;cellular developmental process;cellular process;developmental process;eye photoreceptor cell development;neu
amine metabolic process;carboxylic acid metabolic process;cellular amine metabolic process;cellular amino acid metabolic process;cellular ketone metabolic process;cellular metabolic process;cellular nitrogen compound m
biological regulation;posttranscriptional regulation of gene expression;protein destabilization;regulation of biological process;regulation of biological quality;regulation of gene expression;regulation of macromolecule metaboli
cellular macromolecule metabolic process;cellular metabolic process;cellular nitrogen compound metabolic process;cellular process;macromolecule metabolic process;macromolecule methylation;macromolecule modificatio

biological regulation;biosynthetic process;cellular biosynthetic process;cellular component organization;cellular component organization or biogenesis;cellular macromolecule biosynthetic process;cellular macromolecule met
cellular component assembly;cellular component assembly at cellular level;cellular component biogenesis;cellular component organization;cellular component organization at cellular level;cellular component organization or b
biological regulation;catabolic process;cellular catabolic process;cellular macromolecule catabolic process;cellular macromolecule metabolic process;cellular metabolic process;cellular process;cellular protein metabolic proc
biological regulation;biosynthetic process;carbohydrate metabolic process;carbohydrate transport;cell cycle;cell cycle process;cell surface receptor linked signaling pathway;cellular biosynthetic process;cellular component c
amine metabolic process;aminoglycan catabolic process;aminoglycan metabolic process;carbohydrate catabolic process;carbohydrate metabolic process;catabolic process;chitin catabolic process;chitin metabolic process;
biosynthetic process;cellular biosynthetic process;cellular component disassembly;cellular component disassembly at cellular level;cellular component organization;cellular component organization at cellular level;cellular con
anatomical structure development;apoptosis;autophagic vacuole assembly;autophagy;biological regulation;blood vessel remodeling;cardiac cell development;cardiac muscle cell development;catabolic process;cell communi
biosynthetic process;cellular biosynthetic process;cellular component disassembly;cellular component disassembly at cellular level;cellular component organization;cellular component organization at cellular level;cellular con
biological regulation;biosynthetic process;cellular biosynthetic process;cellular component organization;cellular component organization at cellular level;cellular component organization or biogenesis;cellular component orga
apoptosis;biological regulation;biosynthetic process;cell communication;cell death;cell growth;cellular biosynthetic process;cellular localization;cellular macromolecule biosynthetic process;cellular macromolecule localization
anatomical structure development;biological regulation;biosynthetic process;brain development;cation homeostasis;cellular biosynthetic process;cellular cation homeostasis;cellular chemical homeostasis;cellular homeostasi
biological regulation;catabolic process;cellular catabolic process;cellular macromolecule metabolic process;cellular metabolic process;cellular nitrogen compound catabolic process;cellular nitrogen compound metabolic proc
biological regulation;cellular component assembly;cellular component assembly at cellular level;cellular component biogenesis;cellular component organization;cellular component organization at cellular level;cellular compon
cellular component organization;cellular component organization or biogenesis;cellular membrane organization;cellular process;endoplasmic reticulum membrane organization;establishment of localization;establishment of lo
catabolic process;cellular catabolic process;cellular macromolecule catabolic process;cellular macromolecule metabolic process;cellular metabolic process;cellular process;cellular protein metabolic process;macromolecule c
macromolecule metabolic process;metabolic process;primary metabolic process;protein metabolic process;proteolysis;response to calcium ion;response to chemical stimulus;response to inorganic substance;response to me
cellular metabolic process;cellular process;electron transport chain;generation of precursor metabolites and energy;metabolic process;multicellular organismal process;oxidation-reduction process;respiratory electron transpo
biological regulation;cellular process;cellular response to stimulus;negative regulation of biological process;negative regulation of cell communication;negative regulation of cellular process;negative regulation of Ras protein s
biosynthetic process;cellular biosynthetic process;cellular component disassembly;cellular component disassembly at cellular level;cellular component organization;cellular component organization at cellular level;cellular con
biological regulation;cellular component assembly;cellular component assembly at cellular level;cellular component biogenesis;cellular component organization;cellular component organization at cellular level;cellular compon
biological regulation;cell redox homeostasis;cellular homeostasis;cellular process;homeostatic process;regulation of biological process;regulation of biological quality;regulation of cellular process
anatomical structure formation involved in morphogenesis;anatomical structure morphogenesis;antigen processing and presentation;antigen processing and presentation of exogenous antigen;antigen processing and presen
biosynthetic process;cellular biosynthetic process;cellular metabolic process;cellular process;cofactor biosynthetic process;cofactor metabolic process;iron-sulfur cluster assembly;metabolic process;metallo-sulfur cluster as
biological regulation;cadmium ion transmembrane transport;cadmium ion transport;calcium ion homeostasis;calcium ion import;calcium ion transmembrane transport;calcium ion transport;cation homeostasis;cation transpor
establishment of localization;establishment of protein localization;establishment of RNA localization;mRNA transport;nucleic acid transport;nucleobase-containing compound transport;protein transport;RNA transport;transpo
cellular macromolecule metabolic process;cellular metabolic process;cellular nitrogen compound metabolic process;cellular process;macromolecule metabolic process;metabolic process;mRNA metabolic process;mRNA pro
catabolic process;cellular catabolic process;cellular macromolecule catabolic process;cellular macromolecule metabolic process;cellular metabolic process;cellular nitrogen compound metabolic process;cellular process;mac
biological regulation;cellular metabolic process;cellular process;cellular response to stimulus;lipid metabolic process;metabolic process;oxidation-reduction process;primary metabolic process;regulation of biological process
axon ensheathment;biological regulation;cell cycle process;cellular component assembly;cellular component assembly at cellular level;cellular component organization;cellular component organization at cellular level;cellular
cellular metabolic process;cellular process;electron transport chain;generation of precursor metabolites and energy;metabolic process;mitochondrial electron transport, NADH to ubiquinone;oxidation-reduction process;respi
amine transport;amino acid transmembrane transport;amino acid transport;betaine transport;carboxylic acid transport;carnitine shuttle;carnitine transport;cation transport;cellular lipid metabolic process;cellular metabolic pro
aging;anatomical structure development;anatomical structure morphogenesis;carboxylic acid metabolic process;cardiovascular system development;cell part morphogenesis;cellular component morphogenesis;cellular comp
apoptosis;cell death;cellular component assembly;cellular component assembly at cellular level;cellular component organization;cellular component organization at cellular level;cellular component organization or biogenesis;
cellular metabolic process;cellular process;electron transport chain;generation of precursor metabolites and energy;metabolic process;mitochondrial electron transport, NADH to ubiquinone;oxidation-reduction process;respi
cellular metabolic process;cellular process;electron transport chain;generation of precursor metabolites and energy;metabolic process;mitochondrial electron transport, NADH to ubiquinone;oxidation-reduction process;respi
biological regulation;cellular component organization;cellular component organization or biogenesis;cellular membrane organization;cellular process;endocytosis;establishment of localization;membrane invagination;membran
acute inflammatory response;acute-phase response;anatomical structure homeostasis;biological regulation;defense response;digestive system process;epithelial structure maintenance;homeostatic process;inflammatory res
biological regulation;negative regulation of binding;negative regulation of biological process;negative regulation of molecular function;negative regulation of protein binding;negative regulation of reproductive process;negative
activation of immune response;activation of innate immune response;biological regulation;cell surface receptor linked signaling pathway;cellular component assembly;cellular component assembly at cellular level;cellular com
ATP biosynthetic process;ATP metabolic process;ATP synthesis coupled proton transport;biosynthetic process;cation transport;cellular biosynthetic process;cellular metabolic process;cellular nitrogen compound biosynthetic
activation of phospholipase D activity;biological regulation;cellular component organization;cellular component organization at cellular level;cellular component organization or biogenesis;cellular component organization or bi
alcohol metabolic process;biosynthetic process;carbohydrate biosynthetic process;carbohydrate catabolic process;carbohydrate metabolic process;catabolic process;cellular biosynthetic process;cellular carbohydrate biosy
apoptosis;biological regulation;cell death;cell surface receptor linked signaling pathway;cellular component disassembly;cellular component disassembly at cellular level;cellular component disassembly involved in apoptosis;
biological regulation;cellular component organization;cellular component organization at cellular level;cellular component organization or biogenesis;cellular component organization or biogenesis at cellular level;cellular macro
adherens junction organization;anatomical structure formation involved in morphogenesis;angiogenesis;biological adhesion;biological regulation;calcium-dependent cell-cell adhesion;cell adhesion;cell junction assembly;cell
biological regulation;cell cycle;cell cycle phase;cell cycle process;cell division;cell surface receptor linked signaling pathway;cellular component organization;cellular component organization at cellular level;cellular componen
activation of immune response;activation of innate immune response;apoptosis;biological regulation;biosynthetic process;BMP signaling pathway;catabolic process;cell death;cell surface receptor linked signaling pathway;ce
cellular component organization;cellular component organization at cellular level;cellular component organization or biogenesis;cellular component organization or biogenesis at cellular level;cellular macromolecule metabolic
biological regulation;cellular component assembly;cellular component assembly at cellular level;cellular component organization;cellular component organization at cellular level;cellular component organization or biogenesis;
antibacterial humoral response;antimicrobial humoral response;biosynthetic process;catabolic process;cellular biosynthetic process;cellular catabolic process;cellular component disassembly;cellular component disassembly
biological regulation;biosynthetic process;catabolic process;cell surface receptor linked signaling pathway;cellular biosynthetic process;cellular catabolic process;cellular macromolecule biosynthetic process;cellular macrom
alcohol metabolic process;carbohydrate metabolic process;cellular carbohydrate metabolic process;cellular metabolic process;cellular process;galactose metabolic process;hexose metabolic process;metabolic process;mon
antigen processing and presentation;antigen processing and presentation of exogenous antigen;antigen processing and presentation of exogenous peptide antigen;antigen processing and presentation of exogenous peptide
anatomical structure development;anatomical structure morphogenesis;B cell activation;B cell differentiation;biological regulation;biosynthetic process;cell activation;cell differentiation;cellular biosynthetic process;cellular de
acetyl-CoA metabolic process;alcohol metabolic process;biological regulation;biosynthetic process;cellular biosynthetic process;cellular lipid metabolic process;cellular metabolic process;cellular process;cholesterol biosynth
apoptosis;biological regulation;biosynthetic process;cell death;cellular biosynthetic process;cellular metabolic process;cellular nitrogen compound biosynthetic process;cellular nitrogen compound metabolic process;cellular
anatomical structure development;biological regulation;biosynthetic process;cap-independent translational initiation;cell cycle process;cell surface receptor linked signaling pathway;cellular biosynthetic process;cellular macr
apoptosis;biological regulation;biosynthetic process;cell death;cellular biosynthetic process;cellular macromolecule biosynthetic process;cellular macromolecule metabolic process;cellular metabolic process;cellular nitrogen
biological regulation;biosynthetic process;cellular biosynthetic process;cellular macromolecule biosynthetic process;cellular macromolecule metabolic process;cellular metabolic process;cellular nitrogen compound metabolic
aerobic respiration;biological regulation;cellular component assembly;cellular component assembly at cellular level;cellular component biogenesis;cellular component organization;cellular component organization at cellular le
apoptosis;cell death;cellular component organization;cellular component organization or biogenesis;cellular membrane organization;cellular process;death;endocytosis;establishment of localization;establishment of protein lo
cellular process;endosome transport;establishment of localization;establishment of localization in cell;establishment of protein localization;intracellular protein transport;intracellular transport;protein transport;retrograde trans
biological regulation;cellular process;cellular response to stimulus;establishment of localization;establishment of localization in cell;establishment of protein localization;intracellular protein transport;intracellular signal transdu
cellular macromolecule metabolic process;cellular metabolic process;cellular nitrogen compound metabolic process;cellular process;cleavage in ITS2 between 5.8S rRNA and LSU-rRNA of tricistronic rRNA transcript (SSU-r
actin filament-based movement;actin filament-based process;anatomical structure formation involved in morphogenesis;autophagy;biological regulation;catabolic process;cell projection assembly;cell projection organization;
catabolic process;cellular catabolic process;cellular macromolecule catabolic process;cellular macromolecule metabolic process;cellular metabolic process;cellular nitrogen compound metabolic process;cellular process;DN
biosynthetic process;cellular biosynthetic process;cellular lipid metabolic process;cellular metabolic process;cellular process;ceramide biosynthetic process;ceramide metabolic process;lipid biosynthetic process;lipid metab
autophagic vacuole assembly;autophagy;catabolic process;cellular catabolic process;cellular component assembly;cellular component assembly at cellular level;cellular component organization;cellular component organizati
ameboidal cell migration;biological regulation;cell cycle phase;cell cycle process;cell division;cell migration;cell motility;cellular component assembly;cellular component assembly at cellular level;cellular component movemen
activation of immune response;activation of innate immune response;apoptosis;biological regulation;cell death;cellular process;cellular response to chemical stimulus;cellular response to cytokine stimulus;cellular response t
anatomical structure development;biological regulation;carbohydrate metabolic process;cardiovascular system development;cell surface receptor linked signaling pathway;cellular carbohydrate metabolic process;cellular ma
biological regulation;cellular macromolecule metabolic process;cellular metabolic process;cellular nitrogen compound metabolic process;cellular process;macromolecule metabolic process;maturation of SSU-rRNA;maturatio
biological regulation;biosynthetic process;catabolic process;cell surface receptor linked signaling pathway;cellular biosynthetic process;cellular catabolic process;cellular macromolecule biosynthetic process;cellular macrom
biological regulation;cell cycle checkpoint;mitotic cell cycle checkpoint;regulation of biological process;regulation of cell cycle;regulation of cell cycle arrest;regulation of cell cycle process;regulation of cellular process;regulat
catabolic process;cellular catabolic process;cellular macromolecule catabolic process;cellular macromolecule metabolic process;cellular metabolic process;cellular nitrogen compound metabolic process;cellular process;exo
catabolic process;cell differentiation;cellular developmental process;cellular process;developmental process;epithelial cell differentiation;metabolic process
biological regulation;catabolic process;cellular catabolic process;cellular macromolecule catabolic process;cellular macromolecule metabolic process;cellular metabolic process;cellular nitrogen compound metabolic process
anatomical structure development;cellular macromolecule metabolic process;cellular metabolic process;cellular process;cellular protein metabolic process;developmental process;developmental process involved in reproduc
biosynthetic process;cellular biosynthetic process;cellular component disassembly;cellular component disassembly at cellular level;cellular component organization;cellular component organization at cellular level;cellular con
biosynthetic process;cellular biosynthetic process;cellular component disassembly;cellular component disassembly at cellular level;cellular component organization;cellular component organization at cellular level;cellular con
biological regulation;biosynthetic process;cellular biosynthetic process;cellular macromolecule biosynthetic process;cellular macromolecule metabolic process;cellular metabolic process;cellular nitrogen compound metabolic
biological adhesion;biological regulation;cell adhesion;cell communication;cell junction assembly;cell junction organization;cell-cell adhesion;cell-cell junction assembly;cell-cell junction organization;cell-cell signaling;cellular
behavior;biological regulation;body fluid secretion;carbohydrate homeostasis;cellular chemical homeostasis;cellular glucose homeostasis;cellular homeostasis;cellular ion homeostasis;cellular metabolic process;cellular nitrog
biosynthetic process;cellular biosynthetic process;cellular macromolecule biosynthetic process;cellular macromolecule metabolic process;cellular metabolic process;cellular process;cellular protein metabolic process;macrom
biological regulation;cellular component assembly;cellular component assembly at cellular level;cellular component biogenesis;cellular component organization;cellular component organization at cellular level;cellular compon
cellular component organization;cellular component organization at cellular level;cellular component organization or biogenesis;cellular component organization or biogenesis at cellular level;cellular localization;cellular proces
establishment of localization;lipid transport;organic substance transport;transport
biological regulation;cellular process;cellular response to abiotic stimulus;cellular response to gamma radiation;cellular response to ionizing radiation;cellular response to radiation;cellular response to stimulus;cellular respons
biosynthetic process;cellular biosynthetic process;cellular macromolecule biosynthetic process;cellular macromolecule metabolic process;cellular metabolic process;cellular nitrogen compound metabolic process;cellular pro
cellular macromolecule metabolic process;cellular metabolic process;cellular nitrogen compound metabolic process;cellular process;gene expression;macromolecule metabolic process;metabolic process;mRNA metabolic p
biosynthetic process;cellular biosynthetic process;cellular component disassembly;cellular component disassembly at cellular level;cellular component organization;cellular component organization at cellular level;cellular con
anatomical structure morphogenesis;apoptotic mitochondrial changes;biological regulation;cell part morphogenesis;cellular component assembly;cellular component morphogenesis;cellular component organization;cellular c
biosynthetic process;cellular biosynthetic process;cellular component disassembly;cellular component disassembly at cellular level;cellular component organization;cellular component organization at cellular level;cellular con
activation of MAPKK activity;activation of protein kinase activity;anatomical structure development;biological regulation;cell cycle checkpoint;cell cycle process;cellular macromolecule metabolic process;cellular metabolic pro
cellular macromolecule metabolic process;cellular metabolic process;cellular process;cellular protein metabolic process;cullin deneddylation;macromolecule metabolic process;macromolecule modification;metabolic process
activation of immune response;anatomical structure development;anatomical structure morphogenesis;androgen metabolic process;antigen receptor-mediated signaling pathway;B cell receptor signaling pathway;biological r
cell differentiation;cellular developmental process;cellular process;developmental process;osteoblast differentiation
biological regulation;calcium ion homeostasis;calcium ion transport;carbohydrate metabolic process;cation homeostasis;cation transport;cellular calcium ion homeostasis;cellular carbohydrate metabolic process;cellular catio
glomerular filtration;macromolecule metabolic process;metabolic process;multicellular organismal process;primary metabolic process;protein maturation;protein metabolic process;protein processing;renal system process;sy
biosynthetic process;cellular biosynthetic process;cellular component disassembly;cellular component disassembly at cellular level;cellular component organization;cellular component organization at cellular level;cellular con
cellular metabolic process;cellular process;electron transport chain;generation of precursor metabolites and energy;metabolic process;oxidation-reduction process;respiratory electron transport chain;small molecule metaboli
biological regulation;biosynthetic process;cellular biosynthetic process;cellular component assembly;cellular component assembly at cellular level;cellular component organization;cellular component organization at cellular le
cellular metabolic process;cellular process;electron transport chain;generation of precursor metabolites and energy;metabolic process;mitochondrial electron transport, ubiquinol to cytochrome c;oxidation-reduction process;
anatomical structure development;biosynthetic process;cell development;cellular biosynthetic process;cellular developmental process;cellular macromolecule biosynthetic process;cellular macromolecule metabolic process;c

anatomical structure morphogenesis;apoptotic mitochondrial changes;autophagy;biological regulation;calcium ion homeostasis;calcium-mediated signaling;calcium-mediated signaling using intracellular calcium source;catab
cellular macromolecule metabolic process;cellular metabolic process;cellular process;cellular protein metabolic process;chaperone-mediated protein transport;establishment of localization;establishment of localization in cell;
cellular macromolecule metabolic process;cellular metabolic process;cellular process;cellular protein metabolic process;chaperone-mediated protein transport;establishment of localization;establishment of localization in cell;
anatomical structure morphogenesis;biological regulation;biosynthetic process;cell morphogenesis;cell part morphogenesis;cell proliferation;cellular biosynthetic process;cellular component morphogenesis;cellular componen
alcohol biosynthetic process;alcohol metabolic process;biosynthetic process;carbohydrate biosynthetic process;carbohydrate metabolic process;cellular biosynthetic process;cellular carbohydrate biosynthetic process;cellul
anatomical structure development;biological regulation;cell surface receptor linked signaling pathway;cellular component organization;cellular component organization or biogenesis;cellular membrane organization;cellular pr
catabolic process;cell differentiation;cellular catabolic process;cellular developmental process;cellular macromolecule catabolic process;cellular macromolecule metabolic process;cellular metabolic process;cellular nitrogen c
anatomical structure formation involved in morphogenesis;anatomical structure morphogenesis;axon guidance;biological regulation;cell projection assembly;cell projection organization;cellular component assembly;cellular c
biological regulation;cellular component organization;cellular component organization or biogenesis;cellular localization;cellular macromolecule localization;cellular membrane organization;cellular process;cellular protein local
metabolic process;oxidation-reduction process
cellular process;establishment of localization;transport;vesicle-mediated transport
cellular metabolic process;cellular process;electron transport chain;generation of precursor metabolites and energy;metabolic process;mitochondrial electron transport, NADH to ubiquinone;oxidation-reduction process;respi
anatomical structure development;cellular macromolecule metabolic process;cellular metabolic process;cellular process;cellular protein metabolic process;chaperone-mediated protein transport;developmental process;estab
alternative nuclear mRNA splicing, via spliceosome;biological regulation;biosynthetic process;cell projection organization;cellular biosynthetic process;cellular component organization;cellular component organization at cellul
ATP biosynthetic process;ATP metabolic process;ATP synthesis coupled proton transport;biosynthetic process;cation transport;cellular biosynthetic process;cellular metabolic process;cellular nitrogen compound biosynthetic
cellular metabolic process;cellular process;electron transport chain;generation of precursor metabolites and energy;metabolic process;mitochondrial electron transport, NADH to ubiquinone;oxidation-reduction process;respi
cellular macromolecule metabolic process;cellular metabolic process;cellular nitrogen compound metabolic process;cellular process;macromolecule metabolic process;metabolic process;ncRNA metabolic process;ncRNA pr
biological regulation;cell cycle cytokinesis;cell cycle process;cellular macromolecule metabolic process;cellular metabolic process;cellular process;cellular protein metabolic process;cellular response to stimulus;cytokinesis;c
biological regulation;cellular component assembly;cellular component organization;cellular component organization at cellular level;cellular component organization or biogenesis;cellular component organization or biogenesis
carboxylic acid metabolic process;cellular ketone metabolic process;cellular metabolic process;cellular process;L-ascorbic acid metabolic process;metabolic process;organic acid metabolic process;oxoacid metabolic proce
acute inflammatory response;acute-phase response;biological regulation;cellular component organization;cellular component organization or biogenesis;cellular membrane organization;cellular process;defense response;defe
adult behavior;adult locomotory behavior;behavior;biological regulation;locomotory behavior;multicellular organismal process;negative regulation of biological process;negative regulation of catalytic activity;negative regulatio
biological regulation;carbohydrate catabolic process;carbohydrate metabolic process;catabolic process;cellular carbohydrate catabolic process;cellular carbohydrate metabolic process;cellular catabolic process;cellular lipid
activation of immune response;activation of innate immune response;adaptive immune response;antigen processing and presentation;antigen processing and presentation of exogenous antigen;antigen processing and prese
activation of MAPK activity;adult behavior;anatomical structure development;anatomical structure morphogenesis;axon guidance;behavior;biological regulation;brain development;branching morphogenesis of a tube;carbohy
amine catabolic process;amine metabolic process;anatomical structure development;aromatic amino acid family catabolic process;aromatic amino acid family metabolic process;aromatic compound biosynthetic process;aro
biosynthetic process;cellular biosynthetic process;cellular macromolecule metabolic process;cellular metabolic process;cellular nitrogen compound metabolic process;cellular process;electron transport chain;gene expressio
cellular component organization;cellular component organization at cellular level;cellular component organization or biogenesis;cellular component organization or biogenesis at cellular level;cellular process;mitochondrion or
biological regulation;biosynthetic process;cellular biosynthetic process;cellular macromolecule biosynthetic process;cellular macromolecule metabolic process;cellular metabolic process;cellular nitrogen compound metabolic
anatomical structure development;cardiac muscle tissue development;cellular metabolic process;cellular process;developmental process;electron transport chain;generation of precursor metabolites and energy;metabolic pr
apoptosis;biological regulation;biosynthetic process;catabolic process;cell death;cell differentiation;cellular biosynthetic process;cellular catabolic process;cellular developmental process;cellular macromolecule biosynthetic
activation of immune response;antigen receptor-mediated signaling pathway;B cell activation;B cell differentiation;biological regulation;biosynthetic process;blood coagulation;carbohydrate homeostasis;cell activation;cell dif
anatomical structure homeostasis;base-excision repair;biological regulation;biosynthetic process;catabolic process;cell cycle;cell cycle process;cellular biosynthetic process;cellular catabolic process;cellular component asse
alcohol metabolic process;amine metabolic process;anatomical structure morphogenesis;aromatic amino acid family metabolic process;aromatic compound biosynthetic process;benzene-containing compound metabolic pr
anatomical structure development;anatomical structure morphogenesis;biological regulation;cell development;cell part morphogenesis;cell projection morphogenesis;cell projection organization;cell surface receptor linked sig
aging;amine metabolic process;arachidonic acid metabolic process;biological regulation;carboxylic acid metabolic process;catabolic process;cellular amine metabolic process;cellular amino acid metabolic process;cellular ca
aging;biological regulation;biosynthetic process;carbohydrate metabolic process;carbohydrate transport;cell aging;cell cycle;cell cycle process;cell death;cell surface receptor linked signaling pathway;cellular biosynthetic pro
activation of immune response;adaptive immune response;alcohol metabolic process;anatomical structure development;anatomical structure formation involved in morphogenesis;angiogenesis;antigen receptor-mediated sig
biological regulation;cell proliferation;cell surface receptor linked signaling pathway;cellular component assembly;cellular component assembly at cellular level;cellular component organization;cellular component organization
acute inflammatory response;anatomical structure formation involved in morphogenesis;angiogenesis;angiogenesis involved in wound healing;biological regulation;blood coagulation;blood coagulation, intrinsic pathway;carb
cellular macromolecule metabolic process;cellular metabolic process;cellular process;cellular protein metabolic process;macromolecule metabolic process;macromolecule modification;metabolic process;peptidyl-amino acid
activation of immune response;activation of innate immune response;anatomical structure development;apoptotic mitochondrial changes;biological regulation;cell projection organization;cellular component organization;cellu
amine biosynthetic process;amine metabolic process;apoptotic mitochondrial changes;biological regulation;biosynthetic process;carboxylic acid biosynthetic process;carboxylic acid metabolic process;cellular amine metabo
biosynthetic process;cellular biosynthetic process;cellular lipid metabolic process;cellular metabolic process;cellular process;glycerolipid biosynthetic process;glycerolipid metabolic process;glycerophospholipid biosynthetic
androgen receptor signaling pathway;biological regulation;biosynthetic process;catabolic process;cell cycle;cell cycle arrest;cell cycle process;cell division;cell proliferation;cellular biosynthetic process;cellular catabolic proce
adult behavior;adult locomotory behavior;anatomical structure development;associative learning;behavior;biological regulation;brain development;catabolic process;cation homeostasis;cell development;cellular catabolic pro
acetyl-CoA catabolic process;acetyl-CoA metabolic process;carbohydrate metabolic process;carboxylic acid metabolic process;catabolic process;cellular catabolic process;cellular ketone metabolic process;cellular metabol
ATP biosynthetic process;ATP metabolic process;ATP synthesis coupled proton transport;biosynthetic process;cation transport;cellular biosynthetic process;cellular metabolic process;cellular nitrogen compound biosynthetic
cellular component assembly;cellular component assembly at cellular level;cellular component organization;cellular component organization at cellular level;cellular component organization or biogenesis;cellular component c
alcohol metabolic process;amine metabolic process;anatomical structure development;anatomical structure morphogenesis;benzene-containing compound metabolic process;biological regulation;catecholamine metabolic p
cellular component organization;cellular component organization at cellular level;cellular component organization or biogenesis;cellular component organization or biogenesis at cellular level;cellular process;COPI coating of G
establishment of localization;establishment of localization in cell;establishment of protein localization;intracellular protein transport;intracellular transport;nuclear export;nuclear transport;nucleocytoplasmic transport;protein e
catabolic process;cellular catabolic process;cellular macromolecule catabolic process;cellular macromolecule metabolic process;cellular metabolic process;cellular nitrogen compound metabolic process;cellular process;exo
catabolic process;cellular catabolic process;cellular macromolecule catabolic process;cellular macromolecule metabolic process;cellular metabolic process;cellular nitrogen compound metabolic process;cellular process;exo
90S preribosome assembly;anatomical structure development;anatomical structure morphogenesis;axial mesoderm development;biological regulation;biosynthetic process;catabolic process;cellular biosynthetic process;cell
adiponectin-mediated signaling pathway;anatomical structure morphogenesis;axon guidance;biological regulation;cell cycle;cell surface receptor linked signaling pathway;cellular component assembly;cellular component ass
biosynthetic process;cellular biosynthetic process;cellular component disassembly;cellular component disassembly at cellular level;cellular component organization;cellular component organization at cellular level;cellular cor
biosynthetic process;cellular biosynthetic process;cellular component disassembly;cellular component disassembly at cellular level;cellular component organization;cellular component organization at cellular level;cellular cor
biosynthetic process;cellular biosynthetic process;cellular component disassembly;cellular component disassembly at cellular level;cellular component organization;cellular component organization at cellular level;cellular cor
activation of caspase activity;activation of caspase activity by cytochrome c;apoptosis;biological regulation;biosynthetic process;catabolic process;cell death;cellular biosynthetic process;cellular catabolic process;cellular co
biological regulation;catabolic process;cell cycle checkpoint;cellular catabolic process;cellular component organization;cellular component organization at cellular level;cellular component organization or biogenesis;cellular c
biological regulation;biosynthetic process;body fluid secretion;cellular aromatic compound metabolic process;cellular biosynthetic process;cellular metabolic process;cellular nitrogen compound biosynthetic process;cellular
activation of immune response;apoptosis;biological regulation;biosynthetic process;blood coagulation;blood coagulation, intrinsic pathway;cell death;cellular biosynthetic process;cellular component assembly;cellular compo
biological regulation;blood coagulation;cell activation;cellular component assembly;cellular component assembly at cellular level;cellular component organization;cellular component organization at cellular level;cellular compo
defense response;defense response to virus;immune effector process;immune response;immune system process;innate immune response;multi-organism process;response to biotic stimulus;response to other organism;resp
associative learning;behavior;biological regulation;cellular component organization;cellular component organization or biogenesis;cellular membrane organization;cellular process;cellular response to stimulus;cognition;endoc
anatomical structure development;biological regulation;biosynthetic process;cell development;cellular biosynthetic process;cellular developmental process;cellular macromolecule biosynthetic process;cellular macromolecule
multicellular organismal process;muscle contraction;muscle system process;system process
biological regulation;cell cycle;cellular macromolecule metabolic process;cellular metabolic process;cellular nitrogen compound metabolic process;cellular process;macromolecule metabolic process;macromolecule methylat
aromatic compound catabolic process;catabolic process;cellular aromatic compound metabolic process;cellular catabolic process;cellular metabolic process;cellular process;metabolic process;response to chemical stimulus
biological regulation;biosynthetic process;cellular biosynthetic process;cellular component organization;cellular component organization at cellular level;cellular component organization or biogenesis;cellular component orga
biological regulation;cellular process;cellular response to stimulus;chemotaxis;establishment of localization;intracellular signal transduction;locomotion;metabolic process;positive regulation of catalytic activity;positive regulat
biological regulation;biosynthetic process;cell differentiation;cellular biosynthetic process;cellular developmental process;cellular macromolecule biosynthetic process;cellular macromolecule metabolic process;cellular metab
acyl-CoA biosynthetic process;acyl-CoA metabolic process;acylglycerol biosynthetic process;acylglycerol metabolic process;biological regulation;biosynthetic process;carboxylic acid biosynthetic process;carboxylic acid me
cellular component organization;cellular component organization at cellular level;cellular component organization or biogenesis;cellular component organization or biogenesis at cellular level;cellular process;Golgi organization
biosynthetic process;cellular biosynthetic process;cellular component disassembly;cellular component disassembly at cellular level;cellular component organization;cellular component organization at cellular level;cellular cor
2-oxoglutarate metabolic process;amine catabolic process;amine metabolic process;aromatic amino acid family catabolic process;aromatic amino acid family metabolic process;aromatic compound catabolic process;biosyn
alcohol metabolic process;amine metabolic process;biosynthetic process;cellular amine metabolic process;cellular biogenic amine metabolic process;cellular biosynthetic process;cellular component organization;cellular com
anion transport;B cell activation;B cell differentiation;biological regulation;cell activation;cell differentiation;cell volume homeostasis;cellular developmental process;cellular homeostasis;cellular process;developmental process
cellular macromolecule metabolic process;cellular metabolic process;cellular process;cellular protein metabolic process;developmental process;gamete generation;macromolecule metabolic process;macromolecule modifica
cellular macromolecule metabolic process;cellular metabolic process;cellular nitrogen compound metabolic process;cellular process;macromolecule metabolic process;metabolic process;nitrogen compound metabolic proce
cellular macromolecule metabolic process;cellular metabolic process;cellular nitrogen compound metabolic process;cellular process;macromolecule metabolic process;macromolecule modification;metabolic process;nitroge
cell cycle;cell cycle phase;cell cycle process;cell division;cellular component organization;cellular component organization at cellular level;cellular component organization or biogenesis;cellular component organization or bio
developmental process;multicellular organismal development;multicellular organismal process
macromolecule metabolic process;membrane protein proteolysis;metabolic process;primary metabolic process;protein metabolic process;proteolysis
biological regulation;cell projection assembly;cell projection organization;cellular component assembly;cellular component assembly at cellular level;cellular component organization;cellular component organization at cellular
actin cytoskeleton organization;actin filament-based process;biological regulation;cell motility;cell surface receptor linked signaling pathway;cellular component assembly;cellular component movement;cellular component or
biological regulation;cell surface receptor linked signaling pathway;cellular component organization;cellular component organization or biogenesis;cellular process;cellular response to stimulus;endosome transport;enzyme lin
biological regulation;biosynthetic process;cellular biosynthetic process;cellular macromolecule biosynthetic process;cellular macromolecule metabolic process;cellular metabolic process;cellular nitrogen compound metabolic
biological regulation;developmental process;dorsal/ventral pattern formation;negative regulation of biological process;negative regulation of catalytic activity;negative regulation of cell communication;negative regulation of ce
cellular component organization;cellular component organization or biogenesis;cellular membrane organization;cellular process;endocytosis;establishment of localization;membrane invagination;membrane organization;recep
activation of caspase activity;activation-induced cell death of T cells;aging;apoptosis;biological regulation;biosynthetic process;cell activation;cell aging;cell death;cell differentiation;cellular biosynthetic process;cellular comp
cellular component organization;cellular component organization at cellular level;cellular component organization or biogenesis;cellular component organization or biogenesis at cellular level;cellular macromolecule metabolic
aging;anatomical structure homeostasis;biological regulation;cell aging;cellular component organization;cellular component organization at cellular level;cellular component organization or biogenesis;cellular component orga
establishment of localization;lipid transport;organic substance transport;transport
biological regulation;cellular macromolecule metabolic process;cellular metabolic process;cellular process;cellular protein metabolic process;cellular response to chemical stimulus;cellular response to organic substance;cellu
anatomical structure morphogenesis;anterior/posterior axis specification;axis specification;biological regulation;biosynthetic process;cell cycle;cellular biosynthetic process;cellular component organization;cellular componen
biological regulation;catabolic process;cell cycle arrest;cell cycle process;cell differentiation;cell division;cellular catabolic process;cellular component assembly;cellular component assembly at cellular level;cellular componen
biological regulation;catabolic process;cellular catabolic process;cellular macromolecule catabolic process;cellular macromolecule metabolic process;cellular metabolic process;cellular process;cellular response to chemical s
cellular component organization;cellular component organization at cellular level;cellular component organization or biogenesis;cellular component organization or biogenesis at cellular level;cellular macromolecule metabolic
cellular macromolecule metabolic process;cellular metabolic process;cellular nitrogen compound metabolic process;cellular process;macromolecule metabolic process;maturation of SSU-rRNA;maturation of SSU-rRNA from
ADP catabolic process;ADP metabolic process;catabolic process;cellular catabolic process;cellular metabolic process;cellular nitrogen compound catabolic process;cellular nitrogen compound metabolic process;cellular pro
biological regulation;biosynthetic process;cellular biosynthetic process;cellular component assembly;cellular component organization;cellular component organization or biogenesis;cellular macromolecule biosynthetic proces
antigen processing and presentation;biological regulation;cellular component organization;cellular component organization at cellular level;cellular component organization or biogenesis;cellular component organization or bio
biological regulation;biosynthetic process;canonical Wnt receptor signaling pathway;catabolic process;cell surface receptor linked signaling pathway;cellular biosynthetic process;cellular catabolic process;cellular component
activation of immune response;anatomical structure formation involved in morphogenesis;anatomical structure morphogenesis;angiogenesis;antigen receptor-mediated signaling pathway;biological adhesion;biological regula
anatomical structure development;cranial nerve development;developmental process;nerve development;optic nerve development
biological regulation;cell redox homeostasis;cellular homeostasis;cellular macromolecule metabolic process;cellular metabolic process;cellular process;cellular protein metabolic process;cellular response to stimulus;cellular r
biological regulation;biosynthetic process;cellular biosynthetic process;cellular macromolecule biosynthetic process;cellular macromolecule metabolic process;cellular metabolic process;cellular nitrogen compound metabolic
biological regulation;cellular process;cellular response to stimulus;hippo signaling cascade;intracellular signal transduction;regulation of biological process;regulation of cellular process;response to stimulus;signal transductio
biological regulation;carboxylic acid metabolic process;cellular hormone metabolic process;cellular ketone metabolic process;cellular lipid metabolic process;cellular metabolic process;cellular process;diterpenoid metabolic

behavior;cellular component movement;cellular component organization;cellular component organization at cellular level;cellular component organization or biogenesis;cellular component organization or biogenesis at cellula
biological regulation;catabolic process;cellular catabolic process;cellular macromolecule catabolic process;cellular macromolecule metabolic process;cellular metabolic process;cellular nitrogen compound metabolic process
catabolic process;cellular catabolic process;cellular macromolecule catabolic process;cellular macromolecule metabolic process;cellular metabolic process;cellular nitrogen compound metabolic process;cellular process;defe
cellular process;establishment of localization;transmembrane transport;transport
biosynthetic process;cellular biosynthetic process;cellular metabolic process;cellular nitrogen compound metabolic process;cellular process;coenzyme A biosynthetic process;coenzyme A metabolic process;coenzyme biosy
biosynthetic process;cellular biosynthetic process;cellular component disassembly;cellular component disassembly at cellular level;cellular component organization;cellular component organization at cellular level;cellular cor
cation transport;establishment of localization;ion transport;metal ion transport;transport
cellular component assembly;cellular component organization;cellular component organization or biogenesis;cellular macromolecule metabolic process;cellular metabolic process;cellular nitrogen compound metabolic proce
cellular macromolecule metabolic process;cellular metabolic process;cellular nitrogen compound metabolic process;cellular process;macromolecule metabolic process;macromolecule modification;metabolic process;ncRNA
cation transport;cellular macromolecule metabolic process;cellular metabolic process;cellular nitrogen compound metabolic process;cellular process;establishment of localization;ion transport;macromolecule metabolic proce
activation of JUN kinase activity;activation of MAPK activity;androgen receptor signaling pathway;apoptosis;biological regulation;biosynthetic process;cell cycle cytokinesis;cell cycle process;cell death;cellular biosynthetic p
biological regulation;regulation of autophagic vacuole assembly;regulation of autophagy;regulation of biological process;regulation of catabolic process;regulation of cell communication;regulation of cellular catabolic process
biosynthetic process;cellular biosynthetic process;cellular macromolecule metabolic process;cellular metabolic process;cellular nitrogen compound metabolic process;cellular process;ER to Golgi vesicle-mediated transport;
amine biosynthetic process;amine metabolic process;amino acid salvage;aspartate family amino acid biosynthetic process;aspartate family amino acid metabolic process;biosynthetic process;carboxylic acid biosynthetic pro
biological regulation;catabolic process;cellular catabolic process;cellular macromolecule catabolic process;cellular macromolecule metabolic process;cellular metabolic process;cellular process;cellular protein metabolic proc
biological regulation;negative regulation of appetite;negative regulation of biological process;negative regulation of response to external stimulus;negative regulation of response to extracellular stimulus;negative regulation of
antigen processing and presentation;biological regulation;blood coagulation;cell migration;cell motility;cellular component movement;cellular process;coagulation;hemostasis;immune response;immune system process;leuko
cellular process;establishment of localization;transport;vesicle-mediated transport
catabolic process;cellular catabolic process;cellular macromolecule catabolic process;cellular macromolecule metabolic process;cellular metabolic process;cellular nitrogen compound metabolic process;cellular process;exo
biological regulation;cellular macromolecule metabolic process;cellular metabolic process;cellular nitrogen compound metabolic process;cellular process;humoral immune response;immune response;immune system process
cellular macromolecule metabolic process;cellular metabolic process;cellular nitrogen compound metabolic process;cellular process;macromolecule metabolic process;maturation of SSU-rRNA;maturation of SSU-rRNA from
cellular component organization;cellular component organization at cellular level;cellular component organization or biogenesis;cellular component organization or biogenesis at cellular level;cellular macromolecule metabolic
biological regulation;biosynthetic process;cellular biosynthetic process;cellular macromolecule biosynthetic process;cellular macromolecule metabolic process;cellular metabolic process;cellular nitrogen compound metabolic
amine transport;biological regulation;cellular component assembly;cellular component assembly at cellular level;cellular component organization;cellular component organization at cellular level;cellular component organizatic
cellular macromolecule metabolic process;cellular metabolic process;cellular nitrogen compound metabolic process;cellular process;macromolecule metabolic process;metabolic process;nitrogen compound metabolic proce
biological regulation;cellular macromolecule metabolic process;cellular metabolic process;cellular nitrogen compound metabolic process;cellular process;macromolecule metabolic process;maturation of SSU-rRNA;metabolic
biological regulation;cellular component assembly;cellular component assembly at cellular level;cellular component organization;cellular component organization at cellular level;cellular component organization or biogenesis;
activation of immune response;activation of innate immune response;activation of MAPK activity;activation of MAPKK activity;activation of NF-kappaB-inducing kinase activity;activation of protein kinase activity;anatomical s
biological regulation;cell cycle;cell cycle phase;cell cycle process;cell division;cellular component organization;cellular component organization at cellular level;cellular component organization or biogenesis;cellular componen
cellular component assembly;cellular component assembly at cellular level;cellular component biogenesis;cellular component organization;cellular component organization at cellular level;cellular component organization or b
alcohol metabolic process;biosynthetic process;carbohydrate biosynthetic process;carbohydrate metabolic process;cellular biosynthetic process;cellular carbohydrate metabolic process;cellular lipid metabolic process;cellul
biological regulation;cation homeostasis;cellular cation homeostasis;cellular chemical homeostasis;cellular copper ion homeostasis;cellular homeostasis;cellular ion homeostasis;cellular process;chemical homeostasis;coppe
anatomical structure morphogenesis;biological regulation;cellular macromolecule metabolic process;cellular metabolic process;cellular nitrogen compound metabolic process;cellular process;cellular protein metabolic proces
biological regulation;carboxylic acid metabolic process;catabolic process;cellular catabolic process;cellular ketone metabolic process;cellular lipid metabolic process;cellular macromolecule catabolic process;cellular macrom
biological regulation;cell surface receptor linked signaling pathway;cellular macromolecule metabolic process;cellular metabolic process;cellular process;cellular protein metabolic process;cellular response to chemical stimulu
activation of immune response;activation of innate immune response;anaphase-promoting complex-dependent proteasomal ubiquitin-dependent protein catabolic process;antigen processing and presentation;antigen proces
biological regulation;cellular process;establishment of localization;regulation of biological process;regulation of biosynthetic process;regulation of cellular biosynthetic process;regulation of cellular macromolecule biosynthetic
cellular metabolic process;cellular process;electron transport chain;generation of precursor metabolites and energy;metabolic process;mitochondrial electron transport, NADH to ubiquinone;oxidation-reduction process;respi
cellular metabolic process;cellular process;electron transport chain;generation of precursor metabolites and energy;metabolic process;mitochondrial electron transport, NADH to ubiquinone;oxidation-reduction process;respi
cell proliferation;cell proliferation in external granule layer;cell proliferation in hindbrain;cellular component assembly;cellular component assembly at cellular level;cellular component organization;cellular component organizat
biological regulation;cell redox homeostasis;cellular homeostasis;cellular process;homeostatic process;regulation of biological process;regulation of biological quality;regulation of cellular process
activation of adenylate cyclase activity;activation of immune response;activation of innate immune response;activation of MAPKK activity;activation of protein kinase activity;anatomical structure development;apoptosis;axon
biotin metabolic process;carboxylic acid catabolic process;carboxylic acid metabolic process;catabolic process;cellular amide metabolic process;cellular catabolic process;cellular ketone metabolic process;cellular lipid cata
anatomical structure development;anatomical structure morphogenesis;androgen receptor signaling pathway;apoptosis;attachment of spindle microtubules to chromosome;biological regulation;biosynthetic process;cell cycle
biosynthetic process;cellular biosynthetic process;cellular macromolecule biosynthetic process;cellular macromolecule metabolic process;cellular metabolic process;cellular nitrogen compound metabolic process;cellular pro
alcohol catabolic process;alcohol metabolic process;anatomical structure development;carbohydrate catabolic process;carbohydrate metabolic process;catabolic process;cell development;cellular carbohydrate catabolic pro
biosynthetic process;cellular aromatic compound metabolic process;cellular biosynthetic process;cellular macromolecule metabolic process;cellular metabolic process;cellular nitrogen compound biosynthetic process;cellula
cellular component assembly;cellular component assembly at cellular level;cellular component organization;cellular component organization at cellular level;cellular component organization or biogenesis;cellular component c
3'-phosphoadenosine 5'-phosphosulfate metabolic process;alcohol metabolic process;amine metabolic process;benzene-containing compound metabolic process;biological regulation;catecholamine metabolic process;cate
alcohol metabolic process;biological regulation;biosynthetic process;C21-steroid hormone biosynthetic process;C21-steroid hormone metabolic process;cellular biosynthetic process;cellular hormone metabolic process;cellu
biosynthetic process;cellular biosynthetic process;cellular macromolecule metabolic process;cellular metabolic process;cellular nitrogen compound metabolic process;cellular process;electron transport chain;gene expressio
actin cytoskeleton organization;actin filament bundle assembly;actin filament organization;actin filament-based process;activation of immune response;activation of innate immune response;activation of protein kinase activity
biological regulation;blood coagulation;cell activation;cellular process;coagulation;establishment of localization;establishment of localization in cell;exocytosis;hemostasis;multicellular organismal process;platelet activation;pla
biological regulation;blood coagulation;cell activation;cell surface receptor linked signaling pathway;cellular process;cellular response to stimulus;coagulation;defense response;hemostasis;immune response;immune system
acute inflammatory response;acute-phase response;alpha-beta T cell activation;anatomical structure development;biological regulation;biosynthetic process;brown fat cell differentiation;CD4-positive, alpha-beta T cell activa
B cell activation;B cell differentiation;biological regulation;carbohydrate homeostasis;cell activation;cell differentiation;cell surface receptor linked signaling pathway;cellular developmental process;cellular macromolecule meta
biological regulation;biosynthetic process;cellular biosynthetic process;cellular macromolecule biosynthetic process;cellular macromolecule metabolic process;cellular metabolic process;cellular nitrogen compound metabolic
anatomical structure development;ATP biosynthetic process;ATP metabolic process;ATP synthesis coupled proton transport;biosynthetic process;cation transport;cellular biosynthetic process;cellular metabolic process;cellu
activation of MAPKK activity;activation of protein kinase activity;avoidance of defenses of other organism involved in symbiotic interaction;avoidance of host defenses;biological regulation;biosynthetic process;cellular biosyn
anatomical structure development;anatomical structure formation involved in morphogenesis;biological adhesion;biological regulation;blood coagulation;brain development;cell activation;cell adhesion;cell development;cell ju
ADP biosynthetic process;ADP metabolic process;AMP metabolic process;anatomical structure development;ATP metabolic process;biosynthetic process;brain development;cellular biosynthetic process;cellular metabolic pr
amine transport;amino acid transport;carboxylic acid transport;cellular process;establishment of localization;ion transport;nitrogen compound transport;organic acid transport;organic substance transport;transmembrane tran
actin cytoskeleton organization;actin filament-based process;biological regulation;cellular component organization;cellular component organization at cellular level;cellular component organization or biogenesis;cellular compo
activation of caspase activity;activation of caspase activity by cytochrome c;anatomical structure development;anatomical structure formation involved in morphogenesis;apoptosis;apoptotic mitochondrial changes;B cell hor
apoptosis;apoptosis in response to endoplasmic reticulum stress;biological regulation;cell death;cellular process;death;macromolecule metabolic process;metabolic process;primary metabolic process;programmed cell deat
biosynthetic process;cellular aromatic compound metabolic process;cellular biosynthetic process;cellular metabolic process;cellular nitrogen compound biosynthetic process;cellular nitrogen compound metabolic process;ce
antigen processing and presentation;biological regulation;blood coagulation;cell activation;cell activation involved in immune response;cell differentiation;cellular developmental process;cellular localization;cellular macromole
biological regulation;biosynthetic process;catabolic process;cell cycle;cell cycle process;cellular biosynthetic process;cellular catabolic process;cellular macromolecule biosynthetic process;cellular macromolecule catabolic p
actin cytoskeleton organization;actin filament-based process;biological regulation;cell projection assembly;cell projection organization;cellular component assembly;cellular component assembly at cellular level;cellular compo
abscission;biological regulation;cell division;cellular process;cytokinesis;developmental process;establishment of localization;establishment of protein localization;localization;macromolecule localization;positive regulation of
activation of caspase activity;anatomical structure development;apoptosis;apoptotic mitochondrial changes;biological regulation;brain development;cell death;cellular component assembly;cellular component organization;ce
ATP biosynthetic process;ATP metabolic process;ATP synthesis coupled proton transport;biosynthetic process;cation transport;cellular biosynthetic process;cellular metabolic process;cellular nitrogen compound biosynthetic
cellular component assembly;cellular component assembly at cellular level;cellular component biogenesis;cellular component biogenesis at cellular level;cellular component organization;cellular component organization at ce
cellular component assembly;cellular component assembly at cellular level;cellular component organization;cellular component organization at cellular level;cellular component organization or biogenesis;cellular component c
antigen processing and presentation;antigen processing and presentation of exogenous antigen;antigen processing and presentation of exogenous peptide antigen;antigen processing and presentation of exogenous peptide
antigen processing and presentation;biological regulation;cellular component organization;cellular component organization at cellular level;cellular component organization or biogenesis;cellular component organization or bic
B cell activation;biological regulation;cell activation;cellular process;cellular response to stimulus;immune system process;leukocyte activation;lymphocyte activation;negative regulation of biological process;negative regulatic
biological regulation;negative regulation of biological process;negative regulation of biosynthetic process;negative regulation of cellular biosynthetic process;negative regulation of cellular macromolecule biosynthetic process
actin cytoskeleton organization;actin filament-based process;adult behavior;anatomical structure development;anatomical structure formation involved in morphogenesis;anatomical structure morphogenesis;apoptosis;assoc
anatomical structure development;apoptosis;biological regulation;cell death;cell development;cell division;cellular developmental process;cellular process;cellular process involved in reproduction;death;defense response;dev
amine metabolic process;amine transport;amino acid transmembrane transport;amino acid transport;anatomical structure development;aromatic amino acid transport;biological regulation;blood coagulation;branched-chain a
cellular macromolecule metabolic process;cellular metabolic process;cellular process;cellular protein metabolic process;macromolecule metabolic process;macromolecule modification;metabolic process;primary metabolic p
apoptotic mitochondrial changes;biological regulation;cell fate determination;cellular component organization;cellular component organization at cellular level;cellular component organization or biogenesis;cellular component
catabolic process;cellular catabolic process;cellular component assembly;cellular component organization;cellular component organization or biogenesis;cellular metabolic process;cellular process;macromolecular complex a
biological regulation;biosynthetic process;cellular biosynthetic process;cellular lipid metabolic process;cellular metabolic process;cellular process;cellular response to stimulus;glycerolipid biosynthetic process;glycerolipid me
actin cytoskeleton organization;actin filament-based process;biological regulation;cell chemotaxis;cell migration;cell motility;cellular component movement;cellular component organization;cellular component organization at
biosynthetic process;cellular biosynthetic process;cellular component disassembly;cellular component disassembly at cellular level;cellular component organization;cellular component organization at cellular level;cellular cor
anatomical structure development;biological regulation;biosynthetic process;cell activation;cell differentiation;cell maturation;cellular biosynthetic process;cellular developmental process;cellular macromolecule biosynthetic p
biosynthetic process;cellular biosynthetic process;cellular component disassembly;cellular component disassembly at cellular level;cellular component organization;cellular component organization at cellular level;cellular cor
biological regulation;biosynthetic process;cellular biosynthetic process;cellular component organization;cellular component organization or biogenesis;cellular macromolecule biosynthetic process;cellular macromolecule met
anatomical structure development;biological regulation;cell surface receptor linked signaling pathway;cellular localization;cellular macromolecule localization;cellular macromolecule metabolic process;cellular metabolic proce
biological regulation;biosynthetic process;catabolic process;cellular biosynthetic process;cellular catabolic process;cellular macromolecule biosynthetic process;cellular macromolecule catabolic process;cellular macromolecu
anatomical structure development;anatomical structure morphogenesis;biological regulation;cargo loading into vesicle;cellular component assembly;cellular component assembly at cellular level;cellular component organizati
anatomical structure morphogenesis;biological regulation;biosynthetic process;cellular biosynthetic process;cellular macromolecule biosynthetic process;cellular macromolecule metabolic process;cellular metabolic process;
anatomical structure morphogenesis;biological regulation;catabolic process;cell surface receptor linked signaling pathway;cellular catabolic process;cellular macromolecule catabolic process;cellular macromolecule metaboli
cellular metabolic process;cellular process;electron transport chain;generation of precursor metabolites and energy;metabolic process;mitochondrial electron transport, NADH to ubiquinone;oxidation-reduction process;respi
2'-deoxyribonucleotide biosynthetic process;2'-deoxyribonucleotide metabolic process;alcohol metabolic process;ATP metabolic process;biosynthetic process;carbohydrate metabolic process;cellular biosynthetic process;c
biosynthetic process;carboxylic acid biosynthetic process;carboxylic acid catabolic process;carboxylic acid metabolic process;catabolic process;cellular biosynthetic process;cellular catabolic process;cellular ketone metabo
activation of protein kinase activity;activation of protein kinase C activity by G-protein coupled receptor protein signaling pathway;biological regulation;biosynthetic process;cell surface receptor linked signaling pathway;cellul
anatomical structure development;biological regulation;cellular macromolecule metabolic process;cellular metabolic process;cellular nitrogen compound metabolic process;cellular process;developmental process;hemopoieti
carbohydrate metabolic process;cellular carbohydrate metabolic process;cellular macromolecule metabolic process;cellular metabolic process;cellular process;cellular protein metabolic process;glycosylation;macromolecule
cellular macromolecule metabolic process;cellular metabolic process;cellular process;cellular protein metabolic process;macromolecule metabolic process;macromolecule modification;metabolic process;primary metabolic p
cellular component assembly;cellular component assembly at cellular level;cellular component organization;cellular component organization at cellular level;cellular component organization or biogenesis;cellular component c
cellular macromolecule metabolic process;cellular metabolic process;cellular nitrogen compound metabolic process;cellular process;macromolecule metabolic process;metabolic process;nitrogen compound metabolic proce
apoptosis;biological regulation;cation homeostasis;cation transport;cell cycle;cell death;cellular cation homeostasis;cellular chemical homeostasis;cellular homeostasis;cellular ion homeostasis;cellular iron ion homeostasis;ce
alcohol metabolic process;carbohydrate metabolic process;cellular carbohydrate metabolic process;cellular metabolic process;cellular process;inositol metabolic process;inositol phosphate metabolic process;metabolic proc
activation of MAPKK activity;activation of protein kinase activity;anatomical structure development;biological regulation;cell cycle;cell cycle checkpoint;cellular macromolecule metabolic process;cellular metabolic process;ce
anatomical structure development;biosynthetic process;cellular biosynthetic process;cellular macromolecule biosynthetic process;cellular macromolecule metabolic process;cellular metabolic process;cellular nitrogen compo
biological regulation;biosynthetic process;cellular biosynthetic process;cellular component organization;cellular component organization at cellular level;cellular component organization or biogenesis;cellular component orga
biological regulation;biosynthetic process;cellular biosynthetic process;cellular macromolecule biosynthetic process;cellular macromolecule metabolic process;cellular metabolic process;cellular nitrogen compound metabolic
anatomical structure development;biological regulation;developmental process;liver development;organ development;regulation of biological process;regulation of lipid metabolic process;regulation of metabolic process;regu
anatomical structure development;anatomical structure formation involved in morphogenesis;anatomical structure morphogenesis;axis specification;biological regulation;bone trabecula formation;branching involved in prosta
biosynthetic process;cellular biosynthetic process;cellular component disassembly;cellular component disassembly at cellular level;cellular component organization;cellular component organization at cellular level;cellular cor
biological regulation;biosynthetic process;cellular biosynthetic process;cellular macromolecule biosynthetic process;cellular macromolecule metabolic process;cellular metabolic process;cellular nitrogen compound metabolic
cell differentiation;cellular developmental process;cellular process;developmental process;gamete generation;male gamete generation;multicellular organismal development;multicellular organismal process;multicellular organ
developmental process;keratinization
biological regulation;biosynthetic process;carbohydrate metabolic process;carbohydrate transport;cell cycle;cell cycle process;cell surface receptor linked signaling pathway;cellular biosynthetic process;cellular component c

cell cycle;cell cycle phase;cell cycle process;cell division;cellular component disassembly;cellular component disassembly at cellular level;cellular component organization;cellular component organization at cellular level;cellu
biological regulation;cellular component organization;cellular component organization at cellular level;cellular component organization or biogenesis;cellular component organization or biogenesis at cellular level;cellular proce
anatomical structure development;biosynthetic process;catabolic process;cellular biosynthetic process;cellular catabolic process;cellular macromolecule biosynthetic process;cellular macromolecule catabolic process;cellula
biosynthetic process;cellular biosynthetic process;cellular component disassembly;cellular component disassembly at cellular level;cellular component organization;cellular component organization at cellular level;cellular cor
biological regulation;cell cycle checkpoint;cellular component assembly;cellular component assembly at cellular level;cellular component organization;cellular component organization at cellular level;cellular component orgar
biosynthetic process;cellular biosynthetic process;cellular component disassembly;cellular component disassembly at cellular level;cellular component organization;cellular component organization at cellular level;cellular cor
biological regulation;defense response;immune response;immune system process;innate immune response;regulation of biological process;regulation of cellular metabolic process;regulation of cellular process;regulation of m
alcohol biosynthetic process;alcohol metabolic process;amino sugar biosynthetic process;amino sugar metabolic process;anatomical structure development;biosynthetic process;carbohydrate biosynthetic process;carbohyc
apoptosis;biological regulation;biosynthetic process;cell death;cellular biosynthetic process;cellular macromolecule biosynthetic process;cellular macromolecule metabolic process;cellular metabolic process;cellular nitrogen
biological regulation;cellular process;cellular response to stimulus;cellular response to stress;positive regulation of molecular function;positive regulation of NF-kappaB transcription factor activity;positive regulation of sequen
biological regulation;metabolic process;positive regulation of biological process;positive regulation of biosynthetic process;positive regulation of cellular biosynthetic process;positive regulation of cellular metabolic process;p
cellular macromolecule metabolic process;cellular metabolic process;cellular nitrogen compound metabolic process;cellular process;establishment of localization;establishment of localization in cell;establishment of RNA loca
actin cytoskeleton organization;actin filament organization;actin filament-based process;actin nucleation;Arp2/3 complex-mediated actin nucleation;biological regulation;cellular component organization;cellular component or
cellular macromolecule metabolic process;cellular metabolic process;cellular nitrogen compound metabolic process;cellular process;cellular protein metabolic process;cellular response to stimulus;cellular response to stress;
biological regulation;cell differentiation;cellular component assembly;cellular component assembly at cellular level;cellular component organization;cellular component organization at cellular level;cellular component organizat
autophagic vacuole fusion;biological regulation;body fluid secretion;cellular component assembly;cellular component assembly at cellular level;cellular component organization;cellular component organization at cellular level
catabolic process;cellular catabolic process;cellular component organization;cellular component organization at cellular level;cellular component organization or biogenesis;cellular component organization or biogenesis at ce
biosynthetic process;cellular biosynthetic process;cellular component disassembly;cellular component disassembly at cellular level;cellular component organization;cellular component organization at cellular level;cellular cor
biological regulation;biosynthetic process;cellular biosynthetic process;cellular component organization;cellular component organization at cellular level;cellular component organization or biogenesis;cellular component orgar
cellular macromolecule metabolic process;cellular metabolic process;cellular nitrogen compound metabolic process;cellular process;cellular response to stimulus;cellular response to stress;DNA metabolic process;DNA repai
carbohydrate metabolic process;cation transport;cellular carbohydrate metabolic process;cellular macromolecule metabolic process;cellular metabolic process;cellular process;cellular protein metabolic process;cognition;div
biological regulation;biosynthetic process;cation homeostasis;cellular biosynthetic process;cellular cation homeostasis;cellular chemical homeostasis;cellular homeostasis;cellular ion homeostasis;cellular iron ion homeostasis
cellular component organization;cellular component organization at cellular level;cellular component organization or biogenesis;cellular component organization or biogenesis at cellular level;cellular localization;cellular proces
cellular process;establishment of localization;establishment of localization in cell;establishment of protein localization;intracellular protein transport;intracellular transport;protein transport;transport;vesicle-mediated transport
cellular macromolecule metabolic process;cellular metabolic process;cellular nitrogen compound metabolic process;cellular process;cellular protein metabolic process;macromolecule metabolic process;macromolecule modi
establishment of localization;establishment of localization in cell;establishment of protein localization;intracellular transport;protein transport;transport
biosynthetic process;cellular biosynthetic process;cellular macromolecule biosynthetic process;cellular macromolecule metabolic process;cellular metabolic process;cellular nitrogen compound metabolic process;cellular pro
cellular metabolic process;cellular process;metabolic process;methylation;one-carbon metabolic process;small molecule metabolic process
biological regulation;catabolic process;cellular catabolic process;cellular macromolecule catabolic process;cellular macromolecule metabolic process;cellular metabolic process;cellular process;cellular response to chemical s
apoptosis;biosynthetic process;cell death;cellular biosynthetic process;cellular component disassembly;cellular component disassembly at cellular level;cellular component organization;cellular component organization at cel
biosynthetic process;carboxylic acid biosynthetic process;carboxylic acid metabolic process;cellular biosynthetic process;cellular ketone metabolic process;cellular lipid metabolic process;cellular metabolic process;cellular p
carbohydrate catabolic process;carbohydrate metabolic process;catabolic process;cellular carbohydrate catabolic process;cellular carbohydrate metabolic process;cellular metabolic process;cellular process;glucuronoside c
cellular component organization;cellular component organization or biogenesis;cellular membrane organization;cellular process;endocytosis;establishment of localization;establishment of protein localization;membrane invagi
biological adhesion;biological regulation;cell adhesion;cell division;cell surface receptor linked signaling pathway;cellular component biogenesis;cellular component biogenesis at cellular level;cellular component organization
actin cytoskeleton organization;actin filament-based process;biological regulation;cellular component organization;cellular component organization at cellular level;cellular component organization or biogenesis;cellular compo
cellular component assembly;cellular component assembly at cellular level;cellular component biogenesis;cellular component organization;cellular component organization at cellular level;cellular component organization or b
cellular process;endosome transport;establishment of localization;establishment of localization in cell;establishment of protein localization;intracellular protein transport;intracellular transport;protein transport;retrograde trans
cellular macromolecule metabolic process;cellular metabolic process;cellular nitrogen compound metabolic process;cellular process;macromolecule metabolic process;macromolecule methylation;macromolecule modificatio
biological regulation;catabolic process;cellular catabolic process;cellular component assembly;cellular component assembly at cellular level;cellular component organization;cellular component organization at cellular level;ce
catabolic process;cellular catabolic process;cellular macromolecule catabolic process;cellular macromolecule metabolic process;cellular metabolic process;cellular nitrogen compound metabolic process;cellular process;exo
biological regulation;cellular process;cellular response to stimulus;establishment of localization;establishment of localization in cell;establishment of protein localization;intracellular protein transport;intracellular signal transduc
biosynthetic process;cellular biosynthetic process;cellular macromolecule biosynthetic process;cellular macromolecule metabolic process;cellular metabolic process;cellular process;cellular protein metabolic process;cotrans
biological regulation;regulation of biological process;regulation of catalytic activity;regulation of cellular metabolic process;regulation of cellular process;regulation of dephosphorylation;regulation of hydrolase activity;regulatio
biological adhesion;cell adhesion;cellular process;establishment of localization;ion transmembrane transport;ion transport;transmembrane transport;transport
cellular component assembly;cellular component assembly at cellular level;cellular component organization;cellular component organization at cellular level;cellular component organization or biogenesis;cellular component c
biological regulation;biosynthetic process;cellular biosynthetic process;cellular macromolecule biosynthetic process;cellular macromolecule metabolic process;cellular metabolic process;cellular nitrogen compound metabolic
biosynthetic process;cellular biosynthetic process;cellular ketone metabolic process;cellular metabolic process;cellular process;coenzyme biosynthetic process;coenzyme metabolic process;cofactor biosynthetic process;cof
establishment of localization;transport
cellular metabolic process;cellular nitrogen compound metabolic process;cellular process;metabolic process;nitrogen compound metabolic process;nucleic acid metabolic process;nucleic acid phosphodiester bond hydrolys
biosynthetic process;cellular biosynthetic process;cellular component disassembly;cellular component disassembly at cellular level;cellular component organization;cellular component organization at cellular level;cellular cor
biosynthetic process;cellular biosynthetic process;cellular component disassembly;cellular component disassembly at cellular level;cellular component organization;cellular component organization at cellular level;cellular cor
biological regulation;cation homeostasis;cation transport;cellular cation homeostasis;cellular chemical homeostasis;cellular copper ion homeostasis;cellular homeostasis;cellular ion homeostasis;cellular process;chemical hor
adaptive immune response;biological regulation;biosynthetic process;cell activation;cell chemotaxis;cell migration;cell motility;cell surface receptor linked signaling pathway;cellular biosynthetic process;cellular component as
cellular metabolic process;cellular process;electron transport chain;generation of precursor metabolites and energy;metabolic process;mitochondrial electron transport, NADH to ubiquinone;oxidation-reduction process;respi
biological regulation;cation transport;cellular metabolic process;cellular process;cellular response to chemical stimulus;cellular response to oxidative stress;cellular response to oxygen radical;cellular response to reactive oxy
anatomical structure development;biological regulation;biosynthetic process;cell differentiation;cellular biosynthetic process;cellular component organization;cellular component organization at cellular level;cellular componen
acute inflammatory response;arachidonic acid metabolic process;biological regulation;biosynthetic process;carboxylic acid biosynthetic process;carboxylic acid metabolic process;cellular biosynthetic process;cellular ketone
activation of caspase activity;activation of NF-kappaB-inducing kinase activity;activation of protein kinase activity;apoptosis;apoptosis in response to endoplasmic reticulum stress;biological regulation;cell death;cell surface
biological regulation;biosynthetic process;carbon catabolite regulation of transcription;cell communication;cellular biosynthetic process;cellular component organization;cellular component organization at cellular level;cellular
attachment of spindle microtubules to chromosome;attachment of spindle microtubules to kinetochore;biological regulation;cell cycle checkpoint;cell cycle process;cell division;cellular component organization;cellular compo
viral reproduction
biosynthetic process;cellular biosynthetic process;cellular component assembly;cellular component organization;cellular component organization or biogenesis;cellular macromolecule biosynthetic process;cellular macromole
anatomical structure development;biological regulation;biosynthetic process;cation homeostasis;cation transport;cellular biosynthetic process;cellular cation homeostasis;cellular chemical homeostasis;cellular component as
actin cytoskeleton organization;actin filament organization;actin filament polymerization;actin filament-based process;actin polymerization or depolymerization;biological regulation;cellular component assembly;cellular compo
biological regulation;cellular component organization;cellular component organization at cellular level;cellular component organization or biogenesis;cellular component organization or biogenesis at cellular level;cellular macro
biological regulation;biosynthetic process;cellular biosynthetic process;cellular metabolic process;cellular process;chromosome segregation;cofactor biosynthetic process;cofactor metabolic process;iron-sulfur cluster assem
carboxylic acid metabolic process;cellular ketone metabolic process;cellular lipid metabolic process;cellular metabolic process;cellular process;fatty acid metabolic process;lipid metabolic process;metabolic process;monoca
biological regulation;cellular macromolecule metabolic process;cellular metabolic process;cellular nitrogen compound metabolic process;cellular process;cellular protein metabolic process;cellular response to stimulus;cellula
activation of immune response;activation of innate immune response;adaptive immune response;adaptive immune response based on somatic recombination of immune receptors built from immunoglobulin superfamily doma
anatomical structure morphogenesis;biological regulation;biosynthetic process;cellular biosynthetic process;cellular macromolecule biosynthetic process;cellular macromolecule metabolic process;cellular metabolic process;
bile acid metabolic process;carboxylic acid catabolic process;carboxylic acid metabolic process;catabolic process;cellular catabolic process;cellular ketone metabolic process;cellular lipid catabolic process;cellular lipid meta
biosynthetic process;cellular biosynthetic process;cellular macromolecule biosynthetic process;cellular macromolecule metabolic process;cellular metabolic process;cellular process;cellular protein metabolic process;cotrans
biosynthetic process;cellular biosynthetic process;cellular macromolecule metabolic process;cellular metabolic process;cellular nitrogen compound metabolic process;cellular process;electron transport chain;gene expressio
amine catabolic process;amine metabolic process;branched chain family amino acid catabolic process;branched chain family amino acid metabolic process;carboxylic acid catabolic process;carboxylic acid metabolic proces
adherens junction organization;biological adhesion;biological regulation;cell adhesion;cell junction assembly;cell junction organization;cell-cell junction organization;cellular component assembly;cellular component assembly
behavior;biological regulation;carbohydrate catabolic process;carbohydrate metabolic process;catabolic process;cellular catabolic process;cellular lipid catabolic process;cellular lipid metabolic process;cellular metabolic pro
biological regulation;blood coagulation;cellular component organization;cellular component organization or biogenesis;cellular macromolecule metabolic process;cellular membrane organization;cellular metabolic process;cel
biosynthetic process;cellular biosynthetic process;cellular macromolecule biosynthetic process;cellular macromolecule metabolic process;cellular metabolic process;cellular nitrogen compound metabolic process;cellular pro
apoptosis;biological regulation;cell cycle phase;cell cycle process;cell death;cell proliferation;cellular component organization;cellular component organization at cellular level;cellular component organization or biogenesis;cel
activation of caspase activity;activation-induced cell death of T cells;adaptive immune response;adaptive immune response based on somatic recombination of immune receptors built from immunoglobulin superfamily doma
activation of immune response;activation of innate immune response;anaphase-promoting complex-dependent proteasomal ubiquitin-dependent protein catabolic process;antigen processing and presentation;antigen proces
activation of immune response;activation of innate immune response;anaphase-promoting complex-dependent proteasomal ubiquitin-dependent protein catabolic process;antigen processing and presentation;antigen proces
2'-deoxyribonucleotide metabolic process;base-excision repair;base-excision repair, AP site formation;catabolic process;cellular catabolic process;cellular macromolecule catabolic process;cellular macromolecule metabolic
alcohol catabolic process;alcohol metabolic process;amine catabolic process;amine metabolic process;branched chain family amino acid catabolic process;branched chain family amino acid metabolic process;carbohydrate
cap-independent translational initiation;cellular component assembly;cellular component assembly at cellular level;cellular component disassembly;cellular component disassembly at cellular level;cellular component organiza
actin cytoskeleton organization;actin filament organization;actin filament-based process;anatomical structure development;biological regulation;cell development;cell junction assembly;cell junction organization;cell migration
activation of immune response;activation of innate immune response;anaphase-promoting complex-dependent proteasomal ubiquitin-dependent protein catabolic process;antigen processing and presentation;antigen proces
anatomical structure development;anatomical structure morphogenesis;biological regulation;carbohydrate metabolic process;cardiac chamber morphogenesis;cardiac left ventricle morphogenesis;cardiac ventricle morphoge
biosynthetic process;cellular biosynthetic process;cellular macromolecule biosynthetic process;cellular macromolecule metabolic process;cellular metabolic process;cellular nitrogen compound metabolic process;cellular pro
acrosome assembly;anatomical structure formation involved in morphogenesis;cellular component assembly;cellular component assembly at cellular level;cellular component assembly involved in morphogenesis;cellular com
anion transport;behavioral response to nutrient;biological regulation;blood coagulation;carbohydrate homeostasis;carboxylic acid metabolic process;carboxylic acid transport;cell migration;cell motility;cellular component mo
activation of caspase activity;activation of caspase activity by cytochrome c;apoptosis;biological regulation;cell death;cellular component disassembly;cellular component disassembly at cellular level;cellular component disa
anatomical structure homeostasis;antigen processing and presentation;antigen processing and presentation of exogenous antigen;antigen processing and presentation of exogenous peptide antigen;antigen processing and p
biosynthetic process;catabolic process;cell differentiation;cellular biosynthetic process;cellular catabolic process;cellular component assembly;cellular component assembly at cellular level;cellular component biogenesis;cellu
anatomical structure development;biological regulation;cell surface receptor linked signaling pathway;cellular component organization;cellular component organization at cellular level;cellular component organization or bioge
antigen processing and presentation;antigen processing and presentation of endogenous antigen;antigen processing and presentation of endogenous peptide antigen;antigen processing and presentation of endogenous pep
biological regulation;biosynthetic process;catabolic process;cell proliferation;cellular biosynthetic process;cellular catabolic process;cellular macromolecule biosynthetic process;cellular macromolecule catabolic process;cellu
anaphase-promoting complex-dependent proteasomal ubiquitin-dependent protein catabolic process;biological regulation;catabolic process;cell cycle;cell cycle checkpoint;cell cycle process;cell division;cellular catabolic pr
cellular macromolecule metabolic process;cellular metabolic process;cellular process;cellular protein metabolic process;establishment of localization;establishment of localization in cell;establishment of protein localization;es
antigen processing and presentation;biological regulation;cellular component organization;cellular component organization at cellular level;cellular component organization or biogenesis;cellular component organization or bic
anatomical structure development;apoptosis;biological regulation;camera-type eye development;cell death;cell fate specification;cell surface receptor linked signaling pathway;cellular developmental process;cellular macrom
cellular process;cellular response to cold;cellular response to freezing;cellular response to stimulus;cellular response to stress;response to abiotic stimulus;response to cold;response to freezing;response to stimulus;response
cotranslational protein targeting to membrane;establishment of localization;establishment of localization in cell;establishment of protein localization;intracellular protein transport;intracellular transport;protein targeting;protein
biological regulation;biosynthetic process;cell cycle arrest;cell cycle process;cell surface receptor linked signaling pathway;cellular biosynthetic process;cellular macromolecule metabolic process;cellular metabolic process;c
anatomical structure morphogenesis;biological adhesion;biological regulation;calcium ion-dependent exocytosis;cell adhesion;cell morphogenesis;cell morphogenesis involved in differentiation;cell-substrate adhesion;cellular
alcohol catabolic process;alcohol metabolic process;carbohydrate catabolic process;carbohydrate metabolic process;catabolic process;cellular carbohydrate catabolic process;cellular carbohydrate metabolic process;cellula
cellular process;establishment of localization;establishment of localization in cell;establishment of protein localization;Golgi to plasma membrane transport;Golgi vesicle transport;intracellular transport;post-Golgi vesicle-med
anatomical structure development;anatomical structure morphogenesis;apoptosis;autophagy;biological regulation;brain development;catabolic process;cell death;cell part morphogenesis;cell projection morphogenesis;cell p
apoptosis;cell death;cellular process;death;programmed cell death
cell differentiation;cellular developmental process;cellular process;developmental process;hemopoietic progenitor cell differentiation
biological regulation;cellular macromolecule metabolic process;cellular metabolic process;cellular nitrogen compound metabolic process;cellular process;macromolecule metabolic process;metabolic process;mRNA metaboli
biological regulation;cell cycle;cell division;cellular process;establishment of localization;establishment of localization in cell;exocytosis;regulation of biological process;regulation of cellular process;regulation of establishment
anatomical structure formation involved in morphogenesis;blastocyst formation;cellular macromolecule metabolic process;cellular metabolic process;cellular nitrogen compound metabolic process;cellular process;developme
biological regulation;directional locomotion;locomotion;negative regulation of actin nucleation;negative regulation of biological process;negative regulation of cell migration;negative regulation of cell motility;negative regulation
adipose tissue development;anatomical structure development;biological regulation;carbohydrate homeostasis;carboxylic acid metabolic process;cell differentiation;cellular chemical homeostasis;cellular developmental proc
biological regulation;cellular component organization;cellular component organization at cellular level;cellular component organization or biogenesis;cellular component organization or biogenesis at cellular level;cellular meml
biological regulation;cellular process;cellular response to stimulus;intracellular signal transduction;regulation of biological process;regulation of cellular process;regulation of response to stimulus;regulation of signal transducti

aging;autophagy;biological regulation;catabolic process;cellular catabolic process;cellular metabolic process;cellular process;developmental process;metabolic process;mitochondrion degradation;multicellular organismal ag
cellular process;establishment of localization;transmembrane transport;transport
biological regulation;calcium ion homeostasis;calcium ion transport;calcium-mediated signaling;carbohydrate homeostasis;cation homeostasis;cation transport;cellular calcium ion homeostasis;cellular cation homeostasis;ce
biological regulation;cellular component assembly;cellular component assembly at cellular level;cellular component organization;cellular component organization at cellular level;cellular component organization or biogenesis;
activation of immune response;activation of innate immune response;anaphase-promoting complex-dependent proteasomal ubiquitin-dependent protein catabolic process;antigen processing and presentation;antigen proces
autophagy;biological regulation;catabolic process;cellular catabolic process;cellular metabolic process;cellular process;endosome transport;establishment of localization;establishment of localization in cell;establishment of p
biological regulation;cellular process;cellular response to stimulus;multicellular organismal process;ossification;regulation of biological process;regulation of cellular process;response to stimulus;signal transduction
acyl-CoA biosynthetic process;acyl-CoA metabolic process;amine catabolic process;amine metabolic process;aromatic amino acid family metabolic process;aspartate family amino acid catabolic process;aspartate family am
alcohol catabolic process;alcohol metabolic process;carbohydrate catabolic process;carbohydrate metabolic process;catabolic process;cellular carbohydrate catabolic process;cellular carbohydrate metabolic process;cellula
cellular localization;cellular process;establishment of localization;establishment of localization in cell;establishment of organelle localization;establishment of protein localization;establishment of ribosome localization;intracellu
biosynthetic process;cellular biosynthetic process;cellular lipid metabolic process;cellular metabolic process;cellular process;ceramide biosynthetic process;ceramide metabolic process;lipid biosynthetic process;lipid metabo
actin cytoskeleton organization;actin filament bundle assembly;actin filament organization;actin filament-based process;apoptosis;biological regulation;biosynthetic process;cell death;cellular biosynthetic process;cellular con
biological regulation;cellular component organization;cellular component organization at cellular level;cellular component organization or biogenesis;cellular component organization or biogenesis at cellular level;cellular macr
biological regulation;cellular process;cellular response to stimulus;endosome to lysosome transport;endosome transport;establishment of cell polarity;establishment of localization;establishment of localization in cell;establish
anatomical structure development;anatomical structure formation involved in morphogenesis;biosynthetic process;cellular biosynthetic process;cellular lipid metabolic process;cellular metabolic process;cellular process;deve
3'-UTR-mediated mRNA destabilization;anatomical structure development;axon ensheathment;biological regulation;biosynthetic process;carboxylic acid biosynthetic process;carboxylic acid metabolic process;cell developm
biological regulation;biosynthetic process;cellular biosynthetic process;cellular macromolecule biosynthetic process;cellular macromolecule metabolic process;cellular metabolic process;cellular nitrogen compound metabolic
carbohydrate metabolic process;cellular carbohydrate metabolic process;cellular macromolecule metabolic process;cellular metabolic process;cellular process;cellular protein metabolic process;glycosylation;macromolecule
cell cycle;cell cycle process;cellular component assembly;cellular component organization;cellular component organization at cellular level;cellular component organization or biogenesis;cellular component organization or bio
alanyl-tRNA aminoacylation;amine metabolic process;amino acid activation;carboxylic acid metabolic process;cellular amine metabolic process;cellular amino acid metabolic process;cellular ketone metabolic process;cellula
biosynthetic process;cellular biosynthetic process;cellular lipid metabolic process;cellular metabolic process;cellular process;glycerolipid biosynthetic process;glycerolipid metabolic process;glycerophospholipid biosynthetic
biosynthetic process;carboxylic acid biosynthetic process;carboxylic acid metabolic process;cellular biosynthetic process;cellular ketone metabolic process;cellular lipid metabolic process;cellular metabolic process;cellular p
establishment of localization;lipid transport;organic substance transport;transport
biosynthetic process;cellular biosynthetic process;cellular component disassembly;cellular component disassembly at cellular level;cellular component organization;cellular component organization at cellular level;cellular con
biological regulation;biosynthetic process;catabolic process;cell surface receptor linked signaling pathway;cellular biosynthetic process;cellular catabolic process;cellular component organization;cellular component organizat
anatomical structure development;anatomical structure morphogenesis;biological regulation;biosynthetic process;cell development;cell motility;cell part morphogenesis;cellular biosynthetic process;cellular component morph
cell cycle process;cellular component organization;cellular component organization at cellular level;cellular component organization or biogenesis;cellular component organization or biogenesis at cellular level;cellular macrom
biological regulation;cellular macromolecule metabolic process;cellular metabolic process;cellular nitrogen compound metabolic process;cellular process;induction of apoptosis;induction of apoptosis by extracellular signals;
catabolic process;cellular catabolic process;cellular component assembly;cellular component organization;cellular component organization or biogenesis;cellular metabolic process;cellular nitrogen compound catabolic proce
cell cycle;cellular component organization;cellular component organization at cellular level;cellular component organization or biogenesis;cellular component organization or biogenesis at cellular level;cellular process;mitotic
biological regulation;cellular macromolecule metabolic process;cellular metabolic process;cellular nitrogen compound metabolic process;cellular process;macromolecule metabolic process;metabolic process;mRNA metaboli
biosynthetic process;cellular biosynthetic process;cellular component disassembly;cellular component disassembly at cellular level;cellular component organization;cellular component organization at cellular level;cellular con
amine metabolic process;carboxylic acid metabolic process;cellular amine metabolic process;cellular amino acid metabolic process;cellular ketone metabolic process;cellular metabolic process;cellular modified amino acid m
biological regulation;regulation of biological process;regulation of catabolic process;regulation of cellular catabolic process;regulation of cellular metabolic process;regulation of cellular process;regulation of cellular protein me
biological regulation;cell cycle;cell division;cellular process;cellular response to stimulus;chromosome segregation;intracellular signal transduction;metabolic process;regulation of biological process;regulation of cellular proce
biological regulation;cell cycle phase;cell cycle process;cell division;cell motility;cellular component movement;cellular component organization;cellular component organization at cellular level;cellular component organization
biological regulation;biosynthetic process;cellular biosynthetic process;cellular metabolic process;cellular process;coenzyme biosynthetic process;coenzyme metabolic process;cofactor biosynthetic process;cofactor metabo
biosynthetic process;catabolic process;cellular biosynthetic process;cellular catabolic process;cellular macromolecule biosynthetic process;cellular macromolecule catabolic process;cellular macromolecule metabolic proces
biological regulation;cell differentiation;cell surface receptor linked signaling pathway;cellular component biogenesis;cellular component biogenesis at cellular level;cellular component organization or biogenesis;cellular comp
biological regulation;cell cycle checkpoint;cellular component organization;cellular component organization at cellular level;cellular component organization or biogenesis;cellular component organization or biogenesis at cellu
anatomical structure formation involved in morphogenesis;angiogenesis;biological regulation;biosynthetic process;cellular biosynthetic process;cellular macromolecule biosynthetic process;cellular macromolecule metabolic
biological regulation;cell cycle process;cellular component organization;cellular component organization at cellular level;cellular component organization or biogenesis;cellular component organization or biogenesis at cellular
biological regulation;cell communication;cellular component organization;cellular component organization at cellular level;cellular component organization or biogenesis;cellular component organization or biogenesis at cellula
biosynthetic process;cellular aromatic compound metabolic process;cellular biosynthetic process;cellular metabolic compound salvage;cellular metabolic process;cellular nitrogen compound biosynthetic process;cellular nitr
activation of immune response;activation of innate immune response;biological regulation;cellular process;cellular response to stimulus;cytokine production;defense response;defense response to bacterium;defense respons
antigen processing and presentation;antigen processing and presentation of exogenous antigen;antigen processing and presentation of exogenous peptide antigen;antigen processing and presentation of exogenous peptide
alcohol catabolic process;alcohol metabolic process;carbohydrate catabolic process;carbohydrate metabolic process;catabolic process;cellular carbohydrate catabolic process;cellular carbohydrate metabolic process;cellula
apoptosis;biological regulation;catabolic process;cell death;cellular catabolic process;cellular macromolecule catabolic process;cellular macromolecule metabolic process;cellular metabolic process;cellular process;cytoplas
cellular macromolecule metabolic process;cellular metabolic process;cellular nitrogen compound metabolic process;cellular process;macromolecule metabolic process;metabolic process;ncRNA metabolic process;ncRNA pr
cellular macromolecule metabolic process;cellular metabolic process;cellular nitrogen compound metabolic process;cellular process;gene expression;macromolecule metabolic process;metabolic process;mRNA metabolic p
cellular macromolecule metabolic process;cellular metabolic process;cellular nitrogen compound metabolic process;cellular process;cellular protein metabolic process;macromolecule metabolic process;macromolecule modi
apoptosis;biological regulation;cell death;cellular process;death;metabolic process;negative regulation of anoikis;negative regulation of apoptosis;negative regulation of biological process;negative regulation of cell death;neg
biological regulation;BMP signaling pathway;catabolic process;cell surface receptor linked signaling pathway;cellular catabolic process;cellular component organization;cellular component organization at cellular level;cellular
activation of JUN kinase activity;activation of MAPK activity;activation of MAPKKK activity;activation of protein kinase activity;biological regulation;cellular macromolecule metabolic process;cellular metabolic process;cellular
biological regulation;catabolic process;cellular catabolic process;cellular macromolecule catabolic process;cellular macromolecule metabolic process;cellular metabolic process;cellular nitrogen compound metabolic process
biological regulation;biosynthetic process;catabolic process;cellular biosynthetic process;cellular catabolic process;cellular macromolecule biosynthetic process;cellular macromolecule catabolic process;cellular macromolec
biological regulation;positive regulation of actin filament bundle assembly;positive regulation of biological process;positive regulation of biosynthetic process;positive regulation of cellular biosynthetic process;positive regulati
biological adhesion;biological regulation;calcium ion homeostasis;cation homeostasis;cell adhesion;cell surface receptor linked signaling pathway;cell-cell adhesion;cellular calcium ion homeostasis;cellular cation homeostasi
activation of protein kinase activity;apoptosis;biological regulation;cell death;cellular component disassembly;cellular component disassembly at cellular level;cellular component disassembly involved in apoptosis;cellular co
biological regulation;biosynthetic process;cellular biosynthetic process;cellular component organization;cellular component organization at cellular level;cellular component organization or biogenesis;cellular component orga
cell cycle process;cellular macromolecule metabolic process;cellular metabolic process;cellular process;cellular protein metabolic process;dephosphorylation;G1/S transition of mitotic cell cycle;macromolecule metabolic pro
cellular component organization;cellular component organization or biogenesis;cellular membrane organization;cellular process;endocytosis;establishment of localization;establishment of localization in cell;establishment of p
biological regulation;cellular process;cellular response to chemical stimulus;cellular response to endogenous stimulus;cellular response to hormone stimulus;cellular response to organic substance;cellular response to steroid
amine metabolic process;amino acid activation;biological regulation;carboxylic acid metabolic process;cellular amine metabolic process;cellular amino acid metabolic process;cellular ketone metabolic process;cellular macro
biological regulation;catabolic process;cell differentiation;cell proliferation;cellular catabolic process;cellular developmental process;cellular metabolic process;cellular nitrogen compound catabolic process;cellular nitrogen co
biological regulation;cation homeostasis;cellular cation homeostasis;cellular chemical homeostasis;cellular homeostasis;cellular ion homeostasis;cellular iron ion homeostasis;cellular metal ion homeostasis;cellular process;ch
ATP biosynthetic process;ATP metabolic process;biological regulation;biosynthetic process;cellular biosynthetic process;cellular metabolic process;cellular nitrogen compound biosynthetic process;cellular nitrogen compoun
biological regulation;biosynthetic process;cellular biosynthetic process;cellular component assembly;cellular component assembly at cellular level;cellular component organization;cellular component organization at cellular le
apoptosis;cell death;cellular component organization;cellular component organization at cellular level;cellular component organization or biogenesis;cellular component organization or biogenesis at cellular level;cellular proce
alcohol biosynthetic process;alcohol metabolic process;biosynthetic process;cellular biosynthetic process;cellular lipid metabolic process;cellular metabolic process;cellular process;cholesterol biosynthetic process;cholester
cellular metabolic process;cellular process;electron transport chain;generation of precursor metabolites and energy;metabolic process;mitochondrial electron transport, NADH to ubiquinone;oxidation-reduction process;respi
biological regulation;cell differentiation;cellular developmental process;cellular process;developmental process;multicellular organismal development;multicellular organismal process;regulation of biological process;regulation
biosynthetic process;cellular biosynthetic process;cellular macromolecule metabolic process;cellular metabolic process;cellular nitrogen compound metabolic process;cellular process;electron transport chain;gene expressio
2'-deoxyribonucleotide metabolic process;adenosine catabolic process;adenosine metabolic process;aging;amine transport;anatomical structure development;B cell activation;B cell activation involved in immune response;B
amide biosynthetic process;amine biosynthetic process;amine catabolic process;amine metabolic process;arginine biosynthetic process;arginine biosynthetic process via ornithine;arginine catabolic process;arginine metabol
biosynthetic process;cellular aromatic compound metabolic process;cellular biosynthetic process;cellular metabolic process;cellular nitrogen compound biosynthetic process;cellular nitrogen compound metabolic process;ce
acylglycerol metabolic process;aging;amine metabolic process;anatomical structure development;anatomical structure formation involved in morphogenesis;anatomical structure morphogenesis;angiogenesis;angiogenesis in
adherens junction organization;anatomical structure development;anatomical structure formation involved in morphogenesis;anatomical structure morphogenesis;apoptosis;biological adhesion;biological regulation;calcium-d
biological regulation;biosynthetic process;cellular biosynthetic process;cellular macromolecule biosynthetic process;cellular macromolecule metabolic process;cellular metabolic process;cellular nitrogen compound metabolic
aromatic compound biosynthetic process;biosynthetic process;cellular aromatic compound metabolic process;cellular biosynthetic process;cellular metabolic process;cellular nitrogen compound biosynthetic process;cellular
aging;biological regulation;biosynthetic process;carboxylic acid biosynthetic process;carboxylic acid metabolic process;cell aging;cell proliferation;cell surface receptor linked signaling pathway;cellular biosynthetic process;c
anatomical structure development;biosynthetic process;cellular biosynthetic process;cellular macromolecule metabolic process;cellular metabolic process;cellular nitrogen compound metabolic process;cellular process;devel
amine metabolic process;aminoglycan catabolic process;aminoglycan metabolic process;carbohydrate catabolic process;carbohydrate metabolic process;catabolic process;cellular catabolic process;cellular metabolic proce
activation of immune response;activation of MAPKK activity;activation of phospholipase C activity;activation of protein kinase activity;antigen receptor-mediated signaling pathway;axon guidance;biological regulation;blood c
biosynthetic process;cellular biosynthetic process;cellular macromolecule metabolic process;cellular metabolic process;cellular nitrogen compound metabolic process;cellular process;electron transport chain;gene expressio
amine metabolic process;carboxylic acid catabolic process;carboxylic acid metabolic process;catabolic process;cellular amine metabolic process;cellular amino acid metabolic process;cellular catabolic process;cellular keto
alcohol metabolic process;biological regulation;biosynthetic process;cation homeostasis;cell differentiation;cellular aromatic compound metabolic process;cellular biosynthetic process;cellular component assembly;cellular co
cellular macromolecule metabolic process;cellular metabolic process;cellular process;cellular protein metabolic process;chaperone-mediated protein folding;macromolecule metabolic process;macromolecule modification;me
biological regulation;cell redox homeostasis;cellular chemical homeostasis;cellular homeostasis;cellular ion homeostasis;cellular macromolecule metabolic process;cellular metabolic process;cellular nitrogen compound meta
biological regulation;biosynthetic process;cell cycle arrest;cell cycle process;cell surface receptor linked signaling pathway;cellular biosynthetic process;cellular macromolecule biosynthetic process;cellular macromolecule me
activation of caspase activity;adult behavior;adult locomotory behavior;aging;alcohol biosynthetic process;alcohol metabolic process;amine biosynthetic process;amine metabolic process;amine transport;ATP synthesis coup
carboxylic acid catabolic process;carboxylic acid metabolic process;catabolic process;cellular catabolic process;cellular ketone metabolic process;cellular lipid catabolic process;cellular lipid metabolic process;cellular metab
acid secretion;alcohol metabolic process;amine metabolic process;arachidonic acid metabolic process;arachidonic acid secretion;biological regulation;biosynthetic process;blood coagulation;carboxylic acid biosynthetic pro
3'-UTR-mediated mRNA stabilization;activation of immune response;activation of innate immune response;activation of MAPK activity;anatomical structure development;arachidonic acid metabolic process;biological regulati
cellular macromolecule metabolic process;cellular metabolic process;cellular process;cellular protein metabolic process;macromolecule metabolic process;macromolecule modification;metabolic process;primary metabolic p
biological regulation;biosynthetic process;catabolic process;cellular biosynthetic process;cellular catabolic process;cellular component assembly;cellular component assembly at cellular level;cellular component organization;
biological regulation;establishment of localization;establishment of localization in cell;establishment of protein localization;intracellular protein transport;intracellular transport;protein transport;regulation of actin cytoskeleton o
biological regulation;biosynthetic process;cation homeostasis;cell growth;cellular biosynthetic process;cellular cation homeostasis;cellular chemical homeostasis;cellular homeostasis;cellular ion homeostasis;cellular iron ion h
cellular component biogenesis;cellular component biogenesis at cellular level;cellular component organization or biogenesis;cellular component organization or biogenesis at cellular level;cellular macromolecule metabolic pro
biological regulation;biosynthetic process;cellular biosynthetic process;cellular macromolecule biosynthetic process;cellular macromolecule metabolic process;cellular metabolic process;cellular process;cellular protein metab
apoptosis;apoptosis in response to endoplasmic reticulum stress;biological regulation;catabolic process;cell death;cellular catabolic process;cellular macromolecule catabolic process;cellular macromolecule metabolic proce
biological regulation;biosynthetic process;cellular biosynthetic process;cellular lipid metabolic process;cellular metabolic process;cellular process;cellular response to stimulus;establishment of localization;establishment of pr
biological regulation;cellular macromolecule metabolic process;cellular metabolic process;cellular process;cellular protein metabolic process;macromolecule metabolic process;macromolecule modification;metabolic process
actin cytoskeleton organization;actin filament organization;actin filament-based process;biological regulation;blood coagulation;cell activation;cellular component organization;cellular component organization at cellular level;c
actin cytoskeleton organization;actin filament-based process;anatomical structure development;anatomical structure morphogenesis;antigen processing and presentation;antigen processing and presentation of exogenous a
biosynthetic process;cellular biosynthetic process;cellular component disassembly;cellular component disassembly at cellular level;cellular component organization;cellular component organization at cellular level;cellular con
actin cytoskeleton organization;actin filament-based process;axon guidance;biological regulation;blood coagulation;cell activation;cell chemotaxis;cell migration;cell motility;cellular component movement;cellular component
anatomical structure development;anatomical structure morphogenesis;biological regulation;cell differentiation;cellular developmental process;cellular process;developmental process;muscle organ development;muscle struc
biological regulation;biosynthetic process;cellular biosynthetic process;cellular macromolecule biosynthetic process;cellular macromolecule metabolic process;cellular metabolic process;cellular nitrogen compound metabolic
biosynthetic process;cellular biosynthetic process;cellular macromolecule biosynthetic process;cellular macromolecule metabolic process;cellular metabolic process;cellular nitrogen compound metabolic process;cellular pro
biological regulation;catabolic process;cellular catabolic process;cellular macromolecule catabolic process;cellular macromolecule metabolic process;cellular metabolic process;cellular nitrogen compound metabolic process
activation of caspase activity;activation of NF-kappaB-inducing kinase activity;activation of protein kinase activity;apoptosis;apoptosis in response to endoplasmic reticulum stress;biological regulation;catabolic process;cell c
cellular macromolecule metabolic process;cellular metabolic process;cellular nitrogen compound metabolic process;cellular process;macromolecule metabolic process;maturation of SSU-rRNA;maturation of SSU-rRNA from
biosynthetic process;cellular biosynthetic process;cellular lipid metabolic process;cellular metabolic process;cellular process;dephosphorylation;glycerolipid biosynthetic process;glycerolipid metabolic process;glycerophosph
actin cytoskeleton organization;actin filament-based process;anatomical structure development;cellular component organization;cellular component organization at cellular level;cellular component organization or biogenesis;
antigen processing and presentation;antigen processing and presentation of peptide antigen;antigen processing and presentation of peptide antigen via MHC class I;cellular macromolecule metabolic process;cellular metabo
axon guidance;biological regulation;cell differentiation;cell junction assembly;cell junction organization;cell projection assembly;cell projection organization;cell surface receptor linked signaling pathway;cell-substrate junction
behavior;behavioral response to pain;biological regulation;multicellular organismal process;multicellular organismal response to stress;regulation of biological process;regulation of cellular process;regulation of G-protein coup
actin cytoskeleton organization;actin cytoskeleton reorganization;actin filament-based process;acylglycerol biosynthetic process;acylglycerol metabolic process;alcohol metabolic process;amine biosynthetic process;amine m

activation of immune response;activation of innate immune response;anaphase-promoting complex-dependent proteasomal ubiquitin-dependent protein catabolic process;antigen processing and presentation;antigen proces
activation of immune response;activation of innate immune response;biological adhesion;biological regulation;biosynthetic process;cell adhesion;cell-cell adhesion;cellular biosynthetic process;cellular macromolecule biosynt
biological regulation;biosynthetic process;cellular biosynthetic process;cellular component assembly;cellular component assembly at cellular level;cellular component organization;cellular component organization at cellular le
cellular component assembly;cellular component organization;cellular component organization or biogenesis;macromolecular complex assembly;macromolecular complex subunit organization;macromolecule metabolic proc
amine catabolic process;amine metabolic process;carboxylic acid catabolic process;carboxylic acid metabolic process;catabolic process;cell differentiation;cellular amine metabolic process;cellular amino acid catabolic proc
amine biosynthetic process;amine metabolic process;biosynthetic process;carboxylic acid biosynthetic process;carboxylic acid metabolic process;cellular amine metabolic process;cellular amino acid biosynthetic process;ce
biosynthetic process;catabolic process;cell communication;cellular aromatic compound metabolic process;cellular biosynthetic process;cellular catabolic process;cellular metabolic compound salvage;cellular metabolic proc
biological regulation;regulation of biological process;regulation of catalytic activity;regulation of cellular metabolic process;regulation of cellular process;regulation of dephosphorylation;regulation of hydrolase activity;regulatic
biological regulation;biosynthetic process;cellular biosynthetic process;cellular macromolecule biosynthetic process;cellular macromolecule metabolic process;cellular metabolic process;cellular nitrogen compound metabolic
B cell activation;B cell activation involved in immune response;catabolic process;cell activation;cell activation involved in immune response;cellular catabolic process;cellular macromolecule catabolic process;cellular macrom
cellular metabolic process;cellular process;dephosphorylation;metabolic process;phosphate-containing compound metabolic process;phosphorus metabolic process
biological regulation;cell chemotaxis;cell migration;cell motility;cell surface receptor linked signaling pathway;cellular component movement;cellular process;cellular response to chemical stimulus;cellular response to stimulus
cellular macromolecule metabolic process;cellular metabolic process;cellular nitrogen compound metabolic process;cellular process;macromolecule metabolic process;metabolic process;mRNA metabolic process;mRNA prc
biological regulation;cellular process;cellular response to stimulus;intracellular signal transduction;regulation of biological process;regulation of cellular process;regulation of response to stimulus;regulation of signal transducti
biosynthetic process;cellular biosynthetic process;cellular macromolecule biosynthetic process;cellular macromolecule metabolic process;cellular metabolic process;cellular process;cellular protein metabolic process;macron
catabolic process;lipid catabolic process;lipid metabolic process;metabolic process;primary metabolic process
alcohol metabolic process;carbohydrate metabolic process;cellular carbohydrate metabolic process;cellular component assembly;cellular component organization;cellular component organization or biogenesis;cellular metab
anatomical structure development;behavior;cranial nerve development;developmental process;glossopharyngeal nerve development;locomotory behavior;multicellular organismal process;nerve development;neurological sys
behavior;biological regulation;cellular component organization;cellular component organization at cellular level;cellular component organization or biogenesis;cellular component organization or biogenesis at cellular level;cellu
biological regulation;biosynthetic process;cellular biosynthetic process;cellular macromolecule biosynthetic process;cellular macromolecule metabolic process;cellular metabolic process;cellular nitrogen compound metabolic
anatomical structure formation involved in morphogenesis;angiogenesis;apoptosis;biological regulation;cell death;cellular macromolecule metabolic process;cellular metabolic process;cellular process;cellular protein metabol
activation of caspase activity;apoptosis;biological regulation;cell death;cellular process;cellular response to chemical stimulus;cellular response to hypoxia;cellular response to oxygen levels;cellular response to stimulus;cellul
carboxylic acid metabolic process;cellular ketone metabolic process;cellular lipid metabolic process;cellular metabolic process;cellular process;fatty acid metabolic process;lipid metabolic process;metabolic process;monoca
biological regulation;cellular component assembly;cellular component organization;cellular component organization or biogenesis;macromolecular complex assembly;macromolecular complex subunit organization;protein cor
anatomical structure development;cell differentiation;cellular developmental process;cellular process;developmental process;establishment of localization;establishment of localization in cell;intracellular transport;mitochondri
catabolic process;cellular catabolic process;cellular macromolecule catabolic process;cellular macromolecule metabolic process;cellular metabolic process;cellular nitrogen compound metabolic process;cellular process;exo
attachment of spindle microtubules to chromosome;attachment of spindle microtubules to kinetochore;attachment of spindle microtubules to kinetochore involved in mitotic sister chromatid segregation;biological regulation;c
activation of NF-kappaB-inducing kinase activity;activation of protein kinase activity;biological regulation;cellular macromolecule metabolic process;cellular metabolic process;cellular process;cellular protein metabolic proces
biological regulation;catabolic process;cell cycle;cell differentiation;cell proliferation;cellular catabolic process;cellular developmental process;cellular macromolecule catabolic process;cellular macromolecule metabolic proce
activation of immune response;activation of innate immune response;anaphase-promoting complex-dependent proteasomal ubiquitin-dependent protein catabolic process;antigen processing and presentation;antigen proces
anatomical structure development;biological regulation;cellular macromolecule metabolic process;cellular metabolic process;cellular process;cellular protein metabolic process;'de novo' posttranslational protein folding;'de n
biological regulation;cell cycle;cell cycle phase;cell cycle process;cell division;cell surface receptor linked signaling pathway;cellular component disassembly;cellular component disassembly at cellular level;cellular componen
biological regulation;calcium ion homeostasis;calcium ion import;calcium ion transmembrane transport;calcium ion transport;cation homeostasis;cation transport;cellular calcium ion homeostasis;cellular cation homeostasis;c
biosynthetic process;cellular biosynthetic process;cellular component disassembly;cellular component disassembly at cellular level;cellular component organization;cellular component organization at cellular level;cellular cor
biological regulation;metabolic process;oxidation-reduction process;regulation of biological process;regulation of cell differentiation;regulation of cellular process;regulation of developmental process;regulation of immune sys
developmental process;multicellular organismal development;multicellular organismal process
biological regulation;catabolic process;cellular catabolic process;cellular macromolecule catabolic process;cellular macromolecule metabolic process;cellular metabolic process;cellular nitrogen compound metabolic process
amine transport;amino acid transmembrane transport;amino acid transport;biological regulation;carboxylic acid transport;cell communication;cell-cell signaling;cellular process;establishment of localization;ion transport;neurc
viral reproduction
anatomical structure development;biological regulation;biosynthetic process;cell migration;cell motility;cellular biosynthetic process;cellular component movement;cellular component organization;cellular component organiza
biological regulation;biosynthetic process;cellular biosynthetic process;cellular component biogenesis;cellular component biogenesis at cellular level;cellular component organization;cellular component organization at cellula
biological regulation;cell redox homeostasis;cellular homeostasis;cellular process;homeostatic process;regulation of biological process;regulation of biological quality;regulation of cellular process;response to chemical stimul
anatomical structure development;biological regulation;brain development;cellular component organization;cellular component organization at cellular level;cellular component organization or biogenesis;cellular component o
anatomical structure development;biological regulation;biosynthetic process;cell development;cellular biosynthetic process;cellular developmental process;cellular macromolecule metabolic process;cellular metabolic proces
cellular process;establishment of localization;establishment of localization in cell;establishment of protein localization;intracellular protein transport;intracellular transport;protein transport;transport;vesicle-mediated transport
apoptotic mitochondrial changes;autophagic vacuole assembly;autophagy;catabolic process;cellular catabolic process;cellular component assembly;cellular component assembly at cellular level;cellular component organiza
biosynthetic process;catabolic process;cellular biosynthetic process;cellular catabolic process;cellular lipid catabolic process;cellular lipid metabolic process;cellular metabolic process;cellular process;cellular response to ch
biological regulation;regulation of biological process;regulation of cellular metabolic process;regulation of cellular process;regulation of cellular respiration;regulation of generation of precursor metabolites and energy;regulatic
activation of caspase activity;biological regulation;cellular component organization;cellular component organization or biogenesis;cellular membrane organization;cellular process;establishment of localization;membrane orgar
biosynthetic process;cellular biosynthetic process;cellular component disassembly;cellular component disassembly at cellular level;cellular component organization;cellular component organization at cellular level;cellular cor
biosynthetic process;cellular biosynthetic process;cellular macromolecule biosynthetic process;cellular macromolecule metabolic process;cellular metabolic process;cellular nitrogen compound metabolic process;cellular pro
biological regulation;cell surface receptor linked signaling pathway;cellular component organization;cellular component organization or biogenesis;cellular membrane organization;cellular process;cellular response to stimulus
anatomical structure development;biological regulation;cell communication;cell surface receptor linked signaling pathway;cell-cell signaling;cellular metabolic process;cellular process;cellular response to chemical stimulus;ce
apoptosis;cell death;cell differentiation;cellular developmental process;cellular localization;cellular macromolecule localization;cellular process;cellular protein localization;death;developmental process;ER to Golgi vesicle-med
biosynthetic process;cellular biosynthetic process;cellular component disassembly;cellular component disassembly at cellular level;cellular component organization;cellular component organization at cellular level;cellular cor
anterograde axon cargo transport;anterograde synaptic vesicle transport;axon cargo transport;cellular component movement;cellular process;cytoskeleton-dependent intracellular transport;establishment of localization;estab
biological regulation;metabolic process;negative regulation of catalytic activity;negative regulation of molecular function;negative regulation of monooxygenase activity;negative regulation of nitric-oxide synthase activity;nega
biological regulation;regulation of biological process;regulation of cellular process;regulation of microtubule-based process
establishment of localization;establishment of protein localization;protein transport;transport
biosynthetic process;cellular biosynthetic process;cellular component disassembly;cellular component disassembly at cellular level;cellular component organization;cellular component organization at cellular level;cellular cor
anatomical structure morphogenesis;biological regulation;cell part morphogenesis;cell projection morphogenesis;cell projection organization;cellular component assembly;cellular component assembly at cellular level;cellular
anatomical structure formation involved in morphogenesis;biological regulation;cation homeostasis;cation transport;cell projection assembly;cell projection organization;cell surface receptor linked signaling pathway;cellular c
biosynthetic process;carbohydrate biosynthetic process;carbohydrate metabolic process;cellular carbohydrate metabolic process;cellular macromolecule metabolic process;cellular metabolic process;cellular process;cellular
activation of immune response;activation of innate immune response;activation of MAPK activity;activation of NF-kappaB-inducing kinase activity;activation of protein kinase activity;antigen receptor-mediated signaling pathv
cellular component assembly;cellular component assembly at cellular level;cellular component biogenesis;cellular component organization;cellular component organization at cellular level;cellular component organization or b
activation of immune response;activation of innate immune response;anaphase-promoting complex-dependent proteasomal ubiquitin-dependent protein catabolic process;antigen processing and presentation;antigen proces
apoptosis;biological regulation;carbohydrate metabolic process;cell communication;cell death;cellular carbohydrate metabolic process;cellular component organization;cellular component organization at cellular level;cellular
amine biosynthetic process;amine catabolic process;amine metabolic process;biological regulation;biosynthetic process;branched chain family amino acid biosynthetic process;branched chain family amino acid catabolic prc
cellular macromolecule metabolic process;cellular metabolic process;cellular nitrogen compound metabolic process;cellular process;gene expression;macromolecule metabolic process;metabolic process;mRNA metabolic p
biological regulation;cellular component assembly;cellular component organization;cellular component organization or biogenesis;cellular macromolecule metabolic process;cellular metabolic process;cellular nitrogen compo
cellular component organization;cellular component organization or biogenesis;cellular membrane organization;cellular process;endocytosis;endosome transport;establishment of localization;establishment of localization in ce
biological regulation;biosynthetic process;catabolic process;cell differentiation;cellular biosynthetic process;cellular catabolic process;cellular developmental process;cellular macromolecule biosynthetic process;cellular macr
activation of caspase activity;anatomical structure formation involved in morphogenesis;angiogenesis;biological regulation;cellular process;cellular response to heat;cellular response to stimulus;cellular response to stress;dev
cellular macromolecule metabolic process;cellular metabolic process;cellular process;cellular protein metabolic process;establishment of localization;establishment of localization in cell;establishment of protein localization;es
acidic amino acid transport;amine transport;amino acid transport;anion transport;biological regulation;carboxylic acid transport;cellular chemical homeostasis;cellular homeostasis;cellular ion homeostasis;cellular process;che
amine catabolic process;amine metabolic process;arginine catabolic process;arginine metabolic process;biological regulation;carboxylic acid catabolic process;carboxylic acid metabolic process;catabolic process;cellular an
alcohol biosynthetic process;alcohol metabolic process;amine metabolic process;amino sugar biosynthetic process;amino sugar metabolic process;biosynthetic process;carbohydrate biosynthetic process;carbohydrate meta
actin cytoskeleton organization;actin filament-based process;anatomical structure formation involved in morphogenesis;axon guidance;biological regulation;biosynthetic process;cell projection assembly;cell projection organi
anatomical structure formation involved in morphogenesis;anatomical structure morphogenesis;apoptosis;autophagy;biological regulation;blastocyst formation;blood coagulation;camera-type eye morphogenesis;catabolic pr
adult behavior;adult locomotory behavior;adult walking behavior;amine metabolic process;anatomical structure development;anatomical structure morphogenesis;autophagy;axonogenesis;behavior;biological regulation;carbo
activation of protein kinase activity;biological regulation;cellular hypotonic response;cellular macromolecule metabolic process;cellular metabolic process;cellular process;cellular protein metabolic process;cellular response to
cellular macromolecule metabolic process;cellular metabolic process;cellular process;cellular protein metabolic process;establishment of localization;establishment of localization in cell;establishment of protein localization;es
age-dependent response to oxidative stress;age-dependent response to reactive oxygen species;amine metabolic process;anatomical structure development;apoptotic mitochondrial changes;behavior;biological regulation;b
adult behavior;adult locomotory behavior;alcohol metabolic process;anatomical structure development;anatomical structure morphogenesis;apoptosis;associative learning;axon cargo transport;axon choice point recognition;
axon guidance;biological regulation;blood coagulation;carbohydrate transport;cell differentiation;cell migration;cell motility;cell surface receptor linked signaling pathway;cellular component movement;cellular developmental
biosynthetic process;cellular biosynthetic process;cellular component disassembly;cellular component disassembly at cellular level;cellular component organization;cellular component organization at cellular level;cellular cor
biological regulation;cellular process;cellular response to chemical stimulus;cellular response to endogenous stimulus;cellular response to estradiol stimulus;cellular response to estrogen stimulus;cellular response to hormone
amine metabolic process;carboxylic acid metabolic process;cell differentiation;cellular amine metabolic process;cellular amino acid metabolic process;cellular component assembly;cellular component organization;cellular co
amine metabolic process;carboxylic acid metabolic process;cellular amine metabolic process;cellular amino acid metabolic process;cellular ketone metabolic process;cellular metabolic process;cellular nitrogen compound m
actin cytoskeleton organization;actin filament organization;actin filament-based process;activation of immune response;antigen receptor-mediated signaling pathway;axon guidance;biological regulation;cell activation;cell pro
activation of phospholipase D activity;anatomical structure development;biological regulation;brain development;cell migration;cell motility;cell surface receptor linked signaling pathway;cellular component movement;cellular
anatomical structure development;base-excision repair;cellular macromolecule metabolic process;cellular metabolic process;cellular nitrogen compound metabolic process;cellular process;cellular response to stimulus;cellula
biological regulation;biosynthetic process;cellular biosynthetic process;cellular macromolecule biosynthetic process;cellular macromolecule metabolic process;cellular metabolic process;cellular nitrogen compound metabolic
antigen processing and presentation;biological regulation;cellular component organization;cellular component organization or biogenesis;cellular process;cellular response to stimulus;establishment of localization;establishme
alcohol catabolic process;alcohol metabolic process;amine catabolic process;amine metabolic process;behavior;benzene-containing compound metabolic process;biological regulation;biosynthetic process;catabolic process
adherens junction organization;anatomical structure homeostasis;biological adhesion;biological regulation;canonical Wnt receptor signaling pathway;cell adhesion;cell junction assembly;cell junction organization;cell surface
activation of immune response;activation of innate immune response;anaphase-promoting complex-dependent proteasomal ubiquitin-dependent protein catabolic process;antigen processing and presentation;antigen proces
biological regulation;cellular metabolic process;cellular process;energy derivation by oxidation of organic compounds;energy reserve metabolic process;generation of precursor metabolites and energy;metabolic process;oxic
biosynthetic process;cellular aromatic compound metabolic process;cellular biosynthetic process;cellular metabolic process;cellular nitrogen compound biosynthetic process;cellular nitrogen compound metabolic process;ce
amine biosynthetic process;amine catabolic process;amine metabolic process;betaine biosynthetic process;betaine metabolic process;biosynthetic process;carboxylic acid biosynthetic process;carboxylic acid catabolic proc
actin cytoskeleton organization;actin filament bundle assembly;actin filament capping;actin filament organization;actin filament-based process;anatomical structure homeostasis;anatomical structure morphogenesis;apoptosi
ATP hydrolysis coupled proton transport;biological regulation;cation homeostasis;cation transport;cell surface receptor linked signaling pathway;cellular cation homeostasis;cellular chemical homeostasis;cellular component c
biological regulation;biosynthetic process;catabolic process;cell cycle;cell proliferation;cellular biosynthetic process;cellular catabolic process;cellular component disassembly;cellular component disassembly at cellular level;
acidic amino acid transport;aging;amide biosynthetic process;amine metabolic process;amine transport;amino acid import;amino acid transport;anatomical structure development;anatomical structure formation involved in m
activation of immune response;activation of innate immune response;activation of MAPK activity;biological regulation;cardiac muscle contraction;cellular macromolecule metabolic process;cellular metabolic process;cellular p
activation of immune response;activation of innate immune response;anaphase-promoting complex-dependent proteasomal ubiquitin-dependent protein catabolic process;antigen processing and presentation;antigen proces
adherens junction organization;anatomical structure development;axon guidance;biological regulation;cell differentiation;cell division;cell junction organization;cell surface receptor linked signaling pathway;cell-cell junction or
anatomical structure development;biological regulation;cell cycle;cell cycle process;cell differentiation;cellular component disassembly;cellular component disassembly at cellular level;cellular component organization;cellular
biological regulation;biosynthetic process;cell proliferation;cell surface receptor linked signaling pathway;cellular biosynthetic process;cellular macromolecule biosynthetic process;cellular macromolecule metabolic process;c
apoptotic mitochondrial changes;biosynthetic process;cellular biosynthetic process;cellular component disassembly;cellular component disassembly at cellular level;cellular component organization;cellular component organ
biosynthetic process;cellular biosynthetic process;cellular macromolecule biosynthetic process;cellular macromolecule metabolic process;cellular metabolic process;cellular process;cellular protein metabolic process;cotrans
cellular metabolic process;cellular process;electron transport chain;generation of precursor metabolites and energy;metabolic process;mitochondrial electron transport, NADH to ubiquinone;oxidation-reduction process;respi
anatomical structure morphogenesis;developmental process;metabolic process;organ morphogenesis;oxidation-reduction process

actin cytoskeleton organization;actin filament organization;actin filament polymerization;actin filament-based process;actin polymerization or depolymerization;cellular component assembly;cellular component assembly at ce
activation of immune response;activation of innate immune response;antigen receptor-mediated signaling pathway;biological regulation;catabolic process;cell surface receptor linked signaling pathway;cellular catabolic proce
actin cytoskeleton organization;actin filament-based process;anatomical structure development;apoptosis;biological adhesion;biological regulation;cell adhesion;cell cycle;cell death;cell division;cell projection organization;ce
actin cytoskeleton organization;actin filament organization;actin filament-based process;biological regulation;cellular component organization;cellular component organization at cellular level;cellular component organization c
cellular macromolecule metabolic process;cellular metabolic process;cellular nitrogen compound metabolic process;cellular process;macromolecule metabolic process;metabolic process;ncRNA metabolic process;ncRNA pr
biological regulation;biosynthetic process;cell cycle;cell differentiation;cellular biosynthetic process;cellular developmental process;cellular metabolic process;cellular nitrogen compound biosynthetic process;cellular nitrogen
alcohol metabolic process;anatomical structure development;behavior;behavioral interaction between organisms;biological regulation;cation homeostasis;cell surface receptor linked signaling pathway;cellular cation homeost
biosynthetic process;cellular biosynthetic process;cellular macromolecule biosynthetic process;cellular macromolecule metabolic process;cellular metabolic process;cellular nitrogen compound metabolic process;cellular pro
alcohol biosynthetic process;alcohol metabolic process;biosynthetic process;cellular biosynthetic process;cellular lipid metabolic process;cellular metabolic process;cellular process;cholesterol biosynthetic process;cholester
biological regulation;biosynthetic process;cellular biosynthetic process;cellular metabolic process;cellular nitrogen compound biosynthetic process;cellular nitrogen compound metabolic process;cellular process;metabolic pr
catabolic process;cellular catabolic process;cellular macromolecule catabolic process;cellular macromolecule metabolic process;cellular metabolic process;cellular nitrogen compound metabolic process;cellular process;exo
apoptosis;biological regulation;carbohydrate transport;cell cycle checkpoint;cell death;cellular process;cellular response to stimulus;cellular response to stress;death;DNA damage checkpoint;DNA integrity checkpoint;establi
biological regulation;cell cycle process;cellular component organization;cellular component organization at cellular level;cellular component organization or biogenesis;cellular component organization or biogenesis at cellular
biological adhesion;biological regulation;cell adhesion;cell junction assembly;cell junction organization;cell migration;cell motility;cell surface receptor linked signaling pathway;cell-substrate junction assembly;cellular compor
biological regulation;biosynthetic process;cellular biosynthetic process;cellular macromolecule biosynthetic process;cellular macromolecule metabolic process;cellular metabolic process;cellular nitrogen compound metabolic
biological regulation;biosynthetic process;cellular biosynthetic process;cellular component assembly;cellular component organization;cellular component organization or biogenesis;cellular macromolecule biosynthetic proces
biological regulation;cellular process;chromosome segregation;positive regulation of biological process;positive regulation of cellular metabolic process;positive regulation of cellular process;positive regulation of cellular prote
amine transport;amino acid transport;carboxylic acid transport;cellular process;establishment of localization;extracellular amino acid transport;extracellular transport;glutamine transport;ion transport;neutral amino acid transp
cellular macromolecule metabolic process;cellular metabolic process;cellular nitrogen compound metabolic process;cellular process;developmental process;macromolecule metabolic process;maturation of SSU-rRNA;matur
cellular macromolecule metabolic process;cellular metabolic process;cellular nitrogen compound metabolic process;cellular process;macromolecule metabolic process;metabolic process;mRNA metabolic process;mRNA pro
cellular component organization;cellular component organization at cellular level;cellular component organization or biogenesis;cellular component organization or biogenesis at cellular level;cellular macromolecule metabolic
biological regulation;biosynthetic process;cellular biosynthetic process;cellular macromolecule biosynthetic process;cellular macromolecule metabolic process;cellular metabolic process;cellular nitrogen compound metabolic
cellular macromolecule metabolic process;cellular metabolic process;cellular process;cellular protein metabolic process;cullin deneddylation;macromolecule metabolic process;macromolecule modification;metabolic process
alcohol metabolic process;amino sugar metabolic process;biosynthetic process;carbohydrate biosynthetic process;carbohydrate metabolic process;carboxylic acid metabolic process;cellular carbohydrate metabolic process
cellular component organization;cellular component organization at cellular level;cellular component organization or biogenesis;cellular component organization or biogenesis at cellular level;cellular process;establishment of l
biological regulation;carboxylic acid metabolic process;cellular ketone metabolic process;cellular lipid metabolic process;cellular metabolic process;cellular process;cellular response to stimulus;fatty acid metabolic process;i
developmental process;multicellular organismal development;multicellular organismal process
catabolic process;cellular catabolic process;cellular macromolecule catabolic process;cellular macromolecule metabolic process;cellular metabolic process;cellular process;ER-associated protein catabolic process;macromo
biological adhesion;biological regulation;cell adhesion;cellular macromolecule metabolic process;cellular metabolic process;cellular nitrogen compound metabolic process;cellular process;macromolecule metabolic process;r
biological regulation;calcium ion homeostasis;calcium ion transmembrane transport;calcium ion transport;cation homeostasis;cation transport;cell cycle arrest;cell cycle process;cell proliferation;cellular calcium ion homeosta
ATP biosynthetic process;ATP hydrolysis coupled proton transport;ATP metabolic process;ATP synthesis coupled proton transport;biological regulation;biosynthetic process;cation homeostasis;cation transport;cell surface re
biological regulation;induction of apoptosis;induction of apoptosis by intracellular signals;induction of apoptosis by oxidative stress;induction of programmed cell death;positive regulation of apoptosis;positive regulation of bi
anatomical structure development;biological regulation;cell differentiation;cell proliferation;cellular developmental process;cellular process;developmental process;gamete generation;male gamete generation;maternal placent
cellular macromolecule metabolic process;cellular metabolic process;cellular nitrogen compound metabolic process;cellular process;establishment of localization;establishment of localization in cell;establishment of protein lo
biological regulation;biosynthetic process;cellular biosynthetic process;cellular macromolecule biosynthetic process;cellular macromolecule metabolic process;cellular metabolic process;cellular nitrogen compound metabolic
biological regulation;cell activation;cell activation involved in immune response;cellular process;cytokine secretion;establishment of localization;establishment of localization in cell;establishment of protein localization;exocyto
cellular component assembly;cellular component assembly at cellular level;cellular component organization;cellular component organization at cellular level;cellular component organization or biogenesis;cellular component o
acute inflammatory response;acute-phase response;alcohol metabolic process;cellular macromolecule metabolic process;cellular metabolic process;cellular process;cholesterol metabolic process;defense response;developm
alcohol catabolic process;alcohol metabolic process;carbohydrate catabolic process;carbohydrate metabolic process;catabolic process;cellular carbohydrate catabolic process;cellular carbohydrate metabolic process;cellula
ATP-dependent chromatin remodeling;cellular component organization;cellular component organization at cellular level;cellular component organization or biogenesis;cellular component organization or biogenesis at cellular l
biological regulation;cellular process;cellular response to abiotic stimulus;cellular response to acidity;cellular response to pH;cellular response to stimulus;establishment of localization;establishment of localization in cell;exoc
4-hydroxyproline metabolic process;amine metabolic process;anatomical structure development;anatomical structure formation involved in morphogenesis;anatomical structure morphogenesis;biological regulation;carboxylic
biological regulation;development of secondary female sexual characteristics;development of secondary sexual characteristics;developmental process;developmental process involved in reproduction;negative regulation of b
biosynthetic process;cellular biosynthetic process;cellular component disassembly;cellular component disassembly at cellular level;cellular component organization;cellular component organization at cellular level;cellular com
biosynthetic process;establishment of localization;lipid biosynthetic process;lipid metabolic process;metabolic process;primary metabolic process;steroid biosynthetic process;steroid metabolic process;transport
biological regulation;cellular component organization;cellular component organization or biogenesis;cellular process;cellular response to stimulus;intracellular signal transduction;positive regulation of actin filament polymeriza
establishment of localization;lipid transport;organic substance transport;transport
acidic amino acid transport;amine transport;amino acid transport;anion transport;carboxylic acid transport;establishment of localization;ion transport;L-glutamate transport;nitrogen compound transport;organic acid transpor
biosynthetic process;cellular biosynthetic process;cellular component disassembly;cellular component disassembly at cellular level;cellular component organization;cellular component organization at cellular level;cellular com
amine metabolic process;amino acid activation;carboxylic acid metabolic process;cellular amine metabolic process;cellular amino acid metabolic process;cellular ketone metabolic process;cellular macromolecule metabolic p
amine metabolic process;biosynthetic process;carboxylic acid metabolic process;cellular amine metabolic process;cellular amino acid metabolic process;cellular biosynthetic process;cellular ketone metabolic process;cellula
anatomical structure development;anatomical structure morphogenesis;catabolic process;cellular catabolic process;cellular macromolecule catabolic process;cellular macromolecule metabolic process;cellular metabolic proc
biological regulation;biosynthetic process;carbohydrate homeostasis;cellular biosynthetic process;cellular macromolecule biosynthetic process;cellular macromolecule metabolic process;cellular metabolic process;cellular nit
anatomical structure development;biosynthetic process;blastocyst development;cellular biosynthetic process;cellular macromolecule biosynthetic process;cellular macromolecule metabolic process;cellular metabolic process
apoptosis;biological regulation;biosynthetic process;cell death;cell differentiation;cell proliferation;cellular biosynthetic process;cellular developmental process;cellular macromolecule biosynthetic process;cellular macromolec
ameboidal cell migration;anatomical structure development;associative learning;basement membrane organization;behavior;biological adhesion;biological regulation;cAMP-mediated signaling;cell adhesion;cell migration;cell
biosynthetic process;cellular biosynthetic process;cellular component disassembly;cellular component disassembly at cellular level;cellular component organization;cellular component organization at cellular level;cellular com
defense response;defense response to virus;immune effector process;immune system process;multi-organism process;response to biotic stimulus;response to other organism;response to stimulus;response to stress;respons
anatomical structure development;biological regulation;cell proliferation;developmental process;negative regulation of biological process;negative regulation of cell communication;negative regulation of cellular process;negat
biological regulation;cellular component organization;cellular component organization at cellular level;cellular component organization or biogenesis;cellular component organization or biogenesis at cellular level;cellular proce
cellular macromolecule metabolic process;cellular metabolic process;cellular nitrogen compound metabolic process;cellular process;gene expression;macromolecule metabolic process;metabolic process;mRNA metabolic p
activation of JUN kinase activity;activation of MAPK activity;biological regulation;biosynthetic process;carboxylic acid biosynthetic process;carboxylic acid metabolic process;cellular biosynthetic process;cellular ketone meta
biological regulation;cell cycle;cell differentiation;cellular developmental process;cellular macromolecule metabolic process;cellular metabolic process;cellular nitrogen compound metabolic process;cellular process;cellular re
alcohol metabolic process;anatomical structure development;biological regulation;biosynthetic process;blood vessel development;cell differentiation;cellular developmental process;cellular process;cholesterol biosynthetic pr
alcohol biosynthetic process;alcohol metabolic process;amine catabolic process;amine metabolic process;biosynthetic process;carbohydrate biosynthetic process;carbohydrate metabolic process;carboxylic acid catabolic p
biological regulation;cell differentiation;cellular developmental process;cellular process;cellular response to stimulus;developmental process;gamete generation;male gamete generation;multicellular organismal process;multic
biological regulation;cellular macromolecule metabolic process;cellular metabolic process;cellular process;cellular protein metabolic process;'de novo' posttranslational protein folding;'de novo' protein folding;macromolecule
ATP hydrolysis coupled proton transport;biological regulation;cation homeostasis;cation transport;cell surface receptor linked signaling pathway;cellular cation homeostasis;cellular chemical homeostasis;cellular component c
cellular macromolecule metabolic process;cellular metabolic process;cellular nitrogen compound metabolic process;cellular process;macromolecule metabolic process;metabolic process;mRNA metabolic process;mRNA pro
cellular component organization;cellular component organization at cellular level;cellular component organization or biogenesis;cellular component organization or biogenesis at cellular level;cellular macromolecule metabolic
biosynthetic process;cellular biosynthetic process;cellular component disassembly;cellular component disassembly at cellular level;cellular component organization;cellular component organization at cellular level;cellular com
cellular macromolecule metabolic process;cellular metabolic process;cellular nitrogen compound metabolic process;cellular process;macromolecule metabolic process;metabolic process;ncRNA metabolic process;ncRNA pr
biological regulation;blood vessel endothelial cell migration;blood vessel endothelial cell migration involved in intussusceptive angiogenesis;cell differentiation;cell migration;cell motility;cellular component movement;cellular c
biological regulation;cellular macromolecule metabolic process;cellular metabolic process;cellular nitrogen compound metabolic process;cellular process;cellular response to stimulus;intracellular signal transduction;macromo
cellular component biogenesis;cellular component biogenesis at cellular level;cellular component organization or biogenesis;cellular component organization or biogenesis at cellular level;cellular process;ribonucleoprotein co
cellular macromolecule metabolic process;cellular metabolic process;cellular process;cellular protein metabolic process;cellular response to stimulus;cellular response to stress;macromolecule metabolic process;macromolec
cellular metabolic process;cellular process;electron transport chain;generation of precursor metabolites and energy;metabolic process;mitochondrial electron transport, NADH to ubiquinone;multicellular organismal process;n
biological regulation;catabolic process;cellular catabolic process;cellular macromolecule catabolic process;cellular macromolecule metabolic process;cellular metabolic process;cellular nitrogen compound metabolic process
carbohydrate metabolic process;cellular macromolecule metabolic process;cellular metabolic process;cellular process;cellular protein metabolic process;dephosphorylation;macromolecule metabolic process;macromolecule
biological regulation;biosynthetic process;cellular biosynthetic process;cellular macromolecule biosynthetic process;cellular macromolecule metabolic process;cellular metabolic process;cellular nitrogen compound metabolic
biological regulation;cell surface receptor linked signaling pathway;cellular component assembly;cellular component organization;cellular component organization or biogenesis;cellular process;cellular response to chemical s
biological regulation;biosynthetic process;cell differentiation;cellular biosynthetic process;cellular developmental process;cellular macromolecule biosynthetic process;cellular macromolecule metabolic process;cellular metab
acid secretion;actin cytoskeleton organization;actin filament-based process;activation of adenylate cyclase activity;activation of adenylate cyclase activity by dopamine receptor signaling pathway;activation of adenylate cycla
anatomical structure development;cell development;cellular developmental process;cellular metabolic process;cellular process;central nervous system neuron development;cerebellar Purkinje cell layer development;developm
anatomical structure formation involved in morphogenesis;anatomical structure morphogenesis;biosynthetic process;carboxylic acid biosynthetic process;carboxylic acid metabolic process;cell junction assembly;cell junctio
cellular component organization;cellular component organization at cellular level;cellular component organization or biogenesis;cellular component organization or biogenesis at cellular level;cellular membrane fusion;cellular r
carbohydrate metabolic process;cellular aldehyde metabolic process;cellular metabolic process;cellular process;metabolic process;primary metabolic process;small molecule metabolic process;xenobiotic metabolic process
biological regulation;cell cycle phase;cell cycle process;cellular component organization;cellular component organization at cellular level;cellular component organization or biogenesis;cellular component organization or bioge
establishment of localization;establishment of localization in cell;establishment of RNA localization;intracellular transport;mRNA export from nucleus;mRNA transport;nuclear export;nuclear transport;nucleic acid transport;nuc
anatomical structure maturation;biological regulation;blood vessel maturation;defense response;defense response to bacterium;developmental maturation;developmental process;immune response;immune system process;i
biological regulation;cation homeostasis;cation transport;cell surface receptor linked signaling pathway;cellular cation homeostasis;cellular chemical homeostasis;cellular component organization;cellular component organizat
anatomical structure development;biological regulation;cellular metabolic process;cellular process;developmental process;electron transport chain;generation of precursor metabolites and energy;metabolic process;mitochon
anatomical structure morphogenesis;axon guidance;axonogenesis;biological regulation;biosynthetic process;branchiomotor neuron axon guidance;cell part morphogenesis;cell projection morphogenesis;cell projection organ
cellular component biogenesis;cellular component biogenesis at cellular level;cellular component organization or biogenesis;cellular component organization or biogenesis at cellular level;cellular process;ribonucleoprotein co
catabolic process;cellular catabolic process;cellular ketone metabolic process;cellular metabolic process;cellular process;coenzyme catabolic process;coenzyme metabolic process;cofactor catabolic process;cofactor metab
CAAX-box protein processing;catabolic process;cellular catabolic process;cellular component organization;cellular component organization or biogenesis;cellular macromolecule catabolic process;cellular macromolecule me
antigen processing and presentation;antigen processing and presentation of exogenous antigen;antigen processing and presentation of exogenous peptide antigen;antigen processing and presentation of exogenous peptide
biological regulation;cell redox homeostasis;cellular homeostasis;cellular process;homeostatic process;negative regulation of biological process;negative regulation of cardiac muscle hypertrophy;negative regulation of multic
biological regulation;cellular macromolecule metabolic process;cellular metabolic process;cellular process;cellular protein metabolic process;macromolecule metabolic process;metabolic process;positive regulation of catalyt
anatomical structure development;biological regulation;biosynthetic process;cellular biosynthetic process;cellular macromolecule biosynthetic process;cellular macromolecule metabolic process;cellular metabolic process;ce
alcohol catabolic process;alcohol metabolic process;carbohydrate catabolic process;carbohydrate metabolic process;catabolic process;cellular carbohydrate catabolic process;cellular carbohydrate metabolic process;cellula
amine catabolic process;amine metabolic process;carboxylic acid catabolic process;carboxylic acid metabolic process;catabolic process;cellular amine metabolic process;cellular amino acid catabolic process;cellular amino
anatomical structure development;ATP-dependent chromatin remodeling;biological regulation;biosynthetic process;cellular biosynthetic process;cellular component organization;cellular component organization at cellular lev
acute inflammatory response;acute-phase response;biological regulation;blood coagulation;blood coagulation, intrinsic pathway;calcium ion homeostasis;cation homeostasis;cell activation;cell migration;cell motility;cell surfa
activation of immune response;activation of innate immune response;activation of MAPKK activity;activation of protein kinase activity;axon guidance;behavior;behavioral interaction between organisms;biological regulation;bl
biological regulation;cellular process;cellular response to cadmium ion;cellular response to chemical stimulus;cellular response to inorganic substance;cellular response to metal ion;cellular response to stimulus;cellular respo
biosynthetic process;catabolic process;cellular biosynthetic process;cellular catabolic process;cellular component disassembly;cellular component disassembly at cellular level;cellular component organization;cellular compo
adenine biosynthetic process;adenine metabolic process;adenine salvage;AMP biosynthetic process;AMP metabolic process;AMP salvage;behavior;biological regulation;biosynthetic process;body fluid secretion;cellular aron
biological regulation;cellular macromolecule metabolic process;cellular metabolic process;cellular nitrogen compound metabolic process;cellular process;gene expression;macromolecule metabolic process;metabolic proces
anatomical structure morphogenesis;biological regulation;cell proliferation;cell surface receptor linked signaling pathway;cellular process;cellular response to stimulus;developmental process;epithelial tube morphogenesis;m
biological regulation;biosynthetic process;carbohydrate biosynthetic process;carbohydrate metabolic process;carbohydrate transmembrane transport;carbohydrate transport;carboxylic acid metabolic process;cation transpo
actin cytoskeleton organization;actin cytoskeleton reorganization;actin filament-based process;biological regulation;cell surface receptor linked signaling pathway;cellular component organization;cellular component organizat
autophagic cell death;autophagy;biological regulation;catabolic process;cell death;cellular catabolic process;cellular metabolic process;cellular process;death;establishment of localization;establishment of localization in cell;e
alcohol metabolic process;anatomical structure development;biosynthetic process;carbohydrate biosynthetic process;carbohydrate metabolic process;cellular biosynthetic process;cellular carbohydrate biosynthetic process

aerobic respiration;cellular metabolic process;cellular process;cellular respiration;electron transport chain;energy derivation by oxidation of organic compounds;generation of precursor metabolites and energy;metabolic proce
actin cytoskeleton organization;actin filament organization;actin filament-based process;anatomical structure homeostasis;axon guidance;biological adhesion;biological regulation;blood coagulation;bone resorption;cell activa
anatomical structure development;biological regulation;cell differentiation;cellular developmental process;cellular process;cellular response to stimulus;developmental process;negative regulation of biological process;negativ
anatomical structure formation involved in morphogenesis;angiogenesis;biosynthetic process;catabolic process;cell differentiation;cellular aromatic compound metabolic process;cellular biosynthetic process;cellular catabolic
bile acid biosynthetic process;bile acid metabolic process;biological regulation;biosynthetic process;C21-steroid hormone biosynthetic process;C21-steroid hormone metabolic process;carboxylic acid biosynthetic process;c
anaphase-promoting complex-dependent proteasomal ubiquitin-dependent protein catabolic process;biological regulation;biosynthetic process;blood coagulation;catabolic process;cation transport;cell cycle;cell cycle check
amine catabolic process;amine metabolic process;branched chain family amino acid catabolic process;branched chain family amino acid metabolic process;carboxylic acid catabolic process;carboxylic acid metabolic proces
biological regulation;biosynthetic process;catabolic process;cellular biosynthetic process;cellular catabolic process;cellular macromolecule metabolic process;cellular metabolic process;cellular nitrogen compound metabolic
biosynthetic process;cellular biosynthetic process;cellular metabolic process;cellular process;melanin biosynthetic process;melanin metabolic process;metabolic process;pigment biosynthetic process;pigment metabolic proc
actin cytoskeleton organization;actin filament organization;actin filament-based process;anatomical structure homeostasis;biological adhesion;biological regulation;calcium ion transport;cation transport;cell activation;cell act
alcohol metabolic process;biological regulation;cell differentiation;cellular component assembly;cellular component organization;cellular component organization or biogenesis;cellular developmental process;cellular lipid met
biological regulation;cellular process;cellular response to stimulus;regulation of biological process;regulation of cellular process;response to stimulus;signal transduction
anatomical structure homeostasis;base-excision repair;biological regulation;biosynthetic process;cell cycle;cellular biosynthetic process;cellular component organization;cellular component organization at cellular level;cellula
activation of MAPKK activity;activation of protein kinase activity;biological regulation;blood coagulation;cell activation;cell surface receptor linked signaling pathway;cellular macromolecule metabolic process;cellular metaboli
alcohol metabolic process;amine metabolic process;biosynthetic process;CDP-choline pathway;cellular amine metabolic process;cellular biogenic amine metabolic process;cellular biosynthetic process;cellular lipid metaboli
actin cytoskeleton organization;actin filament-based process;biological regulation;cell chemotaxis;cell junction assembly;cell junction organization;cell migration;cell motility;cell projection organization;cell-cell junction assem
acetyl-CoA metabolic process;alcohol biosynthetic process;alcohol metabolic process;biological regulation;biosynthetic process;carbohydrate biosynthetic process;carbohydrate metabolic process;cellular biosynthetic proce
biological regulation;catabolic process;cellular catabolic process;cellular component organization;cellular component organization at cellular level;cellular component organization or biogenesis;cellular component organizatio
cellular component organization;cellular component organization at cellular level;cellular component organization or biogenesis;cellular component organization or biogenesis at cellular level;cellular process;extracellular matri
anatomical structure morphogenesis;B cell activation;B cell differentiation;cell activation;cell differentiation;cellular developmental process;cellular process;developmental process;establishment of localization;immune system
biological regulation;biosynthetic process;cellular biosynthetic process;cellular macromolecule biosynthetic process;cellular macromolecule metabolic process;cellular metabolic process;cellular process;macromolecule biosy
anatomical structure development;behavior;biological regulation;cellular macromolecule metabolic process;cellular metabolic process;cellular process;cellular protein metabolic process;cellular response to stimulus;cognition
activation of MAPKK activity;activation of protein kinase activity;anatomical structure development;biological regulation;blood coagulation;cell activation;cell development;cell surface receptor linked signaling pathway;cellular
activation of immune response;activation of innate immune response;anaphase-promoting complex-dependent proteasomal ubiquitin-dependent protein catabolic process;biological regulation;catabolic process;cell cycle;ce
anatomical structure development;biological regulation;brain development;catabolic process;developmental process;gamete generation;lipid catabolic process;lipid metabolic process;male gamete generation;metabolic proc
biosynthetic process;cellular biosynthetic process;cellular macromolecule biosynthetic process;cellular macromolecule metabolic process;cellular metabolic process;cellular nitrogen compound metabolic process;cellular pro
acetaldehyde metabolic process;activation of immune response;activation of innate immune response;aging;anatomical structure development;anatomical structure morphogenesis;antigen receptor-mediated signaling pathw
anatomical structure morphogenesis;anterior/posterior pattern specification;cellular macromolecule metabolic process;cellular metabolic process;cellular nitrogen compound metabolic process;cellular process;developmenta
behavior;biological regulation;biosynthetic process;catabolic process;cellular biosynthetic process;cellular catabolic process;cellular component organization;cellular component organization at cellular level;cellular componen
activation of immune response;activation of innate immune response;antigen receptor-mediated signaling pathway;biological regulation;cell differentiation;cell surface receptor linked signaling pathway;cellular developmental
biological regulation;cell cycle;cell surface receptor linked signaling pathway;cellular macromolecule metabolic process;cellular metabolic process;cellular process;cellular protein metabolic process;cellular response to chemi
biological regulation;catabolic process;cellular catabolic process;cellular localization;cellular macromolecule catabolic process;cellular macromolecule localization;cellular macromolecule metabolic process;cellular metabolic
biological regulation;biosynthetic process;cellular biosynthetic process;cellular component organization;cellular component organization at cellular level;cellular component organization or biogenesis;cellular component orga
biological regulation;cell redox homeostasis;cellular homeostasis;cellular macromolecule metabolic process;cellular metabolic process;cellular process;cellular protein metabolic process;cellular response to chemical stimulus
amine metabolic process;amino acid activation;carboxylic acid metabolic process;cellular amine metabolic process;cellular amino acid metabolic process;cellular ketone metabolic process;cellular macromolecule metabolic
cellular component assembly;cellular component assembly at cellular level;cellular component organization;cellular component organization at cellular level;cellular component organization or biogenesis;cellular component c
biological regulation;cell junction assembly;cell junction organization;cellular component assembly;cellular component assembly at cellular level;cellular component organization;cellular component organization at cellular leve
anatomical structure development;biological adhesion;biological regulation;cell adhesion;cellular process;cellular response to chemical stimulus;cellular response to endogenous stimulus;cellular response to hormone stimulu
cation transport;cellular metabolic process;cellular process;electron transport chain;establishment of localization;generation of precursor metabolites and energy;ion transport;metabolic process;metal ion transport;mitochond
alcohol metabolic process;biosynthetic process;cellular metabolic process;cellular process;cholesterol biosynthetic process;cholesterol biosynthetic process via 24,25-dihydrolanosterol;cholesterol metabolic process;demeth
cellular component organization;cellular component organization at cellular level;cellular component organization or biogenesis;cellular component organization or biogenesis at cellular level;cellular process;endosome organi
biosynthetic process;cellular biosynthetic process;cellular metabolic process;cellular nitrogen compound biosynthetic process;cellular nitrogen compound metabolic process;cellular process;coenzyme biosynthetic process;c
amine biosynthetic process;amine metabolic process;biosynthetic process;carboxylic acid biosynthetic process;carboxylic acid metabolic process;cellular amine metabolic process;cellular amino acid biosynthetic process;ce
amine metabolic process;amino acid activation;carboxylic acid metabolic process;cellular amine metabolic process;cellular amino acid metabolic process;cellular ketone metabolic process;cellular macromolecule metabolic
amine catabolic process;amine metabolic process;branched chain family amino acid catabolic process;branched chain family amino acid metabolic process;carboxylic acid catabolic process;carboxylic acid metabolic proces
alkaloid metabolic process;biosynthetic process;cellular amide metabolic process;cellular biosynthetic process;cellular metabolic compound salvage;cellular metabolic process;cellular nitrogen compound biosynthetic proces
activation of protein kinase activity;autophagy;biological regulation;catabolic process;cell migration;cell motility;cell surface receptor linked signaling pathway;cellular catabolic process;cellular component movement;cellular l
biological regulation;cellular macromolecule metabolic process;cellular metabolic process;cellular nitrogen compound metabolic process;cellular process;macromolecule metabolic process;macromolecule methylation;macro
metabolic process;nitrogen compound metabolic process
cell projection organization;cellular component movement;cellular component organization;cellular component organization at cellular level;cellular component organization or biogenesis;cellular component organization or bi
anatomical structure development;biological regulation;calcium ion homeostasis;calcium ion transport;calcium ion transport into cytosol;cation homeostasis;cation transport;cellular calcium ion homeostasis;cellular cation ho
cellular component assembly;cellular component assembly at cellular level;cellular component disassembly;cellular component disassembly at cellular level;cellular component organization;cellular component organization at
acylglycerol biosynthetic process;acylglycerol metabolic process;anatomical structure development;anatomical structure homeostasis;biological regulation;biosynthetic process;carboxylic acid biosynthetic process;carboxyli
biosynthetic process;cellular biosynthetic process;cellular component disassembly;cellular component disassembly at cellular level;cellular component organization;cellular component organization at cellular level;cellular cor
biological regulation;cellular process;cellular response to stimulus;positive regulation of biological process;positive regulation of cell communication;positive regulation of cellular process;positive regulation of I-kappaB kinase
cellular component biogenesis;cellular component biogenesis at cellular level;cellular component organization or biogenesis;cellular component organization or biogenesis at cellular level;cellular macromolecule metabolic pr
apoptosis;cell death;cellular component organization;cellular component organization or biogenesis;cellular membrane organization;cellular process;death;endocytosis;establishment of localization;membrane invagination;me
aromatic compound biosynthetic process;biosynthetic process;cellular aromatic compound metabolic process;cellular biosynthetic process;cellular metabolic process;cellular nitrogen compound biosynthetic process;cellular
acetyl-CoA catabolic process;acetyl-CoA metabolic process;alcohol catabolic process;alcohol metabolic process;carbohydrate catabolic process;carbohydrate metabolic process;catabolic process;cell differentiation;cellular
alcohol biosynthetic process;alcohol metabolic process;biosynthetic process;carbohydrate biosynthetic process;carbohydrate metabolic process;cellular biosynthetic process;cellular carbohydrate biosynthetic process;cellul
anatomical structure development;biological regulation;brain development;cell cycle checkpoint;cell cycle process;cell proliferation;cellular macromolecule metabolic process;cellular metabolic process;cellular process;cellula
biological regulation;cell redox homeostasis;cellular homeostasis;cellular macromolecule metabolic process;cellular metabolic process;cellular process;cellular protein metabolic process;cellular response to stimulus;cellular r
anatomical structure development;biological regulation;cell cycle arrest;cell cycle process;cell differentiation;cell migration;cell motility;cell projection assembly;cell projection organization;cellular component assembly;cellular
biological regulation;biosynthetic process;cellular biosynthetic process;cellular component organization;cellular component organization at cellular level;cellular component organization or biogenesis;cellular component orga
cellular macromolecule metabolic process;cellular metabolic process;cellular process;cellular protein metabolic process;macromolecule metabolic process;metabolic process;primary metabolic process;protein folding;protein
amine catabolic process;amine metabolic process;branched chain family amino acid catabolic process;branched chain family amino acid metabolic process;carboxylic acid catabolic process;carboxylic acid metabolic proces
establishment of localization;transport
biological regulation;cellular macromolecule metabolic process;cellular metabolic process;cellular process;cellular protein metabolic process;cytokine production;defense response;defense response to virus;immune effector
biological regulation;posttranscriptional regulation of gene expression;regulation of biological process;regulation of biosynthetic process;regulation of cellular biosynthetic process;regulation of cellular macromolecule biosynth
apoptosis;biological regulation;cell death;cellular component organization;cellular component organization at cellular level;cellular component organization or biogenesis;cellular component organization or biogenesis at cellul
biological regulation;cellular component organization;cellular component organization at cellular level;cellular component organization or biogenesis;cellular component organization or biogenesis at cellular level;cellular proce
anatomical structure formation involved in morphogenesis;angiogenesis;biological regulation;cell migration;cell motility;cellular component assembly;cellular component assembly at cellular level;cellular component movemen
biological regulation;establishment of localization;establishment of localization in cell;establishment of RNA localization;intracellular transport;mRNA export from nucleus;mRNA transport;nuclear export;nuclear transport;nucle
biological regulation;cell differentiation;cellular developmental process;cellular localization;cellular macromolecule localization;cellular process;cellular protein localization;developmental process;establishment of localization;f
biological regulation;regulation of biological process;regulation of biosynthetic process;regulation of cellular biosynthetic process;regulation of cellular macromolecule biosynthetic process;regulation of cellular metabolic proc
biological regulation;cell redox homeostasis;cellular component organization;cellular component organization or biogenesis;cellular homeostasis;cellular process;homeostatic process;positive regulation of catalytic activity;po
biological regulation;negative regulation of catalytic activity;negative regulation of hydrolase activity;negative regulation of molecular function;negative regulation of phosphatase activity;regulation of biological process;regulat
behavior;exploration behavior;locomotory behavior;locomotory exploration behavior;pigmentation;response to stimulus
cellular component organization;cellular component organization or biogenesis;cellular process;endosome transport;establishment of localization;establishment of localization in cell;establishment of protein localization;intrac
biological regulation;biosynthetic process;cellular biosynthetic process;cellular component organization;cellular component organization at cellular level;cellular component organization or biogenesis;cellular component orga
biological regulation;cellular macromolecule metabolic process;cellular metabolic process;cellular process;cellular protein metabolic process;dephosphorylation;macromolecule metabolic process;macromolecule modification
biological regulation;cellular macromolecule metabolic process;cellular metabolic process;cellular nitrogen compound metabolic process;cellular process;cellular response to chemical stimulus;cellular response to drug;cellula
cellular macromolecule metabolic process;cellular metabolic process;cellular nitrogen compound metabolic process;cellular process;macromolecule metabolic process;maturation of SSU-rRNA;maturation of SSU-rRNA from
anaphase-promoting complex-dependent proteasomal ubiquitin-dependent protein catabolic process;biological regulation;catabolic process;cell cycle;cell cycle checkpoint;cell cycle phase;cell cycle process;cell division;cel
biosynthetic process;cellular biosynthetic process;cellular macromolecule biosynthetic process;cellular macromolecule metabolic process;cellular metabolic process;cellular nitrogen compound metabolic process;cellular pro
cell proliferation
abscission;biological regulation;catabolic process;cell cycle process;cell division;cellular catabolic process;cellular component disassembly;cellular component disassembly at cellular level;cellular component organization;ce
anatomical structure morphogenesis;cell part morphogenesis;cellular component morphogenesis;cellular component organization;cellular component organization at cellular level;cellular component organization or biogenesi
biological regulation;biosynthetic process;cellular biosynthetic process;cellular macromolecule biosynthetic process;cellular macromolecule metabolic process;cellular metabolic process;cellular process;cellular protein metab
biological regulation;biosynthetic process;cellular biosynthetic process;cellular macromolecule biosynthetic process;cellular macromolecule metabolic process;cellular metabolic process;cellular nitrogen compound metabolic
alcohol metabolic process;anatomical structure development;biological regulation;brain development;carbohydrate homeostasis;carbohydrate metabolic process;cell proliferation;cell surface receptor linked signaling pathwa
establishment of localization;establishment of localization in cell;establishment of protein localization;intracellular protein transport;intracellular transport;nuclear import;nuclear transport;nucleocytoplasmic transport;protein in
cell differentiation;cellular developmental process;cellular process;developmental process;epidermal cell differentiation;epithelial cell differentiation;keratinocyte differentiation;macromolecule metabolic process;metabolic proc
amine metabolic process;aromatic compound biosynthetic process;biosynthetic process;carboxylic acid metabolic process;cellular amine metabolic process;cellular amino acid metabolic process;cellular aromatic compound
biological regulation;cellular component organization;cellular component organization at cellular level;cellular component organization or biogenesis;cellular component organization or biogenesis at cellular level;cellular macr
biological regulation;cellular component organization;cellular component organization at cellular level;cellular component organization or biogenesis;cellular component organization or biogenesis at cellular level;cellular memb
antigen processing and presentation;antigen processing and presentation of exogenous antigen;antigen processing and presentation of exogenous peptide antigen;antigen processing and presentation of exogenous peptide
carboxylic acid metabolic process;cellular ketone metabolic process;cellular lipid metabolic process;cellular metabolic process;cellular process;fatty acid metabolic process;lipid metabolic process;metabolic process;monoca
anatomical structure development;antigen processing and presentation;antigen processing and presentation of exogenous antigen;antigen processing and presentation of exogenous peptide antigen;antigen processing and
biological regulation;cellular process;cellular response to stimulus;immune response;immune system process;metabolic process;positive regulation of biological process;positive regulation of catabolic process;positive regula
establishment of localization;establishment of localization in cell;establishment of protein localization;intracellular protein transport;intracellular transport;protein transport;transport
cellular process;establishment of localization;establishment of localization in cell;establishment of protein localization;Golgi vesicle transport;intracellular transport;post-Golgi vesicle-mediated transport;protein transport;respo
biological adhesion;biological regulation;calcium ion import;calcium ion transmembrane transport;calcium ion transport;cation transport;cell adhesion;cell communication;cell-cell signaling;cellular component organization;cel
alcohol biosynthetic process;alcohol metabolic process;amine metabolic process;biosynthetic process;cellular biosynthetic process;cellular lipid metabolic process;cellular metabolic process;cellular nitrogen compound bios
biological regulation;cell cycle;cell cycle checkpoint;cell cycle phase;cell cycle process;cell division;cellular component assembly;cellular component organization;cellular component organization at cellular level;cellular comp
cap-independent translational initiation;cellular component assembly;cellular component assembly at cellular level;cellular component disassembly;cellular component disassembly at cellular level;cellular component organiza
anion transport;biological regulation;cellular localization;cellular macromolecule localization;cellular macromolecule metabolic process;cellular metabolic process;cellular process;cellular protein localization;cellular protein me
biological regulation;biosynthetic process;cellular biosynthetic process;cellular macromolecule biosynthetic process;cellular macromolecule metabolic process;cellular metabolic process;cellular nitrogen compound metabolic
apoptosis;biological regulation;cell death;cell differentiation;cellular developmental process;cellular process;death;developmental process;inner ear receptor cell differentiation;mechanoreceptor differentiation;multicellular org
apoptosis;cell death;cellular component organization;cellular component organization or biogenesis;cellular membrane organization;cellular process;clathrin-mediated endocytosis;death;endocytosis;establishment of localiza
assembly of spliceosomal tri-snRNP;biological regulation;cellular component assembly;cellular component assembly at cellular level;cellular component organization;cellular component organization at cellular level;cellular co
carboxylic acid catabolic process;carboxylic acid metabolic process;catabolic process;cellular catabolic process;cellular ketone metabolic process;cellular lipid catabolic process;cellular lipid metabolic process;cellular metab
cell cycle;cell cycle process;cellular component disassembly;cellular component disassembly at cellular level;cellular component organization;cellular component organization at cellular level;cellular component organization
acidic amino acid transport;alcohol biosynthetic process;alcohol metabolic process;amine transport;amino acid transport;anion transport;aspartate transport;biosynthetic process;carbohydrate biosynthetic process;carbohyd
cellular macromolecule metabolic process;cellular metabolic process;cellular nitrogen compound metabolic process;cellular process;gene expression;macromolecule metabolic process;metabolic process;mRNA metabolic p
ameboidal cell migration;anatomical structure development;anatomical structure morphogenesis;androgen metabolic process;biological regulation;biosynthetic process;carboxylic acid metabolic process;catabolic process;ce
biological regulation;multicellular organismal process;negative regulation of biological process;negative regulation of cellular component organization;negative regulation of cellular process;negative regulation of cytoskeleton
adenine biosynthetic process;adenine metabolic process;adenine salvage;alcohol metabolic process;amine metabolic process;anatomical structure development;anatomical structure morphogenesis;behavior;benzene-conta
antigen processing and presentation;antigen processing and presentation of exogenous antigen;antigen processing and presentation of exogenous peptide antigen;antigen processing and presentation of exogenous peptide
biotin metabolic process;carboxylic acid catabolic process;carboxylic acid metabolic process;catabolic process;cellular amide metabolic process;cellular catabolic process;cellular ketone metabolic process;cellular lipid cata
apoptosis;catabolic process;cell death;cellular catabolic process;cellular component assembly;cellular component assembly at cellular level;cellular component disassembly;cellular component disassembly at cellular level;ce
aerobic respiration;cellular metabolic process;cellular process;cellular respiration;electron transport chain;energy derivation by oxidation of organic compounds;generation of precursor metabolites and energy;metabolic proce
cellular macromolecule metabolic process;cellular metabolic process;cellular nitrogen compound metabolic process;cellular process;gene expression;macromolecule metabolic process;metabolic process;mRNA metabolic p
biological regulation;biosynthetic process;cellular biosynthetic process;cellular component assembly;cellular component assembly at cellular level;cellular component organization;cellular component organization at cellular le
catabolic process;cellular catabolic process;cellular macromolecule catabolic process;cellular macromolecule metabolic process;cellular metabolic process;cellular process;cellular protein metabolic process;macromolecule c

amine catabolic process;amine metabolic process;arginine catabolic process;arginine metabolic process;aromatic amino acid family catabolic process;aromatic amino acid family metabolic process;aromatic compound catab
antigen processing and presentation;biological regulation;cellular component movement;cellular localization;cellular macromolecule localization;cellular macromolecule metabolic process;cellular metabolic process;cellular pr
cell cycle;cell cycle process;cellular component organization;cellular component organization at cellular level;cellular component organization or biogenesis;cellular component organization or biogenesis at cellular level;cellula
activation of immune response;activation of innate immune response;anaphase-promoting complex-dependent proteasomal ubiquitin-dependent protein catabolic process;antigen processing and presentation;antigen proces
catabolic process;cellular catabolic process;cellular metabolic process;cellular nitrogen compound catabolic process;cellular nitrogen compound metabolic process;cellular process;cofactor catabolic process;cofactor metab
biological regulation;catabolic process;cation homeostasis;cellular catabolic process;cellular cation homeostasis;cellular chemical homeostasis;cellular homeostasis;cellular ion homeostasis;cellular iron ion homeostasis;cellu
anatomical structure development;biological regulation;calcium ion transport;cation transport;cellular chemical homeostasis;cellular homeostasis;cellular ion homeostasis;cellular process;cellular response to stimulus;chemica
acyl-CoA metabolic process;carboxylic acid metabolic process;cellular ketone metabolic process;cellular lipid metabolic process;cellular metabolic process;cellular process;coenzyme metabolic process;cofactor metabolic p
anatomical structure development;anatomical structure formation involved in morphogenesis;anatomical structure morphogenesis;axon guidance;biological regulation;canonical Wnt receptor signaling pathway;canonical Wnt
biological regulation;cellular process;cellular response to stimulus;establishment of localization;establishment of localization in cell;establishment of protein localization;intracellular protein transport;intracellular signal transdu
actin cytoskeleton organization;actin filament-based process;biological regulation;calcium ion homeostasis;cation homeostasis;cellular calcium ion homeostasis;cellular cation homeostasis;cellular chemical homeostasis;cellu
biological regulation;biosynthetic process;cellular biosynthetic process;cellular component disassembly;cellular component disassembly at cellular level;cellular component organization;cellular component organization at cell
anatomical structure development;biological regulation;developmental process;hemopoiesis;hemopoietic or lymphoid organ development;organ development;positive regulation of biological process;positive regulation of cell
anatomical structure development;apical protein localization;asymmetric protein localization;biological regulation;brain development;cell communication;cell differentiation;cell-cell signaling;cellular component disassembly;ce
biological regulation;cellular process;cellular response to stimulus;regulation of biological process;regulation of cellular process;response to stimulus;signal transduction
cellular macromolecule metabolic process;cellular metabolic process;cellular nitrogen compound metabolic process;cellular process;macromolecule metabolic process;metabolic process;ncRNA metabolic process;ncRNA pr
biological regulation;cellular metabolic process;cellular process;dephosphorylation;metabolic process;phosphate-containing compound metabolic process;phosphorus metabolic process;regulation of biological process;regu
activation of immune response;activation of innate immune response;biological regulation;cell surface receptor linked signaling pathway;cellular macromolecule metabolic process;cellular metabolic process;cellular process;c
alcohol metabolic process;biological regulation;chemical homeostasis;cholesterol efflux;cholesterol homeostasis;cholesterol metabolic process;cholesterol transport;establishment of localization;establishment of localization
anatomical structure formation involved in morphogenesis;anatomical structure morphogenesis;biological regulation;calcium ion transmembrane transport;calcium ion transport;cardiac muscle tissue morphogenesis;cation tr
aging;anatomical structure formation involved in morphogenesis;anatomical structure morphogenesis;axon guidance;biological regulation;blood coagulation;branching involved in embryonic placenta morphogenesis;cell acti
biological regulation;biosynthetic process;catabolic process;cell proliferation;cellular biosynthetic process;cellular catabolic process;cellular macromolecule biosynthetic process;cellular macromolecule catabolic process;cellu
biological regulation;cation homeostasis;cellular cation homeostasis;cellular chemical homeostasis;cellular divalent inorganic cation homeostasis;cellular homeostasis;cellular ion homeostasis;cellular process;cellular respons
biological regulation;biosynthetic process;cellular biosynthetic process;cellular macromolecule biosynthetic process;cellular macromolecule metabolic process;cellular metabolic process;cellular nitrogen compound metabolic
anatomical structure development;biological regulation;biosynthetic process;cell differentiation;cellular biosynthetic process;cellular developmental process;cellular macromolecule biosynthetic process;cellular macromolecul
acylglycerol metabolic process;anatomical structure development;anatomical structure formation involved in morphogenesis;angiogenesis;biological regulation;blood coagulation;body fluid secretion;calcium ion homeostasis
cellular macromolecule metabolic process;cellular metabolic process;cellular process;cellular protein metabolic process;'de novo' cotranslational protein folding;'de novo' protein folding;macromolecule metabolic process;me
activation of store-operated calcium channel activity;biological regulation;blood coagulation;calcium ion transport;cation transport;coagulation;detection of calcium ion;detection of chemical stimulus;detection of stimulus;div
carbohydrate metabolic process;cellular carbohydrate metabolic process;cellular macromolecule metabolic process;cellular metabolic process;cellular process;cellular protein metabolic process;glycosylation;macromolecule
biological regulation;positive regulation of biological process;positive regulation of cellular component organization;positive regulation of cellular process;positive regulation of establishment of protein localization in plasma m
biological regulation;posttranscriptional regulation of gene expression;regulation of biological process;regulation of biosynthetic process;regulation of cellular biosynthetic process;regulation of cellular macromolecule biosynth
cellular macromolecule metabolic process;cellular metabolic process;cellular nitrogen compound metabolic process;cellular process;DNA metabolic process;DNA recombination;gene expression;macromolecule metabolic pr
anatomical structure development;biosynthetic process;cell cycle phase;cell cycle process;cell development;cell maturation;cellular biosynthetic process;cellular component assembly;cellular component assembly at cellular
3'-UTR-mediated mRNA stabilization;activation of immune response;activation of innate immune response;activation of MAPK activity;aging;alcohol metabolic process;anatomical structure development;anatomical structure
cellular component assembly;cellular component organization;cellular component organization or biogenesis;cellular macromolecule metabolic process;cellular metabolic process;cellular nitrogen compound metabolic proce
biological regulation;cell cycle checkpoint;cellular macromolecule metabolic process;cellular metabolic process;cellular nitrogen compound metabolic process;cellular process;cellular response to stimulus;cellular response to
biological regulation;biosynthetic process;cellular biosynthetic process;cellular component organization;cellular component organization at cellular level;cellular component organization or biogenesis;cellular component orga
biological regulation;biosynthetic process;cellular biosynthetic process;cellular macromolecule biosynthetic process;cellular macromolecule metabolic process;cellular metabolic process;cellular nitrogen compound metabolic
biological regulation;biosynthetic process;carbohydrate metabolic process;carbohydrate transport;cell cycle;cell cycle process;cell surface receptor linked signaling pathway;cellular biosynthetic process;cellular component c
biological regulation;cellular process;cellular response to stimulus;establishment of localization;establishment of protein localization;positive regulation of biological process;positive regulation of cell communication;positive re
apoptosis;apoptosis in response to endoplasmic reticulum stress;biological regulation;catabolic process;cell death;cell redox homeostasis;cellular catabolic process;cellular homeostasis;cellular macromolecule catabolic proc
biological regulation;cellular component movement;cellular homeostasis;cellular process;cellular response to stimulus;cytoskeleton-dependent intracellular transport;establishment of localization;establishment of localization
biological regulation;biosynthetic process;cellular biosynthetic process;cellular macromolecule biosynthetic process;cellular macromolecule metabolic process;cellular metabolic process;cellular nitrogen compound metabolic
acylglycerol biosynthetic process;acylglycerol metabolic process;alcohol catabolic process;alcohol metabolic process;alditol catabolic process;alditol metabolic process;biological regulation;biosynthetic process;carbohydrat
cell projection organization;cellular component organization;cellular component organization at cellular level;cellular component organization or biogenesis;cellular component organization or biogenesis at cellular level;cellula
cell migration;cell motility;cellular component movement;cellular component organization;cellular component organization at cellular level;cellular component organization or biogenesis;cellular component organization or biog
biological regulation;cellular component organization;cellular component organization at cellular level;cellular component organization or biogenesis;cellular component organization or biogenesis at cellular level;cellular proce
biosynthetic process;cellular biosynthetic process;cellular ketone metabolic process;cellular metabolic process;cellular process;coenzyme biosynthetic process;coenzyme metabolic process;cofactor biosynthetic process;cof
ATP-dependent chromatin remodeling;biological regulation;biosynthetic process;cellular biosynthetic process;cellular component disassembly;cellular component disassembly at cellular level;cellular component organization
apoptosis;attachment of GPI anchor to protein;cell death;cell differentiation;cellular developmental process;cellular macromolecule metabolic process;cellular metabolic process;cellular process;cellular protein metabolic proc
cellular process;ER to Golgi vesicle-mediated transport;establishment of localization;establishment of localization in cell;Golgi vesicle transport;intracellular transport;transport;vesicle-mediated transport
cellular macromolecule metabolic process;cellular metabolic process;cellular nitrogen compound metabolic process;cellular process;gene expression;macromolecule metabolic process;metabolic process;mRNA metabolic pr
biosynthetic process;cellular biosynthetic process;cellular component disassembly;cellular component disassembly at cellular level;cellular component organization;cellular component organization at cellular level;cellular cor
biological regulation;biosynthetic process;cellular biosynthetic process;cellular component disassembly;cellular component disassembly at cellular level;cellular component organization;cellular component organization at cell
alternative nuclear mRNA splicing, via spliceosome;cellular macromolecule metabolic process;cellular metabolic process;cellular nitrogen compound metabolic process;cellular process;macromolecule metabolic process;met
base-excision repair;biological regulation;catabolic process;cellular catabolic process;cellular macromolecule catabolic process;cellular macromolecule metabolic process;cellular metabolic process;cellular nitrogen compour
biological regulation;cellular macromolecule metabolic process;cellular metabolic process;cellular process;cellular protein metabolic process;dephosphorylation;dephosphorylation of RNA polymerase II C-terminal domain;ma
cell differentiation;cellular developmental process;cellular process;developmental process;establishment of localization;establishment of protein localization;gamete generation;gene expression;macromolecule metabolic proc
catabolic process;cell cycle;cell division;cell proliferation;cellular catabolic process;cellular component organization;cellular component organization at cellular level;cellular component organization or biogenesis;cellular comp
amine biosynthetic process;amine metabolic process;amino acid salvage;aspartate family amino acid biosynthetic process;aspartate family amino acid metabolic process;biosynthetic process;carboxylic acid biosynthetic pro
biological regulation;positive regulation of catalytic activity;positive regulation of GTPase activity;positive regulation of hydrolase activity;positive regulation of molecular function;regulation of biological process;regulation of ca
biosynthetic process;cellular biosynthetic process;cellular component disassembly;cellular component disassembly at cellular level;cellular component organization;cellular component organization at cellular level;cellular cor
biological regulation;biosynthetic process;cellular biosynthetic process;cellular component assembly;cellular component assembly at cellular level;cellular component organization;cellular component organization at cellular le
asymmetric Golgi ribbon formation;biological adhesion;biological regulation;biosynthetic process;cell adhesion;cell adhesion molecule production;cell migration;cell motility;cell projection assembly;cell projection organization
cellular macromolecule metabolic process;cellular metabolic process;cellular nitrogen compound metabolic process;cellular process;establishment of localization;establishment of localization in cell;establishment of RNA loca
cellular macromolecule metabolic process;cellular metabolic process;cellular nitrogen compound metabolic process;cellular process;macromolecule metabolic process;metabolic process;nitrogen compound metabolic proce
activation of immune response;activation of innate immune response;apoptosis;biological regulation;calcium ion homeostasis;cation homeostasis;cell death;cellular process;cellular response to stimulus;chemical homeostasi
apical protein localization;asymmetric protein localization;biological regulation;cellular component assembly;cellular component organization;cellular component organization at cellular level;cellular component organization or
anatomical structure development;cell differentiation;cellular developmental process;cellular process;developmental process;nervous system development;system development
anatomical structure formation involved in morphogenesis;anatomical structure morphogenesis;angiogenesis;apoptosis;axonal fasciculation;biological regulation;cardiac epithelial to mesenchymal transition;cell death;cell mig
biological regulation;cell activation;cellular component movement;cellular process;cellular response to stimulus;establishment of localization;establishment of localization in cell;establishment of protein localization;immune sy
anatomical structure development;biological regulation;biosynthetic process;cell development;cellular biosynthetic process;cellular developmental process;cellular macromolecule biosynthetic process;cellular macromolecule
biological regulation;blood coagulation;cellular process;coagulation;establishment of localization;establishment of localization in cell;establishment of protein localization;hemostasis;intracellular protein transport;intracellular t
biological regulation;cell surface receptor linked signaling pathway;cellular process;cellular response to stimulus;establishment of localization;establishment of localization in cell;establishment of protein localization;glutamate
actin cytoskeleton organization;actin filament-based process;cellular component biogenesis;cellular component biogenesis at cellular level;cellular component organization;cellular component organization at cellular level;cell
biological regulation;biosynthetic process;cellular biosynthetic process;cellular macromolecule biosynthetic process;cellular macromolecule metabolic process;cellular metabolic process;cellular nitrogen compound metabolic
cellular macromolecule metabolic process;cellular metabolic process;cellular nitrogen compound metabolic process;cellular process;macromolecule metabolic process;metabolic process;mRNA metabolic process;mRNA pro
biological regulation;cellular component organization;cellular component organization at cellular level;cellular component organization or biogenesis;cellular component organization or biogenesis at cellular level;cellular locali
aging;anatomical structure development;biological regulation;cellular macromolecule metabolic process;cellular metabolic process;cellular process;cellular protein metabolic process;cellular response to chemical stimulus;ce
alcohol metabolic process;biosynthetic process;lipid biosynthetic process;lipid metabolic process;metabolic process;primary metabolic process;small molecule metabolic process;steroid biosynthetic process;steroid metabo
developmental process
biological regulation;biosynthetic process;cap-independent translational initiation;cell cycle;cellular biosynthetic process;cellular component assembly;cellular component assembly at cellular level;cellular component disasse
cellular macromolecule metabolic process;cellular metabolic process;cellular nitrogen compound metabolic process;cellular process;macromolecule metabolic process;maturation of SSU-rRNA;maturation of SSU-rRNA from
biological regulation;cell cycle phase;cell cycle process;cellular component organization;cellular component organization at cellular level;cellular component organization or biogenesis;cellular component organization or bioge
biological regulation;biosynthetic process;cellular biosynthetic process;cellular macromolecule biosynthetic process;cellular macromolecule metabolic process;cellular metabolic process;cellular process;cellular protein metab
biological regulation;cell cycle cytokinesis;cell cycle phase;cell cycle process;cellular component assembly;cellular component assembly at cellular level;cellular component organization;cellular component organization at cel
actin cytoskeleton organization;actin filament-based process;actomyosin structure organization;biological adhesion;biological regulation;blood coagulation;cell adhesion;cell junction assembly;cell junction organization;cell m
cellular localization;cellular macromolecule localization;cellular process;cellular protein localization;localization;macromolecule localization;protein localization;protein localization in mitochondrion;protein localization to organe
biological regulation;biosynthetic process;cellular biosynthetic process;cellular component assembly;cellular component assembly at cellular level;cellular component organization;cellular component organization at cellular le
biological regulation;carboxylic acid metabolic process;cellular ketone metabolic process;cellular metabolic process;cellular process;metabolic process;monocarboxylic acid metabolic process;organic acid metabolic process
establishment of localization;transport
activation of immune response;activation of innate immune response;anaphase-promoting complex-dependent proteasomal ubiquitin-dependent protein catabolic process;antigen processing and presentation;antigen proces
biological regulation;cell surface receptor linked signaling pathway;cellular process;cellular response to chemical stimulus;cellular response to cytokine stimulus;cellular response to organic substance;cellular response to stim
cellular component organization;cellular component organization at cellular level;cellular component organization or biogenesis;cellular component organization or biogenesis at cellular level;cellular process;COPI coating of G
axon guidance;biological regulation;cell surface receptor linked signaling pathway;cellular process;cellular response to stimulus;chemotaxis;enzyme linked receptor protein signaling pathway;ephrin receptor signaling pathwa
cellular component assembly;cellular component assembly at cellular level;cellular component organization;cellular component organization at cellular level;cellular component organization or biogenesis;cellular component c
alcohol biosynthetic process;alcohol metabolic process;amine metabolic process;biosynthetic process;cellular biosynthetic process;cellular lipid metabolic process;cellular metabolic process;cellular nitrogen compound biosy
anion transport;biological regulation;blood coagulation;carboxylic acid metabolic process;carboxylic acid transport;cell migration;cell motility;cellular component movement;cellular ketone metabolic process;cellular metaboli
biosynthetic process;cellular biosynthetic process;cellular macromolecule biosynthetic process;cellular macromolecule metabolic process;cellular metabolic process;cellular nitrogen compound metabolic process;cellular pro
anatomical structure development;anatomical structure morphogenesis;biological regulation;blood vessel development;branching involved in embryonic placenta morphogenesis;cellular component organization;cellular comp
biological regulation;cell redox homeostasis;cellular homeostasis;cellular macromolecule metabolic process;cellular metabolic process;cellular process;cellular protein metabolic process;cellular response to chemical stimulus
biological regulation;biosynthetic process;cation transport;cellular biosynthetic process;cellular component organization;cellular component organization at cellular level;cellular component organization or biogenesis;cellular c
biological regulation;establishment of localization;establishment of localization in cell;establishment of protein localization;intracellular protein transport;intracellular transport;maternal process involved in female pregnancy;mu
biological regulation;biosynthetic process;cell cycle process;cell proliferation;cellular biosynthetic process;cellular component organization;cellular component organization at cellular level;cellular component organization or b
actin cytoskeleton organization;actin filament-based process;anatomical structure development;B cell activation;biological regulation;cell activation;cell cycle;cellular component organization;cellular component organization a
amine biosynthetic process;amine metabolic process;apoptotic mitochondrial changes;biosynthetic process;carboxylic acid biosynthetic process;carboxylic acid metabolic process;cellular amine metabolic process;cellular a
biological regulation;cell cycle checkpoint;cellular process;cellular response to stimulus;cellular response to stress;DNA damage checkpoint;DNA integrity checkpoint;negative regulation of catalytic activity;negative regulation
biological regulation;biosynthetic process;cellular biosynthetic process;cellular macromolecule biosynthetic process;cellular macromolecule metabolic process;cellular metabolic process;cellular nitrogen compound metabolic
biological regulation;cellular macromolecule metabolic process;cellular metabolic process;cellular nitrogen compound metabolic process;cellular process;macromolecule metabolic process;metabolic process;mRNA metaboli
cellular macromolecule metabolic process;cellular metabolic process;cellular process;cellular protein metabolic process;chaperone-mediated protein folding;macromolecule metabolic process;macromolecule modification;me
biological regulation;cellular component organization;cellular component organization at cellular level;cellular component organization or biogenesis;cellular component organization or biogenesis at cellular level;cellular macr
antigen processing and presentation;antigen processing and presentation of exogenous antigen;antigen processing and presentation of exogenous peptide antigen;antigen processing and presentation of exogenous peptide
autophagic vacuole fusion;cellular component assembly;cellular component assembly at cellular level;cellular component organization;cellular component organization at cellular level;cellular component organization or bioge
activation of caspase activity;acute inflammatory response;autophagy;biological adhesion;biological regulation;catabolic process;cell adhesion;cell chemotaxis;cell migration;cell motility;cell-cell adhesion;cellular catabolic pr
anatomical structure development;biological regulation;biosynthetic process;catabolic process;cell cycle process;cell surface receptor linked signaling pathway;cellular biosynthetic process;cellular catabolic process;cellular
activation of protein kinase activity;activation of protein kinase B activity;biological regulation;biosynthetic process;cell communication;cell proliferation;cell redox homeostasis;cell-cell signaling;cellular biosynthetic process;c
aging;base-excision repair;biological regulation;biosynthetic process;cell aging;cellular biosynthetic process;cellular component assembly;cellular component assembly at cellular level;cellular component disassembly;cellular
apoptosis;axon guidance;biological regulation;biosynthetic process;cell cycle;cell cycle checkpoint;cell death;cell surface receptor linked signaling pathway;cellular biosynthetic process;cellular macromolecule biosynthetic p
anatomical structure morphogenesis;anterior/posterior pattern specification;biological regulation;camera-type eye morphogenesis;cell differentiation;cellular component organization;cellular component organization at cellular

biological regulation;cellular macromolecule metabolic process;cellular metabolic process;cellular process;cellular protein metabolic process;cellular response to heat;cellular response to stimulus;cellular response to stress;c
anatomical structure morphogenesis;biological regulation;cell morphogenesis;cell morphogenesis involved in differentiation;cellular component morphogenesis;cellular component organization;cellular component organizatio
activation-induced cell death of T cells;aging;alcohol metabolic process;amine metabolic process;aminoglycan metabolic process;anatomical structure development;apoptosis;apoptotic mitochondrial changes;biological regu
2'-deoxyribonucleotide biosynthetic process;2'-deoxyribonucleotide metabolic process;biosynthetic process;catabolic process;cellular aromatic compound metabolic process;cellular biosynthetic process;cellular catabolic p
alpha-beta T cell activation;alpha-beta T cell differentiation;biosynthetic process;catabolic process;cell activation;cell differentiation;cellular biosynthetic process;cellular catabolic process;cellular component disassembly;cell
biological regulation;cellular macromolecule metabolic process;cellular metabolic process;cellular nitrogen compound metabolic process;cellular process;macromolecule metabolic process;metabolic process;mRNA metaboli
biosynthetic process;catabolic process;cellular biosynthetic process;cellular catabolic process;cellular component disassembly;cellular component disassembly at cellular level;cellular component organization;cellular compo
cellular metabolic process;cellular process;electron transport chain;generation of precursor metabolites and energy;metabolic process;oxidation-reduction process;respiratory electron transport chain;response to antibiotic;re
biosynthetic process;carbohydrate metabolic process;carboxylic acid metabolic process;cellular biosynthetic process;cellular component assembly;cellular component organization;cellular component organization or biogene
biological adhesion;cell activation;cell adhesion;cell junction assembly;cell junction organization;cell migration;cell motility;cell proliferation;cell-substrate junction assembly;cellular component assembly;cellular component as
biosynthetic process;cellular biosynthetic process;cellular component disassembly;cellular component disassembly at cellular level;cellular component organization;cellular component organization at cellular level;cellular cor
biological regulation;negative regulation of biological process;negative regulation of biosynthetic process;negative regulation of cellular biosynthetic process;negative regulation of cellular macromolecule biosynthetic process
activation of immune response;activation of innate immune response;anaphase-promoting complex-dependent proteasomal ubiquitin-dependent protein catabolic process;antigen processing and presentation;antigen proces
apoptosis;biological regulation;catabolic process;cell death;cell surface receptor linked signaling pathway;cellular catabolic process;cellular component disassembly;cellular component disassembly at cellular level;cellular co
activation of caspase activity;adaptive immune response;adaptive immune response based on somatic recombination of immune receptors built from immunoglobulin superfamily domains;aging;biological regulation;cell killin
biological regulation;cellular component assembly;cellular component assembly at cellular level;cellular component organization;cellular component organization at cellular level;cellular component organization or biogenesis;
biological regulation;cell cycle;cellular component organization;cellular component organization at cellular level;cellular component organization or biogenesis;cellular component organization or biogenesis at cellular level;cell
biological regulation;biosynthetic process;catabolic process;cellular biosynthetic process;cellular catabolic process;cellular macromolecule biosynthetic process;cellular macromolecule catabolic process;cellular macromolecu
antigen processing and presentation;antigen processing and presentation of exogenous antigen;antigen processing and presentation of exogenous peptide antigen;antigen processing and presentation of exogenous peptide
biosynthetic process;cellular biosynthetic process;cellular component assembly;cellular component assembly at cellular level;cellular component organization;cellular component organization at cellular level;cellular compone
actin cytoskeleton organization;actin filament organization;actin filament polymerization;actin filament-based process;actin polymerization or depolymerization;anatomical structure arrangement;anatomical structure homeosta
biological regulation;biosynthetic process;cellular biosynthetic process;cellular macromolecule biosynthetic process;cellular macromolecule metabolic process;cellular metabolic process;cellular process;cellular protein metab
biological regulation;biosynthetic process;carbohydrate metabolic process;carbohydrate transport;cell cycle;cell cycle process;cell surface receptor linked signaling pathway;cellular biosynthetic process;cellular component c
cellular process;establishment of localization;establishment of localization in cell;exocytosis;secretion;secretion by cell;transport;vesicle-mediated transport
actin cytoskeleton organization;actin filament organization;actin filament-based process;activation of protein kinase activity;adaptive immune response;adaptive immune response based on somatic recombination of immune
biosynthetic process;cellular biosynthetic process;cellular macromolecule biosynthetic process;cellular macromolecule metabolic process;cellular metabolic process;cellular nitrogen compound metabolic process;cellular pro
amine metabolic process;biosynthetic process;carboxylic acid metabolic process;cellular amine metabolic process;cellular amino acid metabolic process;cellular biosynthetic process;cellular ketone metabolic process;cellula
amine metabolic process;amino acid activation;apoptosis;biological regulation;carboxylic acid metabolic process;cell death;cell differentiation;cellular amine metabolic process;cellular amino acid metabolic process;cellular d
biological regulation;biosynthetic process;cellular biosynthetic process;cellular component assembly;cellular component assembly at cellular level;cellular component organization;cellular component organization at cellular le
biological regulation;cell differentiation;cellular component organization;cellular component organization at cellular level;cellular component organization or biogenesis;cellular component organization or biogenesis at cellular
activation of MAPKK activity;activation of protein kinase activity;biological adhesion;biological regulation;cell activation;cell adhesion;cell adhesion mediated by integrin;cell differentiation;cell surface receptor linked signaling
autophagy;biological regulation;catabolic process;cell communication;cell differentiation;cell surface receptor linked signaling pathway;cellular catabolic process;cellular component assembly;cellular component organization;
biological adhesion;cell adhesion;cell differentiation;cell proliferation;cellular developmental process;cellular macromolecule metabolic process;cellular metabolic process;cellular nitrogen compound metabolic process;cellula
anatomical structure development;biological adhesion;biological regulation;cell adhesion;cellular component assembly;cellular component assembly at cellular level;cellular component organization;cellular component organi
alcohol catabolic process;alcohol metabolic process;carbohydrate catabolic process;carbohydrate metabolic process;catabolic process;cellular carbohydrate catabolic process;cellular carbohydrate metabolic process;cellula
biological regulation;cellular component organization;cellular component organization or biogenesis;cellular process;establishment of localization;establishment of localization in cell;Golgi vesicle transport;intracellular transpo
alcohol metabolic process;amyloid precursor protein catabolic process;amyloid precursor protein metabolic process;anatomical structure development;apoptosis;biological regulation;biosynthetic process;catabolic process;c
cellular component assembly;cellular component assembly at cellular level;cellular component organization;cellular component organization at cellular level;cellular component organization or biogenesis;cellular component c
biological regulation;catabolic process;cell proliferation;cellular catabolic process;cellular macromolecule catabolic process;cellular macromolecule metabolic process;cellular metabolic process;cellular nitrogen compound m
ameboidal cell migration;apical junction assembly;apoptosis;biological adhesion;biological regulation;biosynthetic process;cell adhesion;cell cycle;cell death;cell division;cell junction assembly;cell junction organization;cell m
cell differentiation;cellular developmental process;cellular process;developmental process;multicellular organismal development;multicellular organismal process
aging;apoptosis;biological regulation;cell aging;cell death;cellular process;death;developmental process;negative regulation of biological process;negative regulation of biosynthetic process;negative regulation of catalytic act
cellular macromolecule metabolic process;cellular metabolic process;cellular nitrogen compound metabolic process;cellular process;macromolecule metabolic process;metabolic process;mRNA metabolic process;mRNA pro
anatomical structure development;apoptosis;biological regulation;biosynthetic process;cell death;cellular biosynthetic process;cellular metabolic process;cellular process;cofactor biosynthetic process;cofactor metabolic pro
anatomical structure development;biomineral tissue development;developmental process;tissue development
axon extension;biological adhesion;biological regulation;cell adhesion;cell growth;cell-matrix adhesion;cell-substrate adhesion;cellular process;cellular response to stimulus;developmental cell growth;developmental growth;d
anatomical structure morphogenesis;biological regulation;catabolic process;cell part morphogenesis;cellular catabolic process;cellular component maintenance;cellular component maintenance at cellular level;cellular compo
biological regulation;carboxylic acid metabolic process;cellular ketone metabolic process;cellular metabolic process;cellular process;metabolic process;monocarboxylic acid metabolic process;organic acid metabolic process
cellular component assembly;cellular component organization;cellular component organization or biogenesis;cellular metabolic process;cellular nitrogen compound metabolic process;cellular process;coenzyme metabolic pro
cellular macromolecule metabolic process;cellular metabolic process;cellular nitrogen compound metabolic process;cellular process;establishment of localization;establishment of localization in cell;establishment of RNA loca
adaptation of rhodopsin mediated signaling;adaptation of signaling pathway;apocarotenoid metabolic process;biological regulation;cellular aldehyde metabolic process;cellular hormone metabolic process;cellular lipid metab
biological regulation;biosynthetic process;cellular biosynthetic process;cellular macromolecule biosynthetic process;cellular macromolecule metabolic process;cellular metabolic process;cellular nitrogen compound metabolic
behavior;behavioral interaction between organisms;multi-organism process;response to stimulus;social behavior
biological regulation;cell cycle;cellular component assembly;cellular component assembly at cellular level;cellular component organization;cellular component organization at cellular level;cellular component organization or bi
catabolic process;cellular catabolic process;cellular component organization;cellular component organization at cellular level;cellular component organization or biogenesis;cellular component organization or biogenesis at ce
anatomical structure development;ATP-dependent chromatin remodeling;biological regulation;cellular component disassembly;cellular component disassembly at cellular level;cellular component organization;cellular compor
cell cycle;cell cycle process;cellular component organization;cellular component organization at cellular level;cellular component organization or biogenesis;cellular component organization or biogenesis at cellular level;cellula
biological regulation;biosynthetic process;cell differentiation;cell proliferation;cellular biosynthetic process;cellular developmental process;cellular macromolecule biosynthetic process;cellular macromolecule metabolic proces
amine catabolic process;amine metabolic process;biotin metabolic process;branched chain family amino acid catabolic process;branched chain family amino acid metabolic process;carboxylic acid catabolic process;carbox
biological regulation;cell surface receptor linked signaling pathway;cellular process;cellular response to chemical stimulus;cellular response to cytokine stimulus;cellular response to organic substance;cellular response to stim
biological regulation;cellular component organization;cellular component organization at cellular level;cellular component organization or biogenesis;cellular component organization or biogenesis at cellular level;cellular proce
biological regulation;cation homeostasis;chemical homeostasis;homeostatic process;ion homeostasis;iron ion homeostasis;regulation of biological quality
anatomical structure formation involved in morphogenesis;associative learning;behavior;cellular component maintenance;cellular component maintenance at cellular level;cellular component organization;cellular component c
biological regulation;biosynthetic process;cellular biosynthetic process;cellular macromolecule biosynthetic process;cellular macromolecule metabolic process;cellular metabolic process;cellular nitrogen compound metabolic
cellular metabolic process;cellular process;metabolic process;phosphate-containing compound metabolic process;phosphorus metabolic process;phosphorylation
amine catabolic process;amine metabolic process;biotin metabolic process;branched chain family amino acid catabolic process;branched chain family amino acid metabolic process;carboxylic acid catabolic process;carbox
biological regulation;cellular process;establishment of localization;establishment of localization in cell;establishment of protein localization;intracellular protein transport;intracellular transport;positive regulation of catalytic acti
amine metabolic process;amino acid activation;carboxylic acid metabolic process;cellular amine metabolic process;cellular amino acid metabolic process;cellular ketone metabolic process;cellular macromolecule metabolic p
biological regulation;cellular component assembly;cellular component assembly at cellular level;cellular component organization;cellular component organization at cellular level;cellular component organization or biogenesis;
biological regulation;biosynthetic process;cellular biosynthetic process;cellular macromolecule biosynthetic process;cellular macromolecule metabolic process;cellular metabolic process;cellular nitrogen compound metabolic
alternative nuclear mRNA splicing, via spliceosome;anatomical structure development;anatomical structure morphogenesis;appendage morphogenesis;biological regulation;cell differentiation;cellular component assembly;ce
actin cytoskeleton organization;actin cytoskeleton reorganization;actin filament-based process;actin-mediated cell contraction;anatomical structure formation involved in morphogenesis;anatomical structure morphogenesis;a
biosynthetic process;cellular biosynthetic process;cellular component disassembly;cellular component disassembly at cellular level;cellular component organization;cellular component organization at cellular level;cellular cor
anatomical structure development;cell projection organization;cellular component organization;cellular component organization at cellular level;cellular component organization or biogenesis;cellular component organization o
carboxylic acid metabolic process;cellular aldehyde metabolic process;cellular component assembly;cellular component organization;cellular component organization or biogenesis;cellular ketone metabolic process;cellular r
anatomical structure morphogenesis;angiotensin maturation;biological regulation;branching morphogenesis of a tube;cellular macromolecule metabolic process;cellular metabolic process;cellular process;cellular protein meta
establishment of localization;establishment of localization in cell;establishment of RNA localization;gene expression;intracellular transport;macromolecule metabolic process;metabolic process;mRNA export from nucleus;mR
ADP biosynthetic process;ADP metabolic process;AMP metabolic process;biological regulation;biosynthetic process;blood coagulation;cellular biosynthetic process;cellular metabolic process;cellular nitrogen compound bio
multicellular organismal process;neurological system process;sensory perception;sensory perception of light stimulus;system process;visual perception
biological regulation;cellular component organization;cellular component organization at cellular level;cellular component organization or biogenesis;cellular component organization or biogenesis at cellular level;cellular memb
anion transport;biological regulation;cation transport;cell volume homeostasis;cellular homeostasis;cellular process;chloride transport;establishment of localization;homeostatic process;inorganic anion transport;ion transport;
biological regulation;negative regulation of biological process;negative regulation of gene expression;negative regulation of macromolecule metabolic process;negative regulation of metabolic process;positive regulation of bio
biological regulation;cell surface receptor linked signaling pathway;cellular metabolic process;cellular nitrogen compound metabolic process;cellular process;cellular response to chemical stimulus;cellular response to cytokin
adipose tissue development;anatomical structure development;biological regulation;bone development;cell differentiation;cellular component assembly;cellular component disassembly;cellular component disassembly at cell
anatomical structure development;biological adhesion;biological regulation;blood coagulation;brain development;carbohydrate transport;cell activation;cell activation involved in immune response;cell adhesion;cell-cell adhes
axon guidance;chemotaxis;locomotion;response to chemical stimulus;response to external stimulus;response to stimulus;taxis
biosynthetic process;cell proliferation;cellular biosynthetic process;cellular metabolic compound salvage;cellular metabolic process;cellular process;coenzyme biosynthetic process;coenzyme metabolic process;cofactor bios
anatomical structure development;biological regulation;biosynthetic process;cell cycle arrest;cell cycle process;cell development;cell differentiation;cell proliferation;cell surface receptor linked signaling pathway;cellular biosy
actin cytoskeleton organization;actin filament organization;actin filament-based process;actin nucleation;Arp2/3 complex-mediated actin nucleation;axon guidance;biological regulation;cell surface receptor linked signaling pa
biological regulation;cell cycle process;cellular component assembly;cellular component assembly at cellular level;cellular component organization;cellular component organization at cellular level;cellular component organiza
establishment of localization;establishment of localization in cell;intracellular transport;nuclear export;nuclear transport;nucleocytoplasmic transport;transport
catabolic process;cellular catabolic process;cellular macromolecule catabolic process;cellular macromolecule metabolic process;cellular metabolic process;cellular process;ER-associated protein catabolic process;macromo
ATP biosynthetic process;ATP metabolic process;ATP synthesis coupled proton transport;biosynthetic process;cation transport;cellular biosynthetic process;cellular metabolic process;cellular nitrogen compound biosynthetic
cellular component assembly;cellular component assembly at cellular level;cellular component organization;cellular component organization at cellular level;cellular component organization or biogenesis;cellular component c
anatomical structure morphogenesis;biological regulation;cell maturation;cellular developmental process;cellular macromolecule metabolic process;cellular metabolic process;cellular nitrogen compound metabolic process;ce
biological regulation;cation homeostasis;cell surface receptor linked signaling pathway;cellular cation homeostasis;cellular chemical homeostasis;cellular copper ion homeostasis;cellular homeostasis;cellular ion homeostasis
cellular metabolic process;cellular process;electron transport chain;generation of precursor metabolites and energy;metabolic process;oxidation-reduction process;respiratory electron transport chain;response to chemical sti
anatomical structure morphogenesis;axon guidance;biological regulation;cell communication;cell differentiation;cell-cell signaling;cellular developmental process;cellular process;cellular response to stimulus;chemotaxis;deve
amine catabolic process;amine metabolic process;branched chain family amino acid catabolic process;branched chain family amino acid metabolic process;carboxylic acid catabolic process;carboxylic acid metabolic proces
alcohol metabolic process;aldehyde catabolic process;biosynthetic process;carbohydrate catabolic process;carbohydrate metabolic process;carboxylic acid biosynthetic process;carboxylic acid catabolic process;carboxylic
anatomical structure homeostasis;base-excision repair;biological regulation;biosynthetic process;catabolic process;cell cycle;cell cycle checkpoint;cell cycle process;cellular biosynthetic process;cellular catabolic process;ce
activation of immune response;activation of innate immune response;activation of phospholipase C activity;activation of protein kinase A activity;activation of protein kinase activity;acylglycerol catabolic process;acylglycerol
anatomical structure development;biological regulation;cell chemotaxis;cell differentiation;cell migration;cell motility;cellular component movement;cellular component organization;cellular component organization at cellular le
biological regulation;cell cycle phase;cell cycle process;cell division;cellular component assembly;cellular component assembly at cellular level;cellular component organization;cellular component organization at cellular level
biological regulation;cellular component organization;cellular component organization or biogenesis;cellular membrane organization;cellular process;cellular response to stimulus;endocytosis;endosome to lysosome transport
biosynthetic process;cell activation;cell proliferation;cellular aromatic compound metabolic process;cellular biosynthetic process;cellular metabolic process;cellular nitrogen compound biosynthetic process;cellular nitrogen co
ATP hydrolysis coupled proton transport;ATP metabolic process;biological regulation;cation homeostasis;cation transport;cell surface receptor linked signaling pathway;cellular cation homeostasis;cellular chemical homeosta
cellular macromolecule metabolic process;cellular metabolic process;cellular process;cellular protein metabolic process;macromolecule metabolic process;macromolecule methylation;macromolecule modification;metabolic
alcohol metabolic process;biological regulation;biosynthetic process;C21-steroid hormone biosynthetic process;C21-steroid hormone metabolic process;cellular biosynthetic process;cellular hormone metabolic process;cellu
carboxylic acid metabolic process;cellular ketone metabolic process;cellular metabolic process;cellular process;dicarboxylic acid metabolic process;malate metabolic process;metabolic process;organic acid metabolic proce
activation of immune response;activation of innate immune response;anaphase-promoting complex-dependent proteasomal ubiquitin-dependent protein catabolic process;antigen processing and presentation;antigen proces
apoptosis;biological regulation;cell death;cell surface receptor linked signaling pathway;cellular macromolecule metabolic process;cellular metabolic process;cellular process;cellular protein metabolic process;cellular respons
apoptosis;biological adhesion;cell adhesion;cell death;cell-cell adhesion;cellular component disassembly;cellular component disassembly at cellular level;cellular component disassembly involved in apoptosis;cellular compo
biosynthetic process;cellular biosynthetic process;cellular macromolecule biosynthetic process;cellular macromolecule metabolic process;cellular metabolic process;cellular nitrogen compound metabolic process;cellular pro
arachidonic acid metabolic process;carboxylic acid metabolic process;cellular alkene metabolic process;cellular ketone metabolic process;cellular lipid metabolic process;cellular metabolic process;cellular process;cobalami
cellular component organization;cellular component organization at cellular level;cellular component organization or biogenesis;cellular component organization or biogenesis at cellular level;cellular macromolecule metabolic
biological regulation;biosynthetic process;cellular biosynthetic process;cellular component organization;cellular component organization at cellular level;cellular component organization or biogenesis;cellular component orga
alcohol catabolic process;alcohol metabolic process;amino sugar catabolic process;amino sugar metabolic process;carbohydrate catabolic process;carbohydrate metabolic process;catabolic process;cellular carbohydrate c

anatomical structure development;anatomical structure morphogenesis;biological regulation;biosynthetic process;cell differentiation;cell morphogenesis;cell proliferation;cellular biosynthetic process;cellular component morp
ATP biosynthetic process;ATP metabolic process;ATP synthesis coupled proton transport;biosynthetic process;cation transport;cellular biosynthetic process;cellular metabolic process;cellular nitrogen compound biosynthetic
biological regulation;cell cycle phase;cell cycle process;cell division;cell surface receptor linked signaling pathway;cellular component organization;cellular component organization at cellular level;cellular component organiza
biological regulation;biosynthetic process;cellular biosynthetic process;cellular macromolecule biosynthetic process;cellular macromolecule metabolic process;cellular metabolic process;cellular process;cellular protein metab
acetyl-CoA catabolic process;acetyl-CoA metabolic process;carboxylic acid metabolic process;catabolic process;cellular catabolic process;cellular ketone metabolic process;cellular metabolic process;cellular process;coenz
biological regulation;blood coagulation;cation homeostasis;cation transport;cell migration;cell motility;cellular cation homeostasis;cellular chemical homeostasis;cellular component movement;cellular component organization
activation of immune response;activation of innate immune response;anaphase-promoting complex-dependent proteasomal ubiquitin-dependent protein catabolic process;antigen processing and presentation;antigen proces
antigen processing and presentation;antigen processing and presentation of exogenous antigen;antigen processing and presentation of exogenous peptide antigen;antigen processing and presentation of exogenous peptide
biosynthetic process;catabolic process;cellular biosynthetic process;cellular catabolic process;cellular component disassembly;cellular component disassembly at cellular level;cellular component organization;cellular compo
biological regulation;cell differentiation;cellular developmental process;cellular process;cellular response to stimulus;developmental process;intracellular signal transduction;metabolic process;osteoblast differentiation;positive
biological regulation;biosynthetic process;catabolic process;cell differentiation;cellular biosynthetic process;cellular catabolic process;cellular component assembly;cellular component assembly at cellular level;cellular compo
biosynthetic process;catabolic process;cellular biosynthetic process;cellular catabolic process;cellular component disassembly;cellular component disassembly at cellular level;cellular component organization;cellular compo
biosynthetic process;catabolic process;cell proliferation;cellular biosynthetic process;cellular catabolic process;cellular component disassembly;cellular component disassembly at cellular level;cellular component organizatio
activation of immune response;activation of innate immune response;activation of MAPK activity;alcohol metabolic process;anaphase-promoting complex-dependent proteasomal ubiquitin-dependent protein catabolic proce
amine biosynthetic process;amine metabolic process;biosynthetic process;carboxylic acid biosynthetic process;carboxylic acid metabolic process;cellular amine metabolic process;cellular amino acid biosynthetic process;ce
biosynthetic process;cellular biosynthetic process;cellular component disassembly;cellular component disassembly at cellular level;cellular component organization;cellular component organization at cellular level;cellular cor
biosynthetic process;cellular biosynthetic process;cellular component disassembly;cellular component disassembly at cellular level;cellular component organization;cellular component organization at cellular level;cellular cor
biosynthetic process;cellular biosynthetic process;cellular component disassembly;cellular component disassembly at cellular level;cellular component organization;cellular component organization at cellular level;cellular cor
alcohol metabolic process;anatomical structure development;biosynthetic process;brain development;cellular biosynthetic process;cellular lipid metabolic process;cellular metabolic process;cellular process;cellular response
cell differentiation;cellular developmental process;cellular process;developmental process
biological regulation;calcium ion homeostasis;calcium ion transport;calcium-mediated signaling;cation homeostasis;cation transport;cell communication;cell cycle process;cell growth;cell growth involved in cardiac muscle ce
apoptosis;biological regulation;cell cycle checkpoint;cell death;cellular macromolecule metabolic process;cellular metabolic process;cellular process;cellular protein metabolic process;cellular response to stimulus;death;DNA
biological adhesion;cell adhesion;cellular process;establishment of localization;establishment of localization in cell;establishment of protein localization;establishment of protein localization to organelle;establishment of protei
biological regulation;biosynthetic process;cellular biosynthetic process;cellular macromolecule biosynthetic process;cellular macromolecule metabolic process;cellular metabolic process;cellular nitrogen compound metabolic
acetyl-CoA metabolic process;alcohol metabolic process;biosynthetic process;cellular biosynthetic process;cellular lipid metabolic process;cellular metabolic process;cellular process;cholesterol biosynthetic process;cholest
biological regulation;biosynthetic process;cell fate commitment;cell fate commitment involved in formation of primary germ layers;cell surface receptor linked signaling pathway;cellular biosynthetic process;cellular componen
anatomical structure development;cellular macromolecule metabolic process;cellular metabolic process;cellular process;cellular protein metabolic process;chordate embryonic development;developmental process;embryo de
alcohol metabolic process;amine metabolic process;anatomical structure formation involved in morphogenesis;anatomical structure morphogenesis;angiogenesis;catabolic process;cellular amine metabolic process;cellular b
cellular macromolecule metabolic process;cellular metabolic process;cellular nitrogen compound metabolic process;cellular process;enzyme-directed rRNA 2'-O-methylation;macromolecule metabolic process;macromolecul
biological regulation;cell proliferation;cellular macromolecule metabolic process;cellular metabolic process;cellular nitrogen compound metabolic process;cellular process;inner cell mass cell proliferation;macromolecule meta
biosynthetic process;cellular biosynthetic process;cellular metabolic process;cellular nitrogen compound biosynthetic process;cellular nitrogen compound metabolic process;cellular process;coenzyme biosynthetic process;c
activation of immune response;activation of innate immune response;biological regulation;cell cycle;cell surface receptor linked signaling pathway;cellular macromolecule metabolic process;cellular metabolic process;cellular
biological regulation;cellular component movement;cellular component organization;cellular component organization at cellular level;cellular component organization or biogenesis;cellular component organization or biogenes
biological regulation;cellular macromolecule metabolic process;cellular metabolic process;cellular process;cellular protein metabolic process;cellular response to stimulus;dephosphorylation;macromolecule metabolic process
biological regulation;cell surface receptor linked signaling pathway;cellular process;cellular response to chemical stimulus;cellular response to cytokine stimulus;cellular response to organic substance;cellular response to stim
biological regulation;biosynthetic process;cell fate commitment;cell fate commitment involved in formation of primary germ layers;cell surface receptor linked signaling pathway;cellular biosynthetic process;cellular componen
biosynthetic process;cellular biosynthetic process;cellular macromolecule biosynthetic process;cellular macromolecule metabolic process;cellular metabolic process;cellular nitrogen compound metabolic process;cellular pro
response to calcium ion;response to chemical stimulus;response to inorganic substance;response to metal ion;response to stimulus
biological regulation;cellular macromolecule metabolic process;cellular metabolic process;cellular nitrogen compound metabolic process;cellular process;cellular protein metabolic process;cellular response to stimulus;cellula
asymmetric protein localization;basal protein localization;biological adhesion;biological regulation;cell adhesion;cell cycle;cell growth;cell surface receptor linked signaling pathway;cellular component organization;cellular com
multicellular organismal process;neurological system process;sensory perception;sensory perception of pain;system process
cell cycle;cell cycle process;cell division;cellular component organization;cellular component organization at cellular level;cellular component organization or biogenesis;cellular component organization or biogenesis at cellula
adherens junction assembly;adherens junction organization;biological regulation;cell junction assembly;cell junction organization;cell-cell junction assembly;cell-cell junction organization;cellular component assembly;cellular
biosynthetic process;cellular biosynthetic process;cellular component disassembly;cellular component disassembly at cellular level;cellular component organization;cellular component organization at cellular level;cellular cor
biological regulation;cell proliferation;cell surface receptor linked signaling pathway;cellular component biogenesis;cellular component biogenesis at cellular level;cellular component organization or biogenesis;cellular compor
cellular macromolecule metabolic process;cellular metabolic process;cellular nitrogen compound metabolic process;cellular process;macromolecule metabolic process;metabolic process;mRNA metabolic process;mRNA pro
biological regulation;cell cycle process;cellular component assembly;cellular component organization;cellular component organization at cellular level;cellular component organization or biogenesis;cellular component organiz
alcohol metabolic process;anatomical structure development;anatomical structure formation involved in morphogenesis;angiogenesis;anterior/posterior pattern specification;biological regulation;carbohydrate metabolic proc
anatomical structure development;biological regulation;cell development;cellular developmental process;cellular macromolecule metabolic process;cellular metabolic process;cellular process;cellular protein metabolic proces
cation transport;establishment of localization;ion transport;transport
biological regulation;biosynthetic process;cell cycle;cellular biosynthetic process;cellular macromolecule biosynthetic process;cellular macromolecule metabolic process;cellular metabolic process;cellular nitrogen compound
biological regulation;cellular macromolecule metabolic process;cellular metabolic process;cellular process;cellular protein metabolic process;macromolecule metabolic process;metabolic process;negative regulation of cataly
amine biosynthetic process;amine metabolic process;biological regulation;biosynthetic process;carboxylic acid biosynthetic process;carboxylic acid metabolic process;cellular amine metabolic process;cellular amino acid bio
cell cycle;cell division;cellular process
anatomical structure development;axon ensheathment;biological regulation;biosynthetic process;cell development;cellular biosynthetic process;cellular developmental process;cellular macromolecule biosynthetic process;ce
activation of immune response;alpha-beta T cell activation;antigen receptor-mediated signaling pathway;ATP biosynthetic process;ATP metabolic process;ATP synthesis coupled proton transport;biological regulation;biosynth
biological regulation;biosynthetic process;carbohydrate metabolic process;carbohydrate transport;cell cycle;cell cycle process;cell surface receptor linked signaling pathway;cellular biosynthetic process;cellular component c
actin crosslink formation;actin cytoskeleton organization;actin filament bundle assembly;actin filament organization;actin filament-based process;anatomical structure development;anatomical structure morphogenesis;axono
amine metabolic process;amino acid activation;biosynthetic process;carboxylic acid metabolic process;cellular amine metabolic process;cellular amino acid metabolic process;cellular biosynthetic process;cellular ketone me
anatomical structure development;anatomical structure morphogenesis;biological regulation;biosynthetic process;catabolic process;cell surface receptor linked signaling pathway;cellular biosynthetic process;cellular catabol
actin cytoskeleton organization;actin filament-based movement;actin filament-based process;ameboidal cell migration;anatomical structure development;anatomical structure formation involved in morphogenesis;anatomical
axon guidance;biological regulation;chemotaxis;locomotion;regulation of biological process;regulation of cellular process;regulation of establishment of cell polarity;regulation of establishment or maintenance of cell polarity;re
adult behavior;adult locomotory behavior;adult walking behavior;alcohol metabolic process;autophagy;behavior;bile acid metabolic process;biological regulation;carbohydrate metabolic process;carboxylic acid metabolic pro
actin cytoskeleton organization;actin filament organization;actin filament-based process;actin nucleation;Arp2/3 complex-mediated actin nucleation;axon guidance;biological regulation;cell migration;cell motility;cell surface re
biological regulation;cell surface receptor linked signaling pathway;cellular process;cellular response to stimulus;G-protein coupled receptor protein signaling pathway;regulation of biological process;regulation of cellular proc
axon guidance;biological regulation;cell cycle;cell cycle phase;cell cycle process;cell division;cellular component organization;cellular component organization at cellular level;cellular component organization or biogenesis;cel
cellular metabolic process;cellular process;electron transport chain;generation of precursor metabolites and energy;metabolic process;mitochondrial electron transport, NADH to ubiquinone;oxidation-reduction process;respi
cellular process;endosome transport;establishment of localization;establishment of localization in cell;establishment of protein localization;intracellular protein transport;intracellular transport;protein transport;retrograde trans
adult behavior;adult locomotory behavior;aggressive behavior;behavior;behavioral interaction between organisms;biological regulation;cell communication;cell-cell signaling;cellular component organization;cellular componer
catabolic process;cellular catabolic process;cellular macromolecule catabolic process;cellular macromolecule metabolic process;cellular metabolic process;cellular process;ER-associated protein catabolic process;macromo
alcohol metabolic process;biosynthetic process;blood circulation;carboxylic acid metabolic process;cellular ketone metabolic process;cellular metabolic process;cellular process;cholesterol biosynthetic process;cholesterol m
biological regulation;negative regulation of biological process;negative regulation of cell proliferation;negative regulation of cellular process;regulation of biological process;regulation of cell proliferation;regulation of cellular pr
anatomical structure morphogenesis;axonogenesis;biological regulation;cell part morphogenesis;cell projection morphogenesis;cell projection organization;cellular component morphogenesis;cellular component organization
biosynthetic process;cellular biosynthetic process;cellular macromolecule metabolic process;cellular metabolic process;cellular nitrogen compound metabolic process;cellular process;electron transport chain;gene expressio
3'-UTR-mediated mRNA stabilization;anatomical structure development;biological regulation;cellular hyperosmotic response;cellular macromolecule metabolic process;cellular metabolic process;cellular nitrogen compound
activation of immune response;activation of innate immune response;antigen receptor-mediated signaling pathway;apoptosis;biological regulation;biosynthetic process;cell death;cell surface receptor linked signaling pathway
activation of immune response;activation of innate immune response;anaphase-promoting complex-dependent proteasomal ubiquitin-dependent protein catabolic process;antigen processing and presentation;antigen proces
biosynthetic process;catabolic process;cellular biosynthetic process;cellular catabolic process;cellular metabolic process;cellular nitrogen compound catabolic process;cellular nitrogen compound metabolic process;cellular
carboxylic acid catabolic process;carboxylic acid metabolic process;catabolic process;cellular catabolic process;cellular component organization;cellular component organization at cellular level;cellular component organizati
macromolecule metabolic process;metabolic process;primary metabolic process;protein metabolic process;proteolysis
alcohol biosynthetic process;alcohol metabolic process;biological regulation;biosynthetic process;carbohydrate biosynthetic process;carbohydrate metabolic process;catabolic process;cellular biosynthetic process;cellular c
activation of caspase activity;apoptosis;biological regulation;cell death;cellular chemical homeostasis;cellular homeostasis;cellular ion homeostasis;cellular process;cellular response to abiotic stimulus;cellular response to ch
activation of MAPK activity;biological regulation;cellular macromolecule metabolic process;cellular metabolic process;cellular process;cellular protein metabolic process;establishment of localization;establishment of localizati
apoptotic mitochondrial changes;biological regulation;cell death;cellular component organization;cellular component organization at cellular level;cellular component organization or biogenesis;cellular component organizatio
aging;biological regulation;cellular aldehyde metabolic process;cellular metabolic process;cellular process;developmental process;metabolic process;oxidation-reduction process;positive regulation of biological process;posit
biological regulation;biosynthetic process;cellular biosynthetic process;cellular macromolecule biosynthetic process;cellular macromolecule metabolic process;cellular metabolic process;cellular nitrogen compound metabolic
anatomical structure development;anatomical structure morphogenesis;biological regulation;blood coagulation;carboxylic acid metabolic process;cell migration;cell motility;cell surface receptor linked signaling pathway;cellul
biological regulation;blood coagulation;coagulation;hemostasis;multicellular organismal process;negative regulation of biological process;negative regulation of biosynthetic process;negative regulation of cellular biosynthetic
biosynthetic process;catabolic process;cellular biosynthetic process;cellular catabolic process;cellular component disassembly;cellular component disassembly at cellular level;cellular component organization;cellular compo
biosynthetic process;cellular biosynthetic process;cellular macromolecule biosynthetic process;cellular macromolecule metabolic process;cellular metabolic process;cellular process;cellular protein metabolic process;gene ex
anatomical structure development;axon ensheathment;biological regulation;biosynthetic process;cell development;cellular biosynthetic process;cellular developmental process;cellular macromolecule biosynthetic process;ce
adenosine metabolic process;catabolic process;cellular aromatic compound metabolic process;cellular catabolic process;cellular metabolic process;cellular nitrogen compound catabolic process;cellular nitrogen compound
biological regulation;negative regulation of catalytic activity;negative regulation of endopeptidase activity;negative regulation of hydrolase activity;negative regulation of molecular function;negative regulation of peptidase acti
2'-deoxyribonucleotide biosynthetic process;2'-deoxyribonucleotide metabolic process;adenosine metabolic process;AMP biosynthetic process;AMP metabolic process;AMP salvage;biological regulation;biosynthetic proces
actin cytoskeleton organization;actin filament branching;actin filament bundle assembly;actin filament organization;actin filament-based process;adherens junction organization;anatomical structure development;anatomical s
cellular component assembly;cellular component assembly at cellular level;cellular component organization;cellular component organization at cellular level;cellular component organization or biogenesis;cellular component c
biosynthetic process;cellular biosynthetic process;cellular component disassembly;cellular component disassembly at cellular level;cellular component organization;cellular component organization at cellular level;cellular cor
biosynthetic process;cellular biosynthetic process;cellular macromolecule biosynthetic process;cellular macromolecule metabolic process;cellular metabolic process;cellular nitrogen compound metabolic process;cellular pro
biosynthetic process;cellular biosynthetic process;cellular macromolecule biosynthetic process;cellular macromolecule metabolic process;cellular metabolic process;cellular nitrogen compound metabolic process;cellular pro
biological regulation;catabolic process;cellular catabolic process;cellular macromolecule catabolic process;cellular macromolecule metabolic process;cellular metabolic process;cellular process;macromolecule catabolic proc
amine metabolic process;biological regulation;carbohydrate metabolic process;carboxylic acid metabolic process;cell differentiation;cellular aldehyde metabolic process;cellular amine metabolic process;cellular amino acid m
cellular process;establishment of localization;establishment of localization in cell;Golgi vesicle transport;intracellular transport;intra-Golgi vesicle-mediated transport;transport;vesicle-mediated transport
anatomical structure development;astrocyte development;astrocyte differentiation;axon ensheathment;biological regulation;biosynthetic process;cell development;cell differentiation;cellular biosynthetic process;cellular devel
biological regulation;biosynthetic process;cellular biosynthetic process;cellular component organization;cellular component organization at cellular level;cellular component organization or biogenesis;cellular component orgar
anatomical structure development;anatomical structure morphogenesis;axonogenesis;biological regulation;cargo loading into vesicle;cation homeostasis;cell part morphogenesis;cell projection morphogenesis;cell projection
anatomical structure morphogenesis;biological regulation;cell part morphogenesis;cell projection morphogenesis;cell projection organization;cellular component morphogenesis;cellular component organization;cellular compo
axon guidance;biological adhesion;biological regulation;cell adhesion;cellular process;cellular response to stimulus;chemotaxis;locomotion;motor axon guidance;regulation of biological process;regulation of cellular process;r
adaptive immune response;adaptive immune response based on somatic recombination of immune receptors built from immunoglobulin superfamily domains;anatomical structure formation involved in morphogenesis;angiog
cellular component assembly;cellular component assembly at cellular level;cellular component organization;cellular component organization at cellular level;cellular component organization or biogenesis;cellular component c
autophagy;biological regulation;biosynthetic process;catabolic process;cellular biosynthetic process;cellular catabolic process;cellular macromolecule biosynthetic process;cellular macromolecule catabolic process;cellular m
arachidonic acid metabolic process;carboxylic acid metabolic process;cellular alkene metabolic process;cellular ketone metabolic process;cellular lipid metabolic process;cellular metabolic process;cellular process;cyclooxyg
anatomical structure development;brain development;catabolic process;developmental process;gamete generation;lipid catabolic process;lipid metabolic process;male gamete generation;metabolic process;multicellular orga
anatomical structure development;camera-type eye development;chordate embryonic development;developmental process;embryo development;embryo development ending in birth or egg hatching;eye development;in uter
establishment of localization;establishment of localization in cell;establishment of protein localization;establishment of protein localization in mitochondrion;establishment of protein localization to organelle;intracellular protein
catabolic process;cell cycle;cell division;cellular catabolic process;cellular component assembly;cellular component assembly at cellular level;cellular component organization;cellular component organization at cellular level;c
alanyl-tRNA aminoacylation;amine metabolic process;amino acid activation;carboxylic acid metabolic process;cellular amine metabolic process;cellular amino acid metabolic process;cellular component assembly;cellular cor
anatomical structure development;developmental process;embryonic skeletal system development;skeletal system development;system development
cellular macromolecule metabolic process;cellular metabolic process;cellular nitrogen compound metabolic process;cellular process;macromolecule metabolic process;metabolic process;mRNA metabolic process;mRNA pro
biological regulation;cellular hormone metabolic process;cellular lipid metabolic process;cellular metabolic process;cellular process;diterpenoid metabolic process;fat-soluble vitamin metabolic process;hormone metabolic pro
biological regulation;biosynthetic process;cell cycle;cell fate commitment;cell fate commitment involved in formation of primary germ layers;cell surface receptor linked signaling pathway;cellular biosynthetic process;cellular c

biological regulation;biosynthetic process;cellular biosynthetic process;cellular macromolecule biosynthetic process;cellular macromolecule metabolic process;cellular metabolic process;cellular nitrogen compound metabolic
cellular component organization;cellular component organization at cellular level;cellular component organization or biogenesis;cellular component organization or biogenesis at cellular level;cellular macromolecule metabolic
biological regulation;cellular component assembly;cellular component assembly at cellular level;cellular component organization;cellular component organization at cellular level;cellular component organization or biogenesis;
biological regulation;cell cycle process;cell division;cellular component organization;cellular component organization at cellular level;cellular component organization or biogenesis;cellular component organization or biogenes
anatomical structure morphogenesis;biological regulation;biosynthetic process;bone morphogenesis;cellular biosynthetic process;cellular component organization;cellular component organization at cellular level;cellular comp
biosynthetic process;cellular biosynthetic process;cellular macromolecule biosynthetic process;cellular macromolecule metabolic process;cellular metabolic process;cellular nitrogen compound metabolic process;cellular pro
actin cytoskeleton organization;actin filament organization;actin filament-based process;actin polymerization or depolymerization;anatomical structure development;anatomical structure formation involved in morphogenesis;a
cellular process;establishment of localization;establishment of protein localization;protein transport;transport;vesicle-mediated transport
anatomical structure homeostasis;biological regulation;cellular macromolecule metabolic process;cellular metabolic process;cellular nitrogen compound metabolic process;cellular process;homeostatic process;macromolecu
biological regulation;gamete generation;male gamete generation;multicellular organismal process;multicellular organismal reproductive process;positive regulation of biological process;positive regulation of cell proliferation;p
anatomical structure morphogenesis;biological regulation;cell morphogenesis;cellular component morphogenesis;cellular component organization;cellular component organization or biogenesis;cellular developmental proces
biological regulation;biosynthetic process;cellular biosynthetic process;cellular macromolecule biosynthetic process;cellular macromolecule metabolic process;cellular metabolic process;cellular nitrogen compound metabolic
apoptosis;biological regulation;cell death;cellular macromolecule metabolic process;cellular metabolic process;cellular nitrogen compound metabolic process;cellular process;death;gene expression;immune system process;
biological regulation;posttranscriptional regulation of gene expression;protein destabilization;regulation of biological process;regulation of biological quality;regulation of cellular component biogenesis;regulation of cellular co
amine biosynthetic process;amine metabolic process;biosynthetic process;carboxylic acid biosynthetic process;carboxylic acid metabolic process;cellular amine metabolic process;cellular amino acid biosynthetic process;ce
autophagy;biological regulation;catabolic process;cell communication;cell cycle;cell cycle process;cell death;cellular catabolic process;cellular component organization;cellular component organization at cellular level;cellular
biosynthetic process;cellular biosynthetic process;cellular component disassembly;cellular component disassembly at cellular level;cellular component organization;cellular component organization at cellular level;cellular co
attachment of spindle microtubules to chromosome;attachment of spindle microtubules to kinetochore;attachment of spindle microtubules to kinetochore involved in mitotic sister chromatid segregation;biological regulation;
biological regulation;cell death;cellular metabolic process;cellular process;cellular response to stimulus;death;dephosphorylation;intracellular signal transduction;metabolic process;necroptosis;necrotic cell death;phosphate-
biological regulation;catabolic process;cellular catabolic process;cellular macromolecule catabolic process;cellular macromolecule metabolic process;cellular metabolic process;cellular nitrogen compound metabolic process
biosynthetic process;cell cycle phase;cell cycle process;cell division;cellular biosynthetic process;cellular component organization;cellular component organization at cellular level;cellular component organization or biogenesi
cellular component organization;cellular component organization or biogenesis;cellular membrane organization;cellular process;membrane organization;nuclear membrane organization
cellular macromolecule metabolic process;cellular metabolic process;cellular nitrogen compound metabolic process;cellular process;macromolecule metabolic process;maturation of SSU-rRNA;metabolic process;ncRNA me
antigen processing and presentation;antigen processing and presentation of exogenous antigen;antigen processing and presentation of exogenous peptide antigen;antigen processing and presentation of exogenous peptide
cellular macromolecule metabolic process;cellular metabolic process;cellular nitrogen compound metabolic process;cellular process;macromolecule metabolic process;metabolic process;nitrogen compound metabolic proce
biological regulation;cellular macromolecule metabolic process;cellular metabolic process;cellular nitrogen compound metabolic process;cellular process;macromolecule metabolic process;maturation of SSU-rRNA;maturatio
biological regulation;biosynthetic process;carbon catabolite regulation of transcription;carbon catabolite repression of transcription;cell communication;cell cycle process;cell division;cellular biosynthetic process;cellular com
amine catabolic process;amine metabolic process;carboxylic acid catabolic process;carboxylic acid metabolic process;catabolic process;cellular amine metabolic process;cellular amino acid catabolic process;cellular amino
apoptosis;autophagic cell death;biological regulation;catabolic process;cell communication;cell death;cellular catabolic process;cellular component assembly;cellular component organization;cellular component organization
biological regulation;biosynthetic process;cellular biosynthetic process;cellular macromolecule biosynthetic process;cellular macromolecule metabolic process;cellular metabolic process;cellular process;cellular protein metab
cellular component organization;cellular component organization at cellular level;cellular component organization or biogenesis;cellular component organization or biogenesis at cellular level;cellular macromolecule metabolic
biological regulation;cell differentiation;cell proliferation;cellular component assembly;cellular component assembly at cellular level;cellular component organization;cellular component organization at cellular level;cellular com
biological regulation;cellular component assembly;cellular component assembly at cellular level;cellular component organization;cellular component organization at cellular level;cellular component organization or biogenesis;
anatomical structure development;anatomical structure morphogenesis;biological regulation;cell cycle;cell development;cell differentiation;cellular component disassembly;cellular component disassembly at cellular level;cell
3'-phosphoadenosine 5'-phosphosulfate biosynthetic process;3'-phosphoadenosine 5'-phosphosulfate metabolic process;amine metabolic process;aminoglycan metabolic process;anatomical structure development;biosynt
cellular macromolecule metabolic process;cellular metabolic process;cellular nitrogen compound metabolic process;cellular process;macromolecule metabolic process;metabolic process;ncRNA metabolic process;ncRNA pr
alcohol biosynthetic process;alcohol metabolic process;biological regulation;biosynthetic process;carbohydrate biosynthetic process;carbohydrate metabolic process;cell surface receptor linked signaling pathway;cellular bio
biological regulation;biosynthetic process;cell differentiation;cellular biosynthetic process;cellular developmental process;cellular macromolecule biosynthetic process;cellular macromolecule metabolic process;cellular metab
biological regulation;cellular macromolecule metabolic process;cellular metabolic process;cellular process;cellular protein metabolic process;macromolecule metabolic process;metabolic process;positive regulation of biologi
actin filament severing;actin filament-based process;anatomical structure development;anatomical structure morphogenesis;axon guidance;biological adhesion;biological regulation;cell adhesion;cell morphogenesis;cell morp
anatomical structure development;biological regulation;biosynthetic process;Cajal body organization;cell cycle process;cell proliferation;cellular biosynthetic process;cellular component organization;cellular component organ
biological regulation;cellular process;ER to Golgi vesicle-mediated transport;establishment of localization;establishment of localization in cell;establishment of protein localization;Golgi vesicle transport;intracellular transport;p
antigen processing and presentation;antigen processing and presentation of exogenous antigen;antigen processing and presentation of exogenous peptide antigen;antigen processing and presentation of exogenous peptide
activation of signaling protein activity involved in unfolded protein response;biological regulation;biosynthetic process;calcium ion homeostasis;cation homeostasis;cellular biosynthetic process;cellular calcium ion homeostas
cellular metabolic process;cellular process;electron transport chain;generation of precursor metabolites and energy;metabolic process;mitochondrial electron transport, NADH to ubiquinone;oxidation-reduction process;respi
amine metabolic process;biological regulation;biosynthetic process;carboxylic acid metabolic process;cell redox homeostasis;cellular amine metabolic process;cellular amino acid metabolic process;cellular biosynthetic proc
activation of MAPK activity;aging;amine metabolic process;anatomical structure development;anatomical structure homeostasis;anterograde axon cargo transport;auditory receptor cell stereocilium organization;axon cargo tr
actin cytoskeleton organization;actin cytoskeleton reorganization;actin filament-based process;activation of caspase activity;autophagy;biological adhesion;biological regulation;catabolic process;cell adhesion;cell chemotaxi
apical junction assembly;axon guidance;biological regulation;cell junction assembly;cell junction organization;cell migration;cell motility;cell-cell junction assembly;cell-cell junction organization;cellular component assembly;c
amine metabolic process;anatomical structure development;biological regulation;carboxylic acid metabolic process;cell proliferation;cellular amine metabolic process;cellular amino acid metabolic process;cellular ketone met
antigen processing and presentation;antigen processing and presentation of exogenous antigen;antigen processing and presentation of exogenous peptide antigen;antigen processing and presentation of exogenous peptide
acetyl-CoA catabolic process;acetyl-CoA metabolic process;alcohol metabolic process;biological regulation;carbohydrate metabolic process;carboxylic acid metabolic process;catabolic process;cellular carbohydrate metab
amine biosynthetic process;amine metabolic process;biosynthetic process;cellular amine metabolic process;cellular biogenic amine biosynthetic process;cellular biogenic amine metabolic process;cellular biosynthetic proces
biosynthetic process;cellular biosynthetic process;cellular macromolecule biosynthetic process;cellular macromolecule metabolic process;cellular metabolic process;cellular process;cellular protein metabolic process;gene ex
biological regulation;biosynthetic process;cellular biosynthetic process;cellular macromolecule biosynthetic process;cellular macromolecule metabolic process;cellular metabolic process;cellular nitrogen compound metabolic
cell migration;cell motility;cellular component movement;cellular process;endothelial cell migration;locomotion
apoptosis;apoptosis involved in morphogenesis;cell death;cellular developmental process;cellular process;death;developmental process;developmental programmed cell death;programmed cell death
biological regulation;cellular process;cellular response to stimulus;negative regulation of biological process;negative regulation of biosynthetic process;negative regulation of cell proliferation;negative regulation of cellular bios
adhesion to host;adhesion to other organism involved in symbiotic interaction;biological adhesion;biological regulation;cell surface receptor linked signaling pathway;cellular process;cellular response to biotic stimulus;cellula
anatomical structure homeostasis;base-excision repair;biological regulation;biosynthetic process;cell cycle;cellular biosynthetic process;cellular component organization;cellular component organization at cellular level;cellula
anatomical structure homeostasis;base-excision repair;biological regulation;biosynthetic process;cell cycle;cellular biosynthetic process;cellular component organization;cellular component organization at cellular level;cellula
anatomical structure homeostasis;base-excision repair;biological regulation;biosynthetic process;cell cycle;cellular biosynthetic process;cellular component organization;cellular component organization at cellular level;cellula
biological regulation;cellular process;cellular response to stimulus;establishment of localization;establishment of localization in cell;intracellular transport;regulation of biological process;regulation of catalytic activity;regulation
anatomical structure development;apoptosis;apoptosis in response to endoplasmic reticulum stress;biological regulation;brain development;catabolic process;cell cycle process;cell death;cell differentiation;cellular catabolic
amine metabolic process;amino acid activation;biosynthetic process;carboxylic acid metabolic process;cellular amine metabolic process;cellular amino acid metabolic process;cellular biosynthetic process;cellular ketone me
activation of immune response;activation of innate immune response;anaphase-promoting complex-dependent proteasomal ubiquitin-dependent protein catabolic process;antigen processing and presentation;antigen proces
anatomical structure homeostasis;base-excision repair;biological regulation;biosynthetic process;cell cycle checkpoint;cell cycle process;cell proliferation;cellular biosynthetic process;cellular component organization;cellular
biological regulation;biosynthetic process;cell cycle phase;cell cycle process;cellular biosynthetic process;cellular component assembly;cellular component organization;cellular component organization at cellular level;cellula
biological regulation;biosynthetic process;cell differentiation;cell proliferation;cellular biosynthetic process;cellular developmental process;cellular macromolecule biosynthetic process;cellular macromolecule metabolic proces
biosynthetic process;catabolic process;cellular biosynthetic process;cellular catabolic process;cellular component disassembly;cellular component disassembly at cellular level;cellular component organization;cellular compo
cellular component biogenesis;cellular component biogenesis at cellular level;cellular component organization or biogenesis;cellular component organization or biogenesis at cellular level;cellular macromolecule metabolic pro
apoptosis;biological regulation;cell death;cellular component assembly;cellular component organization;cellular component organization at cellular level;cellular component organization or biogenesis;cellular component orga
4-hydroxyproline metabolic process;amine metabolic process;anatomical structure development;biological regulation;carboxylic acid metabolic process;cell redox homeostasis;cellular amine metabolic process;cellular amino
cellular macromolecule metabolic process;cellular metabolic process;cellular nitrogen compound metabolic process;cellular process;cellular protein metabolic process;macromolecule metabolic process;macromolecule modi
alcohol biosynthetic process;alcohol metabolic process;biological adhesion;biosynthetic process;carbohydrate biosynthetic process;carbohydrate metabolic process;cell adhesion;cell death;cell-cell adhesion;cellular biosynt
cellular component biogenesis;cellular component biogenesis at cellular level;cellular component organization or biogenesis;cellular component organization or biogenesis at cellular level;cellular process;metabolic process;ri
biological regulation;biosynthetic process;cellular biosynthetic process;cellular macromolecule biosynthetic process;cellular macromolecule metabolic process;cellular metabolic process;cellular nitrogen compound metabolic
biological regulation;biosynthetic process;cellular biosynthetic process;cellular macromolecule biosynthetic process;cellular macromolecule metabolic process;cellular metabolic process;cellular nitrogen compound metabolic
antigen processing and presentation;biological regulation;cell projection organization;cellular component organization;cellular component organization at cellular level;cellular component organization or biogenesis;cellular co
cellular component assembly;cellular component assembly at cellular level;cellular component organization;cellular component organization at cellular level;cellular component organization or biogenesis;cellular component c
cellular metabolic process;cellular nitrogen compound metabolic process;cellular process;coenzyme metabolic process;cofactor metabolic process;heterocycle metabolic process;metabolic process;NADP metabolic process
biological regulation;cellular component organization;cellular component organization or biogenesis;cellular macromolecule metabolic process;cellular membrane organization;cellular metabolic process;cellular process;cellula
cellular process;endosome transport;establishment of localization;establishment of localization in cell;establishment of protein localization;intracellular transport;protein transport;retrograde transport, endosome to Golgi;trans
response to abiotic stimulus;response to ionizing radiation;response to radiation;response to stimulus;response to X-ray
biological regulation;cell cycle;cell cycle phase;cell cycle process;cell division;cellular component organization;cellular component organization at cellular level;cellular component organization or biogenesis;cellular componen
biological regulation;cellular component organization;cellular component organization at cellular level;cellular component organization or biogenesis;cellular component organization or biogenesis at cellular level;cellular macro
biological regulation;cell differentiation;cellular developmental process;cellular process;developmental process;muscle cell differentiation;negative regulation of biological process;negative regulation of cell communication;neg
cell differentiation;cellular developmental process;cellular process;developmental process;fat cell differentiation
assembly of spliceosomal tri-snRNP;cellular component assembly;cellular component assembly at cellular level;cellular component organization;cellular component organization at cellular level;cellular component organizatio
biological regulation;cell cycle arrest;cell cycle process;cellular metabolic process;cellular process;cellular respiration;energy derivation by oxidation of organic compounds;generation of precursor metabolites and energy;met
biological regulation;cellular component organization;cellular component organization at cellular level;cellular component organization or biogenesis;cellular component organization or biogenesis at cellular level;cellular proce
cellular macromolecule metabolic process;cellular metabolic process;cellular nitrogen compound metabolic process;cellular process;macromolecule metabolic process;metabolic process;nitrogen compound metabolic proce
cellular component assembly;cellular component assembly at cellular level;cellular component organization;cellular component organization at cellular level;cellular component organization or biogenesis;cellular component c
cellular macromolecule metabolic process;cellular metabolic process;cellular nitrogen compound metabolic process;cellular process;macromolecule metabolic process;macromolecule modification;metabolic process;ncRNA
anatomical structure morphogenesis;biological regulation;cell differentiation;cell differentiation in hindbrain;cell part morphogenesis;cell projection morphogenesis;cell projection organization;cellular component assembly;cell
amine biosynthetic process;amine metabolic process;anatomical structure development;aspartate family amino acid biosynthetic process;aspartate family amino acid metabolic process;biosynthetic process;carboxylic acid b
cellular component biogenesis;cellular component biogenesis at cellular level;cellular component organization or biogenesis;cellular component organization or biogenesis at cellular level;cellular process;ribonucleoprotein co
cellular component organization;cellular component organization at cellular level;cellular component organization or biogenesis;cellular component organization or biogenesis at cellular level;cellular macromolecule metabolic
biological regulation;cell proliferation;cellular component biogenesis;cellular component biogenesis at cellular level;cellular component organization or biogenesis;cellular component organization or biogenesis at cellular level
apoptosis;cell death;cellular macromolecule metabolic process;cellular metabolic process;cellular process;cellular protein metabolic process;death;macromolecule metabolic process;macromolecule modification;metabolic p
cellular macromolecule metabolic process;cellular metabolic process;cellular process;cellular protein metabolic process;establishment of localization;establishment of localization in cell;establishment of protein localization;es
anatomical structure development;apoptosis;biological regulation;cell cycle phase;cell cycle process;cell death;cell division;cellular component organization;cellular component organization at cellular level;cellular component
activation of JUN kinase activity;activation of MAPK activity;activation of MAPKK activity;activation of protein kinase activity;biological regulation;cell cycle arrest;cell cycle checkpoint;cell cycle process;cell death;cell differen
anatomical structure formation involved in morphogenesis;angiogenesis;antigen processing and presentation;antigen processing and presentation of endogenous antigen;antigen processing and presentation of endogenous
cellular component biogenesis;cellular component biogenesis at cellular level;cellular component organization or biogenesis;cellular component organization or biogenesis at cellular level;cellular process;ribonucleoprotein co
anatomical structure homeostasis;biological regulation;cell cycle process;cellular component organization;cellular component organization at cellular level;cellular component organization or biogenesis;cellular component org
actin cytoskeleton organization;actin filament depolymerization;actin filament organization;actin filament-based process;actin polymerization or depolymerization;actomyosin structure organization;anatomical structure homeo
apoptosis;biological regulation;biosynthetic process;cell death;cellular biosynthetic process;cellular component assembly;cellular component assembly at cellular level;cellular component organization;cellular component orga
cellular component organization;cellular component organization at cellular level;cellular component organization or biogenesis;cellular component organization or biogenesis at cellular level;cellular membrane organization;ce
biological regulation;cellular macromolecule metabolic process;cellular metabolic process;cellular nitrogen compound metabolic process;cellular process;macromolecule metabolic process;macromolecule modification;metab
acrosome assembly;anatomical structure formation involved in morphogenesis;cellular component assembly;cellular component assembly at cellular level;cellular component assembly involved in morphogenesis;cellular com
apoptosis;biological regulation;catabolic process;cell death;cellular catabolic process;cellular component disassembly;cellular component disassembly at cellular level;cellular component disassembly involved in apoptosis;c
biological regulation;cell surface receptor linked signaling pathway;cellular component organization;cellular component organization or biogenesis;cellular localization;cellular macromolecule localization;cellular membrane org
biological regulation;regulation of biological process;regulation of cell proliferation;regulation of cellular process
anaphase-promoting complex-dependent proteasomal ubiquitin-dependent protein catabolic process;attachment of spindle microtubules to chromosome;attachment of spindle microtubules to kinetochore;biological regulati
anatomical structure development;biological regulation;biosynthetic process;bone development;cellular biosynthetic process;cellular metabolic process;cellular nitrogen compound biosynthetic process;cellular nitrogen comp
activation of JUN kinase activity;activation of MAPK activity;biological regulation;cell differentiation;cellular component assembly;cellular component organization;cellular component organization or biogenesis;cellular develop
actin cytoskeleton organization;actin filament-based process;activation of protein kinase activity;apoptosis;biological regulation;cell cycle;cell death;cell growth;cell migration;cell motility;cell proliferation;cellular component m

alcohol metabolic process;biological regulation;catabolic process;cellular catabolic process;cellular component organization;cellular component organization or biogenesis;cellular lipid metabolic process;cellular macromolec
cellular macromolecule metabolic process;cellular metabolic process;cellular nitrogen compound metabolic process;cellular process;gene expression;macromolecule metabolic process;metabolic process;mRNA metabolic p
actin cytoskeleton organization;actin filament-based process;activation of phospholipase C activity;activation of protein kinase A activity;activation of protein kinase activity;actomyosin structure organization;anatomical struc
acetyl-CoA biosynthetic process;acetyl-CoA biosynthetic process from pyruvate;acetyl-CoA catabolic process;acetyl-CoA metabolic process;alcohol metabolic process;biological regulation;biosynthetic process;carbohydrat
biological regulation;calcium ion homeostasis;calcium ion transport;cation homeostasis;cation transport;cellular calcium ion homeostasis;cellular cation homeostasis;cellular chemical homeostasis;cellular divalent inorganic
ATP hydrolysis coupled proton transport;biological regulation;cation homeostasis;cation transport;cell surface receptor linked signaling pathway;cellular cation homeostasis;cellular chemical homeostasis;cellular component
box C/D snoRNA 3'-end processing;box C/D snoRNA metabolic process;box C/D snoRNA processing;cell differentiation;cellular developmental process;cellular macromolecule metabolic process;cellular metabolic process;
biological regulation;catabolic process;cellular catabolic process;cellular component assembly;cellular component assembly at cellular level;cellular component organization;cellular component organization at cellular level;
biological regulation;blastocyst hatching;cellular process;cellular response to stimulus;developmental process;embryo implantation;hatching;organism emergence from protective structure;positive regulation of biological pro
carboxylic acid catabolic process;carboxylic acid metabolic process;catabolic process;cellular catabolic process;cellular ketone metabolic process;cellular lipid catabolic process;cellular lipid metabolic process;cellular meta
biological regulation;cell surface receptor linked signaling pathway;cellular process;cellular response to chemical stimulus;cellular response to cytokine stimulus;cellular response to interferon-gamma;cellular response to org
adaptive immune response;adaptive immune response based on somatic recombination of immune receptors built from immunoglobulin superfamily domains;biological regulation;cellular component biogenesis;cellular comp
actin filament capping;barbed-end actin filament capping;biological regulation;blood coagulation;cellular component assembly;cellular component movement;cellular component organization;cellular component organization
anatomical structure development;cargo loading into vesicle;cellular component assembly;cellular component assembly at cellular level;cellular component organization;cellular component organization at cellular level;cellula
biological regulation;biosynthetic process;catabolic process;cellular biosynthetic process;cellular catabolic process;cellular macromolecule biosynthetic process;cellular macromolecule metabolic process;cellular metabolic p
biological regulation;cell surface receptor linked signaling pathway;cellular macromolecule metabolic process;cellular metabolic process;cellular nitrogen compound metabolic process;cellular process;cellular response to che
anatomical structure formation involved in morphogenesis;angiogenesis;axon guidance;biological regulation;blood coagulation;cell activation;cell migration;cell motility;cell projection assembly;cell projection organization;cell
amine biosynthetic process;amine metabolic process;aspartate family amino acid metabolic process;biosynthetic process;carboxylic acid metabolic process;cellular amine metabolic process;cellular amino acid metabolic pr
biological regulation;cellular process;cellular response to stimulus;regulation of biological process;regulation of biosynthetic process;regulation of cellular biosynthetic process;regulation of cellular macromolecule biosynthetic
biological regulation;biosynthetic process;cell differentiation;cellular biosynthetic process;cellular developmental process;cellular macromolecule biosynthetic process;cellular macromolecule metabolic process;cellular metab
antigen processing and presentation;antigen processing and presentation of exogenous antigen;antigen processing and presentation of exogenous peptide antigen;antigen processing and presentation of exogenous peptide
activation of immune response;activation of innate immune response;anaphase-promoting complex-dependent proteasomal ubiquitin-dependent protein catabolic process;antigen processing and presentation;antigen proces
actin cytoskeleton organization;actin filament-based process;anatomical structure development;anatomical structure morphogenesis;apical junction assembly;apolipoprotein A-I-mediated signaling pathway;axon guidance;bi
biosynthetic process;catabolic process;cellular biosynthetic process;cellular catabolic process;cellular component disassembly;cellular component disassembly at cellular level;cellular component organization;cellular compo
acylglycerol catabolic process;acylglycerol metabolic process;anatomical structure development;anatomical structure morphogenesis;biological regulation;branching morphogenesis of a tube;carbohydrate metabolic process
acylglycerol catabolic process;acylglycerol metabolic process;biological regulation;carbohydrate metabolic process;catabolic process;cell cycle;cell cycle process;cell division;cell surface receptor linked signaling pathway;ce
biological regulation;biosynthetic process;catabolic process;cellular biosynthetic process;cellular catabolic process;cellular component disassembly;cellular component disassembly at cellular level;cellular component organiz
biological regulation;biosynthetic process;catabolic process;cellular biosynthetic process;cellular catabolic process;cellular component disassembly;cellular component disassembly at cellular level;cellular component organiz
biosynthetic process;catabolic process;cellular biosynthetic process;cellular catabolic process;cellular component biogenesis;cellular component biogenesis at cellular level;cellular component disassembly;cellular componen
biosynthetic process;catabolic process;cellular biosynthetic process;cellular catabolic process;cellular component biogenesis;cellular component biogenesis at cellular level;cellular component disassembly;cellular componen
amine catabolic process;amine metabolic process;anatomical structure development;anatomical structure morphogenesis;biological regulation;biosynthetic process;body fluid secretion;carboxylic acid catabolic process;carb
activation of immune response;activation of innate immune response;anaphase-promoting complex-dependent proteasomal ubiquitin-dependent protein catabolic process;antigen processing and presentation;antigen proces
anatomical structure development;biological regulation;central nervous system development;developmental process;establishment of localization;establishment of RNA localization;mRNA transport;negative regulation of biol
biological regulation;biosynthetic process;cell differentiation;cellular biosynthetic process;cellular developmental process;cellular macromolecule biosynthetic process;cellular macromolecule metabolic process;cellular metab
biosynthetic process;cellular biosynthetic process;cellular macromolecule biosynthetic process;cellular macromolecule metabolic process;cellular metabolic process;cellular nitrogen compound metabolic process;cellular pro
cellular process;endosome transport;establishment of localization;establishment of localization in cell;establishment of protein localization;intracellular transport;protein transport;transport;vesicle-mediated transport
biological regulation;cell surface receptor linked signaling pathway;cellular macromolecule metabolic process;cellular metabolic process;cellular process;cellular protein metabolic process;cellular response to stimulus;enzym
amine biosynthetic process;amine metabolic process;beta-alanine biosynthetic process;beta-alanine metabolic process;biosynthetic process;carboxylic acid biosynthetic process;carboxylic acid metabolic process;catabolic
biological regulation;biosynthetic process;cellular biosynthetic process;cellular macromolecule biosynthetic process;cellular macromolecule metabolic process;cellular metabolic process;cellular nitrogen compound metaboli
biological regulation;biosynthetic process;cellular biosynthetic process;cellular macromolecule biosynthetic process;cellular macromolecule metabolic process;cellular metabolic process;cellular nitrogen compound metaboli
actin filament-based movement;actin filament-based process;anatomical structure morphogenesis;biological regulation;cell chemotaxis;cell migration;cell motility;cell part morphogenesis;cell projection morphogenesis;cell p
cellular macromolecule metabolic process;cellular metabolic process;cellular nitrogen compound metabolic process;cellular process;macromolecule metabolic process;metabolic process;mRNA metabolic process;mRNA pr
anatomical structure development;anatomical structure morphogenesis;biological regulation;cell differentiation;cellular developmental process;cellular process;developmental process;muscle organ development;muscle struc
biological regulation;cellular component assembly;cellular component assembly at cellular level;cellular component organization;cellular component organization at cellular level;cellular component organization or biogenesis;
biological regulation;cell proliferation;cellular component biogenesis;cellular component biogenesis at cellular level;cellular component organization or biogenesis;cellular component organization or biogenesis at cellular level
cellular component assembly;cellular component assembly at cellular level;cellular component organization;cellular component organization at cellular level;cellular component organization or biogenesis;cellular component
biological regulation;biosynthetic process;cellular biosynthetic process;cellular macromolecule biosynthetic process;cellular macromolecule metabolic process;cellular metabolic process;cellular process;cellular protein meta
anatomical structure homeostasis;arachidonic acid metabolic process;biological regulation;biosynthetic process;carboxylic acid biosynthetic process;carboxylic acid metabolic process;cellular biosynthetic process;cellular c
actin cytoskeleton organization;actin filament-based process;actomyosin structure organization;cell differentiation;cellular component organization;cellular component organization at cellular level;cellular component organiza
biological regulation;biosynthetic process;cell differentiation;cellular biosynthetic process;cellular component assembly;cellular component assembly at cellular level;cellular component organization;cellular component organi
actin cytoskeleton organization;actin filament-based process;biological regulation;cell communication;cellular component organization;cellular component organization at cellular level;cellular component organization or bioge
biological regulation;biosynthetic process;cell junction assembly;cell junction organization;cell-substrate junction assembly;cellular biosynthetic process;cellular component assembly;cellular component assembly at cellular l
biological regulation;biosynthetic process;cellular biosynthetic process;cellular macromolecule biosynthetic process;cellular macromolecule metabolic process;cellular metabolic process;cellular nitrogen compound metaboli
anatomical structure development;biological regulation;bone development;cell growth;cellular component organization;cellular component organization at cellular level;cellular component organization or biogenesis;cellular c
alcohol metabolic process;alditol metabolic process;biological regulation;carbohydrate metabolic process;carbohydrate phosphorylation;cellular carbohydrate metabolic process;cellular metabolic process;cellular process;de
apoptotic mitochondrial changes;cellular component organization;cellular component organization at cellular level;cellular component organization or biogenesis;cellular component organization or biogenesis at cellular level;
biological regulation;cellular macromolecule metabolic process;cellular metabolic process;cellular nitrogen compound metabolic process;cellular process;macromolecule metabolic process;metabolic process;mRNA metaboli
carboxylic acid metabolic process;cellular ketone metabolic process;cellular metabolic process;cellular process;metabolic process;organic acid metabolic process;oxoacid metabolic process;small molecule metabolic proces
biological regulation;cellular process;establishment of localization;establishment of localization in cell;establishment of protein localization;establishment of protein localization to organelle;establishment of protein localization
biological regulation;biosynthetic process;cellular biosynthetic process;cellular component organization;cellular component organization at cellular level;cellular component organization or biogenesis;cellular component orga
biological adhesion;cell adhesion;cellular process
biosynthetic process;cellular biosynthetic process;cellular component assembly;cellular component organization;cellular component organization or biogenesis;cellular macromolecule biosynthetic process;cellular macromole
anatomical structure development;anatomical structure morphogenesis;apoptosis;biological regulation;biosynthetic process;camera-type eye development;cardiac chamber morphogenesis;cardiac muscle contraction;cardia
ATP-dependent chromatin remodeling;biosynthetic process;cellular biosynthetic process;cellular component organization;cellular component organization at cellular level;cellular component organization or biogenesis;cellula
biosynthetic process;cellular biosynthetic process;cellular component disassembly;cellular component disassembly at cellular level;cellular component organization;cellular component organization at cellular level;cellular co
cellular macromolecule metabolic process;cellular metabolic process;cellular process;cellular protein metabolic process;macromolecule metabolic process;macromolecule modification;metabolic process;multicellular organis
anatomical structure development;anatomical structure formation involved in morphogenesis;anatomical structure morphogenesis;ATP-dependent chromatin remodeling;biological regulation;biosynthetic process;blood coag
anatomical structure development;biological regulation;cell development;cellular component assembly;cellular component assembly at cellular level;cellular component organization;cellular component organization at cellular
actin cytoskeleton organization;actin filament-based process;biological regulation;cellular component organization;cellular component organization at cellular level;cellular component organization or biogenesis;cellular comp
cellular component organization;cellular component organization at cellular level;cellular component organization or biogenesis;cellular component organization or biogenesis at cellular level;cellular macromolecule metabolic
cell cycle;cell division;cellular process
immune system process
biological regulation;cellular component organization;cellular component organization at cellular level;cellular component organization or biogenesis;cellular component organization or biogenesis at cellular level;cellular macr
anatomical structure development;cell differentiation;cellular developmental process;cellular macromolecule metabolic process;cellular metabolic process;cellular process;cellular protein metabolic process;'de novo' posttran
biological regulation;negative regulation of biological process;negative regulation of cellular component organization;negative regulation of cellular process;negative regulation of developmental process;negative regulation of
biological regulation;biosynthetic process;cellular biosynthetic process;cellular lipid metabolic process;cellular metabolic process;cellular process;cellular response to calcium ion;cellular response to chemical stimulus;cellula
biological regulation;biosynthetic process;carbohydrate metabolic process;carbohydrate transport;cell chemotaxis;cell cycle;cell cycle process;cell migration;cell motility;cell projection assembly;cell projection organization;ce
biological regulation;cellular macromolecule metabolic process;cellular metabolic process;cellular process;cellular protein metabolic process;endosome transport;establishment of localization;establishment of localization in c
cellular process;cellular response to heat;cellular response to stimulus;cellular response to stress;response to abiotic stimulus;response to heat;response to stimulus;response to stress;response to temperature stimulus
biological regulation;cellular macromolecule metabolic process;cellular metabolic process;cellular process;cellular protein metabolic process;dephosphorylation;macromolecule metabolic process;macromolecule modification
biological regulation;biosynthetic process;carboxylic acid biosynthetic process;carboxylic acid metabolic process;cell redox homeostasis;cellular biosynthetic process;cellular homeostasis;cellular ketone metabolic process;c
amine metabolic process;asparagine metabolic process;aspartate family amino acid metabolic process;carboxylic acid metabolic process;cellular amine metabolic process;cellular amino acid metabolic process;cellular keto
acetate biosynthetic process;acetate metabolic process;acetyl-CoA biosynthetic process;acetyl-CoA biosynthetic process from acetate;acetyl-CoA metabolic process;alcohol metabolic process;biosynthetic process;carboxy
actin cytoskeleton organization;actin filament capping;actin filament organization;actin filament-based process;actomyosin structure organization;anatomical structure development;anatomical structure formation involved in
acyl-CoA biosynthetic process;acyl-CoA metabolic process;acylglycerol biosynthetic process;acylglycerol metabolic process;biosynthetic process;carboxylic acid biosynthetic process;carboxylic acid metabolic process;cellu
actin filament-based movement;actin filament-based process;biological regulation;cell cycle process;cellular component movement;cellular component organization;cellular component organization at cellular level;cellular co
actin crosslink formation;actin cytoskeleton organization;actin filament bundle assembly;actin filament organization;actin filament-based process;biological regulation;cell surface receptor linked signaling pathway;cellular con
biological regulation;cellular lipid metabolic process;cellular metabolic process;cellular process;cellular response to stimulus;defense response;inflammatory response;lipid metabolic process;metabolic process;organophosp
biosynthetic process;cellular biosynthetic process;cellular macromolecule biosynthetic process;cellular macromolecule metabolic process;cellular metabolic process;cellular nitrogen compound metabolic process;cellular pro
amine metabolic process;carboxylic acid metabolic process;cell differentiation;cellular amine metabolic process;cellular amino acid metabolic process;cellular developmental process;cellular ketone metabolic process;cellula
cell growth;cellular macromolecule metabolic process;cellular metabolic process;cellular nitrogen compound metabolic process;cellular process;establishment of localization;establishment of localization in cell;establishment
anatomical structure homeostasis;biological regulation;cell surface receptor linked signaling pathway;cellular component assembly;cellular component assembly at cellular level;cellular component organization;cellular comp
biological regulation;cell cycle phase;cell cycle process;cell division;cell projection assembly;cell projection organization;cellular component assembly;cellular component assembly at cellular level;cellular component organiz
cellular macromolecule metabolic process;cellular metabolic process;cellular nitrogen compound metabolic process;cellular process;macromolecule metabolic process;metabolic process;nitrogen compound metabolic proc
biological regulation;biosynthetic process;cellular biosynthetic process;cellular component assembly;cellular component assembly at cellular level;cellular component organization;cellular component organization at cellular l
cellular macromolecule metabolic process;cellular metabolic process;cellular process;cellular protein metabolic process;establishment of localization;establishment of localization in cell;establishment of protein localization;e
2-oxoglutarate metabolic process;acetyl-CoA catabolic process;acetyl-CoA metabolic process;carboxylic acid metabolic process;catabolic process;cellular catabolic process;cellular ketone metabolic process;cellular metab
anatomical structure morphogenesis;cell part morphogenesis;cell projection morphogenesis;cell projection organization;cellular component assembly;cellular component morphogenesis;cellular component organization;cellu
catabolic process;cellular catabolic process;cellular component organization;cellular component organization at cellular level;cellular component organization or biogenesis;cellular component organization or biogenesis at ce
biological regulation;catabolic process;cation transport;cell cycle process;cellular catabolic process;cellular component disassembly;cellular component disassembly at cellular level;cellular component organization;cellular c
cellular macromolecule metabolic process;cellular metabolic process;cellular process;cellular protein metabolic process;establishment of localization;establishment of localization in cell;establishment of protein localization;e
biological regulation;biosynthetic process;cellular biosynthetic process;cellular component assembly;cellular component assembly at cellular level;cellular component organization;cellular component organization at cellular l
alcohol biosynthetic process;alcohol metabolic process;amino sugar biosynthetic process;amino sugar metabolic process;anatomical structure development;biosynthetic process;carbohydrate biosynthetic process;carbohy
biological regulation;positive regulation of ATPase activity;positive regulation of catalytic activity;positive regulation of hydrolase activity;positive regulation of molecular function;regulation of ATPase activity;regulation of biolo
biological regulation;cellular localization;cellular macromolecule localization;cellular process;chemical homeostasis;homeostatic process;interaction with host;interspecies interaction between organisms;intracellular mRNA lo
biological regulation;biosynthetic process;catabolic process;cellular aromatic compound metabolic process;cellular biosynthetic process;cellular catabolic process;cellular metabolic compound salvage;cellular metabolic proc
aging;biosynthetic process;carbohydrate metabolic process;cellular biosynthetic process;cellular carbohydrate metabolic process;cellular macromolecule biosynthetic process;cellular macromolecule metabolic process;cellul
anatomical structure development;biological regulation;biosynthetic process;carbohydrate homeostasis;cellular biosynthetic process;cellular chemical homeostasis;cellular component organization;cellular component organiz
amine metabolic process;aminoglycan catabolic process;aminoglycan metabolic process;anatomical structure development;anatomical structure morphogenesis;biological adhesion;biological regulation;blood coagulation;br
biological adhesion;biological regulation;biosynthetic process;cell adhesion;cell surface receptor linked signaling pathway;cellular biosynthetic process;cellular macromolecule biosynthetic process;cellular macromolecule me
amine transport;amino acid transmembrane transport;amino acid transport;betaine transport;carboxylic acid catabolic process;carboxylic acid metabolic process;carboxylic acid transport;carnitine shuttle;carnitine transport;
2'-deoxyribonucleotide biosynthetic process;2'-deoxyribonucleotide metabolic process;biosynthetic process;cell cycle;cell differentiation;cell proliferation;cellular biosynthetic process;cellular developmental process;cellular
biological regulation;cellular component assembly;cellular component organization;cellular component organization or biogenesis;macromolecular complex assembly;macromolecular complex subunit organization;positive re
anatomical structure development;biological regulation;catabolic process;cell differentiation;cellular catabolic process;cellular chemical homeostasis;cellular component organization;cellular component organization at cellula
biosynthetic process;catabolic process;cellular biosynthetic process;cellular catabolic process;cellular component disassembly;cellular component disassembly at cellular level;cellular component organization;cellular compo
biological regulation;cellular component organization;cellular component organization or biogenesis;cellular macromolecule metabolic process;cellular membrane organization;cellular metabolic process;cellular process;cellul
biosynthetic process;cellular aromatic compound metabolic process;cellular biosynthetic process;cellular metabolic process;cellular nitrogen compound biosynthetic process;cellular nitrogen compound metabolic process;ce
biosynthetic process;carbohydrate metabolic process;cell activation;cellular biosynthetic process;cellular carbohydrate metabolic process;cellular macromolecule biosynthetic process;cellular macromolecule metabolic proce
biosynthetic process;catabolic process;cellular biosynthetic process;cellular catabolic process;cellular component disassembly;cellular component disassembly at cellular level;cellular component organization;cellular compo
anatomical structure homeostasis;antigen processing and presentation;antigen processing and presentation of exogenous antigen;antigen processing and presentation of exogenous peptide antigen;antigen processing and p
alcohol metabolic process;anatomical structure development;cellular aldehyde metabolic process;cellular lipid metabolic process;cellular metabolic process;cellular process;central nervous system development;development
actin cytoskeleton organization;actin filament-based process;biological regulation;cellular component movement;cellular component organization;cellular component organization at cellular level;cellular component organizati
acyl-CoA biosynthetic process;acyl-CoA metabolic process;acylglycerol biosynthetic process;acylglycerol metabolic process;alcohol biosynthetic process;alcohol metabolic process;anion transport;biosynthetic process;carb
biological adhesion;biological regulation;cell adhesion;cell surface receptor linked signaling pathway;cell-cell adhesion;cellular macromolecule metabolic process;cellular metabolic process;cellular process;cellular protein me

biological regulation;biosynthetic process;cellular biosynthetic process;cellular component assembly;cellular component assembly at cellular level;cellular component organization;cellular component organization at cellular le
actin filament-based movement;actin filament-based process;actin-mediated cell contraction;actin-myosin filament sliding;anatomical structure development;axon guidance;biological regulation;cell surface receptor linked si
cellular macromolecule metabolic process;cellular metabolic process;cellular process;cellular protein metabolic process;'de novo' posttranslational protein folding;'de novo' protein folding;macromolecule metabolic process;
biological regulation;biosynthetic process;cellular biosynthetic process;cellular component assembly;cellular component assembly at cellular level;cellular component organization;cellular component organization at cellular le
biological regulation;biosynthetic process;cellular aromatic compound metabolic process;cellular biosynthetic process;cellular metabolic compound salvage;cellular metabolic process;cellular nitrogen compound biosynthetic
biological regulation;biosynthetic process;cellular biosynthetic process;cellular macromolecule biosynthetic process;cellular macromolecule metabolic process;cellular metabolic process;cellular nitrogen compound metabolic
biological regulation;cellular component organization;cellular component organization at cellular level;cellular component organization or biogenesis;cellular component organization or biogenesis at cellular level;cellular proce
biological regulation;cell surface receptor linked signaling pathway;cellular macromolecule metabolic process;cellular metabolic process;cellular nitrogen compound metabolic process;cellular process;cellular response to sti
biological regulation;cellular component organization;cellular component organization or biogenesis;maintenance of location;maintenance of protein location;negative regulation of actin filament polymerization;negative regula
alcohol metabolic process;amine metabolic process;amino acid activation;anatomical structure formation involved in morphogenesis;angiogenesis;apoptosis;biological adhesion;biological regulation;carbohydrate metabolic
3'-UTR-mediated mRNA stabilization;biological regulation;biosynthetic process;cellular biosynthetic process;cellular component organization;cellular component organization or biogenesis;cellular macromolecule biosynthetic
biological regulation;biosynthetic process;cellular biosynthetic process;cellular component assembly;cellular component assembly at cellular level;cellular component organization;cellular component organization at cellular le
biological regulation;cell proliferation;cellular process;cellular response to stimulus;regulation of biological process;regulation of cellular process;response to stimulus;signal transduction
anatomical structure development;anatomical structure formation involved in morphogenesis;anatomical structure morphogenesis;ATP-dependent chromatin remodeling;biological regulation;biosynthetic process;blood coagu
alkene biosynthetic process;biosynthetic process;carboxylic acid biosynthetic process;carboxylic acid metabolic process;cellular alkene metabolic process;cellular biosynthetic process;cellular ketone metabolic process;cellu
actin cytoskeleton organization;actin filament bundle assembly;actin filament organization;actin filament-based process;activation of Janus kinase activity;activation of protein kinase activity;anatomical structure formation inv
alcohol metabolic process;biosynthetic process;cholesterol biosynthetic process;cholesterol metabolic process;lipid biosynthetic process;lipid metabolic process;metabolic process;primary metabolic process;small molecule
anatomical structure formation involved in morphogenesis;biological regulation;cell cycle;cell cycle process;cell projection assembly;cell projection organization;cellular component assembly;cellular component assembly at c
adult behavior;adult locomotory behavior;anatomical structure morphogenesis;axonogenesis;behavior;cell part morphogenesis;cell projection morphogenesis;cell projection organization;cellular component morphogenesis;ce
actin cytoskeleton organization;actin cytoskeleton reorganization;actin filament-based process;biological regulation;cell surface receptor linked signaling pathway;cellular component organization;cellular component organizat
anatomical structure development;anatomical structure formation involved in morphogenesis;biological regulation;cellular process;developmental process;epidermis development;hair cycle process;hair follicle development;
biological regulation;biosynthetic process;cellular biosynthetic process;cellular component organization;cellular component organization at cellular level;cellular component organization or biogenesis;cellular component orga
cellular component assembly;cellular component assembly at cellular level;cellular component organization;cellular component organization at cellular level;cellular component organization or biogenesis;cellular component c
biological adhesion;biological regulation;cell adhesion;cell junction assembly;cell junction organization;cell-cell adhesion;cellular component assembly;cellular component assembly at cellular level;cellular component organiz
biosynthetic process;cellular biosynthetic process;cellular macromolecule biosynthetic process;cellular macromolecule metabolic process;cellular metabolic process;cellular nitrogen compound metabolic process;cellular pro
activation of caspase activity;anatomical structure development;biological regulation;cellular component assembly;cellular component assembly at cellular level;cellular component organization;cellular component organizatio
cellular macromolecule metabolic process;cellular metabolic process;cellular process;cellular protein metabolic process;chaperone-mediated protein folding;macromolecule metabolic process;macromolecule modification;me
biological regulation;biosynthetic process;cellular biosynthetic process;cellular macromolecule biosynthetic process;cellular macromolecule metabolic process;cellular metabolic process;cellular nitrogen compound metabolic
biological regulation;biosynthetic process;carbohydrate metabolic process;carbohydrate transport;cell cycle;cell cycle process;cell surface receptor linked signaling pathway;cellular biosynthetic process;cellular component c
biological regulation;cell projection organization;cell surface receptor linked signaling pathway;cellular component organization;cellular component organization at cellular level;cellular component organization or biogenesis;c
anatomical structure development;biological regulation;cellular component organization;cellular component organization or biogenesis;cellular membrane organization;cellular process;cellular response to stimulus;central nerv
cellular macromolecule metabolic process;cellular metabolic process;cellular nitrogen compound metabolic process;cellular process;macromolecule metabolic process;metabolic process;nitrogen compound metabolic proce
cellular macromolecule metabolic process;cellular metabolic process;cellular process;cellular protein metabolic process;cullin deneddylation;macromolecule metabolic process;macromolecule modification;metabolic process
amine metabolic process;amino acid activation;carboxylic acid metabolic process;cellular amine metabolic process;cellular amino acid metabolic process;cellular ketone metabolic process;cellular macromolecule metabolic
actin cytoskeleton organization;actin filament capping;actin filament-based process;actin modification;barbed-end actin filament capping;biological regulation;cell cycle phase;cell cycle process;cell division;cellular compone
anatomical structure morphogenesis;biological regulation;catabolic process;cell part morphogenesis;cell projection morphogenesis;cell projection organization;cellular catabolic process;cellular component morphogenesis;ce
cellular component assembly;cellular component assembly at cellular level;cellular component biogenesis;cellular component organization;cellular component organization at cellular level;cellular component organization or b
biosynthetic process;cellular biosynthetic process;cellular lipid metabolic process;cellular metabolic process;cellular process;dephosphorylation;glycerolipid biosynthetic process;glycerolipid metabolic process;glycerophosph
anatomical structure formation involved in morphogenesis;angiogenesis;biological regulation;biosynthetic process;cell differentiation;cellular biosynthetic process;cellular chemical homeostasis;cellular component assembly;
acidic amino acid transport;alcohol biosynthetic process;alcohol metabolic process;amine transport;amino acid transport;anion transport;aspartate transport;ATP biosynthetic process;ATP metabolic process;biosynthetic pro
cell death;cellular macromolecule metabolic process;cellular metabolic process;cellular nitrogen compound metabolic process;cellular process;cellular protein metabolic process;cellular response to stimulus;cellular response
5S class rRNA transcription from RNA polymerase III type 1 promoter;biological regulation;biosynthetic process;cellular biosynthetic process;cellular component organization;cellular component organization at cellular level;c
biological regulation;cellular component organization;cellular component organization at cellular level;cellular component organization or biogenesis;cellular component organization or biogenesis at cellular level;cellular memb
biological regulation;cellular process;cellular response to stimulus;cytoplasmic sequestering of NF-kappaB;cytoplasmic sequestering of protein;cytoplasmic sequestering of transcription factor;establishment of localization;es
biological regulation;catabolic process;cell surface receptor linked signaling pathway;cellular catabolic process;cellular macromolecule catabolic process;cellular macromolecule metabolic process;cellular metabolic process;
biological regulation;catabolic process;cellular catabolic process;cellular macromolecule catabolic process;cellular macromolecule metabolic process;cellular metabolic process;cellular process;cellular protein metabolic proc
cellular macromolecule metabolic process;cellular metabolic process;cellular nitrogen compound metabolic process;cellular process;macromolecule metabolic process;metabolic process;mRNA metabolic process;mRNA pro
biosynthetic process;catabolic process;cellular biosynthetic process;cellular catabolic process;cellular component disassembly;cellular component disassembly at cellular level;cellular component organization;cellular compo
biological regulation;cellular macromolecule metabolic process;cellular metabolic process;cellular process;cellular protein metabolic process;demethylation;macromolecule metabolic process;macromolecule modification;met
biological regulation;cell cycle phase;cell cycle process;cell division;cellular component movement;cellular component organization;cellular component organization at cellular level;cellular component organization or biogene
biological regulation;cellular macromolecule metabolic process;cellular metabolic process;cellular process;cellular protein metabolic process;cellular response to stimulus;establishment of localization;establishment of localiza
establishment of localization;establishment of localization in cell;establishment of protein localization;establishment of RNA localization;intracellular protein transport;intracellular transport;nuclear export;nuclear transport;nucl
anatomical structure homeostasis;biological regulation;biosynthetic process;box H/ACA snoRNA 3'-end processing;box H/ACA snoRNA metabolic process;box H/ACA snoRNA processing;cell proliferation;cellular biosynthet
cellular macromolecule metabolic process;cellular metabolic process;cellular nitrogen compound metabolic process;cellular process;gene expression;macromolecule metabolic process;metabolic process;mRNA metabolic p
biological regulation;cell differentiation;cellular developmental process;cellular macromolecule metabolic process;cellular metabolic process;cellular process;cellular protein metabolic process;cellular response to stimulus;cell
cellular macromolecule metabolic process;cellular metabolic process;cellular process;cellular protein metabolic process;cellular response to heat;cellular response to stimulus;cellular response to stress;macromolecule metab
anatomical structure development;biological regulation;brain development;cellular macromolecule metabolic process;cellular metabolic process;cellular process;cellular protein metabolic process;cellular response to abiotic s
anatomical structure development;anatomical structure morphogenesis;anion transport;bicarbonate transport;biological regulation;cation homeostasis;cellular cation homeostasis;cellular chemical homeostasis;cellular home
biological adhesion;biological regulation;blood coagulation;calcium ion homeostasis;calcium-mediated signaling;cardiac muscle contraction;cation homeostasis;cation transport;cell adhesion;cell migration;cell motility;cellula
apoptosis;B cell activation;B cell activation involved in immune response;B cell differentiation;biological regulation;carbohydrate homeostasis;cell activation;cell activation involved in immune response;cell death;cell differenti
anatomical structure development;anatomical structure morphogenesis;biological regulation;biosynthetic process;cell part morphogenesis;cell projection morphogenesis;cell projection organization;cell surface receptor linked
anatomical structure development;anatomical structure homeostasis;base-excision repair;biological regulation;biosynthetic process;cell cycle;cell cycle process;cell differentiation;cell proliferation;cellular biosynthetic process
biological regulation;biosynthetic process;cellular biosynthetic process;cellular component organization;cellular component organization at cellular level;cellular component organization or biogenesis;cellular component orga
anatomical structure development;developmental process;epidermis development;tissue development
alcohol catabolic process;alcohol metabolic process;amine catabolic process;amine metabolic process;benzene-containing compound metabolic process;biological regulation;biosynthetic process;catabolic process;catecho
activation of protein kinase activity;activation of protein kinase C activity by G-protein coupled receptor protein signaling pathway;biological regulation;blood coagulation;cell activation;cell surface receptor linked signaling pa
biosynthetic process;catabolic process;cellular biosynthetic process;cellular catabolic process;cellular component disassembly;cellular component disassembly at cellular level;cellular component organization;cellular compo
alcohol catabolic process;alcohol metabolic process;amine catabolic process;amine metabolic process;anatomical structure development;benzene-containing compound metabolic process;biological regulation;biosynthetic
2'-deoxyribonucleotide biosynthetic process;2'-deoxyribonucleotide metabolic process;benzene-containing compound metabolic process;biosynthetic process;carboxylic acid metabolic process;CDP biosynthetic process;C
acetyl-CoA catabolic process;acetyl-CoA metabolic process;amine catabolic process;amine metabolic process;aspartate family amino acid catabolic process;aspartate family amino acid metabolic process;carboxylic acid ca
biosynthetic process;cellular biosynthetic process;cellular macromolecule biosynthetic process;cellular macromolecule metabolic process;cellular metabolic process;cellular process;cellular protein metabolic process;cotrans
anion transport;biological regulation;establishment of localization;ion transport;negative regulation of biological process;negative regulation of cellular component organization;negative regulation of cellular process;negative r
2-oxoglutarate metabolic process;acetyl-CoA catabolic process;acetyl-CoA metabolic process;carbohydrate metabolic process;carboxylic acid metabolic process;catabolic process;cellular aldehyde metabolic process;cellul
cellular metabolic process;cellular process;electron transport chain;generation of precursor metabolites and energy;metabolic process;mitochondrial electron transport, NADH to ubiquinone;oxidation-reduction process;respi
biological regulation;cell surface receptor linked signaling pathway;cellular component movement;cellular process;cellular response to stimulus;enzyme linked receptor protein signaling pathway;intracellular signal transductic
actin cytoskeleton organization;actin filament organization;actin filament polymerization;actin filament-based process;actin nucleation;actin polymerization or depolymerization;Arp2/3 complex-mediated actin nucleation;axon
anatomical structure formation involved in morphogenesis;anatomical structure morphogenesis;axonogenesis;biological regulation;cell cycle;cell cycle process;cell part morphogenesis;cell projection assembly;cell projection
biosynthetic process;cellular biosynthetic process;cellular component assembly;cellular component assembly at cellular level;cellular component organization;cellular component organization at cellular level;cellular compone
anatomical structure morphogenesis;antigen processing and presentation;axonogenesis;biological regulation;cell differentiation;cell part morphogenesis;cell projection morphogenesis;cell projection organization;cellular comp
biological regulation;cell surface receptor linked signaling pathway;cellular component assembly;cellular component assembly at cellular level;cellular component organization;cellular component organization at cellular level;c
alcohol metabolic process;biological regulation;biosynthetic process;cellular biosynthetic process;cellular macromolecule biosynthetic process;cellular macromolecule metabolic process;cellular metabolic process;cellular nitr
biosynthetic process;catabolic process;cellular biosynthetic process;cellular catabolic process;cellular component disassembly;cellular component disassembly at cellular level;cellular component organization;cellular compo
biological regulation;biosynthetic process;catabolic process;cellular biosynthetic process;cellular catabolic process;cellular component biogenesis;cellular component biogenesis at cellular level;cellular component disassemb
biosynthetic process;catabolic process;cellular biosynthetic process;cellular catabolic process;cellular component disassembly;cellular component disassembly at cellular level;cellular component organization;cellular compo
biological regulation;catabolic process;cell cycle;cell cycle process;cellular catabolic process;cellular component organization;cellular component organization or biogenesis;cellular macromolecule catabolic process;cellular r
axon guidance;biological regulation;biosynthetic process;cell cycle;cell cycle checkpoint;cell surface receptor linked signaling pathway;cellular biosynthetic process;cellular macromolecule biosynthetic process;cellular macro
cellular macromolecule metabolic process;cellular metabolic process;cellular process;cellular protein metabolic process;chaperone-mediated protein folding;macromolecule metabolic process;macromolecule modification;me
amine metabolic process;carboxylic acid metabolic process;cellular amine metabolic process;cellular amino acid metabolic process;cellular ketone metabolic process;cellular metabolic process;cellular nitrogen compound m
cellular macromolecule metabolic process;cellular metabolic process;cellular process;cellular protein metabolic process;dephosphorylation;macromolecule metabolic process;macromolecule modification;metabolic process;
activation of immune response;activation of innate immune response;anatomical structure development;biological regulation;calcineurin-NFAT signaling pathway;calcium ion transport;calcium-mediated signaling;carbohydrat
activation of immune response;adrenergic receptor signaling pathway;aging;anatomical structure development;antigen receptor-mediated signaling pathway;behavior;biological regulation;cAMP catabolic process;cAMP meta
anatomical structure morphogenesis;axon guidance;axonogenesis;biological regulation;cation homeostasis;cell cycle cytokinesis;cell cycle process;cell part morphogenesis;cell projection morphogenesis;cell projection orga
activation of MAPK activity;alcohol metabolic process;autophagy;biological regulation;biosynthetic process;carbohydrate homeostasis;carbohydrate metabolic process;carboxylic acid biosynthetic process;carboxylic acid me
cellular macromolecule metabolic process;cellular metabolic process;cellular nitrogen compound metabolic process;cellular process;cellular protein metabolic process;macromolecule metabolic process;macromolecule modi
biological regulation;biosynthetic process;cell communication;cell surface receptor linked signaling pathway;cellular biosynthetic process;cellular macromolecule biosynthetic process;cellular macromolecule metabolic proces
activation of immune response;activation of innate immune response;anaphase-promoting complex-dependent proteasomal ubiquitin-dependent protein catabolic process;anatomical structure morphogenesis;biological regu
actin crosslink formation;actin cytoskeleton organization;actin filament bundle assembly;actin filament organization;actin filament-based process;axon guidance;biological regulation;cellular component assembly;cellular com
biological regulation;cellular macromolecule metabolic process;cellular metabolic process;cellular nitrogen compound metabolic process;cellular process;macromolecule metabolic process;metabolic process;ncRNA metabo
cell cycle process;cell differentiation;cellular component biogenesis;cellular component biogenesis at cellular level;cellular component organization or biogenesis;cellular component organization or biogenesis at cellular level;
biological regulation;biosynthetic process;cellular biosynthetic process;cellular macromolecule biosynthetic process;cellular macromolecule metabolic process;cellular metabolic process;cellular process;cellular protein metab
cell differentiation;cellular developmental process;cellular macromolecule metabolic process;cellular metabolic process;cellular process;cellular protein metabolic process;developmental process;gamete generation;macromo
actin filament capping;barbed-end actin filament capping;biological regulation;cell communication;cell projection organization;cellular component organization;cellular component organization at cellular level;cellular compone
biological regulation;biosynthetic process;cellular biosynthetic process;cellular macromolecule biosynthetic process;cellular macromolecule metabolic process;cellular metabolic process;cellular nitrogen compound metabolic
anatomical structure development;biological regulation;biosynthetic process;cell cycle phase;cell cycle process;cellular biosynthetic process;cellular component organization;cellular component organization at cellular level;c
biological regulation;biosynthetic process;cellular biosynthetic process;cellular macromolecule biosynthetic process;cellular macromolecule metabolic process;cellular metabolic process;cellular nitrogen compound metabolic
cell death;cellular component assembly;cellular component organization;cellular component organization or biogenesis;cellular process;death;macromolecular complex assembly;macromolecular complex subunit organizatio
ATP-dependent chromatin remodeling;biological regulation;biosynthetic process;cellular biosynthetic process;cellular component disassembly;cellular component disassembly at cellular level;cellular component organization
cellular component assembly;cellular component assembly at cellular level;cellular component organization;cellular component organization at cellular level;cellular component organization or biogenesis;cellular component c
biological regulation;positive regulation of biological process;positive regulation of biosynthetic process;positive regulation of cellular biosynthetic process;positive regulation of cellular metabolic process;positive regulation of
biosynthetic process;cellular biosynthetic process;cellular component organization;cellular component organization at cellular level;cellular component organization or biogenesis;cellular component organization or biogenesis
anatomical structure development;anatomical structure morphogenesis;biological regulation;blood vessel development;branching morphogenesis of a tube;cardiac septum morphogenesis;developmental process;hemopoieti
cellular macromolecule metabolic process;cellular metabolic process;cellular process;cellular protein metabolic process;macromolecule metabolic process;metabolic process;mitochondrial protein processing;primary metabo
biological regulation;cap-independent translational initiation;cell differentiation;cellular developmental process;cellular macromolecule metabolic process;cellular metabolic process;cellular nitrogen compound metabolic proce
biological regulation;biosynthetic process;cellular biosynthetic process;cellular macromolecule biosynthetic process;cellular macromolecule metabolic process;cellular metabolic process;cellular nitrogen compound metabolic
biological regulation;cell cycle phase;cell cycle process;cellular component organization;cellular component organization at cellular level;cellular component organization or biogenesis;cellular component organization or bioge
biosynthetic process;metabolic process
biological regulation;cellular macromolecule metabolic process;cellular metabolic process;cellular nitrogen compound metabolic process;cellular process;cellular protein metabolic process;cellular response to chemical stimu
apoptosis;biological regulation;cell cycle;cell death;cell proliferation;cell surface receptor linked signaling pathway;cellular process;cellular response to chemical stimulus;cellular response to endogenous stimulus;cellular resp
activation of immune response;activation of innate immune response;anaphase-promoting complex-dependent proteasomal ubiquitin-dependent protein catabolic process;antigen processing and presentation;antigen proces

cellular macromolecule metabolic process;cellular metabolic process;cellular nitrogen compound metabolic process;cellular process;establishment of localization;establishment of localization in cell;establishment of protein lo
anatomical structure development;cell differentiation;cellular developmental process;cellular process;developmental process;nervous system development;system development
anterograde axon cargo transport;anterograde synaptic vesicle transport;antigen processing and presentation;antigen processing and presentation of exogenous antigen;antigen processing and presentation of lipid antigen v
biological regulation;biosynthetic process;cellular biosynthetic process;cellular macromolecule biosynthetic process;cellular macromolecule metabolic process;cellular metabolic process;cellular nitrogen compound metabolic
antigen processing and presentation;antigen processing and presentation of exogenous antigen;antigen processing and presentation of exogenous peptide antigen;antigen processing and presentation of exogenous peptide
biological regulation;cell migration;cell motility;cell proliferation;cellular component movement;cellular component organization;cellular component organization or biogenesis;cellular process;locomotion;macromolecule metab
biological regulation;cell differentiation;cellular component organization;cellular component organization at cellular level;cellular component organization or biogenesis;cellular component organization or biogenesis at cellular
cellular component assembly;cellular component assembly at cellular level;cellular component organization;cellular component organization at cellular level;cellular component organization or biogenesis;cellular component c
biological regulation;cellular component assembly;cellular component assembly at cellular level;cellular component organization;cellular component organization at cellular level;cellular component organization or biogenesis;
acetyl-CoA catabolic process;acetyl-CoA metabolic process;carbohydrate metabolic process;catabolic process;cellular carbohydrate metabolic process;cellular catabolic process;cellular metabolic process;cellular process;c
biological regulation;biosynthetic process;cellular biosynthetic process;cellular component assembly;cellular component organization;cellular component organization at cellular level;cellular component organization or bioger
anatomical structure development;astral microtubule organization;biological regulation;cell differentiation;cell proliferation;cellular component organization;cellular component organization at cellular level;cellular component o
biological regulation;cellular component disassembly;cellular component disassembly at cellular level;cellular component organization;cellular component organization at cellular level;cellular component organization or bioge
anatomical structure homeostasis;biological adhesion;biological regulation;cell adhesion;cell surface receptor linked signaling pathway;cell-cell adhesion;cellular component movement;cellular process;cellular response to ch
biological regulation;blood coagulation;cell activation;cell differentiation;cellular developmental process;cellular lipid metabolic process;cellular metabolic process;cellular process;cellular process involved in reproduction;cellu
alcohol biosynthetic process;alcohol metabolic process;biosynthetic process;cellular biosynthetic process;cellular lipid metabolic process;cellular metabolic process;cellular process;cholesterol biosynthetic process;cholester
aging;biological regulation;biosynthetic process;cell communication;cell-cell signaling;cellular biosynthetic process;cellular metabolic process;cellular nitrogen compound biosynthetic process;cellular nitrogen compound met
actin cytoskeleton organization;actin cytoskeleton reorganization;actin filament-based process;activation of signaling protein activity involved in unfolded protein response;biological regulation;blood coagulation;cell activatior
biological adhesion;biological regulation;cell adhesion;cell surface receptor linked signaling pathway;cell-matrix adhesion;cell-substrate adhesion;cellular component organization;cellular component organization at cellular lev
biological regulation;cellular component organization;cellular component organization at cellular level;cellular component organization or biogenesis;cellular component organization or biogenesis at cellular level;cellular macro
anatomical structure development;ATP biosynthetic process;ATP metabolic process;ATP synthesis coupled proton transport;biosynthetic process;cation transport;cellular biosynthetic process;cellular metabolic process;cellu
activation of immune response;activation of innate immune response;anaphase-promoting complex-dependent proteasomal ubiquitin-dependent protein catabolic process;antigen processing and presentation;antigen proces
cell differentiation;cellular developmental process;cellular macromolecule metabolic process;cellular metabolic process;cellular nitrogen compound metabolic process;cellular process;developmental process;epithelial cell diff
alcohol metabolic process;biosynthetic process;cellular biosynthetic process;cellular lipid metabolic process;cellular metabolic process;cellular process;cholesterol biosynthetic process;cholesterol metabolic process;farnesyl
actin filament capping;barbed-end actin filament capping;biological regulation;cell projection assembly;cell projection organization;cellular component assembly;cellular component assembly at cellular level;cellular compone
carbohydrate metabolic process;cellular carbohydrate metabolic process;cellular macromolecule metabolic process;cellular metabolic process;cellular process;cellular protein metabolic process;co-translational protein modif
anatomical structure development;anatomical structure morphogenesis;apocarotenoid metabolic process;behavior;biological regulation;biosynthetic process;carboxylic acid metabolic process;cellular aldehyde metabolic pro
alcohol metabolic process;biosynthetic process;cholesterol biosynthetic process;cholesterol metabolic process;lipid biosynthetic process;lipid metabolic process;metabolic process;primary metabolic process;small molecule
biosynthetic process;cellular biosynthetic process;cellular lipid metabolic process;cellular metabolic process;cellular process;chordate embryonic development;developmental process;embryo development;embryo developm
acylglycerol metabolic process;alcohol metabolic process;amine metabolic process;amine transport;amino acid transmembrane transport;amino acid transport;behavior;betaine metabolic process;betaine transport;biological
biological regulation;cell surface receptor linked signaling pathway;cellular macromolecule metabolic process;cellular metabolic process;cellular process;cellular protein metabolic process;cellular response to stimulus;detecti
biological regulation;biosynthetic process;carbohydrate metabolic process;carbohydrate transport;cell cycle;cell cycle process;cell surface receptor linked signaling pathway;cellular biosynthetic process;cellular component a
amine biosynthetic process;amine metabolic process;apoptosis;biological regulation;biosynthetic process;carboxylic acid biosynthetic process;carboxylic acid metabolic process;cell death;cellular amine metabolic process;c
biological regulation;biosynthetic process;cellular biosynthetic process;cellular macromolecule biosynthetic process;cellular macromolecule metabolic process;cellular metabolic process;cellular nitrogen compound metabolic
biological regulation;biosynthetic process;cellular biosynthetic process;cellular macromolecule biosynthetic process;cellular macromolecule metabolic process;cellular metabolic process;cellular nitrogen compound metabolic
alcohol biosynthetic process;alcohol metabolic process;alpha-ketoglutarate transport;anion transport;biosynthetic process;carbohydrate biosynthetic process;carbohydrate metabolic process;carboxylic acid transport;cellula
biological adhesion;biological regulation;cell adhesion;cellular component organization;cellular component organization or biogenesis;cellular defense response;cellular membrane organization;cellular process;cellular respons
cellular macromolecule metabolic process;cellular metabolic process;cellular process;cellular protein metabolic process;establishment of localization;establishment of localization in cell;establishment of protein localization;es
biological regulation;cellular process;establishment of localization;establishment of localization in cell;establishment of protein localization;Golgi vesicle transport;intracellular transport;positive regulation of biological process;
alternative nuclear mRNA splicing, via spliceosome;biological regulation;biosynthetic process;cellular biosynthetic process;cellular component assembly;cellular component assembly at cellular level;cellular component orgar
biological regulation;cellular process;establishment of localization;establishment of localization in cell;establishment of protein localization;establishment of protein localization in Golgi;establishment of protein localization to c
actin cytoskeleton organization;actin filament bundle assembly;actin filament organization;actin filament-based process;anatomical structure morphogenesis;biological regulation;catabolic process;cell cycle arrest;cell cycle p
carbohydrate metabolic process;cellular carbohydrate metabolic process;cellular macromolecule metabolic process;cellular metabolic process;cellular process;cellular protein metabolic process;glycosylation;macromolecule
biological regulation;cellular macromolecule metabolic process;cellular metabolic process;cellular nitrogen compound metabolic process;cellular process;cellular protein metabolic process;cellular response to stimulus;cellula
biological regulation;cell cycle;cell cycle phase;cell cycle process;cell division;cell proliferation;cellular component organization;cellular component organization at cellular level;cellular component organization or biogenesis;c
alcohol metabolic process;anatomical structure development;biological regulation;biosynthetic process;blood vessel development;cell surface receptor linked signaling pathway;cellular process;cellular response to stimulus;c
activation of immune response;activation of innate immune response;anaphase-promoting complex-dependent proteasomal ubiquitin-dependent protein catabolic process;antigen processing and presentation;antigen proces
biological regulation;cellular component organization;cellular component organization or biogenesis;cellular localization;cellular macromolecule localization;cellular membrane organization;cellular process;cellular protein local
biological regulation;blood coagulation;cellular component assembly;cellular component assembly at cellular level;cellular component organization;cellular component organization at cellular level;cellular component organiza
axon guidance;biological regulation;cell cycle;cell cycle process;cell division;cellular component organization;cellular component organization at cellular level;cellular component organization or biogenesis;cellular component
biological regulation;biosynthetic process;cell differentiation;cellular biosynthetic process;cellular developmental process;cellular macromolecule biosynthetic process;cellular macromolecule metabolic process;cellular metab
actin cytoskeleton organization;actin cytoskeleton reorganization;actin filament-based process;activation of protein kinase activity;anatomical structure morphogenesis;biological regulation;cell communication;cell part morph
carbohydrate metabolic process;catabolic process;cellular carbohydrate metabolic process;cellular catabolic process;cellular macromolecule catabolic process;cellular macromolecule metabolic process;cellular metabolic pr
cellular component assembly;cellular component assembly at cellular level;cellular component organization;cellular component organization at cellular level;cellular component organization or biogenesis;cellular component c
catabolic process;cell cycle;cellular catabolic process;cellular macromolecule catabolic process;cellular macromolecule metabolic process;cellular metabolic process;cellular process;cellular protein metabolic process;macror
cellular component assembly;cellular component assembly at cellular level;cellular component organization;cellular component organization at cellular level;cellular component organization or biogenesis;cellular component c
amine biosynthetic process;amine metabolic process;biosynthetic process;carboxylic acid biosynthetic process;carboxylic acid metabolic process;cellular amine metabolic process;cellular amino acid biosynthetic process;ce
biological adhesion;cell adhesion;cell migration;cell motility;cell proliferation;cellular component movement;cellular process;establishment of cell polarity;establishment of epithelial cell polarity;establishment or maintenance c
apoptosis;biological regulation;cell death;cellular process;death;negative regulation of apoptosis;negative regulation of biological process;negative regulation of cell death;negative regulation of cellular process;negative regula
biological regulation;cellular process;cellular response to stimulus;intracellular signal transduction;positive regulation of catalytic activity;positive regulation of GTPase activity;positive regulation of hydrolase activity;positive re
cellular process;establishment of localization;establishment of localization in cell;establishment of protein localization;Golgi vesicle transport;intracellular protein transport;intracellular transport;intra-Golgi vesicle-mediated tra
biological regulation;cellular macromolecule metabolic process;cellular metabolic process;cellular process;cellular protein metabolic process;chaperone-mediated protein folding;macromolecule metabolic process;metabolic
biological regulation;catabolic process;cellular catabolic process;cellular macromolecule catabolic process;cellular macromolecule metabolic process;cellular metabolic process;cellular process;cellular protein metabolic proc
adenine transport;cellular process;establishment of localization;nitrogen compound transport;nucleobase transport;nucleobase-containing compound transport;purine base transport;purine-containing compound transmemb
activation of protein kinase activity;biological regulation;cell cycle arrest;cell cycle process;cell surface receptor linked signaling pathway;cellular component assembly;cellular component organization;cellular component orga
anatomical structure development;biological regulation;biomineral tissue development;bone marrow development;bone mineralization;cell chemotaxis;cell cycle process;cell migration;cell motility;cell proliferation;cellular com
anatomical structure formation involved in morphogenesis;anatomical structure morphogenesis;angiogenesis;anion transport;apoptosis;biological regulation;branching morphogenesis of a tube;cation homeostasis;cell death;
biological regulation;biosynthetic process;cell activation;cell proliferation;cellular biosynthetic process;cellular component assembly;cellular component assembly at cellular level;cellular component organization;cellular comp
biological regulation;cell cycle arrest;cell cycle process;cellular component assembly;cellular component assembly at cellular level;cellular component organization;cellular component organization at cellular level;cellular com
biological regulation;cell cycle;cell division;cell migration;cell motility;cellular component movement;cellular component organization;cellular component organization at cellular level;cellular component organization or biogene
anatomical structure morphogenesis;axonogenesis;biological regulation;cell part morphogenesis;cell projection morphogenesis;cell projection organization;cellular component assembly;cellular component assembly at cellula
amine biosynthetic process;amine catabolic process;amine metabolic process;behavior;behavioral interaction between organisms;biological regulation;biosynthetic process;carboxylic acid biosynthetic process;carboxylic aci
biological regulation;blood coagulation;blood coagulation, intrinsic pathway;cell activation;cell differentiation;cellular component disassembly;cellular component disassembly at cellular level;cellular component organization;c
acute inflammatory response;acute inflammatory response to antigenic stimulus;adhesion to host;adhesion to other organism involved in symbiotic interaction;aging;anatomical structure development;antigen processing and
cellular component organization;cellular component organization or biogenesis;cellular membrane organization;cellular process;clathrin-mediated endocytosis;endocytosis;establishment of localization;establishment of locali
biological regulation;biosynthetic process;body fluid secretion;cellular aromatic compound metabolic process;cellular biosynthetic process;cellular metabolic process;cellular nitrogen compound biosynthetic process;cellular
actin cytoskeleton organization;actin filament bundle assembly;actin filament organization;actin filament-based process;anatomical structure morphogenesis;biological regulation;cell activation;cell activation involved in immu
activation of phospholipase C activity;activation of protein kinase A activity;activation of protein kinase activity;biological regulation;blood coagulation;cell surface receptor linked signaling pathway;cellular metabolic process;
acetyl-CoA catabolic process;acetyl-CoA metabolic process;aerobic respiration;carboxylic acid metabolic process;catabolic process;cellular catabolic process;cellular ketone metabolic process;cellular metabolic process;cel
androgen receptor signaling pathway;biological regulation;cell motility;cellular component movement;cellular localization;cellular macromolecule localization;cellular macromolecule metabolic process;cellular metabolic proce
cellular macromolecule metabolic process;cellular metabolic process;cellular nitrogen compound metabolic process;cellular process;gene expression;macromolecule metabolic process;metabolic process;mRNA metabolic p
biological regulation;biosynthetic process;cellular biosynthetic process;cellular macromolecule biosynthetic process;cellular macromolecule metabolic process;cellular metabolic process;cellular nitrogen compound metabolic
activation of immune response;activation of innate immune response;anaphase-promoting complex-dependent proteasomal ubiquitin-dependent protein catabolic process;anatomical structure development;antigen processi
biological regulation;regulation of biological process;regulation of cellular component organization;regulation of cellular metabolic process;regulation of cellular process;regulation of chromosome organization;regulation of DN
biological regulation;biosynthetic process;cellular biosynthetic process;cellular macromolecule biosynthetic process;cellular macromolecule metabolic process;cellular metabolic process;cellular process;cellular protein metab
biological regulation;circulatory system process;multicellular organismal process;positive regulation of blood pressure;positive regulation of systemic arterial blood pressure;regulation of anatomical structure size;regulation of
activation of immune response;activation of innate immune response;activation of MAPK activity;alcohol metabolic process;anaphase-promoting complex-dependent proteasomal ubiquitin-dependent protein catabolic proce
anatomical structure development;anatomical structure morphogenesis;anterior/posterior pattern specification;biological regulation;cell differentiation;cell differentiation involved in kidney development;cell morphogenesis;cel
biological regulation;biosynthetic process;cell cycle arrest;cell cycle process;cell proliferation;cell surface receptor linked signaling pathway;cellular biosynthetic process;cellular macromolecule biosynthetic process;cellular m
biological regulation;cellular process;cellular response to stimulus;intracellular signal transduction;positive regulation of biological process;positive regulation of catalytic activity;positive regulation of cell communication;positi
biological regulation;biosynthetic process;carbohydrate metabolic process;carbohydrate transport;cell cycle;cell cycle process;cell surface receptor linked signaling pathway;cellular biosynthetic process;cellular component c
antigen processing and presentation;antigen processing and presentation of exogenous antigen;antigen processing and presentation of exogenous peptide antigen;antigen processing and presentation of exogenous peptide
cellular process;ER to Golgi vesicle-mediated transport;establishment of localization;establishment of localization in cell;establishment of protein localization;Golgi vesicle transport;intracellular protein transport;intracellular tra
biological regulation;cell activation;cell activation involved in immune response;cell killing;cellular process;establishment of localization;establishment of localization in cell;establishment of protein localization;exocytosis;immu
carboxylic acid catabolic process;carboxylic acid metabolic process;catabolic process;cellular catabolic process;cellular component assembly;cellular component organization;cellular component organization or biogenesis;c
actin cytoskeleton organization;actin filament-based process;biological regulation;cellular component organization;cellular component organization at cellular level;cellular component organization or biogenesis;cellular compo
anatomical structure development;biological regulation;cell development;cellular component organization;cellular component organization at cellular level;cellular component organization or biogenesis;cellular component org
cellular component assembly;cellular component organization;cellular component organization at cellular level;cellular component organization or biogenesis;cellular component organization or biogenesis at cellular level;cellu
biological regulation;cellular macromolecule metabolic process;cellular metabolic process;cellular nitrogen compound metabolic process;cellular process;macromolecule metabolic process;metabolic process;mRNA metaboli
adult behavior;adult locomotory behavior;anatomical structure homeostasis;behavior;biological regulation;cell cycle checkpoint;cell differentiation;cell differentiation in spinal cord;cell surface receptor linked signaling pathway
biological regulation;cell cycle;cell cycle phase;cell cycle process;cell division;cellular component organization;cellular component organization at cellular level;cellular component organization or biogenesis;cellular componer
biological regulation;cell activation;cell activation involved in immune response;cell chemotaxis;cell migration;cell motility;cellular component disassembly;cellular component disassembly at cellular level;cellular component n
biological adhesion;cell adhesion;cellular process
biosynthetic process;cellular biosynthetic process;cellular macromolecule biosynthetic process;cellular macromolecule metabolic process;cellular metabolic process;cellular process;cellular protein metabolic process;macron
apoptosis;biological regulation;cell communication;cell death;cell migration;cell motility;cell surface receptor linked signaling pathway;cell-cell signaling;cellular component movement;cellular component organization;cellular c
biological regulation;cell redox homeostasis;cellular homeostasis;cellular macromolecule metabolic process;cellular metabolic process;cellular process;cellular protein metabolic process;cellular response to stimulus;cellular r
biological regulation;cell differentiation;cellular component biogenesis;cellular component biogenesis at cellular level;cellular component organization or biogenesis;cellular component organization or biogenesis at cellular lev
amine metabolic process;amino acid activation;carboxylic acid metabolic process;cellular amine metabolic process;cellular amino acid metabolic process;cellular ketone metabolic process;cellular macromolecule metabolic p
cellular component assembly;cellular component assembly at cellular level;cellular component organization;cellular component organization at cellular level;cellular component organization or biogenesis;cellular component c
biological regulation;cellular component organization;cellular component organization at cellular level;cellular component organization or biogenesis;cellular component organization or biogenesis at cellular level;cellular meml
biological regulation;cell communication;cellular process;cellular response to chemical stimulus;cellular response to external stimulus;cellular response to extracellular stimulus;cellular response to glucose starvation;cellular r
anatomical structure morphogenesis;biological regulation;developmental process;establishment of localization;establishment of RNA localization;gene expression;macromolecule metabolic process;metabolic process;mRNA
anatomical structure morphogenesis;biological regulation;biosynthetic process;cellular biosynthetic process;cellular macromolecule biosynthetic process;cellular macromolecule metabolic process;cellular metabolic process;
biological regulation;cell cycle arrest;cell cycle process;cellular macromolecule metabolic process;cellular metabolic process;cellular process;cellular protein metabolic process;dephosphorylation;macromolecule metabolic pr
antigen processing and presentation;antigen processing and presentation of exogenous antigen;antigen processing and presentation of exogenous peptide antigen;antigen processing and presentation of exogenous peptide
assembly of spliceosomal tri-snRNP;cellular component assembly;cellular component assembly at cellular level;cellular component organization;cellular component organization at cellular level;cellular component organizatic
amine metabolic process;biological regulation;carboxylic acid metabolic process;cellular amine metabolic process;cellular amino acid metabolic process;cellular ketone metabolic process;cellular macromolecule metabolic pr
biological regulation;cellular localization;cellular process;localization;negative regulation of catalytic activity;negative regulation of hydrolase activity;negative regulation of molecular function;negative regulation of phosphatase
cellular component organization;cellular component organization at cellular level;cellular component organization or biogenesis;cellular component organization or biogenesis at cellular level;cellular membrane organization;ce
antigen processing and presentation;antigen processing and presentation of exogenous antigen;antigen processing and presentation of exogenous peptide antigen;antigen processing and presentation of exogenous peptide
activation of immune response;activation of innate immune response;activation of MAPK activity;activation of MAPKK activity;activation of protein kinase activity;aging;anaphase-promoting complex-dependent proteasomal u
cellular metabolic process;cellular process;electron transport chain;generation of precursor metabolites and energy;metabolic process;oxidation-reduction process;respiratory electron transport chain;small molecule metaboli
biological regulation;establishment of localization;lipid transport;modulation of growth of symbiont involved in interaction with host;organic substance transport;positive regulation of biological process;positive regulation of gr
amine metabolic process;AMP biosynthetic process;AMP metabolic process;aspartate family amino acid metabolic process;aspartate metabolic process;biosynthetic process;carboxylic acid metabolic process;cellular amine
acrosome assembly;actin cytoskeleton organization;actin filament-based process;adult behavior;adult locomotory behavior;alcohol metabolic process;ameboidal cell migration;amine metabolic process;anatomical structure c
biosynthetic process;catabolic process;cellular biosynthetic process;cellular catabolic process;cellular component disassembly;cellular component disassembly at cellular level;cellular component organization;cellular compo

amine biosynthetic process;amine metabolic process;apoptotic mitochondrial changes;biological regulation;biosynthetic process;carboxylic acid biosynthetic process;carboxylic acid metabolic process;cell redox homeostasi
biological regulation;blood coagulation;carbohydrate metabolic process;cellular carbohydrate metabolic process;cellular component organization;cellular component organization at cellular level;cellular component organizati
acetyl-CoA catabolic process;acetyl-CoA metabolic process;carbohydrate metabolic process;catabolic process;cellular catabolic process;cellular metabolic process;cellular process;coenzyme catabolic process;coenzyme m
catabolic process;cellular catabolic process;cellular metabolic process;cellular nitrogen compound catabolic process;cellular nitrogen compound metabolic process;cellular process;cofactor catabolic process;cofactor metab
antigen processing and presentation;antigen processing and presentation of exogenous antigen;antigen processing and presentation of exogenous peptide antigen;antigen processing and presentation of exogenous peptide
biosynthetic process;catabolic process;cellular biosynthetic process;cellular catabolic process;cellular component disassembly;cellular component disassembly at cellular level;cellular component organization;cellular compo
anatomical structure homeostasis;ATP-dependent chromatin remodeling;biological regulation;cell cycle;cellular component assembly;cellular component assembly at cellular level;cellular component organization;cellular com
biological regulation;calcium-mediated signaling;carboxylic acid metabolic process;catabolic process;cellular catabolic process;cellular ketone metabolic process;cellular metabolic process;cellular process;cellular response
biosynthetic process;catabolic process;cellular biosynthetic process;cellular catabolic process;cellular component disassembly;cellular component disassembly at cellular level;cellular component organization;cellular compo
activation of immune response;activation of innate immune response;adaptive immune response;adaptive immune response based on somatic recombination of immune receptors built from immunoglobulin superfamily dom
alcohol biosynthetic process;alcohol catabolic process;alcohol metabolic process;alditol catabolic process;alditol metabolic process;biosynthetic process;carbohydrate biosynthetic process;carbohydrate catabolic process;c
biosynthetic process;catabolic process;cellular biosynthetic process;cellular catabolic process;cellular component disassembly;cellular component disassembly at cellular level;cellular component organization;cellular compo
cell communication;cell-cell signaling;cellular process;immune response;immune system process;response to stimulus;signaling
biological regulation;cellular macromolecule metabolic process;cellular metabolic process;cellular process;cellular protein metabolic process;cellular response to chemical stimulus;cellular response to organic substance;cellu
biological regulation;cell cycle arrest;cell cycle process;cellular macromolecule metabolic process;cellular metabolic process;cellular process;cellular protein metabolic process;cellular response to chemical stimulus;cellular re
gamete generation;male gamete generation;multicellular organismal process;multicellular organismal reproductive process;reproductive process;spermatogenesis
biological regulation;catabolic process;cellular catabolic process;cellular macromolecule catabolic process;cellular macromolecule metabolic process;cellular metabolic process;cellular nitrogen compound metabolic process
biological regulation;cation transport;cellular macromolecule metabolic process;cellular metabolic process;cellular process;cellular response to abiotic stimulus;cellular response to acidity;cellular response to pH;cellular respo
biological regulation;biosynthetic process;cellular biosynthetic process;cellular macromolecule biosynthetic process;cellular macromolecule metabolic process;cellular metabolic process;cellular nitrogen compound metabolic
catabolic process;cellular catabolic process;cellular macromolecule catabolic process;cellular macromolecule metabolic process;cellular metabolic process;cellular nitrogen compound metabolic process;cellular process;exo
amine metabolic process;amino acid activation;asparaginyl-tRNA aminoacylation;carboxylic acid metabolic process;cellular amine metabolic process;cellular amino acid metabolic process;cellular ketone metabolic process;c
biological regulation;cell junction assembly;cell junction organization;cell surface receptor linked signaling pathway;cell-cell junction assembly;cell-cell junction organization;cellular component assembly;cellular component as
actin cytoskeleton organization;actin filament organization;actin filament-based process;actin polymerization or depolymerization;activation of immune response;anatomical structure formation involved in morphogenesis;anti
cellular component organization;cellular component organization at cellular level;cellular component organization or biogenesis;cellular component organization or biogenesis at cellular level;cellular process;establishment of l
biological regulation;calcium ion homeostasis;cation homeostasis;cell differentiation;cellular developmental process;cellular process;cellular response to biotic stimulus;cellular response to stimulus;cellular response to stress
developmental process;multicellular organismal development;multicellular organismal process
biological regulation;cellular component organization;cellular component organization or biogenesis;cellular membrane organization;cellular process;endocytosis;establishment of localization;membrane invagination;membran
cellular component assembly;cellular component assembly at cellular level;cellular component organization;cellular component organization at cellular level;cellular component organization or biogenesis;cellular component c
lipid metabolic process;metabolic process;primary metabolic process
antigen processing and presentation;antigen processing and presentation of exogenous antigen;antigen processing and presentation of exogenous peptide antigen;antigen processing and presentation of exogenous peptide
biological regulation;cellular component assembly;cellular component organization;cellular component organization or biogenesis;cellular membrane organization;cellular process;cellular response to chemical stimulus;cellula
biological regulation;cation homeostasis;cation transport;cell differentiation;cellular developmental process;cellular process;chemical homeostasis;developmental process;erythrocyte differentiation;establishment of localizatio
biological regulation;biosynthetic process;cell surface receptor linked signaling pathway;cellular biosynthetic process;cellular macromolecule biosynthetic process;cellular macromolecule metabolic process;cellular metabolic
biosynthetic process;carbohydrate biosynthetic process;carbohydrate metabolic process;cellular biosynthetic process;cellular carbohydrate biosynthetic process;cellular carbohydrate metabolic process;cellular lipid metabol
cellular macromolecule metabolic process;cellular metabolic process;cellular nitrogen compound metabolic process;cellular process;macromolecule metabolic process;metabolic process;mRNA metabolic process;mRNA pro
apoptosis;biological regulation;biosynthetic process;cell death;cellular biosynthetic process;cellular macromolecule biosynthetic process;cellular macromolecule metabolic process;cellular metabolic process;cellular nitrogen
establishment of localization;establishment of localization in cell;establishment of protein localization;establishment of RNA localization;intracellular protein transport;intracellular transport;mRNA transport;nuclear export;nucle
activation of JUN kinase activity;activation of MAPK activity;anatomical structure morphogenesis;apoptosis;biological regulation;cell death;cell part morphogenesis;cell projection morphogenesis;cell projection organization;c
autophagic vacuole assembly;biological regulation;catabolic process;cellular catabolic process;cellular component assembly;cellular component assembly at cellular level;cellular component organization;cellular component
biological regulation;cellular component assembly;cellular component assembly at cellular level;cellular component organization;cellular component organization at cellular level;cellular component organization or biogenesis;
biological regulation;catabolic process;cellular catabolic process;cellular macromolecule catabolic process;cellular macromolecule metabolic process;cellular metabolic process;cellular nitrogen compound metabolic process
biological regulation;cellular component organization;cellular component organization at cellular level;cellular component organization or biogenesis;cellular component organization or biogenesis at cellular level;cellular proce
biosynthetic process;carboxylic acid biosynthetic process;carboxylic acid catabolic process;carboxylic acid metabolic process;catabolic process;cellular biosynthetic process;cellular catabolic process;cellular ketone metabo
anterograde axon cargo transport;anterograde synaptic vesicle transport;antigen processing and presentation;antigen processing and presentation of exogenous antigen;antigen processing and presentation of lipid antigen v
actin cytoskeleton organization;actin filament-based process;axon guidance;biological regulation;cell communication;cell projection assembly;cell projection organization;cell surface receptor linked signaling pathway;cell-cel
biological regulation;cellular process;cellular response to chemical stimulus;cellular response to endogenous stimulus;cellular response to growth factor stimulus;cellular response to organic substance;cellular response to sti
biological regulation;biosynthetic process;cellular biosynthetic process;cellular macromolecule biosynthetic process;cellular macromolecule metabolic process;cellular metabolic process;cellular nitrogen compound metabolic
biological regulation;biosynthetic process;cellular biosynthetic process;cellular component assembly;cellular component assembly at cellular level;cellular component organization;cellular component organization at cellular le
biological regulation;blood coagulation;cell activation;cellular process;coagulation;establishment of localization;establishment of localization in cell;exocytosis;hemostasis;multicellular organismal process;platelet activation;pla
biological regulation;catabolic process;cell proliferation;cellular catabolic process;cellular macromolecule catabolic process;cellular macromolecule metabolic process;cellular metabolic process;cellular nitrogen compound m
3'-phosphoadenosine 5'-phosphosulfate metabolic process;anatomical structure development;cellular lipid metabolic process;cellular metabolic process;cellular nitrogen compound metabolic process;cellular process;depho
biosynthetic process;catabolic process;cellular biosynthetic process;cellular catabolic process;cellular component biogenesis;cellular component biogenesis at cellular level;cellular component disassembly;cellular componen
aromatic compound catabolic process;catabolic process;cellular aromatic compound metabolic process;cellular catabolic process;cellular metabolic process;cellular process;metabolic process;response to chemical stimulus
activation of immune response;activation of innate immune response;anatomical structure development;biological regulation;cell differentiation;cellular component disassembly;cellular component disassembly at cellular leve
biological regulation;biosynthetic process;cellular biosynthetic process;cellular macromolecule biosynthetic process;cellular macromolecule metabolic process;cellular metabolic process;cellular process;cellular protein metab
biological regulation;biosynthetic process;catabolic process;cellular biosynthetic process;cellular catabolic process;cellular component assembly;cellular component assembly at cellular level;cellular component biogenesis;c
biological adhesion;biosynthetic process;catabolic process;cell adhesion;cellular biosynthetic process;cellular catabolic process;cellular component assembly;cellular component assembly at cellular level;cellular component
aldehyde catabolic process;catabolic process;cellular aldehyde metabolic process;cellular catabolic process;cellular metabolic process;cellular process;formaldehyde catabolic process;formaldehyde metabolic process;meta
amine biosynthetic process;amine metabolic process;betaine biosynthetic process;betaine metabolic process;biosynthetic process;carboxylic acid biosynthetic process;carboxylic acid catabolic process;carboxylic acid meta
beta-amyloid metabolic process;cellular macromolecule metabolic process;cellular metabolic process;cellular process;glycoprotein metabolic process;macromolecule metabolic process;metabolic process;primary metabolic
amine biosynthetic process;amine catabolic process;amine metabolic process;biological regulation;biosynthetic process;carboxylic acid biosynthetic process;carboxylic acid catabolic process;carboxylic acid metabolic proc
actin cytoskeleton organization;actin filament bundle assembly;actin filament organization;actin filament-based process;antigen processing and presentation;antigen processing and presentation of exogenous antigen;antiger
anatomical structure development;biosynthetic process;cell proliferation;cellular biosynthetic process;cellular macromolecule biosynthetic process;cellular macromolecule metabolic process;cellular metabolic process;cellular
activation of immune response;activation of innate immune response;anaphase-promoting complex-dependent proteasomal ubiquitin-dependent protein catabolic process;antigen processing and presentation;antigen proces
anatomical structure development;anatomical structure homeostasis;base-excision repair;biological regulation;biosynthetic process;catabolic process;cell cycle;cell cycle checkpoint;cell cycle phase;cell cycle process;cellula
biological regulation;biosynthetic process;catabolic process;cellular biosynthetic process;cellular catabolic process;cellular macromolecule metabolic process;cellular metabolic process;cellular nitrogen compound metabolic
biosynthetic process;catabolic process;cellular biosynthetic process;cellular catabolic process;cellular component disassembly;cellular component disassembly at cellular level;cellular component organization;cellular compo
biological regulation;cellular process;cellular response to abiotic stimulus;cellular response to osmotic stress;cellular response to stimulus;cellular response to stress;multicellular organismal process;negative regulation of cat
alcohol metabolic process;biosynthetic process;carbohydrate biosynthetic process;carbohydrate catabolic process;carbohydrate metabolic process;catabolic process;cellular biosynthetic process;cellular carbohydrate biosy
biological regulation;biosynthetic process;carbohydrate metabolic process;carbohydrate transport;cell cycle;cell cycle process;cell surface receptor linked signaling pathway;cellular biosynthetic process;cellular component c
cellular metabolic process;cellular process;electron transport chain;generation of precursor metabolites and energy;metabolic process;oxidation-reduction process;respiratory electron transport chain;small molecule metaboli
anatomical structure morphogenesis;cellular metabolic process;cellular process;developmental process;metabolic process;methylation;one-carbon metabolic process;organ morphogenesis;organ regeneration;regeneration;re
alcohol catabolic process;alcohol metabolic process;anatomical structure development;androgen biosynthetic process;androgen metabolic process;apocarotenoid metabolic process;arachidonic acid metabolic process;biolo
biosynthetic process;catabolic process;cellular biosynthetic process;cellular catabolic process;cellular component disassembly;cellular component disassembly at cellular level;cellular component organization;cellular compo
antigen processing and presentation;antigen processing and presentation of peptide antigen;antigen processing and presentation of peptide antigen via MHC class I;apoptosis;biological regulation;calcium ion homeostasis;ca
biosynthetic process;catabolic process;cellular biosynthetic process;cellular catabolic process;cellular component assembly;cellular component assembly at cellular level;cellular component disassembly;cellular component c
activation of adenylate cyclase activity;activation of adenylate cyclase activity by adrenergic receptor signaling pathway;activation of adenylate cyclase activity by dopamine receptor signaling pathway;activation of adenylate
biological regulation;blood coagulation;cAMP-mediated signaling;cell activation;cell communication;cell cycle;cell division;cell surface receptor linked signaling pathway;cell-cell signaling;cellular process;cellular response to s
cellular macromolecule metabolic process;cellular metabolic process;cellular nitrogen compound metabolic process;cellular process;macromolecule metabolic process;metabolic process;mRNA metabolic process;mRNA pro
acylglycerol catabolic process;acylglycerol metabolic process;alcohol metabolic process;amine metabolic process;anatomical structure development;biosynthetic process;carbohydrate metabolic process;carbohydrate trans
biological regulation;catabolic process;cellular catabolic process;cellular localization;cellular macromolecule catabolic process;cellular macromolecule localization;cellular macromolecule metabolic process;cellular metabolic
biological regulation;catabolic process;cell communication;cell-cell signaling;cellular process;cellular response to stimulus;inositol phosphate metabolic process;intracellular signal transduction;lipid catabolic process;lipid me
cellular component assembly;cellular component assembly at cellular level;cellular component organization;cellular component organization at cellular level;cellular component organization or biogenesis;cellular component c
activation of immune response;activation of innate immune response;activation of MAPK activity;activation of MAPKK activity;activation of protein kinase activity;aging;anatomical structure development;axon guidance;biolog
abortive mitotic cell cycle;activation of MAPK activity;acylglycerol metabolic process;anatomical structure development;anatomical structure formation involved in morphogenesis;anatomical structure morphogenesis;axon gu
biological regulation;cell cycle;cellular macromolecule metabolic process;cellular metabolic process;cellular process;cellular protein metabolic process;cellular response to stimulus;cellular response to stress;cullin deneddyla
biological adhesion;biological regulation;cell adhesion;cell communication;cell-cell signaling;cell-matrix adhesion;cell-substrate adhesion;cellular process;cellular response to stimulus;establishment of localization;establishme
actin cytoskeleton organization;actin cytoskeleton reorganization;actin filament-based process;anatomical structure development;anatomical structure formation involved in morphogenesis;anatomical structure morphogenes
cell cycle;cell cycle process;cell division;cellular component organization;cellular component organization at cellular level;cellular component organization or biogenesis;cellular component organization or biogenesis at cellula
biological adhesion;biological regulation;cell adhesion;cellular component organization;cellular component organization at cellular level;cellular component organization or biogenesis;cellular component organization or bioger
actin cytoskeleton organization;actin filament organization;actin filament-based process;biological regulation;cell communication;cell communication by chemical coupling;cell communication by electrical coupling;cell prolife
cellular macromolecule metabolic process;cellular metabolic process;cellular nitrogen compound metabolic process;cellular process;localization;macromolecule localization;macromolecule metabolic process;metabolic proce
biosynthetic process;catabolic process;cellular biosynthetic process;cellular catabolic process;cellular component organization;cellular component organization at cellular level;cellular component organization or biogenesis;c
actin cytoskeleton organization;actin filament organization;actin filament-based process;actin nucleation;Arp2/3 complex-mediated actin nucleation;axon guidance;biological regulation;cell surface receptor linked signaling pa
acetyl-CoA catabolic process;acetyl-CoA metabolic process;carboxylic acid metabolic process;catabolic process;cellular catabolic process;cellular ketone metabolic process;cellular metabolic process;cellular process;coenz
biological regulation;cell cycle;cell cycle process;cell division;cell proliferation;cellular component organization;cellular component organization at cellular level;cellular component organization or biogenesis;cellular componen
biological regulation;cell surface receptor linked signaling pathway;cellular process;cellular response to stimulus;G-protein coupled receptor protein signaling pathway;opioid receptor signaling pathway;regulation of biologica
acyl-CoA metabolic process;cellular metabolic process;cellular process;coenzyme metabolic process;cofactor metabolic process;metabolic process;thioester metabolic process
biological regulation;cell proliferation;cellular component biogenesis;cellular component biogenesis at cellular level;cellular component organization;cellular component organization at cellular level;cellular component organiza
cellular macromolecule metabolic process;cellular metabolic process;cellular nitrogen compound metabolic process;cellular process;macromolecule metabolic process;macromolecule modification;metabolic process;ncRNA
biological regulation;biosynthetic process;cellular biosynthetic process;cellular component assembly;cellular component assembly at cellular level;cellular component organization;cellular component organization at cellular le
antigen processing and presentation;antigen processing and presentation of exogenous antigen;antigen processing and presentation of exogenous peptide antigen;antigen processing and presentation of exogenous peptide
amine metabolic process;aminoglycan catabolic process;aminoglycan metabolic process;anatomical structure development;astrocyte cell migration;axon ensheathment;behavior;biological regulation;biosynthetic process;cal
actin cytoskeleton organization;actin filament-based process;anatomical structure formation involved in morphogenesis;biological regulation;blood coagulation;cell activation;cellular component organization;cellular compone
anatomical structure formation involved in morphogenesis;angiogenesis;axon guidance;behavior;biological adhesion;biological regulation;blood coagulation;cell adhesion;cell adhesion mediated by integrin;cell differentiation;
aging;alcohol catabolic process;alcohol metabolic process;aldehyde catabolic process;biological regulation;catabolic process;cellular aldehyde metabolic process;cellular catabolic process;cellular lipid metabolic process;cel
5-phosphoribose 1-diphosphate biosynthetic process;5-phosphoribose 1-diphosphate metabolic process;alcohol biosynthetic process;alcohol metabolic process;AMP biosynthetic process;AMP metabolic process;anatomic
biosynthetic process;catabolic process;cellular biosynthetic process;cellular catabolic process;cellular component biogenesis;cellular component biogenesis at cellular level;cellular component disassembly;cellular componen
aerobic respiration;cellular metabolic process;cellular process;cellular respiration;electron transport chain;energy derivation by oxidation of organic compounds;generation of precursor metabolites and energy;metabolic proce
aging;anatomical structure homeostasis;base-excision repair;biological regulation;biosynthetic process;cell cycle;cell redox homeostasis;cellular biosynthetic process;cellular component organization;cellular component orga
activation of immune response;activation of innate immune response;activation of MAPK activity;activation of MAPKK activity;activation of protein kinase activity;anatomical structure development;anatomical structure morph
aerobic respiration;AMP biosynthetic process;AMP metabolic process;biosynthetic process;cellular aromatic compound metabolic process;cellular biosynthetic process;cellular component assembly;cellular component orga
alcohol catabolic process;alcohol metabolic process;carbohydrate metabolic process;catabolic process;ethanol catabolic process;ethanol metabolic process;metabolic process;primary alcohol catabolic process;primary alco
cellular macromolecule metabolic process;cellular metabolic process;cellular nitrogen compound metabolic process;cellular process;macromolecule metabolic process;maturation of 5.8S rRNA;metabolic process;mRNA met
biological regulation;carboxylic acid catabolic process;carboxylic acid metabolic process;catabolic process;cell differentiation;cellular catabolic process;cellular developmental process;cellular ketone metabolic process;cellul
activation of immune response;activation of innate immune response;anatomical structure development;axon guidance;biological regulation;cell communication;cell cycle;cell surface receptor linked signaling pathway;cell-ce
2'-deoxyribonucleotide metabolic process;ADP biosynthetic process;ADP metabolic process;AMP metabolic process;anatomical structure development;biosynthetic process;brain development;cellular biosynthetic process;c
actin cytoskeleton organization;actin filament depolymerization;actin filament organization;actin filament severing;actin filament-based process;actin polymerization or depolymerization;biological regulation;cellular componen
biosynthetic process;catabolic process;cellular biosynthetic process;cellular catabolic process;cellular component disassembly;cellular component disassembly at cellular level;cellular component organization;cellular compo
biosynthetic process;catabolic process;cellular biosynthetic process;cellular catabolic process;cellular component disassembly;cellular component disassembly at cellular level;cellular component organization;cellular compo
biosynthetic process;catabolic process;cellular biosynthetic process;cellular catabolic process;cellular component disassembly;cellular component disassembly at cellular level;cellular component organization;cellular compo
biological regulation;cellular macromolecule metabolic process;cellular metabolic process;cellular nitrogen compound metabolic process;cellular process;macromolecule metabolic process;metabolic process;mRNA metaboli
anion transport;cellular metabolic process;cellular process;establishment of localization;generation of precursor metabolites and energy;inorganic anion transport;ion transmembrane transport;ion transport;metabolic process
anatomical structure development;cell differentiation;cellular developmental process;cellular process;developmental process;epithelial cell differentiation;muscle organ development;muscle structure development;organ devel
anatomical structure development;anatomical structure morphogenesis;axonogenesis;cell cycle process;cell migration;cell motility;cell part morphogenesis;cell projection morphogenesis;cell projection organization;cellular c
activation of immune response;activation of innate immune response;anaphase-promoting complex-dependent proteasomal ubiquitin-dependent protein catabolic process;antigen processing and presentation;antigen proces
anatomical structure formation involved in morphogenesis;biological regulation;cell cycle phase;cell cycle process;cell division;cell projection assembly;cell projection organization;cell surface receptor linked signaling pathwa

anatomical structure development;anatomical structure morphogenesis;biological regulation;brain development;camera-type eye development;cellular chemical homeostasis;cellular component organization;cellular compone
cellular macromolecule metabolic process;cellular metabolic process;cellular nitrogen compound metabolic process;cellular process;gene expression;macromolecule metabolic process;metabolic process;mRNA metabolic p
3'-UTR-mediated mRNA stabilization;biological regulation;cellular macromolecule metabolic process;cellular metabolic process;cellular nitrogen compound metabolic process;cellular process;developmental process;gene ex
cellular component organization;cellular component organization at cellular level;cellular component organization or biogenesis;cellular component organization or biogenesis at cellular level;cellular process;cytoskeleton orga
adenine nucleotide transport;ATP transport;biological regulation;cellular process;cellular response to calcium ion;cellular response to chemical stimulus;cellular response to inorganic substance;cellular response to metal ion;
aromatic compound biosynthetic process;biosynthetic process;carboxylic acid metabolic process;cellular aromatic compound metabolic process;cellular biosynthetic process;cellular ketone metabolic process;cellular metab
biological regulation;cellular macromolecule metabolic process;cellular metabolic process;cellular process;cellular protein metabolic process;developmental process;endosome transport;establishment of localization;establish
anatomical structure morphogenesis;biological regulation;catabolic process;cell cycle arrest;cell cycle process;cell part morphogenesis;cellular catabolic process;cellular component assembly;cellular component morphogen
anatomical structure development;biological regulation;biosynthetic process;carbohydrate metabolic process;carbohydrate transport;cell cycle;cell cycle process;cell surface receptor linked signaling pathway;cellular biosynt
actin cytoskeleton organization;actin filament-based process;actomyosin structure organization;anatomical structure development;biological regulation;cellular component organization;cellular component organization at cellu
anatomical structure morphogenesis;biological regulation;cell morphogenesis;cell morphogenesis involved in differentiation;cellular component morphogenesis;cellular component organization;cellular component organizatio
biological regulation;establishment of localization;establishment of localization in cell;establishment of protein localization;establishment of protein localization in endoplasmic reticulum membrane;establishment of protein loc
establishment of localization;establishment of localization in cell;establishment of protein localization;establishment of RNA localization;gene expression;intracellular protein transport;intracellular transport;macromolecule met
aging;biological regulation;cardiac cell differentiation;cardiac muscle cell differentiation;cell differentiation;cellular developmental process;cellular process;cellular response to calcium ion;cellular response to chemical stimulus
acetyl-CoA catabolic process;acetyl-CoA metabolic process;biosynthetic process;carboxylic acid metabolic process;catabolic process;cellular biosynthetic process;cellular catabolic process;cellular ketone metabolic proces
biosynthetic process;cellular biosynthetic process;cellular lipid metabolic process;cellular metabolic process;cellular process;ether lipid biosynthetic process;ether lipid metabolic process;glycerol ether biosynthetic process;g
biological regulation;regulation of biological process;regulation of biosynthetic process;regulation of cellular biosynthetic process;regulation of cellular macromolecule biosynthetic process;regulation of cellular metabolic proc
biological regulation;biosynthetic process;cell cycle;cellular biosynthetic process;cellular component organization;cellular component organization at cellular level;cellular component organization or biogenesis;cellular compo
actin cytoskeleton organization;actin filament organization;actin filament-based process;actin nucleation;Arp2/3 complex-mediated actin nucleation;axon guidance;biological regulation;cell surface receptor linked signaling pa
biosynthetic process;cellular biosynthetic process;cellular macromolecule biosynthetic process;cellular macromolecule metabolic process;cellular metabolic process;cellular process;cellular protein metabolic process;cotrans
cell differentiation;cellular developmental process;cellular macromolecule metabolic process;cellular metabolic process;cellular process;cellular protein metabolic process;developmental process;epidermal cell differentiation;
biological regulation;blood coagulation;cAMP-mediated signaling;cell activation;cell communication;cell cycle;cell division;cell surface receptor linked signaling pathway;cell-cell signaling;cellular component organization;cellu
biological adhesion;cell adhesion;cell-cell adhesion;cellular process;homotypic cell-cell adhesion;platelet aggregation
anatomical structure development;behavior;biological adhesion;biological regulation;blood coagulation;cell adhesion;cell differentiation;cell migration;cell motility;cell surface receptor linked signaling pathway;cell-matrix adhe
biological regulation;cell cycle;cell cycle phase;cell cycle process;cellular component organization;cellular component organization at cellular level;cellular component organization or biogenesis;cellular component organizatio
cellular component assembly;cellular component assembly at cellular level;cellular component organization;cellular component organization at cellular level;cellular component organization or biogenesis;cellular component c
ATP biosynthetic process;ATP metabolic process;ATP synthesis coupled proton transport;biosynthetic process;cation transport;cellular biosynthetic process;cellular metabolic process;cellular nitrogen compound biosynthetic
cell differentiation;cellular developmental process;cellular process;developmental process;epithelial cell differentiation
ATP hydrolysis coupled proton transport;ATP metabolic process;biological regulation;cation homeostasis;cation transport;cell surface receptor linked signaling pathway;cellular cation homeostasis;cellular chemical homeosta
acute inflammatory response;acute-phase response;anatomical structure development;astrocyte differentiation;behavior;biological regulation;carbohydrate homeostasis;cell differentiation;cell proliferation;cell surface recepto
alcohol metabolic process;biosynthetic process;carboxylic acid metabolic process;cellular ketone metabolic process;cellular lipid metabolic process;cellular metabolic process;cellular process;cellular response to chemical st
biological regulation;cell cycle;cellular process;cellular response to stimulus;intracellular signal transduction;mitotic cell cycle;negative regulation of biological process;negative regulation of intracellular protein transport;negat
activation of MAPK activity;anatomical structure morphogenesis;biological adhesion;biological regulation;branching morphogenesis of a tube;cell adhesion;cell junction assembly;cell junction organization;cell morphogenesis
anatomical structure development;apoptosis;biological regulation;cell death;cell differentiation;cellular developmental process;cellular process;death;developmental process;muscle organ development;muscle structure devel
biological regulation;cellular macromolecule metabolic process;cellular metabolic process;cellular nitrogen compound metabolic process;cellular process;gene expression;macromolecule metabolic process;metabolic proces
actin filament capping;barbed-end actin filament capping;biological regulation;blood coagulation;cellular component assembly;cellular component movement;cellular component organization;cellular component organization
biological regulation;biosynthetic process;carbohydrate metabolic process;carbohydrate transport;cell cycle;cell cycle process;cell surface receptor linked signaling pathway;cellular biosynthetic process;cellular component a
biological regulation;catabolic process;cell communication;cell-cell signaling;cellular catabolic process;cellular macromolecule catabolic process;cellular macromolecule metabolic process;cellular metabolic process;cellular p
anatomical structure development;antibacterial humoral response;antimicrobial humoral response;defense response;defense response to bacterium;defense response to Gram-positive bacterium;developmental process;hum
5-phosphoribose 1-diphosphate biosynthetic process;5-phosphoribose 1-diphosphate metabolic process;alcohol biosynthetic process;alcohol metabolic process;AMP biosynthetic process;AMP metabolic process;anatomic
anatomical structure morphogenesis;autophagic vacuole assembly;autophagy;biological adhesion;biological regulation;cargo loading into COPII-coated vesicle;cargo loading into vesicle;catabolic process;cell adhesion;cell c
biological regulation;cell communication;cell surface receptor linked signaling pathway;cell-cell signaling;cellular metabolic process;cellular process;cellular response to chemical stimulus;cellular response to endogenous stin
biological regulation;biosynthetic process;cell cycle;cellular biosynthetic process;cellular component assembly;cellular component assembly at cellular level;cellular component disassembly;cellular component disassembly a
anatomical structure development;anatomical structure formation involved in morphogenesis;anatomical structure morphogenesis;angiogenesis;apoptosis;axon guidance;axonogenesis;biological regulation;blood coagulation
carboxylic acid catabolic process;carboxylic acid metabolic process;catabolic process;cellular catabolic process;cellular ketone metabolic process;cellular lipid catabolic process;cellular lipid metabolic process;cellular metab
3'-UTR-mediated mRNA stabilization;biological regulation;cellular macromolecule metabolic process;cellular metabolic process;cellular nitrogen compound metabolic process;cellular process;defense response;gene express
actin cytoskeleton organization;actin filament-based process;activation of immune response;activation of innate immune response;activation of protein kinase activity;antigen receptor-mediated signaling pathway;apoptosis;a
biological regulation;biosynthetic process;cellular biosynthetic process;cellular component assembly;cellular component assembly at cellular level;cellular component organization;cellular component organization at cellular le
amine metabolic process;amino acid activation;carboxylic acid metabolic process;cellular amine metabolic process;cellular amino acid metabolic process;cellular ketone metabolic process;cellular macromolecule metabolic p
activation of immune response;activation of innate immune response;axon guidance;biological regulation;cell communication;cell cycle;cell proliferation;cell surface receptor linked signaling pathway;cell-cell signaling;cellular
actin cytoskeleton organization;actin filament bundle assembly;actin filament organization;actin filament-based process;biological adhesion;biological regulation;cell adhesion;cell communication;cell surface receptor linked s
acetyl-CoA metabolic process;amine catabolic process;amine metabolic process;anthranilate metabolic process;aromatic amino acid family catabolic process;aromatic amino acid family metabolic process;aromatic compou
biological regulation;carboxylic acid catabolic process;carboxylic acid metabolic process;catabolic process;cellular catabolic process;cellular ketone metabolic process;cellular lipid catabolic process;cellular lipid metabolic p
biological regulation;biosynthetic process;cellular biosynthetic process;cellular macromolecule biosynthetic process;cellular macromolecule metabolic process;cellular metabolic process;cellular nitrogen compound metabolic
anatomical structure development;anatomical structure morphogenesis;artery development;blood vessel development;cardiac septum development;developmental process;embryonic morphogenesis;morphogenesis of an ep
apoptosis;biological regulation;biosynthetic process;cell cycle;cell death;cellular biosynthetic process;cellular macromolecule biosynthetic process;cellular macromolecule metabolic process;cellular metabolic process;cellular
biological regulation;cell activation;cellular component organization;cellular component organization or biogenesis;cellular membrane organization;cellular process;cellular response to chemical stimulus;cellular response to hy
anatomical structure morphogenesis;axonogenesis;biological regulation;cell differentiation;cell part morphogenesis;cell projection morphogenesis;cell projection organization;cellular component morphogenesis;cellular compo
establishment of localization;establishment of localization in cell;establishment of protein localization;intracellular protein transport;intracellular transport;nuclear import;nuclear transport;nucleocytoplasmic transport;protein in
activation of protein kinase activity;activation of protein kinase B activity;adult behavior;adult locomotory behavior;alcohol biosynthetic process;alcohol metabolic process;aldehyde catabolic process;amine transport;autopha
actin cytoskeleton organization;actin filament branching;actin filament bundle assembly;actin filament organization;actin filament-based process;biological regulation;cell chemotaxis;cell migration;cell motility;cell projection o
biological regulation;cellular component assembly;cellular component assembly at cellular level;cellular component organization;cellular component organization at cellular level;cellular component organization or biogenesis;
bradykinin catabolic process;catabolic process;cellular catabolic process;cellular metabolic process;cellular process;macromolecule metabolic process;metabolic process;peptide catabolic process;peptide metabolic proces
cell cycle;cell division;cellular component assembly;cellular component organization;cellular component organization or biogenesis;cellular process;macromolecular complex assembly;macromolecular complex subunit organ
macromolecule metabolic process;metabolic process;primary metabolic process;protein metabolic process;proteolysis
biological regulation;cellular macromolecule metabolic process;cellular metabolic process;cellular process;cellular protein metabolic process;macromolecule metabolic process;macromolecule modification;metabolic process
actin cytoskeleton organization;actin filament-based process;anatomical structure morphogenesis;biological regulation;caveola assembly;caveolin-mediated endocytosis;cell part morphogenesis;cell projection morphogenesi
biological regulation;cellular component organization;cellular component organization at cellular level;cellular component organization or biogenesis;cellular component organization or biogenesis at cellular level;cellular meml
activation of immune response;activation of innate immune response;anaphase-promoting complex-dependent proteasomal ubiquitin-dependent protein catabolic process;antigen processing and presentation;antigen proces
biological regulation;biosynthetic process;catabolic process;cellular biosynthetic process;cellular catabolic process;cellular macromolecule biosynthetic process;cellular macromolecule catabolic process;cellular macromolec
anion transport;biological adhesion;biological regulation;cell adhesion;cell-cell adhesion;cellular chemical homeostasis;cellular homeostasis;cellular ion homeostasis;cellular process;cellular response to stimulus;chemical hor
activation of immune response;activation of innate immune response;anaphase-promoting complex-dependent proteasomal ubiquitin-dependent protein catabolic process;antigen processing and presentation;antigen proces
biosynthetic process;cellular biosynthetic process;cellular lipid metabolic process;cellular macromolecule metabolic process;cellular metabolic process;cellular process;cellular protein metabolic process;cellular response to c
biological regulation;cellular macromolecule metabolic process;cellular metabolic process;cellular nitrogen compound metabolic process;cellular process;cleavage involved in rRNA processing;endonucleolytic cleavage in 5'-
activation of protein kinase activity;biological adhesion;biological regulation;cell adhesion;cell cycle;cell-cell adhesion;cellular macromolecule metabolic process;cellular metabolic process;cellular process;cellular protein meta
cellular macromolecule metabolic process;cellular metabolic process;cellular process;cellular protein metabolic process;establishment of localization;establishment of localization in cell;establishment of protein localization;es
activation of immune response;biological regulation;carboxylic acid metabolic process;cell surface receptor linked signaling pathway;cellular ketone metabolic process;cellular lipid metabolic process;cellular metabolic proces
anatomical structure homeostasis;anion transport;behavior;bile acid and bile salt transport;bile acid metabolic process;biological regulation;blood coagulation;carboxylic acid metabolic process;carboxylic acid transport;cell a
adenine transport;apoptotic mitochondrial changes;biological regulation;cellular component organization;cellular component organization at cellular level;cellular component organization or biogenesis;cellular component orga
alcohol biosynthetic process;alcohol metabolic process;biological regulation;biosynthetic process;C21-steroid hormone biosynthetic process;C21-steroid hormone metabolic process;carbohydrate biosynthetic process;carbo
arachidonic acid metabolic process;carboxylic acid metabolic process;cell differentiation;cellular developmental process;cellular ketone metabolic process;cellular lipid metabolic process;cellular metabolic process;cellular pr
amine metabolic process;betaine metabolic process;biological regulation;carboxylic acid metabolic process;carnitine metabolic process;catabolic process;cellular amine metabolic process;cellular amino acid metabolic proce
biological regulation;negative regulation of catalytic activity;negative regulation of endopeptidase activity;negative regulation of hydrolase activity;negative regulation of molecular function;negative regulation of peptidase acti
biological regulation;biosynthetic process;cell differentiation;cellular biosynthetic process;cellular component organization;cellular component organization at cellular level;cellular component organization or biogenesis;cellula
biological regulation;biosynthetic process;cellular biosynthetic process;cellular component organization;cellular component organization at cellular level;cellular component organization or biogenesis;cellular component orga
B cell activation;cell activation;cell proliferation;cellular process;establishment of localization;establishment of localization in cell;exocytosis;immune system process;leukocyte activation;lymphocyte activation;secretion;secret
aging;biological regulation;cell aging;cell junction assembly;cell junction organization;cellular component assembly;cellular component assembly at cellular level;cellular component organization;cellular component organizatio
amine biosynthetic process;amine metabolic process;anatomical structure development;betaine biosynthetic process;betaine metabolic process;biological regulation;biosynthetic process;carboxylic acid biosynthetic process
biological regulation;biosynthetic process;catabolic process;cellular biosynthetic process;cellular catabolic process;cellular component assembly;cellular component assembly at cellular level;cellular component organization;
biological regulation;biosynthetic process;catabolic process;cellular biosynthetic process;cellular catabolic process;cellular component disassembly;cellular component disassembly at cellular level;cellular component organiz
activation of adenylate cyclase activity;activation of adenylate cyclase activity by dopamine receptor signaling pathway;activation of adenylate cyclase activity by G-protein signaling pathway;activation of phospholipase C ac
antigen processing and presentation;antigen processing and presentation of exogenous antigen;antigen processing and presentation of exogenous peptide antigen;antigen processing and presentation of exogenous peptide
cellular component organization;cellular component organization at cellular level;cellular component organization or biogenesis;cellular component organization or biogenesis at cellular level;cellular localization;cellular macro
cell cycle;cell cycle process;cell division;cellular component organization;cellular component organization at cellular level;cellular component organization or biogenesis;cellular component organization or biogenesis at cellula
catabolic process;cellular catabolic process;cellular macromolecule catabolic process;cellular macromolecule metabolic process;cellular metabolic process;cellular nitrogen compound metabolic process;cellular process;mac
cellular macromolecule metabolic process;cellular metabolic process;cellular process;cellular protein metabolic process;cellular response to heat;cellular response to stimulus;cellular response to stress;chaperone cofactor-d
autophagy;biological regulation;catabolic process;cellular catabolic process;cellular component assembly;cellular component assembly at cellular level;cellular component organization;cellular component organization at cellu
cellular macromolecule metabolic process;cellular metabolic process;cellular nitrogen compound metabolic process;cellular process;macromolecule metabolic process;metabolic process;nitrogen compound metabolic proce
establishment of localization;transport
cellular macromolecule metabolic process;cellular metabolic process;cellular nitrogen compound metabolic process;cellular process;macromolecule metabolic process;metabolic process;mRNA metabolic process;mRNA pro
amine biosynthetic process;amine metabolic process;biosynthetic process;carboxylic acid biosynthetic process;carboxylic acid metabolic process;cellular amine metabolic process;cellular amino acid biosynthetic process;ce
biological regulation;cellular macromolecule metabolic process;cellular metabolic process;cellular nitrogen compound metabolic process;cellular process;cellular response to stimulus;cellular response to stress;establishment
biological regulation;cell surface receptor linked signaling pathway;cellular process;cellular response to chemical stimulus;cellular response to cytokine stimulus;cellular response to organic substance;cellular response to stim
cellular macromolecule metabolic process;cellular metabolic process;cellular nitrogen compound metabolic process;cellular process;macromolecule metabolic process;metabolic process;mRNA metabolic process;mRNA pro
developmental process;keratinization
ADP biosynthetic process;ADP metabolic process;AMP metabolic process;ATP metabolic process;biological regulation;biosynthetic process;cell cycle arrest;cell cycle process;cellular biosynthetic process;cellular metabolic
amine biosynthetic process;amine metabolic process;biosynthetic process;carboxylic acid biosynthetic process;carboxylic acid metabolic process;cellular amine metabolic process;cellular amino acid biosynthetic process;ce
beta-amyloid metabolic process;biological regulation;bradykinin catabolic process;catabolic process;cell surface receptor linked signaling pathway;cellular catabolic process;cellular component assembly;cellular component
biological regulation;cellular component assembly;cellular component assembly at cellular level;cellular component organization;cellular component organization at cellular level;cellular component organization or biogenesis;
2-oxoglutarate metabolic process;alcohol biosynthetic process;alcohol metabolic process;alditol biosynthetic process;alditol metabolic process;amine biosynthetic process;amine catabolic process;amine metabolic process;
biological regulation;negative regulation of apoptosis;negative regulation of biological process;negative regulation of catalytic activity;negative regulation of cell death;negative regulation of cellular process;negative regulation
actin cytoskeleton organization;actin filament depolymerization;actin filament organization;actin filament-based process;actin polymerization or depolymerization;ameboidal cell migration;anatomical structure morphogenesis;
biological regulation;cellular process;cellular response to stimulus;establishment of localization;establishment of protein localization;intracellular signal transduction;negative regulation of axonogenesis;negative regulation of b
activation of immune response;activation of innate immune response;activation of MAPK activity;activation of MAPKK activity;activation of protein kinase activity;axon guidance;biological regulation;cell surface receptor linke
biological regulation;cellular macromolecule metabolic process;cellular metabolic process;cellular nitrogen compound metabolic process;cellular process;macromolecule metabolic process;metabolic process;ncRNA metabo
biosynthetic process;catabolic process;cellular biosynthetic process;cellular catabolic process;cellular component disassembly;cellular component disassembly at cellular level;cellular component organization;cellular compo
adherens junction organization;biological adhesion;biological regulation;cell adhesion;cell communication;cell junction assembly;cell junction organization;cell-cell junction organization;cell-cell signaling;cellular component as
adipose tissue development;anatomical structure development;biological regulation;brain development;catabolic process;cellular catabolic process;cellular ketone body metabolic process;cellular ketone metabolic process;c
biosynthetic process;catabolic process;cellular biosynthetic process;cellular catabolic process;cellular component disassembly;cellular component disassembly at cellular level;cellular component organization;cellular compo
biological regulation;cell cycle;cellular process;mitotic cell cycle;negative regulation of binding;negative regulation of molecular function;negative regulation of protein binding;regulation of binding;regulation of molecular funct
ATP-dependent chromatin remodeling;biological regulation;biosynthetic process;cell cycle;cell cycle process;cellular biosynthetic process;cellular component assembly;cellular component assembly at cellular level;cellular co
cellular component assembly;cellular component assembly at cellular level;cellular component organization;cellular component organization at cellular level;cellular component organization or biogenesis;cellular component c
biological regulation;negative regulation of biological process;negative regulation of biosynthetic process;negative regulation of cellular biosynthetic process;negative regulation of cellular macromolecule biosynthetic process
biological regulation;cellular component organization;cellular component organization or biogenesis;cellular membrane organization;cellular process;cellular response to stimulus;endocytosis;establishment of localization;mem
alcohol biosynthetic process;alcohol metabolic process;biological regulation;biosynthetic process;carbohydrate biosynthetic process;carbohydrate homeostasis;carbohydrate metabolic process;carboxylic acid metabolic pro
biological regulation;biosynthetic process;cellular biosynthetic process;cellular macromolecule biosynthetic process;cellular macromolecule metabolic process;cellular metabolic process;cellular nitrogen compound metabolic

cell cycle phase;cell cycle process;cell division;cellular component organization;cellular component organization at cellular level;cellular component organization or biogenesis;cellular component organization or biogenesis at
biological regulation;catabolic process;cellular catabolic process;cellular component organization;cellular component organization at cellular level;cellular component organization or biogenesis;cellular component organizatio
anatomical structure morphogenesis;biological adhesion;biological regulation;cell adhesion;cell junction assembly;cell junction organization;cell morphogenesis;cell morphogenesis involved in differentiation;cell surface recep
biological regulation;biosynthetic process;cellular biosynthetic process;cellular component organization;cellular component organization at cellular level;cellular component organization or biogenesis;cellular component orgar
actin cytoskeleton organization;actin filament-based process;biological regulation;cell differentiation;cellular component organization;cellular component organization at cellular level;cellular component organization or biogen
amine metabolic process;amino acid activation;biosynthetic process;carboxylic acid metabolic process;cellular amine metabolic process;cellular amino acid metabolic process;cellular biosynthetic process;cellular ketone me
B cell activation;B cell activation involved in immune response;cell activation;cell activation involved in immune response;cellular macromolecule metabolic process;cellular metabolic process;cellular nitrogen compound meta
apoptosis;biological regulation;biosynthetic process;cell death;cellular biosynthetic process;cellular macromolecule biosynthetic process;cellular macromolecule metabolic process;cellular metabolic process;cellular nitrogen
anatomical structure development;cellular macromolecule metabolic process;cellular metabolic process;cellular nitrogen compound metabolic process;cellular process;chordate embryonic development;developmental proce
activation of immune response;activation of innate immune response;anaphase-promoting complex-dependent proteasomal ubiquitin-dependent protein catabolic process;antigen processing and presentation;antigen proces
biological regulation;cell cycle;cell cycle phase;cell cycle process;cellular component organization;cellular component organization at cellular level;cellular component organization or biogenesis;cellular component organizatio
anatomical structure development;anatomical structure formation involved in morphogenesis;biological regulation;brain development;cell proliferation;cell surface receptor linked signaling pathway;cellular process;cellular res
actin cytoskeleton organization;actin filament organization;actin filament-based process;actin nucleation;Arp2/3 complex-mediated actin nucleation;biological regulation;cellular component movement;cellular component orga
biological regulation;cellular component organization;cellular component organization at cellular level;cellular component organization or biogenesis;cellular component organization or biogenesis at cellular level;cellular macro
biological regulation;biosynthetic process;cellular biosynthetic process;cellular macromolecule biosynthetic process;cellular macromolecule metabolic process;cellular metabolic process;cellular nitrogen compound metabolic
2-oxoglutarate metabolic process;4-hydroxyproline catabolic process;4-hydroxyproline metabolic process;alcohol biosynthetic process;alcohol metabolic process;amine biosynthetic process;amine catabolic process;amine r
biological regulation;carbohydrate metabolic process;cellular carbohydrate metabolic process;cellular macromolecule metabolic process;cellular metabolic process;cellular process;cellular protein metabolic process;cellular r
autophagy;behavior;behavioral interaction between organisms;biological regulation;calcium ion homeostasis;catabolic process;cation homeostasis;cell differentiation;cell proliferation;cell volume homeostasis;cellular calcium
anatomical structure development;biological regulation;bone development;cellular component organization;cellular component organization at cellular level;cellular component organization or biogenesis;cellular component o
aerobic respiration;cellular metabolic process;cellular process;cellular respiration;electron transport chain;energy derivation by oxidation of organic compounds;generation of precursor metabolites and energy;metabolic proce
acyl-CoA biosynthetic process;acyl-CoA metabolic process;acylglycerol biosynthetic process;acylglycerol metabolic process;adiponectin-mediated signaling pathway;biological regulation;biosynthetic process;carboxylic acic
acetyl-CoA catabolic process;acetyl-CoA metabolic process;alcohol biosynthetic process;alcohol metabolic process;biological regulation;biosynthetic process;carbohydrate biosynthetic process;carbohydrate metabolic proc
alcohol metabolic process;amine biosynthetic process;amine catabolic process;amine metabolic process;aspartate family amino acid catabolic process;aspartate family amino acid metabolic process;betaine biosynthetic pro
biological regulation;cellular component assembly;cellular component organization;cellular component organization or biogenesis;cellular macromolecule metabolic process;cellular metabolic process;cellular process;cellular
biological regulation;biosynthetic process;cell cycle;cellular biosynthetic process;cellular component organization;cellular component organization at cellular level;cellular component organization or biogenesis;cellular compo
biological regulation;cell proliferation;cellular macromolecule metabolic process;cellular metabolic process;cellular process;cellular protein metabolic process;cellular response to chemical stimulus;cellular response to organic
activation of immune response;activation of innate immune response;anaphase-promoting complex-dependent proteasomal ubiquitin-dependent protein catabolic process;antigen processing and presentation;antigen proces
activation of caspase activity;biological regulation;cell differentiation;cellular developmental process;cellular macromolecule metabolic process;cellular metabolic process;cellular process;cellular protein metabolic process;dev
biosynthetic process;catabolic process;cellular biosynthetic process;cellular catabolic process;cellular component biogenesis;cellular component biogenesis at cellular level;cellular component disassembly;cellular componer
activation of caspase activity;anatomical structure morphogenesis;biological regulation;carbohydrate homeostasis;cell cycle;cell differentiation;cellular chemical homeostasis;cellular developmental process;cellular glucose ho
anatomical structure development;apoptosis;biological regulation;catabolic process;cell cycle;cell cycle process;cell death;cell surface receptor linked signaling pathway;cellular catabolic process;cellular component assembl
biological regulation;biosynthetic process;catabolic process;cellular biosynthetic process;cellular catabolic process;cellular component assembly;cellular component assembly at cellular level;cellular component organization;
biological regulation;cellular macromolecule metabolic process;cellular metabolic process;cellular nitrogen compound metabolic process;cellular process;cellular response to stimulus;DNA conformation change;DNA duplex u
actin cytoskeleton organization;actin filament-based process;actin-mediated cell contraction;apical constriction;apoptosis;axon guidance;biological adhesion;biological regulation;bleb assembly;cell adhesion;cell death;cell m
anatomical structure development;apoptosis;biological adhesion;cardiac Purkinje fiber development;cell adhesion;cell death;cell-cell adhesion;cellular component disassembly;cellular component disassembly at cellular level
alcohol biosynthetic process;alcohol metabolic process;amino sugar biosynthetic process;amino sugar metabolic process;biosynthetic process;carbohydrate biosynthetic process;carbohydrate metabolic process;cellular bio
10-formyltetrahydrofolate catabolic process;10-formyltetrahydrofolate metabolic process;aromatic compound catabolic process;biosynthetic process;catabolic process;cellular aromatic compound metabolic process;cellular
apoptosis;cell death;cellular process;death;localization;macromolecule localization;programmed cell death;protein localization
apoptotic cell clearance;biological regulation;cell redox homeostasis;cellular component organization;cellular component organization or biogenesis;cellular homeostasis;cellular macromolecule metabolic process;cellular mer
cellular component assembly;cellular component assembly at cellular level;cellular component organization;cellular component organization at cellular level;cellular component organization or biogenesis;cellular component c
actin cytoskeleton organization;actin filament-based process;actomyosin structure organization;asymmetric Golgi ribbon formation;biological regulation;cell migration;cell motility;cellular component movement;cellular compo
anatomical structure development;anatomical structure morphogenesis;ATP-dependent chromatin remodeling;biological regulation;biosynthetic process;cell surface receptor linked signaling pathway;cellular biosynthetic proc
antigen processing and presentation;antigen processing and presentation of exogenous antigen;antigen processing and presentation of exogenous peptide antigen;antigen processing and presentation of exogenous peptide
biological regulation;biosynthetic process;cell cycle;cell proliferation;cellular biosynthetic process;cellular component organization;cellular component organization at cellular level;cellular component organization or biogenesi
biological regulation;biosynthetic process;cell death;cell projection organization;cellular biosynthetic process;cellular component organization;cellular component organization at cellular level;cellular component organization c
biosynthetic process;catabolic process;cellular biosynthetic process;cellular catabolic process;cellular component disassembly;cellular component disassembly at cellular level;cellular component organization;cellular compo
amine metabolic process;amino acid activation;carboxylic acid metabolic process;cellular amine metabolic process;cellular amino acid metabolic process;cellular ketone metabolic process;cellular macromolecule metabolic p
cellular component assembly;cellular component assembly at cellular level;cellular component organization;cellular component organization at cellular level;cellular component organization or biogenesis;cellular component c
biological adhesion;cell adhesion;cell junction assembly;cell junction organization;cell-cell adhesion;cell-cell junction assembly;cell-cell junction organization;cellular component assembly;cellular component assembly at cellu
anatomical structure development;anatomical structure morphogenesis;axon ensheathment;axonogenesis;biological regulation;cell development;cell part morphogenesis;cell projection morphogenesis;cell projection organiza
catabolic process;cellular catabolic process;cellular macromolecule catabolic process;cellular macromolecule metabolic process;cellular metabolic process;cellular process;cellular protein metabolic process;macromolecule c
cellular component organization;cellular component organization or biogenesis;cellular membrane organization;cellular process;endocytosis;establishment of localization;lipid transport;membrane invagination;membrane orga
biological regulation;biosynthetic process;cellular biosynthetic process;cellular macromolecule biosynthetic process;cellular macromolecule metabolic process;cellular metabolic process;cellular nitrogen compound metabolic
amine metabolic process;amino acid activation;asparaginyl-tRNA aminoacylation;carboxylic acid metabolic process;cellular amine metabolic process;cellular amino acid metabolic process;cellular ketone metabolic process;c
activation of MAPKK activity;activation of protein kinase activity;adenosine receptor signaling pathway;biological regulation;blood coagulation;cAMP-mediated signaling;cell activation;cell communication;cell cycle;cell divisio
adult behavior;adult locomotory behavior;aging;anatomical structure development;anatomical structure morphogenesis;axonogenesis;behavior;biological regulation;catabolic process;cell communication;cell differentiation;ce
catabolic process;cell cycle process;cellular catabolic process;cellular component disassembly;cellular component disassembly at cellular level;cellular component organization;cellular component organization at cellular leve
alcohol catabolic process;alcohol metabolic process;biological regulation;carbohydrate catabolic process;carbohydrate metabolic process;carbohydrate phosphorylation;catabolic process;cellular carbohydrate catabolic proc
activation of caspase activity;aging;anatomical structure morphogenesis;apoptosis;apoptosis in response to endoplasmic reticulum stress;biological regulation;biosynthetic process;branching involved in mammary gland duc
amine biosynthetic process;amine metabolic process;biosynthetic process;carboxylic acid biosynthetic process;carboxylic acid metabolic process;cellular amine metabolic process;cellular amino acid biosynthetic process;ce
anatomical structure development;apoptosis;biological regulation;biosynthetic process;blood circulation;cell death;cell differentiation;cell differentiation involved in kidney development;cell differentiation involved in metaneph
ATP metabolic process;biological regulation;cation transport;cell communication;cell-cell signaling;cellular component organization;cellular component organization or biogenesis;cellular membrane fusion;cellular membrane
biosynthetic process;catabolic process;cellular biosynthetic process;cellular catabolic process;cellular component disassembly;cellular component disassembly at cellular level;cellular component organization;cellular compo
actin cytoskeleton organization;actin filament capping;actin filament-based process;barbed-end actin filament capping;biological regulation;blood coagulation;cellular component movement;cellular component organization;c
actin cytoskeleton organization;actin filament organization;actin filament-based process;actin polymerization or depolymerization;activation of immune response;anatomical structure formation involved in morphogenesis;anti
anatomical structure development;anatomical structure formation involved in morphogenesis;anatomical structure morphogenesis;aortic smooth muscle cell differentiation;appendage morphogenesis;ATP-dependent chroma
activation of immune response;activation of innate immune response;anaphase-promoting complex-dependent proteasomal ubiquitin-dependent protein catabolic process;antigen processing and presentation;antigen proces
actin cytoskeleton organization;actin filament-based process;anatomical structure development;androgen receptor signaling pathway;biological regulation;cell cycle phase;cell cycle process;cell development;cell division;cell
biosynthetic process;catabolic process;cellular biosynthetic process;cellular catabolic process;cellular component biogenesis;cellular component biogenesis at cellular level;cellular component disassembly;cellular componer
biological regulation;biosynthetic process;blood coagulation;cell activation;cell migration;cell motility;cellular biosynthetic process;cellular component assembly;cellular component assembly at cellular level;cellular componer
antigen processing and presentation;antigen processing and presentation of exogenous antigen;antigen processing and presentation of exogenous peptide antigen;antigen processing and presentation of exogenous peptide
biosynthetic process;carbohydrate metabolic process;cellular carbohydrate metabolic process;cellular macromolecule metabolic process;cellular metabolic process;cellular process;cellular protein metabolic process;glycosyl
anatomical structure formation involved in morphogenesis;angiogenesis;biological adhesion;biological regulation;cell adhesion;cell cycle arrest;cell cycle process;cell migration;cell motility;cellular component movement;cellu
biological regulation;biosynthetic process;blood coagulation;catabolic process;cellular biosynthetic process;cellular catabolic process;cellular macromolecule biosynthetic process;cellular macromolecule catabolic process;ce
biological regulation;biosynthetic process;cell proliferation;cellular biosynthetic process;cellular macromolecule biosynthetic process;cellular macromolecule metabolic process;cellular metabolic process;cellular nitrogen com
biological regulation;cell proliferation;cell surface receptor linked signaling pathway;cellular process;cellular response to stimulus;enzyme linked receptor protein signaling pathway;intracellular signal transduction;negative reg
alcohol catabolic process;alcohol metabolic process;biosynthetic process;carbohydrate biosynthetic process;carbohydrate catabolic process;carbohydrate metabolic process;catabolic process;cellular biosynthetic process;c
4-hydroxyproline metabolic process;amine metabolic process;apoptosis;apoptosis in response to endoplasmic reticulum stress;biological regulation;brown fat cell differentiation;calcium ion homeostasis;calcium ion transport
biosynthetic process;cellular biosynthetic process;cellular macromolecule biosynthetic process;cellular macromolecule metabolic process;cellular metabolic process;cellular nitrogen compound metabolic process;cellular pro
biological regulation;biosynthetic process;catabolic process;cell growth;cellular biosynthetic process;cellular catabolic process;cellular macromolecule catabolic process;cellular macromolecule metabolic process;cellular met
cellular component organization;cellular component organization at cellular level;cellular component organization or biogenesis;cellular component organization or biogenesis at cellular level;cellular process;lipid particle orga
assembly of spliceosomal tri-snRNP;biological regulation;biosynthetic process;cell proliferation;cellular component assembly;cellular component assembly at cellular level;cellular component organization;cellular component
antigen processing and presentation;antigen processing and presentation of exogenous antigen;antigen processing and presentation of exogenous peptide antigen;antigen processing and presentation of exogenous peptide
anatomical structure development;biological regulation;cellular component organization;cellular component organization at cellular level;cellular component organization or biogenesis;cellular component organization or bioge
alcohol catabolic process;alcohol metabolic process;anatomical structure homeostasis;biological regulation;carbohydrate catabolic process;carbohydrate homeostasis;carbohydrate metabolic process;carbohydrate phospho
acetyl-CoA biosynthetic process;acetyl-CoA biosynthetic process from pyruvate;acetyl-CoA catabolic process;acetyl-CoA metabolic process;alcohol catabolic process;alcohol metabolic process;biological regulation;biosynth
biological regulation;biosynthetic process;catabolic process;cellular biosynthetic process;cellular catabolic process;cellular component disassembly;cellular component disassembly at cellular level;cellular component organiz
biological regulation;biosynthetic process;cell differentiation;cellular biosynthetic process;cellular developmental process;cellular macromolecule biosynthetic process;cellular macromolecule metabolic process;cellular metab
anatomical structure development;apoptosis;base-excision repair, DNA ligation;biological regulation;catabolic process;cell chemotaxis;cell death;cell migration;cell motility;cellular catabolic process;cellular component assem
antigen processing and presentation;antigen processing and presentation of peptide antigen;antigen processing and presentation of peptide antigen via MHC class I;cellular macromolecule metabolic process;cellular metabo
adherens junction assembly;adherens junction organization;anatomical structure development;anatomical structure formation involved in morphogenesis;anatomical structure homeostasis;anatomical structure morphogenesi
adaptive immune response;adaptive immune response based on somatic recombination of immune receptors built from immunoglobulin superfamily domains;anatomical structure development;B cell activation;B cell activati
biological regulation;cellular macromolecule metabolic process;cellular metabolic process;cellular nitrogen compound metabolic process;cellular process;macromolecule metabolic process;metabolic process;mRNA metaboli
cell cycle;cell division;cellular component organization;cellular component organization or biogenesis;cellular membrane organization;cellular process;endocytosis;establishment of localization;membrane invagination;membra
anatomical structure development;biological regulation;biosynthetic process;catabolic process;cell cycle arrest;cell cycle process;cellular biosynthetic process;cellular catabolic process;cellular macromolecule biosynthetic pr
binding of sperm to zona pellucida;cell recognition;cell-cell recognition;cellular process;cellular process involved in reproduction;reproductive process;sperm-egg recognition
anatomical structure development;anatomical structure formation involved in morphogenesis;androgen receptor signaling pathway;atrial cardiac muscle cell development;biological regulation;biosynthetic process;cardiac cel
biological regulation;biosynthetic process;cell surface receptor linked signaling pathway;cellular biosynthetic process;cellular macromolecule biosynthetic process;cellular macromolecule metabolic process;cellular metabolic
apoptotic mitochondrial changes;biological regulation;cell cycle;cellular component organization;cellular component organization at cellular level;cellular component organization or biogenesis;cellular component organization
biological regulation;cellular macromolecule metabolic process;cellular metabolic process;cellular nitrogen compound metabolic process;cellular process;macromolecule metabolic process;metabolic process;mRNA metaboli
activation of immune response;activation of innate immune response;anaphase-promoting complex-dependent proteasomal ubiquitin-dependent protein catabolic process;antigen processing and presentation;antigen proces
biological adhesion;biological regulation;biosynthetic process;cell adhesion;cellular biosynthetic process;cellular macromolecule biosynthetic process;cellular macromolecule metabolic process;cellular metabolic process;cellu
biosynthetic process;cellular biosynthetic process;cellular macromolecule biosynthetic process;cellular macromolecule metabolic process;cellular metabolic process;cellular process;cellular protein metabolic process;cotrans
activation of immune response;activation of innate immune response;anaphase-promoting complex-dependent proteasomal ubiquitin-dependent protein catabolic process;antigen processing and presentation;antigen proces
anatomical structure development;anatomical structure morphogenesis;basement membrane assembly;basement membrane organization;biological regulation;cell morphogenesis;cell morphogenesis involved in differentiatio
acylglycerol metabolic process;aerobic respiration;aging;alcohol metabolic process;anatomical structure development;biological regulation;catabolic process;cell differentiation;cellular aromatic compound metabolic process;
carbohydrate metabolic process;carboxylic acid metabolic process;cellular ketone metabolic process;cellular metabolic process;cellular nitrogen compound metabolic process;cellular process;coenzyme metabolic process;c
amine transport;amino acid import;amino acid transport;aromatic amino acid transport;biological regulation;blood coagulation;branched-chain aliphatic amino acid transport;calcium ion transport;carbohydrate metabolic proc
4-hydroxyproline metabolic process;amine metabolic process;carboxylic acid metabolic process;cellular amine metabolic process;cellular amino acid metabolic process;cellular component organization;cellular component or
adherens junction assembly;adherens junction organization;anatomical structure development;biological adhesion;biological regulation;cell adhesion;cell junction assembly;cell junction organization;cell migration;cell motility;
amine metabolic process;amino acid activation;anatomical structure formation involved in morphogenesis;angiogenesis;biological regulation;biosynthetic process;carboxylic acid metabolic process;cellular amine metabolic p
biological regulation;blood coagulation;calcium ion homeostasis;calcium ion transmembrane transport;calcium ion transport;cation homeostasis;cation transport;cellular calcium ion homeostasis;cellular cation homeostasis;c
adipose tissue development;amine catabolic process;amine metabolic process;anatomical structure development;biosynthetic process;brain development;branched chain family amino acid catabolic process;branched chain
biological regulation;biosynthetic process;cellular biosynthetic process;cellular macromolecule biosynthetic process;cellular macromolecule metabolic process;cellular metabolic process;cellular nitrogen compound metabolic
alcohol biosynthetic process;alcohol catabolic process;alcohol metabolic process;biosynthetic process;carbohydrate biosynthetic process;carbohydrate catabolic process;carbohydrate metabolic process;catabolic process;c
anatomical structure morphogenesis;biological regulation;catabolic process;cell surface receptor linked signaling pathway;cellular catabolic process;cellular macromolecule catabolic process;cellular macromolecule metaboli
adult behavior;adult locomotory behavior;anatomical structure maturation;behavior;cellular component organization;cellular component organization at cellular level;cellular component organization or biogenesis;cellular comp
amine metabolic process;amino acid activation;carboxylic acid metabolic process;cellular amine metabolic process;cellular amino acid metabolic process;cellular ketone metabolic process;cellular macromolecule metabolic p
cellular process;endosome transport;establishment of localization;establishment of localization in cell;intracellular transport;transport;vesicle-mediated transport
biological regulation;cellular metabolic process;cellular process;cellular response to stimulus;intracellular signal transduction;metabolic process;peptide metabolic process;regulation of biological process;regulation of cellular
biological regulation;C21-steroid hormone metabolic process;carboxylic acid metabolic process;cell differentiation;cell surface receptor linked signaling pathway;cellular developmental process;cellular hormone metabolic pro
biosynthetic process;catabolic process;cellular biosynthetic process;cellular catabolic process;cellular component biogenesis;cellular component biogenesis at cellular level;cellular component disassembly;cellular componer
biosynthetic process;catabolic process;cellular biosynthetic process;cellular catabolic process;cellular component disassembly;cellular component disassembly at cellular level;cellular component organization;cellular compo

activation of immune response;activation of innate immune response;anaphase-promoting complex-dependent proteasomal ubiquitin-dependent protein catabolic process;antigen processing and presentation;antigen proces
amine biosynthetic process;amine metabolic process;anatomical structure development;aspartate family amino acid biosynthetic process;aspartate family amino acid metabolic process;biosynthetic process;carboxylic acid b
cellular macromolecule metabolic process;cellular metabolic process;cellular process;cellular protein metabolic process;macromolecule metabolic process;macromolecule modification;metabolic process;primary metabolic p
biological regulation;cellular macromolecule metabolic process;cellular metabolic process;cellular process;cellular protein metabolic process;establishment of localization;establishment of localization in cell;establishment of p
biological regulation;biosynthetic process;cellular biosynthetic process;cellular macromolecule biosynthetic process;cellular macromolecule metabolic process;cellular metabolic process;cellular nitrogen compound metabolic
biological regulation;regulation of anatomical structure morphogenesis;regulation of biological process;regulation of cell development;regulation of cell differentiation;regulation of cell morphogenesis;regulation of cell morpho
actin cytoskeleton organization;actin filament-based process;biological regulation;blood coagulation;cellular component organization;cellular component organization at cellular level;cellular component organization or biogen
biological regulation;cell cycle;cell cycle phase;cell cycle process;cell division;cell proliferation;cellular component organization;cellular component organization at cellular level;cellular component organization or biogenesis;c
actin cytoskeleton organization;actin filament-based process;ameboidal cell migration;anatomical structure development;anatomical structure formation involved in morphogenesis;anatomical structure morphogenesis;anteri
actin cytoskeleton organization;actin cytoskeleton reorganization;actin filament-based process;actomyosin structure organization;biological regulation;cell migration;cell motility;cellular component movement;cellular compon
2-oxoglutarate metabolic process;acetyl-CoA catabolic process;acetyl-CoA metabolic process;amine metabolic process;anatomical structure development;carbohydrate metabolic process;carboxylic acid metabolic process
activation of protein kinase activity;anatomical structure morphogenesis;biological regulation;cell part morphogenesis;cell projection morphogenesis;cell projection organization;cellular component morphogenesis;cellular con
activation of phospholipase A2 activity;activation of phospholipase A2 activity by calcium-mediated signaling;activation of phospholipase C activity;alkanesulfonate metabolic process;anatomical structure development;anato
biological regulation;biosynthetic process;cellular biosynthetic process;cellular macromolecule biosynthetic process;cellular macromolecule metabolic process;cellular metabolic process;cellular process;cellular protein metab
acetyl-CoA catabolic process;acetyl-CoA metabolic process;amine catabolic process;amine metabolic process;anatomical structure morphogenesis;aspartate family amino acid catabolic process;aspartate family amino acid
cellular component assembly;cellular component assembly at cellular level;cellular component organization;cellular component organization at cellular level;cellular component organization or biogenesis;cellular component c
biological regulation;blood coagulation;cellular component organization;cellular component organization at cellular level;cellular component organization or biogenesis;cellular component organization or biogenesis at cellular
amine metabolic process;B cell activation;B cell proliferation;biosynthetic process;carboxylic acid metabolic process;cell activation;cell proliferation;cellular amine metabolic process;cellular amino acid metabolic process;cell
adenosine biosynthetic process;adenosine metabolic process;AMP catabolic process;AMP metabolic process;biological adhesion;biological regulation;biosynthetic process;catabolic process;cell adhesion;cell-cell adhesion;
biosynthetic process;cellular biosynthetic process;cellular macromolecule biosynthetic process;cellular macromolecule metabolic process;cellular metabolic process;cellular nitrogen compound metabolic process;cellular pro
biological regulation;biosynthetic process;catabolic process;cellular biosynthetic process;cellular catabolic process;cellular component disassembly;cellular component disassembly at cellular level;cellular component organiz
macromolecule metabolic process;metabolic process;primary metabolic process;protein metabolic process;proteolysis
anatomical structure development;ATP-dependent chromatin remodeling;biosynthetic process;blastocyst development;cell cycle;cell proliferation;cellular biosynthetic process;cellular component assembly;cellular componen
biological regulation;biosynthetic process;carbohydrate metabolic process;carbohydrate transport;cell cycle;cell cycle process;cell surface receptor linked signaling pathway;cellular biosynthetic process;cellular component a
anatomical structure morphogenesis;antigen processing and presentation;antigen processing and presentation of exogenous antigen;antigen processing and presentation of exogenous peptide antigen;antigen processing an
biosynthetic process;catabolic process;cellular biosynthetic process;cellular catabolic process;cellular component biogenesis;cellular component biogenesis at cellular level;cellular component disassembly;cellular componen
alcohol biosynthetic process;alcohol catabolic process;alcohol metabolic process;aldonic acid metabolic process;biosynthetic process;carbohydrate biosynthetic process;carbohydrate catabolic process;carbohydrate metab
B cell activation;B cell activation involved in immune response;biological regulation;cell activation;cell activation involved in immune response;cell cycle process;cellular macromolecule metabolic process;cellular metabolic pr
cellular macromolecule metabolic process;cellular metabolic process;cellular nitrogen compound metabolic process;cellular process;gene expression;macromolecule metabolic process;metabolic process;mRNA metabolic p
activation of immune response;activation of innate immune response;anaphase-promoting complex-dependent proteasomal ubiquitin-dependent protein catabolic process;antigen processing and presentation;antigen proces
biosynthetic process;catabolic process;cellular biosynthetic process;cellular catabolic process;cellular component biogenesis;cellular component biogenesis at cellular level;cellular component disassembly;cellular componen
anatomical structure development;axon guidance;biological regulation;biosynthetic process;catabolic process;cell cycle checkpoint;cell cycle process;cellular biosynthetic process;cellular catabolic process;cellular componen
alcohol biosynthetic process;alcohol metabolic process;amine metabolic process;amino sugar biosynthetic process;amino sugar metabolic process;biological regulation;biosynthetic process;carbohydrate biosynthetic proces
actin cytoskeleton organization;actin filament severing;actin filament-based process;biological regulation;biosynthetic process;cellular biosynthetic process;cellular component organization;cellular component organization at
cellular macromolecule metabolic process;cellular metabolic process;cellular nitrogen compound metabolic process;cellular process;macromolecule metabolic process;maturation of 5.8S rRNA;maturation of 5.8S rRNA from
anatomical structure morphogenesis;assembly of spliceosomal tri-snRNP;biological regulation;cell morphogenesis;cell proliferation;cellular component assembly;cellular component assembly at cellular level;cellular compone
amine metabolic process;amino acid activation;biosynthetic process;bis(5'-nucleosidyl) oligophosphate biosynthetic process;bis(5'-nucleosidyl) oligophosphate metabolic process;carboxylic acid metabolic process;cellular a
apoptotic cell clearance;biological regulation;cell redox homeostasis;cellular component organization;cellular component organization or biogenesis;cellular homeostasis;cellular macromolecule metabolic process;cellular me
ATP-dependent chromatin remodeling;biological regulation;biosynthetic process;cell proliferation;cellular biosynthetic process;cellular component assembly;cellular component assembly at cellular level;cellular component or
cellular macromolecule metabolic process;cellular metabolic process;cellular process;cellular protein metabolic process;macromolecule metabolic process;macromolecule modification;metabolic process;primary metabolic p
cellular macromolecule metabolic process;cellular metabolic process;cellular nitrogen compound metabolic process;cellular process;macromolecule metabolic process;metabolic process;nitrogen compound metabolic proce
biological regulation;cellular macromolecule metabolic process;cellular metabolic process;cellular process;cellular protein metabolic process;macromolecule metabolic process;macromolecule modification;metabolic process
amine metabolic process;aminoglycan biosynthetic process;aminoglycan catabolic process;aminoglycan metabolic process;axon guidance;biological regulation;biosynthetic process;carbohydrate biosynthetic process;carbo
4-hydroxyproline metabolic process;amine metabolic process;carboxylic acid metabolic process;cellular amine metabolic process;cellular amino acid metabolic process;cellular ketone metabolic process;cellular macromolec
acyl-CoA biosynthetic process;acyl-CoA metabolic process;acylglycerol biosynthetic process;acylglycerol metabolic process;anatomical structure development;biological regulation;biosynthetic process;carboxylic acid meta
biological regulation;biosynthetic process;cellular biosynthetic process;cellular macromolecule biosynthetic process;cellular macromolecule metabolic process;cellular metabolic process;cellular process;cellular protein metab
activation of caspase activity;apoptosis;apoptosis in response to endoplasmic reticulum stress;biological regulation;catabolic process;cell death;cell differentiation;cell redox homeostasis;cellular catabolic process;cellular co
biosynthetic process;catabolic process;cellular biosynthetic process;cellular catabolic process;cellular component disassembly;cellular component disassembly at cellular level;cellular component organization;cellular compo
activation of immune response;activation of innate immune response;anaphase-promoting complex-dependent proteasomal ubiquitin-dependent protein catabolic process;antigen processing and presentation;antigen proces
actin cytoskeleton organization;actin filament-based process;aging;antigen processing and presentation;antigen processing and presentation of exogenous antigen;antigen processing and presentation of exogenous peptide
catabolic process;cellular catabolic process;cellular lipid catabolic process;cellular lipid metabolic process;cellular metabolic process;cellular process;cellular response to chemical stimulus;cellular response to hydrogen pero
apoptosis;biological regulation;catabolic process;cell cycle;cell cycle process;cell death;cell surface receptor linked signaling pathway;cellular catabolic process;cellular component assembly;cellular component organization;
activation of immune response;activation of innate immune response;anaphase-promoting complex-dependent proteasomal ubiquitin-dependent protein catabolic process;antigen processing and presentation;antigen proces
biological regulation;biosynthetic process;catabolic process;cellular biosynthetic process;cellular catabolic process;cellular component disassembly;cellular component disassembly at cellular level;cellular component organiz
anatomical structure development;androgen metabolic process;bile acid biosynthetic process;bile acid metabolic process;biological regulation;biosynthetic process;carboxylic acid biosynthetic process;carboxylic acid catab
cellular component organization;cellular component organization at cellular level;cellular component organization or biogenesis;cellular component organization or biogenesis at cellular level;cellular process;COPI coating of G
alcohol biosynthetic process;alcohol catabolic process;alcohol metabolic process;biosynthetic process;carbohydrate biosynthetic process;carbohydrate catabolic process;carbohydrate metabolic process;catabolic process;
actin cytoskeleton organization;actin filament organization;actin filament-based process;actin nucleation;anatomical structure formation involved in morphogenesis;Arp2/3 complex-mediated actin nucleation;asymmetric cell
biosynthetic process;catabolic process;cellular biosynthetic process;cellular catabolic process;cellular component disassembly;cellular component disassembly at cellular level;cellular component organization;cellular compo
activation-induced cell death of T cells;anatomical structure development;anatomical structure morphogenesis;apoptosis;biological regulation;biosynthetic process;carbohydrate homeostasis;catabolic process;cell activation
anatomical structure morphogenesis;biosynthetic process;catabolic process;cellular biosynthetic process;cellular catabolic process;cellular component disassembly;cellular component disassembly at cellular level;cellular co
antigen processing and presentation;antigen processing and presentation of exogenous antigen;antigen processing and presentation of exogenous peptide antigen;antigen processing and presentation of exogenous peptide
anatomical structure development;apoptosis;biological regulation;biosynthetic process;C21-steroid hormone catabolic process;C21-steroid hormone metabolic process;catabolic process;cell death;cellular biosynthetic proce
anatomical structure development;anatomical structure homeostasis;biological regulation;biosynthetic process;catabolic process;cell killing;cell proliferation;cellular biosynthetic process;cellular catabolic process;cellular mac
biological regulation;biosynthetic process;cardiac muscle contraction;cellular biosynthetic process;cellular component assembly;cellular component assembly at cellular level;cellular component organization;cellular compone
antigen processing and presentation;antigen processing and presentation of exogenous antigen;antigen processing and presentation of exogenous peptide antigen;antigen processing and presentation of exogenous peptide
aging;ameboidal cell migration;anatomical structure development;anatomical structure formation involved in morphogenesis;anatomical structure morphogenesis;axon ensheathment;biological adhesion;biological regulation;
biological regulation;biosynthetic process;cellular biosynthetic process;cellular macromolecule biosynthetic process;cellular macromolecule metabolic process;cellular metabolic process;cellular nitrogen compound metabolic
anatomical structure development;anatomical structure formation involved in morphogenesis;anatomical structure morphogenesis;apoptosis;apoptosis involved in morphogenesis;astrocyte cell migration;asymmetric protein l
biological regulation;establishment of localization;ion transport;positive regulation of biological process;positive regulation of cell communication;positive regulation of cellular process;positive regulation of response to stimulu
cell cycle;cell cycle phase;cell cycle process;cellular component organization;cellular component organization at cellular level;cellular component organization or biogenesis;cellular component organization or biogenesis at ce
antigen processing and presentation;antigen processing and presentation of exogenous antigen;antigen processing and presentation of exogenous peptide antigen;antigen processing and presentation of exogenous peptide
anatomical structure development;axon guidance;biological regulation;cellular component organization;cellular component organization at cellular level;cellular component organization or biogenesis;cellular component organ
biological regulation;cellular component assembly;cellular component assembly at cellular level;cellular component organization;cellular component organization at cellular level;cellular component organization or biogenesis;
ATP-dependent chromatin remodeling;biological regulation;biosynthetic process;cell cycle phase;cell cycle process;cell division;cellular biosynthetic process;cellular component assembly;cellular component assembly at cellu
cellular process;establishment of localization;transport;vesicle-mediated transport
cell cycle;cellular component organization;cellular component organization at cellular level;cellular component organization or biogenesis;cellular component organization or biogenesis at cellular level;cellular membrane fusio
biological regulation;biosynthetic process;carbohydrate metabolic process;carbohydrate transport;cell cycle;cell cycle process;cell surface receptor linked signaling pathway;cellular biosynthetic process;cellular component c
acyl-CoA biosynthetic process;acyl-CoA metabolic process;acylglycerol biosynthetic process;acylglycerol metabolic process;anatomical structure development;biological regulation;biosynthetic process;brain development;c
actin filament-based movement;actin filament-based process;actin-mediated cell contraction;actin-myosin filament sliding;cellular component movement;cellular process;multicellular organismal process;muscle contraction;
acetyl-CoA catabolic process;acetyl-CoA metabolic process;anatomical structure homeostasis;biological regulation;carboxylic acid metabolic process;catabolic process;cellular catabolic process;cellular ketone metabolic pr
biological regulation;cellular macromolecule metabolic process;cellular metabolic process;cellular nitrogen compound metabolic process;cellular process;gene expression;macromolecule metabolic process;metabolic proces
biological regulation;cell differentiation;cellular developmental process;cellular process;cellular response to stimulus;developmental process;epithelial cell differentiation;negative regulation of apoptosis;negative regulation of b
anion transport;apoptosis;behavior;behavioral defense response;behavioral fear response;cell communication;cell death;cell differentiation;cell-cell signaling;cellular developmental process;cellular process;cognition;death;de
apoptosis;biological regulation;biosynthetic process;catabolic process;cell cycle phase;cell cycle process;cell death;cell division;cellular biosynthetic process;cellular catabolic process;cellular component disassembly;cellula
amine metabolic process;carboxylic acid metabolic process;cellular amine metabolic process;cellular amino acid metabolic process;cellular ketone metabolic process;cellular metabolic process;cellular modified amino acid m
biosynthetic process;catabolic process;cellular biosynthetic process;cellular catabolic process;cellular component disassembly;cellular component disassembly at cellular level;cellular component organization;cellular compo
apoptotic mitochondrial changes;ATP metabolic process;biological regulation;cellular chemical homeostasis;cellular component organization;cellular component organization at cellular level;cellular component organization o
biological regulation;regulation of biological process;regulation of biosynthetic process;regulation of cellular biosynthetic process;regulation of cellular macromolecule biosynthetic process;regulation of cellular metabolic proc
biological regulation;catabolic process;cellular catabolic process;cellular macromolecule catabolic process;cellular macromolecule metabolic process;cellular metabolic process;cellular nitrogen compound metabolic process
biosynthetic process;catabolic process;cellular biosynthetic process;cellular catabolic process;cellular component biogenesis;cellular component biogenesis at cellular level;cellular component disassembly;cellular componen
biosynthetic process;catabolic process;cellular biosynthetic process;cellular catabolic process;cellular component disassembly;cellular component disassembly at cellular level;cellular component organization;cellular compo
activation of immune response;activation of innate immune response;anaphase-promoting complex-dependent proteasomal ubiquitin-dependent protein catabolic process;antigen processing and presentation;antigen proces
acetyl-CoA catabolic process;acetyl-CoA metabolic process;biological regulation;catabolic process;cation transport;cell redox homeostasis;cellular catabolic process;cellular homeostasis;cellular metabolic process;cellular p
biological regulation;cellular component assembly;cellular component assembly at cellular level;cellular component organization;cellular component organization at cellular level;cellular component organization or biogenesis;
biological regulation;biosynthetic process;catabolic process;cellular biosynthetic process;cellular catabolic process;cellular macromolecule biosynthetic process;cellular macromolecule catabolic process;cellular macromolecu
biological regulation;cellular component assembly;cellular component assembly at cellular level;cellular component organization;cellular component organization at cellular level;cellular component organization or biogenesis;
alcohol metabolic process;biosynthetic process;carbohydrate biosynthetic process;carbohydrate metabolic process;carboxylic acid biosynthetic process;carboxylic acid metabolic process;cellular biosynthetic process;cellula
anatomical structure formation involved in morphogenesis;biological regulation;biosynthetic process;cell proliferation;cell redox homeostasis;cellular biosynthetic process;cellular homeostasis;cellular lipid metabolic process;
biological regulation;biosynthetic process;cellular biosynthetic process;cellular macromolecule biosynthetic process;cellular macromolecule metabolic process;cellular metabolic process;cellular nitrogen compound metabolic
biological regulation;biosynthetic process;cellular biosynthetic process;cellular macromolecule biosynthetic process;cellular macromolecule metabolic process;cellular metabolic process;cellular process;cellular protein metab
biological regulation;biosynthetic process;carbohydrate metabolic process;carbohydrate transport;cell cycle;cell cycle process;cell surface receptor linked signaling pathway;cellular biosynthetic process;cellular component a
adherens junction maintenance;adherens junction organization;anatomical structure development;anatomical structure morphogenesis;biological adhesion;biological regulation;cardiac muscle tissue morphogenesis;cell adhe
biological regulation;biosynthetic process;cellular biosynthetic process;cellular macromolecule biosynthetic process;cellular macromolecule metabolic process;cellular metabolic process;cellular nitrogen compound metabolic
anatomical structure development;cell differentiation;cellular developmental process;cellular macromolecule metabolic process;cellular metabolic process;cellular process;cellular protein metabolic process;chaperone-mediat
biological regulation;cell cycle;cell cycle phase;cell cycle process;cell division;cell junction assembly;cell junction organization;cell surface receptor linked signaling pathway;cell-substrate junction assembly;cellular componen
actin cytoskeleton organization;actin filament-based process;ameboidal cell migration;biological regulation;cell migration;cell motility;cellular component movement;cellular component organization;cellular component organiz
amine catabolic process;amine metabolic process;carboxylic acid catabolic process;carboxylic acid metabolic process;catabolic process;cellular amine metabolic process;cellular amino acid catabolic process;cellular amino
aging;anatomical structure formation involved in morphogenesis;apoptosis;axon cargo transport;axon transport of mitochondrion;biological regulation;cell aging;cell death;cellular component movement;cellular component o
actin cytoskeleton organization;actin filament-based process;anatomical structure formation involved in morphogenesis;anatomical structure morphogenesis;axon guidance;biological regulation;cell part morphogenesis;cell p
biological regulation;cell differentiation;cellular developmental process;cellular process;developmental process;osteoblast differentiation;regulation of apoptosis;regulation of biological process;regulation of cell aging;regulatio
ATP-dependent chromatin remodeling;biological regulation;cellular component organization;cellular component organization at cellular level;cellular component organization or biogenesis;cellular component organization or b
anatomical structure formation involved in morphogenesis;anatomical structure morphogenesis;angiogenesis;antigen processing and presentation;antigen processing and presentation of exogenous antigen;antigen processin
biological regulation;catabolic process;cellular catabolic process;cellular macromolecule catabolic process;cellular macromolecule metabolic process;cellular metabolic process;cellular nitrogen compound metabolic process
anatomical structure development;biosynthetic process;cell cycle;cell proliferation;cell proliferation in forebrain;cellular aromatic compound metabolic process;cellular biosynthetic process;cellular component assembly;cellula
alternative nuclear mRNA splicing, via spliceosome;biological regulation;cellular macromolecule metabolic process;cellular metabolic process;cellular nitrogen compound metabolic process;cellular process;gene expression;
anatomical structure development;anatomical structure morphogenesis;aromatic compound biosynthetic process;biosynthetic process;brainstem development;cellular aromatic compound metabolic process;cellular biosynth
alcohol biosynthetic process;alcohol catabolic process;alcohol metabolic process;alditol catabolic process;alditol metabolic process;anatomical structure development;biosynthetic process;camera-type eye development;ca
biosynthetic process;cellular biosynthetic process;cellular component organization;cellular component organization at cellular level;cellular component organization or biogenesis;cellular component organization or biogenesis
alcohol catabolic process;alcohol metabolic process;apoptotic mitochondrial changes;biological regulation;body fluid secretion;carbohydrate catabolic process;carbohydrate homeostasis;carbohydrate metabolic process;ca
anatomical structure development;apoptosis;biological regulation;biosynthetic process;cell cycle;cell cycle process;cell death;cell migration;cell motility;cell surface receptor linked signaling pathway;cellular biosynthetic proc
biosynthetic process;catabolic process;cellular biosynthetic process;cellular catabolic process;cellular component disassembly;cellular component disassembly at cellular level;cellular component organization;cellular compo
biological regulation;biosynthetic process;cellular biosynthetic process;cellular component organization;cellular component organization at cellular level;cellular component organization or biogenesis;cellular component orga
actin filament-based movement;actin filament-based process;anatomical structure development;biological regulation;cell development;cell differentiation;cell surface receptor linked signaling pathway;cellular component mov
cellular component organization;cellular component organization at cellular level;cellular component organization or biogenesis;cellular component organization or biogenesis at cellular level;cellular process;Golgi organization

biological regulation;cell cycle;cell cycle process;cell division;cellular component organization;cellular component organization at cellular level;cellular component organization or biogenesis;cellular component organization or
biological regulation;cellular component assembly;cellular component assembly at cellular level;cellular component organization;cellular component organization at cellular level;cellular component organization or biogenesis;
cellular component organization;cellular component organization at cellular level;cellular component organization or biogenesis;cellular component organization or biogenesis at cellular level;cellular process;extracellular matr
biological regulation;biosynthetic process;carbohydrate metabolic process;carbohydrate transport;cell cycle;cell cycle process;cell surface receptor linked signaling pathway;cellular biosynthetic process;cellular component c
biological regulation;biosynthetic process;cellular biosynthetic process;cellular macromolecule biosynthetic process;cellular macromolecule metabolic process;cellular metabolic process;cellular nitrogen compound metabolic
cellular component assembly;cellular component assembly at cellular level;cellular component organization;cellular component organization at cellular level;cellular component organization or biogenesis;cellular component c
anatomical structure morphogenesis;biological regulation;biosynthetic process;cellular biosynthetic process;cellular component organization;cellular component organization at cellular level;cellular component organization o
biological regulation;cell cycle;cell surface receptor linked signaling pathway;cellular localization;cellular macromolecule localization;cellular process;cellular protein localization;cellular response to stimulus;enzyme linked rece
activation of immune response;activation of innate immune response;anaphase-promoting complex-dependent proteasomal ubiquitin-dependent protein catabolic process;antigen processing and presentation;antigen proces
alcohol metabolic process;amine metabolic process;aminoglycan biosynthetic process;aminoglycan metabolic process;anatomical structure morphogenesis;biosynthetic process;carbohydrate biosynthetic process;carbohyd
cellular macromolecule metabolic process;cellular metabolic process;cellular nitrogen compound metabolic process;cellular process;histone mRNA metabolic process;macromolecule metabolic process;macromolecule modi
alkene biosynthetic process;arachidonic acid metabolic process;biosynthetic process;carboxylic acid biosynthetic process;carboxylic acid metabolic process;catabolic process;cellular alkene metabolic process;cellular biosy
apoptotic chromosome condensation;biological regulation;cell cycle;cell cycle process;cell differentiation;cell division;cellular component organization;cellular component organization at cellular level;cellular component orga
amine metabolic process;carboxylic acid metabolic process;cellular amine metabolic process;cellular amino acid metabolic process;cellular ketone metabolic process;cellular metabolic process;cellular modified amino acid m
anatomical structure development;anatomical structure morphogenesis;axonogenesis;biological regulation;brain development;cell cycle process;cell part morphogenesis;cell projection morphogenesis;cell projection organiza
activation of immune response;activation of innate immune response;anaphase-promoting complex-dependent proteasomal ubiquitin-dependent protein catabolic process;anatomical structure development;antigen processi
anatomical structure development;apoptosis;biological regulation;biosynthetic process;cell death;cellular biosynthetic process;cellular component organization;cellular component organization or biogenesis;cellular macromo
anatomical structure development;apoptosis;apoptotic mitochondrial changes;biological regulation;biosynthetic process;cell death;cell development;cellular biosynthetic process;cellular component organization;cellular comp
anatomical structure morphogenesis;biological regulation;cellular component movement;cellular component organization;cellular component organization at cellular level;cellular component organization or biogenesis;cellular
biological regulation;biosynthetic process;catabolic process;cell chemotaxis;cell differentiation;cell migration;cell motility;cellular biosynthetic process;cellular catabolic process;cellular component assembly;cellular compone
anatomical structure homeostasis;base-excision repair;behavior;biological regulation;biosynthetic process;catabolic process;cell cycle;cellular biosynthetic process;cellular catabolic process;cellular component organization;
amine metabolic process;amino acid activation;biosynthetic process;carboxylic acid metabolic process;cellular amine metabolic process;cellular amino acid metabolic process;cellular biosynthetic process;cellular ketone me
amine metabolic process;biosynthetic process;carboxylic acid metabolic process;cellular amine metabolic process;cellular amino acid metabolic process;cellular aromatic compound metabolic process;cellular biosynthetic p
anatomical structure development;biological regulation;biosynthetic process;cell development;cellular component organization;cellular component organization at cellular level;cellular component organization or biogenesis;c
cellular macromolecule metabolic process;cellular metabolic process;cellular nitrogen compound metabolic process;cellular process;gene expression;macromolecule metabolic process;metabolic process;mRNA metabolic p
amine biosynthetic process;amine metabolic process;biosynthetic process;carboxylic acid biosynthetic process;carboxylic acid metabolic process;cellular amine metabolic process;cellular amino acid biosynthetic process;ce
actin cytoskeleton organization;actin filament organization;actin filament-based process;actin nucleation;anatomical structure morphogenesis;Arp2/3 complex-mediated actin nucleation;asymmetric cell division;axon guidanc
anatomical structure formation involved in morphogenesis;apoptosis;biological regulation;blastocyst formation;carbohydrate homeostasis;cell death;cell junction assembly;cell junction organization;cell-cell junction assembly
anatomical structure morphogenesis;biological regulation;cell cycle phase;cell cycle process;cell part morphogenesis;cell projection morphogenesis;cell projection organization;cellular component assembly;cellular compone
actin cytoskeleton organization;actin filament organization;actin filament-based process;anatomical structure morphogenesis;biological regulation;cell cycle phase;cell cycle process;cell division;cell morphogenesis;cell surfac
cellular process;endosome transport;establishment of localization;establishment of localization in cell;establishment of protein localization;intracellular protein transport;intracellular transport;protein transport;retrograde transp
establishment of localization;lipid transport;organic substance transport;transport
apoptosis;base-excision repair, DNA ligation;biological regulation;catabolic process;cell activation;cell chemotaxis;cell death;cell migration;cell motility;cell projection organization;cellular catabolic process;cellular component
aging;alcohol biosynthetic process;alcohol metabolic process;anatomical structure morphogenesis;apoptosis;biosynthetic process;carbohydrate biosynthetic process;carbohydrate metabolic process;cell death;cell differentia
cell differentiation;cellular developmental process;cellular process;cellular response to calcium ion;cellular response to chemical stimulus;cellular response to inorganic substance;cellular response to metal ion;cellular respons
biological regulation;blood coagulation;cellular process;coagulation;establishment of localization;hemostasis;ion transmembrane transport;ion transport;multicellular organismal process;regulation of biological quality;regulatio
anatomical structure development;biological adhesion;biological regulation;blood coagulation;brown fat cell differentiation;cell adhesion;cell adhesion mediated by integrin;cell communication;cell differentiation;cell junction a
biosynthetic process;catabolic process;cellular biosynthetic process;cellular catabolic process;cellular component disassembly;cellular component disassembly at cellular level;cellular component organization;cellular compo
biological regulation;biosynthetic process;cellular biosynthetic process;cellular macromolecule biosynthetic process;cellular macromolecule metabolic process;cellular metabolic process;cellular nitrogen compound metabolic
activation of MAPKK activity;activation of protein kinase activity;apoptosis;axon guidance;biological regulation;biosynthetic process;cell death;cell surface receptor linked signaling pathway;cellular biosynthetic process;cellul
biosynthetic process;carboxylic acid catabolic process;carboxylic acid metabolic process;cardiolipin acyl-chain remodeling;cardiolipin metabolic process;catabolic process;cellular biosynthetic process;cellular catabolic proc
2-oxoglutarate metabolic process;acetyl-CoA catabolic process;acetyl-CoA metabolic process;alcohol catabolic process;alcohol metabolic process;amine catabolic process;amine metabolic process;anatomical structure dev
biological regulation;biosynthetic process;carbohydrate metabolic process;carbohydrate transport;cell cycle;cell cycle process;cell surface receptor linked signaling pathway;cellular biosynthetic process;cellular component a
cellular component organization;cellular component organization at cellular level;cellular component organization or biogenesis;cellular component organization or biogenesis at cellular level;cellular process;cytoskeleton orga
acetyl-CoA catabolic process;acetyl-CoA metabolic process;carboxylic acid metabolic process;catabolic process;cellular catabolic process;cellular ketone metabolic process;cellular metabolic process;cellular process;citrate
biological regulation;blood coagulation;cell projection organization;cellular component assembly;cellular component organization;cellular component organization at cellular level;cellular component organization or biogenesis
biological regulation;negative regulation of biological process;negative regulation of cell proliferation;negative regulation of cellular process;regulation of biological process;regulation of cell proliferation;regulation of cellular pr
anatomical structure development;biological adhesion;cell adhesion;cell junction assembly;cell junction organization;cell-matrix adhesion;cell-substrate adhesion;cell-substrate junction assembly;cellular component assembly
cellular component movement;cellular component organization;cellular component organization at cellular level;cellular component organization or biogenesis;cellular component organization or biogenesis at cellular level;cel
biological regulation;cellular process;cellular response to stimulus;defense response;establishment of localization;establishment of localization in cell;establishment of protein localization;immune response;immune system pro
alcohol biosynthetic process;alcohol catabolic process;alcohol metabolic process;biological regulation;biosynthetic process;carbohydrate biosynthetic process;carbohydrate catabolic process;carbohydrate metabolic proces
aging;antigen processing and presentation;antigen processing and presentation of exogenous antigen;antigen processing and presentation of exogenous peptide antigen;antigen processing and presentation of exogenous p
anatomical structure development;cellular component organization;cellular component organization at cellular level;cellular component organization or biogenesis;cellular component organization or biogenesis at cellular level
acetyl-CoA catabolic process;acetyl-CoA metabolic process;alcohol biosynthetic process;alcohol metabolic process;biosynthetic process;carbohydrate biosynthetic process;carbohydrate metabolic process;carboxylic acid r
biological regulation;catabolic process;cellular catabolic process;cellular macromolecule catabolic process;cellular macromolecule metabolic process;cellular metabolic process;cellular process;cellular protein metabolic proc
anatomical structure development;androgen receptor signaling pathway;biological regulation;cation transport;cellular component assembly;cellular component assembly at cellular level;cellular component organization;cellula
biological regulation;biosynthetic process;catabolic process;cellular biosynthetic process;cellular catabolic process;cellular component disassembly;cellular component disassembly at cellular level;cellular component organiz
alcohol metabolic process;biosynthetic process;carbohydrate biosynthetic process;carbohydrate metabolic process;cellular biosynthetic process;cellular carbohydrate biosynthetic process;cellular carbohydrate metabolic pr
activation of store-operated calcium channel activity;anatomical structure development;anatomical structure morphogenesis;appendage morphogenesis;biological regulation;calcium ion transmembrane transport;calcium ion
activation of immune response;activation of innate immune response;anatomical structure development;autophagy;biological regulation;catabolic process;cell communication;cell differentiation;cell proliferation;cellular catabo
biological regulation;macromolecule metabolic process;metabolic process;positive regulation of catalytic activity;positive regulation of molecular function;primary metabolic process;protein metabolic process;proteolysis;regu
biological regulation;cell proliferation;cellular process;cellular response to stimulus;intracellular signal transduction;positive regulation of autophagy;positive regulation of biological process;positive regulation of biosynthetic p
biological regulation;defense response;defense response to virus;immune effector process;immune response;immune system process;innate immune response;multi-organism process;negative regulation of biological proces
biological regulation;cellular macromolecule metabolic process;cellular metabolic process;cellular process;cellular protein metabolic process;macromolecule metabolic process;macromolecule modification;metabolic process
biological regulation;biosynthetic process;catabolic process;cellular biosynthetic process;cellular catabolic process;cellular macromolecule biosynthetic process;cellular macromolecule catabolic process;cellular macromolecu
alkene biosynthetic process;biosynthetic process;carboxylic acid biosynthetic process;carboxylic acid metabolic process;cellular alkene metabolic process;cellular biosynthetic process;cellular ketone metabolic process;cellu
anatomical structure development;cellular component organization;cellular component organization at cellular level;cellular component organization or biogenesis;cellular component organization or biogenesis at cellular level
antigen processing and presentation;antigen processing and presentation of exogenous antigen;antigen processing and presentation of exogenous peptide antigen;antigen processing and presentation of exogenous peptide
anatomical structure development;bone development;developmental process
acetyl-CoA catabolic process;acetyl-CoA metabolic process;anatomical structure development;carboxylic acid metabolic process;catabolic process;cellular catabolic process;cellular ketone metabolic process;cellular metab
amine biosynthetic process;amine metabolic process;biological regulation;biosynthetic process;carboxylic acid biosynthetic process;carboxylic acid metabolic process;cellular amine metabolic process;cellular amino acid bio
biosynthetic process;cellular biosynthetic process;cellular macromolecule biosynthetic process;cellular macromolecule metabolic process;cellular metabolic process;cellular process;cellular protein metabolic process;establis
anatomical structure development;biological regulation;biosynthetic process;catabolic process;cell communication;cell cycle arrest;cell cycle process;cell development;cell growth;cell projection organization;cell surface rece
biological regulation;biosynthetic process;catabolic process;cellular biosynthetic process;cellular catabolic process;cellular component disassembly;cellular component disassembly at cellular level;cellular component organiz
amine transport;apoptosis;biological regulation;biosynthetic process;blood coagulation;cell activation;cell death;cellular biosynthetic process;cellular component assembly;cellular component assembly at cellular level;cellula
biological regulation;cell cycle arrest;cell cycle process;cell death;cell surface receptor linked signaling pathway;cellular process;cellular response to chemical stimulus;cellular response to cytokine stimulus;cellular response t
biological adhesion;biological regulation;cell adhesion;cell cycle arrest;cell cycle process;cell junction assembly;cell junction organization;cell motility;cell surface receptor linked signaling pathway;cell-substrate junction asse
cellular macromolecule metabolic process;cellular metabolic process;cellular nitrogen compound metabolic process;cellular process;gene expression;macromolecule metabolic process;metabolic process;mRNA metabolic p
biological regulation;biosynthetic process;cell cycle;cellular biosynthetic process;cellular component organization;cellular component organization at cellular level;cellular component organization or biogenesis;cellular compo
anatomical structure development;anatomical structure morphogenesis;biological regulation;biosynthetic process;branching involved in mammary gland duct morphogenesis;branching morphogenesis of a tube;cellular biosy
apoptosis;biological regulation;biosynthetic process;cell death;cellular biosynthetic process;cellular macromolecule biosynthetic process;cellular macromolecule metabolic process;cellular metabolic process;cellular nitrogen
biological regulation;biosynthetic process;cell cycle arrest;cell cycle process;cell proliferation;cellular biosynthetic process;cellular macromolecule biosynthetic process;cellular macromolecule metabolic process;cellular meta
androgen receptor signaling pathway;biological regulation;biosynthetic process;catabolic process;cellular biosynthetic process;cellular catabolic process;cellular macromolecule catabolic process;cellular macromolecule met
amine biosynthetic process;amine metabolic process;anatomical structure development;aspartate family amino acid metabolic process;biological regulation;biosynthetic process;brain development;carboxylic acid biosynthet
cellular macromolecule metabolic process;cellular metabolic process;cellular nitrogen compound metabolic process;cellular process;gene expression;macromolecule metabolic process;metabolic process;mRNA metabolic p
cellular component organization;cellular component organization at cellular level;cellular component organization or biogenesis;cellular component organization or biogenesis at cellular level;cellular localization;cellular proces
anatomical structure development;biological regulation;catabolic process;cellular catabolic process;cellular macromolecule catabolic process;cellular macromolecule metabolic process;cellular metabolic process;cellular nitro
aging;ATP-dependent chromatin remodeling;biological regulation;cell aging;cell cycle process;cellular component assembly;cellular component assembly at cellular level;cellular component organization;cellular component o
biological regulation;catabolic process;cellular catabolic process;cellular component disassembly;cellular component disassembly at cellular level;cellular component organization;cellular component organization at cellular le
anatomical structure development;biosynthetic process;cell activation;cell proliferation;cellular aromatic compound metabolic process;cellular biosynthetic process;cellular component assembly;cellular component organizati
acetyl-CoA catabolic process;acetyl-CoA metabolic process;biological regulation;carboxylic acid metabolic process;catabolic process;cation homeostasis;cellular catabolic process;cellular cation homeostasis;cellular chemic
biosynthetic process;cellular aromatic compound metabolic process;cellular biosynthetic process;cellular metabolic process;cellular nitrogen compound biosynthetic process;cellular nitrogen compound metabolic process;ce
biosynthetic process;cellular biosynthetic process;cellular macromolecule biosynthetic process;cellular macromolecule metabolic process;cellular metabolic process;cellular process;cellular protein metabolic process;gene ex
biological regulation;cellular macromolecule metabolic process;cellular metabolic process;cellular nitrogen compound metabolic process;cellular process;gene expression;macromolecule metabolic process;metabolic proces
alcohol biosynthetic process;alcohol catabolic process;alcohol metabolic process;biosynthetic process;carbohydrate biosynthetic process;carbohydrate catabolic process;carbohydrate metabolic process;catabolic process;
amine metabolic process;amino acid activation;arginyl-tRNA aminoacylation;carboxylic acid metabolic process;cellular amine metabolic process;cellular amino acid metabolic process;cellular ketone metabolic process;cellula
biosynthetic process;catabolic process;cellular biosynthetic process;cellular catabolic process;cellular component disassembly;cellular component disassembly at cellular level;cellular component organization;cellular compo
axon guidance;cell cycle phase;cell cycle process;cellular component assembly;cellular component assembly at cellular level;cellular component organization;cellular component organization at cellular level;cellular compone
cellular component assembly;cellular component assembly at cellular level;cellular component organization;cellular component organization at cellular level;cellular component organization or biogenesis;cellular component c
cellular component assembly;cellular component assembly at cellular level;cellular component organization;cellular component organization at cellular level;cellular component organization or biogenesis;cellular component c
anatomical structure formation involved in morphogenesis;angiogenesis;biological regulation;biosynthetic process;cell differentiation;cellular biosynthetic process;cellular developmental process;cellular macromolecule biosyn
actin filament-based movement;actin filament-based process;anatomical structure development;anatomical structure morphogenesis;auditory receptor cell differentiation;behavior;biological regulation;cell communication;cel
anatomical structure formation involved in morphogenesis;angiogenesis;ATP biosynthetic process;ATP hydrolysis coupled proton transport;ATP metabolic process;ATP synthesis coupled proton transport;biological regulation
3'-UTR-mediated mRNA stabilization;ATP-dependent chromatin remodeling;biological regulation;cell differentiation;cellular component organization;cellular component organization at cellular level;cellular component organiz
actin cytoskeleton organization;actin filament-based movement;actin filament-based process;actin-mediated cell contraction;actin-myosin filament sliding;actomyosin structure organization;anatomical structure morphogene
amine metabolic process;amino acid activation;biosynthetic process;carboxylic acid metabolic process;cellular amine metabolic process;cellular amino acid metabolic process;cellular biosynthetic process;cellular componen
cellular macromolecule metabolic process;cellular metabolic process;cellular nitrogen compound metabolic process;cellular process;gene expression;macromolecule metabolic process;metabolic process;mRNA metabolic p
anatomical structure development;anatomical structure morphogenesis;axon guidance;biological adhesion;biological regulation;blood coagulation;cell adhesion;cell adhesion mediated by integrin;cell differentiation;cell migra
anatomical structure development;cell activation;cell migration;cell motility;cell proliferation;cellular component movement;cellular process;developmental process;epidermis development;keratinization;keratinocyte activation
binding of sperm to zona pellucida;cell recognition;cell-cell recognition;cellular macromolecule metabolic process;cellular metabolic process;cellular process;cellular process involved in reproduction;cellular protein metabolic
biological regulation;biosynthetic process;catabolic process;cellular biosynthetic process;cellular catabolic process;cellular component disassembly;cellular component disassembly at cellular level;cellular component organiz
biological regulation;cellular macromolecule metabolic process;cellular metabolic process;cellular nitrogen compound metabolic process;cellular process;cellular response to stimulus;gene expression;macromolecule metabo
apoptosis;biological regulation;biosynthetic process;cell cycle;cell cycle process;cell death;cellular biosynthetic process;cellular component organization;cellular component organization at cellular level;cellular component org
actin cytoskeleton organization;actin filament organization;actin filament-based movement;actin filament-based process;actin-mediated cell contraction;actin-myosin filament sliding;actomyosin structure organization;anatom
anatomical structure development;antigen processing and presentation;antigen processing and presentation of exogenous antigen;antigen processing and presentation of exogenous peptide antigen;antigen processing and
catabolic process;cellular catabolic process;cellular macromolecule catabolic process;cellular macromolecule metabolic process;cellular metabolic process;cellular process;ER-associated protein catabolic process;macromo
biological regulation;biosynthetic process;cellular biosynthetic process;cellular component assembly;cellular component assembly at cellular level;cellular component organization;cellular component organization at cellular le
biological regulation;biosynthetic process;cellular biosynthetic process;cellular macromolecule biosynthetic process;cellular macromolecule metabolic process;cellular metabolic process;cellular nitrogen compound metabolic
biological regulation;biosynthetic process;cell proliferation;cellular biosynthetic process;cellular macromolecule biosynthetic process;cellular macromolecule metabolic process;cellular metabolic process;cellular nitrogen com
anatomical structure morphogenesis;apoptosis;apoptotic mitochondrial changes;biological regulation;cell death;cell part morphogenesis;cellular component assembly;cellular component assembly at cellular level;cellular co
amine metabolic process;biosynthetic process;carboxylic acid metabolic process;cellular amine metabolic process;cellular amino acid metabolic process;cellular aromatic compound metabolic process;cellular biosynthetic p
amine metabolic process;amino acid activation;anatomical structure development;brain development;carboxylic acid metabolic process;cellular amine metabolic process;cellular amino acid metabolic process;cellular ketone
biosynthetic process;catabolic process;cellular biosynthetic process;cellular catabolic process;cellular component biogenesis;cellular component biogenesis at cellular level;cellular component disassembly;cellular componen
biological regulation;cellular macromolecule metabolic process;cellular metabolic process;cellular process;cellular protein metabolic process;chaperone-mediated protein folding;macromolecule metabolic process;metabolic
biological regulation;biosynthetic process;cellular biosynthetic process;cellular macromolecule biosynthetic process;cellular macromolecule metabolic process;cellular metabolic process;cellular nitrogen compound metabolic

anatomical structure arrangement;anatomical structure development;axon extension;biological regulation;biosynthetic process;BMP signaling pathway;catabolic process;cell cycle phase;cell cycle process;cell division;cell gr
cellular component organization;cellular component organization at cellular level;cellular component organization or biogenesis;cellular component organization or biogenesis at cellular level;cellular macromolecule metabolic
biological regulation;cell differentiation;cellular developmental process;cellular macromolecule metabolic process;cellular metabolic process;cellular nitrogen compound metabolic process;cellular process;cellular response to
antigen processing and presentation;antigen processing and presentation of exogenous antigen;antigen processing and presentation of exogenous peptide antigen;antigen processing and presentation of exogenous peptide
adenine transport;biological regulation;cellular metabolic process;cellular process;chromosome segregation;energy derivation by oxidation of organic compounds;energy reserve metabolic process;establishment of localizatio
actin cytoskeleton organization;actin filament-based process;actomyosin structure organization;anatomical structure homeostasis;anatomical structure morphogenesis;associative learning;axon extension;axon guidance;B ce
anatomical structure development;anatomical structure formation involved in morphogenesis;biological regulation;blastocyst development;cellular process;cellular response to acid;cellular response to amine stimulus;cellular
biological regulation;biosynthetic process;cellular biosynthetic process;cellular macromolecule biosynthetic process;cellular macromolecule metabolic process;cellular metabolic process;cellular nitrogen compound metaboli
cellular macromolecule metabolic process;cellular metabolic process;cellular nitrogen compound metabolic process;cellular process;gene expression;macromolecule metabolic process;metabolic process;mRNA metabolic p
anatomical structure formation involved in morphogenesis;angiogenesis;biological adhesion;biological regulation;cell adhesion;cell differentiation;cell proliferation;cellular component organization;cellular component organizat
axon extension;biological regulation;cell growth;cell projection assembly;cell projection organization;cell surface receptor linked signaling pathway;cellular component assembly;cellular component assembly at cellular level;c
cell differentiation;cellular developmental process;cellular process;developmental process;hemopoietic progenitor cell differentiation
biological regulation;cellular process;cellular response to acid;cellular response to amine stimulus;cellular response to amino acid stimulus;cellular response to chemical stimulus;cellular response to endogenous stimulus;cell
ameboidal cell migration;anatomical structure development;anatomical structure formation involved in morphogenesis;anatomical structure morphogenesis;angiogenesis;biological adhesion;biological regulation;branching inv
anatomical structure morphogenesis;biological regulation;cell cycle;cell surface receptor linked signaling pathway;cellular component organization;cellular component organization at cellular level;cellular component organizat
anatomical structure development;anatomical structure morphogenesis;axon guidance;biological adhesion;biological regulation;cell adhesion;cell junction assembly;cell junction organization;cell migration;cell morphogenesis
active induction of host immune response by virus;adenine nucleotide transport;ADP transport;apoptosis;ATP transport;biological regulation;cell death;cellular macromolecule metabolic process;cellular metabolic process;cel
amine metabolic process;amino acid activation;aspartyl-tRNA aminoacylation;biosynthetic process;carboxylic acid metabolic process;cellular amine metabolic process;cellular amino acid metabolic process;cellular biosynthe
aging;anatomical structure development;anatomical structure morphogenesis;biological regulation;cell differentiation;cell part morphogenesis;cell projection morphogenesis;cell projection organization;cellular component mor
activation of protein kinase activity;apoptosis;biological regulation;cell death;cellular macromolecule metabolic process;cellular metabolic process;cellular process;cellular protein metabolic process;cellular response to stimul
biological regulation;cell growth;cellular process;growth;negative regulation of apoptosis;negative regulation of biological process;negative regulation of cell death;negative regulation of cellular process;negative regulation of
biological regulation;biosynthetic process;cellular biosynthetic process;cellular component organization;cellular component organization at cellular level;cellular component organization or biogenesis;cellular component orga
assembly of spliceosomal tri-snRNP;biological regulation;cellular component assembly;cellular component assembly at cellular level;cellular component organization;cellular component organization at cellular level;cellular co
actin cytoskeleton organization;actin filament capping;actin filament organization;actin filament polymerization;actin filament severing;actin filament-based process;actin nucleation;actin polymerization or depolymerization;ag
actin filament-based movement;actin filament-based process;actin-mediated cell contraction;actin-myosin filament sliding;biological regulation;cellular component movement;cellular process;multicellular organismal process
anatomical structure development;cell junction assembly;cell junction organization;cell-substrate junction assembly;cellular component assembly;cellular component assembly at cellular level;cellular component organization
actin cytoskeleton organization;actin filament organization;actin filament-based process;adherens junction organization;aging;anatomical structure development;anatomical structure morphogenesis;apical junction assembly;
amine metabolic process;amino acid activation;carboxylic acid metabolic process;cellular amine metabolic process;cellular amino acid metabolic process;cellular ketone metabolic process;cellular macromolecule metabolic
biological regulation;biosynthetic process;calcium ion homeostasis;cation homeostasis;cellular biosynthetic process;cellular calcium ion homeostasis;cellular cation homeostasis;cellular chemical homeostasis;cellular divalen
apoptosis;biological regulation;catabolic process;cell death;cell surface receptor linked signaling pathway;cellular catabolic process;cellular component disassembly;cellular component disassembly at cellular level;cellular co
biological regulation;cellular macromolecule metabolic process;cellular metabolic process;cellular nitrogen compound metabolic process;cellular process;macromolecule metabolic process;metabolic process;mRNA metaboli
biological regulation;biosynthetic process;cellular biosynthetic process;cellular macromolecule biosynthetic process;cellular macromolecule metabolic process;cellular metabolic process;cellular nitrogen compound metaboli
ATP-dependent chromatin remodeling;biological regulation;biosynthetic process;cellular biosynthetic process;cellular component assembly;cellular component assembly at cellular level;cellular component organization;cellul
cell cycle;cell cycle process;cell division;cellular component organization;cellular component organization at cellular level;cellular component organization or biogenesis;cellular component organization or biogenesis at cellula
aging;anatomical structure development;cell differentiation;cell junction assembly;cell junction organization;cell-substrate junction assembly;cellular component assembly;cellular component assembly at cellular level;cellular
amine metabolic process;anatomical structure development;aromatic compound biosynthetic process;biosynthetic process;brainstem development;carboxylic acid metabolic process;cellular amine metabolic process;cellula
aging;biological regulation;catabolic process;cellular catabolic process;cellular component assembly;cellular component assembly at cellular level;cellular component organization;cellular component organization at cellular le
biological regulation;biosynthetic process;cellular biosynthetic process;cellular component assembly;cellular component assembly at cellular level;cellular component organization;cellular component organization at cellular le
cellular component organization;cellular component organization at cellular level;cellular component organization or biogenesis;cellular component organization or biogenesis at cellular level;cellular membrane fusion;cellular
biological regulation;biosynthetic process;cellular biosynthetic process;cellular component organization;cellular component organization at cellular level;cellular component organization or biogenesis;cellular component orga
biological regulation;negative regulation of apoptosis;negative regulation of biological process;negative regulation of cell death;negative regulation of cellular process;negative regulation of programmed cell death;regulation o
alternative nuclear mRNA splicing, via spliceosome;cellular macromolecule metabolic process;cellular metabolic process;cellular nitrogen compound metabolic process;cellular process;establishment of localization;establishm
biological regulation;biosynthetic process;catabolic process;cellular biosynthetic process;cellular catabolic process;cellular macromolecule biosynthetic process;cellular macromolecule catabolic process;cellular macromolec
actin cytoskeleton organization;actin filament-based process;activation of adenylate cyclase activity;ameboidal cell migration;axon guidance;biological regulation;blood coagulation;cell activation;cell migration;cell motility;cel
cellular component movement;cellular process;multicellular organismal process;muscle contraction;muscle system process;system process
acetyl-CoA metabolic process;acyl-CoA biosynthetic process;acyl-CoA metabolic process;acylglycerol biosynthetic process;acylglycerol metabolic process;amine transport;amino acid transmembrane transport;amino acid t
biological regulation;cellular component assembly;cellular component organization;cellular component organization or biogenesis;cellular macromolecule metabolic process;cellular metabolic process;cellular nitrogen compo
cellular macromolecule metabolic process;cellular metabolic process;cellular nitrogen compound metabolic process;cellular process;macromolecule metabolic process;metabolic process;mRNA metabolic process;mRNA pro
apoptosis;biological regulation;cell death;cellular component disassembly;cellular component disassembly at cellular level;cellular component disassembly involved in apoptosis;cellular component organization;cellular comp
biological regulation;cellular component organization;cellular component organization or biogenesis;cellular macromolecule metabolic process;cellular membrane organization;cellular metabolic process;cellular process;cellul
cellular macromolecule metabolic process;cellular metabolic process;cellular nitrogen compound metabolic process;cellular process;macromolecule metabolic process;metabolic process;mRNA metabolic process;mRNA pro
cellular macromolecule metabolic process;cellular metabolic process;cellular process;cellular protein metabolic process;macromolecule metabolic process;metabolic process;primary metabolic process;protein folding;protei
amine biosynthetic process;amine catabolic process;amine metabolic process;anatomical structure development;biological regulation;biosynthetic process;carboxylic acid biosynthetic process;carboxylic acid catabolic proc
biological regulation;cation homeostasis;cation transport;cell differentiation;cellular cation homeostasis;cellular chemical homeostasis;cellular developmental process;cellular homeostasis;cellular ion homeostasis;cellular iron
biological regulation;cell activation;cell activation involved in immune response;cellular component organization;cellular component organization or biogenesis;cellular membrane organization;cellular process;defense respons
biosynthetic process;catabolic process;cellular biosynthetic process;cellular catabolic process;cellular component biogenesis;cellular component biogenesis at cellular level;cellular component disassembly;cellular componen
biosynthetic process;cell cycle;cell cycle process;cellular biosynthetic process;cellular macromolecule biosynthetic process;cellular macromolecule metabolic process;cellular metabolic process;cellular nitrogen compound m
biosynthetic process;catabolic process;cellular biosynthetic process;cellular catabolic process;cellular component biogenesis;cellular component biogenesis at cellular level;cellular component disassembly;cellular componen
apoptosis;cell death;cell proliferation;cellular process;death;establishment of localization;establishment of localization in cell;establishment of protein localization;intracellular protein transport;intracellular transport;nuclear exp
adenosine to inosine editing;anatomical structure development;base conversion or substitution editing;biological regulation;cell differentiation;cell surface receptor linked signaling pathway;cellular component assembly;cellul
anatomical structure morphogenesis;biological regulation;biosynthetic process;catabolic process;cell surface receptor linked signaling pathway;cellular biosynthetic process;cellular catabolic process;cellular macromolecule
actin filament-based movement;actin filament-based process;actin-mediated cell contraction;actin-myosin filament sliding;cell differentiation;cellular component movement;cellular developmental process;cellular process;dev
cell cycle phase;cell cycle process;cell division;cellular component organization;cellular component organization at cellular level;cellular component organization or biogenesis;cellular component organization or biogenesis at
cellular macromolecule metabolic process;cellular metabolic process;cellular process;cellular protein metabolic process;chaperone-mediated protein folding;macromolecule metabolic process;macromolecule modification;me
carbohydrate metabolic process;cellular carbohydrate metabolic process;cellular macromolecule metabolic process;cellular metabolic process;cellular process;cellular protein metabolic process;'de novo' posttranslational pr
anatomical structure morphogenesis;axon guidance;biological adhesion;biological regulation;cell adhesion;cell differentiation;cell morphogenesis;cell morphogenesis involved in differentiation;cell projection organization;cell-
biological regulation;biosynthetic process;cell death;cell division;cellular biosynthetic process;cellular component organization;cellular component organization at cellular level;cellular component organization or biogenesis;ce
alcohol biosynthetic process;alcohol catabolic process;alcohol metabolic process;amine metabolic process;anatomical structure development;biological regulation;biosynthetic process;carbohydrate biosynthetic process;car
alcohol biosynthetic process;alcohol metabolic process;biological regulation;biosynthetic process;biotin metabolic process;carbohydrate biosynthetic process;carbohydrate metabolic process;carboxylic acid metabolic proce
anatomical structure formation involved in morphogenesis;angiogenesis;angiotensin maturation;biological regulation;cell differentiation;cellular developmental process;cellular macromolecule metabolic process;cellular metab
amine metabolic process;amino acid activation;biosynthetic process;bis(5'-nucleosidyl) oligophosphate biosynthetic process;bis(5'-nucleosidyl) oligophosphate metabolic process;carboxylic acid metabolic process;cellular a
binding of sperm to zona pellucida;cell recognition;cell-cell recognition;cellular component assembly;cellular component assembly at cellular level;cellular component organization;cellular component organization at cellular le
5-phosphoribose 1-diphosphate biosynthetic process;5-phosphoribose 1-diphosphate metabolic process;alcohol biosynthetic process;alcohol metabolic process;biological regulation;biosynthetic process;carbohydrate bios
binding of sperm to zona pellucida;biological regulation;cell recognition;cell-cell recognition;cellular component assembly;cellular component assembly at cellular level;cellular component organization;cellular component orga
biological regulation;posttranscriptional regulation of gene expression;regulation of biological process;regulation of gene expression;regulation of macromolecule metabolic process;regulation of metabolic process
biological regulation;blood coagulation;cell activation;cell cycle;cell cycle process;cellular component assembly;cellular component assembly at cellular level;cellular component organization;cellular component organization a
biological regulation;cell cycle;cell cycle process;cell killing;cellular component assembly;cellular component assembly at cellular level;cellular component movement;cellular component organization;cellular component orga
anatomical structure homeostasis;biological regulation;biosynthetic process;catabolic process;cellular biosynthetic process;cellular catabolic process;cellular component organization;cellular component organization at cellul
actin cytoskeleton organization;actin filament bundle assembly;actin filament organization;actin filament-based process;biological regulation;cell projection organization;cellular component assembly;cellular component asser
activation of immune response;anatomical structure homeostasis;biological regulation;complement activation;complement activation, lectin pathway;defense response;fibrinolysis;homeostatic process;humoral immune respo
biological regulation;cell cycle;cell cycle process;cell division;cell killing;cellular component assembly;cellular component assembly at cellular level;cellular component movement;cellular component organization;cellular comp
anatomical structure development;anatomical structure homeostasis;biological regulation;biosynthetic process;brain development;cellular biosynthetic process;cellular component organization;cellular component organizatio
anatomical structure development;anatomical structure homeostasis;biological regulation;biosynthetic process;brain development;cell differentiation;cell proliferation;cellular biosynthetic process;cellular component organizat
biological regulation;cellular macromolecule metabolic process;cellular metabolic process;cellular nitrogen compound metabolic process;cellular process;establishment of localization;establishment of RNA localization;gene e
biological regulation;biosynthetic process;cellular biosynthetic process;cellular component organization;cellular component organization at cellular level;cellular component organization or biogenesis;cellular component orga
alkaloid metabolic process;biological regulation;biosynthetic process;cell communication;cell-cell signaling;cellular amide metabolic process;cellular biosynthetic process;cellular metabolic process;cellular nitrogen compoun
biosynthetic process;cell cycle;cell cycle process;cellular biosynthetic process;cellular component assembly;cellular component assembly at cellular level;cellular component organization;cellular component organization at c
binding of sperm to zona pellucida;cell recognition;cell-cell recognition;cellular macromolecule metabolic process;cellular metabolic process;cellular process;cellular process involved in reproduction;cellular protein metabolic
alcohol metabolic process;carbohydrate metabolic process;carbohydrate phosphorylation;cellular carbohydrate metabolic process;cellular metabolic process;cellular process;fructose 6-phosphate metabolic process;fructose
biological regulation;biosynthetic process;catabolic process;cell surface receptor linked signaling pathway;cellular biosynthetic process;cellular catabolic process;cellular component organization;cellular component organizat
anatomical structure morphogenesis;biological regulation;blood coagulation;bone morphogenesis;cell migration;cell motility;cellular component movement;cellular process;coagulation;developmental process;hemostasis;imn
apoptosis;apoptotic chromosome condensation;biological regulation;cell death;cell differentiation;cellular component disassembly;cellular component disassembly at cellular level;cellular component disassembly involved in
adherens junction organization;anatomical structure development;biological adhesion;biological regulation;biosynthetic process;brain development;cell adhesion;cell junction assembly;cell junction organization;cell surface re
biological regulation;biosynthetic process;catabolic process;cell surface receptor linked signaling pathway;cellular biosynthetic process;cellular catabolic process;cellular macromolecule biosynthetic process;cellular macrom
alanyl-tRNA aminoacylation;amine metabolic process;amino acid activation;anatomical structure development;biological regulation;carboxylic acid metabolic process;cellular amine metabolic process;cellular amino acid meta
antigen processing and presentation;antigen processing and presentation of peptide antigen;antigen processing and presentation of peptide antigen via MHC class I;cellular macromolecule metabolic process;cellular metabo
biological regulation;cell proliferation;establishment of localization;establishment of protein localization;establishment of RNA localization;macromolecule metabolic process;metabolic process;mRNA transport;negative regula
ATP-dependent chromatin remodeling;biological regulation;biosynthetic process;cellular biosynthetic process;cellular component assembly;cellular component assembly at cellular level;cellular component organization;cellul
anatomical structure homeostasis;biological regulation;biosynthetic process;cell cycle process;cellular biosynthetic process;cellular component organization;cellular component organization at cellular level;cellular componen
cell division;cellular component assembly;cellular component assembly at cellular level;cellular component organization;cellular component organization at cellular level;cellular component organization or biogenesis;cellular c
anatomical structure morphogenesis;biological regulation;cell differentiation;cellular developmental process;cellular process;developmental process;morphogenesis of an epithelium;positive regulation of biological process;po
activation of MAPK activity;anatomical structure development;anatomical structure formation involved in morphogenesis;anatomical structure morphogenesis;angiogenesis;behavior;behavioral response to pain;biological adh
viral reproduction
biological regulation;cellular process;cellular response to stimulus;establishment of localization;establishment of protein localization;intracellular signal transduction;protein transport;regulation of biological process;regulation
biosynthetic process;cell cycle;cell cycle process;cellular biosynthetic process;cellular macromolecule biosynthetic process;cellular macromolecule metabolic process;cellular metabolic process;cellular nitrogen compound m
biological regulation;cell differentiation;cellular component assembly;cellular component assembly at cellular level;cellular component organization;cellular component organization at cellular level;cellular component organiza
binding of sperm to zona pellucida;cell recognition;cell-cell recognition;cellular macromolecule metabolic process;cellular metabolic process;cellular process;cellular process involved in reproduction;cellular protein metabolic
cell cycle;cell cycle process;cell division;cellular component organization;cellular component organization at cellular level;cellular component organization or biogenesis;cellular component organization or biogenesis at cellula
biosynthetic process;carbohydrate metabolic process;cellular biosynthetic process;cellular carbohydrate metabolic process;cellular macromolecule biosynthetic process;cellular macromolecule metabolic process;cellular met
biological regulation;blood coagulation;cellular process;cellular response to stimulus;coagulation;hemostasis;multicellular organismal process;negative regulation of apoptosis;negative regulation of biological process;negative
biosynthetic process;cell cycle;cell cycle process;cellular biosynthetic process;cellular macromolecule biosynthetic process;cellular macromolecule metabolic process;cellular metabolic process;cellular nitrogen compound m
amine metabolic process;amino acid activation;apoptosis;biological regulation;carboxylic acid metabolic process;cell death;cellular amine metabolic process;cellular amino acid metabolic process;cellular ketone metabolic p
anatomical structure development;anatomical structure morphogenesis;biological regulation;cellular component organization;cellular component organization at cellular level;cellular component organization or biogenesis;cell
actin cytoskeleton organization;actin filament bundle assembly;actin filament organization;actin filament-based process;cell migration;cell motility;cell proliferation;cellular component assembly;cellular component assembly a
biological regulation;biosynthetic process;catabolic process;cellular biosynthetic process;cellular catabolic process;cellular macromolecule biosynthetic process;cellular macromolecule catabolic process;cellular macromolec
biosynthetic process;cell differentiation;cellular biosynthetic process;cellular developmental process;cellular macromolecule biosynthetic process;cellular macromolecule metabolic process;cellular metabolic process;cellular
amine metabolic process;amino acid activation;biosynthetic process;carboxylic acid metabolic process;cellular amine metabolic process;cellular amino acid metabolic process;cellular biosynthetic process;cellular componen
biological regulation;biosynthetic process;cellular biosynthetic process;cellular component disassembly;cellular component disassembly at cellular level;cellular component organization;cellular component organization at cell
biological regulation;biosynthetic process;cellular biosynthetic process;cellular component assembly;cellular component assembly at cellular level;cellular component organization;cellular component organization at cellular le
alcohol catabolic process;alcohol metabolic process;anatomical structure development;carbohydrate catabolic process;carbohydrate metabolic process;carboxylic acid metabolic process;catabolic process;cell communicati
acute inflammatory response;acute-phase response;aging;amide biosynthetic process;amine biosynthetic process;amine metabolic process;anatomical structure development;arginine biosynthetic process;arginine metaboli
anatomical structure development;biological adhesion;biological regulation;blood coagulation;calcium ion homeostasis;calcium ion transmembrane transport;calcium ion transport;calcium-mediated signaling;cation homeost
binding of sperm to zona pellucida;cell recognition;cell-cell recognition;cellular component assembly;cellular component assembly at cellular level;cellular component organization;cellular component organization at cellular le
apoptosis;biological regulation;cell cycle;cell death;cellular process;cytokinetic cell separation;cytokinetic process;death;establishment of localization;establishment of protein localization;programmed cell death;protein transp
biological regulation;biosynthetic process;cellular biosynthetic process;cellular component assembly;cellular component assembly at cellular level;cellular component organization;cellular component organization at cellular le
actin cytoskeleton organization;actin filament organization;actin filament polymerization;actin filament-based process;actin polymerization or depolymerization;biological regulation;cell projection organization;cellular compone

actin cytoskeleton organization;actin filament organization;actin filament-based process;alcohol biosynthetic process;alcohol catabolic process;alcohol metabolic process;anatomical structure homeostasis;ATP biosynthetic p
actin filament capping;anatomical structure development;apical protein localization;asymmetric protein localization;biological regulation;cell development;cell projection assembly;cell projection organization;cellular componen
cellular component organization;cellular component organization at cellular level;cellular component organization or biogenesis;cellular component organization or biogenesis at cellular level;cellular process;COPI coating of G
binding of sperm to zona pellucida;cell recognition;cell-cell recognition;cellular macromolecule metabolic process;cellular metabolic process;cellular process;cellular process involved in reproduction;cellular protein metabolic
anatomical structure morphogenesis;biological regulation;biosynthetic process;cell morphogenesis;cell morphogenesis involved in differentiation;cellular biosynthetic process;cellular component assembly;cellular component
biological regulation;biosynthetic process;cell communication;cell differentiation;cellular biosynthetic process;cellular developmental process;cellular macromolecule biosynthetic process;cellular macromolecule metabolic pro
biosynthetic process;carboxylic acid catabolic process;carboxylic acid metabolic process;cardiolipin acyl-chain remodeling;cardiolipin metabolic process;catabolic process;cellular biosynthetic process;cellular catabolic proc
adherens junction organization;anatomical structure development;anatomical structure homeostasis;ATP-dependent chromatin remodeling;axon guidance;biological adhesion;biological regulation;blood coagulation;cell adhes
actin cytoskeleton organization;actin cytoskeleton reorganization;actin filament organization;actin filament polymerization;actin filament-based process;actin polymerization or depolymerization;anatomical structure morphoge
biological regulation;cellular component organization;cellular component organization or biogenesis;cellular macromolecule metabolic process;cellular membrane organization;cellular metabolic process;cellular process;cellula
biological regulation;cell cycle;cell cycle phase;cell cycle process;cell division;cellular component organization;cellular component organization at cellular level;cellular component organization or biogenesis;cellular componen
anatomical structure formation involved in morphogenesis;anatomical structure morphogenesis;apoptotic cell clearance;biological regulation;blood vessel remodeling;branching involved in salivary gland morphogenesis;calci
biological regulation;biosynthetic process;cell cycle;cell cycle process;cell proliferation;cellular biosynthetic process;cellular macromolecule biosynthetic process;cellular macromolecule metabolic process;cellular metabolic p
biological regulation;cell cycle;cell cycle phase;cell cycle process;cell division;cellular component organization;cellular component organization at cellular level;cellular component organization or biogenesis;cellular componen
cell cycle;cell division;cellular component assembly;cellular component organization;cellular component organization or biogenesis;cellular process;macromolecular complex assembly;macromolecular complex subunit organ
alcohol catabolic process;alcohol metabolic process;biological regulation;carbohydrate catabolic process;carbohydrate homeostasis;carbohydrate metabolic process;carbohydrate phosphorylation;carbohydrate transport;ca
biosynthetic process;cell cycle;cell cycle process;cellular biosynthetic process;cellular macromolecule biosynthetic process;cellular macromolecule metabolic process;cellular metabolic process;cellular nitrogen compound m
activation of immune response;activation of innate immune response;activation of phospholipase C activity;behavior;biological regulation;blood coagulation;calcium ion homeostasis;calcium ion transport;calcium ion transpo
biological regulation;blood coagulation;cell activation;cellular process;coagulation;establishment of localization;establishment of localization in cell;exocytosis;hemostasis;multicellular organismal process;neurological system
acid secretion;alpha-beta T cell activation;alpha-beta T cell differentiation;anatomical structure development;arachidonic acid secretion;biological regulation;carboxylic acid transport;cell activation;cell differentiation;cell surfa
alcohol biosynthetic process;alcohol metabolic process;apoptosis;biological regulation;biosynthetic process;carbohydrate biosynthetic process;carbohydrate metabolic process;cell death;cellular biosynthetic process;cellular
biological regulation;cell redox homeostasis;cellular homeostasis;cellular macromolecule metabolic process;cellular metabolic process;cellular process;cellular protein metabolic process;cellular response to stimulus;cellular r
biological regulation;biosynthetic process;cell differentiation;cellular biosynthetic process;cellular component assembly;cellular component assembly at cellular level;cellular component organization;cellular component organi
anatomical structure development;biosynthetic process;cellular biosynthetic process;cellular macromolecule biosynthetic process;cellular macromolecule metabolic process;cellular metabolic process;cellular nitrogen compo
biological regulation;cell cycle;cell cycle checkpoint;cell cycle phase;cell cycle process;cell division;cellular component organization;cellular component organization at cellular level;cellular component organization or biogene
binding of sperm to zona pellucida;cell recognition;cell-cell recognition;cellular component assembly;cellular component assembly at cellular level;cellular component organization;cellular component organization at cellular le
catabolic process;cellular catabolic process;cellular macromolecule catabolic process;cellular macromolecule metabolic process;cellular metabolic process;cellular process;cellular protein metabolic process;cellular response
amine metabolic process;amino acid activation;biosynthetic process;carboxylic acid metabolic process;cellular amine metabolic process;cellular amino acid metabolic process;cellular biosynthetic process;cellular ketone me
biological regulation;biosynthetic process;catabolic process;cell differentiation;cellular biosynthetic process;cellular catabolic process;cellular component disassembly;cellular component disassembly at cellular level;cellular c
actin cytoskeleton organization;actin filament-based process;actomyosin structure organization;biological regulation;cell differentiation;cell differentiation involved in embryonic placenta development;cell surface receptor link
cell cycle process;cell division;cellular component movement;cellular component organization;cellular component organization at cellular level;cellular component organization or biogenesis;cellular component organization o
biological regulation;biosynthetic process;cellular biosynthetic process;cellular macromolecule biosynthetic process;cellular macromolecule metabolic process;cellular metabolic process;cellular nitrogen compound metabolic
biological regulation;calcium ion homeostasis;cation homeostasis;cellular calcium ion homeostasis;cellular cation homeostasis;cellular chemical homeostasis;cellular divalent inorganic cation homeostasis;cellular homeostasi
biological regulation;biosynthetic process;cell growth;cellular biosynthetic process;cellular macromolecule biosynthetic process;cellular macromolecule metabolic process;cellular metabolic process;cellular nitrogen compoun
acyl-CoA biosynthetic process;acyl-CoA metabolic process;acylglycerol biosynthetic process;acylglycerol metabolic process;biological regulation;biosynthetic process;carbohydrate metabolic process;carboxylic acid metabo
cellular macromolecule metabolic process;cellular metabolic process;cellular process;cellular protein metabolic process;macromolecule metabolic process;macromolecule modification;metabolic process;primary metabolic p
actin cytoskeleton organization;actin filament bundle assembly;actin filament organization;actin filament-based movement;actin filament-based process;cellular component assembly;cellular component assembly at cellular le
biological regulation;cardiac muscle contraction;cation homeostasis;cation transport;cellular cation homeostasis;cellular chemical homeostasis;cellular homeostasis;cellular ion homeostasis;cellular metal ion homeostasis;cell
10-formyltetrahydrofolate biosynthetic process;10-formyltetrahydrofolate metabolic process;amine biosynthetic process;amine metabolic process;anatomical structure development;anatomical structure formation involved in
axon guidance;biological regulation;cellular chemical homeostasis;cellular component disassembly;cellular component disassembly at cellular level;cellular component movement;cellular component organization;cellular com
ATP biosynthetic process;ATP hydrolysis coupled proton transport;ATP metabolic process;ATP synthesis coupled proton transport;biological regulation;biosynthetic process;cation transport;cellular biosynthetic process;cellu
biosynthetic process;catabolic process;cellular biosynthetic process;cellular catabolic process;cellular component disassembly;cellular component disassembly at cellular level;cellular component organization;cellular compo
carbohydrate metabolic process;cellular carbohydrate metabolic process;cellular macromolecule metabolic process;cellular metabolic process;cellular process;cellular protein metabolic process;glycosylation;macromolecule
biological regulation;biosynthetic process;cellular biosynthetic process;cellular macromolecule biosynthetic process;cellular macromolecule metabolic process;cellular metabolic process;cellular nitrogen compound metabolic
biological regulation;biosynthetic process;cellular biosynthetic process;cellular macromolecule biosynthetic process;cellular macromolecule metabolic process;cellular metabolic process;cellular nitrogen compound metabolic
amine metabolic process;amino acid activation;carboxylic acid metabolic process;cellular amine metabolic process;cellular amino acid metabolic process;cellular ketone metabolic process;cellular macromolecule metabolic p
cellular macromolecule metabolic process;cellular metabolic process;cellular nitrogen compound metabolic process;cellular process;macromolecule metabolic process;metabolic process;mRNA metabolic process;mRNA pro
alcohol biosynthetic process;alcohol catabolic process;alcohol metabolic process;aldehyde biosynthetic process;biological regulation;biosynthetic process;carbohydrate biosynthetic process;carbohydrate catabolic process;
antigen processing and presentation;antigen processing and presentation of exogenous antigen;antigen processing and presentation of exogenous peptide antigen;antigen processing and presentation of exogenous peptide
biological regulation;cellular component organization;cellular component organization or biogenesis;defense response;establishment of localization;establishment of localization in cell;establishment of protein localization;esta
alcohol biosynthetic process;alcohol metabolic process;biosynthetic process;carbohydrate biosynthetic process;carbohydrate metabolic process;cell differentiation;cellular biosynthetic process;cellular carbohydrate biosynth
amine metabolic process;amino acid activation;carboxylic acid metabolic process;cellular amine metabolic process;cellular amino acid metabolic process;cellular ketone metabolic process;cellular macromolecule metabolic p
amide biosynthetic process;amine biosynthetic process;amine catabolic process;amine metabolic process;anatomical structure development;anatomical structure morphogenesis;arginine biosynthetic process;arginine metab
amine metabolic process;amino acid activation;carboxylic acid metabolic process;cell differentiation;cellular amine metabolic process;cellular amino acid metabolic process;cellular developmental process;cellular ketone met
anterior/posterior pattern specification;cellular macromolecule metabolic process;cellular metabolic process;cellular nitrogen compound metabolic process;cellular process;developmental process;gene expression;macromol
4-hydroxyproline metabolic process;amine metabolic process;biological regulation;carboxylic acid metabolic process;cell redox homeostasis;cellular amine metabolic process;cellular amino acid metabolic process;cellular co
actin cytoskeleton organization;actin cytoskeleton reorganization;actin filament bundle assembly;actin filament organization;actin filament-based process;anatomical structure development;axon guidance;biological adhesion;
cell differentiation;cellular component assembly;cellular component assembly at cellular level;cellular component organization;cellular component organization at cellular level;cellular component organization or biogenesis;ce
alcohol biosynthetic process;alcohol metabolic process;aldehyde biosynthetic process;aldehyde catabolic process;anatomical structure formation involved in morphogenesis;angiogenesis;behavior;biological regulation;biosy
apoptosis;cell death;cellular component disassembly;cellular component disassembly at cellular level;cellular component disassembly involved in apoptosis;cellular component organization;cellular component organization a
biological regulation;cellular macromolecule metabolic process;cellular metabolic process;cellular process;cellular protein metabolic process;establishment of localization;establishment of localization in cell;establishment of p
biological regulation;cell differentiation;cellular developmental process;cellular macromolecule metabolic process;cellular metabolic process;cellular nitrogen compound metabolic process;cellular process;circadian regulation
alternative nuclear mRNA splicing, via spliceosome;biological regulation;biosynthetic process;cellular biosynthetic process;cellular component organization;cellular component organization at cellular level;cellular component
anatomical structure development;anatomical structure morphogenesis;biological regulation;cell surface receptor linked signaling pathway;cellular component organization;cellular component organization or biogenesis;cellul
activation of caspase activity;aggresome assembly;biological regulation;biosynthetic process;carbohydrate metabolic process;catabolic process;cellular biosynthetic process;cellular carbohydrate metabolic process;cellular c
biological regulation;biosynthetic process;catabolic process;cell surface receptor linked signaling pathway;cellular biosynthetic process;cellular catabolic process;cellular macromolecule biosynthetic process;cellular macrom
anatomical structure formation involved in morphogenesis;angiogenesis;biological regulation;body fluid secretion;cellular component assembly;cellular component assembly at cellular level;cellular component organization;ce
axon guidance;biological regulation;biosynthetic process;cell communication;cell-cell signaling;cellular biosynthetic process;cellular component disassembly;cellular component disassembly at cellular level;cellular componen
anatomical structure formation involved in morphogenesis;angiogenesis;developmental process
cell cycle phase;cell cycle process;cell proliferation;cellular process;meiosis
activation of signaling protein activity involved in unfolded protein response;anatomical structure arrangement;anatomical structure development;biological regulation;blood coagulation;catabolic process;cell activation;cell co
alcohol metabolic process;carbohydrate catabolic process;carbohydrate metabolic process;catabolic process;cellular carbohydrate catabolic process;cellular carbohydrate metabolic process;cellular glucan metabolic proces
response to stimulus;response to stress
biological regulation;cell differentiation;cellular developmental process;cellular macromolecule metabolic process;cellular metabolic process;cellular nitrogen compound metabolic process;cellular process;cellular response to
biosynthetic process;catabolic process;cellular biosynthetic process;cellular catabolic process;cellular component disassembly;cellular component disassembly at cellular level;cellular component organization;cellular compo
biological regulation;biosynthetic process;cellular biosynthetic process;cellular component movement;cellular macromolecule biosynthetic process;cellular macromolecule metabolic process;cellular metabolic process;cellula
actin filament-based movement;actin filament-based process;ameboidal cell migration;biological regulation;cell migration;cell motility;cellular component movement;cellular component organization;cellular component organi
alcohol biosynthetic process;alcohol metabolic process;biological regulation;biosynthetic process;carbohydrate biosynthetic process;carbohydrate metabolic process;cellular biosynthetic process;cellular carbohydrate biosyn
actin crosslink formation;actin cytoskeleton organization;actin filament bundle assembly;actin filament organization;actin filament-based process;biological regulation;blood coagulation;cell activation;cell junction assembly;ce
actin filament capping;apoptosis;axon guidance;biological regulation;cell death;cellular component disassembly;cellular component disassembly at cellular level;cellular component disassembly involved in apoptosis;cellular
biological regulation;biosynthetic process;cell differentiation;cellular biosynthetic process;cellular developmental process;cellular macromolecule biosynthetic process;cellular macromolecule metabolic process;cellular metab
activation of caspase activity;activation of immune response;activation of innate immune response;adaptive immune response;adaptive immune response based on somatic recombination of immune receptors built from imm
activation of protein kinase activity;activation of protein kinase C activity by G-protein coupled receptor protein signaling pathway;aging;alcohol metabolic process;anatomical structure morphogenesis;aorta morphogenesis;a
biological regulation;calcium ion transport;cation transport;cellular component assembly;cellular component organization;cellular component organization or biogenesis;cellular process;divalent inorganic cation transport;diva
adherens junction assembly;adherens junction organization;anatomical structure morphogenesis;apical junction assembly;axon extension;biological adhesion;biological regulation;blood coagulation;cell activation;cell adhesio
biological regulation;body fluid secretion;cellular component organization;cellular component organization at cellular level;cellular component organization or biogenesis;cellular component organization or biogenesis at cellula
assembly of spliceosomal tri-snRNP;cellular component assembly;cellular component assembly at cellular level;cellular component organization;cellular component organization at cellular level;cellular component organizatio
alcohol metabolic process;cholesterol metabolic process;establishment of localization;lipid metabolic process;lipid transport;metabolic process;organic substance transport;primary metabolic process;small molecule metabo
anatomical structure homeostasis;base-excision repair;biological regulation;biosynthetic process;cell differentiation;cell surface receptor linked signaling pathway;cellular biosynthetic process;cellular component organization
actin cytoskeleton organization;actin filament organization;actin filament-based process;actin rod assembly;activation of immune response;activation of innate immune response;biological regulation;catabolic process;cellular
biological regulation;cellular component movement;cellular process;cellular response to stimulus;intracellular signal transduction;microtubule-based movement;microtubule-based process;regulation of biological process;regu
anatomical structure development;biological adhesion;biological regulation;cell adhesion;cell development;cell migration;cell motility;cell-cell adhesion;cellular component movement;cellular developmental process;cellular pr
cellular component assembly;cellular component assembly at cellular level;cellular component organization;cellular component organization at cellular level;cellular component organization or biogenesis;cellular component c
biological regulation;biosynthetic process;cell differentiation;cellular biosynthetic process;cellular developmental process;cellular macromolecule biosynthetic process;cellular macromolecule metabolic process;cellular metab
alcohol metabolic process;carbohydrate metabolic process;cell death;cellular carbohydrate metabolic process;cellular metabolic process;cellular process;death;glucose metabolic process;hexose metabolic process;metaboli
alternative nuclear mRNA splicing, via spliceosome;cellular macromolecule metabolic process;cellular metabolic process;cellular nitrogen compound metabolic process;cellular process;gene expression;macromolecule metab
anatomical structure development;autophagy;biological adhesion;biological regulation;catabolic process;cell adhesion;cell differentiation;cell junction assembly;cell junction organization;cell motility;cell projection assembly;c
apoptosis;axon guidance;biological regulation;cardiac muscle cell apoptosis;cell cycle;cell cycle process;cell death;cell migration;cell motility;cell surface receptor linked signaling pathway;cellular component assembly;cellula
biological regulation;biosynthetic process;carbohydrate metabolic process;carbohydrate transport;cell cycle;cell cycle process;cell surface receptor linked signaling pathway;cellular biosynthetic process;cellular component c
actin filament-based movement;actin filament-based process;actin-mediated cell contraction;actin-myosin filament sliding;anatomical structure development;apoptosis;astrocyte development;astrocyte differentiation;Bergma
anatomical structure development;biological regulation;cell development;cell surface receptor linked signaling pathway;cellular developmental process;cellular metabolic process;cellular process;cellular response to calcium i
actin cytoskeleton organization;actin filament bundle assembly;actin filament organization;actin filament-based process;biological regulation;blood coagulation;cell activation;cell junction assembly;cell junction organization;c
anatomical structure development;biological adhesion;biological regulation;cell adhesion;cell differentiation;cell junction assembly;cell junction organization;cell-substrate junction assembly;cellular component assembly;cellu
anatomical structure formation involved in morphogenesis;biological regulation;cell cycle arrest;cell cycle process;cell surface receptor linked signaling pathway;cellular process;cellular response to stimulus;developmental pr
anatomical structure development;biological adhesion;brown fat cell differentiation;cell adhesion;cell differentiation;cell junction assembly;cell junction organization;cell-substrate junction assembly;cellular component assemb
actin filament capping;axon guidance;biological regulation;cell cycle cytokinesis;cell cycle process;cellular component assembly;cellular component assembly at cellular level;cellular component organization;cellular compone
anatomical structure development;apoptosis;biological regulation;cardiac cell development;cardiac muscle cell development;cell cycle;cell cycle process;cell death;cell development;cellular component disassembly;cellular c
anatomical structure development;axon guidance;biological regulation;cellular macromolecule metabolic process;cellular metabolic process;cellular process;cellular protein metabolic process;cellular response to chemical sti
biological regulation;biosynthetic process;cellular biosynthetic process;cellular component assembly;cellular component assembly at cellular level;cellular component organization;cellular component organization at cellular le
anatomical structure development;biological adhesion;cell adhesion;cell junction assembly;cell junction organization;cell-substrate junction assembly;cellular component assembly;cellular component assembly at cellular leve
biological regulation;biosynthetic process;cellular biosynthetic process;cellular macromolecule biosynthetic process;cellular macromolecule metabolic process;cellular metabolic process;cellular process;cellular protein metab
base-excision repair;cell differentiation;cellular component organization;cellular component organization at cellular level;cellular component organization or biogenesis;cellular component organization or biogenesis at cellular
anatomical structure formation involved in morphogenesis;biological regulation;blood circulation;cellular component organization;cellular component organization or biogenesis;cellular membrane organization;cellular process
amine metabolic process;amino acid activation;biological regulation;carboxylic acid metabolic process;cellular amine metabolic process;cellular amino acid metabolic process;cellular component assembly;cellular componen
biosynthetic process;cell differentiation;cellular biosynthetic process;cellular developmental process;cellular macromolecule biosynthetic process;cellular macromolecule metabolic process;cellular metabolic process;cellular p
actin cytoskeleton organization;actin filament-based movement;actin filament-based process;actomyosin structure organization;adult heart development;anatomical structure development;anatomical structure formation invo
cell cycle;cell cycle phase;cell cycle process;cell differentiation;cell division;cellular component organization;cellular component organization at cellular level;cellular component organization or biogenesis;cellular component c
biological regulation;biosynthetic process;carbohydrate metabolic process;carbohydrate transport;cell cycle;cell cycle checkpoint;cell cycle phase;cell cycle process;cell division;cell surface receptor linked signaling pathway
antigen processing and presentation;antigen processing and presentation of exogenous antigen;antigen processing and presentation of exogenous peptide antigen;antigen processing and presentation of exogenous peptide
acylglycerol catabolic process;acylglycerol metabolic process;amide biosynthetic process;amine biosynthetic process;amine catabolic process;amine metabolic process;anatomical structure development;anion homeostasis;
actin crosslink formation;actin cytoskeleton organization;actin cytoskeleton reorganization;actin filament organization;actin filament-based process;anatomical structure formation involved in morphogenesis;anatomical structu
actin cytoskeleton organization;actin filament-based process;anatomical structure development;biological regulation;cell differentiation;cell surface receptor linked signaling pathway;cellular component organization;cellular c
acetyl-CoA metabolic process;acyl-CoA biosynthetic process;acyl-CoA metabolic process;acylglycerol biosynthetic process;acylglycerol metabolic process;amine metabolic process;biological regulation;biosynthetic process
actin cytoskeleton organization;actin filament-based process;axon guidance;biological adhesion;biological regulation;blood coagulation;cell activation;cell adhesion;cell junction assembly;cell junction organization;cell-cell ad
adherens junction organization;anatomical structure development;anatomical structure morphogenesis;apoptosis;cardiac muscle tissue morphogenesis;cell death;cell differentiation;cell junction organization;cell-cell junction

antigen processing and presentation;antigen processing and presentation of exogenous antigen;antigen processing and presentation of exogenous peptide antigen;antigen processing and presentation of exogenous peptide
anatomical structure development;anatomical structure formation involved in morphogenesis;anatomical structure homeostasis;anterior/posterior pattern specification;B cell lineage commitment;biological regulation;brain de
actin cytoskeleton organization;actin cytoskeleton reorganization;actin filament-based movement;actin filament-based process;actomyosin structure organization;anatomical structure formation involved in morphogenesis;an
biological regulation;cellular component assembly;cellular component organization;cellular component organization or biogenesis;macromolecular complex assembly;macromolecular complex subunit organization;protein co
apoptosis;cell death;cell junction assembly;cell junction organization;cell-substrate junction assembly;cellular component assembly;cellular component assembly at cellular level;cellular component disassembly;cellular comp

C: GOMF name
binding;cation binding;enzyme activator activity;enzyme regulator activity;GTPase activator activity;GTPase regulator activity;ion binding;metal ion binding;nucleoside-triphosphatase regulator activity;transition metal ion bind
catalytic activity;hydrolase activity;peptidase activity
catalytic activity;methyltransferase activity;N-methyltransferase activity;RNA methyltransferase activity;rRNA (cytosine) methyltransferase activity;rRNA (cytosine-N4-)-methyltransferase activity;rRNA methyltransferase activity
binding;nucleic acid binding;RNA binding;RNA cap binding
binding;p53 binding;protein binding
acetyltransferase activity;binding;C-acetyltransferase activity;C-acyltransferase activity;catalytic activity;cofactor binding;glycine C-acetyltransferase activity;pyridoxal phosphate binding;transferase activity;transferase activity
catalytic activity;NADH dehydrogenase (quinone) activity;NADH dehydrogenase (ubiquinone) activity;NADH dehydrogenase activity;oxidoreductase activity;oxidoreductase activity, acting on NADH or NADPH;oxidoreductase
cation transmembrane transporter activity;hydrogen ion transmembrane transporter activity;inorganic cation transmembrane transporter activity;ion transmembrane transporter activity;monovalent inorganic cation transmemb
binding;enzyme binding;protease binding;protein binding;receptor binding
binding;cation binding;copper chaperone activity;copper ion binding;ion binding;metal ion binding;metallochaperone activity;transition metal ion binding
binding;nucleotide binding
G-protein coupled receptor activity;molecular transducer activity;pheromone receptor activity;receptor activity;signal transducer activity;signaling receptor activity;transmembrane signaling receptor activity
catalytic activity;diphthine synthase activity;methyltransferase activity;S-adenosylmethionine-dependent methyltransferase activity;transferase activity;transferase activity, transferring one-carbon groups
acyl-CoA hydrolase activity;catalytic activity;CoA hydrolase activity;hydrolase activity;hydrolase activity, acting on ester bonds;thiolester hydrolase activity
active transmembrane transporter activity;amine transmembrane transporter activity;amino acid transmembrane transporter activity;carboxylic acid transmembrane transporter activity;L-amino acid transmembrane transporte
binding;cation binding;ion binding;metal ion binding
alcohol binding;binding;carbohydrate binding;catalytic activity;cation binding;CDP-alcohol phosphatidyltransferase activity;CDP-diacylglycerol-inositol 3-phosphatidyltransferase activity;diacylglycerol binding;ion binding;lipic
binding;catalytic activity;GTP binding;GTPase activity;guanyl nucleotide binding;guanyl ribonucleotide binding;hydrolase activity;hydrolase activity, acting on acid anhydrides;hydrolase activity, acting on acid anhydrides, in pl
catalytic activity;NADH dehydrogenase (quinone) activity;NADH dehydrogenase (ubiquinone) activity;NADH dehydrogenase activity;oxidoreductase activity;oxidoreductase activity, acting on NADH or NADPH;oxidoreductase
binding;epidermal growth factor receptor binding;growth factor receptor binding;insulin receptor binding;lipid binding;phosphatidylinositol binding;phospholipid binding;protein binding;protein complex binding;receptor bindin
adenyl nucleotide binding;adenyl ribonucleotide binding;aminoacyl-tRNA ligase activity;ATP binding;binding;catalytic activity;cation binding;ion binding;ligase activity;ligase activity, forming aminoacyl-tRNA and related comp
binding;cation binding;cytokine activity;endopeptidase inhibitor activity;endopeptidase regulator activity;enzyme binding;enzyme inhibitor activity;enzyme regulator activity;ion binding;metal ion binding;metalloendopeptidase
binding;chromatin binding;DNA binding;nucleic acid binding
binding;carbohydrate binding;chemoattractant activity;glycosaminoglycan binding;G-protein-coupled receptor binding;heparin binding;pattern binding;polysaccharide binding;protein binding;receptor binding
binding;protein binding;protein C-terminus binding
carboxylic ester hydrolase activity;catalytic activity;hydrolase activity;hydrolase activity, acting on ester bonds;lipase activity;triglyceride lipase activity
binding;catalytic activity;GDP binding;GTP binding;GTPase activity;guanyl nucleotide binding;guanyl ribonucleotide binding;hydrolase activity;hydrolase activity, acting on acid anhydrides;hydrolase activity, acting on acid anh
transmembrane transporter activity;transporter activity
catalytic activity;NADH dehydrogenase (quinone) activity;NADH dehydrogenase (ubiquinone) activity;NADH dehydrogenase activity;oxidoreductase activity;oxidoreductase activity, acting on NADH or NADPH;oxidoreductase
antibiotic transporter activity;drug transmembrane transporter activity;tetracycline transporter activity;transmembrane transporter activity;transporter activity
C-8 sterol isomerase activity;catalytic activity;cholestenol delta-isomerase activity;drug transmembrane transporter activity;intramolecular oxidoreductase activity;intramolecular oxidoreductase activity, transposing C=C bond
catalytic activity;disulfide oxidoreductase activity;oxidoreductase activity;oxidoreductase activity, acting on a sulfur group of donors;protein disulfide oxidoreductase activity
cation transmembrane transporter activity;ion transmembrane transporter activity;substrate-specific transmembrane transporter activity;substrate-specific transporter activity;transmembrane transporter activity;transporter ac
catalytic activity;hydrolase activity
adenyl nucleotide binding;adenyl ribonucleotide binding;ATP binding;binding;catalytic activity;cob(I)yrinic acid a,c-diamide adenosyltransferase activity;nucleotide binding;purine nucleotide binding;purine ribonucleoside triph
binding;nucleic acid binding;RNA binding;rRNA binding;structural constituent of ribosome;structural molecule activity
binding;identical protein binding;protein binding;transcription factor binding
2'-phosphotransferase activity;5'-nucleotidase activity;binding;catalytic activity;cation binding;hydrolase activity;hydrolase activity, acting on ester bonds;ion binding;magnesium ion binding;metal ion binding;nucleotidase act
binding;identical protein binding;protein binding;protein C-terminus binding;protein dimerization activity;protein heterodimerization activity;protein homodimerization activity
catalytic activity;methyltransferase activity;N-methyltransferase activity;protein binding transcription factor activity;RNA methyltransferase activity;rRNA (adenine) methyltransferase activity;rRNA (adenine-N6,N6-)-dimethyltra
binding;catalytic activity;cation binding;histone methyltransferase activity;histone-lysine N-methyltransferase activity;ion binding;lysine N-methyltransferase activity;metal ion binding;methyltransferase activity;N-methyltransfe
binding;nucleic acid binding;RNA binding;snoRNA binding
binding;catalytic activity;DNA binding;DNA polymerase activity;DNA-directed DNA polymerase activity;nucleic acid binding;nucleotidyltransferase activity;sequence-specific DNA binding;transferase activity;transferase activit
macromolecule transmembrane transporter activity;protein transmembrane transporter activity;protein transporter activity;substrate-specific transmembrane transporter activity;substrate-specific transporter activity;transmem
binding;DNA binding;nucleic acid binding
catalytic activity;cation transmembrane transporter activity;cytochrome-c oxidase activity;heme-copper terminal oxidase activity;hydrogen ion transmembrane transporter activity;inorganic cation transmembrane transporter a
catalytic activity;electron carrier activity;oxidoreductase activity;oxidoreductase activity, acting on paired donors, with incorporation or reduction of molecular oxygen;sphingolipid delta-4 desaturase activity
structural constituent of ribosome;structural molecule activity
catalytic activity;NADH dehydrogenase (quinone) activity;NADH dehydrogenase (ubiquinone) activity;NADH dehydrogenase activity;oxidoreductase activity;oxidoreductase activity, acting on NADH or NADPH;oxidoreductase
binding;carbonate dehydratase activity;carbon-oxygen lyase activity;catalytic activity;cation binding;hydro-lyase activity;ion binding;lyase activity;metal ion binding;transition metal ion binding;zinc ion binding
binding;cation binding;GTP binding;guanyl nucleotide binding;guanyl ribonucleotide binding;ion binding;metal ion binding;nucleotide binding;purine nucleotide binding;purine ribonucleoside triphosphate binding;purine ribonu
binding;enzyme binding;nucleic acid binding;protein binding;RNA binding;RNA cap binding;translation factor activity, nucleic acid binding;translation initiation factor activity;ubiquitin protein ligase binding
4 iron, 4 sulfur cluster binding;binding;catalytic activity;cation binding;cofactor binding;ion binding;iron-sulfur cluster binding;metal cluster binding;metal ion binding;NADH dehydrogenase (quinone) activity;NADH dehydrogen
beta-tubulin binding;binding;cytoskeletal protein binding;microtubule binding;protein binding;protein binding transcription factor activity;protein N-terminus binding;receptor binding;transcription cofactor activity;transcription
binding;GTP binding;guanyl nucleotide binding;guanyl ribonucleotide binding;nucleic acid binding;nucleotide binding;purine nucleotide binding;purine ribonucleoside triphosphate binding;purine ribonucleotide binding;ribonu
binding;cation binding;copper ion binding;ion binding;metal ion binding;transition metal ion binding
binding;chromatin binding;enzyme inhibitor activity;enzyme regulator activity;phosphatase inhibitor activity;phosphatase regulator activity;protein binding transcription factor activity;protein phosphatase inhibitor activity;prote
binding;nucleic acid binding;RNA binding;snRNA binding;U6 snRNA 3'-end binding;U6 snRNA binding
cysteine-type endopeptidase inhibitor activity;endopeptidase inhibitor activity;endopeptidase regulator activity;enzyme inhibitor activity;enzyme regulator activity;kinase inhibitor activity;kinase regulator activity;peptidase inhi
binding;cation binding;cation transmembrane transporter activity;ferric iron binding;ferric iron transmembrane transporter activity;inorganic cation transmembrane transporter activity;ion binding;ion transmembrane transporte
catalytic activity;glutathione transferase activity;transferase activity;transferase activity, transferring alkyl or aryl (other than methyl) groups
catalytic activity;cation transmembrane transporter activity;cytochrome-c oxidase activity;heme-copper terminal oxidase activity;hydrogen ion transmembrane transporter activity;inorganic cation transmembrane transporter a
catalytic activity;NADH dehydrogenase (quinone) activity;NADH dehydrogenase (ubiquinone) activity;NADH dehydrogenase activity;oxidoreductase activity;oxidoreductase activity, acting on NADH or NADPH;oxidoreductase
active transmembrane transporter activity;antiporter activity;binding;binding, bridging;calcium-dependent protein binding;cation transmembrane transporter activity;cation:cation antiporter activity;enzyme binding;hydrogen io
catalytic activity;cation transmembrane transporter activity;cytochrome-c oxidase activity;heme-copper terminal oxidase activity;hydrogen ion transmembrane transporter activity;inorganic cation transmembrane transporter a
androgen binding;benzodiazepine receptor activity;binding;cholesterol binding;hormone binding;lipid binding;molecular transducer activity;neurotransmitter binding;neurotransmitter receptor activity;receptor activity;signal tra
enzyme inhibitor activity;enzyme regulator activity;phosphatase inhibitor activity;phosphatase regulator activity;protein phosphatase inhibitor activity;protein phosphatase regulator activity;protein serine/threonine phosphatas
binding;nucleic acid binding;RNA binding;translation factor activity, nucleic acid binding;translation initiation factor activity
calmodulin-dependent protein kinase activity;catalytic activity;hydrolase activity;hydrolase activity, acting on glycosyl bonds;hydrolase activity, hydrolyzing O-glycosyl compounds;kinase activity;phosphorylase kinase activity;
5'-deoxyribose-5-phosphate lyase activity;AT DNA binding;binding;C2H2 zinc finger domain binding;cAMP response element binding;carbon-oxygen lyase activity;catalytic activity;chromatin binding;chromatin DNA binding;c
binding;catalytic activity;cation binding;enzyme binding;GTPase binding;ion binding;metal ion binding;prenyltransferase activity;protein binding;protein geranylgeranyltransferase activity;protein prenyltransferase activity;Rab g
protein transporter activity;substrate-specific transporter activity;transporter activity
catalytic activity;NADH dehydrogenase (quinone) activity;NADH dehydrogenase (ubiquinone) activity;NADH dehydrogenase activity;oxidoreductase activity;oxidoreductase activity, acting on NADH or NADPH;oxidoreductase
3-keto sterol reductase activity;catalytic activity;estradiol 17-beta-dehydrogenase activity;oxidoreductase activity;oxidoreductase activity, acting on CH-OH group of donors;oxidoreductase activity, acting on the CH-OH grou
binding;nucleic acid binding;RNA binding
binding;catalytic activity;GTPase activity;hydrolase activity;hydrolase activity, acting on acid anhydrides;hydrolase activity, acting on acid anhydrides, in phosphorus-containing anhydrides;molecular transducer activity;nucleo
structural constituent of ribosome;structural molecule activity
3-hydroxybutyrate dehydrogenase activity;binding;catalytic activity;lipid binding;oxidoreductase activity;oxidoreductase activity, acting on CH-OH group of donors;oxidoreductase activity, acting on the CH-OH group of dono
binding;core promoter proximal region DNA binding;core promoter proximal region sequence-specific DNA binding;DNA binding;nucleic acid binding;nucleic acid binding transcription factor activity;protein binding;regulatory
binding;protein binding;receptor binding
binding;DNA binding;identical protein binding;nucleic acid binding;nucleic acid binding transcription factor activity;protein binding;protein binding transcription factor activity;protein dimerization activity;protein homodimeriza
adenyl nucleotide binding;adenyl ribonucleotide binding;AP-2 adaptor complex binding;ATP binding;binding;catalytic activity;kinase activity;Notch binding;nucleotide binding;phosphotransferase activity, alcohol group as acc
catalytic activity;methyltransferase activity;S-adenosylmethionine-dependent methyltransferase activity;transferase activity;transferase activity, transferring one-carbon groups
carboxylic ester hydrolase activity;catalytic activity;hydrolase activity;hydrolase activity, acting on ester bonds;lipase activity;lysophospholipase activity;phospholipase activity
structural constituent of ribosome;structural molecule activity
binding;cation binding;ion binding;metal ion binding
2 iron, 2 sulfur cluster binding;binding;catalytic activity;cation binding;disulfide oxidoreductase activity;electron carrier activity;ion binding;iron-sulfur cluster binding;metal cluster binding;metal ion binding;oxidoreductase acti
binding;calcium ion binding;catalytic activity;cation binding;GTP binding;GTPase activity;guanyl nucleotide binding;guanyl ribonucleotide binding;hydrolase activity;hydrolase activity, acting on acid anhydrides;hydrolase acti
binding;calcium ion binding;cation binding;ion binding;metal ion binding;protein binding;protein dimerization activity;protein heterodimerization activity
catalytic activity;NADH dehydrogenase (quinone) activity;NADH dehydrogenase (ubiquinone) activity;NADH dehydrogenase activity;oxidoreductase activity;oxidoreductase activity, acting on NADH or NADPH;oxidoreductase
cation transmembrane transporter activity;divalent inorganic cation transmembrane transporter activity;inorganic cation transmembrane transporter activity;ion transmembrane transporter activity;magnesium ion transmembra
catalytic activity;oxidoreductase activity
acid-amino acid ligase activity;binding;catalytic activity;cation binding;ion binding;ligase activity;ligase activity, forming carbon-nitrogen bonds;metal ion binding;small conjugating protein ligase activity;transition metal ion bin
catalytic activity;exopeptidase activity;gamma-glutamyl-peptidase activity;hydrolase activity;omega peptidase activity;peptidase activity;peptidase activity, acting on L-amino acid peptides
catalytic activity
catalytic activity;methyltransferase activity;RNA methyltransferase activity;S-adenosylmethionine-dependent methyltransferase activity;transferase activity;transferase activity, transferring one-carbon groups;tRNA (adenine) m

binding;identical protein binding;protein binding;protein dimerization activity;protein homodimerization activity;protein N-terminus binding;structural molecule activity
binding;catalytic activity;cation binding;ion binding;ligase activity;metal ion binding;transition metal ion binding;zinc ion binding
nucleocytoplasmic transporter activity;transporter activity
binding;carbohydrate binding;catalytic activity;chitin binding;chitinase activity;hydrolase activity;hydrolase activity, acting on glycosyl bonds;hydrolase activity, hydrolyzing O-glycosyl compounds;oligosaccharide binding;patt
structural constituent of ribosome;structural molecule activity
binding;nucleic acid binding;ribonucleoprotein binding;ribosomal small subunit binding;RNA binding;translation factor activity, nucleic acid binding;translation initiation factor activity
binding;catalytic activity;cation binding;demethylase activity;histone demethylase activity;histone demethylase activity (H3-K36 specific);histone demethylase activity (H3-K4 specific);ion binding;iron ion binding;metal ion binc
binding;catalytic activity;cation binding;GDP binding;GTP binding;GTPase activity;guanyl nucleotide binding;guanyl ribonucleotide binding;hydrolase activity;hydrolase activity, acting on acid anhydrides;hydrolase activity, act
active transmembrane transporter activity;adenyl nucleotide binding;adenyl ribonucleotide binding;ATP binding;ATPase activity;ATPase activity, coupled;ATPase activity, coupled to movement of substances;ATPase activity, c
armadillo repeat domain binding;binding;calmodulin binding;enzyme binding;phosphatase binding;protein binding;protein complex binding;protein domain specific binding;protein phosphatase 2A binding;protein phosphatas
binding;catalytic activity;cation binding;hydrolase activity;hydrolase activity, acting on carbon-nitrogen (but not peptide) bonds;hydrolase activity, acting on carbon-nitrogen (but not peptide) bonds, in cyclic amidines;ion bindi
binding;protein binding;SNAP receptor activity
acid-amino acid ligase activity;binding;catalytic activity;cation binding;copper ion binding;ion binding;ligase activity;ligase activity, forming carbon-nitrogen bonds;metal ion binding;NEDD8 ligase activity;small conjugating pro
binding;calcium ion binding;calcium-dependent cysteine-type endopeptidase activity;catalytic activity;cation binding;cysteine-type endopeptidase activity;cysteine-type peptidase activity;endopeptidase activity;hydrolase ac
catalytic activity;electron carrier activity;NADH dehydrogenase (quinone) activity;NADH dehydrogenase (ubiquinone) activity;NADH dehydrogenase activity;oxidoreductase activity;oxidoreductase activity, acting on NADH or N
enzyme activator activity;enzyme regulator activity;GTPase activator activity;GTPase regulator activity;nucleoside-triphosphatase regulator activity
structural constituent of ribosome;structural molecule activity
adenyl nucleotide binding;adenyl ribonucleotide binding;ATP binding;ATPase activity;binding;catalytic activity;cytoskeletal protein binding;hydrolase activity;hydrolase activity, acting on acid anhydrides;hydrolase activity, acti
4 iron, 4 sulfur cluster binding;adenyl nucleotide binding;adenyl ribonucleotide binding;ATP binding;binding;cation binding;ion binding;iron-sulfur cluster binding;metal cluster binding;metal ion binding;nucleotide binding;purin
calcium channel inhibitor activity;calcium channel regulator activity;cation transmembrane transporter activity;channel inhibitor activity;channel regulator activity;divalent inorganic cation transmembrane transporter activity;ino
binding;nucleic acid binding;RNA binding
binding;catalytic activity;endonuclease activity;endonuclease activity, active with either ribo- or deoxyribonucleic acids and producing 3'-phosphomonoesters;endoribonuclease activity;endoribonuclease activity, producing 3
antioxidant activity;catalytic activity;glutathione peroxidase activity;glutathione transferase activity;oxidoreductase activity;oxidoreductase activity, acting on peroxide as acceptor;peroxidase activity;transferase activity;transfe
binding;cytoskeletal protein binding;enzyme regulator activity;GTPase regulator activity;guanyl-nucleotide exchange factor activity;kinesin binding;nucleoside-triphosphatase regulator activity;protein binding;Ras guanyl-nucle
catalytic activity;NADH dehydrogenase (quinone) activity;NADH dehydrogenase (ubiquinone) activity;NADH dehydrogenase activity;oxidoreductase activity;oxidoreductase activity, acting on NADH or NADPH;oxidoreductase
catalytic activity;electron carrier activity;NADH dehydrogenase (quinone) activity;NADH dehydrogenase (ubiquinone) activity;NADH dehydrogenase activity;oxidoreductase activity;oxidoreductase activity, acting on NADH or N
binding;enzyme binding;protein binding;small conjugating protein binding;transcription factor binding;ubiquitin binding;ubiquitin protein ligase binding
binding;catalytic activity;cation binding;endonuclease activity;hydrolase activity;hydrolase activity, acting on ester bonds;ion binding;metal ion binding;nuclease activity;nucleic acid binding
catalytic activity;NADH dehydrogenase (quinone) activity;NADH dehydrogenase (ubiquinone) activity;NADH dehydrogenase activity;oxidoreductase activity;oxidoreductase activity, acting on NADH or NADPH;oxidoreductase
catalytic activity;NADH dehydrogenase (quinone) activity;NADH dehydrogenase (ubiquinone) activity;NADH dehydrogenase activity;oxidoreductase activity;oxidoreductase activity, acting on NADH or NADPH;oxidoreductase
binding;catalytic activity;cation binding;coenzyme binding;cofactor binding;ion binding;metal ion binding;NADP binding;NADPH:quinone reductase activity;nucleotide binding;oxidoreductase activity;oxidoreductase activity, a
binding;catalytic activity;hemoglobin binding;protein binding
binding;DNA binding;endopeptidase inhibitor activity;endopeptidase regulator activity;enzyme inhibitor activity;enzyme regulator activity;nucleic acid binding;peptidase inhibitor activity;peptidase regulator activity;serine-type
binding;endopeptidase inhibitor activity;endopeptidase regulator activity;enzyme binding;enzyme inhibitor activity;enzyme regulator activity;peptidase inhibitor activity;peptidase regulator activity;protein binding;serine-type e
binding;DNA binding;nucleic acid binding;nucleic acid binding transcription factor activity;regulatory region DNA binding;regulatory region nucleic acid binding;RNA polymerase II regulatory region DNA binding;RNA polymera
active transmembrane transporter activity;ATPase activity;ATPase activity, coupled;ATPase activity, coupled to movement of substances;ATPase activity, coupled to transmembrane movement of ions;ATPase activity, coupled
binding;catalytic activity;cation binding;enzyme activator activity;enzyme regulator activity;GTP binding;GTPase activity;guanyl nucleotide binding;guanyl ribonucleotide binding;hydrolase activity;hydrolase activity, acting on a
binding;carbohydrate binding;catalytic activity;cation binding;glucose binding;glucosyltransferase activity;glycogenin glucosyltransferase activity;ion binding;metal ion binding;monosaccharide binding;sugar binding;transfera
acetylcholine receptor regulator activity;alpha-tubulin binding;binding;CAAX-protein geranylgeranyltransferase activity;catalytic activity;cytoskeletal protein binding;microtubule binding;prenyltransferase activity;protein binding
binding;calcium-dependent protein serine/threonine phosphatase activity;calmodulin-dependent protein phosphatase activity;catalytic activity;cation binding;hydrolase activity;hydrolase activity, acting on ester bonds;ion bin
adiponectin binding;binding;cadherin binding;calcium ion binding;cation binding;cell adhesion molecule binding;hormone binding;ion binding;lipoprotein particle binding;low-density lipoprotein particle binding;metal ion bindi
binding;channel regulator activity;enzyme binding;enzyme inhibitor activity;enzyme regulator activity;phosphatase binding;phosphatase inhibitor activity;phosphatase regulator activity;potassium channel regulator activity;pro
acid-amino acid ligase activity;adenyl nucleotide binding;adenyl ribonucleotide binding;ATP binding;binding;catalytic activity;ligase activity;ligase activity, forming carbon-nitrogen bonds;nucleotide binding;purine nucleotide b
binding;cation binding;chaperone binding;identical protein binding;ion binding;metal ion binding;protein binding;protein dimerization activity;protein homodimerization activity;protein transporter activity;substrate-specific tran
binding;nucleic acid binding;RNA binding
binding;nucleic acid binding;RNA binding;structural constituent of ribosome;structural molecule activity
acid-amino acid ligase activity;binding;catalytic activity;cation binding;enzyme binding;ion binding;ligase activity;ligase activity, forming carbon-nitrogen bonds;metal ion binding;NEDD8 ligase activity;protein binding;small co
adenyl nucleotide binding;adenyl ribonucleotide binding;ATP binding;binding;carbohydrate kinase activity;catalytic activity;galactokinase activity;kinase activity;N-acetylgalactosamine kinase activity;nucleotide binding;phosp
adenyl nucleotide binding;adenyl ribonucleotide binding;ATP binding;ATPase activity;binding;catalytic activity;hydrolase activity;hydrolase activity, acting on acid anhydrides;hydrolase activity, acting on acid anhydrides, in ph
binding;cation binding;chromatin binding;core promoter binding;core promoter proximal region DNA binding;core promoter proximal region sequence-specific DNA binding;core promoter sequence-specific DNA binding;DNA
adenyl nucleotide binding;adenyl ribonucleotide binding;ATP binding;binding;catalytic activity;identical protein binding;kinase activity;mevalonate kinase activity;nucleotide binding;phosphotransferase activity, alcohol group a
adenyl nucleotide binding;adenyl ribonucleotide binding;ATP binding;binding;catalytic activity;cation binding;ion binding;kinase activity;metal ion binding;nucleobase-containing compound kinase activity;nucleoside diphosph
binding;eukaryotic initiation factor 4E binding;protein binding;translation initiation factor binding;translation regulator activity;translation repressor activity
binding;DNA binding;nucleic acid binding;protein binding transcription factor activity;RNA binding;transcription cofactor activity;transcription corepressor activity;transcription factor binding transcription factor activity
binding;protein binding;transcription factor binding
binding;identical protein binding;protein binding;protein dimerization activity;protein homodimerization activity
binding;enzyme activator activity;enzyme regulator activity;GTPase activator activity;GTPase regulator activity;identical protein binding;nucleoside-triphosphatase regulator activity;protein binding;protein dimerization activity;
protein transporter activity;substrate-specific transporter activity;transporter activity
binding;catalytic activity;GTP binding;GTPase activity;guanyl nucleotide binding;guanyl ribonucleotide binding;hydrolase activity;hydrolase activity, acting on acid anhydrides;hydrolase activity, acting on acid anhydrides, in ph
catalytic activity;cation transmembrane transporter activity;cytochrome-c oxidase activity;heme-copper terminal oxidase activity;hydrogen ion transmembrane transporter activity;inorganic cation transmembrane transporter a
adenyl nucleotide binding;adenyl ribonucleotide binding;ATP binding;binding;catalytic activity;kinase activity;nucleic acid binding;nucleobase-containing compound kinase activity;nucleotide binding;phosphotransferase activ
binding;lipid binding
binding;catalytic activity;cation binding;deoxyribonuclease activity;endodeoxyribonuclease activity;endodeoxyribonuclease activity, producing 5'-phosphomonoesters;endonuclease activity;endonuclease activity, active with
binding;catalytic activity;DNA binding;N-acyltransferase activity;nucleic acid binding;sphingosine N-acyltransferase activity;transferase activity;transferase activity, transferring acyl groups;transferase activity, transferring acyl
binding;nucleic acid binding;RNA binding
carbon-sulfur lyase activity;catalytic activity;lyase activity
binding;cyclic nucleotide binding;cyclic-di-GMP binding;enzyme binding;guanyl nucleotide binding;guanyl ribonucleotide binding;identical protein binding;kinase binding;nucleotide binding;protein binding;protein dimerization
1-phosphatidylinositol binding;binding;cation binding;ion binding;lipid binding;metal ion binding;phosphatidylinositol binding;phospholipid binding;transition metal ion binding;zinc ion binding
catalytic activity;hydrolase activity
catalytic activity;glucosyltransferase activity;protein xylosyltransferase activity;transferase activity;transferase activity, transferring glycosyl groups;transferase activity, transferring hexosyl groups;transferase activity, transferrin
catalytic activity;oxidoreductase activity
binding;identical protein binding;protein binding;protein dimerization activity;protein domain specific binding;protein homodimerization activity
AU-rich element binding;binding;nucleic acid binding;RNA binding
binding;catalytic activity;cation binding;hydrolase activity;hydrolase activity, acting on ester bonds;ion binding;metal ion binding;transition metal ion binding;zinc ion binding
binding;catalytic activity;hydrolase activity;hydrolase activity, acting on acid anhydrides;hydrolase activity, acting on acid anhydrides, in phosphorus-containing anhydrides;m7G(5')pppN diphosphatase activity;nucleic acid bi
structural constituent of ribosome;structural molecule activity
protein binding transcription factor activity;transcription coactivator activity;transcription cofactor activity;transcription factor binding transcription factor activity
nucleobase-containing compound transmembrane transporter activity;nucleoside transmembrane transporter activity;substrate-specific transmembrane transporter activity;substrate-specific transporter activity;transmembra
structural constituent of ribosome;structural molecule activity
catalytic activity;GTPase activity;hydrolase activity;hydrolase activity, acting on acid anhydrides;hydrolase activity, acting on acid anhydrides, in phosphorus-containing anhydrides;nucleoside-triphosphatase activity;pyrophos
catalytic activity;hydrolase activity;hydrolase activity, acting on ester bonds;phosphatase activity;phosphoprotein phosphatase activity;phosphoric ester hydrolase activity;protein tyrosine phosphatase activity;protein tyrosine
binding;catalytic activity;DNA binding;DNA-directed RNA polymerase activity;nucleic acid binding;nucleotidyltransferase activity;RNA polymerase activity;transferase activity;transferase activity, transferring phosphorus-conta
structural constituent of ribosome;structural molecule activity
binding;identical protein binding;protein binding;protein dimerization activity;protein homodimerization activity
5S rRNA binding;binding;nucleic acid binding;RNA binding;rRNA binding;structural constituent of ribosome;structural molecule activity
adenyl nucleotide binding;adenyl ribonucleotide binding;ATP binding;binding;catalytic activity;enzyme inhibitor activity;enzyme regulator activity;kinase activity;kinase inhibitor activity;kinase regulator activity;MAP kinase kina
binding;lipid binding;PDZ domain binding;phosphatidylinositol binding;phosphatidylinositol-3,4-bisphosphate binding;phospholipid binding;protein binding;protein domain specific binding
catalytic activity;oxidoreductase activity
aminopeptidase activity;binding;catalytic activity;cation binding;exopeptidase activity;hydrolase activity;ion binding;manganese ion binding;metal ion binding;metallopeptidase activity;peptidase activity;peptidase activity, acti
structural constituent of ribosome;structural molecule activity
binding;catalytic activity;cation binding;ion binding;ligase activity;metal ion binding;transition metal ion binding;zinc ion binding
binding;nucleic acid binding;ribonucleoprotein binding;ribosome binding;RNA binding;translation factor activity, nucleic acid binding;translation initiation factor activity
catalytic activity;cation transmembrane transporter activity;hydrogen ion transmembrane transporter activity;inorganic cation transmembrane transporter activity;ion transmembrane transporter activity;monovalent inorganic c
nucleic acid binding transcription factor activity;sequence-specific DNA binding transcription factor activity

binding;protein binding;receptor binding
binding;cation binding;ion binding;metal ion binding;transition metal ion binding;zinc ion binding
binding;cation binding;ion binding;metal ion binding;transition metal ion binding;zinc ion binding
binding;catalytic activity;ceramide binding;ceramide transporter activity;kinase activity;lipid binding;lipid transporter activity;phosphatidylinositol binding;phosphatidylinositol-4-phosphate binding;phospholipid binding;phosp
binding;catalytic activity;GTP binding;guanyl nucleotide binding;guanyl ribonucleotide binding;guanylyltransferase activity;mannose-1-phosphate guanylyltransferase activity;mannose-phosphate guanylyltransferase activity;
binding;lipid binding
binding;catalytic activity;deoxyribonuclease activity;deoxyribonuclease II activity;DNA binding;endodeoxyribonuclease activity;endodeoxyribonuclease activity, producing 3'-phosphomonoesters;endonuclease activity;endonu
actin binding;binding;cation binding;cytoskeletal protein binding;ion binding;metal ion binding;protein binding;transition metal ion binding;zinc ion binding
binding;calcium ion binding;cation binding;ion binding;metal ion binding;transition metal ion binding;zinc ion binding
binding;cation binding;heme binding;ion binding;iron ion binding;metal ion binding;tetrapyrrole binding;transition metal ion binding
catalytic activity;NADH dehydrogenase (quinone) activity;NADH dehydrogenase (ubiquinone) activity;NADH dehydrogenase activity;oxidoreductase activity;oxidoreductase activity, acting on NADH or NADPH;oxidoreductase
binding;cation binding;ion binding;metal ion binding
binding;DNA binding;nucleic acid binding;protein binding transcription factor activity;ribonucleoprotein binding;transcription coactivator activity;transcription cofactor activity;transcription factor binding transcription factor ac
cation transmembrane transporter activity;hydrogen ion transmembrane transporter activity;inorganic cation transmembrane transporter activity;ion transmembrane transporter activity;monovalent inorganic cation transmemb
catalytic activity;NADH dehydrogenase (quinone) activity;NADH dehydrogenase (ubiquinone) activity;NADH dehydrogenase activity;oxidoreductase activity;oxidoreductase activity, acting on NADH or NADPH;oxidoreductase
binding;catalytic activity;cation binding;cysteine-type peptidase activity;hydrolase activity;ion binding;metal ion binding;metallopeptidase activity;peptidase activity;peptidase activity, acting on L-amino acid peptides;protein b
binding;identical protein binding;protein binding;protein dimerization activity;protein homodimerization activity
aldo-keto reductase (NADP) activity;binding;catalytic activity;cation binding;cation transmembrane transporter activity;cytochrome-c oxidase activity;enzyme binding;heme binding;heme-copper terminal oxidase activity;hydr
antioxidant activity;binding;catalytic activity;hemoglobin binding;protein binding
binding;cysteine-type endopeptidase inhibitor activity;endopeptidase inhibitor activity;endopeptidase regulator activity;enzyme binding;enzyme inhibitor activity;enzyme regulator activity;peptidase inhibitor activity;peptidase
alpha-galactosidase activity;binding;carbohydrate binding;catalytic activity;galactosidase activity;galactoside binding;hydrolase activity;hydrolase activity, acting on glycosyl bonds;hydrolase activity, hydrolyzing O-glycosyl c
binding;catalytic activity;collagen binding;cysteine-type endopeptidase activity;cysteine-type peptidase activity;endopeptidase activity;fibronectin binding;glycoprotein binding;histone binding;hydrolase activity;peptidase acti
adenyl nucleotide binding;adenyl ribonucleotide binding;ATP binding;binding;catalytic activity;enzyme binding;hepatocyte growth factor-activated receptor activity;kinase activity;molecular transducer activity;nucleotide bindi
6,7-dihydropteridine reductase activity;binding;catalytic activity;coenzyme binding;cofactor binding;electron carrier activity;NAD binding;NADH binding;NADP binding;NADPH binding;nucleotide binding;oxidoreductase activi
catalytic activity;cation transmembrane transporter activity;cytochrome-c oxidase activity;heme-copper terminal oxidase activity;hydrogen ion transmembrane transporter activity;inorganic cation transmembrane transporter a
binding;catalytic activity;DNA binding;DNA-directed RNA polymerase activity;nucleic acid binding;nucleotidyltransferase activity;RNA polymerase activity;transferase activity;transferase activity, transferring phosphorus-conta
2 iron, 2 sulfur cluster binding;binding;catalytic activity;cation binding;electron carrier activity;ion binding;iron-sulfur cluster binding;metal cluster binding;metal ion binding;NADH dehydrogenase (quinone) activity;NADH dehy
3'-5' DNA helicase activity;adenyl deoxyribonucleotide binding;adenyl nucleotide binding;adenyl ribonucleotide binding;ATP binding;ATPase activity;ATPase activity, coupled;ATP-dependent DNA helicase activity;ATP-depend
1-phosphatidylinositol-3-kinase regulator activity;binding;binding, bridging;cytokine receptor binding;enzyme binding;enzyme regulator activity;ErbB-3 class receptor binding;hormone binding;insulin binding;insulin receptor b
binding;damaged DNA binding;DNA binding;nucleic acid binding;single-stranded DNA binding;structure-specific DNA binding
aldo-keto reductase (NADP) activity;binding;catalytic activity;coenzyme binding;cofactor binding;NADP binding;nucleotide binding;oxidoreductase activity;oxidoreductase activity, acting on CH-OH group of donors;oxidoredu
binding;catalytic activity;cation binding;cytoskeletal protein binding;GDP binding;GTP binding;GTPase activity;guanyl nucleotide binding;guanyl ribonucleotide binding;hydrolase activity;hydrolase activity, acting on acid anhy
amine binding;amino acid binding;antioxidant activity;binding;carboxylic acid binding;catalytic activity;glutathione binding;glutathione peroxidase activity;modified amino acid binding;oxidoreductase activity;oxidoreductase a
binding;chromatin binding;hormone receptor binding;nuclear hormone receptor binding;protein binding;protein complex scaffold;protein domain specific binding;receptor binding;receptor signaling complex scaffold activity;S
1-phosphatidylinositol-3-kinase activity;adenyl nucleotide binding;adenyl ribonucleotide binding;ATP binding;binding;catalytic activity;enzyme activator activity;enzyme regulator activity;inositol or phosphatidylinositol kinase a
binding;calcium ion binding;cation binding;ion binding;metal ion binding;polyubiquitin binding;protein binding;small conjugating protein binding;ubiquitin binding
carboxypeptidase activity;catalytic activity;exopeptidase activity;hydrolase activity;peptidase activity;peptidase activity, acting on L-amino acid peptides;serine hydrolase activity;serine-type carboxypeptidase activity;serine-t
binding;catalytic activity;cis-trans isomerase activity;cyclosporin A binding;drug binding;isomerase activity;peptide binding;peptidyl-prolyl cis-trans isomerase activity
adenyl nucleotide binding;adenyl ribonucleotide binding;apoptotic protease activator activity;ATP binding;binding;caspase activator activity;caspase regulator activity;catalytic activity;enzyme activator activity;enzyme regulat
acid-amino acid ligase activity;binding;catalytic activity;enzyme binding;enzyme regulator activity;glutamate-cysteine ligase activity;glutamate-cysteine ligase catalytic subunit binding;ligase activity;ligase activity, forming carb
catalytic activity;transferase activity
adenyl nucleotide binding;adenyl ribonucleotide binding;androgen receptor binding;ATP binding;ATPase activity;ATPase activity, coupled;binding;catalytic activity;cyclin-dependent protein kinase activity;DNA-dependent ATP
acyl-CoA thioesterase activity;catalytic activity;CoA hydrolase activity;hydrolase activity;hydrolase activity, acting on ester bonds;palmitoyl-(protein) hydrolase activity;palmitoyl-CoA hydrolase activity;thiolester hydrolase activ
adenyl nucleotide binding;adenyl ribonucleotide binding;ATP binding;binding;catalytic activity;cation binding;coenzyme binding;cofactor binding;ion binding;isocitrate dehydrogenase (NAD+) activity;isocitrate dehydrogenase
active transmembrane transporter activity;ATPase activity;ATPase activity, coupled;ATPase activity, coupled to movement of substances;ATPase activity, coupled to transmembrane movement of ions;ATPase activity, couplec
binding;enzyme binding;GTPase binding;protein binding;Ran GTPase binding;Ras GTPase binding;small GTPase binding;transporter activity
binding;nucleic acid binding;RNA binding;structural constituent of ribosome;structural molecule activity
binding;cation binding;chromatin binding;enzyme regulator activity;identical protein binding;ion binding;kinase regulator activity;metal ion binding;protein binding;protein domain specific binding;protein kinase regulator activit
structural constituent of ribosome;structural molecule activity
binding;nucleic acid binding;RNA binding;rRNA binding;SSU rRNA binding;structural constituent of ribosome;structural molecule activity
binding;cation binding;electron carrier activity;electron transporter, transferring electrons from CoQH2-cytochrome c reductase complex and cytochrome c oxidase complex activity;heme binding;ion binding;iron ion binding;
binding;bubble DNA binding;damaged DNA binding;DNA binding;DNA insertion or deletion binding;DNA secondary structure binding;double-stranded DNA binding;loop DNA binding;mismatched DNA binding;nucleic acid bi
binding;catalytic activity;coenzyme binding;cofactor binding;dihydroorotate dehydrogenase activity;drug binding;FMN binding;nucleotide binding;oxidoreductase activity;oxidoreductase activity, acting on the CH-CH group o
adrenergic receptor binding;binding;carbohydrate binding;complement binding;complement component C1q binding;glycosaminoglycan binding;G-protein-coupled receptor binding;hyaluronic acid binding;kininogen binding
binding;enzyme activator activity;enzyme regulator activity;lipase activator activity;phospholipase activator activity;protein binding;SNAP receptor activity;SNARE binding;sphingomyelin phosphodiesterase activator activity
binding;nucleic acid binding;RNA binding;single-stranded RNA binding;tRNA binding
enzyme activator activity;enzyme regulator activity;GTPase activator activity;GTPase regulator activity;nucleoside-triphosphatase regulator activity
binding;enzyme regulator activity;GDP binding;GTP binding;guanyl nucleotide binding;guanyl ribonucleotide binding;nucleic acid binding;nucleotide binding;purine nucleotide binding;purine ribonucleoside triphosphate bindi
arylesterase activity;binding;carboxylic ester hydrolase activity;catalytic activity;cation binding;hydrolase activity;hydrolase activity, acting on ester bonds;identical protein binding;ion binding;metal ion binding;protein binding
binding;DNA binding;histone binding;methylated histone residue binding;nucleic acid binding;protein binding;regulatory region DNA binding;regulatory region nucleic acid binding;transcription regulatory region DNA binding
ATPase activity;ATPase activity, coupled;ATPase activity, coupled to movement of substances;binding;catalytic activity;enzyme activator activity;enzyme binding;enzyme regulator activity;GTPase activator activity;GTPase bi
binding;carbohydrate binding;catalytic activity;estradiol 17-beta-dehydrogenase activity;glycosaminoglycan binding;heparin binding;oxidoreductase activity;oxidoreductase activity, acting on CH-OH group of donors;oxidored
structural constituent of ribosome;structural molecule activity
binding;carbon-sulfur lyase activity;catalytic activity;cofactor binding;cysteine-S-conjugate beta-lyase activity;kynurenine-glyoxylate transaminase activity;kynurenine-oxoglutarate transaminase activity;lyase activity;pyridoxal
1-acylglycerol-3-phosphate O-acyltransferase activity;1-acylglycerophosphocholine O-acyltransferase activity;1-alkylglycerophosphocholine O-acetyltransferase activity;acetyltransferase activity;acylglycerol O-acyltransferas
binding;cation binding;DNA binding;identical protein binding;ion binding;metal ion binding;nucleic acid binding;protein binding;transition metal ion binding;zinc ion binding
binding;nucleotide binding
adenyl nucleotide binding;adenyl ribonucleotide binding;ATP binding;ATPase activity;ATPase activity, coupled;ATP-dependent helicase activity;ATP-dependent RNA helicase activity;binding;catalytic activity;helicase activity;h
binding;nucleotide binding
catalytic activity;intramolecular transferase activity;isomerase activity;pseudouridine synthase activity
aspartic endopeptidase activity, intramembrane cleaving;aspartic-type endopeptidase activity;aspartic-type peptidase activity;binding;catalytic activity;endopeptidase activity;enzyme binding;hydrolase activity;identical prote
binding;enzyme binding;enzyme regulator activity;GTPase binding;GTPase regulator activity;GTP-dependent protein binding;guanyl-nucleotide exchange factor activity;lipid binding;nucleoside-triphosphatase regulator activit
binding;protein binding;protein complex binding
binding;binding, bridging;protein binding;protein binding, bridging;SH3/SH2 adaptor activity;signaling adaptor activity
binding;calcium ion binding;cation binding;ion binding;metal ion binding;protein binding;protein domain specific binding;SH3 domain binding
catalytic activity
adenyl nucleotide binding;adenyl ribonucleotide binding;ATP binding;binding;cation binding;chaperone binding;cytokine receptor binding;enzyme binding;interferon-gamma receptor binding;ion binding;kinase binding;metal i
binding;enzyme binding;GTPase binding;kinase binding;protein binding;protein kinase binding;Ral GTPase binding;Ras GTPase binding;small GTPase binding
binding;cation binding;DNA binding;ion binding;metal ion binding;nucleic acid binding;transition metal ion binding;zinc ion binding
adenyl nucleotide binding;adenyl ribonucleotide binding;ATP binding;binding;nucleotide binding;purine nucleotide binding;purine ribonucleoside triphosphate binding;purine ribonucleotide binding;ribonucleotide binding
binding;lipid binding
protein transporter activity;receptor activity;substrate-specific transporter activity;transporter activity
acid-amino acid ligase activity;binding;catalytic activity;cation binding;chromatin binding;ion binding;ligase activity;ligase activity, forming carbon-nitrogen bonds;metal ion binding;protein binding;protein domain specific bind
binding;calcium-dependent protein binding;DNA binding;enzyme binding;identical protein binding;nucleic acid binding;protein binding;protein binding transcription factor activity;protein dimerization activity;protein homodime
binding;enzyme binding;MHC class I protein binding;MHC protein binding;protease binding;protein binding;receptor activity;receptor binding
catalytic activity;methyltransferase activity;N-terminal protein N-methyltransferase activity;protein methyltransferase activity;transferase activity;transferase activity, transferring one-carbon groups
adenosine-diphosphatase activity;ADP-ribose diphosphatase activity;ADP-sugar diphosphatase activity;catalytic activity;hydrolase activity;hydrolase activity, acting on acid anhydrides;hydrolase activity, acting on acid anhyd
binding;nucleic acid binding;nucleotide binding;RNA binding
binding;catalytic activity;enzyme binding;GTP binding;GTPase activity;GTPase binding;guanyl nucleotide binding;guanyl ribonucleotide binding;hydrolase activity;hydrolase activity, acting on acid anhydrides;hydrolase activity
beta-catenin binding;binding;DNA binding;histone binding;nucleic acid binding;protein binding;protein binding transcription factor activity;protein N-terminus binding;regulatory region DNA binding;regulatory region nucleic ac
adenyl nucleotide binding;adenyl ribonucleotide binding;ATP binding;binding;catalytic activity;cation binding;ion binding;kinase activity;metal ion binding;nucleotide binding;phosphotransferase activity, alcohol group as acce
catalytic activity;disulfide oxidoreductase activity;intramolecular oxidoreductase activity;intramolecular oxidoreductase activity, interconverting keto- and enol-groups;intramolecular oxidoreductase activity, transposing S-S b
binding;chromatin binding
binding;cation binding;ion binding;metal ion binding
carboxypeptidase activity;catalytic activity;exopeptidase activity;hydrolase activity;peptidase activity;peptidase activity, acting on L-amino acid peptides;serine hydrolase activity;serine-type carboxypeptidase activity;serine-t

catalytic activity;hydrolase activity;hydrolase activity, acting on acid anhydrides;hydrolase activity, acting on acid anhydrides, in phosphorus-containing anhydrides;microtubule motor activity;motor activity;nucleoside-triphosp
binding;carbohydrate binding
3'-5' exonuclease activity;3'-5'-exoribonuclease activity;AU-rich element binding;binding;catalytic activity;exonuclease activity;exonuclease activity, active with either ribo- or deoxyribonucleic acids and producing 5'-phospho
3'-5' exonuclease activity;3'-5'-exoribonuclease activity;binding;catalytic activity;exonuclease activity;exonuclease activity, active with either ribo- or deoxyribonucleic acids and producing 5'-phosphomonoesters;exoribonucl
active transmembrane transporter activity;adenyl nucleotide binding;adenyl ribonucleotide binding;ATP binding;ATPase activity;ATPase activity, coupled;ATPase activity, coupled to movement of substances;ATPase activity, c
adenyl nucleotide binding;adenyl ribonucleotide binding;ATP binding;binding;catalytic activity;kinase activity;nucleotide binding;pantothenate kinase activity;phosphotransferase activity, alcohol group as acceptor;purine nucl
structural constituent of ribosome;structural molecule activity
binding;cation binding;ion binding;metal ion binding
adenyl nucleotide binding;adenyl ribonucleotide binding;ATP binding;binding;catalytic activity;cation binding;GTP binding;guanyl nucleotide binding;guanyl ribonucleotide binding;guanylyltransferase activity;identical protein b
binding;nucleic acid binding;RNA binding;snoRNA binding
cation channel activity;cation transmembrane transporter activity;channel activity;ion channel activity;ion transmembrane transporter activity;passive transmembrane transporter activity;substrate-specific channel activity;sub
androgen receptor binding;binding;enzyme activator activity;enzyme binding;enzyme regulator activity;heat shock protein binding;histone binding;hormone receptor binding;identical protein binding;kinase activator activity;ki
adenyl nucleotide binding;adenyl ribonucleotide binding;ATP binding;binding;catalytic activity;identical protein binding;kinase activity;nucleotide binding;phosphotransferase activity, alcohol group as acceptor;protein binding;
acireductone synthase activity;binding;catalytic activity;cation binding;hydrolase activity;hydrolase activity, acting on ester bonds;ion binding;magnesium ion binding;metal ion binding;phosphatase activity;phosphoric ester h
acid-amino acid ligase activity;beta-amyloid binding;binding;carbohydrate binding;catalytic activity;ligase activity;ligase activity, forming carbon-nitrogen bonds;protein binding;small conjugating protein ligase activity;ubiquiti
binding;cation binding;heme binding;ion binding;iron ion binding;metal ion binding;tetrapyrrole binding;transition metal ion binding
receptor activity
binding;nucleic acid binding;RNA binding
binding;cation binding;chaperone binding;identical protein binding;ion binding;metal ion binding;protein binding;protein dimerization activity;protein homodimerization activity;transition metal ion binding;transporter activity;zi
binding;DNA binding;nucleic acid binding;protein binding;sequence-specific DNA binding;transcription factor binding
binding;protein binding;SNAP receptor activity;SNARE binding;syntaxin binding
adenyl nucleotide binding;adenyl ribonucleotide binding;ATP binding;binding;catalytic activity;cyclase activity;ligase activity;ligase activity, forming phosphoric ester bonds;nucleic acid binding;nucleotide binding;purine nucle
binding;channel inhibitor activity;channel regulator activity;chloride channel inhibitor activity;chloride channel regulator activity;ion channel inhibitor activity;protein binding;SNAP receptor activity;SNARE binding;syntaxin bind
adenyl nucleotide binding;adenyl ribonucleotide binding;ATP binding;binding;catalytic activity;cation binding;ion binding;kinase activity;magnesium ion binding;MAP kinase kinase activity;MAP kinase kinase kinase activity;me
binding;channel inhibitor activity;channel regulator activity;enzyme binding;enzyme inhibitor activity;enzyme regulator activity;ion channel inhibitor activity;phosphatase binding;phosphatase inhibitor activity;phosphatase regu
catalytic activity;NADH dehydrogenase (quinone) activity;NADH dehydrogenase (ubiquinone) activity;NADH dehydrogenase activity;oxidoreductase activity;oxidoreductase activity, acting on NADH or NADPH;oxidoreductase
alcohol binding;binding;carbohydrate binding;catalytic activity;dolichyl-phosphate beta-D-mannosyltransferase activity;dolichyl-phosphate-mannose-protein mannosyltransferase activity;mannose binding;mannosyltransferas
binding;double-stranded RNA binding;enzyme activator activity;enzyme binding;enzyme regulator activity;identical protein binding;nucleic acid binding;protein binding;protein dimerization activity;protein homodimerization ac
carboxylic ester hydrolase activity;catalytic activity;hydrolase activity;hydrolase activity, acting on ester bonds;lipase activity;lysophospholipase activity;palmitoyl-(protein) hydrolase activity;phospholipase activity;thiolester hy
binding;catalytic activity;cation binding;hydrolase activity;hydrolase activity, acting on ester bonds;ion binding;magnesium ion binding;manganese ion binding;metal ion binding;phosphatase activity;phosphoprotein phosphat
binding;protein binding;transcription factor binding
binding;cofactor binding;pyridoxal phosphate binding;vitamin B6 binding;vitamin binding
binding;cation binding;DNA binding;ion binding;metal ion binding;nucleic acid binding;transition metal ion binding;zinc ion binding
catalytic activity;NADH dehydrogenase (quinone) activity;NADH dehydrogenase (ubiquinone) activity;NADH dehydrogenase activity;oxidoreductase activity;oxidoreductase activity, acting on NADH or NADPH;oxidoreductase
catalytic activity;NADH dehydrogenase (quinone) activity;NADH dehydrogenase (ubiquinone) activity;NADH dehydrogenase activity;oxidoreductase activity;oxidoreductase activity, acting on NADH or NADPH;oxidoreductase
catalytic activity;oxidoreductase activity;oxidoreductase activity, acting on a sulfur group of donors;oxidoreductase activity, acting on a sulfur group of donors, disulfide as acceptor;protein-disulfide reductase (glutathione) act
adenyl nucleotide binding;adenyl ribonucleotide binding;ATP binding;binding;catalytic activity;cation binding;identical protein binding;ion binding;kinase activity;MAP kinase kinase kinase activity;metal ion binding;molecular t
adenyl nucleotide binding;adenyl ribonucleotide binding;ATP binding;binding;catalytic activity;CoA carboxylase activity;ligase activity;ligase activity, forming carbon-carbon bonds;nucleotide binding;propionyl-CoA carboxylas
androgen receptor binding;binding;core promoter binding;DNA binding;enzyme binding;hormone receptor binding;identical protein binding;kinase binding;nuclear hormone receptor binding;nucleic acid binding;nucleic acid b
bisphosphoglycerate 2-phosphatase activity;bisphosphoglycerate mutase activity;bisphosphoglycerate phosphatase activity;catalytic activity;hydrolase activity;hydrolase activity, acting on ester bonds;intramolecular transfer
catalytic activity;hydroxymethylbilane synthase activity;transferase activity;transferase activity, transferring alkyl or aryl (other than methyl) groups
binding;cation binding;identical protein binding;ion binding;metal ion binding;mRNA binding;nucleic acid binding;protein binding;protein dimerization activity;protein homodimerization activity;RNA binding;single-stranded RN
aryl sulfotransferase activity;catalytic activity;sulfotransferase activity;transferase activity;transferase activity, transferring sulfur-containing groups
2 iron, 2 sulfur cluster binding;binding;cation binding;electron carrier activity;ion binding;iron ion binding;iron-sulfur cluster binding;metal cluster binding;metal ion binding;transition metal ion binding
binding;catalytic activity;cation binding;cation transmembrane transporter activity;cytochrome-c oxidase activity;heme-copper terminal oxidase activity;hydrogen ion transmembrane transporter activity;inorganic cation transm
adenyl nucleotide binding;adenyl ribonucleotide binding;ATP binding;binding;binding, bridging;catalytic activity;cation binding;enzyme binding;ephrin receptor binding;growth factor receptor binding;heme binding;hormone re
binding;enzyme binding;protein binding
binding;complement binding;protein binding
binding;chromatin binding;core promoter proximal region DNA binding;core promoter proximal region sequence-specific DNA binding;DNA binding;identical protein binding;nucleic acid binding;nucleic acid binding transcripti
binding;catalytic activity;enzyme binding;hydrolase activity;hydrolase activity, acting on ester bonds;integrin binding;kinase binding;phosphatase activity;phosphoprotein phosphatase activity;phosphoric ester hydrolase activi
binding;nucleic acid binding;nucleotide binding;RNA binding
cation transmembrane transporter activity;hydrogen ion transmembrane transporter activity;inorganic cation transmembrane transporter activity;ion transmembrane transporter activity;monovalent inorganic cation transmemb
adenyl nucleotide binding;adenyl ribonucleotide binding;ATP binding;binding;catalytic activity;double-stranded RNA binding;enzyme regulator activity;eukaryotic translation initiation factor 2alpha kinase activity;kinase activity
adenyl nucleotide binding;adenyl ribonucleotide binding;adenylate kinase activity;ATP binding;binding;catalytic activity;GTP binding;guanyl nucleotide binding;guanyl ribonucleotide binding;kinase activity;nucleobase-contain
active transmembrane transporter activity;amine transmembrane transporter activity;amino acid transmembrane transporter activity;antiporter activity;arginine transmembrane transporter activity;basic amino acid transmemb
actin binding;actin monomer binding;adenyl nucleotide binding;adenyl-nucleotide exchange factor activity;ATPase regulator activity;binding;cytoskeletal protein binding;enzyme regulator activity;lipid binding;nucleoside-triph
aspartic-type endopeptidase activity;aspartic-type peptidase activity;catalytic activity;cyclin-dependent protein kinase inhibitor activity;cyclin-dependent protein kinase regulator activity;cysteine-type endopeptidase activity;c
catalytic activity;cysteine-type endopeptidase activity;cysteine-type peptidase activity;endopeptidase activity;hydrolase activity;peptidase activity;peptidase activity, acting on L-amino acid peptides
binding;catalytic activity;coenzyme binding;cofactor binding;flavin adenine dinucleotide binding;oxidoreductase activity;oxidoreductase activity, acting on the CH-CH group of donors;oxidoreductase activity, acting on the CH
binding;catalytic activity;GDP binding;GTP binding;GTPase activity;guanyl nucleotide binding;guanyl ribonucleotide binding;hydrolase activity;hydrolase activity, acting on acid anhydrides;hydrolase activity, acting on acid anh
catalytic activity;cyclin-dependent protein kinase regulator activity;enzyme regulator activity;kinase activity;kinase regulator activity;protein kinase regulator activity;transferase activity;transferase activity, transferring phospho
binding;enzyme binding;GTP binding;GTPase binding;GTP-dependent protein binding;guanyl nucleotide binding;guanyl ribonucleotide binding;lipid binding;nucleotide binding;phosphatidylinositol binding;phosphatidylinosito
binding;protein binding;protein complex binding;protein domain specific binding
binding;cytokine receptor binding;death receptor binding;protein binding;receptor binding;tumor necrosis factor receptor superfamily binding
cation transmembrane transporter activity;hydrogen ion transmembrane transporter activity;inorganic cation transmembrane transporter activity;ion transmembrane transporter activity;monovalent inorganic cation transmemb
binding;nucleic acid binding;ribonucleoprotein binding;ribosomal large subunit binding;ribosome binding;RNA binding;translation factor activity, nucleic acid binding;translation initiation factor activity
structural constituent of nuclear pore;structural molecule activity
binding;epidermal growth factor binding;growth factor binding;hormone binding;protein binding
binding;catalytic activity;GDP binding;GTP binding;GTPase activity;GTP-dependent protein binding;guanyl nucleotide binding;guanyl ribonucleotide binding;hydrolase activity;hydrolase activity, acting on acid anhydrides;hyd
binding;enzyme binding;enzyme regulator activity;phosphatase binding;phosphatase regulator activity;protein binding;protein phosphatase 2A binding;protein phosphatase binding;protein phosphatase regulator activity;prote
binding;chromatin binding;enzyme binding;protein binding
acetylcholine receptor activator activity;acetylcholine receptor regulator activity;adenyl nucleotide binding;adenyl ribonucleotide binding;ATP binding;binding;catalytic activity;cyclin-dependent protein kinase activity;ErbB-2 cl
AU-rich element binding;binding;nucleic acid binding;nucleotide binding;RNA binding
active transmembrane transporter activity;amine transmembrane transporter activity;amino acid transmembrane transporter activity;antigen binding;antiporter activity;aromatic amino acid transmembrane transporter activity;
catalytic activity;cysteine-type peptidase activity;hydrolase activity;peptidase activity;peptidase activity, acting on L-amino acid peptides;small conjugating protein-specific protease activity;ubiquitin-specific protease activity
BH domain binding;BH3 domain binding;binding;channel activity;death domain binding;identical protein binding;macromolecule transmembrane transporter activity;passive transmembrane transporter activity;protein binding
binding;catalytic activity;cation binding;coenzyme binding;cofactor binding;ion binding;metal ion binding;mRNA 3'-UTR binding;mRNA binding;NADP binding;NADPH binding;NADPH:quinone reductase activity;nucleic acid b
receptor activity
binding;enzyme regulator activity;GTP binding;GTPase regulator activity;guanyl nucleotide binding;guanyl ribonucleotide binding;guanyl-nucleotide exchange factor activity;nucleoside-triphosphatase regulator activity;nucleo
structural constituent of ribosome;structural molecule activity
binding;DNA binding;nucleic acid binding;nucleic acid binding transcription factor activity;protein binding transcription factor activity;sequence-specific DNA binding transcription factor activity;transcription coactivator activit
aminoacyl-tRNA hydrolase activity;binding;carboxylic ester hydrolase activity;catalytic activity;hydrolase activity;hydrolase activity, acting on ester bonds;nucleic acid binding;RNA binding;translation factor activity, nucleic aci
binding;calcium ion binding;cation binding;ion binding;metal ion binding
beta-catenin binding;binding;protein binding
binding;mRNA 3'-UTR binding;mRNA binding;nucleic acid binding;nucleotide binding;RNA binding
binding;catalytic activity;hydrolase activity;hydrolase activity, acting on ester bonds;phosphatase activity;phosphoprotein phosphatase activity;phosphoric ester hydrolase activity;protein binding;protein binding transcription f
binding;enzyme binding;protein binding;ubiquitin protein ligase binding
catalytic activity;NADH dehydrogenase (quinone) activity;NADH dehydrogenase (ubiquinone) activity;NADH dehydrogenase activity;oxidoreductase activity;oxidoreductase activity, acting on NADH or NADPH;oxidoreductase
adenyl nucleotide binding;adenyl ribonucleotide binding;ATP binding;binding;catalytic activity;guanylate kinase activity;kinase activity;nucleobase-containing compound kinase activity;nucleotide binding;nucleotide kinase ac
acid-thiol ligase activity;adenyl nucleotide binding;adenyl ribonucleotide binding;ATP binding;binding;catalytic activity;ligase activity;ligase activity, forming carbon-sulfur bonds;malonyl-CoA synthetase activity;nucleotide bin
acylglycerol kinase activity;adenyl nucleotide binding;adenyl ribonucleotide binding;ATP binding;binding;catalytic activity;ceramide kinase activity;diacylglycerol kinase activity;kinase activity;lipid kinase activity;NAD+ kinase a
acetylglucosaminyltransferase activity;catalytic activity;protein N-acetylglucosaminyltransferase activity;transferase activity;transferase activity, transferring glycosyl groups;transferase activity, transferring hexosyl groups;UDP
binding;enzyme binding;protein binding
binding;catalytic activity;cation binding;ion binding;metal ion binding;nucleotidyltransferase activity;RNA uridylyltransferase activity;transferase activity;transferase activity, transferring phosphorus-containing groups;transition
binding;catalytic activity;cation binding;cupric reductase activity;ferric-chelate reductase activity;ion binding;metal ion binding;oxidoreductase activity;oxidoreductase activity, oxidizing metal ions;oxidoreductase activity, oxid
catalytic activity
acid phosphatase activity;adenyl nucleotide binding;adenyl ribonucleotide binding;ATP binding;binding;catalytic activity;diphosphoinositol-pentakisphosphate kinase activity;hydrolase activity;hydrolase activity, acting on este
adenyl nucleotide binding;adenyl ribonucleotide binding;ATP binding;binding;catalytic activity;kinase activity;MAP kinase kinase kinase activity;molecular transducer activity;nucleotide binding;phosphotransferase activity, alc
binding;catalytic activity;cation binding;ion binding;metal ion binding;oxidoreductase activity;oxidoreductase activity, acting on CH or CH2 groups;oxidoreductase activity, acting on CH or CH2 groups, disulfide as acceptor;ri
catalytic activity;histone methyltransferase activity;histone methyltransferase activity (H3-K36 specific);histone methyltransferase activity (H3-K4 specific);histone-lysine N-methyltransferase activity;lysine N-methyltransferase
binding;nucleic acid binding;nucleotide binding;RNA binding
binding;carbohydrate binding;catalytic activity;cysteine-type endopeptidase activity;cysteine-type peptidase activity;drug binding;endopeptidase activity;frizzled binding;glycosaminoglycan binding;G-protein-coupled recepto
catalytic activity;cysteine-type peptidase activity;hydrolase activity;peptidase activity;peptidase activity, acting on L-amino acid peptides
structural constituent of ribosome;structural molecule activity
binding;nucleic acid binding;RNA binding
adenyl nucleotide binding;adenyl ribonucleotide binding;ATP binding;binding;nucleotide binding;purine nucleotide binding;purine ribonucleoside triphosphate binding;purine ribonucleotide binding;ribonucleotide binding

adenyl nucleotide binding;adenyl ribonucleotide binding;ATP binding;binding;catalytic activity;cation binding;enzyme binding;ion binding;kinase activity;kinase binding;metal ion binding;nucleotide binding;phosphotransferase
catalytic activity;hydrolase activity;hydrolase activity, acting on ester bonds;phosphatase activity;phosphoprotein phosphatase activity;phosphoric ester hydrolase activity
binding;cation binding;enzyme activator activity;enzyme regulator activity;GTPase activator activity;GTPase regulator activity;ion binding;lipid binding;metal ion binding;nucleoside-triphosphatase regulator activity;phosphatid
catalytic activity;cysteine-type peptidase activity;hydrolase activity;peptidase activity;peptidase activity, acting on L-amino acid peptides;small conjugating protein-specific protease activity;ubiquitin-specific protease activity
binding;catalytic activity;GTP binding;GTPase activity;guanyl nucleotide binding;guanyl ribonucleotide binding;hydrolase activity;hydrolase activity, acting on acid anhydrides;hydrolase activity, acting on acid anhydrides, in ph
structural constituent of ribosome;structural molecule activity
enzyme inhibitor activity;enzyme regulator activity;phosphatase inhibitor activity;phosphatase regulator activity;protein phosphatase inhibitor activity;protein phosphatase regulator activity
acetyltransferase activity;binding;carbohydrate binding;catalytic activity;glucosamine 6-phosphate N-acetyltransferase activity;identical protein binding;monosaccharide binding;N-acetyltransferase activity;N-acyltransferase a
binding;DNA binding;nucleic acid binding;RNA binding
binding;cation binding;ion binding;metal ion binding;nucleic acid binding
catalytic activity;hydrolase activity
binding;nucleic acid binding;RNA binding
catalytic activity;deacetylase activity;hydrolase activity;hydrolase activity, acting on glycosyl bonds
ligand-dependent nuclear receptor transcription coactivator activity;protein binding transcription factor activity;transcription coactivator activity;transcription cofactor activity;transcription factor binding transcription factor act
adenyl nucleotide binding;adenyl ribonucleotide binding;ATP binding;binding;catalytic activity;hydrolase activity;hydrolase activity, acting on acid anhydrides;hydrolase activity, acting on acid anhydrides, in phosphorus-conta
binding;channel inhibitor activity;channel regulator activity;chloride channel inhibitor activity;chloride channel regulator activity;ion channel inhibitor activity;protein binding;SNAP receptor activity;SNARE binding
binding;catalytic activity;cation binding;dITP diphosphatase activity;hydrolase activity;hydrolase activity, acting on acid anhydrides;hydrolase activity, acting on acid anhydrides, in phosphorus-containing anhydrides;ion bindi
structural constituent of ribosome;structural molecule activity
binding;identical protein binding;protein binding;protein dimerization activity;protein homodimerization activity
cation transmembrane transporter activity;divalent inorganic cation transmembrane transporter activity;inorganic cation transmembrane transporter activity;ion transmembrane transporter activity;magnesium ion transmembra
2 iron, 2 sulfur cluster binding;4 iron, 4 sulfur cluster binding;binding;cation binding;ferrous iron binding;ion binding;iron ion binding;iron-sulfur cluster binding;metal cluster binding;metal ion binding;protein binding;protein co
binding;cation binding;ion binding;metal ion binding;nucleotide binding;protein binding;SNARE binding;syntaxin binding;transition metal ion binding;zinc ion binding
catalytic activity;cis-trans isomerase activity;isomerase activity;peptidyl-prolyl cis-trans isomerase activity
catalytic activity;cysteine-type peptidase activity;hydrolase activity;peptidase activity;peptidase activity, acting on L-amino acid peptides
binding;enzyme binding;GTPase binding;protein binding;Ran GTPase binding;Ras GTPase binding;R-SMAD binding;SMAD binding;small GTPase binding
catalytic activity;methyltransferase activity;S-adenosylmethionine-dependent methyltransferase activity;transferase activity;transferase activity, transferring one-carbon groups
structural constituent of ribosome;structural molecule activity
carbon-carbon lyase activity;carboxy-lyase activity;catalytic activity;lyase activity;methylmalonyl-CoA decarboxylase activity
2,4-dienoyl-CoA reductase (NADPH) activity;binding;catalytic activity;oxidoreductase activity;oxidoreductase activity, acting on the CH-CH group of donors;oxidoreductase activity, acting on the CH-CH group of donors, NAD
catalytic activity;hydrolase activity;hydrolase activity, acting on glycosyl bonds;hydrolase activity, hydrolyzing O-glycosyl compounds
binding;leucine zipper domain binding;LRR domain binding;nucleic acid binding transcription factor activity;protein binding;protein domain specific binding;sequence-specific DNA binding transcription factor activity
binding;cation binding;ion binding;metal ion binding;transition metal ion binding;zinc ion binding
binding;protein binding;protein complex binding;small conjugating protein binding;ubiquitin binding
binding;nucleic acid binding;RNA binding;snRNA binding;U6 snRNA binding
binding;catalytic activity;GDP binding;GTP binding;GTPase activity;guanyl nucleotide binding;guanyl ribonucleotide binding;hydrolase activity;hydrolase activity, acting on acid anhydrides;hydrolase activity, acting on acid anh
binding;enzyme binding;phosphatase binding;protein binding;protein phosphatase binding
anion channel activity;anion transmembrane transporter activity;binding;catalytic activity;cation binding;channel activity;chloride channel activity;gated channel activity;hydrolase activity;intracellular calcium activated chloride
basal RNA polymerase II transcription machinery binding;basal transcription machinery binding;binding;enzyme binding;identical protein binding;nucleic acid binding;protein binding;RNA binding;RNA polymerase binding;RN
binding;catalytic activity;coenzyme binding;cofactor binding;flavin adenine dinucleotide binding;monooxygenase activity;oxidoreductase activity;oxidoreductase activity, acting on paired donors, with incorporation or reductio
binding;cation binding;ion binding;metal ion binding
3'-5' exonuclease activity;3'-5'-exoribonuclease activity;binding;catalytic activity;exonuclease activity;exonuclease activity, active with either ribo- or deoxyribonucleic acids and producing 5'-phosphomonoesters;exoribonucl
structural constituent of ribosome;structural molecule activity
structural constituent of ribosome;structural molecule activity
binding;catalytic activity;cation binding;hydrolase activity;ion binding;metal ion binding;protein binding;receptor binding
binding;cation binding;cation transmembrane transporter activity;copper chaperone activity;copper ion binding;copper ion transmembrane transporter activity;copper-dependent protein binding;inorganic cation transmembra
1-phosphatidylinositol-3-kinase activity;adenyl nucleotide binding;adenyl ribonucleotide binding;ATP binding;binding;catalytic activity;inositol or phosphatidylinositol kinase activity;kinase activity;lipid binding;lipid kinase acti
binding;catalytic activity;NADH dehydrogenase (quinone) activity;NADH dehydrogenase (ubiquinone) activity;NADH dehydrogenase activity;oxidoreductase activity;oxidoreductase activity, acting on NADH or NADPH;oxidore
antioxidant activity;binding;catalytic activity;cation binding;cation transmembrane transporter activity;copper ion binding;copper ion transmembrane transporter activity;disulfide oxidoreductase activity;inorganic cation transm
adenyl nucleotide binding;adenyl ribonucleotide binding;ATP binding;ATPase activity;ATPase activity, coupled;ATP-dependent DNA helicase activity;ATP-dependent helicase activity;binding;catalytic activity;core promoter bin
amine binding;amino acid binding;binding;carboxylic acid binding;catalytic activity;glutathione binding;intramolecular oxidoreductase activity;isomerase activity;modified amino acid binding;peptide binding;prostaglandin-E sy
binding;protein binding;receptor activity;TRAIL binding
binding;catalytic activity;histone binding;methylated histone residue binding;methyltransferase activity;protein binding;S-adenosylmethionine-dependent methyltransferase activity;transferase activity;transferase activity, trans
binding;carbohydrate binding;cation binding;cytoskeletal protein binding;DNA binding;glycosaminoglycan binding;heparin binding;ion binding;metal ion binding;microtubule binding;nucleic acid binding;nucleic acid binding tr
AU-rich element binding;binding;catalytic activity;enzyme binding;histone deacetylase binding;hydrolase activity;identical protein binding;mRNA binding;nucleic acid binding;protein binding;protein dimerization activity;protei
binding;cation binding;copper ion binding;ion binding;metal ion binding;transition metal ion binding
binding;protein binding;receptor binding
binding;protein binding;protein complex binding
catalytic activity;hydrolase activity
acid-amino acid ligase activity;binding;catalytic activity;cation binding;enzyme binding;enzyme regulator activity;GTPase regulator activity;guanyl-nucleotide exchange factor activity;heme binding;ion binding;iron ion binding;
binding;enzyme binding;kinase binding;NF-kappaB binding;protease binding;protein binding;protein binding transcription factor activity;protein C-terminus binding;protein kinase B binding;protein kinase binding;protein self-a
binding;core promoter proximal region DNA binding;core promoter proximal region sequence-specific DNA binding;DNA binding;double-stranded DNA binding;nucleic acid binding;nucleic acid binding transcription factor act
acetyl-CoA C-acyltransferase activity;acyl-CoA oxidase activity;C-acyltransferase activity;catalytic activity;oxidoreductase activity;oxidoreductase activity, acting on the CH-CH group of donors;oxidoreductase activity, acting
7S RNA binding;binding;nucleic acid binding;RNA binding
catalytic activity;cation transmembrane transporter activity;cytochrome-c oxidase activity;heme-copper terminal oxidase activity;hydrogen ion transmembrane transporter activity;inorganic cation transmembrane transporter a
binding;catalytic activity;dihydrolipoyllysine-residue (2-methylpropanoyl)transferase activity;enzyme binding;protein binding;transferase activity;transferase activity, transferring acyl groups;transferase activity, transferring acyl
binding;cell adhesion molecule binding;protein binding;receptor activity
beta-N-acetylgalactosaminidase activity;beta-N-acetylhexosaminidase activity;catalytic activity;enzyme activator activity;enzyme regulator activity;hexosaminidase activity;hydrolase activity;hydrolase activity, acting on glyco
binding;catalytic activity;GDP binding;GTP binding;GTPase activity;guanyl nucleotide binding;guanyl ribonucleotide binding;hydrolase activity;hydrolase activity, acting on acid anhydrides;hydrolase activity, acting on acid anh
adenyl nucleotide binding;adenyl ribonucleotide binding;ATP binding;binding;catalytic activity;cyclin-dependent protein kinase activity;kinase activity;nucleotide binding;phosphotransferase activity, alcohol group as acceptor
acid phosphatase activity;catalytic activity;hydrolase activity;hydrolase activity, acting on ester bonds;non-membrane spanning protein tyrosine phosphatase activity;phosphatase activity;phosphoprotein phosphatase activity
binding;enzyme binding;identical protein binding;kinase binding;molecular transducer activity;protein binding;receptor activity;signal transducer activity;signaling receptor activity;transmembrane signaling receptor activity
binding;catalytic activity;endopeptidase activity;hydrolase activity;lipopolysaccharide binding;peptidase activity;peptidase activity, acting on L-amino acid peptides;threonine-type endopeptidase activity;threonine-type peptic
catalytic activity;endopeptidase activity;hydrolase activity;peptidase activity;peptidase activity, acting on L-amino acid peptides;threonine-type endopeptidase activity;threonine-type peptidase activity
alkylbase DNA N-glycosylase activity;binding;catalytic activity;damaged DNA binding;DNA binding;DNA N-glycosylase activity;DNA-3-methyladenine glycosylase I activity;DNA-7-methylguanine glycosylase activity;hydrolase
3-hydroxyisobutyrate dehydrogenase activity;binding;catalytic activity;coenzyme binding;cofactor binding;NAD binding;nucleotide binding;oxidoreductase activity;oxidoreductase activity, acting on CH-OH group of donors;ox
binding;nucleic acid binding;receptor activity;RNA binding;translation factor activity, nucleic acid binding;translation initiation factor activity
adenyl nucleotide binding;adenyl ribonucleotide binding;ATP binding;binding;catalytic activity;cation binding;ion binding;kinase activity;lipid binding;metal ion binding;nucleotide binding;phospholipid binding;phosphotransfer
binding;nucleotide binding
catalytic activity;endopeptidase activity;hydrolase activity;peptidase activity;peptidase activity, acting on L-amino acid peptides;threonine-type endopeptidase activity;threonine-type peptidase activity
adenyl nucleotide binding;adenyl ribonucleotide binding;ATP binding;binding;catalytic activity;enzyme binding;kinase activity;kinase binding;nucleotide binding;phosphotransferase activity, alcohol group as acceptor;protein b
adenyl nucleotide binding;adenyl ribonucleotide binding;adenylyltransferase activity;ATP binding;binding;catalytic activity;cation binding;ion binding;magnesium ion binding;manganese ion binding;metal ion binding;nucleic ac
binding;cation binding;DNA binding;ion binding;metal ion binding;nucleic acid binding;RNA binding;transition metal ion binding;zinc ion binding
active transmembrane transporter activity;carboxylic acid transmembrane transporter activity;lactate transmembrane transporter activity;mevalonate transmembrane transporter activity;monocarboxylic acid transmembrane t
catalytic activity;cysteine-type endopeptidase activity;cysteine-type peptidase activity;endopeptidase activity;hydrolase activity;peptidase activity;peptidase activity, acting on L-amino acid peptides
binding;identical protein binding;protein binding
binding;DNA binding;nucleic acid binding;structural constituent of ribosome;structural molecule activity
acid-amino acid ligase activity;binding;catalytic activity;enzyme binding;ligase activity;ligase activity, forming carbon-nitrogen bonds;protein binding;small conjugating protein ligase activity;SUMO ligase activity;transcription f
active transmembrane transporter activity;adenyl nucleotide binding;adenyl ribonucleotide binding;ATP binding;ATPase activity;ATPase activity, coupled;ATPase activity, coupled to movement of substances;ATPase activity, c
binding;catalytic activity;DNA binding;endonuclease activity;enzyme inhibitor activity;enzyme regulator activity;hydrolase activity;hydrolase activity, acting on ester bonds;nuclease activity;nucleic acid binding;phosphatase inh
binding;identical protein binding;protein binding;protein dimerization activity;protein homodimerization activity
AP-1 adaptor complex binding;AP-3 adaptor complex binding;binding;catalytic activity;GTP binding;GTPase activity;GTP-dependent protein binding;guanyl nucleotide binding;guanyl ribonucleotide binding;hydrolase activity
binding;catalytic activity;cation binding;cis-trans isomerase activity;drug binding;FK506 binding;identical protein binding;ion binding;isomerase activity;macrolide binding;metal ion binding;peptidyl-prolyl cis-trans isomerase a
binding;polyubiquitin binding;protein binding;small conjugating protein binding;ubiquitin binding
binding;catalytic activity;cation binding;GDP binding;GTP binding;GTPase activity;guanyl nucleotide binding;guanyl ribonucleotide binding;hydrolase activity;hydrolase activity, acting on acid anhydrides;hydrolase activity, act
binding;protein binding;SNAP receptor activity;SNARE binding
binding;catalytic activity;coenzyme binding;cofactor binding;DNA binding;histone binding;methylated histone residue binding;NAD binding;nucleic acid binding;nucleotide binding;oxidoreductase activity;oxidoreductase activ
binding;calcium ion binding;cation binding;enzyme activator activity;enzyme regulator activity;GTPase activator activity;GTPase regulator activity;ion binding;metal ion binding;nucleoside-triphosphatase regulator activity
actin binding;actin filament binding;beta-tubulin binding;binding;cytoskeletal protein binding;DNA binding;microtubule binding;nucleic acid binding;protein binding;tubulin binding
binding;mRNA binding;nucleic acid binding;nucleotide binding;RNA binding
binding;PDZ domain binding;protein binding;protein domain specific binding
acetoacetate-CoA ligase activity;acid-thiol ligase activity;adenyl nucleotide binding;adenyl ribonucleotide binding;ATP binding;binding;butyrate-CoA ligase activity;catalytic activity;ligase activity;ligase activity, forming carbon
binding;protein binding;SNAP receptor activity;SNARE binding
enzyme activator activity;enzyme regulator activity;GTPase activator activity;GTPase regulator activity;nucleoside-triphosphatase regulator activity

2 iron, 2 sulfur cluster binding;binding;cation binding;identical protein binding;ion binding;iron-sulfur cluster binding;metal cluster binding;metal ion binding;protein binding;protein dimerization activity;protein homodimerizatio
active transmembrane transporter activity;calcium channel activity;cation channel activity;cation transmembrane transporter activity;channel activity;ion channel activity;ion transmembrane transporter activity;passive transme
catalytic activity;hydrolase activity
adenyl nucleotide binding;adenyl ribonucleotide binding;ATP binding;binding;catalytic activity;cation binding;ion binding;kinase activity;metal ion binding;nucleotide binding;phosphotransferase activity, alcohol group as acce
binding;endopeptidase inhibitor activity;endopeptidase regulator activity;enzyme inhibitor activity;enzyme regulator activity;peptidase inhibitor activity;peptidase regulator activity;proteasome binding;protein binding;protein co
AP-2 adaptor complex binding;binding;enzyme activator activity;enzyme regulator activity;GTPase activator activity;GTPase regulator activity;nucleoside-triphosphatase regulator activity;protein binding;protein complex bind
acyl-CoA dehydrogenase activity;binding;carboxylic acid binding;catalytic activity;coenzyme binding;cofactor binding;fatty acid binding;fatty-acyl-CoA binding;flavin adenine dinucleotide binding;glutaryl-CoA dehydrogenase
binding;catalytic activity;cation binding;identical protein binding;ion binding;isomerase activity;metal ion binding;protein binding;protein dimerization activity;protein homodimerization activity;racemase and epimerase activity;
binding;binding, bridging;protein binding;protein binding, bridging;ribonucleoprotein binding;ribosomal large subunit binding
binding;catalytic activity;DNA binding;N-acyltransferase activity;nucleic acid binding;sphingosine N-acyltransferase activity;transferase activity;transferase activity, transferring acyl groups;transferase activity, transferring acyl
actin binding;binding;cytoskeletal protein binding;enzyme binding;leucine zipper domain binding;LRR domain binding;protein binding;protein binding transcription factor activity;protein domain specific binding;transcription c
binding;C2H2 zinc finger domain binding;catalytic activity;cation binding;histone methyltransferase activity;histone methyltransferase activity (H3-K27 specific);histone methyltransferase activity (H3-K9 specific);histone-lysine
binding;lipid binding;phosphatidylinositol binding;phosphatidylinositol-3-phosphate binding;phospholipid binding
binding;cation binding;ion binding;metal ion binding;transition metal ion binding;zinc ion binding
catalytic activity;lysophospholipid acyltransferase activity;transferase activity;transferase activity, transferring acyl groups;transferase activity, transferring acyl groups other than amino-acyl groups
binding;mRNA binding;nucleic acid binding;RNA binding
binding;binding, bridging;estrogen receptor binding;hormone receptor binding;nuclear hormone receptor binding;protein binding;protein binding transcription factor activity;protein binding, bridging;protein complex scaffold;r
catalytic activity;dolichyl-phosphate-mannose-protein mannosyltransferase activity;mannosyltransferase activity;transferase activity;transferase activity, transferring glycosyl groups;transferase activity, transferring hexosyl gr
adenyl nucleotide binding;adenyl ribonucleotide binding;ATP binding;binding;catalytic activity;kinase activity;nucleotide binding;phosphotransferase activity, alcohol group as acceptor;protein kinase activity;protein serine/thre
adenyl nucleotide binding;adenyl ribonucleotide binding;alanine-tRNA ligase activity;aminoacyl-tRNA ligase activity;ATP binding;binding;catalytic activity;cation binding;ion binding;ligase activity;ligase activity, forming aminoa
1-phosphatidylinositol 4-kinase activity;adenyl nucleotide binding;adenyl ribonucleotide binding;AP-3 adaptor complex binding;ATP binding;binding;catalytic activity;cation binding;inositol or phosphatidylinositol kinase activi
binding;catalytic activity;cation binding;ion binding;metal ion binding;oxidoreductase activity;oxidoreductase activity, acting on the CH-CH group of donors;oxidoreductase activity, acting on the CH-CH group of donors, NAD
binding;cholesterol binding;lipid binding;steroid binding;sterol binding
structural constituent of ribosome;structural molecule activity
binding;nucleotide binding
acetyltransferase activity;catalytic activity;H4 histone acetyltransferase activity;histone acetyltransferase activity;lysine N-acetyltransferase activity;N-acetyltransferase activity;N-acyltransferase activity;peptide alpha-N-acetyl
adenyl nucleotide binding;adenyl ribonucleotide binding;ATP binding;ATPase activity;ATPase activity, coupled;ATP-dependent helicase activity;ATP-dependent RNA helicase activity;binding;catalytic activity;helicase activity;h
binding;catalytic activity;cation binding;dCTP diphosphatase activity;deoxyribonucleotide binding;hydrolase activity;hydrolase activity, acting on acid anhydrides;hydrolase activity, acting on acid anhydrides, in phosphorus-c
binding;nucleic acid binding;RNA binding
structural constituent of ribosome;structural molecule activity
binding;catalytic activity;cation binding;holo-[acyl-carrier-protein] synthase activity;ion binding;magnesium ion binding;metal ion binding;phosphotransferase activity, for other substituted phosphate groups;transferase activity
binding;identical protein binding;polyubiquitin binding;protein binding;small conjugating protein binding;ubiquitin binding
alpha-tubulin binding;beta-tubulin binding;binding;catalytic activity;cytoskeletal protein binding;GDP binding;GTP binding;GTPase activity;guanyl nucleotide binding;guanyl ribonucleotide binding;hydrolase activity;hydrolase
binding;catalytic activity;coenzyme binding;cofactor binding;FMN binding;nucleotide binding;oxidoreductase activity;oxidoreductase activity, acting on the CH-NH2 group of donors;oxidoreductase activity, acting on the CH-
adenyl nucleotide binding;adenyl ribonucleotide binding;adenylyltransferase activity;ATP binding;binding;catalytic activity;cation binding;identical protein binding;ion binding;magnesium ion binding;manganese ion binding;me
binding;DNA binding;nucleic acid binding;nucleic acid binding transcription factor activity;protein binding transcription factor activity;sequence-specific DNA binding;sequence-specific DNA binding transcription factor activit
adenyl nucleotide binding;adenyl ribonucleotide binding;ATP binding;binding;catalytic activity;NADH dehydrogenase (quinone) activity;NADH dehydrogenase (ubiquinone) activity;NADH dehydrogenase activity;nucleotide bind
binding;catalytic activity;cation binding;GMP reductase activity;ion binding;metal ion binding;oxidoreductase activity;oxidoreductase activity, acting on NADH or NADPH;oxidoreductase activity, acting on NADH or NADPH, ni
adenyl nucleotide binding;adenyl ribonucleotide binding;ATP binding;binding;catalytic activity;kinase activity;nucleic acid binding;nucleotide binding;phosphoprotein binding;phosphotransferase activity, alcohol group as acce
binding;protein binding;protein N-terminus binding
ADP-ribose diphosphatase activity;ADP-sugar diphosphatase activity;binding;catalytic activity;cation binding;hydrolase activity;hydrolase activity, acting on acid anhydrides;hydrolase activity, acting on acid anhydrides, in ph
binding;enzyme binding;enzyme regulator activity;heat shock protein binding;kinase binding;kinase regulator activity;NF-kappaB binding;protein binding;protein kinase binding;protein kinase regulator activity;small conjugatin
bent DNA binding;binding;catalytic activity;cis-trans isomerase activity;DNA binding;double-stranded DNA binding;isomerase activity;nucleic acid binding;peptidyl-prolyl cis-trans isomerase activity;structure-specific DNA bin
catalytic activity;oxidoreductase activity
binding;nucleotide binding
catalytic activity;cis-trans isomerase activity;isomerase activity;peptidyl-prolyl cis-trans isomerase activity
aminoacyl-tRNA hydrolase activity;carboxylic ester hydrolase activity;catalytic activity;hydrolase activity;hydrolase activity, acting on ester bonds
binding;catalytic activity;cysteine-type endopeptidase activity;cysteine-type peptidase activity;cytokine receptor binding;endopeptidase activity;hydrolase activity;identical protein binding;peptidase activity;peptidase activity,
adenyl nucleotide binding;adenyl ribonucleotide binding;ATP binding;binding;catalytic activity;kinase activity;MAP kinase kinase kinase kinase activity;molecular transducer activity;nucleotide binding;phosphotransferase activ
binding;mRNA binding;nucleic acid binding;nucleotide binding;RNA binding
binding;identical protein binding;protein binding
binding;catalytic activity;cell adhesion molecule binding;fibroblast growth factor receptor binding;growth factor receptor binding;kinase activity;molecular transducer activity;phosphotransferase activity, alcohol group as acce
adenyl nucleotide binding;adenyl ribonucleotide binding;ATP binding;binding;catalytic activity;cation binding;ion binding;kinase activity;metal ion binding;nucleotide binding;phosphotransferase activity, alcohol group as acce
protein binding transcription factor activity;transcription cofactor activity;transcription corepressor activity;transcription factor binding transcription factor activity
binding;catalytic activity;cation binding;hydrolase activity;hydrolase activity, acting on ester bonds;ion binding;metal ion binding;phosphatase activity;phosphoprotein phosphatase activity;phosphoric ester hydrolase activity;p
binding;cation binding;heme binding;ion binding;iron ion binding;ligand-dependent nuclear receptor activity;lipid binding;metal ion binding;molecular transducer activity;receptor activity;signal transducer activity;signaling rec
catalytic activity;deoxyribonucleoside 5'-monophosphate N-glycosidase activity;hydrolase activity;hydrolase activity, acting on glycosyl bonds;hydrolase activity, hydrolyzing N-glycosyl compounds;nucleoside deoxyribosyltra
active transmembrane transporter activity;adenyl nucleotide binding;adenyl ribonucleotide binding;ATP binding;ATPase activity;ATPase activity, coupled;ATPase activity, coupled to movement of substances;ATPase activity, c
binding;nucleic acid binding;RNA binding;translation factor activity, nucleic acid binding;translation initiation factor activity
binding;nucleotide binding
binding;catalytic activity;cation binding;dimethylallyltranstransferase activity;farnesyltranstransferase activity;geranyltranstransferase activity;ion binding;metal ion binding;prenyltransferase activity;transferase activity;transfera
catalytic activity;NADH dehydrogenase (quinone) activity;NADH dehydrogenase (ubiquinone) activity;NADH dehydrogenase activity;oxidoreductase activity;oxidoreductase activity, acting on NADH or NADPH;oxidoreductase
binding;catalytic activity;cation binding;cation transmembrane transporter activity;copper ion binding;cytochrome-c oxidase activity;heme-copper terminal oxidase activity;hydrogen ion transmembrane transporter activity;inc
adenosine deaminase activity;binding;catalytic activity;cation binding;deaminase activity;hydrolase activity;hydrolase activity, acting on carbon-nitrogen (but not peptide) bonds;hydrolase activity, acting on carbon-nitrogen (b
amidine-lyase activity;argininosuccinate lyase activity;carbon-nitrogen lyase activity;catalytic activity;lyase activity
binding;DNA binding;nucleic acid binding
carbon-carbon lyase activity;carboxy-lyase activity;catalytic activity;lyase activity;uroporphyrinogen decarboxylase activity
amine binding;amino acid binding;antioxidant activity;binding;carboxylic acid binding;catalytic activity;glutathione binding;glutathione peroxidase activity;modified amino acid binding;oxidoreductase activity;oxidoreductase a
ankyrin binding;beta-catenin binding;binding;calcium ion binding;cation binding;cell adhesion molecule binding;cytoskeletal protein binding;gamma-catenin binding;glycoprotein binding;GTPase activating protein binding;ion
adenyl nucleotide binding;adenyl ribonucleotide binding;ATP binding;binding;catalytic activity;DNA binding;helicase activity;hydrolase activity;hydrolase activity, acting on acid anhydrides;hydrolase activity, acting on acid anh
anion binding;binding;catalytic activity;cation binding;cyclohydrolase activity;hydrolase activity;hydrolase activity, acting on carbon-nitrogen (but not peptide) bonds;hydrolase activity, acting on carbon-nitrogen (but not peptic
binding;catalytic activity;chemoattractant activity;cytokine activity;cytokine receptor binding;dopachrome isomerase activity;intramolecular oxidoreductase activity;intramolecular oxidoreductase activity, interconverting keto-
catalytic activity;cation transmembrane transporter activity;cytochrome-c oxidase activity;heme-copper terminal oxidase activity;hydrogen ion transmembrane transporter activity;inorganic cation transmembrane transporter a
binding;catalytic activity;cation binding;hydrolase activity;hydrolase activity, acting on ester bonds;ion binding;metal ion binding;N-acetylglucosamine-6-sulfatase activity;sulfuric ester hydrolase activity
binding;calcium ion binding;catalytic activity;cation binding;cytokine receptor binding;enzyme binding;hydrolase activity;hydrolase activity, acting on ester bonds;ion binding;kinase binding;lipase activity;metal ion binding;mo
binding;catalytic activity;cation binding;cation transmembrane transporter activity;cytochrome-c oxidase activity;electron carrier activity;heme-copper terminal oxidase activity;hydrogen ion transmembrane transporter activity
amine binding;amino acid binding;binding;carboxylic acid binding;catalytic activity;cation binding;cobalamin binding;intramolecular transferase activity;ion binding;isomerase activity;metal ion binding;methylmalonyl-CoA mut
2 iron, 2 sulfur cluster binding;binding;catalytic activity;cation binding;ferrochelatase activity;ferrous iron binding;heme binding;ion binding;iron ion binding;iron-responsive element binding;iron-sulfur cluster binding;lyase activ
binding;catalytic activity;cis-trans isomerase activity;drug binding;FK506 binding;isomerase activity;macrolide binding;peptidyl-prolyl cis-trans isomerase activity
binding;catalytic activity;disulfide oxidoreductase activity;electron carrier activity;glutathione disulfide oxidoreductase activity;oxidoreductase activity;oxidoreductase activity, acting on a sulfur group of donors;peptide disulfid
binding;calcium-dependent protein serine/threonine phosphatase activity;calmodulin-dependent protein phosphatase activity;catalytic activity;cation binding;hydrolase activity;hydrolase activity, acting on ester bonds;ion bin
alpha-tubulin binding;binding;calcium ion binding;caspase inhibitor activity;caspase regulator activity;catalytic activity;cation binding;copper ion binding;cysteine-type endopeptidase inhibitor activity;cytoskeletal protein bind
catalytic activity;dodecenoyl-CoA delta-isomerase activity;intramolecular oxidoreductase activity;intramolecular oxidoreductase activity, transposing C=C bonds;isomerase activity
binding;calcium ion binding;calcium-dependent phospholipase A2 activity;calcium-dependent phospholipid binding;carboxylic ester hydrolase activity;catalytic activity;cation binding;hydrolase activity;hydrolase activity, actir
adenyl nucleotide binding;adenyl ribonucleotide binding;ATP binding;binding;calcium-dependent protein kinase activity;calcium-dependent protein serine/threonine kinase activity;calmodulin binding;calmodulin-dependent pr
adenyl nucleotide binding;adenyl ribonucleotide binding;ATP binding;binding;catalytic activity;GTP binding;guanyl nucleotide binding;guanyl ribonucleotide binding;identical protein binding;kinase activity;nucleotide binding;p
binding;nucleic acid binding;nucleotide binding;RNA 7-methylguanosine cap binding;RNA binding;RNA cap binding
binding;lipid binding;phosphatidylinositol binding;phosphatidylinositol-4-phosphate binding;phospholipid binding
4 iron, 4 sulfur cluster binding;adenyl nucleotide binding;adenyl ribonucleotide binding;ATP binding;binding;cation binding;ion binding;iron-sulfur cluster binding;metal cluster binding;metal ion binding;nucleotide binding;purin
binding;box C/D snoRNA binding;nucleic acid binding;RNA binding;snoRNA binding;snRNA binding;U3 snoRNA binding;U4 snRNA binding
catalytic activity;hydrolase activity;peptidase activity
acid-amino acid ligase activity;adenyl nucleotide binding;adenyl ribonucleotide binding;ATP binding;binding;catalytic activity;enzyme binding;ligase activity;ligase activity, forming carbon-nitrogen bonds;nucleotide binding;pr
binding;catalytic activity;GTP binding;GTPase activity;guanyl nucleotide binding;guanyl ribonucleotide binding;hydrolase activity;hydrolase activity, acting on acid anhydrides;hydrolase activity, acting on acid anhydrides, in pl
acid-amino acid ligase activity;binding;catalytic activity;enzyme binding;ligase activity;ligase activity, forming carbon-nitrogen bonds;protein binding;small conjugating protein ligase activity;SUMO ligase activity;ubiquitin prot
catalytic activity;enzyme regulator activity;hydrolase activity;hydrolase activity, acting on acid anhydrides;hydrolase activity, acting on acid anhydrides, in phosphorus-containing anhydrides;motor activity;nitric-oxide synthase
structural constituent of ribosome;structural molecule activity
binding;catalytic activity;GTP binding;GTPase activity;guanyl nucleotide binding;guanyl ribonucleotide binding;hydrolase activity;hydrolase activity, acting on acid anhydrides;hydrolase activity, acting on acid anhydrides, in pl
binding;DNA binding;nucleic acid binding;nucleic acid binding transcription factor activity;regulatory region DNA binding;regulatory region nucleic acid binding;RNA polymerase II regulatory region DNA binding;RNA polymera
binding;cation binding;identical protein binding;ion binding;metal ion binding;nucleic acid binding;nucleotide binding;protein binding;RNA binding;transition metal ion binding;zinc ion binding
binding;nucleotide binding
3'-5' exonuclease activity;3'-5'-exoribonuclease activity;AU-rich element binding;binding;catalytic activity;exonuclease activity;exonuclease activity, active with either ribo- or deoxyribonucleic acids and producing 5'-phosphe
acid-amino acid ligase activity;binding;catalytic activity;cation binding;CD40 receptor binding;cytokine receptor binding;enzyme binding;identical protein binding;ion binding;kinase binding;ligase activity;ligase activity, formin
catalytic activity;hydrolase activity;hydrolase activity, acting on ester bonds;inositol or phosphatidylinositol phosphatase activity;lipid phosphatase activity;phosphatase activity;phosphatidylinositol bisphosphate phosphatase
binding;cation binding;ion binding;metal ion binding;transition metal ion binding;zinc ion binding
aminopeptidase activity;carboxypeptidase activity;catalytic activity;cysteine-type endopeptidase activity;cysteine-type peptidase activity;endopeptidase activity;exopeptidase activity;hydrolase activity;peptidase activity;pept
binding;enzyme binding;enzyme regulator activity;GTPase regulator activity;guanyl-nucleotide exchange factor activity;kinase binding;nucleoside-triphosphatase regulator activity;protein binding;protein kinase binding;Ras gu
binding;cation binding;enzyme activator activity;enzyme regulator activity;GTPase activator activity;GTPase regulator activity;ion binding;metal ion binding;nucleoside-triphosphatase regulator activity;transition metal ion bind
catalytic activity;hydrolase activity;hydrolase activity, acting on ester bonds;phosphatase activity;phosphatidate phosphatase activity;phosphoric ester hydrolase activity;protein binding transcription factor activity;transcriptio

binding;enzyme activator activity;enzyme regulator activity;histone acetyl-lysine binding;histone binding;peptidase activator activity;peptidase regulator activity;protein binding
binding;enzyme binding;kinase binding;mitogen-activated protein kinase binding;protein binding;protein kinase binding
binding;endopeptidase activator activity;endopeptidase regulator activity;enzyme activator activity;enzyme binding;enzyme regulator activity;peptidase activator activity;peptidase regulator activity;protease binding;proteasor
catalytic activity;endopeptidase activity;hydrolase activity;peptidase activity;peptidase activity, acting on L-amino acid peptides;serine hydrolase activity;serine-type endopeptidase activity;serine-type peptidase activity
5S rRNA binding;binding;catalytic activity;nucleic acid binding;RNA binding;rRNA binding;sulfurtransferase activity;thiosulfate sulfurtransferase activity;transferase activity;transferase activity, transferring sulfur-containing gro
binding;catalytic activity;cation binding;hydrolase activity;hydrolase activity, acting on ester bonds;hydroxyacylglutathione hydrolase activity;ion binding;metal ion binding;thiolester hydrolase activity;transition metal ion bindin
catalytic activity;transferase activity;transferase activity, transferring glycosyl groups;transferase activity, transferring pentosyl groups;uridine phosphorylase activity
binding;enzyme binding;phosphatase binding;protein binding;protein phosphatase binding
5'-nucleotidase activity;binding;catalytic activity;cation binding;hydrolase activity;hydrolase activity, acting on ester bonds;ion binding;metal ion binding;nucleotidase activity;phosphatase activity;phosphoric ester hydrolase a
binding;enzyme binding;enzyme regulator activity;GTPase binding;GTPase regulator activity;guanyl-nucleotide exchange factor activity;nucleoside-triphosphatase regulator activity;PDZ domain binding;protein binding;protein
enzyme activator activity;enzyme regulator activity;GTPase activator activity;GTPase regulator activity;nucleoside-triphosphatase regulator activity
structural constituent of ribosome;structural molecule activity
carboxylic ester hydrolase activity;catalytic activity;hydrolase activity;hydrolase activity, acting on ester bonds
catalytic activity;L-xylulose reductase (NADP+) activity;oxidoreductase activity;oxidoreductase activity, acting on CH-OH group of donors;oxidoreductase activity, acting on NADH or NADPH;oxidoreductase activity, acting on
adenyl nucleotide binding;adenyl ribonucleotide binding;ATP binding;binding;carbohydrate binding;catalytic activity;glycosaminoglycan binding;helicase activity;heparin binding;hydrolase activity;hydrolase activity, acting on a
catalytic activity;methyltransferase activity;transferase activity;transferase activity, transferring one-carbon groups
adenyl nucleotide binding;adenyl ribonucleotide binding;ATP binding;ATPase activity;ATPase activity, coupled;binding;catalytic activity;hydrolase activity;hydrolase activity, acting on acid anhydrides;hydrolase activity, acting o
binding;catalytic activity;cation binding;cysteine-type peptidase activity;hydrolase activity;ion binding;metal ion binding;metallopeptidase activity;nucleic acid binding;peptidase activity;peptidase activity, acting on L-amino ac
adenyl nucleotide binding;adenyl ribonucleotide binding;ATP binding;binding;catalytic activity;ligase activity;nucleotide binding;purine nucleotide binding;purine ribonucleoside triphosphate binding;purine ribonucleotide bindi
binding;cytoskeletal protein binding;kinesin binding;protein binding
binding;identical protein binding;nucleic acid binding;protein binding;RNA binding
binding;cation binding;ion binding;metal ion binding;nucleic acid binding
acid-amino acid ligase activity;binding;catalytic activity;cation binding;ion binding;ligase activity;ligase activity, forming carbon-nitrogen bonds;metal ion binding;nucleic acid binding;small conjugating protein ligase activity;ub
acid-amino acid ligase activity;binding;catalytic activity;cation binding;DNA binding;histone binding;ion binding;ligase activity;ligase activity, forming carbon-nitrogen bonds;metal ion binding;nucleic acid binding;protein bindi
catalytic activity;endopeptidase activity;hydrolase activity;peptidase activity;peptidase activity, acting on L-amino acid peptides;threonine-type endopeptidase activity;threonine-type peptidase activity
protein binding transcription factor activity;transcription cofactor activity;transcription corepressor activity;transcription factor binding transcription factor activity
adenyl nucleotide binding;adenyl ribonucleotide binding;ATP binding;binding;catalytic activity;chromatin binding;enzyme binding;histone binding;histone kinase activity;histone kinase activity (H3-S10 specific);histone kinase
binding;calcium ion binding;cation binding;identical protein binding;ion binding;metal ion binding;protein binding;protein dimerization activity;protein heterodimerization activity
antioxidant activity
binding;nucleic acid binding;RNA binding;translation activator activity;translation regulator activity;translation regulator activity, nucleic acid binding
active transmembrane transporter activity;amine transmembrane transporter activity;amino acid transmembrane transporter activity;carboxylic acid transmembrane transporter activity;cation transmembrane transporter activi
acetyltransferase activity;binding;catalytic activity;cation binding;enzyme regulator activity;H3 histone acetyltransferase activity;H4 histone acetyltransferase activity;histone acetyltransferase activity;ion binding;iron-sulfur clus
binding;catalytic activity;cation binding;DNA binding;DNA-directed RNA polymerase activity;ion binding;metal ion binding;nucleic acid binding;nucleoside binding;nucleotidyltransferase activity;ribonucleoside binding;RNA po
1-phosphatidylinositol binding;binding;lipid binding;phosphatidylinositol binding;phosphatidylinositol-3,5-bisphosphate binding;phosphatidylinositol-3-phosphate binding;phosphatidylinositol-4-phosphate binding;phosphatic
antioxidant activity;binding;catalytic activity;coenzyme binding;cofactor binding;flavin adenine dinucleotide binding;oxidoreductase activity;oxidoreductase activity, acting on a sulfur group of donors;oxidoreductase activity, a
binding;catalytic activity;GDP binding;GTP binding;GTPase activity;guanyl nucleotide binding;guanyl ribonucleotide binding;hydrolase activity;hydrolase activity, acting on acid anhydrides;hydrolase activity, acting on acid anh
carbohydrate phosphatase activity;catalytic activity;fructose-2,6-bisphosphate 2-phosphatase activity;hydrolase activity;hydrolase activity, acting on ester bonds;phosphatase activity;phosphoric ester hydrolase activity;suga
binding;GTP binding;guanyl nucleotide binding;guanyl ribonucleotide binding;nucleotide binding;purine nucleotide binding;purine ribonucleoside triphosphate binding;purine ribonucleotide binding;ribonucleotide binding
acid-amino acid ligase activity;Atg12 ligase activity;Atg8 ligase activity;binding;catalytic activity;enzyme binding;ligase activity;ligase activity, forming carbon-nitrogen bonds;protein binding;small conjugating protein ligase act
binding;catalytic activity;cation binding;hydrolase activity;hydrolase activity, acting on ester bonds;ion binding;lipase activity;metal ion binding;phospholipase activity;phosphoric diester hydrolase activity;phosphoric ester hyd
2 iron, 2 sulfur cluster binding;binding;cation binding;ion binding;iron-sulfur cluster binding;metal cluster binding;metal ion binding
structural constituent of ribosome;structural molecule activity
binding;nucleic acid binding;RNA binding
binding;calcium ion binding;cation binding;ion binding;metal ion binding
anion binding;binding;ion binding;molecular transducer activity;PDZ domain binding;phosphate ion binding;protein binding;protein domain specific binding;signal transducer activity
binding;cation binding;ion binding;metal ion binding
binding;nucleic acid binding;RNA binding;structural constituent of ribosome;structural molecule activity
binding;enzyme binding;GTPase binding;protein binding;Rab GTPase binding;Ras GTPase binding;small GTPase binding
structural constituent of ribosome;structural molecule activity
enzyme regulator activity;GTPase regulator activity;guanyl-nucleotide exchange factor activity;nucleoside-triphosphatase regulator activity;Rac guanyl-nucleotide exchange factor activity;Ras guanyl-nucleotide exchange fact
active transmembrane transporter activity;ATPase activity;ATPase activity, coupled;ATPase activity, coupled to movement of substances;ATPase activity, coupled to transmembrane movement of substances;catalytic activity;
catalytic activity;dolichyl-phosphate beta-glucosyltransferase activity;glucosyltransferase activity;oligosaccharyl transferase activity;transferase activity;transferase activity, transferring glycosyl groups;transferase activity, tran
binding;cation binding;enzyme binding;identical protein binding;ion binding;K63-linked polyubiquitin binding;metal ion binding;molecular transducer activity;polyubiquitin binding;protein binding;protein dimerization activity;pr
4 iron, 4 sulfur cluster binding;binding;catalytic activity;cation binding;ion binding;iron-sulfur cluster binding;metal cluster binding;metal ion binding;NADH dehydrogenase (quinone) activity;NADH dehydrogenase (ubiquinone)
bHLH transcription factor binding;binding;protein binding;protein binding transcription factor activity;transcription coactivator activity;transcription cofactor activity;transcription factor binding;transcription factor binding trans
acetylglucosaminyltransferase activity;binding;catalytic activity;enzyme activator activity;enzyme regulator activity;lipid binding;phosphatidylinositol binding;phosphatidylinositol-3,4,5-trisphosphate binding;phospholipid bindi
branched-chain-amino-acid transaminase activity;catalytic activity;L-isoleucine transaminase activity;L-leucine transaminase activity;L-valine transaminase activity;transaminase activity;transferase activity;transferase activity
binding;nucleic acid binding;RNA binding;snRNA binding;U4 snRNA binding;U6 snRNA binding
binding;catalytic activity;cis-trans isomerase activity;cyclosporin A binding;drug binding;isomerase activity;peptide binding;peptidyl-prolyl cis-trans isomerase activity;ribonucleoprotein binding
binding;clathrin binding;protein binding
binding;binding, bridging;calcium ion binding;calcium-dependent cysteine-type endopeptidase activity;calcium-dependent protein binding;catalytic activity;cation binding;cysteine-type endopeptidase activity;cysteine-type p
amine binding;amino acid binding;binding;carboxylic acid binding;catalytic activity;cation binding;dimethylargininase activity;hydrolase activity;hydrolase activity, acting on carbon-nitrogen (but not peptide) bonds;hydrolase a
binding;carbohydrate binding;catalytic activity;glutamine-fructose-6-phosphate transaminase (isomerizing) activity;transaminase activity;transferase activity;transferase activity, transferring nitrogenous groups
binding;cation binding;ion binding;metal ion binding;transition metal ion binding;zinc ion binding
binding;catalytic activity;enzyme binding;GTP binding;GTPase activity;guanyl nucleotide binding;guanyl ribonucleotide binding;hydrolase activity;hydrolase activity, acting on acid anhydrides;hydrolase activity, acting on acid
APG12 activating enzyme activity;APG8 activating enzyme activity;binding;catalytic activity;identical protein binding;protein binding;protein dimerization activity;protein homodimerization activity;small protein activating enzym
adenyl nucleotide binding;adenyl ribonucleotide binding;ATP binding;binding;catalytic activity;cation binding;ion binding;kinase activity;magnesium ion binding;metal ion binding;molecular transducer activity;nucleotide bindir
channel activity;macromolecule transmembrane transporter activity;passive transmembrane transporter activity;porin activity;protein transmembrane transporter activity;protein transporter activity;substrate-specific transmen
antioxidant activity;binding;catalytic activity;cation binding;DNA binding;identical protein binding;ion binding;manganese ion binding;metal ion binding;nucleic acid binding;oxidoreductase activity;oxidoreductase activity, actir
acetylcholine receptor binding;binding;carbohydrate binding;cation binding;DNA binding;endopeptidase inhibitor activity;endopeptidase regulator activity;enzyme activator activity;enzyme binding;enzyme inhibitor activity;enz
adenyl nucleotide binding;adenyl ribonucleotide binding;ATP binding;binding;catalytic activity;enzyme binding;ion channel binding;kinase activity;non-membrane spanning protein tyrosine kinase activity;nucleotide binding;ph
binding;nucleic acid binding;RNA binding;structural constituent of ribosome;structural molecule activity
binding;chromatin binding;chromatin DNA binding;core promoter binding;core promoter proximal region DNA binding;core promoter proximal region sequence-specific DNA binding;core promoter sequence-specific DNA bin
amine binding;amino acid binding;antioxidant activity;binding;carboxylic acid binding;catalytic activity;glutathione binding;glutathione peroxidase activity;glutathione transferase activity;modified amino acid binding;oxidoredu
aminopeptidase activity;binding;carboxypeptidase activity;catalytic activity;cation binding;dipeptidase activity;exopeptidase activity;hydrolase activity;ion binding;manganese ion binding;metal ion binding;metallocarboxypept
binding;binding, bridging;cytoskeletal adaptor activity;cytoskeletal protein binding;enzyme inhibitor activity;enzyme regulator activity;eukaryotic initiation factor eIF2 binding;kinase inhibitor activity;kinase regulator activity;pro
binding;catalytic activity;epidermal growth factor receptor binding;growth factor receptor binding;GTP binding;GTPase activity;guanyl nucleotide binding;guanyl ribonucleotide binding;hydrolase activity;hydrolase activity, acti
binding;damaged DNA binding;DNA binding;enzyme binding;nucleic acid binding;protein binding
binding;catalytic activity;GDP binding;GTP binding;GTPase activity;guanyl nucleotide binding;guanyl ribonucleotide binding;hydrolase activity;hydrolase activity, acting on acid anhydrides;hydrolase activity, acting on acid anh
binding;catalytic activity;catechol O-methyltransferase activity;cation binding;ion binding;magnesium ion binding;metal ion binding;methyltransferase activity;O-methyltransferase activity;S-adenosylmethionine-dependent me
binding;calcium ion binding;cation binding;ion binding;metal ion binding
catalytic activity;endopeptidase activity;hydrolase activity;peptidase activity;peptidase activity, acting on L-amino acid peptides;threonine-type endopeptidase activity;threonine-type peptidase activity
actin binding;actin filament binding;binding;calmodulin binding;cytoskeletal protein binding;protein binding
binding;catalytic activity;cation binding;dCMP deaminase activity;deaminase activity;hydrolase activity;hydrolase activity, acting on carbon-nitrogen (but not peptide) bonds;hydrolase activity, acting on carbon-nitrogen (but no
aldehyde-lyase activity;amine binding;amino acid binding;binding;carbon-carbon lyase activity;carboxylic acid binding;catalytic activity;cofactor binding;glycine hydroxymethyltransferase activity;hydroxymethyl-, formyl- and
actin binding;actin filament binding;binding;cytoskeletal protein binding;identical protein binding;protein binding;protein dimerization activity;protein heterodimerization activity;protein homodimerization activity;spectrin bindin
active transmembrane transporter activity;ATPase activity;ATPase activity, coupled;ATPase activity, coupled to movement of substances;ATPase activity, coupled to transmembrane movement of ions;ATPase activity, couplec
binding;cation binding;DNA binding;ion binding;metal ion binding;nucleic acid binding;structural constituent of ribosome;structural molecule activity;transition metal ion binding;zinc ion binding
beta-tubulin binding;binding;cytoskeletal protein binding;diazepam binding;drug binding;dynactin binding;dynein binding;dynein intermediate chain binding;identical protein binding;ion channel binding;p53 binding;profilin bin
adenyl nucleotide binding;adenyl ribonucleotide binding;ATP binding;binding;catalytic activity;enzyme binding;kinase activity;kinase binding;MAP kinase kinase activity;nucleotide binding;phosphotransferase activity, alcohol
actin binding;beta-tubulin binding;binding;cytoskeletal protein binding;protein binding;tubulin binding
adenyl nucleotide binding;adenyl ribonucleotide binding;ATP binding;binding;catalytic activity;chromatin binding;cyclin-dependent protein kinase activity;DNA binding;kinase activity;nucleic acid binding;nucleotide binding;ph
binding;catalytic activity;NADH dehydrogenase (quinone) activity;NADH dehydrogenase (ubiquinone) activity;NADH dehydrogenase activity;oxidoreductase activity;oxidoreductase activity, acting on NADH or NADPH;oxidored
binding;carbon-sulfur lyase activity;catalytic activity;cation binding;holocytochrome-c synthase activity;ion binding;lyase activity;metal ion binding

actin binding;binding;cytoskeletal protein binding;protein binding
acid-amino acid ligase activity;adenyl nucleotide binding;adenyl ribonucleotide binding;ATP binding;binding;catalytic activity;enzyme binding;ligase activity;ligase activity, forming carbon-nitrogen bonds;nucleotide binding;pr
binding;catalytic activity;enzyme binding;GTP binding;GTPase activity;guanyl nucleotide binding;guanyl ribonucleotide binding;hydrolase activity;hydrolase activity, acting on acid anhydrides;hydrolase activity, acting on acid
catalytic activity;endonuclease activity;endonuclease activity, active with either ribo- or deoxyribonucleic acids and producing 5'-phosphomonoesters;endoribonuclease activity;endoribonuclease activity, producing 5'-phosph
binding;carbohydrate binding;cation binding;DNA binding;endopeptidase inhibitor activity;endopeptidase regulator activity;enzyme inhibitor activity;enzyme regulator activity;glycosaminoglycan binding;heparin binding;identi
binding;catalytic activity;cation binding;hydrolase activity;intramolecular oxidoreductase activity;intramolecular oxidoreductase activity, transposing C=C bonds;ion binding;isomerase activity;isopentenyl-diphosphate delta-is
binding;catalytic activity;cation binding;diphosphotransferase activity;enzyme inhibitor activity;enzyme regulator activity;ion binding;magnesium ion binding;metal ion binding;ribose phosphate diphosphokinase activity;transfe
3'-5' exonuclease activity;3'-5'-exoribonuclease activity;binding;catalytic activity;exonuclease activity;exonuclease activity, active with either ribo- or deoxyribonucleic acids and producing 5'-phosphomonoesters;exoribonucl
adenyl nucleotide binding;adenyl ribonucleotide binding;ATP binding;binding;catalytic activity;cis-trans isomerase activity;enzyme activator activity;enzyme binding;enzyme regulator activity;identical protein binding;isomeras
beta-catenin binding;binding;catalytic activity;enzyme binding;gamma-catenin binding;hydrolase activity;hydrolase activity, acting on ester bonds;kinase binding;molecular transducer activity;phosphatase activity;phosphopr
binding;enzyme binding;protein binding;ubiquitin protein ligase binding
enzyme regulator activity;phosphatase regulator activity;protein phosphatase regulator activity;protein phosphatase type 1 regulator activity
active transmembrane transporter activity;amine transmembrane transporter activity;amino acid transmembrane transporter activity;anion transmembrane transporter activity;anion:cation symporter activity;carboxylic acid tra
binding;nucleic acid binding;RNA binding;rRNA binding;snoRNA binding;U3 snoRNA binding
binding;chromatin binding
binding;cation binding;DNA binding;ion binding;metal ion binding;nucleic acid binding;nucleic acid binding transcription factor activity;regulatory region DNA binding;regulatory region nucleic acid binding;RNA polymerase II c
catalytic activity;cytidylyltransferase activity;N-acylneuraminate cytidylyltransferase activity;nucleotidyltransferase activity;transferase activity;transferase activity, transferring phosphorus-containing groups
binding;enzyme binding;GTPase binding;identical protein binding;protein binding;protein dimerization activity;protein homodimerization activity;Rab GTPase binding;Ras GTPase binding;small GTPase binding
acyl-CoA hydrolase activity;binding;carboxylic ester hydrolase activity;catalytic activity;CoA hydrolase activity;hydrolase activity;hydrolase activity, acting on ester bonds;lipid binding;thiolester hydrolase activity
binding;cytokine activity;protein binding;receptor binding
binding;calcium channel activity;cation channel activity;cation transmembrane transporter activity;channel activity;enzyme binding;ion channel activity;ion transmembrane transporter activity;passive transmembrane transport
active transmembrane transporter activity;ATPase activity;ATPase activity, coupled;ATPase activity, coupled to movement of substances;ATPase activity, coupled to transmembrane movement of ions;ATPase activity, coupled
binding;cation binding;ion binding;metal ion binding;transition metal ion binding;zinc ion binding
binding;nucleic acid binding;nucleotide binding;RNA binding;RNA stem-loop binding
adenyl nucleotide binding;adenyl ribonucleotide binding;adenylyltransferase activity;ATP binding;binding;catalytic activity;nucleic acid binding;nucleotide binding;nucleotidyltransferase activity;purine nucleotide binding;purine
binding;DNA binding;nucleic acid binding
binding;calcium ion binding;cation binding;copper ion binding;fibroblast growth factor binding;growth factor binding;identical protein binding;ion binding;lipid binding;metal ion binding;protein binding;protein dimerization acti
binding;cation binding;ion binding;metal ion binding;transition metal ion binding;zinc ion binding
binding;high-density lipoprotein particle binding;lipid binding;lipoprotein particle binding;protein binding;protein-lipid complex binding;receptor binding
ADP-specific glucokinase activity;binding;catalytic activity;cation binding;ion binding;kinase activity;metal ion binding;phosphotransferase activity, alcohol group as acceptor;transferase activity;transferase activity, transferring
binding;enzyme inhibitor activity;enzyme regulator activity;histone binding;phosphatase inhibitor activity;phosphatase regulator activity;protein binding
binding;cytoskeletal protein binding;enzyme binding;gamma-tubulin binding;GTPase binding;protein binding;Rab GTPase binding;Ras GTPase binding;small GTPase binding;tubulin binding
binding;carboxylic acid binding;catalytic activity;cation binding;enzyme binding;ion binding;iron ion binding;L-ascorbic acid binding;metal ion binding;oxidoreductase activity;oxidoreductase activity, acting on paired donors,
binding;cation binding;ion binding;metal ion binding
catalytic activity;hydrolase activity
carboxypeptidase activity;catalytic activity;exopeptidase activity;hydrolase activity;peptidase activity;peptidase activity, acting on L-amino acid peptides;serine hydrolase activity;serine-type carboxypeptidase activity;serine-t
binding;carboxylic acid binding;coenzyme binding;cofactor binding;fatty acid binding;fatty-acyl-CoA binding;lipid binding;monocarboxylic acid binding
binding;enzyme binding;GTPase binding;GTP-Rho binding;protein binding;Ras GTPase binding;Rho GTPase binding;small GTPase binding
binding;cholesterol binding;lipid binding;steroid binding;sterol binding
acidic amino acid transmembrane transporter activity;active transmembrane transporter activity;amine transmembrane transporter activity;amino acid transmembrane transporter activity;carboxylic acid transmembrane trans
catalytic activity;endonuclease activity;endonuclease activity, active with either ribo- or deoxyribonucleic acids and producing 5'-phosphomonoesters;endoribonuclease activity;endoribonuclease activity, producing 5'-phosph
adenyl nucleotide binding;adenyl ribonucleotide binding;aminoacyl-tRNA ligase activity;ATP binding;binding;catalytic activity;cation binding;cysteine-tRNA ligase activity;ion binding;ligase activity;ligase activity, forming amin
acid-amino acid ligase activity;catalytic activity;ligase activity;ligase activity, forming carbon-nitrogen bonds;phosphopantothenate--cysteine ligase activity
binding;nucleic acid binding;RNA binding
binding;chromatin binding
ligand-dependent nuclear receptor transcription coactivator activity;protein binding transcription factor activity;transcription coactivator activity;transcription cofactor activity;transcription factor binding transcription factor ac
binding;enzyme activator activity;enzyme regulator activity;G-protein alpha-subunit binding;GTPase activator activity;GTPase regulator activity;guanyl-nucleotide exchange factor activity;nucleoside-triphosphatase regulator
binding;protein binding;protein domain specific binding;structural constituent of ribosome;structural molecule activity
binding;nucleotide binding
adenyl nucleotide binding;adenyl ribonucleotide binding;ATP binding;ATPase activity;binding;catalytic activity;hydrolase activity;hydrolase activity, acting on acid anhydrides;hydrolase activity, acting on acid anhydrides, in ph
binding;cytoskeletal protein binding;protein binding
binding;nucleic acid binding;RNA binding
3-hydroxyacyl-CoA dehydratase activity;binding;carbon-oxygen lyase activity;catalytic activity;enzyme activator activity;enzyme binding;enzyme regulator activity;GTPase activator activity;GTPase regulator activity;hydro-lya
enzyme regulator activity;kinase regulator activity
7-dehydrocholesterol reductase activity;catalytic activity;oxidoreductase activity;oxidoreductase activity, acting on the CH-CH group of donors;oxidoreductase activity, acting on the CH-CH group of donors, NAD or NADP as
carboxylic acid transmembrane transporter activity;dicarboxylic acid transmembrane transporter activity;organic acid transmembrane transporter activity;substrate-specific transmembrane transporter activity;substrate-speci
binding;protein binding;protein binding involved in protein folding;unfolded protein binding
active transmembrane transporter activity;ATPase activity;ATPase activity, coupled;ATPase activity, coupled to movement of substances;ATPase activity, coupled to transmembrane movement of ions;ATPase activity, coupled
binding;catalytic activity;cation binding;endonuclease activity;endonuclease activity, active with either ribo- or deoxyribonucleic acids and producing 5'-phosphomonoesters;endoribonuclease activity;endoribonuclease activit
adenyl nucleotide binding;adenyl ribonucleotide binding;ATP binding;ATPase activity;ATPase activity, coupled;ATP-dependent helicase activity;ATP-dependent RNA helicase activity;binding;catalytic activity;helicase activity;h
binding;DNA binding;nucleic acid binding;nucleotide binding
binding;DNA binding;enzyme binding;mRNA 3'-UTR binding;mRNA binding;nucleic acid binding;phosphatase binding;protein binding;RNA binding
acid-amino acid ligase activity;catalytic activity;ligase activity;ligase activity, forming carbon-nitrogen bonds;small conjugating protein ligase activity;UFM1 conjugating enzyme activity
catalytic activity;NADH dehydrogenase (quinone) activity;NADH dehydrogenase (ubiquinone) activity;NADH dehydrogenase activity;oxidoreductase activity;oxidoreductase activity, acting on NADH or NADPH;oxidoreductase
binding;catalytic activity;cation binding;hydrolase activity;hydrolase activity, acting on ester bonds;ion binding;magnesium ion binding;metal ion binding;phosphatase activity;phosphoglycolate phosphatase activity;phosphop
binding;DNA binding;nucleic acid binding
binding;nuclear localization sequence binding;peptide binding;protein transporter activity;signal sequence binding;substrate-specific transporter activity;transporter activity
binding;catalytic activity;cation binding;dioxygenase activity;ion binding;metal ion binding;oxidoreductase activity;oxidoreductase activity, acting on single donors with incorporation of molecular oxygen;oxidoreductase activi
adrenergic receptor binding;beta-2 adrenergic receptor binding;beta-catenin binding;binding;channel regulator activity;chloride channel regulator activity;enzyme binding;G-protein-coupled receptor binding;growth factor rec
catalytic activity;cation transmembrane transporter activity;hydrogen ion transmembrane transporter activity;inorganic cation transmembrane transporter activity;ion transmembrane transporter activity;monovalent inorganic c
acetyltransferase activity;acyl-CoA thioesterase activity;binding;catalytic activity;CoA hydrolase activity;glycerone-phosphate O-acyltransferase activity;hydrolase activity;hydrolase activity, acting on ester bonds;O-acetyltrans
adenyl nucleotide binding;adenyl ribonucleotide binding;ATP binding;binding;nucleotide binding;purine nucleotide binding;purine ribonucleoside triphosphate binding;purine ribonucleotide binding;ribonucleotide binding
binding;catalytic activity;palmitoyltransferase activity;protein binding;protein-cysteine S-acyltransferase activity;protein-cysteine S-palmitoleyltransferase activity;S-acyltransferase activity;SNAP receptor activity;SNARE bindi
alcohol dehydrogenase (NADP+) activity;alditol:NADP+ 1-oxidoreductase activity;aldo-keto reductase (NADP) activity;catalytic activity;electron carrier activity;epoxide hydrolase activity;ether hydrolase activity;hydrolase activ
binding;cation binding;DNA binding;double-stranded DNA binding;ion binding;metal ion binding;nucleic acid binding;structure-specific DNA binding;transition metal ion binding;zinc ion binding
binding;cation binding;ion binding;metal ion binding
binding;chromatin binding
active transmembrane transporter activity;ATPase activity;ATPase activity, coupled;ATPase activity, coupled to movement of substances;ATPase activity, coupled to transmembrane movement of ions;ATPase activity, coupled
catalytic activity;electron carrier activity;NADH dehydrogenase (quinone) activity;NADH dehydrogenase (ubiquinone) activity;NADH dehydrogenase activity;oxidoreductase activity;oxidoreductase activity, acting on NADH or N
acid-amino acid ligase activity;binding;catalytic activity;cation binding;ion binding;ligase activity;ligase activity, forming carbon-nitrogen bonds;metal ion binding;small conjugating protein ligase activity;transition metal ion bin
binding;DNA binding;nucleic acid binding;RNA binding
3-keto sterol reductase activity;binding;carbonyl reductase (NADPH) activity;catalytic activity;coenzyme binding;cofactor binding;NADP binding;NADPH binding;nucleotide binding;oxidoreductase activity;oxidoreductase acti
binding;catalytic activity;cation binding;endopeptidase activity;exopeptidase activity;hydrolase activity;ion binding;metal ion binding;metalloendopeptidase activity;metalloexopeptidase activity;metallopeptidase activity;peptic
structural molecule activity
binding;catalytic activity;cation binding;disulfide oxidoreductase activity;electron carrier activity;ion binding;iron-sulfur cluster binding;metal cluster binding;metal ion binding;oxidoreductase activity;oxidoreductase activity, ac
adenyl nucleotide binding;adenyl ribonucleotide binding;ATP binding;ATPase activity;binding;catalytic activity;cation binding;enzyme activator activity;enzyme regulator activity;hydrolase activity;hydrolase activity, acting on a
binding;cyclin-dependent protein kinase inhibitor activity;cyclin-dependent protein kinase regulator activity;enzyme inhibitor activity;enzyme regulator activity;kinase inhibitor activity;kinase regulator activity;nucleic acid bindi
6-phosphogluconolactonase activity;binding;carbohydrate binding;carboxylic ester hydrolase activity;catalytic activity;hydrolase activity;hydrolase activity, acting on ester bonds;monosaccharide binding;sugar binding
binding;catalytic activity;cation binding;dioxygenase activity;ion binding;iron ion binding;metal ion binding;oxidoreductase activity;oxidoreductase activity, acting on single donors with incorporation of molecular oxygen;oxido
binding;chromatin binding;protein binding transcription factor activity;transcription coactivator activity;transcription cofactor activity;transcription factor binding transcription factor activity
binding;calcium ion binding;catalytic activity;cation binding;endopeptidase activity;growth factor activity;hydrolase activity;ion binding;metal ion binding;molecular transducer activity;peptidase activity;peptidase activity, actin
binding;GDP binding;GMP binding;GTP binding;guanyl nucleotide binding;guanyl ribonucleotide binding;nucleotide binding;protein binding;protein complex binding;purine nucleotide binding;purine ribonucleoside triphospha
binding;cation binding;ion binding;metal ion binding;transition metal ion binding;zinc ion binding
binding;nucleic acid binding;RNA binding;structural constituent of ribosome;structural molecule activity
adenine binding;adenine phosphoribosyltransferase activity;adenyl nucleotide binding;adenyl ribonucleotide binding;AMP binding;binding;catalytic activity;nucleobase binding;nucleotide binding;purine base binding;purine nu
binding;nucleic acid binding;nucleotide binding;RNA binding;snRNA binding
receptor activity
active transmembrane transporter activity;binding;carbohydrate transmembrane transporter activity;cation transmembrane transporter activity;cation:sugar symporter activity;dehydroascorbic acid transporter activity;D-gluco
ATPase binding;binding;catalytic activity;cytoskeletal protein binding;Edg-2 lysophosphatidic acid receptor binding;endothelial differentiation G-protein coupled receptor binding;enzyme binding;GDP binding;G-protein-coupl
binding;enzyme binding;protein binding
binding;carbohydrate binding;catalytic activity;enzyme binding;glucose binding;glucosyltransferase activity;glycogen (starch) synthase activity;kinase binding;monosaccharide binding;protein binding;protein kinase binding;su

binding;catalytic activity;GTP binding;GTPase activity;guanyl nucleotide binding;guanyl ribonucleotide binding;hydrolase activity;hydrolase activity, acting on acid anhydrides;hydrolase activity, acting on acid anhydrides, in pl
binding;protein binding;protein complex binding
binding;catalytic activity;growth factor receptor binding;phosphorylase activity;platelet-derived growth factor receptor binding;protein binding;pyrimidine-nucleoside phosphorylase activity;receptor binding;thymidine phospho
binding;C-acyltransferase activity;carboxylic acid binding;catalytic activity;cholesterol binding;coenzyme binding;cofactor binding;fatty acid binding;fatty-acyl-CoA binding;lipid binding;lipid transporter activity;monocarboxylic
adenyl nucleotide binding;adenyl ribonucleotide binding;ATP binding;binding;catalytic activity;cation binding;cyclin binding;cyclin-dependent protein kinase activity;ion binding;kinase activity;metal ion binding;nucleotide bind
acyl-CoA dehydrogenase activity;binding;catalytic activity;coenzyme binding;cofactor binding;flavin adenine dinucleotide binding;isovaleryl-CoA dehydrogenase activity;oxidoreductase activity;oxidoreductase activity, acting
antioxidant activity;binding;caspase inhibitor activity;caspase regulator activity;catalytic activity;cysteine-type endopeptidase inhibitor activity;DNA binding;endopeptidase inhibitor activity;endopeptidase regulator activity;enz
carbon-carbon lyase activity;carboxy-lyase activity;catalytic activity;D-dopachrome decarboxylase activity;dopachrome isomerase activity;intramolecular oxidoreductase activity;intramolecular oxidoreductase activity, transpo
actin binding;actin filament binding;actin monomer binding;binding;cytoskeletal protein binding;enzyme binding;identical protein binding;kinase binding;myosin binding;myosin heavy chain binding;phosphatidylinositol 3-kina
binding;carboxylic acid binding;catalytic activity;cholesterol binding;cholesterol O-acyltransferase activity;coenzyme binding;cofactor binding;fatty acid binding;fatty-acyl-CoA binding;lipid binding;monocarboxylic acid bindin
binding;calcium ion binding;cation binding;ion binding;metal ion binding
adenyl nucleotide binding;adenyl ribonucleotide binding;ATP binding;binding;DNA binding;enzyme binding;nucleic acid binding;nucleotide binding;protein binding;purine nucleotide binding;purine ribonucleoside triphosphate
binding;ephrin receptor binding;protein binding;protein domain specific binding;receptor binding;SH2 domain binding
binding;catalytic activity;choline-phosphate cytidylyltransferase activity;cytidylyltransferase activity;lipid binding;nucleotidyltransferase activity;transferase activity;transferase activity, transferring phosphorus-containing group
binding;catalytic activity;GTP binding;GTPase activity;guanyl nucleotide binding;guanyl ribonucleotide binding;hydrolase activity;hydrolase activity, acting on acid anhydrides;hydrolase activity, acting on acid anhydrides, in pl
adenyl nucleotide binding;adenyl ribonucleotide binding;ATP binding;binding;carbon-carbon lyase activity;carboxy-lyase activity;catalytic activity;diphosphomevalonate decarboxylase activity;heat shock protein binding;Hsp7
binding;damaged DNA binding;DNA binding;nucleic acid binding;polyubiquitin binding;protein binding;single-stranded DNA binding;small conjugating protein binding;structure-specific DNA binding;ubiquitin binding
binding;calcium ion binding;cation binding;identical protein binding;ion binding;metal ion binding;protein binding;protein dimerization activity;protein heterodimerization activity;protein homodimerization activity
binding;catalytic activity;GTP binding;GTPase activity;guanyl nucleotide binding;guanyl ribonucleotide binding;hydrolase activity;hydrolase activity, acting on acid anhydrides;hydrolase activity, acting on acid anhydrides, in pl
enzyme activator activity;enzyme inhibitor activity;enzyme regulator activity;kinase inhibitor activity;kinase regulator activity;molecular transducer activity;protein kinase inhibitor activity;protein kinase regulator activity;signal t
binding;catalytic activity;enzyme regulator activity;GTP binding;GTPase activity;GTPase regulator activity;guanyl nucleotide binding;guanyl ribonucleotide binding;guanyl-nucleotide exchange factor activity;hydrolase activity;
acid-amino acid ligase activity;catalytic activity;ligase activity;ligase activity, forming carbon-nitrogen bonds;small conjugating protein ligase activity;ubiquitin-protein ligase activity
1-alkyl-2-acetylglycerophosphocholine esterase activity;acetyltransferase activity;carboxylic ester hydrolase activity;catalytic activity;hydrolase activity;hydrolase activity, acting on ester bonds;platelet-activating factor acetylt
binding;cation binding;ion binding;metal ion binding;nucleotide binding
activating transcription factor binding;anion binding;binding;chromatin binding;core promoter proximal region DNA binding;core promoter proximal region sequence-specific DNA binding;DNA binding;enhancer binding;enhan
binding;mRNA binding;nucleic acid binding;nucleotide binding;RNA binding
binding;cation binding;chromatin binding;core promoter binding;core promoter sequence-specific DNA binding;DNA binding;ion binding;metal ion binding;nucleic acid binding;nucleic acid binding transcription factor activity;
acid-amino acid ligase activity;binding;catalytic activity;enzyme binding;ligase activity;ligase activity, forming carbon-nitrogen bonds;protein binding;small conjugating protein ligase activity;ubiquitin protein ligase binding
amine binding;amino acid binding;binding;carboxylic acid binding;catalytic activity;cis-trans isomerase activity;enzyme binding;GTPase activating protein binding;isomerase activity;kinase binding;mitogen-activated protein k
3'-5' exonuclease activity;3'-5'-exoribonuclease activity;7S RNA binding;binding;catalytic activity;exonuclease activity;exonuclease activity, active with either ribo- or deoxyribonucleic acids and producing 5'-phosphomonoes
adenyl nucleotide binding;adenyl ribonucleotide binding;ATP binding;ATPase activity;binding;catalytic activity;cation binding;DNA binding;helicase activity;hydrolase activity;hydrolase activity, acting on acid anhydrides;hydrol
catalytic activity;intramolecular oxidoreductase activity;intramolecular oxidoreductase activity, interconverting keto- and enol-groups;intramolecular oxidoreductase activity, transposing S-S bonds;isomerase activity;oxidored
adenyl nucleotide binding;adenyl ribonucleotide binding;aminoacyl-tRNA editing activity;aminoacyl-tRNA ligase activity;ATP binding;binding;carboxylic ester hydrolase activity;catalytic activity;hydrolase activity;hydrolase act
binding;nucleic acid binding;RNA binding;snoRNA binding
binding;catalytic activity;coenzyme binding;cofactor binding;NADH dehydrogenase (quinone) activity;NADH dehydrogenase (ubiquinone) activity;NADH dehydrogenase activity;oxidoreductase activity;oxidoreductase activity,
binding;catalytic activity;cation binding;demethylase activity;heme binding;ion binding;iron ion binding;metal ion binding;monooxygenase activity;oxidoreductase activity;oxidoreductase activity, acting on paired donors, with
adenyl nucleotide binding;adenyl ribonucleotide binding;ATP binding;binding;catalytic activity;identical protein binding;kinase activity;NAD+ kinase activity;nucleotide binding;phosphotransferase activity, alcohol group as acc
catalytic activity;oxidoreductase activity;oxidoreductase activity, acting on the CH-NH group of donors;oxidoreductase activity, acting on the CH-NH group of donors, NAD or NADP as acceptor;pyrroline-5-carboxylate reduc
adenyl nucleotide binding;adenyl ribonucleotide binding;aminoacyl-tRNA ligase activity;ATP binding;binding;catalytic activity;glutamate-tRNA ligase activity;glutamate-tRNA(Gln) ligase activity;ligase activity;ligase activity, for
3-hydroxyisobutyryl-CoA hydrolase activity;catalytic activity;CoA hydrolase activity;hydrolase activity;hydrolase activity, acting on ester bonds;thiolester hydrolase activity
catalytic activity;ligase activity;nicotinate phosphoribosyltransferase activity;nicotinate-nucleotide diphosphorylase (carboxylating) activity;transferase activity;transferase activity, transferring glycosyl groups;transferase activit
adenyl nucleotide binding;adenyl ribonucleotide binding;ATP binding;binding;catalytic activity;cation binding;enzyme activator activity;enzyme regulator activity;ion binding;kinase activator activity;kinase activity;kinase regula
catalytic activity;methyltransferase activity;RNA methyltransferase activity;S-adenosylmethionine-dependent methyltransferase activity;transferase activity;transferase activity, transferring one-carbon groups
catalytic activity;hydrolase activity;hydrolase activity, acting on carbon-nitrogen (but not peptide) bonds;hydrolase activity, acting on carbon-nitrogen (but not peptide) bonds, in nitriles;nitrilase activity
binding;dynein binding;dynein intermediate chain binding;protein binding
binding;ion channel binding;protein binding
binding;enzyme binding;GTPase binding;protein binding;Ral GTPase binding;Ras GTPase binding;small GTPase binding
1-acylglycerophosphocholine O-acyltransferase activity;1-alkenylglycerophosphocholine O-acyltransferase activity;1-alkylglycerophosphocholine O-acetyltransferase activity;1-alkylglycerophosphocholine O-acyltransferase a
binding;protein binding;protein domain specific binding;structural constituent of ribosome;structural molecule activity
molecular transducer activity;signal transducer activity
binding;catalytic activity;methyltransferase activity;nucleic acid binding;RNA binding;RNA methyltransferase activity;rRNA (pseudouridine) methyltransferase activity;rRNA binding;rRNA methyltransferase activity;S-adenosylm
binding;catalytic activity;cation binding;cofactor binding;ion binding;lyase activity;metal ion binding;molybdenum ion binding;Mo-molybdopterin cofactor sulfurase activity;pyridoxal phosphate binding;transferase activity;trans
binding;catalytic activity;coenzyme binding;cofactor binding;oxidoreductase activity;oxidoreductase activity, acting on the aldehyde or oxo group of donors;oxidoreductase activity, acting on the aldehyde or oxo group of don
catalytic activity;nucleotidyltransferase activity;transferase activity;transferase activity, transferring phosphorus-containing groups
binding;cyclin binding;enzyme binding;kinase binding;NF-kappaB binding;protein binding;protein kinase binding;transcription factor binding
catalytic activity;intramolecular oxidoreductase activity;intramolecular oxidoreductase activity, interconverting keto- and enol-groups;intramolecular oxidoreductase activity, transposing S-S bonds;isomerase activity;protein d
binding;enzyme binding;enzyme inhibitor activity;enzyme regulator activity;phosphatase binding;phosphatase inhibitor activity;phosphatase regulator activity;protein binding;protein phosphatase 1 binding;protein phosphatas
binding;chromatin binding;DNA binding;heat shock protein binding;histone binding;Hsp70 protein binding;nucleic acid binding;protein binding;small conjugating protein binding;ubiquitin binding
binding;protein binding;unfolded protein binding
3-hydroxy-2-methylbutyryl-CoA dehydrogenase activity;3-hydroxyacyl-CoA dehydrogenase activity;catalytic activity;cholate 7-alpha-dehydrogenase activity;oxidoreductase activity;oxidoreductase activity, acting on CH-OH g
acid-amino acid ligase activity;binding;catalytic activity;cation binding;ion binding;ligase activity;ligase activity, forming carbon-nitrogen bonds;metal ion binding;small conjugating protein ligase activity;transition metal ion bin
binding;enzyme binding;enzyme inhibitor activity;enzyme regulator activity;GTP binding;GTPase binding;GTPase inhibitor activity;GTPase regulator activity;GTP-Rho binding;guanyl nucleotide binding;guanyl ribonucleotide b
binding;cation binding;chromatin binding;DNA binding;ion binding;metal ion binding;nucleic acid binding;nucleic acid binding transcription factor activity;sequence-specific DNA binding;sequence-specific DNA binding transc
binding;enzyme binding;identical protein binding;kinase binding;protein binding;protein dimerization activity;protein homodimerization activity;protein kinase binding;protein N-terminus binding
binding;nucleotide binding
binding;cholesterol binding;lipid binding;steroid binding;sterol binding
catalytic activity;exonuclease activity;hydrolase activity;hydrolase activity, acting on ester bonds;nuclease activity;nucleic acid binding transcription factor activity;sequence-specific DNA binding transcription factor activity
binding;catalytic activity;disulfide oxidoreductase activity;electron carrier activity;enzyme activator activity;enzyme regulator activity;GTPase activator activity;GTPase regulator activity;nucleoside-triphosphatase regulator act
binding;enzyme binding;phosphatase binding;protein binding
binding;protein binding;protein C-terminus binding
binding;cation binding;ion binding;metal ion binding;transition metal ion binding;zinc ion binding
binding;calcium channel inhibitor activity;calcium channel regulator activity;catalytic activity;channel inhibitor activity;channel regulator activity;hydrolase activity;hydrolase activity, acting on ester bonds;ion channel binding;io
binding;calcium-dependent protein binding;cation binding;ion binding;metal ion binding;nucleic acid binding;nucleocytoplasmic transporter activity;nucleotide binding;pre-mRNA binding;protein binding;RNA binding;snRNA b
binding;nucleic acid binding;RNA binding;snoRNA binding
binding;enzyme binding;phosphatase binding;protein binding;protein phosphatase binding
5'-3' exonuclease activity;binding;catalytic activity;cation binding;endonuclease activity;endoribonuclease activity;exonuclease activity;hydrolase activity;hydrolase activity, acting on ester bonds;ion binding;metal ion binding;
adenyl nucleotide binding;adenyl ribonucleotide binding;ATP binding;ATPase activity;ATPase activity, coupled;binding;catalytic activity;hydrolase activity;hydrolase activity, acting on acid anhydrides;hydrolase activity, acting o
binding;DNA binding;nucleic acid binding
structural constituent of ribosome;structural molecule activity
binding;enzyme binding;insulin receptor binding;kinase binding;molecular transducer activity;phosphatidylinositol 3-kinase binding;protein binding;protein complex binding;receptor binding;signal transducer activity
receptor activity
catalytic activity;endopeptidase activity;hydrolase activity;peptidase activity;peptidase activity, acting on L-amino acid peptides;serine hydrolase activity;serine-type endopeptidase activity;serine-type peptidase activity
binding;catalytic activity;cation binding;cofactor binding;cysteine desulfurase activity;identical protein binding;ion binding;iron-sulfur cluster binding;metal cluster binding;metal ion binding;protein binding;protein dimerization
ARF guanyl-nucleotide exchange factor activity;binding;enzyme regulator activity;GABA receptor binding;GTPase regulator activity;guanyl-nucleotide exchange factor activity;nucleoside-triphosphatase regulator activity;prote
ARF guanyl-nucleotide exchange factor activity;binding;cytoskeletal protein binding;enzyme regulator activity;GTPase regulator activity;guanyl-nucleotide exchange factor activity;myosin binding;nucleoside-triphosphatase re
binding;cation binding;ion binding;metal ion binding;transition metal ion binding;zinc ion binding
adenyl nucleotide binding;adenyl ribonucleotide binding;ATP binding;ATPase activity;binding;catalytic activity;hydrolase activity;hydrolase activity, acting on acid anhydrides;hydrolase activity, acting on acid anhydrides, in ph
binding;catalytic activity;GTP binding;GTPase activity;guanyl nucleotide binding;guanyl ribonucleotide binding;hydrolase activity;hydrolase activity, acting on acid anhydrides;hydrolase activity, acting on acid anhydrides, in pl
binding;nuclear localization sequence binding;peptide binding;signal sequence binding
binding;enzyme binding;protein binding;ubiquitin protein ligase binding
adenyl nucleotide binding;adenyl ribonucleotide binding;ATP binding;binding;catalytic activity;guanylate kinase activity;kinase activity;nucleobase-containing compound kinase activity;nucleotide binding;nucleotide kinase act
binding;C-acyltransferase activity;catalytic activity;cofactor binding;C-palmitoyltransferase activity;palmitoyltransferase activity;pyridoxal phosphate binding;serine C-palmitoyltransferase activity;transferase activity;transferas
binding;centromeric DNA binding;DNA binding;nucleic acid binding;sequence-specific DNA binding
binding;nucleic acid binding;RNA binding;translation factor activity, nucleic acid binding;translation initiation factor activity
adenyl nucleotide binding;adenyl ribonucleotide binding;anion transmembrane transporter activity;arsenite transmembrane transporter activity;ATP binding;ATPase activity;binding;catalytic activity;cation binding;hydrolase ac
binding;nucleotide binding
binding;lipid binding;phosphatidylinositol binding;phospholipid binding
binding;nucleic acid binding;nucleotide binding;protein binding;protein domain specific binding;RNA binding;RS domain binding;unfolded protein binding
binding;carboxylic acid binding;catalytic activity;coenzyme binding;cofactor binding;dodecenoyl-CoA delta-isomerase activity;fatty acid binding;fatty-acyl-CoA binding;intramolecular oxidoreductase activity;intramolecular ox
binding;DNA binding;nucleic acid binding
acidic amino acid transmembrane transporter activity;active transmembrane transporter activity;amine transmembrane transporter activity;amino acid transmembrane transporter activity;binding;calcium ion binding;carboxyli
aldehyde-lyase activity;binding;carbon-carbon lyase activity;carboxy-lyase activity;catalytic activity;cofactor binding;lyase activity;pyridoxal phosphate binding;sphinganine-1-phosphate aldolase activity;vitamin B6 binding;vi
binding;cytoskeletal protein binding;microtubule binding;protein binding;tubulin binding
binding;catalytic activity;cation binding;guanine phosphoribosyltransferase activity;hypoxanthine phosphoribosyltransferase activity;identical protein binding;ion binding;magnesium ion binding;metal ion binding;nucleotide bi
antigen binding;binding;peptide antigen binding;peptide binding;protein binding;receptor binding
adenyl nucleotide binding;adenyl ribonucleotide binding;ATP binding;binding;biotin binding;biotin carboxylase activity;carboxylic acid binding;catalytic activity;cation binding;CoA carboxylase activity;enzyme binding;ion bind
binding;chromatin binding;chromatin DNA binding;DNA binding;nucleic acid binding;structure-specific DNA binding
catalytic activity;cation transmembrane transporter activity;hydrogen ion transmembrane transporter activity;inorganic cation transmembrane transporter activity;ion transmembrane transporter activity;monovalent inorganic c
binding;nucleic acid binding;nucleotide binding;RNA binding;snRNA binding
binding;histone pre-mRNA DCP binding;nucleic acid binding;RNA binding
binding;catalytic activity;cysteine-type peptidase activity;hydrolase activity;peptidase activity;peptidase activity, acting on L-amino acid peptides;protein binding;small conjugating protein binding;small conjugating protein-sp

binding;catalytic activity;cation binding;fumarylacetoacetase activity;hydrolase activity;hydrolase activity, acting on acid carbon-carbon bonds;hydrolase activity, acting on acid carbon-carbon bonds, in ketonic substances;io
binding;catalytic activity;cytoskeletal protein binding;GTP binding;GTPase activity;guanyl nucleotide binding;guanyl ribonucleotide binding;hydrolase activity;hydrolase activity, acting on acid anhydrides;hydrolase activity, act
binding;catalytic activity;GTP binding;GTPase activity;guanyl nucleotide binding;guanyl ribonucleotide binding;hydrolase activity;hydrolase activity, acting on acid anhydrides;hydrolase activity, acting on acid anhydrides, in p
catalytic activity;endopeptidase activity;hydrolase activity;peptidase activity;peptidase activity, acting on L-amino acid peptides;threonine-type endopeptidase activity;threonine-type peptidase activity
biliverdin reductase activity;catalytic activity;flavin reductase activity;oxidoreductase activity;oxidoreductase activity, acting on the CH-CH group of donors;oxidoreductase activity, acting on the CH-CH group of donors, NAD
binding;catalytic activity;cation binding;heme oxygenase (decyclizing) activity;ion binding;metal ion binding;oxidoreductase activity;oxidoreductase activity, acting on paired donors, with incorporation or reduction of molecula
binding;calcium channel regulator activity;calcium ion binding;calcium-dependent cysteine-type endopeptidase activity;catalytic activity;cation binding;channel regulator activity;cysteine-type endopeptidase activity;cysteine-
acyl-CoA hydrolase activity;acyl-CoA thioesterase activity;binding;carboxylic ester hydrolase activity;catalytic activity;CoA hydrolase activity;hydrolase activity;hydrolase activity, acting on ester bonds;palmitoyl-CoA hydrolas
adenyl nucleotide binding;adenyl ribonucleotide binding;ATP binding;beta-catenin binding;binding;catalytic activity;enzyme binding;kinase activity;kinase binding;NF-kappaB binding;nucleotide binding;p53 binding;phosphot
binding;catalytic activity;GDP binding;GTP binding;GTPase activity;guanyl nucleotide binding;guanyl ribonucleotide binding;hydrolase activity;hydrolase activity, acting on acid anhydrides;hydrolase activity, acting on acid anh
enzyme activator activity;enzyme regulator activity;GTPase activator activity;GTPase regulator activity;nucleoside-triphosphatase regulator activity
binding;nucleic acid binding;RNA binding;structural constituent of ribosome;structural molecule activity
binding;cation binding;ion binding;metal ion binding;transition metal ion binding;zinc ion binding
binding;protein binding;SNARE binding;soluble NSF attachment protein activity;syntaxin binding
binding;GTP binding;guanyl nucleotide binding;guanyl ribonucleotide binding;nucleotide binding;purine nucleotide binding;purine ribonucleoside triphosphate binding;purine ribonucleotide binding;ribonucleotide binding
binding;catalytic activity;cation binding;hydrolase activity;hydrolase activity, acting on ester bonds;ion binding;kinase activity;metal ion binding;molecular transducer activity;NF-kappaB-inducing kinase activity;phosphatase a
acid-amino acid ligase activity;adenyl nucleotide binding;adenyl ribonucleotide binding;ATP binding;binding;catalytic activity;ligase activity;ligase activity, forming carbon-nitrogen bonds;NEDD8 ligase activity;nucleotide bindi
binding;cholesterol binding;enzyme binding;lipid binding;protein binding;steroid binding;sterol binding
activin binding;binding;catalytic activity;cis-trans isomerase activity;cytokine receptor binding;drug binding;FK506 binding;ion channel binding;isomerase activity;macrolide binding;molecular transducer activity;peptidyl-prolyl
binding;binding, bridging;cytokine receptor binding;enzyme binding;ephrin receptor binding;epidermal growth factor receptor binding;growth factor receptor binding;identical protein binding;insulin receptor substrate binding;
acid-amino acid ligase activity;adenyl nucleotide binding;adenyl ribonucleotide binding;ATP binding;binding;catalytic activity;enzyme binding;enzyme regulator activity;ligase activity;ligase activity, forming carbon-nitrogen bo
binding;cation binding;ion binding;metal ion binding;transition metal ion binding;zinc ion binding
binding;nucleic acid binding;nucleotide binding;ribonucleoprotein binding;ribosomal large subunit binding;RNA binding
binding;DNA binding;double-stranded DNA binding;double-stranded telomeric DNA binding;nucleic acid binding;nucleic acid binding transcription factor activity;protein binding;purine-rich negative regulatory element binding
binding;channel inhibitor activity;channel regulator activity;cholesterol binding;enzyme activator activity;enzyme binding;enzyme regulator activity;identical protein binding;inward rectifier potassium channel inhibitor activity;io
adenyl nucleotide binding;adenyl ribonucleotide binding;ATP binding;binding;nucleotide binding;purine nucleotide binding;purine ribonucleoside triphosphate binding;purine ribonucleotide binding;ribonucleotide binding
binding;calcium channel regulator activity;calcium ion binding;cation binding;channel regulator activity;cytoskeletal protein binding;identical protein binding;ion binding;metal ion binding;microtubule binding;microtubule plus-
binding;carbohydrate binding;enzyme binding;protein binding
actin binding;actin filament binding;binding;calcium ion binding;cation binding;cytoskeletal protein binding;ion binding;metal ion binding;protein binding;structural constituent of cytoskeleton;structural molecule activity
binding;DNA binding;mRNA binding;nucleic acid binding;RNA binding
binding;catalytic activity;DNA binding;endonuclease activity;endoribonuclease activity;hydrolase activity;hydrolase activity, acting on ester bonds;mRNA binding;nuclease activity;nucleic acid binding;ribonuclease activity;RN
adenyl nucleotide binding;adenyl ribonucleotide binding;ATP binding;binding;identical protein binding;nucleotide binding;protein binding;protein binding transcription factor activity;purine nucleotide binding;purine ribonucleo
adenyl nucleotide binding;adenyl ribonucleotide binding;ATP binding;binding;catalytic activity;kinase activity;MAP kinase activity;MAP kinase kinase activity;molecular transducer activity;NFAT protein binding;nucleotide bindi
binding;mRNA binding;nucleic acid binding;nucleotide binding;RNA binding
binding;DNA binding;nucleic acid binding
binding;DNA binding;nucleic acid binding;nucleic acid binding transcription factor activity;sequence-specific DNA binding transcription factor activity
nucleocytoplasmic transporter activity;transporter activity
molecular transducer activity;signal transducer activity
binding;enzyme binding;protein binding;ubiquitin protein ligase binding
ATPase activator activity;ATPase binding;ATPase regulator activity;binding;catalytic activity;chaperone binding;disulfide oxidoreductase activity;enzyme activator activity;enzyme binding;enzyme regulator activity;heat shock p
binding;calcium ion binding;catalytic activity;cation binding;GTP binding;GTPase activity;guanyl nucleotide binding;guanyl ribonucleotide binding;hydrolase activity;hydrolase activity, acting on acid anhydrides;hydrolase activ
binding;chromatin binding;protein binding;transcription factor binding
binding;catalytic activity;channel regulator activity;coenzyme binding;cofactor binding;glycerol-3-phosphate dehydrogenase [NAD+] activity;ion channel binding;NAD binding;nucleotide binding;oxidoreductase activity;oxidore
binding;cation binding;enzyme binding;GTPase binding;identical protein binding;ion binding;lipid binding;metal ion binding;phosphatidic acid binding;phospholipid binding;protein binding;Rab GTPase binding;Ras GTPase bi
ATPase activator activity;ATPase binding;ATPase regulator activity;binding;enzyme activator activity;enzyme binding;enzyme regulator activity;nucleoside-triphosphatase regulator activity;protein binding
adenyl nucleotide binding;adenyl ribonucleotide binding;ADP binding;ATP binding;binding;catalytic activity;kinase activity;nucleotide binding;purine nucleotide binding;purine ribonucleoside triphosphate binding;purine ribonu
protein binding transcription factor activity;transcription coactivator activity;transcription cofactor activity;transcription factor binding transcription factor activity
catalytic activity;GPI-anchor transamidase activity;hydrolase activity
binding;nucleic acid binding;ribonucleoprotein binding;ribosomal small subunit binding;RNA binding;rRNA binding
binding;nucleic acid binding;nucleotide binding;RNA binding
binding;catalytic activity;cysteine-type endopeptidase activity;cysteine-type peptidase activity;endopeptidase activity;hydrolase activity;peptidase activity;peptidase activity, acting on L-amino acid peptides;protein binding;pr
binding;catalytic activity;cation binding;DNA binding;endonuclease activity;endoribonuclease activity;hydrolase activity;hydrolase activity, acting on ester bonds;ion binding;metal ion binding;nuclease activity;nucleic acid bin
binding;catalytic activity;cation binding;endonuclease activity;hydrolase activity;hydrolase activity, acting on ester bonds;ion binding;metal ion binding;nuclease activity
binding;catalytic activity;identical protein binding;intramolecular oxidoreductase activity;intramolecular oxidoreductase activity, interconverting aldoses and ketoses;isomerase activity;protein binding;S-methyl-5-thioribose-1-
binding;cadherin binding;cell adhesion molecule binding;enzyme activator activity;enzyme regulator activity;GTPase activator activity;GTPase regulator activity;nucleoside-triphosphatase regulator activity;protein binding
structural constituent of ribosome;structural molecule activity
binding;catalytic activity;DNA binding;DNA-directed RNA polymerase activity;nucleic acid binding;nucleotidyltransferase activity;RNA polymerase activity;transferase activity;transferase activity, transferring phosphorus-conta
binding;enzyme binding;lipid binding;phosphatidylinositol binding;phosphatidylinositol-4-phosphate binding;phospholipid binding;protein binding
adenyl nucleotide binding;adenyl ribonucleotide binding;ATP binding;ATPase activity;ATPase activity, coupled;ATP-dependent helicase activity;ATP-dependent RNA helicase activity;binding;catalytic activity;enzyme binding;h
binding;calcium ion binding;cation binding;chemokine receptor binding;cytokine receptor binding;G-protein-coupled receptor binding;ion binding;metal ion binding;protein binding;receptor binding
binding;ion channel binding;protein binding
receptor activity
14-3-3 protein binding;adenyl nucleotide binding;adenyl ribonucleotide binding;ATP binding;binding;catalytic activity;enzyme binding;hydrolase activity;hydrolase activity, acting on acid anhydrides;hydrolase activity, acting o
binding;catalytic activity;enzyme regulator activity;GTPase regulator activity;guanyl-nucleotide exchange factor activity;nucleic acid binding;nucleoside-triphosphatase regulator activity;nucleotidyltransferase activity;RNA bin
binding;glutamate receptor binding;G-protein-coupled receptor binding;metabotropic glutamate receptor binding;protein binding;receptor binding
binding;cation binding;ion binding;metal ion binding;nucleotide binding
binding;cation binding;enzyme binding;GTPase binding;ion binding;metal ion binding;protein binding;Rab GTPase binding;Ras GTPase binding;small GTPase binding
protein transporter activity;receptor activity;substrate-specific transporter activity;transporter activity
catalytic activity;cytochrome-b5 reductase activity;oxidoreductase activity;oxidoreductase activity, acting on NADH or NADPH;oxidoreductase activity, acting on NADH or NADPH, heme protein as acceptor
binding;nucleic acid binding;RNA binding;translation factor activity, nucleic acid binding;translation initiation factor activity
binding;nucleic acid binding;RNA binding;snoRNA binding
binding;GTP binding;guanyl nucleotide binding;guanyl ribonucleotide binding;nucleotide binding;purine nucleotide binding;purine ribonucleoside triphosphate binding;purine ribonucleotide binding;ribonucleotide binding
1-phosphatidylinositol binding;Arp2/3 complex binding;binding;enzyme binding;identical protein binding;lipid binding;phosphatidylinositol binding;phospholipid binding;protein binding;protein complex binding;protein dimeriz
binding;PDZ domain binding;protein binding;protein domain specific binding
binding;catalytic activity;cysteine-type peptidase activity;hydrolase activity;nucleic acid binding;peptidase activity;peptidase activity, acting on L-amino acid peptides;RNA binding;small conjugating protein-specific protease a
catalytic activity;transferase activity;transferase activity, transferring acyl groups
binding;catalytic activity;cation binding;endopeptidase activator activity;endopeptidase regulator activity;enzyme activator activity;enzyme regulator activity;hydrolase activity;ion binding;metal ion binding;metallopeptidase ac
protein transporter activity;substrate-specific transporter activity;transporter activity
structural molecule activity
binding;calcium ion binding;cation binding;ion binding;metal ion binding
binding;chaperone binding;protein binding;unfolded protein binding
binding;C-acyltransferase activity;catalytic activity;cofactor binding;C-palmitoyltransferase activity;palmitoyltransferase activity;pyridoxal phosphate binding;serine C-palmitoyltransferase activity;transferase activity;transferas
active transmembrane transporter activity;carboxylic acid transmembrane transporter activity;lactate transmembrane transporter activity;monocarboxylic acid transmembrane transporter activity;organic acid transmembrane
binding;catalytic activity;methyltransferase activity;mRNA (guanine-N7-)-methyltransferase activity;mRNA methyltransferase activity;N-methyltransferase activity;nucleic acid binding;RNA binding;RNA methyltransferase activ
endopeptidase inhibitor activity;endopeptidase regulator activity;enzyme inhibitor activity;enzyme regulator activity;peptidase inhibitor activity;peptidase regulator activity;serine-type endopeptidase inhibitor activity
catalytic activity;disulfide oxidoreductase activity;oxidoreductase activity;oxidoreductase activity, acting on a sulfur group of donors;oxidoreductase activity, acting on a sulfur group of donors, disulfide as acceptor;protein dis
binding;enzyme binding;lipid binding;phosphatase binding;phosphatidylinositol binding;phosphatidylinositol-3,5-bisphosphate binding;phosphatidylinositol-3-phosphate binding;phosphatidylinositol-4-phosphate binding;pho
protein transporter activity;substrate-specific transporter activity;transporter activity
binding;chromatin binding;DNA binding;histone acetyl-lysine binding;histone binding;nucleic acid binding;p53 binding;protein binding
binding;nucleic acid binding transcription factor activity;protein binding;protein binding involved in protein folding;sequence-specific DNA binding transcription factor activity
binding;catalytic activity;gamma-glutamylcyclotransferase activity;identical protein binding;protein binding;protein dimerization activity;protein homodimerization activity;transferase activity;transferase activity, transferring acy
binding;cation binding;ion binding;metal ion binding;nucleic acid binding;nucleic acid binding transcription factor activity;RNA binding;sequence-specific DNA binding transcription factor activity;transition metal ion binding;zi
binding;calcium ion binding;catalytic activity;cation binding;cis-trans isomerase activity;drug binding;FK506 binding;ion binding;isomerase activity;macrolide binding;metal ion binding;peptidyl-prolyl cis-trans isomerase activi
binding;cation binding;cytokine binding;DNA binding;growth factor binding;interleukin-1 binding;ion binding;metal ion binding;NACHT domain binding;nucleic acid binding;protein binding;protein domain specific binding;trans
binding;cation binding;ion binding;metal ion binding;transition metal ion binding;zinc ion binding
binding;protein binding;SNAP receptor activity;SNARE binding;syntaxin binding
arachidonic acid binding;binding;calcium ion binding;carboxylic acid binding;cation binding;cytoskeletal protein binding;fatty acid binding;icosanoid binding;icosatetraenoic acid binding;ion binding;lipid binding;metal ion bin
binding;nucleic acid binding;RNA binding;RNA cap binding;translation factor activity, nucleic acid binding;translation initiation factor activity
catalytic activity;disulfide oxidoreductase activity;oxidoreductase activity;oxidoreductase activity, acting on a sulfur group of donors;oxidoreductase activity, acting on a sulfur group of donors, disulfide as acceptor;peptide di
5'-deoxyribose-5-phosphate lyase activity;AT DNA binding;binding;carbon-oxygen lyase activity;catalytic activity;DNA binding;DNA-(apurinic or apyrimidinic site) lyase activity;enzyme binding;hormone receptor binding;ligan
adenyl nucleotide binding;adenyl ribonucleotide binding;ATP binding;binding;catalytic activity;kinase activity;nucleotide binding;phosphotransferase activity, alcohol group as acceptor;protein binding;protein kinase activity;pr
binding;cation binding;DNA binding;DNA secondary structure binding;enhancer binding;enhancer sequence-specific DNA binding;four-way junction DNA binding;ion binding;metal ion binding;nucleic acid binding;nucleic aci

ATPase activator activity;ATPase binding;ATPase regulator activity;binding;chaperone binding;enzyme activator activity;enzyme binding;enzyme regulator activity;heat shock protein binding;Hsp70 protein binding;nucleoside-
binding;calcium ion binding;cation binding;ion binding;metal ion binding;protein binding;RAGE receptor binding;receptor binding
14-3-3 protein binding;adenyl nucleotide binding;adenyl ribonucleotide binding;ATP binding;binding;catalytic activity;enzyme binding;enzyme regulator activity;identical protein binding;kinase activity;lipid binding;nitric-oxide
binding;catalytic activity;cation binding;dUTP diphosphatase activity;hydrolase activity;hydrolase activity, acting on acid anhydrides;hydrolase activity, acting on acid anhydrides, in phosphorus-containing anhydrides;ion bind
binding;carbohydrate binding;glycosaminoglycan binding;heparin binding;nucleic acid binding;pattern binding;polysaccharide binding;RNA binding;structural constituent of ribosome;structural molecule activity
nucleic acid binding transcription factor activity;sequence-specific DNA binding transcription factor activity
binding;mRNA binding;nucleic acid binding;RNA binding;structural constituent of ribosome;structural molecule activity
2 iron, 2 sulfur cluster binding;binding;catalytic activity;cation binding;cation transmembrane transporter activity;hydrogen ion transmembrane transporter activity;inorganic cation transmembrane transporter activity;ion bindi
adenyl nucleotide binding;adenyl ribonucleotide binding;ADP binding;binding;carbon-carbon lyase activity;carboxy-lyase activity;catalytic activity;cation binding;coenzyme binding;cofactor binding;electron carrier activity;ion
binding;integrin binding;protein binding;protein complex binding;receptor binding
structural constituent of ribosome;structural molecule activity
binding;identical protein binding;mRNA binding;nucleic acid binding;protein binding;RNA binding
binding;identical protein binding;protein binding;protein dimerization activity;protein homodimerization activity
binding;nuclear localization sequence binding;peptide binding;protein transporter activity;signal sequence binding;substrate-specific transporter activity;transporter activity
anion binding;apoptotic protease activator activity;binding;caspase activator activity;caspase regulator activity;catalytic activity;chaperone binding;chloride ion binding;cysteine-type endopeptidase activity;cysteine-type pept
binding;calcium ion binding;cation binding;identical protein binding;ion binding;ion channel binding;metal ion binding;protein binding;protein dimerization activity;protein homodimerization activity
binding;catalytic activity;GDP binding;GTP binding;GTPase activity;guanyl nucleotide binding;guanyl ribonucleotide binding;hydrolase activity;hydrolase activity, acting on acid anhydrides;hydrolase activity, acting on acid anh
binding;ribonucleoprotein binding;ribosome binding
binding;enzyme binding;histone pre-mRNA DCP binding;nucleic acid binding;protein binding;RNA binding
binding;catalytic activity;enzyme binding;GDP-dissociation inhibitor binding;GTP binding;GTPase activity;guanyl nucleotide binding;guanyl ribonucleotide binding;hydrolase activity;hydrolase activity, acting on acid anhydride
catalytic activity;hydrolase activity;peptidase activity;peptidase activity, acting on L-amino acid peptides;serine hydrolase activity;serine-type peptidase activity
binding;cytoskeletal protein binding;microtubule binding;nucleic acid binding;protein binding;RNA binding;tubulin binding
catalytic activity;dipeptidase activity;exopeptidase activity;hydrolase activity;peptidase activity;peptidase activity, acting on L-amino acid peptides
binding;catalytic activity;channel regulator activity;cytoskeletal protein binding;enzyme binding;glutamate receptor binding;guanylate kinase activity;hydrolase activity;hydrolase activity, acting on ester bonds;ion channel bind
binding;mRNA binding;nucleic acid binding;RNA binding
adenyl nucleotide binding;adenyl ribonucleotide binding;adenylyltransferase activity;ATP binding;binding;catalytic activity;dephospho-CoA kinase activity;kinase activity;nucleotide binding;nucleotidyltransferase activity;pante
binding;nucleotide binding
binding;catalytic activity;coenzyme binding;cofactor binding;NAD binding;nucleic acid binding transcription factor activity;nucleotide binding;oxidoreductase activity;oxidoreductase activity, acting on CH-OH group of donors
binding;catalytic activity;cation binding;collagen binding;endopeptidase activity;enzyme binding;extracellular matrix binding;hydrolase activity;integrin binding;ion binding;kinase binding;laminin binding;metal ion binding;meta
binding;catalytic activity;cation binding;enzyme binding;identical protein binding;ion binding;kinase activity;kinase binding;metal ion binding;phosphotransferase activity, alcohol group as acceptor;protein binding;protein dom
binding;catalytic activity;coenzyme binding;cofactor binding;identical protein binding;isomerase activity;protein binding;protein dimerization activity;protein homodimerization activity;racemase and epimerase activity;racemas
antigen binding;binding;catalytic activity;coenzyme binding;cofactor binding;delta24(24-1) sterol reductase activity;delta24-sterol reductase activity;enzyme binding;flavin adenine dinucleotide binding;oxidoreductase activity;
binding;cation binding;ion binding;metal ion binding;transition metal ion binding;zinc ion binding
acid-amino acid ligase activity;binding;catalytic activity;enzyme binding;ligase activity;ligase activity, forming carbon-nitrogen bonds;protein binding;small conjugating protein ligase activity;ubiquitin protein ligase binding
adenyl nucleotide binding;adenyl ribonucleotide binding;ATP binding;binding;catalytic activity;enzyme binding;histone deacetylase binding;kinase activity;nucleotide binding;phosphotransferase activity, alcohol group as acce
binding;cation binding;ion binding;metal ion binding;transition metal ion binding;zinc ion binding
binding;nucleic acid binding;RNA binding
2 iron, 2 sulfur cluster binding;binding;cation binding;electron carrier activity;ion binding;iron-sulfur cluster binding;metal cluster binding;metal ion binding
3'-5' RNA helicase activity;adenyl nucleotide binding;adenyl ribonucleotide binding;ATP binding;ATPase activity;ATPase activity, coupled;ATP-dependent helicase activity;ATP-dependent RNA helicase activity;binding;catalyti
catalytic activity;oxidoreductase activity
binding;cation binding;ion binding;metal ion binding;nucleotide binding
binding;nucleic acid binding;RNA binding
1-phosphatidylinositol-5-phosphate 4-kinase activity;adenyl nucleotide binding;adenyl ribonucleotide binding;ATP binding;binding;catalytic activity;identical protein binding;inositol or phosphatidylinositol kinase activity;kinas
alcohol dehydrogenase (NADP+) activity;aldo-keto reductase (NADP) activity;catalytic activity;NADP-retinol dehydrogenase activity;oxidoreductase activity;oxidoreductase activity, acting on CH-OH group of donors;oxidoredu
adenyl nucleotide binding;adenyl ribonucleotide binding;ATP binding;ATPase activity;ATPase activity, coupled;ATP-dependent helicase activity;ATP-dependent RNA helicase activity;binding;catalytic activity;estrogen receptor
binding;DNA binding;nucleic acid binding;nucleic acid binding transcription factor activity;sequence-specific DNA binding transcription factor activity
binding;damaged DNA binding;DNA binding;nucleic acid binding
acetyltransferase activity;binding;catalytic activity;chromatin binding;DNA binding;ligand-dependent nuclear receptor binding;N-acetyltransferase activity;N-acyltransferase activity;nucleic acid binding;protein binding;protein
binding;cytoskeletal protein binding;gamma-tubulin binding;protein binding;structural constituent of cytoskeleton;structural molecule activity;tubulin binding
binding;DNA binding;double-stranded DNA binding;mRNA binding;nucleic acid binding;nucleic acid binding transcription factor activity;protein binding;RNA binding;sequence-specific DNA binding transcription factor activity
adenyl nucleotide binding;adenyl ribonucleotide binding;ATP binding;binding;biotin binding;biotin carboxylase activity;carboxylic acid binding;catalytic activity;cation binding;CoA carboxylase activity;ion binding;ligase activity
antioxidant activity;catalytic activity;electron carrier activity;oxidoreductase activity;oxidoreductase activity, acting on a sulfur group of donors;oxidoreductase activity, acting on a sulfur group of donors, NAD or NADP as acce
binding;protein binding;SNARE binding;syntaxin binding
ion transmembrane transporter activity;substrate-specific transmembrane transporter activity;substrate-specific transporter activity;transmembrane transporter activity;transporter activity
binding;cation binding;chromatin binding;DNA binding;ion binding;metal ion binding;nucleic acid binding
catalytic activity;kinase activity;transferase activity;transferase activity, transferring phosphorus-containing groups
adenyl nucleotide binding;adenyl ribonucleotide binding;ATP binding;binding;catalytic activity;CoA carboxylase activity;ligase activity;ligase activity, forming carbon-carbon bonds;methylcrotonoyl-CoA carboxylase activity;nu
binding;cation binding;enzyme activator activity;enzyme regulator activity;GTPase activator activity;GTPase regulator activity;ion binding;metal ion binding;nucleoside-triphosphatase regulator activity;protein transporter activ
adenyl nucleotide binding;adenyl ribonucleotide binding;aminoacyl-tRNA ligase activity;ATP binding;binding;catalytic activity;ligase activity;ligase activity, forming aminoacyl-tRNA and related compounds;ligase activity, formi
binding;identical protein binding;mRNA binding;nucleic acid binding;protein binding;RNA binding
basal RNA polymerase II transcription machinery binding;basal transcription machinery binding;binding;enzyme binding;protein binding;RNA polymerase binding;RNA polymerase core enzyme binding;RNA polymerase II core
binding;cation binding;double-stranded RNA binding;ion binding;metal ion binding;nucleic acid binding;RNA binding
binding;nucleotide binding
catalytic activity
binding;carboxylic acid binding;catalytic activity;coenzyme binding;cofactor binding;glycerate dehydrogenase activity;glyoxylate reductase (NADP) activity;hydroxypyruvate reductase activity;identical protein binding;NAD bin
catalytic activity;cysteine-type endopeptidase activity;cysteine-type peptidase activity;endopeptidase activity;hydrolase activity;peptidase activity;peptidase activity, acting on L-amino acid peptides
binding;mRNA binding;nucleic acid binding;nucleocytoplasmic transporter activity;nucleotide binding;RNA binding;transporter activity
adenyl nucleotide binding;adenyl ribonucleotide binding;ATP binding;binding;catalytic activity;GTP binding;guanyl nucleotide binding;guanyl ribonucleotide binding;kinase activity;nucleobase-containing compound kinase act
binding;cation binding;ion binding;metal ion binding
binding;cation binding;DEAD/H-box RNA helicase binding;DNA binding;enzyme binding;ion binding;metal ion binding;nucleic acid binding;protein binding
binding;enzyme binding;lipid binding;phosphatidylinositol binding;phospholipid binding;protein binding
active transmembrane transporter activity;anion transmembrane transporter activity;anion:cation symporter activity;binding;cation transmembrane transporter activity;cation:chloride symporter activity;chloride transmembran
2'-5'-oligoadenylate synthetase activity;adenyl nucleotide binding;adenyl ribonucleotide binding;adenylyltransferase activity;ATP binding;binding;catalytic activity;cation binding;double-stranded RNA binding;ion binding;meta
binding;lipid binding;phosphatidylinositol binding;phosphatidylinositol-3,5-bisphosphate binding;phosphatidylinositol-3-phosphate binding;phosphatidylinositol-4-phosphate binding;phosphatidylinositol-5-phosphate binding
binding;protein binding;SNARE binding;syntaxin binding
binding;cation binding;heme binding;ion binding;iron ion binding;lipid binding;metal ion binding;steroid binding;tetrapyrrole binding;transition metal ion binding
adenyl nucleotide binding;adenyl ribonucleotide binding;alkali metal ion binding;ATP binding;binding;catalytic activity;cation binding;cofactor binding;identical protein binding;ion binding;kinase activity;lithium ion binding;mag
binding;DNA binding;nucleic acid binding;nucleic acid binding transcription factor activity;regulatory region DNA binding;regulatory region nucleic acid binding;sequence-specific DNA binding transcription factor activity
structural constituent of cytoskeleton;structural molecule activity
alcohol binding;amine binding;binding;cation binding;ion binding;lipid binding;phosphatidylcholine binding;phospholipid binding;protein binding;protein domain specific binding;quaternary ammonium group binding
cation transmembrane transporter activity;hydrogen ion transmembrane transporter activity;inorganic cation transmembrane transporter activity;ion transmembrane transporter activity;monovalent inorganic cation transmemb
binding;DNA binding;mRNA binding;nucleic acid binding;RNA binding
binding;nucleic acid binding;nucleotide binding;RNA binding
binding;cation binding;drug binding;ion binding;metal ion binding;transition metal ion binding;zinc ion binding
binding;cation binding;electron carrier activity;electron transporter, transferring electrons from CoQH2-cytochrome c reductase complex and cytochrome c oxidase complex activity;heme binding;ion binding;iron ion binding;r
binding;catalytic activity;GDP binding;GTP binding;GTPase activity;guanyl nucleotide binding;guanyl ribonucleotide binding;hydrolase activity;hydrolase activity, acting on acid anhydrides;hydrolase activity, acting on acid anh
3-methyl-2-oxobutanoate dehydrogenase (2-methylpropanoyl-transferring) activity;alpha-ketoacid dehydrogenase activity;binding;carbon-carbon lyase activity;carboxy-lyase activity;catalytic activity;cation binding;ion binding
alcohol dehydrogenase (NADP+) activity;alditol:NADP+ 1-oxidoreductase activity;aldo-keto reductase (NADP) activity;catalytic activity;electron carrier activity;L-glucuronate reductase activity;oxidoreductase activity;oxidored
binding;damaged DNA binding;DNA binding;enzyme binding;nucleic acid binding;phosphatase binding;protein binding;protein phosphatase binding;single-stranded DNA binding;structure-specific DNA binding;ubiquitin prote
adenyl nucleotide binding;adenyl ribonucleotide binding;ATP binding;binding;cAMP-dependent protein kinase activity;catalytic activity;cyclic nucleotide-dependent protein kinase activity;enzyme binding;kinase activity;kinase
binding;carbohydrate binding;chemoattractant activity;extracellular matrix binding;IgE binding;immunoglobulin binding;laminin binding;protein binding;protein complex binding
binding;chromatin binding;chromatin DNA binding;DNA binding;enzyme regulator activity;GTPase regulator activity;guanyl-nucleotide exchange factor activity;histone binding;nucleic acid binding;nucleoside-triphosphatase r
binding;carbohydrate binding;carbohydrate transmembrane transporter activity;hexose transmembrane transporter activity;mannose binding;mannose transmembrane transporter activity;molecular transducer activity;monos
binding;catalytic activity;cation binding;DNA binding;IMP dehydrogenase activity;ion binding;metal ion binding;nucleic acid binding;nucleotide binding;oxidoreductase activity;oxidoreductase activity, acting on CH-OH group
active transmembrane transporter activity;adenyl nucleotide binding;adenyl ribonucleotide binding;ATP binding;ATPase activity;ATPase activity, coupled;ATPase activity, coupled to movement of substances;ATPase activity, c
carboxyl-O-methyltransferase activity;catalytic activity;methyltransferase activity;O-methyltransferase activity;protein carboxyl O-methyltransferase activity;protein methyltransferase activity;protein-L-isoaspartate (D-aspartat
catalytic activity;ferredoxin-NADP+ reductase activity;NADPH-adrenodoxin reductase activity;oxidoreductase activity;oxidoreductase activity, acting on iron-sulfur proteins as donors;oxidoreductase activity, acting on iron-sul
binding;carbon-carbon lyase activity;carboxy-lyase activity;catalytic activity;cation binding;coenzyme binding;cofactor binding;electron carrier activity;ion binding;lyase activity;malate dehydrogenase (decarboxylating) activity
catalytic activity;endopeptidase activity;hydrolase activity;peptidase activity;peptidase activity, acting on L-amino acid peptides;threonine-type endopeptidase activity;threonine-type peptidase activity
catalytic activity;glycylpeptide N-tetradecanoyltransferase activity;myristoyltransferase activity;N-acyltransferase activity;transferase activity;transferase activity, transferring acyl groups;transferase activity, transferring acyl gr
binding;calcium ion binding;cation binding;ion binding;metal ion binding
binding;nucleic acid binding;nucleotide binding;RNA binding
active transmembrane transporter activity;adenyl nucleotide binding;adenyl ribonucleotide binding;ATP binding;ATPase activity;ATPase activity, coupled;ATPase activity, coupled to movement of substances;ATPase activity, c
acetyltransferase activity;catalytic activity;N-acetyltransferase activity;N-acyltransferase activity;transferase activity;transferase activity, transferring acyl groups;transferase activity, transferring acyl groups other than amino-ac
binding;nucleic acid binding;RNA binding;translation elongation factor activity;translation factor activity, nucleic acid binding
catalytic activity;deaminase activity;glucosamine-6-phosphate deaminase activity;hydrolase activity;intramolecular oxidoreductase activity;intramolecular oxidoreductase activity, interconverting aldoses and ketoses;isomeras

binding;chromatin binding;core promoter binding;core promoter sequence-specific DNA binding;DNA binding;nucleic acid binding;protein binding transcription factor activity;regulatory region DNA binding;regulatory region n
binding;cation transmembrane transporter activity;drug binding;hydrogen ion transmembrane transporter activity;hydrogen ion transporting ATP synthase activity, rotational mechanism;inorganic cation transmembrane transp
adenyl nucleotide binding;adenyl ribonucleotide binding;ATP binding;binding;catalytic activity;kinase activity;nucleotide binding;phosphotransferase activity, alcohol group as acceptor;protein kinase activity;protein serine/thre
7S RNA binding;binding;nucleic acid binding;ribonucleoprotein binding;RNA binding;signal recognition particle binding
acid-thiol ligase activity;ATP citrate synthase activity;binding;catalytic activity;CoA-ligase activity;cofactor binding;GDP binding;GTP binding;guanyl nucleotide binding;guanyl ribonucleotide binding;ligase activity;ligase activit
active transmembrane transporter activity;ATPase activator activity;ATPase activity;ATPase activity, coupled;ATPase activity, coupled to movement of substances;ATPase activity, coupled to transmembrane movement of ions
binding;identical protein binding;protein binding
binding;cation binding;ion binding;metal ion binding;structural constituent of ribosome;structural molecule activity
binding;catalytic activity;GTP binding;GTPase activity;guanyl nucleotide binding;guanyl ribonucleotide binding;hydrolase activity;hydrolase activity, acting on acid anhydrides;hydrolase activity, acting on acid anhydrides, in pl
binding;mRNA 5'-UTR binding;mRNA binding;nucleic acid binding;RNA binding;structural constituent of ribosome;structural molecule activity;translation regulator activity
binding;cation binding;ion binding;metal ion binding;structural constituent of ribosome;structural molecule activity;transition metal ion binding;zinc ion binding
binding;nucleic acid binding;nucleotide binding;RNA binding;rRNA binding;structural constituent of ribosome;structural molecule activity
structural constituent of ribosome;structural molecule activity
binding;calcium ion binding;catalytic activity;cation binding;hydrolase activity;hydrolase activity, acting on ester bonds;identical protein binding;ion binding;magnesium ion binding;metal ion binding;phosphatase activity;phos
structural constituent of ribosome;structural molecule activity
structural constituent of ribosome;structural molecule activity
binding;catalytic activity;drug binding;hydroxymethylglutaryl-CoA synthase activity;isomerase activity;organic acid binding;transferase activity;transferase activity, transferring acyl groups;transferase activity, transferring acyl
adenyl nucleotide binding;adenyl ribonucleotide binding;ATP binding;binding;nucleotide binding;purine nucleotide binding;purine ribonucleoside triphosphate binding;purine ribonucleotide binding;ribonucleotide binding
adenyl nucleotide binding;adenyl ribonucleotide binding;ATP binding;binding;calmodulin binding;calmodulin-dependent protein kinase activity;catalytic activity;channel inhibitor activity;channel regulator activity;cytoskeletal p
binding;catalytic activity;enzyme binding;NEDD8 activating enzyme activity;protein binding;protein dimerization activity;protein heterodimerization activity;small protein activating enzyme activity;ubiquitin protein ligase binding
binding;enzyme binding;protein binding;receptor activity
binding;DNA binding;nucleic acid binding
adenyl nucleotide binding;adenyl ribonucleotide binding;ATP binding;binding;catalytic activity;kinase activity;nucleotide binding;phosphomevalonate kinase activity;phosphotransferase activity, phosphate group as acceptor;p
basal RNA polymerase II transcription machinery binding;basal transcription machinery binding;binding;enzyme binding;protein binding;RNA polymerase binding;RNA polymerase core enzyme binding;RNA polymerase II core
catalytic activity;oxidoreductase activity
binding;catalytic activity;cation binding;hydrolase activity;ion binding;metal ion binding;metallopeptidase activity;peptidase activity;peptidase activity, acting on L-amino acid peptides
acid-amino acid ligase activity;binding;catalytic activity;cation binding;ion binding;ligase activity;ligase activity, forming carbon-nitrogen bonds;metal ion binding;small conjugating protein ligase activity;transition metal ion bin
carboxylic ester hydrolase activity;catalytic activity;hydrolase activity;hydrolase activity, acting on ester bonds;lipase activity;lysophospholipase activity;phospholipase activity
catalytic activity;methyltransferase activity;RNA methyltransferase activity;rRNA methyltransferase activity;S-adenosylmethionine-dependent methyltransferase activity;transferase activity;transferase activity, transferring one-
adenyl nucleotide binding;adenyl ribonucleotide binding;adenylyltransferase activity;ATP binding;binding;catalytic activity;FMN adenylyltransferase activity;nucleotide binding;nucleotidyltransferase activity;purine nucleotide b
acid-amino acid ligase activity;adenyl nucleotide binding;adenyl ribonucleotide binding;ATP binding;binding;catalytic activity;ligase activity;ligase activity, forming carbon-nitrogen bonds;NEDD8 activating enzyme activity;nuc
binding;enzyme binding;GTPase binding;protein binding;Rab GTPase binding;Ras GTPase binding;small GTPase binding
enzyme regulator activity;phosphatase regulator activity;protein phosphatase regulator activity;protein phosphatase type 4 regulator activity
binding;cation binding;ion binding;metal ion binding
adenyl nucleotide binding;adenyl ribonucleotide binding;ATP binding;ATPase activity;ATPase activity, coupled;ATP-dependent helicase activity;ATP-dependent RNA helicase activity;binding;catalytic activity;helicase activity;h
catalytic activity;hydrolase activity
binding;calcium ion binding;cation binding;identical protein binding;ion binding;metal ion binding;protein binding;protein dimerization activity;protein homodimerization activity
binding;catalytic activity;cysteine-type peptidase activity;enzyme binding;hydrolase activity;NEDD8-specific protease activity;peptidase activity;peptidase activity, acting on L-amino acid peptides;protein binding;small conjug
binding;ErbB-2 class receptor binding;integrin binding;protein binding;protein complex binding;receptor binding;structural constituent of cytoskeleton;structural molecule activity
binding;neurotransmitter binding
beta-tubulin binding;binding;chaperone binding;cytoskeletal protein binding;enzyme activator activity;enzyme regulator activity;GTPase activator activity;GTPase regulator activity;nucleoside-triphosphatase regulator activity;
binding;ribonucleoprotein binding
binding;nucleotide binding
binding;identical protein binding;protein binding;protein dimerization activity;protein homodimerization activity
catalytic activity;fucosyltransferase activity;peptide-O-fucosyltransferase activity;transferase activity;transferase activity, transferring glycosyl groups;transferase activity, transferring hexosyl groups
adenyl nucleotide binding;adenyl ribonucleotide binding;ATP binding;binding;catalytic activity;channel inhibitor activity;channel regulator activity;chloride channel inhibitor activity;chloride channel regulator activity;enzyme bin
adenyl nucleotide binding;adenyl ribonucleotide binding;ATP binding;ATPase activity;binding;catalytic activity;cation binding;hydrolase activity;hydrolase activity, acting on acid anhydrides;hydrolase activity, acting on acid an
binding;protein binding;protein dimerization activity;protein heterodimerization activity
enzyme inhibitor activity;enzyme regulator activity
binding;enzyme binding;enzyme regulator activity;methionine adenosyltransferase regulator activity;protein binding
binding;GTP binding;guanyl nucleotide binding;guanyl ribonucleotide binding;nucleotide binding;purine nucleotide binding;purine ribonucleoside triphosphate binding;purine ribonucleotide binding;ribonucleotide binding
binding;nucleic acid binding;protein binding;RNA binding;translation factor activity, nucleic acid binding;translation initiation factor activity;translation initiation factor binding
binding;enzyme binding;GTPase binding;protein binding;receptor binding
binding;binding, bridging;cytoskeletal adaptor activity;cytoskeletal protein binding;identical protein binding;proline-rich region binding;protein binding;protein binding, bridging;protein C-terminus binding
adenyl nucleotide binding;adenyl ribonucleotide binding;amine binding;amino acid binding;aminoacyl-tRNA ligase activity;ATP binding;binding;carboxylic acid binding;catalytic activity;identical protein binding;ligase activity;li
binding;catalytic activity;cysteine-type peptidase activity;endopeptidase inhibitor activity;endopeptidase regulator activity;enzyme inhibitor activity;enzyme regulator activity;exopeptidase activity;hydrolase activity;omega pep
actin binding;binding;cytoskeletal protein binding;protein binding;protein complex binding
binding;cholesterol binding;hedgehog receptor activity;lipid binding;lipid transporter activity;molecular transducer activity;receptor activity;signal transducer activity;signaling receptor activity;steroid binding;sterol binding;ster
structural constituent of cytoskeleton;structural molecule activity
amine binding;amino acid binding;binding;carboxylic acid binding;catalytic activity;cation binding;glutathione binding;G-protein coupled receptor activity;ion binding;lipoprotein particle receptor binding;low-density lipoprotein
alpha(1,2)-fucosyltransferase activity;binding;catalytic activity;cytoskeletal protein binding;fucosyltransferase activity;galactoside 2-alpha-L-fucosyltransferase activity;microtubule binding;microtubule plus-end binding;protein
4 iron, 4 sulfur cluster binding;binding;catalytic activity;cation binding;coenzyme binding;cofactor binding;electron carrier activity;enzyme binding;ion binding;iron-sulfur cluster binding;metal cluster binding;metal ion binding;
protein transporter activity;substrate-specific transporter activity;transporter activity
adenyl nucleotide binding;adenyl ribonucleotide binding;ADP binding;AMP binding;binding;catalytic activity;coenzyme binding;cofactor binding;cytochrome-b5 reductase activity;FAD binding;flavin adenine dinucleotide bindi
binding;DNA binding;nucleic acid binding
binding;calcium ion binding;calcium-dependent protein binding;cation binding;cytoskeletal protein binding;identical protein binding;ion binding;ion transmembrane transporter activity;metal ion binding;protein binding;protein
binding;DNA binding;enzyme binding;nucleic acid binding;protein binding
catalytic activity;cation transmembrane transporter activity;cytochrome-c oxidase activity;heme-copper terminal oxidase activity;hydrogen ion transmembrane transporter activity;inorganic cation transmembrane transporter a
binding;DNA binding;double-stranded DNA binding;enzyme binding;GTPase binding;mRNA 3'-UTR binding;mRNA binding;nucleic acid binding;nucleic acid binding transcription factor activity;protein binding;protein binding
binding;chromatin binding;DNA binding;double-stranded DNA binding;enhancer binding;enhancer sequence-specific DNA binding;identical protein binding;nucleic acid binding;nucleic acid binding transcription factor activity
catalytic activity;endopeptidase activity;hydrolase activity;peptidase activity;peptidase activity, acting on L-amino acid peptides;threonine-type endopeptidase activity;threonine-type peptidase activity
3-mercaptopyruvate sulfurtransferase activity;catalytic activity;sulfurtransferase activity;thiosulfate sulfurtransferase activity;transferase activity;transferase activity, transferring sulfur-containing groups
active transmembrane transporter activity;adenyl nucleotide binding;adenyl ribonucleotide binding;ATP binding;ATPase activity;ATPase activity, coupled;ATPase activity, coupled to movement of substances;ATPase activity, c
aminopeptidase activity;binding;catalytic activity;cation binding;exopeptidase activity;hydrolase activity;ion binding;manganese ion binding;metal ion binding;metalloexopeptidase activity;metallopeptidase activity;peptidase a
alkali metal ion binding;binding;catalytic activity;cation binding;hydrolase activity;hydrolase activity, acting on ester bonds;identical protein binding;inositol monophosphate phosphatase activity;inositol or phosphatidylinositol
apoptotic protease activator activity;caspase activator activity;caspase regulator activity;catalytic activity;cysteine-type endopeptidase activity;cysteine-type peptidase activity;endopeptidase activity;enzyme activator activity
binding;chaperone binding;identical protein binding;protein binding;protein dimerization activity;protein homodimerization activity
binding;catalytic activity;cis-trans isomerase activity;cyclosporin A binding;drug binding;isomerase activity;peptide binding;peptidyl-prolyl cis-trans isomerase activity
3-chloroallyl aldehyde dehydrogenase activity;alcohol dehydrogenase (NADP+) activity;aldehyde dehydrogenase (NAD) activity;aldehyde dehydrogenase [NAD(P)+] activity;aldo-keto reductase (NADP) activity;benzaldehyde d
binding;catalytic activity;DNA binding;enzyme activator activity;enzyme regulator activity;nucleic acid binding;phosphatase activator activity;phosphatase regulator activity;protein binding;protein binding transcription factor a
binding;carbohydrate binding;mannose binding;monosaccharide binding;sugar binding
binding;binding, bridging;chromatin binding;histone binding;methylated histone residue binding;protein binding;protein binding, bridging;repressing transcription factor binding;transcription factor binding
binding;nucleic acid binding;RNA binding;structural constituent of ribosome;structural molecule activity
binding;nucleic acid binding;RNA binding;translation factor activity, nucleic acid binding;translation initiation factor activity
adenyl nucleotide binding;adenyl ribonucleotide binding;ATP binding;binding;GTP binding;guanyl nucleotide binding;guanyl ribonucleotide binding;nucleic acid binding;nucleotide binding;purine nucleotide binding;purine ribo
5'-nucleotidase activity;binding;catalytic activity;cation binding;hydrolase activity;hydrolase activity, acting on ester bonds;ion binding;kinase activity;metal ion binding;nucleobase-containing compound kinase activity;nucleos
endopeptidase inhibitor activity;endopeptidase regulator activity;enzyme inhibitor activity;enzyme regulator activity;peptidase inhibitor activity;peptidase regulator activity;serine-type endopeptidase inhibitor activity
binding;cation binding;ion binding;metal ion binding;transition metal ion binding;zinc ion binding
adenosine kinase activity;adenyl nucleotide binding;adenyl ribonucleotide binding;ATP binding;binding;catalytic activity;cation binding;ion binding;kinase activity;metal ion binding;nucleobase-containing compound kinase ac
apolipoprotein A-I receptor binding;apolipoprotein receptor binding;binding;catalytic activity;enzyme binding;GTP binding;GTPase activity;guanyl nucleotide binding;guanyl ribonucleotide binding;hydrolase activity;hydrolase
binding;calcium ion binding;cation binding;DNA binding;ion binding;metal ion binding;nucleic acid binding
structural constituent of ribosome;structural molecule activity
binding;nucleic acid binding;nucleotide binding;RNA binding
binding;nucleotide binding;protein binding transcription factor activity;transcription cofactor activity;transcription corepressor activity;transcription factor binding transcription factor activity
ATPase binding;binding;enzyme binding;K6-linked polyubiquitin binding;polyubiquitin binding;protein binding;small conjugating protein binding;ubiquitin binding;ubiquitin protein ligase binding
binding;carbon-sulfur lyase activity;catalytic activity;cation binding;ion binding;lactoylglutathione lyase activity;lyase activity;metal ion binding;transition metal ion binding;zinc ion binding
transporter activity
binding;enzyme regulator activity;GTPase regulator activity;guanyl-nucleotide exchange factor activity;nucleic acid binding;nucleoside-triphosphatase regulator activity;protein binding;RNA binding;translation factor activity, n
binding;enzyme binding;identical protein binding;protein binding;protein domain specific binding
1-phosphatidylinositol binding;binding;binding, bridging;clathrin adaptor activity;clathrin binding;clathrin heavy chain binding;lipid binding;phosphatidylinositol binding;phospholipid binding;protein binding;protein binding, bri
binding;epidermal growth factor receptor binding;growth factor receptor binding;identical protein binding;insulin receptor binding;lipid binding;phosphatidylinositol binding;phospholipid binding;protein binding;protein comple
binding;protein binding;receptor binding
binding;cytokine activity;protein binding;receptor binding
adenyl nucleotide binding;adenyl ribonucleotide binding;ATP binding;binding;nucleotide binding;purine nucleotide binding;purine ribonucleoside triphosphate binding;purine ribonucleotide binding;ribonucleotide binding
binding;catalytic activity;cysteine-type endopeptidase activity;cysteine-type peptidase activity;endopeptidase activity;hydrolase activity;ion channel binding;p53 binding;peptidase activity;peptidase activity, acting on L-amino
15-oxoprostaglandin 13-oxidase activity;2-alkenal reductase [NAD(P)] activity;binding;catalytic activity;cation binding;ion binding;metal ion binding;oxidoreductase activity;oxidoreductase activity, acting on the CH-CH group
1-alkyl-2-acetylglycerophosphocholine esterase activity;acetyltransferase activity;binding;carboxylic ester hydrolase activity;catalytic activity;hydrolase activity;hydrolase activity, acting on ester bonds;identical protein binding
binding;calcium ion binding;cation binding;ion binding;metal ion binding
binding;heat shock protein binding;protein binding
binding;cation binding;ion binding;metal ion binding;transition metal ion binding;zinc ion binding
adenyl nucleotide binding;adenyl ribonucleotide binding;alanine-tRNA ligase activity;aminoacyl-tRNA ligase activity;ATP binding;binding;catalytic activity;cation binding;ion binding;ligase activity;ligase activity, forming aminoa
3'-5' exonuclease activity;3'-5'-exoribonuclease activity;binding;catalytic activity;cation binding;exonuclease activity;exonuclease activity, active with either ribo- or deoxyribonucleic acids and producing 5'-phosphomonoeste
all-trans-retinol 13,14-reductase activity;catalytic activity;oxidoreductase activity;oxidoreductase activity, acting on the CH-CH group of donors
basal RNA polymerase II transcription machinery binding;basal transcription machinery binding;binding;enzyme binding;protein binding;RNA polymerase binding;RNA polymerase core enzyme binding;RNA polymerase II core

binding;core promoter proximal region DNA binding;core promoter proximal region sequence-specific DNA binding;DNA binding;molecular transducer activity;nucleic acid binding;nucleic acid binding transcription factor acti
catalytic activity;methyltransferase activity;transferase activity;transferase activity, transferring one-carbon groups
binding;nucleic acid binding;RNA binding;translation factor activity, nucleic acid binding;translation initiation factor activity
binding;protein binding;protein domain specific binding
binding;nucleic acid binding;RNA binding
binding;cation binding;ion binding;metal ion binding
arginine N-methyltransferase activity;beta-catenin binding;binding;catalytic activity;DNA binding;histone acetyl-lysine binding;histone binding;histone methyltransferase activity;histone methyltransferase activity (H3-R17 spec
binding;DNA binding;nucleic acid binding
binding;cytoskeletal protein binding;enzyme activator activity;enzyme regulator activity;kinase activator activity;kinase regulator activity;protein binding;protein complex binding;protein kinase activator activity;protein kinase r
binding;cation binding;enzyme activator activity;enzyme regulator activity;GTPase activator activity;GTPase regulator activity;ion binding;metal ion binding;nucleoside-triphosphatase regulator activity;transition metal ion bind
binding;protein binding;SNARE binding;syntaxin binding
binding;enzyme binding;protein binding
catalytic activity;oxidoreductase activity;oxidoreductase activity, acting on the CH-NH group of donors;oxidoreductase activity, acting on the CH-NH group of donors, NAD or NADP as acceptor;pyrroline-5-carboxylate reduc
binding;enzyme binding;GTPase binding;identical protein binding;K63-linked polyubiquitin binding;polyubiquitin binding;protein binding;protein C-terminus binding;Rab GTPase binding;Ras GTPase binding;small conjugating
binding;catalytic activity;enzyme activator activity;enzyme regulator activity;GTPase activator activity;GTPase regulator activity;hydrolase activity;hydrolase activity, acting on ester bonds;molecular transducer activity;nucleos
binding;ribonucleoprotein binding;ribosome binding
binding;chromatin binding
adenyl nucleotide binding;adenyl ribonucleotide binding;ATP binding;ATPase activity;ATPase activity, coupled;ATP-dependent helicase activity;ATP-dependent RNA helicase activity;binding;catalytic activity;helicase activity;h
binding;nucleic acid binding;RNA binding;snoRNA binding
binding;catalytic activity;cation binding;hydrolase activity;identical protein binding;ion binding;metal ion binding;metallopeptidase activity;peptidase activity;peptidase activity, acting on L-amino acid peptides;protein binding;
active transmembrane transporter activity;anion transmembrane transporter activity;ATPase activity;ATPase activity, coupled;ATPase activity, coupled to movement of substances;ATPase activity, coupled to transmembrane r
binding;identical protein binding;lipid binding;protein binding;protein dimerization activity;protein homodimerization activity
binding;catalytic activity;GTP binding;GTPase activity;guanyl nucleotide binding;guanyl ribonucleotide binding;hydrolase activity;hydrolase activity, acting on acid anhydrides;hydrolase activity, acting on acid anhydrides, in pl
binding;cytoskeletal protein binding;enzyme binding;identical protein binding;protein binding;RNA polymerase binding;tau protein binding
acetyltransferase activity;catalytic activity;H4 histone acetyltransferase activity;histone acetyltransferase activity;lysine N-acetyltransferase activity;N-acetyltransferase activity;N-acyltransferase activity;transferase activity;tran
adenyl nucleotide binding;adenyl ribonucleotide binding;ATP binding;binding;catalytic activity;kinase activity;nucleotide binding;phosphotransferase activity, alcohol group as acceptor;protein kinase activity;protein serine/thr
adenyl nucleotide binding;adenyl ribonucleotide binding;adenylylsulfate kinase activity;adenylyltransferase activity;ATP binding;binding;catalytic activity;kinase activity;nucleotide binding;nucleotidyltransferase activity;phosph
binding;nucleic acid binding;RNA binding;snoRNA binding
binding;carbon-oxygen lyase activity;catalytic activity;coenzyme binding;cofactor binding;GDP-mannose 4,6-dehydratase activity;hydro-lyase activity;lyase activity;NADP binding;NADP+ binding;nucleotide binding
binding;DNA binding;nucleic acid binding;protein binding transcription factor activity;sequence-specific DNA binding;transcription coactivator activity;transcription cofactor activity;transcription factor binding transcription fac
adenyl nucleotide binding;adenyl ribonucleotide binding;ATP binding;binding;cation binding;chaperone binding;ion binding;metal ion binding;nucleotide binding;protein binding;purine nucleotide binding;purine ribonucleoside
binding;enzyme activator activity;enzyme binding;enzyme regulator activity;GTPase activator activity;GTPase binding;GTPase regulator activity;identical protein binding;nucleoside-triphosphatase regulator activity;protein bin
binding;cation binding;ion binding;metal ion binding;protein binding;transition metal ion binding;translation initiation factor binding;zinc ion binding
binding;cation binding;ion binding;metal ion binding;transition metal ion binding;zinc ion binding
beta-tubulin binding;binding;cytoskeletal protein binding;enzyme binding;FFAT motif binding;identical protein binding;microtubule binding;protein binding;protein dimerization activity;protein domain specific binding;protein he
adenyl nucleotide binding;adenyl ribonucleotide binding;ATP binding;binding;catalytic activity;kinase activity;NADH dehydrogenase (quinone) activity;NADH dehydrogenase (ubiquinone) activity;NADH dehydrogenase activity;
antioxidant activity;binding;catalytic activity;coenzyme binding;cofactor binding;disulfide oxidoreductase activity;electron carrier activity;flavin adenine dinucleotide binding;glutathione disulfide oxidoreductase activity;glutathi
antioxidant activity;binding;catalytic activity;cation binding;chaperone binding;copper ion binding;enzyme binding;GTPase binding;identical protein binding;ion binding;metal ion binding;oxidoreductase activity;oxidoreductas
antioxidant activity;arachidonic acid binding;binding;calcium ion binding;carboxylic acid binding;cation binding;cytoskeletal protein binding;fatty acid binding;icosanoid binding;icosatetraenoic acid binding;ion binding;lipid bi
binding;GTP binding;guanyl nucleotide binding;guanyl ribonucleotide binding;molecular transducer activity;nucleotide binding;purine nucleotide binding;purine ribonucleoside triphosphate binding;purine ribonucleotide bindi
binding;catalytic activity;dinitrosyl-iron complex binding;enzyme binding;enzyme regulator activity;glutathione transferase activity;JUN kinase binding;kinase binding;kinase regulator activity;nitric oxide binding;protein binding
binding;clathrin binding;clathrin heavy chain binding;peptide binding;protein binding;structural molecule activity
acetyltransferase activity;catalytic activity;dihydrolipoamide S-acyltransferase activity;dihydrolipoyllysine-residue acetyltransferase activity;S-acetyltransferase activity;S-acyltransferase activity;transferase activity;transferase
binding;catalytic activity;identical protein binding;protein binding;protein dimerization activity;protein homodimerization activity;spermidine synthase activity;transferase activity;transferase activity, transferring alkyl or aryl (oth
binding;nucleic acid binding;RNA binding;translation elongation factor activity;translation factor activity, nucleic acid binding
binding;enzyme binding;nucleotide binding;protein binding
binding;calcium ion binding;cation binding;identical protein binding;ion binding;metal ion binding;protein binding
adenyl nucleotide binding;adenyl ribonucleotide binding;ATP binding;binding;endopeptidase inhibitor activity;endopeptidase regulator activity;enzyme binding;enzyme inhibitor activity;enzyme regulator activity;kinase binding
binding;calcium ion binding;calcium-dependent protein binding;cation binding;identical protein binding;ion binding;metal ion binding;protein binding;protein dimerization activity;protein homodimerization activity
binding;catalytic activity;GTP binding;GTPase activity;guanyl nucleotide binding;guanyl ribonucleotide binding;hydrolase activity;hydrolase activity, acting on acid anhydrides;hydrolase activity, acting on acid anhydrides, in pl
adenyl nucleotide binding;adenyl ribonucleotide binding;ATP binding;binding;DNA binding;nucleic acid binding;nucleotide binding;purine nucleotide binding;purine ribonucleoside triphosphate binding;purine ribonucleotide bi
adenyl nucleotide binding;adenyl ribonucleotide binding;ATP binding;ATPase activity;ATPase activity, coupled;binding;catalytic activity;DNA binding;DNA clamp loader activity;DNA-dependent ATPase activity;double-strande
ATPase activity;ATPase activity, coupled;binding;catalytic activity;DNA binding;DNA clamp loader activity;DNA-dependent ATPase activity;hydrolase activity;hydrolase activity, acting on acid anhydrides;hydrolase activity, acti
binding;enzyme activator activity;enzyme binding;enzyme regulator activity;GDP-dissociation inhibitor activity;GTPase activator activity;GTPase binding;GTPase regulator activity;nucleoside-triphosphatase regulator activity;p
binding;enzyme binding;polyubiquitin binding;proteasome binding;protein binding;protein complex binding;ribonucleoprotein binding;ribosome binding;small conjugating protein binding;ubiquitin binding;ubiquitin protein liga
adenyl nucleotide binding;adenyl ribonucleotide binding;aminoacyl-tRNA ligase activity;ATP binding;binding;catalytic activity;histidine-tRNA ligase activity;ligase activity;ligase activity, forming aminoacyl-tRNA and related co
catalytic activity;endopeptidase activity;hydrolase activity;peptidase activity;peptidase activity, acting on L-amino acid peptides;threonine-type endopeptidase activity;threonine-type peptidase activity
3'-5' exonuclease activity;binding;catalytic activity;cation binding;deoxyribonuclease activity;DNA binding;double-stranded DNA binding;endodeoxyribonuclease activity;endonuclease activity;exonuclease activity;hydrolase a
binding;catalytic activity;cation binding;hydrolase activity;hydrolase activity, acting on ester bonds;identical protein binding;ion binding;lipid binding;metal ion binding;molecular transducer activity;nucleic acid binding;phosph
molecular transducer activity;protein binding transcription factor activity;signal transducer activity;transcription cofactor activity;transcription corepressor activity;transcription factor binding transcription factor activity
structural constituent of ribosome;structural molecule activity
binding;enzyme binding;nucleic acid binding;protein binding;RNA binding;snoRNA binding
binding;catalytic activity;cis-trans isomerase activity;cyclosporin A binding;drug binding;estrogen receptor binding;heat shock protein binding;hormone receptor binding;Hsp70 protein binding;Hsp90 protein binding;isomeras
antioxidant activity;catalytic activity;oxidoreductase activity;oxidoreductase activity, acting on paired donors, with incorporation or reduction of molecular oxygen;oxidoreductase activity, acting on peroxide as acceptor;perox
binding;catalytic activity;cis-trans isomerase activity;cyclosporin A binding;drug binding;isomerase activity;peptide binding;peptidyl-prolyl cis-trans isomerase activity
binding;catalytic activity;coenzyme binding;cofactor binding;electron carrier activity;GDP-4-dehydro-D-rhamnose reductase activity;GDP-L-fucose synthase activity;isomerase activity;oxidoreductase activity;oxidoreductase a
binding;catalytic activity;GTP binding;GTPase activity;guanyl nucleotide binding;guanyl ribonucleotide binding;hydrolase activity;hydrolase activity, acting on acid anhydrides;hydrolase activity, acting on acid anhydrides, in pl
binding;nucleotide binding
binding;cation binding;DNA binding;double-stranded DNA binding;ion binding;metal ion binding;nucleic acid binding;nucleotide binding;RNA binding;structure-specific DNA binding;transition metal ion binding;zinc ion bindin
binding;catalytic activity;GDP binding;GTP binding;GTPase activity;guanyl nucleotide binding;guanyl ribonucleotide binding;hydrolase activity;hydrolase activity, acting on acid anhydrides;hydrolase activity, acting on acid anh
structural constituent of cytoskeleton;structural molecule activity
binding;catalytic activity;cation binding;coenzyme binding;cofactor binding;identical protein binding;ion binding;metal ion binding;NADP binding;NADPH binding;NADPH:quinone reductase activity;nucleotide binding;oxidore
binding;lipid binding
binding;enzyme binding;enzyme regulator activity;phosphatase binding;phosphatase regulator activity;protein binding;protein phosphatase 2A binding;protein phosphatase binding;protein phosphatase regulator activity;prote
binding;cation binding;enzyme activator activity;enzyme regulator activity;GTPase activator activity;GTPase regulator activity;ion binding;metal ion binding;nucleoside-triphosphatase regulator activity;transition metal ion bind
binding;chromatin binding
binding;ribonucleoprotein binding;snRNP binding
catalytic activity;kinase activity;phosphotransferase activity, alcohol group as acceptor;protein kinase activity;transferase activity;transferase activity, transferring phosphorus-containing groups
binding;enzyme binding;enzyme inhibitor activity;enzyme regulator activity;lipase binding;lipase inhibitor activity;protein binding;small conjugating protein binding;ubiquitin binding;ubiquitin protein ligase binding
binding;catalytic activity;cation binding;coenzyme binding;cofactor binding;flavin adenine dinucleotide binding;ion binding;metal ion binding;oxidoreductase activity;oxidoreductase activity, acting on the CH-CH group of don
adenyl nucleotide binding;adenyl ribonucleotide binding;ATP binding;ATPase activity;ATPase activity, coupled;ATP-dependent helicase activity;ATP-dependent RNA helicase activity;binding;catalytic activity;helicase activity;h
binding;nucleic acid binding;RNA binding
binding;catalytic activity;enzyme binding;intramolecular transferase activity;isomerase activity;protein binding;pseudouridine synthase activity
binding;epidermal growth factor receptor binding;growth factor receptor binding;nucleic acid binding;protein binding;protein C-terminus binding;receptor binding;RNA binding
5-methyltetrahydrofolate-dependent methyltransferase activity;binding;catalytic activity;cation binding;cobalamin binding;homocysteine S-methyltransferase activity;ion binding;metal ion binding;methionine synthase activity;
3'-tRNA processing endoribonuclease activity;binding;catalytic activity;cation binding;endonuclease activity;endonuclease activity, active with either ribo- or deoxyribonucleic acids and producing 5'-phosphomonoesters;end
binding;catalytic activity;GTP binding;GTPase activity;guanyl nucleotide binding;guanyl ribonucleotide binding;hydrolase activity;hydrolase activity, acting on acid anhydrides;hydrolase activity, acting on acid anhydrides, in ph
acid-amino acid ligase activity;adenyl nucleotide binding;adenyl ribonucleotide binding;ATP binding;binding;catalytic activity;enzyme binding;ligase activity;ligase activity, forming carbon-nitrogen bonds;nucleotide binding;pr
adenyl nucleotide binding;adenyl-nucleotide exchange factor activity;ATPase regulator activity;binding;enzyme regulator activity;nucleoside-triphosphatase regulator activity;nucleotide binding;protein binding;purine nucleoti
acid-amino acid ligase activity;catalytic activity;cysteine-type endopeptidase inhibitor activity;endopeptidase inhibitor activity;endopeptidase regulator activity;enzyme inhibitor activity;enzyme regulator activity;ligase activity;l
binding;catalytic activity;cation binding;ion binding;metal ion binding;methyltransferase activity;N-methyltransferase activity;nucleic acid binding;RNA binding;RNA methyltransferase activity;S-adenosylmethionine-dependent
adenyl nucleotide binding;adenyl ribonucleotide binding;ATP binding;binding;catalytic activity;cation binding;ion binding;kinase activity;magnesium ion binding;MAP kinase kinase kinase activity;metal ion binding;molecular tr
aminopeptidase activity;binding;catalytic activity;cation binding;cytokine receptor binding;exopeptidase activity;growth factor receptor binding;hydrolase activity;interleukin-1 receptor binding;interleukin-1, Type II receptor bi
binding;protein binding;protein domain specific binding
binding;chromatin binding;histone binding;nucleosome binding;protein binding;protein binding transcription factor activity;repressing transcription factor binding;transcription cofactor activity;transcription corepressor activity
binding;cytoskeletal protein binding;dynactin binding;lipid binding;phosphatidylinositol binding;phospholipid binding;protein binding
carbon-oxygen lyase activity;catalytic activity;hydro-lyase activity;intramolecular transferase activity;isomerase activity;ligand-dependent nuclear receptor transcription coactivator activity;lyase activity;protein binding transcri
binding;cation binding;ion binding;metal ion binding
adenyl nucleotide binding;adenyl ribonucleotide binding;ATP binding;ATPase activity;binding;catalytic activity;hydrolase activity;hydrolase activity, acting on acid anhydrides;hydrolase activity, acting on acid anhydrides, in ph
binding;cation binding;ion binding;metal ion binding;protein binding;protein domain specific binding
binding;identical protein binding;protein binding;protein dimerization activity;protein heterodimerization activity;protein homodimerization activity
binding;catalytic activity;cation binding;diphosphotransferase activity;enzyme inhibitor activity;enzyme regulator activity;ion binding;magnesium ion binding;metal ion binding;ribose phosphate diphosphokinase activity;transfe
binding;binding, bridging;cytoskeletal protein binding;enzyme binding;JUN kinase binding;kinase binding;kinesin binding;MAP-kinase scaffold activity;protein binding;protein complex scaffold;protein kinase binding;receptor
adenyl nucleotide binding;adenyl ribonucleotide binding;ATP binding;binding;catalytic activity;enzyme binding;GTPase binding;kinase activity;nucleotide binding;phosphotransferase activity, alcohol group as acceptor;protein

binding;calcium ion binding;cargo receptor activity;cation binding;clathrin binding;clathrin heavy chain binding;glycoprotein binding;ion binding;lipoprotein particle binding;lipoprotein particle receptor activity;low-density lipop
adenyl nucleotide binding;adenyl ribonucleotide binding;AMP binding;binding;cAMP binding;cAMP-dependent protein kinase inhibitor activity;cAMP-dependent protein kinase regulator activity;cyclic nucleotide binding;enzyr
catalytic activity;oxidoreductase activity;oxidoreductase activity, acting on the aldehyde or oxo group of donors;oxidoreductase activity, acting on the aldehyde or oxo group of donors, disulfide as acceptor;pyruvate dehydrog
binding;calcium ion binding;cation binding;ion binding;metal ion binding
active transmembrane transporter activity;ATPase activity;ATPase activity, coupled;ATPase activity, coupled to movement of substances;ATPase activity, coupled to transmembrane movement of ions;ATPase activity, coupled
binding;catalytic activity;methyltransferase activity;nucleic acid binding;RNA binding;RNA methyltransferase activity;rRNA methyltransferase activity;S-adenosylmethionine-dependent methyltransferase activity;transferase ac
adenyl nucleotide binding;adenyl ribonucleotide binding;ATP binding;ATPase activity;ATPase activity, coupled;ATP-dependent helicase activity;ATP-dependent RNA helicase activity;binding;catalytic activity;helicase activity;h
binding;growth factor activity;protein binding;receptor binding
carbon-oxygen lyase activity;catalytic activity;enoyl-CoA hydratase activity;hydro-lyase activity;lyase activity
actin binding;binding;catalytic activity;cytokine binding;cytoskeletal protein binding;enzyme binding;GTP binding;GTPase activity;guanyl nucleotide binding;guanyl ribonucleotide binding;heat shock protein binding;Hsp90 pro
binding;catalytic activity;GTP binding;GTPase activity;guanyl nucleotide binding;guanyl ribonucleotide binding;hydrolase activity;hydrolase activity, acting on acid anhydrides;hydrolase activity, acting on acid anhydrides, in ph
binding;protein binding;SNARE binding;syntaxin binding
binding;catalytic activity;enzyme binding;hydrolase activity;kinase binding;nucleotide binding;protein binding;protein kinase binding;protein kinase C binding
binding;enzyme binding;histone deacetylase binding;nuclear localization sequence binding;peptide binding;protein binding;protein transporter activity;signal sequence binding;substrate-specific transporter activity;transporte
binding;cation binding;enzyme regulator activity;GTPase regulator activity;guanyl-nucleotide exchange factor activity;ion binding;metal ion binding;nucleoside-triphosphatase regulator activity;Ras guanyl-nucleotide exchang
catalytic activity;spermine synthase activity;transferase activity;transferase activity, transferring alkyl or aryl (other than methyl) groups
binding;DNA binding;nucleic acid binding;protein binding transcription factor activity;single-stranded DNA binding;structure-specific DNA binding;transcription coactivator activity;transcription cofactor activity;transcription fa
binding;catalytic activity;chromatin binding;coenzyme binding;cofactor binding;NAD binding;nucleotide binding;oxidoreductase activity;oxidoreductase activity, acting on CH-OH group of donors;oxidoreductase activity, actin
adenyl nucleotide binding;adenyl ribonucleotide binding;ATP binding;binding;nucleotide binding;purine nucleotide binding;purine ribonucleoside triphosphate binding;purine ribonucleotide binding;ribonucleotide binding
binding;endopeptidase activator activity;endopeptidase regulator activity;enzyme activator activity;enzyme regulator activity;identical protein binding;p53 binding;peptidase activator activity;peptidase regulator activity;protein
binding;catalytic activity;cytoskeletal protein binding;GTP binding;GTPase activity;guanyl nucleotide binding;guanyl ribonucleotide binding;hydrolase activity;hydrolase activity, acting on acid anhydrides;hydrolase activity, act
binding;cation binding;ion binding;metal ion binding;nucleic acid binding;RNA binding;rRNA binding;structural constituent of ribosome;structural molecule activity
binding;catalytic activity;cation binding;hydrolase activity;hydrolase activity, acting on ester bonds;ion binding;metal ion binding;phosphatase activity;phosphoprotein phosphatase activity;phosphoric ester hydrolase activity;p
binding;catalytic activity;cation binding;enzyme binding;hydrolase activity;hydrolase activity, acting on ester bonds;ion binding;kinase binding;metal ion binding;myosin phosphatase activity;myosin-light-chain-phosphatase a
binding;nucleic acid binding;RNA binding;structural constituent of ribosome;structural molecule activity
binding;nucleic acid binding;ribonucleoprotein binding;ribosome binding;RNA binding;translation factor activity, nucleic acid binding;translation release factor activity;translation release factor activity, codon specific;translatio
structural constituent of ribosome;structural molecule activity
binding;protein binding;protein N-terminus binding;structural constituent of ribosome;structural molecule activity
4 iron, 4 sulfur cluster binding;amidophosphoribosyltransferase activity;binding;catalytic activity;cation binding;ion binding;iron-sulfur cluster binding;metal cluster binding;metal ion binding;transferase activity;transferase acti
endopeptidase activator activity;endopeptidase regulator activity;enzyme activator activity;enzyme regulator activity;peptidase activator activity;peptidase regulator activity
binding;mRNA binding;nucleic acid binding;RNA binding
binding;nucleotide binding
binding;mRNA 3'-UTR binding;mRNA binding;nucleic acid binding;RNA binding
binding;cAMP-dependent protein kinase activity;catalytic activity;cation binding;cyclic nucleotide-dependent protein kinase activity;enzyme regulator activity;GTPase regulator activity;guanyl-nucleotide exchange factor activ
4 iron, 4 sulfur cluster binding;binding;catalytic activity;cation binding;coenzyme binding;cofactor binding;dihydroorotate dehydrogenase activity;dihydropyrimidine dehydrogenase (NADP+) activity;flavin adenine dinucleotide
activating transcription factor binding;binding;damaged DNA binding;DNA binding;histone binding;methylated histone residue binding;nucleic acid binding;p53 binding;protein binding;protein binding transcription factor activ
adenyl nucleotide binding;adenyl ribonucleotide binding;ATP binding;binding;catalytic activity;DNA binding;double-stranded RNA binding;nucleic acid binding;nucleotide binding;purine nucleotide binding;purine ribonucleosi
actin binding;actin-dependent ATPase activity;adenyl nucleotide binding;adenyl ribonucleotide binding;ADP binding;ATP binding;ATPase activity;ATPase activity, coupled;binding;calmodulin binding;catalytic activity;cation bir
binding;nucleotide binding
binding;cation binding;ion binding;ion channel binding;metal ion binding;protein binding;transition metal ion binding;zinc ion binding
binding;mRNA 3'-UTR binding;mRNA binding;nucleic acid binding;nucleotide binding;RNA binding;rRNA binding;SSU rRNA binding;translation regulator activity;translation repressor activity
binding;ribonucleoprotein binding
binding;clathrin binding;lipid binding;protein binding
catalytic activity;hydrolase activity;peptidase activity
binding;catalytic activity;DNA polymerase activity;intramolecular oxidoreductase activity;isomerase activity;nucleotidyltransferase activity;prostaglandin-E synthase activity;protein binding;RNA-directed DNA polymerase activ
binding;cation binding;ion binding;metal ion binding;nucleic acid binding;protein binding transcription factor activity;RNA binding;transcription cofactor activity;transcription corepressor activity;transcription factor binding trar
binding;identical protein binding;lipid binding;protein binding
binding;cation binding;cytokine receptor binding;enzyme binding;growth factor receptor binding;hormone receptor binding;interleukin-1 receptor binding;ion binding;kinase binding;metal ion binding;nuclear hormone recepto
binding;cation binding;ion binding;metal ion binding;nucleotide binding;transition metal ion binding;zinc ion binding
binding;carboxylic acid binding;catalytic activity;cation binding;ion binding;iron ion binding;L-ascorbic acid binding;metal ion binding;oxidoreductase activity;oxidoreductase activity, acting on paired donors, with incorporatio
adenyl nucleotide binding;adenyl ribonucleotide binding;ATP binding;binding;catalytic activity;cation binding;cyclase activity;FAD-AMP lyase (cyclizing) activity;glycerone kinase activity;ion binding;kinase activity;lyase activity
binding;catalytic activity;cytokine receptor binding;growth factor receptor binding;hydrolase activity;hydrolase activity, acting on ester bonds;interleukin-2 receptor binding;nucleic acid binding;phosphatase activity;phosphop
binding;cation binding;ion binding;metal ion binding;nucleotide binding
binding;carbon-carbon lyase activity;carboxy-lyase activity;catalytic activity;cofactor binding;lyase activity;pyridoxal phosphate binding;vitamin B6 binding;vitamin binding
binding;catalytic activity;cation binding;dioxygenase activity;ion binding;metal ion binding;oxidoreductase activity
binding;nucleotide binding
binding;identical protein binding;protein binding;protein binding transcription factor activity;transcription cofactor activity;transcription corepressor activity;transcription factor binding;transcription factor binding transcription f
binding;cation binding;DNA binding;ion binding;metal ion binding;nucleic acid binding;nucleic acid binding transcription factor activity;sequence-specific DNA binding;sequence-specific DNA binding transcription factor activ
binding;catalytic activity;cation binding;enzyme binding;GTPase binding;ion binding;metal ion binding;prenyltransferase activity;protein binding;protein geranylgeranyltransferase activity;protein prenyltransferase activity;Rab g
binding;catalytic activity;chromatin binding;chromatin DNA binding;core promoter binding;deacetylase activity;DNA binding;enzyme binding;histone deacetylase activity;histone deacetylase activity (H3-K14 specific);histone d
binding;BRE binding;mRNA binding;nucleic acid binding;nucleotide binding;RNA binding;translation regulator activity;translation regulator activity, nucleic acid binding;translation repressor activity;translation repressor activity
binding;enzyme binding;kinase binding;protein binding;protein kinase binding;protein kinase C binding
binding;enzyme binding;GTPase binding;protein binding;protein N-terminus binding;Ral GTPase binding;Ras GTPase binding;small GTPase binding
adenyl nucleotide binding;adenyl ribonucleotide binding;ATP binding;binding;catalytic activity;cation binding;ion binding;kinase activity;magnesium ion binding;metal ion binding;nucleotide binding;phosphotransferase activity
binding;catalytic activity;cation binding;ion binding;metal ion binding;oxidoreductase activity;transition metal ion binding;zinc ion binding
binding;calcium ion binding;calcium-dependent phospholipid binding;catalytic activity;cation binding;endopeptidase activity;hydrolase activity;identical protein binding;ion binding;lipid binding;metal ion binding;NF-kappaB b
acid-amino acid ligase activity;adenyl nucleotide binding;adenyl ribonucleotide binding;ATP binding;binding;catalytic activity;enzyme binding;ligase activity;ligase activity, forming carbon-nitrogen bonds;nucleotide binding;pro
adenyl nucleotide binding;adenyl ribonucleotide binding;ATP binding;binding;nucleotide binding;purine nucleotide binding;purine ribonucleoside triphosphate binding;purine ribonucleotide binding;ribonucleotide binding
binding;catalytic activity;cation binding;hydrolase activity;hydrolase activity, acting on ester bonds;ion binding;metal ion binding;phosphatase activity;phosphoprotein phosphatase activity;phosphoric ester hydrolase activity;p
amine binding;amino acid binding;binding;carboxylic acid binding;catalytic activity;cation binding;disulfide oxidoreductase activity;DNA binding;electron carrier activity;glutathione binding;heme binding;intramolecular oxidore
catalytic activity;hydrolase activity;hydrolase activity, acting on carbon-nitrogen (but not peptide) bonds;hydrolase activity, acting on carbon-nitrogen (but not peptide) bonds, in linear amides;omega-amidase activity
acetate-CoA ligase activity;acid-thiol ligase activity;adenyl nucleotide binding;adenyl ribonucleotide binding;AMP binding;ATP binding;binding;catalytic activity;CoA-ligase activity;ligase activity;ligase activity, forming carbon-
binding;cytoskeletal protein binding;protein binding;tropomyosin binding
catalytic activity;oxidoreductase activity;oxidoreductase activity, acting on the CH-CH group of donors
binding;cytoskeletal protein binding;identical protein binding;lamin binding;microtubule binding;protein binding;tubulin binding
binding;binding, bridging;cytoskeletal adaptor activity;cytoskeletal protein binding;proline-rich region binding;protein binding;protein binding, bridging
enzyme activator activity;enzyme regulator activity;lipase activator activity;phospholipase A2 activator activity;phospholipase activator activity
binding;GTP binding;guanyl nucleotide binding;guanyl ribonucleotide binding;identical protein binding;nucleotide binding;protein binding;purine nucleotide binding;purine ribonucleoside triphosphate binding;purine ribonucle
antioxidant activity;binding;catalytic activity;disulfide oxidoreductase activity;glutathione peroxidase activity;glutathione transferase activity;oxidoreductase activity;oxidoreductase activity, acting on a sulfur group of donors;o
binding;nucleic acid binding;RNA binding;snoRNA binding
binding;protein binding;protein domain specific binding;SH3 domain binding;structural constituent of cytoskeleton;structural molecule activity
binding;catalytic activity;cation binding;coenzyme binding;cofactor binding;ion binding;magnesium ion binding;metal ion binding;thiamine pyrophosphate binding;transferase activity;vitamin binding
binding;nucleotide binding
binding;nucleic acid binding;RNA binding;translation factor activity, nucleic acid binding;translation initiation factor activity
active transmembrane transporter activity;adenyl nucleotide binding;adenyl ribonucleotide binding;ATP binding;binding;chaperone binding;macromolecule transmembrane transporter activity;nucleotide binding;P-P-bond-hy
binding;catalytic activity;cation binding;coenzyme binding;cofactor binding;electron carrier activity;ion binding;isocitrate dehydrogenase (NAD+) activity;isocitrate dehydrogenase activity;magnesium ion binding;metal ion bin
binding;epidermal growth factor receptor binding;growth factor receptor binding;insulin receptor binding;lipid binding;phosphatidylinositol binding;phospholipid binding;protein binding;protein complex binding;receptor bindir
acid-amino acid ligase activity;binding;catalytic activity;cation binding;enzyme binding;identical protein binding;ion binding;ligase activity;ligase activity, forming carbon-nitrogen bonds;metal ion binding;mRNA 3'-UTR binding
adenyl nucleotide binding;adenyl ribonucleotide binding;ATP binding;ATPase activity;ATPase activity, coupled;binding;catalytic activity;hydrolase activity;hydrolase activity, acting on acid anhydrides;hydrolase activity, acting o
binding;catalytic activity;cation binding;endopeptidase activity;hydrolase activity;ion binding;metal ion binding;metalloendopeptidase activity;metallopeptidase activity;peptidase activity;peptidase activity, acting on L-amino a
binding;nucleic acid binding;nucleotide binding;RNA binding;translation factor activity, nucleic acid binding;translation initiation factor activity
binding;catalytic activity;cation binding;intramolecular transferase activity;intramolecular transferase activity, phosphotransferases;ion binding;isomerase activity;magnesium ion binding;metal ion binding;phosphoacetylglucos
ATPase activator activity;ATPase regulator activity;binding;chaperone binding;enzyme activator activity;enzyme regulator activity;nucleoside-triphosphatase regulator activity;protein binding
binding;double-stranded RNA binding;nucleic acid binding;RNA binding
binding;enzyme binding;kinase binding;lipid binding;phosphatidylserine binding;phospholipid binding;protein binding;protein kinase binding;protein kinase C binding
anion binding;binding;catalytic activity;drug binding;ion binding;nucleobase binding;nucleoside binding;phosphate ion binding;purine base binding;purine-nucleoside phosphorylase activity;transferase activity;transferase act
binding;catalytic activity;ribonucleoprotein binding;ribosome binding;transferase activity;transferase activity, transferring glycosyl groups
adenyl nucleotide binding;adenyl ribonucleotide binding;ATP binding;binding;catalytic activity;cation binding;deoxyribonuclease activity;DNA binding;GTP binding;guanyl nucleotide binding;guanyl ribonucleotide binding;hydr
binding;carbohydrate binding;catalytic activity;collagen binding;glycosaminoglycan binding;hexosaminidase activity;hyaluronic acid binding;hyaluronoglucosaminidase activity;hydrolase activity;hydrolase activity, acting on g
adenyl nucleotide binding;adenyl ribonucleotide binding;ATP binding;binding;catalytic activity;cation binding;DNA binding;ion binding;kinase activity;metal ion binding;nucleic acid binding;nucleic acid binding transcription fac
carnitine O-acyltransferase activity;carnitine O-palmitoyltransferase activity;catalytic activity;O-acyltransferase activity;O-palmitoyltransferase activity;palmitoyltransferase activity;transferase activity;transferase activity, transf
adenyl nucleotide binding;adenyl ribonucleotide binding;ATP binding;binding;catalytic activity;kinase activity;nucleobase-containing compound kinase activity;nucleoside phosphate kinase activity;nucleotide binding;nucleoti
binding;enzyme binding;protein binding;RNA polymerase binding
alkyl hydroperoxide reductase activity;antioxidant activity;binding;caspase inhibitor activity;caspase regulator activity;catalytic activity;cysteine-type endopeptidase inhibitor activity;endopeptidase inhibitor activity;endopeptic
structural constituent of ribosome;structural molecule activity
asialoglycoprotein receptor activity;binding;carbohydrate binding;cargo receptor activity;glycosaminoglycan binding;heparin binding;lipoprotein particle receptor binding;low-density lipoprotein particle receptor binding;patter
binding;catalytic activity;coproporphyrinogen oxidase activity;identical protein binding;oxidoreductase activity;oxidoreductase activity, acting on the CH-CH group of donors;oxidoreductase activity, acting on the CH-CH grou
catalytic activity;dolichyl-diphosphooligosaccharide-protein glycotransferase activity;oligosaccharyl transferase activity;transferase activity;transferase activity, transferring glycosyl groups;transferase activity, transferring hex
binding;carbohydrate binding;glycosaminoglycan binding;heparin binding;nucleic acid binding;pattern binding;polysaccharide binding;RNA binding;structural constituent of ribosome;structural molecule activity
binding;catalytic activity;GDP binding;GTP binding;GTPase activity;guanyl nucleotide binding;guanyl ribonucleotide binding;hydrolase activity;hydrolase activity, acting on acid anhydrides;hydrolase activity, acting on acid anh
3-chloroallyl aldehyde dehydrogenase activity;aldehyde dehydrogenase (NAD) activity;aldehyde dehydrogenase [NAD(P)+] activity;catalytic activity;long-chain-alcohol oxidase activity;long-chain-aldehyde dehydrogenase acti
catalytic activity;enzyme activator activity;enzyme regulator activity;GDP-dissociation inhibitor activity;GTPase activator activity;GTPase activity;GTPase regulator activity;hydrolase activity;hydrolase activity, acting on acid an
carboxylic acid transmembrane transporter activity;citrate transmembrane transporter activity;organic acid transmembrane transporter activity;substrate-specific transmembrane transporter activity;substrate-specific transpo
aminopeptidase activity;binding;catalytic activity;cation binding;exopeptidase activity;hydrolase activity;ion binding;metal ion binding;metalloaminopeptidase activity;metalloexopeptidase activity;metallopeptidase activity;pep

actin-dependent ATPase activity;ATPase activity;ATPase activity, coupled;binding;calcium ion binding;catalytic activity;cation binding;hydrolase activity;hydrolase activity, acting on acid anhydrides;hydrolase activity, acting o
binding;chromatin binding;core promoter proximal region DNA binding;core promoter proximal region sequence-specific DNA binding;DNA bending activity;DNA binding;mitochondrial light strand promoter sense binding;nuc
AMP deaminase activity;binding;catalytic activity;cation binding;deaminase activity;hydrolase activity;hydrolase activity, acting on carbon-nitrogen (but not peptide) bonds;hydrolase activity, acting on carbon-nitrogen (but not
binding;core promoter proximal region DNA binding;core promoter proximal region sequence-specific DNA binding;DNA binding;nucleic acid binding;nucleic acid binding transcription factor activity;regulatory region DNA bin
binding;cytoskeletal protein binding;microtubule binding;microtubule minus-end binding;protein binding;tubulin binding
binding;molecular transducer activity;nucleic acid binding;receptor signaling protein activity;RNA binding;signal transducer activity;snoRNA binding
actin binding;actin monomer binding;adenyl nucleotide binding;adenyl ribonucleotide binding;ATP binding;binding;cytoskeletal protein binding;lipid binding;nucleotide binding;phosphatidylinositol binding;phosphatidylinositol
binding;cytokine activity;enzyme binding;GTPase binding;identical protein binding;nucleic acid binding;protein binding;protein dimerization activity;protein homodimerization activity;receptor binding;RNA binding;tRNA bindin
binding;DNA binding;double-stranded DNA binding;identical protein binding;mRNA 3'-UTR binding;mRNA binding;nucleic acid binding;nucleic acid binding transcription factor activity;nucleotide binding;protein binding;RNA
binding;nucleotide binding
activating transcription factor binding;binding;catalytic activity;core promoter binding;deacetylase activity;DNA binding;enzyme binding;histone deacetylase activity;histone deacetylase activity (H3-K14 specific);histone deac
actin binding;binding;cytoskeletal protein binding;enzyme binding;protein binding
adenyl nucleotide binding;adenyl ribonucleotide binding;ATP binding;binding;calmodulin-dependent protein kinase activity;catalytic activity;glutamate receptor activity;ionotropic glutamate receptor activity;kinase activity;mol
binding;catalytic activity;chromo shadow domain binding;DNA binding;lamin binding;nucleic acid binding;oxidoreductase activity;oxidoreductase activity, acting on the CH-CH group of donors;oxidoreductase activity, acting
binding;identical protein binding;protein binding
binding;chaperone binding;protein binding
binding;enzyme binding;kinase binding;protein binding;protein kinase binding;ribonucleoprotein binding;ribosome binding
binding;cytokine binding;endopeptidase inhibitor activity;endopeptidase regulator activity;enzyme inhibitor activity;enzyme regulator activity;growth factor binding;peptidase inhibitor activity;peptidase regulator activity;protein
binding;DNA binding;histone binding;nucleic acid binding;nucleic acid binding transcription factor activity;protein binding;sequence-specific DNA binding transcription factor activity
binding;catalytic activity;GTP binding;GTPase activity;guanyl nucleotide binding;guanyl ribonucleotide binding;hydrolase activity;hydrolase activity, acting on acid anhydrides;hydrolase activity, acting on acid anhydrides, in pl
binding;cation binding;cytoskeletal protein binding;filamin binding;ion binding;metal ion binding;protein binding;transition metal ion binding;zinc ion binding
binding;cation binding;ion binding;metal ion binding;transition metal ion binding;zinc ion binding
binding;GTP binding;guanyl nucleotide binding;guanyl ribonucleotide binding;nucleotide binding;purine nucleotide binding;purine ribonucleoside triphosphate binding;purine ribonucleotide binding;ribonucleotide binding
binding;enzyme binding;histone binding;protein binding;RNA polymerase binding
binding;calcium ion binding;catalytic activity;cation binding;cis-trans isomerase activity;drug binding;FK506 binding;ion binding;isomerase activity;macrolide binding;metal ion binding;peptidyl-prolyl cis-trans isomerase activi
adenyl nucleotide binding;adenyl ribonucleotide binding;ATP binding;binding;catalytic activity;DNA binding;kinase activity;nucleic acid binding;nucleotide binding;phosphotransferase activity, alcohol group as acceptor;protei
transporter activity
arginine N-methyltransferase activity;binding;catalytic activity;histone methyltransferase activity;histone methyltransferase activity (H4-R3 specific);histone-arginine N-methyltransferase activity;identical protein binding;methyl
binding;identical protein binding;lipid binding;protein binding
adenyl nucleotide binding;adenyl ribonucleotide binding;ATP binding;ATPase activity;ATPase activity, coupled;ATP-dependent helicase activity;ATP-dependent RNA helicase activity;binding;catalytic activity;helicase activity;h
adenyl nucleotide binding;adenyl ribonucleotide binding;aminoacyl-tRNA ligase activity;ATP binding;binding;catalytic activity;ligase activity;ligase activity, forming aminoacyl-tRNA and related compounds;ligase activity, formi
actin binding;actin filament binding;binding;cytoskeletal protein binding;enzyme binding;GTPase binding;GTP-Rho binding;myosin binding;myosin II binding;protein binding;Ras GTPase binding;Rho GTPase binding;small GT
binding;catalytic activity;enzyme binding;hydrolase activity;hydrolase activity, acting on ester bonds;kinase binding;phosphatase activity;phosphoprotein phosphatase activity;phosphoric ester hydrolase activity;protein bindin
acyl-CoA dehydrogenase activity;binding;catalytic activity;coenzyme binding;cofactor binding;flavin adenine dinucleotide binding;oxidoreductase activity;oxidoreductase activity, acting on the CH-CH group of donors
catalytic activity;hydrolase activity;hydrolase activity, acting on ester bonds;inositol or phosphatidylinositol phosphatase activity;lipid phosphatase activity;phosphatase activity;phosphatidylinositol bisphosphate phosphatase
angiostatin binding;ATPase binding;ATPase inhibitor activity;ATPase regulator activity;binding;calmodulin binding;enzyme binding;enzyme inhibitor activity;enzyme regulator activity;identical protein binding;nucleoside-triphos
acidic amino acid transmembrane transporter activity;active transmembrane transporter activity;amine transmembrane transporter activity;amino acid transmembrane transporter activity;binding;calcium ion binding;carboxyli
binding;catalytic activity;DNA binding;enzyme binding;NAD+ ADP-ribosyltransferase activity;nucleic acid binding;protein binding;transferase activity;transferase activity, transferring glycosyl groups;transferase activity, transfe
acetyltransferase activity;binding;catalytic activity;DNA binding;enzyme activator activity;enzyme regulator activity;histone acetyltransferase activity;lysine N-acetyltransferase activity;N-acetyltransferase activity;N-acyltransfe
binding;calcium channel inhibitor activity;calcium channel regulator activity;channel inhibitor activity;channel regulator activity;cytoskeletal protein binding;ion channel inhibitor activity;lipid binding;phospholipid binding;protein
binding;nucleotide binding;protein binding;protein complex scaffold;receptor signaling complex scaffold activity;structural molecule activity
acid-amino acid ligase activity;binding;binding, bridging;catalytic activity;enzyme binding;G-protein-coupled receptor binding;heat shock protein binding;Hsp70 protein binding;Hsp90 protein binding;identical protein binding;
binding;DNA binding;enzyme regulator activity;nucleic acid binding;phosphatase regulator activity;protein binding;protein domain specific binding;protein phosphatase regulator activity;protein phosphatase type 1 regulator a
structural constituent of ribosome;structural molecule activity
binding;carboxylic ester hydrolase activity;catalytic activity;enzyme binding;enzyme inhibitor activity;enzyme regulator activity;hydrolase activity;hydrolase activity, acting on ester bonds;phosphatase binding;phosphatase inh
adenyl nucleotide binding;adenyl ribonucleotide binding;ATP binding;binding;catalytic activity;hydrolase activity;hydrolase activity, acting on acid anhydrides;hydrolase activity, acting on acid anhydrides, in phosphorus-conta
binding;GAF domain binding;molecular transducer activity;protein binding;protein binding transcription factor activity;protein domain specific binding;signal transducer activity;transcription coactivator activity;transcription co
binding;nucleic acid binding;RNA binding;tRNA binding
binding;catalytic activity;DNA polymerase activity;intramolecular transferase activity;isomerase activity;nucleic acid binding;nucleotidyltransferase activity;pseudouridine synthase activity;RNA binding;RNA-directed DNA poly
acid-amino acid ligase activity;catalytic activity;ligase activity;ligase activity, forming carbon-nitrogen bonds;small conjugating protein ligase activity;UFM1 conjugating enzyme activity
binding;identical protein binding;protein binding
arylesterase activity;binding;carbonate dehydratase activity;carbon-oxygen lyase activity;carboxylic ester hydrolase activity;catalytic activity;cation binding;hydrolase activity;hydrolase activity, acting on ester bonds;hydro-lya
active transmembrane transporter activity;antigen binding;ATPase activator activity;ATPase activity;ATPase activity, coupled;ATPase activity, coupled to movement of substances;ATPase activity, coupled to transmembrane m
binding;carbohydrate binding;disaccharide binding;galactoside binding;lactose binding;molecular transducer activity;monosaccharide binding;signal transducer activity;sugar binding
binding;cation binding;ion binding;metal ion binding;nucleic acid binding;RNA binding;snRNA binding;U2 snRNA binding
binding;catalytic activity;chromatin binding;damaged DNA binding;dinucleotide insertion or deletion binding;DNA binding;DNA insertion or deletion binding;DNA N-glycosylase activity;DNA polymerase binding;DNA polymera
binding;chromatin binding;DNA binding;nucleic acid binding
binding;protein binding;scaffold protein binding;structural constituent of cytoskeleton;structural molecule activity
catalytic activity;oxidoreductase activity;oxidoreductase activity, acting on the CH-NH2 group of donors;oxidoreductase activity, acting on the CH-NH2 group of donors, oxygen as acceptor;primary amine oxidase activity
adenyl nucleotide binding;adenyl ribonucleotide binding;ATP binding;binding;calcium ion binding;catalytic activity;cation binding;diacylglycerol kinase activity;ion binding;kinase activity;lipid binding;metal ion binding;NAD+ ki
structural constituent of ribosome;structural molecule activity
binding;catalytic activity;coenzyme binding;cofactor binding;electron carrier activity;flavin adenine dinucleotide binding;oxidoreductase activity;oxidoreductase activity, acting on the CH-NH2 group of donors;oxidoreductase
adenyl nucleotide binding;adenyl ribonucleotide binding;ATP binding;binding;catalytic activity;cytidylate kinase activity;kinase activity;nucleobase-containing compound kinase activity;nucleoside diphosphate kinase activity;
catalytic activity;dihydrolipoyllysine-residue succinyltransferase activity;S-acyltransferase activity;S-succinyltransferase activity;succinyltransferase activity;transferase activity;transferase activity, transferring acyl groups;trans
7S RNA binding;binding;endoplasmic reticulum signal peptide binding;nucleic acid binding;peptide binding;RNA binding;signal sequence binding
anion channel activity;anion transmembrane transporter activity;binding;channel activity;gated channel activity;ion channel activity;ion transmembrane transporter activity;nucleotide binding;passive transmembrane transporte
binding;catalytic activity;cation binding;coenzyme binding;cofactor binding;ion binding;isocitrate dehydrogenase (NADP+) activity;isocitrate dehydrogenase activity;magnesium ion binding;metal ion binding;NAD binding;nucl
4 iron, 4 sulfur cluster binding;binding;catalytic activity;cation binding;coenzyme binding;cofactor binding;FMN binding;ion binding;iron-sulfur cluster binding;metal cluster binding;metal ion binding;NAD binding;NADH dehyd
enzyme activator activity;enzyme regulator activity;GDP-dissociation inhibitor activity;GTPase activator activity;GTPase regulator activity;nucleoside-triphosphatase regulator activity;Rho GDP-dissociation inhibitor activity;sm
binding;enzyme binding;protein binding;structural constituent of cytoskeleton;structural molecule activity
binding;catalytic activity;cytoskeletal protein binding;enzyme binding;GDP binding;GTP binding;GTPase activity;GTPase binding;guanyl nucleotide binding;guanyl ribonucleotide binding;hydrolase activity;hydrolase activity, a
7S RNA binding;binding;catalytic activity;drug binding;endoplasmic reticulum signal peptide binding;GDP binding;GTP binding;GTPase activity;guanyl nucleotide binding;guanyl ribonucleotide binding;hydrolase activity;hydro
binding;catalytic activity;cytoskeletal protein binding;GDP binding;GTP binding;GTPase activity;guanyl nucleotide binding;guanyl ribonucleotide binding;hydrolase activity;hydrolase activity, acting on acid anhydrides;hydrolas
binding;catalytic activity;cytoskeletal protein binding;GDP binding;GTP binding;GTPase activity;guanyl nucleotide binding;guanyl ribonucleotide binding;hydrolase activity;hydrolase activity, acting on acid anhydrides;hydrolas
binding;cation binding;DNA binding;ion binding;metal ion binding;nucleic acid binding;nucleic acid binding transcription factor activity;RNA binding;sequence-specific DNA binding transcription factor activity;single-stranded
structural constituent of ribosome;structural molecule activity
binding;nucleotide binding;protein binding;structural constituent of ribosome;structural molecule activity;translation initiation factor binding
binding;nucleic acid binding;RNA binding;structural constituent of ribosome;structural molecule activity
catalytic activity;enzyme regulator activity;hydrolase activity;hydrolase activity, acting on ester bonds;phosphatase activity;phosphatase regulator activity;phosphoprotein phosphatase activity;phosphoric ester hydrolase activ
adenyl nucleotide binding;adenyl ribonucleotide binding;ATP binding;binding;catalytic activity;heat shock protein binding;Hsp90 protein binding;kinase activity;nucleotide binding;phosphotransferase activity, alcohol group as
binding;catalytic activity;cis-trans isomerase activity;drug binding;FK506 binding;isomerase activity;macrolide binding;peptidyl-prolyl cis-trans isomerase activity;receptor activity
aminoacylase activity;binding;catalytic activity;cation binding;hydrolase activity;hydrolase activity, acting on carbon-nitrogen (but not peptide) bonds;hydrolase activity, acting on carbon-nitrogen (but not peptide) bonds, in lin
binding;catalytic activity;hydrolase activity;hydrolase activity, acting on ester bonds;non-membrane spanning protein tyrosine phosphatase activity;phosphatase activity;phosphoprotein phosphatase activity;phosphoric ester
binding;calcium ion binding;calcium-dependent protein serine/threonine phosphatase activity;calmodulin binding;calmodulin-dependent protein phosphatase activity;catalytic activity;cation binding;drug binding;enzyme bind
3',5'-cyclic-AMP phosphodiesterase activity;3',5'-cyclic-nucleotide phosphodiesterase activity;adenyl nucleotide binding;adenyl ribonucleotide binding;adrenergic receptor binding;AMP binding;ATPase binding;beta-2 adrene
binding;binding, bridging;cadherin binding;cell adhesion molecule binding;cytoskeletal protein binding;ion channel binding;protein binding;protein binding, bridging;spectrin binding;structural constituent of cytoskeleton;struc
[acetyl-CoA carboxylase] kinase activity;[hydroxymethylglutaryl-CoA reductase (NADPH)] kinase activity;adenyl nucleotide binding;adenyl ribonucleotide binding;AMP-activated protein kinase activity;ATP binding;binding;cAM
adenyl nucleotide binding;adenyl ribonucleotide binding;ATP binding;binding;catalytic activity;kinase activity;nucleotide binding;phosphotransferase activity, alcohol group as acceptor;protein kinase activity;protein serine/thre
binding;hormone receptor binding;Notch binding;nuclear hormone receptor binding;protein binding;protein binding transcription factor activity;receptor binding;retinoic acid receptor binding;SMAD binding;transcription coact
binding;carbohydrate binding;catalytic activity;chondroitin sulfate binding;glycosaminoglycan binding;hydrolase activity;hydrolase activity, acting on carbon-nitrogen (but not peptide) bonds;pattern binding;polysaccharide bin
binding;nucleic acid binding;nucleotide binding;RNA binding;translation factor activity, nucleic acid binding;translation initiation factor activity
acid-amino acid ligase activity;catalytic activity;ligase activity;ligase activity, forming carbon-nitrogen bonds;small conjugating protein ligase activity;ubiquitin-protein ligase activity
actin binding;actin monomer binding;adenyl nucleotide binding;adenyl ribonucleotide binding;ATP binding;binding;cytoskeletal protein binding;enzyme binding;kinase binding;lipid binding;nucleotide binding;phosphatidylinos
binding;chromatin binding;DNA binding;nucleic acid binding
aminopeptidase activity;binding;catalytic activity;exopeptidase activity;hydrolase activity;identical protein binding;peptidase activity;peptidase activity, acting on L-amino acid peptides;protein binding;serine hydrolase activity;
binding;chromatin binding;DNA binding;nucleic acid binding
binding;cation binding;cytoskeletal protein binding;DNA binding;enzyme binding;histone binding;histone deacetylase binding;ion binding;metal ion binding;nucleic acid binding;phosphoprotein binding;protein binding;ribonuc
aldehyde dehydrogenase (NAD) activity;catalytic activity;oxidoreductase activity;oxidoreductase activity, acting on the aldehyde or oxo group of donors;oxidoreductase activity, acting on the aldehyde or oxo group of donors,
adenyl nucleotide binding;adenyl ribonucleotide binding;ATP binding;binding;catalytic activity;kinase activity;nucleotide binding;phosphotransferase activity, alcohol group as acceptor;protein binding;protein complex binding
binding;chromatin binding;DNA binding;nucleic acid binding;protein binding transcription factor activity;transcription coactivator activity;transcription cofactor activity;transcription factor binding transcription factor activity
binding;cation binding;DNA binding;ion binding;metal ion binding;nucleic acid binding;nucleotide binding;transition metal ion binding;zinc ion binding
binding;nucleic acid binding
binding;calcium ion binding;cation binding;ion binding;metal ion binding
binding;catalytic activity;GTP binding;GTPase activity;guanyl nucleotide binding;guanyl ribonucleotide binding;hydrolase activity;hydrolase activity, acting on acid anhydrides;hydrolase activity, acting on acid anhydrides, in pl
binding;nucleotide binding
binding;catalytic activity;cation binding;endopeptidase activity;hydrolase activity;ion binding;metal ion binding;metalloendopeptidase activity;metallopeptidase activity;peptidase activity;peptidase activity, acting on L-amino a
binding;cation binding;ion binding;metal ion binding;miRNA binding;mRNA 3'-UTR binding;mRNA binding;nucleic acid binding;nucleotide binding;RNA binding;transition metal ion binding;zinc ion binding
binding;catalytic activity;cation binding;endopeptidase activity;hydrolase activity;ion binding;metal ion binding;metalloendopeptidase activity;metallopeptidase activity;peptidase activity;peptidase activity, acting on L-amino a
adenyl nucleotide binding;adenyl ribonucleotide binding;ATP binding;ATPase activity;ATPase activity, coupled;ATP-dependent helicase activity;ATP-dependent RNA helicase activity;binding;catalytic activity;core promoter bin
amine-lyase activity;arylesterase activity;carbon-nitrogen lyase activity;carboxylic ester hydrolase activity;catalytic activity;hydrolase activity;hydrolase activity, acting on ester bonds;lyase activity;strictosidine synthase activity
binding;enzyme binding;identical protein binding;kinase binding;protein binding;protein kinase B binding;protein kinase binding

adenyl nucleotide binding;adenyl ribonucleotide binding;ATP binding;ATPase activity;ATPase activity, coupled;ATP-dependent helicase activity;ATP-dependent RNA helicase activity;binding;catalytic activity;helicase activity;h
transporter activity
binding;DNA binding;nucleic acid binding
binding;cytoskeletal protein binding;enzyme binding;GTPase binding;GTP-dependent protein binding;kinesin binding;protein binding;protein transporter activity;Rab GTPase binding;Ras GTPase binding;small GTPase bindin
binding;catalytic activity;cation binding;endopeptidase activity;epidermal growth factor binding;growth factor binding;hormone binding;hydrolase activity;ion binding;metal ion binding;metalloendopeptidase activity;metallope
binding;chaperone binding;protein binding
binding;chromatin binding;chromatin DNA binding;core promoter binding;core promoter sequence-specific DNA binding;DNA binding;double-stranded DNA binding;double-stranded methylated DNA binding;enzyme binding;
catalytic activity;citrate (Si)-synthase activity;transferase activity;transferase activity, transferring acyl groups;transferase activity, transferring acyl groups, acyl groups converted into alkyl on transfer
enzyme regulator activity;kinase regulator activity;molecular transducer activity;phosphorylase kinase regulator activity;protein kinase regulator activity;signal transducer activity
binding;hormone receptor binding;nuclear hormone receptor binding;protein binding;receptor binding
binding;calcium ion binding;calcium-dependent cysteine-type endopeptidase activity;catalytic activity;cation binding;cysteine-type endopeptidase activity;cysteine-type peptidase activity;endopeptidase activity;hydrolase ac
binding;enzyme binding;enzyme inhibitor activity;enzyme regulator activity;identical protein binding;kinase binding;kinase inhibitor activity;kinase regulator activity;protein binding;protein kinase binding;protein kinase C bindin
binding;enzyme activator activity;enzyme regulator activity;lipid binding
binding;catalytic activity;cation binding;dimethylallyltranstransferase activity;geranyltranstransferase activity;ion binding;metal ion binding;prenyltransferase activity;transferase activity;transferase activity, transferring alkyl or a
antioxidant activity;binding;catalytic activity;cytochrome-b5 reductase activity;identical protein binding;NAD(P)H dehydrogenase (quinone) activity;oxidoreductase activity;oxidoreductase activity, acting on NADH or NADPH;o
binding;catalytic activity;cation binding;enzyme binding;ephrin receptor binding;hydrolase activity;hydrolase activity, acting on ester bonds;ion binding;kinase binding;metal ion binding;phosphatase activity;phosphoprotein ph
receptor activity;viral receptor activity
binding;DNA binding;nucleic acid binding;RNA binding
cation transmembrane transporter activity;hydrogen ion transmembrane transporter activity;inorganic cation transmembrane transporter activity;ion transmembrane transporter activity;monovalent inorganic cation transmemb
catalytic activity;endopeptidase activity;hydrolase activity;peptidase activity;peptidase activity, acting on L-amino acid peptides;threonine-type endopeptidase activity;threonine-type peptidase activity
binding;nucleic acid binding;nucleotide binding;RNA binding
catalytic activity;farnesyl-diphosphate farnesyltransferase activity;farnesyltranstransferase activity;oxidoreductase activity;prenyltransferase activity;squalene synthase activity;transferase activity;transferase activity, transferri
binding;protein binding;protein complex binding;protein domain specific binding
catalytic activity;dolichyl-diphosphooligosaccharide-protein glycotransferase activity;oligosaccharyl transferase activity;transferase activity;transferase activity, transferring glycosyl groups;transferase activity, transferring hex
aldehyde dehydrogenase (NAD) activity;aldehyde dehydrogenase [NAD(P)+] activity;binding;catalytic activity;coenzyme binding;cofactor binding;hormone binding;identical protein binding;NAD binding;NAD+ binding;nucleoti
catalytic activity;intramolecular transferase activity;isomerase activity;lanosterol synthase activity;oxidosqualene cyclase activity
binding;lipid binding
carnitine O-acyltransferase activity;carnitine O-palmitoyltransferase activity;catalytic activity;O-acyltransferase activity;O-palmitoyltransferase activity;palmitoyltransferase activity;transferase activity;transferase activity, transf
aminopeptidase activity;binding;catalytic activity;cation binding;exopeptidase activity;hydrolase activity;ion binding;metal ion binding;metalloaminopeptidase activity;metalloexopeptidase activity;metallopeptidase activity;pep
nucleocytoplasmic transporter activity;structural constituent of nuclear pore;structural molecule activity;transporter activity
binding;nucleic acid binding;protein binding;protein N-terminus binding;ribonucleoprotein binding;ribosome binding;RNA binding;snRNA binding;translation elongation factor activity;translation factor activity, nucleic acid bind
binding;core promoter proximal region DNA binding;core promoter proximal region sequence-specific DNA binding;DNA binding;double-stranded DNA binding;enzyme binding;GTPase binding;nucleic acid binding;nucleic ac
binding;DNA binding;nucleic acid binding
active transmembrane transporter activity;anion transmembrane transporter activity;anion:anion antiporter activity;antiporter activity;C4-dicarboxylate transmembrane transporter activity;carboxylic acid transmembrane trans
cargo receptor activity;receptor activity;scavenger receptor activity
binding;catalytic activity;cation binding;endopeptidase activity;hydrolase activity;ion binding;metal ion binding;metalloendopeptidase activity;metallopeptidase activity;peptidase activity;peptidase activity, acting on L-amino a
binding;carbohydrate binding;cation binding;glycoprotein binding;heat shock protein binding;ion binding;mannose binding;metal ion binding;monosaccharide binding;protein binding;sugar binding
binding;nucleic acid binding;nucleotide binding;pre-mRNA binding;RNA binding
binding;enzyme binding;GTPase binding;protein binding
binding;POZ domain binding;protein binding;protein domain specific binding
catalytic activity;glucosidase activity;hydrolase activity;hydrolase activity, acting on glycosyl bonds;hydrolase activity, hydrolyzing O-glycosyl compounds;mannosyl-oligosaccharide glucosidase activity
acid-amino acid ligase activity;binding;catalytic activity;hormone receptor binding;ligase activity;ligase activity, forming carbon-nitrogen bonds;nuclear hormone receptor binding;protein binding;receptor binding;small conjuga
binding;cytoskeletal protein binding;microtubule binding;microtubule plus-end binding;protein binding;protein C-terminus binding;tubulin binding
3-beta-hydroxy-delta5-steroid dehydrogenase activity;catalytic activity;oxidoreductase activity;oxidoreductase activity, acting on CH-OH group of donors;oxidoreductase activity, acting on the CH-OH group of donors, NAD o
ATPase activator activity;ATPase binding;ATPase regulator activity;binding;cytoskeletal protein binding;enzyme activator activity;enzyme binding;enzyme regulator activity;nucleoside-triphosphatase regulator activity;protein b
binding;DNA binding;histone binding;nucleic acid binding;protein binding
binding;cytoskeletal protein binding;kinetochore binding;microtubule binding;microtubule plus-end binding;protein binding;tubulin binding
binding;nucleotide binding
adenyl nucleotide binding;adenyl ribonucleotide binding;ATP binding;binding;catalytic activity;kinase activity;nucleotide binding;phosphotransferase activity, alcohol group as acceptor;protein kinase activity;protein serine/thre
catalytic activity;dolichyl-diphosphooligosaccharide-protein glycotransferase activity;oligosaccharyl transferase activity;transferase activity;transferase activity, transferring glycosyl groups;transferase activity, transferring hex
binding;DNA binding;nucleic acid binding
binding;carboxypeptidase activity;catalytic activity;cation binding;dipeptidase activity;exopeptidase activity;hydrolase activity;ion binding;metal ion binding;metallopeptidase activity;peptidase activity;peptidase activity, acting
binding;fibroblast growth factor binding;growth factor binding;protein binding
actin binding;binding;cytoskeletal protein binding;protein binding
structural molecule activity
binding;cation binding;heat shock protein binding;Hsp90 protein binding;ion binding;metal ion binding;protein binding;transition metal ion binding;zinc ion binding
catalytic activity;cysteine-type endopeptidase activity;cysteine-type peptidase activity;endopeptidase activity;hydrolase activity;peptidase activity;peptidase activity, acting on L-amino acid peptides;small conjugating protein-
anion channel activity;anion transmembrane transporter activity;binding;channel activity;gated channel activity;ion channel activity;ion transmembrane transporter activity;nucleotide binding;passive transmembrane transporte
binding;catalytic activity;enzyme activator activity;enzyme binding;enzyme regulator activity;kinase activator activity;kinase activity;kinase binding;kinase regulator activity;phosphotransferase activity, alcohol group as accept
binding;cytoskeletal protein binding;microtubule binding;nucleic acid binding;protein binding;ribonucleoprotein binding;ribosome binding;RNA binding;rRNA binding;tubulin binding
anion channel activity;anion transmembrane transporter activity;channel activity;chloride channel activity;gated channel activity;ion channel activity;ion transmembrane transporter activity;passive transmembrane transporter
arginine N-methyltransferase activity;binding;catalytic activity;chromatin binding;core promoter binding;core promoter sequence-specific DNA binding;DNA binding;histone methyltransferase activity;histone-arginine N-methy
binding;enzyme binding;protease binding;protein binding
catalytic activity;glutaminase activity;hydrolase activity;hydrolase activity, acting on carbon-nitrogen (but not peptide) bonds;hydrolase activity, acting on carbon-nitrogen (but not peptide) bonds, in linear amides
binding;calcium-dependent protein binding;chemokine binding;C-X-C chemokine binding;cytokine binding;endopeptidase inhibitor activity;endopeptidase regulator activity;enzyme binding;enzyme inhibitor activity;enzyme re
binding;integrin binding;molecular transducer activity;protein binding;protein complex binding;receptor activity;receptor binding;signal transducer activity;signaling receptor activity;transmembrane signaling receptor activity;v
binding;clathrin binding;clathrin heavy chain binding;peptide binding;protein binding;structural molecule activity
carbon-carbon lyase activity;carboxy-lyase activity;catalytic activity;lyase activity;orotate phosphoribosyltransferase activity;orotidine-5'-phosphate decarboxylase activity;transferase activity;transferase activity, transferring g
actin binding;actin filament binding;binding;calcium ion binding;cation binding;cytoskeletal protein binding;enzyme binding;GTPase binding;ion binding;metal ion binding;protein binding
adenyl nucleotide binding;adenyl ribonucleotide binding;AMP binding;binding;cAMP binding;cAMP-dependent protein kinase inhibitor activity;cAMP-dependent protein kinase regulator activity;cyclic nucleotide binding;enzym
2 iron, 2 sulfur cluster binding;3 iron, 4 sulfur cluster binding;4 iron, 4 sulfur cluster binding;binding;catalytic activity;cation binding;cofactor binding;electron carrier activity;ion binding;iron-sulfur cluster binding;metal cluster b
adenyl nucleotide binding;adenyl ribonucleotide binding;ATP binding;binding;C3HC4-type RING finger domain binding;cation binding;chaperone binding;enzyme binding;G-protein-coupled receptor binding;heat shock protei
binding;cation binding;DNA binding;identical protein binding;ion binding;metal ion binding;nucleic acid binding;nucleotide binding;protein binding;protein binding transcription factor activity;RNA binding;transcription coactiva
adenyl nucleotide binding;adenyl ribonucleotide binding;ATP binding;ATPase activity;binding;catalytic activity;hydrolase activity;hydrolase activity, acting on acid anhydrides;hydrolase activity, acting on acid anhydrides, in ph
nucleic acid binding transcription factor activity;RNA polymerase II core promoter proximal region sequence-specific DNA binding transcription factor activity;RNA polymerase II core promoter proximal region sequence-spec
binding;catalytic activity;GTP binding;GTPase activity;guanyl nucleotide binding;guanyl ribonucleotide binding;hydrolase activity;hydrolase activity, acting on acid anhydrides;hydrolase activity, acting on acid anhydrides, in pl
binding;cation binding;ion binding;metal ion binding;structural constituent of ribosome;structural molecule activity
binding;DNA binding;nucleic acid binding;protein binding;protein binding transcription factor activity;protein domain specific binding;regulatory region DNA binding;regulatory region nucleic acid binding;transcription cofactor
binding;binding, bridging;DNA binding;identical protein binding;mRNA binding;nucleic acid binding;poly(A) RNA binding;poly(U) RNA binding;poly-purine tract binding;poly-pyrimidine tract binding;protein binding;protein bind
binding;binding, bridging;enzyme activator activity;enzyme regulator activity;GTPase activator activity;GTPase regulator activity;nucleoside-triphosphatase regulator activity;protein binding;protein binding, bridging;SH3/SH2
nucleocytoplasmic transporter activity;transporter activity
catalytic activity;hydrolase activity;hydrolase activity, acting on acid anhydrides;hydrolase activity, acting on acid anhydrides, in phosphorus-containing anhydrides;microtubule motor activity;motor activity;nucleoside-triphosp
binding;cation binding;ion binding;metal ion binding;transition metal ion binding;zinc ion binding
binding;protein binding;SNARE binding;syntaxin binding;syntaxin-3 binding
2,4-dienoyl-CoA reductase (NADPH) activity;binding;catalytic activity;coenzyme binding;cofactor binding;NADP binding;NADPH binding;nucleotide binding;oxidoreductase activity;oxidoreductase activity, acting on NADH or
binding;catalytic activity;GTP binding;GTPase activity;guanyl nucleotide binding;guanyl ribonucleotide binding;hydrolase activity;hydrolase activity, acting on acid anhydrides;hydrolase activity, acting on acid anhydrides, in pl
binding;cation binding;ion binding;metal ion binding;mRNA binding;nucleic acid binding;poly(A) RNA binding;poly-purine tract binding;RNA binding;single-stranded RNA binding
binding;chromatin binding
ARF guanyl-nucleotide exchange factor activity;binding;enzyme regulator activity;GTPase regulator activity;guanyl-nucleotide exchange factor activity;lipid binding;nucleoside-triphosphatase regulator activity;phosphatidylinc
binding;cation binding;ion binding;metal ion binding;transition metal ion binding;zinc ion binding
structural constituent of ribosome;structural molecule activity
catalytic activity;intramolecular oxidoreductase activity;intramolecular oxidoreductase activity, interconverting keto- and enol-groups;intramolecular oxidoreductase activity, transposing S-S bonds;isomerase activity;protein d
binding;catalytic activity;GTP binding;GTPase activity;guanyl nucleotide binding;guanyl ribonucleotide binding;hydrolase activity;hydrolase activity, acting on acid anhydrides;hydrolase activity, acting on acid anhydrides, in pl
binding;catalytic activity;cation binding;hydrolase activity;hydrolase activity, acting on acid anhydrides;hydrolase activity, acting on acid anhydrides, in phosphorus-containing anhydrides;hydrolase activity, acting on ester bor
binding;nucleic acid binding;RNA binding;rRNA binding
binding;enzyme binding;phosphatase binding;protein binding;protein complex scaffold;structural molecule activity
binding;enzyme binding;kinase binding;phosphoprotein binding;protein binding;protein kinase binding;translation initiation factor binding
binding;mRNA 3'-UTR binding;mRNA 5'-UTR binding;mRNA binding;nucleic acid binding;nucleotide binding;RNA binding;translation regulator activity
binding;mRNA 3'-UTR binding;mRNA 5'-UTR binding;mRNA binding;nucleic acid binding;nucleotide binding;RNA binding;translation regulator activity
binding;catalytic activity;cation binding;hydrolase activity;hydrolase activity, acting on ester bonds;ion binding;metal ion binding;phosphatase activity;phosphoprotein phosphatase activity;phosphoric ester hydrolase activity;p
adenyl nucleotide binding;adenyl ribonucleotide binding;ATP binding;binding;catalytic activity;hydrolase activity;hydrolase activity, acting on acid anhydrides;hydrolase activity, acting on acid anhydrides, in phosphorus-conta
binding;identical protein binding;protein binding
catalytic activity;hydrolase activity
actin binding;actin filament binding;actin-dependent ATPase activity;adenyl nucleotide binding;adenyl ribonucleotide binding;ATP binding;ATPase activity;ATPase activity, coupled;binding;calmodulin binding;catalytic activity;c
binding;calcium ion binding;cation binding;ion binding;metal ion binding
antigen binding;beta-2-microglobulin binding;binding;peptide antigen binding;peptide binding;protein binding;TAP binding
adenyl nucleotide binding;adenyl ribonucleotide binding;ATP binding;binding;catalytic activity;cyclin-dependent protein kinase activity;histone kinase activity;kinase activity;nucleotide binding;phosphotransferase activity, alco
binding;catalytic activity;coenzyme binding;cofactor binding;electron carrier activity;flavin adenine dinucleotide binding;oxidoreductase activity
binding;lipid binding;oxysterol binding;protein binding;protein domain specific binding;steroid binding;sterol binding
adenylosuccinate synthase activity;anion binding;binding;catalytic activity;cation binding;GTP binding;guanyl nucleotide binding;guanyl ribonucleotide binding;ion binding;ligase activity;ligase activity, forming carbon-nitrogen
binding;carbohydrate binding;cytoskeletal protein binding;dynactin binding;dynein binding;enzyme binding;glycosaminoglycan binding;heparin binding;identical protein binding;microtubule binding;pattern binding;phospholip

acid-amino acid ligase activity;adenyl nucleotide binding;adenyl ribonucleotide binding;ADP binding;amine binding;amino acid binding;ATP binding;binding;carboxylic acid binding;catalytic activity;cation binding;coenzyme b
binding;carbohydrate binding;cation binding;ion binding;mannose binding;metal ion binding;monosaccharide binding;protein binding;sugar binding;unfolded protein binding
binding;catalytic activity;cation binding;coenzyme binding;cofactor binding;ion binding;isocitrate dehydrogenase (NAD+) activity;isocitrate dehydrogenase activity;magnesium ion binding;metal ion binding;NAD binding;nucleo
biliverdin reductase activity;binding;catalytic activity;cation binding;ion binding;metal ion binding;oxidoreductase activity;oxidoreductase activity, acting on the CH-CH group of donors;oxidoreductase activity, acting on the CI
binding;cation binding;ion binding;metal ion binding;transition metal ion binding;zinc ion binding
structural constituent of ribosome;structural molecule activity
binding;catalytic activity;demethylase activity;DNA binding;histone binding;histone demethylase activity;histone demethylase activity (H4-K20 specific);nucleic acid binding;protein binding
antioxidant activity;catalytic activity;disulfide oxidoreductase activity;glutathione dehydrogenase (ascorbate) activity;glutathione disulfide oxidoreductase activity;glutathione transferase activity;methylarsonate reductase activ
structural constituent of ribosome;structural molecule activity
binding;chromatin binding;core promoter proximal region DNA binding;core promoter proximal region sequence-specific DNA binding;DNA binding;nucleic acid binding;nucleic acid binding transcription factor activity;protein
binding;carbohydrate binding;catalytic activity;cation binding;coenzyme binding;cofactor binding;ion binding;L-iditol 2-dehydrogenase activity;metal ion binding;NAD binding;nucleotide binding;oxidoreductase activity;oxidor
binding;nucleic acid binding;RNA binding;structural constituent of ribosome;structural molecule activity
binding;identical protein binding;protein binding
enzyme inhibitor activity;enzyme regulator activity;kinase inhibitor activity;kinase regulator activity;protein kinase inhibitor activity;protein kinase regulator activity
binding;enzyme binding;protein binding;ubiquitin protein ligase binding
binding;DNA binding;enzyme binding;nucleic acid binding;protein binding;RNA binding;ubiquitin protein ligase binding
binding;catalytic activity;cytoskeletal protein binding;GDP binding;GTP binding;GTPase activity;guanyl nucleotide binding;guanyl ribonucleotide binding;hydrolase activity;hydrolase activity, acting on acid anhydrides;hydrolas
basal RNA polymerase II transcription machinery binding;basal transcription machinery binding;binding;cation binding;DNA binding;enzyme binding;ion binding;metal ion binding;nucleic acid binding;protein binding;RNA poly
adenyl nucleotide binding;adenyl ribonucleotide binding;aminoacyl-tRNA ligase activity;aspartate-tRNA ligase activity;aspartate-tRNA(Asn) ligase activity;ATP binding;binding;catalytic activity;identical protein binding;ligase a
binding;double-stranded RNA binding;NF-kappaB binding;nucleic acid binding;protein binding;protein binding transcription factor activity;RNA binding;RNA polymerase II transcription factor binding;transcription coactivator
binding;protein binding;protein domain specific binding;WW domain binding
binding;nuclear localization sequence binding;peptide binding;signal sequence binding
binding;calcium ion binding;cation binding;ion binding;metal ion binding
binding;cation binding;DNA binding;ion binding;metal ion binding;nucleic acid binding;transition metal ion binding;zinc ion binding
binding;cation binding;ion binding;lipid binding;metal ion binding;protein transporter activity;substrate-specific transporter activity;transition metal ion binding;transporter activity;zinc ion binding
binding;protein binding;unfolded protein binding
acetyl-CoA C-acetyltransferase activity;acetyl-CoA C-acyltransferase activity;acetyltransferase activity;C-acetyltransferase activity;C-acyltransferase activity;catalytic activity;transferase activity;transferase activity, transferrin
binding;catalytic activity;cytoskeletal protein binding;hydrolase activity;hydrolase activity, acting on acid anhydrides;hydrolase activity, acting on acid anhydrides, in phosphorus-containing anhydrides;kinesin binding;microtub
adenyl nucleotide binding;adenyl ribonucleotide binding;ATP binding;binding;calcium ion binding;cation binding;GTP binding;guanyl nucleotide binding;guanyl ribonucleotide binding;ion binding;metal ion binding;nucleic acid
ion transmembrane transporter activity;substrate-specific transmembrane transporter activity;substrate-specific transporter activity;transmembrane transporter activity;transporter activity
adenyl nucleotide binding;adenyl ribonucleotide binding;ATP binding;binding;catalytic activity;helicase activity;hydrolase activity;hydrolase activity, acting on acid anhydrides;hydrolase activity, acting on acid anhydrides, in ph
catalytic activity;cytidylyltransferase activity;N-acetylneuraminate synthase activity;N-acylneuraminate cytidylyltransferase activity;N-acylneuraminate-9-phosphate synthase activity;nucleotidyltransferase activity;transferase a
adenyl nucleotide binding;adenyl ribonucleotide binding;ATP binding;ATPase activity;binding;catalytic activity;cation binding;GTP binding;guanyl nucleotide binding;guanyl ribonucleotide binding;hydrolase activity;hydrolase a
binding;nucleotide binding
binding;nucleotide binding
binding;nuclear export signal receptor activity;protein binding
actin binding;actin filament binding;binding;cytoskeletal protein binding;enzyme activator activity;enzyme regulator activity;protein binding
binding;enzyme regulator activity;lipid binding;phosphatase regulator activity;protein binding;protein phosphatase regulator activity;protein phosphatase type 1 regulator activity;small conjugating protein binding;ubiquitin binc
binding;nucleic acid binding;RNA binding;translation factor activity, nucleic acid binding;translation initiation factor activity
3'-5' exonuclease activity;3'-5'-exoribonuclease activity;binding;catalytic activity;endonuclease activity;enzyme regulator activity;exonuclease activity;exonuclease activity, active with either ribo- or deoxyribonucleic acids anc
adenyl nucleotide binding;adenyl ribonucleotide binding;ATP binding;binding;nucleotide binding;purine nucleotide binding;purine ribonucleoside triphosphate binding;purine ribonucleotide binding;ribonucleotide binding
acyl-CoA thioesterase activity;binding;carboxylic acid binding;carboxylic ester hydrolase activity;catalytic activity;CoA hydrolase activity;coenzyme binding;cofactor binding;fatty acid binding;fatty-acyl-CoA binding;hydrolase
binding;enzyme binding;GTP-dependent protein binding;phosphatase binding;protein binding;protein phosphatase binding
binding;binding, bridging;cytokine receptor binding;cytoskeletal adaptor activity;cytoskeletal protein binding;frizzled binding;G-protein-coupled receptor binding;growth factor receptor binding;identical protein binding;interleu
acetyltransferase activator activity;beta-tubulin binding;binding;carbohydrate binding;cytoskeletal protein binding;DNA binding;enzyme activator activity;enzyme regulator activity;glycosaminoglycan binding;heparin binding;n
binding;DNA binding;double-stranded DNA binding;mRNA binding;nucleic acid binding;nucleotide binding;poly(A) RNA binding;poly(G) RNA binding;poly-purine tract binding;RNA binding;single-stranded DNA binding;single-
binding;nucleic acid binding;RNA binding;translation factor activity, nucleic acid binding;translation initiation factor activity
binding;calcium ion binding;cation binding;ion binding;metal ion binding
acid-amino acid ligase activity;binding;catalytic activity;cation binding;ion binding;ligase activity;ligase activity, forming carbon-nitrogen bonds;metal ion binding;nucleic acid binding;RNA binding;small conjugating protein liga
3'(2'),5'-bisphosphate nucleotidase activity;binding;catalytic activity;cation binding;hydrolase activity;hydrolase activity, acting on ester bonds;inositol bisphosphate phosphatase activity;inositol or phosphatidylinositol phosp
binding;nucleic acid binding;RNA binding;structural constituent of ribosome;structural molecule activity
catalytic activity;cis-stilbene-oxide hydrolase activity;epoxide hydrolase activity;ether hydrolase activity;hydrolase activity;hydrolase activity, acting on ether bonds
binding;catalytic activity;collagen binding;cysteine-type endopeptidase activity;cysteine-type peptidase activity;endopeptidase activity;glycoprotein binding;hydrolase activity;peptidase activity;peptidase activity, acting on L-
binding;catalytic activity;GTP binding;GTPase activity;guanyl nucleotide binding;guanyl ribonucleotide binding;hydrolase activity;hydrolase activity, acting on acid anhydrides;hydrolase activity, acting on acid anhydrides, in ph
structural constituent of ribosome;structural molecule activity
binding;laminin receptor activity;receptor activity;ribonucleoprotein binding;ribosome binding;structural constituent of ribosome;structural molecule activity
carboxylic ester hydrolase activity;catalytic activity;deacetylase activity;hydrolase activity;hydrolase activity, acting on ester bonds;methylumbelliferyl-acetate deacetylase activity;S-formylglutathione hydrolase activity;thiolest
acyl-CoA dehydrogenase activity;binding;catalytic activity;coenzyme binding;cofactor binding;flavin adenine dinucleotide binding;identical protein binding;medium-chain-acyl-CoA dehydrogenase activity;oxidoreductase acti
catalytic activity;endopeptidase activity;exopeptidase activity;hydrolase activity;omega peptidase activity;peptidase activity;peptidase activity, acting on L-amino acid peptides;serine hydrolase activity;serine-type endopeptic
acid-ammonia (or amide) ligase activity;adenyl nucleotide binding;adenyl ribonucleotide binding;amine binding;amino acid binding;ammonia ligase activity;ATP binding;binding;carbon-carbon lyase activity;carboxylic acid bin
receptor activity
binding;cation binding;ion binding;metal ion binding;nucleic acid binding;RNA binding;translation factor activity, nucleic acid binding;translation initiation factor activity
catalytic activity;endopeptidase activity;hydrolase activity;peptidase activity;peptidase activity, acting on L-amino acid peptides;threonine-type endopeptidase activity;threonine-type peptidase activity
binding;cation binding;chromatin binding;damaged DNA binding;DNA binding;ion binding;metal ion binding;nucleic acid binding;single-stranded DNA binding;structure-specific DNA binding
antioxidant activity;catalytic activity;oxidoreductase activity;oxidoreductase activity, acting on paired donors, with incorporation or reduction of molecular oxygen;oxidoreductase activity, acting on peroxide as acceptor;perox
binding;nucleic acid binding;RNA binding;rRNA binding;structural constituent of ribosome;structural molecule activity
binding;endopeptidase inhibitor activity;endopeptidase regulator activity;enzyme binding;enzyme inhibitor activity;enzyme regulator activity;peptidase inhibitor activity;peptidase regulator activity;protease binding;protein binc
4-alpha-glucanotransferase activity;alpha-glucosidase activity;amylo-alpha-1,6-glucosidase activity;binding;carbohydrate binding;catalytic activity;glucosidase activity;glycogen debranching enzyme activity;hydrolase activity
nucleocytoplasmic transporter activity;transporter activity
electron carrier activity
catalytic activity;methyltransferase activity;nicotinamide N-methyltransferase activity;N-methyltransferase activity;S-adenosylmethionine-dependent methyltransferase activity;transferase activity;transferase activity, transferrin
15-hydroxyprostaglandin-D dehydrogenase (NADP+) activity;alcohol dehydrogenase (NADP+) activity;alditol:NADP+ 1-oxidoreductase activity;aldo-keto reductase (NADP) activity;androsterone dehydrogenase activity;catalyt
binding;nucleic acid binding;RNA binding;structural constituent of ribosome;structural molecule activity
binding;nucleic acid binding;RNA binding;structural constituent of ribosome;structural molecule activity
adenylate cyclase activity;binding;catalytic activity;cation binding;cyclase activity;GTP binding;GTPase activity;guanyl nucleotide binding;guanyl ribonucleotide binding;hydrolase activity;hydrolase activity, acting on acid anhy
binding;catalytic activity;cation binding;G-protein beta/gamma-subunit complex binding;G-protein-coupled receptor binding;GTP binding;GTPase activity;guanyl nucleotide binding;guanyl ribonucleotide binding;hydrolase ac
binding;cation binding;identical protein binding;ion binding;metal ion binding;nucleotide binding;protein binding;protein complex binding;transition metal ion binding;zinc ion binding
binding;carboxylic acid binding;fatty acid binding;lipid binding;monocarboxylic acid binding;transporter activity
3'-5' exonuclease activity;binding;catalytic activity;exonuclease activity;exoribonuclease activity;hydrolase activity;hydrolase activity, acting on ester bonds;nuclease activity;nucleotide binding;ribonuclease activity
binding;calcium ion binding;calmodulin binding;catalytic activity;cation binding;hydrolase activity;hydrolase activity, acting on ester bonds;ion binding;lipase activity;metal ion binding;molecular transducer activity;phosphatidy
binding;chromatin binding;chromatin DNA binding;DNA binding;nucleic acid binding;structure-specific DNA binding
adenyl nucleotide binding;adenyl ribonucleotide binding;ATP binding;binding;catalytic activity;enzyme activator activity;enzyme regulator activity;hydrolase activity;hydrolase activity, acting on ester bonds;kinase activator act
binding;binding, bridging;catalytic activity;hydrolase activity;hydrolase activity, acting on ester bonds;insulin receptor binding;non-membrane spanning protein tyrosine phosphatase activity;phosphatase activity;phosphoprote
enzyme inhibitor activity;enzyme regulator activity;GTPase inhibitor activity;GTPase regulator activity;nucleoside-triphosphatase regulator activity
molecular transducer activity;signal transducer activity
adenyl nucleotide binding;adenyl ribonucleotide binding;ATP binding;binding;catalytic activity;kinase activity;nucleotide binding;phosphotransferase activity, alcohol group as acceptor;protein kinase activity;protein tyrosine ki
extracellular matrix structural constituent;structural molecule activity
actin binding;binding;cytoskeletal protein binding;profilin binding;protein binding
adenyl nucleotide binding;adenyl ribonucleotide binding;ATP binding;ATPase activity;ATPase activity, coupled;ATP-dependent helicase activity;ATP-dependent RNA helicase activity;binding;catalytic activity;helicase activity;h
binding;cation binding;enzyme binding;ion binding;metal ion binding;protein binding;small conjugating protein binding;transition metal ion binding;ubiquitin binding;ubiquitin protein ligase binding;zinc ion binding
actin binding;actin filament binding;binding;cytoskeletal protein binding;protein binding
acid-thiol ligase activity;adenyl nucleotide binding;adenyl ribonucleotide binding;ATP binding;binding;catalytic activity;CoA-ligase activity;GDP binding;GTP binding;guanyl nucleotide binding;guanyl ribonucleotide binding;liga
adenyl nucleotide binding;adenyl ribonucleotide binding;ATP binding;binding;DNA binding;nucleic acid binding;nucleotide binding;purine nucleotide binding;purine ribonucleoside triphosphate binding;purine ribonucleotide bi
binding;G-protein coupled receptor activity;molecular transducer activity;opioid receptor activity;peptide binding;peptide receptor activity;peptide receptor activity, G-protein coupled;receptor activity;signal transducer activity
acetyl-CoA hydrolase activity;carboxylic ester hydrolase activity;catalytic activity;CoA hydrolase activity;hydrolase activity;hydrolase activity, acting on ester bonds;thiolester hydrolase activity
binding;ribonucleoprotein binding
binding;nucleic acid binding;RNA binding;snoRNA binding
activating transcription factor binding;binding;chromatin binding;DNA binding;nucleic acid binding;protein binding;protein binding transcription factor activity;RNA polymerase II transcription coactivator activity;RNA polymera
aspartic-type endopeptidase activity;aspartic-type peptidase activity;catalytic activity;endopeptidase activity;hydrolase activity;peptidase activity;peptidase activity, acting on L-amino acid peptides
beta-N-acetylhexosaminidase activity;binding;catalytic activity;hexosaminidase activity;hydrolase activity;hydrolase activity, acting on glycosyl bonds;hydrolase activity, hydrolyzing O-glycosyl compounds;identical protein bir
actin binding;actin monomer binding;adenyl nucleotide binding;adenyl-nucleotide exchange factor activity;ATPase regulator activity;binding;cytoskeletal protein binding;enzyme regulator activity;lipid binding;nucleoside-triph
binding;cation binding;cytokine receptor binding;growth factor receptor binding;ion binding;metal ion binding;platelet-derived growth factor receptor binding;protein binding;receptor binding;vascular endothelial growth facto
alcohol dehydrogenase (NAD) activity;binding;carboxylic acid binding;catalytic activity;cation binding;electron carrier activity;fatty acid binding;formaldehyde dehydrogenase activity;ion binding;lipid binding;metal ion binding;
adenyl nucleotide binding;adenyl ribonucleotide binding;ADP binding;AMP binding;ATP binding;binding;carbohydrate binding;catalytic activity;cation binding;diphosphotransferase activity;GDP binding;guanyl nucleotide binc
binding;nucleic acid binding;RNA binding;structural constituent of ribosome;structural molecule activity;tRNA binding
binding;catalytic activity;cation binding;endopeptidase activity;hydrolase activity;ion binding;metal ion binding;metalloendopeptidase activity;metallopeptidase activity;peptidase activity;peptidase activity, acting on L-amino a
3'-5' exonuclease activity;3'-5'-exodeoxyribonuclease activity;binding;carbon-oxygen lyase activity;catalytic activity;cation binding;chromatin binding;chromatin DNA binding;damaged DNA binding;deoxyribonuclease activity
adenyl nucleotide binding;adenyl ribonucleotide binding;ATP binding;binding;catalytic activity;DNA binding;enzyme binding;kinase activity;MAP kinase activity;molecular transducer activity;nucleic acid binding;nucleotide binc
(S)-2-(5-amino-1-(5-phospho-D-ribosyl)imidazole-4-carboxamido)succinate AMP-lyase (fumarate-forming) activity;amidine-lyase activity;carbon-nitrogen lyase activity;catalytic activity;lyase activity;N6-(1,2-dicarboxyethyl)AM
aldehyde dehydrogenase (NAD) activity;catalytic activity;oxidoreductase activity;oxidoreductase activity, acting on the aldehyde or oxo group of donors;oxidoreductase activity, acting on the aldehyde or oxo group of donors,
adenyl nucleotide binding;adenyl ribonucleotide binding;ATP binding;binding;catalytic activity;helicase activity;hydrolase activity;hydrolase activity, acting on acid anhydrides;hydrolase activity, acting on acid anhydrides, in ph
acyl-CoA dehydrogenase activity;binding;catalytic activity;coenzyme binding;cofactor binding;flavin adenine dinucleotide binding;long-chain-acyl-CoA dehydrogenase activity;oxidoreductase activity;oxidoreductase activity, a
adenyl nucleotide binding;adenyl ribonucleotide binding;ATP binding;binding;caspase inhibitor activity;caspase regulator activity;catalytic activity;cation binding;cysteine-type endopeptidase inhibitor activity;endopeptidase in
adenyl nucleotide binding;adenyl ribonucleotide binding;adenylate kinase activity;ATP binding;binding;catalytic activity;kinase activity;nucleobase-containing compound kinase activity;nucleotide binding;nucleotide kinase ac
structural constituent of ribosome;structural molecule activity
binding;nucleic acid binding;RNA binding;structural constituent of ribosome;structural molecule activity
structural constituent of ribosome;structural molecule activity
binding;mRNA binding;nucleic acid binding;nucleotide binding;RNA binding
active transmembrane transporter activity;anion transmembrane transporter activity;binding;inorganic anion transmembrane transporter activity;ion transmembrane transporter activity;phosphate ion carrier activity;phosphate
adenyl nucleotide binding;adenyl ribonucleotide binding;ATP binding;ATPase activity;ATPase activity, coupled;binding;catalytic activity;cation binding;chromatin binding;DNA bending activity;DNA binding;DNA topoisomerase
ATPase activity;catalytic activity;hydrolase activity;hydrolase activity, acting on acid anhydrides;hydrolase activity, acting on acid anhydrides, in phosphorus-containing anhydrides;nucleoside-triphosphatase activity;pyrophos
binding;enzyme regulator activity;GTP binding;guanyl nucleotide binding;guanyl ribonucleotide binding;nucleotide binding;purine nucleotide binding;purine ribonucleoside triphosphate binding;purine ribonucleotide binding;ri

binding;enzyme activator activity;enzyme binding;enzyme regulator activity;GTPase activator activity;GTPase binding;GTPase regulator activity;nucleoside-triphosphatase regulator activity;protein binding;Rab GTPase binding
binding;DNA binding;nucleic acid binding;RNA binding;single-stranded DNA binding;structure-specific DNA binding
AU-rich element binding;binding;double-stranded RNA binding;enzyme binding;kinase binding;mRNA 3'-UTR AU-rich region binding;mRNA 3'-UTR binding;mRNA binding;nucleic acid binding;nucleotide binding;protein bind
structural molecule activity
adenine nucleotide transmembrane transporter activity;ATP transmembrane transporter activity;binding;calcium ion binding;cation binding;ion binding;metal ion binding;nucleobase-containing compound transmembrane tran
adenyl nucleotide binding;adenyl ribonucleotide binding;ATP binding;binding;catalytic activity;formate-tetrahydrofolate ligase activity;identical protein binding;ligase activity;ligase activity, forming carbon-nitrogen bonds;nucle
binding;enzyme binding;GTPase binding;leucine zipper domain binding;LRR domain binding;PDZ domain binding;protein binding;protein domain specific binding;Rab GTPase binding;Ras GTPase binding;small GTPase bind
3'-5' exonuclease activity;3'-5'-exoribonuclease activity;binding;catalytic activity;exonuclease activity;exonuclease activity, active with either ribo- or deoxyribonucleic acids and producing 5'-phosphomonoesters;exoribonucl
nucleocytoplasmic transporter activity;transporter activity
actin binding;binding;cytoskeletal protein binding;protein binding
binding;mRNA binding;nucleic acid binding;nucleotide binding;RNA binding
binding;enzyme activator activity;enzyme binding;enzyme regulator activity;GTPase activator activity;GTPase binding;GTPase regulator activity;nucleoside-triphosphatase regulator activity;protein binding;protein dimerization
binding;enzyme binding;GTPase binding;mRNA binding;nucleic acid binding;pre-miRNA binding;protein binding;protein transporter activity;Ran GTPase binding;Ras GTPase binding;RNA binding;small GTPase binding;subst
binding;identical protein binding;protein binding;protein dimerization activity;protein homodimerization activity
acid-thiol ligase activity;adenyl nucleotide binding;adenyl ribonucleotide binding;ATP binding;binding;catalytic activity;cation binding;CoA-ligase activity;ion binding;ligase activity;ligase activity, forming carbon-sulfur bonds;m
alkylglycerone-phosphate synthase activity;binding;catalytic activity;coenzyme binding;cofactor binding;FAD binding;flavin adenine dinucleotide binding;oxidoreductase activity;oxidoreductase activity, acting on CH-OH grou
binding;cation binding;ion binding;metal ion binding;protein binding transcription factor activity;transcription coactivator activity;transcription cofactor activity;transcription factor binding transcription factor activity;transition r
binding;chromatin binding;enzyme binding;protein binding;protein dimerization activity;protein heterodimerization activity
structural constituent of cytoskeleton;structural molecule activity
7S RNA binding;binding;nucleic acid binding;ribonucleoprotein binding;RNA binding;signal recognition particle binding
binding;binding, bridging;protein binding;protein binding, bridging;structural molecule activity
binding;catalytic activity;cation binding;G-protein beta/gamma-subunit complex binding;G-protein-coupled receptor binding;GTP binding;GTPase activity;guanyl nucleotide binding;guanyl ribonucleotide binding;hydrolase ac
binding;cation binding;ion binding;metal ion binding;transition metal ion binding;zinc ion binding
binding;cation binding;enzyme binding;glycoprotein binding;ion binding;metal ion binding;protease binding;protein binding;protein dimerization activity;protein heterodimerization activity
binding;cation binding;cytoskeletal protein binding;identical protein binding;ion binding;metal ion binding;microtubule binding;microtubule plus-end binding;nucleic acid binding;protein binding;protein dimerization activity;pro
binding;DNA binding;nucleic acid binding
active transmembrane transporter activity;ATPase activity;ATPase activity, coupled;ATPase activity, coupled to movement of substances;ATPase activity, coupled to transmembrane movement of ions;ATPase activity, couplec
active transmembrane transporter activity;adenyl nucleotide binding;adenyl ribonucleotide binding;ATP binding;ATPase activity;ATPase activity, coupled;ATPase activity, coupled to movement of substances;ATPase activity, c
binding;core promoter proximal region DNA binding;core promoter proximal region sequence-specific DNA binding;DNA binding;enzyme binding;kinase binding;ligand-dependent nuclear receptor activity;molecular transduce
acetyl-CoA C-acyltransferase activity;C-acyltransferase activity;catalytic activity;transferase activity;transferase activity, transferring acyl groups;transferase activity, transferring acyl groups other than amino-acyl groups
enzyme activator activity;enzyme regulator activity;GTPase activator activity;GTPase regulator activity;nucleoside-triphosphatase regulator activity
beta-catenin binding;binding;cation binding;cytoskeletal protein binding;ion binding;metal ion binding;protein binding;transition metal ion binding;vinculin binding;zinc ion binding
binding;G-quadruplex RNA binding;mRNA 3'-UTR binding;mRNA binding;nucleic acid binding;RNA binding
binding;nucleic acid binding;nucleotide binding;RNA binding;single-stranded RNA binding
actin binding;binding;cytoskeletal protein binding;protein binding
binding;peptide binding;structural constituent of nuclear pore;structural molecule activity;transporter activity
binding;catalytic activity;cysteine-type endopeptidase activity;cysteine-type peptidase activity;endopeptidase activity;endopeptidase inhibitor activity;endopeptidase regulator activity;enzyme inhibitor activity;enzyme regulato
binding;DNA binding;nucleic acid binding
adenyl nucleotide binding;adenyl ribonucleotide binding;ADP binding;AMP binding;ATP binding;binding;carbohydrate binding;catalytic activity;cation binding;diphosphotransferase activity;GDP binding;guanyl nucleotide binc
binding;catalytic activity;GTP binding;GTPase activity;guanyl nucleotide binding;guanyl ribonucleotide binding;hydrolase activity;hydrolase activity, acting on acid anhydrides;hydrolase activity, acting on acid anhydrides, in pl
binding;calcium channel regulator activity;catalytic activity;channel regulator activity;enzyme binding;GTPase activity;GTPase binding;hydrolase activity;hydrolase activity, acting on acid anhydrides;hydrolase activity, acting o
binding;carbohydrate binding
binding;DNA binding;enzyme inhibitor activity;enzyme regulator activity;histone binding;nucleic acid binding;phosphatase inhibitor activity;phosphatase regulator activity;protein binding;protein phosphatase inhibitor activity;p
actin binding;adenyl nucleotide binding;adenyl ribonucleotide binding;ATP binding;binding;catalytic activity;cytoskeletal protein binding;enzyme binding;JUN kinase binding;kinase activity;kinase binding;molecular transducer
binding;catalytic activity;isomerase activity;protein binding;receptor binding
AU-rich element binding;binding;enzyme binding;kinase binding;nucleic acid binding;nucleotide binding;protein binding;protein kinase binding;RNA binding
adenyl nucleotide binding;adenyl ribonucleotide binding;ATP binding;binding;catalytic activity;enzyme activator activity;enzyme binding;enzyme regulator activity;GTPase binding;identical protein binding;kinase activator activ
binding;nucleic acid binding;RNA binding;translation factor activity, nucleic acid binding;translation initiation factor activity
binding;catalytic activity;cation binding;hydrolase activity;hydrolase activity, acting on acid anhydrides;hydrolase activity, acting on acid anhydrides, in phosphorus-containing anhydrides;inorganic diphosphatase activity;ion b
adenyl nucleotide binding;adenyl ribonucleotide binding;ATP binding;binding;caspase inhibitor activity;caspase regulator activity;catalytic activity;cation binding;cysteine-type endopeptidase inhibitor activity;endopeptidase ir
binding;cation binding;ion binding;metal ion binding;transition metal ion binding;zinc ion binding
binding;catalytic activity;cofactor binding;hydrolase activity;hydrolase activity, acting on acid carbon-carbon bonds;hydrolase activity, acting on acid carbon-carbon bonds, in ketonic substances;identical protein binding;kynu
3-hydroxyacyl-CoA dehydrogenase activity;binding;catalytic activity;coenzyme binding;cofactor binding;NAD binding;NAD+ binding;nucleotide binding;oxidoreductase activity;oxidoreductase activity, acting on CH-OH group
binding;DNA binding;double-stranded RNA binding;identical protein binding;nucleic acid binding;protein binding;protein dimerization activity;protein homodimerization activity;RNA binding
ligand-dependent nuclear receptor transcription coactivator activity;protein binding transcription factor activity;transcription coactivator activity;transcription cofactor activity;transcription factor binding transcription factor act
binding;cadherin binding;cell adhesion molecule binding;cytoskeletal protein binding;enzyme binding;gamma-tubulin binding;GTPase binding;microtubule binding;protein binding;Rab GTPase binding;Ras GTPase binding;sm
binding;enzyme binding;enzyme regulator activity;GTPase binding;GTPase regulator activity;guanyl-nucleotide exchange factor activity;nucleoside-triphosphatase regulator activity;protein binding;Rac GTPase binding;Ras G
binding;histone binding;protein binding;protein transporter activity;substrate-specific transporter activity;transporter activity
androgen receptor binding;binding;catalytic activity;cation binding;copper chaperone activity;copper ion binding;cytokine binding;enzyme binding;glyoxalase III activity;hormone receptor binding;hydrolase activity;identical pr
actin binding;actin filament binding;Arp2/3 complex binding;binding;cytoskeletal protein binding;identical protein binding;protein binding;protein complex binding
binding;nucleic acid binding;ribonucleoprotein binding;ribosome binding;RNA binding;translation factor activity, nucleic acid binding;translation initiation factor activity;tRNA binding
aminopeptidase activity;binding;catalytic activity;cation binding;exopeptidase activity;hydrolase activity;identical protein binding;ion binding;manganese ion binding;metal ion binding;metalloaminopeptidase activity;metalloex
binding;GTP binding;guanyl nucleotide binding;guanyl ribonucleotide binding;nucleotide binding;purine nucleotide binding;purine ribonucleoside triphosphate binding;purine ribonucleotide binding;ribonucleotide binding
binding;catalytic activity;cation binding;dipeptidyl-peptidase activity;exopeptidase activity;hydrolase activity;ion binding;metal ion binding;metallopeptidase activity;peptidase activity;peptidase activity, acting on L-amino acid
ATP-dependent protein binding;binding;catalytic activity;enzyme activator activity;enzyme regulator activity;protein binding;protein C-terminus binding;protein dimerization activity;protein heterodimerization activity;small prot
binding;cytoskeletal protein binding;identical protein binding;lipid binding;phosphatidic acid binding;phospholipid binding;protein binding;transporter activity
binding;cytoskeletal protein binding;dynactin binding;identical protein binding;lipid binding;phosphatidylinositol binding;phospholipid binding;protein binding;protein dimerization activity;protein homodimerization activity
armadillo repeat domain binding;binding;estrogen receptor binding;hormone receptor binding;nuclear hormone receptor binding;protein binding;protein complex scaffold;protein domain specific binding;receptor binding;retin
anion channel activity;anion transmembrane transporter activity;channel activity;chloride channel activity;gated channel activity;ion channel activity;ion transmembrane transporter activity;passive transmembrane transporter
binding;catalytic activity;endopeptidase activity;hydrolase activity;identical protein binding;peptidase activity;peptidase activity, acting on L-amino acid peptides;protein binding;threonine-type endopeptidase activity;threonin
binding;calcium-dependent phospholipid binding;catalytic activity;kinase activity;lipid binding;phospholipid binding;phosphotransferase activity, alcohol group as acceptor;protein kinase activity;protein serine/threonine kinas
adenyl nucleotide binding;adenyl ribonucleotide binding;ATP binding;binding;catalytic activity;identical protein binding;kinase activity;molecular transducer activity;nucleotide binding;phosphotransferase activity, alcohol grou
macromolecule transmembrane transporter activity;protein transmembrane transporter activity;protein transporter activity;substrate-specific transmembrane transporter activity;substrate-specific transporter activity;transmer
binding;C5a anaphylatoxin chemotactic receptor binding;C5L2 anaphylatoxin chemotactic receptor binding;endopeptidase inhibitor activity;endopeptidase regulator activity;enzyme inhibitor activity;enzyme regulator activity;
antioxidant activity;binding;carboxylic acid binding;cation binding;chaperone binding;cofactor binding;copper ion binding;DNA binding;drug binding;fatty acid binding;ion binding;lipid binding;metal ion binding;monocarboxyl
adenine transmembrane transporter activity;nucleobase transmembrane transporter activity;nucleobase-containing compound transmembrane transporter activity;purine base transmembrane transporter activity;substrate-sp
alcohol dehydrogenase (NADP+) activity;alditol:NADP+ 1-oxidoreductase activity;aldo-keto reductase (NADP) activity;catalytic activity;electron carrier activity;glyceraldehyde oxidoreductase activity;oxidoreductase activity;ox
15-hydroxyprostaglandin dehydrogenase (NADP+) activity;carbonyl reductase (NADPH) activity;catalytic activity;oxidoreductase activity;oxidoreductase activity, acting on CH-OH group of donors;oxidoreductase activity, acti
binding;catalytic activity;cation binding;coenzyme binding;cofactor binding;cytochrome-b5 reductase activity;dioxygenase activity;electron carrier activity;flavin adenine dinucleotide binding;FMN binding;hydrolase activity;ion
endopeptidase inhibitor activity;endopeptidase regulator activity;enzyme inhibitor activity;enzyme regulator activity;peptidase inhibitor activity;peptidase regulator activity;serine-type endopeptidase inhibitor activity
binding;complement binding;complement component C3a binding;complement component C3b binding;DNA binding;enzyme binding;G-protein-coupled receptor binding;histone deacetylase binding;nucleic acid binding;nu
binding;DNA binding;histone binding;nucleic acid binding;protein binding
binding;cytokine activity;growth factor receptor binding;high molecular weight B cell growth factor receptor binding;protein binding;receptor binding
binding;cation binding;enzyme binding;ion binding;kinase binding;metal ion binding;protein binding;protein kinase binding;transition metal ion binding;zinc ion binding
1-pyrroline dehydrogenase activity;3-chloroallyl aldehyde dehydrogenase activity;4-trimethylammoniobutyraldehyde dehydrogenase activity;aldehyde dehydrogenase (NAD) activity;amine binding;aminobutyraldehyde dehydr
binding;nucleic acid binding;protein binding;protein N-terminus binding;RNA binding;translation factor activity, nucleic acid binding;translation initiation factor activity
binding;mRNA binding;nucleic acid binding;RNA binding;rRNA binding;SSU rRNA binding;structural constituent of ribosome;structural molecule activity
binding;catalytic activity;enzyme binding;GTPase activity;GTPase binding;hydrolase activity;hydrolase activity, acting on acid anhydrides;hydrolase activity, acting on acid anhydrides, in phosphorus-containing anhydrides;mo
binding;calcium ion binding;cation binding;DNA binding;ion binding;metal ion binding;nucleic acid binding
binding;catalytic activity;cytoskeletal protein binding;hydrolase activity;hydrolase activity, acting on acid anhydrides;hydrolase activity, acting on acid anhydrides, in phosphorus-containing anhydrides;motor activity;nucleosid
binding;protein binding;receptor binding;structural molecule activity
binding;histone binding;protein binding
catalytic activity;endonuclease activity;endonuclease activity, active with either ribo- or deoxyribonucleic acids and producing 5'-phosphomonoesters;endoribonuclease activity;endoribonuclease activity, producing 5'-phosph
binding;heat shock protein binding;protein binding
binding;catalytic activity;GTP binding;GTPase activity;guanyl nucleotide binding;guanyl ribonucleotide binding;hydrolase activity;hydrolase activity, acting on acid anhydrides;hydrolase activity, acting on acid anhydrides, in pl
adenyl nucleotide binding;adenyl ribonucleotide binding;ATP binding;ATPase activity;ATPase activity, coupled;ATP-dependent helicase activity;ATP-dependent RNA helicase activity;binding;catalytic activity;helicase activity;h
adenyl nucleotide binding;adenyl ribonucleotide binding;ATP binding;ATPase activity;binding;catalytic activity;hydrolase activity;hydrolase activity, acting on acid anhydrides;hydrolase activity, acting on acid anhydrides, in ph
binding;nucleic acid binding;nucleotide binding;RNA binding
binding;catalytic activity;cofactor binding;O-phospho-L-serine:2-oxoglutarate aminotransferase activity;pyridoxal phosphate binding;transaminase activity;transferase activity;transferase activity, transferring nitrogenous grou
adenyl nucleotide binding;adenyl ribonucleotide binding;ATP binding;ATPase activity;ATPase activity, coupled;ATP-dependent helicase activity;ATP-dependent RNA helicase activity;binding;catalytic activity;helicase activity;h
binding;nucleic acid binding;RNA binding;RNA cap binding;translation factor activity, nucleic acid binding;translation initiation factor activity
adenyl nucleotide binding;adenyl ribonucleotide binding;ATP binding;ATPase activity;ATPase activity, coupled;ATP-dependent helicase activity;ATP-dependent RNA helicase activity;binding;catalytic activity;helicase activity;h
structural constituent of cytoskeleton;structural molecule activity
adenyl nucleotide binding;adenyl ribonucleotide binding;adenylate kinase activity;ATP binding;binding;catalytic activity;kinase activity;nucleobase-containing compound kinase activity;nucleoside diphosphate kinase activity;r
binding;catalytic activity;cofactor binding;ornithine-oxo-acid transaminase activity;pyridoxal phosphate binding;transaminase activity;transferase activity;transferase activity, transferring nitrogenous groups;vitamin B6 binding
adenyl nucleotide binding;adenyl ribonucleotide binding;ATP binding;ATPase activity;beta-amyloid binding;beta-endorphin binding;binding;catalytic activity;cation binding;endopeptidase activity;glycoprotein binding;hormone
binding;chromatin binding;chromatin DNA binding;DNA binding;enzyme binding;histone deacetylase binding;nucleic acid binding;protein binding;structure-specific DNA binding
binding;carbon-carbon lyase activity;carboxylic acid binding;carboxy-lyase activity;catalytic activity;cofactor binding;L-aspartate:2-oxoglutarate aminotransferase activity;L-cysteine:2-oxoglutarate aminotransferase activity;L
cysteine-type endopeptidase inhibitor activity;endopeptidase inhibitor activity;endopeptidase regulator activity;enzyme inhibitor activity;enzyme regulator activity;peptidase inhibitor activity;peptidase regulator activity
enzyme activator activity;enzyme regulator activity;GDP-dissociation inhibitor activity;GTPase activator activity;GTPase regulator activity;nucleoside-triphosphatase regulator activity;Rab GDP-dissociation inhibitor activity;sm
adenyl nucleotide binding;adenyl ribonucleotide binding;ATP binding;binding;catalytic activity;enzyme activator activity;enzyme regulator activity;kinase activator activity;kinase activity;kinase regulator activity;MAP kinase kin
catalytic activity;methyltransferase activity;S-adenosylmethionine-dependent methyltransferase activity;transferase activity;transferase activity, transferring one-carbon groups
binding;nucleic acid binding;RNA binding;structural constituent of ribosome;structural molecule activity
binding;enzyme binding;GTPase binding;protein binding;protein C-terminus binding;Ras GTPase binding;small GTPase binding
3-oxoacid CoA-transferase activity;binding;catalytic activity;CoA-transferase activity;identical protein binding;protein binding;protein dimerization activity;protein homodimerization activity;transferase activity;transferase activ
structural constituent of ribosome;structural molecule activity
binding;enzyme binding;kinase binding;protein binding;protein kinase binding;SNARE binding;syntaxin binding
binding;enzyme binding;histone binding;histone deacetylase binding;protein binding
binding;cation binding;ion binding;metal ion binding;transition metal ion binding;zinc ion binding
binding;nucleic acid binding;RNA binding
binding;enzyme activator activity;enzyme regulator activity;GTPase activating protein binding;GTPase activator activity;GTPase regulator activity;guanyl-nucleotide exchange factor activity;nucleoside-triphosphatase regulato
binding;carbon-carbon lyase activity;carboxy-lyase activity;catalytic activity;cation binding;GTP binding;guanyl nucleotide binding;guanyl ribonucleotide binding;ion binding;lyase activity;metal ion binding;nucleotide binding;

binding;catalytic activity;cysteine-type endopeptidase activity;cysteine-type peptidase activity;endopeptidase activity;enzyme binding;hydrolase activity;p53 binding;peptidase activity;peptidase activity, acting on L-amino aci
binding;lipid binding;phosphatidylinositol binding;phosphatidylinositol-3,4,5-trisphosphate binding;phospholipid binding
binding;binding, bridging;ligand-dependent nuclear receptor transcription coactivator activity;nucleic acid binding;nucleotide binding;protein binding;protein binding transcription factor activity;protein binding, bridging;RNA b
binding;cation binding;ion binding;metal ion binding;transition metal ion binding;zinc ion binding
adenyl nucleotide binding;adenyl ribonucleotide binding;aminoacyl-tRNA ligase activity;ATP binding;binding;catalytic activity;cation binding;ion binding;ligase activity;ligase activity, forming aminoacyl-tRNA and related comp
adenyl nucleotide binding;adenyl ribonucleotide binding;ATP binding;binding;calcium ion binding;cation binding;DNA binding;ion binding;metal ion binding;nucleic acid binding;nucleotide binding;purine nucleotide binding;pu
binding;DNA binding;identical protein binding;nucleic acid binding;nucleotide binding;protein binding
adenyl nucleotide binding;adenyl ribonucleotide binding;ATP binding;binding;catalytic activity;cation binding;cytoskeletal protein binding;ion binding;ligase activity;ligase activity, forming phosphoric ester bonds;metal ion bin
14-3-3 protein binding;binding;enzyme binding;enzyme inhibitor activity;enzyme regulator activity;kinase binding;molecular transducer activity;phosphatase regulator activity;protein binding;protein kinase binding;signal trans
molecular transducer activity;receptor activity;semaphorin receptor activity;signal transducer activity;signaling receptor activity;transmembrane signaling receptor activity
structural constituent of cytoskeleton;structural molecule activity
binding;chromatin binding;DNA bending activity;DNA binding;double-stranded DNA binding;nucleic acid binding;structure-specific DNA binding
binding;lipid binding;phospholipid binding;transporter activity;vitamin binding;vitamin E binding
binding;catalytic activity;cofactor binding;kynurenine-oxoglutarate transaminase activity;L-aspartate:2-oxoglutarate aminotransferase activity;L-phenylalanine aminotransferase activity;L-phenylalanine:2-oxoglutarate aminotra
binding;calcium ion binding;cation binding;enzyme binding;ion binding;ion channel binding;kinase binding;metal ion binding;phosphoprotein binding;protein binding;protein kinase binding;protein kinase C binding
binding;calcium ion binding;calcium-dependent phospholipid binding;calcium-dependent protein binding;cation binding;integrin binding;ion binding;lipid binding;metal ion binding;phospholipid binding;protein binding;protein
binding;catalytic activity;cis-trans isomerase activity;enzyme binding;isomerase activity;peptide binding;peptidyl-prolyl cis-trans isomerase activity;protein binding;protein complex binding;RNA polymerase binding;unfolded p
binding;catalytic activity;cation binding;cation transmembrane transporter activity;enzyme binding;hydrogen ion transmembrane transporter activity;inorganic cation transmembrane transporter activity;ion binding;ion transme
adenyl nucleotide binding;adenyl ribonucleotide binding;ATP binding;binding;catalytic activity;fatty acid ligase activity;ligase activity;ligase activity, forming carbon-sulfur bonds;long-chain fatty acid-CoA ligase activity;nucleot
binding;catalytic activity;coenzyme binding;cofactor binding;diiodophenylpyruvate reductase activity;L-malate dehydrogenase activity;malate dehydrogenase activity;malic enzyme activity;NAD binding;nucleotide binding;oxi
aldehyde dehydrogenase (NAD) activity;betaine-aldehyde dehydrogenase activity;catalytic activity;L-aminoadipate-semialdehyde dehydrogenase activity;oxidoreductase activity;oxidoreductase activity, acting on the aldehyd
adenyl deoxyribonucleotide binding;adenyl nucleotide binding;binding;binding, bridging;dATP binding;deoxyribonucleotide binding;nucleotide binding;protein binding;protein binding, bridging;purine deoxyribonucleotide bind
binding;chromatin binding;identical protein binding;nucleic acid binding transcription factor activity;protein binding;protein binding transcription factor activity;sequence-specific DNA binding transcription factor activity;transc
binding;carbohydrate binding;DNA binding;glycosaminoglycan binding;heparin binding;nucleic acid binding;nucleotide binding;pattern binding;polysaccharide binding;protein binding transcription factor activity;RNA polymer
binding;catalytic activity;endopeptidase activity;hydrolase activity;NF-kappaB binding;nucleic acid binding;nucleotide binding;peptidase activity;peptidase activity, acting on L-amino acid peptides;protein binding;purine nucle
adenyl nucleotide binding;adenyl ribonucleotide binding;ATP binding;binding;chaperone binding;nucleotide binding;protein binding;purine nucleotide binding;purine ribonucleoside triphosphate binding;purine ribonucleotide b
binding;nucleic acid binding;RNA binding;structural constituent of ribosome;structural molecule activity
apoptotic protease activator activity;binding;binding, bridging;caspase activator activity;caspase regulator activity;channel inhibitor activity;channel regulator activity;enzyme activator activity;enzyme binding;enzyme inhibitor
binding;catalytic activity;cation binding;hydrolase activity;hydrolase activity, acting on ester bonds;ion binding;metal ion binding;phosphatase activity;phosphoprotein phosphatase activity;phosphoric ester hydrolase activity;p
binding;nucleic acid binding;RNA binding;RNA cap binding
adenyl nucleotide binding;adenyl ribonucleotide binding;ATP binding;ATPase activity;ATPase activity, coupled;ATP-dependent DNA helicase activity;ATP-dependent helicase activity;ATP-dependent RNA helicase activity;bind
adenyl nucleotide binding;adenyl ribonucleotide binding;ATP binding;binding;catalytic activity;cation binding;ion binding;kinase activity;metal ion binding;nucleotide binding;phosphotransferase activity, alcohol group as accep
binding;calcium ion binding;cation binding;cell adhesion molecule binding;ion binding;metal ion binding;protein binding
binding;carbohydrate binding;catalytic activity;identical protein binding;nucleotidyltransferase activity;protein binding;transferase activity;transferase activity, transferring phosphorus-containing groups;UDP-N-acetylglucosam
aldehyde dehydrogenase (NAD) activity;catalytic activity;formyltetrahydrofolate dehydrogenase activity;hydroxymethyl-, formyl- and related transferase activity;methyltransferase activity;oxidoreductase activity;oxidoreductas
catalytic activity;intramolecular oxidoreductase activity;intramolecular oxidoreductase activity, interconverting keto- and enol-groups;intramolecular oxidoreductase activity, transposing S-S bonds;isomerase activity;protein d
binding;nucleic acid binding;RNA binding;snRNA binding
actin binding;actin filament binding;adenyl nucleotide binding;adenyl ribonucleotide binding;ADP binding;ATP binding;binding;catalytic activity;cytoskeletal protein binding;DNA binding;hydrolase activity;hydrolase activity, ac
binding;chromatin binding;DNA binding;nucleic acid binding;protein binding;protein binding transcription factor activity;protein N-terminus binding;transcription coactivator activity;transcription cofactor activity;transcription fa
binding;lipid binding;lipoprotein particle receptor binding;low-density lipoprotein particle receptor binding;peptide binding;protein binding;receptor binding;signal sequence binding;transporter activity
acid-amino acid ligase activity;binding;catalytic activity;cation binding;chromatin binding;core promoter proximal region DNA binding;core promoter proximal region sequence-specific DNA binding;DNA binding;histone bindi
binding;cytoskeletal protein binding;FFAT motif binding;microtubule binding;molecular transducer activity;protein binding;protein dimerization activity;protein domain specific binding;protein heterodimerization activity;signal t
structural constituent of ribosome;structural molecule activity
adenyl nucleotide binding;adenyl ribonucleotide binding;aminoacyl-tRNA ligase activity;ATP binding;binding;catalytic activity;ligase activity;ligase activity, forming aminoacyl-tRNA and related compounds;ligase activity, formi
binding;enzyme binding;mRNA binding;nucleic acid binding;protein binding;RNA binding
alpha-catenin binding;binding;cell adhesion molecule binding;protein binding
adenyl nucleotide binding;adenyl ribonucleotide binding;ATP binding;binding;catalytic activity;cation binding;endopeptidase activity;hydrolase activity;ion binding;metal ion binding;metalloendopeptidase activity;metallopepti
adenyl nucleotide binding;adenyl ribonucleotide binding;ATP binding;binding;catalytic activity;FAT10 activating enzyme activity;nucleotide binding;purine nucleotide binding;purine ribonucleoside triphosphate binding;purine r
alcohol binding;amine binding;binding;calcium ion binding;calcium-dependent phospholipid binding;cation binding;identical protein binding;ion binding;lipid binding;metal ion binding;phosphatidylcholine binding;phosphatidy
binding;protein binding;protein binding transcription factor activity;repressing transcription factor binding;RNA polymerase II repressing transcription factor binding;RNA polymerase II transcription cofactor activity;RNA polym
adenyl nucleotide binding;adenyl ribonucleotide binding;aminoacyl-tRNA ligase activity;asparagine-tRNA ligase activity;ATP binding;binding;catalytic activity;ligase activity;ligase activity, forming aminoacyl-tRNA and related c
binding;carboxypeptidase activity;catalytic activity;cation binding;exopeptidase activity;hydrolase activity;ion binding;metal ion binding;metallocarboxypeptidase activity;metalloexopeptidase activity;metallopeptidase activity;
binding;catalytic activity;cation binding;G-protein beta/gamma-subunit complex binding;G-protein-coupled receptor binding;GTP binding;GTPase activity;guanyl nucleotide binding;guanyl ribonucleotide binding;hydrolase ac
2',3'-cyclic-nucleotide 3'-phosphodiesterase activity;binding;catalytic activity;cyclic nucleotide binding;cyclic-nucleotide phosphodiesterase activity;hydrolase activity;hydrolase activity, acting on ester bonds;nucleic acid bin
binding;catalytic activity;GTP binding;GTPase activity;guanyl nucleotide binding;guanyl ribonucleotide binding;hydrolase activity;hydrolase activity, acting on acid anhydrides;hydrolase activity, acting on acid anhydrides, in ph
6-phosphofructokinase activity;adenyl nucleotide binding;adenyl ribonucleotide binding;ATP binding;binding;carbohydrate binding;carbohydrate kinase activity;catalytic activity;cation binding;enzyme binding;fructose binding
binding;cation binding;cobalt ion binding;DNA binding;enzyme binding;identical protein binding;ion binding;metal ion binding;nucleic acid binding;protein binding;protein binding transcription factor activity;protein dimerizatio
adenyl nucleotide binding;adenyl ribonucleotide binding;ATP binding;binding;catalytic activity;cation binding;ion binding;metal ion binding;methionine adenosyltransferase activity;nucleotide binding;purine nucleotide binding;
binding;core promoter binding;core promoter proximal region DNA binding;core promoter proximal region sequence-specific DNA binding;core promoter sequence-specific DNA binding;cytokine receptor binding;DNA bindin
adenyl nucleotide binding;adenyl ribonucleotide binding;ATP binding;ATPase activity;ATPase activity, coupled;binding;catalytic activity;cation binding;enzyme binding;GTPase binding;hydrolase activity;hydrolase activity, acti
binding;nucleic acid binding;RNA binding;structural constituent of ribosome;structural molecule activity
actin binding;actin filament binding;binding;cytoskeletal protein binding;protein binding
binding;profilin binding;protein binding
adenyl nucleotide binding;adenyl ribonucleotide binding;androgen receptor binding;ATP binding;ATPase activity;ATPase activity, coupled;binding;catalytic activity;chromatin binding;DNA binding;DNA polymerase binding;DNA
adenyl nucleotide binding;adenyl ribonucleotide binding;ATP binding;ATPase activity;binding;catalytic activity;hydrolase activity;hydrolase activity, acting on acid anhydrides;hydrolase activity, acting on acid anhydrides, in ph
androgen receptor binding;binding;catalytic activity;chromatin binding;GDP binding;GTP binding;GTPase activity;guanyl nucleotide binding;guanyl ribonucleotide binding;hormone receptor binding;hydrolase activity;hydrolas
binding;nucleic acid binding;RNA binding;rRNA binding;structural constituent of ribosome;structural molecule activity
binding;catalytic activity;cis-trans isomerase activity;isomerase activity;peptide binding;peptidyl-prolyl cis-trans isomerase activity;protein binding;unfolded protein binding;virion binding
catalytic activity;hydrolase activity;hydrolase activity, acting on acid anhydrides;hydrolase activity, acting on acid anhydrides, in phosphorus-containing anhydrides;microtubule motor activity;motor activity;nucleoside-triphos
acetylgalactosaminyltransferase activity;binding;carbohydrate binding;catalytic activity;cation binding;ion binding;manganese ion binding;metal ion binding;polypeptide N-acetylgalactosaminyltransferase activity;transferase a
binding;catalytic activity;dipeptidyl-peptidase activity;endopeptidase activity;enzyme binding;exopeptidase activity;hydrolase activity;identical protein binding;integrin binding;metalloendopeptidase activity;metallopeptidase a
binding;mRNA binding;nucleic acid binding;nucleotide binding;poly(A) RNA binding;poly(U) RNA binding;poly-purine tract binding;poly-pyrimidine tract binding;RNA binding;single-stranded RNA binding
3'-5' DNA helicase activity;adenyl nucleotide binding;adenyl ribonucleotide binding;ATP binding;ATPase activity;ATPase activity, coupled;ATP-dependent 3'-5' DNA helicase activity;ATP-dependent DNA helicase activity;ATP-
enzyme activator activity;enzyme regulator activity;GTPase activator activity;GTPase regulator activity;guanyl-nucleotide exchange factor activity;nucleoside-triphosphatase regulator activity;Ras guanyl-nucleotide exchange
binding;catalytic activity;cation binding;intramolecular transferase activity;intramolecular transferase activity, phosphotransferases;ion binding;isomerase activity;magnesium ion binding;metal ion binding;phosphoglucomutas
catalytic activity;disulfide oxidoreductase activity;intramolecular oxidoreductase activity;intramolecular oxidoreductase activity, interconverting keto- and enol-groups;intramolecular oxidoreductase activity, transposing S-S bo
binding;cation binding;ion binding;metal ion binding;transition metal ion binding;zinc ion binding
5'-3' exonuclease activity;5'-3' exoribonuclease activity;binding;catalytic activity;cation binding;DNA binding;exonuclease activity;exonuclease activity, active with either ribo- or deoxyribonucleic acids and producing 5'-phos
acid-amino acid ligase activity;binding;catalytic activity;identical protein binding;ligase activity;ligase activity, forming carbon-nitrogen bonds;protein binding;small conjugating protein ligase activity;ubiquitin-protein ligase acti
binding;enzyme binding;kinase binding;lipid binding;protein binding;protein kinase binding;protein transporter activity;substrate-specific transporter activity;transporter activity
actin binding;actin filament binding;binding;cytoskeletal protein binding;protein binding
6-phosphofructokinase activity;adenyl nucleotide binding;adenyl ribonucleotide binding;ATP binding;binding;carbohydrate binding;carbohydrate kinase activity;catalytic activity;cation binding;enzyme binding;fructose binding
catalytic activity;oxidoreductase activity;oxidoreductase activity, acting on the aldehyde or oxo group of donors;oxidoreductase activity, acting on the aldehyde or oxo group of donors, disulfide as acceptor;pyruvate dehydro
binding;enzyme binding;fibroblast growth factor binding;growth factor binding;mRNA binding;nucleic acid binding;protein binding;RNA binding;structural constituent of ribosome;structural molecule activity
binding;cation binding;DNA binding;ion binding;metal ion binding;nucleic acid binding;transition metal ion binding;zinc ion binding
binding;chemoattractant activity;chromatin binding;damaged DNA binding;DNA bending activity;DNA binding;double-stranded DNA binding;nucleic acid binding;nucleic acid binding transcription factor activity;protein bindin
aminopeptidase activity;binding;catalytic activity;endopeptidase activity;exopeptidase activity;hydrolase activity;peptidase activity;peptidase activity, acting on L-amino acid peptides;peptide binding;serine hydrolase activity;
activating transcription factor binding;alpha-catenin binding;androgen receptor binding;binding;cadherin binding;cell adhesion molecule binding;DNA binding;double-stranded DNA binding;enzyme binding;estrogen receptor
binding;DNA binding;lamin binding;nucleic acid binding;protein binding
adenyl nucleotide binding;adenyl ribonucleotide binding;ATP binding;ATPase activity;ATPase activity, coupled;binding;catalytic activity;centromeric DNA binding;damaged DNA binding;DNA binding;DNA insertion or deletion
binding;mRNA binding;nucleic acid binding;nucleotide binding;RNA binding
antigen binding;binding;calcium ion binding;calcium-dependent phospholipid binding;calcium-dependent protein binding;cation binding;ion binding;lipid binding;metal ion binding;MHC class II protein complex binding;MHC
androgen receptor binding;binding;cation binding;hormone receptor binding;identical protein binding;ion binding;metal ion binding;nuclear hormone receptor binding;protein binding;protein binding transcription factor activity
acid-amino acid ligase activity;binding;catalytic activity;cation binding;ion binding;ligase activity;ligase activity, forming carbon-nitrogen bonds;metal ion binding;nucleic acid binding transcription factor activity;sequence-spec
basal RNA polymerase II transcription machinery binding;basal transcription machinery binding;binding;enzyme binding;enzyme inhibitor activity;enzyme regulator activity;protein binding;RNA polymerase binding;RNA polym
binding;mRNA 3'-UTR binding;mRNA binding;nucleic acid binding;RNA binding
enzyme regulator activity
binding;DNA binding;nucleic acid binding;structural molecule activity
binding;cation binding;ion binding;metal ion binding
7S RNA binding;binding;endoplasmic reticulum signal peptide binding;nucleic acid binding;peptide binding;ribonucleoprotein binding;RNA binding;signal recognition particle binding;signal sequence binding
binding;carboxylic acid binding;catalytic activity;cation binding;galactosyltransferase activity;glucosyltransferase activity;ion binding;iron ion binding;L-ascorbic acid binding;metal ion binding;oxidoreductase activity;oxidored
aminoacylase activity;antioxidant activity;binding;catalase activity;catalytic activity;cation binding;coenzyme binding;cofactor binding;enzyme binding;heme binding;hydrolase activity;hydrolase activity, acting on carbon-nitro
binding;catalytic activity;coenzyme binding;cofactor binding;lactate dehydrogenase activity;L-lactate dehydrogenase activity;NAD binding;nucleotide binding;oxidoreductase activity;oxidoreductase activity, acting on CH-OH
active transmembrane transporter activity;amine transmembrane transporter activity;amino acid transmembrane transporter activity;antiporter activity;binding;calcium ion transmembrane transporter activity;calcium:cation an
binding;carboxylic acid binding;catalytic activity;cation binding;dioxygenase activity;ion binding;iron ion binding;L-ascorbic acid binding;metal ion binding;oxidoreductase activity;oxidoreductase activity, acting on paired don
alpha-catenin binding;binding;cadherin binding;cell adhesion molecule binding;enzyme binding;identical protein binding;molecular transducer activity;phosphatase binding;protein binding;protein binding transcription factor a
adenyl nucleotide binding;adenyl ribonucleotide binding;aminoacyl-tRNA ligase activity;ATP binding;binding;catalytic activity;ligase activity;ligase activity, forming aminoacyl-tRNA and related compounds;ligase activity, formi
active transmembrane transporter activity;adenyl nucleotide binding;adenyl ribonucleotide binding;ATP binding;ATPase activity;ATPase activity, coupled;ATPase activity, coupled to movement of substances;ATPase activity, c
acetyl-CoA C-acetyltransferase activity;acetyl-CoA C-acyltransferase activity;acetyltransferase activity;binding;C-acetyltransferase activity;C-acyltransferase activity;catalytic activity;cation binding;coenzyme binding;cofactor
activating transcription factor binding;binding;DNA binding;heat shock protein binding;molecular transducer activity;nucleic acid binding;protein binding;RNA binding;signal transducer activity;transcription factor binding;trans
catalytic activity;sedoheptulose-7-phosphate:D-glyceraldehyde-3-phosphate glyceronetransferase activity;transferase activity;transferase activity, transferring aldehyde or ketonic groups
adenyl nucleotide binding;adenyl ribonucleotide binding;ATP binding;ATPase activity;ATPase activity, coupled;ATP-dependent helicase activity;ATP-dependent RNA helicase activity;binding;catalytic activity;helicase activity;h
adenyl nucleotide binding;adenyl ribonucleotide binding;aminoacyl-tRNA ligase activity;ATP binding;binding;catalytic activity;cation binding;cysteine-tRNA ligase activity;identical protein binding;ion binding;ligase activity;liga
binding;lipid binding;phospholipid binding
binding;catalytic activity;cation binding;endopeptidase activity;hydrolase activity;ion binding;metal ion binding;metalloendopeptidase activity;metallopeptidase activity;peptidase activity;peptidase activity, acting on L-amino a
alcohol dehydrogenase (NADP+) activity;alditol:NADP+ 1-oxidoreductase activity;aldo-keto reductase (NADP) activity;bile acid binding;binding;carboxylic acid binding;catalytic activity;ketosteroid monooxygenase activity;lipi
binding;nucleic acid binding;RNA binding;structural constituent of ribosome;structural molecule activity
binding;nucleic acid binding;RNA binding;structural constituent of ribosome;structural molecule activity

adenyl nucleotide binding;adenyl ribonucleotide binding;ATP binding;ATPase activity;binding;binding, bridging;catalytic activity;hydrolase activity;hydrolase activity, acting on acid anhydrides;hydrolase activity, acting on acid
binding;carboxylic acid binding;catalytic activity;cation binding;identical protein binding;ion binding;iron ion binding;L-ascorbic acid binding;metal ion binding;oxidoreductase activity;oxidoreductase activity, acting on paired
binding;chaperone binding;enzyme binding;heat shock protein binding;kinase binding;protein binding;unfolded protein binding
binding;core promoter binding;DNA binding;nucleic acid binding;nucleotide binding;regulatory region DNA binding;regulatory region nucleic acid binding;transcription regulatory region DNA binding
actin binding;actinin binding;binding;cation binding;cytoskeletal protein binding;enzyme binding;ion binding;kinase binding;metal ion binding;protein binding;protein kinase binding;protein kinase C binding;transition metal ion
adenyl nucleotide binding;adenyl ribonucleotide binding;ATP binding;binding;calcium ion binding;catalytic activity;cation binding;GTP binding;guanyl nucleotide binding;guanyl ribonucleotide binding;hydrolase activity;ion bin
binding;protein binding;protein complex binding
binding;carbohydrate binding
adenyl nucleotide binding;adenyl ribonucleotide binding;ATP binding;binding;catalytic activity;cation binding;ion binding;kinase activity;magnesium ion binding;metal ion binding;nucleotide binding;phosphotransferase activity
binding;catalytic activity;cation binding;coenzyme binding;cofactor binding;identical protein binding;ion binding;isocitrate dehydrogenase (NADP+) activity;isocitrate dehydrogenase activity;magnesium ion binding;metal ion b
adenyl nucleotide binding;adenyl ribonucleotide binding;ATP binding;binding;catalytic activity;creatine kinase activity;kinase activity;nucleotide binding;phosphotransferase activity, alcohol group as acceptor;phosphotransfer
actin binding;actin filament binding;adenyl nucleotide binding;adenyl ribonucleotide binding;ATP binding;binding;catalytic activity;chromatin binding;cytoskeletal protein binding;DNA binding;double-stranded DNA binding;enz
binding;nucleic acid binding;ribonucleoprotein binding;ribosome binding;RNA binding;translation factor activity, nucleic acid binding;translation initiation factor activity
binding;catalytic activity;coenzyme binding;cofactor binding;dihydrolipoyl dehydrogenase activity;flavin adenine dinucleotide binding;oxidoreductase activity;oxidoreductase activity, acting on a sulfur group of donors;oxidore
binding;chromatin binding;chromatin DNA binding;DNA binding;nucleic acid binding;structure-specific DNA binding
actin binding;actin filament binding;binding;calcium ion binding;calcium-dependent phospholipid binding;cation binding;cytoskeletal protein binding;ion binding;lipid binding;metal ion binding;phosphatidylinositol binding;pho
adenyl nucleotide binding;adenyl ribonucleotide binding;ATP binding;binding;catalytic activity;CTP synthase activity;ligase activity;ligase activity, forming carbon-nitrogen bonds;nucleotide binding;purine nucleotide binding;p
5'-nucleotidase activity;binding;catalytic activity;cation binding;hydrolase activity;hydrolase activity, acting on ester bonds;ion binding;metal ion binding;nucleotidase activity;nucleotide binding;phosphatase activity;phosphori
3'-5' DNA helicase activity;adenyl nucleotide binding;adenyl ribonucleotide binding;ATP binding;ATPase activity;ATPase activity, coupled;ATP-dependent 3'-5' DNA helicase activity;ATP-dependent DNA helicase activity;ATP-
binding;mRNA binding;nucleic acid binding;RNA binding;structural constituent of ribosome;structural molecule activity
catalytic activity;endopeptidase activity;exopeptidase activity;hydrolase activity;peptidase activity;peptidase activity, acting on L-amino acid peptides;serine hydrolase activity;serine-type endopeptidase activity;serine-type ex
binding;heat shock protein binding;Hsp90 protein binding;protein binding;protein complex binding
binding;cation binding;DNA binding;identical protein binding;ion binding;metal ion binding;nucleic acid binding;nucleocytoplasmic transporter activity;protein anchor;protein binding;structural constituent of nuclear pore;struc
binding;catalytic activity;cytoskeletal protein binding;enzyme binding;GTP binding;GTPase activity;guanyl nucleotide binding;guanyl ribonucleotide binding;hydrolase activity;hydrolase activity, acting on acid anhydrides;hydro
binding;nucleic acid binding;RNA binding;structural constituent of ribosome;structural molecule activity
binding;catalytic activity;coenzyme binding;cofactor binding;NADP binding;nucleotide binding;oxidoreductase activity;oxidoreductase activity, acting on CH-OH group of donors;oxidoreductase activity, acting on the CH-OH
adenyl nucleotide binding;adenyl ribonucleotide binding;ATP binding;ATPase activity;ATPase activity, coupled;binding;catalytic activity;chromatin binding;damaged DNA binding;DNA binding;DNA-dependent ATPase activity;
binding;nucleic acid binding;nucleotide binding;RNA binding
adenyl nucleotide binding;adenyl ribonucleotide binding;ATP binding;ATPase activity;binding;catalytic activity;G-protein-coupled receptor binding;hormone receptor binding;hydrolase activity;hydrolase activity, acting on acid
binding;nucleic acid binding;RNA binding;rRNA binding;structural constituent of ribosome;structural molecule activity
binding;nucleic acid binding;RNA binding;structural constituent of ribosome;structural molecule activity
amine binding;amino acid binding;binding;carbohydrate binding;carboxylic acid binding;catalytic activity;glutamine-fructose-6-phosphate transaminase (isomerizing) activity;transaminase activity;transferase activity;transfera
actin binding;binding;cytoskeletal protein binding;protein binding
binding;protein binding;transcription factor binding
binding;histone binding;nucleic acid binding;nucleotide binding;protein binding;RNA binding;snRNA binding;U4 snRNA binding;U6 snRNA binding;U6atac snRNA binding
adenyl nucleotide binding;adenyl ribonucleotide binding;amine binding;amino acid binding;aminoacyl-tRNA ligase activity;ATP binding;binding;carboxylic acid binding;catalytic activity;cation binding;ion binding;ligase activity
catalytic activity;intramolecular oxidoreductase activity;intramolecular oxidoreductase activity, interconverting keto- and enol-groups;intramolecular oxidoreductase activity, transposing S-S bonds;isomerase activity;protein d
binding;nucleic acid binding;RNA binding
acid-amino acid ligase activity;ATPase activity;binding;catalytic activity;cation binding;hydrolase activity;hydrolase activity, acting on acid anhydrides;hydrolase activity, acting on acid anhydrides, in phosphorus-containing an
adenyl nucleotide binding;adenyl ribonucleotide binding;ATP binding;ATPase activity;ATPase activity, coupled;ATP-dependent helicase activity;ATP-dependent RNA helicase activity;binding;catalytic activity;chromatin binding
acetyltransferase activity;adenyl nucleotide binding;adenyl ribonucleotide binding;ATP binding;binding;catalytic activity;N-acetyltransferase activity;N-acyltransferase activity;nucleotide binding;purine nucleotide binding;purin
adenyl nucleotide binding;adenyl ribonucleotide binding;ATP binding;binding;catalytic activity;cation binding;enzyme activator activity;enzyme regulator activity;ion binding;metal ion binding;nucleotide binding;protein binding
acetylcholine receptor regulator activity;binding;calcium ion binding;carbohydrate binding;cation binding;chondroitin sulfate binding;dystroglycan binding;extracellular matrix binding;glycoprotein binding;glycosaminoglycan b
binding;carboxylic acid binding;catalytic activity;cation binding;dioxygenase activity;electron carrier activity;ion binding;iron ion binding;L-ascorbic acid binding;metal ion binding;oxidoreductase activity;oxidoreductase activit
acid-thiol ligase activity;adenyl nucleotide binding;adenyl ribonucleotide binding;arachidonate-CoA ligase activity;ATP binding;binding;catalytic activity;fatty acid ligase activity;ligase activity;ligase activity, forming carbon-sulf
binding;catalytic activity;GTP binding;GTPase activity;guanyl nucleotide binding;guanyl ribonucleotide binding;hydrolase activity;hydrolase activity, acting on acid anhydrides;hydrolase activity, acting on acid anhydrides, in pl
binding;catalytic activity;coenzyme binding;cofactor binding;DNA binding;electron carrier activity;FAD binding;flavin adenine dinucleotide binding;NAD(P)H oxidase activity;nucleic acid binding;oxidoreductase activity;oxidore
structural constituent of ribosome;structural molecule activity
binding;catalytic activity;endopeptidase activity;hydrolase activity;nucleic acid binding;peptidase activity;peptidase activity, acting on L-amino acid peptides;RNA binding;threonine-type endopeptidase activity;threonine-type
androgen receptor binding;binding;calcium ion binding;carbohydrate binding;cation binding;chaperone binding;complement binding;complement component C1q binding;DNA binding;enzyme binding;glycoprotein binding;ho
antioxidant activity;binding;carboxylic ester hydrolase activity;catalytic activity;enzyme binding;glutathione peroxidase activity;hydrolase activity;hydrolase activity, acting on ester bonds;lipase activity;oxidoreductase activity;
antigen binding;binding;catalytic activity;enzyme regulator activity;hydrolase activity;hydrolase activity, acting on ester bonds;phosphatase activity;phosphatase regulator activity;phosphoprotein phosphatase activity;phospho
adenyl nucleotide binding;adenyl ribonucleotide binding;ATP binding;ATPase activity;binding;catalytic activity;hydrolase activity;hydrolase activity, acting on acid anhydrides;hydrolase activity, acting on acid anhydrides, in ph
structural constituent of ribosome;structural molecule activity
3alpha,7alpha,12alpha-trihydroxy-5beta-cholest-24-enoyl-CoA hydratase activity;3-hydroxyacyl-CoA dehydrogenase activity;binding;carbon-oxygen lyase activity;catalytic activity;hydro-lyase activity;identical protein binding
structural molecule activity
binding;catalytic activity;enzyme binding;intramolecular oxidoreductase activity;intramolecular oxidoreductase activity, interconverting aldoses and ketoses;isomerase activity;protein binding;triose-phosphate isomerase activ
adenyl nucleotide binding;adenyl ribonucleotide binding;ATP binding;binding;nucleotide binding;purine nucleotide binding;purine ribonucleoside triphosphate binding;purine ribonucleotide binding;ribonucleotide binding;struc
binding;nucleic acid binding;RNA binding;rRNA binding;structural constituent of ribosome;structural molecule activity
binding;enzyme binding;kinase binding;protein binding;protein kinase binding;structural constituent of ribosome;structural molecule activity
structural constituent of ribosome;structural molecule activity
binding;clathrin binding;peptide binding;protein binding;protein transporter activity;signal sequence binding;substrate-specific transporter activity;transporter activity
binding;channel regulator activity;enzyme binding;glucocorticoid receptor binding;hormone receptor binding;insulin-like growth factor receptor binding;ion channel binding;nuclear hormone receptor binding;protein binding;pr
antioxidant activity;binding;catalytic activity;cation binding;heme binding;ion binding;iron ion binding;metal ion binding;oxidoreductase activity;oxidoreductase activity, acting on paired donors, with incorporation or reduction
binding;mRNA binding;nucleic acid binding;nucleotide binding;RNA binding
binding;enzyme binding;kinase binding;protein binding;protein kinase binding;protein transporter activity;substrate-specific transporter activity;transporter activity
adenyl nucleotide binding;adenyl ribonucleotide binding;ATP binding;binding;catalytic activity;enzyme binding;kinase activity;kinase binding;molecular transducer activity;nucleotide binding;phosphotransferase activity, alcoh
binding;DNA binding;identical protein binding;nucleic acid binding;nucleotide binding;protein binding
binding;binding, bridging;cation binding;ion binding;ion transmembrane transporter activity;metal ion binding;protein binding;protein binding, bridging;SH3/SH2 adaptor activity;signaling adaptor activity;substrate-specific tra
adenyl nucleotide binding;adenyl ribonucleotide binding;ATP binding;binding;GTP binding;guanyl nucleotide binding;guanyl ribonucleotide binding;nucleotide binding;purine nucleotide binding;purine ribonucleoside triphosph
binding;cation binding;ion binding;metal ion binding;transition metal ion binding;zinc ion binding
catalytic activity;dihydropyrimidinase activity;hydrolase activity;hydrolase activity, acting on carbon-nitrogen (but not peptide) bonds;hydrolase activity, acting on carbon-nitrogen (but not peptide) bonds, in cyclic amides
adenyl nucleotide binding;adenyl ribonucleotide binding;ATP binding;binding;catalytic activity;DNA helicase activity;helicase activity;hydrolase activity;hydrolase activity, acting on acid anhydrides;hydrolase activity, acting on
protein transporter activity;substrate-specific transporter activity;transporter activity
structural constituent of nuclear pore;structural molecule activity;transporter activity
adenyl nucleotide binding;adenyl ribonucleotide binding;ATP binding;binding;catalytic activity;enzyme binding;fatty acid ligase activity;kinase binding;ligase activity;ligase activity, forming carbon-sulfur bonds;long-chain fatty
carbon-oxygen lyase activity;catalytic activity;fumarate hydratase activity;hydro-lyase activity;lyase activity
binding;nucleic acid binding;nucleotide binding;RNA binding
binding;calcium ion binding;calcium-dependent phospholipid binding;calcium-dependent protein binding;cation binding;enzyme inhibitor activity;enzyme regulator activity;identical protein binding;ion binding;lipase inhibitor a
anion channel activity;anion transmembrane transporter activity;binding;channel activity;enzyme binding;gated channel activity;ion channel activity;ion transmembrane transporter activity;kinase binding;passive transmembra
binding;carbon-oxygen lyase activity;catalytic activity;damaged DNA binding;DNA binding;DNA N-glycosylase activity;DNA-(apurinic or apyrimidinic site) lyase activity;enzyme binding;hydrolase activity;hydrolase activity, acti
adenosylhomocysteinase activity;catalytic activity;hydrolase activity;hydrolase activity, acting on ether bonds;trialkylsulfonium hydrolase activity
structural constituent of ribosome;structural molecule activity
2 iron, 2 sulfur cluster binding;4 iron, 4 sulfur cluster binding;binding;catalytic activity;cation binding;electron carrier activity;ion binding;iron-sulfur cluster binding;metal cluster binding;metal ion binding;NADH dehydrogenase
binding;DNA binding;lamin binding;nucleic acid binding;protein binding
adenyl nucleotide binding;adenyl ribonucleotide binding;ATP binding;ATPase activity;binding;catalytic activity;enzyme inhibitor activity;enzyme regulator activity;hydrolase activity;hydrolase activity, acting on acid anhydrides;
binding;nucleic acid binding;RNA binding;structural constituent of ribosome;structural molecule activity
binding;nucleic acid binding;RNA binding;structural constituent of ribosome;structural molecule activity
enzyme regulator activity
binding;catalytic activity;cation transmembrane transporter activity;coenzyme binding;cofactor binding;hydrogen ion transmembrane transporter activity;inorganic cation transmembrane transporter activity;ion transmembran
adenyl nucleotide binding;adenyl ribonucleotide binding;ATP binding;ATPase activity;ATPase activity, coupled;ATP-dependent helicase activity;ATP-dependent protein binding;ATP-dependent RNA helicase activity;binding;ca
binding;DNA binding;nucleic acid binding;nucleotide binding;RNA binding;sequence-specific DNA binding;telomeric DNA binding
binding;nucleic acid binding;RNA binding
binding;carbohydrate binding;catalytic activity;cation binding;glucose binding;identical protein binding;ion binding;metal ion binding;monosaccharide binding;nucleotide binding;nucleotidyltransferase activity;protein binding;
antioxidant activity;binding;catalytic activity;coenzyme binding;cofactor binding;disulfide oxidoreductase activity;electron carrier activity;flavin adenine dinucleotide binding;oxidoreductase activity;oxidoreductase activity, acti
binding;nucleic acid binding;RNA binding;rRNA binding;rRNA primary transcript binding
adenyl nucleotide binding;adenyl ribonucleotide binding;ATP binding;ATPase activity;binding;catalytic activity;hydrolase activity;hydrolase activity, acting on acid anhydrides;hydrolase activity, acting on acid anhydrides, in ph
structural constituent of nuclear pore;structural molecule activity
alpha-catenin binding;binding;channel regulator activity;enzyme binding;intermediate filament binding;ion channel binding;kinase binding;protein binding;protein complex binding;protein complex scaffold;protein kinase bindi
binding;enzyme binding;nucleic acid binding;protein binding;RNA binding
binding;cytoskeletal protein binding;enzyme binding;GTPase binding;kinase binding;microtubule binding;protein binding;protein domain specific binding;protein kinase binding;Rac GTPase binding;Ras GTPase binding;Rho (
actin binding;actin filament binding;binding;cytoskeletal protein binding;protein binding
binding;catalytic activity;cofactor binding;oxidoreductase activity;oxidoreductase activity, acting on a sulfur group of donors;oxidoreductase activity, acting on a sulfur group of donors, quinone or similar compound as accept
structural molecule activity
binding;catalytic activity;cation binding;GTP binding;GTPase activity;guanyl nucleotide binding;guanyl ribonucleotide binding;hydrolase activity;hydrolase activity, acting on acid anhydrides;hydrolase activity, acting on acid ar
adenyl nucleotide binding;adenyl ribonucleotide binding;ATP binding;binding;catalytic activity;cation binding;ion binding;kinase activity;metal ion binding;nucleotide binding;phosphotransferase activity, alcohol group as acce
binding;catalytic activity;cation binding;chromatin binding;deacetylase activity;DNA binding;histone deacetylase activity;hydrolase activity;hydrolase activity, acting on carbon-nitrogen (but not peptide) bonds;hydrolase activit
binding;calcium channel activity;cation binding;cation channel activity;cation transmembrane transporter activity;channel activity;cytokine binding;enzyme binding;extracellular matrix binding;fibronectin binding;gated channe
enzyme inhibitor activity;enzyme regulator activity;ribonuclease inhibitor activity
adenyl nucleotide binding;adenyl ribonucleotide binding;ATP binding;binding;catalytic activity;nucleotide binding;oxidoreductase activity;oxidoreductase activity, acting on CH or CH2 groups;oxidoreductase activity, acting or
binding;nucleic acid binding;nucleotide binding;poly-pyrimidine tract binding;pre-mRNA binding;RNA binding;single-stranded RNA binding
binding;catalytic activity;cyclohydrolase activity;hydrolase activity;hydrolase activity, acting on carbon-nitrogen (but not peptide) bonds;hydrolase activity, acting on carbon-nitrogen (but not peptide) bonds, in cyclic amidines;
binding;calcium ion binding;catalytic activity;cation binding;glycerol-3-phosphate dehydrogenase activity;ion binding;metal ion binding;oxidoreductase activity;oxidoreductase activity, acting on CH-OH group of donors;oxido
binding;catalytic activity;GTP binding;GTPase activity;guanyl nucleotide binding;guanyl ribonucleotide binding;hydrolase activity;hydrolase activity, acting on acid anhydrides;hydrolase activity, acting on acid anhydrides, in pl
adenyl nucleotide binding;adenyl ribonucleotide binding;ATP binding;binding;carbohydrate binding;carbohydrate kinase activity;catalytic activity;fructokinase activity;glucokinase activity;glucose binding;hexokinase activity;ki
amine binding;amino acid binding;antigen binding;binding;carboxylic acid binding;channel regulator activity;enzyme binding;histone deacetylase binding;ion channel binding;MHC class II protein complex binding;MHC protei
binding;nucleic acid binding;RNA binding;structural constituent of ribosome;structural molecule activity
binding;chromatin binding;DNA binding;nucleic acid binding
actin binding;actin filament binding;adenyl nucleotide binding;adenyl ribonucleotide binding;ATP binding;ATPase activity;ATPase activity, coupled;binding;calmodulin binding;catalytic activity;cytoskeletal protein binding;hydro

binding;chromatin binding;DNA binding;nucleic acid binding;nucleosome binding
catalytic activity;galactosyltransferase activity;procollagen galactosyltransferase activity;transferase activity;transferase activity, transferring glycosyl groups;transferase activity, transferring hexosyl groups;UDP-galactosyltran
binding;chromatin binding;DNA binding;nucleic acid binding;protein binding;protein domain specific binding;WD40-repeat domain binding
binding;catalytic activity;GTP binding;GTPase activity;guanyl nucleotide binding;guanyl ribonucleotide binding;hydrolase activity;hydrolase activity, acting on acid anhydrides;hydrolase activity, acting on acid anhydrides, in pl
acetylcholine receptor regulator activity;adenyl nucleotide binding;adenyl ribonucleotide binding;ATP binding;binding;catalytic activity;cation binding;chromatin binding;histone acetyl-lysine binding;histone binding;histone kin
binding;nucleic acid binding;nucleocytoplasmic transporter activity;protein transporter activity;RNA binding;substrate-specific transporter activity;transporter activity
enzyme regulator activity
binding;catalytic activity;coenzyme binding;cofactor binding;electron carrier activity;NAD binding;nucleotide binding;oxidoreductase activity;oxidoreductase activity, acting on CH-OH group of donors;oxidoreductase activity,
binding;mRNA binding;nucleic acid binding;nucleotide binding;RNA binding;tRNA binding
aminopeptidase activity;binding;catalytic activity;cation binding;epoxide hydrolase activity;ether hydrolase activity;exopeptidase activity;hydrolase activity;hydrolase activity, acting on ether bonds;ion binding;leukotriene-A4 h
adenyl nucleotide binding;adenyl ribonucleotide binding;ATP binding;ATPase activity;ATPase activity, coupled;binding;catalytic activity;cation binding;chromatin binding;DNA bending activity;DNA binding;DNA topoisomerase
adenyl nucleotide binding;adenyl ribonucleotide binding;ATP binding;binding;catalytic activity;creatine kinase activity;kinase activity;nucleotide binding;phosphotransferase activity, nitrogenous group as acceptor;purine nucle
binding;cytoskeletal protein binding;molecular transducer activity;protein binding;signal transducer activity;tubulin binding
adenyl nucleotide binding;adenyl ribonucleotide binding;ATP binding;ATPase activity;binding;catalytic activity;hydrolase activity;hydrolase activity, acting on acid anhydrides;hydrolase activity, acting on acid anhydrides, in ph
binding;protein binding;protein N-terminus binding
enzyme inhibitor activity;enzyme regulator activity;kinase inhibitor activity;kinase regulator activity;protein kinase C inhibitor activity;protein kinase inhibitor activity;protein kinase regulator activity;protein serine/threonine kinas
endopeptidase inhibitor activity;endopeptidase regulator activity;enzyme inhibitor activity;enzyme regulator activity;peptidase inhibitor activity;peptidase regulator activity;serine-type endopeptidase inhibitor activity
binding;enzyme binding;fibroblast growth factor binding;growth factor binding;identical protein binding;kinase binding;protein binding;protein dimerization activity;protein homodimerization activity;protein kinase binding;struc
5'-3' exonuclease activity;5'-flap endonuclease activity;binding;catalytic activity;cation binding;damaged DNA binding;deoxyribonuclease activity;DNA binding;double-stranded DNA binding;double-stranded DNA specific exo
adenyl nucleotide binding;adenyl ribonucleotide binding;aminoacyl-tRNA ligase activity;ATP binding;binding;catalytic activity;ligase activity;ligase activity, forming aminoacyl-tRNA and related compounds;ligase activity, formi
adenyl nucleotide binding;adenyl ribonucleotide binding;ATP binding;binding;carbon-nitrogen ligase activity, with glutamine as amido-N-donor;catalytic activity;GMP synthase (glutamine-hydrolyzing) activity;GMP synthase ac
binding;collagen binding;endopeptidase inhibitor activity;endopeptidase regulator activity;enzyme inhibitor activity;enzyme regulator activity;peptidase inhibitor activity;peptidase regulator activity;protein binding;serine-type e
binding;nucleic acid binding;nucleotide binding;RNA binding
adenyl nucleotide binding;adenyl ribonucleotide binding;amino acid kinase activity;ATP binding;binding;catalytic activity;coenzyme binding;cofactor binding;glutamate 5-kinase activity;glutamate-5-semialdehyde dehydrogen
adenyl nucleotide binding;adenyl ribonucleotide binding;ATP binding;binding;nucleotide binding;purine nucleotide binding;purine ribonucleoside triphosphate binding;purine ribonucleotide binding;ribonucleotide binding;struc
binding;GTP binding;guanyl nucleotide binding;guanyl ribonucleotide binding;identical protein binding;nucleotide binding;protein binding;purine nucleotide binding;purine ribonucleoside triphosphate binding;purine ribonucle
adenyl nucleotide binding;adenyl ribonucleotide binding;ATP binding;binding;nucleotide binding;purine nucleotide binding;purine ribonucleoside triphosphate binding;purine ribonucleotide binding;ribonucleotide binding
binding;cation binding;cytoskeletal protein binding;enzyme binding;enzyme regulator activity;GTPase binding;GTPase regulator activity;guanyl-nucleotide exchange factor activity;ion binding;metal ion binding;microtubule bir
protein transporter activity;substrate-specific transporter activity;transporter activity
binding;cation binding;ion binding;lipid binding;metal ion binding;phospholipid binding
binding;cation binding;ion binding;metal ion binding;transition metal ion binding;zinc ion binding
binding;chemoattractant activity;chromatin binding;cytokine activity;damaged DNA binding;DNA bending activity;DNA binding;double-stranded DNA binding;nucleic acid binding;nucleic acid binding transcription factor activi
aldehyde-lyase activity;binding;carbon-carbon lyase activity;catalytic activity;cytoskeletal protein binding;fructose-bisphosphate aldolase activity;lyase activity;protein binding
structural constituent of epidermis;structural molecule activity
active transmembrane transporter activity;adenyl nucleotide binding;adenyl ribonucleotide binding;ATP binding;ATPase activity;ATPase activity, coupled;ATPase activity, coupled to movement of substances;ATPase activity, c
binding;cation binding;ion binding;metal ion binding
binding;nucleic acid binding;RNA binding;structural constituent of ribosome;structural molecule activity
binding;catalytic activity;cation binding;chromatin binding;DNA binding;DNA-directed RNA polymerase activity;ion binding;metal ion binding;nucleic acid binding;nucleoside binding;nucleotidyltransferase activity;ribonucleosi
amine binding;amino acid binding;binding;carboxylic acid binding;enzyme binding;histone deacetylase binding;phosphoprotein binding;phosphoserine binding;protein binding;protein binding transcription factor activity;prote
3-hydroxyacyl-CoA dehydrogenase activity;acetyl-CoA C-acyltransferase activity;binding;C-acyltransferase activity;carbon-oxygen lyase activity;carboxylic acid binding;catalytic activity;coenzyme binding;cofactor binding;en
binding;catalytic activity;cation binding;coenzyme binding;cofactor binding;ion binding;metal ion binding;oxidoreductase activity;oxidoreductase activity, acting on the aldehyde or oxo group of donors;oxidoreductase activity
structural constituent of nuclear pore;structural molecule activity
actinin binding;alpha-actinin binding;binding;cytoskeletal protein binding;muscle alpha-actinin binding;protein binding
3 iron, 4 sulfur cluster binding;4 iron, 4 sulfur cluster binding;aconitate hydratase activity;binding;carbon-oxygen lyase activity;catalytic activity;cation binding;hydro-lyase activity;ion binding;iron ion binding;iron-sulfur cluster
adenyl nucleotide binding;adenyl ribonucleotide binding;ATP binding;binding;calcium ion binding;cation binding;enzyme binding;GTP binding;GTPase binding;guanyl nucleotide binding;guanyl ribonucleotide binding;ion bindi
binding;cation binding;ion binding;metal ion binding;transition metal ion binding;zinc ion binding
structural molecule activity
binding;enzyme binding;enzyme regulator activity;GTPase binding;GTPase regulator activity;nucleoside-triphosphatase regulator activity;protein binding;Ran GTPase binding;Ras GTPase binding;small GTPase binding;transp
binding;bisphosphoglycerate 2-phosphatase activity;bisphosphoglycerate mutase activity;bisphosphoglycerate phosphatase activity;catalytic activity;enzyme binding;hydrolase activity;hydrolase activity, acting on ester bonc
binding;calcium ion binding;carbohydrate binding;cation binding;ion binding;metal ion binding
structural constituent of cytoskeleton;structural molecule activity
catalytic activity;L-malate dehydrogenase activity;malate dehydrogenase (NADP+) activity;malate dehydrogenase activity;oxidoreductase activity;oxidoreductase activity, acting on CH-OH group of donors;oxidoreductase act
binding;catalytic activity;cation binding;cysteine-type endopeptidase activity;cysteine-type peptidase activity;endopeptidase activity;exopeptidase activity;hydrolase activity;ion binding;metal ion binding;omega peptidase act
adenyl nucleotide binding;adenyl ribonucleotide binding;ATP binding;binding;binding, bridging;catalytic activity;cis-trans isomerase activity;drug binding;FK506 binding;GTP binding;guanyl nucleotide binding;guanyl ribonucle
binding;DNA binding;nucleic acid binding;RNA binding;structural constituent of ribosome;structural molecule activity
1,4-alpha-glucan branching enzyme activity;binding;catalytic activity;cation binding;hydrolase activity;hydrolase activity, acting on glycosyl bonds;hydrolase activity, hydrolyzing O-glycosyl compounds;ion binding;transferase
binding;calcium ion binding;catalytic activity;cation binding;electron carrier activity;ion binding;ion channel binding;metal ion binding;oxidoreductase activity;oxidoreductase activity, acting on paired donors, with incorporatior
binding;core promoter binding;core promoter proximal region DNA binding;core promoter proximal region sequence-specific DNA binding;DNA binding;double-stranded DNA binding;identical protein binding;nucleic acid binc
binding;catalytic activity;cation binding;endopeptidase activity;enzyme activator activity;enzyme regulator activity;hydrolase activity;ion binding;metal ion binding;metalloendopeptidase activity;metallopeptidase activity;peptic
binding;eukaryotic initiation factor 4E binding;mRNA 3'-UTR binding;mRNA 5'-UTR binding;mRNA binding;nucleic acid binding;protein binding;RNA binding;RNA cap binding;translation activator activity;translation initiation f
binding;catalytic activity;cation binding;ion binding;metal ion binding;NAD+ ADP-ribosyltransferase activity;transferase activity;transferase activity, transferring glycosyl groups;transferase activity, transferring pentosyl groups
acid-amino acid ligase activity;binding;catalytic activity;cation binding;ion binding;ligase activity;ligase activity, forming carbon-nitrogen bonds;metal ion binding;small conjugating protein ligase activity;transition metal ion bin
adenyl nucleotide binding;adenyl ribonucleotide binding;ATP binding;ATPase activity;ATPase activity, coupled;ATP-dependent helicase activity;ATP-dependent RNA helicase activity;binding;catalytic activity;cation binding;chr
aminopeptidase activity;binding;catalytic activity;cation binding;epoxide hydrolase activity;ether hydrolase activity;exopeptidase activity;hydrolase activity;hydrolase activity, acting on ether bonds;ion binding;metal ion binding
binding;enzyme binding;kinase binding;protein binding;protein kinase binding;protein transporter activity;substrate-specific transporter activity;transporter activity
binding;calcium ion binding;cation binding;ion binding;metal ion binding
binding;catalytic activity;coenzyme binding;cofactor binding;flavin adenine dinucleotide binding;oxidoreductase activity;oxidoreductase activity, acting on the CH-CH group of donors;oxidoreductase activity, acting on the CH
aldehyde-lyase activity;amine binding;amino acid binding;binding;carbon-carbon lyase activity;carboxylic acid binding;catalytic activity;chromatin binding;cofactor binding;glycine hydroxymethyltransferase activity;hydroxyme
binding;catalytic activity;GTP binding;GTPase activity;guanyl nucleotide binding;guanyl ribonucleotide binding;hydrolase activity;hydrolase activity, acting on acid anhydrides;hydrolase activity, acting on acid anhydrides, in pl
5S rDNA binding;adenyl nucleotide binding;adenyl ribonucleotide binding;ATP binding;binding;catalytic activity;DNA binding;drug binding;kinase activity;nucleic acid binding;nucleotide binding;phosphoprotein binding;phosp
binding;nucleic acid binding;RNA binding;rRNA binding;structural constituent of ribosome;structural molecule activity
binding;enzyme binding;identical protein binding;kinase binding;protein binding;protein kinase binding;transcription factor binding;ubiquitin protein ligase binding
binding;nucleic acid binding;RNA binding;translation factor activity, nucleic acid binding;translation initiation factor activity
binding;calcium ion binding;cation binding;cytoskeletal protein binding;identical protein binding;integrin binding;ion binding;metal ion binding;microtubule binding;microtubule plus-end binding;protein binding;protein complex
binding;estrogen receptor binding;hormone receptor binding;nuclear hormone receptor binding;protein binding;protein N-terminus binding;receptor binding;steroid hormone receptor binding
binding;DNA binding;nucleic acid binding
binding;DNA binding;enzyme binding;nucleic acid binding;nucleic acid binding transcription factor activity;protein binding;sequence-specific DNA binding transcription factor activity;ubiquitin protein ligase binding
adenyl nucleotide binding;adenyl ribonucleotide binding;ATP binding;binding;core promoter binding;core promoter sequence-specific DNA binding;DNA binding;hormone receptor binding;ligand-dependent nuclear receptor t
binding;catalytic activity;coenzyme binding;cofactor binding;electron carrier activity;NAD binding;nucleotide binding;oxidoreductase activity;oxidoreductase activity, acting on CH-OH group of donors;oxidoreductase activity,
binding;nucleic acid binding;nucleotide binding;RNA binding
binding;mRNA binding;nucleic acid binding;RNA binding
binding;DNA binding;nucleic acid binding
binding;enzyme binding;enzyme inhibitor activity;enzyme regulator activity;histone binding;identical protein binding;kinase binding;kinase inhibitor activity;kinase regulator activity;NF-kappaB binding;nucleic acid binding;prot
binding;calcium ion binding;calcium-dependent cysteine-type endopeptidase activity;catalytic activity;cation binding;cysteine-type endopeptidase activity;cysteine-type peptidase activity;endopeptidase activity;hydrolase ac
binding;catalytic activity;cation binding;DNA binding;IMP dehydrogenase activity;ion binding;metal ion binding;nucleic acid binding;nucleotide binding;oxidoreductase activity;oxidoreductase activity, acting on CH-OH group
4 iron, 4 sulfur cluster binding;aconitate hydratase activity;binding;carbon-oxygen lyase activity;catalytic activity;cation binding;hydro-lyase activity;ion binding;iron-responsive element binding;iron-sulfur cluster binding;lyase
acid-amino acid ligase activity;adenyl nucleotide binding;adenyl ribonucleotide binding;ATP binding;binding;carbon-carbon lyase activity;carboxy-lyase activity;catalytic activity;identical protein binding;ligase activity;ligase ac
binding;nucleic acid binding;RNA binding;translation elongation factor activity;translation factor activity, nucleic acid binding
binding;nucleic acid binding;nucleotide binding;poly(U) RNA binding;poly-pyrimidine tract binding;RNA binding;single-stranded RNA binding
binding;catalytic activity;cation binding;intramolecular transferase activity;intramolecular transferase activity, phosphotransferases;ion binding;isomerase activity;magnesium ion binding;metal ion binding;phosphoglucomutas
adenyl nucleotide binding;adenyl ribonucleotide binding;amine binding;amino acid binding;aminoacyl-tRNA ligase activity;arginine binding;arginine-tRNA ligase activity;ATP binding;binding;carboxylic acid binding;catalytic ac
binding;nucleic acid binding;RNA binding;rRNA binding;structural constituent of ribosome;structural molecule activity
binding;catalytic activity;GTP binding;GTPase activity;guanyl nucleotide binding;guanyl ribonucleotide binding;hydrolase activity;hydrolase activity, acting on acid anhydrides;hydrolase activity, acting on acid anhydrides, in pl
binding;catalytic activity;GTP binding;GTPase activity;guanyl nucleotide binding;guanyl ribonucleotide binding;hydrolase activity;hydrolase activity, acting on acid anhydrides;hydrolase activity, acting on acid anhydrides, in pl
adenyl nucleotide binding;adenyl ribonucleotide binding;ATP binding;ATPase activity;ATPase activity, coupled;ATP-dependent helicase activity;ATP-dependent RNA helicase activity;binding;catalytic activity;helicase activity;h
acetyltransferase activity;binding;catalytic activity;N-acetyltransferase activity;N-acyltransferase activity;ribonucleoprotein binding;ribosome binding;transferase activity;transferase activity, transferring acyl groups;transferase
actin binding;actin filament binding;adenyl nucleotide binding;adenyl ribonucleotide binding;ADP binding;ATP binding;binding;calmodulin binding;catalytic activity;cytoskeletal protein binding;hydrolase activity;hydrolase activ
active transmembrane transporter activity;adenyl nucleotide binding;adenyl ribonucleotide binding;ATP binding;ATPase activity;ATPase activity, coupled;ATPase activity, coupled to movement of substances;ATPase activity, c
binding;chromatin binding;chromatin DNA binding;core promoter proximal region DNA binding;core promoter proximal region sequence-specific DNA binding;DNA binding;enhancer binding;enhancer sequence-specific DNA
actin binding;binding;cytoskeletal protein binding;protein binding;structural constituent of cytoskeleton;structural constituent of muscle;structural molecule activity
adenyl nucleotide binding;adenyl ribonucleotide binding;aminoacyl-tRNA ligase activity;ATP binding;binding;catalytic activity;histidine-tRNA ligase activity;ligase activity;ligase activity, forming aminoacyl-tRNA and related cor
binding;DNA binding;nucleic acid binding;nucleotide binding;regulatory region DNA binding;regulatory region nucleic acid binding;RNA binding;transcription regulatory region DNA binding
binding;cation binding;collagen binding;ion binding;metal ion binding;protein binding;receptor activity;viral receptor activity
structural constituent of cytoskeleton;structural molecule activity
adenyl nucleotide binding;adenyl ribonucleotide binding;ATP binding;binding;nucleotide binding;protein binding;purine nucleotide binding;purine ribonucleoside triphosphate binding;purine ribonucleotide binding;ribonucleoti
binding;nucleic acid binding;RNA binding;rRNA binding;structural constituent of ribosome;structural molecule activity;translation regulator activity
binding;core promoter proximal region DNA binding;core promoter proximal region sequence-specific DNA binding;DNA binding;nucleic acid binding;nucleic acid binding transcription factor activity;regulatory region DNA bin
binding;enzyme binding;enzyme inhibitor activity;enzyme regulator activity;insulin-like growth factor receptor binding;kinase binding;kinase inhibitor activity;kinase regulator activity;protein binding;protein kinase binding;prote
adenyl nucleotide binding;adenyl ribonucleotide binding;ATP binding;ATPase activity;binding;catalytic activity;cytoskeletal protein binding;hydrolase activity;hydrolase activity, acting on acid anhydrides;hydrolase activity, acti
catalytic activity;hydrolase activity;hydrolase activity, acting on acid anhydrides;hydrolase activity, acting on acid anhydrides, in phosphorus-containing anhydrides;motor activity;nucleoside-triphosphatase activity;pyrophospl
adenyl nucleotide binding;adenyl ribonucleotide binding;ATP binding;ATPase activity;ATPase activity, coupled;ATP-dependent helicase activity;ATP-dependent RNA helicase activity;binding;catalytic activity;chromatin binding
binding;core promoter proximal region DNA binding;core promoter proximal region sequence-specific DNA binding;DNA binding;nucleic acid binding;nucleic acid binding transcription factor activity;regulatory region DNA bin
binding;DNA binding;nucleic acid binding
binding;catalytic activity;enzyme binding;GTP binding;GTPase activity;guanyl nucleotide binding;guanyl ribonucleotide binding;hydrolase activity;hydrolase activity, acting on acid anhydrides;hydrolase activity, acting on acid
adenyl nucleotide binding;adenyl ribonucleotide binding;ATP binding;binding;carbon-nitrogen ligase activity, with glutamine as amido-N-donor;catalytic activity;cation binding;ion binding;ligase activity;ligase activity, forming
adenyl nucleotide binding;adenyl ribonucleotide binding;aminoacyl-tRNA ligase activity;ATP binding;binding;catalytic activity;glutamine-tRNA ligase activity;ligase activity;ligase activity, forming aminoacyl-tRNA and related co
binding;nucleic acid binding;RNA binding;structural constituent of ribosome;structural molecule activity
adenyl nucleotide binding;adenyl ribonucleotide binding;ATP binding;binding;cytokine receptor binding;enzyme binding;kinase binding;nucleotide binding;protein binding;protein kinase binding;purine nucleotide binding;purin
binding;cation binding;ion binding;metal ion binding;nucleic acid binding transcription factor activity;sequence-specific DNA binding transcription factor activity;transition metal ion binding;zinc ion binding

binding;catalytic activity;co-SMAD binding;cysteine-type endopeptidase activity;cysteine-type peptidase activity;endopeptidase activity;hydrolase activity;peptidase activity;peptidase activity, acting on L-amino acid peptides
binding;carboxylic acid binding;catalytic activity;cation binding;ion binding;iron ion binding;L-ascorbic acid binding;metal ion binding;oxidoreductase activity;oxidoreductase activity, acting on paired donors, with incorporatio
binding;mRNA binding;nucleic acid binding;nucleotide binding;poly(A) RNA binding;poly-purine tract binding;RNA binding;single-stranded RNA binding
binding;calcium-dependent protein binding;protein binding
adenine transmembrane transporter activity;binding;enzyme binding;nucleobase transmembrane transporter activity;nucleobase-containing compound transmembrane transporter activity;protein binding;purine base transme
actin binding;binding;cation binding;cell adhesion molecule binding;cytoskeletal protein binding;enzyme binding;ion binding;metal ion binding;peptide binding;protease binding;protein binding;protein dimerization activity;pro
binding;calcium ion binding;calcium-dependent cysteine-type endopeptidase activity;catalytic activity;cation binding;cysteine-type endopeptidase activity;cysteine-type peptidase activity;cytoskeletal protein binding;endope
binding;DNA binding;double-stranded RNA binding;nucleic acid binding;RNA binding
binding;catalytic activity;GTP binding;GTPase activity;guanyl nucleotide binding;guanyl ribonucleotide binding;hydrolase activity;hydrolase activity, acting on acid anhydrides;hydrolase activity, acting on acid anhydrides, in ph
binding;collagen binding;extracellular matrix binding;integrin binding;protein binding;protein complex binding;receptor binding
actin binding;actin filament binding;binding;cytoskeletal protein binding;enzyme binding;GTPase binding;protein binding;protein complex binding;Rac GTPase binding;Ras GTPase binding;Rho GTPase binding;small GTPase
binding;protein binding;protein C-terminus binding
binding;enzyme binding;enzyme inhibitor activity;enzyme regulator activity;GTPase binding;GTPase inhibitor activity;GTPase regulator activity;nucleoside-triphosphatase regulator activity;protein binding;protein transporter a
binding;integrin binding;protein binding;protein complex binding;receptor binding;structural molecule activity
binding;protein binding;scaffold protein binding;structural molecule activity
binding;extracellular matrix structural constituent;glycolipid binding;glycosphingolipid binding;lipid binding;sphingolipid binding;structural molecule activity
active transmembrane transporter activity;antiporter activity;ATP:ADP antiporter activity;secondary active transmembrane transporter activity;solute:solute antiporter activity;transmembrane transporter activity;transporter act
adenyl nucleotide binding;adenyl ribonucleotide binding;aminoacylase activity;aminoacyl-tRNA ligase activity;aspartate-tRNA ligase activity;ATP binding;binding;catalytic activity;hydrolase activity;hydrolase activity, acting on
actin binding;binding;cation binding;cytoskeletal protein binding;enzyme binding;integrin binding;ion binding;kinase binding;metal ion binding;protein binding;protein complex binding;protein kinase binding;receptor binding;t
adenyl nucleotide binding;adenyl ribonucleotide binding;ATP binding;binding;catalytic activity;identical protein binding;kinase activity;nucleotide binding;phosphotransferase activity, alcohol group as acceptor;protein binding
adenyl nucleotide binding;adenyl ribonucleotide binding;ATP binding;binding;nucleotide binding;purine nucleotide binding;purine ribonucleoside triphosphate binding;purine ribonucleotide binding;ribonucleotide binding
5'-3' DNA helicase activity;adenyl nucleotide binding;adenyl ribonucleotide binding;ATP binding;ATPase activity;ATPase activity, coupled;ATP-dependent 5'-3' DNA helicase activity;ATP-dependent DNA helicase activity;ATP-
androgen receptor binding;binding;hormone receptor binding;nuclear hormone receptor binding;nucleic acid binding;protein binding;protein binding transcription factor activity;receptor binding;ribonucleoprotein binding;RNA
actin binding;binding;calcium ion binding;cation binding;cytoskeletal protein binding;ion binding;metal ion binding;myosin binding;myosin II binding;protein binding;protein domain specific binding
actin binding;binding;cytoskeletal protein binding;protein binding;structural constituent of muscle;structural molecule activity
binding;protein binding;scaffold protein binding;structural constituent of cytoskeleton;structural molecule activity
beta-catenin binding;binding;cadherin binding;cell adhesion molecule binding;cytoskeletal protein binding;gamma-catenin binding;protein binding;structural molecule activity;vinculin binding
adenyl nucleotide binding;adenyl ribonucleotide binding;aminoacyl-tRNA ligase activity;ATP binding;binding;catalytic activity;ligase activity;ligase activity, forming aminoacyl-tRNA and related compounds;ligase activity, formi
binding;DNA binding;nucleic acid binding;nucleic acid binding transcription factor activity;sequence-specific DNA binding transcription factor activity
binding;cation binding;enzyme binding;ion binding;metal ion binding;nuclear localization sequence binding;peptide binding;protein binding;protein domain specific binding;protein transporter activity;signal sequence binding;
adenyl nucleotide binding;adenyl ribonucleotide binding;ATP binding;ATPase activity;ATPase activity, coupled;ATP-dependent helicase activity;ATP-dependent RNA helicase activity;binding;catalytic activity;helicase activity;h
binding;DNA binding;nucleic acid binding;nucleic acid binding transcription factor activity;sequence-specific DNA binding transcription factor activity;single-stranded DNA binding;structure-specific DNA binding
adenyl nucleotide binding;adenyl ribonucleotide binding;ATP binding;ATPase activity;binding;catalytic activity;chromatin binding;DNA binding;helicase activity;hydrolase activity;hydrolase activity, acting on acid anhydrides;hy
adenyl nucleotide binding;adenyl ribonucleotide binding;ATP binding;binding;nucleotide binding;protein binding;protein dimerization activity;protein heterodimerization activity;purine nucleotide binding;purine ribonucleoside t
structural constituent of cytoskeleton;structural molecule activity
adenyl nucleotide binding;adenyl ribonucleotide binding;ATP binding;binding;catalytic activity;cation binding;cyclo-ligase activity;hydroxymethyl-, formyl- and related transferase activity;ion binding;ligase activity;ligase activit
adenyl nucleotide binding;adenyl ribonucleotide binding;ADP binding;ATP binding;ATPase activity;ATPase activity, coupled;ATP-dependent peptidase activity;binding;catalytic activity;DNA binding;DNA polymerase binding;en
binding;nucleic acid binding;nucleotide binding;protein binding;protein complex scaffold;RNA binding;structural molecule activity;translation factor activity, nucleic acid binding;translation initiation factor activity
1-phosphatidylinositol binding;binding;calmodulin binding;cation binding;GTP-dependent protein binding;identical protein binding;ion binding;lipid binding;metal ion binding;phosphatidylinositol binding;phospholipid binding;
binding;chromatin binding;core promoter binding;DNA binding;double-stranded DNA binding;nucleic acid binding;nucleotide binding;regulatory region DNA binding;regulatory region nucleic acid binding;structure-specific DN
binding;DNA binding;nucleic acid binding;nucleotide binding;RNA binding;single-stranded DNA binding;single-stranded RNA binding;structure-specific DNA binding
binding;mRNA binding;nucleic acid binding;nucleotide binding;poly(A) RNA binding;poly(U) RNA binding;poly-purine tract binding;poly-pyrimidine tract binding;protein binding;protein C-terminus binding;RNA binding;single-s
actin binding;binding;calmodulin binding;cytoskeletal protein binding;protein binding;tropomyosin binding
acetyl-CoA carboxylase activity;adenyl nucleotide binding;adenyl ribonucleotide binding;ATP binding;binding;biotin carboxylase activity;catalytic activity;cation binding;CoA carboxylase activity;ion binding;ligase activity;ligas
binding;nucleic acid binding;RNA binding
binding;nucleic acid binding;nucleotide binding;RNA binding
binding;binding, bridging;catalytic activity;guanylate kinase activity;kinase activity;nucleobase-containing compound kinase activity;nucleotide kinase activity;phosphotransferase activity, phosphate group as acceptor;protein
adenyl nucleotide binding;adenyl ribonucleotide binding;ATP binding;binding;chaperone binding;nucleotide binding;protein binding;purine nucleotide binding;purine ribonucleoside triphosphate binding;purine ribonucleotide b
adenyl nucleotide binding;adenyl ribonucleotide binding;ATP binding;ATPase activity;ATPase activity, coupled;ATP-dependent helicase activity;ATP-dependent RNA helicase activity;binding;catalytic activity;double-stranded R
adenyl nucleotide binding;adenyl ribonucleotide binding;ATP binding;binding;nucleotide binding;purine nucleotide binding;purine ribonucleoside triphosphate binding;purine ribonucleotide binding;ribonucleotide binding
adenyl nucleotide binding;adenyl ribonucleotide binding;ADP binding;amine binding;amino acid binding;ATP binding;binding;carboxylic acid binding;catalytic activity;coenzyme binding;cofactor binding;glutamate dehydrogen
binding;cargo receptor activity;double-stranded RNA binding;identical protein binding;nucleic acid binding;protein binding;receptor activity;RNA binding;transferrin receptor activity
binding;calcium ion binding;calcium-dependent phospholipid binding;calcium-dependent protein binding;cation binding;enzyme inhibitor activity;enzyme regulator activity;ion binding;lipase inhibitor activity;lipid binding;meta
binding;DNA binding;identical protein binding;mRNA binding;nucleic acid binding;protein binding;protein dimerization activity;protein homodimerization activity;RNA binding;structural constituent of ribosome;structural molec
adenyl nucleotide binding;adenyl ribonucleotide binding;ATP binding;binding;catalytic activity;DNA binding;DNA helicase activity;helicase activity;hydrolase activity;hydrolase activity, acting on acid anhydrides;hydrolase activ
5S rRNA binding;binding;nucleic acid binding;RNA binding;rRNA binding;structural constituent of ribosome;structural molecule activity
binding;importin-alpha export receptor activity;nuclear export signal receptor activity;protein binding;protein transporter activity;substrate-specific transporter activity;transporter activity
adenosine deaminase activity;binding;catalytic activity;cation binding;deaminase activity;DNA binding;double-stranded RNA adenosine deaminase activity;hydrolase activity;hydrolase activity, acting on carbon-nitrogen (but n
adenyl nucleotide binding;adenyl ribonucleotide binding;ATP binding;ATPase activity;ATPase activity, coupled;ATP-dependent helicase activity;ATP-dependent RNA helicase activity;binding;catalytic activity;double-stranded R
binding;calcium ion binding;cation binding;ion binding;metal ion binding;structural constituent of muscle;structural molecule activity
binding;catalytic activity;methyltransferase activity;nucleic acid binding;RNA binding;RNA methyltransferase activity;S-adenosylmethionine-dependent methyltransferase activity;transferase activity;transferase activity, transfe
binding;catalytic activity;cis-trans isomerase activity;drug binding;FK506 binding;heat shock protein binding;isomerase activity;macrolide binding;peptidyl-prolyl cis-trans isomerase activity;protein binding
binding;catalytic activity;glucosyltransferase activity;protein binding;transferase activity;transferase activity, transferring glycosyl groups;transferase activity, transferring hexosyl groups;UDP-glucose:glycoprotein glucosyltrans
binding;extracellular matrix structural constituent;glycolipid binding;glycosphingolipid binding;lipid binding;sphingolipid binding;structural molecule activity
ATPase activity;ATPase activity, coupled;binding;catalytic activity;chromatin binding;core promoter binding;core promoter sequence-specific DNA binding;DNA binding;DNA topoisomerase (ATP-hydrolyzing) activity;DNA topo
binding;carbohydrate binding;catalytic activity;coenzyme binding;cofactor binding;glucose binding;glucose-6-phosphate dehydrogenase activity;identical protein binding;monosaccharide binding;NADP binding;nucleotide bi
adenyl nucleotide binding;adenyl ribonucleotide binding;ATP binding;binding;biotin binding;biotin carboxylase activity;carboxylic acid binding;catalytic activity;cation binding;DNA binding;ion binding;ligase activity;ligase activ
aminopeptidase activity;binding;catalytic activity;cation binding;exopeptidase activity;hydrolase activity;ion binding;metal ion binding;metallopeptidase activity;peptidase activity;peptidase activity, acting on L-amino acid pep
adenyl nucleotide binding;adenyl ribonucleotide binding;aminoacyl-tRNA ligase activity;ATP binding;binding;catalytic activity;glycine-tRNA ligase activity;ligase activity;ligase activity, forming aminoacyl-tRNA and related com
adenyl nucleotide binding;adenyl ribonucleotide binding;ATP binding;binding;nucleotide binding;protein binding;purine nucleotide binding;purine ribonucleoside triphosphate binding;purine ribonucleotide binding;ribonucleoti
adenyl nucleotide binding;adenyl ribonucleotide binding;AMP binding;ATP binding;bile acid binding;binding;carbohydrate binding;carboxylic acid binding;catalytic activity;cofactor binding;drug binding;glucose binding;glycog
adenyl nucleotide binding;adenyl ribonucleotide binding;ATP binding;binding;enzyme binding;nucleotide binding;protein binding;purine nucleotide binding;purine ribonucleoside triphosphate binding;purine ribonucleotide bin
binding;cation binding;ion binding;metal ion binding;nucleotide binding;structural molecule activity;transition metal ion binding;zinc ion binding
binding;catalytic activity;GTP binding;GTPase activity;guanyl nucleotide binding;guanyl ribonucleotide binding;hydrolase activity;hydrolase activity, acting on acid anhydrides;hydrolase activity, acting on acid anhydrides, in ph
binding;catalytic activity;double-stranded RNA binding;GTP binding;GTPase activity;guanyl nucleotide binding;guanyl ribonucleotide binding;hydrolase activity;hydrolase activity, acting on acid anhydrides;hydrolase activity, a
structural molecule activity
ankyrin binding;binding;cytoskeletal protein binding;enzyme binding;protein binding
actin binding;actin filament binding;actin monomer binding;binding;cation binding;cytoskeletal protein binding;ion binding;metal ion binding;protein binding;transition metal ion binding;zinc ion binding
binding;carbohydrate binding;receptor activity;structural molecule activity
binding;catalytic activity;enzyme binding;GTP binding;GTPase activity;guanyl nucleotide binding;guanyl ribonucleotide binding;hydrolase activity;hydrolase activity, acting on acid anhydrides;hydrolase activity, acting on acid
5'-deoxyribose-5-phosphate lyase activity;adenyl nucleotide binding;adenyl ribonucleotide binding;ATP binding;ATPase activity;ATPase activity, coupled;ATP-dependent DNA helicase activity;ATP-dependent helicase activity;
adenyl nucleotide binding;adenyl ribonucleotide binding;ATP binding;ATPase activity;ATPase activity, coupled;ATP-dependent DNA helicase activity;ATP-dependent helicase activity;binding;catalytic activity;damaged DNA bi
binding;DNA binding;nucleic acid binding;nucleotide binding;RNA binding;sequence-specific DNA binding;single-stranded DNA binding;single-stranded telomeric DNA binding;structure-specific DNA binding;telomeric DNA b
binding;catalytic activity;cation binding;chromatin binding;DNA (cytosine-5-)-methyltransferase activity;DNA binding;DNA-methyltransferase activity;ion binding;metal ion binding;methyl-CpG binding;methyltransferase activit
binding;catalytic activity;cytokine activity;drug binding;nicotinamide phosphoribosyltransferase activity;nicotinate-nucleotide diphosphorylase (carboxylating) activity;protein binding;receptor binding;transferase activity;transfe
adenyl nucleotide binding;adenyl ribonucleotide binding;ATP binding;binding;catalytic activity;cation binding;DNA binding;DNA helicase activity;DNA replication origin binding;helicase activity;hydrolase activity;hydrolase activ
adenyl nucleotide binding;adenyl ribonucleotide binding;ATP binding;binding;nucleotide binding;protein binding;purine nucleotide binding;purine ribonucleoside triphosphate binding;purine ribonucleotide binding;ribonucleoti
6-phosphofructokinase activity;adenyl nucleotide binding;adenyl ribonucleotide binding;ATP binding;binding;carbohydrate kinase activity;catalytic activity;cation binding;ion binding;kinase activity;metal ion binding;nucleotide
binding;damaged DNA binding;DNA binding;nucleic acid binding
binding;protein binding;receptor binding
ATPase activity;binding;catalytic activity;enzyme binding;hydrolase activity;hydrolase activity, acting on acid anhydrides;hydrolase activity, acting on acid anhydrides, in phosphorus-containing anhydrides;nucleic acid binding
binding;cadherin binding;cell adhesion molecule binding;protein binding;receptor binding
binding;nucleic acid binding;nucleotide binding;RNA binding;translation factor activity, nucleic acid binding;translation initiation factor activity
adenyl nucleotide binding;adenyl ribonucleotide binding;alanine-tRNA ligase activity;amine binding;amino acid binding;aminoacyl-tRNA editing activity;aminoacyl-tRNA ligase activity;ATP binding;binding;carboxylic acid bindi
aminopeptidase activity;binding;catalytic activity;cation binding;exopeptidase activity;hydrolase activity;ion binding;metal ion binding;metallopeptidase activity;peptidase activity;peptidase activity, acting on L-amino acid pep
binding;enzyme binding;kinase binding;phosphatase binding;protein binding;protein kinase binding;protein phosphatase binding
adenyl nucleotide binding;adenyl ribonucleotide binding;ATP binding;ATPase activity;ATPase activity, coupled;ATP-dependent DNA helicase activity;ATP-dependent helicase activity;binding;catalytic activity;cation binding;DN
adenyl nucleotide binding;adenyl ribonucleotide binding;ATP binding;ATPase activity;binding;binding, bridging;catalytic activity;cation binding;DNA binding;hydrolase activity;hydrolase activity, acting on acid anhydrides;hydro
binding;catalytic activity;GTP binding;GTPase activity;guanyl nucleotide binding;guanyl ribonucleotide binding;hydrolase activity;hydrolase activity, acting on acid anhydrides;hydrolase activity, acting on acid anhydrides, in ph
structural constituent of cytoskeleton;structural molecule activity
binding;calcium ion binding;carbohydrate binding;cation binding;collagen binding;collagen V binding;cytokine binding;extracellular matrix binding;fibrinogen binding;fibroblast growth factor binding;fibronectin binding;glycopr
structural molecule activity
enzyme activator activity;enzyme regulator activity;GDP-dissociation inhibitor activity;GTPase activator activity;GTPase regulator activity;nucleoside-triphosphatase regulator activity;Rab GDP-dissociation inhibitor activity;sm
adenyl nucleotide binding;adenyl ribonucleotide binding;ATP binding;binding;catalytic activity;DNA binding;DNA helicase activity;helicase activity;hydrolase activity;hydrolase activity, acting on acid anhydrides;hydrolase activ
adenyl nucleotide binding;adenyl ribonucleotide binding;ATP binding;binding;nucleotide binding;protein binding;purine nucleotide binding;purine ribonucleoside triphosphate binding;purine ribonucleotide binding;ribonucleoti
adenyl nucleotide binding;adenyl ribonucleotide binding;ATP binding;binding;nucleotide binding;protein binding;protein dimerization activity;protein heterodimerization activity;purine nucleotide binding;purine ribonucleoside t
catalytic activity;dolichyl-diphosphooligosaccharide-protein glycotransferase activity;oligosaccharyl transferase activity;transferase activity;transferase activity, transferring glycosyl groups;transferase activity, transferring hex
binding;calcium ion binding;calcium-dependent phospholipid binding;cation binding;enzyme inhibitor activity;enzyme regulator activity;ion binding;lipase inhibitor activity;lipid binding;metal ion binding;phospholipase inhibitor
adenyl nucleotide binding;adenyl ribonucleotide binding;ATP binding;binding;catalytic activity;chromatin binding;DNA binding;DNA helicase activity;DNA replication origin binding;helicase activity;hydrolase activity;hydrolase a
adenyl nucleotide binding;adenyl ribonucleotide binding;aminoacyl-tRNA ligase activity;ATP binding;binding;catalytic activity;chemokine receptor binding;CXCR chemokine receptor binding;cytokine receptor binding;G-protei
binding;MHC class II protein binding;MHC class II receptor activity;MHC protein binding;molecular transducer activity;protein binding;receptor activity;receptor binding;signal transducer activity;signaling receptor activity;stru
actin binding;actin filament binding;binding;cytoskeletal protein binding;drug binding;protein binding
binding;DNA binding;mRNA binding;nucleic acid binding;RNA binding
adenyl nucleotide binding;adenyl ribonucleotide binding;ATP binding;ATPase activity;ATPase activity, coupled;ATP-dependent helicase activity;ATP-dependent RNA helicase activity;binding;catalytic activity;double-stranded R
adenyl nucleotide binding;adenyl ribonucleotide binding;aminoacyl-tRNA editing activity;aminoacyl-tRNA ligase activity;ATP binding;binding;carboxylic ester hydrolase activity;catalytic activity;hydrolase activity;hydrolase act
adenyl nucleotide binding;adenyl ribonucleotide binding;ATP binding;ATPase activity;ATPase activity, coupled;ATP-dependent DNA helicase activity;ATP-dependent helicase activity;ATP-dependent RNA helicase activity;bind
catalytic activity;lactate dehydrogenase activity;L-lactate dehydrogenase activity;oxidoreductase activity;oxidoreductase activity, acting on CH-OH group of donors;oxidoreductase activity, acting on the CH-OH group of dono
adenyl nucleotide binding;adenyl ribonucleotide binding;amine binding;amino acid binding;argininosuccinate synthase activity;ATP binding;binding;carboxylic acid binding;catalytic activity;identical protein binding;ligase activ
active transmembrane transporter activity;adenyl nucleotide binding;adenyl ribonucleotide binding;ATP binding;ATPase activity;ATPase activity, coupled;ATPase activity, coupled to movement of substances;ATPase activity, c
adenyl nucleotide binding;adenyl ribonucleotide binding;ATP binding;binding;enzyme binding;nucleotide binding;protein binding;purine nucleotide binding;purine ribonucleoside triphosphate binding;purine ribonucleotide bin
structural molecule activity
binding;calcium-dependent protein binding;G-protein-coupled receptor binding;identical protein binding;protein binding;protein dimerization activity;protein homodimerization activity;proteinase activated receptor binding;rec
binding;nucleic acid binding;RNA binding;translation factor activity, nucleic acid binding;translation initiation factor activity
binding;ion channel binding;protein binding;receptor binding

actin binding;aldehyde-lyase activity;binding;carbohydrate binding;carbon-carbon lyase activity;catalytic activity;cytoskeletal protein binding;fructose binding;fructose-bisphosphate aldolase activity;identical protein binding;
structural molecule activity
adenyl nucleotide binding;adenyl ribonucleotide binding;ATP binding;beta-tubulin binding;binding;cytoskeletal protein binding;G-protein beta-subunit binding;nucleotide binding;protein binding;purine nucleotide binding;puri
acid-amino acid ligase activity;binding;catalytic activity;cation binding;chromo shadow domain binding;DNA binding;enzyme binding;ion binding;kinase activity;Krueppel-associated box domain binding;ligase activity;ligase a
binding;catalytic activity;core promoter binding;DNA binding;DNA polymerase activity;DNA-directed DNA polymerase activity;nucleic acid binding;nucleotidyltransferase activity;protein binding;protein binding transcription fa
3-hydroxyacyl-CoA dehydrogenase activity;acetyl-CoA C-acetyltransferase activity;acetyl-CoA C-acyltransferase activity;acetyltransferase activity;binding;C-acetyltransferase activity;C-acyltransferase activity;carbon-oxyge
adenyl nucleotide binding;adenyl ribonucleotide binding;ATP binding;binding;cytoskeletal protein binding;enzyme binding;identical protein binding;kinesin binding;nitric-oxide synthase binding;nucleotide binding;protein bindi
binding;profilin binding;protein binding
adenyl nucleotide binding;adenyl ribonucleotide binding;ATP binding;binding;nucleotide binding;purine nucleotide binding;purine ribonucleoside triphosphate binding;purine ribonucleotide binding;ribonucleotide binding
adenyl nucleotide binding;adenyl ribonucleotide binding;ATP binding;binding;catalytic activity;chromatin binding;dynein binding;hydrolase activity;hydrolase activity, acting on acid anhydrides;hydrolase activity, acting on acid
binding;catalytic activity;cation binding;GTP binding;guanyl nucleotide binding;guanyl ribonucleotide binding;ion binding;metal ion binding;nucleotide binding;protein-glutamine gamma-glutamyltransferase activity;purine nuc
adenyl nucleotide binding;adenyl ribonucleotide binding;ATP binding;binding;catalytic activity;DNA binding;DNA helicase activity;helicase activity;hydrolase activity;hydrolase activity, acting on acid anhydrides;hydrolase activ
binding;catalytic activity;GTP binding;GTPase activity;guanyl nucleotide binding;guanyl ribonucleotide binding;hydrolase activity;hydrolase activity, acting on acid anhydrides;hydrolase activity, acting on acid anhydrides, in p
adenyl nucleotide binding;adenyl ribonucleotide binding;ATP binding;binding;carbohydrate kinase activity;catalytic activity;fructokinase activity;glucokinase activity;hexokinase activity;kinase activity;mannokinase activity;nuc
adenyl nucleotide binding;adenyl ribonucleotide binding;ATP binding;binding;catalytic activity;DNA binding;DNA helicase activity;helicase activity;hydrolase activity;hydrolase activity, acting on acid anhydrides;hydrolase activ
alcohol binding;binding;calcium channel activity;calcium-release channel activity;cation channel activity;cation transmembrane transporter activity;channel activity;gated channel activity;inositol 1,3,4,5 tetrakisphosphate bind
ATPase activity;ATPase activity, coupled;binding;binding, bridging;calcium ion binding;calcium-dependent phospholipid binding;calcium-dependent protein binding;catalytic activity;cation binding;DNA binding;DNA-depende
binding;catalytic activity;coenzyme binding;cofactor binding;cytoskeletal protein binding;glyceraldehyde-3-phosphate dehydrogenase (NAD+) (phosphorylating) activity;identical protein binding;microtubule binding;NAD bindi
catalytic activity;intramolecular oxidoreductase activity;intramolecular oxidoreductase activity, interconverting keto- and enol-groups;intramolecular oxidoreductase activity, transposing S-S bonds;isomerase activity;protein d
binding;chromatin binding;core promoter binding;DNA binding;mRNA binding;nucleic acid binding;nucleotide binding;regulatory region DNA binding;regulatory region nucleic acid binding;RNA binding;single-stranded RNA bi
transporter activity
adenyl nucleotide binding;adenyl ribonucleotide binding;ATP binding;binding;catalytic activity;chromatin binding;hydrolase activity;hydrolase activity, acting on acid anhydrides;hydrolase activity, acting on acid anhydrides, in
adenyl nucleotide binding;adenyl ribonucleotide binding;ATP binding;ATPase activity;ATPase activity, coupled;binding;catalytic activity;hydrolase activity;hydrolase activity, acting on acid anhydrides;hydrolase activity, acting
acid-amino acid ligase activity;adenyl nucleotide binding;adenyl ribonucleotide binding;ATP binding;binding;catalytic activity;ligase activity;ligase activity, forming carbon-nitrogen bonds;nucleotide binding;purine nucleotide b
adenyl nucleotide binding;adenyl ribonucleotide binding;aminoacyl-tRNA ligase activity;ATP binding;binding;catalytic activity;identical protein binding;ligase activity;ligase activity, forming aminoacyl-tRNA and related compou
binding;nucleic acid binding;RNA binding;structural constituent of ribosome;structural molecule activity
binding;protein binding;scaffold protein binding;structural molecule activity
structural molecule activity
binding;catalytic activity;enzyme binding;GTP binding;GTPase activity;guanyl nucleotide binding;guanyl ribonucleotide binding;hydrolase activity;hydrolase activity, acting on acid anhydrides;hydrolase activity, acting on acid
adenyl nucleotide binding;adenyl ribonucleotide binding;androgen receptor binding;ATP binding;ATPase activity;ATPase activity, coupled;ATP-dependent helicase activity;ATP-dependent RNA helicase activity;binding;catalyti
acid-thiol ligase activity;adenyl nucleotide binding;adenyl ribonucleotide binding;ATP binding;ATP citrate synthase activity;binding;catalytic activity;cation binding;CoA-ligase activity;cofactor binding;ion binding;ligase activity
acid-amino acid ligase activity;binding;catalytic activity;cation binding;ion binding;ligase activity;ligase activity, forming carbon-nitrogen bonds;metal ion binding;small conjugating protein ligase activity;transition metal ion bin
actin binding;actin filament binding;adenyl nucleotide binding;adenyl ribonucleotide binding;ATP binding;binding;catalytic activity;cytoskeletal protein binding;hydrolase activity;hydrolase activity, acting on acid anhydrides;hy
active transmembrane transporter activity;adenyl nucleotide binding;adenyl ribonucleotide binding;ADP binding;alkali metal ion binding;ATP binding;ATPase activity;ATPase activity, coupled;ATPase activity, coupled to moven
adenyl nucleotide binding;adenyl ribonucleotide binding;ATP binding;binding;catalytic activity;cyclohydrolase activity;formate-tetrahydrofolate ligase activity;hydrolase activity;hydrolase activity, acting on carbon-nitrogen (but
adenyl nucleotide binding;adenyl ribonucleotide binding;ATP binding;ATPase activity;binding;catalytic activity;cytoskeletal protein binding;hydrolase activity;hydrolase activity, acting on acid anhydrides;hydrolase activity, acti
active transmembrane transporter activity;adenyl nucleotide binding;adenyl ribonucleotide binding;ATP binding;ATPase activity;ATPase activity, coupled;ATPase activity, coupled to movement of substances;ATPase activity, c
binding;nucleic acid binding;RNA binding;structural constituent of ribosome;structural molecule activity
alpha-glucosidase activity;binding;carbohydrate binding;catalytic activity;glucan 1,3-alpha-glucosidase activity;glucosidase activity;hydrolase activity;hydrolase activity, acting on glycosyl bonds;hydrolase activity, hydrolyzing
binding;core promoter binding;DNA binding;identical protein binding;nucleic acid binding;nucleotide binding;protein binding;regulatory region DNA binding;regulatory region nucleic acid binding;transcription regulatory region
adenyl nucleotide binding;adenyl ribonucleotide binding;ATP binding;ATPase activity;ATPase activity, coupled;ATP-dependent helicase activity;ATP-dependent RNA helicase activity;binding;catalytic activity;estrogen receptor
adenyl nucleotide binding;adenyl ribonucleotide binding;aminoacyl-tRNA editing activity;aminoacyl-tRNA ligase activity;ATP binding;binding;carboxylic ester hydrolase activity;catalytic activity;hydrolase activity;hydrolase act
binding;C2H2 zinc finger domain binding;protein binding;protein domain specific binding;protein N-terminus binding
binding;catalytic activity;cation binding;cofactor binding;identical protein binding;ion binding;metal ion binding;protein binding;protein dimerization activity;protein homodimerization activity;transferase activity;transferase acti
catalytic activity;cysteine-type endopeptidase activity;cysteine-type peptidase activity;disulfide oxidoreductase activity;endopeptidase activity;hydrolase activity;hydrolase activity, acting on ester bonds;intramolecular oxidore
actin-dependent ATPase activity;adenyl nucleotide binding;adenyl ribonucleotide binding;ATP binding;ATPase activity;ATPase activity, coupled;binding;catalytic activity;enzyme binding;GTPase binding;hydrolase activity;hydr
adenyl nucleotide binding;adenyl ribonucleotide binding;ATP binding;binding;catalytic activity;kinase activity;nucleotide binding;phosphoglycerate kinase activity;phosphotransferase activity, carboxyl group as acceptor;purin
adenyl nucleotide binding;adenyl ribonucleotide binding;aminoacyl-tRNA editing activity;aminoacyl-tRNA ligase activity;ATP binding;binding;carboxylic ester hydrolase activity;catalytic activity;hydrolase activity;hydrolase act
adenyl nucleotide binding;adenyl ribonucleotide binding;amine binding;amino acid binding;aspartate binding;aspartate carbamoyltransferase activity;ATP binding;binding;carbamoyl-phosphate synthase (ammonia) activity;ca
adenyl nucleotide binding;adenyl ribonucleotide binding;aminoacyl-tRNA editing activity;aminoacyl-tRNA ligase activity;ATP binding;binding;carboxylic ester hydrolase activity;catalytic activity;enzyme binding;GTPase binding
binding;chromatin binding
catalytic activity;endopeptidase activity;hydrolase activity;intramolecular oxidoreductase activity;intramolecular oxidoreductase activity, interconverting keto- and enol-groups;intramolecular oxidoreductase activity, transposin
actin binding;actin filament binding;binding;cell adhesion molecule binding;cytoskeletal protein binding;protein binding
adenyl nucleotide binding;adenyl ribonucleotide binding;ATP binding;ATPase activity;ATPase activity, coupled;ATP-dependent helicase activity;ATP-dependent RNA helicase activity;binding;catalytic activity;helicase activity;h
binding;carbohydrate binding;catalytic activity;glucose-6-phosphate isomerase activity;intramolecular oxidoreductase activity;intramolecular oxidoreductase activity, interconverting aldoses and ketoses;intramolecular transfe
structural molecule activity
adenyl nucleotide binding;adenyl ribonucleotide binding;ATP binding;binding;enzyme binding;nucleotide binding;protein binding;purine nucleotide binding;purine ribonucleoside triphosphate binding;purine ribonucleotide bin
adenyl nucleotide binding;adenyl ribonucleotide binding;ATP binding;binding;core promoter binding;DNA binding;nucleic acid binding;nucleotide binding;purine nucleotide binding;purine ribonucleoside triphosphate binding;
binding;core promoter binding;DNA binding;enzyme binding;histone deacetylase binding;nucleic acid binding;nucleotide binding;protein binding;regulatory region DNA binding;regulatory region nucleic acid binding;sequence
binding;carbohydrate binding;carboxylic acid binding;catalytic activity;glycoprotein binding;G-protein coupled receptor activity;identical protein binding;insulin-like growth factor-activated receptor activity;isoprenoid binding;
adenyl nucleotide binding;adenyl ribonucleotide binding;ADP binding;ATP binding;ATPase activity;binding;catalytic activity;enzyme activator activity;enzyme binding;enzyme regulator activity;hydrolase activity;hydrolase activ
binding;nucleic acid binding;RNA binding;translation factor activity, nucleic acid binding;translation initiation factor activity
binding;calcium ion binding;calcium-dependent phospholipid binding;calcium-dependent protein binding;cation binding;enzyme inhibitor activity;enzyme regulator activity;ion binding;lipase inhibitor activity;lipid binding;meta
adenyl nucleotide binding;adenyl ribonucleotide binding;antigen binding;ATP binding;ATPase activity;ATPase activity, coupled;binding;C3HC4-type RING finger domain binding;catalytic activity;enzyme binding;G-protein-cou
binding;DNA binding;identical protein binding;nucleic acid binding;nucleotide binding;protein binding;protein C-terminus binding;RNA binding;sequence-specific DNA binding;telomeric DNA binding
adenyl nucleotide binding;adenyl ribonucleotide binding;ATP binding;binding;nucleotide binding;protein binding;protein C-terminus binding;purine nucleotide binding;purine ribonucleoside triphosphate binding;purine ribonuc
adenyl nucleotide binding;adenyl ribonucleotide binding;ATP binding;ATPase activity;binding;calcium ion binding;catalytic activity;cation binding;chaperone binding;enzyme binding;glycoprotein binding;hydrolase activity;hyd
binding;catalytic activity;cofactor binding;glycogen phosphorylase activity;phosphorylase activity;pyridoxal phosphate binding;transferase activity;transferase activity, transferring glycosyl groups;transferase activity, transferri
adenyl nucleotide binding;adenyl ribonucleotide binding;ATP binding;ATPase activity;ATPase activity, coupled;ATP-dependent DNA helicase activity;ATP-dependent helicase activity;ATP-dependent RNA helicase activity;bind
binding;nucleic acid binding;RNA binding;structural constituent of ribosome;structural molecule activity
beta-tubulin binding;binding;cytoskeletal protein binding;DNA binding;enzyme binding;microtubule binding;nucleic acid binding;protein binding;RNA binding;single-stranded DNA binding;structure-specific DNA binding;tubuli
actin binding;binding;cytoskeletal protein binding;protein binding
binding;carbon-oxygen lyase activity;catalytic activity;cation binding;DNA binding;enzyme binding;GTPase binding;hydro-lyase activity;ion binding;lyase activity;magnesium ion binding;metal ion binding;nucleic acid binding;
binding;calcium ion binding;cation binding;cytoskeletal protein binding;double-stranded RNA binding;integrin binding;ion binding;ion channel binding;ligand-dependent nuclear receptor transcription coactivator activity;metal
actin binding;binding;calcium ion binding;cation binding;cytoskeletal protein binding;ion binding;metal ion binding;protein binding;structural constituent of cytoskeleton;structural molecule activity
catalytic activity;hydrolase activity;hydrolase activity, acting on ester bonds;nuclease activity;protein binding transcription factor activity;transcription cofactor activity;transcription factor binding transcription factor activity
adenyl nucleotide binding;adenyl ribonucleotide binding;ATP binding;ATPase activity;binding;catalytic activity;chaperone binding;DNA binding;DNA replication origin binding;double-stranded RNA binding;enzyme binding;hyd
apolipoprotein binding;binding;calcium ion binding;cation binding;ion binding;lipoprotein particle receptor binding;lipoprotein transporter activity;metal ion binding;protein binding;protein complex binding;protein transporter a
binding;calcium ion binding;calcium-dependent phospholipid binding;calcium-dependent protein binding;cation binding;channel activity;cholesterol binding;gated channel activity;GTP binding;guanyl nucleotide binding;guan
actin binding;alpha-catenin binding;beta-catenin binding;binding;cadherin binding;cell adhesion molecule binding;cytoskeletal protein binding;dystroglycan binding;enzyme binding;glycoprotein binding;protein binding;structu
structural molecule activity
binding;catalytic activity;K63-linked polyubiquitin binding;nucleic acid binding;polyubiquitin binding;protein binding;RNA binding;second spliceosomal transesterification activity;small conjugating protein binding;snRNA bindi
binding;lipid binding
binding;catalytic activity;cation binding;coenzyme binding;cofactor binding;DNA binding;enzyme binding;identical protein binding;ion binding;kinase binding;metal ion binding;NAD binding;NAD+ ADP-ribosyltransferase activi
adenyl nucleotide binding;adenyl ribonucleotide binding;ATP binding;binding;calcium ion binding;cation binding;enzyme binding;ion binding;lipoprotein particle receptor binding;low-density lipoprotein particle receptor bindin
binding;cell adhesion molecule binding;double-stranded RNA binding;enzyme binding;kinase binding;nucleic acid binding;protein binding;protein kinase binding;receptor binding;RNA binding;structural constituent of cytoske
adenyl nucleotide binding;adenyl ribonucleotide binding;ATP binding;binding;nucleotide binding;purine nucleotide binding;purine ribonucleoside triphosphate binding;purine ribonucleotide binding;ribonucleotide binding
binding;catalytic activity;enzyme binding;GTP binding;GTPase activity;guanyl nucleotide binding;guanyl ribonucleotide binding;hydrolase activity;hydrolase activity, acting on acid anhydrides;hydrolase activity, acting on acid
adenyl nucleotide binding;adenyl ribonucleotide binding;alkali metal ion binding;antigen binding;ATP binding;binding;catalytic activity;cation binding;ion binding;kinase activity;magnesium ion binding;metal ion binding;MHC c
binding;nucleic acid binding;nucleotide binding;protein binding;protein domain specific binding;RNA binding
binding;G-protein-coupled receptor binding;protein binding;receptor activity;receptor binding
adenyl deoxyribonucleotide binding;adenyl nucleotide binding;adenyl ribonucleotide binding;antigen binding;ATP binding;ATPase activity;binding;catalytic activity;CTP binding;dATP binding;deoxyribonucleotide binding;enzy
binding;catalytic activity;cation binding;cis-trans isomerase activity;enzyme binding;GTPase binding;ion binding;isomerase activity;ligase activity;metal ion binding;nucleic acid binding;peptidyl-prolyl cis-trans isomerase activ
binding;double-stranded RNA binding;glycoprotein binding;identical protein binding;nucleic acid binding;protein binding;protein C-terminus binding;RNA binding;scaffold protein binding;structural constituent of cytoskeleton;
binding;calcium ion binding;calmodulin binding;cation binding;enzyme activator activity;enzyme binding;enzyme inhibitor activity;enzyme regulator activity;GTPase activator activity;GTPase inhibitor activity;GTPase regulator
actin binding;actin filament binding;binding;calcium ion binding;cation binding;cytoskeletal protein binding;hormone receptor binding;identical protein binding;integrin binding;ion binding;ligand-dependent nuclear receptor tra
structural molecule activity
actin binding;actin filament binding;ATPase activity;binding;calcium ion binding;catalytic activity;cation binding;cytoskeletal protein binding;hydrolase activity;hydrolase activity, acting on acid anhydrides;hydrolase activity, ac
structural molecule activity
actin binding;ankyrin binding;binding;cytoskeletal protein binding;enzyme binding;GTPase binding;lipid binding;phospholipid binding;protein binding;structural constituent of cytoskeleton;structural molecule activity
structural molecule activity
adenyl nucleotide binding;adenyl ribonucleotide binding;antigen binding;ATP binding;binding;double-stranded RNA binding;enzyme binding;enzyme regulator activity;kinase binding;MHC class II protein complex binding;MH
binding;nucleic acid binding;RNA binding;structural molecule activity;translation factor activity, nucleic acid binding;translation initiation factor activity
binding;carbohydrate binding;glycosaminoglycan binding;heparin binding;pattern binding;polysaccharide binding
binding;nucleic acid binding;RNA binding;translation factor activity, nucleic acid binding
acid-amino acid ligase activity;binding;catalytic activity;DNA binding;ligase activity;ligase activity, forming carbon-nitrogen bonds;nucleic acid binding;small conjugating protein ligase activity;ubiquitin-protein ligase activity
binding;lipid binding;phospholipid binding
adenyl nucleotide binding;adenyl ribonucleotide binding;aminoacyl-tRNA ligase activity;ATP binding;binding;catalytic activity;enzyme binding;glutamate-tRNA ligase activity;GTPase binding;ligase activity;ligase activity, formi
receptor activity
actin binding;actin filament binding;actin-dependent ATPase activity;adenyl nucleotide binding;adenyl ribonucleotide binding;ADP binding;ATP binding;ATPase activity;ATPase activity, coupled;binding;catalytic activity;cytosk
structural molecule activity
binding;chromatin binding;cytoskeletal protein binding;dynein complex binding;enzyme binding;heat shock protein binding;identical protein binding;kinase binding;mitogen-activated protein kinase binding;mRNA binding;nuc
binding;clathrin binding;clathrin light chain binding;double-stranded RNA binding;enzyme binding;kinase binding;nucleic acid binding;protein binding;protein kinase binding;RNA binding;structural molecule activity
adenyl nucleotide binding;adenyl ribonucleotide binding;amine binding;amino acid binding;ATP binding;binding;calcium ion binding;carbamoyl-phosphate synthase (ammonia) activity;carbamoyl-phosphate synthase (glutami
actin binding;actin filament binding;binding;cytoskeletal protein binding;enzyme binding;Fc-gamma receptor I complex binding;glycoprotein binding;GTPase binding;identical protein binding;immunoglobulin receptor binding;
actin binding;binding;cytoskeletal protein binding;identical protein binding;protein binding
[acyl-carrier-protein] S-acetyltransferase activity;[acyl-carrier-protein] S-malonyltransferase activity;3-hydroxyacyl-[acyl-carrier-protein] dehydratase activity;3-hydroxyoctanoyl-[acyl-carrier-protein] dehydratase activity;3-hydr
binding;cytoskeletal protein binding;integrin binding;LIM domain binding;protein binding;protein complex binding;protein domain specific binding;receptor binding;structural constituent of cytoskeleton;structural molecule act
binding;binding, bridging;enzyme binding;kinase binding;protein binding;protein binding, bridging;protein kinase binding;protein kinase C binding;scaffold protein binding;structural constituent of cytoskeleton;structural molec

adenyl nucleotide binding;adenyl ribonucleotide binding;ATP binding;ATPase activity;binding;catalytic activity;hydrolase activity;hydrolase activity, acting on acid anhydrides;hydrolase activity, acting on acid anhydrides, in ph
adenyl nucleotide binding;adenyl ribonucleotide binding;ATP binding;binding;catalytic activity;DNA binding;DNA-dependent protein kinase activity;kinase activity;nucleic acid binding;nucleotide binding;phosphotransferase a
actin binding;actin filament binding;actin-dependent ATPase activity;adenyl nucleotide binding;adenyl ribonucleotide binding;ADP binding;ATP binding;ATPase activity;ATPase activity, coupled;binding;catalytic activity;cytosk
ankyrin binding;binding;cytoskeletal protein binding;protein binding;structural constituent of muscle;structural molecule activity

C: GOCC name	C: KEGG name	C: Organism	N: Protein Probability	N: Combined Total Peptides
cell part;cytoplasmic part;endosomal part;endosome membrane;intracellular organelle part;intracellular part;membrane;organelle membrane;organelle part;recycling endosome membrane	Endocytosis	Homo sapiens	0.9999	1
cell part;cytoplasmic part;endoplasmic reticulum membrane;endoplasmic reticulum part;integral to endoplasmic reticulum membrane;integral to membrane;integral to organelle membrane;intracellular organelle part;intracellu	Protein export	Homo sapiens	0.9999	1
		Homo sapiens	0.9999	2
		Homo sapiens	0.9999	2
cell part;cytoplasm;cytoplasmic mRNA processing body;cytoplasmic part;cytosol;intracellular membrane-bounded organelle;intracellular non-membrane-bounded organelle;intracellular organelle;intracellular part;macromolec	RNA degradation	Homo sapiens	0.9999	2
cell part;cytoplasmic part;intracellular membrane-bounded organelle;intracellular organelle;intracellular organelle part;intracellular part;macromolecular complex;membrane-bounded organelle;membrane-enclosed lumen;mitochondrial intermembrane space;mitochondrial part;mitochondrion;organelle;organelle envelope lumen;or		Homo sapiens	1	2
cell part;cytoplasmic part;intracellular membrane-bounded organelle;intracellular organelle;intracellular organelle part;intracellular part;membrane-bounded organelle;mitochondrion;nuclear part;nucleoplasm;organelle;organel	Glycine, serine and threonine metabolism	Homo sapiens	0.9999	2
cell part;cytoplasmic part;integral to membrane;intracellular membrane-bounded organelle;intracellular organelle;intracellular organelle part;intracellular part;intrinsic to membrane;macromolecular complex;membrane;membra	Alzheimer's disease;Huntington's disease;Oxidative phosphorylation;Parkinson's disease	Homo sapiens	0.9999	2
cell part;cytoplasmic part;extracellular membrane-bounded organelle;extracellular organelle;extracellular region part;extracellular vesicular exosome;integral to membrane;intracellular organelle part;intracellular part;intrinsic to	Alzheimer's disease;Huntington's disease;Oxidative phosphorylation;Parkinson's disease	Homo sapiens	0.9999	2
cell part;extracellular membrane-bounded organelle;extracellular organelle;extracellular region part;extracellular space;extracellular vesicular exosome;integral to membrane;integral to plasma membrane;intrinsic to membrane	Cytokine-cytokine receptor interaction	Homo sapiens	0.9999	2
cell part;cytoplasmic part;endoplasmic reticulum;endoplasmic reticulum membrane;endoplasmic reticulum part;integral to membrane;intracellular membrane-bounded organelle;intracellular organelle;intracellular organelle pa	Protein processing in endoplasmic reticulum	Homo sapiens	0.9999	2
cell part;integral to membrane;intrinsic to membrane;membrane part		Homo sapiens	1	2
cell part;cytoplasmic part;intracellular non-membrane-bounded organelle;intracellular organelle;intracellular organelle part;intracellular part;macromolecular complex;membrane;mitochondrial inner membrane;mitochondrial membrane;mitochondrial part;non-membrane-bounded organelle;organelle;organelle inner membrane;orga		Homo sapiens	0.9998	2
cell part;cytoplasm;cytoplasmic part;intracellular membrane-bounded organelle;intracellular organelle;intracellular organelle part;intracellular part;membrane-bounded organelle;membrane-enclosed lumen;mitochondrial interi	Oxidative phosphorylation	Homo sapiens	1	2
cell part;intracellular organelle part;intracellular part;macromolecular complex;nuclear part;nucleoplasm;organelle part;ribonucleoprotein complex;spliceosomal complex;U12-type spliceosomal complex	Spliceosome	Homo sapiens	0.9999	2
cell part;integral to membrane;intrinsic to membrane;membrane;membrane part;plasma membrane		Homo sapiens	0.9865	2
		Homo sapiens	0.9999	2
cell part;cytoplasmic part;intracellular membrane-bounded organelle;intracellular organelle;intracellular organelle part;intracellular part;macromolecular complex;membrane part;membrane-bounded organelle;mitochondrial membrane part;mitochondrial outer membrane translocase complex;mitochondrial part;mitochondrion;orga		Homo sapiens	0.9999	2
cell part;cytoplasmic part;cytosol;intracellular part		Homo sapiens	0.9998	2
cell part;cytoplasmic part;cytoskeletal part;cytosol;extracellular membrane-bounded organelle;extracellular organelle;extracellular region part;extracellular vesicular exosome;intracellular membrane-bounded organelle;intracellular non-membrane-bounded organelle;intracellular organelle;intracellular organelle part;intracellular par		Homo sapiens	0.9999	2
cell part;cytoplasmic part;endoplasmic reticulum membrane;endoplasmic reticulum part;extracellular membrane-bounded organelle;extracellular organelle;extracellular region part;extracellular vesicular exosome;integral to membrane;intracellular organelle part;intracellular part;intrinsic to membrane;membrane;membrane part;m		Homo sapiens	1	2
cell part;ER membrane protein complex;integral to membrane;intrinsic to membrane;macromolecular complex;membrane part;protein complex		Homo sapiens	0.9999	2
cell part;cis-Golgi network;cytoplasmic part;endoplasmic reticulum exit site;endoplasmic reticulum membrane;endoplasmic reticulum part;endoplasmic reticulum-Golgi intermediate compartment;endoplasmic reticulum-Golgi intermediate compartment membrane;Golgi apparatus;Golgi apparatus part;integral to membrane;intra		Homo sapiens	0.9998	2
		Homo sapiens	1	2
		Homo sapiens	0.9999	3
cell part;cytoplasmic part;endoplasmic reticulum membrane;endoplasmic reticulum part;Golgi apparatus;Golgi apparatus part;Golgi membrane;integral to membrane;intracellular membrane-bounded organelle;intracellular or	Glycerophospholipid metabolism;Inositol phosphate metabolism;Phosphatidylinositol signaling system	Homo sapiens	1	3
cell part;cytoplasm;cytoplasmic membrane-bounded vesicle;cytoplasmic part;cytoplasmic vesicle;cytoskeleton;extracellular membrane-bounded organelle;extracellular organelle;extracellular region part;extracellular vesicular exosome;Golgi apparatus part;intracellular membrane-bounded organelle;intracellular non-membrane-b		Homo sapiens	1	3
cell part;cytoplasmic part;integral to membrane;intracellular membrane-bounded organelle;intracellular organelle;intracellular organelle part;intracellular part;intrinsic to membrane;macromolecular complex;membrane;membra	Alzheimer's disease;Huntington's disease;Oxidative phosphorylation;Parkinson's disease	Homo sapiens	1	3
cell part;cytoplasm;cytoplasmic dynein complex;cytoplasmic part;cytoskeletal part;dynein complex;early endosome membrane;endosomal part;endosome membrane;extrinsic to membrane;intracellular organelle part;intracellular part;macromolecular complex;membrane;membrane part;microtubule associated complex;organelle		Homo sapiens	1	3
cell part;cytoplasmic part;intracellular membrane-bounded organelle;intracellular organelle;intracellular organelle lumen;intracellular organelle part;intracellular part;membrane-bounded organelle;membrane-enclosed lumen;m	Aminoacyl-tRNA biosynthesis	Homo sapiens	1	3
basement membrane;cell part;cytoplasmic membrane-bounded vesicle lumen;cytoplasmic part;cytoplasmic vesicle part;extracellular matrix;extracellular matrix part;extracellular membrane-bounded organelle;extracellular organelle;extracellular region;extracellular region part;extracellular space;extracellular vesicular exosome;in		Homo sapiens	1	3
cell part;chromatin;chromosomal part;cytoplasm;intracellular membrane-bounded organelle;intracellular organelle;intracellular organelle part;intracellular part;membrane-bounded organelle;nucleus;organelle;organelle part		Homo sapiens	1	3
cell part;cytoplasmic membrane-bounded vesicle lumen;cytoplasmic microtubule;cytoplasmic part;cytoplasmic vesicle part;cytoskeletal part;endocytic vesicle lumen;extracellular membrane-bounded organelle;extracellular organelle;extracellular region;extracellular region part;extracellular space;extracellular vesicular exosome;I		Homo sapiens	1	3
adherens junction;anchoring junction;cell junction;cell part;cell surface;cell-substrate adherens junction;cell-substrate junction;cytoplasm;cytoplasmic part;external side of plasma membrane;extracellular membrane-bounded	Cell adhesion molecules (CAMs);ECM-receptor interaction;Malaria	Homo sapiens	1	3
	Retinol metabolism	Homo sapiens	1	3
cell part;cytoplasmic membrane-bounded vesicle;cytoplasmic part;cytoplasmic vesicle;cytoplasmic vesicle membrane;cytoplasmic vesicle part;endocytic vesicle;endocytic vesicle membrane;endoplasmic reticulum membrar	Measles	Homo sapiens	1	3
cell part;cytoplasmic part;extracellular membrane-bounded organelle;extracellular organelle;extracellular region part;extracellular vesicular exosome;integral to membrane;intracellular membrane-bounded organelle;intracellula	Oxidative phosphorylation	Homo sapiens	1	3
cell part;cytoplasmic part;intracellular membrane-bounded organelle;intracellular organelle;intracellular organelle part;intracellular part;macromolecular complex;membrane;membrane part;membrane-bounded organelle;mitoc	Alzheimer's disease;Huntington's disease;Oxidative phosphorylation;Parkinson's disease	Homo sapiens	1	3
cell part;integral to membrane;intrinsic to membrane;membrane part		Homo sapiens	0.9974	3
cell part;cytoplasmic part;endoplasmic reticulum;endoplasmic reticulum membrane;endoplasmic reticulum part;integral to membrane;integral to plasma membrane;intracellular membrane-bounded organelle;intracellular orga	Steroid biosynthesis	Homo sapiens	1	3
cell part;cytoplasm;cytoplasmic part;intracellular part;macromolecular complex;NatC complex;N-terminal protein acetyltransferase complex;protein acetyltransferase complex;protein complex		Homo sapiens	1	3
		Homo sapiens	1	3
		Homo sapiens	1	3
cell part;extracellular membrane-bounded organelle;extracellular organelle;extracellular region part;extracellular vesicular exosome;intracellular organelle part;intracellular part;membrane-bounded organelle;membrane-bounded vesicle;nuclear part;nucleoplasm;organelle;organelle part;vesicle		Homo sapiens	1	3
cell part;cytoplasmic part;intracellular membrane-bounded organelle;intracellular organelle;intracellular organelle part;intracellular part;membrane-bounded organelle;membrane-enclosed lumen;mitochondrial intermembrane space;mitochondrial part;mitochondrion;organelle;organelle envelope lumen;organelle part		Homo sapiens	1	3
cell part;cytoplasm;cytoplasmic membrane-bounded vesicle;cytoplasmic part;cytoplasmic vesicle;extracellular membrane-bounded organelle;extracellular organelle;extracellular region part;extracellular vesicular exosome;Golgi apparatus;integral to membrane;intracellular membrane-bounded organelle;intracellular organelle;intr		Homo sapiens	1	3
cell part;centrosome;cytoplasm;cytoplasmic part;cytoskeletal part;endosomal part;endosome membrane;ESCRT I complex;intracellular membrane-bounded organelle;intracellular non-membrane-bounded organelle;intracellu	Endocytosis	Homo sapiens	1	3
AP-type membrane coat adaptor complex;cell junction;cell part;cell projection;clathrin adaptor complex;clathrin-coated vesicle;coated vesicle;cytoplasm;cytoplasmic membrane-bounded vesicle;cytoplasmic part;cytoplasmic vesicle;intracellular membrane-bounded organelle;intracellular non-membrane-bounded organelle;intra		Homo sapiens	1	3
cell part;cytoplasm;intracellular membrane-bounded organelle;intracellular organelle;intracellular part;membrane-bounded organelle;nucleus;organelle		Homo sapiens	1	3
cell part;cytoplasmic part;intracellular organelle lumen;intracellular organelle part;intracellular part;membrane-enclosed lumen;mitochondrial matrix;mitochondrial part;organelle lumen;organelle part	Porphyrin and chlorophyll metabolism	Homo sapiens	1	3
cell part;cytoplasmic part;intracellular non-membrane-bounded organelle;intracellular organelle;intracellular organelle part;intracellular part;macromolecular complex;membrane;mitochondrial inner membrane;mitochondrial membrane;mitochondrial part;mitochondrial ribosome;non-membrane-bounded organelle;organellar ribos		Homo sapiens	1	3
cell junction;cell part;cytoplasm;cytoskeletal part;intracellular organelle part;intracellular part;membrane;organelle part;postsynaptic density;postsynaptic membrane;synapse part;synaptic membrane		Homo sapiens	0.9941	3
cell part;cytoplasm;cytoplasmic part;extracellular membrane-bounded organelle;extracellular organelle;extracellular region part;extracellular vesicular exosome;intracellular membrane-bounded organelle;intracellular organelle;intracellular organelle part;intracellular part;macromolecular complex;membrane-bounded organelle;me		Homo sapiens	1	3
cell part;cytoplasm;intracellular part;membrane		Homo sapiens	1	3
cell part;cytoplasmic part;intracellular non-membrane-bounded organelle;intracellular organelle;intracellular organelle part;intracellular part;macromolecular complex;membrane;mitochondrial inner membrane;mitochondrial membrane;mitochondrial part;mitochondrial ribosome;non-membrane-bounded organelle;organellar ribos		Homo sapiens	1	3
cell part;cytoplasm;cytoplasmic part;intracellular membrane-bounded organelle;intracellular organelle;intracellular part;membrane-bounded organelle;mitochondrion;nucleus;organelle		Homo sapiens	1	3
cell part;cytoplasm;cytoplasmic part;cytosol;endoplasmic reticulum;intracellular membrane-bounded organelle;intracellular organelle;intracellular part;membrane-bounded organelle;mitochondrion;organelle	Nicotinate and nicotinamide metabolism;Purine metabolism;Pyrimidine metabolism	Homo sapiens	1	3
cell part;cytoplasm;extracellular membrane-bounded organelle;extracellular organelle;extracellular region part;extracellular vesicular exosome;intracellular part;membrane;membrane-bounded organelle;membrane-bounded vesicle;organelle;plasma membrane;vesicle		Homo sapiens	1	3
cell part;integral to membrane;intrinsic to membrane;membrane part		Homo sapiens	1	3
cell part;cytoplasmic part;intracellular non-membrane-bounded organelle;intracellular organelle;intracellular organelle lumen;intracellular organelle part;intracellular part;membrane-enclosed lumen;mitochondrial matrix;mitochondrial nucleoid;mitochondrial part;non-membrane-bounded organelle;nucleoid;organelle;organelle lume		Homo sapiens	1	3
cell part;cytoplasm;intracellular organelle part;intracellular part;nuclear part;nucleoplasm;organelle part		Homo sapiens	1	3
		Homo sapiens	1	3
box H/ACA RNP complex;Cajal body;cell part;intracellular non-membrane-bounded organelle;intracellular organelle;intracellular organelle part;intracellular part;macromolecular complex;non-membrane-bounded organelle;nu	Ribosome biogenesis in eukaryotes	Homo sapiens	1	3
Ada2/Gcn5/Ada3 transcription activator complex;cell part;chromosomal part;DNA polymerase complex;epsilon DNA polymerase complex;histone acetyltransferase complex;intracellular membrane-bounded organelle;intrace	Base excision repair;DNA replication;Nucleotide excision repair;Purine metabolism;Pyrimidine metabolis	Homo sapiens	1	3
cell part;cytoplasmic part;integral to membrane;intracellular membrane-bounded organelle;intracellular organelle;intracellular organelle part;intracellular part;intrinsic to membrane;macromolecular complex;membrane;membrane part;membrane-bounded organelle;mitochondrial inner membrane;mitochondrial membrane;mitocho		Homo sapiens	1	3
cell part;chromatin;chromosomal part;intracellular membrane-bounded organelle;intracellular organelle;intracellular organelle part;intracellular part;membrane-bounded organelle;nucleus;organelle;organelle part		Homo sapiens	0.9999	4
cell part;cytoplasmic part;intracellular organelle part;intracellular part;membrane;membrane part;mitochondrial inner membrane;mitochondrial membrane;mitochondrial membrane part;mitochondrial part;mitochondrial respira	Alzheimer's disease;Cardiac muscle contraction;Huntington's disease;Oxidative phosphorylation;Parkin	Homo sapiens	1	4
cell part;cytoplasmic part;endoplasmic reticulum;endoplasmic reticulum membrane;endoplasmic reticulum part;integral to membrane;integral to plasma membrane;intracellular membrane-bounded organelle;intracellular orga	Sphingolipid metabolism	Homo sapiens	1	4
cell part;cytoplasmic part;intracellular non-membrane-bounded organelle;intracellular organelle;intracellular organelle part;intracellular part;macromolecular complex;membrane;mitochondrial inner membrane;mitochondrial membrane;mitochondrial part;mitochondrial ribosome;non-membrane-bounded organelle;organellar ribos		Homo sapiens	1	4
cell part;cytoplasmic part;intracellular membrane-bounded organelle;intracellular organelle;intracellular organelle part;intracellular part;macromolecular complex;membrane;membrane part;membrane-bounded organelle;mitoc	Alzheimer's disease;Huntington's disease;Oxidative phosphorylation;Parkinson's disease	Homo sapiens	1	4
cell part;integral to membrane;intrinsic to membrane;membrane;membrane part;plasma membrane	Nitrogen metabolism	Homo sapiens	1	4
		Homo sapiens	1	4
		Homo sapiens	1	4
cell part;cytoplasmic part;cytosol;intracellular part;macromolecular complex;mRNA cap binding complex;protein complex;RNA cap binding complex	Insulin signaling pathway;mTOR signaling pathway;RNA transport	Homo sapiens	1	4
cell body;cell part;cell projection;cytoplasmic part;intracellular organelle part;intracellular part;macromolecular complex;membrane;membrane part;mitochondrial inner membrane;mitochondrial membrane;mitochondrial mem	Alzheimer's disease;Huntington's disease;Oxidative phosphorylation;Parkinson's disease	Homo sapiens	1	4
cell part;cytoplasmic part;integral to membrane;intracellular;intracellular membrane-bounded organelle;intracellular non-membrane-bounded organelle;intracellular organelle;intracellular organelle part;intracellular part;intrinsic	Peroxisome	Homo sapiens	1	4
actin cytoskeleton;cell part;cytoplasm;cytoplasmic part;cytoskeleton;intermediate filament cytoskeleton;intracellular membrane-bounded organelle;intracellular non-membrane-bounded organelle;intracellular organelle;intracellular organelle lumen;intracellular organelle part;intracellular part;membrane;membrane-bounded organe		Homo sapiens	1	4
cell part;contractile fiber;cytoplasmic part;intracellular membrane-bounded organelle;intracellular non-membrane-bounded organelle;intracellular organelle;intracellular organelle part;intracellular part;membrane;membrane-bounded organelle;mitochondrial inner membrane;mitochondrial membrane;mitochondrial part;mitochondr		Homo sapiens	1	4
cell part;cell projection;cytoplasm;cytoplasmic part;dendrite;DNA-directed RNA polymerase complex;DNA-directed RNA polymerase II, core complex;intracellular membrane-bounded organelle;intracellular organelle;intracellular organelle part;intracellular part;macromolecular complex;membrane-bounded organelle;mitochondric		Homo sapiens	1	4
cell part;intracellular organelle part;intracellular part;macromolecular complex;nuclear part;organelle part;ribonucleoprotein complex;small nuclear ribonucleoprotein complex;spliceosomal complex;U4/U6 x U5 tri-snRNP com	RNA degradation;Spliceosome	Homo sapiens	1	4
blood microparticle;extracellular membrane-bounded organelle;extracellular organelle;extracellular region;extracellular region part;extracellular space;extracellular vesicular exosome;membrane-bounded organelle;membrane-bounded vesicle;organelle;vesicle		Homo sapiens	1	4
apical plasma membrane;basal part of cell;basal plasma membrane;blood microparticle;cell part;cell surface;coated pit;cytoplasmic membrane-bounded vesicle;cytoplasmic membrane-bounded vesicle lumen;cytoplasmic p	Mineral absorption	Homo sapiens	1	4
cell part;cytoplasm;extracellular membrane-bounded organelle;extracellular organelle;extracellular region part;extracellular vesicular exosome;intracellular part;membrane-bounded organelle;membrane-bounded vesicle;orga	Drug metabolism - cytochrome P450;Glutathione metabolism;Metabolism of xenobiotics by cytochrome	Homo sapiens	1	4
cell part;cytoplasmic part;intracellular organelle part;intracellular part;macromolecular complex;membrane;membrane part;mitochondrial inner membrane;mitochondrial membrane;mitochondrial membrane part;mitochondrial	Alzheimer's disease;Cardiac muscle contraction;Huntington's disease;Oxidative phosphorylation;Parkin	Homo sapiens	1	4
adherens junction;anchoring junction;cell junction;cell part;cell-substrate adherens junction;cell-substrate junction;cytoplasm;focal adhesion;integral to membrane;integral to plasma membrane;intracellular part;intrinsic to me	Cell adhesion molecules (CAMs);Leukocyte transendothelial migration	Homo sapiens	1	4
cell part;cytoplasmic part;intracellular membrane-bounded organelle;intracellular organelle;intracellular organelle part;intracellular part;macromolecular complex;membrane;membrane part;membrane-bounded organelle;mem	Alzheimer's disease;Huntington's disease;Oxidative phosphorylation;Parkinson's disease	Homo sapiens	1	4
adherens junction;anchoring junction;apical plasma membrane;basolateral plasma membrane;cell junction;cell part;cell projection;cell surface;cell-cell contact zone;cell-cell junction;cell-substrate adherens junction;cell-subst	Bile secretion;Cardiac muscle contraction;Gastric acid secretion;Pancreatic secretion;Regulation of acti	Homo sapiens	1	4
cell part;cytoplasmic part;integral to membrane;intracellular organelle part;intracellular part;intrinsic to membrane;membrane;membrane part;mitochondrial inner membrane;mitochondrial membrane;mitochondrial membrane	Alzheimer's disease;Cardiac muscle contraction;Huntington's disease;Oxidative phosphorylation;Parkin	Homo sapiens	1	4
cell part;cytoplasm;cytoplasmic part;extracellular membrane-bounded organelle;extracellular organelle;extracellular region part;extracellular vesicular exosome;integral to membrane;intracellular membrane-bounded organelle	Neuroactive ligand-receptor interaction	Homo sapiens	1	4
		Homo sapiens	1	4
cell part;cytoplasm;intracellular membrane-bounded organelle;intracellular organelle;intracellular part;membrane-bounded organelle;nucleus;organelle	RNA transport	Homo sapiens	1	4
cell part;cytoplasmic part;cytosol;intracellular part;macromolecular complex;membrane;phosphorylase kinase complex;plasma membrane;protein complex	Calcium signaling pathway;Insulin signaling pathway	Homo sapiens	0.9979	4
cell part;chromatin;chromosomal part;chromosome;heterochromatin;intracellular membrane-bounded organelle;intracellular non-membrane-bounded organelle;intracellular organelle;intracellular organelle part;intracellular part;macromolecular complex;membrane-bounded organelle;non-membrane-bounded organelle;nuclear ch		Homo sapiens	1	4
cell part;cytoplasmic part;intracellular part;macromolecular complex;protein complex;Rab-protein geranylgeranyltransferase complex		Homo sapiens	1	4
AP-2 adaptor complex;AP-type membrane coat adaptor complex;cell part;clathrin adaptor complex;clathrin coated vesicle membrane;clathrin-coated endocytic vesicle membrane;coated vesicle membrane;cytoplasmic part;	Endocrine and other factor-regulated calcium reabsorption;Endocytosis;Huntington's disease	Homo sapiens	1	4
cell part;cytoplasmic part;integral to membrane;intracellular organelle part;intracellular part;intrinsic to membrane;macromolecular complex;membrane part;mitochondrial membrane part;mitochondrial part;mitochondrial proton-transporting ATP synthase complex;organelle part;protein complex;proton-transporting ATP synthase		Homo sapiens	1	4
cell part;cytoplasmic part;intracellular organelle part;intracellular part;macromolecular complex;membrane;membrane part;mitochondrial inner membrane;mitochondrial membrane;mitochondrial membrane part;mitochondrial	Alzheimer's disease;Huntington's disease;Oxidative phosphorylation;Parkinson's disease	Homo sapiens	1	4
cell part;cytoplasmic part;endoplasmic reticulum membrane;endoplasmic reticulum part;integral to membrane;intracellular organelle part;intracellular part;intrinsic to membrane;membrane;membrane part;organelle membrane	Steroid biosynthesis;Steroid hormone biosynthesis	Homo sapiens	1	4
cell part;cytoplasmic part;integral to membrane;intracellular membrane-bounded organelle;intracellular organelle;intracellular organelle part;intracellular part;intrinsic to membrane;membrane;membrane part;membrane-bounded organelle;mitochondrial inner membrane;mitochondrial membrane;mitochondrial part;mitochondrion;		Homo sapiens	1	4
catalytic step 2 spliceosome;cell part;cytoplasmic part;cytosol;extracellular membrane-bounded organelle;extracellular organelle;extracellular region part;extracellular vesicular exosome;intracellular membrane-bounded orga	Spliceosome	Homo sapiens	1	4
cell part;extracellular membrane-bounded organelle;extracellular organelle;extracellular region part;extracellular vesicular exosome;extrinsic to internal side of plasma membrane;extrinsic to membrane;extrinsic to plasma me	Chemokine signaling pathway	Homo sapiens	1	4
cell part;cytoplasmic part;intracellular membrane-bounded organelle;intracellular organelle;intracellular organelle part;intracellular part;macromolecular complex;membrane;membrane-bounded organelle;mitochondrial inner membrane;mitochondrial membrane;mitochondrial part;mitochondrial small ribosomal subunit;mitochond		Homo sapiens	1	4
cell part;cytoplasm;cytoplasmic part;intracellular membrane-bounded organelle;intracellular organelle;intracellular organelle lumen;intracellular organelle part;intracellular part;membrane;membrane-bounded organelle;membr	Butanoate metabolism;Synthesis and degradation of ketone bodies	Homo sapiens	1	4
cell junction;cell part;intracellular membrane-bounded organelle;intracellular organelle;intracellular organelle part;intracellular part;membrane-bounded organelle;nuclear part;nucleoplasm;nucleus;organelle;organelle part		Homo sapiens	1	4
cell part;cytoplasmic part;endoplasmic reticulum;endoplasmic reticulum membrane;endoplasmic reticulum part;endoplasmic reticulum-Golgi intermediate compartment;endoplasmic reticulum-Golgi intermediate compartment membrane;Golgi apparatus;integral to membrane;intracellular membrane-bounded organelle;intracellul		Homo sapiens	1	4
cell part;cytoplasm;cytoplasmic part;cytosol;intracellular membrane-bounded organelle;intracellular organelle;intracellular organelle part;intracellular part;membrane-bounded organelle;nuclear part;nucleoplasm;nucleus;orga	Cytosolic DNA-sensing pathway;Hepatitis C;Measles;RIG-I-like receptor signaling pathway;Toll-like rece	Homo sapiens	1	4
axon part;cell leading edge;cell part;cell projection part;clathrin-coated vesicle;coated pit;coated vesicle;cytoplasmic membrane-bounded vesicle;cytoplasmic part;cytoplasmic vesicle;extrinsic to membrane;extrinsic to plasma membrane;intracellular membrane-bounded organelle;intracellular organelle;intracellular part;membrai		Homo sapiens	1	4
		Homo sapiens	1	4
cell part;cytoplasmic part;intracellular membrane-bounded organelle;intracellular organelle;intracellular organelle part;intracellular part;membrane;membrane-bounded organelle;mitochondrial inner membrane;mitochondrial m	Ubiquinone and other terpenoid-quinone biosynthesis	Homo sapiens	1	4
cell part;centrosome;cytoplasm;cytoplasmic part;cytoskeletal part;intracellular non-membrane-bounded organelle;intracellular organelle;intracellular organelle part;intracellular part;membrane;microtubule organizing center;non-membrane-bounded organelle;nuclear part;nucleoplasm;organelle;organelle part;spindle		Homo sapiens	0.9989	4
cell part;cytoplasm;cytoplasmic part;cytosol;extracellular membrane-bounded organelle;extracellular organelle;extracellular region part;extracellular vesicular exosome;intracellular part;membrane-bounded organelle;membrane-bounded vesicle;organelle;vesicle		Homo sapiens	1	4
cell part;cytoplasmic part;intracellular non-membrane-bounded organelle;intracellular organelle;intracellular organelle part;intracellular part;macromolecular complex;membrane;mitochondrial inner membrane;mitochondrial membrane;mitochondrial part;non-membrane-bounded organelle;organelle;organelle inner membrane;orga		Homo sapiens	1	4
cell part;cytoplasmic part;Golgi apparatus;intracellular membrane-bounded organelle;intracellular organelle;intracellular part;membrane-bounded organelle;organelle		Homo sapiens	1	4
cell part;cytoplasmic part;intracellular membrane-bounded organelle;intracellular organelle;intracellular part;membrane-bounded organelle;mitochondrion;organelle		Homo sapiens	1	4
cell part;cytoplasmic part;intracellular membrane-bounded organelle;intracellular organelle;intracellular part;membrane-bounded organelle;mitochondrion;nucleus;organelle		Homo sapiens	1	4
cell part;membrane;plasma membrane		Homo sapiens	1	4
cell part;cytoplasmic part;cytosol;integral to membrane;integral to mitochondrial membrane;integral to mitochondrial outer membrane;integral to organelle membrane;intracellular membrane-bounded organelle;intracellular organelle;intracellular organelle part;intracellular part;intrinsic to membrane;intrinsic to mitochondrial outer		Homo sapiens	1	4
calcium channel complex;cation channel complex;cell part;cytoplasmic part;intracellular membrane-bounded organelle;intracellular organelle;intracellular organelle part;intracellular part;ion channel complex;macromolecular complex;membrane part;membrane-bounded organelle;membrane-enclosed lumen;mitochondrial interm		Homo sapiens	1	4
cell part;cytoplasmic part;intracellular membrane-bounded organelle;intracellular organelle;intracellular part;membrane-bounded organelle;mitochondrion;organelle		Homo sapiens	1	4
cell part;cytoplasmic part;early endosome;early endosome membrane;endosomal part;endosome;endosome membrane;ER membrane protein complex;Golgi apparatus;Golgi apparatus part;Golgi membrane;integral to membrane;intracellular membrane-bounded organelle;intracellular organelle;intracellular organelle part;intrace		Homo sapiens	1	4
cell part;cytoplasmic part;intracellular non-membrane-bounded organelle;intracellular organelle;intracellular organelle part;intracellular part;macromolecular complex;membrane;mitochondrial inner membrane;mitochondrial membrane;mitochondrial part;non-membrane-bounded organelle;organelle;organelle inner membrane;orga		Homo sapiens	1	4
cell part;cytoplasmic part;intracellular organelle part;intracellular part;membrane;mitochondrial inner membrane;mitochondrial membrane;mitochondrial part;organelle inner membrane;organelle membrane;organelle part		Homo sapiens	1	4
		Homo sapiens	1	4
cell part;cytoplasmic membrane-bounded vesicle;cytoplasmic part;cytoplasmic vesicle;cytosol;extracellular membrane-bounded organelle;extracellular organelle;extracellular region part;extracellular space;extracellular vesic	Folate biosynthesis	Homo sapiens	1	4
cell part;cytoplasm;cytoplasmic part;intracellular membrane-bounded organelle;intracellular organelle;intracellular part;membrane-bounded organelle;mitochondrion;nucleus;organelle		Homo sapiens	1	4
cell part;cytoplasmic part;intracellular membrane-bounded organelle;intracellular organelle;intracellular part;macromolecular complex;membrane-bounded organelle;methyltransferase complex;nucleus;organelle;protein complex;tRNA (m1A) methyltransferase complex;tRNA methyltransferase complex		Homo sapiens	1	4

cell part;cytoplasm;cytoplasmic part;cytosol;endosomal part;endosome membrane;ESCRT II complex;extracellular membrane-bounded organelle;extracellular organelle;extracellular region part;extracellular vesicular exosom	Endocytosis	Homo sapiens	1	4
		Homo sapiens	1	4
cell part;cytoplasmic part;intracellular membrane-bounded organelle;intracellular organelle;intracellular organelle part;intracellular part;membrane;membrane-bounded organelle;mitochondrial inner membrane;mitochondrial membrane;mitochondrial part;nucleus;organelle;organelle inner membrane;organelle membrane;organelle		Homo sapiens	1	4
cell part;cytoplasm;intracellular membrane-bounded organelle;intracellular organelle;intracellular part;membrane-bounded organelle;nucleus;organelle		Homo sapiens	1	4
cell part;envelope;intracellular organelle part;intracellular part;macromolecular complex;membrane;membrane part;nuclear envelope;nuclear membrane;nuclear part;nuclear pore;organelle envelope;organelle membrane;orgar	RNA transport	Homo sapiens	1	4
cell part;cytoplasmic part;endosome;extracellular membrane-bounded organelle;extracellular organelle;extracellular region part;extracellular space;extracellular vesicular exosome;Golgi apparatus part;intracellular membrane-bounded organelle;intracellular organelle;intracellular organelle part;intracellular part;late endosome;lyso		Homo sapiens	1	4
cell part;cytoplasmic part;intracellular membrane-bounded organelle;intracellular organelle;intracellular organelle part;intracellular part;large ribosomal subunit;macromolecular complex;membrane;membrane-bounded organelle;mitochondrial inner membrane;mitochondrial membrane;mitochondrial part;mitochondrion;nuclear pa		Homo sapiens	1	4
cell part;intracellular membrane-bounded organelle;intracellular organelle;intracellular part;membrane-bounded organelle;nucleus;organelle		Homo sapiens	1	4
cell part;intracellular membrane-bounded organelle;intracellular organelle;intracellular part;membrane-bounded organelle;nucleus;organelle		Homo sapiens	1	4
Atg12-Atg5-Atg16 complex;autophagic vacuole;axoneme;cell part;cell projection part;cytoplasm;cytoplasmic part;endoplasmic reticulum part;ER-mitochondrion membrane contact site;intracellular membrane-bounded orgar	Regulation of autophagy;RIG-I-like receptor signaling pathway;Shigellosis	Homo sapiens	1	4
cell part;cytoplasmic part;intracellular membrane-bounded organelle;intracellular organelle;intracellular part;membrane-bounded organelle;mitochondrion;organelle		Homo sapiens	1	4
cell part;intracellular membrane-bounded organelle;intracellular non-membrane-bounded organelle;intracellular organelle;intracellular organelle part;intracellular part;membrane-bounded organelle;non-membrane-bounded organelle;nuclear part;nucleolus;nucleoplasm;nucleus;organelle;organelle part		Homo sapiens	1	4
cell part;cytoplasm;cytoplasmic part;EGO complex;intracellular membrane-bounded organelle;intracellular organelle;intracellular organelle part;intracellular part;lysosome;lytic vacuole;macromolecular complex;membrane part;membrane-bounded organelle;nuclear part;nucleoplasm;nucleus;organelle;organelle part;protein comp		Homo sapiens	1	4
ATP-binding cassette (ABC) transporter complex;cell part;cytoplasmic part;endoplasmic reticulum;endosome;envelope;extracellular membrane-bounded organelle;extracellular organelle;extracellular region part;extracellular v	ABC transporters	Homo sapiens	1	4
cell part;cell projection;cell projection part;cytoplasm;dendritic spine;intracellular part;macromolecular complex;membrane;neuron projection;neuron spine;protein complex		Homo sapiens	0.9978	4
cell part;cytoplasm;intracellular membrane-bounded organelle;intracellular organelle;intracellular part;membrane-bounded organelle;nucleus;organelle	Atrazine degradation	Homo sapiens	1	4
cell part;cytoplasmic membrane-bounded vesicle;cytoplasmic part;cytoplasmic vesicle;intracellular membrane-bounded organelle;intracellular organelle;intracellular organelle part;intracellular part;membrane;membrane-bounded organelle;membrane-bounded vesicle;mitochondrial inner membrane;mitochondrial membrane;mitc		Homo sapiens	1	4
cell part;cytoplasm;intracellular membrane-bounded organelle;intracellular organelle;intracellular part;membrane-bounded organelle;nucleus;organelle		Homo sapiens	1	4
cell part;cytoplasmic part;endoplasmic reticulum;endoplasmic reticulum membrane;endoplasmic reticulum part;Golgi apparatus part;Golgi membrane;integral to membrane;intracellular membrane-bounded organelle;intracell	Phagosome;SNARE interactions in vesicular transport	Homo sapiens	1	4
cell part;Cul2-RING ubiquitin ligase complex;Cul3-RING ubiquitin ligase complex;CUL4 RING ubiquitin ligase complex;Cul5-RING ubiquitin ligase complex;Cul7-RING ubiquitin ligase complex;cullin-RING ubiquitin ligase com	Ubiquitin mediated proteolysis	Homo sapiens	1	4
cell part;cytoplasm;extracellular membrane-bounded organelle;extracellular organelle;extracellular region part;extracellular vesicular exosome;intracellular part;membrane;membrane-bounded organelle;membrane-bounded vesicle;organelle;vesicle		Homo sapiens	1	4
cell part;cytoplasm;cytoplasmic part;intracellular membrane-bounded organelle;intracellular organelle;intracellular organelle part;intracellular part;macromolecular complex;membrane;membrane part;membrane-bounded orga	Alzheimer's disease;Huntington's disease;Oxidative phosphorylation;Parkinson's disease	Homo sapiens	1	4
cell part;cytoplasm;intracellular part;intrinsic to internal side of plasma membrane;intrinsic to membrane;intrinsic to plasma membrane;membrane part;plasma membrane part		Homo sapiens	1	4
cell part;cytoplasmic part;intracellular organelle part;intracellular part;macromolecular complex;membrane;mitochondrial inner membrane;mitochondrial membrane;mitochondrial part;mitochondrial small ribosomal subunit;organellar small ribosomal subunit;organelle inner membrane;organelle membrane;organelle part;ribonucleo		Homo sapiens	1	4
cell part;cytoplasmic part;integral to membrane;integral to mitochondrial inner membrane;integral to mitochondrial membrane;integral to organelle membrane;intracellular membrane-bounded organelle;intracellular organelle;intracellular organelle part;intracellular part;intrinsic to membrane;intrinsic to mitochondrial inner membra		Homo sapiens	1	4
cell part;integral to membrane;intrinsic to membrane;membrane part		Homo sapiens	1	4
cell part;cell projection;centrosome;cilium;cytoplasmic part;cytoskeletal part;cytoskeleton;cytosol;extracellular membrane-bounded organelle;extracellular organelle;extracellular region part;extracellular vesicular exosome;intracellular non-membrane-bounded organelle;intracellular organelle;intracellular organelle part;intracellular		Homo sapiens	1	4
cell part;centrosome;cytoplasm;cytoplasmic part;cytoskeletal part;intracellular membrane-bounded organelle;intracellular non-membrane-bounded organelle;intracellular organelle;intracellular organelle part;intracellular part;membrane-bounded organelle;microtubule organizing center;non-membrane-bounded organelle;nucleus;		Homo sapiens	1	4
extracellular membrane-bounded organelle;extracellular organelle;extracellular region part;extracellular vesicular exosome;membrane-bounded organelle;membrane-bounded vesicle;organelle;vesicle		Homo sapiens	1	4
cell part;cytoplasm;cytoplasmic part;endoplasmic reticulum;Golgi apparatus;integral to membrane;intracellular membrane-bounded organelle;intracellular organelle;intracellular organelle part;intracellular part;intrinsic to mem	Mineral absorption	Homo sapiens	1	4
cell part;cytoplasmic part;endoplasmic reticulum membrane;endoplasmic reticulum part;intracellular organelle part;intracellular part;macromolecular complex;membrane;membrane part;nuclear membrane;nuclear part;nuclear pore;organelle membrane;organelle part;pore complex;protein complex		Homo sapiens	1	5
cell part;intracellular organelle part;intracellular part;macromolecular complex;nuclear part;organelle part;ribonucleoprotein complex;small nucleolar ribonucleoprotein complex;spliceosomal complex		Homo sapiens	1	5
		Homo sapiens	1	5
		Homo sapiens	1	5
cell part;cytoplasmic part;endoplasmic reticulum lumen;endoplasmic reticulum part;extracellular membrane-bounded organelle;extracellular organelle;extracellular region;extracellular region part;extracellular space;extracellular vesicular exosome;intracellular membrane-bounded organelle;intracellular organelle;intracellular orgar		Homo sapiens	1	5
cell part;cytoplasmic part;endoplasmic reticulum membrane;endoplasmic reticulum part;envelope;extracellular membrane-bounded organelle;extracellular organelle;extracellular region part;extracellular vesicular exosome;int	Drug metabolism - cytochrome P450;Glutathione metabolism;Metabolism of xenobiotics by cytochrome	Homo sapiens	1	5
cell part;centrosome;cytoplasmic part;cytoskeletal part;cytosol;intracellular non-membrane-bounded organelle;intracellular organelle;intracellular organelle part;intracellular part;microtubule organizing center;non-membrane-bounded organelle;organelle;organelle part		Homo sapiens	1	5
cell part;cytoplasmic part;intracellular organelle part;intracellular part;macromolecular complex;membrane;membrane part;mitochondrial inner membrane;mitochondrial membrane;mitochondrial membrane part;mitochondrial	Alzheimer's disease;Huntington's disease;Oxidative phosphorylation;Parkinson's disease	Homo sapiens	1	5
cell part;cytoplasm;cytoplasmic part;integral to membrane;intracellular membrane-bounded organelle;intracellular organelle;intracellular organelle part;intracellular part;intrinsic to membrane;membrane;membrane part;membrane-bounded organelle;mitochondrial inner membrane;mitochondrial membrane;mitochondrial part;mitc		Homo sapiens	1	5
cell part;cytoplasmic part;intracellular organelle part;intracellular part;macromolecular complex;membrane;membrane part;mitochondrial inner membrane;mitochondrial membrane;mitochondrial membrane part;mitochondrial	Alzheimer's disease;Huntington's disease;Oxidative phosphorylation;Parkinson's disease	Homo sapiens	1	5
Cajal body;cell part;cytoplasm;cytoskeleton;intermediate filament cytoskeleton;intracellular membrane-bounded organelle;intracellular non-membrane-bounded organelle;intracellular organelle;intracellular organelle part;intra	Spliceosome	Homo sapiens	1	5
Cdc48p-Npl4p-Ufd1p AAA ATPase complex;cell part;cytoplasmic part;endoplasmic reticulum part;intracellular organelle part;intracellular part;macromolecular complex;membrane part;nuclear part;nucleoplasm;organelle part;protein complex		Homo sapiens	1	5
cell part;extracellular membrane-bounded organelle;extracellular organelle;extracellular region part;extracellular vesicular exosome;membrane;membrane-bounded organelle;membrane-bounded vesicle;organelle;vesicle	Apoptosis	Homo sapiens	1	5
cell part;cytoplasmic part;integral to membrane;intracellular membrane-bounded organelle;intracellular organelle;intracellular organelle part;intracellular part;intrinsic to membrane;macromolecular complex;membrane;membra	Alzheimer's disease;Huntington's disease;Oxidative phosphorylation;Parkinson's disease	Homo sapiens	1	5
cell part;cytoplasmic part;intracellular organelle part;intracellular part;macromolecular complex;membrane;membrane part;mitochondrial inner membrane;mitochondrial membrane;mitochondrial membrane part;mitochondrial	Alzheimer's disease;Huntington's disease;Oxidative phosphorylation;Parkinson's disease	Homo sapiens	1	5
cell part;cytoplasmic part;cytosol;intracellular part		Homo sapiens	1	5
blood microparticle;extracellular membrane-bounded organelle;extracellular organelle;extracellular region;extracellular region part;extracellular vesicular exosome;high-density lipoprotein particle;macromolecular complex;me	African trypanosomiasis	Homo sapiens	1	5
blood microparticle;cell part;extracellular membrane-bounded organelle;extracellular organelle;extracellular region;extracellular region part;extracellular space;extracellular vesicular exosome;intracellular;intracellular membrane-bounded organelle;intracellular organelle;intracellular part;membrane-bounded organelle;membrane-b		Homo sapiens	1	5
extracellular membrane-bounded organelle;extracellular organelle;extracellular region part;extracellular vesicular exosome;membrane-bounded organelle;membrane-bounded vesicle;organelle;vesicle		Homo sapiens	1	5
cell part;intracellular membrane-bounded organelle;intracellular organelle;intracellular organelle part;intracellular part;membrane-bounded organelle;nuclear part;nucleoplasm;nucleus;organelle;organelle part		Homo sapiens	0.9999	5
cell part;cytoplasmic part;intracellular membrane-bounded organelle;intracellular organelle;intracellular organelle lumen;intracellular organelle part;intracellular part;macromolecular complex;membrane;membrane part;membr	Alzheimer's disease;Huntington's disease;Oxidative phosphorylation;Parkinson's disease	Homo sapiens	1	5
cell part;cytoplasm;cytoplasmic part;extracellular membrane-bounded organelle;extracellular organelle;extracellular region part;extracellular vesicular exosome;Golgi apparatus;Golgi apparatus part;Golgi membrane;intracellular membrane-bounded organelle;intracellular organelle;intracellular organelle part;intracellular part;mem		Homo sapiens	1	5
cell part;cytoplasmic part;cytosol;extracellular membrane-bounded organelle;extracellular organelle;extracellular region part;extracellular vesicular exosome;intracellular organelle lumen;intracellular organelle part;intracellular part;lysosomal lumen;membrane;membrane-bounded organelle;membrane-bounded vesicle;membrane-		Homo sapiens	1	5
CAAX-protein geranylgeranyltransferase complex;cell part;cytoplasm;cytoplasmic part;cytoskeletal part;cytosol;intracellular organelle part;intracellular part;macromolecular complex;microtubule associated complex;organelle part;protein complex;protein farnesyltransferase complex		Homo sapiens	1	5
cell part;cytoplasmic part;cytosol;intracellular part;macromolecular complex;perinuclear region of cytoplasm;protein complex		Homo sapiens	1	5
adherens junction;anchored to membrane;anchoring junction;caveola;cell junction;cell part;cell projection;cell-substrate adherens junction;cell-substrate junction;cytoplasm;external side of plasma membrane;extracellular membrane-bounded organelle;extracellular organelle;extracellular region part;extracellular space;extracellul		Homo sapiens	1	5
cell part;cytoplasm;intracellular organelle part;intracellular part;nuclear part;nucleoplasm;organelle part		Homo sapiens	1	5
cell part;cytoplasmic part;integral to membrane;intracellular membrane-bounded organelle;intracellular organelle;intracellular organelle part;intracellular part;intrinsic to membrane;membrane;membrane part;membrane-bounded organelle;mitochondrial membrane;mitochondrial outer membrane;mitochondrial part;mitochondrion;		Homo sapiens	1	5
cell part;cytoplasmic part;cytosol;endosomal part;endosome membrane;extracellular membrane-bounded organelle;extracellular organelle;extracellular region part;extracellular vesicular exosome;intracellular organelle part;in	Protein processing in endoplasmic reticulum;Ubiquitin mediated proteolysis	Homo sapiens	1	5
cell part;cytoplasmic part;intracellular membrane-bounded organelle;intracellular organelle;intracellular organelle part;intracellular part;macromolecular complex;membrane;membrane part;membrane-bounded organelle;membrane-enclosed lumen;mitochondrial inner membrane;mitochondrial inner membrane presequence trans		Homo sapiens	1	5
catalytic step 2 spliceosome;cell part;cytoplasmic part;cytosol;intracellular membrane-bounded organelle;intracellular organelle;intracellular organelle part;intracellular part;macromolecular complex;membrane-bounded orgar	Spliceosome;Systemic lupus erythematosus	Homo sapiens	1	5
cell part;cytoplasmic part;cytosol;cytosolic small ribosomal subunit;extracellular region part;extracellular space;intracellular organelle part;intracellular part;macromolecular complex;organelle part;ribonucleoprotein complex;small ribosomal subunit		Homo sapiens	1	5
cell part;Cul2-RING ubiquitin ligase complex;Cul3-RING ubiquitin ligase complex;CUL4 RING ubiquitin ligase complex;Cul4A-RING ubiquitin ligase complex;Cul4B-RING ubiquitin ligase complex;Cul5-RING ubiquitin ligase c	Cell cycle;Cell cycle - yeast;Circadian rhythm - mammal;Nucleotide excision repair;Oocyte meiosis;Path	Homo sapiens	1	5
cell part;cytoplasm;intracellular part	Amino sugar and nucleotide sugar metabolism;Galactose metabolism	Homo sapiens	1	5
cell part;chromosomal part;chromosome;chromosome, centromeric region;condensed chromosome kinetochore;condensed chromosome outer kinetochore;condensed chromosome, centromeric region;condensed nuclear chromosome kinetochore;cytoplasm;cytoplasmic part;cytoskeletal part;cytoskeleton;cytosol;intracellular r		Homo sapiens	0.9999	5
cell part;cytoplasm;cytoplasmic part;Golgi apparatus;intracellular membrane-bounded organelle;intracellular organelle;intracellular organelle part;intracellular part;macromolecular complex;membrane;membrane-bounded organelle;nuclear body;nuclear part;nucleoplasm;nucleoplasm part;nucleus;organelle;organelle part;plasma		Homo sapiens	1	5
cell part;cytoplasmic part;cytosol;extracellular membrane-bounded organelle;extracellular organelle;extracellular region part;extracellular vesicular exosome;intracellular part;membrane-bounded organelle;membrane-bounde	Peroxisome;Terpenoid backbone biosynthesis	Homo sapiens	1	5
cell part;cytoplasmic part;cytosol;extracellular membrane-bounded organelle;extracellular organelle;extracellular region part;extracellular vesicular exosome;intracellular membrane-bounded organelle;intracellular organelle;int	Purine metabolism;Pyrimidine metabolism	Homo sapiens	1	5
cell part;cytoplasm;cytoplasmic part;cytosol;intracellular organelle part;intracellular part;macromolecular complex;nuclear part;nucleoplasm;organelle part;protein complex	Acute myeloid leukemia;ErbB signaling pathway;Insulin signaling pathway;mTOR signaling pathway;RN	Homo sapiens	1	5
cell part;cytoplasm;exosome (RNase complex);intracellular membrane-bounded organelle;intracellular non-membrane-bounded organelle;intracellular organelle;intracellular organelle part;intracellular part;macromolecular con	RNA degradation	Homo sapiens	1	5
cell part;Cul3-RING ubiquitin ligase complex;cullin-RING ubiquitin ligase complex;cytoplasm;cytoplasmic part;cytoskeletal part;cytosol;endoplasmic reticulum;intracellular membrane-bounded organelle;intracellular organelle	Ubiquitin mediated proteolysis	Homo sapiens	1	5
cell part;cytoplasmic part;integral to membrane;integral to mitochondrial membrane;integral to organelle membrane;intracellular membrane-bounded organelle;intracellular organelle;intracellular organelle part;intracellular part	Bacterial secretion system;Protein export	Homo sapiens	1	5
cell part;cytoplasmic membrane-bounded vesicle;cytoplasmic part;cytoplasmic vesicle;early endosome;endocytic vesicle;endosome;intracellular membrane-bounded organelle;intracellular organelle;intracellular part;membra	Endocytosis	Homo sapiens	1	5
cell part;cytoplasmic part;cytosol;early endosome;endosome;intracellular membrane-bounded organelle;intracellular organelle;intracellular part;late endosome;macromolecular complex;membrane part;membrane-bounded organelle;organelle;protein complex;retromer complex		Homo sapiens	1	5
cell part;cytoplasmic part;intracellular membrane-bounded organelle;intracellular organelle;intracellular part;membrane-bounded organelle;mitochondrion;organelle		Homo sapiens	1	5
cell part;endomembrane system		Homo sapiens	1	5
cell part;cytoplasmic part;intracellular membrane-bounded organelle;intracellular organelle;intracellular part;membrane-bounded organelle;mitochondrion;organelle		Homo sapiens	1	5
cell part;intracellular non-membrane-bounded organelle;intracellular organelle;intracellular organelle part;intracellular part;membrane;non-membrane-bounded organelle;nuclear part;nucleolus;organelle;organelle part		Homo sapiens	1	5
cell cortex;cell part;cytoplasm;cytoplasmic membrane-bounded vesicle;cytoplasmic part;cytoplasmic vesicle;cytoskeleton;extracellular membrane-bounded organelle;extracellular organelle;extracellular region part;extracellular vesicular exosome;intracellular membrane-bounded organelle;intracellular non-membrane-bounded o		Homo sapiens	1	5
cell part;cytoplasmic part;intracellular membrane-bounded organelle;intracellular organelle;intracellular part;membrane-bounded organelle;mitochondrion;organelle		Homo sapiens	1	5
cell part;intracellular membrane-bounded organelle;intracellular organelle;intracellular part;membrane-bounded organelle;nucleus;organelle		Homo sapiens	1	5
cell part;cytoplasmic part;endoplasmic reticulum;endoplasmic reticulum membrane;endoplasmic reticulum part;integral to membrane;intracellular membrane-bounded organelle;intracellular organelle;intracellular organelle part;intracellular part;intrinsic to membrane;membrane;membrane part;membrane-bounded organelle;nucle		Homo sapiens	1	5
autophagic vacuole;cell part;cytoplasmic part;cytoplasmic vesicle;endoplasmic reticulum membrane;endoplasmic reticulum part;endosomal part;endosome;endosome membrane;Golgi apparatus part;integral to membrane;intracellular membrane-bounded organelle;intracellular organelle;intracellular organelle part;intracellular pa		Homo sapiens	1	5
		Homo sapiens	1	5
cell part;chromosomal part;condensed chromosome kinetochore;cytoplasm;cytoskeletal part;cytoskeleton;intracellular membrane-bounded organelle;intracellular non-membrane-bounded organelle;intracellular organelle;intracellular organelle part;intracellular part;kinetochore;macromolecular complex;membrane-bounded orgar		Homo sapiens	1	5
cell part;cytoplasmic part;cytoplasmic vesicle membrane;cytoplasmic vesicle part;endoplasmic reticulum membrane;endoplasmic reticulum part;Golgi apparatus;integral to membrane;intracellular membrane-bounded organe	Cytosolic DNA-sensing pathway;RIG-I-like receptor signaling pathway	Homo sapiens	1	5
cell part;cytoplasmic part;cytosol;early endosome;endosome;intracellular membrane-bounded organelle;intracellular organelle;intracellular part;membrane-bounded organelle;nucleus;organelle		Homo sapiens	1	5
cell part;extracellular membrane-bounded organelle;extracellular organelle;extracellular region part;extracellular vesicular exosome;intracellular organelle part;intracellular part;membrane-bounded organelle;membrane-bounded vesicle;nuclear part;nucleoplasm;organelle;organelle part;vesicle		Homo sapiens	1	5
		Homo sapiens	1	5
cell part;cytoplasmic part;endoplasmic reticulum lumen;endoplasmic reticulum part;extracellular membrane-bounded organelle;extracellular organelle;extracellular region part;extracellular vesicular exosome;intracellular orgar	Other types of O-glycan biosynthesis	Homo sapiens	1	5
cell part;cytoplasmic part;intracellular membrane-bounded organelle;intracellular organelle;intracellular part;lipid particle;membrane;membrane-bounded organelle;midbody;mitochondrion;nucleus;organelle		Homo sapiens	1	5
axon;cell part;cell projection;chromosomal part;chromosome, centromeric region;cytoplasm;dendrite;filopodium;intracellular non-membrane-bounded organelle;intracellular organelle;intracellular organelle part;intracellular part;macromolecular complex;neuron projection;non-membrane-bounded organelle;nuclear part;nucleolus;		Homo sapiens	1	5
cell part;cytoplasmic part;intracellular membrane-bounded organelle;intracellular organelle;intracellular part;membrane-bounded organelle;mitochondrion;organelle		Homo sapiens	1	5
CCR4-NOT complex;cell part;cytoplasmic mRNA processing body;cytoplasmic part;cytosol;intracellular membrane-bounded organelle;intracellular non-membrane-bounded organelle;intracellular organelle;intracellular organe	RNA degradation	Homo sapiens	1	5
cell part;intracellular membrane-bounded organelle;intracellular organelle;intracellular organelle part;intracellular part;membrane-bounded organelle;nuclear part;nucleoplasm;nucleus;organelle;organelle part		Homo sapiens	1	5
cell part;cytoplasm;cytoplasmic part;cytosol;exosome (RNase complex);intracellular membrane-bounded organelle;intracellular non-membrane-bounded organelle;intracellular organelle;intracellular organelle part;intracellular	RNA degradation	Homo sapiens	1	5
extracellular membrane-bounded organelle;extracellular organelle;extracellular region part;extracellular vesicular exosome;membrane-bounded organelle;membrane-bounded vesicle;organelle;vesicle		Homo sapiens	1	5
cell part;cytoplasm;cytoplasmic part;cytosol;intracellular membrane-bounded organelle;intracellular organelle;intracellular organelle part;intracellular part;membrane-bounded organelle;mitochondrion;nuclear part;nucleoplasr	RNA degradation	Homo sapiens	1	5
cell part;cytoplasmic part;integral to membrane;intracellular membrane-bounded organelle;intracellular organelle;intracellular organelle part;intracellular part;intrinsic to membrane;macromolecular complex;membrane;membrane part;membrane-bounded organelle;mitochondrial inner membrane;mitochondrial membrane;mitocho		Homo sapiens	1	5
cell part;cytoplasmic part;intracellular organelle part;intracellular part;large ribosomal subunit;macromolecular complex;membrane;mitochondrial inner membrane;mitochondrial large ribosomal subunit;mitochondrial membrane;mitochondrial part;mitochondrial small ribosomal subunit;organellar large ribosomal subunit;organellar		Homo sapiens	1	5
cell part;cytoplasmic part;intracellular non-membrane-bounded organelle;intracellular organelle;intracellular organelle part;intracellular part;macromolecular complex;membrane;mitochondrial inner membrane;mitochondrial membrane;mitochondrial part;non-membrane-bounded organelle;organelle;organelle inner membrane;orga		Homo sapiens	1	5
cell part;cytoplasmic part;intracellular membrane-bounded organelle;intracellular organelle;intracellular part;membrane-bounded organelle;mitochondrion;organelle		Homo sapiens	1	5
		Homo sapiens	1	5
cell part;centrosome;cytoplasm;cytoplasmic part;cytoskeletal part;intracellular membrane-bounded organelle;intracellular non-membrane-bounded organelle;intracellular organelle;intracellular organelle part;intracellular part;membrane-bounded organelle;microtubule organizing center;mitochondrion;non-membrane-bounded org		Homo sapiens	1	5
anchoring junction;cell junction;cell part;cell-cell junction;cytoplasm;cytoplasmic part;cytoskeletal part;cytoskeleton;desmosome;internal side of plasma membrane;intracellular non-membrane-bounded organelle;intracellular organelle;intracellular organelle part;intracellular part;membrane;membrane part;midbody;mitotic spindle		Homo sapiens	1	5
apical plasma membrane;basolateral plasma membrane;cell part;integral to membrane;integral to plasma membrane;intrinsic to membrane;intrinsic to plasma membrane;membrane;membrane part;plasma membrane;plasma membrane part		Homo sapiens	1	5
cell part;cytoplasmic part;intracellular non-membrane-bounded organelle;intracellular organelle;intracellular organelle part;intracellular part;macromolecular complex;mitochondrial part;mitochondrial ribosome;non-membrane-bounded organelle;organellar ribosome;organelle;organelle part;ribonucleoprotein complex;ribosome		Homo sapiens	1	5
cell part;cytoplasmic part;intracellular membrane-bounded organelle;intracellular non-membrane-bounded organelle;intracellular organelle;intracellular organelle lumen;intracellular organelle part;intracellular part;membrane;membrane-bounded organelle;membrane-enclosed lumen;mitochondrial inner membrane;mitochondrial ir		Homo sapiens	1	5
cell part;cytoplasm;cytoplasmic part;intracellular organelle part;intracellular part;membrane;mitochondrial membrane;mitochondrial outer membrane;mitochondrial part;organelle membrane;organelle outer membrane;organelle part;outer membrane		Homo sapiens	1	5
cell part;cytoplasmic part;extracellular region part;extracellular space;high-density lipoprotein particle;integral to membrane;intracellular membrane-bounded organelle;intracellular organelle;intracellular part;intrinsic to membrane;low-density lipoprotein particle;macromolecular complex;membrane part;membrane-bounded orgar		Homo sapiens	1	5
cell part;cytoplasmic part;endoplasmic reticulum membrane;endoplasmic reticulum part;extracellular membrane-bounded organelle;extracellular organelle;extracellular region part;extracellular vesicular exosome;integral to membrane;intracellular organelle part;intracellular part;intrinsic to membrane;membrane;membrane part;m		Homo sapiens	1	5
cell part;cytoplasmic part;cytosol;extracellular membrane-bounded organelle;extracellular organelle;extracellular region part;extracellular vesicular exosome;intracellular membrane-bounded organelle;intracellular organelle;intracellular part;membrane-bounded organelle;membrane-bounded vesicle;nucleus;organelle;vesicle		Homo sapiens	1	5
cell part;intracellular membrane-bounded organelle;intracellular organelle;intracellular part;membrane-bounded organelle;nucleus;organelle		Homo sapiens	1	5
cell part;intracellular membrane-bounded organelle;intracellular organelle;intracellular organelle part;intracellular part;macromolecular complex;membrane-bounded organelle;nuclear part;nucleoplasm;nucleus;organelle;organ	Spliceosome	Homo sapiens	1	5
cell part;cytoplasmic part;intracellular non-membrane-bounded organelle;intracellular organelle;intracellular organelle part;intracellular part;large ribosomal subunit;macromolecular complex;membrane;mitochondrial inner membrane;mitochondrial membrane;mitochondrial part;mitochondrial ribosome;non-membrane-bounded or		Homo sapiens	1	5
cell junction;cell part;clathrin-coated vesicle;coated vesicle;cytoplasmic membrane-bounded vesicle;cytoplasmic part;cytoplasmic vesicle;integral to membrane;integral to mitochondrial membrane;integral to organelle membrane;intracellular membrane-bounded organelle;intracellular organelle;intracellular organelle part;intracell		Homo sapiens	1	5
cell part;cytoplasmic part;extracellular region part;extracellular space;intracellular membrane-bounded organelle;intracellular non-membrane-bounded organelle;intracellular organelle;intracellular organelle part;intracellular part;macromolecular complex;membrane;membrane-bounded organelle;mitochondrial inner membrane;mi		Homo sapiens	1	5
cell part;cytoplasm;intracellular membrane-bounded organelle;intracellular organelle;intracellular part;membrane;membrane-bounded organelle;nucleus;organelle;plasma membrane	MAPK signaling pathway	Homo sapiens	1	5
cell part;cytoplasm;intracellular organelle part;intracellular part;macromolecular complex;nuclear part;nucleoplasm;organelle part;protein complex;signalosome		Homo sapiens	1	5
cell part;cell projection membrane;cell projection part;cytoplasm;extracellular membrane-bounded organelle;extracellular organelle;extracellular region part;extracellular vesicular exosome;intracellular organelle part;intracellular part;leading edge membrane;membrane;membrane part;membrane-bounded organelle;membrane-bo		Homo sapiens	1	5
cell part;cytoplasmic part;endoplasmic reticulum;endoplasmic reticulum membrane;endoplasmic reticulum part;intracellular membrane-bounded organelle;intracellular organelle;intracellular organelle part;intracellular part;lysosomal membrane;membrane;membrane part;membrane-bounded organelle;mitochondrion;nucleus;orga		Homo sapiens	1	5
cell part;cytoplasmic part;early endosome membrane;endosomal part;endosome membrane;Golgi apparatus;Golgi apparatus part;Golgi membrane;integral to membrane;intracellular membrane-bounded organelle;intracellular organelle;intracellular organelle part;intracellular part;intrinsic to membrane;late endosome membrane;l		Homo sapiens	1	5
cell part;cytoplasmic part;extracellular membrane-bounded organelle;extracellular organelle;extracellular region part;extracellular vesicular exosome;intracellular membrane-bounded organelle;intracellular organelle;intracellular part;membrane-bounded organelle;membrane-bounded vesicle;mitochondrion;organelle;vesicle		Homo sapiens	1	5
cell part;cytoplasmic part;intracellular membrane-bounded organelle;intracellular organelle;intracellular organelle part;intracellular part;large ribosomal subunit;macromolecular complex;membrane;membrane-bounded organelle;mitochondrial inner membrane;mitochondrial membrane;mitochondrial part;mitochondrion;organelle;c		Homo sapiens	1	5
cell part;cytoplasmic part;integral to membrane;intracellular membrane-bounded organelle;intracellular organelle;intracellular organelle part;intracellular part;intrinsic to membrane;macromolecular complex;membrane;membra	Alzheimer's disease;Huntington's disease;Oxidative phosphorylation;Parkinson's disease	Homo sapiens	1	5
		Homo sapiens	1	5
cell part;cytoplasm;cytoplasmic part;cytosol;eukaryotic 43S preinitiation complex;eukaryotic 48S preinitiation complex;eukaryotic translation initiation factor 3 complex;extracellular membrane-bounded organelle;extracellular organelle;extracellular region part;extracellular vesicular exosome;intracellular membrane-bounded orga		Homo sapiens	1	5
cell part;cytoplasmic part;extracellular membrane-bounded organelle;extracellular organelle;extracellular region part;extracellular vesicular exosome;intracellular organelle part;intracellular part;macromolecular complex;memb	Alzheimer's disease;Cardiac muscle contraction;Huntington's disease;Oxidative phosphorylation;Parkin	Homo sapiens	1	5
cell part;chromatin;chromosomal part;cytoplasmic part;intracellular membrane-bounded organelle;intracellular non-membrane-bounded organelle;intracellular organelle;intracellular organelle part;intracellular part;macromolecular complex;membrane-bounded organelle;mitochondrion;non-membrane-bounded organelle;nuclear p		Homo sapiens	1	5

cell part;cytoplasmic part;intracellular membrane-bounded organelle;intracellular non-membrane-bounded organelle;intracellular organelle;intracellular organelle part;intracellular part;membrane-bounded organelle;mitochondrion;non-membrane-bounded organelle;nuclear part;nucleolus;nucleus;organelle;organelle part		Homo sapiens	1	5
		Homo sapiens	1	5
cell part;cytoplasmic part;integral to membrane;integral to mitochondrial membrane;integral to mitochondrial outer membrane;integral to organelle membrane;integral to peroxisomal membrane;intracellular membrane-bounded organelle;intracellular organelle;intracellular organelle part;intracellular part;intrinsic to membrane;intrir		Homo sapiens	1	5
cell part;cytoplasmic part;extracellular region part;extracellular space;intracellular organelle part;intracellular part;macromolecular complex;membrane;mitochondrial inner membrane;mitochondrial intermembrane space protein transporter complex;mitochondrial membrane;mitochondrial part;organelle inner membrane;organelle		Homo sapiens	1	5
cell part;cytoplasmic part;intracellular membrane-bounded organelle;intracellular non-membrane-bounded organelle;intracellular organelle;intracellular organelle part;intracellular part;macromolecular complex;membrane;membrane-bounded organelle;mitochondrial inner membrane;mitochondrial intermembrane space protein tra		Homo sapiens	1	5
cell part;cytoplasmic part;cytosol;endoplasmic reticulum membrane;endoplasmic reticulum part;Golgi apparatus;intracellular membrane-bounded organelle;intracellular organelle;intracellular organelle part;intracellular part;membrane;membrane part;membrane-bounded organelle;mitochondrion;nuclear part;nucleoplasm;organel		Homo sapiens	1	5
cell part;cytoplasm;cytoplasmic part;extracellular membrane-bounded organelle;extracellular organelle;extracellular region part;extracellular vesicular exosome;intracellular membrane-bounded organelle;intracellular organelle	Amino sugar and nucleotide sugar metabolism;Fructose and mannose metabolism	Homo sapiens	1	5
cell part;coated pit;cytoplasm;intracellular membrane-bounded organelle;intracellular organelle;intracellular part;membrane;membrane part;membrane-bounded organelle;nucleus;organelle;plasma membrane	Endocytosis	Homo sapiens	1	5
cell part;cytoplasmic part;extracellular membrane-bounded organelle;extracellular organelle;extracellular region part;extracellular vesicular exosome;intracellular;intracellular membrane-bounded organelle;intracellular organel	Lysosome	Homo sapiens	1	6
actin cytoskeleton;actin filament bundle;actomyosin;cell part;cell projection;cytoplasm;cytoskeletal part;cytoskeleton;intracellular non-membrane-bounded organelle;intracellular organelle;intracellular organelle part;intracellul	Axon guidance	Homo sapiens	1	6
cell part;cell surface;cytoplasmic part;endoplasmic reticulum-Golgi intermediate compartment;Golgi apparatus;Golgi apparatus part;integral to Golgi membrane;integral to membrane;integral to organelle membrane;intracellular membrane-bounded organelle;intracellular organelle;intracellular organelle part;intracellular part;intrins		Homo sapiens	1	6
		Homo sapiens	1	6
cell part;cytoplasmic part;integral to membrane;intracellular organelle part;intracellular part;intrinsic to membrane;membrane;membrane part;mitochondrial membrane;mitochondrial outer membrane;mitochondrial part;organelle membrane;organelle outer membrane;organelle part;outer membrane		Homo sapiens	1	6
cell part;cytoplasmic part;endoplasmic reticulum;Golgi apparatus;intracellular membrane-bounded organelle;intracellular organelle;intracellular part;membrane-bounded organelle;organelle		Homo sapiens	1	6
cell part;cytoplasmic part;integral to membrane;intracellular membrane-bounded organelle;intracellular organelle;intracellular organelle part;intracellular part;intrinsic to membrane;macromolecular complex;membrane;membra	Alzheimer's disease;Huntington's disease;Oxidative phosphorylation;Parkinson's disease	Homo sapiens	1	6
cell part;cytoplasmic part;intracellular membrane-bounded organelle;intracellular organelle;intracellular organelle part;intracellular part;membrane;membrane-bounded organelle;membrane-enclosed lumen;mitochondrial inner membrane;mitochondrial intermembrane space;mitochondrial membrane;mitochondrial part;mitochond		Homo sapiens	1	6
cell part;cell projection part;cytoplasm;cytoplasmic part;intracellular membrane-bounded organelle;intracellular non-membrane-bounded organelle;intracellular organelle;intracellular organelle part;intracellular part;macromole	Spliceosome	Homo sapiens	1	6
cell part;cytoplasmic part;extracellular membrane-bounded organelle;extracellular organelle;extracellular region part;extracellular vesicular exosome;intracellular membrane-bounded organelle;intracellular organelle;intracellul	Oxidative phosphorylation	Homo sapiens	1	6
cell part;cytoplasmic part;endoplasmic reticulum;integral to membrane;intracellular membrane-bounded organelle;intracellular organelle;intracellular organelle part;intracellular part;intrinsic to membrane;macromolecular com	Alzheimer's disease;Huntington's disease;Oxidative phosphorylation;Parkinson's disease	Homo sapiens	1	6
cell part;intracellular membrane-bounded organelle;intracellular non-membrane-bounded organelle;intracellular organelle;intracellular organelle part;intracellular part;macromolecular complex;membrane-bounded organelle;non-membrane-bounded organelle;nuclear part;nucleolus;nucleus;organelle;organelle part;ribonucleoprote		Homo sapiens	1	6
cell division site part;cell part;cleavage furrow;cytoplasm;cytoplasmic part;early endosome;endosome;extracellular membrane-bounded organelle;extracellular organelle;extracellular region part;extracellular vesicular exosome	Endocytosis	Homo sapiens	1	6
cell part;cytoplasmic part;extracellular membrane-bounded organelle;extracellular organelle;extracellular region part;extracellular vesicular exosome;integral to membrane;integral to organelle membrane;integral to peroxisom	Peroxisome	Homo sapiens	1	6
cell part;cytoplasmic part;endoplasmic reticulum membrane;endoplasmic reticulum part;extracellular membrane-bounded organelle;extracellular organelle;extracellular region part;extracellular vesicular exosome;integral to membrane;intracellular organelle part;intracellular part;intrinsic to membrane;membrane;membrane part;m		Homo sapiens	1	6
blood microparticle;cell part;cytoplasmic membrane-bounded vesicle lumen;cytoplasmic part;cytoplasmic vesicle part;endocytic vesicle lumen;extracellular membrane-bounded organelle;extracellular organelle;extracellular region;extracellular region part;extracellular space;extracellular vesicular exosome;haptoglobin-hemoglob		Homo sapiens	1	6
cell part;cytoplasm;extracellular membrane-bounded organelle;extracellular organelle;extracellular region part;extracellular space;extracellular vesicular exosome;intracellular non-membrane-bounded organelle;intracellular organelle;intracellular organelle part;intracellular part;membrane-bounded organelle;membrane-bounded v		Homo sapiens	1	6
cell part;cytoplasm;cytoplasmic part;extracellular membrane-bounded organelle;extracellular organelle;extracellular region;extracellular region part;extracellular vesicular exosome;Golgi apparatus;intracellular membrane-bou	Galactose metabolism;Glycerolipid metabolism;Glycosphingolipid biosynthesis - globo series;Lysosome	Homo sapiens	1	6
cell part;cytoplasmic part;extracellular membrane-bounded organelle;extracellular organelle;extracellular region;extracellular region part;extracellular space;extracellular vesicular exosome;intracellular membrane-bounded org	Antigen processing and presentation;Lysosome;Phagosome;Rheumatoid arthritis	Homo sapiens	1	6
basal plasma membrane;cell part;cell surface;extracellular region;integral to membrane;integral to plasma membrane;intrinsic to membrane;intrinsic to plasma membrane;membrane;membrane part;plasma membrane;plasma	Adherens junction;Axon guidance;Bacterial invasion of epithelial cells;Cytokine-cytokine receptor intera	Homo sapiens	1	6
cell part;cell projection;cytoplasm;cytoplasmic part;cytosol;extracellular membrane-bounded organelle;extracellular organelle;extracellular region part;extracellular vesicular exosome;intracellular membrane-bounded organell	Folate biosynthesis	Homo sapiens	1	6
cell part;cytoplasmic part;integral to membrane;intracellular membrane-bounded organelle;intracellular organelle;intracellular organelle part;intracellular part;intrinsic to membrane;membrane;membrane part;membrane-bound	Alzheimer's disease;Cardiac muscle contraction;Huntington's disease;Oxidative phosphorylation;Parkin	Homo sapiens	1	6
cell part;cytoplasmic part;integral to membrane;integral to mitochondrial inner membrane;integral to mitochondrial membrane;integral to organelle membrane;integral to plasma membrane;intracellular organelle part;intracellular part;intrinsic to membrane;intrinsic to mitochondrial inner membrane;intrinsic to organelle membrane;		Homo sapiens	1	6
cell part;cytoplasmic part;cytosol;DNA-directed RNA polymerase complex;DNA-directed RNA polymerase I complex;DNA-directed RNA polymerase II, core complex;DNA-directed RNA polymerase III complex;intracellular m	Cytosolic DNA-sensing pathway;Huntington's disease;Purine metabolism;Pyrimidine metabolism;RNA p	Homo sapiens	1	6
cell part;cytoplasmic part;intracellular membrane-bounded organelle;intracellular organelle;intracellular organelle part;intracellular part;macromolecular complex;membrane;membrane part;membrane-bounded organelle;mito	Alzheimer's disease;Huntington's disease;Oxidative phosphorylation;Parkinson's disease	Homo sapiens	1	6
carboxy-terminal domain protein kinase complex;cell part;core TFIIH complex;holo TFIIH complex;intracellular membrane-bounded organelle;intracellular organelle;intracellular organelle part;intracellular part;macromolecular	Basal transcription factors;Nucleotide excision repair	Homo sapiens	1	6
1-phosphatidylinositol-4-phosphate 3-kinase, class IA complex;cell junction;cell part;cell-cell junction;cytoplasm;cytoplasmic part;cytosol;cytosolic part;intracellular membrane-bounded organelle;intracellular organelle;intrac	Acute myeloid leukemia;Aldosterone-regulated sodium reabsorption;Amoebiasis;Apoptosis;B cell recep	Homo sapiens	1	6
cell part;chromosomal part;DNA replication factor A complex;intracellular organelle part;intracellular part;macromolecular complex;nuclear chromosome part;nuclear part;nucleoplasm;organelle part;protein complex	DNA replication;Homologous recombination;Mismatch repair;Nucleotide excision repair	Homo sapiens	1	6
cell part;cytoplasm;cytoplasmic part;cytosol;extracellular membrane-bounded organelle;extracellular organelle;extracellular region part;extracellular vesicular exosome;intracellular membrane-bounded organelle;intracellular o	Folate biosynthesis	Homo sapiens	1	6
cell part;cell projection;centrosome;cilium;cytoplasmic microtubule;cytoplasmic part;cytoskeletal part;extracellular membrane-bounded organelle;extracellular organelle;extracellular region part;extracellular vesicular exosome;Golgi apparatus;Golgi apparatus part;Golgi membrane;intracellular membrane-bounded organelle;intrac		Homo sapiens	1	6
cell part;cytoplasmic part;cytosol;extracellular membrane-bounded organelle;extracellular organelle;extracellular region part;extracellular vesicular exosome;intracellular membrane-bounded organelle;intracellular organelle;int	Glutathione metabolism	Homo sapiens	1	6
cell part;cytoplasm;cytoskeletal part;envelope;intracellular membrane-bounded organelle;intracellular organelle;intracellular organelle part;intracellular part;macromolecular complex;membrane;membrane part;membrane-bou	RNA transport	Homo sapiens	1	6
1-phosphatidylinositol-4-phosphate 3-kinase, class IA complex;cell part;cell projection;cytoplasm;cytoplasmic part;cytosol;cytosolic part;intracellular part;lamellipodium;macromolecular complex;membrane;phosphatidylinos	Acute myeloid leukemia;Aldosterone-regulated sodium reabsorption;Amoebiasis;Apoptosis;B cell recep	Homo sapiens	1	6
AP-2 adaptor complex;AP-type membrane coat adaptor complex;cell part;cell projection membrane;cell projection part;cilium membrane;cilium part;clathrin adaptor complex;coated pit;cytoplasm;cytoplasmic part;cytoplasm	Endocytosis	Homo sapiens	1	6
basal part of cell;cell part;cytoplasmic part;extracellular membrane-bounded organelle;extracellular organelle;extracellular region part;extracellular vesicular exosome;intracellular membrane-bounded organelle;intracellular org	Protein digestion and absorption	Homo sapiens	1	6
cell part;cytoplasm;extracellular membrane-bounded organelle;extracellular organelle;extracellular region part;extracellular vesicular exosome;intracellular part;membrane-bounded organelle;membrane-bounded vesicle;organelle;vesicle		Homo sapiens	1	6
cell part;cytoplasmic part;cytosol;intracellular membrane-bounded organelle;intracellular organelle;intracellular organelle part;intracellular part;membrane-bounded organelle;mitochondrion;nuclear part;nucleoplasm;organelle	Adipocytokine signaling pathway;Chagas disease (American trypanosomiasis);Colorectal cancer;Epithel	Homo sapiens	1	6
cell part;cytoplasmic part;cytosol;glutamate-cysteine ligase complex;intracellular part;macromolecular complex;protein complex	Glutathione metabolism	Homo sapiens	1	6
cell part;cytoplasmic part;endoplasmic reticulum membrane;endoplasmic reticulum part;integral to membrane;intracellular organelle part;intracellular part;intrinsic to membrane;membrane;membrane part;organelle membrane	Glycerophospholipid metabolism	Homo sapiens	1	6
cell part;cytoplasm;cytoplasmic part;holo TFIIH complex;intracellular membrane-bounded organelle;intracellular organelle;intracellular organelle part;intracellular part;macromolecular complex;membrane-bounded organelle;n	Basal transcription factors;Cell cycle;Nucleotide excision repair	Homo sapiens	1	6
axon;cell body;cell part;cell projection;clathrin-coated vesicle;coated vesicle;cytoplasmic membrane-bounded vesicle;cytoplasmic part;cytoplasmic vesicle;cytosol;dendrite;extracellular membrane-bounded organelle;extrace	Fatty acid elongation in mitochondria;Lysosome	Homo sapiens	1	6
cell part;cytoplasmic part;intracellular membrane-bounded organelle;intracellular non-membrane-bounded organelle;intracellular organelle;intracellular organelle lumen;intracellular organelle part;intracellular part;membrane-b	Citrate cycle (TCA cycle)	Homo sapiens	1	6
cell part;cytoplasmic part;intracellular organelle lumen;intracellular organelle part;intracellular part;macromolecular complex;membrane part;membrane-enclosed lumen;mitochondrial matrix;mitochondrial membrane part;mitc	Alzheimer's disease;Huntington's disease;Oxidative phosphorylation;Parkinson's disease	Homo sapiens	1	6
cell part;cytoplasm;cytoplasmic part;cytosol;extracellular membrane-bounded organelle;extracellular organelle;extracellular region part;extracellular vesicular exosome;intracellular non-membrane-bounded organelle;intracellu	RNA transport	Homo sapiens	1	6
axon;cell part;cell projection;cytoplasmic part;cytoskeletal part;cytosol;extracellular membrane-bounded organelle;extracellular organelle;extracellular region part;extracellular vesicular exosome;F-actin capping protein complex;intracellular membrane-bounded organelle;intracellular organelle;intracellular organelle part;intracellul		Homo sapiens	1	6
cell part;COPI vesicle coat;cytoplasmic part;cytoplasmic vesicle part;cytosol;Golgi apparatus part;intracellular organelle part;intracellular part;macromolecular complex;membrane coat;membrane part;organelle part;protein complex;vesicle coat		Homo sapiens	1	6
cell part;cytoplasmic part;cytosol;extracellular membrane-bounded organelle;extracellular organelle;extracellular region part;extracellular vesicular exosome;intracellular organelle part;intracellular part;macromolecular complex;membrane part;membrane-bounded organelle;membrane-bounded vesicle;nuclear part;nuclear pore;c		Homo sapiens	1	6
catalytic step 2 spliceosome;cell part;cytoplasmic part;cytosol;intracellular membrane-bounded organelle;intracellular organelle;intracellular organelle part;intracellular part;macromolecular complex;membrane-bounded orga	RNA degradation;Spliceosome	Homo sapiens	1	6
cell part;cytoplasmic mRNA processing body;cytoplasmic part;cytosol;extracellular membrane-bounded organelle;extracellular organelle;extracellular region part;extracellular vesicular exosome;intracellular non-membrane-b	RNA degradation;Spliceosome	Homo sapiens	1	6
adherens junction;anchoring junction;cell junction;cell part;cell-substrate adherens junction;cell-substrate junction;cytoplasmic part;cytosol;cytosolic large ribosomal subunit;eukaryotic 80S initiation complex;focal adhesion;ir	Ribosome	Homo sapiens	1	6
cell part;cytoplasm;cytoplasmic part;cytosol;extracellular membrane-bounded organelle;extracellular organelle;extracellular region part;extracellular vesicular exosome;intracellular membrane-bounded organelle;intracellular c	Adherens junction;Circadian rhythm - plant;Measles;Ribosome biogenesis in eukaryotes;Tight junction;\	Homo sapiens	1	6
cell part;cytoplasmic part;intracellular membrane-bounded organelle;intracellular non-membrane-bounded organelle;intracellular organelle;intracellular organelle part;intracellular part;macromolecular complex;membrane;membrane-bounded organelle;mitochondrial inner membrane;mitochondrial membrane;mitochondrial part;m		Homo sapiens	1	6
cell part;cytoplasm;cytoplasmic part;intracellular membrane-bounded organelle;intracellular organelle;intracellular organelle part;intracellular part;macromolecular complex;membrane;membrane-bounded organelle;mitochondrial inner membrane;mitochondrial membrane;mitochondrial part;mitochondrial small ribosomal subunit;		Homo sapiens	1	6
cell part;cytoplasmic part;intracellular organelle part;intracellular part;macromolecular complex;membrane;mitochondrial inner membrane;mitochondrial membrane;mitochondrial part;mitochondrial small ribosomal subunit;organellar small ribosomal subunit;organelle inner membrane;organelle membrane;organelle part;ribonucle		Homo sapiens	1	6
cell part;cytoplasmic part;cytosol;intracellular membrane-bounded organelle;intracellular organelle;intracellular organelle part;intracellular part;macromolecular complex;membrane;membrane part;membrane-bounded organe	Alzheimer's disease;Amyotrophic lateral sclerosis (ALS);Apoptosis;Colorectal cancer;Huntington's disea	Homo sapiens	1	6
cell part;cytoplasm;extracellular membrane-bounded organelle;extracellular organelle;extracellular region part;extracellular vesicular exosome;intracellular membrane-bounded organelle;intracellular non-membrane-bounded	Nucleotide excision repair	Homo sapiens	1	6
cell body;cell part;cytoplasmic part;integral to membrane;intracellular membrane-bounded organelle;intracellular organelle;intracellular organelle part;intracellular part;intrinsic to membrane;intrinsic to mitochondrial inner men	Pyrimidine metabolism	Homo sapiens	1	6
cell part;cell surface;cytoplasm;cytoplasmic part;cytosol;extracellular region part;extracellular space;intracellular membrane-bounded organelle;intracellular non-membrane-bounded organelle;intracellular organelle;intracellular organelle lumen;intracellular organelle part;intracellular part;membrane;membrane-bounded organelle;r		Homo sapiens	1	6
basolateral plasma membrane;cell part;cell projection;cell projection part;cell surface;clathrin-coated vesicle;coated vesicle;cytoplasmic membrane-bounded vesicle;cytoplasmic part;cytoplasmic vesicle;cytosol;dendritic spi	SNARE interactions in vesicular transport;Vasopressin-regulated water reabsorption	Homo sapiens	1	6
actin cytoskeleton;apical part of cell;cell part;cell projection membrane;cell projection part;cytoskeleton;intracellular non-membrane-bounded organelle;intracellular organelle;intracellular part;leading edge membrane;membrane part;non-membrane-bounded organelle;organelle;plasma membrane part;ruffle membrane		Homo sapiens	1	6
cell body;cell part;cell projection;cytoplasm;cytoskeleton;dendrite;intracellular non-membrane-bounded organelle;intracellular organelle;intracellular part;membrane;neuron projection;neuronal cell body;non-membrane-bounded organelle;organelle;plasma membrane		Homo sapiens	1	6
cell part;cytoplasm;cytoplasmic part;cytosol;eukaryotic translation initiation factor 2B complex;intracellular part;macromolecular complex;membrane;plasma membrane;protein complex	RNA transport	Homo sapiens	1	6
cell part;cytoplasmic part;cytoskeletal part;endoplasmic reticulum;integral to membrane;integral to plasma membrane;intracellular membrane-bounded organelle;intracellular organelle;intracellular organelle part;intracellular part;intrinsic to membrane;intrinsic to plasma membrane;membrane;membrane part;membrane-bounded		Homo sapiens	1	6
cell part;intracellular membrane-bounded organelle;intracellular organelle;intracellular organelle part;intracellular part;membrane;membrane-bounded organelle;nuclear body;nuclear membrane;nuclear part;nuclear speck;nucleoplasm;nucleoplasm part;nucleus;organelle;organelle membrane;organelle part		Homo sapiens	1	6
cell part;cytoplasmic part;extracellular region;intracellular membrane-bounded organelle;intracellular organelle;intracellular part;lysosome;lytic vacuole;membrane;membrane-bounded organelle;mitochondrion;nucleus;organe	Aminobenzoate degradation;Bisphenol degradation	Homo sapiens	1	6
cell part;chromatin remodeling complex;histone methyltransferase complex;intracellular membrane-bounded organelle;intracellular non-membrane-bounded organelle;intracellular organelle;intracellular organelle part;intracellular part;macromolecular complex;membrane-bounded organelle;methyltransferase complex;MLL1 comp		Homo sapiens	1	6
cell part;cytoplasmic part;cytosol;intracellular part;membrane	Pancreatic cancer;Pathways in cancer	Homo sapiens	1	6
cell part;cytoplasm;extracellular membrane-bounded organelle;extracellular organelle;extracellular region part;extracellular vesicular exosome;intracellular membrane-bounded organelle;intracellular organelle;intracellular organelle part;intracellular part;membrane-bounded organelle;membrane-bounded vesicle;nuclear part;nucle		Homo sapiens	1	6
cell part;intracellular non-membrane-bounded organelle;intracellular organelle;intracellular organelle part;intracellular part;non-membrane-bounded organelle;nuclear part;nucleolus;organelle;organelle part		Homo sapiens	1	6
cell part;cytoplasmic part;endoplasmic reticulum membrane;endoplasmic reticulum part;extracellular matrix;extracellular region part;integral to membrane;intracellular organelle part;intracellular part;intrinsic to membrane;me	Biosynthesis of unsaturated fatty acids;Steroid hormone biosynthesis	Homo sapiens	1	6
cell part;intracellular membrane-bounded organelle;intracellular non-membrane-bounded organelle;intracellular organelle;intracellular organelle part;intracellular part;membrane-bounded organelle;non-membrane-bounded organelle;nuclear part;nucleolus;nucleus;organelle;organelle part		Homo sapiens	1	6
cell junction;cell part;cell-cell junction;cytoplasmic part;endosome;Golgi apparatus;Golgi apparatus part;intracellular membrane-bounded organelle;intracellular organelle;intracellular organelle part;intracellular part;membrane	Tight junction	Homo sapiens	1	6
cell part;cytoplasmic part;intracellular non-membrane-bounded organelle;intracellular organelle;intracellular organelle part;intracellular part;macromolecular complex;membrane;mitochondrial inner membrane;mitochondrial membrane;mitochondrial part;non-membrane-bounded organelle;organelle;organelle inner membrane;orga		Homo sapiens	1	6
adherens junction;anchoring junction;cell junction;cell part;cell-substrate adherens junction;cell-substrate junction;focal adhesion;integral to membrane;intrinsic to membrane;membrane part		Homo sapiens	1	6
cell part;cytoplasmic part;integral to membrane;intracellular organelle part;intracellular part;intrinsic to membrane;membrane;membrane part;mitochondrial inner membrane;mitochondrial membrane;mitochondrial part;organelle inner membrane;organelle membrane;organelle part		Homo sapiens	1	6
cell part;cytoplasmic part;intracellular membrane-bounded organelle;intracellular organelle;intracellular part;membrane-bounded organelle;mitochondrion;organelle	Selenocompound metabolism;Tryptophan metabolism	Homo sapiens	1	6
cell part;cytoplasmic part;endoplasmic reticulum;endoplasmic reticulum membrane;endoplasmic reticulum part;Golgi apparatus part;Golgi membrane;Golgi stack;integral to membrane;intracellular membrane-bounded organ	Ether lipid metabolism;Glycerophospholipid metabolism	Homo sapiens	1	6
		Homo sapiens	1	6
cell part;cell surface;ion channel complex;macromolecular complex;membrane;membrane part;plasma membrane;protein complex		Homo sapiens	1	6
cell part;chromatin;chromosomal part;heterochromatin;intracellular membrane-bounded organelle;intracellular organelle;intracellular organelle part;intracellular part;macromolecular complex;membrane-bounded organelle;nuclear part;nucleoplasm;nucleus;organelle;organelle part;PcG protein complex;PRC1 complex;protein com		Homo sapiens	1	6
		Homo sapiens	1	6
cell part;cytoplasm;intracellular non-membrane-bounded organelle;intracellular organelle;intracellular organelle part;intracellular part;non-membrane-bounded organelle;nuclear part;nucleolus;organelle;organelle part		Homo sapiens	1	6
cell part;cytoplasm;intracellular membrane-bounded organelle;intracellular organelle;intracellular part;membrane-bounded organelle;nucleus;organelle		Homo sapiens	1	6
		Homo sapiens	1	6
apical part of cell;cell part;centriole;centrosome;cytoplasmic part;cytoskeletal part;cytosol;intracellular non-membrane-bounded organelle;intracellular organelle;intracellular organelle part;intracellular part;membrane;microtubule organizing center;microtubule organizing center part;non-membrane-bounded organelle;nuclear part		Homo sapiens	1	6
cell part;integral to membrane;intrinsic to membrane;lateral plasma membrane;membrane part;plasma membrane part	Wnt signaling pathway	Homo sapiens	1	6
cell part;cell surface;cytoplasmic part;endoplasmic reticulum;endoplasmic reticulum part;integral to cytosolic side of endoplasmic reticulum membrane;integral to endoplasmic reticulum membrane;integral to lumenal side of endoplasmic reticulum membrane;integral to membrane;integral to organelle membrane;intracellular mer		Homo sapiens	1	6
apical plasma membrane;cell part;cytoplasmic membrane-bounded vesicle;cytoplasmic part;cytoplasmic vesicle;cytosol;endocytic vesicle;intracellular membrane-bounded organelle;intracellular organelle;intracellular part;membrane;membrane part;membrane-bounded organelle;membrane-bounded vesicle;organelle;plasma m		Homo sapiens	1	6
cell part;cell projection;cytoplasmic part;cytoskeleton;cytosol;extracellular membrane-bounded organelle;extracellular organelle;extracellular region part;extracellular vesicular exosome;intracellular non-membrane-bounded c	Regulation of actin cytoskeleton	Homo sapiens	1	6
cell part;cytoplasmic part;cytosol;early endosome membrane;endosomal part;endosome membrane;intracellular organelle part;intracellular part;membrane;organelle membrane;organelle part	Endocytosis;Jak-STAT signaling pathway	Homo sapiens	1	6
cell part;intracellular non-membrane-bounded organelle;intracellular organelle;intracellular organelle part;intracellular part;macromolecular complex;non-membrane-bounded organelle;nuclear part;nucleolar part;nucleolus;org	Ribosome biogenesis in eukaryotes	Homo sapiens	1	6
cell part;cytoplasm;intracellular part		Homo sapiens	1	6
cell part;coated pit;membrane;membrane part;plasma membrane		Homo sapiens	1	6
cell part;cytoplasmic part;intracellular membrane-bounded organelle;intracellular organelle;intracellular part;membrane-bounded organelle;mitochondrion;organelle		Homo sapiens	1	6
cell part;cytoplasmic part;integral to membrane;intracellular membrane-bounded organelle;intracellular organelle;intracellular organelle part;intracellular part;intrinsic to membrane;membrane part;membrane-bounded organelle;mitochondrion;nuclear part;nucleoplasm;organelle;organelle part		Homo sapiens	1	6
cell junction;cell part;cytoplasm;cytoplasmic part;cytosol;extrinsic to membrane;extrinsic to plasma membrane;intracellular membrane-bounded organelle;intracellular non-membrane-bounded organelle;intracellular organelle;intracellular organelle lumen;intracellular organelle part;intracellular part;membrane;membrane part;mem		Homo sapiens	1	6
		Homo sapiens	1	6
cell cortex part;cell part;cytoplasmic part;cytosol;exocyst;intracellular membrane-bounded organelle;intracellular organelle;intracellular part;macromolecular complex;membrane;membrane-bounded organelle;organelle;plasma membrane;protein complex		Homo sapiens	1	6
cell part;intracellular membrane-bounded organelle;intracellular organelle;intracellular part;membrane-bounded organelle;nucleus;organelle	Spliceosome	Homo sapiens	1	6
cell part;chromosomal part;chromosome, telomeric region;intracellular;intracellular membrane-bounded organelle;intracellular non-membrane-bounded organelle;intracellular organelle;intracellular organelle part;intracellular part;macromolecular complex;membrane-bounded organelle;non-membrane-bounded organelle;nuclear b		Homo sapiens	1	6
cell part;cytoplasm;cytoplasmic part;endosomal part;endosome membrane;Golgi apparatus;intracellular membrane-bounded organelle;intracellular organelle;intracellular organelle part;intracellular part;late endosome membrane;membrane;membrane-bounded organelle;organelle;organelle membrane;organelle part		Homo sapiens	1	6
		Homo sapiens	1	6
aggresome;cell part;cytoplasmic part;cytoskeleton;cytosol;endoplasmic reticulum;endoplasmic reticulum membrane;endoplasmic reticulum part;inclusion body;integral to membrane;intermediate filament cytoskeleton;intrace	Protein export;Protein processing in endoplasmic reticulum	Homo sapiens	1	6
cell part;chromatin;chromosomal part;euchromatin;heterochromatin;histone methyltransferase complex;intracellular membrane-bounded organelle;intracellular organelle;intracellular organelle part;intracellular part;macromolecular complex;membrane-bounded organelle;methyltransferase complex;MLL1 complex;nuclear body;nu		Homo sapiens	1	6
cell part;cytoplasm;cytoplasmic part;early endosome;endosomal part;endosome;endosome membrane;ESCRT I complex;extracellular membrane-bounded organelle;extracellular organelle;extracellular region part;extracellula	Endocytosis	Homo sapiens	1	6
		Homo sapiens	1	6
		Homo sapiens	1	6
cell part;cytoplasmic part;cytoplasmic ubiquitin ligase complex;early endosome;endoplasmic reticulum;endoplasmic reticulum part;endosome;ER ubiquitin ligase complex;Hrd1p ubiquitin ligase complex;integral to endoplasr	Amyotrophic lateral sclerosis (ALS);Protein processing in endoplasmic reticulum	Homo sapiens	1	6
cell part;cytoplasm;intracellular membrane-bounded organelle;intracellular organelle;intracellular organelle part;intracellular part;membrane-bounded organelle;nuclear part;nucleoplasm;nucleus;organelle;organelle part		Homo sapiens	1	6
cell part;cytoplasmic part;integral to membrane;intracellular membrane-bounded organelle;intracellular non-membrane-bounded organelle;intracellular organelle;intracellular organelle part;intracellular part;intrinsic to membrane;macromolecular complex;membrane;membrane part;membrane-bounded organelle;Noc complex;No		Homo sapiens	1	6
		Homo sapiens	1	6
cell part;cytoplasmic part;extracellular membrane-bounded organelle;extracellular organelle;extracellular region part;extracellular vesicular exosome;intracellular;intracellular organelle lumen;intracellular organelle part;intracell	Purine metabolism	Homo sapiens	1	6
cell part;chromosome;condensed chromosome;condensed nuclear chromosome;cytoplasm;intracellular membrane-bounded organelle;intracellular non-membrane-bounded organelle;intracellular organelle;intracellular organelle part;intracellular part;membrane-bounded organelle;non-membrane-bounded organelle;nuclear chro		Homo sapiens	1	6
cell part;cytoplasmic membrane-bounded vesicle;cytoplasmic part;cytoplasmic vesicle;cytoplasmic vesicle membrane;cytoplasmic vesicle part;endocytic vesicle;endocytic vesicle membrane;extracellular membrane-bounded organelle;extracellular organelle;extracellular region part;extracellular vesicular exosome;Golgi apparatu		Homo sapiens	1	6
cell part;cytoskeletal part;intracellular membrane-bounded organelle;intracellular organelle;intracellular organelle part;intracellular part;macromolecular complex;membrane-bounded organelle;microtubule;nuclear part;nucleop	Wnt signaling pathway	Homo sapiens	1	6
cell part;cytoplasm;cytoplasmic part;Golgi apparatus;intracellular membrane-bounded organelle;intracellular organelle;intracellular organelle part;intracellular part;membrane;membrane-bounded organelle;nuclear part;nucleoplasm;nucleus;organelle;organelle part;plasma membrane		Homo sapiens	1	6
cell part;cytoplasmic part;integral to membrane;intracellular membrane-bounded organelle;intracellular organelle;intracellular organelle part;intracellular part;intrinsic to membrane;membrane;membrane part;membrane-bounded organelle;mitochondrial inner membrane;mitochondrial membrane;mitochondrial part;mitochondrion;		Homo sapiens	1	6
cell part;cytoplasmic part;endoplasmic reticulum;endoplasmic reticulum membrane;endoplasmic reticulum part;integral to membrane;intracellular membrane-bounded organelle;intracellular non-membrane-bounded organelle;intracellular organelle;intracellular organelle part;intracellular part;intrinsic to membrane;membrane;mem		Homo sapiens	1	6
cell part;cytoplasm;intracellular organelle part;intracellular part;macromolecular complex;NELF complex;nuclear part;nucleoplasm;nucleoplasm part;organelle part;protein complex;transcription elongation factor complex		Homo sapiens	1	6
cell part;cytoplasmic part;cytosol;extracellular membrane-bounded organelle;extracellular organelle;extracellular region part;extracellular vesicular exosome;intracellular part;membrane-bounded organelle;membrane-bounde	Cell cycle - yeast	Homo sapiens	0.9999	6
cell part;cytoplasmic part;cytosol;extracellular membrane-bounded organelle;extracellular organelle;extracellular region part;extracellular vesicular exosome;intracellular part;membrane-bounded organelle;membrane-bounded vesicle;organelle;vesicle		Homo sapiens	1	6

cell part;cell projection;centrosome;cilium;cytoplasm;cytoplasmic dynein complex;cytoplasmic part;cytoskeletal part;dynein complex;intracellular non-membrane-bounded organelle;intracellular organelle;intracellular organelle part;intracellular part;macromolecular complex;membrane;microtubule;microtubule associated complex		Homo sapiens	1	6
cell part;ER membrane protein complex;integral to membrane;intrinsic to membrane;macromolecular complex;membrane part;protein complex		Homo sapiens	1	6
cell part;chromatin;chromosomal part;cytoplasm;cytoplasmic exosome (RNase complex);cytoplasmic part;cytosol;exosome (RNase complex);intracellular membrane-bounded organelle;intracellular non-membrane-bounded	RNA degradation	Homo sapiens	1	6
cell part;chromatin;chromosomal part;cytoplasm;cytoplasmic exosome (RNase complex);cytoplasmic part;cytosol;exosome (RNase complex);intracellular membrane-bounded organelle;intracellular non-membrane-bounded	RNA degradation	Homo sapiens	1	6
cell part;cytoplasmic part;integral to membrane;integral to mitochondrial membrane;integral to organelle membrane;intracellular organelle part;intracellular part;intrinsic to membrane;intrinsic to organelle membrane;membran	ABC transporters	Homo sapiens	1	6
cell part;cytoplasm;intracellular part	Pantothenate and CoA biosynthesis	Homo sapiens	1	6
cell part;cytoplasmic part;intracellular organelle part;intracellular part;macromolecular complex;membrane;mitochondrial inner membrane;mitochondrial membrane;mitochondrial part;mitochondrial small ribosomal subunit;organellar small ribosomal subunit;organelle inner membrane;organelle membrane;organelle part;ribonuclec		Homo sapiens	1	6
cell part;cytoplasmic part;intracellular membrane-bounded organelle;intracellular organelle;intracellular part;membrane-bounded organelle;mitochondrion;organelle		Homo sapiens	1	6
cell part;cytoplasmic part;intracellular membrane-bounded organelle;intracellular organelle;intracellular part;membrane-bounded organelle;mitochondrion;organelle		Homo sapiens	1	6
Cajal body;cell part;cytoplasm;intracellular non-membrane-bounded organelle;intracellular organelle;intracellular organelle part;intracellular part;macromolecular complex;non-membrane-bounded organelle;nuclear body;nucl	Ribosome biogenesis in eukaryotes	Homo sapiens	1	6
box H/ACA RNP complex;box H/ACA snoRNP complex;Cajal body;cell part;intracellular membrane-bounded organelle;intracellular non-membrane-bounded organelle;intracellular organelle;intracellular organelle part;intracell	Ribosome biogenesis in eukaryotes	Homo sapiens	1	6
cell part;chromatin;chromatin remodeling complex;chromosomal part;chromosome, centromeric region;cytoplasm;cytoplasmic part;cytosol;heterochromatin;intracellular membrane-bounded organelle;intracellular non-memb	Amyotrophic lateral sclerosis (ALS);MAPK signaling pathway	Homo sapiens	1	6
cell part;cytoplasm;intracellular membrane-bounded organelle;intracellular organelle;intracellular part;membrane;membrane-bounded organelle;nucleus;organelle;plasma membrane	Protein processing in endoplasmic reticulum	Homo sapiens	1	6
cell cortex;cell part;cell projection;cytoplasmic part;endomembrane system;intracellular organelle part;intracellular part;lamellipodium;membrane;nuclear part;nucleoplasm;organelle part;perinuclear region of cytoplasm		Homo sapiens	1	6
cell part;cytoplasmic part;cytosol;extracellular membrane-bounded organelle;extracellular organelle;extracellular region part;extracellular vesicular exosome;intracellular membrane-bounded organelle;intracellular organelle;int	Cysteine and methionine metabolism	Homo sapiens	1	6
cell part;cell projection;cell projection part;cullin-RING ubiquitin ligase complex;cytoplasmic part;cytosol;dendritic spine;endoplasmic reticulum;extracellular membrane-bounded organelle;extracellular organelle;extracellular r	Protein processing in endoplasmic reticulum;Ubiquitin mediated proteolysis	Homo sapiens	1	6
cell part;endomembrane system;extracellular region part;extracellular space;intracellular membrane-bounded organelle;intracellular organelle;intracellular organelle part;intracellular part;membrane;membrane-bounded organelle;nuclear part;nucleoplasm;nucleus;organelle;organelle part		Homo sapiens	1	6
adherens junction;anchoring junction;cell junction;cell part;cell surface;cell-substrate adherens junction;cell-substrate junction;centrosome;cytoplasmic part;cytoskeletal part;extracellular membrane-bounded organelle;extracellular organelle;extracellular region;extracellular region part;extracellular vesicular exosome;focal adhesi		Homo sapiens	1	6
cell part;cytoplasmic part;endoplasmic reticulum membrane;endoplasmic reticulum part;endoplasmic reticulum-Golgi intermediate compartment membrane;Golgi apparatus;integral to membrane;intracellular membrane-bounded organelle;intracellular organelle;intracellular organelle part;intracellular part;intrinsic to membrane;m		Homo sapiens	1	6
cell part;cytoplasm;cytoplasmic part;cytosol;exosome (RNase complex);intracellular membrane-bounded organelle;intracellular non-membrane-bounded organelle;intracellular organelle;intracellular organelle part;intracellular	RNA degradation	Homo sapiens	1	6
cell part;cytoplasmic mRNA processing body;cytoplasmic part;intracellular non-membrane-bounded organelle;intracellular organelle;intracellular part;macromolecular complex;non-membrane-bounded organelle;organelle;ribonucleoprotein complex;RNA granule		Homo sapiens	1	6
cell part;intracellular membrane-bounded organelle;intracellular non-membrane-bounded organelle;intracellular organelle;intracellular organelle part;intracellular part;macromolecular complex;membrane-bounded organelle;nc	Ribosome biogenesis in eukaryotes	Homo sapiens	1	6
cell part;cytoplasmic part;intracellular membrane-bounded organelle;intracellular organelle;intracellular organelle part;intracellular part;macromolecular complex;membrane;membrane-bounded organelle;membrane-enclosed lumen;mitochondrial inner membrane;mitochondrial intermembrane space;mitochondrial intermembrane		Homo sapiens	1	6
cell part;cytoplasmic part;endoplasmic reticulum membrane;endoplasmic reticulum part;extracellular membrane-bounded organelle;extracellular organelle;extracellular region part;extracellular vesicular exosome;integral to membrane;intracellular organelle part;intracellular part;intrinsic to membrane;membrane;membrane part;m		Homo sapiens	1	6
cell part;cytoplasmic part;intracellular membrane-bounded organelle;intracellular organelle;intracellular part;membrane-bounded organelle;mitochondrion;nucleus;organelle		Homo sapiens	1	6
adherens junction;anchoring junction;azurophil granule;cell junction;cell part;cell projection;cell-substrate adherens junction;cell-substrate junction;cytoplasm;cytoplasmic membrane-bounded vesicle;cytoplasmic part;cytopla	SNARE interactions in vesicular transport	Homo sapiens	1	7
cell part;intracellular membrane-bounded organelle;intracellular organelle;intracellular organelle part;intracellular part;membrane-bounded organelle;nuclear part;nucleoplasm;nucleus;organelle;organelle part		Homo sapiens	1	7
cell part;chromosome;intracellular non-membrane-bounded organelle;intracellular organelle;intracellular organelle part;intracellular part;macromolecular complex;Mpp10 complex;non-membrane-bounded organelle;nuclear p	Ribosome biogenesis in eukaryotes	Homo sapiens	1	7
azurophil granule;cell part;cytoplasmic membrane-bounded vesicle;cytoplasmic part;cytoplasmic vesicle;early endosome;early endosome membrane;endocytic vesicle;endomembrane system;endosomal part;endosome;end	Phagosome;SNARE interactions in vesicular transport	Homo sapiens	1	7
Ada2/Gcn5/Ada3 transcription activator complex;cell part;cytoplasm;cytoplasmic part;cytosol;endosomal part;endosome membrane;histone acetyltransferase complex;intracellular membrane-bounded organelle;intracellular	Adherens junction;Leishmaniasis;MAPK signaling pathway;Measles;NOD-like receptor signaling pathwa	Homo sapiens	1	7
cell part;cytoplasm;cytoplasmic part;ER membrane protein complex;intracellular membrane-bounded organelle;intracellular organelle;intracellular part;macromolecular complex;membrane;membrane-bounded organelle;mitochondrion;nucleus;organelle;protein complex		Homo sapiens	1	7
cell part;cytoplasm;intracellular organelle part;intracellular part;nuclear part;nucleoplasm;organelle part		Homo sapiens	1	7
cell part;cytoplasmic part;intracellular membrane-bounded organelle;intracellular organelle;intracellular organelle part;intracellular part;macromolecular complex;membrane;membrane part;membrane-bounded organelle;mem	Alzheimer's disease;Huntington's disease;Oxidative phosphorylation;Parkinson's disease	Homo sapiens	1	7
cell part;cytoplasmic part;dolichol-phosphate-mannose synthase complex;endoplasmic reticulum;endoplasmic reticulum membrane;endoplasmic reticulum part;intracellular membrane-bounded organelle;intracellular organel	N-Glycan biosynthesis	Homo sapiens	1	7
cell part;cytoplasmic part;endosome;intracellular membrane-bounded organelle;intracellular organelle;intracellular part;membrane-bounded organelle;organelle		Homo sapiens	1	7
cell part;cytoplasm;cytoplasmic part;cytosol;intracellular organelle part;intracellular part;membrane;nuclear part;nucleoplasm;organelle part;perinuclear region of cytoplasm		Homo sapiens	1	7
cell part;cytoplasmic part;cytosol;extracellular membrane-bounded organelle;extracellular organelle;extracellular region part;extracellular vesicular exosome;intracellular membrane-bounded organelle;intracellular organelle;int	Glycerophospholipid metabolism	Homo sapiens	1	7
cell part;cytoplasmic part;cytosol;intracellular part;membrane	MAPK signaling pathway	Homo sapiens	1	7
cell part;cytoplasm;cytoplasmic part;cytoskeleton;cytosol;intermediate filament cytoskeleton;intracellular membrane-bounded organelle;intracellular non-membrane-bounded organelle;intracellular organelle;intracellular organelle part;intracellular part;macromolecular complex;membrane-bounded organelle;non-membrane-bounc		Homo sapiens	1	7
cell part;cytoplasm;cytoplasmic part;extracellular membrane-bounded organelle;extracellular organelle;extracellular region part;extracellular vesicular exosome;intracellular;intracellular membrane-bounded organelle;intracellular organelle;intracellular part;membrane-bounded organelle;membrane-bounded vesicle;mitochondrion;		Homo sapiens	1	7
cell part;cytoplasmic part;Golgi apparatus;integral to membrane;intracellular membrane-bounded organelle;intracellular organelle;intracellular part;intrinsic to membrane;membrane part;membrane-bounded organelle;nucleus;organelle		Homo sapiens	1	7
cell part;cytoplasmic part;extracellular membrane-bounded organelle;extracellular organelle;extracellular region part;extracellular vesicular exosome;integral to membrane;intracellular membrane-bounded organelle;intracellul	Alzheimer's disease;Huntington's disease;Oxidative phosphorylation;Parkinson's disease	Homo sapiens	1	7
cell part;cytoplasm;cytoplasmic part;integral to membrane;intracellular organelle part;intracellular part;intrinsic to membrane;macromolecular complex;membrane;membrane part;mitochondrial inner membrane;mitochondrial	Alzheimer's disease;Huntington's disease;Oxidative phosphorylation;Parkinson's disease	Homo sapiens	1	7
cell part;cytoplasm;cytoplasmic part;endoplasmic reticulum;intracellular membrane-bounded organelle;intracellular organelle;intracellular part;membrane-bounded organelle;nucleus;organelle		Homo sapiens	1	7
cell part;cytoplasmic part;endoplasmic reticulum lumen;endoplasmic reticulum part;intracellular organelle lumen;intracellular organelle part;intracellular part;membrane-enclosed lumen;organelle lumen;organelle part	Glutathione metabolism	Homo sapiens	1	7
cell part;cell projection;cytoplasm;cytoplasmic part;cytosol;Golgi apparatus;intracellular membrane-bounded organelle;intracellular organelle;intracellular organelle part;intracellular part;membrane;membrane-bounded organe	Acute myeloid leukemia;B cell receptor signaling pathway;Bladder cancer;Chemokine signaling pathway	Homo sapiens	1	7
cell part;cytoplasmic part;cytosol;intracellular membrane-bounded organelle;intracellular organelle;intracellular organelle lumen;intracellular organelle part;intracellular part;membrane-bounded organelle;membrane-enclosed l	Carbon fixation pathways in prokaryotes;Glyoxylate and dicarboxylate metabolism;Propanoate metabol	Homo sapiens	1	7
cell part;chromatin;chromatin remodeling complex;chromosomal part;cytoskeletal part;intracellular membrane-bounded organelle;intracellular non-membrane-bounded organelle;intracellular organelle;intracellular organelle pa	Bladder cancer;Cell cycle;Chronic myeloid leukemia;Glioma;Melanoma;Non-small cell lung cancer;Panc	Homo sapiens	1	7
cell part;cytoplasm;extracellular membrane-bounded organelle;extracellular organelle;extracellular region part;extracellular vesicular exosome;intracellular membrane-bounded organelle;intracellular organelle;intracellular organelle part;intracellular part;membrane-bounded organelle;membrane-bounded vesicle;nuclear part;nucle		Homo sapiens	1	7
extracellular membrane-bounded organelle;extracellular organelle;extracellular region part;extracellular vesicular exosome;membrane-bounded organelle;membrane-bounded vesicle;organelle;vesicle	Glycolysis / Gluconeogenesis	Homo sapiens	1	7
cell part;cytoplasmic part;cytosol;intracellular part	Porphyrin and chlorophyll metabolism	Homo sapiens	1	7
Cajal body;cell part;commitment complex;intracellular organelle part;intracellular part;macromolecular complex;nuclear body;nuclear part;nucleoplasm;nucleoplasm part;organelle part;prespliceosome;ribonucleoprotein comp	Spliceosome	Homo sapiens	1	7
cell part;cytoplasmic part;cytosol;intracellular part	Sulfur metabolism	Homo sapiens	0.9981	7
cell part;cytoplasmic part;intracellular membrane-bounded organelle;intracellular organelle;intracellular organelle lumen;intracellular organelle part;intracellular part;membrane-bounded organelle;membrane-enclosed lumen;mitochondrial matrix;mitochondrial part;mitochondrion;organelle;organelle lumen;organelle part		Homo sapiens	1	7
cell part;cytoplasmic part;extracellular membrane-bounded organelle;extracellular organelle;extracellular region part;extracellular vesicular exosome;intracellular membrane-bounded organelle;intracellular organelle;intracellul	Alzheimer's disease;Cardiac muscle contraction;Huntington's disease;Oxidative phosphorylation;Parkin	Homo sapiens	1	7
actin filament;caveola;cell part;cell projection;cell projection membrane;cell projection part;cytoplasm;cytoplasmic part;cytoskeletal part;cytosol;endosome;extracellular membrane-bounded organelle;extracellular organelle;ex	Adherens junction;Bacterial invasion of epithelial cells;Endocytosis;Epithelial cell signaling in Helicobact	Homo sapiens	1	7
cell part;cytoplasmic part;cytoplasmic vesicle membrane;cytoplasmic vesicle part;endocytic vesicle membrane;endosomal part;endosome;endosome membrane;extracellular membrane-bounded organelle;extracellular orgar	ko05152;Lysosome;Phagosome	Homo sapiens	1	7
adherens junction;anchored to external side of plasma membrane;anchored to membrane;anchored to plasma membrane;anchoring junction;cell junction;cell part;cell surface;cell-substrate adherens junction;cell-substrate ju	Complement and coagulation cascades;Hematopoietic cell lineage	Homo sapiens	1	7
cell part;chromatin;chromosomal part;chromosome, centromeric region;condensed chromosome, centromeric region;cytoplasm;intracellular membrane-bounded organelle;intracellular organelle;intracellular organelle part;intr	ko05152	Homo sapiens	1	7
cell part;cytoplasmic part;endoplasmic reticulum;endoplasmic reticulum-Golgi intermediate compartment;intracellular membrane-bounded organelle;intracellular organelle;intracellular organelle part;intracellular part;membrane;membrane-bounded organelle;nuclear part;nucleoplasm;nucleus;organelle;organelle part;plasma mem		Homo sapiens	1	7
cell part;cytoplasmic part;intracellular membrane-bounded organelle;intracellular organelle;intracellular organelle part;intracellular part;macromolecular complex;membrane-bounded organelle;mitochondrion;NELF complex;nuclear part;nucleoplasm;nucleoplasm part;nucleus;organelle;organelle part;protein complex;transcription		Homo sapiens	1	7
cell part;cytoplasmic part;intracellular membrane-bounded organelle;intracellular organelle;intracellular organelle part;intracellular part;macromolecular complex;membrane;membrane part;membrane-bounded organelle;mitc	Alzheimer's disease;Huntington's disease;Oxidative phosphorylation;Parkinson's disease	Homo sapiens	1	7
cell part;cytoplasm;cytoplasmic part;cytosol;intracellular membrane-bounded organelle;intracellular non-membrane-bounded organelle;intracellular organelle;intracellular part;macromolecular complex;membrane;membrane-	Hepatitis C;Measles;Protein processing in endoplasmic reticulum	Homo sapiens	1	7
adherens junction;anchoring junction;apical plasma membrane;cell junction;cell part;cell-substrate adherens junction;cell-substrate junction;clathrin coated vesicle membrane;clathrin-coated endocytic vesicle membrane;coa	Hematopoietic cell lineage	Homo sapiens	1	7
cell part;cytoplasmic part;extracellular membrane-bounded organelle;extracellular organelle;extracellular region part;extracellular vesicular exosome;intracellular membrane-bounded organelle;intracellular organelle;intracellul	Purine metabolism	Homo sapiens	1	7
cell part;integral to membrane;integral to plasma membrane;intrinsic to membrane;intrinsic to plasma membrane;membrane;membrane part;plasma membrane;plasma membrane part		Homo sapiens	1	7
axon part;cell part;cell projection part;cytoplasm;cytoskeleton;extracellular membrane-bounded organelle;extracellular organelle;extracellular region part;extracellular vesicular exosome;intracellular non-membrane-bounded c	Regulation of actin cytoskeleton;Shigellosis	Homo sapiens	1	7
cell part;cytoplasmic part;cytosol;intracellular membrane-bounded organelle;intracellular organelle;intracellular organelle part;intracellular part;membrane;membrane-bounded organelle;nuclear part;nucleoplasm;nucleus;orga	Alzheimer's disease;Amoebiasis;Amyotrophic lateral sclerosis (ALS);Apoptosis;Colorectal cancer;Epithe	Homo sapiens	1	7
cell part;cytoplasm;cytoplasmic part;endoplasmic reticulum;endoplasmic reticulum membrane;endoplasmic reticulum part;intracellular membrane-bounded organelle;intracellular organelle;intracellular organelle part;intracellular part;IPAF inflammasome complex;macromolecular complex;membrane;membrane part;membrane-bc		Homo sapiens	1	7
cell part;cytoplasmic part;integral to membrane;integral to mitochondrial inner membrane;integral to mitochondrial membrane;integral to organelle membrane;intracellular organelle part;intracellular part;intrinsic to membrane;	Porphyrin and chlorophyll metabolism	Homo sapiens	1	7
apical plasma membrane;cell part;cell projection;cilium;cytoplasmic membrane-bounded vesicle;cytoplasmic part;cytoplasmic vesicle;cytoplasmic vesicle membrane;cytoplasmic vesicle part;dendrite;endosomal part;endosome;endosome membrane;exocytic vesicle;extracellular membrane-bounded organelle;extracellular orgar		Homo sapiens	1	7
cell part;cyclin-dependent protein kinase activating kinase holoenzyme complex;holo TFIIH complex;intracellular membrane-bounded organelle;intracellular organelle;intracellular organelle part;intracellular part;macromolecul	Basal transcription factors;Cell cycle;Nucleotide excision repair	Homo sapiens	1	7
cell cortex;cell part;cell projection;cytoplasm;cytoplasmic part;Golgi apparatus part;Golgi membrane;intracellular organelle part;intracellular part;membrane;organelle membrane;organelle part;plasma membrane;ruffle;trans-Golgi network membrane		Homo sapiens	1	7
cell part;centrosome;cytoplasmic membrane-bounded vesicle;cytoplasmic part;cytoplasmic vesicle;cytoskeletal part;cytosol;endoplasmic reticulum-Golgi intermediate compartment;extracellular membrane-bounded organelle;extracellular organelle;extracellular region part;extracellular vesicular exosome;intracellular membrane-		Homo sapiens	1	7
cell part;cytoplasmic part;cytosol;extracellular membrane-bounded organelle;extracellular organelle;extracellular region part;extracellular vesicular exosome;integral to membrane;integral to mitochondrial membrane;integral t	Alzheimer's disease;Amyotrophic lateral sclerosis (ALS);Apoptosis;ko05152;Natural killer cell mediated c	Homo sapiens	1	7
cell part;cytoplasmic part;intracellular membrane-bounded organelle;intracellular organelle;intracellular organelle part;intracellular part;macromolecular complex;membrane;membrane part;membrane-bounded organelle;mitc	Oxidative phosphorylation	Homo sapiens	1	7
cell part;cytoplasm;cytoskeletal part;extracellular membrane-bounded organelle;extracellular organelle;extracellular region part;extracellular vesicular exosome;intermediate filament;intracellular membrane-bounded organelle	Ribosome biogenesis in eukaryotes	Homo sapiens	1	7
cell part;cytoplasmic membrane-bounded vesicle;cytoplasmic part;cytoplasmic vesicle;endoplasmic reticulum;extracellular membrane-bounded organelle;extracellular organelle;extracellular region part;extracellular vesicular exosome;intracellular membrane-bounded organelle;intracellular organelle;intracellular organelle part;intr		Homo sapiens	1	7
cell part;cytoplasmic part;cytosol;endoplasmic reticulum;endoplasmic reticulum membrane;endoplasmic reticulum part;endoplasmic reticulum Sec complex;integral to membrane;intracellular membrane-bounded organelle;ir	Phagosome;Protein export;Protein processing in endoplasmic reticulum;Vibrio cholerae infection	Homo sapiens	1	7
cell part;cytoplasmic membrane-bounded vesicle;cytoplasmic part;cytoplasmic vesicle;early endosome;early endosome membrane;endocytic vesicle;endosomal part;endosome;endosome membrane;extracellular membrane	Amoebiasis;Endocytosis;ko05152;Phagosome;Vasopressin-regulated water reabsorption	Homo sapiens	1	7
cell part;cytoplasm;cytoplasmic part;cytoskeletal part;cytosol;intracellular organelle part;intracellular part;macromolecular complex;microtubule;organelle part;protein complex		Homo sapiens	1	7
cell part;centromeric heterochromatin;chromatin;chromocenter;chromosomal part;chromosome, centromeric region;cytoskeletal part;female pronucleus;heterochromatin;intracellular membrane-bounded organelle;intracellular non-membrane-bounded organelle;intracellular organelle;intracellular organelle part;intracellular part;ma		Homo sapiens	1	7
axon;cell body;cell junction;cell part;cell projection;cell projection part;cyclin-dependent protein kinase 5 holoenzyme complex;cyclin-dependent protein kinase holoenzyme complex;cytoplasm;cytoplasmic part;cytoskeletal p	Alzheimer's disease;Axon guidance	Homo sapiens	1	7
cell part;cytoplasm;cytoplasmic part;extracellular membrane-bounded organelle;extracellular organelle;extracellular region part;extracellular vesicular exosome;intracellular membrane-bounded organelle;intracellular non-membrane-bounded organelle;intracellular organelle;intracellular part;lysosome;lytic vacuole;macromolecular		Homo sapiens	1	7
apical plasma membrane;cell part;cytoplasm;cytoplasmic part;cytosol;extracellular membrane-bounded organelle;extracellular organelle;extracellular region part;extracellular vesicular exosome;integral to membrane;integral to plasma membrane;intracellular membrane-bounded organelle;intracellular non-membrane-bounded or		Homo sapiens	1	7
		Homo sapiens	1	7
Bcl-2 family protein complex;cell part;cytoplasm;cytoplasmic part;cytosol;integral to membrane;intracellular membrane-bounded organelle;intracellular organelle;intracellular organelle lumen;intracellular organelle part;intracellular part;intrinsic to membrane;macromolecular complex;membrane;membrane part;membrane-bounde		Homo sapiens	1	7
cell part;cytoplasm;cytoplasmic part;cytosol;extracellular membrane-bounded organelle;extracellular organelle;extracellular region part;extracellular vesicular exosome;Golgi apparatus;intracellular membrane-bounded organelle;intracellular organelle;intracellular part;membrane-bounded organelle;membrane-bounded vesicle;org		Homo sapiens	1	7
cell part;cytoplasmic part;early endosome membrane;endoplasmic reticulum;endosomal part;endosome membrane;Golgi apparatus part;Golgi membrane;intracellular membrane-bounded organelle;intracellular organelle;intracellular organelle part;intracellular part;late endosome membrane;macromolecular complex;membrane;r		Homo sapiens	1	7
		Homo sapiens	1	7
cell part;cytoplasm;cytoplasmic part;intracellular membrane-bounded organelle;intracellular non-membrane-bounded organelle;intracellular organelle;intracellular organelle part;intracellular part;macromolecular complex;membrane;membrane-bounded organelle;mitochondrial inner membrane;mitochondrial membrane;mitochon		Homo sapiens	1	7
cell part;intracellular membrane-bounded organelle;intracellular organelle;intracellular part;membrane;membrane-bounded organelle;nucleus;organelle		Homo sapiens	1	7
cell part;cytoplasmic part;intracellular membrane-bounded organelle;intracellular organelle;intracellular organelle lumen;intracellular organelle part;intracellular part;large ribosomal subunit;macromolecular complex;membrane;membrane-bounded organelle;membrane-enclosed lumen;mitochondrial inner membrane;mitochondrial		Homo sapiens	1	7
cell part;cytoplasmic part;endoplasmic reticulum;endoplasmic reticulum lumen;endoplasmic reticulum part;intracellular membrane-bounded organelle;intracellular non-membrane-bounded organelle;intracellular organelle;intracellular organelle lumen;intracellular organelle part;intracellular part;membrane-bounded organelle;mem		Homo sapiens	1	7
cell part;cell projection membrane;cell projection part;cytoplasm;intracellular membrane-bounded organelle;intracellular organelle;intracellular part;leading edge membrane;membrane part;membrane-bounded organelle;nucleus;organelle;plasma membrane part;ruffle membrane		Homo sapiens	1	7
cell part;cytoplasmic part;endoplasmic reticulum;intracellular membrane-bounded organelle;intracellular organelle;intracellular part;membrane;membrane-bounded organelle;organelle;plasma membrane		Homo sapiens	1	7
cell part;cytoplasm;cytoplasmic part;cytosol;intracellular membrane-bounded organelle;intracellular organelle;intracellular organelle part;intracellular part;membrane-bounded organelle;nuclear body;nuclear part;nuclear specl	mRNA surveillance pathway;RNA transport	Homo sapiens	1	7
cell part;coated vesicle;coated vesicle membrane;COPI coated vesicle membrane;COPI-coated vesicle;cytoplasmic membrane-bounded vesicle;cytoplasmic part;cytoplasmic vesicle;cytoplasmic vesicle membrane;cytoplasmic vesicle part;endoplasmic reticulum;endoplasmic reticulum membrane;endoplasmic reticulum part;en		Homo sapiens	1	7
cell part;cytoplasm;cytoskeleton;intracellular membrane-bounded organelle;intracellular non-membrane-bounded organelle;intracellular organelle;intracellular organelle part;intracellular part;membrane-bounded organelle;non-membrane-bounded organelle;nuclear part;nucleoplasm;nucleus;organelle;organelle part		Homo sapiens	1	7
cell part;cytoplasmic part;cytosol;extracellular membrane-bounded organelle;extracellular organelle;extracellular region part;extracellular vesicular exosome;intracellular membrane-bounded organelle;intracellular organelle;intracellular part;membrane-bounded organelle;membrane-bounded vesicle;nucleus;organelle;vesicle		Homo sapiens	1	7
cell part;cytoplasmic part;intracellular organelle part;intracellular part;macromolecular complex;membrane;membrane part;mitochondrial inner membrane;mitochondrial membrane;mitochondrial membrane part;mitochondrial	Alzheimer's disease;Huntington's disease;Oxidative phosphorylation;Parkinson's disease	Homo sapiens	1	7
cell part;cytoplasmic part;cytosol;intracellular part	Purine metabolism	Homo sapiens	1	7
cell part;intracellular organelle part;intracellular part;nuclear part;nucleoplasm;organelle part		Homo sapiens	1	7
cell part;cytoplasmic part;intracellular membrane-bounded organelle;intracellular organelle;intracellular part;membrane-bounded organelle;mitochondrion;organelle		Homo sapiens	1	7
cell part;cytoplasmic part;intracellular membrane-bounded organelle;intracellular organelle;intracellular organelle part;intracellular part;membrane;membrane-bounded organelle;mitochondrial membrane;mitochondrial part;mi	Glycerolipid metabolism	Homo sapiens	1	7
extracellular membrane-bounded organelle;extracellular organelle;extracellular region part;extracellular vesicular exosome;membrane-bounded organelle;membrane-bounded vesicle;organelle;vesicle		Homo sapiens	1	7
		Homo sapiens	1	7
cell part;cytoplasmic part;endoplasmic reticulum lumen;endoplasmic reticulum part;intracellular organelle lumen;intracellular organelle part;intracellular part;membrane-enclosed lumen;organelle lumen;organelle part		Homo sapiens	1	7
cell part;CUL4 RING ubiquitin ligase complex;cullin-RING ubiquitin ligase complex;cytoplasm;intracellular membrane-bounded organelle;intracellular organelle;intracellular part;macromolecular complex;membrane-bounded organelle;nucleus;organelle;protein complex;ubiquitin ligase complex		Homo sapiens	1	7
cell part;centrosome;contractile fiber;cytoplasm;cytoplasmic part;cytoskeletal part;Golgi apparatus;intracellular membrane-bounded organelle;intracellular non-membrane-bounded organelle;intracellular organelle;intracellular organelle part;intracellular part;membrane-bounded organelle;microtubule organizing center;myofibril;nc		Homo sapiens	1	7
		Homo sapiens	1	7
		Homo sapiens	1	7
cell part;cytoplasmic part;endosomal part;endosome;endosome membrane;integral to membrane;integral to plasma membrane;intracellular membrane-bounded organelle;intracellular organelle;intracellular organelle part;intra	p53 signaling pathway	Homo sapiens	1	7
		Homo sapiens	1	7
cell part;cytoplasm;cytoplasmic part;cytosol;intracellular organelle part;intracellular part;membrane;nuclear part;nucleoplasm;organelle part;plasma membrane		Homo sapiens	1	7
cell part;intracellular non-membrane-bounded organelle;intracellular organelle;intracellular organelle part;intracellular part;non-membrane-bounded organelle;nuclear part;nucleolus;organelle;organelle part		Homo sapiens	1	7
cell part;cytoplasm;cytoplasmic part;cytosol;extracellular membrane-bounded organelle;extracellular organelle;extracellular region part;extracellular vesicular exosome;intracellular membrane-bounded organelle;intracellular c	MAPK signaling pathway	Homo sapiens	1	7
cell part;cytoplasm;extracellular membrane-bounded organelle;extracellular organelle;extracellular region part;extracellular vesicular exosome;intracellular organelle part;intracellular part;membrane-bounded organelle;membr	Glutathione metabolism;p53 signaling pathway;Purine metabolism;Pyrimidine metabolism	Homo sapiens	1	7
cell part;chromatin;chromosomal part;intracellular organelle part;intracellular part;nuclear chromatin;nuclear chromosome part;nuclear part;organelle part		Homo sapiens	1	7
cell part;cytoplasm;intracellular membrane-bounded organelle;intracellular organelle;intracellular organelle part;intracellular part;macromolecular complex;membrane-bounded organelle;nuclear part;nucleoplasm;nucleus;orga	mRNA surveillance pathway	Homo sapiens	1	7
		Homo sapiens	1	7
cell part;chylomicron;extracellular membrane-bounded organelle;extracellular organelle;extracellular region part;extracellular vesicular exosome;integral to membrane;intrinsic to membrane;low-density lipoprotein particle;macromolecular complex;membrane;membrane part;membrane-bounded organelle;membrane-bounded ve		Homo sapiens	1	7
cell part;cell surface;cytoplasmic part;cytosol;extracellular matrix;extracellular membrane-bounded organelle;extracellular organelle;extracellular region;extracellular region part;extracellular space;extracellular vesicular exoso	Wnt signaling pathway	Homo sapiens	1	7
		Homo sapiens	1	7
cell part;cytoplasmic part;intracellular non-membrane-bounded organelle;intracellular organelle;intracellular organelle part;intracellular part;macromolecular complex;membrane;mitochondrial inner membrane;mitochondrial membrane;mitochondrial part;mitochondrial ribosome;non-membrane-bounded organelle;organellar ribosc		Homo sapiens	1	7
cell part;cytoplasm;intracellular organelle part;intracellular part;macromolecular complex;nuclear part;nucleoplasm;nucleoplasm part;organelle part;protein complex;transcription factor complex		Homo sapiens	1	7
cell part;cytoplasm;cytoplasmic part;intracellular membrane-bounded organelle;intracellular organelle;intracellular part;membrane-bounded organelle;mitochondrion;organelle	Ribosome biogenesis in eukaryotes	Homo sapiens	1	7
cell part;cytoplasm;cytoplasmic part;Golgi apparatus;intracellular membrane-bounded organelle;intracellular organelle;intracellular organelle part;intracellular part;membrane-bounded organelle;nuclear part;nucleoplasm;organelle;organelle part		Homo sapiens	1	7
cell part;cytoskeleton;envelope;intermediate filament cytoskeleton;intracellular membrane-bounded organelle;intracellular non-membrane-bounded organelle;intracellular organelle;intracellular organelle part;intracellular part;r	RNA transport	Homo sapiens	1	7

cell part;cytoplasmic part;cytosol;intracellular membrane-bounded organelle;intracellular organelle;intracellular part;membrane-bounded organelle;nucleus;organelle		Homo sapiens	1	7
cell part;intracellular membrane-bounded organelle;intracellular non-membrane-bounded organelle;intracellular organelle;intracellular organelle part;intracellular part;membrane-bounded organelle;non-membrane-bounded organelle;nuclear part;nucleolus;nucleus;organelle;organelle part		Homo sapiens	1	7
cell part;cell projection;cytoplasm;cytoplasmic part;cytoskeleton;cytosol;intracellular non-membrane-bounded organelle;intracellular organelle;intracellular part;lamellipodium;membrane;non-membrane-bounded organelle;org	Endocytosis	Homo sapiens	1	7
cell part;cytoplasmic part;cytosol;intracellular membrane-bounded organelle;intracellular organelle;intracellular organelle part;intracellular part;membrane-bounded organelle;nuclear part;nucleoplasm;nucleus;organelle;organe	Protein processing in endoplasmic reticulum	Homo sapiens	1	7
cell part;cytoplasmic part;intracellular organelle lumen;intracellular organelle part;intracellular part;membrane-enclosed lumen;mitochondrial matrix;mitochondrial part;organelle lumen;organelle part		Homo sapiens	1	7
cell part;intracellular membrane-bounded organelle;intracellular organelle;intracellular part;membrane-bounded organelle;nucleus;organelle		Homo sapiens	1	7
cell part;cytoplasmic part;intracellular non-membrane-bounded organelle;intracellular organelle;intracellular organelle part;intracellular part;macromolecular complex;membrane;mitochondrial inner membrane;mitochondrial membrane;mitochondrial part;non-membrane-bounded organelle;organelle;organelle inner membrane;orga		Homo sapiens	1	7
cell part;cytoplasm;intracellular part		Homo sapiens	1	7
cell part;cytoplasmic part;cytosol;endoplasmic reticulum-Golgi intermediate compartment;endosomal part;endosome;endosome membrane;Golgi apparatus part;Golgi membrane;intracellular membrane-bounded organelle;ir	Amino sugar and nucleotide sugar metabolism	Homo sapiens	1	7
cell part;cytoplasm;extracellular region part;intercellular bridge;intracellular membrane-bounded organelle;intracellular organelle;intracellular organelle part;intracellular part;macromolecular complex;membrane-bounded orgar	RNA transport;Spliceosome	Homo sapiens	1	7
cell part;intracellular membrane-bounded organelle;intracellular non-membrane-bounded organelle;intracellular organelle;intracellular organelle part;intracellular part;membrane-bounded organelle;non-membrane-bounded organelle;nuclear body;nuclear part;nuclear speck;nucleolus;nucleoplasm;nucleoplasm part;nucleus;organ		Homo sapiens	1	7
cell part;cytoplasmic part;endoplasmic reticulum;endoplasmic reticulum membrane;endoplasmic reticulum part;intracellular membrane-bounded organelle;intracellular organelle;intracellular organelle part;intracellular part;membrane;membrane part;membrane-bounded organelle;organelle;organelle membrane;organelle part		Homo sapiens	1	7
cell part;cytoplasm;cytoplasmic part;cytosol;extracellular membrane-bounded organelle;extracellular organelle;extracellular region part;extracellular vesicular exosome;intracellular membrane-bounded organelle;intracellular non-membrane-bounded organelle;intracellular organelle;intracellular organelle part;intracellular part;mem		Homo sapiens	1	7
cell part;cytoplasmic part;Golgi apparatus;intracellular membrane-bounded organelle;intracellular organelle;intracellular organelle part;intracellular part;macromolecular complex;membrane-bounded organelle;nuclear body;nu	RNA transport;Spliceosome	Homo sapiens	1	7
adherens junction;anchoring junction;Arp2/3 protein complex;cell junction;cell part;cell-substrate adherens junction;cell-substrate junction;cytoplasm;cytoskeletal part;extracellular membrane-bounded organelle;extracellular	Bacterial invasion of epithelial cells;Fc gamma R-mediated phagocytosis;Pathogenic Escherichia coli inf	Homo sapiens	1	7
cell part;cytoplasm;intracellular membrane-bounded organelle;intracellular non-membrane-bounded organelle;intracellular organelle;intracellular organelle part;intracellular part;membrane-bounded organelle;non-membrane-bounded organelle;nuclear part;nucleolus;nucleus;organelle;organelle part		Homo sapiens	1	7
cell part;cytoplasmic part;intracellular membrane-bounded organelle;intracellular organelle;intracellular part;membrane-bounded organelle;mitochondrion;nucleus;organelle		Homo sapiens	1	7
cell part;cytoplasm;cytoplasmic part;cytosol;Golgi apparatus;intracellular membrane-bounded organelle;intracellular organelle;intracellular organelle part;intracellular part;macromolecular complex;membrane-bounded organelle;methylosome;methyltransferase complex;nuclear part;nucleoplasm;nucleus;organelle;organelle part;p		Homo sapiens	1	7
cell part;extracellular membrane-bounded organelle;extracellular organelle;extracellular region part;extracellular vesicular exosome;membrane;membrane-bounded organelle;membrane-bounded vesicle;organelle;vesicle	Purine metabolism;Thiamine metabolism	Homo sapiens	1	7
cell part;cytoplasmic part;endoplasmic reticulum membrane;endoplasmic reticulum part;integral to membrane;intracellular organelle part;intracellular part;intrinsic to membrane;membrane;membrane part;organelle membrane;organelle part		Homo sapiens	1	7
azurophil granule membrane;cell part;cell surface;cytoplasm;cytoplasmic part;cytoplasmic vesicle membrane;cytoplasmic vesicle part;cytosol;early endosome;endosomal part;endosome;endosome membrane;extracellular m	SNARE interactions in vesicular transport	Homo sapiens	1	7
cell part;cytoplasm;cytoplasmic part;cytosol;intracellular part	Drug metabolism - other enzymes;Purine metabolism;Pyrimidine metabolism	Homo sapiens	1	7
cell part;cytoplasmic part;intracellular non-membrane-bounded organelle;intracellular organelle;intracellular organelle part;intracellular part;large ribosomal subunit;macromolecular complex;membrane;mitochondrial inner me	Ribosome	Homo sapiens	1	7
cell part;chromatin remodeling complex;cytoplasmic part;Golgi apparatus;Golgi apparatus part;histone methyltransferase complex;intracellular membrane-bounded organelle;intracellular organelle;intracellular organelle part;intracellular part;macromolecular complex;membrane-bounded organelle;methyltransferase complex;nuc		Homo sapiens	1	7
cell part;collagen;extracellular matrix part;extracellular region part;intracellular membrane-bounded organelle;intracellular non-membrane-bounded organelle;intracellular organelle;intracellular organelle part;intracellular part;macromolecular complex;membrane-bounded organelle;mRNA cleavage and polyadenylation specificity f		Homo sapiens	1	7
cell part;cytoplasmic part;endoplasmic reticulum part;integral to membrane;integral to plasma membrane;intracellular organelle part;intracellular part;intrinsic to membrane;intrinsic to plasma membrane;macromolecular complex;membrane;membrane part;oligosaccharyltransferase complex;organelle part;plasma membrane;plas		Homo sapiens	1	7
cell part;cytoplasm;cytoplasmic part;cytosol;intracellular membrane-bounded organelle;intracellular organelle;intracellular organelle lumen;intracellular organelle part;intracellular part;membrane-bounded organelle;membrane-enclosed lumen;mitochondrial matrix;mitochondrial part;mitochondrion;nucleus;organelle;organelle lume		Homo sapiens	1	7
cell part;cytoplasm;cytoplasmic membrane-bounded vesicle;cytoplasmic part;cytoplasmic vesicle;endosomal part;endosome;endosome membrane;intracellular membrane-bounded organelle;intracellular organelle;intracellular organelle part;intracellular part;late endosome;late endosome membrane;lysosomal membrane;lysoso		Homo sapiens	1	7
cell part;cytoplasmic membrane-bounded vesicle;cytoplasmic part;cytoplasmic vesicle;endocytic vesicle;endosomal part;endosome;endosome membrane;HOPS complex;intracellular membrane-bounded organelle;intracellular organelle;intracellular organelle part;intracellular part;late endosome;late endosome membrane;lysoso		Homo sapiens	1	7
catalytic step 2 spliceosome;cell part;intracellular organelle part;intracellular part;macromolecular complex;nuclear part;organelle part;ribonucleoprotein complex;spliceosomal complex		Homo sapiens	1	7
cell part;cytoplasm;histone methyltransferase complex;intracellular membrane-bounded organelle;intracellular non-membrane-bounded organelle;intracellular organelle;intracellular organelle part;intracellular part;macromolecular complex;membrane-bounded organelle;methyltransferase complex;MLL1 complex;non-membrane-		Homo sapiens	1	7
cell part;cytoplasm;intracellular membrane-bounded organelle;intracellular organelle;intracellular organelle part;intracellular part;membrane-bounded organelle;nuclear part;nucleoplasm;nucleus;organelle;organelle part		Homo sapiens	1	7
cell part;intracellular non-membrane-bounded organelle;intracellular organelle;intracellular organelle part;intracellular part;macromolecular complex;non-membrane-bounded organelle;nuclear part;nucleolar part;nucleolus;organelle;organelle part;protein complex;t-UTP complex		Homo sapiens	1	7
cell part;cytoplasm;cytoplasmic part;endoplasmic reticulum;extracellular membrane-bounded organelle;extracellular organelle;extracellular region part;extracellular vesicular exosome;intracellular membrane-bounded organelle;intracellular organelle;intracellular part;lipid particle;membrane;membrane-bounded organelle;membra		Homo sapiens	1	7
cell part;cytoplasmic part;endoplasmic reticulum lumen;endoplasmic reticulum part;intracellular organelle lumen;intracellular organelle part;intracellular part;membrane;membrane-enclosed lumen;organelle lumen;organelle part		Homo sapiens	1	7
cell part;integral to membrane;intrinsic to membrane;membrane part		Homo sapiens	1	7
cell part;cytoplasmic part;intracellular non-membrane-bounded organelle;intracellular organelle;intracellular organelle part;intracellular part;macromolecular complex;membrane;mitochondrial inner membrane;mitochondrial membrane;mitochondrial part;non-membrane-bounded organelle;organelle;organelle inner membrane;orga		Homo sapiens	1	7
cell part;cytoplasmic part;cytosol;extracellular membrane-bounded organelle;extracellular organelle;extracellular region part;extracellular vesicular exosome;intracellular part;membrane-bounded organelle;membrane-bounded vesicle;organelle;vesicle		Homo sapiens	1	7
cell part;cytoplasmic part;intracellular membrane-bounded organelle;intracellular organelle;intracellular organelle part;intracellular part;membrane;membrane-bounded organelle;microbody;microbody membrane;microbody p	Peroxisome	Homo sapiens	1	7
cell part;cytoplasmic part;cytosol;intracellular membrane-bounded organelle;intracellular organelle;intracellular part;membrane-bounded organelle;mitochondrion;organelle		Homo sapiens	1	7
cell part;clathrin coat;clathrin vesicle coat;coated pit;cytoplasmic part;cytoplasmic vesicle part;intracellular;intracellular organelle part;intracellular part;macromolecular complex;membrane;membrane coat;membrane part;organelle part;plasma membrane;protein complex;vesicle coat		Homo sapiens	1	7
adherens junction;anchoring junction;cell junction;cell part;cell-substrate adherens junction;cell-substrate junction;centrosome;cytoplasm;cytoplasmic part;cytoskeletal part;focal adhesion;Golgi apparatus;intracellular membrane-bounded organelle;intracellular non-membrane-bounded organelle;intracellular organelle;intracellula		Homo sapiens	1	7
cell part;cell projection membrane;cell projection part;cytoplasm;cytoskeleton;intracellular non-membrane-bounded organelle;intracellular organelle;intracellular part;lamellipodium membrane;leading edge membrane;membrane part;non-membrane-bounded organelle;organelle;plasma membrane part		Homo sapiens	1	7
cell part;cytoplasmic part;intracellular membrane-bounded organelle;intracellular organelle;intracellular organelle part;intracellular part;membrane;membrane-bounded organelle;mitochondrial membrane;mitochondrial part;mitochondrion;organelle;organelle membrane;organelle part		Homo sapiens	1	7
cell part;cytoplasm;cytoplasmic part;cytosol;early endosome;endosomal part;endosome;endosome membrane;extracellular membrane-bounded organelle;extracellular organelle;extracellular region part;extracellular vesicular exosome;intracellular membrane-bounded organelle;intracellular organelle;intracellular organelle part;int		Homo sapiens	1	7
cell part;cytoplasmic part;intracellular membrane-bounded organelle;intracellular organelle;intracellular part;macromolecular complex;membrane-bounded organelle;methyltransferase complex;nucleus;organelle;protein complex;tRNA (m1A) methyltransferase complex;tRNA methyltransferase complex		Homo sapiens	1	7
cell part;cytoplasm;cytoplasmic part;cytosol;early endosome;endosomal part;endosome;endosome membrane;ESCRT I complex;extracellular membrane-bounded organelle;extracellular organelle;extracellular region part;ext	Endocytosis	Homo sapiens	1	7
cell part;cytoplasmic part;cytosol;intracellular organelle part;intracellular part;macromolecular complex;nuclear part;organelle part;ribonucleoprotein complex;small nucleolar ribonucleoprotein complex;spliceosomal complex	RNA degradation;Spliceosome	Homo sapiens	1	7
adherens junction;anchoring junction;cell division site part;cell junction;cell part;cell-substrate adherens junction;cell-substrate junction;cleavage furrow;cytoplasmic part;cytoplasmic vesicle membrane;cytoplasmic vesicle part;endoplasmic reticulum membrane;endoplasmic reticulum part;endosome;extracellular membrane-bou		Homo sapiens	1	7
cell part;cytoplasmic part;endoplasmic reticulum part;integral to endoplasmic reticulum membrane;integral to membrane;integral to organelle membrane;intracellular organelle part;intracellular part;intrinsic to endoplasmic ret	Protein processing in endoplasmic reticulum	Homo sapiens	1	7
cell part;cytoplasm;intracellular part		Homo sapiens	1	7
basal plasma membrane;cell junction;cell part;extracellular region;integral to membrane;integral to plasma membrane;intrinsic to membrane;intrinsic to plasma membrane;membrane;membrane part;plasma membrane;plasm	Olfactory transduction;Pancreatic secretion	Homo sapiens	1	7
cell part;intracellular non-membrane-bounded organelle;intracellular organelle;intracellular organelle part;intracellular part;non-membrane-bounded organelle;nuclear part;nucleolus;organelle;organelle part		Homo sapiens	1	7
cell part;cytoplasm;cytoplasmic part;cytoskeletal part;cytosol;intracellular membrane-bounded organelle;intracellular organelle;intracellular organelle part;intracellular part;macromolecular complex;membrane-bounded organelle;microtubule organizing center;nuclear part;nucleoplasm;nucleus;organelle;organelle part;perinuclear		Homo sapiens	1	7
cell part;cell projection;cytoplasmic part;Golgi apparatus;intracellular membrane-bounded organelle;intracellular organelle;intracellular organelle part;intracellular part;membrane;membrane-bounded organelle;mitochondrial ir	Ubiquinone and other terpenoid-quinone biosynthesis	Homo sapiens	1	7
cell body;cell part;cell projection;cell projection part;cytoplasm;cytoplasmic part;cytosol;dendritic spine;Golgi apparatus;Golgi apparatus part;Golgi cisterna membrane;Golgi membrane;intracellular membrane-bounded organelle;intracellular organelle;intracellular organelle part;intracellular part;membrane;membrane-bounded or		Homo sapiens	1	7
adherens junction;anchoring junction;cell junction;cell part;cell-substrate adherens junction;cell-substrate junction;cytoplasmic part;focal adhesion;intracellular membrane-bounded organelle;intracellular non-membrane-bour	Ribosome biogenesis in eukaryotes	Homo sapiens	1	7
cell part;cytoplasmic part;cytoskeleton;intermediate filament cytoskeleton;intracellular membrane-bounded organelle;intracellular non-membrane-bounded organelle;intracellular organelle;intracellular organelle part;intracellular part;macromolecular complex;membrane;membrane-bounded organelle;mitochondrial inner membran		Homo sapiens	1	7
cell junction;cell part;cytoplasmic part;intracellular membrane-bounded organelle;intracellular organelle;intracellular organelle part;intracellular part;macromolecular complex;membrane;membrane-bounded organelle;mitochondrial inner membrane;mitochondrial membrane;mitochondrial part;mitochondrial small ribosomal subun		Homo sapiens	1	7
cell part;cytoplasmic part;intracellular membrane-bounded organelle;intracellular organelle;intracellular part;membrane-bounded organelle;microbody;mitochondrion;organelle;peroxisome	Peroxisome	Homo sapiens	1	8
cell part;cytoplasmic part;cytosol;intracellular part	Mineral absorption	Homo sapiens	1	8
cell part;clathrin-coated vesicle;coated vesicle;cytoplasm;cytoplasmic membrane-bounded vesicle;cytoplasmic part;cytoplasmic vesicle;cytosol;cytosolic part;extracellular membrane-bounded organelle;extracellular organell	Inositol phosphate metabolism;Phosphatidylinositol signaling system	Homo sapiens	1	8
cell part;cytoplasmic part;extracellular membrane-bounded organelle;extracellular organelle;extracellular region part;extracellular vesicular exosome;intracellular membrane-bounded organelle;intracellular organelle;intracellula	Alzheimer's disease;Huntington's disease;Oxidative phosphorylation;Parkinson's disease	Homo sapiens	1	8
cell part;cytoplasm;cytoplasmic part;cytosol;intracellular membrane-bounded organelle;intracellular organelle;intracellular part;membrane-bounded organelle;nucleus;organelle	Amyotrophic lateral sclerosis (ALS)	Homo sapiens	1	8
cell part;extracellular membrane-bounded organelle;extracellular organelle;extracellular region part;extracellular vesicular exosome;intracellular membrane-bounded organelle;intracellular non-membrane-bounded organelle;intracellular organelle;intracellular organelle part;intracellular part;membrane-bounded organelle;membrane		Homo sapiens	1	8
cell part;cytoplasmic part;endoplasmic reticulum membrane;endoplasmic reticulum part;integral to membrane;intracellular organelle part;intracellular part;intrinsic to membrane;membrane;membrane part;membrane-enclose	Arachidonic acid metabolism	Homo sapiens	1	8
cell part;cell surface;integral to membrane;intrinsic to membrane;membrane;membrane part;plasma membrane	Apoptosis;Cytokine-cytokine receptor interaction;Measles;Natural killer cell mediated cytotoxicity	Homo sapiens	1	8
cell part;chromatin;chromatin remodeling complex;chromatin silencing complex;chromosomal part;cytoplasm;heterochromatin;intracellular membrane-bounded organelle;intracellular non-membrane-bounded organelle;intracellular organelle;intracellular organelle part;intracellular part;macromolecular complex;membrane;membra		Homo sapiens	1	8
cell part;chromosomal part;condensed chromosome kinetochore;cytoskeletal part;intracellular membrane-bounded organelle;intracellular non-membrane-bounded organelle;intracellular organelle;intracellular organelle part;intracellular part;kinetochore;macromolecular complex;membrane-bounded organelle;microtubule;non-me		Homo sapiens	1	8
cell part;cytoplasm;intracellular part		Homo sapiens	1	8
cell part;centrosome;cytoplasmic part;cytoskeletal part;intracellular membrane-bounded organelle;intracellular non-membrane-bounded organelle;intracellular organelle;intracellular organelle part;intracellular part;macromolec	mRNA surveillance pathway	Homo sapiens	1	8
cell part;contractile fiber;cytoplasmic part;intracellular membrane-bounded organelle;intracellular non-membrane-bounded organelle;intracellular organelle;intracellular organelle lumen;intracellular organelle part;intracellular part;membrane;membrane-bounded organelle;membrane-enclosed lumen;mitochondrial inner membrane		Homo sapiens	1	8
cell part;cytoplasmic part;cytosol;early endosome;endosome;Golgi apparatus;intracellular membrane-bounded organelle;intracellular non-membrane-bounded organelle;intracellular organelle;intracellular organelle part;intrac	Regulation of actin cytoskeleton	Homo sapiens	1	8
cell part;cytoplasmic part;endoplasmic reticulum;endoplasmic reticulum lumen;endoplasmic reticulum part;extracellular matrix;extracellular region part;extracellular space;intracellular membrane-bounded organelle;intracellular organelle;intracellular organelle lumen;intracellular organelle part;intracellular part;macromolecular com		Homo sapiens	1	8
cell part;cytoskeletal part;intracellular organelle part;intracellular part;macromolecular complex;MMXD complex;organelle part;protein complex		Homo sapiens	1	8
cell part;cytoplasm;extracellular membrane-bounded organelle;extracellular organelle;extracellular region part;extracellular vesicular exosome;intracellular part;membrane-bounded organelle;membrane-bounded vesicle;orga	Glycerophospholipid metabolism	Homo sapiens	1	8
cell part;centriole;cytoplasm;cytoplasmic part;cytoskeletal part;intracellular membrane-bounded organelle;intracellular non-membrane-bounded organelle;intracellular organelle;intracellular organelle part;intracellular part;mer	Ubiquitin mediated proteolysis	Homo sapiens	1	8
CBM complex;cell part;cytoplasm;cytoplasmic microtubule;cytoplasmic part;cytoskeletal part;cytosol;immunological synapse;intracellular membrane-bounded organelle;intracellular organelle;intracellular organelle part;intrac	B cell receptor signaling pathway;ko05152;T cell receptor signaling pathway	Homo sapiens	1	8
cell part;intracellular membrane-bounded organelle;intracellular non-membrane-bounded organelle;intracellular organelle;intracellular organelle part;intracellular part;membrane-bounded organelle;non-membrane-bounded organelle;nuclear part;nucleolus;nucleoplasm;nucleus;organelle;organelle part		Homo sapiens	1	8
cell part;cytoplasmic part;intracellular membrane-bounded organelle;intracellular organelle;intracellular organelle lumen;intracellular organelle part;intracellular part;membrane;membrane-bounded organelle;membrane-enclos	Benzoate degradation;Biosynthesis of unsaturated fatty acids;Fatty acid metabolism;Peroxisome;PPAR	Homo sapiens	1	8
cell part;cytoplasm;cytoplasmic part;cytosol;intracellular membrane-bounded organelle;intracellular non-membrane-bounded organelle;intracellular organelle;intracellular organelle part;intracellular part;macromolecular comp	Protein export	Homo sapiens	1	8
cell part;cytoplasmic part;integral to membrane;intracellular membrane-bounded organelle;intracellular organelle;intracellular organelle part;intracellular part;intrinsic to membrane;membrane;membrane part;membrane-bounc	Alzheimer's disease;Cardiac muscle contraction;Huntington's disease;Oxidative phosphorylation;Parkin	Homo sapiens	1	8
cell part;cytoplasmic part;dihydrolipoyl dehydrogenase complex;intracellular membrane-bounded organelle;intracellular non-membrane-bounded organelle;intracellular organelle;intracellular organelle lumen;intracellular orgar	Valine, leucine and isoleucine degradation	Homo sapiens	1	8
adherens junction;anchoring junction;cell junction;cell part;cell surface;cell-substrate adherens junction;cell-substrate junction;cytoplasm;extracellular membrane-bounded organelle;extracellular organelle;extracellular region	Cell adhesion molecules (CAMs)	Homo sapiens	1	8
apical cortex;cell cortex part;cell part;cytoplasmic part;extracellular membrane-bounded organelle;extracellular organelle;extracellular region part;extracellular vesicular exosome;hydrogen:potassium-exchanging ATPase com	Lysosome	Homo sapiens	1	8
actin cytoskeleton;axon;axon part;axon terminus;cell body;cell part;cell projection;cell projection part;clathrin-coated vesicle;coated vesicle;cytoplasm;cytoplasmic membrane-bounded vesicle;cytoplasmic part;cytoplasmic v	Amoebiasis;Amyotrophic lateral sclerosis (ALS);Endocytosis;ko05152;Phagosome;Vasopressin-regulate	Homo sapiens	1	8
cell part;intracellular membrane-bounded organelle;intracellular organelle;intracellular part;membrane-bounded organelle;nucleus;organelle		Homo sapiens	1	8
cell part;cytoplasm;intracellular membrane-bounded organelle;intracellular organelle;intracellular part;membrane-bounded organelle;nucleus;organelle		Homo sapiens	1	8
cell part;cytoplasm;extracellular membrane-bounded organelle;extracellular organelle;extracellular region part;extracellular vesicular exosome;internal side of plasma membrane;intracellular part;membrane part;membrane-bo	Riboflavin metabolism	Homo sapiens	1	8
CD95 death-inducing signaling complex;cell part;cell surface;cytoplasm;cytoplasmic part;cytosol;death-inducing signaling complex;external side of plasma membrane;extracellular membrane-bounded organelle;extracellular	African trypanosomiasis;Allograft rejection;Alzheimer's disease;Apoptosis;Autoimmune thyroid disease;(	Homo sapiens	1	8
cell part;cytoplasm;cytoplasmic part;cytosol;extracellular membrane-bounded organelle;extracellular organelle;extracellular region part;extracellular vesicular exosome;intracellular membrane-bounded organelle;intracellular c	Proteasome	Homo sapiens	1	8
cell part;cytoplasm;cytoplasmic part;cytosol;extracellular membrane-bounded organelle;extracellular organelle;extracellular region part;extracellular vesicular exosome;Golgi apparatus;intracellular membrane-bounded organe	Proteasome	Homo sapiens	1	8
cell part;cytoplasmic part;intracellular non-membrane-bounded organelle;intracellular organelle;intracellular organelle part;intracellular part;mitochondrial nucleoid;mitochondrial part;non-membrane-bounded organelle;nuclea	Base excision repair	Homo sapiens	1	8
cell part;cytoplasmic part;intracellular organelle lumen;intracellular organelle part;intracellular part;membrane-enclosed lumen;mitochondrial matrix;mitochondrial part;organelle lumen;organelle part	Valine, leucine and isoleucine degradation	Homo sapiens	1	8
cell part;cytoplasm;intracellular membrane-bounded organelle;intracellular organelle;intracellular organelle part;intracellular part;membrane-bounded organelle;nuclear part;nucleoplasm;nucleus;organelle;organelle part		Homo sapiens	1	8
apical plasma membrane;cell junction;cell leading edge;cell part;cell-cell junction;cytoplasm;cytoplasmic part;cytoskeleton;cytosol;endosome;extracellular membrane-bounded organelle;extracellular organelle;extracellular re	Endocytosis;Insulin signaling pathway;Tight junction	Homo sapiens	1	8
cell part;intracellular membrane-bounded organelle;intracellular non-membrane-bounded organelle;intracellular organelle;intracellular organelle part;intracellular part;membrane-bounded organelle;non-membrane-bounded organelle;nuclear part;nucleolus;nucleus;organelle;organelle part		Homo sapiens	1	8
actin cytoskeleton;cell part;cytoplasm;cytoplasmic part;cytoskeleton;cytosol;extracellular membrane-bounded organelle;extracellular organelle;extracellular region part;extracellular vesicular exosome;Golgi apparatus;intracel	Proteasome	Homo sapiens	1	8
beta-catenin destruction complex;cell part;cytoplasmic part;cytosol;intracellular part;macromolecular complex;protein complex	Chemokine signaling pathway	Homo sapiens	1	8
cell part;cytoplasm;intracellular membrane-bounded organelle;intracellular organelle;intracellular organelle part;intracellular part;membrane-bounded organelle;nuclear part;nucleoplasm;nucleus;organelle;organelle part	mRNA surveillance pathway	Homo sapiens	1	8
cell body;cell part;cell projection;cytoplasmic membrane-bounded vesicle;cytoplasmic part;cytoplasmic vesicle;intracellular membrane-bounded organelle;intracellular organelle;intracellular organelle part;intracellular part;macromolecular complex;membrane part;membrane-bounded organelle;membrane-bounded vesicle;neuro		Homo sapiens	1	8
cell part;centrosome;cytoplasmic part;cytoskeletal part;extracellular membrane-bounded organelle;extracellular organelle;extracellular region part;extracellular vesicular exosome;integral to membrane;integral to plasma membrane;intracellular membrane-bounded organelle;intracellular non-membrane-bounded organelle;intrace		Homo sapiens	1	8
cell part;cytoplasm;cytoplasmic part;cytosol;intracellular membrane-bounded organelle;intracellular organelle;intracellular organelle part;intracellular part;membrane-bounded organelle;nuclear part;nucleoplasm;nucleus;orga	Alzheimer's disease;Apoptosis	Homo sapiens	1	8
adherens junction;anchoring junction;cell junction;cell part;cell-substrate adherens junction;cell-substrate junction;coated vesicle membrane;cytoplasm;cytoplasmic part;cytoplasmic vesicle membrane;cytoplasmic vesicle pa	Antigen processing and presentation	Homo sapiens	1	8
adherens junction;anchoring junction;cell junction;cell part;cell-substrate adherens junction;cell-substrate junction;cytoplasm;cytoplasmic part;cytosol;cytosolic small ribosomal subunit;focal adhesion;intracellular membrane-	Ribosome	Homo sapiens	1	8
cell part;cell projection;cytoplasm;dendrite;fibrillar center;intracellular membrane-bounded organelle;intracellular non-membrane-bounded organelle;intracellular organelle;intracellular organelle part;intracellular part;macromol	RNA transport	Homo sapiens	1	8
cell part;cytoplasmic part;endoplasmic reticulum membrane;endoplasmic reticulum part;integral to endoplasmic reticulum membrane;integral to membrane;integral to organelle membrane;intracellular organelle part;intracellu	ABC transporters;Antigen processing and presentation;Phagosome;Primary immunodeficiency	Homo sapiens	1	8
cell part;cytoplasm;intracellular membrane-bounded organelle;intracellular organelle;intracellular organelle part;intracellular part;macromolecular complex;membrane-bounded organelle;nuclear body;nuclear part;nuclear speck;nucleoplasm;nucleoplasm part;nucleus;organelle;organelle part;ribonucleoprotein complex;spliceosom		Homo sapiens	1	8
cell part;chromosomal part;condensed chromosome kinetochore;cytoplasmic part;cytoskeletal part;cytosol;intracellular membrane-bounded organelle;intracellular non-membrane-bounded organelle;intracellular organelle;inti	Cell cycle;Cell cycle - yeast;Meiosis - yeast;Oocyte meiosis;Progesterone-mediated oocyte maturation	Homo sapiens	1	8
cell part;cytoplasmic part;integral to membrane;intracellular organelle part;intracellular part;intrinsic to membrane;membrane;membrane part;mitochondrial membrane;mitochondrial outer membrane;mitochondrial part;organelle membrane;organelle outer membrane;organelle part;outer membrane		Homo sapiens	1	8
cell part;cytoplasmic membrane-bounded vesicle;cytoplasmic part;cytoplasmic vesicle;cytoplasmic vesicle membrane;cytoplasmic vesicle part;early endosome;endocytic vesicle;endocytic vesicle membrane;endosome;Golgi apparatus part;intracellular membrane-bounded organelle;intracellular organelle;intracellular organelle p		Homo sapiens	1	8
cell part;cytoplasmic part;endoplasmic reticulum membrane;endoplasmic reticulum part;integral to endoplasmic reticulum membrane;integral to membrane;integral to organelle membrane;intracellular organelle part;intracellular part;intrinsic to endoplasmic reticulum membrane;intrinsic to membrane;intrinsic to organelle membra		Homo sapiens	1	8
BRISC complex;cell part;cytoplasm;intracellular organelle part;intracellular part;macromolecular complex;nuclear part;organelle part;protein complex		Homo sapiens	1	8
cell part;cytoplasmic part;cytosol;endoplasmic reticulum part;integral to membrane;intracellular organelle part;intracellular part;intrinsic to membrane;macromolecular complex;membrane;membrane part;organelle part;protein complex;Sec61 translocon complex;translocon complex		Homo sapiens	1	8
cell body;cell part;cell projection;cytoplasmic part;cytosol;dendrite;endoplasmic reticulum membrane;endoplasmic reticulum part;extracellular membrane-bounded organelle;extracellular organelle;extracellular region part;ext	Insulin signaling pathway;mTOR signaling pathway	Homo sapiens	1	8
apical plasma membrane;cell junction;cell part;cell projection;cell surface;clathrin coated vesicle membrane;clathrin-coated vesicle;coated vesicle;coated vesicle membrane;cytoplasmic membrane-bounded vesicle;cytoplasr	Phagosome;SNARE interactions in vesicular transport	Homo sapiens	1	8
cell part;cytoplasm;cytoplasmic part;Golgi apparatus;intracellular membrane-bounded organelle;intracellular organelle;intracellular organelle part;intracellular part;membrane-bounded organelle;nuclear part;nucleoplasm;organelle;organelle part		Homo sapiens	1	8
cell part;cytoplasmic part;early endosome;endosome;intracellular membrane-bounded organelle;intracellular organelle;intracellular part;membrane-bounded organelle;organelle		Homo sapiens	1	8
cell part;integral to membrane;intrinsic to membrane;membrane part		Homo sapiens	1	8
cell body;cell junction;cell part;cell projection;cytoplasm;cytoplasmic part;cytoskeletal part;cytosol;dendrite;intracellular membrane-bounded organelle;intracellular non-membrane-bounded organelle;intracellular organelle;intracellular organelle part;intracellular part;macromolecular complex;membrane-bounded organelle;microtu		Homo sapiens	1	8
cell part;cytoplasm;cytoplasmic microtubule;cytoplasmic part;cytoskeletal part;endoplasmic reticulum;endoplasmic reticulum membrane;endoplasmic reticulum part;Golgi apparatus;integral to membrane;intracellular membrane-bounded organelle;intracellular organelle;intracellular organelle part;intracellular part;intrinsic to mem		Homo sapiens	1	8
		Homo sapiens	1	8
cell part;intracellular membrane-bounded organelle;intracellular organelle;intracellular part;membrane-bounded organelle;nucleus;organelle		Homo sapiens	1	8
cell part;cytoplasm;intracellular membrane-bounded organelle;intracellular organelle;intracellular organelle part;intracellular part;membrane-bounded organelle;nuclear part;nucleoplasm;organelle;organelle part	Tight junction	Homo sapiens	1	8
cell part;intracellular membrane-bounded organelle;intracellular non-membrane-bounded organelle;intracellular organelle;intracellular organelle part;intracellular part;membrane-bounded organelle;non-membrane-bounded organelle;nuclear part;nucleolus;nucleus;organelle;organelle part		Homo sapiens	1	8
cell part;cell projection;lamellipodium		Homo sapiens	1	8
cell part;cytoplasmic part;cytosol;intracellular part	Butanoate metabolism	Homo sapiens	1	8
cell part;cytoplasmic membrane-bounded vesicle;cytoplasmic part;cytoplasmic vesicle;endocytic vesicle;endomembrane system;endosomal part;endosome membrane;extracellular membrane-bounded organelle;extracellula	Phagosome	Homo sapiens	1	8
cell part;cytoplasmic part;cytosol;intracellular part		Homo sapiens	1	8

cell part;cytoplasmic part;endoplasmic reticulum;endoplasmic reticulum membrane;endoplasmic reticulum part;integral to membrane;intracellular membrane-bounded organelle;intracellular organelle;intracellular organelle part;intracellular part;intrinsic to membrane;macromolecular complex;membrane;membrane part;membrane		Homo sapiens	1	8
cell part;integral to membrane;intrinsic to membrane;membrane part		Homo sapiens	1	8
calcium channel complex;cation channel complex;cell part;cytoplasmic part;integral to membrane;integral to mitochondrial inner membrane;integral to mitochondrial membrane;integral to organelle membrane;intracellular membrane-bounded organelle;intracellular organelle;intracellular organelle part;intracellular part;intrinsic to		Homo sapiens	1	8
cell part;cytoplasmic part;intracellular membrane-bounded organelle;intracellular organelle;intracellular part;membrane-bounded organelle;mitochondrion;organelle		Homo sapiens	1	8
cell part;cytoplasm;cytoplasmic part;cytoskeletal part;intracellular organelle part;intracellular part;macromolecular complex;microtubule;microtubule organizing center;nuclear part;nucleoplasm;organelle part;protein complex;spindle pole		Homo sapiens	1	8
cell part;chromosomal part;condensed chromosome kinetochore;cytoplasm;cytoplasmic part;cytoskeletal part;cytoskeleton;extracellular membrane-bounded organelle;extracellular organelle;extracellular region part;extracellular vesicular exosome;intracellular;intracellular non-membrane-bounded organelle;intracellular organelle		Homo sapiens	1	8
cell part;cytoplasm;cytoplasmic part;cytosol;endoplasmic reticulum;intracellular membrane-bounded organelle;intracellular organelle;intracellular organelle part;intracellular part;macromolecular complex;membrane;membran	Proteasome	Homo sapiens	1	8
cell part;integral to membrane;intrinsic to membrane;membrane part		Homo sapiens	1	8
autophagic vacuole;cell part;cytoplasmic part;cytoplasmic vesicle;endosomal part;endosome membrane;intracellular membrane-bounded organelle;intracellular organelle;intracellular organelle part;intracellular part;macromolecular complex;membrane;membrane part;membrane-bounded organelle;organelle;organelle membrane		Homo sapiens	1	8
cell part;cytoplasm;extracellular membrane-bounded organelle;extracellular organelle;extracellular region part;extracellular vesicular exosome;intracellular;intracellular part;membrane-bounded organelle;membrane-bounded vesicle;organelle;vesicle		Homo sapiens	1	8
cell part;cytoplasmic part;intracellular membrane-bounded organelle;intracellular organelle;intracellular organelle lumen;intracellular organelle part;intracellular part;membrane;membrane-bounded organelle;membrane-enclos	Benzoate degradation;Fatty acid metabolism;Lysine degradation;Tryptophan metabolism	Homo sapiens	1	8
cell part;cytoplasmic part;cytosol;extracellular membrane-bounded organelle;extracellular organelle;extracellular region part;extracellular vesicular exosome;intracellular part;membrane-bounded organelle;membrane-bounde	Carbon fixation in photosynthetic organisms;Pentose and glucuronate interconversions;Pentose phosph	Homo sapiens	1	8
cell part;cytoplasm;intracellular membrane-bounded organelle;intracellular non-membrane-bounded organelle;intracellular organelle;intracellular organelle part;intracellular part;membrane;membrane-bounded organelle;non-r	Ribosome biogenesis in eukaryotes;RNA transport	Homo sapiens	1	8
cell part;cytoplasmic part;endoplasmic reticulum;endoplasmic reticulum membrane;endoplasmic reticulum part;integral to membrane;intracellular membrane-bounded organelle;intracellular organelle;intracellular organelle part;intracellular part;intrinsic to membrane;membrane;membrane part;membrane-bounded organelle;nucle		Homo sapiens	1	8
actin cytoskeleton;cell part;cytoplasm;cytoskeleton;intracellular membrane-bounded organelle;intracellular non-membrane-bounded organelle;intracellular organelle;intracellular part;membrane;membrane-bounded organelle;non-membrane-bounded organelle;nucleus;organelle;plasma membrane		Homo sapiens	1	8
cell part;chromatin;chromosomal part;cytoskeleton;intermediate filament cytoskeleton;intracellular membrane-bounded organelle;intracellular non-membrane-bounded organelle;intracellular organelle;intracellular organelle pa	Lysine degradation	Homo sapiens	1	8
cell part;cytoplasm;cytoplasmic part;cytosol;early endosome;early endosome membrane;endosomal part;endosome;endosome membrane;immunological synapse;intracellular membrane-bounded organelle;intracellular organelle;intracellular organelle part;intracellular part;membrane;membrane part;membrane-bounded organel		Homo sapiens	1	8
cell part;cytoplasm;intracellular membrane-bounded organelle;intracellular organelle;intracellular part;membrane-bounded organelle;nucleus;organelle		Homo sapiens	1	8
cell part;cytoplasmic part;Golgi apparatus;intracellular membrane-bounded organelle;intracellular organelle;intracellular part;membrane-bounded organelle;organelle		Homo sapiens	1	8
cell part;cytoplasmic part;endoplasmic reticulum membrane;endoplasmic reticulum part;integral to membrane;intracellular organelle part;intracellular part;intrinsic to membrane;membrane;membrane part;organelle membrane	Glycerophospholipid metabolism	Homo sapiens	1	8
cell part;cytoplasm;intracellular membrane-bounded organelle;intracellular organelle;intracellular part;membrane-bounded organelle;nucleus;organelle		Homo sapiens	1	8
cell part;cytoplasm;cytoskeletal part;holo TFIIH complex;intracellular membrane-bounded organelle;intracellular non-membrane-bounded organelle;intracellular organelle;intracellular organelle part;intracellular part;macromolecular complex;membrane;membrane-bounded organelle;MMXD complex;non-membrane-bounded orga		Homo sapiens	1	8
cell part;extracellular region part;extracellular space;membrane		Homo sapiens	1	8
		Homo sapiens	1	8
	Ribosome biogenesis in eukaryotes	Homo sapiens	1	8
cell part;centrosome;cytoplasmic microtubule;cytoplasmic part;cytoskeletal part;cytosol;intracellular non-membrane-bounded organelle;intracellular organelle;intracellular organelle part;intracellular part;macromolecular complex;membrane;microtubule;microtubule organizing center;non-membrane-bounded organelle;nuclear pa		Homo sapiens	1	8
cell part;cytoplasm;intracellular membrane-bounded organelle;intracellular organelle;intracellular part;membrane-bounded organelle;nucleus;organelle		Homo sapiens	1	8
cell body;cell junction;cell part;cell pole;cell projection;cell tip;cytoplasmic membrane-bounded vesicle;cytoplasmic part;cytoplasmic vesicle;cytosol;dendrite;endosome;extracellular region part;Golgi apparatus;growing cell ti	Inositol phosphate metabolism;Phosphatidylinositol signaling system	Homo sapiens	1	8
cell part;cytoplasmic part;intracellular membrane-bounded organelle;intracellular organelle;intracellular part;membrane-bounded organelle;mitochondrion;nucleus;organelle	Fatty acid elongation in mitochondria	Homo sapiens	1	8
		Homo sapiens	1	8
cell part;cytoplasmic part;intracellular membrane-bounded organelle;intracellular non-membrane-bounded organelle;intracellular organelle;intracellular organelle part;intracellular part;macromolecular complex;membrane;membrane-bounded organelle;mitochondrial inner membrane;mitochondrial membrane;mitochondrial part;m		Homo sapiens	1	8
Cdc73/Paf1 complex;cell part;chromatin;chromosomal part;cytoplasm;cytoplasmic part;intracellular membrane-bounded organelle;intracellular organelle;intracellular organelle part;intracellular part;macromolecular complex;r	RNA degradation	Homo sapiens	1	8
acrosomal vesicle;cell part;cytoplasmic membrane-bounded vesicle;cytoplasmic part;cytoplasmic vesicle;intracellular membrane-bounded organelle;intracellular organelle;intracellular part;macromolecular complex;membrane-bounded organelle;membrane-bounded vesicle;mitochondrion;nucleus;organelle;perinuclear region of		Homo sapiens	1	8
cell part;cytoplasm;extracellular membrane-bounded organelle;extracellular organelle;extracellular region part;extracellular vesicular exosome;intracellular part;membrane-bounded organelle;membrane-bounded vesicle;organelle;vesicle		Homo sapiens	1	8
cell part;cytoplasm;intracellular non-membrane-bounded organelle;intracellular organelle;intracellular organelle part;intracellular part;membrane;non-membrane-bounded organelle;nuclear part;nucleolus;organelle;organelle part		Homo sapiens	1	8
cell part;cytoplasmic part;cytosol;intracellular part		Homo sapiens	1	8
cell part;cytoplasmic part;Golgi apparatus;Golgi apparatus part;Golgi membrane;intracellular membrane-bounded organelle;intracellular organelle;intracellular organelle part;intracellular part;membrane;membrane-bounded organelle;organelle;organelle membrane;organelle part		Homo sapiens	1	8
catalytic step 2 spliceosome;cell part;cytoplasm;cytoplasmic part;Golgi apparatus;intracellular membrane-bounded organelle;intracellular organelle;intracellular organelle part;intracellular part;macromolecular complex;membrane-bounded organelle;nuclear body;nuclear part;nuclear speck;nucleoplasm;nucleoplasm part;organell		Homo sapiens	1	8
cell part;cytoplasmic part;intracellular membrane-bounded organelle;intracellular organelle;intracellular organelle part;intracellular part;large ribosomal subunit;macromolecular complex;membrane;membrane-bounded organelle;mitochondrial inner membrane;mitochondrial large ribosomal subunit;mitochondrial membrane;mitoch		Homo sapiens	1	8
cell part;cytoplasmic part;cytosol;extracellular membrane-bounded organelle;extracellular organelle;extracellular region part;extracellular vesicular exosome;intracellular part;membrane-bounded organelle;membrane-bounde	Pantothenate and CoA biosynthesis	Homo sapiens	1	8
cell part;cytoplasm;cytoplasmic part;cytosol;endoplasmic reticulum membrane;endoplasmic reticulum part;intracellular membrane-bounded organelle;intracellular organelle;intracellular organelle part;intracellular part;membra	Protein processing in endoplasmic reticulum	Homo sapiens	1	8
cell part;cytoplasm;cytoplasmic part;cytoskeletal part;endosomal part;endosome membrane;extracellular membrane-bounded organelle;extracellular organelle;extracellular region part;extracellular vesicular exosome;intracellular organelle part;intracellular part;late endosome membrane;lysosomal membrane;membrane;membrar		Homo sapiens	1	8
cell part;cytoplasm;intracellular membrane-bounded organelle;intracellular organelle;intracellular part;membrane-bounded organelle;nucleus;organelle		Homo sapiens	1	8
cell part;cytoplasm;cytoplasmic part;cytosol;extracellular membrane-bounded organelle;extracellular organelle;extracellular region part;extracellular vesicular exosome;intracellular organelle part;intracellular part;membrane-b	Vitamin B6 metabolism	Homo sapiens	1	8
cell part;cytoplasmic part;intracellular membrane-bounded organelle;intracellular organelle;intracellular part;membrane-bounded organelle;mitochondrion;organelle		Homo sapiens	1	8
cell part;intracellular membrane-bounded organelle;intracellular non-membrane-bounded organelle;intracellular organelle;intracellular organelle part;intracellular part;membrane-bounded organelle;non-membrane-bounded organelle;nuclear part;nucleolus;nucleus;organelle;organelle part		Homo sapiens	1	8
BRCA1-A complex;BRISC complex;cell part;cytoplasm;intracellular membrane-bounded organelle;intracellular organelle;intracellular organelle part;intracellular part;macromolecular complex;membrane-bounded organelle;nuclear part;nucleoplasm;nucleus;organelle;organelle part;protein complex		Homo sapiens	1	8
cell part;cytoplasm;intracellular membrane-bounded organelle;intracellular organelle;intracellular organelle part;intracellular part;membrane-bounded organelle;nuclear part;nucleoplasm;nucleus;organelle;organelle part		Homo sapiens	1	8
cell part;cytoplasmic part;cytosol;endosomal part;endosome membrane;extracellular membrane-bounded organelle;extracellular organelle;extracellular region part;extracellular vesicular exosome;intracellular membrane-bour	Endocytosis	Homo sapiens	1	8
cell part;cytoplasm;cytoplasmic part;extracellular membrane-bounded organelle;extracellular organelle;extracellular region part;extracellular vesicular exosome;integral to membrane;intracellular membrane-bounded organelle	Alzheimer's disease;Huntington's disease;Oxidative phosphorylation;Parkinson's disease	Homo sapiens	1	8
cell part;cytoplasmic part;cytosol;intracellular part	Purine metabolism	Homo sapiens	1	8
cell part;cytoplasm;cytoplasmic part;cytosol;endosomal part;endosome membrane;intracellular organelle part;intracellular part;membrane;organelle membrane;organelle part	Cytosolic DNA-sensing pathway;Hepatitis C;Measles;RIG-I-like receptor signaling pathway;Toll-like rece	Homo sapiens	1	8
cell part;centrosome;cytoplasm;cytoplasmic dynein complex;cytoplasmic part;cytoskeletal part;cytosol;dynactin complex;dynein complex;intracellular membrane-bounded organelle;intracellular non-membrane-bounded orga	Huntington's disease;Vasopressin-regulated water reabsorption	Homo sapiens	1	8
cell part;cytoplasmic part;cytosol;extracellular membrane-bounded organelle;extracellular organelle;extracellular region part;extracellular vesicular exosome;intracellular;intracellular membrane-bounded organelle;intracellular	Purine metabolism	Homo sapiens	1	8
CD95 death-inducing signaling complex;Cdc48p-Npl4p-Ufd1p AAA ATPase complex;cell part;cytoplasmic part;cytosol;death-inducing signaling complex;endoplasmic reticulum part;envelope;intracellular membrane-bounded organelle;intracellular organelle;intracellular organelle part;intracellular part;macromolecular complex;m		Homo sapiens	1	8
cell part;cytoplasm;cytoplasmic part;cytoskeletal part;extracellular membrane-bounded organelle;extracellular organelle;extracellular region part;extracellular vesicular exosome;intracellular membrane-bounded organelle;intracellular non-membrane-bounded organelle;intracellular organelle;intracellular organelle lumen;intracellula		Homo sapiens	1	8
cell part;membrane		Homo sapiens	1	8
cell part;intracellular organelle part;intracellular part;macromolecular complex;nuclear part;nucleoplasm;organelle part;ribonucleoprotein complex;spliceosomal complex;U12-type spliceosomal complex	Spliceosome	Homo sapiens	1	8
catalytic step 2 spliceosome;cell part;extracellular membrane-bounded organelle;extracellular organelle;extracellular region part;extracellular vesicular exosome;intracellular organelle part;intracellular part;macromolecular cor	Spliceosome	Homo sapiens	1	8
cell part;cytoplasmic part;cytosol;intracellular membrane-bounded organelle;intracellular organelle;intracellular part;membrane;membrane-bounded organelle;mitochondrion;organelle		Homo sapiens	1	8
cell part;cytoplasm;intracellular membrane-bounded organelle;intracellular organelle;intracellular part;membrane-bounded organelle;nucleus;organelle		Homo sapiens	1	8
cell part;cytoplasm;intracellular part;membrane;plasma membrane		Homo sapiens	1	8
cell part;cytoplasmic part;cytosol;intracellular organelle part;intracellular part;macromolecular complex;nuclear part;organelle part;ribonucleoprotein complex;small nuclear ribonucleoprotein complex;spliceosomal complex;U	RNA degradation;Spliceosome	Homo sapiens	1	8
catalytic step 2 spliceosome;cell part;cytoplasm;cytoplasmic part;cytosol;exon-exon junction complex;intracellular membrane-bounded organelle;intracellular organelle;intracellular organelle part;intracellular part;macromolec	mRNA surveillance pathway;RNA transport;Spliceosome	Homo sapiens	1	8
bleb;cell part;cell projection;cytoplasm;cytoskeleton;intracellular membrane-bounded organelle;intracellular non-membrane-bounded organelle;intracellular organelle;intracellular part;membrane;membrane-bounded organelle;non-membrane-bounded organelle;nucleus;organelle		Homo sapiens	1	8
cell part;cell surface;integral to membrane;intrinsic to membrane;membrane;membrane part;presynaptic membrane;synapse part;synaptic membrane		Homo sapiens	1	8
cell part;cytoplasm;cytoplasmic part;cytosol;extracellular membrane-bounded organelle;extracellular organelle;extracellular region part;extracellular vesicular exosome;intracellular membrane-bounded organelle;intracellular non-membrane-bounded organelle;intracellular organelle;intracellular organelle part;intracellular part;mem		Homo sapiens	1	8
cell part;chromatin remodeling complex;cytoplasm;histone deacetylase complex;intracellular organelle part;intracellular part;macromolecular complex;nuclear body;nuclear part;nuclear speck;nucleoplasm;nucleoplasm part;c	mRNA surveillance pathway;RNA transport	Homo sapiens	1	9
cell part;cytoplasmic part;cytosol;intracellular part		Homo sapiens	1	9
cell part;clathrin coated vesicle membrane;clathrin-coated vesicle;coated vesicle;coated vesicle membrane;cytoplasmic membrane-bounded vesicle;cytoplasmic part;cytoplasmic vesicle;cytoplasmic vesicle membrane;cytoplasmic vesicle part;endosomal part;endosome membrane;Golgi apparatus part;integral to membrane;int		Homo sapiens	1	9
cell part;endomembrane system;integral to membrane;intrinsic to membrane;membrane;membrane part		Homo sapiens	1	9
cell part;cytoplasm;cytoplasmic part;cytosol;extracellular membrane-bounded organelle;extracellular organelle;extracellular region part;extracellular vesicular exosome;intracellular membrane-bounded organelle;intracellular organelle;intracellular part;membrane-bounded organelle;membrane-bounded vesicle;nucleus;organelle;v		Homo sapiens	1	9
cell part;cytoplasm;extracellular membrane-bounded organelle;extracellular organelle;extracellular region part;extracellular vesicular exosome;intracellular membrane-bounded organelle;intracellular organelle;intracellular organelle part;intracellular part;membrane-bounded organelle;membrane-bounded vesicle;nuclear part;nucle		Homo sapiens	1	9
cell part;cytoplasmic part;integral to membrane;intracellular membrane-bounded organelle;intracellular organelle;intracellular organelle part;intracellular part;intrinsic to membrane;membrane;membrane part;membrane-bounc	ABC transporters	Homo sapiens	1	9
cell part;cytoplasmic part;integral to membrane;integral to plasma membrane;intracellular membrane-bounded organelle;intracellular organelle;intracellular part;intrinsic to membrane;intrinsic to plasma membrane;membrane;membrane part;membrane-bounded organelle;mitochondrion;organelle;plasma membrane part		Homo sapiens	1	9
cell part;cytoplasmic part;cytosol;eukaryotic 43S preinitiation complex;eukaryotic 48S preinitiation complex;eukaryotic translation initiation factor 3 complex;intracellular part;macromolecular complex;protein complex;ribonuc	RNA transport	Homo sapiens	1	9
cell part;intracellular organelle part;intracellular part;nuclear part;nucleoplasm;organelle part		Homo sapiens	1	9
cell part;cytoplasmic part;endoplasmic reticulum;endoplasmic reticulum membrane;endoplasmic reticulum part;extracellular membrane-bounded organelle;extracellular organelle;extracellular region part;extracellular space;extracellular vesicular exosome;Golgi apparatus part;Golgi membrane;integral to membrane;intracellular m		Homo sapiens	1	9
cell part;cytoplasmic part;cytosol;intracellular part	Terpenoid backbone biosynthesis	Homo sapiens	1	9
cell part;cytoplasmic part;extracellular membrane-bounded organelle;extracellular organelle;extracellular region part;extracellular vesicular exosome;intracellular organelle part;intracellular part;macromolecular complex;memb	Alzheimer's disease;Huntington's disease;Oxidative phosphorylation;Parkinson's disease	Homo sapiens	1	9
cell part;external side of plasma membrane;integral to membrane;integral to plasma membrane;intrinsic to membrane;intrinsic to plasma membrane;membrane;membrane part;plasma membrane part		Homo sapiens	1	9
cell part;cytoplasmic part;extracellular membrane-bounded organelle;extracellular organelle;extracellular region part;extracellular vesicular exosome;integral to membrane;intracellular membrane-bounded organelle;intracellula	Alzheimer's disease;Cardiac muscle contraction;Huntington's disease;Oxidative phosphorylation;Parkin	Homo sapiens	1	9
cell body;cell junction;cell part;cell projection cytoplasm;cell projection part;cell surface;cytoplasm;cytoplasmic membrane-bounded vesicle lumen;cytoplasmic part;cytoplasmic vesicle part;cytosol;dendrite cytoplasm;extern	Primary immunodeficiency;Purine metabolism	Homo sapiens	1	9
cell part;cytoplasm;cytoplasmic part;cytosol;extracellular membrane-bounded organelle;extracellular organelle;extracellular region part;extracellular vesicular exosome;intracellular part;membrane-bounded organelle;membra	Alanine, aspartate and glutamate metabolism;Arginine and proline metabolism	Homo sapiens	1	9
cell part;chromatin;chromosomal part;cytoplasm;intracellular membrane-bounded organelle;intracellular organelle;intracellular organelle part;intracellular part;membrane-bounded organelle;nucleus;organelle;organelle part		Homo sapiens	1	9
cell part;cytoplasm;cytoplasmic part;cytosol;intracellular organelle part;intracellular part;nuclear part;nucleoplasm;organelle part	Porphyrin and chlorophyll metabolism	Homo sapiens	1	9
cell part;cytoplasm;cytoplasmic part;cytosol;extracellular membrane-bounded organelle;extracellular organelle;extracellular region part;extracellular vesicular exosome;intracellular membrane-bounded organelle;intracellular c	Arachidonic acid metabolism;Glutathione metabolism	Homo sapiens	1	9
actin cytoskeleton;adherens junction;aggresome;anchoring junction;apical junction complex;apical part of cell;axon part;axon terminus;basolateral plasma membrane;catenin complex;cell junction;cell part;cell projection part	Adherens junction;Bacterial invasion of epithelial cells;Bladder cancer;Cell adhesion molecules (CAMs);	Homo sapiens	1	9
cell part;cytoskeleton;intracellular membrane-bounded organelle;intracellular non-membrane-bounded organelle;intracellular organelle;intracellular organelle part;intracellular part;macromolecular complex;membrane-bounde	Basal transcription factors	Homo sapiens	1	9
cell part;cytoplasmic part;extracellular region part;extracellular space;intracellular membrane-bounded organelle;intracellular organelle;intracellular part;membrane-bounded organelle;mitochondrion;organelle	One carbon pool by folate	Homo sapiens	1	9
cell part;cell surface;cytoplasm;extracellular membrane-bounded organelle;extracellular organelle;extracellular region;extracellular region part;extracellular space;extracellular vesicular exosome;intracellular organelle part;intra	Phenylalanine metabolism;Tyrosine metabolism	Homo sapiens	1	9
cell part;cytoplasmic part;intracellular membrane-bounded organelle;intracellular organelle;intracellular organelle part;intracellular part;membrane;membrane-bounded organelle;membrane-enclosed lumen;mitochondrial inne	Alzheimer's disease;Cardiac muscle contraction;Huntington's disease;Oxidative phosphorylation;Parkin	Homo sapiens	1	9
cell part;cytoplasmic part;extracellular membrane-bounded organelle;extracellular organelle;extracellular region part;extracellular vesicular exosome;intracellular organelle lumen;intracellular organelle part;intracellular part;lyso	Glycosaminoglycan degradation;Lysosome	Homo sapiens	1	9
cell junction;cell part;cell projection;cell-cell junction;cytoplasm;cytoplasmic part;cytosol;intracellular organelle part;intracellular part;lamellipodium;macromolecular complex;membrane;nuclear part;organelle part;plasma mem	Calcium signaling pathway;Epithelial cell signaling in Helicobacter pylori infection;ErbB signaling pathwa	Homo sapiens	1	9
cell part;cytoplasmic part;extracellular membrane-bounded organelle;extracellular organelle;extracellular region part;extracellular vesicular exosome;intracellular organelle part;intracellular part;membrane;membrane-bounded	Alzheimer's disease;Cardiac muscle contraction;Huntington's disease;Oxidative phosphorylation;Parkin	Homo sapiens	1	9
cell part;cytoplasmic part;intracellular membrane-bounded organelle;intracellular organelle;intracellular organelle lumen;intracellular organelle part;intracellular part;membrane-bounded organelle;membrane-enclosed lumen;m	Carbon fixation pathways in prokaryotes;Glyoxylate and dicarboxylate metabolism;Propanoate metabol	Homo sapiens	1	9
cell part;cytoplasmic part;intracellular membrane-bounded organelle;intracellular organelle;intracellular organelle lumen;intracellular organelle part;intracellular part;membrane;membrane-bounded organelle;membrane-enclos	Porphyrin and chlorophyll metabolism	Homo sapiens	1	9
cell part;cytoplasmic part;endoplasmic reticulum;endoplasmic reticulum membrane;endoplasmic reticulum part;extracellular membrane-bounded organelle;extracellular organelle;extracellular region part;extracellular vesicular exosome;intracellular membrane-bounded organelle;intracellular organelle;intracellular organelle part;int		Homo sapiens	1	9
cell part;cytoplasmic part;cytosol;extracellular membrane-bounded organelle;extracellular organelle;extracellular region part;extracellular vesicular exosome;intracellular membrane-bounded organelle;intracellular organelle;intracellular part;membrane-bounded organelle;membrane-bounded vesicle;mitochondrion;nucleus;organe		Homo sapiens	1	9
calcium channel complex;cation channel complex;cell part;cell projection;cytoplasmic part;cytosol;intracellular membrane-bounded organelle;intracellular organelle;intracellular organelle part;intracellular part;ion channel com	MAPK signaling pathway	Homo sapiens	1	9
actin cytoskeleton;axon;axon part;cell cortex;cell junction;cell part;cell projection;cell projection part;clathrin-coated vesicle;coated vesicle;cytoplasm;cytoplasmic membrane-bounded vesicle;cytoplasmic part;cytoplasmic ve	Alzheimer's disease;Parkinson's disease	Homo sapiens	1	9
cell part;cytoplasmic part;extracellular membrane-bounded organelle;extracellular organelle;extracellular region part;extracellular vesicular exosome;intracellular membrane-bounded organelle;intracellular organelle;intracellula	Fatty acid metabolism	Homo sapiens	1	9
cell part;cytoplasm;cytoplasmic membrane-bounded vesicle;cytoplasmic part;cytoplasmic vesicle;cytosol;endoplasmic reticulum membrane;endoplasmic reticulum part;Golgi apparatus;intracellular membrane-bounded orga	alpha-Linolenic acid metabolism;Arachidonic acid metabolism;Ether lipid metabolism;Fat digestion and	Homo sapiens	1	9
cell part;centrosome;cytoplasm;cytoplasmic part;cytoskeletal part;cytosol;extracellular membrane-bounded organelle;extracellular organelle;extracellular region part;extracellular vesicular exosome;intracellular membrane-bo	MAPK signaling pathway;Neurotrophin signaling pathway;VEGF signaling pathway	Homo sapiens	1	9
cell part;cytoplasm;intracellular organelle part;intracellular part;membrane;nuclear membrane;nuclear part;organelle membrane;organelle part;plasma membrane	Selenocompound metabolism	Homo sapiens	1	9
cell part;cytoplasm;cytoplasmic part;cytosol;intracellular membrane-bounded organelle;intracellular organelle;intracellular organelle part;intracellular part;macromolecular complex;membrane-bounded organelle;mRNA cap bi	mRNA surveillance pathway;RNA transport;Spliceosome	Homo sapiens	1	9
cell part;cytoplasmic part;cytosol;Golgi apparatus part;Golgi membrane;intracellular organelle part;intracellular part;membrane;organelle membrane;organelle part;trans-Golgi network membrane		Homo sapiens	1	9
cell part;cytoplasm;cytoplasmic part;cytosol;extracellular membrane-bounded organelle;extracellular organelle;extracellular region part;extracellular vesicular exosome;intracellular part;membrane;membrane-bounded organelle;membrane-bounded vesicle;organelle;plasma membrane;vesicle		Homo sapiens	1	9
box C/D snoRNP complex;cell part;intracellular membrane-bounded organelle;intracellular non-membrane-bounded organelle;intracellular organelle;intracellular organelle part;intracellular part;macromolecular complex;memb	Ribosome biogenesis in eukaryotes;Spliceosome	Homo sapiens	1	9
cell part;cytoplasmic part;endoplasmic reticulum;endoplasmic reticulum membrane;endoplasmic reticulum part;integral to membrane;intracellular membrane-bounded organelle;intracellular organelle;intracellular organelle pa	Protein export	Homo sapiens	1	9
actin cytoskeleton;cell part;cytoplasm;cytoskeleton;extracellular membrane-bounded organelle;extracellular organelle;extracellular region part;extracellular vesicular exosome;intermediate filament cytoskeleton;intracellular m	Ubiquitin mediated proteolysis	Homo sapiens	1	9
cell part;cytoplasmic part;extracellular membrane-bounded organelle;extracellular organelle;extracellular region part;extracellular vesicular exosome;Golgi apparatus part;Golgi membrane;intracellular organelle part;intracellular part;membrane;membrane-bounded organelle;membrane-bounded vesicle;organelle;organelle membr		Homo sapiens	1	9
cell part;intracellular membrane-bounded organelle;intracellular organelle;intracellular organelle part;intracellular part;membrane-bounded organelle;nuclear body;nuclear part;nucleoplasm;nucleoplasm part;nucleus;organelle;	RNA transport	Homo sapiens	1	9
cell part;cytoplasmic membrane-bounded vesicle lumen;cytoplasmic part;cytoplasmic vesicle part;cytoskeleton;cytosol;extracellular region;intracellular membrane-bounded organelle;intracellular non-membrane-bounded org	Regulation of actin cytoskeleton	Homo sapiens	1	9
cell part;cell projection;centrosome;chromosomal part;cilium;cytoplasm;cytoplasmic dynein complex;cytoplasmic part;cytoskeletal part;cytosol;dynein complex;extracellular membrane-bounded organelle;extracellular organe	Vasopressin-regulated water reabsorption	Homo sapiens	1	9
cell part;cytoplasmic part;intracellular membrane-bounded organelle;intracellular organelle;intracellular organelle part;intracellular part;macromolecular complex;membrane;membrane-bounded organelle;mitochondrial inner membrane;mitochondrial membrane;mitochondrial part;mitochondrial small ribosomal subunit;mitochond		Homo sapiens	1	9
adherens junction;anchoring junction;cell junction;cell part;cell-substrate adherens junction;cell-substrate junction;cytoplasmic part;cytosol;endoplasmic reticulum membrane;endoplasmic reticulum part;extracellular membra	Bacterial invasion of epithelial cells;Shigellosis	Homo sapiens	1	9
cell part;intracellular membrane-bounded organelle;intracellular non-membrane-bounded organelle;intracellular organelle;intracellular organelle part;intracellular part;membrane-bounded organelle;non-membrane-bounded organelle;nuclear part;nucleolus;nucleoplasm;nucleus;organelle;organelle part		Homo sapiens	1	9
cell part;cytoplasm;intracellular membrane-bounded organelle;intracellular non-membrane-bounded organelle;intracellular organelle;intracellular organelle part;intracellular part;membrane;membrane-bounded organelle;non-membrane-bounded organelle;nuclear part;nucleolus;nucleoplasm;nucleus;organelle;organelle part;plasm		Homo sapiens	1	9
cell part;intracellular membrane-bounded organelle;intracellular organelle;intracellular organelle part;intracellular part;membrane-bounded organelle;nuclear part;nucleoplasm;nucleus;organelle;organelle part		Homo sapiens	1	9
cell part;chromosome;cytoplasm;cytoplasmic part;cytoskeleton;cytosol;exosome (RNase complex);extracellular membrane-bounded organelle;extracellular organelle;extracellular region part;extracellular vesicular exosome;ir	RNA degradation	Homo sapiens	1	9
CD40 receptor complex;cell cortex;cell part;cytoplasm;cytoplasmic part;cytosol;internal side of plasma membrane;intracellular part;macromolecular complex;membrane;membrane part;membrane raft;organelle membrane;or	Adipocytokine signaling pathway;Apoptosis;Hepatitis C;MAPK signaling pathway;Osteoclast differentiat	Homo sapiens	1	9
cell part;cytoplasm;extracellular region part;intercellular bridge;intracellular membrane-bounded organelle;intracellular non-membrane-bounded organelle;intracellular organelle;intracellular organelle part;intracellular part;macromolecular complex;membrane;membrane-bounded organelle;non-membrane-bounded organelle;nucle		Homo sapiens	1	9
cell part;cytoplasm;cytoplasmic part;cytosol;intracellular part;membrane;plasma membrane	Fructose and mannose metabolism	Homo sapiens	1	9
actin filament bundle;actomyosin;adherens junction;anchoring junction;cell junction;cell part;cell-substrate adherens junction;cell-substrate junction;contractile fiber part;cytoplasmic part;cytoskeletal part;focal adhesion;intracellular membrane-bounded organelle;intracellular organelle;intracellular organelle part;intracellular part;n		Homo sapiens	1	9
cell part;cytoplasm;cytoplasmic part;cytosol;extracellular membrane-bounded organelle;extracellular organelle;extracellular region part;extracellular vesicular exosome;intracellular membrane-bounded organelle;intracellular organelle;intracellular organelle part;intracellular part;membrane-bounded organelle;membrane-bounded v		Homo sapiens	1	9
adherens junction;anchoring junction;cell body;cell cortex;cell junction;cell part;cell projection;cell-substrate adherens junction;cell-substrate junction;cytoplasmic part;cytosol;focal adhesion;intracellular part;lamellipodium;m	Regulation of actin cytoskeleton	Homo sapiens	1	9
adherens junction;anchoring junction;cell junction;cell part;cell-substrate adherens junction;cell-substrate junction;focal adhesion;intracellular organelle part;intracellular part;nuclear part;nucleoplasm;organelle part	Endocytosis	Homo sapiens	1	9
cell part;cytoplasm;cytoplasmic part;cytosol;endoplasmic reticulum membrane;endoplasmic reticulum part;envelope;intracellular membrane-bounded organelle;intracellular organelle;intracellular organelle part;intracellular part;macromolecular complex;membrane;membrane part;membrane-bounded organelle;mitochondrial mer		Homo sapiens	1	9

cell part;cytoplasmic part;cytosol;intracellular membrane-bounded organelle;intracellular organelle;intracellular organelle part;intracellular part;membrane-bounded organelle;nuclear body;nuclear part;nuclear speck;nucleopla	Proteasome	Homo sapiens	1	9
cell part;cytoplasm;cytoplasmic part;endoplasmic reticulum;ER membrane protein complex;intracellular membrane-bounded organelle;intracellular non-membrane-bounded organelle;intracellular organelle;intracellular organelle part;intracellular part;macromolecular complex;membrane-bounded organelle;mitochondrion;non-me		Homo sapiens	1	9
cell part;cytoplasm;intracellular;intracellular membrane-bounded organelle;intracellular organelle;intracellular part;membrane-bounded organelle;nucleus;organelle		Homo sapiens	1	9
cell part;cytoplasm;integral to membrane;integral to plasma membrane;intracellular organelle part;intracellular part;intrinsic to membrane;intrinsic to plasma membrane;macromolecular complex;membrane;membrane part;nuclear part;nucleoplasm;organelle part;plasma membrane;plasma membrane part;proteasome complex;p		Homo sapiens	1	9
cell part;cytoplasmic part;endopeptidase Clp complex;intracellular membrane-bounded organelle;intracellular organelle;intracellular organelle lumen;intracellular organelle part;intracellular part;macromolecular complex;memb	Cell cycle - Caulobacter	Homo sapiens	1	9
cell part;cytoplasmic part;extracellular membrane-bounded organelle;extracellular organelle;extracellular region part;extracellular space;extracellular vesicular exosome;intracellular membrane-bounded organelle;intracellular	Cysteine and methionine metabolism;Sulfur relay system	Homo sapiens	1	9
cell part;cytoplasm;cytoplasmic part;extracellular membrane-bounded organelle;extracellular organelle;extracellular region part;extracellular vesicular exosome;intracellular organelle lumen;intracellular organelle part;intracellu	Pyruvate metabolism	Homo sapiens	1	9
cell part;cytoplasmic part;cytosol;intracellular part	Drug metabolism - other enzymes;Pyrimidine metabolism	Homo sapiens	1	9
cell part;cytoplasm;intracellular organelle part;intracellular part;membrane;nuclear part;nucleoplasm;organelle part;plasma membrane		Homo sapiens	1	9
cell part;intracellular membrane-bounded organelle;intracellular non-membrane-bounded organelle;intracellular organelle;intracellular organelle part;intracellular part;membrane-bounded organelle;non-membrane-bounded organelle;nuclear part;nucleolus;nucleus;organelle;organelle part		Homo sapiens	1	9
cell part;cytoplasmic exosome (RNase complex);cytoplasmic part;cytosol;exosome (RNase complex);intracellular non-membrane-bounded organelle;intracellular organelle;intracellular organelle part;intracellular part;macromo	RNA degradation	Homo sapiens	1	9
		Homo sapiens	1	9
cell part;cytoplasmic part;cytosol;intracellular part		Homo sapiens	1	9
cell part;intracellular membrane-bounded organelle;intracellular non-membrane-bounded organelle;intracellular organelle;intracellular organelle part;intracellular part;membrane-bounded organelle;non-membrane-bounded organelle;nuclear body;nuclear part;nuclear speck;nucleolus;nucleoplasm part;nucleus;organelle;organelle		Homo sapiens	1	9
cell junction;cell part;cell-cell junction;cytoplasmic part;cytosol;intracellular part;membrane;occluding junction;plasma membrane;tight junction		Homo sapiens	1	9
cell part;cytoplasmic part;intracellular non-membrane-bounded organelle;intracellular organelle;intracellular part;macromolecular complex;non-membrane-bounded organelle;organelle;ribonucleoprotein complex;ribosome	Ribosome	Homo sapiens	1	9
cell part;cytoplasmic part;endoplasmic reticulum;integral to membrane;intracellular membrane-bounded organelle;intracellular organelle;intracellular part;intrinsic to membrane;membrane;membrane part;membrane-bounded	Bile secretion	Homo sapiens	1	9
brush border;cell part;cell projection;cytoplasmic microtubule;cytoplasmic part;cytoskeletal part;extracellular membrane-bounded organelle;extracellular organelle;extracellular region part;extracellular vesicular exosome;intra	Pentose and glucuronate interconversions	Homo sapiens	1	9
cell part;extracellular matrix;extracellular region part;interstitial matrix;intracellular organelle part;intracellular part;nuclear part;nucleoplasm;organelle part;proteinaceous extracellular matrix		Homo sapiens	1	9
		Homo sapiens	1	9
		Homo sapiens	1	9
cell junction;cell part;cytoplasmic part;intracellular membrane-bounded organelle;intracellular organelle;intracellular organelle part;intracellular part;membrane;membrane-bounded organelle;microbody;microbody membrane;microbody part;mitochondrion;nucleus;organelle;organelle membrane;organelle part;peroxisomal membr		Homo sapiens	1	9
cell junction;cell part;clathrin-coated vesicle;coated vesicle;cytoplasm;cytoplasmic membrane-bounded vesicle;cytoplasmic part;cytoplasmic vesicle;eukaryotic translation initiation factor 3 complex;intracellular membrane-bounded organelle;intracellular organelle;intracellular organelle part;intracellular part;macromolecular comp		Homo sapiens	1	9
cell part;cytoplasmic part;endoplasmic reticulum lumen;endoplasmic reticulum part;endoplasmic reticulum-Golgi intermediate compartment;extracellular membrane-bounded organelle;extracellular organelle;extracellular region part;extracellular space;extracellular vesicular exosome;intracellular membrane-bounded organelle;int		Homo sapiens	1	9
cell part;cytoplasm;cytoplasmic part;extracellular membrane-bounded organelle;extracellular organelle;extracellular region part;extracellular vesicular exosome;integral to membrane;intracellular membrane-bounded organelle;intracellular organelle;intracellular part;intrinsic to membrane;membrane part;membrane-bounded orga		Homo sapiens	1	9
cell part;cytoplasmic part;intracellular organelle part;intracellular part;membrane-enclosed lumen;mitochondrial intermembrane space;mitochondrial part;organelle envelope lumen;organelle part		Homo sapiens	1	9
cell part;cytoplasm;cytoplasmic part;intracellular membrane-bounded organelle;intracellular organelle;intracellular part;membrane-bounded organelle;mitochondrion;organelle		Homo sapiens	1	9
cell part;cytoplasmic part;intracellular membrane-bounded organelle;intracellular organelle;intracellular part;membrane-bounded organelle;mitochondrion;organelle		Homo sapiens	1	9
cell junction;cell part;extracellular membrane-bounded organelle;extracellular organelle;extracellular region part;extracellular vesicular exosome;macromolecular complex;membrane;membrane-bounded organelle;membrane-bounded vesicle;organelle;postsynaptic membrane;presynaptic membrane;protein complex;receptor co		Homo sapiens	1	9
cell part;cytoplasmic part;cytoskeleton;intracellular membrane-bounded organelle;intracellular non-membrane-bounded organelle;intracellular organelle;intracellular part;membrane-bounded organelle;mitochondrion;non-membrane-bounded organelle;organelle		Homo sapiens	1	9
cell part;cytoplasmic mRNA processing body;cytoplasmic part;cytosol;intracellular non-membrane-bounded organelle;intracellular organelle;intracellular part;macromolecular complex;membrane;non-membrane-bounded or	RNA degradation	Homo sapiens	1	9
cell part;intracellular membrane-bounded organelle;intracellular organelle;intracellular part;macromolecular complex;membrane-bounded organelle;nucleus;organelle;protein complex;ubiquitin ligase complex		Homo sapiens	1	9
cell part;chromosomal part;chromosome;condensed chromosome;condensed chromosome kinetochore;cytoplasm;cytoskeletal part;intracellular membrane-bounded organelle;intracellular non-membrane-bounded organelle;intracellular organelle;intracellular organelle part;intracellular part;kinetochore;macromolecular complex;		Homo sapiens	1	9
cell part;intracellular membrane-bounded organelle;intracellular organelle;intracellular part;membrane-bounded organelle;nucleus;organelle		Homo sapiens	1	9
cell part;chromatin;chromosomal part;heterochromatin;intracellular membrane-bounded organelle;intracellular organelle;intracellular organelle part;intracellular part;membrane-bounded organelle;nuclear chromatin;nuclear chromosome part;nuclear heterochromatin;nuclear part;nucleoplasm;nucleus;organelle;organelle part		Homo sapiens	1	9
cell part;cytoplasm;cytoplasmic part;cytosol;extracellular membrane-bounded organelle;extracellular organelle;extracellular region part;extracellular vesicular exosome;intracellular membrane-bounded organelle;intracellular	Proteasome	Homo sapiens	1	9
cell part;cytoplasm;cytoplasmic part;cytosolic part;intracellular membrane-bounded organelle;intracellular organelle;intracellular part;macromolecular complex;membrane-bounded organelle;nucleus;organelle;prefoldin complex;protein complex		Homo sapiens	1	9
cell part;cytoplasm;cytoplasmic part;cytoskeletal part;cytosol;Golgi apparatus part;Golgi stack;intracellular membrane-bounded organelle;intracellular non-membrane-bounded organelle;intracellular organelle;intracellular organelle part;intracellular part;membrane-bounded organelle;non-membrane-bounded organelle;nuclear pa		Homo sapiens	1	9
calcium channel complex;cation channel complex;cell part;cytoplasmic part;integral to membrane;integral to mitochondrial membrane;integral to organelle membrane;intracellular;intracellular membrane-bounded organelle;intracellular organelle;intracellular organelle part;intracellular part;intrinsic to membrane;intrinsic to organel		Homo sapiens	1	9
cell part;cytoplasmic part;intracellular membrane-bounded organelle;intracellular non-membrane-bounded organelle;intracellular organelle;intracellular organelle part;intracellular part;macromolecular complex;membrane;membrane-bounded organelle;mitochondrial inner membrane;mitochondrial membrane;mitochondrial part;m		Homo sapiens	1	9
cell part;cytoplasm;cytoplasmic part;extracellular membrane-bounded organelle;extracellular organelle;extracellular region part;extracellular vesicular exosome;intracellular membrane-bounded organelle;intracellular organelle;intracellular part;membrane-bounded organelle;membrane-bounded vesicle;mitochondrion;organelle;ve		Homo sapiens	1	9
cell part;cytoplasm;intracellular organelle part;intracellular part;nuclear part;nucleoplasm;organelle part		Homo sapiens	1	9
cell part;intracellular membrane-bounded organelle;intracellular non-membrane-bounded organelle;intracellular organelle;intracellular organelle part;intracellular part;membrane-bounded organelle;non-membrane-bounded organelle;nuclear part;nucleolus;organelle;organelle part		Homo sapiens	1	9
cell part;intracellular membrane-bounded organelle;intracellular organelle;intracellular part;membrane-bounded organelle;nucleus;organelle		Homo sapiens	1	9
cell part;cytoplasm;cytoplasmic part;cytosol;intracellular part	RNA transport	Homo sapiens	1	9
cell part;extracellular membrane-bounded organelle;extracellular organelle;extracellular region part;extracellular vesicular exosome;integral to membrane;integral to plasma membrane;intrinsic to membrane;intrinsic to plasma membrane;membrane;membrane part;membrane-bounded organelle;membrane-bounded vesicle;orga		Homo sapiens	1	9
cell part;intracellular membrane-bounded organelle;intracellular non-membrane-bounded organelle;intracellular organelle;intracellular organelle part;intracellular part;membrane-bounded organelle;non-membrane-bounded organelle;nuclear part;nucleolus;nucleoplasm;nucleus;organelle;organelle part		Homo sapiens	1	9
		Homo sapiens	1	9
cell part;cytoplasm;Elongator holoenzyme complex;histone acetyltransferase complex;intracellular non-membrane-bounded organelle;intracellular organelle;intracellular organelle part;intracellular part;macromolecular complex;non-membrane-bounded organelle;nuclear part;nucleolus;nucleoplasm part;organelle;organelle part;p		Homo sapiens	1	9
cell part;cytoplasm;DNA-directed RNA polymerase complex;DNA-directed RNA polymerase I complex;intracellular non-membrane-bounded organelle;intracellular organelle;intracellular organelle part;intracellular part;macrom	Purine metabolism;Pyrimidine metabolism;RNA polymerase	Homo sapiens	1	9
cell part;cytoplasmic part;cytosol;intracellular part;membrane		Homo sapiens	1	9
cell part;cytoplasmic part;intracellular membrane-bounded organelle;intracellular organelle;intracellular organelle lumen;intracellular organelle part;intracellular part;membrane-bounded organelle;membrane-enclosed lumen;m	Pyrimidine metabolism;Selenocompound metabolism	Homo sapiens	1	9
cell part;cytoplasmic part;endoplasmic reticulum part;endoplasmic reticulum tubular network;extracellular membrane-bounded organelle;extracellular organelle;extracellular region part;extracellular vesicular exosome;Golgi apparatus;intracellular;intracellular membrane-bounded organelle;intracellular organelle;intracellular organ		Homo sapiens	1	9
cell part;cytoplasmic part;cytosol;intracellular;intracellular part	Fructose and mannose metabolism	Homo sapiens	1	9
cell part;cytoplasmic part;endoplasmic reticulum;extracellular membrane-bounded organelle;extracellular organelle;extracellular region part;extracellular vesicular exosome;Golgi apparatus;intracellular membrane-bounded o	Protein processing in endoplasmic reticulum	Homo sapiens	1	9
cell part;intracellular membrane-bounded organelle;intracellular non-membrane-bounded organelle;intracellular organelle;intracellular organelle part;intracellular part;membrane-bounded organelle;non-membrane-bounded organelle;nuclear part;nucleolus;nucleus;organelle;organelle part		Homo sapiens	1	9
cell part;cytoplasmic part;cytoplasmic ubiquitin ligase complex;cytosol;intracellular part;macromolecular complex;protein complex;ubiquitin ligase complex	Regulation of autophagy	Homo sapiens	1	9
extracellular membrane-bounded organelle;extracellular organelle;extracellular region part;extracellular vesicular exosome;membrane-bounded organelle;membrane-bounded vesicle;organelle;vesicle		Homo sapiens	1	9
cell part;cytoplasmic part;endoplasmic reticulum;endoplasmic reticulum membrane;endoplasmic reticulum part;Golgi apparatus;Golgi apparatus part;Golgi membrane;integral to membrane;intracellular membrane-bounded o	Sphingolipid metabolism	Homo sapiens	1	9
cell part;cytoplasm;histone methyltransferase complex;integral to membrane;intracellular non-membrane-bounded organelle;intracellular organelle;intracellular organelle part;intracellular part;intrinsic to membrane;macromolecular complex;membrane;membrane part;methyltransferase complex;MLL1 complex;non-membrane-bo		Homo sapiens	1	9
cell part;cytoplasmic part;extracellular membrane-bounded organelle;extracellular organelle;extracellular region part;extracellular vesicular exosome;integral to membrane;intracellular membrane-bounded organelle;intracellular organelle;intracellular organelle part;intracellular part;intrinsic to membrane;membrane;membrane part		Homo sapiens	1	9
cell part;cytoplasmic part;integral to membrane;intracellular;intracellular membrane-bounded organelle;intracellular organelle;intracellular organelle part;intracellular part;intrinsic to membrane;membrane;membrane part;membrane-bounded organelle;mitochondrial inner membrane;mitochondrial membrane;mitochondrial part;mit		Homo sapiens	1	9
cell part;cytoplasmic part;intracellular membrane-bounded organelle;intracellular organelle;intracellular organelle part;intracellular part;large ribosomal subunit;macromolecular complex;membrane;membrane-bounded organelle;mitochondrial inner membrane;mitochondrial large ribosomal subunit;mitochondrial membrane;mitoch		Homo sapiens	1	9
		Homo sapiens	1	9
cell part;intracellular organelle part;intracellular part;macromolecular complex;membrane;mRNA cleavage and polyadenylation specificity factor complex;mRNA cleavage factor complex;nuclear part;nucleoplasm;nucleoplasm	mRNA surveillance pathway	Homo sapiens	1	9
cell part;clathrin coat;clathrin coat of coated pit;cytoplasmic part;intracellular membrane-bounded organelle;intracellular organelle;intracellular part;macromolecular complex;membrane;membrane coat;membrane part;membrane-bounded organelle;nucleus;organelle;plasma membrane;plasma membrane part;protein complex		Homo sapiens	1	9
actin filament;cell part;cytoskeletal part;extracellular membrane-bounded organelle;extracellular organelle;extracellular region part;extracellular vesicular exosome;extrinsic to internal side of plasma membrane;extrinsic to me	Chemokine signaling pathway;MAPK signaling pathway;Regulation of actin cytoskeleton	Homo sapiens	1	9
cell part;cytoplasmic part;endoplasmic reticulum membrane;endoplasmic reticulum part;integral to membrane;intracellular organelle part;intracellular part;intrinsic to membrane;membrane;membrane part;organelle membrane;organelle part		Homo sapiens	1	9
cell part;cytoplasmic part;endoplasmic reticulum;endoplasmic reticulum membrane;endoplasmic reticulum part;Golgi apparatus part;Golgi membrane;integral to membrane;intracellular membrane-bounded organelle;intracellular organelle;intracellular organelle part;intracellular part;intrinsic to membrane;membrane;membrane pa		Homo sapiens	1	9
		Homo sapiens	1	9
cell part;cytoplasmic part;cytosolic small ribosomal subunit;intracellular organelle part;intracellular part;macromolecular complex;membrane;mitochondrial inner membrane;mitochondrial membrane;mitochondrial part;organelle inner membrane;organelle membrane;organelle part;ribonucleoprotein complex;small ribosomal subu		Homo sapiens	1	9
AP-type membrane coat adaptor complex;cell part;clathrin adaptor complex;cytoplasmic part;cytoplasmic vesicle membrane;cytoplasmic vesicle part;Golgi apparatus;intracellular membrane-bounded organelle;intracellular	Lysosome	Homo sapiens	1	9
cell part;cytoplasm;cytoplasmic part;cytosol;Golgi apparatus part;Golgi membrane;intracellular membrane-bounded organelle;intracellular organelle;intracellular organelle part;intracellular part;membrane;membrane-bounded organelle;nuclear part;nucleoplasm;nucleus;organelle;organelle membrane;organelle part		Homo sapiens	1	9
cell part;cytoplasmic part;cytosol;intracellular membrane-bounded organelle;intracellular organelle;intracellular part;membrane-bounded organelle;nucleus;organelle		Homo sapiens	1	9
cell part;COPI vesicle coat;COPII vesicle coat;cytoplasmic part;cytoplasmic vesicle part;endoplasmic reticulum;endoplasmic reticulum membrane;endoplasmic reticulum part;endoplasmic reticulum-Golgi intermediate compartment;endoplasmic reticulum-Golgi intermediate compartment membrane;extracellular membrane-bour		Homo sapiens	1	9
cell part;cytoplasmic part;intracellular non-membrane-bounded organelle;intracellular organelle;intracellular organelle part;intracellular part;macromolecular complex;membrane;mitochondrial inner membrane;mitochondrial membrane;mitochondrial part;mitochondrial ribosome;non-membrane-bounded organelle;organellar ribos		Homo sapiens	1	9
cell junction;cell part;cell projection;cell projection part;cytoplasm;cytoplasmic part;cytoskeleton;cytosol;dendrite;dendritic spine;extrinsic to internal side of plasma membrane;extrinsic to membrane;extrinsic to plasma membrane;filopodium;intracellular non-membrane-bounded organelle;intracellular organelle;intracellular part;n		Homo sapiens	1	9
cell part;cytoplasmic part;cytosol;extracellular membrane-bounded organelle;extracellular organelle;extracellular region part;extracellular vesicular exosome;intracellular organelle part;intracellular part;lysosomal membrane;m	Collecting duct acid secretion;Epithelial cell signaling in Helicobacter pylori infection;Oxidative phospho	Homo sapiens	1	9
cell part;cytoplasmic part;endoplasmic reticulum membrane;endoplasmic reticulum part;integral to membrane;intracellular organelle part;intracellular part;intrinsic to membrane;membrane;membrane part;organelle membran	N-Glycan biosynthesis	Homo sapiens	1	9
cell part;cytoplasm;cytoplasmic part;cytoskeletal part;cytosol;cytosolic part;IkappaB kinase complex;intracellular;intracellular membrane-bounded organelle;intracellular non-membrane-bounded organelle;intracellular organe	Acute myeloid leukemia;Adipocytokine signaling pathway;Apoptosis;B cell receptor signaling pathway;(	Homo sapiens	1	9
cell part;cytoplasmic part;intracellular membrane-bounded organelle;intracellular organelle;intracellular organelle part;intracellular part;macromolecular complex;membrane;membrane part;membrane-bounded organelle;mito	Alzheimer's disease;Huntington's disease;Oxidative phosphorylation;Parkinson's disease	Homo sapiens	1	10
cell part;cytoplasm;cytoplasmic part;cytosol;intracellular membrane-bounded organelle;intracellular organelle;intracellular organelle part;intracellular part;macromolecular complex;membrane-bounded organelle;nuclear part;nucleoplasm;nucleus;organelle;organelle part;proteasome regulatory particle;protein complex		Homo sapiens	1	10
cell part;cytoplasm;cytoplasmic part;cytoskeletal part;cytosol;histone acetyltransferase complex;histone methyltransferase complex;intracellular membrane-bounded organelle;intracellular organelle;intracellular organelle part	Other types of O-glycan biosynthesis	Homo sapiens	1	10
cell part;cytoplasmic part;intracellular membrane-bounded organelle;intracellular organelle;intracellular organelle lumen;intracellular organelle part;intracellular part;membrane-bounded organelle;membrane-enclosed lumen;m	Pantothenate and CoA biosynthesis;Valine, leucine and isoleucine biosynthesis;Valine, leucine and isole	Homo sapiens	1	10
Cajal body;cell part;intracellular membrane-bounded organelle;intracellular organelle;intracellular organelle part;intracellular part;macromolecular complex;membrane-bounded organelle;nuclear body;nuclear part;nuclear spe	Spliceosome	Homo sapiens	1	10
cell part;cytoplasm;intracellular organelle part;intracellular part;macromolecular complex;nuclear body;nuclear part;nuclear speck;nucleoplasm part;organelle part;ribonucleoprotein complex;small nuclear ribonucleoprotein co	Spliceosome	Homo sapiens	1	10
cell part;cytoplasm;cytoplasmic part;cytosol;early endosome;endosome;extracellular membrane-bounded organelle;extracellular organelle;extracellular region part;extracellular vesicular exosome;intracellular membrane-bounded organelle;intracellular organelle;intracellular part;membrane;membrane-bounded organelle;membra		Homo sapiens	1	10
CCR4-NOT complex;cell part;cytoplasmic mRNA processing body;cytoplasmic part;cytosol;intracellular membrane-bounded organelle;intracellular non-membrane-bounded organelle;intracellular organelle;intracellular organ	RNA degradation	Homo sapiens	1	10
cell part;cytoplasm;cytoplasmic part;cytoplasmic vesicle;endoplasmic reticulum;endoplasmic reticulum exit site;endoplasmic reticulum membrane;endoplasmic reticulum part;endosome;extracellular membrane-bounded organelle;extracellular organelle;extracellular region part;extracellular vesicular exosome;intracellular membr		Homo sapiens	1	10
cell part;cytoplasmic part;intracellular organelle part;intracellular part;membrane;mitochondrial membrane;mitochondrial outer membrane;mitochondrial part;organelle membrane;organelle outer membrane;organelle part;outer membrane		Homo sapiens	1	10
cell part;cytoplasmic part;endoplasmic reticulum;endoplasmic reticulum membrane;endoplasmic reticulum part;extracellular membrane-bounded organelle;extracellular organelle;extracellular region part;extracellular vesicular exosome;integral to membrane;intracellular membrane-bounded organelle;intracellular organelle;intrace		Homo sapiens	1	10
cell part;cytoplasmic part;extracellular membrane-bounded organelle;extracellular organelle;extracellular region part;extracellular vesicular exosome;intracellular membrane-bounded organelle;intracellular organelle;intracellular part;membrane-bounded organelle;membrane-bounded vesicle;mitochondrion;organelle;vesicle		Homo sapiens	1	10
cell part;cytoplasmic part;cytosol;intracellular part	Alanine, aspartate and glutamate metabolism;Amino sugar and nucleotide sugar metabolism	Homo sapiens	1	10
actin filament bundle;actomyosin;cell part;cell projection;cytoplasm;cytoskeletal part;intracellular organelle part;intracellular part;lamellipodium;organelle part;stress fiber	Axon guidance	Homo sapiens	1	10
cell part;cytoplasmic part;cytoskeleton;cytosol;integral to membrane;intracellular membrane-bounded organelle;intracellular non-membrane-bounded organelle;intracellular organelle;intracellular organelle part;intracellular part;intrinsic to membrane;intrinsic to mitochondrial outer membrane;intrinsic to organelle membrane;memb		Homo sapiens	1	10
axoneme;cell part;cell projection part;cytoplasm;cytoplasmic part;cytosol;intracellular part;organelle part;pre-autophagosomal structure	Regulation of autophagy	Homo sapiens	1	10
cell part;cytoplasm;extracellular membrane-bounded organelle;extracellular organelle;extracellular region part;extracellular vesicular exosome;intracellular part;membrane-bounded organelle;membrane-bounded vesicle;organelle;vesicle		Homo sapiens	1	10
cell part;cytoplasm;cytoplasmic part;extracellular membrane-bounded organelle;extracellular organelle;extracellular region part;extracellular vesicular exosome;integral to membrane;integral to mitochondrial membrane;integr	Amyotrophic lateral sclerosis (ALS)	Homo sapiens	1	10
cell part;cytoplasmic part;extracellular membrane-bounded organelle;extracellular organelle;extracellular region part;extracellular vesicular exosome;intracellular membrane-bounded organelle;intracellular non-membrane-bou	Huntington's disease;Peroxisome	Homo sapiens	1	10
apical part of cell;axon;cell junction;cell part;cell projection;cell projection part;cell surface;cell-cell junction;ciliary rootlet;cilium part;coated pit;coated vesicle;cytoplasm;cytoplasmic membrane-bounded vesicle;cytoplasmic r	Alzheimer's disease	Homo sapiens	1	10
actin filament;adherens junction;anchoring junction;cell junction;cell part;cell-substrate adherens junction;cell-substrate junction;cytoplasm;cytoplasmic part;cytoskeletal part;cytosol;extracellular membrane-bounded organel	Adherens junction;Tight junction	Homo sapiens	1	10
cell part;cytoplasmic part;intracellular organelle part;intracellular part;large ribosomal subunit;macromolecular complex;membrane;mitochondrial inner membrane;mitochondrial large ribosomal subunit;mitochondrial membrane;mitochondrial part;organellar large ribosomal subunit;organelle inner membrane;organelle membrane;		Homo sapiens	1	10
cell part;chromatin;chromosomal part;euchromatin;extracellular membrane-bounded organelle;extracellular organelle;extracellular region part;extracellular vesicular exosome;heterochromatin;intracellular membrane-bounded	Systemic lupus erythematosus	Homo sapiens	1	10
apical part of cell;cell part;cytoplasmic part;endoplasmic reticulum;endoplasmic reticulum membrane;endoplasmic reticulum part;integral to membrane;intracellular membrane-bounded organelle;intracellular organelle;intrace	Drug metabolism - cytochrome P450;Glutathione metabolism;Metabolism of xenobiotics by cytochrome	Homo sapiens	1	10
extracellular membrane-bounded organelle;extracellular organelle;extracellular region part;extracellular vesicular exosome;membrane-bounded organelle;membrane-bounded vesicle;organelle;vesicle		Homo sapiens	1	10
cell junction;cell part;cell-cell junction;cytoplasm;cytoplasmic part;cytosol;endoplasmic reticulum;intracellular membrane-bounded organelle;intracellular non-membrane-bounded organelle;intracellular organelle;intracellular p	ErbB signaling pathway;Pathogenic Escherichia coli infection;T cell receptor signaling pathway	Homo sapiens	1	10
cell part;cell projection membrane;cell projection part;cytoplasmic part;cytosol;extracellular membrane-bounded organelle;extracellular organelle;extracellular region part;extracellular vesicular exosome;Golgi apparatus;intracellular membrane-bounded organelle;intracellular organelle;intracellular part;leading edge membrane;me		Homo sapiens	1	10
cell part;intracellular membrane-bounded organelle;intracellular organelle;intracellular organelle part;intracellular part;membrane-bounded organelle;nuclear part;nucleoplasm;nucleus;organelle;organelle part	Base excision repair	Homo sapiens	1	10
cell part;cytoplasm;intracellular membrane-bounded organelle;intracellular organelle;intracellular part;membrane-bounded organelle;nucleus;organelle		Homo sapiens	1	10
cell part;cytoplasmic membrane-bounded vesicle;cytoplasmic part;cytoplasmic vesicle;endosome;extracellular membrane-bounded organelle;extracellular organelle;extracellular region part;extracellular vesicular exosome;in	Endocytosis	Homo sapiens	1	10
axon;cell body;cell part;cell projection;cell projection part;cytoplasmic part;cytosol;dendritic spine;extracellular membrane-bounded organelle;extracellular organelle;extracellular region part;extracellular vesicular exosome;int	Betalain biosynthesis;Steroid hormone biosynthesis;Tyrosine metabolism	Homo sapiens	1	10
adherens junction;anchoring junction;cell junction;cell part;cell-cell adherens junction;cell-cell junction;cytoplasm;integral to membrane;intracellular part;intrinsic to membrane;membrane;membrane part;plasma membrane	Cell adhesion molecules (CAMs)	Homo sapiens	1	10
cell part;centrosome;cytoplasm;cytoplasmic part;cytoskeletal part;cytosol;extracellular membrane-bounded organelle;extracellular organelle;extracellular region part;extracellular vesicular exosome;intracellular membrane-bo	Proteasome	Homo sapiens	1	10
actin cytoskeleton;adherens junction;anchoring junction;cell cortex;cell junction;cell part;cell-substrate adherens junction;cell-substrate junction;centrosome;cytoplasmic part;cytoskeletal part;cytoskeleton;extracellular memb	Fc gamma R-mediated phagocytosis	Homo sapiens	1	10
cell part;cytoplasmic part;cytosol;extracellular membrane-bounded organelle;extracellular organelle;extracellular region part;extracellular vesicular exosome;intracellular part;membrane-bounded organelle;membrane-bounde	Pyrimidine metabolism	Homo sapiens	1	10
cell part;cytoplasm;cytoplasmic part;cytosol;extracellular membrane-bounded organelle;extracellular organelle;extracellular region part;extracellular vesicular exosome;intracellular membrane-bounded organelle;intracellular	Cyanoamino acid metabolism;Glycine, serine and threonine metabolism;Methane metabolism;One carb	Homo sapiens	1	10
adherens junction;anchoring junction;cell junction;cell part;cell projection;cell projection part;cell-substrate adherens junction;cell-substrate junction;cytoplasmic part;cytoskeletal part;cytoskeleton;cytosol;dendritic spine;F-actin capping protein complex;focal adhesion;intermediate filament cytoskeleton;intracellular membrane-b		Homo sapiens	1	10
apical plasma membrane;cell part;cell projection;cytoplasmic part;cytosol;endosome;extracellular membrane-bounded organelle;extracellular organelle;extracellular region part;extracellular vesicular exosome;intracellular me	Collecting duct acid secretion;Epithelial cell signaling in Helicobacter pylori infection;Oxidative phospho	Homo sapiens	1	10
cell part;cytoplasmic part;cytosol;cytosolic small ribosomal subunit;intracellular membrane-bounded organelle;intracellular non-membrane-bounded organelle;intracellular organelle;intracellular organelle part;intracellular part	Ribosome	Homo sapiens	1	10
autophagic vacuole;axon;cell part;cell projection;cytoplasm;cytoplasmic part;cytoplasmic vesicle membrane;cytoplasmic vesicle part;cytosol;dendrite;endoplasmic reticulum;endosome;Golgi apparatus;inclusion body;intrace	Huntington's disease	Homo sapiens	1	10
cell part;cytoplasm;cytoplasmic part;cytosol;intracellular organelle part;intracellular part;membrane;nuclear part;nucleoplasm;organelle part	Amyotrophic lateral sclerosis (ALS);Fc epsilon RI signaling pathway;GnRH signaling pathway;MAPK sigr	Homo sapiens	1	10
cell part;cytoplasmic part;cytosol;extracellular membrane-bounded organelle;extracellular organelle;extracellular region part;extracellular vesicular exosome;intracellular membrane-bounded organelle;intracellular organelle;int	Proteasome	Homo sapiens	1	10
adherens junction;anchoring junction;apical part of cell;basolateral plasma membrane;cell junction;cell part;cell-substrate adherens junction;cell-substrate junction;clathrin-coated vesicle;coated vesicle;cytoplasmic membrar	Notch signaling pathway	Homo sapiens	1	10
cell part;cytoplasmic part;cytoskeletal part;endoplasmic reticulum;envelope;integral to membrane;intracellular membrane-bounded organelle;intracellular organelle;intracellular organelle part;intracellular part;intrinsic to memb	Arrhythmogenic right ventricular cardiomyopathy (ARVC);Dilated cardiomyopathy;Hypertrophic cardiom	Homo sapiens	1	10
carboxy-terminal domain protein kinase complex;cell part;cytoplasm;intracellular membrane-bounded organelle;intracellular organelle;intracellular organelle part;intracellular part;macromolecular complex;membrane;membrane-bounded organelle;nuclear body;nuclear part;nucleoplasm;nucleoplasm part;organelle;organelle part;		Homo sapiens	1	10
cell part;cytoplasmic part;intracellular membrane-bounded organelle;intracellular non-membrane-bounded organelle;intracellular organelle;intracellular organelle part;intracellular part;macromolecular complex;membrane;membrane-bounded organelle;mitochondrial inner membrane;mitochondrial membrane;mitochondrial part;m		Homo sapiens	1	10
cell part;cytoplasmic part;endoplasmic reticulum part;extracellular membrane-bounded organelle;extracellular organelle;extracellular region part;extracellular vesicular exosome;integral to membrane;intracellular organelle par	Protein processing in endoplasmic reticulum	Homo sapiens	1	10
cell part;cytoplasmic part;intracellular membrane-bounded organelle;intracellular organelle;intracellular organelle part;intracellular part;macromolecular complex;membrane;membrane part;membrane-bounded organelle;mem	Alzheimer's disease;Huntington's disease;Oxidative phosphorylation;Parkinson's disease	Homo sapiens	1	10
cell part;cytoplasmic part;intracellular membrane-bounded organelle;intracellular organelle;intracellular organelle part;intracellular part;membrane;membrane-bounded organelle;mitochondrial inner membrane;mitochondrial m	Porphyrin and chlorophyll metabolism	Homo sapiens	1	10

cell part;cytoplasmic membrane-bounded vesicle;cytoplasmic part;cytoplasmic vesicle;cytosol;Golgi apparatus;Golgi apparatus part;Golgi membrane;integral to membrane;intracellular membrane-bounded organelle;intracellular organelle;intracellular organelle part;intracellular part;intrinsic to membrane;membrane;membrane pa		Homo sapiens	1	10
cell part;cytoplasm;cytoplasmic part;cytosol;extracellular membrane-bounded organelle;extracellular organelle;extracellular region part;extracellular vesicular exosome;intracellular membrane-bounded organelle;intracellular c	Ubiquitin mediated proteolysis	Homo sapiens	1	10
adherens junction;anchoring junction;cell cortex;cell division site part;cell junction;cell part;cell projection;cell projection membrane;cell projection part;cell-substrate adherens junction;cell-substrate junction;cleavage furrow;c	Endocytosis;Fc gamma R-mediated phagocytosis	Homo sapiens	1	10
cell part;cytoplasm;cytoskeleton;intracellular non-membrane-bounded organelle;intracellular organelle;intracellular part;non-membrane-bounded organelle;organelle		Homo sapiens	1	10
cell part;intracellular membrane-bounded organelle;intracellular organelle;intracellular organelle part;intracellular part;macromolecular complex;membrane-bounded organelle;multimeric ribonuclease P complex;nuclear part;n	Ribosome biogenesis in eukaryotes;RNA transport	Homo sapiens	1	10
cell part;membrane;midbody		Homo sapiens	1	10
cell part;extracellular membrane-bounded organelle;extracellular organelle;extracellular region part;extracellular vesicular exosome;integral to membrane;intracellular membrane-bounded organelle;intracellular organelle;intracellular part;intrinsic to membrane;membrane;membrane part;membrane-bounded organelle;membrane-b		Homo sapiens	1	10
cell part;cytoplasmic part;Golgi apparatus;intracellular membrane-bounded organelle;intracellular non-membrane-bounded organelle;intracellular organelle;intracellular organelle part;intracellular part;membrane-bounded organelle;non-membrane-bounded organelle;nuclear part;nucleolus;nucleus;organelle;organelle part		Homo sapiens	1	10
cell part;cytoplasmic part;cytosol;intracellular membrane-bounded organelle;intracellular organelle;intracellular part;membrane-bounded organelle;microbody;mitochondrion;organelle;peroxisome	Terpenoid backbone biosynthesis	Homo sapiens	1	10
macromolecular complex;protein complex;ribose phosphate diphosphokinase complex		Homo sapiens	1	10
cell part;cytoplasmic part;cytosol;exosome (RNase complex);intracellular membrane-bounded organelle;intracellular non-membrane-bounded organelle;intracellular organelle;intracellular organelle part;intracellular part;macro	RNA degradation	Homo sapiens	1	10
cell part;cytoplasm;cytoskeletal part;intracellular organelle part;intracellular part;macromolecular complex;microtubule associated complex;organelle part;protein complex		Homo sapiens	1	10
calcium channel complex;cation channel complex;cell part;cytoplasm;extracellular membrane-bounded organelle;extracellular organelle;extracellular region part;extracellular vesicular exosome;intracellular membrane-bounded organelle;intracellular organelle;intracellular organelle part;intracellular part;ion channel complex;macro		Homo sapiens	1	10
adherens junction;anchoring junction;axon;cell body;cell junction;cell part;cell projection;cell surface;cell-cell junction;cilium;dendrite;integral to membrane;integral to plasma membrane;intrinsic to membrane;intrinsic to plasma membrane;leading edge membrane;membrane part;neuron projection;neuronal cell body;nonmotile p		Homo sapiens	1	10
cell part;cytoplasmic part;cytosol;elongin complex;intracellular organelle part;intracellular part;macromolecular complex;nuclear part;nucleoplasm;nucleoplasm part;organelle part;protein complex;transcription elongation fact	Pathways in cancer;Renal cell carcinoma;Ubiquitin mediated proteolysis	Homo sapiens	1	10
cell part;Cul2-RING ubiquitin ligase complex;Cul5-RING ubiquitin ligase complex;cullin-RING ubiquitin ligase complex;cytoplasmic part;cytoplasmic ubiquitin ligase complex;cytosol;elongin complex;extracellular membrane-b	Pathways in cancer;Renal cell carcinoma;Ubiquitin mediated proteolysis	Homo sapiens	1	10
cell part;chromosome;cytoplasm;extracellular membrane-bounded organelle;extracellular organelle;extracellular region part;extracellular vesicular exosome;intracellular membrane-bounded organelle;intracellular non-membrane-bounded organelle;intracellular organelle;intracellular part;membrane-bounded organelle;membrane-		Homo sapiens	1	10
cell part;cytoplasmic membrane-bounded vesicle;cytoplasmic part;cytoplasmic vesicle;extracellular membrane-bounded organelle;extracellular organelle;extracellular region part;extracellular vesicular exosome;Golgi apparat	Protein digestion and absorption	Homo sapiens	1	10
cell part;extracellular membrane-bounded organelle;extracellular organelle;extracellular region part;extracellular vesicular exosome;membrane;membrane-bounded organelle;membrane-bounded vesicle;organelle;vesicle		Homo sapiens	1	10
cell part;intracellular non-membrane-bounded organelle;intracellular organelle;intracellular organelle part;intracellular part;macromolecular complex;non-membrane-bounded organelle;nuclear part;nucleolus;organelle;organelle part;preribosome;ribonucleoprotein complex;small-subunit processome		Homo sapiens	1	10
		Homo sapiens	1	10
cell part;cytoplasmic part;intracellular organelle part;intracellular part;lysosomal membrane;membrane;organelle membrane;organelle part;vacuolar membrane;vacuolar part		Homo sapiens	1	10
cell part;intracellular organelle part;intracellular part;macromolecular complex;mRNA cleavage and polyadenylation specificity factor complex;mRNA cleavage factor complex;nuclear part;nucleoplasm;nucleoplasm part;organ	mRNA surveillance pathway	Homo sapiens	1	10
cell part;chromatin;chromatin remodeling complex;chromosomal part;histone methyltransferase complex;intracellular organelle part;intracellular part;macromolecular complex;methyltransferase complex;nuclear part;nucleoplasm part;organelle part;protein complex;protein serine/threonine phosphatase complex;PTW/PP1 phosp		Homo sapiens	1	10
cell part;integral to membrane;intracellular membrane-bounded organelle;intracellular organelle;intracellular part;intrinsic to membrane;membrane;membrane part;membrane-bounded organelle;nucleus;organelle		Homo sapiens	1	10
cell part;cytoplasm;extracellular membrane-bounded organelle;extracellular organelle;extracellular region part;extracellular vesicular exosome;intracellular organelle part;intracellular part;macromolecular complex;membrane-bounded organelle;membrane-bounded vesicle;nuclear part;nucleoplasm;organelle;organelle part;protein		Homo sapiens	1	10
		Homo sapiens	1	10
cell part;cytoplasm;cytoskeleton;intermediate filament cytoskeleton;intracellular non-membrane-bounded organelle;intracellular organelle;intracellular part;membrane;non-membrane-bounded organelle;organelle;plasma membrane		Homo sapiens	1	10
cell part;intracellular membrane-bounded organelle;intracellular organelle;intracellular organelle part;intracellular part;membrane;membrane-bounded organelle;nuclear part;nucleoplasm;nucleus;organelle;organelle part	Amino sugar and nucleotide sugar metabolism	Homo sapiens	1	10
cell part;cis-Golgi network;cytoplasmic part;Golgi apparatus;Golgi apparatus part;Golgi cisterna;Golgi membrane;integral to membrane;intracellular membrane-bounded organelle;intracellular organelle;intracellular organelle part;intracellular part;intrinsic to membrane;membrane;membrane part;membrane-bounded organelle;org		Homo sapiens	1	10
extracellular region		Homo sapiens	1	10
cell part;cytoplasm;extracellular membrane-bounded organelle;extracellular organelle;extracellular region part;extracellular vesicular exosome;intracellular part;membrane-bounded organelle;membrane-bounded vesicle;organelle;vesicle		Homo sapiens	1	10
cell part;cytoplasmic membrane-bounded vesicle;cytoplasmic part;cytoplasmic vesicle;extracellular membrane-bounded organelle;extracellular organelle;extracellular region;extracellular region part;extracellular vesicular exosome;Golgi apparatus;intracellular membrane-bounded organelle;intracellular organelle;intracellular part;		Homo sapiens	1	10
cell part;cytoplasm;cytoplasmic part;endoplasmic reticulum;endoplasmic reticulum membrane;endoplasmic reticulum part;envelope;intracellular membrane-bounded organelle;intracellular organelle;intracellular organelle part;intracellular part;membrane;membrane part;membrane-bounded organelle;nuclear envelope;nuclear par		Homo sapiens	1	10
cell junction;cell part;cell-cell junction;cytoplasm;cytoskeleton;intracellular non-membrane-bounded organelle;intracellular organelle;intracellular organelle part;intracellular part;membrane;non-membrane-bounded organelle;n	mRNA surveillance pathway;Tight junction	Homo sapiens	1	10
cell part;Cul5-RING ubiquitin ligase complex;cullin-RING ubiquitin ligase complex;cytoplasmic part;cytosol;intracellular membrane-bounded organelle;intracellular organelle;intracellular part;macromolecular complex;membra	Ubiquitin mediated proteolysis	Homo sapiens	1	10
cell part;cytoplasm;cytoplasmic membrane-bounded vesicle;cytoplasmic part;cytoplasmic vesicle;cytoplasmic vesicle membrane;cytoplasmic vesicle part;endocytic vesicle membrane;endosomal part;endosome membrane;	Collecting duct acid secretion;Epithelial cell signaling in Helicobacter pylori infection;ko05152;Lysosome	Homo sapiens	1	10
cell part;cytoplasm;cytoplasmic part;Golgi apparatus;intracellular membrane-bounded organelle;intracellular non-membrane-bounded organelle;intracellular organelle;intracellular organelle part;intracellular part;membrane-bounded organelle;non-membrane-bounded organelle;nuclear part;nucleolus;organelle;organelle part		Homo sapiens	1	10
cell part;centrosome;cytoplasmic part;cytoskeletal part;intracellular membrane-bounded organelle;intracellular non-membrane-bounded organelle;intracellular organelle;intracellular organelle part;intracellular part;macromolecular complex;membrane-bounded organelle;microtubule;microtubule organizing center;mitochondrion;n		Homo sapiens	1	10
cell part;cytoplasm;intracellular organelle part;intracellular part;nuclear part;nucleoplasm;organelle part	mRNA surveillance pathway	Homo sapiens	1	10
cell part;cytoplasmic part;intracellular;intracellular membrane-bounded organelle;intracellular organelle;intracellular part;membrane-bounded organelle;mitochondrion;organelle	RNA transport	Homo sapiens	1	10
cell part;cytoplasm;intracellular membrane-bounded organelle;intracellular organelle;intracellular organelle part;intracellular part;membrane-bounded organelle;nuclear part;nucleoplasm;nucleus;organelle;organelle part		Homo sapiens	1	10
cell part;cytoplasm;cytoplasmic part;cytosol;extracellular membrane-bounded organelle;extracellular organelle;extracellular region part;extracellular space;extracellular vesicular exosome;intracellular membrane-bounded organelle;intracellular organelle;intracellular part;lysosome;lytic vacuole;mast cell granule;membrane-bounde		Homo sapiens	1	10
cell part;intracellular membrane-bounded organelle;intracellular non-membrane-bounded organelle;intracellular organelle;intracellular organelle part;intracellular part;membrane-bounded organelle;non-membrane-bounded organelle;nuclear part;nucleolus;nucleoplasm;nucleus;organelle;organelle part		Homo sapiens	1	10
cell part;cytoplasmic part;endoplasmic reticulum membrane;endoplasmic reticulum part;extracellular region;intracellular organelle part;intracellular part;membrane;membrane part;organelle membrane;organelle part		Homo sapiens	1	10
cell part;cytoplasmic part;endoplasmic reticulum;extracellular region;intracellular membrane-bounded organelle;intracellular organelle;intracellular part;membrane;membrane-bounded organelle;organelle	Glycolysis / Gluconeogenesis	Homo sapiens	1	10
cell part;chromatin remodeling complex;chromosomal part;cytoplasmic membrane-bounded vesicle;cytoplasmic part;cytoplasmic vesicle;intracellular membrane-bounded organelle;intracellular organelle;intracellular organelle part;intracellular part;macromolecular complex;membrane-bounded organelle;membrane-bounded ves		Homo sapiens	1	10
cell part;cytoplasmic membrane-bounded vesicle;cytoplasmic part;cytoplasmic vesicle;cytoplasmic vesicle membrane;cytoplasmic vesicle part;cytoskeletal part;early endosome;early endosome membrane;endosomal part;e	Endocytosis	Homo sapiens	1	10
cell part;cytoplasm;cytoplasmic part;cytosol;intracellular membrane-bounded organelle;intracellular organelle;intracellular part;membrane-bounded organelle;nucleus;organelle	Pathways in cancer;Renal cell carcinoma	Homo sapiens	1	10
cell part;extracellular region part;extracellular space;intracellular membrane-bounded organelle;intracellular organelle;intracellular organelle part;intracellular part;membrane-bounded organelle;nuclear part;nucleoplasm;nucleus;organelle;organelle part		Homo sapiens	1	10
cell junction;cell part;cytoplasmic part;intracellular membrane-bounded organelle;intracellular non-membrane-bounded organelle;intracellular organelle;intracellular organelle part;intracellular part;macromolecular complex;membrane;membrane-bounded organelle;mitochondrial inner membrane;mitochondrial membrane;mitocho		Homo sapiens	1	10
extracellular membrane-bounded organelle;extracellular organelle;extracellular region part;extracellular vesicular exosome;membrane-bounded organelle;membrane-bounded vesicle;organelle;vesicle		Homo sapiens	1	10
extracellular membrane-bounded organelle;extracellular organelle;extracellular region part;extracellular vesicular exosome;membrane-bounded organelle;membrane-bounded vesicle;organelle;vesicle		Homo sapiens	1	10
cell part;cytoplasmic part;Golgi apparatus;Golgi apparatus part;Golgi membrane;integral to membrane;intracellular membrane-bounded organelle;intracellular organelle;intracellular organelle part;intracellular part;intrinsic to membrane;membrane;membrane part;membrane-bounded organelle;mitochondrion;organelle;organelle m		Homo sapiens	1	10
actin cytoskeleton;cell part;cytoplasm;cytoskeleton;endomembrane system;intracellular non-membrane-bounded organelle;intracellular organelle;intracellular part;membrane;microtubule cytoskeleton;non-membrane-bounded organelle;organelle;plasma membrane		Homo sapiens	1	10
cell part;cytoplasmic part;cytosol;endoplasmic reticulum part;intracellular organelle part;intracellular part;membrane;nuclear membrane;nuclear part;organelle membrane;organelle part;perinuclear endoplasmic reticulum;plasma membrane		Homo sapiens	1	10
cell part;cytoplasmic part;integral to membrane;intracellular membrane-bounded organelle;intracellular organelle;intracellular organelle part;intracellular part;intrinsic to membrane;membrane;membrane part;membrane-bounded organelle;mitochondrial inner membrane;mitochondrial membrane;mitochondrial part;mitochondrion;		Homo sapiens	1	10
cell part;cytoplasmic part;intracellular non-membrane-bounded organelle;intracellular organelle;intracellular organelle part;intracellular part;macromolecular complex;membrane;mitochondrial inner membrane;mitochondrial membrane;mitochondrial part;non-membrane-bounded organelle;organelle;organelle inner membrane;orga		Homo sapiens	1	10
cell part;cytoplasmic part;cytosol;intracellular organelle lumen;intracellular organelle part;intracellular part;membrane-enclosed lumen;mitochondrial matrix;mitochondrial part;organelle lumen;organelle part	Aminoacyl-tRNA biosynthesis	Homo sapiens	1	10
cell part;cytoplasmic part;cytosol;extracellular membrane-bounded organelle;extracellular organelle;extracellular region part;extracellular vesicular exosome;intracellular part;membrane-bounded organelle;membrane-bounde	Pantothenate and CoA biosynthesis	Homo sapiens	1	10
cell part;cytoplasm;cytoplasmic part;cytosol;exon-exon junction complex;intracellular membrane-bounded organelle;intracellular organelle;intracellular organelle part;intracellular part;macromolecular complex;membrane-bou	mRNA surveillance pathway;RNA transport	Homo sapiens	1	10
actin cytoskeleton;cell part;cytoskeleton;extracellular region part;extracellular space;intracellular membrane-bounded organelle;intracellular non-membrane-bounded organelle;intracellular organelle;intracellular organelle part;intracellular part;membrane-bounded organelle;non-membrane-bounded organelle;nuclear part;nucleop		Homo sapiens	1	10
catalytic step 2 spliceosome;cell part;intracellular membrane-bounded organelle;intracellular organelle;intracellular organelle part;intracellular part;macromolecular complex;membrane;membrane-bounded organelle;nuclear p	Spliceosome	Homo sapiens	1	10
cell part;cytoplasm;cytoskeleton;extracellular region part;intercellular bridge;intracellular membrane-bounded organelle;intracellular non-membrane-bounded organelle;intracellular organelle;intracellular organelle part;intracellular part;macromolecular complex;membrane;membrane-bounded organelle;microtubule cytoskeleton;n		Homo sapiens	1	10
cell part;cytoplasm;intracellular part;membrane;plasma membrane		Homo sapiens	1	10
cell part;cytoplasmic part;intracellular organelle part;intracellular part;large ribosomal subunit;macromolecular complex;membrane;mitochondrial inner membrane;mitochondrial large ribosomal subunit;mitochondrial membrane;mitochondrial part;organellar large ribosomal subunit;organelle inner membrane;organelle membrane;c		Homo sapiens	1	10
cell part;intracellular organelle part;intracellular part;nuclear part;nucleoplasm;organelle part		Homo sapiens	1	10
cell part;membrane		Homo sapiens	1	10
cell part;intracellular non-membrane-bounded organelle;intracellular organelle;intracellular organelle part;intracellular part;membrane;non-membrane-bounded organelle;nuclear part;nucleolus;nucleoplasm;organelle;organelle part;plasma membrane		Homo sapiens	1	10
cell part;cytoplasmic part;cytoskeletal part;cytosol;intermediate filament;intracellular membrane-bounded organelle;intracellular organelle;intracellular organelle part;intracellular part;macromolecular complex;membrane-bounded organelle;nucleus;organelle;organelle part;protein complex		Homo sapiens	1	10
catalytic step 2 spliceosome;cell part;intracellular membrane-bounded organelle;intracellular organelle;intracellular organelle part;intracellular part;macromolecular complex;membrane-bounded organelle;nuclear part;nucleop	Spliceosome	Homo sapiens	1	10
cell part;cytoplasm;intracellular membrane-bounded organelle;intracellular non-membrane-bounded organelle;intracellular organelle;intracellular organelle part;intracellular part;membrane-bounded organelle;non-membrane-bounded organelle;nuclear part;nucleolus;nucleus;organelle;organelle part		Homo sapiens	1	10
adherens junction;anchoring junction;cell junction;cell part;cell-substrate adherens junction;cell-substrate junction;cytoplasmic part;endoplasmic reticulum;endoplasmic reticulum membrane;endoplasmic reticulum part;focal adhesion;integral to membrane;intracellular membrane-bounded organelle;intracellular organelle;intracell		Homo sapiens	1	10
cell part;cyclin-dependent protein kinase holoenzyme complex;intracellular membrane-bounded organelle;intracellular organelle;intracellular organelle part;intracellular part;macromolecular complex;membrane-bounded organelle;nuclear cyclin-dependent protein kinase holoenzyme complex;nuclear part;nucleus;organelle;organ		Homo sapiens	1	10
cell part;cytoplasmic part;endoplasmic reticulum;endoplasmic reticulum membrane;endoplasmic reticulum part;integral to membrane;intracellular membrane-bounded organelle;intracellular organelle;intracellular organelle pa	Steroid biosynthesis	Homo sapiens	1	10
cell part;cytoplasmic part;integral to membrane;intracellular membrane-bounded organelle;intracellular organelle;intracellular organelle part;intracellular part;intrinsic to membrane;membrane;membrane part;membrane-bounc	Proximal tubule bicarbonate reclamation	Homo sapiens	1	10
cell part;cytoplasm;extracellular membrane-bounded organelle;extracellular organelle;extracellular region part;extracellular vesicular exosome;intracellular part;membrane-bounded organelle;membrane-bounded vesicle;organelle;vesicle		Homo sapiens	1	10
cell part;cytoplasm;cytoplasmic part;cytosolic part;extracellular membrane-bounded organelle;extracellular organelle;extracellular region part;extracellular vesicular exosome;intracellular membrane-bounded organelle;intracellular non-membrane-bounded organelle;intracellular organelle;intracellular organelle part;intracellular par		Homo sapiens	1	10
cell part;cytoplasmic part;cytosol;extracellular membrane-bounded organelle;extracellular organelle;extracellular region part;extracellular vesicular exosome;intracellular organelle part;intracellular part;lysosomal membrane;m	Epithelial cell signaling in Helicobacter pylori infection;ko05152;Lysosome;Oxidative phosphorylation;Ph	Homo sapiens	1	10
cell part;intracellular membrane-bounded organelle;intracellular organelle;intracellular part;membrane-bounded organelle;nucleus;organelle		Homo sapiens	1	10
cell cortex part;cell part;cell projection part;centriolar satellite;cytoplasmic part;cytoskeletal part;cytosol;exocyst;growth cone membrane;intracellular organelle part;intracellular part;macromolecular complex;membrane;memb	Insulin signaling pathway	Homo sapiens	1	10
cell part;cytoplasmic part;intracellular membrane-bounded organelle;intracellular organelle;intracellular organelle part;intracellular part;macromolecular complex;membrane;membrane-bounded organelle;mitochondrial inner membrane;mitochondrial membrane;mitochondrial part;mitochondrial small ribosomal subunit;mitochond		Homo sapiens	1	10
cell part;cytoplasm;intracellular membrane-bounded organelle;intracellular non-membrane-bounded organelle;intracellular organelle;intracellular organelle part;intracellular part;membrane;membrane-bounded organelle;non-membrane-bounded organelle;nuclear part;nucleolus;nucleus;organelle;organelle part		Homo sapiens	1	10
cell part;integral to membrane;integral to nuclear inner membrane;integral to organelle membrane;intracellular organelle part;intracellular part;intrinsic to membrane;intrinsic to nuclear inner membrane;intrinsic to organelle membrane;membrane;membrane part;nuclear inner membrane;nuclear membrane;nuclear membrane part;r		Homo sapiens	1	10
cell part;cytoplasmic mRNA processing body;cytoplasmic part;cytosol;extracellular membrane-bounded organelle;extracellular organelle;extracellular region part;extracellular vesicular exosome;intracellular non-membrane-bounded organelle;intracellular organelle;intracellular part;macromolecular complex;membrane-bounded c		Homo sapiens	1	10
cell part;cytoplasmic part;intracellular membrane-bounded organelle;intracellular non-membrane-bounded organelle;intracellular organelle;intracellular organelle part;intracellular part;membrane-bounded organelle;mitochondrion;non-membrane-bounded organelle;nuclear part;nucleolus;nucleus;organelle;organelle part		Homo sapiens	1	10
cell part;intracellular membrane-bounded organelle;intracellular non-membrane-bounded organelle;intracellular organelle;intracellular organelle part;intracellular part;membrane-bounded organelle;non-membrane-bounded organelle;nuclear part;nucleolus;nucleus;organelle;organelle part		Homo sapiens	1	10
extracellular membrane-bounded organelle;extracellular organelle;extracellular region part;extracellular vesicular exosome;membrane-bounded organelle;membrane-bounded vesicle;organelle;vesicle		Homo sapiens	1	10
cell part;cytoplasmic part;extracellular membrane-bounded organelle;extracellular organelle;extracellular region part;extracellular vesicular exosome;intracellular membrane-bounded organelle;intracellular organelle;intracellula	Alzheimer's disease;Huntington's disease;Oxidative phosphorylation;Parkinson's disease	Homo sapiens	1	10
cell part;cytoplasm;cytoplasmic part;Golgi apparatus;intracellular membrane-bounded organelle;intracellular non-membrane-bounded organelle;intracellular organelle;intracellular organelle part;intracellular part;membrane;membrane-bounded organelle;non-membrane-bounded organelle;nuclear membrane;nuclear part;nucleolu		Homo sapiens	1	11
	Glyoxylate and dicarboxylate metabolism	Homo sapiens	1	11
cell part;cytoplasm;intracellular membrane-bounded organelle;intracellular organelle;intracellular part;membrane-bounded organelle;nucleus;organelle		Homo sapiens	1	11
		Homo sapiens	1	11
cell part;cytoplasmic part;cytosol;intracellular organelle part;intracellular part;macromolecular complex;membrane part;nuclear part;nuclear pore;nucleoplasm;organelle part;pore complex;protein complex		Homo sapiens	1	11
cell part;cytoplasm;intracellular membrane-bounded organelle;intracellular organelle;intracellular part;membrane-bounded organelle;nucleus;organelle		Homo sapiens	1	11
actin cytoskeleton;apical plasma membrane;brush border membrane;cell part;cell periphery;cell projection;cell projection membrane;cell projection part;centrosome;cytoplasm;cytoplasmic part;cytoskeletal part;cytoskeleton;endomembrane system;extracellular membrane-bounded organelle;extracellular organelle;extracellular r		Homo sapiens	1	11
cell part;cytoplasmic part;intracellular membrane-bounded organelle;intracellular organelle;intracellular organelle part;intracellular part;membrane;membrane part;membrane-bounded organelle;mitochondrial inner membrane	Alzheimer's disease;Cardiac muscle contraction;Huntington's disease;Oxidative phosphorylation;Parkin	Homo sapiens	1	11
cell part;cytoplasmic part;intracellular membrane-bounded organelle;intracellular organelle;intracellular organelle lumen;intracellular organelle part;intracellular part;membrane;membrane-bounded organelle;membrane-enclos	Glycerophospholipid metabolism;Peroxisome	Homo sapiens	1	11
cell part;cytoplasm;cytoplasmic part;intracellular membrane-bounded organelle;intracellular non-membrane-bounded organelle;intracellular organelle;intracellular organelle part;intracellular part;membrane;membrane-bounde	Ribosome biogenesis in eukaryotes	Homo sapiens	1	11
cell part;cytoplasm;cytoplasmic part;cytoplasmic vesicle membrane;cytoplasmic vesicle part;cytosol;endoplasmic reticulum;endosome;extracellular membrane-bounded organelle;extracellular organelle;extracellular region pa	SNARE interactions in vesicular transport	Homo sapiens	1	11
cell part;cytoplasmic part;cytosol;extracellular membrane-bounded organelle;extracellular organelle;extracellular region part;extracellular vesicular exosome;Golgi apparatus;intracellular membrane-bounded organelle;intracellular organelle;intracellular part;membrane-bounded organelle;membrane-bounded vesicle;mitochondrio		Homo sapiens	1	11
cell part;chromosome;condensed chromosome;cytoplasmic part;female pronucleus;Golgi apparatus;intracellular membrane-bounded organelle;intracellular non-membrane-bounded organelle;intracellular organelle;intracellular organelle part;intracellular part;membrane;membrane-bounded organelle;mitochondrion;non-membra		Homo sapiens	1	11
		Homo sapiens	1	11
cell part;chromatin;chromosomal part;cytoplasm;intracellular membrane-bounded organelle;intracellular organelle;intracellular organelle part;intracellular part;membrane;membrane-bounded organelle;nucleus;organelle;organelle part		Homo sapiens	1	11
cell part;cytoplasmic part;cytosol;extracellular membrane-bounded organelle;extracellular organelle;extracellular region part;extracellular vesicular exosome;intracellular organelle part;intracellular part;lysosomal membrane;m	Collecting duct acid secretion;Epithelial cell signaling in Helicobacter pylori infection;Oxidative phospho	Homo sapiens	1	11
cell part;cytoplasmic part;intracellular membrane-bounded organelle;intracellular organelle;intracellular organelle part;intracellular part;macromolecular complex;membrane;membrane part;membrane-bounded organelle;mitoc	Alzheimer's disease;Huntington's disease;Oxidative phosphorylation;Parkinson's disease	Homo sapiens	1	11
axon;cell part;cell projection;cytoskeleton;intracellular membrane-bounded organelle;intracellular non-membrane-bounded organelle;intracellular organelle;intracellular part;membrane;membrane-bounded organelle;microtubule cytoskeleton;neuron projection;non-membrane-bounded organelle;nucleus;organelle		Homo sapiens	1	11
cell part;granular component;intracellular non-membrane-bounded organelle;intracellular organelle;intracellular organelle part;intracellular part;non-membrane-bounded organelle;nuclear part;nucleolar part;nucleolus;nucleoplasm;organelle;organelle part		Homo sapiens	1	11
cell part;cytoplasm;cytoplasmic part;cytosol;extracellular region part;extracellular space;intracellular organelle part;intracellular part;nuclear part;nucleoplasm;organelle part	Arachidonic acid metabolism	Homo sapiens	1	11
cell part;cytoplasmic part;endoplasmic reticulum membrane;endoplasmic reticulum part;extracellular membrane-bounded organelle;extracellular organelle;extracellular region part;extracellular vesicular exosome;integral to membrane;intracellular organelle part;intracellular part;intrinsic to membrane;membrane;membrane part;m		Homo sapiens	1	11
cell division site part;cell part;centrosome;chromosomal part;cleavage furrow;condensed chromosome kinetochore;cytoplasmic part;cytoskeletal part;cytosol;dynactin complex;intracellular non-membrane-bounded organelle;intracellular organelle;intracellular organelle part;intracellular part;kinetochore;macromolecular complex;		Homo sapiens	1	11
cell cortex;cell part;contractile fiber part;cytoplasmic part;extracellular membrane-bounded organelle;extracellular organelle;extracellular region part;extracellular vesicular exosome;intracellular part;membrane-bounded organelle;membrane-bounded vesicle;organelle;organelle part;vesicle;Z disc		Homo sapiens	1	11
cell part;cytoplasm;cytoplasmic part;endopeptidase Clp complex;intracellular membrane-bounded organelle;intracellular non-membrane-bounded organelle;intracellular organelle;intracellular organelle lumen;intracellular orga	Cell cycle - Caulobacter	Homo sapiens	1	11
cell part;cytoplasm;intracellular membrane-bounded organelle;intracellular organelle;intracellular organelle part;intracellular part;membrane-bounded organelle;nuclear part;nucleoplasm;nucleus;organelle;organelle part		Homo sapiens	1	11
cell part;cytoplasmic part;cytosol;extracellular membrane-bounded organelle;extracellular organelle;extracellular region part;extracellular vesicular exosome;intracellular part;membrane-bounded organelle;membrane-bounde	Pentose phosphate pathway	Homo sapiens	1	11
cell part;cytoplasm;cytoplasmic part;intracellular membrane-bounded organelle;intracellular organelle;intracellular organelle lumen;intracellular organelle part;intracellular part;membrane-bounded organelle;membrane-enclosed lumen;mitochondrial matrix;mitochondrial part;mitochondrion;nuclear part;nucleoplasm;organelle;orga		Homo sapiens	1	11
cell part;chromatin;chromatin remodeling complex;chromosomal part;DNA helicase complex;H4/H2A histone acetyltransferase complex;histone acetyltransferase complex;Ino80 complex;intracellular membrane-bounded organelle;intracellular organelle;intracellular organelle part;intracellular part;macromolecular complex;membr		Homo sapiens	1	11
blood microparticle;cell part;cytoplasmic part;endoplasmic reticulum lumen;endoplasmic reticulum part;extracellular membrane-bounded organelle;extracellular organelle;extracellular region;extracellular region part;extracellu	Complement and coagulation cascades;Neuroactive ligand-receptor interaction;Regulation of actin cyto	Homo sapiens	1	11
adherens junction;anchoring junction;cell junction;cell part;cell-substrate adherens junction;cell-substrate junction;cytoplasm;cytoplasmic part;cytosol;extrinsic to internal side of plasma membrane;extrinsic to membrane;ext	Acute myeloid leukemia;Aldosterone-regulated sodium reabsorption;Axon guidance;B cell receptor sign	Homo sapiens	1	11
cell part;cytoplasm;cytoplasmic part;intracellular membrane-bounded organelle;intracellular organelle;intracellular part;membrane-bounded organelle;nucleus;organelle;perinuclear region of cytoplasm	Mineral absorption	Homo sapiens	1	11
adherens junction;anchoring junction;cell junction;cell part;cell-substrate adherens junction;cell-substrate junction;cytoplasmic part;cytosol;cytosolic large ribosomal subunit;extracellular membrane-bounded organelle;extrac	Ribosome	Homo sapiens	1	11
cell part;cytoplasm;cytoplasmic part;cytosol;extracellular membrane-bounded organelle;extracellular organelle;extracellular region part;extracellular vesicular exosome;intracellular organelle part;intracellular part;membrane-b	Purine metabolism	Homo sapiens	1	11
catalytic step 2 spliceosome;cell part;intracellular non-membrane-bounded organelle;intracellular organelle;intracellular organelle part;intracellular part;macromolecular complex;non-membrane-bounded organelle;nuclear par	Spliceosome	Homo sapiens	1	11
basal plasma membrane;cell part;cytoplasmic part;cytosol;extracellular membrane-bounded organelle;extracellular organelle;extracellular region part;extracellular space;extracellular vesicular exosome;integral to membrane;integral to plasma membrane;intracellular membrane-bounded organelle;intracellular organelle;intracellula		Homo sapiens	1	11
		Homo sapiens	1	11
basolateral plasma membrane;blood microparticle;caveola;cell cortex part;cell junction;cell part;cell-cell junction;cortical actin cytoskeleton;cortical cytoskeleton;cytoplasmic membrane-bounded vesicle;cytoplasmic part;cyto	Adipocytokine signaling pathway;Bile secretion;Pathways in cancer;Renal cell carcinoma	Homo sapiens	1	11
adherens junction;anchoring junction;cell division site part;cell junction;cell part;cell surface;cell-substrate adherens junction;cell-substrate junction;cleavage furrow;cytoplasmic part;cytoplasmic vesicle membrane;cytoplasm	Pancreatic cancer;Pathways in cancer	Homo sapiens	1	11
cell body;cell part;cell projection;cytolytic granule;cytoplasm;cytoplasmic membrane-bounded vesicle;cytoplasmic part;cytoplasmic vesicle;dendrite;endosomal part;endosome;endosome membrane;external side of plasma r	ko05152;Lysosome;Phagosome	Homo sapiens	1	11
cell part;cytoplasm;cytoplasmic part;cytosol;inclusion body;intracellular part;membrane	Insulin signaling pathway;Starch and sucrose metabolism	Homo sapiens	1	11

cell part;cytoplasmic part;intracellular organelle part;intracellular part;macromolecular complex;membrane;membrane part;mitochondrial inner membrane;mitochondrial membrane;mitochondrial membrane part;mitochondrial	Alzheimer's disease;Cardiac muscle contraction;Huntington's disease;Oxidative phosphorylation;Parkin	Homo sapiens	1	11
adherens junction;anchoring junction;cell junction;cell part;cell projection;cell-substrate adherens junction;cell-substrate junction;cytoplasmic part;cytoplasmic vesicle membrane;cytoplasmic vesicle part;cytosol;endocytic ve	Adherens junction;Axon guidance;B cell receptor signaling pathway;Chemokine signaling pathway;Colo	Homo sapiens	1	11
apical plasma membrane;basolateral plasma membrane;cell junction;cell part;cell surface;cell-cell junction;extracellular membrane-bounded organelle;extracellular organelle;extracellular region part;extracellular vesicular exosome;integral to membrane;intrinsic to membrane;lateral plasma membrane;membrane;membrane part;r		Homo sapiens	1	11
cell part;cytoplasmic part;cytosol;intracellular part	Bladder cancer;Drug metabolism - other enzymes;Pyrimidine metabolism	Homo sapiens	1	11
cell part;cytoplasm;cytoplasmic part;extracellular membrane-bounded organelle;extracellular organelle;extracellular region part;extracellular vesicular exosome;intracellular membrane-bounded organelle;intracellular organelle	Peroxisome;PPAR signaling pathway;Primary bile acid biosynthesis	Homo sapiens	1	11
Cajal body;cell part;centrosome;chromosomal part;chromosome;chromosome, telomeric region;condensed chromosome;cyclin-dependent protein kinase holoenzyme complex;cytoplasm;cytoplasmic part;cytoskeletal part;c	Cell cycle;Measles;Oocyte meiosis;p53 signaling pathway;Pathways in cancer;Progesterone-mediated c	Homo sapiens	1	11
cell part;cytoplasmic part;intracellular membrane-bounded organelle;intracellular organelle;intracellular organelle lumen;intracellular organelle part;intracellular part;membrane-bounded organelle;membrane-enclosed lumen;m	Valine, leucine and isoleucine degradation	Homo sapiens	1	11
cell part;cytoplasm;cytoplasmic part;cytoplasmic vesicle;cytosol;extracellular membrane-bounded organelle;extracellular organelle;extracellular region part;extracellular space;extracellular vesicular exosome;intracellular men	Peroxisome	Homo sapiens	1	11
cell part;cytoplasm;extracellular membrane-bounded organelle;extracellular organelle;extracellular region part;extracellular vesicular exosome;intracellular part;membrane-bounded organelle;membrane-bounded vesicle;organelle;vesicle		Homo sapiens	1	11
actin filament;axon;cell cortex part;cell junction;cell part;cell projection;cell-cell junction;cortical actin cytoskeleton;cortical cytoskeleton;cytoplasm;cytoplasmic membrane-bounded vesicle;cytoplasmic part;cytoplasmic vesic	ko05152;Phagosome	Homo sapiens	1	11
cell part;cytoplasmic part;endoplasmic reticulum;endoplasmic reticulum membrane;endoplasmic reticulum part;integral to membrane;intracellular membrane-bounded organelle;intracellular organelle;intracellular organelle pa	Steroid biosynthesis	Homo sapiens	1	11
extracellular membrane-bounded organelle;extracellular organelle;extracellular region part;extracellular vesicular exosome;membrane-bounded organelle;membrane-bounded vesicle;organelle;vesicle		Homo sapiens	1	11
cell part;chromosomal part;DNA replication factor C complex;intracellular membrane-bounded organelle;intracellular organelle;intracellular organelle part;intracellular part;macromolecular complex;membrane-bounded organe	DNA replication;Mismatch repair;Nucleotide excision repair	Homo sapiens	1	11
actin cytoskeleton;cell part;cytoplasm;cytoplasmic part;cytoskeleton;cytosol;extracellular membrane-bounded organelle;extracellular organelle;extracellular region part;extracellular vesicular exosome;intracellular membrane-	Bacterial invasion of epithelial cells;Chemokine signaling pathway;Chronic myeloid leukemia;ErbB signa	Homo sapiens	1	11
cell part;cytoplasmic part;cytosol;endoplasmic reticulum membrane;endoplasmic reticulum part;glycogen granule;intracellular organelle part;intracellular part;membrane;membrane part;organelle membrane;organelle part	Glycerophospholipid metabolism;Phosphonate and phosphinate metabolism	Homo sapiens	1	11
cell junction;cell part;cell projection;cell-cell junction;cytoplasm;cytoplasmic membrane-bounded vesicle;cytoplasmic part;cytoplasmic vesicle;cytoplasmic vesicle membrane;cytoplasmic vesicle part;endocytic vesicle;endos	Tight junction	Homo sapiens	1	11
cell part;cytoplasmic part;cytosol;intracellular part	Terpenoid backbone biosynthesis	Homo sapiens	1	11
cell part;cytoplasm;intracellular membrane-bounded organelle;intracellular organelle;intracellular organelle part;intracellular part;macromolecular complex;membrane-bounded organelle;nuclear part;nucleoplasm;nucleotide-e	Nucleotide excision repair;Protein processing in endoplasmic reticulum	Homo sapiens	1	11
extracellular matrix part;extracellular region;extracellular region part;fibril;microfibril		Homo sapiens	1	11
cell part;cytoplasm;cytoplasmic part;endoplasmic reticulum;intracellular membrane-bounded organelle;intracellular organelle;intracellular part;membrane-bounded organelle;organelle;perinuclear region of cytoplasm		Homo sapiens	1	11
cell part;cytoplasmic part;intracellular membrane-bounded organelle;intracellular organelle;intracellular part;macromolecular complex;membrane-bounded organelle;mitochondrion;nucleus;organelle;ribonucleoprotein complex		Homo sapiens	1	11
cell part;intracellular		Homo sapiens	1	11
cell junction;cell part;cell projection;cytoplasm;cytoplasmic part;cytosol;early endosome;endosome;extracellular membrane-bounded organelle;extracellular organelle;extracellular region part;extracellular vesicular exosome;g	Chemokine signaling pathway;Focal adhesion;Leukocyte transendothelial migration;Long-term potentia	Homo sapiens	1	11
cell part;Cul7-RING ubiquitin ligase complex;cullin-RING ubiquitin ligase complex;cytoplasmic part;cytosol;extracellular membrane-bounded organelle;extracellular organelle;extracellular region part;extracellular vesicular exo	Cell cycle;Cell cycle - yeast;Circadian rhythm - mammal;Oocyte meiosis;Protein processing in endoplas	Homo sapiens	1	11
cell part;cytoplasm;cytoplasmic part;cytosol;extracellular membrane-bounded organelle;extracellular organelle;extracellular region part;extracellular vesicular exosome;intracellular non-membrane-bounded organelle;intracellu	Ether lipid metabolism	Homo sapiens	1	11
Cajal body;catalytic step 2 spliceosome;cell part;intracellular organelle part;intracellular part;macromolecular complex;nuclear body;nuclear part;nuclear speck;nucleoplasm;nucleoplasm part;organelle part;ribonucleoprotein c	Shigellosis;Spliceosome	Homo sapiens	1	11
cell part;cytoplasm;cytoplasmic part;cytosol;I-kappaB/NF-kappaB complex;intracellular membrane-bounded organelle;intracellular organelle;intracellular organelle part;intracellular part;macromolecular complex;membrane-b	Acute myeloid leukemia;Adipocytokine signaling pathway;Amoebiasis;Apoptosis;B cell receptor signalir	Homo sapiens	1	11
cell part;cytoplasm;cytoplasmic part;intracellular membrane-bounded organelle;intracellular non-membrane-bounded organelle;intracellular organelle;intracellular organelle part;intracellular part;membrane-bounded organelle;mitochondrial nucleoid;mitochondrial part;mitochondrion;non-membrane-bounded organelle;nucleoid;or		Homo sapiens	1	11
cell part;chromatin remodeling complex;cytoplasm;cytoskeletal part;envelope;histone deacetylase complex;intracellular membrane-bounded organelle;intracellular organelle;intracellular organelle part;intracellular part;macromolecular complex;membrane-bounded organelle;microtubule;nuclear envelope;nuclear part;nucleoplasm		Homo sapiens	1	11
cell part;cytoplasm;cytoplasmic part;cytosol;extracellular membrane-bounded organelle;extracellular organelle;extracellular region part;extracellular vesicular exosome;intracellular membrane-bounded organelle;intracellular organelle;intracellular part;macromolecular complex;membrane-bounded organelle;membrane-bounded v		Homo sapiens	1	11
cell part;cytoplasm;intracellular membrane-bounded organelle;intracellular organelle;intracellular organelle part;intracellular part;membrane-bounded organelle;midbody;nuclear body;nuclear part;nuclear speck;nucleoplasm;r	RIG-I-like receptor signaling pathway	Homo sapiens	1	11
cell part;cytoplasm;cytoplasmic exosome (RNase complex);cytoplasmic part;cytosol;exosome (RNase complex);intracellular membrane-bounded organelle;intracellular non-membrane-bounded organelle;intracellular organell	RNA degradation	Homo sapiens	1	11
cell part;cytoplasm;intracellular membrane-bounded organelle;intracellular non-membrane-bounded organelle;intracellular organelle;intracellular organelle part;intracellular part;membrane;membrane-bounded organelle;non-membrane-bounded organelle;nuclear part;nucleolus;nucleoplasm;nucleus;organelle;organelle part		Homo sapiens	1	11
cell part;cytoplasmic part;endoplasmic reticulum;endoplasmic reticulum lumen;endoplasmic reticulum membrane;endoplasmic reticulum part;intracellular membrane-bounded organelle;intracellular organelle;intracellular organelle lumen;intracellular organelle part;intracellular part;membrane;membrane part;membrane-bounded c		Homo sapiens	1	11
cell part;cytoplasmic part;intracellular membrane-bounded organelle;intracellular organelle;intracellular organelle lumen;intracellular organelle part;intracellular part;membrane-bounded organelle;membrane-enclosed lumen;m	Aminoacyl-tRNA biosynthesis;Valine, leucine and isoleucine biosynthesis	Homo sapiens	1	11
cell part;cytoplasm;intracellular non-membrane-bounded organelle;intracellular organelle;intracellular organelle part;intracellular part;macromolecular complex;non-membrane-bounded organelle;nuclear part;nucleolar part;nu	Ribosome biogenesis in eukaryotes	Homo sapiens	1	11
adherens junction;anchoring junction;cell junction;cell part;cell-substrate adherens junction;cell-substrate junction;cytoplasmic part;cytosol;extracellular membrane-bounded organelle;extracellular organelle;extracellular region part;extracellular vesicular exosome;focal adhesion;intracellular part;membrane-bounded organelle;me		Homo sapiens	1	11
extracellular membrane-bounded organelle;extracellular organelle;extracellular region;extracellular region part;extracellular space;extracellular vesicular exosome;membrane-bounded organelle;membrane-bounded vesicle;organelle;vesicle		Homo sapiens	1	11
cell part;cytoplasmic part;intracellular membrane-bounded organelle;intracellular organelle;intracellular organelle lumen;intracellular organelle part;intracellular part;macromolecular complex;membrane;membrane part;membr	Alzheimer's disease;Huntington's disease;Oxidative phosphorylation;Parkinson's disease	Homo sapiens	1	11
cell part;cytoplasmic part;endoplasmic reticulum;endoplasmic reticulum membrane;endoplasmic reticulum part;integral to membrane;intracellular membrane-bounded organelle;intracellular organelle;intracellular organelle pa	Steroid biosynthesis	Homo sapiens	1	11
cell part;cytoplasmic part;early endosome;endosome;intracellular membrane-bounded organelle;intracellular organelle;intracellular organelle part;intracellular part;macromolecular complex;membrane-bounded organelle;nuclear part;nucleoplasm;organelle;organelle part;protein complex;WASH complex		Homo sapiens	1	11
cell part;cytoplasmic part;intracellular membrane-bounded organelle;intracellular organelle;intracellular part;membrane-bounded organelle;mitochondrion;organelle		Homo sapiens	1	11
	Arginine and proline metabolism	Homo sapiens	1	11
cell part;cytoplasmic part;intracellular membrane-bounded organelle;intracellular organelle;intracellular organelle lumen;intracellular organelle part;intracellular part;membrane-bounded organelle;membrane-enclosed lumen;m	Aminoacyl-tRNA biosynthesis;Porphyrin and chlorophyll metabolism	Homo sapiens	1	11
cell part;cytoplasmic part;extracellular membrane-bounded organelle;extracellular organelle;extracellular region part;extracellular vesicular exosome;intracellular organelle lumen;intracellular organelle part;intracellular part;me	beta-Alanine metabolism;Propanoate metabolism;Valine, leucine and isoleucine degradation	Homo sapiens	1	11
cell part;cytoplasm;cytoplasmic part;cytosol;extracellular membrane-bounded organelle;extracellular organelle;extracellular region part;extracellular vesicular exosome;Golgi apparatus;intracellular membrane-bounded organe	Nicotinate and nicotinamide metabolism	Homo sapiens	1	11
actin filament;basal cortex;cell cortex part;cell part;cytoplasmic part;cytoskeletal part;intracellular membrane-bounded organelle;intracellular organelle;intracellular organelle part;intracellular part;lateral plasma membrane;macromolecular complex;membrane;membrane part;membrane-bounded organelle;mitochondrion;nucleus;		Homo sapiens	1	11
		Homo sapiens	1	11
cell part;cytoplasmic part;extracellular membrane-bounded organelle;extracellular organelle;extracellular region part;extracellular vesicular exosome;intracellular membrane-bounded organelle;intracellular organelle;intracellular part;membrane-bounded organelle;membrane-bounded vesicle;mitochondrion;nucleus;organelle;vesic		Homo sapiens	1	11
cell part;cytoplasm;intracellular part		Homo sapiens	1	11
cell part;cytoplasm;cytoplasmic part;endoplasmic reticulum membrane;endoplasmic reticulum part;intracellular organelle part;intracellular part;membrane;membrane part;organelle membrane;organelle part;perinuclear region	Spliceosome	Homo sapiens	1	11
cell cortex part;cell part;cell projection part;cytoplasm;cytoplasmic part;cytosol;endosome;exocyst;growth cone;intracellular membrane-bounded organelle;intracellular organelle;intracellular part;late endosome;macromolecular complex;membrane;membrane-bounded organelle;organelle;plasma membrane;protein complex;site		Homo sapiens	1	11
cell part;cytoplasmic part;endoplasmic reticulum;endoplasmic reticulum membrane;endoplasmic reticulum part;Golgi apparatus;Golgi apparatus part;Golgi membrane;integral to membrane;intracellular membrane-bounded c	Ether lipid metabolism;Glycerophospholipid metabolism	Homo sapiens	1	11
cell part;cytoplasmic part;intracellular membrane-bounded organelle;intracellular non-membrane-bounded organelle;intracellular organelle;intracellular organelle part;intracellular part;macromolecular complex;membrane;membrane-bounded organelle;mitochondrial inner membrane;mitochondrial membrane;mitochondrial part;m		Homo sapiens	1	11
cell part;cytoplasm;extracellular membrane-bounded organelle;extracellular organelle;extracellular region part;extracellular vesicular exosome;intracellular part;membrane-bounded organelle;membrane-bounded vesicle;orga	Pathways in cancer;Thyroid cancer	Homo sapiens	1	11
cell part;cytoplasm;intracellular membrane-bounded organelle;intracellular non-membrane-bounded organelle;intracellular organelle;intracellular organelle part;intracellular part;membrane-bounded organelle;non-membrane-b	Ribosome biogenesis in eukaryotes	Homo sapiens	1	11
cell part;cytoplasm;cytoskeleton;intracellular non-membrane-bounded organelle;intracellular organelle;intracellular part;non-membrane-bounded organelle;organelle		Homo sapiens	1	11
cell part;cytoplasmic part;cytosol;intracellular part		Homo sapiens	1	11
cell part;cytoplasmic part;intracellular membrane-bounded organelle;intracellular organelle;intracellular part;macromolecular complex;membrane-bounded organelle;mitochondrion;organelle;oxoglutarate dehydrogenase complex;protein complex		Homo sapiens	1	11
cell part;cytoplasm;extracellular membrane-bounded organelle;extracellular organelle;extracellular region part;extracellular vesicular exosome;intracellular part;membrane-bounded organelle;membrane-bounded vesicle;orga	Amino sugar and nucleotide sugar metabolism;Fructose and mannose metabolism	Homo sapiens	1	11
cell part;centrosome;cytoplasm;cytoplasmic part;cytoskeletal part;endomembrane system;intracellular membrane-bounded organelle;intracellular non-membrane-bounded organelle;intracellular organelle;intracellular organelle part;intracellular part;macromolecular complex;membrane;membrane-bounded organelle;microtubule c		Homo sapiens	1	11
cell part;cell surface;cytoplasmic part;endoplasmic reticulum;endoplasmic reticulum membrane;endoplasmic reticulum part;integral to membrane;intracellular membrane-bounded organelle;intracellular organelle;intracellular organelle part;intracellular part;intrinsic to membrane;membrane;membrane part;membrane-bounded org		Homo sapiens	1	11
adherens junction;anchoring junction;cell junction;cell part;cell projection;cell projection membrane;cell projection part;cytoplasm;cytoplasmic part;cytoskeleton;dendritic spine;filopodium;intracellular non-membrane-bounded organelle;intracellular organelle;intracellular organelle part;intracellular part;lamellipodium;leading edge		Homo sapiens	1	11
cell part;cytoplasm;cytoplasmic part;cytosol;intracellular membrane-bounded organelle;intracellular organelle;intracellular organelle part;intracellular part;membrane;membrane-bounded organelle;nuclear membrane;nuclear part;nucleoplasm;nucleus;organelle;organelle membrane;organelle part		Homo sapiens	1	11
cell part;cytoplasm;cytoplasmic part;intracellular membrane-bounded organelle;intracellular organelle;intracellular organelle part;intracellular part;membrane;membrane-bounded organelle;microbody;microbody membrane;microbody part;organelle;organelle membrane;organelle part;peroxisomal membrane;peroxisomal part		Homo sapiens	1	11
cell part;cytoplasm;cytoplasmic part;endoplasmic reticulum;intracellular membrane-bounded organelle;intracellular organelle;intracellular organelle lumen;intracellular organelle part;intracellular part;membrane;membrane-bou	Alzheimer's disease;Valine, leucine and isoleucine degradation	Homo sapiens	1	11
cell part;cytoplasmic part;endosomal part;endosome;endosome membrane;extracellular membrane-bounded organelle;extracellular organelle;extracellular region part;extracellular vesicular exosome;integral to membrane;integral to plasma membrane;intracellular membrane-bounded organelle;intracellular organelle;intracellular c		Homo sapiens	1	11
cell part;cytoplasm;cytoplasmic part;cytosol;intracellular part		Homo sapiens	1	11
cell part;cytoplasmic part;intracellular membrane-bounded organelle;intracellular organelle;intracellular organelle part;intracellular part;membrane-bounded organelle;mitochondrion;nuclear part;nucleoplasm;nucleus;organelle;organelle part		Homo sapiens	1	11
cell part;cytoplasmic part;cytosol;intracellular part		Homo sapiens	1	11
cell part;intracellular membrane-bounded organelle;intracellular organelle;intracellular organelle part;intracellular part;membrane-bounded organelle;nuclear part;nucleoplasm;nucleus;organelle;organelle part		Homo sapiens	1	11
cell part;cytoplasm;cytoplasmic part;cytosol;extracellular membrane-bounded organelle;extracellular organelle;extracellular region part;extracellular vesicular exosome;Golgi apparatus;Golgi apparatus part;Golgi membrane;intracellular membrane-bounded organelle;intracellular organelle;intracellular organelle part;intracellular pa		Homo sapiens	1	11
		Homo sapiens	1	11
cell part;cytoplasm;intracellular membrane-bounded organelle;intracellular organelle;intracellular organelle part;intracellular part;membrane-bounded organelle;nuclear body;nuclear part;nuclear speck;nucleoplasm;nucleoplasm part;organelle;organelle part		Homo sapiens	1	11
cell part;intracellular organelle part;intracellular part;membrane;nuclear membrane;nuclear part;organelle membrane;organelle part		Homo sapiens	1	11
cell part;intracellular membrane-bounded organelle;intracellular non-membrane-bounded organelle;intracellular organelle;intracellular organelle part;intracellular part;membrane-bounded organelle;non-membrane-bounded organelle;nuclear part;nucleolus;nucleus;organelle;organelle part		Homo sapiens	1	11
cell part;cell projection;cytoplasm;extracellular membrane-bounded organelle;extracellular organelle;extracellular region part;extracellular vesicular exosome;intracellular membrane-bounded organelle;intracellular organelle;intracellular part;lamellipodium;membrane-bounded organelle;membrane-bounded vesicle;nucleus;organe		Homo sapiens	1	11
cell part;cytoplasm;intracellular part		Homo sapiens	1	11
cell part;cytoplasmic part;extracellular membrane-bounded organelle;extracellular organelle;extracellular region part;extracellular vesicular exosome;intracellular membrane-bounded organelle;intracellular organelle;intracellular organelle part;intracellular part;membrane-bounded organelle;membrane-bounded vesicle;mitochondr		Homo sapiens	1	11
cell part;cytoplasmic part;extracellular membrane-bounded organelle;extracellular organelle;extracellular region part;extracellular vesicular exosome;intracellular membrane-bounded organelle;intracellular organelle;intracellular part;membrane-bounded organelle;membrane-bounded vesicle;mitochondrion;organelle;vesicle		Homo sapiens	1	11
cell part;cytoplasmic part;cytosol;endosomal part;endosome membrane;extracellular membrane-bounded organelle;extracellular organelle;extracellular region part;extracellular vesicular exosome;intracellular organelle part;in	Endocytosis	Homo sapiens	1	11
cell part;intracellular membrane-bounded organelle;intracellular non-membrane-bounded organelle;intracellular organelle;intracellular organelle part;intracellular part;membrane-bounded organelle;non-membrane-bounded organelle;nuclear part;nucleolus;nucleus;organelle;organelle part		Homo sapiens	1	11
ACF complex;cell part;chromatin accessibility complex;chromatin assembly complex;chromatin remodeling complex;chromosome;intracellular non-membrane-bounded organelle;intracellular organelle;intracellular organelle part;intracellular part;macromolecular complex;non-membrane-bounded organelle;nuclear chromosome;r		Homo sapiens	1	11
cell part;cytoplasmic part;cytosol;extracellular membrane-bounded organelle;extracellular organelle;extracellular region part;extracellular vesicular exosome;intracellular part;membrane-bounded organelle;membrane-bounde	Fructose and mannose metabolism	Homo sapiens	1	11
catalytic step 2 spliceosome;cell part;cytoplasm;intracellular membrane-bounded organelle;intracellular organelle;intracellular organelle part;intracellular part;macromolecular complex;membrane-bounded organelle;nuclear p	Spliceosome	Homo sapiens	1	11
		Homo sapiens	1	11
cell part;intracellular non-membrane-bounded organelle;intracellular organelle;intracellular organelle part;intracellular part;macromolecular complex;non-membrane-bounded organelle;nuclear part;nucleolar part;nucleolus;org	Ribosome biogenesis in eukaryotes	Homo sapiens	1	11
anaphase-promoting complex;cell part;cullin-RING ubiquitin ligase complex;cytoplasmic part;cytosol;intracellular membrane-bounded organelle;intracellular organelle;intracellular organelle part;intracellular part;macromolecu	Cell cycle;Cell cycle - yeast;Meiosis - yeast;Oocyte meiosis;Progesterone-mediated oocyte maturation;U	Homo sapiens	1	11
cell part;intracellular organelle part;intracellular part;macromolecular complex;mRNA cleavage and polyadenylation specificity factor complex;mRNA cleavage factor complex;nuclear part;nucleoplasm;nucleoplasm part;organ	mRNA surveillance pathway	Homo sapiens	1	11
cell part;cytoplasm;intracellular non-membrane-bounded organelle;intracellular organelle;intracellular organelle part;intracellular part;membrane;non-membrane-bounded organelle;nuclear part;nucleolus;organelle;organelle part;plasma membrane		Homo sapiens	1	11
cell part;centrosome;cytoplasm;cytoplasmic part;cytoskeletal part;cytosol;early endosome;endosomal part;endosome;endosome membrane;extracellular membrane-bounded organelle;extracellular organelle;extracellular reg	Endocytosis	Homo sapiens	1	11
cell part;cytoplasmic part;intracellular membrane-bounded organelle;intracellular non-membrane-bounded organelle;intracellular organelle;intracellular organelle part;intracellular part;membrane-bounded organelle;mitochondrial nucleoid;mitochondrial part;mitochondrion;non-membrane-bounded organelle;nucleoid;nucleus;orga		Homo sapiens	1	11
cell part;cytoplasmic part;intracellular membrane-bounded organelle;intracellular organelle;intracellular organelle part;intracellular part;macromolecular complex;membrane-bounded organelle;mitochondrial part;mitochondrial small ribosomal subunit;mitochondrion;organellar small ribosomal subunit;organelle;organelle part;ribon		Homo sapiens	1	11
cell part;intracellular membrane-bounded organelle;intracellular non-membrane-bounded organelle;intracellular organelle;intracellular organelle part;intracellular part;macromolecular complex;membrane-bounded organelle;non-membrane-bounded organelle;nuclear body;nuclear part;nuclear speck;nucleolus;nucleoplasm;nucle		Homo sapiens	1	11
cell part;cytoplasmic part;cytosol;intracellular part;membrane;plasma membrane	Adipocytokine signaling pathway;Aldosterone-regulated sodium reabsorption;Insulin signaling pathway;	Homo sapiens	1	11
cell part;cytoplasm;intracellular membrane-bounded organelle;intracellular organelle;intracellular part;membrane-bounded organelle;nucleus;organelle		Homo sapiens	1	11
basolateral plasma membrane;cell part;extracellular membrane-bounded organelle;extracellular organelle;extracellular region part;extracellular space;extracellular vesicular exosome;extrinsic to membrane;extrinsic to plasma membrane;integral to membrane;integral to plasma membrane;intrinsic to membrane;intrinsic to plasma		Homo sapiens	1	11
cell part;cytoplasm;cytoplasmic part;cytosol;intracellular membrane-bounded organelle;intracellular non-membrane-bounded organelle;intracellular organelle;intracellular organelle lumen;intracellular organelle part;intracellula	Sulfur relay system;Thiamine metabolism	Homo sapiens	1	11
asymmetric synapse;axonemal microtubule;axoneme part;cell junction;cell part;cell projection;cell projection part;cytoplasmic membrane-bounded vesicle;cytoplasmic part;cytoplasmic vesicle;cytoskeletal part;cytosol;dendritic spine;endosome;Golgi apparatus part;Golgi membrane;intracellular membrane-bounded organelle;in		Homo sapiens	1	11
cell part;cytoplasm;cytoplasmic part;cytosol;Golgi apparatus;Golgi apparatus part;Golgi membrane;intracellular membrane-bounded organelle;intracellular non-membrane-bounded organelle;intracellular organelle;intracellular organelle part;intracellular part;macromolecular complex;membrane;membrane-bounded organelle;non		Homo sapiens	1	11
AP-type membrane coat adaptor complex;cell part;clathrin adaptor complex;clathrin coated vesicle membrane;clathrin-coated endocytic vesicle membrane;coated vesicle membrane;cytoplasmic part;cytoplasmic vesicle me	Lysosome	Homo sapiens	1	11
cell part;cytoplasm;cytoplasmic part;cytosol;intracellular membrane-bounded organelle;intracellular organelle;intracellular organelle part;intracellular part;membrane-bounded organelle;nuclear part;nucleoplasm;nucleus;organelle;organelle part		Homo sapiens	1	11
cell part;centrosome;cytoplasmic part;cytoskeletal part;cytosol;intracellular non-membrane-bounded organelle;intracellular organelle;intracellular organelle part;intracellular part;kinesin complex;macromolecular complex;membrane;microtubule;microtubule associated complex;microtubule organizing center;non-membrane-boun		Homo sapiens	1	12
cell part;cytoplasmic exosome (RNase complex);cytoplasmic part;cytosol;exosome (RNase complex);intracellular part;macromolecular complex;membrane;protein complex		Homo sapiens	1	12
cell part;cytoplasm;intracellular membrane-bounded organelle;intracellular organelle;intracellular part;membrane-bounded organelle;nucleus;organelle		Homo sapiens	1	12
cell part;cytoplasmic part;extracellular membrane-bounded organelle;extracellular organelle;extracellular region part;extracellular vesicular exosome;Golgi apparatus part;Golgi membrane;integral to membrane;intracellular organelle part;intracellular part;intrinsic to membrane;membrane;membrane part;membrane-bounded orga		Homo sapiens	1	12
actin cytoskeleton;adherens junction;anchoring junction;basement membrane;basolateral plasma membrane;cell junction;cell part;cell projection membrane;cell projection part;cell-cell junction;cell-substrate adherens junctio	Tight junction	Homo sapiens	1	12
cell part;cytoplasmic part;endoplasmic reticulum membrane;endoplasmic reticulum palmitoyltransferase complex;endoplasmic reticulum part;integral to membrane;intracellular membrane-bounded organelle;intracellular orga	Sphingolipid metabolism	Homo sapiens	1	12
cell part;chromosomal part;condensed chromosome kinetochore;cytoplasm;cytoplasmic part;cytoskeletal part;cytosol;endoplasmic reticulum;endoplasmic reticulum membrane;endoplasmic reticulum part;intracellular membrane-bounded organelle;intracellular non-membrane-bounded organelle;intracellular organelle;intracellula		Homo sapiens	1	12
		Homo sapiens	1	12
cell part;cytoplasm;cytoplasmic part;endoplasmic reticulum membrane;endoplasmic reticulum part;extracellular membrane-bounded organelle;extracellular organelle;extracellular region part;extracellular vesicular exosome;intracellular membrane-bounded organelle;intracellular non-membrane-bounded organelle;intracellular org		Homo sapiens	1	12
cell part;intracellular membrane-bounded organelle;intracellular organelle;intracellular organelle part;intracellular part;membrane-bounded organelle;nuclear part;nucleoplasm;nucleus;organelle;organelle part		Homo sapiens	1	12
cell part;cytoplasm;intracellular part		Homo sapiens	1	12
cell part;clathrin coated vesicle membrane;clathrin-coated vesicle;coated pit;coated vesicle;coated vesicle membrane;cytoplasm;cytoplasmic membrane-bounded vesicle;cytoplasmic part;cytoplasmic vesicle;cytoplasmic vesicle membrane;cytoplasmic vesicle part;cytoskeleton;intracellular membrane-bounded organelle;intrace		Homo sapiens	1	12
cell part;cytoplasm;intracellular membrane-bounded organelle;intracellular organelle;intracellular organelle part;intracellular part;membrane-bounded organelle;nuclear body;nuclear part;nuclear speck;nucleoplasm;nucleoplas	Spliceosome	Homo sapiens	1	12
cell part;cytoplasmic part;intracellular membrane-bounded organelle;intracellular organelle;intracellular organelle lumen;intracellular organelle part;intracellular part;membrane;membrane-bounded organelle;membrane-enclos	Fatty acid metabolism;Peroxisome	Homo sapiens	1	12
cell part;chromosome;cytoplasm;cytoplasmic part;cytosol;extracellular membrane-bounded organelle;extracellular organelle;extracellular region part;extracellular vesicular exosome;intracellular membrane-bounded organelle;intracellular non-membrane-bounded organelle;intracellular organelle;intracellular organelle part;intracell		Homo sapiens	1	12
cell part;cytoplasmic part;integral to membrane;intracellular membrane-bounded organelle;intracellular organelle;intracellular organelle part;intracellular part;intrinsic to membrane;membrane;membrane part;membrane-bounded organelle;mitochondrial inner membrane;mitochondrial membrane;mitochondrial part;mitochondrion;		Homo sapiens	1	12
cell junction;cell part;intracellular membrane-bounded organelle;intracellular non-membrane-bounded organelle;intracellular organelle;intracellular organelle part;intracellular part;macromolecular complex;membrane-boundec	Spliceosome	Homo sapiens	1	12
cell part;cytoplasmic part;endoplasmic reticulum;endoplasmic reticulum membrane;endoplasmic reticulum part;integral to endoplasmic reticulum membrane;integral to membrane;integral to organelle membrane;intracellular	Sphingolipid metabolism	Homo sapiens	1	12
cell part;cytoplasm;cytoskeletal part;intracellular non-membrane-bounded organelle;intracellular organelle;intracellular organelle part;intracellular part;macromolecular complex;microtubule;microtubule associated complex;non-membrane-bounded organelle;organelle;organelle part;protein complex;spindle		Homo sapiens	1	12
cell part;cytoplasm;cytoplasmic part;cytosol;extracellular membrane-bounded organelle;extracellular organelle;extracellular region part;extracellular vesicular exosome;intracellular part;membrane-bounded organelle;membra	Drug metabolism - other enzymes;Purine metabolism	Homo sapiens	1	12
cell part;cell surface;coated vesicle membrane;cytoplasmic part;cytoplasmic vesicle membrane;cytoplasmic vesicle part;early endosome membrane;endocytic vesicle membrane;endoplasmic reticulum;endoplasmic reticulun	Allograft rejection;Antigen processing and presentation;Autoimmune thyroid disease;Cell adhesion mole	Homo sapiens	1	12
cell part;cytoplasmic part;cytosol;intracellular organelle lumen;intracellular organelle part;intracellular part;membrane-enclosed lumen;mitochondrial matrix;mitochondrial part;organelle lumen;organelle part	Carbon fixation pathways in prokaryotes;Glyoxylate and dicarboxylate metabolism;Propanoate metabol	Homo sapiens	1	12
actin cytoskeleton;cell part;chromatin;chromosomal part;cytoplasmic part;cytoskeleton;euchromatin;Golgi apparatus;intracellular membrane-bounded organelle;intracellular non-membrane-bounded organelle;intracellular organelle;intracellular organelle part;intracellular part;macromolecular complex;membrane-bounded organel		Homo sapiens	1	12
cell part;cytoplasmic part;intracellular membrane-bounded organelle;intracellular organelle;intracellular organelle part;intracellular part;membrane;membrane part;membrane-bounded organelle;mitochondrial inner membrane	Alzheimer's disease;Cardiac muscle contraction;Huntington's disease;Oxidative phosphorylation;Parkin	Homo sapiens	1	12
cell part;cytoplasm;intracellular non-membrane-bounded organelle;intracellular organelle;intracellular organelle part;intracellular part;macromolecular complex;non-membrane-bounded organelle;nuclear part;nucleolus;nucleo	Spliceosome	Homo sapiens	1	12
catalytic step 2 spliceosome;cell part;cytoplasmic part;cytosol;extracellular membrane-bounded organelle;extracellular organelle;extracellular region part;extracellular vesicular exosome;histone pre-mRNA 3'end processing c	Spliceosome;Systemic lupus erythematosus	Homo sapiens	1	12
cell part;cytoplasm;extracellular membrane-bounded organelle;extracellular organelle;extracellular region part;extracellular vesicular exosome;intracellular membrane-bounded organelle;intracellular organelle;intracellular organelle part;intracellular part;membrane-bounded organelle;membrane-bounded vesicle;nuclear part;nucle		Homo sapiens	1	12

cell part;cytoplasmic part;cytosol;extracellular membrane-bounded organelle;extracellular organelle;extracellular region part;extracellular vesicular exosome;intracellular part;membrane-bounded organelle;membrane-bounde	Styrene degradation;Tyrosine metabolism	Homo sapiens	1	12
cell part;cytoplasmic part;cytoplasmic vesicle;cytosol;extracellular membrane-bounded organelle;extracellular organelle;extracellular region part;extracellular vesicular exosome;Golgi apparatus;Golgi apparatus part;Golgi membrane;intracellular membrane-bounded organelle;intracellular organelle;intracellular organelle part;intrac		Homo sapiens	1	12
cell part;centrosome;chromosome;condensed chromosome;condensed nuclear chromosome;cytoplasm;cytoplasmic part;cytoskeletal part;cytosol;endosome;gamma-tubulin complex;intracellular membrane-bounded organelle;intracellular non-membrane-bounded organelle;intracellular organelle;intracellular organelle part;intrac		Homo sapiens	1	12
cell part;cytoplasm;cytoplasmic mRNA processing body;cytoplasmic part;cytosol;extracellular membrane-bounded organelle;extracellular organelle;extracellular region part;extracellular vesicular exosome;intracellular membr	Proteasome	Homo sapiens	1	12
cell part;cytoplasm;cytoplasmic part;cytosol;extracellular membrane-bounded organelle;extracellular organelle;extracellular region part;extracellular vesicular exosome;intracellular organelle part;intracellular part;membrane;m	Porphyrin and chlorophyll metabolism;Riboflavin metabolism	Homo sapiens	1	12
cell part;cytoplasmic part;endoplasmic reticulum membrane;endoplasmic reticulum part;intracellular organelle part;intracellular part;membrane;membrane part;organelle membrane;organelle part;plasma membrane	Mineral absorption;Porphyrin and chlorophyll metabolism	Homo sapiens	1	12
axon part;axon terminus;cell part;cell projection part;contractile fiber part;cytoplasm;cytoplasmic part;cytosol;dendritic spine neck;endoplasmic reticulum;endoplasmic reticulum membrane;endoplasmic reticulum part;extracellular membrane-bounded organelle;extracellular organelle;extracellular region part;extracellular vesicula		Homo sapiens	1	12
cell part;cytoplasmic part;extracellular membrane-bounded organelle;extracellular organelle;extracellular region part;extracellular vesicular exosome;intracellular membrane-bounded organelle;intracellular organelle;intracellula	Biosynthesis of unsaturated fatty acids	Homo sapiens	1	12
beta-catenin destruction complex;cell body;cell part;cell projection;cell projection part;centrosome;cytoplasm;cytoplasmic part;cytoskeletal part;cytosol;dendritic shaft;dendritic spine;growth cone;intracellular membrane-bou	Alzheimer's disease;Axon guidance;B cell receptor signaling pathway;Basal cell carcinoma;Cell cycle;Cl	Homo sapiens	1	12
cell part;cytoplasmic membrane-bounded vesicle;cytoplasmic part;cytoplasmic vesicle;early endosome;early endosome membrane;endocytic vesicle;endosomal part;endosome;endosome membrane;extracellular membrane	Amoebiasis;Endocytosis;ko05152;Phagosome;Vasopressin-regulated water reabsorption	Homo sapiens	1	12
cell part;cytoplasmic part;cytosol;endoplasmic reticulum;extracellular membrane-bounded organelle;extracellular organelle;extracellular region part;extracellular vesicular exosome;intracellular membrane-bounded organelle;intracellular organelle;intracellular part;membrane-bounded organelle;membrane-bounded vesicle;mitoch		Homo sapiens	1	12
cell part;cytoplasmic part;intracellular membrane-bounded organelle;intracellular organelle;intracellular organelle part;intracellular part;large ribosomal subunit;macromolecular complex;membrane;membrane-bounded organelle;mitochondrial inner membrane;mitochondrial large ribosomal subunit;mitochondrial membrane;mitoch		Homo sapiens	1	12
cell cortex;cell part;cytoplasmic part;intracellular part		Homo sapiens	1	12
cell part;cytoplasmic part;cytosol;extracellular membrane-bounded organelle;extracellular organelle;extracellular region part;extracellular vesicular exosome;intracellular organelle part;intracellular part;macromolecular complex;membrane;membrane part;membrane-bounded organelle;membrane-bounded vesicle;organelle;organ		Homo sapiens	1	12
cell part;cytoplasm;cytoplasmic part;intracellular membrane-bounded organelle;intracellular organelle;intracellular part;membrane;membrane-bounded organelle;mitochondrion;organelle		Homo sapiens	1	12
cell part;intracellular membrane-bounded organelle;intracellular non-membrane-bounded organelle;intracellular organelle;intracellular organelle part;intracellular part;macromolecular complex;membrane-bounded organelle;non-membrane-bounded organelle;nuclear part;nucleolus;nucleus;organelle;organelle part;preribosome;pr		Homo sapiens	1	12
cell part;cytoplasm;cytoplasmic part;cytoskeletal part;intracellular membrane-bounded organelle;intracellular organelle;intracellular organelle part;intracellular part;macromolecular complex;membrane;membrane-bounded organelle;microtubule organizing center;nuclear part;nucleoplasm;nucleus;organelle;organelle part;plasma r		Homo sapiens	1	12
cell part;cytoplasm;cytoplasmic part;cytosol;extracellular membrane-bounded organelle;extracellular organelle;extracellular region part;extracellular vesicular exosome;intracellular part;membrane-bounded organelle;membra	Ubiquitin mediated proteolysis	Homo sapiens	1	12
cell part;cytoplasmic part;endoplasmic reticulum;extracellular membrane-bounded organelle;extracellular organelle;extracellular region part;extracellular vesicular exosome;intracellular membrane-bounded organelle;intracellu	Lysosome	Homo sapiens	1	12
cell part;contractile fiber part;cytoplasm;cytoplasmic part;cytosol;endoplasmic reticulum membrane;endoplasmic reticulum part;extracellular membrane-bounded organelle;extracellular organelle;extracellular region part;extracellular vesicular exosome;intracellular organelle part;intracellular part;membrane;membrane part;memb		Homo sapiens	1	12
cell junction;cell part;cell-cell junction;cytoplasm;cytoplasmic part;cytosol;endosome;extracellular membrane-bounded organelle;extracellular organelle;extracellular region part;extracellular vesicular exosome;Golgi apparatu	Acute myeloid leukemia;B cell receptor signaling pathway;Chemokine signaling pathway;Chronic myelo	Homo sapiens	1	12
cell part;cytoplasm;extracellular membrane-bounded organelle;extracellular organelle;extracellular region part;extracellular vesicular exosome;intracellular membrane-bounded organelle;intracellular organelle;intracellular part	Parkinson's disease;Ubiquitin mediated proteolysis	Homo sapiens	1	12
cell part;cytoplasm;cytoplasmic part;intracellular membrane-bounded organelle;intracellular organelle;intracellular part;membrane-bounded organelle;nucleus;organelle;perinuclear region of cytoplasm	Mineral absorption	Homo sapiens	1	12
cell part;cell projection;cytoplasm;dendrite;intracellular membrane-bounded organelle;intracellular organelle;intracellular part;membrane-bounded organelle;neuron projection;nucleus;organelle		Homo sapiens	1	12
cell body;cell part;cell projection;chromosomal part;chromosome, telomeric region;cytoplasm;dendrite;DNA replication factor A complex;intracellular membrane-bounded organelle;intracellular organelle;intracellular organelle part;intracellular part;macromolecular complex;membrane-bounded organelle;neuron projection;neurona		Homo sapiens	1	12
cell part;extracellular membrane-bounded organelle;extracellular organelle;extracellular region part;extracellular vesicular exosome;integral to membrane;intrinsic to membrane;membrane part;membrane-bounded organelle;membrane-bounded vesicle;organelle;vesicle		Homo sapiens	1	12
acrosomal membrane;adherens junction;anchoring junction;apical plasma membrane;basolateral plasma membrane;caveola;cell cortex;cell junction;cell part;cell projection;cell-substrate adherens junction;cell-substrate junc	Bacterial invasion of epithelial cells;Endocytosis;Focal adhesion;Viral myocarditis	Homo sapiens	1	12
cell part;cytoplasmic part;cytosol;intracellular part;membrane		Homo sapiens	1	12
adherens junction;anchoring junction;cell junction;cell part;cell-substrate adherens junction;cell-substrate junction;cytoplasm;cytoplasmic part;cytoplasmic vesicle membrane;cytoplasmic vesicle part;cytoskeletal part;dystrophin-associated glycoprotein complex;extracellular membrane-bounded organelle;extracellular organelle;		Homo sapiens	1	12
cell cortex part;cell part;cortical endoplasmic reticulum;cytoplasmic part;cytoskeletal part;endoplasmic reticulum;endoplasmic reticulum membrane;endoplasmic reticulum part;integral to endoplasmic reticulum membrane;integral to membrane;integral to organelle membrane;integral to plasma membrane;intracellular membrane		Homo sapiens	1	12
cell part;cytoplasmic part;endoplasmic reticulum;endoplasmic reticulum membrane;endoplasmic reticulum part;integral to membrane;intracellular membrane-bounded organelle;intracellular organelle;intracellular organelle part;intracellular part;intrinsic to membrane;membrane;membrane part;membrane-bounded organelle;orga		Homo sapiens	1	12
brush border;cell part;cell projection;cytoplasm;extracellular membrane-bounded organelle;extracellular organelle;extracellular region part;extracellular vesicular exosome;intracellular part;membrane-bounded organelle;membrane-bounded vesicle;organelle;vesicle		Homo sapiens	1	12
cell part;cytoplasmic part;endoplasmic reticulum;intracellular membrane-bounded organelle;intracellular non-membrane-bounded organelle;intracellular organelle;intracellular organelle part;intracellular part;membrane-bounded organelle;non-membrane-bounded organelle;nuclear part;nucleolus;organelle;organelle part		Homo sapiens	1	12
cell part;cytoplasm;cytoplasmic part;cytosol;intracellular membrane-bounded organelle;intracellular organelle;intracellular part;membrane-bounded organelle;nucleus;organelle		Homo sapiens	1	12
cell part;germ cell nucleus;intracellular membrane-bounded organelle;intracellular organelle;intracellular part;male germ cell nucleus;membrane-bounded organelle;nucleus;organelle		Homo sapiens	1	12
cell part;cytoplasm;cytoplasmic part;cytoskeletal part;cytosol;extracellular membrane-bounded organelle;extracellular organelle;extracellular region part;extracellular vesicular exosome;intracellular membrane-bounded organ	Amyotrophic lateral sclerosis (ALS);Chagas disease (American trypanosomiasis);Epithelial cell signaling	Homo sapiens	1	12
cell part;intracellular membrane-bounded organelle;intracellular organelle;intracellular organelle part;intracellular part;macromolecular complex;membrane;membrane-bounded organelle;mRNA cleavage factor complex;nucle	mRNA surveillance pathway	Homo sapiens	1	12
cell part;chromosomal part;intracellular membrane-bounded organelle;intracellular organelle;intracellular organelle part;intracellular part;macromolecular complex;membrane-bounded organelle;nucleosome;nucleus;organelle;organelle part;protein-DNA complex		Homo sapiens	1	12
		Homo sapiens	1	12
cell part;integrator complex;intracellular membrane-bounded organelle;intracellular organelle;intracellular organelle part;intracellular part;macromolecular complex;membrane-bounded organelle;nuclear part;nucleoplasm part;nucleus;organelle;organelle part;protein complex;SOSS complex		Homo sapiens	1	12
cell part;cytoplasm;Elongator holoenzyme complex;histone acetyltransferase complex;intracellular organelle part;intracellular part;macromolecular complex;nuclear part;nucleoplasm part;organelle part;protein complex;transcription elongation factor complex		Homo sapiens	1	12
cell part;intracellular organelle part;intracellular part;macromolecular complex;nuclear body;nuclear matrix;nuclear part;nuclear speck;nucleoplasm;nucleoplasm part;organelle part;ribonucleoprotein complex;small nuclear rib	Spliceosome	Homo sapiens	1	12
cell part;cytoplasm;envelope;intracellular organelle part;intracellular part;macromolecular complex;membrane;membrane part;nuclear envelope;nuclear membrane;nuclear part;nuclear pore;nucleoplasm;organelle envelope;or	RNA transport	Homo sapiens	1	12
cell part;cytoplasmic part;endoplasmic reticulum membrane;endoplasmic reticulum part;extracellular membrane-bounded organelle;extracellular organelle;extracellular region part;extracellular vesicular exosome;integral to membrane;intracellular organelle part;intracellular part;intrinsic to membrane;membrane;membrane part;m		Homo sapiens	1	12
cell part;cytoplasm;intracellular part		Homo sapiens	1	12
cell part;cytoplasmic part;endoplasmic reticulum;endoplasmic reticulum chaperone complex;endoplasmic reticulum lumen;endoplasmic reticulum part;intracellular membrane-bounded organelle;intracellular organelle;intracel	Protein processing in endoplasmic reticulum	Homo sapiens	1	12
cell part;cytoplasmic part;cytosol;extracellular membrane-bounded organelle;extracellular organelle;extracellular region part;extracellular vesicular exosome;integral to membrane;integral to mitochondrial membrane;integral to mitochondrial outer membrane;integral to organelle membrane;intracellular organelle part;intracellular p		Homo sapiens	1	12
cell part;chromatin;chromosomal part;cytoplasm;histone methyltransferase complex;intracellular membrane-bounded organelle;intracellular non-membrane-bounded organelle;intracellular organelle;intracellular organelle part;intracellular part;macromolecular complex;membrane;membrane-bounded organelle;methyltransferase		Homo sapiens	1	12
cell part;cytoplasmic part;cytosol;extracellular membrane-bounded organelle;extracellular organelle;extracellular region part;extracellular vesicular exosome;glycerol-3-phosphate dehydrogenase complex;intracellular part;ma	Glycerophospholipid metabolism	Homo sapiens	1	12
cell part;membrane		Homo sapiens	1	12
cell part;cytoplasmic part;endosomal part;endosome;endosome membrane;extrinsic to membrane;intracellular membrane-bounded organelle;intracellular organelle;intracellular organelle part;intracellular part;late endosome;late endosome membrane;membrane;membrane part;membrane-bounded organelle;organelle;organelle		Homo sapiens	1	12
cell part;intracellular part;macromolecular complex;protein complex;tRNA-splicing ligase complex		Homo sapiens	1	12
cell part;cytoplasm;cytoskeletal part;intracellular organelle part;intracellular part;macromolecular complex;microtubule;organelle part;protein complex		Homo sapiens	1	12
cell part;cytoplasm;cytoplasmic part;endoplasmic reticulum;endoplasmic reticulum membrane;endoplasmic reticulum part;integral to membrane;intracellular membrane-bounded organelle;intracellular organelle;intracellular organelle part;intracellular part;intrinsic to membrane;membrane;membrane part;membrane-bounded orga		Homo sapiens	1	12
cell part;cytoplasmic part;integral to membrane;intracellular membrane-bounded organelle;intracellular organelle;intracellular part;intrinsic to membrane;membrane part;membrane-bounded organelle;mitochondrion;organelle		Homo sapiens	1	12
cell part;chromatin;chromatin remodeling complex;chromosomal part;intracellular membrane-bounded organelle;intracellular organelle;intracellular organelle part;intracellular part;macromolecular complex;membrane-bounded organelle;nuclear chromatin;nuclear chromosome part;nuclear part;nucleus;organelle;organelle part;pro		Homo sapiens	1	12
cell part;cytoplasmic membrane-bounded vesicle;cytoplasmic part;cytoplasmic vesicle;endoplasmic reticulum membrane;endoplasmic reticulum part;GPI-anchor transamidase complex;integral to endoplasmic reticulum mer	Glycosylphosphatidylinositol(GPI)-anchor biosynthesis	Homo sapiens	1	12
cell part;cytoplasmic part;endoplasmic reticulum membrane;endoplasmic reticulum part;endoplasmic reticulum-Golgi intermediate compartment;endoplasmic reticulum-Golgi intermediate compartment membrane;Golgi apparatus part;Golgi membrane;integral to membrane;intracellular membrane-bounded organelle;intracellula		Homo sapiens	1	12
cell part;cytoplasm;cytoplasmic part;extracellular membrane-bounded organelle;extracellular organelle;extracellular region part;extracellular vesicular exosome;Golgi apparatus;intracellular membrane-bounded organelle;intracellular organelle;intracellular organelle part;intracellular part;membrane-bounded organelle;membrane-b		Homo sapiens	1	12
catalytic step 2 spliceosome;cell part;cytoplasm;intracellular organelle part;intracellular part;macromolecular complex;nuclear part;nucleoplasm;organelle part;ribonucleoprotein complex;small nuclear ribonucleoprotein comp	Spliceosome	Homo sapiens	1	12
cell part;cytoplasmic part;intracellular organelle lumen;intracellular organelle part;intracellular part;membrane-enclosed lumen;mitochondrial matrix;mitochondrial part;organelle lumen;organelle part		Homo sapiens	1	12
cell part;cytoplasmic part;intracellular membrane-bounded organelle;intracellular organelle;intracellular organelle part;intracellular part;membrane;membrane-bounded organelle;mitochondrial inner membrane;mitochondrial membrane;mitochondrial part;mitochondrion;organelle;organelle inner membrane;organelle membrane;org		Homo sapiens	1	12
cell part;intracellular organelle part;intracellular part;macromolecular complex;nuclear part;nucleoplasm;organelle part;ribonucleoprotein complex;spliceosomal complex	Spliceosome	Homo sapiens	1	12
cell part;cullin-RING ubiquitin ligase complex;cytoplasm;intracellular membrane-bounded organelle;intracellular organelle;intracellular part;macromolecular complex;membrane-bounded organelle;nucleus;organelle;protein complex;SCF ubiquitin ligase complex;ubiquitin ligase complex		Homo sapiens	1	12
cell part;DNA-directed RNA polymerase II, holoenzyme;intracellular organelle part;intracellular part;macromolecular complex;nuclear part;nucleoplasm part;organelle part;protein complex		Homo sapiens	1	12
cell part;cytoplasm;cytoplasmic part;cytosol;Golgi apparatus;intracellular membrane-bounded organelle;intracellular organelle;intracellular part;membrane-bounded organelle;nucleus;organelle;perinuclear region of cytoplasm		Homo sapiens	1	12
cell part;cytoplasm;intracellular membrane-bounded organelle;intracellular organelle;intracellular part;membrane-bounded organelle;nucleus;organelle	mRNA surveillance pathway	Homo sapiens	1	12
cell part;cell projection;cytoplasm;cytoplasmic part;cytosol;intracellular non-membrane-bounded organelle;intracellular organelle;intracellular organelle part;intracellular part;non-membrane-bounded organelle;nuclear part;nu	Cysteine and methionine metabolism	Homo sapiens	1	12
cell junction;cell part;cytoplasmic membrane-bounded vesicle;cytoplasmic part;cytoplasmic vesicle;intracellular membrane-bounded organelle;intracellular organelle;intracellular part;membrane-bounded organelle;membrane-bounded vesicle;organelle;vesicle		Homo sapiens	1	12
cell part;cytoplasmic part;intracellular membrane-bounded organelle;intracellular non-membrane-bounded organelle;intracellular organelle;intracellular organelle part;intracellular part;macromolecular complex;membrane;membrane-bounded organelle;mitochondrial inner membrane;mitochondrial membrane;mitochondrial part;m		Homo sapiens	1	12
cell part;DNA-directed RNA polymerase complex;DNA-directed RNA polymerase I complex;intracellular membrane-bounded organelle;intracellular non-membrane-bounded organelle;intracellular organelle;intracellular organe	Purine metabolism;Pyrimidine metabolism;RNA polymerase	Homo sapiens	1	12
cell part;cytoplasmic part;cytosol;endosome;Golgi apparatus;Golgi apparatus part;Golgi cisterna;Golgi cisterna membrane;Golgi membrane;intracellular membrane-bounded organelle;intracellular organelle;intracellular organelle part;intracellular part;membrane;membrane-bounded organelle;membrane-enclosed lumen;mitochon		Homo sapiens	1	12
cell part;chromosome;condensed chromosome;condensed nuclear chromosome;CURI complex;cytoplasmic part;intracellular membrane-bounded organelle;intracellular non-membrane-bounded organelle;intracellular organe	Ribosome biogenesis in eukaryotes	Homo sapiens	1	12
cell part;cytoplasmic part;endoplasmic reticulum;intracellular membrane-bounded organelle;intracellular organelle;intracellular part;membrane-bounded organelle;nucleus;organelle		Homo sapiens	1	12
cell junction;cell part;cytoplasm;cytoplasmic part;cytoskeleton;extracellular membrane-bounded organelle;extracellular organelle;extracellular region part;extracellular vesicular exosome;intracellular non-membrane-bounded organelle;intracellular organelle;intracellular organelle part;intracellular part;membrane;membrane-bound		Homo sapiens	1	12
cell junction;cell part;cell projection;clathrin-coated vesicle;coated vesicle;cytoplasm;cytoplasmic membrane-bounded vesicle;cytoplasmic part;cytoplasmic vesicle;cytoplasmic vesicle membrane;cytoplasmic vesicle part;cytoskeletal part;dendrite;Golgi apparatus;Golgi apparatus part;Golgi membrane;Golgi-associated vesicle;G		Homo sapiens	1	12
cell part;integral to membrane;intrinsic to membrane;membrane part		Homo sapiens	1	12
cell body;cell part;cell projection;cytoplasmic part;endoplasmic reticulum;endoplasmic reticulum part;envelope;extracellular membrane-bounded organelle;extracellular organelle;extracellular region part;extracellular vesicular exosome;integral to endoplasmic reticulum membrane;integral to membrane;integral to organelle memb		Homo sapiens	1	12
axon;cell part;cell projection;cytoplasm;cytoskeletal part;intracellular organelle part;intracellular part;kinesin complex;macromolecular complex;microtubule;microtubule associated complex;neuron projection;organelle part;protein complex		Homo sapiens	1	12
cell part;cytoplasm;cytoplasmic part;cytosol;eukaryotic translation initiation factor 2B complex;intracellular part;macromolecular complex;protein complex	RNA transport	Homo sapiens	1	12
cell part;cytoplasmic part;endosomal part;endosome membrane;Golgi apparatus;Golgi apparatus part;Golgi membrane;integral to membrane;intracellular membrane-bounded organelle;intracellular organelle;intracellular orga	Endocytosis	Homo sapiens	1	12
basal part of cell;cell junction;cell part;cytoplasm;cytoplasmic part;cytosol;intracellular part;membrane;plasma membrane;postsynaptic membrane;synapse part;synaptic membrane		Homo sapiens	1	12
cell part;cytoplasmic part;extracellular membrane-bounded organelle;extracellular organelle;extracellular region part;extracellular vesicular exosome;intracellular organelle part;intracellular part;membrane;membrane-bounded organelle;membrane-bounded vesicle;mitochondrial inner membrane;mitochondrial membrane;mitocho		Homo sapiens	1	12
cell part;intracellular membrane-bounded organelle;intracellular non-membrane-bounded organelle;intracellular organelle;intracellular organelle part;intracellular part;membrane-bounded organelle;non-membrane-bounded organelle;nuclear part;nucleolus;nucleus;organelle;organelle part		Homo sapiens	1	12
cell part;cytoplasmic part;intracellular membrane-bounded organelle;intracellular organelle;intracellular organelle part;intracellular part;membrane-bounded organelle;mitochondrion;nuclear part;nucleoplasm;organelle;organelle part		Homo sapiens	1	12
cell part;cytoplasmic part;endosome;intracellular membrane-bounded organelle;intracellular organelle;intracellular part;membrane;membrane-bounded organelle;mitochondrion;organelle		Homo sapiens	1	12
cell part;cytoplasm;intracellular organelle part;intracellular part;nuclear body;nuclear part;nuclear speck;nucleoplasm;nucleoplasm part;organelle part		Homo sapiens	1	12
cell part;cytoplasmic part;early endosome;endosomal part;endosome;endosome membrane;extracellular membrane-bounded organelle;extracellular organelle;extracellular region part;extracellular vesicular exosome;intracellular membrane-bounded organelle;intracellular organelle;intracellular organelle part;intracellular part;lysos		Homo sapiens	1	12
cell part;cytoplasmic part;cytosol;endoplasmic reticulum;endoplasmic reticulum membrane;endoplasmic reticulum part;integral to membrane;intracellular membrane-bounded organelle;intracellular organelle;intracellular orga	Protein export;Protein processing in endoplasmic reticulum	Homo sapiens	1	12
cell part;cytoplasmic part;extracellular membrane-bounded organelle;extracellular organelle;extracellular region part;extracellular vesicular exosome;integral to membrane;intracellular membrane-bounded organelle;intracellul	Amino sugar and nucleotide sugar metabolism	Homo sapiens	1	12
cell part;intracellular membrane-bounded organelle;intracellular organelle;intracellular part;membrane-bounded organelle;nucleus;organelle		Homo sapiens	1	12
cell part;cytoplasm;intracellular part;membrane;plasma membrane		Homo sapiens	1	12
cell part;intracellular membrane-bounded organelle;intracellular non-membrane-bounded organelle;intracellular organelle;intracellular organelle part;intracellular part;macromolecular complex;membrane;membrane-bounded	Ribosome biogenesis in eukaryotes	Homo sapiens	1	12
cell part;chromosomal part;cytoplasm;cytoplasmic part;cytosol;intracellular membrane-bounded organelle;intracellular non-membrane-bounded organelle;intracellular organelle;intracellular organelle part;intracellular part;kine	NOD-like receptor signaling pathway;Plant-pathogen interaction	Homo sapiens	1	12
cell part;cytoplasm;cytoplasmic microtubule;cytoplasmic part;cytoskeletal part;endoplasmic reticulum membrane;endoplasmic reticulum part;integral to membrane;intracellular organelle part;intracellular part;intrinsic to mem	Protein export	Homo sapiens	1	12
cell part;cell projection;clathrin-coated vesicle;coated vesicle;cytoplasm;cytoplasmic membrane-bounded vesicle;cytoplasmic part;cytoplasmic vesicle;cytoplasmic vesicle membrane;cytoplasmic vesicle part;endosome;extracellular membrane-bounded organelle;extracellular organelle;extracellular region part;extracellular vesicu		Homo sapiens	1	12
cell junction;cell part;cell-cell junction;cytoplasmic part;cytoplasmic vesicle;cytoskeleton;extracellular membrane-bounded organelle;extracellular organelle;extracellular region part;extracellular vesicular exosome;integral to m	Cell adhesion molecules (CAMs);Epithelial cell signaling in Helicobacter pylori infection;Leukocyte transe	Homo sapiens	1	12
cell part;cytoplasmic part;extracellular membrane-bounded organelle;extracellular organelle;extracellular region part;extracellular vesicular exosome;integral to membrane;intracellular membrane-bounded organelle;intracellular organelle;intracellular organelle part;intracellular part;intrinsic to membrane;membrane;membrane part		Homo sapiens	1	12
cell part;cytoplasmic part;cytosol;eukaryotic 43S preinitiation complex;eukaryotic 48S preinitiation complex;eukaryotic translation initiation factor 3 complex;intracellular part;macromolecular complex;membrane;protein comp	RNA transport	Homo sapiens	1	13
cell part;cytoplasmic part;intracellular organelle lumen;intracellular organelle part;intracellular part;membrane-enclosed lumen;mitochondrial matrix;mitochondrial part;organelle lumen;organelle part	Citrate cycle (TCA cycle);Glycolysis / Gluconeogenesis;Pyruvate metabolism	Homo sapiens	1	13
cell part;cis-Golgi network;cytoplasmic membrane-bounded vesicle;cytoplasmic part;cytoplasmic vesicle;endocytic vesicle;endosomal part;endosome membrane;Golgi apparatus;Golgi apparatus part;Golgi cisterna membrane;Golgi lumen;Golgi membrane;integral to membrane;intracellular membrane-bounded organelle;intrace		Homo sapiens	1	13
cell part;cytoplasmic part;cytosol;extracellular membrane-bounded organelle;extracellular organelle;extracellular region part;extracellular vesicular exosome;intracellular membrane-bounded organelle;intracellular organelle;int	Proteasome	Homo sapiens	1	13
cell part;cytoplasmic part;cytosol;extracellular membrane-bounded organelle;extracellular organelle;extracellular region part;extracellular vesicular exosome;intracellular organelle part;intracellular part;membrane-bounded organelle;membrane-bounded vesicle;nuclear part;nucleoplasm;organelle;organelle part;vesicle		Homo sapiens	1	13
cell part;COPI vesicle coat;cytoplasmic part;cytoplasmic vesicle part;cytosol;Golgi apparatus;Golgi apparatus part;intracellular membrane-bounded organelle;intracellular organelle;intracellular organelle part;intracellular part;macromolecular complex;membrane coat;membrane part;membrane-bounded organelle;nuclear part;nu		Homo sapiens	1	13
actin filament bundle;actomyosin;apical part of cell;cell part;contractile fiber part;cytoplasmic part;cytoskeletal part;cytosol;extracellular membrane-bounded organelle;extracellular organelle;extracellular region part;extracellu	Focal adhesion;Leukocyte transendothelial migration;Regulation of actin cytoskeleton;Tight junction	Homo sapiens	1	13
cell part;cytoplasm;cytoplasmic part;cytosolic part;intracellular part;macromolecular complex;prefoldin complex;protein complex		Homo sapiens	1	13
cell part;cytoplasmic part;endoplasmic reticulum membrane;endoplasmic reticulum palmitoyltransferase complex;endoplasmic reticulum part;integral to membrane;intracellular organelle part;intracellular part;intrinsic to mem	Sphingolipid metabolism	Homo sapiens	1	13
actin cytoskeleton;cell part;cytoskeleton;integral to membrane;integral to plasma membrane;intracellular non-membrane-bounded organelle;intracellular organelle;intracellular organelle part;intracellular part;intrinsic to membrane;intrinsic to plasma membrane;membrane;membrane part;non-membrane-bounded organelle;nuclea		Homo sapiens	1	13
cell part;intracellular membrane-bounded organelle;intracellular non-membrane-bounded organelle;intracellular organelle;intracellular organelle part;intracellular part;macromolecular complex;membrane-bounded organelle;m	mRNA surveillance pathway	Homo sapiens	1	13
cell part;extracellular membrane-bounded organelle;extracellular organelle;extracellular region;extracellular region part;extracellular space;extracellular vesicular exosome;membrane;membrane-bounded organelle;membrane-bounded vesicle;organelle;vesicle		Homo sapiens	1	13
cell part;cytoplasm;extracellular membrane-bounded organelle;extracellular organelle;extracellular region part;extracellular vesicular exosome;intracellular membrane-bounded organelle;intracellular organelle;intracellular part;macromolecular complex;membrane-bounded organelle;membrane-bounded vesicle;nucleus;organelle;		Homo sapiens	1	13
cell part;clathrin-coated vesicle;coated vesicle;cytoplasm;cytoplasmic membrane-bounded vesicle;cytoplasmic part;cytoplasmic vesicle;cytosol;early endosome;early endosome membrane;early phagosome;endocytic vesicle;endosomal part;endosome;endosome membrane;extracellular membrane-bounded organelle;extracell		Homo sapiens	1	13
cell part;cytoplasm;intracellular membrane-bounded organelle;intracellular organelle;intracellular part;membrane;membrane-bounded organelle;nucleus;organelle		Homo sapiens	1	13
carboxy-terminal domain protein kinase complex;cell part;chromatin;chromosomal part;chromosome;condensed chromosome;condensed nuclear chromosome;cytoplasm;intracellular membrane-bounded organelle;intracellular non-membrane-bounded organelle;intracellular organelle;intracellular organelle part;intracellular part;		Homo sapiens	1	13
cell part;cytoplasmic part;cytosolic part;intracellular part;macromolecular complex;prefoldin complex;protein complex		Homo sapiens	1	13
cell part;cytoplasmic part;cytosol;extracellular membrane-bounded organelle;extracellular organelle;extracellular region part;extracellular vesicular exosome;intracellular part;membrane-bounded organelle;membrane-bounde	Glutathione metabolism	Homo sapiens	1	13
cell part;cytoplasm;intracellular membrane-bounded organelle;intracellular organelle;intracellular part;membrane-bounded organelle;organelle		Homo sapiens	1	13
		Homo sapiens	1	13
cell part;intracellular membrane-bounded organelle;intracellular organelle;intracellular organelle part;intracellular part;membrane-bounded organelle;nuclear part;nucleoplasm;nucleus;organelle;organelle part		Homo sapiens	1	13
cell part;cytoplasmic part;endoplasmic reticulum;endoplasmic reticulum membrane;endoplasmic reticulum part;intracellular membrane-bounded organelle;intracellular organelle;intracellular organelle part;intracellular part;membrane;membrane part;membrane-bounded organelle;organelle;organelle membrane;organelle part		Homo sapiens	1	13
cell part;cytoplasm;intracellular organelle part;intracellular part;nuclear body;nuclear part;nucleoplasm part;organelle part;PML body		Homo sapiens	1	13
cell part;coated vesicle membrane;COPII vesicle coat;cytoplasmic part;cytoplasmic vesicle membrane;cytoplasmic vesicle part;cytosol;endoplasmic reticulum membrane;endoplasmic reticulum part;ER to Golgi transport ves	Protein processing in endoplasmic reticulum	Homo sapiens	1	13
cell junction;cell part;cell projection;centrosome;cytoplasm;cytoplasmic part;cytoskeletal part;intracellular non-membrane-bounded organelle;intracellular organelle;intracellular organelle part;intracellular part;macromolecular	SNARE interactions in vesicular transport	Homo sapiens	1	13
cell part;cytoplasmic part;cytoskeleton;cytosol;extracellular membrane-bounded organelle;extracellular organelle;extracellular region;extracellular region part;extracellular space;extracellular vesicular exosome;intracellular membrane-bounded organelle;intracellular non-membrane-bounded organelle;intracellular organelle;intrace		Homo sapiens	1	13
cell part;chromatoid body;cytoplasm;cytoplasmic mRNA processing body;cytoplasmic part;cytosol;eukaryotic translation initiation factor 4F complex;extracellular membrane-bounded organelle;extracellular organelle;extrace	Insulin signaling pathway;mTOR signaling pathway;RNA transport	Homo sapiens	1	13
cell part;cytoplasm;cytoplasmic part;cytosol;extracellular membrane-bounded organelle;extracellular organelle;extracellular region part;extracellular vesicular exosome;intracellular membrane-bounded organelle;intracellular organelle;intracellular organelle part;intracellular part;membrane-bounded organelle;membrane-bounded		Homo sapiens	1	13
adherens junction;anchoring junction;cell junction;cell part;cell-substrate adherens junction;cell-substrate junction;chromatin;chromosomal part;cytoplasmic part;cytosol;focal adhesion;heterochromatin;intracellular membrane-bounded organelle;intracellular organelle;intracellular organelle part;intracellular part;macromolecular c		Homo sapiens	1	13
cell part;cytoplasmic part;cytosol;intracellular membrane-bounded organelle;intracellular organelle;intracellular part;membrane-bounded organelle;nucleus;organelle	Adherens junction;Circadian rhythm - plant;Measles;Ribosome biogenesis in eukaryotes;Tight junction;V	Homo sapiens	1	13
cell part;chromatin remodeling complex;chromosomal part;DNA helicase complex;Ino80 complex;intracellular membrane-bounded organelle;intracellular organelle;intracellular organelle part;intracellular part;macromolecular complex;membrane-bounded organelle;nuclear chromosome part;nuclear matrix;nuclear part;nucleoplas		Homo sapiens	1	13

cell part;cytoplasmic part;cytosol;extracellular membrane-bounded organelle;extracellular organelle;extracellular region part;extracellular vesicular exosome;intracellular membrane-bounded organelle;intracellular non-membr	Protein processing in endoplasmic reticulum	Homo sapiens		1	13
cell part;cell projection;cytoplasmic part;extracellular membrane-bounded organelle;extracellular organelle;extracellular region part;extracellular space;extracellular vesicular exosome;intracellular membrane-bounded organelle;intracellular organelle;intracellular part;membrane-bounded organelle;membrane-bounded vesicle;neu		Homo sapiens		1	13
cell junction;cell part;cell-cell junction;cytoplasm;cytoplasmic part;cytoskeletal part;cytoskeleton;cytosol;intracellular membrane-bounded organelle;intracellular non-membrane-bounded organelle;intracellular organelle;intrac	Acute myeloid leukemia;Adipocytokine signaling pathway;Apoptosis;B cell receptor signaling pathway;C	Homo sapiens		1	13
cell part;cytoplasmic part;extracellular membrane-bounded organelle;extracellular organelle;extracellular region part;extracellular vesicular exosome;intracellular membrane-bounded organelle;intracellular organelle;intracellula	Pyrimidine metabolism	Homo sapiens		1	13
adherens junction;anchoring junction;cell junction;cell part;cell-substrate adherens junction;cell-substrate junction;cytoplasm;cytoplasmic part;cytosol;cytosolic large ribosomal subunit;extracellular membrane-bounded orga	Ribosome	Homo sapiens		1	13
cell part;intracellular membrane-bounded organelle;intracellular organelle;intracellular organelle part;intracellular part;macromolecular complex;membrane-bounded organelle;nuclear part;nucleus;organelle;organelle part;ribo	Spliceosome	Homo sapiens		1	13
cell part;cytoplasm;cytoplasmic part;cytosol;cytosolic large ribosomal subunit;intracellular non-membrane-bounded organelle;intracellular organelle;intracellular organelle part;intracellular part;large ribosomal subunit;macrom	Ribosome	Homo sapiens		1	13
cell part;cytoplasmic part;intracellular membrane-bounded organelle;intracellular organelle;intracellular organelle part;intracellular part;macromolecular complex;membrane;membrane part;membrane-bounded organelle;mito	Alzheimer's disease;Cardiac muscle contraction;Huntington's disease;Oxidative phosphorylation;Parkin	Homo sapiens		1	13
cell part;cytoplasmic part;cytosol;intracellular membrane-bounded organelle;intracellular organelle;intracellular part;membrane-bounded organelle;mitochondrion;organelle	Carbon fixation in photosynthetic organisms;Pyruvate metabolism	Homo sapiens		1	13
adherens junction;anchoring junction;basement membrane;cell junction;cell part;cell-substrate adherens junction;cell-substrate junction;cytoplasmic part;cytosol;extracellular matrix part;extracellular region part;focal adhesion;integral to membrane;integral to plasma membrane;intracellular part;intrinsic to membrane;intrinsic to p		Homo sapiens		1	13
cell part;cytoplasmic part;intracellular membrane-bounded organelle;intracellular non-membrane-bounded organelle;intracellular organelle;intracellular organelle part;intracellular part;macromolecular complex;membrane;membrane-bounded organelle;mitochondrial inner membrane;mitochondrial membrane;mitochondrial part;no		Homo sapiens		1	13
cell part;cytoplasm;cytosolic large ribosomal subunit;extracellular membrane-bounded organelle;extracellular organelle;extracellular region part;extracellular vesicular exosome;intracellular membrane-bounded organelle;intracellular organelle;intracellular organelle part;intracellular part;large ribosomal subunit;macromolecular cor		Homo sapiens		1	13
cell part;cytoplasmic part;cytosol;extracellular membrane-bounded organelle;extracellular organelle;extracellular region part;extracellular vesicular exosome;intracellular membrane-bounded organelle;intracellular organelle;int	Proteasome	Homo sapiens		1	13
cell part;cell projection;cytoplasm;cytoplasmic part;cytosol;dendrite;intracellular membrane-bounded organelle;intracellular organelle;intracellular organelle part;intracellular part;macromolecular complex;membrane part;membrane-bounded organelle;neuron projection;nuclear part;nuclear pore;nucleoplasm;nucleus;organelle;org		Homo sapiens		1	13
cell part;cytoplasmic part;endoplasmic reticulum;extracellular membrane-bounded organelle;extracellular organelle;extracellular region part;extracellular space;extracellular vesicular exosome;Golgi apparatus;intracellular me	Lysosome	Homo sapiens		1	13
cell part;extracellular membrane-bounded organelle;extracellular organelle;extracellular region part;extracellular vesicular exosome;extrinsic to membrane;extrinsic to plasma membrane;membrane part;membrane-bounded organelle;membrane-bounded vesicle;organelle;plasma membrane part;vesicle		Homo sapiens		1	13
cell part;cytoplasmic membrane-bounded vesicle;cytoplasmic part;cytoplasmic vesicle;endoplasmic reticulum membrane;endoplasmic reticulum part;endoplasmic reticulum-Golgi intermediate compartment membrane;extracellular membrane-bounded organelle;extracellular organelle;extracellular region part;extracellular vesicu		Homo sapiens		1	13
catalytic step 2 spliceosome;cell part;cytoplasmic part;cytosol;exon-exon junction complex;intracellular membrane-bounded organelle;intracellular organelle;intracellular organelle part;intracellular part;macromolecular compl	mRNA surveillance pathway;RNA transport;Spliceosome	Homo sapiens		1	13
cell part;cytoplasmic part;cytosol;endoplasmic reticulum;endoplasmic reticulum part;integral to endoplasmic reticulum membrane;integral to membrane;integral to organelle membrane;intracellular membrane-bounded organ	Phagosome;Protein export;Protein processing in endoplasmic reticulum;Vibrio cholerae infection	Homo sapiens		1	13
catalytic step 2 spliceosome;cell part;cytoplasm;cytoplasmic part;cytosol;extracellular membrane-bounded organelle;extracellular organelle;extracellular region part;extracellular vesicular exosome;intracellular organelle part;i	Spliceosome;Systemic lupus erythematosus	Homo sapiens		1	13
adherens junction;anchoring junction;cell junction;cell part;cell projection;cell projection membrane;cell projection part;cell-substrate adherens junction;cell-substrate junction;cytoplasm;cytoplasmic membrane-bounded vesi	Adherens junction;Amyotrophic lateral sclerosis (ALS);Axon guidance;B cell receptor signaling pathway;	Homo sapiens		1	13
cell part;cytoplasmic part;endoplasmic reticulum membrane;endoplasmic reticulum part;extracellular membrane-bounded organelle;extracellular organelle;extracellular region part;extracellular vesicular exosome;integral to m	Protein export	Homo sapiens		1	13
cell part;cytoplasm;cytoskeleton;envelope;intracellular membrane-bounded organelle;intracellular non-membrane-bounded organelle;intracellular organelle;intracellular organelle part;intracellular part;macromolecular complex	RNA transport	Homo sapiens		1	13
cell part;cytoplasm;intracellular membrane-bounded organelle;intracellular organelle;intracellular organelle part;intracellular part;membrane;membrane-bounded organelle;nuclear membrane;nuclear part;nucleus;organelle;organelle membrane;organelle part		Homo sapiens		1	13
axon part;basal lamina;basolateral plasma membrane;cell junction;cell part;cell projection membrane;cell projection part;cell-cell contact zone;cell-cell junction;cytoplasm;cytoplasmic part;cytoskeletal part;cytosol;endoplasm	T cell receptor signaling pathway	Homo sapiens		1	13
cell part;intracellular membrane-bounded organelle;intracellular organelle;intracellular organelle part;intracellular part;membrane-bounded organelle;nuclear part;nucleoplasm;nucleus;organelle;organelle part	mRNA surveillance pathway	Homo sapiens		1	13
cell part;cytoplasm;cytoplasmic part;extracellular membrane-bounded organelle;extracellular organelle;extracellular region part;extracellular vesicular exosome;intracellular organelle lumen;intracellular organelle part;intracellu	Pantothenate and CoA biosynthesis	Homo sapiens		1	13
cell part;cytoplasmic part;cytosol;intracellular membrane-bounded organelle;intracellular organelle;intracellular part;membrane;membrane-bounded organelle;nucleus;organelle		Homo sapiens		1	13
cell part;cytoplasm;intracellular membrane-bounded organelle;intracellular non-membrane-bounded organelle;intracellular organelle;intracellular organelle part;intracellular part;membrane-bounded organelle;non-membrane-b	Spliceosome	Homo sapiens		1	13
cell part;cytoplasmic part;cytosol;intracellular membrane-bounded organelle;intracellular organelle;intracellular organelle part;intracellular part;macromolecular complex;membrane-bounded organelle;nuclear part;nucleoplasn	Chronic myeloid leukemia;Notch signaling pathway;Pathways in cancer;Wnt signaling pathway	Homo sapiens		1	13
adherens junction;anchoring junction;basolateral plasma membrane;cell junction;cell part;cell surface;cell-substrate adherens junction;cell-substrate junction;cytoplasm;extracellular membrane-bounded organelle;extracellular organelle;extracellular region part;extracellular space;extracellular vesicular exosome;focal adhesion;int		Homo sapiens		1	13
aggresome;autophagic vacuole;cell part;cytoplasm;cytoplasmic mRNA processing body;cytoplasmic part;cytoplasmic vesicle;cytosol;endoplasmic reticulum;endosome;extracellular membrane-bounded organelle;extracellula	Osteoclast differentiation	Homo sapiens		1	13
apical part of cell;cell part;cytoplasm;intracellular membrane-bounded organelle;intracellular non-membrane-bounded organelle;intracellular organelle;intracellular organelle part;intracellular part;membrane;membrane-bounded organelle;non-membrane-bounded organelle;nuclear part;nucleolus;nucleus;organelle;organelle part		Homo sapiens		1	13
acrosomal membrane;adherens junction;anchoring junction;caveola;cell junction;cell part;cell-substrate adherens junction;cell-substrate junction;cytoplasmic membrane-bounded vesicle;cytoplasmic part;cytoplasmic vesicle	Insulin signaling pathway	Homo sapiens		1	13
cell part;cytoplasmic part;cytosol;extracellular membrane-bounded organelle;extracellular organelle;extracellular region part;extracellular vesicular exosome;intracellular part;membrane-bounded organelle;membrane-bounde	Amino sugar and nucleotide sugar metabolism;Galactose metabolism	Homo sapiens		1	13
cell part;cytoplasmic part;cytosol;intracellular part		Homo sapiens		1	13
cell part;cytoplasmic part;cytoskeleton;cytosol;endoplasmic reticulum;endoplasmic reticulum membrane;endoplasmic reticulum part;Golgi apparatus part;Golgi membrane;integral to membrane;intracellular membrane-bounc	Steroid biosynthesis	Homo sapiens		1	13
catalytic step 2 spliceosome;cell part;intracellular organelle part;intracellular part;macromolecular complex;nuclear part;nucleoplasm;organelle part;ribonucleoprotein complex;small nuclear ribonucleoprotein complex;spliceos	Spliceosome	Homo sapiens		1	13
cell part;cytoplasm;extracellular membrane-bounded organelle;extracellular organelle;extracellular region part;extracellular vesicular exosome;intracellular membrane-bounded organelle;intracellular organelle;intracellular part;macromolecular complex;membrane-bounded organelle;membrane-bounded vesicle;nucleus;organelle;		Homo sapiens		1	13
apical junction complex;cell division site part;cell junction;cell part;cell projection;cell-cell junction;centrosome;cleavage furrow;cytoplasm;cytoplasmic part;cytoskeletal part;cytoskeleton;cytosol;intermediate filament cytoskeleton;intracellular membrane-bounded organelle;intracellular non-membrane-bounded organelle;intracell		Homo sapiens		1	13
adherens junction;anchoring junction;cell junction;cell part;cell-substrate adherens junction;cell-substrate junction;focal adhesion;intracellular membrane-bounded organelle;intracellular organelle;intracellular part;membrane-bounded organelle;nucleus;organelle		Homo sapiens		1	13
cell part;cytoplasm;intracellular membrane-bounded organelle;intracellular organelle;intracellular part;macromolecular complex;membrane-bounded organelle;nucleus;organelle;protein complex;tRNA-splicing ligase complex		Homo sapiens		1	13
cell part;cytoplasm;cytoplasmic part;cytosol;intracellular membrane-bounded organelle;intracellular organelle;intracellular organelle part;intracellular part;membrane-bounded organelle;nuclear part;nucleoplasm;nucleus;organelle;organelle part		Homo sapiens		1	13
cell part;cytoplasm;intracellular membrane-bounded organelle;intracellular organelle;intracellular part;membrane-bounded organelle;organelle		Homo sapiens		1	13
cell part;intracellular organelle part;intracellular part;macromolecular complex;nuclear part;organelle part;precatalytic spliceosome;ribonucleoprotein complex;spliceosomal complex	Spliceosome	Homo sapiens		1	13
cell part;cytoplasm;cytoplasmic part;intracellular non-membrane-bounded organelle;intracellular organelle;intracellular organelle part;intracellular part;membrane-enclosed lumen;mitochondrial intermembrane space;mitochondrial part;non-membrane-bounded organelle;nuclear part;nucleolus;nucleoplasm;organelle;organelle env		Homo sapiens		1	13
cell part;chromatin;chromosomal part;cytoplasm;cytoplasmic part;intracellular membrane-bounded organelle;intracellular organelle;intracellular organelle part;intracellular part;macromolecular complex;membrane-bounded o	RNA degradation	Homo sapiens		1	13
		Homo sapiens		1	13
cell part;cell projection;cytoplasm;cytoskeleton;filopodium;intracellular non-membrane-bounded organelle;intracellular organelle;intracellular part;lamellipodium;membrane;non-membrane-bounded organelle;organelle;plasma membrane		Homo sapiens		1	13
cell part;cytoplasmic exosome (RNase complex);cytoplasmic part;exosome (RNase complex);intracellular membrane-bounded organelle;intracellular non-membrane-bounded organelle;intracellular organelle;intracellular organelle lumen;intracellular organelle part;intracellular part;macromolecular complex;membrane-bounded org		Homo sapiens		1	13
cell part;cytoplasm;cytoplasmic part;intracellular organelle lumen;intracellular organelle part;intracellular part;membrane-enclosed lumen;mitochondrial matrix;mitochondrial part;organelle lumen;organelle part		Homo sapiens		1	13
cell body;cell part;cell projection;cilium;cytoplasm;cytoplasmic part;extracellular membrane-bounded organelle;extracellular organelle;extracellular region;extracellular region part;extracellular space;extracellular vesicular exosome;intracellular membrane-bounded organelle;intracellular organelle;intracellular organelle part;intracel		Homo sapiens		1	13
cell part;intracellular organelle part;intracellular part;macromolecular complex;nuclear body;nuclear part;nuclear speck;nucleoplasm;nucleoplasm part;organelle part;protein complex;THO complex;THO complex part of transc	RNA transport;Spliceosome	Homo sapiens		1	13
cell part;cytoplasm;extracellular membrane-bounded organelle;extracellular organelle;extracellular region part;extracellular vesicular exosome;intracellular part;membrane;membrane-bounded organelle;membrane-bounded v	Inositol phosphate metabolism;Phosphatidylinositol signaling system;Regulation of actin cytoskeleton	Homo sapiens		1	13
cell part;cytoplasmic part;endoplasmic reticulum membrane;endoplasmic reticulum part;integral to membrane;intracellular;intracellular organelle part;intracellular part;intrinsic to membrane;membrane;membrane part;organel	Retinol metabolism	Homo sapiens		1	13
cell part;cytoplasm;intracellular membrane-bounded organelle;intracellular non-membrane-bounded organelle;intracellular organelle;intracellular organelle part;intracellular part;membrane;membrane-bounded organelle;non-membrane-bounded organelle;nuclear part;nucleolus;nucleus;organelle;organelle part		Homo sapiens		1	13
cell part;intracellular membrane-bounded organelle;intracellular organelle;intracellular part;membrane-bounded organelle;nucleus;organelle		Homo sapiens		1	13
cell part;chromosomal part;condensed chromosome kinetochore;cytoplasmic part;cytosol;extracellular membrane-bounded organelle;extracellular organelle;extracellular region part;extracellular vesicular exosome;intracellular membrane-bounded organelle;intracellular non-membrane-bounded organelle;intracellular organelle;in		Homo sapiens		1	13
cell junction;cell part;CUL4 RING ubiquitin ligase complex;Cul4B-RING ubiquitin ligase complex;cullin-RING ubiquitin ligase complex;intracellular organelle part;intracellular part;macromolecular complex;nuclear part;nucleop	Nucleotide excision repair;p53 signaling pathway;Ubiquitin mediated proteolysis	Homo sapiens		1	13
cell part;chromatin;chromatin remodeling complex;chromosomal part;chromosome;intracellular membrane-bounded organelle;intracellular non-membrane-bounded organelle;intracellular organelle;intracellular organelle part;intracellular part;macromolecular complex;membrane-bounded organelle;nBAF complex;non-membrane-		Homo sapiens		1	13
cell part;centriole;centrosome;cytoplasm;cytoplasmic part;cytoskeletal part;cytosol;intracellular non-membrane-bounded organelle;intracellular organelle;intracellular organelle part;intracellular part;macromolecular complex;membrane;microtubule;microtubule organizing center;microtubule organizing center part;non-membrane-		Homo sapiens		1	13
cell part;chromosomal part;DNA replication factor A complex;intracellular membrane-bounded organelle;intracellular organelle;intracellular organelle part;intracellular part;macromolecular complex;membrane-bounded organelle;nuclear chromosome part;nuclear part;nucleoplasm;nucleus;organelle;organelle part;protein complex		Homo sapiens		1	13
cell part;cytoplasmic part;cytosol;intracellular membrane-bounded organelle;intracellular organelle;intracellular organelle lumen;intracellular organelle part;intracellular part;membrane;membrane-bounded organelle;membrane	Valine, leucine and isoleucine degradation	Homo sapiens		1	13
cell part;cytoplasmic part;cytosol;extracellular membrane-bounded organelle;extracellular organelle;extracellular region part;extracellular vesicular exosome;intracellular part;membrane-bounded organelle;membrane-bounded vesicle;organelle;vesicle		Homo sapiens		1	13
cell part;clathrin-coated vesicle;coated vesicle;cytoplasmic membrane-bounded vesicle;cytoplasmic part;cytoplasmic vesicle;endoplasmic reticulum;endoplasmic reticulum membrane;endoplasmic reticulum part;endoplasmic reticulum-Golgi intermediate compartment;endoplasmic reticulum-Golgi intermediate compartment me		Homo sapiens		1	13
cell part;cytoplasm;extracellular membrane-bounded organelle;extracellular organelle;extracellular region part;extracellular vesicular exosome;intracellular part;membrane-bounded organelle;membrane-bounded vesicle;organelle;vesicle		Homo sapiens		1	13
cell part;cytoplasmic part;integral to membrane;intracellular membrane-bounded organelle;intracellular organelle;intracellular organelle part;intracellular part;intrinsic to membrane;membrane;membrane part;membrane-bounded organelle;mitochondrial membrane;mitochondrial part;mitochondrion;organelle;organelle membrane;o		Homo sapiens		1	13
cell part;cytoplasmic part;intracellular membrane-bounded organelle;intracellular organelle;intracellular part;membrane-bounded organelle;mitochondrion;organelle		Homo sapiens		1	13
		Homo sapiens		1	13
cell part;cell projection;cilium;cytoplasm;cytoplasmic part;cytoskeleton;integral to membrane;intracellular membrane-bounded organelle;intracellular non-membrane-bounded organelle;intracellular organelle;intracellular organelle part;intracellular part;intrinsic to membrane;lysosomal membrane;membrane;membrane part;memb		Homo sapiens		1	13
axon part;axon terminus;cell body;cell junction;cell part;cell projection;cell projection part;cytoskeletal part;dendrite;intracellular organelle part;intracellular part;membrane;neuron projection;neuron projection terminus;neuronal cell body;organelle part;postsynaptic density;postsynaptic membrane;synapse part;synaptic membran		Homo sapiens		1	13
cell part;extracellular region part;extracellular space;intracellular membrane-bounded organelle;intracellular organelle;intracellular part;membrane-bounded organelle;nucleus;organelle		Homo sapiens		1	13
cell part;cytoplasm;intracellular organelle part;intracellular part;nuclear part;nucleoplasm;organelle part		Homo sapiens		1	13
		Homo sapiens		1	13
cell part;cytoplasmic part;cytosol;intracellular membrane-bounded organelle;intracellular organelle;intracellular organelle lumen;intracellular organelle part;intracellular part;membrane-bounded organelle;membrane-enclosed l	Valine, leucine and isoleucine degradation	Homo sapiens		1	13
cell part;cytoplasm;cytoplasmic part;cytosol;Golgi apparatus;Golgi apparatus part;Golgi membrane;intracellular membrane-bounded organelle;intracellular organelle;intracellular organelle part;intracellular part;membrane;mer	Endocytosis	Homo sapiens		1	13
cell part;cytoplasmic part;intracellular membrane-bounded organelle;intracellular organelle;intracellular organelle lumen;intracellular organelle part;intracellular part;membrane-bounded organelle;membrane-enclosed lumen;m	Aminoacyl-tRNA biosynthesis	Homo sapiens		1	13
cell part;intracellular organelle part;intracellular part;macromolecular complex;nuclear part;organelle part;ribonucleoprotein complex;small nuclear ribonucleoprotein complex;U1 snRNP		Homo sapiens		1	13
cell part;DNA-directed RNA polymerase II, holoenzyme;intracellular membrane-bounded organelle;intracellular organelle;intracellular organelle part;intracellular part;macromolecular complex;membrane-bounded organelle;nuclear part;nucleoplasm part;nucleus;organelle;organelle part;protein complex		Homo sapiens		1	13
cell part;centrosome;cytoplasm;cytoplasmic part;cytoskeletal part;intracellular membrane-bounded organelle;intracellular non-membrane-bounded organelle;intracellular organelle;intracellular organelle part;intracellular part;macromolecular complex;membrane-bounded organelle;microtubule organizing center;non-membrane-b		Homo sapiens		1	13
actin cytoskeleton;adherens junction;anchoring junction;cell junction;cell part;cell-substrate adherens junction;cell-substrate junction;contractile fiber part;cytoplasm;cytoplasmic part;cytoskeleton;cytosol;focal adhesion;intra	Focal adhesion	Homo sapiens		1	13
cell part;cytoplasmic part;intracellular non-membrane-bounded organelle;intracellular organelle;intracellular organelle part;intracellular part;macromolecular complex;membrane;mitochondrial inner membrane;mitochondrial membrane;mitochondrial part;mitochondrial ribosome;non-membrane-bounded organelle;organellar ribos		Homo sapiens		1	13
cell part;cytoplasm;extracellular membrane-bounded organelle;extracellular organelle;extracellular region part;extracellular vesicular exosome;intracellular membrane-bounded organelle;intracellular organelle;intracellular part;membrane;membrane-bounded organelle;membrane-bounded vesicle;nucleus;organelle;vesicle		Homo sapiens		1	13
cell body;cell part;cell projection;cytoplasm;cytoplasmic part;cytosol;dendrite;extracellular region part;extracellular space;intracellular part;membrane;neuron projection;neuronal cell body;perinuclear region of cytoplasm;plasma membrane		Homo sapiens		1	13
cell part;cytoplasm;cytoplasmic part;cytosol;extracellular membrane-bounded organelle;extracellular organelle;extracellular region part;extracellular vesicular exosome;intracellular organelle lumen;intracellular organelle part;i	Glyoxylate and dicarboxylate metabolism;Pyruvate metabolism	Homo sapiens		1	13
cell part;cytoplasmic part;endoplasmic reticulum;extracellular membrane-bounded organelle;extracellular organelle;extracellular region part;extracellular space;extracellular vesicular exosome;intracellular membrane-boundec	Lysosome	Homo sapiens		1	13
cell part;cytoplasmic part;cytosol;inclusion body;intracellular organelle part;intracellular part;macromolecular complex;membrane part;nuclear body;nuclear inclusion body;nuclear part;nuclear pore;nuclear speck;nucleoplasn	mRNA surveillance pathway;Ribosome biogenesis in eukaryotes;RNA transport	Homo sapiens		1	13
cell part;cytoplasmic part;intracellular membrane-bounded organelle;intracellular organelle;intracellular organelle lumen;intracellular organelle part;intracellular part;membrane-bounded organelle;membrane-enclosed lumen;m	Pyrimidine metabolism	Homo sapiens		1	13
cell part;intracellular membrane-bounded organelle;intracellular organelle;intracellular part;membrane-bounded organelle;nucleus;organelle	Ribosome biogenesis in eukaryotes	Homo sapiens		1	13
cell part;cytoplasm;intracellular membrane-bounded organelle;intracellular organelle;intracellular organelle part;intracellular part;membrane-bounded organelle;nuclear matrix;nuclear part;nucleoplasm;nucleus;organelle;organelle part		Homo sapiens		1	13
cell part;cytoplasmic part;early endosome;endosome;extracellular membrane-bounded organelle;extracellular organelle;extracellular region part;extracellular vesicular exosome;extrinsic to membrane;intracellular membrane-bounded organelle;intracellular organelle;intracellular part;membrane part;membrane-bounded organelle;		Homo sapiens		1	13
cell part;cytoplasmic part;integral to membrane;integral to plasma membrane;intracellular organelle part;intracellular part;intrinsic to membrane;intrinsic to plasma membrane;lysosomal membrane;membrane;membrane part;organelle membrane;organelle part;plasma membrane;plasma membrane part;vacuolar membrane;vacu		Homo sapiens		1	13
cell part;cytoplasmic part;endoplasmic reticulum;integral to membrane;integral to plasma membrane;intracellular membrane-bounded organelle;intracellular organelle;intracellular part;intrinsic to membrane;intrinsic to plasma membrane;membrane part;membrane-bounded organelle;organelle;plasma membrane part		Homo sapiens		1	13
cell part;cytoplasm;cytoplasmic part;cytosol;extracellular region part;extracellular space;intracellular membrane-bounded organelle;intracellular organelle;intracellular organelle part;intracellular part;membrane;membrane-bou	Hepatitis C;Measles	Homo sapiens		1	13
cell junction;cell part;cell projection;cytoplasm;cytoskeletal part;intracellular non-membrane-bounded organelle;intracellular organelle;intracellular organelle part;intracellular part;macromolecular complex;non-membrane-bounded organelle;organelle;organelle part;podosome;protein complex		Homo sapiens		1	14
apical plasma membrane;basolateral plasma membrane;cell part;cytoplasmic membrane-bounded vesicle;cytoplasmic part;cytoplasmic vesicle;cytosol;extracellular membrane-bounded organelle;extracellular organelle;extracellular region part;extracellular vesicular exosome;intracellular membrane-bounded organelle;intracellula		Homo sapiens		1	14
cell part;cytoplasmic part;endoplasmic reticulum;endoplasmic reticulum membrane;endoplasmic reticulum part;extracellular membrane-bounded organelle;extracellular organelle;extracellular region part;extracellular vesicular exosome;integral to membrane;intracellular membrane-bounded organelle;intracellular non-membrane-		Homo sapiens		1	14
cell part;cytoplasm;cytoplasmic part;cytosol;extracellular membrane-bounded organelle;extracellular organelle;extracellular region part;extracellular vesicular exosome;intracellular membrane-bounded organelle;intracellular o	Vitamin B6 metabolism	Homo sapiens		1	14
cell part;cytoplasm;cytoplasmic part;cytosol;extracellular membrane-bounded organelle;extracellular organelle;extracellular region part;extracellular vesicular exosome;intracellular membrane-bounded organelle;intracellular organelle;intracellular part;membrane-bounded organelle;membrane-bounded vesicle;nucleus;organelle;v		Homo sapiens		1	14
actin cytoskeleton;adherens junction;anchoring junction;Arp2/3 protein complex;cell junction;cell part;cell projection;cell-substrate adherens junction;cell-substrate junction;cytoplasmic part;cytoskeletal part;cytoskeleton;cyto	Bacterial invasion of epithelial cells;Fc gamma R-mediated phagocytosis;Pathogenic Escherichia coli inf	Homo sapiens		1	14
cell part;cytoplasmic part;cytosol;endosomal part;endosome membrane;extracellular membrane-bounded organelle;extracellular organelle;extracellular region part;extracellular vesicular exosome;intracellular organelle part;in	Endocytosis	Homo sapiens		1	14
cell part;intracellular membrane-bounded organelle;intracellular organelle;intracellular part;membrane-bounded organelle;nucleus;organelle		Homo sapiens		1	14
cell part;cytoplasmic part;endoplasmic reticulum;endoplasmic reticulum membrane;endoplasmic reticulum part;integral to membrane;intracellular membrane-bounded organelle;intracellular organelle;intracellular organelle part;intracellular part;intrinsic to membrane;macromolecular complex;membrane;membrane part;membrane		Homo sapiens		1	14
cell part;cytoplasm;cytoplasmic part;extracellular membrane-bounded organelle;extracellular organelle;extracellular region part;extracellular vesicular exosome;intracellular membrane-bounded organelle;intracellular organelle	Alzheimer's disease;Huntington's disease;Oxidative phosphorylation;Parkinson's disease	Homo sapiens		1	14
cell part;intracellular membrane-bounded organelle;intracellular organelle;intracellular organelle part;intracellular part;macromolecular complex;membrane-bounded organelle;nuclear body;nuclear part;nuclear speck;nucleoplasm;nucleoplasm part;nucleus;organelle;organelle part;ribonucleoprotein complex;small nuclear ribonucl		Homo sapiens		1	14
cell part;intracellular membrane-bounded organelle;intracellular organelle;intracellular part;membrane-bounded organelle;nucleus;organelle		Homo sapiens		1	14
cell part;cytoplasm;cytoplasmic part;cytosol;intracellular membrane-bounded organelle;intracellular organelle;intracellular part;membrane-bounded organelle;nucleus;organelle;perinuclear region of cytoplasm	Mineral absorption	Homo sapiens		1	14
cell part;cytoplasmic part;integral to membrane;intracellular membrane-bounded organelle;intracellular organelle;intracellular organelle part;intracellular part;intrinsic to membrane;membrane;membrane part;membrane-bounc	Alzheimer's disease;Cardiac muscle contraction;Huntington's disease;Oxidative phosphorylation;Parkin	Homo sapiens		1	14
adherens junction;anchoring junction;cell junction;cell part;cell-substrate adherens junction;cell-substrate junction;extracellular membrane-bounded organelle;extracellular organelle;extracellular region part;extracellular vesicu	MAPK signaling pathway;Regulation of actin cytoskeleton;Tight junction	Homo sapiens		1	14
cell part;cytoplasmic part;dihydrolipoyl dehydrogenase complex;intracellular membrane-bounded organelle;intracellular organelle;intracellular organelle lumen;intracellular organelle part;intracellular part;macromolecular comp	Valine, leucine and isoleucine degradation	Homo sapiens		1	14
apical plasma membrane;cell part;cytoplasmic part;cytosol;extracellular membrane-bounded organelle;extracellular organelle;extracellular region part;extracellular space;extracellular vesicular exosome;intracellular part;mem	Caprolactam degradation;Glycerolipid metabolism;Glycolysis / Gluconeogenesis	Homo sapiens		1	14
cell part;chromatin;chromosomal part;DNA replication factor A complex;intracellular membrane-bounded organelle;intracellular organelle;intracellular organelle part;intracellular part;macromolecular complex;membrane-bounc	DNA replication;Homologous recombination;Mismatch repair;Nucleotide excision repair	Homo sapiens		1	14
AMP-activated protein kinase complex;calcium channel complex;cAMP-dependent protein kinase complex;cation channel complex;cell part;cell projection;centrosome;cilium;cytoplasmic part;cytoskeletal part;cytosol;extrac	Amoebiasis;Apoptosis;Bile secretion;Calcium signaling pathway;Chemokine signaling pathway;Dilated c	Homo sapiens		1	14
cell part;cytoplasm;cytoplasmic part;external side of plasma membrane;extracellular matrix;extracellular membrane-bounded organelle;extracellular organelle;extracellular region part;extracellular space;extracellular vesicular exosome;immunological synapse;intracellular membrane-bounded organelle;intracellular organelle;intrac		Homo sapiens		1	14
cell part;chromatin;chromosomal part;chromosome;condensed chromosome;condensed nuclear chromosome;cytoplasm;intracellular membrane-bounded organelle;intracellular non-membrane-bounded organelle;intracellular organelle;intracellular organelle part;intracellular part;membrane;membrane-bounded organelle;non-me		Homo sapiens		1	14
cell part;cytoplasmic part;endosome;integral to membrane;integral to plasma membrane;intracellular membrane-bounded organelle;intracellular organelle;intracellular organelle part;intracellular part;intrinsic to membrane;intri	Lysosome;Phagosome	Homo sapiens		1	14
cell junction;cell part;cytoplasm;cytoplasmic part;cytosol;intracellular membrane-bounded organelle;intracellular organelle;intracellular organelle part;intracellular part;membrane-bounded organelle;nuclear part;nucleoplasm;r	Drug metabolism - other enzymes;Purine metabolism	Homo sapiens		1	14
cell part;cell projection;cytoplasmic membrane-bounded vesicle;cytoplasmic part;cytoplasmic vesicle;cytosol;endomembrane system;extracellular membrane-bounded organelle;extracellular organelle;extracellular region par	Collecting duct acid secretion;Epithelial cell signaling in Helicobacter pylori infection;Oxidative phospho	Homo sapiens		1	14
cell part;cytoplasm;cytoplasmic part;endoplasmic reticulum;extracellular membrane-bounded organelle;extracellular organelle;extracellular region part;extracellular vesicular exosome;intracellular membrane-bounded organelle;intracellular organelle;intracellular part;membrane-bounded organelle;membrane-bounded vesicle;orga		Homo sapiens		1	14
cell part;cytoplasmic part;intracellular membrane-bounded organelle;intracellular organelle;intracellular organelle lumen;intracellular organelle part;intracellular part;membrane-bounded organelle;membrane-enclosed lumen;mitochondrial matrix;mitochondrial part;mitochondrion;organelle;organelle lumen;organelle part		Homo sapiens		1	14
cell part;cytoplasmic part;intracellular membrane-bounded organelle;intracellular organelle;intracellular organelle lumen;intracellular organelle part;intracellular part;membrane-bounded organelle;membrane-enclosed lumen;m	Pyruvate metabolism;Two-component system	Homo sapiens		1	14
cell part;cytoplasm;cytoplasmic part;cytosol;extracellular membrane-bounded organelle;extracellular organelle;extracellular region part;extracellular vesicular exosome;intracellular membrane-bounded organelle;intracellular o	Proteasome	Homo sapiens		1	14
actin cytoskeleton;cell junction;cell part;cytoplasm;cytoplasmic part;cytoskeleton;cytosol;extrinsic to membrane;intracellular non-membrane-bounded organelle;intracellular organelle;intracellular part;membrane;membrane part;non-membrane-bounded organelle;organelle;plasma membrane		Homo sapiens		1	14
anchoring junction;cell junction;cell part;cell-cell junction;cytoplasmic part;cytosol;desmosome;extracellular membrane-bounded organelle;extracellular organelle;extracellular region part;extracellular vesicular exosome;integral to membrane;intracellular part;intrinsic to membrane;membrane;membrane part;membrane-bounded o		Homo sapiens		1	14
cell part;cleavage body;intracellular membrane-bounded organelle;intracellular organelle;intracellular organelle part;intracellular part;macromolecular complex;membrane-bounded organelle;mRNA cleavage and polyadenylati	mRNA surveillance pathway	Homo sapiens		1	14
basolateral plasma membrane;cell part;extracellular membrane-bounded organelle;extracellular organelle;extracellular region part;extracellular vesicular exosome;integral to membrane;integral to plasma membrane;intrinsic to	ABC transporters;Vitamin digestion and absorption	Homo sapiens		1	14
cell part;cytoplasm;cytoplasmic part;cytosolic part;cytosolic ribosome;intracellular;intracellular membrane-bounded organelle;intracellular non-membrane-bounded organelle;intracellular organelle;intracellular organelle part;intracellular part;macromolecular complex;membrane;membrane-bounded organelle;NatA complex;non-m		Homo sapiens		1	14
cell part;cytoplasmic part;intracellular membrane-bounded organelle;intracellular organelle;intracellular organelle part;intracellular part;membrane-bounded organelle;mitochondrion;nuclear part;nucleoplasm;nucleus;organelle;organelle part		Homo sapiens		1	14
cell part;cytoplasm;extracellular membrane-bounded organelle;extracellular organelle;extracellular region part;extracellular vesicular exosome;intracellular part;membrane-bounded organelle;membrane-bounded vesicle;orga	Amino sugar and nucleotide sugar metabolism	Homo sapiens		1	14

cell junction;cell part;cytoplasm;cytoplasmic part;cytosol;intracellular membrane-bounded organelle;intracellular organelle;intracellular organelle part;intracellular part;macromolecular complex;membrane;membrane-bounded organelle;nuclear part;nucleoplasm;nucleoplasm part;nucleus;organelle;organelle part;protein complex;t		Homo sapiens	1	14
cell part;cytoplasmic part;extracellular membrane-bounded organelle;extracellular organelle;extracellular region part;extracellular vesicular exosome;intracellular membrane-bounded organelle;intracellular organelle;intracellula	Alzheimer's disease;Huntington's disease;Oxidative phosphorylation;Parkinson's disease	Homo sapiens	1	14
cell part;cell projection;centrosome;chromosomal part;cilium;condensed chromosome kinetochore;cytoplasm;cytoplasmic part;cytoskeletal part;cytosol;intracellular non-membrane-bounded organelle;intracellular organelle;ir	Hedgehog signaling pathway;Wnt signaling pathway	Homo sapiens	1	14
cell part;cytoplasmic part;cytosol;endoplasmic reticulum part;extracellular membrane-bounded organelle;extracellular organelle;extracellular region part;extracellular vesicular exosome;intracellular organelle part;intracellular p	Protein export	Homo sapiens	1	14
cell part;cytoplasm;cytoplasmic part;extracellular membrane-bounded organelle;extracellular organelle;extracellular region part;extracellular vesicular exosome;intracellular membrane-bounded organelle;intracellular organelle	Citrate cycle (TCA cycle);Propanoate metabolism	Homo sapiens	1	14
caveola;cell part;cytoplasmic membrane-bounded vesicle;cytoplasmic part;cytoplasmic vesicle;extracellular membrane-bounded organelle;extracellular organelle;extracellular region part;extracellular vesicular exosome;intrac	Aldosterone-regulated sodium reabsorption;Bile secretion;Carbohydrate digestion and absorption;Cardi	Homo sapiens	1	14
cell part;cytoplasm;cytoplasmic part;cytosol;intracellular organelle part;intracellular part;macromolecular complex;nuclear part;nucleoplasm;organelle part;proteasome accessory complex;proteasome complex;proteasome re	Proteasome	Homo sapiens	1	14
cell part;coated vesicle membrane;cytoplasmic part;cytoplasmic vesicle membrane;cytoplasmic vesicle part;cytosol;endoplasmic reticulum membrane;endoplasmic reticulum part;envelope;ER to Golgi transport vesicle mem	Protein processing in endoplasmic reticulum;RNA transport	Homo sapiens	1	14
adherens junction;anchoring junction;cell junction;cell part;cell-substrate adherens junction;cell-substrate junction;cytoplasmic part;cytosol;cytosolic large ribosomal subunit;extracellular membrane-bounded organelle;extrac	Ribosome	Homo sapiens	1	14
adherens junction;anchoring junction;cell junction;cell part;cell-substrate adherens junction;cell-substrate junction;cytoplasmic part;endoplasmic reticulum;extracellular membrane-bounded organelle;extracellular organelle;ex	MAPK signaling pathway;Regulation of actin cytoskeleton;Tight junction	Homo sapiens	1	14
adherens junction;anchoring junction;cell junction;cell part;cell-substrate adherens junction;cell-substrate junction;cytoplasm;cytoplasmic part;cytosol;cytosolic small ribosomal subunit;extracellular membrane-bounded orga	Ribosome	Homo sapiens	1	14
adherens junction;anchoring junction;cell junction;cell part;cell-substrate adherens junction;cell-substrate junction;cytoplasm;cytoplasmic part;cytosol;cytosolic small ribosomal subunit;extracellular membrane-bounded orga	Ribosome	Homo sapiens	1	14
cell part;cytoplasm;cytoplasmic part;cytosol;cytosolic large ribosomal subunit;extracellular membrane-bounded organelle;extracellular organelle;extracellular region part;extracellular vesicular exosome;intracellular membrane	Ribosome	Homo sapiens	1	14
cell part;cytoplasmic part;cytoplasmic vesicle membrane;cytoplasmic vesicle part;cytosol;cytosolic large ribosomal subunit;endocytic vesicle membrane;endosomal part;endosome membrane;extracellular membrane-bounde	Ribosome	Homo sapiens	1	14
cell part;cell projection;cytoplasm;cytoplasmic part;cytosol;intracellular part;neuron projection	Glycine, serine and threonine metabolism;Methane metabolism	Homo sapiens	1	14
cell part;cytoplasmic part;intracellular membrane-bounded organelle;intracellular non-membrane-bounded organelle;intracellular organelle;intracellular organelle part;intracellular part;macromolecular complex;membrane;membrane-bounded organelle;mitochondrial inner membrane;mitochondrial membrane;mitochondrial part;m		Homo sapiens	1	14
cell part;cytoplasmic part;intracellular membrane-bounded organelle;intracellular organelle;intracellular organelle part;intracellular part;macromolecular complex;membrane;membrane-bounded organelle;mitochondrial inner membrane;mitochondrial membrane;mitochondrial part;mitochondrial small ribosomal subunit;mitochond		Homo sapiens	1	14
cell part;cytoplasmic part;intracellular membrane-bounded organelle;intracellular non-membrane-bounded organelle;intracellular organelle;intracellular organelle part;intracellular part;macromolecular complex;membrane;membrane-bounded organelle;mitochondrial inner membrane;mitochondrial membrane;mitochondrial part;m		Homo sapiens	1	14
cell part;cytoplasm;cytoplasmic part;cytosol;intracellular membrane-bounded organelle;intracellular organelle;intracellular organelle part;intracellular part;membrane;membrane-bounded organelle;nuclear part;nucleoplasm;nu	Butanoate metabolism;Synthesis and degradation of ketone bodies;Terpenoid backbone biosynthesis;V	Homo sapiens	1	14
cell part;intracellular membrane-bounded organelle;intracellular organelle;intracellular part;membrane-bounded organelle;nucleus;organelle		Homo sapiens	1	14
axon part;calcium channel complex;cation channel complex;cell body;cell junction;cell part;cell projection part;cell-cell contact zone;cell-cell junction;cytoplasm;cytoplasmic part;cytoplasmic vesicle membrane;cytoplasmic v	Calcium signaling pathway;ErbB signaling pathway;Gastric acid secretion;Glioma;GnRH signaling pathw	Homo sapiens	1	14
cell part;cytoplasm;cytoplasmic part;cytosol;intracellular membrane-bounded organelle;intracellular organelle;intracellular part;membrane;membrane-bounded organelle;nucleus;organelle;plasma membrane	Alzheimer's disease	Homo sapiens	1	14
adherens junction;anchoring junction;cell junction;cell part;cell-substrate adherens junction;cell-substrate junction;cytoplasmic part;extracellular membrane-bounded organelle;extracellular organelle;extracellular region part;e	Lysosome	Homo sapiens	1	14
cell part;cytoplasm;elongin complex;extracellular region part;extracellular space;integral to membrane;intracellular membrane-bounded organelle;intracellular organelle;intracellular organelle part;intracellular part;intrinsic to membrane;macromolecular complex;membrane part;membrane-bounded organelle;nuclear part;nucleopla		Homo sapiens	1	14
cell part;cytoplasmic part;cytosol;extracellular membrane-bounded organelle;extracellular organelle;extracellular region part;extracellular vesicular exosome;intracellular membrane-bounded organelle;intracellular organelle;in	Peroxisome;Terpenoid backbone biosynthesis	Homo sapiens	1	14
cell part;integral to membrane;intrinsic to membrane;membrane part		Homo sapiens	1	14
Cdc73/Paf1 complex;cell part;chromatin;chromosomal part;intracellular organelle part;intracellular part;macromolecular complex;nuclear body;nuclear part;nuclear speck;nucleoplasm part;organelle part;protein complex;transcription elongation factor complex;transcriptionally active chromatin		Homo sapiens	1	14
cell part;cytoplasmic part;intracellular membrane-bounded organelle;intracellular organelle;intracellular part;membrane;membrane-bounded organelle;microbody;mitochondrion;organelle;peroxisome		Homo sapiens	1	14
cell part;cytoplasmic part;endoplasmic reticulum membrane;endoplasmic reticulum part;integral to membrane;intracellular organelle part;intracellular part;intrinsic to membrane;membrane;membrane part;organelle membrane;organelle part		Homo sapiens	1	14
cell part;chromosome;cytoplasm;cytoplasmic part;cytoskeletal part;intracellular non-membrane-bounded organelle;intracellular organelle;intracellular organelle part;intracellular part;microtubule organizing center;non-membrane-bounded organelle;nuclear part;nucleolus;nucleoplasm;organelle;organelle part		Homo sapiens	1	14
cell part;cytoplasmic part;endoplasmic reticulum;endoplasmic reticulum membrane;endoplasmic reticulum part;integral to membrane;intracellular membrane-bounded organelle;intracellular organelle;intracellular organelle pa	Glycerophospholipid metabolism	Homo sapiens	1	14
cell part;intracellular membrane-bounded organelle;intracellular non-membrane-bounded organelle;intracellular organelle;intracellular organelle part;intracellular part;macromolecular complex;membrane-bounded organelle;non-membrane-bounded organelle;nuclear part;nucleolus;nucleus;organelle;organelle part;preribosome;pr		Homo sapiens	1	14
cell part;integral to membrane;integrator complex;intracellular organelle part;intracellular part;intrinsic to membrane;macromolecular complex;membrane;membrane part;nuclear membrane;nuclear part;nucleoplasm part;organelle membrane;organelle part;protein complex		Homo sapiens	1	14
cell part;cytoplasm;cytoplasmic part;cytosol;intracellular organelle lumen;intracellular organelle part;intracellular part;membrane;membrane-enclosed lumen;mitochondrial matrix;mitochondrial part;organelle lumen;organelle p	Riboflavin metabolism	Homo sapiens	1	14
cell part;cytoplasmic part;cytosol;intracellular membrane-bounded organelle;intracellular organelle;intracellular part;membrane-bounded organelle;nucleus;organelle	Ubiquitin mediated proteolysis	Homo sapiens	1	14
cell part;cytoplasm;cytoplasmic part;cytoplasmic vesicle;cytoskeleton;Golgi apparatus;intracellular membrane-bounded organelle;intracellular non-membrane-bounded organelle;intracellular organelle;intracellular part;membrane;membrane-bounded organelle;non-membrane-bounded organelle;organelle;plasma membrane;vesi		Homo sapiens	1	14
cell part;intracellular part;macromolecular complex;protein complex;protein phosphatase 4 complex;protein serine/threonine phosphatase complex		Homo sapiens	1	14
cell part;cytoplasm;intracellular non-membrane-bounded organelle;intracellular organelle;intracellular organelle part;intracellular part;non-membrane-bounded organelle;nuclear part;nucleolus;organelle;organelle part		Homo sapiens	1	14
Cdc73/Paf1 complex;cell part;intracellular non-membrane-bounded organelle;intracellular organelle;intracellular organelle part;intracellular part;macromolecular complex;non-membrane-bounded organelle;nuclear part;nucleolus;nucleoplasm;nucleoplasm part;organelle;organelle part;protein complex;transcription elongation fact		Homo sapiens	1	14
catalytic step 2 spliceosome;cell part;cytoplasm;intracellular membrane-bounded organelle;intracellular organelle;intracellular organelle part;intracellular part;macromolecular complex;membrane;membrane-bounded organell	Spliceosome	Homo sapiens	1	14
cell part;cytoplasmic part;cytosol;extracellular membrane-bounded organelle;extracellular organelle;extracellular region part;extracellular vesicular exosome;intracellular part;membrane-bounded organelle;membrane-bounde	Chlorocyclohexane and chlorobenzene degradation;Fluorobenzoate degradation;Toluene degradation	Homo sapiens	1	14
cell part;cytoplasm;cytoplasmic part;cytosol;extracellular membrane-bounded organelle;extracellular organelle;extracellular region part;extracellular vesicular exosome;intracellular non-membrane-bounded organelle;intracellular organelle;intracellular organelle part;intracellular part;membrane;membrane-bounded organelle;meml		Homo sapiens	1	14
cell part;cytoplasm;extracellular membrane-bounded organelle;extracellular organelle;extracellular region part;extracellular vesicular exosome;intracellular part;membrane-bounded organelle;membrane-bounded vesicle;organelle;vesicle		Homo sapiens	1	14
basal plasma membrane;basement membrane;cell junction;cell part;cell-substrate junction;cytoplasm;extracellular matrix part;extracellular region part;hemidesmosome;intracellular membrane-bounded organelle;intracellular	NOD-like receptor signaling pathway	Homo sapiens	1	14
cell part;cytoplasmic part;intracellular membrane-bounded organelle;intracellular organelle;intracellular organelle part;intracellular part;membrane;membrane-bounded organelle;mitochondrial inner membrane;mitochondrial membrane;mitochondrial part;mitochondrion;organelle;organelle inner membrane;organelle membrane;org		Homo sapiens	1	14
actin cytoskeleton;cell part;centrosome;chromosomal part;condensin complex;cytoplasm;cytoplasmic part;cytoskeletal part;cytoskeleton;cytosol;intracellular membrane-bounded organelle;intracellular non-membrane-bounc	Cell cycle - yeast	Homo sapiens	1	14
adherens junction;anchoring junction;cell junction;cell part;cell-cell junction;cytoplasm;cytoskeletal part;intracellular organelle part;intracellular part;lateral plasma membrane;macromolecular complex;membrane part;microtubule;occluding junction;organelle part;plasma membrane part;protein complex;tight junction		Homo sapiens	1	14
cell part;cytoplasmic part;intracellular membrane-bounded organelle;intracellular organelle;intracellular organelle part;intracellular part;macromolecular complex;membrane;membrane-bounded organelle;mitochondrial inner membrane;mitochondrial membrane;mitochondrial part;mitochondrial small ribosomal subunit;mitochond		Homo sapiens	1	14
cell part;intracellular non-membrane-bounded organelle;intracellular organelle;intracellular organelle part;intracellular part;macromolecular complex;non-membrane-bounded organelle;nuclear part;nucleolar part;nucleolus;nucleoplasm;organelle;organelle part;PeBoW complex;preribosome;preribosome, large subunit precursor;pr		Homo sapiens	1	14
cell part;intracellular;intracellular membrane-bounded organelle;intracellular organelle;intracellular part;membrane-bounded organelle;nucleus;organelle	mRNA surveillance pathway	Homo sapiens	1	14
cell part;cytoplasm;cytoplasmic part;cytosol;endosomal part;endosome;endosome membrane;ESCRT III complex;extracellular membrane-bounded organelle;extracellular organelle;extracellular region part;extracellular vesicu	Endocytosis	Homo sapiens	1	14
cell part;cytoplasmic part;endoplasmic reticulum;intracellular membrane-bounded organelle;intracellular organelle;intracellular part;membrane;membrane-bounded organelle;organelle	Other types of O-glycan biosynthesis	Homo sapiens	1	14
cell part;cytoplasm;intracellular part		Homo sapiens	1	14
cell part;cytoplasmic part;endoplasmic reticulum membrane;endoplasmic reticulum part;integral to membrane;intracellular organelle part;intracellular part;intrinsic to membrane;membrane;membrane part;organelle membrane;organelle part		Homo sapiens	1	14
cell part;cytoplasmic part;cytosol;intracellular organelle part;intracellular part;membrane;nuclear membrane;nuclear part;organelle membrane;organelle part		Homo sapiens	1	14
	Protein processing in endoplasmic reticulum	Homo sapiens	1	14
cell part;cytoplasmic part;cytosol;extracellular membrane-bounded organelle;extracellular organelle;extracellular region part;extracellular vesicular exosome;intracellular;intracellular membrane-bounded organelle;intracellular	Cysteine and methionine metabolism	Homo sapiens	1	14
cell part;cytoplasm;cytoskeleton;intracellular non-membrane-bounded organelle;intracellular organelle;intracellular part;non-membrane-bounded organelle;organelle		Homo sapiens	1	14
cell part;cytoplasm;cytoplasmic part;cytosol;eukaryotic translation initiation factor 2B complex;intracellular part;macromolecular complex;protein complex	RNA transport	Homo sapiens	1	14
cell part;cytoplasmic part;cytoskeleton;extrinsic to membrane;extrinsic to plasma membrane;intracellular non-membrane-bounded organelle;intracellular organelle;intracellular organelle part;intracellular part;membrane;membrane part;membrane raft;membrane-enclosed lumen;mitochondrial inner membrane;mitochondrial intern		Homo sapiens	1	14
cell part;cytoplasm;intracellular organelle part;intracellular part;macromolecular complex;membrane;membrane part;nuclear membrane;nuclear part;nuclear pore;nucleoplasm;organelle membrane;organelle part;pore complex	RNA transport	Homo sapiens	1	14
cell part;cell projection;cytoplasm;cytoplasmic part;cytoskeleton;cytosol;extracellular membrane-bounded organelle;extracellular organelle;extracellular region part;extracellular vesicular exosome;filopodium;intracellular non-	Adherens junction;Regulation of actin cytoskeleton	Homo sapiens	1	14
cell part;cytoplasmic part;cytosol;intracellular membrane-bounded organelle;intracellular organelle;intracellular organelle lumen;intracellular organelle part;intracellular part;membrane-bounded organelle;membrane-enclosed l	Aminoacyl-tRNA biosynthesis	Homo sapiens	1	14
cell part;chromatin remodeling complex;chromosomal part;cytoplasmic part;cytosol;DNA helicase complex;Ino80 complex;intracellular membrane-bounded organelle;intracellular organelle;intracellular organelle part;intracellular part;macromolecular complex;membrane-bounded organelle;nuclear chromosome part;nuclear part;r		Homo sapiens	1	14
actin cytoskeleton;cell junction;cell part;cell projection;cell-cell junction;cytoplasmic part;cytoskeleton;cytosol;early endosome;endosome;extracellular membrane-bounded organelle;extracellular organelle;extracellular region	Adherens junction;Bacterial invasion of epithelial cells;Fc gamma R-mediated phagocytosis;Regulation	Homo sapiens	1	14
axon part;axonal growth cone;cell part;cell projection part;growth cone;site of polarized growth		Homo sapiens	1	15
cell part;cytoplasmic part;endoplasmic reticulum;endosomal part;endosome membrane;envelope;extracellular membrane-bounded organelle;extracellular organelle;extracellular region;extracellular region part;extracellular ve	Lysosome	Homo sapiens	1	15
actin cytoskeleton;adherens junction;anchoring junction;Arp2/3 protein complex;cell junction;cell part;cell projection;cell-substrate adherens junction;cell-substrate junction;cytoplasm;cytoplasmic part;cytoskeletal part;cytosl	Bacterial invasion of epithelial cells;Fc gamma R-mediated phagocytosis;Pathogenic Escherichia coli inf	Homo sapiens	1	15
cell part;cytoplasm;extracellular membrane-bounded organelle;extracellular organelle;extracellular region part;extracellular vesicular exosome;integral to membrane;integral to plasma membrane;intracellular membrane-bounded organelle;intracellular organelle;intracellular part;intrinsic to membrane;intrinsic to plasma membrane		Homo sapiens	1	15
cell cortex;cell part;cell projection membrane;cell projection part;chromosomal part;condensed chromosome kinetochore;cytoplasm;cytoplasmic microtubule;cytoplasmic part;cytoskeletal part;cytosol;Golgi apparatus;Golgi apparatus part;intracellular membrane-bounded organelle;intracellular non-membrane-bounded organelle		Homo sapiens	1	15
cell part;cytoplasmic part;intracellular membrane-bounded organelle;intracellular organelle;intracellular organelle part;intracellular part;macromolecular complex;membrane;membrane part;membrane-bounded organelle;mito	Alzheimer's disease;Huntington's disease;Oxidative phosphorylation;Parkinson's disease	Homo sapiens	1	15
cell part;cytoplasmic part;cytosol;early endosome;endosomal part;endosome;endosome membrane;extracellular membrane-bounded organelle;extracellular organelle;extracellular region part;extracellular vesicular exosome;intracellular membrane-bounded organelle;intracellular organelle;intracellular organelle part;intracellular p		Homo sapiens	1	15
axon part;axon terminus;cell body;cell part;cell projection part;cytoplasm;cytoplasmic part;cytoskeletal part;extracellular membrane-bounded organelle;extracellular organelle;extracellular region part;extracellular vesicular exosome;intracellular non-membrane-bounded organelle;intracellular organelle;intracellular organelle part;ir		Homo sapiens	1	15
cell part;cytoplasm;cytoplasmic part;endoplasmic reticulum;endoplasmic reticulum membrane;endoplasmic reticulum part;extracellular membrane-bounded organelle;extracellular organelle;extracellular region part;extracellular vesicular exosome;integral to membrane;intracellular membrane-bounded organelle;intracellular orgar		Homo sapiens	1	15
cell part;cytoplasm;cytoplasmic part;cytosolic part;endoplasmic reticulum;endoplasmic reticulum membrane;endoplasmic reticulum part;extracellular membrane-bounded organelle;extracellular organelle;extracellular region p	Amino sugar and nucleotide sugar metabolism	Homo sapiens	1	15
cell part;chromosomal part;extracellular membrane-bounded organelle;extracellular organelle;extracellular region part;extracellular vesicular exosome;intracellular membrane-bounded organelle;intracellular organelle;intracellu	Systemic lupus erythematosus	Homo sapiens	1	15
cell part;cell projection;cytoplasm;cytoplasmic part;cytosol;envelope;extracellular membrane-bounded organelle;extracellular organelle;extracellular region part;extracellular vesicular exosome;extrinsic to internal side of plasma membrane;extrinsic to membrane;extrinsic to plasma membrane;intracellular membrane-bounded org		Homo sapiens	1	15
cell part;chromosomal part;extracellular membrane-bounded organelle;extracellular organelle;extracellular region part;extracellular vesicular exosome;intracellular membrane-bounded organelle;intracellular organelle;intracellu	Systemic lupus erythematosus	Homo sapiens	1	15
cell part;cytoplasmic part;extracellular membrane-bounded organelle;extracellular organelle;extracellular region part;extracellular vesicular exosome;intracellular membrane-bounded organelle;intracellular organelle;intracellula	Alzheimer's disease;Cardiac muscle contraction;Huntington's disease;Oxidative phosphorylation;Parkin	Homo sapiens	1	15
cell junction;cell part;cell-cell junction;cytoplasm;cytoplasmic part;intracellular membrane-bounded organelle;intracellular organelle;intracellular part;macromolecular complex;membrane-bounded organelle;nucleus;occluding	Tight junction	Homo sapiens	1	15
cell part;cytoplasm;cytoplasmic part;cytosol;I-kappaB/NF-kappaB complex;intracellular membrane-bounded organelle;intracellular organelle;intracellular organelle part;intracellular part;macromolecular complex;membrane-b	Acute myeloid leukemia;Adipocytokine signaling pathway;Amoebiasis;Apoptosis;B cell receptor signalir	Homo sapiens	1	15
cell part;cytoplasm;cytoplasmic part;cytosol;extracellular membrane-bounded organelle;extracellular organelle;extracellular region part;extracellular vesicular exosome;intracellular membrane-bounded organelle;intracellular c	Proteasome	Homo sapiens	1	15
cell junction;cell part;cell projection;cytoplasmic part;extracellular membrane-bounded organelle;extracellular organelle;extracellular region part;extracellular vesicular exosome;intracellular organelle part;intracellular part;mem	Cysteine and methionine metabolism;Sulfur relay system	Homo sapiens	1	15
cell part;cytoplasmic part;cytosol;integral to membrane;intracellular membrane-bounded organelle;intracellular organelle;intracellular organelle lumen;intracellular organelle part;intracellular part;intrinsic to membrane;membra	ABC transporters;Peroxisome	Homo sapiens	1	15
adherens junction;anchoring junction;cell junction;cell part;cell-substrate adherens junction;cell-substrate junction;cytoplasm;cytoplasmic part;extracellular membrane-bounded organelle;extracellular organelle;extracellular re	Arginine and proline metabolism;Glutathione metabolism	Homo sapiens	1	15
cell part;cytoplasm;cytoplasmic part;cytosol;extracellular membrane-bounded organelle;extracellular organelle;extracellular region part;extracellular vesicular exosome;intracellular part;membrane-bounded organelle;membra	Inositol phosphate metabolism;Phosphatidylinositol signaling system;Streptomycin biosynthesis	Homo sapiens	1	15
cell part;cytoplasm;cytoplasmic part;cytosol;extracellular region;intracellular part;IPAF inflammasome complex;macromolecular complex;NALP1 inflammasome complex;NALP3 inflammasome complex;protein complex	Amyotrophic lateral sclerosis (ALS);Cytosolic DNA-sensing pathway;NOD-like receptor signaling pathwa	Homo sapiens	1	15
cell part;cell surface;cytoplasmic membrane-bounded vesicle;cytoplasmic part;cytoplasmic vesicle;endoplasmic reticulum;endoplasmic reticulum lumen;endoplasmic reticulum part;extracellular membrane-bounded organelle	Protein processing in endoplasmic reticulum	Homo sapiens	1	15
cell part;cytoplasmic part;intracellular organelle lumen;intracellular organelle part;intracellular part;membrane;membrane-enclosed lumen;mitochondrial inner membrane;mitochondrial matrix;mitochondrial membrane;mitocho	Toxoplasmosis	Homo sapiens	1	15
cell part;cytoplasm;cytoplasmic part;cytosol;endoplasmic reticulum;extracellular region part;extracellular space;intracellular membrane-bounded organelle;intracellular organelle;intracellular organelle part;intracellular part;me	beta-Alanine metabolism;Drug metabolism - cytochrome P450;Glycolysis / Gluconeogenesis;Histidine r	Homo sapiens	1	15
cell junction;cell part;intracellular membrane-bounded organelle;intracellular organelle;intracellular organelle part;intracellular part;macromolecular complex;membrane-bounded organelle;nuclear part;nucleoplasm;nucleoplas	Basal transcription factors	Homo sapiens	1	15
acrosomal membrane;adherens junction;anchoring junction;cell junction;cell part;cell-substrate adherens junction;cell-substrate junction;cytoplasmic membrane-bounded vesicle;cytoplasmic part;cytoplasmic vesicle;cytoplasmic vesicle membrane;cytoplasmic vesicle part;extracellular membrane-bounded organelle;extracellular		Homo sapiens	1	15
cell part;centromeric heterochromatin;chromatin;chromatin remodeling complex;chromocenter;chromosomal part;envelope;heterochromatin;histone deacetylase complex;histone methyltransferase complex;intracellular membrane-bounded organelle;intracellular non-membrane-bounded organelle;intracellular organelle;intracellu		Homo sapiens	1	15
cell part;cytoplasmic part;cytosol;cytosolic large ribosomal subunit;intracellular organelle part;intracellular part;large ribosomal subunit;macromolecular complex;membrane;organelle part;ribonucleoprotein complex	Ribosome	Homo sapiens	1	15
cell part;cytoplasmic part;cytosol;intracellular part	RNA transport	Homo sapiens	1	15
cell part;cytoplasm;cytoplasmic part;cytosol;eukaryotic translation initiation factor 2B complex;intracellular part;macromolecular complex;protein complex	RNA transport	Homo sapiens	1	15
cell part;cytoplasmic part;cytosol;intracellular part	Nicotinate and nicotinamide metabolism;Purine metabolism;Pyrimidine metabolism	Homo sapiens	1	15
cell part;cytoplasmic part;cytosol;extracellular membrane-bounded organelle;extracellular organelle;extracellular region part;extracellular space;extracellular vesicular exosome;intracellular part;membrane-bounded organelle;membrane-bounded vesicle;organelle;vesicle		Homo sapiens	1	15
		Homo sapiens	1	15
cell part;cytoplasm;cytoplasmic part;cytosol;intracellular organelle part;intracellular part;nuclear part;nucleoplasm;organelle part	Purine metabolism	Homo sapiens	1	15
adherens junction;anchoring junction;apical part of cell;cell body;cell junction;cell part;cell projection;cell-cell junction;cell-substrate adherens junction;cell-substrate junction;cytoplasm;cytoplasmic membrane-bounded vesic	Adherens junction;Axon guidance;Bacterial invasion of epithelial cells;Chemokine signaling pathway;En	Homo sapiens	1	15
catalytic step 2 spliceosome;cell part;cytoplasmic part;cytosol;extracellular membrane-bounded organelle;extracellular organelle;extracellular region part;extracellular vesicular exosome;intracellular organelle part;intracellular	Spliceosome	Homo sapiens	1	15
cell part;cytoplasmic part;cytosol;endoplasmic reticulum;endoplasmic reticulum-Golgi intermediate compartment;envelope;extracellular membrane-bounded organelle;extracellular organelle;extracellular region part;extracellular space;extracellular vesicular exosome;Golgi apparatus;intracellular membrane-bounded organelle;int		Homo sapiens	1	15
cell part;cytoplasmic part;intracellular membrane-bounded organelle;intracellular non-membrane-bounded organelle;intracellular organelle;intracellular organelle part;intracellular part;macromolecular complex;membrane;membrane-bounded organelle;mitochondrial inner membrane;mitochondrial membrane;mitochondrial part;m		Homo sapiens	1	15
cell part;cytoplasm;intracellular organelle part;intracellular part;nuclear part;nucleoplasm;organelle part	Spliceosome	Homo sapiens	1	15
cell part;cytoplasm;extracellular membrane-bounded organelle;extracellular organelle;extracellular region part;extracellular vesicular exosome;intracellular membrane-bounded organelle;intracellular organelle;intracellular orga	Spliceosome	Homo sapiens	1	15
Cdc48p-Npl4p-Ufd1p AAA ATPase complex;cell body;cell part;cell projection;cytoplasm;cytoplasmic part;dendrite;endoplasmic reticulum part;intracellular membrane-bounded organelle;intracellular organelle;intracellular organelle part;intracellular part;macromolecular complex;membrane part;membrane-bounded organelle;neu		Homo sapiens	1	15
cell part;cytoplasm;extracellular membrane-bounded organelle;extracellular organelle;extracellular region part;extracellular vesicular exosome;intracellular part;membrane-bounded organelle;membrane-bounded vesicle;orga	MAPK signaling pathway - yeast;Pyruvate metabolism	Homo sapiens	1	15
cell part;cytoplasmic part;cytosol;endoplasmic reticulum-Golgi intermediate compartment;Golgi apparatus;Golgi apparatus part;Golgi cisterna membrane;Golgi membrane;Golgi transport complex;intracellular membrane-bounded organelle;intracellular non-membrane-bounded organelle;intracellular organelle;intracellular organe		Homo sapiens	1	15
cell part;cytoplasm;cytoplasmic part;cytosol;eukaryotic translation initiation factor 2B complex;intracellular membrane-bounded organelle;intracellular organelle;intracellular part;macromolecular complex;membrane-bounded	RNA transport	Homo sapiens	1	15
cell part;centromeric heterochromatin;chromatin;chromosomal part;chromosome, centromeric region;condensed chromosome, centromeric region;cytoskeletal part;envelope;euchromatin;heterochromatin;intracellular membrane-bounded organelle;intracellular non-membrane-bounded organelle;intracellular organelle;intracellula		Homo sapiens	1	15
cell body;cell part;clathrin-coated vesicle;coated pit;coated vesicle;cytoplasmic membrane-bounded vesicle;cytoplasmic part;cytoplasmic vesicle;Golgi apparatus;intracellular membrane-bounded organelle;intracellular organelle;intracellular part;membrane;membrane part;membrane-bounded organelle;membrane-bounded ves		Homo sapiens	1	15
cell part;cell projection;cytoplasm;cytoplasmic part;cytosol;early endosome membrane;endosomal part;endosome membrane;extrinsic to membrane;Golgi apparatus;intracellular membrane-bounded organelle;intracellular organelle;intracellular organelle part;intracellular part;lamellipodium;macromolecular complex;membrane;m		Homo sapiens	1	15
adherens junction;anchoring junction;axon;cell body;cell junction;cell part;cell projection;cell-substrate adherens junction;cell-substrate junction;external side of plasma membrane;extracellular membrane-bounded organelle;	Cell adhesion molecules (CAMs)	Homo sapiens	1	15
cell part;cytoplasmic part;cytosol;extracellular membrane-bounded organelle;extracellular organelle;extracellular region;extracellular region part;extracellular space;extracellular vesicular exosome;intracellular part;membrane-	African trypanosomiasis;Cytokine-cytokine receptor interaction;Cytosolic DNA-sensing pathway;ko0515	Homo sapiens	1	15
cell part;intracellular membrane-bounded organelle;intracellular non-membrane-bounded organelle;intracellular organelle;intracellular organelle part;intracellular part;membrane-bounded organelle;non-membrane-bounded or	Ribosome biogenesis in eukaryotes	Homo sapiens	1	15
cell part;cytoplasm;cytoplasmic part;early endosome;endosome;intracellular membrane-bounded organelle;intracellular organelle;intracellular organelle part;intracellular part;membrane-bounded organelle;nuclear part;nucleoplasm;nucleus;organelle;organelle part		Homo sapiens	1	15
cell part;cytoplasm;extracellular membrane-bounded organelle;extracellular organelle;extracellular region part;extracellular vesicular exosome;intracellular part;membrane-bounded organelle;membrane-bounded vesicle;organelle;vesicle		Homo sapiens	1	15
cell part;intracellular non-membrane-bounded organelle;intracellular organelle;intracellular organelle part;intracellular part;non-membrane-bounded organelle;nuclear part;nucleolus;organelle;organelle part	Ribosome biogenesis in eukaryotes	Homo sapiens	1	15
cell part;cytoplasm;cytoplasmic part;cytosol;extracellular membrane-bounded organelle;extracellular organelle;extracellular region part;extracellular vesicular exosome;intracellular part;membrane;membrane-bounded organe	Ether lipid metabolism	Homo sapiens	1	15
cell part;cytoplasmic part;endoplasmic reticulum;endoplasmic reticulum lumen;endoplasmic reticulum part;intracellular membrane-bounded organelle;intracellular organelle;intracellular organelle lumen;intracellular organelle part;intracellular part;membrane-bounded organelle;membrane-enclosed lumen;organelle;organelle lumer		Homo sapiens	1	15
cell part;cytoplasm;cytoplasmic part;integral to membrane;intracellular membrane-bounded organelle;intracellular organelle;intracellular organelle part;intracellular part;intrinsic to membrane;membrane;membrane part;membrane-bounded organelle;mitochondrial membrane;mitochondrial outer membrane;mitochondrial part;nuc		Homo sapiens	1	15
cell part;cytoplasm;intracellular membrane-bounded organelle;intracellular organelle;intracellular part;membrane-bounded organelle;nucleus;organelle		Homo sapiens	1	15
cell part;intracellular organelle part;intracellular part;macromolecular complex;nuclear part;organelle part;ribonucleoprotein complex;spliceosomal complex	Spliceosome	Homo sapiens	1	15
cell part;cytoplasmic part;intracellular membrane-bounded organelle;intracellular organelle;intracellular part;membrane-bounded organelle;mitochondrion;organelle	Aminoacyl-tRNA biosynthesis	Homo sapiens	1	15
cell part;cytoplasm;intracellular part		Homo sapiens	1	15
cell part;cytoplasmic part;intracellular organelle lumen;intracellular organelle part;intracellular part;membrane-enclosed lumen;mitochondrial matrix;mitochondrial part;organelle lumen;organelle part		Homo sapiens	1	15
cell part;cytoplasmic part;endoplasmic reticulum membrane;endoplasmic reticulum part;intracellular organelle part;intracellular part;membrane;membrane part;nuclear membrane;nuclear outer membrane;nuclear part;organe	Retinol metabolism	Homo sapiens	1	15
Cdc73/Paf1 complex;cell part;intracellular membrane-bounded organelle;intracellular organelle;intracellular organelle part;intracellular part;macromolecular complex;membrane-bounded organelle;nuclear part;nucleoplasm;nucleoplasm part;nucleus;organelle;organelle part;protein complex;transcription elongation factor complex		Homo sapiens	1	15

cell part;cytoplasm;cytoplasmic part;cytoskeletal part;extracellular membrane-bounded organelle;extracellular organelle;extracellular region part;extracellular vesicular exosome;intracellular membrane-bounded organelle;intracellular organelle;intracellular organelle part;intracellular part;membrane;membrane-bounded organelle;r		Homo sapiens	1	15
cell part;cytoplasmic part;intracellular membrane-bounded organelle;intracellular non-membrane-bounded organelle;intracellular organelle;intracellular organelle part;intracellular part;membrane-bounded organelle;mitochondrial nucleoid;mitochondrial part;mitochondrion;non-membrane-bounded organelle;nuclear part;nucleoid;		Homo sapiens	1	15
cell part;cytoplasmic part;eukaryotic 43S preinitiation complex;eukaryotic 48S preinitiation complex;eukaryotic translation initiation factor 3 complex;eukaryotic translation initiation factor 3 complex, eIF3m;intracellular part;macromolecular complex;protein complex;ribonucleoprotein complex;translation preinitiation complex		Homo sapiens	1	15
cell part;cytoplasmic part;cytosol;endosomal part;endosome membrane;ESCRT III complex;extracellular membrane-bounded organelle;extracellular organelle;extracellular region part;extracellular vesicular exosome;intracellu	Endocytosis	Homo sapiens	1	15
cell part;cytoplasm;cytoplasmic part;intracellular membrane-bounded organelle;intracellular non-membrane-bounded organelle;intracellular organelle;intracellular part;macromolecular complex;membrane;membrane-bounded organelle;non-membrane-bounded organelle;nucleus;organelle;polysomal ribosome;ribonucleoprotein (		Homo sapiens	1	15
cell part;intracellular organelle part;intracellular part;nuclear part;nucleoplasm;organelle part		Homo sapiens	1	15
cell part;cytoplasm;cytoplasmic part;cytosol;intracellular membrane-bounded organelle;intracellular organelle;intracellular organelle part;intracellular part;membrane-bounded organelle;nuclear part;nucleoplasm;nucleus;organelle;organelle part		Homo sapiens	1	15
catalytic step 2 spliceosome;cell part;cytoplasmic part;cytosol;intracellular membrane-bounded organelle;intracellular organelle;intracellular organelle part;intracellular part;macromolecular complex;membrane-bounded orgar	mRNA surveillance pathway;RNA transport	Homo sapiens	1	15
cell junction;cell part;cell projection;cell projection part;cytoplasmic part;cytoskeletal part;cytoskeleton;cytosol;endoplasmic reticulum;extracellular membrane-bounded organelle;extracellular organelle;extracellular region part;extracellular vesicular exosome;filopodium;growth cone;intracellular;intracellular membrane-bounded o		Homo sapiens	1	15
cell part;cytoplasm;cytoplasmic part;Golgi apparatus;Golgi apparatus part;Golgi membrane;intracellular membrane-bounded organelle;intracellular non-membrane-bounded organelle;intracellular organelle;intracellular organe	Endocytosis	Homo sapiens	1	15
cell part;intracellular non-membrane-bounded organelle;intracellular organelle;intracellular organelle part;intracellular part;macromolecular complex;non-membrane-bounded organelle;nuclear part;nucleolar part;nucleolus;org	Ribosome biogenesis in eukaryotes	Homo sapiens	1	15
cell part;cytoplasm;integral to membrane;intracellular part;intrinsic to membrane;membrane;membrane part;plasma membrane		Homo sapiens	1	15
cell part;cis-Golgi network;cytoplasmic membrane-bounded vesicle;cytoplasmic part;cytoplasmic vesicle;endoplasmic reticulum membrane;endoplasmic reticulum part;Golgi apparatus part;Golgi cisterna membrane;Golgi membrane;Golgi transport complex;Golgi-associated vesicle;intracellular membrane-bounded organelle;in		Homo sapiens	1	15
cell part;cytoplasm;intracellular organelle part;intracellular part;macromolecular complex;NELF complex;nuclear part;nucleoplasm;nucleoplasm part;organelle part;protein complex;transcription elongation factor complex		Homo sapiens	1	15
cell part;cytoplasm;intracellular membrane-bounded organelle;intracellular organelle;intracellular organelle part;intracellular part;macromolecular complex;membrane;membrane-bounded organelle;nuclear part;nucleoplasm;n	Spliceosome	Homo sapiens	1	15
cell part;cytoplasmic part;endoplasmic reticulum membrane;endoplasmic reticulum part;integral to membrane;intracellular organelle part;intracellular part;intrinsic to membrane;membrane;membrane part;organelle membrane;organelle part		Homo sapiens	1	15
cell part;cytoplasm;intracellular part	Arginine and proline metabolism	Homo sapiens	1	15
autophagic vacuole;cell part;cytoplasm;cytoplasmic membrane-bounded vesicle;cytoplasmic part;cytoplasmic vesicle;cytosol;endosome;Golgi apparatus;Golgi apparatus part;Golgi membrane;intracellular membrane-bounded organelle;intracellular organelle;intracellular organelle part;intracellular part;membrane;membrane-bou		Homo sapiens	1	15
cell part;cytoplasmic part;intracellular non-membrane-bounded organelle;intracellular organelle;intracellular organelle part;intracellular part;macromolecular complex;membrane;mitochondrial inner membrane;mitochondrial membrane;mitochondrial part;non-membrane-bounded organelle;organelle;organelle inner membrane;orga		Homo sapiens	1	15
cell part;chromosomal part;condensed chromosome kinetochore;cytoplasmic part;cytosol;envelope;intracellular non-membrane-bounded organelle;intracellular organelle;intracellular organelle part;intracellular part;kinetocho	RNA transport	Homo sapiens	1	15
cell part;cytoplasmic part;integral to membrane;intracellular membrane-bounded organelle;intracellular organelle;intracellular organelle part;intracellular part;intrinsic to membrane;membrane;membrane part;membrane-bounded organelle;mitochondrial membrane;mitochondrial outer membrane;mitochondrial part;mitochondrion;		Homo sapiens	1	15
cell part;cytoplasm;exon-exon junction complex;intracellular non-membrane-bounded organelle;intracellular organelle;intracellular organelle part;intracellular part;macromolecular complex;non-membrane-bounded organelle;	mRNA surveillance pathway;RNA transport	Homo sapiens	1	15
cell part;cytoplasm;intracellular membrane-bounded organelle;intracellular organelle;intracellular organelle part;intracellular part;membrane;membrane-bounded organelle;nuclear part;nucleoplasm;nucleus;organelle;organelle part;plasma membrane		Homo sapiens	1	15
cell part;cytoplasmic part;endoplasmic reticulum lumen;endoplasmic reticulum part;Golgi apparatus;integral to membrane;integral to nuclear inner membrane;integral to organelle membrane;intracellular membrane-bounded organelle;intracellular organelle;intracellular organelle lumen;intracellular organelle part;intracellular part;ir		Homo sapiens	1	15
cell part;intracellular non-membrane-bounded organelle;intracellular organelle;intracellular organelle part;intracellular part;macromolecular complex;non-membrane-bounded organelle;nuclear part;nucleolus;organelle;organell	Ribosome biogenesis in eukaryotes	Homo sapiens	1	15
AP-type membrane coat adaptor complex;cell part;clathrin adaptor complex;clathrin coated vesicle membrane;clathrin-coated endocytic vesicle membrane;coated vesicle membrane;cytoplasmic part;cytoplasmic vesicle me	Lysosome	Homo sapiens	1	15
cell part;cytoplasm;intracellular non-membrane-bounded organelle;intracellular organelle;intracellular organelle part;intracellular part;membrane;non-membrane-bounded organelle;nuclear part;nucleolus;organelle;organelle part		Homo sapiens	1	15
90S preribosome;cell part;cytoplasmic part;intracellular membrane-bounded organelle;intracellular non-membrane-bounded organelle;intracellular organelle;intracellular organelle part;intracellular part;macromolecular comple	Ribosome biogenesis in eukaryotes	Homo sapiens	1	15
cell part;chromosome;condensed chromosome;condensed nuclear chromosome;cytoplasmic part;cytoskeletal part;early endosome;endomembrane system;endosomal part;endosome;ESCRT III complex;extracellular membrane-bounded organelle;extracellular organelle;extracellular region part;extracellular vesicular exosome;ir		Homo sapiens	1	15
cell part;cytoplasmic part;extracellular membrane-bounded organelle;extracellular organelle;extracellular region part;extracellular vesicular exosome;intracellular membrane-bounded organelle;intracellular organelle;intracellular organelle part;intracellular part;lysosome;lytic vacuole;macromolecular complex;membrane;membrane		Homo sapiens	1	15
autophagic vacuole membrane;cell part;cytoplasm;cytoplasmic part;extracellular membrane-bounded organelle;extracellular organelle;extracellular region part;extracellular vesicular exosome;Golgi apparatus part;Golgi mem	Endocytosis	Homo sapiens	1	15
cell part;extracellular membrane-bounded organelle;extracellular organelle;extracellular region part;extracellular vesicular exosome;membrane;membrane-bounded organelle;membrane-bounded vesicle;organelle;vesicle	mRNA surveillance pathway	Homo sapiens	1	15
cell part;cytoplasmic part;extracellular membrane-bounded organelle;extracellular organelle;extracellular region part;extracellular vesicular exosome;integral to membrane;intracellular membrane-bounded organelle;intracellular organelle;intracellular organelle part;intracellular part;intrinsic to membrane;macromolecular complex;r		Homo sapiens	1	15
		Homo sapiens	1	16
actin cytoskeleton;axon;axon part;axon terminus;cell part;cell projection;cell projection part;cerebellar mossy fiber;clathrin-coated vesicle;coated vesicle;contractile fiber part;cytoplasm;cytoplasmic membrane-bounded vesicle;cytoplasmic part;cytoplasmic vesicle;cytoskeleton;I band;initial segment;intracellular membrane-boun		Homo sapiens	1	16
cell part;chromatin;chromosomal part;cytoplasm;intracellular membrane-bounded organelle;intracellular organelle;intracellular organelle part;intracellular part;macromolecular complex;membrane-bounded organelle;nuclear chromatin;nuclear chromosome part;nuclear matrix;nuclear part;nucleoplasm;nucleus;organelle;organelle		Homo sapiens	1	16
adherens junction;anchoring junction;cell junction;cell part;cell-substrate adherens junction;cell-substrate junction;cytoplasmic part;focal adhesion;Golgi apparatus;intracellular membrane-bounded organelle;intracellular organelle;intracellular part;membrane;membrane-bounded organelle;organelle;perinuclear region of cytoplasr		Homo sapiens	1	16
cell part;cytoplasmic part;cytosol;intracellular part	Purine metabolism;Selenocompound metabolism;Sulfur metabolism	Homo sapiens	1	16
box C/D snoRNP complex;cell part;intracellular membrane-bounded organelle;intracellular non-membrane-bounded organelle;intracellular organelle;intracellular organelle part;intracellular part;macromolecular complex;membrane-bounded organelle;non-membrane-bounded organelle;nuclear part;nucleolar part;nucleolus;nucleu		Homo sapiens	1	16
cell part;cytoplasm;extracellular membrane-bounded organelle;extracellular organelle;extracellular region part;extracellular vesicular exosome;intracellular part;membrane-bounded organelle;membrane-bounded vesicle;orgai	Amino sugar and nucleotide sugar metabolism;Fructose and mannose metabolism	Homo sapiens	1	16
cell part;cytoplasm;extracellular membrane-bounded organelle;extracellular organelle;extracellular region part;extracellular vesicular exosome;intracellular;intracellular membrane-bounded organelle;intracellular non-membrane-bounded organelle;intracellular organelle;intracellular organelle part;intracellular part;membrane-bounc		Homo sapiens	1	16
cell part;cytoplasmic part;cytosol;extracellular membrane-bounded organelle;extracellular organelle;extracellular region part;extracellular vesicular exosome;intracellular part;membrane;membrane-bounded organelle;membra	Protein processing in endoplasmic reticulum	Homo sapiens	1	16
cell junction;cell part;cell projection;cell projection part;cytoplasm;cytoplasmic membrane-bounded vesicle;cytoplasmic part;cytoplasmic vesicle;cytoskeletal part;cytosol;dendritic spine head;endocytic vesicle;intracellular m	Axon guidance	Homo sapiens	1	16
axon;Cajal body;cell body;cell part;cell projection;cell projection part;cytoplasm;cytoplasmic part;growth cone;intracellular membrane-bounded organelle;intracellular non-membrane-bounded organelle;intracellular organelle;intracellular organelle part;intracellular part;macromolecular complex;membrane-bounded organelle;neur		Homo sapiens	1	16
cell part;cytoplasmic membrane-bounded vesicle;cytoplasmic part;cytoplasmic vesicle;endoplasmic reticulum membrane;endoplasmic reticulum part;endoplasmic reticulum-Golgi intermediate compartment;endoplasmic reti	Phagosome;SNARE interactions in vesicular transport	Homo sapiens	1	16
cell part;coated vesicle membrane;COPII vesicle coat;cytoplasmic part;cytoplasmic vesicle membrane;cytoplasmic vesicle part;cytosol;endoplasmic reticulum membrane;endoplasmic reticulum part;ER to Golgi transport ves	Protein processing in endoplasmic reticulum	Homo sapiens	1	16
cell part;cytoplasmic part;endoplasmic reticulum;endoplasmic reticulum membrane;endoplasmic reticulum part;Golgi apparatus;Golgi apparatus part;Golgi membrane;integral to membrane;intracellular membrane-bounded organelle;intracellular organelle;intracellular organelle part;intracellular part;intrinsic to membrane;membra		Homo sapiens	1	16
cell part;cytoplasmic part;intracellular organelle lumen;intracellular organelle part;intracellular part;macromolecular complex;membrane;membrane part;membrane-enclosed lumen;mitochondrial inner membrane;mitochondria	Alzheimer's disease;Huntington's disease;Oxidative phosphorylation;Parkinson's disease	Homo sapiens	1	16
cell part;cytoplasmic part;cytosol;external side of plasma membrane;extracellular membrane-bounded organelle;extracellular organelle;extracellular region part;extracellular vesicular exosome;intracellular organelle lumen;intr	Glutathione metabolism	Homo sapiens	1	16
cell body;cell part;cell projection cytoplasm;cell projection part;cytoplasm;cytoplasmic membrane-bounded vesicle;cytoplasmic part;cytoplasmic vesicle;cytosol;dendrite cytoplasm;dense core granule;extracellular matrix;ext	Amyotrophic lateral sclerosis (ALS);Huntington's disease;Peroxisome;Prion diseases	Homo sapiens	1	16
cell part;cytoplasmic part;cytoskeleton;cytosol;extracellular membrane-bounded organelle;extracellular organelle;extracellular region;extracellular region part;extracellular space;extracellular vesicular exosome;intracellular membrane-bounded organelle;intracellular non-membrane-bounded organelle;intracellular organelle;intrace		Homo sapiens	1	16
cell division site part;cell part;cleavage furrow;cytoplasmic part;cytosol;extracellular membrane-bounded organelle;extracellular organelle;extracellular region part;extracellular vesicular exosome;intracellular membrane-bounded organelle;intracellular organelle;intracellular part;membrane;membrane-bounded organelle;membrane		Homo sapiens	1	16
cell part;cytoplasm;cytoplasmic part;cytosol;extracellular membrane-bounded organelle;extracellular organelle;extracellular region part;extracellular space;extracellular vesicular exosome;intracellular;intracellular membrane-b	Drug metabolism - cytochrome P450;Glutathione metabolism;Metabolism of xenobiotics by cytochrome	Homo sapiens	1	16
cell part;clathrin coat;clathrin coat of coated pit;clathrin coat of trans-Golgi network vesicle;clathrin coated vesicle membrane;clathrin complex;clathrin vesicle coat;clathrin-coated endocytic vesicle membrane;coated vesicle	Bacterial invasion of epithelial cells;Endocrine and other factor-regulated calcium reabsorption;Endocyto	Homo sapiens	1	16
cell part;cytoplasmic part;intracellular membrane-bounded organelle;intracellular organelle;intracellular organelle lumen;intracellular organelle part;intracellular part;macromolecular complex;membrane-bounded organelle;mer	Citrate cycle (TCA cycle);Glycolysis / Gluconeogenesis;Pyruvate metabolism	Homo sapiens	1	16
cell part;cytoplasmic part;cytosol;intracellular part	Arginine and proline metabolism;beta-Alanine metabolism;Cysteine and methionine metabolism;Glutath	Homo sapiens	1	16
cell part;cytoplasm;cytoplasmic part;cytosol;eukaryotic translation elongation factor 1 complex;intracellular part;macromolecular complex;protein complex		Homo sapiens	1	16
cell part;intracellular organelle part;intracellular part;macromolecular complex;nuclear body;nuclear part;nuclear speck;nucleoplasm;nucleoplasm part;organelle part;ribonucleoprotein complex;spliceosomal complex	Spliceosome	Homo sapiens	1	16
		Homo sapiens	1	16
cell part;cytoplasm;extracellular membrane-bounded organelle;extracellular organelle;extracellular region part;extracellular vesicular exosome;intracellular membrane-bounded organelle;intracellular organelle;intracellular part;membrane-bounded organelle;membrane-bounded vesicle;nucleus;organelle;vesicle		Homo sapiens	1	16
cell part;extracellular membrane-bounded organelle;extracellular organelle;extracellular region part;extracellular vesicular exosome;membrane part;membrane raft;membrane-bounded organelle;membrane-bounded vesicle;o	Cell cycle - yeast;Chagas disease (American trypanosomiasis);Hepatitis C;Long-term depression;Meios	Homo sapiens	1	16
cell part;cell projection;cytoplasm;extracellular membrane-bounded organelle;extracellular organelle;extracellular region part;extracellular space;extracellular vesicular exosome;intracellular membrane-bounded organelle;intracellular organelle;intracellular organelle part;intracellular part;membrane-bounded organelle;membrane-b		Homo sapiens	1	16
cell part;cytoplasmic part;cytoplasmic vesicle;cytosol;extracellular region part;Golgi apparatus part;Golgi membrane;host cell part;intracellular membrane-bounded organelle;intracellular organelle;intracellular organelle part;intracellular part;membrane;membrane-bounded organelle;nucleus;organelle;organelle membrane;organell		Homo sapiens	1	16
cell part;chromosomal part;cytoplasm;DNA replication factor C complex;intracellular membrane-bounded organelle;intracellular organelle;intracellular organelle part;intracellular part;macromolecular complex;membrane-bour	DNA replication;Mismatch repair;Nucleotide excision repair	Homo sapiens	1	16
cell junction;cell part;chromosomal part;cytoplasmic part;DNA replication factor C complex;extracellular membrane-bounded organelle;extracellular organelle;extracellular region part;extracellular vesicular exosome;Golgi app	DNA replication;Mismatch repair;Nucleotide excision repair	Homo sapiens	1	16
cell part;chromosomal part;DNA replication factor C complex;intracellular membrane-bounded organelle;intracellular organelle;intracellular organelle part;intracellular part;macromolecular complex;membrane-bounded organe	DNA replication;Mismatch repair;Nucleotide excision repair	Homo sapiens	1	16
cell part;cytoplasm;envelope;intracellular membrane-bounded organelle;intracellular organelle;intracellular organelle part;intracellular part;membrane-bounded organelle;nuclear envelope;nuclear part;nucleus;organelle;organelle envelope;organelle part		Homo sapiens	1	16
BAT3 complex;cell part;cytoplasm;cytoplasmic part;cytosol;cytosolic part;ER membrane insertion complex;intracellular membrane-bounded organelle;intracellular organelle;intracellular organelle part;intracellular part;macromolecular complex;membrane-bounded organelle;nuclear part;nucleoplasm;nucleus;organelle;organelle p		Homo sapiens	1	16
cell part;cytoplasmic part;intracellular membrane-bounded organelle;intracellular organelle;intracellular organelle lumen;intracellular organelle part;intracellular part;membrane-bounded organelle;membrane-enclosed lumen;r	Aminoacyl-tRNA biosynthesis	Homo sapiens	1	16
cell part;cytoplasm;cytoplasmic part;cytosol;extracellular membrane-bounded organelle;extracellular organelle;extracellular region part;extracellular vesicular exosome;intracellular membrane-bounded organelle;intracellular c	Proteasome	Homo sapiens	1	16
cell part;chromatin;chromosomal part;cytoplasmic part;cytosol;intracellular membrane-bounded organelle;intracellular organelle;intracellular organelle part;intracellular part;macromolecular complex;membrane-bounded orga	Homologous recombination;Non-homologous end-joining	Homo sapiens	1	16
cell body;cell part;cell projection;cytoplasm;cytoplasmic part;cytosol;intracellular membrane-bounded organelle;intracellular organelle;intracellular organelle part;intracellular part;membrane;membrane-bounded organelle;neu	MAPK signaling pathway	Homo sapiens	1	16
cell part;cytoplasm;intracellular organelle part;intracellular part;macromolecular complex;nuclear part;organelle part;protein complex;signalosome		Homo sapiens	1	16
cell part;cytoplasm;cytoplasmic part;cytosol;cytosolic small ribosomal subunit;intracellular non-membrane-bounded organelle;intracellular organelle;intracellular organelle part;intracellular part;macromolecular complex;memb	Ribosome	Homo sapiens	1	16
90S preribosome;cell part;cytoplasmic part;intracellular membrane-bounded organelle;intracellular non-membrane-bounded organelle;intracellular organelle;intracellular organelle part;intracellular part;macromolecular complex;membrane;membrane-bounded organelle;mitochondrion;Noc complex;Noc4p-Nop14p complex;non-r		Homo sapiens	1	16
cell part;cytoplasm;intracellular membrane-bounded organelle;intracellular non-membrane-bounded organelle;intracellular organelle;intracellular organelle part;intracellular part;membrane-bounded organelle;non-membrane-t	Calcium signaling pathway;Huntington's disease;Parkinson's disease	Homo sapiens	1	16
cell part;cytoplasmic part;cytosol;endoplasmic reticulum;extracellular membrane-bounded organelle;extracellular organelle;extracellular region part;extracellular space;extracellular vesicular exosome;intracellular membrane-bounded organelle;intracellular organelle;intracellular part;membrane-bounded organelle;membrane-bour		Homo sapiens	1	16
cell part;cytoplasm;intracellular membrane-bounded organelle;intracellular organelle;intracellular organelle part;intracellular part;membrane-bounded organelle;nuclear body;nuclear matrix;nuclear part;nuclear speck;nucleoplasm;nucleoplasm part;nucleus;organelle;organelle part		Homo sapiens	1	16
cell part;cytoplasm;extracellular membrane-bounded organelle;extracellular organelle;extracellular region part;extracellular vesicular exosome;intracellular part;membrane-bounded organelle;membrane-bounded vesicle;orgai	Amino sugar and nucleotide sugar metabolism;Fructose and mannose metabolism	Homo sapiens	1	16
cell part;intracellular membrane-bounded organelle;intracellular non-membrane-bounded organelle;intracellular organelle;intracellular organelle part;intracellular part;membrane;membrane-bounded organelle;non-membrane-	Ribosome biogenesis in eukaryotes	Homo sapiens	1	16
cell part;cytoplasmic part;cytoskeletal part;cytoskeleton;intracellular non-membrane-bounded organelle;intracellular organelle;intracellular organelle part;intracellular part;microtubule cytoskeleton;microtubule organizing center;non-membrane-bounded organelle;nuclear body;nuclear part;nuclear speck;nucleoplasm;nucleoplasm		Homo sapiens	1	16
cell part;cytoplasm;intracellular membrane-bounded organelle;intracellular organelle;intracellular organelle part;intracellular part;membrane-bounded organelle;nuclear body;nuclear part;nuclear speck;nucleoplasm;nucleoplasm part;organelle;organelle part		Homo sapiens	1	16
cell part;cell projection membrane;cell projection part;clathrin-coated endocytic vesicle;clathrin-coated vesicle;coated pit;coated vesicle;cytoplasmic membrane-bounded vesicle;cytoplasmic part;cytoplasmic vesicle;endocytic vesicle;endosomal part;endosome membrane;extracellular membrane-bounded organelle;extracellular		Homo sapiens	1	16
cell part;cytoplasm;cytoskeleton;intracellular non-membrane-bounded organelle;intracellular organelle;intracellular part;non-membrane-bounded organelle;organelle	Pathways in cancer;Thyroid cancer	Homo sapiens	1	16
cell part;intracellular non-membrane-bounded organelle;intracellular organelle;intracellular organelle part;intracellular part;non-membrane-bounded organelle;nuclear part;nucleolus;organelle;organelle part		Homo sapiens	1	16
blood microparticle;cell part;cytoplasmic part;early endosome;endosome;extracellular region part;intracellular membrane-bounded organelle;intracellular organelle;intracellular part;membrane-bounded organelle;organelle		Homo sapiens	1	16
extracellular membrane-bounded organelle;extracellular organelle;extracellular region part;extracellular vesicular exosome;membrane-bounded organelle;membrane-bounded vesicle;organelle;vesicle	p53 signaling pathway	Homo sapiens	1	16
actin filament;axon;cell part;cell projection;cell projection part;cytoplasmic part;cytoskeletal part;early endosome;endosome;growth cone;intracellular membrane-bounded organelle;intracellular organelle;intracellular organelle part;intracellular part;macromolecular complex;membrane;membrane-bounded organelle;neuron projec		Homo sapiens	1	16
cell part;cytoplasmic part;early endosome;early endosome membrane;endosomal part;endosome;endosome membrane;intracellular membrane-bounded organelle;intracellular organelle;intracellular organelle part;intracellular part;macromolecular complex;membrane;membrane-bounded organelle;organelle;organelle membrane;		Homo sapiens	1	16
cell part;cytoplasmic part;endoplasmic reticulum;endoplasmic reticulum membrane;endoplasmic reticulum part;integral to membrane;intracellular membrane-bounded organelle;intracellular organelle;intracellular organelle part;intracellular part;intrinsic to membrane;membrane;membrane part;membrane-bounded organelle;orgar		Homo sapiens	1	16
cell part;cytoplasmic part;endoplasmic reticulum membrane;endoplasmic reticulum part;integral to endoplasmic reticulum membrane;integral to membrane;integral to organelle membrane;intracellular organelle part;intracellular part;intrinsic to endoplasmic reticulum membrane;intrinsic to membrane;intrinsic to organelle membra		Homo sapiens	1	16
cell part;cytoplasmic part;cytoplasmic vesicle membrane;cytoplasmic vesicle part;cytosol;Golgi apparatus part;Golgi membrane;Golgi-associated vesicle membrane;intracellular organelle part;intracellular part;membrane;orga	Endocytosis	Homo sapiens	1	16
cell part;integral to membrane;integral to nuclear inner membrane;integral to organelle membrane;intracellular organelle part;intracellular part;intrinsic to membrane;intrinsic to nuclear inner membrane;intrinsic to organelle membrane;membrane;membrane part;nuclear membrane;nuclear membrane part;nuclear part;organelle me		Homo sapiens	1	16
cell part;intracellular membrane-bounded organelle;intracellular non-membrane-bounded organelle;intracellular organelle;intracellular organelle part;intracellular part;membrane-bounded organelle;non-membrane-bounded organelle;nuclear body;nuclear part;nuclear speck;nucleolus;nucleoplasm part;nucleus;organelle;organelle		Homo sapiens	1	16
Cajal body;cell part;histone methyltransferase complex;intracellular membrane-bounded organelle;intracellular organelle;intracellular organelle part;intracellular part;macromolecular complex;membrane-bounded organelle;me	Spliceosome	Homo sapiens	1	16
cell part;cytoplasmic part;intracellular membrane-bounded organelle;intracellular organelle;intracellular part;membrane-bounded organelle;mitochondrion;organelle		Homo sapiens	1	16
Cdc48p-Npl4p-Ufd1p AAA ATPase complex;cell part;cytoplasmic part;endoplasmic reticulum;endoplasmic reticulum part;intracellular membrane-bounded organelle;intracellular organelle;intracellular organelle part;intracellular part;lipid particle;macromolecular complex;membrane part;membrane-bounded organelle;organelle;or		Homo sapiens	1	16
		Homo sapiens	1	16
cell part;cytoplasm;intracellular non-membrane-bounded organelle;intracellular organelle;intracellular organelle part;intracellular part;non-membrane-bounded organelle;nuclear part;nucleolus;organelle;organelle part		Homo sapiens	1	16
cell part;intracellular membrane-bounded organelle;intracellular organelle;intracellular organelle part;intracellular part;membrane-bounded organelle;nuclear body;nuclear part;nucleoplasm part;nucleus;organelle;organelle part		Homo sapiens	1	16
		Homo sapiens	1	16
cell part;cytoplasm;cytoplasmic part;Golgi apparatus;Golgi apparatus part;intracellular membrane-bounded organelle;intracellular non-membrane-bounded organelle;intracellular organelle;intracellular organelle part;intracellular part;macromolecular complex;membrane;membrane-bounded organelle;non-membrane-bounded org		Homo sapiens	1	16
cell part;cytoplasm;cytoplasmic part;cytosol;intracellular part	Cysteine and methionine metabolism;One carbon pool by folate;Selenocompound metabolism	Homo sapiens	1	16
cell part;intracellular membrane-bounded organelle;intracellular non-membrane-bounded organelle;intracellular organelle;intracellular organelle part;intracellular part;membrane;membrane-bounded organelle;non-membrane-bounded organelle;nuclear part;nucleolus;nucleus;organelle;organelle part		Homo sapiens	1	16
cell part;cytoplasmic part;intracellular membrane-bounded organelle;intracellular organelle;intracellular organelle part;intracellular part;membrane-bounded organelle;mitochondrion;nuclear part;nucleoplasm;nucleus;organelle	RNA transport	Homo sapiens	1	16
cell part;extracellular region part;extracellular space;intracellular membrane-bounded organelle;intracellular non-membrane-bounded organelle;intracellular organelle;intracellular organelle part;intracellular part;membrane;mer	Ribosome biogenesis in eukaryotes	Homo sapiens	1	16
cell part;cytoplasm;intracellular membrane-bounded organelle;intracellular organelle;intracellular part;membrane-bounded organelle;nucleus;organelle		Homo sapiens	1	16
cell part;cytoplasm;intracellular organelle part;intracellular part;nuclear part;nucleoplasm;organelle part	Ubiquitin mediated proteolysis	Homo sapiens	1	16
cell part;cytoplasmic part;intracellular membrane-bounded organelle;intracellular organelle;intracellular organelle lumen;intracellular organelle part;intracellular part;membrane-bounded organelle;membrane-enclosed lumen;mitochondrial matrix;mitochondrial part;mitochondrion;organelle;organelle lumen;organelle part		Homo sapiens	1	16
cell part;cytoplasmic part;cytoskeletal part;endosome;Golgi apparatus part;intracellular membrane-bounded organelle;intracellular organelle;intracellular organelle part;intracellular part;membrane;membrane-bounded organe	Ubiquitin mediated proteolysis	Homo sapiens	1	16
cell part;cytoplasm;intracellular membrane-bounded organelle;intracellular organelle;intracellular organelle part;intracellular part;membrane-bounded organelle;nuclear part;nucleoplasm;nucleus;organelle;organelle part	Endocytosis	Homo sapiens	1	16
cell part;cytoplasmic part;intracellular membrane-bounded organelle;intracellular organelle;intracellular part;membrane-bounded organelle;mitochondrion;nucleus;organelle		Homo sapiens	1	16
cell part;cytoplasm;intracellular membrane-bounded organelle;intracellular organelle;intracellular part;membrane-bounded organelle;nucleus;organelle	MAPK signaling pathway;Tight junction	Homo sapiens	1	16
cell part;cytoplasmic part;cytosol;endoplasmic reticulum;endoplasmic reticulum lumen;endoplasmic reticulum membrane;endoplasmic reticulum part;extracellular membrane-bounded organelle;extracellular organelle;extracellular region;extracellular region part;extracellular space;extracellular vesicular exosome;integral to memb		Homo sapiens	1	16
cell part;cytoplasm;intracellular membrane-bounded organelle;intracellular non-membrane-bounded organelle;intracellular organelle;intracellular organelle part;intracellular part;membrane;membrane-bounded organelle;non-membrane-bounded organelle;nuclear membrane;nuclear part;nucleolus;nucleoplasm;nucleus;organelle;c		Homo sapiens	1	16
cell part;cytoplasm;cytoplasmic part;cytosol;endosomal part;endosome membrane;ESCRT III complex;extracellular membrane-bounded organelle;extracellular organelle;extracellular region part;extracellular vesicular exosom	Endocytosis	Homo sapiens	1	16
actin cytoskeleton;cell part;contractile fiber part;cytoplasmic part;cytoskeleton;extracellular membrane-bounded organelle;extracellular organelle;extracellular region part;extracellular space;extracellular vesicular exosome;I b	Axon guidance;Fc gamma R-mediated phagocytosis;Regulation of actin cytoskeleton	Homo sapiens	1	16
cell part;intracellular membrane-bounded organelle;intracellular non-membrane-bounded organelle;intracellular organelle;intracellular organelle part;intracellular part;membrane-bounded organelle;non-membrane-bounded organelle;nuclear part;nucleolus;nucleoplasm;nucleus;organelle;organelle part		Homo sapiens	1	16
cell part;cell projection;cytoplasmic part;cytoplasmic vesicle membrane;cytoplasmic vesicle part;early endosome membrane;endosomal part;endosome membrane;extrinsic to endosome membrane;extrinsic to internal side of plasma membrane;extrinsic to membrane;extrinsic to organelle membrane;extrinsic to plasma membra		Homo sapiens	1	16
cell part;chromatin;chromosomal part;cytoplasmic part;intracellular membrane-bounded organelle;intracellular non-membrane-bounded organelle;intracellular organelle;intracellular organelle part;intracellular part;macromolecular complex;membrane-bounded organelle;mitochondrion;non-membrane-bounded organelle;nuclear c		Homo sapiens	1	16
cell part;cis-Golgi network;coated vesicle membrane;cytoplasm;cytoplasmic part;cytoplasmic vesicle membrane;cytoplasmic vesicle part;endoplasmic reticulum exit site;endoplasmic reticulum part;endoplasmic reticulum-Golgi intermediate compartment;ER to Golgi transport vesicle membrane;Golgi apparatus part;intracellular		Homo sapiens	1	16
cell part;cytoplasmic part;extracellular region;intracellular membrane-bounded organelle;intracellular organelle;intracellular part;membrane-bounded organelle;mitochondrion;organelle		Homo sapiens	1	17
cell part;chromatin;chromosomal part;cytoplasm;cytoplasmic part;cytosol;intracellular membrane-bounded organelle;intracellular organelle;intracellular organelle part;intracellular part;membrane-bounded organelle;nuclear cl	Apoptosis	Homo sapiens	1	17
cell part;cytoplasm;cytoplasmic membrane-bounded vesicle;cytoplasmic part;cytoplasmic vesicle;cytosol;early endosome;early endosome membrane;endosomal part;endosome;endosome membrane;extracellular membran	Endocytosis;Phagosome	Homo sapiens	1	17
cell part;cytoplasm;cytoplasmic part;intracellular part;perinuclear region of cytoplasm		Homo sapiens	1	17
cell part;chromosomal part;condensed chromosome kinetochore;cytoplasmic part;cytosol;intracellular non-membrane-bounded organelle;intracellular organelle;intracellular organelle part;intracellular part;kinetochore;macron	Cell cycle;Cell cycle - yeast	Homo sapiens	1	17
macromolecular complex;protein complex;ribose phosphate diphosphokinase complex		Homo sapiens	1	17
acrosomal vesicle;cell part;cytoplasm;cytoplasmic membrane-bounded vesicle;cytoplasmic part;cytoplasmic vesicle;cytosol;extracellular membrane-bounded organelle;extracellular organelle;extracellular region part;extracellular vesicular exosome;integral to membrane;intracellular membrane-bounded organelle;intracellular org		Homo sapiens	1	17
adherens junction;anchoring junction;cell junction;cell part;cell-substrate adherens junction;cell-substrate junction;cytoplasmic part;focal adhesion;Golgi apparatus;intracellular membrane-bounded organelle;intracellular orga	Axon guidance;ErbB signaling pathway;Focal adhesion;Regulation of actin cytoskeleton;Renal cell carci	Homo sapiens	1	17

apical part of cell;basolateral plasma membrane;cell part;cell surface;clathrin coated vesicle membrane;clathrin-coated endocytic vesicle membrane;coated pit;coated vesicle membrane;cytoplasmic part;cytoplasmic vesicle	Bile secretion;Endocytosis;Hepatitis C;Toxoplasmosis	Homo sapiens		1	17
catalytic step 2 spliceosome;cell part;intracellular organelle part;intracellular part;macromolecular complex;nuclear part;nucleoplasm;organelle part;ribonucleoprotein complex;small nuclear ribonucleoprotein complex;spliceos	Spliceosome	Homo sapiens		1	17
AMP-activated protein kinase complex;cAMP-dependent protein kinase complex;cell part;cytoplasmic part;cytosol;intracellular part;macromolecular complex;membrane;neuromuscular junction;plasma membrane;protein co	Apoptosis;Insulin signaling pathway	Homo sapiens		1	17
cell part;cytoplasmic part;extracellular membrane-bounded organelle;extracellular organelle;extracellular region part;extracellular vesicular exosome;intracellular membrane-bounded organelle;intracellular organelle;intracellula	Butanoate metabolism;Citrate cycle (TCA cycle);Glycolysis / Gluconeogenesis;Pyruvate metabolism;Vali	Homo sapiens		1	17
cell part;cytoplasm;cytoplasmic part;endosome;extracellular membrane-bounded organelle;extracellular organelle;extracellular region part;extracellular space;extracellular vesicular exosome;intracellular membrane-bounded organelle;intracellular organelle;intracellular part;late endosome;membrane-bounded organelle;membran		Homo sapiens		1	17
apical part of cell;cell part;cytoplasmic part;cytoplasmic vesicle;cytosol;extracellular membrane-bounded organelle;extracellular organelle;extracellular region part;extracellular vesicular exosome;intracellular organelle;intracel	Collecting duct acid secretion;Epithelial cell signaling in Helicobacter pylori infection;Oxidative phospho	Homo sapiens		1	17
box C/D snoRNP complex;Cajal body;cell part;extracellular membrane-bounded organelle;extracellular organelle;extracellular region part;extracellular vesicular exosome;granular component;intracellular membrane-bounded	Ribosome biogenesis in eukaryotes	Homo sapiens		1	17
cell part;cytoplasm;cytoplasmic mRNA processing body;cytoplasmic part;cytosol;intracellular membrane-bounded organelle;intracellular non-membrane-bounded organelle;intracellular organelle;intracellular organelle part;int	RNA degradation	Homo sapiens		1	17
cell part;cytoplasmic part;extracellular membrane-bounded organelle;extracellular organelle;extracellular region part;extracellular space;extracellular vesicular exosome;intracellular membrane-bounded organelle;intracellular organelle;intracellular part;membrane-bounded organelle;membrane-bounded vesicle;mitochondrion;org		Homo sapiens		1	17
cell part;cytoplasmic part;extracellular membrane-bounded organelle;extracellular organelle;extracellular region part;extracellular vesicular exosome;intracellular membrane-bounded organelle;intracellular organelle;intracellula	Aminobenzoate degradation;Benzoate degradation;beta-Alanine metabolism;Butanoate metabolism;Ca	Homo sapiens		1	17
cell part;cytoplasmic part;cytosol;extracellular region;Golgi apparatus part;Golgi membrane;intracellular organelle part;intracellular part;membrane;organelle membrane;organelle part		Homo sapiens		1	17
cell part;extracellular region part;extracellular space;intracellular membrane-bounded organelle;intracellular organelle;intracellular part;membrane-bounded organelle;nucleus;organelle		Homo sapiens		1	17
actin cytoskeleton;cell cortex part;cell part;cortical cytoskeleton;cytoplasmic part;cytoskeletal part;cytoskeleton;cytosol;extracellular membrane-bounded organelle;extracellular organelle;extracellular region;extracellular region part;extracellular vesicular exosome;F-actin capping protein complex;intracellular non-membrane-bou		Homo sapiens		1	17
cell part;cis-Golgi network;clathrin-coated vesicle;coated vesicle;COPI-coated vesicle;cytoplasmic membrane-bounded vesicle;cytoplasmic part;cytoplasmic vesicle;cytoplasmic vesicle membrane;cytoplasmic vesicle part;endoplasmic reticulum;endoplasmic reticulum membrane;endoplasmic reticulum part;endoplasmic reticulu		Homo sapiens		1	17
cell part;chromatin remodeling complex;cytoplasm;cytoskeleton;extracellular membrane-bounded organelle;extracellular organelle;extracellular region part;extracellular vesicular exosome;histone deacetylase complex;intracellular membrane-bounded organelle;intracellular non-membrane-bounded organelle;intracellular organell		Homo sapiens		1	17
cell part;cytoplasm;cytoplasmic part;cytosol;intracellular organelle part;intracellular part;membrane;nuclear part;nucleoplasm;organelle part		Homo sapiens		1	17
cell part;cytoplasmic part;cytosol;intracellular part;membrane;plasma membrane	B cell receptor signaling pathway;Chemokine signaling pathway;Fc epsilon RI signaling pathway;Fc gan	Homo sapiens		1	17
cell part;cytoplasmic part;cytosol;extracellular membrane-bounded organelle;extracellular organelle;extracellular region part;extracellular vesicular exosome;intracellular part;membrane-bounded organelle;membrane-bounde	Arginine and proline metabolism;beta-Alanine metabolism;Cysteine and methionine metabolism;Glutath	Homo sapiens		1	17
cell part;extracellular membrane-bounded organelle;extracellular organelle;extracellular region part;extracellular vesicular exosome;intracellular membrane-bounded organelle;intracellular non-membrane-bounded organelle;intracellular organelle;intracellular organelle part;intracellular part;macromolecular complex;membrane-bou		Homo sapiens		1	17
cell junction;cell part;intracellular membrane-bounded organelle;intracellular organelle;intracellular organelle part;intracellular part;macromolecular complex;membrane-bounded organelle;nuclear part;nucleoplasm part;nucleu	Chronic myeloid leukemia;Notch signaling pathway;Pathways in cancer;Wnt signaling pathway	Homo sapiens		1	17
cell part;centrosome;cytoplasm;cytoplasmic part;cytoskeletal part;cytosol;dynactin complex;extracellular membrane-bounded organelle;extracellular organelle;extracellular region part;extracellular vesicular exosome;intracellular non-membrane-bounded organelle;intracellular organelle;intracellular organelle part;intracellular part;		Homo sapiens		1	17
cell part;cytoplasm;cytoplasmic part;cytosol;intracellular membrane-bounded organelle;intracellular organelle;intracellular organelle part;intracellular part;macromolecular complex;membrane;membrane-bounded organelle;nu	Antigen processing and presentation;Hepatitis C;Proteasome	Homo sapiens		1	17
adherens junction;anchoring junction;apical junction complex;cell cortex;cell division site part;cell junction;cell part;cell projection;cell-cell junction;cell-substrate adherens junction;cell-substrate junction;cleavage furrow;cyto	Adherens junction;Axon guidance;Bacterial invasion of epithelial cells;Chemokine signaling pathway;Co	Homo sapiens		1	17
cell part;cytoplasmic part;cytosol;cytosolic large ribosomal subunit;intracellular organelle part;intracellular part;large ribosomal subunit;macromolecular complex;organelle part;ribonucleoprotein complex	Ribosome	Homo sapiens		1	17
cell part;cell projection;cell projection part;cytoplasm;cytoplasmic part;cytosol;dendritic spine;extracellular membrane-bounded organelle;extracellular organelle;extracellular region part;extracellular vesicular exosome;glycog	Focal adhesion;Insulin signaling pathway;Long-term potentiation;Meiosis - yeast;Oocyte meiosis;Regula	Homo sapiens		1	17
adherens junction;anchoring junction;cell junction;cell part;cell-substrate adherens junction;cell-substrate junction;cytoplasmic part;cytosol;extracellular membrane-bounded organelle;extracellular organelle;extracellular regio	Focal adhesion;Insulin signaling pathway;Long-term potentiation;Meiosis - yeast;Oocyte meiosis;Regula	Homo sapiens		1	17
cell part;cytoplasm;cytoplasmic part;cytosol;cytosolic small ribosomal subunit;extracellular membrane-bounded organelle;extracellular organelle;extracellular region part;extracellular vesicular exosome;intracellular organelle	Ribosome	Homo sapiens		1	17
cell part;cytoplasm;cytoplasmic part;cytosol;intracellular part;macromolecular complex;protein complex;translation release factor complex	mRNA surveillance pathway	Homo sapiens		1	17
cell part;cytoplasm;cytoplasmic part;cytosol;cytosolic small ribosomal subunit;extracellular membrane-bounded organelle;extracellular organelle;extracellular region part;extracellular vesicular exosome;intracellular organelle	Ribosome	Homo sapiens		1	17
cell part;cytoplasm;cytoplasmic part;cytosol;cytosolic small ribosomal subunit;intracellular organelle part;intracellular part;macromolecular complex;organelle part;ribonucleoprotein complex;small ribosomal subunit	Ribosome	Homo sapiens		1	17
cell part;cytoplasmic part;cytosol;intracellular part	Alanine, aspartate and glutamate metabolism;Purine metabolism	Homo sapiens		1	17
cell part;cytoplasm;cytoplasmic part;cytosol;extracellular membrane-bounded organelle;extracellular organelle;extracellular region part;extracellular vesicular exosome;intracellular organelle part;intracellular part;macromolec	Antigen processing and presentation;Proteasome	Homo sapiens		1	17
cell body;cell part;cell projection;cell projection part;cytoplasm;cytoplasmic part;cytoskeletal part;dendritic shaft;dendritic spine;intracellular non-membrane-bounded organelle;intracellular organelle;intracellular organelle part;intracellular part;macromolecular complex;membrane;microtubule;mRNA cap binding complex;neuron p		Homo sapiens		1	17
cell part;intracellular membrane-bounded organelle;intracellular organelle;intracellular organelle part;intracellular part;membrane-bounded organelle;nuclear body;nuclear part;nuclear speck;nucleoplasm;nucleoplasm part;nuc	Spliceosome	Homo sapiens		1	17
cell part;intracellular organelle part;intracellular part;macromolecular complex;mRNA cleavage and polyadenylation specificity factor complex;mRNA cleavage factor complex;nuclear part;nucleoplasm;nucleoplasm part;orgar	mRNA surveillance pathway	Homo sapiens		1	17
cell part;cytoplasmic part;cytosol;early endosome;endoplasmic reticulum;endosome;intracellular membrane-bounded organelle;intracellular organelle;intracellular part;macromolecular complex;membrane-bounded organelle;organelle;protein complex;WASH complex		Homo sapiens		1	17
cell part;cytoplasm;cytoplasmic part;cytosol;intracellular membrane-bounded organelle;intracellular organelle;intracellular part;membrane;membrane-bounded organelle;nucleus;organelle;perinuclear region of cytoplasm		Homo sapiens		1	17
cell part;cytoplasm;cytoplasmic part;cytosol;intracellular part	beta-Alanine metabolism;Drug metabolism - other enzymes;Pantothenate and CoA biosynthesis;Pyrimic	Homo sapiens		1	17
cell part;chromosomal part;condensed chromosome kinetochore;cytoplasm;intracellular membrane-bounded organelle;intracellular non-membrane-bounded organelle;intracellular organelle;intracellular organelle part;intracellular part;kinetochore;macromolecular complex;membrane-bounded organelle;non-membrane-bounded		Homo sapiens		1	17
cell part;cytoplasm;intracellular membrane-bounded organelle;intracellular non-membrane-bounded organelle;intracellular organelle;intracellular organelle part;intracellular part;macromolecular complex;membrane;membrane-bounded organelle;non-membrane-bounded organelle;nuclear part;nucleolus;nucleoplasm;nucleus;orga		Homo sapiens		1	17
actin cytoskeleton;cell cortex;cell part;cell projection;cell projection part;cytoplasm;cytoplasmic part;cytoskeletal part;cytoskeleton;cytosol;filamentous actin;filopodium tip;intracellular non-membrane-bounded organelle;intracellular organelle;intracellular organelle part;intracellular part;lamellipodium;macromolecular complex;me		Homo sapiens		1	17
cell part;intracellular membrane-bounded organelle;intracellular non-membrane-bounded organelle;intracellular organelle;intracellular organelle part;intracellular part;membrane-bounded organelle;non-membrane-bounded or	Spliceosome	Homo sapiens		1	17
adherens junction;anchoring junction;cell junction;cell part;cell-substrate adherens junction;cell-substrate junction;cytoplasm;cytoplasmic part;cytosol;focal adhesion;intracellular membrane-bounded organelle;intracellular organelle;intracellular part;membrane;membrane-bounded organelle;nucleus;organelle;plasma membrane		Homo sapiens		1	17
cell part;cytoplasm;cytoplasmic part;intracellular membrane-bounded organelle;intracellular non-membrane-bounded organelle;intracellular organelle;intracellular organelle part;intracellular part;macromolecular complex;membrane-bounded organelle;non-membrane-bounded organelle;nuclear part;nucleoplasm;nucleus;organell		Homo sapiens		1	17
cell part;intracellular organelle part;intracellular part;macromolecular complex;nuclear part;nucleolar part;nucleoplasm;organelle part;PeBoW complex;preribosome;preribosome, large subunit precursor;protein complex;ribonucleoprotein complex		Homo sapiens		1	17
cell part;clathrin-coated vesicle;coated vesicle;cytoplasmic membrane-bounded vesicle;cytoplasmic part;cytoplasmic vesicle;cytosol;Golgi apparatus;intracellular membrane-bounded organelle;intracellular organelle;intracellular organelle part;intracellular part;membrane;membrane-bounded organelle;membrane-bounded vesicl		Homo sapiens		1	17
cell part;cytoplasm;cytoplasmic part;Golgi apparatus;intracellular membrane-bounded organelle;intracellular organelle;intracellular organelle part;intracellular part;membrane-bounded organelle;nuclear part;nucleoplasm;organelle;organelle part		Homo sapiens		1	17
cell part;cytoplasmic part;endoplasmic reticulum membrane;endoplasmic reticulum part;integral to membrane;intracellular organelle part;intracellular part;intrinsic to membrane;macromolecular complex;membrane;membran	Protein export	Homo sapiens		1	17
cell part;chromosomal part;chromosome, telomeric region;cytoplasm;cytoplasmic part;cytosol;extracellular membrane-bounded organelle;extracellular organelle;extracellular region part;extracellular vesicular exosome;intracellular membrane-bounded organelle;intracellular organelle;intracellular organelle part;intracellular part;ma		Homo sapiens		1	17
actin cytoskeleton;adherens junction;anchoring junction;cell junction;cell part;cell-substrate adherens junction;cell-substrate junction;cytoplasm;cytoskeleton;focal adhesion;intracellular non-membrane-bounded organelle;intracellular organelle;intracellular part;non-membrane-bounded organelle;organelle		Homo sapiens		1	17
cell part;cytoplasmic part;intracellular membrane-bounded organelle;intracellular non-membrane-bounded organelle;intracellular organelle;intracellular organelle part;intracellular part;macromolecular complex;membrane-bounded organelle;non-membrane-bounded organelle;nuclear part;nucleoplasm;nucleus;organelle;organelle		Homo sapiens		1	17
cell cortex;cell part;cell projection;cytoplasm;cytoplasmic part;cytoskeleton;cytosol;extracellular membrane-bounded organelle;extracellular organelle;extracellular region part;extracellular vesicular exosome;Golgi apparatus;i	Insulin signaling pathway	Homo sapiens		1	17
adherens junction;anchoring junction;cell junction;cell part;cell-substrate adherens junction;cell-substrate junction;cytoplasm;cytoskeleton;focal adhesion;intracellular membrane-bounded organelle;intracellular non-membran	NOD-like receptor signaling pathway	Homo sapiens		1	17
cell part;cytoplasm;extracellular membrane-bounded organelle;extracellular organelle;extracellular region part;extracellular vesicular exosome;intracellular membrane-bounded organelle;intracellular organelle;intracellular orga	Spliceosome	Homo sapiens		1	17
cell part;cytoplasmic part;endoplasmic reticulum;endoplasmic reticulum lumen;endoplasmic reticulum part;extracellular matrix;extracellular membrane-bounded organelle;extracellular organelle;extracellular region part;extracellular vesicular exosome;intracellular membrane-bounded organelle;intracellular organelle;intracellular o		Homo sapiens		1	17
cell part;cytoplasmic part;cytosol;extracellular membrane-bounded organelle;extracellular organelle;extracellular region part;extracellular vesicular exosome;intracellular membrane-bounded organelle;intracellular organelle;int	Glycerolipid metabolism;Methane metabolism;RIG-I-like receptor signaling pathway	Homo sapiens		1	17
cell part;cytoplasmic part;integral to membrane;intracellular membrane-bounded organelle;intracellular organelle;intracellular organelle part;intracellular part;intrinsic to membrane;macromolecular complex;membrane;membrane part;membrane-bounded organelle;mitochondrial inner membrane;mitochondrial inner membrane pr		Homo sapiens		1	17
cell part;cytoplasmic part;endosome;intracellular membrane-bounded organelle;intracellular organelle;intracellular organelle part;intracellular part;membrane;membrane-bounded organelle;mitochondrial inner membrane;mitochondrial membrane;mitochondrial part;organelle;organelle inner membrane;organelle membrane;organe		Homo sapiens		1	17
		Homo sapiens		1	17
cell part;cytoplasmic part;Golgi apparatus;intracellular membrane-bounded organelle;intracellular organelle;intracellular part;membrane-bounded organelle;organelle		Homo sapiens		1	17
cell part;extracellular membrane-bounded organelle;extracellular organelle;extracellular region part;extracellular vesicular exosome;extrinsic to membrane;intracellular;membrane part;membrane-bounded organelle;membrane-bounded vesicle;organelle;vesicle		Homo sapiens		1	17
cell part;intracellular organelle part;intracellular part;nuclear part;nucleoplasm;organelle part		Homo sapiens		1	17
adherens junction;anchoring junction;cell junction;cell part;cell-substrate adherens junction;cell-substrate junction;focal adhesion;membrane;plasma membrane		Homo sapiens		1	17
cell part;intracellular membrane-bounded organelle;intracellular organelle;intracellular organelle part;intracellular part;macromolecular complex;membrane;membrane-bounded organelle;mRNA cleavage factor complex;nuclea	mRNA surveillance pathway	Homo sapiens		1	17
cell junction;cell part;cytoplasm;extracellular region part;intercellular bridge;intracellular membrane-bounded organelle;intracellular organelle;intracellular part;membrane-bounded organelle;nucleus;organelle		Homo sapiens		1	17
cell part;chromatin;chromosomal part;intracellular organelle part;intracellular part;macromolecular complex;nuclear body;nuclear chromatin;nuclear chromosome part;nuclear part;nuclear speck;nucleoplasm;nucleoplasm part;organelle part;protein complex		Homo sapiens		1	17
cell part;cytoplasmic part;intracellular non-membrane-bounded organelle;intracellular organelle;intracellular organelle part;intracellular part;macromolecular complex;membrane;mitochondrial inner membrane;mitochondrial membrane;mitochondrial part;non-membrane-bounded organelle;organelle;organelle inner membrane;orga		Homo sapiens		1	17
cell part;cytoplasmic part;intracellular part;macromolecular complex;protein complex;Rab-protein geranylgeranyltransferase complex		Homo sapiens		1	17
cell part;chromatin;chromatin remodeling complex;chromosomal part;cytoplasm;ESC/E(Z) complex;heterochromatin;histone deacetylase complex;histone methyltransferase complex;intracellular membrane-bounded organell	Cell cycle;Chronic myeloid leukemia;Huntington's disease;Notch signaling pathway;Pathways in cancer	Homo sapiens		1	17
cell part;cytoplasm;intracellular membrane-bounded organelle;intracellular organelle;intracellular organelle part;intracellular part;macromolecular complex;membrane;membrane-bounded organelle;nuclear part;nucleoplasm;nucleus;organelle;organelle part;ribonucleoprotein complex		Homo sapiens		1	17
caveola;cell part;cytoplasm;intracellular part;macromolecular complex;membrane part;membrane raft;plasma membrane part;protein complex		Homo sapiens		1	17
cell cortex part;cell part;cell projection part;cytoplasm;cytoplasmic part;cytosol;exocyst;growth cone membrane;intracellular part;macromolecular complex;membrane;membrane part;myelin sheath abaxonal region;plasma m	Tight junction	Homo sapiens		1	17
cell part;cytoplasm;cytoplasmic part;cytoskeletal part;intracellular organelle part;intracellular part;membrane;microtubule organizing center;midbody;organelle part;plasma membrane		Homo sapiens		1	17
cell part;cytoplasm;intracellular membrane-bounded organelle;intracellular organelle;intracellular organelle part;intracellular part;membrane-bounded organelle;nuclear part;nucleoplasm;nucleus;organelle;organelle part		Homo sapiens		1	17
cell part;cytoplasm;cytoplasmic part;endoplasmic reticulum;intracellular membrane-bounded organelle;intracellular organelle;intracellular organelle part;intracellular part;membrane;membrane-bounded organelle;nuclear matrix;nuclear part;nucleoplasm;nucleus;organelle;organelle part;plasma membrane		Homo sapiens		1	17
cell part;cytoplasm;cytoskeletal part;cytoskeleton;intracellular non-membrane-bounded organelle;intracellular organelle;intracellular organelle part;intracellular part;macromolecular complex;microtubule;microtubule cytoskeleton;non-membrane-bounded organelle;nuclear part;nucleoplasm;organelle;organelle part;protein comple		Homo sapiens		1	17
cell part;cytoplasmic part;extracellular membrane-bounded organelle;extracellular organelle;extracellular region part;extracellular vesicular exosome;integral to membrane;intracellular organelle part;intracellular part;intrinsic to membrane;membrane;membrane part;membrane-bounded organelle;membrane-bounded vesicle;mito		Homo sapiens		1	17
cell part;cytoplasm;cytoplasmic part;cytosol;extracellular membrane-bounded organelle;extracellular organelle;extracellular region part;extracellular vesicular exosome;intracellular membrane-bounded organelle;intracellular organelle;intracellular part;membrane;membrane-bounded organelle;membrane-bounded vesicle;nucleus		Homo sapiens		1	17
cell part;chromosomal part;condensed chromosome kinetochore;cytoplasmic part;cytoskeletal part;cytosol;envelope;intracellular non-membrane-bounded organelle;intracellular organelle;intracellular organelle part;intracellul	RNA transport	Homo sapiens		1	17
cell part;cytoplasm;intracellular membrane-bounded organelle;intracellular organelle;intracellular organelle part;intracellular part;membrane-bounded organelle;nuclear part;nucleoplasm;nucleus;organelle;organelle part	Ubiquitin mediated proteolysis	Homo sapiens		1	17
cell part;cytoplasmic part;intracellular membrane-bounded organelle;intracellular organelle;intracellular part;membrane-bounded organelle;mitochondrion;organelle		Homo sapiens		1	17
cell part;cytoplasm;intracellular part		Homo sapiens		1	17
cell part;cytoplasmic part;cytosol;Golgi apparatus part;Golgi membrane;integral to membrane;intracellular membrane-bounded organelle;intracellular organelle;intracellular organelle part;intracellular part;intrinsic to membran	Arachidonic acid metabolism	Homo sapiens		1	17
cell part;centrosome;cytoplasm;cytoplasmic part;cytoskeletal part;extracellular membrane-bounded organelle;extracellular organelle;extracellular region part;extracellular vesicular exosome;intracellular membrane-bounded c	Alanine, aspartate and glutamate metabolism	Homo sapiens		1	17
cell part;cytoplasm;cytoplasmic part;cytosol;intracellular membrane-bounded organelle;intracellular organelle;intracellular organelle part;intracellular part;membrane-bounded organelle;nuclear part;nucleoplasm;organelle;orga	Carbon fixation pathways in prokaryotes;Glycolysis / Gluconeogenesis;Methane metabolism;Propanoat	Homo sapiens		1	17
cell part;cytoplasm;intracellular membrane-bounded organelle;intracellular non-membrane-bounded organelle;intracellular organelle;intracellular organelle part;intracellular part;macromolecular complex;membrane-bounded organelle;non-membrane-bounded organelle;nuclear part;nucleolus;nucleus;organelle;organelle part;prote		Homo sapiens		1	17
cell part;contractile fiber part;cytoplasmic part;cytoskeletal part;intracellular organelle part;intracellular part;organelle part;striated muscle thin filament		Homo sapiens		1	17
cell part;cytoplasmic part;endoplasmic reticulum;endoplasmic reticulum membrane;endoplasmic reticulum part;integral to endoplasmic reticulum membrane;integral to membrane;integral to organelle membrane;intracellular	Biosynthesis of unsaturated fatty acids	Homo sapiens		1	17
cell part;chromosomal part;chromosome;chromosome, telomeric region;condensed chromosome;condensed nuclear chromosome;cytoplasmic part;endosomal part;endosome membrane;envelope;integral to membrane;integral to nuclear inner membrane;integral to organelle membrane;intracellular non-membrane-bounded org		Homo sapiens		1	17
cell part;cytoplasm;cytoplasmic part;cytoskeleton;cytosol;extracellular membrane-bounded organelle;extracellular organelle;extracellular region part;extracellular vesicular exosome;intracellular non-membrane-bounded organelle;intracellular organelle;intracellular organelle part;intracellular part;membrane;membrane-bounded or		Homo sapiens		1	17
extracellular membrane-bounded organelle;extracellular organelle;extracellular region part;extracellular vesicular exosome;membrane-bounded organelle;membrane-bounded vesicle;organelle;vesicle	Protein processing in endoplasmic reticulum	Homo sapiens		1	17
cell part;cytoplasm;intracellular part;macromolecular complex;membrane;polysome;ribonucleoprotein complex		Homo sapiens		1	17
cell part;cytoplasmic part;extracellular membrane-bounded organelle;extracellular organelle;extracellular region part;extracellular vesicular exosome;intracellular;intracellular membrane-bounded organelle;intracellular organel	Drug metabolism - cytochrome P450;Glutathione metabolism;Metabolism of xenobiotics by cytochrome	Homo sapiens		1	17
box C/D snoRNP complex;Cajal body;cell part;cytoplasm;intracellular membrane-bounded organelle;intracellular non-membrane-bounded organelle;intracellular organelle;intracellular organelle part;intracellular part;macromo	Ribosome biogenesis in eukaryotes	Homo sapiens		1	17
cell part;cytoplasm;cytoplasmic part;cytosol;intracellular membrane-bounded organelle;intracellular organelle;intracellular organelle part;intracellular part;macromolecular complex;membrane;membrane-bounded organelle;nu	RNA transport	Homo sapiens		1	17
actin cytoskeleton;cell junction;cell part;cell projection;cell-cell junction;cytoplasm;cytoskeletal part;cytoskeleton;extracellular membrane-bounded organelle;extracellular organelle;extracellular region part;extracellular vesicul	Bacterial invasion of epithelial cells	Homo sapiens		1	17
cell part;integral to membrane;intrinsic to membrane;membrane;membrane part		Homo sapiens		1	18
cell part;intracellular membrane-bounded organelle;intracellular organelle;intracellular organelle part;intracellular part;membrane-bounded organelle;nuclear part;nucleoplasm;nucleus;organelle;organelle part	Spliceosome	Homo sapiens		1	18
cell part;cytoplasmic part;cytosol;eukaryotic 43S preinitiation complex;eukaryotic 48S preinitiation complex;eukaryotic translation initiation factor 3 complex;eukaryotic translation initiation factor 3 complex, eIF3m;extracellula	Measles;RNA transport	Homo sapiens		1	18
cell part;cytoplasmic part;intracellular membrane-bounded organelle;intracellular organelle;intracellular organelle lumen;intracellular organelle part;intracellular part;membrane;membrane-bounded organelle;membrane-enclosed lumen;mitochondrial inner membrane;mitochondrial matrix;mitochondrial membrane;mitochondrial pa		Homo sapiens		1	18
cell part;cytoplasmic part;intracellular membrane-bounded organelle;intracellular organelle;intracellular organelle lumen;intracellular organelle part;intracellular part;membrane-bounded organelle;membrane-enclosed lumen;m	Citrate cycle (TCA cycle)	Homo sapiens		1	18
cell part;cell projection;cytoplasm;cytoplasmic part;early endosome membrane;endosomal part;endosome membrane;extracellular membrane-bounded organelle;extracellular organelle;extracellular region part;extracellular vesicular exosome;extrinsic to membrane;intracellular membrane-bounded organelle;intracellular organelle		Homo sapiens		1	18
cell part;cell projection;chromosomal part;HULC complex;intracellular membrane-bounded organelle;intracellular organelle;intracellular organelle part;intracellular part;macromolecular complex;membrane;membrane-bounded organelle;neuron projection;nuclear part;nucleoplasm;nucleus;organelle;organelle part;protein complex;		Homo sapiens		1	18
cell part;centrosome;cytoplasm;cytoplasmic part;cytoskeletal part;cytosol;endosomal part;endosome;endosome membrane;extracellular membrane-bounded organelle;extracellular organelle;extracellular region part;extracell	Endocytosis	Homo sapiens		1	18
cell part;cytoplasmic part;intracellular organelle lumen;intracellular organelle part;intracellular part;membrane;membrane-enclosed lumen;mitochondrial inner membrane;mitochondrial matrix;mitochondrial membrane;mitochondrial part;organelle inner membrane;organelle lumen;organelle membrane;organelle part		Homo sapiens		1	18
cell part;cytoplasm;cytoplasmic part;cytosol;eukaryotic 43S preinitiation complex;eukaryotic 48S preinitiation complex;eukaryotic translation initiation factor 3 complex;intracellular part;macromolecular complex;perinuclear re	RNA transport	Homo sapiens		1	18
cell part;cytoplasmic part;cytosol;intracellular part	Amino sugar and nucleotide sugar metabolism	Homo sapiens		1	18
cell part;cytoplasm;cytoplasmic part;cytosol;endoplasmic reticulum;extracellular membrane-bounded organelle;extracellular organelle;extracellular region part;extracellular vesicular exosome;intracellular membrane-bounded organelle;intracellular organelle;intracellular part;membrane-bounded organelle;membrane-bounded ves		Homo sapiens		1	18
cell body;cell part;cell projection;cytoplasm;cytoplasmic part;cytoskeletal part;dendrite;endoplasmic reticulum;extracellular membrane-bounded organelle;extracellular organelle;extracellular region part;extracellular vesicular exosome;intracellular membrane-bounded organelle;intracellular non-membrane-bounded organelle;intra		Homo sapiens		1	18
caveola;cell part;cytoplasm;cytoplasmic part;cytosol;intracellular part;membrane part;membrane raft;plasma membrane part		Homo sapiens		1	18
cell part;cytoplasm;cytoplasmic part;cytoskeleton;cytosol;extracellular membrane-bounded organelle;extracellular organelle;extracellular region part;extracellular vesicular exosome;intracellular;intracellular non-membrane-bo	Nicotinate and nicotinamide metabolism;Purine metabolism;Pyrimidine metabolism	Homo sapiens		1	18
autophagic vacuole membrane;cell part;cytoplasmic part;endoplasmic reticulum;endoplasmic reticulum membrane;endoplasmic reticulum part;integral to membrane;intracellular membrane-bounded organelle;intracellular org	N-Glycan biosynthesis;Protein processing in endoplasmic reticulum;Various types of N-glycan biosynthe	Homo sapiens		1	18
cell part;cell projection membrane;cell projection part;centrosome;cytoplasm;cytoplasmic part;cytoskeletal part;cytosol;extracellular membrane-bounded organelle;extracellular organelle;extracellular region part;extracellular v	Purine metabolism;Pyrimidine metabolism	Homo sapiens		1	18
adherens junction;anchoring junction;basolateral plasma membrane;cell junction;cell part;cell surface;cell-substrate adherens junction;cell-substrate junction;cytoplasm;cytoplasmic part;external side of plasma membrane;ex	ECM-receptor interaction;Hematopoietic cell lineage;Shigellosis	Homo sapiens		1	18
cell part;cell periphery;cell projection;cytoplasm;cytoplasmic part;cytosol;extracellular membrane-bounded organelle;extracellular organelle;extracellular region part;extracellular vesicular exosome;intracellular membrane-bou	Purine metabolism;Pyrimidine metabolism	Homo sapiens		1	18
cell part;cytoplasmic part;intracellular membrane-bounded organelle;intracellular non-membrane-bounded organelle;intracellular organelle;intracellular organelle part;intracellular part;membrane;membrane-bounded organelle	Fatty acid metabolism;PPAR signaling pathway	Homo sapiens		1	18
cell part;cytoplasmic part;cytosol;intracellular membrane-bounded organelle;intracellular organelle;intracellular organelle lumen;intracellular organelle part;intracellular part;membrane-bounded organelle;membrane-enclosed l	Pyrimidine metabolism	Homo sapiens		1	18
blood microparticle;cell part;cytoplasm;cytoplasmic membrane-bounded vesicle;cytoplasmic part;cytoplasmic vesicle;cytoskeleton;endoplasmic reticulum;extracellular membrane-bounded organelle;extracellular organelle;extracellular region part;extracellular space;extracellular vesicular exosome;integral to membrane;integral t		Homo sapiens		1	18
cell part;cytoplasm;cytoplasmic part;cytosol;early endosome;endosome;extracellular membrane-bounded organelle;extracellular organelle;extracellular region part;extracellular vesicular exosome;intracellular membrane-bounded organelle;intracellular organelle;intracellular organelle lumen;intracellular organelle part;intracellular p		Homo sapiens		1	18
adherens junction;anchoring junction;cell junction;cell part;cell-substrate adherens junction;cell-substrate junction;cytoplasmic part;cytosol;cytosolic large ribosomal subunit;extracellular membrane-bounded organelle;extrac	Ribosome	Homo sapiens		1	18
cell part;cell surface;cytoplasmic part;endoplasmic reticulum;endoplasmic reticulum lumen;endoplasmic reticulum part;extracellular region;integral to membrane;intracellular membrane-bounded organelle;intracellular organelle;intracellular organelle lumen;intracellular organelle part;intracellular part;intrinsic to membrane;membra		Homo sapiens		1	18
cell part;cytoplasm;cytoplasmic part;intracellular membrane-bounded organelle;intracellular organelle;intracellular organelle part;intracellular part;membrane;membrane-bounded organelle;membrane-enclosed lumen;mitocho	Porphyrin and chlorophyll metabolism	Homo sapiens		1	18
cell part;cytoplasmic part;endoplasmic reticulum;endoplasmic reticulum membrane;endoplasmic reticulum part;integral to membrane;intracellular membrane-bounded organelle;intracellular organelle;intracellular organelle pa	N-Glycan biosynthesis;Protein processing in endoplasmic reticulum;Various types of N-glycan biosynthe	Homo sapiens		1	18
cell part;cytoplasmic part;cytosol;cytosolic large ribosomal subunit;intracellular organelle part;intracellular part;large ribosomal subunit;macromolecular complex;membrane;organelle part;ribonucleoprotein complex	Ribosome	Homo sapiens		1	18
cell part;cytoplasmic membrane-bounded vesicle;cytoplasmic part;cytoplasmic vesicle;cytoplasmic vesicle membrane;cytoplasmic vesicle part;endocytic vesicle;endocytic vesicle membrane;endosome;extracellular membra	Amoebiasis;Endocytosis;ko05152;Phagosome	Homo sapiens		1	18
cell part;cytoplasmic part;endoplasmic reticulum membrane;endoplasmic reticulum part;extracellular membrane-bounded organelle;extracellular organelle;extracellular region part;extracellular vesicular exosome;integral to m	Arginine and proline metabolism;Ascorbate and aldarate metabolism;beta-Alanine metabolism;Chloroalk	Homo sapiens		1	18
cell part;cytoplasm;cytoplasmic membrane-bounded vesicle;cytoplasmic part;cytoplasmic vesicle;cytoskeleton;cytosol;extracellular membrane-bounded organelle;extracellular organelle;extracellular region part;extracellular v	Neurotrophin signaling pathway;Vasopressin-regulated water reabsorption	Homo sapiens		1	18
cell part;cytoplasmic part;extracellular membrane-bounded organelle;extracellular organelle;extracellular region part;extracellular vesicular exosome;integral to membrane;intracellular membrane-bounded organelle;intracellular organelle;intracellular organelle part;intracellular part;intrinsic to membrane;membrane;membrane part		Homo sapiens		1	18
cell part;cytoplasm;cytoplasmic part;cytosol;cytosolic part;cytosolic ribosome;intracellular membrane-bounded organelle;intracellular non-membrane-bounded organelle;intracellular organelle;intracellular part;macromolecular complex;membrane-bounded organelle;non-membrane-bounded organelle;nucleus;organelle;ribonucle		Homo sapiens		1	18

cell part;cytoplasmic membrane-bounded vesicle;cytoplasmic part;cytoplasmic vesicle;intracellular membrane-bounded organelle;intracellular organelle;intracellular part;melanosome;membrane;membrane-bounded organelle;membrane-bounded vesicle;nucleus;organelle;pigment granule;vesicle		Homo sapiens	1	18
brush border;cell part;cell projection;cytoplasmic part;cytoskeletal part;cytosol;extracellular membrane-bounded organelle;extracellular organelle;extracellular region part;extracellular vesicular exosome;intracellular organelle	Vascular smooth muscle contraction	Homo sapiens	1	18
cell part;cytoplasm;cytoplasmic part;cytosolic part;intracellular membrane-bounded organelle;intracellular organelle;intracellular part;macromolecular complex;membrane-bounded organelle;nucleus;organelle;prefoldin complex;protein complex		Homo sapiens	1	18
cell part;cytoplasmic part;cytosol;intracellular membrane-bounded organelle;intracellular non-membrane-bounded organelle;intracellular organelle;intracellular organelle lumen;intracellular organelle part;intracellular part;mem	Huntington's disease	Homo sapiens	1	18
cell part;cytoplasmic part;cytosol;intracellular part	Purine metabolism	Homo sapiens	1	18
cell part;intracellular membrane-bounded organelle;intracellular organelle;intracellular part;membrane-bounded organelle;nucleus;organelle		Homo sapiens	1	18
cell part;cytoplasm;intracellular part		Homo sapiens	1	18
cell part;intracellular membrane-bounded organelle;intracellular non-membrane-bounded organelle;intracellular organelle;intracellular organelle part;intracellular part;macromolecular complex;membrane-bounded organelle;nc	Ribosome biogenesis in eukaryotes	Homo sapiens	1	18
actin cytoskeleton;adherens junction;anchoring junction;cell junction;cell part;cell projection;cell-cell junction;cell-substrate adherens junction;cell-substrate junction;contractile fiber;cytoplasm;cytoplasmic part;cytoskeleton;filopodium;focal adhesion;intracellular non-membrane-bounded organelle;intracellular organelle;intracellu		Homo sapiens	1	18
aminoacyl-tRNA synthetase multienzyme complex;cell part;cell surface;cytoplasm;cytoplasmic membrane-bounded vesicle;cytoplasmic part;cytoplasmic vesicle;cytosol;endoplasmic reticulum;extracellular region part;extracellular space;Golgi apparatus;intracellular membrane-bounded organelle;intracellular organelle;intracellula		Homo sapiens	1	18
cell part;intracellular membrane-bounded organelle;intracellular organelle;intracellular organelle part;intracellular part;membrane-bounded organelle;nuclear part;nucleoplasm;nucleus;organelle;organelle part		Homo sapiens	1	18
cell part;intracellular non-membrane-bounded organelle;intracellular organelle;intracellular organelle part;intracellular part;non-membrane-bounded organelle;nuclear part;nucleolus;nucleoplasm;organelle;organelle part	Spliceosome	Homo sapiens	1	18
		Homo sapiens	1	18
cell part;chromatin;chromatin remodeling complex;chromosomal part;cytoplasm;cytoplasmic part;cytosol;histone deacetylase complex;intracellular membrane-bounded organelle;intracellular organelle;intracellular organelle p	Cell cycle;Chronic myeloid leukemia;Huntington's disease;Notch signaling pathway;Pathways in cancer	Homo sapiens	1	18
cell part;cytoplasm;cytoskeleton;extracellular membrane-bounded organelle;extracellular organelle;extracellular region part;extracellular vesicular exosome;intracellular non-membrane-bounded organelle;intracellular organelle;intracellular organelle part;intracellular part;membrane;membrane-bounded organelle;membrane-bound		Homo sapiens	1	18
adherens junction;anchoring junction;axon;cell body;cell cortex;cell junction;cell part;cell projection;cell projection part;cell-substrate adherens junction;cell-substrate junction;cytoplasm;cytoplasmic part;cytoskeletal part;cyto	Calcium signaling pathway;Chemokine signaling pathway;GnRH signaling pathway;Leukocyte transend	Homo sapiens	1	18
cell part;envelope;integral to membrane;integral to nuclear inner membrane;integral to organelle membrane;intracellular organelle part;intracellular part;intrinsic to membrane;intrinsic to nuclear inner membrane;intrinsic to organelle membrane;membrane;membrane part;nuclear envelope;nuclear membrane;nuclear membrane pa		Homo sapiens	1	18
cell part;centriolar satellite;centrosome;cytoplasm;cytoplasmic part;cytoskeletal part;cytosol;intracellular non-membrane-bounded organelle;intracellular organelle;intracellular organelle part;intracellular part;macromolecular complex;membrane;microtubule organizing center;microtubule organizing center part;non-membrane-bou		Homo sapiens	1	18
cell part;cytoplasm;cytoskeletal part;intracellular organelle part;intracellular part;macromolecular complex;microtubule;organelle part;protein complex		Homo sapiens	1	18
cell part;cytoplasmic part;cytosol;intracellular part;macromolecular complex;protein complex;TORC2 complex	mTOR signaling pathway	Homo sapiens	1	18
anchored to membrane;cell part;cell surface;extracellular region part;extracellular space;intrinsic to membrane;membrane;membrane part;plasma membrane		Homo sapiens	1	18
cell part;intracellular membrane-bounded organelle;intracellular organelle;intracellular part;membrane-bounded organelle;nucleus;organelle		Homo sapiens	1	18
	Ribosome biogenesis in eukaryotes	Homo sapiens	1	18
cell part;ER membrane protein complex;integral to membrane;intrinsic to membrane;macromolecular complex;membrane part;protein complex		Homo sapiens	1	18
actin filament bundle;actomyosin;adherens junction;anchoring junction;cell cortex;cell junction;cell part;cell-substrate adherens junction;cell-substrate junction;cytoplasmic part;cytoskeletal part;cytosol;focal adhesion;intracellular organelle part;intracellular part;organelle part;stress fiber		Homo sapiens	1	18
cell part;intracellular membrane-bounded organelle;intracellular organelle;intracellular part;membrane-bounded organelle;nucleus;organelle		Homo sapiens	1	18
cell part;cytoplasm;cytoskeleton;intracellular non-membrane-bounded organelle;intracellular organelle;intracellular part;non-membrane-bounded organelle;organelle		Homo sapiens	1	18
cell part;cytoplasm;extracellular membrane-bounded organelle;extracellular organelle;extracellular region part;extracellular vesicular exosome;intracellular membrane-bounded organelle;intracellular organelle;intracellular part;membrane-bounded organelle;membrane-bounded vesicle;nucleus;organelle;vesicle		Homo sapiens	1	18
cell part;cytoplasmic part;endoplasmic reticulum lumen;endoplasmic reticulum membrane;endoplasmic reticulum part;intracellular organelle lumen;intracellular organelle part;intracellular part;membrane;membrane part;membrane-enclosed lumen;organelle lumen;organelle membrane;organelle part		Homo sapiens	1	18
cell part;cis-Golgi network;COPI vesicle coat;cytoplasm;cytoplasmic part;cytoplasmic vesicle part;cytoskeletal part;endoplasmic reticulum-Golgi intermediate compartment;Golgi apparatus;Golgi apparatus part;intracellular membrane-bounded organelle;intracellular organelle;intracellular organelle part;intracellular part;macromo		Homo sapiens	1	18
cell part;cytoplasmic part;cytosol;intracellular organelle part;intracellular part;macromolecular complex;membrane part;nuclear part;nuclear pore;nucleoplasm;organelle part;pore complex;protein complex	RNA transport	Homo sapiens	1	18
cell part;cytoplasm;cytoplasmic part;cytosol;intracellular membrane-bounded organelle;intracellular organelle;intracellular organelle part;intracellular part;membrane-bounded organelle;nuclear part;nucleoplasm;nucleus;organelle;organelle part		Homo sapiens	1	18
cell junction;cell part;cell projection;cytoplasm;cytoplasmic part;cytoskeletal part;early endosome membrane;endosomal part;endosome membrane;intracellular non-membrane-bounded organelle;intracellular organelle;intrac	Endocytosis	Homo sapiens	1	18
cell part;cytoplasm;intracellular non-membrane-bounded organelle;intracellular organelle;intracellular organelle part;intracellular part;membrane;non-membrane-bounded organelle;nuclear part;nucleolus;organelle;organelle part;plasma membrane		Homo sapiens	1	18
cell junction;cell part;clathrin-coated vesicle;coated vesicle;cytoplasmic membrane-bounded vesicle;cytoplasmic part;cytoplasmic vesicle;extracellular membrane-bounded organelle;extracellular organelle;extracellular region part;extracellular vesicular exosome;intracellular membrane-bounded organelle;intracellular organelle;in		Homo sapiens	1	18
cell part;cytoplasmic part;intracellular organelle lumen;intracellular organelle part;intracellular part;membrane-enclosed lumen;mitochondrial matrix;mitochondrial part;organelle lumen;organelle part	Aminoacyl-tRNA biosynthesis	Homo sapiens	1	18
actin cytoskeleton;adherens junction;anchoring junction;cell junction;cell part;cell-substrate adherens junction;cell-substrate junction;cytoplasm;cytoplasmic part;cytoskeletal part;cytoskeleton;focal adhesion;intracellular membrane-bounded organelle;intracellular non-membrane-bounded organelle;intracellular organelle;intracell		Homo sapiens	1	18
cell part;cytoplasm;cytoplasmic membrane-bounded vesicle;cytoplasmic part;cytoplasmic vesicle;early endosome;endosome;extracellular membrane-bounded organelle;extracellular organelle;extracellular region part;extracellular vesicular exosome;intracellular membrane-bounded organelle;intracellular organelle;intracellular or		Homo sapiens	1	18
cell part;cell projection;cytoplasmic part;dendrite;intracellular membrane-bounded organelle;intracellular organelle;intracellular part;membrane-bounded organelle;mitochondrion;neuron projection;nucleus;organelle	Geraniol degradation;Naphthalene degradation	Homo sapiens	1	18
alpha-amino-3-hydroxy-5-methyl-4-isoxazolepropionic acid selective glutamate receptor complex;cell part;cytoplasmic part;endoplasmic reticulum membrane;endoplasmic reticulum part;Golgi apparatus;Golgi apparatus part;Golgi membrane;integral to endoplasmic reticulum membrane;integral to membrane;integral to organel		Homo sapiens	1	18
cell part;cell projection;cilium;extracellular membrane-bounded organelle;extracellular organelle;extracellular region part;extracellular vesicular exosome;membrane-bounded organelle;membrane-bounded vesicle;organelle;vesicle		Homo sapiens	1	18
cell part;cell surface;cytoplasmic part;intracellular membrane-bounded organelle;intracellular organelle;intracellular part;membrane-bounded organelle;mitochondrion;organelle		Homo sapiens	1	18
cell part;cytoplasmic part;integral to membrane;integral to plasma membrane;intracellular membrane-bounded organelle;intracellular organelle;intracellular organelle part;intracellular part;intrinsic to membrane;intrinsic to plasma membrane;membrane;membrane part;membrane-bounded organelle;mitochondrial inner membrane		Homo sapiens	1	18
cell part;cytoplasm;cytoskeletal part;extracellular membrane-bounded organelle;extracellular organelle;extracellular region part;extracellular vesicular exosome;intracellular membrane-bounded organelle;intracellular organelle	Base excision repair	Homo sapiens	1	18
cell part;intracellular organelle part;intracellular part;macromolecular complex;nuclear part;nucleoplasm;nucleoplasm part;organelle part;protein complex;transcription factor complex;transcription factor TFIIIC complex		Homo sapiens	1	18
cell part;cytoplasm;cytoplasmic part;endosome;extracellular membrane-bounded organelle;extracellular organelle;extracellular region part;extracellular vesicular exosome;intracellular membrane-bounded organelle;intracellular organelle;intracellular organelle part;intracellular part;membrane;membrane-bounded organelle;membr		Homo sapiens	1	18
cell part;cytoplasm;intracellular part		Homo sapiens	1	18
cell part;cytoplasm;cytoplasmic part;cytoskeleton;cytosol;endoplasmic reticulum;extracellular membrane-bounded organelle;extracellular organelle;extracellular region part;extracellular vesicular exosome;inclusion body;inter	Protein processing in endoplasmic reticulum;Ubiquitin mediated proteolysis	Homo sapiens	1	18
cell part;cytoplasm;intracellular membrane-bounded organelle;intracellular organelle;intracellular organelle part;intracellular part;macromolecular complex;membrane-bounded organelle;nuclear part;nucleoplasm;nucleus;organelle;organelle part;protein complex;signalosome		Homo sapiens	1	18
cell part;cytoplasm;intracellular membrane-bounded organelle;intracellular organelle;intracellular organelle part;intracellular part;membrane-bounded organelle;nuclear body;nuclear part;nuclear speck;nucleoplasm;nucleoplas	Spliceosome	Homo sapiens	1	18
cell part;cytoplasm;cytoplasmic part;cytosol;cytosolic large ribosomal subunit;intracellular non-membrane-bounded organelle;intracellular organelle;intracellular organelle part;intracellular part;large ribosomal subunit;macrom	Ribosome	Homo sapiens	1	18
		Homo sapiens	1	18
cell part;centrosome;chromosomal part;condensed chromosome kinetochore;cytoplasm;cytoplasmic dynein complex;cytoplasmic part;cytoskeletal part;dynein complex;intracellular non-membrane-bounded organelle;intrace	Phagosome;Vasopressin-regulated water reabsorption	Homo sapiens	1	18
cell part;cytoplasm;cytoplasmic part;cytosol;intracellular organelle part;intracellular part;membrane;nuclear part;nucleoplasm;organelle part		Homo sapiens	1	19
cell part;cytoplasm;intracellular organelle part;intracellular part;nuclear part;nucleoplasm;organelle part	RNA transport	Homo sapiens	1	19
box H/ACA RNP complex;box H/ACA snoRNP complex;Cajal body;cell part;cytoplasm;intracellular membrane-bounded organelle;intracellular non-membrane-bounded organelle;intracellular organelle;intracellular organelle p	Ribosome biogenesis in eukaryotes	Homo sapiens	1	19
cell part;intracellular organelle part;intracellular part;nuclear part;nucleoplasm;organelle part		Homo sapiens	1	19
cell part;cytoplasm;cytoplasmic part;cytosol;endoplasmic reticulum;endoplasmic reticulum membrane;endoplasmic reticulum part;intracellular membrane-bounded organelle;intracellular organelle;intracellular organelle part;intracellular part;macromolecular complex;membrane;membrane part;membrane-bounded organelle;nucl		Homo sapiens	1	19
cell part;cytoplasmic part;cytosol;intracellular part	Protein processing in endoplasmic reticulum	Homo sapiens	1	19
cell part;cell projection;contractile fiber part;cytoplasm;cytoplasmic part;cytosol;intracellular part;membrane;neuron projection;organelle part;plasma membrane;Z disc		Homo sapiens	1	19
apical part of cell;axon;basolateral plasma membrane;cell part;cell projection;cytoplasm;cytoplasmic part;cytosol;extracellular membrane-bounded organelle;extracellular organelle;extracellular region part;extracellular space;	Nitrogen metabolism	Homo sapiens	1	19
apical plasma membrane;basolateral plasma membrane;caveola;cell junction;cell part;cell-cell contact zone;cell-cell junction;extracellular membrane-bounded organelle;extracellular organelle;extracellular region part;extracel	Aldosterone-regulated sodium reabsorption;Bile secretion;Carbohydrate digestion and absorption;Cardi	Homo sapiens	1	19
cell part;cell surface;cytoplasm;extracellular matrix;extracellular membrane-bounded organelle;extracellular organelle;extracellular region part;extracellular space;extracellular vesicular exosome;intracellular;intracellular membrane-bounded organelle;intracellular organelle;intracellular part;membrane-bounded organelle;membran		Homo sapiens	1	19
cell part;cytoplasm;intracellular organelle part;intracellular part;macromolecular complex;nuclear part;nucleoplasm;organelle part;ribonucleoprotein complex	Systemic lupus erythematosus	Homo sapiens	1	19
cell part;centrosome;chromosomal part;cytoplasm;cytoplasmic part;cytoskeletal part;DNA replication factor C complex;extracellular membrane-bounded organelle;extracellular organelle;extracellular region part;extracellular v	Base excision repair;Cell cycle;DNA replication;Mismatch repair;Nucleotide excision repair	Homo sapiens	1	19
cell part;intracellular non-membrane-bounded organelle;intracellular organelle;intracellular organelle part;intracellular part;non-membrane-bounded organelle;nuclear part;nucleolus;nucleoplasm;organelle;organelle part		Homo sapiens	1	19
cell part;cytoskeletal part;extracellular membrane-bounded organelle;extracellular organelle;extracellular region part;extracellular vesicular exosome;intermediate filament;intracellular membrane-bounded organelle;intracellular organelle;intracellular organelle part;intracellular part;macromolecular complex;membrane-bounded or		Homo sapiens	1	19
cell part;cytoplasmic part;integral to membrane;intracellular membrane-bounded organelle;intracellular organelle;intracellular organelle part;intracellular part;intrinsic to membrane;membrane;membrane part;membrane-bounc	Arginine and proline metabolism;Drug metabolism - cytochrome P450;Glycine, serine and threonine me	Homo sapiens	1	19
cell part;cytoplasmic part;cytosol;intracellular part;membrane;plasma membrane	Glycerolipid metabolism;Glycerophospholipid metabolism;Phosphatidylinositol signaling system	Homo sapiens	1	19
cell part;cytoplasmic part;cytosol;cytosolic small ribosomal subunit;intracellular organelle part;intracellular part;macromolecular complex;membrane;organelle part;ribonucleoprotein complex;small ribosomal subunit	Ribosome	Homo sapiens	1	19
cell part;cytoplasmic part;envelope;extracellular membrane-bounded organelle;extracellular organelle;extracellular region part;extracellular vesicular exosome;integral to membrane;intracellular membrane-bounded organelle;	Arginine and proline metabolism;Drug metabolism - cytochrome P450;Glycine, serine and threonine me	Homo sapiens	1	19
cell part;cytoplasm;cytoplasmic part;cytosol;extracellular membrane-bounded organelle;extracellular organelle;extracellular region part;extracellular vesicular exosome;intracellular membrane-bounded organelle;intracellular r	Pyrimidine metabolism	Homo sapiens	1	19
cell part;cytoplasmic part;extracellular membrane-bounded organelle;extracellular organelle;extracellular region part;extracellular vesicular exosome;intracellular membrane-bounded organelle;intracellular organelle;intracellula	Citrate cycle (TCA cycle);Lysine degradation	Homo sapiens	1	19
cell part;cytoplasm;cytoplasmic part;cytosol;extracellular membrane-bounded organelle;extracellular organelle;extracellular region part;extracellular vesicular exosome;intercellular bridge;intracellular membrane-bounded orga	Protein export	Homo sapiens	1	19
cell part;cytoplasmic part;extracellular membrane-bounded organelle;extracellular organelle;extracellular region part;extracellular vesicular exosome;intracellular membrane-bounded organelle;intracellular non-membrane-bou	Calcium signaling pathway;Huntington's disease;Parkinson's disease	Homo sapiens	1	19
cell part;cytoplasmic part;extracellular membrane-bounded organelle;extracellular organelle;extracellular region part;extracellular vesicular exosome;intracellular membrane-bounded organelle;intracellular organelle;intracellula	Carbon fixation pathways in prokaryotes;Citrate cycle (TCA cycle);Glutathione metabolism;Peroxisome	Homo sapiens	1	19
cell part;cytoplasmic part;intracellular organelle part;intracellular part;macromolecular complex;membrane;membrane part;mitochondrial inner membrane;mitochondrial membrane;mitochondrial membrane part;mitochondrial	Alzheimer's disease;Huntington's disease;Oxidative phosphorylation;Parkinson's disease	Homo sapiens	1	19
cell part;cytoplasmic part;cytoskeleton;cytosol;extracellular membrane-bounded organelle;extracellular organelle;extracellular region part;extracellular vesicular exosome;immunological synapse;intracellular non-membrane-b	Neurotrophin signaling pathway;Vasopressin-regulated water reabsorption	Homo sapiens	1	19
Arp2/3 protein complex;cell part;cell projection;cytoplasmic part;cytoskeletal part;cytosol;extracellular membrane-bounded organelle;extracellular organelle;extracellular region part;extracellular vesicular exosome;intracellula	Bacterial invasion of epithelial cells;Fc gamma R-mediated phagocytosis;Pathogenic Escherichia coli in	Homo sapiens	1	19
cell body;cell part;cell projection;centrosome;cilium;clathrin-coated vesicle;coated vesicle;cytoplasmic membrane-bounded vesicle;cytoplasmic part;cytoplasmic vesicle;cytoplasmic vesicle membrane;cytoplasmic vesicle pa	Pancreatic secretion	Homo sapiens	1	19
cell part;cytoplasm;cytoplasmic part;cytosol;intracellular membrane-bounded organelle;intracellular non-membrane-bounded organelle;intracellular organelle;intracellular organelle part;intracellular part;macromolecular comp	Bacterial secretion system;Protein export	Homo sapiens	1	19
adherens junction;anchoring junction;cell junction;cell part;cell projection;cell-substrate adherens junction;cell-substrate junction;cilium;cytoplasmic membrane-bounded vesicle;cytoplasmic part;cytoplasmic vesicle;cytoplasmic vesicle membrane;cytoplasmic vesicle part;endocytic vesicle membrane;endoplasmic reticulum mem		Homo sapiens	1	19
cell part;clathrin-coated vesicle;coated vesicle;cytoplasmic membrane-bounded vesicle;cytoplasmic part;cytoplasmic vesicle;cytoplasmic vesicle membrane;cytoplasmic vesicle part;cytosol;early endosome;early endosome membrane;endocytic vesicle;endocytic vesicle membrane;endoplasmic reticulum;endosomal part;endos		Homo sapiens	1	19
cell part;cytoplasmic part;cytosol;endoplasmic reticulum;intracellular membrane-bounded organelle;intracellular organelle;intracellular part;membrane-bounded organelle;nucleus;organelle		Homo sapiens	1	19
adherens junction;anchoring junction;cell junction;cell part;cell-substrate adherens junction;cell-substrate junction;cytoplasm;cytoplasmic part;cytosol;cytosolic large ribosomal subunit;extracellular membrane-bounded orgar	Ribosome	Homo sapiens	1	19
cell part;cytoplasm;cytoplasmic part;cytosol;cytosolic small ribosomal subunit;intracellular membrane-bounded organelle;intracellular non-membrane-bounded organelle;intracellular organelle;intracellular organelle part;intrac	Ribosome	Homo sapiens	1	19
adherens junction;anchoring junction;cell junction;cell part;cell-substrate adherens junction;cell-substrate junction;cytoplasm;cytoplasmic part;cytosol;cytosolic large ribosomal subunit;extracellular membrane-bounded orgar	Ribosome	Homo sapiens	1	19
cell part;cytoplasmic part;cytosol;intracellular organelle part;intracellular part;macromolecular complex;nuclear part;nucleoplasm;organelle part;protein complex;protein phosphatase type 2A complex;protein serine/threonine	Cell cycle - yeast;Chagas disease (American trypanosomiasis);Hepatitis C;mRNA surveillance pathway;T	Homo sapiens	1	19
cell part;chromatin remodeling complex;chromosomal part;cytoplasmic part;cytosol;histone deacetylase complex;intracellular membrane-bounded organelle;intracellular organelle;intracellular organelle part;intracellular part;n	Adherens junction;Circadian rhythm - plant;Measles;Ribosome biogenesis in eukaryotes;Tight junction;V	Homo sapiens	1	19
cell part;cytoplasmic part;endoplasmic reticulum membrane;endoplasmic reticulum part;intracellular membrane-bounded organelle;intracellular organelle;intracellular organelle part;intracellular part;membrane;membrane part;membrane-bounded organelle;nucleus;organelle;organelle membrane;organelle part		Homo sapiens	1	19
cell part;cytoplasmic part;cytosol;extracellular membrane-bounded organelle;extracellular organelle;extracellular region part;extracellular vesicular exosome;intracellular part;membrane-bounded organelle;membrane-bounde	Arginine and proline metabolism	Homo sapiens	1	19
cell junction;cell part;cell projection;cytoplasm;cytoplasmic part;cytoskeletal part;cytosol;intracellular non-membrane-bounded organelle;intracellular organelle;intracellular organelle part;intracellular part;macromolecular complex;membrane;non-membrane-bounded organelle;organelle;organelle part;plasma membrane;podosom		Homo sapiens	1	19
calcineurin complex;cell part;contractile fiber part;cytoplasm;cytoplasmic part;cytosol;intracellular membrane-bounded organelle;intracellular organelle;intracellular organelle part;intracellular part;macromolecular complex;me	Alzheimer's disease;Amyotrophic lateral sclerosis (ALS);Apoptosis;Axon guidance;B cell receptor signal	Homo sapiens	1	19
apical plasma membrane;calcium channel complex;cation channel complex;cell part;centrosome;contractile fiber;cytoplasmic part;cytoskeletal part;cytosol;Golgi apparatus;intracellular membrane-bounded organelle;intracel	Purine metabolism	Homo sapiens	1	19
axon part;basolateral plasma membrane;cell junction;cell part;cell projection;cell projection part;cell surface;cell-cell contact zone;cell-cell junction;contractile fiber part;costamere;cytoplasmic part;cytoskeleton;dendrite;endoplasmic reticulum;Golgi apparatus;initial segment;intercalated disc;intracellular membrane-bounded orga		Homo sapiens	1	19
AMP-activated protein kinase complex;apical plasma membrane;cell part;cytoplasm;cytoplasmic part;cytosol;intracellular;intracellular membrane-bounded organelle;intracellular organelle;intracellular organelle part;intracellul	Adipocytokine signaling pathway;Hypertrophic cardiomyopathy (HCM);Insulin signaling pathway;mTOR	Homo sapiens	1	19
catalytic step 2 spliceosome;cell part;chromosome;intracellular non-membrane-bounded organelle;intracellular organelle;intracellular organelle part;intracellular part;macromolecular complex;non-membrane-bounded organelle;nuclear part;nucleoplasm;organelle;organelle part;ribonucleoprotein complex;spliceosomal complex		Homo sapiens	1	19
catalytic step 2 spliceosome;cell part;chromatin;chromosomal part;intracellular membrane-bounded organelle;intracellular organelle;intracellular organelle part;intracellular part;macromolecular complex;membrane-bounded c	Notch signaling pathway;Spliceosome	Homo sapiens	1	19
cell part;cullin-RING ubiquitin ligase complex;cytoplasmic part;cytosol;intracellular organelle part;intracellular part;macromolecular complex;nuclear part;nucleoplasm;organelle part;protein complex;SCF ubiquitin ligase comp	Cell cycle;Cell cycle - yeast;Circadian rhythm - mammal;Oocyte meiosis;Protein processing in endoplas	Homo sapiens	1	19
cell body;cell part;cell projection;cell projection part;cytoplasmic membrane-bounded vesicle;cytoplasmic part;cytoplasmic vesicle;cytoskeletal part;cytosol;exocytic vesicle;extracellular region part;extracellular space;filamentous actin;growth cone;intracellular membrane-bounded organelle;intracellular organelle;intracellular orga		Homo sapiens	1	19
cell part;chromatin;chromosomal part;cytoplasmic part;cytosol;euchromatin;heterochromatin;intracellular membrane-bounded organelle;intracellular non-membrane-bounded organelle;intracellular organelle;intracellular organelle part;intracellular part;macromolecular complex;membrane-bounded organelle;non-membrane-boun		Homo sapiens	1	19
cell part;chromosome;condensed chromosome;condensed nuclear chromosome;cytoplasmic part;endoplasmic reticulum;intracellular membrane-bounded organelle;intracellular non-membrane-bounded organelle;intracellular organelle;intracellular organelle part;intracellular part;membrane-bounded organelle;non-membrane-bo		Homo sapiens	1	19
cell part;cytoplasmic part;cytosol;eukaryotic translation initiation factor 4F complex;intracellular part;macromolecular complex;membrane;perinuclear region of cytoplasm;protein complex		Homo sapiens	1	19
cell part;cytoplasm;cytoplasmic part;cytosol;intracellular membrane-bounded organelle;intracellular organelle;intracellular part;membrane-bounded organelle;nucleus;organelle	Ubiquitin mediated proteolysis	Homo sapiens	1	19
cell part;cell projection;cell projection part;contractile fiber;cytoplasm;cytoplasmic part;cytoskeleton;extracellular membrane-bounded organelle;extracellular organelle;extracellular region part;extracellular vesicular exosome;filopodium;growth cone;intracellular non-membrane-bounded organelle;intracellular organelle;intracellula		Homo sapiens	1	19
cell part;cytoplasmic part;intracellular membrane-bounded organelle;intracellular organelle;intracellular organelle part;intracellular part;membrane-bounded organelle;mitochondrion;nuclear part;nucleoplasm;nucleus;organelle;organelle part		Homo sapiens	1	19
cell part;cytoplasm;cytoplasmic part;cytosol;intracellular membrane-bounded organelle;intracellular organelle;intracellular part;membrane;membrane-bounded organelle;nucleus;organelle		Homo sapiens	1	19
cell part;chromosomal part;chromosome;intracellular non-membrane-bounded organelle;intracellular organelle;intracellular organelle part;intracellular part;kinetochore;macromolecular complex;non-membrane-bounded organelle;nuclear chromosome;nuclear part;organelle;organelle part;protein complex		Homo sapiens	1	19
cell part;chromosomal part;condensed chromosome kinetochore;intracellular membrane-bounded organelle;intracellular non-membrane-bounded organelle;intracellular organelle;intracellular organelle part;intracellular part;kinetochore;macromolecular complex;membrane-bounded organelle;non-membrane-bounded organelle;n		Homo sapiens	1	19
cell part;extracellular membrane-bounded organelle;extracellular organelle;extracellular region part;extracellular vesicular exosome;membrane;membrane-bounded organelle;membrane-bounded vesicle;organelle;vesicle		Homo sapiens	1	19
cell part;cytoplasm;cytoplasmic part;cytosol;intracellular part;membrane;plasma membrane		Homo sapiens	1	19
cell part;chromatin;chromatin remodeling complex;chromosomal part;intracellular organelle part;intracellular part;macromolecular complex;nBAF complex;npBAF complex;nuclear chromatin;nuclear chromosome part;nuclear part;nucleoplasm;organelle part;protein complex;SWI/SNF complex;SWI/SNF-type complex		Homo sapiens	1	19
cell part;intracellular membrane-bounded organelle;intracellular non-membrane-bounded organelle;intracellular organelle;intracellular organelle part;intracellular part;membrane-bounded organelle;non-membrane-bounded organelle;nuclear part;nucleolus;nucleus;organelle;organelle part		Homo sapiens	1	19
cell part;cytoplasm;intracellular membrane-bounded organelle;intracellular organelle;intracellular organelle part;intracellular part;membrane-bounded organelle;nuclear part;nucleoplasm;nucleus;organelle;organelle part	Basal transcription factors	Homo sapiens	1	19
cell part;intracellular organelle part;intracellular part;nuclear part;nucleoplasm;organelle part		Homo sapiens	1	19
cell part;membrane part;membrane raft		Homo sapiens	1	19
cell part;cytoplasmic part;intracellular organelle lumen;intracellular organelle part;intracellular part;membrane-enclosed lumen;mitochondrial matrix;mitochondrial part;organelle lumen;organelle part		Homo sapiens	1	19
cell part;intracellular organelle part;intracellular part;membrane;nuclear membrane;nuclear part;nucleoplasm;organelle membrane;organelle part		Homo sapiens	1	19
cell part;cytoplasmic part;intracellular membrane-bounded organelle;intracellular organelle;intracellular organelle lumen;intracellular organelle part;intracellular part;membrane-bounded organelle;membrane-enclosed lumen;mitochondrial matrix;mitochondrial part;mitochondrion;organelle;organelle lumen;organelle part		Homo sapiens	1	19
cell part;cytoplasm;cytoplasmic part;intracellular membrane-bounded organelle;intracellular non-membrane-bounded organelle;intracellular organelle;intracellular organelle part;intracellular part;macromolecular complex;membrane-bounded organelle;non-membrane-bounded organelle;nuclear body;nuclear part;nuclear speck;n		Homo sapiens	1	19
cell part;cytoplasmic part;intracellular organelle part;intracellular part;membrane;membrane-enclosed lumen;mitochondrial intermembrane space;mitochondrial part;organelle envelope lumen;organelle part;plasma membrane	Renin-angiotensin system	Homo sapiens	1	19
cell part;chromosomal part;chromosome, telomeric region;cytoplasm;cytoplasmic part;cytosol;extracellular membrane-bounded organelle;extracellular organelle;extracellular region part;extracellular vesicular exosome;intrace	RNA degradation	Homo sapiens	1	19
cell part;extracellular region;integral to membrane;intrinsic to membrane;membrane;membrane part;plasma membrane		Homo sapiens	1	19
cell part;cytoplasm;intracellular membrane-bounded organelle;intracellular organelle;intracellular part;membrane;membrane-bounded organelle;nucleus;organelle;plasma membrane		Homo sapiens	1	19
cell part;cytoplasm;cytoskeletal part;intracellular non-membrane-bounded organelle;intracellular organelle;intracellular organelle part;intracellular part;macromolecular complex;membrane;microtubule;mitotic spindle;non-membrane-bounded organelle;organelle;organelle part;protein complex;spindle		Homo sapiens	1	19
cell part;cell surface;extracellular membrane-bounded organelle;extracellular organelle;extracellular region part;extracellular vesicular exosome;integral to membrane;intrinsic to membrane;membrane;membrane part;membrane-bounded organelle;membrane-bounded vesicle;organelle;vesicle		Homo sapiens	1	19
cell part;cytoplasmic part;endoplasmic reticulum;endoplasmic reticulum lumen;endoplasmic reticulum part;intracellular membrane-bounded organelle;intracellular organelle;intracellular organelle lumen;intracellular organelle p	Protein processing in endoplasmic reticulum	Homo sapiens	1	19
cell part;cytoplasm;cytoplasmic part;cytosol;early endosome membrane;endosomal part;endosome membrane;extracellular membrane-bounded organelle;extracellular organelle;extracellular region part;extracellular vesicula	Colorectal cancer;Pathways in cancer	Homo sapiens	1	19
cell part;cytoplasmic part;cytosol;extracellular membrane-bounded organelle;extracellular organelle;extracellular region part;extracellular vesicular exosome;intracellular organelle part;intracellular part;macromolecular comple	Antigen processing and presentation;Proteasome	Homo sapiens	1	19

cell part;cytoplasm;envelope;extracellular membrane-bounded organelle;extracellular organelle;extracellular region part;extracellular vesicular exosome;intracellular non-membrane-bounded organelle;intracellular organelle;intracellular organelle part;intracellular part;macromolecular complex;membrane;membrane part;membran		Homo sapiens	1	19
		Homo sapiens	1	19
cell part;membrane		Homo sapiens	1	19
axon part;cell part;cell projection part;cytoplasmic part;endosomal part;endosome membrane;Golgi apparatus;Golgi apparatus part;Golgi membrane;intracellular membrane-bounded organelle;intracellular organelle;intracellu	Lysosome	Homo sapiens	1	20
cell part;cytoplasmic part;endoplasmic reticulum;intracellular membrane-bounded organelle;intracellular non-membrane-bounded organelle;intracellular organelle;intracellular organelle part;intracellular part;membrane-bounded organelle;non-membrane-bounded organelle;nuclear part;nucleolus;nucleus;organelle;organelle part		Homo sapiens	1	20
AP-type membrane coat adaptor complex;cell part;clathrin adaptor complex;clathrin coated vesicle membrane;clathrin-coated endocytic vesicle membrane;clathrin-coated vesicle;coated vesicle;coated vesicle membrane;cy	Lysosome	Homo sapiens	1	20
cell part;cell surface;cytoplasmic part;cytosol;intracellular part		Homo sapiens	1	20
cell part;cytoplasmic part;early endosome membrane;endosomal part;endosome membrane;extracellular membrane-bounded organelle;extracellular organelle;extracellular region part;extracellular vesicular exosome;intracellular organelle part;intracellular part;lysosomal membrane;membrane;membrane-bounded organelle;mem		Homo sapiens	1	20
cell part;cytoplasm;cytoskeletal part;cytoskeleton;extracellular membrane-bounded organelle;extracellular organelle;extracellular region part;extracellular vesicular exosome;intracellular non-membrane-bounded organelle;intracellular organelle;intracellular organelle part;intracellular part;macromolecular complex;membrane-boun		Homo sapiens	1	20
Barr body;cell part;centromeric heterochromatin;chromatin;chromosomal part;chromosome;condensed chromosome;extracellular membrane-bounded organelle;extracellular organelle;extracellular region part;extracellular ve	Systemic lupus erythematosus	Homo sapiens	1	20
cell part;cytoplasmic part;extracellular membrane-bounded organelle;extracellular organelle;extracellular region part;extracellular vesicular exosome;intracellular membrane-bounded organelle;intracellular organelle;intracellula	Citrate cycle (TCA cycle);Glyoxylate and dicarboxylate metabolism	Homo sapiens	1	20
cell part;cytoplasm;Elongator holoenzyme complex;histone acetyltransferase complex;intracellular non-membrane-bounded organelle;intracellular organelle;intracellular organelle part;intracellular part;macromolecular complex;non-membrane-bounded organelle;nuclear part;nucleolus;nucleoplasm part;organelle;organelle part;pr		Homo sapiens	1	20
cell part;cytoplasm;cytoplasmic part;cytoskeletal part;cytoskeleton;intracellular membrane-bounded organelle;intracellular non-membrane-bounded organelle;intracellular organelle;intracellular organelle part;intracellular part;membrane-bounded organelle;microtubule cytoskeleton;microtubule organizing center;non-membrane-b		Homo sapiens	1	20
cell part;cytoplasm;cytoplasmic part;cytosol;extracellular membrane-bounded organelle;extracellular organelle;extracellular region part;extracellular vesicular exosome;intracellular part;membrane;membrane-bounded organelle;membrane-bounded vesicle;organelle;plasma membrane;vesicle		Homo sapiens	1	20
adherens junction;anchoring junction;cell junction;cell part;cell-substrate adherens junction;cell-substrate junction;contractile fiber part;cytoplasm;cytoplasmic part;cytoskeletal part;cytoskeleton;cytosol;extracellular membra	Amoebiasis;MAPK signaling pathway;VEGF signaling pathway	Homo sapiens	1	20
cell part;cytoplasmic part;extracellular membrane-bounded organelle;extracellular organelle;extracellular region;extracellular region part;extracellular space;extracellular vesicular exosome;Golgi apparatus;integral to membra	Lysosome	Homo sapiens	1	20
cell part;cytoplasm;cytoplasmic part;cytosol;intracellular membrane-bounded organelle;intracellular organelle;intracellular organelle part;intracellular part;membrane-bounded organelle;mitochondrion;nuclear part;nucleoplasr	Terpenoid backbone biosynthesis	Homo sapiens	1	20
cell body;cell part;cytoplasm;cytoplasmic part;cytosol;extracellular membrane-bounded organelle;extracellular organelle;extracellular region part;extracellular vesicular exosome;intracellular part;membrane-bounded organelle;membrane-bounded vesicle;neuronal cell body;organelle;vesicle		Homo sapiens	1	20
cell part;cytoplasmic part;cytoplasmic vesicle;cytosol;early endosome;endoplasmic reticulum;endosome;intracellular membrane-bounded organelle;intracellular organelle;intracellular part;membrane-bounded organelle;organ	Adherens junction;Insulin signaling pathway	Homo sapiens	1	20
cell part;external side of plasma membrane;extracellular membrane-bounded organelle;extracellular organelle;extracellular region part;extracellular vesicular exosome;integrin complex;macromolecular complex;membrane;m	Arrhythmogenic right ventricular cardiomyopathy (ARVC);Dilated cardiomyopathy;ECM-receptor interact	Homo sapiens	1	20
cell part;intracellular organelle part;intracellular part;nuclear body;nuclear part;nuclear speck;nucleoplasm;nucleoplasm part;organelle part		Homo sapiens	1	20
cell part;cytoplasmic part;extracellular membrane-bounded organelle;extracellular organelle;extracellular region part;extracellular vesicular exosome;intracellular membrane-bounded organelle;intracellular organelle;intracellula	Alzheimer's disease;Huntington's disease;Oxidative phosphorylation;Parkinson's disease	Homo sapiens	1	20
cell part;cytoplasm;cytoplasmic mRNA processing body;cytoplasmic part;cytosol;extracellular membrane-bounded organelle;extracellular organelle;extracellular region part;extracellular vesicular exosome;intracellular membr	Proteasome	Homo sapiens	1	20
cell part;intracellular membrane-bounded organelle;intracellular organelle;intracellular organelle part;intracellular part;macromolecular complex;membrane-bounded organelle;nuclear part;nucleoplasm;nucleus;organelle;organelle part;ribonucleoprotein complex;spliceosomal complex		Homo sapiens	1	20
cell part;cytoplasmic part;endoplasmic reticulum;endoplasmic reticulum membrane;endoplasmic reticulum part;integral to membrane;intracellular membrane-bounded organelle;intracellular organelle;intracellular organelle pa	Steroid biosynthesis	Homo sapiens	1	20
cell part;centriole;cytoplasm;cytoplasmic membrane-bounded vesicle;cytoplasmic part;cytoplasmic vesicle;cytoskeletal part;extracellular membrane-bounded organelle;extracellular organelle;extracellular region part;extracellular vesicular exosome;F-actin capping protein complex;intracellular membrane-bounded organelle;intra		Homo sapiens	1	20
cell part;cytoplasmic part;endoplasmic reticulum membrane;endoplasmic reticulum part;integral to membrane;intracellular organelle part;intracellular part;intrinsic to membrane;macromolecular complex;membrane;membran	N-Glycan biosynthesis;Protein processing in endoplasmic reticulum;Various types of N-glycan biosynthe	Homo sapiens	1	20
cell part;cytoplasm;cytoplasmic part;cytosol;extracellular membrane-bounded organelle;extracellular organelle;extracellular region part;extracellular vesicular exosome;intracellular part;membrane-bounded organelle;membra	beta-Alanine metabolism;Drug metabolism - cytochrome P450;Glycolysis / Gluconeogenesis;Histidine r	Homo sapiens	1	20
cell part;cytoplasmic part;endoplasmic reticulum membrane;endoplasmic reticulum part;intracellular organelle part;intracellular part;lipid particle;membrane;membrane part;organelle membrane;organelle part	Steroid biosynthesis	Homo sapiens	1	20
cell part;cytoplasmic part;endoplasmic reticulum membrane;endoplasmic reticulum part;extracellular membrane-bounded organelle;extracellular organelle;extracellular region part;extracellular vesicular exosome;Golgi apparatus part;Golgi membrane;intracellular organelle part;intracellular part;membrane;membrane part;membra		Homo sapiens	1	20
cell part;cytoplasmic part;integral to membrane;integral to mitochondrial membrane;integral to mitochondrial outer membrane;integral to organelle membrane;intracellular membrane-bounded organelle;intracellular organelle;	Adipocytokine signaling pathway;Fatty acid metabolism;PPAR signaling pathway	Homo sapiens	1	20
cell part;cytoplasm;cytoplasmic part;cytosol;intracellular part;membrane;plasma membrane		Homo sapiens	1	20
cell part;centrosome;chromosomal part;condensed chromosome kinetochore;cytoplasmic part;cytoskeletal part;cytosol;envelope;intracellular non-membrane-bounded organelle;intracellular organelle;intracellular organelle pa	RNA transport	Homo sapiens	1	20
annulate lamellae;cell part;cytoplasm;cytoplasmic part;cytosol;endoplasmic reticulum membrane;endoplasmic reticulum part;extracellular membrane-bounded organelle;extracellular organelle;extracellular region part;extracellular vesicular exosome;intracellular membrane-bounded organelle;intracellular organelle;intracellular org		Homo sapiens	1	20
cell part;CRD-mediated mRNA stability complex;cytoplasm;cytoplasmic part;extracellular membrane-bounded organelle;extracellular organelle;extracellular region part;extracellular vesicular exosome;histone pre-mRNA 3'end processing complex;intracellular membrane-bounded organelle;intracellular non-membrane-bounded c		Homo sapiens	1	20
cell part;intracellular membrane-bounded organelle;intracellular organelle;intracellular organelle part;intracellular part;macromolecular complex;membrane-bounded organelle;nuclear body;nuclear part;nuclear speck;nucleoplasm;nucleoplasm part;nucleus;organelle;organelle part;protein complex;transcription export complex		Homo sapiens	1	20
cell part;cytoplasmic part;integral to membrane;integral to plasma membrane;intracellular membrane-bounded organelle;intracellular organelle;intracellular organelle part;intracellular part;intrinsic to membrane;intrinsic to plasma membrane;membrane;membrane part;membrane-bounded organelle;mitochondrial inner membrane		Homo sapiens	1	20
blood microparticle;cell part;extracellular matrix;extracellular membrane-bounded organelle;extracellular organelle;extracellular region part;extracellular space;extracellular vesicular exosome;membrane;membrane-bounded organelle;membrane-bounded vesicle;organelle;proteinaceous extracellular matrix;vesicle		Homo sapiens	1	20
cell part;cytoplasmic part;extracellular region part;extracellular space;intracellular membrane-bounded organelle;intracellular organelle;intracellular organelle lumen;intracellular organelle part;intracellular part;membrane;membrane-bounded organelle;membrane-enclosed lumen;mitochondrial inner membrane;mitochondrial matri		Homo sapiens	1	20
cell part;cell surface;cytoplasmic part;endoplasmic reticulum membrane;endoplasmic reticulum part;endoplasmic reticulum-Golgi intermediate compartment;endoplasmic reticulum-Golgi intermediate compartment membran	Protein processing in endoplasmic reticulum	Homo sapiens	1	20
cell part;intracellular organelle part;intracellular part;nuclear body;nuclear part;nuclear speck;nucleoplasm;nucleoplasm part;organelle part	Spliceosome	Homo sapiens	1	20
cell part;cytoplasm;cytoplasmic part;extracellular membrane-bounded organelle;extracellular organelle;extracellular region part;extracellular vesicular exosome;Golgi apparatus;Golgi apparatus part;Golgi membrane;intracellular membrane-bounded organelle;intracellular organelle;intracellular organelle part;intracellular part;mem		Homo sapiens	1	20
cell part;Cul3-RING ubiquitin ligase complex;cullin-RING ubiquitin ligase complex;cytoplasmic part;cytoskeletal part;cytosol;extracellular membrane-bounded organelle;extracellular organelle;extracellular region part;extracell	Ubiquitin mediated proteolysis	Homo sapiens	1	20
cell part;cytoplasmic part;endoplasmic reticulum;endoplasmic reticulum membrane;endoplasmic reticulum part;extracellular membrane-bounded organelle;extracellular organelle;extracellular region part;extracellular vesicula	N-Glycan biosynthesis;Protein processing in endoplasmic reticulum	Homo sapiens	1	20
cell part;cytoplasm;intracellular membrane-bounded organelle;intracellular organelle;intracellular organelle part;intracellular part;membrane-bounded organelle;nuclear part;nucleoplasm;nucleus;organelle;organelle part	Ubiquitin mediated proteolysis	Homo sapiens	1	20
cell cortex part;cell part;cell projection membrane;cell projection part;centrosome;cortical cytoskeleton;cortical microtubule cytoskeleton;cytoplasm;cytoplasmic microtubule;cytoplasmic part;cytoskeletal part;cytoskeleton;cytosol;Golgi apparatus;intracellular membrane-bounded organelle;intracellular non-membrane-bounded o		Homo sapiens	1	20
cell part;cytoplasmic part;endoplasmic reticulum;endoplasmic reticulum membrane;endoplasmic reticulum part;integral to membrane;intracellular membrane-bounded organelle;intracellular organelle;intracellular organelle pa	Steroid biosynthesis	Homo sapiens	1	20
cell part;cytoplasmic part;cytosol;intracellular organelle part;intracellular part;macromolecular complex;nuclear part;nucleoplasm;organelle part;proteasome accessory complex;proteasome complex;protein complex		Homo sapiens	1	20
cell part;integral to membrane;intracellular membrane-bounded organelle;intracellular organelle;intracellular organelle part;intracellular part;intrinsic to membrane;membrane;membrane part;membrane-bounded organelle;nuclear inner membrane;nuclear membrane;nuclear part;nucleus;organelle;organelle inner membrane;organe		Homo sapiens	1	20
cell part;chromosomal part;extracellular membrane-bounded organelle;extracellular organelle;extracellular region;extracellular region part;extracellular vesicular exosome;intracellular membrane-bounded organelle;intracellula	Systemic lupus erythematosus	Homo sapiens	1	20
cell cortex;cell cortex part;cell part;centrosomal corona;centrosome;chromosomal part;condensed chromosome kinetochore;cortical cytoskeleton;cortical microtubule cytoskeleton;cytoplasmic microtubule;cytoplasmic part;cytoskeletal part;cytoskeleton;cytosol;extracellular membrane-bounded organelle;extracellular organelle;		Homo sapiens	1	20
catalytic step 2 spliceosome;cell part;cytoplasmic part;cytosol;extracellular membrane-bounded organelle;extracellular organelle;extracellular region part;extracellular vesicular exosome;intracellular membrane-bounded orga	mRNA surveillance pathway;RNA transport;Spliceosome	Homo sapiens	1	20
cell part;cytoplasm;intracellular part		Homo sapiens	1	20
axon;cell junction;cell part;cell projection;cytoplasm;cytoplasmic part;cytoskeletal part;cytosol;dendrite;extracellular membrane-bounded organelle;extracellular organelle;extracellular region part;extracellular vesicular exosome;Golgi apparatus;intracellular membrane-bounded organelle;intracellular organelle;intracellular organell		Homo sapiens	1	20
cell part;cytoplasmic part;endoplasmic reticulum;endoplasmic reticulum part;integral to membrane;intracellular membrane-bounded organelle;intracellular organelle;intracellular organelle part;intracellular part;intrinsic to mem	N-Glycan biosynthesis;Protein processing in endoplasmic reticulum;Various types of N-glycan biosynthe	Homo sapiens	1	20
cell part;chromatin;chromosomal part;cytoplasm;intracellular organelle part;intracellular part;macromolecular complex;membrane;nuclear chromatin;nuclear chromosome part;nuclear part;organelle part;protein complex		Homo sapiens	1	20
cell part;intracellular membrane-bounded organelle;intracellular organelle;intracellular organelle part;intracellular part;membrane-bounded organelle;nuclear part;nucleoplasm;nucleus;organelle;organelle part		Homo sapiens	1	20
cell part;chromosomal part;intracellular membrane-bounded organelle;intracellular non-membrane-bounded organelle;intracellular organelle;intracellular organelle part;intracellular part;macromolecular complex;membrane-bounded organelle;non-membrane-bounded organelle;nuclear part;nucleolus;nucleosome;nucleus;organell		Homo sapiens	1	20
cell part;cytoplasmic part;cytosol;extracellular membrane-bounded organelle;extracellular organelle;extracellular region part;extracellular vesicular exosome;intracellular part;membrane-bounded organelle;membrane-bounded vesicle;organelle;vesicle		Homo sapiens	1	20
adherens junction;anchoring junction;cell junction;cell part;cell projection membrane;cell projection part;cell-substrate adherens junction;cell-substrate junction;cytoplasmic part;cytoskeletal part;cytosol;filamentous actin;focal adhesion;intracellular organelle part;intracellular part;leading edge membrane;macromolecular complex		Homo sapiens	1	20
cell part;cytoplasm;intracellular membrane-bounded organelle;intracellular organelle;intracellular organelle part;intracellular part;macromolecular complex;membrane;membrane-bounded organelle;nuclear part;nucleoplasm;nucleus;organelle;organelle part;ribonucleoprotein complex;spliceosomal complex		Homo sapiens	1	20
cell part;cell projection membrane;cell projection part;cytoplasm;extracellular membrane-bounded organelle;extracellular organelle;extracellular region part;extracellular vesicular exosome;intracellular part;leading edge membrane;macromolecular complex;membrane;membrane part;membrane-bounded organelle;membrane-bo		Homo sapiens	1	20
cell part;COPI vesicle coat;cytoplasmic part;cytoplasmic vesicle part;cytosol;Golgi apparatus part;intracellular organelle part;intracellular part;macromolecular complex;membrane coat;membrane part;organelle part;protein complex;vesicle coat		Homo sapiens	1	20
		Homo sapiens	1	20
		Homo sapiens	1	20
cell part;cytoplasmic part;extracellular membrane-bounded organelle;extracellular organelle;extracellular region part;extracellular vesicular exosome;intracellular membrane-bounded organelle;intracellular organelle;intracellula	Calcium signaling pathway;Huntington's disease;Parkinson's disease	Homo sapiens	1	20
cell part;cytoplasmic part;cytosol;extracellular membrane-bounded organelle;extracellular organelle;extracellular region part;extracellular vesicular exosome;intracellular part;membrane-bounded organelle;membrane-bounde	mTOR signaling pathway	Homo sapiens	1	20
cell part;cytoplasm;cytoskeletal part;intracellular membrane-bounded organelle;intracellular non-membrane-bounded organelle;intracellular organelle;intracellular organelle part;intracellular part;membrane-bounded organelle	Ribosome biogenesis in eukaryotes	Homo sapiens	1	20
actin cytoskeleton;apical part of cell;cell junction;cell part;cell projection;cell surface;cell-cell junction;centrosome;chloride channel complex;cytoplasm;cytoplasmic part;cytoplasmic vesicle membrane;cytoplasmic vesicle part;cytoskeletal part;cytoskeleton;cytosol;extracellular membrane-bounded organelle;extracellular organell		Homo sapiens	1	20
cell part;cytoplasm;cytoplasmic part;cytosol;intracellular membrane-bounded organelle;intracellular organelle;intracellular organelle part;intracellular part;macromolecular complex;membrane-bounded organelle;methylosome	Cell cycle - yeast;RNA transport	Homo sapiens	1	21
Cajal body;catalytic step 2 spliceosome;cell part;cytoplasm;cytoplasmic part;cytosol;Golgi apparatus;intracellular membrane-bounded organelle;intracellular organelle;intracellular organelle part;intracellular part;macromolecu	Spliceosome	Homo sapiens	1	21
cell part;cytoplasm;intracellular organelle part;intracellular part;membrane;nuclear body;nuclear matrix;nuclear part;nuclear speck;nucleoplasm;nucleoplasm part;organelle part	Spliceosome	Homo sapiens	1	21
adherens junction;anchoring junction;caveola;cell junction;cell part;cell-substrate adherens junction;cell-substrate junction;centriolar satellite;cytoplasmic membrane-bounded vesicle;cytoplasmic part;cytoplasmic vesicle;cyto	Insulin signaling pathway	Homo sapiens	1	21
cell part;cytoplasmic part;cytosol;intracellular membrane-bounded organelle;intracellular organelle;intracellular organelle lumen;intracellular organelle part;intracellular part;membrane-bounded organelle;membrane-enclosed l	Alanine, aspartate and glutamate metabolism;Arginine and proline metabolism;D-Glutamine and D-gluta	Homo sapiens	1	21
blood microparticle;cell part;cytoplasmic membrane-bounded vesicle lumen;cytoplasmic part;cytoplasmic vesicle part;cytosol;extracellular membrane-bounded organelle;extracellular organelle;extracellular region;extracellula	Complement and coagulation cascades	Homo sapiens	1	21
adherens junction;anchoring junction;cell junction;cell part;cell surface;cell-substrate adherens junction;cell-substrate junction;external side of plasma membrane;extracellular membrane-bounded organelle;extracellular orgar	African trypanosomiasis;Cell adhesion molecules (CAMs);Leukocyte transendothelial migration;Malaria;I	Homo sapiens	1	21
cell part;cell projection membrane;cell projection part;cilium membrane;cilium part;clathrin coat;clathrin coat of coated pit;clathrin coat of trans-Golgi network vesicle;clathrin vesicle coat;cytoplasm;cytoplasmic part;cytoplasi	Bacterial invasion of epithelial cells;Endocrine and other factor-regulated calcium reabsorption;Endocyto	Homo sapiens	1	21
cell part;cytoplasm;cytoplasmic part;cytosol;intracellular membrane-bounded organelle;intracellular organelle;intracellular part;membrane-bounded organelle;nucleus;organelle	Drug metabolism - other enzymes;Pyrimidine metabolism	Homo sapiens	1	21
actin cytoskeleton;actin filament;actin filament bundle;adherens junction;anchoring junction;cell junction;cell part;cell projection;cell projection membrane;cell projection part;cell-substrate adherens junction;cell-substrate junction;cytoplasm;cytoplasmic part;cytoskeletal part;cytoskeleton;cytosol;extracellular membrane-bounde		Homo sapiens	1	21
adherens junction;AMP-activated protein kinase complex;anchoring junction;cAMP-dependent protein kinase complex;cell junction;cell part;cell-substrate adherens junction;cell-substrate junction;centrosome;cytoplasm;cyto	Apoptosis;Insulin signaling pathway	Homo sapiens	1	21
cell part;cytoplasmic part;extracellular membrane-bounded organelle;extracellular organelle;extracellular region part;extracellular vesicular exosome;fumarate reductase complex;intracellular membrane-bounded organelle;int	Alzheimer's disease;Citrate cycle (TCA cycle);Huntington's disease;Oxidative phosphorylation;Parkinsor	Homo sapiens	1	21
cell part;cytoplasmic part;cytosol;extracellular membrane-bounded organelle;extracellular organelle;extracellular region part;extracellular vesicular exosome;intracellular membrane-bounded organelle;intracellular organelle;int	Protein processing in endoplasmic reticulum	Homo sapiens	1	21
cell part;cytoplasm;intracellular membrane-bounded organelle;intracellular organelle;intracellular organelle part;intracellular part;membrane-bounded organelle;nuclear part;nucleoplasm;nucleus;organelle;organelle part		Homo sapiens	1	21
cell part;cytoplasm;cytoplasmic part;endoplasmic reticulum;intracellular membrane-bounded organelle;intracellular organelle;intracellular organelle part;intracellular part;membrane-bounded organelle;nuclear part;nucleoplasm;nucleus;organelle;organelle part;perinuclear region of cytoplasm		Homo sapiens	1	21
cell part;cytoplasm;cytoplasmic part;cytosol;cytosolic part;cytosolic proteasome complex;inclusion body;intracellular membrane-bounded organelle;intracellular organelle;intracellular organelle part;intracellular part;macromo	Proteasome	Homo sapiens	1	21
cell part;cytoplasm;intracellular membrane-bounded organelle;intracellular organelle;intracellular organelle part;intracellular part;membrane-bounded organelle;nuclear body;nuclear part;nuclear speck;nucleoplasm;nucleoplasm part;nucleus;organelle;organelle part		Homo sapiens	1	21
cell part;cytoplasm;cytoplasmic part;cytosol;intracellular part;membrane;plasma membrane	RNA transport	Homo sapiens	1	21
cell part;cytoplasmic part;endoplasmic reticulum;extracellular region part;extracellular space;intracellular membrane-bounded organelle;intracellular organelle;intracellular part;membrane-bounded organelle;nucleus;organelle;perinuclear region of cytoplasm		Homo sapiens	1	21
cell part;cytoplasm;cytoplasmic part;cytoplasmic vesicle membrane;cytoplasmic vesicle part;cytosol;cytosolic small ribosomal subunit;endocytic vesicle membrane;endosomal part;endosome membrane;extracellular membr	Ribosome	Homo sapiens	1	21
cell junction;cell part;cell projection part;cytoplasm;cytoskeleton;extracellular membrane-bounded organelle;extracellular organelle;extracellular region part;extracellular vesicular exosome;growth cone;intracellular membrane-bounded organelle;intracellular non-membrane-bounded organelle;intracellular organelle;intracellular org		Homo sapiens	1	21
cell part;cytoplasm;Grb2-Sos complex;intracellular membrane-bounded organelle;intracellular organelle;intracellular organelle part;intracellular part;macromolecular complex;membrane;membrane part;membrane-bounded organelle;nuclear part;nucleoplasm;nucleus;organelle;organelle part;plasma membrane part;protein compl		Homo sapiens	1	21
cell part;cell projection;cytoplasm;cytoplasmic part;cytosol;extracellular membrane-bounded organelle;extracellular organelle;extracellular region part;extracellular vesicular exosome;intracellular part;membrane;membrane-bounded organelle;membrane-bounded vesicle;organelle;plasma membrane;ruffle;vesicle		Homo sapiens	1	21
cell part;cytoplasmic part;cytosol;envelope;intracellular organelle part;intracellular part;macromolecular complex;membrane part;nuclear envelope;nuclear part;nuclear pore;Nup107-160 complex;organelle envelope;organelle	RNA transport	Homo sapiens	1	21
cell part;centrosome;cytoplasm;cytoplasmic dynein complex;cytoplasmic part;cytoskeletal part;cytosol;dynein complex;intracellular non-membrane-bounded organelle;intracellular organelle;intracellular organelle part;intracel	Phagosome;Vasopressin-regulated water reabsorption	Homo sapiens	1	21
cell part;COPII vesicle coat;cytoplasmic part;cytoplasmic vesicle part;endomembrane system;endoplasmic reticulum membrane;endoplasmic reticulum part;endoplasmic reticulum-Golgi intermediate compartment membran	Protein processing in endoplasmic reticulum	Homo sapiens	1	21
apical plasma membrane;azurophil granule;cell part;cytolytic granule;cytoplasmic membrane-bounded vesicle;cytoplasmic part;cytoplasmic vesicle;cytosol;extracellular membrane-bounded organelle;extracellular organelle;extracellular region part;extracellular vesicular exosome;intracellular membrane-bounded organelle;intrace		Homo sapiens	1	21
cell part;cytoplasm;cytoplasmic part;extracellular membrane-bounded organelle;extracellular organelle;extracellular region part;extracellular vesicular exosome;intracellular membrane-bounded organelle;intracellular organelle;intracellular organelle lumen;intracellular organelle part;intracellular part;membrane-bounded organelle;r		Homo sapiens	1	21
cell part;cytoplasm;cytoplasmic part;endoplasmic reticulum;intracellular membrane-bounded organelle;intracellular organelle;intracellular part;membrane-bounded organelle;nucleus;organelle;perinuclear region of cytoplasm		Homo sapiens	1	21
cell part;cytoplasmic part;endoplasmic reticulum membrane;endoplasmic reticulum part;integral to membrane;intracellular membrane-bounded organelle;intracellular organelle;intracellular organelle part;intracellular part;intrinsic to membrane;membrane;membrane part;membrane-bounded organelle;organelle;organelle membrar		Homo sapiens	1	21
cell part;cytoplasmic part;endoplasmic reticulum;endoplasmic reticulum membrane;endoplasmic reticulum part;integral to membrane;intracellular membrane-bounded organelle;intracellular organelle;intracellular organelle part;intracellular part;intrinsic to membrane;membrane;membrane part;membrane-bounded organelle;orgar		Homo sapiens	1	21
cell part;cytoplasm;cytoskeleton;intracellular non-membrane-bounded organelle;intracellular organelle;intracellular part;non-membrane-bounded organelle;organelle		Homo sapiens	1	21
cell part;cytoplasm;intracellular membrane-bounded organelle;intracellular non-membrane-bounded organelle;intracellular organelle;intracellular organelle part;intracellular part;membrane-bounded organelle;non-membrane-bounded organelle;nuclear body;nuclear part;nuclear speck;nucleolus;nucleoplasm;nucleoplasm part;nuc		Homo sapiens	1	21
cell part;cytoplasm;intracellular part;membrane		Homo sapiens	1	21
actin cytoskeleton;cell part;chromatin;chromosomal part;chromosome;chromosome, centromeric region;cytoplasmic part;cytoskeleton;cytosol;intermediate filament cytoskeleton;intracellular membrane-bounded organelle;in	Cell cycle;Cell cycle - yeast;Meiosis - yeast	Homo sapiens	1	21
cell leading edge;cell part;cis-Golgi network;cytoplasmic part;endoplasmic reticulum lumen;endoplasmic reticulum part;endoplasmic reticulum-Golgi intermediate compartment;Golgi apparatus;Golgi apparatus part;Golgi membrane;Golgi stack;intracellular membrane-bounded organelle;intracellular organelle;intracellular organel		Homo sapiens	1	21
adherens junction;anchoring junction;cell junction;cell part;cell-substrate adherens junction;cell-substrate junction;cytoplasm;focal adhesion;intracellular membrane-bounded organelle;intracellular organelle;intracellular part;membrane;membrane-bounded organelle;nucleus;organelle;plasma membrane		Homo sapiens	1	21
cell part;cytosolic large ribosomal subunit;intracellular organelle part;intracellular part;large ribosomal subunit;macromolecular complex;organelle part;ribonucleoprotein complex	Ribosome	Homo sapiens	1	21
adherens junction;anchoring junction;cell junction;cell part;cell projection;cell-cell junction;cell-substrate adherens junction;cell-substrate junction;cytoplasmic part;cytoplasmic vesicle membrane;cytoplasmic vesicle part;cyto	Endocytosis	Homo sapiens	1	21
cell part;cell surface;cytoplasmic part;endoplasmic reticulum lumen;endoplasmic reticulum membrane;endoplasmic reticulum part;endoplasmic reticulum-Golgi intermediate compartment;extracellular membrane-bounded organelle;extracellular organelle;extracellular region part;extracellular vesicular exosome;intracellular memb		Homo sapiens	1	21
cell part;cytoplasm;cytoplasmic part;Golgi apparatus;intracellular membrane-bounded organelle;intracellular non-membrane-bounded organelle;intracellular organelle;intracellular organelle part;intracellular part;membrane;me	Ribosome biogenesis in eukaryotes	Homo sapiens	1	21
cell part;cytoplasmic part;extracellular membrane-bounded organelle;extracellular organelle;extracellular region part;extracellular vesicular exosome;intracellular organelle lumen;intracellular organelle part;intracellular part;me	Oxidative phosphorylation	Homo sapiens	1	21
cell part;intracellular membrane-bounded organelle;intracellular non-membrane-bounded organelle;intracellular organelle;intracellular organelle part;intracellular part;membrane-bounded organelle;non-membrane-bounded organelle;nuclear part;nucleolus;nucleus;organelle;organelle part		Homo sapiens	1	21
cell part;cytoplasm;cytoplasmic part;extracellular membrane-bounded organelle;extracellular organelle;extracellular region part;extracellular vesicular exosome;intracellular membrane-bounded organelle;intracellular organelle;intracellular organelle part;intracellular part;membrane;membrane-bounded organelle;membrane-bound		Homo sapiens	1	21
cell part;cytoplasmic part;endoplasmic reticulum;endoplasmic reticulum part;integral to endoplasmic reticulum membrane;integral to membrane;integral to organelle membrane;intracellular membrane-bounded organelle;intracellular organelle;intracellular organelle part;intracellular part;intrinsic to endoplasmic reticulum membrar		Homo sapiens	1	21
cell part;cytoplasm;cytoplasmic part;cytoskeleton;cytosol;intracellular membrane-bounded organelle;intracellular non-membrane-bounded organelle;intracellular organelle;intracellular part;membrane-bounded organelle;non-membrane-bounded organelle;nucleus;organelle		Homo sapiens	1	21
cell part;cytoplasm;cytoplasmic part;cytosol;intracellular membrane-bounded organelle;intracellular organelle;intracellular part;membrane-bounded organelle;nucleus;organelle		Homo sapiens	1	22
cell part;cytoplasm;intracellular membrane-bounded organelle;intracellular organelle;intracellular organelle part;intracellular part;membrane-bounded organelle;nuclear part;nucleoplasm;nucleus;organelle;organelle part		Homo sapiens	1	22
cell part;centrosome;cytoplasmic dynein complex;cytoplasmic part;cytoskeletal part;cytosol;dynein complex;intracellular non-membrane-bounded organelle;intracellular organelle;intracellular organelle part;intracellular part;m	Phagosome;Vasopressin-regulated water reabsorption	Homo sapiens	1	22
Cajal body;cell part;cytoplasm;intracellular membrane-bounded organelle;intracellular organelle;intracellular organelle part;intracellular part;macromolecular complex;membrane-bounded organelle;nuclear body;nuclear part;r	Spliceosome	Homo sapiens	1	22
cell part;cytoplasmic part;cytosol;endoplasmic reticulum;extracellular membrane-bounded organelle;extracellular organelle;extracellular region part;extracellular vesicular exosome;intracellular membrane-bounded organelle;i	Cysteine and methionine metabolism	Homo sapiens	1	22
axolemma;axon;axon part;basolateral plasma membrane;brush border;cell body;cell part;cell projection;cell projection membrane;cell projection part;cytoplasmic part;cytoplasmic vesicle;cytoskeletal part;endoplasmic reticulum;endosome;extracellular membrane-bounded organelle;extracellular organelle;extracellular region par		Homo sapiens	1	22
cell part;cytoplasmic part;integral to membrane;intracellular membrane-bounded organelle;intracellular organelle;intracellular organelle part;intracellular part;intrinsic to membrane;membrane;membrane part;membrane-bounded organelle;mitochondrial inner membrane;mitochondrial membrane;mitochondrial part;mitochondrion;		Homo sapiens	1	22
cell part;cell surface;coated vesicle membrane;cytoplasmic part;cytoplasmic vesicle membrane;cytoplasmic vesicle part;early endosome membrane;endocytic vesicle membrane;endoplasmic reticulum;endoplasmic reticulun	Allograft rejection;Antigen processing and presentation;Autoimmune thyroid disease;Cell adhesion mole	Homo sapiens	1	22
cell part;centrosome;cytoplasm;cytoplasmic part;cytoskeletal part;cytosol;extracellular membrane-bounded organelle;extracellular organelle;extracellular region part;extracellular vesicular exosome;intracellular membrane-bo	Cell cycle;Gap junction;Oocyte meiosis;p53 signaling pathway;Progesterone-mediated oocyte maturatic	Homo sapiens	1	22
cell part;cytoplasmic part;extracellular membrane-bounded organelle;extracellular organelle;extracellular region part;extracellular vesicular exosome;intracellular membrane-bounded organelle;intracellular organelle;intracellular organelle lumen;intracellular organelle part;intracellular part;membrane-bounded organelle;membrane-		Homo sapiens	1	22
cell junction;cell part;cytoplasm;cytoplasmic part;Golgi apparatus;Golgi apparatus part;Golgi membrane;intracellular membrane-bounded organelle;intracellular non-membrane-bounded organelle;intracellular organelle;intracellular organelle part;intracellular part;membrane;membrane-bounded organelle;non-membrane-boundec		Homo sapiens	1	22
cell part;cytoplasm;cytoplasmic part;cytosol;extracellular membrane-bounded organelle;extracellular organelle;extracellular region part;extracellular vesicular exosome;intracellular part;membrane;membrane-bounded organe	Alanine, aspartate and glutamate metabolism;Purine metabolism	Homo sapiens	1	22
astral microtubule;axon;cell body;cell cortex;cell leading edge;cell part;cell projection;cell projection part;centrosome;chromosomal part;cilium;cytoplasmic microtubule;cytoplasmic part;cytoskeletal part;cytosol;envelope;ext	Ether lipid metabolism	Homo sapiens	1	22
adherens junction;anchoring junction;cell junction;cell part;cell-substrate adherens junction;cell-substrate junction;cytoplasm;cytoplasmic part;cytosol;cytosolic small ribosomal subunit;extracellular membrane-bounded orgai	Ribosome	Homo sapiens	1	22

cell part;cytoplasmic part;cytosol;glutamate-cysteine ligase complex;intracellular part;macromolecular complex;protein complex	Glutathione metabolism	Homo sapiens	1	22
cell part;cytoplasm;extracellular membrane-bounded organelle;extracellular organelle;extracellular region part;extracellular vesicular exosome;intracellular membrane-bounded organelle;intracellular organelle;intracellular part;membrane;membrane-bounded organelle;membrane-bounded vesicle;nucleus;organelle;vesicle		Homo sapiens	1	22
cell part;coated vesicle membrane;contractile fiber part;cytoplasmic part;cytoplasmic vesicle membrane;cytoplasmic vesicle part;endoplasmic reticulum membrane;endoplasmic reticulum part;endoplasmic reticulum-Golgi in	Protein processing in endoplasmic reticulum	Homo sapiens	1	22
cell part;cytoplasmic part;intracellular membrane-bounded organelle;intracellular organelle;intracellular organelle lumen;intracellular organelle part;intracellular part;membrane-bounded organelle;membrane-enclosed lumen;m	Citrate cycle (TCA cycle)	Homo sapiens	1	22
cell part;cytoplasmic part;cytosol;extracellular membrane-bounded organelle;extracellular organelle;extracellular region part;extracellular vesicular exosome;intracellular part;membrane-bounded organelle;membrane-bounde	Porphyrin and chlorophyll metabolism	Homo sapiens	1	22
cell part;coated vesicle membrane;COPII vesicle coat;cytoplasmic part;cytoplasmic vesicle membrane;cytoplasmic vesicle part;cytosol;endoplasmic reticulum membrane;endoplasmic reticulum part;ER to Golgi transport ves	Protein processing in endoplasmic reticulum	Homo sapiens	1	22
cell part;cytoplasm;cytoplasmic part;cytosol;cytosolic small ribosomal subunit;extracellular membrane-bounded organelle;extracellular organelle;extracellular region part;extracellular vesicular exosome;intracellular organelle	Ribosome	Homo sapiens	1	22
cell part;chromosomal part;chromosome;extracellular membrane-bounded organelle;extracellular organelle;extracellular region;extracellular region part;extracellular vesicular exosome;intracellular membrane-bounded organe	Systemic lupus erythematosus	Homo sapiens	1	22
cell part;cytoplasm;cytoplasmic part;cytosol;extracellular membrane-bounded organelle;extracellular organelle;extracellular region part;extracellular vesicular exosome;intracellular part;membrane-bounded organelle;membra	Drug metabolism - cytochrome P450;Glutathione metabolism;Metabolism of xenobiotics by cytochrome	Homo sapiens	1	22
cell part;cytoplasmic part;cytosol;cytosolic large ribosomal subunit;intracellular non-membrane-bounded organelle;intracellular organelle;intracellular organelle part;intracellular part;large ribosomal subunit;macromolecular co	Ribosome	Homo sapiens	1	22
Bcl3/NF-kappaB2 complex;cell part;cytoplasm;cytoplasmic part;cytosol;I-kappaB/NF-kappaB complex;intracellular membrane-bounded organelle;intracellular organelle;intracellular organelle part;intracellular part;macromole	MAPK signaling pathway;Osteoclast differentiation;Pathways in cancer	Homo sapiens	1	22
cell part;cell projection;cilium;cytoplasmic part;cytosol;extracellular membrane-bounded organelle;extracellular organelle;extracellular region part;extracellular space;extracellular vesicular exosome;intracellular organelle part;	Fructose and mannose metabolism	Homo sapiens	1	22
adherens junction;anchoring junction;cell junction;cell part;cell-substrate adherens junction;cell-substrate junction;cytoplasm;cytoplasmic part;cytosol;cytosolic large ribosomal subunit;focal adhesion;intracellular membrane-	Ribosome	Homo sapiens	1	22
cell part;extracellular region part;extracellular space;intracellular non-membrane-bounded organelle;intracellular organelle;intracellular organelle part;intracellular part;non-membrane-bounded organelle;nuclear part;nucleolus;nucleoplasm;organelle;organelle part		Homo sapiens	1	22
cell part;cytoplasm;cytoplasmic part;endoplasmic reticulum;endoplasmic reticulum lumen;endoplasmic reticulum part;endoplasmic reticulum Sec complex;extracellular membrane-bounded organelle;extracellular organelle;ex	Protein processing in endoplasmic reticulum	Homo sapiens	1	22
cell part;Cul2-RING ubiquitin ligase complex;cullin-RING ubiquitin ligase complex;cytoplasmic part;cytosol;intracellular organelle part;intracellular part;macromolecular complex;nuclear part;nucleoplasm;organelle part;protein	Pathways in cancer;Renal cell carcinoma;Ubiquitin mediated proteolysis	Homo sapiens	1	22
cell part;intracellular membrane-bounded organelle;intracellular organelle;intracellular organelle part;intracellular part;membrane-bounded organelle;nuclear part;nucleoplasm;nucleus;organelle;organelle part		Homo sapiens	1	22
adherens junction;anchoring junction;cell junction;cell part;cell-substrate adherens junction;cell-substrate junction;cytoplasm;cytoplasmic part;cytosol;extracellular membrane-bounded organelle;extracellular organelle;extracellular region part;extracellular vesicular exosome;focal adhesion;intracellular membrane-bounded organe		Homo sapiens	1	22
cell junction;cell part;clathrin coated vesicle membrane;clathrin-coated vesicle;coated vesicle;coated vesicle membrane;cytoplasmic membrane-bounded vesicle;cytoplasmic part;cytoplasmic vesicle;cytoplasmic vesicle mer	Endocytosis;Vasopressin-regulated water reabsorption	Homo sapiens	1	22
cell part;intracellular membrane-bounded organelle;intracellular organelle;intracellular organelle part;intracellular part;membrane-bounded organelle;nuclear matrix;nuclear part;nucleoplasm;organelle;organelle part		Homo sapiens	1	22
cell part;cytoplasm;cytoplasmic mRNA processing body;cytoplasmic part;cytosol;intracellular membrane-bounded organelle;intracellular non-membrane-bounded organelle;intracellular organelle;intracellular organelle part;int	RNA degradation	Homo sapiens	1	22
cell part;cytoplasmic part;intracellular membrane-bounded organelle;intracellular organelle;intracellular organelle lumen;intracellular organelle part;intracellular part;membrane-bounded organelle;membrane-enclosed lumen;m	Aminoacyl-tRNA biosynthesis	Homo sapiens	1	22
apical plasma membrane;cell junction;cell part;cell-cell junction;cytoplasm;cytoplasmic part;endoplasmic reticulum;endoplasmic reticulum membrane;endoplasmic reticulum part;integral to membrane;intercellular canaliculus;intracellular membrane-bounded organelle;intracellular non-membrane-bounded organelle;intracellular c		Homo sapiens	1	22
actin filament bundle;actomyosin;adherens junction;anchoring junction;cell junction;cell part;cell projection;cell-substrate adherens junction;cell-substrate junction;cytoplasm;cytoplasmic part;cytoskeletal part;cytosol;filopodi	Regulation of actin cytoskeleton	Homo sapiens	1	22
cell part;cell projection;cilium;cytoplasm;cytoplasmic part;cytosol;extracellular membrane-bounded organelle;extracellular organelle;extracellular region part;extracellular vesicular exosome;intracellular membrane-bounded organelle;intracellular organelle;intracellular part;membrane-bounded organelle;membrane-bounded vesicl		Homo sapiens	1	22
cell part;cytoplasmic part;endoplasmic reticulum;integral to membrane;intracellular membrane-bounded organelle;intracellular organelle;intracellular part;intrinsic to membrane;membrane;membrane part;membrane-bounded organelle;organelle		Homo sapiens	1	22
cell part;chromosome;cytoplasm;intracellular non-membrane-bounded organelle;intracellular organelle;intracellular organelle part;intracellular part;non-membrane-bounded organelle;nuclear part;nucleoplasm;organelle;organelle part		Homo sapiens	1	22
cell part;cytoplasm;cytoplasmic part;early endosome membrane;endosomal part;endosome membrane;intracellular membrane-bounded organelle;intracellular organelle;intracellular organelle part;intracellular part;membrane;	Endocytosis	Homo sapiens	1	22
cell part;cytoplasm;intracellular membrane-bounded organelle;intracellular organelle;intracellular part;membrane-bounded organelle;nucleus;organelle		Homo sapiens	1	22
cell part;cytoplasm;extracellular membrane-bounded organelle;extracellular organelle;extracellular region part;extracellular vesicular exosome;intracellular membrane-bounded organelle;intracellular non-membrane-bounded	Benzoate degradation;Butanoate metabolism;Carbon fixation pathways in prokaryotes;Fatty acid metab	Homo sapiens	1	22
cell part;cell projection;cell projection part;ciliary rootlet;cilium part;cytoplasmic part;cytoskeletal part;cytosol;intracellular organelle part;intracellular part;kinesin complex;kinesin I complex;macromolecular complex;membrane;microtubule;microtubule associated complex;neuron projection;organelle part;protein complex		Homo sapiens	1	22
cell part;cytoplasmic part;early endosome membrane;endoplasmic reticulum;endosomal part;endosome membrane;extracellular membrane-bounded organelle;extracellular organelle;extracellular region part;extracellular vesi	Endocytosis	Homo sapiens	1	22
cell part;cytoplasmic part;integral to membrane;intracellular membrane-bounded organelle;intracellular organelle;intracellular organelle part;intracellular part;intrinsic to membrane;membrane;membrane part;membrane-bounded organelle;mitochondrial inner membrane;mitochondrial membrane;mitochondrial part;mitochondrion;		Homo sapiens	1	22
cell part;cytoplasmic mRNA processing body;cytoplasmic part;cytosol;extracellular region part;extracellular space;intracellular non-membrane-bounded organelle;intracellular organelle;intracellular part;macromolecular complex;non-membrane-bounded organelle;organelle;ribonucleoprotein complex;RNA granule		Homo sapiens	1	22
cell part;cytoplasm;cytoplasmic part;cytosol;extracellular membrane-bounded organelle;extracellular organelle;extracellular region part;extracellular vesicular exosome;intracellular part;membrane-bounded organelle;membra	Amino sugar and nucleotide sugar metabolism	Homo sapiens	1	22
cell part;centrosome;cytoplasm;cytoplasmic part;cytoskeletal part;extracellular membrane-bounded organelle;extracellular organelle;extracellular region part;extracellular vesicular exosome;intracellular non-membrane-bounded organelle;intracellular organelle;intracellular organelle part;intracellular part;membrane;membrane-bo		Homo sapiens	1	22
cell part;intracellular non-membrane-bounded organelle;intracellular organelle;intracellular organelle part;intracellular part;macromolecular complex;non-membrane-bounded organelle;nuclear part;nucleolus;organelle;organell	Ribosome biogenesis in eukaryotes	Homo sapiens	1	22
cell part;intracellular organelle part;intracellular part;nuclear part;nucleoplasm;organelle part		Homo sapiens	1	22
cell part;cytoplasm;intracellular membrane-bounded organelle;intracellular organelle;intracellular organelle part;intracellular part;macromolecular complex;membrane part;membrane-bounded organelle;nuclear part;nuclear pore;nucleus;organelle;organelle part;pore complex;protein complex		Homo sapiens	1	22
cell cortex;cell junction;cell part;cell projection;clathrin coated vesicle membrane;coated vesicle membrane;cytoplasm;cytoplasmic part;cytoplasmic vesicle membrane;cytoplasmic vesicle part;cytoskeletal part;cytosol;dendrite;extracellular membrane-bounded organelle;extracellular organelle;extracellular region part;extracellula		Homo sapiens	1	22
cell part;chromosome;cytoplasm;cytoplasmic part;cytoskeleton;cytosol;Golgi apparatus part;Golgi stack;intermediate filament cytoskeleton;intracellular non-membrane-bounded organelle;intracellular organelle;intracellular o	Protein processing in endoplasmic reticulum	Homo sapiens	1	22
cell part;cytoplasmic part;eukaryotic 43S preinitiation complex;eukaryotic 48S preinitiation complex;eukaryotic translation initiation factor 3 complex;fibrillar center;intracellular organelle part;intracellular part;macromolecular complex;membrane;nuclear part;nucleolar part;nucleoplasm;organelle part;protein complex;ribonucleopr		Homo sapiens	1	22
cell part;cytoplasmic exosome (RNase complex);cytoplasmic part;cytosol;exosome (RNase complex);intracellular organelle part;intracellular part;macromolecular complex;membrane;nuclear exosome (RNase complex);nuclea	RNA degradation	Homo sapiens	1	22
Barr body;cell part;chromosome;intracellular non-membrane-bounded organelle;intracellular organelle;intracellular organelle part;intracellular part;non-membrane-bounded organelle;nuclear chromosome;nuclear part;organelle;organelle part;sex chromosome;X chromosome		Homo sapiens	1	23
cell body;cell part;cell projection;cytoplasm;cytoplasmic part;cytosol;extracellular membrane-bounded organelle;extracellular organelle;extracellular region part;extracellular vesicular exosome;intracellular membrane-bounde	Biosynthesis of unsaturated fatty acids	Homo sapiens	1	23
AP-3 adaptor complex;AP-type membrane coat adaptor complex;cell part;clathrin coated vesicle membrane;coated vesicle membrane;cytoplasmic part;cytoplasmic vesicle membrane;cytoplasmic vesicle part;Golgi apparat	Lysosome	Homo sapiens	1	23
adherens junction;anchoring junction;blood microparticle;cell junction;cell part;cell-substrate adherens junction;cell-substrate junction;cytoplasm;cytoplasmic membrane-bounded vesicle;cytoplasmic part;cytoplasmic vesicle;cytoskeleton;cytosol;endoplasmic reticulum membrane;endoplasmic reticulum part;extracellular membr		Homo sapiens	1	23
cell part;cytoplasm;extracellular membrane-bounded organelle;extracellular organelle;extracellular region part;extracellular vesicular exosome;intracellular membrane-bounded organelle;intracellular organelle;intracellular part;membrane-bounded organelle;membrane-bounded vesicle;nucleus;organelle;vesicle		Homo sapiens	1	23
cell part;cytoplasm;extracellular membrane-bounded organelle;extracellular organelle;extracellular region part;extracellular vesicular exosome;intracellular membrane-bounded organelle;intracellular organelle;intracellular organelle part;intracellular part;macromolecular complex;membrane-bounded organelle;membrane-bounded		Homo sapiens	1	23
cell part;cytoplasmic part;cytosol;eukaryotic 43S preinitiation complex;eukaryotic 48S preinitiation complex;eukaryotic translation initiation factor 3 complex;eukaryotic translation initiation factor 3 complex, eIF3m;intracellula	RNA transport	Homo sapiens	1	23
cell part;cytoplasmic membrane-bounded vesicle;cytoplasmic part;cytoplasmic vesicle;endoplasmic reticulum;endoplasmic reticulum lumen;endoplasmic reticulum part;extracellular region;Golgi apparatus;intracellular membrane-bounded organelle;intracellular organelle;intracellular organelle lumen;intracellular organelle part;int		Homo sapiens	1	23
cell part;cytoplasm;intracellular membrane-bounded organelle;intracellular organelle;intracellular organelle part;intracellular part;membrane;membrane-bounded organelle;nuclear part;nucleoplasm;nucleus;organelle;organelle	Ubiquitin mediated proteolysis	Homo sapiens	1	23
cell part;cytoplasmic part;cytosol;extracellular membrane-bounded organelle;extracellular organelle;extracellular region part;extracellular vesicular exosome;intracellular part;membrane-bounded organelle;membrane-bounde	Sulfur metabolism	Homo sapiens	1	23
adherens junction;anchoring junction;cell junction;cell part;cell-substrate adherens junction;cell-substrate junction;cytoplasm;cytoplasmic part;cytosol;cytosolic large ribosomal subunit;extracellular membrane-bounded orgar	Ribosome	Homo sapiens	1	23
cell part;cytoplasmic part;endoplasmic reticulum membrane;endoplasmic reticulum part;integral to membrane;intracellular organelle part;intracellular part;intrinsic to membrane;membrane;membrane part;organelle membrane	Bile secretion;Metabolism of xenobiotics by cytochrome P450	Homo sapiens	1	23
cell part;cytoplasmic membrane-bounded vesicle;cytoplasmic part;cytoplasmic vesicle;extracellular membrane-bounded organelle;extracellular organelle;extracellular region;extracellular region part;extracellular space;extrac	Antigen processing and presentation;Lysosome	Homo sapiens	1	23
cell part;cytoplasmic part;endoplasmic reticulum;endoplasmic reticulum membrane;endoplasmic reticulum part;extracellular membrane-bounded organelle;extracellular organelle;extracellular region part;extracellular vesicula	Protein export	Homo sapiens	1	23
adherens junction;anchoring junction;cell junction;cell part;cell-substrate adherens junction;cell-substrate junction;cytoplasmic part;cytosol;cytosolic small ribosomal subunit;extracellular membrane-bounded organelle;extrac	Ribosome	Homo sapiens	1	23
90S preribosome;cell part;cytoplasm;cytoplasmic part;cytosol;cytosolic small ribosomal subunit;extracellular membrane-bounded organelle;extracellular organelle;extracellular region part;extracellular vesicular exosome;intra	Ribosome	Homo sapiens	1	23
cell part;cytoplasm;cytoplasmic membrane-bounded vesicle;cytoplasmic part;cytoplasmic vesicle;extracellular membrane-bounded organelle;extracellular organelle;extracellular region part;extracellular vesicular exosome;Go	Methane metabolism	Homo sapiens	1	23
axon;cell part;cell projection;cytoplasmic part;extracellular membrane-bounded organelle;extracellular organelle;extracellular region part;extracellular vesicular exosome;intracellular membrane-bounded organelle;intracellular	beta-Alanine metabolism;Fatty acid metabolism;PPAR signaling pathway;Propanoate metabolism;Valine	Homo sapiens	1	23
cell part;cytoplasm;extracellular membrane-bounded organelle;extracellular organelle;extracellular region part;extracellular vesicular exosome;intracellular organelle part;intracellular part;membrane;membrane-bounded organelle;membrane-bounded vesicle;nuclear membrane;nuclear part;organelle;organelle membrane;organelle		Homo sapiens	1	23
axon part;axon terminus;cell part;cell projection part;cytoplasm;cytoplasmic part;cytosol;endoplasmic reticulum;extracellular membrane-bounded organelle;extracellular organelle;extracellular region part;extracellular vesicula	Alanine, aspartate and glutamate metabolism;Arginine and proline metabolism;Nitrogen metabolism;Two	Homo sapiens	1	23
adherens junction;alphav-beta5 integrin complex;anchoring junction;cell junction;cell leading edge;cell part;cell surface;cell-substrate adherens junction;cell-substrate junction;cytoplasmic membrane-bounded vesicle;cytopla	Arrhythmogenic right ventricular cardiomyopathy (ARVC);Dilated cardiomyopathy;ECM-receptor interact	Homo sapiens	1	23
cell part;cytoplasm;cytoplasmic part;cytosol;eukaryotic translation initiation factor 2 complex;intracellular part;macromolecular complex;protein complex	RNA transport	Homo sapiens	1	23
cell part;cytoplasm;cytoplasmic part;cytosol;extracellular membrane-bounded organelle;extracellular organelle;extracellular region part;extracellular vesicular exosome;intracellular membrane-bounded organelle;intracellular c	Proteasome	Homo sapiens	1	23
cell part;chromosomal part;DNA replication factor A complex;germ cell nucleus;intracellular membrane-bounded organelle;intracellular organelle;intracellular organelle part;intracellular part;lateral element;macromolecular cor	DNA replication;Homologous recombination;Mismatch repair;Nucleotide excision repair	Homo sapiens	1	23
cell part;cytoplasm;cytoplasmic part;cytosol;extracellular membrane-bounded organelle;extracellular organelle;extracellular region part;extracellular vesicular exosome;intracellular part;membrane-bounded organelle;membrane-bounded vesicle;organelle;vesicle		Homo sapiens	1	23
adherens junction;anchoring junction;cell junction;cell part;cell-substrate adherens junction;cell-substrate junction;cytoplasm;cytoplasmic part;cytosol;cytosolic large ribosomal subunit;focal adhesion;intracellular membrane-	Ribosome	Homo sapiens	1	23
cell part;cytoplasm;cytoplasmic part;cytosol;extracellular membrane-bounded organelle;extracellular organelle;extracellular region part;extracellular space;extracellular vesicular exosome;intracellular part;macromolecular cor	Amoebiasis	Homo sapiens	1	23
cell part;cytoplasm;cytoplasmic part;cytosol;endoplasmic reticulum;inclusion body;intracellular membrane-bounded organelle;intracellular organelle;intracellular organelle part;intracellular part;isoamylase complex;macromole	Starch and sucrose metabolism	Homo sapiens	1	23
adherens junction;anchoring junction;cell junction;cell part;cell-substrate adherens junction;cell-substrate junction;cytoplasmic part;cytosol;focal adhesion;intracellular membrane-bounded organelle;intracellular organelle;intra	RNA transport	Homo sapiens	1	23
cell part;cytoplasmic part;extracellular membrane-bounded organelle;extracellular organelle;extracellular region part;extracellular vesicular exosome;intracellular membrane-bounded organelle;intracellular organelle;intracellular organelle lumen;intracellular organelle part;intracellular part;membrane-bounded organelle;membrane-		Homo sapiens	1	23
cell part;cytoplasmic part;cytosol;intracellular part	Nicotinate and nicotinamide metabolism	Homo sapiens	1	23
cell part;cytoplasm;cytoplasmic part;cytosol;extracellular membrane-bounded organelle;extracellular organelle;extracellular region part;extracellular vesicular exosome;intracellular;intracellular membrane-bounded organelle;i	Arachidonic acid metabolism;Metabolism of xenobiotics by cytochrome P450;Steroid hormone biosynth	Homo sapiens	1	23
cell part;cytoplasm;cytoplasmic part;cytosol;cytosolic large ribosomal subunit;extracellular membrane-bounded organelle;extracellular organelle;extracellular region part;extracellular vesicular exosome;intracellular non-memb	Ribosome	Homo sapiens	1	23
cell part;clathrin-coated vesicle;coated vesicle;cytoplasmic membrane-bounded vesicle;cytoplasmic part;cytoplasmic vesicle;cytosol;endoplasmic reticulum;endoplasmic reticulum membrane;endoplasmic reticulum part;end	Protein processing in endoplasmic reticulum	Homo sapiens	1	23
cell part;cytoplasm;cytoplasmic part;cytosol;cytosolic small ribosomal subunit;extracellular membrane-bounded organelle;extracellular organelle;extracellular region part;extracellular vesicular exosome;intracellular membrane	Ribosome	Homo sapiens	1	23
cell part;cytoplasm;cytoplasmic part;cytosol;extracellular membrane-bounded organelle;extracellular organelle;extracellular region part;extracellular vesicular exosome;extrinsic to internal side of plasma membrane;extrinsic t	Amoebiasis;Bile secretion;Calcium signaling pathway;Chagas disease (American trypanosomiasis);Dilate	Homo sapiens	1	23
cell part;centrosome;cytoplasm;cytoplasmic part;cytoskeletal part;extracellular membrane-bounded organelle;extracellular organelle;extracellular region part;extracellular vesicular exosome;extrinsic to internal side of plasma	Axon guidance;Chagas disease (American trypanosomiasis);Chemokine signaling pathway;Gap junction	Homo sapiens	1	23
cell part;intracellular membrane-bounded organelle;intracellular organelle;intracellular organelle part;intracellular part;membrane-bounded organelle;nuclear part;nucleoplasm;nucleus;organelle;organelle part		Homo sapiens	1	23
cell part;cytoplasm;cytoplasmic part;cytosol;extracellular membrane-bounded organelle;extracellular organelle;extracellular region part;extracellular vesicular exosome;intracellular organelle part;intracellular part;membrane-b	PPAR signaling pathway	Homo sapiens	1	23
cell part;chromatin;chromosomal part;cytoplasm;exosome (RNase complex);intracellular membrane-bounded organelle;intracellular non-membrane-bounded organelle;intracellular organelle;intracellular organelle part;intracel	RNA degradation	Homo sapiens	1	23
cell part;cytoplasmic part;cytosol;intracellular organelle part;intracellular part;macromolecular complex;membrane;nuclear part;nucleoplasm;organelle part;protein complex	African trypanosomiasis;Alzheimer's disease;Amoebiasis;Calcium signaling pathway;Chagas disease (A	Homo sapiens	1	23
cell part;chromatin;chromosomal part;euchromatin;intracellular organelle part;intracellular part;macromolecular complex;nuclear chromatin;nuclear chromosome part;nuclear euchromatin;nuclear part;nucleosome;organelle part;protein-DNA complex		Homo sapiens	1	23
adherens junction;anchoring junction;axon;cell cortex;cell junction;cell part;cell projection;cell projection cytoplasm;cell projection part;cell-substrate adherens junction;cell-substrate junction;cytoplasm;cytoplasmic part;cytos	Acute myeloid leukemia;B cell receptor signaling pathway;Bladder cancer;Chemokine signaling pathway	Homo sapiens	1	23
cell part;cytoplasm;cytoplasmic part;cytosol;intracellular membrane-bounded organelle;intracellular organelle;intracellular part;macromolecular complex;membrane-bounded organelle;mitochondrion;nucleus;organelle;protein	Adipocytokine signaling pathway;Chronic myeloid leukemia;Epithelial cell signaling in Helicobacter pylor	Homo sapiens	1	23
cell part;cytoplasm;intracellular membrane-bounded organelle;intracellular organelle;intracellular organelle part;intracellular part;macromolecular complex;membrane-bounded organelle;nuclear part;nucleus;organelle;organelle part;protein complex;signalosome		Homo sapiens	1	23
adherens junction;anchoring junction;cell junction;cell part;cell-substrate adherens junction;cell-substrate junction;cytoplasm;cytoplasmic part;cytosol;focal adhesion;intracellular part;presynaptic active zone;synapse part		Homo sapiens	1	23
adherens junction;anchoring junction;cell junction;cell part;cell-cell junction;cell-substrate adherens junction;cell-substrate junction;focal adhesion;integral to membrane;integral to plasma membrane;intrinsic to membrane;intrinsic to plasma membrane;membrane part;plasma membrane part		Homo sapiens	1	23
cell part;chromosomal part;condensin complex;cytoplasm;cytoplasmic part;cytosol;intracellular membrane-bounded organelle;intracellular organelle;intracellular organelle part;intracellular part;macromolecular complex;mem	Cell cycle - yeast	Homo sapiens	1	23
basal lamina;basement membrane;extracellular matrix part;extracellular membrane-bounded organelle;extracellular organelle;extracellular region;extracellular region part;extracellular vesicular exosome;laminin complex;lamir	African trypanosomiasis;Amoebiasis;ECM-receptor interaction;Focal adhesion;Pathways in cancer;Sma	Homo sapiens	1	23
actin cytoskeleton;actomyosin;cell cortex;cell junction;cell part;cell projection;cell-cell junction;cytoplasm;cytoplasmic part;cytoskeletal part;cytoskeleton;dendrite;gap junction;intracellular non-membrane-bounded organelle;intracellular organelle;intracellular organelle part;intracellular part;membrane;neuron projection;non-meml		Homo sapiens	1	23
Cajal body;cell part;cytoplasm;intracellular membrane-bounded organelle;intracellular non-membrane-bounded organelle;intracellular organelle;intracellular organelle part;intracellular part;membrane;membrane-bounded orga	Spliceosome	Homo sapiens	1	23
cell part;cytoplasmic part;cytosol;endoplasmic reticulum;intracellular membrane-bounded organelle;intracellular organelle;intracellular organelle part;intracellular part;membrane;membrane part;membrane-bounded organelle	Protein processing in endoplasmic reticulum	Homo sapiens	1	23
actin cytoskeleton;Arp2/3 protein complex;cell part;cytoplasmic part;cytoskeletal part;cytoskeleton;cytosol;extracellular membrane-bounded organelle;extracellular organelle;extracellular region part;extracellular vesicular exc	Bacterial invasion of epithelial cells;Fc gamma R-mediated phagocytosis;Pathogenic Escherichia coli int	Homo sapiens	1	23
cell part;cytoplasmic part;intracellular membrane-bounded organelle;intracellular organelle;intracellular organelle lumen;intracellular organelle part;intracellular part;macromolecular complex;membrane;membrane-bounded or	Citrate cycle (TCA cycle);Propanoate metabolism	Homo sapiens	1	23
cell part;chromatin;chromosomal part;chromosome;chromosome, centromeric region;cytoplasmic part;cytosol;intracellular membrane-bounded organelle;intracellular non-membrane-bounded organelle;intracellular organelle;intracellular organelle part;intracellular part;membrane-bounded organelle;non-membrane-bounded orga		Homo sapiens	1	23
cell part;cytoplasm;intracellular membrane-bounded organelle;intracellular organelle;intracellular part;membrane;membrane-bounded organelle;nucleus;organelle		Homo sapiens	1	23
cell part;cytoplasmic part;intracellular membrane-bounded organelle;intracellular organelle;intracellular part;membrane-bounded organelle;mitochondrion;organelle		Homo sapiens	1	23
cell part;chromosome;condensed chromosome;cytoplasm;intracellular membrane-bounded organelle;intracellular non-membrane-bounded organelle;intracellular organelle;intracellular organelle part;intracellular part;macromolecular complex;membrane;membrane-bounded organelle;non-membrane-bounded organelle;nuclear p		Homo sapiens	1	24
box C/D snoRNP complex;cell part;cytoplasm;intracellular non-membrane-bounded organelle;intracellular organelle;intracellular organelle part;intracellular part;macromolecular complex;membrane;non-membrane-bounded (	Ribosome biogenesis in eukaryotes	Homo sapiens	1	24
cell part;chromatin;chromosomal part;cytoplasmic part;cytosol;heterochromatin;intracellular membrane-bounded organelle;intracellular organelle;intracellular organelle part;intracellular part;membrane-bounded organelle;nuclear chromatin;nuclear chromosome part;nuclear heterochromatin;nuclear part;nuclear periphery;nucleop		Homo sapiens	1	24
cell part;cytoplasmic membrane-bounded vesicle;cytoplasmic part;cytoplasmic vesicle;extracellular membrane-bounded organelle;extracellular organelle;extracellular region;extracellular region part;extracellular space;extrac	ko05152;Lysosome	Homo sapiens	1	24
acrosomal vesicle;cell part;cytoplasmic membrane-bounded vesicle;cytoplasmic part;cytoplasmic vesicle;extracellular membrane-bounded organelle;extracellular organelle;extracellular region part;extracellular vesicular exos	Amino sugar and nucleotide sugar metabolism;Glycosaminoglycan degradation;Glycosphingolipid biosy	Homo sapiens	1	24
adherens junction;anchoring junction;blood microparticle;cell cortex;cell junction;cell part;cell projection;cell-substrate adherens junction;cell-substrate junction;cytoplasm;cytoplasmic part;cytoskeleton;cytosol;extracellular r	Regulation of actin cytoskeleton;Shigellosis	Homo sapiens	1	24
adherens junction;alphav-beta3 integrin-vitronectin complex;anchoring junction;cell junction;cell part;cell projection;cell surface;cell-substrate adherens junction;cell-substrate junction;cytoplasm;external side of plasma mem	Arrhythmogenic right ventricular cardiomyopathy (ARVC);Bacterial invasion of epithelial cells;Dilated car	Homo sapiens	1	24
cell part;cytoplasmic part;extracellular membrane-bounded organelle;extracellular organelle;extracellular region part;extracellular vesicular exosome;intracellular membrane-bounded organelle;intracellular organelle;intracellula	Chloroalkane and chloroalkene degradation;Drug metabolism - cytochrome P450;Fatty acid metabolism	Homo sapiens	1	24
extracellular membrane-bounded organelle;extracellular organelle;extracellular region part;extracellular vesicular exosome;macromolecular complex;membrane-bounded organelle;membrane-bounded vesicle;organelle;protei	Pentose phosphate pathway;Purine metabolism	Homo sapiens	1	24
cell part;cytoplasmic part;cytosol;cytosolic large ribosomal subunit;extracellular membrane-bounded organelle;extracellular organelle;extracellular region part;extracellular vesicular exosome;intracellular membrane-bounded	Ribosome	Homo sapiens	1	24
cell part;cytoplasmic part;extracellular membrane-bounded organelle;extracellular organelle;extracellular region part;extracellular vesicular exosome;intracellular membrane-bounded organelle;intracellular organelle;intracellula	Alzheimer's disease;Cardiac muscle contraction;Huntington's disease;Oxidative phosphorylation;Parkin	Homo sapiens	1	24
cell part;centrosome;cytoplasm;cytoplasmic part;cytoskeletal part;endoplasmic reticulum;intracellular membrane-bounded organelle;intracellular non-membrane-bounded organelle;intracellular organelle;intracellular organelle	Base excision repair	Homo sapiens	1	24
adherens junction;anchoring junction;axon;caveola;cell junction;cell part;cell projection;cell projection cytoplasm;cell projection part;cell-substrate adherens junction;cell-substrate junction;cytoplasm;cytoplasmic part;cytoske	Acute myeloid leukemia;Adherens junction;Aldosterone-regulated sodium reabsorption;Alzheimer's dise	Homo sapiens	1	24
cell part;cytoplasmic part;cytosol;intracellular membrane-bounded organelle;intracellular organelle;intracellular part;membrane-bounded organelle;mitochondrion;organelle	Alanine, aspartate and glutamate metabolism;Purine metabolism	Homo sapiens	1	24
cell part;cytoplasmic part;intracellular membrane-bounded organelle;intracellular organelle;intracellular organelle lumen;intracellular organelle part;intracellular part;membrane-bounded organelle;membrane-enclosed lumen;m	Arginine and proline metabolism;Ascorbate and aldarate metabolism;beta-Alanine metabolism;Chloroall	Homo sapiens	1	24
catalytic step 2 spliceosome;cell part;intracellular membrane-bounded organelle;intracellular non-membrane-bounded organelle;intracellular organelle;intracellular organelle part;intracellular part;macromolecular complex;mer	RNA degradation	Homo sapiens	1	24
cell part;cytoplasm;cytoplasmic part;intracellular membrane-bounded organelle;intracellular non-membrane-bounded organelle;intracellular organelle;intracellular organelle lumen;intracellular organelle part;intracellular part;m	Fatty acid metabolism	Homo sapiens	1	24
cell part;cytoplasm;cytoplasmic part;cytosol;intracellular membrane-bounded organelle;intracellular organelle;intracellular organelle part;intracellular part;membrane-bounded organelle;nuclear part;nucleoplasm;nucleus;orgar	Long-term potentiation;MAPK signaling pathway;mTOR signaling pathway;Neurotrophin signaling pathw	Homo sapiens	1	24
cell part;cytoplasmic part;cytosol;extracellular membrane-bounded organelle;extracellular organelle;extracellular region part;extracellular vesicular exosome;intracellular organelle part;intracellular part;membrane;membrane-b	Purine metabolism	Homo sapiens	1	24
actin cytoskeleton;cell cortex part;cell part;cortical actin cytoskeleton;cortical cytoskeleton;cytoplasmic part;cytoskeletal part;cytoskeleton;extracellular membrane-bounded organelle;extracellular organelle;extracellular region part;extracellular vesicular exosome;intracellular non-membrane-bounded organelle;intracellular organe		Homo sapiens	1	24
adherens junction;anchoring junction;cell junction;cell part;cell-substrate adherens junction;cell-substrate junction;cytoplasmic part;cytosol;cytosolic large ribosomal subunit;extracellular membrane-bounded organelle;extrac	Ribosome	Homo sapiens	1	24
adherens junction;anchoring junction;cell junction;cell part;cell-substrate adherens junction;cell-substrate junction;cytoplasmic part;cytosol;cytosolic large ribosomal subunit;extracellular membrane-bounded organelle;extrac	Ribosome	Homo sapiens	1	24
cell part;centrosome;cytoplasm;cytoplasmic part;cytoskeletal part;cytosol;cytosolic large ribosomal subunit;intracellular non-membrane-bounded organelle;intracellular organelle;intracellular organelle part;intracellular part;lar	Ribosome	Homo sapiens	1	24
cell part;intracellular membrane-bounded organelle;intracellular organelle;intracellular part;membrane-bounded organelle;nucleus;organelle	Spliceosome	Homo sapiens	1	24
cell part;cytoplasmic part;extracellular membrane-bounded organelle;extracellular organelle;extracellular region part;extracellular vesicular exosome;integral to membrane;integral to plasma membrane;intracellular membrane-bounded organelle;intracellular organelle;intracellular organelle part;intracellular part;intrinsic to membra		Homo sapiens	1	24
cell part;cytoplasm;intracellular part		Homo sapiens	1	24
cell part;cytoplasm;cytoplasmic part;cytosol;DNA topoisomerase complex (ATP-hydrolyzing);intracellular membrane-bounded organelle;intracellular organelle;intracellular organelle part;intracellular part;macromolecular complex;membrane-bounded organelle;nuclear part;nucleoplasm;nucleus;organelle;organelle part;protein com		Homo sapiens	1	24
cell part;cytoplasmic part;cytosol;extracellular membrane-bounded organelle;extracellular organelle;extracellular region part;extracellular vesicular exosome;intracellular organelle part;intracellular part;macromolecular comple	Proteasome	Homo sapiens	1	24
actin cytoskeleton;cell cortex part;cell division site part;cell part;cell projection membrane;cell projection part;chromosomal part;ciliary transition zone;cilium membrane;cilium part;cleavage furrow;condensed chromosome kinetochore;cytoplasm;cytoplasmic part;cytoskeletal part;cytoskeleton;exocyst;extracellular membrane-bo		Homo sapiens	1	24

cell part;cytoplasm;cytoplasmic part;endoplasmic reticulum part;endoplasmic reticulum tubular network;extracellular membrane-bounded organelle;extracellular organelle;extracellular region part;extracellular vesicular exosome;intracellular organelle part;intracellular part;macromolecular complex;membrane-bounded organelle;r		Homo sapiens	1	24
cell part;cytoplasm;extracellular membrane-bounded organelle;extracellular organelle;extracellular region part;extracellular vesicular exosome;intracellular membrane-bounded organelle;intracellular organelle;intracellular orga	Spliceosome	Homo sapiens	1	24
cell part;cytoplasm;cytoplasmic part;cytosol;intracellular membrane-bounded organelle;intracellular organelle;intracellular organelle part;intracellular part;membrane;membrane-bounded organelle;nuclear part;nucleoplasm;nucleus;organelle;organelle part		Homo sapiens	1	24
cell part;cytoplasm;cytoskeletal part;intracellular non-membrane-bounded organelle;intracellular organelle;intracellular organelle part;intracellular part;macromolecular complex;microtubule;non-membrane-bounded organelle;organelle;organelle part;protein complex;spindle		Homo sapiens	1	24
cell part;cytoplasm;cytoplasmic part;integral to membrane;intracellular membrane-bounded organelle;intracellular organelle;intracellular organelle part;intracellular part;intrinsic to membrane;membrane;membrane part;membrane-bounded organelle;mitochondrial inner membrane;mitochondrial membrane;mitochondrial part;mitc		Homo sapiens	1	24
cell part;cytoplasmic part;intracellular membrane-bounded organelle;intracellular organelle;intracellular part;membrane;membrane-bounded organelle;mitochondrion;organelle	One carbon pool by folate	Homo sapiens	1	24
actin cytoskeleton;adherens junction;anchoring junction;cell junction;cell part;cell-substrate adherens junction;cell-substrate junction;cytoplasm;cytoskeleton;focal adhesion;intracellular non-membrane-bounded organelle;intracellular organelle;intracellular part;non-membrane-bounded organelle;organelle		Homo sapiens	1	24
cell part;cytoplasm;cytoplasmic part;cytosolic part;Golgi apparatus part;Golgi membrane;IkappaB kinase complex;intracellular organelle part;intracellular part;macromolecular complex;membrane;organelle membrane;organelle part;presynaptic active zone;presynaptic membrane;protein complex;synapse;synapse part;synaptic r		Homo sapiens	1	24
cell part;cytoplasm;cytoplasmic exosome (RNase complex);cytoplasmic part;exosome (RNase complex);intracellular membrane-bounded organelle;intracellular organelle;intracellular organelle part;intracellular part;macromol	Purine metabolism;Pyrimidine metabolism;RNA degradation	Homo sapiens	1	24
cell part;chromosomal part;condensed chromosome kinetochore;cytoplasmic part;cytosol;envelope;intracellular non-membrane-bounded organelle;intracellular organelle;intracellular organelle part;intracellular part;kinetocho	RNA transport	Homo sapiens	1	24
actin filament bundle;actomyosin;adherens junction;anchoring junction;cell junction;cell part;cell-cell junction;cell-substrate adherens junction;cell-substrate junction;cytoskeletal part;cytoskeleton;extracellular membrane-bounded organelle;extracellular organelle;extracellular region part;extracellular vesicular exosome;focal adhe		Homo sapiens	1	24
cell part;cytoplasm;intracellular membrane-bounded organelle;intracellular organelle;intracellular organelle part;intracellular part;macromolecular complex;membrane-bounded organelle;nuclear part;nucleoplasm;nucleus;organelle;organelle part;ribonucleoprotein complex		Homo sapiens	1	24
cell part;cytoplasm;intracellular part;macromolecular complex;membrane;plasma membrane;protein complex		Homo sapiens	1	24
cell part;cytoplasm;cytoplasmic part;cytosol;intracellular membrane-bounded organelle;intracellular organelle;intracellular organelle part;intracellular part;macromolecular complex;membrane-bounded organelle;nuclear part;r	RNA transport	Homo sapiens	1	24
beta-catenin destruction complex;cell body;cell part;cell projection;cytoplasm;cytoplasmic part;extracellular membrane-bounded organelle;extracellular organelle;extracellular region part;extracellular vesicular exosome;intrac	Wnt signaling pathway	Homo sapiens	1	24
cell part;cytoplasmic part;extracellular membrane-bounded organelle;extracellular organelle;extracellular region part;extracellular vesicular exosome;intracellular membrane-bounded organelle;intracellular organelle;intracellula	Citrate cycle (TCA cycle);Propanoate metabolism	Homo sapiens	1	24
cell part;cytoplasmic part;intracellular membrane-bounded organelle;intracellular non-membrane-bounded organelle;intracellular organelle;intracellular organelle lumen;intracellular organelle part;intracellular part;membrane;m	Ether lipid metabolism;Peroxisome	Homo sapiens	1	25
adherens junction;anchoring junction;cell junction;cell part;cell-substrate adherens junction;cell-substrate junction;cytoplasm;cytoskeleton;focal adhesion;intracellular non-membrane-bounded organelle;intracellular organelle;intracellular organelle part;intracellular part;macromolecular complex;non-membrane-bounded organelle		Homo sapiens	1	25
cell part;DSIF complex;intracellular membrane-bounded organelle;intracellular organelle;intracellular organelle part;intracellular part;macromolecular complex;membrane-bounded organelle;nuclear part;nucleoplasm;nucleoplasm part;nucleus;organelle;organelle part;protein complex;transcription elongation factor complex		Homo sapiens	1	25
actin cytoskeleton;adherens junction;anchoring junction;Arp2/3 protein complex;cell junction;cell leading edge;cell part;cell projection;cell-substrate adherens junction;cell-substrate junction;cytoplasm;cytoplasmic part;cytos	Bacterial invasion of epithelial cells;Fc gamma R-mediated phagocytosis;Pathogenic Escherichia coli inf	Homo sapiens	1	25
cell part;cytoplasm;cytoplasmic part;cytosol;endoplasmic reticulum;intracellular membrane-bounded organelle;intracellular non-membrane-bounded organelle;intracellular organelle;intracellular organelle part;intracellular part	Protein export	Homo sapiens	1	25
cell part;cornified envelope;cytoplasm;cytoskeleton;extracellular membrane-bounded organelle;extracellular organelle;extracellular region part;extracellular vesicular exosome;intracellular non-membrane-bounded organelle;intracellular organelle;intracellular part;membrane-bounded organelle;membrane-bounded vesicle;non-me		Homo sapiens	1	25
cell part;centrosome;cytoplasm;cytoplasmic membrane-bounded vesicle;cytoplasmic part;cytoplasmic vesicle;cytoskeletal part;extracellular membrane-bounded organelle;extracellular organelle;extracellular region part;extra	Axon guidance;Chagas disease (American trypanosomiasis);Chemokine signaling pathway;Gap junction	Homo sapiens	1	25
adherens junction;anchoring junction;cell junction;cell part;cell-substrate adherens junction;cell-substrate junction;extracellular membrane-bounded organelle;extracellular organelle;extracellular region part;extracellular vesicular exosome;focal adhesion;intracellular membrane-bounded organelle;intracellular organelle;intracellula		Homo sapiens	1	25
adherens junction;alpha3-beta1 integrin complex;anchoring junction;basolateral plasma membrane;cell junction;cell part;cell periphery;cell projection membrane;cell projection part;cell surface;cell-substrate adherens junctio	Arrhythmogenic right ventricular cardiomyopathy (ARVC);Dilated cardiomyopathy;ECM-receptor interact	Homo sapiens	1	25
cell part;cell projection;centrosome;chromosomal part;cytoplasm;cytoplasmic microtubule;cytoplasmic part;cytoplasmic vesicle membrane;cytoplasmic vesicle part;cytoskeletal part;cytoskeleton;cytosol;endosome;intermediate filament;intracellular membrane-bounded organelle;intracellular non-membrane-bounded organelle;ir		Homo sapiens	1	25
[truncated: 650,616 more chars]
