# Supplementary material for: Extracellular Vesicles Bearing Vimentin Drive Epithelial–Mesenchymal Transition
Source: Mol Cell Proteomics. 2025 Jul 4;24(12):101028. doi: 10.1016/j.mcpro.2025.101028 (PMC12719745; doi:10.1016/j.mcpro.2025.101028)
Supplement: Supplemental Data 9 [file mmc12.pdf]

Sheet 9.Supplementary data for Fig1

**Fig1C HDF Evs and HDF Vim -/- Evs group (n=668)**					
Biological process					
Enrichment FDR	nGenes	Pathway Genes	Fold Enrichment	Pathway	URL
3.57514444591713E-73	80	178	16.3751520790095	GO:0002181 cytoplasmic translation	http://amigo.geneontology.org/amigo/term/GO:0002181
3.71135883762984E-21	44	240	6.6796974522293	GO:0022618 ribonucleoprotein complex assembly	http://amigo.geneontology.org/amigo/term/GO:0022618
1.3818869800738E-20	44	248	6.46422334086706	GO:0071826 ribonucleoprotein complex subunit organization	http://amigo.geneontology.org/amigo/term/GO:0071826
2.16963702468326E-49	117	784	5.43732329715326	GO:0006412 translation	http://amigo.geneontology.org/amigo/term/GO:0006412
8.32014818671021E-48	117	814	5.23693054664392	GO:0043043 peptide biosynthetic proc.	http://amigo.geneontology.org/amigo/term/GO:0043043
7.31693868142031E-22	64	509	4.58118203546357	GO:0022613 ribonucleoprotein complex biogenesis	http://amigo.geneontology.org/amigo/term/GO:0022613
8.62278168355583E-45	125	999	4.55889807004457	GO:0006518 peptide metabolic proc.	http://amigo.geneontology.org/amigo/term/GO:0006518
4.16485774325969E-42	119	959	4.52109582035427	GO:0043604 amide biosynthetic proc.	http://amigo.geneontology.org/amigo/term/GO:0043604
3.88609970345142E-36	130	1301	3.64067082156303	GO:0043603 cellular amide metabolic proc.	http://amigo.geneontology.org/amigo/term/GO:0043603
1.13102756071606E-33	124	1259	3.58848646433576	GO:0034645 cellular macromolecule biosynthetic proc.	http://amigo.geneontology.org/amigo/term/GO:0034645
4.05303947204803E-28	145	1888	2.79821686413689	GO:1901566 organonitrogen compound biosynthetic proc.	http://amigo.geneontology.org/amigo/term/GO:1901566
4.69865808097095E-25	147	2069	2.5886432412963	GO:0043933 protein-containing complex organization	http://amigo.geneontology.org/amigo/term/GO:0043933
1.34625259267335E-21	131	1859	2.56748114697649	GO:0065003 protein-containing complex assembly	http://amigo.geneontology.org/amigo/term/GO:0065003
1.61501750302301E-33	227	3630	2.27842422487761	GO:0044085 cellular component biogenesis	http://amigo.geneontology.org/amigo/term/GO:0044085
7.36182148044374E-28	205	3365	2.21964821457302	GO:0022607 cellular component assembly	http://amigo.geneontology.org/amigo/term/GO:0022607
Cellular Component					
Enrichment FDR	nGenes	Pathway Genes	Fold Enrichment	Pathway	URL
7.54777158387269E-50	41	57	26.2074254106604	GO:0022625 cytosolic large ribosomal subunit	http://amigo.geneontology.org/amigo/term/GO:0022625
1.68470873673497E-68	66	122	19.7105826459225	GO:0022626 cytosolic ribosome	http://amigo.geneontology.org/amigo/term/GO:0022626
6.64197588581541E-49	67	222	10.9960621449475	GO:0044391 ribosomal subunit	http://amigo.geneontology.org/amigo/term/GO:0044391
1.18376832076594E-43	69	283	8.88337534604218	GO:0005840 ribosome	http://amigo.geneontology.org/amigo/term/GO:0005840
1.0589603229958E-48	92	484	6.92560667473812	GO:0030055 cell-substrate junction	http://amigo.geneontology.org/amigo/term/GO:0030055
1.6802449133958E-47	90	475	6.90341937646664	GO:0005925 focal adhesion	http://amigo.geneontology.org/amigo/term/GO:0005925
1.09509595551498E-55	124	807	5.5983946203206	GO:1990904 ribonucleoprotein complex	http://amigo.geneontology.org/amigo/term/GO:1990904
3.56882541231459E-142	312	2316	4.90830335632487	GO:0070062 extracellular exosome	http://amigo.geneontology.org/amigo/term/GO:0070062
3.56882541231459E-142	313	2342	4.8693703187977	GO:1903561 extracellular vesicle	http://amigo.geneontology.org/amigo/term/GO:1903561
3.56882541231459E-142	313	2343	4.86729205575083	GO:0043230 extracellular organelle	http://amigo.geneontology.org/amigo/term/GO:0043230
3.56882541231459E-142	313	2343	4.86729205575083	GO:0065010 extracellular membrane-bounded organelle	http://amigo.geneontology.org/amigo/term/GO:0065010
6.87347066237206E-36	107	926	4.21005867301317	GO:0070161 anchoring junction	http://amigo.geneontology.org/amigo/term/GO:0070161
2.78191730402219E-108	336	3577	3.42243882982038	GO:0005615 extracellular space	http://amigo.geneontology.org/amigo/term/GO:0005615
7.31912233906033E-111	376	4466	3.06749937960129	GO:0031982 vesicle	http://amigo.geneontology.org/amigo/term/GO:0031982
6.1475816612537E-93	361	4673	2.81466542449442	GO:0005576 extracellular region	http://amigo.geneontology.org/amigo/term/GO:0005576
Fig1D cluster 1,n=343					
Biological process					
Enrichment FDR	nGenes	Pathway Genes	Fold Enrichment	Pathway	URL
3.34219921585093E-06	17	209	5.65694216198136	GO:0000070 mitotic sister chromatid segregation	http://amigo.geneontology.org/amigo/term/GO:0000070
3.57595490602388E-06	18	238	5.25986564838701	GO:0000819 sister chromatid segregation	http://amigo.geneontology.org/amigo/term/GO:0000819
1.48604220665802E-06	36	825	3.03478308925117	GO:1903047 mitotic cell cycle proc.	http://amigo.geneontology.org/amigo/term/GO:1903047
1.78115395253096E-06	40	996	2.79305672538177	GO:0000278 mitotic cell cycle	http://amigo.geneontology.org/amigo/term/GO:0000278
1.78115395253096E-06	42	1082	2.69961065009635	GO:0006396 RNA processing	http://amigo.geneontology.org/amigo/term/GO:0006396
1.43409400944614E-09	67	1795	2.59590893312223	GO:0045184 establishment of protein localization	http://amigo.geneontology.org/amigo/term/GO:0045184
1.06937483339316E-08	62	1680	2.56661962657403	GO:0015031 protein transport	http://amigo.geneontology.org/amigo/term/GO:0015031
9.4784307040316E-08	60	1709	2.44167744161519	GO:0046907 intracellular transport	http://amigo.geneontology.org/amigo/term/GO:0046907
2.80566236925216E-07	68	2175	2.17434650455927	GO:0071705 nitrogen compound transport	http://amigo.geneontology.org/amigo/term/GO:0071705
9.74484198606787E-08	74	2388	2.15514502604206	GO:0051649 establishment of localization in cell	http://amigo.geneontology.org/amigo/term/GO:0051649
9.74484198606787E-08	79	2642	2.07956922199034	GO:0008104 protein localization	http://amigo.geneontology.org/amigo/term/GO:0008104
9.4784307040316E-08	89	3120	1.98387596446107	GO:0051641 cellular localization	http://amigo.geneontology.org/amigo/term/GO:0051641
1.35780525137282E-07	88	3133	1.95344586551437	GO:0033036 macromolecule localization	http://amigo.geneontology.org/amigo/term/GO:0033036
4.83222757647645E-06	73	2618	1.93924339561743	GO:0071702 organic substance transport	http://amigo.geneontology.org/amigo/term/GO:0071702
2.19455993382035E-07	107	4206	1.76926795849611	GO:0006996 organelle organization	http://amigo.geneontology.org/amigo/term/GO:0006996
Cellular Component					
Enrichment FDR	nGenes	Pathway Genes	Fold Enrichment	Pathway	URL
0.00523990982708971	4	18	15.4549138804458	GO:0032433 filopodium tip	http://amigo.geneontology.org/amigo/term/GO:0032433
0.00620194318460758	4	20	13.9094224924012	GO:0042575 DNA polymerase complex	http://amigo.geneontology.org/amigo/term/GO:0042575
0.00234543730515741	11	172	4.44778044815155	GO:0000776 kinetochore	http://amigo.geneontology.org/amigo/term/GO:0000776
0.00359130954624609	11	182	4.20339690704432	GO:0000779 condensed chromosome centromeric region	http://amigo.geneontology.org/amigo/term/GO:0000779
7.85103068802927E-05	17	287	4.11951537231395	GO:0000793 condensed chromosome	http://amigo.geneontology.org/amigo/term/GO:0000793
0.00203762001285629	14	265	3.67418707346447	GO:0000775 chromosome centromeric region	http://amigo.geneontology.org/amigo/term/GO:0000775
6.73041042273601E-05	31	807	2.67157433249342	GO:1990904 ribonucleoprotein complex	http://amigo.geneontology.org/amigo/term/GO:1990904
2.17083247589611E-09	57	1489	2.66231390888808	GO:0140513 nuclear protein-containing complex	http://amigo.geneontology.org/amigo/term/GO:0140513
8.37422828973311E-07	43	1126	2.65588440130219	GO:0005730 nucleolus	http://amigo.geneontology.org/amigo/term/GO:0005730
0.000342419033843857	31	884	2.43886932841877	GO:0140535 intracellular protein-containing complex	http://amigo.geneontology.org/amigo/term/GO:0140535
2.09818327577277E-05	52	1695	2.13359873039783	GO:1902494 catalytic complex	http://amigo.geneontology.org/amigo/term/GO:1902494
5.19705424373064E-18	147	4973	2.05578635268749	GO:0031981 nuclear lumen	http://amigo.geneontology.org/amigo/term/GO:0031981
6.73041042273601E-05	53	1830	2.01420598933679	GO:0005739 mitochondrion	http://amigo.geneontology.org/amigo/term/GO:0005739
9.88515274951996E-15	132	4581	2.00397704540162	GO:0005654 nucleoplasm	http://amigo.geneontology.org/amigo/term/GO:0005654
0.00620194318460758	49	2003	1.70135222697868	GO:0005694 chromosome	http://amigo.geneontology.org/amigo/term/GO:0005694
Fig1D cluster 2, n=919					
Biological process					
Enrichment FDR	nGenes	Pathway Genes	Fold Enrichment	Pathway	URL
1.44384504507409E-27	81	475	4.51598684210526	GO:0042060 wound healing	http://amigo.geneontology.org/amigo/term/GO:0042060
7.61180981706409E-25	84	563	3.95122853759621	GO:0030335 positive reg. of cell migration	http://amigo.geneontology.org/amigo/term/GO:0030335
1.32868932485959E-28	122	977	3.30694160127374	GO:0030334 reg. of cell migration	http://amigo.geneontology.org/amigo/term/GO:0030334
3.24233338382316E-27	123	1038	3.13811616891458	GO:2000145 reg. of cell motility	http://amigo.geneontology.org/amigo/term/GO:2000145
1.06119337795534E-25	123	1085	3.00217933947773	GO:0040012 reg. of locomotion	http://amigo.geneontology.org/amigo/term/GO:0040012
6.71937413703928E-26	125	1109	2.98496831479812	GO:0051270 reg. of cellular component movement	http://amigo.geneontology.org/amigo/term/GO:0051270
5.94381087519808E-28	161	1602	2.66148867734776	GO:0016477 cell migration	http://amigo.geneontology.org/amigo/term/GO:0016477
5.13681698117223E-28	167	1695	2.60920394952475	GO:0016192 vesicle-mediated transport	http://amigo.geneontology.org/amigo/term/GO:0016192
6.41920706318956E-27	166	1729	2.54257840113103	GO:0007155 cell adhesion	http://amigo.geneontology.org/amigo/term/GO:0007155
3.72186841531266E-26	194	2254	2.2793398156364	GO:0006928 movement of cell or subcellular component	http://amigo.geneontology.org/amigo/term/GO:0006928
3.30801740118497E-32	243	2867	2.2446045517963	GO:0009653 anatomical structure morphogenesis	http://amigo.geneontology.org/amigo/term/GO:0009653
6.8134865450896E-27	217	2642	2.17514482925393	GO:0008104 protein localization	http://amigo.geneontology.org/amigo/term/GO:0008104
6.18880263361836E-28	236	2945	2.12220807394831	GO:0032879 reg. of localization	http://amigo.geneontology.org/amigo/term/GO:0032879
1.03943651457797E-26	242	3133	2.04557887363904	GO:0033036 macromolecule localization	http://amigo.geneontology.org/amigo/term/GO:0033036
1.03943651457797E-26	267	3630	1.94789657943067	GO:0044085 cellular component biogenesis	http://amigo.geneontology.org/amigo/term/GO:0044085
Cellular Component					
Enrichment FDR	nGenes	Pathway Genes	Fold Enrichment	Pathway	URL
1.25817047577214E-49	107	484	5.8546329775023	GO:0030055 cell-substrate junction	http://amigo.geneontology.org/amigo/term/GO:0030055
1.06576600165646E-48	105	475	5.85405701754386	GO:0005925 focal adhesion	http://amigo.geneontology.org/amigo/term/GO:0005925
1.45380215358449E-43	97	453	5.67067543536914	GO:0062023 collagen-containing extracellular matrix	http://amigo.geneontology.org/amigo/term/GO:0062023
2.25524370400402E-36	102	602	4.48709163898117	GO:0031012 extracellular matrix	http://amigo.geneontology.org/amigo/term/GO:0031012
2.42091993417036E-36	102	603	4.47965035931454	GO:0030312 external encapsulating structure	http://amigo.geneontology.org/amigo/term/GO:0030312
1.92414503900814E-36	128	926	3.66066714662827	GO:0070161 anchoring junction	http://amigo.geneontology.org/amigo/term/GO:0070161
1.85988206096412E-94	312	2342	3.52800313122687	GO:1903561 extracellular vesicle	http://amigo.geneontology.org/amigo/term/GO:1903561
1.85988206096412E-94	312	2343	3.52649736804666	GO:0043230 extracellular organelle	http://amigo.geneontology.org/amigo/term/GO:0043230
1.85988206096412E-94	312	2343	3.52649736804666	GO:0065010 extracellular membrane-bounded organelle	http://amigo.geneontology.org/amigo/term/GO:0065010
5.84450012115368E-93	308	2316	3.5218708021493	GO:0070062 extracellular exosome	http://amigo.geneontology.org/amigo/term/GO:0070062
1.78738007032711E-77	362	3577	2.68009932283416	GO:0005615 extracellular space	http://amigo.geneontology.org/amigo/term/GO:0005615
1.85456822393362E-72	401	4466	2.37786345598846	GO:0031982 vesicle	http://amigo.geneontology.org/amigo/term/GO:0031982
3.13769216551184E-34	242	2849	2.24949056199056	GO:0031410 cytoplasmic vesicle	http://amigo.geneontology.org/amigo/term/GO:0031410
3.28477375374541E-34	242	2851	2.2479125258194	GO:0097708 intracellular vesicle	http://amigo.geneontology.org/amigo/term/GO:0097708
1.23429533946413E-61	392	4673	2.22152673752289	GO:0005576 extracellular region	http://amigo.geneontology.org/amigo/term/GO:0005576
Fig1D cluster 3, n=81					
Biological process					
Enrichment FDR	nGenes	Pathway Genes	Fold Enrichment	Pathway	URL
4.10018545990273E-07	9	149	17.9490107208228	GO:0030833 reg. of actin filament polymerization	http://amigo.geneontology.org/amigo/term/GO:0030833
4.10018545990273E-07	10	206	14.425040978439	GO:0120032 reg. of plasma membrane bounded cell projectio	http://amigo.geneontology.org/amigo/term/GO:0120032
4.10018545990273E-07	10	208	14.2863386613387	GO:0060491 reg. of cell projection assembly	http://amigo.geneontology.org/amigo/term/GO:0060491
5.17351578954335E-07	10	215	13.8212020537602	GO:0008154 actin polymerization or depolymerization	http://amigo.geneontology.org/amigo/term/GO:0008154
1.76243479992814E-10	17	438	11.5334460060487	GO:0043254 reg. of protein-containing complex assembly	http://amigo.geneontology.org/amigo/term/GO:0043254
5.75307737624923E-07	11	288	11.3497023809524	GO:0110053 reg. of actin filament organization	http://amigo.geneontology.org/amigo/term/GO:0110053
9.28459770111727E-09	15	418	10.6634872304729	GO:0032970 reg. of actin filament-based proc.	http://amigo.geneontology.org/amigo/term/GO:0032970
6.87385627055299E-07	12	376	9.48369715390992	GO:0032956 reg. of actin cytoskeleton organization	http://amigo.geneontology.org/amigo/term/GO:0032956
2.19779015921027E-07	14	481	8.64902664902665	GO:0007015 actin filament organization	http://amigo.geneontology.org/amigo/term/GO:0007015
2.38333588575793E-08	19	857	6.58805255421358	GO:0030029 actin filament-based proc.	http://amigo.geneontology.org/amigo/term/GO:0030029
6.87385627055299E-07	16	769	6.18269636735176	GO:0030036 actin cytoskeleton organization	http://amigo.geneontology.org/amigo/term/GO:0030036

1.06568749763388E-08	21	1025	6.08807095343681	GO:0044087 reg. of cellular component biogenesis	http://amigo.geneontology.org/amigo/term/GO:0044087
2.42997762280055E-07	25	1859	3.9961786465283	GO:0065003 protein-containing complex assembly	http://amigo.geneontology.org/amigo/term/GO:0065003
1.03896292839858E-07	27	2069	3.87781913591483	GO:0043933 protein-containing complex organization	http://amigo.geneontology.org/amigo/term/GO:0043933
7.13900522054498E-08	30	2509	3.55307904530702	GO:0051128 reg. of cellular component organization	http://amigo.geneontology.org/amigo/term/GO:0051128
Cellular Component					
Enrichment FDR	nGenes	Pathway Genes	Fold Enrichment	Pathway	URL
4.66565398525576E-09	11	216	15.1329365079365	GO:0030027 lamellipodium	http://amigo.geneontology.org/amigo/term/GO:0030027
2.11543858324834E-09	15	475	9.38386876281613	GO:0005925 focal adhesion	http://amigo.geneontology.org/amigo/term/GO:0005925
6.96995379425166E-09	14	446	9.32776192417448	GO:0031252 cell leading edge	http://amigo.geneontology.org/amigo/term/GO:0031252
2.47543128277809E-09	15	484	9.20937533540839	GO:0030055 cell-substrate junction	http://amigo.geneontology.org/amigo/term/GO:0030055
1.02770927830428E-09	16	532	8.9370178693487	GO:0015629 actin cytoskeleton	http://amigo.geneontology.org/amigo/term/GO:0015629
4.66565398525576E-09	19	926	6.09715015006592	GO:0070161 anchoring junction	http://amigo.geneontology.org/amigo/term/GO:0070161
4.48318412550014E-21	43	2316	5.51714218424063	GO:0070062 extracellular exosome	http://amigo.geneontology.org/amigo/term/GO:0070062
4.48318412550014E-21	43	2342	5.45589295418501	GO:1903561 extracellular vesicle	http://amigo.geneontology.org/amigo/term/GO:1903561
4.48318412550014E-21	43	2343	5.45356436137486	GO:0043230 extracellular organelle	http://amigo.geneontology.org/amigo/term/GO:0043230
4.48318412550014E-21	43	2343	5.45356436137486	GO:0065010 extracellular membrane-bounded organelle	http://amigo.geneontology.org/amigo/term/GO:0065010
3.34531503675987E-09	29	2293	3.75818555626667	GO:0030054 cell junction	http://amigo.geneontology.org/amigo/term/GO:0030054
5.9260701680227E-15	44	3577	3.65525779783538	GO:0005615 extracellular space	http://amigo.geneontology.org/amigo/term/GO:0005615
3.63118454063848E-07	27	2477	3.23908267751627	GO:0042995 cell projection	http://amigo.geneontology.org/amigo/term/GO:0042995
1.35728345123495E-14	48	4466	3.19379321976725	GO:0031982 vesicle	http://amigo.geneontology.org/amigo/term/GO:0031982
1.6424698652602E-11	45	4673	2.86154782516863	GO:0005576 extracellular region	http://amigo.geneontology.org/amigo/term/GO:0005576
Fig1D cluster 4, n=1262					
Biological process					
Enrichment FDR	nGenes	Pathway Genes	Fold Enrichment	Pathway	URL
9.57926739682441E-78	101	178	10.6157312557994	GO:0002181 cytoplasmic translation	http://amigo.geneontology.org/amigo/term/GO:0002181
4.41227774664707E-43	99	341	5.43161976103184	GO:0000377 RNA splicing via transesterification reactions wit	http://amigo.geneontology.org/amigo/term/GO:0000377
4.41227774664707E-43	99	341	5.43161976103184	GO:0000398 mRNA splicing via spliceosome	http://amigo.geneontology.org/amigo/term/GO:0000398
2.26168558015473E-43	100	345	5.42287319136834	GO:0000375 RNA splicing via transesterification reactions	http://amigo.geneontology.org/amigo/term/GO:0000375
1.68438497813094E-79	201	784	4.79654517162548	GO:0006412 translation	http://amigo.geneontology.org/amigo/term/GO:0006412
5.05294043473181E-79	204	814	4.68872008855656	GO:0043043 peptide biosynthetic proc.	http://amigo.geneontology.org/amigo/term/GO:0043043
5.0267357335967E-45	122	495	4.61108550756956	GO:0008380 RNA splicing	http://amigo.geneontology.org/amigo/term/GO:0008380
2.26168558015473E-43	125	537	4.35496101262122	GO:0006397 mRNA processing	http://amigo.geneontology.org/amigo/term/GO:0006397
5.69337857651322E-80	228	999	4.26990195428462	GO:0006518 peptide metabolic proc.	http://amigo.geneontology.org/amigo/term/GO:0006518
3.60897845454908E-74	215	959	4.19438601636858	GO:0043604 amide biosynthetic proc.	http://amigo.geneontology.org/amigo/term/GO:0043604
5.55310752142835E-76	255	1301	3.6670043736405	GO:0043603 cellular amide metabolic proc.	http://amigo.geneontology.org/amigo/term/GO:0043603
2.58566054579476E-43	157	823	3.56901490170676	GO:0016071 mRNA metabolic proc.	http://amigo.geneontology.org/amigo/term/GO:0016071
6.24121276772192E-58	206	1082	3.5619556165485	GO:0006396 RNA processing	http://amigo.geneontology.org/amigo/term/GO:0006396
6.24121276772192E-58	224	1259	3.32867069284309	GO:0034645 cellular macromolecule biosynthetic proc.	http://amigo.geneontology.org/amigo/term/GO:0034645
1.98703911162634E-66	297	1888	2.94308634297435	GO:1901566 organonitrogen compound biosynthetic proc.	http://amigo.geneontology.org/amigo/term/GO:1901566
Cellular Component					
Enrichment FDR	nGenes	Pathway Genes	Fold Enrichment	Pathway	URL
5.18209615135762E-43	44	57	14.4419675517494	GO:0022625 cytosolic large ribosomal subunit	http://amigo.geneontology.org/amigo/term/GO:0022625
8.58594690117165E-62	75	122	11.5013806415292	GO:0022626 cytosolic ribosome	http://amigo.geneontology.org/amigo/term/GO:0022626
4.89560853307553E-44	81	222	6.82622483481028	GO:0044391 ribosomal subunit	http://amigo.geneontology.org/amigo/term/GO:0044391
3.14444466106616E-44	91	283	6.01594006512399	GO:0005840 ribosome	http://amigo.geneontology.org/amigo/term/GO:0005840
7.0866507852943E-30	66	220	5.61267375306623	GO:0005681 spliceosomal complex	http://amigo.geneontology.org/amigo/term/GO:0005681
4.6138991030757E-94	220	807	5.10032311307134	GO:1990904 ribonucleoprotein complex	http://amigo.geneontology.org/amigo/term/GO:1990904
3.74141115086848E-29	97	475	3.82055687050824	GO:0005925 focal adhesion	http://amigo.geneontology.org/amigo/term/GO:0005925
1.56882214816924E-76	351	2316	2.8354180876889	GO:0070062 extracellular exosome	http://amigo.geneontology.org/amigo/term/GO:0070062
1.60695705419613E-75	351	2342	2.80394034632258	GO:1903561 extracellular vesicle	http://amigo.geneontology.org/amigo/term/GO:1903561
1.60695705419613E-75	351	2343	2.80274361548762	GO:0043230 extracellular organelle	http://amigo.geneontology.org/amigo/term/GO:0043230
1.60695705419613E-75	351	2343	2.80274361548762	GO:0065010 extracellular membrane-bounded organelle	http://amigo.geneontology.org/amigo/term/GO:0065010
4.83931606638523E-72	543	4973	2.04281912186806	GO:0031981 nuclear lumen	http://amigo.geneontology.org/amigo/term/GO:0031981
9.36035735548061E-59	488	4581	1.99300355926386	GO:0005654 nucleoplasm	http://amigo.geneontology.org/amigo/term/GO:0005654
1.61605995371122E-41	378	3577	1.97706707544407	GO:0005615 extracellular space	http://amigo.geneontology.org/amigo/term/GO:0005615
1.17835354736815E-48	457	4466	1.91445880366567	GO:0031982 vesicle	http://amigo.geneontology.org/amigo/term/GO:0031982

Genes
RPL31 EIF3I RPL6 RPLP0 EIF4H EIF3A RPL24 RPL22 RPL36 EIF3M RPL8 RPL26 RPL29 RPL9 CNBP RPL15 RPL35A RPL17 RPS20 RPL18 RPS5 EIF3L RPL3 EIF3D RPS16 RPS19 RPL18A EIF3B RPL28
STRAP RPS5 RPL6 RPLP0 SNRPD3 RPL3 RPS19 EIF4H EIF3A PTGES3 SF3B1 RPL5 SRSF6 SNRPD2 LSM4 SNRNP200 RPL10 RPS14 SNRPD1 RPL38 RUVBL2 RPL23A RPS28 EIF3I SF3B2 EIF3L EIF3D
STRAP RPS5 RPL6 RPLP0 SNRPD3 RPL3 RPS19 EIF4H EIF3A PTGES3 SF3B1 RPL5 SRSF6 SNRPD2 LSM4 SNRNP200 RPL10 RPS14 SNRPD1 RPL38 RUVBL2 RPL23A RPS28 EIF3I SF3B2 EIF3L EIF3D
SARS1 KARS1 RPL31 RPS5 EIF3I RPL6 RPLP0 AARS1 EIF3L EIF3D RBM3 GARS1 EIF3B EIF4H EIF3A TARS1 RARS1 RPL24 FXR1 DARS1 FARSB RPL22 RPL36 LARS1 NARS1 EPRS1 WARS1 RPS2 RPL
SARS1 KARS1 RPL31 RPS5 EIF3I RPL6 RPLP0 AARS1 EIF3L EIF3D RBM3 GARS1 EIF3B EIF4H EIF3A TARS1 RARS1 RPL24 FXR1 DARS1 FARSB RPL22 RPL36 LARS1 NARS1 EPRS1 WARS1 RPS2 RPL
STRAP XPO1 RPS5 RPL6 RPLP0 SNRPD3 RPL3 RPS16 RPS19 EIF4H EIF3A UTP6 PTGES3 SF3B1 RPL5 SRSF6 SNRPD2 LSM4 RAN RPL35 RPS8 SNRNP200 RPL10 RPL7 RPL26 ABCE1 RPS14 SNRPD1
SARS1 KARS1 RPL31 RPS5 EIF3I RPL6 RPLP0 AARS1 EIF3L EIF3D RBM3 GARS1 EIF3B EIF4H EIF3A TARS1 RARS1 RPL24 FXR1 DARS1 FARSB RPL22 RPL36 LARS1 NARS1 EPRS1 WARS1 RPS2 NPE
SARS1 KARS1 RPL31 RPS5 EIF3I RPL6 RPLP0 AARS1 EIF3L EIF3D RBM3 GARS1 EIF3B EIF4H EIF3A TARS1 RARS1 RPL24 FXR1 DARS1 FARSB RPL22 RPL36 LARS1 NARS1 EPRS1 WARS1 RPS2 RPL
SARS1 KARS1 RPL31 RPS5 EIF3I RPL6 RPLP0 AARS1 ACOT7 EIF3L EIF3D RBM3 GARS1 EIF3B EIF4H EIF3A TARS1 RARS1 RPL24 FXR1 DARS1 FARSB RPL22 RPL36 LARS1 NARS1 EPRS1 WARS1 RP
SARS1 KARS1 RPL31 RPS5 EIF3I RPL6 RPLP0 AARS1 EIF3L EIF3D RBM3 GARS1 EIF3B EIF4H EIF3A TARS1 RARS1 RPL24 FXR1 DARS1 FARSB RPL22 RPL36 LARS1 NARS1 EPRS1 WARS1 RPS2 RPL
SARS1 KARS1 RPL31 MOXD1 RPS5 EIF3I RPL6 RPLP0 AARS1 EIF3L EIF3D SMS RBM3 NAMPT GARS1 EIF3B EIF4H EIF3A UGDH ATP5F1B TARS1 RARS1 RPL24 FXR1 DARS1 FARSB RPL22 SRM URO
STRAP TMSB10 VAMP3 WDR1 SAR1A RPS5 RPL6 RPLP0 SNRPD3 RPL3 PSMC6 COTL1 RPS19 NAPA EIF4H EIF3A PSMD9 PTGES3 CAND1 SF3B1 RPL5 SRSF6 SNRPD2 VASP DSTN LSM4 TUBG1 DBN
STRAP TMSB10 VAMP3 SAR1A RPS5 RPL6 RPLP0 SNRPD3 RPL3 PSMC6 COTL1 RPS19 NAPA EIF4H EIF3A PSMD9 PTGES3 CAND1 SF3B1 RPL5 SRSF6 SNRPD2 VASP LSM4 TUBG1 DBNL SNRNP200
ANLN STRAP EHD2 TMSB10 VAMP3 LIMA1 NCKAP1 WDR1 NTN4 FSCN1 SAR1A XPO1 RPS5 NSFL1C DYNLL1 RPL6 RPLP0 SNRPD3 RPL3 PSMC6 MYL9 MAPRE1 COTL1 EHD4 RPS16 RPS19 NAPA EI
ANLN STRAP EHD2 TMSB10 VAMP3 LIMA1 NCKAP1 WDR1 NTN4 FSCN1 SAR1A RPS5 NSFL1C DYNLL1 RPL6 RPLP0 SNRPD3 RPL3 PSMC6 MYL9 MAPRE1 COTL1 EHD4 RPS19 NAPA EIF4H EIF3A PS

Genes
RPL18 RPL31 RPL6 RPLP0 RPL3 RPL18A RPL28 RPL19 RPL34 RPL24 RPL22 RPL21 RPL5 RPL23 RPL36 RPL27 RPL35 RPL13A RPL11 RPL32 RPL10 RPL7 RPL30 RPL8 RPL26 RPL29 RPL9 RPL27A RP
RPS20 RPL18 RPL31 RPS5 RPL6 RPLP0 RPL3 RPS16 RPS19 RPL18A RPL28 RPL19 RPL34 RPS13 RPS12 RPL24 RPL22 RPL21 RPL5 RPS10 RPL23 RPL36 RPL27 RPS15A RPL35 RPS6 RPS24 RPS2
RPS20 RPL18 RPL31 RPS5 RPL6 RPLP0 RPL3 RPS16 RPS19 RPL18A RPL28 RPL19 RPL34 RPS13 RPS12 RPL24 RPL22 RPL21 RPL5 RPS10 RPL23 RPL36 RPL27 RPS15A RPL35 RPS6 RPS24 RPS2
RPS20 RPL18 RPL31 RPS5 RPL6 RPLP0 RPL3 RPS16 RPS19 RPL18A RPL28 RPL19 RPL34 RPS13 RPS12 RPL24 RPL22 RPL21 RPL5 RPS10 RPL23 RPL36 RPL27 RPS15A RPL35 RPS6 RPS24 RPS2
LAP3 RALA MRC2 TSPAN9 VIM LIMA1 NCKAP1 RPL18 PABPC1 RPL31 PVR FERMT2 RAB21 RPS5 RPL6 RPLP0 PXN NRP1 TRIOBP PACSIN2 RPL3 RPS16 RPS19 PDLIM1 RPL19 RPS13 CORO1C ARPC
LAP3 RALA MRC2 TSPAN9 VIM LIMA1 NCKAP1 RPL18 PABPC1 RPL31 PVR FERMT2 RAB21 RPS5 RPL6 RPLP0 PXN NRP1 TRIOBP PACSIN2 RPL3 RPS16 RPS19 PDLIM1 RPL19 RPS13 CORO1C ARPC
RPS20 RPL18 RPL31 RPS5 RPL6 RPLP0 RPL3 RPS16 RPS19 RPL18A RPL28 RPL19 RPL34 RPS13 RPS12 RPL24 RPL22 RPL21 RPL5 RPS10 RPL23 RPL36 RPL27 RPS15A RPL35 RPS6 RPS24 RPS2
TSPAN6 LAP3 RALA RPS20 VTA1 PTBP1 MVP HEBP1 GPRC5A ERP44 EHD2 VIM SARS1 PSMA4 AKR7A2 THRAP3 PHPT1 SLC2A3 NCKAP1 SLK DIP2B VPS35 DNAJA2 RAB27A SLC44A1 PABPC1 RPL31
TSPAN6 LAP3 RALA RPS20 VTA1 PTBP1 MVP HEBP1 GPRC5A ERP44 EHD2 VIM SARS1 PSMA4 AKR7A2 THRAP3 PHPT1 SLC2A3 NCKAP1 SLK DIP2B VPS35 DNAJA2 RAB27A SLC44A1 PABPC1 RPL31
TSPAN6 LAP3 RALA RPS20 VTA1 PTBP1 MVP HEBP1 GPRC5A ERP44 EHD2 VIM SARS1 PSMA4 AKR7A2 THRAP3 PHPT1 SLC2A3 NCKAP1 SLK DIP2B VPS35 DNAJA2 RAB27A SLC44A1 PABPC1 RPL31
TSPAN6 LAP3 RALA RPS20 VTA1 PTBP1 MVP HEBP1 GPRC5A ERP44 EHD2 VIM SARS1 PSMA4 AKR7A2 THRAP3 PHPT1 SLC2A3 NCKAP1 SLK DIP2B VPS35 DNAJA2 RAB27A SLC44A1 PABPC1 RPL31
LAP3 RALA MRC2 TSPAN9 VIM LIMA1 NCKAP1 RPL18 PABPC1 RPL31 PVR FERMT2 RAB21 RPS5 RPL6 RPLP0 PXN NRP1 TRIOBP PACSIN2 RPL3 RPS16 RPS19 PDLIM1 RPL19 RPS13 CORO1C ARPC
TSPAN6 LAP3 RALA RPS20 VTA1 PTBP1 MVP HEBP1 GPRC5A ERP44 EHD2 VIM SARS1 PSMA4 AKR7A2 THRAP3 PHPT1 SLC2A3 NCKAP1 SLK DIP2B VPS35 DNAJA2 RAB27A SLC44A1 PABPC1 RPL31
TSPAN6 LAP3 RALA RPS20 VTA1 PTBP1 MVP HEBP1 GPRC5A ERP44 EHD2 VIM SARS1 PSMA4 AKR7A2 THRAP3 PHPT1 SLC2A3 NCKAP1 SLK DIP2B VPS35 DNAJA2 RAB27A SLC44A1 PABPC1 RPL31
TSPAN6 LAP3 RALA RPS20 VTA1 PTBP1 MVP HEBP1 GPRC5A ERP44 EHD2 VIM SARS1 PSMA4 AKR7A2 THRAP3 PHPT1 SLC2A3 NCKAP1 SLK DIP2B VPS35 DNAJA2 RAB27A SLC44A1 PABPC1 RPL31

Genes
SPDL1 NDC80 NUSAP1 KIF23 NUF2 NCAPG2 RAD21 DPF2 ACTL6A CHMP5 AKAP8 PCID2 PSMG2 TACC3 SPAG5 EML4 CENPF
SPDL1 NDC80 TOP2A NUSAP1 KIF23 NUF2 NCAPG2 RAD21 DPF2 ACTL6A CHMP5 AKAP8 PCID2 PSMG2 TACC3 SPAG5 EML4 CENPF
SPDL1 NDC80 TPX2 POLA1 CENPF NUSAP1 KIF23 NUF2 NCAPG2 MKI67 CLASP2 RAD21 GINS3 DPF2 ACTL6A EXOC7 MNAT1 NES CDC73 TACC3 CHMP5 AKAP8 KIF20A CKS2 PCID2 PSMG2 PLRG1
SPDL1 NDC80 TPX2 POLA1 CENPF CKS2 NUSAP1 KIF23 NUF2 NCAPG2 MKI67 CLASP2 RAD21 GINS3 DPF2 ACTL6A EXOC7 MNAT1 NES CDC73 TACC3 CHMP5 AKAP8 NUP88 KIF20A RNF2 PCID2 PS
CLNS1A EXOSC7 XAB2 DIMT1 ALKBH5 POP1 PIH1D1 PTBP3 AHNAK RNF113A SAFB2 THUMPD3 CDC73 RBM17 MPHOSPH6 SNRPF CWC15 SSU72 NOL9 PRPF18 TSR1 PLRG1 POP7 CSTF2T GEMIN4
ARIH2 RANBP3 SCAMP1 ZFAND6 RAB3D ATP13A1 NUP88 ZPR1 WLS RAB32 RAB22A NAPG TIMM10 OS9 RANBP6 RAB5A MIA3 ARFIP1 MFF RAB43 MTX1 KRT18 CENPF PTPN14 MMGT1 NUP160 AP2
ARIH2 RANBP3 SCAMP1 ZFAND6 RAB3D NUP88 ZPR1 WLS RAB32 RAB22A NAPG TIMM10 OS9 RANBP6 RAB5A MIA3 ARFIP1 MFF RAB43 MTX1 KRT18 CENPF PTPN14 NUP160 AP2S1 CHMP5 AAAS
ARIH2 RANBP3 TBC1D23 CHMP5 ZFAND6 ALKBH5 AAAS NUP88 GOSR1 ZPR1 TFG CCDC88A WLS RAB32 RAB9A RAB22A PCID2 NAPG TIMM10 OS9 LTV1 RANBP6 RHOT2 RAB5A MIA3 ATP6V0D1 IW
ARIH2 RANBP3 SCAMP1 ZFAND6 ALKBH5 RAB3D NUP88 ZPR1 WLS RAB32 RAB22A PCID2 NAPG TIMM10 OS9 RANBP6 RAB5A MIA3 IWS1 THOC7 ARFIP1 PHAX MFF RAB43 SLC25A22 MTX1 RALBP
ARIH2 RANBP3 TBC1D23 SPDL1 NDC80 CHMP5 ZFAND6 ALKBH5 AAAS NUP88 GOSR1 ZPR1 TFG CCDC88A WLS CENPF RAB32 RAB9A RAB22A PCID2 NAPG TIMM10 OS9 LTV1 RANBP6 NUSAP1 RH
ARIH2 RANBP3 SPDL1 SCAMP1 ZFAND6 RAB3D ATP13A1 NUP88 ZPR1 WLS RAB32 OPTN RAB22A NAPG PPHLN1 TIMM10 OS9 RANBP6 RAB5A LIN7C MIA3 ARFIP1 MFF RAB43 MTX1 SP100 KRT18
ARIH2 RANBP3 TBC1D23 SPDL1 NDC80 CHMP5 ZFAND6 ALKBH5 AAAS RAB3D ATP13A1 NUP88 GOSR1 ZPR1 TFG CCDC88A WLS CENPF RAB32 OPTN RAB9A RAB22A PCID2 NAPG PPHLN1 TIMM10
ARIH2 RANBP3 SPDL1 SCAMP1 ZFAND6 ALKBH5 RAB3D ATP13A1 NUP88 ZPR1 WLS RAB32 OPTN RAB22A PCID2 NAPG PPHLN1 TIMM10 OS9 RANBP6 RAB5A LIN7C MIA3 IWS1 THOC7 ARFIP1 PHA
ARIH2 RANBP3 SCAMP1 ZFAND6 ALKBH5 RAB3D NUP88 ZPR1 WLS RAB32 RAB22A PCID2 NAPG TIMM10 OS9 RANBP6 RAB5A MIA3 IWS1 THOC7 ARFIP1 PHAX MFF RAB43 SLC25A22 NFKB1 MTX1
MBD3 ACTL6A ARIH2 TACC3 SPDL1 NDC80 CHMP5 ZFAND6 TPX2 GOSR1 ZPR1 KRT18 CCDC88A RND3 RAB32 SLIRP OPTN ATPAF1 PCID2 TOP2A NES PPHLN1 TIMM10 KRT7 NUSAP1 KIF23 DIAPH3

Genes
ABI1 ABI2 MYO5A AP2A1
POLA1 POLD2 POLE3 CHRAC1
SPDL1 NDC80 NUF2 ACTL6A NUP160 SPAG5 CHMP5 CENPF NUP43 CLASP2 ERCC6L
SPDL1 NDC80 NUF2 ACTL6A NUP160 SPAG5 CHMP5 CENPF NUP43 CLASP2 ERCC6L
SPDL1 NDC80 NUF2 RAD21 ACTL6A NUP160 SPAG5 CHMP5 CENPF NUP43 MKI67 AKAP8 TOP2A NCAPG2 ADD3 CLASP2 ERCC6L
SPDL1 NDC80 CENPF NUF2 ACTL6A NUP160 SPAG5 CHMP5 NUP43 CHRAC1 TOP2A CLASP2 RAD21 ERCC6L
RPL39 CLNS1A XAB2 POP1 PIH1D1 NUFIP2 RNF113A LTV1 MRPS9 SNRPF CWC15 PRPF18 TSR1 CD2BP2 PLRG1 MRPS22 MAK16 PPIL3 MRPS23 MRPL20 NSRP1 TOP2A NAA35 PHAX POP7 EIF3J RB
PAF1 MNAT1 NUP160 RANBP3 CLNS1A EXOSC7 XAB2 AAAS POLA1 POLR2C POP1 CHRAC1 POLD2 NUP88 POLR1G NUP43 RNF2 MORF4L2 RNF113A PCID2 DPF2 CDC73 ACTL6A POLR1E SNRPF CC
NOP16 POP1 CCDC86 DDX55 POLR1G POLR1E NUSAP1 MAK16 EXOSC7 HSPB6 MRI1 SP100 DIMT1 POLA1 STK24 PIH1D1 AKAP8 GRWD1 RBM28 ZPR1 UCHL5 MORF4L2 TOP2A THUMPD3 MPHOSPH
PAF1 MNAT1 POLA1 POLR2C POLD2 CUL2 RNF7 POLR1G RNF2 MORF4L2 CKS2 CDC73 NAA35 ACTL6A POLR1E PPP2R1B CNOT9 POLE3 RNF20 UBE2L6 ARIH2 UBQLN4 PATL1 USP47 UCHL5 UBE4B
PAF1 MNAT1 NDUFB4 EXOSC7 XAB2 POLA1 POLR2C POP1 CHRAC1 POLD2 CUL2 RNF7 POLR1G RNF2 MORF4L2 CKS2 DAD1 NDUFA2 DPF2 CDC73 NAA35 ACTL6A POLR1E PPP2R1B SNRPF POLE3
PAF1 MNAT1 NOP16 EXOSC7 POLA1 POLR2C POP1 AKAP8 POLD2 NUFIP2 ZPR1 CCDC86 DDX55 POLR1G MORF4L2 PPHLN1 CDC73 ACTL6A POLR1E NUSAP1 POLE3 DTX3L RAD21 TBL1XR1 GINS3
NDUFB4 SLIRP MTX1 APOO ME1 DIMT1 CYB5B BCAT2 PITRM1 RAB32 ATPAF1 MPST TST NDUFA2 TIMM10 MRPS9 KYAT3 PPA2 RHOT2 DCK HK2 NAXE NDUFB6 MICU2 NDUFV1 GFM1 MFF TMEM12
PAF1 MNAT1 POLR2C NUFIP2 ZPR1 MORF4L2 PPHLN1 CDC73 ACTL6A DTX3L TBL1XR1 GINS3 SUPT5H MBD3 RANBP3 RFC1 MRI1 NOP16 NDUFB4 SP100 RNF126 CLNS1A NCBP3 MGLL SPAG5 XAB
SP100 MBD3 DPF2 CDC73 ACTL6A PAF1 SPDL1 NDC80 POLA1 CHRAC1 POLD2 CENPF NUF2 NCAPG2 POLE3 MKI67 RNF20 RAD21 GINS3 NUP160 RFC1 SPAG5 CHMP5 CCDC86 UCHL5 NUP43 MOF

Genes
FERMT3 ILK SERPINE1 FN1 PLAU F13A1 F10 ANXA1 MYOF AXL FGB F2 ANXA6 F5 PLAUR FAP PLAT VTN F3 SERPINE2 EMILIN1 ITGB1 ITGA5 ANXA2 S100A10 ITGB3 TFPI CD9 FBLN1 CHMP2B GNAS
CD99 SEMA3B SEMA3C CXCL12 PDGFRB ANXA1 FBLN1 LAMB1 SIRT1 CEMIP AKT2 COL1A1 VTN WNT5B SASH1 THBS4 PDCD10 FN1 GLIPR2 PLAU PODXL THBS1 CDH13 AKT1 EPHA2 CCN1 FERMT
CD99 SEMA3B SEMA3C CXCL12 PDGFRB AMOTL2 ANXA1 THY1 RHOG PLXNB2 SRGAP2 TNC ATP2B4 ROCK1 FBLN1 LAMB1 SIRT1 CEMIP SFRP1 AKT2 SERPINE1 C5 ENG COL1A1 VTN BST1 WNT5B
CD99 SEMA3B SEMA3C CXCL12 PDGFRB AMOTL2 ANXA1 THY1 RHOG PLXNB2 SRGAP2 TNC ATP2B4 ROCK1 FBLN1 LAMB1 SIRT1 CEMIP SFRP1 AKT2 SERPINE1 C5 ENG COL1A1 VTN BST1 WNT5B
CD99 SEMA3B SEMA3C CXCL12 PDGFRB AMOTL2 ANXA1 THY1 RHOG PLXNB2 SRGAP2 TNC F3 ATP2B4 ROCK1 FBLN1 LAMB1 SIRT1 CEMIP SFRP1 AKT2 SERPINE1 C5 ENG COL1A1 VTN BST1 WN
CD99 SEMA3B SEMA3C CXCL12 PDGFRB AMOTL2 ANXA1 THY1 RHOG PLXNB2 SRGAP2 TNC HDAC6 ATP2B4 ROCK1 FBLN1 LAMB1 SIRT1 CEMIP SFRP1 AKT2 SERPINE1 C5 ENG COL1A1 VTN BST1
CD99 ITGA3 PIK3C2A SEMA3B CTNNA1 RHOA NTN4 SEMA3C ITGB5 LAMB1 ITGA6 CORO1A PLAT ENG CXCL12 PDGFRB AMOTL2 ITGA4 PODXL ANXA1 LAMC1 SH3KBP1 ITGB1 THY1 RHOC AXL LAM
ITGB3 AP2B1 RALA HEATR5B VTA1 EHD3 EHD2 RABEP1 KIF1B GOLGA5 RAB27A EXOC5 SCARB1 TSG101 RAB7A PTPN23 DNM2 AP4E1 CHMP2B RAB10 EZR PACSIN2 CHMP4B RP2 COG4 RAB11A E
FERMT3 ILK CD99 ITGA3 CLDN11 CNTN1 CD44 CTNNA1 LIMS2 PVR FERMT2 NTN4 ITGB5 NID2 ICAM1 LAMB1 ITGA6 CDH23 VTN PTK7 ITGA4 FN1 CCN2 TGFBI PLAU PODXL NECTIN2 POSTN ANXA1
CD99 ITGA3 PIK3C2A SEMA3B CNTN1 CTNNA1 KIF1B ELMO2 RHOA NTN4 SEMA3C ITGB5 LAMB1 ITGA6 CORO1A PLAT ENG CXCL12 PDGFRB AMOTL2 ITGA4 VASP PODXL MYO1B EPHB2 ARL8B NC
SEMA3B CNTN1 RHOA ROCK1 MAP4K4 LIMS2 NTN4 SEMA3C FRYL MCAM APLP2 LAMB1 EZR SERPINE1 ENG CXCL12 VAT1 COL12A1 PDGFRB AMOTL2 VASP NECTIN2 MAP1S EPHB2 MEIS2 NOTCH
M6PR HEATR5B EHD3 EHD2 NUP133 PTPN23 RAB21 CHMP2B RAB10 IPO11 EZR NUP188 TIMM13 RAB11A EHD4 RAB5C EHD1 RAB23 TTK STXBP3 RAB14 CHMP2A CHMP1A VPS25 ARFIP2 FLOT2 A
ITGB3 NOP10 CD99 RALA HFE SEMA3B HOMER3 WNK1 RAB27A SEMA3C RAB10 EZR PACSIN2 CXCL12 RAB5C PDGFRB AMOTL2 AAK1 RAP1A RAP1B ANXA1 RAB13 ARL6IP5 CASK THY1 CALM3 RA
NOP10 M6PR HEATR5B EHD3 EHD2 NUP133 PTPN23 RAB21 CHMP2B RAB10 IPO11 EZR NUP188 TIMM13 PLTP RAB11A EHD4 RAB5C EHD1 RAB23 TTK STXBP3 RAB14 THOC2 LDLR CHMP2A CHMP
LAS1L HCCS EHD3 CLDN11 EHD2 ATG5 RHOA ROCK1 CLASP1 NTN4 RAB7A LAMB1 EZR CRNKL1 COG4 EHD4 ERI1 CEP41 FTSJ3 UTP6 RAB34 GAR1 ATG2A EHD1 RAB23 FN1 UTP25 RAB14 HELLS

Genes
LAP3 CD99 ITGA3 RALA CD9 TSPAN9 PLAUR EHD3 FHL1 CD44 TNC CTNNA1 RHOA LIMS2 PVR FERMT2 MCAM FAP DNM2 EPB41L2 RAB21 ITGB5 RAB10 ICAM1 ITGA6 EZR NRP1 PACSIN2 PROCR
LAP3 CD99 ITGA3 RALA CD9 TSPAN9 PLAUR EHD3 FHL1 CD44 TNC CTNNA1 RHOA LIMS2 PVR FERMT2 MCAM FAP DNM2 EPB41L2 RAB21 ITGB5 RAB10 ICAM1 ITGA6 EZR NRP1 PACSIN2 PROCR
SEMA3B TIMP2 TNC LTBP1 ITIH4 ITIH1 COL11A1 FBLN1 MMP2 NID2 LTBP4 ICAM1 LAMB1 TIMP3 SRPX SFRP1 COMP PCOLCE SERPINE1 OGN CXCL12 LGALS3BP COL1A1 VTN WNT5B COL12A1 LAM
SEMA3B TIMP2 TNC LTBP1 ITIH4 ITIH1 COL11A1 FBLN1 MMP2 NID2 LTBP4 ICAM1 LAMB1 TIMP3 SRPX SFRP1 COMP PCOLCE SERPINE1 OGN CXCL12 LGALS3BP COL1A1 VTN WNT5B COL12A1 LAM
SEMA3B TIMP2 TNC LTBP1 ITIH4 ITIH1 COL11A1 FBLN1 MMP2 NID2 LTBP4 ICAM1 LAMB1 TIMP3 SRPX SFRP1 COMP PCOLCE SERPINE1 OGN CXCL12 LGALS3BP COL1A1 VTN WNT5B COL12A1 LAM
LAP3 CD99 ITGA3 RALA CD9 TSPAN9 PLAUR EHD3 FHL1 CD44 TNC CTNNA1 RHOA LIMS2 PVR FERMT2 MCAM FAP DNM2 EPB41L2 RAB21 ITGB5 RAB10 ICAM1 ITGA6 EZR NRP1 PACSIN2 PROCR
TSPAN6 LAP3 ITGA3 RALA VTA1 CD9 PIK3C2A MVP GPRC5A CNTN1 EHD2 CD44 FUT8 ATP6V0A1 ITIH4 ITIH1 SLC2A3 MON2 GNAI3 DIP2B PFKP RHOA ACSL4 ATP1B3 RAB27A SLC44A1 ATP2B1 CYB
TSPAN6 LAP3 ITGA3 RALA VTA1 CD9 PIK3C2A MVP GPRC5A CNTN1 EHD2 CD44 FUT8 ATP6V0A1 ITIH4 ITIH1 SLC2A3 MON2 GNAI3 DIP2B PFKP RHOA ACSL4 ATP1B3 RAB27A SLC44A1 ATP2B1 CYB
TSPAN6 LAP3 ITGA3 RALA VTA1 CD9 PIK3C2A MVP GPRC5A CNTN1 EHD2 CD44 FUT8 ATP6V0A1 ITIH4 ITIH1 SLC2A3 MON2 GNAI3 DIP2B PFKP RHOA ACSL4 ATP1B3 RAB27A SLC44A1 ATP2B1 CYB
TSPAN6 LAP3 ITGA3 RALA VTA1 CD9 PIK3C2A MVP GPRC5A CNTN1 EHD2 CD44 FUT8 ATP6V0A1 ITIH4 ITIH1 SLC2A3 MON2 GNAI3 DIP2B PFKP RHOA ACSL4 ATP1B3 RAB27A SLC44A1 ATP2B1 CYB
TSPAN6 LAP3 ITGA3 RALA VTA1 CD9 ELOA PIK3C2A MVP GPRC5A CNTN1 EHD2 CD44 FUT8 ATP6V0A1 TNC ITIH4 ITIH1 SLC2A3 MON2 GNAI3 DIP2B PFKP RHOA ACSL4 ATP1B3 RAB27A SLC44A1 A
TSPAN6 LAP3 ITGA3 RALA VTA1 CD9 PIK3C2A MVP GPRC5A CNTN1 EHD2 CD44 FUT8 ATP6V0A1 ITIH4 ITIH1 SLC2A3 MON2 GNAI3 DIP2B PFKP RHOA ACSL4 ATP1B3 RAB27A SLC44A1 ATP2B1 CYB
CD63 HEATR5B VTA1 EHD3 EHD2 RABEP1 KIF1B RHOA RAB27A TSG101 RAB7A PTPN23 MOXD1 RAB21 CHMP2B RAB10 PACSIN2 CHMP4B RAB11A EHD4 RAB5C EHD1 RAB35 RAB23 AMOTL2 STXB
CD63 HEATR5B VTA1 EHD3 EHD2 RABEP1 KIF1B RHOA RAB27A TSG101 RAB7A PTPN23 MOXD1 RAB21 CHMP2B RAB10 PACSIN2 CHMP4B RAB11A EHD4 RAB5C EHD1 RAB35 RAB23 AMOTL2 STXB
TSPAN6 LAP3 ITGA3 RALA VTA1 CD9 ELOA PIK3C2A MVP GPRC5A CNTN1 EHD2 CD44 FUT8 ATP6V0A1 TNC LTBP1 ITIH4 ITIH1 SLC2A3 MON2 GNAI3 DIP2B PFKP RHOA ACSL4 ATP1B3 RAB27A SLC

Genes
CAPG PFN2 CAPZB TWF2 ARPC3 RDX SPTAN1 ARPC4 NCKAP1
CAPZB TWF2 NCKAP1 FSCN1 CAV1 ACTR3 PFN2 GDI2 SEPTIN7 RDX
CAPZB TWF2 NCKAP1 FSCN1 CAV1 ACTR3 PFN2 GDI2 SEPTIN7 RDX
CAPG PFN2 CAPZB CFL1 TWF2 ARPC3 RDX SPTAN1 ARPC4 NCKAP1
CAPG PFN2 CAPZB NAPA TWF2 LAMP2 FSCN1 STUB1 SUMO1 ISG15 ARPC3 RDX MSN SPTAN1 ARPC4 FBLIM1 NCKAP1
CAPG PFN2 CAPZB GMFB TWF2 CORO1B NCKAP1 ARPC3 RDX SPTAN1 ARPC4
CAPG PFN2 CAPZB GMFB TWF2 CAV1 CAMK2D CORO1B NCKAP1 FSCN1 ARPC3 SUMO1 RDX SPTAN1 ARPC4
CAPG PFN2 CAPZB GMFB TWF2 CORO1B NCKAP1 FSCN1 ARPC3 RDX SPTAN1 ARPC4
CAPG PFN2 FSCN1 CAPZB ARPC3 ACTR3 CORO1B CFL1 GMFB ARPC4 TWF2 NCKAP1 RDX SPTAN1
CAPG NCKAP1 PFN2 FSCN1 CAPZB ARPC3 ACTR3 CORO1B CFL1 GMFB SPTAN1 ARPC4 TWF2 CAV1 CAMK2D ACTB SUMO1 RDX CAVIN3
CAPG NCKAP1 PFN2 FSCN1 CAPZB ARPC3 ACTR3 CORO1B CFL1 GMFB SPTAN1 ARPC4 TWF2 ACTB RDX CAVIN3

CAPG PFN2 CAPZB NAPA RDX MSN TWF2 LAMP2 NCKAP1 FSCN1 STUB1 CAV1 ACTR3 SUMO1 ISG15 ARPC3 SPTAN1 ARPC4 GDI2 SEPTIN7 FBLIM1
CAPG VAMP3 PFN2 CAPZB NAPA CLTB TWF2 DDX23 LAMP2 FSCN1 STUB1 UBE2S SUMO1 TK1 ISG15 ARPC3 RDX MSN AP2M1 RRM1 SPTAN1 ARPC4 RTN4 FBLIM1 NCKAP1
LAMP2 CAPG VAMP3 PFN2 CAPZB NAPA CFL1 CLTB TWF2 DDX23 FSCN1 STUB1 UBE2S SUMO1 TK1 ISG15 ARPC3 RDX MSN AP2M1 RRM1 SPTAN1 ARPC4 RTN4 FBLIM1 NCKAP1 APEH
CAPG PFN2 CAPZB NAPA RDX MSN GMFB TWF2 ACTB CORO1B LAMP2 NCKAP1 FSCN1 STUB1 CAV1 ACTR3 RTN4 SUMO1 ISG15 VIM ARPC3 AP2M1 SPTAN1 ARPC4 GDI2 SEPTIN7 FBLIM1 CFL1 C
Genes
FSCN1 CFL1 ACTB ARPC3 ACTR3 CORO1B CAPG NCKAP1 CAPZB TWF2 RDX
MRC2 VIM GDI2 NCKAP1 ACTB CAV1 ARPC3 ACTR3 YWHAQ RDX MSN FBLIM1 CORO1B CFL1 REXO2
FSCN1 CFL1 ACTB ARPC3 ACTR3 S100A11 CORO1B VIM CAPG NCKAP1 CAPZB ATP6V1B2 TWF2 RDX
MRC2 VIM GDI2 NCKAP1 ACTB CAV1 ARPC3 ACTR3 YWHAQ RDX MSN FBLIM1 CORO1B CFL1 REXO2
CAPG FSCN1 ACTB CAPZB ARPC3 ACTR3 FBLIM1 CFL1 SPTAN1 ARPC4 TWF2 SEPTIN7 CORO1B NCKAP1 RDX CLIC4
MRC2 VIM GDI2 NCKAP1 ACTB CAV1 ARPC3 ACTR3 YWHAQ RDX MSN FBLIM1 S100A11 CORO1B CFL1 FSCN1 REXO2 TNKS1BP1 CLIC4
LAMP2 HEBP1 MDH1 VIM CAPG GDI2 NCKAP1 PFN2 FSCN1 ACTB CAPZB NAPA ARPC3 MOB1A ACTR3 PCYOX1 SEPTIN7 PEPD NAGK DUT TXNDC17 YWHAQ ATP6V1G1 RDX UACA ATIC MSN ATP6V
LAMP2 HEBP1 MDH1 VIM CAPG GDI2 NCKAP1 PFN2 FSCN1 ACTB CAPZB NAPA ARPC3 MOB1A ACTR3 PCYOX1 SEPTIN7 PEPD NAGK DUT TXNDC17 YWHAQ ATP6V1G1 RDX UACA ATIC MSN ATP6V
LAMP2 HEBP1 MDH1 VIM CAPG GDI2 NCKAP1 PFN2 FSCN1 ACTB CAPZB NAPA ARPC3 MOB1A ACTR3 PCYOX1 SEPTIN7 PEPD NAGK DUT TXNDC17 YWHAQ ATP6V1G1 RDX UACA ATIC MSN ATP6V
LAMP2 HEBP1 MDH1 VIM CAPG GDI2 NCKAP1 PFN2 FSCN1 ACTB CAPZB NAPA ARPC3 MOB1A ACTR3 PCYOX1 SEPTIN7 PEPD NAGK DUT TXNDC17 YWHAQ ATP6V1G1 RDX UACA ATIC MSN ATP6V
ACTB MRC2 VIM GDI2 NCKAP1 CAV1 ARPC3 ACTR3 YWHAQ RDX MSN FBLIM1 S100A11 CORO1B CFL1 CLTB SPTAN1 FSCN1 REXO2 RTN4 TNKS1BP1 CLIC4 CAPG VAMP3 PFN2 CAPZB NAPA ATP6V
LAMP2 HEBP1 MDH1 VIM CAPG GDI2 NCKAP1 PFN2 FSCN1 ACTB CAPZB NAPA ARPC3 MOB1A ACTR3 PCYOX1 SEPTIN7 PEPD NAGK DUT TXNDC17 YWHAQ ATP6V1G1 RDX UACA ATIC MSN ATP6V
VIM FSCN1 ACTB RDX CAMK2D MSN CFL1 SPTAN1 ARPC3 ACTR3 SEPTIN7 S100A11 CLIC4 CORO1B TWF2 TRIO CAPG VAMP3 NCKAP1 CAPZB CAV1 RTN4 STAT1 ATP6V1B2 CLTB S100A4 ARPC4
LAMP2 HEBP1 MDH1 VIM CAPG GDI2 NCKAP1 PFN2 FSCN1 ACTB CAPZB NAPA ARPC3 MOB1A ACTR3 PCYOX1 SEPTIN7 PEPD NAGK DUT TXNDC17 YWHAQ ATP6V1G1 RDX UACA ATIC MSN ATP6V
LAMP2 HEBP1 MDH1 VIM CAPG GDI2 NCKAP1 PFN2 FSCN1 ACTB CAPZB NAPA ARPC3 MOB1A ACTR3 PCYOX1 SEPTIN7 PEPD NAGK DUT TXNDC17 YWHAQ ATP6V1G1 RDX UACA ATIC MSN ATP6V
Genes
EIF4B ZC3H15 EIF2B3 RPL31 EIF3I RPL6 RPLP0 EIF5 EIF4H EIF3A RPL24 RPL22 ETF1 EIF2S2 RPL36 EIF2S3 DENR EIF3M RPL8 EIF4A1 RPL26 RPL29 RPL9 RPSA CNBP RPL15 RPL35A DRG1 RPL17 F
LUC7L STRAP GPKOW DAZAP1 WBP11 SF3B2 CDC5L CIRBP HNRNPM SNRPD3 DDX17 PHF5A SRSF5 PQBP1 RBM3 SNRNP70 SF3A2 EFTUD2 PRPF19 SRSF9 SRSF3 QKI IK FXR1 NCL SF3B6 SF3B1
LUC7L STRAP GPKOW DAZAP1 WBP11 SF3B2 CDC5L CIRBP HNRNPM SNRPD3 DDX17 PHF5A SRSF5 PQBP1 RBM3 SNRNP70 SF3A2 EFTUD2 PRPF19 SRSF9 SRSF3 QKI IK FXR1 NCL SF3B6 SF3B1
LUC7L STRAP GPKOW DAZAP1 WBP11 SF3B2 CDC5L CIRBP HNRNPM SNRPD3 DDX17 PHF5A SRSF5 PQBP1 RBM3 SNRNP70 SF3A2 EFTUD2 PRPF19 SRSF9 SRSF3 QKI IK FXR1 NCL SF3B6 SF3B1
EIF4B KARS1 ZC3H15 IARS2 EIF2B3 RPL31 RPS5 EIF3I RPL6 RPLP0 AARS1 EIF3L EIF5 RBM3 EIF3E GARS1 EIF3B EIF4H EIF3A RPL24 FXR1 FARSB RPL22 DARS2 ETF1 TSFM CNOT1 EIF2S2 FXR2 RF
EIF4B KARS1 ZC3H15 IARS2 EIF2B3 RPL31 RPS5 EIF3I RPL6 RPLP0 AARS1 EIF3L EIF5 RBM3 EIF3E GARS1 EIF3B EIF4H EIF3A RPL24 FXR1 FARSB RPL22 DARS2 ETF1 TSFM CNOT1 EIF2S2 FXR2 RF
LUC7L PTBP1 STRAP GPKOW DAZAP1 WBP11 RTRAF SF3B2 CDC5L HNRNPH3 CIRBP HNRNPM SNRPD3 DDX17 PHF5A SRSF5 PQBP1 RBM3 SNRNP70 SF3A2 EFTUD2 PRPF19 SRSF9 SRSF3 QKI IK
LUC7L STRAP GPKOW DAZAP1 WBP11 SF3B2 CDC5L CIRBP HNRNPM SNRPD3 DDX17 PHF5A SRSF5 CSTF2 PQBP1 RBM3 SNRNP70 SF3A2 EFTUD2 PRPF19 CPSF6 SRSF9 SRSF3 QKI IK FXR1 NCL
EIF4B HAGH KARS1 ZC3H15 IARS2 EIF2B3 RPL31 RPS5 GSTP1 EIF3I RPL6 RPLP0 AARS1 EIF3L EIF5 RBM3 EIF3E GSR ETHE1 GARS1 EIF3B EIF4H EIF3A LTA4H RPL24 FXR1 FARSB RPL22 DARS2 SF
EIF4B KARS1 ZC3H15 IARS2 EIF2B3 RPL31 RPS5 EIF3I RPL6 RPLP0 AARS1 EIF3L EIF5 RBM3 EIF3E GARS1 EIF3B EIF4H EIF3A RPL24 FXR1 FARSB RPL22 DARS2 ETF1 TSFM CNOT1 EIF2S2 FXR2 RF
HEXB EIF4B HAGH KARS1 ZC3H15 IARS2 EIF2B3 RPL31 RPS5 GSTP1 EIF3I RPL6 RPLP0 AARS1 EIF3L EIF5 RBM3 EIF3E GSR ETHE1 GARS1 EIF3B EIF4H EIF3A LTA4H VNN1 GNPDA1 RPL24 FXR1 FA
NCBP1 LUC7L STRAP EDC4 MRTO4 GPKOW DAZAP1 WBP11 SF3B2 XRN2 CDC5L CIRBP HNRNPM SNRPD3 DDX17 PHF5A SRSF5 CSTF2 PQBP1 RBM3 SNRNP70 SF3A2 EFTUD2 PRPF19 CPSF6 SRSF
ELAC2 LUC7L PTBP1 STRAP MTREX MRTO4 THUMPD1 GPKOW DAZAP1 WBP11 SRRT RTRAF SF3B2 DDX18 AARS1 CDC5L HNRNPH3 CIRBP HNRNPM SNRPD3 PES1 DDX17 PHF5A SRSF5 CSTF2 PQ
DPM1 EIF4B KARS1 ZC3H15 IARS2 EIF2B3 RPL31 RPS5 EIF3I RPL6 RPLP0 AARS1 EIF3L EIF5 POFUT1 RBM3 EIF3E GARS1 EIF3B EIF4H EIF3A RPL24 FXR1 GBE1 MOGS FARSB RPL22 DARS2 RPN2
DPM1 ADSS2 EIF4B KARS1 ZC3H15 IARS2 COASY ASNS EIF2B3 RPL31 RPS5 EIF3I SEPHS1 RPL6 RPLP0 AARS1 ATP5F1D MTAP EIF3L EIF5 POFUT1 SMS RBM3 EIF3E NAMPT GARS1 EIF3B EIF4H EI
Genes
RPL18 RPL31 RPL6 RPLP0 RPL3 RPL18A RPL28 RPL19 RPL34 RPL24 RPL22 RPL21 RPL5 RPL23 RPL36 RPL27 RPL35 RPL13A RPL11 RPL32 RPL37 RPL10 RPL7 RPL30 RPL8 RPL26 RPL29 RPL9 RPL
RPS20 RPL18 RPL31 RPS5 RPL6 RPLP0 RPL3 RPS16 RPS19 RPL18A RPL28 RPL19 RPL34 RPS13 RPS12 RPL24 RPS15 RPL22 RPL21 RPL5 RPS10 RPL23 RPL36 RPL27 RPS15A RPL35 RPS6 RPS24
RPS20 RPL18 RPL31 RPS5 RPL6 RPLP0 RPL3 RPS16 RPS19 RPL18A RPL28 RPL19 RPL34 RPS13 RPS12 RPL24 RPS15 RPL22 RPL21 RPL5 RPS10 RPL23 RPL36 RPL27 RPS15A RPL35 RPS6 RPS24
RPS20 RPL18 RPL31 RPS5 RPL6 RPLP0 RPL3 RPS16 RPS19 RPL18A RPL28 RPL19 RPL34 RPS13 RPS12 RPL24 RPS15 RPL22 RPL21 RPL5 RPS10 RPL23 RPL36 RPL27 RPS15A RPL35 RPS6 RPS24
LUC7L SNRNP40 U2AF2 GPKOW WBP11 SF3B2 CDC5L CIRBP HNRNPM SF3A1 SNRPD3 PHF5A RBM3 SNRNP70 SF3A2 EFTUD2 DHX15 PRPF19 NCL SF3B6 SF3B1 BCAS2 SMU1 LSM8 LSM7 TRA2B
RPS20 RPL18 RPL31 RPS5 RPL6 RPLP0 RPL3 RPS16 RPS19 RPL18A RPL28 RPL19 RPL34 RPS13 RPS12 RPL24 RPS15 RPL22 RPL21 RPL5 RPS10 RPL23 RPL36 RPL27 RPS15A RPL35 RPS6 RPS24
LASP1 CAPN1 VCL HSPA5 LIMA1 PPP1R12A RPL18 CNN2 RPL31 ACTN1 RPS5 TRIP6 RPL6 RPLP0 RPL3 ARHGEF7 RPS16 RPS19 HSPB1 PDLIM1 RPL19 PFN1 HSPA8 RPS13 CORO1C HSPA9 PRKAR2
ARF5 AK2 GGCT SPAG9 RPS20 PTBP1 LTF CAPN1 SERPINB1 ERP44 GRN VCL ADSS2 CUL3 PSMA4 HSPA5 HEXB AKR7A2 THRAP3 PHPT1 CS GPC1 CTSA COASY NUCB2 RPL31 WDR1 ACTN1 LMAN
ARF5 AK2 GGCT SPAG9 RPS20 PTBP1 LTF CAPN1 SERPINB1 ERP44 GRN VCL ADSS2 CUL3 PSMA4 HSPA5 HEXB AKR7A2 THRAP3 PHPT1 CS GPC1 CTSA COASY NUCB2 RPL31 WDR1 ACTN1 LMAN
ARF5 AK2 GGCT SPAG9 RPS20 PTBP1 LTF CAPN1 SERPINB1 ERP44 GRN VCL ADSS2 CUL3 PSMA4 HSPA5 HEXB AKR7A2 THRAP3 PHPT1 CS GPC1 CTSA COASY NUCB2 RPL31 WDR1 ACTN1 LMAN
ARF5 AK2 GGCT SPAG9 RPS20 PTBP1 LTF CAPN1 SERPINB1 ERP44 GRN VCL ADSS2 CUL3 PSMA4 HSPA5 HEXB AKR7A2 THRAP3 PHPT1 CS GPC1 CTSA COASY NUCB2 RPL31 WDR1 ACTN1 LMAN
RPL3 RPL5 RPL36 RPS11 RPL13A RPL11 RPS3A RPL7 RPL4 RPL23A KPNA6 MRTO4 POLD1 U2AF2 TCOF1 DIS3 SRRT DDX18 SUPT16H HNRNPH3 PES1 POLDIP3 SRSF5 SMCHD1 RBBP7 PQBP1 KPN
KPNA6 U2AF2 SRRT SUPT16H HNRNPH3 POLDIP3 SRSF5 RBBP7 PQBP1 KPNA3 ELOB BABAM1 CHD4 SRSF9 SRSF3 KPNA1 PPM1G SRSF7 SRSF4 HDAC1 SRSF11 RBBP5 RAD23B TARDBP NCOA5 S
ARF5 AK2 GGCT SPAG9 RPS20 PTBP1 LTF CAPN1 SERPINB1 ERP44 GRN VCL ADSS2 CUL3 PSMA4 HSPA5 HEXB AKR7A2 THRAP3 PHPT1 CS GPC1 CTSA MTHFD2 COASY GRIPAP1 NUCB2 RPL31 W
ARF5 AK2 GGCT SPAG9 RPS20 PTBP1 LTF CAPN1 SERPINB1 ERP44 GRN VCL ADSS2 CUL3 PSMA4 HSPA5 HEXB AKR7A2 THRAP3 PHPT1 CS GPC1 CTSA COASY NUCB2 RPL31 WDR1 ACTN1 LMAN
