# Supplementary material for: Extracellular Vesicles Bearing Vimentin Drive Epithelial–Mesenchymal Transition
Source: Mol Cell Proteomics. 2025 Jul 4;24(12):101028. doi: 10.1016/j.mcpro.2025.101028 (PMC12719745; doi:10.1016/j.mcpro.2025.101028)
Supplement: Supplemental Data 10 [file mmc13.pdf]

Sheet 10.Supplementary data 1(Fig2)

**HDF EV only (n=376)**					
KEGG					
Enrichment FDR	nGenes	Pathway Genes	Fold Enrichment	Pathway	URL
2.82874510745527E-09	16	108	9.57564344005022	Path:hsa03013 Nucleocytoplasmic transport	http://www.genome.jp/kegg-bin/show_pathway?hsa03013
2.82874510745527E-09	17	131	8.38782507439513	Path:hsa03040 Spliceosome	http://www.genome.jp/kegg-bin/show_pathway?hsa03040
0.00507520891743017	6	58	6.6864406779661	Path:hsa00240 Pyrimidine metabolism	http://www.genome.jp/kegg-bin/show_pathway?hsa00240
0.00193378690142216	8	85	6.08334995014955	Path:hsa01232 Nucleotide metabolism	http://www.genome.jp/kegg-bin/show_pathway?hsa01232
0.00454179144306598	7	77	5.87596302003082	Path:hsa03008 Ribosome biogenesis in eukaryotes	http://www.genome.jp/kegg-bin/show_pathway?hsa03008
0.00210544607422987	8	89	5.80994096362598	Path:hsa05235 PD-L1 expression and PD-1 checkpoint pathway in cancer	http://www.genome.jp/kegg-bin/show_pathway?hsa05235
0.00488790693614831	7	79	5.72720446256168	Path:hsa01521 EGFR tyrosine kinase inhibitor resistance	http://www.genome.jp/kegg-bin/show_pathway?hsa01521
0.000720385519347555	12	169	4.58950957777555	Path:hsa04141 Protein processing in endoplasmic reticulum	http://www.genome.jp/kegg-bin/show_pathway?hsa04141
0.00152808831699531	11	157	4.52860844218936	Path:hsa05160 Hepatitis C	http://www.genome.jp/kegg-bin/show_pathway?hsa05160
0.000720385519347555	14	224	4.03972457627119	Path:hsa05163 Human cytomegalovirus infection	http://www.genome.jp/kegg-bin/show_pathway?hsa05163
0.00296525889368753	12	210	3.69346246973366	Path:hsa05170 Human immunodeficiency virus 1 infection	http://www.genome.jp/kegg-bin/show_pathway?hsa05170
0.00507520891743017	11	197	3.6090940376839	Path:hsa05130 Pathogenic Escherichia coli infection	http://www.genome.jp/kegg-bin/show_pathway?hsa05130
0.00573336897673445	11	202	3.51976002685014	Path:hsa05205 Proteoglycans in cancer	http://www.genome.jp/kegg-bin/show_pathway?hsa05205
0.00193378690142216	17	364	3.01869528776308	Path:hsa05014 Amyotrophic lateral sclerosis	http://www.genome.jp/kegg-bin/show_pathway?hsa05014
0.000720385519347555	46	1538	1.9331841925459	Path:hsa01100 Metabolic pathways	http://www.genome.jp/kegg-bin/show_pathway?hsa01100
Biological process					
Enrichment FDR	nGenes	Pathway Genes	Fold Enrichment	Pathway	URL
3.55272149134636E-08	32	537	3.85165546191964	GO:0006397 mRNA processing	http://amigo.geneontology.org/amigo/term/GO:0006397
2.22073209870814E-09	50	1082	2.98685735768664	GO:0006396 RNA processing	http://amigo.geneontology.org/amigo/term/GO:0006396
1.86772817917227E-07	42	934	2.90652560519726	GO:0006886 intracellular protein transport	http://amigo.geneontology.org/amigo/term/GO:0006886
1.63119675865749E-12	74	1709	2.79873253265365	GO:0046907 intracellular transport	http://amigo.geneontology.org/amigo/term/GO:0046907
1.31070995862318E-09	67	1680	2.57772901533495	GO:0015031 protein transport	http://amigo.geneontology.org/amigo/term/GO:0015031
1.48498309181028E-09	69	1795	2.48459940512724	GO:0045184 establishment of protein localization	http://amigo.geneontology.org/amigo/term/GO:0045184
1.48498309181028E-09	78	2175	2.31796610169492	GO:0071705 nitrogen compound transport	http://amigo.geneontology.org/amigo/term/GO:0071705
1.84899418866351E-07	63	1782	2.28509673001198	GO:0034613 cellular protein localization	http://amigo.geneontology.org/amigo/term/GO:0034613
1.86772817917227E-07	63	1791	2.27361383187122	GO:0070727 cellular macromolecule localization	http://amigo.geneontology.org/amigo/term/GO:0070727
8.08320508415054E-09	80	2388	2.16534650654402	GO:0051649 establishment of localization in cell	http://amigo.geneontology.org/amigo/term/GO:0051649
2.22073209870814E-09	87	2642	2.12842415222161	GO:0008104 protein localization	http://amigo.geneontology.org/amigo/term/GO:0008104
1.48498309181028E-09	98	3120	2.03022055627988	GO:0051641 cellular localization	http://amigo.geneontology.org/amigo/term/GO:0051641
1.63045480968855E-09	98	3133	2.02179640459407	GO:0033036 macromolecule localization	http://amigo.geneontology.org/amigo/term/GO:0033036
1.86772817917227E-07	85	2805	1.95865434001027	GO:0009056 catabolic proc.	http://amigo.geneontology.org/amigo/term/GO:0009056
1.48498309181028E-09	120	4206	1.84409681085133	GO:0006996 organelle organization	http://amigo.geneontology.org/amigo/term/GO:0006996
Cellular Component					
Enrichment FDR	nGenes	Pathway Genes	Fold Enrichment	Pathway	URL
2.82800646808139E-05	9	63	9.23365617433414	GO:0071005 U2-type precatalytic spliceosome	http://amigo.geneontology.org/amigo/term/GO:0071005
3.43130187367353E-05	9	65	8.94954367666232	GO:0071011 precatalytic spliceosome	http://amigo.geneontology.org/amigo/term/GO:0071011
3.63434830526161E-05	11	106	6.70746722097857	GO:0005684 U2-type spliceosomal complex	http://amigo.geneontology.org/amigo/term/GO:0005684
2.1581579241043E-05	16	220	4.70077041602465	GO:0005681 spliceosomal complex	http://amigo.geneontology.org/amigo/term/GO:0005681
0.000108158967808211	23	489	3.04011992651901	GO:0016607 nuclear speck	http://amigo.geneontology.org/amigo/term/GO:0016607
3.69268124635697E-06	35	807	2.80327851637158	GO:1990904 ribonucleoprotein complex	http://amigo.geneontology.org/amigo/term/GO:1990904
1.87719944380835E-05	37	952	2.51209763566444	GO:0016604 nuclear body	http://amigo.geneontology.org/amigo/term/GO:0016604
4.48415451396832E-11	83	2342	2.29067217646803	GO:1903561 extracellular vesicle	http://amigo.geneontology.org/amigo/term/GO:1903561
4.48415451396832E-11	83	2343	2.28969451015285	GO:0043230 extracellular organelle	http://amigo.geneontology.org/amigo/term/GO:0043230
4.48415451396832E-11	83	2343	2.28969451015285	GO:0065010 extracellular membrane-bounded organelle	http://amigo.geneontology.org/amigo/term/GO:0065010
5.57520044507929E-11	82	2316	2.2884795527063	GO:0070062 extracellular exosome	http://amigo.geneontology.org/amigo/term/GO:0070062
1.49465314402434E-12	134	4581	1.89067223128693	GO:0005654 nucleoplasm	http://amigo.geneontology.org/amigo/term/GO:0005654
3.09630798160986E-13	144	4973	1.87161178840314	GO:0031981 nuclear lumen	http://amigo.geneontology.org/amigo/term/GO:0031981
9.25906218382349E-09	120	4466	1.73673783843275	GO:0031982 vesicle	http://amigo.geneontology.org/amigo/term/GO:0031982
1.14194508571014E-05	93	3577	1.68048928417432	GO:0005615 extracellular space	http://amigo.geneontology.org/amigo/term/GO:0005615

Genes
ALYREF XPO7 KPNA3 MAGOH NCBP1 NUP98 NMD3 TMEM33 IPO9 RANBP2 SEC13 SUMO2 TPR SUMO1 NUP93 RBM8A
ALYREF SF3A1 SRSF10 TCERG1 SF3A3 LSM6 DDX42 U2AF2 U2SURP RBMX MAGOH NCBP1 LSM7 SNRPF PRPF4 EFTUD2 RBM8A
DCTD DTYMK NT5C2 NME1 RRM2 ASMTL
ADSS2 DCTD DTYMK AK2 NT5C2 NME1 RRM2 ASMTL
CSNK2A2 XRN2 REXO2 NOB1 EIF6 NMD3 SBDS
CSNK2A2 MTOR JAK1 PPP3CA MAPK3 MAP2K2 RELA STAT3
MTOR GRB2 JAK1 AXL MAPK3 MAP2K2 STAT3
PDIA6 SEC61B LMAN2 HSPBP1 UBXN1 PRKCSH RAD23B RRBP1 SEC13 SSR4 BAG2 SEC24C
PSME3 GRB2 IFIT1 JAK1 MX1 MAPK3 MAP2K2 RELA STAT3 CASP3 RIPK1
MTOR GRB2 JAK1 PLCB3 PPP3CA MAPK3 MAP2K2 PTK2 RELA ROCK1 STAT3 CASP3 RIPK1 ROCK2
AP1B1 AP1G1 MTOR PPP3CA MAPK3 MAP2K2 PTK2 RELA BST2 CASP3 RIPK1 AP1M1
ABCF2 WASF2 TUBB3 MYO1D MAPK3 RELA ROCK1 CASP3 RIPK1 ROCK2 SEC24C
MTOR GRB2 ITGB5 MAPK3 MAP2K2 PTK2 ROCK1 STAT3 CAMK2D CASP3 ROCK2
ALYREF PSMD14 TUBB3 ATXN2L COX6B1 MTOR NCBP1 NUP98 PPP3CA PSMB2 RANBP2 SEC13 TPR CASP3 VAPB NUP93 MATR3
GNPDA1 AKR1A1 BPNT1 SPTLC1 NUDT5 CKB COX6B1 ADSS2 DCTD DTYMK AK2 ACSL3 NT5C2 GALE HIBCH GCLM GSR GYG1 GMPPA GYS1 HSD17B10 HK1 IMPA1 ACADVL LTA4H MARS1 MTAP
Genes
DAZAP1 CSTF2 EFTUD2 DDX39A PRPF4 NCBP1 SNRPF RBMX MBNL1 SAFB LSM6 NUDT21 SF1 SF3A3 SRSF10 UBL5 SUPT4H1 RBM14 U2AF2 SF3A1 LSM7 MAGOH SRRM2 HNRNPF RBM8A TCERG1
DAZAP1 RTRAF DDX18 CSTF2 EFTUD2 SMU1 DDX39A PRPF4 NCBP1 SNRPF NOB1 RBMX MBNL1 SAFB MAGOH LSM6 NUDT21 SF1 HNRNPF RSL1D1 SF3A3 SRSF10 UBL5 SUPT4H1 RBM14 EIF6
CSNK2A2 SAE1 UBL5 LAMP2 SCFD1 KPNA3 NUP93 STX4 SGTA SEC61B SPCS2 VPS26A SPCS3 XPO7 SRPRB RANBP2 SEC13 TMED9 NACA IPO9 TOMM70 AIP SUMO1 STAT3 SNX1 TPR ELAVL1
CSNK2A2 SAE1 UBL5 LAMP2 EHD3 SNX1 TPR CHMP5 SCFD1 KPNA3 NUP93 STX4 SGTA COPE SEC61B TFG SPCS2 VPS26A DDX39A COPB1 SPCS3 SNX6 XPO7 WASHC4 NCBP1 ANP32A SRPRB
CSNK2A2 SAE1 UBL5 LAMP2 SCFD1 KPNA3 NUP93 STX4 SGTA RAB3D SEC61B SPCS2 VPS26A SPCS3 XPO7 SRPRB RANBP2 SEC13 TMED9 NACA ARRDC1 IPO9 AP1B1 TOMM70 AP1G1 C1QTNF3
CSNK2A2 SAE1 UBL5 LAMP2 SCFD1 KPNA3 NUP93 STX4 SGTA RAB3D SEC61B SPCS2 VPS26A SPCS3 XPO7 SRPRB RANBP2 SEC13 TMED9 NACA ARRDC1 IPO9 AP1B1 TOMM70 AP1G1 C1QTNF3
CSNK2A2 SAE1 UBL5 LAMP2 TPR SCFD1 PLTP KPNA3 NUP93 STX4 SGTA RAB3D SEC61B SPCS2 VPS26A DDX39A SPCS3 XPO7 NCBP1 SRPRB RANBP2 SEC13 TMED9 NACA ARRDC1 IPO9 AP1B1
CSNK2A2 SAE1 UBL5 LAMP2 EHD3 SCFD1 KPNA3 NUP93 STX4 SGTA RAB3D SEC61B SPCS2 VPS26A TM9SF2 SPCS3 XPO7 SRPRB RANBP2 SEC13 TMED9 NACA IPO9 TWF2 TOMM70 H2AC4
CSNK2A2 SAE1 UBL5 LAMP2 EHD3 SCFD1 KPNA3 NUP93 STX4 SGTA RAB3D SEC61B SPCS2 VPS26A TM9SF2 SPCS3 XPO7 SRPRB RANBP2 SEC13 TMED9 NACA IPO9 TWF2 TOMM70 H2AC4
CSNK2A2 SAE1 UBL5 LAMP2 EHD3 SNX1 TPR PICALM CHMP5 SCFD1 KPNA3 NUP93 STX4 SGTA COPE SEC61B TFG SPCS2 VPS26A DDX39A COPB1 SPCS3 SNX6 XPO7 WASHC4 NCBP1 NUMA1
CSNK2A2 SAE1 UBL5 LAMP2 EHD3 SCFD1 KPNA3 NUP93 STX4 SGTA RAB3D SEC61B EXOC8 SPCS2 TJP2 VPS26A TM9SF2 SPCS3 XPO7 SRPRB RANBP2 SEC13 TMED9 NACA ARRDC1 IPO9 TWF2
CSNK2A2 SAE1 UBL5 LAMP2 EHD3 SNX1 TPR PICALM CHMP5 SCFD1 KPNA3 NUP93 STX4 SGTA RAB3D COPE SEC61B TFG EXOC8 SPCS2 VPS26A DDX39A VAPB TM9SF2 COPB1 SPCS3 SNX6
CSNK2A2 SAE1 UBL5 LAMP2 EHD3 TPR SCFD1 PLTP KPNA3 NUP93 STX4 SGTA RAB3D SEC61B EXOC8 SPCS2 TJP2 VPS26A DDX39A TM9SF2 SPCS3 XPO7 NCBP1 SRPRB RANBP2 SEC13 TMED9
CSNK2A2 LAMP2 UBA6 PSME4 UBE2K PCNP XRN2 RBX1 SGTA LTA4H GNPDA1 PSMD14 PCYOX1 GALE RAD23B PSMB2 PRDX5 PSME3 IMPA1 NCBP1 SORD ATG3 QDPR HK1 UBE2L6 WDR26 HIBCH
CSNK2A2 SAE1 UBL5 ZNF207 NCAPD2 EHD3 CAPG TPR ROCK1 PSME4 PICALM CHMP5 ADD1 STX4 MTPN SMC3 RAB34 TMEM33 DBN1 RND3 PTPA NCAPH CIT VAPB MAP1B TOP2A RPA1 NES
Genes
SF3A1 EFTUD2 SMU1 LSM7 PRPF4 SNRPF LSM6 SRRM2 SF3A3
SMU1 SF3A1 EFTUD2 LSM7 PRPF4 SNRPF LSM6 SRRM2 SF3A3
U2AF2 SF3A1 EFTUD2 LSM7 SMU1 PRPF4 SNRPF LSM6 SRRM2 SF3A3 RBM8A
U2AF2 SF3A1 EFTUD2 SMU1 LSM7 SNRPF RBMX MAGOH SF3A3 PRPF4 LSM6 SRRM2 SF1 HNRNPF RBM8A API5
U2AF2 SRSF10 RBM14 BCLAF1 SF3A1 EFTUD2 DDX39A PRPF4 UNC45A IFI16 API5 SRRM2 ATXN2L SF3A3 ALYREF DDX42 RBM8A PSME4 PABPN1 SUMO1 SMU1 MAGOH TCERG1
RPS27 RPS26 U2AF2 SF3A1 EFTUD2 SMU1 RPS4Y1 LSM7 PRPF4 SNRPF NOB1 EIF2A RBMX VBP1 MAGOH LSM6 HNRNPF RSL1D1 SF3A3 EIF6 HSD17B10 PABPN1 PIN4 TOP2A NCBP1 SRRM2 SF1
USP7 U2AF2 SUMO1 SRSF10 RBM14 BCLAF1 PCNP ADD1 SF3A1 CSTF2 EFTUD2 NUP98 DDX39A RPA1 PRPF4 UNC45A PARP1 PALM2AKAP2 IFI16 API5 NUDT21 SRRM2 ATXN2L SF3A3 ALYREF DRG1
AK2 LAMP2 GGCT ADSS2 CAPG AP1M1 C1QTNF3 ITGB5 CHMP5 BLVRB MTAP NDRG3 EEA1 STX4 GSR PDCD5 RAB3D PLOD3 RAB34 TMEM109 LTA4H GNPDA1 PPP1R7 PCYOX1 P3H1 AKR1A1 CTSD
AK2 LAMP2 GGCT ADSS2 CAPG AP1M1 C1QTNF3 ITGB5 CHMP5 BLVRB MTAP NDRG3 EEA1 STX4 GSR PDCD5 RAB3D PLOD3 RAB34 TMEM109 LTA4H GNPDA1 PPP1R7 PCYOX1 P3H1 AKR1A1 CTSD
AK2 LAMP2 GGCT ADSS2 CAPG AP1M1 C1QTNF3 ITGB5 CHMP5 BLVRB MTAP NDRG3 EEA1 STX4 GSR PDCD5 RAB3D PLOD3 RAB34 TMEM109 LTA4H GNPDA1 PPP1R7 PCYOX1 P3H1 AKR1A1 CTSD
AK2 LAMP2 GGCT ADSS2 CAPG AP1M1 C1QTNF3 ITGB5 CHMP5 BLVRB MTAP NDRG3 EEA1 STX4 GSR PDCD5 RAB3D PLOD3 RAB34 TMEM109 LTA4H GNPDA1 PPP1R7 PCYOX1 P3H1 AKR1A1 CTSD
USP7 U2AF2 KPNA3 PPM1G SUMO1 RAD23B PSME3 SSRP1 MBNL1 NOLC1 HNRNPF BANF1 SRSF10 SUPT4H1 RBM14 ZNF207 NCAPD2 BCLAF1 RAI14 CAPG ELAVL1 PSME4 NUCKS1 DAZAP1
USP7 U2AF2 DDX18 KPNA3 SMC3 PPM1G SUMO1 RAD23B PSME3 RPA1 PARP1 SSRP1 MBNL1 IFI16 LSM6 NOLC1 HNRNPF BANF1 SRSF10 SUPT4H1 RBM14 EIF6 ZNF207 NCAPD2 BCLAF1 RAI14
AK2 LAMP2 GGCT ADSS2 CAPG AP1M1 C1QTNF3 ITGB5 CHMP5 BLVRB MTAP NDRG3 EEA1 STX4 GSR PDCD5 RAB3D PLOD3 RAB34 TMEM109 LTA4H GNPDA1 PPP1R7 PCYOX1 P3H1 AKR1A1 CTSD
AK2 LAMP2 GGCT ADSS2 CAPG AP1M1 C1QTNF3 ITGB5 CHMP5 BLVRB MTAP PLTP NDRG3 EEA1 STX4 GSR PDCD5 RAB3D PLOD3 RAB34 TMEM109 LTA4H GNPDA1 PPP1R7 PCYOX1 P3H1 AKR1A1
