# Supplementary material for: Extracellular Vesicles Bearing Vimentin Drive Epithelial–Mesenchymal Transition
Source: Mol Cell Proteomics. 2025 Jul 4;24(12):101028. doi: 10.1016/j.mcpro.2025.101028 (PMC12719745; doi:10.1016/j.mcpro.2025.101028)
Supplement: Supplemental Data 11 [file mmc14.pdf]

Sheet 11.GO_MCF7

**Fig3 cluster 1, n=411**					
Biological process					
Enrichment FDR	nGenes	Pathway Genes	Fold Enrichment	Pathway	URL
9.24035618983372E-12	12	28	25.0156705539359	GO:0018126 protein hydroxylation	http://amigo.geneontology.org/amigo/term/GO:0018126
1.89932774586492E-09	17	110	9.02080241187384	GO:0009135 purine nucleoside diphosphate metabolic proc.	http://amigo.geneontology.org/amigo/term/GO:0009135
9.36943064828131E-10	18	120	8.75548469387755	GO:0009185 ribonucleoside diphosphate metabolic proc.	http://amigo.geneontology.org/amigo/term/GO:0009185
1.94393954666385E-11	27	250	6.30394897959184	GO:0019318 hexose metabolic proc.	http://amigo.geneontology.org/amigo/term/GO:0019318
3.53863807693746E-12	29	269	6.29266557924285	GO:0005996 monosaccharide metabolic proc.	http://amigo.geneontology.org/amigo/term/GO:0005996
9.17865567550313E-12	43	627	4.0030392539791	GO:0005975 carbohydrate metabolic proc.	http://amigo.geneontology.org/amigo/term/GO:0005975
1.79626241222472E-09	40	660	3.53756957328386	GO:0055086 nucleobase-containing small molecule metabolic proc.	http://amigo.geneontology.org/amigo/term/GO:0055086
2.06629615078474E-11	54	983	3.20648473020948	GO:0019752 carboxylic acid metabolic proc.	http://amigo.geneontology.org/amigo/term/GO:0019752
5.3372814570173E-11	54	1009	3.12385975202767	GO:0043436 oxoacid metabolic proc.	http://amigo.geneontology.org/amigo/term/GO:0043436
9.3799868411213E-11	54	1028	3.06612304454856	GO:0006082 organic acid metabolic proc.	http://amigo.geneontology.org/amigo/term/GO:0006082
9.67736571899105E-14	87	1936	2.6230274392815	GO:0044281 small molecule metabolic proc.	http://amigo.geneontology.org/amigo/term/GO:0044281
5.90838199476618E-14	94	2160	2.54017148526077	GO:0033554 cellular response to stress	http://amigo.geneontology.org/amigo/term/GO:0033554
3.3353205111627E-12	93	2312	2.34792409610903	GO:1901575 organic substance catabolic proc.	http://amigo.geneontology.org/amigo/term/GO:1901575
6.83957413933257E-11	101	2805	2.10173251118629	GO:0009056 catabolic proc.	http://amigo.geneontology.org/amigo/term/GO:0009056
1.02871830386349E-13	146	4424	1.92631218400561	GO:0006950 response to stress	http://amigo.geneontology.org/amigo/term/GO:0006950
Cellular Component					
Enrichment FDR	nGenes	Pathway Genes	Fold Enrichment	Pathway	URL
1.38990196531498E-10	20	154	7.58050622846541	GO:0005793 endoplasmic reticulum-Golgi intermediate compartment	http://amigo.geneontology.org/amigo/term/GO:0005793
1.33355020738911E-19	39	329	6.91922802555673	GO:0005788 endoplasmic reticulum lumen	http://amigo.geneontology.org/amigo/term/GO:0005788
5.69345804956545E-10	29	368	4.59980174134871	GO:0034774 secretory granule lumen	http://amigo.geneontology.org/amigo/term/GO:0034774
5.94925483866502E-10	29	371	4.56260657901975	GO:0060205 cytoplasmic vesicle lumen	http://amigo.geneontology.org/amigo/term/GO:0060205
6.32933974622899E-10	29	373	4.53814220057996	GO:0031983 vesicle lumen	http://amigo.geneontology.org/amigo/term/GO:0031983
9.44901401228633E-44	144	2316	3.62921645342075	GO:0070062 extracellular exosome	http://amigo.geneontology.org/amigo/term/GO:0070062
9.42449355528303E-44	145	2342	3.61384936126457	GO:1903561 extracellular vesicle	http://amigo.geneontology.org/amigo/term/GO:1903561
9.42449355528303E-44	145	2343	3.61230695863493	GO:0043230 extracellular organelle	http://amigo.geneontology.org/amigo/term/GO:0043230
9.42449355528303E-44	145	2343	3.61230695863493	GO:0065010 extracellular membrane-bounded organelle	http://amigo.geneontology.org/amigo/term/GO:0065010
2.26993005717715E-30	158	3577	2.57826219668745	GO:0005615 extracellular space	http://amigo.geneontology.org/amigo/term/GO:0005615
1.90759285515507E-16	98	2262	2.52884615384615	GO:0005783 endoplasmic reticulum	http://amigo.geneontology.org/amigo/term/GO:0005783
2.61076802083711E-30	179	4466	2.33950106016433	GO:0031982 vesicle	http://amigo.geneontology.org/amigo/term/GO:0031982
9.71368435084716E-24	171	4673	2.13594105521515	GO:0005576 extracellular region	http://amigo.geneontology.org/amigo/term/GO:0005576
7.93568024185375E-12	149	4973	1.74886684012032	GO:0031981 nuclear lumen	http://amigo.geneontology.org/amigo/term/GO:0031981
5.94925483866502E-10	135	4581	1.72013451746121	GO:0005654 nucleoplasm	http://amigo.geneontology.org/amigo/term/GO:0005654
Fig3 cluster 2, n=640					
Biological process					
Enrichment FDR	nGenes	Pathway Genes	Fold Enrichment	Pathway	URL
1.08166892758396E-49	62	178	13.4171490182726	GO:0002181 cytoplasmic translation	http://amigo.geneontology.org/amigo/term/GO:0002181
1.33187335609785E-53	119	784	5.84681637806638	GO:0006412 translation	http://amigo.geneontology.org/amigo/term/GO:0006412
5.58936688325964E-53	120	814	5.67865386047204	GO:0043043 peptide biosynthetic proc.	http://amigo.geneontology.org/amigo/term/GO:0043043
6.24524188234815E-19	47	327	5.53654279801069	GO:0042254 ribosome biogenesis	http://amigo.geneontology.org/amigo/term/GO:0042254
5.33468781691539E-52	128	959	5.1413825428424	GO:0043604 amide biosynthetic proc.	http://amigo.geneontology.org/amigo/term/GO:0043604
2.49652322175785E-49	127	999	4.89696261918484	GO:0006518 peptide metabolic proc.	http://amigo.geneontology.org/amigo/term/GO:0006518
4.39617177854453E-23	64	509	4.84340457621401	GO:0022613 ribonucleoprotein complex biogenesis	http://amigo.geneontology.org/amigo/term/GO:0022613
1.11699918076705E-47	142	1301	4.20435717668615	GO:0043603 cellular amide metabolic proc.	http://amigo.geneontology.org/amigo/term/GO:0043603
2.01007119881475E-38	127	1259	3.88567566049695	GO:0034645 cellular macromolecule biosynthetic proc.	http://amigo.geneontology.org/amigo/term/GO:0034645
4.31051119317443E-15	60	645	3.58327460653042	GO:0034660 ncRNA metabolic proc.	http://amigo.geneontology.org/amigo/term/GO:0034660
1.24399551119376E-44	167	1888	3.40724244564287	GO:1901566 organonitrogen compound biosynthetic proc.	http://amigo.geneontology.org/amigo/term/GO:1901566
6.78520086907657E-13	72	983	2.8214186627208	GO:0019752 carboxylic acid metabolic proc.	http://amigo.geneontology.org/amigo/term/GO:0019752
6.78520086907657E-13	74	1028	2.77285500923633	GO:0006082 organic acid metabolic proc.	http://amigo.geneontology.org/amigo/term/GO:0006082
3.63255350582227E-15	116	1936	2.30802863344186	GO:0044281 small molecule metabolic proc.	http://amigo.geneontology.org/amigo/term/GO:0044281
7.00117855050709E-17	181	3630	1.92070428805966	GO:0044085 cellular component biogenesis	http://amigo.geneontology.org/amigo/term/GO:0044085
Cellular Component					
Enrichment FDR	nGenes	Pathway Genes	Fold Enrichment	Pathway	URL
5.91352595178124E-36	33	57	22.3011695906433	GO:0022625 cytosolic large ribosomal subunit	http://amigo.geneontology.org/amigo/term/GO:0022625
8.61002799574993E-42	48	122	15.1554893194237	GO:0022626 cytosolic ribosome	http://amigo.geneontology.org/amigo/term/GO:0022626
9.2471007158026E-37	44	121	14.0073461891644	GO:0015934 large ribosomal subunit	http://amigo.geneontology.org/amigo/term/GO:0015934
5.40534643083995E-47	65	222	11.2784375284375	GO:0044391 ribosomal subunit	http://amigo.geneontology.org/amigo/term/GO:0044391
1.35257407925932E-45	70	283	9.52796516400757	GO:0005840 ribosome	http://amigo.geneontology.org/amigo/term/GO:0005840
1.42131106113939E-32	72	475	5.83885167464115	GO:0005925 focal adhesion	http://amigo.geneontology.org/amigo/term/GO:0005925
7.29706467794732E-33	73	484	5.80986518073295	GO:0030055 cell-substrate junction	http://amigo.geneontology.org/amigo/term/GO:0030055
5.11687175051662E-44	108	807	5.15511997296384	GO:1990904 ribonucleoprotein complex	http://amigo.geneontology.org/amigo/term/GO:1990904
6.4769384719184E-23	63	520	4.66687062937063	GO:0005759 mitochondrial matrix	http://amigo.geneontology.org/amigo/term/GO:0005759
1.12248491260629E-24	88	926	3.66066714662827	GO:0070161 anchoring junction	http://amigo.geneontology.org/amigo/term/GO:0070161
3.21465658674085E-40	178	2316	2.96053366131086	GO:0070062 extracellular exosome	http://amigo.geneontology.org/amigo/term/GO:0070062
9.9331959119213E-40	178	2342	2.92766693407172	GO:1903561 extracellular vesicle	http://amigo.geneontology.org/amigo/term/GO:1903561
9.9331959119213E-40	178	2343	2.92641739632777	GO:0043230 extracellular organelle	http://amigo.geneontology.org/amigo/term/GO:0043230
9.9331959119213E-40	178	2343	2.92641739632777	GO:0065010 extracellular membrane-bounded organelle	http://amigo.geneontology.org/amigo/term/GO:0065010
4.47061008646563E-23	192	3577	2.06762057251294	GO:0005615 extracellular space	http://amigo.geneontology.org/amigo/term/GO:0005615
Reference List and cluster 2, n=76					
Biological process					
Enrichment FDR	nGenes	Pathway Genes	Fold Enrichment	Pathway	URL
0.0334281492264904	2	5	128.907042253521	GO:1905821 positive reg. of chromosome condensation	http://amigo.geneontology.org/amigo/term/GO:1905821
0.0334281492264904	2	6	107.422535211268	GO:0010032 meiotic chromosome condensation	http://amigo.geneontology.org/amigo/term/GO:0010032
0.0380111029086678	2	8	80.5669014084507	GO:0046719 reg. by virus of viral protein levels in host cell	http://amigo.geneontology.org/amigo/term/GO:0046719
0.00699973258223892	4	39	33.0530877573131	GO:1902745 positive reg. of lamellipodium organization	http://amigo.geneontology.org/amigo/term/GO:1902745
0.0334281492264904	3	31	31.1871876419809	GO:0010592 positive reg. of lamellipodium assembly	http://amigo.geneontology.org/amigo/term/GO:0010592
0.0351112778600162	4	94	13.7135151333533	GO:0097581 lamellipodium organization	http://amigo.geneontology.org/amigo/term/GO:0097581
0.0351112778600162	5	172	9.36824434981985	GO:0016241 reg. of macroautophagy	http://amigo.geneontology.org/amigo/term/GO:0016241
0.0334281492264904	11	959	3.69650016889659	GO:0043604 amide biosynthetic proc.	http://amigo.geneontology.org/amigo/term/GO:0043604
0.0161937118275416	14	1269	3.55535577531382	GO:0033043 reg. of organelle organization	http://amigo.geneontology.org/amigo/term/GO:0033043
0.0334281492264904	13	1301	3.22019898019941	GO:0043603 cellular amide metabolic proc.	http://amigo.geneontology.org/amigo/term/GO:0043603
0.0363640574040678	16	1936	2.66336864160168	GO:0044281 small molecule metabolic proc.	http://amigo.geneontology.org/amigo/term/GO:0044281
0.00582296214349997	30	4206	2.2986277149344	GO:0006996 organelle organization	http://amigo.geneontology.org/amigo/term/GO:0006996
0.0225675706855425	25	3630	2.2194738680014	GO:0044085 cellular component biogenesis	http://amigo.geneontology.org/amigo/term/GO:0044085
0.0334281492264904	23	3365	2.20272063286106	GO:0022607 cellular component assembly	http://amigo.geneontology.org/amigo/term/GO:0022607
Cellular Component					
Enrichment FDR	nGenes	Pathway Genes	Fold Enrichment	Pathway	URL
0.00224113166450191	2	5	128.907042253521	GO:0005850 eukaryotic translation initiation factor 2 complex	http://amigo.geneontology.org/amigo/term/GO:0005850
0.00089635698490621	3	17	56.870753935377	GO:0000221 vacuolar proton-transporting V-type ATPase V1 domain	http://amigo.geneontology.org/amigo/term/GO:0000221
0.00215356756587306	3	25	38.6721126760563	GO:0033178 proton-transporting two-sector ATPase complex catalytic	http://amigo.geneontology.org/amigo/term/GO:0033178
0.00191291327407068	4	64	20.1417253521127	GO:0022627 cytosolic small ribosomal subunit	http://amigo.geneontology.org/amigo/term/GO:0022627
0.00215356756587306	4	74	17.4198705748002	GO:0001725 stress fiber	http://amigo.geneontology.org/amigo/term/GO:0001725
0.00215356756587306	4	74	17.4198705748002	GO:0097517 contractile actin filament bundle	http://amigo.geneontology.org/amigo/term/GO:0097517
0.00241043822379078	4	82	15.720371006527	GO:0032432 actin filament bundle	http://amigo.geneontology.org/amigo/term/GO:0032432
0.00241043822379078	4	82	15.720371006527	GO:0042641 actomyosin	http://amigo.geneontology.org/amigo/term/GO:0042641
0.00241043822379078	10	770	4.18529357965978	GO:0048471 perinuclear region of cytoplasm	http://amigo.geneontology.org/amigo/term/GO:0048471
9.37544910499402E-05	22	2316	3.06126395679778	GO:0070062 extracellular exosome	http://amigo.geneontology.org/amigo/term/GO:0070062
9.37544910499402E-05	22	2342	3.02727895983931	GO:1903561 extracellular vesicle	http://amigo.geneontology.org/amigo/term/GO:1903561
9.37544910499402E-05	22	2343	3.02598690735965	GO:0043230 extracellular organelle	http://amigo.geneontology.org/amigo/term/GO:0043230
9.37544910499402E-05	22	2343	3.02598690735965	GO:0065010 extracellular membrane-bounded organelle	http://amigo.geneontology.org/amigo/term/GO:0065010
0.00215356756587306	28	4466	2.02048655569782	GO:0031982 vesicle	http://amigo.geneontology.org/amigo/term/GO:0031982
KEGG					
Enrichment FDR	nGenes	Pathway Genes	Fold Enrichment	Pathway	URL
0.00276265172522663	4	50	25.7814084507042	Path:hsa05110 Vibrio cholerae infection	http://www.genome.jp/kegg-bin/show_pathway?hsa0511
0.0414626572614547	2	27	23.8716744913928	Path:hsa04966 Collecting duct acid secretion	http://www.genome.jp/kegg-bin/show_pathway?hsa0496
0.00476658688334802	4	77	16.7411743186391	Path:hsa03008 Ribosome biogenesis in eukaryotes	http://www.genome.jp/kegg-bin/show_pathway?hsa0300
0.0265010737079227	3	70	13.8114688128773	Path:hsa05120 Epithelial cell signaling in Helicobacter pylori infection	http://www.genome.jp/kegg-bin/show_pathway?hsa0512
0.0289472193604925	3	78	12.3949079089924	Path:hsa04721 Synaptic vesicle cycle	http://www.genome.jp/kegg-bin/show_pathway?hsa0472
0.0383395003416263	3	89	10.8629529988922	Path:hsa05235 PD-L1 expression and PD-1 checkpoint pathway in ca	http://www.genome.jp/kegg-bin/show_pathway?hsa0523
0.00476658688334802	5	155	10.3957292139936	Path:hsa04150 mTOR signaling pathway	http://www.genome.jp/kegg-bin/show_pathway?hsa0415
0.0265010737079227	4	151	8.53689021546498	Path:hsa04145 Phagosome	http://www.genome.jp/kegg-bin/show_pathway?hsa0414
0.0265010737079227	4	153	8.42529687931511	Path:hsa01240 Biosynthesis of cofactors	http://www.genome.jp/kegg-bin/show_pathway?hsa0124
0.0277487670695818	4	161	8.00664858717523	Path:hsa05206 MicroRNAs in cancer	http://www.genome.jp/kegg-bin/show_pathway?hsa0520
0.00476658688334802	7	331	6.81532700736139	Path:hsa05165 Human papillomavirus infection	http://www.genome.jp/kegg-bin/show_pathway?hsa0516
0.00729770075677038	14	1538	2.93351526584736	Path:hsa01100 Metabolic pathways	http://www.genome.jp/kegg-bin/show_pathway?hsa0110

Genes
P4HA2 PLOD1 PLOD3 P4HA1 EGLN1 PLOD2 ASPH P4HB P3H1 PRDX4 FKBP10 ERO1A
PFKP PKM ENO1 PGK1 ALDOC GAPDH TPI1 ENO2 PFKL ALDOA HK2 PGAM1 AK2 GUK1 AK4 NCOR1 PGM1
PFKP PKM ENO1 PGK1 ALDOC GAPDH TPI1 ENO2 PFKL ALDOA HK2 RRM1 PGAM1 AK2 GUK1 AK4 NCOR1 PGM1
FUCA2 PFKP PDK3 PGK1 BCKDK TPI1 GALE PFKL GALM FUCA1 GAA ENO1 PGM1 USP7 PKM MAN2B1 ALDOC GAPDH ENO2 IGFBP4 AKT1 IGFBP3 TKFC HK2 FABP5 DDB1 ALDOA
FUCA2 PFKP PDK3 PGK1 BCKDK TPI1 GALE PFKL GALM FUCA1 GAA ENO1 PGM1 USP7 PKM MAN2B1 ALDOC GAPDH ENO2 IGFBP4 AKT1 IGFBP3 TKFC HK2 FABP5 DDB1 ALDOA SLC2A3 SLC2A1
FUCA2 PFKP PKM PDK3 ENO1 PGM1 PYGL PGK1 BCKDK GYS1 ALDOC GAPDH TPI1 ENO2 GBE1 GALE PFKL GALM TKFC ALDOA HK2 UGP2 GAA FUCA1 SNCA PGAM1 USP7 HEXB GANAB MAN2B
PFKP PKM ENO1 PGK1 NAMPT ALDOC GAPDH TPI1 ENO2 GMDS PDE4D AMPD2 GFPT2 MACROD1 PFKL ALDOA HK2 AK4 RRM1 UGP2 ADSS1 DPYD SDHA SDHB UGDH HSD17B4 GUK1 PGAM1 DP
CPS1 PFKP PKM PTGS2 ENO1 PTGS1 PGK1 DECR1 BCAT2 BLMH ALDOC GAPDH TPI1 ENO2 DARS1 KYNU ACO1 ASL HACL1 HSD17B4 PFKL PYCR2 ALDOA HK2 AKR1C3 BCKDHA ATP2B4 SDHA U
CPS1 PFKP PKM PTGS2 ENO1 PTGS1 PGK1 DECR1 BCAT2 BLMH ALDOC GAPDH TPI1 ENO2 DARS1 KYNU ACO1 ASL HACL1 HSD17B4 PFKL PYCR2 ALDOA HK2 AKR1C3 BCKDHA ATP2B4 SDHA U
CPS1 PFKP PKM PTGS2 ENO1 PTGS1 PGK1 DECR1 BCAT2 BLMH ALDOC GAPDH TPI1 ENO2 DARS1 KYNU ACO1 ASL HACL1 HSD17B4 PFKL PYCR2 ALDOA HK2 AKR1C3 BCKDHA ATP2B4 SDHA U
DPM1 FUCA2 CPS1 PFKP PKM PDK3 PTGS2 CA12 ENO1 PTGS1 PGK1 BCKDK DECR1 BCAT2 NAMPT CA9 BLMH ALDOC GAPDH TPI1 ENO2 GMDS PDE4D DARS1 KYNU AMPD2 GALE ACO1 ASL HA
RECQL UBA6 HSPA5 POLD3 XRCC5 PDS5B PSMC6 MPG UBR5 RPA3 ERLIN1 TMEM109 RAD50 FAM162A RPA2 WRNIP1 PRDX5 RPA1 MACROD1 EGLN1 UGGT1 ERLIN2 PDIA4 CUL4B ANKZF1 NIPBL H
FUCA2 UBA6 HSPA5 HEXB PFKP PKM ENO1 PYGL PSMC6 PSMA3 PSMA6 CTSZ PSMA7 PGK1 DECR1 BLMH ALDOC CTSC DCPS GAPDH TPI1 ENO2 PDE4D KYNU PCYOX1 PLA2G4A GALE PSMA1 H
FUCA2 UBA6 HSPA5 HEXB PFKP PKM ENO1 PYGL PSMC6 PSMA3 PSMA6 CTSZ PSMA7 PGK1 DECR1 BLMH ALDOC CTSC DCPS GAPDH TPI1 ENO2 PDE4D KYNU PCYOX1 PLA2G4A GALE CAT LRP
RECQL UBA6 HSPA5 POLD3 XRCC5 PDS5B PSMC6 MPG UBR5 RPA3 ERLIN1 TMEM109 RAD50 FAM162A RPA2 PPL CAT SLPI WRNIP1 PRDX5 BST2 RPA1 MACROD1 EGLN1 UGGT1 ERLIN2 PDIA4 CU

Genes
NUCB2 LMAN1 TMED1 TMED7 TMED4 LRPAP1 LMAN2 ERP44 HSPA5 ERGIC1 PDIA6 P4HB MYDGF ERGIC3 UGGT1 BCAP31 CTSZ CTSC COL7A1 CD55
HSPA5 LRPAP1 GANAB PRKCSH ERP44 TXNDC12 COLGALT1 HSP90B1 PDIA5 P4HA2 PLOD3 VTN COL7A1 P4HA1 CALU COL5A1 UGGT1 FKBP10 PDIA6 PDIA4 PPIB PDIA3 POGLUT3 P4HB FUCA2 PLA
FUCA2 MVP SERPINB1 ERP44 HEXB PKM RAB27A XRCC5 PYGL CTSZ MAN2B1 ALDOC CTSC CTSD CAT PRDX4 SLPI GNS GGH SRP14 PFKL ALDOA FABP5 PGAM1 DPP7 FUCA1 ARHGAP45 PPIA X
FUCA2 MVP SERPINB1 ERP44 HEXB PKM RAB27A XRCC5 PYGL CTSZ MAN2B1 ALDOC CTSC CTSD CAT PRDX4 SLPI GNS GGH SRP14 PFKL ALDOA FABP5 PGAM1 DPP7 FUCA1 ARHGAP45 PPIA X
FUCA2 MVP SERPINB1 ERP44 HEXB PKM RAB27A XRCC5 PYGL CTSZ MAN2B1 ALDOC CTSC CTSD CAT PRDX4 SLPI GNS GGH SRP14 PFKL ALDOA FABP5 PGAM1 DPP7 FUCA1 ARHGAP45 PPIA X
FUCA2 AK2 RALA MVP SERPINB1 ERP44 EHD2 HSPA5 HEXB SYNE2 SLC2A3 PFKP PKM RAB27A NUCB2 LMAN1 ENO1 TTC38 PGM1 PLOD1 GANAB ICAM1 GLG1 PTGS1 LGALS1 PACSIN2 PYGL PSM
FUCA2 AK2 RALA MVP SERPINB1 ERP44 EHD2 HSPA5 HEXB SYNE2 SLC2A3 PFKP PKM RAB27A NUCB2 LMAN1 ENO1 TTC38 PGM1 PLOD1 GANAB ICAM1 GLG1 PTGS1 LGALS1 PACSIN2 PYGL PSM
FUCA2 AK2 RALA MVP SERPINB1 ERP44 EHD2 HSPA5 HEXB SYNE2 SLC2A3 PFKP PKM RAB27A NUCB2 LMAN1 ENO1 TTC38 PGM1 PLOD1 GANAB ICAM1 GLG1 PTGS1 LGALS1 PACSIN2 PYGL PSM
FUCA2 AK2 RALA MVP SERPINB1 ERP44 EHD2 HSPA5 HEXB SYNE2 SLC2A3 PFKP PKM RAB27A NUCB2 LMAN1 ENO1 TTC38 PGM1 PLOD1 GANAB ICAM1 GLG1 PTGS1 LGALS1 PACSIN2 PYGL PSM
FUCA2 AK2 RALA MVP SERPINB1 ERP44 EHD2 HSPA5 HEXB SYNE2 SLC2A3 PFKP PKM RAB27A NUCB2 LMAN1 ENO1 TTC38 PGM1 PLOD1 GANAB ICAM1 GLG1 PTGS1 LGALS1 PACSIN2 PYGL PSM
DPM1 HSPA5 PDIA5 P4HA2 LMAN1 PLOD1 GANAB TMED1 PLOD3 ERLIN1 ERGIC1 PLA2G4A P3H1 TXNDC12 ESYT2 P4HA1 PRDX4 RRBP1 ERGIC3 CALU PRKCSH TMED7 CKAP4 UGGT1 USO1 ERLIN2
FUCA2 AK2 RALA MVP SERPINB1 ERP44 EHD2 HSPA5 HEXB SYNE2 SLC2A3 PFKP PKM RAB27A NUCB2 LMAN1 ENO1 TTC38 PGM1 PLOD1 GANAB ICAM1 GLG1 PTGS1 LGALS1 PACSIN2 PYGL PSM
FUCA2 AK2 RALA MVP SERPINB1 ERP44 EHD2 HSPA5 HEXB SYNE2 SLC2A3 PFKP PKM RAB27A NUCB2 LMAN1 ENO1 TTC38 PGM1 PLOD1 GANAB ICAM1 GLG1 PTGS1 LGALS1 PACSIN2 PYGL PSM
USP7 POLD3 SMCHD1 RPA3 POLD2 DDX50 SMC3 SRSF9 RAD50 RPA2 KDM3B TARDBP MORF4L2 RPA1 MACROD1 ARGLU1 NAT10 NARF HNRNPU GTF2E1 SRSF2 WDR43 POLR2H LSM6 PCBP1 RBM
USP7 SRSF9 KDM3B TARDBP MORF4L2 MACROD1 ARGLU1 HNRNPU GTF2E1 SRSF2 POLR2H PCBP1 RBM4 NPM1 RBM12B SRSF10 CTR9 HNRNPUL2 TAF6L KDM1A RECQL ANLN LRRC7 SMC1A P4H

Genes
EIF4B RPL31 EIF3I RPL6 RPL24 EIF2S2 RPL36 EIF2S3 RPL8 EIF4A1 RPL26 RPL9 CNBP RPL15 RPL35A RPL17 RPS20 RPL18 RPS5 EIF3L RPL3 EIF3D EIF3E RPL18A RPL28 RPL19 RPL34 RPL21 RPL5
LARS2 SARS1 EIF4B RPL31 RPS5 EIF3I RPL6 AARS1 EIF3L EIF3D EEF1A2 EIF3E GARS1 EXOSC3 HARS2 TARS1 RPL24 DARS2 MRPS7 EIF2S2 RPL36 EIF2S3 LARS1 NARS1 EPRS1 WARS1 RPS2 RPL1
LARS2 GCLM SARS1 EIF4B RPL31 RPS5 EIF3I RPL6 AARS1 EIF3L EIF3D EEF1A2 EIF3E GARS1 EXOSC3 HARS2 TARS1 RPL24 DARS2 MRPS7 EIF2S2 RPL36 EIF2S3 LARS1 NARS1 EPRS1 WARS1 RPS2
LSG1 RPS5 RPL6 RPL3 EXOSC3 GTPBP4 BYSL RPL5 DDX54 RRP36 RPP40 MRPS7 DDX31 ERAL1 LTV1 RPL35 WDR12 NOB1 NVL RPL10 RPL7 RPL26 NSA2 RPS14 POP5 NMD3 RPS7 ZNF622 RPS27
LARS2 GCLM SARS1 EIF4B RPL31 RPS5 EIF3I RPL6 SPTLC1 AARS1 EIF3L EIF3D EEF1A2 EIF3E GARS1 EXOSC3 HARS2 TARS1 RPL24 DARS2 MRPS7 EIF2S2 RPL36 ASS1 EIF2S3 ACLY PDHA1 LARS1
LARS2 GCLM SARS1 EIF4B RPL31 RPS5 EIF3I RPL6 AARS1 EIF3L EIF3D EEF1A2 EIF3E GSR GARS1 EXOSC3 HARS2 TARS1 RPL24 ECE1 DARS2 IDE MRPS7 EIF2S2 RPL36 EIF2S3 LARS1 NARS1 EPRS
LSG1 EIF4B RPS5 RPL6 SRPK1 RPL3 EXOSC3 GTPBP4 LUC7L3 BYSL RPL5 DDX54 RRP36 RPP40 MRPS7 DDX31 EIF2S2 EIF2S3 ERAL1 LTV1 RPL35 WDR12 NOB1 NVL RPL10 RPL7 RPL26 NSA2 RPS
LARS2 GCLM SARS1 EIF4B ACSL4 RPL31 RPS5 EIF3I RPL6 SPTLC1 AARS1 ACOT7 EIF3L EIF3D EEF1A2 EIF3E GSR GARS1 EXOSC3 HARS2 TARS1 RPL24 ECE1 DARS2 ACOT2 IDE ACOT9 ACSL3 MR
LARS2 SARS1 EIF4B RPL31 RPS5 EIF3I B4GALT1 RPL6 AARS1 EIF3L EIF3D EEF1A2 EIF3E GARS1 EXOSC3 HARS2 TARS1 RPL24 DARS2 MRPS7 EIF2S2 RPL36 EIF2S3 LARS1 NARS1 EPRS1 WARS1 RP
ELAC2 LARS2 SARS1 DIS3 AARS1 TRMT1 GARS1 EXOSC3 BYSL HARS2 TARS1 DARS2 DDX54 RRP36 RPP40 LARS1 THUMPD3 NARS1 EPRS1 RPL35 POLR1E WDR12 YARS2 WARS1 NOB1 RPL7 RPP
LARS2 GCLM SARS1 ADSS2 EIF4B ASNS RPL31 RPS5 EIF3I CAD B4GALT1 RPL6 SPTLC1 AARS1 EIF3L EIF3D EEF1A2 EIF3E GARS1 EXOSC3 HARS2 TARS1 RPL24 UMPS GLS CTH DARS2 MRPS7 EIF
LARS2 SARS1 ME1 IDH3G ACSL4 ASNS HSD17B10 CAD AARS1 ACOT7 IDH3B GARS1 ENO3 CPT1A HARS2 TARS1 GLS CTH ABCD3 DARS2 ACOT2 ACAT2 ACSL3 MPST ASS1 ACLY PDHA1 LARS1 NA
LARS2 SARS1 ME1 IDH3G ACSL4 ASNS HSD17B10 CAD AARS1 ACOT7 IDH3B GARS1 ENO3 CPT1A HARS2 TARS1 GLS CTH ABCD3 DARS2 ACOT2 ACAT2 ACSL3 MPST ASS1 ACLY PDHA1 LARS1 NA
LARS2 DERA SARS1 ADSS2 ME1 MTHFD2 IDH3G ACSL4 ASNS HSD17B10 CAD SPTLC1 AARS1 ACOT7 MTHFD1 SAMHD1 IDH3B GARS1 ENO3 CPT1A HARS2 TARS1 UMPS GLS CTH ABCD3 DARS2 A
HCCS BAIAP2L1 LSG1 LIMA1 EIF4B WDR1 CLASP1 FSCN1 RPS5 RPL6 LAMB1 NUBP2 SRPK1 RPL3 PLS3 COTL1 NAPA LSR EXOSC3 GTPBP4 LUC7L3 RAB34 EHD1 NDUFS8 BYSL PLS1 RPL5 CALD1

Genes
RPL18 RPL31 RPL6 RPL3 RPL18A RPL28 RPL19 RPL34 RPL24 RPL21 RPL5 RPL23 RPL36 RPL35 RPL13A RPL11 RPL37 RPL10 RPL7 RPL8 RPL26 RPL9 RPL27A RPL13 RPL4 RPL15 RPL35A RPL14 RP
RPS20 RPL18 RPL31 RPS5 RPL6 RPL3 RPL18A RPL28 RPL19 RPL34 RPL24 RPL21 RPL5 RPS10 RPL23 RPL36 RPL35 RPS24 RPS2 RPS11 RPL13A RPL11 RPS3A RPL37 RPL10 RPL7 RPL8 RPL26 RPL
RPL18 RPL31 RPL6 RPL3 RPL18A RPL28 RPL19 RPL34 RPL24 RPL21 RPL5 RPL23 RPL36 RPL35 RPL13A RPL11 RPL37 RPL10 RPL7 RPL8 RPL26 RPL9 RPL27A RPL13 RPL4 RPL15 RPL35A RPL14 RP
RPS20 RPL18 RPL31 RPS5 RPL6 RPL3 RPL18A RPL28 RPL19 RPL34 RPL24 RPL21 RPL5 RPS10 RPL23 RPL36 RPL35 RPS24 RPS2 RPS11 RPL13A RPL11 RPS3A RPL37 RPL10 RPL7 RPL8 RPL26 RPL
RPS20 RPL18 RPL31 RPS5 RPL6 RPL3 RPL18A RPL28 RPL19 RPL34 RPL24 RPL21 RPL5 RPS10 RPL23 RPL36 RPL35 RPS24 RPS2 RPS11 RPL13A RPL11 RPS3A RPL37 RPL10 RPL7 RPL8 RPL26 RPL
CD99 VIM VCL CTNNA1 LIMA1 PPP1R12A RPL18 CNN2 RPL31 FERMT2 MCAM RPS5 TRIP6 RPL6 PXN RPL3 PROCR HSPB1 ENG PDLIM1 RPL19 FHL2 ARHGEF2 RPL5 RPS10 AHNAK RPL23 CDC42EP
CD99 VIM VCL CTNNA1 LIMA1 PPP1R12A RPL18 CNN2 RPL31 FERMT2 MCAM RPS5 TRIP6 RPL6 PXN RPL3 PROCR HSPB1 ENG PDLIM1 RPL19 FHL2 ARHGEF2 RPL5 RPS10 AHNAK RPL23 CDC42EP
RPS20 RPL18 RPL31 RPS5 RPL6 RPL3 RPL18A RPL28 RPL19 RPL34 RPL24 RPL21 RPL5 RPS10 RPL23 RPL36 RPL35 RPS24 RPS2 RPS11 RPL13A RPL11 RPS3A RPL37 RPL10 RPL7 RPL8 RPL26 RPL
CS MRPL32 MRPS30 MRPL3 MRPL19 MRPS2 MRPS7 MRPS26 ERAL1 MRPS9 TBRG4 MRPL15 MRPS5 MRPL13 MRPL11 MRPS22 MRPL48 SHMT2 MRPL53 POLRMT MRPL4 MRPL18 CLPP PMPCA SLC
CD99 BAIAP2L1 VIM VCL CTNNA1 LIMA1 PPP1R12A RPL18 CNN2 RPL31 FERMT2 MCAM RPS5 TRIP6 RPL6 PXN RPL3 PROCR HSPB1 ENG PDLIM1 RPL19 EIF4G2 KRT18 FHL2 ARHGEF2 RPL5 RPS10
BAIAP2L1 RPS20 WIZ VIM SARS1 VCL ADSS2 ATP6V1H CS DIP2B ACSL4 ATP1B3 RPL31 WDR1 TFRC FSCN1 FBLN1 CARMIL1 TNPO1 RPS5 EIF3I CAD CPNE3 B4GALT1 MTMR2 AARS1 LAMB1 PHGDH
BAIAP2L1 RPS20 WIZ VIM SARS1 VCL ADSS2 ATP6V1H CS DIP2B ACSL4 ATP1B3 RPL31 WDR1 TFRC FSCN1 FBLN1 CARMIL1 TNPO1 RPS5 EIF3I CAD CPNE3 B4GALT1 MTMR2 AARS1 LAMB1 PHGDH
BAIAP2L1 RPS20 WIZ VIM SARS1 VCL ADSS2 ATP6V1H CS DIP2B ACSL4 ATP1B3 RPL31 WDR1 TFRC FSCN1 FBLN1 CARMIL1 TNPO1 RPS5 EIF3I CAD CPNE3 B4GALT1 MTMR2 AARS1 LAMB1 PHGDH
BAIAP2L1 RPS20 WIZ VIM SARS1 VCL ADSS2 ATP6V1H CS DIP2B ACSL4 ATP1B3 RPL31 WDR1 TFRC FSCN1 FBLN1 CARMIL1 TNPO1 RPS5 EIF3I CAD CPNE3 B4GALT1 MTMR2 AARS1 LAMB1 PHGDH
BAIAP2L1 RPS20 WIZ VIM SARS1 VCL ADSS2 ATP6V1H CS MTHFD2 DIP2B ACSL4 ATP1B3 RPL31 WDR1 TFRC FSCN1 FBLN1 CARMIL1 TNPO1 RPS5 EIF3I CAD CPNE3 B4GALT1 MTMR2 AARS1 LAMB

Genes
NCAPD2 NCAPH
NCAPD2 NCAPH
IFIT1 STAT1
FSCN1 CORO1B MTOR TWF2
FSCN1 MTOR TWF2
TWF2 FSCN1 CORO1B MTOR
MTOR SPTLC1 ATP6V1E1 ATP6V1G1 ATP6V1B2
GCLM SPTLC1 EIF2S2 MARS1 RPS27 RPS26 MTOR EIF2A VIM ROCK2 ACSL3
MAP1B TWF2 CORO1B NCAPD2 FSCN1 TMEM33 NCAPH ROCK2 MTOR OPA1 RAP1GDS1 INF2 PARP1 MAP2K2
GCLM SPTLC1 GSR ACSL3 EIF2S2 MARS1 RPS27 RPS26 MTOR NAGK EIF2A VIM ROCK2
ADSS2 HSD17B10 SPTLC1 ACSL3 DUT DDAH1 PDXK MARS1 MTOR GCLM OPA1 TJP2 NAGK PARP1 REXO2 PEPD
NCAPD2 VIM PSME4 FSCN1 RAB34 TMEM33 NCAPH MAP1B ROCK2 DYNC1LI2 ENAH ATXN2L CORO1B RPS27 FHL3 OPA1 TWF2 AP1B1 MTOR SBDS HAT1 GCLM RAP1GDS1 INF2 HSD17B10 UFC1 P
FSCN1 NAPA RAB34 EIF2S2 ROCK2 NOB1 ATXN2L NMD3 RPS27 TWF2 AP1B1 HSD17B10 SBDS HAT1 CORO1B ISG15 OPA1 MAP1B RAP1GDS1 PARP1 MTOR ACSL3 EIF2A TRIP6 KPNA3
FSCN1 NAPA RAB34 EIF2S2 ROCK2 ATXN2L RPS27 TWF2 AP1B1 HSD17B10 SBDS HAT1 CORO1B ISG15 OPA1 MAP1B RAP1GDS1 PARP1 MTOR ACSL3 EIF2A TRIP6 KPNA3

Genes
EIF2S2 EIF2A
ATP6V1E1 ATP6V1G1 ATP6V1B2
ATP6V1E1 ATP6V1G1 ATP6V1B2
RPS27 RPS26 EIF2A ISG15
TRIP6 FHL3 FSCN1 CORO1B
TRIP6 FHL3 FSCN1 CORO1B
TRIP6 FHL3 FSCN1 CORO1B
TRIP6 FHL3 FSCN1 CORO1B
S100A4 RAB34 STAT1 ACSL3 MAP2K2 CORO1B INF2 MAP1B UNC45A TWF2
VIM ADSS2 FSCN1 GSR NAPA RAB34 PEPD NAGK DUT ATP6V1E1 ATP6V1G1 RAP1GDS1 UFC1 GMPPA ATP6V1B2 DDAH1 PDXK MARS1 CORO1B S100A4 RPS26 TWF2
VIM ADSS2 FSCN1 GSR NAPA RAB34 PEPD NAGK DUT ATP6V1E1 ATP6V1G1 RAP1GDS1 UFC1 GMPPA ATP6V1B2 DDAH1 PDXK MARS1 CORO1B S100A4 RPS26 TWF2
VIM ADSS2 FSCN1 GSR NAPA RAB34 PEPD NAGK DUT ATP6V1E1 ATP6V1G1 RAP1GDS1 UFC1 GMPPA ATP6V1B2 DDAH1 PDXK MARS1 CORO1B S100A4 RPS26 TWF2
VIM ADSS2 FSCN1 GSR NAPA RAB34 PEPD NAGK DUT ATP6V1E1 ATP6V1G1 RAP1GDS1 UFC1 GMPPA ATP6V1B2 DDAH1 PDXK MARS1 CORO1B S100A4 RPS26 TWF2
VIM ADSS2 FSCN1 GSR NAPA RAB34 PEPD NAGK DUT ATP6V1E1 ATP6V1G1 RAP1GDS1 UFC1 GMPPA ATP6V1B2 DDAH1 PDXK MARS1 CORO1B S100A4 RPS26 TWF2 AP1B1 TMEM33 MTOR RABAC1

Genes
ATP6V1B2 ATP6V1E1 TJP2 ATP6V1G1
ATP6V1B2 ATP6V1E1
REXO2 NOB1 NMD3 SBDS
ATP6V1B2 ATP6V1E1 ATP6V1G1
ATP6V1B2 ATP6V1E1 NAPA
MTOR MAP2K2 STAT1
MTOR ATP6V1B2 ATP6V1E1 MAP2K2 ATP6V1G1
DYNC1LI2 ATP6V1B2 ATP6V1E1 ATP6V1G1
ADSS2 GCLM GMPPA PDXK
MTOR MAP2K2 FSCN1 VIM
MTOR ATP6V1B2 ATP6V1E1 MAP2K2 STAT1 ATP6V1G1 ISG15
SPTLC1 ADSS2 DUT ACSL3 GCLM GSR GMPPA HSD17B10 MARS1 ATP6V1B2 ATP6V1E1 NAGK PDXK ATP6V1G1
