# Supplementary material for: Extracellular Vesicles Bearing Vimentin Drive Epithelial–Mesenchymal Transition
Source: Mol Cell Proteomics. 2025 Jul 4;24(12):101028. doi: 10.1016/j.mcpro.2025.101028 (PMC12719745; doi:10.1016/j.mcpro.2025.101028)
Supplement: Supplemental Data 12 [file mmc15.pdf]

Sheet 12.MCF10A_GO

**Fig3 cluster 1, n=87**					
Biological process					
Enrichment FDR	nGenes	Pathway Genes	Fold Enrichment	Pathway	URL
1.83010709544294E-08	14	385	10.0245345016429	GO:0031589 cell-substrate adhesion	http://amigo.geneontology.org/amigo/term/GO:0031589
8.46921995894346E-10	20	769	7.16969307659768	GO:0030036 actin cytoskeleton organization	http://amigo.geneontology.org/amigo/term/GO:0030036
2.4549246561313E-10	22	857	7.07683007408865	GO:0030029 actin filament-based proc.	http://amigo.geneontology.org/amigo/term/GO:0030029
8.46921995894346E-10	23	1071	5.92018494144646	GO:0000902 cell morphogenesis	http://amigo.geneontology.org/amigo/term/GO:0000902
2.4549246561313E-10	29	1602	4.99036595821488	GO:0016477 cell migration	http://amigo.geneontology.org/amigo/term/GO:0016477
1.07608799058131E-09	28	1729	4.46436759182479	GO:0007155 cell adhesion	http://amigo.geneontology.org/amigo/term/GO:0007155
8.46921995894346E-10	29	1806	4.42667013569227	GO:0048870 cell motility	http://amigo.geneontology.org/amigo/term/GO:0048870
8.46921995894346E-10	29	1806	4.42667013569227	GO:0051674 localization of cell	http://amigo.geneontology.org/amigo/term/GO:0051674
1.06032973831401E-09	30	1992	4.15172739149369	GO:0040011 locomotion	http://amigo.geneontology.org/amigo/term/GO:0040011
3.36812757964261E-10	33	2254	4.03605370906875	GO:0006928 movement of cell or subcellular component	http://amigo.geneontology.org/amigo/term/GO:0006928
8.46921995894346E-10	32	2239	3.93996889747467	GO:0048468 cell development	http://amigo.geneontology.org/amigo/term/GO:0048468
2.69353299447051E-09	35	2867	3.36540441500918	GO:0009653 anatomical structure morphogenesis	http://amigo.geneontology.org/amigo/term/GO:0009653
2.4549246561313E-10	47	4617	2.80630514259768	GO:0048731 system development	http://amigo.geneontology.org/amigo/term/GO:0048731
1.06032973831401E-09	45	4552	2.72525515065535	GO:0030154 cell differentiation	http://amigo.geneontology.org/amigo/term/GO:0030154
1.07608799058131E-09	45	4576	2.71096185441065	GO:0048869 cellular developmental proc.	http://amigo.geneontology.org/amigo/term/GO:0048869
Cellular Component					
Enrichment FDR	nGenes	Pathway Genes	Fold Enrichment	Pathway	URL
2.7530811610257E-25	29	484	16.5176988947526	GO:0030055 cell-substrate junction	http://amigo.geneontology.org/amigo/term/GO:0030055
2.11957210770789E-24	28	475	16.2502980342422	GO:0005925 focal adhesion	http://amigo.geneontology.org/amigo/term/GO:0005925
2.5612863836978E-09	11	192	15.7938629518072	GO:0001726 ruffle	http://amigo.geneontology.org/amigo/term/GO:0001726
1.40018704004912E-07	10	216	12.7627175368139	GO:0030027 lamellipodium	http://amigo.geneontology.org/amigo/term/GO:0030027
9.45586038347949E-13	18	446	11.1258847047382	GO:0031252 cell leading edge	http://amigo.geneontology.org/amigo/term/GO:0031252
1.54899977940205E-22	33	926	9.8242603242343	GO:0070161 anchoring junction	http://amigo.geneontology.org/amigo/term/GO:0070161
1.90483223377995E-09	16	532	8.29096838481746	GO:0015629 actin cytoskeleton	http://amigo.geneontology.org/amigo/term/GO:0015629
7.14428738571582E-17	40	2293	4.80897860959757	GO:0030054 cell junction	http://amigo.geneontology.org/amigo/term/GO:0030054
4.86603505443943E-15	38	2316	4.52315999750297	GO:0070062 extracellular exosome	http://amigo.geneontology.org/amigo/term/GO:0070062
4.86603505443943E-15	38	2342	4.4729455825008	GO:1903561 extracellular vesicle	http://amigo.geneontology.org/amigo/term/GO:1903561
4.86603505443943E-15	38	2343	4.47103651481727	GO:0043230 extracellular organelle	http://amigo.geneontology.org/amigo/term/GO:0043230
4.86603505443943E-15	38	2343	4.47103651481727	GO:0065010 extracellular membrane-bounded organelle	http://amigo.geneontology.org/amigo/term/GO:0065010
1.2920191841357E-13	44	3577	3.3910222943774	GO:0005615 extracellular space	http://amigo.geneontology.org/amigo/term/GO:0005615
1.32568316057253E-11	46	4466	2.8394617430762	GO:0031982 vesicle	http://amigo.geneontology.org/amigo/term/GO:0031982
3.16622568510641E-10	45	4673	2.65468894624077	GO:0005576 extracellular region	http://amigo.geneontology.org/amigo/term/GO:0005576
Fig3 cluster 2, n=117					
Biological process					
Enrichment FDR	nGenes	Pathway Genes	Fold Enrichment	Pathway	URL
4.74335785633895E-05	4	12	68.0982142857143	GO:0000727 double-strand break repair via break-induced replication	http://amigo.geneontology.org/amigo/term/GO:0000727
1.70629721293503E-05	5	23	44.4118788819876	GO:0006268 DNA unwinding involved in DNA replication	http://amigo.geneontology.org/amigo/term/GO:0006268
1.49033361090499E-05	9	153	12.0173319327731	GO:0006275 reg. of DNA replication	http://amigo.geneontology.org/amigo/term/GO:0006275
1.38031980283156E-06	12	248	9.88522465437788	GO:0006364 rRNA processing	http://amigo.geneontology.org/amigo/term/GO:0006364
8.63166415452626E-07	13	288	9.22163318452381	GO:0016072 rRNA metabolic proc.	http://amigo.geneontology.org/amigo/term/GO:0016072
2.48211247135132E-06	13	327	8.12180537352556	GO:0042254 ribosome biogenesis	http://amigo.geneontology.org/amigo/term/GO:0042254
8.97087515607632E-06	12	303	8.09087694483734	GO:0006260 DNA replication	http://amigo.geneontology.org/amigo/term/GO:0006260
2.70024533730821E-05	11	281	7.99729918657855	GO:0033044 reg. of chromosome organization	http://amigo.geneontology.org/amigo/term/GO:0033044
2.93340173650334E-08	18	483	7.61346495119787	GO:0034470 ncRNA processing	http://amigo.geneontology.org/amigo/term/GO:0034470
1.38031980283156E-06	16	509	6.42183553185518	GO:0022613 ribonucleoprotein complex biogenesis	http://amigo.geneontology.org/amigo/term/GO:0022613
3.67902976608187E-08	20	645	6.33471760797342	GO:0034660 ncRNA metabolic proc.	http://amigo.geneontology.org/amigo/term/GO:0034660
4.18045350615405E-08	25	1082	4.7203013599155	GO:0006396 RNA processing	http://amigo.geneontology.org/amigo/term/GO:0006396
2.67639350345453E-08	27	1173	4.70243423456339	GO:0051276 chromosome organization	http://amigo.geneontology.org/amigo/term/GO:0051276
6.30532406344433E-05	38	3403	2.28128017295663	GO:0051173 positive reg. of nitrogen compound metabolic proc.	http://amigo.geneontology.org/amigo/term/GO:0051173
1.49033361090499E-05	45	4206	2.18574867536173	GO:0006996 organelle organization	http://amigo.geneontology.org/amigo/term/GO:0006996
Cellular Component					
Enrichment FDR	nGenes	Pathway Genes	Fold Enrichment	Pathway	URL
5.78247989237286E-06	4	11	74.288961038961	GO:0042555 MCM complex	http://amigo.geneontology.org/amigo/term/GO:0042555
5.78247989237286E-06	4	11	74.288961038961	GO:0071162 CMG complex	http://amigo.geneontology.org/amigo/term/GO:0071162
8.02377607484559E-06	4	12	68.0982142857143	GO:0031261 DNA replication preinitiation complex	http://amigo.geneontology.org/amigo/term/GO:0031261
1.45159862317482E-08	12	187	13.1098166539343	GO:0000781 chromosome telomeric region	http://amigo.geneontology.org/amigo/term/GO:0000781
3.86487869699001E-09	17	423	8.21042299898683	GO:0098687 chromosomal region	http://amigo.geneontology.org/amigo/term/GO:0098687
2.49844563292113E-05	10	270	7.56646825396825	GO:0000228 nuclear chromosome	http://amigo.geneontology.org/amigo/term/GO:0000228
4.39858609892878E-06	21	1126	3.81011323268206	GO:0005730 nucleolus	http://amigo.geneontology.org/amigo/term/GO:0005730
7.09115199477403E-07	26	1489	3.56726710160223	GO:0140513 nuclear protein-containing complex	http://amigo.geneontology.org/amigo/term/GO:0140513
4.53755310610122E-08	31	1830	3.46072892271663	GO:0005739 mitochondrion	http://amigo.geneontology.org/amigo/term/GO:0005739
2.11658632809396E-06	32	2316	2.82272390821614	GO:0070062 extracellular exosome	http://amigo.geneontology.org/amigo/term/GO:0070062
2.11658632809396E-06	32	2342	2.79138709283884	GO:1903561 extracellular vesicle	http://amigo.geneontology.org/amigo/term/GO:1903561
2.11658632809396E-06	32	2343	2.79019571977318	GO:0043230 extracellular organelle	http://amigo.geneontology.org/amigo/term/GO:0043230
2.11658632809396E-06	32	2343	2.79019571977318	GO:0065010 extracellular membrane-bounded organelle	http://amigo.geneontology.org/amigo/term/GO:0065010
1.53363859325838E-13	61	4581	2.72036088502198	GO:0005654 nucleoplasm	http://amigo.geneontology.org/amigo/term/GO:0005654
3.27570968585595E-14	65	4973	2.67024970555284	GO:0031981 nuclear lumen	http://amigo.geneontology.org/amigo/term/GO:0031981
Reference List and cluster 2, n=76					
Biological process					
Enrichment FDR	nGenes	Pathway Genes	Fold Enrichment	Pathway	URL
0.0433416768672177	1	7	544.785714285714	GO:0032532 reg. of microvillus length	http://amigo.geneontology.org/amigo/term/GO:0032532
0.0433416768672177	1	8	476.6875	GO:0002036 reg. of L-glutamate import across plasma membrane	http://amigo.geneontology.org/amigo/term/GO:0002036
0.0433416768672177	1	12	317.791666666667	GO:0010917 negative reg. of mitochondrial membrane potential	http://amigo.geneontology.org/amigo/term/GO:0010917
0.0433416768672177	1	13	293.346153846154	GO:0032530 reg. of microvillus organization	http://amigo.geneontology.org/amigo/term/GO:0032530
0.0433416768672177	1	13	293.346153846154	GO:0032536 reg. of cell projection size	http://amigo.geneontology.org/amigo/term/GO:0032536
0.0433416768672177	2	149	51.1879194630872	GO:0030833 reg. of actin filament polymerization	http://amigo.geneontology.org/amigo/term/GO:0030833
0.0433416768672177	2	168	45.3988095238095	GO:0008064 reg. of actin polymerization or depolymerization	http://amigo.geneontology.org/amigo/term/GO:0008064
0.0433416768672177	2	169	45.1301775147929	GO:0030832 reg. of actin filament length	http://amigo.geneontology.org/amigo/term/GO:0030832
0.0433416768672177	2	185	41.227027027027	GO:0030041 actin filament polymerization	http://amigo.geneontology.org/amigo/term/GO:0030041
0.0433416768672177	2	215	35.4744186046512	GO:0008154 actin polymerization or depolymerization	http://amigo.geneontology.org/amigo/term/GO:0008154
0.0433416768672177	2	221	34.5113122171946	GO:0032271 reg. of protein polymerization	http://amigo.geneontology.org/amigo/term/GO:0032271
0.0433416768672177	2	376	20.2845744680851	GO:0032956 reg. of actin cytoskeleton organization	http://amigo.geneontology.org/amigo/term/GO:0032956
0.0433416768672177	2	383	19.9138381201044	GO:0032535 reg. of cellular component size	http://amigo.geneontology.org/amigo/term/GO:0032535
0.0433416768672177	4	2642	5.77365632096896	GO:0008104 protein localization	http://amigo.geneontology.org/amigo/term/GO:0008104
Cellular Component					
Enrichment FDR	nGenes	Pathway Genes	Fold Enrichment	Pathway	URL
0.000788213731468621	2	21	363.190476190476	GO:0030130 clathrin coat of trans-Golgi network vesicle	http://amigo.geneontology.org/amigo/term/GO:0030130
0.000788213731468621	2	23	331.608695652174	GO:0012510 trans-Golgi network transport vesicle membrane	http://amigo.geneontology.org/amigo/term/GO:0012510
0.00123407462379557	2	35	217.914285714286	GO:0030125 clathrin vesicle coat	http://amigo.geneontology.org/amigo/term/GO:0030125
0.00158370883149584	2	51	149.549019607843	GO:0030118 clathrin coat	http://amigo.geneontology.org/amigo/term/GO:0030118
0.00167560348537719	2	60	127.116666666667	GO:0030660 Golgi-associated vesicle membrane	http://amigo.geneontology.org/amigo/term/GO:0030660
0.00167560348537719	2	62	123.016129032258	GO:0030120 vesicle coat	http://amigo.geneontology.org/amigo/term/GO:0030120
0.00305672643184298	2	95	80.2842105263158	GO:0005798 Golgi-associated vesicle	http://amigo.geneontology.org/amigo/term/GO:0005798
0.00305672643184298	2	100	76.27	GO:0030117 membrane coat	http://amigo.geneontology.org/amigo/term/GO:0030117
0.00305672643184298	2	100	76.27	GO:0048475 coated membrane	http://amigo.geneontology.org/amigo/term/GO:0048475
0.00905876682396362	2	181	42.1381215469613	GO:0030665 clathrin-coated vesicle membrane	http://amigo.geneontology.org/amigo/term/GO:0030665
0.00911057684667476	4	2316	6.58635578583765	GO:0070062 extracellular exosome	http://amigo.geneontology.org/amigo/term/GO:0070062
0.00911057684667476	4	2343	6.51045667947076	GO:0043230 extracellular organelle	http://amigo.geneontology.org/amigo/term/GO:0043230
0.00911057684667476	4	2343	6.51045667947076	GO:0065010 extracellular membrane-bounded organelle	http://amigo.geneontology.org/amigo/term/GO:0065010
0.00911057684667476	5	4466	4.26948051948052	GO:0031982 vesicle	http://amigo.geneontology.org/amigo/term/GO:0031982
KEGG					
Enrichment FDR	nGenes	Pathway Genes	Fold Enrichment	Pathway	URL
0.00232698383486805	2	77	99.051948051948	Path:hsa05100 Bacterial invasion of epithelial cells	http://www.genome.jp/kegg-bin/show_pathway?hsa0510
0.0483674184302628	1	53	71.9528301886792	Path:hsa04961 Endocrine and other factor-regulated calcium reabsorp	http://www.genome.jp/kegg-bin/show_pathway?hsa0496
0.00341597662797227	2	132	57.780303030303	Path:hsa04142 Lysosome	http://www.genome.jp/kegg-bin/show_pathway?hsa0414
0.00814980831723254	2	251	30.3864541832669	Path:hsa04144 Endocytosis	http://www.genome.jp/kegg-bin/show_pathway?hsa0414

Genes
ITGB6 ITGAV ITGA5 ILK LAMB3 PARVA VCL TLN1 KANK1 PTK2B SRC CRK FLNA COL17A1
WDR1 PLEK2 PLS3 KANK1 CALD1 IQGAP1 SH3KBP1 GSN PDLIM7 PARVA ARPC4 TWF2 HSP90B1 CRK FLNA PTK2B PALLD TLN1 ABLIM3 SRC
WDR1 PLEK2 PLS3 KANK1 CALD1 IQGAP1 SH3KBP1 GSN PDLIM7 PARVA ARPC4 TWF2 FLNA HSP90B1 CRK SUN2 PTK2B PALLD TLN1 ABLIM3 SRC ATP2A2
LAMA3 LAMC2 PALLD ILK LAMB3 PARVA KANK1 ITGAV VCL WDR1 ITGB6 PTK2B YWHAH NUMB EGFR ALCAM RAPH1 SRC IQGAP1 SH3KBP1 CRK FLNA TWF2
CD99 LAMA3 ITGB6 NAV1 SH3KBP1 VAV2 CRK LAMB3 ITGA5 PTK2B FLNA VCL WDR1 STK10 SUN2 LAMA4 PALLD ITGAV IQGAP1 EGFR SGPL1 ILK SRC PARVA NUMB HSPA5 LAMC2 HSPB1 KANK1
VCL HSPB1 TLN1 ILK FLNA CD99 LAMA3 ITGB6 TGFBI PTK2B PALLD ITGAV ITGA5 ALCAM LAMB3 SRC PARVA KANK1 IGFBP7 LAMC2 STK10 LAMA4 CRK ANXA2 TGM2 EGFR COL17A1 ATP2A2
CD99 LAMA3 ITGB6 NAV1 SH3KBP1 VAV2 CRK LAMB3 ITGA5 PTK2B FLNA VCL WDR1 STK10 SUN2 LAMA4 PALLD ITGAV IQGAP1 EGFR SGPL1 ILK SRC PARVA NUMB HSPA5 LAMC2 HSPB1 KANK1
CD99 LAMA3 ITGB6 NAV1 SH3KBP1 VAV2 CRK LAMB3 ITGA5 PTK2B FLNA VCL WDR1 STK10 SUN2 LAMA4 PALLD ITGAV IQGAP1 EGFR SGPL1 ILK SRC PARVA NUMB HSPA5 LAMC2 HSPB1 KANK1
CD99 LAMA3 LAMC2 WDR1 ITGB6 PALLD NAV1 SH3KBP1 VAV2 CRK LAMB3 ITGA5 PTK2B FLNA VCL STK10 SUN2 LAMA4 ITGAV IQGAP1 EGFR SGPL1 ILK ALCAM SRC PARVA NUMB HSPA5 HSPB1
CD99 LAMA3 LAMC2 ITGB6 PALLD NAV1 SH3KBP1 VAV2 CRK LAMB3 ITGA5 FLNA PTK2B VCL WDR1 STK10 SUN2 LAMA4 ITGAV IQGAP1 EGFR GSN SGPL1 ILK ALCAM SRC PARVA NUMB HSPA5 M
MAP4 LAMA3 LAMC2 WDR1 PALLD ILK LAMB3 SRC PARVA TWF2 FLNA VCL KANK1 ITGAV ANXA2 TGM2 HSPA5 AKR1B1 NDRG1 FHL2 IRF6 PTK2B YWHAH NUMB CUL4B CRK ALCAM BPGM RAPH1
LAMA3 LAMC2 WDR1 CALD1 PALLD ITGAV ITGA5 ILK LAMB3 PARVA KANK1 FLNA VCL HSPA5 PLEKHA1 ITGB6 FHL2 PTK2B YWHAH NUMB EGFR VAV2 SGPL1 ALCAM RAPH1 ANXA2 SRC TGM2 SE
HSPA5 YWHAH MAP4 LAMA3 LAMC2 CALD1 PALLD NAV1 ITGAV GSN ITGA5 PDLIM7 SRC TWF2 FLNA KANK1 ANXA2 TGM2 VCL WDR1 AKR1B1 SUN2 NDRG1 PLEKHA1 LAMA4 ITGB6 FHL2 TGFBI P
MAP4 LAMA3 LAMC2 WDR1 PTK2B PALLD NAV1 EGFR ILK LAMB3 SRC PARVA TWF2 FLNA VCL KANK1 ITGAV ANXA2 TGM2 HSPA5 AKR1B1 SUN2 NDRG1 PLEKHA1 LAMA4 ITGB6 FHL2 IRF6 TGFBI
MAP4 LAMA3 LAMC2 WDR1 PTK2B PALLD NAV1 EGFR ILK LAMB3 SRC PARVA TWF2 FLNA VCL KANK1 ITGAV ANXA2 TGM2 HSPA5 AKR1B1 SUN2 NDRG1 PLEKHA1 LAMA4 ITGB6 FHL2 IRF6 TGFBI

Genes
CD99 VCL HSPA5 HSPB1 FHL2 PALLD NUMB TES TLN1 ITGAV IQGAP1 EGFR GSN ITGA5 ANXA5 ILK HSP90B1 PDIA3 ALCAM FLNA IGF2R PARVA TGM2 COL17A1 ITGB6 PTK2B PDLIM7 SH3KBP1 SR
CD99 VCL HSPA5 HSPB1 FHL2 PALLD NUMB TES TLN1 ITGAV IQGAP1 EGFR GSN ITGA5 ANXA5 ILK HSP90B1 PDIA3 ALCAM FLNA IGF2R PARVA TGM2 ITGB6 PTK2B PDLIM7 SH3KBP1 SRC
KANK1 PLEKHA1 ITGAV IQGAP1 EGFR ITGA5 PALLD TLN1 GSN PDLIM7 SRC
PTK2B PALLD ITGAV ABLIM3 PLEK2 GSN ILK RAPH1 PARVA TWF2
KANK1 PLEKHA1 PTK2B PALLD ITGAV IQGAP1 EGFR ITGA5 ABLIM3 PLEK2 TLN1 GSN ILK RAPH1 PDLIM7 SRC PARVA TWF2
CD99 VCL HSPA5 HSPB1 FHL2 PALLD NUMB TES TLN1 ITGAV IQGAP1 EGFR GSN ITGA5 ANXA5 ILK HSP90B1 PDIA3 ALCAM ANXA2 FLNA IGF2R PARVA TGM2 COL17A1 ITGB6 PTK2B PDLIM7 YWHA
WDR1 PLS3 CALD1 GSN ILK ABLIM3 PDLIM7 PARVA ARPC4 TWF2 FLNA PALLD VCL IQGAP1 CRK SRC
CD99 VCL HSPA5 HSPB1 FHL2 PALLD NUMB TES TLN1 ITGAV IQGAP1 EGFR GSN ITGA5 ANXA5 ILK HSP90B1 PDIA3 ALCAM ANXA2 FLNA IGF2R PARVA TGM2 COL17A1 ITGB6 PTK2B CLTB PDLIM7
VCL HSPA5 LAMA3 PFKP WDR1 STK10 AP1M1 PLOD1 AKR1B1 TOM1 NDRG1 HSPB1 PLEKHA1 LAMA4 IRF6 TGFBI YWHAH TXNDC17 TLN1 ITGAV IQGAP1 GSN PLOD2 CUL4B IGFBP7 ANXA5 HSP90B
VCL HSPA5 LAMA3 PFKP WDR1 STK10 AP1M1 PLOD1 AKR1B1 TOM1 NDRG1 HSPB1 PLEKHA1 LAMA4 IRF6 TGFBI YWHAH TXNDC17 TLN1 ITGAV IQGAP1 GSN PLOD2 CUL4B IGFBP7 ANXA5 HSP90B
VCL HSPA5 LAMA3 PFKP WDR1 STK10 AP1M1 PLOD1 AKR1B1 TOM1 NDRG1 HSPB1 PLEKHA1 LAMA4 IRF6 TGFBI YWHAH TXNDC17 TLN1 ITGAV IQGAP1 GSN PLOD2 CUL4B IGFBP7 ANXA5 HSP90B
VCL HSPA5 LAMA3 PFKP WDR1 STK10 AP1M1 PLOD1 AKR1B1 TOM1 NDRG1 HSPB1 PLEKHA1 LAMA4 IRF6 TGFBI YWHAH TXNDC17 TLN1 ITGAV IQGAP1 GSN PLOD2 CUL4B IGFBP7 ANXA5 HSP90B
VCL HSPA5 LAMA3 PFKP WDR1 STK10 AP1M1 PLOD1 AKR1B1 TOM1 NDRG1 HSPB1 PLEKHA1 LAMA4 IRF6 TGFBI YWHAH TXNDC17 TLN1 ITGAV IQGAP1 GSN PLOD2 CUL4B IGFBP7 ANXA5 HSP90B
VCL HSPA5 LAMA3 PFKP WDR1 STK10 AP1M1 PLOD1 AKR1B1 TOM1 NDRG1 HSPB1 PLEKHA1 LAMA4 IRF6 TGFBI YWHAH TXNDC17 TLN1 ITGAV IQGAP1 GSN PLOD2 CUL4B IGFBP7 ANXA5 HSP90B
VCL HSPA5 LAMA3 PFKP WDR1 STK10 AP1M1 PLOD1 AKR1B1 TOM1 NDRG1 HSPB1 PLEKHA1 LAMA4 IRF6 TGFBI YWHAH TXNDC17 TLN1 ITGAV IQGAP1 GSN PLOD2 CUL4B IGFBP7 ANXA5 HSP90B

Genes
MCM6 MCM5 MCM4 MCM3
MCM6 MCM4 RPA1 MCM5 MCM3
MCM6 MCM5 MCM4 MCM3 PCNA LPIN1 CDK1 GTPBP4 UCHL5
MTREX MRTO4 PES1 UTP25 RRP1B NSA2 METTL15 EXOSC10 RPS28 GTPBP4 RPP30 DDX21
MTREX MRTO4 PES1 UTP25 RRP1B NSA2 METTL15 EXOSC10 RPS28 GTPBP4 MACROH2A1 RPP30 DDX21
MTREX MRTO4 PES1 GTPBP4 UTP25 RRP1B NSA2 METTL15 EXOSC10 RPS28 RPP30 DDX21 GLUL
MCM6 MCM5 MCM4 MCM3 RPA1 PCNA DDX21 SUPT16H LPIN1 CDK1 GTPBP4 UCHL5
BUB3 MAD2L1 ANAPC7 NCAPD2 MACROH2A1 SMC4 XRCC1 UCHL5 DNMT1 EXOSC10 USP7
ELAC2 NSUN2 MTREX MRTO4 ELP1 PES1 UTP25 RPP30 RRP1B NSA2 METTL15 EXOSC10 RPS28 DUS3L GTPBP4 GRSF1 DDX21 DDX5
MTREX MRTO4 PES1 GTPBP4 UTP25 SNRPD2 RRP1B NSA2 METTL15 EXOSC10 RPS28 SART3 HSP90AB1 RPP30 DDX21 GLUL
ELAC2 NSUN2 MTREX MRTO4 ELP1 PES1 UTP25 RPP30 RRP1B NSA2 METTL15 EXOSC10 RPS28 PNPT1 DUS3L GTPBP4 MACROH2A1 GRSF1 DDX21 DDX5
ELAC2 NSUN2 MTREX MRTO4 ELP1 SART3 PES1 RBM3 UTP25 SNRPD2 GRSF1 TBRG4 PNPT1 RPP30 RRP1B NSA2 METTL15 EXOSC10 RPS28 DDX5 DUS3L GTPBP4 APP EIF4A1 DDX21
CHD4 NCAPD2 MCM6 SUPT16H MCM4 SMC4 RPA1 BUB3 MAD2L1 ANAPC7 DDX21 MTA3 SART3 HSP90AB1 MCM5 APEX1 MCM3 MACROH2A1 UCHL5 HLTF XRCC1 DNMT1 PCNA CDK1 EXOSC10 IFI1
SUPT16H RBM3 PSME3 LPIN1 SDCBP LARP1 CNBP MTA3 HSP90AB1 CHD4 DDX21 SART3 APEX1 NAMPT FAM162A EPCAM GRSF1 PCNA PNPT1 APP S100A8 S100A9 IFI16 NAA15 NKRF USP7 HLTF
CHD4 NCAPD2 MCM6 PCM1 SUPT16H DSP MCM4 SMC4 FAM162A PPL PEX11B CLUH RPA1 BUB3 SQSTM1 MAD2L1 KRT15 KRT5 ANAPC7 RPS28 EPPK1 TIMM8B DDX21 MTA3 SART3 HSP90AB1 MC

Genes
MCM6 MCM5 MCM4 MCM3
MCM6 MCM5 MCM4 MCM3
MCM6 MCM5 MCM4 MCM3
MCM6 MCM5 MCM4 MCM3 MACROH2A1 RPA1 PCNA CDK1 APEX1 POLR2B CHD4 XRCC1
MCM6 MCM5 MCM4 MCM3 MACROH2A1 RPA1 PCNA CDK1 NCAPD2 BUB3 MAD2L1 APEX1 POLR2B CHD4 XRCC1 SMC4 DNMT1
RPA1 NCAPD2 MACROH2A1 UCHL5 PCNA SMC4 MCM6 MCM5 MCM4 MCM3
MRTO4 PES1 GTPBP4 UTP25 RPP30 IFI16 DDX21 EXOSC10 NKRF NSUN2 XRCC1 APEX1 UBTF DDX5 MACROH2A1 UCHL5 RRP1B MTREX HLTF EIF4A1 NSA2
POLR2B MTA3 SUPT16H PES1 RBM3 CHD4 SNRPD2 RPA1 PCNA RPP30 BUB3 EXOSC10 ANAPC7 HLTF MTREX SART3 HSP90AB1 DDX5 UCHL5 MAD2L1 DDX21 XRCC1 MCM6 MCM5 MCM4 MCM3
UQCRC1 HSP90AB1 BCKDHA ELAC2 ALDH18A1 MTHFD2 GSR FAM162A SDHB IVD LPIN1 TBRG4 PNPT1 GLUD1 COQ8A NDUFA7 TIMM8B NSUN2 APEX1 GRSF1 GLUL ALDH1B1 ACSL1 COA6 METTL1
ITGA3 FKBP5 HSP90AB1 DSP GSR NAMPT CPVL DDX5 MACROH2A1 PPL EPCAM SNRPD2 ACLY PCNA DSG3 GLUL SDCBP PPP2R1B APP S100A8 SQSTM1 EIF4A1 S100A9 FASN CDK1 KRT15 IMPDH
ITGA3 FKBP5 HSP90AB1 DSP GSR NAMPT CPVL DDX5 MACROH2A1 PPL EPCAM SNRPD2 ACLY PCNA DSG3 GLUL SDCBP PPP2R1B APP S100A8 SQSTM1 EIF4A1 S100A9 FASN CDK1 KRT15 IMPDH
ITGA3 FKBP5 HSP90AB1 DSP GSR NAMPT CPVL DDX5 MACROH2A1 PPL EPCAM SNRPD2 ACLY PCNA DSG3 GLUL SDCBP PPP2R1B APP S100A8 SQSTM1 EIF4A1 S100A9 FASN CDK1 KRT15 IMPDH
ITGA3 FKBP5 HSP90AB1 DSP GSR NAMPT CPVL DDX5 MACROH2A1 PPL EPCAM SNRPD2 ACLY PCNA DSG3 GLUL SDCBP PPP2R1B APP S100A8 SQSTM1 EIF4A1 S100A9 FASN CDK1 KRT15 IMPDH
USP7 POLR2B MTA3 SART3 SUPT16H CHD4 PSME3 GRSF1 MAD2L1 KPNA2 NKRF ELAC2 NCAPD2 MTREX HLTF XRCC1 MCM6 PCM1 FKBP5 PES1 MCM5 APEX1 RBM3 MCM4 NAMPT GTPBP4 DDX5
USP7 POLR2B MRTO4 MTA3 SART3 SUPT16H PES1 GTPBP4 CHD4 UTP25 PSME3 RPA1 GRSF1 RPP30 IFI16 MAD2L1 DDX21 EXOSC10 KPNA2 NKRF HLTF ELAC2 NCAPD2 NSUN2 MTREX XRCC1 MC

Genes
TWF2
ARL6IP5
ARL6IP5
TWF2
TWF2
TWF2 ARPC4
TWF2 ARPC4
TWF2 ARPC4
TWF2 ARPC4
TWF2 ARPC4
TWF2 ARPC4
TWF2 ARPC4
TWF2 ARPC4
TWF2 ARL6IP5 AP1M1 CLTB

Genes
AP1M1 CLTB
AP1M1 CLTB
CLTB AP1M1
CLTB AP1M1
AP1M1 CLTB
CLTB AP1M1
AP1M1 CLTB
CLTB AP1M1
CLTB AP1M1
CLTB AP1M1
AP1M1 TXNDC17 ARPC4 TWF2
AP1M1 TXNDC17 ARPC4 TWF2
AP1M1 TXNDC17 ARPC4 TWF2
AP1M1 TXNDC17 ARPC4 TWF2 CLTB

Genes
ARPC4 CLTB
CLTB
CLTB AP1M1
ARPC4 CLTB
