# Supplementary material for: Extracellular Vesicles Bearing Vimentin Drive Epithelial–Mesenchymal Transition
Source: Mol Cell Proteomics. 2025 Jul 4;24(12):101028. doi: 10.1016/j.mcpro.2025.101028 (PMC12719745; doi:10.1016/j.mcpro.2025.101028)
Supplement: Supplemental Data 13 [file mmc16.pdf]

Sheet 13.reference list

Pasted	Symbol	Ensembl Gene ID	Entrez	Type	Species	Chr	Position (Mbp)	Description	percentage_gc_content	transcript_count	genomeSpan	cds_length	transcript_length	FiveUTR	ThreeUTR	nExons
ISG15	ISG15	ENSG00000187608	9636	protein_coding	Human	1	1.001138	ISG15 ubiquitin like modifier [Source:HGNC Symbol;Acc:HGNC:4053]	56.44	3	13403	468	694	79	62	3
LRRC47	LRRC47	ENSG00000130764	57470	protein_coding	Human	1	3.778559	leucine rich repeat containing 47 [Source:HGNC Symbol;Acc:HGNC:29207]	54.7	3	17940	952	1771	22	1300	5
VAMP3	VAMP3	ENSG00000049245	9341	protein_coding	Human	1	7.771296	vesicle associated membrane protein 3 [Source:HGNC Symbol;Acc:HGNC:12644]	42.69	3	10137	261	1135	140	974	4
SRM	SRM	ENSG00000116649	6723	protein_coding	Human	1	11.054584	spermidine synthase [Source:HGNC Symbol;Acc:HGNC:11296]	59.85	6	5437	440	668	135	193	4
MTOR	MTOR	ENSG00000198793	2475	protein_coding	Human	1	11.106535	mechanistic target of rapamycin kinase [Source:HGNC Symbol;Acc:HGNC:3942]	44.55	16	156022	4374	4117	74	424	26
EFHD2	EFHD2	ENSG00000142634	79180	protein_coding	Human	1	15.409888	EF-hand domain family member D2 [Source:HGNC Symbol;Acc:HGNC:28670]	56.77	2	20452	630	1660	84	988	4
FBLIM1	FBLIM1	ENSG00000162458	54751	protein_coding	Human	1	15.756607	filamin binding LIM protein 1 [Source:HGNC Symbol;Acc:HGNC:24686]	53.53	15	29988	461	1055	93	1185	5
CAPZB	CAPZB	ENSG00000077549	832	protein_coding	Human	1	19.338775	capping actin protein of muscle Z-line subunit beta [Source:HGNC Symbol;Acc:HGNC:1491]	49.21	15	146765	603	2776	145	1887	7
GALE	GALE	ENSG00000117308	2582	protein_coding	Human	1	23.795599	UDP-galactose-4-epimerase [Source:HGNC Symbol;Acc:HGNC:4116]	60.06	16	5183	712	1040	77	348	7
SRSF10	SRSF10	ENSG00000188529	10772	protein_coding	Human	1	23.964347	serine and arginine rich splicing factor 10 [Source:HGNC Symbol;Acc:HGNC:16713]	38.94	16	16581	505	1939	61	1648	5
CLIC4	CLIC4	ENSG00000169504	25932	protein_coding	Human	1	24.745382	chloride intracellular channel 4 [Source:HGNC Symbol;Acc:HGNC:13518]	41.66	4	98940	762	2022	140	2139	5
HMGN2	HMGN2	ENSG00000198830	3151	protein_coding	Human	1	26.47244	high mobility group nucleosomal binding domain 2 [Source:HGNC Symbol;Acc:HGNC:4986]	47.23	10	4203	273	1028	100	1158	5
WASF2	WASF2	ENSG00000158195	10163	protein_coding	Human	1	27.40423	WASP family member 2 [Source:HGNC Symbol;Acc:HGNC:12733]	44.01	2	85938	1172	3382	84	2044	8
AK2	AK2	ENSG00000004455	204	protein_coding	Human	1	33.007986	adenylate kinase 2 [Source:HGNC Symbol;Acc:HGNC:362]	46.32	24	73011	395	3242	51	1317	6
PSMB2	PSMB2	ENSG00000126067	5690	protein_coding	Human	1	35.599541	proteasome 20S subunit beta 2 [Source:HGNC Symbol;Acc:HGNC:9539]	40.99	3	41986	430	3257	87	3724	5
SF3A3	SF3A3	ENSG00000183431	10946	protein_coding	Human	1	37.956975	splicing factor 3a subunit 3 [Source:HGNC Symbol;Acc:HGNC:10767]	44.37	8	33101	1506	939	57	1211	7
FHL3	FHL3	ENSG00000183386	2275	protein_coding	Human	1	37.99677	four and a half LIM domains 3 [Source:HGNC Symbol;Acc:HGNC:3704]	56.84	5	8837	843	1141	89	635	5
P3H1	P3H1	ENSG00000117385	64175	protein_coding	Human	1	42.746335	prolyl 3-hydroxylase 1 [Source:HGNC Symbol;Acc:HGNC:19316]	50.04	14	20750	1602	1586	60	361	8
EIF2B3	EIF2B3	ENSG00000070785	8891	protein_coding	Human	1	44.850522	eukaryotic translation initiation factor 2B subunit gamma [Source:HGNC Symbol;Acc:HGNC:3259]	42.55	10	136201	972	1041	71	288	7
AKR1A1	AKR1A1	ENSG00000117448	10327	protein_coding	Human	1	45.550543	aldo-keto reductase family 1 member A1 [Source:HGNC Symbol;Acc:HGNC:380]	49.1	14	19507	693	871	118	210	5
TXNDC12	TXNDC12	ENSG00000117862	51060	protein_coding	Human	1	52.020131	thioredoxin domain containing 12 [Source:HGNC Symbol;Acc:HGNC:24626]	42.81	5	35061	286	867	90	550	5
CZIB	CZIB	ENSG00000162384	54987	protein_coding	Human	1	53.214099	CXXC motif containing zinc binding protein [Source:HGNC Symbol;Acc:HGNC:26059]	46.99	7	6536	483	1140	59	560	4
MAGOH	MAGOH	ENSG00000162385	4116	protein_coding	Human	1	53.2269	mago homolog, exon junction complex subunit [Source:HGNC Symbol;Acc:HGNC:6815]	41.43	4	11619	386	647	60	132	4
DHCR24	DHCR24	ENSG00000116133	1718	protein_coding	Human	1	54.849627	24-dehydrocholesterol reductase [Source:HGNC Symbol;Acc:HGNC:2859]	47.67	12	37569	853	2621	75	1158	8
JAK1	JAK1	ENSG00000162434	3716	protein_coding	Human	1	64.833223	Janus kinase 1 [Source:HGNC Symbol;Acc:HGNC:6190]	42.71	31	234532	2593	4203	124	1032	20
RABGGTB	RABGGTB	ENSG00000137955	5876	protein_coding	Human	1	75.786197	Rab geranylgeranyltransferase subunit beta [Source:HGNC Symbol;Acc:HGNC:9796]	37.85	14	8890	728	1221	28	224	6
DDAH1	DDAH1	ENSG00000153904	23576	protein_coding	Human	1	85.318481	dimethylarginine dimethylaminohydrolase 1 [Source:HGNC Symbol;Acc:HGNC:2715]	39.19	3	259883	655	2888	142	2969	6
GCLM	GCLM	ENSG00000023909	2730	protein_coding	Human	1	93.885199	glutamate-cysteine ligase modifier subunit [Source:HGNC Symbol;Acc:HGNC:4312]	36.37	4	24258	792	2275	280	2874	5
CNN3	CNN3	ENSG00000117519	1266	protein_coding	Human	1	94.896949	calponin 3 [Source:HGNC Symbol;Acc:HGNC:2157]	42.96	7	30275	816	1216	196	790	6
COL11A1	COL11A1	ENSG00000060718	1301	protein_coding	Human	1	102.876467	collagen type XI alpha 1 chain [Source:HGNC Symbol;Acc:HGNC:2186]	33.18	14	232406	3151	3462	296	604	33
RBM8A	RBM8A	ENSG00000265241	9939	protein_coding	Human	1	145.921556	RNA binding motif protein 8A [Source:HGNC Symbol;Acc:HGNC:9905]	44	8	6123	524	1518	41	1261	5
S100A11	S100A11	ENSG00000163191	6282	protein_coding	Human	1	152.032506	S100 calcium binding protein A11 [Source:HGNC Symbol;Acc:HGNC:10488]	45.08	2	15402	318	560	89	156	3
S100A4	S100A4	ENSG00000196154	6275	protein_coding	Human	1	153.543613	S100 calcium binding protein A4 [Source:HGNC Symbol;Acc:HGNC:10494]	56.3	6	6524	306	579	44	142	3
S100A16	S100A16	ENSG00000188643	140576	protein_coding	Human	1	153.606886	S100 calcium binding protein A16 [Source:HGNC Symbol;Acc:HGNC:20441]	57.24	5	6260	312	972	118	507	3
RPS27	RPS27	ENSG00000177954	6232	protein_coding	Human	1	153.990762	ribosomal protein S27 [Source:HGNC Symbol;Acc:HGNC:10416]	45.91	5	1394	250	503	34	78	4
UBAP2L	UBAP2L	ENSG00000143569	9898	protein_coding	Human	1	154.220179	ubiquitin associated protein 2 like [Source:HGNC Symbol;Acc:HGNC:29877]	43.58	20	51332	1828	1635	57	467	12
NES	NES	ENSG00000132688	10763	protein_coding	Human	1	156.668763	nestin [Source:HGNC Symbol;Acc:HGNC:7756]	59.01	1	8645	4866	5568	143	559	4
HDGF	HDGF	ENSG00000143321	3068	protein_coding	Human	1	156.742109	heparin binding growth factor [Source:HGNC Symbol;Acc:HGNC:4856]	51.35	10	24817	730	1340	158	843	6
IFI16	IFI16	ENSG00000163565	3428	protein_coding	Human	1	158.999968	interferon gamma inducible protein 16 [Source:HGNC Symbol;Acc:HGNC:5395]	38.34	13	55188	1315	2059	143	245	8
NCSTN	NCSTN	ENSG00000162736	23385	protein_coding	Human	1	160.343294	nicastrin [Source:HGNC Symbol;Acc:HGNC:17091]	46.83	45	15659	1088	2728	61	383	13
UFC1	UFC1	ENSG00000143222	51506	protein_coding	Human	1	161.152776	ubiquitin-fold modifier conjugating enzyme 1 [Source:HGNC Symbol;Acc:HGNC:26941]	46.11	6	6081	504	671	20	364	4
UAP1	UAP1	ENSG00000117143	6675	protein_coding	Human	1	162.561722	UDP-N-acetylglucosamine pyrophosphorylase 1 [Source:HGNC Symbol;Acc:HGNC:12457]	39.26	5	39519	1544	1800	56	1877	5
NUF2	NUF2	ENSG00000143228	83540	protein_coding	Human	1	163.266576	NUF2 component of NDC80 kinetochore complex [Source:HGNC Symbol;Acc:HGNC:14621]	36.44	12	89189	875	1010	112	233	8
TIPRL	TIPRL	ENSG00000143155	261726	protein_coding	Human	1	168.178962	TOR signaling pathway regulator [Source:HGNC Symbol;Acc:HGNC:30231]	38.53	2	23148	678	1862	70	1115	6
TPR	TPR	ENSG00000047410	7175	protein_coding	Human	1	186.311652	translocated promoter region, nuclear basket protein [Source:HGNC Symbol;Acc:HGNC:12017]	34.16	9	64042	3241	2272	196	2319	12
NEK7	NEK7	ENSG00000151414	140609	protein_coding	Human	1	198.156994	NIMA related kinase 7 [Source:HGNC Symbol;Acc:HGNC:13386]	35.84	8	165427	451	1105	135	1470	6
IPO9	IPO9	ENSG00000198700	55705	protein_coding	Human	1	201.829149	importin 9 [Source:HGNC Symbol;Acc:HGNC:19425]	41.9	4	55143	1797	3392	53	4189	10
RABIF	RABIF	ENSG00000183155	5877	protein_coding	Human	1	202.878282	RAB interacting factor [Source:HGNC Symbol;Acc:HGNC:9797]	45.5	1	10868	372	3119	51	2696	2
NUCKS1	NUCKS1	ENSG00000069275	64710	protein_coding	Human	1	205.712822	nuclear casein kinase and cyclin dependent kinase substrate 1 [Source:HGNC Symbol;Acc:HGNC:29923]	39.53	2	37361	732	3314	209	5458	4
BPNT1	BPNT1	ENSG00000162813	10380	protein_coding	Human	1	220.057482	3'(2'), 5'-bisphosphate nucleotidase 1 [Source:HGNC Symbol;Acc:HGNC:1096]	40.42	11	32981	663	1324	91	1125	7
WDR26	WDR26	ENSG00000162923	80232	protein_coding	Human	1	224.385146	WD repeat domain 26 [Source:HGNC Symbol;Acc:HGNC:21208]	38.39	14	51888	1412	3098	401	2496	9
ENAH	ENAH	ENSG00000154380	55740	protein_coding	Human	1	225.486765	ENAH actin regulator [Source:HGNC Symbol;Acc:HGNC:18271]	38.55	10	166378	1560	5000	189	6773	9
ACBD3	ACBD3	ENSG00000182827	64746	protein_coding	Human	1	226.144679	acyl-CoA binding domain containing 3 [Source:HGNC Symbol;Acc:HGNC:15453]	41.45	2	42063	1587	1939	66	1931	5
PARP1	PARP1	ENSG00000143799	142	protein_coding	Human	1	226.36021	poly(ADP-ribose) polymerase 1 [Source:HGNC Symbol;Acc:HGNC:270]	46.86	26	47945	1660	3276	166	413	13
EXOC8	EXOC8	ENSG00000116903	149371	protein_coding	Human	1	231.332753	exocyst complex component 8 [Source:HGNC Symbol;Acc:HGNC:24659]	43.25	1	5100	2178	5100	107	2815	1
ADSS2	ADSS2	ENSG00000035687	159	protein_coding	Human	1	244.408494	adenylosuccinate synthase 2 [Source:HGNC Symbol;Acc:HGNC:292]	38.09	3	43416	1371	1431	92	1092	7
YWHAQ	YWHAQ	ENSG00000134308	10971	protein_coding	Human	2	9.583967	tyrosine 3-monooxygenase/tryptophan 5-monooxygenase activation protein theta [Source:HGNC Symbol;Acc:HGNC:12854]	40.32	5	47031	642	1226	114	1316	4
RRM2	RRM2	ENSG00000171848	6241	protein_coding	Human	2	10.120698	ribonucleotide reductase regulatory subunit M2 [Source:HGNC Symbol;Acc:HGNC:10452]	50.79	17	91028	1000	1580	144	1483	6
PDIA6	PDIA6	ENSG00000143870	10130	protein_coding	Human	2	10.783391	protein disulfide isomerase family A member 6 [Source:HGNC Symbol;Acc:HGNC:30168]	46.27	8	54587	1205	2017	192	787	12
ROCK2	ROCK2	ENSG00000134318	9475	protein_coding	Human	2	11.179759	Rho associated coiled-coil containing protein kinase 2 [Source:HGNC Symbol;Acc:HGNC:10252]	36.75	12	168572	2791	3790	310	1880	17
PPM1G	PPM1G	ENSG00000115241	5496	protein_coding	Human	2	27.381195	protein phosphatase, Mg2+/Mn2+ dependent 1G [Source:HGNC Symbol;Acc:HGNC:9278]	45.39	3	28397	1641	2266	169	400	7
EHD3	EHD3	ENSG00000013016	30845	protein_coding	Human	2	31.234152	EH domain containing 3 [Source:HGNC Symbol;Acc:HGNC:3244]	50.24	1	35300	1608	4825	470	2747	6
PSME4	PSME4	ENSG00000068878	23198	protein_coding	Human	2	53.864069	proteasome activator subunit 4 [Source:HGNC Symbol;Acc:HGNC:20635]	38.04	9	106925	3624	2376	132	450	14
RTN4	RTN4	ENSG00000115310	57142	protein_coding	Human	2	54.972187	reticulon 4 [Source:HGNC Symbol;Acc:HGNC:14085]	38.6	14	140435	1781	2290	143	836	6
MDH1	MDH1	ENSG00000014641	4190	protein_coding	Human	2	63.588609	malate dehydrogenase 1 [Source:HGNC Symbol;Acc:HGNC:6970]	38.05	16	18589	644	942	160	162	5
UGP2	UGP2	ENSG00000169764	7360	protein_coding	Human	2	63.840952	UDP-glucose pyrophosphorylase 2 [Source:HGNC Symbol;Acc:HGNC:12527]	38.65	39	50611	530	1148	97	185	7
PCYOX1	PCYOX1	ENSG00000116005	51449	protein_coding	Human	2	70.257386	prenylcysteine oxidase 1 [Source:HGNC Symbol;Acc:HGNC:20588]	45.24	6	23800	776	1846	77	2927	5
NAGK	NAGK	ENSG00000124357	55577	protein_coding	Human	2	71.064344	N-acetylglucosamine kinase [Source:HGNC Symbol;Acc:HGNC:17174]	49.56	29	15465	643	1222	132	377	6
MOB1A	MOB1A	ENSG00000114978	55233	protein_coding	Human	2	74.152528	MOB kinase activator 1A [Source:HGNC Symbol;Acc:HGNC:16015]	41.02	6	26371	651	1532	205	4040	4
CAPG	CAPG	ENSG00000042493	822	protein_coding	Human	2	85.394753	capping actin protein, gelsolin like [Source:HGNC Symbol;Acc:HGNC:1474]	51.32	15	23680	821	816	49	118	7
NCAPH	NCAPH	ENSG00000121152	23397	protein_coding	Human	2	96.335766	non-SMC condensin I complex subunit H [Source:HGNC Symbol;Acc:HGNC:1112]	45.16	7	41326	1543	2317	65	1198	13
RANBP2	RANBP2	ENSG00000153201	5903	protein_coding	Human	2	108.719482	RAN binding protein 2 [Source:HGNC Symbol;Acc:HGNC:9848]	39.01	19	66329	4111	4384	89	1455	9
ACTR3	ACTR3	ENSG00000115091	10096	protein_coding	Human	2	113.890063	actin related protein 3 [Source:HGNC Symbol;Acc:HGNC:170]	36.57	8	72534	569	2171	163	1377	6
DDX18	DDX18	ENSG00000088205	8886	protein_coding	Human	2	117.814691	DEAD-box helicase 18 [Source:HGNC Symbol;Acc:HGNC:2741]	39.2	6	17687	1328	2086	87	1653	6
RND3	RND3	ENSG00000115963	390	protein_coding	Human	2	150.468195	Rho family GTPase 3 [Source:HGNC Symbol;Acc:HGNC:671]	38.66	9	70817	503	1219	106	1787	4
PSMD14	PSMD14	ENSG00000115233	10213	protein_coding	Human	2	161.308425	proteasome 26S subunit, non-ATPase 14 [Source:HGNC Symbol;Acc:HGNC:16889]	35.34	5	103293	593	1860	107	317	6
HAT1	HAT1	ENSG00000128708	8520	protein_coding	Human	2	171.922448	histone acetyltransferase 1 [Source:HGNC Symbol;Acc:HGNC:4821]	39.93	6	61239	593	2150	44	250	8
NCKAP1	NCKAP1	ENSG00000061676	10787	protein_coding	Human	2	182.909115	NCK associated protein 1 [Source:HGNC Symbol;Acc:HGNC:7666]	35.82	8	129744	3396	3609	558	8706	10
COL5A2	COL5A2	ENSG00000204262	1290	protein_coding	Human	2	189.031898	collagen type V alpha 2 chain [Source:HGNC Symbol;Acc:HGNC:2210]	34.93	4	193415	2830	3697	124	2164	30
HIBCH	HIBCH	ENSG00000198130	26275	protein_coding	Human	2	190.189735	3-hydroxyisobutyryl-CoA hydrolase [Source:HGNC Symbol;Acc:HGNC:4908]	38.94	14	154459	570	1007	95	493	6
STAT1	STAT1	ENSG00000115415	6772	protein_coding	Human	2	190.90846	signal transducer and activator of transcription 1 [Source:HGNC Symbol;Acc:HGNC:11362]	38.26	41	112501	1560	3236	109	681	19
NIF3L1	NIF3L1	ENSG00000196290	60491	protein_coding	Human	2	200.889327	NGG1 interacting factor 3 like 1 [Source:HGNC Symbol;Acc:HGNC:13390]	41.02	9	14612	797	1205	105	264	6
SUMO1	SUMO1	ENSG00000116030	7341	protein_coding	Human	2	202.206182	small ubiquitin like modifier 1 [Source:HGNC Symbol;Acc:HGNC:12502]	41.66	10	32418	255	870	115	443	5
ABI2	ABI2	ENSG00000138443	10152	protein_coding	Human	2	203.32828	abl interactor 2 [Source:HGNC Symbol;Acc:HGNC:24011]	39.49	24	119449	802	2541	129	2241	7
ATIC	ATIC	ENSG00000138363	471	protein_coding	Human	2	215.311956	5-aminoimidazole-4-carboxamide ribonucleotide formyltransferase/IMP cyclohydrolase [Source:HGNC Symbol;Acc:HGNC:79	41.05	14	37818	754	1006	140	87	7
GMPPA	GMPPA	ENSG00000144591	29926	protein_coding	Human	2	219.498865	GDP-mannose pyrophosphorylase A [Source:HGNC Symbol;Acc:HGNC:22923]	57.41	48	15034	712	1926	134	189	11
ACSL3	ACSL3	ENSG00000123983	2181	protein_coding	Human	2	222.860942	acyl-CoA synthetase long chain family member 3 [Source:HGNC Symbol;Acc:HGNC:3570]	39.46	36	83698	1742	4327	115	2124	13
PTMA	PTMA	ENSG00000187514	5757	protein_coding	Human	2	231.706895	prothymosin alpha [Source:HGNC Symbol;Acc:HGNC:9623]	61.69	12	6657	325	877	193	387	4
PPP1R7	PPP1R7	ENSG00000115685	5510	protein_coding	Human	2	241.149576	protein phosphatase 1 regulatory subunit 7 [Source:HGNC Symbol;Acc:HGNC:9295]	48.38	20	34077	784	887	164	203	7
DTYMK	DTYMK	ENSG00000168393	1841	protein_coding	Human	2	241.675747	deoxythymidylate kinase [Source:HGNC Symbol;Acc:HGNC:3061]	51.06	7	11198	456	767	96	233	4
ARPC4	ARPC4	ENSG00000241553	10093	protein_coding	Human	3	9.792495	actin related protein 2/3 complex subunit 4 [Source:HGNC Symbol;Acc:HGNC:707]	48.66	8	14607	336	872	53	361	6
SEC13	SEC13	ENSG00000157020	6396	protein_coding	Human	3	10.293131	SEC13 homolog, nuclear pore and COPII coat complex component [Source:HGNC Symbol;Acc:HGNC:10697]	46.78	19	27982	593	987	178	289	5
APEH	APEH	ENSG00000164062	327	protein_coding	Human	3	49.674014	acylaminoacyl-peptide hydrolase [Source:HGNC Symbol;Acc:HGNC:586]	58.07	16	9958	836	1073	42	198	9
TWF2	TWF2	ENSG00000247596	11344	protein_coding	Human	3	52.228612	twinfilin actin binding protein 2 [Source:HGNC Symbol;Acc:HGNC:9621]	57.04	18	18177	683	1896	126	417	8
ARL6IP5	ARL6IP5	ENSG00000144746	10550	protein_coding	Human	3	69.084937	ADP ribosylation factor like GTPase 6 interacting protein 5 [Source:HGNC Symbol;Acc:HGNC:16937]	42.48	5	21156	324	1020	44	707	3
TOMM70	TOMM70	ENSG00000154174	9868	protein_coding	Human	3	100.363431	translocase of outer mitochondrial membrane 70 [Source:HGNC Symbol;Acc:HGNC:11985]	38.43	3	37659	1827	1887	140	2133	5
TFG	TFG	ENSG00000114354	10342	protein_coding	Human	3	100.709295	trafficking from ER to golgi regulator [Source:HGNC Symbol;Acc:HGNC:11758]	36.42	36	39670	998	2108	126	459	8
PCNP	PCNP	ENSG00000081154	57092	protein_coding	Human	3	101.57418	PEST proteolytic signal containing nuclear protein [Source:HGNC Symbol;Acc:HGNC:30023]	39.69	8	20286	225	1188	33	1011	4
CIP2A	CIP2A	ENSG00000163507	57650	protein_coding	Human	3	108.549864	cellular inhibitor of PP2A [Source:HGNC Symbol;Acc:HGNC:29302]	34.15	7	39781	1124	2005	79	666	12
ATG3	ATG3	ENSG00000144848	64422	protein_coding	Human	3	112.53251	autophagy related 3 [Source:HGNC Symbol;Acc:HGNC:20962]	36.32	9	29537	589	1330	256	651	7
ITGB5	ITGB5	ENSG00000082781	3693	protein_coding	Human	3	124.761948	integrin subunit beta 5 [Source:HGNC Symbol;Acc:HGNC:6160]	46.61	13	139471	790	1120	180	931	5
CDV3	CDV3	ENSG00000091527	55573	protein_coding	Human	3	133.573686	CDV3 homolog [Source:HGNC Symbol;Acc:HGNC:26928]	42.48	10	16576	424	1531	156	1090	5
SRPRB	SRPRB	ENSG00000144867	58477	protein_coding	Human	3	133.784023	SRP receptor subunit beta [Source:HGNC Symbol;Acc:HGNC:24085]	41.15	6	41750	598	1481	138	1223	6
U2SURP	U2SURP	ENSG00000163714	23350	protein_coding	Human	3	142.964497	U2 snRNP associated SURP domain containing [Source:HGNC Symbol;Acc:HGNC:30855]	38.44	17	96229	1362	1867	140	1076	12
GYG1	GYG1	ENSG00000163754	2992	protein_coding	Human	3	148.991408	glycogenin 1 [Source:HGNC Symbol;Acc:HGNC:4699]	41.21	14	40368	671	1239	147	1356	5
PFN2	PFN2	ENSG00000070087	5217	protein_coding	Human	3	149.964904	profilin 2 [Source:HGNC Symbol;Acc:HGNC:8882]	39.59	18	85885	291	997	66	552	3
EIF2A	EIF2A	ENSG00000144895	83939	protein_coding	Human	3	150.546678	eukaryotic translation initiation factor 2A [Source:HGNC Symbol;Acc:HGNC:3254]	37.63	16	39339	942	1205	30	390	8
MBNL1	MBNL1	ENSG00000152601	4154	protein_coding	Human	3	152.243828	muscleblind like splicing regulator 1 [Source:HGNC Symbol;Acc:HGNC:6923]	35.71	24	221953	911	3154	562	1435	7
NMD3	NMD3	ENSG00000169251	51068	protein_coding	Human	3	161.104696	NMD3 ribosome export adaptor [Source:HGNC Symbol;Acc:HGNC:24250]	37.99	10	148837	897	1332	134	1052	9
AP2M1	AP2M1	ENSG00000161203	1173	protein_coding	Human	3	184.174689	adaptor related protein complex 2 subunit mu 1 [Source:HGNC Symbol;Acc:HGNC:564]	54.09	34	9526	1053	2249	91	395	9
LPP	LPP	ENSG00000145012	4026	protein_coding	Human	3	188.153284	LIM domain containing preferred translocation partner in lipoma [Source:HGNC Symbol;Acc:HGNC:6679]	39.17	22	737388	725	3192	84	10621	5
CCDC50	CCDC50	ENSG00000152492	152137	protein_coding	Human	3	191.329085	coiled-coil domain containing 50 [Source:HGNC Symbol;Acc:HGNC:18111]	36.59	3	69575	1185	3658	436	3657	8

OPA1	OPA1	ENSG00000198836	4976	protein_coding	Human	3	193.593144	OPA1 mitochondrial dynamin like GTPase [Source:HGNC Symbol;Acc:HGNC:8140]	36.98	32	104668	1824	3902	159	783	21
ADD1	ADD1	ENSG00000087274	118	protein_coding	Human	4	2.843844	adducin 1 [Source:HGNC Symbol;Acc:HGNC:243]	44.84	29	86233	1448	2023	90	1116	8
HGFAC	HGFAC	ENSG00000109758	3083	protein_coding	Human	4	3.441968	HGF activator [Source:HGNC Symbol;Acc:HGNC:4894]	67.43	4	7519	1978	1680	30	67	10
LRPAP1	LRPAP1	ENSG00000163956	4043	protein_coding	Human	4	3.503612	LDL receptor related protein associated protein 1 [Source:HGNC Symbol;Acc:HGNC:6701]	57.26	6	28835	744	2602	7	3222	6
QDPR	QDPR	ENSG00000151552	5860	protein_coding	Human	4	17.460261	quinoid dihydropteridine reductase [Source:HGNC Symbol;Acc:HGNC:9752]	45.07	9	51946	469	1020	50	410	5
FAM114A1	FAM114A1	ENSG00000197712	92689	protein_coding	Human	4	38.867677	family with sequence similarity 114 member A1 [Source:HGNC Symbol;Acc:HGNC:25087]	42.57	6	78063	1055	1370	78	1092	7
UBE2K	UBE2K	ENSG00000078140	3093	protein_coding	Human	4	39.698109	ubiquitin conjugating enzyme E2 K [Source:HGNC Symbol;Acc:HGNC:4914]	41.99	7	84684	406	1787	182	1582	6
TMEM33	TMEM33	ENSG00000109133	55161	protein_coding	Human	4	41.935129	transmembrane protein 33 [Source:HGNC Symbol;Acc:HGNC:25541]	35.28	8	25675	540	2388	160	1653	6
UBA6	UBA6	ENSG00000033178	55236	protein_coding	Human	4	67.612652	ubiquitin like modifier activating enzyme 6 [Source:HGNC Symbol;Acc:HGNC:25581]	35.51	6	88504	1352	2568	35	2328	12
RAP1GDS1	RAP1GDS1	ENSG00000138698	5910	protein_coding	Human	4	98.261384	Rap1 GTPase-GDP dissociation stimulator 1 [Source:HGNC Symbol;Acc:HGNC:9859]	36.01	21	182475	920	1145	118	446	8
PPP3CA	PPP3CA	ENSG00000138814	5530	protein_coding	Human	4	101.023409	protein phosphatase 3 catalytic subunit alpha [Source:HGNC Symbol;Acc:HGNC:9314]	35.79	9	324870	943	2377	406	1589	9
BDH2	BDH2	ENSG00000164039	56898	protein_coding	Human	4	103.077592	3-hydroxybutyrate dehydrogenase 2 [Source:HGNC Symbol;Acc:HGNC:32389]	38.27	9	22279	389	1376	55	758	8
CAMK2D	CAMK2D	ENSG00000145349	817	protein_coding	Human	4	113.418054	calcium/calmodulin dependent protein kinase II delta [Source:HGNC Symbol;Acc:HGNC:1462]	36.23	35	343874	1312	3922	294	1731	17
SLC7A11	SLC7A11	ENSG00000151012	23657	protein_coding	Human	4	138.164097	solute carrier family 7 member 11 [Source:HGNC Symbol;Acc:HGNC:11059]	35.52	2	78253	867	5221	280	4001	10
LSM 6.00	LSM 6.00	ENSG00000164167	11157	protein_coding	Human	4	146.175703	LSM6 homolog, U6 small nuclear RNA and mRNA degradation associated [Source:HGNC Symbol;Acc:HGNC:17017]	39.7	8	24298	243	860	112	518	4
SPCS3	SPCS3	ENSG00000129128	60559	protein_coding	Human	4	176.319966	signal peptidase complex subunit 3 [Source:HGNC Symbol;Acc:HGNC:26212]	36.76	4	12280	543	2073	111	3915	4
DCTD	DCTD	ENSG00000129187	1635	protein_coding	Human	4	182.89006	dCMP deaminase [Source:HGNC Symbol;Acc:HGNC:2710]	46.1	17	27877	272	956	88	441	5
CASP3	CASP3	ENSG00000164305	836	protein_coding	Human	4	184.627696	caspase 3 [Source:HGNC Symbol;Acc:HGNC:1504]	41.13	11	22367	607	2457	81	1101	7
TRIO	TRIO	ENSG00000038382	7204	protein_coding	Human	5	14.143342	trio Rho guanine nucleotide exchange factor [Source:HGNC Symbol;Acc:HGNC:12303]	43.38	26	388787	4981	2798	397	795	15
C1QTNF3	C1QTNF3	ENSG00000082196	114899	protein_coding	Human	5	34.017858	C1q and TNF related 3 [Source:HGNC Symbol;Acc:HGNC:14326]	39.58	6	25356	850	1630	88	1928	4
RAI14	RAI14	ENSG00000039560	26064	protein_coding	Human	5	34.656328	retinoic acid induced 14 [Source:HGNC Symbol;Acc:HGNC:14873]	42.11	28	176285	1174	1273	93	410	8
MAP1B	MAP1B	ENSG00000131711	4131	protein_coding	Human	5	72.107234	microtubule associated protein 1B [Source:HGNC Symbol;Acc:HGNC:6836]	42.29	6	102332	2361	3205	78	2386	5
GLRX	GLRX	ENSG00000173221	2745	protein_coding	Human	5	95.751319	glutaredoxin [Source:HGNC Symbol;Acc:HGNC:4330]	41.08	7	71408	321	1297	58	214	3
CAST	CAST	ENSG00000153113	831	protein_coding	Human	5	96.525267	calpastatin [Source:HGNC Symbol;Acc:HGNC:1515]	39.41	59	254329	1423	2125	99	513	18
LOX	LOX	ENSG00000113083	4015	protein_coding	Human	5	122.063195	lysyl oxidase [Source:HGNC Symbol;Acc:HGNC:6664]	38.02	6	15219	613	2329	265	1183	6
ALDH7A1	ALDH7A1	ENSG00000164904	501	protein_coding	Human	5	126.5312	aldehyde dehydrogenase 7 family member A1 [Source:HGNC Symbol;Acc:HGNC:877]	43.04	37	64163	887	1683	44	508	11
HNRNPA0	HNRNPA0	ENSG00000177733	10949	protein_coding	Human	5	137.745651	heterogeneous nuclear ribonucleoprotein A0 [Source:HGNC Symbol;Acc:HGNC:5030]	41.02	1	8713	918	8713	297	7498	1
MATR3	MATR3	ENSG00000280987	9782	protein_coding	Human	5	139.273752	matrin 3 [Source:NCBI gene (formerly Entrezgene);Acc:9782]	40.63	11	57920	1190	2100	99	936	11
PFDN1	PFDN1	ENSG00000113068	5201	protein_coding	Human	5	140.245035	prefoldin subunit 1 [Source:HGNC Symbol;Acc:HGNC:8866]	40.78	7	58079	277	921	26	480	3
HARS1	HARS1	ENSG00000170445	3035	protein_coding	Human	5	140.673035	histidyl-tRNA synthetase 1 [Source:HGNC Symbol;Acc:HGNC:4816]	45.84	30	18503	1032	2601	81	525	10
GNPDA1	GNPDA1	ENSG00000113552	10007	protein_coding	Human	5	141.991749	glucosamine-6-phosphate deaminase 1 [Source:HGNC Symbol;Acc:HGNC:4417]	44.88	13	21293	687	1227	123	1106	6
TCERG1	TCERG1	ENSG00000113649	10915	protein_coding	Human	5	146.447311	transcription elongation regulator 1 [Source:HGNC Symbol;Acc:HGNC:15630]	36.35	17	64651	3244	1797	19	807	9
ATOX1	ATOX1	ENSG00000177556	475	protein_coding	Human	5	151.742316	antioxidant 1 copper chaperone [Source:HGNC Symbol;Acc:HGNC:798]	46.3	7	30217	200	1109	148	776	3
CLTB	CLTB	ENSG00000175416	1212	protein_coding	Human	5	176.392501	clathrin light chain B [Source:HGNC Symbol;Acc:HGNC:2091]	53.08	7	24039	471	827	176	264	4
LMAN2	LMAN2	ENSG00000169223	10960	protein_coding	Human	5	177.315805	lectin, mannose binding 2 [Source:HGNC Symbol;Acc:HGNC:16986]	49.4	11	36036	842	1857	63	1108	6
DBN1	DBN1	ENSG00000113758	1627	protein_coding	Human	5	177.456608	drebrin 1 [Source:HGNC Symbol;Acc:HGNC:2695]	59.54	11	17794	1253	1579	135	660	8
TMED9	TMED9	ENSG00000184840	54732	protein_coding	Human	5	177.592203	transmembrane p24 trafficking protein 9 [Source:HGNC Symbol;Acc:HGNC:24878]	52.72	6	5040	708	1038	12	1826	4
SERPINB9	SERPINB9	ENSG00000170542	5272	protein_coding	Human	6	2.88727	serpin family B member 9 [Source:HGNC Symbol;Acc:HGNC:8955]	43.78	1	16040	1131	4143	60	2893	7
SERPINB6	SERPINB6	ENSG00000124570	5269	protein_coding	Human	6	2.948159	serpin family B member 6 [Source:HGNC Symbol;Acc:HGNC:8950]	47.23	25	24007	732	1713	336	134	6
RIPK1	RIPK1	ENSG00000137275	8737	protein_coding	Human	6	3.063824	receptor interacting serine/threonine kinase 1 [Source:HGNC Symbol;Acc:HGNC:10019]	45.37	19	51364	837	3163	140	698	9
H2AC4	H2AC4	ENSG00000278463	8335	protein_coding	Human	6	26.033092	H2A clustered histone 4 [Source:HGNC Symbol;Acc:HGNC:4734]	55.22	1	527	393	527	50	84	1
BTN2A1	BTN2A1	ENSG00000112763	11120	protein_coding	Human	6	26.457904	butyrophilin subfamily 2 member A1 [Source:HGNC Symbol;Acc:HGNC:1136]	45.31	7	18718	847	2114	127	902	7
BAG2	BAG2	ENSG00000112208	9532	protein_coding	Human	6	57.172326	BAG cochaperone 2 [Source:HGNC Symbol;Acc:HGNC:938]	38.77	1	17508	636	6651	372	5643	3
SERINC1	SERINC1	ENSG00000111897	57515	protein_coding	Human	6	122.443351	serine incorporator 1 [Source:HGNC Symbol;Acc:HGNC:13464]	35.93	1	28457	1362	3125	70	1693	10
VNN 1.00	VNN 1.00	ENSG00000112299	8876	protein_coding	Human	6	132.680849	vanin 1 [Source:HGNC Symbol;Acc:HGNC:12705]	38.11	1	33207	1542	3853	20	2291	7
BCLAF1	BCLAF1	ENSG00000029363	9774	protein_coding	Human	6	136.256627	BCL2 associated transcription factor 1 [Source:HGNC Symbol;Acc:HGNC:16863]	35.59	24	33225	1976	2830	75	1313	8
FBXO30	FBXO30	ENSG00000118496	84085	protein_coding	Human	6	145.793502	F-box protein 30 [Source:HGNC Symbol;Acc:HGNC:15600]	35.27	1	21294	2238	9051	104	6604	3
ACTB	ACTB	ENSG00000075624	60	protein_coding	Human	7	5.526409	actin beta [Source:HGNC Symbol;Acc:HGNC:132]	56.77	23	37494	711	1290	76	509	5
FSCN1	FSCN1	ENSG00000075618	6624	protein_coding	Human	7	5.592816	fascin actin-bundling protein 1 [Source:HGNC Symbol;Acc:HGNC:11148]	59.62	5	13840	664	1368	114	1181	4
GGCT	GGCT	ENSG00000006625	79017	protein_coding	Human	7	30.496621	gamma-glutamylcyclotransferase [Source:HGNC Symbol;Acc:HGNC:21705]	40.54	8	8221	359	929	108	352	4
SEPTIN7	SEPTIN7	ENSG00000122545	989	protein_coding	Human	7	35.800932	septin 7 [Source:HGNC Symbol;Acc:HGNC:1717]	36.13	18	106179	760	2166	122	1884	8
SBDS	SBDS	ENSG00000126524	51119	protein_coding	Human	7	66.98768	SBDS ribosome maturation factor [Source:HGNC Symbol;Acc:HGNC:19440]	43.01	13	8014	497	1482	136	478	5
POR	POR	ENSG00000127948	5447	protein_coding	Human	7	75.8992	cytochrome p450 oxidoreductase [Source:HGNC Symbol;Acc:HGNC:9208]	50.98	24	87656	717	1016	96	288	6
SLC25A13	SLC25A13	ENSG00000004864	10165	protein_coding	Human	7	96.12022	solute carrier family 25 member 13 [Source:HGNC Symbol;Acc:HGNC:10983]	39.76	8	201928	1427	1177	171	675	8
PDAP1	PDAP1	ENSG00000106244	11333	protein_coding	Human	7	99.392048	PDGFA associated protein 1 [Source:HGNC Symbol;Acc:HGNC:14634]	53.84	5	16550	262	813	46	1080	4
COPS6	COPS6	ENSG00000168090	10980	protein_coding	Human	7	100.088969	COP9 signalosome subunit 6 [Source:HGNC Symbol;Acc:HGNC:21749]	52.66	10	3219	616	829	26	229	6
TRIP6	TRIP6	ENSG00000087077	7205	protein_coding	Human	7	100.867387	thyroid hormone receptor interactor 6 [Source:HGNC Symbol;Acc:HGNC:12311]	56.08	9	6068	577	1018	109	173	6
PLOD3	PLOD3	ENSG00000106397	8985	protein_coding	Human	7	101.205977	procollagen-lysine,2-oxoglutarate 5-dioxygenase 3 [Source:HGNC Symbol;Acc:HGNC:9083]	58.5	16	12444	734	844	148	183	5
DNAJC2	DNAJC2	ENSG00000105821	27000	protein_coding	Human	7	103.312289	DnaJ heat shock protein family (Hsp40) member C2 [Source:HGNC Symbol;Acc:HGNC:13192]	40.48	11	32542	955	1498	144	733	9
CAV1	CAV1	ENSG00000105974	857	protein_coding	Human	7	116.524994	caveolin 1 [Source:HGNC Symbol;Acc:HGNC:1527]	41.69	9	36186	424	1455	103	962	3
FAM3C	FAM3C	ENSG00000196937	10447	protein_coding	Human	7	121.348878	FAM3 metabolism regulating signaling molecule C [Source:HGNC Symbol;Acc:HGNC:18664]	35.92	5	47487	489	982	77	1583	6
MTPN	MTPN	ENSG00000105887	136319	protein_coding	Human	7	135.92676	myotrophin [Source:HGNC Symbol;Acc:HGNC:15667]	36.4	2	50600	258	2330	256	1816	3
ABCF2	ABCF2	ENSG00000033050	10061	protein_coding	Human	7	151.211484	ATP binding cassette subfamily F member 2 [Source:HGNC Symbol;Acc:HGNC:71]	48.3	5	15722	1152	1479	107	2570	7
ATP6V1B2	ATP6V1B2	ENSG00000147416	526	protein_coding	Human	8	20.197381	ATPase H+ transporting V1 subunit B2 [Source:HGNC Symbol;Acc:HGNC:854]	39.5	6	29439	530	2065	14	347	8
XPO7	XPO7	ENSG00000130227	23039	protein_coding	Human	8	21.919662	exportin 7 [Source:HGNC Symbol;Acc:HGNC:14108]	39.92	9	86924	1953	1589	112	1347	9
GSR	GSR	ENSG00000104687	2936	protein_coding	Human	8	30.678066	glutathione-disulfide reductase [Source:HGNC Symbol;Acc:HGNC:4623]	44.66	8	49781	1092	1669	11	600	12
HOOK3	HOOK3	ENSG00000168172	84376	protein_coding	Human	8	42.896946	hook microtubule tethering protein 3 [Source:HGNC Symbol;Acc:HGNC:23576]	40.46	6	133590	760	3181	154	4259	10
ARMC1	ARMC1	ENSG00000104442	55156	protein_coding	Human	8	65.602458	armadillo repeat containing 1 [Source:HGNC Symbol;Acc:HGNC:17684]	42.61	6	31760	529	1496	148	1331	5
IMPA1	IMPA1	ENSG00000133731	3612	protein_coding	Human	8	81.656914	inositol monophosphatase 1 [Source:HGNC Symbol;Acc:HGNC:6050]	37.43	13	29418	583	952	53	501	7
ATP6V1C1	ATP6V1C1	ENSG00000155097	528	protein_coding	Human	8	103.021063	ATPase H+ transporting V1 subunit C1 [Source:HGNC Symbol;Acc:HGNC:856]	38.39	6	51989	1036	2550	80	2342	10
HAS2	HAS2	ENSG00000170961	3037	protein_coding	Human	8	121.612116	hyaluronan synthase 2 [Source:HGNC Symbol;Acc:HGNC:4819]	39.12	1	29325	1659	4240	588	1993	4
PTK2	PTK2	ENSG00000169398	5747	protein_coding	Human	8	140.6579	protein tyrosine kinase 2 [Source:HGNC Symbol;Acc:HGNC:9611]	40.07	58	344317	1008	1424	166	569	10
MTAP	MTAP	ENSG00000099810	4507	protein_coding	Human	9	21.802636	methylthioadenosine phosphorylase [Source:HGNC Symbol;Acc:HGNC:7413]	40.07	13	135016	592	1864	119	2481	5
SMU1	SMU1	ENSG00000122692	55234	protein_coding	Human	9	33.041765	SMU1 DNA replication regulator and spliceosomal factor [Source:HGNC Symbol;Acc:HGNC:18247]	41.17	1	34910	1542	7136	66	5528	12
CHMP5	CHMP5	ENSG00000086065	51510	protein_coding	Human	9	33.264879	charged multivesicular body protein 5 [Source:HGNC Symbol;Acc:HGNC:26942]	41.65	3	17192	588	1478	115	1226	6
TJP2	TJP2	ENSG00000119139	9414	protein_coding	Human	9	69.121264	tight junction protein 2 [Source:HGNC Symbol;Acc:HGNC:11828]	43.41	31	153352	2340	2853	135	872	13
SPTLC1	SPTLC1	ENSG00000090054	10558	protein_coding	Human	9	92.000087	serine palmitoyltransferase long chain base subunit 1 [Source:HGNC Symbol;Acc:HGNC:11277]	41.69	25	115327	520	2283	22	515	13
NCBP1	NCBP1	ENSG00000136937	4686	protein_coding	Human	9	97.633668	nuclear cap binding protein subunit 1 [Source:HGNC Symbol;Acc:HGNC:7658]	37.74	5	40081	834	1430	138	973	8
ANP32B	ANP32B	ENSG00000136938	10541	protein_coding	Human	9	97.983341	acidic nuclear phosphoprotein 32 family member B [Source:HGNC Symbol;Acc:HGNC:16677]	43.52	3	32603	756	857	215	512	5
TBC1D2	TBC1D2	ENSG00000095383	55357	protein_coding	Human	9	98.199011	TBC1 domain family member 2 [Source:HGNC Symbol;Acc:HGNC:18026]	48.96	6	56639	2333	2600	153	396	10
SEC61B	SEC61B	ENSG00000106803	10952	protein_coding	Human	9	99.222064	SEC61 translocon subunit beta [Source:HGNC Symbol;Acc:HGNC:16993]	42.24	3	8552	210	613	86	191	3
RAD23B	RAD23B	ENSG00000119318	5887	protein_coding	Human	9	107.283137	RAD23 homolog B, nucleotide excision repair protein [Source:HGNC Symbol;Acc:HGNC:9813]	38	5	49056	654	1986	200	1845	7
AKAP2	PALM2AKAF	ENSG00000157654	445815	protein_coding	Human	9	109.498325	PALM2 and AKAP2 fusion [Source:HGNC Symbol;Acc:HGNC:33529]	41.98	17	674188	1754	2946	109	2544	5
PRPF4	PRPF4	ENSG00000136875	9128	protein_coding	Human	9	113.275642	pre-mRNA processing factor 4 [Source:HGNC Symbol;Acc:HGNC:17349]	42.31	3	18368	1568	2495	94	1797	10
ATP6V1G1	ATP6V1G1	ENSG00000136888	9550	protein_coding	Human	9	114.587706	ATPase H+ transporting V1 subunit G1 [Source:HGNC Symbol;Acc:HGNC:864]	44.1	8	11210	232	1146	80	583	3
PSMD5	PSMD5	ENSG00000095261	5711	protein_coding	Human	9	120.815496	proteasome 26S subunit, non-ATPase 5 [Source:HGNC Symbol;Acc:HGNC:9563]	40.53	8	27456	736	966	19	568	4
RABGAP1	RABGAP1	ENSG00000011454	23637	protein_coding	Human	9	122.940833	RAB GTPase activating protein 1 [Source:HGNC Symbol;Acc:HGNC:17155]	38.49	11	164034	1387	1431	77	628	9
PPP6C	PPP6C	ENSG00000119414	5537	protein_coding	Human	9	125.146573	protein phosphatase 6 catalytic subunit [Source:HGNC Symbol;Acc:HGNC:9323]	42.35	4	43367	794	3298	156	3099	7
GAPVD1	GAPVD1	ENSG00000165219	26130	protein_coding	Human	9	125.261794	GTPase activating protein and VPS9 domains 1 [Source:HGNC Symbol;Acc:HGNC:23375]	42.11	19	105414	3282	3427	75	1799	15
SPTAN1	SPTAN1	ENSG00000197694	6709	protein_coding	Human	9	128.552558	spectrin alpha, non-erythrocytic 1 [Source:HGNC Symbol;Acc:HGNC:11273]	46.82	39	81105	2587	1787	46	211	11
PTPA	PTPA	ENSG00000119383	5524	protein_coding	Human	9	129.11095	protein phosphatase 2 phosphatase activator [Source:HGNC Symbol;Acc:HGNC:9308]	52.59	24	37997	518	1340	147	844	6
FUBP3	FUBP3	ENSG00000107164	8939	protein_coding	Human	9	130.578965	far upstream element binding protein 3 [Source:HGNC Symbol;Acc:HGNC:4005]	47.12	8	59388	697	1970	88	577	10
ARRDC1	ARRDC1	ENSG00000197070	92714	protein_coding	Human	9	137.605685	arrestin domain containing 1 [Source:HGNC Symbol;Acc:HGNC:28633]	60.75	12	9676	755	1001	23	233	5
GDI2	GDI2	ENSG00000057608	2665	protein_coding	Human	10	5.765223	GDP dissociation inhibitor 2 [Source:HGNC Symbol;Acc:HGNC:4227]	43.74	9	76910	702	1159	131	352	6
NUDT5	NUDT5	ENSG00000165609	11164	protein_coding	Human	10	12.16533	nudix hydrolase 5 [Source:HGNC Symbol;Acc:HGNC:8052]	44.24	9	30815	547	1125	85	629	8
VIM	VIM	ENSG00000026025	7431	protein_coding	Human	10	17.228241	vimentin [Source:HGNC Symbol;Acc:HGNC:12692]	46.3	10	9353	927	1371	240	434	5
HNRNPF	HNRNPF	ENSG00000169813	3185	protein_coding	Human	10	43.385617	heterogeneous nuclear ribonucleoprotein F [Source:HGNC Symbol;Acc:HGNC:5039]	47.68	8	23570	1112	2103	99	952	4
VPS26A	VPS26A	ENSG00000122958	9559	protein_coding	Human	10	69.123512	VPS26, retromer complex component A [Source:HGNC Symbol;Acc:HGNC:12711]	39.83	9	50901	719	1571	149	1337	7
HK1	HK1	ENSG00000156515	3098	protein_coding	Human	10	69.269984	hexokinase 1 [Source:HGNC Symbol;Acc:HGNC:4922]	47.64	18	131901	1591	1932	114	605	12
PSAP	PSAP	ENSG00000197746	5660	protein_coding	Human	10	71.816298	prosaposin [Source:HGNC Symbol;Acc:HGNC:9498]	47.65	4	34954	1038	1042	30	1143	6
DNAJC9	DNAJC9	ENSG00000213551	23234	protein_coding	Human	10	73.183362	DnaJ heat shock protein family (Hsp40) member C9 [Source:HGNC Symbol;Acc:HGNC:19123]	39.91	3	63894	783	1140	66	1446	4
SEC24C	SEC24C	ENSG00000176986	9632	protein_coding	Human	10	73.744372	SEC24 homolog C, COPII coat complex component [Source:HGNC Symbol;Acc:HGNC:10705]	47.03	7	27790	2158	2744	44	750	15
IFIT3	IFIT3	ENSG00000119917	3437	protein_coding	Human	10	89.327307	interferon induced protein with tetratricopeptide repeats 3 [Source:HGNC Symbol;Acc:HGNC:5411]	42.34	15	50167	1041	2231	126	1082	3
IFIT1	IFIT1	ENSG00000185745	3434	protein_coding	Human	10	89.392546	interferon induced protein with tetratricopeptide repeats 1 [Source:HGNC Symbol;Acc:HGNC:5407]	38.55	2	13942	1390	4458	128	2774	2
KIF11	KIF11	ENSG00000138160	3832	protein_coding	Human	10	92.574105	kinesin family member 11 [Source:HGNC Symbol;Acc:HGNC:6388]	40.82	5	81291	2545	4946	192	1097	22
ERLIN1	ERLIN1	ENSG00000107566	10613	protein_coding	Human	10	100.150094	ER lipid raft associated 1 [Source:HGNC Symbol;Acc:HGNC:16947]	38.15	3	35940	973	1967	171	1180	11
OGA	OGA	ENSG00000198408	10724	protein_coding	Human	10	101.784443	O-GlcNAcase [Source:HGNC Symbol;Acc:HGNC:7056]	41.92	11	34023	2139	2089	414	1302	8
NOLC1	NOLC1	ENSG00000166197	9221	protein_coding	Human	10	102.152176	nucleolar and coiled-body phosphoprotein 1 [Source:HGNC Symbol;Acc:HGNC:15608]	47.22	9	11696	1305	2405	48	954	10
NT5C2	NT5C2	ENSG00000076685	22978	protein_coding	Human	10	103.087185	5'-nucleotidase, cytosolic II [Source:HGNC Symbol;Acc:HGNC:8022]	42.4	27	190421	904	2384	110	772	14
SMC3	SMC3	ENSG00000108055	9126	protein_coding	Human	10	110.567684	structural maintenance of chromosomes 3 [Source:HGNC Symbol;Acc:HGNC:2468]	36.7	13	38365	3654	3573	122	1746	14
SEC23IP	SEC23IP	ENSG00000107651	11196	protein_coding	Human	10	119.89273	SEC23 interacting protein [Source:HGNC Symbol;Acc:HGNC:17018]	40.1	7	51928	1348	1480	53	1184	5
CTBP2	CTBP2	ENSG00000175029	1488	protein_coding	Human	10	124.984317	C-terminal binding protein 2 [Source:HGNC Symbol;Acc:HGNC:2495]	50.11	14	176854	1277	1963	164	1677	6
CTSD	CTSD	ENSG00000117984	1509	protein_coding	Human	11	1.752752	cathepsin D [Source:HGNC Symbol;Acc:HGNC:2529]	64.73	16	11822	1100	1853	72	494	8
NUP98	NUP98	ENSG00000110713	4928	protein_coding	Human	11	3.671083	nucleoporin 98 and 96 precursor [Source:HGNC Symbol;Acc:HGNC:8068]	41.69	36	126710	3333	3825	99	782	18
RRM1	RRM1	ENSG00000167325	6240	protein_coding	Human	11	4.094707	ribonucleotide reductase catalytic subunit M1 [Source:HGNC Symbol;Acc:HGNC:10451]	40.37	14	44226	839	1330	163	208	9

CAVIN3	CAVIN3	ENSG00000170955	112464	protein_coding	Human	11	6.318946	caveolae associated protein 3 [Source:HGNC Symbol;Acc:HGNC:9400]	64.65	4	1587	834	904	40	118	2
CYB5R2	CYB5R2	ENSG00000166394	51700	protein_coding	Human	11	7.6651	cytochrome b5 reductase 2 [Source:HGNC Symbol;Acc:HGNC:24376]	49.61	14	12123	606	1291	130	264	5
COPB1	COPB1	ENSG00000129083	1315	protein_coding	Human	11	14.44344	COPI coat complex subunit beta 1 [Source:HGNC Symbol;Acc:HGNC:2231]	38.27	12	56588	1598	1134	109	315	7
RCN1	RCN1	ENSG00000049449	5954	protein_coding	Human	11	32.091074	reticulocalbin 1 [Source:HGNC Symbol;Acc:HGNC:9934]	46.64	7	14649	328	1419	134	556	4
API5	API5	ENSG00000166181	8539	protein_coding	Human	11	43.311963	apoptosis inhibitor 5 [Source:HGNC Symbol;Acc:HGNC:594]	37.75	11	32567	1253	1538	118	884	8
ARHGAP1	ARHGAP1	ENSG00000175220	392	protein_coding	Human	11	46.67708	Rho GTPase activating protein 1 [Source:HGNC Symbol;Acc:HGNC:673]	53.08	7	23540	966	1547	108	1957	7
TNKS1BP1	TNKS1BP1	ENSG00000149115	85456	protein_coding	Human	11	57.299638	tankyrase 1 binding protein 1 [Source:HGNC Symbol;Acc:HGNC:19081]	55.55	7	25315	2479	2885	136	379	6
SSRP1	SSRP1	ENSG00000149136	6749	protein_coding	Human	11	57.325986	structure specific recognition protein 1 [Source:HGNC Symbol;Acc:HGNC:11327]	50.56	5	9907	898	2280	137	419	9
UBE2L6	UBE2L6	ENSG00000156587	9246	protein_coding	Human	11	57.551656	ubiquitin conjugating enzyme E2 L6 [Source:HGNC Symbol;Acc:HGNC:12490]	48.66	5	16629	326	939	167	699	4
OSBP	OSBP	ENSG00000110048	5007	protein_coding	Human	11	59.574398	oxysterol binding protein [Source:HGNC Symbol;Acc:HGNC:8503]	42.65	3	41377	1470	2192	110	1149	8
TMEM109	TMEM109	ENSG00000110108	79073	protein_coding	Human	11	60.914158	transmembrane protein 109 [Source:HGNC Symbol;Acc:HGNC:28771]	51.06	3	9286	732	1604	38	1244	3
UBXN1	UBXN1	ENSG00000162191	51035	protein_coding	Human	11	62.676498	UBX domain protein 1 [Source:HGNC Symbol;Acc:HGNC:18402]	52.86	18	2620	768	971	109	110	6
ATL3	ATL3	ENSG00000184743	25923	protein_coding	Human	11	63.624087	atlastin GTPase 3 [Source:HGNC Symbol;Acc:HGNC:24526]	42.43	4	47835	1235	2590	129	2649	8
STIP1	STIP1	ENSG00000168439	10963	protein_coding	Human	11	64.185272	stress induced phosphoprotein 1 [Source:HGNC Symbol;Acc:HGNC:11387]	50.06	12	19272	1084	1060	163	288	7
PLCB3	PLCB3	ENSG00000149782	5331	protein_coding	Human	11	64.25153	phospholipase C beta 3 [Source:HGNC Symbol;Acc:HGNC:9056]	60.31	4	17621	3638	3270	99	355	24
PRDX5	PRDX5	ENSG00000126432	25824	protein_coding	Human	11	64.318121	peroxiredoxin 5 [Source:HGNC Symbol;Acc:HGNC:9355]	57.3	3	3691	512	640	54	73	5
SF1	SF1	ENSG00000168066	7536	protein_coding	Human	11	64.764606	splicing factor 1 [Source:HGNC Symbol;Acc:HGNC:12950]	49.33	21	14181	1235	1565	223	603	7
SCYL1	SCYL1	ENSG00000142186	57410	protein_coding	Human	11	65.525077	SCY1 like pseudokinase 1 [Source:HGNC Symbol;Acc:HGNC:14372]	57.59	14	13628	1901	1568	75	162	10
RELA	RELA	ENSG00000173039	5970	protein_coding	Human	11	65.653599	RELA proto-oncogene, NF-kB subunit [Source:HGNC Symbol;Acc:HGNC:9955]	55.64	26	9492	667	1092	103	557	6
CFL1	CFL1	ENSG00000172757	1072	protein_coding	Human	11	65.823022	cofilin 1 [Source:HGNC Symbol;Acc:HGNC:1874]	50.18	15	39005	434	955	153	353	3
BANF1	BANF1	ENSG00000175334	8815	protein_coding	Human	11	66.002228	BAF nuclear assembly factor 1 [Source:HGNC Symbol;Acc:HGNC:17397]	57.86	8	1922	233	662	150	309	3
CD248	CD248	ENSG00000174807	57124	protein_coding	Human	11	66.314494	CD248 molecule [Source:HGNC Symbol;Acc:HGNC:18219]	65.39	1	2551	2274	2551	17	260	1
DPP3	DPP3	ENSG00000254986	10072	protein_coding	Human	11	66.480013	dipeptidyl peptidase 3 [Source:HGNC Symbol;Acc:HGNC:3008]	52.26	16	29645	1228	1115	97	328	8
RBM14	RBM14	ENSG00000239306	10432	protein_coding	Human	11	66.616626	RNA binding motif protein 14 [Source:HGNC Symbol;Acc:HGNC:14219]	49.61	9	13309	742	1228	72	914	3
CORO1B	CORO1B	ENSG00000172725	57175	protein_coding	Human	11	67.43551	coronin 1B [Source:HGNC Symbol;Acc:HGNC:2253]	64.53	10	8312	973	1730	42	1022	7
AIP	AIP	ENSG00000110711	9049	protein_coding	Human	11	67.468174	aryl hydrocarbon receptor interacting protein [Source:HGNC Symbol;Acc:HGNC:358]	52.61	11	22981	816	1195	110	185	5
NUMA1	NUMA1	ENSG00000137497	4926	protein_coding	Human	11	72.002864	nuclear mitotic apparatus protein 1 [Source:HGNC Symbol;Acc:HGNC:8059]	46.62	33	77830	1671	1932	85	651	10
PPME1	PPME1	ENSG00000214517	51400	protein_coding	Human	11	74.171267	protein phosphatase methylesterase 1 [Source:HGNC Symbol;Acc:HGNC:30178]	38.61	9	83437	1182	1894	116	1193	7
SPCS2	SPCS2	ENSG00000118363	9789	protein_coding	Human	11	74.949261	signal peptidase complex subunit 2 [Source:HGNC Symbol;Acc:HGNC:28962]	39.25	8	29773	538	822	42	613	4
PICALM	PICALM	ENSG00000073921	8301	protein_coding	Human	11	85.957175	phosphatidylinositol binding clathrin assembly protein [Source:HGNC Symbol;Acc:HGNC:15514]	37.29	24	112708	892	1256	156	413	10
YAP1	YAP1	ENSG00000137693	10413	protein_coding	Human	11	102.110447	Yes1 associated transcriptional regulator [Source:HGNC Symbol;Acc:HGNC:16262]	38.64	11	122978	1320	2868	363	1932	7
RDX	RDX	ENSG00000137710	5962	protein_coding	Human	11	109.864295	radixin [Source:HGNC Symbol;Acc:HGNC:9944]	39.02	22	432418	912	1848	110	500	9
NNMT	NNMT	ENSG00000166741	4837	protein_coding	Human	11	114.257787	nicotinamide N-methyltransferase [Source:HGNC Symbol;Acc:HGNC:7861]	43.32	8	55750	795	877	123	934	3
REXO2	REXO2	ENSG00000076043	25996	protein_coding	Human	11	114.439435	RNA exonuclease 2 [Source:HGNC Symbol;Acc:HGNC:17851]	39.48	19	10845	407	709	43	168	4
PAFAH1B2	PAFAH1B2	ENSG00000168092	5049	protein_coding	Human	11	117.144284	platelet activating factor acetylhydrolase 1b catalytic subunit 2 [Source:HGNC Symbol;Acc:HGNC:8575]	43.37	8	32611	539	1495	61	1247	5
NCAPD2	NCAPD2	ENSG00000010292	9918	protein_coding	Human	12	6.493356	non-SMC condensin I complex subunit D2 [Source:HGNC Symbol;Acc:HGNC:24305]	46.34	13	38600	1891	1378	45	342	9
HEBP1	HEBP1	ENSG00000013583	50865	protein_coding	Human	12	12.97487	heme binding protein 1 [Source:HGNC Symbol;Acc:HGNC:17176]	43.51	5	25396	446	1038	151	613	3
DDX23	DDX23	ENSG00000174243	9416	protein_coding	Human	12	48.829756	DEAD-box helicase 23 [Source:HGNC Symbol;Acc:HGNC:17347]	49.65	23	23087	1106	1760	77	408	8
MYG1	MYG1	ENSG00000139637	60314	protein_coding	Human	12	53.299695	MYG1 exonuclease [Source:HGNC Symbol;Acc:HGNC:17590]	51.2	10	7483	768	846	91	25	4
RPS26	RPS26	ENSG00000197728	6231	protein_coding	Human	12	56.041351	ribosomal protein S26 [Source:HGNC Symbol;Acc:HGNC:10414]	47.33	4	3347	348	1060	184	378	4
ESYT1	ESYT1	ENSG00000139641	23344	protein_coding	Human	12	56.11825	extended synaptotagmin 1 [Source:HGNC Symbol;Acc:HGNC:29534]	50.49	10	26425	2308	1248	66	496	10
NACA	NACA	ENSG00000196531	4666	protein_coding	Human	12	56.712305	nascent polypeptide associated complex subunit alpha [Source:HGNC Symbol;Acc:HGNC:7629]	47.53	26	19324	941	1254	132	134	7
STAT6	STAT6	ENSG00000166888	6778	protein_coding	Human	12	57.095408	signal transducer and activator of transcription 6 [Source:HGNC Symbol;Acc:HGNC:11368]	50.56	25	36732	1315	1813	120	435	12
MARS1	MARS1	ENSG00000166986	4141	protein_coding	Human	12	57.475445	methionyl-tRNA synthetase 1 [Source:HGNC Symbol;Acc:HGNC:6898]	49.33	35	42125	549	871	55	120	6
EEA1	EEA1	ENSG00000102189	8411	protein_coding	Human	12	92.770637	early endosome antigen 1 [Source:HGNC Symbol;Acc:HGNC:3185]	36.99	5	158695	1155	2828	206	1875	12
SNRPF	SNRPF	ENSG00000139343	6636	protein_coding	Human	12	95.858952	small nuclear ribonucleoprotein polypeptide F [Source:HGNC Symbol;Acc:HGNC:11162]	39.08	5	44877	283	673	112	211	3
LTA4H	LTA4H	ENSG00000111144	4048	protein_coding	Human	12	96.000753	leukotriene A4 hydrolase [Source:HGNC Symbol;Acc:HGNC:6710]	39.07	10	42768	1382	1864	109	150	12
WASHC4	WASHC4	ENSG00000136051	23325	protein_coding	Human	12	105.107324	WASH complex subunit 4 [Source:HGNC Symbol;Acc:HGNC:29174]	35.27	14	61807	1204	1673	87	641	12
PWP1	PWP1	ENSG00000136045	11137	protein_coding	Human	12	107.685799	PWP1 homolog, endonuclein [Source:HGNC Symbol;Acc:HGNC:17015]	40.53	5	27364	891	1247	90	412	9
ARPC3	ARPC3	ENSG00000111229	10094	protein_coding	Human	12	110.434823	actin related protein 2/3 complex subunit 3 [Source:HGNC Symbol;Acc:HGNC:706]	45.85	9	15600	363	577	61	332	4
CIT	CIT	ENSG00000122966	11113	protein_coding	Human	12	119.685791	citron rho-interacting serine/threonine kinase [Source:HGNC Symbol;Acc:HGNC:1985]	43.74	40	191530	2677	4733	126	1250	22
KPNA3	KPNA3	ENSG00000102753	3839	protein_coding	Human	13	49.69932	karyopherin subunit alpha 3 [Source:HGNC Symbol;Acc:HGNC:6396]	38.63	2	93363	920	2405	176	1396	10
SUGT1	SUGT1	ENSG00000165416	10910	protein_coding	Human	13	52.652709	SGT1 homolog, MIS12 kinetochore complex assembly cochaperone [Source:HGNC Symbol;Acc:HGNC:16987]	36.99	4	48201	1050	4739	84	6556	10
LMO7	LMO7	ENSG00000136153	4008	protein_coding	Human	13	75.620434	LIM domain 7 [Source:HGNC Symbol;Acc:HGNC:6646]	38.67	27	239437	2191	2422	313	1332	12
TM9SF2	TM9SF2	ENSG00000125304	9375	protein_coding	Human	13	99.446311	transmembrane 9 superfamily member 2 [Source:HGNC Symbol;Acc:HGNC:11865]	40.68	6	117738	1341	1717	271	933	9
PIP4P1	PIP4P1	ENSG00000165782	90809	protein_coding	Human	14	20.457681	phosphatidylinositol-4,5-bisphosphate 4-phosphatase 1 [Source:HGNC Symbol;Acc:HGNC:19299]	50.54	7	3785	539	1365	132	684	4
PABPN1	PABPN1	ENSG00000100836	8106	protein_coding	Human	14	23.321457	poly(A) binding protein nuclear 1 [Source:HGNC Symbol;Acc:HGNC:8565]	50.18	8	4707	629	1170	222	578	5
STXBP6	STXBP6	ENSG00000168952	29091	protein_coding	Human	14	24.809454	syntaxin binding protein 6 [Source:HGNC Symbol;Acc:HGNC:19666]	39.21	7	240694	527	2393	133	1240	6
SCFD1	SCFD1	ENSG00000092108	23256	protein_coding	Human	14	30.622291	sec1 family domain containing 1 [Source:HGNC Symbol;Acc:HGNC:20726]	36.13	72	115404	1087	2487	53	495	19
SNX6	SNX6	ENSG00000129515	58533	protein_coding	Human	14	34.561093	sorting nexin 6 [Source:HGNC Symbol;Acc:HGNC:14970]	44.32	11	69068	641	1473	46	881	9
RTRAF	RTRAF	ENSG00000087302	51637	protein_coding	Human	14	51.989514	RNA transcription, translation and transport factor [Source:HGNC Symbol;Acc:HGNC:23169]	36.67	7	21181	478	1580	82	1697	5
GMFB	GMFB	ENSG00000197045	2764	protein_coding	Human	14	54.474484	glia maturation factor beta [Source:HGNC Symbol;Acc:HGNC:4373]	36.63	10	14543	276	1885	67	2137	5
SEL1L	SEL1L	ENSG00000071537	6400	protein_coding	Human	14	81.471547	SEL1L adaptor subunit of ERAD E3 ubiquitin ligase [Source:HGNC Symbol;Acc:HGNC:10717]	38.48	6	62307	1260	2020	101	3030	7
CKB	CKB	ENSG00000166165	1152	protein_coding	Human	14	103.519667	creatine kinase B [Source:HGNC Symbol;Acc:HGNC:1991]	70.41	18	3167	556	796	77	169	4
INF2	INF2	ENSG00000203485	64423	protein_coding	Human	14	104.681146	inverted formin 2 [Source:HGNC Symbol;Acc:HGNC:23791]	62.41	50	41390	2012	2612	140	546	12
FAM98B	FAM98B	ENSG00000171262	283742	protein_coding	Human	15	38.454127	family with sequence similarity 98 member B [Source:HGNC Symbol;Acc:HGNC:26773]	36.34	4	33584	867	2101	22	2580	6
SORD	SORD	ENSG00000140263	6652	protein_coding	Human	15	45.023147	sorbitol dehydrogenase [Source:HGNC Symbol;Acc:HGNC:11184]	45.27	12	54039	487	3076	98	1134	6
DUT	DUT	ENSG00000128951	1854	protein_coding	Human	15	48.331011	deoxyuridine triphosphatase [Source:HGNC Symbol;Acc:HGNC:3078]	39.96	11	12363	520	959	109	580	6
COPS2	COPS2	ENSG00000166200	9318	protein_coding	Human	15	49.106068	COP9 signalosome subunit 2 [Source:HGNC Symbol;Acc:HGNC:30747]	35.44	8	49594	889	1665	34	1602	8
TMOD3	TMOD3	ENSG00000138594	29766	protein_coding	Human	15	51.829628	tropomodulin 3 [Source:HGNC Symbol;Acc:HGNC:11873]	41.02	11	117668	591	1361	124	2383	5
SNX1	SNX1	ENSG00000028528	6642	protein_coding	Human	15	64.094123	sorting nexin 1 [Source:HGNC Symbol;Acc:HGNC:11172]	43.68	14	51968	680	2053	44	1316	8
ANP32A	ANP32A	ENSG00000140350	8125	protein_coding	Human	15	68.778535	acidic nuclear phosphoprotein 32 family member A [Source:HGNC Symbol;Acc:HGNC:13233]	49.39	11	42363	508	1315	132	634	4
UACA	UACA	ENSG00000137831	55075	protein_coding	Human	15	70.654554	uveal autoantigen with coiled-coil domains and ankyrin repeats [Source:HGNC Symbol;Acc:HGNC:15947]	37.35	14	109005	3161	2187	233	740	10
UNC45A	UNC45A	ENSG00000140553	55898	protein_coding	Human	15	90.93018	unc-45 myosin chaperone A [Source:HGNC Symbol;Acc:HGNC:30594]	53.82	17	23914	1941	1665	100	307	9
STUB1	STUB1	ENSG00000103266	10273	protein_coding	Human	16	0.680224	STIP1 homology and U-box containing protein 1 [Source:HGNC Symbol;Acc:HGNC:11427]	64.94	11	2647	578	905	173	182	5
JPT2	JPT2	ENSG00000206053	90861	protein_coding	Human	16	1.678256	Jupiter microtubule associated homolog 2 [Source:HGNC Symbol;Acc:HGNC:14137]	47.78	14	24025	437	1102	64	867	5
SRRM2	SRRM2	ENSG00000167978	23524	protein_coding	Human	16	2.752626	serine/arginine repetitive matrix 2 [Source:HGNC Symbol;Acc:HGNC:16639]	52.69	31	19913	1647	1270	103	227	5
USP7	USP7	ENSG00000187555	7874	protein_coding	Human	16	8.892097	ubiquitin specific peptidase 7 [Source:HGNC Symbol;Acc:HGNC:12630]	46.42	23	83232	1267	1619	142	560	12
RSL1D1	RSL1D1	ENSG00000171490	26156	protein_coding	Human	16	11.83385	ribosomal L1 domain containing 1 [Source:HGNC Symbol;Acc:HGNC:24534]	45.24	12	17731	524	1194	36	783	6
ATXN2L	ATXN2L	ENSG00000168488	11273	protein_coding	Human	16	28.822999	ataxin 2 like [Source:HGNC Symbol;Acc:HGNC:31326]	52.72	26	14239	1752	1863	170	419	10
MAPK3	MAPK3	ENSG00000102882	5595	protein_coding	Human	16	30.114105	mitogen-activated protein kinase 3 [Source:HGNC Symbol;Acc:HGNC:6877]	56.36	15	9402	816	1144	199	241	6
BOLA2	BOLA2B	ENSG00000169627	552900	protein_coding	Human	16	30.192932	bolA family member 2B [Source:HGNC Symbol;Acc:HGNC:32479]	63.13	5	1375	265	543	189	148	3
STX4	STX4	ENSG00000103496	6810	protein_coding	Human	16	31.032889	syntaxin 4 [Source:HGNC Symbol;Acc:HGNC:11439]	55.52	17	10087	507	952	192	147	6
NUDT21	NUDT21	ENSG00000167005	11051	protein_coding	Human	16	56.429133	nudix hydrolase 21 [Source:HGNC Symbol;Acc:HGNC:13870]	39.54	7	23067	449	1077	94	3579	3
MT1E	MT1E	ENSG00000169715	4493	protein_coding	Human	16	56.625475	metallothionein 1E [Source:HGNC Symbol;Acc:HGNC:7397]	57.57	3	1638	230	594	173	191	2
NUP93	NUP93	ENSG00000102900	9688	protein_coding	Human	16	56.730118	nucleoporin 93 [Source:HGNC Symbol;Acc:HGNC:28958]	42.43	21	120169	864	1515	86	1518	8
CSNK2A2	CSNK2A2	ENSG00000070770	1459	protein_coding	Human	16	58.157907	casein kinase 2 alpha 2 [Source:HGNC Symbol;Acc:HGNC:2459]	41.51	8	40200	882	2426	370	197	9
DYNC1LI2	DYNC1LI2	ENSG00000135720	1783	protein_coding	Human	16	66.720893	dynein cytoplasmic 1 light intermediate chain 2 [Source:HGNC Symbol;Acc:HGNC:2966]	44.86	15	30717	592	1138	10	520	6
NAE1	NAE1	ENSG00000159593	8883	protein_coding	Human	16	66.802875	NEDD8 activating enzyme E1 subunit 1 [Source:HGNC Symbol;Acc:HGNC:621]	46.31	22	70382	655	849	76	120	8
ATP6V0D1	ATP6V0D1	ENSG00000159720	9114	protein_coding	Human	16	67.438014	ATPase H+ transporting V0 subunit d1 [Source:HGNC Symbol;Acc:HGNC:13724]	53.77	17	43168	467	1201	68	149	6
SLC12A4	SLC12A4	ENSG00000124067	6560	protein_coding	Human	16	67.943474	solute carrier family 12 member 4 [Source:HGNC Symbol;Acc:HGNC:10913]	56.91	19	26128	2028	2039	71	526	12
NOB1	NOB1	ENSG00000141101	28987	protein_coding	Human	16	69.741871	NIN1 (RPN12) binding protein 1 homolog [Source:HGNC Symbol;Acc:HGNC:29540]	48.25	6	13056	547	833	11	229	6
AP1G1	AP1G1	ENSG00000166747	164	protein_coding	Human	16	71.729	adaptor related protein complex 1 subunit gamma 1 [Source:HGNC Symbol;Acc:HGNC:555]	40.73	22	80202	826	1590	164	934	9
TUBB3	TUBB3	ENSG00000258947	10381	protein_coding	Human	16	89.921392	tubulin beta 3 class III [Source:HGNC Symbol;Acc:HGNC:20772]	58.5	16	17370	461	1334	73	267	4
RPA1	RPA1	ENSG00000132383	6117	protein_coding	Human	17	1.829702	replication protein A1 [Source:HGNC Symbol;Acc:HGNC:10289]	44.49	7	70381	906	1676	92	2907	7
TXNDC17	TXNDC17	ENSG00000129235	84817	protein_coding	Human	17	6.640985	thioredoxin domain containing 17 [Source:HGNC Symbol;Acc:HGNC:28218]	41.89	10	3557	285	806	45	548	3
ACADVL	ACADVL	ENSG00000072778	37	protein_coding	Human	17	7.217125	acyl-CoA dehydrogenase very long chain [Source:HGNC Symbol;Acc:HGNC:92]	58.51	39	8142	837	869	82	154	7
RAB34	RAB34	ENSG00000109113	83871	protein_coding	Human	17	28.714281	RAB34, member RAS oncogene family [Source:HGNC Symbol;Acc:HGNC:16519]	58.35	26	4149	595	944	132	201	7
MYO18A	MYO18A	ENSG00000196535	399687	protein_coding	Human	17	29.071122	myosin XVIIIA [Source:HGNC Symbol;Acc:HGNC:31104]	53.73	24	109277	2698	2185	86	1086	10
ZNF207	ZNF207	ENSG00000010244	7756	protein_coding	Human	17	32.350132	zinc finger protein 207 [Source:HGNC Symbol;Acc:HGNC:12998]	38.46	17	31754	894	2118	152	2648	7
MYO1D	MYO1D	ENSG00000176658	4642	protein_coding	Human	17	32.492522	myosin ID [Source:HGNC Symbol;Acc:HGNC:7598]	40.83	16	384656	2022	1460	241	1027	8
TOP2A	TOP2A	ENSG00000131747	7153	protein_coding	Human	17	40.388525	DNA topoisomerase II alpha [Source:HGNC Symbol;Acc:HGNC:11989]	39.79	5	29372	1707	1626	87	581	10
FKBP10	FKBP10	ENSG00000141756	60681	protein_coding	Human	17	41.81268	FKBP prolyl isomerase 10 [Source:HGNC Symbol;Acc:HGNC:18169]	56.17	9	10534	771	1275	44	648	5
STAT3	STAT3	ENSG00000168610	6774	protein_coding	Human	17	42.313324	signal transducer and activator of transcription 3 [Source:HGNC Symbol;Acc:HGNC:11364]	45.02	50	75217	2162	3514	102	1408	19
PSME3	PSME3	ENSG00000131467	10197	protein_coding	Human	17	42.824385	proteasome activator subunit 3 [Source:HGNC Symbol;Acc:HGNC:9570]	46.03	16	19376	523	1499	198	1262	9
EFTUD2	EFTUD2	ENSG00000108883	9343	protein_coding	Human	17	44.849948	elongation factor Tu GTP binding domain containing 2 [Source:HGNC Symbol;Acc:HGNC:30858]	46.17	26	49498	1360	1320	78	339	9
NME1	NME1	ENSG00000239672	4830	protein_coding	Human	17	51.153559	NME/NM23 nucleoside diphosphate kinase 1 [Source:HGNC Symbol;Acc:HGNC:7849]	46.38	10	8870	375	914	114	407	4
SUPT4H1	SUPT4H1	ENSG00000213246	6827	protein_coding	Human	17	58.345175	SPT4 homolog, DSIF elongation factor subunit [Source:HGNC Symbol;Acc:HGNC:11467]	47.01	7	7919	279	947	96	479	4
MRC2	MRC2	ENSG00000011028	9902	protein_coding	Human	17	62.62767	mannose receptor C type 2 [Source:HGNC Symbol;Acc:HGNC:16875]	52.16	7	65928	1514	2224	286	784	11
DDX42	DDX42	ENSG00000198231	11325	protein_coding	Human	17	63.773603	DEAD-box helicase 42 [Source:HGNC Symbol;Acc:HGNC:18676]	42	18	45715	1527	1757	127	527	7
JPT1	JPT1	ENSG00000189159	51155	protein_coding	Human	17	75.135248	Jupiter microtubule associated homolog 1 [Source:HGNC Symbol;Acc:HGNC:14569]	46.27	12	33034	339	994	197	477	4
SUMO2	SUMO2	ENSG00000188612	6613	protein_coding	Human	17	75.165586	small ubiquitin like modifier 2 [Source:HGNC Symbol;Acc:HGNC:11125]	46.28	3	17374	221	1488	131	1088	4
GRB2	GRB2	ENSG00000177885	2885	protein_coding	Human	17	75.318076	growth factor receptor bound protein 2 [Source:HGNC Symbol;Acc:HGNC:4566]	44.22	12	87634	512	1773	184	1496	4
TK1	TK1	ENSG00000167900	7083	protein_coding	Human	17	78.174091	thymidine kinase 1 [Source:HGNC Symbol;Acc:HGNC:11830]	53.19	6	13143	576	950	90	388	6
ALYREF	ALYREF	ENSG00000183684	10189	protein_coding	Human	17	81.887835	Aly/REF export factor [Source:HGNC Symbol;Acc:HGNC:19071]	57.54	4	3752	795	581	6	296	4

GPS1	GPS1	ENSG00000169727	2873	protein_coding	Human	17	82.050691	G protein pathway suppressor 1 [Source:HGNC Symbol;Acc:HGNC:4549]	66.61	28	6780	734	1132	61	255	7
ROCK1	ROCK1	ENSG00000067900	6093	protein_coding	Human	18	20.946906	Rho associated coiled-coil containing protein kinase 1 [Source:HGNC Symbol;Acc:HGNC:10251]	36.91	7	164908	2593	2573	506	1706	11
SERPINB2	SERPINB2	ENSG00000197632	5055	protein_coding	Human	18	63.871692	serpin family B member 2 [Source:HGNC Symbol;Acc:HGNC:8584]	38.08	6	32197	744	1179	109	578	6
DAZAP1	DAZAP1	ENSG00000071626	26528	protein_coding	Human	19	1.407569	DAZ associated protein 1 [Source:HGNC Symbol;Acc:HGNC:2683]	58.75	11	28119	1157	2039	137	816	8
LSM 7.00	LSM 7.00	ENSG00000130332	51690	protein_coding	Human	19	2.32152	LSM7 homolog, U6 small nuclear RNA and mRNA degradation associated [Source:HGNC Symbol;Acc:HGNC:20470]	57.7	7	7092	188	570	66	161	3
SGTA	SGTA	ENSG00000104969	6449	protein_coding	Human	19	2.754715	small glutamine rich tetratricopeptide repeat co-chaperone alpha [Source:HGNC Symbol;Acc:HGNC:10819]	57.8	18	28648	739	2124	108	554	9
THOP1	THOP1	ENSG00000172009	7064	protein_coding	Human	19	2.785503	thimet oligopeptidase 1 [Source:HGNC Symbol;Acc:HGNC:11793]	59.07	14	30305	955	1315	247	560	6
MAP2K2	MAP2K2	ENSG00000126934	5605	protein_coding	Human	19	4.090321	mitogen-activated protein kinase kinase 2 [Source:HGNC Symbol;Acc:HGNC:6842]	57.41	15	33802	758	1393	247	139	6
MYDGF	MYDGF	ENSG00000074842	56005	protein_coding	Human	19	4.641374	myeloid derived growth factor [Source:HGNC Symbol;Acc:HGNC:16948]	54.73	4	28989	449	625	18	261	4
SAFB	SAFB	ENSG00000160633	6294	protein_coding	Human	19	5.623035	scaffold attachment factor B [Source:HGNC Symbol;Acc:HGNC:10520]	47.35	14	45444	1930	1707	111	143	10
ELAVL1	ELAVL1	ENSG00000066044	1994	protein_coding	Human	19	7.958573	ELAV like RNA binding protein 1 [Source:HGNC Symbol;Acc:HGNC:3312]	52.21	5	47087	706	2012	85	1455	5
UBL5	UBL5	ENSG00000198258	59286	protein_coding	Human	19	9.827892	ubiquitin like 5 [Source:HGNC Symbol;Acc:HGNC:13736]	52.38	8	2224	194	563	50	112	4
KANK2	KANK2	ENSG00000197256	25959	protein_coding	Human	19	11.16427	KN motif and ankyrin repeat domains 2 [Source:HGNC Symbol;Acc:HGNC:29300]	52.98	13	33522	1160	1617	152	1584	5
RAB3D	RAB3D	ENSG00000105514	9545	protein_coding	Human	19	11.322068	RAB3D, member RAS oncogene family [Source:HGNC Symbol;Acc:HGNC:9779]	51.11	2	24203	660	2582	114	1694	5
PRKCSH	PRKCSH	ENSG00000130175	5589	protein_coding	Human	19	11.435284	protein kinase C substrate 80K-H [Source:HGNC Symbol;Acc:HGNC:9411]	56.85	22	15685	918	980	116	214	8
DDX39A	DDX39A	ENSG00000123136	10212	protein_coding	Human	19	14.408798	DExD-box helicase 39A [Source:HGNC Symbol;Acc:HGNC:17821]	55.55	21	10586	623	963	58	128	5
AP1M1	AP1M1	ENSG00000072958	8907	protein_coding	Human	19	16.197854	adaptor related protein complex 1 subunit mu 1 [Source:HGNC Symbol;Acc:HGNC:13667]	51.53	15	48053	696	1624	108	2228	6
BST2	BST2	ENSG00000130303	684	protein_coding	Human	19	17.402939	bone marrow stromal cell antigen 2 [Source:HGNC Symbol;Acc:HGNC:1119]	58.4	3	2692	423	780	55	197	4
COPE	COPE	ENSG00000105669	11316	protein_coding	Human	19	18.899514	COPI coat complex subunit epsilon [Source:HGNC Symbol;Acc:HGNC:2234]	57.05	11	19874	782	844	28	119	6
PDCD5	PDCD5	ENSG00000105185	9141	protein_coding	Human	19	32.58119	programmed cell death 5 [Source:HGNC Symbol;Acc:HGNC:8764]	45.21	8	6264	254	640	50	312	4
PEPD	PEPD	ENSG00000124299	5184	protein_coding	Human	19	33.38695	peptidase D [Source:HGNC Symbol;Acc:HGNC:8840]	53.89	35	134874	981	1398	56	326	11
UBA2	UBA2	ENSG00000126261	10054	protein_coding	Human	19	34.428352	ubiquitin like modifier activating enzyme 2 [Source:HGNC Symbol;Acc:HGNC:30661]	42.17	10	42900	840	1229	92	613	8
COX6B1	COX6B1	ENSG00000126267	1340	protein_coding	Human	19	35.648323	cytochrome c oxidase subunit 6B1 [Source:HGNC Symbol;Acc:HGNC:2280]	48.83	5	10460	213	532	212	129	3
TBCB	TBCB	ENSG00000105254	1155	protein_coding	Human	19	36.114289	tubulin folding cofactor B [Source:HGNC Symbol;Acc:HGNC:1989]	55.29	15	11659	457	703	116	161	4
BLVRB	BLVRB	ENSG00000090013	645	protein_coding	Human	19	40.447765	biliverdin reductase B [Source:HGNC Symbol;Acc:HGNC:1063]	49.72	6	18000	536	870	62	161	4
AXL	AXL	ENSG00000167601	558	protein_coding	Human	19	41.219223	AXL receptor tyrosine kinase [Source:HGNC Symbol;Acc:HGNC:905]	50.86	5	42544	2408	2546	141	851	14
HNRNPUL1	HNRNPUL1	ENSG00000105323	11100	protein_coding	Human	19	41.262496	heterogeneous nuclear ribonucleoprotein U like 1 [Source:HGNC Symbol;Acc:HGNC:17011]	48	21	45292	1298	1714	105	511	9
RABAC1	RABAC1	ENSG00000105404	10567	protein_coding	Human	19	41.956681	Rab acceptor 1 [Source:HGNC Symbol;Acc:HGNC:9794]	61.23	10	2641	414	700	55	160	3
CLPTM1	CLPTM1	ENSG00000104853	1209	protein_coding	Human	19	44.954585	CLPTM1 regulator of GABA type A receptor forward trafficking [Source:HGNC Symbol;Acc:HGNC:2087]	52.83	12	38757	1048	1216	157	254	7
PPP5C	PPP5C	ENSG00000011485	5536	protein_coding	Human	19	46.347087	protein phosphatase 5 catalytic subunit [Source:HGNC Symbol;Acc:HGNC:9322]	50.7	15	45895	1358	1481	82	450	6
SAE1	SAE1	ENSG00000142230	10055	protein_coding	Human	19	47.113274	SUMO1 activating enzyme subunit 1 [Source:HGNC Symbol;Acc:HGNC:30660]	47.18	14	97363	594	1690	81	651	8
NAPA	NAPA	ENSG00000105402	8775	protein_coding	Human	19	47.487637	NSF attachment protein alpha [Source:HGNC Symbol;Acc:HGNC:7641]	55.43	22	27455	437	978	96	284	6
GYS1	GYS1	ENSG00000104812	2997	protein_coding	Human	19	48.96813	glycogen synthase 1 [Source:HGNC Symbol;Acc:HGNC:4706]	52.54	7	25181	1526	1381	198	801	7
ETFB	ETFB	ENSG00000105379	2109	protein_coding	Human	19	51.345169	electron transfer flavoprotein subunit beta [Source:HGNC Symbol;Acc:HGNC:3482]	51.45	5	21220	781	1689	857	42	4
HSPBP1	HSPBP1	ENSG00000133265	23640	protein_coding	Human	19	55.262223	HSPA (Hsp70) binding protein 1 [Source:HGNC Symbol;Acc:HGNC:24989]	51.9	9	18159	629	957	123	294	5
UBE2S	UBE2S	ENSG00000108106	27338	protein_coding	Human	19	55.399745	ubiquitin conjugating enzyme E2 S [Source:HGNC Symbol;Acc:HGNC:17895]	54.64	4	8044	507	1009	170	864	4
U2AF2	U2AF2	ENSG00000063244	11338	protein_coding	Human	19	55.654146	U2 small nuclear RNA auxiliary factor 2 [Source:HGNC Symbol;Acc:HGNC:23156]	56.03	7	20571	872	1522	660	401	8
UBE2M	UBE2M	ENSG00000130725	9040	protein_coding	Human	19	58.555712	ubiquitin conjugating enzyme E2 M [Source:HGNC Symbol;Acc:HGNC:12491]	61.64	5	3243	369	602	168	190	4
RRBP1	RRBP1	ENSG00000125844	6238	protein_coding	Human	20	17.613678	ribosome binding protein 1 [Source:HGNC Symbol;Acc:HGNC:10448]	52.51	10	68618	2403	2666	139	378	15
RBBP9	RBBP9	ENSG00000089050	10741	protein_coding	Human	20	18.48654	RB binding protein 9, serine hydrolase [Source:HGNC Symbol;Acc:HGNC:9892]	41.62	3	10686	561	1865	58	3224	4
XRN2	XRN2	ENSG00000088930	22803	protein_coding	Human	20	21.303331	5'-3' exoribonuclease 2 [Source:HGNC Symbol;Acc:HGNC:12836]	37.21	1	86495	2853	3408	68	487	30
EIF2S2	EIF2S2	ENSG00000125977	8894	protein_coding	Human	20	34.088309	eukaryotic translation initiation factor 2 subunit beta [Source:HGNC Symbol;Acc:HGNC:3266]	42.17	1	23935	1002	2556	133	1421	9
DYNLRB1	DYNLRB1	ENSG00000125971	83658	protein_coding	Human	20	34.516395	dynein light chain roadblock-type 1 [Source:HGNC Symbol;Acc:HGNC:15468]	48.33	5	24564	244	998	84	519	4
EIF6	EIF6	ENSG00000242372	3692	protein_coding	Human	20	35.278907	eukaryotic translation initiation factor 6 [Source:HGNC Symbol;Acc:HGNC:6159]	52.05	9	6079	536	874	64	224	5
NDRG3	NDRG3	ENSG00000101079	57446	protein_coding	Human	20	36.651766	NDRG family member 3 [Source:HGNC Symbol;Acc:HGNC:14462]	44.5	5	94325	958	2340	68	1600	14
PLTP	PLTP	ENSG00000100979	5360	protein_coding	Human	20	45.898621	phospholipid transfer protein [Source:HGNC Symbol;Acc:HGNC:9093]	51.86	5	13535	1341	1765	158	227	15
VAPB	VAPB	ENSG00000124164	9217	protein_coding	Human	20	58.389229	VAMP associated protein B and C [Source:HGNC Symbol;Acc:HGNC:12649]	42.8	6	61873	416	3362	157	2856	4
HSPA13	HSPA13	ENSG00000155304	6782	protein_coding	Human	21	14.371115	heat shock protein family A (Hsp70) member 13 [Source:HGNC Symbol;Acc:HGNC:11375]	36.7	2	12370	1416	2282	27	2502	4
MX1	MX1	ENSG00000157601	4599	protein_coding	Human	21	41.42002	MX dynamin like GTPase 1 [Source:HGNC Symbol;Acc:HGNC:7532]	47.98	48	50052	1948	3059	92	792	15
PDXK	PDXK	ENSG00000160209	8566	protein_coding	Human	21	43.719094	pyridoxal kinase [Source:HGNC Symbol;Acc:HGNC:8819]	57.26	19	43214	636	2056	166	3920	6
ATP6V1E1	ATP6V1E1	ENSG00000131100	529	protein_coding	Human	22	17.592136	ATPase H+ transporting V1 subunit E1 [Source:HGNC Symbol;Acc:HGNC:857]	45.03	9	36614	624	861	109	436	6
PITPNB	PITPNB	ENSG00000180957	23760	protein_coding	Human	22	27.851669	phosphatidylinositol transfer protein beta [Source:HGNC Symbol;Acc:HGNC:9002]	39.71	10	68466	664	1157	84	1007	7
AP1B1	AP1B1	ENSG00000100280	162	protein_coding	Human	22	29.32768	adaptor related protein complex 1 subunit beta 1 [Source:HGNC Symbol;Acc:HGNC:554]	51.4	9	60904	2250	2626	91	1094	13
SF3A1	SF3A1	ENSG00000099995	10291	protein_coding	Human	22	30.331988	splicing factor 3a subunit 1 [Source:HGNC Symbol;Acc:HGNC:10765]	49.02	10	24932	867	1059	107	942	4
DRG1	DRG1	ENSG00000185721	4733	protein_coding	Human	22	31.399604	developmentally regulated GTP binding protein 1 [Source:HGNC Symbol;Acc:HGNC:3029]	44.4	7	131031	444	675	77	217	5
RBX1	RBX1	ENSG00000100387	9978	protein_coding	Human	22	40.951347	ring-box 1 [Source:HGNC Symbol;Acc:HGNC:9928]	44.92	3	21963	327	1205	21	821	3
ARFGAP3	ARFGAP3	ENSG00000242247	26286	protein_coding	Human	22	42.796502	ADP ribosylation factor GTPase activating protein 3 [Source:HGNC Symbol;Acc:HGNC:661]	45	6	61605	904	1136	115	630	8
ATXN10	ATXN10	ENSG00000130638	25814	protein_coding	Human	22	45.671798	ataxin 10 [Source:HGNC Symbol;Acc:HGNC:10549]	40.57	12	173510	965	1077	184	864	6
ASMTL	ASMTL	ENSG00000169093	8623	protein_coding	Human		1.403139	acetylserotonin O-methyltransferase like [Source:HGNC Symbol;Acc:HGNC:751]	52.23	6	50624	1792	1408	90	129	8
RPS4Y1	RPS4Y1	ENSG00000129824	6192	protein_coding	Human		2.841602	ribosomal protein S4 Y-linked 1 [Source:HGNC Symbol;Acc:HGNC:10425]	40.56	4	90399	790	863	24	374	5
HSD17B10	HSD17B10	ENSG00000072506	3028	protein_coding	Human		53.431258	hydroxysteroid 17-beta dehydrogenase 10 [Source:HGNC Symbol;Acc:HGNC:4800]	51.98	9	3113	677	1098	16	269	4
MSN	MSN	ENSG00000147065	4478	protein_coding	Human		65.588377	moesin [Source:HGNC Symbol;Acc:HGNC:7373]	44.63	16	153555	1210	2526	136	1689	8
SNX12	SNX12	ENSG00000147164	29934	protein_coding	Human		71.056332	sorting nexin 12 [Source:HGNC Symbol;Acc:HGNC:14976]	42.55	7	17095	494	1228	88	1227	4
PIN4	PIN4	ENSG00000102309	5303	protein_coding	Human		72.181353	peptidylprolyl cis/trans isomerase, NIMA-interacting 4 [Source:HGNC Symbol;Acc:HGNC:8992]	44.57	9	121574	333	1226	93	754	4
CSTF2	CSTF2	ENSG00000101811	1478	protein_coding	Human		100.820359	cleavage stimulation factor subunit 2 [Source:HGNC Symbol;Acc:HGNC:2484]	38.71	4	21162	1380	1774	55	214	12
PGRMC1	PGRMC1	ENSG00000101856	10857	protein_coding	Human		119.236245	progesterone receptor membrane component 1 [Source:HGNC Symbol;Acc:HGNC:16090]	42.58	2	8222	510	1820	99	1212	2
LAMP2	LAMP2	ENSG00000005893	3920	protein_coding	Human		120.426148	lysosomal associated membrane protein 2 [Source:HGNC Symbol;Acc:HGNC:6501]	38.61	4	43218	1120	3431	153	2160	8
RBMX	RBMX	ENSG00000147274	27316	protein_coding	Human		136.848004	RNA binding motif protein X-linked [Source:HGNC Symbol;Acc:HGNC:9910]	43.01	12	32761	614	1636	90	498	6
SSR4	SSR4	ENSG00000180879	6748	protein_coding	Human		153.793516	signal sequence receptor subunit 4 [Source:HGNC Symbol;Acc:HGNC:11326]	61.52	12	4984	503	1031	164	65	5
VBP1	VBP1	ENSG00000155959	7411	protein_coding	Human		155.197007	VHL binding protein 1 [Source:HGNC Symbol;Acc:HGNC:12662]	40.41	4	42835	584	1173	52	723	6
BAG6	NA	Not mapped		NA	NA		NA	NA	NA							
CEP170	NA	Not mapped		NA	NA		NA	NA	NA							
CTSB	NA	Not mapped		NA	NA		NA	NA	NA							
DDT	NA	Not mapped		NA	NA		NA	NA	NA							
DNAJC8	NA	Not mapped		NA	NA		NA	NA	NA							
GCN1	NA	Not mapped		NA	NA		NA	NA	NA							
GMPR2	NA	Not mapped		NA	NA		NA	NA	NA							
GNL1	NA	Not mapped		NA	NA		NA	NA	NA							
GPI	NA	Not mapped		NA	NA		NA	NA	NA							
LSS	NA	Not mapped		NA	NA		NA	NA	NA							
NAP1L4	NA	Not mapped		NA	NA		NA	NA	NA							
NAPRT	NA	Not mapped		NA	NA		NA	NA	NA							
NEDD8	NA	Not mapped		NA	NA		NA	NA	NA							
NHERF1	NA	Not mapped		NA	NA		NA	NA	NA							
PFDN6	NA	Not mapped		NA	NA		NA	NA	NA							
PPP1R18	NA	Not mapped		NA	NA		NA	NA	NA							
PUF60	NA	Not mapped		NA	NA		NA	NA	NA							
QNG1	NA	Not mapped		NA	NA		NA	NA	NA							
RNH1	NA	Not mapped		NA	NA		NA	NA	NA							
SCAMP3	NA	Not mapped		NA	NA		NA	NA	NA							
SHMT1	NA	Not mapped		NA	NA		NA	NA	NA							
SSBP1	NA	Not mapped		NA	NA		NA	NA	NA							
TBCE	NA	Not mapped		NA	NA		NA	NA	NA							
TJP1	NA	Not mapped		NA	NA		NA	NA	NA							
VASN	NA	Not mapped		NA	NA		NA	NA	NA							
