# Supplementary material for: Extracellular Vesicles Bearing Vimentin Drive Epithelial–Mesenchymal Transition
Source: Mol Cell Proteomics. 2025 Jul 4;24(12):101028. doi: 10.1016/j.mcpro.2025.101028 (PMC12719745; doi:10.1016/j.mcpro.2025.101028)
Supplement: Supplemental Data 14 [file mmc17.docx]

Figure legends for supplementary Table 2

Table 2.1. List of top 100 proteins frequently detected in EVs (data from 2023/10/17) used to build Venn diagrams

Table 2.2. List of all proteins detected in Evs (data from 2023/10/17) used to build Venn diagrams

Table 2.3. List of identified proteins in HDF EV including label free quantitation of all samples. This table is a direct output of FragPipe and contains missing values. Venn diagrams are based on this table.

Table 2.4. List of identified proteins in HDF EV after imputation based on normal distribution. This table includes the results from ANOVA statistical testing and was used for hierarchical clustering.

Table 2.5. List of identified proteins in MCF7, including label free quantitation of all samples. This table is a direct output of FragPipe and contains missing values. Venn diagrams are based on this table.

Table 2.6. List of identified proteins in MCF7 after imputation based on normal distribution. This table includes the results from Student t-test statistical testing and was used for hierarchical clustering.

Table 2.7. List of identified proteins in MCF10 after imputation based on normal distribution. This table includes the results from Student t-test statistical testing and was used for hierarchical clustering.

Table 2.8. List of identified proteins in MCF10, including label free quantitation of all samples. This table is a direct output of FragPipe and contains missing values. Venn diagrams are based on this table.

Table 2.9. Summary of the top15 enriched biological processes and cellular components from data of Figure 7. This is a direct output of ShinyGO.

Table 2.10. Summary of the top15 enriched KEGG pathways, biological processes, cellular components from data of Figure 8. This is a direct output of ShinyGO.

Table 2.11. Summary of the enriched KEGG pathways and top15 biological processes and cellular components from data of Figure 9. This is a direct output of ShinyGO.

Table 2.12. Summary of the enriched KEGG pathways and top15 biological processes and cellular components from data of Figure 10. This is a direct output of ShinyGO.

Table 2.13. Reference list of proteins used for the Venn diagrams in Figs. 9F and 10F. It contains proteins found in only WT EVs (Fig. 8A) and proteins from cluster 3 (enriched in WT samples) of Fig. 7D.
