# Supplementary material for: Extracellular Vesicles Bearing Vimentin Drive Epithelial–Mesenchymal Transition
Source: Mol Cell Proteomics. 2025 Jul 4;24(12):101028. doi: 10.1016/j.mcpro.2025.101028 (PMC12719745; doi:10.1016/j.mcpro.2025.101028)
Supplement: Supplemental Data 2 [file mmc5.pdf]

Sheet 2

PRIMARY ID	ENTREZ GENE ID	SPECIES	GENE SYMBOL	GENE NAME	SYNONYMS	HGNC	MIM
1	30970	Drosophila melanogaster	CG3038	CG3038 gene product from transcript CG3038-RB	DmelCG3038|EG:BACR37P7.1	0	0
2	30971	Drosophila melanogaster	G9a	CG2995 gene product from transcript CG2995-RA	CG2995|DmelCG2995|EG:BACR37P7.2|EHMT|cab65850 G9a Dm|dG9a	0	0
3	30972	Drosophila melanogaster	CG13377	CG13377 gene product from transcript CG13377-RA	BG01596|DmelCG13377|EG:BACR37P7.9	0	0
4	30973	Drosophila melanogaster	cin	cinnamon	CG2945|CIN|DmelCG2945|EG:BACR37P7.3|fs(1)M50|l(1)1Ae|l(1)G0142	0	0
5	30975	Drosophila melanogaster	ewg	erect wing	CG3114|DmelCG3114|EC3|EG:BACR37P7.7|EWG|NRF-1|l(1)1Ag|l(1)DA659|l(1)EC3|l(1)EC3f|l(1)VA720|l[EC3]	0	0
6	30976	Drosophila melanogaster	CG13375	CG13375 gene product from transcript CG13375-RA	DmelCG13375|EG:BACR37P7.8	0	0
7	30977	Drosophila melanogaster	CG12470	CG12470 gene product from transcript CG12470-RA	DmelCG12470|EG:BACR37P7.5|NEST:bs07e10|NEST:bs17h08	0	0
8	30978	Drosophila melanogaster	Or1a	Odorant receptor 1a	1A.1|1a|CG17885|DOR68|DmelCG17885|OR1a	0	0
9	30979	Drosophila melanogaster	CG3777	CG3777 gene product from transcript CG3777-RB	BEST:CK01670|CK01670|DmelCG3777|EG:125H10.1	0	0
10	30980	Drosophila melanogaster	y	yellow	CG3757|DmelCG3757|EG:125H10.2|T6	0	0
11	30981	Drosophila melanogaster	ac	achaete	990 E5 F1|AC|AS-C T5|AS-C T5ac|ASC|Ac|Ac/Sc|CG3796|DmelCG3796|EG:125H10.3|Hw|T5|ascT5|sc/T5	0	0
12	30982	Drosophila melanogaster	sc	scute	AS-C T4|AS-C T4sc|Ac/Sc|CG3827|DROACS2|DmelCG3827|EG:198A6.1|Hw|Sc|T4|T4 AS-C|ascT4|bHLHc28|l(1)1Ba|sc/T4|scalpha|scute/sisterlessB|sis b|sis-b|sisB|sisb	0	0
13	30983	Drosophila melanogaster	l(1)sc	lethal of scute	AS-C T3|AS-T3|ASC-T3|CG3839|Df(1)sc|DmelCG3839|EG:198A6.2|L'Sc|L'sc|L(1)sc|T3|ascT3|bHLHc28|l'sc|l(1)1Ba|l-sc|lsc|sc/T3	0	0
14	30984	Drosophila melanogaster	pcl	pepsinogen-like	CG13374|DmelCG13374|EG:EG0001.1|PCL|T2|scbeta	0	0
15	30985	Drosophila melanogaster	ase	asense	AS-C T8|AS-C T8ase|AS-T8|ASC|Ase|CG3258|DmelCG3258|EG:165H7.2|T1|T1a|T8|as-T8|ascT8|bHLHc28|sc/T8	0	0
16	30986	Drosophila melanogaster	Cyp4g1	Cytochrome P450-4g1	4g1|ASC-T1|CG3972|Cyt-P450-A1|DmelCG3972|EG:165H7.1|P-450|P-450-A|P450|P450-A|P450A|T1|anon-WO0140519.102|cyp4g1|scgamma	0	0
17	30988	Drosophila melanogaster	CG13373	CG13373 gene product from transcript CG13373-RB	DmelCG13373	0	0
18	30990	Drosophila melanogaster	CG3176	CG3176 gene product from transcript CG3176-RA	BcDNA:RH59219|DmelCG3176	0	0
19	30991	Drosophila melanogaster	CG18273	CG18273 gene product from transcript CG18273-RA	CG13372|DmelCG18273|EG:171D11.6	0	0
20	30994	Drosophila melanogaster	CG3156	CG3156 gene product from transcript CG3156-RA	DmelCG3156|EG:171D11.2	0	0
21	30995	Drosophila melanogaster	CG17896	CG17896 gene product from transcript CG17896-RB	153323_at|DmelCG17896|EG:171D11.1	0	0
22	30996	Drosophila melanogaster	CG17778	CG17778 gene product from transcript CG17778-RA	BcDNA:RH02374|DmelCG17778|EG:171D11.5	0	0
23	30998	Drosophila melanogaster	svr	silver	CG18503|CG32818|CG4122|CP|CPD|CpepE|CypD|DmelCG4122|EG:171D11.3|EP(X)0356|cc|dwp|l(1)1Bd|poi|slv	0	0
24	31000	Drosophila melanogaster	elav	embryonic lethal abnormal vision	44C11|9F8A9|CG4262|DmelCG4262|EC7|EG:65F1.2|ELAV|ElaV|Elav|Elav-9F8A9|elav-1|elav-2|elav-3|fliJ|l(1)1Be|l(1)EC7|l(1)G0031|l(1)G0319|weg	0	0
25	31001	Drosophila melanogaster	CG4293	CG4293 gene product from transcript CG4293-RD	154711_at|DmelCG4293|EG:65F1.1	0	0
26	31002	Drosophila melanogaster	Appl	beta amyloid protein precursor-like	APP|APP-like|APPL|Abeta|BcDNA:GH04413|CG7727|DmelCG7727|EG:65F1.5|appl	0	0
27	31003	Drosophila melanogaster	vnd	ventral nervous system defective	CG6172|Dm vnd|Dm-NK2|DmelCG6172|EC6|EG:118B3.1|HDC16822|NK-2|NK2|Nkx2|VND|Vnd|dNK-2|l(1)1Bf|l(1)EA142|l(1)EC6|l(1)GA100|l(1)GA122|l(1)RC24|l(1)VA208|l(1)VE769|lab|nk-2|vnd/NK-2	0	0
28	31004	Drosophila melanogaster	CG13366	CG13366 gene product from transcript CG13366-RA	AAD55419|DmelCG13366|EG:118B3.2	0	0
29	31005	Drosophila melanogaster	CG13365	CG13365 gene product from transcript CG13365-RA	BcDNA:LP07417|DmelCG13365	0	0
30	31006	Drosophila melanogaster	mod(r)	modifier of rudimentary	CG17828|DmelCG17828|EG:115C2.5|anon-WO0153538.78|vps37A	0	0
31	31007	Drosophila melanogaster	CG13369	CG13369 gene product from transcript CG13369-RA	DmelCG13369|EG:115C2.1	0	0
32	31009	Drosophila melanogaster	RpL36	Ribosomal protein L36	BcDNA:AT29875|CG7622|DmelCG7622|EG:115C2.7|L36a|M(1)1B|M(1)Bld|Vi|anon-EST:Posey274|l(1)1Bj|l(1)G0471	0	0
33	31010	Drosophila melanogaster	l(1)1Bi	lethal (1) 1Bi	CG6189|DmelCG6189|EG:115C2.2|HM-419|HM-52|l(1)Bi|l(1)lBi	0	0
34	31011	Drosophila melanogaster	Dredd	Death related ced-3/Nedd2-like protein	CG7486|DCP-2|DCP-2/DREDD|DCP2|DREDD|Dcp-2|Dcp-2/Dredd|Dcp2|Dedd|DmelCG7486|Dredd/DCP-2|EG:115C2.9|Redd|anon-1BCa|dcp-2/dredd|dredd	0	0
35	31012	Drosophila melanogaster	su(s)	suppressor of sable	CG6222|DmelCG6222|EG:115C2.3|su-s|su-v	0	0
36	31013	Drosophila melanogaster	CG13367	CG13367 gene product from transcript CG13367-RA	DmelCG13367|EG:115C2.8|anon-1BCb	0	0
37	31014	Drosophila melanogaster	Roc1a	CG16982 gene product from transcript CG16982-RC	BEST:CK01110|CG16982|CK01110|DmelCG16982|EG:115C2.11|ROC1|Rbx1|Rbx1/Roc|Roc1alpha|anon-EST:Posey65|dRbx1|dRoc1|dRoc1a	0	0
38	31015	Drosophila melanogaster	Hmt4-20	Histone methyltransferase 4-20	BcDNA:LD36415|CG13363|DmelCG13363|EG:115C2.10|SUV4-20|Su(var)4-20|Suv4-20|Suv4.20|hmt4-20	0	0
39	31016	Drosophila melanogaster	skpA	CG16983 gene product from transcript CG16983-RD	CG16983|DmelCG16983|EG:115C2.4|SKP1|Skp|Skp1|SkpA|anon-WO03040301.234|anon-WO03040301.236|anon-WO03040301.238|dSkp-1|dSkpA|dskpA|l(1)G0037|l(1)G0058|l(1)G0109|l(1)G0389|spkA	0	0
40	31017	Drosophila melanogaster	sdk	sidekick	BcDNA:LD22322|BcDNA:RE63453|CG5227|CT16627|DmelCG5227|EG:BACR19J1.1	0	0
41	31018	Drosophila melanogaster	CG13362	CG13362 gene product from transcript CG13362-RA	DmelCG13362	0	0
42	31019	Drosophila melanogaster	CG13361	CG13361 gene product from transcript CG13361-RA	DmelCG13361	0	0
43	31020	Drosophila melanogaster	CG5254	CG5254 gene product from transcript CG5254-RA	CT16777|DmelCG5254|EG:BACR19J1.2	0	0
44	31021	Drosophila melanogaster	CG5273	CG5273 gene product from transcript CG5273-RA	DmelCG5273|EG:BACR19J1.3	0	0
45	31022	Drosophila melanogaster	RpL22	Ribosomal protein L22	CG7434|DmelCG7434|EG:BACR19J1.4|L22|PBP-12|RL22|RPL22|Rpl22|anon-EST:Posey53|rpL22|rpl22	0	0
46	31023	Drosophila melanogaster	fz3	frizzled 3	CG16785|DFz3|Dfz3|Dm Fz3|DmelCG16785|EG:34F3.6|Fz3|dFz3|dfz3	0	0
47	31024	Drosophila melanogaster	tw	Protein O-mannosyltransferase 2	CG12311|DPOMT2|DmPOMT2|DmelCG12311|EG:34F3.7|POMT2|Pomt2|dPOMT2	0	0
48	31025	Drosophila melanogaster	CG13360	CG13360 gene product from transcript CG13360-RA	DmelCG13360|EG:34F3.5	0	0
49	31026	Drosophila melanogaster	CG16989	CG16989 gene product from transcript CG16989-RA	DmelCG16989|EG:34F3.4	0	0
50	31027	Drosophila melanogaster	Rbf	Retinoblastoma-family protein	CG7413|DmelCG7413|EG:34F3.3|FBF|RB|RBF|RBF1|Rb|Rb1|RbF|Rbf1|dRBF|pRb|rb|rbf|rbf1	0	0
51	31028	Drosophila melanogaster	CG13359	CG13359 gene product from transcript CG13359-RA	DmelCG13359|EG:34F3.9	0	0
52	31029	Drosophila melanogaster	CG13358	CG13358 gene product from transcript CG13358-RC	DmelCG13358|EG:34F3.10	0	0
53	31030	Drosophila melanogaster	Sec22	CG7359 gene product from transcript CG7359-RA	34F3.8|CG7359|DmelCG7359|EG:34F3.8|SEC22|Sec22p	0	0
54	31031	Drosophila melanogaster	CG42248	CG42248 gene product from transcript CG42248-RG	CG12467|CG14633|DmelCG42248|Dmel_CG12467|Dmel_CG14633|EG:34F3.1|EG:34F3.2	0	0
55	31032	Drosophila melanogaster	CG14635	CG14635 gene product from transcript CG14635-RA	DmelCG14635	0	0
56	31033	Drosophila melanogaster	CG11664	CG11664 gene product from transcript CG11664-RA	DmelCG11664|EG:BACR7A4.3|SP193	0	0
57	31035	Drosophila melanogaster	CG14632	CG14632 gene product from transcript CG14632-RB	DmelCG14632	0	0
58	31036	Drosophila melanogaster	CG11663	CG11663 gene product from transcript CG11663-RA	BcDNA:AT05678|DmelCG11663	0	0
59	31037	Drosophila melanogaster	CG3711	CG3711 gene product from transcript CG3711-RA	DmelCG3711|EG:BACR7A4.19	0	0
60	31038	Drosophila melanogaster	MED22	Mediator complex subunit 22	144358_at|CG18830|CG3034|DmelCG3034|EG:BACR7A4.6|Med22|Med24|Srb6/Surf5|anon-EST:Liang-1.82|clone 1.82|dMED24	0	0
61	31039	Drosophila melanogaster	CG3708	CG3708 gene product from transcript CG3708-RA	152685_at|DmelCG3708|EG:BACR7A4.18	0	0
62	31042	Drosophila melanogaster	TRAM	CG11642 gene product from transcript CG11642-RB	CG11642|CG18830|DmelCG11642|EG:BACR7A4.5	0	0
63	31043	Drosophila melanogaster	CG3704	CG3704 gene product from transcript CG3704-RA	DmelCG3704|EG:BACR7A4.17	0	0
64	31044	Drosophila melanogaster	mus81	CG3026 gene product from transcript CG3026-RA	CG3026|DmelCG3026|EG:BACR7A4.16	0	0
65	31045	Drosophila melanogaster	CG3703	CG3703 gene product from transcript CG3703-RA	BACR7A4.15|DmelCG3703|EG:BACR7A4.15	0	0
66	31046	Drosophila melanogaster	CG3699	CG3699 gene product from transcript CG3699-RA	CT12411|DmelCG3699|EG:BACR7A4.14	0	0
67	31047	Drosophila melanogaster	CG3690	CG3690 gene product from transcript CG3690-RA	DmelCG3690|EG:BACR7A4.13|anon-WO0140519.250	0	0
68	31048	Drosophila melanogaster	TfIIA-S-2	CG11639 gene product from transcript CG11639-RA	CG11639|DmelCG11639|EG:BACR7A4.7|TFIIA|tfiia-s-2	0	0
69	31049	Drosophila melanogaster	CG14631	CG14631 gene product from transcript CG14631-RA	DmelCG14631	0	0
70	31050	Drosophila melanogaster	CG11638	CG11638 gene product from transcript CG11638-RA	DmelCG11638|EG:BACR7A4.12	0	0
71	31052	Drosophila melanogaster	CDC45L	CG3658 gene product from transcript CG3658-RA	CDC45|CG3658|Cdc45|D|DmCdc45|DmelCG3658|EG:BACR7A4.11|anon-1Ec|dCDC45	0	0
72	31054	Drosophila melanogaster	su(w[a])	suppressor of white-apricot	CG3019|DmSWAP|DmelCG3019|EG:BACR7A4.10|SWAP|su(w)|su(w[a])-1|su(w[a])-2|su(wa)|su[wa]	0	0
73	31055	Drosophila melanogaster	CG14629	CG14629 gene product from transcript CG14629-RA	DmelCG14629|EG:103E12.2	0	0
74	31056	Drosophila melanogaster	CG3655	CG3655 gene product from transcript CG3655-RB	DmelCG3655|EG:103E12.3|RE126B2	0	0
75	31057	Drosophila melanogaster	CG14628	CG14628 gene product from transcript CG14628-RA	DmelCG14628|EG:BACR42I17.12|cg14628	0	0
76	31059	Drosophila melanogaster	eIF4E-7	CG32859 gene product from transcript CG32859-RA	CG11392|CG11393|CG32859|CT31794|DmelCG32859|EG:BACR42I17.1|eIF4E	0	0
77	31060	Drosophila melanogaster	CG11378	CG11378 gene product from transcript CG11378-RA	DmelCG11378|EG:BACR42I17.2	0	0
78	31061	Drosophila melanogaster	CG11384	CG11384 gene product from transcript CG11384-RA	DmelCG11384|EG:BACR42I17.3	0	0
79	31062	Drosophila melanogaster	CG11379	CG11379 gene product from transcript CG11379-RA	DmelCG11379|EG:BACR42I17.4|bs31h12.y1	0	0
80	31063	Drosophila melanogaster	CG14627	CG14627 gene product from transcript CG14627-RA	DmelCG14627|EG:BACR42I17.5	0	0
81	31064	Drosophila melanogaster	CG14626	CG14626 gene product from transcript CG14626-RA	DmelCG14626|EG:BACR42I17.6	0	0
82	31065	Drosophila melanogaster	CG11380	CG11380 gene product from transcript CG11380-RA	DmelCG11380|EG:BACR42I17.7	0	0
83	31066	Drosophila melanogaster	CG14625	CG14625 gene product from transcript CG14625-RB	DmelCG14625|EG:BACR42I17.8	0	0
84	31067	Drosophila melanogaster	CG11381	CG11381 gene product from transcript CG11381-RA	DmelCG11381	0	0
85	31068	Drosophila melanogaster	CG14624	CG14624 gene product from transcript CG14624-RA	BACR42I17.9|DmelCG14624|EG:BACR42I17.9	0	0
86	31069	Drosophila melanogaster	CG11382	CG11382 gene product from transcript CG11382-RB	DmelCG11382|EG:BACR42I17.10	0	0
87	31070	Drosophila melanogaster	CG11398	CG11398 gene product from transcript CG11398-RA	DmelCG11398|EG:BACR42I17.11	0	0
88	31071	Drosophila melanogaster	CG3638	CG3638 gene product from transcript CG3638-RE	BG00376|DmelCG3638|EG:33C11.3	0	0
89	31072	Drosophila melanogaster	CG11403	CG11403 gene product from transcript CG11403-RA	DmelCG11403|EG:33C11.2|cg11403	0	0
90	31074	Drosophila melanogaster	CG32812	CG32812 gene product from transcript CG32812-RA	CG11408|DmelCG32812|EG:114D9.1	0	0
91	31075	Drosophila melanogaster	DAAM	Dishevelled Associated Activator of Morphogenesis	CG14622|Daam|DmelCG14622|EG:114D9.2|anon-EST:Posey148|dDAAM|daam	0	0
92	31078	Drosophila melanogaster	CG11409	CG11409 gene product from transcript CG11409-RB	DmelCG11409|EG:8D8.2	0	0
93	31079	Drosophila melanogaster	CG11412	CG11412 gene product from transcript CG11412-RC	DmAAF45606|DmelCG11412|EG:8D8.6	0	0
94	31080	Drosophila melanogaster	Tsp2A	Tetraspanin 2A	CG11415|D8.7|Dm.Tsp2A|DmelCG11415|EG:8D8.7	0	0
95	31081	Drosophila melanogaster	CG11418	CG11418 gene product from transcript CG11418-RA	DmNP_569904|DmelCG11418|EG:8D8.8	0	0
96	31082	Drosophila melanogaster	CG12773	CG12773 gene product from transcript CG12773-RA	DmelCG12773|EG:8D8.3	0	0
97	31083	Drosophila melanogaster	CG11417	CG11417 gene product from transcript CG11417-RA	DmelCG11417|EG:8D8.4	0	0
98	31084	Drosophila melanogaster	png	pan gu	CG11420|DmelCG11420|EG:8D8.5|PNG|Png|fs(1)M2|pan-gnu	0	0
99	31085	Drosophila melanogaster	CG14770	CG14770 gene product from transcript CG14770-RA	DmelCG14770	0	0
100	31086	Drosophila melanogaster	ssx	sister-of-Sex-lethal	CG3056|DmelCG3056|EG:132E8.1|anon-EST:Posey102|cg3056	0	0
101	31089	Drosophila melanogaster	CG32813	CG32813 gene product from transcript CG32813-RE	CG11446|CG14771|DmelCG32813|anon-WO0153538.75	0	0
102	31090	Drosophila melanogaster	CG11448	CG11448 gene product from transcript CG11448-RA	DmelCG11448|EG:132E8.4	0	0
103	31093	Drosophila melanogaster	Gr2a	Gustatory receptor 2a	CG18531|DmelCG18531|EG:BACN32G11.1|GR2B1|Gr2B1	0	0
104	31094	Drosophila melanogaster	CG14785	CG14785 gene product from transcript CG14785-RA	DmelCG14785|EG:BACN32G11.2	0	0
105	31095	Drosophila melanogaster	CG14786	CG14786 gene product from transcript CG14786-RA	DmelCG14786|EG:BACN32G11.3	0	0
106	31096	Drosophila melanogaster	CG14787	CG14787 gene product from transcript CG14787-RA	DmelCG14787|EG:BACN32G11.4	0	0
107	31097	Drosophila melanogaster	ns3	nucleostemin 3	BACN32G11.5|CG14788|DmelCG14788|EG:BACN32G11.5|dNS3|dmNuc3|l(1)G0431	0	0
108	31098	Drosophila melanogaster	O-fut2	O-fucosyltransferase 2	CG14789|DmelCG14789|EG:BACN32G11.6|O-FUT2|O-fut|Ofut2	0	0
109	31099	Drosophila melanogaster	CG14777	CG14777 gene product from transcript CG14777-RB	BcDNA:RH63812|DmelCG14777|EG:80H7.10	0	0

<table>
<tr><td>110</td><td>31100</td><td>Drosophila melanogaster</td><td>CG14778</td><td>CG14778 gene product from transcript CG14778-RA</td><td>DmelCG14778</td><td>0</td><td>0</td></tr>
<tr><td>111</td><td>31101</td><td>Drosophila melanogaster</td><td>pck</td><td>pickel</td><td>CG14779|DmelCG14779|EG:80H7.2|Mega|l(1)G0044|mega</td><td>0</td><td>0</td></tr>
<tr><td>112</td><td>31102</td><td>Drosophila melanogaster</td><td>CG14780</td><td>CG14780 gene product from transcript CG14780-RA</td><td>DmelCG14780|EG:80H7.3|SP95</td><td>0</td><td>0</td></tr>
<tr><td>113</td><td>31103</td><td>Drosophila melanogaster</td><td>Rab27</td><td>CG14791 gene product from transcript CG14791-RB</td><td>AAF45634|AAF45635|CG14791|DmRab27|DmelCG14791|EG:80H7.4|dm-Rab27</td><td>0</td><td>0</td></tr>
<tr><td>114</td><td>31104</td><td>Drosophila melanogaster</td><td>mei-38</td><td>meiotic 38</td><td>CG14781|DmelCG14781|EG:80H7.11|Ssp1|ssp1</td><td>0</td><td>0</td></tr>
<tr><td>115</td><td>31105</td><td>Drosophila melanogaster</td><td>CG14782</td><td>CG14782 gene product from transcript CG14782-RA</td><td>DmelCG14782|EG:80H7.5</td><td>0</td><td>0</td></tr>
<tr><td>116</td><td>31106</td><td>Drosophila melanogaster</td><td>sta</td><td>stubarista</td><td>37LRP/p40|CG14792|DmelCG14792|EG:80H7.6|Lam-r|P40|Rp40|RpSA|STA|Sa|anon-EST:Liang-2.56|clone 2.56|k14|l(1)2Af|l(1)G0130|l(1)G0448</td><td>0</td><td>0</td></tr>
<tr><td>117</td><td>31107</td><td>Drosophila melanogaster</td><td>Nmdar2</td><td>NMDA receptor 2</td><td>79G8T|CG14793|CG14794|CG33513|CT34603|DmelCG33513|DrNR2|EG:80H7.7|ESTS:79G8T|NMDAR2|anon-sts34|dNR2|dnmdar-II</td><td>0</td><td>0</td></tr>
<tr><td>118</td><td>31109</td><td>Drosophila melanogaster</td><td>CG14795</td><td>CG14795 gene product from transcript CG14795-RB</td><td>DmelCG14795|EG:196F3.3</td><td>0</td><td>0</td></tr>
<tr><td>119</td><td>31110</td><td>Drosophila melanogaster</td><td>CG32810</td><td>CG32810 gene product from transcript CG32810-RB</td><td>CG14783|CG14784|DmelCG32810|EG:196F3.2</td><td>0</td><td>0</td></tr>
<tr><td>120</td><td>31111</td><td>Drosophila melanogaster</td><td>Mur2B</td><td>Mucin related 2B</td><td>CG14796|DmelCG14796|EG:56G7.1</td><td>0</td><td>0</td></tr>
<tr><td>121</td><td>31115</td><td>Drosophila melanogaster</td><td>CG11509</td><td>CG11509 gene product from transcript CG11509-RA</td><td>DmelCG11509</td><td>0</td><td>0</td></tr>
<tr><td>122</td><td>31118</td><td>Drosophila melanogaster</td><td>dor</td><td>deep orange</td><td>CG3093|Deep-orange|DmelCG3093|Dor|EG:171E4.1|Vps18|dor1|l(1)2Be|l(1)2Bw|l(1)7|l(1)76</td><td>0</td><td>0</td></tr>
<tr><td>123</td><td>31119</td><td>Drosophila melanogaster</td><td>CG3740</td><td>CG3740 gene product from transcript CG3740-RA</td><td>DmelCG3740|EG:171E4.4</td><td>0</td><td>0</td></tr>
<tr><td>124</td><td>31120</td><td>Drosophila melanogaster</td><td>hfw</td><td>halfway</td><td>CG3095|CT10406|DmelCG3095|EG:171E4.2|SWI|l(1)2Bf|l(1)nfw|swi</td><td>0</td><td>0</td></tr>
<tr><td>125</td><td>31121</td><td>Drosophila melanogaster</td><td>CG32809</td><td>CG32809 gene product from transcript CG32809-RG</td><td>CG3737|CG3783|CG3791|DmelCG32809|EG:171E4.3|EG:73D1.1|EG:9D2.2</td><td>0</td><td>0</td></tr>
<tr><td>126</td><td>31123</td><td>Drosophila melanogaster</td><td>CG14797</td><td>CG14797 gene product from transcript CG14797-RC</td><td>DmelCG14797</td><td>0</td><td>0</td></tr>
<tr><td>127</td><td>31124</td><td>Drosophila melanogaster</td><td>b6</td><td>CG3100 gene product from transcript CG3100-RA</td><td>CG3100|DmelCG3100|EG:9D2.1</td><td>0</td><td>0</td></tr>
<tr><td>128</td><td>31126</td><td>Drosophila melanogaster</td><td>CG14798</td><td>CG14798 gene product from transcript CG14798-RA</td><td>BcDNA:RE57135|DmelCG14798</td><td>0</td><td>0</td></tr>
<tr><td>129</td><td>31127</td><td>Drosophila melanogaster</td><td>a6</td><td>CG3771 gene product from transcript CG3771-RA</td><td>CG3771|DmelCG3771|EG:9D2.3</td><td>0</td><td>0</td></tr>
<tr><td>130</td><td>31128</td><td>Drosophila melanogaster</td><td>CG3795</td><td>CG3795 gene product from transcript CG3795-RA</td><td>BcDNA:GH24985|DmelCG3795|EG:9D2.4|SP160</td><td>0</td><td>0</td></tr>
<tr><td>131</td><td>31129</td><td>Drosophila melanogaster</td><td>Scgdelta</td><td>Sarcoglycan delta</td><td>BEST:GH21860|CG14808|CT34621|DmSCG-gammadelta|DmelCG14808|EG:4F1.1|GH21860|Sgcd</td><td>0</td><td>0</td></tr>
<tr><td>132</td><td>31130</td><td>Drosophila melanogaster</td><td>Adar</td><td>Adenosine deaminase acting on RNA</td><td>ADAR|ADAR1|CG12598|DmelCG12598|EG:BACN35H14.1|adar|cg12598|dADAR|dAdar|hypnos-2</td><td>0</td><td>0</td></tr>
<tr><td>133</td><td>31131</td><td>Drosophila melanogaster</td><td>CG14810</td><td>CG14810 gene product from transcript CG14810-RB</td><td>DmelCG14810</td><td>0</td><td>0</td></tr>
<tr><td>134</td><td>31132</td><td>Drosophila melanogaster</td><td>CG14811</td><td>CG14811 gene product from transcript CG14811-RB</td><td>DmelCG14811</td><td>0</td><td>0</td></tr>
<tr><td>135</td><td>31136</td><td>Drosophila melanogaster</td><td>CG42666</td><td>CG42666 gene product from transcript CG42666-RG</td><td>CG14799|CG14800|CG14801|CG17968|DmelCG42666|Dmel_CG14799|Dmel_CG14801|EG:131F2.2|EG:137E7.1</td><td>0</td><td>0</td></tr>
<tr><td>136</td><td>31137</td><td>Drosophila melanogaster</td><td>CG14812</td><td>CG14812 gene product from transcript CG14812-RA</td><td>BcDNA:RE59545|DmelCG14812|EG:131F2.3</td><td>0</td><td>0</td></tr>
<tr><td>137</td><td>31139</td><td>Drosophila melanogaster</td><td>CG14814</td><td>CG14814 gene product from transcript CG14814-RA</td><td>DmelCG14814|EG:63B12.6</td><td>0</td><td>0</td></tr>
<tr><td>138</td><td>31140</td><td>Drosophila melanogaster</td><td>MED18</td><td>Mediator complex subunit 18</td><td>CG14802|DmelCG14802|EG:63B12.13|Med18|Srb5/Med28b|dp28b|p28|p28/CG14802</td><td>0</td><td>0</td></tr>
<tr><td>139</td><td>31141</td><td>Drosophila melanogaster</td><td>CG14815</td><td>CG14815 gene product from transcript CG14815-RA</td><td>DmelCG14815|EG:63B12.5</td><td>0</td><td>0</td></tr>
<tr><td>140</td><td>31142</td><td>Drosophila melanogaster</td><td>CG14803</td><td>CG14803 gene product from transcript CG14803-RA</td><td>DmelCG14803|EG:63B12.9</td><td>0</td><td>0</td></tr>
<tr><td>141</td><td>31143</td><td>Drosophila melanogaster</td><td>Pgam5</td><td>Phosphoglycerate mutase 5</td><td>CG14816|DPGAM5|DmelCG14816|EG:63B12.4|dPGAM5</td><td>0</td><td>0</td></tr>
<tr><td>142</td><td>31144</td><td>Drosophila melanogaster</td><td>Vps26</td><td>Vacuolar protein sorting 26</td><td>131F2S|CG14804|DmelCG14804|Dvps26|EG:63B12.8|ESTS:131F2S|anon-sts13</td><td>0</td><td>0</td></tr>
<tr><td>143</td><td>31145</td><td>Drosophila melanogaster</td><td>CG14817</td><td>CG14817 gene product from transcript CG14817-RA</td><td>DmelCG14817|EG:63B12.11</td><td>0</td><td>0</td></tr>
<tr><td>144</td><td>31146</td><td>Drosophila melanogaster</td><td>CG14805</td><td>CG14805 gene product from transcript CG14805-RA</td><td>DmelCG14805|EG:63B12.7|i207</td><td>0</td><td>0</td></tr>
<tr><td>145</td><td>31147</td><td>Drosophila melanogaster</td><td>CG14818</td><td>CG14818 gene product from transcript CG14818-RA</td><td>BcDNA:RE09176|BcDNA:RH52516|CT34631|DmelCG14818|EG:63B12.12</td><td>0</td><td>0</td></tr>
<tr><td>146</td><td>31148</td><td>Drosophila melanogaster</td><td>CG14806</td><td>CG14806 gene product from transcript CG14806-RA</td><td>DmelCG14806</td><td>0</td><td>0</td></tr>
<tr><td>147</td><td>31149</td><td>Drosophila melanogaster</td><td>trr</td><td>trithorax-related</td><td>CG3848|DmelCG3848|EG:63B12.3|TRR|Trr|anon-2Bb</td><td>0</td><td>0</td></tr>
<tr><td>148</td><td>31150</td><td>Drosophila melanogaster</td><td>mRpL16</td><td>mitochondrial ribosomal protein L16</td><td>CG3109|D-15|D15|DmelCG3109|EG:63B12.2|L16|anon-2Bd</td><td>0</td><td>0</td></tr>
<tr><td>149</td><td>31151</td><td>Drosophila melanogaster</td><td>arm</td><td>armadillo</td><td>ARM|Arm|CG11579|Dm Arm|DmelCG11579|EG:86E4.6|beta-Cat|beta-cat|beta-cat-arm|beta-catenin/Arm|betacat|l(1)2Bv|l(1)G0192|l(1)G0234|l(1)G0410|l(1)arm|t12687 ALR Dm</td><td>0</td><td>0</td></tr>
<tr><td>150</td><td>31152</td><td>Drosophila melanogaster</td><td>CG32801</td><td>CG32801 gene product from transcript CG32801-RA</td><td>CG14819|DmelCG32801</td><td>0</td><td>0</td></tr>
<tr><td>151</td><td>31153</td><td>Drosophila melanogaster</td><td>mip130</td><td>Myb-interacting protein 130</td><td>86E4.4|CG3480|DmelCG3480|EG:86E4.4|Mip130|Mip130/TWIT|TWIT|Twit|twit</td><td>0</td><td>0</td></tr>
<tr><td>152</td><td>31154</td><td>Drosophila melanogaster</td><td>Edem1</td><td>CG3810 gene product from transcript CG3810-RC</td><td>CG3810|DM-pI|DmelCG3810|EDEM1|EG.86E4.2|EG:86E4.2</td><td>0</td><td>0</td></tr>
<tr><td>153</td><td>31155</td><td>Drosophila melanogaster</td><td>Rbcn-3B</td><td>Rabconnectin-3B</td><td>CG17766|DmelCG17766|EG:86E4.3|FK39</td><td>0</td><td>0</td></tr>
<tr><td>154</td><td>31156</td><td>Drosophila melanogaster</td><td>eIF2B-epsilon</td><td>CG3806 gene product from transcript CG3806-RB</td><td>CG3806|DmelCG3806|EG:86E4.1|eIF-2bepsilon|eIF2Bepsilon|elf2B-epsilon</td><td>0</td><td>0</td></tr>
<tr><td>155</td><td>31157</td><td>Drosophila melanogaster</td><td>Ocrl</td><td>Oculocerebrorenal syndrome of Lowe 1 ortholog</td><td>CG3573|DmelCG3573|EG:86E4.5|dOCRL</td><td>0</td><td>0</td></tr>
<tr><td>156</td><td>31158</td><td>Drosophila melanogaster</td><td>CG11596</td><td>CG11596 gene product from transcript CG11596-RC</td><td>DmelCG11596|EG:39E1.1</td><td>0</td><td>0</td></tr>
<tr><td>157</td><td>31160</td><td>Drosophila melanogaster</td><td>CG3587</td><td>CG3587 gene product from transcript CG3587-RA</td><td>DmelCG3587|EG:39E1.2</td><td>0</td><td>0</td></tr>
<tr><td>158</td><td>31161</td><td>Drosophila melanogaster</td><td>CG3600</td><td>CG3600 gene product from transcript CG3600-RA</td><td>DmelCG3600|EG:BACH61I5.1|EP(X)1232|EP1232</td><td>0</td><td>0</td></tr>
<tr><td>159</td><td>31162</td><td>Drosophila melanogaster</td><td>Hr4</td><td>CG16902 gene product from transcript CG16902-RC</td><td>CG16902|DHR4|DmelCG16902|EG:133E12.2|GRF|NR6A2</td><td>0</td><td>0</td></tr>
<tr><td>160</td><td>31163</td><td>Drosophila melanogaster</td><td>CG4406</td><td>CG4406 gene product from transcript CG4406-RA</td><td>DmelCG4406|EG:133E12.3</td><td>0</td><td>0</td></tr>
<tr><td>161</td><td>31165</td><td>Drosophila melanogaster</td><td>usp</td><td>ultraspiracle</td><td>2C|CF1|CF1/USP|CF1/Usp|CG4380|Cf1|DmUSP|DmUsp|DmelCG4380|EG:22E5.1|NR2B4|USP|Usp|XR2C|cf1|dUSP|dmUSP|l(1)2Cf|l(1)usp</td><td>0</td><td>0</td></tr>
<tr><td>162</td><td>31166</td><td>Drosophila melanogaster</td><td>Actn</td><td>alpha actinin</td><td>CG4376|CT14163|CT14232|DmelCG4376|EG:133E12.1|GA17|HM-29|actn|alpha-actinin|fliA|l(1)2Cb|l(1)EA82|y</td><td>0</td><td>0</td></tr>
<tr><td>163</td><td>31167</td><td>Drosophila melanogaster</td><td>CG4325</td><td>CG4325 gene product from transcript CG4325-RA</td><td>DmelCG4325|EG:22E5.12</td><td>0</td><td>0</td></tr>
<tr><td>164</td><td>31168</td><td>Drosophila melanogaster</td><td>moody</td><td>CG4322 gene product from transcript CG4322-RD</td><td>1631|CG4322|DmelCG4322|EG:22E5.11|EP1631|Moody</td><td>0</td><td>0</td></tr>
<tr><td>165</td><td>31169</td><td>Drosophila melanogaster</td><td>CG4313</td><td>CG4313 gene product from transcript CG4313-RA</td><td>DmelCG4313|EG:22E5.10</td><td>0</td><td>0</td></tr>
<tr><td>166</td><td>31170</td><td>Drosophila melanogaster</td><td>SIK2</td><td>Salt-induced kinase 2</td><td>CG4290|DmelCG4290|EG:22E5.8|SIK</td><td>0</td><td>0</td></tr>
<tr><td>167</td><td>31171</td><td>Drosophila melanogaster</td><td>CG4281</td><td>CG4281 gene product from transcript CG4281-RA</td><td>DmelCG4281|EG:22E5.7</td><td>0</td><td>0</td></tr>
<tr><td>168</td><td>31172</td><td>Drosophila melanogaster</td><td>CG14054</td><td>CG14054 gene product from transcript CG14054-RA</td><td>BcDNA:AT13882|DmelCG14054</td><td>0</td><td>0</td></tr>
<tr><td>169</td><td>31173</td><td>Drosophila melanogaster</td><td>CG4194</td><td>CG4194 gene product from transcript CG4194-RA</td><td>DmelCG4194|EG:22E5.6</td><td>0</td><td>0</td></tr>
<tr><td>170</td><td>31174</td><td>Drosophila melanogaster</td><td>CG4199</td><td>CG4199 gene product from transcript CG4199-RG</td><td>143005_at|DmelCG4199|EG:22E5.5</td><td>0</td><td>0</td></tr>
<tr><td>171</td><td>31175</td><td>Drosophila melanogaster</td><td>CG4061</td><td>CG4061 gene product from transcript CG4061-RA</td><td>DmelCG4061|EG:22E5.3|RTC1_DROME</td><td>0</td><td>0</td></tr>
<tr><td>172</td><td>31176</td><td>Drosophila melanogaster</td><td>CG4045</td><td>CG4045 gene product from transcript CG4045-RA</td><td>DmelCG4045|EG:22E5.4</td><td>0</td><td>0</td></tr>
<tr><td>173</td><td>31177</td><td>Drosophila melanogaster</td><td>CG4025</td><td>CG4025 gene product from transcript CG4025-RA</td><td>DmelCG4025|EG:22E5.9|anon-WO0140519.169</td><td>0</td><td>0</td></tr>
<tr><td>174</td><td>31178</td><td>Drosophila melanogaster</td><td>CG16903</td><td>CG16903 gene product from transcript CG16903-RA</td><td>DmelCG16903|EG:67A9.2|anon-WO03040301.211</td><td>0</td><td>0</td></tr>
<tr><td>175</td><td>31179</td><td>Drosophila melanogaster</td><td>Unc-76</td><td>CG3981 gene product from transcript CG3981-RB</td><td>BcDNA:GH10260|CG18851|CG3981|DmelCG3981|Dunc-76|EG:67A9.1|UNC-76|l(1)G0066|l(1)G0158|l(1)G0310|l(1)G0333|l(1)G0360|unc-76</td><td>0</td><td>0</td></tr>
<tr><td>176</td><td>31181</td><td>Drosophila melanogaster</td><td>ph-p</td><td>polyhomeotic proximal</td><td>CG18412|CG18414|DmelCG18412|EG:87B1.5|PH|PH-p|PHP|Ph|Ph-p|PhP|ph|ph-P|ph[P]|ph[p]|phm|php</td><td>0</td><td>0</td></tr>
<tr><td>177</td><td>31184</td><td>Drosophila melanogaster</td><td>CG3835</td><td>CG3835 gene product from transcript CG3835-RB</td><td>DmelCG3835|EG87B1|EG:87B1.3</td><td>0</td><td>0</td></tr>
<tr><td>178</td><td>31185</td><td>Drosophila melanogaster</td><td>Pgd</td><td>Phosphogluconate dehydrogenase</td><td>6-PGD|6-Pgd|6-pgd|6PGD|6PGDH|6Pgd|6Pgdh|6pgdh|A7|CG3724|DmelCG3724|EG:87B1.4|N1|PGD|Pdg|l(1)2Dc|l(1)2De|l(1)A7|l(1)G0385|l(1)N1|l(1)N3[90]|l(1)Pgd|l(1)Pgd-A|l35|pgd</td><td>0</td><td>0</td></tr>
<tr><td>179</td><td>31186</td><td>Drosophila melanogaster</td><td>bcn92</td><td>CG3717 gene product from transcript CG3717-RA</td><td>BcDNA:RE57896|CG3717|DmelCG3717|EG:87B1.6|NEST:bs15a04</td><td>0</td><td>0</td></tr>
<tr><td>180</td><td>31187</td><td>Drosophila melanogaster</td><td>wapl</td><td>wings apart-like</td><td>C204|CG3707|DmelCG3707|EG:87B1.2|N2|RNA D|RNAD|l(1)2Dd|l(1)2Df|l(1)C204|l(1)N2|l(1)N5[72]|l74|pasc|wap</td><td>0</td><td>0</td></tr>
<tr><td>181</td><td>31188</td><td>Drosophila melanogaster</td><td>Cyp4d1</td><td>Cytochrome P450-4d1</td><td>4d1|CG3656|CYP4D1|CYP6D1|Cyp4D1|Cyt-P450-4D1|DmelCG3656|EG:87B1.1|P-450|P450|cyp4d1</td><td>0</td><td>0</td></tr>
<tr><td>182</td><td>31189</td><td>Drosophila melanogaster</td><td>CG3630</td><td>CG3630 gene product from transcript CG3630-RA</td><td>152A3.3|DmelCG3630|EG:152A3.3</td><td>0</td><td>0</td></tr>
<tr><td>183</td><td>31190</td><td>Drosophila melanogaster</td><td>CG3621</td><td>CG3621 gene product from transcript CG3621-RA</td><td>152A3.7|BcDNA:RE75088|DmelCG3621|EG:152A3.7|RNAA|anon-2D|anon-2Db</td><td>0</td><td>0</td></tr>
<tr><td>184</td><td>31192</td><td>Drosophila melanogaster</td><td>Cyp4d2</td><td>Cytochrome P450-4d2</td><td>4d2|CG3466|CYP4D2|Cyt-P450-4D2|DmelCG3466|EG:152A3.4|P-450|P450</td><td>0</td><td>0</td></tr>
<tr><td>185</td><td>31193</td><td>Drosophila melanogaster</td><td>Cyp4ae1</td><td>Cytochrome P450-4ae1</td><td>4ae1|CG10755|CYP4E4|Cyp4e4|Cypae1|DmelCG10755|EG:152A3.6|I|P-450|P450|TcD20|anon-2Ea</td><td>0</td><td>0</td></tr>
<tr><td>186</td><td>31194</td><td>Drosophila melanogaster</td><td>pn</td><td>prune</td><td>CG3461|DmelCG3461|EG:152A3.5|N3|N4|TcD37</td><td>0</td><td>0</td></tr>
<tr><td>187</td><td>31195</td><td>Drosophila melanogaster</td><td>Nmd3</td><td>Nonsense-mediated mRNA 3</td><td>CG3460|DmelCG3460|EG:152A3.1|II|TcD12|TcD13|anon-2Eb|dNmd3</td><td>0</td><td>0</td></tr>
<tr><td>188</td><td>31196</td><td>Drosophila melanogaster</td><td>CG3457</td><td>CG3457 gene product from transcript CG3457-RA</td><td>DmelCG3457|EG:17E2.1</td><td>0</td><td>0</td></tr>
<tr><td>189</td><td>31197</td><td>Drosophila melanogaster</td><td>CG14053</td><td>CG14053 gene product from transcript CG14053-RA</td><td>BcDNA:AT10359|DmelCG14053</td><td>0</td><td>0</td></tr>
<tr><td>190</td><td>31198</td><td>Drosophila melanogaster</td><td>Mct1</td><td>Monocarboxylate transporter 1</td><td>CG3456|DmelCG3456|EG:103B4.3</td><td>0</td><td>0</td></tr>
<tr><td>191</td><td>31200</td><td>Drosophila melanogaster</td><td>msta</td><td>CG33548 gene product from transcript CG33548-RB</td><td>CG18033|CG32799|CG32800|CG33548|DmelCG33548|EG:103B4.4|EG:103B4.5|MSTA|Msta</td><td>0</td><td>0</td></tr>
<tr><td>192</td><td>31201</td><td>Drosophila melanogaster</td><td>Vinc</td><td>Vinculin</td><td>CG3299|CT11081|DmelCG3299|Dmvincp|EG:103B4.1|Vin|Vin2EF|vinc</td><td>0</td><td>0</td></tr>
<tr><td>193</td><td>31203</td><td>Drosophila melanogaster</td><td>CG14052</td><td>CG14052 gene product from transcript CG14052-RA</td><td>DmelCG14052</td><td>0</td><td>0</td></tr>
<tr><td>194</td><td>31204</td><td>Drosophila melanogaster</td><td>pcx</td><td>pecanex</td><td>CG3443|DMPCX|DmelCG3443|EG:30B8.4|fs(1)12-1012|fs(1)12-1743|fs(1)12-2135|fs(1)12-3014|fs(1)12-3102|fs(1)12-4169|fs(1)14-1153|fs(1)14-567|fs(1)A387|fs(1)M10|fs(1)pcx|mel(1)R|mel(1)R1</td><td>0</td><td>0</td></tr>
<tr><td>195</td><td>31205</td><td>Drosophila melanogaster</td><td>kz</td><td>kurz</td><td>CG3228|DmelCG3228|EG:30B8.2|N1|N8|l(1)2Eb|l(1)N4|l67</td><td>0</td><td>0</td></tr>
<tr><td>196</td><td>31207</td><td>Drosophila melanogaster</td><td>Or2a</td><td>Odorant receptor 2a</td><td>2F.1|2a|AN4|CG3206|DOR2F.1|DOR62|DmelCG3206|Dor62|EG:30B8.7|Or2F.1|Or62|dor62</td><td>0</td><td>0</td></tr>
<tr><td>197</td><td>31208</td><td>Drosophila melanogaster</td><td>crn</td><td>crooked neck</td><td>62D9.a|CG18842|CG3193|Crn|DmelCG3193|EG:30B8.1|N10|N5|RC63|l(1)2|l(1)2Fa|l(1)CRN|l(1)N7|l(1)crn|l30|yok</td><td>0</td><td>0</td></tr>
<tr><td>198</td><td>31209</td><td>Drosophila melanogaster</td><td>CG3191</td><td>CG3191 gene product from transcript CG3191-RA</td><td>62D9.a|BEST:GM01072|CG18842|DmelCG3191|EG:30B8.3</td><td>0</td><td>0</td></tr>
<tr><td>199</td><td>31210</td><td>Drosophila melanogaster</td><td>CG3091</td><td>CG3091 gene product from transcript CG3091-RA</td><td>DmelCG3091</td><td>0</td><td>0</td></tr>
<tr><td>200</td><td>31211</td><td>Drosophila melanogaster</td><td>CG3078</td><td>CG3078 gene product from transcript CG3078-RA</td><td>DmelCG3078|EG:30B8.6</td><td>0</td><td>0</td></tr>
<tr><td>201</td><td>31212</td><td>Drosophila melanogaster</td><td>l(1)G0144</td><td>lethal (1) G0144</td><td>CG3073|DmelCG3073</td><td>0</td><td>0</td></tr>
<tr><td>202</td><td>31213</td><td>Drosophila melanogaster</td><td>CG3071</td><td>CG3071 gene product from transcript CG3071-RA</td><td>DmelCG3071|EG:25E8.3</td><td>0</td><td>0</td></tr>
<tr><td>203</td><td>31214</td><td>Drosophila melanogaster</td><td>CG2924</td><td>CG2924 gene product from transcript CG2924-RA</td><td>DmelCG2924|EG:25E8.2|NICE5</td><td>0</td><td>0</td></tr>
<tr><td>204</td><td>31215</td><td>Drosophila melanogaster</td><td>CG2918</td><td>CG2918 gene product from transcript CG2918-RA</td><td>CT9894|DmelCG2918|EG:25E8.1</td><td>0</td><td>0</td></tr>
<tr><td>205</td><td>31217</td><td>Drosophila melanogaster</td><td>CG2865</td><td>CG2865 gene product from transcript CG2865-RA</td><td>DmelCG2865|EG:25E8.4|anon-WO03040301.165</td><td>0</td><td>0</td></tr>
<tr><td>206</td><td>31218</td><td>Drosophila melanogaster</td><td>CG14050</td><td>CG14050 gene product from transcript CG14050-RB</td><td>DmelCG14050|EG:BACH48C10.1|H48C10.1</td><td>0</td><td>0</td></tr>
<tr><td>207</td><td>31219</td><td>Drosophila melanogaster</td><td>CG2854</td><td>CG2854 gene product from transcript CG2854-RA</td><td>DmelCG2854|EG:BACH48C10.2</td><td>0</td><td>0</td></tr>
<tr><td>208</td><td>31220</td><td>Drosophila melanogaster</td><td>Ilp6</td><td>Insulin-like peptide 6</td><td>CG14049|DILP|DILP6|Dilp|Dilp 6|DmelCG14049|Ilp|Ilp-6|dILP6|dilp|dilp-6|dilp6</td><td>0</td><td>0</td></tr>
<tr><td>209</td><td>31221</td><td>Drosophila melanogaster</td><td>phl</td><td>pole hole</td><td>11-29|C110|CG2845|D-RAF|D-Raf|D-raf|D-raf1|DRaf|DRaf1|DmelCG2845|Draf|Draf-1|Draf1|EG:BACH48C10.3|Phl|RAF|Raf|Raf/phl|Raf1|draf|l(1)2Fe|l(1)G0475|l(1)ph|l(1)phl|l(1)pole hole|l(1)polehole|l(1)poleh</td><td>0</td><td>0</td></tr>
<tr><td>210</td><td>31222</td><td>Drosophila melanogaster</td><td>mRpL14</td><td>mitochondrial ribosomal protein L14</td><td>BcDNA:RH18819|CG14048|DmelCG14048|EG:BACH48C10.6|L14|mRPL14</td><td>0</td><td>0</td></tr>
<tr><td>211</td><td>31224</td><td>Drosophila melanogaster</td><td>CG14047</td><td>CG14047 gene product from transcript CG14047-RH</td><td>DmelCG14047|EG:BACH48C10.4</td><td>0</td><td>0</td></tr>
<tr><td>212</td><td>31225</td><td>Drosophila melanogaster</td><td>PsGEF</td><td>Protostome-specific GEF</td><td>CG14045|DmPsGEF|DmelCG14045|EG:BACH7M4.1|EG:BACH7M4.2</td><td>0</td><td>0</td></tr>
<tr><td>213</td><td>31226</td><td>Drosophila melanogaster</td><td>CG12496</td><td>CG12496 gene product from transcript CG12496-RA</td><td>DmelCG12496|EG:BACH7M4.4</td><td>0</td><td>0</td></tr>
<tr><td>214</td><td>31227</td><td>Drosophila melanogaster</td><td>gt</td><td>giant</td><td>CG7952|DmelCG7952|EG:BACH7M4.5|GIAN_DROME|GT|Giant|Gt|l(1)3Aa|l(1)giant</td><td>0</td><td>0</td></tr>
<tr><td>215</td><td>31228</td><td>Drosophila melanogaster</td><td>tko</td><td>technical knockout</td><td>CG7925|DmelCG7925|EG:BACH59J11.1|MRP-S12|MRPS12|S12|l(1)3Ab|l(1)tko|mRpS12|mt-rps12|rps12</td><td>0</td><td>0</td></tr>
<tr><td>216</td><td>31229</td><td>Drosophila melanogaster</td><td>boi</td><td>brother of ihog</td><td>Boi|CG13756|CG32796|CG7894|CT23737|CT33235|DmelCG32796|EG:BACH59J11.2</td><td>0</td><td>0</td></tr>
<tr><td>217</td><td>31230</td><td>Drosophila melanogaster</td><td>z</td><td>zeste</td><td>CG7803|DmelCG7803|EG:BACH59J11.3|Z|e(bx)|en-bx</td><td>0</td><td>0</td></tr>
<tr><td>218</td><td>31234</td><td>Drosophila melanogaster</td><td>Pdfr</td><td>Pigment-dispersing factor receptor</td><td>BACR25B3.3|CG13758|DmeCG13758|DmelCG13758|EG:BACR25B3.3|Han|PDF-R1|PDFR|Pdf-R1|PdfR|anon-WO0170980.157|anon-WO0170980.158|gop|han|pdfr</td><td>0</td><td>0</td></tr>
<tr><td>219</td><td>31235</td><td>Drosophila melanogaster</td><td>Vha36-3</td><td>Vacuolar H[+] ATPase subunit 36-3</td><td>CG8310|DmelCG8310|EG:BACR25B3.4|vha36-3</td><td>0</td><td>0</td></tr>
<tr><td>220</td><td>31236</td><td>Drosophila melanogaster</td><td>CG13759</td><td>CG13759 gene product from transcript CG13759-RB</td><td>BcDNA:AT19803|CT33239|DmelCG13759|EG:BACR25B3.5</td><td>0</td><td>0</td></tr>
</table>

<table>
<tr><td>221</td><td>31237</td><td>Drosophila melanogaster</td><td>CG13760</td><td>CG13760 gene product from transcript CG13760-RD</td><td>DmelCG13760|EG:BACR25B3.6</td><td>0</td><td>0</td></tr>
<tr><td>222</td><td>31239</td><td>Drosophila melanogaster</td><td>egh</td><td>egghead</td><td>CG9659|DmelCG9659|EG:BACR25B3.8|ZW-4|l(1)3Ae|l(1)zw4|zw-4|zw4</td><td>0</td><td>0</td></tr>
<tr><td>223</td><td>31240</td><td>Drosophila melanogaster</td><td>Klp3A</td><td>Kinesin-like protein at 3A</td><td>3A7Kin|BcDNA:LD21815|CG8590|DmKLP3A|DmKlp3A|DmelCG8590|EG:BACR25B3.9|KIF4|KIF4A|KLP-3A|KLP3A|Klp3a|fs(1)M4|klp3A|mei-352</td><td>0</td><td>0</td></tr>
<tr><td>224</td><td>31243</td><td>Drosophila melanogaster</td><td>CG8636</td><td>CG8636 gene product from transcript CG8636-RA</td><td>DmelCG8636|cg8636|eIF s4|eIF-3|eIF3-S4</td><td>0</td><td>0</td></tr>
<tr><td>225</td><td>31244</td><td>Drosophila melanogaster</td><td>Tsp3A</td><td>Tetraspanin 3A</td><td>BACR7C10.6|CG10742|Dm.Tsp3A|DmelCG10742|EG:BACR7C10.6</td><td>0</td><td>0</td></tr>
<tr><td>226</td><td>31245</td><td>Drosophila melanogaster</td><td>Seipin</td><td>CG9904 gene product from transcript CG9904-RA</td><td>CG9904|DmelCG9904|EG:BACR7C10.1</td><td>0</td><td>0</td></tr>
<tr><td>227</td><td>31246</td><td>Drosophila melanogaster</td><td>brv3</td><td>brivido-3</td><td>CG13762|CT33244|DmelCG13762|EG:BACR7C10.7</td><td>0</td><td>0</td></tr>
<tr><td>228</td><td>31247</td><td>Drosophila melanogaster</td><td>PI4KIIIalpha</td><td>Phosphatidylinositol 4-kinase III alpha</td><td>CG10260|DmelCG10260|EG:BACR7C10.2|PI4K|PI4KIII[[alpha]]|Pi4KIIIalpha|stt4</td><td>0</td><td>0</td></tr>
<tr><td>229</td><td>31248</td><td>Drosophila melanogaster</td><td>sgg</td><td>shaggy</td><td>CG2621|DMSGG3|DMZ3K25Z|Dm Zw3|DmelCG2621|Dmsgg3|EG:155E2.3|EG:BACR7C10.8|GSK|GSK-3|GSK-3B|GSK-3beta|GSK-3betaSgg|GSK3|GSK3-beta|GSK3Beta|GSK3beta|GSK3beta/SGG|GSK</td><td>0</td><td>0</td></tr>
<tr><td>230</td><td>31249</td><td>Drosophila melanogaster</td><td>HLH3B</td><td>Helix loop helix protein 3B</td><td>CG2655|DmelCG2655|DroSCL|EG:155E2.2|HLH3b|bHLHa20</td><td>0</td><td>0</td></tr>
<tr><td>231</td><td>31250</td><td>Drosophila melanogaster</td><td>CG2652</td><td>CG2652 gene product from transcript CG2652-RA</td><td>DmelCG2652|EG:155E2.5</td><td>0</td><td>0</td></tr>
<tr><td>232</td><td>31251</td><td>Drosophila melanogaster</td><td>per</td><td>period</td><td>CG2647|Clk|DmelCG2647|EG:155E2.4|PER|Per|clk-6|dPER|dper|unamed</td><td>0</td><td>0</td></tr>
<tr><td>233</td><td>31252</td><td>Drosophila melanogaster</td><td>CG2650</td><td>CG2650 gene product from transcript CG2650-RA</td><td>0.9|0.9 gene|DmelCG2650|EG:155E2.1|anon-3B1.2</td><td>0</td><td>0</td></tr>
<tr><td>234</td><td>31253</td><td>Drosophila melanogaster</td><td>CG2658</td><td>CG2658 gene product from transcript CG2658-RB</td><td>Dm100G10.aj|DmelCG2658|EG:100G10.7|anon-3Ba</td><td>0</td><td>0</td></tr>
<tr><td>235</td><td>31254</td><td>Drosophila melanogaster</td><td>CG2662</td><td>CG2662 gene product from transcript CG2662-RA</td><td>DmelCG2662|EG:100G10.6|MAD|snuts</td><td>0</td><td>0</td></tr>
<tr><td>236</td><td>31255</td><td>Drosophila melanogaster</td><td>Csat</td><td>CG2675 gene product from transcript CG2675-RA</td><td>CG2675|CSAT|DmNST|DmUGT|DmelCG2675|EG:100G10.5|UDP-Gal/UDP-GalNAc|anon-3Bb|dNST-1|dNST-2|ugt</td><td>0</td><td>0</td></tr>
<tr><td>237</td><td>31256</td><td>Drosophila melanogaster</td><td>eIF2B-beta</td><td>CG2677 gene product from transcript CG2677-RA</td><td>CG2677|DMC100G10.3|DmelCG2677|EG:100G10.3|eIF2Bbeta</td><td>0</td><td>0</td></tr>
<tr><td>238</td><td>31257</td><td>Drosophila melanogaster</td><td>CG2680</td><td>CG2680 gene product from transcript CG2680-RB</td><td>DmelCG2680|EG:100G10.4|Phos2680</td><td>0</td><td>0</td></tr>
<tr><td>239</td><td>31258</td><td>Drosophila melanogaster</td><td>CG2681</td><td>CG2681 gene product from transcript CG2681-RA</td><td>100G10.2|DmelCG2681|EG:100G10.2</td><td>0</td><td>0</td></tr>
<tr><td>240</td><td>31259</td><td>Drosophila melanogaster</td><td>CG2685</td><td>CG2685 gene product from transcript CG2685-RA</td><td>DmelCG2685|EG:100G10.1</td><td>0</td><td>0</td></tr>
<tr><td>241</td><td>31260</td><td>Drosophila melanogaster</td><td>CG2694</td><td>CG2694 gene product from transcript CG2694-RA</td><td>DmelCG2694|EG:100G10.8|EG:95B7.10</td><td>0</td><td>0</td></tr>
<tr><td>242</td><td>31261</td><td>Drosophila melanogaster</td><td>CG2701</td><td>CG2701 gene product from transcript CG2701-RA</td><td>DmelCG2701|EG:95B7.9|II|anon-3Bd</td><td>0</td><td>0</td></tr>
<tr><td>243</td><td>31262</td><td>Drosophila melanogaster</td><td>fs(1)Yb</td><td>female sterile (1) Yb</td><td>CG2706|DmelCG2706|EG:95B7.8|YB|Yb|fs(1)M104|fs(1)Y[b]|nov</td><td>0</td><td>0</td></tr>
<tr><td>244</td><td>31263</td><td>Drosophila melanogaster</td><td>fs(1)Ya</td><td>female sterile (1) Young arrest</td><td>CG2707|DmelCG2707|EG:95B7.4|YA|Ya|fs(1)73|fs(1)M12|fs(1)Y2|fs(1)Y[a]</td><td>0</td><td>0</td></tr>
<tr><td>245</td><td>31264</td><td>Drosophila melanogaster</td><td>CG2709</td><td>CG2709 gene product from transcript CG2709-RA</td><td>DmelCG2709|EG:95B7.5</td><td>0</td><td>0</td></tr>
<tr><td>246</td><td>31265</td><td>Drosophila melanogaster</td><td>dwg</td><td>deformed wings</td><td>CG2711|CG2714|DWG|DmelCG2711|EG:95B7.6|SBP|Sbp|ZW-5|ZW5|Zw-5|Zw5|l(1)3Be|l(1)zw5|ves|zw-5|zw5</td><td>0</td><td>0</td></tr>
<tr><td>247</td><td>31266</td><td>Drosophila melanogaster</td><td>ttm50</td><td>tiny tim 50</td><td>CG2713|DmelCG2713|EG:95B7.3|Gp99|i238|i90</td><td>0</td><td>0</td></tr>
<tr><td>248</td><td>31267</td><td>Drosophila melanogaster</td><td>CG2712</td><td>CG2712 gene product from transcript CG2712-RA</td><td>DmelCG2712|EG:95B7.7|III|anon-3Be</td><td>0</td><td>0</td></tr>
<tr><td>249</td><td>31268</td><td>Drosophila melanogaster</td><td>crm</td><td>cramped</td><td>CG2714|DmelCG2714|EG:95B7.2|ZW-9|l(1)3C1|l(1)3Ca|l(1)zw9|sa|sta[P]|swa|zw-9|zw9</td><td>0</td><td>0</td></tr>
<tr><td>250</td><td>31269</td><td>Drosophila melanogaster</td><td>Syx4</td><td>Syntaxin 4</td><td>CG2715|Dm Syx4|DmSyx4|DmelCG2715|EG:95B7.1|EG:95B7.11|Syx|Syx-4|syx4</td><td>0</td><td>0</td></tr>
<tr><td>251</td><td>31270</td><td>Drosophila melanogaster</td><td>CG32795</td><td>CG32795 gene product from transcript CG32795-RD</td><td>CG2716|CG2766|DmelCG32795|EG:BACN33B1.2</td><td>0</td><td>0</td></tr>
<tr><td>252</td><td>31271</td><td>Drosophila melanogaster</td><td>w</td><td>white</td><td>BACN33B1.1|CG2759|DMWHITE|DmelCG2759|EG:BACN33B1.1|c23|e(g)|m(g)|mw|w(AT)[[13]]</td><td>0</td><td>0</td></tr>
<tr><td>253</td><td>31272</td><td>Drosophila melanogaster</td><td>CG12498</td><td>CG12498 gene product from transcript CG12498-RA</td><td>DmelCG12498|EG:BACR43E12.1</td><td>0</td><td>0</td></tr>
<tr><td>254</td><td>31273</td><td>Drosophila melanogaster</td><td>CG14416</td><td>CG14416 gene product from transcript CG14416-RA</td><td>DmelCG14416|EG:BACR43E12.7</td><td>0</td><td>0</td></tr>
<tr><td>255</td><td>31274</td><td>Drosophila melanogaster</td><td>CG14417</td><td>CG14417 gene product from transcript CG14417-RA</td><td>DmelCG14417|EG:BACR43E12.6</td><td>0</td><td>0</td></tr>
<tr><td>256</td><td>31275</td><td>Drosophila melanogaster</td><td>CG14418</td><td>CG14418 gene product from transcript CG14418-RA</td><td>DmelCG14418|EG:BACR43E12.5</td><td>0</td><td>0</td></tr>
<tr><td>257</td><td>31276</td><td>Drosophila melanogaster</td><td>CG14419</td><td>CG14419 gene product from transcript CG14419-RA</td><td>DmelCG14419</td><td>0</td><td>0</td></tr>
<tr><td>258</td><td>31277</td><td>Drosophila melanogaster</td><td>CG3526</td><td>CG3526 gene product from transcript CG3526-RC</td><td>DmelCG3526|EG:BACR43E12.4</td><td>0</td><td>0</td></tr>
<tr><td>259</td><td>31278</td><td>Drosophila melanogaster</td><td>CG3588</td><td>CG3588 gene product from transcript CG3588-RH</td><td>DmelCG3588|EG:100G7.6</td><td>0</td><td>0</td></tr>
<tr><td>260</td><td>31279</td><td>Drosophila melanogaster</td><td>CG14424</td><td>CG14424 gene product from transcript CG14424-RA</td><td>DmelCG14424|EG:100G7.5</td><td>0</td><td>0</td></tr>
<tr><td>261</td><td>31280</td><td>Drosophila melanogaster</td><td>CG32793</td><td>CG32793 gene product from transcript CG32793-RA</td><td>CG18089|CG18852|CG3591|DmelCG32793|EG:100G7.1|EG:100G7.2|anon-3Ca|anon-3Cb</td><td>0</td><td>0</td></tr>
<tr><td>262</td><td>31282</td><td>Drosophila melanogaster</td><td>CG3592</td><td>CG3592 gene product from transcript CG3592-RA</td><td>DmelCG3592|frag7</td><td>0</td><td>0</td></tr>
<tr><td>263</td><td>31283</td><td>Drosophila melanogaster</td><td>CG3598</td><td>CG3598 gene product from transcript CG3598-RA</td><td>DmelCG3598|EG:100G7.3</td><td>0</td><td>0</td></tr>
<tr><td>264</td><td>31284</td><td>Drosophila melanogaster</td><td>CG14420</td><td>CG14420 gene product from transcript CG14420-RA</td><td>DmelCG14420</td><td>0</td><td>0</td></tr>
<tr><td>265</td><td>31285</td><td>Drosophila melanogaster</td><td>CG14421</td><td>CG14421 gene product from transcript CG14421-RA</td><td>DmelCG14421</td><td>0</td><td>0</td></tr>
<tr><td>266</td><td>31286</td><td>Drosophila melanogaster</td><td>CG14422</td><td>CG14422 gene product from transcript CG14422-RA</td><td>DmelCG14422</td><td>0</td><td>0</td></tr>
<tr><td>267</td><td>31287</td><td>Drosophila melanogaster</td><td>CG14423</td><td>CG14423 gene product from transcript CG14423-RA</td><td>DmelCG14423</td><td>0</td><td>0</td></tr>
<tr><td>268</td><td>31288</td><td>Drosophila melanogaster</td><td>CG17959</td><td>CG17959 gene product from transcript CG17959-RA</td><td>DmelCG17959</td><td>0</td><td>0</td></tr>
<tr><td>269</td><td>31289</td><td>Drosophila melanogaster</td><td>CG3603</td><td>CG3603 gene product from transcript CG3603-RA</td><td>BcDNA:RH09070|DmelCG3603</td><td>0</td><td>0</td></tr>
<tr><td>270</td><td>31290</td><td>Drosophila melanogaster</td><td>rst</td><td>roughest</td><td>CG4125|CT13684|DmelCG4125|IREE-C|IRREC/RST|Irre-C|IrreC|IrreC-Rst|IrreC-rst|IrreC/Rst|IrrecC|Rst|UB883|irreC|irreC-rst|irrecC-rst|rst-irreC|rst-irrecC|rst/irrecC</td><td>0</td><td>0</td></tr>
<tr><td>271</td><td>31291</td><td>Drosophila melanogaster</td><td>CG4116</td><td>CG4116 gene product from transcript CG4116-RA</td><td>DmelCG4116</td><td>0</td><td>0</td></tr>
<tr><td>272</td><td>31292</td><td>Drosophila melanogaster</td><td>kirre</td><td>kin of irre</td><td>CG3653|CT12279|DUF|DUF/KIRRE|DmelCG3653|Duf|Duf/Kirre|EG:163A10.1|Kirre|duf|duf/kirre</td><td>0</td><td>0</td></tr>
<tr><td>273</td><td>31293</td><td>Drosophila melanogaster</td><td>N</td><td>Notch</td><td>1.1|16-178|16-55|Ax|CG3936|CT13012|Chp|Co|DmelCG3936|EG:140G11.1|EG:163A10.2|anon-EST:Liang-1.12|clone 1.12|co|dNotch|fa|l(1)3Cb|l(1)Ax|l(1)N|n[fah]|nd|notch|shd|spl|swb</td><td>0</td><td>0</td></tr>
<tr><td>274</td><td>31294</td><td>Drosophila melanogaster</td><td>Fcp3C</td><td>Follicle cell protein 3C</td><td>CG4015|DmelCG4015|EG:140G11.2|Ecp3C|VMP3C</td><td>0</td><td>0</td></tr>
<tr><td>275</td><td>31295</td><td>Drosophila melanogaster</td><td>CG3939</td><td>CG3939 gene product from transcript CG3939-RA</td><td>BEST:SD07039|DmelCG3939|EG:140G11.5</td><td>0</td><td>0</td></tr>
<tr><td>276</td><td>31297</td><td>Drosophila melanogaster</td><td>CG14265</td><td>CG14265 gene product from transcript CG14265-RB</td><td>BcDNA:RE43371|DmelCG14265|EG:96G10.8</td><td>0</td><td>0</td></tr>
<tr><td>277</td><td>31298</td><td>Drosophila melanogaster</td><td>ng2</td><td>new glue 2</td><td>BcDNA:RE05239|CG14266|DmelCG14266|EG:96G10.4|ng|ng-2</td><td>0</td><td>0</td></tr>
<tr><td>278</td><td>31299</td><td>Drosophila melanogaster</td><td>ng3</td><td>new glue 3</td><td>BcDNA:LP03924|CG10788|DmelCG10788|EG:96G10.3|ng|ng-3</td><td>0</td><td>0</td></tr>
<tr><td>279</td><td>31300</td><td>Drosophila melanogaster</td><td>ng1</td><td>new glue 1</td><td>CG10781|DmelCG10781|EG:96G10.5|SC1|ng|ng-1|ngp</td><td>0</td><td>0</td></tr>
<tr><td>280</td><td>31301</td><td>Drosophila melanogaster</td><td>ng4</td><td>new glue 4</td><td>CG10789|DmelCG10789|EG:96G10.2|ng|ng-4</td><td>0</td><td>0</td></tr>
<tr><td>281</td><td>31303</td><td>Drosophila melanogaster</td><td>Pig1</td><td>Pre-intermoult gene 1</td><td>CG10790|DmelCG10790|EG:96G10.1|Pig-1|gsg|pig1</td><td>0</td><td>0</td></tr>
<tr><td>282</td><td>31304</td><td>Drosophila melanogaster</td><td>Sgs4</td><td>Salivary gland secretion 4</td><td>CG12181|DmelCG12181|EG:96G10.6|SGS-4|SGS4|Sgs|Sgs-4|sgs-4|sgs4</td><td>0</td><td>0</td></tr>
<tr><td>283</td><td>31308</td><td>Drosophila melanogaster</td><td>CG10793</td><td>CG10793 gene product from transcript CG10793-RA</td><td>DmelCG10793</td><td>0</td><td>0</td></tr>
<tr><td>284</td><td>31309</td><td>Drosophila melanogaster</td><td>dnc</td><td>dunce</td><td>CG10791|CG10792|CG10797|CG14267|CG14268|CG32498|DmelCG32498|EG:140G11.4|EG:96G10.7|EG:BACN05I09.3|EG:BACN5I9.2|PDE4|PDEII|fs(1)M42</td><td>0</td><td>0</td></tr>
<tr><td>285</td><td>31310</td><td>Drosophila melanogaster</td><td>dm</td><td>diminutive</td><td>CG10798|D-Myc|DMYc|Dm|DmelCG10798|Dmyc|EG:BACN5I9.1|MYC|Myc|anon-WO03040301.171|bHLHe57|c-MYC|c-Myc|c-myc|d-myc|dMYC|dMyc|dMyc1|dm/dMyc|dm/myc|dmyc|dmyc1|l(1)G0139|l(</td><td>0</td><td>0</td></tr>
<tr><td>286</td><td>31311</td><td>Drosophila melanogaster</td><td>CG12535</td><td>CG12535 gene product from transcript CG12535-RB</td><td>DmelCG12535</td><td>0</td><td>0</td></tr>
<tr><td>287</td><td>31312</td><td>Drosophila melanogaster</td><td>CG14269</td><td>CG14269 gene product from transcript CG14269-RA</td><td>DmelCG14269</td><td>0</td><td>0</td></tr>
<tr><td>288</td><td>31313</td><td>Drosophila melanogaster</td><td>CG16782</td><td>CG16782 gene product from transcript CG16782-RA</td><td>DmelCG16782</td><td>0</td><td>0</td></tr>
<tr><td>289</td><td>31314</td><td>Drosophila melanogaster</td><td>CG10801</td><td>CG10801 gene product from transcript CG10801-RA</td><td>DmelCG10801</td><td>0</td><td>0</td></tr>
<tr><td>290</td><td>31315</td><td>Drosophila melanogaster</td><td>CG16781</td><td>CG16781 gene product from transcript CG16781-RA</td><td>DmelCG16781</td><td>0</td><td>0</td></tr>
<tr><td>291</td><td>31316</td><td>Drosophila melanogaster</td><td>CG12206</td><td>CG12206 gene product from transcript CG12206-RA</td><td>DmelCG12206</td><td>0</td><td>0</td></tr>
<tr><td>292</td><td>31317</td><td>Drosophila melanogaster</td><td>CG10804</td><td>CG10804 gene product from transcript CG10804-RB</td><td>DmelCG10804</td><td>0</td><td>0</td></tr>
<tr><td>293</td><td>31318</td><td>Drosophila melanogaster</td><td>CG10802</td><td>CG10802 gene product from transcript CG10802-RA</td><td>DmelCG10802|anon-EST:Posey113</td><td>0</td><td>0</td></tr>
<tr><td>294</td><td>31319</td><td>Drosophila melanogaster</td><td>CG14270</td><td>CG14270 gene product from transcript CG14270-RA</td><td>DmelCG14270</td><td>0</td><td>0</td></tr>
<tr><td>295</td><td>31320</td><td>Drosophila melanogaster</td><td>CG10803</td><td>CG10803 gene product from transcript CG10803-RA</td><td>DmelCG10803</td><td>0</td><td>0</td></tr>
<tr><td>296</td><td>31321</td><td>Drosophila melanogaster</td><td>Gas8</td><td>Growth arrest specific protein 8</td><td>CG14271|DmelCG14271</td><td>0</td><td>0</td></tr>
<tr><td>297</td><td>31322</td><td>Drosophila melanogaster</td><td>CG32791</td><td>CG32791 gene product from transcript CG32791-RA</td><td>CG13019|CG13020|DmelCG32791</td><td>0</td><td>0</td></tr>
<tr><td>298</td><td>31323</td><td>Drosophila melanogaster</td><td>CG13021</td><td>CG13021 gene product from transcript CG13021-RA</td><td>BcDNA:AT30881|DmelCG13021</td><td>0</td><td>0</td></tr>
<tr><td>299</td><td>31326</td><td>Drosophila melanogaster</td><td>CG2875</td><td>CG2875 gene product from transcript CG2875-RB</td><td>DmelCG2875</td><td>0</td><td>0</td></tr>
<tr><td>300</td><td>31328</td><td>Drosophila melanogaster</td><td>Ilp7</td><td>Insulin-like peptide 7</td><td>CG13317|DILP|DILP7|Dilp|Dilp 7|DmelCG13317|ILP7|Ilp|Ilp-7|dilp|dilp-7|dilp7</td><td>0</td><td>0</td></tr>
<tr><td>301</td><td>31329</td><td>Drosophila melanogaster</td><td>Parg</td><td>Poly(ADP-ribose) glycohydrolase</td><td>CG2864|DmelCG2864|EG:114E2.1|HD10914|PARG|parg</td><td>0</td><td>0</td></tr>
<tr><td>302</td><td>31331</td><td>Drosophila melanogaster</td><td>Mnt</td><td>CG13316 gene product from transcript CG13316-RD</td><td>CG13316|CG2856|DmMnt|DmelCG13316|EG:114E2.2|MNT|Mad|Mad-3E|bHLHe43|dMad|dMnt|dmnt|mnt</td><td>0</td><td>0</td></tr>
<tr><td>303</td><td>31332</td><td>Drosophila melanogaster</td><td>Rala</td><td>Ras-related protein</td><td>CG2849|D-RalA|DRal|DRala|Dm RalA|DmelCG2849|Dral|DralA|Drala|RALA|Ral|Ral-a|RalA|anon-EST:fe1H4|dRal|l(1)G0174|l(1)G0373|l(1)G0501|ral|ralA</td><td>0</td><td>0</td></tr>
<tr><td>304</td><td>31335</td><td>Drosophila melanogaster</td><td>HIP-R</td><td>Hsc/Hsp70-interacting protein related</td><td>CG2947|DmelCG2947|Hip|anon-EST:fe1A9</td><td>0</td><td>0</td></tr>
<tr><td>305</td><td>31336</td><td>Drosophila melanogaster</td><td>CG32779</td><td>CG32779 gene product from transcript CG32779-RA</td><td>CG15238|CG18771|DmelCG32779</td><td>0</td><td>0</td></tr>
<tr><td>306</td><td>31338</td><td>Drosophila melanogaster</td><td>CG2901</td><td>CG2901 gene product from transcript CG2901-RA</td><td>DmelCG2901</td><td>0</td><td>0</td></tr>
<tr><td>307</td><td>31339</td><td>Drosophila melanogaster</td><td>ec</td><td>echinus</td><td>CG2904|DmelCG2904|EG:EG0002.3</td><td>0</td><td>0</td></tr>
<tr><td>308</td><td>31340</td><td>Drosophila melanogaster</td><td>yin</td><td>CG2913 gene product from transcript CG2913-RA</td><td>CG2913|DmelCG2913|EG:EG0002.1|OPT1|OpT1|Yin|opt|opt1</td><td>0</td><td>0</td></tr>
<tr><td>309</td><td>31341</td><td>Drosophila melanogaster</td><td>CG2930</td><td>CG2930 gene product from transcript CG2930-RD</td><td>BcDNA:GH06717|CG32780|CG32787|DmelCG2930</td><td>0</td><td>0</td></tr>
<tr><td>310</td><td>31342</td><td>Drosophila melanogaster</td><td>VhaAC39-1</td><td>Vacuolar H[+] ATPase subunit AC39-1</td><td>CG2934|DmelCG2934|V-ATPase|VhaAC39|Vhaac39|anon-EST:GressD5|vhaAC39|vhaAC39-1</td><td>0</td><td>0</td></tr>
<tr><td>311</td><td>31343</td><td>Drosophila melanogaster</td><td>CG15239</td><td>CG15239 gene product from transcript CG15239-RA</td><td>DmelCG15239</td><td>0</td><td>0</td></tr>
<tr><td>312</td><td>31344</td><td>Drosophila melanogaster</td><td>CG42541</td><td>CG42541 gene product from transcript CG42541-RD</td><td>BcDNA:RH43809|CG15240|CG16828|CG16829|CG32776|DmelCG42541|Dmel_CG15240|Dmel_CG32776</td><td>0</td><td>0</td></tr>
<tr><td>313</td><td>31347</td><td>Drosophila melanogaster</td><td>CG2938</td><td>CG2938 gene product from transcript CG2938-RB</td><td>DmelCG2938</td><td>0</td><td>0</td></tr>
<tr><td>314</td><td>31348</td><td>Drosophila melanogaster</td><td>CG2941</td><td>CG2941 gene product from transcript CG2941-RA</td><td>DmelCG2941</td><td>0</td><td>0</td></tr>
<tr><td>315</td><td>31349</td><td>Drosophila melanogaster</td><td>Vap-33-1</td><td>CG5014 gene product from transcript CG5014-RB</td><td>BcDNA:GM03307|CG5014|DVAP|DVAP-33A|DVAP33A|DmelCG5014|VAP|VAP-33|VAP-33-1|VAP33-A|VAPB|Vap33-1|anon-EST:Liang-2.42|anon-WO03040301.124|clone 2.42|dVAP|dVAP33A|dvap33a|l(1)(</td><td>0</td><td>0</td></tr>
<tr><td>316</td><td>31350</td><td>Drosophila melanogaster</td><td>lva</td><td>lava lamp</td><td>ABP2|CG#6450|CG6450|DmelCG6450|LVA|Lava|Lva|Lvl|lava|lvl</td><td>0</td><td>0</td></tr>
<tr><td>317</td><td>31351</td><td>Drosophila melanogaster</td><td>CG6428</td><td>CG6428 gene product from transcript CG6428-RA</td><td>CG-6428|DmelCG6428</td><td>0</td><td>0</td></tr>
<tr><td>318</td><td>31352</td><td>Drosophila melanogaster</td><td>CG6414</td><td>CG6414 gene product from transcript CG6414-RA</td><td>DmelCG6414</td><td>0</td><td>0</td></tr>
<tr><td>319</td><td>31353</td><td>Drosophila melanogaster</td><td>CG43284</td><td>CG43284 gene product from transcript CG43284-RB</td><td>CG32778|CG32790|CG4985|DmelCG43284|Dmel_CG32778|Dmel_CG32790</td><td>0</td><td>0</td></tr>
<tr><td>320</td><td>31354</td><td>Drosophila melanogaster</td><td>CG15376</td><td>CG15376 gene product from transcript CG15376-RA</td><td>DmelCG15376</td><td>0</td><td>0</td></tr>
<tr><td>321</td><td>31355</td><td>Drosophila melanogaster</td><td>CG6379</td><td>CG6379 gene product from transcript CG6379-RA</td><td>DmelCG6379</td><td>0</td><td>0</td></tr>
<tr><td>322</td><td>31356</td><td>Drosophila melanogaster</td><td>CG15375</td><td>CG15375 gene product from transcript CG15375-RA</td><td>DmelCG15375</td><td>0</td><td>0</td></tr>
<tr><td>323</td><td>31357</td><td>Drosophila melanogaster</td><td>Xpac</td><td>Xeroderma pigmentosum group A-like</td><td>CG6358|DhR14|DhXPA|DmXPA|DmelCG6358|Dxpa|EG:EG0007.8|XPAC|XPA_DROME|Xpa|dmXPA|dxpa</td><td>0</td><td>0</td></tr>
<tr><td>324</td><td>31358</td><td>Drosophila melanogaster</td><td>brn</td><td>brainiac</td><td>CG4934|DmelCG4934|EG:EG0007.6|brc|fs(1)107|fs(1)A107|l(1)6P6</td><td>0</td><td>0</td></tr>
<tr><td>325</td><td>31359</td><td>Drosophila melanogaster</td><td>cib</td><td>ciboulot</td><td>CG4944|DmelCG4944|EG0007-11|EG:EG0007.11</td><td>0</td><td>0</td></tr>
<tr><td>326</td><td>31361</td><td>Drosophila melanogaster</td><td>dgt4</td><td>dim gamma-tubulin 4</td><td>BcDNA:SD26403|CG4865|Dgt4|DmelCG4865|EG0007.10|EG:EG0007.10|msd3</td><td>0</td><td>0</td></tr>
<tr><td>327</td><td>31362</td><td>Drosophila melanogaster</td><td>Tip60</td><td>CG6121 gene product from transcript CG6121-RA</td><td>6121|CG6121|DmEG0007.7|Dmel/TIP60|DmelCG6121|DmelTIP60|EG0007.7|EG:EG0007.7|TIP60|dTIP60|dTip60|dmHAG405</td><td>0</td><td>0</td></tr>
<tr><td>328</td><td>31363</td><td>Drosophila melanogaster</td><td>tyf</td><td>twenty-four</td><td>CG4857|DmelCG4857|EG:EG0007.12|EG:EG0007.4</td><td>0</td><td>0</td></tr>
<tr><td>329</td><td>31364</td><td>Drosophila melanogaster</td><td>Fas2</td><td>Fasciclin 2</td><td>1D4|Ab 1D4|CG3665|CT12301|DmelCG3665|EG:EG0007.3|FAS 11|FAS II|FAS2|FASII|Fas|Fas II|FasII|FascII|Fasii|MAB1D4|anon-EST:Liang-1.60|clone 1.60|fas II|fas-II|fas2|fasII|l(1)G0032|l(1)G0048|l(1)G008</td><td>0</td><td>0</td></tr>
<tr><td>330</td><td>31365</td><td>Drosophila melanogaster</td><td>CG15571</td><td>CG15571 gene product from transcript CG15571-RA</td><td>DmelCG15571</td><td>0</td><td>0</td></tr>
<tr><td>331</td><td>31366</td><td>Drosophila melanogaster</td><td>CG15570</td><td>CG15570 gene product from transcript CG15570-RA</td><td>DmelCG15570</td><td>0</td><td>0</td></tr>
</table>

332	31367	Drosophila melanogaster	Femcoat	CG15573 gene product from transcript CG15573-RB	CG15573|DmelCG15573	0	0
333	31368	Drosophila melanogaster	Muc4B	Mucin 4B	CG15574|CG15575|CG32774|DmelCG32774	0	0
334	31369	Drosophila melanogaster	CG15576	CG15576 gene product from transcript CG15576-RB	DmelCG15576	0	0
335	31370	Drosophila melanogaster	CG12691	CG12691 gene product from transcript CG12691-RA	DmelCG12691	0	0
336	31371	Drosophila melanogaster	CG15572	CG15572 gene product from transcript CG15572-RA	DmelCG15572	0	0
337	31372	Drosophila melanogaster	CG12692	CG12692 gene product from transcript CG12692-RB	DmelCG12692	0	0
338	31373	Drosophila melanogaster	mei-9	meiotic 9	CG3697|DhR1|DmXPF|DmelCG3697|MEI-9|Rad1p|mei9|mus(1)110|mus110|xpf/mei-9	0	0
339	31374	Drosophila melanogaster	CG2982	CG2982 gene product from transcript CG2982-RB	DmelCG2982	0	0
340	31375	Drosophila melanogaster	CG12693	CG12693 gene product from transcript CG12693-RB	DmelCG12693	0	0
341	31376	Drosophila melanogaster	norpA	no receptor potential A	CG3620|CdkA|DIP2|DmelCG3620|MRE18|NORPA|NorpA|PI-PLC|PLC|PLC-beta|PLCbeta|anon-EST:Posey221|nofC|rof|x12|x13|x16|x24	0	0
342	31377	Drosophila melanogaster	CG3626	CG3626 gene product from transcript CG3626-RA	DmelCG3626	0	0
343	31378	Drosophila melanogaster	CG12688	CG12688 gene product from transcript CG12688-RB	DmelCG12688	0	0
344	31379	Drosophila melanogaster	bi	bifid	Bl|CG3578|Dm-OMB|Dm-omb|DmelCG3578|OMB|Omb|Qd|T3|dm-omb|l(1)bi|l(1)omb|omb	0	0
345	31380	Drosophila melanogaster	CG3556	CG3556 gene product from transcript CG3556-RB	DmelCG3556	0	0
346	31381	Drosophila melanogaster	rb	ruby	AP-3beta|CG11427|DmelCG11427|Rb|beta3|cg11427	0	0
347	31382	Drosophila melanogaster	CHOp24	CG3564 gene product from transcript CG3564-RA	CG3564|DmelCG3564|Emp24|p24	0	0
348	31383	Drosophila melanogaster	CG3568	CG3568 gene product from transcript CG3568-RA	DmelCG3568	0	0
349	31384	Drosophila melanogaster	Usf	CG17592 gene product from transcript CG17592-RC	CG17592|Dm Usf|DmUsf|DmelCG17592|bHLHb13	0	0
350	31385	Drosophila melanogaster	CG15912	CG15912 gene product from transcript CG15912-RA	DmelCG15912	0	0
351	31386	Drosophila melanogaster	CG11436	CG11436 gene product from transcript CG11436-RA	DmelCG11436	0	0
352	31387	Drosophila melanogaster	CG3527	CG3527 gene product from transcript CG3527-RA	DmelCG3527	0	0
353	31388	Drosophila melanogaster	CG11444	CG11444 gene product from transcript CG11444-RA	DmelCG11444	0	0
354	31389	Drosophila melanogaster	CG3546	CG3546 gene product from transcript CG3546-RA	DmelCG3546	0	0
355	31390	Drosophila melanogaster	CG12684	CG12684 gene product from transcript CG12684-RA	DmelCG12684	0	0
356	31391	Drosophila melanogaster	peb	pebbled	CG12212|DmelCG12212|EG:66A1.1|EP55|HNT|Hind|Hnt|PEB|Peb|anon-4Cg|hnd|hnt	0	0
357	31392	Drosophila melanogaster	CG12184	CG12184 gene product from transcript CG12184-RA	DmelCG12184|EG:66A1.3|GH15731P	0	0
358	31393	Drosophila melanogaster	CG12179	CG12179 gene product from transcript CG12179-RD	DmelCG12179|EG:66A1.2	0	0
359	31394	Drosophila melanogaster	pon	partner of numb	CG3346|DmelCG3346|PON|Pon	0	0
360	31395	Drosophila melanogaster	CG3081	CG3081 gene product from transcript CG3081-RA	DmelCG3081|EG:84H4.4	0	0
361	31396	Drosophila melanogaster	CG3062	CG3062 gene product from transcript CG3062-RA	DmelCG3062|EG:84H4.5	0	0
362	31397	Drosophila melanogaster	HLH4C	Helix loop helix protein 4C	CG3052|DmelCG3052|DroNHLH|EG:84H4.2|bHLH36	0	0
363	31398	Drosophila melanogaster	mRpL30	mitochondrial ribosomal protein L30	CG7038|DmelCG7038|L30|mRPL30	0	0
364	31399	Drosophila melanogaster	torp4a	CG3024 gene product from transcript CG3024-RA	CG3024|DmTorp4a|DmelCG3024|EG:84H4.1|TORP4A|Torp4a	0	0
365	31401	Drosophila melanogaster	CG15473	CG15473 gene product from transcript CG15473-RA	DmelCG15473	0	0
366	31402	Drosophila melanogaster	CG3009	CG3009 gene product from transcript CG3009-RA	DmelCG3009	0	0
367	31404	Drosophila melanogaster	Pp2C1	Protein phosphatase 2C	CG2984|DmelCG2984|PP2C|PP2C1|anon-WO03040301.132|dpp2c1	0	0
368	31405	Drosophila melanogaster	ctp	cut up	CG6998|Cdlc1|Ctp|DLC8|Ddlc-1|Ddlc1|Dlc|DmelCG6998|LC8|anon-WO03040301.128|ddlc1|dlc|l(1)G0153|l(1)G0204|l(1)G0207|l(1)G0244|l(1)G0248|l(1)G0338|l(1)G0371|l(1)G0387|l(1)G0432|l(1)G0445	0	0
369	31406	Drosophila melanogaster	l(1)G0334	lethal (1) G0334	CG7010|DmelCG7010|L(1)g0334	0	0
370	31407	Drosophila melanogaster	CG7024	CG7024 gene product from transcript CG7024-RA	DmelCG7024	0	0
371	31408	Drosophila melanogaster	Proc-R	Proctolin receptor	CG6986|DmelCG6986|anon-WO0170980.73|anon-WO0170980.74	0	0
372	31409	Drosophila melanogaster	CG15472	CG15472 gene product from transcript CG15472-RA	DmelCG15472	0	0
373	31410	Drosophila melanogaster	Bteb2	CG2932 gene product from transcript CG2932-RA	BteB2|CG2932|DmelCG2932	0	0
374	31411	Drosophila melanogaster	CG2871	CG2871 gene product from transcript CG2871-RA	DmelCG2871	0	0
375	31412	Drosophila melanogaster	CG15471	CG15471 gene product from transcript CG15471-RB	DmelCG15471	0	0
376	31413	Drosophila melanogaster	CG6978	CG6978 gene product from transcript CG6978-RA	DmelCG6978	0	0
377	31414	Drosophila melanogaster	CG2861	CG2861 gene product from transcript CG2861-RA	DmelCG2861	0	0
378	31415	Drosophila melanogaster	CG12682	CG12682 gene product from transcript CG12682-RA	DmelCG12682	0	0
379	31416	Drosophila melanogaster	CG12681	CG12681 gene product from transcript CG12681-RA	DmelCG12681	0	0
380	31419	Drosophila melanogaster	CG6927	CG6927 gene product from transcript CG6927-RA	DmelCG6927	0	0
381	31420	Drosophila melanogaster	CG32772	CG32772 gene product from transcript CG32772-RC	CG15469|CG6911|DmelCG32772	0	0
382	31422	Drosophila melanogaster	CG4041	CG4041 gene product from transcript CG4041-RB	DmelCG4041	0	0
383	31423	Drosophila melanogaster	CG6903	CG6903 gene product from transcript CG6903-RA	DmelCG6903	0	0
384	31424	Drosophila melanogaster	CG4068	CG4068 gene product from transcript CG4068-RC	DmelCG4068|hp-CG4068B	0	0
385	31425	Drosophila melanogaster	Ptp4E	Protein tyrosine phosphatase 4E	CG6899|CT21187|DPTP4E|DmelCG6899|PTP4E|PtP4E|R-PTP 4E	0	0
386	31426	Drosophila melanogaster	SIP3	Syntaxin Interacting Protein 3	CG15468|DmelCG15468	0	0
387	31427	Drosophila melanogaster	CG12680	CG12680 gene product from transcript CG12680-RB	DmelCG12680	0	0
388	31429	Drosophila melanogaster	ovo	CG6824 gene product from transcript CG6824-RE	CG15467|CG6824|DmelCG6824|Fs(1)K1103|Fs(1)K1237|Fs(1)K155|OVO|Ovo|Ovo-D|Shv|Svb|Svb/Ovo|fs(1)K1237|fs(1)M1|fs(1)M38|ovo/shavenbaby|ovo/svb|ovoD|svb	0	0
389	31430	Drosophila melanogaster	CG32767	CG32767 gene product from transcript CG32767-RD	BcDNA:GM08530|CG5086|CG5113|DmelCG32767|LD34931|i7	0	0
390	31433	Drosophila melanogaster	CG6789	CG6789 gene product from transcript CG6789-RA	DmelCG6789	0	0
391	31434	Drosophila melanogaster	CG15465	CG15465 gene product from transcript CG15465-RA	DmelCG15465	0	0
392	31435	Drosophila melanogaster	CG5062	CG5062 gene product from transcript CG5062-RA	BcDNA:AT13663|DmelCG5062|FBgn0029747	0	0
393	31436	Drosophila melanogaster	CG15464	CG15464 gene product from transcript CG15464-RB	DmelCG15464	0	0
394	31438	Drosophila melanogaster	CG42749	CG42749 gene product from transcript CG42749-RB	CG15785|CG15786|DmelCG42749|Dmel_CG15785|Dmel_CG15786	0	0
395	31439	Drosophila melanogaster	CG3323	CG3323 gene product from transcript CG3323-RA	DmelCG3323	0	0
396	31440	Drosophila melanogaster	CG17764	CG17764 gene product from transcript CG17764-RA	DmelCG17764	0	0
397	31441	Drosophila melanogaster	Cdk7	Cyclin-dependent kinase 7	CD 7|CDK7|CG3319|DmCDK7|DmCdk7|DmMO15|DmMo15|Dmcdk7|DmelCG3319|Mo15|cdk-7|cdk7|dCdk7|p40[MO15]	0	0
398	31442	Drosophila melanogaster	snf	sans fille	CG4528|D25|DmelCG4528|SNF|SNF/D25|Snf|U1-A|U2 snRNP|U2 snRNP B'' protein|fs(1)1621|fs(1)A16121|fs(1)A1621|liz|snRNP B''|snf1621	0	0
399	31443	Drosophila melanogaster	TrxT	Thioredoxin T	3315|CG3315|DmTrxT|DmelTrxT|DmelCG3315	0	0
400	31444	Drosophila melanogaster	dhd	deadhead	4193|CG4193|DmTrx-1|Dmdhd|DmelCG4193|Dmeldhd|Trx-1	0	0
401	31445	Drosophila melanogaster	CG4198	CG4198 gene product from transcript CG4198-RA	DmelCG4198	0	0
402	31446	Drosophila melanogaster	CG15930	CG15930 gene product from transcript CG15930-RB	DmelCG15930	0	0
403	31447	Drosophila melanogaster	Sas10	CG4202 gene product from transcript CG4202-RA	CG4202|DmelCG4202|dSAS10|l(1)G0106|sas-10|sas10	0	0
404	31448	Drosophila melanogaster	Rnp4F	RNA-binding protein 4F	4F-rnp|4f-rnp|CG3312|DmelCG3312|RNP-4F|rnp-4f	0	0
405	31449	Drosophila melanogaster	Mcm3	Minichromosome maintenance 3	CG4206|DmMCM3|DmMcm3|DmeMCM3|DmelCG4206|MCM3|McM3|Mcp-PCR2|PCR2|dMCM3	0	0
406	31450	Drosophila melanogaster	CG3309	CG3309 gene product from transcript CG3309-RA	BEST:LD39762|DmelCG3309	0	0
407	31451	Drosophila melanogaster	XRCC1	CG4208 gene product from transcript CG4208-RA	4208|CG4208|DmelCG4208|Xrcc1	0	0
408	31456	Drosophila melanogaster	SK	small conductance calcium-activated potassium chan	BcDNA:GM01744|CG10706|CG12733|CG4179|DmelCG10706|dSK	0	0
409	31457	Drosophila melanogaster	NAAT1	Nutrient Amino Acid Transporter 1	CG3252|DmNAT1|DmelCG3252|anon-WO0149848.3|dmNAT1	0	0
410	31458	Drosophila melanogaster	CG4165	CG4165 gene product from transcript CG4165-RD	DmelCG4165	0	0
411	31459	Drosophila melanogaster	yu	CG3249 gene product from transcript CG3249-RC	BcDNA:GM04319|CG3249|DmelCG3249|PKAAP	0	0
412	31460	Drosophila melanogaster	CG16756	CG16756 gene product from transcript CG16756-RA	BcDNA:RH17440|DmelCG16756	0	0
413	31461	Drosophila melanogaster	CG15784	CG15784 gene product from transcript CG15784-RA	DmelCG15784	0	0
414	31463	Drosophila melanogaster	SPR	Sex peptide receptor	CG12731|CG16752|DmelCG16752	0	0
415	31464	Drosophila melanogaster	CG3239	CG3239 gene product from transcript CG3239-RB	BEST:GH09377|DmelCG3239	0	0
416	31465	Drosophila melanogaster	CG4151	CG4151 gene product from transcript CG4151-RA	DmelCG4151	0	0
417	31466	Drosophila melanogaster	CG12730	CG12730 gene product from transcript CG12730-RA	BcDNA:RE01432|DmelCG12730	0	0
418	31469	Drosophila melanogaster	Vsx2	Visual system homeobox 2 ortholog	CG15781|CG15782|CG15783|CG33980|Chx2|DChx2|DmelCG33980|dChx2|dVsx2	0	0
419	31470	Drosophila melanogaster	Vsx1	Visual system homeobox 1 ortholog	CG4136|Chx1|DChx1|Dchx1|DmelCG4136|dChx1|dVsx1	0	0
420	31471	Drosophila melanogaster	CG34435	CG34435 gene product from transcript CG34435-RA	CG15780|CG17763|DmelCG34435|Dmel_CG15780|Dmel_CG17763	0	0
421	31472	Drosophila melanogaster	CG34434	CG34434 gene product from transcript CG34434-RB	CG15780|CG17763|DmelCG34434|Dmel_CG15780|Dmel_CG17763	0	0
422	31473	Drosophila melanogaster	RhoGAP5A	CG3208 gene product from transcript CG3208-RB	CG3208|DmelCG3208	0	0
423	31474	Drosophila melanogaster	Mlc-c	Myosin light chain cytoplasmic	CG3201|DmMLC_C|DmelCG3201|MyoII|mlc-c|nmELC	0	0
424	31475	Drosophila melanogaster	l(1)G0045	lethal (1) G0045	BcDNA:LD21623|CG32763|CG4129|DmelCG32763|l(1)G0312	0	0
425	31480	Drosophila melanogaster	Sirt4	CG3187 gene product from transcript CG3187-RC	CG3187|D.mel3|DmelCG3187|dSIRT4|dmSRT407	0	0
426	31481	Drosophila melanogaster	CG4119	CG4119 gene product from transcript CG4119-RA	BcDNA:LD23634|DmelCG4119|cg4119	0	0
427	31483	Drosophila melanogaster	RpL35	Ribosomal protein L35	BcDNA:RE09547|CG4111|DmelCG4111|M(1)5A|RpL35e|anon-EST:Posey157|anon-EST:fe1C12	0	0
428	31486	Drosophila melanogaster	CG42449	CG42449 gene product from transcript CG42449-RB	CG15778|DmelCG42449|Dmel_CG15778	0	0
429	31487	Drosophila melanogaster	CG3160	CG3160 gene product from transcript CG3160-RA	DmelCG3160	0	0
430	31489	Drosophila melanogaster	CG3149	CG3149 gene product from transcript CG3149-RA	BcDNA:GH08388|DmelCG3149	0	0
431	31490	Drosophila melanogaster	CG4096	CG4096 gene product from transcript CG4096-RB	CT13580|DmelCG4096	0	0
432	31491	Drosophila melanogaster	CG33080	CG33080 gene product from transcript CG33080-RA	CG15774|CG15775|CG3141|DmelCG33080	0	0
433	31493	Drosophila melanogaster	CG32758	CG32758 gene product from transcript CG32758-RA	CG11465|CG3130|CG3138|DmelCG32758	0	0
434	31494	Drosophila melanogaster	CG15773	CG15773 gene product from transcript CG15773-RB	DmelCG15773	0	0
435	31496	Drosophila melanogaster	IntS6	Integrator 6	CG3125|DmelCG3125|Int6	0	0
436	31497	Drosophila melanogaster	CG4078	CG4078 gene product from transcript CG4078-RA	DmelCG4078	0	0
437	31498	Drosophila melanogaster	CG15772	CG15772 gene product from transcript CG15772-RA	DmelCG15772	0	0
438	31499	Drosophila melanogaster	lin-52	CG15929 gene product from transcript CG15929-RA	CG15929|DmelCG15929|Lin-52|Lin52|dLIN-52|dLin-52|dLin52|dlin-52	0	0
439	31500	Drosophila melanogaster	CG15771	CG15771 gene product from transcript CG15771-RA	CR33074|DmelCG15771	0	0
440	31503	Drosophila melanogaster	CG3097	CG3097 gene product from transcript CG3097-RA	DmelCG3097	0	0
441	31504	Drosophila melanogaster	CG42264	CG42264 gene product from transcript CG42264-RB	CG15768|CG15769|DmelCG42264|Dmel_CG15768|Dmel_CG15769	0	0
442	31506	Drosophila melanogaster	CG3108	CG3108 gene product from transcript CG3108-RA	DmelCG3108	0	0

443	31507	Drosophila melanogaster	CG42699	CG42699 gene product from transcript CG42699-RC	CG15770|CG4064|DmelCG42699|Dmel_CG15770|Dmel_CG4064	0	0
444	31508	Drosophila melanogaster	CG15767	CG15767 gene product from transcript CG15767-RA	DmelCG15767	0	0
445	31509	Drosophila melanogaster	CG12239	CG12239 gene product from transcript CG12239-RA	DmelCG12239	0	0
446	31510	Drosophila melanogaster	Cpr5C	Cuticular protein 5C	CG4052|DmelCG4052	0	0
447	31511	Drosophila melanogaster	Prosbeta2R1	Proteasome beta2R1 subunit	CG18341|DmelCG18341	0	0
448	31512	Drosophila melanogaster	CG15766	CG15766 gene product from transcript CG15766-RA	DmelCG15766	0	0
449	31513	Drosophila melanogaster	CG15765	CG15765 gene product from transcript CG15765-RA	DmelCG15765	0	0
450	31515	Drosophila melanogaster	CG12729	CG12729 gene product from transcript CG12729-RA	DmelCG12729	0	0
451	31516	Drosophila melanogaster	CG15764	CG15764 gene product from transcript CG15764-RA	BcDNA:AT16792|DmelCG15764	0	0
452	31517	Drosophila melanogaster	CG3033	CG3033 gene product from transcript CG3033-RA	DmelCG3033	0	0
453	31518	Drosophila melanogaster	mof	males absent on the first	CG3025|DmelCG3025|MOF|Mof|dMOF|dmMOF|max	0	0
454	31519	Drosophila melanogaster	CG3016	CG3016 gene product from transcript CG3016-RA	DmelCG3016	0	0
455	31520	Drosophila melanogaster	CG16721	CG16721 gene product from transcript CG16721-RA	DmelCG16721	0	0
456	31521	Drosophila melanogaster	Act5C	Actin 5C	A|ACT5C|Ac5C|Act|Act-5C|Act5|Act5c|Actin|Actin/BAP47|Actin5C|BAP47|Bap47|CG4027|DmelCG4027|M32055|T11|act|act 5C|act5C|actin|actin5C|anon-EST:fe2D2|beta-actin|beta-actin/Bap47|cyt5C|l(1)	0	0
457	31522	Drosophila melanogaster	CG4020	CG4020 gene product from transcript CG4020-RA	DmelCG4020	0	0
458	31523	Drosophila melanogaster	CG12236	CG12236 gene product from transcript CG12236-RA	DmelCG12236	0	0
459	31524	Drosophila melanogaster	CG3011	CG3011 gene product from transcript CG3011-RA	144561_at|DmelCG3011|anon-WO03040301.100	0	0
460	31525	Drosophila melanogaster	CG3726	CG3726 gene product from transcript CG3726-RA	DmelCG3726	0	0
461	31526	Drosophila melanogaster	CG12728	CG12728 gene product from transcript CG12728-RA	DmelCG12728	0	0
462	31527	Drosophila melanogaster	CG6041	CG6041 gene product from transcript CG6041-RA	DmelCG6041|SP86	0	0
463	31528	Drosophila melanogaster	CG6048	CG6048 gene product from transcript CG6048-RA	DmelCG6048|SP108	0	0
464	31529	Drosophila melanogaster	CG6067	CG6067 gene product from transcript CG6067-RA	DmelCG6067	0	0
465	31532	Drosophila melanogaster	CG5966	CG5966 gene product from transcript CG5966-RA	141233_at|DmelCG5966	0	0
466	31533	Drosophila melanogaster	CG4766	CG4766 gene product from transcript CG4766-RA	BcDNA:GH11415|DmelCG4766|gh11415	0	0
467	31535	Drosophila melanogaster	rux	roughex	CG4336|DmelCG4336|Rux	0	0
468	31536	Drosophila melanogaster	CG5941	CG5941 gene product from transcript CG5941-RA	DmelCG5941|anon-WO03040301.150|anon-WO03040301.151	0	0
469	31537	Drosophila melanogaster	CG5937	CG5937 gene product from transcript CG5937-RD	DmelCG5937	0	0
470	31538	Drosophila melanogaster	CG5921	CG5921 gene product from transcript CG5921-RD	DmelCG5921	0	0
471	31539	Drosophila melanogaster	CG5928	CG5928 gene product from transcript CG5928-RB	DmelCG5928	0	0
472	31540	Drosophila melanogaster	Tsp5D	Tetraspanin 5D	CG4690|Dm.Tsp5D|DmelCG4690	0	0
473	31541	Drosophila melanogaster	CG4666	CG4666 gene product from transcript CG4666-RA	DmelCG4666	0	0
474	31542	Drosophila melanogaster	CG4660	CG4660 gene product from transcript CG4660-RA	BcDNA:RH58440|DmelCG4660	0	0
475	31543	Drosophila melanogaster	raptor	CG4320 gene product from transcript CG4320-RA	4320|CG4320|DmelCG4320|Raptor|dRap|dRaptor|raptor/CG4320	0	0
476	31544	Drosophila melanogaster	Mipp2	Multiple inositol polyphosphate phosphatase 2	CG4317|DmelCG4317|l(1)G0050|l(1)G0303	0	0
477	31547	Drosophila melanogaster	Nep1	Neprilysin 1	CG5894|CG5905|CG5914|DmeNEP1|DmelCG5905|NEP|NEP1|dNEP1	0	0
478	31550	Drosophila melanogaster	Ca-alpha1T	CG15899 gene product from transcript CG15899-RE	CG12544|CG15899|CG4222|Caalpha1T|DmelCG15899	0	0
479	31551	Drosophila melanogaster	vanin-like	CG32754 gene product from transcript CG32754-RA	CG32754|CG3648|DmelCG32754|Vanin-like	0	0
480	31552	Drosophila melanogaster	Btd	Biotinidase	BTD|CG3599|CT12113|DmelCG3599	0	0
481	31553	Drosophila melanogaster	Efr	ER GDP-fucose transporter	CG3774|DmelCG3774	0	0
482	31554	Drosophila melanogaster	sqh	spaghetti squash	CG3595|DmMRLC_C|DmelCG3595|MLC|MLRC|MRLC|Myo|Myo II|MyoII|NMM|RLC|RMLC|Sqh|Squ|myoII|sgh|spg|sqh/RLC|squ|ssq	0	0
483	31555	Drosophila melanogaster	CG14446	CG14446 gene product from transcript CG14446-RC	DmelCG14446|MRE2	0	0
484	31556	Drosophila melanogaster	CG14445	CG14445 gene product from transcript CG14445-RA	DmelCG14445	0	0
485	31557	Drosophila melanogaster	Rbcn-3A	Rabconnectin-3A	CG3585|DmX|DmelCG3585|Dmx|FV10|X|anon-X	0	0
486	31558	Drosophila melanogaster	Spx	Spliceosomal protein on the X	CG3780|DmelCG3780|SPX|spx	0	0
487	31560	Drosophila melanogaster	CG3781	CG3781 gene product from transcript CG3781-RA	DmelCG3781	0	0
488	31562	Drosophila melanogaster	CG3566	CG3566 gene product from transcript CG3566-RB	BcDNA:RH45308|DmelCG3566	0	0
489	31565	Drosophila melanogaster	Top3beta	Topoisomerase 3beta	CG3458|DmelCG3458|TOP3|Top3|Topo IIIbeta|top3[beta]|top3beta|topo III[beta]	0	0
490	31566	Drosophila melanogaster	wuho	CG15897 gene product from transcript CG15897-RA	CG15897|DmelCG15897|wh	0	0
491	31567	Drosophila melanogaster	Rpt4	CG3455 gene product from transcript CG3455-RB	CG3455|Dm_Rpt4a|Dm_Rpt4b|DmelCG3455|Dmp42D|l(1)G0114|l(1)G0227|l(1)G0345|p42D	0	0
492	31568	Drosophila melanogaster	CG15896	CG15896 gene product from transcript CG15896-RB	DmelCG15896	0	0
493	31571	Drosophila melanogaster	CG3815	CG3815 gene product from transcript CG3815-RA	DmelCG3815|PF1|Pf1	0	0
494	31572	Drosophila melanogaster	CG12219	CG12219 gene product from transcript CG12219-RA	BEST:LD26135|DmelCG12219	0	0
495	31573	Drosophila melanogaster	CG3823	CG3823 gene product from transcript CG3823-RA	DmelCG3823	0	0
496	31574	Drosophila melanogaster	CG15894	CG15894 gene product from transcript CG15894-RB	DmelCG15894	0	0
497	31576	Drosophila melanogaster	CG3842	CG3842 gene product from transcript CG3842-RA	DmelCG3842	0	0
498	31577	Drosophila melanogaster	CG3847	CG3847 gene product from transcript CG3847-RA	DmelCG3847	0	0
499	31578	Drosophila melanogaster	CG3446	CG3446 gene product from transcript CG3446-RA	DmelCG3446	0	0
500	31579	Drosophila melanogaster	kdn	knockdown	BEST:GM01832|CG3861|CS|Csyn|DmelCG3861|l(1)G0030|l(1)G0033|l(1)G0159	0	0
501	31580	Drosophila melanogaster	swa	swallow	CG3429|DmelCG3429|Swa|anon-WO0140519.70|fs(1)1497|fs(1)1502|fs(1)M11-999|fs(1)M13C82|fs(1)M44|fs(1)m44|sww	0	0
502	31581	Drosophila melanogaster	Marf	Mitochondrial assembly regulatory factor	CG3869|DmelCG3869|MARF|Mfn|anon-WO0125274.3|dMfn|dmfn|marf|mfn|mfn2	0	0
503	31582	Drosophila melanogaster	PpV	Protein phosphatase V	CG12217|DmPpV-6A|DmelCG12217|PPPV6A|PPV|PPV 6A	0	0
504	31585	Drosophila melanogaster	CG3918	CG3918 gene product from transcript CG3918-RA	DmelCG3918	0	0
505	31586	Drosophila melanogaster	CG3342	CG3342 gene product from transcript CG3342-RA	DmelCG3342	0	0
506	31587	Drosophila melanogaster	Spat	Serine pyruvate aminotransferase	CG3926|Dm-Spat|DmelCG3926|spa|spat	0	0
507	31588	Drosophila melanogaster	RpL7A	Ribosomal protein L7A	CG3314|DmelCG3314|L7A|L7a|M(1)5D6A|Surf-3/rpL7a|Surf3/rpL7a	0	0
508	31589	Drosophila melanogaster	dx	deltex	CG3929|DMDELTEX|DmelCG3929|Dx	0	0
509	31591	Drosophila melanogaster	CG34417	CG34417 gene product from transcript CG34417-RI	BcDNA:GH23906|CG3950|CG3960|DmelCG34417|Dmel_CG3950|Dmel_CG3960	0	0
510	31592	Drosophila melanogaster	CG17717	CG17717 gene product from transcript CG17717-RA	BcDNA:AT19932|DmelCG17717	0	0
511	31593	Drosophila melanogaster	Pat1	Protein interacting with APP tail-1	CG10695|DmelCG10695|PAT1	0	0
512	31594	Drosophila melanogaster	Apc7	Anaphase promoting complex 7	APC7|CG14444|DmelCG14444	0	0
513	31595	Drosophila melanogaster	CG14443	CG14443 gene product from transcript CG14443-RB	DmelCG14443|cg14443	0	0
514	31596	Drosophila melanogaster	pigs	pickled eggs	CG3973|DmelCG3973	0	0
515	31597	Drosophila melanogaster	CG3226	CG3226 gene product from transcript CG3226-RA	DmelCG3226|Q9W3Y3	0	0
516	31598	Drosophila melanogaster	l(1)G0148	lethal (1) G0148	CG32742|CG8655|CG8656|CT25076|DmelCG32742|anon-WO03040301.250|cdc7-related|l(1)G0149|l(1)G0461	0	0
517	31600	Drosophila melanogaster	CG3224	CG3224 gene product from transcript CG3224-RA	DmelCG3224	0	0
518	31601	Drosophila melanogaster	Ctr1A	Copper transporter 1A	BcDNA:RH42635|CG3977|DmCtr1A|DmelCG3977|ctr1A	0	0
519	31602	Drosophila melanogaster	CG3198	CG3198 gene product from transcript CG3198-RA	DmelCG3198	0	0
520	31603	Drosophila melanogaster	Mcm6	Minichromosome maintenance 6	CG4039|DmMCM6|DmeMCM6|DmelCG4039|MCM6|McM6|fs(1)K1214|mcm6	0	0
521	31604	Drosophila melanogaster	CG3192	CG3192 gene product from transcript CG3192-RA	DmelCG3192	0	0
522	31605	Drosophila melanogaster	l(1)G0255	lethal (1) G0255	CG4094|DmelCG4094|FBgn0029889|l(1)G0290	0	0
523	31606	Drosophila melanogaster	CG4095	CG4095 gene product from transcript CG4095-RA	DmelCG4095	0	0
524	31607	Drosophila melanogaster	Pink1	PTEN-induced putative kinase 1	BEST:GH23468|CG4523|DmelCG4523|PINK|PINK1|dPINK1|dPink1|pink1	0	0
525	31608	Drosophila melanogaster	CG3184	CG3184 gene product from transcript CG3184-RA	DmelCG3184	0	0
526	31609	Drosophila melanogaster	CG14442	CG14442 gene product from transcript CG14442-RB	DmelCG14442	0	0
527	31610	Drosophila melanogaster	CG14440	CG14440 gene product from transcript CG14440-RA	DmelCG14440	0	0
528	31611	Drosophila melanogaster	CG14441	CG14441 gene product from transcript CG14441-RA	DmelCG14441	0	0
529	31612	Drosophila melanogaster	CG3168	CG3168 gene product from transcript CG3168-RC	BEST:LP06294|DmelCG3168|LP06294.5prime	0	0
530	31613	Drosophila melanogaster	RpL17	Ribosomal protein L17	CG3203|DmelCG3203|Rpl17|anon-EST:Posey276|mRpL17	0	0
531	31614	Drosophila melanogaster	CG14439	CG14439 gene product from transcript CG14439-RB	DmelCG14439	0	0
532	31615	Drosophila melanogaster	CG14438	CG14438 gene product from transcript CG14438-RA	DmelCG14438	0	0
533	31617	Drosophila melanogaster	shf	shifted	CG3134|CG3135|CT10514|D-Wif|DmWIF|DmWif|DmelCG3135|Shf|shf-ov	0	0
534	31618	Drosophila melanogaster	C3G	CG42328 gene product from transcript CG42328-RC	C(3)G|CG14436|CG3126|CG42328|DC3-G|DC3G|DmelCG42328|Dmel_CG14436|Dmel_CG3126	0	0
535	31620	Drosophila melanogaster	pod1	CG4532 gene product from transcript CG4532-RA	BEST:LD37601|BcDNA:LD08951|CG4532|DmelCG4532|Dpod1|POD-1|coronin|dmPod	0	0
536	31621	Drosophila melanogaster	iav	inactive	CG4536|CT14708|DmelCG4536|IAV|TRPV|hypoB	0	0
537	31622	Drosophila melanogaster	Nf-YC	Nuclear factor Y-box C	CG3075|DmelCG3075|NF-YC|nf-yc	0	0
538	31623	Drosophila melanogaster	CG4542	CG4542 gene product from transcript CG4542-RA	Dalg8|DmelCG4542	0	0
539	31624	Drosophila melanogaster	Atx-1	Ataxin 1	CG4547|DmelCG4547|dAtx-1|dAtx1	0	0
540	31625	Drosophila melanogaster	Smg1	CG32743 gene product from transcript CG32743-RA	CG32743|CG4549|CG4555|DmelCG32743|SMG1|Smg|anon-EST:Liang-1.24|anon-WO0170980.133|clone 1.24|dSmg1|rst(1a)cyr|smg1	0	0
541	31626	Drosophila melanogaster	CG12796	CG12796 gene product from transcript CG12796-RA	DmelCG12796	0	0
542	31628	Drosophila melanogaster	CG14435	CG14435 gene product from transcript CG14435-RA	DmelCG14435	0	0
543	31629	Drosophila melanogaster	CG4557	CG4557 gene product from transcript CG4557-RA	DmelCG4557	0	0
544	31630	Drosophila melanogaster	Cht11	CG3044 gene product from transcript CG3044-RA	CG3044|DmelCG3044	0	0
545	31631	Drosophila melanogaster	CG4558	CG4558 gene product from transcript CG4558-RA	DmelCG4558	0	0
546	31632	Drosophila melanogaster	CG14434	CG14434 gene product from transcript CG14434-RA	DmelCG14434	0	0
547	31638	Drosophila melanogaster	CG32732	CG32732 gene product from transcript CG32732-RA	CG12542|CG3042|DmelCG32732	0	0
548	31639	Drosophila melanogaster	CG14431	CG14431 gene product from transcript CG14431-RB	DmelCG14431	0	0
549	31641	Drosophila melanogaster	CG4586	CG4586 gene product from transcript CG4586-RA	DmelCG4586	0	0
550	31642	Drosophila melanogaster	CG3040	CG3040 gene product from transcript CG3040-RA	CT9706|DmelCG3040	0	0
551	31644	Drosophila melanogaster	bou	boudin	BcDNA:RE28342|CG14430|DmelCG14430|JNXL4|jnL4|l(1)6Ea|l(1)jnL4	0	0
552	31645	Drosophila melanogaster	inx6	CG17063 gene product from transcript CG17063-RA	CG17063|Dm-inx6|DmelCG17063|inx-6|prp6	0	0
553	31646	Drosophila melanogaster	inx2	innexin 2	CG4590|D-inx-2|Dm-inx|Dm-inx2|DmelCG4590|INX-2|Inx2|Ix2|inx-2|kropf|l(1)G0035|l(1)G0036|l(1)G0043|l(1)G0059|l(1)G0118|l(1)G0157|l(1)G0317|l(1)G0364|prp33	0	0

554	31647	Drosophila melanogaster	cm	carmine	AP-3|CG3035|CG32731|DmelCG3035|Dmu3|Mu3|ap-3|cg3035|l(1)VEM119|mu3		0	0
555	31648	Drosophila melanogaster	CG3032	CG3032 gene product from transcript CG3032-RA	DmelCG3032		0	0
556	31649	Drosophila melanogaster	CG4593	CG4593 gene product from transcript CG4593-RA	DmelCG4593		0	0
557	31650	Drosophila melanogaster	CG12541	CG12541 gene product from transcript CG12541-RD	DmelCG12541		0	0
558	31651	Drosophila melanogaster	CG14427	CG14427 gene product from transcript CG14427-RA	DmelCG14427		0	0
559	31652	Drosophila melanogaster	nullo	CG14426 gene product from transcript CG14426-RB	BcDNA:RE47733|CG14426|DmelCG14426|Nullo		0	0
560	31653	Drosophila melanogaster	CG4607	CG4607 gene product from transcript CG4607-RA	DmelCG4607		0	0
561	31656	Drosophila melanogaster	CG4615	CG4615 gene product from transcript CG4615-RA	DmelCG4615		0	0
562	31657	Drosophila melanogaster	CG4617	CG4617 gene product from transcript CG4617-RA	DmelCG4617		0	0
563	31658	Drosophila melanogaster	CG8300	CG8300 gene product from transcript CG8300-RA	DmelCG8300		0	0
564	31659	Drosophila melanogaster	fz4	frizzled 4	CG4626|DFz4|Dfz4|Dm Fz4|DmelCG4626|Fz4|anon-WO0170980.10|anon-WO0170980.11		0	0
565	31660	Drosophila melanogaster	CG9650	CG9650 gene product from transcript CG9650-RG	DmelCG9650		0	0
566	31661	Drosophila melanogaster	CG1958	CG1958 gene product from transcript CG1958-RA	DmelCG1958		0	0
567	31662	Drosophila melanogaster	CG1677	CG1677 gene product from transcript CG1677-RB	DmelCG1677		0	0
568	31663	Drosophila melanogaster	CG2059	CG2059 gene product from transcript CG2059-RB	DmelCG2059		0	0
569	31664	Drosophila melanogaster	unc-119	CG1659 gene product from transcript CG1659-RA	CG1659|DmUNC-119|DmelCG1659|Unc-119|Unc119|anon-EST:Posey123|dm unc-119		0	0
570	31665	Drosophila melanogaster	brk	brinker	Brk|CG9653|Dm brk|DmelCG9653|ssg-1		0	0
571	31666	Drosophila melanogaster	Atg5	Autophagy-specific gene 5	ATG5|CG1643|DmAtg5|DmelCG1643|atg5		0	0
572	31667	Drosophila melanogaster	Dok	Downstream of kinase	CG2079|Ddok|DmelCG2079|l(1)G0331		0	0
573	31668	Drosophila melanogaster	CG18155	CG18155 gene product from transcript CG18155-RB	DmelCG18155|FBgn0029945		0	0
574	31669	Drosophila melanogaster	CBP	sarcoplasmic calcium-binding protein	BcDNA.GH12350|BcDNA:GH12350|CG1435|DmelCG1435		0	0
575	31670	Drosophila melanogaster	CG15034	CG15034 gene product from transcript CG15034-RA	DmelCG15034		0	0
576	31671	Drosophila melanogaster	CG1999	CG1999 gene product from transcript CG1999-RA	DmelCG1999|anon-WO0140519.160		0	0
577	31672	Drosophila melanogaster	CheA7a	Chemosensory protein A 7a	CG15033|DmelCG15033		0	0
578	31673	Drosophila melanogaster	CG15035	CG15035 gene product from transcript CG15035-RA	DmelCG15035		0	0
579	31674	Drosophila melanogaster	CG9657	CG9657 gene product from transcript CG9657-RA	DmelCG9657		0	0
580	31675	Drosophila melanogaster	CG11369	CG11369 gene product from transcript CG11369-RA	DmelCG11369		0	0
581	31676	Drosophila melanogaster	CG12689	CG12689 gene product from transcript CG12689-RB	DmelCG12689		0	0
582	31678	Drosophila melanogaster	CHES-1-like	Checkpoint suppressor homologue	CG12690|Ches-1-like|Ches1|Dmches-1|DmelCG12690|FoxN|anon-EST:Posey7|ches1		0	0
583	31679	Drosophila melanogaster	CG15478	CG15478 gene product from transcript CG15478-RA	DmelCG15478		0	0
584	31680	Drosophila melanogaster	Hira	CG12153 gene product from transcript CG12153-RA	CG12153|Dhh|DmelCG12153|HIRA|dHira|dhira|hira|ssm		0	0
585	31681	Drosophila melanogaster	NELF-B	CG32721 gene product from transcript CG32721-RA	BcDNA:GH10333|CG12152|CG32721|DmelCG32721|NELF		0	0
586	31682	Drosophila melanogaster	CG12155	CG12155 gene product from transcript CG12155-RA	DmelCG12155		0	0
587	31683	Drosophila melanogaster	Pdp	Pyruvate dehydrogenase phosphatase	153332_at|CG12151|DmelCG12151|PDP|pdp		0	0
588	31684	Drosophila melanogaster	Rab39	CG12156 gene product from transcript CG12156-RB	AAF46271|CG12156|DmRab39|DmelCG12156		0	0
589	31686	Drosophila melanogaster	Ir7a	Ionotropic receptor 7a	CG12663|DmelCG12663|IR7a		0	0
590	31687	Drosophila melanogaster	CG1402	CG1402 gene product from transcript CG1402-RB	DmelCG1402		0	0
591	31688	Drosophila melanogaster	CG10920	CG10920 gene product from transcript CG10920-RA	DmelCG10920		0	0
592	31689	Drosophila melanogaster	CG1409	CG1409 gene product from transcript CG1409-RB	BcDNA:AT29287|D.M.BLPp|DmelCG1409		0	0
593	31690	Drosophila melanogaster	Ir7b	Ionotropic receptor 7b	CG15326|DmelCG15326|IR7b		0	0
594	31691	Drosophila melanogaster	dec-1	defective chorion 1	CG2175|DmelCG2175|Fc|Fc1|Fcp7C|dec|dec1|dec[[1]]|fs(1)11-549|fs(1)12-1873|fs(1)12-2514|fs(1)12-3512|fs(1)12-365|fs(1)12-3907|fs(1)12-403|fs(1)12-4860|fs(1)13-453|fs(1)13C-57|fs(1)13C-73|fs(1)14-96		0	0
595	31692	Drosophila melanogaster	Ir7c	Ionotropic receptor 7c	CG15324|CT35324|DmelCG15324|IR7c		0	0
596	31694	Drosophila melanogaster	Ir7g	Ionotropic receptor 7g	CG15325|DmelCG15325|IR7g		0	0
597	31695	Drosophila melanogaster	CG10932	CG10932 gene product from transcript CG10932-RA	154229_at|DmelCG10932		0	0
598	31696	Drosophila melanogaster	Nek2	CG17256 gene product from transcript CG17256-RA	CG17256|Dm Nek2|DmNek2|DmelCG17256|NEK2		0	0
599	31697	Drosophila melanogaster	CG18624	CG18624 gene product from transcript CG18624-RC	CG15328|DmelCG18624|Dromel_CG18624_FBtr0071140_mORF		0	0
600	31698	Drosophila melanogaster	p115	CG1422 gene product from transcript CG1422-RA	CG1422|DmelCG1422|dp115|l(1)G0306		0	0
601	31700	Drosophila melanogaster	RpS6	Ribosomal protein S6	(Rp)S6|CG10944|DS6|DmelCG10944|M(1)7BC|M(1)7C|RPS6|Rps6|S6|air8|air[8]|anon-WO02059370.61|hen|l(1)air8|l(1)air[8]|l(1)hen|pp30|rpS6|rps6		0	0
602	31701	Drosophila melanogaster	bys	by S6	CG1430|DmelCG1430		0	0
603	31702	Drosophila melanogaster	dpr14	CG10946 gene product from transcript CG10946-RB	CG10946|CT30653|DmelCG10946|Dpr-14		0	0
604	31703	Drosophila melanogaster	CG1444	CG1444 gene product from transcript CG1444-RA	DmelCG1444		0	0
605	31704	Drosophila melanogaster	snz	snazarus	CG1514|DmelCG1514		0	0
606	31705	Drosophila melanogaster	hdm	hold'em	CG15329|DmelCG15329		0	0
607	31706	Drosophila melanogaster	Ykt6	CG1515 gene product from transcript CG1515-RA	CG1515|DmelCG1515|YKT6|Ykt6p|l(1)G0155		0	0
608	31707	Drosophila melanogaster	CG10777	CG10777 gene product from transcript CG10777-RB	DmRH1|DmelCG10777|cg10777		0	0
609	31708	Drosophila melanogaster	CG10778	CG10778 gene product from transcript CG10778-RA	DmelCG10778		0	0
610	31712	Drosophila melanogaster	l(1)G0193	lethal (1) G0193	CG2206|DmelCG2206|l(1)G0327		0	0
611	31713	Drosophila melanogaster	CG42593	CG42593 gene product from transcript CG42593-RA	CG1530|CG1531|DmelCG42593|Dmel_CG1530|Dmel_CG1531		0	0
612	31714	Drosophila melanogaster	CG15332	CG15332 gene product from transcript CG15332-RA	DmelCG15332		0	0
613	31715	Drosophila melanogaster	CG15330	CG15330 gene product from transcript CG15330-RA	DmelCG15330		0	0
614	31716	Drosophila melanogaster	sws	swiss cheese	CG2212|DmelCG2212|Sws|olfE		0	0
615	31717	Drosophila melanogaster	sn	singed	CG15331|CG1536|CG32858|DmelCG32858|SN|Sn|fs(1)A1057|fs(1)K1421|fs(1)K418|fs(1)K473|fs(1)K743|fs(1)M45		0	0
616	31718	Drosophila melanogaster	Tbh	Tyramine beta hydroxylase	CG1543|DBH|Dbh|DmelCG1543|T beta h|TBH|TBetaH|TBetah|TbetaH|Tbetah|tbh		0	0
617	31720	Drosophila melanogaster	CG15333	CG15333 gene product from transcript CG15333-RA	CG18293|DmelCG15333		0	0
618	31721	Drosophila melanogaster	CG2233	CG2233 gene product from transcript CG2233-RA	DmelCG2233		0	0
619	31722	Drosophila melanogaster	fs(1)h	female sterile (1) homeotic	CG2252|DmelCG2252|Fs(1)hL|Fs(1)hS|N72|ORE-1|anon-EST:Liang-1.81|anon-EST:fe1G2|clone 1.81|fs(1)1456|fs(1)26/26A|fs(1)M16|fs(1)R10.4|fsh|fst(1)h|l(1)7Da|l(1)G0093|l(1)G0495|rnc		0	0
620	31724	Drosophila melanogaster	Upf2	CG2253 gene product from transcript CG2253-RA	CG2253|DmelCG2253|UPF2|upf2		0	0
621	31725	Drosophila melanogaster	CG1571	CG1571 gene product from transcript CG1571-RA	DmelCG1571		0	0
622	31726	Drosophila melanogaster	CG2254	CG2254 gene product from transcript CG2254-RA	DmelCG2254		0	0
623	31727	Drosophila melanogaster	CG2256	CG2256 gene product from transcript CG2256-RC	DmelCG2256		0	0
624	31728	Drosophila melanogaster	Ubc-E2H	CG2257 gene product from transcript CG2257-RA	CG2257|DmelCG2257|UbcE2H|dUbc-E2H		0	0
625	31729	Drosophila melanogaster	CG2258	CG2258 gene product from transcript CG2258-RA	DmelCG2258		0	0
626	31731	Drosophila melanogaster	CG1575	CG1575 gene product from transcript CG1575-RA	DmelCG1575		0	0
627	31732	Drosophila melanogaster	CG2260	CG2260 gene product from transcript CG2260-RA	DmelCG2260		0	0
628	31733	Drosophila melanogaster	cyr	cypher	CG15335|CG15338|DmelCG15335		0	0
629	31735	Drosophila melanogaster	CG2116	CG2116 gene product from transcript CG2116-RA	DmelCG2116		0	0
630	31736	Drosophila melanogaster	CG10958	CG10958 gene product from transcript CG10958-RA	DmelCG10958		0	0
631	31737	Drosophila melanogaster	CG2120	CG2120 gene product from transcript CG2120-RB	DmelCG2120		0	0
632	31738	Drosophila melanogaster	Smox	Smad on X	CG2262|DSMAD2|DSmad2|DmelCG2262|SMAD2|SMOX|Sad|Smad2|dSMAD2|dSmad2|dsmad2|l(1)G0348|sad|smad2|smox|ted|tmp		0	0
633	31739	Drosophila melanogaster	CG17982	CG17982 gene product from transcript CG17982-RA	DmelCG17982		0	0
634	31740	Drosophila melanogaster	CG2263	CG2263 gene product from transcript CG2263-RA	DmelCG2263		0	0
635	31741	Drosophila melanogaster	CG2129	CG2129 gene product from transcript CG2129-RA	DmelCG2129		0	0
636	31742	Drosophila melanogaster	CG15336	CG15336 gene product from transcript CG15336-RC	DmelCG15336		0	0
637	31743	Drosophila melanogaster	CG10959	CG10959 gene product from transcript CG10959-RA	DmelCG10959		0	0
638	31744	Drosophila melanogaster	Gbeta5	CG10763 gene product from transcript CG10763-RA	CG10763|DmelCG10763|G[[beta]]|Gbeta|Gbeta 5|Gbetagamma		0	0
639	31745	Drosophila melanogaster	CG18262	CG18262 gene product from transcript CG18262-RA	DmelCG18262		0	0
640	31746	Drosophila melanogaster	Traf6	TNF-receptor-associated factor 6	CG10961|DTRAF2|DmelCG10961|TRAF|TRAF2|TRAF6|Traf2|dTRAF2|dTRAF6|dTraf2|dtraf2		0	0
641	31747	Drosophila melanogaster	GIIIspla2	CG1583 gene product from transcript CG1583-RB	BEST:HL07869|CG1583|DmelCG1583|Giiispla2		0	0
642	31748	Drosophila melanogaster	CG15337	CG15337 gene product from transcript CG15337-RA	DmelCG15337		0	0
643	31749	Drosophila melanogaster	CG10761	CG10761 gene product from transcript CG10761-RA	DmelCG10761		0	0
644	31750	Drosophila melanogaster	Or7a	Odorant receptor 7a	7D.1|7a|CG10759|DOR30|DmOR7a|DmelCG10759		0	0
645	31751	Drosophila melanogaster	CG2278	CG2278 gene product from transcript CG2278-RA	DmelCG2278		0	0
646	31759	Drosophila melanogaster	CG2147	CG2147 gene product from transcript CG2147-RA	BcDNA:LP02728|DmelCG2147		0	0
647	31760	Drosophila melanogaster	Trxr-1	Thioredoxin reductase-1	2151|CG2151|DTR|Dm-TrxR|DmTR|DmTrx|DmTrxR|DmTrxR-1|DmelCG2151|GR|Gr|Trx|TrxR|TrxR-1|Trxr1|anon-WO03040301.185|anon-WO03040301.187|dTrxR|dmtrxr-1|gr|l(1)G0154|l(1)G0379|l(1)G0477|l(		0	0
648	31761	Drosophila melanogaster	sni	sniffer	CG10964|DmelCG10964|SNI|anon-WO03040301.183		0	0
649	31762	Drosophila melanogaster	ND75	NADH:ubiquinone reductase 75kD subunit precursor	CG2286|DmelCG2286		0	0
650	31763	Drosophila melanogaster	CG1632	CG1632 gene product from transcript CG1632-RA	DmelCG1632|SPH198		0	0
651	31764	Drosophila melanogaster	Corp	Companion of reaper	CG10965|DmelCG10965|corp		0	0
652	31765	Drosophila melanogaster	CG15343	CG15343 gene product from transcript CG15343-RA	DmelCG15343		0	0
653	31766	Drosophila melanogaster	CG1636	CG1636 gene product from transcript CG1636-RA	DmelCG1636		0	0
654	31767	Drosophila melanogaster	nAcRalpha-7E	nicotinic Acetylcholine Receptor alpha 7E	Acr7E|CG2302|D[a3]|Da3|Dalpha3|DmelCG2302|alpha3|nACRalpha-7E|nAChR|nAcRe		0	0
655	31769	Drosophila melanogaster	CG15344	CG15344 gene product from transcript CG15344-RA	DmelCG15344		0	0
656	31770	Drosophila melanogaster	CG1387	CG1387 gene product from transcript CG1387-RA	DmelCG1387		0	0
657	31771	Drosophila melanogaster	CG10555	CG10555 gene product from transcript CG10555-RA	DmelCG10555		0	0
658	31772	Drosophila melanogaster	CG11190	CG11190 gene product from transcript CG11190-RA	Dm*AAF46367/27-522|DmelCG11190		0	0
659	31773	Drosophila melanogaster	Trf2	TATA box binding protein-related factor 2	CG11195|CG18009|DmelCG18009|TLF|TRF2|dTRF2|dTrf2|l(1)G0039|l(1)G0071|l(1)G0295|l(1)G0425|p79/TRF2|trf2		0	0
660	31775	Drosophila melanogaster	CG12125	CG12125 gene product from transcript CG12125-RA	DmelCG12125		0	0
661	31776	Drosophila melanogaster	CG1440	CG1440 gene product from transcript CG1440-RA	DmelCG1440		0	0
662	31777	Drosophila melanogaster	CG12123	CG12123 gene product from transcript CG12123-RA	DmelCG12123		0	0
663	31778	Drosophila melanogaster	org-1	optomotor-blind-related-gene-1	CG11202|Dm-ORG-1|DmelCG11202|Org-1		0	0
664	31779	Drosophila melanogaster	CG15347	CG15347 gene product from transcript CG15347-RA	DmelCG15347		0	0

<table>
<tr><th></th><th></th><th></th><th></th><th></th><th></th><th></th><th></th></tr>
<tr><td>665</td><td>31780</td><td>Drosophila melanogaster</td><td>Es2</td><td>CG1474 gene product from transcript CG1474-RA</td><td>CG1474|DmelCG1474|ES2|es</td><td>0</td><td>0</td></tr>
<tr><td>666</td><td>31781</td><td>Drosophila melanogaster</td><td>Sptr</td><td>Sepiapterin reductase</td><td>CG12117|DmelCG12117|SR|sptr</td><td>0</td><td>0</td></tr>
<tr><td>667</td><td>31782</td><td>Drosophila melanogaster</td><td>CG12116</td><td>CG12116 gene product from transcript CG12116-RA</td><td>144701_at|DmelCG12116</td><td>0</td><td>0</td></tr>
<tr><td>668</td><td>31785</td><td>Drosophila melanogaster</td><td>Cp7Fb</td><td>Chorion protein b at 7F</td><td>B|C|CG15350|DmelCG15350|cp7Fb</td><td>0</td><td>0</td></tr>
<tr><td>669</td><td>31786</td><td>Drosophila melanogaster</td><td>Cp7Fc</td><td>Chorion protein c at 7F</td><td>CG15351|D|DmelCG15351|E|s37</td><td>0</td><td>0</td></tr>
<tr><td>670</td><td>31787</td><td>Drosophila melanogaster</td><td>Cp36</td><td>Chorion protein 36</td><td>CG1478|CP36|DmelCG1478|Dms36|E4|S36|cp36|dec-2|dec2|dec[[2]]|fs(1)C2|s36|s36-1|s365</td><td>0</td><td>0</td></tr>
<tr><td>671</td><td>31788</td><td>Drosophila melanogaster</td><td>Cp38</td><td>Chorion protein 38</td><td>ACE1|CG11213|CP38|DmelCG11213|Dms38|E3|S38|cp38|s38|s38-1</td><td>0</td><td>0</td></tr>
<tr><td>672</td><td>31789</td><td>Drosophila melanogaster</td><td>otu</td><td>ovarian tumor</td><td>CG12743|DROOTUA|DmelCG12743|K|OTU|Otu|fs(1)231|fs(1)23l|fs(1)M101|fs(1)jA265|fs(1)otu</td><td>0</td><td>0</td></tr>
<tr><td>673</td><td>31791</td><td>Drosophila melanogaster</td><td>PIP82</td><td>CG11219 gene product from transcript CG11219-RA</td><td>CG11219|DmelCG11219</td><td>0</td><td>0</td></tr>
<tr><td>674</td><td>31792</td><td>Drosophila melanogaster</td><td>Nrg</td><td>Neuroglian</td><td>CG1634|CT4318|DmelCG1634|NRG|Ngl|RA35|ceb|l(1)7Fa|l(1)G0099|l(1)G0413|l(1)G0488|nrg</td><td>0</td><td>0</td></tr>
<tr><td>675</td><td>31793</td><td>Drosophila melanogaster</td><td>IntS4</td><td>Integrator 4</td><td>CG12113|DmelCG12113|Int4|l(1)G0095</td><td>0</td><td>0</td></tr>
<tr><td>676</td><td>31794</td><td>Drosophila melanogaster</td><td>CG12112</td><td>CG12112 gene product from transcript CG12112-RA</td><td>DmelCG12112</td><td>0</td><td>0</td></tr>
<tr><td>677</td><td>31795</td><td>Drosophila melanogaster</td><td>Trf4-1</td><td>CG11265 gene product from transcript CG11265-RE</td><td>AAF46390|CG11265|DmTRF4-1|DmelCG11265</td><td>0</td><td>0</td></tr>
<tr><td>678</td><td>31796</td><td>Drosophila melanogaster</td><td>CG12111</td><td>CG12111 gene product from transcript CG12111-RA</td><td>DmelCG12111</td><td>0</td><td>0</td></tr>
<tr><td>679</td><td>31797</td><td>Drosophila melanogaster</td><td>spirit</td><td>Serine Protease Immune Response Integrator</td><td>CG2056|DmelCG2056|SP33|c-SP33</td><td>0</td><td>0</td></tr>
<tr><td>680</td><td>31798</td><td>Drosophila melanogaster</td><td>CG12065</td><td>CG12065 gene product from transcript CG12065-RF</td><td>DmelCG12065</td><td>0</td><td>0</td></tr>
<tr><td>681</td><td>31799</td><td>Drosophila melanogaster</td><td>CG12081</td><td>CG12081 gene product from transcript CG12081-RA</td><td>DmelCG12081</td><td>0</td><td>0</td></tr>
<tr><td>682</td><td>31800</td><td>Drosophila melanogaster</td><td>Crag</td><td>Calmodulin-binding protein related to a Rab3 GDP/GT</td><td>CG12737|CJ|CRAG|DmelCG12737</td><td>0</td><td>0</td></tr>
<tr><td>683</td><td>31801</td><td>Drosophila melanogaster</td><td>Caf1-180</td><td>CG12109 gene product from transcript CG12109-RB</td><td>CAF-1 p180|CAF1-180|CG12109|DmelCG12109|NURF|p180</td><td>0</td><td>0</td></tr>
<tr><td>684</td><td>31802</td><td>Drosophila melanogaster</td><td>oc</td><td>ocelliless</td><td>CG12154|DmelCG12154|Oc|Otd|Otx|l(1)7Ff|l(1)8Ac|ort|otd|uvi</td><td>0</td><td>0</td></tr>
<tr><td>685</td><td>31803</td><td>Drosophila melanogaster</td><td>CG12772</td><td>CG12772 gene product from transcript CG12772-RA</td><td>DmelCG12772|FBgn 30055</td><td>0</td><td>0</td></tr>
<tr><td>686</td><td>31804</td><td>Drosophila melanogaster</td><td>CG11284</td><td>CG11284 gene product from transcript CG11284-RA</td><td>DmelCG11284</td><td>0</td><td>0</td></tr>
<tr><td>687</td><td>31805</td><td>Drosophila melanogaster</td><td>Ppt1</td><td>Palmitoyl-protein thioesterase 1</td><td>CG12108|DmPpt1|DmelCG12108|PPT1|ppt1</td><td>0</td><td>0</td></tr>
<tr><td>688</td><td>31806</td><td>Drosophila melanogaster</td><td>Ogg1</td><td>CG1795 gene product from transcript CG1795-RA</td><td>1795|BcDNA:LD19945|CG1795|DmelCG1795|dOGG1</td><td>0</td><td>0</td></tr>
<tr><td>689</td><td>31807</td><td>Drosophila melanogaster</td><td>CG11294</td><td>CG11294 gene product from transcript CG11294-RA</td><td>CT31519|DmelCG11294</td><td>0</td><td>0</td></tr>
<tr><td>690</td><td>31808</td><td>Drosophila melanogaster</td><td>Hexo2</td><td>Hexosaminidase 2</td><td>CG1787|DmHex2|DmelCG1787|HEX 2|HEX2|HEXO2</td><td>0</td><td>0</td></tr>
<tr><td>691</td><td>31809</td><td>Drosophila melanogaster</td><td>CG2004</td><td>CG2004 gene product from transcript CG2004-RA</td><td>DmelCG2004</td><td>0</td><td>0</td></tr>
<tr><td>692</td><td>31810</td><td>Drosophila melanogaster</td><td>CG1785</td><td>CG1785 gene product from transcript CG1785-RA</td><td>DmelCG1785</td><td>0</td><td>0</td></tr>
<tr><td>693</td><td>31811</td><td>Drosophila melanogaster</td><td>l(1)G0020</td><td>lethal (1) G0020</td><td>CG1994|DmelCG1994|p110</td><td>0</td><td>0</td></tr>
<tr><td>694</td><td>31812</td><td>Drosophila melanogaster</td><td>CG1789</td><td>CG1789 gene product from transcript CG1789-RA</td><td>DmelCG1789</td><td>0</td><td>0</td></tr>
<tr><td>695</td><td>31813</td><td>Drosophila melanogaster</td><td>Lim1</td><td>CG11354 gene product from transcript CG11354-RA</td><td>BcDNA:GH04929|CG10760|CG11354|Dlim1|DmelCG11354|Lim|dLim1|dlim-1|dlim1|lim1</td><td>0</td><td>0</td></tr>
<tr><td>696</td><td>31815</td><td>Drosophila melanogaster</td><td>CG12075</td><td>CG12075 gene product from transcript CG12075-RC</td><td>DmelCG12075</td><td>0</td><td>0</td></tr>
<tr><td>697</td><td>31816</td><td>Drosophila melanogaster</td><td>Moe</td><td>Moesin</td><td>CG10701|D17|DmelCG10701|Dmoe|EMR1|ERM|Emr1|anon-WO03040301.155|anon-WO03040301.157|anon-WO03040301.159|anon-WO03040301.161|dMoe|l(1)G0067|l(1)G0323|l(1)G0404|l(1)G0415|mo</td><td>0</td><td>0</td></tr>
<tr><td>698</td><td>31817</td><td>Drosophila melanogaster</td><td>CG1885</td><td>CG1885 gene product from transcript CG1885-RA</td><td>DmelCG1885</td><td>0</td><td>0</td></tr>
<tr><td>699</td><td>31818</td><td>Drosophila melanogaster</td><td>Rbm13</td><td>RNA-binding motif protein 13</td><td>CG10648|DmelCG10648|RBM13</td><td>0</td><td>0</td></tr>
<tr><td>700</td><td>31822</td><td>Drosophila melanogaster</td><td>CG12661</td><td>CG12661 gene product from transcript CG12661-RA</td><td>DmelCG12661</td><td>0</td><td>0</td></tr>
<tr><td>701</td><td>31824</td><td>Drosophila melanogaster</td><td>CG10962</td><td>CG10962 gene product from transcript CG10962-RB</td><td>BcDNA:RE29909|CG7090|DmelCG10962|EP(X)1565|EP1565</td><td>0</td><td>0</td></tr>
<tr><td>702</td><td>31825</td><td>Drosophila melanogaster</td><td>CG12662</td><td>CG12662 gene product from transcript CG12662-RA</td><td>DmelCG12662</td><td>0</td><td>0</td></tr>
<tr><td>703</td><td>31826</td><td>Drosophila melanogaster</td><td>rdgA</td><td>retinal degeneration A</td><td>CG10966|CG12660|CG15352|CG34344|CG42667|DGK|DGK2|DmelCG42667|Dmel_CG10966|Dmel_CG12660|Dmel_CG15352|Dmel_CG34344|dDGK2|x35</td><td>0</td><td>0</td></tr>
<tr><td>704</td><td>31829</td><td>Drosophila melanogaster</td><td>CG15365</td><td>CG15365 gene product from transcript CG15365-RA</td><td>DmelCG15365</td><td>0</td><td>0</td></tr>
<tr><td>705</td><td>31830</td><td>Drosophila melanogaster</td><td>CG10970</td><td>CG10970 gene product from transcript CG10970-RA</td><td>BcDNA:HL08157|DmelCG10970</td><td>0</td><td>0</td></tr>
<tr><td>706</td><td>31831</td><td>Drosophila melanogaster</td><td>CG7267</td><td>CG7267 gene product from transcript CG7267-RB</td><td>DmelCG7267</td><td>0</td><td>0</td></tr>
<tr><td>707</td><td>31832</td><td>Drosophila melanogaster</td><td>CG15366</td><td>CG15366 gene product from transcript CG15366-RA</td><td>DmelCG15366</td><td>0</td><td>0</td></tr>
<tr><td>708</td><td>31833</td><td>Drosophila melanogaster</td><td>CG7246</td><td>CG7246 gene product from transcript CG7246-RA</td><td>DmelCG7246</td><td>0</td><td>0</td></tr>
<tr><td>709</td><td>31834</td><td>Drosophila melanogaster</td><td>HP1b</td><td>CG7041 gene product from transcript CG7041-RA</td><td>CG7041|DmelCG7041|HP1|HP1B|Hp1b</td><td>0</td><td>0</td></tr>
<tr><td>710</td><td>31835</td><td>Drosophila melanogaster</td><td>APC4</td><td>Anaphase Promoting Complex 4</td><td>CG32707|CG4350|DmelCG32707</td><td>0</td><td>0</td></tr>
<tr><td>711</td><td>31837</td><td>Drosophila melanogaster</td><td>CG6999</td><td>CG6999 gene product from transcript CG6999-RA</td><td>DmelCG6999</td><td>0</td><td>0</td></tr>
<tr><td>712</td><td>31838</td><td>Drosophila melanogaster</td><td>CG7033</td><td>CG7033 gene product from transcript CG7033-RA</td><td>CCT2|Cct2|DmelCG7033|SD02216p|TCP-1beta</td><td>0</td><td>0</td></tr>
<tr><td>713</td><td>31839</td><td>Drosophila melanogaster</td><td>CG7766</td><td>CG7766 gene product from transcript CG7766-RC</td><td>DmelCG7766</td><td>0</td><td>0</td></tr>
<tr><td>714</td><td>31840</td><td>Drosophila melanogaster</td><td>Bx42</td><td>CG8264 gene product from transcript CG8264-RA</td><td>BX42|CG8264|DmelCG8264|SKIP|Skip</td><td>0</td><td>0</td></tr>
<tr><td>715</td><td>31841</td><td>Drosophila melanogaster</td><td>CG7039</td><td>CG7039 gene product from transcript CG7039-RA</td><td>DmelCG7039</td><td>0</td><td>0</td></tr>
<tr><td>716</td><td>31842</td><td>Drosophila melanogaster</td><td>AP-1gamma</td><td>CG9113 gene product from transcript CG9113-RD</td><td>AP-1|AP-1[gamma]|CG9113|DmelCG9113|cg9113|gamma|gamma-Ada</td><td>0</td><td>0</td></tr>
<tr><td>717</td><td>31843</td><td>Drosophila melanogaster</td><td>fend</td><td>forked end</td><td>BcDNA:RH56795|CG12664|DmelCG12664|Dromel_CG12664_FBtr0071349_ld14_mORF|LD14|ld14</td><td>0</td><td>0</td></tr>
<tr><td>718</td><td>31844</td><td>Drosophila melanogaster</td><td>CG7065</td><td>CG7065 gene product from transcript CG7065-RA</td><td>DmelCG7065</td><td>0</td><td>0</td></tr>
<tr><td>719</td><td>31845</td><td>Drosophila melanogaster</td><td>fh</td><td>frataxin homolog</td><td>BcDNA:AT09528|CG8971|DFH|Dfh|DmelCG8971|X25|dfh</td><td>0</td><td>0</td></tr>
<tr><td>720</td><td>31846</td><td>Drosophila melanogaster</td><td>dalao</td><td>CG7055 gene product from transcript CG7055-RA</td><td>BAF57|BAP111|Bap111|CG7055|DmelCG7055|anon-EST:Liang-1.83|clone 1.83</td><td>0</td><td>0</td></tr>
<tr><td>721</td><td>31848</td><td>Drosophila melanogaster</td><td>His3.3B</td><td>Histone H3.3B</td><td>CG8989|DmelCG8989|H3.3|H3.3B|His3.3|HisH3-3Q|PH3|anon-EST:fe1B6|dH3.3B</td><td>0</td><td>0</td></tr>
<tr><td>722</td><td>31849</td><td>Drosophila melanogaster</td><td>Ost48</td><td>Oligosaccharyltransferase 48kD subunit</td><td>CG9022|DmOST50|DmelCG9022|DrOST|OST48|OST48/WBP1|Ots48|anon-EST:Liang-1.11|clone 1.11|ost48</td><td>0</td><td>0</td></tr>
<tr><td>723</td><td>31851</td><td>Drosophila melanogaster</td><td>Zpr1</td><td>CG9060 gene product from transcript CG9060-RA</td><td>CG9060|DmelCG9060</td><td>0</td><td>0</td></tr>
<tr><td>724</td><td>31853</td><td>Drosophila melanogaster</td><td>CG12115</td><td>CG12115 gene product from transcript CG12115-RA</td><td>BcDNA:LP02922|DmelCG12115</td><td>0</td><td>0</td></tr>
<tr><td>725</td><td>31854</td><td>Drosophila melanogaster</td><td>CG12057</td><td>CG12057 gene product from transcript CG12057-RA</td><td>BcDNA:LP05863|DmelCG12057</td><td>0</td><td>0</td></tr>
<tr><td>726</td><td>31855</td><td>Drosophila melanogaster</td><td>CG12056</td><td>CG12056 gene product from transcript CG12056-RA</td><td>DmelCG12056</td><td>0</td><td>0</td></tr>
<tr><td>727</td><td>31856</td><td>Drosophila melanogaster</td><td>CG12106</td><td>CG12106 gene product from transcript CG12106-RA</td><td>BcDNA:GH03263|DmelCG12106</td><td>0</td><td>0</td></tr>
<tr><td>728</td><td>31857</td><td>Drosophila melanogaster</td><td>CG12118</td><td>CG12118 gene product from transcript CG12118-RB</td><td>DmelCG12118</td><td>0</td><td>0</td></tr>
<tr><td>729</td><td>31858</td><td>Drosophila melanogaster</td><td>su(r)</td><td>suppressor of rudimentary</td><td>CG2194|DmelCG2194|Dreg-3|Pyd1|Reg-3</td><td>0</td><td>0</td></tr>
<tr><td>730</td><td>31859</td><td>Drosophila melanogaster</td><td>CG12119</td><td>CG12119 gene product from transcript CG12119-RA</td><td>DmelCG12119</td><td>0</td><td>0</td></tr>
<tr><td>731</td><td>31860</td><td>Drosophila melanogaster</td><td>Obp8a</td><td>Odorant-binding protein 8a</td><td>8a|CG12665|DmelCG12665</td><td>0</td><td>0</td></tr>
<tr><td>732</td><td>31861</td><td>Drosophila melanogaster</td><td>CG15368</td><td>CG15368 gene product from transcript CG15368-RA</td><td>DmelCG15368</td><td>0</td><td>0</td></tr>
<tr><td>733</td><td>31862</td><td>Drosophila melanogaster</td><td>CG15369</td><td>CG15369 gene product from transcript CG15369-RA</td><td>DmelCG15369</td><td>0</td><td>0</td></tr>
<tr><td>734</td><td>31863</td><td>Drosophila melanogaster</td><td>t</td><td>tan</td><td>CG12120|DmelCG12120</td><td>0</td><td>0</td></tr>
<tr><td>735</td><td>31864</td><td>Drosophila melanogaster</td><td>CG15370</td><td>CG15370 gene product from transcript CG15370-RA</td><td>DmelCG15370</td><td>0</td><td>0</td></tr>
<tr><td>736</td><td>31865</td><td>Drosophila melanogaster</td><td>Gr8a</td><td>Gustatory receptor 8a</td><td>CG15371|DmelCG15371|Gr8D1</td><td>0</td><td>0</td></tr>
<tr><td>737</td><td>31866</td><td>Drosophila melanogaster</td><td>CG12121</td><td>CG12121 gene product from transcript CG12121-RA</td><td>DmelCG12121</td><td>0</td><td>0</td></tr>
<tr><td>738</td><td>31867</td><td>Drosophila melanogaster</td><td>Ir8a</td><td>Ionotropic receptor 8a</td><td>CG12122|CG12666|CG32704|CT7438|DmelCG32704|IR8a</td><td>0</td><td>0</td></tr>
<tr><td>739</td><td>31869</td><td>Drosophila melanogaster</td><td>mxc</td><td>multi sex combs</td><td>CG12058|CG12124|DmelCG12124|Dmel_CG12058|l(1)mbn</td><td>0</td><td>0</td></tr>
<tr><td>740</td><td>31872</td><td>Drosophila melanogaster</td><td>Dsor1</td><td>Downstream of raf1</td><td>CG15793|D-MEK|D-MEK/Dsor|D-Mek|D-SOR|D-Sor|D-mek|D-sor|D-sor-1|DMEK-1|DRODSOR1|DSOR1|DSORT|DSor|DSor1|Dmek|DmelCG15793|Dsor|EK1-1|MAPKK|MEK|MEK/Dsor1|MEK1/2|Mek|SOR|</td><td>0</td><td>0</td></tr>
<tr><td>741</td><td>31873</td><td>Drosophila melanogaster</td><td>CG17754</td><td>CG17754 gene product from transcript CG17754-RC</td><td>BcDNA:LP02641|DmelCG17754</td><td>0</td><td>0</td></tr>
<tr><td>742</td><td>31874</td><td>Drosophila melanogaster</td><td>ric8a</td><td>CG15797 gene product from transcript CG15797-RA</td><td>CG15797|DmRic8|DmelCG15797|RIC-8|Ric-8|l(1)G0397|ric-8|ric-8a</td><td>0</td><td>0</td></tr>
<tr><td>743</td><td>31877</td><td>Drosophila melanogaster</td><td>CG32703</td><td>CG32703 gene product from transcript CG32703-RB</td><td>CG17441|CG2309|DmelCG32703|ERK7</td><td>0</td><td>0</td></tr>
<tr><td>744</td><td>31879</td><td>Drosophila melanogaster</td><td>CG17440</td><td>CG17440 gene product from transcript CG17440-RA</td><td>DmelCG17440</td><td>0</td><td>0</td></tr>
<tr><td>745</td><td>31880</td><td>Drosophila melanogaster</td><td>CG17446</td><td>CG17446 gene product from transcript CG17446-RB</td><td>DmelCG17446</td><td>0</td><td>0</td></tr>
<tr><td>746</td><td>31881</td><td>Drosophila melanogaster</td><td>CG16892</td><td>CG16892 gene product from transcript CG16892-RA</td><td>DmelCG16892</td><td>0</td><td>0</td></tr>
<tr><td>747</td><td>31883</td><td>Drosophila melanogaster</td><td>lz</td><td>lozenge</td><td>CG1689|DmelCG1689|Lz|amx|fs(1)A1569|fs(1)M69|spe</td><td>0</td><td>0</td></tr>
<tr><td>748</td><td>31887</td><td>Drosophila melanogaster</td><td>CG34449</td><td>CG34449 gene product from transcript CG34449-RB</td><td>CG12136|CG15372|CG17251|CG32705|DmelCG34449|Dmel_CG32705|Pen16|anon-Pen16|p16|pen16</td><td>0</td><td>0</td></tr>
<tr><td>749</td><td>31890</td><td>Drosophila melanogaster</td><td>BCL7-like</td><td>CG17252 gene product from transcript CG17252-RA</td><td>Bcl7-like|CG17252|DmelCG17252</td><td>0</td><td>0</td></tr>
<tr><td>750</td><td>31896</td><td>Drosophila melanogaster</td><td>CG32700</td><td>CG32700 gene product from transcript CG32700-RA</td><td>BcDNA:GH19985|CG12651|CG12652|CG18429|CG2994|DmelCG32700|Tes89</td><td>0</td><td>0</td></tr>
<tr><td>751</td><td>31897</td><td>Drosophila melanogaster</td><td>RpS28b</td><td>Ribosomal protein S28b</td><td>CG2998|DmelCG2998|M(1)8F</td><td>0</td><td>0</td></tr>
<tr><td>752</td><td>31898</td><td>Drosophila melanogaster</td><td>CG15317</td><td>CG15317 gene product from transcript CG15317-RA</td><td>DmelCG15317</td><td>0</td><td>0</td></tr>
<tr><td>753</td><td>31899</td><td>Drosophila melanogaster</td><td>CG32699</td><td>CG32699 gene product from transcript CG32699-RB</td><td>CG15318|CG18535|CG2996|CT7882|DmelCG32699|tok-like</td><td>0</td><td>0</td></tr>
<tr><td>754</td><td>31902</td><td>Drosophila melanogaster</td><td>Gga</td><td>Golgi-localized, gamma-adaptin ear containing, ARF b</td><td>CG3002|DmelCG3002|GGA|dGGA|dmGGA</td><td>0</td><td>0</td></tr>
<tr><td>755</td><td>31903</td><td>Drosophila melanogaster</td><td>CG3004</td><td>CG3004 gene product from transcript CG3004-RA</td><td>DmelCG3004</td><td>0</td><td>0</td></tr>
<tr><td>756</td><td>31904</td><td>Drosophila melanogaster</td><td>Aats-lys</td><td>Lysyl-tRNA synthetase</td><td>CG12141|DmelCG12141|KRS|LysRS</td><td>0</td><td>0</td></tr>
<tr><td>757</td><td>31905</td><td>Drosophila melanogaster</td><td>CG42797</td><td>CG42797 gene product from transcript CG42797-RE</td><td>CG3003|CG3099|DmelCG42797|Dmel_CG3003|Dmel_CG3099</td><td>0</td><td>0</td></tr>
<tr><td>758</td><td>31907</td><td>Drosophila melanogaster</td><td>l(1)G0232</td><td>lethal (1) G0232</td><td>CG3101|CG3102|CG32697|DmelCG32697|L(1)G0232|MEG2</td><td>0</td><td>0</td></tr>
<tr><td>759</td><td>31909</td><td>Drosophila melanogaster</td><td>CG3106</td><td>CG3106 gene product from transcript CG3106-RA</td><td>DmelCG3106</td><td>0</td><td>0</td></tr>
<tr><td>760</td><td>31912</td><td>Drosophila melanogaster</td><td>btd</td><td>buttonhead</td><td>BTD|Btd|CG12653|DmelCG12653</td><td>0</td><td>0</td></tr>
<tr><td>761</td><td>31913</td><td>Drosophila melanogaster</td><td>Sp1</td><td>CG1343 gene product from transcript CG1343-RD</td><td>CG1343|D-Sp1|D-Sp8|D.Sp1|DmelCG1343|SP1|dSp1|sp1</td><td>0</td><td>0</td></tr>
<tr><td>762</td><td>31914</td><td>Drosophila melanogaster</td><td>CG1354</td><td>CG1354 gene product from transcript CG1354-RC</td><td>DmelCG1354</td><td>0</td><td>0</td></tr>
<tr><td>763</td><td>31915</td><td>Drosophila melanogaster</td><td>CG32698</td><td>CG32698 gene product from transcript CG32698-RB</td><td>CG9678|CG9683|DmelCG32698</td><td>0</td><td>0</td></tr>
<tr><td>764</td><td>31917</td><td>Drosophila melanogaster</td><td>Ser7</td><td>CG2045 gene product from transcript CG2045-RA</td><td>CG2045|DmelCG2045|SP#2|SP2|SP3|c-SP3</td><td>0</td><td>0</td></tr>
<tr><td>765</td><td>31918</td><td>Drosophila melanogaster</td><td>CG32695</td><td>CG32695 gene product from transcript CG32695-RA</td><td>BcDNA:RH68724|CG15246|DmelCG32695</td><td>0</td><td>0</td></tr>
<tr><td>766</td><td>31919</td><td>Drosophila melanogaster</td><td>CG15247</td><td>CG15247 gene product from transcript CG15247-RA</td><td>DmelCG15247</td><td>0</td><td>0</td></tr>
<tr><td>767</td><td>31920</td><td>Drosophila melanogaster</td><td>CG1468</td><td>CG1468 gene product from transcript CG1468-RA</td><td>BcDNA:GH18955|DmelCG1468</td><td>0</td><td>0</td></tr>
<tr><td>768</td><td>31921</td><td>Drosophila melanogaster</td><td>CG9686</td><td>CG9686 gene product from transcript CG9686-RA</td><td>BcDNA:RH74092|CT6802|DmelCG9686|anon-EST:Posey3</td><td>0</td><td>0</td></tr>
<tr><td>769</td><td>31922</td><td>Drosophila melanogaster</td><td>CG9689</td><td>CG9689 gene product from transcript CG9689-RA</td><td>BcDNA:GH05731|DmelCG9689</td><td>0</td><td>0</td></tr>
<tr><td>770</td><td>31923</td><td>Drosophila melanogaster</td><td>CG9691</td><td>CG9691 gene product from transcript CG9691-RB</td><td>BcDNA:RH12290|CT27394|DmelCG9691</td><td>0</td><td>0</td></tr>
<tr><td>771</td><td>31924</td><td>Drosophila melanogaster</td><td>CG15249</td><td>CG15249 gene product from transcript CG15249-RB</td><td>DmelCG15249</td><td>0</td><td>0</td></tr>
<tr><td>772</td><td>31925</td><td>Drosophila melanogaster</td><td>CG1986</td><td>CG1986 gene product from transcript CG1986-RA</td><td>DmelCG1986</td><td>0</td><td>0</td></tr>
<tr><td>773</td><td>31926</td><td>Drosophila melanogaster</td><td>Idgf4</td><td>Imaginal disc growth factor 4</td><td>CG1780|DmIDGF4|DmelCG1780|IDGF|IDGF-4|IDGF4|idgf4</td><td>0</td><td>0</td></tr>
<tr><td>774</td><td>31927</td><td>Drosophila melanogaster</td><td>CG1791</td><td>CG1791 gene product from transcript CG1791-RA</td><td>DmelCG1791|FBpp0071364</td><td>0</td><td>0</td></tr>
<tr><td>775</td><td>31928</td><td>Drosophila melanogaster</td><td>CG1889</td><td>CG1889 gene product from transcript CG1889-RA</td><td>DmelCG1889|FBpp0071343|FBpp0071344</td><td>0</td><td>0</td></tr>
</table>

776	31929	Drosophila melanogaster	CG43386	CG43386 gene product from transcript CG43386-RB	BcDNA:RE65233|CG15250|CG15251|DmelCG43386|Dmel_CG15250|Dmel_CG15251	0	0
777	31931	Drosophila melanogaster	CG15252	CG15252 gene product from transcript CG15252-RB	DmelCG15252	0	0
778	31933	Drosophila melanogaster	CG32694	CG32694 gene product from transcript CG32694-RC	BcDNA:RE02049|CG12634|CG12649|CG18512|DmelCG32694	0	0
779	31934	Drosophila melanogaster	CG2990	CG2990 gene product from transcript CG2990-RB	DmelCG2990	0	0
780	31935	Drosophila melanogaster	Cht6	CG43374 gene product from transcript CG43374-RC	CG15313|CG15314|CG2989|CG43374|CT5624|DmelCG43374|Dmel_CG15313|Dmel_CG15314|Dmel_CG2989	0	0
781	31938	Drosophila melanogaster	Yp2	Yolk protein 2	CG2979|DmelCG2979|VG2|VIT2Dm|YP|YP-2|YP2|YPII|YP[[2]]|fs(1)A59|fs(1)K313|fs(1)M35|fs(1)m35|yp|yp2	0	0
782	31939	Drosophila melanogaster	Yp1	Yolk protein 1	CG2985|DmYP1|DmelCG2985|VG1|VIT1Dm|YP|YP-1|YP1|YPI|YP[[1]]|fs(1)1163|fs(1)A1163|yp|yp-1|yp1	0	0
783	31940	Drosophila melanogaster	CG15312	CG15312 gene product from transcript CG15312-RE	CT35293|DmelCG15312	0	0
784	31942	Drosophila melanogaster	CG32685	CG32685 gene product from transcript CG32685-RC	CG12646|CG12647|CG2971|DmelCG32685	0	0
785	31943	Drosophila melanogaster	CG2972	CG2972 gene product from transcript CG2972-RA	DmelCG2972	0	0
786	31944	Drosophila melanogaster	CG2974	CG2974 gene product from transcript CG2974-RB	DmelCG2974	0	0
787	31945	Drosophila melanogaster	CG34104	CG34104 gene product from transcript CG34104-RB	CG12094|CG12102|DmelCG34104	0	0
788	31947	Drosophila melanogaster	CG12645	CG12645 gene product from transcript CG12645-RB	DmelCG12645	0	0
789	31948	Drosophila melanogaster	CG15311	CG15311 gene product from transcript CG15311-RA	DmelCG15311	0	0
790	31949	Drosophila melanogaster	CG15309	CG15309 gene product from transcript CG15309-RA	BcDNA:GH10478|DmelCG15309	0	0
791	31950	Drosophila melanogaster	l(1)G0230	lethal (1) G0230	CG2968|DmelCG2968|anon-WO03040301.109|anon-WO03040301.110	0	0
792	31952	Drosophila melanogaster	CG2962	CG2962 gene product from transcript CG2962-RA	DmelCG2962	0	0
793	31953	Drosophila melanogaster	CG34408	CG34408 gene product from transcript CG34408-RB	BcDNA:GH09358|CG2967|CG32689|DmelCG34408|Dmel_CG2967|Dmel_CG32689	0	0
794	31954	Drosophila melanogaster	Ipod	Interaction partner of Dnmt2	CG2961|DmelCG2961|IPOD	0	0
795	31955	Drosophila melanogaster	Hk	Hyperkinetic	CG17779|CG2287|CG32688|CG34319|CG43388|DmelCG43388|Dmel_CG32688|Dmel_CG34319	0	0
796	31957	Drosophila melanogaster	alpha-Man-I	alpha Mannosidase I	CG17809|CG18799|CG32682|CG32684|CG42275|DM-GI|DmelCG42275|Dmel_CG32682|Dmel_CG32684|MA12|MAN-1|Man'ase|alpha-Man-I|mas-1|mas1	0	0
797	31958	Drosophila melanogaster	CG2909	CG2909 gene product from transcript CG2909-RA	DmelCG2909	0	0
798	31960	Drosophila melanogaster	Gip	GIP-like	CG2227|DmelCG2227|T15	0	0
799	31961	Drosophila melanogaster	CG15306	CG15306 gene product from transcript CG15306-RA	DmelCG15306	0	0
800	31962	Drosophila melanogaster	CG15307	CG15307 gene product from transcript CG15307-RA	DmelCG15307	0	0
801	31963	Drosophila melanogaster	CG12641	CG12641 gene product from transcript CG12641-RA	DmelCG12641	0	0
802	31964	Drosophila melanogaster	l(1)G0289	lethal (1) G0289	CG2221|DmelCG2221	0	0
803	31965	Drosophila melanogaster	CG17841	CG17841 gene product from transcript CG17841-RA	BcDNA:GH12326|DmelCG17841|anon-EST:Liang-2.12|clone 2.12	0	0
804	31966	Drosophila melanogaster	CG2898	CG2898 gene product from transcript CG2898-RA	DmelCG2898	0	0
805	31967	Drosophila melanogaster	Psf3	CG2222 gene product from transcript CG2222-RA	CG2222|DmelCG2222	0	0
806	31970	Drosophila melanogaster	CG32683	CG32683 gene product from transcript CG32683-RA	CG15303|CG2881|CG2883|DmelCG32683	0	0
807	31971	Drosophila melanogaster	RabX2	CG2885 gene product from transcript CG2885-RA	CG2885|DmRabX2|DmelCG2885	0	0
808	31973	Drosophila melanogaster	CG12640	CG12640 gene product from transcript CG12640-RA	DmelCG12640	0	0
809	31975	Drosophila melanogaster	Or9a	Odorant receptor 9a	9E.1|9a|CG15302|DOR95|DmelCG15302|OR9a|or9a	0	0
810	31976	Drosophila melanogaster	nocte	no circadian temperature entrainment	CG17255|DmelCG17255	0	0
811	31977	Drosophila melanogaster	CG2889	CG2889 gene product from transcript CG2889-RA	DmelCG2889	0	0
812	31978	Drosophila melanogaster	CG2887	CG2887 gene product from transcript CG2887-RA	DmelCG2887	0	0
813	31979	Drosophila melanogaster	PPP4R2r	Protein phosphatase 4 regulatory subunit 2-related pro	BcDNA:LD29930|CG2890|DmelCG2890|PP4R|PP4R2|PPP4R2|R2	0	0
814	31980	Drosophila melanogaster	CG32687	CG32687 gene product from transcript CG32687-RA	BcDNA:RH29237|CG2143|CG2892|CT6984|CT7080|Cg32687|DmelCG32687	0	0
815	31986	Drosophila melanogaster	CG15296	CG15296 gene product from transcript CG15296-RA	BcDNA:AT26906|DmelCG15296	0	0
816	31987	Drosophila melanogaster	spri	sprint	CG12638|CG15297|CG15298|CG15299|CG15300|CG15301|CG32674|CG32680|CG33175|CG34414|DmelCG34414|Dmel_CG32680|Dmel_CG33175|Rin1|Spri|anon-WO03040301.275	0	0
817	31988	Drosophila melanogaster	Hmr	Hybrid male rescue	BcDNA:LD22117|CG1619|DmelCG1619|Hmr-mel|hmr	0	0
818	31989	Drosophila melanogaster	CG2124	CG2124 gene product from transcript CG2124-RA	DmelCG2124	0	0
819	31990	Drosophila melanogaster	CG1628	CG1628 gene product from transcript CG1628-RB	DmelCG1628	0	0
820	31991	Drosophila melanogaster	CG43347	CG43347 gene product from transcript CG43347-RB	BcDNA:RH11383|CG15295|CG9817|DmelCG43347|Dmel_CG15295|Dmel_CG9817	0	0
821	31993	Drosophila melanogaster	Rab9Db	Rab GTPase 9Db	CG9807|DmelCG9807	0	0
822	31994	Drosophila melanogaster	CG9806	CG9806 gene product from transcript CG9806-RA	DmelCG9806	0	0
823	31995	Drosophila melanogaster	CG2111	CG2111 gene product from transcript CG2111-RA	DmelCG2111|pepCG2111	0	0
824	31996	Drosophila melanogaster	CG12637	CG12637 gene product from transcript CG12637-RA	DmelCG12637	0	0
825	31997	Drosophila melanogaster	X11Lbeta	CG32677 gene product from transcript CG32677-RA	CG1852|CG1861|CG32677|DmelCG32677|Dmint2|X11B|x11Lbeta	0	0
826	32000	Drosophila melanogaster	CG1826	CG1826 gene product from transcript CG1826-RA	DmelCG1826	0	0
827	32001	Drosophila melanogaster	Atg8a	Autophagy-specific gene 8a	ATG8a|Atg8|Atg8/LC3|Atg8A|BcDNA:LD05816|CG1534|CG32672|DmelCG32672|DrAtg8a|atg8|atg8a	0	0
828	32002	Drosophila melanogaster	Rph	Rabphilin	CG11556|DmelCG11556	0	0
829	32005	Drosophila melanogaster	CG32676	CG32676 gene product from transcript CG32676-RA	CG9725|CG9732|DmelCG32676	0	0
830	32006	Drosophila melanogaster	CG15211	CG15211 gene product from transcript CG15211-RC	DmelCG15211	0	0
831	32007	Drosophila melanogaster	sesB	stress-sensitive B	A/A-T|A/A-T/sesB|ADP/ATP translocase|ADT2|ANT|CG16944|DmelCG16944|EM12|Hmr|S12|anon-WO02059370.55|dANT|jive|l(1)9Ed|l(1)9Fa|l(1)DC701|l(1)EM31|l(1)G0126|l(1)G0247|l(1)G0386|l(1)S12|orang	0	0
832	32008	Drosophila melanogaster	Ant2	Adenine nucleotide translocase 2	ANT2|CG1683|DmelCG1683|l(1)G0126|l(1)G0247|l(1)G0386	0	0
833	32009	Drosophila melanogaster	Imp	IGF-II mRNA-binding protein	CG1691|DmelCG1691|IGF-II|IMP|IMP-1|MRE11|ORE-9|anon-EST:fe2B3|anon-WO0140519.39|cg1691|dIMP|imp|l(1)G0072	0	0
834	32011	Drosophila melanogaster	CG15209	CG15209 gene product from transcript CG15209-RA	BcDNA:SD22308|DmelCG15209|anon-EST:Posey262	0	0
835	32013	Drosophila melanogaster	CG17333	CG17333 gene product from transcript CG17333-RA	DmelCG17333	0	0
836	32014	Drosophila melanogaster	CG2202	CG2202 gene product from transcript CG2202-RA	DmelCG2202	0	0
837	32015	Drosophila melanogaster	feo	fascetto	CG11207|DmelCG11207|EA86|Feo|Q54|l(1)9Fe|l(1)BP1	0	0
838	32016	Drosophila melanogaster	sofe	sister of feo	CG1655|DmelCG1655	0	0
839	32017	Drosophila melanogaster	CG2186	CG2186 gene product from transcript CG2186-RA	DmelCG2186	0	0
840	32018	Drosophila melanogaster	CG2157	CG2157 gene product from transcript CG2157-RA	DmelCG2157	0	0
841	32019	Drosophila melanogaster	CG1637	CG1637 gene product from transcript CG1637-RC	DmelCG1637	0	0
842	32020	Drosophila melanogaster	Ork1	Open rectifier K[+] channel 1	CG1615|DmelCG1615|KCNK0|K[[2P]]|ORK|ORK1|anon-WO0161006.1|anon-WO0161006.22|anon-WO0161006.23|d-ORK1|dORK|ork1	0	0
843	32021	Drosophila melanogaster	CG1582	CG1582 gene product from transcript CG1582-RA	DmelCG1582|cg1582	0	0
844	32022	Drosophila melanogaster	CG15208	CG15208 gene product from transcript CG15208-RA	DmelCG15208|anon-WO0140519.64	0	0
845	32023	Drosophila melanogaster	CG15207	CG15207 gene product from transcript CG15207-RA	DmelCG15207	0	0
846	32024	Drosophila melanogaster	CG11203	CG11203 gene product from transcript CG11203-RB	DmelCG11203	0	0
847	32026	Drosophila melanogaster	v	vermilion	152559_at|CG2155|DmelCG2155|NP_511113|Ver|ver|vm	0	0
848	32027	Drosophila melanogaster	CG2145	CG2145 gene product from transcript CG2145-RA	DmelCG2145	0	0
849	32028	Drosophila melanogaster	Myo10A	unconventional myosin class XV	10A|AAF47980|CG2136|CG2174|DmXV|DmelCG2174|Syph	0	0
850	32029	Drosophila melanogaster	Rab9Fb	Rab GTPase 9Fb	CG2532|CG32670|DmRabX3|DmelCG32670|RabX3	0	0
851	32030	Drosophila melanogaster	CG16922	CG16922 gene product from transcript CG16922-RA	DmelCG16922	0	0
852	32032	Drosophila melanogaster	C901	CG1567 gene product from transcript CG1567-RA	10|CG1567|CT4052|DmelCG1567|anon-10Ab|c901|cDNA 901	0	0
853	32033	Drosophila melanogaster	CG42339	CG42339 gene product from transcript CG42339-RB	CG12626|CG15204|DmelCG42339|Dmel_CG12626|Dmel_CG15204	0	0
854	32034	Drosophila melanogaster	CG11160	CG11160 gene product from transcript CG11160-RB	DmelCG11160	0	0
855	32035	Drosophila melanogaster	CG1552	CG1552 gene product from transcript CG1552-RB	DmelCG1552|anon-EST:Posey230	0	0
856	32036	Drosophila melanogaster	CG1545	CG1545 gene product from transcript CG1545-RB	BcDNA:GH01560|DmelCG1545|anon-EST:Posey256	0	0
857	32037	Drosophila melanogaster	CG1537	CG1537 gene product from transcript CG1537-RA	BcDNA:GH02938|DmelCG1537	0	0
858	32038	Drosophila melanogaster	CG15203	CG15203 gene product from transcript CG15203-RA	144845_at|DIGF-1|DmelCG15203	0	0
859	32039	Drosophila melanogaster	sev	sevenless	7LES|7less|CG18085|CT3980|DROSEV|DROSEV1|DmHD-265|DmelCG18085|HD-265|SEV|Sev|sev(AC)[[14]]	0	0
860	32040	Drosophila melanogaster	Vago	CG2081 gene product from transcript CG2081-RC	BcDNA:RH70969|CG2081|DIGF-2|DmelCG2081	0	0
861	32041	Drosophila melanogaster	CG2076	CG2076 gene product from transcript CG2076-RA	DmelCG2076	0	0
862	32042	Drosophila melanogaster	CG2061	CG2061 gene product from transcript CG2061-RC	BcDNA:LD28247|DmelCG2061	0	0
863	32043	Drosophila melanogaster	CG42249	CG42249 gene product from transcript CG42249-RC	CG11126|CG1961|DmelCG42249|Dmel_CG11126|Dmel_CG1961	0	0
864	32045	Drosophila melanogaster	Hsp60	Heat shock protein 60	12|BP5|CG12101|Cpn60|DmelCG12101|Dmhsp60|G62|HSP60|HSP60A|Hsp60A|IEF16|Mmp-P1|SSP 7506|cpn60|d-hsp60|hsp60|hsp60A|l(1)10Ac|l(1)BP5|l(1)G8|l(1)HM21|l(1)L12|l(1)dp025	0	0
865	32046	Drosophila melanogaster	CG11122	CG11122 gene product from transcript CG11122-RA	DmelCG11122	0	0
866	32047	Drosophila melanogaster	Rpt3	CG16916 gene product from transcript CG16916-RA	16916|CG16916|Dm_Rpt3a|DmelCG16916|Dmp48A|S6B|TBP7|l(1)G0052|p48A	0	0
867	32049	Drosophila melanogaster	Klp10A	CG1453 gene product from transcript CG1453-RA	CG1453|DmCG1453|DmKLP10A|DmKlp10A|DmelCG1453|KIF2A|KLP10A|KLP10a|Kif2A|anon-WO03040301.142|klp10A	0	0
868	32050	Drosophila melanogaster	CG18292	CG18292 gene product from transcript CG18292-RA	CDK2AP1|DOC1|DmelCG18292|anon-WO03040301.144	0	0
869	32051	Drosophila melanogaster	CG15199	CG15199 gene product from transcript CG15199-RA	DIGF-3|DmelCG15199	0	0
870	32052	Drosophila melanogaster	CG15202	CG15202 gene product from transcript CG15202-RB	BcDNA:GH13342|DIGF-4|DmelCG15202	0	0
871	32053	Drosophila melanogaster	CG15201	CG15201 gene product from transcript CG15201-RA	BcDNA:RH06304|DIGF-5|DmelCG15201	0	0
872	32055	Drosophila melanogaster	CG1908	CG1908 gene product from transcript CG1908-RB	DmelCG1908	0	0
873	32056	Drosophila melanogaster	rtv	retroactive	1|CG1397|DmelCG1397|L1|l(1)10Ag|l(1)G13|l(1)L1	0	0
874	32057	Drosophila melanogaster	Dlic	Dynein light intermediate chain	CG1938|D-LIC|DLIC|DLIC2|Dlic2|DmelCG1938|dlic|dlic2|l(1)G0065|l(1)G0190	0	0
875	32058	Drosophila melanogaster	antdh	CG1386 gene product from transcript CG1386-RA	AntDH|Antdh|CG1386|DmelCG1386	0	0
876	32059	Drosophila melanogaster	CG1394	CG1394 gene product from transcript CG1394-RA	BEST:GH24085|DmelCG1394|anon-WO0140519.177|bs30g07.y1	0	0
877	32060	Drosophila melanogaster	CG15200	CG15200 gene product from transcript CG15200-RA	DmelCG15200	0	0
878	32062	Drosophila melanogaster	CG11106	CG11106 gene product from transcript CG11106-RA	BcDNA:AT24116|DmelCG11106	0	0
879	32063	Drosophila melanogaster	CG42683	CG42683 gene product from transcript CG42683-RB	CG11105|CG12625|DmelCG42683|Dmel_CG11105|Dmel_CG12625	0	0
880	32065	Drosophila melanogaster	CG15198	CG15198 gene product from transcript CG15198-RA	DmelCG15198	0	0
881	32067	Drosophila melanogaster	Ir10a	Ionotropic receptor 10a	CG12623|CG12624|CG34143|DmelCG34143|IR10a	0	0
882	32068	Drosophila melanogaster	sisA	sisterless A	CG1641|DmelCG1641|sis A|sis a|sis-A|sis-a|sisa	0	0
883	32069	Drosophila melanogaster	l(1)10Bb	lethal (1) 10Bb	10Ba|CG1639|DmelCG1639|l(1)G0169|l(1)G14|l(1)GLM14	0	0
884	32070	Drosophila melanogaster	CG1657	CG1657 gene product from transcript CG1657-RA	DmelCG1657	0	0
885	32071	Drosophila melanogaster	Kap3	Kinesin associated protein 3	CG11759|DmKAP|DmKap|DmelCG11759|KAP3|Kap|dKAP3|kap3	0	0
886	32072	Drosophila melanogaster	CG34348	CG34348 gene product from transcript CG34348-RA	CG11757|CG15197|DmelCG34348|Dmel_CG11757	0	0

887	32073	Drosophila melanogaster	CG1597	CG1597 gene product from transcript CG1597-RB	DmelCG1597	0	0
888	32074	Drosophila melanogaster	CG11756	CG11756 gene product from transcript CG11756-RA	BcDNA:AT06648|DmelCG11756	0	0
889	32075	Drosophila melanogaster	CG1738	CG1738 gene product from transcript CG1738-RA	BcDNA:RE11532|DmelCG1738	0	0
890	32076	Drosophila melanogaster	CG11752	CG11752 gene product from transcript CG11752-RA	BcDNA:RH44935|DmelCG11752|anon-EST:Posey214	0	0
891	32077	Drosophila melanogaster	CG1737	CG1737 gene product from transcript CG1737-RA	DmelCG1737	0	0
892	32078	Drosophila melanogaster	dsh	dishevelled	CG1720|CG18361|DSH|Dm Dsh|DmelCG18361|Dsh|Dvl|l(1)10Bd|l(1)G0267|l(1)G17|l(1)dsh	0	0
893	32079	Drosophila melanogaster	CG11750	CG11750 gene product from transcript CG11750-RA	BcDNA:RE09126|BcDNA:RH21749|DmelCG11750	0	0
894	32080	Drosophila melanogaster	hop	hopscotch	4|CG1594|Dm JAK|DmHD-160|DmelCG1594|HD-160|HOP|Hop|Hop1|JAK|Jak|L4|Tum|Tum-1|d-jak|jak|l(1)10Be|l(1)G18|l(1)L4|l(1)hop|msvl	0	0
895	32081	Drosophila melanogaster	Tim8	CG1728 gene product from transcript CG1728-RA	BcDNA:SD06593|CG1728|DmelCG1728	0	0
896	32083	Drosophila melanogaster	dlg1	discs large 1	11|CG1725|CG1730|CPD|DLG|DLG-A|Discs-large|Dlg|Dlg-A|Dlg1|DlgA|DmelCG1725|Drodlg|PSD95|SAP97|anon-EST:Posey93|anon-WO03040301.258|anon-WO03040301.260|anon-WO03040301.268|d.	0	0
897	32084	Drosophila melanogaster	CG15196	CG15196 gene product from transcript CG15196-RB	DmelCG15196	0	0
898	32085	Drosophila melanogaster	Gr10b	Gustatory receptor 10b	CG12622|DmelCG12622|Gr10B2	0	0
899	32086	Drosophila melanogaster	Or10a	Odorant receptor 10a	10B.1|10a|CG17867|DOR92|DmelCG17867	0	0
900	32087	Drosophila melanogaster	Gs2	Glutamine synthetase 2	CG1743|DmelCG1743|GS2|GsII|gs-c|gs2	0	0
901	32088	Drosophila melanogaster	CG11727	CG11727 gene product from transcript CG11727-RA	BcDNA:GH08923|BcDNA:LD11130|DmelCG11727|MRE21	0	0
902	32089	Drosophila melanogaster	Sk1	Sphingosine kinase 1	CG1747|DmelCG1747|SK1|Sphk1	0	0
903	32090	Drosophila melanogaster	HP5	Heterochromatin protein 5	CG1745|DmelCG1745	0	0
904	32092	Drosophila melanogaster	CG43155	CG43155 gene product from transcript CG43155-RC	CG15194|CG1756|DmelCG43155|Dmel_CG15194|Dmel_CG1756|anon-WO0109301.51|anon-WO0109301.52|anon-WO0109301.9	0	0
905	32093	Drosophila melanogaster	Cyp4g15	CG11715 gene product from transcript CG11715-RA	152658_at|4g15|CG11715|DmelCG11715	0	0
906	32094	Drosophila melanogaster	CG1749	CG1749 gene product from transcript CG1749-RA	DmelCG1749	0	0
907	32095	Drosophila melanogaster	Spase25	Spase 25-subunit	CG1751|DmelCG1751	0	0
908	32097	Drosophila melanogaster	Drak	Death-associated protein kinase related	CG11713|CG1760|CG32666|DmelCG32666|EP(X)1452|EP1452|drak	0	0
909	32098	Drosophila melanogaster	CG1572	CG1572 gene product from transcript CG1572-RA	DmelCG1572	0	0
910	32099	Drosophila melanogaster	PGRP-SA	Peptidoglycan recognition protein SA	BcDNA:AT30827|CG11709|Dm PGRP-SA|DmelCG11709|SA|SEMMELWEIS|seml	0	0
911	32100	Drosophila melanogaster	RpII215	RNA polymerase II 215kD subunit	5|8WG16|CG1554|CTD|DmelCG1554|H5|II|L5|POL|Pol II|Pol II CTD|Pol II0[ser2]|Pol II0[ser5]|Pol II[ser2]|Pol IIa|Pol IIo|Pol IIo[Ser2]|Pol IIo[ser2]|Pol IIo[ser5]|Pol-IIa|PolII|PolIIo|RNA Pol II|RNA Pol	0	0
912	32101	Drosophila melanogaster	CG11699	CG11699 gene product from transcript CG11699-RA	DmelCG11699	0	0
913	32102	Drosophila melanogaster	Kmn1	kinetochore Mis12-Ndc80 network component 1	CG1558|CT4038|DmelCG1558|Nsl1|anon-WO03040301.201|dmNsl1R|l(1)G0237	0	0
914	32103	Drosophila melanogaster	CG11697	CG11697 gene product from transcript CG11697-RA	DmelCG11697|a|anon-WO03040301.203	0	0
915	32104	Drosophila melanogaster	CG11696	CG11696 gene product from transcript CG11696-RA	DmelCG11696|b	0	0
916	32106	Drosophila melanogaster	CG11695	CG11695 gene product from transcript CG11695-RB	DmelCG11695|c	0	0
917	32107	Drosophila melanogaster	nod	no distributive disjunction	CG1763|DmNod|DmelCG1763|DnMod|KIF22|NOD|Nod|l(1)TW6	0	0
918	32108	Drosophila melanogaster	CG1561	CG1561 gene product from transcript CG1561-RB	DmelCG1561|e	0	0
919	32109	Drosophila melanogaster	rho-4	rhomboid-4	CG1697|DmelCG1697|Rho-4|Rho-related [10C6]|rho 4|rho4	0	0
920	32110	Drosophila melanogaster	CG2533	CG2533 gene product from transcript CG2533-RA	BcDNA:AT28768|DmelCG2533	0	0
921	32111	Drosophila melanogaster	CG2247	CG2247 gene product from transcript CG2247-RA	DmelCG2247	0	0
922	32112	Drosophila melanogaster	CG1703	CG1703 gene product from transcript CG1703-RA	DmelCG1703	0	0
923	32113	Drosophila melanogaster	CG15220	CG15220 gene product from transcript CG15220-RA	BcDNA:RH55360|DmelCG15220	0	0
924	32114	Drosophila melanogaster	Met	Methoprene-tolerant	CG1705|DmMet|DmelCG1705|Mett|Rst(1)JH|bHLHe59|met	0	0
925	32115	Drosophila melanogaster	Ptp10D	Protein tyrosine phosphatase 10D	CG1817|CT4920|DPTP|DPTP10D|DPTP[[10D]]|DmelCG1817|PTP10D|Ptp10|R-PTP 10D|chi|dptp|ptp10D	0	0
926	32116	Drosophila melanogaster	CG2371	CG2371 gene product from transcript CG2371-RB	CG2442|CG2443|DmelCG2371	0	0
927	32119	Drosophila melanogaster	bif	bifocal	CG1822|DmelCG1822	0	0
928	32120	Drosophila melanogaster	PhKgamma	Phosphorylase kinase gamma	CG1830|DPhK-gamma|DmelCG1830|EP779|ESTS:194B3T|Phkgamma|anon-sts6	0	0
929	32121	Drosophila melanogaster	CG2444	CG2444 gene product from transcript CG2444-RA	BcDNA:LP01642|DIGF-6|DmelCG2444	0	0
930	32122	Drosophila melanogaster	FucT6	alpha1,6-fucosyltransferase	6-FucT|BEST:CK00490|CG2448|CK00490|Dm alpha1|DmelCG2448|Fuc-TVIII	0	0
931	32123	Drosophila melanogaster	Amun	CG2446 gene product from transcript CG2446-RB	CG2446|DmelCG2446|EP1503|anon-WO03040301.104|anon-WO03040301.105|anon-WO03040301.107	0	0
932	32124	Drosophila melanogaster	prtp	pretaporter	CG1837|DmelCG1837|anon-EST:Posey253|p47	0	0
933	32125	Drosophila melanogaster	Tango10	Transport and Golgi organization 10	CG1841|DmelCG1841|TANGO10	0	0
934	32126	Drosophila melanogaster	inaF-D	CG42563 gene product from transcript CG42563-RB	CG2457|CG42563|DmelCG42563|Dmel_CG2457|INAF|InaF|anon-WO0172774.148|inaF	0	0
935	32127	Drosophila melanogaster	CG15221	CG15221 gene product from transcript CG15221-RB	DmelCG15221	0	0
936	32128	Drosophila melanogaster	CG9360	CG9360 gene product from transcript CG9360-RA	DmelCG9360	0	0
937	32130	Drosophila melanogaster	dy	dusky	And|CG9355|DmelCG9355|ala|dy1	0	0
938	32131	Drosophila melanogaster	Karl	CG4139 gene product from transcript CG4139-RB	CG4139|DmelCG4139|karl	0	0
939	32132	Drosophila melanogaster	CkIIbeta	Casein kinase II beta subunit	And|CCK2|CG15224|CK II|CK-2|CK-II beta|CK-IIbeta|CK2|CK2-beta|CK2beta|CKII|CKII beta|CKII-beta1|CKIIb|CKIIbeta|Cask-II-b|Ck-IIbeta|CkII|CkII-beta|CkIIbeta1|DmCK2beta|DmCKIIbeta|DmCkIIbeta|D	0	0
940	32133	Drosophila melanogaster	Hsc70-3	Heat shock protein cognate 3	BiP|Bip|CG1579|CG4147|DmelCG4147|Grp78|HSC-70|HSC3|HSC70|HSC70-3|Hsc-3|Hsc-70-3|Hsc3|Hsc3p|Hsc70|anon-WO0138581.7|bip|dhsc70|dmHsc72|hsc3|hsc70|hsc70-3|hsc72|l(1)G0102|l(1)G01	0	0
941	32135	Drosophila melanogaster	CG1578	CG1578 gene product from transcript CG1578-RA	DmelCG1578	0	0
942	32136	Drosophila melanogaster	CG43154	CG43154 gene product from transcript CG43154-RD	CG1905|CG32662|CG32663|DmelCG43154|Dmel_CG32662|Dmel_CG32663|anon-EST:Liang-2.41|clone 2.41	0	0
943	32137	Drosophila melanogaster	CG15741	CG15741 gene product from transcript CG15741-RA	DmelCG15741	0	0
944	32138	Drosophila melanogaster	Cyp28c1	CG1895 gene product from transcript CG1895-RA	28c1|CG1895|DmelCG1895	0	0
945	32139	Drosophila melanogaster	CG15740	CG15740 gene product from transcript CG15740-RB	DmelCG15740|i175	0	0
946	32140	Drosophila melanogaster	p24-1	p24-related-1	CG1967|DmelCG1967|anon-EST:Liang-1.45|clone 1.45|p24|p24.1	0	0
947	32141	Drosophila melanogaster	CG10347	CG10347 gene product from transcript CG10347-RB	DmelCG10347|HSP20|Q9VYT5	0	0
948	32142	Drosophila melanogaster	ATP7	CG1886 gene product from transcript CG1886-RB	CG1886|DmATP7|DmelCG1886|dmATP7	0	0
949	32143	Drosophila melanogaster	CG2025	CG2025 gene product from transcript CG2025-RA	DmelCG2025	0	0
950	32144	Drosophila melanogaster	CG1847	CG1847 gene product from transcript CG1847-RA	DmelCG1847	0	0
951	32145	Drosophila melanogaster	CG11802	CG11802 gene product from transcript CG11802-RA	DmelCG11802	0	0
952	32146	Drosophila melanogaster	CG15739	CG15739 gene product from transcript CG15739-RA	DmelCG15739|Phos15739	0	0
953	32147	Drosophila melanogaster	CG10352	CG10352 gene product from transcript CG10352-RA	DmelCG10352|Phos10352	0	0
954	32148	Drosophila melanogaster	CG10353	CG10353 gene product from transcript CG10353-RE	DmelCG10353	0	0
955	32149	Drosophila melanogaster	SelG	Selenoprotein G	BcDNA:GH03581|CG1844|DmelCG1844|G-rich|SelK|dselG|dselK	0	0
956	32150	Drosophila melanogaster	CG1840	CG1840 gene product from transcript CG1840-RA	AAF48112|BcDNA:LD12750|DmelCG1840	0	0
957	32151	Drosophila melanogaster	CG15738	CG15738 gene product from transcript CG15738-RA	DmelCG15738	0	0
958	32152	Drosophila melanogaster	wisp	wispy	CG15737|DmCG15737|DmelCG15737|TAMP|fs(1)M19	0	0
959	32153	Drosophila melanogaster	Upf1	CG1559 gene product from transcript CG1559-RA	CG1559|Dm-Upf1|DmUpf1|DmelCG1559|UPF1|Upf1p|upf1	0	0
960	32154	Drosophila melanogaster	pot	papillote	CG2467|DmelCG2467	0	0
961	32156	Drosophila melanogaster	Sclp	CG2471 gene product from transcript CG2471-RA	CG2471|CT8171|DmelCG2471|dSCLP	0	0
962	32157	Drosophila melanogaster	CG10362	CG10362 gene product from transcript CG10362-RA	DmelCG10362	0	0
963	32158	Drosophila melanogaster	cac	cacophony	13|CG1522|CG15928|CG43368|Ca-alpha1A|Ca[[V]]2.1|Cac|Dmca1A|Dmcalpha1A|DmelCG43368|Dmel_CG1522|Dmel_CG15928|EE171|H18|H47|L13|P18|alpha1A|cac1|l(1)11Aa|l(1)13|l(1)L13|ldg|nbA|nonB	0	0
964	32159	Drosophila melanogaster	gd	gastrulation-defective	CG1505|DmelCG1505|GD|SP186|fs(1)18|fs(1)190-5|fs(1)573|fs(1)A573|fs(1)M18|fs(1)gd|gas	0	0
965	32160	Drosophila melanogaster	tsg	twisted gastrulation	CG1502|DmelCG1502|RF32|Tsg|dTsg|l(1)11Ab|l(1)11Ac|l(1)RF32|l(1)tsg	0	0
966	32161	Drosophila melanogaster	CG18130	CG18130 gene product from transcript CG18130-RA	DmelCG18130	0	0
967	32162	Drosophila melanogaster	fw	furrowed	CG1500|CT3745|DmelCG1500|fw-like|wr	0	0
968	32163	Drosophila melanogaster	CG1806	CG1806 gene product from transcript CG1806-RA	DmelCG1806	0	0
969	32164	Drosophila melanogaster	CG1492	CG1492 gene product from transcript CG1492-RA	DmelCG1492	0	0
970	32165	Drosophila melanogaster	regucalcin	CG1803 gene product from transcript CG1803-RA	CG1803|DmelCG1803|Regucalcin|T1	0	0
971	32166	Drosophila melanogaster	Chrac-16	CG15736 gene product from transcript CG15736-RA	15736|BcDNA:RE59557|CG15736|CHRAC|CHRAC-16|CHRAC16|Chrac16|DmelCG15736|joey	0	0
972	32167	Drosophila melanogaster	CG15735	CG15735 gene product from transcript CG15735-RA	BcDNA:LD05893|DmelCG15735	0	0
973	32168	Drosophila melanogaster	Tango4	Transport and Golgi organization 4	CG1796|DmelCG1796|TANGO4	0	0
974	32169	Drosophila melanogaster	Usp7	ubiquitin-specific protease 7	CG1490|DmelCG1490|USP7	0	0
975	32170	Drosophila melanogaster	Cyp311a1	CG1488 gene product from transcript CG1488-RB	311a1|CG1488|DmelCG1488	0	0
976	32172	Drosophila melanogaster	Cyp318a1	CG1786 gene product from transcript CG1786-RA	318a1|CG11364|CG1786|DmelCG1786	0	0
977	32173	Drosophila melanogaster	CG1950	CG1950 gene product from transcript CG1950-RA	DmelCG1950	0	0
978	32174	Drosophila melanogaster	Muc11A	Mucin 11A	CG2779|CG32656|DmelCG32656	0	0
979	32176	Drosophila melanogaster	CG12721	CG12721 gene product from transcript CG12721-RA	BcDNA:RE60404|DmelCG12721	0	0
980	32177	Drosophila melanogaster	CG15734	CG15734 gene product from transcript CG15734-RD	DmelCG15734	0	0
981	32178	Drosophila melanogaster	CG11356	CG11356 gene product from transcript CG11356-RA	DmelCG11356	0	0
982	32179	Drosophila melanogaster	CG2750	CG2750 gene product from transcript CG2750-RB	DmelCG2750|anon-WO0140519.124	0	0
983	32180	Drosophila melanogaster	CG1924	CG1924 gene product from transcript CG1924-RA	DmelCG1924	0	0
984	32183	Drosophila melanogaster	Ten-a	Tenascin accessory	1.2|CG11270|CG12720|CG15733|CG18182|CG2578|CG2590|CG32659|CG42338|DmelCG42338|Dmel_CG12720|Dmel_CG15733|Dmel_CG32659|DmtenA|Dmtena|Ten11A|Ten[a]|ten-a|tenA|tenA(AT)[[14]]|t	0	0
985	32188	Drosophila melanogaster	CG2577	CG2577 gene product from transcript CG2577-RA	DmelCG2577	0	0
986	32189	Drosophila melanogaster	Ir11a	Ionotropic receptor 11a	CG15732|CT35969|DmelCG15732|IR11a	0	0
987	32190	Drosophila melanogaster	CG2574	CG2574 gene product from transcript CG2574-RA	DmelCG2574|bs21b01.y1	0	0
988	32191	Drosophila melanogaster	Pkcdelta	Protein kinase C delta	CG10524|CG11245|CG42349|DmelCG42349|Dmel_CG10524|Dmel_CG11245|PKCd	0	0
989	32193	Drosophila melanogaster	CG15927	CG15927 gene product from transcript CG15927-RA	DmelCG15927	0	0
990	32194	Drosophila melanogaster	CG15731	CG15731 gene product from transcript CG15731-RA	DmelCG15731	0	0
991	32195	Drosophila melanogaster	Rab40	CG1900 gene product from transcript CG1900-RA	CG1900|DmRab40|DmelCG1900|rab40	0	0
992	32196	Drosophila melanogaster	CG42258	CG42258 gene product from transcript CG42258-RC	BcDNA:RE29468|CG15926|CG17788|DmelCG42258|Dmel_CG15926|Dmel_CG17788	0	0
993	32198	Drosophila melanogaster	Cpr11A	Cuticular protein 11A	CG2560|DmelCG2560|L12	0	0
994	32199	Drosophila melanogaster	Lsp1alpha	Larval serum protein 1 alpha	CG2559|DmeLSP1a|DmelCG2559|LSP 1|LSP-1|LSP-1 alpha|LSP-1alpha|LSP1|LSP1-alpha|LSP1alpha|Lsp-1alpha|Lsp1|Lsp1-alpha	0	0
995	32200	Drosophila melanogaster	CG15730	CG15730 gene product from transcript CG15730-RA	DmelCG15730	0	0
996	32201	Drosophila melanogaster	CG2556	CG2556 gene product from transcript CG2556-RA	DmelCG2556	0	0
997	32202	Drosophila melanogaster	CG42251	CG42251 gene product from transcript CG42251-RC	BcDNA:GH02424|CG11146|CG32653|DmelCG42251|Dmel_CG11146|Dmel_CG32653	0	0

998	32203	Drosophila melanogaster	Cpr11B	Cuticular protein 11B	CG2555|DmelCpr11B|DmelCG2555	0	0
999	32204	Drosophila melanogaster	CG15729	CG15729 gene product from transcript CG15729-RA	DmelCG15729	0	0
1000	32205	Drosophila melanogaster	CG11138	CG11138 gene product from transcript CG11138-RC	BEST:RE18684|BcDNA:RE48052|CG1855|DmelCG11138|EP(X)1313|RE18684	0	0
1001	32207	Drosophila melanogaster	LIMK1	LIM-kinase1	CG1848|D-LIMK1|D-Limk|DLIMK|DLIMK1|Dlimk|DmelCG1848|LIM-kinase 1|LIMK|LimK|Limk|dLIMK|dLIMK1|limk	0	0
1002	32208	Drosophila melanogaster	CG1824	CG1824 gene product from transcript CG1824-RA	DmelCG1824	0	0
1003	32209	Drosophila melanogaster	Sec16	CG32654 gene product from transcript CG32654-RE	BcDNA:RE70141|CG18079|CG2551|CG32654|DmelCG32654|dSec16	0	0
1004	32211	Drosophila melanogaster	CG1463	CG1463 gene product from transcript CG1463-RA	DmelCG1463	0	0
1005	32212	Drosophila melanogaster	CG2543	CG2543 gene product from transcript CG2543-RA	DmelCG2543	0	0
1006	32213	Drosophila melanogaster	CG11085	CG11085 gene product from transcript CG11085-RA	DmelCG11085	0	0
1007	32214	Drosophila melanogaster	CG15728	CG15728 gene product from transcript CG15728-RB	DmelCG15728	0	0
1008	32215	Drosophila melanogaster	Aven	Aven ortholog	CG15727|DmelCG15727|dAven	0	0
1009	32216	Drosophila melanogaster	CG2540	CG2540 gene product from transcript CG2540-RA	DmelCG2540	0	0
1010	32217	Drosophila melanogaster	tomosyn	CG17762 gene product from transcript CG17762-RI	BcDNA:RH19475|CG11056|CG17762|CG2205|DmelCG17762|Tomosyn	0	0
1011	32221	Drosophila melanogaster	CkIalpha	Casein kinase Ialpha	CG2028|CK I|CK1|CK1a|CK1alpha|CKI|CKI alpha|CKIa|CKIalpha|CkIa|DmelCG2028|PKA-C|anon-WO03040301.93|anon-WO03040301.95|ck1a|ck1alpha|dmCK1|dmckI|l(1)G0492	0	0
1012	32222	Drosophila melanogaster	Tis11	Tis11 homolog	CG4070|DTIS11|DmelCG4070|TIS11|TIS11cc1|TIScc1|anon-EST:Liang-1.28|clone 1.28|dTTP|dTis11|l(1)G0124	0	0
1013	32224	Drosophila melanogaster	CG15725	CG15725 gene product from transcript CG15725-RA	DmelCG15725	0	0
1014	32225	Drosophila melanogaster	Smr	Smrter	CG4013|DmelCG4013|E52|SMR|SMRTER|anon-WO0153538.73|l(1)G0361|smr	0	0
1015	32226	Drosophila melanogaster	CG4004	CG4004 gene product from transcript CG4004-RA	CG18137|DmelCG4004	0	0
1016	32228	Drosophila melanogaster	ade5	CG3989 gene product from transcript CG3989-RA	AIRc-SAICARs|Ade5|CG3989|DmelCG3989	0	0
1017	32229	Drosophila melanogaster	CG12717	CG12717 gene product from transcript CG12717-RA	DmelCG12717	0	0
1018	32230	Drosophila melanogaster	CG3812	CG3812 gene product from transcript CG3812-RB	AGPAT2|DmelCG3812	0	0
1019	32233	Drosophila melanogaster	Pde9	Phosphodiesterase 9	CG1627|CG32648|CG3754|CG3761|CG3765|CG42276|DmelCG42276|Dmel_CG32648|Dmel_CG3754|PDE9	0	0
1020	32234	Drosophila melanogaster	CG3775	CG3775 gene product from transcript CG3775-RA	BEST:GH06882|DmelCG3775	0	0
1021	32238	Drosophila melanogaster	CG4661	CG4661 gene product from transcript CG4661-RA	DmelCG4661	0	0
1022	32239	Drosophila melanogaster	CG32649	CG32649 gene product from transcript CG32649-RA	CG4410|DmelCG32649	0	0
1023	32240	Drosophila melanogaster	CG4407	CG4407 gene product from transcript CG4407-RA	DmelCG4407|Fad1	0	0
1024	32241	Drosophila melanogaster	CG4404	CG4404 gene product from transcript CG4404-RA	DmelCG4404	0	0
1025	32242	Drosophila melanogaster	mRpL49	mitochondrial ribosomal protein L49	BcDNA:RE35556|CG4647|DmelCG4647|MRP-L49|mRpl49	0	0
1026	32243	Drosophila melanogaster	CG4400	CG4400 gene product from transcript CG4400-RA	BRMS1L|DmelCG4400	0	0
1027	32244	Drosophila melanogaster	CG4645	CG4645 gene product from transcript CG4645-RA	DmelCG4645	0	0
1028	32245	Drosophila melanogaster	fne	found in neurons	BEST:HL02010|BcDNA:HL02010|BcDNA:RE25290|CG4396|CR32646|DmelCG4396|Dmel_CR32646|Fne|MRE28|cg4396	0	0
1029	32246	Drosophila melanogaster	CG4395	CG4395 gene product from transcript CG4395-RA	DmelCG4395|anon-WO0170980.1|anon-WO0170980.2	0	0
1030	32247	Drosophila melanogaster	CG15721	CG15721 gene product from transcript CG15721-RB	DmelCG15721	0	0
1031	32248	Drosophila melanogaster	CG12716	CG12716 gene product from transcript CG12716-RA	DmelCG12716	0	0
1032	32249	Drosophila melanogaster	CG15719	CG15719 gene product from transcript CG15719-RC	DmelCG15719	0	0
1033	32250	Drosophila melanogaster	CG2209	CG2209 gene product from transcript CG2209-RA	DmelCG2209|anon-WO0140519.110	0	0
1034	32251	Drosophila melanogaster	rad	radish	CG15720|CG42628|CG4346|DmelCG42628|Dmel_CG15720|Dmel_CG4346|rsh	0	0
1035	32252	Drosophila melanogaster	CG12715	CG12715 gene product from transcript CG12715-RA	DmelCG12715	0	0
1036	32253	Drosophila melanogaster	CG42629	CG42629 gene product from transcript CG42629-RB	CG15720|CG18646|CG4368|DmelCG42629|Dmel_CG18646	0	0
1037	32256	Drosophila melanogaster	hep	hemipterous	CG2190|CG4353|D-MKK7|DHEP/MKK7|DJNKK|DMKK7|DmelCG4353|HEP|HEP/MKK7|Hep|Hep/Mkk7|JNKK|MAPKK|MKK7|Mkk7|dJNKK|hem|hp|l(1)7P1|l(1)G0107|l(1)G0208	0	0
1038	32257	Drosophila melanogaster	lic	licorne	CG12244|D-MKK3|DMEK3|DMKK3|DMKK3/lic|DmMKK3|DmelCG12244|MEK3|MEK3/MKK3|MKK3|Mek3|Mkk3|Mpk3|dMKK3|l(1)G0252|p38|p38MAPKK	0	0
1039	32258	Drosophila melanogaster	CG2200	CG2200 gene product from transcript CG2200-RA	DmelCG2200	0	0
1040	32259	Drosophila melanogaster	CG43313	CG43313 gene product from transcript CG43313-RA	CG12714|CG4351|DmelCG43313|Dmel_CG12714|Dmel_CG4351	0	0
1041	32260	Drosophila melanogaster	Fer3HCH	Ferritin 3 heavy chain homologue	CG4349|DmelCG4349	0	0
1042	32261	Drosophila melanogaster	CG42237	CG42237 gene product from transcript CG42237-RA	C133|CG4346|DmelCG42237|Dmel_CG4346|PLA2	0	0
1043	32262	Drosophila melanogaster	CG15717	CG15717 gene product from transcript CG15717-RA	DmelCG15717	0	0
1044	32263	Drosophila melanogaster	CG4330	CG4330 gene product from transcript CG4330-RA	AA202479|DmelCG4330	0	0
1045	32264	Drosophila melanogaster	CG32645	CG32645 gene product from transcript CG32645-RB	CG15716|CG4331|DmelCG32645	0	0
1046	32266	Drosophila melanogaster	CG4318	CG4318 gene product from transcript CG4318-RB	DmelCG4318	0	0
1047	32267	Drosophila melanogaster	CG4332	CG4332 gene product from transcript CG4332-RA	DmelCG4332	0	0
1048	32268	Drosophila melanogaster	Bap60	Brahma associated protein 60kD	BAP60|BAPp60|CG4303|DmelCG4303|i127|i133	0	0
1049	32269	Drosophila melanogaster	CG12096	CG12096 gene product from transcript CG12096-RA	12096|DmelCG12096	0	0
1050	32271	Drosophila melanogaster	CG12723	CG12723 gene product from transcript CG12723-RA	DmelCG12723	0	0
1051	32272	Drosophila melanogaster	CG2453	CG2453 gene product from transcript CG2453-RA	DmelCG2453	0	0
1052	32273	Drosophila melanogaster	sno	strawberry notch	CG1903|DmelCG1903|Sno|g-l|l(1)11Ea	0	0
1053	32274	Drosophila melanogaster	REG	CG1591 gene product from transcript CG1591-RA	CG1591|DmelCG1591|REGgamma|dREG|dREGgamma	0	0
1054	32275	Drosophila melanogaster	mew	multiple edematous wings	CG1771|CT5254|DmelCG1771|PS1|PS1alpha|PSalpha1|aPS1|alpha-PS1|alpha1Int|alphaPS1|alpha[[PS1]]|l(1)G0429|l(1)G0443	0	0
1055	32276	Drosophila melanogaster	CG15742	CG15742 gene product from transcript CG15742-RB	DmelCG15742	0	0
1056	32278	Drosophila melanogaster	HDAC4	CG1770 gene product from transcript CG1770-RD	CG1770|DHDAC4|DmelCG1770|GC1770|HDAC|HDAC4a|dHDAC4|dmHDA405	0	0
1057	32279	Drosophila melanogaster	CG15743	CG15743 gene product from transcript CG15743-RA	DmelCG15743	0	0
1058	32281	Drosophila melanogaster	CG1764	CG1764 gene product from transcript CG1764-RA	DmelCG1764|cg1764	0	0
1059	32282	Drosophila melanogaster	CG1622	CG1622 gene product from transcript CG1622-RA	DmelCG1622	0	0
1060	32283	Drosophila melanogaster	CG15745	CG15745 gene product from transcript CG15745-RB	DmelCG15745	0	0
1061	32285	Drosophila melanogaster	IP3K2	Inositol 1,4,5-triphosphate kinase 2	CG12724|CG1630|CG34359|D-IP3K2|DIP3K2|DmelCG34359|Dmel_CG12724|Dmel_CG1630|IP[[3]]K-2|IP[[3]]K2|NP2758|dmIP3K2|dmIP3Kbeta|dmIP[[3]]beta	0	0
1062	32288	Drosophila melanogaster	CG15747	CG15747 gene product from transcript CG15747-RA	DmelCG15747	0	0
1063	32290	Drosophila melanogaster	Syt12	Synaptotagmin 12	CG10617|DmelCG10617|Syt 12|dsyt12|syt12	0	0
1064	32291	Drosophila melanogaster	dmrt11E	doublesex-Mab related 11E	CG15749|DmelCG15749|Dmrt11E	0	0
1065	32292	Drosophila melanogaster	CG1640	CG1640 gene product from transcript CG1640-RB	DmelCG1640	0	0
1066	32293	Drosophila melanogaster	Rbp1-like	CG1987 gene product from transcript CG1987-RA	BEST:HL02433|BcDNA:RE47308|CG1987|DmelCG1987|RBP1-like|i159	0	0
1067	32294	Drosophila melanogaster	Tim9a	CG1660 gene product from transcript CG1660-RA	CG1660|DmelCG1660|Tim9	0	0
1068	32295	Drosophila melanogaster	Yippee	CG1989 gene product from transcript CG1989-RA	CG1989|DmelCG1989	0	0
1069	32296	Drosophila melanogaster	CG1662	CG1662 gene product from transcript CG1662-RA	DmelCG1662	0	0
1070	32297	Drosophila melanogaster	CG1673	CG1673 gene product from transcript CG1673-RA	DmelCG1673	0	0
1071	32298	Drosophila melanogaster	CG12725	CG12725 gene product from transcript CG12725-RA	DmelCG12725	0	0
1072	32299	Drosophila melanogaster	CG1681	CG1681 gene product from transcript CG1681-RA	DmelCG1681	0	0
1073	32300	Drosophila melanogaster	CG1998	CG1998 gene product from transcript CG1998-RA	DmelCG1998	0	0
1074	32301	Drosophila melanogaster	Set2	CG1716 gene product from transcript CG1716-RA	CG1716|DmelCG1716|Hypb|dHypb|dSet2|set2	0	0
1075	32302	Drosophila melanogaster	CG34324	CG34324 gene product from transcript CG34324-RA	CG12726|DmelCG34324	0	0
1076	32303	Drosophila melanogaster	CG32635	CG32635 gene product from transcript CG32635-RA	CG12727|CG15751|CG15752|CT36003|DmelCG32635|tld-like	0	0
1077	32306	Drosophila melanogaster	CG15753	CG15753 gene product from transcript CG15753-RB	DmelCG15753	0	0
1078	32307	Drosophila melanogaster	CG15754	CG15754 gene product from transcript CG15754-RA	DmelCG15754	0	0
1079	32308	Drosophila melanogaster	CG15756	CG15756 gene product from transcript CG15756-RA	DmelCG15756	0	0
1080	32309	Drosophila melanogaster	Cpr12A	Cuticular protein 12A	BcDNA:LP12385|CG15757|DmelCG15757	0	0
1081	32310	Drosophila melanogaster	CG11816	CG11816 gene product from transcript CG11816-RA	DmelCG11816	0	0
1082	32311	Drosophila melanogaster	CG32631	CG32631 gene product from transcript CG32631-RA	CG1733|DmelCG32631	0	0
1083	32312	Drosophila melanogaster	Tango13	Transport and Golgi organization 13	CG1573|CG15758|CG15759|CG32629|CG32629/CG32632|CG32632|DmelCG32632|Dmel_CG15758|Dmel_CG32629|TANGO13	0	0
1084	32314	Drosophila melanogaster	up	upheld	BcDNA:LD08591|CG7107|DmTpnT|DmelCG7107|FBgn0004169|Tn-T|TnT|TpnT|dro TnT|int|tnT|upheld2|wupB	0	0
1085	32315	Drosophila melanogaster	CG11178	CG11178 gene product from transcript CG11178-RA	DmelCG11178	0	0
1086	32316	Drosophila melanogaster	Ndc80	CG9938 gene product from transcript CG9938-RA	CG9938|DmelCG9938|Hec1|dmNdc80|hec1	0	0
1087	32317	Drosophila melanogaster	BthD	BthD selenoprotein	CG11177|DmelCG11177|SelH|dselH|dselM	0	0
1088	32318	Drosophila melanogaster	tth	toothrin	CG12175|DmelCG12175|anon-EST:fe2G7	0	0
1089	32319	Drosophila melanogaster	Tango2	Transport and Golgi organization 2	CG11176|DmelCG11176|TANGO2	0	0
1090	32320	Drosophila melanogaster	CG2691	CG2691 gene product from transcript CG2691-RA	DmelCG2691	0	0
1091	32321	Drosophila melanogaster	NFAT	NFAT homolog	CG11172|DmelCG11172|EP1335|MESR1|NF-AT5|NFAT5|dNFAT	0	0
1092	32322	Drosophila melanogaster	Lig4	Ligase4	CG12176|DmelCG12176|RE37186|lig4	0	0
1093	32323	Drosophila melanogaster	CG11164	CG11164 gene product from transcript CG11164-RA	DmelCG11164	0	0
1094	32324	Drosophila melanogaster	CG15760	CG15760 gene product from transcript CG15760-RA	DmelCG15760	0	0
1095	32325	Drosophila melanogaster	CG11162	CG11162 gene product from transcript CG11162-RA	DmelCG11162	0	0
1096	32326	Drosophila melanogaster	CG12177	CG12177 gene product from transcript CG12177-RA	DmelCG12177	0	0
1097	32327	Drosophila melanogaster	CG11158	CG11158 gene product from transcript CG11158-RA	DmelCG11158	0	0
1098	32328	Drosophila melanogaster	CG9940	CG9940 gene product from transcript CG9940-RB	145027_at|DmelCG9940|Q9VYA0	0	0
1099	32329	Drosophila melanogaster	NnaD	Drosophila Nna1 ortholog	CG11428|CG32627|DmelCG32627	0	0
1100	32330	Drosophila melanogaster	CG9941	CG9941 gene product from transcript CG9941-RA	A|DmelCG9941|x93	0	0
1101	32334	Drosophila melanogaster	CG11134	CG11134 gene product from transcript CG11134-RA	1|DmelCG11134	0	0
1102	32335	Drosophila melanogaster	CG11151	CG11151 gene product from transcript CG11151-RA	2|DmelCG11151	0	0
1103	32337	Drosophila melanogaster	CG10990	CG10990 gene product from transcript CG10990-RB	DmelCG10990	0	0
1104	32338	Drosophila melanogaster	Rtc1	CG11130 gene product from transcript CG11130-RA	CG11130|DmelCG11130	0	0
1105	32339	Drosophila melanogaster	Yp3	Yolk protein 3	CG11129|DmelCG11129|VG3|VIT3Dm|YP|YP-3|YP3|YP[[3]]|fs(1)1526|fs(1)A1526|yp|yp3	0	0
1106	32340	Drosophila melanogaster	rdgB	retinal degeneration B	CG11111|DmelCG11111|DrdgB|DrdgBalpha|PITP|RdgB|RdgBalpha|hypoF|ota1|x36	0	0
1107	32341	Drosophila melanogaster	CG10992	CG10992 gene product from transcript CG10992-RA	CG-10992|DmelCG10992|anon-EST:ParkEST132	0	0
1108	32342	Drosophila melanogaster	CG11103	CG11103 gene product from transcript CG11103-RB	BcDNA:LP03404|DmelCG11103	0	0

<table>
<tr><td>1109</td><td>32343</td><td>Drosophila melanogaster</td><td>inaE</td><td>inactivation no afterpotential E</td><td>CG11096|CG11102|CG11104|CG15761|CG32630|CG33174|DmelCG33174|EP(X)1101</td><td>0</td><td>0</td></tr>
<tr><td>1110</td><td>32344</td><td>Drosophila melanogaster</td><td>CG10993</td><td>CG10993 gene product from transcript CG10993-RA</td><td>BcDNA:GH23064|DmelCG10993</td><td>0</td><td>0</td></tr>
<tr><td>1111</td><td>32345</td><td>Drosophila melanogaster</td><td>CG10996</td><td>CG10996 gene product from transcript CG10996-RA</td><td>DmelCG10996</td><td>0</td><td>0</td></tr>
<tr><td>1112</td><td>32348</td><td>Drosophila melanogaster</td><td>CG11095</td><td>CG11095 gene product from transcript CG11095-RA</td><td>DmelCG11095</td><td>0</td><td>0</td></tr>
<tr><td>1113</td><td>32349</td><td>Drosophila melanogaster</td><td>Clic</td><td>Chloride intracellular channel</td><td>CG10997|DCLIC|DmCLIC|DmelCG10997|l(1)G0053|l(1)G0472</td><td>0</td><td>0</td></tr>
<tr><td>1114</td><td>32350</td><td>Drosophila melanogaster</td><td>CG11092</td><td>CG11092 gene product from transcript CG11092-RA</td><td>BcDNA:LD21129|DmelCG11092|nup93</td><td>0</td><td>0</td></tr>
<tr><td>1115</td><td>32351</td><td>Drosophila melanogaster</td><td>jub</td><td>Ajuba LIM protein</td><td>CG11063|DmelCG11063|Jub|djub</td><td>0</td><td>0</td></tr>
<tr><td>1116</td><td>32352</td><td>Drosophila melanogaster</td><td>CG32626</td><td>CG32626 gene product from transcript CG32626-RI</td><td>CG11058|CG11065|CG15762|DmelCG32626</td><td>0</td><td>0</td></tr>
<tr><td>1117</td><td>32353</td><td>Drosophila melanogaster</td><td>CG11071</td><td>CG11071 gene product from transcript CG11071-RA</td><td>DmelCG11071</td><td>0</td><td>0</td></tr>
<tr><td>1118</td><td>32354</td><td>Drosophila melanogaster</td><td>mamo</td><td>maternal gene required for meiosis</td><td>CG11072|CG11075|CG11082|CG32606|CG32611|CG34346|DmelCG34346|Dmel_CG11072|Dmel_CG32606|Dmel_CG32611</td><td>0</td><td>0</td></tr>
<tr><td>1119</td><td>32357</td><td>Drosophila melanogaster</td><td>CG11068</td><td>CG11068 gene product from transcript CG11068-RA</td><td>DmelCG11068</td><td>0</td><td>0</td></tr>
<tr><td>1120</td><td>32358</td><td>Drosophila melanogaster</td><td>ben</td><td>bendless</td><td>BEN|Ben|Bend|CG18319|DmelCG18319|Ubc13|UbcD3|anon-WO03040301.242|nj-262|ubc13</td><td>0</td><td>0</td></tr>
<tr><td>1121</td><td>32359</td><td>Drosophila melanogaster</td><td>CG12480</td><td>CG12480 gene product from transcript CG12480-RB</td><td>DmelCG12480</td><td>0</td><td>0</td></tr>
<tr><td>1122</td><td>32360</td><td>Drosophila melanogaster</td><td>betaNACtes1</td><td>CG13402 gene product from transcript CG13402-RA</td><td>CG13402|DmelCG13402</td><td>0</td><td>0</td></tr>
<tr><td>1123</td><td>32361</td><td>Drosophila melanogaster</td><td>CG1368</td><td>CG1368 gene product from transcript CG1368-RA</td><td>DmelCG1368</td><td>0</td><td>0</td></tr>
<tr><td>1124</td><td>32362</td><td>Drosophila melanogaster</td><td>CG11581</td><td>CG11581 gene product from transcript CG11581-RA</td><td>DmelCG11581</td><td>0</td><td>0</td></tr>
<tr><td>1125</td><td>32363</td><td>Drosophila melanogaster</td><td>CG11584</td><td>CG11584 gene product from transcript CG11584-RB</td><td>DmelCG11584</td><td>0</td><td>0</td></tr>
<tr><td>1126</td><td>32364</td><td>Drosophila melanogaster</td><td>CG12481</td><td>CG12481 gene product from transcript CG12481-RB</td><td>DmelCG12481</td><td>0</td><td>0</td></tr>
<tr><td>1127</td><td>32365</td><td>Drosophila melanogaster</td><td>CG11585</td><td>CG11585 gene product from transcript CG11585-RB</td><td>DmelCG11585</td><td>0</td><td>0</td></tr>
<tr><td>1128</td><td>32366</td><td>Drosophila melanogaster</td><td>CG13403</td><td>CG13403 gene product from transcript CG13403-RA</td><td>DmelCG13403</td><td>0</td><td>0</td></tr>
<tr><td>1129</td><td>32367</td><td>Drosophila melanogaster</td><td>yl</td><td>yolkless</td><td>CG1372|CT3114|DmYPR|DmelCG1372|LR13|YL|Yl|fs(1)148|fs(1)5|fs(1)A148|fs(1)K184|fs(1)L186|fs(1)L193|fs(1)L196|fs(1)L211|fs(1)M205|fs(1)M25|fs(1)yl|fs(1)yolkless</td><td>0</td><td>0</td></tr>
<tr><td>1130</td><td>32368</td><td>Drosophila melanogaster</td><td>CG11590</td><td>CG11590 gene product from transcript CG11590-RA</td><td>BcDNA:GM24986|DmelCG11590</td><td>0</td><td>0</td></tr>
<tr><td>1131</td><td>32371</td><td>Drosophila melanogaster</td><td>l(1)G0469</td><td>lethal (1) G0469</td><td>CG11595|CG12482|CG32597|DmelCG32597|anon-WO03040301.232</td><td>0</td><td>0</td></tr>
<tr><td>1132</td><td>32373</td><td>Drosophila melanogaster</td><td>l(1)G0007</td><td>lethal (1) G0007</td><td>CG1375|CG1405|CG32604|DmelCG32604|EP764|EP784|Prp16|cg1405|l(1)G0028|l(1)G0103|l(1)G0176|l(1)G0308|l(1)G0416|l(1)G0476|l(1)G0491|prp16</td><td>0</td><td>0</td></tr>
<tr><td>1133</td><td>32374</td><td>Drosophila melanogaster</td><td>CG11674</td><td>CG11674 gene product from transcript CG11674-RA</td><td>DmelCG11674</td><td>0</td><td>0</td></tr>
<tr><td>1134</td><td>32375</td><td>Drosophila melanogaster</td><td>mRpL38</td><td>mitochondrial ribosomal protein L38</td><td>CG15871|DmelCG15871|MRP-L3|mRpL3a</td><td>0</td><td>0</td></tr>
<tr><td>1135</td><td>32377</td><td>Drosophila melanogaster</td><td>CG1434</td><td>CG1434 gene product from transcript CG1434-RA</td><td>DmelCG1434</td><td>0</td><td>0</td></tr>
<tr><td>1136</td><td>32378</td><td>Drosophila melanogaster</td><td>Fbxl4</td><td>F box and leucine-rich-repeat gene 4</td><td>CG1839|DmelCG1839|dFbxl4</td><td>0</td><td>0</td></tr>
<tr><td>1137</td><td>32379</td><td>Drosophila melanogaster</td><td>mRNA-cap</td><td>mRNA-capping-enzyme</td><td>CG1810|DmelCG1810|PIR</td><td>0</td><td>0</td></tr>
<tr><td>1138</td><td>32381</td><td>Drosophila melanogaster</td><td>CG1461</td><td>CG1461 gene product from transcript CG1461-RA</td><td>DmelCG1461</td><td>0</td><td>0</td></tr>
<tr><td>1139</td><td>32382</td><td>Drosophila melanogaster</td><td>CG13404</td><td>CG13404 gene product from transcript CG13404-RB</td><td>DmelCG13404</td><td>0</td><td>0</td></tr>
<tr><td>1140</td><td>32384</td><td>Drosophila melanogaster</td><td>Muc12Ea</td><td>Mucin 12Ea</td><td>CG32602|CG5228|DmelCG32602</td><td>0</td><td>0</td></tr>
<tr><td>1141</td><td>32385</td><td>Drosophila melanogaster</td><td>CG9400</td><td>CG9400 gene product from transcript CG9400-RB</td><td>DmelCG9400|anon-WO0140519.10</td><td>0</td><td>0</td></tr>
<tr><td>1142</td><td>32386</td><td>Drosophila melanogaster</td><td>betaNACtes2</td><td>CG18157 gene product from transcript CG18157-RA</td><td>CG18157|DmelCG18157</td><td>0</td><td>0</td></tr>
<tr><td>1143</td><td>32387</td><td>Drosophila melanogaster</td><td>dpr8</td><td>CG32600 gene product from transcript CG32600-RA</td><td>CG11477|CG14415|CG32600|CG5291|CT16867|DmelCG32600|Dpr-8</td><td>0</td><td>0</td></tr>
<tr><td>1144</td><td>32389</td><td>Drosophila melanogaster</td><td>betaNACtes4</td><td>CG18313 gene product from transcript CG18313-RA</td><td>CG18313|DmelCG18313</td><td>0</td><td>0</td></tr>
<tr><td>1145</td><td>32392</td><td>Drosophila melanogaster</td><td>CG9411</td><td>CG9411 gene product from transcript CG9411-RA</td><td>DmelCG9411</td><td>0</td><td>0</td></tr>
<tr><td>1146</td><td>32393</td><td>Drosophila melanogaster</td><td>CG12540</td><td>CG12540 gene product from transcript CG12540-RA</td><td>BcDNA:RE49992|DmelCG12540</td><td>0</td><td>0</td></tr>
<tr><td>1147</td><td>32394</td><td>Drosophila melanogaster</td><td>CG14414</td><td>CG14414 gene product from transcript CG14414-RD</td><td>BcDNA:AT01764|DmelCG14414|cg14414</td><td>0</td><td>0</td></tr>
<tr><td>1148</td><td>32395</td><td>Drosophila melanogaster</td><td>mRpS25</td><td>mitochondrial ribosomal protein S25</td><td>25mRps|CG14413|DmelCG14413|MRP-S25|MrpS25</td><td>0</td><td>0</td></tr>
<tr><td>1149</td><td>32396</td><td>Drosophila melanogaster</td><td>nmdyn-D6</td><td>CG5310 gene product from transcript CG5310-RA</td><td>CG5310|DmelCG5310</td><td>0</td><td>0</td></tr>
<tr><td>1150</td><td>32397</td><td>Drosophila melanogaster</td><td>CG9413</td><td>CG9413 gene product from transcript CG9413-RA</td><td>DmelCG9413</td><td>0</td><td>0</td></tr>
<tr><td>1151</td><td>32398</td><td>Drosophila melanogaster</td><td>NetA</td><td>Netrin-A</td><td>CG18657|CT27014|DmelCG18657|NetrinA|netA|netrin|netrin A</td><td>0</td><td>0</td></tr>
<tr><td>1152</td><td>32399</td><td>Drosophila melanogaster</td><td>CG5321</td><td>CG5321 gene product from transcript CG5321-RB</td><td>DmelCG5321|anon-EST:Posey66</td><td>0</td><td>0</td></tr>
<tr><td>1153</td><td>32400</td><td>Drosophila melanogaster</td><td>NetB</td><td>Netrin-B</td><td>CG10521|CT29512|DmelCG10521|NETB|netB|netrin|netrin B</td><td>0</td><td>0</td></tr>
<tr><td>1154</td><td>32401</td><td>Drosophila melanogaster</td><td>CG15890</td><td>CG15890 gene product from transcript CG15890-RA</td><td>DmelCG15890</td><td>0</td><td>0</td></tr>
<tr><td>1155</td><td>32402</td><td>Drosophila melanogaster</td><td>CG5334</td><td>CG5334 gene product from transcript CG5334-RA</td><td>DmelCG5334</td><td>0</td><td>0</td></tr>
<tr><td>1156</td><td>32403</td><td>Drosophila melanogaster</td><td>CG5347</td><td>CG5347 gene product from transcript CG5347-RA</td><td>DmelCG5347</td><td>0</td><td>0</td></tr>
<tr><td>1157</td><td>32406</td><td>Drosophila melanogaster</td><td>rut</td><td>rutabaga</td><td>AC|Ac12F|CG9533|DmelCG9533|EP1603|RUT|Rut|Rut-AC</td><td>0</td><td>0</td></tr>
<tr><td>1158</td><td>32407</td><td>Drosophila melanogaster</td><td>CG14408</td><td>CG14408 gene product from transcript CG14408-RA</td><td>DmelCG14408</td><td>0</td><td>0</td></tr>
<tr><td>1159</td><td>32408</td><td>Drosophila melanogaster</td><td>CG14411</td><td>CG14411 gene product from transcript CG14411-RA</td><td>DmelCG14411</td><td>0</td><td>0</td></tr>
<tr><td>1160</td><td>32409</td><td>Drosophila melanogaster</td><td>CG14410</td><td>CG14410 gene product from transcript CG14410-RA</td><td>DmelCG14410</td><td>0</td><td>0</td></tr>
<tr><td>1161</td><td>32410</td><td>Drosophila melanogaster</td><td>CG14407</td><td>CG14407 gene product from transcript CG14407-RA</td><td>BcDNA:RH03087|DmelCG14407</td><td>0</td><td>0</td></tr>
<tr><td>1162</td><td>32412</td><td>Drosophila melanogaster</td><td>CG12539</td><td>CG12539 gene product from transcript CG12539-RA</td><td>Dm GMCiota2|DmelCG12539</td><td>0</td><td>0</td></tr>
<tr><td>1163</td><td>32413</td><td>Drosophila melanogaster</td><td>CG9522</td><td>CG9522 gene product from transcript CG9522-RA</td><td>Dm GMCiota1|DmelCG9522</td><td>0</td><td>0</td></tr>
<tr><td>1164</td><td>32414</td><td>Drosophila melanogaster</td><td>CG9521</td><td>CG9521 gene product from transcript CG9521-RA</td><td>Dm GMCtheta2|DmelCG9521</td><td>0</td><td>0</td></tr>
<tr><td>1165</td><td>32415</td><td>Drosophila melanogaster</td><td>CG9519</td><td>CG9519 gene product from transcript CG9519-RA</td><td>Dm GMCtheta1|DmelCG9519</td><td>0</td><td>0</td></tr>
<tr><td>1166</td><td>32416</td><td>Drosophila melanogaster</td><td>CG9518</td><td>CG9518 gene product from transcript CG9518-RA</td><td>Dm GMCzeta1|DmelCG9518</td><td>0</td><td>0</td></tr>
<tr><td>1167</td><td>32417</td><td>Drosophila melanogaster</td><td>CG9517</td><td>CG9517 gene product from transcript CG9517-RA</td><td>Dm GMCepsilon1|DmelCG9517</td><td>0</td><td>0</td></tr>
<tr><td>1168</td><td>32418</td><td>Drosophila melanogaster</td><td>CG9514</td><td>CG9514 gene product from transcript CG9514-RA</td><td>Dm GMCdelta1|DmelCG9514</td><td>0</td><td>0</td></tr>
<tr><td>1169</td><td>32419</td><td>Drosophila melanogaster</td><td>CG9512</td><td>CG9512 gene product from transcript CG9512-RA</td><td>CT26924|DmelCG9512</td><td>0</td><td>0</td></tr>
<tr><td>1170</td><td>32420</td><td>Drosophila melanogaster</td><td>CG9509</td><td>CG9509 gene product from transcript CG9509-RA</td><td>Dm GMCbeta2|DmelCG9509</td><td>0</td><td>0</td></tr>
<tr><td>1171</td><td>32421</td><td>Drosophila melanogaster</td><td>CG14406</td><td>CG14406 gene product from transcript CG14406-RA</td><td>DmelCG14406</td><td>0</td><td>0</td></tr>
<tr><td>1172</td><td>32422</td><td>Drosophila melanogaster</td><td>CG12398</td><td>CG12398 gene product from transcript CG12398-RA</td><td>Dm GMCgamma1|DmelCG12398</td><td>0</td><td>0</td></tr>
<tr><td>1173</td><td>32423</td><td>Drosophila melanogaster</td><td>Eo</td><td>Ecdysone oxidase</td><td>CG9504|DmEO|DmelCG9504|EObeta1</td><td>0</td><td>0</td></tr>
<tr><td>1174</td><td>32424</td><td>Drosophila melanogaster</td><td>CG9503</td><td>CG9503 gene product from transcript CG9503-RA</td><td>Dm GMCalpha1|DmelCG9503</td><td>0</td><td>0</td></tr>
<tr><td>1175</td><td>32425</td><td>Drosophila melanogaster</td><td>Flo-2</td><td>flotillin 2</td><td>CG11547|CG14409|CG32593|DmFlo2|DmelCG32593|FLO2|FLO[Dm]-2|flo2|reggie1/flo2</td><td>0</td><td>0</td></tr>
<tr><td>1176</td><td>32426</td><td>Drosophila melanogaster</td><td>CG9009</td><td>CG9009 gene product from transcript CG9009-RB</td><td>BcDNA:GH02901|DmelCG9009</td><td>0</td><td>0</td></tr>
<tr><td>1177</td><td>32427</td><td>Drosophila melanogaster</td><td>CG9030</td><td>CG9030 gene product from transcript CG9030-RB</td><td>DmelCG9030</td><td>0</td><td>0</td></tr>
<tr><td>1178</td><td>32428</td><td>Drosophila melanogaster</td><td>eag</td><td>ether a go-go</td><td>CG10952|CIKE|DmelCG10952|EAG|Eag</td><td>0</td><td>0</td></tr>
<tr><td>1179</td><td>32429</td><td>Drosophila melanogaster</td><td>hiw</td><td>highwire</td><td>CG32592|CG9041|CG9049|DmelCG32592|Hiw</td><td>0</td><td>0</td></tr>
<tr><td>1180</td><td>32430</td><td>Drosophila melanogaster</td><td>be</td><td>ben</td><td>BEST:LD10566|CG15026|CG32594|CG5530|DmelCG32594|E|anon-EST:CL1a5</td><td>0</td><td>0</td></tr>
<tr><td>1181</td><td>32434</td><td>Drosophila melanogaster</td><td>CG5548</td><td>CG5548 gene product from transcript CG5548-RA</td><td>DmelCG5548</td><td>0</td><td>0</td></tr>
<tr><td>1182</td><td>32435</td><td>Drosophila melanogaster</td><td>CG9053</td><td>CG9053 gene product from transcript CG9053-RA</td><td>DmelCG9053|p24</td><td>0</td><td>0</td></tr>
<tr><td>1183</td><td>32436</td><td>Drosophila melanogaster</td><td>dob</td><td>doppelganger von brummer</td><td>CG5560|DmelCG5560</td><td>0</td><td>0</td></tr>
<tr><td>1184</td><td>32437</td><td>Drosophila melanogaster</td><td>Lsd-2</td><td>Lipid storage droplet-2</td><td>145098_at|CG9057|DmelCG9057|LSD-2|LSD2|Lsd2|plin2</td><td>0</td><td>0</td></tr>
<tr><td>1185</td><td>32438</td><td>Drosophila melanogaster</td><td>CG33177</td><td>CG33177 gene product from transcript CG33177-RA</td><td>CG5579|DmelCG33177</td><td>0</td><td>0</td></tr>
<tr><td>1186</td><td>32439</td><td>Drosophila melanogaster</td><td>CG9065</td><td>CG9065 gene product from transcript CG9065-RA</td><td>DmelCG9065</td><td>0</td><td>0</td></tr>
<tr><td>1187</td><td>32440</td><td>Drosophila melanogaster</td><td>CG15027</td><td>CG15027 gene product from transcript CG15027-RA</td><td>BcDNA:SD22530|DmelCG15027</td><td>0</td><td>0</td></tr>
<tr><td>1188</td><td>32441</td><td>Drosophila melanogaster</td><td>CG5599</td><td>CG5599 gene product from transcript CG5599-RA</td><td>DmelCG5599</td><td>0</td><td>0</td></tr>
<tr><td>1189</td><td>32442</td><td>Drosophila melanogaster</td><td>rab3-GEF</td><td>CG5627 gene product from transcript CG5627-RC</td><td>BEST:GH21304|CG5627|DmelCG5627|anon-EST:Posey246</td><td>0</td><td>0</td></tr>
<tr><td>1190</td><td>32443</td><td>Drosophila melanogaster</td><td>CG9072</td><td>CG9072 gene product from transcript CG9072-RA</td><td>DmelCG9072</td><td>0</td><td>0</td></tr>
<tr><td>1191</td><td>32444</td><td>Drosophila melanogaster</td><td>Cyp4s3</td><td>CG9081 gene product from transcript CG9081-RA</td><td>4s3|CG9081|DmelCG9081</td><td>0</td><td>0</td></tr>
<tr><td>1192</td><td>32446</td><td>Drosophila melanogaster</td><td>RpL37a</td><td>Ribosomal protein L37a</td><td>CG9091|DmelCG9091|M(1)13A|RpL37</td><td>0</td><td>0</td></tr>
<tr><td>1193</td><td>32447</td><td>Drosophila melanogaster</td><td>CG9095</td><td>CG9095 gene product from transcript CG9095-RA</td><td>CT26106|DmelCG9095</td><td>0</td><td>0</td></tr>
<tr><td>1194</td><td>32448</td><td>Drosophila melanogaster</td><td>CG15028</td><td>CG15028 gene product from transcript CG15028-RD</td><td>DmelCG15028</td><td>0</td><td>0</td></tr>
<tr><td>1195</td><td>32449</td><td>Drosophila melanogaster</td><td>CG15029</td><td>CG15029 gene product from transcript CG15029-RA</td><td>DmelCG15029</td><td>0</td><td>0</td></tr>
<tr><td>1196</td><td>32450</td><td>Drosophila melanogaster</td><td>CG5662</td><td>CG5662 gene product from transcript CG5662-RA</td><td>DmelCG5662</td><td>0</td><td>0</td></tr>
<tr><td>1197</td><td>32452</td><td>Drosophila melanogaster</td><td>CG9101</td><td>CG9101 gene product from transcript CG9101-RA</td><td>DmelCG9101</td><td>0</td><td>0</td></tr>
<tr><td>1198</td><td>32453</td><td>Drosophila melanogaster</td><td>PPYR1</td><td>Protein phosphatase Y regulator 1</td><td>BcDNA:AT19571|CG15031|DmelCG15031</td><td>0</td><td>0</td></tr>
<tr><td>1199</td><td>32454</td><td>Drosophila melanogaster</td><td>CG9106</td><td>CG9106 gene product from transcript CG9106-RA</td><td>DmelCG9106</td><td>0</td><td>0</td></tr>
<tr><td>1200</td><td>32455</td><td>Drosophila melanogaster</td><td>CG5877</td><td>CG5877 gene product from transcript CG5877-RA</td><td>DmelCG5877</td><td>0</td><td>0</td></tr>
<tr><td>1201</td><td>32457</td><td>Drosophila melanogaster</td><td>gce</td><td>germ cell-expressed bHLH-PAS</td><td>CG15032|CG42739|CG6211|DmelCG42739|Dmel_CG15032|Dmel_CG6211|Dmgce</td><td>0</td><td>0</td></tr>
<tr><td>1202</td><td>32458</td><td>Drosophila melanogaster</td><td>Top1</td><td>Topoisomerase 1</td><td>CG6146|DmelCG6146|TOP1|TopI|Topo I|TopoI|dTopoI|l(1)G0134|l(1)G0201|l(1)G0229|l(1)G0278|l(1)top1|top1|topo I|topoI</td><td>0</td><td>0</td></tr>
<tr><td>1203</td><td>32459</td><td>Drosophila melanogaster</td><td>dah</td><td>discontinuous actin hexagon</td><td>Apd|CG6157|Dah|DmelCG6157|fs(1)dah</td><td>0</td><td>0</td></tr>
<tr><td>1204</td><td>32460</td><td>Drosophila melanogaster</td><td>CG9114</td><td>CG9114 gene product from transcript CG9114-RA</td><td>DmelCG9114</td><td>0</td><td>0</td></tr>
<tr><td>1205</td><td>32461</td><td>Drosophila melanogaster</td><td>HDAC6</td><td>CG6170 gene product from transcript CG6170-RA</td><td>CG6170|DHDAC2|DmHDAC2|DmelCG6170|HDAC|HDAC2|dHDAC2|dHDAC6|dmHDA404</td><td>0</td><td>0</td></tr>
<tr><td>1206</td><td>32462</td><td>Drosophila melanogaster</td><td>CG9123</td><td>CG9123 gene product from transcript CG9123-RA</td><td>DmelCG9123</td><td>0</td><td>0</td></tr>
<tr><td>1207</td><td>32463</td><td>Drosophila melanogaster</td><td>CG12608</td><td>CG12608 gene product from transcript CG12608-RA</td><td>DmelCG12608</td><td>0</td><td>0</td></tr>
<tr><td>1208</td><td>32464</td><td>Drosophila melanogaster</td><td>CG6227</td><td>CG6227 gene product from transcript CG6227-RA</td><td>DmRH29|DmelCG6227|Prp5|cg6227</td><td>0</td><td>0</td></tr>
<tr><td>1209</td><td>32467</td><td>Drosophila melanogaster</td><td>CG9164</td><td>CG9164 gene product from transcript CG9164-RC</td><td>DmelCG9164</td><td>0</td><td>0</td></tr>
<tr><td>1210</td><td>32468</td><td>Drosophila melanogaster</td><td>cngl</td><td>CNG channel-like</td><td>CG18210|CG43395|CG9176|DmelCG43395|Dmel_CG18210|Dmel_CG9176</td><td>0</td><td>0</td></tr>
<tr><td>1211</td><td>32470</td><td>Drosophila melanogaster</td><td>Scamp</td><td>CG9195 gene product from transcript CG9195-RA</td><td>BcDNA:GM04934|CG9195|DmelCG9195|SCAMP|anon-EST:fe1F7|dmSC|dmSCAMP</td><td>0</td><td>0</td></tr>
<tr><td>1212</td><td>32471</td><td>Drosophila melanogaster</td><td>Ahcy13</td><td>Adenosylhomocysteinase at 13</td><td>CG11654|DmelCG11654|SAHH|ahcY</td><td>0</td><td>0</td></tr>
<tr><td>1213</td><td>32472</td><td>Drosophila melanogaster</td><td>CG11655</td><td>CG11655 gene product from transcript CG11655-RA</td><td>DmelCG11655</td><td>0</td><td>0</td></tr>
<tr><td>1214</td><td>32473</td><td>Drosophila melanogaster</td><td>shtd</td><td>shattered</td><td>APC1|Apc1|CG9198|DmelCG9198|Shtd</td><td>0</td><td>0</td></tr>
<tr><td>1215</td><td>32474</td><td>Drosophila melanogaster</td><td>CG6294</td><td>CG6294 gene product from transcript CG6294-RB</td><td>DmelCG6294</td><td>0</td><td>0</td></tr>
<tr><td>1216</td><td>32475</td><td>Drosophila melanogaster</td><td>CG6299</td><td>CG6299 gene product from transcript CG6299-RB</td><td>DmelCG6299</td><td>0</td><td>0</td></tr>
<tr><td>1217</td><td>32476</td><td>Drosophila melanogaster</td><td>CG15642</td><td>CG15642 gene product from transcript CG15642-RA</td><td>DmelCG15642</td><td>0</td><td>0</td></tr>
<tr><td>1218</td><td>32477</td><td>Drosophila melanogaster</td><td>CG15641</td><td>CG15641 gene product from transcript CG15641-RA</td><td>DmelCG15641</td><td>0</td><td>0</td></tr>
<tr><td>1219</td><td>32478</td><td>Drosophila melanogaster</td><td>Grip128</td><td>CG9201 gene product from transcript CG9201-RB</td><td>CG9201|DGrip128|Dgrip128|Dgrips128|DmelCG9201|GCP5|grip128</td><td>0</td><td>0</td></tr>
</table>

<table>
<tr><td>1220</td><td>32479</td><td>Drosophila melanogaster</td><td>CG6308</td><td>CG6308 gene product from transcript CG6308-RA</td><td>DmelCG6308</td><td>0</td><td>0</td></tr>
<tr><td>1221</td><td>32480</td><td>Drosophila melanogaster</td><td>CG9203</td><td>CG9203 gene product from transcript CG9203-RA</td><td>DmelCG9203</td><td>0</td><td>0</td></tr>
<tr><td>1222</td><td>32481</td><td>Drosophila melanogaster</td><td>CG6324</td><td>CG6324 gene product from transcript CG6324-RA</td><td>DmelCG6324</td><td>0</td><td>0</td></tr>
<tr><td>1223</td><td>32482</td><td>Drosophila melanogaster</td><td>CG6340</td><td>CG6340 gene product from transcript CG6340-RC</td><td>DmelCG6340</td><td>0</td><td>0</td></tr>
<tr><td>1224</td><td>32483</td><td>Drosophila melanogaster</td><td>Gmap</td><td>Golgi microtubule-associated protein</td><td>CG32587|CG33206|CG6350|CG7821|DmelCG33206|dGMAP|l(1)G0168</td><td>0</td><td>0</td></tr>
<tr><td>1225</td><td>32485</td><td>Drosophila melanogaster</td><td>Ac13E</td><td>Adenylyl cyclase 35C</td><td>AC 13E|Ac35C|CG9210|DAC9|DmelCG9210</td><td>0</td><td>0</td></tr>
<tr><td>1226</td><td>32487</td><td>Drosophila melanogaster</td><td>CG42299</td><td>CG42299 gene product from transcript CG42299-RA</td><td>CG15644|CG18620|CG32584|DmelCG42299|Dmel_CG32584</td><td>0</td><td>0</td></tr>
<tr><td>1227</td><td>32488</td><td>Drosophila melanogaster</td><td>CG7860</td><td>CG7860 gene product from transcript CG7860-RA</td><td>DmelCG7860</td><td>0</td><td>0</td></tr>
<tr><td>1228</td><td>32489</td><td>Drosophila melanogaster</td><td>CG15643</td><td>CG15643 gene product from transcript CG15643-RA</td><td>CG32583|DmelCG15643</td><td>0</td><td>0</td></tr>
<tr><td>1229</td><td>32490</td><td>Drosophila melanogaster</td><td>CG9213</td><td>CG9213 gene product from transcript CG9213-RA</td><td>CG32585|DmelCG9213</td><td>0</td><td>0</td></tr>
<tr><td>1230</td><td>32492</td><td>Drosophila melanogaster</td><td>cerv</td><td>cervantes</td><td>CG15645|DmelCG15645</td><td>0</td><td>0</td></tr>
<tr><td>1231</td><td>32493</td><td>Drosophila melanogaster</td><td>CG7872</td><td>CG7872 gene product from transcript CG7872-RA</td><td>DmelCG7872</td><td>0</td><td>0</td></tr>
<tr><td>1232</td><td>32494</td><td>Drosophila melanogaster</td><td>CG9215</td><td>CG9215 gene product from transcript CG9215-RA</td><td>DmelCG9215</td><td>0</td><td>0</td></tr>
<tr><td>1233</td><td>32495</td><td>Drosophila melanogaster</td><td>CG8097</td><td>CG8097 gene product from transcript CG8097-RA</td><td>DmelCG8097</td><td>0</td><td>0</td></tr>
<tr><td>1234</td><td>32496</td><td>Drosophila melanogaster</td><td>CG8105</td><td>CG8105 gene product from transcript CG8105-RA</td><td>DmelCG8105|FBgn0030661</td><td>0</td><td>0</td></tr>
<tr><td>1235</td><td>32497</td><td>Drosophila melanogaster</td><td>CG9220</td><td>CG9220 gene product from transcript CG9220-RC</td><td>DmelCG9220</td><td>0</td><td>0</td></tr>
<tr><td>1236</td><td>32498</td><td>Drosophila melanogaster</td><td>sog</td><td>short gastrulation</td><td>CG9224|Dm sog|DmelCG9224|SOG|Sog|l(1)G0160|l(1)G0395|l(1)G0479</td><td>0</td><td>0</td></tr>
<tr><td>1237</td><td>32499</td><td>Drosophila melanogaster</td><td>CG8117</td><td>CG8117 gene product from transcript CG8117-RA</td><td>DmelCG8117|dCrsp70</td><td>0</td><td>0</td></tr>
<tr><td>1238</td><td>32500</td><td>Drosophila melanogaster</td><td>CG8119</td><td>CG8119 gene product from transcript CG8119-RA</td><td>DmelCG8119</td><td>0</td><td>0</td></tr>
<tr><td>1239</td><td>32501</td><td>Drosophila melanogaster</td><td>CG15646</td><td>CG15646 gene product from transcript CG15646-RA</td><td>DmelCG15646</td><td>0</td><td>0</td></tr>
<tr><td>1240</td><td>32502</td><td>Drosophila melanogaster</td><td>CG12708</td><td>CG12708 gene product from transcript CG12708-RA</td><td>DmelCG12708</td><td>0</td><td>0</td></tr>
<tr><td>1241</td><td>32503</td><td>Drosophila melanogaster</td><td>CG15599</td><td>CG15599 gene product from transcript CG15599-RB</td><td>DmelCG15599</td><td>0</td><td>0</td></tr>
<tr><td>1242</td><td>32504</td><td>Drosophila melanogaster</td><td>CG8128</td><td>CG8128 gene product from transcript CG8128-RA</td><td>DmelCG8128</td><td>0</td><td>0</td></tr>
<tr><td>1243</td><td>32505</td><td>Drosophila melanogaster</td><td>CG9240</td><td>CG9240 gene product from transcript CG9240-RA</td><td>DmelCG9240|anon-EST:Posey267</td><td>0</td><td>0</td></tr>
<tr><td>1244</td><td>32506</td><td>Drosophila melanogaster</td><td>Pis</td><td>Phosphatidylinositol synthase</td><td>CG9245|DmelCG9245|anon-WO02059370.38|dPIS|dpis</td><td>0</td><td>0</td></tr>
<tr><td>1245</td><td>32507</td><td>Drosophila melanogaster</td><td>CG8134</td><td>CG8134 gene product from transcript CG8134-RA</td><td>DmelCG8134</td><td>0</td><td>0</td></tr>
<tr><td>1246</td><td>32508</td><td>Drosophila melanogaster</td><td>CG9281</td><td>CG9281 gene product from transcript CG9281-RB</td><td>BEST:LD38280|DmelCG9281</td><td>0</td><td>0</td></tr>
<tr><td>1247</td><td>32509</td><td>Drosophila melanogaster</td><td>CG15601</td><td>CG15601 gene product from transcript CG15601-RA</td><td>DmelCG15601</td><td>0</td><td>0</td></tr>
<tr><td>1248</td><td>32510</td><td>Drosophila melanogaster</td><td>CG8184</td><td>CG8184 gene product from transcript CG8184-RB</td><td>DmelCG8184|URE-B1/CG8184</td><td>0</td><td>0</td></tr>
<tr><td>1249</td><td>32511</td><td>Drosophila melanogaster</td><td>CG8191</td><td>CG8191 gene product from transcript CG8191-RA</td><td>DmelCG8191</td><td>0</td><td>0</td></tr>
<tr><td>1250</td><td>32512</td><td>Drosophila melanogaster</td><td>CG12379</td><td>CG12379 gene product from transcript CG12379-RB</td><td>DmelCG12379</td><td>0</td><td>0</td></tr>
<tr><td>1251</td><td>32513</td><td>Drosophila melanogaster</td><td>l(1)G0136</td><td>lethal (1) G0136</td><td>BcDNA:AT26381|CG8198|DmelCG8198</td><td>0</td><td>0</td></tr>
<tr><td>1252</td><td>32514</td><td>Drosophila melanogaster</td><td>Actr13E</td><td>Actin-related protein 13E</td><td>ARP|ARP(13E)|ARP13E|Acrp|Arp13E|Arp4|CG11678|DmelCG11678|arp4</td><td>0</td><td>0</td></tr>
<tr><td>1253</td><td>32515</td><td>Drosophila melanogaster</td><td>CG11679</td><td>CG11679 gene product from transcript CG11679-RA</td><td>DmelCG11679</td><td>0</td><td>0</td></tr>
<tr><td>1254</td><td>32516</td><td>Drosophila melanogaster</td><td>CG8206</td><td>CG8206 gene product from transcript CG8206-RA</td><td>DmelCG8206</td><td>0</td><td>0</td></tr>
<tr><td>1255</td><td>32517</td><td>Drosophila melanogaster</td><td>CG8944</td><td>CG8944 gene product from transcript CG8944-RB</td><td>DmelCG8944</td><td>0</td><td>0</td></tr>
<tr><td>1256</td><td>32518</td><td>Drosophila melanogaster</td><td>Tcp-1zeta</td><td>T-cp1zeta</td><td>CCT6|CG8231|DmelCG8231|L(1)g0022|Rcd3|TCP-1-zeta|TCP-1zeta|Tcp-1-zeta|l(1)G0022|l(1)G0027|l(1)G0057</td><td>0</td><td>0</td></tr>
<tr><td>1257</td><td>32520</td><td>Drosophila melanogaster</td><td>CG8239</td><td>CG8239 gene product from transcript CG8239-RA</td><td>DmelCG8239</td><td>0</td><td>0</td></tr>
<tr><td>1258</td><td>32521</td><td>Drosophila melanogaster</td><td>CG8260</td><td>CG8260 gene product from transcript CG8260-RB</td><td>DmelCG8260</td><td>0</td><td>0</td></tr>
<tr><td>1259</td><td>32522</td><td>Drosophila melanogaster</td><td>Graf</td><td>CG8948 gene product from transcript CG8948-RB</td><td>CG8948|D-Graf|DmelCG8948</td><td>0</td><td>0</td></tr>
<tr><td>1260</td><td>32523</td><td>Drosophila melanogaster</td><td>mRpL3</td><td>mitochondrial ribosomal protein L3</td><td>CG8288|DmelCG8288|L3</td><td>0</td><td>0</td></tr>
<tr><td>1261</td><td>32524</td><td>Drosophila melanogaster</td><td>CG17209</td><td>CG17209 gene product from transcript CG17209-RC</td><td>DmelCG17209</td><td>0</td><td>0</td></tr>
<tr><td>1262</td><td>32525</td><td>Drosophila melanogaster</td><td>CG8952</td><td>CG8952 gene product from transcript CG8952-RA</td><td>DmelCG8952|SP52</td><td>0</td><td>0</td></tr>
<tr><td>1263</td><td>32526</td><td>Drosophila melanogaster</td><td>CG33172</td><td>CG33172 gene product from transcript CG33172-RB</td><td>CG8473|DmelCG33172</td><td>0</td><td>0</td></tr>
<tr><td>1264</td><td>32528</td><td>Drosophila melanogaster</td><td>CG8959</td><td>CG8959 gene product from transcript CG8959-RA</td><td>DmelCG8959</td><td>0</td><td>0</td></tr>
<tr><td>1265</td><td>32529</td><td>Drosophila melanogaster</td><td>Paf-AHalpha</td><td>Platelet-activating factor acetylhydrolase alpha</td><td>AAC83820|CG8962|Dmaalpha[[PAFAH]]|DmelCG8962|PAFAHalpha</td><td>0</td><td>0</td></tr>
<tr><td>1266</td><td>32530</td><td>Drosophila melanogaster</td><td>mRpS30</td><td>mitochondrial ribosomal protein S30</td><td>CG8470|DmelCG8470|MRP-S30|mRSp30</td><td>0</td><td>0</td></tr>
<tr><td>1267</td><td>32531</td><td>Drosophila melanogaster</td><td>Rhp</td><td>Rhophilin</td><td>CG8497|DmelCG8497</td><td>0</td><td>0</td></tr>
<tr><td>1268</td><td>32532</td><td>Drosophila melanogaster</td><td>CG8974</td><td>CG8974 gene product from transcript CG8974-RD</td><td>DmelCG8974</td><td>0</td><td>0</td></tr>
<tr><td>1269</td><td>32533</td><td>Drosophila melanogaster</td><td>CG15602</td><td>CG15602 gene product from transcript CG15602-RA</td><td>DmelCG15602</td><td>0</td><td>0</td></tr>
<tr><td>1270</td><td>32534</td><td>Drosophila melanogaster</td><td>PGRP-LE</td><td>Peptidoglycan recognition protein LE</td><td>CG8995|DmelCG8995|PGRP|anon-WO0153538.66</td><td>0</td><td>0</td></tr>
<tr><td>1271</td><td>32535</td><td>Drosophila melanogaster</td><td>CG8509</td><td>CG8509 gene product from transcript CG8509-RA</td><td>DmelCG8509</td><td>0</td><td>0</td></tr>
<tr><td>1272</td><td>32536</td><td>Drosophila melanogaster</td><td>sd</td><td>scalloped</td><td>CG8544|DROEXO|DROEXO2|DmelCG8544|EP(X)1088|EP1088|SD|Sd|TEAD|anon-EST:Liang-2.14|clone 2.14|l(1)G0239|l(1)G0262|l(1)G0309|l(1)G0315|l(1)G0483|l(1)HF394|l(1)lll|sp</td><td>0</td><td>0</td></tr>
<tr><td>1273</td><td>32537</td><td>Drosophila melanogaster</td><td>Chc</td><td>Clathrin heavy chain</td><td>CG9012|CHC|CLH|Cla|D-Chc|DmelCG9012|chc|dCHC|l(1)13Fb|l(1)G0438|l(1)VI</td><td>0</td><td>0</td></tr>
<tr><td>1274</td><td>32538</td><td>Drosophila melanogaster</td><td>CG8565</td><td>CG8565 gene product from transcript CG8565-RA</td><td>DmelCG8565|SPRK2</td><td>0</td><td>0</td></tr>
<tr><td>1275</td><td>32539</td><td>Drosophila melanogaster</td><td>Aats-arg</td><td>Arginyl-tRNA synthetase</td><td>ArgRS|CG9020|DmelCG9020|RRS</td><td>0</td><td>0</td></tr>
<tr><td>1276</td><td>32540</td><td>Drosophila melanogaster</td><td>CG8578</td><td>CG8578 gene product from transcript CG8578-RA</td><td>DmelCG8578</td><td>0</td><td>0</td></tr>
<tr><td>1277</td><td>32541</td><td>Drosophila melanogaster</td><td>UBL3</td><td>CG9038 gene product from transcript CG9038-RC</td><td>CG9038|DmelCG9038|HCG-1|MUB|Ubl3</td><td>0</td><td>0</td></tr>
<tr><td>1278</td><td>32542</td><td>Drosophila melanogaster</td><td>CG15914</td><td>CG15914 gene product from transcript CG15914-RA</td><td>DmelCG15914|PIG</td><td>0</td><td>0</td></tr>
<tr><td>1279</td><td>32543</td><td>Drosophila melanogaster</td><td>Myb</td><td>Myb oncogene-like</td><td>CG9045|D-myb|DMyb|Dm Myb|Dm myb|Dm-Myb|Dm-myb|DmMYB|DmMyb|DmelCG9045|Dmyb|MYB|c-Myb|c-myb|dMyb|dmMYB|dmyb|l(1)XVI|myb|p85</td><td>0</td><td>0</td></tr>
<tr><td>1280</td><td>32544</td><td>Drosophila melanogaster</td><td>Gbeta13F</td><td>G protein beta-subunit 13F</td><td>CG10545|DmelCG10545|G b13F|G-beta|G-betab|G[[beta]]Gb13F|Gbb|Gbeta|anon-EST:Liang-1.22|clone 1.22|dgbeta</td><td>0</td><td>0</td></tr>
<tr><td>1281</td><td>32545</td><td>Drosophila melanogaster</td><td>Gapdh2</td><td>Glyceraldehyde 3 phosphate dehydrogenase 2</td><td>CG8893|DmelCG8893|GA3PDH|GADPH|GAP|GAPDH|GAPDH II|GAPDH2|Gapd|Gapdh|Gapdh-2|Gapdh13F</td><td>0</td><td>0</td></tr>
<tr><td>1282</td><td>32546</td><td>Drosophila melanogaster</td><td>CG16952</td><td>CG16952 gene product from transcript CG16952-RC</td><td>DmelCG16952</td><td>0</td><td>0</td></tr>
<tr><td>1283</td><td>32547</td><td>Drosophila melanogaster</td><td>tay</td><td>tay bridge</td><td>CG9056|DmelCG9056|XI|l(1)13Ff|l(1)14Ac|l(1)XI</td><td>0</td><td>0</td></tr>
<tr><td>1284</td><td>32548</td><td>Drosophila melanogaster</td><td>MSBP</td><td>membrane steroid binding protein</td><td>CG9066|DmelCG9066|dm_MSBP</td><td>0</td><td>0</td></tr>
<tr><td>1285</td><td>32549</td><td>Drosophila melanogaster</td><td>CG15916</td><td>CG15916 gene product from transcript CG15916-RA</td><td>BcDNA:RH08992|DmelCG15916</td><td>0</td><td>0</td></tr>
<tr><td>1286</td><td>32551</td><td>Drosophila melanogaster</td><td>CycD</td><td>Cyclin D</td><td>CDI3|CG9096|CYCD|Cdi3|DmcycD|DmelCG9096|cdi3|cycD|cyclinD|dCycD</td><td>0</td><td>0</td></tr>
<tr><td>1287</td><td>32552</td><td>Drosophila melanogaster</td><td>CG8909</td><td>CG8909 gene product from transcript CG8909-RC</td><td>CT25574|Dm CG8909|DmelCG8909</td><td>0</td><td>0</td></tr>
<tr><td>1288</td><td>32553</td><td>Drosophila melanogaster</td><td>CG8916</td><td>CG8916 gene product from transcript CG8916-RB</td><td>DmelCG8916</td><td>0</td><td>0</td></tr>
<tr><td>1289</td><td>32554</td><td>Drosophila melanogaster</td><td>Lcch3</td><td>Ligand-gated chloride channel homolog 3</td><td>CG17336|DmGABAbeta|DmGA_b|DmelCG17336|LCCH3</td><td>0</td><td>0</td></tr>
<tr><td>1290</td><td>32556</td><td>Drosophila melanogaster</td><td>Stim</td><td>Stromal interaction molecule</td><td>CG9126|D-STIM|D-Stim|DmelCG9126|STIM|dSTIM|dStim|dstim|stim1</td><td>0</td><td>0</td></tr>
<tr><td>1291</td><td>32557</td><td>Drosophila melanogaster</td><td>CG8924</td><td>CG8924 gene product from transcript CG8924-RB</td><td>DmelCG8924</td><td>0</td><td>0</td></tr>
<tr><td>1292</td><td>32558</td><td>Drosophila melanogaster</td><td>CG8928</td><td>CG8928 gene product from transcript CG8928-RA</td><td>DmelCG8928</td><td>0</td><td>0</td></tr>
<tr><td>1293</td><td>32561</td><td>Drosophila melanogaster</td><td>mmd</td><td>mind-meld</td><td>CG15603|CG15604|CG42252|CG9163|CT26192|DmelCG42252|Dmel_CG15603|Dmel_CG15604|Dmel_CG9163</td><td>0</td><td>0</td></tr>
<tr><td>1294</td><td>32562</td><td>Drosophila melanogaster</td><td>Or13a</td><td>Odorant receptor 13a</td><td>13F.1|13a|CG12697|DOR38|DmelCG12697|dOr13a</td><td>0</td><td>0</td></tr>
<tr><td>1295</td><td>32563</td><td>Drosophila melanogaster</td><td>CG9170</td><td>CG9170 gene product from transcript CG9170-RB</td><td>DmelCG9170|anon-WO0140519.214</td><td>0</td><td>0</td></tr>
<tr><td>1296</td><td>32564</td><td>Drosophila melanogaster</td><td>CG8931</td><td>CG8931 gene product from transcript CG8931-RA</td><td>DmelCG8931</td><td>0</td><td>0</td></tr>
<tr><td>1297</td><td>32565</td><td>Drosophila melanogaster</td><td>CG9172</td><td>CG9172 gene product from transcript CG9172-RA</td><td>20 kDa|DmelCG9172</td><td>0</td><td>0</td></tr>
<tr><td>1298</td><td>32566</td><td>Drosophila melanogaster</td><td>eIF5</td><td>CG9177 gene product from transcript CG9177-RE</td><td>CG9177|DmelCG9177|anon-EST:fe3C6</td><td>0</td><td>0</td></tr>
<tr><td>1299</td><td>32567</td><td>Drosophila melanogaster</td><td>exd</td><td>extradenticle</td><td>CG8933|DExd|Dm-EXD|DmelCG8933|Dpbx|EXD|Exd|Pbx1|anon-EST:fe1H3|l(1)IV|td48</td><td>0</td><td>0</td></tr>
<tr><td>1300</td><td>32568</td><td>Drosophila melanogaster</td><td>CG8939</td><td>CG8939 gene product from transcript CG8939-RA</td><td>DmelCG8939</td><td>0</td><td>0</td></tr>
<tr><td>1301</td><td>32569</td><td>Drosophila melanogaster</td><td>vap</td><td>vacuolar peduncle</td><td>CG9209|D-RasGAP|DmelCG9209|RAS-GAP|RasGAP|RasGap|rasGap</td><td>0</td><td>0</td></tr>
<tr><td>1302</td><td>32570</td><td>Drosophila melanogaster</td><td>CG12698</td><td>CG12698 gene product from transcript CG12698-RA</td><td>DmelCG12698</td><td>0</td><td>0</td></tr>
<tr><td>1303</td><td>32571</td><td>Drosophila melanogaster</td><td>CG12395</td><td>CG12395 gene product from transcript CG12395-RA</td><td>DmelCG12395</td><td>0</td><td>0</td></tr>
<tr><td>1304</td><td>32572</td><td>Drosophila melanogaster</td><td>dpr18</td><td>CG14948 gene product from transcript CG14948-RA</td><td>CG14948|CT34788|DmelCG14948|Dpr-18</td><td>0</td><td>0</td></tr>
<tr><td>1305</td><td>32573</td><td>Drosophila melanogaster</td><td>Nipsnap</td><td>CG9212 gene product from transcript CG9212-RC</td><td>CG9212|DmelCG9212</td><td>0</td><td>0</td></tr>
<tr><td>1306</td><td>32574</td><td>Drosophila melanogaster</td><td>Tob</td><td>CG9214 gene product from transcript CG9214-RA</td><td>CG9214|DmelCG9214</td><td>0</td><td>0</td></tr>
<tr><td>1307</td><td>32575</td><td>Drosophila melanogaster</td><td>CG8958</td><td>CG8958 gene product from transcript CG8958-RA</td><td>DmelCG8958</td><td>0</td><td>0</td></tr>
<tr><td>1308</td><td>32576</td><td>Drosophila melanogaster</td><td>CG42353</td><td>CG42353 gene product from transcript CG42353-RA</td><td>CG9216|DmelCG42353|Dmel_CG9216</td><td>0</td><td>0</td></tr>
<tr><td>1309</td><td>32579</td><td>Drosophila melanogaster</td><td>disco</td><td>disconnected</td><td>CG9908|Disco|DmelCG9908|disc</td><td>0</td><td>0</td></tr>
<tr><td>1310</td><td>32580</td><td>Drosophila melanogaster</td><td>CG12507</td><td>CG12507 gene product from transcript CG12507-RB</td><td>DmelCG12507</td><td>0</td><td>0</td></tr>
<tr><td>1311</td><td>32581</td><td>Drosophila melanogaster</td><td>kat80</td><td>katanin 80</td><td>CG13956|CG9910|DmelCG13956|Kat80|Katanin80</td><td>0</td><td>0</td></tr>
<tr><td>1312</td><td>32582</td><td>Drosophila melanogaster</td><td>Mfe2</td><td>peroxisomal Multifunctional enzyme type 2</td><td>CG3415|DmMFE-2|DmelCG3415|MFE-2</td><td>0</td><td>0</td></tr>
<tr><td>1313</td><td>32584</td><td>Drosophila melanogaster</td><td>Pros28.1</td><td>Proteasome 28kD subunit 1</td><td>28.1Pros|3422|CG3422|DmelCG3422|PROS-28.1|PROS-Dm28.1|PROSA-28.1|PSA4_DROME|Pros-Dm28.1|Pros28|Prosalpha4|a4|alpha4|alpha4_dm|pros28.1</td><td>0</td><td>0</td></tr>
<tr><td>1314</td><td>32585</td><td>Drosophila melanogaster</td><td>eas</td><td>easily shocked</td><td>CG3525|DmelCG3525|EP770|EtnK|PC80|RE10142|RH11|ala</td><td>0</td><td>0</td></tr>
<tr><td>1315</td><td>32586</td><td>Drosophila melanogaster</td><td>CG3560</td><td>CG3560 gene product from transcript CG3560-RA</td><td>BcDNA:RH44664|DmelCG3560|anon-EST:Posey167</td><td>0</td><td>0</td></tr>
<tr><td>1316</td><td>32587</td><td>Drosophila melanogaster</td><td>caz</td><td>cabeza</td><td>BcDNA:GM09207|CG3606|DmelCG3606|P19|Pen19|SARFH|Sarfh|TFIID|anon-Pen19|cas|p19|pen19</td><td>0</td><td>0</td></tr>
<tr><td>1317</td><td>32588</td><td>Drosophila melanogaster</td><td>CG9911</td><td>CG9911 gene product from transcript CG9911-RD</td><td>CG9912|DmelCG9911|LD41494p</td><td>0</td><td>0</td></tr>
<tr><td>1318</td><td>32589</td><td>Drosophila melanogaster</td><td>CG3632</td><td>CG3632 gene product from transcript CG3632-RH</td><td>DmelCG3632</td><td>0</td><td>0</td></tr>
<tr><td>1319</td><td>32591</td><td>Drosophila melanogaster</td><td>CG3679</td><td>CG3679 gene product from transcript CG3679-RA</td><td>BcDNA:LD21675|DmelCG3679</td><td>0</td><td>0</td></tr>
<tr><td>1320</td><td>32592</td><td>Drosophila melanogaster</td><td>CG9914</td><td>CG9914 gene product from transcript CG9914-RA</td><td>DmelCG9914</td><td>0</td><td>0</td></tr>
<tr><td>1321</td><td>32593</td><td>Drosophila melanogaster</td><td>CG9915</td><td>CG9915 gene product from transcript CG9915-RB</td><td>DmelCG9915</td><td>0</td><td>0</td></tr>
<tr><td>1322</td><td>32594</td><td>Drosophila melanogaster</td><td>CG13957</td><td>CG13957 gene product from transcript CG13957-RA</td><td>DmelCG13957|p116</td><td>0</td><td>0</td></tr>
<tr><td>1323</td><td>32595</td><td>Drosophila melanogaster</td><td>Cyp1</td><td>Cyclophilin 1</td><td>BcDNA:GM09255|CG9916|Cpy1|CyP-1|Cyp-1|DmelCG9916|cyp-1|cyp1</td><td>0</td><td>0</td></tr>
<tr><td>1324</td><td>32596</td><td>Drosophila melanogaster</td><td>CG9917</td><td>CG9917 gene product from transcript CG9917-RA</td><td>DmelCG9917|anon-WO0140519.229</td><td>0</td><td>0</td></tr>
<tr><td>1325</td><td>32597</td><td>Drosophila melanogaster</td><td>CalpC</td><td>Calpain C</td><td>CG3692|DmelCG3692</td><td>0</td><td>0</td></tr>
<tr><td>1326</td><td>32598</td><td>Drosophila melanogaster</td><td>CG9919</td><td>CG9919 gene product from transcript CG9919-RA</td><td>DmelCG9919</td><td>0</td><td>0</td></tr>
<tr><td>1327</td><td>32599</td><td>Drosophila melanogaster</td><td>CG9921</td><td>CG9921 gene product from transcript CG9921-RA</td><td>DmelCG9921</td><td>0</td><td>0</td></tr>
<tr><td>1328</td><td>32600</td><td>Drosophila melanogaster</td><td>bss</td><td>bang senseless</td><td>bas</td><td>0</td><td>0</td></tr>
<tr><td>1329</td><td>32601</td><td>Drosophila melanogaster</td><td>sl</td><td>small wing</td><td>CG4200|DmelCG4200|PLC|PLC&ygr;|PLC-gamma|PLC-gamma D|PLC-gammaD|PLCgamma|PLCgammaDmel|Plcgamma|p145|plc-gamma|plc-gammad</td><td>0</td><td>0</td></tr>
<tr><td>1330</td><td>32602</td><td>Drosophila melanogaster</td><td>U2af50</td><td>U2 small nuclear riboprotein auxiliary factor 50</td><td>9-21|CG9998|DU2AF50|DmU2AF[50]|DmelCG9998|U2AF|U2AF-50|U2AF50|U2af|dU2AF50|dU2AF[50]|dUTAf[50]|l(1)14Ca|l(1)14Cc|l(1)9-21</td><td>0</td><td>0</td></tr>
</table>

<table>
<tr><td>1331</td><td>32603</td><td>Drosophila melanogaster</td><td>nonA</td><td>no on or off transient A</td><td>Bj6|Bj6-1|CG4211|DmelCG4211|NonA|diss|non-A|nonA/diss|opm2|p54[nrb]|x14</td><td>0</td><td>0</td></tr>
<tr><td>1332</td><td>32604</td><td>Drosophila melanogaster</td><td>Fur2</td><td>Furin 2</td><td>154521_at|CG18734|CG4235|DMH#11|Dfur2|Dfurin2|DmelCG18734|FUR2|Furin|fur2|furin</td><td>0</td><td>0</td></tr>
<tr><td>1333</td><td>32605</td><td>Drosophila melanogaster</td><td>CG9992</td><td>CG9992 gene product from transcript CG9992-RB</td><td>DmelCG9992</td><td>0</td><td>0</td></tr>
<tr><td>1334</td><td>32606</td><td>Drosophila melanogaster</td><td>CG4239</td><td>CG4239 gene product from transcript CG4239-RA</td><td>DmelCG4239</td><td>0</td><td>0</td></tr>
<tr><td>1335</td><td>32607</td><td>Drosophila melanogaster</td><td>TH1</td><td>CG9984 gene product from transcript CG9984-RA</td><td>CG9984|DmelCG9984|NELF|NELF-D</td><td>0</td><td>0</td></tr>
<tr><td>1336</td><td>32608</td><td>Drosophila melanogaster</td><td>mei-41</td><td>meiotic 41</td><td>4252|ATR|CG4252|DmATR|DmelCG4252|Dmmei41|MEI41|MEI41/FRP1|Mei-41|Mei-41/DATR|atr|fs(1)M37|mei-195|mei41|meiP41|mus(1)103|mus(1)104|mus-103|mus-104|mus103|mus104</td><td>0</td><td>0</td></tr>
<tr><td>1337</td><td>32609</td><td>Drosophila melanogaster</td><td>CG9981</td><td>CG9981 gene product from transcript CG9981-RA</td><td>DmelCG9981</td><td>0</td><td>0</td></tr>
<tr><td>1338</td><td>32610</td><td>Drosophila melanogaster</td><td>CG4301</td><td>CG4301 gene product from transcript CG4301-RC</td><td>DmelCG4301</td><td>0</td><td>0</td></tr>
<tr><td>1339</td><td>32611</td><td>Drosophila melanogaster</td><td>Traf-like</td><td>TNF-receptor-associated factor-like</td><td>CG4394|DmelCG4394|TRAF3|Traf-related|Traf3|dTRAF3</td><td>0</td><td>0</td></tr>
<tr><td>1340</td><td>32612</td><td>Drosophila melanogaster</td><td>Anxb11</td><td>Annexin B11</td><td>AnxB11|BcDNA:GH16395|CG9968|DmelCG9968|annexin</td><td>0</td><td>0</td></tr>
<tr><td>1341</td><td>32613</td><td>Drosophila melanogaster</td><td>hang</td><td>hangover</td><td>BcDNA:GM07804|BcDNA:LD22118|CG32575|CG4411|CG4416|DmelCG32575|LD22118</td><td>0</td><td>0</td></tr>
<tr><td>1342</td><td>32615</td><td>Drosophila melanogaster</td><td>CG9947</td><td>CG9947 gene product from transcript CG9947-RA</td><td>DmelCG9947</td><td>0</td><td>0</td></tr>
<tr><td>1343</td><td>32616</td><td>Drosophila melanogaster</td><td>rngo</td><td>rings lost</td><td>CG4420|DmelCG4420</td><td>0</td><td>0</td></tr>
<tr><td>1344</td><td>32617</td><td>Drosophila melanogaster</td><td>eIF-2alpha</td><td>eukaryotic translation Initiation Factor 2alpha</td><td>CG9946|DmelCG9946|Eif2alpha|IF2A_DROME|M(1)14C|M(1)19-153|M(1)8e3-10|deIF2alpha|eIF-2|eIF-2 a|eIF-2 alpha|eIF2 alpha|eIF2-alpha|eIF2a|eIF2alpha|eiF-2alpha|eIF2alpha|l(1)14Cf|l(1)19-153</td><td>0</td><td>0</td></tr>
<tr><td>1345</td><td>32619</td><td>Drosophila melanogaster</td><td>para</td><td>paralytic</td><td>CG9907|DmNa[[V]]|DmNa[[v]]|DmNa[[v]]1|DmelCG9907|Ocd|l(1)14Da|l(1)ESHS48|olfD|sbl|sbl-1</td><td>0</td><td>0</td></tr>
<tr><td>1346</td><td>32620</td><td>Drosophila melanogaster</td><td>CG9906</td><td>CG9906 gene product from transcript CG9906-RA</td><td>DmelCG9906</td><td>0</td><td>0</td></tr>
<tr><td>1347</td><td>32621</td><td>Drosophila melanogaster</td><td>CG9903</td><td>CG9903 gene product from transcript CG9903-RA</td><td>DmelCG9903</td><td>0</td><td>0</td></tr>
<tr><td>1348</td><td>32622</td><td>Drosophila melanogaster</td><td>CG9902</td><td>CG9902 gene product from transcript CG9902-RA</td><td>DmelCG9902</td><td>0</td><td>0</td></tr>
<tr><td>1349</td><td>32623</td><td>Drosophila melanogaster</td><td>Arp14D</td><td>Actin-related protein 14D</td><td>ARP14D|ARP2|Actr14D|Arp2|CG9901|DmelCG9901|arp2</td><td>0</td><td>0</td></tr>
<tr><td>1350</td><td>32624</td><td>Drosophila melanogaster</td><td>Pp2B-14D</td><td>Protein phosphatase 2B at 14D</td><td>CG9842|CanA14D|CnA|CnnA14D|D33|DmPp2B-14D|DmelCG9842|PP2B|PP2B 14D|PP2B-14D|Pp12-14D|Pp2B-14DF|PpD33|canA</td><td>0</td><td>0</td></tr>
<tr><td>1351</td><td>32625</td><td>Drosophila melanogaster</td><td>P5CDh2</td><td>delta-1-Pyrroline-5-carboxylate dehydrogenase 2</td><td>CG33092|CG6670|DmP5CDh2|DmelCG33092</td><td>0</td><td>0</td></tr>
<tr><td>1352</td><td>32626</td><td>Drosophila melanogaster</td><td>CG13014</td><td>CG13014 gene product from transcript CG13014-RA</td><td>DmelCG13014</td><td>0</td><td>0</td></tr>
<tr><td>1353</td><td>32627</td><td>Drosophila melanogaster</td><td>Cap</td><td>Chromosome-associated protein</td><td>CG9802|DmSMC3|DmSMC3/Cap|DmelCG9802|SMC3|Smc3|cap|dSMC3</td><td>0</td><td>0</td></tr>
<tr><td>1354</td><td>32629</td><td>Drosophila melanogaster</td><td>CG9784</td><td>CG9784 gene product from transcript CG9784-RA</td><td>DmelCG9784|IPP</td><td>0</td><td>0</td></tr>
<tr><td>1355</td><td>32630</td><td>Drosophila melanogaster</td><td>Nup153</td><td>Nucleoporin 153</td><td>CG4453|DmelCG4453|NUP153|Nup 153|dNup153|dmNup153|nup153</td><td>0</td><td>0</td></tr>
<tr><td>1356</td><td>32631</td><td>Drosophila melanogaster</td><td>mbt</td><td>mushroom bodies tiny</td><td>CG18582|DmMBT|DmPAK2|DmelCG18582|Mbt|Pak2|STE20|Ste20|anon-EST:fe1G7|dPAK2</td><td>0</td><td>0</td></tr>
<tr><td>1357</td><td>32632</td><td>Drosophila melanogaster</td><td>CG9782</td><td>CG9782 gene product from transcript CG9782-RA</td><td>DmelCG9782</td><td>0</td><td>0</td></tr>
<tr><td>1358</td><td>32633</td><td>Drosophila melanogaster</td><td>CG9777</td><td>CG9777 gene product from transcript CG9777-RA</td><td>DmelCG9777</td><td>0</td><td>0</td></tr>
<tr><td>1359</td><td>32635</td><td>Drosophila melanogaster</td><td>RpS19a</td><td>Ribosomal protein S19a</td><td>BcDNA:GM26647|CG4464|DmelCG4464|M(1)14E|RpS19|S19|S19a</td><td>0</td><td>0</td></tr>
<tr><td>1360</td><td>32636</td><td>Drosophila melanogaster</td><td>SmG</td><td>Small ribonucleoprotein particle protein SmG</td><td>BcDNA:RE61341|BcDNA:RH35475|CG9742|DmelCG9742|G|HD10913|dG|snRNPG</td><td>0</td><td>0</td></tr>
<tr><td>1361</td><td>32637</td><td>Drosophila melanogaster</td><td>mthl1</td><td>methuselah-like 1</td><td>CG4521|DmelCG4521|Mth-1|anon-WO0170980.22|anon-WO0170980.23</td><td>0</td><td>0</td></tr>
<tr><td>1362</td><td>32638</td><td>Drosophila melanogaster</td><td>CG32573</td><td>CG32573 gene product from transcript CG32573-RA</td><td>CG13013|CG4524|DmelCG32573|Dromel_CG32573_FBtr0074315_mORF</td><td>0</td><td>0</td></tr>
<tr><td>1363</td><td>32639</td><td>Drosophila melanogaster</td><td>CG9723</td><td>CG9723 gene product from transcript CG9723-RA</td><td>DmelCG9723</td><td>0</td><td>0</td></tr>
<tr><td>1364</td><td>32640</td><td>Drosophila melanogaster</td><td>r</td><td>rudimentary</td><td>ACT|CAD|CG18572|CG4601|CPS|DHO|DRORUD|DmelCG18572|GAT|PYR1|Su(b)|csp2|fs(1)829|fs(1)M34|l(1)Ab</td><td>0</td><td>0</td></tr>
<tr><td>1365</td><td>32641</td><td>Drosophila melanogaster</td><td>CG15865</td><td>CG15865 gene product from transcript CG15865-RA</td><td>DmelCG15865|anon-15Ab</td><td>0</td><td>0</td></tr>
<tr><td>1366</td><td>32642</td><td>Drosophila melanogaster</td><td>CG13012</td><td>CG13012 gene product from transcript CG13012-RB</td><td>DmelCG13012</td><td>0</td><td>0</td></tr>
<tr><td>1367</td><td>32643</td><td>Drosophila melanogaster</td><td>CG13010</td><td>CG13010 gene product from transcript CG13010-RA</td><td>DmelCG13010</td><td>0</td><td>0</td></tr>
<tr><td>1368</td><td>32644</td><td>Drosophila melanogaster</td><td>sing</td><td>singles bar</td><td>CG13011|DmelCG13011|singles-bar</td><td>0</td><td>0</td></tr>
<tr><td>1369</td><td>32645</td><td>Drosophila melanogaster</td><td>Axs</td><td>Abnormal X segregation</td><td>CG9703|DmelCG9703|axs</td><td>0</td><td>0</td></tr>
<tr><td>1370</td><td>32646</td><td>Drosophila melanogaster</td><td>Sep4</td><td>Septin 4</td><td>CG9699|DmelCG9699|sep4</td><td>0</td><td>0</td></tr>
<tr><td>1371</td><td>32647</td><td>Drosophila melanogaster</td><td>CG9676</td><td>CG9676 gene product from transcript CG9676-RA</td><td>DmelCG9676|SP99</td><td>0</td><td>0</td></tr>
<tr><td>1372</td><td>32648</td><td>Drosophila melanogaster</td><td>spheroide</td><td>CG9675 gene product from transcript CG9675-RA</td><td>CG9675|DmelCG9675|SPH114|Spheroide|anon-EST:ParkEST153|anon-WO0140519.154</td><td>0</td><td>0</td></tr>
<tr><td>1373</td><td>32649</td><td>Drosophila melanogaster</td><td>CG9673</td><td>CG9673 gene product from transcript CG9673-RA</td><td>DmelCG9673|SPH81</td><td>0</td><td>0</td></tr>
<tr><td>1374</td><td>32650</td><td>Drosophila melanogaster</td><td>CG4653</td><td>CG4653 gene product from transcript CG4653-RA</td><td>DmelCG4653|SPH179</td><td>0</td><td>0</td></tr>
<tr><td>1375</td><td>32651</td><td>Drosophila melanogaster</td><td>CG9672</td><td>CG9672 gene product from transcript CG9672-RA</td><td>BcDNA:GH16384|DmelCG9672|SPH161</td><td>0</td><td>0</td></tr>
<tr><td>1376</td><td>32652</td><td>Drosophila melanogaster</td><td>CG4678</td><td>CG4678 gene product from transcript CG4678-RE</td><td>DmelCG4678</td><td>0</td><td>0</td></tr>
<tr><td>1377</td><td>32654</td><td>Drosophila melanogaster</td><td>CG13008</td><td>CG13008 gene product from transcript CG13008-RA</td><td>CG13009|DmelCG13008</td><td>0</td><td>0</td></tr>
<tr><td>1378</td><td>32656</td><td>Drosophila melanogaster</td><td>CG34325</td><td>CG34325 gene product from transcript CG34325-RA</td><td>CG18358|DmelCG34325</td><td>0</td><td>0</td></tr>
<tr><td>1379</td><td>32657</td><td>Drosophila melanogaster</td><td>CG32572</td><td>CG32572 gene product from transcript CG32572-RA</td><td>CG13006|CG13007|DmelCG32572</td><td>0</td><td>0</td></tr>
<tr><td>1380</td><td>32659</td><td>Drosophila melanogaster</td><td>CG32568</td><td>CG32568 gene product from transcript CG32568-RB</td><td>CG4724|DmelCG32568</td><td>0</td><td>0</td></tr>
<tr><td>1381</td><td>32660</td><td>Drosophila melanogaster</td><td>CG9634</td><td>CG9634 gene product from transcript CG9634-RA</td><td>BcDNA:LD21405|DmelCG9634</td><td>0</td><td>0</td></tr>
<tr><td>1382</td><td>32661</td><td>Drosophila melanogaster</td><td>if</td><td>inflated</td><td>CG9623|CT27194|DmelCG9623|If|PS 2|PS2|PS2alpha|PSalpha2|aPS2|alpha2Int|alphaPS2|alphaPS2C|alpha[[PS2(ms8)]]|alpha[[PS2]]|in|inf</td><td>0</td><td>0</td></tr>
<tr><td>1383</td><td>32662</td><td>Drosophila melanogaster</td><td>mRpL22</td><td>mitochondrial ribosomal protein L22</td><td>CG4742|DmelCG4742|L22</td><td>0</td><td>0</td></tr>
<tr><td>1384</td><td>32663</td><td>Drosophila melanogaster</td><td>CG9609</td><td>CG9609 gene product from transcript CG9609-RA</td><td>DmelCG9609</td><td>0</td><td>0</td></tr>
<tr><td>1385</td><td>32664</td><td>Drosophila melanogaster</td><td>Sap30</td><td>SIN3-associated polypeptide 30</td><td>CG4756|DmelCG4756|SAP30</td><td>0</td><td>0</td></tr>
<tr><td>1386</td><td>32665</td><td>Drosophila melanogaster</td><td>Rrp45</td><td>CG9606 gene product from transcript CG9606-RA</td><td>CG9606|DmelCG9606|dRrp45|rrp45</td><td>0</td><td>0</td></tr>
<tr><td>1387</td><td>32666</td><td>Drosophila melanogaster</td><td>CG4768</td><td>CG4768 gene product from transcript CG4768-RB</td><td>DmelCG4768</td><td>0</td><td>0</td></tr>
<tr><td>1388</td><td>32668</td><td>Drosophila melanogaster</td><td>CG4789</td><td>CG4789 gene product from transcript CG4789-RA</td><td>DmelCG4789</td><td>0</td><td>0</td></tr>
<tr><td>1389</td><td>32670</td><td>Drosophila melanogaster</td><td>CG13005</td><td>CG13005 gene product from transcript CG13005-RA</td><td>DmelCG13005</td><td>0</td><td>0</td></tr>
<tr><td>1390</td><td>32671</td><td>Drosophila melanogaster</td><td>ppk28</td><td>pickpocket 28</td><td>CG4805|DmelCG4805|PPK28</td><td>0</td><td>0</td></tr>
<tr><td>1391</td><td>32672</td><td>Drosophila melanogaster</td><td>CG4829</td><td>CG4829 gene product from transcript CG4829-RB</td><td>DmelCG4829</td><td>0</td><td>0</td></tr>
<tr><td>1392</td><td>32673</td><td>Drosophila melanogaster</td><td>CG13004</td><td>CG13004 gene product from transcript CG13004-RD</td><td>DmelCG13004</td><td>0</td><td>0</td></tr>
<tr><td>1393</td><td>32674</td><td>Drosophila melanogaster</td><td>RSG7</td><td>Regulator of G-protein signalling 7</td><td>CG9108|DmelCG9108|RGS7|dRGS7</td><td>0</td><td>0</td></tr>
<tr><td>1394</td><td>32675</td><td>Drosophila melanogaster</td><td>CG13003</td><td>CG13003 gene product from transcript CG13003-RB</td><td>DmelCG13003</td><td>0</td><td>0</td></tr>
<tr><td>1395</td><td>32676</td><td>Drosophila melanogaster</td><td>CG4872</td><td>CG4872 gene product from transcript CG4872-RA</td><td>DmelCG4872</td><td>0</td><td>0</td></tr>
<tr><td>1396</td><td>32677</td><td>Drosophila melanogaster</td><td>CG9104</td><td>CG9104 gene product from transcript CG9104-RA</td><td>DmelCG9104</td><td>0</td><td>0</td></tr>
<tr><td>1397</td><td>32678</td><td>Drosophila melanogaster</td><td>Rcp</td><td>Receptor component protein</td><td>CG4875|DmelCG4875|dRCP</td><td>0</td><td>0</td></tr>
<tr><td>1398</td><td>32679</td><td>Drosophila melanogaster</td><td>CG9099</td><td>CG9099 gene product from transcript CG9099-RA</td><td>DmelCG9099</td><td>0</td><td>0</td></tr>
<tr><td>1399</td><td>32680</td><td>Drosophila melanogaster</td><td>CG4880</td><td>CG4880 gene product from transcript CG4880-RA</td><td>DmelCG4880</td><td>0</td><td>0</td></tr>
<tr><td>1400</td><td>32681</td><td>Drosophila melanogaster</td><td>CG13002</td><td>CG13002 gene product from transcript CG13002-RA</td><td>DmelCG13002</td><td>0</td><td>0</td></tr>
<tr><td>1401</td><td>32682</td><td>Drosophila melanogaster</td><td>CG4928</td><td>CG4928 gene product from transcript CG4928-RA</td><td>BcDNA:GH10120|DmelCG4928</td><td>0</td><td>0</td></tr>
<tr><td>1402</td><td>32683</td><td>Drosophila melanogaster</td><td>wus</td><td>wurst</td><td>CG9089|DmelCG9089|l(1)G0162</td><td>0</td><td>0</td></tr>
<tr><td>1403</td><td>32684</td><td>Drosophila melanogaster</td><td>CG13001</td><td>CG13001 gene product from transcript CG13001-RA</td><td>DmelCG13001</td><td>0</td><td>0</td></tr>
<tr><td>1404</td><td>32685</td><td>Drosophila melanogaster</td><td>CG13000</td><td>CG13000 gene product from transcript CG13000-RA</td><td>DmelCG13000</td><td>0</td><td>0</td></tr>
<tr><td>1405</td><td>32686</td><td>Drosophila melanogaster</td><td>RhoGAP15B</td><td>CG4937 gene product from transcript CG4937-RC</td><td>CG4937|DmelCG4937|gap20</td><td>0</td><td>0</td></tr>
<tr><td>1406</td><td>32687</td><td>Drosophila melanogaster</td><td>CG9086</td><td>CG9086 gene product from transcript CG9086-RB</td><td>DmelCG9086|Ubr1</td><td>0</td><td>0</td></tr>
<tr><td>1407</td><td>32688</td><td>Drosophila melanogaster</td><td>CG9059</td><td>CG9059 gene product from transcript CG9059-RD</td><td>BcDNA:GH06235|DmelCG9059</td><td>0</td><td>0</td></tr>
<tr><td>1408</td><td>32689</td><td>Drosophila melanogaster</td><td>CG12433</td><td>CG12433 gene product from transcript CG12433-RC</td><td>DmelCG12433</td><td>0</td><td>0</td></tr>
<tr><td>1409</td><td>32690</td><td>Drosophila melanogaster</td><td>CG8949</td><td>CG8949 gene product from transcript CG8949-RD</td><td>BP1032|CG34014|DmelCG8949</td><td>0</td><td>0</td></tr>
<tr><td>1410</td><td>32691</td><td>Drosophila melanogaster</td><td>CG4949</td><td>CG4949 gene product from transcript CG4949-RB</td><td>DmelCG4949</td><td>0</td><td>0</td></tr>
<tr><td>1411</td><td>32692</td><td>Drosophila melanogaster</td><td>CG4955</td><td>CG4955 gene product from transcript CG4955-RA</td><td>DmelCG4955|anon-WO0140519.97</td><td>0</td><td>0</td></tr>
<tr><td>1412</td><td>32693</td><td>Drosophila melanogaster</td><td>CG8945</td><td>CG8945 gene product from transcript CG8945-RC</td><td>Cg8945|DmelCG8945</td><td>0</td><td>0</td></tr>
<tr><td>1413</td><td>32694</td><td>Drosophila melanogaster</td><td>CG16700</td><td>CG16700 gene product from transcript CG16700-RA</td><td>DmelCG16700</td><td>0</td><td>0</td></tr>
<tr><td>1414</td><td>32695</td><td>Drosophila melanogaster</td><td>CG4991</td><td>CG4991 gene product from transcript CG4991-RB</td><td>CT15971|DmelCG4991</td><td>0</td><td>0</td></tr>
<tr><td>1415</td><td>32696</td><td>Drosophila melanogaster</td><td>Arpc3B</td><td>CG8936 gene product from transcript CG8936-RA</td><td>Arpc3|BcDNA:SD24339|CG8936|DmelCG8936|P21-ARC</td><td>0</td><td>0</td></tr>
<tr><td>1416</td><td>32698</td><td>Drosophila melanogaster</td><td>CG5004</td><td>CG5004 gene product from transcript CG5004-RA</td><td>DmelCG5004|anon-15Db</td><td>0</td><td>0</td></tr>
<tr><td>1417</td><td>32700</td><td>Drosophila melanogaster</td><td>RpS5a</td><td>Ribosomal protein S5a</td><td>CG8922|DmelCG8922|M(1)15D|M(1)34i28|M(1)o|M[[o]]|RS5|RpS5|S5a|rpS5|rps5</td><td>0</td><td>0</td></tr>
<tr><td>1418</td><td>32701</td><td>Drosophila melanogaster</td><td>CG5010</td><td>CG5010 gene product from transcript CG5010-RA</td><td>BcDNA:RE73921|DmelCG5010|anon-15Dd</td><td>0</td><td>0</td></tr>
<tr><td>1419</td><td>32703</td><td>Drosophila melanogaster</td><td>baz</td><td>bazooka</td><td>Baz|Baz/Par-3|Bazooka|CG5055|CG5505|D-Par3|D-par3|DmelCG5055|PAR-3|PAR3|Par-3|Par3|dPar-3|l(1)G0484|par-3</td><td>0</td><td>0</td></tr>
<tr><td>1420</td><td>32704</td><td>Drosophila melanogaster</td><td>CG8918</td><td>CG8918 gene product from transcript CG8918-RA</td><td>DmelCG8918</td><td>0</td><td>0</td></tr>
<tr><td>1421</td><td>32705</td><td>Drosophila melanogaster</td><td>CG5070</td><td>CG5070 gene product from transcript CG5070-RA</td><td>DmelCG5070</td><td>0</td><td>0</td></tr>
<tr><td>1422</td><td>32706</td><td>Drosophila melanogaster</td><td>CG32563</td><td>CG32563 gene product from transcript CG32563-RA</td><td>CG5095|DmelCG32563</td><td>0</td><td>0</td></tr>
<tr><td>1423</td><td>32707</td><td>Drosophila melanogaster</td><td>CG12995</td><td>CG12995 gene product from transcript CG12995-RA</td><td>DmelCG12995</td><td>0</td><td>0</td></tr>
<tr><td>1424</td><td>32708</td><td>Drosophila melanogaster</td><td>CG18258</td><td>CG18258 gene product from transcript CG18258-RA</td><td>CT41369|DmelCG18258</td><td>0</td><td>0</td></tr>
<tr><td>1425</td><td>32709</td><td>Drosophila melanogaster</td><td>CG5162</td><td>CG5162 gene product from transcript CG5162-RA</td><td>DmelCG5162</td><td>0</td><td>0</td></tr>
<tr><td>1426</td><td>32710</td><td>Drosophila melanogaster</td><td>CG12998</td><td>CG12998 gene product from transcript CG12998-RA</td><td>BcDNA:RH61745|CT32204|DmelCG12998</td><td>0</td><td>0</td></tr>
<tr><td>1427</td><td>32711</td><td>Drosophila melanogaster</td><td>CG5172</td><td>CG5172 gene product from transcript CG5172-RC</td><td>BcDNA:RH06401|CT16557|DmelCG5172</td><td>0</td><td>0</td></tr>
<tr><td>1428</td><td>32713</td><td>Drosophila melanogaster</td><td>CG10598</td><td>CG10598 gene product from transcript CG10598-RA</td><td>DmelCG10598|GCR 17|GCR-11|GCR11|GCR17|Pen15EF|anon-Pen15EF|pen15EF</td><td>0</td><td>0</td></tr>
<tr><td>1429</td><td>32714</td><td>Drosophila melanogaster</td><td>CG10597</td><td>CG10597 gene product from transcript CG10597-RA</td><td>DmelCG10597</td><td>0</td><td>0</td></tr>
<tr><td>1430</td><td>32715</td><td>Drosophila melanogaster</td><td>CG8915</td><td>CG8915 gene product from transcript CG8915-RA</td><td>DmelCG8915|cg8915</td><td>0</td><td>0</td></tr>
<tr><td>1431</td><td>32716</td><td>Drosophila melanogaster</td><td>CG8675</td><td>CG8675 gene product from transcript CG8675-RA</td><td>DmelCG8675</td><td>0</td><td>0</td></tr>
<tr><td>1432</td><td>32718</td><td>Drosophila melanogaster</td><td>f</td><td>forked</td><td>CG12996|CG42864|CG5424|DmelCG42864|Dmel_CG12996|Dmel_CG5424|X1</td><td>0</td><td>0</td></tr>
<tr><td>1433</td><td>32719</td><td>Drosophila melanogaster</td><td>CG8664</td><td>CG8664 gene product from transcript CG8664-RA</td><td>DmelCG8664</td><td>0</td><td>0</td></tr>
<tr><td>1434</td><td>32720</td><td>Drosophila melanogaster</td><td>CG8661</td><td>CG8661 gene product from transcript CG8661-RA</td><td>BcDNA:LP01183|DmelCG8661</td><td>0</td><td>0</td></tr>
<tr><td>1435</td><td>32721</td><td>Drosophila melanogaster</td><td>Fim</td><td>Fimbrin</td><td>CG8649|DmelCG8649|FIM</td><td>0</td><td>0</td></tr>
<tr><td>1436</td><td>32722</td><td>Drosophila melanogaster</td><td>CG5445</td><td>CG5445 gene product from transcript CG5445-RD</td><td>DmelCG5445</td><td>0</td><td>0</td></tr>
<tr><td>1437</td><td>32723</td><td>Drosophila melanogaster</td><td>B-H2</td><td>BarH2</td><td>B|BH2|Bar|Bar-H2|Barh2|CG5488|DmelCG5488|bar-h2</td><td>0</td><td>0</td></tr>
<tr><td>1438</td><td>32724</td><td>Drosophila melanogaster</td><td>B-H1</td><td>BarH1</td><td>BH1|Bar|Bar H1|Bar-H1|BarHI|Barh1|CG5529|DmelCG5529|bar-h1|barH1|barh1</td><td>0</td><td>0</td></tr>
<tr><td>1439</td><td>32725</td><td>Drosophila melanogaster</td><td>CG8611</td><td>CG8611 gene product from transcript CG8611-RA</td><td>BcDNA:GH02833|DmRH11|DmelCG8611|cg8611</td><td>0</td><td>0</td></tr>
<tr><td>1440</td><td>32726</td><td>Drosophila melanogaster</td><td>CG5613</td><td>CG5613 gene product from transcript CG5613-RA</td><td>DmelCG5613</td><td>0</td><td>0</td></tr>
<tr><td>1441</td><td>32727</td><td>Drosophila melanogaster</td><td>p-cup</td><td>presidents-cup</td><td>CG12993|DmelCG12993</td><td>0</td><td>0</td></tr>
</table>

<table>
<tr><td>1442</td><td>32728</td><td>Drosophila melanogaster</td><td>CG8568</td><td>CG8568 gene product from transcript CG8568-RA</td><td>DmelCG8568</td><td>0</td><td>0</td></tr>
<tr><td>1443</td><td>32729</td><td>Drosophila melanogaster</td><td>CG8557</td><td>CG8557 gene product from transcript CG8557-RB</td><td>DmelCG8557</td><td>0</td><td>0</td></tr>
<tr><td>1444</td><td>32730</td><td>Drosophila melanogaster</td><td>CG12432</td><td>CG12432 gene product from transcript CG12432-RA</td><td>DmelCG12432</td><td>0</td><td>0</td></tr>
<tr><td>1445</td><td>32731</td><td>Drosophila melanogaster</td><td>ppk23</td><td>pickpocket 23</td><td>CG8527|DmelCG8527|PPK23</td><td>0</td><td>0</td></tr>
<tr><td>1446</td><td>32732</td><td>Drosophila melanogaster</td><td>l(1)G0222</td><td>lethal (1) G0222</td><td>CG8465|DmelCG8465|anon-WO03040301.175|anon-WO03040301.177|anon-WO03040301.179|l(1)G0316</td><td>0</td><td>0</td></tr>
<tr><td>1447</td><td>32733</td><td>Drosophila melanogaster</td><td>CG12992</td><td>CG12992 gene product from transcript CG12992-RB</td><td>DmelCG12992</td><td>0</td><td>0</td></tr>
<tr><td>1448</td><td>32734</td><td>Drosophila melanogaster</td><td>CG12991</td><td>CG12991 gene product from transcript CG12991-RA</td><td>DmelCG12991</td><td>0</td><td>0</td></tr>
<tr><td>1449</td><td>32737</td><td>Drosophila melanogaster</td><td>CG8408</td><td>CG8408 gene product from transcript CG8408-RA</td><td>DmelCG8408</td><td>0</td><td>0</td></tr>
<tr><td>1450</td><td>32738</td><td>Drosophila melanogaster</td><td>CG8326</td><td>CG8326 gene product from transcript CG8326-RA</td><td>BEST:LD29336|DmelCG8326</td><td>0</td><td>0</td></tr>
<tr><td>1451</td><td>32739</td><td>Drosophila melanogaster</td><td>CG8316</td><td>CG8316 gene product from transcript CG8316-RA</td><td>DmelCG8316</td><td>0</td><td>0</td></tr>
<tr><td>1452</td><td>32740</td><td>Drosophila melanogaster</td><td>CG5703</td><td>CG5703 gene product from transcript CG5703-RA</td><td>BEST:LP01573|DmelCG5703</td><td>0</td><td>0</td></tr>
<tr><td>1453</td><td>32741</td><td>Drosophila melanogaster</td><td>CG8289</td><td>CG8289 gene product from transcript CG8289-RA</td><td>DmelCG8289</td><td>0</td><td>0</td></tr>
<tr><td>1454</td><td>32742</td><td>Drosophila melanogaster</td><td>CG5800</td><td>CG5800 gene product from transcript CG5800-RA</td><td>DmRH14|DmelCG5800|cg5800</td><td>0</td><td>0</td></tr>
<tr><td>1455</td><td>32743</td><td>Drosophila melanogaster</td><td>RhoGAPp190</td><td>CG32555 gene product from transcript CG32555-RB</td><td>8267|BcDNA:GH17919|BcDNA:GM05133|CG32555|CG8240|CG8267|D-p190|DRhoGAP|DmelCG32555|RhoGAP|RhoGAP P190|RhoGAP-16B12|p190 RhoGAP|p190-RhoGAP|p190RhoGAP</td><td>0</td><td>0</td></tr>
<tr><td>1456</td><td>32745</td><td>Drosophila melanogaster</td><td>IntS2</td><td>Integrator 2</td><td>CG8211|DmelCG8211|Int2</td><td>0</td><td>0</td></tr>
<tr><td>1457</td><td>32746</td><td>Drosophila melanogaster</td><td>beta-Spec</td><td>beta Spectrin</td><td>CG5870|DmelCG5870|SPEC8|Spec|Spec-beta|Specbeta|anon-EST:fe1B3|b spectrin|b-Spec|beta|beta-spec|betaSpec|betaspec|i168|i6|kus|l(1)G0074|l(1)G0108|l(1)G0198</td><td>0</td><td>0</td></tr>
<tr><td>1458</td><td>32747</td><td>Drosophila melanogaster</td><td>CG12990</td><td>CG12990 gene product from transcript CG12990-RA</td><td>DmelCG12990</td><td>0</td><td>0</td></tr>
<tr><td>1459</td><td>32748</td><td>Drosophila melanogaster</td><td>chas</td><td>chascon</td><td>CG12987|CG12988|CG12989|CG32556|DmelCG32556|EP-631</td><td>0</td><td>0</td></tr>
<tr><td>1460</td><td>32751</td><td>Drosophila melanogaster</td><td>CG8188</td><td>CG8188 gene product from transcript CG8188-RB</td><td>DmelCG8188|Ubc</td><td>0</td><td>0</td></tr>
<tr><td>1461</td><td>32752</td><td>Drosophila melanogaster</td><td>par-6</td><td>CG5884 gene product from transcript CG5884-RA</td><td>CG5884|DPar-6|DPar6|Dm-Par-6|DmPAR-6|DmPar-6|DmPar6|DmelCG5884|Dmpar6|PAR-6|PAR6|Par|Par-6|Par6|dPAR-6|dPar-6|dmPar-6|par|par6</td><td>0</td><td>0</td></tr>
<tr><td>1462</td><td>32753</td><td>Drosophila melanogaster</td><td>CG8173</td><td>CG8173 gene product from transcript CG8173-RA</td><td>DmelCG8173</td><td>0</td><td>0</td></tr>
<tr><td>1463</td><td>32754</td><td>Drosophila melanogaster</td><td>CG42684</td><td>CG42684 gene product from transcript CG42684-RE</td><td>CG15816|CG32559|CG32560|CG42270|CG5898|CG5960|DmelCG42684|Dmel_CG15816|Dmel_CG32559|Dmel_CG32560|Dmel_CG42270</td><td>0</td><td>0</td></tr>
<tr><td>1464</td><td>32757</td><td>Drosophila melanogaster</td><td>unc-4</td><td>CG6269 gene product from transcript CG6269-RA</td><td>CG6269|DPHD-1|DmelCG6269|Dunc-4</td><td>0</td><td>0</td></tr>
<tr><td>1465</td><td>32758</td><td>Drosophila melanogaster</td><td>OdsH</td><td>Ods-site homeobox</td><td>CG6352|DmelCG6352|OdsH[mel]|odsH</td><td>0</td><td>0</td></tr>
<tr><td>1466</td><td>32759</td><td>Drosophila melanogaster</td><td>CG12986</td><td>CG12986 gene product from transcript CG12986-RA</td><td>DmelCG12986</td><td>0</td><td>0</td></tr>
<tr><td>1467</td><td>32760</td><td>Drosophila melanogaster</td><td>Socs16D</td><td>Suppressor of Cytokine Signaling at 16D</td><td>CG8146|DmelCG8146|SOCS|SOCS16D|Socs 16D|Socs16E|dmSocs16D|socs16D</td><td>0</td><td>0</td></tr>
<tr><td>1468</td><td>32761</td><td>Drosophila melanogaster</td><td>CG6398</td><td>CG6398 gene product from transcript CG6398-RA</td><td>DmelCG6398</td><td>0</td><td>0</td></tr>
<tr><td>1469</td><td>32762</td><td>Drosophila melanogaster</td><td>e(y)1</td><td>enhancer of yellow 1</td><td>CG6474|DmelCG6474|E(y)1|E(y)1/dTAF9|TAF40|TAF40/42|TAF40/42/e(y)1|TAF42|TAF9|TAFII40|TAFII40(42)|TAF[II]40|TAF[[II]]|TAF[[II]]40|TAF[[II]]40/42|TAF[[II]]42|TFIID|TFIID 42|TFIID-p42|Taf40|Taf42|Taf9|Ta</td><td>0</td><td>0</td></tr>
<tr><td>1470</td><td>32763</td><td>Drosophila melanogaster</td><td>CG8142</td><td>CG8142 gene product from transcript CG8142-RA</td><td>DmRFC2|DmelCG8142|RFC2|RfC</td><td>0</td><td>0</td></tr>
<tr><td>1471</td><td>32764</td><td>Drosophila melanogaster</td><td>Ucp4A</td><td>CG6492 gene product from transcript CG6492-RA</td><td>CG6492|DmUCP4A|DmelCG6492|UCP4A|Ucp4a|dmUCP4a</td><td>0</td><td>0</td></tr>
<tr><td>1472</td><td>32765</td><td>Drosophila melanogaster</td><td>CG15814</td><td>CG15814 gene product from transcript CG15814-RB</td><td>DmelCG15814</td><td>0</td><td>0</td></tr>
<tr><td>1473</td><td>32766</td><td>Drosophila melanogaster</td><td>CG6506</td><td>CG6506 gene product from transcript CG6506-RA</td><td>DmelCG6506|Spt7|dSpt7</td><td>0</td><td>0</td></tr>
<tr><td>1474</td><td>32767</td><td>Drosophila melanogaster</td><td>CG32554</td><td>CG32554 gene product from transcript CG32554-RA</td><td>CG7871|DmelCG32554</td><td>0</td><td>0</td></tr>
<tr><td>1475</td><td>32768</td><td>Drosophila melanogaster</td><td>CG6762</td><td>CG6762 gene product from transcript CG6762-RD</td><td>DmelCG6762</td><td>0</td><td>0</td></tr>
<tr><td>1476</td><td>32769</td><td>Drosophila melanogaster</td><td>Arp8</td><td>Actin-related protein 8</td><td>CG7846|DmelCG7846|dArp8</td><td>0</td><td>0</td></tr>
<tr><td>1477</td><td>32770</td><td>Drosophila melanogaster</td><td>CG6769</td><td>CG6769 gene product from transcript CG6769-RA</td><td>DmelCG6769</td><td>0</td><td>0</td></tr>
<tr><td>1478</td><td>32771</td><td>Drosophila melanogaster</td><td>mnb</td><td>minibrain</td><td>CG42273|CG7826|CG7835|DYRK1|DmelCG42273|Dmel_CG7826|Dmel_CG7835|Dyrk1|ME-IV|MNB|Mnb|min</td><td>0</td><td>0</td></tr>
<tr><td>1479</td><td>32773</td><td>Drosophila melanogaster</td><td>CG6788</td><td>CG6788 gene product from transcript CG6788-RA</td><td>DmelCG6788|FBpp0074269</td><td>0</td><td>0</td></tr>
<tr><td>1480</td><td>32774</td><td>Drosophila melanogaster</td><td>CG12985</td><td>CG12985 gene product from transcript CG12985-RA</td><td>DmelCG12985</td><td>0</td><td>0</td></tr>
<tr><td>1481</td><td>32775</td><td>Drosophila melanogaster</td><td>GS</td><td>Glutathione Synthetase</td><td>6835|CG32497|CG33065|CG6835|DmelCG6835</td><td>0</td><td>0</td></tr>
<tr><td>1482</td><td>32776</td><td>Drosophila melanogaster</td><td>CG7772</td><td>CG7772 gene product from transcript CG7772-RA</td><td>DmelCG7772|bs13c01.y1</td><td>0</td><td>0</td></tr>
<tr><td>1483</td><td>32777</td><td>Drosophila melanogaster</td><td>Vps4</td><td>Vacuolar protein sorting 4</td><td>BcDNA:GH02678|CG6842|DmBcDNA.GH02678|DmelCG6842|bcDNA:GH02678|dvps4|vps4</td><td>0</td><td>0</td></tr>
<tr><td>1484</td><td>32778</td><td>Drosophila melanogaster</td><td>CG6847</td><td>CG6847 gene product from transcript CG6847-RA</td><td>DmelCG6847</td><td>0</td><td>0</td></tr>
<tr><td>1485</td><td>32780</td><td>Drosophila melanogaster</td><td>Sh</td><td>Shaker</td><td>BcDNA:GH03046|CG12348|CG17860|CG7640|DmelCG12348|EKO|Eko|F-c|SH|Sha|Shw|V-I|minisleep|sh</td><td>0</td><td>0</td></tr>
<tr><td>1486</td><td>32781</td><td>Drosophila melanogaster</td><td>CG12672</td><td>CG12672 gene product from transcript CG12672-RA</td><td>DmelCG12672</td><td>0</td><td>0</td></tr>
<tr><td>1487</td><td>32782</td><td>Drosophila melanogaster</td><td>CG6867</td><td>CG6867 gene product from transcript CG6867-RA</td><td>CT21241|DmelCG6867</td><td>0</td><td>0</td></tr>
<tr><td>1488</td><td>32784</td><td>Drosophila melanogaster</td><td>CG15373</td><td>CG15373 gene product from transcript CG15373-RB</td><td>DmelCG15373</td><td>0</td><td>0</td></tr>
<tr><td>1489</td><td>32785</td><td>Drosophila melanogaster</td><td>Dhc16F</td><td>Dynein heavy chain at 16F</td><td>CG7092|DHC|Dhc|DmelCG7092|dhc</td><td>0</td><td>0</td></tr>
<tr><td>1490</td><td>32786</td><td>Drosophila melanogaster</td><td>CG7536</td><td>CG7536 gene product from transcript CG7536-RB</td><td>DmelCG7536</td><td>0</td><td>0</td></tr>
<tr><td>1491</td><td>32787</td><td>Drosophila melanogaster</td><td>dik</td><td>diskette</td><td>Ada3|CG7098|DADA3|DmelCG7098|HL-XIV|dAda3</td><td>0</td><td>0</td></tr>
<tr><td>1492</td><td>32788</td><td>Drosophila melanogaster</td><td>CG7206</td><td>CG7206 gene product from transcript CG7206-RB</td><td>DmelCG7206</td><td>0</td><td>0</td></tr>
<tr><td>1493</td><td>32789</td><td>Drosophila melanogaster</td><td>scu</td><td>scully</td><td>17beta-HSD10|CG7113|DmelCG7113|HL-X|HLII|Scu|l(1)16Fd|l(1)17Ad</td><td>0</td><td>0</td></tr>
<tr><td>1494</td><td>32790</td><td>Drosophila melanogaster</td><td>RhoGAP16F</td><td>CG7122 gene product from transcript CG7122-RB</td><td>CG7122|DmelCG7122</td><td>0</td><td>0</td></tr>
<tr><td>1495</td><td>32791</td><td>Drosophila melanogaster</td><td>CG7192</td><td>CG7192 gene product from transcript CG7192-RA</td><td>DmelCG7192</td><td>0</td><td>0</td></tr>
<tr><td>1496</td><td>32792</td><td>Drosophila melanogaster</td><td>Taf8</td><td>TBP-associated factor 8</td><td>CG7128|DmelCG7128|HL-VIII|Prodos|TAF|TAF8|TAF[[II]]|TFIID|dmTAF8|l(1)16Fg|pds|pro|prod</td><td>0</td><td>0</td></tr>
<tr><td>1497</td><td>32793</td><td>Drosophila melanogaster</td><td>CG7135</td><td>CG7135 gene product from transcript CG7135-RA</td><td>DmelCG7135</td><td>0</td><td>0</td></tr>
<tr><td>1498</td><td>32794</td><td>Drosophila melanogaster</td><td>wupA</td><td>wings up A</td><td>CG7178|DROTROPONI|DmTpnI|DmelCG7178|HDP|HL-I|HL08042|HLI|Tn I|TnI|TpnI|WupA|hdp|wup-A</td><td>0</td><td>0</td></tr>
<tr><td>1499</td><td>32796</td><td>Drosophila melanogaster</td><td>ari-1</td><td>ariadne</td><td>ARI1_DROME|Ari-1|CG5659|DmelCG5659|HL-V|ari|l(1)16Fj</td><td>0</td><td>0</td></tr>
<tr><td>1500</td><td>32797</td><td>Drosophila melanogaster</td><td>Frq1</td><td>Frequenin 1</td><td>CG5744|DmelCG5744|Frq|HL-II|frq|frq1</td><td>0</td><td>0</td></tr>
<tr><td>1501</td><td>32798</td><td>Drosophila melanogaster</td><td>Andorra</td><td>CG12611 gene product from transcript CG12611-RA</td><td>CG12611|DmelCG12611|and|andorra</td><td>0</td><td>0</td></tr>
<tr><td>1502</td><td>32799</td><td>Drosophila melanogaster</td><td>Frq2</td><td>Frequenin 2</td><td>CG5907|DmelCG5907|Frq|frq|frq2</td><td>0</td><td>0</td></tr>
<tr><td>1503</td><td>32800</td><td>Drosophila melanogaster</td><td>Her</td><td>HES-related</td><td>CG5927|DmelCG5927|bHLHc21</td><td>0</td><td>0</td></tr>
<tr><td>1504</td><td>32801</td><td>Drosophila melanogaster</td><td>Him</td><td>Holes in muscle</td><td>CG15064|DmelCG15064|him</td><td>0</td><td>0</td></tr>
<tr><td>1505</td><td>32803</td><td>Drosophila melanogaster</td><td>CG33639</td><td>CG33639 gene product from transcript CG33639-RC</td><td>CG16958|CG5936|DmelCG33639|anon-WO0170980.40|anon-WO0170980.41</td><td>0</td><td>0</td></tr>
<tr><td>1506</td><td>32805</td><td>Drosophila melanogaster</td><td>upd2</td><td>unpaired 2</td><td>CG5988|DmelCG5988|UPD2|Upd2|cg5988</td><td>0</td><td>0</td></tr>
<tr><td>1507</td><td>32806</td><td>Drosophila melanogaster</td><td>CG15059</td><td>CG15059 gene product from transcript CG15059-RB</td><td>DmelCG15059</td><td>0</td><td>0</td></tr>
<tr><td>1508</td><td>32813</td><td>Drosophila melanogaster</td><td>os</td><td>outstretched</td><td>CG5993|DmelCG5993|OS|Os|UPD|Upd|Upd1|bdw|l(1)YC43|l(1)YM55|od|odsy|sis-C|sis-c|sisC|sy|unp|upa|upd|upd1</td><td>0</td><td>0</td></tr>
<tr><td>1509</td><td>32814</td><td>Drosophila melanogaster</td><td>CG6023</td><td>CG6023 gene product from transcript CG6023-RA</td><td>DmelCG6023</td><td>0</td><td>0</td></tr>
<tr><td>1510</td><td>32815</td><td>Drosophila melanogaster</td><td>CG6123</td><td>CG6123 gene product from transcript CG6123-RB</td><td>DmelCG6123</td><td>0</td><td>0</td></tr>
<tr><td>1511</td><td>32816</td><td>Drosophila melanogaster</td><td>CG6106</td><td>CG6106 gene product from transcript CG6106-RA</td><td>DmelCG6106</td><td>0</td><td>0</td></tr>
<tr><td>1512</td><td>32817</td><td>Drosophila melanogaster</td><td>CrebB-17A</td><td>Cyclic-AMP response element binding protein B at 17</td><td>CG6103|CRE-BP|CREB|CREB-B|CREB-b|CREB2|CREB2a|CREB2b|CREBB-17A|Creb|Creb17A|Creb2|Creb2-b|Creb2b|CrebB|CrebB17-A|DmelCG6103|S162|dCREB|dCREB-2|dCREB-2a|dCREB-B|dCRE</td><td>0</td><td>0</td></tr>
<tr><td>1513</td><td>32818</td><td>Drosophila melanogaster</td><td>por</td><td>porcupine</td><td>15175|CG6205|DmelCG6205|Porc|dmPorc|l(1)17Ac|poc|porc</td><td>0</td><td>0</td></tr>
<tr><td>1514</td><td>32819</td><td>Drosophila melanogaster</td><td>CG6179</td><td>CG6179 gene product from transcript CG6179-RA</td><td>DmelCG6179|NOSIPL</td><td>0</td><td>0</td></tr>
<tr><td>1515</td><td>32820</td><td>Drosophila melanogaster</td><td>betaCop</td><td>beta-coatomer protein</td><td>BcDNA.GH09317|BcDNA:GH09317|Beta Cop|BetaCop|CG6223|DmelCG6223|bcop|beta-COP|beta-Cop|beta-CopII|betaCOP</td><td>0</td><td>0</td></tr>
<tr><td>1516</td><td>32821</td><td>Drosophila melanogaster</td><td>Tsf1</td><td>Transferrin 1</td><td>143958_at|CG6186|DmelCG6186|TSF1|Tf|Trf|anon-EST:Posey265</td><td>0</td><td>0</td></tr>
<tr><td>1517</td><td>32822</td><td>Drosophila melanogaster</td><td>CG32549</td><td>CG32549 gene product from transcript CG32549-RD</td><td>CG6247|CG6267|CG6278|DmelCG32549</td><td>0</td><td>0</td></tr>
<tr><td>1518</td><td>32824</td><td>Drosophila melanogaster</td><td>CG15056</td><td>CG15056 gene product from transcript CG15056-RA</td><td>DmelCG15056</td><td>0</td><td>0</td></tr>
<tr><td>1519</td><td>32825</td><td>Drosophila melanogaster</td><td>CG42323</td><td>CG42323 gene product from transcript CG42323-RE</td><td>CG15055|CG32550|DmelCG42323|Dmel_CG15055|Dmel_CG32550</td><td>0</td><td>0</td></tr>
<tr><td>1520</td><td>32826</td><td>Drosophila melanogaster</td><td>CG32548</td><td>CG32548 gene product from transcript CG32548-RB</td><td>CG15051|CG6306|DmelCG32548|anon-WO0140519.88</td><td>0</td><td>0</td></tr>
<tr><td>1521</td><td>32827</td><td>Drosophila melanogaster</td><td>CG6290</td><td>CG6290 gene product from transcript CG6290-RA</td><td>BcDNA:GH02837|DmelCG6290</td><td>0</td><td>0</td></tr>
<tr><td>1522</td><td>32831</td><td>Drosophila melanogaster</td><td>CG6361</td><td>CG6361 gene product from transcript CG6361-RA</td><td>DmelCG6361|SP31|c-SP31</td><td>0</td><td>0</td></tr>
<tr><td>1523</td><td>32832</td><td>Drosophila melanogaster</td><td>psh</td><td>persephone</td><td>CG6367|DmelCG6367|Psh|SP28|c-SP28</td><td>0</td><td>0</td></tr>
<tr><td>1524</td><td>32833</td><td>Drosophila melanogaster</td><td>CG15046</td><td>CG15046 gene product from transcript CG15046-RA</td><td>DmelCG15046</td><td>0</td><td>0</td></tr>
<tr><td>1525</td><td>32834</td><td>Drosophila melanogaster</td><td>CG15044</td><td>CG15044 gene product from transcript CG15044-RA</td><td>BcDNA:RE30049|DmelCG15044</td><td>0</td><td>0</td></tr>
<tr><td>1526</td><td>32835</td><td>Drosophila melanogaster</td><td>CG15043</td><td>CG15043 gene product from transcript CG15043-RA</td><td>BcDNA:RE41194|DmelCG15043</td><td>0</td><td>0</td></tr>
<tr><td>1527</td><td>32836</td><td>Drosophila melanogaster</td><td>GalNAc-T2</td><td>UDP-N-acetyl-alpha-D-galactosamine:polypeptide N-</td><td>CG6394|DmelCG6394|PGANT7|anon-WO02059370.15|dGalNAc-T2|pgant7</td><td>0</td><td>0</td></tr>
<tr><td>1528</td><td>32837</td><td>Drosophila melanogaster</td><td>Rad51D</td><td>CG6318 gene product from transcript CG6318-RA</td><td>CG6318|Dm CG6318|DmelCG6318</td><td>0</td><td>0</td></tr>
<tr><td>1529</td><td>32838</td><td>Drosophila melanogaster</td><td>Wnt5</td><td>Wnt oncogene analog 5</td><td>CG6407|DWnt-3|DWnt-3/5|DWnt-5|DWnt3|DWnt3/5|DWnt5|Dm DWnt3/5|Dm-3|DmelCG6407|Dwnt-3|Dwnt-5|Dwnt5|Wnt|Wnt-3|Wnt3|Wnt3/5|anon-WO03040301.228|wnt3|wnt5</td><td>0</td><td>0</td></tr>
<tr><td>1530</td><td>32839</td><td>Drosophila melanogaster</td><td>Ggt-1</td><td>gamma-glutamyl transpeptidase</td><td>CG6461|DmelCG6461|GGT-1</td><td>0</td><td>0</td></tr>
<tr><td>1531</td><td>32840</td><td>Drosophila melanogaster</td><td>CG6470</td><td>CG6470 gene product from transcript CG6470-RA</td><td>DmelCG6470|anon-WO0140519.137</td><td>0</td><td>0</td></tr>
<tr><td>1532</td><td>32841</td><td>Drosophila melanogaster</td><td>Aats-his</td><td>Histidyl-tRNA synthetase</td><td>CG6335|DmelCG6335|HRS|HisRS|l(1)G0165|l(1)G0358</td><td>0</td><td>0</td></tr>
<tr><td>1533</td><td>32842</td><td>Drosophila melanogaster</td><td>CG15048</td><td>CG15048 gene product from transcript CG15048-RB</td><td>DmelCG15048</td><td>0</td><td>0</td></tr>
<tr><td>1534</td><td>32843</td><td>Drosophila melanogaster</td><td>CG6481</td><td>CG6481 gene product from transcript CG6481-RA</td><td>DmelCG6481</td><td>0</td><td>0</td></tr>
<tr><td>1535</td><td>32844</td><td>Drosophila melanogaster</td><td>CG15042</td><td>CG15042 gene product from transcript CG15042-RA</td><td>DmelCG15042</td><td>0</td><td>0</td></tr>
<tr><td>1536</td><td>32845</td><td>Drosophila melanogaster</td><td>CG15047</td><td>CG15047 gene product from transcript CG15047-RA</td><td>DmelCG15047</td><td>0</td><td>0</td></tr>
<tr><td>1537</td><td>32846</td><td>Drosophila melanogaster</td><td>Bx</td><td>Beadex</td><td>Bd|CG6500|DLMO|Dlmo|DmelCG6500|LMO|Lmo|Ptd|Rho|beadex/dLMO|dLMO|dLmo|dlmo|dttg|fliH|hdp|hdp-a|hld</td><td>0</td><td>0</td></tr>
<tr><td>1538</td><td>32848</td><td>Drosophila melanogaster</td><td>CG15040</td><td>CG15040 gene product from transcript CG15040-RA</td><td>DmelCG15040</td><td>0</td><td>0</td></tr>
<tr><td>1539</td><td>32849</td><td>Drosophila melanogaster</td><td>wgn</td><td>wengen</td><td>CG6531|DmelCG6531|Wgn|vader</td><td>0</td><td>0</td></tr>
<tr><td>1540</td><td>32850</td><td>Drosophila melanogaster</td><td>Rip11</td><td>Rab11 interacting protein</td><td>CG6606|DmelCG6606|dRip11|l(1)G0003|l(1)G0070|l(1)G0092|l(1)G0205|l(1)G0297|l(1)G0434</td><td>0</td><td>0</td></tr>
<tr><td>1541</td><td>32851</td><td>Drosophila melanogaster</td><td>CG6540</td><td>CG6540 gene product from transcript CG6540-RA</td><td>DmelCG6540</td><td>0</td><td>0</td></tr>
<tr><td>1542</td><td>32852</td><td>Drosophila melanogaster</td><td>CG6617</td><td>CG6617 gene product from transcript CG6617-RA</td><td>DmelCG6617|anon-EST:Posey180</td><td>0</td><td>0</td></tr>
<tr><td>1543</td><td>32853</td><td>Drosophila melanogaster</td><td>Ing3</td><td>CG6632 gene product from transcript CG6632-RA</td><td>CG6632|DmelCG6632|dIng3</td><td>0</td><td>0</td></tr>
<tr><td>1544</td><td>32854</td><td>Drosophila melanogaster</td><td>CG6659</td><td>CG6659 gene product from transcript CG6659-RA</td><td>DmelCG6659</td><td>0</td><td>0</td></tr>
<tr><td>1545</td><td>32855</td><td>Drosophila melanogaster</td><td>fu</td><td>fused</td><td>CG6551|Dm fu|DmelCG6551|FU|Fu|dFu|fu[mel]|l(1)fu</td><td>0</td><td>0</td></tr>
<tr><td>1546</td><td>32856</td><td>Drosophila melanogaster</td><td>CG6696</td><td>CG6696 gene product from transcript CG6696-RA</td><td>DmelCG6696</td><td>0</td><td>0</td></tr>
<tr><td>1547</td><td>32857</td><td>Drosophila melanogaster</td><td>phm</td><td>phantom</td><td>306a1|CG6578|CYP306A1|Cyp306a1|Cyp306a1-Dm|DmelCG6578|exo</td><td>0</td><td>0</td></tr>
<tr><td>1548</td><td>32858</td><td>Drosophila melanogaster</td><td>Cyp18a1</td><td>Cytochrome P450-18a1</td><td>181H6T|18a1|CG6816|CT20826|CYP18|CYP18A1-Dm|Cyp18|Cyt-P450-17C|CytP450|DmelCG6816|ESTS:181H6T|Eig17-1|P-450|P450|cyp18|cyp18a1</td><td>0</td><td>0</td></tr>
<tr><td>1549</td><td>32859</td><td>Drosophila melanogaster</td><td>Cyp308a1</td><td>CG6585 gene product from transcript CG6585-RA</td><td>308a1|CG6585|DmelCG6585</td><td>0</td><td>0</td></tr>
<tr><td>1550</td><td>32860</td><td>Drosophila melanogaster</td><td>CCKLR-17D1</td><td>CCK-like receptor at 17D1</td><td>CG42301|CG6857|Dm CG6857|DmelCG42301|Dmel_CG6857|anon-WO0170980.61|anon-WO0170980.62</td><td>0</td><td>0</td></tr>
<tr><td>1551</td><td>32861</td><td>Drosophila melanogaster</td><td>CG6873</td><td>CG6873 gene product from transcript CG6873-RA</td><td>DmelCG6873|adf</td><td>0</td><td>0</td></tr>
<tr><td>1552</td><td>32862</td><td>Drosophila melanogaster</td><td>CG12609</td><td>CG12609 gene product from transcript CG12609-RA</td><td>DmelCG12609</td><td>0</td><td>0</td></tr>
</table>

<table>
<tr><td>1553</td><td>32864</td><td>Drosophila melanogaster</td><td>CCKLR-17D3</td><td>CCK-like receptor at 17D3</td><td>CG32540|CG6861|CG6881|CG6894|DSK-R1|DmelCG32540|anon-WO0131005.21|anon-WO0170980.64|anon-WO0170980.65</td><td>0</td><td>0</td></tr>
<tr><td>1554</td><td>32865</td><td>Drosophila melanogaster</td><td>CG6891</td><td>CG6891 gene product from transcript CG6891-RA</td><td>DmelCG6891</td><td>0</td><td>0</td></tr>
<tr><td>1555</td><td>32866</td><td>Drosophila melanogaster</td><td>CG18259</td><td>CG18259 gene product from transcript CG18259-RA</td><td>DmelCG18259</td><td>0</td><td>0</td></tr>
<tr><td>1556</td><td>32868</td><td>Drosophila melanogaster</td><td>CG6900</td><td>CG6900 gene product from transcript CG6900-RB</td><td>DmelCG6900</td><td>0</td><td>0</td></tr>
<tr><td>1557</td><td>32869</td><td>Drosophila melanogaster</td><td>CG6961</td><td>CG6961 gene product from transcript CG6961-RA</td><td>DmelCG6961|cg6961</td><td>0</td><td>0</td></tr>
<tr><td>1558</td><td>32871</td><td>Drosophila melanogaster</td><td>CG7053</td><td>CG7053 gene product from transcript CG7053-RA</td><td>DmelCG7053</td><td>0</td><td>0</td></tr>
<tr><td>1559</td><td>32872</td><td>Drosophila melanogaster</td><td>bnb</td><td>bangles and beads</td><td>Bnb|CG7088|DmelCG7088|GAP43|Gap43</td><td>0</td><td>0</td></tr>
<tr><td>1560</td><td>32873</td><td>Drosophila melanogaster</td><td>CG7058</td><td>CG7058 gene product from transcript CG7058-RB</td><td>DmelCG7058</td><td>0</td><td>0</td></tr>
<tr><td>1561</td><td>32874</td><td>Drosophila melanogaster</td><td>CG42450</td><td>CG42450 gene product from transcript CG42450-RA</td><td>CG32543|CG7095|DmelCG42450|Dmel_CG32543|Dmel_CG7095</td><td>0</td><td>0</td></tr>
<tr><td>1562</td><td>32875</td><td>Drosophila melanogaster</td><td>CG7101</td><td>CG7101 gene product from transcript CG7101-RA</td><td>DmelCG7101</td><td>0</td><td>0</td></tr>
<tr><td>1563</td><td>32876</td><td>Drosophila melanogaster</td><td>Pvf1</td><td>PDGF- and VEGF-related factor 1</td><td>CG7103|DmVEGF-1|DmelCG7103|EP1624|PVF|PVF1|PVFs|Pvf|VEGF|VEGF2-B|Vegf17E|Vegf17e-a|Vegf17e-b|l(1)G0146|pvf1|vegf1</td><td>0</td><td>0</td></tr>
<tr><td>1564</td><td>32877</td><td>Drosophila melanogaster</td><td>CG34422</td><td>CG34422 gene product from transcript CG34422-RA</td><td>ARID4B|CG7274|CG7282|DmelCG34422|Dmel_CG7274|Dmel_CG7282</td><td>0</td><td>0</td></tr>
<tr><td>1565</td><td>32878</td><td>Drosophila melanogaster</td><td>CG7280</td><td>CG7280 gene product from transcript CG7280-RA</td><td>DmelCG7280</td><td>0</td><td>0</td></tr>
<tr><td>1566</td><td>32880</td><td>Drosophila melanogaster</td><td>CG7322</td><td>CG7322 gene product from transcript CG7322-RA</td><td>DmelCG7322</td><td>0</td><td>0</td></tr>
<tr><td>1567</td><td>32881</td><td>Drosophila melanogaster</td><td>CG7288</td><td>CG7288 gene product from transcript CG7288-RA</td><td>DmelCG7288</td><td>0</td><td>0</td></tr>
<tr><td>1568</td><td>32882</td><td>Drosophila melanogaster</td><td>CG7326</td><td>CG7326 gene product from transcript CG7326-RA</td><td>DmelCG7326</td><td>0</td><td>0</td></tr>
<tr><td>1569</td><td>32885</td><td>Drosophila melanogaster</td><td>CG7332</td><td>CG7332 gene product from transcript CG7332-RA</td><td>DmelCG7332</td><td>0</td><td>0</td></tr>
<tr><td>1570</td><td>32886</td><td>Drosophila melanogaster</td><td>CG7358</td><td>CG7358 gene product from transcript CG7358-RA</td><td>DmelCG7358</td><td>0</td><td>0</td></tr>
<tr><td>1571</td><td>32887</td><td>Drosophila melanogaster</td><td>CG7349</td><td>CG7349 gene product from transcript CG7349-RA</td><td>DmelCG7349</td><td>0</td><td>0</td></tr>
<tr><td>1572</td><td>32888</td><td>Drosophila melanogaster</td><td>CG7378</td><td>CG7378 gene product from transcript CG7378-RB</td><td>BcDNA:RH25447|DmelCG7378</td><td>0</td><td>0</td></tr>
<tr><td>1573</td><td>32889</td><td>Drosophila melanogaster</td><td>CG32541</td><td>CG32541 gene product from transcript CG32541-RA</td><td>CG12527|CG14188|DmelCG32541|EP(X)1644|LD15404</td><td>0</td><td>0</td></tr>
<tr><td>1574</td><td>32891</td><td>Drosophila melanogaster</td><td>CG14190</td><td>CG14190 gene product from transcript CG14190-RA</td><td>DmelCG14190</td><td>0</td><td>0</td></tr>
<tr><td>1575</td><td>32892</td><td>Drosophila melanogaster</td><td>CG7406</td><td>CG7406 gene product from transcript CG7406-RA</td><td>DmelCG7406</td><td>0</td><td>0</td></tr>
<tr><td>1576</td><td>32893</td><td>Drosophila melanogaster</td><td>CG14191</td><td>CG14191 gene product from transcript CG14191-RA</td><td>BcDNA:RE09269|DmelCG14191</td><td>0</td><td>0</td></tr>
<tr><td>1577</td><td>32894</td><td>Drosophila melanogaster</td><td>CG7423</td><td>CG7423 gene product from transcript CG7423-RA</td><td>DmelCG7423</td><td>0</td><td>0</td></tr>
<tr><td>1578</td><td>32895</td><td>Drosophila melanogaster</td><td>CG15882</td><td>CG15882 gene product from transcript CG15882-RA</td><td>DmelCG15882</td><td>0</td><td>0</td></tr>
<tr><td>1579</td><td>32896</td><td>Drosophila melanogaster</td><td>tgy</td><td>twiggy</td><td>CG7440|DmelCG7440</td><td>0</td><td>0</td></tr>
<tr><td>1580</td><td>32897</td><td>Drosophila melanogaster</td><td>Obp18a</td><td>Odorant-binding protein 18a</td><td>18a|BcDNA:RH25665|CG15883|DmelCG15883</td><td>0</td><td>0</td></tr>
<tr><td>1581</td><td>32898</td><td>Drosophila melanogaster</td><td>RhoGAP18B</td><td>CG42274 gene product from transcript CG42274-RC</td><td>5-131|CG17015|CG42274|CG7481|CG7502|CG7531|DmelCG42274|Dmel_CG7481|Dmel_CG7502|whir</td><td>0</td><td>0</td></tr>
<tr><td>1582</td><td>32901</td><td>Drosophila melanogaster</td><td>inx5</td><td>CG7537 gene product from transcript CG7537-RB</td><td>CG7537|Dm-inx5|DmelCG7537|inx-5</td><td>0</td><td>0</td></tr>
<tr><td>1583</td><td>32902</td><td>Drosophila melanogaster</td><td>CG7556</td><td>CG7556 gene product from transcript CG7556-RA</td><td>DmelCG7556</td><td>0</td><td>0</td></tr>
<tr><td>1584</td><td>32903</td><td>Drosophila melanogaster</td><td>CG7453</td><td>CG7453 gene product from transcript CG7453-RA</td><td>DmelCG7453</td><td>0</td><td>0</td></tr>
<tr><td>1585</td><td>32905</td><td>Drosophila melanogaster</td><td>Mec2</td><td>CG7635 gene product from transcript CG7635-RA</td><td>CG7635|DMec2|DmelCG7635</td><td>0</td><td>0</td></tr>
<tr><td>1586</td><td>32906</td><td>Drosophila melanogaster</td><td>CG14193</td><td>CG14193 gene product from transcript CG14193-RA</td><td>DmelCG14193</td><td>0</td><td>0</td></tr>
<tr><td>1587</td><td>32907</td><td>Drosophila melanogaster</td><td>CG7914</td><td>CG7914 gene product from transcript CG7914-RA</td><td>DmelCG7914</td><td>0</td><td>0</td></tr>
<tr><td>1588</td><td>32908</td><td>Drosophila melanogaster</td><td>CG14194</td><td>CG14194 gene product from transcript CG14194-RA</td><td>DmelCG14194</td><td>0</td><td>0</td></tr>
<tr><td>1589</td><td>32909</td><td>Drosophila melanogaster</td><td>CG7990</td><td>CG7990 gene product from transcript CG7990-RA</td><td>DmelCG7990</td><td>0</td><td>0</td></tr>
<tr><td>1590</td><td>32910</td><td>Drosophila melanogaster</td><td>Ulp1</td><td>CG12359 gene product from transcript CG12359-RA</td><td>BcDNA:GH02751|CG12359|DmUlp1|DmelCG12359|ulp1</td><td>0</td><td>0</td></tr>
<tr><td>1591</td><td>32911</td><td>Drosophila melanogaster</td><td>CG14195</td><td>CG14195 gene product from transcript CG14195-RA</td><td>DmelCG14195</td><td>0</td><td>0</td></tr>
<tr><td>1592</td><td>32912</td><td>Drosophila melanogaster</td><td>Mur18B</td><td>Mucin related 18B</td><td>CG7874|DmelCG7874|anon-WO0140519.120</td><td>0</td><td>0</td></tr>
<tr><td>1593</td><td>32913</td><td>Drosophila melanogaster</td><td>Muc18B</td><td>Mucin 18B</td><td>CG7876|DmelCG7876</td><td>0</td><td>0</td></tr>
<tr><td>1594</td><td>32914</td><td>Drosophila melanogaster</td><td>CG7884</td><td>CG7884 gene product from transcript CG7884-RA</td><td>DmelCG7884</td><td>0</td><td>0</td></tr>
<tr><td>1595</td><td>32915</td><td>Drosophila melanogaster</td><td>CG14196</td><td>CG14196 gene product from transcript CG14196-RA</td><td>DmelCG14196</td><td>0</td><td>0</td></tr>
<tr><td>1596</td><td>32916</td><td>Drosophila melanogaster</td><td>CG7889</td><td>CG7889 gene product from transcript CG7889-RA</td><td>DmelCG7889</td><td>0</td><td>0</td></tr>
<tr><td>1597</td><td>32917</td><td>Drosophila melanogaster</td><td>CG7992</td><td>CG7992 gene product from transcript CG7992-RA</td><td>DmelCG7992</td><td>0</td><td>0</td></tr>
<tr><td>1598</td><td>32918</td><td>Drosophila melanogaster</td><td>Hs3st-B</td><td>Heparan sulfate 3-O sulfotransferase-B</td><td>CG7890|DmelCG7890|dHs3st-B</td><td>0</td><td>0</td></tr>
<tr><td>1599</td><td>32919</td><td>Drosophila melanogaster</td><td>rictor</td><td>rapamycin-insensitive companion of Tor</td><td>8002|CG8002|DmelCG8002|Rictor|dRic|dRictor</td><td>0</td><td>0</td></tr>
<tr><td>1600</td><td>32920</td><td>Drosophila melanogaster</td><td>vav</td><td>CG7893 gene product from transcript CG7893-RB</td><td>BcDNA:GH01128|CG7893|DmelCG7893|DroVav|Vav</td><td>0</td><td>0</td></tr>
<tr><td>1601</td><td>32921</td><td>Drosophila melanogaster</td><td>CG8010</td><td>CG8010 gene product from transcript CG8010-RA</td><td>DmelCG8010</td><td>0</td><td>0</td></tr>
<tr><td>1602</td><td>32922</td><td>Drosophila melanogaster</td><td>CG32536</td><td>CG32536 gene product from transcript CG32536-RA</td><td>CG8020|DmelCG32536</td><td>0</td><td>0</td></tr>
<tr><td>1603</td><td>32923</td><td>Drosophila melanogaster</td><td>CG8028</td><td>CG8028 gene product from transcript CG8028-RA</td><td>DmelCG8028|MCT</td><td>0</td><td>0</td></tr>
<tr><td>1604</td><td>32924</td><td>Drosophila melanogaster</td><td>CG8034</td><td>CG8034 gene product from transcript CG8034-RA</td><td>DmelCG8034</td><td>0</td><td>0</td></tr>
<tr><td>1605</td><td>32925</td><td>Drosophila melanogaster</td><td>CG8051</td><td>CG8051 gene product from transcript CG8051-RA</td><td>DmelCG8051|EP(X)1550|anon-WO03002137.1</td><td>0</td><td>0</td></tr>
<tr><td>1606</td><td>32926</td><td>Drosophila melanogaster</td><td>out</td><td>outsiders</td><td>BcDNA:LD28120|CG8062|DmelCG8062</td><td>0</td><td>0</td></tr>
<tr><td>1607</td><td>32928</td><td>Drosophila melanogaster</td><td>gfA</td><td>giant fibre A</td><td>CG32538|CG8082|CG8109|CT24218|Da7|Dalpha7|DmelCG32538|gfa|jpl|nAChR|nAcRalpha-18C|nj507|nj520|nj522|nj54|nj75</td><td>0</td><td>0</td></tr>
<tr><td>1608</td><td>32930</td><td>Drosophila melanogaster</td><td>kek5</td><td>kekkon5</td><td>CG12199|CT10486|DmelCG12199|Kek5</td><td>0</td><td>0</td></tr>
<tr><td>1609</td><td>32932</td><td>Drosophila melanogaster</td><td>CG12200</td><td>CG12200 gene product from transcript CG12200-RA</td><td>DmelCG12200</td><td>0</td><td>0</td></tr>
<tr><td>1610</td><td>32933</td><td>Drosophila melanogaster</td><td>CG32533</td><td>CG32533 gene product from transcript CG32533-RA</td><td>CG12211|CG14198|DmelCG32533|cg12211</td><td>0</td><td>0</td></tr>
<tr><td>1611</td><td>32934</td><td>Drosophila melanogaster</td><td>Nat1</td><td>CG12202 gene product from transcript CG12202-RA</td><td>CG12202|DmelCG12202|dNat1|l(1)G0311</td><td>0</td><td>0</td></tr>
<tr><td>1612</td><td>32935</td><td>Drosophila melanogaster</td><td>pcm</td><td>pacman</td><td>CG3291|DmelCG3291|Pcm|XRN1|XRN1/pcm|Xrn1|Xrn1p</td><td>0</td><td>0</td></tr>
<tr><td>1613</td><td>32936</td><td>Drosophila melanogaster</td><td>CG12203</td><td>CG12203 gene product from transcript CG12203-RA</td><td>DmelCG12203</td><td>0</td><td>0</td></tr>
<tr><td>1614</td><td>32937</td><td>Drosophila melanogaster</td><td>CG12204</td><td>CG12204 gene product from transcript CG12204-RA</td><td>DmelCG12204</td><td>0</td><td>0</td></tr>
<tr><td>1615</td><td>32938</td><td>Drosophila melanogaster</td><td>Pfrx</td><td>6-phosphofructo-2-kinase</td><td>CG3400|DmelCG3400</td><td>0</td><td>0</td></tr>
<tr><td>1616</td><td>32939</td><td>Drosophila melanogaster</td><td>CG14200</td><td>CG14200 gene product from transcript CG14200-RA</td><td>DmelCG14200</td><td>0</td><td>0</td></tr>
<tr><td>1617</td><td>32940</td><td>Drosophila melanogaster</td><td>l(1)G0156</td><td>lethal (1) G0156</td><td>CG12233|DmelCG12233|FBgn0031024</td><td>0</td><td>0</td></tr>
<tr><td>1618</td><td>32941</td><td>Drosophila melanogaster</td><td>CoRest</td><td>CG42687 gene product from transcript CG42687-RC</td><td>BcDNA:GH01486|BcDNA:LD11215|CG32534|CG32535|CG33525|CG3878|CG42687|CoREST|DmelCG42687|Dmel_CG32534|Dmel_CG33525|dCoREST|i169</td><td>0</td><td>0</td></tr>
<tr><td>1619</td><td>32942</td><td>Drosophila melanogaster</td><td>CG12231</td><td>CG12231 gene product from transcript CG12231-RA</td><td>DmelCG12231</td><td>0</td><td>0</td></tr>
<tr><td>1620</td><td>32943</td><td>Drosophila melanogaster</td><td>CG32532</td><td>CG32532 gene product from transcript CG32532-RA</td><td>CG14201|CG14202|CG14203|DmelCG32532</td><td>0</td><td>0</td></tr>
<tr><td>1621</td><td>32946</td><td>Drosophila melanogaster</td><td>Grip84</td><td>gamma-tubulin ring protein 84</td><td>CG3917|DGrip84|Dgrip84|Dgrips84|DmelCG3917|GCP2|grip84|l(1)[[t]]PG36|l(1)[[t]]PG66</td><td>0</td><td>0</td></tr>
<tr><td>1622</td><td>32947</td><td>Drosophila melanogaster</td><td>car</td><td>carnation</td><td>CG12230|Car|Dm-Vps33|DmelCG12230|VPS33a|Vps33|Vps33/carnation|Vps33A|Vps33a|Vps33p|carn|l(1)G0447</td><td>0</td><td>0</td></tr>
<tr><td>1623</td><td>32948</td><td>Drosophila melanogaster</td><td>Tao-1</td><td>CG14217 gene product from transcript CG14217-RF</td><td>AAF48973|CG14217|DmelCG14217|MARKK|TAO1|dTao-1|tao-1|tao1</td><td>0</td><td>0</td></tr>
<tr><td>1624</td><td>32949</td><td>Drosophila melanogaster</td><td>CG14218</td><td>CG14218 gene product from transcript CG14218-RC</td><td>DmelCG14218|SPH211</td><td>0</td><td>0</td></tr>
<tr><td>1625</td><td>32950</td><td>Drosophila melanogaster</td><td>CG14204</td><td>CG14204 gene product from transcript CG14204-RA</td><td>DmelCG14204</td><td>0</td><td>0</td></tr>
<tr><td>1626</td><td>32951</td><td>Drosophila melanogaster</td><td>CG14219</td><td>CG14219 gene product from transcript CG14219-RA</td><td>DmelCG14219</td><td>0</td><td>0</td></tr>
<tr><td>1627</td><td>32952</td><td>Drosophila melanogaster</td><td>CG14205</td><td>CG14205 gene product from transcript CG14205-RA</td><td>DmelCG14205</td><td>0</td><td>0</td></tr>
<tr><td>1628</td><td>32953</td><td>Drosophila melanogaster</td><td>RpS10b</td><td>Ribosomal protein S10b</td><td>CG14206|CT33819|DmelCG14206|GM19176|M(1)18C|M(1)36f|M(1)n|M[[n]]|S10b|i3|i73|l(1)carot13</td><td>0</td><td>0</td></tr>
<tr><td>1629</td><td>32954</td><td>Drosophila melanogaster</td><td>CG14220</td><td>CG14220 gene product from transcript CG14220-RB</td><td>DmelCG14220|SDS3|Sds3</td><td>0</td><td>0</td></tr>
<tr><td>1630</td><td>32955</td><td>Drosophila melanogaster</td><td>CG14207</td><td>CG14207 gene product from transcript CG14207-RB</td><td>DmelCG14207|HspB8|dHspB8</td><td>0</td><td>0</td></tr>
<tr><td>1631</td><td>32958</td><td>Drosophila melanogaster</td><td>CG14210</td><td>CG14210 gene product from transcript CG14210-RA</td><td>BcDNA:RE23450|DmelCG14210</td><td>0</td><td>0</td></tr>
<tr><td>1632</td><td>32961</td><td>Drosophila melanogaster</td><td>CG14221</td><td>CG14221 gene product from transcript CG14221-RB</td><td>DmelCG14221</td><td>0</td><td>0</td></tr>
<tr><td>1633</td><td>32962</td><td>Drosophila melanogaster</td><td>CG14222</td><td>CG14222 gene product from transcript CG14222-RA</td><td>DmAAF48987|DmelCG14222</td><td>0</td><td>0</td></tr>
<tr><td>1634</td><td>32963</td><td>Drosophila melanogaster</td><td>MKP-4</td><td>MAPK Phosphatase 4</td><td>CG14211|DMKP-4|DmelCG14211|Mkp4</td><td>0</td><td>0</td></tr>
<tr><td>1635</td><td>32964</td><td>Drosophila melanogaster</td><td>CG14212</td><td>CG14212 gene product from transcript CG14212-RA</td><td>DmelCG14212</td><td>0</td><td>0</td></tr>
<tr><td>1636</td><td>32965</td><td>Drosophila melanogaster</td><td>e(y)3</td><td>enhancer of yellow 3</td><td>CG12238|DmelCG12238|SAYP|e(y)[3]|l(1)G0084|l(1)G0266|l(1)G0283|l(1)G0381|l(1)G0409|sayp</td><td>0</td><td>0</td></tr>
<tr><td>1637</td><td>32966</td><td>Drosophila melanogaster</td><td>Rcd-1</td><td>Required for cell differentiation 1 ortholog</td><td>CAF40|CG14213|DmelCG14213|Drcd-1</td><td>0</td><td>0</td></tr>
<tr><td>1638</td><td>32967</td><td>Drosophila melanogaster</td><td>CG12237</td><td>CG12237 gene product from transcript CG12237-RA</td><td>DmelCG12237</td><td>0</td><td>0</td></tr>
<tr><td>1639</td><td>32968</td><td>Drosophila melanogaster</td><td>Sec61gamma</td><td>CG14214 gene product from transcript CG14214-RA</td><td>CG14214|DmelCG14214|Sec61</td><td>0</td><td>0</td></tr>
<tr><td>1640</td><td>32969</td><td>Drosophila melanogaster</td><td>Arp11</td><td>CG12235 gene product from transcript CG12235-RA</td><td>ARP11|CG12235|DmelCG12235</td><td>0</td><td>0</td></tr>
<tr><td>1641</td><td>32970</td><td>Drosophila melanogaster</td><td>Ranbp21</td><td>CG12234 gene product from transcript CG12234-RA</td><td>CG12234|DmelCG12234|Exp-5|Exp5|RanBP21|dmExp5</td><td>0</td><td>0</td></tr>
<tr><td>1642</td><td>32971</td><td>Drosophila melanogaster</td><td>CG14215</td><td>CG14215 gene product from transcript CG14215-RA</td><td>DmelCG14215</td><td>0</td><td>0</td></tr>
<tr><td>1643</td><td>32972</td><td>Drosophila melanogaster</td><td>CG14223</td><td>CG14223 gene product from transcript CG14223-RA</td><td>DmelCG14223</td><td>0</td><td>0</td></tr>
<tr><td>1644</td><td>32973</td><td>Drosophila melanogaster</td><td>CG14216</td><td>CG14216 gene product from transcript CG14216-RA</td><td>BEST:CK01830|DmelCG14216|Ssu72</td><td>0</td><td>0</td></tr>
<tr><td>1645</td><td>32974</td><td>Drosophila melanogaster</td><td>Zw</td><td>Zwischenferment</td><td>CG12529|DmelCG12529|G-6-PD|G-6-pdh|G-6PD|G-6pd|G6-PD|G6-pd|G6PD|G6PDH|G6pd|G6pdh|g6pd|zw</td><td>0</td><td>0</td></tr>
<tr><td>1646</td><td>32975</td><td>Drosophila melanogaster</td><td>et</td><td>eye transformer</td><td>CG14225|DmelCG14225|ET</td><td>0</td><td>0</td></tr>
<tr><td>1647</td><td>32976</td><td>Drosophila melanogaster</td><td>dome</td><td>domeless</td><td>142932_at|CG14226|CT33841|DOME|DmelCG14226|Dome|anon-18DEb|l(1)G0217|l(1)G0218|l(1)G0264|l(1)G0282|l(1)G0321|l(1)G0367|l(1)G0405|l(1)G0441|l(1)G0468|mom|vsp</td><td>0</td><td>0</td></tr>
<tr><td>1648</td><td>32977</td><td>Drosophila melanogaster</td><td>Ubqn</td><td>Ubiquilin</td><td>BEST:LD26923|CG14224|Chap|DSK2|DmelCG14224|Dsk2|anon-18DEa|clone 5.42|dUbqln|ubqn</td><td>0</td><td>0</td></tr>
<tr><td>1649</td><td>32978</td><td>Drosophila melanogaster</td><td>CG14227</td><td>CG14227 gene product from transcript CG14227-RB</td><td>DmelCG14227|SPH158</td><td>0</td><td>0</td></tr>
<tr><td>1650</td><td>32979</td><td>Drosophila melanogaster</td><td>Mer</td><td>Merlin</td><td>BG01543|CG14228|D-Mer|DmelCG14228|Dmerlin|EMR2|Emr2|mer</td><td>0</td><td>0</td></tr>
<tr><td>1651</td><td>32980</td><td>Drosophila melanogaster</td><td>CG14229</td><td>CG14229 gene product from transcript CG14229-RA</td><td>BcDNA:RE31307|DmelCG14229</td><td>0</td><td>0</td></tr>
<tr><td>1652</td><td>32981</td><td>Drosophila melanogaster</td><td>Cdc42</td><td>CG12530 gene product from transcript CG12530-RC</td><td>CDC42|CG12530|Cdc42Dm|D-CDC42|D-Cdc42|D-cdc42|DCDC42|DCdc42|Dcdc42|Dm Cdc42|DmCDC42|Dmcdc42|DmelCG12530|cdc42</td><td>0</td><td>0</td></tr>
<tr><td>1653</td><td>32982</td><td>Drosophila melanogaster</td><td>CG14231</td><td>CG14231 gene product from transcript CG14231-RB</td><td>DmelCG14231</td><td>0</td><td>0</td></tr>
<tr><td>1654</td><td>32983</td><td>Drosophila melanogaster</td><td>CG14232</td><td>CG14232 gene product from transcript CG14232-RB</td><td>DmelCG14232|anon-18DEc</td><td>0</td><td>0</td></tr>
<tr><td>1655</td><td>32984</td><td>Drosophila melanogaster</td><td>CG14230</td><td>CG14230 gene product from transcript CG14230-RA</td><td>DmelCG14230|Nol8|anon-18DEe|cg14230|clone 9.42</td><td>0</td><td>0</td></tr>
<tr><td>1656</td><td>32986</td><td>Drosophila melanogaster</td><td>CG12531</td><td>CG12531 gene product from transcript CG12531-RA</td><td>DmelCG12531</td><td>0</td><td>0</td></tr>
<tr><td>1657</td><td>32987</td><td>Drosophila melanogaster</td><td>Bap</td><td>Beta Adaptin</td><td>AP-2beta|AP-beta1|BAD1|CG12532|DmelCG12532|bap|beta1/2|cg12532</td><td>0</td><td>0</td></tr>
<tr><td>1658</td><td>32988</td><td>Drosophila melanogaster</td><td>CG14234</td><td>CG14234 gene product from transcript CG14234-RA</td><td>DmelCG14234</td><td>0</td><td>0</td></tr>
<tr><td>1659</td><td>32989</td><td>Drosophila melanogaster</td><td>CG14235</td><td>CG14235 gene product from transcript CG14235-RA</td><td>DmelCG14235</td><td>0</td><td>0</td></tr>
<tr><td>1660</td><td>32990</td><td>Drosophila melanogaster</td><td>CG32528</td><td>CG32528 gene product from transcript CG32528-RA</td><td>CG12533|DmelCG32528|parvin</td><td>0</td><td>0</td></tr>
<tr><td>1661</td><td>32991</td><td>Drosophila melanogaster</td><td>Alr</td><td>Augmenter of liver regeneration</td><td>CG12534|DmelCG12534|alr</td><td>0</td><td>0</td></tr>
<tr><td>1662</td><td>32992</td><td>Drosophila melanogaster</td><td>CG12703</td><td>CG12703 gene product from transcript CG12703-RA</td><td>DmelCG12703|i17</td><td>0</td><td>0</td></tr>
<tr><td>1663</td><td>32993</td><td>Drosophila melanogaster</td><td>CG12702</td><td>CG12702 gene product from transcript CG12702-RA</td><td>DmelCG12702</td><td>0</td><td>0</td></tr>
</table>

1664	32994	Drosophila melanogaster	vfl	vielfaltig	CG12701|DmelCG12701|EP1344|l(1)G0225|l(1)G0353|l(1)G0427|zld	0	0
1665	32995	Drosophila melanogaster	skpD	CG12700 gene product from transcript CG12700-RA	BcDNA:AT18217|CG12700|DmelCG12700|Skp|dskpD	0	0
1666	32996	Drosophila melanogaster	skpC	CG11941 gene product from transcript CG11941-RA	CG11941|DmelCG11941|Skp|SkpC|dskpC	0	0
1667	32997	Drosophila melanogaster	skpE	CG11942 gene product from transcript CG11942-RA	CG11942|DmelCG11942|Skp	0	0
1668	32999	Drosophila melanogaster	amn	amnesiac	Amn|CG11937|DmelCG11937|Drm-amn|EP(X)1442|PACAP|PACAP-38|PACAP-like|PACAP38|PACAP38-like|Pacap38|chpd	0	0
1669	33000	Drosophila melanogaster	CG32529	CG32529 gene product from transcript CG32529-RA	CG11936|CG15619|DmelCG32529	0	0
1670	33001	Drosophila melanogaster	CG15618	CG15618 gene product from transcript CG15618-RB	DmelCG15618	0	0
1671	33002	Drosophila melanogaster	CG11943	CG11943 gene product from transcript CG11943-RB	DmelCG11943|Ov4|anon-EST:Posey281|nup205	0	0
1672	33003	Drosophila melanogaster	pico	CG11940 gene product from transcript CG11940-RA	BcDNA:LP01106|CG11940|DmelCG11940|Pico|jog	0	0
1673	33004	Drosophila melanogaster	CG12655	CG12655 gene product from transcript CG12655-RA	DmelCG12655	0	0
1674	33005	Drosophila melanogaster	Nep3	Neprilysin 3	CEC|CG9565|DmeNEP3|DmelCG9565|NEP3|dNEP3	0	0
1675	33006	Drosophila melanogaster	CG17003	CG17003 gene product from transcript CG17003-RA	DmelCG17003	0	0
1676	33007	Drosophila melanogaster	D2R	Dopamine 2-like receptor	CG17004|CG33517|CG9569|DD2R|DmelCG33517|Dop2R|anon-WO0170980.130|anon-WO0170980.131|anon-WO0170980.31|anon-WO0170980.32	0	0
1677	33009	Drosophila melanogaster	CG9570	CG9570 gene product from transcript CG9570-RA	DmelCG9570	0	0
1678	33010	Drosophila melanogaster	fd19B	forkhead domain 19B	CG9571|Dmcg9571|DmelCG9571	0	0
1679	33011	Drosophila melanogaster	CG34331	CG34331 gene product from transcript CG34331-RB	DmelCG34331	0	0
1680	33012	Drosophila melanogaster	CG15322	CG15322 gene product from transcript CG15322-RB	DmelCG15322	0	0
1681	33013	Drosophila melanogaster	CG9572	CG9572 gene product from transcript CG9572-RA	CT17356|DmelCG9572	0	0
1682	33014	Drosophila melanogaster	Rab35	CG9575 gene product from transcript CG9575-RA	CG9575|DRab35|DmRab35|DmelCG9575|dRab35	0	0
1683	33015	Drosophila melanogaster	CG9576	CG9576 gene product from transcript CG9576-RA	DmelCG9576	0	0
1684	33016	Drosophila melanogaster	CG9577	CG9577 gene product from transcript CG9577-RA	DmelCG9577	0	0
1685	33017	Drosophila melanogaster	CG9581	CG9581 gene product from transcript CG9581-RA	DmelCG9581	0	0
1686	33018	Drosophila melanogaster	CG9578	CG9578 gene product from transcript CG9578-RA	DmelCG9578	0	0
1687	33019	Drosophila melanogaster	AnnX	Annexin X	Ann|Annx|CG9579|DmelCG9579|annexin|anxX	0	0
1688	33022	Drosophila melanogaster	obst-A	obstructor-A	CG17052|DmelCG17052|obst	0	0
1689	33023	Drosophila melanogaster	Peritrophin-A	Peritrophin A	CG17058|DmelCG17058|PeriA|obst-D	0	0
1690	33024	Drosophila melanogaster	CG17068	CG17068 gene product from transcript CG17068-RA	DmelCG17068	0	0
1691	33025	Drosophila melanogaster	Rab10	Rab-protein 10	AAF50924|CG17060|DRAB10|DRab10|DmRab10|DmelCG17060|Rab-r10|rab10	0	0
1692	33026	Drosophila melanogaster	CG17065	CG17065 gene product from transcript CG17065-RA	DmelCG17065	0	0
1693	33027	Drosophila melanogaster	inx7	innexin 7	CG2977|CG32731|D-inx-7|Dm-inx7|DmelCG2977|inx-7|prp7	0	0
1694	33028	Drosophila melanogaster	CG1504	CG1504 gene product from transcript CG1504-RB	CT3673|DmelCG1504	0	0
1695	33029	Drosophila melanogaster	CG1631	CG1631 gene product from transcript CG1631-RA	DmelCG1631	0	0
1696	33031	Drosophila melanogaster	CG12679	CG12679 gene product from transcript CG12679-RA	DmelCG12679	0	0
1697	33032	Drosophila melanogaster	CG15460	CG15460 gene product from transcript CG15460-RC	DmelCG15460	0	0
1698	33033	Drosophila melanogaster	l(1)G0004	lethal (1) G0004	CG11738|DmelCG11738	0	0
1699	33034	Drosophila melanogaster	Syx16	Syntaxin 16	CG1467|Dm Syx16|DmSyx16|DmelCG1467|SYX16|Syx|dSyx 16|dSyx16	0	0
1700	33035	Drosophila melanogaster	HERC2	CG11734 gene product from transcript CG11734-RC	CG11734|DmelCG11734	0	0
1701	33036	Drosophila melanogaster	CG15459	CG15459 gene product from transcript CG15459-RA	DmelCG15459	0	0
1702	33037	Drosophila melanogaster	Obp19a	Odorant-binding protein 19a	19a|CG11748|DmelCG11748|OBP19a	0	0
1703	33038	Drosophila melanogaster	Obp19b	Odorant-binding protein 19b	19b|CG1670|DmelCG1670|anon-WO0140519.23	0	0
1704	33039	Drosophila melanogaster	Obp19c	Odorant-binding protein 19c	19c|CG15457|DmelCG15457	0	0
1705	33040	Drosophila melanogaster	Pbprp2	Pheromone-binding protein-related protein 2	19d|BcDNA:HL07789|BcDNA:RH68082|CG1668|DmelCG1668|Obp19d|PB-PRP2|PBP2|PBPRP-2|PBPRP2|pbprp-2|pbprp2	0	0
1706	33043	Drosophila melanogaster	cactin	CG1676 gene product from transcript CG1676-RA	CG1676|DmelCG1676	0	0
1707	33044	Drosophila melanogaster	CG11710	CG11710 gene product from transcript CG11710-RA	DmelCG11710	0	0
1708	33045	Drosophila melanogaster	mal	maroon-like	5|CG1692|DmelCG1692|SRF|bz|ma-l	0	0
1709	33046	Drosophila melanogaster	CG1695	CG1695 gene product from transcript CG1695-RB	DmelCG1695	0	0
1710	33047	Drosophila melanogaster	CG1702	CG1702 gene product from transcript CG1702-RB	DmelCG1702|cg1702	0	0
1711	33048	Drosophila melanogaster	RhoGAP19D	CG1412 gene product from transcript CG1412-RB	1412|CG1412|DmelCG1412	0	0
1712	33049	Drosophila melanogaster	CG1812	CG1812 gene product from transcript CG1812-RA	DmelCG1812	0	0
1713	33051	Drosophila melanogaster	RunxB	CG42267 gene product from transcript CG42267-RA	CG15454|CG15455|CG42267|DmelCG42267|Dmel_CG15454|Dmel_CG15455	0	0
1714	33056	Drosophila melanogaster	Cyp6v1	CG1829 gene product from transcript CG1829-RA	6v1|CG1829|DmelCG1829	0	0
1715	33057	Drosophila melanogaster	CG1835	CG1835 gene product from transcript CG1835-RB	BcDNA:GH25431|DmelCG1835|anon-WO0140519.219	0	0
1716	33058	Drosophila melanogaster	hydra	CG1338 gene product from transcript CG1338-RA	CG1338|DmelCG1338	0	0
1717	33059	Drosophila melanogaster	run	runt	1|2|AA33|CG1849|DmelCG1849|LB5|P235|RUN|Run|Runt|l(1)19Ea|l(1)AA33|l(1)B2/13.1|l(1)LB9|lLB5|leg	0	0
1718	33060	Drosophila melanogaster	CG1324	CG1324 gene product from transcript CG1324-RA	DmelCG1324|anon-WO0140519.192	0	0
1719	33061	Drosophila melanogaster	CG15452	CG15452 gene product from transcript CG15452-RA	DmelCG15452	0	0
1720	33062	Drosophila melanogaster	shakB	shaking B	2|3|6|CG12678|CG1321|CG15451|CG32508|CG34358|DmelCG34358|Dmel_CG12678|Dmel_CG32508|E81|Pas|R-9-29|R9-29|Shak B|Shak-B|ShakB|W3|inx8|l(1)19Eb|l(1)LB21|l(1)R-10-14|l(1)R-10-3|l(1)R-1	0	0
1721	33064	Drosophila melanogaster	CG15450	CG15450 gene product from transcript CG15450-RA	DmelCG15450	0	0
1722	33066	Drosophila melanogaster	CG1314	CG1314 gene product from transcript CG1314-RA	DmelCG1314|anon-WO0140519.251	0	0
1723	33068	Drosophila melanogaster	CG42707	CG42707 gene product from transcript CG42707-RB	CG11233|DmelCG42707|Dmel_CG11233	0	0
1724	33071	Drosophila melanogaster	CG11227	CG11227 gene product from transcript CG11227-RC	DmelCG11227	0	0
1725	33072	Drosophila melanogaster	Npc1b	Niemann-Pick type C-1b	151|4|7|AF2/19|CG12092|DmelCG12092|Dmel_CG11233|NPC1b|Q256|R-9-28|R9-28|dncp1b|l(1)19Ec|l(1)2-3|l(1)R-9-28|l151|l152|lN5|lt2-14a|npc1b	0	0
1726	33073	Drosophila melanogaster	Ser6	Serine protease 6	CG2071|DmelCG2071|SP47	0	0
1727	33074	Drosophila melanogaster	CG1304	CG1304 gene product from transcript CG1304-RA	DmelCG1304|SP46	0	0
1728	33075	Drosophila melanogaster	r-cup	ryder cup	CG10998|DmelCG10998	0	0
1729	33076	Drosophila melanogaster	CG1532	CG1532 gene product from transcript CG1532-RA	DmelCG1532	0	0
1730	33077	Drosophila melanogaster	CG1529	CG1529 gene product from transcript CG1529-RA	DmelCG1529	0	0
1731	33078	Drosophila melanogaster	Ntf-2	Nuclear transport factor-2	BcDNA:GM08921|CG1740|DNTF-2|DmelCG1740|Dntf-2|Dntf2|NTF-2|Ntf2|dmNTF2|l(1)G0086|l(1)G0125|l(1)G0337|l(1)G0428|ntf-2|ntf2	0	0
1732	33079	Drosophila melanogaster	CG15449	CG15449 gene product from transcript CG15449-RA	DmelCG15449	0	0
1733	33081	Drosophila melanogaster	CG1753	CG1753 gene product from transcript CG1753-RA	DmelCG1753	0	0
1734	33082	Drosophila melanogaster	CG1518	CG1518 gene product from transcript CG1518-RA	DmelCG1518	0	0
1735	33083	Drosophila melanogaster	bves	CG32513 gene product from transcript CG32513-RA	BcDNA:SD21670|CG15448|CG1754|CG32513|DmelCG32513	0	0
1736	33085	Drosophila melanogaster	CG32512	CG32512 gene product from transcript CG32512-RA	CG15447|CG1739|DmelCG32512	0	0
1737	33088	Drosophila melanogaster	CG15446	CG15446 gene product from transcript CG15446-RC	DmelCG15446	0	0
1738	33090	Drosophila melanogaster	CG1503	CG1503 gene product from transcript CG1503-RA	DmelCG1503	0	0
1739	33093	Drosophila melanogaster	unc	uncoordinated	3|4|5|CG1501|DmelCG1501|UNC|Unc|W5|l(1)19Ef|l(1)6-3-212|lLV7	0	0
1740	33094	Drosophila melanogaster	CG15445	CG15445 gene product from transcript CG15445-RA	DmelCG15445	0	0
1741	33096	Drosophila melanogaster	CG14579	CG14579 gene product from transcript CG14579-RA	DmelCG14579	0	0
1742	33097	Drosophila melanogaster	CG1724	CG1724 gene product from transcript CG1724-RA	BcDNA:AT13347|DmelCG1724	0	0
1743	33098	Drosophila melanogaster	CG32521	CG32521 gene product from transcript CG32521-RA	CG1726|CG1735|DmelCG32521	0	0
1744	33101	Drosophila melanogaster	CG1722	CG1722 gene product from transcript CG1722-RA	DmelCG1722	0	0
1745	33102	Drosophila melanogaster	CG1494	CG1494 gene product from transcript CG1494-RA	DmelCG1494	0	0
1746	33103	Drosophila melanogaster	CG1718	CG1718 gene product from transcript CG1718-RB	DmelCG1718	0	0
1747	33104	Drosophila melanogaster	CG1801	CG1801 gene product from transcript CG1801-RC	DmelCG1801	0	0
1748	33105	Drosophila melanogaster	Pros45	CG1489 gene product from transcript CG1489-RA	1489|CG1489|DUG|Dm_Rpt6a|DmelCG1489|Dug|PRS8_DROME|Rpt6|Ug|dRpt6|p42C|pros45|sug-1|sug1	0	0
1749	33107	Drosophila melanogaster	Dd	Dullard	BcDNA:LD08201|CG1696|DmelCG1696|anon-WO03040301.167|ddd|l(1)G0269	0	0
1750	33108	Drosophila melanogaster	CG1486	CG1486 gene product from transcript CG1486-RA	DmelCG1486	0	0
1751	33109	Drosophila melanogaster	tty	tweety	CG1693|DmelCG1693|twe	0	0
1752	33110	Drosophila melanogaster	fliI	flightless I	15|BcDNA:LD21753|CG1484|CT3691|DCA3-19|DmelCG1484|EN3|FLI-I|Fli-I|W2|fli|fli I|fli-I|fltI|fltO|l(1)18-80|l(1)19Fd|l(1)ESHS53|l(1)W-2|lDCB2-19|sdby|w-2	0	0
1753	33111	Drosophila melanogaster	dod	dodo	CG17051|DmelCG17051|Pin1	0	0
1754	33112	Drosophila melanogaster	pen	penguin	CG1685|DmelCG1685	0	0
1755	33114	Drosophila melanogaster	Sep1	Septin-1	CG1403|Diff6|DmelCG1403|anon-19Fa|iby|sep1|tu1	0	0
1756	33117	Drosophila melanogaster	slgA	sluggish A	CG1417|DmelCG1417|EE85|PRODH|sl|slg|tu3	0	0
1757	33118	Drosophila melanogaster	Hlc	Helicase	11P1|14|7|A112|CG1666|DmRH10|DmelCG1666|HLC|HM-44|hlc|l(1)11P1|l(1)19Ff|l(1)8-1|l(1)A112|l(1)S-19F6|l11P1|lA112	0	0
1758	33119	Drosophila melanogaster	mst	misato	CG1424|DmelCG1424|LB-20|LB20|l(1)19Fg|l(1)LB14|l(1)LB20|lLB14	0	0
1759	33120	Drosophila melanogaster	lcs	la costa	CG12794|DmelCG12794	0	0
1760	33121	Drosophila melanogaster	CG1678	CG1678 gene product from transcript CG1678-RA	BcDNA:GH04760|DmelCG1678	0	0
1761	33123	Drosophila melanogaster	CG10918	CG10918 gene product from transcript CG10918-RA	DmelCG10918	0	0
1762	33125	Drosophila melanogaster	CG42343	CG42343 gene product from transcript CG42343-RF	BP1062|BcDNA:RE23430|CG12564|CG12565|CG14583|CG34019|CT34319|DmelCG42343|Dmel_CG12564|Dmel_CG12565|Dmel_CG14583|Dmel_CG34019|pp-CT34319|pp-CT34320|pp-CT34321	0	0
1763	33126	Drosophila melanogaster	Ir20a	Ionotropic receptor 20a	CG14584|DmelCG14584|IR20a	0	0
1764	33127	Drosophila melanogaster	Cyp6t1	CG1644 gene product from transcript CG1644-RA	6t1|CG1644|DmelCG1644	0	0
1765	33128	Drosophila melanogaster	CG14621	CG14621 gene product from transcript CG14621-RA	DmelCG14621	0	0
1766	33129	Drosophila melanogaster	CG14615	CG14615 gene product from transcript CG14615-RA	DmelCG14615	0	0
1767	33130	Drosophila melanogaster	tilB	touch insensitive larva B	CG14620|DmelCG14620	0	0
1768	33131	Drosophila melanogaster	CG14614	CG14614 gene product from transcript CG14614-RB	BcDNA:GM08142|DmelCG14614	0	0
1769	33132	Drosophila melanogaster	CG14619	CG14619 gene product from transcript CG14619-RG	DmelCG14619	0	0
1770	33133	Drosophila melanogaster	CG14613	CG14613 gene product from transcript CG14613-RA	ADP/ATP translocase|CT34370|DmelCG14613	0	0
1771	33134	Drosophila melanogaster	CG14618	CG14618 gene product from transcript CG14618-RA	DmelCG14618	0	0
1772	33135	Drosophila melanogaster	CG12576	CG12576 gene product from transcript CG12576-RA	DmelCG12576|anon-WO0140519.40	0	0
1773	33136	Drosophila melanogaster	Cp110	CG14617 gene product from transcript CG14617-RB	CG14617|CG32501|DCP110|DmelCG14617	0	0
1774	33137	Drosophila melanogaster	l(1)G0196	lethal (1) G0196	CG14616|CG32502|DmelCG14616	0	0

<table>
<tr><td>1775</td><td>33139</td><td>Drosophila melanogaster</td><td>S6kII</td><td>Ribosomal protein S6 kinase II</td><td>CG17596|DmelCG17596|RSK|Rsk|S6KII|dRSK|ign|rsk|rsk2</td><td>0</td><td>0</td></tr>
<tr><td>1776</td><td>33140</td><td>Drosophila melanogaster</td><td>CG17598</td><td>CG17598 gene product from transcript CG17598-RA</td><td>DmelCG17598|PP2C</td><td>0</td><td>0</td></tr>
<tr><td>1777</td><td>33141</td><td>Drosophila melanogaster</td><td>CG17600</td><td>CG17600 gene product from transcript CG17600-RA</td><td>CG17602|DmelCG17600</td><td>0</td><td>0</td></tr>
<tr><td>1778</td><td>33142</td><td>Drosophila melanogaster</td><td>CG17599</td><td>CG17599 gene product from transcript CG17599-RA</td><td>DmelCG17599</td><td>0</td><td>0</td></tr>
<tr><td>1779</td><td>33143</td><td>Drosophila melanogaster</td><td>CG17601</td><td>CG17601 gene product from transcript CG17601-RA</td><td>DmelCG17601</td><td>0</td><td>0</td></tr>
<tr><td>1780</td><td>33144</td><td>Drosophila melanogaster</td><td>Cda4</td><td>Chitin deacetylase-like 4</td><td>BcDNA:RE51076|CG12447|CG13238|CG32499|CG9557|CG9558|DmCDA4|DmelCG32499</td><td>0</td><td>0</td></tr>
<tr><td>1781</td><td>33147</td><td>Drosophila melanogaster</td><td>CG12446</td><td>CG12446 gene product from transcript CG12446-RA</td><td>DmelCG12446</td><td>0</td><td>0</td></tr>
<tr><td>1782</td><td>33148</td><td>Drosophila melanogaster</td><td>fog</td><td>folded gastrulation</td><td>114|CG9559|CR41108|CR41109|CR41110|DmelCG9559|M67|l(1)114|l(1)16-3-190|l(1)20Ba|l114</td><td>0</td><td>0</td></tr>
<tr><td>1783</td><td>33155</td><td>Drosophila melanogaster</td><td>CG11023</td><td>CG11023 gene product from transcript CG11023-RB</td><td>DmelCG11023</td><td>0</td><td>0</td></tr>
<tr><td>1784</td><td>33156</td><td>Drosophila melanogaster</td><td>l(2)gl</td><td>lethal (2) giant larvae</td><td>CG2671|D-LGL|DmelCG2671|L(2)GL|L(2)gl|LGL|Lgl|MENE (2L)-B|dlgl|gl|l(2)|l(2) giant larva|l(2)giant larvae|l-gl|l[[2]]gl|lgl|p127|p127l(2)gl</td><td>0</td><td>0</td></tr>
<tr><td>1785</td><td>33157</td><td>Drosophila melanogaster</td><td>Ir21a</td><td>Ionotropic receptor 21a</td><td>CG2657|CT8983|DmelCG2657|IR21a</td><td>0</td><td>0</td></tr>
<tr><td>1786</td><td>33158</td><td>Drosophila melanogaster</td><td>Cda5</td><td>Chitin deacetylase-like 5</td><td>BcDNA:RH43162|CG2761|CG2776|CG31973|DmCDA5|DmelCG31973</td><td>0</td><td>0</td></tr>
<tr><td>1787</td><td>33161</td><td>Drosophila melanogaster</td><td>dbr</td><td>debra</td><td>BcDNA:LD26519|CG11371|Dbr|DmelCG11371|EP0456|EP456</td><td>0</td><td>0</td></tr>
<tr><td>1788</td><td>33162</td><td>Drosophila melanogaster</td><td>galectin</td><td>CG11372 gene product from transcript CG11372-RD</td><td>CG11372|DmelCG11372|Dmgal</td><td>0</td><td>0</td></tr>
<tr><td>1789</td><td>33163</td><td>Drosophila melanogaster</td><td>CG11374</td><td>CG11374 gene product from transcript CG11374-RC</td><td>DmelCG11374</td><td>0</td><td>0</td></tr>
<tr><td>1790</td><td>33165</td><td>Drosophila melanogaster</td><td>CG11376</td><td>CG11376 gene product from transcript CG11376-RA</td><td>Dm zir|DmelCG11376|FBgn 31216|anon-EST:Posey54</td><td>0</td><td>0</td></tr>
<tr><td>1791</td><td>33166</td><td>Drosophila melanogaster</td><td>CG11377</td><td>CG11377 gene product from transcript CG11377-RA</td><td>CT31762|DmelCG11377</td><td>0</td><td>0</td></tr>
<tr><td>1792</td><td>33167</td><td>Drosophila melanogaster</td><td>Nhe1</td><td>Na[+]/H[+] hydrogen exchanger 1</td><td>CG12178|DmNHE1|DmelCG12178|NHE1|nhe1</td><td>0</td><td>0</td></tr>
<tr><td>1793</td><td>33169</td><td>Drosophila melanogaster</td><td>CG13694</td><td>CG13694 gene product from transcript CG13694-RA</td><td>DmelCG13694</td><td>0</td><td>0</td></tr>
<tr><td>1794</td><td>33170</td><td>Drosophila melanogaster</td><td>CG4822</td><td>CG4822 gene product from transcript CG4822-RB</td><td>DmelCG4822</td><td>0</td><td>0</td></tr>
<tr><td>1795</td><td>33171</td><td>Drosophila melanogaster</td><td>CG3164</td><td>CG3164 gene product from transcript CG3164-RA</td><td>BEST:CK02656|CK02656|DmelCG3164</td><td>0</td><td>0</td></tr>
<tr><td>1796</td><td>33172</td><td>Drosophila melanogaster</td><td>Gs1</td><td>Glutamine synthetase 1</td><td>CG2718|DmelCG2718|GS1|GSI|GsI|fs(2)P52|fs(2)throng|gs-m|gsI|l(2)21Bc|mt-gs|thg</td><td>0</td><td>0</td></tr>
<tr><td>1797</td><td>33174</td><td>Drosophila melanogaster</td><td>CG31974</td><td>CG31974 gene product from transcript CG31974-RB</td><td>CG18501|DmelCG31974</td><td>0</td><td>0</td></tr>
<tr><td>1798</td><td>33175</td><td>Drosophila melanogaster</td><td>CG11454</td><td>CG11454 gene product from transcript CG11454-RA</td><td>DmelCG11454|cg11454</td><td>0</td><td>0</td></tr>
<tr><td>1799</td><td>33176</td><td>Drosophila melanogaster</td><td>CG42399</td><td>CG42399 gene product from transcript CG42399-RB</td><td>CG11620|CG4648|DmelCG42399|Dmel_CG11620|Dmel_CG4648</td><td>0</td><td>0</td></tr>
<tr><td>1800</td><td>33178</td><td>Drosophila melanogaster</td><td>CG3709</td><td>CG3709 gene product from transcript CG3709-RA</td><td>DmelCG3709|anon-WO0257455.7</td><td>0</td><td>0</td></tr>
<tr><td>1801</td><td>33179</td><td>Drosophila melanogaster</td><td>CG11455</td><td>CG11455 gene product from transcript CG11455-RF</td><td>DmelCG11455</td><td>0</td><td>0</td></tr>
<tr><td>1802</td><td>33180</td><td>Drosophila melanogaster</td><td>CG3436</td><td>CG3436 gene product from transcript CG3436-RA</td><td>DmelCG3436</td><td>0</td><td>0</td></tr>
<tr><td>1803</td><td>33182</td><td>Drosophila melanogaster</td><td>mRpL10</td><td>mitochondrial ribosomal protein L10</td><td>CG11488|DmelCG11488|L10</td><td>0</td><td>0</td></tr>
<tr><td>1804</td><td>33183</td><td>Drosophila melanogaster</td><td>CG11617</td><td>CG11617 gene product from transcript CG11617-RA</td><td>DmelCG11617|Mkx</td><td>0</td><td>0</td></tr>
<tr><td>1805</td><td>33184</td><td>Drosophila melanogaster</td><td>CG11490</td><td>CG11490 gene product from transcript CG11490-RA</td><td>DmelCG11490</td><td>0</td><td>0</td></tr>
<tr><td>1806</td><td>33185</td><td>Drosophila melanogaster</td><td>kis</td><td>kismet</td><td>136/31|2532|5841|BEST:GM02209|CG18326|CG3660|CG3696|DmelCG3696|EK2-4|GM02209|KIS|KIS-L|Kis|Su(Pc)21AB|anon-WO0172774.164|anon-WO0257455.5|kiss|l(2)07812|l(2)k08827|l(2)k11324|l(2</td><td>0</td><td>0</td></tr>
<tr><td>1807</td><td>33187</td><td>Drosophila melanogaster</td><td>CG13693</td><td>CG13693 gene product from transcript CG13693-RA</td><td>DmelCG13693</td><td>0</td><td>0</td></tr>
<tr><td>1808</td><td>33190</td><td>Drosophila melanogaster</td><td>CG3645</td><td>CG3645 gene product from transcript CG3645-RA</td><td>DmelCG3645</td><td>0</td><td>0</td></tr>
<tr><td>1809</td><td>33191</td><td>Drosophila melanogaster</td><td>CG17075</td><td>CG17075 gene product from transcript CG17075-RB</td><td>DmelCG17075</td><td>0</td><td>0</td></tr>
<tr><td>1810</td><td>33192</td><td>Drosophila melanogaster</td><td>CG3345</td><td>CG3345 gene product from transcript CG3345-RA</td><td>DmelCG3345|anon-WO0140519.207</td><td>0</td><td>0</td></tr>
<tr><td>1811</td><td>33193</td><td>Drosophila melanogaster</td><td>CG11555</td><td>CG11555 gene product from transcript CG11555-RA</td><td>DmelCG11555|anon-21Ca</td><td>0</td><td>0</td></tr>
<tr><td>1812</td><td>33194</td><td>Drosophila melanogaster</td><td>mbm</td><td>mushroom body miniature</td><td>CG11604|DmelCG11604|mus</td><td>0</td><td>0</td></tr>
<tr><td>1813</td><td>33195</td><td>Drosophila melanogaster</td><td>CG17078</td><td>CG17078 gene product from transcript CG17078-RB</td><td>DmelCG17078|anon-21Cb</td><td>0</td><td>0</td></tr>
<tr><td>1814</td><td>33196</td><td>Drosophila melanogaster</td><td>smo</td><td>smoothened</td><td>CG11561|DmelCG11561|SMO|Smo|dSmo</td><td>0</td><td>0</td></tr>
<tr><td>1815</td><td>33197</td><td>Drosophila melanogaster</td><td>CG11601</td><td>CG11601 gene product from transcript CG11601-RA</td><td>DmelCG11601</td><td>0</td><td>0</td></tr>
<tr><td>1816</td><td>33198</td><td>Drosophila melanogaster</td><td>CG3625</td><td>CG3625 gene product from transcript CG3625-RD</td><td>DmelCG3625</td><td>0</td><td>0</td></tr>
<tr><td>1817</td><td>33199</td><td>Drosophila melanogaster</td><td>CG11592</td><td>CG11592 gene product from transcript CG11592-RA</td><td>BEST:CK02467|CK02467|DmelCG11592</td><td>0</td><td>0</td></tr>
<tr><td>1818</td><td>33200</td><td>Drosophila melanogaster</td><td>CG11562</td><td>CG11562 gene product from transcript CG11562-RA</td><td>BcDNA:RE44650|DmelCG11562</td><td>0</td><td>0</td></tr>
<tr><td>1819</td><td>33201</td><td>Drosophila melanogaster</td><td>U2af38</td><td>U2 small nuclear riboprotein auxiliary factor 38</td><td>CG3582|DU2AF38|DmelCG3582|U2AF|U2AF38|dU2AF38|dU2AF[38]|l(2)06751</td><td>0</td><td>0</td></tr>
<tr><td>1820</td><td>33202</td><td>Drosophila melanogaster</td><td>Hop</td><td>Hsp70/Hsp90 organizing protein homolog</td><td>CG2720|DmelCG2720|EP(2)0418|Sti1|dHop|dSTI1|dhop|dmSTI1|l(2)k00616</td><td>0</td><td>0</td></tr>
<tr><td>1821</td><td>33203</td><td>Drosophila melanogaster</td><td>Pi3K21B</td><td>CG2699 gene product from transcript CG2699-RA</td><td>CG2699|DmelCG2699|Dp60|PI(3)K|PI3K|PI3K21B|PI[[3]]K|Pi3Kp60|dP60|dPI3K|droPIK57|p60</td><td>0</td><td>0</td></tr>
<tr><td>1822</td><td>33204</td><td>Drosophila melanogaster</td><td>Plc21C</td><td>Phospholipase C at 21C</td><td>CG4574|DmelCG4574|PLC|PLC-21|PLC-21C|PLC21C|PLC21c|PLC[[Beta]]|Plc21|Plcbeta21C|plc-21|plc21|plc21C|plcbeta21c</td><td>0</td><td>0</td></tr>
<tr><td>1823</td><td>33205</td><td>Drosophila melanogaster</td><td>CG11912</td><td>CG11912 gene product from transcript CG11912-RA</td><td>DmelCG11912|SP140|anon-WO0140519.112</td><td>0</td><td>0</td></tr>
<tr><td>1824</td><td>33206</td><td>Drosophila melanogaster</td><td>CG11911</td><td>CG11911 gene product from transcript CG11911-RA</td><td>DmelCG11911|SP103</td><td>0</td><td>0</td></tr>
<tr><td>1825</td><td>33207</td><td>Drosophila melanogaster</td><td>Ent1</td><td>Equilibrative nucleoside transporter 1</td><td>CG11907|DmENT1|DmelCG11907|anon-EST:fe2G2|dENT1</td><td>0</td><td>0</td></tr>
<tr><td>1826</td><td>33208</td><td>Drosophila melanogaster</td><td>al</td><td>aristaless</td><td>A1|AL|CG3935|DmelCG3935|L13-4|lambda13-4</td><td>0</td><td>0</td></tr>
<tr><td>1827</td><td>33209</td><td>Drosophila melanogaster</td><td>CG4213</td><td>CG4213 gene product from transcript CG4213-RA</td><td>DmelCG4213</td><td>0</td><td>0</td></tr>
<tr><td>1828</td><td>33210</td><td>Drosophila melanogaster</td><td>RpI135</td><td>RNA polymerase I 135kD subunit</td><td>CG4033|DmRP135|DmelCG4033|RP135|RpIII135|l(2)k16513</td><td>0</td><td>0</td></tr>
<tr><td>1829</td><td>33211</td><td>Drosophila melanogaster</td><td>alpha-Adaptin</td><td>CG4260 gene product from transcript CG4260-RA</td><td>AP-2|AP-2alpha|AP2|Alpha-Adaptin|CG31654|CG4260|D-Ada|D-alphaAda|DmelCG4260|MENE (2L)-A|MENE(2L)-A|ada|alpha|alpha-Ada|alpha-ada|alpha-adaptin|cg4260|dAP-2a|l(2)06694</td><td>0</td><td>0</td></tr>
<tr><td>1830</td><td>33212</td><td>Drosophila melanogaster</td><td>ebi</td><td>CG4063 gene product from transcript CG4063-RA</td><td>CG4063|DmelCG4063|Ebi|Tb11|Tbl1|l(2)k16213</td><td>0</td><td>0</td></tr>
<tr><td>1831</td><td>33213</td><td>Drosophila melanogaster</td><td>CG13690</td><td>CG13690 gene product from transcript CG13690-RA</td><td>DmelCG13690</td><td>0</td><td>0</td></tr>
<tr><td>1832</td><td>33214</td><td>Drosophila melanogaster</td><td>RpLP1</td><td>Ribosomal protein LP1</td><td>CG4087|DmelCG4087|LP1|M(2)21C|M(2)21C1-2|RLA1_DROME|RpA2|RpP2|Rplp1|rp21C|rpA2|rpP2|rpa2</td><td>0</td><td>0</td></tr>
<tr><td>1833</td><td>33215</td><td>Drosophila melanogaster</td><td>CG11885</td><td>CG11885 gene product from transcript CG11885-RA</td><td>BcDNA:AT13539|DmelCG11885</td><td>0</td><td>0</td></tr>
<tr><td>1834</td><td>33216</td><td>Drosophila melanogaster</td><td>CG13692</td><td>CG13692 gene product from transcript CG13692-RA</td><td>DmelCG13692</td><td>0</td><td>0</td></tr>
<tr><td>1835</td><td>33217</td><td>Drosophila melanogaster</td><td>BBS8</td><td>CG13691 gene product from transcript CG13691-RA</td><td>BBS2|CG13691|DmelCG13691</td><td>0</td><td>0</td></tr>
<tr><td>1836</td><td>33218</td><td>Drosophila melanogaster</td><td>ex</td><td>expanded</td><td>1270|CG4114|DmelCG4114|Ex|brl|l(2)01270|l(2)ey</td><td>0</td><td>0</td></tr>
<tr><td>1837</td><td>33219</td><td>Drosophila melanogaster</td><td>crq</td><td>croquemort</td><td>CD36|CG31655|CG4280|CRQ|Cd36|Cqr|Crq|D-CD36|DmelCG4280|anon-WO0140519.8|cqm|cqr</td><td>0</td><td>0</td></tr>
<tr><td>1838</td><td>33220</td><td>Drosophila melanogaster</td><td>CG4164</td><td>CG4164 gene product from transcript CG4164-RA</td><td>DmelCG4164</td><td>0</td><td>0</td></tr>
<tr><td>1839</td><td>33221</td><td>Drosophila melanogaster</td><td>CG4133</td><td>CG4133 gene product from transcript CG4133-RA</td><td>DmelCG4133</td><td>0</td><td>0</td></tr>
<tr><td>1840</td><td>33222</td><td>Drosophila melanogaster</td><td>CG4297</td><td>CG4297 gene product from transcript CG4297-RA</td><td>DmelCG4297</td><td>0</td><td>0</td></tr>
<tr><td>1841</td><td>33223</td><td>Drosophila melanogaster</td><td>MED15</td><td>Mediator complex subunit 15</td><td>Arc105|BcDNA:GH03922|CG4184|DmelCG4184|Med15|Med9/ARC105|dARC105|med15</td><td>0</td><td>0</td></tr>
<tr><td>1842</td><td>33224</td><td>Drosophila melanogaster</td><td>cbt</td><td>cabut</td><td>CG4427|Cabot|Cabut|DmelCG4427|EP(2)2237|EP2237|dTIEG</td><td>0</td><td>0</td></tr>
<tr><td>1843</td><td>33225</td><td>Drosophila melanogaster</td><td>ush</td><td>u-shaped</td><td>CG2762|DmelCG2762|FOG|Ush|dFOG|l(2)19|u-sh|ushaped</td><td>0</td><td>0</td></tr>
<tr><td>1844</td><td>33226</td><td>Drosophila melanogaster</td><td>lwr</td><td>lesswright</td><td>CG3018|DUbc9|DmUbc9|DmelCG3018|Dmubc9|FBgn0010602|Lwr|UBC9|Ubc 9|Ubc-9|Ubc9|dUBC9|dUbc9|dip4|hbl|i105|i56|l(2)01519|l(2)02858|l(2)05486|l(2)05487|semi|ubc9</td><td>0</td><td>0</td></tr>
<tr><td>1845</td><td>33227</td><td>Drosophila melanogaster</td><td>Spp</td><td>Signal peptide protease</td><td>CG11840|DmelCG11840|SPP|spp</td><td>0</td><td>0</td></tr>
<tr><td>1846</td><td>33228</td><td>Drosophila melanogaster</td><td>nAcRbeta-21C</td><td>nicotinic acetylcholine receptor beta 21C</td><td>CG11822|CT33131|Dbeta3|DmelCG11822|nAChR|nAcR-21Beta</td><td>0</td><td>0</td></tr>
<tr><td>1847</td><td>33229</td><td>Drosophila melanogaster</td><td>Ets21C</td><td>Ets at 21C</td><td>CG2914|D-Ets-6|D-Ets6|D-ets-6|DmelCG2914|ETS6|Ets|Ets6|ets21C|ets21c</td><td>0</td><td>0</td></tr>
<tr><td>1848</td><td>33230</td><td>Drosophila melanogaster</td><td>rempA</td><td>reduced mechanoreceptor potential A</td><td>CG11838|CHE-11|Che-11|DmelCG11838|IFT140|IFT40|OSEG|OSEG3|Oseg3|REMPA|oseg3|remp</td><td>0</td><td>0</td></tr>
<tr><td>1849</td><td>33231</td><td>Drosophila melanogaster</td><td>CG2789</td><td>CG2789 gene product from transcript CG2789-RA</td><td>DmelCG2789|anon-EST:Posey116</td><td>0</td><td>0</td></tr>
<tr><td>1850</td><td>33232</td><td>Drosophila melanogaster</td><td>CG11835</td><td>CG11835 gene product from transcript CG11835-RA</td><td>DmelCG11835</td><td>0</td><td>0</td></tr>
<tr><td>1851</td><td>33233</td><td>Drosophila melanogaster</td><td>CG2794</td><td>CG2794 gene product from transcript CG2794-RA</td><td>DmelCG2794</td><td>0</td><td>0</td></tr>
<tr><td>1852</td><td>33234</td><td>Drosophila melanogaster</td><td>Nle</td><td>Notchless</td><td>CG2863|DmelCG2863|anon-WO0026364|l(2)k13714</td><td>0</td><td>0</td></tr>
<tr><td>1853</td><td>33235</td><td>Drosophila melanogaster</td><td>CG2807</td><td>CG2807 gene product from transcript CG2807-RA</td><td>DmelCG2807|anon-21Cd</td><td>0</td><td>0</td></tr>
<tr><td>1854</td><td>33236</td><td>Drosophila melanogaster</td><td>Ipk2</td><td>CG13688 gene product from transcript CG13688-RA</td><td>CG13688|DmelCG13688|dmIpk2</td><td>0</td><td>0</td></tr>
<tr><td>1855</td><td>33237</td><td>Drosophila melanogaster</td><td>cold</td><td>coiled</td><td>CG2813|DmelCG2813|cg2813|leaky4</td><td>0</td><td>0</td></tr>
<tr><td>1856</td><td>33238</td><td>Drosophila melanogaster</td><td>Ptth</td><td>Prothoracicotropic hormone</td><td>CG13687|DmelCG13687|PTTH|ptth</td><td>0</td><td>0</td></tr>
<tr><td>1857</td><td>33239</td><td>Drosophila melanogaster</td><td>Pph13</td><td>PvuII-PstI homology 13</td><td>60Mun1|CG2819|DmelCG2819|Mu|PPH13</td><td>0</td><td>0</td></tr>
<tr><td>1858</td><td>33240</td><td>Drosophila melanogaster</td><td>Gsc</td><td>Goosecoid</td><td>60Mun1|60Mun2|CG2851|D-Gsc|D-gsc|DmelCG2851|M72|PPH25|Pph25|gsc|l(2)05341</td><td>0</td><td>0</td></tr>
<tr><td>1859</td><td>33241</td><td>Drosophila melanogaster</td><td>CG13689</td><td>CG13689 gene product from transcript CG13689-RA</td><td>DmelCG13689</td><td>0</td><td>0</td></tr>
<tr><td>1860</td><td>33242</td><td>Drosophila melanogaster</td><td>lectin-21Cb</td><td>CG13686 gene product from transcript CG13686-RB</td><td>AC 004573B|AC004573b|CG13686|DmelCG13686</td><td>0</td><td>0</td></tr>
<tr><td>1861</td><td>33244</td><td>Drosophila melanogaster</td><td>CG2839</td><td>CG2839 gene product from transcript CG2839-RA</td><td>DmelCG2839</td><td>0</td><td>0</td></tr>
<tr><td>1862</td><td>33245</td><td>Drosophila melanogaster</td><td>ds</td><td>dachsous</td><td>CG17941|CT39575|DmelCG17941|Ds|EP-822|cad2|dachs|l(2)05142</td><td>0</td><td>0</td></tr>
<tr><td>1863</td><td>33247</td><td>Drosophila melanogaster</td><td>Eaat2</td><td>Excitatory amino acid transporter 2</td><td>CG3159|DmelCG3159|EAAT|dEAAT2|dEaat2</td><td>0</td><td>0</td></tr>
<tr><td>1864</td><td>33248</td><td>Drosophila melanogaster</td><td>GABA-B-R3</td><td>metabotropic GABA-B receptor subtype 3</td><td>CG3022|D-GABA[[B]]R3|D-Gaba3|DmelCG3022|GABA[[B]]-R3|GB3|anon-WO0170980.13|anon-WO0170980.14</td><td>0</td><td>0</td></tr>
<tr><td>1865</td><td>33249</td><td>Drosophila melanogaster</td><td>CG12506</td><td>CG12506 gene product from transcript CG12506-RA</td><td>DmelCG12506|cg12506</td><td>0</td><td>0</td></tr>
<tr><td>1866</td><td>33250</td><td>Drosophila melanogaster</td><td>CG13947</td><td>CG13947 gene product from transcript CG13947-RA</td><td>DmelCG13947</td><td>0</td><td>0</td></tr>
<tr><td>1867</td><td>33251</td><td>Drosophila melanogaster</td><td>Gr21a</td><td>Gustatory receptor 21a</td><td>(GR) 21a|21D.1|CG13948|DmGr21a|DmelCG13948|GR21D.1|GR21D1|GR21a|Gr21D1</td><td>0</td><td>0</td></tr>
<tr><td>1868</td><td>33252</td><td>Drosophila melanogaster</td><td>CG3544</td><td>CG3544 gene product from transcript CG3544-RA</td><td>DmelCG3544</td><td>0</td><td>0</td></tr>
<tr><td>1869</td><td>33253</td><td>Drosophila melanogaster</td><td>Pkg21D</td><td>cGMP-dependent protein kinase 21D</td><td>CG3324|DG1|Dg1|DmelCG3324|PKG|PKG 21D|PKG-21D|Pkg1|cGMP|dg1</td><td>0</td><td>0</td></tr>
<tr><td>1870</td><td>33254</td><td>Drosophila melanogaster</td><td>KH1</td><td>CG3561 gene product from transcript CG3561-RA</td><td>CG3561|DmRH9|DmelCG3561|cg3561</td><td>0</td><td>0</td></tr>
<tr><td>1871</td><td>33255</td><td>Drosophila melanogaster</td><td>Saf6</td><td>SAGA factor-like TAF6</td><td>CG3883|DmelCG3883|SAF6|saf6</td><td>0</td><td>0</td></tr>
<tr><td>1872</td><td>33256</td><td>Drosophila melanogaster</td><td>pex12</td><td>peroxin 12</td><td>CG3639|DmelCG3639</td><td>0</td><td>0</td></tr>
<tr><td>1873</td><td>33257</td><td>Drosophila melanogaster</td><td>CG15880</td><td>CG15880 gene product from transcript CG15880-RA</td><td>DmelCG15880</td><td>0</td><td>0</td></tr>
<tr><td>1874</td><td>33258</td><td>Drosophila melanogaster</td><td>CG3876</td><td>CG3876 gene product from transcript CG3876-RA</td><td>DmelCG3876</td><td>0</td><td>0</td></tr>
<tr><td>1875</td><td>33259</td><td>Drosophila melanogaster</td><td>Clp</td><td>Clipper</td><td>CG3642|CLP|CPSF30|DmelCG3642|Ssb-c6a|c6a|clp</td><td>0</td><td>0</td></tr>
<tr><td>1876</td><td>33260</td><td>Drosophila melanogaster</td><td>CG3662</td><td>CG3662 gene product from transcript CG3662-RB</td><td>DmelCG3662</td><td>0</td><td>0</td></tr>
<tr><td>1877</td><td>33261</td><td>Drosophila melanogaster</td><td>CG3862</td><td>CG3862 gene product from transcript CG3862-RA</td><td>DmelCG3862</td><td>0</td><td>0</td></tr>
<tr><td>1878</td><td>33262</td><td>Drosophila melanogaster</td><td>dock</td><td>dreadlocks</td><td>CG3727|Dm0447|DmelCG3727|Dock|Nck|dck|doc|l(2)04723</td><td>0</td><td>0</td></tr>
<tr><td>1879</td><td>33263</td><td>Drosophila melanogaster</td><td>drongo</td><td>CG3365 gene product from transcript CG3365-RH</td><td>CG3365|DmelCG3365</td><td>0</td><td>0</td></tr>
<tr><td>1880</td><td>33264</td><td>Drosophila melanogaster</td><td>CG4291</td><td>CG4291 gene product from transcript CG4291-RA</td><td>DmelCG4291</td><td>0</td><td>0</td></tr>
<tr><td>1881</td><td>33265</td><td>Drosophila melanogaster</td><td>kraken</td><td>CG3943 gene product from transcript CG3943-RA</td><td>CG3943|DmelCG3943</td><td>0</td><td>0</td></tr>
<tr><td>1882</td><td>33266</td><td>Drosophila melanogaster</td><td>CG13949</td><td>CG13949 gene product from transcript CG13949-RA</td><td>BcDNA:AT06839|DmelCG13949</td><td>0</td><td>0</td></tr>
<tr><td>1883</td><td>33267</td><td>Drosophila melanogaster</td><td>CG13950</td><td>CG13950 gene product from transcript CG13950-RA</td><td>DmelCG13950</td><td>0</td><td>0</td></tr>
<tr><td>1884</td><td>33268</td><td>Drosophila melanogaster</td><td>aru</td><td>arouser</td><td>CG4276|DmelCG4276</td><td>0</td><td>0</td></tr>
<tr><td>1885</td><td>33269</td><td>Drosophila melanogaster</td><td>dbe</td><td>dribble</td><td>CG4258|DBE|DmelCG4258|l(2)k05428|l(2)k06708</td><td>0</td><td>0</td></tr>
</table>

<table>
<tr><td>1886</td><td>33270</td><td>Drosophila melanogaster</td><td>PNUTS</td><td>CG33526 gene product from transcript CG33526-RD</td><td>CG31657|CG33526|CG4124|DmelCG33526|PNUTS-RB|PNUTSDm</td><td>0</td><td>0</td></tr>
<tr><td>1887</td><td>33271</td><td>Drosophila melanogaster</td><td>ninaA</td><td>neither inactivation nor afterpotential A</td><td>CG3966|DmelCG3966|NinaA|ninA</td><td>0</td><td>0</td></tr>
<tr><td>1888</td><td>33272</td><td>Drosophila melanogaster</td><td>CG15824</td><td>CG15824 gene product from transcript CG15824-RA</td><td>DmelCG15824</td><td>0</td><td>0</td></tr>
<tr><td>1889</td><td>33274</td><td>Drosophila melanogaster</td><td>Lsp1beta</td><td>Larval serum protein 1 beta</td><td>CG4178|DmeLSP1b|DmelCG4178|LSP 1|LSP-1|LSP-1 beta|LSP-1beta|LSP1|LSP1beta|Lsp-1b|Lsp-1beta|Lsp1|Lsp1-beta|Lsp1[beta]|Lsp1b</td><td>0</td><td>0</td></tr>
<tr><td>1890</td><td>33275</td><td>Drosophila melanogaster</td><td>GluRIIC</td><td>Glutamate receptor IIC</td><td>CG4226|CT13021|DGluR-IIC|DGluRIIC|DGluRIII|DmelCG4226|GluR|GluR3|GluRII-C|GluRIIC/III|GluRIII|GlutRIII|glutamate receptor III</td><td>0</td><td>0</td></tr>
<tr><td>1891</td><td>33276</td><td>Drosophila melanogaster</td><td>CG4341</td><td>CG4341 gene product from transcript CG4341-RA</td><td>BcDNA:GH12144|DmelCG4341</td><td>0</td><td>0</td></tr>
<tr><td>1892</td><td>33277</td><td>Drosophila melanogaster</td><td>IA-2</td><td>IA-2 ortholog</td><td>CG11344|CG31795|CG4355|DmelCG31795|R-PTPX/1A2|anon-WO0153538.69|ia2</td><td>0</td><td>0</td></tr>
<tr><td>1893</td><td>33278</td><td>Drosophila melanogaster</td><td>CG4375</td><td>CG4375 gene product from transcript CG4375-RA</td><td>BcDNA:AT13819|DmelCG4375</td><td>0</td><td>0</td></tr>
<tr><td>1894</td><td>33279</td><td>Drosophila melanogaster</td><td>CG4415</td><td>CG4415 gene product from transcript CG4415-RA</td><td>DmelCG4415</td><td>0</td><td>0</td></tr>
<tr><td>1895</td><td>33281</td><td>Drosophila melanogaster</td><td>S</td><td>Star</td><td>CG4385|DmelCG4385|E(Raf)2B|EK2-2|ES2-4|EY2-4|Star/asteroid|fltr|l(2)05671|l(2)07056|l(2)21Eb|l(2)c00080|l(2)k09538|l(2)rK134|star</td><td>0</td><td>0</td></tr>
<tr><td>1896</td><td>33282</td><td>Drosophila melanogaster</td><td>ast</td><td>asteroid</td><td>CG4426|DmelCG4426|S[r]|Star/asteroid</td><td>0</td><td>0</td></tr>
<tr><td>1897</td><td>33283</td><td>Drosophila melanogaster</td><td>Atg4</td><td>Autophagy-specific gene 4</td><td>CG4428|DmelCG4428|atg4</td><td>0</td><td>0</td></tr>
<tr><td>1898</td><td>33284</td><td>Drosophila melanogaster</td><td>CG4629</td><td>CG4629 gene product from transcript CG4629-RA</td><td>DmelCG4629|anon-WO0257455.15</td><td>0</td><td>0</td></tr>
<tr><td>1899</td><td>33285</td><td>Drosophila melanogaster</td><td>mtRNApol</td><td>mitochondrial RNA polymerase</td><td>CG4644|DmelCG4644|mt RNA pol|mtRNA-pol</td><td>0</td><td>0</td></tr>
<tr><td>1900</td><td>33286</td><td>Drosophila melanogaster</td><td>CG14339</td><td>CG14339 gene product from transcript CG14339-RA</td><td>DmelCG14339</td><td>0</td><td>0</td></tr>
<tr><td>1901</td><td>33287</td><td>Drosophila melanogaster</td><td>CG14340</td><td>CG14340 gene product from transcript CG14340-RA</td><td>DmelCG14340</td><td>0</td><td>0</td></tr>
<tr><td>1902</td><td>33288</td><td>Drosophila melanogaster</td><td>Pino</td><td>Pinocchio</td><td>CG4710|DmelCG4710|anon-WO0118547.131|smi21F</td><td>0</td><td>0</td></tr>
<tr><td>1903</td><td>33289</td><td>Drosophila melanogaster</td><td>CG4552</td><td>CG4552 gene product from transcript CG4552-RA</td><td>DmelCG4552</td><td>0</td><td>0</td></tr>
<tr><td>1904</td><td>33290</td><td>Drosophila melanogaster</td><td>Iris</td><td>CG4715 gene product from transcript CG4715-RA</td><td>CG4715|CG4715-env|Dm-cF|DmelCG4715</td><td>0</td><td>0</td></tr>
<tr><td>1905</td><td>33291</td><td>Drosophila melanogaster</td><td>CG4577</td><td>CG4577 gene product from transcript CG4577-RA</td><td>DmelCG4577</td><td>0</td><td>0</td></tr>
<tr><td>1906</td><td>33292</td><td>Drosophila melanogaster</td><td>CG4726</td><td>CG4726 gene product from transcript CG4726-RA</td><td>DmelCG4726</td><td>0</td><td>0</td></tr>
<tr><td>1907</td><td>33293</td><td>Drosophila melanogaster</td><td>CG4749</td><td>CG4749 gene product from transcript CG4749-RA</td><td>DmelCG4749|anon-WO0118547.81</td><td>0</td><td>0</td></tr>
<tr><td>1908</td><td>33294</td><td>Drosophila melanogaster</td><td>Tfb4</td><td>CG5041 gene product from transcript CG5041-RA</td><td>CG5041|DmelCG5041|TFB4</td><td>0</td><td>0</td></tr>
<tr><td>1909</td><td>33295</td><td>Drosophila melanogaster</td><td>CG4764</td><td>CG4764 gene product from transcript CG4764-RA</td><td>DmelCG4764</td><td>0</td><td>0</td></tr>
<tr><td>1910</td><td>33298</td><td>Drosophila melanogaster</td><td>Tango14</td><td>Transport and Golgi organization 14</td><td>BcDNA:SD10843|CG4775|DmelCG4775|TANGO14|l(2)k00619</td><td>0</td><td>0</td></tr>
<tr><td>1911</td><td>33299</td><td>Drosophila melanogaster</td><td>CG5080</td><td>CG5080 gene product from transcript CG5080-RB</td><td>CT16297|DmelCG5080</td><td>0</td><td>0</td></tr>
<tr><td>1912</td><td>33300</td><td>Drosophila melanogaster</td><td>CG4785</td><td>CG4785 gene product from transcript CG4785-RA</td><td>DmelCG4785|Q9VPY0</td><td>0</td><td>0</td></tr>
<tr><td>1913</td><td>33301</td><td>Drosophila melanogaster</td><td>CG14341</td><td>CG14341 gene product from transcript CG14341-RB</td><td>DmelCG14341</td><td>0</td><td>0</td></tr>
<tr><td>1914</td><td>33303</td><td>Drosophila melanogaster</td><td>CG5118</td><td>CG5118 gene product from transcript CG5118-RA</td><td>DmelCG5118|anon-WO0140519.11</td><td>0</td><td>0</td></tr>
<tr><td>1915</td><td>33304</td><td>Drosophila melanogaster</td><td>CG4887</td><td>CG4887 gene product from transcript CG4887-RA</td><td>DmelCG4887|cg4887</td><td>0</td><td>0</td></tr>
<tr><td>1916</td><td>33305</td><td>Drosophila melanogaster</td><td>CG4896</td><td>CG4896 gene product from transcript CG4896-RC</td><td>DmelCG4896|cg4896</td><td>0</td><td>0</td></tr>
<tr><td>1917</td><td>33306</td><td>Drosophila melanogaster</td><td>CG5126</td><td>CG5126 gene product from transcript CG5126-RA</td><td>DmelCG5126</td><td>0</td><td>0</td></tr>
<tr><td>1918</td><td>33307</td><td>Drosophila melanogaster</td><td>Tgt</td><td>tRNA-guanine transglycosylase</td><td>CG4947|DmelCG4947|TGT</td><td>0</td><td>0</td></tr>
<tr><td>1919</td><td>33308</td><td>Drosophila melanogaster</td><td>CG5001</td><td>CG5001 gene product from transcript CG5001-RB</td><td>DmelCG5001</td><td>0</td><td>0</td></tr>
<tr><td>1920</td><td>33309</td><td>Drosophila melanogaster</td><td>CG5139</td><td>CG5139 gene product from transcript CG5139-RA</td><td>DmelCG5139</td><td>0</td><td>0</td></tr>
<tr><td>1921</td><td>33310</td><td>Drosophila melanogaster</td><td>CG14342</td><td>CG14342 gene product from transcript CG14342-RB</td><td>DmelCG14342</td><td>0</td><td>0</td></tr>
<tr><td>1922</td><td>33312</td><td>Drosophila melanogaster</td><td>CG42329</td><td>CG42329 gene product from transcript CG42329-RA</td><td>CG14343|CG5156|CT16503|DmelCG42329|Dmel_CG14343|Dmel_CG5156</td><td>0</td><td>0</td></tr>
<tr><td>1923</td><td>33313</td><td>Drosophila melanogaster</td><td>CG5397</td><td>CG5397 gene product from transcript CG5397-RA</td><td>CT16527|DmelCG5397</td><td>0</td><td>0</td></tr>
<tr><td>1924</td><td>33314</td><td>Drosophila melanogaster</td><td>robo3</td><td>CG5423 gene product from transcript CG5423-RA</td><td>CG14345|CG5423|CT17158|DmelCG5423|ROBO3|Robo 3|Robo-3|Robo3|dRobo-3</td><td>0</td><td>0</td></tr>
<tr><td>1925</td><td>33317</td><td>Drosophila melanogaster</td><td>a5</td><td>antennal protein 5</td><td>A5|CG5430|DmelCG5430</td><td>0</td><td>0</td></tr>
<tr><td>1926</td><td>33318</td><td>Drosophila melanogaster</td><td>CG5440</td><td>CG5440 gene product from transcript CG5440-RB</td><td>DmelCG5440</td><td>0</td><td>0</td></tr>
<tr><td>1927</td><td>33319</td><td>Drosophila melanogaster</td><td>Cdlc2</td><td>Cytoplasmic dynein light chain 2</td><td>CG5450|Cdcl2|Dlc|DmelCG5450|anon-WO0140519.117|dlc</td><td>0</td><td>0</td></tr>
<tr><td>1928</td><td>33320</td><td>Drosophila melanogaster</td><td>CG5556</td><td>CG5556 gene product from transcript CG5556-RA</td><td>DmelCG5556</td><td>0</td><td>0</td></tr>
<tr><td>1929</td><td>33321</td><td>Drosophila melanogaster</td><td>CG5561</td><td>CG5561 gene product from transcript CG5561-RA</td><td>DmelCG5561</td><td>0</td><td>0</td></tr>
<tr><td>1930</td><td>33323</td><td>Drosophila melanogaster</td><td>CG5565</td><td>CG5565 gene product from transcript CG5565-RA</td><td>DmelCG5565</td><td>0</td><td>0</td></tr>
<tr><td>1931</td><td>33324</td><td>Drosophila melanogaster</td><td>NLaz</td><td>Neural Lazarillo</td><td>BcDNA:RE67583|CG16933|CG33126|CT33980|DmelCG33126|Nlaz</td><td>0</td><td>0</td></tr>
<tr><td>1932</td><td>33325</td><td>Drosophila melanogaster</td><td>CG14346</td><td>CG14346 gene product from transcript CG14346-RA</td><td>DmelCG14346</td><td>0</td><td>0</td></tr>
<tr><td>1933</td><td>33330</td><td>Drosophila melanogaster</td><td>CG31661</td><td>CG31661 gene product from transcript CG31661-RA</td><td>CG10872|DmelCG31661</td><td>0</td><td>0</td></tr>
<tr><td>1934</td><td>33331</td><td>Drosophila melanogaster</td><td>CG18131</td><td>CG18131 gene product from transcript CG18131-RF</td><td>BcDNA:AT12853|DmelCG18131</td><td>0</td><td>0</td></tr>
<tr><td>1935</td><td>33332</td><td>Drosophila melanogaster</td><td>CG7420</td><td>CG7420 gene product from transcript CG7420-RA</td><td>DmelCG7420</td><td>0</td><td>0</td></tr>
<tr><td>1936</td><td>33333</td><td>Drosophila melanogaster</td><td>CG18132</td><td>CG18132 gene product from transcript CG18132-RA</td><td>DmelCG18132</td><td>0</td><td>0</td></tr>
<tr><td>1937</td><td>33334</td><td>Drosophila melanogaster</td><td>halo</td><td>CG7428 gene product from transcript CG7428-RA</td><td>CG7428|DmelCG7428</td><td>0</td><td>0</td></tr>
<tr><td>1938</td><td>33335</td><td>Drosophila melanogaster</td><td>Or22a</td><td>Odorant receptor 22a</td><td>22A.1|22a|AN11|CG12193|DOR22A.1|DOR22a|DOR53|Dmel Or22a|Dmel22a|DmelCG12193|Dor53|OR22a|Or22A.1|Or53|dOr22a|dor53</td><td>0</td><td>0</td></tr>
<tr><td>1939</td><td>33336</td><td>Drosophila melanogaster</td><td>Or22b</td><td>Odorant receptor 22b</td><td>22A.2|22b|AN12|CG4231|DOR22A.2|DOR22b|DOR67|DmelCG4231|Dor67|OR22b|Or22A.2|Or67|dOr22b|dor67</td><td>0</td><td>0</td></tr>
<tr><td>1940</td><td>33337</td><td>Drosophila melanogaster</td><td>CG10869</td><td>CG10869 gene product from transcript CG10869-RA</td><td>DmelCG10869</td><td>0</td><td>0</td></tr>
<tr><td>1941</td><td>33338</td><td>Drosophila melanogaster</td><td>CG31935</td><td>CG31935 gene product from transcript CG31935-RA</td><td>AAF51358|CG10870|CG7373|DmelCG31935</td><td>0</td><td>0</td></tr>
<tr><td>1942</td><td>33339</td><td>Drosophila melanogaster</td><td>haf</td><td>hattifattener</td><td>CG14351|CT33985|DmelCG14351|tartan/capricious-like</td><td>0</td><td>0</td></tr>
<tr><td>1943</td><td>33341</td><td>Drosophila melanogaster</td><td>CG14352</td><td>CG14352 gene product from transcript CG14352-RA</td><td>DmelCG14352</td><td>0</td><td>0</td></tr>
<tr><td>1944</td><td>33343</td><td>Drosophila melanogaster</td><td>chinmo</td><td>Chronologically inappropriate morphogenesis</td><td>CG10871|CG17156|CG17649|CG31666|Chinmo|DmelCG31666|F|l(2)04111</td><td>0</td><td>0</td></tr>
<tr><td>1945</td><td>33346</td><td>Drosophila melanogaster</td><td>cpb</td><td>capping protein beta</td><td>CAPB_DROME|CG17158|CP|CP-beta|CPB|CapZ|Cpb|DmelCG17158|J|bnd</td><td>0</td><td>0</td></tr>
<tr><td>1946</td><td>33347</td><td>Drosophila melanogaster</td><td>CG17660</td><td>CG17660 gene product from transcript CG17660-RA</td><td>DmelCG17660</td><td>0</td><td>0</td></tr>
<tr><td>1947</td><td>33348</td><td>Drosophila melanogaster</td><td>mRpL48</td><td>mitochondrial ribosomal protein L48</td><td>CG17642|DmelCG17642|MRP-L48</td><td>0</td><td>0</td></tr>
<tr><td>1948</td><td>33349</td><td>Drosophila melanogaster</td><td>frtz</td><td>fritz</td><td>CG17657|DmelCG17657|anon-WO0118547.105</td><td>0</td><td>0</td></tr>
<tr><td>1949</td><td>33350</td><td>Drosophila melanogaster</td><td>CG18317</td><td>CG18317 gene product from transcript CG18317-RD</td><td>DmelCG18317</td><td>0</td><td>0</td></tr>
<tr><td>1950</td><td>33351</td><td>Drosophila melanogaster</td><td>Eno</td><td>Enolase</td><td>BEST:LD15491|CG17654|CT32526|DmelCG17654|ENOA|Eno2|T18|anon-WO0153538.76|eno</td><td>0</td><td>0</td></tr>
<tr><td>1951</td><td>33353</td><td>Drosophila melanogaster</td><td>CG17652</td><td>CG17652 gene product from transcript CG17652-RA</td><td>DmelCG17652</td><td>0</td><td>0</td></tr>
<tr><td>1952</td><td>33354</td><td>Drosophila melanogaster</td><td>CG17646</td><td>CG17646 gene product from transcript CG17646-RB</td><td>ABCG1|BEST:GH05015|CG17647|DmelCG17646</td><td>0</td><td>0</td></tr>
<tr><td>1953</td><td>33356</td><td>Drosophila melanogaster</td><td>CG17712</td><td>CG17712 gene product from transcript CG17712-RA</td><td>BcDNA:GH03377|DmelCG17712</td><td>0</td><td>0</td></tr>
<tr><td>1954</td><td>33357</td><td>Drosophila melanogaster</td><td>CG17648</td><td>CG17648 gene product from transcript CG17648-RA</td><td>BcDNA:GH02930|DmelCG17648</td><td>0</td><td>0</td></tr>
<tr><td>1955</td><td>33358</td><td>Drosophila melanogaster</td><td>CG17650</td><td>CG17650 gene product from transcript CG17650-RA</td><td>DmelCG17650</td><td>0</td><td>0</td></tr>
<tr><td>1956</td><td>33359</td><td>Drosophila melanogaster</td><td>CG31664</td><td>CG31664 gene product from transcript CG31664-RB</td><td>CG15622|DmelCG31664</td><td>0</td><td>0</td></tr>
<tr><td>1957</td><td>33360</td><td>Drosophila melanogaster</td><td>c-cup</td><td>calcutta cup</td><td>CG15623|DmelCG15623</td><td>0</td><td>0</td></tr>
<tr><td>1958</td><td>33361</td><td>Drosophila melanogaster</td><td>wry</td><td>weary</td><td>CG12704|CG15621|CG31665|CT35769|DmelCG31665</td><td>0</td><td>0</td></tr>
<tr><td>1959</td><td>33363</td><td>Drosophila melanogaster</td><td>CG31663</td><td>CG31663 gene product from transcript CG31663-RB</td><td>BcDNA:GH27779|CG15359|CG15360|DmelCG31663</td><td>0</td><td>0</td></tr>
<tr><td>1960</td><td>33365</td><td>Drosophila melanogaster</td><td>CG7295</td><td>CG7295 gene product from transcript CG7295-RA</td><td>DmelCG7295</td><td>0</td><td>0</td></tr>
<tr><td>1961</td><td>33366</td><td>Drosophila melanogaster</td><td>CG15358</td><td>CG15358 gene product from transcript CG15358-RA</td><td>DmelCG15358</td><td>0</td><td>0</td></tr>
<tr><td>1962</td><td>33367</td><td>Drosophila melanogaster</td><td>CG7337</td><td>CG7337 gene product from transcript CG7337-RA</td><td>DmelCG7337</td><td>0</td><td>0</td></tr>
<tr><td>1963</td><td>33369</td><td>Drosophila melanogaster</td><td>Der-1</td><td>Derlin-1</td><td>CG10908|DmelCG10908</td><td>0</td><td>0</td></tr>
<tr><td>1964</td><td>33370</td><td>Drosophila melanogaster</td><td>CG15356</td><td>CG15356 gene product from transcript CG15356-RA</td><td>DmelCG15356|EMSY</td><td>0</td><td>0</td></tr>
<tr><td>1965</td><td>33371</td><td>Drosophila melanogaster</td><td>CG15362</td><td>CG15362 gene product from transcript CG15362-RA</td><td>DmelCG15362</td><td>0</td><td>0</td></tr>
<tr><td>1966</td><td>33372</td><td>Drosophila melanogaster</td><td>CG7289</td><td>CG7289 gene product from transcript CG7289-RA</td><td>DmelCG7289</td><td>0</td><td>0</td></tr>
<tr><td>1967</td><td>33373</td><td>Drosophila melanogaster</td><td>Got2</td><td>Glutamate oxaloacetate transaminase 2</td><td>152801_at|CG4233|DmelCG4233|Got|Got-2|got2</td><td>0</td><td>0</td></tr>
<tr><td>1968</td><td>33374</td><td>Drosophila melanogaster</td><td>Npc2a</td><td>Niemann-Pick type C-2a</td><td>BEST:GH08376|BcDNA:GH22047|BcDNA:RH53228|CG7291|CT22505|DmML3|DmelCG7291|NPC2|dnpc2a|npc2a</td><td>0</td><td>0</td></tr>
<tr><td>1969</td><td>33377</td><td>Drosophila melanogaster</td><td>CG4238</td><td>CG4238 gene product from transcript CG4238-RF</td><td>DmelCG4238</td><td>0</td><td>0</td></tr>
<tr><td>1970</td><td>33379</td><td>Drosophila melanogaster</td><td>Su(dx)</td><td>Suppressor of deltex</td><td>CG4244|DmelCG4244|Su(Dx)|Su(deltex)|anon-WO0073329.1|anon-WO0073329.2|anon-WO0073329.4|su(dx)</td><td>0</td><td>0</td></tr>
<tr><td>1971</td><td>33381</td><td>Drosophila melanogaster</td><td>Or22c</td><td>Odorant receptor 22c</td><td>22C.1|22c|CG15377|DOR16|DOR22C.1|DmelCG15377|Or22C.1</td><td>0</td><td>0</td></tr>
<tr><td>1972</td><td>33384</td><td>Drosophila melanogaster</td><td>CG12674</td><td>CG12674 gene product from transcript CG12674-RA</td><td>DmelCG12674</td><td>0</td><td>0</td></tr>
<tr><td>1973</td><td>33385</td><td>Drosophila melanogaster</td><td>CG4259</td><td>CG4259 gene product from transcript CG4259-RA</td><td>DmelCG4259|SPH173</td><td>0</td><td>0</td></tr>
<tr><td>1974</td><td>33386</td><td>Drosophila melanogaster</td><td>GlyP</td><td>Glycogen phosphorylase</td><td>BcDNA:LD24485|CG7254|DGPH|DmelCG7254|GP|Glp1|anon-WO0257455.33|l(2)k07918</td><td>0</td><td>0</td></tr>
<tr><td>1975</td><td>33388</td><td>Drosophila melanogaster</td><td>CG11723</td><td>CG11723 gene product from transcript CG11723-RA</td><td>DmelCG11723</td><td>0</td><td>0</td></tr>
<tr><td>1976</td><td>33389</td><td>Drosophila melanogaster</td><td>CG7261</td><td>CG7261 gene product from transcript CG7261-RA</td><td>BcDNA:LD22320|DmelCG7261</td><td>0</td><td>0</td></tr>
<tr><td>1977</td><td>33390</td><td>Drosophila melanogaster</td><td>AIF</td><td>Apoptosis inducing factor</td><td>CG7263|DmAIF|DmelCG7263</td><td>0</td><td>0</td></tr>
<tr><td>1978</td><td>33391</td><td>Drosophila melanogaster</td><td>CG15382</td><td>CG15382 gene product from transcript CG15382-RA</td><td>BcDNA:RE26473|DmelCG15382</td><td>0</td><td>0</td></tr>
<tr><td>1979</td><td>33392</td><td>Drosophila melanogaster</td><td>aop</td><td>anterior open</td><td>AOP|Aop|Aop/Yan|CG3166|DROYANET|DROYANETSB|DmelCG3166|SK2-1|YAN|Yan|aop/yan|fus6|pok|yan|yan/pok</td><td>0</td><td>0</td></tr>
<tr><td>1980</td><td>33394</td><td>Drosophila melanogaster</td><td>CG10874</td><td>CG10874 gene product from transcript CG10874-RB</td><td>DmelCG10874|FBgn 31395</td><td>0</td><td>0</td></tr>
<tr><td>1981</td><td>33395</td><td>Drosophila melanogaster</td><td>CG31668</td><td>CG31668 gene product from transcript CG31668-RC</td><td>CG10879|CG31668-PB|DmelCG31668</td><td>0</td><td>0</td></tr>
<tr><td>1982</td><td>33396</td><td>Drosophila melanogaster</td><td>CG15385</td><td>CG15385 gene product from transcript CG15385-RA</td><td>DmelCG15385</td><td>0</td><td>0</td></tr>
<tr><td>1983</td><td>33397</td><td>Drosophila melanogaster</td><td>Uch</td><td>Ubiquitin carboxy-terminal hydrolase</td><td>CG4265|DmelCG4265|UBL|UCH|UCH-D|UCH-D19|uch|uch-D</td><td>0</td><td>0</td></tr>
<tr><td>1984</td><td>33398</td><td>Drosophila melanogaster</td><td>CG10880</td><td>CG10880 gene product from transcript CG10880-RA</td><td>DmelCG10880</td><td>0</td><td>0</td></tr>
<tr><td>1985</td><td>33399</td><td>Drosophila melanogaster</td><td>mio</td><td>missing oocyte</td><td>CG15384|CG7074|DmelCG7074|Mio</td><td>0</td><td>0</td></tr>
<tr><td>1986</td><td>33401</td><td>Drosophila melanogaster</td><td>CG7082</td><td>CG7082 gene product from transcript CG7082-RA</td><td>DmelCG7082|cg7082</td><td>0</td><td>0</td></tr>
<tr><td>1987</td><td>33402</td><td>Drosophila melanogaster</td><td>l(2)s5379</td><td>lethal (2) s5379</td><td>BEST:LD28579|BcDNA:LD10657|CG7085|DmelCG7085</td><td>0</td><td>0</td></tr>
<tr><td>1988</td><td>33403</td><td>Drosophila melanogaster</td><td>CG15387</td><td>CG15387 gene product from transcript CG15387-RA</td><td>DmelCG15387</td><td>0</td><td>0</td></tr>
<tr><td>1989</td><td>33404</td><td>Drosophila melanogaster</td><td>CG31679</td><td>CG31679 gene product from transcript CG31679-RA</td><td>CG17006|DmelCG31679</td><td>0</td><td>0</td></tr>
<tr><td>1990</td><td>33405</td><td>Drosophila melanogaster</td><td>CG4267</td><td>CG4267 gene product from transcript CG4267-RA</td><td>DmelCG4267</td><td>0</td><td>0</td></tr>
<tr><td>1991</td><td>33406</td><td>Drosophila melanogaster</td><td>Ser12</td><td>Serine protease 12</td><td>CG17240|DmelCG17240|SP92</td><td>0</td><td>0</td></tr>
<tr><td>1992</td><td>33407</td><td>Drosophila melanogaster</td><td>CG17012</td><td>CG17012 gene product from transcript CG17012-RA</td><td>DmelCG17012|SP163</td><td>0</td><td>0</td></tr>
<tr><td>1993</td><td>33408</td><td>Drosophila melanogaster</td><td>CG4270</td><td>CG4270 gene product from transcript CG4270-RA</td><td>BcDNA:AT04879|DmelCG4270</td><td>0</td><td>0</td></tr>
<tr><td>1994</td><td>33409</td><td>Drosophila melanogaster</td><td>gho</td><td>ghost</td><td>BcDNA:LP05220|CG10882|DmelCG10882|FBgn0031408|Sec24|Sec24CD|sten</td><td>0</td><td>0</td></tr>
<tr><td>1995</td><td>33410</td><td>Drosophila melanogaster</td><td>CG4271</td><td>CG4271 gene product from transcript CG4271-RA</td><td>DmelCG4271|SPH164</td><td>0</td><td>0</td></tr>
<tr><td>1996</td><td>33411</td><td>Drosophila melanogaster</td><td>CG17237</td><td>CG17237 gene product from transcript CG17237-RA</td><td>BcDNA:AT15471|DmAAF51272|DmelCG17237</td><td>0</td><td>0</td></tr>
</table>

1997	33412	Drosophila melanogaster	robl22E	CG10838 gene product from transcript CG10838-RA	CG10838|DmelCG10838	0	0
1998	33413	Drosophila melanogaster	CG16995	CG16995 gene product from transcript CG16995-RA	DmelCG16995	0	0
1999	33414	Drosophila melanogaster	CG9967	CG9967 gene product from transcript CG9967-RA	DmelCG9967	0	0
2000	33418	Drosophila melanogaster	Rab5	Rab-protein 5	AAF51265|BAA88244|CG3664|DRab5|Dm Rab5|DmRab5|DmelCG3664|Drab5|RAB5|RAB5a|Rab|Rab-5|dRab5|l(2)k08232|rab5	0	0
2001	33419	Drosophila melanogaster	Axud1	CG4272 gene product from transcript CG4272-RA	BcDNA:GH09817|C676|CG4272|DAxud1|DmelCG4272	0	0
2002	33420	Drosophila melanogaster	CG3597	CG3597 gene product from transcript CG3597-RA	BVRA|DmelCG3597	0	0
2003	33421	Drosophila melanogaster	CG3609	CG3609 gene product from transcript CG3609-RA	CT12127|DmelCG3609	0	0
2004	33422	Drosophila melanogaster	CG15390	CG15390 gene product from transcript CG15390-RA	BcDNA:RE18748|DmelCG15390	0	0
2005	33423	Drosophila melanogaster	CG9866	CG9866 gene product from transcript CG9866-RA	DmelCG9866	0	0
2006	33424	Drosophila melanogaster	CG9867	CG9867 gene product from transcript CG9867-RA	DmelCG9867	0	0
2007	33425	Drosophila melanogaster	CG9870	CG9870 gene product from transcript CG9870-RA	DmelCG9870	0	0
2008	33426	Drosophila melanogaster	CG3557	CG3557 gene product from transcript CG3557-RA	DmelCG3557	0	0
2009	33427	Drosophila melanogaster	VGlut	Vesicular glutamate transporter	CG9887|DVGLUT|DVGluT|DVGlut|DmelCG9887|DvGluT|VGLUT|VGluT|dvGluT|dvglut|vGLUT|vGluT	0	0
2010	33429	Drosophila melanogaster	CG18641	CG18641 gene product from transcript CG18641-RC	CG11535|DmelCG18641	0	0
2011	33431	Drosophila melanogaster	CG9886	CG9886 gene product from transcript CG9886-RA	DmelCG9886	0	0
2012	33432	Drosophila melanogaster	dpp	decapentaplegic	BMP|CG9885|DPP|DPP-C|Dm-DPP|DmelCG9885|Dpp|Hin-d|M(2)23AB|M(2)LS1|TGF-b|TGF-beta|TGFbeta|Tg|blk|ho|l(2)10638|l(2)22Fa|l(2)k17036|shv	0	0
2013	33433	Drosophila melanogaster	CG15393	CG15393 gene product from transcript CG15393-RA	DmelCG15393	0	0
2014	33434	Drosophila melanogaster	Slh	SLY-1 homologous	CG3539|DmelCG3539|SLY1|Sly|Sly1|Sly1p|d-sly|slh	0	0
2015	33435	Drosophila melanogaster	oaf	out at first	CG9884|DmelCG9884|l(2)22Fb|l(2)ND1|tnd	0	0
2016	33436	Drosophila melanogaster	CG3528	CG3528 gene product from transcript CG3528-RA	DmelCG3528	0	0
2017	33437	Drosophila melanogaster	CG3515	CG3515 gene product from transcript CG3515-RA	DmelCG3515	0	0
2018	33438	Drosophila melanogaster	Cyp309a1	CG9964 gene product from transcript CG9964-RA	309a1|CG9964|DmelCG9964	0	0
2019	33439	Drosophila melanogaster	Cyp309a2	CG18559 gene product from transcript CG18559-RA	309a2|CG18559|DmelCG18559	0	0
2020	33440	Drosophila melanogaster	Rad1	Radiation insensitive 1	CG3240|DmelCG3240|Dromel_CG3240_FBtr0077747_rad1_mORF|Rad1[Dm]|rad1	0	0
2021	33441	Drosophila melanogaster	CG3227	CG3227 gene product from transcript CG3227-RA	BcDNA:RE55538|DmelCG3227|insv	0	0
2022	33442	Drosophila melanogaster	CG9883	CG9883 gene product from transcript CG9883-RA	DmelCG9883	0	0
2023	33443	Drosophila melanogaster	CG3214	CG3214 gene product from transcript CG3214-RB	B17.2|BcDNA:GH12382|DmelCG3214|anon-EST:Posey62	0	0
2024	33444	Drosophila melanogaster	p16-ARC	CG9881 gene product from transcript CG9881-RA	Arpc5|CG9881|DmelCG9881|P16-ARC|p16	0	0
2025	33445	Drosophila melanogaster	Drp1	Dynamin related protein 1	CG3210|DRP1|DmelCG3210|Dnm1/Drp1|Drp|Drp-1|drp-1|drp1|frat|l(2)22Fc|l(2)22Fd|l(2)ND2|l(2)ND3	0	0
2026	33446	Drosophila melanogaster	CG15394	CG15394 gene product from transcript CG15394-RB	DmelCG15394|anon-SAGE:Wang-060	0	0
2027	33447	Drosophila melanogaster	CG15395	CG15395 gene product from transcript CG15395-RB	DmelCG15395	0	0
2028	33448	Drosophila melanogaster	CG9962	CG9962 gene product from transcript CG9962-RA	DmelCG9962	0	0
2029	33449	Drosophila melanogaster	Prosbeta4R1	Proteasome beta4R1 subunit	BcDNA:AT30033|CG17301|DmelCG17301	0	0
2030	33450	Drosophila melanogaster	Or23a	Odorant receptor 23a	23A.1|23a|AN5|CG9880|DOR23A.1|DOR64|DmelCG9880|Dor64|Or23A.1|Or64|dor64	0	0
2031	33451	Drosophila melanogaster	Prosbeta4R2	Proteasome beta4R2 subunit	CG17302|DmelCG17302|anon-WO0140519.122	0	0
2032	33452	Drosophila melanogaster	CG9879	CG9879 gene product from transcript CG9879-RA	DmelCG9879	0	0
2033	33453	Drosophila melanogaster	Gr23a	Gustatory receptor 23a	23A.1a|23A1.b|23A1a|23A1b|CG15396|DmelCG15396|GR23A.1|Gr23A1a|Gr23A1b|Gr23a.a|Gr23a.b|Gr23aA|Gr23aB	0	0
2034	33454	Drosophila melanogaster	CG15398	CG15398 gene product from transcript CG15398-RB	DmelCG15398	0	0
2035	33455	Drosophila melanogaster	CG31690	CG31690 gene product from transcript CG31690-RD	CG11542|CG15397|DmelCG31690	0	0
2036	33458	Drosophila melanogaster	Hrs	Hepatocyte growth factor regulated tyrosine kinase su	CG2903|DmelCG2903|HRS|HRS-2|Vps27|hrs|l(2)23AB5|l(2)23Ad|l(2)23Ad(Hrs)|vps27	0	0
2037	33459	Drosophila melanogaster	CG9961	CG9961 gene product from transcript CG9961-RA	DmelCG9961	0	0
2038	33460	Drosophila melanogaster	Cwc25	CG2843 gene product from transcript CG2843-RA	CG2843|CWC25|DmelCG2843	0	0
2039	33461	Drosophila melanogaster	Pgk	Phosphoglycerate kinase	169C9S|CG3127|DmelCG3127|ESTS:169C9S|PGK|anon-sts18|l(2)23AB3|l(2)23Ac|pgk	0	0
2040	33462	Drosophila melanogaster	CG9894	CG9894 gene product from transcript CG9894-RC	DmelCG9894|anon-EST:Liang-2.54|clone 2.54	0	0
2041	33465	Drosophila melanogaster	Trn-SR	Transportin-Serine/Arginine rich	CG2848|DmelCG2848|anon-EST:Posey294|dTRN-SR	0	0
2042	33466	Drosophila melanogaster	CG3077	CG3077 gene product from transcript CG3077-RA	DmelCG3077	0	0
2043	33467	Drosophila melanogaster	aph-1	anterior pharynx defective 1	Aph-1|CG2855|Dm APH-1|DmelCG2855|Pen-1|aph|aph1|dAph-1|l(2)23AB6|l(2)23Ag|pen-1	0	0
2044	33468	Drosophila melanogaster	Taf10b	TBP-associated factor 10b	CG3069|DmelCG3069|TAF|TAF10b|TAFII16|TAF[[II]]|TAF[[II]]16|TFIID|Taf16|TafII16|dTAF10b|dTAF[[II]]16|dmTAF10b|dtafII16	0	0
2045	33469	Drosophila melanogaster	Taf10	TBP-associated factor 10	BcDNA:RE73934|CG2859|DmelCG2859|TAF|TAF10|TAF[[II]]|TAF[[II]]24|TFIID|Taf24|TafII24|dTAF10|dTAFII24|dTAF[[II]]24|dmTAF10|dmTaf10|dtafII24|taf24	0	0
2046	33470	Drosophila melanogaster	colt	congested-like trachea	CG3057|DmelCG3057	0	0
2047	33471	Drosophila melanogaster	CG2862	CG2862 gene product from transcript CG2862-RA	DmelCG2862	0	0
2048	33472	Drosophila melanogaster	CG15399	CG15399 gene product from transcript CG15399-RA	DmelCG15399	0	0
2049	33473	Drosophila melanogaster	Syt1	Synaptotagmin 1	CG3139|D. Syt I|DSYT|DSYT2|DSypt|DSyt|DmelCG3139|Droso1|Dsyt2|SYT|SYT I|SYT1|Syt|Syt 1|Syt I|Syt-1|SytI|dSYT|dsyt1|dsytI|l(2)23AB1|l(2)23Ba|l(2)k05909|syt|syt 1|syt I|syt-1|syt1|sytI	0	0
2050	33474	Drosophila melanogaster	daw	dawdle	ALP23B|Alp|Alp23B|Alp23b|CG16987|DAW|Daw|DmelCG16987|cg16987	0	0
2051	33475	Drosophila melanogaster	CG2964	CG2964 gene product from transcript CG2964-RA	DmelCG2964	0	0
2052	33476	Drosophila melanogaster	CG15400	CG15400 gene product from transcript CG15400-RB	DmelCG15400|G6pase	0	0
2053	33477	Drosophila melanogaster	Duox	Dual oxidase	CG3131|DUOX|DmelCG3131|dDuox	0	0
2054	33478	Drosophila melanogaster	CG3123	CG3123 gene product from transcript CG3123-RA	DmelCG3123	0	0
2055	33479	Drosophila melanogaster	CG3119	CG3119 gene product from transcript CG3119-RA	DmelCG3119	0	0
2056	33480	Drosophila melanogaster	Cpr23B	Cuticular protein 23B	CG2973|DmelCpr23B|DmelCG2973	0	0
2057	33481	Drosophila melanogaster	CG2975	CG2975 gene product from transcript CG2975-RA	DmelCG2975	0	0
2058	33482	Drosophila melanogaster	CG18558	CG18558 gene product from transcript CG18558-RB	DmelCG18558	0	0
2059	33483	Drosophila melanogaster	CG18557	CG18557 gene product from transcript CG18557-RA	DmelCG18557|c-SPH125	0	0
2060	33484	Drosophila melanogaster	CG3117	CG3117 gene product from transcript CG3117-RB	DmelCG3117|SPH94|c-SPH94	0	0
2061	33485	Drosophila melanogaster	CG2983	CG2983 gene product from transcript CG2983-RA	DmelCG2983	0	0
2062	33486	Drosophila melanogaster	CG3104	CG3104 gene product from transcript CG3104-RA	DmelCG3104	0	0
2063	33487	Drosophila melanogaster	RpS21	Ribosomal protein S21	CG2986|DmelCG2986|M(2)23B|l(2)03575|l(2)168/14|l(2)k16814|oho23|oho23B|rpS21	0	0
2064	33488	Drosophila melanogaster	CG2991	CG2991 gene product from transcript CG2991-RA	CT10107|CT42388|DmelCG2991	0	0
2065	33489	Drosophila melanogaster	CG31694	CG31694 gene product from transcript CG31694-RA	CG15401|CG3098|DmelCG31694	0	0
2066	33490	Drosophila melanogaster	CG8813	CG8813 gene product from transcript CG8813-RA	DmelCG8813|anon-WO0140519.96	0	0
2067	33492	Drosophila melanogaster	CG8814	CG8814 gene product from transcript CG8814-RA	DmelCG8814	0	0
2068	33493	Drosophila melanogaster	Prx6005	Peroxiredoxin 6005	153181_at|CG3083|DPx-6005|DPx6005|DmelCG3083	0	0
2069	33495	Drosophila melanogaster	NTPase	CG3059 gene product from transcript CG3059-RD	CG3059|DmelCG3059|NTPDase6|dmNTPase	0	0
2070	33496	Drosophila melanogaster	lilli	lilliputian	154454_at|189Y|BEST:GH09955|BcDNA:GM02019|CG8817|DmelCG8817|Lilli|S(ls)4|SS2-1|SY2-1|Su(Raf)2A|Su(Raf)2a|l(2)00632|l(2)23Cc|l(2)K00632|l(2)K07920|l(2)k07920|l(2)k16124	0	0
2071	33498	Drosophila melanogaster	Rbp9	RNA-binding protein 9	CG15402|CG3151|DmelCG3151|RBP9|RBP9-2|RRM10|RRM9|Rbp10|Rpb9|fes|fs(2)B|rbp|rbp9|rbp9a|rrm10|rrm9	0	0
2072	33499	Drosophila melanogaster	Ts	CG3181 gene product from transcript CG3181-RA	CG3181|DmelCG3181|FBgn0024920|TS|anon-23Ca|c1.5	0	0
2073	33500	Drosophila melanogaster	Rrp1	Recombination repair protein 1	3178|CG3178|DmelCG3178|RRP1|RRP1_DROME|Rrp-1|rrp	0	0
2074	33501	Drosophila melanogaster	gammaTub23C	gamma-Tubulin at 23C	CG 3157|CG3157|D.m.GAMMA-23C|DmelCG3157|GAMMA23C|GTU88*|GTUB|Tub23|g-Tubulin|gamma tubulin|gamma-Tub|gamma-Tub23C|gamma-tub|gamma-tub 23C|gamma-tub23|gamma-tub23C|ga	0	0
2075	33502	Drosophila melanogaster	CG9641	CG9641 gene product from transcript CG9641-RA	DmelCG9641	0	0
2076	33503	Drosophila melanogaster	CG3165	CG3165 gene product from transcript CG3165-RA	DmelCG3165	0	0
2077	33504	Drosophila melanogaster	CG9643	CG9643 gene product from transcript CG9643-RA	DmelCG9643	0	0
2078	33505	Drosophila melanogaster	Chd1	Chromodomain-helicase-DNA-binding protein 1	CG3733|CHD-1|CHD1|DmCHD1|DmelCG3733|chd1|dCHD1|l(2)23Cd	0	0
2079	33507	Drosophila melanogaster	okr	okra	CG3736|DhR54|DmRAD54|DmRad54|DmelCG3736|Dmrad54|RAD54|RAD54L|Rad54|Ras54|rad54	0	0
2080	33509	Drosophila melanogaster	CG17265	CG17265 gene product from transcript CG17265-RA	DmelCG17265	0	0
2081	33510	Drosophila melanogaster	CG17224	CG17224 gene product from transcript CG17224-RC	DmelCG17224	0	0
2082	33511	Drosophila melanogaster	CG17264	CG17264 gene product from transcript CG17264-RA	DmelCG17264	0	0
2083	33512	Drosophila melanogaster	alpha4GT1	CG17223 gene product from transcript CG17223-RA	CG17223|DmelCG17223	0	0
2084	33513	Drosophila melanogaster	CG3542	CG3542 gene product from transcript CG3542-RA	DmelCG3542	0	0
2085	33514	Drosophila melanogaster	CG3605	CG3605 gene product from transcript CG3605-RA	DmelCG3605	0	0
2086	33515	Drosophila melanogaster	CG17219	CG17219 gene product from transcript CG17219-RA	DmelCG17219	0	0
2087	33516	Drosophila melanogaster	GABPI	beta4GalNAcTB pilot	CG17257|DmelCG17257	0	0
2088	33517	Drosophila melanogaster	CG17258	CG17258 gene product from transcript CG17258-RB	DmelCG17258	0	0
2089	33518	Drosophila melanogaster	CG17259	CG17259 gene product from transcript CG17259-RA	DmSRS1|DmelCG17259|SerRS	0	0
2090	33519	Drosophila melanogaster	CG17260	CG17260 gene product from transcript CG17260-RA	DmelCG17260	0	0
2091	33520	Drosophila melanogaster	cnir	cornichon related	CG17262|Cnir|DmelCG17262	0	0
2092	33521	Drosophila melanogaster	CG17221	CG17221 gene product from transcript CG17221-RB	DM_7295851|DmelCG17221	0	0
2093	33522	Drosophila melanogaster	CG17261	CG17261 gene product from transcript CG17261-RA	DmelCG17261	0	0
2094	33524	Drosophila melanogaster	CG3523	CG3523 gene product from transcript CG3523-RA	BcDNA:GH07626|BcDNA:gh07626|DM_7295848|DmelCG3523|FAS|Fatty acid synthase|anon-EST:Posey235|anon-EST:Posey43|anon-WO0138581.1|dFAS|fas	0	0
2095	33526	Drosophila melanogaster	toc	toucan	BA34|BcDNA:LD27161|CG9660|DmelCG9660|Toc|l(2)01361|l(2)05527|l(2)K08224|l(2)k08224	0	0
2096	33527	Drosophila melanogaster	CG15403	CG15403 gene product from transcript CG15403-RA	DmelCG15403	0	0
2097	33528	Drosophila melanogaster	CG12400	CG12400 gene product from transcript CG12400-RA	BcDNA:RE29623|DmelCG12400	0	0
2098	33529	Drosophila melanogaster	Mad	Mothers against dpp	2/23|CG12399|DmelCG12399|E(zen)2|En(vvl)|MAD|Mat|P-Mad|P-mad|PMad|Smad|Smad1|apg|c28|dMad|l(2)K00237|l(2)k00237|mad|p-Mad|pMAD|pMad|pSmad	0	0
2099	33530	Drosophila melanogaster	gkt	glaikit	CG8825|CG8826|DmelCG8825|Tdp1|anon-23Db|c22	0	0
2100	33532	Drosophila melanogaster	Hydr2	alpha/beta hydrolase2	C24|CG3488|CT11757|DMU29170|DmelCG3488|HYDR2|anon-23D|anon-23Da|c24|cg3488	0	0
2101	33534	Drosophila melanogaster	CG34393	CG34393 gene product from transcript CG34393-RA	CG15405|CG8829|DmelCG34393|Dmel_CG15405|Dmel_CG8829	0	0
2102	33536	Drosophila melanogaster	CG34406	CG34406 gene product from transcript CG34406-RA	CG18555|CG3485|DmelCG34406|Dmel_CG18555|Dmel_CG3485|NAT1	0	0
2103	33537	Drosophila melanogaster	CG15404	CG15404 gene product from transcript CG15404-RA	DmelCG15404	0	0
2104	33538	Drosophila melanogaster	CG3347	CG3347 gene product from transcript CG3347-RB	DmelCG3347	0	0
2105	33539	Drosophila melanogaster	CG3332	CG3332 gene product from transcript CG3332-RB	DmelCG3332	0	0
2106	33540	Drosophila melanogaster	CG9664	CG9664 gene product from transcript CG9664-RA	DmelCG9664	0	0
2107	33541	Drosophila melanogaster	CG9663	CG9663 gene product from transcript CG9663-RA	DmelCG9663	0	0

<table>
<tr><td>2108</td><td>33542</td><td>Drosophila melanogaster</td><td>CG15406</td><td>CG15406 gene product from transcript CG15406-RA</td><td>DmelCG15406</td><td>0</td><td>0</td></tr>
<tr><td>2109</td><td>33543</td><td>Drosophila melanogaster</td><td>CG3277</td><td>CG3277 gene product from transcript CG3277-RC</td><td>DmelCG3277</td><td>0</td><td>0</td></tr>
<tr><td>2110</td><td>33544</td><td>Drosophila melanogaster</td><td>CG3326</td><td>CG3326 gene product from transcript CG3326-RA</td><td>DmCG3326|DmelCG3326</td><td>0</td><td>0</td></tr>
<tr><td>2111</td><td>33545</td><td>Drosophila melanogaster</td><td>CG8837</td><td>CG8837 gene product from transcript CG8837-RA</td><td>DmelCG8837</td><td>0</td><td>0</td></tr>
<tr><td>2112</td><td>33547</td><td>Drosophila melanogaster</td><td>CG3285</td><td>CG3285 gene product from transcript CG3285-RA</td><td>DmelCG3285</td><td>0</td><td>0</td></tr>
<tr><td>2113</td><td>33548</td><td>Drosophila melanogaster</td><td>CG15408</td><td>CG15408 gene product from transcript CG15408-RA</td><td>DmelCG15408</td><td>0</td><td>0</td></tr>
<tr><td>2114</td><td>33551</td><td>Drosophila melanogaster</td><td>CG8838</td><td>CG8838 gene product from transcript CG8838-RA</td><td>DmelCG8838|anon-WO0140519.135</td><td>0</td><td>0</td></tr>
<tr><td>2115</td><td>33552</td><td>Drosophila melanogaster</td><td>CG34394</td><td>CG34394 gene product from transcript CG34394-RF</td><td>BcDNA:RE58243|CG15411|CG3304|DmelCG34394|Dmel_CG15411|Dmel_CG3304</td><td>0</td><td>0</td></tr>
<tr><td>2116</td><td>33553</td><td>Drosophila melanogaster</td><td>CG15412</td><td>CG15412 gene product from transcript CG15412-RB</td><td>DmelCG15412</td><td>0</td><td>0</td></tr>
<tr><td>2117</td><td>33554</td><td>Drosophila melanogaster</td><td>CG9662</td><td>CG9662 gene product from transcript CG9662-RA</td><td>BcDNA:AT03617|DmelCG9662</td><td>0</td><td>0</td></tr>
<tr><td>2118</td><td>33555</td><td>Drosophila melanogaster</td><td>Ptpa</td><td>Phosphotyrosyl phosphatase activator</td><td>CG3289|DPTPA|DmelCG3289|PTPA</td><td>0</td><td>0</td></tr>
<tr><td>2119</td><td>33556</td><td>Drosophila melanogaster</td><td>pgant2</td><td>polypeptide GalNAc transferase 2</td><td>CG3254|DmelCG3254</td><td>0</td><td>0</td></tr>
<tr><td>2120</td><td>33557</td><td>Drosophila melanogaster</td><td>CG8840</td><td>CG8840 gene product from transcript CG8840-RA</td><td>DmelCG8840</td><td>0</td><td>0</td></tr>
<tr><td>2121</td><td>33559</td><td>Drosophila melanogaster</td><td>CG2772</td><td>CG2772 gene product from transcript CG2772-RA</td><td>BcDNA:RE45077|DmelCG2772</td><td>0</td><td>0</td></tr>
<tr><td>2122</td><td>33560</td><td>Drosophila melanogaster</td><td>CG2774</td><td>CG2774 gene product from transcript CG2774-RA</td><td>DmelCG2774</td><td>0</td><td>0</td></tr>
<tr><td>2123</td><td>33561</td><td>Drosophila melanogaster</td><td>CG12795</td><td>CG12795 gene product from transcript CG12795-RB</td><td>DmelCG12795</td><td>0</td><td>0</td></tr>
<tr><td>2124</td><td>33562</td><td>Drosophila melanogaster</td><td>Cog3</td><td>CG3248 gene product from transcript CG3248-RA</td><td>CG 3248|CG3248|COG3|DmelCG3248|SEC34|dCOG3</td><td>0</td><td>0</td></tr>
<tr><td>2125</td><td>33563</td><td>Drosophila melanogaster</td><td>sec5</td><td>CG8843 gene product from transcript CG8843-RA</td><td>CG8843|DmelCG8843|Sec-5|Sec5|Sec5p|dsec5</td><td>0</td><td>0</td></tr>
<tr><td>2126</td><td>33564</td><td>Drosophila melanogaster</td><td>CG3246</td><td>CG3246 gene product from transcript CG3246-RA</td><td>DmelCG3246</td><td>0</td><td>0</td></tr>
<tr><td>2127</td><td>33565</td><td>Drosophila melanogaster</td><td>msl-2</td><td>male-specific lethal 2</td><td>CG3241|DmelCG3241|MSL|MSL-2|MSL2|Msl 1-3|Msl-2|Msl2|km(2)A|kmA|msl|msl2</td><td>0</td><td>0</td></tr>
<tr><td>2128</td><td>33567</td><td>Drosophila melanogaster</td><td>CG3238</td><td>CG3238 gene product from transcript CG3238-RA</td><td>DmelCG3238</td><td>0</td><td>0</td></tr>
<tr><td>2129</td><td>33568</td><td>Drosophila melanogaster</td><td>CG31776</td><td>CG31776 gene product from transcript CG31776-RA</td><td>CG8845|CG8845a|DmelCG31776</td><td>0</td><td>0</td></tr>
<tr><td>2130</td><td>33569</td><td>Drosophila melanogaster</td><td>Thor</td><td>CG8846 gene product from transcript CG8846-RA</td><td>153432_at|43-BP|4E-BP|4E-BP1|4EBP|4e-bp|4eBP|4ebp|BcDNA.HL08053|BcDNA:HL08053|CG8846|CT8705|Dm 4E-BP|DmelCG8846|Phas1|anon-WO0172774.158|d4E-BP|d4E-BP1|d4EBP|d4e-bp|d4el</td><td>0</td><td>0</td></tr>
<tr><td>2131</td><td>33570</td><td>Drosophila melanogaster</td><td>CG15414</td><td>CG15414 gene product from transcript CG15414-RA</td><td>DmelCG15414</td><td>0</td><td>0</td></tr>
<tr><td>2132</td><td>33571</td><td>Drosophila melanogaster</td><td>tim</td><td>timeless</td><td>CG3234|DmelCG3234|Ritsu|TIM|Tim|dTIM|dTim|dtim|rit|tim1</td><td>0</td><td>0</td></tr>
<tr><td>2133</td><td>33572</td><td>Drosophila melanogaster</td><td>CG31954</td><td>CG31954 gene product from transcript CG31954-RA</td><td>CG3229|DmelCG31954|SP107</td><td>0</td><td>0</td></tr>
<tr><td>2134</td><td>33573</td><td>Drosophila melanogaster</td><td>CG17593</td><td>CG17593 gene product from transcript CG17593-RA</td><td>DmelCG17593</td><td>0</td><td>0</td></tr>
<tr><td>2135</td><td>33574</td><td>Drosophila melanogaster</td><td>CG3213</td><td>CG3213 gene product from transcript CG3213-RA</td><td>DmelCG3213|bs30c03.y1</td><td>0</td><td>0</td></tr>
<tr><td>2136</td><td>33575</td><td>Drosophila melanogaster</td><td>CG8851</td><td>CG8851 gene product from transcript CG8851-RA</td><td>DmelCG8851</td><td>0</td><td>0</td></tr>
<tr><td>2137</td><td>33576</td><td>Drosophila melanogaster</td><td>Sr-CIV</td><td>Scavenger receptor class C, type IV</td><td>CG3212|CT10789|DmelCG3212|SR-CIV</td><td>0</td><td>0</td></tr>
<tr><td>2138</td><td>33577</td><td>Drosophila melanogaster</td><td>CG8852</td><td>CG8852 gene product from transcript CG8852-RA</td><td>CT9284|DmelCG8852</td><td>0</td><td>0</td></tr>
<tr><td>2139</td><td>33578</td><td>Drosophila melanogaster</td><td>Spindly</td><td>CG15415 gene product from transcript CG15415-RB</td><td>CG15415|DmelCG15415</td><td>0</td><td>0</td></tr>
<tr><td>2140</td><td>33579</td><td>Drosophila melanogaster</td><td>CG8853</td><td>CG8853 gene product from transcript CG8853-RA</td><td>CHE-13|DmelCG8853|Hippi|IFT57</td><td>0</td><td>0</td></tr>
<tr><td>2141</td><td>33581</td><td>Drosophila melanogaster</td><td>sob</td><td>sister of odd and bowl</td><td>CG3242|DmelCG3242|Sob|org1</td><td>0</td><td>0</td></tr>
<tr><td>2142</td><td>33583</td><td>Drosophila melanogaster</td><td>odd</td><td>odd skipped</td><td>CG3851|DmelCG3851|ODD|Odd|l(2)01863|ods</td><td>0</td><td>0</td></tr>
<tr><td>2143</td><td>33585</td><td>Drosophila melanogaster</td><td>CG15418</td><td>CG15418 gene product from transcript CG15418-RA</td><td>DmelCG15418</td><td>0</td><td>0</td></tr>
<tr><td>2144</td><td>33589</td><td>Drosophila melanogaster</td><td>CG16704</td><td>CG16704 gene product from transcript CG16704-RA</td><td>BcDNA:RH31195|CT37177|DmelCG16704</td><td>0</td><td>0</td></tr>
<tr><td>2145</td><td>33590</td><td>Drosophila melanogaster</td><td>CG3513</td><td>CG3513 gene product from transcript CG3513-RA</td><td>DmelCG3513</td><td>0</td><td>0</td></tr>
<tr><td>2146</td><td>33591</td><td>Drosophila melanogaster</td><td>CG16713</td><td>CG16713 gene product from transcript CG16713-RA</td><td>CT37195|DmelCG16713</td><td>0</td><td>0</td></tr>
<tr><td>2147</td><td>33592</td><td>Drosophila melanogaster</td><td>CG16712</td><td>CG16712 gene product from transcript CG16712-RA</td><td>BcDNA:RH05411|BcDNA:RH38008|CT37183|DmelCG16712</td><td>0</td><td>0</td></tr>
<tr><td>2148</td><td>33593</td><td>Drosophila melanogaster</td><td>CG3604</td><td>CG3604 gene product from transcript CG3604-RA</td><td>BcDNA:LP04037|DmelCG3604</td><td>0</td><td>0</td></tr>
<tr><td>2149</td><td>33594</td><td>Drosophila melanogaster</td><td>CG10031</td><td>CG10031 gene product from transcript CG10031-RA</td><td>DmelCG10031</td><td>0</td><td>0</td></tr>
<tr><td>2150</td><td>33595</td><td>Drosophila melanogaster</td><td>CG2816</td><td>CG2816 gene product from transcript CG2816-RB</td><td>DmelCG2816</td><td>0</td><td>0</td></tr>
<tr><td>2151</td><td>33597</td><td>Drosophila melanogaster</td><td>CG2818</td><td>CG2818 gene product from transcript CG2818-RA</td><td>DmelCG2818</td><td>0</td><td>0</td></tr>
<tr><td>2152</td><td>33599</td><td>Drosophila melanogaster</td><td>Shaw</td><td>Shaker cognate w</td><td>CG15419|CG2822|DmelCG2822|Shaw2|dShaw|dShaw2|shaw</td><td>0</td><td>0</td></tr>
<tr><td>2153</td><td>33600</td><td>Drosophila melanogaster</td><td>CG10019</td><td>CG10019 gene product from transcript CG10019-RB</td><td>BEST:GH25970|DmelCG10019|GH25970</td><td>0</td><td>0</td></tr>
<tr><td>2154</td><td>33601</td><td>Drosophila melanogaster</td><td>CG31772</td><td>CG31772 gene product from transcript CG31772-RA</td><td>CG10020|DmelCG31772</td><td>0</td><td>0</td></tr>
<tr><td>2155</td><td>33602</td><td>Drosophila melanogaster</td><td>bowl</td><td>brother of odd with entrails limited</td><td>17-29-5|Bowl|CG10021|DmelCG10021|Su(tor)2-1|l(2)c|org2</td><td>0</td><td>0</td></tr>
<tr><td>2156</td><td>33603</td><td>Drosophila melanogaster</td><td>CG31958</td><td>CG31958 gene product from transcript CG31958-RA</td><td>CG10022|DmelCG31958</td><td>0</td><td>0</td></tr>
<tr><td>2157</td><td>33604</td><td>Drosophila melanogaster</td><td>CG3921</td><td>CG3921 gene product from transcript CG3921-RB</td><td>DmelCG3921</td><td>0</td><td>0</td></tr>
<tr><td>2158</td><td>33606</td><td>Drosophila melanogaster</td><td>CG3407</td><td>CG3407 gene product from transcript CG3407-RA</td><td>DmelCG3407|cg3407</td><td>0</td><td>0</td></tr>
<tr><td>2159</td><td>33607</td><td>Drosophila melanogaster</td><td>slp1</td><td>sloppy paired 1</td><td>CG16738|DmelCG16738|Dmslp1|FD6|FoxG|SLP1|Slp|Slp-1|Slp1|l(2)05965|sko1|slp|slp-1|slp-2</td><td>0</td><td>0</td></tr>
<tr><td>2160</td><td>33608</td><td>Drosophila melanogaster</td><td>slp2</td><td>sloppy paired 2</td><td>CG2939|DmelCG2939|Dmslp2|FD7|FoxG|Slp|slp</td><td>0</td><td>0</td></tr>
<tr><td>2161</td><td>33609</td><td>Drosophila melanogaster</td><td>CG3964</td><td>CG3964 gene product from transcript CG3964-RB</td><td>DmelCG3964</td><td>0</td><td>0</td></tr>
<tr><td>2162</td><td>33610</td><td>Drosophila melanogaster</td><td>Cep97</td><td>CG3980 gene product from transcript CG3980-RC</td><td>CG3980|CT13231|DCep97|DmelCG3980</td><td>0</td><td>0</td></tr>
<tr><td>2163</td><td>33611</td><td>Drosophila melanogaster</td><td>capu</td><td>cappuccino</td><td>CG15420|CG3399|Capu|DmelCG3399|capp</td><td>0</td><td>0</td></tr>
<tr><td>2164</td><td>33613</td><td>Drosophila melanogaster</td><td>fred</td><td>friend of echinoid</td><td>CG12675|CG31774|CG3390|CG3393|CT11413|DmelCG31774|Fred</td><td>0</td><td>0</td></tr>
<tr><td>2165</td><td>33615</td><td>Drosophila melanogaster</td><td>CG15422</td><td>CG15422 gene product from transcript CG15422-RA</td><td>DmelCG15422</td><td>0</td><td>0</td></tr>
<tr><td>2166</td><td>33616</td><td>Drosophila melanogaster</td><td>CG15423</td><td>CG15423 gene product from transcript CG15423-RA</td><td>DmelCG15423</td><td>0</td><td>0</td></tr>
<tr><td>2167</td><td>33617</td><td>Drosophila melanogaster</td><td>CG10039</td><td>CG10039 gene product from transcript CG10039-RA</td><td>DmelCG10039</td><td>0</td><td>0</td></tr>
<tr><td>2168</td><td>33619</td><td>Drosophila melanogaster</td><td>ed</td><td>echinoid</td><td>1X5|CG12676|CG15424|CG16842|CT13476|CT35484|DmelCG12676|E(Elp)24D|E(Elp)[24D]|Ed|l(2)k01102</td><td>0</td><td>0</td></tr>
<tr><td>2169</td><td>33621</td><td>Drosophila melanogaster</td><td>Sr-CI</td><td>Scavenger receptor class C, type I</td><td>CG4099|CT9876|D-SR-CI|DSR-CI|DmelCG4099|SR-C1|SR-CI|Sr-C1|Sr-CI|dSR-C1|dSR-CI|dSRC-I|dScR-I</td><td>0</td><td>0</td></tr>
<tr><td>2170</td><td>33622</td><td>Drosophila melanogaster</td><td>CG2955</td><td>CG2955 gene product from transcript CG2955-RA</td><td>DmelCG2955|anon-WO0140519.175</td><td>0</td><td>0</td></tr>
<tr><td>2171</td><td>33623</td><td>Drosophila melanogaster</td><td>Or24a</td><td>Odorant receptor 24a</td><td>24D.1|24a|CG11767|DOR24D.1|DOR48|DmelCG11767|Or24D.1</td><td>0</td><td>0</td></tr>
<tr><td>2172</td><td>33626</td><td>Drosophila melanogaster</td><td>CG3714</td><td>CG3714 gene product from transcript CG3714-RC</td><td>AAF51037|DmelCG3714</td><td>0</td><td>0</td></tr>
<tr><td>2173</td><td>33627</td><td>Drosophila melanogaster</td><td>ft</td><td>fat</td><td>79/18|CG3352|CT11259|Dm Fat|DmelCG3352|Fat|Ft|l(2)24Da|l(2)79/18|l(2)fat|l(2)fd|l(2)ft|l(2)gd-1|l(2)gd-l|l(2)gd2|l(2)k07918</td><td>0</td><td>0</td></tr>
<tr><td>2174</td><td>33628</td><td>Drosophila melanogaster</td><td>CG3702</td><td>CG3702 gene product from transcript CG3702-RA</td><td>DmelCG3702|cg3702</td><td>0</td><td>0</td></tr>
<tr><td>2175</td><td>33629</td><td>Drosophila melanogaster</td><td>RpL40</td><td>Ribosomal protein L40</td><td>BcDNA:RE10554|CEP52|CG2960|DUb52|DmelCG2960|Dub52|M(2)24D|Rpl40|Ub52|Ubi-f|Ubi-f52|Ubiq</td><td>0</td><td>0</td></tr>
<tr><td>2176</td><td>33630</td><td>Drosophila melanogaster</td><td>CG15425</td><td>CG15425 gene product from transcript CG15425-RA</td><td>DmelCG15425</td><td>0</td><td>0</td></tr>
<tr><td>2177</td><td>33631</td><td>Drosophila melanogaster</td><td>Art2</td><td>Arginine methyltransferase 2</td><td>CG3675|DART2|DmelCG3675</td><td>0</td><td>0</td></tr>
<tr><td>2178</td><td>33635</td><td>Drosophila melanogaster</td><td>CG16857</td><td>CG16857 gene product from transcript CG16857-RA</td><td>BcDNA:GH11322|CT35491|DmelCG16857|GH11322</td><td>0</td><td>0</td></tr>
<tr><td>2179</td><td>33636</td><td>Drosophila melanogaster</td><td>Atet</td><td>ABC transporter expressed in trachea</td><td>CG2969|DmelCG2969</td><td>0</td><td>0</td></tr>
<tr><td>2180</td><td>33637</td><td>Drosophila melanogaster</td><td>CG15429</td><td>CG15429 gene product from transcript CG15429-RA</td><td>BcDNA:AT23465|DmelCG15429</td><td>0</td><td>0</td></tr>
<tr><td>2181</td><td>33638</td><td>Drosophila melanogaster</td><td>Traf4</td><td>TNF-receptor-associated factor 4</td><td>CG3048|DTRAF|DTRAF1|DmelCG3048|TRAF|TRAF1|Traf1|dTRAF|dTRAF1|dTraf1|dtraf1|traf1|traf4</td><td>0</td><td>0</td></tr>
<tr><td>2182</td><td>33639</td><td>Drosophila melanogaster</td><td>CG17612</td><td>CG17612 gene product from transcript CG17612-RB</td><td>DmelCG17612</td><td>0</td><td>0</td></tr>
<tr><td>2183</td><td>33640</td><td>Drosophila melanogaster</td><td>CG3338</td><td>CG3338 gene product from transcript CG3338-RA</td><td>DmelCG3338</td><td>0</td><td>0</td></tr>
<tr><td>2184</td><td>33641</td><td>Drosophila melanogaster</td><td>Psf2</td><td>CG18013 gene product from transcript CG18013-RA</td><td>CG18013|DmelCG18013|P{lacW}l(2)SH0805[SH0805]|l(2)SH0805|l(2)SH2 0805</td><td>0</td><td>0</td></tr>
<tr><td>2185</td><td>33642</td><td>Drosophila melanogaster</td><td>Tps1</td><td>Trehalose-6-phosphate synthase 1</td><td>BcDNA:GH08860|CG4104|CG4104 PA|DmelCG4104|TPS|TreS|dtps1|jf5|l(2)24Ea|l(2)jf4|l(2)jf5|l(2)k08903|tps1</td><td>0</td><td>0</td></tr>
<tr><td>2186</td><td>33643</td><td>Drosophila melanogaster</td><td>CG3652</td><td>CG3652 gene product from transcript CG3652-RA</td><td>DmelCG3652</td><td>0</td><td>0</td></tr>
<tr><td>2187</td><td>33644</td><td>Drosophila melanogaster</td><td>l(2)k05819</td><td>lethal (2) k05819</td><td>CG3054|DmelCG3054|anon-WO0140519.156</td><td>0</td><td>0</td></tr>
<tr><td>2188</td><td>33645</td><td>Drosophila melanogaster</td><td>Dim1</td><td>CG3058 gene product from transcript CG3058-RA</td><td>BcDNA:RE13747|CG3058|DmelCG3058|U5-15kD</td><td>0</td><td>0</td></tr>
<tr><td>2189</td><td>33646</td><td>Drosophila melanogaster</td><td>CG15431</td><td>CG15431 gene product from transcript CG15431-RA</td><td>DmelCG15431</td><td>0</td><td>0</td></tr>
<tr><td>2190</td><td>33647</td><td>Drosophila melanogaster</td><td>CG15432</td><td>CG15432 gene product from transcript CG15432-RA</td><td>DmelCG15432</td><td>0</td><td>0</td></tr>
<tr><td>2191</td><td>33648</td><td>Drosophila melanogaster</td><td>morgue</td><td>modifier of rpr and grim, ubiquitously expressed</td><td>BcDNA:GH02435|CG15437|DmelCG15437|Morgue</td><td>0</td><td>0</td></tr>
<tr><td>2192</td><td>33649</td><td>Drosophila melanogaster</td><td>Elp3</td><td>Elongator complex protein 3</td><td>CG15433|D-elp3|Dmel/ELP3|DmelCG15433|DmelELP3|ELP3|HAT|dmHAG408|elp-3|elp3</td><td>0</td><td>0</td></tr>
<tr><td>2193</td><td>33650</td><td>Drosophila melanogaster</td><td>CG15438</td><td>CG15438 gene product from transcript CG15438-RA</td><td>BEST:CK02623|CK02623|DmelCG15438</td><td>0</td><td>0</td></tr>
<tr><td>2194</td><td>33651</td><td>Drosophila melanogaster</td><td>CG15439</td><td>CG15439 gene product from transcript CG15439-RA</td><td>DmelCG15439</td><td>0</td><td>0</td></tr>
<tr><td>2195</td><td>33652</td><td>Drosophila melanogaster</td><td>CG15440</td><td>CG15440 gene product from transcript CG15440-RA</td><td>DmelCG15440|Secp43|cg15440</td><td>0</td><td>0</td></tr>
<tr><td>2196</td><td>33653</td><td>Drosophila melanogaster</td><td>Gs1l</td><td>GS1-like</td><td>CG15441|DmelCG15441|GS1l|gs1-like</td><td>0</td><td>0</td></tr>
<tr><td>2197</td><td>33654</td><td>Drosophila melanogaster</td><td>RpL27A</td><td>Ribosomal protein L27A</td><td>CG15442|DmelCG15442|M|M(2)24F|M(2)24F-1|M(2)Z|M(2)z|M(2L)z|M[[z]]|Mz|RpL27Aa|RpL27Ab|RpL27a|RpL27a2|anon-EST:Posey257|l(2)24EFh|l(2)24Fb|rpL27a</td><td>0</td><td>0</td></tr>
<tr><td>2198</td><td>33655</td><td>Drosophila melanogaster</td><td>CG15435</td><td>CG15435 gene product from transcript CG15435-RA</td><td>DmelCG15435</td><td>0</td><td>0</td></tr>
<tr><td>2199</td><td>33656</td><td>Drosophila melanogaster</td><td>CG15443</td><td>CG15443 gene product from transcript CG15443-RA</td><td>DmelCG15443</td><td>0</td><td>0</td></tr>
<tr><td>2200</td><td>33657</td><td>Drosophila melanogaster</td><td>CG15436</td><td>CG15436 gene product from transcript CG15436-RA</td><td>DmelCG15436</td><td>0</td><td>0</td></tr>
<tr><td>2201</td><td>33658</td><td>Drosophila melanogaster</td><td>CG17840</td><td>CG17840 gene product from transcript CG17840-RA</td><td>DmelCG17840</td><td>0</td><td>0</td></tr>
<tr><td>2202</td><td>33659</td><td>Drosophila melanogaster</td><td>ine</td><td>inebriated</td><td>CG15444|CG15444d|DMINE|DmelCG15444|ine/rosA|rosA</td><td>0</td><td>0</td></tr>
<tr><td>2203</td><td>33661</td><td>Drosophila melanogaster</td><td>HP6</td><td>Heterochromatin protein 6</td><td>CG15636|DmelCG15636|Umbrea|umbrea</td><td>0</td><td>0</td></tr>
<tr><td>2204</td><td>33665</td><td>Drosophila melanogaster</td><td>CG15635</td><td>CG15635 gene product from transcript CG15635-RB</td><td>DmelCG15635</td><td>0</td><td>0</td></tr>
<tr><td>2205</td><td>33667</td><td>Drosophila melanogaster</td><td>CG3355</td><td>CG3355 gene product from transcript CG3355-RB</td><td>DmelCG3355|SP21</td><td>0</td><td>0</td></tr>
<tr><td>2206</td><td>33668</td><td>Drosophila melanogaster</td><td>CG11929</td><td>CG11929 gene product from transcript CG11929-RA</td><td>DmelCG11929</td><td>0</td><td>0</td></tr>
<tr><td>2207</td><td>33669</td><td>Drosophila melanogaster</td><td>Bsg25A</td><td>Blastoderm-specific gene 25A</td><td>CG12205|DmelCG12205|anon-EST:Liang-1.70|bsg25A|clone 1.70</td><td>0</td><td>0</td></tr>
<tr><td>2208</td><td>33670</td><td>Drosophila melanogaster</td><td>CG15634</td><td>CG15634 gene product from transcript CG15634-RA</td><td>DmelCG15634</td><td>0</td><td>0</td></tr>
<tr><td>2209</td><td>33671</td><td>Drosophila melanogaster</td><td>CG3251</td><td>CG3251 gene product from transcript CG3251-RA</td><td>DmelCG3251</td><td>0</td><td>0</td></tr>
<tr><td>2210</td><td>33672</td><td>Drosophila melanogaster</td><td>Taf12L</td><td>TBP-associated factor 30kD subunit alpha-2</td><td>CG15632|DmelCG15632|TAF|TAF12L|TAF30alpha-2|TAF[[II]]|TFIID|Taf30alpha-2|dTAF12|dTAF12L|dTAF[[II]]30a|dTAF[[II]]30alpha-2|dmTAF12L|dmTAF12b|rye</td><td>0</td><td>0</td></tr>
<tr><td>2211</td><td>33675</td><td>Drosophila melanogaster</td><td>CG15631</td><td>CG15631 gene product from transcript CG15631-RA</td><td>DmelCG15631</td><td>0</td><td>0</td></tr>
<tr><td>2212</td><td>33676</td><td>Drosophila melanogaster</td><td>CG15630</td><td>CG15630 gene product from transcript CG15630-RA</td><td>CG31970|CT35785|DmelCG15630</td><td>0</td><td>0</td></tr>
<tr><td>2213</td><td>33677</td><td>Drosophila melanogaster</td><td>CG3294</td><td>CG3294 gene product from transcript CG3294-RB</td><td>DmelCG3294|cg3294</td><td>0</td><td>0</td></tr>
<tr><td>2214</td><td>33678</td><td>Drosophila melanogaster</td><td>Clect27</td><td>C-type lectin 27kD</td><td>CG3244|DmelCG3244</td><td>0</td><td>0</td></tr>
<tr><td>2215</td><td>33679</td><td>Drosophila melanogaster</td><td>CG15629</td><td>CG15629 gene product from transcript CG15629-RA</td><td>DmelCG15629</td><td>0</td><td>0</td></tr>
<tr><td>2216</td><td>33680</td><td>Drosophila melanogaster</td><td>CG3225</td><td>CG3225 gene product from transcript CG3225-RA</td><td>DmelCG3225|cg3225</td><td>0</td><td>0</td></tr>
<tr><td>2217</td><td>33681</td><td>Drosophila melanogaster</td><td>CG15628</td><td>CG15628 gene product from transcript CG15628-RA</td><td>DmelCG15628|anon-WO02059370.23</td><td>0</td><td>0</td></tr>
<tr><td>2218</td><td>33682</td><td>Drosophila melanogaster</td><td>CG2976</td><td>CG2976 gene product from transcript CG2976-RA</td><td>DmelCG2976</td><td>0</td><td>0</td></tr>
</table>

2219	33683	Drosophila melanogaster	Ir25a	Ionotropic receptor 25a	CG15627|CT35778|DmelCG15627|IR25a	0	0
2220	33684	Drosophila melanogaster	CG15626	CG15626 gene product from transcript CG15626-RA	DmelCG15626	0	0
2221	33685	Drosophila melanogaster	CG12194	CG12194 gene product from transcript CG12194-RA	DmelCG12194|anon-EST:Posey105	0	0
2222	33686	Drosophila melanogaster	CG2950	CG2950 gene product from transcript CG2950-RD	BcDNA:GH06479|DmelCG2950|cg2950	0	0
2223	33687	Drosophila melanogaster	CG11927	CG11927 gene product from transcript CG11927-RA	DmelCG11927	0	0
2224	33688	Drosophila melanogaster	mRpS2	mitochondrial ribosomal protein S2	CG2937|DmelCG2937|MRP-S2	0	0
2225	33689	Drosophila melanogaster	CG11926	CG11926 gene product from transcript CG11926-RA	DmelCG11926	0	0
2226	33690	Drosophila melanogaster	pog	poor gastrulation	CG11923|CG11925|CG31660|DmelCG31660|anon-WO0170980.181|anon-WO0170980.182|pg	0	0
2227	33692	Drosophila melanogaster	Cf2	Chorion factor 2	BcDNA:GM09668|CF2|CF2.5|CG11924|DmelCG11924|cf2	0	0
2228	33693	Drosophila melanogaster	CG3008	CG3008 gene product from transcript CG3008-RA	DmelCG3008	0	0
2229	33694	Drosophila melanogaster	CG15625	CG15625 gene product from transcript CG15625-RA	DmelCG15625	0	0
2230	33695	Drosophila melanogaster	CG3036	CG3036 gene product from transcript CG3036-RA	151989_at|CT10168|DmelCG3036	0	0
2231	33696	Drosophila melanogaster	CG2837	CG2837 gene product from transcript CG2837-RB	DmelCG2837	0	0
2232	33700	Drosophila melanogaster	hoe2	hoepel2	CG15624|DmelCG15624	0	0
2233	33701	Drosophila melanogaster	Sgs1	Salivary gland secretion 1	CG3047|DmelCG3047|Muc25B|Sgs|Sgs-1|sgs-1|sgs1	0	0
2234	33702	Drosophila melanogaster	CG14044	CG14044 gene product from transcript CG14044-RA	DmelCG14044	0	0
2235	33703	Drosophila melanogaster	mRpL24	mitochondrial ribosomal protein L24	CG8849|DmelCG8849|L24|RpL24	0	0
2236	33704	Drosophila melanogaster	betaggt-I	beta subunit of type I geranylgeranyl transferase	CG3469|DmelCG3469|GGT1|SR2-2|beta GGT-I|beta-GGT|betaGGT-I|betaGGTI|bro3|ggt-1|ggt1	0	0
2237	33705	Drosophila melanogaster	jet	jetlag	CG8873|DmelCG8873|JET	0	0
2238	33706	Drosophila melanogaster	Jon25Biii	Jonah 25Biii	25B|CG8871|DmelCG8871|Jon25B|SP177	0	0
2239	33707	Drosophila melanogaster	Jon25Bii	Jonah 25Bii	25B|CG8869|DmelCG8869|Jon25B|SP112	0	0
2240	33708	Drosophila melanogaster	Jon25Bi	Jonah 25Bi	25B|CG8867|DmelCG8867|Jon25B|SP137|Ser4	0	0
2241	33709	Drosophila melanogaster	Marcal1	CG3753 gene product from transcript CG3753-RA	CG3753|DmelCG3753|Marca1	0	0
2242	33710	Drosophila melanogaster	Trip1	CG8882 gene product from transcript CG8882-RA	CG8882|DmTRIP|DmelCG8882|eIF-3beta|eIF3-S2	0	0
2243	33711	Drosophila melanogaster	Scox	Synthesis of cytochrome c oxidase	CG8885|DmelCG8885|SCO|SCOX|dSco1|l(2)SH1783|l(2)SH2 1783|scox	0	0
2244	33712	Drosophila melanogaster	CG3756	CG3756 gene product from transcript CG3756-RA	DmelCG3756	0	0
2245	33714	Drosophila melanogaster	CG14043	CG14043 gene product from transcript CG14043-RA	DmelCG14043	0	0
2246	33715	Drosophila melanogaster	mRpL28	mitochondrial ribosomal protein L28	CG3782|DmelCG3782|MRP-L28	0	0
2247	33716	Drosophila melanogaster	Gmd	GDP-mannose 4,6-dehydratase	CG8890|Dm-gmd|DmGMD|DmelCG8890|GMD|gmd|l(2)SH1606|l(2)SH2 1606	0	0
2248	33717	Drosophila melanogaster	CG3792	CG3792 gene product from transcript CG3792-RA	DmelCG3792|anon-EST:Posey8	0	0
2249	33718	Drosophila melanogaster	CG8891	CG8891 gene product from transcript CG8891-RA	DmelCG8891	0	0
2250	33719	Drosophila melanogaster	CG8892	CG8892 gene product from transcript CG8892-RC	DmelCG8892	0	0
2251	33721	Drosophila melanogaster	Rtnl1	CG33113 gene product from transcript CG33113-RF	142893_at|CG18623|CG33113|CG8895|DmelCG33113|G9|Rtnl-1|rtnl1	0	0
2252	33725	Drosophila melanogaster	CG3887	CG3887 gene product from transcript CG3887-RA	DmelCG3887	0	0
2253	33726	Drosophila melanogaster	vkg	viking	1209|6072|CG16858|CT25584|Coll IValpha2|DmColA2|DmelCG16858|Vkg|alpha(IV)2/vkg|coll. IV|l(2)01209	0	0
2254	33727	Drosophila melanogaster	Cg25C	Collagen type IV	CG25C|CG4145|CT12803|Cgc25|Cgc25C|Coll|Coll IV|DCg1|Dcg|Dcg1|DmelCG4145|alpha(IV)1/Cg25C|alpha1|cg25c|gp125	0	0
2255	33732	Drosophila melanogaster	CG9121	CG9121 gene product from transcript CG9121-RA	DmelCG9121	0	0
2256	33734	Drosophila melanogaster	CG14040	CG14040 gene product from transcript CG14040-RA	DmelCG14040	0	0
2257	33735	Drosophila melanogaster	CG14036	CG14036 gene product from transcript CG14036-RA	DmelCG14036	0	0
2258	33736	Drosophila melanogaster	His3.3A	Histone H3.3A	CG5825|DmelCG5825|H3.3|H3.3A|His-3.3A|His3.3|PH3|dH3.3A	0	0
2259	33738	Drosophila melanogaster	Rpn11	CG18174 gene product from transcript CG18174-RA	37B|CG18174|DmS13|DmelCG18174|RPN11|RPn11|S13|p37B|yip5	0	0
2260	33739	Drosophila melanogaster	qtc	quick-to-court	CG14039|DmelCG14039	0	0
2261	33742	Drosophila melanogaster	CG5828	CG5828 gene product from transcript CG5828-RA	DmelCG5828	0	0
2262	33743	Drosophila melanogaster	CG4230	CG4230 gene product from transcript CG4230-RA	DmelCG4230	0	0
2263	33744	Drosophila melanogaster	CG8680	CG8680 gene product from transcript CG8680-RA	DmelCG8680	0	0
2264	33748	Drosophila melanogaster	Cyp28d2	CG6081 gene product from transcript CG6081-RA	28d2|CG6081|DmelCG6081	0	0
2265	33749	Drosophila melanogaster	Cyp28d1	CG10833 gene product from transcript CG10833-RA	28d1|CG10833|DmelCG10833	0	0
2266	33750	Drosophila melanogaster	CG7742	CG7742 gene product from transcript CG7742-RA	DmelCG7742	0	0
2267	33751	Drosophila melanogaster	CG14034	CG14034 gene product from transcript CG14034-RB	DmelCG14034|anon-SAGE:Wang-122	0	0
2268	33752	Drosophila melanogaster	TpnC25D	Troponin C at 25D	BcDNA:RE53289|CG6514|DmTpnC25D|DmTpnCII|DmelCG6514|TNC25D|TnC|TpnC II|TpnCII|anon-EST:Posey103	0	0
2269	33753	Drosophila melanogaster	tkv	thickveins	Atkv|Atr25D|Brk25D|Brk25D1|Brk25D2|CG14026|DmelCG14026|Dtfr|STK-A|TKV|TKV1|Tkv|dtfr|kv|l(2)04415|l(2)25Da|str|tkv1	0	0
2270	33754	Drosophila melanogaster	Cyp4ac1	CG14032 gene product from transcript CG14032-RA	4ac1|CG14032|DmelCG14032|cyp4ac1	0	0
2271	33755	Drosophila melanogaster	Cyp4ac2	CG17970 gene product from transcript CG17970-RB	4ac2|CG17970|DmelCG17970	0	0
2272	33756	Drosophila melanogaster	Cyp4ac3	CG14031 gene product from transcript CG14031-RA	4ac3|CG14031|DmelCG14031	0	0
2273	33757	Drosophila melanogaster	Bsg25D	Blastoderm-specific gene 25D	CG14025|DmelCG14025|brg25D|bsg25D|bsq25D	0	0
2274	33758	Drosophila melanogaster	Bub1	Bub1 homologue	BUB1|BUB1-like|BubR1|BubRI|CG14030|CG14030/Bub1|DmelCG14030|bub1	0	0
2275	33759	Drosophila melanogaster	vri	vrille	CG14029|DmelCG14029|VRI|argo|jf23|l(2)25Db|l(2)jf23|mat(2)ea-G|mat(2)earlyRS32	0	0
2276	33760	Drosophila melanogaster	CG14024	CG14024 gene product from transcript CG14024-RA	DmelCG14024	0	0
2277	33761	Drosophila melanogaster	CG14023	CG14023 gene product from transcript CG14023-RD	DmelCG14023	0	0
2278	33763	Drosophila melanogaster	CG14022	CG14022 gene product from transcript CG14022-RA	BcDNA:RH62365|DmelCG14022	0	0
2279	33764	Drosophila melanogaster	TotM	Turandot M	CG14027|DmelCG14027|tot|totM	0	0
2280	33765	Drosophila melanogaster	fusl	fuseless	CG14021|DmelCG14021	0	0
2281	33766	Drosophila melanogaster	CG12512	CG12512 gene product from transcript CG12512-RA	DmelCG12512	0	0
2282	33768	Drosophila melanogaster	nompC	no mechanoreceptor potential C	CG11020|CG17468|CT30855|DmelCG11020|NOMPC|NompC|ORE-13|TRPN|TRPN1|jf24|l(2)25Dc|l(2)jf24|nomp	0	0
2283	33769	Drosophila melanogaster	H15	CG6604 gene product from transcript CG6604-RA	CG6604|Dm-H15|DmelCG6604|Nmr|Nmr-1|Tbx20|dm-H15|nmr|nmr1|nmr[H15]	0	0
2284	33770	Drosophila melanogaster	mid	midline	CG6634|DmelCG6634|H15r|H15r/nmr2|Mid(H15)|extra|los|nmr2	0	0
2285	33771	Drosophila melanogaster	CG14020	CG14020 gene product from transcript CG14020-RA	DmelCG14020	0	0
2286	33772	Drosophila melanogaster	CG7382	CG7382 gene product from transcript CG7382-RA	DmelCG7382	0	0
2287	33774	Drosophila melanogaster	CG7371	CG7371 gene product from transcript CG7371-RA	DmelCG7371	0	0
2288	33775	Drosophila melanogaster	CG6907	CG6907 gene product from transcript CG6907-RA	DmelCG6907	0	0
2289	33776	Drosophila melanogaster	CG31648	CG31648 gene product from transcript CG31648-RA	CG6922|DmelCG31648	0	0
2290	33777	Drosophila melanogaster	CG7277	CG7277 gene product from transcript CG7277-RA	DmelCG7277	0	0
2291	33778	Drosophila melanogaster	Cap-D3	Chromosome associated protein D3	BcDNA:RE74832|CAP-D3|CG14019|CG31989|CG6922|DmelCG31989|dCap-D3	0	0
2292	33779	Drosophila melanogaster	tomb	tombola	CG14016|DmelCG14016|Tomb	0	0
2293	33780	Drosophila melanogaster	CG14015	CG14015 gene product from transcript CG14015-RA	DmelCG14015	0	0
2294	33781	Drosophila melanogaster	Hel25E	Helicase at 25E	CG7269|Dbp25F|DmRH4|DmelCG7269|HEL|Hel|UAP56|WM6|Wm6|hel|jf26|l(2)25Eb|l(2)gdh-12|l(2)gdh12|l(2)jf26|l(2)k11511|sz|uap56	0	0
2295	33782	Drosophila melanogaster	Lam	Lamin	2459|74/76|CG6944|D5|DM[[O]]|Dm|Dm(0)|Dm0|Dm1|Dm2|DmLamin|DmO|Dm[[0]]|Dm[[1]]|Dm[[2]]|Dm[[mit]]|Dm[[o]]|DmelCG6944|Dmo|LAM|Lam Dm[[0]]|Lam(Dm0)|LamDm[[0]]|Lam[[Dm0]]|Lamin Dm[[0]]|f2	0	0
2296	33783	Drosophila melanogaster	Oscillin	CG6957 gene product from transcript CG6957-RC	CG6957|DmelCG6957	0	0
2297	33784	Drosophila melanogaster	CG14014	CG14014 gene product from transcript CG14014-RB	DmelCG14014	0	0
2298	33785	Drosophila melanogaster	CG18269	CG18269 gene product from transcript CG18269-RA	DmelCG18269	0	0
2299	33786	Drosophila melanogaster	CG14013	CG14013 gene product from transcript CG14013-RA	DmelCG14013	0	0
2300	33787	Drosophila melanogaster	CG14017	CG14017 gene product from transcript CG14017-RA	DmelCG14017	0	0
2301	33788	Drosophila melanogaster	GluRIIA	Glutamate receptor IIA	CG6992|D-GluRIIA|DGluR-II|DGluR-IIA|DGluR2a|DGluRIIA|DGluRIIa|DgluRII|DmelCG6992|Glu-RII|Glu-RIIA|GluR|GluR-IIA|GluR2a|GluRII|GluRII-A|GluRIIa|GlurIIA|dGluRII|dGluRIIA|dglur-IIA|dglurIIA|glurIIA|g	0	0
2302	33789	Drosophila melanogaster	GluRIIB	Glutamate receptor IIB	CG7234|DGluIIB|DGluR-IIB|DGluRIIB|DmelCG7234|Glu-RIIB|GluRIIb|dGluRIIB|dglurIIB|glurIIB	0	0
2303	33790	Drosophila melanogaster	CG14011	CG14011 gene product from transcript CG14011-RC	DmelCG14011	0	0
2304	33791	Drosophila melanogaster	CG7251	CG7251 gene product from transcript CG7251-RA	DmelCG7251|anon-WO0140519.103|bs11h10.y1	0	0
2305	33792	Drosophila melanogaster	CG18266	CG18266 gene product from transcript CG18266-RA	DmelCG18266|anon-WO0140519.54	0	0
2306	33793	Drosophila melanogaster	CG14010	CG14010 gene product from transcript CG14010-RC	CT33567|DmelCG14010	0	0
2307	33795	Drosophila melanogaster	CG31646	CG31646 gene product from transcript CG31646-RA	CG14008|CG14009|CT33566|DmelCG31646	0	0
2308	33796	Drosophila melanogaster	Hsp60C	CG7235 gene product from transcript CG7235-RA	CG7235|DmelCG7235|bs36d10.y1	0	0
2309	33797	Drosophila melanogaster	CG12511	CG12511 gene product from transcript CG12511-RC	DmelCG12511	0	0
2310	33798	Drosophila melanogaster	CG7236	CG7236 gene product from transcript CG7236-RA	AC018104|DmelCG7236|KKIALRE	0	0
2311	33799	Drosophila melanogaster	CG14007	CG14007 gene product from transcript CG14007-RB	DmelCG14007	0	0
2312	33800	Drosophila melanogaster	CG11149	CG11149 gene product from transcript CG11149-RD	DmelCG11149	0	0
2313	33802	Drosophila melanogaster	CG14006	CG14006 gene product from transcript CG14006-RA	DmelCG14006	0	0
2314	33803	Drosophila melanogaster	CG11147	CG11147 gene product from transcript CG11147-RA	DmelCG11147	0	0
2315	33804	Drosophila melanogaster	CG11029	CG11029 gene product from transcript CG11029-RA	DmelCG11029	0	0
2316	33805	Drosophila melanogaster	CG11030	CG11030 gene product from transcript CG11030-RA	DmelCG11030	0	0
2317	33806	Drosophila melanogaster	obst-E	obstructor-E	CG11142|DmelCG11142	0	0
2318	33807	Drosophila melanogaster	CG9171	CG9171 gene product from transcript CG9171-RC	DmelCG9171	0	0
2319	33808	Drosophila melanogaster	CG14005	CG14005 gene product from transcript CG14005-RA	DmelCG14005	0	0
2320	33809	Drosophila melanogaster	CG7239	CG7239 gene product from transcript CG7239-RA	DmelCG7239|anon-EST:Posey250	0	0
2321	33810	Drosophila melanogaster	CG11034	CG11034 gene product from transcript CG11034-RB	DmelCG11034	0	0
2322	33812	Drosophila melanogaster	CG34381	CG34381 gene product from transcript CG34381-RB	CG14002|CG14003|CG14004|CG31645|DmelCG34381|Dmel_CG14004|Dmel_CG31645|anon-WO0170980.172|anon-WO0170980.173	0	0
2323	33814	Drosophila melanogaster	CG8965	CG8965 gene product from transcript CG8965-RA	DmelCG8965	0	0
2324	33815	Drosophila melanogaster	CG9029	CG9029 gene product from transcript CG9029-RA	DmelCG9029	0	0
2325	33816	Drosophila melanogaster	Acp26Ab	Accessory gland-specific peptide 26Ab	Acp|Acp26A|Acp26Aab|Acp26a|CG9024|DmelCG9024|Mst26A|Mst26Ab|acp26Ab|msP355b|mst 355b|mst355b	0	0
2326	33817	Drosophila melanogaster	Acp26Aa	Accessory gland-specific peptide 26Aa	Acp|Acp26A|Acp26Aab|Acp26a|CG8982|DmelCG8982|Mst26A|Mst26Aa|Ovulin|msP355a|mst 355a|mst355a	0	0
2327	33818	Drosophila melanogaster	CG9021	CG9021 gene product from transcript CG9021-RA	DmelCG9021	0	0
2328	33819	Drosophila melanogaster	bchs	blue cheese	BcDNA:SD12984|Bchs|Beach1|CG14001|CG9011|DBEACH1|DmelCG14001|EP2299|alfy|bchd	0	0
2329	33820	Drosophila melanogaster	CG14000	CG14000 gene product from transcript CG14000-RA	DmelCG14000	0	0

2330	33822	Drosophila melanogaster	CG9016	CG9016 gene product from transcript CG9016-RB	BcDNA:AT19175|DmelCG9016|NEST:bs09a09|NEST:bs20d08|NEST:bs24f02|NEST:bs36a03|bs03c03.y1|bs05g03.y1|bs07e11.y1|bs09c06.y1|bs10d11.y1|bs10f09.y1|bs13h06.y1|bs14b11.y1|bs16c10.y1|b	0	0
2331	33823	Drosophila melanogaster	dsf	dissatisfaction	CG9019|DSF|DmelCG9019|NR2E4	0	0
2332	33824	Drosophila melanogaster	Gpdh	Glycerol 3 phosphate dehydrogenase	CG9042|DROGPDHA|DmG3PDH|DmelCG9042|G3PDH|GAPDH|GPD|GPDA|GPDH|GPDH-1|Gdh|Gpd|alpha-GPD|alpha-GPDH|alpha-GPDH-1|alpha-Gpdh|alphaGPDH|alphaGpd|alphaGpdh|alphaGpdh-1|	0	0
2333	33825	Drosophila melanogaster	CG9044	CG9044 gene product from transcript CG9044-RB	CT25972|DmelCG9044	0	0
2334	33826	Drosophila melanogaster	CG13999	CG13999 gene product from transcript CG13999-RA	DmelCG13999	0	0
2335	33827	Drosophila melanogaster	Vm26Ab	Vitelline membrane 26Ab	CG9046|Dm sV23|DmelCG9046|Sv23|TU-4|VM26A.2|Vm26A|fs(2)QJ42|sV23|sV26|sv23	0	0
2336	33828	Drosophila melanogaster	Vm26Ac	Vitelline membrane 26Aac	CG13997|DmelCG13997|Fcp26Ac|TU-3|fc20	0	0
2337	33829	Drosophila melanogaster	Vm26Aa	Vitelline membrane 26Aa	CG9048|DmelCG9048|TU-2|VM26A.1|VM26Aa|Vm26A|Vm26A.1|Vm26A1|sV17	0	0
2338	33830	Drosophila melanogaster	psd	palisade	BcDNA:GM32356|CG9050|DmelCG9050|Fcp26Aa|TU-1	0	0
2339	33831	Drosophila melanogaster	CG13992	CG13992 gene product from transcript CG13992-RC	DmelCG13992	0	0
2340	33832	Drosophila melanogaster	Ucp4C	CG9064 gene product from transcript CG9064-RA	CG9064|DmUCP4C|DmelCG9064|UCP4c|Ucp4c|anon-WO02079478.1|anon-WO02079478.2|anon-WO0242455.3|anon-WO0242455.4|anon-WO03037362.7|anon-WO03061681.1|anon-WO03061681.2|d	0	0
2341	33833	Drosophila melanogaster	Ucp4B	CG18340 gene product from transcript CG18340-RA	CG18340|DmUCP4B|DmelCG18340|dmUCP4b	0	0
2342	33834	Drosophila melanogaster	chic	chickadee	CG9553|Chi|Chic|D88|DmelCG9553|GH4|chi|l(2)27/7|profilin|sand	0	0
2343	33835	Drosophila melanogaster	eIF-4a	Eukaryotic initiation factor 4a	2439|9075|CG9075|Dm-eIF4A|DmRH2|DmelCG9075|EIF-4A|EiF4A|Eif-4a|Eif4a|L(2L)162|Su(dpp)YE9|anon-26Aa|anon26A|deIF4A|eIF 4A|eIF-4A|eIF-4a-I|eIF4A|eIF4A-I|eIF4AI|eIF4a|eif-4a|eif4A|l(2)02439|l(2)	0	0
2344	33836	Drosophila melanogaster	ifc	infertile crescent	CG9078|DES-1|DmelCG9078|des|des-1|des1|l(2)26Ab|l(2)gdh-2|ms(2)04875	0	0
2345	33837	Drosophila melanogaster	lid	little imaginal discs	CG9088|DmelCG9088|KDM5|LID|Lid|dJARID1|l(2)10424	0	0
2346	33838	Drosophila melanogaster	Tsp26A	Tetraspanin 26A	CG9093|Dm.Tsp26A|DmelCG9093	0	0
2347	33839	Drosophila melanogaster	Gal	beta galactosidase	CG9092|DmelCG9092|beta-GAL|beta-Gal-1|beta-gal|gal|lacZ-1	0	0
2348	33840	Drosophila melanogaster	CG9098	CG9098 gene product from transcript CG9098-RA	DmelCG9098	0	0
2349	33841	Drosophila melanogaster	H2.0	Homeodomain protein 2.0	CG11607|DmelCG11607	0	0
2350	33842	Drosophila melanogaster	CG13996	CG13996 gene product from transcript CG13996-RA	DmelCG13996	0	0
2351	33843	Drosophila melanogaster	CG9107	CG9107 gene product from transcript CG9107-RA	DmelCG9107|Rrp7	0	0
2352	33844	Drosophila melanogaster	CG9109	CG9109 gene product from transcript CG9109-RA	DmelCG9109	0	0
2353	33845	Drosophila melanogaster	mtm	myotubularin	CG9115|DmelCG9115|MTM1|dMTMH1	0	0
2354	33846	Drosophila melanogaster	CG9117	CG9117 gene product from transcript CG9117-RA	DmelCG9117	0	0
2355	33847	Drosophila melanogaster	ade2	adenosine 2	153122_at|Ade2|CG9127|DmelCG9127|pym	0	0
2356	33849	Drosophila melanogaster	CG12393	CG12393 gene product from transcript CG12393-RB	DmelCG12393	0	0
2357	33850	Drosophila melanogaster	CG9135	CG9135 gene product from transcript CG9135-RB	DmelCG9135	0	0
2358	33851	Drosophila melanogaster	CG13995	CG13995 gene product from transcript CG13995-RA	DmelCG13995|anon-WO0170980.169|anon-WO0170980.170	0	0
2359	33852	Drosophila melanogaster	CG9140	CG9140 gene product from transcript CG9140-RA	DmelCG9140	0	0
2360	33853	Drosophila melanogaster	CG13994	CG13994 gene product from transcript CG13994-RA	BcDNA:AT31406|DmelCG13994	0	0
2361	33854	Drosophila melanogaster	Fbw5	CG9144 gene product from transcript CG9144-RA	CG9144|DmelCG9144|FBgn 31773|dFbw5	0	0
2362	33855	Drosophila melanogaster	CG9147	CG9147 gene product from transcript CG9147-RB	DmelCG9147	0	0
2363	33856	Drosophila melanogaster	CG9150	CG9150 gene product from transcript CG9150-RB	DmelCG9150	0	0
2364	33857	Drosophila melanogaster	CG13993	CG13993 gene product from transcript CG13993-RA	DmelCG13993|anon-EST:Posey288	0	0
2365	33858	Drosophila melanogaster	CG9154	CG9154 gene product from transcript CG9154-RA	DmelCG9154	0	0
2366	33859	Drosophila melanogaster	Kr-h2	Kruppel homolog 2	CG9159|DmelCG9159|Kr-H2|Kr-h	0	0
2367	33861	Drosophila melanogaster	Kr-h1	Kruppel homolog 1	44/11|CG18783|CG9167|DmelCG18783|Kr h|Kr-H1|Kr-h|P381|Pow|l(2)05208|l(2)10642	0	0
2368	33862	Drosophila melanogaster	CG9175	CG9175 gene product from transcript CG9175-RB	DmelCG9175	0	0
2369	33863	Drosophila melanogaster	stai	stathmin	BEST:LD33989|CG31641|CG5981|DmelCG31641|LD33989|SCG10-like	0	0
2370	33864	Drosophila melanogaster	Arc-p20	CG5972 gene product from transcript CG5972-RA	Arp-p20|Arpc4|BcDNA:RE43724|CG5972|DmelCG5972|P-20 ARC|anon-EST:Posey49|arc-p20|p20|p20Arc	0	0
2371	33865	Drosophila melanogaster	WDR79	WD repeat domain 79 homolog	CG9226|DmelCG9226|dWDR79|p70	0	0
2372	33866	Drosophila melanogaster	tectonic	CG42731 gene product from transcript CG42731-RA	CG42731|CG9227|DmelCG42731|Dmel_CG9227|Tectonic	0	0
2373	33867	Drosophila melanogaster	CG9222	CG9222 gene product from transcript CG9222-RA	DmelCG9222	0	0
2374	33868	Drosophila melanogaster	CG13991	CG13991 gene product from transcript CG13991-RA	DmelCG13991	0	0
2375	33869	Drosophila melanogaster	CG13989	CG13989 gene product from transcript CG13989-RA	BcDNA:AT09383|DmelCG13989	0	0
2376	33874	Drosophila melanogaster	CG9486	CG9486 gene product from transcript CG9486-RA	DmelCG9486	0	0
2377	33875	Drosophila melanogaster	CG13983	CG13983 gene product from transcript CG13983-RA	DmelCG13983	0	0
2378	33879	Drosophila melanogaster	CG13984	CG13984 gene product from transcript CG13984-RA	DmelCG13984	0	0
2379	33881	Drosophila melanogaster	Gef26	CG9491 gene product from transcript CG9491-RB	CG9491|DmelCG9491|GEF|PDF-GEF Dizzy|PDZ-GEF|dPDZ-GEF|dizzy|dzy|l(2)k13720	0	0
2380	33882	Drosophila melanogaster	Pez	CG9493 gene product from transcript CG9493-RA	CG9493|DmelCG9493|PTPD1	0	0
2381	33883	Drosophila melanogaster	Cpr	Cytochrome P450 reductase	CCR|CG11567|CPR|DMR|DmCPR|DmelCG11567|NCPR|P450|cpr	0	0
2382	33884	Drosophila melanogaster	CG9497	CG9497 gene product from transcript CG9497-RA	DmelCG9497	0	0
2383	33885	Drosophila melanogaster	CG9498	CG9498 gene product from transcript CG9498-RA	DmelCG9498	0	0
2384	33886	Drosophila melanogaster	ppk7	pickpocket 7	CG9499|DmelCG9499|PPK7	0	0
2385	33887	Drosophila melanogaster	ppk14	pickpocket 14	CG9501|DmelCG9501|PPK14	0	0
2386	33888	Drosophila melanogaster	CG9500	CG9500 gene product from transcript CG9500-RA	DmelCG9500|FBpp0078910	0	0
2387	33889	Drosophila melanogaster	CG9505	CG9505 gene product from transcript CG9505-RA	DmelCG9505	0	0
2388	33890	Drosophila melanogaster	slam	slow as molasses	CG9506|DmelCG9506|Slam|wdo	0	0
2389	33892	Drosophila melanogaster	CG9507	CG9507 gene product from transcript CG9507-RA	DmelCG9507	0	0
2390	33893	Drosophila melanogaster	CG42370	CG42370 gene product from transcript CG42370-RA	CG13981|CG33295|CG9508|CG9511|CT26920|DmelCG42370|Dmel_CG9508|Dmel_CG9511	0	0
2391	33894	Drosophila melanogaster	CG42369	CG42369 gene product from transcript CG42369-RA	CG13981|CG33295|CG9508|CG9511|CT26922|DmelCG42369|Dmel_CG9508|Dmel_CG9511	0	0
2392	33895	Drosophila melanogaster	CG13982	CG13982 gene product from transcript CG13982-RA	DmelCG13982	0	0
2393	33896	Drosophila melanogaster	Tig	Tiggrin	CG11527|CT36389|DmelCG11527|TIG|tig	0	0
2394	33897	Drosophila melanogaster	l(2)k11101	lethal (2) k11101	CG9523|DmelCG9523	0	0
2395	33898	Drosophila melanogaster	CG9527	CG9527 gene product from transcript CG9527-RA	DmelCG9527	0	0
2396	33899	Drosophila melanogaster	retm	real-time	CG9528|DmelCG9528	0	0
2397	33900	Drosophila melanogaster	frj	farjavit	CG9526|CT26954|DmelCG9526|Frj	0	0
2398	33901	Drosophila melanogaster	CG16947	CG16947 gene product from transcript CG16947-RA	DmelCG16947|anon-EST:Posey20	0	0
2399	33902	Drosophila melanogaster	CG9531	CG9531 gene product from transcript CG9531-RA	DmelCG9531	0	0
2400	33903	Drosophila melanogaster	mmy	mummy	BcDNA:LD24639|CG9535|DmelCG9535|LD44264|anon-WO0140519.242|cao|cys|cyst|cystic|l(2)k13717b|mum	0	0
2401	33904	Drosophila melanogaster	CG9536	CG9536 gene product from transcript CG9536-RB	DmelCG9536	0	0
2402	33905	Drosophila melanogaster	Sec61alpha	CG9539 gene product from transcript CG9539-RA	0455|BEST:LD29847|BcDNA:LD29847|CG9539|DSec61alpha|DmelCG9539|E(br)165|LD29847|SEC61-alpha|SEC61ALPHA|Sec61|Sec61-alpha|Sec61a|anon-WO0118547.195|anon-WO0118547.277|dSec	0	0
2403	33906	Drosophila melanogaster	DLP	Daxx-like protein	CG9537|Daxx|DmelCG9537	0	0
2404	33907	Drosophila melanogaster	CG9542	CG9542 gene product from transcript CG9542-RA	DmelCG9542	0	0
2405	33908	Drosophila melanogaster	epsilonCOP	CG9543 gene product from transcript CG9543-RA	BcDNA:LD29885|CG9543|DmelCG9543|bcDNA:LD29885|epsilon-Cop	0	0
2406	33909	Drosophila melanogaster	CG9548	CG9548 gene product from transcript CG9548-RA	BcDNA:RH02789|DmelCG9548	0	0
2407	33910	Drosophila melanogaster	CG31638	CG31638 gene product from transcript CG31638-RA	CG13764|CG9545|DmelCG31638	0	0
2408	33911	Drosophila melanogaster	CG9547	CG9547 gene product from transcript CG9547-RA	152078_at|DmelCG9547	0	0
2409	33913	Drosophila melanogaster	CG9550	CG9550 gene product from transcript CG9550-RA	DmelCG9550	0	0
2410	33914	Drosophila melanogaster	CG31637	CG31637 gene product from transcript CG31637-RA	CG13765|CG9551|DmelCG31637|anon-WO0118547.107	0	0
2411	33916	Drosophila melanogaster	eya	eyes absent	BcDNA:LD16029|CG9554|CLIFT|DmelCG9554|EY2-1|EYA|Eya|cli|cli/eya|del1|eEya|eay|ey-2	0	0
2412	33917	Drosophila melanogaster	osm-6	CG9595 gene product from transcript CG9595-RA	CG9595|DmelCG9595|IFT52|NGD5|OSM-6	0	0
2413	33918	Drosophila melanogaster	CG11015	CG11015 gene product from transcript CG11015-RA	BcDNA:RH49324|DmelCG11015|Vb|anon-EST:Posey143|anon-WO0118547.187	0	0
2414	33919	Drosophila melanogaster	CG11043	CG11043 gene product from transcript CG11043-RA	DmelCG11043|Vb	0	0
2415	33920	Drosophila melanogaster	CG9596	CG9596 gene product from transcript CG9596-RB	DmelCG9596	0	0
2416	33921	Drosophila melanogaster	Ent2	Equilibrative nucleoside transporter 2	BEST:LD04971|CG11045|CG31911|DmENT2|DmelCG31911|LD04971|ent2	0	0
2417	33922	Drosophila melanogaster	CG13766	CG13766 gene product from transcript CG13766-RA	DmelCG13766	0	0
2418	33923	Drosophila melanogaster	CG11319	CG11319 gene product from transcript CG11319-RA	DmelCG11319	0	0
2419	33924	Drosophila melanogaster	CG11050	CG11050 gene product from transcript CG11050-RA	BcDNA:LD22339|DmelCG11050	0	0
2420	33925	Drosophila melanogaster	CG11320	CG11320 gene product from transcript CG11320-RB	CT31597|DmelCG11320	0	0
2421	33927	Drosophila melanogaster	Oatp26F	Organic anion transporting polypeptide 26F	CG11332|CG13767|CG18784|CG31634|DmelCG31634|oatp 26F|oatp26F	0	0
2422	33928	Drosophila melanogaster	CG31635	CG31635 gene product from transcript CG31635-RB	BcDNA:GM03875|CG13768|CG13769|CT33252|DmelCG31635	0	0
2423	33930	Drosophila melanogaster	Tango1	Transport and Golgi organization 1	CG11098|DmelCG11098|TANGO1	0	0
2424	33931	Drosophila melanogaster	CG31633	CG31633 gene product from transcript CG31633-RA	CG13770|DmelCG31633	0	0
2425	33932	Drosophila melanogaster	CG11070	CG11070 gene product from transcript CG11070-RA	BcDNA:LD34475|CG 11070|DmelCG11070|UFD2a	0	0
2426	33933	Drosophila melanogaster	CG13771	CG13771 gene product from transcript CG13771-RA	DmelCG13771	0	0
2427	33934	Drosophila melanogaster	cup	CG11181 gene product from transcript CG11181-RB	CG11181|CUP|Cup|DmelCG11181|fs(1)cup|fs(2)cup|i239	0	0
2428	33935	Drosophila melanogaster	Galt	Galactose-1-phosphate uridylyltransferase	CG9232|DmelCG9232|GALT|dGALT	0	0
2429	33937	Drosophila melanogaster	cort	cortex	CG11330|Cort|DmelCG11330|cor	0	0
2430	33938	Drosophila melanogaster	CG11329	CG11329 gene product from transcript CG11329-RA	DmelCG11329|EST2|GM14348|anon-WO0118547.82	0	0
2431	33939	Drosophila melanogaster	Nhe3	Na[+]/H[+] hydrogen exchanger 3	CG11328|DmNHE3|DmelCG11328|NHE3|nhe3	0	0
2432	33940	Drosophila melanogaster	CG11327	CG11327 gene product from transcript CG11327-RA	DmelCG11327	0	0
2433	33941	Drosophila melanogaster	Tsp	Thrombospondin	CG11326|CT31613|D-TSP|DTSP|DmelCG11326|SP295|tsp	0	0
2434	33942	Drosophila melanogaster	GRHR	Gonadotropin-releasing hormone receptor	AKHR|Akhr|BEST:GH19447|CG11325|DAKHR|DAKHR-1|DGRHR|DmelCG11325|GRHR/CG11325|GnRHR|akhr	0	0
2435	33943	Drosophila melanogaster	CG11188	CG11188 gene product from transcript CG11188-RA	DmelCG11188|anon-WO0118547.190	0	0
2436	33944	Drosophila melanogaster	homer	CG11324 gene product from transcript CG11324-RC	BEST:LD03829|CG11324|Dhom|Dhomer|Dm Homer|DmelCG11324|Dvh|Hom|Homer|LD03829|dhom	0	0
2437	33946	Drosophila melanogaster	TTLL3B	Tubulin tyrosine ligase-like 3B	CG11201|DmelCG11201|dmTTLL3B	0	0
2438	33947	Drosophila melanogaster	TTLL3A	Tubulin tyrosine ligase-like 3A	CG11323|DmelCG11323|dmTTLL3A	0	0
2439	33948	Drosophila melanogaster	CG11221	CG11221 gene product from transcript CG11221-RA	DmelCG11221|PKN	0	0
2440	33949	Drosophila melanogaster	CG11322	CG11322 gene product from transcript CG11322-RB	DmelCG11322	0	0

2441	33950	Drosophila melanogaster	CG11321	CG11321 gene product from transcript CG11321-RB	DmelCG11321	0	0
2442	33951	Drosophila melanogaster	CG17378	CG17378 gene product from transcript CG17378-RA	DmelCG17378	0	0
2443	33952	Drosophila melanogaster	nrv1	nervana 1	42kDa|CG9258|DmelCG9258|K-ATPase|Na|NaK ATPase|Na[+]/K[+] ATPase|Na[+]/K[+]-ATPase|Na[+]/K[+]ATPase|Nrv|Nrv1	0	0
2444	33953	Drosophila melanogaster	nrv2	nervana 2	42kDa|CG9261|DmelCG9261|K-ATPase|NEST:bs13c10|Na|NaK ATPase|Na[+]/K[+] ATPase|Na[+]/K[+]-ATPase|Na[+]/K[+]ATPase|Nrv|Nrv2|Nrv2.2|ecta|l(2)SH0329|l(2)SH2 0329|l(2)k04223|l(2)k13315|nrv2.2	0	0
2445	33954	Drosophila melanogaster	CG17377	CG17377 gene product from transcript CG17377-RB	BEST:LP06279|DmelCG17377|anon-WO0140519.32	0	0
2446	33955	Drosophila melanogaster	CG11236	CG11236 gene product from transcript CG11236-RA	DmelCG11236	0	0
2447	33956	Drosophila melanogaster	CG17375	CG17375 gene product from transcript CG17375-RB	DmelCG17375	0	0
2448	33957	Drosophila melanogaster	sens-2	senseless-2	CG11243|CG17373|CG31632|D.m Sens-2|DmelCG31632|sens2	0	0
2449	33959	Drosophila melanogaster	Rca1	Regulator of cyclin A1	152549_at|CG10800|CLD4|DmelCG10800|Emi1|P3300|RCA1|S(rux)2A|l(2)03300|rca1	0	0
2450	33960	Drosophila melanogaster	l(2)k09022	lethal (2) k09022	CG10805|DmelCG10805|anon-WO0118547.160	0	0
2451	33961	Drosophila melanogaster	Nha1	Na[+]/H[+] hydrogen antiporter 1	CG10806|DmNHA1|DmelCG10806	0	0
2452	33962	Drosophila melanogaster	neuroligin	CG13772 gene product from transcript CG13772-RB	CG13772|DmelCG13772|dnl2|nl2	0	0
2453	33963	Drosophila melanogaster	CG13773	CG13773 gene product from transcript CG13773-RA	BEST:GH23590|DmelCG13773	0	0
2454	33964	Drosophila melanogaster	Rat1	CG10354 gene product from transcript CG10354-RA	CG10354|DmelCG10354|RAT1|XRN2|Xrn2|dRAT1|dhp1	0	0
2455	33965	Drosophila melanogaster	wee	CG4488 gene product from transcript CG4488-RA	CG4488|DmelCG4488|Dwee|Dwee1|WEE1|Wee|Wee 1|Wee1|dWee1|dwee1|wee1	0	0
2456	33966	Drosophila melanogaster	nop5	CG10206 gene product from transcript CG10206-RA	2/30|CG10206|DNOP5|DmelCG10206|Dnop5|FBgn0026196|NOP5|Nop5	0	0
2457	33967	Drosophila melanogaster	x16	CG10203 gene product from transcript CG10203-RA	2/30|9G8|CG10203|DmelCG10203|Dx16|Dxl6|RBP1-like|TN166|X16/9G8|XL6|Xl6|anon-WO0118547.78|d9G8|dx16|dxl6|l(2)k00230|xl6	0	0
2458	33968	Drosophila melanogaster	Hrb27C	Heterogeneous nuclear ribonucleoprotein at 27C	10280|CG10377|DmelCG10377|HRP48|Hrb27-C|Hrb27c|Hrb48|Hrp48|Ov5|RRM7|Rbp7|hnRNP27C|hnRNP48|hrb27C|hrb27c|hrp48|l(2)02647|l(2)10280|l(2)2647|linha|p50|rrm7	0	0
2459	33969	Drosophila melanogaster	CG18304	CG18304 gene product from transcript CG18304-RA	CG13774|DmelCG18304	0	0
2460	33971	Drosophila melanogaster	CG10158	CG10158 gene product from transcript CG10158-RA	DmelCG10158|FGFR1OP2|FGOP2	0	0
2461	33972	Drosophila melanogaster	ihog	interference hedgehog	CG9211|CT26314|DmelCG9211|Ihog|iHog	0	0
2462	33973	Drosophila melanogaster	Gas41	CG9207 gene product from transcript CG9207-RA	CG9207|DmelCG9207|GAS41|dGas41	0	0
2463	33974	Drosophila melanogaster	SA	Stromalin	CG3423|DSA|DSA1|DmSA|DmelCG3423|ESTS:92H2T|SCC3|Scc3	0	0
2464	33975	Drosophila melanogaster	CG13775	CG13775 gene product from transcript CG13775-RA	DmelCG13775	0	0
2465	33976	Drosophila melanogaster	CG3430	CG3430 gene product from transcript CG3430-RA	DmelCG3430	0	0
2466	33977	Drosophila melanogaster	Atac1	ATAC component 1	CG9200|DmelCG9200	0	0
2467	33978	Drosophila melanogaster	CG10399	CG10399 gene product from transcript CG10399-RA	145934_at|DmelCG10399	0	0
2468	33979	Drosophila melanogaster	sip2	septin interacting protein 2	CG9188|DmelCG9188	0	0
2469	33981	Drosophila melanogaster	smt3	CG4494 gene product from transcript CG4494-RA	CG4494|Dm0342|DmSUMO-1|DmSmt3|DmelCG4494|Dmsmt3|SMT3|SUMO|Smt3|Sumo|anon-EST:Posey240|dSmt3|l(2)04493|sumo	0	0
2470	33982	Drosophila melanogaster	snRNP-U1-70K	small ribonucleoprotein particle U1 subunit 70K	70K|CG8749|Dm70K|DmelCG8749|U1 70K|U1 70K snRNP|U1 70k|U1 snRNP|U1-70K|U1-70k|U170K|U1snRNP-70K|dU1-70K|l(2)02107|snRNP|snRNP27D|snRNP70K	0	0
2471	33983	Drosophila melanogaster	uif	uninflatable	CG9138|CT26172|DmelCG9138|SP1070|poly-EGF	0	0
2472	33984	Drosophila melanogaster	CG43322	CG43322 gene product from transcript CG43322-RA	CG13776|DmelCG43322	0	0
2473	33985	Drosophila melanogaster	Pcp	Pupal cuticle protein	CG3440|DMCPGART|DmelPcp|DmelCG3440|Gart|Pcpgart	0	0
2474	33986	Drosophila melanogaster	ade3	adenosine 3	143062_at|ADE8|Ade3|CG31628|CG8761|D|DmelCG31628|GART|Gart|Gart/ade3|gart|l(2)27Da	0	0
2475	33987	Drosophila melanogaster	CG3476	CG3476 gene product from transcript CG3476-RA	DmelCG3476	0	0
2476	33988	Drosophila melanogaster	Rab30	CG9100 gene product from transcript CG9100-RB	AAF52477|CG9100|DmRab30|DmelCG9100	0	0
2477	33989	Drosophila melanogaster	CG11266	CG11266 gene product from transcript CG11266-RG	CAPER|CC1.3|Caper|DmelCG11266|HCC1|cg11266	0	0
2478	33991	Drosophila melanogaster	Mnn1	Menin 1	CG13778|CG33266|DmelCG13778|MEN1|Menin1|menin|mnn1	0	0
2479	33992	Drosophila melanogaster	Nuf2	CG8902 gene product from transcript CG8902-RA	CG8902|DmelCG8902|anon-WO0118547.214|dmNuf2|l(2)SH2 2276	0	0
2480	33993	Drosophila melanogaster	CG11289	CG11289 gene product from transcript CG11289-RB	DmelCG11289	0	0
2481	33994	Drosophila melanogaster	Pvf2	PDGF- and VEGF-related factor 2	CG13780|DmVEGF-2|DmelCG13780|PV2|PVF|PVF2|PVFs|Pvf|VEGF|VEGF-2|VEGF27Cb|Vegf27Cb|pvf2	0	0
2482	33995	Drosophila melanogaster	Pvf3	PDGF- and VEGF-related factor 3	CG13781|CG13782|CG13783|CG31629|CG34378|CG4492|DmVEGF-3|DmelCG34378|Dmel_CG13783|Dmel_CG31629|PVF|PVF3|PVFs|Pvf|VEGF|VEGF27Ca|Vegf27Ca|pvf3	0	0
2483	33999	Drosophila melanogaster	CG4495	CG4495 gene product from transcript CG4495-RA	DmelCG4495	0	0
2484	34000	Drosophila melanogaster	CG4496	CG4496 gene product from transcript CG4496-RA	DmelCG4496	0	0
2485	34001	Drosophila melanogaster	CG4497	CG4497 gene product from transcript CG4497-RB	DmelCG4497	0	0
2486	34002	Drosophila melanogaster	CG4502	CG4502 gene product from transcript CG4502-RB	DmelCG4502|anon-WO0118547.403	0	0
2487	34003	Drosophila melanogaster	CG13784	CG13784 gene product from transcript CG13784-RC	BcDNA:SD10385|DmelCG13784	0	0
2488	34004	Drosophila melanogaster	ico	iconoclast	B|CG4567|DmelCG4567|G|GFM1|dEF-G1|l(2)27Cb|l(2)27Ea	0	0
2489	34005	Drosophila melanogaster	Ndae1	Na[+]-driven anion exchanger 1	153385_at|CG31906|CG42253|CG4675|DmelCG42253|Dmel_CG31906|Dmel_CG4675|NDAE1|Ndae-1|anon-WO0257455.13|l(2)k10316|ndae1	0	0
2490	34006	Drosophila melanogaster	CG13786	CG13786 gene product from transcript CG13786-RA	DmelCG13786	0	0
2491	34007	Drosophila melanogaster	Wnt4	Wnt oncogene analog 4	CG4698|DWnt-4|DWnt4|Dm DWnt4|DmelCG4698|Dwnt4|Wnt|Wnt-4|anon-EST:Liang-2.4|clone 2.4|wnt-4|wnt4	0	0
2492	34009	Drosophila melanogaster	wg	wingless	Br|CG4889|DWint-1|DWnt-1|Dint-1|Dm Wg|Dm-1|DmelCG4889|Gla|l|Sp|WG|WNT|Wg|Wnt|Wnt-1|Wnt/Wg|Wnt1|dWnt|fg|int-1|l(2)02657|l(2)rO727|l(2)wg|spd|wgl|wnt|wnt1	0	0
2493	34010	Drosophila melanogaster	Wnt6	CG4969 gene product from transcript CG4969-RB	CG4969|D-Wnt-6|DWnt-6|DWnt6|DmelCG4969|wnt6	0	0
2494	34011	Drosophila melanogaster	Wnt10	CG4971 gene product from transcript CG4971-RB	CG4971|D-Wnt-10|DWnt-10|DWnt10|Dm DWnt10|DmelCG4971|wnt10	0	0
2495	34012	Drosophila melanogaster	ninaC	neither inactivation nor afterpotential C	2.2|CG5125|CG54125|CT16120|CT42491|DRONINAC|Dm NinaC|DmelCG5125|NINA C|NINAC|Nina C|NinaC|ninac	0	0
2496	34013	Drosophila melanogaster	CG5149	CG5149 gene product from transcript CG5149-RA	DmelCG5149	0	0
2497	34014	Drosophila melanogaster	CG5155	CG5155 gene product from transcript CG5155-RA	DmelCG5155	0	0
2498	34015	Drosophila melanogaster	CG5160	CG5160 gene product from transcript CG5160-RA	DmelCG5160	0	0
2499	34016	Drosophila melanogaster	CG5171	CG5171 gene product from transcript CG5171-RC	DmelCG5171	0	0
2500	34017	Drosophila melanogaster	CG5177	CG5177 gene product from transcript CG5177-RA	DmelCG5177	0	0
2501	34018	Drosophila melanogaster	CG5181	CG5181 gene product from transcript CG5181-RA	DmelCG5181	0	0
2502	34019	Drosophila melanogaster	CG15818	CG15818 gene product from transcript CG15818-RA	DmelCG15818	0	0
2503	34021	Drosophila melanogaster	CG5261	CG5261 gene product from transcript CG5261-RB	DLAT|DmelCG5261|EP(2)0816|EP816|anon-WO0118547.121	0	0
2504	34022	Drosophila melanogaster	CG5958	CG5958 gene product from transcript CG5958-RA	DmelCG5958	0	0
2505	34023	Drosophila melanogaster	CG5973	CG5973 gene product from transcript CG5973-RA	DmelCG5973	0	0
2506	34024	Drosophila melanogaster	santa-maria	scavenger receptor acting in neural tissue and majority	BEST:CK01577|CG12789|CK01577|DmelCG12789|Santa Maria|santa maria	0	0
2507	34027	Drosophila melanogaster	CG6055	CG6055 gene product from transcript CG6055-RA	DmelCG6055	0	0
2508	34029	Drosophila melanogaster	CG6441	CG6441 gene product from transcript CG6441-RA	DmelCG6441|anon-WO0140519.45	0	0
2509	34030	Drosophila melanogaster	CG42533	CG42533 gene product from transcript CG42533-RF	BcDNA:RH71439|CG15819|CG6630|Dm ziz|DmelCG42533|Dmel_CG15819|Dmel_CG6630	0	0
2510	34031	Drosophila melanogaster	Pbprp5	Pheromone-binding protein-related protein 5	28a|CG6641|DmelCG6641|Obp28a|PB-PRP5|PBP5|PBPRP-5|PBPRP5|obp28a|pbprp-5|pbprp5	0	0
2511	34032	Drosophila melanogaster	Rapgap1	CG34374 gene product from transcript CG34374-RD	CG13789|CG13790|CG33529|CG34374|CG6682|DmelCG34374|Dmel_CG13790|Dmel_CG33529|RapGap1|rapgap1	0	0
2512	34034	Drosophila melanogaster	CG13791	CG13791 gene product from transcript CG13791-RA	DmelCG13791	0	0
2513	34035	Drosophila melanogaster	Spn7	Serine protease inhibitor 7	CG6717|DmelCG6717|Spn28B	0	0
2514	34036	Drosophila melanogaster	Cyp4d21	CG6730 gene product from transcript CG6730-RA	4d21|CG6730|DmelCG6730|cyp4d21|sxe1	0	0
2515	34037	Drosophila melanogaster	CG6739	CG6739 gene product from transcript CG6739-RA	DmelCG6739	0	0
2516	34038	Drosophila melanogaster	CG13792	CG13792 gene product from transcript CG13792-RA	DmelCG13792	0	0
2517	34039	Drosophila melanogaster	Slob	Slowpoke binding protein	CG6772|DmelCG6772|slob	0	0
2518	34041	Drosophila melanogaster	CG18585	CG18585 gene product from transcript CG18585-RA	DmelCG18585	0	0
2519	34042	Drosophila melanogaster	CG7025	CG7025 gene product from transcript CG7025-RA	DmelCG7025	0	0
2520	34044	Drosophila melanogaster	TepII	Thiolester containing protein II	145970_at|145971_at|CG18589|CG7052|DmelCG7052|T13|TEP2|Tep II|Tep2|dTEPII|tepII	0	0
2521	34045	Drosophila melanogaster	TepIII	Thiolester containing protein III	CG7068|DmelCG7068|Tep3|dTEPIII	0	0
2522	34046	Drosophila melanogaster	Ntl	Neurotransmitter transporter-like	CG7075|DmelCG7075|ntl	0	0
2523	34047	Drosophila melanogaster	CG13793	CG13793 gene product from transcript CG13793-RB	DmelCG13793	0	0
2524	34048	Drosophila melanogaster	CG13794	CG13794 gene product from transcript CG13794-RA	DmelCG13794	0	0
2525	34049	Drosophila melanogaster	CG13795	CG13795 gene product from transcript CG13795-RB	CT33284|DmelCG13795	0	0
2526	34051	Drosophila melanogaster	CG13796	CG13796 gene product from transcript CG13796-RD	CG13796-PA|CG18590|CT42497|DmelCG13796	0	0
2527	34052	Drosophila melanogaster	Acp1	Adult cuticle protein 1	CG7216|CG7216-PA|Dacp-1|DmelCG7216|acp1	0	0
2528	34053	Drosophila melanogaster	CG7214	CG7214 gene product from transcript CG7214-RA	DmelCG7214	0	0
2529	34054	Drosophila melanogaster	CG7211	CG7211 gene product from transcript CG7211-RA	DmelCG7211	0	0
2530	34055	Drosophila melanogaster	CG7203	CG7203 gene product from transcript CG7203-RB	DmelCG7203	0	0
2531	34056	Drosophila melanogaster	CG14538	CG14538 gene product from transcript CG14538-RA	DmelCG14538	0	0
2532	34057	Drosophila melanogaster	CG7196	CG7196 gene product from transcript CG7196-RA	DmelCG7196	0	0
2533	34058	Drosophila melanogaster	CG7191	CG7191 gene product from transcript CG7191-RA	DmelCG7191	0	0
2534	34059	Drosophila melanogaster	CG7179	CG7179 gene product from transcript CG7179-RB	CT22169|DmelCG7179|Opa|anon-28Cg|tld-like	0	0
2535	34060	Drosophila melanogaster	Uro	Urate oxidase	CG7171|Dm UO|DmelCG7171|OU|UO|UOX|Uo|anon-WO0140519.210|uro	0	0
2536	34061	Drosophila melanogaster	CG7164	CG7164 gene product from transcript CG7164-RA	DmelCG7164	0	0
2537	34062	Drosophila melanogaster	CG7154	CG7154 gene product from transcript CG7154-RA	DmelCG7154	0	0
2538	34063	Drosophila melanogaster	CG7149	CG7149 gene product from transcript CG7149-RA	DmelCG7149|cpt	0	0
2539	34064	Drosophila melanogaster	LKR	lysine ketoglutarate reductase	BEST:CK02318|CG7144|CK02318|DmelCG7144|SDH|anon-WO0149856.1|dLKR	0	0
2540	34065	Drosophila melanogaster	Herp	Homocysteine-induced endoplasmic reticulum protein	CG1453|CG14536|DmelCG14536|dHerp	0	0
2541	34066	Drosophila melanogaster	r2d2	CG7138 gene product from transcript CG7138-RB	CG7138|DmelCG7138|R2D2|R2d2|cg7138	0	0
2542	34067	Drosophila melanogaster	cdc14	CG7134 gene product from transcript CG7134-RB	CDC-14|CDC14|CG7134|DmelCG7134	0	0
2543	34068	Drosophila melanogaster	LanB1	CG7123 gene product from transcript CG7123-RA	BcDNA:LD21794|CG7123|CT22015|DmelCG7123|EP-600|LM-B1/beta|Lam-B1|LamB1|Lan B1|beta1|lamB1|mspl	0	0
2544	34069	Drosophila melanogaster	CG7115	CG7115 gene product from transcript CG7115-RA	BcDNA:LD21794|DmelCG7115|PP2C	0	0
2545	34070	Drosophila melanogaster	Rack1	Receptor of activated protein kinase C 1	CG7111|DmelCG7111|RACK1|dRack1|i173|rack1	0	0
2546	34072	Drosophila melanogaster	CG14537	CG14537 gene product from transcript CG14537-RA	BcDNA:AT11469|DmelCG14537	0	0
2547	34073	Drosophila melanogaster	CG14535	CG14535 gene product from transcript CG14535-RA	DmelCG14535	0	0
2548	34075	Drosophila melanogaster	TwdlE	TweedleE	CG14534|DmelCG14534	0	0
2549	34077	Drosophila melanogaster	spz3	spatzle 3	CG14533|CG7104|CT21967|CT34263|DmelCG7104|Spz3	0	0
2550	34079	Drosophila melanogaster	CG7102	CG7102 gene product from transcript CG7102-RA	CG17973|DmelCG7102	0	0
2551	34080	Drosophila melanogaster	SmE	Small ribonucleoprotein particle protein SmE	BcDNA:GM19936|CG18591|DmelCG18591|E|dE|snRNPE	0	0

2552	34082	Drosophila melanogaster	Spn28Da	Serpin 28Da	CG12318|CG31902|DmelCG31902	0	0
2553	34086	Drosophila melanogaster	CG7231	CG7231 gene product from transcript CG7231-RE	DmelCG7231	0	0
2554	34087	Drosophila melanogaster	pes	peste	CG7228|DmelCG7228|Pes	0	0
2555	34088	Drosophila melanogaster	CG7227	CG7227 gene product from transcript CG7227-RA	DmelCG7227	0	0
2556	34089	Drosophila melanogaster	CG7224	CG7224 gene product from transcript CG7224-RB	BcDNA:GH18422|DmelCG7224|anon-EST:Posey12	0	0
2557	34090	Drosophila melanogaster	Wwox	WW domain containing oxidoreductase	CG7221|DmWWOX|DmelCG7221|anon-EST:Posey177	0	0
2558	34091	Drosophila melanogaster	Spn28D	Serpin 28D	CG7219|Dm-Spn28D|DmelCG7219|Spn28Dc|spn28D	0	0
2559	34092	Drosophila melanogaster	CG12560	CG12560 gene product from transcript CG12560-RB	DmelCG12560	0	0
2560	34093	Drosophila melanogaster	Tg	Transglutaminase	CG7356|DmelCG7356|TG	0	0
2561	34094	Drosophila melanogaster	CG7367	CG7367 gene product from transcript CG7367-RB	DmelCG7367|TL	0	0
2562	34095	Drosophila melanogaster	baf	barrier to autointegration factor	BAF|CG7380|DmelCG7380|anon-WO0172774.178|dBAF	0	0
2563	34096	Drosophila melanogaster	Cka	Connector of kinase to AP-1	5836|CG7392|CKA|Dcka|DmelCG7392|anon-WO0172774.174|anon-WO0172774.175|anon-WO0172774.177|anon-WO0172774.179|cka|l(2)05836|l(2)s1883	0	0
2564	34097	Drosophila melanogaster	CG7429	CG7429 gene product from transcript CG7429-RA	DmelCG7429	0	0
2565	34098	Drosophila melanogaster	RpL36A	Ribosomal protein L36A	CG7424|DmelCG7424|M(2)28DE|anon-EST:fe1B2	0	0
2566	34099	Drosophila melanogaster	CG7466	CG7466 gene product from transcript CG7466-RA	DmelCG7466	0	0
2567	34102	Drosophila melanogaster	Trf	TBP-related factor	CG7562|DmelCG7562|TRF|TRF1|Trf1|X70838|dTRF-1|dTRF1|trf	0	0
2568	34105	Drosophila melanogaster	CG8673	CG8673 gene product from transcript CG8673-RA	DmelCG8673	0	0
2569	34106	Drosophila melanogaster	CG12375	CG12375 gene product from transcript CG12375-RA	DmelCG12375	0	0
2570	34107	Drosophila melanogaster	CG8668	CG8668 gene product from transcript CG8668-RA	DmelCG8668	0	0
2571	34109	Drosophila melanogaster	CG8552	CG8552 gene product from transcript CG8552-RA	CG14278|DmelCG8552|Sec23-IP	0	0
2572	34110	Drosophila melanogaster	Rbsn-5	Rabenosyn-5	CG8506|DmelCG8506|EEA1|MENE (2L)-C|MENE(2L)-C|Rbsn|rbsn|rbsn-5	0	0
2573	34111	Drosophila melanogaster	CG8498	CG8498 gene product from transcript CG8498-RA	DmelCG8498	0	0
2574	34112	Drosophila melanogaster	CG8486	CG8486 gene product from transcript CG8486-RE	DmelCG8486|anon-EST:Liang-1.32|clone 1.32	0	0
2575	34113	Drosophila melanogaster	fos28F	fos-related gene at 28F	CG18103|CG31608|DmelCG18103|OrfKD|fos500	0	0
2576	34114	Drosophila melanogaster	CG8475	CG8475 gene product from transcript CG8475-RA	DmelCG8475	0	0
2577	34115	Drosophila melanogaster	CG8460	CG8460 gene product from transcript CG8460-RA	DmelCG8460	0	0
2578	34116	Drosophila melanogaster	CG8455	CG8455 gene product from transcript CG8455-RA	DmelCG8455	0	0
2579	34117	Drosophila melanogaster	CG8451	CG8451 gene product from transcript CG8451-RA	CG8451-PA|DmelCG8451	0	0
2580	34118	Drosophila melanogaster	CG8419	CG8419 gene product from transcript CG8419-RA	DmelCG8419	0	0
2581	34119	Drosophila melanogaster	Su(var)205	Suppressor of variegation 205	CG8409|DmHP-1|DmHP1|DmelCG8409|E(var)29|E(var)29A|FBgn0003607|H3|HP-1|HP1|HP1-VS|HP1A|HP1a|HP1alpha|Hp1|Hp1a|SU(VAR)205|Su(Var)2-5|Su(var)|Su(var)2-05|Su(var)2-5|Su(var)2-501|Su(va	0	0
2582	34120	Drosophila melanogaster	Ssb-c31a	Single stranded-binding protein c31A	C31A|CG8396|DmelCG8396|PC4-like|Ssb-C31a|p11	0	0
2583	34121	Drosophila melanogaster	CG8372	CG8372 gene product from transcript CG8372-RA	DmelCG8372	0	0
2584	34122	Drosophila melanogaster	CG8360	CG8360 gene product from transcript CG8360-RA	BcDNA:LP03131|DmelCG8360	0	0
2585	34123	Drosophila melanogaster	CG8353	CG8353 gene product from transcript CG8353-RA	DmelCG8353	0	0
2586	34124	Drosophila melanogaster	CG8349	CG8349 gene product from transcript CG8349-RA	DmelCG8349	0	0
2587	34125	Drosophila melanogaster	CG8292	CG8292 gene product from transcript CG8292-RA	DmelCG8292	0	0
2588	34126	Drosophila melanogaster	Snx6	CG8282 gene product from transcript CG8282-RA	CG8282|DmelCG8282|Dsnx6	0	0
2589	34127	Drosophila melanogaster	Pvr	PDGF- and VEGF-receptor related	8222|CG8222|CT24332|DmVEGFR|DmelCG8222|PVR|VEGFR|VEGFR-A|VGR1|Vegf|Vegfr|Vegfr-b|Vegfr-c|Vgr1|pvr|stai|vgr1	0	0
2590	34129	Drosophila melanogaster	CG14277	CG14277 gene product from transcript CG14277-RA	DmelCG14277	0	0
2591	34131	Drosophila melanogaster	CG8086	CG8086 gene product from transcript CG8086-RF	CG8113|DmelCG8086	0	0
2592	34132	Drosophila melanogaster	Btk29A	Btk family kinase at 29A	C-src2|C-src4|CG18355|CG8049|CT2415|CT41718|DSrc28|DTec29|Dm SRC2|DmelCG8049|Dsrc28C|Dsrc29A|S13|SRC 29A|Src2|Src29A|Tec|Tec29|Tec29A|btk|btk29A|btk29a|c-src/fps|dsrc29A|fic|src-4|s	0	0
2593	34134	Drosophila melanogaster	wol	wollknaeuel	ALG5|CG7870|DmelCG7870|alg5	0	0
2594	34135	Drosophila melanogaster	Scgalpha	Sarcoglycan alpha	CG7851|DmSCG-alphaepsilon|DmelCG7851|Scga	0	0
2595	34136	Drosophila melanogaster	CG7840	CG7840 gene product from transcript CG7840-RA	DmelCG7840|cg7840	0	0
2596	34137	Drosophila melanogaster	CG7830	CG7830 gene product from transcript CG7830-RA	DmelCG7830	0	0
2597	34138	Drosophila melanogaster	CG7818	CG7818 gene product from transcript CG7818-RA	DmelCG7818	0	0
2598	34139	Drosophila melanogaster	CG7810	CG7810 gene product from transcript CG7810-RA	DmelCG7810	0	0
2599	34140	Drosophila melanogaster	CG7806	CG7806 gene product from transcript CG7806-RA	Dme_CG7806|DmelCG7806	0	0
2600	34141	Drosophila melanogaster	mtsh	mitoshell	CG7795|DmelCG7795	0	0
2601	34142	Drosophila melanogaster	CG7787	CG7787 gene product from transcript CG7787-RA	BcDNA:RE45155|DmelCG7787	0	0
2602	34143	Drosophila melanogaster	CG7781	CG7781 gene product from transcript CG7781-RA	DmelCG7781	0	0
2603	34144	Drosophila melanogaster	CG14275	CG14275 gene product from transcript CG14275-RA	BcDNA:RE73208|CT33900|DmelCG14275	0	0
2604	34145	Drosophila melanogaster	CG14274	CG14274 gene product from transcript CG14274-RA	DmelCG14274	0	0
2605	34146	Drosophila melanogaster	CG14273	CG14273 gene product from transcript CG14273-RA	DmelCG14273	0	0
2606	34147	Drosophila melanogaster	CG7778	CG7778 gene product from transcript CG7778-RA	DmelCG7778	0	0
2607	34148	Drosophila melanogaster	CG7627	CG7627 gene product from transcript CG7627-RB	Dme_CG7627|DmelCG7627	0	0
2608	34149	Drosophila melanogaster	RpS13	Ribosomal protein S13	CG13389|DmelCG13389|M(2)29B|M(2)32|M(2)32A|RPS13|Rp17|S13|l(2)k09614	0	0
2609	34152	Drosophila melanogaster	CG17292	CG17292 gene product from transcript CG17292-RB	DmelCG17292	0	0
2610	34153	Drosophila melanogaster	CG17293	CG17293 gene product from transcript CG17293-RA	DmelCG17293	0	0
2611	34154	Drosophila melanogaster	CG13390	CG13390 gene product from transcript CG13390-RA	DmelCG13390	0	0
2612	34155	Drosophila melanogaster	CG17294	CG17294 gene product from transcript CG17294-RA	DmelCG17294|Phos17294	0	0
2613	34156	Drosophila melanogaster	Aats-ala	Alanyl-tRNA synthetase	ARS|AlaRS|CG13391|DmelCG13391|alaS	0	0
2614	34157	Drosophila melanogaster	CG13392	CG13392 gene product from transcript CG13392-RA	DmelCG13392	0	0
2615	34158	Drosophila melanogaster	Rcd4	Reduction in Cnn dots 4	BcDNA:SD16838|CG17295|DmelCG17295	0	0
2616	34159	Drosophila melanogaster	CG13393	CG13393 gene product from transcript CG13393-RA	BcDNA:RE23864|DmelCG13393	0	0
2617	34160	Drosophila melanogaster	CG13384	CG13384 gene product from transcript CG13384-RJ	DmelCG13384	0	0
2618	34161	Drosophila melanogaster	CG42820	CG42820 gene product from transcript CG42820-RA	CG13394|DmelCG42820|Dmel_CG13394	0	0
2619	34162	Drosophila melanogaster	Acp29AB	Accessory gland-specific peptide 29AB	AC 004423B|AC004423b|Acp29|Acp29AB-II|Acp29B|CG17797|DmelCG17797	0	0
2620	34164	Drosophila melanogaster	CG13385	CG13385 gene product from transcript CG13385-RA	DmelCG13385	0	0
2621	34165	Drosophila melanogaster	CG13386	CG13386 gene product from transcript CG13386-RA	DmelCG13386	0	0
2622	34166	Drosophila melanogaster	fy	fuzzy	CG13396|DmelCG13396	0	0
2623	34167	Drosophila melanogaster	emb	embargoed	CG13387|CRM1|Crm1|DCRM1|DmelCG13387|Emb|XPO-1|XPO1|Xpo1|crm1|dCRM1|l(2)k16715	0	0
2624	34169	Drosophila melanogaster	CG13398	CG13398 gene product from transcript CG13398-RA	DmelCG13398	0	0
2625	34170	Drosophila melanogaster	Akap200	A kinase anchor protein 200	AKAP200|Akap 200|BcDNA:LP02734|CG13388|DAKAP200|DmelCG13388|EP(2)2254|MESR2|akap200|anon-WO0118547.134|anon-WO0118547.213|dpo	0	0
2626	34171	Drosophila melanogaster	grk	gurken	CG17610|CT32746|DmelCG17610|GRK|Grk|s-Grk	0	0
2627	34172	Drosophila melanogaster	D12	CG13400 gene product from transcript CG13400-RA	CG13400|DmelCG13400	0	0
2628	34175	Drosophila melanogaster	U26	CG13401 gene product from transcript CG13401-RA	CG13401|DmelCG13401	0	0
2629	34176	Drosophila melanogaster	fu12	CG17608 gene product from transcript CG17608-RA	AGPAT1|CG17608|DmelCG17608	0	0
2630	34177	Drosophila melanogaster	fu2	CG9233 gene product from transcript CG9233-RA	CG9233|DmelCG9233|FU12	0	0
2631	34179	Drosophila melanogaster	d	dachs	29C3-D1|29CD|AAF52683|CG10595|CG13087|CG31610|CG42840|D|DmelCG42840|Dmel_CG10595|Dmel_CG13087|Myo29D|anon-EST:Liang-1.78|clone 1.78|l(2)c00146	0	0
2632	34180	Drosophila melanogaster	CG13088	CG13088 gene product from transcript CG13088-RA	DmelCG13088	0	0
2633	34181	Drosophila melanogaster	Dh31	Diuretic hormone 31	CG13094|DH 31|DH-II|DH31|DH[[31]|DH[[31]]|Dh[[31]]|DmelCG13094|Drm-DH[[31]]|Drome-DH[[31]]|anon-EST:Posey114|anon-WO02059370.45|dh31|drome-DH31	0	0
2634	34182	Drosophila melanogaster	Bace	beta-site APP-cleaving enzyme	CG13095|DmelCG13095|anon-WO0140519.142|dBACE	0	0
2635	34183	Drosophila melanogaster	CG13096	CG13096 gene product from transcript CG13096-RA	DmelCG13096	0	0
2636	34184	Drosophila melanogaster	CG13097	CG13097 gene product from transcript CG13097-RA	DmelCG13097	0	0
2637	34185	Drosophila melanogaster	CG13089	CG13089 gene product from transcript CG13089-RA	DmelCG13089	0	0
2638	34186	Drosophila melanogaster	mRpL51	mitochondrial ribosomal protein L51	CG13098|DmelCG13098|MRP-L51	0	0
2639	34187	Drosophila melanogaster	CG13090	CG13090 gene product from transcript CG13090-RA	DmelCG13090	0	0
2640	34188	Drosophila melanogaster	CG13091	CG13091 gene product from transcript CG13091-RA	CT32316|DmelCG13091	0	0
2641	34189	Drosophila melanogaster	Acer	Angiotensin-converting enzyme-related	ACE|ACER|CG10593|DmelCG10593|l(2)k07704	0	0
2642	34191	Drosophila melanogaster	CG17834	CG17834 gene product from transcript CG17834-RA	BcDNA:LP07538|DmelCG17834|anon-290a|anon-29Da	0	0
2643	34192	Drosophila melanogaster	Sema-1a	CG18405 gene product from transcript CG18405-RD	BcDNA:RE36155|CG18405|CT26404|D-Sema 1a|D-Sema I|D-Sema-I|D-SemaI|D-semaI|DmelCG18405|Dsema 1a|Dsema-I|Fas-IV|FasIV|Sema 1a|Sema-I|Sema1A|Sema1a|SemaI|d-sema I|d-semaI|l(2)k13	0	0
2644	34193	Drosophila melanogaster	Glt	Glutactin	CG9280|CT26441|DmelCG9280	0	0
2645	34194	Drosophila melanogaster	CG9287	CG9287 gene product from transcript CG9287-RA	DmelCG9287	0	0
2646	34195	Drosophila melanogaster	CG9289	CG9289 gene product from transcript CG9289-RA	CT26467|DmelCG9289	0	0
2647	34196	Drosophila melanogaster	PrBP	Prenyl-binding protein	CG9296|DmPrBP|DmPrBP/delta|DmelCG9296	0	0
2648	34197	Drosophila melanogaster	Trs23	CG9298 gene product from transcript CG9298-RA	CG9298|DmelCG9298|TRS23|Trs23p	0	0
2649	34198	Drosophila melanogaster	CG9314	CG9314 gene product from transcript CG9314-RA	CT26521|DmelCG9314|anon-WO0140519.249	0	0
2650	34202	Drosophila melanogaster	CG12438	CG12438 gene product from transcript CG12438-RA	DmelCG12438	0	0
2651	34203	Drosophila melanogaster	CG9463	CG9463 gene product from transcript CG9463-RA	DmelCG9463	0	0
2652	34204	Drosophila melanogaster	CG9465	CG9465 gene product from transcript CG9465-RB	DmelCG9465	0	0
2653	34205	Drosophila melanogaster	CG9466	CG9466 gene product from transcript CG9466-RA	DmelCG9466	0	0
2654	34206	Drosophila melanogaster	CG9468	CG9468 gene product from transcript CG9468-RA	DmelCG9468	0	0
2655	34208	Drosophila melanogaster	CG32986	CG32986 gene product from transcript CG32986-RA	CG15866|DmelCG32986	0	0
2656	34209	Drosophila melanogaster	CG9483	CG9483 gene product from transcript CG9483-RA	DmelCG9483|anon-WO0140519.161	0	0
2657	34211	Drosophila melanogaster	Tsp29Fa	Tetraspanin 29Fa	CG9494|Dm.Tsp29Fa|DmelCG9494	0	0
2658	34212	Drosophila melanogaster	Tsp29Fb	Tetraspanin 29Fb	CG9496|Dm.Tsp29Fb|DmelCG9496|anon-EST:ParkEST130	0	0
2659	34215	Drosophila melanogaster	C1GalTA	Core 1 Galactosyltransferase A	CG9520|DmelCG9520|anon-WO0144478.18|dC1beta3GalT1	0	0
2660	34217	Drosophila melanogaster	CG9525	CG9525 gene product from transcript CG9525-RA	DmelCG9525	0	0
2661	34218	Drosophila melanogaster	CG32984	CG32984 gene product from transcript CG32984-RA	CG17789|DmelCG32984	0	0
2662	34219	Drosophila melanogaster	CG18088	CG18088 gene product from transcript CG18088-RA	DmelCG18088	0	0

2663	34220	Drosophila melanogaster	CG9541	CG9541 gene product from transcript CG9541-RA	DmelCG9541	0	0
2664	34221	Drosophila melanogaster	CG13101	CG13101 gene product from transcript CG13101-RA	DmelCG13101	0	0
2665	34222	Drosophila melanogaster	rost	rolling stone	CG9552|DmelCG9552	0	0
2666	34223	Drosophila melanogaster	CG9555	CG9555 gene product from transcript CG9555-RB	DmelCG9555	0	0
2667	34224	Drosophila melanogaster	CG17906	CG17906 gene product from transcript CG17906-RA	DmelCG17906	0	0
2668	34225	Drosophila melanogaster	alien	CG9556 gene product from transcript CG9556-RB	Alien|BcDNA:LD10463|CG9556|CH2|CSN2|Csn2|DCH2|Dch2|DmelCG9556	0	0
2669	34226	Drosophila melanogaster	Try29F	Trypsin 29F	CG9564|DmelCG9564|SP106|try29F|try29F.2	0	0
2670	34227	Drosophila melanogaster	CG9568	CG9568 gene product from transcript CG9568-RA	DmelCG9568|FBgn 32087|anon-WO0140519.183	0	0
2671	34228	Drosophila melanogaster	CG13102	CG13102 gene product from transcript CG13102-RB	BcDNA:RE57454|DmelCG13102	0	0
2672	34229	Drosophila melanogaster	Rcd-1r	Rcd-1 related	CG9573|DmelCG9573|Drcd-1r	0	0
2673	34230	Drosophila melanogaster	CG9582	CG9582 gene product from transcript CG9582-RA	DmelCG9582	0	0
2674	34231	Drosophila melanogaster	CG31708	CG31708 gene product from transcript CG31708-RC	CG13103|CG13104|CG13105|CT32337|CT32338|DmelCG31708	0	0
2675	34234	Drosophila melanogaster	CG12439	CG12439 gene product from transcript CG12439-RA	BcDNA:AT16114|DmelCG12439	0	0
2676	34235	Drosophila melanogaster	Toll-4	CG18241 gene product from transcript CG18241-RA	CG18241|CT29238|DmelCG18241|TL4|Tl-4|dTLR4|dToll4|toll	0	0
2677	34236	Drosophila melanogaster	Or30a	Odorant receptor 30a	30A.1|30a|CG13106|DOR56|DmelCG13106	0	0
2678	34237	Drosophila melanogaster	CG32982	CG32982 gene product from transcript CG32982-RE	CG18566|CG9584|CG9585|DmelCG32982	0	0
2679	34240	Drosophila melanogaster	CG13108	CG13108 gene product from transcript CG13108-RA	DmelCG13108	0	0
2680	34241	Drosophila melanogaster	CG9586	CG9586 gene product from transcript CG9586-RB	DmelCG9586	0	0
2681	34242	Drosophila melanogaster	tai	taiman	58/2|CG13109|CG18494|DAIB1|DmTaiman|DmelCG13109|EP(2)2630|NEST:bs13g05|TAI|Tai|bs13g05.y1|l(2)01351|l(2)k05802|l(2)k05809	0	0
2682	34244	Drosophila melanogaster	aust	australin	CG17009|DmelCG17009	0	0
2683	34245	Drosophila melanogaster	borr	borealin-related	Bor|Borr|CG4454|DmelCG4454|dBor	0	0
2684	34249	Drosophila melanogaster	CG17005	CG17005 gene product from transcript CG17005-RB	DmelCG17005	0	0
2685	34250	Drosophila melanogaster	Cks30A	Cyclin-dependent kinase subunit 30A	CDI2|CG3738|Cdi2|Cks|DmCks1|DmelCG3738|U40077|cdi2|cks|cks30A|fs(2)rem|rem	0	0
2686	34251	Drosophila melanogaster	Eaat1	Excitatory amino acid transporter 1	AF001784|CG3747|Dglt-1|Dglut-1|DmelCG3747|EAAT|EAAT1|dEAAT|dEAAT1|dEaat1	0	0
2687	34252	Drosophila melanogaster	CG3748	CG3748 gene product from transcript CG3748-RB	BcDNA:AT30262|DmelCG3748|anon-WO0140519.143	0	0
2688	34253	Drosophila melanogaster	CG13110	CG13110 gene product from transcript CG13110-RA	BcDNA:AT19071|DmelCG13110	0	0
2689	34256	Drosophila melanogaster	Aldh	Aldehyde dehydrogenase	146084_at|ACDH|ALDH|CG3752|DmelCG3752	0	0
2690	34257	Drosophila melanogaster	CG4438	CG4438 gene product from transcript CG4438-RB	DmelCG4438	0	0
2691	34258	Drosophila melanogaster	CG3759	CG3759 gene product from transcript CG3759-RB	DmelCG3759|MCO1	0	0
2692	34259	Drosophila melanogaster	Fbp2	Fat body protein 2	CG3763|DmelCG3763|Fbp-2|P6|T19	0	0
2693	34260	Drosophila melanogaster	FucTB	CG4435 gene product from transcript CG4435-RA	CG4435|CT14430|DmelCG4435	0	0
2694	34262	Drosophila melanogaster	CG3769	CG3769 gene product from transcript CG3769-RA	DmelCG3769	0	0
2695	34263	Drosophila melanogaster	numb	CG3779 gene product from transcript CG3779-RB	CG3779|DmelCG3779|N7-1|Nb|Numb|d-numb|dNumb|l(2)03235|l(2)3235|l(2)s2201|nb|numbs	0	0
2696	34264	Drosophila melanogaster	Gdi	GDP dissociation inhibitor	CG4422|DmelCG4422|GDI|Gdl|N7-3|dGDI|l(2)N7-3|rab GDI	0	0
2697	34268	Drosophila melanogaster	Oatp30B	Organic anion transporting polypeptide 30B	CG3811|DmelCG3811|oatp 30B	0	0
2698	34269	Drosophila melanogaster	CG17855	CG17855 gene product from transcript CG17855-RA	DmelCG17855	0	0
2699	34270	Drosophila melanogaster	Cpr30B	Cuticular protein 30B	CG3818|DmelCpr30B|DmelCG3818	0	0
2700	34271	Drosophila melanogaster	CG13113	CG13113 gene product from transcript CG13113-RA	DmelCG13113	0	0
2701	34272	Drosophila melanogaster	CG13114	CG13114 gene product from transcript CG13114-RB	DmelCG13114	0	0
2702	34274	Drosophila melanogaster	jp	junctophilin	CG13115|CG4405|DmelCG4405|JP	0	0
2703	34275	Drosophila melanogaster	CG3838	CG3838 gene product from transcript CG3838-RA	DmelCG3838|NEST:bs09e08|anon-WO0118547.445|bs09e08.y1	0	0
2704	34276	Drosophila melanogaster	CG4389	CG4389 gene product from transcript CG4389-RA	BcDNA:GH12558|DmelCG4389	0	0
2705	34277	Drosophila melanogaster	gcm	glial cells missing	CG12245|DmelCG12245|GCM|GCM1|Gcm|N7-4|glide|glide/gcm|l(2)N7-4|ucc	0	0
2706	34278	Drosophila melanogaster	CG3841	CG3841 gene product from transcript CG3841-RB	DmelCG3841	0	0
2707	34279	Drosophila melanogaster	CG4382	CG4382 gene product from transcript CG4382-RB	DmelCG4382	0	0
2708	34280	Drosophila melanogaster	gcm2	CG3858 gene product from transcript CG3858-RA	CG3858|DmelCG3858|FBgn0019809|Gcm2|dGCM2	0	0
2709	34281	Drosophila melanogaster	Trx-2	thioredoxin-2	31884|CG31884|CG3864|DmTrx-2|DmelTrx-2|DmelCG31884|Trx|anon-WO0118547.188|dmtrx-2	0	0
2710	34282	Drosophila melanogaster	GlcAT-S	CG3881 gene product from transcript CG3881-RA	CG3881|DGlcAT-S|DmGlcAT-BSI|DmelCG3881	0	0
2711	34283	Drosophila melanogaster	CG15828	CG15828 gene product from transcript CG15828-RC	CT39214|DmelCG15828	0	0
2712	34284	Drosophila melanogaster	Pka-C1	cAMP-dependent protein kinase 1	6353|C|CG4379|CdkA|Cos|Cos-1|Cos1|DC0|DCO|Dc0|Dco|Dcpk|DmelCG4379|PKA|PKA CI|PKA CI|PKA-C1|PKAC1|PKAc|PKAcA|Pka|Pka-C|PkaC1|cos1|dPKA|dcO|dco|l(2)01272|l(2)cos[[1]]|l(2)s4402|pKA	0	0
2713	34286	Drosophila melanogaster	pelo	pelota	CG3959|DmelCG3959|ms(2)01559	0	0
2714	34287	Drosophila melanogaster	CG4364	CG4364 gene product from transcript CG4364-RA	DmelCG4364	0	0
2715	34288	Drosophila melanogaster	CG13116	CG13116 gene product from transcript CG13116-RA	DmelCG13116	0	0
2716	34289	Drosophila melanogaster	CG13117	CG13117 gene product from transcript CG13117-RA	BcDNA:SD27505|DmelCG13117	0	0
2717	34291	Drosophila melanogaster	Cyp4e3	Cytochrome P450-4e3	4e3|CG4105|Cyp4E3|DmelCG4105|P-450|P450|cyp4e3	0	0
2718	34292	Drosophila melanogaster	zf30C	Zinc finger protein 30C	CG3998|DmelCG3998|Zf30c|Zn30C|anon-EST:Liang-1.52|clone 1.52|l(2)k02506|zf30c	0	0
2719	34293	Drosophila melanogaster	Taf11	TBP-associated factor 11	CG4079|DmelCG4079|TAF|TAF11|TAF30b|TAF30beta|TAF[II]30beta|TAF[[II]]|TAF[[II]]30beta|TFIID|TFIID 28 beta|TFIID-p28beta|Taf30beta|Taf[[II]]30beta|d30beta|dTAFII30|dTAFII30 beta|dTAFII30beta|dTAF[[II	0	0
2720	34294	Drosophila melanogaster	und	uninitiated	CG4008|DMAP2|DmelCG4008|MAP2	0	0
2721	34295	Drosophila melanogaster	CG13120	CG13120 gene product from transcript CG13120-RA	DmelCG13120	0	0
2722	34296	Drosophila melanogaster	CG4017	CG4017 gene product from transcript CG4017-RA	DmelCG4017	0	0
2723	34297	Drosophila melanogaster	CG17633	CG17633 gene product from transcript CG17633-RA	DmelCG17633	0	0
2724	34298	Drosophila melanogaster	CG13121	CG13121 gene product from transcript CG13121-RA	DmelCG13121	0	0
2725	34299	Drosophila melanogaster	ppk11	pickpocket 11	CG34058|CG4110|DmelCG34058|PPK11	0	0
2726	34300	Drosophila melanogaster	IP3K1	Inositol 1,4,5-triphosphate kinase 1	CG4026|D-IP3K1|DmelCG4026|IP3KI|IP[[3]]K-1|UY530|anon-WO0118547.189|dmIP3Kalpha|dmIP[[3]]Kalpha	0	0
2727	34302	Drosophila melanogaster	CG4036	CG4036 gene product from transcript CG4036-RC	DmelCG4036	0	0
2728	34303	Drosophila melanogaster	CG13123	CG13123 gene product from transcript CG13123-RA	DmelCG13123	0	0
2729	34304	Drosophila melanogaster	nAcRalpha-30D	nicotinic Acetylcholine Receptor alpha 30D	BcDNA:GH01410|CG4128|CT13662|Dalpha6|DmelCG4128|alpha6|nAChR|nAChR alpha 6|rsn	0	0
2730	34306	Drosophila melanogaster	CG4537	CG4537 gene product from transcript CG4537-RB	DmelCG4537	0	0
2731	34307	Drosophila melanogaster	CG5924	CG5924 gene product from transcript CG5924-RA	DmelCG5924	0	0
2732	34309	Drosophila melanogaster	RpS2	Ribosomal protein S2	CG5920|Dm01335|DmelCG5920|S2|anon-EST:Posey202|dS2|l(2)03848|l(2)k04603|sop	0	0
2733	34310	Drosophila melanogaster	CG13124	CG13124 gene product from transcript CG13124-RE	DmelCG13124	0	0
2734	34311	Drosophila melanogaster	Etl1	Etl1 homologue	CG5899|DmelCG5899	0	0
2735	34312	Drosophila melanogaster	Srp54	CG4602 gene product from transcript CG4602-RA	CG4602|DmelCG4602|SRp54|anon-EST:Liang-2.16|clone 2.16|dSRp54|p75	0	0
2736	34313	Drosophila melanogaster	yip2	yippee interacting protein 2	CG4600|DmelCG4600|Yip2	0	0
2737	34314	Drosophila melanogaster	CG5885	CG5885 gene product from transcript CG5885-RA	AI945207|BEST:CK01296|CK01296|DmelCG5885	0	0
2738	34315	Drosophila melanogaster	CG4598	CG4598 gene product from transcript CG4598-RA	DmelCG4598	0	0
2739	34316	Drosophila melanogaster	CG4594	CG4594 gene product from transcript CG4594-RA	DmelCG4594	0	0
2740	34317	Drosophila melanogaster	CG4592	CG4592 gene product from transcript CG4592-RA	DmelCG4592	0	0
2741	34318	Drosophila melanogaster	CG13125	CG13125 gene product from transcript CG13125-RA	CT32363|DmelCG13125|mel CG13125	0	0
2742	34319	Drosophila melanogaster	CG42366	CG42366 gene product from transcript CG42366-RA	CG31711|CG31878|CG4588|DmelCG42366|Dmel_CG31711|Dmel_CG31878|IME2|MAK	0	0
2743	34320	Drosophila melanogaster	CG31712	CG31712 gene product from transcript CG31712-RA	CG5879|DmelCG31712	0	0
2744	34321	Drosophila melanogaster	CG4619	CG4619 gene product from transcript CG4619-RA	CG31879|DmelCG4619	0	0
2745	34322	Drosophila melanogaster	CG5853	CG5853 gene product from transcript CG5853-RA	DmelCG5853	0	0
2746	34323	Drosophila melanogaster	CG13126	CG13126 gene product from transcript CG13126-RA	DmelCG13126|anon-WO0118547.208	0	0
2747	34324	Drosophila melanogaster	CG4709	CG4709 gene product from transcript CG4709-RA	DmelCG4709	0	0
2748	34325	Drosophila melanogaster	CG4658	CG4658 gene product from transcript CG4658-RA	DmelCG4658	0	0
2749	34326	Drosophila melanogaster	CG5846	CG5846 gene product from transcript CG5846-RA	DmelCG5846	0	0
2750	34327	Drosophila melanogaster	CG5850	CG5850 gene product from transcript CG5850-RD	DmelCG5850	0	0
2751	34328	Drosophila melanogaster	Dref	DNA replication-related element factor	CG5838|DREF|DmDREF|DmelCG5838|Dmel_DREF|dDREF|dref|p80|p80/DREF	0	0
2752	34329	Drosophila melanogaster	RpL13	Ribosomal protein L13	Bbc1|CG4651|Dmbbc1|DmelCG4651|L13|M(2)31A|Rpl13|anon-EST:fe1D1|bbc1|rpL13	0	0
2753	34330	Drosophila melanogaster	bib	big brain	BIB_DROME|Bib|CG4722|DmelCG4722|EP(2)2278|l(2)neo66|l(2)neo91|m-bib	0	0
2754	34331	Drosophila melanogaster	CG31755	CG31755 gene product from transcript CG31755-RC	CG5831|DmelCG31755	0	0
2755	34333	Drosophila melanogaster	Trp1	Translocation protein 1	CG4758|DmelCG4758|Dtrp1|Tp1|sec62	0	0
2756	34334	Drosophila melanogaster	CG13131	CG13131 gene product from transcript CG13131-RB	DmelCG13131	0	0
2757	34335	Drosophila melanogaster	CG13127	CG13127 gene product from transcript CG13127-RA	DmelCG13127	0	0
2758	34336	Drosophila melanogaster	obst-B	obstructor-B	BEST:CK00178|BcDNA:GH02976|CG4778|CK00178|DmelCG4778	0	0
2759	34338	Drosophila melanogaster	Pen	Pendulin	2.1|CG4799|DPend|Dalpha2|Dimp-alpha2|DmelCG4799|IMPalpha2|Imp-alpha2|Kap alpha2|Kap-alpha2|Kpna2|OHO31|Oho31|Rch1|alpha2|alpha2A-Kap|anon-WO0140519.258|bs29g06.y1|imp alpha2|imp	0	0
2760	34339	Drosophila melanogaster	Spn31A	Serpin 31A	CG4804|DmelCG4804	0	0
2761	34341	Drosophila melanogaster	CG31714	CG31714 gene product from transcript CG31714-RB	CG13132|CG5833|DmelCG31714	0	0
2762	34342	Drosophila melanogaster	CG13133	CG13133 gene product from transcript CG13133-RA	DmelCG13133	0	0
2763	34345	Drosophila melanogaster	CG13135	CG13135 gene product from transcript CG13135-RA	DmelCG13135	0	0
2764	34348	Drosophila melanogaster	CG4839	CG4839 gene product from transcript CG4839-RB	DmelCG4839	0	0
2765	34349	Drosophila melanogaster	CG13137	CG13137 gene product from transcript CG13137-RB	DmelCG13137	0	0
2766	34350	Drosophila melanogaster	Sur	Sulfonylurea receptor	BEST:CK00325|CG5772|CK00325|CYO|Dme_CG5772|DmelCG5772|Dsur|SUR|cyo|dSUR|dSur|dsur|sur	0	0
2767	34351	Drosophila melanogaster	Ripalpha	RPA-interacting protein alpha	CG18145|DmelCG18145|Tes83|dRIPalpha	0	0
2768	34352	Drosophila melanogaster	RpL7	Ribosomal protein L7	BEST:GH04411|CG4897|DmelCG4897|L7|M(2)31A|RPL7	0	0
2769	34353	Drosophila melanogaster	CG34159	CG34159 gene product from transcript CG34159-RA	CG5739|DmelCG34159	0	0
2770	34354	Drosophila melanogaster	CG5734	CG5734 gene product from transcript CG5734-RA	DmelCG5734	0	0
2771	34355	Drosophila melanogaster	CG5731	CG5731 gene product from transcript CG5731-RA	DmelCG5731	0	0
2772	34356	Drosophila melanogaster	CG5727	CG5727 gene product from transcript CG5727-RA	BcDNA:LD02427|DmelCG5727	0	0
2773	34357	Drosophila melanogaster	CG4901	CG4901 gene product from transcript CG4901-RA	DmelCG4901|cg4901	0	0

2774	34358	Drosophila melanogaster	Npc1a	Niemann-Pick type C-1a	CG5722|DmNPC|DmelCG5722|NPC1|NPC1a|Npc1|Npc1_Dm|dNPC1|dNPC1a|dmNPC1|dncp1a|dnpc1a|npc1|npc1a	0	0
2775	34359	Drosophila melanogaster	Pros35	Proteasome 35kD subunit	143314_at|CG4904|DmelCG4904|PROS-35|PROS-Dm35|Pros-35|Pros-Dm35|Prosalpha6|alpha_dm|anon-SAGE:Wang-116|pros 35|pros35	0	0
2776	34360	Drosophila melanogaster	CG4908	CG4908 gene product from transcript CG4908-RB	DmelCG4908	0	0
2777	34361	Drosophila melanogaster	CG5708	CG5708 gene product from transcript CG5708-RB	DmelCG5708	0	0
2778	34362	Drosophila melanogaster	CG5694	CG5694 gene product from transcript CG5694-RA	DmelCG5694	0	0
2779	34363	Drosophila melanogaster	eEF1delta	CG4912 gene product from transcript CG4912-RB	CG4912|DmelCG4912|eEF1d	0	0
2780	34364	Drosophila melanogaster	me31B	maternal expression at 31B	CG4916|DDX6|Dhh1p/Me31b|DmRH3|DmelCG4916|ME31B|Mat31B|Me31B|Me31b|l(2)k06607	0	0
2781	34367	Drosophila melanogaster	Ror	CG4926 gene product from transcript CG4926-RA	CG4926|DRor|DmHD-2|DmelCG4926|Dror|HD-2|ROR|ROR1	0	0
2782	34368	Drosophila melanogaster	CG5676	CG5676 gene product from transcript CG5676-RA	BEST:LD31953|DmelCG5676|LD31953	0	0
2783	34370	Drosophila melanogaster	Rsf1	Repressor splicing factor 1	CG5655|DmelCG5655|ROX21|RSF1|Rox21|rox21	0	0
2784	34371	Drosophila melanogaster	CG18619	CG18619 gene product from transcript CG18619-RD	BcDNA:RH73910|DmelCG18619	0	0
2785	34372	Drosophila melanogaster	Mob3	CG4946 gene product from transcript CG4946-RA	CG4946|DMob3|DmelCG4946|Dmob3|dmob3	0	0
2786	34373	Drosophila melanogaster	CG4953	CG4953 gene product from transcript CG4953-RA	DmelCG4953	0	0
2787	34374	Drosophila melanogaster	CG4957	CG4957 gene product from transcript CG4957-RA	DmelCG4957	0	0
2788	34376	Drosophila melanogaster	trk	trunk	CG5619|DmelCG5619|fs(2)trunk	0	0
2789	34377	Drosophila melanogaster	Utx	CG5640 gene product from transcript CG5640-RD	CG5640|DmelCG5640|anon-31BCa|dUTX|dUtx	0	0
2790	34378	Drosophila melanogaster	CG5604	CG5604 gene product from transcript CG5604-RA	DmelCG5604	0	0
2791	34379	Drosophila melanogaster	Hand	CG18144 gene product from transcript CG18144-RA	CG18144|Dhand|DmelCG18144|HAND|bHLHa28|dHAND|dHand|hand|hand-C	0	0
2792	34380	Drosophila melanogaster	CYLD	cylindromatosis	CG5603|DmelCG5603|dCYLD	0	0
2793	34381	Drosophila melanogaster	CG13138	CG13138 gene product from transcript CG13138-RB	DmelCG13138	0	0
2794	34383	Drosophila melanogaster	CG5390	CG5390 gene product from transcript CG5390-RA	BEST:GH06341|DmelCG5390|SPH35|c-SPH35|gh06341	0	0
2795	34384	Drosophila melanogaster	CG4968	CG4968 gene product from transcript CG4968-RA	DmelCG4968	0	0
2796	34385	Drosophila melanogaster	Cdk5alpha	Cdk5 activator-like protein	CG5387|Cdk5a|D-p35|DmelCG5387|Dp35|P35|dCdk5alpha|p25|p35|p35-21C|p35-31C	0	0
2797	34386	Drosophila melanogaster	CG5385	CG5385 gene product from transcript CG5385-RA	DmelCG5385	0	0
2798	34387	Drosophila melanogaster	CG5384	CG5384 gene product from transcript CG5384-RA	DmelCG5384	0	0
2799	34388	Drosophila melanogaster	CG4972	CG4972 gene product from transcript CG4972-RA	DmelCG4972	0	0
2800	34389	Drosophila melanogaster	CG5381	CG5381 gene product from transcript CG5381-RA	DmelCG5381	0	0
2801	34390	Drosophila melanogaster	CG4995	CG4995 gene product from transcript CG4995-RB	CG18626|DmelCG4995	0	0
2802	34392	Drosophila melanogaster	RnrL	Ribonucleoside diphosphate reductase large subunit	CG5371|DmRNR1|DmelCG5371|RNR1|RnrM1|RntL|l(2)k06709	0	0
2803	34393	Drosophila melanogaster	CG5375	CG5375 gene product from transcript CG5375-RB	DmelCG5375	0	0
2804	34394	Drosophila melanogaster	CG5037	CG5037 gene product from transcript CG5037-RA	DmelCG5037	0	0
2805	34395	Drosophila melanogaster	GATAd	CG5034 gene product from transcript CG5034-RA	CG5034|DmelCG5034|dGATA-D|dGATAd	0	0
2806	34396	Drosophila melanogaster	SamDC	S-adenosylmethionine decarboxylase	CG5029|DmelCG5029|l(2)31Dc|l(2)31Dd|l(2)31De	0	0
2807	34397	Drosophila melanogaster	Sps2	Selenophosphate synthetase 2	CG5025|DmelCG5025|Dsps2|SPS2|dSPS2|dsps2|sps2	0	0
2808	34398	Drosophila melanogaster	CG5022	CG5022 gene product from transcript CG5022-RA	DmelCG5022	0	0
2809	34401	Drosophila melanogaster	CG5367	CG5367 gene product from transcript CG5367-RA	DmelCG5367	0	0
2810	34402	Drosophila melanogaster	CG5045	CG5045 gene product from transcript CG5045-RA	DmelCG5045	0	0
2811	34403	Drosophila melanogaster	Cand1	Cullin-associated and neddylation-dissociated 1	BcDNA:GH07774|CG5366|DmelCG5366	0	0
2812	34404	Drosophila melanogaster	pim	pimples	CG5052|DmelCG5052|PIM|frag2|l(2)31Df	0	0
2813	34405	Drosophila melanogaster	lft	lowfat	CG13139|DmelCG13139	0	0
2814	34406	Drosophila melanogaster	CG5056	CG5056 gene product from transcript CG5056-RA	DmelCG5056	0	0
2815	34408	Drosophila melanogaster	dpr19	CG13140 gene product from transcript CG13140-RA	BcDNA:LP09705|CG13140|DmelCG13140|Dpr-19	0	0
2816	34409	Drosophila melanogaster	gny	garnysstan	CG5091|Dalg6|DmelCG5091|alg6	0	0
2817	34410	Drosophila melanogaster	CG5096	CG5096 gene product from transcript CG5096-RA	CT16355|DmelCG5096	0	0
2818	34411	Drosophila melanogaster	cdc2	CG5363 gene product from transcript CG5363-RA	5363|CDC2|CDCDm|CDK1|CDK1/CDC2|CG5363|Cdc2|Cdk-1|Cdk1|Dcdc2|Dm cdc2|DmCdc2|DmCdk1|Dmcdc2|DmelCG5363|cdc|cdc2Dm|cdk1|group 4|l(2)31Eh	0	0
2819	34412	Drosophila melanogaster	mRpS7	mitochondrial ribosomal protein S7	CG5108|DmelCG5108|MRP-S7|RpS7|mRPS7	0	0
2820	34413	Drosophila melanogaster	da	daughterless	5102|CG5102|DA|Da|DmelCG5102|bHLHb22|l(2)31Ea	0	0
2821	34414	Drosophila melanogaster	Mdh1	Malate dehydrogenase 1	CG5362|CT17038|DmelCG5362|MDH|MDH-1|Mdh|Mdh-1|Mdh2|MdhD|cMdh|sMdh	0	0
2822	34416	Drosophila melanogaster	CG31716	CG31716 gene product from transcript CG31716-RH	CG5244|CG5251|DmelCG31716|NOT4|Not4|cg5251|not3/5|not4	0	0
2823	34419	Drosophila melanogaster	Ip259	Intronic Protein 259	CG5277|DmelCG5277|IP259	0	0
2824	34420	Drosophila melanogaster	RpS27A	Ribosomal protein S27A	CG5271|DUb80|DmelCG5271|Dub80|M(2)31E|UB3-D|Ubi-f80|Ubi-m|Ubiq|l(2)04820|l(2)31Ei|mfs(2)31|mfs(2)48|mfs48	0	0
2825	34421	Drosophila melanogaster	CG5355	CG5355 gene product from transcript CG5355-RA	DmelCG5355	0	0
2826	34422	Drosophila melanogaster	Klp31E	CG5300 gene product from transcript CG5300-RD	CG5300|DmCG5300|DmKlp31D|DmKlp31E|DmelCG5300|KIF21A	0	0
2827	34423	Drosophila melanogaster	RfC3	Replication factor C subunit 3	CG5313|DRFC|DmRFC3|DmelCG5313|Rfc3	0	0
2828	34425	Drosophila melanogaster	CG5168	CG5168 gene product from transcript CG5168-RB	DmelCG5168	0	0
2829	34426	Drosophila melanogaster	SmB	Small ribonucleoprotein particle protein SmB	B|CG5352|DmelCG5352|SYM10|Sm B|l(2)SH0509|l(2)SH2 0509|snRNP B	0	0
2830	34427	Drosophila melanogaster	KdelR	KDEL receptor	BEST:CK00230|CG5183|CK00230|DmelCG5183|ERD2|Erd2|dmErd2|l(2)k00311	0	0
2831	34428	Drosophila melanogaster	CG5188	CG5188 gene product from transcript CG5188-RA	DmelCG5188|MAP1B	0	0
2832	34429	Drosophila melanogaster	CG5343	CG5343 gene product from transcript CG5343-RA	DmelCG5343	0	0
2833	34430	Drosophila melanogaster	TfIIB	Transcription factor IIB	CG5193|DmelCG5193|TFIIB|Tfiib|TfIIB|dTFIIB|dTfIIB|dmTFIIB	0	0
2834	34431	Drosophila melanogaster	CG5337	CG5337 gene product from transcript CG5337-RA	DmelCG5337	0	0
2835	34432	Drosophila melanogaster	CG5198	CG5198 gene product from transcript CG5198-RA	DmelCG5198	0	0
2836	34433	Drosophila melanogaster	CHIP	CG5203 gene product from transcript CG5203-RA	CG5203|Chip|DmelCG5203|dCHIP	0	0
2837	34434	Drosophila melanogaster	CG13142	CG13142 gene product from transcript CG13142-RB	DmelCG13142	0	0
2838	34435	Drosophila melanogaster	CG6232	CG6232 gene product from transcript CG6232-RB	CT19510|DmelCG6232	0	0
2839	34436	Drosophila melanogaster	CG5322	CG5322 gene product from transcript CG5322-RA	DmelCG5322	0	0
2840	34437	Drosophila melanogaster	CG6206	CG6206 gene product from transcript CG6206-RA	BcDNA:GH02419|DmelCG6206|dLM	0	0
2841	34438	Drosophila melanogaster	RluA-1	CG31719 gene product from transcript CG31719-RA	CG13143|CG31719|CG7475|DmelCG31719	0	0
2842	34440	Drosophila melanogaster	RluA-2	CG6187 gene product from transcript CG6187-RA	CG6187|DmelCG6187|yumo	0	0
2843	34441	Drosophila melanogaster	Grip75	CG6176 gene product from transcript CG6176-RA	75p|CG6176|DGrip75|Dgrip75|Dgrip75/d75p|Dgrip75s|DmelCG6176|d75p	0	0
2844	34442	Drosophila melanogaster	CG7456	CG7456 gene product from transcript CG7456-RA	DmelCG7456	0	0
2845	34443	Drosophila melanogaster	CG6144	CG6144 gene product from transcript CG6144-RB	DmelCG6144	0	0
2846	34444	Drosophila melanogaster	CG13144	CG13144 gene product from transcript CG13144-RA	DmelCG13144	0	0
2847	34445	Drosophila melanogaster	Myo31DF	Myosin 31DF	CG7438|Dm IA|DmIA|DmelCG7438|DroMIA|IA|MYOIA|Myo-IA|Myo1A|MyoIA|myoID|souther	0	0
2848	34446	Drosophila melanogaster	CG6094	CG6094 gene product from transcript CG6094-RA	DmelCG6094|FBgn 32261	0	0
2849	34447	Drosophila melanogaster	CG7384	CG7384 gene product from transcript CG7384-RA	DmelCG7384	0	0
2850	34449	Drosophila melanogaster	Lrr47	Leucine-rich repeat 47	CG6098|CT19173|DmelCG6098|LRR47|Lrr-47	0	0
2851	34450	Drosophila melanogaster	Lip4	Lipase 4	CG6113|DmelCG6113|dLip4	0	0
2852	34451	Drosophila melanogaster	CG18301	CG18301 gene product from transcript CG18301-RA	DmelCG18301	0	0
2853	34452	Drosophila melanogaster	CG18302	CG18302 gene product from transcript CG18302-RA	DmelCG18302	0	0
2854	34453	Drosophila melanogaster	Trim9	CG31721 gene product from transcript CG31721-RB	CG13145|CG31721|CG6256|DmelCG31721	0	0
2855	34454	Drosophila melanogaster	CG6138	CG6138 gene product from transcript CG6138-RA	BcDNA:AT25107|DmelCG6138	0	0
2856	34455	Drosophila melanogaster	w-cup	world cup	BcDNA:AT31112|CG7363|DmelCG7363	0	0
2857	34457	Drosophila melanogaster	CG7329	CG7329 gene product from transcript CG7329-RA	DmelCG7329|ORE-4	0	0
2858	34458	Drosophila melanogaster	CG31872	CG31872 gene product from transcript CG31872-RA	CG17093|CG17101|DmelCG31872	0	0
2859	34460	Drosophila melanogaster	CG18284	CG18284 gene product from transcript CG18284-RB	Acp|BEST:GH10507|DmelCG18284	0	0
2860	34461	Drosophila melanogaster	CG17097	CG17097 gene product from transcript CG17097-RB	DmelCG17097	0	0
2861	34462	Drosophila melanogaster	CG17098	CG17098 gene product from transcript CG17098-RA	DmelCG17098	0	0
2862	34463	Drosophila melanogaster	CG31871	CG31871 gene product from transcript CG31871-RA	CG17087|CG17099|DmelCG31871	0	0
2863	34465	Drosophila melanogaster	CG17104	CG17104 gene product from transcript CG17104-RA	BEST:LD16579|DmelCG17104	0	0
2864	34466	Drosophila melanogaster	CG17105	CG17105 gene product from transcript CG17105-RA	DmelCG17105	0	0
2865	34467	Drosophila melanogaster	CG17107	CG17107 gene product from transcript CG17107-RA	DmelCG17107	0	0
2866	34468	Drosophila melanogaster	CG7299	CG7299 gene product from transcript CG7299-RB	BcDNA:RE24635|CT22515|DmelCG7299	0	0
2867	34469	Drosophila melanogaster	CG7296	CG7296 gene product from transcript CG7296-RA	BcDNA:RH68528|DmelCG7296	0	0
2868	34470	Drosophila melanogaster	CG7294	CG7294 gene product from transcript CG7294-RA	BcDNA:RE28280|DmelCG7294	0	0
2869	34471	Drosophila melanogaster	CG17108	CG17108 gene product from transcript CG17108-RA	BEST:GH23489|CT33647|DmelCG17108|gh23489	0	0
2870	34472	Drosophila melanogaster	CG7300	CG7300 gene product from transcript CG7300-RC	DmelCG7300	0	0
2871	34474	Drosophila melanogaster	CG6415	CG6415 gene product from transcript CG6415-RA	151967_at|DmelCG6415|anon-32Aa	0	0
2872	34476	Drosophila melanogaster	CG6431	CG6431 gene product from transcript CG6431-RC	DmelCG6431|chr2L_10724193_10724543.0	0	0
2873	34477	Drosophila melanogaster	CG6443	CG6443 gene product from transcript CG6443-RA	DmelCG6443	0	0
2874	34478	Drosophila melanogaster	CG17118	CG17118 gene product from transcript CG17118-RA	DmelCG17118	0	0
2875	34479	Drosophila melanogaster	CG6750	CG6750 gene product from transcript CG6750-RA	DmelCG6750	0	0
2876	34480	Drosophila melanogaster	Dpy-30L1	Dpy-30-like 1	CG6444|DmelCG6444|dpy-30L1	0	0
2877	34481	Drosophila melanogaster	Nup107	Nucleoporin 107	BcDNA:LD18761|CG6743|DmelCG6743|Nup 107|Nup170|dmNup107|nup107	0	0
2878	34482	Drosophila melanogaster	Vha16-5	Vacuolar H[+] ATPase subunit 16-5	CG6737|DmelCG6737|vha16-5	0	0
2879	34483	Drosophila melanogaster	CG12299	CG12299 gene product from transcript CG12299-RA	DmelCG12299	0	0
2880	34484	Drosophila melanogaster	CG6729	CG6729 gene product from transcript CG6729-RA	DmelCG6729	0	0
2881	34485	Drosophila melanogaster	CG17124	CG17124 gene product from transcript CG17124-RB	DmelCG17124|anon-WO0118547.153	0	0
2882	34486	Drosophila melanogaster	CG6495	CG6495 gene product from transcript CG6495-RA	BcDNA:GH10711|DmelCG6495|GH10711	0	0
2883	34487	Drosophila melanogaster	UbcD2	Ubiquitin conjugating enzyme 2	CG6720|DmelCG6720|l(2)k13206|ubcD2	0	0
2884	34488	Drosophila melanogaster	CG6724	CG6724 gene product from transcript CG6724-RA	DmelCG6724	0	0

2885	34489	Drosophila melanogaster	CG17127	CG17127 gene product from transcript CG17127-RB	DmelCG17127	0	0
2886	34490	Drosophila melanogaster	CG31869	CG31869 gene product from transcript CG31869-RA	CG17130|CG18499|DmelCG31869	0	0
2887	34493	Drosophila melanogaster	CG6508	CG6508 gene product from transcript CG6508-RA	DmelCG6508	0	0
2888	34494	Drosophila melanogaster	CG17134	CG17134 gene product from transcript CG17134-RA	DmelCG17134	0	0
2889	34495	Drosophila melanogaster	Nos	Nitric oxide synthase	CG6713|DNOS|DNOS1|DmelCG6713|NOS|dNOS|dNos|drNOSoxy|nNOS	0	0
2890	34496	Drosophila melanogaster	CG6700	CG6700 gene product from transcript CG6700-RA	DmelCG6700	0	0
2891	34497	Drosophila melanogaster	CG17139	CG17139 gene product from transcript CG17139-RA	CG31722|CG31722-A|DmelCG17139	0	0
2892	34499	Drosophila melanogaster	Porin2	CG17137 gene product from transcript CG17137-RA	BEST:LP07660|CG17137|DmPorin2|DmelCG17137|LP07660.3prime|Porin 2	0	0
2893	34500	Drosophila melanogaster	porin	CG6647 gene product from transcript CG6647-RA	CG6647|DVDAC|DmPorin1|DmVDAC|DmelCG6647|POR-1|Porin|VDAC|Vdac|anon-WO02059370.56|cg6647|l(2)k05123	0	0
2894	34502	Drosophila melanogaster	CG43129	CG43129 gene product from transcript CG43129-RC	CG17085|CG17086|DmelCG43129|Dmel_CG17085|Dmel_CG17086	0	0
2895	34503	Drosophila melanogaster	Dnz1	DNZDHHC/NEW1 zinc finger protein 11	CG6627|DNZ1|DmelCG6627	0	0
2896	34504	Drosophila melanogaster	ial	Ipll-aurora-like kinase	AurB|Aurora B|Aurora-B|CG6620|DmAIRK2|DmAurB|DmAuroraB|DmelCG6620|IAL|IAL1|Ial|aurB|aurora B	0	0
2897	34505	Drosophila melanogaster	Stam	Signal transducing adaptor molecule	CG6521|DmelCG6521|Dstam|STAM|stam	0	0
2898	34506	Drosophila melanogaster	CG12517	CG12517 gene product from transcript CG12517-RA	DmelCG12517	0	0
2899	34507	Drosophila melanogaster	CG14071	CG14071 gene product from transcript CG14071-RB	DmelCG14071	0	0
2900	34508	Drosophila melanogaster	CG14070	CG14070 gene product from transcript CG14070-RA	DmelCG14070	0	0
2901	34509	Drosophila melanogaster	CG7309	CG7309 gene product from transcript CG7309-RC	DmelCG7309	0	0
2902	34510	Drosophila melanogaster	CG14069	CG14069 gene product from transcript CG14069-RA	DmelCG14069	0	0
2903	34513	Drosophila melanogaster	CG14072	CG14072 gene product from transcript CG14072-RB	DmelCG14072	0	0
2904	34514	Drosophila melanogaster	CG33129	CG33129 gene product from transcript CG33129-RE	CG6087|CG6089|DmelCG33129	0	0
2905	34516	Drosophila melanogaster	YL-1	CG4621 gene product from transcript CG4621-RA	CG4621|DmelCG4621|dYL-1	0	0
2906	34517	Drosophila melanogaster	CG16743	CG16743 gene product from transcript CG16743-RA	BcDNA:RH23514|DmelCG16743	0	0
2907	34519	Drosophila melanogaster	SCAR	CG4636 gene product from transcript CG4636-RA	BEST:SD02991|CG4636|D-SCAR|DWave|DmSCAR|DmelCG4636|Dscar|SCAR/WAVE|Scar|WAVE|Wave|dWAVE|l(2)k03107|l(2)k13811|scar|wave	0	0
2908	34521	Drosophila melanogaster	piwi	CG6122 gene product from transcript CG6122-RA	CG6122|DmelCG6122|PIWI|Piwi	0	0
2909	34524	Drosophila melanogaster	aub	aubergine	AUB|Aub|CG6137|DmelCG6137|ms(2)32D|sti|sting	0	0
2910	34526	Drosophila melanogaster	RpL9	Ribosomal protein L9	CG6141|DmelCG6141|L9|M(2)32D|Rpl9|anon-EST:fe3A6|anon-WO0153538.25|anon-WO0153538.26|anon-WO0153538.27|rpL9	0	0
2911	34527	Drosophila melanogaster	Nup154	Nucleoporin 154	BcDNA:LD21772|CG4579|DmelCG4579|Nup32D|l(2)01501|l(2)10432|ms(2)zk|nup154|nup32D|tip|tlp|zk	0	0
2912	34528	Drosophila melanogaster	Art8	Arginine methyltransferase 8	CG16840|DART8|DmelCG16840	0	0
2913	34529	Drosophila melanogaster	dUTPase	Deoxyuridine triphosphatase	BcDNA:LD08534|CG4584|DmelCG4584|LD08534|UTPase|anon-SAGE:Wang-077	0	0
2914	34530	Drosophila melanogaster	Samuel	SAM-motif ubiquitously expressed punctatedly localiz	78BP|CG12291|CG14917|CG14918|CG31868|DmelCG31868|Moses|moses	0	0
2915	34531	Drosophila melanogaster	Acp32CD	Accessory gland-specific peptide 32CD	CG4605|DmelCG4605	0	0
2916	34532	Drosophila melanogaster	CG14913	CG14913 gene product from transcript CG14913-RB	DmelCG14913	0	0
2917	34536	Drosophila melanogaster	CG14915	CG14915 gene product from transcript CG14915-RA	DmelCG14915	0	0
2918	34537	Drosophila melanogaster	Ast-C	Allatostatin C	ASC|AST-C|ASTC|Ast2|AstC|BEST:GH06087|BcDNA:RH36507|CG14919|CG149199|DAP-C|DmelCG14919|Drm-AST-C|FLT|M-ASH|ast2|flt|gh06087	0	0
2919	34538	Drosophila melanogaster	Ast-CC	Allatostatin double C	ASCC|ASTCC|CG14920|DmelCG14920	0	0
2920	34539	Drosophila melanogaster	CG16854	CG16854 gene product from transcript CG16854-RB	DmelCG16854	0	0
2921	34540	Drosophila melanogaster	CG4705	CG4705 gene product from transcript CG4705-RB	DmelCG4705	0	0
2922	34541	Drosophila melanogaster	Ge-1	CG6181 gene product from transcript CG6181-RA	CG6181|DmelCG6181|EDC4|GE-1	0	0
2923	34542	Drosophila melanogaster	Reps	CG6192 gene product from transcript CG6192-RB	CG6192|DmelCG6192|REPS	0	0
2924	34543	Drosophila melanogaster	l(2)gd1	lethal (2) giant discs 1	CG4713|DmelCG4713|SH495|gd|l(2)SH0495|l(2)SH2 0495|l(2)gd|l(2)giant disc|l[[2]]gd|lgd	0	0
2925	34544	Drosophila melanogaster	CG6201	CG6201 gene product from transcript CG6201-RA	DmelCG6201	0	0
2926	34545	Drosophila melanogaster	Gr32a	Gustatory receptor 32a	32D.1|32D1|CG14916|DmelCG14916|GR32D.1|GR32D1|GR32a|Gr32A|Gr32D1|Gr32d	0	0
2927	34546	Drosophila melanogaster	CG6230	CG6230 gene product from transcript CG6230-RA	BcDNA:GH06032|DmelCG6230|dCOD1	0	0
2928	34547	Drosophila melanogaster	CG14921	CG14921 gene product from transcript CG14921-RA	DmelCG14921	0	0
2929	34548	Drosophila melanogaster	Csl4	CG6249 gene product from transcript CG6249-RA	CG6249|CSL4|DmelCG6249|dCsl4	0	0
2930	34549	Drosophila melanogaster	Nup160	Nucleoporin 160	CG4738|DmelCG4738|Nup160[mel]|l(2)SH2 2055|l(2)SH2055|nup160	0	0
2931	34550	Drosophila melanogaster	RfC38	Replication factor C 38kD subunit	BcDNA:LD06837|CG6258|DmRFC5|DmelCG6258|Rfc38|l(2)k13807|l(2)rfc38|n(2)k13807	0	0
2932	34551	Drosophila melanogaster	CG4751	CG4751 gene product from transcript CG4751-RA	DmelCG4751	0	0
2933	34552	Drosophila melanogaster	hgo	homogentisate 1,2-dioxygenase	CG4779|DmelCG4779	0	0
2934	34553	Drosophila melanogaster	Gyc32E	Guanyl cyclase at 32E	CG33114|CG6275|Cyg1|DMRGC|DmelCG33114|GC|GYC 32E|Gyc-r	0	0
2935	34554	Drosophila melanogaster	CG6287	CG6287 gene product from transcript CG6287-RA	DmelCG6287|GH26	0	0
2936	34557	Drosophila melanogaster	Ca-beta	Ca2+-channel-protein-beta-subunit	CG14922|CG14923|CG33305|CG42403|CG6320|CaBeta|DmCa1beta|DmelCG42403|Dmel_CG33305|Dmel_CG6320|dmcab1	0	0
2937	34558	Drosophila melanogaster	Vm32E	Vitelline membrane 32E	CG16874|DmelCG16874|VM321|VM32E|VMP32E|Vm3|Vm32Ea|Vm32Ec	0	0
2938	34559	Drosophila melanogaster	CG4788	CG4788 gene product from transcript CG4788-RA	DmelCG4788	0	0
2939	34560	Drosophila melanogaster	ab	abrupt	Ab|B2149|CG4807|DmelCG4807|clu|l(2)k02807|ptd	0	0
2940	34564	Drosophila melanogaster	Ppt2	Palmitoyl-protein Thioesterase 2	CG4851|DmelCG4851|PPT-2	0	0
2941	34565	Drosophila melanogaster	mre11	meiotic recombination 11	16928|CG16928|DmelCG16928|MRE11|Mre11	0	0
2942	34566	Drosophila melanogaster	Osi21	Osiris 21	CG14925|DmelCG14925	0	0
2943	34567	Drosophila melanogaster	CG14926	CG14926 gene product from transcript CG14926-RA	DmelCG14926|bs34f02.y1	0	0
2944	34568	Drosophila melanogaster	salr	spalt-related	32F|CG4881|Cf3|DmelCG4881|Sal-r|Spalt|sal|sal-r|salR|spalt	0	0
2945	34569	Drosophila melanogaster	salm	spalt major	3602|B1164|CG6464|DmelCG6464|SAL|SPALT|Sal|SalM|Salm|Spalt|l(2)03602|sal|salII|salM|slam|spalt	0	0
2946	34570	Drosophila melanogaster	sala	spalt-adjacent	BcDNA:RE40068|CG4922|DmelCG4922|nsal|sal	0	0
2947	34571	Drosophila melanogaster	CG6488	CG6488 gene product from transcript CG6488-RA	DmelCG6488	0	0
2948	34572	Drosophila melanogaster	spz4	spatzle 4	CG14928|CT34755|DmelCG14928|Spz4	0	0
2949	34573	Drosophila melanogaster	CG6509	CG6509 gene product from transcript CG6509-RB	Dlg5|DmelCG6509|LD32687	0	0
2950	34576	Drosophila melanogaster	CG14930	CG14930 gene product from transcript CG14930-RA	DmelCG14930	0	0
2951	34577	Drosophila melanogaster	CG31705	CG31705 gene product from transcript CG31705-RA	BcDNA:GH07269|CG6528|DmelCG31705	0	0
2952	34578	Drosophila melanogaster	Mst33A	CG6541 gene product from transcript CG6541-RB	CG6541|DmelCG6541	0	0
2953	34581	Drosophila melanogaster	spag4	sperm-associated antigen 4	CG6589|DmelCG6589|Dspag4|dspag|giac	0	0
2954	34582	Drosophila melanogaster	kek2	kekkon-2	CG4977|CT15962|DmelCG4977|Kek2|NB7	0	0
2955	34583	Drosophila melanogaster	CG6614	CG6614 gene product from transcript CG6614-RA	DmelCG6614	0	0
2956	34584	Drosophila melanogaster	CG12307	CG12307 gene product from transcript CG12307-RA	DmelCG12307	0	0
2957	34585	Drosophila melanogaster	CG4983	CG4983 gene product from transcript CG4983-RA	DmelCG4983|anon-WO0140519.134	0	0
2958	34586	Drosophila melanogaster	CG4988	CG4988 gene product from transcript CG4988-RA	DmelCG4988	0	0
2959	34587	Drosophila melanogaster	Vha100-5	Vacuolar H[+] ATPase subunit 100-5	CG12602|DmelCG12602|vha100-5	0	0
2960	34588	Drosophila melanogaster	CG14931	CG14931 gene product from transcript CG14931-RB	DmelCG14931	0	0
2961	34589	Drosophila melanogaster	CG14932	CG14932 gene product from transcript CG14932-RC	DmelCG14932	0	0
2962	34590	Drosophila melanogaster	Tsp33B	Tetraspanin 33B	CG14936|Dm.Tsp33B|DmelCG14936	0	0
2963	34591	Drosophila melanogaster	CG14937	CG14937 gene product from transcript CG14937-RA	DmelCG14937	0	0
2964	34592	Drosophila melanogaster	crol	crooked legs	CG14938|Crol|DmelCG14938|ORE-15|anon-EST:Liang-1.74|anon-EST:Liang-2.48|clone 1.74|clone 2.48|crol alpha|crol beta|crol gamma|l(2)04418|l(2)s2346	0	0
2965	34593	Drosophila melanogaster	CycY	Cyclin Y	CG14939|Dcyclin Y|DmelCG14939|anon-WO0118547.165|i182	0	0
2966	34594	Drosophila melanogaster	Pde1c	Phosphodiesterase 1c	CG14940|CG14942|CG14943|CG14944|CG31757|CG31758|CG42325|DmelCG42325|Dmel_CG14940|Dmel_CG14943|Dmel_CG14944|Dmel_CG31757|Dmel_CG31758|PDE1C|PDE1c	0	0
2967	34595	Drosophila melanogaster	esc	extra sexcombs	CG14941|DmelCG14941|ESC|Esc|L41867|dESC|sc	0	0
2968	34597	Drosophila melanogaster	Mal-B1	Maltase B1	CG14934|DmelCG14934|Drome_B1|mal_B1	0	0
2969	34598	Drosophila melanogaster	Mal-B2	Maltase B2	CG14935|DmelCG14935|Drome_B2|FBgn0032382|mal_B2	0	0
2970	34601	Drosophila melanogaster	Or33a	Odorant receptor 33a	33B.1|33a|AN3|CG16960|DOR33B.1|DOR73|DmelCG16960|Or33B.1|Or73|dor73	0	0
2971	34602	Drosophila melanogaster	Or33b	Odorant receptor 33b	33B.2|33b|AN1|CG16961|DOR33B.2|DOR72|DmelCG16961|Or33B.2|Or72|dor72	0	0
2972	34603	Drosophila melanogaster	Or33c	Odorant receptor 33c	33B.3|33c|AN2|CG5006|DOR33B.3|DOR71|DmelCG5006|Or33B.3|Or71|dor71	0	0
2973	34604	Drosophila melanogaster	Cry	Crystallin	CG16963|DmelCry|DmelCG16963|c|dcy	0	0
2974	34605	Drosophila melanogaster	CG16964	CG16964 gene product from transcript CG16964-RA	BcDNA:GH05159|DmelCG16964	0	0
2975	34607	Drosophila melanogaster	CG16965	CG16965 gene product from transcript CG16965-RB	DmelCG16965	0	0
2976	34608	Drosophila melanogaster	CG6686	CG6686 gene product from transcript CG6686-RB	DmelCG6686|SART1	0	0
2977	34609	Drosophila melanogaster	zuc	zucchini	CG12314|DmelCG12314|Zuc	0	0
2978	34610	Drosophila melanogaster	dgt2	dim gamma-tubulin 2	CG16969|Dgt2|DmelCG16969	0	0
2979	34611	Drosophila melanogaster	escl	CG5202 gene product from transcript CG5202-RA	CG5202|CG5202-PA|DmelCG5202|ESC|ESC-LIKE|ESC1|ESCL	0	0
2980	34613	Drosophila melanogaster	CG12264	CG12264 gene product from transcript CG12264-RA	CG12264-PA|DmelCG12264	0	0
2981	34614	Drosophila melanogaster	CG6746	CG6746 gene product from transcript CG6746-RA	DmelCG6746	0	0
2982	34615	Drosophila melanogaster	Rh5	Rhodopsin 5	CG5279|DMELRH5|Dm Rh5|DmelCG5279|RH5|Rh|rh5	0	0
2983	34616	Drosophila melanogaster	CG6734	CG6734 gene product from transcript CG6734-RA	DmelCG6734	0	0
2984	34618	Drosophila melanogaster	Tom70	Translocase of outer membrane 70	CG6756|DmelCG6756|dtom70	0	0
2985	34619	Drosophila melanogaster	CG6766	CG6766 gene product from transcript CG6766-RA	DmelCG6766	0	0
2986	34620	Drosophila melanogaster	CG6785	CG6785 gene product from transcript CG6785-RA	DmelCG6785	0	0
2987	34621	Drosophila melanogaster	CG6770	CG6770 gene product from transcript CG6770-RA	CT21019|DmelCG6770|anon-WO02059370.25|p8	0	0
2988	34622	Drosophila melanogaster	CG6792	CG6792 gene product from transcript CG6792-RA	DmelCG6792	0	0
2989	34623	Drosophila melanogaster	CG14945	CG14945 gene product from transcript CG14945-RB	DmelCG14945	0	0
2990	34624	Drosophila melanogaster	Jhl-21	CG12317 gene product from transcript CG12317-RB	CG12317|DmelCG12317|Jhl-21	0	0
2991	34625	Drosophila melanogaster	RpL7-like	Ribosomal protein L7-like	CG5317|DmelCG5317	0	0
2992	34626	Drosophila melanogaster	rab3-GAP	CG7061 gene product from transcript CG7061-RA	BcDNA:LD23336|CG7061|DmelCG7061|Rab3-GAP	0	0
2993	34627	Drosophila melanogaster	CG14946	CG14946 gene product from transcript CG14946-RD	DmelCG14946	0	0
2994	34628	Drosophila melanogaster	CG14947	CG14947 gene product from transcript CG14947-RA	DmelCG14947	0	0
2995	34629	Drosophila melanogaster	prd	paired	CG6716|DmelCG6716|PRD|Prd|pr	0	0

2996	34630	Drosophila melanogaster	CG5325	CG5325 gene product from transcript CG5325-RB	BEST:GH03076|DmelCG5325	0	0
2997	34631	Drosophila melanogaster	CG6712	CG6712 gene product from transcript CG6712-RA	DmelCG6712|ORF2	0	0
2998	34632	Drosophila melanogaster	Mt2	Methyltransferase 2	CG10692|DNMT|DNMT2|DmMT2|DmelCG10692|Dnmt|Dnmt2|dDNMT|dDNMT2|dDnmt2|dnmt2|nmt2	0	0
2999	34633	Drosophila melanogaster	Ced-12	CG5336 gene product from transcript CG5336-RA	CG5336|Ced12|Dced-12|DmelCG5336|ELMO|Elmo|ced-12|dced-12|elmo	0	0
3000	34634	Drosophila melanogaster	Patsas	CG6618 gene product from transcript CG6618-RA	CG6618|DmelCG6618	0	0
3001	34636	Drosophila melanogaster	Rab6	Rab-protein 6	136/6|AAF53168|CG6601|DRab6|Dm Rab6|DmRab6|DmelCG6601|Drab6|dRab6|drab6|l(2)08323|rab6|wrt	0	0
3002	34637	Drosophila melanogaster	Phae1	Phaedra 1	CG16996|DmelCG16996|SP134	0	0
3003	34638	Drosophila melanogaster	Phae2	Phaedra 2	CG16997|DmelCG16997|SP147	0	0
3004	34639	Drosophila melanogaster	CG17211	CG17211 gene product from transcript CG17211-RA	DmelCG17211	0	0
3005	34640	Drosophila melanogaster	rho-6	rhomboid-6	CG17212|DmelCG17212|Rho-related [33C1]|rho 6|rho6	0	0
3006	34641	Drosophila melanogaster	Gr33a	Gustatory receptor 33a	CG17213|DmelCG17213|GR33a|Gr33C1	0	0
3007	34642	Drosophila melanogaster	CG31760	CG31760 gene product from transcript CG31760-RB	BcDNA:RE52162|CG17214|CG17215|DmelCG31760	0	0
3008	34644	Drosophila melanogaster	CG17217	CG17217 gene product from transcript CG17217-RA	DmelCG17217	0	0
3009	34645	Drosophila melanogaster	CG6583	CG6583 gene product from transcript CG6583-RA	DmelCG6583	0	0
3010	34646	Drosophila melanogaster	crok	crooked	BcDNA:RE65321|CG17218|DmelCG17218	0	0
3011	34647	Drosophila melanogaster	atilla	CG6579 gene product from transcript CG6579-RB	CG6579|DmelCG6579	0	0
3012	34648	Drosophila melanogaster	aret	arrest	Aret|BcDNA:GM15173|Bru|Bruno|CG31762|CG6319|Dm Bru|DmelCG31762|NEST:bs01g04|NEST:bs34h11|aret/bruno1|arrest/Bruno|bru|bruno|ms(2)01284	0	0
3013	34650	Drosophila melanogaster	CG17010	CG17010 gene product from transcript CG17010-RA	DmelCG17010	0	0
3014	34651	Drosophila melanogaster	Pkd2	Polycystic kidney disease gene-2	AMO|Amo|CG6504|DmelCG6504|Dmpkd2|PKD2|TRPP|amo|pkd2	0	0
3015	34652	Drosophila melanogaster	vir-1	virus-induced RNA 1	BEST:GH02216|CG15493|CG31764|CG5453|DmelCG31764|Vir-1|vir1	0	0
3016	34654	Drosophila melanogaster	CG6405	CG6405 gene product from transcript CG6405-RA	DmelCG6405	0	0
3017	34655	Drosophila melanogaster	CG5446	CG5446 gene product from transcript CG5446-RA	BcDNA:RE36920|DmelCG5446	0	0
3018	34656	Drosophila melanogaster	CG6388	CG6388 gene product from transcript CG6388-RA	DmelCG6388	0	0
3019	34657	Drosophila melanogaster	CG5435	CG5435 gene product from transcript CG5435-RA	BcDNA:AT11334|DmelCG5435	0	0
3020	34658	Drosophila melanogaster	Elf	Ef1alpha-like factor	CG6382|DSUP35|DmelCG6382|Dsup35|delf|dsup35|eRF3	0	0
3021	34660	Drosophila melanogaster	Oatp33Ea	Organic anion transporting polypeptide 33Ea	CG5427|DmelCG5427|oatp 33Ea	0	0
3022	34661	Drosophila melanogaster	CG5421	CG5421 gene product from transcript CG5421-RA	DmelCG5421	0	0
3023	34662	Drosophila melanogaster	Oatp33Eb	Organic anion transporting polypeptide 33Eb	CG6417|DmelCG6417|oatp 33Eb	0	0
3024	34663	Drosophila melanogaster	CG5418	CG5418 gene product from transcript CG5418-RA	DmelCG5418	0	0
3025	34664	Drosophila melanogaster	CG17024	CG17024 gene product from transcript CG17024-RA	DmelCG17024	0	0
3026	34665	Drosophila melanogaster	bun	bunched	1550|Bun|BunA|CG31857|CG42281|CG5461|DmelCG42281|Dmel_CG31857|Dmel_CG5461|EP(2)0488|TSC-22|Tsc22|bnc|l(2)00255|l(2)02687|l(2)05479|l(2)06475|l(2)k02903|l(2)rI043|shs	0	0
3027	34667	Drosophila melanogaster	ref2	CG17031 gene product from transcript CG17031-RB	CG17031|DmelCG17031|REF2|cg17031	0	0
3028	34669	Drosophila melanogaster	nub	nubbin	CG15488|CG15489|CG34395|CG6246|DmelCG34395|Dmel_CG15488|Dmel_CG15489|Dmel_CG6246|Nb|Nub|Oct-1|Oct1|PDM-1|PDM1|POU33F1|Pdm|Pdm-1|Pdm1|dOct1|dPOU-19|dPOU-19/pdm-1|dP	0	0
3029	34671	Drosophila melanogaster	CG15485	CG15485 gene product from transcript CG15485-RA	DmelCG15485	0	0
3030	34673	Drosophila melanogaster	pdm2	POU domain protein 2	CG12287|CG15486|CG15487|DmelCG12287|Oct-2|Oct2|Pdm|Pdm-2|Pdm2|dOct2|dPOU-28|dPOU28|dPOU28/pdm-2|dim|dpou28|miti|pdm|pdm-2	0	0
3031	34674	Drosophila melanogaster	CG5525	CG5525 gene product from transcript CG5525-RA	Cct4|DmelCG5525|Tcp-1delta	0	0
3032	34675	Drosophila melanogaster	CG6153	CG6153 gene product from transcript CG6153-RB	6153|DmelCG6153	0	0
3033	34676	Drosophila melanogaster	CG5780	CG5780 gene product from transcript CG5780-RB	DmelCG5780	0	0
3034	34677	Drosophila melanogaster	PICK1	CG6167 gene product from transcript CG6167-RA	CG6167|DmelCG6167	0	0
3035	34678	Drosophila melanogaster	CG5781	CG5781 gene product from transcript CG5781-RA	DmelCG5781	0	0
3036	34679	Drosophila melanogaster	CG17036	CG17036 gene product from transcript CG17036-RA	DmelCG17036	0	0
3037	34680	Drosophila melanogaster	CG5776	CG5776 gene product from transcript CG5776-RA	DmelCG5776	0	0
3038	34681	Drosophila melanogaster	spict	spichthyin	CG12292|DmelCG12292|Spict|spg6	0	0
3039	34682	Drosophila melanogaster	CG15484	CG15484 gene product from transcript CG15484-RA	DmelCG15484	0	0
3040	34683	Drosophila melanogaster	CG6180	CG6180 gene product from transcript CG6180-RA	DmelCG6180	0	0
3041	34684	Drosophila melanogaster	CG5787	CG5787 gene product from transcript CG5787-RA	DmelCG5787	0	0
3042	34685	Drosophila melanogaster	CG5792	CG5792 gene product from transcript CG5792-RA	DmelCG5792	0	0
3043	34686	Drosophila melanogaster	MRP	Multidrug-Resistance like Protein 1	CG6214|DmelCG6214|dMRP|dMRP/CG6214	0	0
3044	34687	Drosophila melanogaster	CG15483	CG15483 gene product from transcript CG15483-RA	DmelCG15483	0	0
3045	34688	Drosophila melanogaster	kek1	kekkon-1	BEST:GM02380|CG12283|CT18186|DmelCG12283|Dmkek1|GM02380|KEK1|Kek|Kek-1|Kek1|NB1|SD01674|kek|kek-1|kekon|l(2)01433|n(2)k07332|uns	0	0
3046	34689	Drosophila melanogaster	ACXC	CG5983 gene product from transcript CG5983-RB	AC|Acxc|CG5983|DACXC|DmelCG5983	0	0
3047	34693	Drosophila melanogaster	CG16800	CG16800 gene product from transcript CG16800-RB	DmelCG16800	0	0
3048	34695	Drosophila melanogaster	Vha68-3	Vacuolar H[+] ATPase subunit 68-3	CG5075|DmelCG5075|vha68-3	0	0
3049	34696	Drosophila melanogaster	CG12404	CG12404 gene product from transcript CG12404-RA	DmelCG12404	0	0
3050	34699	Drosophila melanogaster	CG9934	CG9934 gene product from transcript CG9934-RB	CG 9934|DmelCG9934|UFD2b|dUfd2|ufd2	0	0
3051	34700	Drosophila melanogaster	A16	CG9933 gene product from transcript CG9933-RA	CG9933|DmelCG9933	0	0
3052	34701	Drosophila melanogaster	CG9932	CG9932 gene product from transcript CG9932-RC	DmelCG9932|EP2317|Ef4A|eIF-4A	0	0
3053	34702	Drosophila melanogaster	CG5142	CG5142 gene product from transcript CG5142-RC	DmelCG5142	0	0
3054	34703	Drosophila melanogaster	CG5122	CG5122 gene product from transcript CG5122-RA	DmelCG5122	0	0
3055	34704	Drosophila melanogaster	CG9928	CG9928 gene product from transcript CG9928-RA	BcDNA:RH70154|CT27834|DmelCG9928	0	0
3056	34705	Drosophila melanogaster	Pk34A	CG5182 gene product from transcript CG5182-RA	CG5182|DmelCG5182|GSK3a	0	0
3057	34706	Drosophila melanogaster	CG5204	CG5204 gene product from transcript CG5204-RA	DmelCG5204|anon-WO0118547.212	0	0
3058	34707	Drosophila melanogaster	DnaJ-H	DnaJ homolog	CG9828|DmelCG9828|NEST:bs08d07|dDnaJ-H|dHdj2	0	0
3059	34708	Drosophila melanogaster	Sir2	CG5216 gene product from transcript CG5216-RA	BEST:LD38188|CG5216|D.mel1|DSir2|DmelCG5216|SIR2|Sir2L|dSIR2|dSir2|dmSRT406|dsir2|l(2)05326|sir2	0	0
3060	34709	Drosophila melanogaster	Sfmbt	Scm-related gene containing four mbt domains	CG16975|DmelCG16975|SFMBT|dSFMBT|dSfmbt	0	0
3061	34710	Drosophila melanogaster	CG5439	CG5439 gene product from transcript CG5439-RA	DmelCG5439	0	0
3062	34711	Drosophila melanogaster	CG5287	CG5287 gene product from transcript CG5287-RA	DmelCG5287	0	0
3063	34712	Drosophila melanogaster	CG5458	CG5458 gene product from transcript CG5458-RA	DmelCG5458	0	0
3064	34713	Drosophila melanogaster	CG16974	CG16974 gene product from transcript CG16974-RA	CT35580|DmelCG16974|Dromel_CG16974_FBtr0080489_mORF	0	0
3065	34714	Drosophila melanogaster	Edem2	CG5682 gene product from transcript CG5682-RA	CG5682|DmelCG5682|EDEM2	0	0
3066	34715	Drosophila melanogaster	CG16972	CG16972 gene product from transcript CG16972-RA	DmelCG16972|anon-WO0140519.52	0	0
3067	34716	Drosophila melanogaster	Pect	Phosphoethanolamine cytidylyltransferase	CG5547|DmelCG5547|PECT|pect	0	0
3068	34717	Drosophila melanogaster	CG15482	CG15482 gene product from transcript CG15482-RA	DmelCG15482	0	0
3069	34718	Drosophila melanogaster	kek4	kekkon4	CG9431|CT26742|DmelCG9431	0	0
3070	34719	Drosophila melanogaster	CG9426	CG9426 gene product from transcript CG9426-RA	DmelCG9426|lpp	0	0
3071	34720	Drosophila melanogaster	CG5705	CG5705 gene product from transcript CG5705-RA	DmelCG5705	0	0
3072	34721	Drosophila melanogaster	Ski6	CG15481 gene product from transcript CG15481-RA	CG15481|DmelCG15481|dRrp41|dSki6	0	0
3073	34722	Drosophila melanogaster	CG16812	CG16812 gene product from transcript CG16812-RA	DmelCG16812	0	0
3074	34723	Drosophila melanogaster	CG15480	CG15480 gene product from transcript CG15480-RA	DmelCG15480	0	0
3075	34724	Drosophila melanogaster	CG16813	CG16813 gene product from transcript CG16813-RA	DmelCG16813	0	0
3076	34725	Drosophila melanogaster	CG16815	CG16815 gene product from transcript CG16815-RB	DmelCG16815	0	0
3077	34726	Drosophila melanogaster	Prosalpha6T	Proteasome alpha6T subunit	5648|Alpha6T|CG5648|DmelCG5648|prosalpha6T	0	0
3078	34727	Drosophila melanogaster	CG15479	CG15479 gene product from transcript CG15479-RA	DmelCG15479	0	0
3079	34728	Drosophila melanogaster	CG5867	CG5867 gene product from transcript CG5867-RA	BcDNA:GH05536|CT18390|DmelCG5867	0	0
3080	34729	Drosophila melanogaster	CG5945	CG5945 gene product from transcript CG5945-RA	CT18564|DmelCG5945|cg5945	0	0
3081	34730	Drosophila melanogaster	CG16820	CG16820 gene product from transcript CG16820-RA	DmelCG16820|cg16820	0	0
3082	34731	Drosophila melanogaster	CG31728	CG31728 gene product from transcript CG31728-RC	CG16821|DmelCG31728|SP36|c-SP36	0	0
3083	34732	Drosophila melanogaster	CG6043	CG6043 gene product from transcript CG6043-RG	DmelCG6043	0	0
3084	34733	Drosophila melanogaster	CG6108	CG6108 gene product from transcript CG6108-RA	DmelCG6108	0	0
3085	34734	Drosophila melanogaster	Drep-4	DNA fragmentation factor-related protein 4	CAD|CG9414|DREP-4|DmelCG9414|Drep4|Rep4|dCAD|drep4	0	0
3086	34735	Drosophila melanogaster	CG6116	CG6116 gene product from transcript CG6116-RA	DmelCG6116|UVRAG	0	0
3087	34736	Drosophila melanogaster	CG31729	CG31729 gene product from transcript CG31729-RB	CG16823|CG6263|DmelCG31729	0	0
3088	34738	Drosophila melanogaster	CG15639	CG15639 gene product from transcript CG15639-RA	DmelCG15639	0	0
3089	34739	Drosophila melanogaster	CG16825	CG16825 gene product from transcript CG16825-RA	BcDNA:AT09390|DmelCG16825	0	0
3090	34740	Drosophila melanogaster	CG16970	CG16970 gene product from transcript CG16970-RB	DmelCG16970|FBgn 32504	0	0
3091	34741	Drosophila melanogaster	CG16826	CG16826 gene product from transcript CG16826-RA	DmelCG16826	0	0
3092	34742	Drosophila melanogaster	CG9395	CG9395 gene product from transcript CG9395-RB	DmelCG9395	0	0
3093	34743	Drosophila melanogaster	CG9377	CG9377 gene product from transcript CG9377-RA	DmelCG9377|SPH166|c-SPH166	0	0
3094	34745	Drosophila melanogaster	CG6523	CG6523 gene product from transcript CG6523-RA	DmelCG6523	0	0
3095	34747	Drosophila melanogaster	CG9306	CG9306 gene product from transcript CG9306-RA	DmelCG9306	0	0
3096	34748	Drosophila melanogaster	CG9305	CG9305 gene product from transcript CG9305-RA	Bdp1|DmelCG9305	0	0
3097	34749	Drosophila melanogaster	CG6565	CG6565 gene product from transcript CG6565-RA	DmelCG6565	0	0
3098	34750	Drosophila melanogaster	CG9302	CG9302 gene product from transcript CG9302-RA	DmelCG9302|anon-WO0118547.80	0	0
3099	34751	Drosophila melanogaster	loqs	loquacious	CG6866|DmelCG6866|Loqs|R3D1|R3D1-L|R3D1-S|TRBP|cg6866|dRax|loq|r3d1	0	0
3100	34752	Drosophila melanogaster	CG9293	CG9293 gene product from transcript CG9293-RB	DmelCG9293	0	0
3101	34753	Drosophila melanogaster	CG7099	CG7099 gene product from transcript CG7099-RA	DmelCG7099|FBgn 32517	0	0
3102	34754	Drosophila melanogaster	RpL24	Ribosomal protein L24	CG9282|DmelCG9282|M(2)34BC|anon-EST:Posey242|anon-EST:Posey37	0	0
3103	34755	Drosophila melanogaster	CG16957	CG16957 gene product from transcript CG16957-RA	DmelCG16957	0	0
3104	34756	Drosophila melanogaster	CG10859	CG10859 gene product from transcript CG10859-RA	DmelCG10859	0	0
3105	34757	Drosophila melanogaster	CG7110	CG7110 gene product from transcript CG7110-RB	DmelCG7110	0	0
3106	34758	Drosophila melanogaster	Vm34Ca	Vitelline membrane 34Ca	CG9271|DmelCG9271|VM26A.1|VM34C|VM34C.1|Vm34C|Vm34Cc	0	0

<table>
<tr><td>3107</td><td>34759</td><td>Drosophila melanogaster</td><td>CG16848</td><td>CG16848 gene product from transcript CG16848-RA</td><td>DmelCG16848|FBgn0032522</td><td>0</td><td>0</td></tr>
<tr><td>3108</td><td>34760</td><td>Drosophila melanogaster</td><td>CG16956</td><td>CG16956 gene product from transcript CG16956-RA</td><td>DmelCG16956</td><td>0</td><td>0</td></tr>
<tr><td>3109</td><td>34761</td><td>Drosophila melanogaster</td><td>Tehao</td><td>CG7121 gene product from transcript CG7121-RA</td><td>CG7121|CT22017|DmelCG7121|Tho|Tl-5|Toll 5|Toll-5|dTLR5|dToll5|tehao|toll</td><td>0</td><td>0</td></tr>
<tr><td>3110</td><td>34762</td><td>Drosophila melanogaster</td><td>CG9267</td><td>CG9267 gene product from transcript CG9267-RA</td><td>DmelCG9267|Q9VJX8</td><td>0</td><td>0</td></tr>
<tr><td>3111</td><td>34763</td><td>Drosophila melanogaster</td><td>Hsp60D</td><td>CG16954 gene product from transcript CG16954-RA</td><td>CG16954|DmelCG16954</td><td>0</td><td>0</td></tr>
<tr><td>3112</td><td>34764</td><td>Drosophila melanogaster</td><td>CG42810</td><td>CG42810 gene product from transcript CG42810-RA</td><td>CG16849|DmelCG42810|Dmel_CG16849</td><td>0</td><td>0</td></tr>
<tr><td>3113</td><td>34767</td><td>Drosophila melanogaster</td><td>B4</td><td>CG9239 gene product from transcript CG9239-RA</td><td>B4/Susi|BG:DS07660.4|CG9239|CG9260|DmelCG9239|Susi|n(2)05337</td><td>0</td><td>0</td></tr>
<tr><td>3114</td><td>34768</td><td>Drosophila melanogaster</td><td>CG15638</td><td>CG15638 gene product from transcript CG15638-RA</td><td>BG:BACR48E02.1|DmelCG15638</td><td>0</td><td>0</td></tr>
<tr><td>3115</td><td>34769</td><td>Drosophila melanogaster</td><td>CG16852</td><td>CG16852 gene product from transcript CG16852-RA</td><td>BG:BACR48E02.2|BcDNA:AT28681|DmelCG16852</td><td>0</td><td>0</td></tr>
<tr><td>3116</td><td>34770</td><td>Drosophila melanogaster</td><td>CG9263</td><td>CG9263 gene product from transcript CG9263-RA</td><td>DmelCG9263</td><td>0</td><td>0</td></tr>
<tr><td>3117</td><td>34771</td><td>Drosophila melanogaster</td><td>CG16853</td><td>CG16853 gene product from transcript CG16853-RA</td><td>BG:BACR48E02.3|DmelCG16853</td><td>0</td><td>0</td></tr>
<tr><td>3118</td><td>34772</td><td>Drosophila melanogaster</td><td>kuz</td><td>kuzbanian</td><td>11410|34Da|ADAM10|BG:DS07660.3|CG7147|CT22079|DmelCG7147|GS11410|Kuz|br38|l(2)03782|l(2)34Da|l(2)br38|l(2)c00136|l(2)k01403|l34Da</td><td>0</td><td>0</td></tr>
<tr><td>3119</td><td>34773</td><td>Drosophila melanogaster</td><td>CG9254</td><td>CG9254 gene product from transcript CG9254-RA</td><td>BG:DS07660.1|DS07660.1|DmelCG9254</td><td>0</td><td>0</td></tr>
<tr><td>3120</td><td>34774</td><td>Drosophila melanogaster</td><td>ics</td><td>icarus</td><td>BG:BACR48E02.4|CG9031|CT25926|DmelCG9031|RSU-1|Rsu-1</td><td>0</td><td>0</td></tr>
<tr><td>3121</td><td>34775</td><td>Drosophila melanogaster</td><td>CG18507</td><td>CG18507 gene product from transcript CG18507-RB</td><td>BG:DS01368.1|DS01368.1|DmelCG18507|bg:ds01368</td><td>0</td><td>0</td></tr>
<tr><td>3122</td><td>34776</td><td>Drosophila melanogaster</td><td>CG7311</td><td>CG7311 gene product from transcript CG7311-RD</td><td>BG:DS08249.2|DmelCG7311|GPO|GPO-2|Gpo|alpha-GPO</td><td>0</td><td>0</td></tr>
<tr><td>3123</td><td>34777</td><td>Drosophila melanogaster</td><td>CG9014</td><td>CG9014 gene product from transcript CG9014-RA</td><td>BG:DS08249.3|DS08249.3|DmelCG9014</td><td>0</td><td>0</td></tr>
<tr><td>3124</td><td>34778</td><td>Drosophila melanogaster</td><td>TM9SF4</td><td>CG7364 gene product from transcript CG7364-RA</td><td>BG:DS00797.1|CG16861|CG7364|DmelCG7364|n(2)k07245</td><td>0</td><td>0</td></tr>
<tr><td>3125</td><td>34779</td><td>Drosophila melanogaster</td><td>CG9008</td><td>CG9008 gene product from transcript CG9008-RA</td><td>BG:DS00797.2|DmelCG9008</td><td>0</td><td>0</td></tr>
<tr><td>3126</td><td>34780</td><td>Drosophila melanogaster</td><td>p38b</td><td>CG7393 gene product from transcript CG7393-RA</td><td>186F5S|BG:DS00797.3|CG7393|D-p38|D-p38 MAPK|D-p38b|Dm p38b|DmelCG7393|Dp38|ESTS:186F5S|Mpk34C|anon-sts23|dp38b|p38|p38 MAPK|p38 beta|p38B|p38beta</td><td>0</td><td>0</td></tr>
<tr><td>3127</td><td>34781</td><td>Drosophila melanogaster</td><td>CG16890</td><td>CG16890 gene product from transcript CG16890-RB</td><td>BG:DS00797.4|DmelCG16890</td><td>0</td><td>0</td></tr>
<tr><td>3128</td><td>34783</td><td>Drosophila melanogaster</td><td>CG31731</td><td>CG31731 gene product from transcript CG31731-RB</td><td>BG:DS00797.5|CG7491|CG7559|D00797.5|DS00797.5|DmelCG31731</td><td>0</td><td>0</td></tr>
<tr><td>3129</td><td>34784</td><td>Drosophila melanogaster</td><td>CG16863</td><td>CG16863 gene product from transcript CG16863-RA</td><td>BG:DS00797.6|DS00797.6|DmelCG16863</td><td>0</td><td>0</td></tr>
<tr><td>3130</td><td>34785</td><td>Drosophila melanogaster</td><td>sec71</td><td>CG7578 gene product from transcript CG7578-RA</td><td>BG:DS00797.7|BIG1|CG7578|DmelCG7578</td><td>0</td><td>0</td></tr>
<tr><td>3131</td><td>34786</td><td>Drosophila melanogaster</td><td>CAH1</td><td>Carbonic anhydrase 1</td><td>BG:DS00941.1|CAH|CG7820|DmelCG7820|Gh7</td><td>0</td><td>0</td></tr>
<tr><td>3132</td><td>34787</td><td>Drosophila melanogaster</td><td>adat</td><td>CG16889 gene product from transcript CG16889-RA</td><td>Adat|BG:DS00941.2|CG16889|DmelCG16889|dADAT1</td><td>0</td><td>0</td></tr>
<tr><td>3133</td><td>34788</td><td>Drosophila melanogaster</td><td>CG16865</td><td>CG16865 gene product from transcript CG16865-RA</td><td>BG:DS00941.3|DmelCG16865</td><td>0</td><td>0</td></tr>
<tr><td>3134</td><td>34789</td><td>Drosophila melanogaster</td><td>CG16888</td><td>CG16888 gene product from transcript CG16888-RA</td><td>DmelCG16888</td><td>0</td><td>0</td></tr>
<tr><td>3135</td><td>34790</td><td>Drosophila melanogaster</td><td>Sos</td><td>Son of sevenless</td><td>2.3|34Ea|BG:DS00941.4|CG7793|DmelCG7793|E(sev)2A|E(var)189|EK2-8|EY2-3|SOS|Sos1|Su(tor)2-2|br24|br25|dSos|dme-SOS|l(2)34Ea|l(2)Sos|l(2)br24|l(2)br25|l(2)k05224|l(2)k06321|sos</td><td>0</td><td>0</td></tr>
<tr><td>3136</td><td>34791</td><td>Drosophila melanogaster</td><td>b</td><td>black</td><td>BG:DS00941.5|CG7811|DGad2|DmGad2|DmelCG7811|GAD[[2]]|Gad2|anon-34Db|cDNA 1</td><td>0</td><td>0</td></tr>
<tr><td>3137</td><td>34792</td><td>Drosophila melanogaster</td><td>tam</td><td>tamas</td><td>34Dc|AAC47658|BG:DS00941.6|CG8987|D-pol gamma-alpha|DNApol-gamma|DNApol-gamma125|DmelCG8987|MtPolA|Polgamma|Tam|anon-34Dc|br5|cDNA 2|l(2)34Dc|l(2)br14|l(2)br5|l(2)brS|mtDNA pol|</td><td>0</td><td>0</td></tr>
<tr><td>3138</td><td>34793</td><td>Drosophila melanogaster</td><td>Sop2</td><td>Suppressor of profilin 2</td><td>34Dd|ARC-P41|ARPC1|ARPC1/p41|Arc41|ArcP41|ArpC1|Arpc1|BG:DS00941.7|CG8978|D-sop2|DSop2|DmelCG8978|Dsop2|Spo2|arc41|arpc1|br32|l(2)34Dd|l(2)Sop2|l(2)br32|p42|sop|sop2|sop2/arc41</td><td>0</td><td>0</td></tr>
<tr><td>3139</td><td>34794</td><td>Drosophila melanogaster</td><td>Orc5</td><td>Origin recognition complex subunit 5</td><td>BG:DS00941.8|CG7833|D-orc5|DmORC5|DmelCG7833|ORC|ORC5|br39|dORC5|l(2)34Df|l(2)br39|orc5</td><td>0</td><td>0</td></tr>
<tr><td>3140</td><td>34796</td><td>Drosophila melanogaster</td><td>RpII33</td><td>RNA polymerase II 33kD subunit</td><td>152117_at|BG:DS00941.10|CG7885|D-rpII33|DmelCG7885|Pol II|Pol II RPII33|PolII|PolIIo|RNA Pol II|RNA pol II|RNA polII|RNAP|RPB3|Rpb3|RpII33|br17|l(2)34Dg|l(2)br17|l(2)k05605|l34Dg|pol II|polII|rpII33</td><td>0</td><td>0</td></tr>
<tr><td>3141</td><td>34799</td><td>Drosophila melanogaster</td><td>CG8997</td><td>CG8997 gene product from transcript CG8997-RA</td><td>BG:DS00941.12|CT25774|DS00941.12|DmelCG8997</td><td>0</td><td>0</td></tr>
<tr><td>3142</td><td>34800</td><td>Drosophila melanogaster</td><td>CG7916</td><td>CG7916 gene product from transcript CG7916-RA</td><td>BG:DS00941.13|CT23894|DmelCG7916|LP05416</td><td>0</td><td>0</td></tr>
<tr><td>3143</td><td>34801</td><td>Drosophila melanogaster</td><td>CG7953</td><td>CG7953 gene product from transcript CG7953-RA</td><td>BG:DS00941.14|DS00941.14|DmelCG7953|anon-WO0140519.73</td><td>0</td><td>0</td></tr>
<tr><td>3144</td><td>34802</td><td>Drosophila melanogaster</td><td>CG7968</td><td>CG7968 gene product from transcript CG7968-RA</td><td>BG:DS00941.15|DS00941.15|DmelCG7968</td><td>0</td><td>0</td></tr>
<tr><td>3145</td><td>34803</td><td>Drosophila melanogaster</td><td>cenG1A</td><td>centaurin gamma 1A</td><td>BG:DS08220.1|CG16866|CG16867|CG31811|CG7982|CenG1A|DmelCG31811|cenG1a|ceng1A</td><td>0</td><td>0</td></tr>
<tr><td>3146</td><td>34804</td><td>Drosophila melanogaster</td><td>Smg5</td><td>CG8954 gene product from transcript CG8954-RA</td><td>BG:DS08220.2|CG8954|DmelCG8954|SMG5|anon-34Ea|dSMG5</td><td>0</td><td>0</td></tr>
<tr><td>3147</td><td>34805</td><td>Drosophila melanogaster</td><td>Ance</td><td>Angiotensin converting enzyme</td><td>ACE|ANCE|AnCE|BG:DS08220.3|CG8827|DmelCG8827|RACE|Race|ance|anon-EST:fe3D10|br31|l(2)34Eb|l(2)br31|l34Eb|race</td><td>0</td><td>0</td></tr>
<tr><td>3148</td><td>34806</td><td>Drosophila melanogaster</td><td>Ance-2</td><td>CG16869 gene product from transcript CG16869-RA</td><td>CG16869|CG31844|DmelCG16869</td><td>0</td><td>0</td></tr>
<tr><td>3149</td><td>34807</td><td>Drosophila melanogaster</td><td>Acyp</td><td>Acylphosphatase</td><td>ACPDro|AcPDro|BG:DS00180.1|CG16870|DmelCG16870</td><td>0</td><td>0</td></tr>
<tr><td>3150</td><td>34808</td><td>Drosophila melanogaster</td><td>Ance-3</td><td>CG17988 gene product from transcript CG17988-RB</td><td>BG:DS00180.5|CG17988|CG31766|CG31843|CT40163|DS00180.5|DmelCG17988</td><td>0</td><td>0</td></tr>
<tr><td>3151</td><td>34809</td><td>Drosophila melanogaster</td><td>CG16886</td><td>CG16886 gene product from transcript CG16886-RB</td><td>BG:DS00180.2|DmelCG16886</td><td>0</td><td>0</td></tr>
<tr><td>3152</td><td>34811</td><td>Drosophila melanogaster</td><td>CG16885</td><td>CG16885 gene product from transcript CG16885-RA</td><td>CG18634|DmelCG16885</td><td>0</td><td>0</td></tr>
<tr><td>3153</td><td>34812</td><td>Drosophila melanogaster</td><td>CG16884</td><td>CG16884 gene product from transcript CG16884-RA</td><td>BG:DS00180.3|DmelCG16884</td><td>0</td><td>0</td></tr>
<tr><td>3154</td><td>34813</td><td>Drosophila melanogaster</td><td>nimB1</td><td>nimrod B1</td><td>BG:DS00180.12|BcDNA:GH07762|CG33119|CG8855|CT25428|DS00180.12|DmelCG33119|NimB1</td><td>0</td><td>0</td></tr>
<tr><td>3155</td><td>34814</td><td>Drosophila melanogaster</td><td>nimB4</td><td>nimrod B4</td><td>BG:DS00180.8|CG16882|CG33115|CT37470|DS00180.8|DmelCG33115|NimB4</td><td>0</td><td>0</td></tr>
<tr><td>3156</td><td>34815</td><td>Drosophila melanogaster</td><td>nimB5</td><td>nimrod B5</td><td>BG:DS00180.9|CG16873|DmelCG16873|NimB5</td><td>0</td><td>0</td></tr>
<tr><td>3157</td><td>34816</td><td>Drosophila melanogaster</td><td>nimC1</td><td>nimrod C1</td><td>BG:DS00180.10|CG8942|CT25688|DmelCG8942|Nim|NimC|NimC1|P1|nimrod</td><td>0</td><td>0</td></tr>
<tr><td>3158</td><td>34817</td><td>Drosophila melanogaster</td><td>Cyp28a5</td><td>CG8864 gene product from transcript CG8864-RA</td><td>28a5|BEST:GH10483|BG:DS00180.11|CG8864|DS00180.11|DmelCG8864|cyp28a5|gh10483</td><td>0</td><td>0</td></tr>
<tr><td>3159</td><td>34818</td><td>Drosophila melanogaster</td><td>nimC2</td><td>nimrod C2</td><td>BG:DS00180.14|CG18146|CG18146-PB|CT40902|DS00180.14|DmelCG18146|NimC2</td><td>0</td><td>0</td></tr>
<tr><td>3160</td><td>34819</td><td>Drosophila melanogaster</td><td>rk</td><td>rickets</td><td>BG:DS00180.13|CG8930|CT25644|DLGR-2|DLGR2|DmelCG8930|LGR2|rk/CG8930</td><td>0</td><td>0</td></tr>
<tr><td>3161</td><td>34822</td><td>Drosophila melanogaster</td><td>CG4500</td><td>CG4500 gene product from transcript CG4500-RA</td><td>2a9|BG:DS05899.1|DmelCG4500|l|anon-EST:CL2a9</td><td>0</td><td>0</td></tr>
<tr><td>3162</td><td>34823</td><td>Drosophila melanogaster</td><td>CG18095</td><td>CG18095 gene product from transcript CG18095-RB</td><td>BG:DS05899.7|DS05899.7|DmelCG18095</td><td>0</td><td>0</td></tr>
<tr><td>3163</td><td>34824</td><td>Drosophila melanogaster</td><td>nimC4</td><td>nimrod C4</td><td>BG:DS05899.3|CG16876|CT35247|DmelCG16876|NimC4|simu</td><td>0</td><td>0</td></tr>
<tr><td>3164</td><td>34826</td><td>Drosophila melanogaster</td><td>nAcRalpha-34E</td><td>nicotinic Acetylcholine Receptor alpha 34E</td><td>BG:DS05899.4|BG:DS05899.5|CG16878|CG32975|CG4498|CT14633|Dalpha5|DmelCG32975|anon-WO02059370.44|nAChR</td><td>0</td><td>0</td></tr>
<tr><td>3165</td><td>34827</td><td>Drosophila melanogaster</td><td>CG16879</td><td>CG16879 gene product from transcript CG16879-RA</td><td>BG:DS01759.1|DS01759.1|DmelCG16879</td><td>0</td><td>0</td></tr>
<tr><td>3166</td><td>34828</td><td>Drosophila melanogaster</td><td>CG15293</td><td>CG15293 gene product from transcript CG15293-RA</td><td>BG:DS01759.2|CT35241|DmelCG15293|HL03474</td><td>0</td><td>0</td></tr>
<tr><td>3167</td><td>34829</td><td>Drosophila melanogaster</td><td>CG17341</td><td>CG17341 gene product from transcript CG17341-RB</td><td>BG:DS01523.1|DmelCG17341</td><td>0</td><td>0</td></tr>
<tr><td>3168</td><td>34830</td><td>Drosophila melanogaster</td><td>CG43333</td><td>CG43333 gene product from transcript CG43333-RA</td><td>BG:DS01523.2|CG15291|CG32972|DmelCG43333|Dmel_CG32972</td><td>0</td><td>0</td></tr>
<tr><td>3169</td><td>34831</td><td>Drosophila melanogaster</td><td>smi35A</td><td>smell impaired 35A</td><td>BG:DS01523.3|CG4551|DYRK|DYRK2|DmelCG4551|Dyrk2|dDYRK2</td><td>0</td><td>0</td></tr>
<tr><td>3170</td><td>34833</td><td>Drosophila melanogaster</td><td>ms(2)34Fe</td><td>male sterile (2) 34Fe</td><td>BG:DS01068.1|CG15287|DmelCG15287|br40|l(2)34Fe|ms(2)34Fh|ms(2)br40|ms34Fe</td><td>0</td><td>0</td></tr>
<tr><td>3171</td><td>34834</td><td>Drosophila melanogaster</td><td>CG15286</td><td>CG15286 gene product from transcript CG15286-RA</td><td>BG:DS01068.11|DmelCG15286</td><td>0</td><td>0</td></tr>
<tr><td>3172</td><td>34835</td><td>Drosophila melanogaster</td><td>CG33090</td><td>CG33090 gene product from transcript CG33090-RB</td><td>BG:DS01068.2|CG15287|CG31837|CG7469|DmelCG33090</td><td>0</td><td>0</td></tr>
<tr><td>3173</td><td>34836</td><td>Drosophila melanogaster</td><td>CG18125</td><td>CG18125 gene product from transcript CG18125-RA</td><td>BG:DS01068.10|DS01068.10|DmelCG18125|SPH190</td><td>0</td><td>0</td></tr>
<tr><td>3174</td><td>34837</td><td>Drosophila melanogaster</td><td>mTTF</td><td>mitochondrial transcription termination factor</td><td>BG:DS01068.4|CG18124|DS01068.4|DmTTF|DmelCG18124</td><td>0</td><td>0</td></tr>
<tr><td>3175</td><td>34838</td><td>Drosophila melanogaster</td><td>l(2)34Fc</td><td>lethal (2) 34Fc</td><td>A2|BG:DS01068.5|BcDNA:RE60882|CG7532|DS01068.5|DmelCG7532|anon-EST:ParkEST060|br8|l(2)br8|l34Fc</td><td>0</td><td>0</td></tr>
<tr><td>3176</td><td>34839</td><td>Drosophila melanogaster</td><td>l(2)34Fd</td><td>lethal (2) 34Fd</td><td>A4|BG:DS01068.6|CG7516|DmelCG7516|br15|l(2)br15</td><td>0</td><td>0</td></tr>
<tr><td>3177</td><td>34840</td><td>Drosophila melanogaster</td><td>Rab14</td><td>Rab-protein 14</td><td>57H4T|AAF53390|BG:DS01068.7|CG4212|DRAB14|DRab14|DmRab14|DmelCG4212|ESTS:57H4T|Rab-r14|anon-sts33|rab14</td><td>0</td><td>0</td></tr>
<tr><td>3178</td><td>34842</td><td>Drosophila melanogaster</td><td>spel1</td><td>spellchecker1</td><td>BG:DS01068.9|CG4215|DmelCG4215|Msh2|SPEL1|spel|spel-1</td><td>0</td><td>0</td></tr>
<tr><td>3179</td><td>34843</td><td>Drosophila melanogaster</td><td>ppk</td><td>pickpocket</td><td>BG:DS06238.1|CG3478|DmelCG3478|PPK|PPK1|dmdNaC1|dmdNaCl|mdNaC1|pkt|ppk1</td><td>0</td><td>0</td></tr>
<tr><td>3180</td><td>34844</td><td>Drosophila melanogaster</td><td>elB</td><td>elbow B</td><td>BG:DS06238.3|CG4220|DmelCG4220|P10|el|el[B]|elb|elbow</td><td>0</td><td>0</td></tr>
<tr><td>3181</td><td>34845</td><td>Drosophila melanogaster</td><td>pburs</td><td>partner of burs</td><td>Bursbeta|CG15284|DmelCG15284|bur &gr;|burs-beta|bursbeta</td><td>0</td><td>0</td></tr>
<tr><td>3182</td><td>34846</td><td>Drosophila melanogaster</td><td>Cpr35B</td><td>Cuticular protein 35B</td><td>BG:DS06238.4|CG3474|DS06283.4|DmelCpr35B|DmelCG3474</td><td>0</td><td>0</td></tr>
<tr><td>3183</td><td>34847</td><td>Drosophila melanogaster</td><td>noc</td><td>no ocelli</td><td>BG:DS04641.1|CG4491|DmelCG4491|Noc|br22|el|l(2)35Ba|l(2)35Ba/nocA|l(2)br22|l35Ba|nocA|noc[A]</td><td>0</td><td>0</td></tr>
<tr><td>3184</td><td>34848</td><td>Drosophila melanogaster</td><td>CG4218</td><td>CG4218 gene product from transcript CG4218-RA</td><td>BG:DS04641.8|DmelCG4218|anon-SAGE:Wang-118|anon-SAGE:Wang-133</td><td>0</td><td>0</td></tr>
<tr><td>3185</td><td>34849</td><td>Drosophila melanogaster</td><td>CG3473</td><td>CG3473 gene product from transcript CG3473-RA</td><td>BG:DS01486.1|BcDNA:AT16033|DmelCG3473</td><td>0</td><td>0</td></tr>
<tr><td>3186</td><td>34850</td><td>Drosophila melanogaster</td><td>osp</td><td>outspread</td><td>BG:DS01486.5|BG:DS01486.7|BG:DS07721.2|CG3479|DmelCG3479|alw|anon-WO02059370.75|l(2)rJ571|v(2)rJ571</td><td>0</td><td>0</td></tr>
<tr><td>3187</td><td>34853</td><td>Drosophila melanogaster</td><td>CG15282</td><td>CG15282 gene product from transcript CG15282-RA</td><td>BG:DS07721.3|BcDNA:RE15191|DS07721.3|DmelCG15282</td><td>0</td><td>0</td></tr>
<tr><td>3188</td><td>34854</td><td>Drosophila melanogaster</td><td>CG42685</td><td>CG42685 gene product from transcript CG42685-RA</td><td>BG:DS07721.6|CG12636|CG33482|DS07721.6|DmelCG42685|Dmel_CG12636|Dmel_CG33482</td><td>0</td><td>0</td></tr>
<tr><td>3189</td><td>34856</td><td>Drosophila melanogaster</td><td>CG4691</td><td>CG4691 gene product from transcript CG4691-RA</td><td>BG:DS06874.1|DmelCG4691|anon-WO0140519.47</td><td>0</td><td>0</td></tr>
<tr><td>3190</td><td>34857</td><td>Drosophila melanogaster</td><td>CSN1a</td><td>COP9 complex homolog subunit 1 a</td><td>BG:DS06874.2|CG4697|CH1-1|COP9|CSN1|CSN1b|Csn1A|Dch1|Dch1-1|DmelCG4697|csn1</td><td>0</td><td>0</td></tr>
<tr><td>3191</td><td>34858</td><td>Drosophila melanogaster</td><td>CG4701</td><td>CG4701 gene product from transcript CG4701-RA</td><td>BG:DS06874.3|DmDM19DC4Z|DmelCG4701</td><td>0</td><td>0</td></tr>
<tr><td>3192</td><td>34859</td><td>Drosophila melanogaster</td><td>CG4650</td><td>CG4650 gene product from transcript CG4650-RB</td><td>DmelCG4650|SPH197</td><td>0</td><td>0</td></tr>
<tr><td>3193</td><td>34863</td><td>Drosophila melanogaster</td><td>CG15279</td><td>CG15279 gene product from transcript CG15279-RB</td><td>BG:DS03431.1|DS03431.1|DmNAT6|DmelCG15279|anon-WO0149848.9</td><td>0</td><td>0</td></tr>
<tr><td>3194</td><td>34864</td><td>Drosophila melanogaster</td><td>CG4480</td><td>CG4480 gene product from transcript CG4480-RB</td><td>DmelCG4480</td><td>0</td><td>0</td></tr>
<tr><td>3195</td><td>34865</td><td>Drosophila melanogaster</td><td>CG15278</td><td>CG15278 gene product from transcript CG15278-RA</td><td>DmelCG15278|NEST:bs32h08|bs32h08.y1</td><td>0</td><td>0</td></tr>
<tr><td>3196</td><td>34866</td><td>Drosophila melanogaster</td><td>Mst35Ba</td><td>Male-specific-transcript-35Ba</td><td>BG:DS03431.2|BcDNA:AT31403|CG4479|DmelCG4479|ProtA|mst35B|mst35Bb|mst35b|protA</td><td>0</td><td>0</td></tr>
<tr><td>3197</td><td>34867</td><td>Drosophila melanogaster</td><td>Mst35Bb</td><td>Male-specific-transcript-35Bb</td><td>BG:DS03431.3|BcDNA:GH11850|CG4478|DmelCG4478|ProtB|mst35b|prot-B</td><td>0</td><td>0</td></tr>
<tr><td>3198</td><td>34870</td><td>Drosophila melanogaster</td><td>CG3491</td><td>CG3491 gene product from transcript CG3491-RA</td><td>BG:DS03323.1|DmelCG3491</td><td>0</td><td>0</td></tr>
<tr><td>3199</td><td>34872</td><td>Drosophila melanogaster</td><td>mol</td><td>moladietz</td><td>35Bb|B1|BG:DS01219.1|CG15268|CG4482|DmelCG4482|br3|l(2)35Bb|l(2)SH1330|l(2)SH2 1330|l(2)br23|l(2)br3|l35Bb|nip</td><td>0</td><td>0</td></tr>
<tr><td>3200</td><td>34873</td><td>Drosophila melanogaster</td><td>dyn-p25</td><td>dynactin-subunit-p25</td><td>BG:DS00929.1|C4|CG10846|DS00929.1|DmelCG10846|br2|l(2)35Bf|l(2)br2|p25</td><td>0</td><td>0</td></tr>
<tr><td>3201</td><td>34874</td><td>Drosophila melanogaster</td><td>l(2)35Be</td><td>lethal (2) 35Be</td><td>BG:DS00929.2|C3|CG4140|DmelCG4140|br10|l(2)br10</td><td>0</td><td>0</td></tr>
<tr><td>3202</td><td>34875</td><td>Drosophila melanogaster</td><td>NC2beta</td><td>CG4185 gene product from transcript CG4185-RA</td><td>BG:DS00929.3|CG4185|DmelCG4185|Dr1|NC2 beta|Nc2b|dDr1|dNC2</td><td>0</td><td>0</td></tr>
<tr><td>3203</td><td>34876</td><td>Drosophila melanogaster</td><td>l(2)35Bc</td><td>lethal (2) 35Bc</td><td>BG:DS00929.4|C1|CG4103|DmelCG4103|br4|l(2)br|l(2)br11|l(2)br13|l(2)br4|l(2)k08808</td><td>0</td><td>0</td></tr>
<tr><td>3204</td><td>34877</td><td>Drosophila melanogaster</td><td>l(2)35Bd</td><td>lethal (2) 35Bd</td><td>BG:DS00929.5|C2|CG3688|DmelCG3688|br9|l(2)10408|l(2)br9</td><td>0</td><td>0</td></tr>
<tr><td>3205</td><td>34878</td><td>Drosophila melanogaster</td><td>GABA-B-R1</td><td>metabotropic GABA-B receptor subtype 1</td><td>BG:DS00929.6|CG15274|D-GABA[[B]]R1|D-Gaba1|DmelCG15274|GABA[[B]]|GABA[[B]]R1|GB1|anon-WO0170980.175|anon-WO0170980.176</td><td>0</td><td>0</td></tr>
<tr><td>3206</td><td>34879</td><td>Drosophila melanogaster</td><td>yellow-c</td><td>CG4182 gene product from transcript CG4182-RA</td><td>BG:DS00929.8|CG4182|DmelCG4182|anon-EST:ParkEST125</td><td>0</td><td>0</td></tr>
<tr><td>3207</td><td>34881</td><td>Drosophila melanogaster</td><td>Su(H)</td><td>Suppressor of Hairless</td><td>BG:DS00929.10|C|CBF1|CG3497|D|DmelCG3497|E(H)|FTZ-F2|RBJK_DROME|RBP-J kappa|RBP-J[[Kappa]]|RBP-Jkappa|SU(H)|SUH|Su(h)|Su)(H)|SuH|anon-WO0257455.3|br7|dRBP-JK|dRBP-J[[Kappa]]|(</td><td>0</td><td>0</td></tr>
<tr><td>3208</td><td>34882</td><td>Drosophila melanogaster</td><td>ck</td><td>crinkled</td><td>53D10S|BG:DS00929.11|CG7595|D|D2|DM7a|DMyoVIIa|Dm 35B|DmVIIA|DmVIIa|DmelCG7595|Dro35B|ESTS:53D10S|Mhc35BC|MyoVIIA|anon-35Bb|br27|ck/MyoVIIA|l(1)35Ca|l(2)07130|l(2)35Ca|l(2)br27|l3</td><td>0</td><td>0</td></tr>
<tr><td>3209</td><td>34883</td><td>Drosophila melanogaster</td><td>TfIIS</td><td>RNA polymerase II elongation factor</td><td>BG:DS00929.12|CG3710|DmS-II|DmSII|DmelCG3710|RnpSII|TFIIS|TFIISA|TFS-II|br52|l(2)35Cf|l(2)35cF|l(2)br52|l35Cf</td><td>0</td><td>0</td></tr>
<tr><td>3210</td><td>34884</td><td>Drosophila melanogaster</td><td>vas</td><td>vasa</td><td>BG:DS00929.14|BG:DS00929.15|CG33678|CG3506|CG43081|DmRH25|DmelCG43081|Dmel_CG33678|Dmel_CG3506|EP(2)0812|VAS|VASA|Vas|Vasa|cgt|fs(2)ltoRJ36|pf7|solo</td><td>0</td><td>0</td></tr>
<tr><td>3211</td><td>34885</td><td>Drosophila melanogaster</td><td>vig</td><td>vasa intronic gene</td><td>BG:DS00929.13|CG4170|DmelCG4170|EP(2)0812|VIG|Vig</td><td>0</td><td>0</td></tr>
<tr><td>3212</td><td>34886</td><td>Drosophila melanogaster</td><td>CG15270</td><td>CG15270 gene product from transcript CG15270-RC</td><td>BG:DS04929.1|DmelCG15270</td><td>0</td><td>0</td></tr>
<tr><td>3213</td><td>34887</td><td>Drosophila melanogaster</td><td>CG15269</td><td>CG15269 gene product from transcript CG15269-RA</td><td>BG:DS04929.3|DS04929.3|DmelCG15269</td><td>0</td><td>0</td></tr>
<tr><td>3214</td><td>34888</td><td>Drosophila melanogaster</td><td>stc</td><td>shuttle craft</td><td>151662_s_at|BG:DS04929.4|BcDNA.LD22726|BcDNA:LD22726|CG3647|Cf4|DmelCG3647|LD22726|br33|l(2)35Cb|l(2)SH1565|l(2)SH2 1565|l(2)br33|l35Cb</td><td>0</td><td>0</td></tr>
<tr><td>3215</td><td>34889</td><td>Drosophila melanogaster</td><td>CG4168</td><td>CG4168 gene product from transcript CG4168-RB</td><td>BG:DS03192.2|DmelCG4168</td><td>0</td><td>0</td></tr>
<tr><td>3216</td><td>34890</td><td>Drosophila melanogaster</td><td>ZnT35C</td><td>CG3994 gene product from transcript CG3994-RA</td><td>BG:DS07295.1|CG3994|DmelCG3994</td><td>0</td><td>0</td></tr>
<tr><td>3217</td><td>34891</td><td>Drosophila melanogaster</td><td>dao</td><td>down and out</td><td>BG:DS07295.2|CG15267|DmelCG15267|anon-WO0140519.126</td><td>0</td><td>0</td></tr>
</table>

3218	34892	Drosophila melanogaster	CG3975	CG3975 gene product from transcript CG3975-RA	BG:DS07295.3|BcDNA:GH08865|DmelCG3975	0	0
3219	34893	Drosophila melanogaster	l(2)35Cc	lethal (2) 35Cc	BG:DS07295.5|CG15266|DmelCG15266|br50|l(2)br50|l35Cc	0	0
3220	34894	Drosophila melanogaster	yuri	yuri gagarin	BG:DS05639.1|BG:DS07851.11|CG11860|CG12635|CG31732|DmelCG31732	0	0
3221	34896	Drosophila melanogaster	Cul-3	Cullin-3	BG:DS07851.2|CG11861|CG31829|CG42616|CUL-3|CUL3|Cul3|DMcul-3|DmelCG42616|Dmel_CG11861|Dmel_CG31829|br34|cul-3|cul3|dCul-3|dCul3|gft|gft/dCul-3|guftagu|l(2)06430|l(2)35Cd|l(2)br34|l35	0	0
3222	34897	Drosophila melanogaster	UK114	CG15261 gene product from transcript CG15261-RA	BG:DS07851.3|BcDNA:GM01181|CG15261|DUK 114|DUK114|DmelCG15261	0	0
3223	34898	Drosophila melanogaster	CG15263	CG15263 gene product from transcript CG15263-RA	BG:DS07851.4|DmelCG15263	0	0
3224	34899	Drosophila melanogaster	CG15260	CG15260 gene product from transcript CG15260-RB	BG:DS07851.8|DmelCG15260	0	0
3225	34901	Drosophila melanogaster	CG15262	CG15262 gene product from transcript CG15262-RA	BG:DS07851.5|DmelCG15262	0	0
3226	34902	Drosophila melanogaster	nht	no hitter	BG:DS07851.6|CG15259|DS07851.6|DmelCG15259|Nht|TAF4|TFIID|dTAF4L|dTAF[[II]]110|dmTAF4b	0	0
3227	34903	Drosophila melanogaster	esg	escargot	4B7|BG:DS07851.7|CG3758|DmelCG3758|Esg|br43|dgl|flg|l(2)07082|l(2)35Ce|l(2)4B7|l(2)br43|l(2)esg|l35Ce|shof|wiz	0	0
3228	34904	Drosophila melanogaster	CG15258	CG15258 gene product from transcript CG15258-RA	DmelCG15258	0	0
3229	34906	Drosophila melanogaster	wor	worniu	BG:DS03023.1|CG4158|DmelCG4158|Wor|br35|l(2)35Da|l(2)br35|l35Da	0	0
3230	34907	Drosophila melanogaster	CG4161	CG4161 gene product from transcript CG4161-RA	BG:DS03023.2|DmelCG4161	0	0
3231	34908	Drosophila melanogaster	sna	snail	BG:DS01845.1|CG3956|DmelCG3956|SNA|Sco|Sna|br28|br29|l(2)35Db|l(2)br28|l(2)br29|l(3)br28|l35Db|sn	0	0
3232	34910	Drosophila melanogaster	lace	CG4162 gene product from transcript CG4162-RA	BG:DS01845.3|CG4162|DmelCG4162|Lace|SPT-II|anon-EST:Posey289|br36|l(2)35Dc|l(2)br36|l35Dc	0	0
3233	34911	Drosophila melanogaster	CG15256	CG15256 gene product from transcript CG15256-RC	BG:DS04862.2|DmelCG15256	0	0
3234	34912	Drosophila melanogaster	kek3	kekkon-3	BG:DS04862.1|CG4192|CT12629|DmelCG4192	0	0
3235	34913	Drosophila melanogaster	CG15255	CG15255 gene product from transcript CG15255-RA	BG:BACR44L22.1|DmelCG15255	0	0
3236	34914	Drosophila melanogaster	CG11864	CG11864 gene product from transcript CG11864-RA	BACR44L22.8|BG:BACR44L22.8|DmelCG11864	0	0
3237	34915	Drosophila melanogaster	CG15254	CG15254 gene product from transcript CG15254-RA	BACcr44L22.2|BG:BACR44L22.2|DmelCG15254	0	0
3238	34916	Drosophila melanogaster	CG15253	CG15253 gene product from transcript CG15253-RA	BACR44L22.3|BG:BACR44L22.3|DmelCG15253	0	0
3239	34917	Drosophila melanogaster	CG11865	CG11865 gene product from transcript CG11865-RA	BACR44L22.4|BG:BACR44L22.4|DmelCG11865	0	0
3240	34918	Drosophila melanogaster	Or35a	Odorant receptor 35a	35a|36E.1|BACR44L22.5|BG:BACR44L22.5|CG17868|DOR91|DmelCG17868|OR35a	0	0
3241	34919	Drosophila melanogaster	CG7631	CG7631 gene product from transcript CG7631-RA	BG:BACR44L22.6|DmelCG7631	0	0
3242	34920	Drosophila melanogaster	CG18480	CG18480 gene product from transcript CG18480-RA	BG:DS07108.4|BcDNA:LP11031|CT42128|DmelCG18480	0	0
3243	34921	Drosophila melanogaster	CG4587	CG4587 gene product from transcript CG4587-RD	BG:DS07108.2|DS07108.2|DmelCG4587	0	0
3244	34922	Drosophila melanogaster	CG18477	CG18477 gene product from transcript CG18477-RA	BG:DS07108.1|DmelCG18477|SPH69	0	0
3245	34923	Drosophila melanogaster	CG18478	CG18478 gene product from transcript CG18478-RA	BG:DS07108.5|DS07108.5|DmelCG18478|SPH157	0	0
3246	34924	Drosophila melanogaster	CycE	Cyclin E	3938|BG:DS07108.3|CDI7|CG3938|CYCE|Cdi7|Cyc E|CycIE|CyeE|D-CycE|DmCycE|DmcycE|DmcyclinE|DmelCG3938|br37|cdi7|cycE|cyclinE|cycline|dm-cycE|fond|l(2)05206|l(2)35Dd|l(2)br37|l(2)k02514|l(2)	0	0
3247	34925	Drosophila melanogaster	l(2)35Di	lethal (2) 35Di	BG:DS09217.1|BcDNA:GM23292|CG13240|DmelCG13240	0	0
3248	34927	Drosophila melanogaster	Gli	Gliotactin	BG:DS09217.3|CG3903|DmelCG3903|gli|l(2)35Dg|l(2)br45|n(2)k09033	0	0
3249	34928	Drosophila melanogaster	CG3793	CG3793 gene product from transcript CG3793-RB	BG:DS09217.4|DmelCG3793	0	0
3250	34930	Drosophila melanogaster	Ku80	CG18801 gene product from transcript CG18801-RA	BG:DS09217.6|CG18801|DmKu80|DmelCG18801|KU80|Ku|ku80	0	0
3251	34931	Drosophila melanogaster	CG18109	CG18109 gene product from transcript CG18109-RA	BG:DS02252.3|DS02252.3|DmelCG18109	0	0
3252	34932	Drosophila melanogaster	CG18518	CG18518 gene product from transcript CG18518-RA	BG:DS02252.4|DS02252.4|DmelCG18518	0	0
3253	34933	Drosophila melanogaster	CG13243	CG13243 gene product from transcript CG13243-RA	BG:DS02252.1|DmelCG13243	0	0
3254	34934	Drosophila melanogaster	Tektin-A	Tektin A	BG:DS02252.2|CG4767|DmelCG4767|anon-WO0140519.235	0	0
3255	34935	Drosophila melanogaster	CG7653	CG7653 gene product from transcript CG7653-RB	BG:DS00365.1|DmelCG7653	0	0
3256	34937	Drosophila melanogaster	TepI	Thiolester containing protein I	BG:DS00365.2|CG18096|DmelCG18096|Tep I|Tep-1|Tep1|dTEPI|tep1	0	0
3257	34938	Drosophila melanogaster	CG31821	CG31821 gene product from transcript CG31821-RA	BG:DS00365.3|CG7647|DmelCG31821	0	0
3258	34939	Drosophila melanogaster	beat-Ib	beaten path Ib	BG:DS00365.4|BcDNA:RE51929|CG7644|CT23325|DmelCG7644|beat Ib|beat-B	0	0
3259	34940	Drosophila melanogaster	CG13244	CG13244 gene product from transcript CG13244-RA	BcDNA:GH27987|DmelCG13244	0	0
3260	34941	Drosophila melanogaster	CG4793	CG4793 gene product from transcript CG4793-RD	BG:DS07486.3|DmelCG4793|SPH208	0	0
3261	34942	Drosophila melanogaster	CG12448	CG12448 gene product from transcript CG12448-RC	BG:DS07486.4|DmelCG12448	0	0
3262	34943	Drosophila melanogaster	CG18063	CG18063 gene product from transcript CG18063-RC	BG:DS07486.5|DS07486.5|DmelCG18063	0	0
3263	34944	Drosophila melanogaster	CG10839	CG10839 gene product from transcript CG10839-RB	BG:DS07486.2|DmelCG10839	0	0
3264	34945	Drosophila melanogaster	beat-Ic	beaten path Ic	BG:DS00913.1|CG4838|CT15531|DmelCG4838|beat Ic|beat-C	0	0
3265	34946	Drosophila melanogaster	BicC	Bicaudal C	BG:DS00913.2|Bic-C|CG4824|DmelCG4824|Dromel_CG4824_FBtr0080804_bicc_mORF|br34|l(2)br34	0	0
3266	34947	Drosophila melanogaster	beat-Ia	beaten path Ia	BG:DS00913.3|Beat Ia|CCT|CG4846|CT15567|Cct|DmelCG4846|TRiC|beat|beat Ia|beat1a|l(2)35Ea|l(2)35Eb|l(2)br47|l(2)br48|tric	0	0
3267	34948	Drosophila melanogaster	CG4891	CG4891 gene product from transcript CG4891-RA	BG:DS04095.1|DS04095.1|DmelCG4891	0	0
3268	34949	Drosophila melanogaster	CG4892	CG4892 gene product from transcript CG4892-RA	BG:DS04095.2|BcDNA:AT24169|DS04095.2|DmelCG4892	0	0
3269	34950	Drosophila melanogaster	Ca-alpha1D	Ca[2+]-channel protein alpha[[1]] subunit D	BG:DS02795.1|CG4894|Ca-_1D|Ca-a1D|Ca-alpha1|Caalpha1D|DmCa1D|DmCa1alpha1|Dmca1D|DmelCG4894|DroCa1|alpha1|alpha1D|l(2)35Fa|l35Fa	0	0
3270	34951	Drosophila melanogaster	CG42817	CG42817 gene product from transcript CG42817-RA	BG:DS07473.1|CG12455|DS07473.1A|DmelCG42817|Dmel_CG12455	0	0
3271	34952	Drosophila melanogaster	PRL-1	CG4993 gene product from transcript CG4993-RB	BG:DS07473.3|BcDNA:RE40268|CG4993|DmelCG4993|PRL|PrL-1	0	0
3272	34953	Drosophila melanogaster	EndoGI	Endonuclease G inhibitor	BG:DS07473.2|CG4930|DmelCG4930|endoGI	0	0
3273	34954	Drosophila melanogaster	twe	twine	BG:DS02740.1|CG4965|Cdc25|DmelCG4965|TWINE|Twe|cdc25|l(2)35Fh|mat(2)syn-A|mat(2)synHB5|mat(2)syn[HB5]|twn	0	0
3274	34955	Drosophila melanogaster	CG4935	CG4935 gene product from transcript CG4935-RA	BG:DS02740.2|DmelCG4935|MET30	0	0
3275	34956	Drosophila melanogaster	crp	cropped	35F(AT)[[17]]|AP-4|AP4|BG:DS02740.3|CG7664|DmelCG7664|MS:35F.AT17|bHLHe63|dAP-4|l(2)00232|l(2)35Fd|l(2)SH1614|l(2)SH2 1614|l(2)k00809|l(2)k03505	0	0
3276	34957	Drosophila melanogaster	pkaap	CG4132 gene product from transcript CG4132-RA	BG:DS02740.4|CG4132|DS02740.4|DmelCG4132	0	0
3277	34958	Drosophila melanogaster	CG17329	CG17329 gene product from transcript CG17329-RA	BG:DS02740.5|BcDNA:AT18988|DS02740.5|DmelCG17329	0	0
3278	34959	Drosophila melanogaster	CG13258	CG13258 gene product from transcript CG13258-RA	DmelCG13258	0	0
3279	34961	Drosophila melanogaster	heix	heixuedian	124/1|BG:DS02740.7|CG5876|DmelCG5876|ipa-6d|l(2)35Fc|l(2)k12401	0	0
3280	34962	Drosophila melanogaster	CG17328	CG17328 gene product from transcript CG17328-RA	BG:DS02740.8|DS02740.8|DmelCG17328	0	0
3281	34963	Drosophila melanogaster	CG5869	CG5869 gene product from transcript CG5869-RA	BG:DS02740.9|BcDNA:SD03793|DS02740.9|DmelCG5869	0	0
3282	34964	Drosophila melanogaster	c(2)M	crossover suppressor on 2 of Manheim	BG:DS02740.10|C(2)M|CG4249|DS02740.10|DmelCG4249|MEI-910|c(2)m|mei-910	0	0
3283	34965	Drosophila melanogaster	CG5861	CG5861 gene product from transcript CG5861-RA	BG:DS02740.11|DmelCG5861|III|anon-35Fa|anon-35Fc	0	0
3284	34966	Drosophila melanogaster	Syx5	Syntaxin 5	BG:DS02740.12|CG4214|DmSyx5|DmelCG4214|II|SED5|SYN5|Sed5|Synx5|Syx|Syx5A|anon-35Fb|anon-35Fd|dSed5|dSed5p|dSyx5|dsed5|l(2)35Ff|sed5|syn5|syx5	0	0
3285	34967	Drosophila melanogaster	cni	cornichon	BG:DS02740.13|CG5855|Cni|DmelCG5855|IV|anon-35Fe|spnG	0	0
3286	34968	Drosophila melanogaster	fzy	fizzy	BG:DS02740.14|CDC20|CG4274|Cdc20|Cdc20/Fizzy|Cdc20/Fzy|Cdc20[Fzy]|Cdc20[fizzy]|DmelCG4274|FZY|Fizzy|Fzy|Fzy/Cdc20|cdc20|cdc20/fizzy|dCDC20|dFZY	0	0
3287	34969	Drosophila melanogaster	cact	cactus	5848|BG:DS02740.15|CACT|CG5848|Cact|DmelCG5848|cac|dip6|fs(2)ltoRN48|n(2)k17003	0	0
3288	34970	Drosophila melanogaster	CG4278	CG4278 gene product from transcript CG4278-RA	BG:DS02740.16|DmelCG4278|anon-35F/36A|un5	0	0
3289	34974	Drosophila melanogaster	chif	chiffon	BG:DS09218.1|BG:DS09218.2|CG5813|CG5817|Chif|DS09218.1|Dbf4|DmelCG5813|chf|fs(2)chiffon	0	0
3290	34975	Drosophila melanogaster	CG4455	CG4455 gene product from transcript CG4455-RA	BEST:LD27171|BG:DS09218.3|DmelCG4455|LD27171	0	0
3291	34976	Drosophila melanogaster	CaBP1	CG5809 gene product from transcript CG5809-RA	AAL28897|BG:DS09218.4|CG5809|DmCaBP1|DmelCG5809	0	0
3292	34977	Drosophila melanogaster	jhamt	juvenile hormone acid methyltransferase	BG:DS09218.5|CG17330|DmJHAMT|DmelCG17330|JAHMT|JHAMT|JHamt|Jhamt	0	0
3293	34978	Drosophila melanogaster	Idgf1	Imaginal disc growth factor 1	BG:DS02780.5|CG4472|DmIDGF1|DmelCG4472|IDGF|IDGF-1|IDGF1|idgf1	0	0
3294	34979	Drosophila melanogaster	Idgf2	Imaginal disc growth factor 2	BG:DS02780.4|CG4475|DmIDGF2|DmelCG4475|IDGF|IDGF-2|IDGF2|idgf2	0	0
3295	34980	Drosophila melanogaster	CG5888	CG5888 gene product from transcript CG5888-RA	BG:DS02780.1|CT18477|DmelCG5888	0	0
3296	34981	Drosophila melanogaster	Idgf3	Imaginal disc growth factor 3	BG:DS02780.2|CG4559|DmIDGF3|DmelCG4559|IDGF|IDGF-3|IDGF3|idgf3	0	0
3297	34982	Drosophila melanogaster	dac	dachshund	BG:DS02780.3|CG4952|DAC|Dac|Dac2-3|Dach|Dm-DAC|DmelCG4952|dach|l(2)36Ae|l(2)rK364|spn	0	0
3298	34983	Drosophila melanogaster	CG4580	CG4580 gene product from transcript CG4580-RA	DmelCG4580	0	0
3299	34984	Drosophila melanogaster	Tpr2	Tetratricopeptide repeat protein 2	BEST:LD24941|CG4599|DmelCG4599|EU3220|Tpr-2|Trp2|dTPR2|dTpr2|dtpr2|dtrpr2|tpr2	0	0
3300	34985	Drosophila melanogaster	CG5953	CG5953 gene product from transcript CG5953-RA	DmelCG5953	0	0
3301	34986	Drosophila melanogaster	CG5968	CG5968 gene product from transcript CG5968-RA	BcDNA:AT27976|DmelCG5968	0	0
3302	34987	Drosophila melanogaster	CG42389	CG42389 gene product from transcript CG42389-RE	CG13260|CG13261|CG31738|CG4668|CT15043|CT15049|DmelCG42389|Dmel_CG13260|Dmel_CG31738|anon-EST:Liang-1.16|clone 1.16	0	0
3303	34988	Drosophila melanogaster	CG4631	CG4631 gene product from transcript CG4631-RA	DmelCG4631	0	0
3304	34991	Drosophila melanogaster	trpgamma	CG5996 gene product from transcript CG5996-RB	CG5996|DmelCG5996|TRPgamma|dTRPg|dTRPgamma|trp-gamma	0	0
3305	34992	Drosophila melanogaster	her	hermaphrodite	CG4694|DmelCG4694|FBgn 1185|l(2)mat	0	0
3306	34993	Drosophila melanogaster	grp	grapes	1C|CG17161|CHK1|CHK1/Grapes|Chk1|DmChk1|DmelCG17161|Grp|Grp/DChk1|Pk36A|Pk?1|chk1|dChk1|grp(chk)|grps|l(2)k15102|lemp	0	0
3307	34995	Drosophila melanogaster	Cyt-c-d	Cytochrome c distal	CG13263|Cyt C|Cyt c|Cyt-c1|CytC-1|CytC1|Cytc|Cytc-d|DC3|DmelCG13263|bln|bs30f07.y1|cyt c|cyt c-d|cyt-c-d|cyt.c|cytochrome c|dc3|ms(2)04445|pDMc02	0	0
3308	34996	Drosophila melanogaster	Cyt-c-p	Cytochrome c proximal	CG17903|Cyt C|Cyt c|Cyt-c2|CytC-2|Cytc|Cytc-p|DC4|DMc01|DmelCG17903|cyt c|cyt c-p|cyt-c-p|cyt-c2|cyt.c|cytochrome c|dc4|l(2)SH1590|pDMc01	0	0
3309	34997	Drosophila melanogaster	VhaSFD	Vacuolar H[+]-ATPase SFD subunit	144191_at|CG17332|DmelCG17332|V-ATPase|vhaSFD	0	0
3310	34998	Drosophila melanogaster	CG17996	CG17996 gene product from transcript CG17996-RA	BcDNA:GM20557|DmelCG17996	0	0
3311	34999	Drosophila melanogaster	CG17331	CG17331 gene product from transcript CG17331-RA	17331|DmelCG17331|GC17331|Prosbeta4|Psmb2	0	0
3312	35000	Drosophila melanogaster	CG17904	CG17904 gene product from transcript CG17904-RA	DmelCG17904	0	0
3313	35001	Drosophila melanogaster	glu	gluon	BcDNA.LD20207|BcDNA:LD20207|CG11397|DmSMC4|DmSMC4/gluon|DmelCG11397|Gluon|SMC4|anon-WO0118547.156|dSMC4|l(2)k08819	0	0
3314	35002	Drosophila melanogaster	ChLD3	CG17905 gene product from transcript CG17905-RA	CG17905|DmCDA3|DmChLD3|DmelCG17905	0	0
3315	35003	Drosophila melanogaster	LSm7	CG13277 gene product from transcript CG13277-RB	BcDNA:RH73529|CG13277|DmelCG13277	0	0
3316	35004	Drosophila melanogaster	CG17912	CG17912 gene product from transcript CG17912-RD	BcDNA:LD27810|DmelCG17912	0	0
3317	35005	Drosophila melanogaster	yellow-b	CG17914 gene product from transcript CG17914-RA	CG17914|DmelCG17914|Yellow-b	0	0
3318	35006	Drosophila melanogaster	CG13278	CG13278 gene product from transcript CG13278-RA	DmelCG13278	0	0
3319	35007	Drosophila melanogaster	Mhc	Myosin heavy chain	Bsh|CG17927|DROMHC|Dm II|DmMHC|DmelCG17927|DroMII|FBgn0002741|Ifm(2)2|MHC|MRP|Mhc36B|Mrp|MyHC|Myo|Nup|Sht|Stp|ifm(2)2|l(2)36Ae|l(2)M66|l(2)k10423|mMHC|mhc|sMHC	0	0
3320	35008	Drosophila melanogaster	Cyt-b5-r	Cytochrome b5-related	CG13279|Cyt-b|Cyt-b5|Cytb|Cytb5|DmelCG13279|TU-36B|TU36B	0	0
3321	35009	Drosophila melanogaster	CG17928	CG17928 gene product from transcript CG17928-RA	DmelCG17928|anon-WO0118547.16	0	0
3322	35013	Drosophila melanogaster	CG13272	CG13272 gene product from transcript CG13272-RA	CG33312|DmelCG13272|Dmel_CG33312	0	0
3323	35015	Drosophila melanogaster	CG13280	CG13280 gene product from transcript CG13280-RA	DmelCG13280	0	0
3324	35016	Drosophila melanogaster	Cas	CAS/CSE1 segregation protein	BcDNA:LD14270|CAS|CG13281|Dcas|DmelCG13281|cas|dcas|l(2)k03902	0	0
3325	35019	Drosophila melanogaster	CG13282	CG13282 gene product from transcript CG13282-RA	DmelCG13282	0	0
3326	35020	Drosophila melanogaster	CG13283	CG13283 gene product from transcript CG13283-RA	DmelCG13283	0	0
3327	35021	Drosophila melanogaster	CG13284	CG13284 gene product from transcript CG13284-RA	DmelCG13284	0	0
3328	35022	Drosophila melanogaster	CG6012	CG6012 gene product from transcript CG6012-RA	DmelCG6012	0	0

3329	35026	Drosophila melanogaster	tweek	CG42555 gene product from transcript CG42555-RH	CG15133|CG15134|CG42555|CG42706|CG4841|DmelCG42555|Dmel_CG15133|Dmel_CG15134|Dmel_CG4841|Tweek	0	0
3330	35027	Drosophila melanogaster	CG12288	CG12288 gene product from transcript CG12288-RA	DmelCG12288|cg12288	0	0
3331	35029	Drosophila melanogaster	ApepP	Aminopeptidase P	AP-P|CG6291|DAP-P|Daminopep-p|DmelCG6291	0	0
3332	35031	Drosophila melanogaster	beat-IIIb	CG33179 gene product from transcript CG33179-RC	CG15135|CG33179|CG4855|DmelCG33179|beat IIIb	0	0
3333	35032	Drosophila melanogaster	CG6304	CG6304 gene product from transcript CG6304-RA	DmelCG6304|anon-WO0140519.248	0	0
3334	35033	Drosophila melanogaster	CG15136	CG15136 gene product from transcript CG15136-RA	DmelCG15136	0	0
3335	35034	Drosophila melanogaster	CG12620	CG12620 gene product from transcript CG12620-RA	DmelCG12620	0	0
3336	35035	Drosophila melanogaster	beat-IIIa	CG12621 gene product from transcript CG12621-RD	CG12621|DmelCG12621|beat IIIa	0	0
3337	35037	Drosophila melanogaster	beat-IIIc	CG15138 gene product from transcript CG15138-RA	BcDNA:RE14414|Beat-IIIC|CG15138|CG6365|DmelCG15138|beat IIIc	0	0
3338	35040	Drosophila melanogaster	CG6380	CG6380 gene product from transcript CG6380-RA	DmelCG6380	0	0
3339	35041	Drosophila melanogaster	Lrch	Leucine-rich-repeats and calponin homology domain p	CG6860|CT21229|DmelCG6860|dLRCH	0	0
3340	35042	Drosophila melanogaster	CLIP-190	Cytoplasmic linker protein 190	CG5020|CLIP190|Clip- 190|Clip-190|Clip190|D-CLIP-19|D-CLIP-190|D-CLIP-195|D-CLIP-90|D-Clip-190|DmelCG5020	0	0
3341	35043	Drosophila melanogaster	Rpb11	CG6840 gene product from transcript CG6840-RA	BcDNA:GM15177|CG6840|DmelCG6840|RPB11	0	0
3342	35044	Drosophila melanogaster	CG15141	CG15141 gene product from transcript CG15141-RA	BEST:LD24839|DmelCG15141	0	0
3343	35045	Drosophila melanogaster	Dif	Dorsal-related immunity factor	6794|CG6794|DIF|DmelCG6794|dif	0	0
3344	35046	Drosophila melanogaster	CG5043	CG5043 gene product from transcript CG5043-RA	DmelCG5043	0	0
3345	35047	Drosophila melanogaster	dl	dorsal	CG6667|DL|Dl|DmelCG6667|GSd447|anon-EST:GressD7|d|dL|fs(2)k10816|mat(2)dorsal	0	0
3346	35048	Drosophila melanogaster	CG5050	CG5050 gene product from transcript CG5050-RA	DmelCG5050	0	0
3347	35049	Drosophila melanogaster	CG6639	CG6639 gene product from transcript CG6639-RA	DmelCG6639|SPH93|c-SPH93	0	0
3348	35050	Drosophila melanogaster	CG18563	CG18563 gene product from transcript CG18563-RB	DmelCG18563|SPH156	0	0
3349	35051	Drosophila melanogaster	BicD	Bicaudal D	BIC-D|Bic-D|Bic[D]|CG6605|DmelCG6605|anon-EST:fe2A11	0	0
3350	35052	Drosophila melanogaster	Sgt	small glutamine-rich tetratricopeptide containing prote	CG5094|DmelCG5094|dsgt	0	0
3351	35053	Drosophila melanogaster	Aac11	CG6582 gene product from transcript CG6582-RA	Api5|BcDNA.LD09852|BcDNA:LD09852|CG6582|DmelCG6582|l(2)k06710	0	0
3352	35054	Drosophila melanogaster	fws	four way stop	CG6549|COG5|Cog5|DmelCG6549|Fws|anon-EST:Posey162|anon-EST:Posey186|dCOG5	0	0
3353	35055	Drosophila melanogaster	CG5110	CG5110 gene product from transcript CG5110-RB	BcDNA:RE09173|DmelCG5110|MP1|dMP1	0	0
3354	35056	Drosophila melanogaster	CG6453	CG6453 gene product from transcript CG6453-RA	DmelCG6453	0	0
3355	35057	Drosophila melanogaster	CG5131	CG5131 gene product from transcript CG5131-RA	DmelCG5131	0	0
3356	35058	Drosophila melanogaster	qua	quail	CG6433|DmelCG6433|Qua|anon-WO0140519.71|fs(2)A12	0	0
3357	35059	Drosophila melanogaster	CG15142	CG15142 gene product from transcript CG15142-RA	DmelCG15142	0	0
3358	35060	Drosophila melanogaster	CG6412	CG6412 gene product from transcript CG6412-RA	DmelCG6412|mtEF-Ts	0	0
3359	35061	Drosophila melanogaster	Dhc36C	Dynein heavy chain at 36C	CG5526|DHC|Dhc|DmelCG5526|dhc	0	0
3360	35062	Drosophila melanogaster	CG15143	CG15143 gene product from transcript CG15143-RA	DmelCG15143	0	0
3361	35063	Drosophila melanogaster	CG15144	CG15144 gene product from transcript CG15144-RB	DmelCG15144	0	0
3362	35064	Drosophila melanogaster	CG15145	CG15145 gene product from transcript CG15145-RA	DmelCG15145	0	0
3363	35065	Drosophila melanogaster	CG7094	CG7094 gene product from transcript CG7094-RA	DmelCG7094	0	0
3364	35066	Drosophila melanogaster	Oli	Olig family	CG5545|DmelCG5545|Doli|Olig|bHLHb23	0	0
3365	35067	Drosophila melanogaster	CG6870	CG6870 gene product from transcript CG6870-RA	DmelCG6870	0	0
3366	35068	Drosophila melanogaster	Sytalpha	Synaptotagmin alpha	CG5559|DmelCG5559|SYT3|Syt alpha|Syt3|dsytalpha|sytalpha	0	0
3367	35069	Drosophila melanogaster	CG15147	CG15147 gene product from transcript CG15147-RB	DmelCG15147	0	0
3368	35070	Drosophila melanogaster	CadN	Cadherin-N	B|CG7100|CT21941|D-cad|DN|DN-CAD|DN-Cad|DN-cad|DNCad|DNcad|DmelCG7100|N-Cad|N-Cadh|N-cad|NCAD|NCad|Ncad|S(DmcycE[JP])2.12|anon-EST:CL32|cad1|cadN|l(2)36Da|l(2)Bld|ncad	0	0
3369	35071	Drosophila melanogaster	CadN2	Cadherin-N2	CG42829|CG7527|CT23007|DN-Cad2|DN-cad2|DN-like|DNcad2|DmNCad2|DmelCG42829|Dmel_CG7527|N-Cad2|NCad2|cadN2	0	0
3370	35072	Drosophila melanogaster	CG5674	CG5674 gene product from transcript CG5674-RB	DmelCG5674	0	0
3371	35073	Drosophila melanogaster	btv	beethoven	CG15148|DHC|DHC36D|Dhc36D|DmelCG15148	0	0
3372	35074	Drosophila melanogaster	CG5681	CG5681 gene product from transcript CG5681-RA	DmelCG5681	0	0
3373	35075	Drosophila melanogaster	CG42635	CG42635 gene product from transcript CG42635-RA	CG15149|DmelCG42635|Dmel_CG15149	0	0
3374	35076	Drosophila melanogaster	elfless	CG15150 gene product from transcript CG15150-RB	BcDNA:AT24563|CG15150|DmelCG15150	0	0
3375	35077	Drosophila melanogaster	rdo	reduced ocelli	CG15151|CT35050|DmelCG15151|PFE	0	0
3376	35078	Drosophila melanogaster	Arr1	Arrestin 1	3H10|ARR1|Arr|ArrA|CG5711|DA|DAI|DS-Ag|DmArr1|Dmarrestin|DmelCG5711|Dro.-Arr.I|FBgn0000120|PRII|arr1	0	0
3377	35079	Drosophila melanogaster	CG31741	CG31741 gene product from transcript CG31741-RA	CG5750|DmelCG31741	0	0
3378	35080	Drosophila melanogaster	CG15153	CG15153 gene product from transcript CG15153-RA	DmelCG15153	0	0
3379	35081	Drosophila melanogaster	CG5755	CG5755 gene product from transcript CG5755-RA	DmelCG5755|anon-WO0140519.163	0	0
3380	35082	Drosophila melanogaster	CG15152	CG15152 gene product from transcript CG15152-RA	DmelCG15152	0	0
3381	35083	Drosophila melanogaster	CG5758	CG5758 gene product from transcript CG5758-RC	DmelCG5758	0	0
3382	35084	Drosophila melanogaster	kel	kelch	CG7210|Dm.Ring_canal/KELC_DROME|DmelCG7210|Kel|Kelch	0	0
3383	35085	Drosophila melanogaster	Socs36E	Suppressor of cytokine signaling at 36E	CG15154|DmelCG15154|SOCS|SOCS36E|Socs|anon-EST:Liang-2.2|clone 2.2|dSOCS|dmSocs36E|socs36E|socs60E1	0	0
3384	35086	Drosophila melanogaster	CG17681	CG17681 gene product from transcript CG17681-RA	DmelCG17681	0	0
3385	35087	Drosophila melanogaster	CG15155	CG15155 gene product from transcript CG15155-RA	CT35055|DmelCG15155	0	0
3386	35088	Drosophila melanogaster	CG5783	CG5783 gene product from transcript CG5783-RA	DmelCG5783	0	0
3387	35089	Drosophila melanogaster	CG7200	CG7200 gene product from transcript CG7200-RA	DmelCG7200	0	0
3388	35090	Drosophila melanogaster	CG42750	CG42750 gene product from transcript CG42750-RB	CG15157|CG15158|CG31746|CG31786|CG42400|CG7167|CG7198|DmelCG42750|Dmel_CG15157|Dmel_CG31746|Dmel_CG31786|Dmel_CG42400	0	0
3389	35091	Drosophila melanogaster	CG7180	CG7180 gene product from transcript CG7180-RA	DmelCG7180|R-PTPk	0	0
3390	35095	Drosophila melanogaster	CG5790	CG5790 gene product from transcript CG5790-RA	DmelCG5790	0	0
3391	35096	Drosophila melanogaster	Acp36DE	Accessory gland peptide 36DE	Acp|Acp36D|CG7157|CT22109|DmelCG7157|anon-WO0140519.55	0	0
3392	35097	Drosophila melanogaster	Fas3	Fasciclin 3	CG5803|DmelCG5803|FAS III|FAS3|FASIII|Fas|Fas III|Fas-3|Fas-III|FasIII|FasIIIface|FascIII|fas III|fas-III|fas3|fasIII|fascIII	0	0
3393	35098	Drosophila melanogaster	RpS26	Ribosomal protein S26	CG10305|DmelCG10305|M(2)36F|M(2)S13|M(2)m|M[[s]]|Rps26|anon-EST:fe2A3|l(2)04553|l(2)36Fa|rpS26|rpS31	0	0
3394	35099	Drosophila melanogaster	ncm	nucampholin	CG12750|DmelCG12750|E-2a|l(2)SH0931|l(2)SH2 0931	0	0
3395	35100	Drosophila melanogaster	bsf	bicoid stability factor	BSF|CG10302|DmelCG10302	0	0
3396	35101	Drosophila melanogaster	Ntf-2r	Nuclear transport factor-2-related	CG10174|DmelCG10174|Dntf-2|Dntf-2R|Dntf-2r	0	0
3397	35102	Drosophila melanogaster	CG10283	CG10283 gene product from transcript CG10283-RD	DmelCG10283	0	0
3398	35103	Drosophila melanogaster	CG10176	CG10176 gene product from transcript CG10176-RB	DmelCG10176	0	0
3399	35104	Drosophila melanogaster	kon	kon-tiki	CG10275|CT28869|DmelCG10275|perd	0	0
3400	35105	Drosophila melanogaster	CG10178	CG10178 gene product from transcript CG10178-RB	DmelCG10178	0	0
3401	35106	Drosophila melanogaster	CG10211	CG10211 gene product from transcript CG10211-RA	DmelCG10211	0	0
3402	35107	Drosophila melanogaster	Pde11	Phosphodiesterase 11	CG10231|CG15159|CG34341|DmelCG34341|Dmel_CG10231|Dmel_CG15159|PDE11	0	0
3403	35109	Drosophila melanogaster	CG15160	CG15160 gene product from transcript CG15160-RA	DmelCG15160	0	0
3404	35110	Drosophila melanogaster	amos	absent MD neurons and olfactory sensilla	Amos|CG10393|DmelCG10393|Roi|Tft|asolo|bHLHa10|rolo	0	0
3405	35111	Drosophila melanogaster	CG10413	CG10413 gene product from transcript CG10413-RA	DmelCG10413	0	0
3406	35112	Drosophila melanogaster	CG10333	CG10333 gene product from transcript CG10333-RA	DmRH19|DmelCG10333|cg10333	0	0
3407	35113	Drosophila melanogaster	Atac2	ATAC complex component 2	CG10414|DmelCG10414|KAT14	0	0
3408	35114	Drosophila melanogaster	CG15161	CG15161 gene product from transcript CG15161-RA	DmelCG15161|IFT46	0	0
3409	35115	Drosophila melanogaster	Cyp310a1	CG10391 gene product from transcript CG10391-RA	310a1|CG10391|Cyp310|Cyp310a|DmelCG10391|H|anon-EST:CL1d7	0	0
3410	35116	Drosophila melanogaster	MESR3	Misexpression suppressor of ras 3	CG15162|DmelCG15162	0	0
3411	35117	Drosophila melanogaster	CG43338	CG43338 gene product from transcript CG43338-RB	CG15165|CG31790|CG42627|DmelCG43338|Dmel_CG31790	0	0
3412	35118	Drosophila melanogaster	CG31751	CG31751 gene product from transcript CG31751-RA	CG15163|CG15164|DmelCG31751|anon-ct|ct	0	0
3413	35119	Drosophila melanogaster	tos	tosca	CG10387|DmTosca|DmelCG10387|Tosca	0	0
3414	35121	Drosophila melanogaster	msl-1	male-specific lethal 1	CG10385|DmelCG10385|MSL|MSL-1|MSL1|MSL3|Msl 1-3|Msl-1|Msl1|i94|km(2)B|kmB|mls-1|msl|msl1	0	0
3415	35122	Drosophila melanogaster	CG10336	CG10336 gene product from transcript CG10336-RC	DmelCG10336	0	0
3416	35123	Drosophila melanogaster	CG10383	CG10383 gene product from transcript CG10383-RA	DmelCG10383	0	0
3417	35124	Drosophila melanogaster	CG10338	CG10338 gene product from transcript CG10338-RA	DmelCG10338	0	0
3418	35125	Drosophila melanogaster	CG10341	CG10341 gene product from transcript CG10341-RA	DmelCG10341	0	0
3419	35126	Drosophila melanogaster	CG10376	CG10376 gene product from transcript CG10376-RA	DmelCG10376|PP2C	0	0
3420	35127	Drosophila melanogaster	CG10343	CG10343 gene product from transcript CG10343-RA	DmelCG10343|anon-WO0118547.123	0	0
3421	35128	Drosophila melanogaster	Jwa	Jwa ortholog	CG10373|DmelCG10373|djwa	0	0
3422	35129	Drosophila melanogaster	Faf	Fas-associated factor	CG10372|DmelCG10372|FFAF|GT#49	0	0
3423	35130	Drosophila melanogaster	Grip71	CG10346 gene product from transcript CG10346-RA	CG10346|Dgp71WD|Dgrip71|DmelCG10346|Grip71wd	0	0
3424	35131	Drosophila melanogaster	Irk3	Inwardly rectifying potassium channel 3	CG10369|DmelCG10369|dKirIII|irk3	0	0
3425	35132	Drosophila melanogaster	CG10348	CG10348 gene product from transcript CG10348-RB	DmelCG10348	0	0
3426	35134	Drosophila melanogaster	CG15167	CG15167 gene product from transcript CG15167-RA	DmelCG15167	0	0
3427	35135	Drosophila melanogaster	ham	hamlet	CG10568|CG15906|CG15907|CG31753|DmelCG31753|Ham	0	0
3428	35137	Drosophila melanogaster	CG17324	CG17324 gene product from transcript CG17324-RB	BEST:GH06505|DmelCG17324|GH06505	0	0
3429	35138	Drosophila melanogaster	CG17323	CG17323 gene product from transcript CG17323-RD	CT35071|DmelCG17323	0	0
3430	35139	Drosophila melanogaster	CG17322	CG17322 gene product from transcript CG17322-RB	BEST:LD25345|DmelCG17322|LD25345	0	0
3431	35140	Drosophila melanogaster	CG17597	CG17597 gene product from transcript CG17597-RA	DmelCG17597	0	0
3432	35142	Drosophila melanogaster	CG10600	CG10600 gene product from transcript CG10600-RA	DmelCG10600	0	0
3433	35143	Drosophila melanogaster	CG31752	CG31752 gene product from transcript CG31752-RA	CG10599|DmelCG31752	0	0
3434	35144	Drosophila melanogaster	CG17321	CG17321 gene product from transcript CG17321-RA	DmelCG17321	0	0
3435	35145	Drosophila melanogaster	mRpL13	mitochondrial ribosomal protein L13	CG10602|CG10603|DmelCG10603|L13|anon-EST:fe3C4	0	0
3436	35146	Drosophila melanogaster	CG10602	CG10602 gene product from transcript CG10602-RE	DmelCG10602|pepCG10602	0	0
3437	35147	Drosophila melanogaster	tup	tailup	CG10619|DmelCG10619|Isl|Tup|isl|islet|l(2)37Aa|l(2)E41	0	0
3438	35149	Drosophila melanogaster	ssp3	short spindle 3	CG10666|CG18397|DmelCG18397|Ssp3	0	0
3439	35150	Drosophila melanogaster	CG10428	CG10428 gene product from transcript CG10428-RA	DmelCG10428	0	0

3440	35151	Drosophila melanogaster	Nedd8	CG10679 gene product from transcript CG10679-RA	CG10679|DmelCG10679|nedd8	0	0
3441	35152	Drosophila melanogaster	CG10621	CG10621 gene product from transcript CG10621-RA	BEST:LD26123|DmelCG10621|anon-WO0140519.13	0	0
3442	35153	Drosophila melanogaster	CG10623	CG10623 gene product from transcript CG10623-RA	DmelCG10623	0	0
3443	35154	Drosophila melanogaster	Nak	Numb-associated kinase	CG10637|DmelCG10637|nak	0	0
3444	35155	Drosophila melanogaster	Tango6	Transport and Golgi organization 6	CG18398|DmelCG18398|TANGO6	0	0
3445	35156	Drosophila melanogaster	CG10639	CG10639 gene product from transcript CG10639-RA	DmelCG10639	0	0
3446	35157	Drosophila melanogaster	CG10431	CG10431 gene product from transcript CG10431-RA	DmelCG10431|dmCG10431	0	0
3447	35158	Drosophila melanogaster	CG10641	CG10641 gene product from transcript CG10641-RA	DmelCG10641|EFhd2|efhd|efhd2	0	0
3448	35159	Drosophila melanogaster	CG15168	CG15168 gene product from transcript CG15168-RA	BcDNA:RE09053|DmelCG15168	0	0
3449	35160	Drosophila melanogaster	CG15170	CG15170 gene product from transcript CG15170-RA	DmelCG15170	0	0
3450	35161	Drosophila melanogaster	CG15169	CG15169 gene product from transcript CG15169-RA	DmelCG15169	0	0
3451	35162	Drosophila melanogaster	CG10650	CG10650 gene product from transcript CG10650-RA	BEST:GH09876|DmelCG10650	0	0
3452	35163	Drosophila melanogaster	CG31792	CG31792 gene product from transcript CG31792-RA	CG17338|DmelCG31792	0	0
3453	35166	Drosophila melanogaster	robl37BC	CG15171 gene product from transcript CG15171-RA	CG15171|DmelCG15171	0	0
3454	35167	Drosophila melanogaster	CG15172	CG15172 gene product from transcript CG15172-RA	DmelCG15172	0	0
3455	35168	Drosophila melanogaster	Side	similar to Deadpan	CG10446|DmelCG10446|Sidestep|bHLHe51|side	0	0
3456	35169	Drosophila melanogaster	hk	hook	CG10653|DmelCG10653|dHk	0	0
3457	35170	Drosophila melanogaster	mib2	mind bomb 2	Be|CG17492|D-mibl|DmelCG17492|i115|l(2)37Be|suelto	0	0
3458	35172	Drosophila melanogaster	CG15173	CG15173 gene product from transcript CG15173-RA	DmelCG15173	0	0
3459	35173	Drosophila melanogaster	Acn	Acinus	Ba|CG10437|CG10473|DmelCG10473|dAcn|dacn|hkl|l(2)37Ba	0	0
3460	35174	Drosophila melanogaster	CG10470	CG10470 gene product from transcript CG10470-RA	B2|DmelCG10470|TU37B2|TUB2|TuB2|anon-37B-2	0	0
3461	35176	Drosophila melanogaster	Rpn3	Regulatory particle non-ATPase 3	CG10484|CG42641|DXA2_DROME|DmelCG42641|Dmel_CG10484|Dox|Dox-A2|DoxA2|l(2)37Bf|l(2)37Bf[+]|p58	0	0
3462	35177	Drosophila melanogaster	CG10492	CG10492 gene product from transcript CG10492-RA	B1|DmelCG10492|TU37B1|TuB1|anon-37B-1	0	0
3463	35178	Drosophila melanogaster	Phlpp	PH domain leucine-rich repeat protein phosphatase	CG10493|CT29458|DmelCG10493|PP2C|dPHLPP	0	0
3464	35179	Drosophila melanogaster	CG10495	CG10495 gene product from transcript CG10495-RA	DmelCG10495|anon-WO0118547.200	0	0
3465	35180	Drosophila melanogaster	CG17343	CG17343 gene product from transcript CG17343-RA	DmelCG17343	0	0
3466	35181	Drosophila melanogaster	CG10702	CG10702 gene product from transcript CG10702-RD	DmelCG10702	0	0
3467	35182	Drosophila melanogaster	CG17572	CG17572 gene product from transcript CG17572-RA	DmelCG17572|SPH64|c-SPH64	0	0
3468	35183	Drosophila melanogaster	CG10700	CG10700 gene product from transcript CG10700-RA	DmelCG10700	0	0
3469	35184	Drosophila melanogaster	Lim3	CG10699 gene product from transcript CG10699-RF	CG10699|CG15174|DmelCG10699|Lim-3A|l(2)37Bd|l(2)Bd|lim3	0	0
3470	35185	Drosophila melanogaster	CG17344	CG17344 gene product from transcript CG17344-RA	BcDNA:AT10305|DmelCG17344	0	0
3471	35188	Drosophila melanogaster	amd	alpha methyl dopa-resistant	AMD-r|CG10501|CG17345|DmelCG10501|X04695|fAMD|l(2)37Bk|l(2)amd|l(2)amd alpha-methyldopa hypersensitive|l(2)amd[H]	0	0
3472	35189	Drosophila melanogaster	CG10561	CG10561 gene product from transcript CG10561-RA	C2|Cs|DmelCG10561|ORF1|ORF2|ORF3|TU37C1|TU37C2|TuC1|anon-37Cb|anon-37Cc|anon-37Cd|anon-37Cs|cs|l(2)37Cs	0	0
3473	35190	Drosophila melanogaster	Ddc	Dopa decarboxylase	5-HT|AADC|CG10697|DDC|DdcDm|DmelCG10697|HL9|ddc|fDDC|l(2)37Bl|l(2)37Ch|l(2)k02104	0	0
3474	35192	Drosophila melanogaster	l(2)37Cb	lethal (2) 37Cb	CG10689|Cb|DmelCG10689|cg10689|l(2)E104	0	0
3475	35193	Drosophila melanogaster	l(2)37Cd	lethal (2) 37Cd	CG10563|Cd|DmelCG10563	0	0
3476	35194	Drosophila melanogaster	Aats-asn	Asparaginyl-tRNA synthetase	AsnRS|BcDNA:GH06451|CG10687|Ca|DmelCG10687|NRS|l(2)37Ca|l(2)37Ca)E11|l(2)E11	0	0
3477	35195	Drosophila melanogaster	l(2)37Ce	lethal (2) 37Ce	CG17347|Ce|DmelCG17347|l(2)37Ce)E62|l(2)E62	0	0
3478	35196	Drosophila melanogaster	l(2)37Cg	lethal (2) 37Cg	CG10685|Cg|DmelCG10685	0	0
3479	35197	Drosophila melanogaster	brat	brain tumor	BRAT|Brat|CG10719|Cf|DmelCG10719|E60|anon-37CDa|fs(2)ltoPM43|l(2)37Cf|l(2)E60|l(2)brat	0	0
3480	35198	Drosophila melanogaster	CG17568	CG17568 gene product from transcript CG17568-RA	DmelCG17568	0	0
3481	35199	Drosophila melanogaster	gammaTub37C	gamma-Tubulin at 37C	CG17566|D.m.GAMMA-37C|DmelCG17566|GTU88*|PL10|Rb1011|TW1|fs(2)TW1|fs(2)TWl|fsTW1|g-Tubulin|gamma Tub37CD|gamma tubulin|gamma-Tub|gamma-Tub37C|gamma-tub37CD|gamma-tubulin	0	0
3482	35200	Drosophila melanogaster	pigeon	CG10739 gene product from transcript CG10739-RB	CG10739|DmelCG10739|lio|pio	0	0
3483	35203	Drosophila melanogaster	CG31797	CG31797 gene product from transcript CG31797-RA	CG15175|CG15176|CT35089|DmelCG31797	0	0
3484	35205	Drosophila melanogaster	CG17564	CG17564 gene product from transcript CG17564-RB	DmelCG17564	0	0
3485	35206	Drosophila melanogaster	CG10750	CG10750 gene product from transcript CG10750-RA	DmelCG10750	0	0
3486	35207	Drosophila melanogaster	dnt	doughnut on 2	CG17559|DmelCG17559	0	0
3487	35208	Drosophila melanogaster	CG13086	CG13086 gene product from transcript CG13086-RA	DmelCG13086	0	0
3488	35209	Drosophila melanogaster	CG17349	CG17349 gene product from transcript CG17349-RA	DmelCG17349|anon-WO0140519.204	0	0
3489	35210	Drosophila melanogaster	CG17350	CG17350 gene product from transcript CG17350-RA	DmelCG17350	0	0
3490	35211	Drosophila melanogaster	fon	fondue	CG15825|DmelCG15825	0	0
3491	35212	Drosophila melanogaster	CG17549	CG17549 gene product from transcript CG17549-RB	DmelCG17549	0	0
3492	35213	Drosophila melanogaster	CG17544	CG17544 gene product from transcript CG17544-RB	DmelCG17544	0	0
3493	35215	Drosophila melanogaster	Pax	Paxillin	CG18061|CG18576|CG31794|CT40481|CT42454|DPaxillin|DPxn|DPxn37|DmelCG31794|Dpax|DpaxA|PDLP|pax	0	0
3494	35216	Drosophila melanogaster	Lectin-galC1	Galactose-specific C-type lectin	AC007082a|CG9976|DL1|Dgal-1|DmelCG9976|lectin-galC1	0	0
3495	35218	Drosophila melanogaster	CG16771	CG16771 gene product from transcript CG16771-RC	DmelCG16771	0	0
3496	35219	Drosophila melanogaster	CG13085	CG13085 gene product from transcript CG13085-RA	DmelCG13085	0	0
3497	35220	Drosophila melanogaster	CG9987	CG9987 gene product from transcript CG9987-RA	DmelCG9987	0	0
3498	35221	Drosophila melanogaster	Rab9	CG9994 gene product from transcript CG9994-RA	146494_at|CG9994|DmRab9|DmelCG9994	0	0
3499	35222	Drosophila melanogaster	CG10237	CG10237 gene product from transcript CG10237-RD	DmelCG10237	0	0
3500	35223	Drosophila melanogaster	RanGap	Ran GTPase activating protein	CG9999|CT28175|DmelCG9999|Ran|RanGAP|RanGap1|SD|Scl|Sd|Sd-RanGAP|Sd-RanGap|dRanGAP|ranGAP|ranGap|rangap	0	0
3501	35225	Drosophila melanogaster	Top2	Topoisomerase 2	CG10223|CHRAC|DmelCG10223|TOP2|TOPOII|TopII|Topo II|TopoII|dCHRAC|dTopoII|top2|topo II|topoII	0	0
3502	35226	Drosophila melanogaster	CG10026	CG10026 gene product from transcript CG10026-RA	DmelCG10026	0	0
3503	35227	Drosophila melanogaster	tj	traffic jam	CG10034|Dmaf|DmelCG10034|PL3|TJ|Tj|fs(2)eo-B|fs(2)eo2|fs(2)eoPL3|maf-L|maf1	0	0
3504	35228	Drosophila melanogaster	CG10195	CG10195 gene product from transcript CG10195-RB	DmelCG10195	0	0
3505	35229	Drosophila melanogaster	CG13084	CG13084 gene product from transcript CG13084-RA	DmelCG13084	0	0
3506	35230	Drosophila melanogaster	CG13083	CG13083 gene product from transcript CG13083-RB	DmelCG13083	0	0
3507	35231	Drosophila melanogaster	CG10194	CG10194 gene product from transcript CG10194-RA	DmelCG10194	0	0
3508	35232	Drosophila melanogaster	CG18094	CG18094 gene product from transcript CG18094-RA	CG10048|DmelCG18094	0	0
3509	35234	Drosophila melanogaster	CG10189	CG10189 gene product from transcript CG10189-RA	DmelCG10189|i180|i199	0	0
3510	35235	Drosophila melanogaster	swm	second mitotic wave missing	CG10084|DmelCG10084|E1|S(Rux)2B|S(rux)2B|Swm|l(2)37Dh|l(2)E1	0	0
3511	35236	Drosophila melanogaster	Top3alpha	Topoisomerase 3alpha	CG10123|DmTop3alpha|DmelCG10123|Top3a|Topo IIIalpha|dTopoIIIa|top3alpha|topo IIIalpha	0	0
3512	35237	Drosophila melanogaster	CG10188	CG10188 gene product from transcript CG10188-RB	DmelCG10188	0	0
3513	35238	Drosophila melanogaster	CG10186	CG10186 gene product from transcript CG10186-RH	CT28647|DmelCG10186|anon-EST:Posey189	0	0
3514	35239	Drosophila melanogaster	CG10132	CG10132 gene product from transcript CG10132-RA	DmelCG10132	0	0
3515	35240	Drosophila melanogaster	CG10166	CG10166 gene product from transcript CG10166-RA	DmelCG10166|Dol-P-ManTase	0	0
3516	35241	Drosophila melanogaster	CG10137	CG10137 gene product from transcript CG10137-RA	DmelCG10137	0	0
3517	35242	Drosophila melanogaster	CG10165	CG10165 gene product from transcript CG10165-RC	DmelCG10165	0	0
3518	35243	Drosophila melanogaster	CG31697	CG31697 gene product from transcript CG31697-RA	CG10355|DmelCG31697	0	0
3519	35244	Drosophila melanogaster	CG13082	CG13082 gene product from transcript CG13082-RA	DmelCG13082	0	0
3520	35245	Drosophila melanogaster	CG13081	CG13081 gene product from transcript CG13081-RA	DmelCG13081	0	0
3521	35246	Drosophila melanogaster	ref(2)P	refractory to sigma P	CG10360|DmelCG10360|Ref(2)P|Ref(2)p|Ref2P|p62|ref|ref(2)Pn|ref(2)Po2|ref(2)p|ref2p	0	0
3522	35247	Drosophila melanogaster	CG10337	CG10337 gene product from transcript CG10337-RA	DmelCG10337	0	0
3523	35248	Drosophila melanogaster	TepIV	Thiolester containing protein IV	CG10363|CT29102|DmelCG10363|TEP IV|Tep4|dTEPIV|tep4|tepIV	0	0
3524	35250	Drosophila melanogaster	CG13079	CG13079 gene product from transcript CG13079-RA	CG13080|DmelCG13079	0	0
3525	35251	Drosophila melanogaster	CG13078	CG13078 gene product from transcript CG13078-RA	DmelCG13078	0	0
3526	35252	Drosophila melanogaster	CG13077	CG13077 gene product from transcript CG13077-RA	DmelCG13077	0	0
3527	35253	Drosophila melanogaster	spi	spitz	CG10334|CT29014|DmelCG10334|EP(2)2378|Spi|Spitz|anon-WO0118547.158|l(2)01068|l(2)37Fa|l(2)s3547|s-spi	0	0
3528	35254	Drosophila melanogaster	msb1l	CG10364 gene product from transcript CG10364-RA	CG10364|DmelCG10364|msh1l	0	0
3529	35255	Drosophila melanogaster	CG10268	CG10268 gene product from transcript CG10268-RA	BcDNA:SD12705|DmelCG10268	0	0
3530	35256	Drosophila melanogaster	Hakai	CG10263 gene product from transcript CG10263-RC	CG10263|DmelCG10263|hakai	0	0
3531	35257	Drosophila melanogaster	CG10262	CG10262 gene product from transcript CG10262-RA	DmPCNA2|DmelCG10262|PCNA2	0	0
3532	35258	Drosophila melanogaster	CG10366	CG10366 gene product from transcript CG10366-RA	DmelCG10366	0	0
3533	35259	Drosophila melanogaster	Lar	Leukocyte-antigen-related-like	CG10443|DLAR|DLar|Dlar|DmelCG10443|LAR|PTP38A|dlar	0	0
3534	35260	Drosophila melanogaster	CG10462	CG10462 gene product from transcript CG10462-RB	BcDNA:SD07008|DmelCG10462	0	0
3535	35261	Drosophila melanogaster	Nf-YB	Nuclear factor Y-box B	BcDNA:RH50436|CG10447|DmelCG10447|NF-YB|dNF-YB	0	0
3536	35262	Drosophila melanogaster	CG10631	CG10631 gene product from transcript CG10631-RA	DmelCG10631	0	0
3537	35263	Drosophila melanogaster	CG10628	CG10628 gene product from transcript CG10628-RA	DmelCG10628	0	0
3538	35264	Drosophila melanogaster	CG10463	CG10463 gene product from transcript CG10463-RA	DmelCG10463	0	0
3539	35265	Drosophila melanogaster	fbp	fructose-1,6-bisphosphatase	CG10611|CG31692|DmelCG31692|FbPase	0	0
3540	35266	Drosophila melanogaster	bsh	brain-specific homeobox	BSH|Bsh|CG10604|DmelCG10604|E86|Hox11-310|lambdaE86	0	0
3541	35267	Drosophila melanogaster	CdGAPr	CG10538 gene product from transcript CG10538-RA	10538|CG10538|DmelCG10538|FBgn 32821|GAP|d-CdGAPr	0	0
3542	35268	Drosophila melanogaster	CG10466	CG10466 gene product from transcript CG10466-RA	DmelCG10466|anon-WO0140519.255|cg10466	0	0
3543	35270	Drosophila melanogaster	CG13962	CG13962 gene product from transcript CG13962-RA	DmelCG13962	0	0
3544	35271	Drosophila melanogaster	CG13958	CG13958 gene product from transcript CG13958-RA	DmelCG13958	0	0
3545	35273	Drosophila melanogaster	CG10481	CG10481 gene product from transcript CG10481-RA	DmelCG10481	0	0
3546	35275	Drosophila melanogaster	Victoria	CG33117 gene product from transcript CG33117-RA	CG13963|CG33117|DmelCG33117|TotE	0	0
3547	35277	Drosophila melanogaster	sick	sickie	CG10662|CG13959|CG13960|CG13961|CG13964|CG33316|CG34343|CG42589|DmelCG42589|Dmel_CG10662|Dmel_CG13964|Dmel_CG33316|Dmel_CG34343	0	0
3548	35279	Drosophila melanogaster	CG10664	CG10664 gene product from transcript CG10664-RB	COX|COX IV|COXIV|DmelCG10664|IV|cox4	0	0
3549	35280	Drosophila melanogaster	CG13965	CG13965 gene product from transcript CG13965-RA	DmelCG13965	0	0
3550	35281	Drosophila melanogaster	CG16772	CG16772 gene product from transcript CG16772-RA	DmelCG16772|anon-WO0138581.5	0	0

3551	35282	Drosophila melanogaster	CG10680	CG10680 gene product from transcript CG10680-RA	DmelCG10680	0	0
3552	35284	Drosophila melanogaster	CG13966	CG13966 gene product from transcript CG13966-RB	DmelCG13966	0	0
3553	35285	Drosophila melanogaster	CG10659	CG10659 gene product from transcript CG10659-RA	DmelCG10659	0	0
3554	35286	Drosophila melanogaster	sNPF	short neuropeptide F precursor	38B.2|CG13968|DmelCG13968|Drm-sNPF|Drm-sNPF-1|Drm-sNPF-2|Drm-sNPF-3|Drm-sNPF-4|Drm-sNPF-AP1|LRLRFamide|lrlrfa|s-NPF|sNPF-1|sNPF-2|sNPF-2s|sNPF-3|sNPF-4|sNPF1|sNPF3|snpf	0	0
3555	35287	Drosophila melanogaster	barr	barren	38B.3|Barr|Barren|CAP-H|CG10726|Cap-H|DmelCG10726|dCap-H	0	0
3556	35288	Drosophila melanogaster	lok	loki	10895|38B.4|CG10895|CHK-2|CHK2|Chk2|DmCHk2|DmChk2|Dmchk2|DmelCG10895|Dmnk|Dmnk-L|Dmnk-S|Dmnk/DChk2|LOKI/CHK2|MNK|chk|chk2|chk2/lok|mnk	0	0
3557	35289	Drosophila melanogaster	vls	valois	38B.5|CG107278|CG10728|DmelCG10728|val	0	0
3558	35291	Drosophila melanogaster	CG10730	CG10730 gene product from transcript CG10730-RA	38B.7|DmelCG10730	0	0
3559	35292	Drosophila melanogaster	pr	purple	38B.9|CG16784|DmelCG16784|anon-38Bb	0	0
3560	35293	Drosophila melanogaster	neb	nebbish	38B.10|38B.12|CG10718|Dm0332|DmKlp38B|DmelCG10718|KIF14|KLP 38B|KLP-38B|KLP38B|Klp38|Klp38B|Mot|Neb|klp38B|l(2)03552|l(2)k00802|l(2)k07614|sl(2)ry|sl(2)ry3|tio	0	0
3561	35295	Drosophila melanogaster	CG10747	CG10747 gene product from transcript CG10747-RA	38B.13|DmelCG10747	0	0
3562	35296	Drosophila melanogaster	CG10721	CG10721 gene product from transcript CG10721-RA	38B.14|DmelCG10721	0	0
3563	35297	Drosophila melanogaster	Taf13	TBP-associated factor 13	38B.15|CG10756|DmelCG10756|TAF|TAF13|TAF18|TAF[[II]]|TAF[[II]]18|TFIID|Taf18|dTAF[[II]]18	0	0
3564	35298	Drosophila melanogaster	nesd	nessun dorma	38B.16|CG10722|DmelCG10722	0	0
3565	35299	Drosophila melanogaster	mRpS18B	mitochondrial ribosomal protein S18B	38B.17|CG10757|DmelCG10757|MRP-S18-(2)|mRpS18b	0	0
3566	35300	Drosophila melanogaster	Kua	CG10723 gene product from transcript CG10723-RA	38B.18|CG10723|DmelCG10723	0	0
3567	35303	Drosophila melanogaster	CG10651	CG10651 gene product from transcript CG10651-RA	38C.2|DmelCG10651	0	0
3568	35305	Drosophila melanogaster	La	La autoantigen-like	38C.38|BEST:LD24519|CG10922|D-La|DLa/SS-B|DmelCG10922|dmLa	0	0
3569	35306	Drosophila melanogaster	rtGEF	rho-type guanine exchange factor	38C.40|ARHGEF7|CG10043|DmelCG10043|DrhoGEF|DrtGEF|Rho/RacGEF|RhoGEF|beta-PIX|betaPIX|dPix|dpix|rhoGEF	0	0
3570	35307	Drosophila melanogaster	Ugt37a1	UDP-glycosyltransferase 37a1	38C.41|CG11012|DmUgt37a1|DmeUgt37a1|DmelCG11012	0	0
3571	35308	Drosophila melanogaster	CG16798	CG16798 gene product from transcript CG16798-RA	38C.41|DmelCG16798	0	0
3572	35309	Drosophila melanogaster	CG10947	CG10947 gene product from transcript CG10947-RC	38C.42|DmelCG10947	0	0
3573	35310	Drosophila melanogaster	CG10949	CG10949 gene product from transcript CG10949-RA	38C.46|DmelCG10949|FBgn0032858	0	0
3574	35311	Drosophila melanogaster	Arc-p34	CG10954 gene product from transcript CG10954-RA	38C.47|ARC-P34|ARC-p34|ARPC2/p34|ArcP34|Arpc2|CG10954|DmelCG10954|anon-WO0118547.154|p34	0	0
3575	35312	Drosophila melanogaster	CG15130	CG15130 gene product from transcript CG15130-RB	38C.48|DmelCG15130	0	0
3576	35314	Drosophila melanogaster	CG31688	CG31688 gene product from transcript CG31688-RD	38C.49|38C.52|CG11016|CG11017|CG11019|CG18597|CG31688-PA|DmelCG31688|Dmel_CG11019	0	0
3577	35315	Drosophila melanogaster	cdc23	CG2508 gene product from transcript CG2508-RA	38C.54|APC8|Apc8|CG2508|CG2508-PA|Cdc23|DmelCG2508	0	0
3578	35316	Drosophila melanogaster	CG2493	CG2493 gene product from transcript CG2493-RA	38C.55|DmelCG2493	0	0
3579	35319	Drosophila melanogaster	CG15475	CG15475 gene product from transcript CG15475-RA	38D.19|DmelCG15475	0	0
3580	35320	Drosophila melanogaster	CG17472	CG17472 gene product from transcript CG17472-RA	38D.23|BcDNA:GH20534|DmelCG17472	0	0
3581	35321	Drosophila melanogaster	CG17470	CG17470 gene product from transcript CG17470-RA	38D.25|DmelCG17470|anon-WO0140519.68|anon-WO0153538.51	0	0
3582	35322	Drosophila melanogaster	phr6-4	(6-4)-photolyase	38D.25|64PHR|CG2488|D6-4|Dm 64|DmelCG2488|phr(6-4)	0	0
3583	35323	Drosophila melanogaster	CG2608	CG2608 gene product from transcript CG2608-RA	38D.26|DmelCG2608	0	0
3584	35324	Drosophila melanogaster	CG2611	CG2611 gene product from transcript CG2611-RA	38D.27|DmelCG2611	0	0
3585	35325	Drosophila melanogaster	bru	brunelleschi	38D.28|CG2478|DmelCG2478	0	0
3586	35326	Drosophila melanogaster	CG2614	CG2614 gene product from transcript CG2614-RA	38D.29|DmelCG2614	0	0
3587	35327	Drosophila melanogaster	CG31678	CG31678 gene product from transcript CG31678-RB	38D.30|CG17465|CG17466|DmelCG31678	0	0
3588	35329	Drosophila melanogaster	ik2	IkappaB kinase-like 2	38D.31|APTX7|CG2615|DIK2|Dik2|DmIKKepsilon|DmIKKepsilon/dIK2|DmelCG2615|Dmik2|IK2|IKK|IKKepsilon|Ik2|Ik2/DmIKK|dIKK|dik2|l(2)38Ea	0	0
3589	35330	Drosophila melanogaster	Cen	Centrocortin	38E.1|CG1962|DmelCG1962|cen	0	0
3590	35331	Drosophila melanogaster	CG2617	CG2617 gene product from transcript CG2617-RA	38E.2|DmelCG2617	0	0
3591	35332	Drosophila melanogaster	Hr38	Hormone receptor-like in 38	38E.3|38E.7|CG1864|DHR38|Dhr38|DmelCG1864|HR38|NR4A4|dHR38|hr38|l(2)02306	0	0
3592	35333	Drosophila melanogaster	CG9316	CG9316 gene product from transcript CG9316-RA	38E.9|DmelCG9316	0	0
3593	35334	Drosophila melanogaster	CG9317	CG9317 gene product from transcript CG9317-RB	38E.10|DmelCG9317	0	0
3594	35335	Drosophila melanogaster	CG9318	CG9318 gene product from transcript CG9318-RA	38E.10|DmelCG9318|TM9SF2	0	0
3595	35336	Drosophila melanogaster	Fs(2)Ket	Female sterile (2) Ketel	38E.11|CG2637|DmelCG2637|Fs(2)Hon|Fs(2)Sz6|Fs(2)Sz7|Hon|IMPbeta1|Impbeta|Ket|Ketel|Kpnb1|MRE26|dKap-beta|fs(2)lo-B|fs(2)lto2|fs(2)ltoHD43|imp-beta|ketel|kettle	0	0
3596	35337	Drosophila melanogaster	CG9319	CG9319 gene product from transcript CG9319-RA	38E.13|DmelCG9319	0	0
3597	35338	Drosophila melanogaster	ns4	nucleostemin 4	38E.14|CG9320|DmelCG9320|dmNS4	0	0
3598	35339	Drosophila melanogaster	CG9323	CG9323 gene product from transcript CG9323-RA	38E.15|DmelCG9323|cg9323	0	0
3599	35340	Drosophila melanogaster	dia	diaphanous	38E.16|CG1768|DIA|Dia|Dias|DmelCG1768|l(2)k07135|ms(2)04138	0	0
3600	35341	Drosophila melanogaster	cad	caudal	38E.19|CAD|CG1759|Cad|DmelCG1759|S67|anon-WO2004063362.83|cd	0	0
3601	35342	Drosophila melanogaster	Pomp	CG9324 gene product from transcript CG9324-RA	38E.20|CG9324|DmelCG9324|POMP|pomp	0	0
3602	35343	Drosophila melanogaster	vari	varicose	38E.21|CG9326|DmelCG9326|Vari|l(2)03953|l(2)03953b|l(2)38EFa|szar	0	0
3603	35344	Drosophila melanogaster	CG9328	CG9328 gene product from transcript CG9328-RB	38E.22|BEST:LD01639|BcDNA:SD01241|DmelCG9328|LD01639	0	0
3604	35346	Drosophila melanogaster	CheB38c	Chemosensory protein B 38c	CG14405|DmelCG14405	0	0
3605	35347	Drosophila melanogaster	CG9331	CG9331 gene product from transcript CG9331-RF	DmelCG9331	0	0
3606	35348	Drosophila melanogaster	CG31673	CG31673 gene product from transcript CG31673-RA	BcDNA:LD14730|CCG31673|CG9332|DmelCG31673	0	0
3607	35349	Drosophila melanogaster	Oseg5	CG9333 gene product from transcript CG9333-RB	CG9333|CHE-2|Che-2|DmelCG9333|IFT80|OSEG|OSEG5	0	0
3608	35351	Drosophila melanogaster	CG31676	CG31676 gene product from transcript CG31676-RA	BcDNA:RH34376|CG14403|CG14404|DmelCG31676	0	0
3609	35352	Drosophila melanogaster	CG14402	CG14402 gene product from transcript CG14402-RA	BcDNA:AT08210|DmelCG14402	0	0
3610	35353	Drosophila melanogaster	CG9335	CG9335 gene product from transcript CG9335-RA	BcDNA:LD40063|DmelCG9335	0	0
3611	35354	Drosophila melanogaster	CG14400	CG14400 gene product from transcript CG14400-RA	DmelCG14400	0	0
3612	35355	Drosophila melanogaster	CG9336	CG9336 gene product from transcript CG9336-RB	BcDNA:RE71975|DmelCG9336	0	0
3613	35357	Drosophila melanogaster	CG9338	CG9338 gene product from transcript CG9338-RA	BcDNA:GH07967|DmelCG9338	0	0
3614	35358	Drosophila melanogaster	CG14401	CG14401 gene product from transcript CG14401-RA	DmelCG14401	0	0
3615	35359	Drosophila melanogaster	sky	skywalker	CG14399|CG9339|CG9340|DmelCG9339|anon-WO0118547.455	0	0
3616	35362	Drosophila melanogaster	Mtp	Microsomal triacylglycerol transfer protein	CG9342|CT3751|DmelCG9342|MTP|dMTP	0	0
3617	35364	Drosophila melanogaster	RPA2	Replication protein A2	153194_at|CG9273|DmelCG9273|dRPA2	0	0
3618	35365	Drosophila melanogaster	CG9272	CG9272 gene product from transcript CG9272-RA	9272|DmelCG9272	0	0
3619	35366	Drosophila melanogaster	CG9270	CG9270 gene product from transcript CG9270-RC	Dme_CG9270|DmelCG9270	0	0
3620	35368	Drosophila melanogaster	betaInt-nu	beta[nu] integrin	Beta[[nu]]|CG1762|CT5192|DmelCG1762|betaInt[nu]|beta[[v]]|beta[nu]-integrin|betanu|betav	0	0
3621	35369	Drosophila melanogaster	CG9265	CG9265 gene product from transcript CG9265-RA	DmelCG9265	0	0
3622	35370	Drosophila melanogaster	tadr	torn and diminished rhabdomeres	CG9264|DmelCG9264	0	0
3623	35372	Drosophila melanogaster	CG9259	CG9259 gene product from transcript CG9259-RA	DmelCG9259	0	0
3624	35374	Drosophila melanogaster	CG12050	CG12050 gene product from transcript CG12050-RB	DmelCG12050	0	0
3625	35375	Drosophila melanogaster	CG9257	CG9257 gene product from transcript CG9257-RA	DmelCG9257	0	0
3626	35376	Drosophila melanogaster	Nhe2	Na[+]/H[+] hydrogen exchanger 2	CG9255|CG9256|DmNHE2|DmelCG9256|NHE2|nhe2	0	0
3627	35378	Drosophila melanogaster	Dap160	Dynamin associated protein 160	CG1099|DAP-160|DAP160|Dap160/intersectin|DmelCG1099|dap160|noncoding_4110|p160	0	0
3628	35379	Drosophila melanogaster	CG9253	CG9253 gene product from transcript CG9253-RA	DmRH24|DmelCG9253|anon-EST:GressD10|cg9253	0	0
3629	35380	Drosophila melanogaster	del	deadlock	CG9252|DmelCG9252	0	0
3630	35381	Drosophila melanogaster	E2f2	E2F transcription factor 2	CG1071|DmelCG1071|E2F|E2F2|dE2F-2|dE2F2|dE2f2|de2f2|e2f2	0	0
3631	35382	Drosophila melanogaster	Mpp6	M-phase phosphoprotein 6	BcDNA:RE45276|CG9250|DmelCG9250|MPP6|Mpps|dMPP6|l(2)k16403|mpp6	0	0
3632	35383	Drosophila melanogaster	CG9249	CG9249 gene product from transcript CG9249-RA	DmelCG9249	0	0
3633	35384	Drosophila melanogaster	CG9248	CG9248 gene product from transcript CG9248-RB	DmelCG9248	0	0
3634	35385	Drosophila melanogaster	CG9247	CG9247 gene product from transcript CG9247-RA	DmelCG9247	0	0
3635	35386	Drosophila melanogaster	CG9246	CG9246 gene product from transcript CG9246-RA	DmelCG9246	0	0
3636	35387	Drosophila melanogaster	CG43345	CG43345 gene product from transcript CG43345-RB	CG9243|DmelCG43345|Dmel_CG9243	0	0
3637	35390	Drosophila melanogaster	Mcm10	Sensitized chromosome inheritance modifier 19	CG9241|DmMCM10|DmelCG9241|Scim19|mcm10	0	0
3638	35393	Drosophila melanogaster	CG31624	CG31624 gene product from transcript CG31624-RA	CG17401|DmelCG31624|Tes88	0	0
3639	35394	Drosophila melanogaster	clumsy	CG8681 gene product from transcript CG8681-RB	CG8681|CT9037|DmelCG8681|GluR39B	0	0
3640	35395	Drosophila melanogaster	CG8679	CG8679 gene product from transcript CG8679-RA	DmelCG8679|dLEM3	0	0
3641	35396	Drosophila melanogaster	CG8678	CG8678 gene product from transcript CG8678-RA	Atg18-like|DmelCG8678|l(2)k09410	0	0
3642	35397	Drosophila melanogaster	CG8677	CG8677 gene product from transcript CG8677-RA	BEST:LD14959|BEST:LD19244|DmelCG8677|LD14959|dRsf-1	0	0
3643	35398	Drosophila melanogaster	Hr39	Hormone receptor-like in 39	CG8676|DHR30|DHR39|DHR39/Ftz-F1beta|DmelCG8676|EP(2)2490|FTZ-F1|FTZ-F1B|FTZ-F1beta|HR39|NR5B1|betaFTZ-E1|betaFTZ-F1|dFTZ-F1beta|fs(2)04443|fs(2)neo8|ftz-F1|ftz-F1beta|ftz-f1beta|i70	0	0
3644	35399	Drosophila melanogaster	l(2)k14505	lethal (2) k14505	CG8674|DmelCG8674	0	0
3645	35400	Drosophila melanogaster	CG8671	CG8671 gene product from transcript CG8671-RA	DmelCG8671|anon-WO0118547.182	0	0
3646	35402	Drosophila melanogaster	Mio	Mlx interactor	154826_at|CG18362|DmelCG18362|anon-39Da|bHLHd12|dmondo	0	0
3647	35404	Drosophila melanogaster	dimm	dimmed	CG8667|DIMM|Dimm|DmelCG8667|Mistr|bHLHa16|c929|dim	0	0
3648	35405	Drosophila melanogaster	Tsp39D	Tetraspanin 39D	CG8666|Dm.Tsp39D|DmelCG8666	0	0
3649	35407	Drosophila melanogaster	CG8665	CG8665 gene product from transcript CG8665-RA	DmelCG8665	0	0
3650	35408	Drosophila melanogaster	nrv3	nervana 3	CG8663|DmelCG8663|JYbeta1|Nrv3	0	0
3651	35409	Drosophila melanogaster	CG17571	CG17571 gene product from transcript CG17571-RA	38C.22|DmelCG17571|SP83|anon-38C.22	0	0
3652	35410	Drosophila melanogaster	CG17570	CG17570 gene product from transcript CG17570-RA	38C.24|DmelCG17570|anon-38C.24	0	0
3653	35411	Drosophila melanogaster	Lamp1	CG3305 gene product from transcript CG3305-RA	CG3305|DmelCG3305|LAMP1|Lamp|Lamp-1	0	0
3654	35414	Drosophila melanogaster	nompB	no mechanoreceptor potential B	146590_s_at|CG12548|CG14465|CG15190|CG4137|DmelCG12548|IFT88|NOMPB|OSM-5|Tg737|nomp|osm-5	0	0
3655	35417	Drosophila melanogaster	CG2201	CG2201 gene product from transcript CG2201-RF	DmelCG2201	0	0
3656	35418	Drosophila melanogaster	Df31	Decondensation factor 31	BEST:LD04967|CG2207|DF 31|DF31|DmelCG2207|LD04967|anon-EST:Liang-1.46|anon-EST:Posey126|anon-EST:fe1A4|anon-WO0153538.53|anon1A4|clone 1.46|l(2)k05815	0	0
3657	35419	Drosophila melanogaster	Ac3	CG1506 gene product from transcript CG1506-RA	AC 3|CG1506|DAC3|DAC39E|DmelCG1506	0	0
3658	35420	Drosophila melanogaster	Cul-2	Cullin-2	CG1512|CUL2|Cul2|DMcul-2|DmelCG1512|cul-2|l(2)02074	0	0
3659	35421	Drosophila melanogaster	CG2225	CG2225 gene product from transcript CG2225-RC	DmelCG2225	0	0
3660	35422	Drosophila melanogaster	Ef2b	Elongation factor 2b	CG2238|DmelCG2238|EF-2|EF-2b|EF2|Ef2|anon-EST:Liang-1.44|clone 1.44|eEF2|eF2	0	0
3661	35424	Drosophila melanogaster	CG31619	CG31619 gene product from transcript CG31619-RC	CG13236|CG2122|CG2131|CT6940|DmelCG31619	0	0

3662	35425	Drosophila melanogaster	step	steppke	CG11628|CG11633|CYH1|DmelCG11628|GPH|GRP1|GRP1/cytohesin 1|Grp1|cytohesin/GRP1|l(2)SH0323|l(2)SH2 0323|l(2)k08110|stepk	0	0
3663	35426	Drosophila melanogaster	CG1416	CG1416 gene product from transcript CG1416-RA	DmelCG1416	0	0
3664	35427	Drosophila melanogaster	CG31612	CG31612 gene product from transcript CG31612-RA	CG11631|CG11632|DmelCG31612	0	0
3665	35429	Drosophila melanogaster	CG11630	CG11630 gene product from transcript CG11630-RA	DmelCG11630	0	0
3666	35430	Drosophila melanogaster	tsh	teashirt	CG1374|DmelCG1374|T shirt|Tsh|ae|ae[l]|l(2)04319|l(2)B4-2-12	0	0
3667	35431	Drosophila melanogaster	CG11629	CG11629 gene product from transcript CG11629-RA	DmelCG11629	0	0
3668	35432	Drosophila melanogaster	CG1421	CG1421 gene product from transcript CG1421-RA	BcDNA:LP11549|DmelCG1421	0	0
3669	35433	Drosophila melanogaster	CG1428	CG1428 gene product from transcript CG1428-RA	DmelCG1428	0	0
3670	35434	Drosophila melanogaster	CG11634	CG11634 gene product from transcript CG11634-RA	BcDNA:AT09142|DmelCG11634	0	0
3671	35435	Drosophila melanogaster	CG2528	CG2528 gene product from transcript CG2528-RB	DmelCG2528	0	0
3672	35437	Drosophila melanogaster	ttm3	tiny tim 3	CG6691|DmelCG6691	0	0
3673	35438	Drosophila melanogaster	CG10834	CG10834 gene product from transcript CG10834-RA	DmelCG10834	0	0
3674	35439	Drosophila melanogaster	CG6675	CG6675 gene product from transcript CG6675-RB	DmelCG6675	0	0
3675	35440	Drosophila melanogaster	CG3651	CG3651 gene product from transcript CG3651-RA	DmelCG3651	0	0
3676	35442	Drosophila melanogaster	CG42748	CG42748 gene product from transcript CG42748-RG	CG15216|CG15217|CG6448|DmelCG42748|Dmel_CG15216|Dmel_CG6448|anon-WO0140519.28	0	0
3677	35445	Drosophila melanogaster	CG1832	CG1832 gene product from transcript CG1832-RA	DmelCG1832	0	0
3678	35447	Drosophila melanogaster	CG3635	CG3635 gene product from transcript CG3635-RB	DmelCG3635	0	0
3679	35449	Drosophila melanogaster	Ir40a	Ionotropic receptor 40a	CG42352|CG5922|CG5929|DmelCG42352|Dmel_CG5922|Dmel_CG5929|IR40a	0	0
3680	35452	Drosophila melanogaster	CG3262	CG3262 gene product from transcript CG3262-RD	DmelCG3262	0	0
3681	35453	Drosophila melanogaster	RpL21	Ribosomal protein L21	BcDNA:RH06526|CG12775|DmelCG12775|M(2)39F|RPL21|anon-EST:Posey18|anon-EST:Posey30	0	0
3682	35454	Drosophila melanogaster	Tif-IA	CG3278 gene product from transcript CG3278-RA	CG16938|CG3278|CG5951|DmelCG3278|TIF-1A|TIF-IA|Tif-1A|TifIA|dTIF-IA	0	0
3683	35473	Drosophila melanogaster	TpnC41C	Troponin C at 41C	CG2981|DmTnC1|DmTpnC41C|DmTpnCIIIa|DmelCG2981|TNC41C|TPNC|TnC|TnC1|TnC41C|TpnC IIIa|TpnCIIIa	0	0
3684	35475	Drosophila melanogaster	CG3107	CG3107 gene product from transcript CG3107-RA	DmelCG3107	0	0
3685	35478	Drosophila melanogaster	gus	gustavus	A11|CG2944|CG2946|DmelCG2944|GUS|Gus|dmSSB4|i25	0	0
3686	35480	Drosophila melanogaster	Atf6	CG3136 gene product from transcript CG3136-RA	ATF6|CG3136|DmelCG3136	0	0
3687	35483	Drosophila melanogaster	Nipped-A	CG33554 gene product from transcript CG33554-RE	41Ah|CG10549|CG2905|CG33554|DmelCG33554|GroupV|Nip-A|NipA|Nipped A|Nipped[A]|TRRAP|Tra-1|Tra1|dTRRAP|dTra1|dmTRRAP|l(2)41Ah|l(2)C123|l(2)EMS34-12|tra1	0	0
3688	35485	Drosophila melanogaster	d4	CG2682 gene product from transcript CG2682-RB	CG2682|D4|Dd4|DmelCG2682|dd4	0	0
3689	35486	Drosophila melanogaster	sxc	super sex combs	BcDNA:GH04245|CG10392|DmelCG10392|OGT|Ogt|P1201|SXC|Sxc|l(2)02637|l(2)NC130|sxc/Ogt	0	0
3690	35488	Drosophila melanogaster	CG10465	CG10465 gene product from transcript CG10465-RA	DmelCG10465	0	0
3691	35490	Drosophila melanogaster	CG10395	CG10395 gene product from transcript CG10395-RA	DmelCG10395	0	0
3692	35491	Drosophila melanogaster	CG10396	CG10396 gene product from transcript CG10396-RA	BcDNA:AT07685|COX IV|COXIV|DmelCG10396|IV|cox4	0	0
3693	35492	Drosophila melanogaster	CG10417	CG10417 gene product from transcript CG10417-RA	DmelCG10417|PP2Cg	0	0
3694	35494	Drosophila melanogaster	CG42345	CG42345 gene product from transcript CG42345-RE	CG10398|CG10408|CG30437|CG32838|CG5342|DmelCG42345|Dmel_CG30437|Dmel_CG32838|MCO2	0	0
3695	35496	Drosophila melanogaster	CG30440	CG30440 gene product from transcript CG30440-RA	CG10412|CG10416|CG12183|DmelCG30440	0	0
3696	35498	Drosophila melanogaster	TpnC4	Troponin C isoform 4	BcDNA:RH03361|CG12408|DmTnC4|DmTpnC41F|DmTpnCIIIb|DmelCG12408|TNC41F6|TnC|TnC4|TpnC IIIb|TpnC41F|TpnCIIIb|tnC4	0	0
3697	35499	Drosophila melanogaster	CG11665	CG11665 gene product from transcript CG11665-RA	DmelCG11665	0	0
3698	35500	Drosophila melanogaster	CG1344	CG1344 gene product from transcript CG1344-RA	BcDNA:LD22679|DmelCG1344	0	0
3699	35501	Drosophila melanogaster	l(2)NC136	lethal (2) NC136	CG8426|CNOT3|DmelCG8426|NOT3|NOT3/5|Not3|not3|not3/5	0	0
3700	35503	Drosophila melanogaster	CG8245	CG8245 gene product from transcript CG8245-RA	DmelCG8245	0	0
3701	35504	Drosophila melanogaster	kune	kune-kune	CG1298|DmelCG1298	0	0
3702	35505	Drosophila melanogaster	scaf	scarface	CG11066|CG15900|DmelCG11066|SPH142|scarf	0	0
3703	35507	Drosophila melanogaster	CG17337	CG17337 gene product from transcript CG17337-RA	Dm52|DmelCG17337|Y|anon-41Fb	0	0
3704	35509	Drosophila melanogaster	ap	apterous	AP|Ap|Apt|CG8376|DmelCG8376|LIM|S-2a|Xa|blt|trw	0	0
3705	35511	Drosophila melanogaster	CG7791	CG7791 gene product from transcript CG7791-RA	DmelCG7791	0	0
3706	35512	Drosophila melanogaster	gp210	CG7897 gene product from transcript CG7897-RA	CG14467|CG7897|DmelCG7897|GP210|gp120	0	0
3707	35513	Drosophila melanogaster	CG11163	CG11163 gene product from transcript CG11163-RD	BEST:CK02137|CK02137|DmelCG11163	0	0
3708	35514	Drosophila melanogaster	Or42a	Odorant receptor 42a	41E.2|42a|CG17250|DOR117|DmelCG17250|OR42a|Or42A	0	0
3709	35515	Drosophila melanogaster	Tsp42A	Tetraspanin 42A	CG14468|Dm.Tsp42A|DmelCG14468	0	0
3710	35516	Drosophila melanogaster	Or42b	Odorant receptor 42b	41E.1|42b|CG12754|DOR118|DmelCG12754	0	0
3711	35519	Drosophila melanogaster	CG43366	CG43366 gene product from transcript CG43366-RB	CG12551|CG14470|DmelCG43366|Dmel_CG12551|Dmel_CG14470	0	0
3712	35520	Drosophila melanogaster	CG7882	CG7882 gene product from transcript CG7882-RA	DmelCG7882	0	0
3713	35521	Drosophila melanogaster	CG7881	CG7881 gene product from transcript CG7881-RA	DmelCG7881	0	0
3714	35522	Drosophila melanogaster	BubR1	Bub1-related kinase	31/13|BUB1|BUBR1|BcDNA:LD23835|Bub1|CG 7838|CG7838|DmBUB1|Dmbub1|DmelCG7838|Mad3|bub1|bubR1|bubr1|dBUB1|l(2)k03113|l(2)k06109	0	0
3715	35523	Drosophila melanogaster	mle	maleless	CG11680|DmelCG11680|MLE|mak|mll|mls|nap|nap[ts]	0	0
3716	35524	Drosophila melanogaster	Src42A	Src oncogene at 42A	CG7873|DSrc42A|Dm SRC41|DmHD-29|DmelCG7873|Dsrc41|Dsrc42A|Dtk5|HD-29|SK2-4|SRC 42A|SRC42A|Src|Src41|Src42|Src42a|Su(D-raf)1|Su(Raf)1|Su(phl)1|Su1|Tk5|dSrc|dtk-5|dtk5|l(2)k10108|src|sr	0	0
3717	35525	Drosophila melanogaster	CG14471	CG14471 gene product from transcript CG14471-RB	DmelCG14471	0	0
3718	35526	Drosophila melanogaster	Act42A	Actin 42A	42A|A|AFFX-Dros-ACTIN_M_r_at|Act|Act42|Act42a|Actin|Actin/BAP47|BAP47|C|CG12051|DmelCG12051|act 42A|act42A|actin	0	0
3719	35527	Drosophila melanogaster	Pngl	PNGase-like	CG7865|DmelCG7865|PNGase	0	0
3720	35528	Drosophila melanogaster	dream	CG7863 gene product from transcript CG7863-RA	CG7863|DREAM|DmelCG7863|Dream|Dream/Strica|STRICA|STRICA/DREAM|Strica|Strica/Dream|strica	0	0
3721	35529	Drosophila melanogaster	SCAP	SREBP cleavage activating protein	CG14592|CG33131|CG8356|DmelCG33131|Scap|Scap_Dm|dSCAP|dScap|dscap	0	0
3722	35531	Drosophila melanogaster	CG14591	CG14591 gene product from transcript CG14591-RD	DmelCG14591	0	0
3723	35532	Drosophila melanogaster	tbce	tubulin-specific chaperone E	CG7861|DmelCG7861	0	0
3724	35533	Drosophila melanogaster	CG7856	CG7856 gene product from transcript CG7856-RA	DmelCG7856	0	0
3725	35535	Drosophila melanogaster	CCHa2r	CCHamide-2 receptor	CG14593|CG14594|DmelCG14593|anon-WO0131005.23	0	0
3726	35536	Drosophila melanogaster	CG7845	CG7845 gene product from transcript CG7845-RB	DmelCG7845|WDR74	0	0
3727	35537	Drosophila melanogaster	CG7849	CG7849 gene product from transcript CG7849-RB	DmelCG7849	0	0
3728	35538	Drosophila melanogaster	CG14590	CG14590 gene product from transcript CG14590-RA	DmelCG14590	0	0
3729	35539	Drosophila melanogaster	Ars2	CG7843 gene product from transcript CG7843-RA	CG7843|DmelCG7843|anon-WO0118547.166	0	0
3730	35540	Drosophila melanogaster	EcR	Ecdysone receptor	CG1765|CG8347|DEcR|Dhr23|DmEcR|DmelCG1765|ECR|EcR-A|EcR-B|EcR-B1|EcRB-1|EcRB1|EcdR|Ecr|NR1H1|anon-WO0229075.1|dECR|dmEcR|ecr|lie|ms(2)06410|ms(2)42A|snt	0	0
3731	35541	Drosophila melanogaster	CG14589	CG14589 gene product from transcript CG14589-RA	DmelCG14589	0	0
3732	35543	Drosophila melanogaster	Cyp6w1	CG8345 gene product from transcript CG8345-RA	6w1|AntP450|CG8345|CYP6W1|DmelCG8345	0	0
3733	35544	Drosophila melanogaster	CG8343	CG8343 gene product from transcript CG8343-RA	BEST:GH10831|DmelCG8343|GH10831|GH10831-Inc	0	0
3734	35545	Drosophila melanogaster	CG11211	CG11211 gene product from transcript CG11211-RA	BcDNA:RH26557|DmelCG11211	0	0
3735	35546	Drosophila melanogaster	Ptr	Patched-related	CG11212|DmelCG11212|Ptr_Dm|ptc-related-gene|ptr	0	0
3736	35547	Drosophila melanogaster	CG8335	CG8335 gene product from transcript CG8335-RA	DmelCG8335|eIF3-S5	0	0
3737	35549	Drosophila melanogaster	CG30431	CG30431 gene product from transcript CG30431-RA	CG8324|CG8341|DmelCG30431	0	0
3738	35550	Drosophila melanogaster	CG17994	CG17994 gene product from transcript CG17994-RA	DmelCG17994	0	0
3739	35552	Drosophila melanogaster	bin3	bicoid-interacting protein 3	Bin3|CG8276|DmelCG8276|l(2)SH1014|l(2)SH2 1014	0	0
3740	35553	Drosophila melanogaster	tomboy40	CG8330 gene product from transcript CG8330-RA	CG8330|DmelCG8330	0	0
3741	35554	Drosophila melanogaster	Pld	Phospholipase D	CG12110|DmelCG12110|PLD|dPLD|dPld|pld|pld1	0	0
3742	35555	Drosophila melanogaster	jing	CG9397 gene product from transcript CG9397-RI	1.28|CG9397|CG9403|CT9093|DmelCG9397|Dmel_CG9403|l(2)01094|l(2)22F3	0	0
3743	35556	Drosophila melanogaster	CG15233	CG15233 gene product from transcript CG15233-RB	BEST:LD11861|DmelCG15233	0	0
3744	35559	Drosophila melanogaster	Trap1	CG3152 gene product from transcript CG3152-RA	CG3152|DmelCG3152|TRAP1|TRAP1dr|dtrap-1|trap1	0	0
3745	35560	Drosophila melanogaster	Bap170	Brahma associated protein 170kD	BAP170|BcDNA:GH12174|CG3274|DmelCG3274|bap170|hfl	0	0
3746	35561	Drosophila melanogaster	Fmo-2	Flavin-containing monooxygenase 2	CG3174|DmFMO-2|DmelCG3174|fmo2	0	0
3747	35563	Drosophila melanogaster	geminin	CG3183 gene product from transcript CG3183-RA	CG3183|Dm Geminin|Dm geminin|DmelCG3183|Gem|Geminin|anon-WO0257455.1|gem|l(2)k14019|v(2)k09107	0	0
3748	35564	Drosophila melanogaster	sced	scrambled	CG3273|CT9143|DmelCG3273	0	0
3749	35565	Drosophila melanogaster	Dpit47	DNA polymerase interacting tpr containing protein of 4	CG3189|DmelCG3189	0	0
3750	35567	Drosophila melanogaster	CG15908	CG15908 gene product from transcript CG15908-RB	DmelCG15908	0	0
3751	35568	Drosophila melanogaster	CG9410	CG9410 gene product from transcript CG9410-RD	DmelCG9410	0	0
3752	35569	Drosophila melanogaster	CG3194	CG3194 gene product from transcript CG3194-RB	DmelCG3194	0	0
3753	35570	Drosophila melanogaster	CG3271	CG3271 gene product from transcript CG3271-RA	DmelCG3271	0	0
3754	35571	Drosophila melanogaster	CG17266	CG17266 gene product from transcript CG17266-RA	DmelCG17266	0	0
3755	35572	Drosophila melanogaster	CG15909	CG15909 gene product from transcript CG15909-RA	DmelCG15909	0	0
3756	35573	Drosophila melanogaster	Tdc1	Tyrosine decarboxylase 1	CG30445|CG3686|DmelCG30445|dTdc1|tdc|tdc1	0	0
3757	35574	Drosophila melanogaster	CG9422	CG9422 gene product from transcript CG9422-RC	BcDNA:RE35072|DmelCG9422	0	0
3758	35575	Drosophila melanogaster	CG3270	CG3270 gene product from transcript CG3270-RA	DmelCG3270	0	0
3759	35576	Drosophila melanogaster	Mob4	CG3403 gene product from transcript CG3403-RA	CG3403|DMob4|DmelCG3403|Dmob4|dmob4	0	0
3760	35577	Drosophila melanogaster	Rab2	Rab-protein 2	CG3269|DRAB2|DRab2|DmRab2|DmelCG3269|Drab2|rab2	0	0
3761	35578	Drosophila melanogaster	CG3409	CG3409 gene product from transcript CG3409-RB	DmelCG3409	0	0
3762	35579	Drosophila melanogaster	ZIP1	Zinc/iron regulated transporter-related protein 1	CG9428|DmelCG9428|Zip1|dZIP1	0	0
3763	35580	Drosophila melanogaster	CG9430	CG9430 gene product from transcript CG9430-RB	DmelCG9430	0	0
3764	35583	Drosophila melanogaster	phtf	CG3268 gene product from transcript CG3268-RA	BcDNA.GH08636|BcDNA:GH08636|CG3268|DmelCG3268|d-phtf	0	0
3765	35584	Drosophila melanogaster	Eb1	CG3265 gene product from transcript CG3265-RF	BcDNA.LD08743|BcDNA:LD08743|CG3265|Dm EB1|DmEB1|DmelCG3265|EB-1|EB1|S(DmcycE[JP])2.5|anon-EST:Liang-2.33|anon-EST:Liang-2.52|clone 2.33|clone 2.52|dEB-1|dEB1|dEb1|eb1|l(2)04524|l(	0	0
3766	35585	Drosophila melanogaster	CG3420	CG3420 gene product from transcript CG3420-RA	BcDNA:RE40412|DmelCG3420	0	0
3767	35586	Drosophila melanogaster	CG9436	CG9436 gene product from transcript CG9436-RA	DmelCG9436	0	0
3768	35587	Drosophila melanogaster	Cyp6a2	Cytochrome P450-6a2	143127_at|6a2|91-R|91R|CG9438|CYP6A2|CYP6a2|Cyp6A2|Cyp6a|Cyt-P450-B1|DmelCG9438|P-450|P-450-B|P450|P450-B|P450-B1|cyp6a2	0	0
3769	35588	Drosophila melanogaster	Epac	CG34392 gene product from transcript CG34392-RC	CG15235|CG3427|CG34392|D-EPAC|D-Epac|DmelCG34392|Dmel_CG15235|Dmel_CG3427|epac	0	0
3770	35590	Drosophila melanogaster	SdhB	Succinate dehydrogenase B	CG3283|DmelCG3283|Ip|SDH|SDH-IP|SDH-Ip|SDHb|sdhB	0	0
3771	35591	Drosophila melanogaster	CG15237	CG15237 gene product from transcript CG15237-RA	DmelCG15237	0	0
3772	35592	Drosophila melanogaster	ubl	ubiquitin like	CG3450|DmelCG3450|dubl|l(2)k03203|mut182	0	0

<table>
<tr><td>3773</td><td>35594</td><td>Drosophila melanogaster</td><td>koi</td><td>klaroid</td><td>CG18584|DmelCG18584</td><td>0</td><td>0</td></tr>
<tr><td>3774</td><td>35595</td><td>Drosophila melanogaster</td><td>CG15236</td><td>CG15236 gene product from transcript CG15236-RB</td><td>DmelCG15236</td><td>0</td><td>0</td></tr>
<tr><td>3775</td><td>35596</td><td>Drosophila melanogaster</td><td>coro</td><td>CG9446 gene product from transcript CG9446-RC</td><td>CG9446|DmelCG9446</td><td>0</td><td>0</td></tr>
<tr><td>3776</td><td>35597</td><td>Drosophila melanogaster</td><td>CG9447</td><td>CG9447 gene product from transcript CG9447-RB</td><td>DmelCG9447</td><td>0</td><td>0</td></tr>
<tr><td>3777</td><td>35599</td><td>Drosophila melanogaster</td><td>Spn42Db</td><td>Serpin 42Db</td><td>CG9454|DmelCG9454</td><td>0</td><td>0</td></tr>
<tr><td>3778</td><td>35600</td><td>Drosophila melanogaster</td><td>Spn42Dc</td><td>Serpin 42Dc</td><td>CG9455|DmelCG9455</td><td>0</td><td>0</td></tr>
<tr><td>3779</td><td>35602</td><td>Drosophila melanogaster</td><td>Spn42De</td><td>Serpin 42De</td><td>CG9460|DmelCG9460</td><td>0</td><td>0</td></tr>
<tr><td>3780</td><td>35604</td><td>Drosophila melanogaster</td><td>CG3358</td><td>CG3358 gene product from transcript CG3358-RD</td><td>DmelCG3358</td><td>0</td><td>0</td></tr>
<tr><td>3781</td><td>35608</td><td>Drosophila melanogaster</td><td>Cyp6u1</td><td>CG3567 gene product from transcript CG3567-RA</td><td>6u1|CG3567|DmelCG3567</td><td>0</td><td>0</td></tr>
<tr><td>3782</td><td>35609</td><td>Drosophila melanogaster</td><td>vimar</td><td>visceral mesodermal armadillo-repeats</td><td>CG3572|DmelCG3572|Vimar|anon-EST:Posey213|l(2)k16722</td><td>0</td><td>0</td></tr>
<tr><td>3783</td><td>35610</td><td>Drosophila melanogaster</td><td>CG17002</td><td>CG17002 gene product from transcript CG17002-RB</td><td>DmelCG17002</td><td>0</td><td>0</td></tr>
<tr><td>3784</td><td>35611</td><td>Drosophila melanogaster</td><td>CG30159</td><td>CG30159 gene product from transcript CG30159-RC</td><td>CG3364|DmelCG30159</td><td>0</td><td>0</td></tr>
<tr><td>3785</td><td>35612</td><td>Drosophila melanogaster</td><td>Tsp42Ec</td><td>Tetraspanin 42Ec</td><td>CG12847|Dm.Tsp42Ec|DmelCG12847</td><td>0</td><td>0</td></tr>
<tr><td>3786</td><td>35613</td><td>Drosophila melanogaster</td><td>Tsp42Ed</td><td>Tetraspanin 42Ed</td><td>BcDNA:GH07074|CG12846|Dm.Tsp42Ed|DmelCG12846|GH07074</td><td>0</td><td>0</td></tr>
<tr><td>3787</td><td>35614</td><td>Drosophila melanogaster</td><td>Tsp42Ee</td><td>Tetraspanin 42Ee</td><td>BcDNA:LD16071|CG10106|Dm.Tsp42Ee|DmelCG10106|anon-WO0118547.151</td><td>0</td><td>0</td></tr>
<tr><td>3788</td><td>35615</td><td>Drosophila melanogaster</td><td>Tsp42Ef</td><td>Tetraspanin 42Ef</td><td>CG12845|Dm.Tsp42Ef|DmelCG12845</td><td>0</td><td>0</td></tr>
<tr><td>3789</td><td>35616</td><td>Drosophila melanogaster</td><td>Tsp42Eg</td><td>Tetraspanin 42Eg</td><td>BcDNA:GM06962|CG12142|Dm.Tsp42Eg|DmelCG12142</td><td>0</td><td>0</td></tr>
<tr><td>3790</td><td>35617</td><td>Drosophila melanogaster</td><td>Tsp42Eh</td><td>Tetraspanin 42Eh</td><td>CG12844|Dm.Tsp42Eh|DmelCG12844</td><td>0</td><td>0</td></tr>
<tr><td>3791</td><td>35618</td><td>Drosophila melanogaster</td><td>Tsp42Ei</td><td>Tetraspanin 42Ei</td><td>CG12843|Dm.Tsp42Ei|DmelCG12843|Tsp42EI</td><td>0</td><td>0</td></tr>
<tr><td>3792</td><td>35620</td><td>Drosophila melanogaster</td><td>Tsp42Ej</td><td>Tetraspanin 42Ej</td><td>CG12143|Dm.Tsp42Ej|DmelCG12143|Sun|sun|tsp42ej</td><td>0</td><td>0</td></tr>
<tr><td>3793</td><td>35621</td><td>Drosophila melanogaster</td><td>Tsp42Ek</td><td>Tetraspanin 42Ek</td><td>CG12841|Dm.Tsp42Ek|DmelCG12841</td><td>0</td><td>0</td></tr>
<tr><td>3794</td><td>35622</td><td>Drosophila melanogaster</td><td>Tsp42El</td><td>Tetraspanin 42El</td><td>CG12840|Dm.Tsp42El|DmelCG12840|Tsp42E1|anon-EST:Posey244</td><td>0</td><td>0</td></tr>
<tr><td>3795</td><td>35623</td><td>Drosophila melanogaster</td><td>lbm</td><td>late bloomer</td><td>CG2374|Dm.Tsp42Em|DmelCG2374|lbm|Lbm|lbl</td><td>0</td><td>0</td></tr>
<tr><td>3796</td><td>35624</td><td>Drosophila melanogaster</td><td>Tsp42En</td><td>Tetraspanin 42En</td><td>BcDNA:RH02160|CG12839|Dm.Tsp42En|DmelCG12839</td><td>0</td><td>0</td></tr>
<tr><td>3797</td><td>35625</td><td>Drosophila melanogaster</td><td>Tsp42Eo</td><td>Tetraspanin 42Eo</td><td>BcDNA:RH51444|CG12838|Dm.Tsp42Eo|DmelCG12838</td><td>0</td><td>0</td></tr>
<tr><td>3798</td><td>35626</td><td>Drosophila melanogaster</td><td>Tsp42Ep</td><td>Tetraspanin 42Ep</td><td>CG4471|Dm.Tsp42Ep|DmelCG4471</td><td>0</td><td>0</td></tr>
<tr><td>3799</td><td>35627</td><td>Drosophila melanogaster</td><td>pgant3</td><td>polypeptide GalNAc transferase 3</td><td>BcDNA:GH09147|CG4445|DmelCG4445|anon-WO0118547.119</td><td>0</td><td>0</td></tr>
<tr><td>3800</td><td>35628</td><td>Drosophila melanogaster</td><td>Tsp42Eq</td><td>Tetraspanin 42Eq</td><td>CG12832|Dm.Tsp42Eq|DmelCG12832</td><td>0</td><td>0</td></tr>
<tr><td>3801</td><td>35629</td><td>Drosophila melanogaster</td><td>Tsp42Er</td><td>Tetraspanin 42Er</td><td>CG12837|Dm.Tsp42Er|DmelCG12837</td><td>0</td><td>0</td></tr>
<tr><td>3802</td><td>35630</td><td>Drosophila melanogaster</td><td>CG12836</td><td>CG12836 gene product from transcript CG12836-RA</td><td>DmelCG12836</td><td>0</td><td>0</td></tr>
<tr><td>3803</td><td>35631</td><td>Drosophila melanogaster</td><td>CG12831</td><td>CG12831 gene product from transcript CG12831-RB</td><td>DmelCG12831</td><td>0</td><td>0</td></tr>
<tr><td>3804</td><td>35632</td><td>Drosophila melanogaster</td><td>CG12835</td><td>CG12835 gene product from transcript CG12835-RA</td><td>DmelCG12835</td><td>0</td><td>0</td></tr>
<tr><td>3805</td><td>35634</td><td>Drosophila melanogaster</td><td>Cyp9b1</td><td>Cytochrome P450-9b1</td><td>9b1|CG4485|DmelCG4485|P-450|P450</td><td>0</td><td>0</td></tr>
<tr><td>3806</td><td>35635</td><td>Drosophila melanogaster</td><td>Cyp9b2</td><td>Cytochrome P450-9b2</td><td>9b2|BEST:GH08116|CG4486|DmelCG4486|P-450|P-450 Cyp9b2|P450|cyp9b2</td><td>0</td><td>0</td></tr>
<tr><td>3807</td><td>35639</td><td>Drosophila melanogaster</td><td>Spn43Ad</td><td>Serpin 43Ad</td><td>CG1859|DmelCG1859</td><td>0</td><td>0</td></tr>
<tr><td>3808</td><td>35641</td><td>Drosophila melanogaster</td><td>CG11060</td><td>CG11060 gene product from transcript CG11060-RA</td><td>DmelCG11060</td><td>0</td><td>0</td></tr>
<tr><td>3809</td><td>35642</td><td>Drosophila melanogaster</td><td>CG33140</td><td>CG33140 gene product from transcript CG33140-RA</td><td>BcDNA:AT21895|CG11074|DmelCG33140</td><td>0</td><td>0</td></tr>
<tr><td>3810</td><td>35644</td><td>Drosophila melanogaster</td><td>Or43a</td><td>Odorant receptor 43a</td><td>43B.1|43B1|43a|AN14|CG1854|DOR43B.1|DOR43a|DOR87|DmelCG1854|Dor87|OR43a|Or|Or43A|Or43B.1|Or87|dor87|or43a</td><td>0</td><td>0</td></tr>
<tr><td>3811</td><td>35646</td><td>Drosophila melanogaster</td><td>Gadd45</td><td>CG11086 gene product from transcript CG11086-RA</td><td>BcDNA:RH70774|CG11086|D-GADD45|DmelCG11086|GADD45|GG1086</td><td>0</td><td>0</td></tr>
<tr><td>3812</td><td>35647</td><td>Drosophila melanogaster</td><td>CG1850</td><td>CG1850 gene product from transcript CG1850-RA</td><td>DmelCG1850</td><td>0</td><td>0</td></tr>
<tr><td>3813</td><td>35648</td><td>Drosophila melanogaster</td><td>Br140</td><td>CG1845 gene product from transcript CG1845-RA</td><td>CG1845|DmelCG1845</td><td>0</td><td>0</td></tr>
<tr><td>3814</td><td>35649</td><td>Drosophila melanogaster</td><td>Incenp</td><td>Inner centromere protein</td><td>CG12165|DmINCENP|DmelCG12165|INCENP|anon-WO0118547.171|dIncenp|incenp|mat(2)ea-C|mat(2)earlyQA26</td><td>0</td><td>0</td></tr>
<tr><td>3815</td><td>35651</td><td>Drosophila melanogaster</td><td>CG12164</td><td>CG12164 gene product from transcript CG12164-RA</td><td>DmelCG12164</td><td>0</td><td>0</td></tr>
<tr><td>3816</td><td>35652</td><td>Drosophila melanogaster</td><td>Dscam</td><td>Down syndrome cell adhesion molecule</td><td>43Bc|CG17800|CT39257|DSCAM|DScam|DmDscam|DmelCG17800|Dscam1|FBgn0033159|Neu1|dScam|dscam|l(2)05518|l(2)43Bc|p270</td><td>0</td><td>0</td></tr>
<tr><td>3817</td><td>35653</td><td>Drosophila melanogaster</td><td>cos</td><td>costa</td><td>CG1708|COS2|COS[2]|Cos-2|Cos2|Cos[2]|DmelCG1708|cos-2|cos2|l(2)161/28|l(2)cos[[2]]|l(2)k16101|l(2)k16128</td><td>0</td><td>0</td></tr>
<tr><td>3818</td><td>35654</td><td>Drosophila melanogaster</td><td>CG11107</td><td>CG11107 gene product from transcript CG11107-RA</td><td>DmelCG11107|anon-EST:Liang-1.18|cg11107|clone 1.18</td><td>0</td><td>0</td></tr>
<tr><td>3819</td><td>35655</td><td>Drosophila melanogaster</td><td>Gr43a</td><td>Gustatory receptor 43a</td><td>43C.1|43C1|CG1712|DmGr43a|DmelCG1712|GR43C.1|Gr43C1</td><td>0</td><td>0</td></tr>
<tr><td>3820</td><td>35656</td><td>Drosophila melanogaster</td><td>CG1707</td><td>CG1707 gene product from transcript CG1707-RA</td><td>DmelCG1707</td><td>0</td><td>0</td></tr>
<tr><td>3821</td><td>35658</td><td>Drosophila melanogaster</td><td>CG11112</td><td>CG11112 gene product from transcript CG11112-RC</td><td>CT8227|DmelCG11112</td><td>0</td><td>0</td></tr>
<tr><td>3822</td><td>35659</td><td>Drosophila melanogaster</td><td>CG11113</td><td>CG11113 gene product from transcript CG11113-RB</td><td>DmelCG11113</td><td>0</td><td>0</td></tr>
<tr><td>3823</td><td>35660</td><td>Drosophila melanogaster</td><td>Eaf</td><td>ELL-associated factor</td><td>CG11166|CG1706|DmelCG11166|EP(2)2475|LD35207|dEaf</td><td>0</td><td>0</td></tr>
<tr><td>3824</td><td>35661</td><td>Drosophila melanogaster</td><td>CG1701</td><td>CG1701 gene product from transcript CG1701-RB</td><td>DmelCG1701</td><td>0</td><td>0</td></tr>
<tr><td>3825</td><td>35662</td><td>Drosophila melanogaster</td><td>so</td><td>sine oculis</td><td>CG11121|DmelCG11121|Drl|Mdu|SO|So|ami|mda|med|old|somda</td><td>0</td><td>0</td></tr>
<tr><td>3826</td><td>35663</td><td>Drosophila melanogaster</td><td>CG11145</td><td>CG11145 gene product from transcript CG11145-RA</td><td>BcDNA:AT20622|DmelCG11145</td><td>0</td><td>0</td></tr>
<tr><td>3827</td><td>35664</td><td>Drosophila melanogaster</td><td>CG11123</td><td>CG11123 gene product from transcript CG11123-RA</td><td>DmelCG11123</td><td>0</td><td>0</td></tr>
<tr><td>3828</td><td>35665</td><td>Drosophila melanogaster</td><td>sPLA2</td><td>secretory Phospholipase A2</td><td>CG11124|CG14507|DmelCG11124|sPLA[[2]]|secretory phospholipase A[[2]]</td><td>0</td><td>0</td></tr>
<tr><td>3829</td><td>35667</td><td>Drosophila melanogaster</td><td>kappaB-Ras</td><td>CG1669 gene product from transcript CG1669-RB</td><td>BcDNA:RH38596|CG1669|DmelCG1669|dkappaB-Ras</td><td>0</td><td>0</td></tr>
<tr><td>3830</td><td>35669</td><td>Drosophila melanogaster</td><td>CG11125</td><td>CG11125 gene product from transcript CG11125-RA</td><td>DmelCG11125</td><td>0</td><td>0</td></tr>
<tr><td>3831</td><td>35670</td><td>Drosophila melanogaster</td><td>CG30502</td><td>CG30502 gene product from transcript CG30502-RB</td><td>CG1642|CG1645|DmelCG30502</td><td>0</td><td>0</td></tr>
<tr><td>3832</td><td>35671</td><td>Drosophila melanogaster</td><td>Inos</td><td>CG11143 gene product from transcript CG11143-RA</td><td>CG11143|DmelCG11143|INOS|bs36h12.y1</td><td>0</td><td>0</td></tr>
<tr><td>3833</td><td>35672</td><td>Drosophila melanogaster</td><td>CG11141</td><td>CG11141 gene product from transcript CG11141-RB</td><td>DmelCG11141</td><td>0</td><td>0</td></tr>
<tr><td>3834</td><td>35673</td><td>Drosophila melanogaster</td><td>CG11127</td><td>CG11127 gene product from transcript CG11127-RA</td><td>DmelCG11127</td><td>0</td><td>0</td></tr>
<tr><td>3835</td><td>35674</td><td>Drosophila melanogaster</td><td>p47</td><td>CG11139 gene product from transcript CG11139-RA</td><td>CG11139|DmelCG11139|anon-WO0118547.216|dp47</td><td>0</td><td>0</td></tr>
<tr><td>3836</td><td>35677</td><td>Drosophila melanogaster</td><td>Coop</td><td>Corepressor of Pangolin</td><td>CG1621|DmelCG1621</td><td>0</td><td>0</td></tr>
<tr><td>3837</td><td>35678</td><td>Drosophila melanogaster</td><td>CG1620</td><td>CG1620 gene product from transcript CG1620-RA</td><td>146745_at|DmelCG1620</td><td>0</td><td>0</td></tr>
<tr><td>3838</td><td>35679</td><td>Drosophila melanogaster</td><td>dpa</td><td>disc proliferation abnormal</td><td>43Ca|CG1616|DmMCM4|DmMcm4|DmeMCM4|DmelCG1616|Dpa|MCM4|MCM4_DROME|Mcm4|l(2)43Ca|mcm4</td><td>0</td><td>0</td></tr>
<tr><td>3839</td><td>35680</td><td>Drosophila melanogaster</td><td>didum</td><td>dilute class unconventional myosin</td><td>43CD|CG2146|Didum|DmV|DmelCG2146|M5|MYOV|MyoV|MyoV43CD|NEST:bs14c05|NMC7|myoV|soy</td><td>0</td><td>0</td></tr>
<tr><td>3840</td><td>35681</td><td>Drosophila melanogaster</td><td>CG12736</td><td>CG12736 gene product from transcript CG12736-RA</td><td>DmelCG12736</td><td>0</td><td>0</td></tr>
<tr><td>3841</td><td>35682</td><td>Drosophila melanogaster</td><td>az2</td><td>CG1605 gene product from transcript CG1605-RA</td><td>CG1605|DmelCG1605|zf43C</td><td>0</td><td>0</td></tr>
<tr><td>3842</td><td>35683</td><td>Drosophila melanogaster</td><td>CG1603</td><td>CG1603 gene product from transcript CG1603-RA</td><td>DmelCG1603</td><td>0</td><td>0</td></tr>
<tr><td>3843</td><td>35684</td><td>Drosophila melanogaster</td><td>CG1602</td><td>CG1602 gene product from transcript CG1602-RA</td><td>DmelCG1602</td><td>0</td><td>0</td></tr>
<tr><td>3844</td><td>35685</td><td>Drosophila melanogaster</td><td>CG2144</td><td>CG2144 gene product from transcript CG2144-RA</td><td>DmelCG2144</td><td>0</td><td>0</td></tr>
<tr><td>3845</td><td>35686</td><td>Drosophila melanogaster</td><td>Orc1</td><td>Origin recognition complex subunit 1</td><td>CG10667|DmORC1|DmOrc1p|DmelCG10667|ORC|ORC1|dORC1|orc1|rDmORC</td><td>0</td><td>0</td></tr>
<tr><td>3846</td><td>35687</td><td>Drosophila melanogaster</td><td>CG1600</td><td>CG1600 gene product from transcript CG1600-RA</td><td>DmelCG1600</td><td>0</td><td>0</td></tr>
<tr><td>3847</td><td>35688</td><td>Drosophila melanogaster</td><td>Cyt-b5</td><td>CG2140 gene product from transcript CG2140-RB</td><td>1857|BcDNA:GH02503|BcDNA:RE66521|CG2140|CYT-B5|CYTB5|DmelCG2140|l(2)01857</td><td>0</td><td>0</td></tr>
<tr><td>3848</td><td>35689</td><td>Drosophila melanogaster</td><td>CG2137</td><td>CG2137 gene product from transcript CG2137-RA</td><td>146747_at|DmelCG2137|GPO|GPO-3|Gpo|alpha-GPO|gpo-3</td><td>0</td><td>0</td></tr>
<tr><td>3849</td><td>35690</td><td>Drosophila melanogaster</td><td>CG1598</td><td>CG1598 gene product from transcript CG1598-RA</td><td>BEST:GH10483|BcDNA:GM18141|DmelCG1598|gh10483</td><td>0</td><td>0</td></tr>
<tr><td>3850</td><td>35691</td><td>Drosophila melanogaster</td><td>Corin</td><td>CG2105 gene product from transcript CG2105-RB</td><td>CG2105|DmelCG2105|SP75</td><td>0</td><td>0</td></tr>
<tr><td>3851</td><td>35692</td><td>Drosophila melanogaster</td><td>CG30499</td><td>CG30499 gene product from transcript CG30499-RA</td><td>CG1364|DmelCG30499</td><td>0</td><td>0</td></tr>
<tr><td>3852</td><td>35693</td><td>Drosophila melanogaster</td><td>Vps13</td><td>Vacuolar protein sorting 13</td><td>BEST:LD02743|CG2093|DmelCG2093</td><td>0</td><td>0</td></tr>
<tr><td>3853</td><td>35694</td><td>Drosophila melanogaster</td><td>blow</td><td>blown fuse</td><td>Blow|CG1363|DmelCG1363|l(2)43Eb</td><td>0</td><td>0</td></tr>
<tr><td>3854</td><td>35695</td><td>Drosophila melanogaster</td><td>CG1360</td><td>CG1360 gene product from transcript CG1360-RA</td><td>DmelCG1360</td><td>0</td><td>0</td></tr>
<tr><td>3855</td><td>35696</td><td>Drosophila melanogaster</td><td>scra</td><td>scraps</td><td>ABP8|Abp8|Ani|Anil|Anillin|CG2092|DmelCG2092|Scra|ani|anil|anillin|l(2)03427|l(2)43Ec|l(2)k08255</td><td>0</td><td>0</td></tr>
<tr><td>3856</td><td>35697</td><td>Drosophila melanogaster</td><td>CG1358</td><td>CG1358 gene product from transcript CG1358-RD</td><td>DmelCG1358</td><td>0</td><td>0</td></tr>
<tr><td>3857</td><td>35698</td><td>Drosophila melanogaster</td><td>CG43340</td><td>CG43340 gene product from transcript CG43340-RH</td><td>BcDNA:GH02712|BcDNA:HL07907|CG17983|CG17984|CG2088|CG30492|CG30494|CT6764|DmelCG43340|Dmel_CG30492|Dmel_CG30494</td><td>0</td><td>0</td></tr>
<tr><td>3858</td><td>35701</td><td>Drosophila melanogaster</td><td>Rpt1</td><td>CG1341 gene product from transcript CG1341-RA</td><td>1341|CG1341|Dm_Rpt1|DmelCG1341|Dmp48B|Rpt1/p48B|anon-WO0118547.90|dRpt1|l(2)05643|l(2)43Ed|p48B</td><td>0</td><td>0</td></tr>
<tr><td>3859</td><td>35702</td><td>Drosophila melanogaster</td><td>CG17985</td><td>CG17985 gene product from transcript CG17985-RA</td><td>DmelCG17985</td><td>0</td><td>0</td></tr>
<tr><td>3860</td><td>35704</td><td>Drosophila melanogaster</td><td>CG30491</td><td>CG30491 gene product from transcript CG30491-RA</td><td>CG17986|CG2074|DmelCG30491</td><td>0</td><td>0</td></tr>
<tr><td>3861</td><td>35705</td><td>Drosophila melanogaster</td><td>Gr43b</td><td>CG1339 gene product from transcript CG1339-RA</td><td>CG1339|DmelCG1339</td><td>0</td><td>0</td></tr>
<tr><td>3862</td><td>35706</td><td>Drosophila melanogaster</td><td>CG2070</td><td>CG2070 gene product from transcript CG2070-RA</td><td>DmelCG2070</td><td>0</td><td>0</td></tr>
<tr><td>3863</td><td>35707</td><td>Drosophila melanogaster</td><td>CG2065</td><td>CG2065 gene product from transcript CG2065-RA</td><td>DmelCG2065</td><td>0</td><td>0</td></tr>
<tr><td>3864</td><td>35708</td><td>Drosophila melanogaster</td><td>CG2064</td><td>CG2064 gene product from transcript CG2064-RA</td><td>BcDNA:SD07613|DmelCG2064</td><td>0</td><td>0</td></tr>
<tr><td>3865</td><td>35709</td><td>Drosophila melanogaster</td><td>CG12042</td><td>CG12042 gene product from transcript CG12042-RA</td><td>DmelCG12042</td><td>0</td><td>0</td></tr>
<tr><td>3866</td><td>35710</td><td>Drosophila melanogaster</td><td>CG12826</td><td>CG12826 gene product from transcript CG12826-RA</td><td>DmelCG12826</td><td>0</td><td>0</td></tr>
<tr><td>3867</td><td>35711</td><td>Drosophila melanogaster</td><td>mRpL52</td><td>mitochondrial ribosomal protein L52</td><td>BcDNA:RH01479|CG1577|DmelCG1577|MRP-L52</td><td>0</td><td>0</td></tr>
<tr><td>3868</td><td>35712</td><td>Drosophila melanogaster</td><td>CG12107</td><td>CG12107 gene product from transcript CG12107-RA</td><td>DmelCG12107</td><td>0</td><td>0</td></tr>
<tr><td>3869</td><td>35713</td><td>Drosophila melanogaster</td><td>U2A</td><td>CG1406 gene product from transcript CG1406-RA</td><td>BcDNA:RE56869|CG1406|DmelCG1406|U2A'|dU2A'|l(2)43Ee</td><td>0</td><td>0</td></tr>
<tr><td>3870</td><td>35715</td><td>Drosophila melanogaster</td><td>CG1399</td><td>CG1399 gene product from transcript CG1399-RD</td><td>CG1576|DmelCG1399</td><td>0</td><td>0</td></tr>
<tr><td>3871</td><td>35717</td><td>Drosophila melanogaster</td><td>tor</td><td>torso</td><td>CG1389|DmelCG1389|TOR|Tor|Torso|splc</td><td>0</td><td>0</td></tr>
<tr><td>3872</td><td>35718</td><td>Drosophila melanogaster</td><td>CG1941</td><td>CG1941 gene product from transcript CG1941-RA</td><td>DmelCG1941</td><td>0</td><td>0</td></tr>
<tr><td>3873</td><td>35719</td><td>Drosophila melanogaster</td><td>CG1942</td><td>CG1942 gene product from transcript CG1942-RA</td><td>DmelCG1942</td><td>0</td><td>0</td></tr>
<tr><td>3874</td><td>35720</td><td>Drosophila melanogaster</td><td>CG1946</td><td>CG1946 gene product from transcript CG1946-RA</td><td>DmelCG1946</td><td>0</td><td>0</td></tr>
<tr><td>3875</td><td>35724</td><td>Drosophila melanogaster</td><td>cn</td><td>cinnabar</td><td>CG1555|DmelCG1555|Dmelcn</td><td>0</td><td>0</td></tr>
<tr><td>3876</td><td>35726</td><td>Drosophila melanogaster</td><td>CG12825</td><td>CG12825 gene product from transcript CG12825-RA</td><td>DmelCG12825|dmCG12825</td><td>0</td><td>0</td></tr>
<tr><td>3877</td><td>35727</td><td>Drosophila melanogaster</td><td>CG12824</td><td>CG12824 gene product from transcript CG12824-RA</td><td>DmelCG12824</td><td>0</td><td>0</td></tr>
<tr><td>3878</td><td>35728</td><td>Drosophila melanogaster</td><td>Gapdh1</td><td>Glyceraldehyde 3 phosphate dehydrogenase 1</td><td>BEST:GH12586|CG12055|DmelCG12055|GA3PDH|GADPH|GAP|GAPDH|GAPDH I|GAPDH-1|GAPDHI|Gapd|Gapdh|Gapdh-1|Gapdh43E|gadph|gapdh|gapdh-1|gh12586</td><td>0</td><td>0</td></tr>
<tr><td>3879</td><td>35730</td><td>Drosophila melanogaster</td><td>CG1553</td><td>CG1553 gene product from transcript CG1553-RA</td><td>DmelCG1553</td><td>0</td><td>0</td></tr>
<tr><td>3880</td><td>35731</td><td>Drosophila melanogaster</td><td>sax</td><td>saxophone</td><td>Bkr43E|Brk43E|CG1891|DmelCG1891|SAX|STK-B|Sa|Sax</td><td>0</td><td>0</td></tr>
<tr><td>3881</td><td>35732</td><td>Drosophila melanogaster</td><td>CG1550</td><td>CG1550 gene product from transcript CG1550-RA</td><td>DmelCG1550</td><td>0</td><td>0</td></tr>
<tr><td>3882</td><td>35733</td><td>Drosophila melanogaster</td><td>CG1882</td><td>CG1882 gene product from transcript CG1882-RA</td><td>DmelCG1882</td><td>0</td><td>0</td></tr>
<tr><td>3883</td><td>35735</td><td>Drosophila melanogaster</td><td>phr</td><td>photorepair</td><td>C2PHR|CG11205|CG11205-PA|Dm CPD|DmelCG11205|Phl|anon-OV1|anonOV1</td><td>0</td><td>0</td></tr>
</table>

3884	35737	Drosophila melanogaster	CG12822	CG12822 gene product from transcript CG12822-RA	CG12822-PA|DmelCG12822	0	0
3885	35738	Drosophila melanogaster	Dgk	Diacyl glycerol kinase	CG12820|CG1535|CG18654|CG34361|DGKb|DmelCG34361|Dmel_CG12820|Dmel_CG18654|dDGK|dgk	0	0
3886	35741	Drosophila melanogaster	CG12159	CG12159 gene product from transcript CG12159-RB	DmelCG12159	0	0
3887	35742	Drosophila melanogaster	lin19	lin-19-like	CG1877|CUL1|Cul-1|Cul1|Cullin1|DmelCG1877|cul-1|cul1|dCul-1|dCul1|l(2)02074|l(2)k01207|lin-19/cul-1|lin19-like	0	0
3888	35743	Drosophila melanogaster	Or43b	Odorant receptor 43b	25A.1/43|43b|AN7|CG17853|DOR25A.1|DOR81|DmelCG17853|OR43b|Or25A.1	0	0
3889	35744	Drosophila melanogaster	Kdm4A	Histone demethylase 4A	CG15835|DmelCG15835|dJMJD2(1)|dKDM4A	0	0
3890	35745	Drosophila melanogaster	CG8791	CG8791 gene product from transcript CG8791-RA	DmelCG8791|anon-WO0140519.99	0	0
3891	35746	Drosophila melanogaster	rnh1	ribonuclease H1	74/9|CG8729|DmelCG8729|RNase H1|RnH1|l(2)43F1-5|l(2)43Fa|l(2)k07409|l(2)k07624|rnhl	0	0
3892	35747	Drosophila melanogaster	drosha	CG8730 gene product from transcript CG8730-RA	CG8730|DmelCG8730|Drosha	0	0
3893	35748	Drosophila melanogaster	CG8728	CG8728 gene product from transcript CG8728-RA	DmelCG8728	0	0
3894	35749	Drosophila melanogaster	CG14764	CG14764 gene product from transcript CG14764-RA	CG2923|DmelCG14764	0	0
3895	35751	Drosophila melanogaster	CG11165	CG11165 gene product from transcript CG11165-RA	BcDNA:AT10229|BcDNA:AT23738|DmelCG11165	0	0
3896	35753	Drosophila melanogaster	CG2906	CG2906 gene product from transcript CG2906-RE	DmelCG2906	0	0
3897	35754	Drosophila melanogaster	CG2915	CG2915 gene product from transcript CG2915-RB	DmelCG2915	0	0
3898	35756	Drosophila melanogaster	nito	spenito	BcDNA:GH11110|CG2910|Dm44A/spenito|DmSSp|DmelCG2910|cg2910|dOTT	0	0
3899	35757	Drosophila melanogaster	CG14763	CG14763 gene product from transcript CG14763-RA	DmelCG14763	0	0
3900	35758	Drosophila melanogaster	CG8726	CG8726 gene product from transcript CG8726-RB	DmelCG8726	0	0
3901	35759	Drosophila melanogaster	CSN4	COP9 complex homolog subunit 4	CG8725|CH4|Cops4|Csn4|DCH4|Dch4|DmelCG8725|csn4|l(2)k08018	0	0
3902	35761	Drosophila melanogaster	ACC	Acetyl-CoA carboxylase	CG11198|CG8723|DmACC|DmelCG11198|FBgn0043811|dACC	0	0
3903	35762	Drosophila melanogaster	Nup44A	Nucleoporin 44A	CG8722|DmelCG8722|FBgn 33247|Nup44A/Seh1|nup44A|seh1	0	0
3904	35763	Drosophila melanogaster	Dic3	Dicarboxylate carrier 3	CG11196|Dic3p|DmelCG11196	0	0
3905	35764	Drosophila melanogaster	Hey	Hairy/E(spl)-related with YRPW motif	CG11194|DmelCG11194|Hesr-1|bHLHe44|d-hey|dHey|hesr-1|hey	0	0
3906	35765	Drosophila melanogaster	CG11191	CG11191 gene product from transcript CG11191-RA	DmelCG11191	0	0
3907	35766	Drosophila melanogaster	Odc1	Ornithine decarboxylase 1	CG8721|DmelCG8721|dodc1	0	0
3908	35767	Drosophila melanogaster	Odc2	Ornithine decarboxylase 2	CG8719|DmelCG8719|dodc2	0	0
3909	35768	Drosophila melanogaster	CG14762	CG14762 gene product from transcript CG14762-RA	CT34565|DmelCG14762	0	0
3910	35770	Drosophila melanogaster	CG12769	CG12769 gene product from transcript CG12769-RB	DmelCG12769	0	0
3911	35771	Drosophila melanogaster	lig	lingerer	CG8715|DmelCG8715	0	0
3912	35773	Drosophila melanogaster	slv	saliva	CG8717|DmelCG8717	0	0
3913	35774	Drosophila melanogaster	sut1	sugar transporter 1	CG8714|DmelCG8714	0	0
3914	35777	Drosophila melanogaster	CG8713	CG8713 gene product from transcript CG8713-RA	DmelCG8713|anon-WO0109301.39|anon-WO0109301.40|anon-WO0109301.41|anon-WO0109301.42|anon-WO0109301.43|anon-WO0109301.44|anon-WO0109301.45|anon-WO0109301.46|anon-WO01	0	0
3915	35778	Drosophila melanogaster	CG8712	CG8712 gene product from transcript CG8712-RA	DmelCG8712	0	0
3916	35779	Drosophila melanogaster	CG11210	CG11210 gene product from transcript CG11210-RA	CT31310|DmelCG11210	0	0
3917	35780	Drosophila melanogaster	Cul-4	Cullin-4	CG8711|CUL4|Cul4|DMcul-4|DmelCG8711|cul-4|dCul4	0	0
3918	35781	Drosophila melanogaster	CG18316	CG18316 gene product from transcript CG18316-RA	DmelCG18316	0	0
3919	35783	Drosophila melanogaster	CG30372	CG30372 gene product from transcript CG30372-RC	CG14758|CG2226|DmelCG30372	0	0
3920	35784	Drosophila melanogaster	Nup50	Nucleoporin 50	CG2158|DmelCG2158|nup50	0	0
3921	35785	Drosophila melanogaster	coil	coilin	CG8710|Coilin|DmelCG8710	0	0
3922	35786	Drosophila melanogaster	Socs44A	Suppressor of Cytokine Signaling at 44A	CG2160|DmelCG2160|SOCS|SOCS44A|Socs44E|dmSocs44A|socs44A	0	0
3923	35787	Drosophila melanogaster	Pbp49	PSEA-binding protein 49kD	CG11508|CG42515|DMPBP49|DmPBP|DmPBP49|DmSNAP50|DmelCG42515|Dmel_CG11508|Dromel_CG11508_FBtr0088817_mORF	0	0
3924	35788	Drosophila melanogaster	Pabp2	CG2163 gene product from transcript CG2163-RA	CG2163|DmelCG2163|PABP2|PABPN1|ROX2|Rox2|dpabII|pAbp2|pabp2|rox2	0	0
3925	35789	Drosophila melanogaster	Obp44a	CG2297 gene product from transcript CG2297-RA	44a|CG2297|DmelCG2297|anon-EST:ParkEST107|bs11e05.y1	0	0
3926	35790	Drosophila melanogaster	Lpin	Lipin	CG8709|DmLpin|DmelCG8709|EP2431|anon-WO0118547.133	0	0
3927	35792	Drosophila melanogaster	CG8708	CG8708 gene product from transcript CG8708-RC	DmelCG8708|anon-WO0140519.114|anon-WO0144478.10	0	0
3928	35793	Drosophila melanogaster	RagC	CG8707 gene product from transcript CG8707-RA	CG8707|DmelCG8707|Gtr2|dRagC	0	0
3929	35795	Drosophila melanogaster	CG2183	CG2183 gene product from transcript CG2183-RA	BEST:LD05795|DmelCG2183	0	0
3930	35796	Drosophila melanogaster	Mlh1	CG11482 gene product from transcript CG11482-RA	CG11482|DmelCG11482|dmlh-1|dmlh1	0	0
3931	35797	Drosophila melanogaster	CG14757	CG14757 gene product from transcript CG14757-RA	146793_at|DmelCG14757	0	0
3932	35798	Drosophila melanogaster	CG14756	CG14756 gene product from transcript CG14756-RA	DmelCG14756	0	0
3933	35799	Drosophila melanogaster	LRP1	LDL receptor protein 1	CG30368|CG33087|CG8706|CT9297|Dm CG33087|DmelCG33087|LDLR|lrp1	0	0
3934	35800	Drosophila melanogaster	dpn	deadpan	44C|CG8704|DPN|DmelCG8704|Dpn|anon-EST:fe1B12|bHLHe50	0	0
3935	35801	Drosophila melanogaster	pnut	peanut	CG8705|DmelCG8705|E(sina)44C|E(sina)8|PNUT|Pnut|anon-EST:Liang-2.18|clone 2.18|l(2)02502	0	0
3936	35802	Drosophila melanogaster	CG14760	CG14760 gene product from transcript CG14760-RA	DmelCG14760|SP120	0	0
3937	35803	Drosophila melanogaster	CG14759	CG14759 gene product from transcript CG14759-RA	DmelCG14759	0	0
3938	35804	Drosophila melanogaster	CG2291	CG2291 gene product from transcript CG2291-RA	BcDNA:AT16234|DmelCG2291	0	0
3939	35805	Drosophila melanogaster	CG12126	CG12126 gene product from transcript CG12126-RA	BcDNA:AT16918|DmelCG12126	0	0
3940	35806	Drosophila melanogaster	CG30371	CG30371 gene product from transcript CG30371-RA	CG2280|DmelCG30371|SP80	0	0
3941	35808	Drosophila melanogaster	CG11635	CG11635 gene product from transcript CG11635-RA	DmelCG11635	0	0
3942	35809	Drosophila melanogaster	boly	borrelly	CG2149|CG30362|DmelCG30362|NEST:bs34d07	0	0
3943	35810	Drosophila melanogaster	CG18449	CG18449 gene product from transcript CG18449-RA	DmelCG18449	0	0
3944	35811	Drosophila melanogaster	CG2127	CG2127 gene product from transcript CG2127-RA	DmelCG2127|NEST:bs19a07|anon-WO0140519.25|bs19a07.y1	0	0
3945	35812	Drosophila melanogaster	CG8701	CG8701 gene product from transcript CG8701-RA	DmelCG8701|anon-WO0140519.78	0	0
3946	35813	Drosophila melanogaster	pdm3	pou domain motif 3	BcDNA:AT16994|CG11641|CG14755|CG42698|DmelCG42698|Dmel_CG11641|Dmel_CG14755	0	0
3947	35814	Drosophila melanogaster	CG2121	CG2121 gene product from transcript CG2121-RB	CG8698|DmelCG2121	0	0
3948	35816	Drosophila melanogaster	CSN7	COP9 complex homolog subunit 7	CG2038|CH7|CSN7b|Dch7|DmelCG2038|csn7	0	0
3949	35817	Drosophila melanogaster	Lcp1	Larval cuticle protein 1	1|CG11650|CP1|DMLCP1|DmelLcp1|DmelCG11650|I|LCP-1|LCP1|L[[3]]CP1|lcp1	0	0
3950	35818	Drosophila melanogaster	Lcp2	Larval cuticle protein 2	2|CG8697|CP2|DmelLcp2|DmelCG8697|II|LCP-2|LCP2|L[[3]]CP2|lcp2	0	0
3951	35819	Drosophila melanogaster	Lcp3	Larval cuticle protein 3	3|CG2043|CP3|DMLCP3|DmelLcp3|DmelCG2043|III|LCP-3|LCP3|L[[3]]CP3|LcpIII|lcp3	0	0
3952	35820	Drosophila melanogaster	Lcp4	Larval cuticle protein 4	4|CG2044|DmelLcp4|DmelCG2044|IV|LCP-4|LCP4|L[[3]]CP4|lcp4	0	0
3953	35821	Drosophila melanogaster	Cyp4ad1	CG2110 gene product from transcript CG2110-RA	4ad1|BEST:LD02646|CG2110|DmelCG2110	0	0
3954	35822	Drosophila melanogaster	Cyp4e2	Cytochrome P450-4e2	4e2|CG2060|CYP4E2|Cyt-P450-4E2|DmelCG2060|P-450|P450	0	0
3955	35824	Drosophila melanogaster	Mal-A1	Maltase A1	CG8696|DmelCG8696|Drome_A1|H|LvpH|V|lvpH|mal_A1	0	0
3956	35825	Drosophila melanogaster	Mal-A2	Maltase A2	CG8694|D|DmelCG8694|Drome_A2|LvpD|lvpD|mal_A2	0	0
3957	35826	Drosophila melanogaster	Mal-A3	Maltase A3	CG8695|DmelCG8695|Drome_A3|L|LvpL|V|lvpL|mal_A3	0	0
3958	35827	Drosophila melanogaster	Mal-A4	Maltase A4	CG8693|DmelCG8693|Drome_A4|mal_A4	0	0
3959	35828	Drosophila melanogaster	Mal-A5	Maltase A5	CG30359|CG8689|DmelCG30359|Drome_A5|mal_A5	0	0
3960	35829	Drosophila melanogaster	Mal-A7	Maltase A7	CG11669|CT36683|DmelCG11669|Drome_A7|mal_A7	0	0
3961	35830	Drosophila melanogaster	Mal-A8	Maltase A8	CG8690|CT6532|DmelCG8690|Drome_A8|mal_A8	0	0
3962	35832	Drosophila melanogaster	mtt	mangetout	CG18447|CG30361|CG8692|DmGluBR|DmGluRB|DmXR|DmelCG30361|Glu-RB|GluRB|anon-WO0170980.4|anon-WO0170980.5|mXr	0	0
3963	35834	Drosophila melanogaster	CG12780	CG12780 gene product from transcript CG12780-RA	DmelCG12780|GNBP-like	0	0
3964	35837	Drosophila melanogaster	Cyp6a13	CG2397 gene product from transcript CG2397-RA	6a13|CG2397|DmelCG2397|cyp6a13	0	0
3965	35838	Drosophila melanogaster	CG42326	CG42326 gene product from transcript CG42326-RC	CG14747|CG14748|DmelCG42326|Dmel_CG14747|Dmel_CG14748	0	0
3966	35840	Drosophila melanogaster	CG14752	CG14752 gene product from transcript CG14752-RA	DmelCG14752	0	0
3967	35841	Drosophila melanogaster	CG8736	CG8736 gene product from transcript CG8736-RB	BcDNA:RH08259|DmelCG8736	0	0
3968	35842	Drosophila melanogaster	CG8735	CG8735 gene product from transcript CG8735-RA	DmelCG8735	0	0
3969	35843	Drosophila melanogaster	rgr	regular	CG14751|CG8643|DmelCG8643	0	0
3970	35845	Drosophila melanogaster	CG8642	CG8642 gene product from transcript CG8642-RA	DmelCG8642|FBpp0087808	0	0
3971	35846	Drosophila melanogaster	Cirl	CG8639 gene product from transcript CG8639-RH	BcDNA:GH07331|CG8639|CIRL|DmelCG8639|anon-WO0170980.7|anon-WO0170980.8|dcirl	0	0
3972	35847	Drosophila melanogaster	Vps25	Vacuolar protein sorting 25	CG14750|DmelCG14750|ESCRT-I|ESCRT-II|VPS25|dVps25|l(2)44Db|l(2)k08904|vps25	0	0
3973	35848	Drosophila melanogaster	beta3GalTII	Galactosyltransferase II	CG8734|DmelCG8734|GalTII|dbeta3GalTII	0	0
3974	35849	Drosophila melanogaster	CG14749	CG14749 gene product from transcript CG14749-RA	DmelCG14749|GLE1	0	0
3975	35850	Drosophila melanogaster	CG8635	CG8635 gene product from transcript CG8635-RA	DmelCG8635	0	0
3976	35851	Drosophila melanogaster	ptc	patched	BcDNA:RH36596|CG2411|Conf|DmelCG2411|PTC|Ptc|Ptc_Dm|Ptch|com4|dPtc|l(2)k02507|pat|ptch|rubr|tuf	0	0
3977	35853	Drosophila melanogaster	Jon44E	Jonah 44E	CG8579|DmelCG8579|Jon43E|SP100	0	0
3978	35854	Drosophila melanogaster	CG8586	CG8586 gene product from transcript CG8586-RA	DmelCG8586|SPH58|c-SPH58	0	0
3979	35855	Drosophila melanogaster	CG8738	CG8738 gene product from transcript CG8738-RA	DmelCG8738|SPH121|c-SPH121	0	0
3980	35856	Drosophila melanogaster	CG8584	CG8584 gene product from transcript CG8584-RA	BEST:LP01468|DmelCG8584	0	0
3981	35857	Drosophila melanogaster	CG12376	CG12376 gene product from transcript CG12376-RA	DmelCG12376|anon-WO0140519.77	0	0
3982	35858	Drosophila melanogaster	CG14744	CG14744 gene product from transcript CG14744-RC	DmelCG14744	0	0
3983	35859	Drosophila melanogaster	PGRP-SC1a	CG14746 gene product from transcript CG14746-RA	CG14746|DmelCG14746|PGRP SC|PGRP-SC|PGRP-SC1|PGRP-SC1A|picky	0	0
3984	35860	Drosophila melanogaster	CG14743	CG14743 gene product from transcript CG14743-RB	DmelCG14743	0	0
3985	35861	Drosophila melanogaster	PGRP-SC1b	CG8577 gene product from transcript CG8577-RA	CG8577|CT8705|DmelCG8577|PGRP SC|PGRP-SC|PGRP-SC1|PGRP-SC1B|SC1B	0	0
3986	35862	Drosophila melanogaster	PGRP-SC2	CG14745 gene product from transcript CG14745-RA	CG14745|CG14745 PGRP|DmelCG14745|PGRP-SC	0	0
3987	35864	Drosophila melanogaster	gcl	germ cell-less	CG8411|DmelCG8411|GCL|gcl-1	0	0
3988	35865	Drosophila melanogaster	stmA	stambha A	CG8739|DmelCG8739|cmp44E|rbo|stambhA|stm-A	0	0
3989	35866	Drosophila melanogaster	CG8740	CG8740 gene product from transcript CG8740-RD	BcDNA:GH05582|DmelCG8740	0	0
3990	35867	Drosophila melanogaster	CG8746	CG8746 gene product from transcript CG8746-RA	DmelCG8746	0	0
3991	35875	Drosophila melanogaster	CG8272	CG8272 gene product from transcript CG8272-RA	CT8327|DmelCG8272	0	0
3992	35877	Drosophila melanogaster	sec31	CG8266 gene product from transcript CG8266-RB	CG8266|DmelCG8266|SEC31|Sec31|dSec31p	0	0
3993	35878	Drosophila melanogaster	ana2	anastral spindle 2	Ana2|BcDNA:LD22033|CG8262|DmelCG8262	0	0
3994	35879	Drosophila melanogaster	CG13751	CG13751 gene product from transcript CG13751-RA	BcDNA:RH09719|DmelCG13751	0	0

3995	35880	Drosophila melanogaster	MrgBP	CG13746 gene product from transcript CG13746-RA	CG13746|DmelCG13746|dMrgB|dMrgBP	0	0
3996	35881	Drosophila melanogaster	Ggamma1	G protein gamma 1	CG8261|D-G[[gamma]]1|DmelCG8261|G[[gamma]]|Gbetagamma|Gg1|Ggamma|Ggamma-1|bro4|dg1alpha|l(2)k08017	0	0
3997	35882	Drosophila melanogaster	CG8258	CG8258 gene product from transcript CG8258-RA	CCT8|DmelCG8258|TCP-1theta	0	0
3998	35884	Drosophila melanogaster	CG8252	CG8252 gene product from transcript CG8252-RA	DmelCG8252	0	0
3999	35885	Drosophila melanogaster	CG30349	CG30349 gene product from transcript CG30349-RA	CG13750|CG2396|DmelCG30349	0	0
4000	35886	Drosophila melanogaster	Pgi	Phosphoglucose isomerase	CG8251|DmelCG8251|GPI|Gpi|PGI|pgi	0	0
4001	35888	Drosophila melanogaster	CG8248	CG8248 gene product from transcript CG8248-RA	DmelCG8248	0	0
4002	35889	Drosophila melanogaster	CG8247	CG8247 gene product from transcript CG8247-RB	DmelCG8247	0	0
4003	35890	Drosophila melanogaster	CG8243	CG8243 gene product from transcript CG8243-RA	DmelCG8243|gap40	0	0
4004	35891	Drosophila melanogaster	CG8237	CG8237 gene product from transcript CG8237-RA	DmelCG8237	0	0
4005	35892	Drosophila melanogaster	CG8235	CG8235 gene product from transcript CG8235-RA	DmelCG8235	0	0
4006	35893	Drosophila melanogaster	CG8232	CG8232 gene product from transcript CG8232-RA	DmelCG8232|PAN2|Pan2|pan2	0	0
4007	35894	Drosophila melanogaster	CG13749	CG13749 gene product from transcript CG13749-RB	DmelCG13749	0	0
4008	35895	Drosophila melanogaster	FANCI	Fanconi anemia complementation group I homologue	CG13745|DmelCG13745|fanci	0	0
4009	35896	Drosophila melanogaster	CG13748	CG13748 gene product from transcript CG13748-RA	DmelCG13748	0	0
4010	35897	Drosophila melanogaster	CG8230	CG8230 gene product from transcript CG8230-RA	BcDNA:GH02536|DmelCG8230	0	0
4011	35898	Drosophila melanogaster	CG8229	CG8229 gene product from transcript CG8229-RE	DmelCG8229	0	0
4012	35899	Drosophila melanogaster	Tom7	Translocase of outer membrane 7	BcDNA:AT23266|CG8226|DmelCG8226|dtom7	0	0
4013	35900	Drosophila melanogaster	babo	baboon	ATR-1|ATR-I|Atf1|Atr-1|Atr-I|Atr45A|AtrI|BABO|Bab|Babo|CG8224|DmelCG8224|Gprk-3|Gprk3|STK-E|atr-I|l(2)44Fd|l(2)k16912	0	0
4014	35901	Drosophila melanogaster	CG8216	CG8216 gene product from transcript CG8216-RA	DmelCG8216	0	0
4015	35902	Drosophila melanogaster	CG8213	CG8213 gene product from transcript CG8213-RB	DmelCG8213|SP44|c-SP44	0	0
4016	35904	Drosophila melanogaster	CG34350	CG34350 gene product from transcript CG34350-RB	CG11824|CG8181|DmelCG34350|Dmel_CG11824|Dmel_CG8181|SP59	0	0
4017	35905	Drosophila melanogaster	CG8172	CG8172 gene product from transcript CG8172-RD	DmelCG8172|SP32|c-SP32	0	0
4018	35906	Drosophila melanogaster	CG13744	CG13744 gene product from transcript CG13744-RA	DmelCG13744|SP67|c-SP67	0	0
4019	35907	Drosophila melanogaster	CG13747	CG13747 gene product from transcript CG13747-RA	DmelCG13747|dmCG13747	0	0
4020	35908	Drosophila melanogaster	CG8170	CG8170 gene product from transcript CG8170-RB	DmelCG8170|SP54	0	0
4021	35909	Drosophila melanogaster	Ance-4	CG8196 gene product from transcript CG8196-RA	CG8196|DmelCG8196	0	0
4022	35910	Drosophila melanogaster	CG8193	CG8193 gene product from transcript CG8193-RA	DmePPO2|DmelCG8193|PO45|PPO2|ProPO45|proPO45	0	0
4023	35911	Drosophila melanogaster	CG13743	CG13743 gene product from transcript CG13743-RA	DmelCG13743	0	0
4024	35912	Drosophila melanogaster	CG8197	CG8197 gene product from transcript CG8197-RA	DmelCG8197	0	0
4025	35913	Drosophila melanogaster	ana	anachronism	CG8084|DmelCG8084|med	0	0
4026	35914	Drosophila melanogaster	CG8083	CG8083 gene product from transcript CG8083-RA	BEST:CK00459|CK00459|DmelCG8083	0	0
4027	35915	Drosophila melanogaster	CG11778	CG11778 gene product from transcript CG11778-RB	DmelCG11778	0	0
4028	35916	Drosophila melanogaster	CG13742	CG13742 gene product from transcript CG13742-RB	BcDNA:LD16446|DmelCG13742	0	0
4029	35917	Drosophila melanogaster	Dbp45A	DEAD box protein 45A	CG12759|DBP45A|Dbp45|DmRH18|DmelCG12759|dbp45A	0	0
4030	35918	Drosophila melanogaster	CG8080	CG8080 gene product from transcript CG8080-RA	CG30346|DmelCG8080	0	0
4031	35919	Drosophila melanogaster	CG13741	CG13741 gene product from transcript CG13741-RA	CG30346|DmelCG13741	0	0
4032	35920	Drosophila melanogaster	CG8078	CG8078 gene product from transcript CG8078-RA	DmelCG8078|anon-WO0118547.76	0	0
4033	35921	Drosophila melanogaster	CG8777	CG8777 gene product from transcript CG8777-RA	DmelCG8777|SKI3|ski3	0	0
4034	35922	Drosophila melanogaster	Vang	Van Gogh	CG8075|DmelCG8075|Stb|Stbm|Vang/Stbm|stb|stbm|stbm/vang|strabismus/Van Gogh|vang|vang/stbm|vangogh/strabismus	0	0
4035	35923	Drosophila melanogaster	Pmm45A	Phosphomannomutase 45A	CG8073|DmelCG8073	0	0
4036	35924	Drosophila melanogaster	tsu	tsunagi	CG8781|DmelCG8781|Tsu|Y14|Y14/Tsunagi|cg8781|y14	0	0
4037	35925	Drosophila melanogaster	Mys45A	Mystery 45A	CG8070|DmelCG8070|Mys-45A|anon-WO0118547.170	0	0
4038	35926	Drosophila melanogaster	Phax	Phosphorylated adaptor for RNA export	BEST:GH22533|CG8069|DmelCG8069|dPHAX	0	0
4039	35927	Drosophila melanogaster	Su(var)2-10	Suppressor of variegation 2-10	CG8068|CLOT2057|DPIAS|DmelCG8068|Dpias|PIAS|SU(VAR)2-10|Su(Var)2-10|Su(var)-10|Su-var(2)10|Suvar(2)10|ZIMP|ZimpA|ZimpB|dPIAS|dpias|i184|l(2)03697|pias|zimp	0	0
4040	35928	Drosophila melanogaster	CG11784	CG11784 gene product from transcript CG11784-RB	BcDNA:LD22317|DmelCG11784	0	0
4041	35929	Drosophila melanogaster	CG18659	CG18659 gene product from transcript CG18659-RC	BcDNA:GH08789|CG30347|CG30348|CG8059|DmelCG18659|anon-WO0118547.140|anon-WO0118547.145	0	0
4042	35930	Drosophila melanogaster	Hydr1	alpha/beta hydrolase 1	CG8058|Dm.HYDR1|DmelCG8058	0	0
4043	35931	Drosophila melanogaster	alc	alicorn	8057|CG8057|DmelCG8057|FBgn0033383|betaAMPK|l(2)45Ad	0	0
4044	35932	Drosophila melanogaster	CG8788	CG8788 gene product from transcript CG8788-RA	BEST:HL05962|DmelCG8788|n(2)k04512	0	0
4045	35933	Drosophila melanogaster	shrb	shrub	CG8055|DmelCG8055|ESCRT-III|Shrb|Snf7|Snf7/Vps32|Vps32|anon-WO0118547.180|vps32|vps32/snf7	0	0
4046	35934	Drosophila melanogaster	Prp38	pre-mRNA processing factor 38	CG30342|CG8054|DmelCG30342|dprp38	0	0
4047	35935	Drosophila melanogaster	CG8008	CG8008 gene product from transcript CG8008-RB	DmelCG8008	0	0
4048	35936	Drosophila melanogaster	CG8046	CG8046 gene product from transcript CG8046-RA	DmelCG8046	0	0
4049	35937	Drosophila melanogaster	Rad51C	CG2412 gene product from transcript CG2412-RB	CG2412|Dm CG2412|DmelCG2412	0	0
4050	35938	Drosophila melanogaster	CG42382	CG42382 gene product from transcript CG42382-RA	CG8016|DmelCG42382|Dmel_CG8016	0	0
4051	35939	Drosophila melanogaster	Rme-8	Receptor mediated endocytosis 8	CG8014|DmelCG8014|E(shi)2-1|l(2)45Ba|rme-8	0	0
4052	35940	Drosophila melanogaster	ltd	lightoid	CG8024|DRABR1|DmRab32|DmelCG8024|DrabRP1|RAB-RP1|Rab-RP1|Rab-RP1/CG8024|Rab-r1|Rab32|Rab32/RP1|RabRP1|anon-WO0118547.106	0	0
4053	35941	Drosophila melanogaster	CG8026	CG8026 gene product from transcript CG8026-RD	DmelCG8026|Dromel_CG8026_FBtr0088611_mORF|anon-WO0242455.53|anon-WO0242455.6|anon-WO0242455.7	0	0
4054	35942	Drosophila melanogaster	unpg	unplugged	CG1650|DmelCG1650|Gbx|anon-45C|unp|upg	0	0
4055	35943	Drosophila melanogaster	CG8027	CG8027 gene product from transcript CG8027-RA	Cg8027|DmelCG8027	0	0
4056	35944	Drosophila melanogaster	VhaAC45	Vacuolar H[+] ATPase accessory protein AC45	CG8029|DmelCG8029|anon-WO0118547.117|l(2)SH1634|l(2)SH2 1634|vhaAC45	0	0
4057	35946	Drosophila melanogaster	Cyp4p2	CG1944 gene product from transcript CG1944-RA	4p2|CG1944|DmelCG1944	0	0
4058	35948	Drosophila melanogaster	Cyp4p3	CG10843 gene product from transcript CG10843-RA	4p3|CG10843|DmelCG10843	0	0
4059	35949	Drosophila melanogaster	hig	hikaru genki	CG2040|CT6282|DmelCG2040|HIG	0	0
4060	35950	Drosophila melanogaster	Pkn	Protein kinase related to protein kinase N	3-2|CG2049|CG2055|CT6637|CT6660|DPKN|DmelCG2049|Dpkn|PKN|PRK2|Pk45C|Pk?3|dPKN|l(2)06736|l(2)45Ca|v(2)rG232	0	0
4061	35952	Drosophila melanogaster	CG2063	CG2063 gene product from transcript CG2063-RA	DmelCG2063	0	0
4062	35953	Drosophila melanogaster	CG1968	CG1968 gene product from transcript CG1968-RA	DmelCG1968	0	0
4063	35954	Drosophila melanogaster	Mad1	CG2072 gene product from transcript CG2072-RA	CG2072|DmelCG2072|TXBP181-like	0	0
4064	35955	Drosophila melanogaster	Drep-2	DNA fragmentation factor-related protein 2	CG1975|DREP-2|DmelCG1975|Drep2|Rep2|anon-WO0118547.162|anon-WO0118547.168|drep2	0	0
4065	35956	Drosophila melanogaster	Myd88	CG2078 gene product from transcript CG2078-RA	CG2078|DMMYD88|DmMyD88|DmMyd88|DmelCG2078|EP(2)2535|Kra|LD20892|MYD88|MyD88|Myd88F|dMyD88|dMyd88|kra|myd88	0	0
4066	35957	Drosophila melanogaster	CG13739	CG13739 gene product from transcript CG13739-RA	BcDNA:GH21254|DmelCG13739	0	0
4067	35958	Drosophila melanogaster	Or45a	Odorant receptor 45a	45C.1|45a|CG1978|DOR58|DmelCG1978	0	0
4068	35959	Drosophila melanogaster	CG13954	CG13954 gene product from transcript CG13954-RB	DmelCG13954	0	0
4069	35962	Drosophila melanogaster	CG8800	CG8800 gene product from transcript CG8800-RA	DmelCG8800	0	0
4070	35963	Drosophila melanogaster	CG8801	CG8801 gene product from transcript CG8801-RA	BcDNA:LD23830|CG30341|DmelCG8801|LD23830	0	0
4071	35966	Drosophila melanogaster	wun	wunen	CG8804|DmelCG8804|LPP-like|Wun|Wunen|l(2)k16806	0	0
4072	35968	Drosophila melanogaster	CG13955	CG13955 gene product from transcript CG13955-RA	DmelCG13955	0	0
4073	35969	Drosophila melanogaster	prel	preli-like	BcDNA:LD36516|CG8806|DmelCG8806	0	0
4074	35970	Drosophila melanogaster	Pdk	Pyruvate dehydrogenase kinase	BcDNA:LD09837|CG8808|DmPDK|DmPdk|DmelCG8808|pdk	0	0
4075	35971	Drosophila melanogaster	ced-6	CG11804 gene product from transcript CG11804-RD	CG11804|DmelCG11804|ced6|dCed-6|dced-6|drCed-6|drced-6	0	0
4076	35974	Drosophila melanogaster	Camta	Calmodulin-binding transcription activator	CG13952|CG13953|CG42332|CG8809|DmCAMTA|DmelCG42332|Dmel_CG13952|Dmel_CG13953|Dmel_CG8809|camta|dCAMTA	0	0
4077	35975	Drosophila melanogaster	Wnt2	Wnt oncogene analog 2	CG1916|D-wnt-2|DWnt-2|DWnt2|Dm DWnt2|Dm-2|DmelCG1916|Dwnt-2|Wnt|Wnt-2|dWnt2|wnt2	0	0
4078	35977	Drosophila melanogaster	brp	bruchpilot	BRP|Brp|CG12932|CG12933|CG1931|CG30336|CG30337|CG34146|CG42344|Cast|DmelCG42344|Dmel_CG12932|Dmel_CG34146|MAbnc82|Mabnc82|NC82|Nc82|mAb nc82|mAbnc82|nc-82|nc82	0	0
4079	35979	Drosophila melanogaster	CG1888	CG1888 gene product from transcript CG1888-RA	DmelCG1888	0	0
4080	35980	Drosophila melanogaster	Or45b	Odorant receptor 45b	45F.1|45b|CG12931|DOR107|DmelCG12931	0	0
4081	35981	Drosophila melanogaster	CG1809	CG1809 gene product from transcript CG1809-RA	DmelCG1809	0	0
4082	35984	Drosophila melanogaster	CG1814	CG1814 gene product from transcript CG1814-RC	DmelCG1814	0	0
4083	35985	Drosophila melanogaster	CG1868	CG1868 gene product from transcript CG1868-RB	DmelCG1868	0	0
4084	35986	Drosophila melanogaster	Updo	CG1818 gene product from transcript CG1818-RA	CG1818|DmelCG1818	0	0
4085	35987	Drosophila melanogaster	CG12929	CG12929 gene product from transcript CG12929-RA	DmelCG12929	0	0
4086	35988	Drosophila melanogaster	RpL31	Ribosomal protein L31	BcDNA:RE59131|CG1821|DmelCG1821|M(2)45F|anon-EST:Posey68	0	0
4087	35989	Drosophila melanogaster	CG1827	CG1827 gene product from transcript CG1827-RC	DmelCG1827|cg1827	0	0
4088	35991	Drosophila melanogaster	Map60	Microtubule-associated protein 60	CG1825|CP-60|CP60|Cp60|DMAP-60|DMAP60|DmelCG1825	0	0
4089	35993	Drosophila melanogaster	CG1902	CG1902 gene product from transcript CG1902-RA	DmelCG1902	0	0
4090	35995	Drosophila melanogaster	CG30000	CG30000 gene product from transcript CG30000-RA	BcDNA:RE63575|CG12930|CG12930B|DmelCG30000	0	0
4091	35996	Drosophila melanogaster	CG12926	CG12926 gene product from transcript CG12926-RA	DmelCG12926	0	0
4092	35997	Drosophila melanogaster	Mmp2	Matrix metalloproteinase 2	2-MMP|CG1794|DM2-MMP|Dm2-MMP|DmelCG1794|MMP2|anon-WO0118547.84|dm-2MMP|l(2)02353|mmp2	0	0
4093	35998	Drosophila melanogaster	Uba1	Ubiquitin activating enzyme 1	2/31|CG1782|DmUba1|DmelCG1782|E1|UBA-1|Uba 1|l(2)03405|l(2)05642|l(2)s3484|uba-1|uba1	0	0
4094	35999	Drosophila melanogaster	CG1773	CG1773 gene product from transcript CG1773-RA	DmelCG1773|SP74	0	0
4095	36000	Drosophila melanogaster	CG10459	CG10459 gene product from transcript CG10459-RA	DmelCG10459	0	0
4096	36001	Drosophila melanogaster	dap	dacapo	CDI4|CDKN2B|CG1772|CIB1|Cdi4|Dac|Dap|Decapo|DmelCG1772|E(Sev-CycE)2B|P15|cdi4|dacapo/cyclin-dependent kinase interactor 4|l(2)04454|p21|p21[dacapo]|p27|p27[Dap]	0	0
4097	36002	Drosophila melanogaster	CG42347	CG42347 gene product from transcript CG42347-RC	CG1776|CG30004|DmelCG42347|Dmel_CG1776|Dmel_CG30004	0	0
4098	36003	Drosophila melanogaster	trpl	trp-like	CG1694|CG18345|DmTRPL|DmelCG18345|TRPL|TrpL|Trpl|dTRPL|dTrpL|trp-l	0	0
4099	36004	Drosophila melanogaster	CG1690	CG1690 gene product from transcript CG1690-RA	DmelCG1690	0	0
4100	36005	Drosophila melanogaster	CG1688	CG1688 gene product from transcript CG1688-RA	BcDNA:GH04802|DmelCG1688|anon-WO0109301.12|anon-WO0109301.47|anon-WO0109301.48|anon-WO0109301.49|anon-WO0109301.50	0	0
4101	36006	Drosophila melanogaster	CG1698	CG1698 gene product from transcript CG1698-RA	DmNAT5|DmelCG1698	0	0
4102	36009	Drosophila melanogaster	CG1648	CG1648 gene product from transcript CG1648-RA	DmelCG1648	0	0
4103	36010	Drosophila melanogaster	dila	dilatory	CG1625|CG1626|DmelCG1625	0	0
4104	36011	Drosophila melanogaster	hebe	CG1623 gene product from transcript CG1623-RD	CG1623|DmelCG1623	0	0
4105	36012	Drosophila melanogaster	CG1663	CG1663 gene product from transcript CG1663-RA	DmelCG1663	0	0

<table>
<tr><td>4106</td><td>36013</td><td>Drosophila melanogaster</td><td>Lsm11</td><td>CG12924 gene product from transcript CG12924-RA</td><td>CG12924|DmelCG12924|LSM11|dLsm11</td><td>0</td><td>0</td></tr>
<tr><td>4107</td><td>36014</td><td>Drosophila melanogaster</td><td>CG1665</td><td>CG1665 gene product from transcript CG1665-RA</td><td>DmelCG1665</td><td>0</td><td>0</td></tr>
<tr><td>4108</td><td>36015</td><td>Drosophila melanogaster</td><td>CG1599</td><td>CG1599 gene product from transcript CG1599-RA</td><td>DmelCG1599</td><td>0</td><td>0</td></tr>
<tr><td>4109</td><td>36016</td><td>Drosophila melanogaster</td><td>CG1667</td><td>CG1667 gene product from transcript CG1667-RA</td><td>DmelCG1667</td><td>0</td><td>0</td></tr>
<tr><td>4110</td><td>36017</td><td>Drosophila melanogaster</td><td>Orc6</td><td>Origin recognition complex subunit 6</td><td>CG1584|DmORC 6|DmORC6|DmelCG1584|ORC|ORC6|orc6|rDmORC</td><td>0</td><td>0</td></tr>
<tr><td>4111</td><td>36018</td><td>Drosophila melanogaster</td><td>Prosalpha7</td><td>Proteasome alpha7 subunit</td><td>1519|Alpha7|CG1519|DmelCG1519|PSA3_DROME|alpha7|alpha7_dm</td><td>0</td><td>0</td></tr>
<tr><td>4112</td><td>36019</td><td>Drosophila melanogaster</td><td>CG1671</td><td>CG1671 gene product from transcript CG1671-RA</td><td>DmelCG1671</td><td>0</td><td>0</td></tr>
<tr><td>4113</td><td>36020</td><td>Drosophila melanogaster</td><td>CG1516</td><td>CG1516 gene product from transcript CG1516-RP</td><td>BcDNA|BcDNA:GH06348|DmelCG1516|PC</td><td>0</td><td>0</td></tr>
<tr><td>4114</td><td>36022</td><td>Drosophila melanogaster</td><td>CG1675</td><td>CG1675 gene product from transcript CG1675-RA</td><td>DmelCG1675</td><td>0</td><td>0</td></tr>
<tr><td>4115</td><td>36023</td><td>Drosophila melanogaster</td><td>CG18446</td><td>CG18446 gene product from transcript CG18446-RA</td><td>DmelCG18446</td><td>0</td><td>0</td></tr>
<tr><td>4116</td><td>36024</td><td>Drosophila melanogaster</td><td>CG12744</td><td>CG12744 gene product from transcript CG12744-RA</td><td>DmelCG12744</td><td>0</td><td>0</td></tr>
<tr><td>4117</td><td>36025</td><td>Drosophila melanogaster</td><td>sec24</td><td>CG1472 gene product from transcript CG1472-RA</td><td>CG1472|DmelCG1472|SEC24|Sec24|Sec24AB</td><td>0</td><td>0</td></tr>
<tr><td>4118</td><td>36026</td><td>Drosophila melanogaster</td><td>CG12923</td><td>CG12923 gene product from transcript CG12923-RA</td><td>DmelCG12923</td><td>0</td><td>0</td></tr>
<tr><td>4119</td><td>36027</td><td>Drosophila melanogaster</td><td>CG30007</td><td>CG30007 gene product from transcript CG30007-RB</td><td>CG12138|DmelCG30007</td><td>0</td><td>0</td></tr>
<tr><td>4120</td><td>36028</td><td>Drosophila melanogaster</td><td>CG1513</td><td>CG1513 gene product from transcript CG1513-RA</td><td>DmelCG1513</td><td>0</td><td>0</td></tr>
<tr><td>4121</td><td>36029</td><td>Drosophila melanogaster</td><td>CG1441</td><td>CG1441 gene product from transcript CG1441-RB</td><td>DmelCG1441</td><td>0</td><td>0</td></tr>
<tr><td>4122</td><td>36030</td><td>Drosophila melanogaster</td><td>Fmrf</td><td>FMRFamide-related</td><td>CG2346|DPKQDFMRF-amide|DPKQDFMRFamide|DmelCG2346|Drm-FMRF-1|Drm-FMRF-2|Drm-FMRF-3|Drm-FMRF-4|Drm-FMRF-5|Drm-FMRFa1|Drm-FMRFa2|Drm-FMRFa3|Drm-FMRFa4|FMRF|FMRI</td><td>0</td><td>0</td></tr>
<tr><td>4123</td><td>36031</td><td>Drosophila melanogaster</td><td>CG12140</td><td>CG12140 gene product from transcript CG12140-RA</td><td>DmelCG12140</td><td>0</td><td>0</td></tr>
<tr><td>4124</td><td>36032</td><td>Drosophila melanogaster</td><td>Mef2</td><td>Myocyte enhancer factor 2</td><td>22.21|BEST:SD04091|C|CG1429|D-MEF2|D-Mef2|D-mef2|DMEF-2|DMEF2|DMedf2|DMef-2|DMef2|Dmef|Dmef-2|Dmef2|DmelCG1429|MEF-2|MEF2|Mef|Mef-2|SD04091|dMEF2|dMef2|dmef2|l(2)46CFr|mb2</td><td>0</td><td>0</td></tr>
<tr><td>4125</td><td>36033</td><td>Drosophila melanogaster</td><td>Pal1</td><td>Peptidyl-alpha-hydroxyglycine-alpha-amidating lyase</td><td>CG12130|DmelCG12130|PAL1|anon-WO0149856.3|dPAL1</td><td>0</td><td>0</td></tr>
<tr><td>4126</td><td>36034</td><td>Drosophila melanogaster</td><td>CG15863</td><td>CG15863 gene product from transcript CG15863-RA</td><td>DmelCG15863</td><td>0</td><td>0</td></tr>
<tr><td>4127</td><td>36035</td><td>Drosophila melanogaster</td><td>CG1418</td><td>CG1418 gene product from transcript CG1418-RA</td><td>DmelCG1418</td><td>0</td><td>0</td></tr>
<tr><td>4128</td><td>36036</td><td>Drosophila melanogaster</td><td>CG12133</td><td>CG12133 gene product from transcript CG12133-RA</td><td>DmelCG12133|SP15</td><td>0</td><td>0</td></tr>
<tr><td>4129</td><td>36037</td><td>Drosophila melanogaster</td><td>Adam</td><td>CG12131 gene product from transcript CG12131-RA</td><td>5.3|CG12131|DmelCG12131|E|eIF3-S1|l(2)46CFd|l(2)46Cf|l(2)k13906</td><td>0</td><td>0</td></tr>
<tr><td>4130</td><td>36038</td><td>Drosophila melanogaster</td><td>CG12134</td><td>CG12134 gene product from transcript CG12134-RA</td><td>DmelCG12134</td><td>0</td><td>0</td></tr>
<tr><td>4131</td><td>36039</td><td>Drosophila melanogaster</td><td>eve</td><td>even skipped</td><td>10.5|10.9|14.10|20.35|CG2328|DmelCG2328|E(eve)|EVE|Eve|F|V|VI|eve2|l(2)46CFg|l(2)46CFh|l(2)46CFj|l(2)46CFp|l(2)46Ce|l(2)46Cg</td><td>0</td><td>0</td></tr>
<tr><td>4132</td><td>36040</td><td>Drosophila melanogaster</td><td>TER94</td><td>CG2331 gene product from transcript CG2331-RA</td><td>22.26|7.12|Apple|BcDNA.GM02885|BcDNA:GM02885|CDC48|CG2331|DmTER94|DmelCG2331|l|Ter94|Tera|VCP|VCP/p97|Vcp|anon-WO2004063362.65|anon-WO2004063362.67|dTERA|dVCP|dmTERA|l(</td><td>0</td><td>0</td></tr>
<tr><td>4133</td><td>36041</td><td>Drosophila melanogaster</td><td>Pka-R2</td><td>cAMP-dependent protein kinase R2</td><td>BcDNA:GM01761|CG15862|Cos|Cos-1|Cos1|DmelCG15862|Epa|PKA|PKA RII|PKA-R2|PKA-RII|PKa-R2|Pka-RII|RII|RII[[DR]]|cos1|pkA|pka-RII</td><td>0</td><td>0</td></tr>
<tr><td>4134</td><td>36042</td><td>Drosophila melanogaster</td><td>CG12128</td><td>CG12128 gene product from transcript CG12128-RB</td><td>DmelCG12128</td><td>0</td><td>0</td></tr>
<tr><td>4135</td><td>36043</td><td>Drosophila melanogaster</td><td>CG1407</td><td>CG1407 gene product from transcript CG1407-RB</td><td>DmelCG1407</td><td>0</td><td>0</td></tr>
<tr><td>4136</td><td>36044</td><td>Drosophila melanogaster</td><td>CG12129</td><td>CG12129 gene product from transcript CG12129-RA</td><td>DmelCG12129|cg12129</td><td>0</td><td>0</td></tr>
<tr><td>4137</td><td>36045</td><td>Drosophila melanogaster</td><td>oys</td><td>oysgedart</td><td>CG18445|DmelCG18445|Oys</td><td>0</td><td>0</td></tr>
<tr><td>4138</td><td>36046</td><td>Drosophila melanogaster</td><td>sel</td><td>seele</td><td>CG12918|DmelCG12918|Sle</td><td>0</td><td>0</td></tr>
<tr><td>4139</td><td>36047</td><td>Drosophila melanogaster</td><td>Def</td><td>Defensin</td><td>143607_at|CG1385|DEF|DmelCG1385|def</td><td>0</td><td>0</td></tr>
<tr><td>4140</td><td>36048</td><td>Drosophila melanogaster</td><td>magu</td><td>CG2264 gene product from transcript CG2264-RD</td><td>CG2264|CT7504|DmelCG2264|PENT|pent</td><td>0</td><td>0</td></tr>
<tr><td>4141</td><td>36049</td><td>Drosophila melanogaster</td><td>CG2292</td><td>CG2292 gene product from transcript CG2292-RA</td><td>DmelCG2292</td><td>0</td><td>0</td></tr>
<tr><td>4142</td><td>36050</td><td>Drosophila melanogaster</td><td>mRpL42</td><td>mitochondrial ribosomal protein L42</td><td>BcDNA:RE72116|CG12921|DmelCG12921|MRP-S32|MRPL31|mRpS32</td><td>0</td><td>0</td></tr>
<tr><td>4143</td><td>36051</td><td>Drosophila melanogaster</td><td>cdc2rk</td><td>cdc2-related-kinase</td><td>CG1362|Dcdrk|DmelCG1362</td><td>0</td><td>0</td></tr>
<tr><td>4144</td><td>36052</td><td>Drosophila melanogaster</td><td>CG12920</td><td>CG12920 gene product from transcript CG12920-RA</td><td>DmelCG12920</td><td>0</td><td>0</td></tr>
<tr><td>4145</td><td>36053</td><td>Drosophila melanogaster</td><td>CG1371</td><td>CG1371 gene product from transcript CG1371-RA</td><td>DmelCG1371</td><td>0</td><td>0</td></tr>
<tr><td>4146</td><td>36054</td><td>Drosophila melanogaster</td><td>egr</td><td>eiger</td><td>BcDNA:RH51659|CG12919|DmelCG12919|Ect1|Egr|Regg1|darth|dt1|regg1</td><td>0</td><td>0</td></tr>
<tr><td>4147</td><td>36055</td><td>Drosophila melanogaster</td><td>sut4</td><td>sugar transporter 4</td><td>CG1380|DmelCG1380</td><td>0</td><td>0</td></tr>
<tr><td>4148</td><td>36056</td><td>Drosophila melanogaster</td><td>CG2269</td><td>CG2269 gene product from transcript CG2269-RD</td><td>DmelCG2269|anon-WO0118547.141</td><td>0</td><td>0</td></tr>
<tr><td>4149</td><td>36057</td><td>Drosophila melanogaster</td><td>Jra</td><td>Jun-related antigen</td><td>AP-1|AP1|CG2275|D-Jun|D-jun|D-jun/Jra|DJUN|DJun|Djun|DmelCG2275|JRA|JUN|Jun|V|c-Jun|cJun|d-JRA|d-Jun|d-jun|dAP-1|dJRA|dJUN|dJra|dJun|djun|dm-Jun|jra|jun|l(2)46Ef|l(2)IA109|l(2R)IA109</td><td>0</td><td>0</td></tr>
<tr><td>4150</td><td>36058</td><td>Drosophila melanogaster</td><td>RpLP0-like</td><td>Ribosomal protein LP0-like</td><td>CG1381|DmelCG1381|FBgn0033485</td><td>0</td><td>0</td></tr>
<tr><td>4151</td><td>36059</td><td>Drosophila melanogaster</td><td>14-3-3zeta</td><td>CG17870 gene product from transcript CG17870-RB</td><td>14-3-3|14-3-3 zeta|14-3-3-zeta|14-3-3ZETA|2G1|4-3-3 zeta|5.11|549|BEST:GH05075|CG17870|D14-3-3|D14-3-3zeta|DmelCG17870|K|LEO|Leo|PAR-5|PAR5|Par-5|d14-3-3zeta|l(2)07103|l(2)46CFe|l(2)46Ee</td><td>0</td><td>0</td></tr>
<tr><td>4152</td><td>36060</td><td>Drosophila melanogaster</td><td>Pfk</td><td>Phosphofructokinase</td><td>BcDNA.GH12192|BcDNA:GH12192|CG4001|DmelCG4001|PFK|l(2)06339|pfk</td><td>0</td><td>0</td></tr>
<tr><td>4153</td><td>36061</td><td>Drosophila melanogaster</td><td>CG11866</td><td>CG11866 gene product from transcript CG11866-RB</td><td>DmelCG11866</td><td>0</td><td>0</td></tr>
<tr><td>4154</td><td>36064</td><td>Drosophila melanogaster</td><td>gem</td><td>gemini</td><td>BcDNA:HL07889|CG11867|CG30011|CG3459|DmelCG30011|anon-AE003830.1|dCP2</td><td>0</td><td>0</td></tr>
<tr><td>4155</td><td>36065</td><td>Drosophila melanogaster</td><td>CG12917</td><td>CG12917 gene product from transcript CG12917-RB</td><td>DmelCG12917</td><td>0</td><td>0</td></tr>
<tr><td>4156</td><td>36068</td><td>Drosophila melanogaster</td><td>CG18011</td><td>CG18011 gene product from transcript CG18011-RA</td><td>CG17847|DmelCG18011</td><td>0</td><td>0</td></tr>
<tr><td>4157</td><td>36071</td><td>Drosophila melanogaster</td><td>KCNQ</td><td>KCNQ potassium channel</td><td>CG12215|CG12915|CG33135|DKCNQ|DmelCG33135|dKCNQ|kcnq1</td><td>0</td><td>0</td></tr>
<tr><td>4158</td><td>36072</td><td>Drosophila melanogaster</td><td>CG12214</td><td>CG12214 gene product from transcript CG12214-RB</td><td>DmelCG12214</td><td>0</td><td>0</td></tr>
<tr><td>4159</td><td>36073</td><td>Drosophila melanogaster</td><td>Hr46</td><td>Hormone receptor-like in 46</td><td>12.6|2.4|CG11823|CG12208|CG33183|D|DHR3|Dhr3|DmelCG33183|HR3|Hr3|NR1F4|dHR3|hr46|l(2)46CFi|l(2)k09242</td><td>0</td><td>0</td></tr>
<tr><td>4160</td><td>36075</td><td>Drosophila melanogaster</td><td>CG12912</td><td>CG12912 gene product from transcript CG12912-RA</td><td>DmelCG12912</td><td>0</td><td>0</td></tr>
<tr><td>4161</td><td>36076</td><td>Drosophila melanogaster</td><td>Hdc</td><td>Histidine decarboxylase</td><td>CG3454|DmelCG3454|JK910|anon-WO0140519.259|hdc</td><td>0</td><td>0</td></tr>
<tr><td>4162</td><td>36077</td><td>Drosophila melanogaster</td><td>CG12209</td><td>CG12209 gene product from transcript CG12209-RA</td><td>BcDNA:AT13439|DmelCG12209</td><td>0</td><td>0</td></tr>
<tr><td>4163</td><td>36078</td><td>Drosophila melanogaster</td><td>CG12914</td><td>CG12914 gene product from transcript CG12914-RA</td><td>DmelCG12914</td><td>0</td><td>0</td></tr>
<tr><td>4164</td><td>36079</td><td>Drosophila melanogaster</td><td>CG12913</td><td>CG12913 gene product from transcript CG12913-RA</td><td>DmelCG12913</td><td>0</td><td>0</td></tr>
<tr><td>4165</td><td>36080</td><td>Drosophila melanogaster</td><td>Syb</td><td>Synaptobrevin</td><td>21.15|CG12210|DmelCG12210|J|SYB|VAMP|VAMP2|Vamp|c-Syb|l(2)46CFq|l(2)46El|syb</td><td>0</td><td>0</td></tr>
<tr><td>4166</td><td>36081</td><td>Drosophila melanogaster</td><td>CG12911</td><td>CG12911 gene product from transcript CG12911-RB</td><td>DmelCG12911</td><td>0</td><td>0</td></tr>
<tr><td>4167</td><td>36082</td><td>Drosophila melanogaster</td><td>CG12910</td><td>CG12910 gene product from transcript CG12910-RA</td><td>DmelCG12910</td><td>0</td><td>0</td></tr>
<tr><td>4168</td><td>36084</td><td>Drosophila melanogaster</td><td>CAP</td><td>CG18408 gene product from transcript CG18408-RI</td><td>CAP/Vinexin|CG18408|CG18409|CG3451|DCAP|DmelCG18408|Rexin|cap|rexin</td><td>0</td><td>0</td></tr>
<tr><td>4169</td><td>36086</td><td>Drosophila melanogaster</td><td>JhI-1</td><td>Juvenile hormone-inducible protein 1</td><td>CG3298|DmelCG3298|JHI-1|dRNase Z|dRNaseZ|dtRNaseZ|tRNase Z</td><td>0</td><td>0</td></tr>
<tr><td>4170</td><td>36087</td><td>Drosophila melanogaster</td><td>CG12909</td><td>CG12909 gene product from transcript CG12909-RA</td><td>DmelCG12909</td><td>0</td><td>0</td></tr>
<tr><td>4171</td><td>36088</td><td>Drosophila melanogaster</td><td>Obp46a</td><td>Odorant-binding protein 46a</td><td>CG12905|DmelCG12905</td><td>0</td><td>0</td></tr>
<tr><td>4172</td><td>36089</td><td>Drosophila melanogaster</td><td>Ndg</td><td>Nidogen/entactin</td><td>BEST:CK00552|CG12908|CT32053|DmelCG12908|ndg</td><td>0</td><td>0</td></tr>
<tr><td>4173</td><td>36091</td><td>Drosophila melanogaster</td><td>CG12907</td><td>CG12907 gene product from transcript CG12907-RB</td><td>BcDNA:AT31822|DmelCG12907|anon-SAGE:Wang-109|anon-WO0140519.79</td><td>0</td><td>0</td></tr>
<tr><td>4174</td><td>36092</td><td>Drosophila melanogaster</td><td>CG12902</td><td>CG12902 gene product from transcript CG12902-RC</td><td>BcDNA:AT11401|DmelCG12902</td><td>0</td><td>0</td></tr>
<tr><td>4175</td><td>36093</td><td>Drosophila melanogaster</td><td>CG42733</td><td>CG42733 gene product from transcript CG42733-RD</td><td>CG12901|DmelCG42733|Dmel_CG12901|Tes85-2</td><td>0</td><td>0</td></tr>
<tr><td>4176</td><td>36094</td><td>Drosophila melanogaster</td><td>Gr47a</td><td>Gustatory receptor 47a</td><td>47A.1|47A1|CG12906|DmelCG12906|GR47A.1|Gr47A|Gr47A1</td><td>0</td><td>0</td></tr>
<tr><td>4177</td><td>36095</td><td>Drosophila melanogaster</td><td>Ir47a</td><td>Ionotropic receptor 47a</td><td>CG12900|DmelCG12900|IR47a</td><td>0</td><td>0</td></tr>
<tr><td>4178</td><td>36096</td><td>Drosophila melanogaster</td><td>CG12898</td><td>CG12898 gene product from transcript CG12898-RA</td><td>DmelCG12898</td><td>0</td><td>0</td></tr>
<tr><td>4179</td><td>36098</td><td>Drosophila melanogaster</td><td>Prx2540-2</td><td>Peroxiredoxin 2540-2</td><td>CG11765|DPx-2540-2|DPx2540|DmelCG11765|Prx2540</td><td>0</td><td>0</td></tr>
<tr><td>4180</td><td>36099</td><td>Drosophila melanogaster</td><td>CG11825</td><td>CG11825 gene product from transcript CG11825-RB</td><td>BcDNA:RE25483|DmelCG11825</td><td>0</td><td>0</td></tr>
<tr><td>4181</td><td>36101</td><td>Drosophila melanogaster</td><td>CG12896</td><td>CG12896 gene product from transcript CG12896-RA</td><td>BEST:LD11278|DPx-2540-1 variant|DmelCG12896|RH54193</td><td>0</td><td>0</td></tr>
<tr><td>4182</td><td>36102</td><td>Drosophila melanogaster</td><td>RanBPM</td><td>Ran-binding protein M</td><td>CG11763|CG42236|DmelCG42236|Dmel_CG11763|l(2)k05201</td><td>0</td><td>0</td></tr>
<tr><td>4183</td><td>36103</td><td>Drosophila melanogaster</td><td>CG12895</td><td>CG12895 gene product from transcript CG12895-RA</td><td>BcDNA:RE29446|DmelCG12895</td><td>0</td><td>0</td></tr>
<tr><td>4184</td><td>36104</td><td>Drosophila melanogaster</td><td>G-oalpha47A</td><td>G protein oalpha 47A</td><td>Bkh|CG2204|DG[[o]]|DGalpha0|DGalpha[[o]]|DROGPAD|DmelCG2204|G(o)alpha47A|G-alpha-47A|G-oalpha|G-oalpha47a|G[[o]]alpha|G[[oalpha]]|Galpha47A|Galpha[[047A]]|Galpha[[0]]|Galpha[[o]]|Galphao|(</td><td>0</td><td>0</td></tr>
<tr><td>4185</td><td>36105</td><td>Drosophila melanogaster</td><td>Cyp49a1</td><td>CG18377 gene product from transcript CG18377-RA</td><td>49a1|CG12894|CG18377|Cyp49a1p|DmelCG18377</td><td>0</td><td>0</td></tr>
<tr><td>4186</td><td>36107</td><td>Drosophila melanogaster</td><td>Caf1-105</td><td>CG12892 gene product from transcript CG12892-RA</td><td>CAF p105|CAF1-105|CG12892|Caf1-10|DmelCG12892|p105</td><td>0</td><td>0</td></tr>
<tr><td>4187</td><td>36108</td><td>Drosophila melanogaster</td><td>CG11777</td><td>CG11777 gene product from transcript CG11777-RA</td><td>BcDNA:RE61564|DmelCG11777</td><td>0</td><td>0</td></tr>
<tr><td>4188</td><td>36109</td><td>Drosophila melanogaster</td><td>whd</td><td>withered</td><td>BEST:GH27794|CG12891|CPT1|CPTI|DmelCG12891|gh27794</td><td>0</td><td>0</td></tr>
<tr><td>4189</td><td>36110</td><td>Drosophila melanogaster</td><td>trsn</td><td>translin</td><td>CG11761|DmelCG11761|TB-RBP</td><td>0</td><td>0</td></tr>
<tr><td>4190</td><td>36111</td><td>Drosophila melanogaster</td><td>CG17765</td><td>CG17765 gene product from transcript CG17765-RA</td><td>DmelCG17765</td><td>0</td><td>0</td></tr>
<tr><td>4191</td><td>36118</td><td>Drosophila melanogaster</td><td>psq</td><td>pipsqueak</td><td>BTB-V|BtbV|CG2368|DmelCG2368|PSQ|Psq|eyeful|pip|psk|psq-1|rum|zep</td><td>0</td><td>0</td></tr>
<tr><td>4192</td><td>36121</td><td>Drosophila melanogaster</td><td>CG11883</td><td>CG11883 gene product from transcript CG11883-RA</td><td>DmelCG11883</td><td>0</td><td>0</td></tr>
<tr><td>4193</td><td>36122</td><td>Drosophila melanogaster</td><td>Git</td><td>CG16728 gene product from transcript CG16728-RA</td><td>CG16728|CT37209|DmelCG16728|arfgap2|dgit</td><td>0</td><td>0</td></tr>
<tr><td>4194</td><td>36123</td><td>Drosophila melanogaster</td><td>Elp2</td><td>Elongator complex protein 2</td><td>CG1187|CG11887|D-elp2|DmelCG11887|StIP|Stip|elp-2</td><td>0</td><td>0</td></tr>
<tr><td>4195</td><td>36124</td><td>Drosophila melanogaster</td><td>CG12934</td><td>CG12934 gene product from transcript CG12934-RA</td><td>DmelCG12934</td><td>0</td><td>0</td></tr>
<tr><td>4196</td><td>36125</td><td>Drosophila melanogaster</td><td>stan</td><td>starry night</td><td>CG11895|CT20776|DmelCG11895|Flam|Fmi|Stan|fmi|fmi/stan</td><td>0</td><td>0</td></tr>
<tr><td>4197</td><td>36126</td><td>Drosophila melanogaster</td><td>CG12309</td><td>CG12309 gene product from transcript CG12309-RA</td><td>DmelCG12309</td><td>0</td><td>0</td></tr>
<tr><td>4198</td><td>36127</td><td>Drosophila melanogaster</td><td>Rab3</td><td>Rab-protein 3</td><td>AAF58762|CG7576|DRAB3|DRab3|DmRab3|DmelCG7576|Drab3|Rab3A|dm-Rab3|drab3|rab3</td><td>0</td><td>0</td></tr>
<tr><td>4199</td><td>36128</td><td>Drosophila melanogaster</td><td>CG12338</td><td>CG12338 gene product from transcript CG12338-RA</td><td>DmelCG12338</td><td>0</td><td>0</td></tr>
<tr><td>4200</td><td>36129</td><td>Drosophila melanogaster</td><td>CG7220</td><td>CG7220 gene product from transcript CG7220-RD</td><td>BEST:GH28095|BcDNA:GH28095|DmelCG7220</td><td>0</td><td>0</td></tr>
<tr><td>4201</td><td>36130</td><td>Drosophila melanogaster</td><td>Mat1</td><td>CG7614 gene product from transcript CG7614-RA</td><td>AT-1|CG7614|DmelCG7614|MAT1</td><td>0</td><td>0</td></tr>
<tr><td>4202</td><td>36131</td><td>Drosophila melanogaster</td><td>CG33144</td><td>CG33144 gene product from transcript CG33144-RB</td><td>CG12339|CG7621|DmelCG33144</td><td>0</td><td>0</td></tr>
<tr><td>4203</td><td>36133</td><td>Drosophila melanogaster</td><td>wde</td><td>windei</td><td>BcDNA:LD26050|CG12340|DmelCG12340|LD26050|Wde|i10|i119</td><td>0</td><td>0</td></tr>
<tr><td>4204</td><td>36134</td><td>Drosophila melanogaster</td><td>CG12935</td><td>CG12935 gene product from transcript CG12935-RA</td><td>DmelCG12935</td><td>0</td><td>0</td></tr>
<tr><td>4205</td><td>36135</td><td>Drosophila melanogaster</td><td>CG7637</td><td>CG7637 gene product from transcript CG7637-RA</td><td>DmelCG7637</td><td>0</td><td>0</td></tr>
<tr><td>4206</td><td>36136</td><td>Drosophila melanogaster</td><td>mms4</td><td>CG12936 gene product from transcript CG12936-RA</td><td>CG12936|DmelCG12936</td><td>0</td><td>0</td></tr>
<tr><td>4207</td><td>36137</td><td>Drosophila melanogaster</td><td>CG12341</td><td>CG12341 gene product from transcript CG12341-RA</td><td>BcDNA:SD17342|DmelCG12341</td><td>0</td><td>0</td></tr>
<tr><td>4208</td><td>36138</td><td>Drosophila melanogaster</td><td>CG7222</td><td>CG7222 gene product from transcript CG7222-RA</td><td>DmelCG7222</td><td>0</td><td>0</td></tr>
<tr><td>4209</td><td>36139</td><td>Drosophila melanogaster</td><td>dgo</td><td>diego</td><td>CG12342|Dgo|DmelCG12342|EP(2)2619|EP2619</td><td>0</td><td>0</td></tr>
<tr><td>4210</td><td>36141</td><td>Drosophila melanogaster</td><td>Lsm10</td><td>CG12938 gene product from transcript CG12938-RA</td><td>CG12938|DmelCG12938|dLsm10</td><td>0</td><td>0</td></tr>
<tr><td>4211</td><td>36142</td><td>Drosophila melanogaster</td><td>RpS15Ab</td><td>Ribosomal protein S15Ab</td><td>CG12324|CT22283|DmelCG12324|RpS15A</td><td>0</td><td>0</td></tr>
<tr><td>4212</td><td>36143</td><td>Drosophila melanogaster</td><td>CG12343</td><td>CG12343 gene product from transcript CG12343-RA</td><td>DmelCG12343|Syf2</td><td>0</td><td>0</td></tr>
<tr><td>4213</td><td>36144</td><td>Drosophila melanogaster</td><td>CG12325</td><td>CG12325 gene product from transcript CG12325-RA</td><td>DmelCG12325</td><td>0</td><td>0</td></tr>
<tr><td>4214</td><td>36145</td><td>Drosophila melanogaster</td><td>CG12344</td><td>CG12344 gene product from transcript CG12344-RA</td><td>DmelCG12344</td><td>0</td><td>0</td></tr>
<tr><td>4215</td><td>36146</td><td>Drosophila melanogaster</td><td>CG7686</td><td>CG7686 gene product from transcript CG7686-RA</td><td>BcDNA:LD21529|DmelCG7686</td><td>0</td><td>0</td></tr>
<tr><td>4216</td><td>36147</td><td>Drosophila melanogaster</td><td>CG30015</td><td>CG30015 gene product from transcript CG30015-RA</td><td>CG7699|CG7701|DmelCG30015|anon-EST:Posey2</td><td>0</td><td>0</td></tr>
</table>

4217	36150	Drosophila melanogaster	nclb	no child left behind	CG6751|DmelCG6751	0	0
4218	36154	Drosophila melanogaster	CG18004	CG18004 gene product from transcript CG18004-RB	DmelCG18004	0	0
4219	36156	Drosophila melanogaster	CG30020	CG30020 gene product from transcript CG30020-RA	BEST:LD40262|CG12941|CG18353|DmelCG30020|p190	0	0
4220	36157	Drosophila melanogaster	cag	CG12346 gene product from transcript CG12346-RA	CG12346|DmelCG12346	0	0
4221	36158	Drosophila melanogaster	CG12942	CG12942 gene product from transcript CG12942-RA	DmelCG12942	0	0
4222	36159	Drosophila melanogaster	CG7712	CG7712 gene product from transcript CG7712-RA	BcDNA:RE25411|DmelCG7712	0	0
4223	36160	Drosophila melanogaster	Rpb5	CG11979 gene product from transcript CG11979-RA	CG11979|DmRPB5|DmelCG11979|RPB5	0	0
4224	36161	Drosophila melanogaster	CG12943	CG12943 gene product from transcript CG12943-RA	DmelCG12943	0	0
4225	36162	Drosophila melanogaster	Obp47a	Odorant-binding protein 47a	47a|CG12944|DmelCG12944	0	0
4226	36163	Drosophila melanogaster	Spn47C	Serpin 47C	CG7722|DmelCG7722|spn47C	0	0
4227	36167	Drosophila melanogaster	BBS4	CG13232 gene product from transcript CG13232-RA	CG13232|DmelCG13232	0	0
4228	36168	Drosophila melanogaster	CG13229	CG13229 gene product from transcript CG13229-RA	Dm CG13229|DmelCG13229|anon-WO0170980.142|anon-WO0170980.143	0	0
4229	36169	Drosophila melanogaster	CG13231	CG13231 gene product from transcript CG13231-RA	DmelCG13231	0	0
4230	36170	Drosophila melanogaster	CG12391	CG12391 gene product from transcript CG12391-RA	DmelCG12391|anon-WO0140519.201	0	0
4231	36171	Drosophila melanogaster	shn	schnurri	BEST:SD06302|CG7734|DmelCG7734|EP(2)2359|EP2359|SD06302|SHN|Shn|l(2)04738|quo	0	0
4232	36172	Drosophila melanogaster	CG9084	CG9084 gene product from transcript CG9084-RB	DmelCG9084	0	0
4233	36173	Drosophila melanogaster	Syx7	Syntaxin 7	Avl|CG5081|Dm Syx7|DmSyx7|DmelCG5081|SYX7|Syx|Syx7/Avl|avl|dSyn7|syx7|syx7/avl	0	0
4234	36174	Drosophila melanogaster	CG7737	CG7737 gene product from transcript CG7737-RA	DmelCG7737	0	0
4235	36176	Drosophila melanogaster	metro	menage a trois	CG13219|CG18338|CG30021|DmelCG30021|FBgn0050021|Metro|skf	0	0
4236	36177	Drosophila melanogaster	CG13218	CG13218 gene product from transcript CG13218-RA	DmelCG13218	0	0
4237	36178	Drosophila melanogaster	CG13228	CG13228 gene product from transcript CG13228-RA	DmelCG13228	0	0
4238	36179	Drosophila melanogaster	CG13227	CG13227 gene product from transcript CG13227-RA	DmelCG13227	0	0
4239	36180	Drosophila melanogaster	CG13217	CG13217 gene product from transcript CG13217-RA	BcDNA:RE32413|DmelCG13217	0	0
4240	36181	Drosophila melanogaster	CG13216	CG13216 gene product from transcript CG13216-RA	DmelCG13216	0	0
4241	36182	Drosophila melanogaster	CG13215	CG13215 gene product from transcript CG13215-RA	DmelCG13215	0	0
4242	36183	Drosophila melanogaster	Listericin	CG9080 gene product from transcript CG9080-RA	146991_at|BcDNA:RH62530|CG9080|DmelCG9080	0	0
4243	36184	Drosophila melanogaster	CG13226	CG13226 gene product from transcript CG13226-RA	DmelCG13226	0	0
4244	36186	Drosophila melanogaster	CG34227	CG34227 gene product from transcript CG34227-RA	CG7738|DmelCG34227	0	0
4245	36187	Drosophila melanogaster	Or47a	Odorant receptor 47a	47E.1|47a|AN10|CG13225|DOR24|DOR47E.1|DmelCG13225|OR47a|Or24|Or47E.1|dor24	0	0
4246	36188	Drosophila melanogaster	Cpr47Ea	Cuticular protein 47Ea	CG9079|DmelCpr47Ea|DmelCG9079	0	0
4247	36189	Drosophila melanogaster	Cpr47Eb	Cuticular protein 47Eb	CG13224|DmelCpr47Eb|DmelCG13224	0	0
4248	36190	Drosophila melanogaster	CG13223	CG13223 gene product from transcript CG13223-RA	DmelCG13223	0	0
4249	36191	Drosophila melanogaster	Cpr47Ec	Cuticular protein 47Ec	CG9077|Cpr47eg|DmelCpr47Ec|DmelCG9077	0	0
4250	36192	Drosophila melanogaster	Cpr47Ed	Cuticular protein 47Ed	CG9076|DmelCpr47Ed|DmelCG9076	0	0
4251	36193	Drosophila melanogaster	Cpr47Ee	Cuticular protein 47Ee	CG13222|DmelCpr47Ee|DmelCG13222	0	0
4252	36194	Drosophila melanogaster	Cpr47Ef	Cuticular protein 47Ef	CG13214|DmelCpr47Ef|DmelCG13214	0	0
4253	36195	Drosophila melanogaster	TpnC47D	Troponin C at 47D	BcDNA:RE34610|CG9073|DmTnC2|DmTpnC47D|DmTpnCIb|DmelCG9073|TNC|TNC47D|TnC|TnC47D|TpnC Ib|TpnCIb	0	0
4254	36196	Drosophila melanogaster	Cpr47Eg	Cuticular protein 47Eg	BcDNA:RE09259|CG9070|DmelCG9070|Lcp2a	0	0
4255	36197	Drosophila melanogaster	CG9067	CG9067 gene product from transcript CG9067-RA	DmelCG9067	0	0
4256	36199	Drosophila melanogaster	CG9062	CG9062 gene product from transcript CG9062-RB	DmelCG9062	0	0
4257	36200	Drosophila melanogaster	CG13220	CG13220 gene product from transcript CG13220-RA	DmelCG13220	0	0
4258	36201	Drosophila melanogaster	fbl6	CG13213 gene product from transcript CG13213-RB	CG13213|DmelCG13213	0	0
4259	36202	Drosophila melanogaster	CG18335	CG18335 gene product from transcript CG18335-RA	DmelCG18335	0	0
4260	36203	Drosophila melanogaster	dare	defective in the avoidance of repellents	AR|CG12390|Dare|DmelCG12390|W8	0	0
4261	36205	Drosophila melanogaster	CG42336	CG42336 gene product from transcript CG42336-RB	BcDNA:AT13707|CG13210|CG13211|DmelCG42336|Dmel_CG13210|Dmel_CG13211	0	0
4262	36207	Drosophila melanogaster	Obp47b	Odorant-binding protein 47b	CG13208|DmelCG13208	0	0
4263	36208	Drosophila melanogaster	CG7741	CG7741 gene product from transcript CG7741-RA	DmelCG7741	0	0
4264	36209	Drosophila melanogaster	Fpps	Farnesyl pyrophosphate synthase	CG12389|DmFPPS|DmelCG12389|FPS|fpps|fps|l(2)k06103|v(2)k03514	0	0
4265	36210	Drosophila melanogaster	CG7745	CG7745 gene product from transcript CG7745-RA	DmelCG7745	0	0
4266	36212	Drosophila melanogaster	Or47b	Odorant receptor 47b	47E.2|47b|CG13206|DOR25|DOR47E.2|DmelCG13206|OR 47b|OR47b|Or47E.2	0	0
4267	36213	Drosophila melanogaster	sha	shavenoid	CG13209|DmelCG13209|koj	0	0
4268	36214	Drosophila melanogaster	lambdaTry	CG12350 gene product from transcript CG12350-RA	CG12350|DmelCG12350|SP76	0	0
4269	36215	Drosophila melanogaster	kappaTry	CG12388 gene product from transcript CG12388-RB	CG12388|DmelCG12388|SPH109	0	0
4270	36216	Drosophila melanogaster	zetaTry	zetaTrypsin	CG12387|DmZetaTry|DmelCG12387|SP110	0	0
4271	36217	Drosophila melanogaster	etaTry	etaTrypsin	169C4S|CG12386|DmEtaTry|DmelCG12386|ESTS:169C4S|SP57|anon-sts17	0	0
4272	36218	Drosophila melanogaster	thetaTry	thetaTrypsin	CG12385|DmThetaTry|DmelCG12385|SP139	0	0
4273	36221	Drosophila melanogaster	gammaTry	gammaTrypsin	BcDNA:RE40325|CG30028|DmGammaTry|DmelCG30028|SPH180|gamma|gamma-Try	0	0
4274	36222	Drosophila melanogaster	CG13202	CG13202 gene product from transcript CG13202-RA	DmelCG13202	0	0
4275	36223	Drosophila melanogaster	iotaTry	iotaTrypsin	143775_at|CG7754|DmelCG7754|SP129	0	0
4276	36224	Drosophila melanogaster	CG12384	CG12384 gene product from transcript CG12384-RA	BcDNA:GH16986|BcDNA:RH17411|DmelCG12384	0	0
4277	36227	Drosophila melanogaster	CG13204	CG13204 gene product from transcript CG13204-RA	DmelCG13204	0	0
4278	36229	Drosophila melanogaster	CG13203	CG13203 gene product from transcript CG13203-RC	DmelCG13203|anon-EST:Posey175	0	0
4279	36230	Drosophila melanogaster	Tsp47F	Tetraspanin 47F	CG9033|Dm.Tsp47F|DmelCG9033	0	0
4280	36232	Drosophila melanogaster	CG9027	CG9027 gene product from transcript CG9027-RD	CG13200|CT25938|DmelCG9027|SOD3	0	0
4281	36233	Drosophila melanogaster	CG30022	CG30022 gene product from transcript CG30022-RB	BcDNA:RE56416|CG9026|DmelCG30022	0	0
4282	36234	Drosophila melanogaster	CG7759	CG7759 gene product from transcript CG7759-RA	DmelCG7759	0	0
4283	36235	Drosophila melanogaster	CG7763	CG7763 gene product from transcript CG7763-RA	BEST:CK02422|CK02422A|CK02422a|DmelCG7763	0	0
4284	36236	Drosophila melanogaster	Drip	CG9023 gene product from transcript CG9023-RA	CG9023|DRIP|DmelCG9023|I|anon-48Aa|drip	0	0
4285	36237	Drosophila melanogaster	CG7777	CG7777 gene product from transcript CG7777-RB	Aqp7777|DmelCG7777	0	0
4286	36238	Drosophila melanogaster	E(Pc)	Enhancer of Polycomb	CG7776|DmelCG7776|E(PC)|GH05739|GH14582|III|anon-48Ac|e(Pc)|l(2)28-28-12	0	0
4287	36239	Drosophila melanogaster	inv	invected	CG17835|DmelCG17835|Engrailed/Invected|IV|Inv|er|in	0	0
4288	36240	Drosophila melanogaster	en	engrailed	153867_at|Apa|CG9015|DmelCG9015|EN|En|EnR|Eng|Engrailed|Engrailed/Invected|Es|V|en1|spa2|spt	0	0
4289	36241	Drosophila melanogaster	tou	toutatis	CG10897|Dm Tou|DmelCG10897|EP(2)0622|EP0622|EP622|VI|anon-48Ad|gene VI	0	0
4290	36242	Drosophila melanogaster	Egm	Enigma	CG9006|DmelCG9006|egm|l(2)k14708	0	0
4291	36243	Drosophila melanogaster	CG9005	CG9005 gene product from transcript CG9005-RC	BcDNA:GH05710|DmelCG9005	0	0
4292	36244	Drosophila melanogaster	CG9003	CG9003 gene product from transcript CG9003-RD	DmelCG9003	0	0
4293	36245	Drosophila melanogaster	CG13198	CG13198 gene product from transcript CG13198-RA	DmelCG13198	0	0
4294	36246	Drosophila melanogaster	Roc2	CG8998 gene product from transcript CG8998-RA	BcDNA:RE61847|CG8998|DmelCG8998|Roc|dRoc2|roc2	0	0
4295	36248	Drosophila melanogaster	Tret1-1	Trehalose transporter 1-1	CG30035|CG7797|CG7801|DmTret1-1|DmelCG30035	0	0
4296	36249	Drosophila melanogaster	Tret1-2	Trehalose transporter 1-2	CG8234|DmTret1-2|DmelCG8234	0	0
4297	36250	Drosophila melanogaster	CG13196	CG13196 gene product from transcript CG13196-RA	DmelCG13196	0	0
4298	36251	Drosophila melanogaster	Buffy	CG8238 gene product from transcript CG8238-RA	BUFFY|Bcl-2-48AE|Buffy/Borg2|Buffy/DBorg-2|Buffy/dBorg-2|CG8238|Dbx|DmelCG8238|Drob2|buffy|dBORG-2/BUFFY|dBorg-2|dBorg2|dbuffy	0	0
4299	36252	Drosophila melanogaster	wal	walrus	BcDNA.LD07532|BcDNA:GH09945|BcDNA:LD07532|CG8996|DmelCG8996|EP2253|Etf|P447|l(2)02516	0	0
4300	36253	Drosophila melanogaster	CG13197	CG13197 gene product from transcript CG13197-RA	BcDNA:RE27552|DmelCG13197	0	0
4301	36254	Drosophila melanogaster	Ir48b	Ionotropic receptor 48b	CG13195|DmelCG13195|IR48b	0	0
4302	36255	Drosophila melanogaster	pyr	pyramus	CG13194|DFGF8-2|DmPyr|DmelCG13194|FGF8-like2|Pyr|fgf8-like	0	0
4303	36256	Drosophila melanogaster	CG13193	CG13193 gene product from transcript CG13193-RA	DmelCG13193	0	0
4304	36257	Drosophila melanogaster	Tango3	Transport and Golgi organization 3	CG12444|DmelCG12444|IR48c|TANGO3	0	0
4305	36258	Drosophila melanogaster	ths	thisbe	CG12443|DFGF8-1|DmThs|DmelCG12443|FGF8-like1|Neu4|Ths|fgf8-like	0	0
4306	36259	Drosophila melanogaster	CG13192	CG13192 gene product from transcript CG13192-RA	DmelCG13192	0	0
4307	36260	Drosophila melanogaster	Sobp	Sine oculis-binding protein	CG8991|DmelCG8991|Sbp	0	0
4308	36262	Drosophila melanogaster	S2P	site-2 protease	CG8988|DmelCG8988|anon-WO0118547.346|dS2P|ds2p	0	0
4309	36263	Drosophila melanogaster	Sln	Silnoon	CG8271|DmelCG8271	0	0
4310	36264	Drosophila melanogaster	Mtor	Megator	Bx34|CG8274|DmelCG8274|MTOR|TPR|Tpr|l(2)k03905	0	0
4311	36265	Drosophila melanogaster	TwdlBeta	CG8986 gene product from transcript CG8986-RA	CG8986|CT25838|DmelCG8986	0	0
4312	36266	Drosophila melanogaster	Damm	Death associated molecule related to Mch2	CG18188|DAMM|Damm/Daydream|Daydream|DmelCG18188|damm	0	0
4313	36268	Drosophila melanogaster	CG13185	CG13185 gene product from transcript CG13185-RB	CG18329|DmelCG13185	0	0
4314	36269	Drosophila melanogaster	cuff	cutoff	CG13190|DmelCG13190	0	0
4315	36270	Drosophila melanogaster	ERp60	CG8983 gene product from transcript CG8983-RA	CG8983|D-ERp60|DmelCG8983|Erp60|Ov6|anon-EST:fe3D9	0	0
4316	36271	Drosophila melanogaster	Ef1alpha48D	Elongation factor 1alpha48D	1275|CG8280|Dm0084|DmEF1|DmelCG8280|EF|EF-1|EF-1[a]|EF-1a|EF-1alpha|EF-1alpha F1|EF-1alphaF|EF-1alphaF1|EF-1alphaF[[1]]|EF1-alpha|EF1alpha|EF1alpha 48D|EF1alpha48D|Ef1a10|Ef1a48D|Ef1	0	0
4317	36272	Drosophila melanogaster	MCPH1	Microcephalin	CG13184|CG30038|CG42572|CG8981|Dmcph1|DmelCG42572|Dmel_CG30038|Dmel_CG8981|awol|mcph1	0	0
4318	36273	Drosophila melanogaster	CG13189	CG13189 gene product from transcript CG13189-RA	DmelCG13189	0	0
4319	36275	Drosophila melanogaster	CG13183	CG13183 gene product from transcript CG13183-RA	DmelCG13183	0	0
4320	36276	Drosophila melanogaster	CG13188	CG13188 gene product from transcript CG13188-RB	DmelCG13188	0	0
4321	36277	Drosophila melanogaster	CG8979	CG8979 gene product from transcript CG8979-RC	8979|DmelCG8979|cg8979	0	0
4322	36278	Drosophila melanogaster	CG8290	CG8290 gene product from transcript CG8290-RA	BEST:LD29214|DmelCG8290|LD29214	0	0
4323	36279	Drosophila melanogaster	CG33145	CG33145 gene product from transcript CG33145-RA	CG8976|DmelCG33145	0	0
4324	36280	Drosophila melanogaster	RnrS	Ribonucleoside diphosphate reductase small subunit	CG8975|DmRNR2|DmelCG8975|RNR2|RNrS|Rnr2|Rnrs|anon-WO0118547.148|dRNR2|rnr2	0	0
4325	36281	Drosophila melanogaster	rho-7	rhomboid-7	CG8972|DmelCG8972|PARL|Rhomboid-7|anon-WO0138581.3	0	0
4326	36282	Drosophila melanogaster	CG8298	CG8298 gene product from transcript CG8298-RC	DmelCG8298	0	0
4327	36283	Drosophila melanogaster	otk	off-track	CG8967|CT25769|DTrk|DmelCG8967|Dtkr|Dtrk|IGGtyk|OFT/TRK|OTK|Otk|Tk48D|Trk48D|anon-WO2004063362.81|dTrk	0	0

4328	36284	Drosophila melanogaster	CG8964	CG8964 gene product from transcript CG8964-RA	CT25760|DmelCG8964	0	0
4329	36285	Drosophila melanogaster	CG8889	CG8889 gene product from transcript CG8889-RC	11471|DmelCG8889|GS11471	0	0
4330	36286	Drosophila melanogaster	pds5	CG17509 gene product from transcript CG17509-RB	CG17509|DmelCG17509|Pds5|anon-WO0118547.183|dPds5|l(2)k13312	0	0
4331	36287	Drosophila melanogaster	CG8321	CG8321 gene product from transcript CG8321-RA	DmelCG8321	0	0
4332	36288	Drosophila melanogaster	128up	upstream of RpIII128	CG8340|DmelCG8340|GTP-bp|X71866	0	0
4333	36289	Drosophila melanogaster	RpIII128	RNA polymerase III 128kD subunit	CG8344|DmRP128|DmelCG8344|RP128	0	0
4334	36290	Drosophila melanogaster	Drep-1	DNA fragmentation factor-related protein 1	CG8357|DREP-1|DREP-1/dICAD|DmelCG8357|Drep1|ICAD|ICAD/DREP-1|Rep1|dICAD|drep1	0	0
4335	36292	Drosophila melanogaster	Drep-3	DNA fragmentation factor-related protein 3	BcDNA:AT08574|CG13187|CG8364|DREP-3|DmelCG8364|Drep3|Rep3|drep3	0	0
4336	36293	Drosophila melanogaster	CG8888	CG8888 gene product from transcript CG8888-RA	DmelCG8888	0	0
4337	36294	Drosophila melanogaster	CG13186	CG13186 gene product from transcript CG13186-RA	AAF58585|BcDNA:AT11581|DmelCG13186	0	0
4338	36295	Drosophila melanogaster	jeb	jelly belly	CG13180|CG13182|CG30040|DmelCG30040|Jeb|l(2)SH0422|l(2)k05644	0	0
4339	36297	Drosophila melanogaster	CG18343	CG18343 gene product from transcript CG18343-RA	DmelCG18343	0	0
4340	36298	Drosophila melanogaster	skpB	CG8881 gene product from transcript CG8881-RA	CG8881|DmelCG8881|Skp|dskpB	0	0
4341	36299	Drosophila melanogaster	CG8378	CG8378 gene product from transcript CG8378-RA	BcDNA:LD29892|DmelCG8378	0	0
4342	36300	Drosophila melanogaster	CG13178	CG13178 gene product from transcript CG13178-RA	DmelCG13178	0	0
4343	36301	Drosophila melanogaster	Hen1	CG12367 gene product from transcript CG12367-RA	CG12367|DmHen1|DmelCG12367|FBgn 33686|Pimet|hen-1|hen1	0	0
4344	36302	Drosophila melanogaster	CG8878	CG8878 gene product from transcript CG8878-RA	BcDNA:LD23371|DmelCG8878|VRK	0	0
4345	36303	Drosophila melanogaster	CG8407	CG8407 gene product from transcript CG8407-RB	DmelCG8407	0	0
4346	36304	Drosophila melanogaster	Prp8	pre-mRNA processing factor 8	CG8877|DmelCG8877|E-2e|prp8	0	0
4347	36306	Drosophila melanogaster	SmD3	Small ribonucleoprotein particle protein SmD3	CG8427|DmelCG8427|SYM10|Sm D3|dD3|guf|guf2|l(2)131/7|l(2)154/1|l(2)90/37|smD3|snRNP Sm D3	0	0
4348	36307	Drosophila melanogaster	Oda	Ornithine decarboxylase antizyme	AZ|CG16747|D-AZ|DmelCG16747|ODA|guf|l(2)02833|l(2)k09037|oda	0	0
4349	36308	Drosophila melanogaster	Cct5	T-complex Chaperonin 5	BcDNA.GM12270|BcDNA:GM12270|CCT5|CG8439|DmelCG8439|TCP-1epsilon|l(2)06444	0	0
4350	36309	Drosophila melanogaster	EndoG	Endonuclease G	CG8862|DmelCG8862	0	0
4351	36310	Drosophila melanogaster	CG8860	CG8860 gene product from transcript CG8860-RA	BcDNA:GM14157|DmelCG8860	0	0
4352	36311	Drosophila melanogaster	wash	washout	CG13176|DmelCG13176|p63|p65	0	0
4353	36314	Drosophila melanogaster	SmF	Small ribonucleoprotein particle protein SmF	BcDNA:GM23968|CG16792|Deb|Deb-B|DebB|DmelCG16792|dF|deb-b|snRNP-F	0	0
4354	36316	Drosophila melanogaster	Cyp6g1	CG8453 gene product from transcript CG8453-RA	6g1|CG8453|CYP6G1|Cyp6-like|Cyp6gl|DDT-R|DmelCG8453|RDDT|Rl|Rl[DDT]|Rl[II]|Rst(2)DDT|anon-WO03025223.16|anon-WO03025223.17|cyp6g1	0	0
4355	36317	Drosophila melanogaster	Cyp6g2	CG8859 gene product from transcript CG8859-RA	6g2|CG8859|DmelCG8859	0	0
4356	36318	Drosophila melanogaster	Cyp6t3	CG8457 gene product from transcript CG8457-RA	6t3|CG8457|DmelCG8457	0	0
4357	36319	Drosophila melanogaster	CG8858	CG8858 gene product from transcript CG8858-RA	DmelCG8858	0	0
4358	36321	Drosophila melanogaster	RpS11	Ribosomal protein S11	CG8857|DmelCG8857|M(2)48E|S11|anon-EST:fe1D2|anon-MMS23|clone 23	0	0
4359	36323	Drosophila melanogaster	CG13171	CG13171 gene product from transcript CG13171-RA	DmelCG13171	0	0
4360	36324	Drosophila melanogaster	CG8854	CG8854 gene product from transcript CG8854-RB	DmelCG8854	0	0
4361	36325	Drosophila melanogaster	CG13170	CG13170 gene product from transcript CG13170-RA	DmelCG13170	0	0
4362	36326	Drosophila melanogaster	CG43316	CG43316 gene product from transcript CG43316-RA	CG13169|DmelCG43316|Dmel_CG13169	0	0
4363	36327	Drosophila melanogaster	CG13168	CG13168 gene product from transcript CG13168-RC	DmelCG13168	0	0
4364	36328	Drosophila melanogaster	Vha36-2	Vacuolar H[+] ATPase subunit 36-2	CG13167|DmelCG13167|vha36-2	0	0
4365	36329	Drosophila melanogaster	Cam	Calmodulin	3909|CAM|CG8472|CaM|Cal|Cal49A|Cam1|D-CaM|DmCalm|DmelCG8472|anon-EST:Posey59|cam|dCaM|l(2)03909|l(2)3909	0	0
4366	36330	Drosophila melanogaster	CG42700	CG42700 gene product from transcript CG42700-RC	CG13165|CG34233|DmelCG42700|Dmel_CG13165|Dmel_CG34233|ORE-16	0	0
4367	36331	Drosophila melanogaster	CG8850	CG8850 gene product from transcript CG8850-RA	DmNAT4|DmelCG8850	0	0
4368	36332	Drosophila melanogaster	SIP2	Syntaxin Interacting Protein 2	CG13164|DmelCG13164|Sip2	0	0
4369	36333	Drosophila melanogaster	CG17739	CG17739 gene product from transcript CG17739-RA	CT39321|DmelCG17739|anon-WO0153538.35	0	0
4370	36334	Drosophila melanogaster	CG30046	CG30046 gene product from transcript CG30046-RB	CG17740|CT39323|DmelCG30046	0	0
4371	36335	Drosophila melanogaster	CG13163	CG13163 gene product from transcript CG13163-RA	DmelCG13163	0	0
4372	36336	Drosophila melanogaster	CG8841	CG8841 gene product from transcript CG8841-RC	DmelCG8841	0	0
4373	36337	Drosophila melanogaster	garz	gartenzwerg	CG 8487|CG8487|DmelCG8487|GBF1	0	0
4374	36338	Drosophila melanogaster	CG8490	CG8490 gene product from transcript CG8490-RA	DmelCG8490	0	0
4375	36339	Drosophila melanogaster	Den1	Deneddylase 1	CG8493|DEN1|DmelCG8493	0	0
4376	36340	Drosophila melanogaster	CG8839	CG8839 gene product from transcript CG8839-RA	DmelCG8839	0	0
4377	36341	Drosophila melanogaster	ana3	anastral spindle 3	Ana3|CG13162|DmelCG13162	0	0
4378	36342	Drosophila melanogaster	CG33012	CG33012 gene product from transcript CG33012-RA	CG13161|CG30041|DmelCG33012	0	0
4379	36343	Drosophila melanogaster	CG13160	CG13160 gene product from transcript CG13160-RA	DmelCG13160	0	0
4380	36344	Drosophila melanogaster	CG13159	CG13159 gene product from transcript CG13159-RA	BcDNA:RE25177|DmelCG13159	0	0
4381	36345	Drosophila melanogaster	Cpr49Ab	Cuticular protein 49Ab	CG30042|CG8496|DmelCpr49Ab|DmelCG30042	0	0
4382	36346	Drosophila melanogaster	CG13155	CG13155 gene product from transcript CG13155-RA	DmelCG13155	0	0
4383	36347	Drosophila melanogaster	CG8501	CG8501 gene product from transcript CG8501-RA	DmelCG8501	0	0
4384	36348	Drosophila melanogaster	Cpr49Ac	Cuticular protein 49Ac	CG8502|DmelCG8502	0	0
4385	36349	Drosophila melanogaster	Cpr49Ad	Cuticular protein 49Ad	CG8836|DmelCG8836	0	0
4386	36350	Drosophila melanogaster	Or49a	Odorant receptor 49a	49A.1|49a|CG13158|DOR10|DmelCG13158|OR49a	0	0
4387	36351	Drosophila melanogaster	Cpr49Ae	Cuticular protein 49Ae	CG8505|DmelCpr49Ae|DmelCG8505	0	0
4388	36352	Drosophila melanogaster	Cpr49Af	Cuticular protein 49Af	CG8510|DmelCpr49Af|DmelCG8510	0	0
4389	36353	Drosophila melanogaster	Cpr49Ag	Cuticular protein 49Ag	CG8511|DmelCpr49Ag|DmelCG8511	0	0
4390	36354	Drosophila melanogaster	Cpr49Ah	Cuticular protein 49Ah	CG8515|DmelCpr49Ah|DmelCG8515	0	0
4391	36355	Drosophila melanogaster	CG13157	CG13157 gene product from transcript CG13157-RA	DmelCG13157	0	0
4392	36356	Drosophila melanogaster	CG8834	CG8834 gene product from transcript CG8834-RB	DmelCG8834	0	0
4393	36357	Drosophila melanogaster	CG8520	CG8520 gene product from transcript CG8520-RA	DmelCG8520|FBgn 33734	0	0
4394	36358	Drosophila melanogaster	CG8525	CG8525 gene product from transcript CG8525-RA	DmelCG8525	0	0
4395	36359	Drosophila melanogaster	CG13154	CG13154 gene product from transcript CG13154-RA	DmelCG13154	0	0
4396	36360	Drosophila melanogaster	Nup54	Nucleoporin 54	CG8831|DmelCG8831|nup54	0	0
4397	36361	Drosophila melanogaster	CG8830	CG8830 gene product from transcript CG8830-RB	DmelCG8830	0	0
4398	36362	Drosophila melanogaster	Dyb	Dystrobrevin-like	CG8529|DmDYB|DmelCG8529	0	0
4399	36363	Drosophila melanogaster	Lac	Lachesin	CG12369|CT24921|DmelCG12369|bulb|l(2)k11012|l(2)k11012b|lac	0	0
4400	36364	Drosophila melanogaster	dgt5	dim gamma-tubulin 5	CG8828|Dgt5|DmelCG8828|msd2	0	0
4401	36365	Drosophila melanogaster	CG8545	CG8545 gene product from transcript CG8545-RA	DmelCG8545	0	0
4402	36366	Drosophila melanogaster	CG8550	CG8550 gene product from transcript CG8550-RA	DmelCG8550	0	0
4403	36368	Drosophila melanogaster	Dh44-R2	Diuretic hormone 44 receptor 2	CG12370|CG13156|DH44-R2|DmelCG12370|anon-WO0170980.103|anon-WO0170980.104	0	0
4404	36371	Drosophila melanogaster	s-cup	stanley-cup	CG13152|CG13153|CG30044|DmelCG30044	0	0
4405	36372	Drosophila melanogaster	vis	vismay	CG8821|DmelCG8821|TGIFa|achi/vis|dTGIFa|zaa	0	0
4406	36373	Drosophila melanogaster	achi	achintya	Achi|CG8819|DmelCG8819|ach|achi/vis|dTGIFb|zaa	0	0
4407	36374	Drosophila melanogaster	CG13151	CG13151 gene product from transcript CG13151-RA	DmelCG13151	0	0
4408	36375	Drosophila melanogaster	CG8818	CG8818 gene product from transcript CG8818-RA	DmelCG8818	0	0
4409	36376	Drosophila melanogaster	CG8569	CG8569 gene product from transcript CG8569-RA	DmelCG8569	0	0
4410	36377	Drosophila melanogaster	fra	frazzled	CG8581|CT24981|DCC|DmelCG8581|Fra	0	0
4411	36378	Drosophila melanogaster	Cyp301a1	CG8587 gene product from transcript CG8587-RA	301a1|CG8587|DmelCG8587	0	0
4412	36379	Drosophila melanogaster	Ak6	Adenylate kinase 6	CG8816|DAK6|DmelCG8816	0	0
4413	36380	Drosophila melanogaster	stil	stand still	152072_at|CG8592|DmelCG8592|Y10276	0	0
4414	36381	Drosophila melanogaster	ClC-b	Chloride channel-b	CG8594|DmClC-b|DmClCb|DmelCG8594	0	0
4415	36382	Drosophila melanogaster	Sin3A	CG8815 gene product from transcript CG8815-RC	159A11T|27/3|97/15|CG8815|DmelCG8815|ES2-3|ESTS:159A11T|EY2-9|SIN3|SIN3A|Sin 3A|Sin3|Sin3a|dSin3|dSin3A|l(2)08269|l(2)k02703|l(2)k05415|l(2)k09715|ld|sin3|sin3A|sni3	0	0
4416	36383	Drosophila melanogaster	Amph	Amphiphysin	Amp|CG8604|DAMP|Damp|DmelCG8604|amph|dAmph|damph	0	0
4417	36384	Drosophila melanogaster	Galpha49B	G protein alpha49B	CG17759|DGalpha[[q]]|DGalphaq|DGq|DGqalpha|Dgq|DmGalpha[[q]]|DmelCG17759|Dromel_CG17759_galpha49b_mORF|G alpha 49B|G?q|G[[a]]q|G[[alphaq]]|G[[q]]|G[[q]]alpha|G[[qalpha]]|G[q]|Galpha|Gal	0	0
4418	36385	Drosophila melanogaster	CG17760	CG17760 gene product from transcript CG17760-RB	BcDNA:SD21019|DmelCG17760	0	0
4419	36386	Drosophila melanogaster	muskelin	CG8811 gene product from transcript CG8811-RB	CG8811|DmMK|DmelCG8811	0	0
4420	36387	Drosophila melanogaster	spt4	CG12372 gene product from transcript CG12372-RA	142904_at|CG12372|DSIF|DmelCG12372|Dspt4|SPT4|Spt4|dDSIF|dspt4|l(2)k05316	0	0
4421	36390	Drosophila melanogaster	Iswi	Imitation SWI	ACF|CG8625|CHRAC|DmelCG8625|ISW|ISWI|NURF|NURF-140|Nurf-140|SWI|anon-EP1279744.124|dCHRAC|dISWI|dNURF|iswi|p140|p140/ISWI	0	0
4422	36391	Drosophila melanogaster	CG8785	CG8785 gene product from transcript CG8785-RA	DmelCG8785	0	0
4423	36392	Drosophila melanogaster	CG8778	CG8778 gene product from transcript CG8778-RA	DmelCG8778	0	0
4424	36393	Drosophila melanogaster	CG8632	CG8632 gene product from transcript CG8632-RA	DmelCG8632	0	0
4425	36394	Drosophila melanogaster	CG8646	CG8646 gene product from transcript CG8646-RB	DmelCG8646	0	0
4426	36395	Drosophila melanogaster	nemy	no extended memory	CG13147|CG8776|CytB561|DmelCG8776|Dmel_CG8772|anon-WO0140519.146	0	0
4427	36396	Drosophila melanogaster	CG42708	CG42708 gene product from transcript CG42708-RA	CG8772|CG8872|DmelCG42708|Dmel_CG8772	0	0
4428	36397	Drosophila melanogaster	CG8771	CG8771 gene product from transcript CG8771-RB	DmelCG8771	0	0
4429	36398	Drosophila melanogaster	CG13148	CG13148 gene product from transcript CG13148-RA	DmelCG13148	0	0
4430	36399	Drosophila melanogaster	Obp49a	Odorant-binding protein 49a	BcDNA:GH25962|CG30052|CG8769|DmelCG30052	0	0
4431	36400	Drosophila melanogaster	CG8768	CG8768 gene product from transcript CG8768-RA	DmelCG8768	0	0
4432	36401	Drosophila melanogaster	wuc	Wake-up-call	147114_at|BcDNA:AT27450|CG12442|DmelCG12442	0	0
4433	36403	Drosophila melanogaster	CG42663	CG42663 gene product from transcript CG42663-RB	BcDNA:SD15892|CG12488|CG13486|CG18356|DmelCG42663|Dmel_CG12488|Dmel_CG18356	0	0
4434	36404	Drosophila melanogaster	mos	CG8767 gene product from transcript CG8767-RA	CG8767|D-mos|DMOS|DmelCG8767|MOS|dmos	0	0
4435	36405	Drosophila melanogaster	Taz	Tafazzin	CG8766|DmelCG8766|TAZ|dTAZ|tafazzin	0	0
4436	36406	Drosophila melanogaster	mRpL18	mitochondrial ribosomal protein L18	CG12373|DmelCG12373|HL|MRP-L18|anon-49Ca	0	0
4437	36408	Drosophila melanogaster	Dgkepsilon	Diacyl glycerol kinase epsilon	CG8657|DGKe|DGKepsilon|DmelCG8657	0	0
4438	36409	Drosophila melanogaster	Nacalpha	Nascent polypeptide associated complex protein alph	CG8759|DmelCG8759|NAC|NACalpha|aic|alpha-NAC|alphaNAC|l(2)04329	0	0

<table>
<tr><td>4439</td><td>36410</td><td>Drosophila melanogaster</td><td>CG12374</td><td>CG12374 gene product from transcript CG12374-RA</td><td>DmelCG12374|anon-WO0140519.63</td><td>0</td><td>0</td></tr>
<tr><td>4440</td><td>36411</td><td>Drosophila melanogaster</td><td>sca</td><td>scabrous</td><td>CG17579|DmelCG17579|FBpp0086968|FBpp0086969|Sca|Sca1b|anon-EST:Liang-38|clone 38|mAb sca1</td><td>0</td><td>0</td></tr>
<tr><td>4441</td><td>36412</td><td>Drosophila melanogaster</td><td>Cyp9h1</td><td>CG17577 gene product from transcript CG17577-RA</td><td>9h1|CG17577|DmelCG17577</td><td>0</td><td>0</td></tr>
<tr><td>4442</td><td>36413</td><td>Drosophila melanogaster</td><td>Or49b</td><td>Odorant receptor 49b</td><td>49D.1|49b|AN13|CG17584|DOR105|DmelCG17584</td><td>0</td><td>0</td></tr>
<tr><td>4443</td><td>36414</td><td>Drosophila melanogaster</td><td>CG17575</td><td>CG17575 gene product from transcript CG17575-RA</td><td>DmelCG17575|anon-SAGE:Wang-113</td><td>0</td><td>0</td></tr>
<tr><td>4444</td><td>36415</td><td>Drosophila melanogaster</td><td>CG17574</td><td>CG17574 gene product from transcript CG17574-RA</td><td>BcDNA:RH03863|DmelCG17574</td><td>0</td><td>0</td></tr>
<tr><td>4445</td><td>36417</td><td>Drosophila melanogaster</td><td>CG3790</td><td>CG3790 gene product from transcript CG3790-RA</td><td>DmelCG3790</td><td>0</td><td>0</td></tr>
<tr><td>4446</td><td>36418</td><td>Drosophila melanogaster</td><td>CG3814</td><td>CG3814 gene product from transcript CG3814-RA</td><td>BEST:CK02213|CK02213|DmelCG3814</td><td>0</td><td>0</td></tr>
<tr><td>4447</td><td>36419</td><td>Drosophila melanogaster</td><td>Nmda1</td><td>N-methyl-D-aspartate receptor-associated protein</td><td>CG3798|DRONMDA|DmelCG3798|NMDARA1|cDNA2|dNMDARA1</td><td>0</td><td>0</td></tr>
<tr><td>4448</td><td>36420</td><td>Drosophila melanogaster</td><td>Aats-asp</td><td>Aspartyl-tRNA synthetase</td><td>Aats-Asp|AspRS|CG3821|DRS|DmelCG3821|aats-asp|anon-EST:Liang-2.49|cDNA1|clone 2.49|l(2)49Db|l(2)k04508|l(2)v27|l(2)vr1|poney|vr1</td><td>0</td><td>0</td></tr>
<tr><td>4449</td><td>36421</td><td>Drosophila melanogaster</td><td>vg</td><td>vestigial</td><td>CG3830|DmelCG3830|VG|Vg|vg21</td><td>0</td><td>0</td></tr>
<tr><td>4450</td><td>36423</td><td>Drosophila melanogaster</td><td>CG13319</td><td>CG13319 gene product from transcript CG13319-RA</td><td>BcDNA:GH19585|DmelCG13319</td><td>0</td><td>0</td></tr>
<tr><td>4451</td><td>36424</td><td>Drosophila melanogaster</td><td>sug</td><td>sugarbabe</td><td>CG3850|DmelCG3850</td><td>0</td><td>0</td></tr>
<tr><td>4452</td><td>36425</td><td>Drosophila melanogaster</td><td>CG17019</td><td>CG17019 gene product from transcript CG17019-RA</td><td>DmelCG17019</td><td>0</td><td>0</td></tr>
<tr><td>4453</td><td>36426</td><td>Drosophila melanogaster</td><td>CG13322</td><td>CG13322 gene product from transcript CG13322-RA</td><td>DmelCG13322|anon-WO0118547.137</td><td>0</td><td>0</td></tr>
<tr><td>4454</td><td>36427</td><td>Drosophila melanogaster</td><td>Sans</td><td>Sans ortholog</td><td>CG13320|DSANS|DmelCG13320</td><td>0</td><td>0</td></tr>
<tr><td>4455</td><td>36428</td><td>Drosophila melanogaster</td><td>Mdr49</td><td>Multi drug resistance 49</td><td>CG3879|Dm5|DmelCG3879|MDR49|MDR5|Mes5|mdr49|mrd49</td><td>0</td><td>0</td></tr>
<tr><td>4456</td><td>36429</td><td>Drosophila melanogaster</td><td>CG3884</td><td>CG3884 gene product from transcript CG3884-RA</td><td>DmelCG3884</td><td>0</td><td>0</td></tr>
<tr><td>4457</td><td>36430</td><td>Drosophila melanogaster</td><td>CG13321</td><td>CG13321 gene product from transcript CG13321-RA</td><td>DmelCG13321</td><td>0</td><td>0</td></tr>
<tr><td>4458</td><td>36431</td><td>Drosophila melanogaster</td><td>Psc</td><td>Posterior sex combs</td><td>49Ea|CG3886|D-Bmi|DmelCG3886|PSC|Psc1|Su(z)2-C|dPSC|l(2)49Ea|l(2)Psc|l(2)vr14|psc|vr14</td><td>0</td><td>0</td></tr>
<tr><td>4459</td><td>36432</td><td>Drosophila melanogaster</td><td>Su(z)2</td><td>Suppressor of zeste 2</td><td>Arp|CG3905|DmelCG3905|SU(Z)2|SUZ2|Su(z)[[2]]|l(2)k06344|su(z)2</td><td>0</td><td>0</td></tr>
<tr><td>4460</td><td>36433</td><td>Drosophila melanogaster</td><td>CG13323</td><td>CG13323 gene product from transcript CG13323-RA</td><td>DmelCG13323|FBgn0033788</td><td>0</td><td>0</td></tr>
<tr><td>4461</td><td>36434</td><td>Drosophila melanogaster</td><td>CG13324</td><td>CG13324 gene product from transcript CG13324-RA</td><td>DmelCG13324</td><td>0</td><td>0</td></tr>
<tr><td>4462</td><td>36436</td><td>Drosophila melanogaster</td><td>Drl-2</td><td>Derailed 2</td><td>CG12463|CG3915|DNT-like|DmelCG3915|dnl|drl-2</td><td>0</td><td>0</td></tr>
<tr><td>4463</td><td>36437</td><td>Drosophila melanogaster</td><td>CG13325</td><td>CG13325 gene product from transcript CG13325-RA</td><td>DmelCG13325</td><td>0</td><td>0</td></tr>
<tr><td>4464</td><td>36438</td><td>Drosophila melanogaster</td><td>CG3955</td><td>CG3955 gene product from transcript CG3955-RA</td><td>DmelCG3955</td><td>0</td><td>0</td></tr>
<tr><td>4465</td><td>36439</td><td>Drosophila melanogaster</td><td>CG13326</td><td>CG13326 gene product from transcript CG13326-RA</td><td>DmelCG13326</td><td>0</td><td>0</td></tr>
<tr><td>4466</td><td>36440</td><td>Drosophila melanogaster</td><td>Cap-G</td><td>CG34438 gene product from transcript CG34438-RF</td><td>CAP-G|CG13327|CG17054|CG30057|CG34438|CapG|DCAP-G|DmCAP-G|DmelCG34438|Dmel_CG17054|cap-G|cap-g|dCAP-G|dCap-G|dcap-g|l(2)49Ff|l(2)vr9|vr-9|vr9</td><td>0</td><td>0</td></tr>
<tr><td>4467</td><td>36442</td><td>Drosophila melanogaster</td><td>PR2</td><td>Fak-like tyrosine kinase</td><td>CG3969|DPR2|DRODPR2|DmHD-11|DmelCG3969|HD-11|PR2/ACK|Rp2|dACK</td><td>0</td><td>0</td></tr>
<tr><td>4468</td><td>36444</td><td>Drosophila melanogaster</td><td>TppII</td><td>tripeptidyl-peptidase II</td><td>CG3991|DmelCG3991|TPP II|TPP2|TPPII|anon-WO0118547.127|dTPP II|dTPPII|l(2)k14301</td><td>0</td><td>0</td></tr>
<tr><td>4469</td><td>36445</td><td>Drosophila melanogaster</td><td>Nrk</td><td>Neurospecific receptor kinase</td><td>CG4007|DNRK|DmHD-434|DmelCG4007|Dnrk|HD-434|ROR2|anon-WO2004063362.79|l(2)k14301|nrk|nrtk_dros</td><td>0</td><td>0</td></tr>
<tr><td>4470</td><td>36446</td><td>Drosophila melanogaster</td><td>CG15870</td><td>CG15870 gene product from transcript CG15870-RA</td><td>DmelCG15870</td><td>0</td><td>0</td></tr>
<tr><td>4471</td><td>36447</td><td>Drosophila melanogaster</td><td>GLaz</td><td>Glial Lazarillo</td><td>CG4604|DmelCG4604</td><td>0</td><td>0</td></tr>
<tr><td>4472</td><td>36448</td><td>Drosophila melanogaster</td><td>Spt-I</td><td>Serine palmitoyltransferase subunit I</td><td>CG4016|DmelCG4016|SPT-I|Spt1|l(2)SH1626|l(2)SH2 1626|lcb1</td><td>0</td><td>0</td></tr>
<tr><td>4473</td><td>36449</td><td>Drosophila melanogaster</td><td>CG33137</td><td>CG33137 gene product from transcript CG33137-RA</td><td>CG4023|DmelCG33137</td><td>0</td><td>0</td></tr>
<tr><td>4474</td><td>36454</td><td>Drosophila melanogaster</td><td>lat</td><td>latheo</td><td>CG4088|DmORC3|DmelCG4088|LAT|ORC|ORC3|Orc3|l(2)49Fd|l(2)vr6|vr-6|vr6</td><td>0</td><td>0</td></tr>
<tr><td>4475</td><td>36455</td><td>Drosophila melanogaster</td><td>FLASH</td><td>FLASH ortholog</td><td>CG4616|DmelCG4616|dFLASH</td><td>0</td><td>0</td></tr>
<tr><td>4476</td><td>36456</td><td>Drosophila melanogaster</td><td>AQP</td><td>aquaporin</td><td>AQP-dr|CG12251|DmelCG12251</td><td>0</td><td>0</td></tr>
<tr><td>4477</td><td>36457</td><td>Drosophila melanogaster</td><td>CG4627</td><td>CG4627 gene product from transcript CG4627-RA</td><td>DmelCG4627</td><td>0</td><td>0</td></tr>
<tr><td>4478</td><td>36458</td><td>Drosophila melanogaster</td><td>CG4630</td><td>CG4630 gene product from transcript CG4630-RA</td><td>DmelCG4630</td><td>0</td><td>0</td></tr>
<tr><td>4479</td><td>36459</td><td>Drosophila melanogaster</td><td>CG4646</td><td>CG4646 gene product from transcript CG4646-RA</td><td>BcDNA:SD14289|DmelCG4646</td><td>0</td><td>0</td></tr>
<tr><td>4480</td><td>36460</td><td>Drosophila melanogaster</td><td>Fsn</td><td>CG4643 gene product from transcript CG4643-RA</td><td>BEST:GH20696|CG4643|DFsn|DmelCG4643|Wals</td><td>0</td><td>0</td></tr>
<tr><td>4481</td><td>36461</td><td>Drosophila melanogaster</td><td>Dp</td><td>DP transcription factor</td><td>49Fk|CG4654|DDP1|DP|DP1|DmDP|DmelCG4654|Dp1|TFPD2|dDP|dDP1|dDp|dp|l(2)49Fk|l(2)vr10|vr10</td><td>0</td><td>0</td></tr>
<tr><td>4482</td><td>36462</td><td>Drosophila melanogaster</td><td>pex13</td><td>peroxin 13</td><td>CG4663|DmelCG4663</td><td>0</td><td>0</td></tr>
<tr><td>4483</td><td>36463</td><td>Drosophila melanogaster</td><td>fsd</td><td>fates-shifted</td><td>CG12765|DmelCG12765</td><td>0</td><td>0</td></tr>
<tr><td>4484</td><td>36464</td><td>Drosophila melanogaster</td><td>CG4670</td><td>CG4670 gene product from transcript CG4670-RA</td><td>DmQSOX1|DmelCG4670|QSOX1</td><td>0</td><td>0</td></tr>
<tr><td>4485</td><td>36465</td><td>Drosophila melanogaster</td><td>CG4676</td><td>CG4676 gene product from transcript CG4676-RA</td><td>DmelCG4676</td><td>0</td><td>0</td></tr>
<tr><td>4486</td><td>36466</td><td>Drosophila melanogaster</td><td>CG4679</td><td>CG4679 gene product from transcript CG4679-RA</td><td>DmelCG4679</td><td>0</td><td>0</td></tr>
<tr><td>4487</td><td>36467</td><td>Drosophila melanogaster</td><td>CG4688</td><td>CG4688 gene product from transcript CG4688-RA</td><td>DmelCG4688</td><td>0</td><td>0</td></tr>
<tr><td>4488</td><td>36468</td><td>Drosophila melanogaster</td><td>Mp20</td><td>Muscle protein 20</td><td>CG4696|DMMP20|DmelCG4696|Dmmp20|Tpn|mp20</td><td>0</td><td>0</td></tr>
<tr><td>4489</td><td>36469</td><td>Drosophila melanogaster</td><td>CG4712</td><td>CG4712 gene product from transcript CG4712-RB</td><td>DmelCG4712</td><td>0</td><td>0</td></tr>
<tr><td>4490</td><td>36470</td><td>Drosophila melanogaster</td><td>CG4714</td><td>CG4714 gene product from transcript CG4714-RA</td><td>DmelCG4714|anon-WO0140519.159</td><td>0</td><td>0</td></tr>
<tr><td>4491</td><td>36471</td><td>Drosophila melanogaster</td><td>CG4716</td><td>CG4716 gene product from transcript CG4716-RA</td><td>BEST:GH18008|DmelCG4716|GH16|gh18008</td><td>0</td><td>0</td></tr>
<tr><td>4492</td><td>36472</td><td>Drosophila melanogaster</td><td>CG10799</td><td>CG10799 gene product from transcript CG10799-RA</td><td>CT30256|DmelCG10799</td><td>0</td><td>0</td></tr>
<tr><td>4493</td><td>36473</td><td>Drosophila melanogaster</td><td>CG42319</td><td>CG42319 gene product from transcript CG42319-RE</td><td>CG17041|CG17042|DmelCG42319|Dmel_CG17041|Dmel_CG17042</td><td>0</td><td>0</td></tr>
<tr><td>4494</td><td>36475</td><td>Drosophila melanogaster</td><td>Dh31-R1</td><td>Diuretic hormone 31 receptor 1</td><td>CG17043|CG17415|CG32843|DH31|DH31-R|DH31-R1|DH31R|DmeCG32843|DmelCG32843|anon-WO0170980.136|anon-WO0170980.137</td><td>0</td><td>0</td></tr>
<tr><td>4495</td><td>36477</td><td>Drosophila melanogaster</td><td>CG4734</td><td>CG4734 gene product from transcript CG4734-RA</td><td>DmelCG4734</td><td>0</td><td>0</td></tr>
<tr><td>4496</td><td>36478</td><td>Drosophila melanogaster</td><td>CG17047</td><td>CG17047 gene product from transcript CG17047-RA</td><td>DmelCG17047</td><td>0</td><td>0</td></tr>
<tr><td>4497</td><td>36479</td><td>Drosophila melanogaster</td><td>CG17048</td><td>CG17048 gene product from transcript CG17048-RB</td><td>DmelCG17048</td><td>0</td><td>0</td></tr>
<tr><td>4498</td><td>36481</td><td>Drosophila melanogaster</td><td>CG10814</td><td>CG10814 gene product from transcript CG10814-RA</td><td>DmelCG10814</td><td>0</td><td>0</td></tr>
<tr><td>4499</td><td>36482</td><td>Drosophila melanogaster</td><td>CG17050</td><td>CG17050 gene product from transcript CG17050-RA</td><td>DmelCG17050</td><td>0</td><td>0</td></tr>
<tr><td>4500</td><td>36483</td><td>Drosophila melanogaster</td><td>CG17049</td><td>CG17049 gene product from transcript CG17049-RA</td><td>DmelCG17049</td><td>0</td><td>0</td></tr>
<tr><td>4501</td><td>36484</td><td>Drosophila melanogaster</td><td>AttC</td><td>Attacin-C</td><td>Att|CG4740|DIM 16|DIM-16|DmelCG4740|MPAC|att|att C|attC</td><td>0</td><td>0</td></tr>
<tr><td>4502</td><td>36485</td><td>Drosophila melanogaster</td><td>CG4744</td><td>CG4744 gene product from transcript CG4744-RA</td><td>DmelCG4744</td><td>0</td><td>0</td></tr>
<tr><td>4503</td><td>36486</td><td>Drosophila melanogaster</td><td>IM10</td><td>Immune induced molecule 10</td><td>CG18279|DIM 10|DIM 12|DIM 13|DIM 24|DIM-10|DIM-12|DIM-13|DIM-24|DIM10|DIM12|DIM13|DmelCG18279|im10</td><td>0</td><td>0</td></tr>
<tr><td>4504</td><td>36487</td><td>Drosophila melanogaster</td><td>CG18278</td><td>CG18278 gene product from transcript CG18278-RA</td><td>DmelCG18278</td><td>0</td><td>0</td></tr>
<tr><td>4505</td><td>36488</td><td>Drosophila melanogaster</td><td>CG42321</td><td>CG42321 gene product from transcript CG42321-RM</td><td>CG17034|CG30061|DmelCG42321|Dmel_CG17034|Dmel_CG30061|anon-WO0153538.54</td><td>0</td><td>0</td></tr>
<tr><td>4506</td><td>36489</td><td>Drosophila melanogaster</td><td>Ser8</td><td>CG4812 gene product from transcript CG4812-RA</td><td>CG4812|DmelCG4812|SP#3|SP3|SP90</td><td>0</td><td>0</td></tr>
<tr><td>4507</td><td>36491</td><td>Drosophila melanogaster</td><td>cnn</td><td>centrosomin</td><td>CG 4832|CG18370|CG4832|CNN|Cnn|Cnn1|DmelCG4832|cen|mat(2)syn-C|mat(2)synHK21|mfs|obv</td><td>0</td><td>0</td></tr>
<tr><td>4508</td><td>36492</td><td>Drosophila melanogaster</td><td>cbs</td><td>centrosomin's beautiful sister</td><td>CG4840|Cbs|DmelCG4840|dem</td><td>0</td><td>0</td></tr>
<tr><td>4509</td><td>36494</td><td>Drosophila melanogaster</td><td>cbc</td><td>crowded by cid</td><td>CG5970|DmelCG5970|cg5970</td><td>0</td><td>0</td></tr>
<tr><td>4510</td><td>36495</td><td>Drosophila melanogaster</td><td>cid</td><td>centromere identifier</td><td>BcDNA:RE21270|CENP-A|CENP-A/CID|CENP-A/Cid|CENP-C|CENPA|CG13329|CID|CID/CENP-A|CenH3|CenH3/CID|CenH3[CID]|CenH3[Cid]|Cenp-A|CenpA|CenpA/CID|Cid|Cid[Mel]|DmelCG13329|cenH</td><td>0</td><td>0</td></tr>
<tr><td>4511</td><td>36496</td><td>Drosophila melanogaster</td><td>bbc</td><td>bb in a boxcar</td><td>CG6016|DmelCG6016|cept</td><td>0</td><td>0</td></tr>
<tr><td>4512</td><td>36497</td><td>Drosophila melanogaster</td><td>drk</td><td>downstream of receptor kinase</td><td>24/1|CG6033|DRK|DmelCG6033|Drk|Drk/Grb2|E(sev)2B|GRB2|Grb-2|Grb/drk|Grb2|Grb2/drk|P1112|Su(sev)R1|crkl|l(2)10626|l(2)k13809</td><td>0</td><td>0</td></tr>
<tr><td>4513</td><td>36498</td><td>Drosophila melanogaster</td><td>mars</td><td>CG17064 gene product from transcript CG17064-RB</td><td>CG17064|DmelCG17064|HURP|Mars|dhrp/gkap|gkap</td><td>0</td><td>0</td></tr>
<tr><td>4514</td><td>36499</td><td>Drosophila melanogaster</td><td>mip120</td><td>Myb-interacting protein 120</td><td>CG6061|DmelCG6061|Mip120|l(3)L4569|p120</td><td>0</td><td>0</td></tr>
<tr><td>4515</td><td>36501</td><td>Drosophila melanogaster</td><td>CG13330</td><td>CG13330 gene product from transcript CG13330-RA</td><td>DmelCG13330</td><td>0</td><td>0</td></tr>
<tr><td>4516</td><td>36503</td><td>Drosophila melanogaster</td><td>CG13331</td><td>CG13331 gene product from transcript CG13331-RB</td><td>DmelCG13331</td><td>0</td><td>0</td></tr>
<tr><td>4517</td><td>36504</td><td>Drosophila melanogaster</td><td>CG13332</td><td>CG13332 gene product from transcript CG13332-RA</td><td>DmelCG13332</td><td>0</td><td>0</td></tr>
<tr><td>4518</td><td>36506</td><td>Drosophila melanogaster</td><td>CG6145</td><td>CG6145 gene product from transcript CG6145-RE</td><td>CT19307|DmelCG6145</td><td>0</td><td>0</td></tr>
<tr><td>4519</td><td>36507</td><td>Drosophila melanogaster</td><td>CG33156</td><td>CG33156 gene product from transcript CG33156-RE</td><td>CG30063|CG6152|DmelCG33156</td><td>0</td><td>0</td></tr>
<tr><td>4520</td><td>36508</td><td>Drosophila melanogaster</td><td>Roe1</td><td>CG6155 gene product from transcript CG6155-RA</td><td>BcDNA:GM03203|CG6155|DROE1|DmelCG6155|Droe1|cg6155|droe1</td><td>0</td><td>0</td></tr>
<tr><td>4521</td><td>36509</td><td>Drosophila melanogaster</td><td>CG13333</td><td>CG13333 gene product from transcript CG13333-RA</td><td>DmelCG13333</td><td>0</td><td>0</td></tr>
<tr><td>4522</td><td>36510</td><td>Drosophila melanogaster</td><td>CG13334</td><td>CG13334 gene product from transcript CG13334-RB</td><td>DmelCG13334|NEST:bs27e02</td><td>0</td><td>0</td></tr>
<tr><td>4523</td><td>36511</td><td>Drosophila melanogaster</td><td>CG42807</td><td>CG42807 gene product from transcript CG42807-RA</td><td>CG13335|CG6183|DmelCG42807|Dmel_CG13335</td><td>0</td><td>0</td></tr>
<tr><td>4524</td><td>36513</td><td>Drosophila melanogaster</td><td>CG6191</td><td>CG6191 gene product from transcript CG6191-RA</td><td>BcDNA:GH06193|DmelCG6191|Dromel_CG6191_FBtr0087678_mORF</td><td>0</td><td>0</td></tr>
<tr><td>4525</td><td>36514</td><td>Drosophila melanogaster</td><td>CG6197</td><td>CG6197 gene product from transcript CG6197-RA</td><td>DmelCG6197|XAB2|Xab2</td><td>0</td><td>0</td></tr>
<tr><td>4526</td><td>36515</td><td>Drosophila melanogaster</td><td>S-Lap5</td><td>Sperm-Leucylaminopeptidase 5</td><td>CG18369|DmelCG18369|S-LAP 5</td><td>0</td><td>0</td></tr>
<tr><td>4527</td><td>36516</td><td>Drosophila melanogaster</td><td>CG12464</td><td>CG12464 gene product from transcript CG12464-RA</td><td>DmelCG12464</td><td>0</td><td>0</td></tr>
<tr><td>4528</td><td>36517</td><td>Drosophila melanogaster</td><td>CG6209</td><td>CG6209 gene product from transcript CG6209-RA</td><td>DmelCG6209|mst101(2)</td><td>0</td><td>0</td></tr>
<tr><td>4529</td><td>36518</td><td>Drosophila melanogaster</td><td>CG13337</td><td>CG13337 gene product from transcript CG13337-RA</td><td>DmelCG13337</td><td>0</td><td>0</td></tr>
<tr><td>4530</td><td>36519</td><td>Drosophila melanogaster</td><td>CG18368</td><td>CG18368 gene product from transcript CG18368-RA</td><td>DmelCG18368</td><td>0</td><td>0</td></tr>
<tr><td>4531</td><td>36520</td><td>Drosophila melanogaster</td><td>CG6220</td><td>CG6220 gene product from transcript CG6220-RA</td><td>DmelCG6220</td><td>0</td><td>0</td></tr>
<tr><td>4532</td><td>36521</td><td>Drosophila melanogaster</td><td>fas</td><td>faint sausage</td><td>CG17716|CT39263|DmelCG17716|l(2)05488|l(2)5488</td><td>0</td><td>0</td></tr>
<tr><td>4533</td><td>36522</td><td>Drosophila melanogaster</td><td>CG6280</td><td>CG6280 gene product from transcript CG6280-RA</td><td>DmelCG6280</td><td>0</td><td>0</td></tr>
<tr><td>4534</td><td>36523</td><td>Drosophila melanogaster</td><td>Cpr50Ca</td><td>Cuticular protein 50Ca</td><td>CG13338|DmelCG13338</td><td>0</td><td>0</td></tr>
<tr><td>4535</td><td>36524</td><td>Drosophila melanogaster</td><td>S-Lap7</td><td>Sperm-Leucylaminopeptidase 7</td><td>CG13340|DmelCG13340|S-LAP 7</td><td>0</td><td>0</td></tr>
<tr><td>4536</td><td>36525</td><td>Drosophila melanogaster</td><td>Cpr50Cb</td><td>Cuticular protein 50Cb</td><td>CG6305|DmelCpr50Cb|DmelCG6305</td><td>0</td><td>0</td></tr>
<tr><td>4537</td><td>36526</td><td>Drosophila melanogaster</td><td>stj</td><td>straightjacket</td><td>CG12295|DmelCG12295|alpha[[2]]delta|dalpha[[2]]delta-3|l(2)k10814</td><td>0</td><td>0</td></tr>
<tr><td>4538</td><td>36527</td><td>Drosophila melanogaster</td><td>fl(2)d</td><td>female lethal d</td><td>CG6315|DmelCG6315|v(2)k16105</td><td>0</td><td>0</td></tr>
<tr><td>4539</td><td>36528</td><td>Drosophila melanogaster</td><td>CG13339</td><td>CG13339 gene product from transcript CG13339-RA</td><td>BcDNA:RE69336|DmelCG13339</td><td>0</td><td>0</td></tr>
<tr><td>4540</td><td>36529</td><td>Drosophila melanogaster</td><td>CG6329</td><td>CG6329 gene product from transcript CG6329-RA</td><td>DmelCG6329</td><td>0</td><td>0</td></tr>
<tr><td>4541</td><td>36530</td><td>Drosophila melanogaster</td><td>CG6337</td><td>CG6337 gene product from transcript CG6337-RA</td><td>DmelCG6337</td><td>0</td><td>0</td></tr>
<tr><td>4542</td><td>36531</td><td>Drosophila melanogaster</td><td>CG6347</td><td>CG6347 gene product from transcript CG6347-RA</td><td>DmelCG6347</td><td>0</td><td>0</td></tr>
<tr><td>4543</td><td>36532</td><td>Drosophila melanogaster</td><td>CG6357</td><td>CG6357 gene product from transcript CG6357-RA</td><td>DmelCG6357</td><td>0</td><td>0</td></tr>
<tr><td>4544</td><td>36533</td><td>Drosophila melanogaster</td><td>synaptogyrin</td><td>CG10808 gene product from transcript CG10808-RA</td><td>CG10808|DmelCG10808|Synaptogyrin|sng-1</td><td>0</td><td>0</td></tr>
<tr><td>4545</td><td>36534</td><td>Drosophila melanogaster</td><td>CG30484</td><td>CG30484 gene product from transcript CG30484-RA</td><td>CG13342|DmelCG30484</td><td>0</td><td>0</td></tr>
<tr><td>4546</td><td>36536</td><td>Drosophila melanogaster</td><td>CG6543</td><td>CG6543 gene product from transcript CG6543-RA</td><td>CG18645|DmelCG6543</td><td>0</td><td>0</td></tr>
<tr><td>4547</td><td>36537</td><td>Drosophila melanogaster</td><td>CG6553</td><td>CG6553 gene product from transcript CG6553-RA</td><td>DmelCG6553</td><td>0</td><td>0</td></tr>
<tr><td>4548</td><td>36538</td><td>Drosophila melanogaster</td><td>tum</td><td>tumbleweed</td><td>13345|AcGAP|CG13345|DRacGAP|DmelCG13345|RacGAP|RacGAP50C|RacGAP50c|RacGap|RacGap50C|RhoGAP-50C14|TUM|acGAP|eon|i249|racGAP50|racGAP50C|racGAP50c</td><td>0</td><td>0</td></tr>
<tr><td>4549</td><td>36539</td><td>Drosophila melanogaster</td><td>CG13343</td><td>CG13343 gene product from transcript CG13343-RA</td><td>DmelCG13343</td><td>0</td><td>0</td></tr>
</table>

4550	36540	Drosophila melanogaster	CG16935	CG16935 gene product from transcript CG16935-RA	DM_7303260|DmelCG16935	0	0
4551	36541	Drosophila melanogaster	CG13344	CG13344 gene product from transcript CG13344-RA	DmelCG13344|anon-EST:GreesD1|anon-EST:GressD1	0	0
4552	36542	Drosophila melanogaster	shot	short stop	42/4|CG18076|CG18637|DmelCG18076|Grv|Shot|grv|kak|kop|l(2)CA4|l(2)k03010|l(2)k04204|l(2)k05434|l(2)k05821|l(2)k10821|l(2)k15606|shortstop/kakapo	0	0
4553	36543	Drosophila melanogaster	DJ-1alpha	CG6646 gene product from transcript CG6646-RA	CG6646|DJ-1|DJ-1A|DJ-1a|DmelCG6646	0	0
4554	36544	Drosophila melanogaster	AGO1	Argonaute-1	Ago|Ago-1|Ago1|CG6671|Dm Ago1|DmelCG6671|MRE20|ago1|ago1-1|anon-WO0257455.29|dAGO1|dAgo1|l(2)04845|l(2)4845|l(2)k00208|l(2)k08121	0	0
4555	36545	Drosophila melanogaster	CG13349	CG13349 gene product from transcript CG13349-RA	DmelCG13349|GC13349|p42E	0	0
4556	36546	Drosophila melanogaster	Cp1	Cysteine proteinase-1	CG6692|CP1|CT20780|CysP-1|DCP1|Dcp-1|Dcp1|DmelCG6692|cp1|fs(2)50Ca	0	0
4557	36547	Drosophila melanogaster	Aats-phe	Phenylalanyl-tRNA synthetase	CG13348|DmelCG13348|FRS|PheRS|Pts	0	0
4558	36548	Drosophila melanogaster	CG6704	CG6704 gene product from transcript CG6704-RA	DmelCG6704	0	0
4559	36549	Drosophila melanogaster	CG18568	CG18568 gene product from transcript CG18568-RA	DmelCG18568|anon-WO0140519.113	0	0
4560	36550	Drosophila melanogaster	CG6701	CG6701 gene product from transcript CG6701-RD	DmelCG6701|EP(2)2054|EP2054	0	0
4561	36551	Drosophila melanogaster	Ctf4	CG13350 gene product from transcript CG13350-RA	CG13350|DmelCG13350|anon-WO0140519.180	0	0
4562	36552	Drosophila melanogaster	CG8067	CG8067 gene product from transcript CG8067-RA	DmelCG8067	0	0
4563	36554	Drosophila melanogaster	RN-tre	tre oncogene-related protein	CG13351|CG8085|DRN-tre|DmelCG8085	0	0
4564	36555	Drosophila melanogaster	mam	mastermind	CG8118|Dm0064|DmelCG8118|Mam|N-2G|SR2-4|dMam|l(2)03505|l(2)04615|mam(CAG)[[7]]	0	0
4565	36556	Drosophila melanogaster	CG18371	CG18371 gene product from transcript CG18371-RA	DmelCG18371	0	0
4566	36559	Drosophila melanogaster	CG42288	CG42288 gene product from transcript CG42288-RA	CG13353|DmelCG42288|Dmel_CG13353	0	0
4567	36560	Drosophila melanogaster	Rcd1	Reduction in Cnn dots 1	CG8233|DmelCG8233|NSL3	0	0
4568	36561	Drosophila melanogaster	pea	peanuts	CG8241|DmelCG8241|Prp22|cg8241|prp22	0	0
4569	36562	Drosophila melanogaster	CG13016	CG13016 gene product from transcript CG13016-RA	DmelCG13016	0	0
4570	36563	Drosophila melanogaster	CG8257	CG8257 gene product from transcript CG8257-RA	DmelCG8257	0	0
4571	36564	Drosophila melanogaster	O-fut1	O-fucosyltransferase 1	4R6|AAF58290.1|CG12366|DmelCG12366|O-FUT1|O-fut|OFUT1|Ofut1|l(2)SH2 2260|l(2)SH2260|ntc|nti	0	0
4572	36565	Drosophila melanogaster	Tango7	Transport and Golgi organization 7	CG8309|DmelCG8309|TANGO7|Tango 7|anon-EST:Posey67|anon-EST:Posey76	0	0
4573	36566	Drosophila melanogaster	CG8323	CG8323 gene product from transcript CG8323-RA	DmelCG8323	0	0
4574	36567	Drosophila melanogaster	CG18327	CG18327 gene product from transcript CG18327-RA	DmelCG18327	0	0
4575	36568	Drosophila melanogaster	CG18324	CG18324 gene product from transcript CG18324-RB	DmelCG18324	0	0
4576	36569	Drosophila melanogaster	CG8331	CG8331 gene product from transcript CG8331-RD	CT24597|DmelCG8331	0	0
4577	36570	Drosophila melanogaster	mRpS16	mitochondrial ribosomal protein S16	147189_at|BcDNA:SD15809|CG8338|DmelCG8338|MRP-S16	0	0
4578	36571	Drosophila melanogaster	cg	combgap	CG8367|DmelCG8367|l(2)07659|l(2)k11504|mig	0	0
4579	36573	Drosophila melanogaster	CG30069	CG30069 gene product from transcript CG30069-RA	CG13017|CG8381|DmelCG30069	0	0
4580	36574	Drosophila melanogaster	VGAT	Vesicular GABA Transporter	CG8394|CG8394.2|DmelCG8394|VIAAT|vGAT	0	0
4581	36575	Drosophila melanogaster	Sox15	Sox box protein 15	CG13945|CG8404|DSox-15|DmelCG8404|Dsox-15|SOX15|SOX50E|SOXF|Sox F|Sox50E|SoxE|SoxF	0	0
4582	36576	Drosophila melanogaster	RpS23	Ribosomal protein S23	CG8415|CT24703|DmelCG8415|M(2)50E|anon-EST:Posey158|anon-EST:Posey63	0	0
4583	36577	Drosophila melanogaster	CG8468	CG8468 gene product from transcript CG8468-RC	DmelCG8468	0	0
4584	36578	Drosophila melanogaster	opa1-like	optic atrophy 1-like	CG8479|DmelCG8479|OPA1|OPA1-like|Opa1|Opa1-like|dOPA1|dOpa1|l(2)s3475|opa1	0	0
4585	36579	Drosophila melanogaster	CG8485	CG8485 gene product from transcript CG8485-RA	152964_at|DmelCG8485|SNRK	0	0
4586	36580	Drosophila melanogaster	CG8494	CG8494 gene product from transcript CG8494-RA	DmelCG8494	0	0
4587	36581	Drosophila melanogaster	CG8503	CG8503 gene product from transcript CG8503-RA	DmelCG8503	0	0
4588	36582	Drosophila melanogaster	Mdr50	Multi drug resistance 50	CG8523|Dm2|Dm3|DmelCG8523|MDR50|mdr50	0	0
4589	36583	Drosophila melanogaster	Hsc70-5	Heat shock protein cognate 5	CG8542|DmelCG8542|HSC-70|HSC5|HSC70|Hsc-5|Hsc5|Hsc70|dhsc70|hsc70|hsc70-5|l(2)k04907	0	0
4590	36584	Drosophila melanogaster	CG8531	CG8531 gene product from transcript CG8531-RA	DmelCG8531	0	0
4591	36585	Drosophila melanogaster	beta4GalNAcTA	CG8536 gene product from transcript CG8536-RA	BcDNA:GH13356|CG8536|DmGalT|DmelCG8536|anon-WO0118547.128|b4GalNAcTA|beta4Gal-NAcTA|beta4GalNAcT1|beta4GalNacTA|beta4GalTA|dbeta4GalTA	0	0
4592	36586	Drosophila melanogaster	CG8547	CG8547 gene product from transcript CG8547-RC	DmelCG8547	0	0
4593	36587	Drosophila melanogaster	SelD	Selenide,water dikinase	142926_at|CG8553|Dm SelD|Dm-SelD|DmelCG8553|SPS1|SeS|anon-WO0172774.145|anon-WO0172774.146|dSPS1|dsps1|l(2)SH1599|l(2)SH2 1599|l(2)k12303|ptf1|ptuf|ptuf1|selD|selD/sps1	0	0
4594	36588	Drosophila melanogaster	conv	convoluted	ALS|Als|CG8561|CT24963|DmelCG8561|dALS|l(2)k06507b|tartan/capricious-like	0	0
4595	36589	Drosophila melanogaster	Ih	I[[h]] channel	BcDNA:GH25778|CG8585|CR30068|DMIH|DmelCG8585|Dmel_CR30068	0	0
4596	36590	Drosophila melanogaster	tej	tejas	CG8589|DmelCG8589|anon-EST:fe1D11	0	0
4597	36592	Drosophila melanogaster	Shroom	CG34379 gene product from transcript CG34379-RD	CG13942|CG13944|CG34379|CG8603|DmelCG34379|Dmel_CG13942|Dmel_CG8603|Shrm|dShrm|dmShroom	0	0
4598	36593	Drosophila melanogaster	CG8613	CG8613 gene product from transcript CG8613-RA	DmelCG8613	0	0
4599	36594	Drosophila melanogaster	CG8617	CG8617 gene product from transcript CG8617-RA	DmelCG8617	0	0
4600	36595	Drosophila melanogaster	Arc1	Activity-regulated cytoskeleton associated protein 1	CG12505|DmelCG12505|anon-WO0153538.31|anon-WO0153538.40|anon-WO0153538.41|anon-WO0153538.43|anon-WO0153538.45|dARC1|dArc1	0	0
4601	36597	Drosophila melanogaster	Arc2	CG13941 gene product from transcript CG13941-RA	BcDNA:SD11637|CG13941|DmelCG13941|anon-WO0118547.456|dARC2|dArc2	0	0
4602	36598	Drosophila melanogaster	Tfb1	CG8151 gene product from transcript CG8151-RB	CG8151|DmelCG8151|Dmp62|TFB1|TfBI|l(2)06949|l(2)06950|p62	0	0
4603	36600	Drosophila melanogaster	Obp50e	Odorant-binding protein 50e	CG13939|DmelCG13939	0	0
4604	36601	Drosophila melanogaster	Dh44-R1	Diuretic hormone 44 receptor 1	CG8422|DH44-R1|DH[[44]]-R1|DmeCG8422|DmelCG8422|anon-WO0170980.100|anon-WO0170980.101	0	0
4605	36602	Drosophila melanogaster	CG10104	CG10104 gene product from transcript CG10104-RA	DmelCG10104	0	0
4606	36603	Drosophila melanogaster	CG17385	CG17385 gene product from transcript CG17385-RA	DmelCG17385	0	0
4607	36604	Drosophila melanogaster	Sin1	SAPK-interacting protein 1	CG10105|DmelCG10105|dSin1|sin1	0	0
4608	36605	Drosophila melanogaster	CG17386	CG17386 gene product from transcript CG17386-RA	CT33484|DmelCG17386	0	0
4609	36606	Drosophila melanogaster	phyl	phyllopod	CG10108|D(DmcycE[JP])2.2|DmelCG10108|PHYL|Phyl|SR2-3|Su(Raf)2C	0	0
4610	36609	Drosophila melanogaster	Oaz	O/E-associated zinc finger protein	CG17388|CG17389|CG17390|CG42702|DmelCG42702|Dmel_CG17388|Dmel_CG17389|Dmel_CG17390|dmOAZ	0	0
4611	36612	Drosophila melanogaster	Asx	Additional sex combs	ASX|CG8787|DmelCG8787|xen	0	0
4612	36613	Drosophila melanogaster	Cpr51A	Cuticular protein 51A	CG10112|DmelCG10112	0	0
4613	36614	Drosophila melanogaster	ttv	tout-velu	CG10117|DEXT1|DmelCG10117|Ext1|P1.15|Ttv|l(2)00681|l(2)05282|l(2)k00115|l(2)k03617	0	0
4614	36615	Drosophila melanogaster	LamC	Lamin C	CG10119|DmelCG10119|G-IF|LAMC|lamC|pG-IF	0	0
4615	36616	Drosophila melanogaster	CG12869	CG12869 gene product from transcript CG12869-RA	DmelCG12869	0	0
4616	36617	Drosophila melanogaster	RpI1	RNA polymerase I subunit	153129_at|CG10122|DmRPA1|DmelCG10122|RPA1	0	0
4617	36619	Drosophila melanogaster	tra2	transformer 2	CG10128|DmelCG10128|TRA 2|TRA-2|TRA2|Tra-2|Tra2|tra-2	0	0
4618	36620	Drosophila melanogaster	CG12868	CG12868 gene product from transcript CG12868-RB	CT32008|DmelCG12868	0	0
4619	36621	Drosophila melanogaster	Su(var)2-HP2	CG12864 gene product from transcript CG12864-RB	CG12864|DmelCG12864|HP2|SU(VAR)2-HP2	0	0
4620	36623	Drosophila melanogaster	CG12863	CG12863 gene product from transcript CG12863-RA	DmelCG12863	0	0
4621	36624	Drosophila melanogaster	CG10131	CG10131 gene product from transcript CG10131-RA	DmelCG10131	0	0
4622	36625	Drosophila melanogaster	CG12862	CG12862 gene product from transcript CG12862-RA	DmelCG12862	0	0
4623	36626	Drosophila melanogaster	CG10139	CG10139 gene product from transcript CG10139-RA	DmelCG10139	0	0
4624	36627	Drosophila melanogaster	Adgf-E	Adenosine deaminase-related growth factor E	ADGF-E|CG10143|DmelCG10143|adgfe	0	0
4625	36628	Drosophila melanogaster	CG12861	CG12861 gene product from transcript CG12861-RA	DmelCG12861	0	0
4626	36629	Drosophila melanogaster	CG12860	CG12860 gene product from transcript CG12860-RA	DmelCG12860|anon-WO0140519.75|bs33f06.y1	0	0
4627	36630	Drosophila melanogaster	CG12866	CG12866 gene product from transcript CG12866-RB	DmelCG12866	0	0
4628	36631	Drosophila melanogaster	CG30479	CG30479 gene product from transcript CG30479-RA	CG18373|DmelCG30479	0	0
4629	36632	Drosophila melanogaster	mspo	M-spondin	CG10145|CT28539|DmelCG10145|Mspo|m-spo	0	0
4630	36633	Drosophila melanogaster	CG12865	CG12865 gene product from transcript CG12865-RA	DmelCG12865	0	0
4631	36634	Drosophila melanogaster	CG12858	CG12858 gene product from transcript CG12858-RB	DmelCG12858	0	0
4632	36635	Drosophila melanogaster	Dro	Drosocin	143609_at|BcDNA:RH31634|CG10816|DIM 11|DIM 15|DIM 9|DIM-15|DROC|DmelCG10816|Drc|Dros|Drs|IM15|drc|dro|drosocin	0	0
4633	36636	Drosophila melanogaster	AttA	Attacin-A	ATT|Att|Att A|BcDNA:LP05763|CG10146|DmelCG10146|anon-WO0140519.5|att|att A|attA	0	0
4634	36637	Drosophila melanogaster	AttB	Attacin-B	Att|AttB1|AttB2|AttBn|CG18372|DmelCG18372|att|att B1|attB	0	0
4635	36638	Drosophila melanogaster	Rpn6	Proteasome p44.5 subunit	BcDNA.LD18931|BcDNA:LD18931|CG10149|DmelCG10149|Pros44.5|l(2)k00103|p42B	0	0
4636	36639	Drosophila melanogaster	CG10151	CG10151 gene product from transcript CG10151-RA	DmelCG10151	0	0
4637	36640	Drosophila melanogaster	CG12859	CG12859 gene product from transcript CG12859-RA	DmelCG12859|anon-EST:fe3B12	0	0
4638	36641	Drosophila melanogaster	CG10153	CG10153 gene product from transcript CG10153-RA	DmelCG10153	0	0
4639	36642	Drosophila melanogaster	CG12857	CG12857 gene product from transcript CG12857-RA	DmelCG12857	0	0
4640	36643	Drosophila melanogaster	Spred	Sprouty-related protein with EVH-1 domain	AE33|CG10155|Dm AE33|DmelCG10155|dSPRED|spred	0	0
4641	36644	Drosophila melanogaster	CG42254	CG42254 gene product from transcript CG42254-RD	CG12854|CR30478|DmelCG42254|Dmel_CG12854|Dmel_CR30478	0	0
4642	36645	Drosophila melanogaster	BEAF-32	Boundary element-associated factor of 32kD	BEAF|BEAF-32A|BEAF-32B|BEAF32|BEAF32A|BEAF32B|BEAF32b|Beaf-32|CG10159|DmelCG10159|beaf|bs04c01.y1	0	0
4643	36648	Drosophila melanogaster	CG10200	CG10200 gene product from transcript CG10200-RB	DmelCG10200	0	0
4644	36649	Drosophila melanogaster	CG10202	CG10202 gene product from transcript CG10202-RA	DmelCG10202|anon-WO0140519.148	0	0
4645	36650	Drosophila melanogaster	CG10205	CG10205 gene product from transcript CG10205-RA	CT28709|DmelCG10205	0	0
4646	36651	Drosophila melanogaster	NaPi-T	Na[+]-dependent inorganic phosphate cotransporter	CG10207|DmelCG10207|Dropo|Na/Pi-cotransporter	0	0
4647	36652	Drosophila melanogaster	CG10209	CG10209 gene product from transcript CG10209-RA	DmelCG10209	0	0
4648	36653	Drosophila melanogaster	SMC2	CG10212 gene product from transcript CG10212-RA	CG10212|DmSMC2|DmelCG10212|dSMC2	0	0
4649	36654	Drosophila melanogaster	Ercc1	CG10215 gene product from transcript CG10215-RA	CG10215|DmelCG10215|ERCC1	0	0
4650	36655	Drosophila melanogaster	Ciao1	CG12797 gene product from transcript CG12797-RA	CG12797|DmelCG12797	0	0
4651	36656	Drosophila melanogaster	HPS1	Hermansky-Pudlak Syndrome 1 ortholog	CG12855|DmelCG12855|HPS|dHPS1	0	0
4652	36658	Drosophila melanogaster	Pcf11	CG10228 gene product from transcript CG10228-RE	CG10228|DmelCG10228|anon-WO0118547.146|dPcf11|l(2)k08015|pcf11	0	0
4653	36661	Drosophila melanogaster	Cyp6a23	CG10242 gene product from transcript CG10242-RA	6a23|CG10242|Cypa23|DmelCG10242	0	0
4654	36662	Drosophila melanogaster	Cyp6a19	CG10243 gene product from transcript CG10243-RA	6a19|CG10243|DmelCG10243	0	0
4655	36663	Drosophila melanogaster	Cyp6a9	Cytochrome P450-6a9	6a9|CG10246|CYP6A9|Cyt-P450-rAF4|DmelCG10246|P-450|P450	0	0
4656	36664	Drosophila melanogaster	Cyp6a20	CG10245 gene product from transcript CG10245-RB	6a20|CG10245|DmelCG10245	0	0
4657	36665	Drosophila melanogaster	Cyp6a21	CG10247 gene product from transcript CG10247-RA	6a21|CG10247|DmelCG10247|cyp6a21	0	0
4658	36666	Drosophila melanogaster	Cyp6a8	Cytochrome P450-6a8	6a8|CG10248|CYP6A8|Cyt-P450-rAF5|DmelCG10248|P-450|P450	0	0
4659	36667	Drosophila melanogaster	Cyp317a1	CG17453 gene product from transcript CG17453-RA	317a1|CG17453|DmelCG17453	0	0
4660	36668	Drosophila melanogaster	CG10249	CG10249 gene product from transcript CG10249-RJ	BEST:GH19041|BcDNA:GH03482|DmelCG10249	0	0

4661	36669	Drosophila melanogaster	CG10253	CG10253 gene product from transcript CG10253-RD	DmelCG10253	0	0
4662	36670	Drosophila melanogaster	Lap1	CG10255 gene product from transcript CG10255-RA	CG10255|CT28815|DmelCG10255|dLAP1|dlap1	0	0
4663	36671	Drosophila melanogaster	CG10257	CG10257 gene product from transcript CG10257-RA	BcDNA:RH51312|DmelCG10257	0	0
4664	36673	Drosophila melanogaster	CG12424	CG12424 gene product from transcript CG12424-RC	BcDNA:GM06913|DmelCG12424	0	0
4665	36674	Drosophila melanogaster	pcs	parcas	CG7761|DmelCG7761|prt	0	0
4666	36675	Drosophila melanogaster	CG7639	CG7639 gene product from transcript CG7639-RB	DmelCG7639	0	0
4667	36676	Drosophila melanogaster	CG10265	CG10265 gene product from transcript CG10265-RA	CG30474|DmelCG10265	0	0
4668	36678	Drosophila melanogaster	chn	charlatan	CG11798|Chn|DmelCG11798|EPIL#6|EPIL6|anon-EST:Liang-1.13|clone 1.13|l(2)02064|l(2)k04218|taco	0	0
4669	36679	Drosophila melanogaster	CG8089	CG8089 gene product from transcript CG8089-RA	DmelCG8089	0	0
4670	36680	Drosophila melanogaster	CG7544	CG7544 gene product from transcript CG7544-RA	DmelCG7544	0	0
4671	36681	Drosophila melanogaster	igl	igloo	CG18285|DmelCG18285	0	0
4672	36682	Drosophila melanogaster	CG8090	CG8090 gene product from transcript CG8090-RB	DmelCG8090	0	0
4673	36683	Drosophila melanogaster	CG11807	CG11807 gene product from transcript CG11807-RA	CT14564|DmelCG11807	0	0
4674	36685	Drosophila melanogaster	row	relative of woc	CG8092|DmelCG8092|Row|anon-WO0118547.194	0	0
4675	36686	Drosophila melanogaster	CG8093	CG8093 gene product from transcript CG8093-RA	CT22063|DmelCG8093	0	0
4676	36687	Drosophila melanogaster	CG11808	CG11808 gene product from transcript CG11808-RA	DmelCG11808	0	0
4677	36688	Drosophila melanogaster	mRpL41	mitochondrial ribosomal protein L41	BcDNA:RE19416|CG12954|DmelCG12954|YmL27	0	0
4678	36689	Drosophila melanogaster	CG8079	CG8079 gene product from transcript CG8079-RA	DmelCG8079	0	0
4679	36692	Drosophila melanogaster	scb	scab	CG8095|CT21280|CT36929|Dm0620|DmelCG8095|E(Sev-CycE)E93|E(sev-cycE)[e93]|PS3|Vol|aPS3|alpha-PS3|alphaInt3|alphaPS3|alpha[[PS3]]|beta[[PS3]]|l(2)01288|vol	0	0
4680	36693	Drosophila melanogaster	alphaPS4	CG16827 gene product from transcript CG16827-RA	CG16827|CT37393|DmelCG16827|alpha-ps4	0	0
4681	36694	Drosophila melanogaster	trpm	CG34123 gene product from transcript CG34123-RG	BcDNA:GH04950|CG16805|CG30078|CG30079|CG34123|DmelCG34123|TRPM|TrpM|dTRPM	0	0
4682	36695	Drosophila melanogaster	CG8102	CG8102 gene product from transcript CG8102-RB	DmelCG8102	0	0
4683	36696	Drosophila melanogaster	CG8152	CG8152 gene product from transcript CG8152-RA	DmelCG8152	0	0
4684	36697	Drosophila melanogaster	mus210	mutagen-sensitive 210	CG8153|DhR4|DhXPC|DmXPC|DmelCG8153|XPC[DM]|Xpcc|mus(2)201|mus(2)201G1|mus(2)201[GI]|mus201[G1]|mus212	0	0
4685	36698	Drosophila melanogaster	CG8155	CG8155 gene product from transcript CG8155-RA	DmelCG8155	0	0
4686	36699	Drosophila melanogaster	Arf51F	ADP ribosylation factor 51F	AFR51F|ARF3|ARF6|ARFIII|Arf51f|Arf6|CG8156|Dm Arf51F|DmelCG8156|EP(2)2612|LD12493|arf6|dARFIII|dArf6	0	0
4687	36700	Drosophila melanogaster	CG8157	CG8157 gene product from transcript CG8157-RA	BcDNA:RE07473|DmelCG8157	0	0
4688	36701	Drosophila melanogaster	CG8160	CG8160 gene product from transcript CG8160-RA	DmelCG8160	0	0
4689	36702	Drosophila melanogaster	Hr51	Hormone receptor 51	CG16801|CG16801/NR2E3|DHR51|DmelCG16801|HR51|NR2E3|PNR|fdx|unf	0	0
4690	36703	Drosophila melanogaster	unc-5	CG8166 gene product from transcript CG8166-RA	AAF7419|CG8166|CG8168|CT20824|DmelCG8166|Dunc5|UNC-5|UNC5|UNC5D|Unc-5|Unc5|Unc5d|unc5	0	0
4691	36705	Drosophila melanogaster	Pms2	CG8169 gene product from transcript CG8169-RA	CG8169|DmelCG8169|Mlh2|dmlh-2|dmlh2|dpms2|pms2	0	0
4692	36706	Drosophila melanogaster	SRPK	CG8174 gene product from transcript CG8174-RA	BcDNA:SD03158|CG8174|DmelCG8174|dSRPK|dSRPK1|srpk	0	0
4693	36708	Drosophila melanogaster	Mtk	Metchnikowin	BcDNA:RH07954|CG8175|DIM 17|DmelCG8175|METCH|Metc|Metch|metch|metk|mtk	0	0
4694	36712	Drosophila melanogaster	CG8179	CG8179 gene product from transcript CG8179-RA	DmelCG8179	0	0
4695	36713	Drosophila melanogaster	CG8180	CG8180 gene product from transcript CG8180-RA	DmelCG8180	0	0
4696	36714	Drosophila melanogaster	CG12964	CG12964 gene product from transcript CG12964-RB	DmelCG12964	0	0
4697	36715	Drosophila melanogaster	Ir52a	Ionotropic receptor 52a	CG12960|DmelCG12960|IR52a	0	0
4698	36716	Drosophila melanogaster	Ir52d	Ionotropic receptor 52d	CG12961|CG30464|DmelCG30464|IR52d	0	0
4699	36717	Drosophila melanogaster	GalNAc-T1	CG8182 gene product from transcript CG8182-RA	CG8182|DmelCG8182|pgant1	0	0
4700	36718	Drosophila melanogaster	Khc-73	Kinesin-73	CG8183|DmKIN73|DmKlp73|DmelCG8183|Khc73|anon-EST:fe3C9	0	0
4701	36719	Drosophila melanogaster	CG30467	CG30467 gene product from transcript CG30467-RA	CG8185|DmelCG30467	0	0
4702	36720	Drosophila melanogaster	CG8187	CG8187 gene product from transcript CG8187-RA	DmelCG8187	0	0
4703	36722	Drosophila melanogaster	eIF2B-gamma	CG8190 gene product from transcript CG8190-RA	CG8190|DmelCG8190|eIF2Bgamma	0	0
4704	36723	Drosophila melanogaster	CG8192	CG8192 gene product from transcript CG8192-RA	DmelCG8192	0	0
4705	36724	Drosophila melanogaster	CG12963	CG12963 gene product from transcript CG12963-RD	BcDNA:AT28470|DmelCG12963	0	0
4706	36725	Drosophila melanogaster	CG8195	CG8195 gene product from transcript CG8195-RA	DmelCG8195	0	0
4707	36726	Drosophila melanogaster	Flo	flotillin	CG8200|CK02126|DmelCG8200|FLO1|FLO[Dm]|Flo[Dm]|flo|reggie-2|reggie2/flo1	0	0
4708	36727	Drosophila melanogaster	Cdk5	Cyclin-dependent kinase 5	CDK5|CG8203|DmCdk5|DmelCG8203|cdk5	0	0
4709	36728	Drosophila melanogaster	CG8204	CG8204 gene product from transcript CG8204-RA	BcDNA:AT23078|DmelCG8204	0	0
4710	36730	Drosophila melanogaster	CG8207	CG8207 gene product from transcript CG8207-RA	DmelCG8207	0	0
4711	36731	Drosophila melanogaster	Vha14-1	Vacuolar H[+] ATPase subunit 14-1	ATPase|CG8210|DmelCG8210|V-ATPase|VAF1_DROME|Vha14|l(2)SH1248|l(2)SH2 1248|vha14|vha14-1	0	0
4712	36732	Drosophila melanogaster	Ranbp11	CG33139 gene product from transcript CG33139-RA	CG33139|CG8212|DmelCG33139|SP49|imp-beta11|impbeta11	0	0
4713	36733	Drosophila melanogaster	CG8214	CG8214 gene product from transcript CG8214-RA	DmelCG8214	0	0
4714	36736	Drosophila melanogaster	CG30089	CG30089 gene product from transcript CG30089-RA	CG12305|CG12966|CG12967|DmelCG30089	0	0
4715	36740	Drosophila melanogaster	Zasp52	Z band alternatively spliced PDZ-motif protein 52	CG12968|CG12969|CG30084|CG8242|DmelCG30084|Zasp|tun|tungus|zasp	0	0
4716	36741	Drosophila melanogaster	Poxn	Pox neuro	CG8246|DmelCG8246|P4|Pox-n|PoxN|neuro|pox-n|poxn	0	0
4717	36742	Drosophila melanogaster	CG8249	CG8249 gene product from transcript CG8249-RC	DmelCG8249	0	0
4718	36743	Drosophila melanogaster	tun	tungus	BcDNA:LD38553|CG8253|DmelCG8253|Ntaq1	0	0
4719	36744	Drosophila melanogaster	CG12970	CG12970 gene product from transcript CG12970-RC	DmelCG12970	0	0
4720	36746	Drosophila melanogaster	sli	slit	CG8355|CT21700|CT37068|DmelCG8355|Slit|dSlit|fus3|l(2)05248|wdh	0	0
4721	36747	Drosophila melanogaster	bdg	bedraggled	CG8291|DmelCG8291	0	0
4722	36748	Drosophila melanogaster	Iap2	Inhibitor of apoptosis 2	CG8293|D-IAP2|D-iap2|DIAP|DIAP-2|DIAP2|DIAPII|DIHA|Diap|Diap2|Diha|DmelCG8293|IAP|IAP2|Ilp|dIAP|dIAP2|dILP|diap2	0	0
4723	36749	Drosophila melanogaster	CG8297	CG8297 gene product from transcript CG8297-RA	DmelCG8297	0	0
4724	36750	Drosophila melanogaster	Mlf	Myelodysplasia/myeloid leukemia factor	BcDNA:SD18148|CG8295|DmelCG8295|MLF|dMLF|dmlf	0	0
4725	36751	Drosophila melanogaster	CG8299	CG8299 gene product from transcript CG8299-RA	DmelCG8299|SP65	0	0
4726	36752	Drosophila melanogaster	Cyp4aa1	CG8302 gene product from transcript CG8302-RB	4aa1|CG8302|DmelCG8302	0	0
4727	36753	Drosophila melanogaster	Strn-Mlck	Stretchin-Mlck	143736_at|A(225)|BEST:GH22543|BcDNA:RH74685|CG18255|CG8304|CT41348|DmelCG18255|MLCK|MLCK-1|MLCK-2|MLCK-3|Mlc-k|Mlck|mlck|strn-mlck	0	0
4728	36754	Drosophila melanogaster	CG8366	CG8366 gene product from transcript CG8366-RA	DmelCG8366	0	0
4729	36756	Drosophila melanogaster	CG15699	CG15699 gene product from transcript CG15699-RB	DmelCG15699	0	0
4730	36757	Drosophila melanogaster	CG8314	CG8314 gene product from transcript CG8314-RA	DmelCG8314|anon-WO0118547.93	0	0
4731	36758	Drosophila melanogaster	CG8315	CG8315 gene product from transcript CG8315-RA	DmelCG8315	0	0
4732	36759	Drosophila melanogaster	CG8320	CG8320 gene product from transcript CG8320-RA	DmelCG8320	0	0
4733	36760	Drosophila melanogaster	ATPCL	ATP citrate lyase	BcDNA:LD21334|CG8322|DmATPCL|DmelCG8322|anon-WO0140519.179|l(2)01466|l(2)k09217|n(2)k09217	0	0
4734	36761	Drosophila melanogaster	CG8370	CG8370 gene product from transcript CG8370-RA	DmelCG8370	0	0
4735	36762	Drosophila melanogaster	CG8386	CG8386 gene product from transcript CG8386-RA	DmelCG8386	0	0
4736	36763	Drosophila melanogaster	CG8388	CG8388 gene product from transcript CG8388-RA	DmelCG8388	0	0
4737	36764	Drosophila melanogaster	CG8389	CG8389 gene product from transcript CG8389-RB	DmelCG8389	0	0
4738	36766	Drosophila melanogaster	Rrp42	CG8395 gene product from transcript CG8395-RA	CG8395|DmelCG8395|anon-EST:Posey140|anon-EST:Posey187|dRrp42	0	0
4739	36767	Drosophila melanogaster	CG8397	CG8397 gene product from transcript CG8397-RA	DmelCG8397	0	0
4740	36768	Drosophila melanogaster	CG8399	CG8399 gene product from transcript CG8399-RA	DmelCG8399|anon-EST:ParkEST009	0	0
4741	36769	Drosophila melanogaster	casp	caspar	CG8400|DmelCG8400	0	0
4742	36770	Drosophila melanogaster	CG8401	CG8401 gene product from transcript CG8401-RB	DmelCG8401	0	0
4743	36771	Drosophila melanogaster	SP2353	CG8403 gene product from transcript CG8403-RA	BcDNA:GH12319|CG8403|CT18665|DmelCG8403	0	0
4744	36772	Drosophila melanogaster	CG8405	CG8405 gene product from transcript CG8405-RA	DmelCG8405	0	0
4745	36773	Drosophila melanogaster	Dg	Dystroglycan	CG18250|CT41273|DG|DmDG|DmelCG18250|atu|dg|dgn|dys	0	0
4746	36774	Drosophila melanogaster	CG8414	CG8414 gene product from transcript CG8414-RA	DmelCG8414	0	0
4747	36775	Drosophila melanogaster	Rho1	CG8416 gene product from transcript CG8416-RB	8416|AAF01186|CG8416|D-Rho1|DRho|DRho1|DRhoA|Dm Rho1|DmRHO-A|DmelCG8416|Drho1|DrhoA|E3.10/J3.8|RHO|RHO1|Rho|Rho A|Rho GTPase|Rho kinase|RhoA|RhoN19|dRho1|dRhoA|dnRho|l(2)	0	0
4748	36776	Drosophila melanogaster	Ric	Ras which interacts with Calmodulin	CG8418|DmelCG8418|RIC	0	0
4749	36778	Drosophila melanogaster	Asph	Aspartyl beta-hydroxylase	BAH/humbug|CG18658|CG8421|DmelCG8421	0	0
4750	36779	Drosophila melanogaster	Jhedup	Juvenile hormone esterase duplication	CG8424|DmelCG8424|JHEdup	0	0
4751	36780	Drosophila melanogaster	Jhe	Juvenile hormone esterase	CG8425|DmJhe|DmelCG8425|JH|JHE|jhe	0	0
4752	36782	Drosophila melanogaster	Got1	Glutamate oxaloacetate transaminase 1	BEST:GH07823|CG8430|DmelCG8430|FBgn 1124|GOT-1|Got-1|Got2	0	0
4753	36784	Drosophila melanogaster	Aats-cys	Cysteinyl-tRNA synthetase	BcDNA:LD21177|CG8431|CRS|CysRS|DmelCG8431	0	0
4754	36787	Drosophila melanogaster	CG10734	CG10734 gene product from transcript CG10734-RA	DmelCG10734	0	0
4755	36788	Drosophila melanogaster	lbk	lambik	CG8434|DmelCG8434|Lbk	0	0
4756	36789	Drosophila melanogaster	CG8435	CG8435 gene product from transcript CG8435-RA	DmelCG8435	0	0
4757	36790	Drosophila melanogaster	Ptp52F	CG18243 gene product from transcript CG18243-RB	CG18243|CT18044|DPTP52F|DmelCG18243|PTP52F	0	0
4758	36791	Drosophila melanogaster	Lis-1	Lissencephaly-1	CG8440|D-Lis1|DLIS-1|DLIS1|DLis-1|DLis1|Dlis-1|Dlis1|DmLIS1|DmelCG8440|LIS-1|Lis 1|Lis1|dLis1|l(2)k13209|l(2R)W8|lis-1|lis1|n(2)k11702	0	0
4759	36792	Drosophila melanogaster	CG8441	CG8441 gene product from transcript CG8441-RB	DmelCG8441	0	0
4760	36793	Drosophila melanogaster	clu	clueless	CG8443|DmelCG8443|anon-WO0118547.143	0	0
4761	36794	Drosophila melanogaster	calypso	CG8445 gene product from transcript CG8445-RB	BAP1|CG8445|DmelCG8445	0	0
4762	36795	Drosophila melanogaster	CG8446	CG8446 gene product from transcript CG8446-RC	DmelCG8446	0	0
4763	36797	Drosophila melanogaster	mrj	CG8448 gene product from transcript CG8448-RD	CG8448|DmelCG8448|dMRJ|dmrj	0	0
4764	36798	Drosophila melanogaster	CG7798	CG7798 gene product from transcript CG7798-RA	DmelCG7798	0	0
4765	36799	Drosophila melanogaster	CG15706	CG15706 gene product from transcript CG15706-RA	DmelCG15706	0	0
4766	36800	Drosophila melanogaster	Tsf3	Transferrin 3	CG3666|DmelCG3666	0	0
4767	36801	Drosophila melanogaster	CG15701	CG15701 gene product from transcript CG15701-RA	DmelCG15701	0	0
4768	36802	Drosophila melanogaster	CG7786	CG7786 gene product from transcript CG7786-RA	DmelCG7786	0	0
4769	36803	Drosophila melanogaster	CG3687	CG3687 gene product from transcript CG3687-RA	BcDNA:AT22273|DmelCG3687	0	0
4770	36804	Drosophila melanogaster	krimp	krimper	CG15707|DmelCG15707|mtc	0	0
4771	36805	Drosophila melanogaster	CG15708	CG15708 gene product from transcript CG15708-RA	DmelCG15708	0	0

4772	36806	Drosophila melanogaster	Cng	Cyclic-nucleotide-gated ion channel protein	CG15709|CG42701|CG7779|CNG|DmCNGC|DmelCG42701|Dmel_CG15709|Dmel_CG7779|cng|dCNCalpha	0	0
4773	36808	Drosophila melanogaster	fidipidine	CG7773 gene product from transcript CG7773-RA	CG7773|DmelCG7773	0	0
4774	36809	Drosophila melanogaster	csul	capsuleen	CG3730|Csul|DART5|Dart5|DmelCG3730|JBP1|PRMT5|dPRMT5|dart5|dprmt5	0	0
4775	36810	Drosophila melanogaster	Khc	Kinesin heavy chain	CG7765|DK|DKH|DmK|DmKHC|DmelCG7765|Dmkin|KHC|KIF5A|KIN|Kin|khc|kin|kinesin|l(2)W12|l(2)k13219|l(2)k13314|l(2R)W12|pgs	0	0
4776	36811	Drosophila melanogaster	CG33017	CG33017 gene product from transcript CG33017-RB	CG15702|CG15703|DmelCG33017	0	0
4777	36813	Drosophila melanogaster	cato	cousin of atonal	CG7760|Cato|DATH-B|DmelCG7760|bHLHa12	0	0
4778	36814	Drosophila melanogaster	CG15704	CG15704 gene product from transcript CG15704-RA	BcDNA:RH57845|DmelCG15704	0	0
4779	36815	Drosophila melanogaster	CG15705	CG15705 gene product from transcript CG15705-RA	DmelCG15705	0	0
4780	36816	Drosophila melanogaster	CG7755	CG7755 gene product from transcript CG7755-RA	DmelCG7755	0	0
4781	36817	Drosophila melanogaster	CG9068	CG9068 gene product from transcript CG9068-RA	DmelCG9068	0	0
4782	36818	Drosophila melanogaster	CG30099	CG30099 gene product from transcript CG30099-RA	CG7750|DmelCG30099	0	0
4783	36819	Drosophila melanogaster	JhI-26	Juvenile hormone-inducible protein 26	CG3767|DmelCG3767	0	0
4784	36820	Drosophila melanogaster	CG7747	CG7747 gene product from transcript CG7747-RA	DmelCG7747	0	0
4785	36821	Drosophila melanogaster	Atg9	Autophagy-specific gene 9	CG3615|DmelCG3615|atg9	0	0
4786	36824	Drosophila melanogaster	CG8060	CG8060 gene product from transcript CG8060-RA	DmelCG8060	0	0
4787	36825	Drosophila melanogaster	CG4282	CG4282 gene product from transcript CG4282-RA	DmelCG4282	0	0
4788	36826	Drosophila melanogaster	Vha44	Vacuolar H[+] ATPase 44kD C subunit	6072|CG8048|DmelCG8048|V-ATPase|l(2)6072|l(2)SH1339|l(2)SH2 1339|vha44	0	0
4789	36829	Drosophila melanogaster	CG7997	CG7997 gene product from transcript CG7997-RB	CT24026|DmelCG7997	0	0
4790	36830	Drosophila melanogaster	Nup62	Nucleoporin 62	CG6251|DmelCG6251|p62	0	0
4791	36831	Drosophila melanogaster	wcd	wicked	CG7989|DmelCG7989|l(2)k07824	0	0
4792	36832	Drosophila melanogaster	CG15710	CG15710 gene product from transcript CG15710-RA	DmelCG15710	0	0
4793	36833	Drosophila melanogaster	CG6262	CG6262 gene product from transcript CG6262-RB	DmelCG6262	0	0
4794	36834	Drosophila melanogaster	CG15711	CG15711 gene product from transcript CG15711-RA	DmelCG15711	0	0
4795	36838	Drosophila melanogaster	CG4398	CG4398 gene product from transcript CG4398-RA	DmelCG4398	0	0
4796	36839	Drosophila melanogaster	CG7848	CG7848 gene product from transcript CG7848-RA	DmelCG7848	0	0
4797	36840	Drosophila melanogaster	CG4409	CG4409 gene product from transcript CG4409-RB	DmelCG4409	0	0
4798	36841	Drosophila melanogaster	CG15925	CG15925 gene product from transcript CG15925-RA	DmelCG15925	0	0
4799	36843	Drosophila melanogaster	CG15712	CG15712 gene product from transcript CG15712-RA	DmelCG15712	0	0
4800	36844	Drosophila melanogaster	S-Lap8	Sperm-Leucylaminopeptidase 8	CG4439|DmelCG4439|S-LAP 8|anon-WO0140519.41	0	0
4801	36845	Drosophila melanogaster	CG7813	CG7813 gene product from transcript CG7813-RA	DmelCG7813	0	0
4802	36846	Drosophila melanogaster	Sema-2a	CG4700 gene product from transcript CG4700-RC	112/40|BcDNA:LD09561|CG4700|CT14922|D-Sema II|D-Sema-II|D-Sema2a|D-SemaII|D-semaII|DmelCG4700|Dsema-II|Sema2a|SemaII|dSema-II|l(2)03021|sema 2a|sema II|sema-2a|sema-II|sema2a|sema	0	0
4803	36847	Drosophila melanogaster	loopin-1	CG4750 gene product from transcript CG4750-RA	CG4750|DmelCG4750|S-LAP 6|anon-WO0140519.81|kl3 lp-1|kl3lp1|loopin	0	0
4804	36848	Drosophila melanogaster	Syn2	Syntrophin-like 2	CG4905|DmSYN-2|DmelCG4905	0	0
4805	36849	Drosophila melanogaster	DAT	Dopamine transporter	CG8380|DmDAT|DmelCG8380|dDAT|fmn	0	0
4806	36850	Drosophila melanogaster	CG4945	CG4945 gene product from transcript CG4945-RA	CT15864|DmelCG4945|PKN	0	0
4807	36851	Drosophila melanogaster	RpS15	Ribosomal protein S15	CG8332|DmelCG8332|M(2)53|anon-EST:Posey137|anon-EST:Posey185	0	0
4808	36852	Drosophila melanogaster	CG4927	CG4927 gene product from transcript CG4927-RC	DmelCG4927|SP42	0	0
4809	36853	Drosophila melanogaster	CG8317	CG8317 gene product from transcript CG8317-RA	DmelCG8317	0	0
4810	36854	Drosophila melanogaster	Cdk4	Cyclin-dependent kinase 4	8-6|CDK4|CDK4/6|CG5072|Cdk4/6|DmCdk4|DmelCG5072|Pk53C|Pk?7|cdk|cdk4|cdk4/6|dCdk4|l(2)05428|l(2)0671|l(2)k06503|l(2)s4639|l(2)sh0671	0	0
4811	36855	Drosophila melanogaster	RpLP2	Ribosomal protein LP2	BcDNA:SD22208|CG4918|DmelCG4918|LP2|M(2)53|RLA2_DROME|RpA1|RpP1|RplP2|T1|Tp1|bs34h02.y1|rpA1	0	0
4812	36856	Drosophila melanogaster	CG8311	CG8311 gene product from transcript CG8311-RA	DmelCG8311	0	0
4813	36857	Drosophila melanogaster	CG8306	CG8306 gene product from transcript CG8306-RA	DmelCG8306	0	0
4814	36858	Drosophila melanogaster	CG8303	CG8303 gene product from transcript CG8303-RB	DmelCG8303	0	0
4815	36859	Drosophila melanogaster	CG5089	CG5089 gene product from transcript CG5089-RA	DmelCG5089|anon-WO0140519.170	0	0
4816	36860	Drosophila melanogaster	CG5065	CG5065 gene product from transcript CG5065-RA	DmelCG5065	0	0
4817	36862	Drosophila melanogaster	gprs	CG18471 gene product from transcript CG18471-RA	CG18471|CG5967|DmelCG18471|Gprs	0	0
4818	36863	Drosophila melanogaster	Amyrel	CG8221 gene product from transcript CG8221-RA	CG8221|DmelCG8221|amyrel	0	0
4819	36864	Drosophila melanogaster	Nach	CG8178 gene product from transcript CG8178-RA	CG8178|DmelCG8178|PPK4|ppk4	0	0
4820	36865	Drosophila melanogaster	Picot	CG8098 gene product from transcript CG8098-RA	CG8098|DmelCG8098|picot	0	0
4821	36868	Drosophila melanogaster	CG5210	CG5210 gene product from transcript CG5210-RA	Chit|Chit13|Cht13|DS47|DmDS47|DmelCG5210|chit|ds47	0	0
4822	36871	Drosophila melanogaster	CG15617	CG15617 gene product from transcript CG15617-RA	CT35755|DmelCG15617	0	0
4823	36872	Drosophila melanogaster	Acp53C14a	CG8626 gene product from transcript CG8626-RA	CG8626|DmelCG8626	0	0
4824	36873	Drosophila melanogaster	Acp53C14b	CG15616 gene product from transcript CG15616-RA	CG15616|DmelCG15616	0	0
4825	36874	Drosophila melanogaster	Acp53Ea	Accessory gland-specific peptide 53Ea	ACP53EA|Acp53E|BcDNA:AT09943|CG8622|DmelCG8622	0	0
4826	36875	Drosophila melanogaster	CG5267	CG5267 gene product from transcript CG5267-RA	DmelCG5267	0	0
4827	36876	Drosophila melanogaster	unc-104	CG8566 gene product from transcript CG8566-RB	CG8566|DmCG8566|DmKlp53D|DmelCG8566|Dunc104|KIF1B|Klp53D|Unc-104|Unc104|imac	0	0
4828	36877	Drosophila melanogaster	CG5348	CG5348 gene product from transcript CG5348-RC	DmelCG5348	0	0
4829	36878	Drosophila melanogaster	Sod2	Superoxide dismutase 2 (Mn)	CG8905|DmelCG8905|Mito SOD|Mn SOD|Mn-SOD|Mn-SOD2|MnSOD|MnSODII|Mn[2+]SOD|SOD|SOD-2|SOD2|Sod-2|dSOD2|mitSOD2|mnSOD	0	0
4830	36879	Drosophila melanogaster	Arp53D	Actin-related protein 53D	ARP53D|Actr53D|CG5409|DmelCG5409|arp5|arp53D	0	0
4831	36880	Drosophila melanogaster	resilin	CG15920 gene product from transcript CG15920-RA	CG15920|DmelCG15920	0	0
4832	36881	Drosophila melanogaster	CG5522	CG5522 gene product from transcript CG5522-RA	DmelCG5522|RalGPS	0	0
4833	36882	Drosophila melanogaster	CG15615	CG15615 gene product from transcript CG15615-RB	DmelCG15615	0	0
4834	36883	Drosophila melanogaster	CG5550	CG5550 gene product from transcript CG5550-RB	DmelCG5550|FBpp0086184	0	0
4835	36886	Drosophila melanogaster	IntS8	Integrator 8	CG5859|DmelCG5859|EG:EG0003.5|Int8	0	0
4836	36887	Drosophila melanogaster	Fen1	Flap endonuclease 1	CG8648|DmFEN-1|DmelCG8648|EG:EG0003.3|FEN1	0	0
4837	36889	Drosophila melanogaster	Psi	P-element somatic inhibitor	CG8912|DmelCG8912|EG:EG0003.2|PSI	0	0
4838	36891	Drosophila melanogaster	CG6426	CG6426 gene product from transcript CG6426-RA	BcDNA:RH01665|DmelCG6426|l(2)k04810|n(2)k04810	0	0
4839	36893	Drosophila melanogaster	CG6429	CG6429 gene product from transcript CG6429-RA	BcDNA:RH62928|DmelCG6429	0	0
4840	36894	Drosophila melanogaster	CG6435	CG6435 gene product from transcript CG6435-RA	DmelCG6435	0	0
4841	36895	Drosophila melanogaster	CG6472	CG6472 gene product from transcript CG6472-RA	DmelCG6472	0	0
4842	36896	Drosophila melanogaster	CG8910	CG8910 gene product from transcript CG8910-RA	DmelCG8910|EG:EG0003.1|anon-WO0140519.220	0	0
4843	36897	Drosophila melanogaster	inaC	inactivation no afterpotential C	CG6518|DmelCG6518|Dpkc2|INAC|InaC|PKC|PKC 53E|PKCi|Pkc2|dPKC53E|dPKC53E(ey)|ePKC|eye PKC|eye-PKC|pkc-2	0	0
4844	36899	Drosophila melanogaster	CG15614	CG15614 gene product from transcript CG15614-RA	DmelCG15614	0	0
4845	36900	Drosophila melanogaster	Vha16-4	Vacuolar H[+] ATPase subunit 16-4	CG9013|DmelCG9013|V-ATPase|vha16-4	0	0
4846	36903	Drosophila melanogaster	CG6665	CG6665 gene product from transcript CG6665-RA	DmelCG6665	0	0
4847	36904	Drosophila melanogaster	CG9010	CG9010 gene product from transcript CG9010-RA	DmelCG9010|anon-WO0140519.132	0	0
4848	36905	Drosophila melanogaster	Cbp53E	Calbindin 53E	CG6702|Calb|Cbp33|DCBP|Dcbp|Dcp|DmelCG6702|cbn|cbp53E	0	0
4849	36907	Drosophila melanogaster	ste24b	ste24b prenyl protease type I	CG9001|DmelCG9001|DmelSte24|DmelSte24b	0	0
4850	36908	Drosophila melanogaster	ste24a	prenyl protease type I	CG9000|DmelCG9000|DmelSte24|DmelSte24a	0	0
4851	36909	Drosophila melanogaster	CG6796	CG6796 gene product from transcript CG6796-RA	DmelCG6796	0	0
4852	36911	Drosophila melanogaster	CG6805	CG6805 gene product from transcript CG6805-RA	BEST:CK01299|CK01299|DmelCG6805|IPP	0	0
4853	36912	Drosophila melanogaster	CG15609	CG15609 gene product from transcript CG15609-RB	DmelCG15609|dEHBP-1	0	0
4854	36913	Drosophila melanogaster	CG8963	CG8963 gene product from transcript CG8963-RD	DmelCG8963	0	0
4855	36914	Drosophila melanogaster	Ark	Apaf-1-related-killer	APAF1|ARK|Apaf-1|Apaf1|CG6829|D-Apaf-1|DARK|Dapaf-1|Dapaf-1/HAC-1|Dark|Dark/Apaf-I|Dark/Dapaf-1/HAC1|Dark/Hac-1/dApaf-1|Dark/Hac-1/dApaf1|DmelCG6829|Hac-1|Hac-1/Dark|Hac1|T1|anon-	0	0
4856	36915	Drosophila melanogaster	RhoGEF2	CG9635 gene product from transcript CG9635-RF	CG9635|DRhoGEF|DRhoGEF2|DmelCG9635|DrhoGEF2|Gef2|RG2|Rho-GEF2|RhoGEF|RhoGef2|Su(Rho1)2B|T2|dRhoGEF|dRhoGEF2|l(2)04291|rhoGEF2|rhogef2|shar	0	0
4857	36916	Drosophila melanogaster	CG9640	CG9640 gene product from transcript CG9640-RA	DmelCG9640	0	0
4858	36917	Drosophila melanogaster	CG9642	CG9642 gene product from transcript CG9642-RA	DmelCG9642	0	0
4859	36918	Drosophila melanogaster	CG9646	CG9646 gene product from transcript CG9646-RA	DmelCG9646	0	0
4860	36919	Drosophila melanogaster	fat-spondin	CG6953 gene product from transcript CG6953-RB	CG6953|CT21501|DmelCG6953|Fat-spondin	0	0
4861	36920	Drosophila melanogaster	tef	teflon	CG8961|DmelCG8961|l(2)k15914	0	0
4862	36921	Drosophila melanogaster	CG8950	CG8950 gene product from transcript CG8950-RB	DmelCG8950	0	0
4863	36922	Drosophila melanogaster	CG6967	CG6967 gene product from transcript CG6967-RD	CT21565|DmelCG6967	0	0
4864	36924	Drosophila melanogaster	CG30460	CG30460 gene product from transcript CG30460-RE	CG15607|CG15608|DmelCG30460|anon-WO0140519.237	0	0
4865	36926	Drosophila melanogaster	CG6984	CG6984 gene product from transcript CG6984-RA	DmelCG6984	0	0
4866	36927	Drosophila melanogaster	GstS1	Glutathione S transferase S1	CG8938|DmGST-2|DmGST2|DmGSTS1|DmGSTS1-1|DmGst-2|DmGstS1-1|DmelCG8938|Est2|GST|GST S1|GST-2|GST2|GSTII|GSTS1|Gst2|GstS1-1|S1|gstD2|gsts1|l(2)04227|l(2)k08805|n(2)06253	0	0
4867	36929	Drosophila melanogaster	CG30456	CG30456 gene product from transcript CG30456-RB	CG15612|CG15613|DmelCG30456	0	0
4868	36930	Drosophila melanogaster	CG15611	CG15611 gene product from transcript CG15611-RB	DmelCG15611	0	0
4869	36931	Drosophila melanogaster	Spn53F	Serpin 53F	CG10956|DmelCG10956	0	0
4870	36932	Drosophila melanogaster	Amy-d	Amylase distal	AMY|Amy|Amy(d)|Amy-distal|Amy1|Amy3|Amy6|AmyB|AmyD|CG17876|DmelCG17876|alpha-AMY|alphaAmy|amy|amy-d|amyB(3)	0	0
4871	36933	Drosophila melanogaster	CG15605	CG15605 gene product from transcript CG15605-RA	BcDNA:AT23338|DmelCG15605	0	0
4872	36934	Drosophila melanogaster	Cda9	Chitin deacetylase-like 9	CG15918|DmCDA9|DmelCG15918	0	0
4873	36935	Drosophila melanogaster	CG11400	CG11400 gene product from transcript CG11400-RA	DmelCG11400	0	0
4874	36936	Drosophila melanogaster	CG15917	CG15917 gene product from transcript CG15917-RA	DmelCG15917	0	0
4875	36937	Drosophila melanogaster	CG11395	CG11395 gene product from transcript CG11395-RA	DmelCG11395	0	0
4876	36938	Drosophila melanogaster	CG17290	CG17290 gene product from transcript CG17290-RA	BcDNA:RE15976|DmelCG17290	0	0
4877	36939	Drosophila melanogaster	CG17287	CG17287 gene product from transcript CG17287-RA	DmelCG17287	0	0
4878	36940	Drosophila melanogaster	CG30457	CG30457 gene product from transcript CG30457-RA	CG17288|CG30457-PA|DmelCG30457	0	0
4879	36941	Drosophila melanogaster	CG10953	CG10953 gene product from transcript CG10953-RA	CG10953-PA|DmelCG10953	0	0
4880	36942	Drosophila melanogaster	CG10950	CG10950 gene product from transcript CG10950-RA	DmelCG10950	0	0
4881	36943	Drosophila melanogaster	CG18469	CG18469 gene product from transcript CG18469-RA	BcDNA:AT30190|DmelCG18469	0	0
4882	36944	Drosophila melanogaster	CG12699	CG12699 gene product from transcript CG12699-RB	BEST:GH14656|DmelCG12699|GH14656|NEST:bs24f01|anon-WO0140519.144|bs24f01.y1	0	0

4883	36945	Drosophila melanogaster	mbl	muscleblind	CG10941|CG14477|CG33197|DmelCG33197|Mbl|chaste|chst|cht|l(2)01038|l(2)k01212|l(2)k05507|mm|n(2)k07110	0	0
4884	36948	Drosophila melanogaster	CG6568	CG6568 gene product from transcript CG6568-RA	DmelCG6568	0	0
4885	36950	Drosophila melanogaster	CG30101	CG30101 gene product from transcript CG30101-RA	CG15901|CG6564|DmelCG30101|FBgn0050101	0	0
4886	36951	Drosophila melanogaster	Prosalpha5	Proteasome alpha5 subunit	154644_at|CG10938|DmelCG10938|PRCZ|PSA5_DROME|PSMA5|ProsMA5|alpha5_dm|prosMA5	0	0
4887	36952	Drosophila melanogaster	cnk	connector enhancer of ksr	CG6556|CNK|Cnk|DmelCG6556|EK2-3|anon-WO0140519.129|anon-WO0257455.27|l(2)k16314	0	0
4888	36953	Drosophila melanogaster	l(2)k01209	lethal (2) k01209	CG4798|DmelCG4798|l(2)K01209|l(2)k08901	0	0
4889	36954	Drosophila melanogaster	CG6550	CG6550 gene product from transcript CG6550-RA	DmelCG6550	0	0
4890	36955	Drosophila melanogaster	CG4802	CG4802 gene product from transcript CG4802-RA	DmelCG4802	0	0
4891	36956	Drosophila melanogaster	Bap55	Brahma associated protein 55kD	BAF53|BAP55|Baf53|CG6546|DmelCG6546|bap55	0	0
4892	36957	Drosophila melanogaster	Lhr	Lethal hybrid rescue	CG18468|DmelCG18468|HP3|LHR	0	0
4893	36958	Drosophila melanogaster	CG18467	CG18467 gene product from transcript CG18467-RA	DmelCG18467	0	0
4894	36959	Drosophila melanogaster	EDTP	Egg-derived tyrosine phosphatase	BcDNA:LD23181|CG6542|D-EDTP|DmelCG6542|anon-WO0118547.124	0	0
4895	36960	Drosophila melanogaster	mthl4	methuselah-like 4	CG6536|DmelCG6536|anon-WO0170980.55|anon-WO0170980.56	0	0
4896	36961	Drosophila melanogaster	mthl3	methuselah-like 3	104/8|BEST:GM02553|CG6530|DmelCG6530|GENE 1|Mth-3|anon-WO0170980.52|anon-WO0170980.53|gene 1|l(2)k10408	0	0
4897	36962	Drosophila melanogaster	CG10764	CG10764 gene product from transcript CG10764-RA	DmelCG10764|FBgn0034221|GENE 2|SP77|anon-54Ba|gene 2	0	0
4898	36963	Drosophila melanogaster	robl	roadblock	CG10751|DLC|DmelCG10751|LC7|l(2)k10408	0	0
4899	36964	Drosophila melanogaster	CG14478	CG14478 gene product from transcript CG14478-RA	BEST:LD13441|DmelCG14478|GENE 4|gene 4	0	0
4900	36965	Drosophila melanogaster	CG6522	CG6522 gene product from transcript CG6522-RA	DmelCG6522	0	0
4901	36966	Drosophila melanogaster	qkr54B	quaking related 54B	CG4816|DmelCG4816|QKR54B|SAM|SAM50|Sam50|p62GAP|qrk54B	0	0
4902	36967	Drosophila melanogaster	CG6520	CG6520 gene product from transcript CG6520-RA	DmelCG6520	0	0
4903	36968	Drosophila melanogaster	veil	CG4827 gene product from transcript CG4827-RA	CG4827|DmelCG4827	0	0
4904	36969	Drosophila melanogaster	CG30103	CG30103 gene product from transcript CG30103-RA	BcDNA:AT08275|CG4837|DmelCG30103	0	0
4905	36973	Drosophila melanogaster	CG4847	CG4847 gene product from transcript CG4847-RA	BEST:GH19547|DmelCG4847	0	0
4906	36974	Drosophila melanogaster	CG4853	CG4853 gene product from transcript CG4853-RA	DmelCG4853	0	0
4907	36975	Drosophila melanogaster	Apc10	Anaphase promoting complex subunit 10	APC10|CG11419|DmelCG11419	0	0
4908	36976	Drosophila melanogaster	CG4866	CG4866 gene product from transcript CG4866-RB	DmelCG4866|mRpS4	0	0
4909	36978	Drosophila melanogaster	Patronin	CG33130 gene product from transcript CG33130-RD	BcDNA:LD17191|CG18459|CG18460|CG18462|CG30102|CG33130|CG6516|DmelCG33130|Ssp4|l(2)k07433|ssp4	0	0
4910	36981	Drosophila melanogaster	eIF3-S9	CG4878 gene product from transcript CG4878-RA	CG4878|DmelCG4878|cg4878|eIF3 S9	0	0
4911	36982	Drosophila melanogaster	CG43370	CG43370 gene product from transcript CG43370-RD	CG18432|CG18631|DmelCG43370|Dmel_CG18432|Dmel_CG18631	0	0
4912	36984	Drosophila melanogaster	cyp33	cyclophilin-33	CG4886|Cyp33|Dcyp33|DmelCG4886	0	0
4913	36985	Drosophila melanogaster	RpL18A	Ribosomal protein L18A	CG6510|DmelCG6510|L18|L18A|L18a|RPL18a|RpL18a|anon-EST:Posey75	0	0
4914	36986	Drosophila melanogaster	MESR4	Misexpression suppressor of ras 4	CG4903|DmelCG4903|EP(2)0988|EP0386|EP0988|EP988|Mesr4	0	0
4915	36988	Drosophila melanogaster	CG14480	CG14480 gene product from transcript CG14480-RA	DmelCG14480	0	0
4916	36989	Drosophila melanogaster	ns2	nucleostemin 2	CG6501|DmelCG6501|NS2|Ngp|Ngp1|dmNS2	0	0
4917	36990	Drosophila melanogaster	POSH	Plenty of SH3s	155116_at|BEST:GM09451|CG4909|DPOSH|DmelCG4909|GM09451|dPOSH|l(2)k15815|posh	0	0
4918	36991	Drosophila melanogaster	CG14482	CG14482 gene product from transcript CG14482-RA	DmelCG14482	0	0
4919	36992	Drosophila melanogaster	Rab4	Rab-protein 4	CG 4921|CG4921|DRAB4|DmRab4|DmelCG4921|RAB4c|rab4	0	0
4920	36993	Drosophila melanogaster	Dcr-2	Dicer-2	CG6493|DCR-2|DCR2|DICER|Dcr|Dcr2|Dicer|DmelCG6493|cg6493|dcr-2|dcr2|dic2|dicer|dicer2	0	0
4921	36994	Drosophila melanogaster	CG6484	CG6484 gene product from transcript CG6484-RA	DmelCG6484	0	0
4922	36995	Drosophila melanogaster	CG14483	CG14483 gene product from transcript CG14483-RA	DmelCG14483	0	0
4923	36996	Drosophila melanogaster	RhoGAP54D	CG6477 gene product from transcript CG6477-RA	CG6477|DmelCG6477	0	0
4924	36997	Drosophila melanogaster	icln	CG4924 gene product from transcript CG4924-RA	CG4924|DmelCG4924	0	0
4925	36998	Drosophila melanogaster	CG11423	CG11423 gene product from transcript CG11423-RA	DmelCG11423	0	0
4926	36999	Drosophila melanogaster	lack	lethal with a checkpoint kinase	CG4943|D-smurf|DSmurf|DmelCG4943|Dsmurf|Lack|Smurf|Smurf ubiquitin ligase|d-smurf|dSmurf|dSmurf1	0	0
4927	37000	Drosophila melanogaster	CG10936	CG10936 gene product from transcript CG10936-RB	DmelCG10936	0	0
4928	37002	Drosophila melanogaster	CG18186	CG18186 gene product from transcript CG18186-RA	DmelCG18186	0	0
4929	37004	Drosophila melanogaster	CCHa1r	CCHamide-1 receptor	CG14484|CG18192|CG30106|DmelCG30106	0	0
4930	37005	Drosophila melanogaster	eIF3-S8	CG4954 gene product from transcript CG4954-RA	CG4954|DmelCG4954	0	0
4931	37006	Drosophila melanogaster	CG6459	CG6459 gene product from transcript CG6459-RA	DmelCG6459	0	0
4932	37007	Drosophila melanogaster	Sema-1b	CG6446 gene product from transcript CG6446-RA	BcDNA:GH03186|CG6446|D-semaIII|DmelCG6446|Sema 1b|Sema III|Sema1b|sema-III	0	0
4933	37008	Drosophila melanogaster	CG42562	CG42562 gene product from transcript CG42562-RA	CG14481|DmelCG42562|Dmel_CG14481	0	0
4934	37009	Drosophila melanogaster	HPS4	Hermansky-Pudlak Syndrome 4 ortholog	CG4966|DmelCG4966|dHPS4	0	0
4935	37010	Drosophila melanogaster	swi2	CG14485 gene product from transcript CG14485-RA	CG14485|DmelCG14485	0	0
4936	37011	Drosophila melanogaster	rdgBbeta	CG17818 gene product from transcript CG17818-RA	BcDNA.LD14189|BcDNA:LD14189|CG17818|DmelCG17818|DrdgBbeta|RdgB-beta|RdgBbeta	0	0
4937	37012	Drosophila melanogaster	CG6424	CG6424 gene product from transcript CG6424-RA	BcDNA:GH05095|DmelCG6424|GH05095|anon-WO0118547.96|anon-WO0153538.32|anon-WO0153538.33|anon-WO0153538.34	0	0
4938	37013	Drosophila melanogaster	CG10934	CG10934 gene product from transcript CG10934-RA	DmelCG10934|anon-WO0140519.130	0	0
4939	37014	Drosophila melanogaster	CG10933	CG10933 gene product from transcript CG10933-RA	DmelCG10933	0	0
4940	37015	Drosophila melanogaster	Snx16	Sorting nexin 16	CG6410|DmelCG6410	0	0
4941	37016	Drosophila melanogaster	CG4975	CG4975 gene product from transcript CG4975-RC	DmelCG4975	0	0
4942	37017	Drosophila melanogaster	CG4984	CG4984 gene product from transcript CG4984-RD	DmelCG4984	0	0
4943	37018	Drosophila melanogaster	CG34195	CG34195 gene product from transcript CG34195-RA	CG4989|DmelCG34195	0	0
4944	37019	Drosophila melanogaster	CG6406	CG6406 gene product from transcript CG6406-RA	DmelCG6406	0	0
4945	37020	Drosophila melanogaster	CG6401	CG6401 gene product from transcript CG6401-RA	Dm*AAF57802/5-442|DmelCG6401	0	0
4946	37021	Drosophila melanogaster	CG4996	CG4996 gene product from transcript CG4996-RA	DmelCG4996	0	0
4947	37022	Drosophila melanogaster	Ir54a	Ionotropic receptor 54a	CG14487|DmelCG14487|IR54a	0	0
4948	37024	Drosophila melanogaster	CG10931	CG10931 gene product from transcript CG10931-RA	DmelCG10931	0	0
4949	37025	Drosophila melanogaster	CG5002	CG5002 gene product from transcript CG5002-RA	DmelCG5002	0	0
4950	37026	Drosophila melanogaster	CG6385	CG6385 gene product from transcript CG6385-RA	DmelCG6385	0	0
4951	37027	Drosophila melanogaster	HLH54F	CG5005 gene product from transcript CG5005-RA	CG5005|DmelCG5005|HLH102C|HLH54F-Dm|bHLH54F|bHLHa9	0	0
4952	37028	Drosophila melanogaster	CG5009	CG5009 gene product from transcript CG5009-RA	BcDNA:GH07485|DmelCG5009|GH07485	0	0
4953	37029	Drosophila melanogaster	CG6370	CG6370 gene product from transcript CG6370-RA	DmelCG6370	0	0
4954	37030	Drosophila melanogaster	CG14488	CG14488 gene product from transcript CG14488-RA	DmelCG14488	0	0
4955	37031	Drosophila melanogaster	CG18635	CG18635 gene product from transcript CG18635-RA	DmelCG18635	0	0
4956	37032	Drosophila melanogaster	CG6362	CG6362 gene product from transcript CG6362-RA	DmelCG6362|EG:52C10.6	0	0
4957	37033	Drosophila melanogaster	fab1	CG6355 gene product from transcript CG6355-RA	CG6355|DmelCG6355|EG:52C10.5|Fab1|PIKfyve|fab-1	0	0
4958	37034	Drosophila melanogaster	aft	adrift	CG5032|DmelCG5032|EG:52C10.7	0	0
4959	37035	Drosophila melanogaster	UbcD10	Ubiquitin conjugating enzyme 10	CG5788|DmelCG5788|EG:52C10.4|ubc|ubcD10	0	0
4960	37036	Drosophila melanogaster	CG5033	CG5033 gene product from transcript CG5033-RA	DmelCG5033|EG:52C10.1	0	0
4961	37037	Drosophila melanogaster	CG5036	CG5036 gene product from transcript CG5036-RB	DmelCG5036|EG:52C10.2	0	0
4962	37038	Drosophila melanogaster	grh	grainy head	BcDNA:LD38807|CG30111|CG42311|CG5058|DREB|DmelCG42311|Dmel_CG30111|Dmel_CG5058|EG:191D12.1|Elf-1|Elf-1/NTF-1|Elf1|GRH|Gh|Grh|NTF|NTF-1|NTF-1/Elf-1|NTF1|Ntf|Ntf1|elf1|elf1(CAG)[[7	0	0
4963	37039	Drosophila melanogaster	olf186-M	CG14489 gene product from transcript CG14489-RA	CG14489|DmelCG14489	0	0
4964	37040	Drosophila melanogaster	olf186-F	CG11430 gene product from transcript CG11430-RB	BcDNA:LD03241|CG11430|CRACM1|DmelCG11430|Olf186-F|Orai|SPH206|dOrai|dOrai1|dorai|l(2)k11505	0	0
4965	37041	Drosophila melanogaster	CG14490	CG14490 gene product from transcript CG14490-RA	DmelCG14490	0	0
4966	37042	Drosophila melanogaster	thr	three rows	CG5785|DmelCG5785|THR|Thr|ana|anch|frag1	0	0
4967	37043	Drosophila melanogaster	Mapmodulin	CG5784 gene product from transcript CG5784-RB	Anp32a|CG5784|CT18148|CT42180|DmelCG5784|anon-EST:fe3A2	0	0
4968	37044	Drosophila melanogaster	proPO-A1	prophenol oxidase A1	A1|A[[1]]|Bc|CG42639|CG5779|DmePPOA1|DmelCG42639|Dmel_CG5779|Dox-A1|DoxA1|Mox|PO A1|PPO1|Phox|ProPO A1|i12|pro-A|pro-PO|pro-POA[[1]]|proPO|proPO A[[1]]|proPO54	0	0
4969	37045	Drosophila melanogaster	CG14492	CG14492 gene product from transcript CG14492-RA	DmelCG14492	0	0
4970	37046	Drosophila melanogaster	CG14491	CG14491 gene product from transcript CG14491-RA	DmelCG14491	0	0
4971	37047	Drosophila melanogaster	elk	eag-like K[+] channel	CG5076|DmelCG5076	0	0
4972	37051	Drosophila melanogaster	CG5084	CG5084 gene product from transcript CG5084-RA	DmelCG5084	0	0
4973	37052	Drosophila melanogaster	CG10910	CG10910 gene product from transcript CG10910-RB	DmelCG10910	0	0
4974	37053	Drosophila melanogaster	CG5773	CG5773 gene product from transcript CG5773-RA	BcDNA:RH46075|DmelCG5773	0	0
4975	37054	Drosophila melanogaster	CG5770	CG5770 gene product from transcript CG5770-RA	BcDNA:LP06072|DmelCG5770	0	0
4976	37055	Drosophila melanogaster	CG5767	CG5767 gene product from transcript CG5767-RA	CT18118|DmelCG5767	0	0
4977	37056	Drosophila melanogaster	CG14495	CG14495 gene product from transcript CG14495-RA	DmelCG14495	0	0
4978	37057	Drosophila melanogaster	Muc55B	Mucin 55B	CG5765|DmelCG5765	0	0
4979	37058	Drosophila melanogaster	CG10911	CG10911 gene product from transcript CG10911-RA	DmelCG10911|anon-WO0140519.211	0	0
4980	37059	Drosophila melanogaster	CG10912	CG10912 gene product from transcript CG10912-RA	DmelCG10912	0	0
4981	37062	Drosophila melanogaster	CG5757	CG5757 gene product from transcript CG5757-RA	DmelCG5757	0	0
4982	37063	Drosophila melanogaster	CG5098	CG5098 gene product from transcript CG5098-RA	DmelCG5098	0	0
4983	37064	Drosophila melanogaster	CG5756	CG5756 gene product from transcript CG5756-RB	DmelCG5756	0	0
4984	37065	Drosophila melanogaster	stau	staufen	CG5753|DmelCG5753|Dmstau|STAU|Stau|Stauf|dStau	0	0
4985	37066	Drosophila melanogaster	Sema-5c	Semaphorin-5c	BEST:CK02567|BG01245|CG5661|CK02567|CT17880|DmelCG5661|Sema 5c|Sema-5C|sema 5c|sema-5c	0	0
4986	37068	Drosophila melanogaster	Hsf	Heat shock factor	CG5748|D-HSF|Dm-Hsf|DmHSF|DmelCG5748|HSF|HSF1|dHSF|dHSFa|hsf|l(2)03091	0	0
4987	37069	Drosophila melanogaster	Pcl	Polycomblike	CG5109|DmelCG5109|PCL|l(2)s1859|pcl	0	0
4988	37070	Drosophila melanogaster	pAbp	polyA-binding protein	BcDNA:LD24412|CG5119|DmelCG5119|PABP|PABP55B|PABPC1|PAbp|Pabp|anon-EST:Posey247|anon-EST:Posey249|dPABP|duo|l(2)SH1908|l(2)SH2 1908|l(2)k10109|pAB|pABP|pabp	0	0
4989	37071	Drosophila melanogaster	CG17680	CG17680 gene product from transcript CG17680-RA	BcDNA:RE55001|DmelCG17680	0	0
4990	37072	Drosophila melanogaster	CG5742	CG5742 gene product from transcript CG5742-RA	DmelCG5742	0	0
4991	37073	Drosophila melanogaster	adp	adipose	CG5124|DmelCG5124|anon-WO0196371.1|anon-WO0196371.27|anon-WO0196371.3	0	0
4992	37075	Drosophila melanogaster	CG10914	CG10914 gene product from transcript CG10914-RA	DmelCG10914	0	0
4993	37076	Drosophila melanogaster	CG10915	CG10915 gene product from transcript CG10915-RA	DmelCG10915	0	0

<table>
<tr><td>4994</td><td>37078</td><td>Drosophila melanogaster</td><td>Nup75</td><td>Nucleoporin 75</td><td>CG5733|DmelCG5733|Nup 75|nup75</td><td>0</td><td>0</td></tr>
<tr><td>4995</td><td>37079</td><td>Drosophila melanogaster</td><td>MED9</td><td>Mediator complex subunit 9</td><td>BcDNA:LD07740|BcDNA:RH42446|CG30112|CG30113|CG42517|CG5134|DmelCG42517|Dmel_CG5134</td><td>0</td><td>0</td></tr>
<tr><td>4996</td><td>37080</td><td>Drosophila melanogaster</td><td>Dgp-1</td><td>CG5729 gene product from transcript CG5729-RA</td><td>CG5729|DmelCG5729</td><td>0</td><td>0</td></tr>
<tr><td>4997</td><td>37081</td><td>Drosophila melanogaster</td><td>CG10916</td><td>CG10916 gene product from transcript CG10916-RA</td><td>DmelCG10916</td><td>0</td><td>0</td></tr>
<tr><td>4998</td><td>37082</td><td>Drosophila melanogaster</td><td>CG5726</td><td>CG5726 gene product from transcript CG5726-RA</td><td>DmelCG5726</td><td>0</td><td>0</td></tr>
<tr><td>4999</td><td>37083</td><td>Drosophila melanogaster</td><td>nopo</td><td>no poles</td><td>CG5140|DmelCG5140|Nopo|TRIP</td><td>0</td><td>0</td></tr>
<tr><td>5000</td><td>37084</td><td>Drosophila melanogaster</td><td>CG5721</td><td>CG5721 gene product from transcript CG5721-RA</td><td>DmelCG5721</td><td>0</td><td>0</td></tr>
<tr><td>5001</td><td>37087</td><td>Drosophila melanogaster</td><td>CG14500</td><td>CG14500 gene product from transcript CG14500-RA</td><td>DmelCG14500</td><td>0</td><td>0</td></tr>
<tr><td>5002</td><td>37088</td><td>Drosophila melanogaster</td><td>CG33958</td><td>CG33958 gene product from transcript CG33958-RA</td><td>CG14501|CG5719|DmelCG33958|GYC</td><td>0</td><td>0</td></tr>
<tr><td>5003</td><td>37089</td><td>Drosophila melanogaster</td><td>fj</td><td>four-jointed</td><td>CG10917|DmelCG10917|Fj</td><td>0</td><td>0</td></tr>
<tr><td>5004</td><td>37090</td><td>Drosophila melanogaster</td><td>Ote</td><td>Otefin</td><td>CG5581|DmelCG5581|ote|otefin</td><td>0</td><td>0</td></tr>
<tr><td>5005</td><td>37092</td><td>Drosophila melanogaster</td><td>CG14502</td><td>CG14502 gene product from transcript CG14502-RC</td><td>DmelCG14502</td><td>0</td><td>0</td></tr>
<tr><td>5006</td><td>37093</td><td>Drosophila melanogaster</td><td>CG18536</td><td>CG18536 gene product from transcript CG18536-RA</td><td>DmelCG18536</td><td>0</td><td>0</td></tr>
<tr><td>5007</td><td>37094</td><td>Drosophila melanogaster</td><td>CG18537</td><td>CG18537 gene product from transcript CG18537-RA</td><td>DmelCG18537</td><td>0</td><td>0</td></tr>
<tr><td>5008</td><td>37095</td><td>Drosophila melanogaster</td><td>CG18538</td><td>CG18538 gene product from transcript CG18538-RA</td><td>DmelCG18538</td><td>0</td><td>0</td></tr>
<tr><td>5009</td><td>37096</td><td>Drosophila melanogaster</td><td>CG18539</td><td>CG18539 gene product from transcript CG18539-RB</td><td>DmelCG18539</td><td>0</td><td>0</td></tr>
<tr><td>5010</td><td>37097</td><td>Drosophila melanogaster</td><td>CG18540</td><td>CG18540 gene product from transcript CG18540-RB</td><td>DmelCG18540</td><td>0</td><td>0</td></tr>
<tr><td>5011</td><td>37098</td><td>Drosophila melanogaster</td><td>CG14505</td><td>CG14505 gene product from transcript CG14505-RB</td><td>DmelCG14505</td><td>0</td><td>0</td></tr>
<tr><td>5012</td><td>37099</td><td>Drosophila melanogaster</td><td>IM23</td><td>Immune induced molecule 23</td><td>BcDNA:RH67033|CG15066|DIM 23|DIM-23|DmelCG15066</td><td>0</td><td>0</td></tr>
<tr><td>5013</td><td>37100</td><td>Drosophila melanogaster</td><td>IM1</td><td>Immune induced molecule 1</td><td>BcDNA:RH08745|CG18108|DIM 1|DIM-1|DmelCG18108|IM2-like</td><td>0</td><td>0</td></tr>
<tr><td>5014</td><td>37101</td><td>Drosophila melanogaster</td><td>CG18107</td><td>CG18107 gene product from transcript CG18107-RA</td><td>BcDNA:RH09147|DmelCG18107</td><td>0</td><td>0</td></tr>
<tr><td>5015</td><td>37102</td><td>Drosophila melanogaster</td><td>CG15067</td><td>CG15067 gene product from transcript CG15067-RA</td><td>DmelCG15067|anon-EST:Posey85</td><td>0</td><td>0</td></tr>
<tr><td>5016</td><td>37104</td><td>Drosophila melanogaster</td><td>Idgf5</td><td>Imaginal disc growth factor 5</td><td>CG5154|Cht14|DmIDGF5|DmelCG5154|IDGF|IDGF-5|IDGF5</td><td>0</td><td>0</td></tr>
<tr><td>5017</td><td>37105</td><td>Drosophila melanogaster</td><td>GstE10</td><td>Glutathione S transferase E10</td><td>CG17522|DmGSTE10|DmelCG17522|GSTE10</td><td>0</td><td>0</td></tr>
<tr><td>5018</td><td>37106</td><td>Drosophila melanogaster</td><td>GstE1</td><td>Glutathione S transferase E1</td><td>CG5164|CT16545|DmGST-3|DmGSTE1|DmGSTE1-1|DmelCG5164|GST-3|GST-E1|GST3|GSTE1|Gst-3|Gst-theta 55F|Gst3|Gst55F|gst-3|gstE1</td><td>0</td><td>0</td></tr>
<tr><td>5019</td><td>37107</td><td>Drosophila melanogaster</td><td>GstE2</td><td>Glutathione S transferase E2</td><td>CG17523|DmGSTE2|DmelCG17523|GSTE2</td><td>0</td><td>0</td></tr>
<tr><td>5020</td><td>37108</td><td>Drosophila melanogaster</td><td>GstE3</td><td>Glutathione S transferase E3</td><td>BEST:GH09055|CG17524|DmGSTE3|DmelCG17524|E3|GST-E3|GSTE3</td><td>0</td><td>0</td></tr>
<tr><td>5021</td><td>37109</td><td>Drosophila melanogaster</td><td>GstE4</td><td>Glutathione S transferase E4</td><td>CG17525|DmGSTE4|DmelCG17525|GSTE4</td><td>0</td><td>0</td></tr>
<tr><td>5022</td><td>37110</td><td>Drosophila melanogaster</td><td>GstE5</td><td>Glutathione S transferase E5</td><td>CG17527|DmGSTE5|DmelCG17527|GSTE5</td><td>0</td><td>0</td></tr>
<tr><td>5023</td><td>37111</td><td>Drosophila melanogaster</td><td>GstE6</td><td>Glutathione S transferase E6</td><td>CG17530|CT38747|DmGSTE6|DmelCG17530|E6|GSTE6</td><td>0</td><td>0</td></tr>
<tr><td>5024</td><td>37112</td><td>Drosophila melanogaster</td><td>GstE7</td><td>Glutathione S transferase E7</td><td>CG17531|DmGSTE7|DmelCG17531|E7|GSTE7|gstE7</td><td>0</td><td>0</td></tr>
<tr><td>5025</td><td>37113</td><td>Drosophila melanogaster</td><td>GstE8</td><td>Glutathione S transferase E8</td><td>BcDNA:GM08326|CG17533|DmGSTE8|DmelCG17533|GST3-2|GSTE8|Gst3-2</td><td>0</td><td>0</td></tr>
<tr><td>5026</td><td>37116</td><td>Drosophila melanogaster</td><td>Dp1</td><td>Dodeca-satellite-binding protein 1</td><td>BcDNA:LD21383|BcDNA:LD21677|CG5170|DDP1|DmelCG5170|anon-WO0118547.516|anon-WO0172774.168|ddp1</td><td>0</td><td>0</td></tr>
<tr><td>5027</td><td>37117</td><td>Drosophila melanogaster</td><td>CG5174</td><td>CG5174 gene product from transcript CG5174-RM</td><td>BcDNA:RE18604|BcDNA:RH27395|DmelCG5174</td><td>0</td><td>0</td></tr>
<tr><td>5028</td><td>37118</td><td>Drosophila melanogaster</td><td>CG12263</td><td>CG12263 gene product from transcript CG12263-RA</td><td>DmelCG12263|anon-WO0140519.205</td><td>0</td><td>0</td></tr>
<tr><td>5029</td><td>37119</td><td>Drosophila melanogaster</td><td>CG30118</td><td>CG30118 gene product from transcript CG30118-RB</td><td>CG16859|CG5544|DmelCG30118</td><td>0</td><td>0</td></tr>
<tr><td>5030</td><td>37121</td><td>Drosophila melanogaster</td><td>slim</td><td>scruin like at the midline</td><td>CG5186|DmelCG5186|l(2)s1866</td><td>0</td><td>0</td></tr>
<tr><td>5031</td><td>37122</td><td>Drosophila melanogaster</td><td>CG5189</td><td>CG5189 gene product from transcript CG5189-RB</td><td>DmelCG5189|dp14|p14</td><td>0</td><td>0</td></tr>
<tr><td>5032</td><td>37123</td><td>Drosophila melanogaster</td><td>Prp19</td><td>CG5519 gene product from transcript CG5519-RA</td><td>BcDNA:LD02793|CG5519|DmelCG5519|GPB|Gbp|l(2)07838|prp19</td><td>0</td><td>0</td></tr>
<tr><td>5033</td><td>37124</td><td>Drosophila melanogaster</td><td>CG5190</td><td>CG5190 gene product from transcript CG5190-RA</td><td>DmelCG5190</td><td>0</td><td>0</td></tr>
<tr><td>5034</td><td>37125</td><td>Drosophila melanogaster</td><td>CG17669</td><td>CG17669 gene product from transcript CG17669-RA</td><td>DmelCG17669</td><td>0</td><td>0</td></tr>
<tr><td>5035</td><td>37126</td><td>Drosophila melanogaster</td><td>CG30116</td><td>CG30116 gene product from transcript CG30116-RC</td><td>BcDNA:GH04922|CG17671|CG5512|DmelCG30116</td><td>0</td><td>0</td></tr>
<tr><td>5036</td><td>37128</td><td>Drosophila melanogaster</td><td>CG5224</td><td>CG5224 gene product from transcript CG5224-RA</td><td>DmelCG5224|cg5224</td><td>0</td><td>0</td></tr>
<tr><td>5037</td><td>37129</td><td>Drosophila melanogaster</td><td>CG43066</td><td>CG43066 gene product from transcript CG43066-RA</td><td>CG42510|CG5226|DmelCG43066|Dmel_CG42510|Dmel_CG5226|anon-WO0149848.1</td><td>0</td><td>0</td></tr>
<tr><td>5038</td><td>37130</td><td>Drosophila melanogaster</td><td>CG10924</td><td>CG10924 gene product from transcript CG10924-RA</td><td>DmelCG10924</td><td>0</td><td>0</td></tr>
<tr><td>5039</td><td>37131</td><td>Drosophila melanogaster</td><td>Pepck</td><td>Phosphoenolpyruvate carboxykinase</td><td>143299_at|CG10924|CG17725|DmelCG17725|Dromel_CG17725_FBtr0086701_pepck_mORF|PEPCK|dPEPCK|pepck</td><td>0</td><td>0</td></tr>
<tr><td>5040</td><td>37132</td><td>Drosophila melanogaster</td><td>Rgk2</td><td>CG34390 gene product from transcript CG34390-RA</td><td>CG15069|CG30119|CG34390|DmelCG34390|Dmel_CG15069|Dmel_CG30119</td><td>0</td><td>0</td></tr>
<tr><td>5041</td><td>37134</td><td>Drosophila melanogaster</td><td>CG30115</td><td>CG30115 gene product from transcript CG30115-RE</td><td>CG18430|CG5503|DmelCG30115</td><td>0</td><td>0</td></tr>
<tr><td>5042</td><td>37135</td><td>Drosophila melanogaster</td><td>CG10927</td><td>CG10927 gene product from transcript CG10927-RA</td><td>DmelCG10927</td><td>0</td><td>0</td></tr>
<tr><td>5043</td><td>37136</td><td>Drosophila melanogaster</td><td>mRpS28</td><td>mitochondrial ribosomal protein S28</td><td>BcDNA:RH13070|CG5497|DmelCG5497|MRP-S35</td><td>0</td><td>0</td></tr>
<tr><td>5044</td><td>37137</td><td>Drosophila melanogaster</td><td>CG5323</td><td>CG5323 gene product from transcript CG5323-RA</td><td>DmelCG5323</td><td>0</td><td>0</td></tr>
<tr><td>5045</td><td>37138</td><td>Drosophila melanogaster</td><td>CG5327</td><td>CG5327 gene product from transcript CG5327-RA</td><td>DmelCG5327|NEST:bs33e07</td><td>0</td><td>0</td></tr>
<tr><td>5046</td><td>37139</td><td>Drosophila melanogaster</td><td>CG5493</td><td>CG5493 gene product from transcript CG5493-RB</td><td>DmelCG5493</td><td>0</td><td>0</td></tr>
<tr><td>5047</td><td>37140</td><td>Drosophila melanogaster</td><td>CG5335</td><td>CG5335 gene product from transcript CG5335-RA</td><td>DmelCG5335</td><td>0</td><td>0</td></tr>
<tr><td>5048</td><td>37141</td><td>Drosophila melanogaster</td><td>Atg7</td><td>Autophagy-specific gene 7</td><td>CG5489|DmelCG5489|atg-7|atg7</td><td>0</td><td>0</td></tr>
<tr><td>5049</td><td>37142</td><td>Drosophila melanogaster</td><td>sec6</td><td>CG5341 gene product from transcript CG5341-RA</td><td>CG5341|DmelCG5341|Dsec6|Sec6|Sec6p|dsec6|sec 6</td><td>0</td><td>0</td></tr>
<tr><td>5050</td><td>37143</td><td>Drosophila melanogaster</td><td>Eip55E</td><td>CG5345 gene product from transcript CG5345-RA</td><td>CG5345|DmelCG5345|EIP40|Eip40|Eip55BD</td><td>0</td><td>0</td></tr>
<tr><td>5051</td><td>37144</td><td>Drosophila melanogaster</td><td>CG5482</td><td>CG5482 gene product from transcript CG5482-RA</td><td>DmelCG5482</td><td>0</td><td>0</td></tr>
<tr><td>5052</td><td>37146</td><td>Drosophila melanogaster</td><td>CG30122</td><td>CG30122 gene product from transcript CG30122-RB</td><td>CG18610|CG5477|DmelCG30122|LD11002|SAF-A</td><td>0</td><td>0</td></tr>
<tr><td>5053</td><td>37147</td><td>Drosophila melanogaster</td><td>SP2637</td><td>CG5473 gene product from transcript CG5473-RC</td><td>BEST:GH24992|CG5473|DmelCG5473|dm-ntan1</td><td>0</td><td>0</td></tr>
<tr><td>5054</td><td>37148</td><td>Drosophila melanogaster</td><td>Gint3</td><td>GDI interacting protein 3</td><td>CG5469|DmelCG5469|Dromel_CG5469_FBtr0086654_gint3_mORF</td><td>0</td><td>0</td></tr>
<tr><td>5055</td><td>37149</td><td>Drosophila melanogaster</td><td>edl</td><td>ETS-domain lacking</td><td>CG15085|DmelCG15085|EDL|Edl|MAE|Mae|anon-WO02077276.2|anon-WO02077276.3|edl/mae|l(2)k06602|mae</td><td>0</td><td>0</td></tr>
<tr><td>5056</td><td>37150</td><td>Drosophila melanogaster</td><td>CG15086</td><td>CG15086 gene product from transcript CG15086-RF</td><td>DmelCG15086</td><td>0</td><td>0</td></tr>
<tr><td>5057</td><td>37152</td><td>Drosophila melanogaster</td><td>CG42856</td><td>CG42856 gene product from transcript CG42856-RC</td><td>CG15072|CG18604|DmelCG42856|Dmel_CG15072|Dmel_CG18604|EMK/KIAA0999</td><td>0</td><td>0</td></tr>
<tr><td>5058</td><td>37153</td><td>Drosophila melanogaster</td><td>CG15071</td><td>CG15071 gene product from transcript CG15071-RA</td><td>DmelCG15071</td><td>0</td><td>0</td></tr>
<tr><td>5059</td><td>37155</td><td>Drosophila melanogaster</td><td>CG15073</td><td>CG15073 gene product from transcript CG15073-RA</td><td>DmelCG15073</td><td>0</td><td>0</td></tr>
<tr><td>5060</td><td>37156</td><td>Drosophila melanogaster</td><td>CG15087</td><td>CG15087 gene product from transcript CG15087-RA</td><td>DmelCG15087</td><td>0</td><td>0</td></tr>
<tr><td>5061</td><td>37157</td><td>Drosophila melanogaster</td><td>List</td><td>Lithium-inducible SLC6 transporter</td><td>CG15088|DmNAT2|DmelCG15088</td><td>0</td><td>0</td></tr>
<tr><td>5062</td><td>37158</td><td>Drosophila melanogaster</td><td>CG18609</td><td>CG18609 gene product from transcript CG18609-RA</td><td>CT42569|DmelCG18609</td><td>0</td><td>0</td></tr>
<tr><td>5063</td><td>37159</td><td>Drosophila melanogaster</td><td>CG17821</td><td>CG17821 gene product from transcript CG17821-RA</td><td>DmelCG17821</td><td>0</td><td>0</td></tr>
<tr><td>5064</td><td>37161</td><td>Drosophila melanogaster</td><td>Hs3st-A</td><td>Heparan sulfate 3-O sulfotransferase-A</td><td>CG15074|CG15075|CG15076|CG30123|CG30124|CG33147|DmelCG33147</td><td>0</td><td>0</td></tr>
<tr><td>5065</td><td>37163</td><td>Drosophila melanogaster</td><td>Cyp12b2</td><td>CG15077 gene product from transcript CG15077-RA</td><td>12b2|CG15077|DmelCG15077</td><td>0</td><td>0</td></tr>
<tr><td>5066</td><td>37164</td><td>Drosophila melanogaster</td><td>CG42351</td><td>CG42351 gene product from transcript CG42351-RG</td><td>141242_at|BP1008|CG1509|CG15092|CG34020|DmelCG42351|Dmel_CG15092|Dmel_CG34020|anon-WO0118547.167</td><td>0</td><td>0</td></tr>
<tr><td>5067</td><td>37165</td><td>Drosophila melanogaster</td><td>Mctp</td><td>Multiple C2 domain and transmembrane region proteir</td><td>CG15078|CG33146|CG33148|DmMCTP|DmelCG15078</td><td>0</td><td>0</td></tr>
<tr><td>5068</td><td>37166</td><td>Drosophila melanogaster</td><td>CG15093</td><td>CG15093 gene product from transcript CG15093-RB</td><td>CT34968|DmelCG15093</td><td>0</td><td>0</td></tr>
<tr><td>5069</td><td>37167</td><td>Drosophila melanogaster</td><td>CG15080</td><td>CG15080 gene product from transcript CG15080-RA</td><td>DmelCG15080</td><td>0</td><td>0</td></tr>
<tr><td>5070</td><td>37168</td><td>Drosophila melanogaster</td><td>CG15094</td><td>CG15094 gene product from transcript CG15094-RA</td><td>DmelCG15094</td><td>0</td><td>0</td></tr>
<tr><td>5071</td><td>37170</td><td>Drosophila melanogaster</td><td>CG15096</td><td>CG15096 gene product from transcript CG15096-RA</td><td>CT42567|DmelCG15096</td><td>0</td><td>0</td></tr>
<tr><td>5072</td><td>37172</td><td>Drosophila melanogaster</td><td>CG15097</td><td>CG15097 gene product from transcript CG15097-RC</td><td>Dm.CG15097|DmelCG15097</td><td>0</td><td>0</td></tr>
<tr><td>5073</td><td>37173</td><td>Drosophila melanogaster</td><td>CG15082</td><td>CG15082 gene product from transcript CG15082-RA</td><td>DmelCG15082</td><td>0</td><td>0</td></tr>
<tr><td>5074</td><td>37174</td><td>Drosophila melanogaster</td><td>CG15098</td><td>CG15098 gene product from transcript CG15098-RA</td><td>DmelCG15098|dmCG15098</td><td>0</td><td>0</td></tr>
<tr><td>5075</td><td>37175</td><td>Drosophila melanogaster</td><td>CG15083</td><td>CG15083 gene product from transcript CG15083-RA</td><td>BcDNA:RH17222|DmelCG15083</td><td>0</td><td>0</td></tr>
<tr><td>5076</td><td>37176</td><td>Drosophila melanogaster</td><td>CG15099</td><td>CG15099 gene product from transcript CG15099-RA</td><td>DmelCG15099</td><td>0</td><td>0</td></tr>
<tr><td>5077</td><td>37177</td><td>Drosophila melanogaster</td><td>CG15100</td><td>CG15100 gene product from transcript CG15100-RA</td><td>DmelCG15100|MRS|anon-EST:Liang-2.15|clone 2.15|p120</td><td>0</td><td>0</td></tr>
<tr><td>5078</td><td>37178</td><td>Drosophila melanogaster</td><td>CG15084</td><td>CG15084 gene product from transcript CG15084-RA</td><td>DmelCG15084</td><td>0</td><td>0</td></tr>
<tr><td>5079</td><td>37179</td><td>Drosophila melanogaster</td><td>CG18190</td><td>CG18190 gene product from transcript CG18190-RB</td><td>DmelCG18190</td><td>0</td><td>0</td></tr>
<tr><td>5080</td><td>37181</td><td>Drosophila melanogaster</td><td>Jheh2</td><td>Juvenile hormone epoxide hydrolase 2</td><td>CG15102|CT34977|DmEH|DmelCG15102|jheh2</td><td>0</td><td>0</td></tr>
<tr><td>5081</td><td>37182</td><td>Drosophila melanogaster</td><td>Jheh3</td><td>Juvenile hormone epoxide hydrolase 3</td><td>CG15106|DmelCG15106</td><td>0</td><td>0</td></tr>
<tr><td>5082</td><td>37183</td><td>Drosophila melanogaster</td><td>Dpt</td><td>Diptericin</td><td>143443_at|CG12763|DIPT|DPT|Dep|Dip|Dipt|DmelCG12763|DptA|dip|dipt|dpt</td><td>0</td><td>0</td></tr>
<tr><td>5083</td><td>37184</td><td>Drosophila melanogaster</td><td>DptB</td><td>Diptericin B</td><td>147473_at|BcDNA:RH29451|CG10794|DiptB|Diptericin|DmelCG10794|dpt B|dptlp</td><td>0</td><td>0</td></tr>
<tr><td>5084</td><td>37185</td><td>Drosophila melanogaster</td><td>sano</td><td>serrano</td><td>CG12758|DmelCG12758</td><td>0</td><td>0</td></tr>
<tr><td>5085</td><td>37187</td><td>Drosophila melanogaster</td><td>prod</td><td>proliferation disrupter</td><td>CG18608|DmelCG18608|PROD|Prod|l(2)88/10|l(2)k08810</td><td>0</td><td>0</td></tr>
<tr><td>5086</td><td>37188</td><td>Drosophila melanogaster</td><td>Topors</td><td>Topoisomerase I-interacting protein</td><td>CG15104|DmelCG15104|TOPORS|dTopors|dtopors</td><td>0</td><td>0</td></tr>
<tr><td>5087</td><td>37189</td><td>Drosophila melanogaster</td><td>CG18605</td><td>CG18605 gene product from transcript CG18605-RB</td><td>DmelCG18605</td><td>0</td><td>0</td></tr>
<tr><td>5088</td><td>37190</td><td>Drosophila melanogaster</td><td>abba</td><td>another B-box affiliate</td><td>CG15105|DmelCG15105</td><td>0</td><td>0</td></tr>
<tr><td>5089</td><td>37191</td><td>Drosophila melanogaster</td><td>5-HT1B</td><td>Serotonin receptor 1B</td><td>5-HT-dro2B|5-HT1BDro|5-HT[1B]Dro|5-HT[[1BDro]]|5-HT[[dro2B]]|5HT-R2B|5HT-dro2B|5HT-dro[[2B]]|5HT1B|5htdro2b|CG15113|CG30126|DRO2B|Dm5HTdro2B|DmelCG15113|Dmel_CG30126|d5-HT1B</td><td>0</td><td>0</td></tr>
<tr><td>5090</td><td>37192</td><td>Drosophila melanogaster</td><td>CG15115</td><td>CG15115 gene product from transcript CG15115-RA</td><td>DmelCG15115</td><td>0</td><td>0</td></tr>
<tr><td>5091</td><td>37194</td><td>Drosophila melanogaster</td><td>CG15116</td><td>CG15116 gene product from transcript CG15116-RA</td><td>DmelCG15116</td><td>0</td><td>0</td></tr>
<tr><td>5092</td><td>37195</td><td>Drosophila melanogaster</td><td>CG15109</td><td>CG15109 gene product from transcript CG15109-RC</td><td>DmelCG15109</td><td>0</td><td>0</td></tr>
<tr><td>5093</td><td>37196</td><td>Drosophila melanogaster</td><td>5-HT1A</td><td>Serotonin receptor 1A</td><td>5-HT-dro2A|5-HT1ADro|5-HT[1A]Dro|5-HT[[1ADro]]|5HT-R2A|5HT-dro2A|5HT-drp[[2A]]|5HT1A|5HT[[1ADro]]|5htdro2a|CG16720|DRO2A|Dm5HTdro2A|DmelCG16720|d5-HT1A</td><td>0</td><td>0</td></tr>
<tr><td>5094</td><td>37197</td><td>Drosophila melanogaster</td><td>CG15117</td><td>CG15117 gene product from transcript CG15117-RA</td><td>DmelCG15117</td><td>0</td><td>0</td></tr>
<tr><td>5095</td><td>37198</td><td>Drosophila melanogaster</td><td>botv</td><td>brother of tout-velu</td><td>BcDNA:LD21192|Botv|CG15110|DEXT3|DEXTL3|Dextl3|DmelCG15110|Extl3</td><td>0</td><td>0</td></tr>
<tr><td>5096</td><td>37199</td><td>Drosophila melanogaster</td><td>CG15118</td><td>CG15118 gene product from transcript CG15118-RC</td><td>DmelCG15118|anon-EST:fe1H12</td><td>0</td><td>0</td></tr>
<tr><td>5097</td><td>37200</td><td>Drosophila melanogaster</td><td>CG15111</td><td>CG15111 gene product from transcript CG15111-RB</td><td>DmelCG15111</td><td>0</td><td>0</td></tr>
<tr><td>5098</td><td>37201</td><td>Drosophila melanogaster</td><td>ena</td><td>enabled</td><td>CG15112|DmelCG15112|ENA/VASP|Ena|Enb|VASP|enb|l(2)02029</td><td>0</td><td>0</td></tr>
<tr><td>5099</td><td>37202</td><td>Drosophila melanogaster</td><td>CG10737</td><td>CG10737 gene product from transcript CG10737-RS</td><td>10737|DmelCG10737</td><td>0</td><td>0</td></tr>
<tr><td>5100</td><td>37203</td><td>Drosophila melanogaster</td><td>hppy</td><td>happyhour</td><td>CG7097|DmelCG7097|EP-704|GLK/KHS1|MAP4K3|anon-WO0153538.68|l(2)SH1261|l(2)SH2 1261</td><td>0</td><td>0</td></tr>
<tr><td>5101</td><td>37204</td><td>Drosophila melanogaster</td><td>CG7137</td><td>CG7137 gene product from transcript CG7137-RA</td><td>DmelCG7137</td><td>0</td><td>0</td></tr>
<tr><td>5102</td><td>37205</td><td>Drosophila melanogaster</td><td>cora</td><td>coracle</td><td>CG11949|COR|Cor|Cora|D4.1|DmelCG11949|cor|l(2)k08713</td><td>0</td><td>0</td></tr>
<tr><td>5103</td><td>37206</td><td>Drosophila melanogaster</td><td>wbl</td><td>windbeutel</td><td>CG7225|DmelCG7225|Wind|Windb|wbt|wind</td><td>0</td><td>0</td></tr>
<tr><td>5104</td><td>37207</td><td>Drosophila melanogaster</td><td>CG7229</td><td>CG7229 gene product from transcript CG7229-RA</td><td>7229|DmelCG7229</td><td>0</td><td>0</td></tr>
</table>

5105	37209	Drosophila melanogaster	CG11906	CG11906 gene product from transcript CG11906-RA	11906|DmelCG11906	0	0
5106	37210	Drosophila melanogaster	CG10476	CG10476 gene product from transcript CG10476-RA	DmelCG10476	0	0
5107	37211	Drosophila melanogaster	CG10474	CG10474 gene product from transcript CG10474-RA	DmelCG10474	0	0
5108	37212	Drosophila melanogaster	CG18606	CG18606 gene product from transcript CG18606-RB	DmelCG18606	0	0
5109	37213	Drosophila melanogaster	CG18607	CG18607 gene product from transcript CG18607-RA	DmelCG18607	0	0
5110	37214	Drosophila melanogaster	FK506-bp2	FK506-binding protein 2	11001|143729_at|CG11001|DmelCG11001|DrFKBP12|FKBP|FKBP12|Fk506-bp2|dFKBP12	0	0
5111	37215	Drosophila melanogaster	mip40	Myb-interacting protein 40	CG15119|DmelCG15119|Mip40|p40	0	0
5112	37216	Drosophila melanogaster	Tab2	TAK1-associated binding protein 2	7417|CG7417|DmelCG7417|ORF1|TAB|TAB2|cg7417|dTAB2|galere	0	0
5113	37217	Drosophila melanogaster	CG7461	CG7461 gene product from transcript CG7461-RB	DmelCG7461|cg7461	0	0
5114	37218	Drosophila melanogaster	endoB	endophilin B	CG9834|D-EndoB|D-endoB|DmelCG9834|EndoB	0	0
5115	37219	Drosophila melanogaster	Rgk1	CG9811 gene product from transcript CG9811-RB	CG9811|CT27512|DmelCG9811	0	0
5116	37220	Drosophila melanogaster	CG9975	CG9975 gene product from transcript CG9975-RA	DmelCG9975	0	0
5117	37221	Drosophila melanogaster	CG11961	CG11961 gene product from transcript CG11961-RB	CG11961-PA|DmelCG11961	0	0
5118	37222	Drosophila melanogaster	CG10051	CG10051 gene product from transcript CG10051-RA	DmelCG10051	0	0
5119	37223	Drosophila melanogaster	CG9416	CG9416 gene product from transcript CG9416-RA	CG9416-PA|DmelCG9416	0	0
5120	37224	Drosophila melanogaster	CG10062	CG10062 gene product from transcript CG10062-RA	DmelCG10062|anon-WO0140519.226	0	0
5121	37225	Drosophila melanogaster	CG10073	CG10073 gene product from transcript CG10073-RB	DmelCG10073	0	0
5122	37226	Drosophila melanogaster	CG10081	CG10081 gene product from transcript CG10081-RA	DmelCG10081	0	0
5123	37227	Drosophila melanogaster	CG11257	CG11257 gene product from transcript CG11257-RA	DmelCG11257	0	0
5124	37228	Drosophila melanogaster	SdhA	Succinate dehydrogenase A	CG17246|DmelCG17246|Scs-fp|Sdh|Sdha|scs-fp|sdh	0	0
5125	37229	Drosophila melanogaster	cer	crammer	CG10460|D/CTLA-2|DmelCG10460	0	0
5126	37230	Drosophila melanogaster	hts	hu li tai shao	1B1|ADD-87|Add|Add-hts|Adducin|CG34197|CG43443|CG9325|DmelCG43443|Dmel_CG34197|Dmel_CG9325|EST D|HTS|HTS-R1|HTS-RC|Hts|Hts-RC|HtsF|HtsRC|Ovhts|Ovhts-RC|add-like|adducin|anor	0	0
5127	37232	Drosophila melanogaster	CalpA	Calpain-A	CG18152|CG7563|DmelCG7563|EST C	0	0
5128	37233	Drosophila melanogaster	Fak56D	Focal Adhesion Kinase	CG10023|CT28129|DFAK|DFak|DFak56|Dfak|Dfak56|DmFAK|DmelCG10023|FAK|Fak|Fak56|fak|fak56	0	0
5129	37235	Drosophila melanogaster	RpL11	Ribosomal protein L11	BcDNA:LD17235|CG7726|DL11|DmelCG7726|EST A|L11|M(2)56CD|RPL11|l(2)k16914	0	0
5130	37236	Drosophila melanogaster	CG7735	CG7735 gene product from transcript CG7735-RA	DmelCG7735	0	0
5131	37237	Drosophila melanogaster	Elongin-C	Elongin C	BcDNA:RH71704|CG9291|DmelCG9291|l(2)SH1299	0	0
5132	37238	Drosophila melanogaster	betaTub56D	beta-Tubulin at 56D	1t|B1t|BETA 56D|CG9277|DTB2|Dmbeta1|DmelCG9277|T|Tub|Tubulin|beta-Tub|beta-Tub56D|beta-tub|beta-tubulin56D|beta1|beta1-Tubulin|beta1-tub|beta1Tub|beta1t|beta1tub|beta56D|betaTub|betaTub1	0	0
5133	37239	Drosophila melanogaster	CG7744	CG7744 gene product from transcript CG7744-RA	DmelCG7744	0	0
5134	37244	Drosophila melanogaster	CG11242	CG11242 gene product from transcript CG11242-RA	DmelCG11242	0	0
5135	37245	Drosophila melanogaster	Oseg6	CG11237 gene product from transcript CG11237-RA	CG11237|DmelCG11237|OSEG|OSEG6|Wdr19	0	0
5136	37246	Drosophila melanogaster	Rep	Rab escort protein	CG8432|DmelCG8432|REP	0	0
5137	37247	Drosophila melanogaster	hpo	hippo	CG11228|DmelCG11228|Hpo|MST|MST2|dMST	0	0
5138	37248	Drosophila melanogaster	CG15120	CG15120 gene product from transcript CG15120-RA	DmelCG15120	0	0
5139	37249	Drosophila melanogaster	CG11007	CG11007 gene product from transcript CG11007-RA	DmelCG11007	0	0
5140	37250	Drosophila melanogaster	Ir56b	Ionotropic receptor 56b	CG15121|DmelCG15121|IR56b	0	0
5141	37251	Drosophila melanogaster	Ir56c	Ionotropic receptor 56c	CG15122|DmelCG15122|IR56c	0	0
5142	37252	Drosophila melanogaster	Ir56d	Ionotropic receptor 56d	CG15904|DmelCG15904|IR56d	0	0
5143	37253	Drosophila melanogaster	CG16716	CG16716 gene product from transcript CG16716-RD	DmelCG16716|anon-WO0140519.27	0	0
5144	37254	Drosophila melanogaster	sm	smooth	BcDNA:GH10856|CG9218|DmelCG9218|l(2)05338|smo	0	0
5145	37255	Drosophila melanogaster	CG18367	CG18367 gene product from transcript CG18367-RA	CG15123|DmelCG18367	0	0
5146	37256	Drosophila melanogaster	CG15124	CG15124 gene product from transcript CG15124-RA	DmelCG15124	0	0
5147	37257	Drosophila melanogaster	CG15905	CG15905 gene product from transcript CG15905-RB	DmelCG15905	0	0
5148	37258	Drosophila melanogaster	CG15125	CG15125 gene product from transcript CG15125-RA	DmelCG15125	0	0
5149	37259	Drosophila melanogaster	CG11018	CG11018 gene product from transcript CG11018-RA	DmelCG11018|anon-WO0140519.121	0	0
5150	37261	Drosophila melanogaster	isopeptidase-T-3	CG11025 gene product from transcript CG11025-RA	CG11025|DmelCG11025|ISOT-3|IsoT-3	0	0
5151	37263	Drosophila melanogaster	CG15128	CG15128 gene product from transcript CG15128-RA	DmelCG15128	0	0
5152	37264	Drosophila melanogaster	Obp56a	Odorant-binding protein 56a	56a|BcDNA:RE46170|CG11797|DmelCG11797|obp56a	0	0
5153	37265	Drosophila melanogaster	Obp56b	Odorant-binding protein 56b	56b|CG15129|CG30129|DmelCG30129|OBP56b	0	0
5154	37266	Drosophila melanogaster	Obp56d	Odorant-binding protein 56d	56d|BEST:GH09027|BcDNA:RE22207|CG11218|CT31326|DmelCG11218|OBP56d|obp56d	0	0
5155	37267	Drosophila melanogaster	Obp56e	Odorant-binding protein 56e	56e|CG8462|DmelCG8462	0	0
5156	37268	Drosophila melanogaster	CG8517	CG8517 gene product from transcript CG8517-RA	8517|DmelCG8517|mtr	0	0
5157	37269	Drosophila melanogaster	Or56a	Odorant receptor 56a	56E.1|56a|CG12501|DOR59|DmelCG12501	0	0
5158	37270	Drosophila melanogaster	Obp56g	Odorant-binding protein 56g	56g|CG13873|DmelCG13873|OBP56g	0	0
5159	37271	Drosophila melanogaster	Obp56h	Odorant-binding protein 56h	56h|CG13874|DmelCG13874|OBP56h	0	0
5160	37272	Drosophila melanogaster	Toll-7	CG8595 gene product from transcript CG8595-RA	CG8595|CT24947|DmelCG8595|TOLL 7|Tl-7|dTLR7|dToll7|toll	0	0
5161	37273	Drosophila melanogaster	CG13872	CG13872 gene product from transcript CG13872-RA	DmelCG13872	0	0
5162	37274	Drosophila melanogaster	CG10822	CG10822 gene product from transcript CG10822-RA	DmelCG10822	0	0
5163	37275	Drosophila melanogaster	CG8654	CG8654 gene product from transcript CG8654-RC	DmelCG8654	0	0
5164	37276	Drosophila melanogaster	CG16898	CG16898 gene product from transcript CG16898-RA	DmelCG16898	0	0
5165	37277	Drosophila melanogaster	18w	18 wheeler	18-wheeler|18W|CG8896|CT25100|DmelCG8896|l(2)00053|tlr|toll	0	0
5166	37278	Drosophila melanogaster	CG11041	CG11041 gene product from transcript CG11041-RB	DmelCG11041	0	0
5167	37280	Drosophila melanogaster	CG16894	CG16894 gene product from transcript CG16894-RA	DmelCG16894	0	0
5168	37281	Drosophila melanogaster	CG11044	CG11044 gene product from transcript CG11044-RA	DmelCG11044	0	0
5169	37282	Drosophila melanogaster	CG11099	CG11099 gene product from transcript CG11099-RA	CT31033|DmelCG11099	0	0
5170	37283	Drosophila melanogaster	CG13869	CG13869 gene product from transcript CG13869-RA	DmelCG13869	0	0
5171	37284	Drosophila melanogaster	CG11048	CG11048 gene product from transcript CG11048-RA	DmelCG11048	0	0
5172	37285	Drosophila melanogaster	CG11208	CG11208 gene product from transcript CG11208-RA	DmelCG11208|anon-WO0118547.196	0	0
5173	37286	Drosophila melanogaster	ppk6	pickpocket 6	CG11209|DmelCG11209|PPK6	0	0
5174	37287	Drosophila melanogaster	CG9864	CG9864 gene product from transcript CG9864-RA	CT27832|DmelCG9864	0	0
5175	37288	Drosophila melanogaster	Ate1	CG9204 gene product from transcript CG9204-RC	ATE1|Ate|CG9204|Dm-Ate1|DmelCG9204|ate1|l(2)k10809	0	0
5176	37289	Drosophila melanogaster	CG11055	CG11055 gene product from transcript CG11055-RB	A|DmelCG11055|anon-56Ea	0	0
5177	37290	Drosophila melanogaster	mus209	mutagen-sensitive 209	53/13|CG9193|DmPCNA|DmelCG9193|MUS209|Mus209|PCNA|PCNA/mus209|Pcna|dPCNA|l(2)02448|l(2)56F|l(2)56Fa|l(2)s1534|pcna	0	0
5178	37292	Drosophila melanogaster	RpS18	Ribosomal protein S18	B|BcDNA:RH43343|CG8900|DmelCG8900|M(2)56F|RS18_DROME|Rps18|S18|anon-56Fa|anon-EST:Posey27|anon-EST:fe2D3|rpS18	0	0
5179	37293	Drosophila melanogaster	CG8908	CG8908 gene product from transcript CG8908-RB	DmelCG8908	0	0
5180	37294	Drosophila melanogaster	CG10444	CG10444 gene product from transcript CG10444-RA	DmelCG10444	0	0
5181	37295	Drosophila melanogaster	CG11788	CG11788 gene product from transcript CG11788-RA	DmelCG11788	0	0
5182	37296	Drosophila melanogaster	CG9143	CG9143 gene product from transcript CG9143-RA	DmelCG9143|cg9143	0	0
5183	37297	Drosophila melanogaster	CG9090	CG9090 gene product from transcript CG9090-RA	DmelCG9090	0	0
5184	37298	Drosophila melanogaster	CG16868	CG16868 gene product from transcript CG16868-RA	DmelCG16868	0	0
5185	37299	Drosophila melanogaster	Cpr56F	Cuticular protein 56F	CG9036|DmelCpr56F|DmelCG9036	0	0
5186	37300	Drosophila melanogaster	CkIIbeta2	Casein kinase II beta2 subunit	CG8914|CK2|CK2beta'|CKIIb2|CKIIbeta'|DmCK2beta'|DmCKIIbeta'|DmelCG8914	0	0
5187	37301	Drosophila melanogaster	CG11200	Carbonyl reductase	DmelCG11200	0	0
5188	37302	Drosophila melanogaster	CG13868	CG13868 gene product from transcript CG13868-RA	DmelCG13868|anon-WO0153538.60	0	0
5189	37303	Drosophila melanogaster	CG13871	CG13871 gene product from transcript CG13871-RA	DmelCG13871	0	0
5190	37304	Drosophila melanogaster	CG8920	CG8920 gene product from transcript CG8920-RB	BcDNA:LD21403|DmelCG8920	0	0
5191	37305	Drosophila melanogaster	MED8	Mediator complex subunit 8	ARC32|Arc32|CG13867|CG30140|DmelCG13867|Med8/ARC32|dARC32	0	0
5192	37306	Drosophila melanogaster	CG8929	CG8929 gene product from transcript CG8929-RA	DmelCG8929|anon-WO0118547.91	0	0
5193	37307	Drosophila melanogaster	CG16739	CG16739 gene product from transcript CG16739-RA	DmelCG16739	0	0
5194	37308	Drosophila melanogaster	CG13870	CG13870 gene product from transcript CG13870-RA	DmelCG13870	0	0
5195	37309	Drosophila melanogaster	CG11192	CG11192 gene product from transcript CG11192-RB	DmelCG11192|SP82	0	0
5196	37310	Drosophila melanogaster	CG12484	CG12484 gene product from transcript CG12484-RB	CG30143|CG33041|CT32777|DmelCG12484|Dmel_CG30143|Dmel_CG33041	0	0
5197	37311	Drosophila melanogaster	Obp57c	Odorant-binding protein 57c	57c|BcDNA:RH68952|CG13421|DmelCG13421|Obp57a	0	0
5198	37312	Drosophila melanogaster	CG13426	CG13426 gene product from transcript CG13426-RA	DmelCG13426	0	0
5199	37313	Drosophila melanogaster	CG13422	CG13422 gene product from transcript CG13422-RA	DmelCG13422|GNBP-like|GNBPlike 3	0	0
5200	37314	Drosophila melanogaster	CG18067	CG18067 gene product from transcript CG18067-RA	CT40493|DmelCG18067	0	0
5201	37315	Drosophila melanogaster	CG13423	CG13423 gene product from transcript CG13423-RA	DmelCG13423	0	0
5202	37316	Drosophila melanogaster	CG13427	CG13427 gene product from transcript CG13427-RA	DmelCG13427	0	0
5203	37317	Drosophila melanogaster	CG13428	CG13428 gene product from transcript CG13428-RB	DmelCG13428	0	0
5204	37318	Drosophila melanogaster	CG30148	CG30148 gene product from transcript CG30148-RA	BcDNA:AT18611|CG13429|DmelCG30148	0	0
5205	37319	Drosophila melanogaster	Cpr57A	Cuticular protein 57A	CG18066|DmelCG18066	0	0
5206	37322	Drosophila melanogaster	lms	lateral muscles scarcer	CG13424|DmelCG13424	0	0
5207	37323	Drosophila melanogaster	Mgat1	UDP-GlcNAc:a-3-D-mannoside-beta-1,2-N-acetylgluc	CG13431|DmelCG13431|GlcNAc-TI|GlcNAcT1|MGAT1|dMGAT1	0	0
5208	37325	Drosophila melanogaster	Nnf1a	CG13434 gene product from transcript CG13434-RA	CG13434|DmelCG13434|dmNnf1R-2	0	0
5209	37328	Drosophila melanogaster	Hil	Hillarin	CG10439|CG13435|CG30147|D-hil|DmelCG30147	0	0
5210	37329	Drosophila melanogaster	CG9945	CG9945 gene product from transcript CG9945-RB	DmelCG9945	0	0
5211	37330	Drosophila melanogaster	CG11180	CG11180 gene product from transcript CG11180-RA	DmelCG11180	0	0
5212	37331	Drosophila melanogaster	CG16742	CG16742 gene product from transcript CG16742-RA	DmelCG16742	0	0
5213	37332	Drosophila melanogaster	Rcd6	Reduction in Cnn dots 6	CG11175|DmelCG11175	0	0
5214	37334	Drosophila melanogaster	CG13436	CG13436 gene product from transcript CG13436-RA	DmelCG13436	0	0
5215	37335	Drosophila melanogaster	rig	rigor mortis	CG11171|CG13440|CG30149|DmelCG30149|Rig|anon-SAGE:Wang-132|l(2)05056	0	0

<table>
<tr><td>5216</td><td>37336</td><td>Drosophila melanogaster</td><td>maf-S</td><td>CG9954 gene product from transcript CG9954-RA</td><td>CG9954|DmelCG9954|Maf-S|anon-EST:Posey173|maf2</td><td>0</td><td>0</td></tr>
<tr><td>5217</td><td>37337</td><td>Drosophila melanogaster</td><td>CG11110</td><td>CG11110 gene product from transcript CG11110-RA</td><td>DmelCG11110</td><td>0</td><td>0</td></tr>
<tr><td>5218</td><td>37339</td><td>Drosophila melanogaster</td><td>DMAP1</td><td>CG11132 gene product from transcript CG11132-RA</td><td>CG11132|DMA|DmelCG11132|dDMAP1</td><td>0</td><td>0</td></tr>
<tr><td>5219</td><td>37340</td><td>Drosophila melanogaster</td><td>CG16799</td><td>CG16799 gene product from transcript CG16799-RA</td><td>DmelCG16799</td><td>0</td><td>0</td></tr>
<tr><td>5220</td><td>37341</td><td>Drosophila melanogaster</td><td>CG11159</td><td>CG11159 gene product from transcript CG11159-RA</td><td>DmelCG11159</td><td>0</td><td>0</td></tr>
<tr><td>5221</td><td>37342</td><td>Drosophila melanogaster</td><td>Lrt</td><td>Leucine-rich tendon-specific protein</td><td>CG11136|CT31131|DmelCG11136|lrt|tartan/capricious-like</td><td>0</td><td>0</td></tr>
<tr><td>5222</td><td>37343</td><td>Drosophila melanogaster</td><td>CG13437</td><td>CG13437 gene product from transcript CG13437-RA</td><td>DmelCG13437</td><td>0</td><td>0</td></tr>
<tr><td>5223</td><td>37344</td><td>Drosophila melanogaster</td><td>Fem-1</td><td>CG9025 gene product from transcript CG9025-RA</td><td>CG9025|DmelCG9025|dFEM-1</td><td>0</td><td>0</td></tr>
<tr><td>5224</td><td>37345</td><td>Drosophila melanogaster</td><td>exu</td><td>exuperantia</td><td>CG8994|DmelCG8994|Exu|bs30h09.y1|exu1</td><td>0</td><td>0</td></tr>
<tr><td>5225</td><td>37347</td><td>Drosophila melanogaster</td><td>Gr57a</td><td>Gustatory receptor 57a</td><td>57B.1|CG13441|DmelCG13441|GR57B.1|Gr57B1</td><td>0</td><td>0</td></tr>
<tr><td>5226</td><td>37348</td><td>Drosophila melanogaster</td><td>CG13438</td><td>CG13438 gene product from transcript CG13438-RA</td><td>DmelCG13438</td><td>0</td><td>0</td></tr>
<tr><td>5227</td><td>37349</td><td>Drosophila melanogaster</td><td>CG13442</td><td>CG13442 gene product from transcript CG13442-RA</td><td>DmelCG13442</td><td>0</td><td>0</td></tr>
<tr><td>5228</td><td>37351</td><td>Drosophila melanogaster</td><td>CG13443</td><td>CG13443 gene product from transcript CG13443-RA</td><td>DmelCG13443</td><td>0</td><td>0</td></tr>
<tr><td>5229</td><td>37353</td><td>Drosophila melanogaster</td><td>CG15226</td><td>CG15226 gene product from transcript CG15226-RA</td><td>DmelCG15226</td><td>0</td><td>0</td></tr>
<tr><td>5230</td><td>37354</td><td>Drosophila melanogaster</td><td>CG15225</td><td>CG15225 gene product from transcript CG15225-RA</td><td>DmelCG15225</td><td>0</td><td>0</td></tr>
<tr><td>5231</td><td>37355</td><td>Drosophila melanogaster</td><td>insc</td><td>inscuteable</td><td>25/17|CG11312|DmelCG11312|Ins|Insc|fam|l(2)05475|l(2)k12405|nem</td><td>0</td><td>0</td></tr>
<tr><td>5232</td><td>37356</td><td>Drosophila melanogaster</td><td>sktl</td><td>skittles</td><td>25/17|CG9985|DmelCG9985|PIP5-kinase|PIP5K|PIP5K 57B6|Sktl|fam|l(2)05475|l(2)k12405|ski</td><td>0</td><td>0</td></tr>
<tr><td>5233</td><td>37357</td><td>Drosophila melanogaster</td><td>CG17999</td><td>CG17999 gene product from transcript CG17999-RA</td><td>DmelCG17999</td><td>0</td><td>0</td></tr>
<tr><td>5234</td><td>37358</td><td>Drosophila melanogaster</td><td>CG9993</td><td>CG9993 gene product from transcript CG9993-RA</td><td>DmelCG9993</td><td>0</td><td>0</td></tr>
<tr><td>5235</td><td>37359</td><td>Drosophila melanogaster</td><td>CG15227</td><td>CG15227 gene product from transcript CG15227-RA</td><td>DmelCG15227</td><td>0</td><td>0</td></tr>
<tr><td>5236</td><td>37360</td><td>Drosophila melanogaster</td><td>Ipk1</td><td>CG30295 gene product from transcript CG30295-RB</td><td>CG15228|CG15229|CG15230|CG30295|DmelCG30295|dmIpk1</td><td>0</td><td>0</td></tr>
<tr><td>5237</td><td>37363</td><td>Drosophila melanogaster</td><td>CG9236</td><td>CG9236 gene product from transcript CG9236-RB</td><td>DmelCG9236</td><td>0</td><td>0</td></tr>
<tr><td>5238</td><td>37364</td><td>Drosophila melanogaster</td><td>otp</td><td>orthopedia</td><td>BcDNA:RE58095|CG10036|CG2965|DmelCG10036|Otp|W26|bk24|w26</td><td>0</td><td>0</td></tr>
<tr><td>5239</td><td>37366</td><td>Drosophila melanogaster</td><td>CG9235</td><td>CG9235 gene product from transcript CG9235-RA</td><td>DmelCG9235</td><td>0</td><td>0</td></tr>
<tr><td>5240</td><td>37367</td><td>Drosophila melanogaster</td><td>Rx</td><td>Retinal Homeobox</td><td>CG10052|DRx|DmelCG10052|Drx|E97|PPH17|W60|bk50|drx|wom</td><td>0</td><td>0</td></tr>
<tr><td>5241</td><td>37368</td><td>Drosophila melanogaster</td><td>Act57B</td><td>Actin 57B</td><td>Act57A|Act57b|Actin|BEST:RE38067|C|CG10067|CG30294|DmelCG10067|M|T10|act 57A|act57A|act57B|act57b|actin|anon-EST:CL2c12</td><td>0</td><td>0</td></tr>
<tr><td>5242</td><td>37371</td><td>Drosophila melanogaster</td><td>CG15649</td><td>CG15649 gene product from transcript CG15649-RB</td><td>BcDNA:GH08880|DmelCG15649</td><td>0</td><td>0</td></tr>
<tr><td>5243</td><td>37372</td><td>Drosophila melanogaster</td><td>CG9344</td><td>CG9344 gene product from transcript CG9344-RA</td><td>BcDNA:RE43665|DmelCG9344</td><td>0</td><td>0</td></tr>
<tr><td>5244</td><td>37373</td><td>Drosophila melanogaster</td><td>CG15650</td><td>CG15650 gene product from transcript CG15650-RA</td><td>DmelCG15650</td><td>0</td><td>0</td></tr>
<tr><td>5245</td><td>37374</td><td>Drosophila melanogaster</td><td>CG9313</td><td>CG9313 gene product from transcript CG9313-RA</td><td>DmelCG9313</td><td>0</td><td>0</td></tr>
<tr><td>5246</td><td>37375</td><td>Drosophila melanogaster</td><td>CG15651</td><td>CG15651 gene product from transcript CG15651-RA</td><td>DmelCG15651</td><td>0</td><td>0</td></tr>
<tr><td>5247</td><td>37376</td><td>Drosophila melanogaster</td><td>CG3216</td><td>CG3216 gene product from transcript CG3216-RD</td><td>DmelCG3216|GYC</td><td>0</td><td>0</td></tr>
<tr><td>5248</td><td>37377</td><td>Drosophila melanogaster</td><td>dgt3</td><td>dim gamma-tubulin 3</td><td>CG3221|Dgt3|DmelCG3221</td><td>0</td><td>0</td></tr>
<tr><td>5249</td><td>37378</td><td>Drosophila melanogaster</td><td>Pros29</td><td>Proteasome 29kD subunit</td><td>CG9327|DmelCG9327|PROS-29|PSB3_DROME|Pros20S-alpha3|Prosalpha3|alpha3_dm|pros29</td><td>0</td><td>0</td></tr>
<tr><td>5250</td><td>37379</td><td>Drosophila melanogaster</td><td>CG10543</td><td>CG10543 gene product from transcript CG10543-RA</td><td>DmelCG10543</td><td>0</td><td>0</td></tr>
<tr><td>5251</td><td>37381</td><td>Drosophila melanogaster</td><td>CG9346</td><td>CG9346 gene product from transcript CG9346-RA</td><td>DmelCG9346|cg9346</td><td>0</td><td>0</td></tr>
<tr><td>5252</td><td>37382</td><td>Drosophila melanogaster</td><td>CG3295</td><td>CG3295 gene product from transcript CG3295-RA</td><td>DmelCG3295</td><td>0</td><td>0</td></tr>
<tr><td>5253</td><td>37384</td><td>Drosophila melanogaster</td><td>RIC-3</td><td>CG30296 gene product from transcript CG30296-RE</td><td>CG15652|CG30296|CG9349|DmRIC-3|DmelCG30296</td><td>0</td><td>0</td></tr>
<tr><td>5254</td><td>37385</td><td>Drosophila melanogaster</td><td>CG9350</td><td>CG9350 gene product from transcript CG9350-RA</td><td>BcDNA:RH51452|DmelCG9350</td><td>0</td><td>0</td></tr>
<tr><td>5255</td><td>37386</td><td>Drosophila melanogaster</td><td>shg</td><td>shotgun</td><td>CADH|CG3722|CT12481|Cad|CadE|Cadh|D-cad|DCAD2|DCad|DCad2|DE|DE Cadh|DE-CAD|DE-CAD2|DE-Cad|DE-Cadherin|DE-cad|DE-cadh|DE-cadherin|DEC|DECad|DE[cyto]|DEcad|DmelCG3722|E-CA</td><td>0</td><td>0</td></tr>
<tr><td>5256</td><td>37387</td><td>Drosophila melanogaster</td><td>cpa</td><td>capping protein alpha</td><td>CG10540|Cpa|DmelCG10540|dMiro1|miro|scrd</td><td>0</td><td>0</td></tr>
<tr><td>5257</td><td>37388</td><td>Drosophila melanogaster</td><td>CG15653</td><td>CG15653 gene product from transcript CG15653-RA</td><td>DmelCG15653</td><td>0</td><td>0</td></tr>
<tr><td>5258</td><td>37389</td><td>Drosophila melanogaster</td><td>mRpL54</td><td>mitochondrial ribosomal protein L54</td><td>BcDNA:SD09147|CG9353|DmelCG9353|MRP-L54</td><td>0</td><td>0</td></tr>
<tr><td>5259</td><td>37390</td><td>Drosophila melanogaster</td><td>Cht8</td><td>CG9357 gene product from transcript CG9357-RA</td><td>CG9357|DmelCG9357</td><td>0</td><td>0</td></tr>
<tr><td>5260</td><td>37392</td><td>Drosophila melanogaster</td><td>Cht9</td><td>CG10531 gene product from transcript CG10531-RA</td><td>CG10531|DmCHT9|DmelCG10531</td><td>0</td><td>0</td></tr>
<tr><td>5261</td><td>37393</td><td>Drosophila melanogaster</td><td>CG10527</td><td>CG10527 gene product from transcript CG10527-RA</td><td>DmelCG10527|FAMeT</td><td>0</td><td>0</td></tr>
<tr><td>5262</td><td>37395</td><td>Drosophila melanogaster</td><td>CG4030</td><td>CG4030 gene product from transcript CG4030-RA</td><td>DmelCG4030</td><td>0</td><td>0</td></tr>
<tr><td>5263</td><td>37397</td><td>Drosophila melanogaster</td><td>CG4038</td><td>CG4038 gene product from transcript CG4038-RA</td><td>CG4038-PA|DmelCG4038|GCR 101|GCR101|GCR101 SnRNP|Pen101|anon-Pen101|dGAR1</td><td>0</td><td>0</td></tr>
<tr><td>5264</td><td>37398</td><td>Drosophila melanogaster</td><td>CG34396</td><td>CG34396 gene product from transcript CG34396-RC</td><td>CG15654|CG15655|DmelCG34396|Dmel_CG15654|Dmel_CG15655|anon-WO0109301.1|anon-WO0109301.16|anon-WO0109301.17</td><td>0</td><td>0</td></tr>
<tr><td>5265</td><td>37399</td><td>Drosophila melanogaster</td><td>CG9394</td><td>CG9394 gene product from transcript CG9394-RA</td><td>DmelCG9394|anon-EST:Posey227</td><td>0</td><td>0</td></tr>
<tr><td>5266</td><td>37400</td><td>Drosophila melanogaster</td><td>king-tubby</td><td>king tubby</td><td>87D3S|CG9398|D-tulp|DmelCG9398|Dtulp|ESTS:87D3S|TULP|Tulp</td><td>0</td><td>0</td></tr>
<tr><td>5267</td><td>37401</td><td>Drosophila melanogaster</td><td>CG4050</td><td>CG4050 gene product from transcript CG4050-RA</td><td>DmelCG4050|anon-DM192|anon-j</td><td>0</td><td>0</td></tr>
<tr><td>5268</td><td>37402</td><td>Drosophila melanogaster</td><td>mago</td><td>mago nashi</td><td>CG9401|Dm MGN|DmMago|DmelCG9401|MAGOH|MGN|Mago|Magoh|l(2)57Ca|mgn</td><td>0</td><td>0</td></tr>
<tr><td>5269</td><td>37403</td><td>Drosophila melanogaster</td><td>Magi</td><td>CG30388 gene product from transcript CG30388-RA</td><td>CG15656|CG30388|CG4117|D-MAGI|DmelCG30388|Ima|ima</td><td>0</td><td>0</td></tr>
<tr><td>5270</td><td>37405</td><td>Drosophila melanogaster</td><td>CG9406</td><td>CG9406 gene product from transcript CG9406-RA</td><td>DmelCG9406</td><td>0</td><td>0</td></tr>
<tr><td>5271</td><td>37407</td><td>Drosophila melanogaster</td><td>CG9418</td><td>CG9418 gene product from transcript CG9418-RA</td><td>BEST:LD07122|DmelCG9418|LD07122</td><td>0</td><td>0</td></tr>
<tr><td>5272</td><td>37408</td><td>Drosophila melanogaster</td><td>CG15657</td><td>CG15657 gene product from transcript CG15657-RA</td><td>BcDNA:AT24036|DmelCG15657</td><td>0</td><td>0</td></tr>
<tr><td>5273</td><td>37410</td><td>Drosophila melanogaster</td><td>CG30389</td><td>CG30389 gene product from transcript CG30389-RA</td><td>BcDNA:AT02070|CG10518|CG4126|DmelCG30389</td><td>0</td><td>0</td></tr>
<tr><td>5274</td><td>37411</td><td>Drosophila melanogaster</td><td>CG4266</td><td>CG4266 gene product from transcript CG4266-RA</td><td>DmelCG4266|cg4266</td><td>0</td><td>0</td></tr>
<tr><td>5275</td><td>37412</td><td>Drosophila melanogaster</td><td>CG9437</td><td>CG9437 gene product from transcript CG9437-RA</td><td>DmelCG9437</td><td>0</td><td>0</td></tr>
<tr><td>5276</td><td>37413</td><td>Drosophila melanogaster</td><td>LSm1</td><td>CG4279 gene product from transcript CG4279-RA</td><td>CG4279|DmelCG4279|Lsm1</td><td>0</td><td>0</td></tr>
<tr><td>5277</td><td>37414</td><td>Drosophila melanogaster</td><td>Xpd</td><td>Xeroderma pigmentosum D</td><td>CG9433|DhR3|DhXPD|DmXPD|DmelCG9433|ERCC2|XPD|XPD/ERCC2|l(2)SH2 2137|l(2)SH2137|xpd</td><td>0</td><td>0</td></tr>
<tr><td>5278</td><td>37415</td><td>Drosophila melanogaster</td><td>Pu</td><td>Punch</td><td>CG9441|DmelCG9441|GCH1|GTPC|GTPCH|Gr|l(2)57Cf|p[Gr]|upi</td><td>0</td><td>0</td></tr>
<tr><td>5279</td><td>37416</td><td>Drosophila melanogaster</td><td>CG4286</td><td>CG4286 gene product from transcript CG4286-RA</td><td>DmelCG4286</td><td>0</td><td>0</td></tr>
<tr><td>5280</td><td>37417</td><td>Drosophila melanogaster</td><td>tud</td><td>tudor</td><td>CG9450|DmelCG9450|TUD|Tud|tu</td><td>0</td><td>0</td></tr>
<tr><td>5281</td><td>37418</td><td>Drosophila melanogaster</td><td>CG15658</td><td>CG15658 gene product from transcript CG15658-RA</td><td>CT35842|DmelCG15658</td><td>0</td><td>0</td></tr>
<tr><td>5282</td><td>37419</td><td>Drosophila melanogaster</td><td>Glycogenin</td><td>CG9480 gene product from transcript CG9480-RB</td><td>CG9480|DmelCG9480|FBgn0034603|NEST:bs16a02</td><td>0</td><td>0</td></tr>
<tr><td>5283</td><td>37420</td><td>Drosophila melanogaster</td><td>CG4302</td><td>CG4302 gene product from transcript CG4302-RA</td><td>BEST:GH09393|DmelCG4302|GH09393</td><td>0</td><td>0</td></tr>
<tr><td>5284</td><td>37421</td><td>Drosophila melanogaster</td><td>CG15661</td><td>CG15661 gene product from transcript CG15661-RA</td><td>DmelCG15661</td><td>0</td><td>0</td></tr>
<tr><td>5285</td><td>37422</td><td>Drosophila melanogaster</td><td>ASPP</td><td>Ankyrin-repeat, SH3-domain, and Proline-rich-region c</td><td>CG18375|DmelCG18375|dASPP</td><td>0</td><td>0</td></tr>
<tr><td>5286</td><td>37423</td><td>Drosophila melanogaster</td><td>CG30391</td><td>CG30391 gene product from transcript CG30391-RA</td><td>CG15662|DmelCG30391</td><td>0</td><td>0</td></tr>
<tr><td>5287</td><td>37427</td><td>Drosophila melanogaster</td><td>CG10069</td><td>CG10069 gene product from transcript CG10069-RA</td><td>DmelCG10069</td><td>0</td><td>0</td></tr>
<tr><td>5288</td><td>37428</td><td>Drosophila melanogaster</td><td>CG10505</td><td>CG10505 gene product from transcript CG10505-RA</td><td>Dme_CG10505|DmelCG10505</td><td>0</td><td>0</td></tr>
<tr><td>5289</td><td>37429</td><td>Drosophila melanogaster</td><td>Sgf29</td><td>CG30390 gene product from transcript CG30390-RA</td><td>CG10509|CG30390|DmelCG30390|dSgf29</td><td>0</td><td>0</td></tr>
<tr><td>5290</td><td>37430</td><td>Drosophila melanogaster</td><td>RpL29</td><td>Ribosomal protein L29</td><td>BcDNA:RH58777|CG10071|DL43|DmelCG10071|L29|RpL43|anon-EST:Posey204|anon-EST:Posey73</td><td>0</td><td>0</td></tr>
<tr><td>5291</td><td>37431</td><td>Drosophila melanogaster</td><td>CG9752</td><td>CG9752 gene product from transcript CG9752-RA</td><td>DmelCG9752</td><td>0</td><td>0</td></tr>
<tr><td>5292</td><td>37433</td><td>Drosophila melanogaster</td><td>CG42672</td><td>CG42672 gene product from transcript CG42672-RD</td><td>CG10074|CG15665|CG30386|CG30387|DmelCG42672|Dmel_CG30386|Dmel_CG30387</td><td>0</td><td>0</td></tr>
<tr><td>5293</td><td>37434</td><td>Drosophila melanogaster</td><td>CG9754</td><td>CG9754 gene product from transcript CG9754-RA</td><td>DmelCG9754</td><td>0</td><td>0</td></tr>
<tr><td>5294</td><td>37435</td><td>Drosophila melanogaster</td><td>CG9485</td><td>CG9485 gene product from transcript CG9485-RD</td><td>BEST:GH22856|DmelCG9485|FBgn0034618</td><td>0</td><td>0</td></tr>
<tr><td>5295</td><td>37437</td><td>Drosophila melanogaster</td><td>CG30394</td><td>CG30394 gene product from transcript CG30394-RB</td><td>CG10500|CG15670|DmelCG30394|anon-WO0172774.187|anon-WO0172774.188</td><td>0</td><td>0</td></tr>
<tr><td>5296</td><td>37439</td><td>Drosophila melanogaster</td><td>CG15666</td><td>CG15666 gene product from transcript CG15666-RD</td><td>BcDNA:RH67628|DmelCG15666</td><td>0</td><td>0</td></tr>
<tr><td>5297</td><td>37440</td><td>Drosophila melanogaster</td><td>CG9822</td><td>CG9822 gene product from transcript CG9822-RA</td><td>Agr-like|DmelCG9822</td><td>0</td><td>0</td></tr>
<tr><td>5298</td><td>37441</td><td>Drosophila melanogaster</td><td>CG17974</td><td>CG17974 gene product from transcript CG17974-RA</td><td>DmelCG17974</td><td>0</td><td>0</td></tr>
<tr><td>5299</td><td>37443</td><td>Drosophila melanogaster</td><td>CG10795</td><td>CG10795 gene product from transcript CG10795-RA</td><td>DmelCG10795|LD27358</td><td>0</td><td>0</td></tr>
<tr><td>5300</td><td>37444</td><td>Drosophila melanogaster</td><td>EfSec</td><td>CG9841 gene product from transcript CG9841-RA</td><td>CG9841|DmelCG9841|EFSec|EFsec|Efsec|Eftu|SelB/eEFsec|deEFsec|eEFsec</td><td>0</td><td>0</td></tr>
<tr><td>5301</td><td>37445</td><td>Drosophila melanogaster</td><td>Acox57D-p</td><td>acyl-Coenzyme A oxidase at 57D proximal</td><td>Acox57Dp|CG9707|D-acox|DmelCG9707|acox</td><td>0</td><td>0</td></tr>
<tr><td>5302</td><td>37446</td><td>Drosophila melanogaster</td><td>Acox57D-d</td><td>acyl-Coenzyme A oxidase at 57D distal</td><td>Acox57Dd|CG9709|D-acox|DmelCG9709|acox</td><td>0</td><td>0</td></tr>
<tr><td>5303</td><td>37447</td><td>Drosophila melanogaster</td><td>Sdc</td><td>Syndecan</td><td>CG10497|CT27296|DmelCG10497|Dsyn|Syd|Syn|dSdc|l(2)10608|sdc|syd|syn</td><td>0</td><td>0</td></tr>
<tr><td>5304</td><td>37449</td><td>Drosophila melanogaster</td><td>Fkbp13</td><td>CG9847 gene product from transcript CG9847-RC</td><td>BcDNA.GM07659|BcDNA:GH08925|BcDNA:GM07659|CG9847|DmelCG9847|FKB13|FKBP13|GM07659|fkbp13|l(2)00734</td><td>0</td><td>0</td></tr>
<tr><td>5305</td><td>37450</td><td>Drosophila melanogaster</td><td>CG10496</td><td>CG10496 gene product from transcript CG10496-RA</td><td>DmelCG10496</td><td>0</td><td>0</td></tr>
<tr><td>5306</td><td>37451</td><td>Drosophila melanogaster</td><td>MESK2</td><td>Misexpression suppressor of KSR 2</td><td>BcDNA:GH12032|BcDNA:LD27504|BcDNA:RH10174|CG15668|CG15669|DmelCG15669|Mesk2|l(2)01467|mesk2</td><td>0</td><td>0</td></tr>
<tr><td>5307</td><td>37453</td><td>Drosophila melanogaster</td><td>CG10494</td><td>CG10494 gene product from transcript CG10494-RA</td><td>DmelCG10494</td><td>0</td><td>0</td></tr>
<tr><td>5308</td><td>37454</td><td>Drosophila melanogaster</td><td>CG30283</td><td>CG30283 gene product from transcript CG30283-RB</td><td>CG10450|DmelCG30283|SP37|SPH37</td><td>0</td><td>0</td></tr>
<tr><td>5309</td><td>37455</td><td>Drosophila melanogaster</td><td>Egfr</td><td>Epidermal growth factor receptor</td><td>C-erb|CG10079|D-EGFR|D-Egf|DEGFR|DER|DER flb|DER/EGFR|DER/faint little ball|DER/top|DER/torpedo|DER1|Degfr|Der|DmHD-33|DmelCG10079|EFG-R|EGF-R|EGFR|EGFr|EK2-6|Egf|Egf-r|EgfR|El|Elp|</td><td>0</td><td>0</td></tr>
<tr><td>5310</td><td>37456</td><td>Drosophila melanogaster</td><td>CG10440</td><td>CG10440 gene product from transcript CG10440-RB</td><td>DmelCG10440</td><td>0</td><td>0</td></tr>
<tr><td>5311</td><td>37458</td><td>Drosophila melanogaster</td><td>CG10433</td><td>CG10433 gene product from transcript CG10433-RA</td><td>DmelCG10433</td><td>0</td><td>0</td></tr>
<tr><td>5312</td><td>37459</td><td>Drosophila melanogaster</td><td>CG15673</td><td>CG15673 gene product from transcript CG15673-RB</td><td>DmelCG15673</td><td>0</td><td>0</td></tr>
<tr><td>5313</td><td>37461</td><td>Drosophila melanogaster</td><td>PTP-ER</td><td>Protein tyrosine phosphatase-ERK/Enhancer of Ras1</td><td>CG9856|DmelCG9856|ER2-5|ptp-er</td><td>0</td><td>0</td></tr>
<tr><td>5314</td><td>37462</td><td>Drosophila melanogaster</td><td>mahj</td><td>mahjong</td><td>BcDNA:SD08722|CG10080|DmelCG10080|VprBP|anon-WO0172774.151</td><td>0</td><td>0</td></tr>
<tr><td>5315</td><td>37463</td><td>Drosophila melanogaster</td><td>CG15674</td><td>CG15674 gene product from transcript CG15674-RA</td><td>DmelCG15674</td><td>0</td><td>0</td></tr>
<tr><td>5316</td><td>37464</td><td>Drosophila melanogaster</td><td>CG10321</td><td>CG10321 gene product from transcript CG10321-RA</td><td>DmelCG10321</td><td>0</td><td>0</td></tr>
<tr><td>5317</td><td>37465</td><td>Drosophila melanogaster</td><td>CG10082</td><td>CG10082 gene product from transcript CG10082-RA</td><td>DmelCG10082|anon-WO0172774.149</td><td>0</td><td>0</td></tr>
<tr><td>5318</td><td>37466</td><td>Drosophila melanogaster</td><td>CG10320</td><td>CG10320 gene product from transcript CG10320-RE</td><td>10320|DmelCG10320|anon-EST:Posey47|anon-EST:Posey84</td><td>0</td><td>0</td></tr>
<tr><td>5319</td><td>37467</td><td>Drosophila melanogaster</td><td>Rae1</td><td>CG9862 gene product from transcript CG9862-RA</td><td>CG9862|DmelCG9862|dmrae1</td><td>0</td><td>0</td></tr>
<tr><td>5320</td><td>37468</td><td>Drosophila melanogaster</td><td>pirk</td><td>poor Imd response upon knock-in</td><td>BcDNA:SD16010|CG15678|DmelCG15678|Pims|Pirk|pims|rudra</td><td>0</td><td>0</td></tr>
<tr><td>5321</td><td>37469</td><td>Drosophila melanogaster</td><td>CG42365</td><td>CG42365 gene product from transcript CG42365-RA</td><td>CG15675|DmelCG42365|Dmel_CG15675</td><td>0</td><td>0</td></tr>
<tr><td>5322</td><td>37470</td><td>Drosophila melanogaster</td><td>CG9865</td><td>CG9865 gene product from transcript CG9865-RA</td><td>DmelCG9865|Dromel_CG9865_FBtr0071680_cx9c_morf</td><td>0</td><td>0</td></tr>
<tr><td>5323</td><td>37471</td><td>Drosophila melanogaster</td><td>NC2alpha</td><td>CG10318 gene product from transcript CG10318-RA</td><td>CG10318|DmelCG10318|Drap1|NC2|dDrap|dDrap1|dDrap1/dNC2a|dNC2</td><td>0</td><td>0</td></tr>
<tr><td>5324</td><td>37472</td><td>Drosophila melanogaster</td><td>CG15676</td><td>CG15676 gene product from transcript CG15676-RA</td><td>DmelCG15676</td><td>0</td><td>0</td></tr>
<tr><td>5325</td><td>37473</td><td>Drosophila melanogaster</td><td>CG30263</td><td>CG30263 gene product from transcript CG30263-RB</td><td>CG15677|CG15679|CT35864|DmelCG30263</td><td>0</td><td>0</td></tr>
<tr><td>5326</td><td>37475</td><td>Drosophila melanogaster</td><td>CG10306</td><td>CG10306 gene product from transcript CG10306-RA</td><td>DmelCG10306</td><td>0</td><td>0</td></tr>
</table>

5327	37476	Drosophila melanogaster	Tbp	TATA binding protein	CG9874|DmelCG9874|RBP|TBP|TBP38|TFIID|TFIIDtau|TfIID|dTBP|dTFIID|dTbp|dtbp	0	0
5328	37477	Drosophila melanogaster	CG10307	CG10307 gene product from transcript CG10307-RA	CT28947|DmelCG10307	0	0
5329	37478	Drosophila melanogaster	Tim10	Translocase of inner membrane 10	CG9878|DmelCG9878|Dromel_CG9878_FBtr0071676_tim10_mORF|bs31d12.y1|tim10	0	0
5330	37479	Drosophila melanogaster	CG17922	CG17922 gene product from transcript CG17922-RA	DmelCG17922	0	0
5331	37480	Drosophila melanogaster	HmgZ	HMG protein Z	BcDNA:RE28596|CG17921|DmelCG17921|HMG-Z|anon-EST:Liang-1.58|clone 1.58|dHMG-Z|hmg-Z	0	0
5332	37481	Drosophila melanogaster	HmgD	High mobility group protein D	CG17950|Dm-HMG-D100|DmelCG17950|HMG D|HMG-D|HMG-D100|HMGD|Hmg-D|anon-EST:Liang-1.9|clone 1.9|dHMG-D|hmg-D	0	0
5333	37482	Drosophila melanogaster	LBR	Lamin B receptor	CG17952|DmelCG17952|dLBR	0	0
5334	37483	Drosophila melanogaster	Grx-1	CG7975 gene product from transcript CG7975-RA	CG7975|DmelCG7975	0	0
5335	37484	Drosophila melanogaster	CG4021	CG4021 gene product from transcript CG4021-RA	DmelCG4021|cg4021	0	0
5336	37485	Drosophila melanogaster	lox2	lysyl oxidase-like 2	CG4402|DmelCG4402|Dmloxl-2|dmlox-2	0	0
5337	37486	Drosophila melanogaster	CG4386	CG4386 gene product from transcript CG4386-RA	DmelCG4386|SP17	0	0
5338	37487	Drosophila melanogaster	CG13492	CG13492 gene product from transcript CG13492-RD	DmelCG13492	0	0
5339	37488	Drosophila melanogaster	CG4363	CG4363 gene product from transcript CG4363-RA	BcDNA:LP06375|CT14230|DmelCG4363	0	0
5340	37489	Drosophila melanogaster	CG4377	CG4377 gene product from transcript CG4377-RA	CT14296|DmelCG4377	0	0
5341	37490	Drosophila melanogaster	CG4372	CG4372 gene product from transcript CG4372-RA	DmelCG4372	0	0
5342	37491	Drosophila melanogaster	CG9294	CG9294 gene product from transcript CG9294-RB	DmelCG9294|SP20	0	0
5343	37492	Drosophila melanogaster	comr	cookie monster	CG13493|Comr|DmelCG13493|Z2-1340|zab	0	0
5344	37493	Drosophila melanogaster	PpN58A	Protein phosphatase N at 58A	CG3245|DmPpN-58A|DmelCG3245|PPN|PPN 58A|PpN-58A	0	0
5345	37496	Drosophila melanogaster	CG13488	CG13488 gene product from transcript CG13488-RB	DmelCG13488	0	0
5346	37497	Drosophila melanogaster	CG13494	CG13494 gene product from transcript CG13494-RA	BcDNA:AT17606|DmelCG13494	0	0
5347	37498	Drosophila melanogaster	CG34369	CG34369 gene product from transcript CG34369-RA	CG13489|CG13490|DmelCG34369|Dmel_CG13489|Dmel_CG13490	0	0
5348	37500	Drosophila melanogaster	CG9304	CG9304 gene product from transcript CG9304-RA	DmelCG9304	0	0
5349	37501	Drosophila melanogaster	Gr58c	Gustatory receptor 58c	58A.3|58A3|CG13491|DmelCG13491|GR58A.3|Gr58A3	0	0
5350	37502	Drosophila melanogaster	Gr58b	Gustatory receptor 58b	58A.2|58A2|CG13495|DmelCG13495|GR58A.2|Gr58A2	0	0
5351	37507	Drosophila melanogaster	CG9308	CG9308 gene product from transcript CG9308-RA	DmelCG9308|p24	0	0
5352	37509	Drosophila melanogaster	CG13500	CG13500 gene product from transcript CG13500-RA	DmelCG13500	0	0
5353	37510	Drosophila melanogaster	CG13501	CG13501 gene product from transcript CG13501-RA	DmelCG13501	0	0
5354	37511	Drosophila melanogaster	Snp	Snipper	65K|CG11478|CG30327|CG42257|CG6393|DmelCG42257|Dmel_CG30327|Dmel_CG6393|cg11478	0	0
5355	37513	Drosophila melanogaster	CG11475	CG11475 gene product from transcript CG11475-RA	DmelCG11475	0	0
5356	37514	Drosophila melanogaster	CG11474	CG11474 gene product from transcript CG11474-RC	DmelCG11474	0	0
5357	37515	Drosophila melanogaster	CG2921	CG2921 gene product from transcript CG2921-RA	DmelCG2921	0	0
5358	37516	Drosophila melanogaster	GlcT-1	CG6437 gene product from transcript CG6437-RA	CG6437|DGlcT-1|DmelCG6437	0	0
5359	37517	Drosophila melanogaster	synj	synaptojanin	CG6562|DmelCG6562|IPP|Synj	0	0
5360	37518	Drosophila melanogaster	CG13502	CG13502 gene product from transcript CG13502-RA	DmelCG13502	0	0
5361	37519	Drosophila melanogaster	CG11073	CG11073 gene product from transcript CG11073-RB	DmelCG11073	0	0
5362	37520	Drosophila melanogaster	CG6613	CG6613 gene product from transcript CG6613-RA	DmelCG6613	0	0
5363	37521	Drosophila melanogaster	Vrp1	Verprolin 1	CG13503|D-WIP|Dm_Vrp1|DmelCG13503|WIP|dWIP|sltr|solas|vrp1|wip	0	0
5364	37522	Drosophila melanogaster	CG30280	CG30280 gene product from transcript CG30280-RB	CG6676|DmelCG30280|FBpp0071673	0	0
5365	37523	Drosophila melanogaster	mei-S332	meiotic from via Salaria 332	BEST:LD13774|CG5303|DmelCG5303|MEI-S322|MEI-S332|Mei-S332|MeiS332|mei-S322|mei-s332|meiS322|meiS332	0	0
5366	37524	Drosophila melanogaster	GM130	CG11061 gene product from transcript CG11061-RB	CG11061|DmelCG11061|dGM130|dgm130	0	0
5367	37526	Drosophila melanogaster	CG34205	CG34205 gene product from transcript CG34205-RA	CG13504|DmelCG34205	0	0
5368	37527	Drosophila melanogaster	CG11269	CG11269 gene product from transcript CG11269-RA	DmelCG11269	0	0
5369	37528	Drosophila melanogaster	Fmr1	CG6203 gene product from transcript CG6203-RC	AT24755|BcDNA:GM08679|CG6203|DmelCG6203|EP(3)3517|FMR|FMR1|FMRP|FMRp|FXR|Fmrp|cg6203|dFMR|dFMR1|dFMRP|dFXR|dFXR1|dFXRP|dFmr1|dFmrp|dfmr|dfmr1|dfxr|dfxr1|dmfr1|fmr|fmr1	0	0
5370	37531	Drosophila melanogaster	CG3045	CG3045 gene product from transcript CG3045-RA	DmelCG3045	0	0
5371	37532	Drosophila melanogaster	CG6758	CG6758 gene product from transcript CG6758-RA	DmelCG6758	0	0
5372	37533	Drosophila melanogaster	CG11170	CG11170 gene product from transcript CG11170-RB	DmelCG11170	0	0
5373	37534	Drosophila melanogaster	CG11275	CG11275 gene product from transcript CG11275-RA	DmelCG11275|i188	0	0
5374	37535	Drosophila melanogaster	MED16	Mediator complex subunit 16	CG5465|DmelCG5465|Med16|Sin4/TRAP95|Trap95|dTRAP95|med16	0	0
5375	37536	Drosophila melanogaster	Vps35	Vacuolar protein sorting 35	CG5625|DmelCG5625|Dvps35|vps35	0	0
5376	37537	Drosophila melanogaster	CG3074	CG3074 gene product from transcript CG3074-RB	DmelCG3074|dmw	0	0
5377	37538	Drosophila melanogaster	CG3292	CG3292 gene product from transcript CG3292-RA	DmelCG3292	0	0
5378	37539	Drosophila melanogaster	CG3290	CG3290 gene product from transcript CG3290-RA	DmelCG3290	0	0
5379	37540	Drosophila melanogaster	CG3264	CG3264 gene product from transcript CG3264-RA	DmelCG3264	0	0
5380	37541	Drosophila melanogaster	CG11291	CG11291 gene product from transcript CG11291-RA	DmelCG11291|Phos11291	0	0
5381	37542	Drosophila melanogaster	ari-2	ariadne 2	CG5709|DmelCG5709|ari2|l(2)07768	0	0
5382	37543	Drosophila melanogaster	Oatp58Da	Organic anion transporting polypeptide 58Da	CG30277|CG3387|DmelCG30277|oatp 58Da	0	0
5383	37544	Drosophila melanogaster	Oatp58Db	Organic anion transporting polypeptide 58Db	58Db|CG3382|DmelCG3382|oatp 58Db	0	0
5384	37545	Drosophila melanogaster	Oatp58Dc	Organic anion transporting polypeptide 58Dc	CG3380|DmelCG3380|OATP|oatp 58Dc	0	0
5385	37546	Drosophila melanogaster	dve	defective proventriculus	CG5799|DmelCG5799|Dve|Dve-s|dep|l(2)01738	0	0
5386	37547	Drosophila melanogaster	CG5819	CG5819 gene product from transcript CG5819-RC	CT18212|DmelCG5819|tartan/capricious-like	0	0
5387	37548	Drosophila melanogaster	wdp	windpipe	CG30504|CG3413|CT11415|CT39092|DmelCG3413	0	0
5388	37549	Drosophila melanogaster	Gp150	CG5820 gene product from transcript CG5820-RA	CG5820|CT18251|DmelCG5820|GP150|anon-EST:fe2A1|gp150|l(2)52A|p150	0	0
5389	37551	Drosophila melanogaster	T3dh	Type III alcohol dehydrogenase	CG3425|DmelCG3425	0	0
5390	37552	Drosophila melanogaster	Liprin-gamma	CG11206 gene product from transcript CG11206-RA	CG11206|DmelCG11206|Liprin CG11206	0	0
5391	37553	Drosophila melanogaster	CG11298	CG11298 gene product from transcript CG11298-RA	BcDNA:AT10824|DmelCG11298	0	0
5392	37554	Drosophila melanogaster	Rtf1	CG10955 gene product from transcript CG10955-RA	CG10955|DmelCG10955|dRtf1	0	0
5393	37555	Drosophila melanogaster	wrapper	CG10382 gene product from transcript CG10382-RA	CG10382|CT19059|DmelCG10382	0	0
5394	37556	Drosophila melanogaster	CG13506	CG13506 gene product from transcript CG13506-RA	CT32874|DmelCG13506	0	0
5395	37557	Drosophila melanogaster	CG3624	CG3624 gene product from transcript CG3624-RA	CT12183|DmelCG3624	0	0
5396	37558	Drosophila melanogaster	CG6044	CG6044 gene product from transcript CG6044-RA	BcDNA:RH44966|DmelCG6044	0	0
5397	37559	Drosophila melanogaster	qkr58E-3	quaking related 58E-3	CG3584|DmelCG3584|KEP1|kep1|qrk58E-3	0	0
5398	37560	Drosophila melanogaster	Mes4	CG11301 gene product from transcript CG11301-RB	11301|BcDNA:GM21739|CG11301|DmelCG11301|NF-Yc	0	0
5399	37561	Drosophila melanogaster	qkr58E-1	quaking related 58E-1	CG3613|DmelCG3613|QKR58E-1|qkr-58-E|qrk58E-1	0	0
5400	37562	Drosophila melanogaster	qkr58E-2	quaking related 58E-2	CG5821|DmelCG5821|qrk58E-2	0	0
5401	37563	Drosophila melanogaster	mRpS29	mitochondrial ribosomal protein S29	CG3633|DAP3|DmelCG3633|MRP-S29|anon-WO0118547.102	0	0
5402	37564	Drosophila melanogaster	rad50	CG6339 gene product from transcript CG6339-RE	6339|CG6339|DmelCG6339|Dmrad50|RAD50|Rad50	0	0
5403	37565	Drosophila melanogaster	CG10344	CG10344 gene product from transcript CG10344-RA	DmelCG10344	0	0
5404	37566	Drosophila melanogaster	ppk12	pickpocket 12	CG10972|DmelCG10972|PPK12	0	0
5405	37567	Drosophila melanogaster	CG10384	CG10384 gene product from transcript CG10384-RB	DmelCG10384|cg10384	0	0
5406	37568	Drosophila melanogaster	CG3701	CG3701 gene product from transcript CG3701-RA	DmelCG3701	0	0
5407	37569	Drosophila melanogaster	CG4752	CG4752 gene product from transcript CG4752-RA	DmelCG4752	0	0
5408	37570	Drosophila melanogaster	CG4554	CG4554 gene product from transcript CG4554-RA	DmelCG4554	0	0
5409	37571	Drosophila melanogaster	CG4610	CG4610 gene product from transcript CG4610-RA	DmelCG4610	0	0
5410	37572	Drosophila melanogaster	CG6018	CG6018 gene product from transcript CG6018-RA	DmalphaE1-O|DmelCG6018|alphaE1-O	0	0
5411	37573	Drosophila melanogaster	px	plexus	CG4444|DmelCG4444|l(2)k08134|l(2)k08316	0	0
5412	37574	Drosophila melanogaster	CG11362	CG11362 gene product from transcript CG11362-RA	DmelCG11362	0	0
5413	37575	Drosophila melanogaster	dnr1	defense repressor 1	CG12489|DmelCG12489|Dnr1	0	0
5414	37576	Drosophila melanogaster	CG3927	CG3927 gene product from transcript CG3927-RA	BcDNA:AT29209|DmelCG3927|cg3927	0	0
5415	37577	Drosophila melanogaster	nsr	novel spermatogenesis regulator	CG3875|DmelCG3875|cg3875	0	0
5416	37578	Drosophila melanogaster	CG4269	CG4269 gene product from transcript CG4269-RA	DmelCG4269	0	0
5417	37579	Drosophila melanogaster	CG4294	CG4294 gene product from transcript CG4294-RB	DmelCG4294|FBgn0034742	0	0
5418	37580	Drosophila melanogaster	RpS16	Ribosomal protein S16	CG4046|DmelCG4046|M(2)58F|S16|bs32d05.y1	0	0
5419	37581	Drosophila melanogaster	Vps20	Vacuolar protein sorting 20	CG4071|DmelCG4071|ESCRT-III|anon-EST:Posey40|vps20	0	0
5420	37582	Drosophila melanogaster	CG4329	CG4329 gene product from transcript CG4329-RB	DmelCG4329|anon-WO0140519.244	0	0
5421	37583	Drosophila melanogaster	CG33143	CG33143 gene product from transcript CG33143-RC	CG13509|CG4232|DmelCG33143	0	0
5422	37585	Drosophila melanogaster	CG17807	CG17807 gene product from transcript CG17807-RA	DmelCG17807	0	0
5423	37586	Drosophila melanogaster	Cdk9	Cyclin-dependent kinase 9	CDK9|CG5179|DmelCG5179|P-TEF|P-TEFb|PTefb|cdk9	0	0
5424	37587	Drosophila melanogaster	bonsai	CG4207 gene product from transcript CG4207-RA	CG4207|DmMRPS15|DmelCG4207|MRP-S15|mRpS15	0	0
5425	37588	Drosophila melanogaster	CG3732	CG3732 gene product from transcript CG3732-RA	DmelCG3732	0	0
5426	37589	Drosophila melanogaster	RpS24	Ribosomal protein S24	BcDNA:RE59324|CG3751|DmelCG3751|M(2)58F|S24|l(2)SH2 2053|l(2)SH2053	0	0
5427	37590	Drosophila melanogaster	Ugt58Fa	CG4414 gene product from transcript CG4414-RA	AC 004377A|CG4414|DmelCG4414	0	0
5428	37591	Drosophila melanogaster	CG2852	CG2852 gene product from transcript CG2852-RA	DmelCG2852	0	0
5429	37592	Drosophila melanogaster	CG30195	CG30195 gene product from transcript CG30195-RA	CG13513|DmelCG30195	0	0
5430	37593	Drosophila melanogaster	CG3746	CG3746 gene product from transcript CG3746-RA	DmelCG3746	0	0
5431	37594	Drosophila melanogaster	Cyp6d2	CG4373 gene product from transcript CG4373-RA	6d2|CG4373|DmelCG4373	0	0
5432	37595	Drosophila melanogaster	CG30196	CG30196 gene product from transcript CG30196-RA	CG13514|DmelCG30196	0	0
5433	37596	Drosophila melanogaster	CG13510	CG13510 gene product from transcript CG13510-RB	DmelCG13510	0	0
5434	37597	Drosophila melanogaster	CG13511	CG13511 gene product from transcript CG13511-RC	DmelCG13511	0	0
5435	37598	Drosophila melanogaster	CG42565	CG42565 gene product from transcript CG42565-RA	CG13512|DmelCG42565|Dmel_CG13512	0	0
5436	37599	Drosophila melanogaster	CG4250	CG4250 gene product from transcript CG4250-RB	BcDNA:RH52355|CT13934|DmelCG4250	0	0
5437	37600	Drosophila melanogaster	CG30269	CG30269 gene product from transcript CG30269-RA	CG13515|DmelCG30269	0	0

<table>
<tr><td></td><td></td><td></td><td></td><td></td><td></td><td></td><td></td></tr>
<tr><td>5438</td><td>37601</td><td>Drosophila melanogaster</td><td>RYBP</td><td>Ring and YY1 Binding Protein</td><td>CG12190|DmelCG12190|dRYBP</td><td>0</td><td>0</td></tr>
<tr><td>5439</td><td>37602</td><td>Drosophila melanogaster</td><td>ppa</td><td>partner of paired</td><td>CG9952|DmelCG9952|I-55|Ppa</td><td>0</td><td>0</td></tr>
<tr><td>5440</td><td>37603</td><td>Drosophila melanogaster</td><td>robo</td><td>roundabout</td><td>BEST:GM07780|CG13521|CT32892|D-Robo1|DmelCG13521|ROBO|Robo|Robo-1|Robo1|dRobo|dRobo1|robo1</td><td>0</td><td>0</td></tr>
<tr><td>5441</td><td>37605</td><td>Drosophila melanogaster</td><td>Obp59a</td><td>Odorant-binding protein 59a</td><td>59a|CG13517|DmelCG13517|Obp58a</td><td>0</td><td>0</td></tr>
<tr><td>5442</td><td>37606</td><td>Drosophila melanogaster</td><td>CG30259</td><td>CG30259 gene product from transcript CG30259-RA</td><td>CG13522|CG13523|DmelCG30259</td><td>0</td><td>0</td></tr>
<tr><td>5443</td><td>37607</td><td>Drosophila melanogaster</td><td>Obp58b</td><td>Odorant-binding protein 58b</td><td>CG13518|DmelCG13518</td><td>0</td><td>0</td></tr>
<tr><td>5444</td><td>37608</td><td>Drosophila melanogaster</td><td>Obp58c</td><td>Odorant-binding protein 58c</td><td>AAF46892|CG13524|DmelCG13524</td><td>0</td><td>0</td></tr>
<tr><td>5445</td><td>37609</td><td>Drosophila melanogaster</td><td>Obp58d</td><td>Odorant-binding protein 58d</td><td>CG13519|DmelCG13519</td><td>0</td><td>0</td></tr>
<tr><td>5446</td><td>37610</td><td>Drosophila melanogaster</td><td>CG30268</td><td>CG30268 gene product from transcript CG30268-RB</td><td>CG13520|DmelCG30268</td><td>0</td><td>0</td></tr>
<tr><td>5447</td><td>37613</td><td>Drosophila melanogaster</td><td>CG13526</td><td>CG13526 gene product from transcript CG13526-RA</td><td>BcDNA:AT13807|DmelCG13526</td><td>0</td><td>0</td></tr>
<tr><td>5448</td><td>37614</td><td>Drosophila melanogaster</td><td>CG42260</td><td>CG42260 gene product from transcript CG42260-RB</td><td>CG13530|CG3536|DmelCG42260|Dmel_CG13530|Dmel_CG3536</td><td>0</td><td>0</td></tr>
<tr><td>5449</td><td>37615</td><td>Drosophila melanogaster</td><td>CG13527</td><td>CG13527 gene product from transcript CG13527-RA</td><td>DmelCG13527|SPH182</td><td>0</td><td>0</td></tr>
<tr><td>5450</td><td>37617</td><td>Drosophila melanogaster</td><td>blw</td><td>bellwether</td><td>A2(2nd)27|ATPsyn-alpha|Blw|CG3612|DmelCG3612|l(2)01410|l(2)03972|l(2)59ABa|l(2)k00212|ms(2)01504|ms(2)Pry58F</td><td>0</td><td>0</td></tr>
<tr><td>5451</td><td>37618</td><td>Drosophila melanogaster</td><td>CycB</td><td>Cyclin B</td><td>2g|3510|CG3510|CYCB|Cyc B|Cycb|DmcycB|DmelCG3510|cyc B|cyc-B|cycB|cyclin B|cyclinB|mAbF2F4</td><td>0</td><td>0</td></tr>
<tr><td>5452</td><td>37619</td><td>Drosophila melanogaster</td><td>stl</td><td>stall</td><td>BcDNA:GM08694|CG3622|DmelCG3622|fs(2)A16</td><td>0</td><td>0</td></tr>
<tr><td>5453</td><td>37621</td><td>Drosophila melanogaster</td><td>CG42284</td><td>CG42284 gene product from transcript CG42284-RC</td><td>CG13528|CG13529|CG30264|CG30266|DmelCG42284|Dmel_CG30264|Dmel_CG30266</td><td>0</td><td>0</td></tr>
<tr><td>5454</td><td>37622</td><td>Drosophila melanogaster</td><td>CG30265</td><td>CG30265 gene product from transcript CG30265-RA</td><td>CG11682|DmelCG30265</td><td>0</td><td>0</td></tr>
<tr><td>5455</td><td>37623</td><td>Drosophila melanogaster</td><td>CG12490</td><td>CG12490 gene product from transcript CG12490-RB</td><td>DmelCG12490</td><td>0</td><td>0</td></tr>
<tr><td>5456</td><td>37624</td><td>Drosophila melanogaster</td><td>CG9825</td><td>CG9825 gene product from transcript CG9825-RA</td><td>DmelCG9825</td><td>0</td><td>0</td></tr>
<tr><td>5457</td><td>37625</td><td>Drosophila melanogaster</td><td>CG9826</td><td>CG9826 gene product from transcript CG9826-RB</td><td>DmelCG9826</td><td>0</td><td>0</td></tr>
<tr><td>5458</td><td>37626</td><td>Drosophila melanogaster</td><td>CG3649</td><td>CG3649 gene product from transcript CG3649-RB</td><td>DmelCG3649</td><td>0</td><td>0</td></tr>
<tr><td>5459</td><td>37627</td><td>Drosophila melanogaster</td><td>CG13531</td><td>CG13531 gene product from transcript CG13531-RB</td><td>DmelCG13531</td><td>0</td><td>0</td></tr>
<tr><td>5460</td><td>37628</td><td>Drosophila melanogaster</td><td>RpL23</td><td>Ribosomal protein L23</td><td>CG3661|DmelCG3661|L17|L23|RL17|RPL17A|RpL17A|Rpl17A</td><td>0</td><td>0</td></tr>
<tr><td>5461</td><td>37629</td><td>Drosophila melanogaster</td><td>inaD</td><td>inactivation no afterpotential D</td><td>CG3504|DmelCG3504|INAD|Ina-D|InaD</td><td>0</td><td>0</td></tr>
<tr><td>5462</td><td>37631</td><td>Drosophila melanogaster</td><td>fd59A</td><td>forkhead domain 59A</td><td>CG3668|DmelCG3668|Dmfd3|FD3|fd3</td><td>0</td><td>0</td></tr>
<tr><td>5463</td><td>37632</td><td>Drosophila melanogaster</td><td>CG13532</td><td>CG13532 gene product from transcript CG13532-RA</td><td>CT32907|DmelCG13532</td><td>0</td><td>0</td></tr>
<tr><td>5464</td><td>37633</td><td>Drosophila melanogaster</td><td>PIP5K59B</td><td>CG3682 gene product from transcript CG3682-RG</td><td>CG17281|CG3682|DmelCG3682|PI4P5K|PIP5K 59B</td><td>0</td><td>0</td></tr>
<tr><td>5465</td><td>37635</td><td>Drosophila melanogaster</td><td>CG3501</td><td>CG3501 gene product from transcript CG3501-RA</td><td>BcDNA:SD01117|DmelCG3501</td><td>0</td><td>0</td></tr>
<tr><td>5466</td><td>37636</td><td>Drosophila melanogaster</td><td>CG3499</td><td>CG3499 gene product from transcript CG3499-RB</td><td>DmCG3499|DmelCG3499</td><td>0</td><td>0</td></tr>
<tr><td>5467</td><td>37637</td><td>Drosophila melanogaster</td><td>asrij</td><td>CG13533 gene product from transcript CG13533-RA</td><td>CG13533|Dmasrij|DmelCG13533|FBgn 34793</td><td>0</td><td>0</td></tr>
<tr><td>5468</td><td>37638</td><td>Drosophila melanogaster</td><td>Gmer</td><td>GDP-4-keto-6-deoxy-D-mannose 3,5-epimerase/4-re</td><td>CG3495|DmGMER|DmelCG3495|GER|gmer</td><td>0</td><td>0</td></tr>
<tr><td>5469</td><td>37639</td><td>Drosophila melanogaster</td><td>MED23</td><td>Mediator complex subunit 23</td><td>CG3695|DmelCG3695|Gal11/SUR2|Med23|Trap150|Trap150beta|dSUR2|dTRAP150beta</td><td>0</td><td>0</td></tr>
<tr><td>5470</td><td>37640</td><td>Drosophila melanogaster</td><td>CG3700</td><td>CG3700 gene product from transcript CG3700-RB</td><td>DmelCG3700|SP48|c-SP48</td><td>0</td><td>0</td></tr>
<tr><td>5471</td><td>37641</td><td>Drosophila melanogaster</td><td>nahoda</td><td>CG12781 gene product from transcript CG12781-RA</td><td>CG12781|CT36793|DmelCG12781</td><td>0</td><td>0</td></tr>
<tr><td>5472</td><td>37643</td><td>Drosophila melanogaster</td><td>CG42678</td><td>CG42678 gene product from transcript CG42678-RG</td><td>BcDNA:LD42159|BcDNA:RE29641|CG30190|CG30193|CG9848|DmelCG42678|Dmel_CG30190|Dmel_CG30193|anon-WO0118547.113</td><td>0</td><td>0</td></tr>
<tr><td>5473</td><td>37644</td><td>Drosophila melanogaster</td><td>CG3788</td><td>CG3788 gene product from transcript CG3788-RB</td><td>DmelCG3788</td><td>0</td><td>0</td></tr>
<tr><td>5474</td><td>37646</td><td>Drosophila melanogaster</td><td>CG3800</td><td>CG3800 gene product from transcript CG3800-RA</td><td>BEST:GM06241|CG11716|DmelCG3800|anon-EST:fe1B4|anon-EST:fe2B6</td><td>0</td><td>0</td></tr>
<tr><td>5475</td><td>37647</td><td>Drosophila melanogaster</td><td>CG9849</td><td>CG9849 gene product from transcript CG9849-RA</td><td>DmelCG9849</td><td>0</td><td>0</td></tr>
<tr><td>5476</td><td>37648</td><td>Drosophila melanogaster</td><td>CG3831</td><td>CG3831 gene product from transcript CG3831-RA</td><td>DmelCG3831</td><td>0</td><td>0</td></tr>
<tr><td>5477</td><td>37650</td><td>Drosophila melanogaster</td><td>CG32833</td><td>CG32833 gene product from transcript CG32833-RA</td><td>BcDNA:GH11992|CG30331|CG9898|DmelCG32833|SPH196</td><td>0</td><td>0</td></tr>
<tr><td>5478</td><td>37651</td><td>Drosophila melanogaster</td><td>CG9897</td><td>CG9897 gene product from transcript CG9897-RA</td><td>DmelCG9897|SPH195</td><td>0</td><td>0</td></tr>
<tr><td>5479</td><td>37652</td><td>Drosophila melanogaster</td><td>CG9896</td><td>CG9896 gene product from transcript CG9896-RA</td><td>DmelCG9896</td><td>0</td><td>0</td></tr>
<tr><td>5480</td><td>37654</td><td>Drosophila melanogaster</td><td>CG42741</td><td>CG42741 gene product from transcript CG42741-RA</td><td>CG34209|CG9895|DmelCG42741|Dmel_CG34209|Dmel_CG9895</td><td>0</td><td>0</td></tr>
<tr><td>5481</td><td>37655</td><td>Drosophila melanogaster</td><td>twi</td><td>twist</td><td>CG2956|DTwist|DmelCG2956|EC9|TWI|Tw|Twi|bHLHb17|dip5</td><td>0</td><td>0</td></tr>
<tr><td>5482</td><td>37657</td><td>Drosophila melanogaster</td><td>CG30194</td><td>CG30194 gene product from transcript CG30194-RD</td><td>CG13536|CG3037|DmelCG30194</td><td>0</td><td>0</td></tr>
<tr><td>5483</td><td>37659</td><td>Drosophila melanogaster</td><td>CG9890</td><td>CG9890 gene product from transcript CG9890-RA</td><td>DmelCG9890|anon-EST:Posey169|Id46465</td><td>0</td><td>0</td></tr>
<tr><td>5484</td><td>37661</td><td>Drosophila melanogaster</td><td>l(2)k09913</td><td>lethal (2) k09913</td><td>1(2)k09913|CG3082|DmelCG3082</td><td>0</td><td>0</td></tr>
<tr><td>5485</td><td>37662</td><td>Drosophila melanogaster</td><td>Fib</td><td>Fibrillarin</td><td>CG9888|DmelCG9888|Fibri|GCR-6|GCR6|Pen59C5|fib|pen59C5</td><td>0</td><td>0</td></tr>
<tr><td>5486</td><td>37663</td><td>Drosophila melanogaster</td><td>CG3085</td><td>CG3085 gene product from transcript CG3085-RA</td><td>DmelCG3085|anon-WO0140519.123</td><td>0</td><td>0</td></tr>
<tr><td>5487</td><td>37664</td><td>Drosophila melanogaster</td><td>Art7</td><td>Arginine methyltransferase 7</td><td>CG9882|DART7|DmelCG9882|dart7</td><td>0</td><td>0</td></tr>
<tr><td>5488</td><td>37666</td><td>Drosophila melanogaster</td><td>CG9877</td><td>CG9877 gene product from transcript CG9877-RA</td><td>BcDNA:RE26296|DmelCG9877</td><td>0</td><td>0</td></tr>
<tr><td>5489</td><td>37667</td><td>Drosophila melanogaster</td><td>CG13538</td><td>CG13538 gene product from transcript CG13538-RA</td><td>DmelCG13538</td><td>0</td><td>0</td></tr>
<tr><td>5490</td><td>37668</td><td>Drosophila melanogaster</td><td>CG9876</td><td>CG9876 gene product from transcript CG9876-RA</td><td>DmelCG9876</td><td>0</td><td>0</td></tr>
<tr><td>5491</td><td>37669</td><td>Drosophila melanogaster</td><td>RpL37b</td><td>Ribosomal protein L37b</td><td>CG9873|DmelCG9873</td><td>0</td><td>0</td></tr>
<tr><td>5492</td><td>37671</td><td>Drosophila melanogaster</td><td>Klp59C</td><td>CG3219 gene product from transcript CG3219-RA</td><td>CG3219|DmCG3219|DmKLP59C|DmKlp59C|DmelCG3219|KIF2B|KLP59C</td><td>0</td><td>0</td></tr>
<tr><td>5493</td><td>37672</td><td>Drosophila melanogaster</td><td>CG3215</td><td>CG3215 gene product from transcript CG3215-RA</td><td>DmelCG3215|gpdh-2</td><td>0</td><td>0</td></tr>
<tr><td>5494</td><td>37673</td><td>Drosophila melanogaster</td><td>CG13544</td><td>CG13544 gene product from transcript CG13544-RA</td><td>DmelCG13544</td><td>0</td><td>0</td></tr>
<tr><td>5495</td><td>37674</td><td>Drosophila melanogaster</td><td>Klp59D</td><td>CG12192 gene product from transcript CG12192-RA</td><td>CG12192|DmKlp59D|DmelCG12192|KLP59D</td><td>0</td><td>0</td></tr>
<tr><td>5496</td><td>37675</td><td>Drosophila melanogaster</td><td>CG13545</td><td>CG13545 gene product from transcript CG13545-RB</td><td>DmelCG13545</td><td>0</td><td>0</td></tr>
<tr><td>5497</td><td>37676</td><td>Drosophila melanogaster</td><td>CG9899</td><td>CG9899 gene product from transcript CG9899-RA</td><td>DmelCG9899</td><td>0</td><td>0</td></tr>
<tr><td>5498</td><td>37679</td><td>Drosophila melanogaster</td><td>CG34371</td><td>CG34371 gene product from transcript CG34371-RA</td><td>CG13546|CG13547|CG13548|CG30188|CT32921|CT32922|DmelCG34371|Dmel_CG13548|Dmel_CG30188</td><td>0</td><td>0</td></tr>
<tr><td>5499</td><td>37680</td><td>Drosophila melanogaster</td><td>CG13539</td><td>CG13539 gene product from transcript CG13539-RA</td><td>DmelCG13539</td><td>0</td><td>0</td></tr>
<tr><td>5500</td><td>37681</td><td>Drosophila melanogaster</td><td>LS2</td><td>Large Subunit 2</td><td>CG3162|DmelCG3162|cg3162</td><td>0</td><td>0</td></tr>
<tr><td>5501</td><td>37682</td><td>Drosophila melanogaster</td><td>CG3092</td><td>CG3092 gene product from transcript CG3092-RA</td><td>DmelCG3092|anon-WO0140519.90</td><td>0</td><td>0</td></tr>
<tr><td>5502</td><td>37683</td><td>Drosophila melanogaster</td><td>yip3</td><td>yippee interacting protein 3</td><td>BcDNA:AT20887|CG13549|DmelCG13549|NEST:bs18b12|bs18b12.y1</td><td>0</td><td>0</td></tr>
<tr><td>5503</td><td>37684</td><td>Drosophila melanogaster</td><td>RpL22-like</td><td>Ribosomal protein L22-like</td><td>CG9871|DmelCG9871|rpL22-like</td><td>0</td><td>0</td></tr>
<tr><td>5504</td><td>37685</td><td>Drosophila melanogaster</td><td>CG12782</td><td>CG12782 gene product from transcript CG12782-RA</td><td>DmelCG12782</td><td>0</td><td>0</td></tr>
<tr><td>5505</td><td>37686</td><td>Drosophila melanogaster</td><td>CG13540</td><td>CG13540 gene product from transcript CG13540-RA</td><td>DmelCG13540</td><td>0</td><td>0</td></tr>
<tr><td>5506</td><td>37687</td><td>Drosophila melanogaster</td><td>ord</td><td>orientation disruptor</td><td>CG3134|DmelCG3134|ORD|Ord</td><td>0</td><td>0</td></tr>
<tr><td>5507</td><td>37688</td><td>Drosophila melanogaster</td><td>CG3124</td><td>CG3124 gene product from transcript CG3124-RA</td><td>DmelCG3124|anon-WO0140519.230</td><td>0</td><td>0</td></tr>
<tr><td>5508</td><td>37689</td><td>Drosophila melanogaster</td><td>CG13541</td><td>CG13541 gene product from transcript CG13541-RA</td><td>DmelCG13541</td><td>0</td><td>0</td></tr>
<tr><td>5509</td><td>37690</td><td>Drosophila melanogaster</td><td>Prosbeta5R</td><td>Proteasome beta5R subunit</td><td>CG9868|DmelCG9868|Prosbeta5R1</td><td>0</td><td>0</td></tr>
<tr><td>5510</td><td>37691</td><td>Drosophila melanogaster</td><td>CG30412</td><td>CG30412 gene product from transcript CG30412-RA</td><td>CG9803|DmelCG30412|anon-WO0140519.195</td><td>0</td><td>0</td></tr>
<tr><td>5511</td><td>37692</td><td>Drosophila melanogaster</td><td>CG9861</td><td>CG9861 gene product from transcript CG9861-RA</td><td>DmelCG9861</td><td>0</td><td>0</td></tr>
<tr><td>5512</td><td>37694</td><td>Drosophila melanogaster</td><td>CG9863</td><td>CG9863 gene product from transcript CG9863-RB</td><td>BcDNA:AT26616|DmelCG9863</td><td>0</td><td>0</td></tr>
<tr><td>5513</td><td>37695</td><td>Drosophila melanogaster</td><td>CG3502</td><td>CG3502 gene product from transcript CG3502-RB</td><td>BEST:LD34564|DmelCG3502</td><td>0</td><td>0</td></tr>
<tr><td>5514</td><td>37696</td><td>Drosophila melanogaster</td><td>CG30409</td><td>CG30409 gene product from transcript CG30409-RB</td><td>CG9872|DmelCG30409</td><td>0</td><td>0</td></tr>
<tr><td>5515</td><td>37697</td><td>Drosophila melanogaster</td><td>CG3500</td><td>CG3500 gene product from transcript CG3500-RA</td><td>BcDNA:AT03803|DmelCG3500</td><td>0</td><td>0</td></tr>
<tr><td>5516</td><td>37698</td><td>Drosophila melanogaster</td><td>CG9875</td><td>CG9875 gene product from transcript CG9875-RA</td><td>BcDNA:GH23338|DmelCG9875</td><td>0</td><td>0</td></tr>
<tr><td>5517</td><td>37699</td><td>Drosophila melanogaster</td><td>CG34424</td><td>CG34424 gene product from transcript CG34424-RA</td><td>CG11079|DmelCG34424|anon-WO0118547.139</td><td>0</td><td>0</td></tr>
<tr><td>5518</td><td>37701</td><td>Drosophila melanogaster</td><td>CG13550</td><td>CG13550 gene product from transcript CG13550-RA</td><td>DmelCG13550</td><td>0</td><td>0</td></tr>
<tr><td>5519</td><td>37702</td><td>Drosophila melanogaster</td><td>CG3493</td><td>CG3493 gene product from transcript CG3493-RA</td><td>DmelCG3493|dGolgin-245</td><td>0</td><td>0</td></tr>
<tr><td>5520</td><td>37703</td><td>Drosophila melanogaster</td><td>yellow-d</td><td>CG9889 gene product from transcript CG9889-RA</td><td>BcDNA:GH20107|CG9889|DmelCG9889</td><td>0</td><td>0</td></tr>
<tr><td>5521</td><td>37704</td><td>Drosophila melanogaster</td><td>yellow-d2</td><td>CG9891 gene product from transcript CG9891-RA</td><td>CG9891|CT27880|DmelCG9891</td><td>0</td><td>0</td></tr>
<tr><td>5522</td><td>37705</td><td>Drosophila melanogaster</td><td>CG30414</td><td>CG30414 gene product from transcript CG30414-RA</td><td>CG3487|DmelCG30414|SP62</td><td>0</td><td>0</td></tr>
<tr><td>5523</td><td>37706</td><td>Drosophila melanogaster</td><td>eIF2B-delta</td><td>CG10315 gene product from transcript CG10315-RA</td><td>CG10315|DmelCG10315|eIF2Bdelta</td><td>0</td><td>0</td></tr>
<tr><td>5524</td><td>37707</td><td>Drosophila melanogaster</td><td>CG3530</td><td>CG3530 gene product from transcript CG3530-RC</td><td>BcDNA|BcDNA:GH04637|CG18093|DmelCG3530</td><td>0</td><td>0</td></tr>
<tr><td>5525</td><td>37708</td><td>Drosophila melanogaster</td><td>CG3520</td><td>CG3520 gene product from transcript CG3520-RA</td><td>DmelCG3520</td><td>0</td><td>0</td></tr>
<tr><td>5526</td><td>37709</td><td>Drosophila melanogaster</td><td>CG9812</td><td>CG9812 gene product from transcript CG9812-RB</td><td>BcDNA:RH38235|DmelCG9812</td><td>0</td><td>0</td></tr>
<tr><td>5527</td><td>37710</td><td>Drosophila melanogaster</td><td>CG9815</td><td>CG9815 gene product from transcript CG9815-RB</td><td>DmelCG9815</td><td>0</td><td>0</td></tr>
<tr><td>5528</td><td>37711</td><td>Drosophila melanogaster</td><td>Or59a</td><td>Odorant receptor 59a</td><td>59D.1|59a|AN6|CG9820|DOR46|DOR59D.1|DmelCG9820|Or46|Or59D.1|dor46</td><td>0</td><td>0</td></tr>
<tr><td>5529</td><td>37712</td><td>Drosophila melanogaster</td><td>CG5357</td><td>CG5357 gene product from transcript CG5357-RA</td><td>DmelCG5357</td><td>0</td><td>0</td></tr>
<tr><td>5530</td><td>37713</td><td>Drosophila melanogaster</td><td>skpF</td><td>CG12227 gene product from transcript CG12227-RA</td><td>CG12227|DmelCG12227|Skp</td><td>0</td><td>0</td></tr>
<tr><td>5531</td><td>37715</td><td>Drosophila melanogaster</td><td>Or59b</td><td>Odorant receptor 59b</td><td>59E.1|59b|CG3569|DOR119|DmelCG3569</td><td>0</td><td>0</td></tr>
<tr><td>5532</td><td>37716</td><td>Drosophila melanogaster</td><td>Or59c</td><td>Odorant receptor 59c</td><td>59E.2|59c|CG17226|DOR120|DmelCG17226</td><td>0</td><td>0</td></tr>
<tr><td>5533</td><td>37717</td><td>Drosophila melanogaster</td><td>CG13557</td><td>CG13557 gene product from transcript CG13557-RA</td><td>DmelCG13557</td><td>0</td><td>0</td></tr>
<tr><td>5534</td><td>37719</td><td>Drosophila melanogaster</td><td>CG13558</td><td>CG13558 gene product from transcript CG13558-RA</td><td>DmelCG13558</td><td>0</td><td>0</td></tr>
<tr><td>5535</td><td>37720</td><td>Drosophila melanogaster</td><td>CG13559</td><td>CG13559 gene product from transcript CG13559-RA</td><td>DmelCG13559</td><td>0</td><td>0</td></tr>
<tr><td>5536</td><td>37721</td><td>Drosophila melanogaster</td><td>CG3906</td><td>CG3906 gene product from transcript CG3906-RA</td><td>DmelCG3906</td><td>0</td><td>0</td></tr>
<tr><td>5537</td><td>37723</td><td>Drosophila melanogaster</td><td>mi</td><td>minus</td><td>CG5360|DmelCG5360|l(2)SH0818|l(2)SH2 0818</td><td>0</td><td>0</td></tr>
<tr><td>5538</td><td>37724</td><td>Drosophila melanogaster</td><td>bw</td><td>brown</td><td>CG17632|DmelCG17632|Pm|Su(w[co2])|Su(w[coJ])|ptm</td><td>0</td><td>0</td></tr>
<tr><td>5539</td><td>37725</td><td>Drosophila melanogaster</td><td>Gr59e</td><td>Gustatory receptor 59e</td><td>59E.2|59E2|CG15872|CG33151|DmelCG33151|GR59E.2|Gr59E2</td><td>0</td><td>0</td></tr>
<tr><td>5540</td><td>37726</td><td>Drosophila melanogaster</td><td>Gr59f</td><td>Gustatory receptor 59f</td><td>59E.1|CG33150|CG5365|DmelCG33150|GR59E.1|Gr59E1</td><td>0</td><td>0</td></tr>
<tr><td>5541</td><td>37727</td><td>Drosophila melanogaster</td><td>wmd</td><td>wing morphogenesis defect</td><td>CG3957|CG3957/wmd|DmelCG3957|pter</td><td>0</td><td>0</td></tr>
<tr><td>5542</td><td>37728</td><td>Drosophila melanogaster</td><td>levy</td><td>CG17280 gene product from transcript CG17280-RA</td><td>BcDNA:RH03295|CG17280|COX6A|DmelCG17280|VIa</td><td>0</td><td>0</td></tr>
<tr><td>5543</td><td>37729</td><td>Drosophila melanogaster</td><td>Dcp-1</td><td>Death caspase-1</td><td>CG5370|DCP-1|DCP1|Dcp1|DmelCG5370|ccp1|dcp-1|dcp1|l(2)01862|l(2)02132</td><td>0</td><td>0</td></tr>
<tr><td>5544</td><td>37730</td><td>Drosophila melanogaster</td><td>pita</td><td>CG3941 gene product from transcript CG3941-RA</td><td>CG3941|DmelCG3941|spdk</td><td>0</td><td>0</td></tr>
<tr><td>5545</td><td>37731</td><td>Drosophila melanogaster</td><td>Rrp4</td><td>CG3931 gene product from transcript CG3931-RA</td><td>CG3931|DmelCG3931|RRP4|dRrp4</td><td>0</td><td>0</td></tr>
<tr><td>5546</td><td>37732</td><td>Drosophila melanogaster</td><td>alphaPS5</td><td>CG5372 gene product from transcript CG5372-RA</td><td>CG5372|CT17068|DmelCG5372</td><td>0</td><td>0</td></tr>
<tr><td>5547</td><td>37733</td><td>Drosophila melanogaster</td><td>Pi3K59F</td><td>Phosphotidylinositol 3 kinase 59F</td><td>CG5373|DmVps34|DmelCG5373|PI(3)K|PI3K|PI3K-59F|PI3K_59F|PI[[3]]K|Pi3K_59F|VPS34|Vps34|dPI3K|dVps34|dVps34/PI3K59F|vps34</td><td>0</td><td>0</td></tr>
<tr><td>5548</td><td>37734</td><td>Drosophila melanogaster</td><td>apt</td><td>apontic</td><td>3041|CG5393|DmelCG5393|TDF|TDF/APT|l(2)03041|l(2)06369|l(2)09049|l(2)59Ea|l(2)k11531|tdf</td><td>0</td><td>0</td></tr>
</table>

5549	37736	Drosophila melanogaster	CG5398	CG5398 gene product from transcript CG5398-RA	Aqp5398|DmelCG5398|anon-WO0140519.151	0	0
5550	37737	Drosophila melanogaster	CG17664	CG17664 gene product from transcript CG17664-RA	Aqp17664|DmelCG17664	0	0
5551	37738	Drosophila melanogaster	CG17662	CG17662 gene product from transcript CG17662-RB	Aqp17662|DmelCG17662	0	0
5552	37739	Drosophila melanogaster	CG4019	CG4019 gene product from transcript CG4019-RA	Aqp4019|DmelCG4019	0	0
5553	37741	Drosophila melanogaster	Pde8	Phosphodiesterase 8	CG5411|DmelCG5411|PDE8|pde8	0	0
5554	37742	Drosophila melanogaster	CG5428	CG5428 gene product from transcript CG5428-RB	DmelCG5428	0	0
5555	37743	Drosophila melanogaster	CG5431	CG5431 gene product from transcript CG5431-RA	DmelCG5431	0	0
5556	37744	Drosophila melanogaster	l(2)efl	lethal (2) essential for life	CG4533|Cryab|DmelCG4533|L(2)efl|el(2)13-1|l(2)13-1|l(2)59Fa|l(2)Efl	0	0
5557	37745	Drosophila melanogaster	CG11293	CG11293 gene product from transcript CG11293-RB	DmelCG11293	0	0
5558	37746	Drosophila melanogaster	Pal2	Peptidyl-alpha-hydroxyglycine-alpha-amidating lyase 2	CG5472|DmelCG5472|PAL|PAL2|Pal|anon-WO0149856.5|dPAL2	0	0
5559	37748	Drosophila melanogaster	angel	CG12273 gene product from transcript CG12273-RA	CG12273|DmelCG12273	0	0
5560	37749	Drosophila melanogaster	CG30183	CG30183 gene product from transcript CG30183-RA	CG4093|CG4530|DmelCG30183	0	0
5561	37750	Drosophila melanogaster	mRpL43	mitochondrial ribosomal protein L43	BcDNA:RE35686|CG5479|CI-B8|DmelCG5479|mRpL-CI-B8	0	0
5562	37751	Drosophila melanogaster	CG4091	CG4091 gene product from transcript CG4091-RA	DmelCG4091	0	0
5563	37754	Drosophila melanogaster	l(2)not	lethal (2) neighbor of tid	1(2) not|CG4084|DmelCG4084|cDNA 10F|ntid	0	0
5564	37755	Drosophila melanogaster	Sesn	Sestrin	BEST:GH20113|BcDNA:GH20113|CG11299|DmelCG11299|dSesn	0	0
5565	37756	Drosophila melanogaster	CG18128	CG18128 gene product from transcript CG18128-RA	DmelCG18128	0	0
5566	37757	Drosophila melanogaster	egl	egalitarian	CG4051|DmelCG4051|EGL|Egl|egal	0	0
5567	37758	Drosophila melanogaster	CG13560	CG13560 gene product from transcript CG13560-RA	DmelCG13560	0	0
5568	37759	Drosophila melanogaster	CG12491	CG12491 gene product from transcript CG12491-RA	DmelCG12491	0	0
5569	37760	Drosophila melanogaster	CG11300	CG11300 gene product from transcript CG11300-RA	BcDNA:LP08443|DmelCG11300	0	0
5570	37761	Drosophila melanogaster	CG5532	CG5532 gene product from transcript CG5532-RA	DmelCG5532	0	0
5571	37762	Drosophila melanogaster	CG9850	CG9850 gene product from transcript CG9850-RA	CG11169|Dimp|DmelCG9850|dimp	0	0
5572	37763	Drosophila melanogaster	CG15800	CG15800 gene product from transcript CG15800-RA	DmelCG15800	0	0
5573	37765	Drosophila melanogaster	CG13561	CG13561 gene product from transcript CG13561-RA	DmelCG13561	0	0
5574	37766	Drosophila melanogaster	CG5539	CG5539 gene product from transcript CG5539-RA	DmelCG5539	0	0
5575	37767	Drosophila melanogaster	Ssrp	Structure specific recognition protein	CG4817|Cf5|DmelCG4817|DssRP|SSRP|SSRP1|dSSRP|dSSRP1|dSsrp|ssrp	0	0
5576	37768	Drosophila melanogaster	CG5543	CG5543 gene product from transcript CG5543-RA	DmelCG5543|anon-WO0118547.181	0	0
5577	37769	Drosophila melanogaster	Nxt1	NTF2-related export protein 1	CG12752|DmNXT1|DmelCG12752|Nxt|dNxt|dmNXT|dmNXT1|dmP15|nxt1|p15	0	0
5578	37770	Drosophila melanogaster	CG4797	CG4797 gene product from transcript CG4797-RA	DmelCG4797	0	0
5579	37771	Drosophila melanogaster	CG4763	CG4763 gene product from transcript CG4763-RA	DmelCG4763	0	0
5580	37772	Drosophila melanogaster	CG5549	CG5549 gene product from transcript CG5549-RB	DmelCG5549	0	0
5581	37774	Drosophila melanogaster	usnp	ubisnap	CG11173|DmelCG11173|SNAP-29|Ubisnap	0	0
5582	37775	Drosophila melanogaster	CG5554	CG5554 gene product from transcript CG5554-RA	DmelCG5554	0	0
5583	37776	Drosophila melanogaster	eIF6	CG17611 gene product from transcript CG17611-RA	CG17611|DeIF6|DmelCG17611|anon-WO0172774.153|anon-WO0172774.155|eif6|l(2)k13214	0	0
5584	37778	Drosophila melanogaster	gbb	glass bottom boat	60A|BMP|CG5562|Dm-GBB|DmelCG5562|GBB|Gbb|Gbb-60A|SixtyA|TGF-b|TGFbeta-60A|Tgfb-60|Tgfbeta-60A|gbb-60A|gcn|l(2)60A-J|tgfb-60A|vgr/60A	0	0
5585	37780	Drosophila melanogaster	wibg	within bgcn	BEST:HL05757|CG10330|CG30176|DmelCG30176|PYM|Pym|UD3	0	0
5586	37781	Drosophila melanogaster	TBPH	CG10327 gene product from transcript CG10327-RC	CG10327|DmelCG10327|TDP-43|TDP43|dtar	0	0
5587	37782	Drosophila melanogaster	CG4585	CG4585 gene product from transcript CG4585-RA	CK00282|DmelCG4585|UD2|anon-60Ab	0	0
5588	37783	Drosophila melanogaster	CG5569	CG5569 gene product from transcript CG5569-RA	BcDNA:SD18780|DmelCG5569	0	0
5589	37784	Drosophila melanogaster	Thiolase	CG4581 gene product from transcript CG4581-RA	CG4581|DmelCG4581|l(2)k09828|thiolase	0	0
5590	37785	Drosophila melanogaster	ken	ken and barbie	2970|3907|5029|8253|BCL-6/KEN|CG5575|DmelCG5575|KEN|Ken|P420|l(2)00628|l(2)02970|l(2)05029|ok|okina	0	0
5591	37786	Drosophila melanogaster	TM4SF	Transmembrane 4 superfamily	CG11303|Dm.Tsp60A|DmelCG11303	0	0
5592	37787	Drosophila melanogaster	CG4882	CG4882 gene product from transcript CG4882-RA	DmelCG4882|GM14313|LD24471|UD1|anon-60Aa	0	0
5593	37788	Drosophila melanogaster	PHDP	Putative homeodomain protein	CG11182|DmelCG11182|anon-60Ac	0	0
5594	37789	Drosophila melanogaster	CG5597	CG5597 gene product from transcript CG5597-RA	CT17698|DmelCG5597	0	0
5595	37790	Drosophila melanogaster	Dcp1	Decapping protein 1	CG11183|DCP1|Dcp-1|Dcp1p|DmelCG11183|anon-WO0118547.163|dDCP1|dDcp1	0	0
5596	37791	Drosophila melanogaster	DNA-ligI	DNA ligase I	CG5602|DmDNA Lig I|DmelCG5602	0	0
5597	37792	Drosophila melanogaster	Upf3	CG11184 gene product from transcript CG11184-RB	CG11184|DmelCG11184|UPF3	0	0
5598	37793	Drosophila melanogaster	CG17658	CG17658 gene product from transcript CG17658-RA	DmelCG17658	0	0
5599	37794	Drosophila melanogaster	CG5339	CG5339 gene product from transcript CG5339-RA	DmelCG5339	0	0
5600	37795	Drosophila melanogaster	CG5591	CG5591 gene product from transcript CG5591-RA	DmelCG5591	0	0
5601	37797	Drosophila melanogaster	CG13562	CG13562 gene product from transcript CG13562-RC	DmelCG13562	0	0
5602	37798	Drosophila melanogaster	Nap1	Nucleosome assembly protein 1	CG5330|DmelCG5330|NAP-1|NAP1|Nap-1|dNAP-1|dNAP1|dNap|dNap1|p56/dCAF-4	0	0
5603	37800	Drosophila melanogaster	kcc	kazachoc	BEST:CK01510|CG2768|CG5594|CK01510|DmelCG5594|E(sda)J|KCC|Line J|l(2)60A-C	0	0
5604	37801	Drosophila melanogaster	CG2812	CG2812 gene product from transcript CG2812-RA	DmelCG2812	0	0
5605	37803	Drosophila melanogaster	CG3735	CG3735 gene product from transcript CG3735-RA	DmelCG3735	0	0
5606	37804	Drosophila melanogaster	Tal	CG2827 gene product from transcript CG2827-RA	154176_at|CG2827|CT9666|DmelCG2827|tal	0	0
5607	37805	Drosophila melanogaster	G-salpha60A	G protein salpha 60A	CG2835|DGs|DGsalpha|DmelCG2835|G-5alpha|G-alpha-60A|GSalpha|G[[S]]alpha|G[[alphas]]|G[[s]]-alpha-60A|G[[s]]alpha|Galpha60A|GalphaS|Galpha[[s]]|Galphas|Gs|Gs-alpha|Gs-alpha60A|Gsalpha|Gsal	0	0
5608	37806	Drosophila melanogaster	CG13565	CG13565 gene product from transcript CG13565-RA	BcDNA:RH09340|DmelCG13565	0	0
5609	37807	Drosophila melanogaster	CG2970	CG2970 gene product from transcript CG2970-RA	DmelCG2970	0	0
5610	37808	Drosophila melanogaster	fzr2	fizzy-related 2	CG16783|DmelCG16783|FZR2	0	0
5611	37809	Drosophila melanogaster	CG3803	CG3803 gene product from transcript CG3803-RA	DmelCG3803	0	0
5612	37810	Drosophila melanogaster	thoc5	CG2980 gene product from transcript CG2980-RA	CG2980|DmelCG2980|THOC5|garm	0	0
5613	37811	Drosophila melanogaster	CG16787	CG16787 gene product from transcript CG16787-RA	DmelCG16787	0	0
5614	37813	Drosophila melanogaster	CG13566	CG13566 gene product from transcript CG13566-RB	DmelCG13566	0	0
5615	37814	Drosophila melanogaster	Fmo-1	Flavin-containing monooxygenase 1	CG3006|DmFMO-1|DmelCG3006|FMO-1	0	0
5616	37815	Drosophila melanogaster	Alas	Aminolevulinate synthase	ALAS|CG3017|DmelCG3017|alas	0	0
5617	37817	Drosophila melanogaster	CG10904	CG10904 gene product from transcript CG10904-RA	DmelCG10904	0	0
5618	37818	Drosophila melanogaster	CG3065	CG3065 gene product from transcript CG3065-RA	DmelCG3065	0	0
5619	37820	Drosophila melanogaster	Gadd34	CG3825 gene product from transcript CG3825-RA	CG3825|Dm-GADD34|DmelCG3825|GADD34|dGADD34	0	0
5620	37822	Drosophila melanogaster	Sox14	Sox box protein 14	CG17263|CG3090|DSox-14|DSox14|DSox60B|DmelCG3090|Dsox-14|SOX14|SOX60B|SOXC|sox14	0	0
5621	37823	Drosophila melanogaster	Phm	Peptidylglycine-alpha-hydroxylating monooxygenase	CG3832|DmelCG3832|PHM|dPHM	0	0
5622	37824	Drosophila melanogaster	Pask	PAS kinase	CG3105|DmelCG3105|pask	0	0
5623	37825	Drosophila melanogaster	CG3860	CG3860 gene product from transcript CG3860-RA	DmelCG3860	0	0
5624	37830	Drosophila melanogaster	CG4324	CG4324 gene product from transcript CG4324-RA	DmelCG4324	0	0
5625	37831	Drosophila melanogaster	CG3121	CG3121 gene product from transcript CG3121-RA	DmelCG3121|anon-WO0140519.109	0	0
5626	37832	Drosophila melanogaster	CG3907	CG3907 gene product from transcript CG3907-RB	DmelCG3907	0	0
5627	37833	Drosophila melanogaster	CG11388	CG11388 gene product from transcript CG11388-RA	DmelCG11388|anon-EST:Posey287	0	0
5628	37834	Drosophila melanogaster	Adk2	Adenylate kinase-2	ADK-2|BcDNA:SD09634|CG3140|Dak2|DmelCG3140|anon-Dak2	0	0
5629	37836	Drosophila melanogaster	CG3163	CG3163 gene product from transcript CG3163-RA	DmelCG3163	0	0
5630	37837	Drosophila melanogaster	Chi	Chip	CG3924|CHIP|DmelCG3924|LDB|Ldb|chip|dLDB/Chip|dLdb|l(2)04405|l(2)k04405	0	0
5631	37838	Drosophila melanogaster	MAN1	CG3167 gene product from transcript CG3167-RA	BcDNA:LD24895|CG3167|DmelCG3167|dMAN1	0	0
5632	37839	Drosophila melanogaster	CG13567	CG13567 gene product from transcript CG13567-RB	DmelCG13567	0	0
5633	37840	Drosophila melanogaster	IntS1	Integrator 1	CG3173|DmelCG3173|Int1	0	0
5634	37841	Drosophila melanogaster	tsr	twinstar	12/8|ADF|ADF/cofilin|Adf|CG4254|Cadf|D-cof|D61|DmelCG4254|T8|Tsr|cofilin|cofilin/ADF|cofilin/actin depolymerizing factor homolog|l(2)03101|l(2)k03101|l(2)k05633|l(2)k13409|ntf	0	0
5635	37842	Drosophila melanogaster	gammaSnap	gamma-soluble NSF attachment protein	CG3988|DmelCG3988|gSnap|gamma SNAP|gamma-SNAP|gamma-Snap|gammaSNAP-2	0	0
5636	37843	Drosophila melanogaster	sei	seizure	CG3182|Derg|DmelCG3182|erg	0	0
5637	37844	Drosophila melanogaster	CG13568	CG13568 gene product from transcript CG13568-RG	DmelCG13568	0	0
5638	37845	Drosophila melanogaster	CG13563	CG13563 gene product from transcript CG13563-RB	DmelCG13563	0	0
5639	37846	Drosophila melanogaster	eIF-5A	CG3186 gene product from transcript CG3186-RA	1296|CG3186|DmelCG3186|anon-EST:fe2H10|eIF5A|eIF5a|l(2)01296	0	0
5640	37849	Drosophila melanogaster	RpL39	Ribosomal protein L39	BcDNA:RH27094|CG3997|DmelCG3997|L39|M(2)60B|RPl46|Rp46|RpL46|anon-EST:Posey16|rp46	0	0
5641	37850	Drosophila melanogaster	Rap2l	Ras-associated protein 2-like	CG3204|Dm Rap21|DmelCG3204|Rap2|Rap21|Rap2L	0	0
5642	37851	Drosophila melanogaster	yki	yorkie	CG4005|DmelCG4005|YKI|Yki|yap	0	0
5643	37852	Drosophila melanogaster	CG3209	CG3209 gene product from transcript CG3209-RC	DmelCG3209	0	0
5644	37853	Drosophila melanogaster	Mlp60A	Muscle LIM protein at 60A	BcDNA:RE14544|BcDNA:RH74176|CG30174|CG30179|CG3220|CG33149|CG42309|DLim-2|DMLP1|DMlp1|DmLIM-2|DmelCG42309|Dmel_CG30174|Dmel_CG30179|Dmel_CG33149|MLP|MLP1|Mlp1|dM	0	0
5645	37854	Drosophila melanogaster	CG10339	CG10339 gene product from transcript CG10339-RA	DmelCG10339	0	0
5646	37855	Drosophila melanogaster	CG13564	CG13564 gene product from transcript CG13564-RA	DmelCG13564	0	0
5647	37856	Drosophila melanogaster	CG16786	CG16786 gene product from transcript CG16786-RA	DmelCG16786	0	0
5648	37857	Drosophila melanogaster	snama	something that sticks like glue	BCDNA:LD21643|BcDNA:LD21643|CG3231|DmelCG3231|SNAMA|l(2)rQ313|mnm	0	0
5649	37858	Drosophila melanogaster	gek	genghis khan	CG4012|DmelCG4012|Gek|MRCKalpha|MdaPk|Mrck|anon-WO0200864.1|dMRCK|l(2)09373	0	0
5650	37859	Drosophila melanogaster	enok	enoki mushroom	CG11290|Cg11290|DmelCG11290|Enok|S1|anon-60Ba|anon-WO0200864.1|anon-WO0200864.2|dmHAG406|rot	0	0
5651	37860	Drosophila melanogaster	CG4049	CG4049 gene product from transcript CG4049-RA	Arip4|BEST:LD04705|DmelCG4049	0	0
5652	37861	Drosophila melanogaster	CG3253	CG3253 gene product from transcript CG3253-RB	BEST:GH04269|DmelCG3253	0	0
5653	37862	Drosophila melanogaster	CG3257	CG3257 gene product from transcript CG3257-RB	DmelCG3257|anon-EST:Posey117	0	0
5654	37863	Drosophila melanogaster	CG13569	CG13569 gene product from transcript CG13569-RB	DmelCG13569	0	0
5655	37864	Drosophila melanogaster	tamo	CG4057 gene product from transcript CG4057-RA	60B|CG4057|DmelCG4057	0	0
5656	37866	Drosophila melanogaster	CG4065	CG4065 gene product from transcript CG4065-RB	DmelCG4065|anon-EST:Nelson1	0	0
5657	37867	Drosophila melanogaster	Dat	Dopamine N acetyltransferase	AA-NAT1|Aanat1|CG3318|Dat1|DmelCG3318|NAT1|aaNAT|aaNAT1|aaNat	0	0
5658	37869	Drosophila melanogaster	DnaJ-60	DnaJ-like-60	CG12240|CG42567|DmJ60|DmelCG42567|Dmel_CG12240|DnaJ60|anon-WO0172774.147	0	0
5659	37871	Drosophila melanogaster	CG3328	CG3328 gene product from transcript CG3328-RA	DmelCG3328	0	0

<table>
<tr><td>5660</td><td>37872</td><td>Drosophila melanogaster</td><td>mRpS17</td><td>mitochondrial ribosomal protein S17</td><td>CG4326|DmelCG4326|MRP-S17</td><td>0</td><td>0</td></tr>
<tr><td>5661</td><td>37873</td><td>Drosophila melanogaster</td><td>Nop60B</td><td>Nucleolar protein at 60B</td><td>CG333|CG3333|DmelCG3333|FBgn0023184|MFL/Nop60B|Nop60|Nop60b|Uhg6|dUhg6|l(2)k05318|mfl|nop60B</td><td>0</td><td>0</td></tr>
<tr><td>5662</td><td>37874</td><td>Drosophila melanogaster</td><td>CG3363</td><td>CG3363 gene product from transcript CG3363-RB</td><td>DmelCG3363</td><td>0</td><td>0</td></tr>
<tr><td>5663</td><td>37875</td><td>Drosophila melanogaster</td><td>CG3362</td><td>CG3362 gene product from transcript CG3362-RA</td><td>DmelCG3362</td><td>0</td><td>0</td></tr>
<tr><td>5664</td><td>37876</td><td>Drosophila melanogaster</td><td>CG3356</td><td>CG3356 gene product from transcript CG3356-RA</td><td>DmelCG3356</td><td>0</td><td>0</td></tr>
<tr><td>5665</td><td>37877</td><td>Drosophila melanogaster</td><td>CG11406</td><td>CG11406 gene product from transcript CG11406-RB</td><td>DmelCG11406</td><td>0</td><td>0</td></tr>
<tr><td>5666</td><td>37879</td><td>Drosophila melanogaster</td><td>CG30419</td><td>CG30419 gene product from transcript CG30419-RA</td><td>CG13572|CG13573|DmelCG30419</td><td>0</td><td>0</td></tr>
<tr><td>5667</td><td>37881</td><td>Drosophila melanogaster</td><td>Ir60a</td><td>Ionotropic receptor 60a</td><td>CG13576|DmelCG13576|IR60a</td><td>0</td><td>0</td></tr>
<tr><td>5668</td><td>37882</td><td>Drosophila melanogaster</td><td>nord</td><td>CG30418 gene product from transcript CG30418-RA</td><td>CG13574|CG30418|CG3372|DmelCG30418</td><td>0</td><td>0</td></tr>
<tr><td>5669</td><td>37883</td><td>Drosophila melanogaster</td><td>CG13575</td><td>CG13575 gene product from transcript CG13575-RA</td><td>DmelCG13575|anon-WO0170980.148|anon-WO0170980.149</td><td>0</td><td>0</td></tr>
<tr><td>5670</td><td>37884</td><td>Drosophila melanogaster</td><td>CG3376</td><td>CG3376 gene product from transcript CG3376-RB</td><td>DmelCG3376|aSMase</td><td>0</td><td>0</td></tr>
<tr><td>5671</td><td>37885</td><td>Drosophila melanogaster</td><td>CG13577</td><td>CG13577 gene product from transcript CG13577-RB</td><td>DmelCG13577</td><td>0</td><td>0</td></tr>
<tr><td>5672</td><td>37886</td><td>Drosophila melanogaster</td><td>nvy</td><td>nervy</td><td>CG3385|DmelCG3385|Nvy</td><td>0</td><td>0</td></tr>
<tr><td>5673</td><td>37887</td><td>Drosophila melanogaster</td><td>CG3394</td><td>CG3394 gene product from transcript CG3394-RA</td><td>DmelCG3394</td><td>0</td><td>0</td></tr>
<tr><td>5674</td><td>37888</td><td>Drosophila melanogaster</td><td>betaTub60D</td><td>beta-Tubulin at 60D</td><td>143391_i_at|3t|B3t|BETA 60D|CG3401|D.m.BETA-60D|DTB3|Dmbeta3|DmelCG3401|T|Tub|Tub60D|beta-Tub60D|beta-Tub6D|beta-tub|beta3|beta3 TU|beta3-Tub|beta3TUB|beta3Tub|beta3t|beta60C|betaT</td><td>0</td><td>0</td></tr>
<tr><td>5675</td><td>37889</td><td>Drosophila melanogaster</td><td>slbo</td><td>slow border cells</td><td>C/EBP|CG4354|DC/EBP|Dm-c/EBP|DmC/EBP|DmelCG4354|Slbo|fs(2)7|fs(2)8|fs(2)ry7|fs(2)ry8|slobo</td><td>0</td><td>0</td></tr>
<tr><td>5676</td><td>37890</td><td>Drosophila melanogaster</td><td>bs</td><td>blistered</td><td>BS|Bs|CG3411|D-SRF|DSRF|DSrf|DSrf/bs|DmSRF|DmelCG3411|Group IIc|Mal|SRF|Serf|Srf|ba|bs/DSRF|bs/Dsrf|dSRF|dsrf|l(2)03267|pruned/DSRF|px</td><td>0</td><td>0</td></tr>
<tr><td>5677</td><td>37891</td><td>Drosophila melanogaster</td><td>CG13578</td><td>CG13578 gene product from transcript CG13578-RA</td><td>DmelCG13578</td><td>0</td><td>0</td></tr>
<tr><td>5678</td><td>37892</td><td>Drosophila melanogaster</td><td>mAcR-60C</td><td>muscarinic Acetylcholine Receptor 60C</td><td>Acr60C|AcrC|CG4356|DM1|Dm1|DmelCG4356|mAChR|mAChR-1|mAcR60C</td><td>0</td><td>0</td></tr>
<tr><td>5679</td><td>37893</td><td>Drosophila melanogaster</td><td>slik</td><td>Sterile20-like kinase</td><td>CG4527|DPlkk1|DmelCG4527|PLKK1|Plkk/Slik|Plkk1|SLK|Slik|Slik1|Slk|dPlkk/Slik|slk</td><td>0</td><td>0</td></tr>
<tr><td>5680</td><td>37894</td><td>Drosophila melanogaster</td><td>Mov34</td><td>CG3416 gene product from transcript CG3416-RA</td><td>CG3416|DmelCG3416|PRSC_DROME|Rpn8|l(2)k08003|p39B</td><td>0</td><td>0</td></tr>
<tr><td>5681</td><td>37895</td><td>Drosophila melanogaster</td><td>SerT</td><td>Serotonin transporter</td><td>CG4545|DmSERT|DmSerT|DmelCG4545|SERT|Sert|dSERT|dSERT1|dSerT|dSert|dsert</td><td>0</td><td>0</td></tr>
<tr><td>5682</td><td>37896</td><td>Drosophila melanogaster</td><td>CG3419</td><td>CG3419 gene product from transcript CG3419-RB</td><td>DmelCG3419</td><td>0</td><td>0</td></tr>
<tr><td>5683</td><td>37897</td><td>Drosophila melanogaster</td><td>CG42383</td><td>CG42383 gene product from transcript CG42383-RA</td><td>CG4556|DmelCG42383|Dmel_CG4556|eyc|p47</td><td>0</td><td>0</td></tr>
<tr><td>5684</td><td>37898</td><td>Drosophila melanogaster</td><td>CG15873</td><td>CG15873 gene product from transcript CG15873-RA</td><td>DmelCG15873|SP184|SPH184</td><td>0</td><td>0</td></tr>
<tr><td>5685</td><td>37899</td><td>Drosophila melanogaster</td><td>Pgam5-2</td><td>Phosphoglycerate mutase 5-2</td><td>CG15874|DmelCG15874|dPGAM5-2</td><td>0</td><td>0</td></tr>
<tr><td>5686</td><td>37900</td><td>Drosophila melanogaster</td><td>CG3483</td><td>CG3483 gene product from transcript CG3483-RA</td><td>DmelCG3483|anon-WO0140519.111</td><td>0</td><td>0</td></tr>
<tr><td>5687</td><td>37901</td><td>Drosophila melanogaster</td><td>CG4563</td><td>CG4563 gene product from transcript CG4563-RA</td><td>DmelCG4563</td><td>0</td><td>0</td></tr>
<tr><td>5688</td><td>37902</td><td>Drosophila melanogaster</td><td>CG3492</td><td>CG3492 gene product from transcript CG3492-RA</td><td>DmelCG3492|anon-WO0140519.253</td><td>0</td><td>0</td></tr>
<tr><td>5689</td><td>37903</td><td>Drosophila melanogaster</td><td>CG3494</td><td>CG3494 gene product from transcript CG3494-RA</td><td>CT11751|DmelCG3494</td><td>0</td><td>0</td></tr>
<tr><td>5690</td><td>37904</td><td>Drosophila melanogaster</td><td>CG16837</td><td>CG16837 gene product from transcript CG16837-RA</td><td>DmelCG16837</td><td>0</td><td>0</td></tr>
<tr><td>5691</td><td>37905</td><td>Drosophila melanogaster</td><td>CG13579</td><td>CG13579 gene product from transcript CG13579-RA</td><td>DmelCG13579|anon-WO0170980.151|anon-WO0170980.152</td><td>0</td><td>0</td></tr>
<tr><td>5692</td><td>37906</td><td>Drosophila melanogaster</td><td>CG13589</td><td>CG13589 gene product from transcript CG13589-RA</td><td>DmelCG13589</td><td>0</td><td>0</td></tr>
<tr><td>5693</td><td>37907</td><td>Drosophila melanogaster</td><td>CG13590</td><td>CG13590 gene product from transcript CG13590-RA</td><td>DmelCG13590</td><td>0</td><td>0</td></tr>
<tr><td>5694</td><td>37909</td><td>Drosophila melanogaster</td><td>Ssl</td><td>Suppressor of Stellate-like</td><td>CG13591|CK2betates|CKIIb1|DmCK2betates|DmelCG13591|NEST:bs30a09|SSL|Su(Ste)l|betaCK2tes</td><td>0</td><td>0</td></tr>
<tr><td>5695</td><td>37910</td><td>Drosophila melanogaster</td><td>Pros28.1B</td><td>Proteasome 28kD subunit 1B</td><td>CG4569|DmelCG4569|Prosalpha4T2|a4-t2|alpha4t2|alpha4t2_dm|dalpha7|pros28.1B</td><td>0</td><td>0</td></tr>
<tr><td>5696</td><td>37911</td><td>Drosophila melanogaster</td><td>CG13581</td><td>CG13581 gene product from transcript CG13581-RA</td><td>BcDNA:AT03240|DmelCG13581</td><td>0</td><td>0</td></tr>
<tr><td>5697</td><td>37912</td><td>Drosophila melanogaster</td><td>Letm1</td><td>CG4589 gene product from transcript CG4589-RB</td><td>B4D|CG4589|DmLETM1|DmelCG4589|anon-60Da|anon60Da|dLetm1</td><td>0</td><td>0</td></tr>
<tr><td>5698</td><td>37914</td><td>Drosophila melanogaster</td><td>CG4612</td><td>CG4612 gene product from transcript CG4612-RC</td><td>DmelCG4612|cg4612</td><td>0</td><td>0</td></tr>
<tr><td>5699</td><td>37916</td><td>Drosophila melanogaster</td><td>Brca2</td><td>Breast cancer 2, early onset homolog</td><td>30169|BcDNA:SD25109|CG13583|CG13584|CG30169|DmelCG30169|brca2|dmbrca2</td><td>0</td><td>0</td></tr>
<tr><td>5700</td><td>37917</td><td>Drosophila melanogaster</td><td>Ir60e</td><td>Ionotropic receptor 60e</td><td>CG13592|DmelCG13592|IR60e</td><td>0</td><td>0</td></tr>
<tr><td>5701</td><td>37918</td><td>Drosophila melanogaster</td><td>CG13585</td><td>CG13585 gene product from transcript CG13585-RA</td><td>BcDNA:AT28250|DmelCG13585</td><td>0</td><td>0</td></tr>
<tr><td>5702</td><td>37919</td><td>Drosophila melanogaster</td><td>CG4622</td><td>CG4622 gene product from transcript CG4622-RC</td><td>DmelCG4622</td><td>0</td><td>0</td></tr>
<tr><td>5703</td><td>37920</td><td>Drosophila melanogaster</td><td>CG11413</td><td>CG11413 gene product from transcript CG11413-RA</td><td>BcDNA:RH33060|CT31865|DmelCG11413</td><td>0</td><td>0</td></tr>
<tr><td>5704</td><td>37921</td><td>Drosophila melanogaster</td><td>itp</td><td>ion transport peptide</td><td>BcDNA:SD05282|CG13586|CHH-like|DmelCG13586|DrmITP|DrmITPL1|DrmITPL2|ITP|chgh</td><td>0</td><td>0</td></tr>
<tr><td>5705</td><td>37922</td><td>Drosophila melanogaster</td><td>Nurf-38</td><td>Nucleosome remodeling factor - 38kD</td><td>38|CG4634|DmelCG4634|N38|NURF|NURF-38|NURF38|Nurf 38|Nurf38|dNURF|l(2)k16102|p38|p38/NURF-38</td><td>0</td><td>0</td></tr>
<tr><td>5706</td><td>37923</td><td>Drosophila melanogaster</td><td>CG11414</td><td>CG11414 gene product from transcript CG11414-RA</td><td>DmelCG11414</td><td>0</td><td>0</td></tr>
<tr><td>5707</td><td>37924</td><td>Drosophila melanogaster</td><td>uri</td><td>unconventional prefoldin RPB5 interactor</td><td>CG11416|DmelCG11416|dURI|ori</td><td>0</td><td>0</td></tr>
<tr><td>5708</td><td>37925</td><td>Drosophila melanogaster</td><td>Fcp1</td><td>CG12252 gene product from transcript CG12252-RA</td><td>CG12252|DmelCG12252|Dmfcp1|fcp1</td><td>0</td><td>0</td></tr>
<tr><td>5709</td><td>37926</td><td>Drosophila melanogaster</td><td>CG3511</td><td>CG3511 gene product from transcript CG3511-RA</td><td>DmelCG3511</td><td>0</td><td>0</td></tr>
<tr><td>5710</td><td>37927</td><td>Drosophila melanogaster</td><td>Start1</td><td>CG3522 gene product from transcript CG3522-RB</td><td>CG3522|DmelCG3522|Start|start1</td><td>0</td><td>0</td></tr>
<tr><td>5711</td><td>37928</td><td>Drosophila melanogaster</td><td>CG4681</td><td>CG4681 gene product from transcript CG4681-RA</td><td>DmelCG4681|anon-WO0140519.29</td><td>0</td><td>0</td></tr>
<tr><td>5712</td><td>37929</td><td>Drosophila melanogaster</td><td>pio</td><td>piopio</td><td>CG30167|CG3541|DmelCG3541|Pio</td><td>0</td><td>0</td></tr>
<tr><td>5713</td><td>37930</td><td>Drosophila melanogaster</td><td>CG13587</td><td>CG13587 gene product from transcript CG13587-RA</td><td>DmelCG13587</td><td>0</td><td>0</td></tr>
<tr><td>5714</td><td>37931</td><td>Drosophila melanogaster</td><td>CG4692</td><td>CG4692 gene product from transcript CG4692-RB</td><td>DmelCG4692|anon-EST:Posey45</td><td>0</td><td>0</td></tr>
<tr><td>5715</td><td>37932</td><td>Drosophila melanogaster</td><td>CG3548</td><td>CG3548 gene product from transcript CG3548-RA</td><td>DmelCG3548</td><td>0</td><td>0</td></tr>
<tr><td>5716</td><td>37933</td><td>Drosophila melanogaster</td><td>CG3565</td><td>CG3565 gene product from transcript CG3565-RA</td><td>DmelCG3565|anon-WO0140519.176</td><td>0</td><td>0</td></tr>
<tr><td>5717</td><td>37934</td><td>Drosophila melanogaster</td><td>CG3570</td><td>CG3570 gene product from transcript CG3570-RA</td><td>DmelCG3570</td><td>0</td><td>0</td></tr>
<tr><td>5718</td><td>37935</td><td>Drosophila melanogaster</td><td>CG4707</td><td>CG4707 gene product from transcript CG4707-RA</td><td>Cg4707|DmelCG4707</td><td>0</td><td>0</td></tr>
<tr><td>5719</td><td>37936</td><td>Drosophila melanogaster</td><td>CG42361</td><td>CG42361 gene product from transcript CG42361-RA</td><td>CG3579|DmelCG42361|Dmel_CG13588|Dmel_CG3579</td><td>0</td><td>0</td></tr>
<tr><td>5720</td><td>37937</td><td>Drosophila melanogaster</td><td>CG42360</td><td>CG42360 gene product from transcript CG42360-RA</td><td>CG13588|CG3579|DmelCG42360|Dmel_CG13588|Dmel_CG3579</td><td>0</td><td>0</td></tr>
<tr><td>5721</td><td>37938</td><td>Drosophila melanogaster</td><td>CG3608</td><td>CG3608 gene product from transcript CG3608-RA</td><td>DmelCG3608</td><td>0</td><td>0</td></tr>
<tr><td>5722</td><td>37939</td><td>Drosophila melanogaster</td><td>CG4741</td><td>CG4741 gene product from transcript CG4741-RB</td><td>DmelCG4741</td><td>0</td><td>0</td></tr>
<tr><td>5723</td><td>37940</td><td>Drosophila melanogaster</td><td>CG13594</td><td>CG13594 gene product from transcript CG13594-RA</td><td>DmelCG13594</td><td>0</td><td>0</td></tr>
<tr><td>5724</td><td>37941</td><td>Drosophila melanogaster</td><td>Cyp9c1</td><td>Cytochrome P450-9c1</td><td>9c1|CG3616|DmelCG3616|P-450|P450</td><td>0</td><td>0</td></tr>
<tr><td>5725</td><td>37942</td><td>Drosophila melanogaster</td><td>CG3640</td><td>CG3640 gene product from transcript CG3640-RA</td><td>DmelCG3640</td><td>0</td><td>0</td></tr>
<tr><td>5726</td><td>37943</td><td>Drosophila melanogaster</td><td>CG4781</td><td>CG4781 gene product from transcript CG4781-RA</td><td>CT15357|DmelCG4781</td><td>0</td><td>0</td></tr>
<tr><td>5727</td><td>37944</td><td>Drosophila melanogaster</td><td>CG3663</td><td>CG3663 gene product from transcript CG3663-RA</td><td>DmelCG3663</td><td>0</td><td>0</td></tr>
<tr><td>5728</td><td>37945</td><td>Drosophila melanogaster</td><td>CG30161</td><td>CG30161 gene product from transcript CG30161-RA</td><td>BcDNA:AT15604|CG4856|DmelCG30161</td><td>0</td><td>0</td></tr>
<tr><td>5729</td><td>37946</td><td>Drosophila melanogaster</td><td>CG3683</td><td>CG3683 gene product from transcript CG3683-RC</td><td>DmelCG3683</td><td>0</td><td>0</td></tr>
<tr><td>5730</td><td>37947</td><td>Drosophila melanogaster</td><td>Pof</td><td>Painting of fourth</td><td>CG3691|DmelCG3691|POF|cg3691|pof</td><td>0</td><td>0</td></tr>
<tr><td>5731</td><td>37948</td><td>Drosophila melanogaster</td><td>CG4806</td><td>CG4806 gene product from transcript CG4806-RA</td><td>DmelCG4806|cg4806</td><td>0</td><td>0</td></tr>
<tr><td>5732</td><td>37949</td><td>Drosophila melanogaster</td><td>Mmp1</td><td>Matrix metalloproteinase 1</td><td>CG4859|Dm1-MMP|DmelCG4859|MMP1|Mmp 1|dMMP1|dm1-MMP|l(2)k04809|mmp1</td><td>0</td><td>0</td></tr>
<tr><td>5733</td><td>37950</td><td>Drosophila melanogaster</td><td>ST6Gal</td><td>Sialyltransferase</td><td>CG4871|D.SiaT|D.SialT|DSiaT|DmPST|DmelCG4871|dSiaT</td><td>0</td><td>0</td></tr>
<tr><td>5734</td><td>37953</td><td>Drosophila melanogaster</td><td>CG33988</td><td>CG33988 gene product from transcript CG33988-RA</td><td>CG13595|CG13596|CG18510|DmelCG33988|cg13596</td><td>0</td><td>0</td></tr>
<tr><td>5735</td><td>37954</td><td>Drosophila melanogaster</td><td>CG30421</td><td>CG30421 gene product from transcript CG30421-RA</td><td>CG3872|CG9189|DmelCG30421</td><td>0</td><td>0</td></tr>
<tr><td>5736</td><td>37956</td><td>Drosophila melanogaster</td><td>spz6</td><td>spatzle 6</td><td>BcDNA:LP10725|CG9196|CT26280|DmelCG9196|Spz6</td><td>0</td><td>0</td></tr>
<tr><td>5737</td><td>37957</td><td>Drosophila melanogaster</td><td>CG3880</td><td>CG3880 gene product from transcript CG3880-RA</td><td>DmelCG3880</td><td>0</td><td>0</td></tr>
<tr><td>5738</td><td>37959</td><td>Drosophila melanogaster</td><td>CG3894</td><td>CG3894 gene product from transcript CG3894-RC</td><td>CG9197|DmelCG3894</td><td>0</td><td>0</td></tr>
<tr><td>5739</td><td>37960</td><td>Drosophila melanogaster</td><td>CG16936</td><td>CG16936 gene product from transcript CG16936-RA</td><td>BcDNA:GH04753|DmelCG16936|GST</td><td>0</td><td>0</td></tr>
<tr><td>5740</td><td>37961</td><td>Drosophila melanogaster</td><td>Eps-15</td><td>Epidermal growth factor receptor pathway substrate c</td><td>CG16932|DmelCG16932|EH1|EPS-15|Eps15|anon-WO0118547.85|eps-15|eps15|i31</td><td>0</td><td>0</td></tr>
<tr><td>5741</td><td>37962</td><td>Drosophila melanogaster</td><td>egg</td><td>eggless</td><td>AAF47268 Dm|BcDNA:AT13877|CG12196|CG30422|CG30426|Dm Set1|Dm-setdb1|DmSetdb1|DmelCG12196|Dmel_CG30422|Dmel_CG30426|Dmsetdb1|Egg|SETDB1|Set1|SetDB1|dEset|dSETDB1|dSet</td><td>0</td><td>0</td></tr>
<tr><td>5742</td><td>37963</td><td>Drosophila melanogaster</td><td>Lcp9</td><td>Larval cuticle protein 9</td><td>CG16914|DmelLcp9|DmelCG16914|LCP9|lcp9</td><td>0</td><td>0</td></tr>
<tr><td>5743</td><td>37964</td><td>Drosophila melanogaster</td><td>Eap</td><td>Exu-associated protein</td><td>CG3594|DmelCG3594|cg3594</td><td>0</td><td>0</td></tr>
<tr><td>5744</td><td>37965</td><td>Drosophila melanogaster</td><td>CG16912</td><td>CG16912 gene product from transcript CG16912-RA</td><td>DmelCG16912|anon-EST:fe1E12</td><td>0</td><td>0</td></tr>
<tr><td>5745</td><td>37966</td><td>Drosophila melanogaster</td><td>CG3589</td><td>CG3589 gene product from transcript CG3589-RA</td><td>DmelCG3589</td><td>0</td><td>0</td></tr>
<tr><td>5746</td><td>37967</td><td>Drosophila melanogaster</td><td>key</td><td>kenny</td><td>CG16910|DmIKK-gamma|DmIKKgamma|DmelCG16910|Dmikkgamma|IKK|IKK-gamma|IKKg|IKKgamma|KEY|Kenny|Key|dIKK|dIKK-gamma|dmIKKgamma</td><td>0</td><td>0</td></tr>
<tr><td>5747</td><td>37968</td><td>Drosophila melanogaster</td><td>Reg-5</td><td>Rhythmically expressed gene 5</td><td>CG2928|DREG-5|DmelCG2928|Dreg|Dreg-5|Dreg5|dreg-5|reg-5</td><td>0</td><td>0</td></tr>
<tr><td>5748</td><td>37970</td><td>Drosophila melanogaster</td><td>Orc4</td><td>Origin recognition complex subunit 4</td><td>CG2917|DmORC4|DmelCG2917|ORC|ORC4|orc4|rDmORC</td><td>0</td><td>0</td></tr>
<tr><td>5749</td><td>37971</td><td>Drosophila melanogaster</td><td>CG12849</td><td>CG12849 gene product from transcript CG12849-RA</td><td>DmelCG12849</td><td>0</td><td>0</td></tr>
<tr><td>5750</td><td>37972</td><td>Drosophila melanogaster</td><td>CG3611</td><td>CG3611 gene product from transcript CG3611-RA</td><td>BcDNA:AT12780|DmelCG3611</td><td>0</td><td>0</td></tr>
<tr><td>5751</td><td>37973</td><td>Drosophila melanogaster</td><td>Dll</td><td>Distal-less</td><td>2.7|Art|Ba|BcDNA:LP01770|CG3629|DLL|DmelCG3629|E(Arp)|En(Arp)|dl|dll|l(2)01092|l(2)387</td><td>0</td><td>0</td></tr>
<tr><td>5752</td><td>37974</td><td>Drosophila melanogaster</td><td>CG3650</td><td>CG3650 gene product from transcript CG3650-RA</td><td>DmelCG3650|SP159</td><td>0</td><td>0</td></tr>
<tr><td>5753</td><td>37977</td><td>Drosophila melanogaster</td><td>CG16896</td><td>CG16896 gene product from transcript CG16896-RA</td><td>DmelCG16896</td><td>0</td><td>0</td></tr>
<tr><td>5754</td><td>37978</td><td>Drosophila melanogaster</td><td>Atf-2</td><td>Activating transcription factor-2</td><td>ATF-2|ATF2|CG12850|CG30420|CG3749|DmelCG30420|dATF-2</td><td>0</td><td>0</td></tr>
<tr><td>5755</td><td>37979</td><td>Drosophila melanogaster</td><td>NKAIN</td><td>Na,K-ATPase Interacting</td><td>CG32836|CG34413|CG9047|DmelCG34413|Dmel_CG32836|Dmel_CG9047|anon-WO0118547.94|dNKAIN</td><td>0</td><td>0</td></tr>
<tr><td>5756</td><td>37980</td><td>Drosophila melanogaster</td><td>Ance-5</td><td>CG10142 gene product from transcript CG10142-RA</td><td>CG10142|DmelCG10142</td><td>0</td><td>0</td></tr>
<tr><td>5757</td><td>37981</td><td>Drosophila melanogaster</td><td>NaCP60E</td><td>Na channel protein 60E</td><td>CG18506|CG34405|CG9071|CT24831|DIC60|DSC|DSC1|DSCI|DmelCG34405|Dmel_CG18506|Dmel_CG9071|Na-CP|dsc1|smi60E</td><td>0</td><td>0</td></tr>
<tr><td>5758</td><td>37982</td><td>Drosophila melanogaster</td><td>CG9083</td><td>CG9083 gene product from transcript CG9083-RB</td><td>DmelCG9083</td><td>0</td><td>0</td></tr>
<tr><td>5759</td><td>37983</td><td>Drosophila melanogaster</td><td>Tpc2</td><td>Thiamine pyrophosphate carrier protein 2</td><td>CG2857|DmTpc2|DmelCG2857</td><td>0</td><td>0</td></tr>
<tr><td>5760</td><td>37985</td><td>Drosophila melanogaster</td><td>pain</td><td>painless</td><td>CG15860|CT31987|DmelCG15860|EP2251|Pain</td><td>0</td><td>0</td></tr>
<tr><td>5761</td><td>37986</td><td>Drosophila melanogaster</td><td>CG30427</td><td>CG30427 gene product from transcript CG30427-RC</td><td>BEST:LD36843|CG18680|CG2858|DmelCG30427</td><td>0</td><td>0</td></tr>
<tr><td>5762</td><td>37987</td><td>Drosophila melanogaster</td><td>CG3760</td><td>CG3760 gene product from transcript CG3760-RC</td><td>DmelCG3760|anon-EST:fe2C9|anon-fe2C9|anon2C9</td><td>0</td><td>0</td></tr>
<tr><td>5763</td><td>37988</td><td>Drosophila melanogaster</td><td>CG2811</td><td>CG2811 gene product from transcript CG2811-RA</td><td>BcDNA:RE67445|DmelCG2811</td><td>0</td><td>0</td></tr>
<tr><td>5764</td><td>37989</td><td>Drosophila melanogaster</td><td>Tina-1</td><td>Troponin C-akin-1</td><td>BEST:GM14784|CG2803|DmelCG2803</td><td>0</td><td>0</td></tr>
<tr><td>5765</td><td>37990</td><td>Drosophila melanogaster</td><td>CG15861</td><td>CG15861 gene product from transcript CG15861-RA</td><td>DmelCG15861</td><td>0</td><td>0</td></tr>
<tr><td>5766</td><td>37991</td><td>Drosophila melanogaster</td><td>CG3770</td><td>CG3770 gene product from transcript CG3770-RA</td><td>CT12588|DmelCG3770</td><td>0</td><td>0</td></tr>
<tr><td>5767</td><td>37992</td><td>Drosophila melanogaster</td><td>CG2790</td><td>CG2790 gene product from transcript CG2790-RA</td><td>BcDNA:GH03108|DmelCG2790</td><td>0</td><td>0</td></tr>
<tr><td>5768</td><td>37993</td><td>Drosophila melanogaster</td><td>CG12851</td><td>CG12851 gene product from transcript CG12851-RB</td><td>DmelCG12851</td><td>0</td><td>0</td></tr>
<tr><td>5769</td><td>37994</td><td>Drosophila melanogaster</td><td>CG2765</td><td>CG2765 gene product from transcript CG2765-RF</td><td>DmelCG2765</td><td>0</td><td>0</td></tr>
<tr><td>5770</td><td>37995</td><td>Drosophila melanogaster</td><td>RpL19</td><td>Ribosomal protein L19</td><td>CG2746|DmelCG2746|E(Dl)KP135|L19|M(2)60E|M(2)a|M(2)c|M60E|anon-EST:Posey33|l(2)k03704|rpL19|rpl19</td><td>0</td><td>0</td></tr>
</table>

5771	37996	Drosophila melanogaster	CG3776	CG3776 gene product from transcript CG3776-RA	DmP29|DmelCG3776|EP(2)0835|Jhebp29|P29	0	0
5772	37997	Drosophila melanogaster	Phk-3	Pherokine 3	BcDNA:RE09339|CG9358|DmelCG9358|PHK-3	0	0
5773	37998	Drosophila melanogaster	CG2736	CG2736 gene product from transcript CG2736-RA	DmelCG2736|cg2736	0	0
5774	37999	Drosophila melanogaster	emp	epithelial membrane protein	CG2727|DmelCG2727	0	0
5775	38000	Drosophila melanogaster	CG3829	CG3829 gene product from transcript CG3829-RA	DmelCG3829	0	0
5776	38001	Drosophila melanogaster	zip	zipper	CG15792|DROMHC|Dm nmII|DmelCG15792|DmnmII|Dronm-MII|E(br)|MHC|Mhc-c|Mhc-c[1]|Myo|Myo II|Myo-II|Myo-II HC|Myo2|MyoII|NMM|Zip|anon-WO0140519.37|l(2)02957|myoII|nmMHC|nmy-2|zip/My	0	0
5777	38002	Drosophila melanogaster	uzip	unzipped	CG3533|DmelCG3533|nzip|zip	0	0
5778	38003	Drosophila melanogaster	Nplp1	Neuropeptide-like precursor 1	CG3441|DmelCG3441|IPNa|IPNamide|MTY-amide|MTYa|NPLP1|nplp1	0	0
5779	38004	Drosophila melanogaster	gsb-n	gooseberry-neuro	BSH-4|BSH4|CG2692|DmelCG2692|Gsb-p|GsbN|Gsbn|gsb|gsb-neuro|gsb-p|gsbN|gsbn|gsbp	0	0
5780	38005	Drosophila melanogaster	gsb	gooseberry	BSH-9|BSH9|BSH9c2|CG3388|DmelCG3388|GSB-D|Gsb-d|gsb-2|gsb-d|gsb-distal|gsbBSH9|gsbd|l(2)01155|l(2)gsb	0	0
5781	38006	Drosophila melanogaster	gol	goliath	CG2679|DmelCG2679|Gol|anon-G1|g1|meso	0	0
5782	38007	Drosophila melanogaster	CG16778	CG16778 gene product from transcript CG16778-RB	BTB-III|BtbIII|CG2672|DmelCG16778|Lov|Tkr|anon-G2|dTKR|g2|tkr	0	0
5783	38009	Drosophila melanogaster	Peb	Protein ejaculatory bulb	BcDNA:GH06048|CG2668|DmelCG2668|P38|PEB|PEB-me|anon-WO0140519.140|peb	0	0
5784	38011	Drosophila melanogaster	CG9380	CG9380 gene product from transcript CG9380-RC	DmelCG9380	0	0
5785	38012	Drosophila melanogaster	Kr	Kruppel	CG3340|Dm-Kr|DmelCG3340|If|KR|kr	0	0
5786	38013	Drosophila melanogaster	mthl8	methuselah-like 8	CG11869|CG13406|CG32475|DmelCG32475|Mth-8|anon-WO0170980.145|anon-WO0170980.146	0	0
5787	38015	Drosophila melanogaster	Lsp1gamma	Larval serum protein 1 gamma	CG6821|DmeLSP1g|DmelCG6821|LSP 1|LSP-1 gamma|LSP-1gamma|LSP1|Lsp-1gamma|Lsp1|Lsp1-gamma|Lsp1[gamma]|Lsp1gama	0	0
5788	38016	Drosophila melanogaster	CG13405	CG13405 gene product from transcript CG13405-RB	BcDNA:AT11049|DmelCG13405	0	0
5789	38017	Drosophila melanogaster	Pdk1	Phosphoinositide-dependent kinase 1	1210|CG1201|CG1210|DSTPK61|DmelCG1210|Dstpk61|PDK|PDK-1|PDK1|PDK1/Pk61C|PK61C|Pk61C|Pk61C/PDK1|dPDK-1|dPDK1|dSTPK61	0	0
5790	38019	Drosophila melanogaster	CG6845	CG6845 gene product from transcript CG6845-RE	BEST:LD22345|DmelCG6845	0	0
5791	38020	Drosophila melanogaster	CG7051	CG7051 gene product from transcript CG7051-RA	DmelCG7051	0	0
5792	38021	Drosophila melanogaster	p130CAS	CG1212 gene product from transcript CG1212-RD	CG1212|CT1293|DCas|Dcas|DmelCG1212	0	0
5793	38022	Drosophila melanogaster	CG7049	CG7049 gene product from transcript CG7049-RA	DmelCG7049	0	0
5794	38023	Drosophila melanogaster	Vdup1	Vitamin D[[3]] up-regulated protein 1	CG7047|DmelCG7047|VDUP1|dvdup1	0	0
5795	38024	Drosophila melanogaster	CG13875	CG13875 gene product from transcript CG13875-RA	DmelCG13875	0	0
5796	38026	Drosophila melanogaster	Mtch	Mitochondrial carrier homolog 1	CG6851|DmelCG6851	0	0
5797	38027	Drosophila melanogaster	rno	rhinoceros	CG7036|DmelCG7036	0	0
5798	38028	Drosophila melanogaster	mri	mrityu	CG1216|DmelCG1216	0	0
5799	38029	Drosophila melanogaster	NitFhit	Nitrilase and fragile histidine triad fusion protein	CG7067|DmelCG7067	0	0
5800	38031	Drosophila melanogaster	CG13876	CG13876 gene product from transcript CG13876-RA	DmelCG13876	0	0
5801	38032	Drosophila melanogaster	CG7028	CG7028 gene product from transcript CG7028-RA	BcDNA:GH04978|DmelCG7028|FBgn0027587|PRP4|prp4	0	0
5802	38033	Drosophila melanogaster	thoc7	CG17143 gene product from transcript CG17143-RB	CG17143|DmelCG17143|THOC7	0	0
5803	38034	Drosophila melanogaster	CG16940	CG16940 gene product from transcript CG16940-RB	DmelCG16940	0	0
5804	38036	Drosophila melanogaster	CG13877	CG13877 gene product from transcript CG13877-RC	DmelCG13877	0	0
5805	38037	Drosophila melanogaster	pyx	pyrexia	CG17142|CT33412|DmelCG17142|Pyx	0	0
5806	38044	Drosophila melanogaster	wac	wee Augmin	CG13879|DmelCG13879|Wac|was	0	0
5807	38045	Drosophila melanogaster	Tudor-SN	CG7008 gene product from transcript CG7008-RA	CG7008|DmelCG7008|SND1|TSN|TSN-1|dP100|p100|tudor staphylococcus/micrococcal nuclease|tudor-SN	0	0
5808	38046	Drosophila melanogaster	mRpL17	mitochondrial ribosomal protein L17	BcDNA:RE43278|CG13880|DmelCG13880|L17|mRPL17	0	0
5809	38047	Drosophila melanogaster	miple	CG1221 gene product from transcript CG1221-RA	CG1221|DmelCG1221|Miple|anon-EST:Posey203|miple1	0	0
5810	38049	Drosophila melanogaster	ttm2	tiny tim 2	CG12313|DmelCG12313	0	0
5811	38050	Drosophila melanogaster	CG42377	CG42377 gene product from transcript CG42377-RA	CG13881|CG13882|DmelCG42377|Dmel_CG13881|Dmel_CG13882	0	0
5812	38051	Drosophila melanogaster	RhoGEF3	CG42378 gene product from transcript CG42378-RI	CG1225|CG13882|CG13883|CG42378|DmelCG42378|Dmel_CG1225|Dmel_CG13883|DrhoGEF3|RhoGEF|rhoGEF3	0	0
5813	38055	Drosophila melanogaster	Atac3	ATAC complex component 3	CG32343|CG6994|DmelCG32343|cg6994	0	0
5814	38056	Drosophila melanogaster	mthl9	methuselah-like 9	CG17084|DmelCG17084|Mth-9|anon-WO0170980.163|anon-WO0170980.164	0	0
5815	38057	Drosophila melanogaster	mthl10	methuselah-like 10	CG17061|DmelCG17061|anon-WO0170980.166|anon-WO0170980.167|mth2	0	0
5816	38058	Drosophila melanogaster	mth	methuselah	CG6936|DmelCG6936|MTH|Mth	0	0
5817	38059	Drosophila melanogaster	Ptpmeg	CG1228 gene product from transcript CG1228-RH	CG1228|DmelCG1228|MEG1|Meg|PTP-meg|anon-WO0118547.211|dMEG1|ptpmeg|scc	0	0
5818	38060	Drosophila melanogaster	CG1231	CG1231 gene product from transcript CG1231-RA	DmelCG1231	0	0
5819	38062	Drosophila melanogaster	CG6905	CG6905 gene product from transcript CG6905-RA	DmelCG6905|cdc5-like|cef1	0	0
5820	38063	Drosophila melanogaster	CG1233	CG1233 gene product from transcript CG1233-RB	DmelCG1233	0	0
5821	38064	Drosophila melanogaster	CG13884	CG13884 gene product from transcript CG13884-RA	BcDNA:AT18596|DmelCG13884	0	0
5822	38065	Drosophila melanogaster	trh	trachealess	3.1|BP1081|CG13885|CG42865|CG6883|DMU42699|DmelCG42865|Dmel_CG13885|Dmel_CG6883|HDC07747|TRH|Trh|bHLHe13|l(3)10512	0	0
5823	38066	Drosophila melanogaster	CG13891	CG13891 gene product from transcript CG13891-RA	DmelCG13891	0	0
5824	38067	Drosophila melanogaster	klar	klarsicht	CG17046|DmelCG17046|Klar|kls|marb	0	0
5825	38068	Drosophila melanogaster	CG17180	CG17180 gene product from transcript CG17180-RA	DmelCG17180	0	0
5826	38069	Drosophila melanogaster	Cypl	Cyclophilin-like	CG13892|DmelCG13892|cycl	0	0
5827	38070	Drosophila melanogaster	hipk	homeodomain interacting protein kinase	CG17090|DmelCG17090|HIPK|dhipk2	0	0
5828	38071	Drosophila melanogaster	Ppm1	CG12169 gene product from transcript CG12169-RA	CG12169|DmelCG12169|PP2C|dPPM1	0	0
5829	38072	Drosophila melanogaster	CG17181	CG17181 gene product from transcript CG17181-RA	DmelCG17181	0	0
5830	38073	Drosophila melanogaster	MED14	Mediator complex subunit 14	CG12031|DmelCG12031|Med14|Trap170|dTRAP170|p160	0	0
5831	38074	Drosophila melanogaster	CG13893	CG13893 gene product from transcript CG13893-RA	DmelCG13893	0	0
5832	38075	Drosophila melanogaster	Reg-2	Rhythmically expressed gene 2	CG3200|DmelCG3200|Dreg-2	0	0
5833	38076	Drosophila melanogaster	Gale	UDP-galactose 4'-epimerase	CG12030|DmelCG12030|GALE|dGALE	0	0
5834	38077	Drosophila melanogaster	CG3402	CG3402 gene product from transcript CG3402-RA	BcDNA:LD06317|DmelCG3402	0	0
5835	38078	Drosophila melanogaster	MED30	Mediator complex subunit 30	CG17183|DmelCG17183|Med11/TRAP25|Med20|Med30|Trap25|dMED20|dTRAP25|med30	0	0
5836	38079	Drosophila melanogaster	Rev1	CG12189 gene product from transcript CG12189-RA	AAF47401|CG12189|DmREV1|DmRev1|DmelCG12189|REV1|dREV1|drev1	0	0
5837	38080	Drosophila melanogaster	CG17129	CG17129 gene product from transcript CG17129-RC	DmelCG17129	0	0
5838	38081	Drosophila melanogaster	CG3386	CG3386 gene product from transcript CG3386-RA	DmelCG3386	0	0
5839	38082	Drosophila melanogaster	ebd1	earthbound 1	CG3371|DmelCG3371|EP3592	0	0
5840	38083	Drosophila melanogaster	CG3344	CG3344 gene product from transcript CG3344-RA	DmelCG3344	0	0
5841	38084	Drosophila melanogaster	RabX6	CG12015 gene product from transcript CG12015-RA	CG12015|DmRabX6|DmelCG12015	0	0
5842	38085	Drosophila melanogaster	Vti1	CG3279 gene product from transcript CG3279-RA	CG3279|DmelCG3279|VTI1	0	0
5843	38086	Drosophila melanogaster	CG13894	CG13894 gene product from transcript CG13894-RA	DmelCG13894|dmCG13894	0	0
5844	38087	Drosophila melanogaster	CG13895	CG13895 gene product from transcript CG13895-RA	DmelCG13895	0	0
5845	38088	Drosophila melanogaster	CkIIalpha-i3	CKII-alpha subunit interactor-3	CG3217|CKIIalpha-i3|DmelCG3217	0	0
5846	38089	Drosophila melanogaster	CG13896	CG13896 gene product from transcript CG13896-RB	DmelCG13896	0	0
5847	38090	Drosophila melanogaster	CG13897	CG13897 gene product from transcript CG13897-RA	DmelCG13897|anon-WO0118547.557	0	0
5848	38091	Drosophila melanogaster	emc	extra macrochaetae	0094/26|0203/10|0587/01|0977/09|Ach|CG1007|Dm0688|DmelCG1007|Emc|bHLHb28|gov|l(3)04322|l(3)05592|l(3)61Da|l(3)j4E11|ms(3)61CD	0	0
5849	38092	Drosophila melanogaster	CG13898	CG13898 gene product from transcript CG13898-RA	DmelCG13898	0	0
5850	38093	Drosophila melanogaster	CG13900	CG13900 gene product from transcript CG13900-RA	DmelCG13900|SF3B3	0	0
5851	38094	Drosophila melanogaster	CG42553	CG42553 gene product from transcript CG42553-RA	CG13886|CT33419|DmelCG42553|Dmel_CG13886	0	0
5852	38095	Drosophila melanogaster	CG13901	CG13901 gene product from transcript CG13901-RA	DmelCG13901	0	0
5853	38096	Drosophila melanogaster	CG13887	CG13887 gene product from transcript CG13887-RC	DmelCG13887	0	0
5854	38097	Drosophila melanogaster	CG13902	CG13902 gene product from transcript CG13902-RB	DmelCG13902	0	0
5855	38098	Drosophila melanogaster	Gr61a	Gustatory receptor 61a	CG13888|Dmel61a|DmelCG13888|Gr61D1	0	0
5856	38099	Drosophila melanogaster	CG13889	CG13889 gene product from transcript CG13889-RA	DmelCG13889	0	0
5857	38100	Drosophila melanogaster	CG13890	CG13890 gene product from transcript CG13890-RA	DmelCG13890	0	0
5858	38101	Drosophila melanogaster	dpr20	CG12191 gene product from transcript CG12191-RA	CG12191|CT9818|DmelCG12191|Dpr-20	0	0
5859	38102	Drosophila melanogaster	CG12502	CG12502 gene product from transcript CG12502-RB	DmelCG12502	0	0
5860	38103	Drosophila melanogaster	CG32479	CG32479 gene product from transcript CG32479-RA	CG12503|CG13903|DmelCG32479	0	0
5861	38104	Drosophila melanogaster	CG13907	CG13907 gene product from transcript CG13907-RA	DmelCG13907	0	0
5862	38106	Drosophila melanogaster	CG34056	CG34056 gene product from transcript CG34056-RA	CG13904|CG13904-1|DmelCG34056	0	0
5863	38107	Drosophila melanogaster	CG13905	CG13905 gene product from transcript CG13905-RB	DmelCG13905	0	0
5864	38109	Drosophila melanogaster	Glut1	Glucose transporter 1	0074/12|CG1086|DmGLUT1|DmGluT1|DmelCG1086|Dmglut1|GLU|glut1|l(3)61Ea|l(3)L1H|l(3)S007412	0	0
5865	38110	Drosophila melanogaster	CG13908	CG13908 gene product from transcript CG13908-RB	DmelCG13908	0	0
5866	38113	Drosophila melanogaster	CG9205	CG9205 gene product from transcript CG9205-RA	DmelCG9205	0	0
5867	38116	Drosophila melanogaster	bab1	bric a brac 1	BAB1|Bab1|CG13910|CG9097|DmelCG9097|anon-WO0118547.639|bab|bab-I	0	0
5868	38120	Drosophila melanogaster	CG13912	CG13912 gene product from transcript CG13912-RA	DmelCG13912	0	0
5869	38121	Drosophila melanogaster	Trh	Tryptophan hydroxylase	CG9122|CT9937|DTRH|DmelCG9122|TRH|TRHn	0	0
5870	38122	Drosophila melanogaster	LysX	Lysozyme X	BcDNA:LP09906|CG9120|DmelCG9120|lysX	0	0
5871	38124	Drosophila melanogaster	CG9119	CG9119 gene product from transcript CG9119-RA	CT9987|DmelCG9119	0	0
5872	38125	Drosophila melanogaster	LysB	Lysozyme B	CG1179|DmelCG1179|Lys B|lysB	0	0
5873	38126	Drosophila melanogaster	LysC	Lysozyme C	CG9111|DmelCG9111|Lys C|lysC	0	0
5874	38127	Drosophila melanogaster	LysD	Lysozyme D	CG9118|DmelCG9118|lysD	0	0
5875	38128	Drosophila melanogaster	LysE	Lysozyme E	BcDNA:LP07339|CG1180|DmelCG1180|Lys E|lysE	0	0
5876	38129	Drosophila melanogaster	LysP	Lysozyme P	CG9116|DmelCG9116|lysP	0	0
5877	38130	Drosophila melanogaster	LysS	Lysozyme S	BcDNA:LP06719|CG1165|DmelCG1165|lysS	0	0
5878	38131	Drosophila melanogaster	mwh	multiple wing hairs	CG13913|DmelCG13913	0	0
5879	38133	Drosophila melanogaster	CG9194	CG9194 gene product from transcript CG9194-RA	DmelCG9194|anon-WO0109301.19|anon-WO0109301.20|anon-WO0109301.21|anon-WO0109301.22|anon-WO0109301.23|anon-WO0109301.24|anon-WO0109301.25|anon-WO0109301.26|anon-WO01	0	0
5880	38134	Drosophila melanogaster	CG9192	CG9192 gene product from transcript CG9192-RA	DmelCG9192	0	0
5881	38135	Drosophila melanogaster	Klp61F	Kinesin-like protein at 61F	6345|CG9191|CG9191-PA|DmKLP61F|DmKRP130|DmKlp61F|DmelCG9191|KIP8|KLP2|KLP61|KLP61F|KLP61f|KRP130|KRP[(130)]|KRP[[130]]|Klp2|Klp61F/KRP130|Klp61f|Krp130|klp61F|klp61f|l(3)07012	0	0

5882	38136	Drosophila melanogaster	Psf1	CG9187 gene product from transcript CG9187-RA	CG9187|CG9187-PA|DmelCG9187|psf1	0	0
5883	38137	Drosophila melanogaster	Sac1	CG9128 gene product from transcript CG9128-RB	CG9128|DmelCG9128|dsac1|l(3)2107|sac1	0	0
5884	38138	Drosophila melanogaster	CG9129	CG9129 gene product from transcript CG9129-RA	DmelCG9129	0	0
5885	38141	Drosophila melanogaster	CG9134	CG9134 gene product from transcript CG9134-RB	DmelCG9134	0	0
5886	38142	Drosophila melanogaster	CG32333	CG32333 gene product from transcript CG32333-RA	CG9137|CG9146|DmelCG32333	0	0
5887	38144	Drosophila melanogaster	Rabex-5	CG9139 gene product from transcript CG9139-RA	CG9139|DmelCG9139|RabGEF1	0	0
5888	38145	Drosophila melanogaster	scf	supercoiling factor	BEST:CK02682|CG9148|CK02682|DCB-45|DmelCG9148|SCF|anon-EST:Posey14	0	0
5889	38146	Drosophila melanogaster	Rac1	CG2248 gene product from transcript CG2248-RA	2248|CG2248|D-Rac|D-Rac 1|D-Rac1|DRac|DRac1|DRacA|Dm Rac1|DmRAC|DmRAC1|DmelCG2248|Dmrac1|Drac|Drac1|Drac1a|DracA|GTPase Rac1|RAC1|Rac|Rac GTPase|Rac-1|RacA|dRac|dRac1|rac	0	0
5890	38147	Drosophila melanogaster	CG9149	CG9149 gene product from transcript CG9149-RA	DmelCG9149	0	0
5891	38148	Drosophila melanogaster	CG2277	CG2277 gene product from transcript CG2277-RA	DmelCG2277	0	0
5892	38149	Drosophila melanogaster	CG2469	CG2469 gene product from transcript CG2469-RB	DmelCG2469	0	0
5893	38150	Drosophila melanogaster	CG9186	CG9186 gene product from transcript CG9186-RB	DmelCG9186	0	0
5894	38151	Drosophila melanogaster	CG9153	CG9153 gene product from transcript CG9153-RB	DmelCG9153|FBgn0035207	0	0
5895	38152	Drosophila melanogaster	CG9184	CG9184 gene product from transcript CG9184-RB	DmelCG9184	0	0
5896	38153	Drosophila melanogaster	Myo61F	Myosin 61F	CG9155|Dm IB|DmIB|DmelCG9155|DroMIB|IB|MIB|MYO61F|MYOIB|Myo-IB|Myo1B	0	0
5897	38154	Drosophila melanogaster	mtacp1	mitochondrial acyl carrier protein 1	CG9160|DmelCG9160|ND-AcC|l(3)1303|l(3)j4A6|mtACP|mtACP1	0	0
5898	38155	Drosophila melanogaster	msd1	mitotic spindle density 1	CG13914|DmelCG13914	0	0
5899	38156	Drosophila melanogaster	msd5	mitotic spindle density 5	CG2213|DmelCG2213	0	0
5900	38157	Drosophila melanogaster	CG2211	CG2211 gene product from transcript CG2211-RA	DmelCG2211|anon-WO0118547.230	0	0
5901	38159	Drosophila melanogaster	CG2199	CG2199 gene product from transcript CG2199-RB	DmelCG2199	0	0
5902	38160	Drosophila melanogaster	Ptp61F	Protein tyrosine phosphatase 61F	BEST:LP01280|CG9178|CG9181|DPTP61F|DmelCG9181|PTP61F|R-PTP 61F|anon-WO0118547.297|anon-WO0140519.98|dPTP61F|dptp61F|ptp61F	0	0
5903	38161	Drosophila melanogaster	312	CG9166 gene product from transcript CG9166-RA	BEST:CK01660|CG9166|DmelCG9166	0	0
5904	38163	Drosophila melanogaster	CG9168	CG9168 gene product from transcript CG9168-RA	DmelCG9168	0	0
5905	38164	Drosophila melanogaster	FucTD	CG9169 gene product from transcript CG9169-RA	CG9169|DmelCG9169	0	0
5906	38165	Drosophila melanogaster	CG9173	CG9173 gene product from transcript CG9173-RA	DmelCG9173	0	0
5907	38167	Drosophila melanogaster	SA-2	Stromalin-2	CG1|CG13916|DmelCG13916|SNM|anon-WO0109301.35|pf-1|snm	0	0
5908	38168	Drosophila melanogaster	rho	rhomboid	CG1004|DMRHO|DMRHOa|DMRHOb|DRORHO|DmelCG1004|Dmel		
	0	0					
5909	38169	Drosophila melanogaster	stet	stem cell tumor	B-Rho|Brho|CG12083|CG12504|CG33166|DmelCG33166|Rho-2|Rho2|brho|rho-2|rho2	0	0
5910	38170	Drosophila melanogaster	robl62A	CG1014 gene product from transcript CG1014-RA	CG1014|DmelCG1014|rob162A	0	0
5911	38173	Drosophila melanogaster	pUf68	poly U binding factor 68kD	CG12085|DPUF68|DmelCG12085|Fir|HFP|Hfp|PUF60|Puf60|anon-WO0118547.408|dFIR|hfp|pUbsf	0	0
5912	38174	Drosophila melanogaster	cue	cueball	CG12086|CT5570|DmelCG12086|ms(3)02509	0	0
5913	38175	Drosophila melanogaster	Psa	Puromycin sensitive aminopeptidase	CG1009|DmelCG1009|PSA|dPSA|dPsa|l(3)06226	0	0
5914	38176	Drosophila melanogaster	CG12090	CG12090 gene product from transcript CG12090-RB	DmelCG12090	0	0
5915	38177	Drosophila melanogaster	CG12091	CG12091 gene product from transcript CG12091-RA	DmelCG12091	0	0
5916	38178	Drosophila melanogaster	CG7852	CG7852 gene product from transcript CG7852-RA	DmelCG7852	0	0
5917	38180	Drosophila melanogaster	Cct2	CTP:phosphocholine cytidylyltransferase 2	CCT2|CG18330|CT1187|DmelCG18330|cct2	0	0
5918	38181	Drosophila melanogaster	CG12099	CG12099 gene product from transcript CG12099-RB	DmelCG12099	0	0
5919	38182	Drosophila melanogaster	pex10	peroxin 10	CG7864|DmelCG7864	0	0
5920	38183	Drosophila melanogaster	CG12003	CG12003 gene product from transcript CG12003-RA	DmelCG12003	0	0
5921	38184	Drosophila melanogaster	CG7879	CG7879 gene product from transcript CG7879-RB	DmelCG7879|cg7879	0	0
5922	38185	Drosophila melanogaster	CG12004	CG12004 gene product from transcript CG12004-RC	DmelCG12004|anon-EST:fe3A8	0	0
5923	38186	Drosophila melanogaster	CG13917	CG13917 gene product from transcript CG13917-RB	DmelCG13917	0	0
5924	38187	Drosophila melanogaster	CG12104	CG12104 gene product from transcript CG12104-RA	DmelCG12104	0	0
5925	38190	Drosophila melanogaster	CG12105	CG12105 gene product from transcript CG12105-RA	DmelCG12105	0	0
5926	38192	Drosophila melanogaster	CG13926	CG13926 gene product from transcript CG13926-RA	DmelCG13926	0	0
5927	38193	Drosophila melanogaster	ABCB7	CG7955 gene product from transcript CG7955-RC	CG7955|DmelCG7955|dABCB7	0	0
5928	38194	Drosophila melanogaster	GC	gamma-glutamyl carboxylase	CG13927|DgammaC|DmelCG13927|dGC|gammaC	0	0
5929	38195	Drosophila melanogaster	CG13928	CG13928 gene product from transcript CG13928-RA	DmelCG13928	0	0
5930	38196	Drosophila melanogaster	n-syb	n-synaptobrevin	CG17248|DmelCG17248|Dn-syb|N-SYB|N-Syb|N-syb|Nsyb|SYB|Syb62A|n-Syb|nSYB|nSyb|nsyb|syb	0	0
5931	38197	Drosophila melanogaster	metl	methyltransferase-like	CG13929|DmelCG13929|anon-EST:Posey109	0	0
5932	38198	Drosophila melanogaster	Bgb	Big brother	CG7959|DmelCG7959|bgb|en(lz)D/9|rpbeta2	0	0
5933	38199	Drosophila melanogaster	alphaCop	alpha-coatomer protein	CG7961|DmelCG7961|alpha-COP|alphaCOP	0	0
5934	38200	Drosophila melanogaster	CG13919	CG13919 gene product from transcript CG13919-RA	DmelCG13919	0	0
5935	38201	Drosophila melanogaster	CG17249	CG17249 gene product from transcript CG17249-RA	DmelCG17249	0	0
5936	38202	Drosophila melanogaster	Bro	Brother	CG7960|DmelCG7960|bro|rpbeta1	0	0
5937	38203	Drosophila melanogaster	CG13920	CG13920 gene product from transcript CG13920-RA	BEST:CK00246|CK00246|DmelCG13920	0	0
5938	38204	Drosophila melanogaster	CG7967	CG7967 gene product from transcript CG7967-RA	DmelCG7967	0	0
5939	38205	Drosophila melanogaster	CG7970	CG7970 gene product from transcript CG7970-RA	DmelCG7970|anon-WO0118547.268	0	0
5940	38206	Drosophila melanogaster	CG7971	CG7971 gene product from transcript CG7971-RC	BcDNA:GH04236|DmelCG7971|anon-EST:fe1A7	0	0
5941	38207	Drosophila melanogaster	CG7974	CG7974 gene product from transcript CG7974-RA	DmelCG7974	0	0
5942	38208	Drosophila melanogaster	RpL23A	Ribosomal protein L23A	153215_at|CG7977|DmelCG7977|L23A|L23a|M(3)62A|PBP-3|RPL23a|RpL23a|RpL25|anon-EST:Liang-1.4|anon-EST:fe2D1|clone 1.4|rpL25|rpl23a	0	0
5943	38209	Drosophila melanogaster	RabX5	CG7980 gene product from transcript CG7980-RB	CG7980|DmRabX5|DmelCG7980	0	0
5944	38210	Drosophila melanogaster	CG13930	CG13930 gene product from transcript CG13930-RA	DmelCG13930	0	0
5945	38211	Drosophila melanogaster	CG12011	CG12011 gene product from transcript CG12011-RA	DmelCG12011|anon-EST:Liang-1.72|clone 1.72	0	0
5946	38212	Drosophila melanogaster	CG13931	CG13931 gene product from transcript CG13931-RA	DmelCG13931	0	0
5947	38214	Drosophila melanogaster	CG7991	CG7991 gene product from transcript CG7991-RB	DmelCG7991	0	0
5948	38216	Drosophila melanogaster	CG18171	CG18171 gene product from transcript CG18171-RA	DmelCG18171	0	0
5949	38217	Drosophila melanogaster	CG12035	CG12035 gene product from transcript CG12035-RA	DmelCG12035	0	0
5950	38218	Drosophila melanogaster	drpr	draper	BcDNA:GH03529|CG18172|CG2086|CT41022|CT6730|DmelCG2086|Drpr	0	0
5951	38219	Drosophila melanogaster	Oseg4	CG2069 gene product from transcript CG2069-RA	CG2069|DmelCG2069|OSEG|OSEG4|Wdr35|cytC40	0	0
5952	38220	Drosophila melanogaster	CG18173	CG18173 gene product from transcript CG18173-RA	DmelCG18173	0	0
5953	38221	Drosophila melanogaster	CG7995	CG7995 gene product from transcript CG7995-RC	DmelCG7995	0	0
5954	38222	Drosophila melanogaster	CG13921	CG13921 gene product from transcript CG13921-RB	DmelCG13921	0	0
5955	38223	Drosophila melanogaster	Cht2	Chitinase 2	CG2054|CHT2|DmCHT2|DmelCG2054|cht2	0	0
5956	38224	Drosophila melanogaster	CG8001	CG8001 gene product from transcript CG8001-RA	DmelCG8001	0	0
5957	38226	Drosophila melanogaster	Dhc62B	Dynein heavy chain at 62B	CG15804|CG18613|DHC|Dhc|DmelCG15804|dhc	0	0
5958	38227	Drosophila melanogaster	CG13933	CG13933 gene product from transcript CG13933-RA	DmelCG13933|dmASBa	0	0
5959	38228	Drosophila melanogaster	CG12018	CG12018 gene product from transcript CG12018-RA	12018|BcDNA:HL03874|DmelCG12018	0	0
5960	38229	Drosophila melanogaster	CG2021	CG2021 gene product from transcript CG2021-RA	DmelCG2021	0	0
5961	38230	Drosophila melanogaster	mRpL46	mitochondrial ribosomal protein L46	CG13922|DmelCG13922|MRP-L46	0	0
5962	38231	Drosophila melanogaster	alpha-Spec	alpha Spectrin	3A9|CG1977|DmelCG1977|Spec|alfa-Spec|alpha|alpha-Sp|alpha-Spc|alpha-Spe|alpha-Spect|alpha-spec|alpha-spectrin|alphaSp|alphaSpec|anon-EST:fe2E2|dre3|klo|l(3)04276|l(3)62Bd|l(3)alpha-Spec|l(3)c	0	0
5963	38232	Drosophila melanogaster	Cdc37	CG12019 gene product from transcript CG12019-RA	CG12019|CPDR-1|CPDR-2|DROCPDR|DmelCG12019|E(sev)3B|cdc37	0	0
5964	38233	Drosophila melanogaster	CG12020	CG12020 gene product from transcript CG12020-RA	DmelCG12020	0	0
5965	38235	Drosophila melanogaster	JTBR	CG1935 gene product from transcript CG1935-RA	BcDNA:RH07980|CG1935|DmelCG1935|anon-EST:fe2G4|dJTB-like|dJTBR	0	0
5966	38238	Drosophila melanogaster	GV1	CG12023 gene product from transcript CG12023-RA	BcDNA:RH49123|CG12023|DIP1|DmelCG12023	0	0
5967	38239	Drosophila melanogaster	Cpr62Ba	Cuticular protein 62Ba	CG13934|DmelCpr62Ba|DmelCG13934	0	0
5968	38240	Drosophila melanogaster	Cpr62Bb	Cuticular protein 62Bb	CG13935|DmelCpr62Bb|DmelCG13935	0	0
5969	38241	Drosophila melanogaster	Cpr62Bc	Cuticular protein 62Bc	CG1919|DmelCpr62Bc|DmelCG1919	0	0
5970	38242	Drosophila melanogaster	CG13936	CG13936 gene product from transcript CG13936-RC	DmelCG13936	0	0
5971	38243	Drosophila melanogaster	CG12024	CG12024 gene product from transcript CG12024-RA	DmelCG12024	0	0
5972	38244	Drosophila melanogaster	R	Roughened	BEST:GH18528|C-ras3|CG1956|D-Rap1|DRap|DRap1|DmelCG1956|Dras3|Dras62B|E(faf)|RAP1|Rap|Rap1|Ras3|RasIII|l(3)62Bf|l(3)R|rap1|ras 3|ras3	0	0
5973	38246	Drosophila melanogaster	CG12025	CG12025 gene product from transcript CG12025-RA	DmelCG12025	0	0
5974	38247	Drosophila melanogaster	CG13924	CG13924 gene product from transcript CG13924-RC	DmelCG13924	0	0
5975	38248	Drosophila melanogaster	dre4	CG1828 gene product from transcript CG1828-RB	CG1828|DRE4/dSPT16|DmelCG1828|Spt16|dSPT16|dSpt16|dspt16|l(3)62Bg|l(3)dre4|spt16	0	0
5976	38249	Drosophila melanogaster	CG13937	CG13937 gene product from transcript CG13937-RD	DmelCG13937	0	0
5977	38251	Drosophila melanogaster	Tmhs	Tetraspan membrane protein in hair cell stereocilia orth	CG12026|DmelCG12026|TMHS|l(3)62Be|l(3)neo7|neo7	0	0
5978	38252	Drosophila melanogaster	CG1887	CG1887 gene product from transcript CG1887-RD	DmelCG1887	0	0
5979	38254	Drosophila melanogaster	Dbx	CG42234 gene product from transcript CG42234-RC	CG12361|CG13925|CG42234|DmelCG42234|Dmel_CG12361|Dmel_CG13925|dDbx|dbx	0	0
5980	38255	Drosophila melanogaster	CG5687	CG5687 gene product from transcript CG5687-RA	DmelCG5687|anon-EST:Posey42	0	0
5981	38256	Drosophila melanogaster	Mfap1	Microfibril-associated protein 1	CG1017|DmelCG1017|dmfap1	0	0
5982	38257	Drosophila melanogaster	mu2	mutator 2	CG1960|DmelCG1960|MU2|cDNA D|mu-2|mutator[2]	0	0
5983	38258	Drosophila melanogaster	CG5690	CG5690 gene product from transcript CG5690-RA	DmelCG5690|anon-62BCa|cDNA C	0	0
5984	38259	Drosophila melanogaster	CG42863	CG42863 gene product from transcript CG42863-RB	CG11814|CG5691|DmelCG42863|Dmel_CG11814|Dmel_CG5691	0	0
5985	38261	Drosophila melanogaster	CG1140	CG1140 gene product from transcript CG1140-RB	BcDNA:RH54340|CT1116|DmelCG1140|cDNA B	0	0
5986	38262	Drosophila melanogaster	CG1927	CG1927 gene product from transcript CG1927-RA	BcDNA:GH11112|DmelCG1927|anon-62BCb|anon-WO0118547.340|cDNA A	0	0
5987	38263	Drosophila melanogaster	CG11815	CG11815 gene product from transcript CG11815-RA	DmelCG11815	0	0
5988	38264	Drosophila melanogaster	CG1139	CG1139 gene product from transcript CG1139-RA	DmelCG1139	0	0
5989	38273	Drosophila melanogaster	CG15822	CG15822 gene product from transcript CG15822-RD	DmelCG15822	0	0
5990	38274	Drosophila melanogaster	CG15879	CG15879 gene product from transcript CG15879-RA	DmelCG15879	0	0
5991	38277	Drosophila melanogaster	CG15820	CG15820 gene product from transcript CG15820-RA	BcDNA:SD08776|DmelCG15820	0	0

5992	38278	Drosophila melanogaster	CG13810	CG13810 gene product from transcript CG13810-RB	DmelCG13810	0	0
5993	38280	Drosophila melanogaster	CG8960	CG8960 gene product from transcript CG8960-RA	DmelCG8960	0	0
5994	38281	Drosophila melanogaster	CG15878	CG15878 gene product from transcript CG15878-RA	DmelCG15878	0	0
5995	38282	Drosophila melanogaster	osm-1	CG13809 gene product from transcript CG13809-RA	CG13809|DmelCG13809|IFT172|OSEG|OSEG2|OSM-1|Oseg2	0	0
5996	38283	Drosophila melanogaster	CG9018	CG9018 gene product from transcript CG9018-RA	DmelCG9018	0	0
5997	38284	Drosophila melanogaster	ACXD	CG5712 gene product from transcript CG5712-RA	AC|CG5712|DACXD|DmelCG5712	0	0
5998	38285	Drosophila melanogaster	CG32301	CG32301 gene product from transcript CG32301-RB	AC|CG8970|DmelCG32301	0	0
5999	38286	Drosophila melanogaster	CG1275	CG1275 gene product from transcript CG1275-RD	DmelCG1275|anon-WO0118547.260	0	0
6000	38287	Drosophila melanogaster	CG2034	CG2034 gene product from transcript CG2034-RA	DmelCG2034|anon-i1	0	0
6001	38288	Drosophila melanogaster	oxt	peptide O-xylosyltransferase	CG17771|CG17772|CG32300|DmelCG32300|OXT|anon-i2|dOXT	0	0
6002	38290	Drosophila melanogaster	CG13807	CG13807 gene product from transcript CG13807-RA	DmelCG13807	0	0
6003	38291	Drosophila melanogaster	ecd	ecdysoneless	CG5714|DmelCG5714|ecd-1|ecd1|ecdl|l(3)62Db|l(3)ecd|l(3)ecd-1|l(3)ecd1	0	0
6004	38292	Drosophila melanogaster	CG13806	CG13806 gene product from transcript CG13806-RA	DmelCG13806	0	0
6005	38293	Drosophila melanogaster	CG32302	CG32302 gene product from transcript CG32302-RA	CG13805|DmelCG32302	0	0
6006	38294	Drosophila melanogaster	yellow-g	CG5717 gene product from transcript CG5717-RA	CG5717|DmelCG5717	0	0
6007	38295	Drosophila melanogaster	yellow-g2	CG13804 gene product from transcript CG13804-RA	CG13804|DmelCG13804|yg2	0	0
6008	38296	Drosophila melanogaster	DmsR-2	Dromyosuppressin receptor 2	CG13803|DMSR-2|Dm CG13803|DmelCG13803|anon-WO0170980.160|anon-WO0170980.161	0	0
6009	38297	Drosophila melanogaster	CG13802	CG13802 gene product from transcript CG13802-RB	DmelCG13802	0	0
6010	38298	Drosophila melanogaster	DmsR-1	Dromyosuppressin receptor 1	CG8985|DMSR-1|Dm CG8985|DmelCG8985|anon-WO0170980.106|anon-WO0170980.107	0	0
6011	38299	Drosophila melanogaster	CG13801	CG13801 gene product from transcript CG13801-RA	DmelCG13801	0	0
6012	38300	Drosophila melanogaster	CG1317	CG1317 gene product from transcript CG1317-RC	DmelCG1317	0	0
6013	38301	Drosophila melanogaster	CG8993	CG8993 gene product from transcript CG8993-RA	8993|DmelCG8993	0	0
6014	38302	Drosophila melanogaster	mRpL23	mitochondrial ribosomal protein L23	BcDNA:RE49852|CG1320|DmelCG1320|L23	0	0
6015	38303	Drosophila melanogaster	CG9004	CG9004 gene product from transcript CG9004-RA	DmelCG9004	0	0
6016	38304	Drosophila melanogaster	CG15877	CG15877 gene product from transcript CG15877-RA	DmelCG15877	0	0
6017	38305	Drosophila melanogaster	CG13800	CG13800 gene product from transcript CG13800-RB	DmelCG13800	0	0
6018	38306	Drosophila melanogaster	CG42669	CG42669 gene product from transcript CG42669-RM	CG12790|CG13797|CG13798|CG13799|CG16763|CG32306|CG33231|DmelCG42669|Dmel_CG32306|Dmel_CG33231	0	0
6019	38310	Drosophila melanogaster	CG16762	CG16762 gene product from transcript CG16762-RA	DmelCG16762	0	0
6020	38311	Drosophila melanogaster	Cyp4d20	CG16761 gene product from transcript CG16761-RA	4d20|CG16761|DmelCG16761|RH59895	0	0
6021	38313	Drosophila melanogaster	CG1146	CG1146 gene product from transcript CG1146-RB	DmelCG1146	0	0
6022	38315	Drosophila melanogaster	CG16758	CG16758 gene product from transcript CG16758-RB	CG16760|DmelCG16758|c62E-15	0	0
6023	38321	Drosophila melanogaster	dos	daughter of sevenless	141511_at|CG1044|DOS|DmelCG1044|Dos|E(csw)3A|E(csw[CS])3A|Gab-1|Su(sev)3A|Su(sev[S11])3A	0	0
6024	38322	Drosophila melanogaster	CG16984	CG16984 gene product from transcript CG16984-RA	BcDNA:AT01695|DmelCG16984	0	0
6025	38323	Drosophila melanogaster	CG16985	CG16985 gene product from transcript CG16985-RA	BcDNA:RE30174|DmelCG16985	0	0
6026	38324	Drosophila melanogaster	CG12182	CG12182 gene product from transcript CG12182-RA	BcDNA:GH02340|DmelCG12182	0	0
6027	38325	Drosophila melanogaster	CG16986	CG16986 gene product from transcript CG16986-RA	DmelCG16986	0	0
6028	38326	Drosophila melanogaster	Pxn	Peroxidasin	CG12002|CT1239|DmelCG12002|Dpxn|pxn	0	0
6029	38327	Drosophila melanogaster	MEP-1	CG1244 gene product from transcript CG1244-RG	CG1244|DmelCG1244|MEP1|anon-EST:Liang-1.37|anon-WO0118547.584|clone 1.37|dMEP-1	0	0
6030	38328	Drosophila melanogaster	CG14949	CG14949 gene product from transcript CG14949-RA	DmelCG14949	0	0
6031	38329	Drosophila melanogaster	CG1143	CG1143 gene product from transcript CG1143-RA	DmelCG1143	0	0
6032	38330	Drosophila melanogaster	CG1246	CG1246 gene product from transcript CG1246-RB	DmelCG1246	0	0
6033	38331	Drosophila melanogaster	Aats-pro	Prolyl-tRNA synthetase	CG12186|DmelCG12186|PRS|ProRS	0	0
6034	38332	Drosophila melanogaster	CG42304	CG42304 gene product from transcript CG42304-RA	CG1248|CG32297|DmelCG42304|Dmel_CG32297	0	0
6035	38334	Drosophila melanogaster	CG14950	CG14950 gene product from transcript CG14950-RB	DmelCG14950	0	0
6036	38337	Drosophila melanogaster	CG43375	CG43375 gene product from transcript CG43375-RC	CG12187|CG16976|DmelCG43375|Dmel_CG12187|Dmel_CG16976|anon-EST:Posey171	0	0
6037	38338	Drosophila melanogaster	Mrtf	Myocardin-related transcription factor	CG12188|CG14951|CG32296|DMRTF|DmelCG32296|MAL-D|MRTF|Mal-D|dMRTF|dMrtf|mal|mal-d	0	0
6038	38340	Drosophila melanogaster	Acp62F	Accessory gland peptide 62F	Acp62|CG1262|CT2528|DmelCG1262	0	0
6039	38341	Drosophila melanogaster	CG1240	CG1240 gene product from transcript CG1240-RA	DmelCG1240	0	0
6040	38342	Drosophila melanogaster	CG9977	CG9977 gene product from transcript CG9977-RA	DmelCG9977	0	0
6041	38343	Drosophila melanogaster	CG12093	CG12093 gene product from transcript CG12093-RA	DmelCG12093	0	0
6042	38344	Drosophila melanogaster	Atg2	Autophagy-specific gene 2	ATG2|CG1241|DmelCG1241|EP3697|atg2	0	0
6043	38345	Drosophila melanogaster	mRpS35	mitochondrial ribosomal protein S35	CG2101|DmelCG2101|MRP-S28	0	0
6044	38346	Drosophila melanogaster	pgant6	polypeptide GalNAc transferase 6	CG2103|DmelCG2103	0	0
6045	38347	Drosophila melanogaster	CG43444	CG43444 gene product from transcript CG43444-RA	CG2083|CG9973|DmelCG43444|Dmel_CG2083|Dmel_CG9973|Idax	0	0
6046	38350	Drosophila melanogaster	spz5	spatzle 5	CG9972|CT28115|DNT2|DmelCG9972|NT2|Spz5	0	0
6047	38351	Drosophila melanogaster	CG9970	CG9970 gene product from transcript CG9970-RA	DmelCG9970	0	0
6048	38352	Drosophila melanogaster	Shab	Shaker cognate b	CG1066|CG43128|CG9965|DmelCG43128|Dmel_CG1066|Dmel_CG9965|Kv2|dShab|shab	0	0
6049	38354	Drosophila melanogaster	Or63a	Odorant receptor 63a	63B.1|63a|CG9969|DOR28|DmelCG9969	0	0
6050	38355	Drosophila melanogaster	CG2107	CG2107 gene product from transcript CG2107-RA	DmelCG2107	0	0
6051	38356	Drosophila melanogaster	CG2113	CG2113 gene product from transcript CG2113-RA	BcDNA:AT18037|DmelCG2113	0	0
6052	38357	Drosophila melanogaster	FR	Fmrf Receptor	CG2114|DmelCG2114|DrmFMRFa-R|FMRFaR	0	0
6053	38358	Drosophila melanogaster	CG32487	CG32487 gene product from transcript CG32487-RA	CG2077|DmelCG32487|Phos32487	0	0
6054	38360	Drosophila melanogaster	CG2162	CG2162 gene product from transcript CG2162-RA	DmelCG2162|anon-EST:Posey170	0	0
6055	38362	Drosophila melanogaster	scramb2	scramblase 2	CG1893|DmelCG1893|Scramb2	0	0
6056	38363	Drosophila melanogaster	CG32485	CG32485 gene product from transcript CG32485-RA	CG2159|DmelCG32485	0	0
6057	38364	Drosophila melanogaster	CG1271	CG1271 gene product from transcript CG1271-RC	DmelCG1271	0	0
6058	38365	Drosophila melanogaster	CG16753	CG16753 gene product from transcript CG16753-RA	DmelCG16753	0	0
6059	38367	Drosophila melanogaster	CG32486	CG32486 gene product from transcript CG32486-RD	CG14954|CG16745|DmelCG32486|anon-WO0118547.267	0	0
6060	38369	Drosophila melanogaster	CG11486	CG11486 gene product from transcript CG11486-RG	DmelCG11486	0	0
6061	38370	Drosophila melanogaster	Cht7	CG1869 gene product from transcript CG1869-RA	CG1869|DmCHT7|DmelCG1869	0	0
6062	38371	Drosophila melanogaster	CG14955	CG14955 gene product from transcript CG14955-RD	DmelCG14955	0	0
6063	38372	Drosophila melanogaster	prominin-like	CG7740 gene product from transcript CG7740-RD	CG7740|DmelCG7740|bs29e02.y1	0	0
6064	38373	Drosophila melanogaster	CG11537	CG11537 gene product from transcript CG11537-RB	DmelCG11537	0	0
6065	38374	Drosophila melanogaster	CG1291	CG1291 gene product from transcript CG1291-RA	DmelCG1291	0	0
6066	38375	Drosophila melanogaster	CG12082	CG12082 gene product from transcript CG12082-RB	DmelCG12082	0	0
6067	38376	Drosophila melanogaster	BtbVII	BTB-protein-VII	BTB-VII|BcDNA:GH07729|CG11494|CG14956|CG43365|DmelCG43365|Dmel_CG11494|Dmel_CG14956	0	0
6068	38378	Drosophila melanogaster	CG12079	CG12079 gene product from transcript CG12079-RA	DmelCG12079	0	0
6069	38379	Drosophila melanogaster	pfk	piefke	CG15812|DmelCG15812	0	0
6070	38381	Drosophila melanogaster	CG14962	CG14962 gene product from transcript CG14962-RA	DmelCG14962	0	0
6071	38382	Drosophila melanogaster	CG32278	CG32278 gene product from transcript CG32278-RA	CG12032|DmelCG32278	0	0
6072	38383	Drosophila melanogaster	CG14963	CG14963 gene product from transcript CG14963-RA	DmelCG14963	0	0
6073	38384	Drosophila melanogaster	CG14964	CG14964 gene product from transcript CG14964-RB	CT34810|DmelCG14964	0	0
6074	38385	Drosophila melanogaster	Girdin	CG12734 gene product from transcript CG12734-RA	CG12734|DmelCG12734	0	0
6075	38386	Drosophila melanogaster	CG14957	CG14957 gene product from transcript CG14957-RA	DmelCG14957	0	0
6076	38387	Drosophila melanogaster	CG14958	CG14958 gene product from transcript CG14958-RA	DmelCG14958	0	0
6077	38388	Drosophila melanogaster	CG14965	CG14965 gene product from transcript CG14965-RA	63BC-T3|DmelCG14965|ORF1|anon-63BC-T3|dmLD47616p	0	0
6078	38389	Drosophila melanogaster	Hsp83	Heat shock protein 83	143198_at|83|83K HSP|CG1242|DMHSP82|DmelCG1242|E(sev)3A|E(sina)2|HSP82|HSP83|HSP90|Hsp82|Hsp90|ORF1|Su(Raf)3A|anon-EST:Liang-2.53|anon-WO0068693|anon-WO0140519.209|clone 2.53	0	0
6079	38390	Drosophila melanogaster	CG14966	CG14966 gene product from transcript CG14966-RA	DmelCG14966	0	0
6080	38391	Drosophila melanogaster	gry	gryzun	CG17569|DmelCG17569	0	0
6081	38392	Drosophila melanogaster	CG32271	CG32271 gene product from transcript CG32271-RA	CG11532|DmelCG32271|SP176	0	0
6082	38393	Drosophila melanogaster	CG32277	CG32277 gene product from transcript CG32277-RA	CG11531|DmelCG32277|SP183	0	0
6083	38395	Drosophila melanogaster	CG14967	CG14967 gene product from transcript CG14967-RB	DmelCG14967	0	0
6084	38396	Drosophila melanogaster	CG12034	CG12034 gene product from transcript CG12034-RA	DmelCG12034|nSMase	0	0
6085	38397	Drosophila melanogaster	RpL28	Ribosomal protein L28	BcDNA:GH04183|CG12740|DmelCG12740|L28|M(3)63B|anon-WO0118547.321	0	0
6086	38398	Drosophila melanogaster	CG17737	CG17737 gene product from transcript CG17737-RA	BcDNA:RE14985|DmelCG17737|anon-EST:Posey272	0	0
6087	38399	Drosophila melanogaster	CG11505	CG11505 gene product from transcript CG11505-RD	DmelCG11505	0	0
6088	38400	Drosophila melanogaster	CG17746	CG17746 gene product from transcript CG17746-RB	Cg17746|DmelCG17746|PP2C	0	0
6089	38401	Drosophila melanogaster	CG12078	CG12078 gene product from transcript CG12078-RB	DmelCG12078	0	0
6090	38402	Drosophila melanogaster	ckd	cracked	BcDNA:RH52423|CG14959|DmelCG14959|EP(2)3139|EP(3)0714|EP3139|EP714	0	0
6091	38403	Drosophila melanogaster	CG14960	CG14960 gene product from transcript CG14960-RA	DmelCG14960	0	0
6092	38404	Drosophila melanogaster	CG12017	CG12017 gene product from transcript CG12017-RB	DmelCG12017	0	0
6093	38405	Drosophila melanogaster	CG12009	CG12009 gene product from transcript CG12009-RB	DmelCG12009	0	0
6094	38406	Drosophila melanogaster	CG14968	CG14968 gene product from transcript CG14968-RB	DmelCG14968|anon-WO0107627.1|anon-WO0107627.2|anon-WO0107627.3	0	0
6095	38407	Drosophila melanogaster	ZnT63C	CG17723 gene product from transcript CG17723-RD	CG17723|DmelCG17723|ZnT1|dZnT1	0	0
6096	38408	Drosophila melanogaster	dro2	drosomycin-2	CG10813|CG32279|CT30310|DmelCG32279|Dro-D|Dro2|Drs-ID|dmy2	0	0
6097	38409	Drosophila melanogaster	dro5	drosomycin-5	BcDNA:GH09576|CG10812|DmelCG10812|Dro-G|Dro5|Drs-IG|dmy5	0	0
6098	38410	Drosophila melanogaster	CG12077	CG12077 gene product from transcript CG12077-RB	DmelCG12077	0	0
6099	38411	Drosophila melanogaster	CG12016	CG12016 gene product from transcript CG12016-RA	DmelCG12016|Dromel_CG12016_FBtr0073063_mORF	0	0
6100	38412	Drosophila melanogaster	CG11526	CG11526 gene product from transcript CG11526-RB	DmelCG11526	0	0
6101	38413	Drosophila melanogaster	PHGPx	CG12013 gene product from transcript CG12013-RC	CG12013|DmPHGPx|DmelCG12013|GPx|anon-WO0257455.17	0	0
6102	38414	Drosophila melanogaster	CG14961	CG14961 gene product from transcript CG14961-RA	DmelCG14961	0	0

6103	38415	Drosophila melanogaster	CG14969	CG14969 gene product from transcript CG14969-RA	DmelCG14969	0	0
6104	38416	Drosophila melanogaster	dro6	drosomycin-6	CG10815|CG11520|CG32268|DmelCG32268|Dro-II|Dro6|Drs-II	0	0
6105	38418	Drosophila melanogaster	kst	karst	CG12008|DmelCG12008|Spec-beta[[H]]|beta-Spectrin|betaH|betaH-Spec|betaHS|beta[[H]]|beta[[H]]-SPEC|beta[[H]]-Sp|beta[[H]]-Spec|beta[[H]]-Spectrin|beta[[H]]spectrin|beta[[Heavy]]spectrin|beta[[heavy	0	0
6106	38419	Drosophila melanogaster	Drs	Drosomycin	BcDNA:LP03851|CG10810|Crp|DIM 19|DRO|DROM|DRS|DmelCG10810|Drm|Drom|Dros|Droso|dmy1|drm|drom|drs|drsm	0	0
6107	38420	Drosophila melanogaster	YT521-B	CG12076 gene product from transcript CG12076-RB	BcDNA:GH01918|CG12076|DmelCG12076|dYT521-B	0	0
6108	38421	Drosophila melanogaster	CG12010	CG12010 gene product from transcript CG12010-RA	DmelCG12010	0	0
6109	38422	Drosophila melanogaster	CG12012	CG12012 gene product from transcript CG12012-RA	DmelCG12012	0	0
6110	38423	Drosophila melanogaster	CG12014	CG12014 gene product from transcript CG12014-RA	DmelCG12014|anon-EST:Posey282	0	0
6111	38424	Drosophila melanogaster	sty	sprouty	CG1921|CS3-3|DmelCG1921|STY|Spry|Sry|Sty|dSPRY|spry	0	0
6112	38427	Drosophila melanogaster	armi	armitage	APC16|Armi|CG11513|DmelCG11513	0	0
6113	38428	Drosophila melanogaster	CycJ	Cyclin J	CDI5|CG10308|Cdi5|DmelCG10308|cdi5|cycJ|cyclinJ	0	0
6114	38429	Drosophila melanogaster	CG14971	CG14971 gene product from transcript CG14971-RA	DmelCG14971	0	0
6115	38430	Drosophila melanogaster	CG42324	CG42324 gene product from transcript CG42324-RD	CG14972|CG14973|DmelCG42324|Dmel_CG14972|Dmel_CG14973	0	0
6116	38432	Drosophila melanogaster	ImpE2	Ecdysone-inducible gene E2	CG1934|DmelCG1934|IMP-E2|Imp-E2	0	0
6117	38433	Drosophila melanogaster	Eip63E	Ecdysone-induced protein 63E	CG10579|DmelCG10579|EIP63E|L63|PFTAIRE|cdc2-63E	0	0
6118	38434	Drosophila melanogaster	CG10359	CG10359 gene product from transcript CG10359-RE	DmelCG10359|FBpp0072995	0	0
6119	38435	Drosophila melanogaster	CG10357	CG10357 gene product from transcript CG10357-RC	DmelCG10357	0	0
6120	38436	Drosophila melanogaster	dar1	dendritic arbor reduction 1	CG12029|DAR1|Dar1|DmelCG12029|l(3)63Ed|l(3)SH5	0	0
6121	38437	Drosophila melanogaster	CG10862	CG10862 gene product from transcript CG10862-RA	DmelCG10862	0	0
6122	38438	Drosophila melanogaster	CG32264	CG32264 gene product from transcript CG32264-RK	CG10857|CG10860|CG12604|CG14978|DmelCG32264	0	0
6123	38440	Drosophila melanogaster	CG10858	CG10858 gene product from transcript CG10858-RB	DmelCG10858	0	0
6124	38443	Drosophila melanogaster	ntc	nutcracker	CG10855|DmelCG10855	0	0
6125	38444	Drosophila melanogaster	IntS10	Integrator 10	CG1120|DmelCG1120|Int10	0	0
6126	38445	Drosophila melanogaster	CG32262	CG32262 gene product from transcript CG32262-RA	CG10854|DmelCG32262	0	0
6127	38446	Drosophila melanogaster	CG12006	CG12006 gene product from transcript CG12006-RA	DmelCG12006	0	0
6128	38447	Drosophila melanogaster	Scsalpha	Succinyl coenzyme A synthetase alpha subunit	CG1065|DmelCG1065|scsalpha	0	0
6129	38448	Drosophila melanogaster	Acp63F	Accessory gland peptide 63F	Acp63|Acp63F/64A|Acp63f|CG10852|DmelCG10852|acp63F	0	0
6130	38451	Drosophila melanogaster	Awh	Arrowhead	CG1072|DmelCG1072|awh|l(3)63Ea|l(3)63Ea/Awh|l(3)64Ea|l(3)SH9|l63Ea	0	0
6131	38452	Drosophila melanogaster	Fie	Fire exit	BEST:HL04053|CG1079|DmelCG1079|HL04053	0	0
6132	38453	Drosophila melanogaster	Gr63a	Gustatory receptor 63a	CG14979|DmGr63a|DmelCG14979|GR63a|Gr63F1	0	0
6133	38454	Drosophila melanogaster	CG14977	CG14977 gene product from transcript CG14977-RA	BcDNA:RH34338|DmelCG14977	0	0
6134	38455	Drosophila melanogaster	CG14980	CG14980 gene product from transcript CG14980-RB	DmelCG14980	0	0
6135	38456	Drosophila melanogaster	Ubi-p63E	Ubiquitin-63E	CG11624|DmUb|DmUbi-p63E|DmelCG11624|Ub|Ubi-P63e|Ubi-p|Ubi63E|Ubi63p|Ubip|Ubiq|Ubp|ubi|ubi-p63E|ubi63E	0	0
6136	38457	Drosophila melanogaster	Sc2	CG10849 gene product from transcript CG10849-RA	CG10849|DmelCG10849|dSc2|l(3)05634|l(3)63Eb|l(3)SH4	0	0
6137	38459	Drosophila melanogaster	mge	maggie	BcDNA:LP07226|CG14981|DmelCG14981|l(3)63Fc|l(3)SH3	0	0
6138	38461	Drosophila melanogaster	CG10866	CG10866 gene product from transcript CG10866-RA	DmelCG10866	0	0
6139	38462	Drosophila melanogaster	CG12766	CG12766 gene product from transcript CG12766-RA	DmelCG12766	0	0
6140	38463	Drosophila melanogaster	CG10863	CG10863 gene product from transcript CG10863-RA	BcDNA:GH10614|DmelCG10863|dm1/AKR2E3	0	0
6141	38464	Drosophila melanogaster	CG14982	CG14982 gene product from transcript CG14982-RB	DmelCG14982	0	0
6142	38465	Drosophila melanogaster	CG10853	CG10853 gene product from transcript CG10853-RA	BcDNA:LP09837|DmelCG10853	0	0
6143	38466	Drosophila melanogaster	CG14983	CG14983 gene product from transcript CG14983-RA	DmelCG14983	0	0
6144	38467	Drosophila melanogaster	CG14984	CG14984 gene product from transcript CG14984-RB	DmelCG14984	0	0
6145	38468	Drosophila melanogaster	CG12605	CG12605 gene product from transcript CG12605-RB	DmelCG12605|anon-WO0140519.93	0	0
6146	38469	Drosophila melanogaster	scrt	scratch	BcDNA:RH71479|CG1130|DmelCG1130|Scrt|anon-EST:Liang-1.31|anon-EST:Liang-1.67|anon-WO02059370.57|clone 1.31|clone 1.67	0	0
6147	38470	Drosophila melanogaster	CG14985	CG14985 gene product from transcript CG14985-RB	DmelCG14985	0	0
6148	38471	Drosophila melanogaster	fd64A	forkhead domain 64A	CG1132|DmelCG1132|Dmfd2|FD2|FoxL1|fd2	0	0
6149	38472	Drosophila melanogaster	CG1134	CG1134 gene product from transcript CG1134-RA	DmelCG1134	0	0
6150	38473	Drosophila melanogaster	CG11594	CG11594 gene product from transcript CG11594-RC	DmelCG11594	0	0
6151	38477	Drosophila melanogaster	CG11593	CG11593 gene product from transcript CG11593-RB	DmelCG11593|anon-EST:Posey64	0	0
6152	38478	Drosophila melanogaster	Rcd5	Reduction in Cnn dots 5	CG1135|DmelCG1135|MCRS1|MCRS2|dMCRS1|dMCRS2|p78	0	0
6153	38479	Drosophila melanogaster	CG1136	CG1136 gene product from transcript CG1136-RA	DmelCG1136|novel	0	0
6154	38480	Drosophila melanogaster	Dpy-30L2	Dpy-30-like 2	CG11591|DmelCG11591|dpy-30L2	0	0
6155	38484	Drosophila melanogaster	Gad1	Glutamic acid decarboxylase 1	CG14994|DGAD1|DGad1|DmelCG14994|GAD|GAD-1|GAD1|GAD[[1]]|Gad|Glb|dGad1|dgad1|gad|gad1|l(3)64An|l(3)SH10	0	0
6156	38485	Drosophila melanogaster	CG14989	CG14989 gene product from transcript CG14989-RE	BcDNA:GH06302|DmelCG14989	0	0
6157	38486	Drosophila melanogaster	CG14990	CG14990 gene product from transcript CG14990-RA	DmelCG14990|SPH97	0	0
6158	38487	Drosophila melanogaster	CG14995	CG14995 gene product from transcript CG14995-RA	CT34848|DmelCG14995|NEST:bs15f01|NEST:bs17f06|anon-WO0140519.252|bs17f06.y1	0	0
6159	38488	Drosophila melanogaster	Fit1	Fermitin 1	CG14991|DmelCG14991|anon-WO0118547.421	0	0
6160	38489	Drosophila melanogaster	Ack	CG14992 gene product from transcript CG14992-RA	ACK|ACK2|BcDNA:GH10777|CG14992|DACK|DAck|DmelCG14992|p145	0	0
6161	38490	Drosophila melanogaster	Chd64	CG14996 gene product from transcript CG14996-RB	CG-14996|CG14996|CT34849|DmelCG14996|anon-EST:Liang-1.80|clone 1.80	0	0
6162	38491	Drosophila melanogaster	ens	ensconsin	CG14998|Dm-308|DmelCG14998	0	0
6163	38492	Drosophila melanogaster	RfC4	Replication factor C subunit 4	154910_at|BEST:GH24992|CG14999|DmRFC4|DmelCG14999|RfC40|Rfc4|Rfc40|l(3)64Ai|l(3)A1|l(3)Rfc4|l(3)SG13|l(3)SH17|l(3)dsl-1|l(3)dsl1|l(3)e20|l(3)rfc40|rfc|rfc4|rfc40|rfc[40]	0	0
6164	38493	Drosophila melanogaster	Rop	Ras opposite	CG15811|DmelCG15811|ROP|SEC1|cs1|l(3)64Ah|l(3)SH16|ras2|rop|rop(Sec1)	0	0
6165	38494	Drosophila melanogaster	Ras64B	Ras oncogene at 64B	BcDNA:RE36103|C-ras2|CG1167|D-ras-2|D-ras2|DRas2|Dm-ras-64B|DmelCG1167|Dmras64B|Dras2|Dras64B|RAS|RAS2|Ras|Ras2|c-ras2|ras|ras-2|ras2	0	0
6166	38495	Drosophila melanogaster	Akh	Adipokinetic hormone	AKH|CG1171|DmelCG1171|Drm-AKH|Hrth|akh|dAkh	0	0
6167	38496	Drosophila melanogaster	CG1299	CG1299 gene product from transcript CG1299-RA	DmelCG1299|SP18|c-SP18	0	0
6168	38499	Drosophila melanogaster	mas	masquerade	CG15002|DmelCG15002|SPH79|c-SPH79	0	0
6169	38500	Drosophila melanogaster	Ero1L	Endoplasmic reticulum oxidoreductin-1-like	CG1333|DmelCG1333|ERO1-L|kiga|l(3)64Ag|l(3)SH22	0	0
6170	38501	Drosophila melanogaster	Teh4	tipE homolog 4	CG15003|DmelCG15003|TEH4|anon-WO0172774.111	0	0
6171	38502	Drosophila melanogaster	Teh2	tipE homolog 2	CG15004|DmelCG15004|TEH2	0	0
6172	38503	Drosophila melanogaster	VhaM9.7-c	Vacuolar H[+] ATPase subunit M9.7-c	CG11589|DmelCG11589|V-ATPase|VhaM9.7-1|vhaM9.2-1|vhaM9.7-1|vhaM9.7-3	0	0
6173	38504	Drosophila melanogaster	tipE	temperature-induced paralytic E	CG1232|DmelCG1232|TipE|tip-E	0	0
6174	38505	Drosophila melanogaster	CG43367	CG43367 gene product from transcript CG43367-RE	CG1295|CG1308|CG1332|DmelCG43367|Dmel_CG1295|Dmel_CG1308|Dmel_CG1332	0	0
6175	38506	Drosophila melanogaster	CG15005	CG15005 gene product from transcript CG15005-RA	DmelCG15005	0	0
6176	38508	Drosophila melanogaster	Cpr64Aa	Cuticular protein 64Aa	CG15006|DmelCpr64Aa|DmelCG15006	0	0
6177	38509	Drosophila melanogaster	Cpr64Ab	Cuticular protein 64Ab	CG15007|DmelCpr64Ab|DmelCG15007	0	0
6178	38510	Drosophila melanogaster	Cpr64Ac	Cuticular protein 64Ac	CG15008|DmelCpr64Ac|DmelCG15008	0	0
6179	38511	Drosophila melanogaster	Cpr64Ad	Cuticular protein 64Ad	CG1259|DmelCpr64Ad|DmelCG1259	0	0
6180	38513	Drosophila melanogaster	ImpL2	Ecdysone-inducible gene L2	CG15009|CT34862|DmelCG15009|GH28|IMP-L2|IMPL2|Imp-12|Imp-L2|ImpL-2	0	0
6181	38514	Drosophila melanogaster	CG14997	CG14997 gene product from transcript CG14997-RB	BcDNA:GH04863|DmelCG14997	0	0
6182	38515	Drosophila melanogaster	pav	pavarotti	0934/13|0973/08|2A12|2A12MEL|CG1258|DmKLP1/pav|DmMKLP1|DmelCG1258|KIF23|Mklp1|PAV|PAV-KLP|PAV-MKLP|PAV/KLP|Pav|Pav KLP|Pav-KLP|Pav-Klp|Pav-klp|PavKLP|anon-EST:fe2A12|anon2/	0	0
6183	38516	Drosophila melanogaster	ago	archipelago	Ago|CG15010|DmFbw7|DmelCG15010|SCF[Ago]|anon-WO0118547.345|cdc4	0	0
6184	38517	Drosophila melanogaster	CG1265	CG1265 gene product from transcript CG1265-RB	BcDNA:RE09466|DmelCG1265	0	0
6185	38518	Drosophila melanogaster	CG15011	CG15011 gene product from transcript CG15011-RA	DmelCG15011	0	0
6186	38519	Drosophila melanogaster	CG1309	CG1309 gene product from transcript CG1309-RA	DmelCG1309	0	0
6187	38520	Drosophila melanogaster	CG11586	CG11586 gene product from transcript CG11586-RA	DmelCG11586	0	0
6188	38521	Drosophila melanogaster	VhaM9.7-a	Vacuolar H[+] ATPase subunit M9.7-a	CG1268|DmelCG1268|vhaM9.7-1	0	0
6189	38522	Drosophila melanogaster	CG1273	CG1273 gene product from transcript CG1273-RB	DmelCG1273	0	0
6190	38523	Drosophila melanogaster	CG1311	CG1311 gene product from transcript CG1311-RA	DmelCG1311	0	0
6191	38525	Drosophila melanogaster	srw	shrew	CG11582|DmelCG11582	0	0
6192	38526	Drosophila melanogaster	CG1316	CG1316 gene product from transcript CG1316-RA	DmelCG1316|cg1316	0	0
6193	38527	Drosophila melanogaster	TfIIEbeta	Transcription factor IIEbeta	CG1276|DmelCG1276|E[[S]]|TFIIE|TfIIE|dTFIIE-S|dTFIIES	0	0
6194	38528	Drosophila melanogaster	Hexo1	Hexosaminidase 1	CG1318|DmHex1|DmelCG1318|HEX 1|HEX1|HEXO1	0	0
6195	38529	Drosophila melanogaster	CG15012	CG15012 gene product from transcript CG15012-RB	DmelCG15012	0	0
6196	38530	Drosophila melanogaster	CG1319	CG1319 gene product from transcript CG1319-RB	BcDNA:RE08888|BcDNA:RH09920|DmelCG1319	0	0
6197	38531	Drosophila melanogaster	dyl	dusky-like	CG15013|DmelCG15013	0	0
6198	38532	Drosophila melanogaster	CG32250	CG32250 gene product from transcript CG32250-RA	BcDNA:RE36975|CG1326|DmelCG32250	0	0
6199	38533	Drosophila melanogaster	CG15014	CG15014 gene product from transcript CG15014-RA	DmelCG15014	0	0
6200	38534	Drosophila melanogaster	Cip4	CG15015 gene product from transcript CG15015-RG	CG11341|CG15015|DCIP4|DmelCG15015|cip4|dCIP4	0	0
6201	38535	Drosophila melanogaster	mRpS6	mitochondrial ribosomal protein S6	BcDNA:RH10862|CG15016|DmelCG15016|MRP-S6|Mrps6	0	0
6202	38538	Drosophila melanogaster	CG11342	CG11342 gene product from transcript CG11342-RA	DmelCG11342	0	0
6203	38539	Drosophila melanogaster	DopEcR	Dopamine/Ecdysteroid receptor	CG18314|DmDopEcR|DmelCG18314|anon-WO0170980.139|anon-WO0170980.140	0	0
6204	38540	Drosophila melanogaster	slow	slowdown	CG7447|CT22877|DmelCG7447|Slow|blr|slomo	0	0
6205	38541	Drosophila melanogaster	Syx17	Syntaxin 17	CG7452|Dm Syx17|DmSyx17|DmelCG7452|Syx	0	0
6206	38542	Drosophila melanogaster	CG15019	CG15019 gene product from transcript CG15019-RB	DmelCG15019	0	0
6207	38543	Drosophila melanogaster	DOR	CG11347 gene product from transcript CG11347-RE	CG11347|DmelCG11347|dDOR	0	0
6208	38544	Drosophila melanogaster	CG15020	CG15020 gene product from transcript CG15020-RA	DmelCG15020	0	0
6209	38545	Drosophila melanogaster	nAcRbeta-64B	nicotinic Acetylcholine Receptor beta 64B	AChR|AChR64B|ARD|AchR64B|Acr64B|AcrD|Ard|CG11348|CG11348-PA|CG12606|Dbeta1|DmelCG11348|ard|beta 64B|nAChR	0	0
6210	38546	Drosophila melanogaster	CG15021	CG15021 gene product from transcript CG15021-RA	CT34875|DmelCG15021	0	0
6211	38547	Drosophila melanogaster	CG12607	CG12607 gene product from transcript CG12607-RB	BcDNA:RE67729|DmelCG12607	0	0
6212	38548	Drosophila melanogaster	CG11345	CG11345 gene product from transcript CG11345-RA	DmelCG11345	0	0
6213	38549	Drosophila melanogaster	CG15022	CG15022 gene product from transcript CG15022-RA	BcDNA:RE24507|DmelCG15022	0	0

6214	38550	Drosophila melanogaster	CG15023	CG15023 gene product from transcript CG15023-RB	DmelCG15023	0	0
6215	38552	Drosophila melanogaster	CG11349	CG11349 gene product from transcript CG11349-RB	DmelCG11349	0	0
6216	38553	Drosophila melanogaster	CG7465	CG7465 gene product from transcript CG7465-RA	DmelCG7465	0	0
6217	38554	Drosophila melanogaster	CG11350	CG11350 gene product from transcript CG11350-RB	CG1135|DmelCG11350	0	0
6218	38555	Drosophila melanogaster	CG13722	CG13722 gene product from transcript CG13722-RA	DmelCG13722	0	0
6219	38556	Drosophila melanogaster	CG13721	CG13721 gene product from transcript CG13721-RA	DmelCG13721	0	0
6220	38557	Drosophila melanogaster	CG13720	CG13720 gene product from transcript CG13720-RA	DmelCG13720	0	0
6221	38558	Drosophila melanogaster	NT1	Neurotrophin 1	CG18318|CG32242|CG32244|CG42576|CT30097|DNT1|DmelCG42576|Dmel_CG32242|Dmel_CG32244|Spz2|spz2	0	0
6222	38559	Drosophila melanogaster	Tie	Tie-like receptor tyrosine kinase	CG7525|DPR1|DmelCG7525|RTK|tie	0	0
6223	38560	Drosophila melanogaster	CG11353	CG11353 gene product from transcript CG11353-RA	DmelCG11353|anon-WO0172774.162	0	0
6224	38561	Drosophila melanogaster	CG11357	CG11357 gene product from transcript CG11357-RD	BcDNA:GM04393|DmelCG11357	0	0
6225	38562	Drosophila melanogaster	CG42540	CG42540 gene product from transcript CG42540-RF	CG13718|CG13719|CG32245|CG7468|DmelCG42540|Dmel_CG13719|Dmel_CG32245	0	0
6226	38565	Drosophila melanogaster	Rpd3	CG7471 gene product from transcript CG7471-RA	CG7471|DHDAC1|DRpd3|DmHDAC1|DmelCG7471|E(var)3-64BC|HDAC|HDAC-1|HDAC1|Hdac1|RPD3|Rpd3/HDAC|Su(var)3-26|Su(var)326|Su(var)328|dHDAC-1|dHDAC1|dRPD3|dRpd3|dmHDA401|drpd3	0	0
6227	38567	Drosophila melanogaster	CG13716	CG13716 gene product from transcript CG13716-RA	DmelCG13716|HL1	0	0
6228	38571	Drosophila melanogaster	CG7514	CG7514 gene product from transcript CG7514-RA	DmelCG7514	0	0
6229	38572	Drosophila melanogaster	CG18418	CG18418 gene product from transcript CG18418-RA	DmelCG18418|anon-WO0140519.190	0	0
6230	38573	Drosophila melanogaster	CG15876	CG15876 gene product from transcript CG15876-RA	DmelCG15876|HL3	0	0
6231	38574	Drosophila melanogaster	CG13712	CG13712 gene product from transcript CG13712-RA	DmelCG13712|HL4	0	0
6232	38575	Drosophila melanogaster	CG12493	CG12493 gene product from transcript CG12493-RA	DmelCG12493|cg12493	0	0
6233	38576	Drosophila melanogaster	CG13711	CG13711 gene product from transcript CG13711-RA	DmelCG13711|HL5	0	0
6234	38578	Drosophila melanogaster	Gef64C	Guanine nucleotide exchange factor GEF64C	CG13709|CG13710|CG32239|DmelCG32239|EP(3)3035|EP3035|GEF|GEF(64C)|GEF64C|RhoGEF|RhoGEF64C	0	0
6235	38579	Drosophila melanogaster	CG7509	CG7509 gene product from transcript CG7509-RA	CT1509|DmelCG7509|tartan/capricious-like	0	0
6236	38580	Drosophila melanogaster	Dhc64C	Dynein heavy chain 64C	CD|CG7507|Cdhc|DHC|DHC64C|Dhc|Dhc46C|Dhc64|Dhc64c|DmelCG7507|Dynein|Fs(3)Lab|Fs(3)Laborc|Fs(3)Sz18|HMW MAP|Lab|Su(Gl)77|cDhc|cDhc64C|dhc|dhc64C|l(3)64Ca	0	0
6237	38581	Drosophila melanogaster	Aats-leu	Leucyl-tRNA synthetase	CG7479|DmelCG7479|LRS|LeuRS	0	0
6238	38582	Drosophila melanogaster	CG13708	CG13708 gene product from transcript CG13708-RA	CT33170|DmelCG13708	0	0
6239	38583	Drosophila melanogaster	CG13707	CG13707 gene product from transcript CG13707-RB	DmelCG13707	0	0
6240	38584	Drosophila melanogaster	CG32237	CG32237 gene product from transcript CG32237-RA	BcDNA:RE17910|CG13703|CG13706|DmelCG32237	0	0
6241	38586	Drosophila melanogaster	CG17150	CG17150 gene product from transcript CG17150-RD	DmelCG17150	0	0
6242	38587	Drosophila melanogaster	CG13705	CG13705 gene product from transcript CG13705-RA	CG13705-PA|DmelCG13705	0	0
6243	38588	Drosophila melanogaster	CG13704	CG13704 gene product from transcript CG13704-RA	BcDNA:RE40159|DmelCG13704	0	0
6244	38589	Drosophila melanogaster	Rh50	CG7499 gene product from transcript CG7499-RA	BcDNA:GH03016|CG7499|DmelCG7499|Rh-like	0	0
6245	38590	Drosophila melanogaster	Con	Connectin	CG7503|CON|CT1840|DmelCG7503|Ubx-t35|anon-64C|con|conn	0	0
6246	38591	Drosophila melanogaster	CG17030	CG17030 gene product from transcript CG17030-RA	DmelCG17030	0	0
6247	38592	Drosophila melanogaster	CG12027	CG12027 gene product from transcript CG12027-RA	DmelCG12027	0	0
6248	38593	Drosophila melanogaster	Srp54k	Signal recognition particle protein 54k	BcDNA.GM09489|BcDNA:GM09489|CG4659|DmelCG4659|Srp54|l(3)01418	0	0
6249	38594	Drosophila melanogaster	Gen	XPG-like endonuclease	BcDNA:GM10765|CG10670|DmGEN|DmelCG10670	0	0
6250	38595	Drosophila melanogaster	Aats-ala-m	mitochondrial alanyl-tRNA synthetase	AlaS|CG4633|Dm mt AlaRS|DmelCG4633	0	0
6251	38596	Drosophila melanogaster	CG10671	CG10671 gene product from transcript CG10671-RB	DmelCG10671|FIT2|i136	0	0
6252	38597	Drosophila melanogaster	CG4623	CG4623 gene product from transcript CG4623-RA	DmelCG4623|GDAP1	0	0
6253	38598	Drosophila melanogaster	CG10672	CG10672 gene product from transcript CG10672-RA	DmelCG10672	0	0
6254	38599	Drosophila melanogaster	CHMP2B	Charged multivesicular body protein 2b	CG4618|DmelCG4618|cg4618	0	0
6255	38600	Drosophila melanogaster	CG10673	CG10673 gene product from transcript CG10673-RA	DmelCG10673	0	0
6256	38601	Drosophila melanogaster	CG4611	CG4611 gene product from transcript CG4611-RA	DmelCG4611	0	0
6257	38602	Drosophila melanogaster	CG10674	CG10674 gene product from transcript CG10674-RA	DmelCG10674	0	0
6258	38603	Drosophila melanogaster	CG4603	CG4603 gene product from transcript CG4603-RA	DmelCG4603	0	0
6259	38604	Drosophila melanogaster	CG4597	CG4597 gene product from transcript CG4597-RA	DmelCG4597	0	0
6260	38605	Drosophila melanogaster	shep	alan shepard	BcDNA:LD40028|BcDNA:RH63980|CG10647|CG10649|CG10668|CG32423|DmelCG32423|alan|alan-shepard|anon-EST:Posey83|anon-WO0118547.198|anon-WO0172774.41|cg10668	0	0
6261	38608	Drosophila melanogaster	CG4669	CG4669 gene product from transcript CG4669-RA	DmelCG4669|anon-WO0140519.194	0	0
6262	38610	Drosophila melanogaster	lama	lamina ancestor	CG10645|DmelCG10645	0	0
6263	38611	Drosophila melanogaster	Klp64D	Kinesin-like protein at 64D	CG10642|DmKlp64D|DmelCG10642|KIF3A|KLP4|KLP64D|KLP64Ddm|Kif3A|Klp4|klp64D	0	0
6264	38612	Drosophila melanogaster	CG4769	CG4769 gene product from transcript CG4769-RA	DmelCG4769|anon-EST:Posey141	0	0
6265	38613	Drosophila melanogaster	Uev1A	CG10640 gene product from transcript CG10640-RA	CG10640|DmUEV|DmelCG10640|UEV|UEV1A|UEV1a|Uev1a|dUEV1a|dUev1A|uev1a	0	0
6266	38614	Drosophila melanogaster	membrin	CG4780 gene product from transcript CG4780-RA	BcDNA:SD22572|CG4780|DmelCG4780	0	0
6267	38615	Drosophila melanogaster	CG10635	CG10635 gene product from transcript CG10635-RA	BcDNA:SD11994|DmelCG10635	0	0
6268	38616	Drosophila melanogaster	Ir64a	Ionotropic receptor 64a	CG10633|CT29782|DmelCG10633|IR64a	0	0
6269	38619	Drosophila melanogaster	CG4835	CG4835 gene product from transcript CG4835-RA	DmelCG4835	0	0
6270	38620	Drosophila melanogaster	blanks	CG10630 gene product from transcript CG10630-RA	CG10630|DmelCG10630	0	0
6271	38622	Drosophila melanogaster	Lkr	Leucokinin receptor	CG10626|DLKR|DmelCG10626|LK-R|LKR|LkR|anon-WO0131005.17|anon-WO0170980.118|anon-WO0170980.119|dLKR|lkr	0	0
6272	38623	Drosophila melanogaster	CG13285	CG13285 gene product from transcript CG13285-RB	DmelCG13285	0	0
6273	38624	Drosophila melanogaster	CG10625	CG10625 gene product from transcript CG10625-RJ	DmelCG10625|anon-WO0118547.236	0	0
6274	38628	Drosophila melanogaster	bc10	CG4867 gene product from transcript CG4867-RA	CG4867|DmelCG4867|dbc10	0	0
6275	38629	Drosophila melanogaster	Msr-110	CG10596 gene product from transcript CG10596-RB	CG10596|DmelCG10596	0	0
6276	38630	Drosophila melanogaster	l(3)psg2	lethal (3) persistent salivary gland 2	CG5146|DmelCG5146|anon-EST:Liang-1.47|clone 1.47	0	0
6277	38631	Drosophila melanogaster	spo	spook	307a1|CG10594|CYP307A1|CYP307A1-Dm|Cyp307a1|DmelCG10594|Dmspo|Spo	0	0
6278	38632	Drosophila melanogaster	CG10592	CG10592 gene product from transcript CG10592-RA	DmelCG10592	0	0
6279	38633	Drosophila melanogaster	CG5150	CG5150 gene product from transcript CG5150-RA	DmelCG5150	0	0
6280	38634	Drosophila melanogaster	CG10591	CG10591 gene product from transcript CG10591-RA	DmelCG10591	0	0
6281	38635	Drosophila melanogaster	CG10590	CG10590 gene product from transcript CG10590-RA	DmelCG10590|TM9SF3	0	0
6282	38636	Drosophila melanogaster	mthl2	methuselah-like 2	CG17795|DmelCG17795|Mth-2|anon-WO0170980.28|anon-WO0170980.29	0	0
6283	38637	Drosophila melanogaster	Eaf6	CG12756 gene product from transcript CG12756-RA	CG12756|DmelCG12756|dEaf6	0	0
6284	38638	Drosophila melanogaster	Blimp-1	CG5249 gene product from transcript CG5249-RA	CG5249|DmelCG5249|blimp-1|dBlimp-1|dBlimp1	0	0
6285	38639	Drosophila melanogaster	lin-28	CG17334 gene product from transcript CG17334-RA	BcDNA:RE05342|CG17334|DmelCG17334|EP(3)0915	0	0
6286	38640	Drosophila melanogaster	Sse	Separase	CG10583|DmelCG10583|SSE|l(3)13m-281|l(3)SG14|sse	0	0
6287	38643	Drosophila melanogaster	DnaJ-1	DnaJ-like-1	CG10578|DNAJ-1|DNAJ1|DROJ1|DmelCG10578|DnaJ1 64EF|DroJ1|EU3500|HSP40|HSP40/HDJ1|Hdj1|Hsp-40|Hsp40|anon-WO0140519.166|anon-WO0172774.135|dHDJ1|dHDJ1/HSP40|dHdj1|dhdJ1|d	0	0
6288	38644	Drosophila melanogaster	Ubp64E	Ubiquitin-specific protease 64E	BcDNA:LD12764|CG5486|D-UBP-64E|D-Ubp-64E|DmelCG5486|E(var)1|E(var)3-64E|E-var(3)64E|UBP|Ubp64|ubp64|ubp64E	0	0
6289	38645	Drosophila melanogaster	CG10576	CG10576 gene product from transcript CG10576-RA	BEST:CK00496|DmelCG10576	0	0
6290	38646	Drosophila melanogaster	Txl	Thioredoxin-like	5495|CG5495|DmelCG5495|anon-WO0118547.248|txl	0	0
6291	38647	Drosophila melanogaster	Ppat-Dpck	Bifunctional Phosphopantetheine adenylyltransferase	CG10575|DPCK/PPAT|DmelCG10575|PPAT-DPCK|dPPAT-DPCK	0	0
6292	38648	Drosophila melanogaster	scny	scrawny	2331|CG5505|DmelCG5505|Usp36|anon-WO0118547.247|dUSP36|et|l(3)02331|mule	0	0
6293	38649	Drosophila melanogaster	Myt1	CG32417 gene product from transcript CG32417-RA	CG10569|CG32417|DmelCG32417|Dmyt1|MYT 1|MYT1|dMyt1|myt1	0	0
6294	38651	Drosophila melanogaster	Cralbp	Cellular retinaldehyde binding protein	CG10546|CRALBP|DmelCG10546	0	0
6295	38652	Drosophila melanogaster	Bre1	CG10542 gene product from transcript CG10542-RA	BRE1|Bre-1|CG10542|DmelCG10542|bre1|dBre1|l(3)01640	0	0
6296	38653	Drosophila melanogaster	Tektin-C	Tektin C	CG10541|DmelCG10541	0	0
6297	38654	Drosophila melanogaster	S6k	RPS6-p70-protein kinase	10539|7084|CG10539|DS6K|DmelCG10539|Dp70S6k|Dp70[s6k]|Dp70s6k|S6K|S6K1|dS6K|dS6k|dp70[S6k]|dp70s6k|dps6k|ds6k|fs(3)07084|l(3)07084|p70 S6K|p70/S6K|p70S6K|p70[S6 kinase]|p70[S6K]|p	0	0
6298	38655	Drosophila melanogaster	CG5537	CG5537 gene product from transcript CG5537-RA	DmelCG5537|anon-EST:Posey275|uprt	0	0
6299	38656	Drosophila melanogaster	mad2	CG17498 gene product from transcript CG17498-RA	CG17498|DmelCG17498|MAD2|Mad2	0	0
6300	38657	Drosophila melanogaster	vn	vein	CG10491|CT29452|Dm vn|DmelCG10491|P1749|Vein|Vn|ddd|l(3)10567|l(3)10568|l(3)L6A|l(3)ddd|l(3)rF264|l(3)vn10567|wvn	0	0
6301	38658	Drosophila melanogaster	CG5568	CG5568 gene product from transcript CG5568-RA	DmelCG5568	0	0
6302	38659	Drosophila melanogaster	CG18586	CG18586 gene product from transcript CG18586-RB	DmelCG18586	0	0
6303	38660	Drosophila melanogaster	CG13287	CG13287 gene product from transcript CG13287-RA	DmelCG13287	0	0
6304	38661	Drosophila melanogaster	Pole2	CG10489 gene product from transcript CG10489-RA	10489|CG10489|DNApol-epsilon|DmelCG10489|POLE2	0	0
6305	38662	Drosophila melanogaster	CG5592	CG5592 gene product from transcript CG5592-RA	DmelCG5592	0	0
6306	38663	Drosophila melanogaster	QC	Glutaminyl cyclase	CG10487|CG32412|DmelCG32412|DromeQC	0	0
6307	38664	Drosophila melanogaster	CG10486	CG10486 gene product from transcript CG10486-RC	DmelCG10486	0	0
6308	38665	Drosophila melanogaster	CG13288	CG13288 gene product from transcript CG13288-RB	DmelCG13288|dmCG13288	0	0
6309	38666	Drosophila melanogaster	CG10483	CG10483 gene product from transcript CG10483-RA	DmelCG10483	0	0
6310	38667	Drosophila melanogaster	CG32407	CG32407 gene product from transcript CG32407-RA	BcDNA:AT29938|CG10727|DmelCG32407	0	0
6311	38668	Drosophila melanogaster	CG33523	CG33523 gene product from transcript CG33523-RE	BcDNA:GH09028|CG10727|CG10977|CG32410|DmelCG33523	0	0
6312	38669	Drosophila melanogaster	Bj1	Bj1 protein	BJ1|CG10480|CG18640 (Bj1)|DmelCG10480|RCC1|RanGEF|Rcc1|dRCC1|inxs|sdt	0	0
6313	38674	Drosophila melanogaster	CG10479	CG10479 gene product from transcript CG10479-RA	DmelCG10479	0	0
6314	38675	Drosophila melanogaster	CG10478	CG10478 gene product from transcript CG10478-RA	CG11040|DmelCG10478|g7295403	0	0
6315	38677	Drosophila melanogaster	CG32406	CG32406 gene product from transcript CG32406-RA	CG13292|CG6062|DmelCG32406	0	0
6316	38679	Drosophila melanogaster	CG10477	CG10477 gene product from transcript CG10477-RA	DmelCG10477|SP51|anon-WO0140519.232	0	0
6317	38680	Drosophila melanogaster	yip7	yippee interacting protein 7	CG6457|DmelCG6457|MAC|SP116|SPH116|mac	0	0
6318	38681	Drosophila melanogaster	CG6462	CG6462 gene product from transcript CG6462-RA	DmelCG6462|SP127	0	0
6319	38682	Drosophila melanogaster	Jon65Aiv	Jonah 65Aiv	BEST:GH23808|CG6467|DmelCG6467|Jon65A|SP117	0	0
6320	38683	Drosophila melanogaster	Jon65Aiii	Jonah 65Aiii	CG6483|CG6484|DmelCG6483|Jon65A|SP98|anon-EST:GressD16|anon-WO0140519.59	0	0
6321	38684	Drosophila melanogaster	Jon65Aii	Jonah 65Aii	CG6580|DmelCG6580|Jon65A|SP153	0	0
6322	38685	Drosophila melanogaster	Jon65Ai	Jonah 65Ai	CG10475|CT29408|DmelCG10475|Jon65A|SP145	0	0
6323	38687	Drosophila melanogaster	CG6592	CG6592 gene product from transcript CG6592-RA	DmelCG6592|SP105	0	0
6324	38688	Drosophila melanogaster	CG10472	CG10472 gene product from transcript CG10472-RA	DmelCG10472|SP78|anon-WO0140519.101	0	0

<table>
<tr><td>6325</td><td>38689</td><td>Drosophila melanogaster</td><td>CG42269</td><td>CG42269 gene product from transcript CG42269-RE</td><td>CG6596|CG6600|DmelCG42269|Dmel_CG6596|Dmel_CG6600</td><td>0</td><td>0</td></tr>
<tr><td>6326</td><td>38691</td><td>Drosophila melanogaster</td><td>CG6602</td><td>CG6602 gene product from transcript CG6602-RA</td><td>DmelCG6602</td><td>0</td><td>0</td></tr>
<tr><td>6327</td><td>38692</td><td>Drosophila melanogaster</td><td>CG13295</td><td>CG13295 gene product from transcript CG13295-RA</td><td>DmelCG13295</td><td>0</td><td>0</td></tr>
<tr><td>6328</td><td>38693</td><td>Drosophila melanogaster</td><td>CG6610</td><td>CG6610 gene product from transcript CG6610-RA</td><td>BcDNA:RE60135|DmelCG6610</td><td>0</td><td>0</td></tr>
<tr><td>6329</td><td>38694</td><td>Drosophila melanogaster</td><td>CG6619</td><td>CG6619 gene product from transcript CG6619-RA</td><td>DmelCG6619</td><td>0</td><td>0</td></tr>
<tr><td>6330</td><td>38695</td><td>Drosophila melanogaster</td><td>CG13293</td><td>CG13293 gene product from transcript CG13293-RA</td><td>DmelCG13293</td><td>0</td><td>0</td></tr>
<tr><td>6331</td><td>38696</td><td>Drosophila melanogaster</td><td>CG10469</td><td>CG10469 gene product from transcript CG10469-RA</td><td>DmelCG10469|SP123</td><td>0</td><td>0</td></tr>
<tr><td>6332</td><td>38697</td><td>Drosophila melanogaster</td><td>CG10467</td><td>CG10467 gene product from transcript CG10467-RA</td><td>DmelCG10467</td><td>0</td><td>0</td></tr>
<tr><td>6333</td><td>38698</td><td>Drosophila melanogaster</td><td>loj</td><td>logjam</td><td>CG10733|DmelCG10733|l(3)04026|p24</td><td>0</td><td>0</td></tr>
<tr><td>6334</td><td>38700</td><td>Drosophila melanogaster</td><td>Ets65A</td><td>Ets at 65A</td><td>CG13294|CG6882|CG7018|D-Ets-3|D-ets-3|DmelCG7018|ETS3|Ets|Ets3|ets65A</td><td>0</td><td>0</td></tr>
<tr><td>6335</td><td>38701</td><td>Drosophila melanogaster</td><td>l(3)mbn</td><td>lethal (3) malignant blood neoplasm</td><td>CG12755|DmelCG12755|MBN|l(3)malignant blood neoplasm1|l(3)mbn-1|l(3)mbn1</td><td>0</td><td>0</td></tr>
<tr><td>6336</td><td>38702</td><td>Drosophila melanogaster</td><td>Lcp65Ag2</td><td>CG10534 gene product from transcript CG10534-RA</td><td>BcDNA:RE01644|CG10534|DCP8-beta|DmelCG10534|LCP8|L[[3]]CP8|Lcp-g2|Lcp8</td><td>0</td><td>0</td></tr>
<tr><td>6337</td><td>38703</td><td>Drosophila melanogaster</td><td>Lcp65Ag1</td><td>CG10530 gene product from transcript CG10530-RA</td><td>CG10530|DCP8-alpha|DmelCG10530|LCP8|L[[3]]CP8|Lcp-g1|Lcp8</td><td>0</td><td>0</td></tr>
<tr><td>6338</td><td>38706</td><td>Drosophila melanogaster</td><td>Cpr65Aw</td><td>Cuticular protein 65Aw</td><td>C32404|CG10461|CG32404|DmelCpr65Aw|DmelCG32404</td><td>0</td><td>0</td></tr>
<tr><td>6339</td><td>38707</td><td>Drosophila melanogaster</td><td>Lcp65Ad</td><td>CG6955 gene product from transcript CG6955-RA</td><td>BcDNA:LP05231|CG6955|DCP1|DMCLP65d|DmelLcp65Ad|DmelCG6955|Lcp-d</td><td>0</td><td>0</td></tr>
<tr><td>6340</td><td>38708</td><td>Drosophila melanogaster</td><td>Lcp65Ac</td><td>CG6956 gene product from transcript CG6956-RA</td><td>CG6956|DCP2|DMLCP65c|DmelCG6956|LCP65Ac|Lcp-c</td><td>0</td><td>0</td></tr>
<tr><td>6341</td><td>38709</td><td>Drosophila melanogaster</td><td>Lcp65Aa</td><td>CG7287 gene product from transcript CG7287-RA</td><td>CG7287|DCP4|DMLCP65a|DmelLCp65Aa|DmelCG7287|Lcp-a</td><td>0</td><td>0</td></tr>
<tr><td>6342</td><td>38710</td><td>Drosophila melanogaster</td><td>Acp65Aa</td><td>CG10297 gene product from transcript CG10297-RA</td><td>ACP65A|Acp65A|BcDNA:RH61147|CG10297|DCP5|DMACP65A|DmelAcp65Aa|DmelCG10297</td><td>0</td><td>0</td></tr>
<tr><td>6343</td><td>38711</td><td>Drosophila melanogaster</td><td>CG13297</td><td>CG13297 gene product from transcript CG13297-RA</td><td>BcDNA:RE64858|DmelCG13297</td><td>0</td><td>0</td></tr>
<tr><td>6344</td><td>38712</td><td>Drosophila melanogaster</td><td>Cpr65Az</td><td>Cuticular protein 65Az</td><td>CG12330|DmelCpr65Az|DmelCG12330</td><td>0</td><td>0</td></tr>
<tr><td>6345</td><td>38713</td><td>Drosophila melanogaster</td><td>CG13296</td><td>CG13296 gene product from transcript CG13296-RA</td><td>DmelCG13296</td><td>0</td><td>0</td></tr>
<tr><td>6346</td><td>38714</td><td>Drosophila melanogaster</td><td>CG10289</td><td>CG10289 gene product from transcript CG10289-RA</td><td>DmelCG10289</td><td>0</td><td>0</td></tr>
<tr><td>6347</td><td>38715</td><td>Drosophila melanogaster</td><td>CG7376</td><td>CG7376 gene product from transcript CG7376-RA</td><td>DmelCG7376|SHPRH</td><td>0</td><td>0</td></tr>
<tr><td>6348</td><td>38716</td><td>Drosophila melanogaster</td><td>CG10274</td><td>CG10274 gene product from transcript CG10274-RA</td><td>DmelCG10274</td><td>0</td><td>0</td></tr>
<tr><td>6349</td><td>38717</td><td>Drosophila melanogaster</td><td>D19B</td><td>CG10270 gene product from transcript CG10270-RA</td><td>CG10270|DmelCG10270</td><td>0</td><td>0</td></tr>
<tr><td>6350</td><td>38718</td><td>Drosophila melanogaster</td><td>D19A</td><td>CG10269 gene product from transcript CG10269-RA</td><td>CG10269|DmelCG10269</td><td>0</td><td>0</td></tr>
<tr><td>6351</td><td>38719</td><td>Drosophila melanogaster</td><td>CG7386</td><td>CG7386 gene product from transcript CG7386-RA</td><td>DmelCG7386</td><td>0</td><td>0</td></tr>
<tr><td>6352</td><td>38720</td><td>Drosophila melanogaster</td><td>CG13298</td><td>CG13298 gene product from transcript CG13298-RA</td><td>DmelCG13298|cg13298</td><td>0</td><td>0</td></tr>
<tr><td>6353</td><td>38721</td><td>Drosophila melanogaster</td><td>Trn</td><td>Transportin</td><td>CG7398|DmelCG7398|IMPbeta2|TRN|Tm|Trn1|dTRN</td><td>0</td><td>0</td></tr>
<tr><td>6354</td><td>38722</td><td>Drosophila melanogaster</td><td>CG8219</td><td>CG8219 gene product from transcript CG8219-RA</td><td>148274_at|DmelCG8219|EP(3)3072</td><td>0</td><td>0</td></tr>
<tr><td>6355</td><td>38723</td><td>Drosophila melanogaster</td><td>LanA</td><td>Laminin A</td><td>5]]|CG10236|CT28769|DmelCG10236|FBgn0002526|LM-A/alpha1|Lam|Lam A|Lam-A|LamA|Lan|alpha3/5|alpha[[3|hdln|lamA|laminin alpha3/5|lanA</td><td>0</td><td>0</td></tr>
<tr><td>6356</td><td>38724</td><td>Drosophila melanogaster</td><td>CG13299</td><td>CG13299 gene product from transcript CG13299-RA</td><td>DmelCG13299</td><td>0</td><td>0</td></tr>
<tr><td>6357</td><td>38725</td><td>Drosophila melanogaster</td><td>CG10226</td><td>CG10226 gene product from transcript CG10226-RA</td><td>AMT|DmelCG10226</td><td>0</td><td>0</td></tr>
<tr><td>6358</td><td>38726</td><td>Drosophila melanogaster</td><td>Mdr65</td><td>Multiple drug resistance 65</td><td>CG10181|DmelCG10181|MDR65|Mdr65A|mdr65</td><td>0</td><td>0</td></tr>
<tr><td>6359</td><td>38727</td><td>Drosophila melanogaster</td><td>Best2</td><td>Bestrophin 2</td><td>CG10173|DmelCG10173|dbest2</td><td>0</td><td>0</td></tr>
<tr><td>6360</td><td>38728</td><td>Drosophila melanogaster</td><td>CG10163</td><td>CG10163 gene product from transcript CG10163-RA</td><td>DmelCG10163</td><td>0</td><td>0</td></tr>
<tr><td>6361</td><td>38730</td><td>Drosophila melanogaster</td><td>CG13300</td><td>CG13300 gene product from transcript CG13300-RA</td><td>DmelCG13300</td><td>0</td><td>0</td></tr>
<tr><td>6362</td><td>38732</td><td>Drosophila melanogaster</td><td>CG42747</td><td>CG42747 gene product from transcript CG42747-RC</td><td>CG14910|CG14911|DmelCG42747|Dmel_CG14910|Dmel_CG14911</td><td>0</td><td>0</td></tr>
<tr><td>6363</td><td>38733</td><td>Drosophila melanogaster</td><td>CG10147</td><td>CG10147 gene product from transcript CG10147-RA</td><td>DmelCG10147</td><td>0</td><td>0</td></tr>
<tr><td>6364</td><td>38734</td><td>Drosophila melanogaster</td><td>CG8270</td><td>CG8270 gene product from transcript CG8270-RB</td><td>DmelCG8270</td><td>0</td><td>0</td></tr>
<tr><td>6365</td><td>38735</td><td>Drosophila melanogaster</td><td>CG10144</td><td>CG10144 gene product from transcript CG10144-RA</td><td>DmelCG10144|anon-WO0118547.365</td><td>0</td><td>0</td></tr>
<tr><td>6366</td><td>38736</td><td>Drosophila melanogaster</td><td>sfl</td><td>sulfateless</td><td>CG8339|CT24567|DmelCG8339|NDST|dw|l(3)03844</td><td>0</td><td>0</td></tr>
<tr><td>6367</td><td>38738</td><td>Drosophila melanogaster</td><td>ndl</td><td>nudel</td><td>CG10129|DmelCG10129|Ndl|SP89|mat(3)2|mel(3)5|nud</td><td>0</td><td>0</td></tr>
<tr><td>6368</td><td>38740</td><td>Drosophila melanogaster</td><td>CG8368</td><td>CG8368 gene product from transcript CG8368-RB</td><td>DmelCG8368</td><td>0</td><td>0</td></tr>
<tr><td>6369</td><td>38741</td><td>Drosophila melanogaster</td><td>CG8398</td><td>CG8398 gene product from transcript CG8398-RA</td><td>DmelCG8398|anon-EST:Posey31</td><td>0</td><td>0</td></tr>
<tr><td>6370</td><td>38742</td><td>Drosophila melanogaster</td><td>Glu-RI</td><td>Glutamate receptor I</td><td>CG8442|DGluR-I|DGluR-IA|DGluRI|DmelCG8442|Glu-R1|dGluRI|dglur-IA</td><td>0</td><td>0</td></tr>
<tr><td>6371</td><td>38743</td><td>Drosophila melanogaster</td><td>eIF4E-4</td><td>CG10124 gene product from transcript CG10124-RA</td><td>BEST:GH01027|CG10124|CT28485|DmelCG10124|NEST:bs04e06|eIF4E</td><td>0</td><td>0</td></tr>
<tr><td>6372</td><td>38744</td><td>Drosophila melanogaster</td><td>SP1173</td><td>CG10121 gene product from transcript CG10121-RC</td><td>CG10121|DmelCG10121</td><td>0</td><td>0</td></tr>
<tr><td>6373</td><td>38745</td><td>Drosophila melanogaster</td><td>CG8519</td><td>CG8519 gene product from transcript CG8519-RA</td><td>DmelCG8519</td><td>0</td><td>0</td></tr>
<tr><td>6374</td><td>38746</td><td>Drosophila melanogaster</td><td>ple</td><td>pale</td><td>CG10118|DH65B|DTH|DmelCG10118|Pale|TH|Th|VII|dTH1|dTH65B|pale/ple</td><td>0</td><td>0</td></tr>
<tr><td>6375</td><td>38747</td><td>Drosophila melanogaster</td><td>dikar</td><td>CG42799 gene product from transcript CG42799-RC</td><td>CG10115|CG32393|CG32394|CG42799|DmelCG42799|Dmel_CG32393|Dmel_CG32394</td><td>0</td><td>0</td></tr>
<tr><td>6376</td><td>38748</td><td>Drosophila melanogaster</td><td>CG10107</td><td>CG10107 gene product from transcript CG10107-RA</td><td>DmelCG10107</td><td>0</td><td>0</td></tr>
<tr><td>6377</td><td>38749</td><td>Drosophila melanogaster</td><td>CG8549</td><td>CG8549 gene product from transcript CG8549-RA</td><td>DmelCG8549</td><td>0</td><td>0</td></tr>
<tr><td>6378</td><td>38750</td><td>Drosophila melanogaster</td><td>CG10103</td><td>CG10103 gene product from transcript CG10103-RA</td><td>DmelCG10103</td><td>0</td><td>0</td></tr>
<tr><td>6379</td><td>38751</td><td>Drosophila melanogaster</td><td>CG32392</td><td>CG32392 gene product from transcript CG32392-RB</td><td>CG10099|CG14912|DmelCG32392</td><td>0</td><td>0</td></tr>
<tr><td>6380</td><td>38752</td><td>Drosophila melanogaster</td><td>vvl</td><td>ventral veins lacking</td><td>CG10037|Cf1-a|Cf1a|Cf1a/drifter|Cfla|DFR|Dfr/Vvl|DmelCG10037|Drf|VVL|Vv1|dfr|dfr-vvl|dfr/vvl|drf|prd3|sep|ut|vvl/dfr|vvl/dfr/ut</td><td>0</td><td>0</td></tr>
<tr><td>6381</td><td>38753</td><td>Drosophila melanogaster</td><td>Prat2</td><td>Phosphoribosylamidotransferase 2</td><td>CG10078|DmelCG10078</td><td>0</td><td>0</td></tr>
<tr><td>6382</td><td>38754</td><td>Drosophila melanogaster</td><td>CG14820</td><td>CG14820 gene product from transcript CG14820-RA</td><td>DmelCG14820</td><td>0</td><td>0</td></tr>
<tr><td>6383</td><td>38755</td><td>Drosophila melanogaster</td><td>tow</td><td>target of wingless</td><td>CG14821|DmelCG14821</td><td>0</td><td>0</td></tr>
<tr><td>6384</td><td>38756</td><td>Drosophila melanogaster</td><td>CG10077</td><td>CG10077 gene product from transcript CG10077-RA</td><td>BcDNA:HL01868|BcDNA:HL07910|DmRH5|DmelCG10077|cg10077|p90</td><td>0</td><td>0</td></tr>
<tr><td>6385</td><td>38757</td><td>Drosophila melanogaster</td><td>CG9948</td><td>CG9948 gene product from transcript CG9948-RA</td><td>DmelCG9948</td><td>0</td><td>0</td></tr>
<tr><td>6386</td><td>38758</td><td>Drosophila melanogaster</td><td>CG10075</td><td>CG10075 gene product from transcript CG10075-RA</td><td>DmelCG10075</td><td>0</td><td>0</td></tr>
<tr><td>6387</td><td>38760</td><td>Drosophila melanogaster</td><td>sgl</td><td>sugarless</td><td>8310|BG01214|CG10072|DmelCG10072|UDP-GlcDH|UDPGDH|XIII|kiwi|l(3)05007|l(3)08310|l(3)10503|l(3)65CEd|l(3)SG9|ska|sl</td><td>0</td><td>0</td></tr>
<tr><td>6388</td><td>38761</td><td>Drosophila melanogaster</td><td>CG10064</td><td>CG10064 gene product from transcript CG10064-RA</td><td>DmelCG10064</td><td>0</td><td>0</td></tr>
<tr><td>6389</td><td>38762</td><td>Drosophila melanogaster</td><td>Mis12</td><td>CG18156 gene product from transcript CG18156-RA</td><td>BcDNA:RE19545|CG18156|DmelCG18156|Mis-12|dmMis12</td><td>0</td><td>0</td></tr>
<tr><td>6390</td><td>38763</td><td>Drosophila melanogaster</td><td>CG9953</td><td>CG9953 gene product from transcript CG9953-RA</td><td>DmelCG9953</td><td>0</td><td>0</td></tr>
<tr><td>6391</td><td>38764</td><td>Drosophila melanogaster</td><td>CG10063</td><td>CG10063 gene product from transcript CG10063-RA</td><td>BcDNA:AT30027|DmelCG10063</td><td>0</td><td>0</td></tr>
<tr><td>6392</td><td>38765</td><td>Drosophila melanogaster</td><td>G-ialpha65A</td><td>G protein alphai subunit 65A</td><td>CG10060|DG[[alpha1]]|DGalpha1|DmelCG10060|G-alpha-65A|G-ia65A|G-ialpha65a|G-oalpha65A|G[[alphai]]|G[[i]]alpha|Gai|Galpha i|Galpha65A|Galpha[[i65A]]|Galpha[[i]]|Galphai|Galphaiota65A|Gi|Gi-alpha</td><td>0</td><td>0</td></tr>
<tr><td>6393</td><td>38766</td><td>Drosophila melanogaster</td><td>bin</td><td>biniou</td><td>Bin|CG18647|DmFoxF|Dmbin|DmelCG18647|FOXF|FoxF|XIV|binI|l(3)65CEe</td><td>0</td><td>0</td></tr>
<tr><td>6394</td><td>38768</td><td>Drosophila melanogaster</td><td>CG17744</td><td>CG17744 gene product from transcript CG17744-RA</td><td>DmelCG17744</td><td>0</td><td>0</td></tr>
<tr><td>6395</td><td>38769</td><td>Drosophila melanogaster</td><td>mp</td><td>multiplexin</td><td>CG14822|CG33171|CG42543|CG8645|CG8647|CT14872|DmelCG42543|Dmel_CG33171|Dmel_CG8647|Dmp</td><td>0</td><td>0</td></tr>
<tr><td>6396</td><td>38771</td><td>Drosophila melanogaster</td><td>CG8641</td><td>CG8641 gene product from transcript CG8641-RA</td><td>DmelCG8641</td><td>0</td><td>0</td></tr>
<tr><td>6397</td><td>38772</td><td>Drosophila melanogaster</td><td>CG14823</td><td>CG14823 gene product from transcript CG14823-RD</td><td>DmelCG14823</td><td>0</td><td>0</td></tr>
<tr><td>6398</td><td>38773</td><td>Drosophila melanogaster</td><td>Cpr65Ea</td><td>Cuticular protein 65Ea</td><td>CG8640|DmelCpr65Ea|DmelCG8640</td><td>0</td><td>0</td></tr>
<tr><td>6399</td><td>38774</td><td>Drosophila melanogaster</td><td>Cpr65Eb</td><td>Cuticular protein 65Eb</td><td>BcDNA:RE28679|CG8638|DmelCpr65Eb|DmelCG8638</td><td>0</td><td>0</td></tr>
<tr><td>6400</td><td>38775</td><td>Drosophila melanogaster</td><td>Cpr65Ec</td><td>Cuticular protein 65Ec</td><td>CG8634|DmelCpr65Ec|DmelCG8634</td><td>0</td><td>0</td></tr>
<tr><td>6401</td><td>38779</td><td>Drosophila melanogaster</td><td>msl-3</td><td>male-specific lethal 3</td><td>CG8631|DmMsl-3|DmelCG8631|MSL|MSL-3|MSL3|Msl 1-3|Msl3|mle(3)132|mle3|mls3|msl|msl3</td><td>0</td><td>0</td></tr>
<tr><td>6402</td><td>38780</td><td>Drosophila melanogaster</td><td>BBS1</td><td>CG14825 gene product from transcript CG14825-RA</td><td>CG14825|DmelCG14825</td><td>0</td><td>0</td></tr>
<tr><td>6403</td><td>38781</td><td>Drosophila melanogaster</td><td>CG8629</td><td>CG8629 gene product from transcript CG8629-RA</td><td>BcDNA:RE33457|DmelCG8629</td><td>0</td><td>0</td></tr>
<tr><td>6404</td><td>38782</td><td>Drosophila melanogaster</td><td>CG15829</td><td>CG15829 gene product from transcript CG15829-RA</td><td>DmelCG15829</td><td>0</td><td>0</td></tr>
<tr><td>6405</td><td>38783</td><td>Drosophila melanogaster</td><td>CG8628</td><td>CG8628 gene product from transcript CG8628-RA</td><td>DmelCG8628|anon-SAGE:Wang-073</td><td>0</td><td>0</td></tr>
<tr><td>6406</td><td>38784</td><td>Drosophila melanogaster</td><td>Dbi</td><td>Diazepam-binding inhibitor</td><td>ACBP|BcDNA:RH39533|CG8627|DBI|DmelCG8627|anon-WO0172774.131</td><td>0</td><td>0</td></tr>
<tr><td>6407</td><td>38785</td><td>Drosophila melanogaster</td><td>melt</td><td>melted</td><td>1441/14|CG8624|DmelCG8624|anon-WO03014152.10|dMelt</td><td>0</td><td>0</td></tr>
<tr><td>6408</td><td>38786</td><td>Drosophila melanogaster</td><td>corn</td><td>cornetto</td><td>CG32386|CG32390|CG42278|CG8621|DmelCG42278|Dmel_CG32386|Dmel_CG32390</td><td>0</td><td>0</td></tr>
<tr><td>6409</td><td>38788</td><td>Drosophila melanogaster</td><td>Dscam2</td><td>Down syndrome cell adhesion molecule 2</td><td>BcDNA:AT03867|CG14828|CG17742|CG32387|CG32389|CG42256|CG8618|CG8619|CT14580|CT14584|CT34644|DmelCG42256|Dmel_CG32387|Dmel_CG32389|Dscam-like</td><td>0</td><td>0</td></tr>
<tr><td>6410</td><td>38791</td><td>Drosophila melanogaster</td><td>CG14826</td><td>CG14826 gene product from transcript CG14826-RB</td><td>DmelCG14826</td><td>0</td><td>0</td></tr>
<tr><td>6411</td><td>38792</td><td>Drosophila melanogaster</td><td>CG14829</td><td>CG14829 gene product from transcript CG14829-RB</td><td>DmelCG14829</td><td>0</td><td>0</td></tr>
<tr><td>6412</td><td>38793</td><td>Drosophila melanogaster</td><td>BHD</td><td>Birt-Hogg-Dube homolog</td><td>CG8616|DBHD|DmelCG8616</td><td>0</td><td>0</td></tr>
<tr><td>6413</td><td>38794</td><td>Drosophila melanogaster</td><td>RpL18</td><td>Ribosomal protein L18</td><td>CG8615|DmelCG8615|M(3)65F|RpL18e</td><td>0</td><td>0</td></tr>
<tr><td>6414</td><td>38795</td><td>Drosophila melanogaster</td><td>Neos</td><td>Neosin</td><td>BcDNA.HL05936|BcDNA:HL05936|CG8614|DmelCG8614|l(3)L2249</td><td>0</td><td>0</td></tr>
<tr><td>6415</td><td>38796</td><td>Drosophila melanogaster</td><td>mRpL50</td><td>mitochondrial ribosomal protein L50</td><td>ANON-65Eb|BcDNA:GM06185|BcDNA:RE06140|CG8612|DmelCG8612|MRP-L50|anon-65Eb</td><td>0</td><td>0</td></tr>
<tr><td>6416</td><td>38797</td><td>Drosophila melanogaster</td><td>mei-P22</td><td>meiotic P22</td><td>CG14827|DmelCG14827|MEI-P22|Mei-P22</td><td>0</td><td>0</td></tr>
<tr><td>6417</td><td>38798</td><td>Drosophila melanogaster</td><td>Cdc27</td><td>CG8610 gene product from transcript CG8610-RA</td><td>APC3|Apc3|CDC27|CDC27Dm|CG8610|Dmcdc27|DmelCG8610|cdc27|l(3)L7123|mks</td><td>0</td><td>0</td></tr>
<tr><td>6418</td><td>38799</td><td>Drosophila melanogaster</td><td>MED4</td><td>Mediator complex subunit 4</td><td>CG8609|DmelCG8609|Med4|Med4/TRAP36|Trap36|dTRAP36|dp34|p34</td><td>0</td><td>0</td></tr>
<tr><td>6419</td><td>38800</td><td>Drosophila melanogaster</td><td>CG14830</td><td>CG14830 gene product from transcript CG14830-RC</td><td>DmelCG14830</td><td>0</td><td>0</td></tr>
<tr><td>6420</td><td>38801</td><td>Drosophila melanogaster</td><td>unc-13-4A</td><td>CG32381 gene product from transcript CG32381-RB</td><td>CG14831|CG14832|CG14833|CG32381|CG8608|DmelCG32381|dunc-13-4A</td><td>0</td><td>0</td></tr>
<tr><td>6421</td><td>38805</td><td>Drosophila melanogaster</td><td>CG8607</td><td>CG8607 gene product from transcript CG8607-RA</td><td>DmelCG8607</td><td>0</td><td>0</td></tr>
<tr><td>6422</td><td>38806</td><td>Drosophila melanogaster</td><td>RhoGEF4</td><td>CG8606 gene product from transcript CG8606-RA</td><td>CG8606|DRhoGEF4|DmelCG8606</td><td>0</td><td>0</td></tr>
<tr><td>6423</td><td>38807</td><td>Drosophila melanogaster</td><td>CG8605</td><td>CG8605 gene product from transcript CG8605-RA</td><td>DmelCG8605</td><td>0</td><td>0</td></tr>
<tr><td>6424</td><td>38808</td><td>Drosophila melanogaster</td><td>CG8602</td><td>CG8602 gene product from transcript CG8602-RA</td><td>DmelCG8602|anon-WO0118547.349|anon-WO0118547.382</td><td>0</td><td>0</td></tr>
<tr><td>6425</td><td>38809</td><td>Drosophila melanogaster</td><td>mus312</td><td>mutagen-sensitive 312</td><td>CG8601|DmelCG8601|mus(3)312</td><td>0</td><td>0</td></tr>
<tr><td>6426</td><td>38810</td><td>Drosophila melanogaster</td><td>CG8600</td><td>CG8600 gene product from transcript CG8600-RB</td><td>DmelCG8600</td><td>0</td><td>0</td></tr>
<tr><td>6427</td><td>38811</td><td>Drosophila melanogaster</td><td>lark</td><td>CG8597 gene product from transcript CG8597-RD</td><td>CG8597|DmelCG8597|LARK|Lark|dLARK</td><td>0</td><td>0</td></tr>
<tr><td>6428</td><td>38812</td><td>Drosophila melanogaster</td><td>eco</td><td>CG8598 gene product from transcript CG8598-RA</td><td>CG8598|Deco|DmelCG8598|deco</td><td>0</td><td>0</td></tr>
<tr><td>6429</td><td>38813</td><td>Drosophila melanogaster</td><td>CG8596</td><td>CG8596 gene product from transcript CG8596-RA</td><td>DmelCG8596</td><td>0</td><td>0</td></tr>
<tr><td>6430</td><td>38814</td><td>Drosophila melanogaster</td><td>CG14834</td><td>CG14834 gene product from transcript CG14834-RA</td><td>CT34650|DmelCG14834</td><td>0</td><td>0</td></tr>
<tr><td>6431</td><td>38815</td><td>Drosophila melanogaster</td><td>Srp19</td><td>Signal recognition particle protein 19</td><td>CG4457|DmelCG4457|SRP19|srp19</td><td>0</td><td>0</td></tr>
<tr><td>6432</td><td>38816</td><td>Drosophila melanogaster</td><td>qm</td><td>quemao</td><td>CG8593|DmelCG8593|GGPPS|GGPPS/qm|bro2</td><td>0</td><td>0</td></tr>
<tr><td>6433</td><td>38817</td><td>Drosophila melanogaster</td><td>CTCF</td><td>CG8591 gene product from transcript CG8591-RA</td><td>CG8591|DmelCG8591|dCTCF</td><td>0</td><td>0</td></tr>
<tr><td>6434</td><td>38818</td><td>Drosophila melanogaster</td><td>pst</td><td>pastrel</td><td>CG8588|Dm-65|DmelCG8588</td><td>0</td><td>0</td></tr>
<tr><td>6435</td><td>38819</td><td>Drosophila melanogaster</td><td>sec63</td><td>CG8583 gene product from transcript CG8583-RA</td><td>CG8583|DmelCG8583|anon-WO0118547.333|peg</td><td>0</td><td>0</td></tr>
</table>

<table>
<tr><td>6436</td><td>38820</td><td>Drosophila melanogaster</td><td>Sh3beta</td><td>CG8582 gene product from transcript CG8582-RD</td><td>CG8582|DmelCG8582|LD12912|SH3beta</td><td>0</td><td>0</td></tr>
<tr><td>6437</td><td>38821</td><td>Drosophila melanogaster</td><td>akirin</td><td>CG8580 gene product from transcript CG8580-RD</td><td>Akirin|CG8580|DmAkirin|DmelCG8580|EP(3)0906|anon-WO0118547.397|bhr</td><td>0</td><td>0</td></tr>
<tr><td>6438</td><td>38823</td><td>Drosophila melanogaster</td><td>SMSr</td><td>CG32380 gene product from transcript CG32380-RA</td><td>CG32380|CG8572|CG8576|Css3beta|DmelCG32380|dSMSr|dmSMSr</td><td>0</td><td>0</td></tr>
<tr><td>6439</td><td>38824</td><td>Drosophila melanogaster</td><td>smid</td><td>smallminded</td><td>CG8571|DmelCG8571|Smid|l(3)j6B8|l(3)j8A6|l(3)s2898</td><td>0</td><td>0</td></tr>
<tr><td>6440</td><td>38825</td><td>Drosophila melanogaster</td><td>CG8564</td><td>CG8564 gene product from transcript CG8564-RA</td><td>DmelCG8564</td><td>0</td><td>0</td></tr>
<tr><td>6441</td><td>38826</td><td>Drosophila melanogaster</td><td>CG8563</td><td>CG8563 gene product from transcript CG8563-RA</td><td>DmelCG8563</td><td>0</td><td>0</td></tr>
<tr><td>6442</td><td>38828</td><td>Drosophila melanogaster</td><td>CG8562</td><td>CG8562 gene product from transcript CG8562-RA</td><td>CT15463|DmelCG8562</td><td>0</td><td>0</td></tr>
<tr><td>6443</td><td>38829</td><td>Drosophila melanogaster</td><td>CG18417</td><td>CG18417 gene product from transcript CG18417-RA</td><td>DmelCG18417</td><td>0</td><td>0</td></tr>
<tr><td>6444</td><td>38830</td><td>Drosophila melanogaster</td><td>CG8560</td><td>CG8560 gene product from transcript CG8560-RA</td><td>CT14378|DmelCG8560</td><td>0</td><td>0</td></tr>
<tr><td>6445</td><td>38831</td><td>Drosophila melanogaster</td><td>Rac2</td><td>CG8556 gene product from transcript CG8556-RA</td><td>CG8556|D-Rac 2|D-Rac2|DRac2|DRacB|DmRAC2|DmelCG8556|Drac1b|Drac2|Rac|Rac GTPase|Rac-2|Rac1b|RacB|rac2</td><td>0</td><td>0</td></tr>
<tr><td>6446</td><td>38832</td><td>Drosophila melanogaster</td><td>CG14835</td><td>CG14835 gene product from transcript CG14835-RA</td><td>DmelCG14835</td><td>0</td><td>0</td></tr>
<tr><td>6447</td><td>38833</td><td>Drosophila melanogaster</td><td>sphinx2</td><td>CG32382 gene product from transcript CG32382-RB</td><td>CG32382|CG8555|DmelCG32382|SPH194|Sphinx2</td><td>0</td><td>0</td></tr>
<tr><td>6448</td><td>38835</td><td>Drosophila melanogaster</td><td>CG8546</td><td>CG8546 gene product from transcript CG8546-RB</td><td>DmelCG8546</td><td>0</td><td>0</td></tr>
<tr><td>6449</td><td>38836</td><td>Drosophila melanogaster</td><td>Tsp66A</td><td>Tetraspanin 66A</td><td>CG1699|CG16991|Dm.Tsp66A|DmelCG16991</td><td>0</td><td>0</td></tr>
<tr><td>6450</td><td>38837</td><td>Drosophila melanogaster</td><td>CG8543</td><td>CG8543 gene product from transcript CG8543-RA</td><td>DmelCG8543</td><td>0</td><td>0</td></tr>
<tr><td>6451</td><td>38838</td><td>Drosophila melanogaster</td><td>CG8541</td><td>CG8541 gene product from transcript CG8541-RA</td><td>DmelCG8541</td><td>0</td><td>0</td></tr>
<tr><td>6452</td><td>38839</td><td>Drosophila melanogaster</td><td>mthl6</td><td>methuselah-like 6</td><td>CG16992|DmelCG16992|anon-WO0170980.127|anon-WO0170980.128</td><td>0</td><td>0</td></tr>
<tr><td>6453</td><td>38840</td><td>Drosophila melanogaster</td><td>Cyp316a1</td><td>CG8540 gene product from transcript CG8540-RA</td><td>316a1|CG8540|DmelCG8540|cyp316a1</td><td>0</td><td>0</td></tr>
<tr><td>6454</td><td>38841</td><td>Drosophila melanogaster</td><td>Cyp4d8</td><td>Cytochrome P450-4d8</td><td>4d8|CG4321|DmelCG4321|P-450|P450</td><td>0</td><td>0</td></tr>
<tr><td>6455</td><td>38842</td><td>Drosophila melanogaster</td><td>CG8539</td><td>CG8539 gene product from transcript CG8539-RA</td><td>DmelCG8539</td><td>0</td><td>0</td></tr>
<tr><td>6456</td><td>38843</td><td>Drosophila melanogaster</td><td>CG7548</td><td>CG7548 gene product from transcript CG7548-RA</td><td>DmelCG7548</td><td>0</td><td>0</td></tr>
<tr><td>6457</td><td>38844</td><td>Drosophila melanogaster</td><td>CG7546</td><td>CG7546 gene product from transcript CG7546-RD</td><td>DmelCG7546|anon-EST:Liang-2.45|clone 2.45</td><td>0</td><td>0</td></tr>
<tr><td>6458</td><td>38845</td><td>Drosophila melanogaster</td><td>ltl</td><td>larval translucida</td><td>CG14836|CG32372|CG7545|CT15760|DmelCG32372|F|anon-EST:CL1a11</td><td>0</td><td>0</td></tr>
<tr><td>6459</td><td>38846</td><td>Drosophila melanogaster</td><td>lqf</td><td>liquid facets</td><td>0110/27|CG8532|DmelCG8532|E(faf)bE25|Epsin|LqF|Lqf|anon-WO0118547.239|dEpsin|epsin|l(3)S011027</td><td>0</td><td>0</td></tr>
<tr><td>6460</td><td>38847</td><td>Drosophila melanogaster</td><td>CG16998</td><td>CG16998 gene product from transcript CG16998-RA</td><td>DmelCG16998|SP104</td><td>0</td><td>0</td></tr>
<tr><td>6461</td><td>38848</td><td>Drosophila melanogaster</td><td>CG32374</td><td>CG32374 gene product from transcript CG32374-RA</td><td>CG8528|DmelCG32374|SP148</td><td>0</td><td>0</td></tr>
<tr><td>6462</td><td>38849</td><td>Drosophila melanogaster</td><td>CG14837</td><td>CG14837 gene product from transcript CG14837-RC</td><td>DmelCG14837</td><td>0</td><td>0</td></tr>
<tr><td>6463</td><td>38850</td><td>Drosophila melanogaster</td><td>CG7526</td><td>CG7526 gene product from transcript CG7526-RC</td><td>CT16044|DmelCG7526</td><td>0</td><td>0</td></tr>
<tr><td>6464</td><td>38851</td><td>Drosophila melanogaster</td><td>CG14838</td><td>CG14838 gene product from transcript CG14838-RA</td><td>DmelCG14838</td><td>0</td><td>0</td></tr>
<tr><td>6465</td><td>38852</td><td>Drosophila melanogaster</td><td>CG7716</td><td>CG7716 gene product from transcript CG7716-RA</td><td>DmelCG7716</td><td>0</td><td>0</td></tr>
<tr><td>6466</td><td>38857</td><td>Drosophila melanogaster</td><td>CG7506</td><td>CG7506 gene product from transcript CG7506-RA</td><td>DmelCG7506</td><td>0</td><td>0</td></tr>
<tr><td>6467</td><td>38858</td><td>Drosophila melanogaster</td><td>PGRP-SD</td><td>CG7496 gene product from transcript CG7496-RA</td><td>CG7496|Dm PGRP-SD|DmelCG7496|pgrp-sd</td><td>0</td><td>0</td></tr>
<tr><td>6468</td><td>38859</td><td>Drosophila melanogaster</td><td>CG7492</td><td>CG7492 gene product from transcript CG7492-RA</td><td>DPLG4|DmelCG7492|anon-EST:fe1A11</td><td>0</td><td>0</td></tr>
<tr><td>6469</td><td>38863</td><td>Drosophila melanogaster</td><td>Ank2</td><td>Ankyrin 2</td><td>CG18025|CG18227|CG18768|CG32373|CG32375|CG32377|CG34121|CG34158|CG34416|CG42734|CG7462|CG7493|DAnk2|Dank2|DmelCG42734|Dmel_CG32375|Dmel_CG32377|Dmel_CG34121|Dmel</td><td>0</td><td>0</td></tr>
<tr><td>6470</td><td>38864</td><td>Drosophila melanogaster</td><td>CG12262</td><td>CG12262 gene product from transcript CG12262-RA</td><td>DmelCG12262</td><td>0</td><td>0</td></tr>
<tr><td>6471</td><td>38865</td><td>Drosophila melanogaster</td><td>CG7457</td><td>CG7457 gene product from transcript CG7457-RA</td><td>CT13960|DmelCG7457</td><td>0</td><td>0</td></tr>
<tr><td>6472</td><td>38866</td><td>Drosophila melanogaster</td><td>CG8492</td><td>CG8492 gene product from transcript CG8492-RD</td><td>DmelCG8492</td><td>0</td><td>0</td></tr>
<tr><td>6473</td><td>38868</td><td>Drosophila melanogaster</td><td>Snmp2</td><td>Sensory neuron membrane protein 2</td><td>148358_at|CG16832|CG7422|DmelCG7422|SNMP2</td><td>0</td><td>0</td></tr>
<tr><td>6474</td><td>38869</td><td>Drosophila melanogaster</td><td>CG13685</td><td>CG13685 gene product from transcript CG13685-RA</td><td>DmelCG13685</td><td>0</td><td>0</td></tr>
<tr><td>6475</td><td>38870</td><td>Drosophila melanogaster</td><td>CG7409</td><td>CG7409 gene product from transcript CG7409-RA</td><td>CT16169|DmelCG7409</td><td>0</td><td>0</td></tr>
<tr><td>6476</td><td>38871</td><td>Drosophila melanogaster</td><td>Hn</td><td>Henna</td><td>CG7399|DTPH|DmelCG7399|PAH|Pah|TPH|TRH|Tph|bu|pah</td><td>0</td><td>0</td></tr>
<tr><td>6477</td><td>38872</td><td>Drosophila melanogaster</td><td>Clk</td><td>Clock</td><td>CG7391|CLK|CLOCK|DmelCG7391|Jerk|Jrk|PAS1|bHLHe10|clk|clock|dCLK|dCLK/JRK|dCLOCK|dClck|dClk|dClock|dclk|jrk</td><td>0</td><td>0</td></tr>
<tr><td>6478</td><td>38873</td><td>Drosophila melanogaster</td><td>CG32369</td><td>CG32369 gene product from transcript CG32369-RC</td><td>CG13684|CG7388|DmelCG32369</td><td>0</td><td>0</td></tr>
<tr><td>6479</td><td>38876</td><td>Drosophila melanogaster</td><td>CG32365</td><td>CG32365 gene product from transcript CG32365-RA</td><td>CG13682|CG13683|CG8294|DmelCG32365</td><td>0</td><td>0</td></tr>
<tr><td>6480</td><td>38878</td><td>Drosophila melanogaster</td><td>eIF4E-5</td><td>CG8277 gene product from transcript CG8277-RA</td><td>CG8277|CT24507|DmelCG8277|eIF4E</td><td>0</td><td>0</td></tr>
<tr><td>6481</td><td>38879</td><td>Drosophila melanogaster</td><td>pbl</td><td>pebble</td><td>0083/20|0293/09|0542/03|CG8114|DmelCG8114|ECT2|PBL|Pbl|anon-WO0172774.22|anon-WO0172774.25|l(3)09645</td><td>0</td><td>0</td></tr>
<tr><td>6482</td><td>38880</td><td>Drosophila melanogaster</td><td>CG8281</td><td>CG8281 gene product from transcript CG8281-RA</td><td>DmelCG8281</td><td>0</td><td>0</td></tr>
<tr><td>6483</td><td>38881</td><td>Drosophila melanogaster</td><td>CG8111</td><td>CG8111 gene product from transcript CG8111-RA</td><td>DmelCG8111</td><td>0</td><td>0</td></tr>
<tr><td>6484</td><td>38883</td><td>Drosophila melanogaster</td><td>Srp9</td><td>Signal recognition particle protein 9</td><td>CG8268|DmelCG8268</td><td>0</td><td>0</td></tr>
<tr><td>6485</td><td>38884</td><td>Drosophila melanogaster</td><td>exex</td><td>extra-extra</td><td>CG8254|DHB9|DmelCG8254|HB9|Hb9|dHb9|dhb9|hb9</td><td>0</td><td>0</td></tr>
<tr><td>6486</td><td>38885</td><td>Drosophila melanogaster</td><td>RNaseX25</td><td>Ribonuclease X25</td><td>CG8194|DmRNase-66B|DmRNase-X25|DmelCG8194</td><td>0</td><td>0</td></tr>
<tr><td>6487</td><td>38886</td><td>Drosophila melanogaster</td><td>CG8042</td><td>CG8042 gene product from transcript CG8042-RB</td><td>BcDNA:GH10229|DmelCG8042|anon-WO0140519.245|anon-WO0172774.24</td><td>0</td><td>0</td></tr>
<tr><td>6488</td><td>38887</td><td>Drosophila melanogaster</td><td>HP4</td><td>Heterochromatin protein 4</td><td>BcDNA:GM01628|CG8044|DmelCG8044|Hip|hip</td><td>0</td><td>0</td></tr>
<tr><td>6489</td><td>38888</td><td>Drosophila melanogaster</td><td>CG8209</td><td>CG8209 gene product from transcript CG8209-RA</td><td>DmelCG8209|anon-WO0172774.138</td><td>0</td><td>0</td></tr>
<tr><td>6490</td><td>38889</td><td>Drosophila melanogaster</td><td>CG8038</td><td>CG8038 gene product from transcript CG8038-RA</td><td>BcDNA:LD45688|DmelCG8038|anon-WO0172774.38</td><td>0</td><td>0</td></tr>
<tr><td>6491</td><td>38890</td><td>Drosophila melanogaster</td><td>nmo</td><td>nemo</td><td>CG7892|DmelCG7892|NEMO|NEMO/NLK|Nemo|Nlk|Nmo|ORE-6|adk|adk1|anon-EST:Gibbs3|anon-EST:Liang-2.43|anon-WO0118547.332|anon-WO0140519.256|anon-WO0172774.138|clone 2.43</td><td>0</td><td>0</td></tr>
<tr><td>6492</td><td>38891</td><td>Drosophila melanogaster</td><td>bip1</td><td>CG7574 gene product from transcript CG7574-RA</td><td>Bip1|CG7574|DmelCG7574|anon-WO0118547.246|bip-I</td><td>0</td><td>0</td></tr>
<tr><td>6493</td><td>38893</td><td>Drosophila melanogaster</td><td>CG7565</td><td>CG7565 gene product from transcript CG7565-RA</td><td>CT23145|DmelCG7565</td><td>0</td><td>0</td></tr>
<tr><td>6494</td><td>38895</td><td>Drosophila melanogaster</td><td>Ect4</td><td>CG43119 gene product from transcript CG43119-RI</td><td>CG13680|CG13681|CG34373|CG43119|CG7905|CG7915|DmelCG43119|Dmel_CG13680|Dmel_CG13681|Dmel_CG34373|Dmel_CG7915|anon-WO0118547.272</td><td>0</td><td>0</td></tr>
<tr><td>6495</td><td>38897</td><td>Drosophila melanogaster</td><td>CG7927</td><td>CG7927 gene product from transcript CG7927-RA</td><td>BcDNA:GH11023|DmelCG7927|anon-WO0172774.138</td><td>0</td><td>0</td></tr>
<tr><td>6496</td><td>38898</td><td>Drosophila melanogaster</td><td>Arp66B</td><td>Actin-related protein 66B</td><td>ARP3|AcTr66B|Actr66B|Arp2/3|Arp3|CG7558|DmelCG7558|anon-WO0172774.138|arp3|arp66B|schwachling</td><td>0</td><td>0</td></tr>
<tr><td>6497</td><td>38900</td><td>Drosophila melanogaster</td><td>ldbr</td><td>lariat debranching enzyme</td><td>CG7942|DBR1|DmelCG7942|Lbdr|Ldbr</td><td>0</td><td>0</td></tr>
<tr><td>6498</td><td>38901</td><td>Drosophila melanogaster</td><td>CG7550</td><td>CG7550 gene product from transcript CG7550-RA</td><td>DmelCG7550</td><td>0</td><td>0</td></tr>
<tr><td>6499</td><td>38904</td><td>Drosophila melanogaster</td><td>CG7504</td><td>CG7504 gene product from transcript CG7504-RC</td><td>DmelCG7504</td><td>0</td><td>0</td></tr>
<tr><td>6500</td><td>38905</td><td>Drosophila melanogaster</td><td>mus301</td><td>mutagen-sensitive 301</td><td>CG7972|DmelCG7972|SpnC|anon-WO0118547.354|cg7972|mus(3)301|spn-C|spnC</td><td>0</td><td>0</td></tr>
<tr><td>6501</td><td>38906</td><td>Drosophila melanogaster</td><td>CG13676</td><td>CG13676 gene product from transcript CG13676-RA</td><td>DmelCG13676</td><td>0</td><td>0</td></tr>
<tr><td>6502</td><td>38907</td><td>Drosophila melanogaster</td><td>CG13675</td><td>CG13675 gene product from transcript CG13675-RC</td><td>DmelCG13675</td><td>0</td><td>0</td></tr>
<tr><td>6503</td><td>38909</td><td>Drosophila melanogaster</td><td>Nmt</td><td>N-myristoyl transferase</td><td>153290_at|CG7436|DmelCG7436|Nmt1|dNMT|l(3)j1C7|l(3)j1c7|nmt</td><td>0</td><td>0</td></tr>
<tr><td>6504</td><td>38910</td><td>Drosophila melanogaster</td><td>mthl7</td><td>methuselah-like 7</td><td>CG7476|DmelCG7476|anon-WO0170980.85|anon-WO0170980.86</td><td>0</td><td>0</td></tr>
<tr><td>6505</td><td>38911</td><td>Drosophila melanogaster</td><td>CG7979</td><td>CG7979 gene product from transcript CG7979-RA</td><td>DmelCG7979</td><td>0</td><td>0</td></tr>
<tr><td>6506</td><td>38912</td><td>Drosophila melanogaster</td><td>ERR</td><td>estrogen-related receptor</td><td>CG7404|DmelCG7404|NR3B4|dERR</td><td>0</td><td>0</td></tr>
<tr><td>6507</td><td>38913</td><td>Drosophila melanogaster</td><td>Atg18</td><td>Autophagy-specific gene 18</td><td>ATG18|CG7986|DmelCG7986|atg18</td><td>0</td><td>0</td></tr>
<tr><td>6508</td><td>38914</td><td>Drosophila melanogaster</td><td>MED24</td><td>Mediator complex subunit 24</td><td>CG7999|DmelCG7999|Med24|Trap100|dTRAP100|l(3)psg5|med24</td><td>0</td><td>0</td></tr>
<tr><td>6509</td><td>38915</td><td>Drosophila melanogaster</td><td>CG7387</td><td>CG7387 gene product from transcript CG7387-RA</td><td>DmelCG7387|anon-WO0140519.72</td><td>0</td><td>0</td></tr>
<tr><td>6510</td><td>38916</td><td>Drosophila melanogaster</td><td>CG7375</td><td>CG7375 gene product from transcript CG7375-RA</td><td>DmelCG7375|UbcD12|anon-WO0118547.309</td><td>0</td><td>0</td></tr>
<tr><td>6511</td><td>38917</td><td>Drosophila melanogaster</td><td>CG8005</td><td>CG8005 gene product from transcript CG8005-RA</td><td>DmelCG8005</td><td>0</td><td>0</td></tr>
<tr><td>6512</td><td>38918</td><td>Drosophila melanogaster</td><td>CG7366</td><td>CG7366 gene product from transcript CG7366-RA</td><td>DmelCG7366|anon-WO0140519.185</td><td>0</td><td>0</td></tr>
<tr><td>6513</td><td>38919</td><td>Drosophila melanogaster</td><td>CG13679</td><td>CG13679 gene product from transcript CG13679-RA</td><td>BcDNA:RE17113|DmelCG13679</td><td>0</td><td>0</td></tr>
<tr><td>6514</td><td>38920</td><td>Drosophila melanogaster</td><td>CG8006</td><td>CG8006 gene product from transcript CG8006-RA</td><td>DmelCG8006</td><td>0</td><td>0</td></tr>
<tr><td>6515</td><td>38921</td><td>Drosophila melanogaster</td><td>CG13674</td><td>CG13674 gene product from transcript CG13674-RA</td><td>DmelCG13674</td><td>0</td><td>0</td></tr>
<tr><td>6516</td><td>38922</td><td>Drosophila melanogaster</td><td>CG13678</td><td>CG13678 gene product from transcript CG13678-RA</td><td>BcDNA:RE24439|DmelCG13678</td><td>0</td><td>0</td></tr>
<tr><td>6517</td><td>38923</td><td>Drosophila melanogaster</td><td>eIF4E-3</td><td>CG8023 gene product from transcript CG8023-RA</td><td>CG8023|DmelCG8023|anon-WO0140519.227|eIF4E</td><td>0</td><td>0</td></tr>
<tr><td>6518</td><td>38924</td><td>Drosophila melanogaster</td><td>CG7213</td><td>CG7213 gene product from transcript CG7213-RA</td><td>DmelCG7213</td><td>0</td><td>0</td></tr>
<tr><td>6519</td><td>38928</td><td>Drosophila melanogaster</td><td>cert</td><td>ceramide transfer protein</td><td>BcDNA:GH07688|CERT|CG7207|Dcert|DmelCG7207|dcert</td><td>0</td><td>0</td></tr>
<tr><td>6520</td><td>38929</td><td>Drosophila melanogaster</td><td>CG7201</td><td>CG7201 gene product from transcript CG7201-RA</td><td>DmelCG7201</td><td>0</td><td>0</td></tr>
<tr><td>6521</td><td>38930</td><td>Drosophila melanogaster</td><td>Rab-RP3</td><td>Rab-related protein 3</td><td>AAF50452|CG7062|DRABR3|DRabRP3|DmRab19|DmelCG7062|Rab-r3|Rab19|Rab19/RP3</td><td>0</td><td>0</td></tr>
<tr><td>6522</td><td>38931</td><td>Drosophila melanogaster</td><td>CG7197</td><td>CG7197 gene product from transcript CG7197-RA</td><td>DmelCG7197</td><td>0</td><td>0</td></tr>
<tr><td>6523</td><td>38932</td><td>Drosophila melanogaster</td><td>CG13671</td><td>CG13671 gene product from transcript CG13671-RA</td><td>DmelCG13671</td><td>0</td><td>0</td></tr>
<tr><td>6524</td><td>38933</td><td>Drosophila melanogaster</td><td>CG7194</td><td>CG7194 gene product from transcript CG7194-RA</td><td>BEST:CK00048|BEST:CK01174|CK01174|DmelCG7194|ck00048</td><td>0</td><td>0</td></tr>
<tr><td>6525</td><td>38934</td><td>Drosophila melanogaster</td><td>Sbp2</td><td>SECIS-binding protein 2</td><td>CG7066|DmelCG7066|SBP2</td><td>0</td><td>0</td></tr>
<tr><td>6526</td><td>38935</td><td>Drosophila melanogaster</td><td>Gr66a</td><td>Gustatory receptor 66a</td><td>CG7189|DmelCG7189|GR66A|GR66C1|GR66a|Gr66A|Gr66C1</td><td>0</td><td>0</td></tr>
<tr><td>6527</td><td>38936</td><td>Drosophila melanogaster</td><td>CG7188</td><td>CG7188 gene product from transcript CG7188-RA</td><td>DmelCG7188|d-BI-1</td><td>0</td><td>0</td></tr>
<tr><td>6528</td><td>38937</td><td>Drosophila melanogaster</td><td>CG7185</td><td>CG7185 gene product from transcript CG7185-RB</td><td>CFIm-68k|DmelCG7185|cg7185</td><td>0</td><td>0</td></tr>
<tr><td>6529</td><td>38938</td><td>Drosophila melanogaster</td><td>CG13670</td><td>CG13670 gene product from transcript CG13670-RA</td><td>DmelCG13670</td><td>0</td><td>0</td></tr>
<tr><td>6530</td><td>38940</td><td>Drosophila melanogaster</td><td>Cpr66Cb</td><td>Cuticular protein 66Cb</td><td>CG7076|DmelCpr66Cb|DmelCG7076</td><td>0</td><td>0</td></tr>
<tr><td>6531</td><td>38941</td><td>Drosophila melanogaster</td><td>pex2</td><td>peroxin 2</td><td>CG7081|DmelCG7081</td><td>0</td><td>0</td></tr>
<tr><td>6532</td><td>38942</td><td>Drosophila melanogaster</td><td>DNApol-alpha50</td><td>DNA polymerase alpha 50kD</td><td>CG7108|DNA polalpha|DNApol-alpha|DNApolalpha|DNApolalpha50|DmelCG7108|Dmpol alpha|Dpri50|POLA|PRI1|Pol|Polalpha|anon-WO0140519.257|pol alpha|polalpha|polalpha50</td><td>0</td><td>0</td></tr>
<tr><td>6533</td><td>38943</td><td>Drosophila melanogaster</td><td>CG7083</td><td>CG7083 gene product from transcript CG7083-RA</td><td>DmelCG7083</td><td>0</td><td>0</td></tr>
<tr><td>6534</td><td>38944</td><td>Drosophila melanogaster</td><td>CG7182</td><td>CG7182 gene product from transcript CG7182-RA</td><td>DmelCG7182</td><td>0</td><td>0</td></tr>
<tr><td>6535</td><td>38945</td><td>Drosophila melanogaster</td><td>CG7112</td><td>CG7112 gene product from transcript CG7112-RB</td><td>DmelCG7112|FBgn 35879</td><td>0</td><td>0</td></tr>
<tr><td>6536</td><td>38946</td><td>Drosophila melanogaster</td><td>CG17352</td><td>CG17352 gene product from transcript CG17352-RA</td><td>CT33100|DmelCG17352</td><td>0</td><td>0</td></tr>
<tr><td>6537</td><td>38952</td><td>Drosophila melanogaster</td><td>Jon66Ci</td><td>Jonah 66Ci</td><td>66C|CG7118|DmelCG7118|Jon66C|SP171</td><td>0</td><td>0</td></tr>
<tr><td>6538</td><td>38953</td><td>Drosophila melanogaster</td><td>Jon66Cii</td><td>Jonah 66Cii</td><td>66C|CCG7170|CG7170|DmelCG7170|Jon66C|SP165</td><td>0</td><td>0</td></tr>
<tr><td>6539</td><td>38954</td><td>Drosophila melanogaster</td><td>CG7120</td><td>CG7120 gene product from transcript CG7120-RD</td><td>BcDNA:AT15424|DmelCG7120</td><td>0</td><td>0</td></tr>
<tr><td>6540</td><td>38955</td><td>Drosophila melanogaster</td><td>mkg-p</td><td>monkey king protein</td><td>CG7163|DmelCG7163|mkg|mkg-r|mkgr</td><td>0</td><td>0</td></tr>
<tr><td>6541</td><td>38956</td><td>Drosophila melanogaster</td><td>CG13667</td><td>CG13667 gene product from transcript CG13667-RA</td><td>DmelCG13667</td><td>0</td><td>0</td></tr>
<tr><td>6542</td><td>38957</td><td>Drosophila melanogaster</td><td>Oseg1</td><td>CG7161 gene product from transcript CG7161-RA</td><td>CG7161|DAF-10|DmelCG7161|IFT122|OSEG|OSEG1|OSM-4|Wdr10|oseg1</td><td>0</td><td>0</td></tr>
<tr><td>6543</td><td>38958</td><td>Drosophila melanogaster</td><td>mtrm</td><td>matrimony</td><td>CG18543|D52|DmelCG18543|anon-D52|anonD52</td><td>0</td><td>0</td></tr>
<tr><td>6544</td><td>38959</td><td>Drosophila melanogaster</td><td>exo70</td><td>CG7127 gene product from transcript CG7127-RA</td><td>CG7127|DmelCG7127|Exo70|Exo70p</td><td>0</td><td>0</td></tr>
<tr><td>6545</td><td>38961</td><td>Drosophila melanogaster</td><td>Cbl</td><td>CG7037 gene product from transcript CG7037-RB</td><td>CBL|CG7037|CG7043|D-Cbl|D-Cbl L|D-cbl|D-cblL|D-cblS|DCbl|Dcbl|DmelCG7037|Dv-Cbl|Dv-cbl|F165|anon-WO0118547.68|cbl|d-cbl|dCbl</td><td>0</td><td>0</td></tr>
<tr><td>6546</td><td>38963</td><td>Drosophila melanogaster</td><td>Unr</td><td>Upstream of N-ras</td><td>BcDNA:LD13080|CG7015|CR32028|DmelCG7015|Dmel_CR32028|MRE30|UNR|dUNR</td><td>0</td><td>0</td></tr>
</table>

6547	38964	Drosophila melanogaster	CG6983	CG6983 gene product from transcript CG6983-RA	DmelCG6983	0	0
6548	38966	Drosophila melanogaster	CG43163	CG43163 gene product from transcript CG43163-RC	CG6902|CG6915|DmelCG43163|Dmel_CG6902|Dmel_CG6915	0	0
6549	38968	Drosophila melanogaster	ZC3H3	CG6694 gene product from transcript CG6694-RA	CG6694|DmelCG6694|dZC3H3	0	0
6550	38969	Drosophila melanogaster	CG6745	CG6745 gene product from transcript CG6745-RA	CT20937|DmelCG6745	0	0
6551	38970	Drosophila melanogaster	CG6683	CG6683 gene product from transcript CG6683-RA	DmelCG6683	0	0
6552	38971	Drosophila melanogaster	CG6765	CG6765 gene product from transcript CG6765-RA	DmelCG6765	0	0
6553	38972	Drosophila melanogaster	CG6776	CG6776 gene product from transcript CG6776-RA	DmelCG6776|T14	0	0
6554	38973	Drosophila melanogaster	se	sepia	CG6781|DmelCG6781|cg6781	0	0
6555	38974	Drosophila melanogaster	CG6673	CG6673 gene product from transcript CG6673-RA	CG6673-A|CG6673-B|CG6673A|CG6673B|DmelCG6673|anon-EST:Posey29	0	0
6556	38975	Drosophila melanogaster	CG6662	CG6662 gene product from transcript CG6662-RA	DmelCG6662	0	0
6557	38976	Drosophila melanogaster	foi	fear-of-intimacy	CG6817|DmelCG6817|FOI|dFOI|kas|l(3)j8E8|l(3)neo13	0	0
6558	38978	Drosophila melanogaster	rhea	CG6831 gene product from transcript CG6831-RB	CG6831|CT21159|DmelCG6831|Rhea|Talin|Tln|talin	0	0
6559	38979	Drosophila melanogaster	CG6638	CG6638 gene product from transcript CG6638-RA	DmelCG6638	0	0
6560	38981	Drosophila melanogaster	CG43078	CG43078 gene product from transcript CG43078-RJ	CG17926|CG32352|CG32353|CG32355|CG6239|DmelCG43078|Dmel_CG32352|Dmel_CG32353|Dmel_CG32355	0	0
6561	38982	Drosophila melanogaster	Nelf-E	Negative elongation factor E	CG5994|DmelCG5994|NELF|NELF-E|Nelf-e|anon-66Da|cg5994|dNelf-E	0	0
6562	38983	Drosophila melanogaster	RpL14	Ribosomal protein L14	CG6253|DmelCG6253|L14|M(3)|M(3)66|M(3)66D|M66D|RPL14|anon-EST:Posey193U|anon-EST:Posey6|rpl14	0	0
6563	38984	Drosophila melanogaster	CG5989	CG5989 gene product from transcript CG5989-RA	DmelCG5989|anon-66Db	0	0
6564	38985	Drosophila melanogaster	CG6282	CG6282 gene product from transcript CG6282-RC	DmelCG6282	0	0
6565	38986	Drosophila melanogaster	S-Lap1	Sperm-Leucylaminopeptidase 1	CG6372|DmelCG6372|S-LAP 1|anon-WO0140519.89|bs33g03.y1|bs34e01.y1	0	0
6566	38987	Drosophila melanogaster	GAPsec	CG5978 gene product from transcript CG5978-RA	BEST:LD24460|CG5978|DmelCG5978|dGAPsec	0	0
6567	38988	Drosophila melanogaster	Zasp66	Z band alternatively spliced PDZ-motif protein 66	CG6416|DmelCG6416|anon-WO0153538.30	0	0
6568	38989	Drosophila melanogaster	Cdc6	CG5971 gene product from transcript CG5971-RA	CG5971|DmCdc6|DmelCG5971	0	0
6569	38990	Drosophila melanogaster	Cpr66D	Cuticular protein 66D	CG32029|CG6458|CG6469|DmelCpr66D|DmelCG32029	0	0
6570	38992	Drosophila melanogaster	CG13305	CG13305 gene product from transcript CG13305-RC	BcDNA:RE33542|CT32600|DmelCG13305	0	0
6571	38993	Drosophila melanogaster	Arr2	Arrestin 2	49|AH|ARR2|Arr|Arr-2|ArrB|ArrB-ml|CG5962|DAII|DmelCG5962|Dro.-Arr.II|PRI|arr|arr2	0	0
6572	38994	Drosophila melanogaster	CG6486	CG6486 gene product from transcript CG6486-RA	DmelCG6486	0	0
6573	38995	Drosophila melanogaster	h	hairy	8247|CG6494|DmelCG6494|HRY|Hairy|bHLHb39|bHLHc14|brr|dDr1|l(3)08247|l(3)rM384	0	0
6574	38997	Drosophila melanogaster	CG6511	CG6511 gene product from transcript CG6511-RA	DmelCG6511	0	0
6575	38998	Drosophila melanogaster	Cp18	Chorion protein 18	ACE3|CG6517|CP18|DmelCG6517|Dms18|E5|S-18|S18|cp18|s-18|s18|s18-1	0	0
6576	38999	Drosophila melanogaster	Cp15	Chorion protein 15	C.s15|CG6519|Cs15|DmelCG6519|Dms15|E6|S15|cp15|melcp15|s-15|s15|s15-1	0	0
6577	39000	Drosophila melanogaster	Cp19	Chorion protein 19	C.s19|CG6524|CP19|DmelCG6524|Dms19|S19|cp19|s19|s19-1	0	0
6578	39001	Drosophila melanogaster	Cp16	Chorion protein 16	CG6533|CP16|DmelCG6533|Dms16|Ov31|S16|X16715|cp16|melcp16|s16|s16-1	0	0
6579	39002	Drosophila melanogaster	Prm	Paramyosin	0106/05|CG5939|DmelCG5939|FBgn0003149|PM|PM/mPM|Para|l(3)10631|l(3)S010605|mPM|pm|prm	0	0
6580	39003	Drosophila melanogaster	CG6576	CG6576 gene product from transcript CG6576-RA	DmelCG6576	0	0
6581	39004	Drosophila melanogaster	Fhos	CG42610 gene product from transcript CG42610-RC	CG32025|CG32030|CG42610|CG5775|CG5797|DmelCG42610|Dmel_CG32025|Dmel_CG32030|dFHOS|desert	0	0
6582	39005	Drosophila melanogaster	CG5804	CG5804 gene product from transcript CG5804-RA	BcDNA:RE05521|DmelCG5804	0	0
6583	39007	Drosophila melanogaster	CG13310	CG13310 gene product from transcript CG13310-RA	DmelCG13310	0	0
6584	39008	Drosophila melanogaster	CG13311	CG13311 gene product from transcript CG13311-RA	BcDNA:RE29808|DmelCG13311	0	0
6585	39010	Drosophila melanogaster	CG13312	CG13312 gene product from transcript CG13312-RA	DmelCG13312	0	0
6586	39011	Drosophila melanogaster	CG13308	CG13308 gene product from transcript CG13308-RB	DmelCG13308	0	0
6587	39012	Drosophila melanogaster	CG13309	CG13309 gene product from transcript CG13309-RA	CT32604|DmelCG13309	0	0
6588	39013	Drosophila melanogaster	dally	division abnormally delayed	CG4974|CT15952|Dally|DmelCG4974|anon-EST:Liang-1.63|clone 1.63|gem|l(3)06464	0	0
6589	39014	Drosophila melanogaster	Mcm7	Minichromosome maintenance 7	CG4978|DmMCM7|DmeMCM7|DmelCG4978|MCM7|McM7|Mcp-PCR1|PCR1|anon-EST:Liang-1.66|clone 1.66|mcm7	0	0
6590	39015	Drosophila melanogaster	TrpA1	Transient receptor potential A1	ANKTM1|Anktm1|CG5751|CG5761|CT18073|DmelCG5751|TRPA|TRPA1|TrpA|dANKTM1|dTRPA|dTRPA1|dTrpA1|dtrpA1|trpA1	0	0
6591	39016	Drosophila melanogaster	mfr	misfire	CG5747|DmelCG5747	0	0
6592	39017	Drosophila melanogaster	Tsp66E	Tetraspanin 66E	CG4999|Dm.Tsp66E|DmelCG4999	0	0
6593	39018	Drosophila melanogaster	orb2	CG43113 gene product from transcript CG43113-RF	CG43113|CG5735|CG5741|DmelCG43113|Dmel_CG5735|Dmel_CG5741|Orb2|anon-WO0140519.222|cg5735	0	0
6594	39020	Drosophila melanogaster	GNBP3	Gram-negative bacteria binding protein 3	CG5008|DGNBP-3|DmelCG5008|GNBP|GNBP-3|Hades|gnbp3	0	0
6595	39022	Drosophila melanogaster	CG13313	CG13313 gene product from transcript CG13313-RA	DmelCG13313	0	0
6596	39023	Drosophila melanogaster	CG5660	CG5660 gene product from transcript CG5660-RA	DmelCG5660	0	0
6597	39024	Drosophila melanogaster	CG5653	CG5653 gene product from transcript CG5653-RA	DmelCG5653	0	0
6598	39025	Drosophila melanogaster	CG5021	CG5021 gene product from transcript CG5021-RC	DmelCG5021	0	0
6599	39026	Drosophila melanogaster	CG5026	CG5026 gene product from transcript CG5026-RB	DmelCG5026	0	0
6600	39027	Drosophila melanogaster	pix	pixie	BEST:GH18088|CG5651|DmelCG5651|E3-c	0	0
6601	39028	Drosophila melanogaster	Srp68	Signal recognition particle protein 68	CG5064|DmelCG5064	0	0
6602	39029	Drosophila melanogaster	CG5644	CG5644 gene product from transcript CG5644-RA	DmelCG5644|anon-EST:Posey104	0	0
6603	39030	Drosophila melanogaster	CG13314	CG13314 gene product from transcript CG13314-RB	DmelCG13314	0	0
6604	39031	Drosophila melanogaster	Galk	Galactokinase	CG5288|CT16885|DmelCG5288|GALK|cg5288|dGALK	0	0
6605	39032	Drosophila melanogaster	CG5068	CG5068 gene product from transcript CG5068-RA	DmelCG5068	0	0
6606	39033	Drosophila melanogaster	CG5280	CG5280 gene product from transcript CG5280-RA	DmelCG5280	0	0
6607	39034	Drosophila melanogaster	smg	smaug	CG5263|D69|DmelCG5263|SMG|Smg|anon-D69|anonD69	0	0
6608	39035	Drosophila melanogaster	CG5087	CG5087 gene product from transcript CG5087-RA	DmelCG5087	0	0
6609	39036	Drosophila melanogaster	Doc3	Dorsocross3	CG5093|Dm-DOC3|DmelCG5093|doc3	0	0
6610	39037	Drosophila melanogaster	CG5194	CG5194 gene product from transcript CG5194-RA	DmelCG5194|anon-WO0140519.239	0	0
6611	39038	Drosophila melanogaster	Doc2	Dorsocross2	CG5187|Dm-DOC2|DmelCG5187|doc2	0	0
6612	39039	Drosophila melanogaster	Doc1	Dorsocross1	CG5133|Dm-DOC1|DmelCG5133|Doc|Ect2|Tb66F2|doc|doc1	0	0
6613	39040	Drosophila melanogaster	CG5144	CG5144 gene product from transcript CG5144-RA	DmelCG5144	0	0
6614	39041	Drosophila melanogaster	Argk	Arginine kinase	AK|AK-1|ARK|ArgK|CG32031|CG4929|CG5173|DmelCG32031|argK|argk	0	0
6615	39043	Drosophila melanogaster	CG4911	CG4911 gene product from transcript CG4911-RA	DmelCG4911	0	0
6616	39044	Drosophila melanogaster	CG4942	CG4942 gene product from transcript CG4942-RA	DmelCG4942	0	0
6617	39048	Drosophila melanogaster	Tequila	CG4821 gene product from transcript CG4821-RE	BEST:CK00198|BEST:GH18038|CG18153|CG18403|CG4821|CG4948|CK00198|DmelCG4821|SP72|Teq|anon-EST:ParkEST161|graal|teq	0	0
6618	39049	Drosophila melanogaster	bol	boule	BcDNA:AT13332|Bol|Boule|CG4727|CG4760|DmelCG4760|ms(3)07272	0	0
6619	39050	Drosophila melanogaster	Dhpr	Dihydropteridine reductase	CG4665|DHPR|DmelCG4665	0	0
6620	39051	Drosophila melanogaster	Use1	CG14181 gene product from transcript CG14181-RA	CG14181|DmelCG14181|FBgn 35965	0	0
6621	39052	Drosophila melanogaster	nwk	nervous wreck	CG43479|CG4641|CG4684|DmelCG43479|Dmel_CG4641|Dmel_CG4684|NWK|Nwk	0	0
6622	39054	Drosophila melanogaster	Rdl	Resistant to dieldrin	CG10537|DmRDL|DmRdl|DmelCG10537|GABA|GABA[[A]]|GABA[[A]] receptor|GABAr|LCCH1|RDL|Rd1|gaba|rdl	0	0
6623	39055	Drosophila melanogaster	Slc45-1	Slc45 ortholog 1	CG4484|DmelCG4484|scrt	0	0
6624	39056	Drosophila melanogaster	CG4476	CG4476 gene product from transcript CG4476-RB	DmNAT3|DmelCG4476|anon-WO0149848.7	0	0
6625	39057	Drosophila melanogaster	CG4483	CG4483 gene product from transcript CG4483-RA	DmelCG4483	0	0
6626	39058	Drosophila melanogaster	CG4477	CG4477 gene product from transcript CG4477-RB	DmelCG4477	0	0
6627	39062	Drosophila melanogaster	PGRP-LA	Peptidoglycan recognition protein LA	CG18614|CG32042|CG4361|CG4384|DmelCG32042|PGRPLA	0	0
6628	39063	Drosophila melanogaster	PGRP-LC	Peptidoglycan recognition protein LC	(PGRP)-LC|CG4432|Dm PGRP-LC|DmelCG4432|PGRP-LCa|PGRP-LCx|PGRPLC|Pgrp-LC|ird7|tot	0	0
6629	39064	Drosophila melanogaster	PGRP-LF	Peptidoglycan recognition protein LF	CG4437|DmelCG4437|PGRP-LCa|PGRP-like|PGRPLF|anon-WO0118547.581	0	0
6630	39065	Drosophila melanogaster	UGP	CG4347 gene product from transcript CG4347-RE	CG4347|DmelCG4347|UDPGPP|UGPase	0	0
6631	39066	Drosophila melanogaster	CG34455	CG34455 gene product from transcript CG34455-RC	CG4446|DmelCG34455|Dmel_CG4446	0	0
6632	39067	Drosophila melanogaster	CG4447	CG4447 gene product from transcript CG4447-RA	DmelCG4447	0	0
6633	39068	Drosophila melanogaster	Klp67A	Kinesin-like protein at 67A	0427/05|CG10923|DmKlp67A|DmelCG10923|KIF18A|KLP3|KLP67A|KRP67A|Kin-8|Klp3|Klp67|anon-WO0140519.224|anon-WO0172774.120|klp67A|l(3)S042705	0	0
6634	39069	Drosophila melanogaster	CG4452	CG4452 gene product from transcript CG4452-RB	DmelCG4452|anon-WO0172774.118|cg4452	0	0
6635	39070	Drosophila melanogaster	Fdxh	Ferredoxin	BcDNA:RH67819|CG4205|DmelCG4205	0	0
6636	39071	Drosophila melanogaster	Hsp67Bc	Heat shock gene 67Bc	CG4190|Dm-HSP67Bc|DmelCG4190|HSP67|HSP67Bc|HSPB8|Hsp-G3|Hspg3|gene 3|gene-3|gene3|gene5|hsp67B|small hsp locus 67B	0	0
6637	39074	Drosophila melanogaster	CG4461	CG4461 gene product from transcript CG4461-RA	148460_at|BEST:GH12586|DmelCG4461|Hsp20|gh12586	0	0
6638	39075	Drosophila melanogaster	Hsp26	Heat shock protein 26	26|26K|CG4183|DmHsp26|DmelCG4183|HSP26|hsp 26|hsp26|small hsp locus 67B	0	0
6639	39076	Drosophila melanogaster	Hsp67Ba	Heat shock gene 67Ba	CG4167|DROHSP|DROHSP1|DmelCG4167|Hsp-G1|gene 1|gene1|small hsp locus 67B	0	0
6640	39077	Drosophila melanogaster	Hsp23	Heat shock protein 23	23|CG4463|DmHsp23|DmelCG4463|HSP23|HSP23/IAP|Hsp-23|hsp 22K|hsp 23|hsp 23K|hsp23|hsp23/22|small hsp locus 67B	0	0
6641	39078	Drosophila melanogaster	Hsp27	Heat shock protein 27	27K|28|CG4466|Dhsp27|DmHsp27|DmelCG4466|HSP27|HSP28|Hsp28|anon-WO0140519.69|hsp 27|hsp-27|hsp26|hsp27|hsp27/26|hsp28|small hsp locus 67B	0	0
6642	39079	Drosophila melanogaster	CG4080	CG4080 gene product from transcript CG4080-RB	DmelCG4080	0	0
6643	39081	Drosophila melanogaster	Cpr67B	Cuticular protein 67B	CG3672|DmelCG3672	0	0
6644	39082	Drosophila melanogaster	CG4022	CG4022 gene product from transcript CG4022-RC	DmelCG4022	0	0
6645	39083	Drosophila melanogaster	CG3689	CG3689 gene product from transcript CG3689-RB	DmelCG3689	0	0
6646	39084	Drosophila melanogaster	CG3982	CG3982 gene product from transcript CG3982-RA	DmelCG3982|bs12d10.y1	0	0
6647	39085	Drosophila melanogaster	aay	astray	0423/14|CG3705|DmelCG3705|anon-WO0172774.117	0	0
6648	39086	Drosophila melanogaster	CG3967	CG3967 gene product from transcript CG3967-RC	DmelCG3967	0	0
6649	39088	Drosophila melanogaster	RpS17	Ribosomal protein S17	BcDNA:RE44119|CG3922|D9|DmelCG3922|M|M(3)67|M(3)67C|M(3)RpS17|M(3)S33|M(3)i|M(3)i(55)|M(3)i[55]|M(3)q|M(3L)i|M(3L)i[55]|Minute|RPS17|Rps17|anon-EST:Posey48|l(3)67BDo|l(3)dtOA4|rpS17	0	0
6650	39089	Drosophila melanogaster	MTF-1	Metal response element-binding Transcription Factor-	CG3743|DmelCG3743|anon-WO0118547.411|dMTF-1|mtf-1	0	0
6651	39090	Drosophila melanogaster	Bet3	CG3911 gene product from transcript CG3911-RA	BET3|Bet3p|CG3911|DmelCG3911|dBet3	0	0
6652	39091	Drosophila melanogaster	Nf-YA	Nuclear factor Y-box A	CG3891|DmelCG3891|NF-YA|dNF-YA	0	0
6653	39092	Drosophila melanogaster	CG33926	CG33926 gene product from transcript CG33926-RA	DmelCG33926	0	0
6654	39093	Drosophila melanogaster	CG3529	CG3529 gene product from transcript CG3529-RB	DmelCG3529	0	0
6655	39094	Drosophila melanogaster	CG3448	CG3448 gene product from transcript CG3448-RB	DmelCG3448	0	0
6656	39095	Drosophila melanogaster	phol	pleiohomeotic like	CG3445|DmelCG3445|PHOL|Phol	0	0
6657	39096	Drosophila melanogaster	CG3437	CG3437 gene product from transcript CG3437-RB	DmelCG3437	0	0

<table>
<tr><td>6658</td><td>39097</td><td>Drosophila melanogaster</td><td>CG3552</td><td>CG3552 gene product from transcript CG3552-RD</td><td>DmelCG3552</td><td>0</td><td>0</td></tr>
<tr><td>6659</td><td>39098</td><td>Drosophila melanogaster</td><td>CG3434</td><td>CG3434 gene product from transcript CG3434-RA</td><td>DmelCG3434</td><td>0</td><td>0</td></tr>
<tr><td>6660</td><td>39099</td><td>Drosophila melanogaster</td><td>CG32043</td><td>CG32043 gene product from transcript CG32043-RA</td><td>CG18632|CG3628|DmelCG32043|anon-WO0118547.368|anon-WO0118547.427</td><td>0</td><td>0</td></tr>
<tr><td>6661</td><td>39101</td><td>Drosophila melanogaster</td><td>CG14180</td><td>CG14180 gene product from transcript CG14180-RA</td><td>DmelCG14180</td><td>0</td><td>0</td></tr>
<tr><td>6662</td><td>39102</td><td>Drosophila melanogaster</td><td>Uch-L3</td><td>Ubiquitin C-terminal hydrolase</td><td>BcDNA.LD24440|BcDNA:LD24440|CG3431|DmelCG3431|UCH|dUCH37|l(3)j2B8|p37A|uch-L3</td><td>0</td><td>0</td></tr>
<tr><td>6663</td><td>39103</td><td>Drosophila melanogaster</td><td>Jarid2</td><td>Jumonji, AT rich interactive domain 2</td><td>CG3654|DmelCG3654|dJARID2|dJmj|djmj</td><td>0</td><td>0</td></tr>
<tr><td>6664</td><td>39104</td><td>Drosophila melanogaster</td><td>pall</td><td>pallbearer</td><td>CG3428|DmelCG3428</td><td>0</td><td>0</td></tr>
<tr><td>6665</td><td>39105</td><td>Drosophila melanogaster</td><td>CG32036</td><td>CG32036 gene product from transcript CG32036-RC</td><td>CG3426|DmelCG32036</td><td>0</td><td>0</td></tr>
<tr><td>6666</td><td>39106</td><td>Drosophila melanogaster</td><td>path</td><td>pathetic</td><td>CG3424|DmelCG3424|anon-WO0118547.271|anon-WO0118547.370|pth</td><td>0</td><td>0</td></tr>
<tr><td>6667</td><td>39107</td><td>Drosophila melanogaster</td><td>CG3408</td><td>CG3408 gene product from transcript CG3408-RA</td><td>CT11439|DmelCG3408</td><td>0</td><td>0</td></tr>
<tr><td>6668</td><td>39108</td><td>Drosophila melanogaster</td><td>RpS9</td><td>Ribosomal protein S9</td><td>CG3395|DmelCG3395|M(3)67C|RpL11|S9</td><td>0</td><td>0</td></tr>
<tr><td>6669</td><td>39109</td><td>Drosophila melanogaster</td><td>Or67a</td><td>Odorant receptor 67a</td><td>67B.1|67a|CG12526|DOR84|DmelCG12526</td><td>0</td><td>0</td></tr>
<tr><td>6670</td><td>39110</td><td>Drosophila melanogaster</td><td>Ir67a</td><td>Ionotropic receptor 67a</td><td>CG12525|DmelCG12525|IR67a</td><td>0</td><td>0</td></tr>
<tr><td>6671</td><td>39111</td><td>Drosophila melanogaster</td><td>CG42673</td><td>CG42673 gene product from transcript CG42673-RD</td><td>BcDNA:RE71517|BcDNA:RE71585|CG14178|CG17357|CG3179|CG32048|CG34272|DmelCG42673|Dmel_CG32048|Dmel_CG34272</td><td>0</td><td>0</td></tr>
<tr><td>6672</td><td>39113</td><td>Drosophila melanogaster</td><td>CG14177</td><td>CG14177 gene product from transcript CG14177-RA</td><td>DmelCG14177</td><td>0</td><td>0</td></tr>
<tr><td>6673</td><td>39114</td><td>Drosophila melanogaster</td><td>CG3222</td><td>CG3222 gene product from transcript CG3222-RA</td><td>DmelCG3222|NEST:bs17c12|bs17c12.y1</td><td>0</td><td>0</td></tr>
<tr><td>6674</td><td>39115</td><td>Drosophila melanogaster</td><td>CG3088</td><td>CG3088 gene product from transcript CG3088-RA</td><td>DmelCG3088|SPH188</td><td>0</td><td>0</td></tr>
<tr><td>6675</td><td>39116</td><td>Drosophila melanogaster</td><td>CG3306</td><td>CG3306 gene product from transcript CG3306-RA</td><td>DmelCG3306</td><td>0</td><td>0</td></tr>
<tr><td>6676</td><td>39117</td><td>Drosophila melanogaster</td><td>CG3280</td><td>CG3280 gene product from transcript CG3280-RD</td><td>DmelCG3280</td><td>0</td><td>0</td></tr>
<tr><td>6677</td><td>39118</td><td>Drosophila melanogaster</td><td>LanB2</td><td>Laminin B2</td><td>CG3322|CT11145|DROLAMB2A|DmelCG3322|LAMB2A|LM-B2/gamma|LMG1|Lam-B2|anon-EST:fe2H3|gamma1|lamB2</td><td>0</td><td>0</td></tr>
<tr><td>6678</td><td>39119</td><td>Drosophila melanogaster</td><td>CG3335</td><td>CG3335 gene product from transcript CG3335-RA</td><td>Dm-RBD-1|DmelCG3335|Q9VT19|cg3335</td><td>0</td><td>0</td></tr>
<tr><td>6679</td><td>39120</td><td>Drosophila melanogaster</td><td>Or67b</td><td>Odorant receptor 67b</td><td>67B.2|67b|CG14176|DmelCG14176</td><td>0</td><td>0</td></tr>
<tr><td>6680</td><td>39121</td><td>Drosophila melanogaster</td><td>CG8336</td><td>CG8336 gene product from transcript CG8336-RB</td><td>DmelCG8336</td><td>0</td><td>0</td></tr>
<tr><td>6681</td><td>39122</td><td>Drosophila melanogaster</td><td>fry</td><td>furry</td><td>CG14171|CG32045|CG6774|CG6780|DmelCG32045|Fry|SS3-7|l(3)02240|yeti</td><td>0</td><td>0</td></tr>
<tr><td>6682</td><td>39123</td><td>Drosophila melanogaster</td><td>CG8329</td><td>CG8329 gene product from transcript CG8329-RA</td><td>DmelCG8329|SP170</td><td>0</td><td>0</td></tr>
<tr><td>6683</td><td>39124</td><td>Drosophila melanogaster</td><td>CG18179</td><td>CG18179 gene product from transcript CG18179-RA</td><td>BEST:LP02275|DmelCG18179|SP152</td><td>0</td><td>0</td></tr>
<tr><td>6684</td><td>39125</td><td>Drosophila melanogaster</td><td>CG18180</td><td>CG18180 gene product from transcript CG18180-RA</td><td>DmelCG18180|SP151</td><td>0</td><td>0</td></tr>
<tr><td>6685</td><td>39127</td><td>Drosophila melanogaster</td><td>CG33696</td><td>CG33696 gene product from transcript CG33696-RA</td><td>CG16726|CG32047|Dm CG33696|DmelCG33696</td><td>0</td><td>0</td></tr>
<tr><td>6686</td><td>39129</td><td>Drosophila melanogaster</td><td>CG16717</td><td>CG16717 gene product from transcript CG16717-RA</td><td>DmelCG16717</td><td>0</td><td>0</td></tr>
<tr><td>6687</td><td>39130</td><td>Drosophila melanogaster</td><td>alphaTub67C</td><td>alpha-Tubulin at 67C</td><td>4t|ALPHA 67C|CG8308|D.m.ALPHA-67C|DTA2|DmelCG8308|Fs(3)Kav|Fs(3)Kavar|Fs(3)Sz14|Fs(3)Sz22|Fs(3)Tom|Fs(3)Tomaj|Kavar|T|Tom|Tub|aTub67C|alpha-Tub|alpha-Tub67C|alpha-tub67C|alpha-tubulin|</td><td>0</td><td>0</td></tr>
<tr><td>6688</td><td>39131</td><td>Drosophila melanogaster</td><td>CG16719</td><td>CG16719 gene product from transcript CG16719-RA</td><td>DmelCG16719</td><td>0</td><td>0</td></tr>
<tr><td>6689</td><td>39132</td><td>Drosophila melanogaster</td><td>CG6767</td><td>CG6767 gene product from transcript CG6767-RA</td><td>DmelCG6767</td><td>0</td><td>0</td></tr>
<tr><td>6690</td><td>39133</td><td>Drosophila melanogaster</td><td>UbcD4</td><td>Ubiquitin conjugating enzyme 4</td><td>CG8284|DmelCG8284|E2</td><td>0</td><td>0</td></tr>
<tr><td>6691</td><td>39134</td><td>Drosophila melanogaster</td><td>CG6761</td><td>CG6761 gene product from transcript CG6761-RA</td><td>DmelCG6761</td><td>0</td><td>0</td></tr>
<tr><td>6692</td><td>39135</td><td>Drosophila melanogaster</td><td>CG16711</td><td>CG16711 gene product from transcript CG16711-RB</td><td>DmelCG16711</td><td>0</td><td>0</td></tr>
<tr><td>6693</td><td>39136</td><td>Drosophila melanogaster</td><td>SH3PX1</td><td>CG6757 gene product from transcript CG6757-RA</td><td>CG6757|DSH3PX|DSH3PX1|DmelCG6757|dSH3PX1|p63</td><td>0</td><td>0</td></tr>
<tr><td>6694</td><td>39137</td><td>Drosophila melanogaster</td><td>vsg</td><td>visgun</td><td>BEST:GH15083|CG16707|DmelCG16707|FBgn0045823|GH15083|Vsg|anon-WO0118547.164</td><td>0</td><td>0</td></tr>
<tr><td>6695</td><td>39138</td><td>Drosophila melanogaster</td><td>CG18178</td><td>CG18178 gene product from transcript CG18178-RA</td><td>DmelCG18178</td><td>0</td><td>0</td></tr>
<tr><td>6696</td><td>39139</td><td>Drosophila melanogaster</td><td>CG14174</td><td>CG14174 gene product from transcript CG14174-RA</td><td>DmelCG14174</td><td>0</td><td>0</td></tr>
<tr><td>6697</td><td>39141</td><td>Drosophila melanogaster</td><td>defl</td><td>deflated</td><td>CG18176|DmelCG18176|Int7|IntS7</td><td>0</td><td>0</td></tr>
<tr><td>6698</td><td>39142</td><td>Drosophila melanogaster</td><td>Naa60</td><td>N(alpha)-acetyltransferase 60</td><td>CG18177|DmAAF50213|DmelCG18177|NatF|dNAA60|dNaa60|dNatF</td><td>0</td><td>0</td></tr>
<tr><td>6699</td><td>39143</td><td>Drosophila melanogaster</td><td>ATPsyn-b</td><td>ATP synthase, subunit b</td><td>CG8189|DmelCG8189</td><td>0</td><td>0</td></tr>
<tr><td>6700</td><td>39144</td><td>Drosophila melanogaster</td><td>CG6749</td><td>CG6749 gene product from transcript CG6749-RA</td><td>DmelCG6749</td><td>0</td><td>0</td></tr>
<tr><td>6701</td><td>39145</td><td>Drosophila melanogaster</td><td>CG42268</td><td>CG42268 gene product from transcript CG42268-RD</td><td>CG14170|CG32044|CG32046|CG6740|DmelCG42268|Dmel_CG32044|Dmel_CG32046</td><td>0</td><td>0</td></tr>
<tr><td>6702</td><td>39147</td><td>Drosophila melanogaster</td><td>CG8177</td><td>CG8177 gene product from transcript CG8177-RG</td><td>BcDNA:RE59468|DmelCG8177|anon-EST:Posey259</td><td>0</td><td>0</td></tr>
<tr><td>6703</td><td>39148</td><td>Drosophila melanogaster</td><td>CG14168</td><td>CG14168 gene product from transcript CG14168-RB</td><td>DmelCG14168</td><td>0</td><td>0</td></tr>
<tr><td>6704</td><td>39149</td><td>Drosophila melanogaster</td><td>Ilp1</td><td>Insulin-like peptide 1</td><td>CG14173|DILP|DILP1|Dilp|Dilp 1|Dilp-1|Dilp1|DmelCG14173|Ilp|Ilp-1|dilp|dilp-1|dilp1</td><td>0</td><td>0</td></tr>
<tr><td>6705</td><td>39150</td><td>Drosophila melanogaster</td><td>Ilp2</td><td>Insulin-like peptide 2</td><td>CG8167|DILP|DILP2|Dilp|Dilp 2|Dilp-2|Dilp2|DmelCG8167|ILP2|IRP|Ilp|Ilp-2|dILP-2|dILP2|dIlp2|dilp|dilp-2|dilp2|ilp2</td><td>0</td><td>0</td></tr>
<tr><td>6706</td><td>39151</td><td>Drosophila melanogaster</td><td>Ilp3</td><td>Insulin-like peptide 3</td><td>CG14167|DILP|DILP3|Dilp|Dilp 3|Dilp-3|Dilp3|DmelCG14167|Ilp|Ilp-3|dILP-3|dILP3|dIlp3|dilp|dilp-3|dilp3|ilp3</td><td>0</td><td>0</td></tr>
<tr><td>6707</td><td>39152</td><td>Drosophila melanogaster</td><td>Ilp4</td><td>Insulin-like peptide 4</td><td>A|BcDNA:RE27042|CG6736|DILP|DILP4|Dilp|Dilp 4|Dirlp|DmelCG6736|Ilp|Ilp-4|Mes3|anon-EST:CLB3|dilp|dilp-4|dilp4|ilp4|mes3</td><td>0</td><td>0</td></tr>
<tr><td>6708</td><td>39153</td><td>Drosophila melanogaster</td><td>CG33205</td><td>CG33205 gene product from transcript CG33205-RC</td><td>BcDNA:RE55923|BcDNA:RH19933|CG14172|CG32049|CG32051|CG8154|CG8163|DmelCG33205</td><td>0</td><td>0</td></tr>
<tr><td>6709</td><td>39154</td><td>Drosophila melanogaster</td><td>CG32050</td><td>CG32050 gene product from transcript CG32050-RA</td><td>CG14172|DmelCG32050</td><td>0</td><td>0</td></tr>
<tr><td>6710</td><td>39156</td><td>Drosophila melanogaster</td><td>I-2</td><td>Inhibitor-2</td><td>CG10574|Dm I-2|DmelCG10574|I-2Dm|I-2PP1|SET</td><td>0</td><td>0</td></tr>
<tr><td>6711</td><td>39157</td><td>Drosophila melanogaster</td><td>Cdk8</td><td>Cyclin-dependent kinase 8</td><td>CDK8|CG10572|DmCdk8|DmelCG10572|cdk8|dTRAP56|p58</td><td>0</td><td>0</td></tr>
<tr><td>6712</td><td>39158</td><td>Drosophila melanogaster</td><td>Gap1</td><td>GTPase-activating protein 1</td><td>CG6721|DmelCG6721|GAP|GAP1|Gap|Gap 1|Ras-GAP|gap|gap1|mip|rI533|rasGAP|sxt</td><td>0</td><td>0</td></tr>
<tr><td>6713</td><td>39159</td><td>Drosophila melanogaster</td><td>CG10809</td><td>CG10809 gene product from transcript CG10809-RA</td><td>DmelCG10809</td><td>0</td><td>0</td></tr>
<tr><td>6714</td><td>39160</td><td>Drosophila melanogaster</td><td>iPLA2-VIA</td><td>calcium-independent phospholipase A2 VIA</td><td>CG6718|DmelCG6718</td><td>0</td><td>0</td></tr>
<tr><td>6715</td><td>39161</td><td>Drosophila melanogaster</td><td>CG8108</td><td>CG8108 gene product from transcript CG8108-RA</td><td>BcDNA:GH07921|DmelCG8108</td><td>0</td><td>0</td></tr>
<tr><td>6716</td><td>39164</td><td>Drosophila melanogaster</td><td>Taf2</td><td>TBP-associated factor 2</td><td>CG6711|DmelCG6711|TAF|TAF150|TAF2|TAFII150|TAF[II]150|TAF[[II]]|TAF[[II]]150|TFIID|Taf150|TafII150|Taf[[II]]150|dTAF II 150|dTAF150|dTAFII150|dTAF[[II]]150</td><td>0</td><td>0</td></tr>
<tr><td>6717</td><td>39165</td><td>Drosophila melanogaster</td><td>CalpB</td><td>Calpain-B</td><td>CALPB|CG8107|DmelCG8107|calpB</td><td>0</td><td>0</td></tr>
<tr><td>6718</td><td>39166</td><td>Drosophila melanogaster</td><td>CG6709</td><td>CG6709 gene product from transcript CG6709-RB</td><td>DmelCG6709</td><td>0</td><td>0</td></tr>
<tr><td>6719</td><td>39167</td><td>Drosophila melanogaster</td><td>CG14164</td><td>CG14164 gene product from transcript CG14164-RB</td><td>DmelCG14164</td><td>0</td><td>0</td></tr>
<tr><td>6720</td><td>39168</td><td>Drosophila melanogaster</td><td>CG6707</td><td>CG6707 gene product from transcript CG6707-RA</td><td>DmelCG6707</td><td>0</td><td>0</td></tr>
<tr><td>6721</td><td>39169</td><td>Drosophila melanogaster</td><td>nudE</td><td>CG8104 gene product from transcript CG8104-RB</td><td>CG8104|DmelCG8104|DnudE|NudE</td><td>0</td><td>0</td></tr>
<tr><td>6722</td><td>39172</td><td>Drosophila melanogaster</td><td>CG6685</td><td>CG6685 gene product from transcript CG6685-RA</td><td>DmelCG6685</td><td>0</td><td>0</td></tr>
<tr><td>6723</td><td>39173</td><td>Drosophila melanogaster</td><td>Nc</td><td>Nedd2-like caspase</td><td>CG8090|CG8091|DRONC|DmelCG8091|Dronc|Dronc/Casp9|NcDronc|anon-EST:GreesD6|anon-EST:GressD6|anon-WO0118547.313|caspase-9|dronc</td><td>0</td><td>0</td></tr>
<tr><td>6724</td><td>39174</td><td>Drosophila melanogaster</td><td>CG6674</td><td>CG6674 gene product from transcript CG6674-RA</td><td>DmelCG6674</td><td>0</td><td>0</td></tr>
<tr><td>6725</td><td>39175</td><td>Drosophila melanogaster</td><td>vnc</td><td>variable nurse cells</td><td>ARD1|Ard1|CG11989|DmAAF50178|DmelCG11989|Dromel_CG11989_FBtr0076348_ard1_mORF|ard1|dArd1|l(3)67BDk</td><td>0</td><td>0</td></tr>
<tr><td>6726</td><td>39177</td><td>Drosophila melanogaster</td><td>CG14160</td><td>CG14160 gene product from transcript CG14160-RA</td><td>DmelCG14160</td><td>0</td><td>0</td></tr>
<tr><td>6727</td><td>39178</td><td>Drosophila melanogaster</td><td>CG32053</td><td>CG32053 gene product from transcript CG32053-RA</td><td>CG6645|DmelCG32053</td><td>0</td><td>0</td></tr>
<tr><td>6728</td><td>39179</td><td>Drosophila melanogaster</td><td>CG42825</td><td>CG42825 gene product from transcript CG42825-RA</td><td>CG6640|DmelCG42825|Dmel_CG6640|GH19118|anon-WO0140519.30</td><td>0</td><td>0</td></tr>
<tr><td>6729</td><td>39180</td><td>Drosophila melanogaster</td><td>dpr10</td><td>CG32057 gene product from transcript CG32057-RB</td><td>BcDNA:RE37920|CG14158|CG14159|CG32057|CT33762|DmelCG32057|Dpr-10</td><td>0</td><td>0</td></tr>
<tr><td>6730</td><td>39181</td><td>Drosophila melanogaster</td><td>CG8072</td><td>CG8072 gene product from transcript CG8072-RA</td><td>DmelCG8072</td><td>0</td><td>0</td></tr>
<tr><td>6731</td><td>39183</td><td>Drosophila melanogaster</td><td>CG6628</td><td>CG6628 gene product from transcript CG6628-RA</td><td>DmelCG6628</td><td>0</td><td>0</td></tr>
<tr><td>6732</td><td>39186</td><td>Drosophila melanogaster</td><td>CG8065</td><td>CG8065 gene product from transcript CG8065-RB</td><td>DmelCG8065</td><td>0</td><td>0</td></tr>
<tr><td>6733</td><td>39188</td><td>Drosophila melanogaster</td><td>CG32055</td><td>CG32055 gene product from transcript CG32055-RA</td><td>CG6590|CT20492|DmelCG32055</td><td>0</td><td>0</td></tr>
<tr><td>6734</td><td>39189</td><td>Drosophila melanogaster</td><td>Or67c</td><td>Odorant receptor 67c</td><td>67D.1|67c|CG14156|DOR77|DmelCG14156</td><td>0</td><td>0</td></tr>
<tr><td>6735</td><td>39191</td><td>Drosophila melanogaster</td><td>Or67d</td><td>Odorant receptor 67d</td><td>67d|CG14157|DmelCG14157</td><td>0</td><td>0</td></tr>
<tr><td>6736</td><td>39193</td><td>Drosophila melanogaster</td><td>CG12362</td><td>CG12362 gene product from transcript CG12362-RB</td><td>DmelCG12362</td><td>0</td><td>0</td></tr>
<tr><td>6737</td><td>39194</td><td>Drosophila melanogaster</td><td>Ir67b</td><td>Ionotropic receptor 67b</td><td>CG12303|DmelCG12303|IR67b</td><td>0</td><td>0</td></tr>
<tr><td>6738</td><td>39195</td><td>Drosophila melanogaster</td><td>Gem3</td><td>Gemin3</td><td>BcDNA.LD05563|BcDNA:LD05563|CG6539|DHH1|Dhh1|DmRH22|DmelCG6539|dGem3|l(3)rL562</td><td>0</td><td>0</td></tr>
<tr><td>6739</td><td>39196</td><td>Drosophila melanogaster</td><td>CG32061</td><td>CG32061 gene product from transcript CG32061-RA</td><td>CG8040|DmelCG32061</td><td>0</td><td>0</td></tr>
<tr><td>6740</td><td>39197</td><td>Drosophila melanogaster</td><td>CG6527</td><td>CG6527 gene product from transcript CG6527-RA</td><td>BcDNA:AT13283|DmelCG6527</td><td>0</td><td>0</td></tr>
<tr><td>6741</td><td>39198</td><td>Drosophila melanogaster</td><td>A2bp1</td><td>Ataxin-2 binding protein 1</td><td>A2BP1|BcDNA:GM06073|CG14155|CG18441|CG32062|DmelCG32062|cg18441|dA2BP1</td><td>0</td><td>0</td></tr>
<tr><td>6742</td><td>39201</td><td>Drosophila melanogaster</td><td>CG14151</td><td>CG14151 gene product from transcript CG14151-RA</td><td>DmelCG14151</td><td>0</td><td>0</td></tr>
<tr><td>6743</td><td>39202</td><td>Drosophila melanogaster</td><td>hay</td><td>haywire</td><td>CG8019|DhR25|DhXPB|DmXPB|DmelCG8019|ERCC3|Hay|TFIIH|XPB|Xpb/hay|hwr|i50|ms(3)nc2|nc2</td><td>0</td><td>0</td></tr>
<tr><td>6744</td><td>39203</td><td>Drosophila melanogaster</td><td>E(z)</td><td>Enhancer of zeste</td><td>CG6502|DmelCG6502|E(Z)|E(z)1|EZ|E[z]|Ez|Su(z)301|l(3)1902|l(3)67Fa|l(3)B12|l(3)SG17|l(3)ds12|pco|pco/E(z)</td><td>0</td><td>0</td></tr>
<tr><td>6745</td><td>39204</td><td>Drosophila melanogaster</td><td>CG8009</td><td>CG8009 gene product from transcript CG8009-RB</td><td>BcDNA:LD14173|DmelCG8009</td><td>0</td><td>0</td></tr>
<tr><td>6746</td><td>39205</td><td>Drosophila melanogaster</td><td>CG18628</td><td>CG18628 gene product from transcript CG18628-RA</td><td>BcDNA:GH08251|DmelCG18628</td><td>0</td><td>0</td></tr>
<tr><td>6747</td><td>39206</td><td>Drosophila melanogaster</td><td>CG32066</td><td>CG32066 gene product from transcript CG32066-RC</td><td>CG6487|CG6491|DmelCG32066</td><td>0</td><td>0</td></tr>
<tr><td>6748</td><td>39207</td><td>Drosophila melanogaster</td><td>CG14154</td><td>CG14154 gene product from transcript CG14154-RA</td><td>DmelCG14154</td><td>0</td><td>0</td></tr>
<tr><td>6749</td><td>39208</td><td>Drosophila melanogaster</td><td>CG14153</td><td>CG14153 gene product from transcript CG14153-RA</td><td>DmelCG14153</td><td>0</td><td>0</td></tr>
<tr><td>6750</td><td>39210</td><td>Drosophila melanogaster</td><td>CG8003</td><td>CG8003 gene product from transcript CG8003-RA</td><td>DmelCG8003</td><td>0</td><td>0</td></tr>
<tr><td>6751</td><td>39212</td><td>Drosophila melanogaster</td><td>simj</td><td>simjang</td><td>CG32067|CG7983|CG8000|DmelCG32067|anon-WO0118547.361|dp66|l(3)01814|l(3)j4A5|l(3)rN672|p66|p66/68-like</td><td>0</td><td>0</td></tr>
<tr><td>6752</td><td>39213</td><td>Drosophila melanogaster</td><td>CG11811</td><td>CG11811 gene product from transcript CG11811-RA</td><td>DmelCG11811</td><td>0</td><td>0</td></tr>
<tr><td>6753</td><td>39214</td><td>Drosophila melanogaster</td><td>CG6463</td><td>CG6463 gene product from transcript CG6463-RA</td><td>BcDNA:RH11203|DmelCG6463</td><td>0</td><td>0</td></tr>
<tr><td>6754</td><td>39215</td><td>Drosophila melanogaster</td><td>NijA</td><td>Ninjurin A</td><td>CG6449|DmelCG6449|ninA</td><td>0</td><td>0</td></tr>
<tr><td>6755</td><td>39216</td><td>Drosophila melanogaster</td><td>CG12523</td><td>CG12523 gene product from transcript CG12523-RA</td><td>BcDNA:RE74758|DmelCG12523</td><td>0</td><td>0</td></tr>
<tr><td>6756</td><td>39217</td><td>Drosophila melanogaster</td><td>tna</td><td>tonalli</td><td>0583/02|CG7958|DmelCG7958|anon-WO0118547.590|l(3)L6731|l(3)S058302|l(3)rI075</td><td>0</td><td>0</td></tr>
<tr><td>6757</td><td>39218</td><td>Drosophila melanogaster</td><td>CG6418</td><td>CG6418 gene product from transcript CG6418-RB</td><td>DmRH27|DmelCG6418|cg6418</td><td>0</td><td>0</td></tr>
<tr><td>6758</td><td>39219</td><td>Drosophila melanogaster</td><td>blos4</td><td>BLOC-1 subunit 4</td><td>CG14149|Dm Blos4|DmelCG14149</td><td>0</td><td>0</td></tr>
<tr><td>6759</td><td>39220</td><td>Drosophila melanogaster</td><td>CG6409</td><td>CG6409 gene product from transcript CG6409-RA</td><td>DmelCG6409</td><td>0</td><td>0</td></tr>
<tr><td>6760</td><td>39221</td><td>Drosophila melanogaster</td><td>CG7949</td><td>CG7949 gene product from transcript CG7949-RA</td><td>DmelCG7949</td><td>0</td><td>0</td></tr>
<tr><td>6761</td><td>39222</td><td>Drosophila melanogaster</td><td>CG6404</td><td>CG6404 gene product from transcript CG6404-RA</td><td>BcDNA:GH02220|DmelCG6404</td><td>0</td><td>0</td></tr>
<tr><td>6762</td><td>39223</td><td>Drosophila melanogaster</td><td>Cpr67Fa1</td><td>Cuticular protein 67Fa1</td><td>BcDNA:RE57116|CG7941|DmelCpr67Fa1|DmelCG7941</td><td>0</td><td>0</td></tr>
<tr><td>6763</td><td>39224</td><td>Drosophila melanogaster</td><td>Cpr67Fa2</td><td>Cuticular protein 67Fa2</td><td>BcDNA:LP01241|CG18349|DmelCpr67Fa2|DmelCG18349</td><td>0</td><td>0</td></tr>
<tr><td>6764</td><td>39225</td><td>Drosophila melanogaster</td><td>Cpr67Fb</td><td>Cuticular protein 67Fb</td><td>CG18348|DmelCpr67Fb|DmelCG18348</td><td>0</td><td>0</td></tr>
<tr><td>6765</td><td>39226</td><td>Drosophila melanogaster</td><td>Aps</td><td>CG6391 gene product from transcript CG6391-RC</td><td>CG6391|DmelCG6391|anon-WO0118547.266</td><td>0</td><td>0</td></tr>
<tr><td>6766</td><td>39227</td><td>Drosophila melanogaster</td><td>CG14147</td><td>CG14147 gene product from transcript CG14147-RA</td><td>BcDNA:RE16005|DmelCG14147</td><td>0</td><td>0</td></tr>
<tr><td>6767</td><td>39228</td><td>Drosophila melanogaster</td><td>klu</td><td>klumpfuss</td><td>CG12296|DmelCG12296|Klu|P09036|l(3)09036|l(3)10052</td><td>0</td><td>0</td></tr>
<tr><td>6768</td><td>39230</td><td>Drosophila melanogaster</td><td>CG32079</td><td>CG32079 gene product from transcript CG32079-RB</td><td>CG11806|DmelCG32079</td><td>0</td><td>0</td></tr>
</table>

6769	39231	Drosophila melanogaster	CG6327	CG6327 gene product from transcript CG6327-RD	DmelCG6327	0	0
6770	39232	Drosophila melanogaster	CG7888	CG7888 gene product from transcript CG7888-RC	DmelCG7888	0	0
6771	39233	Drosophila melanogaster	CG6321	CG6321 gene product from transcript CG6321-RA	DmelCG6321	0	0
6772	39234	Drosophila melanogaster	blos2	BLOC-1 subunit 2	CG14145|DmBlos2|DmelCG14145	0	0
6773	39235	Drosophila melanogaster	CG32069	CG32069 gene product from transcript CG32069-RA	CG6316|DmelCG32069	0	0
6774	39237	Drosophila melanogaster	CG6310	CG6310 gene product from transcript CG6310-RA	BcDNA:AT03632|DmelCG6310	0	0
6775	39238	Drosophila melanogaster	Mocs1	Molybdenum cofactor synthesis 1 ortholog	CG33048|CG7858|DmelCG33048|MOCS1A|MOCS1B|Mocs1A|Mocs1A-B|Mocs1B|Mocsl|Mosc1A+Mosc1B|lxd|mocsl	0	0
6776	39240	Drosophila melanogaster	CG7839	CG7839 gene product from transcript CG7839-RA	DmelCG7839|anon-EST:fe2H6	0	0
6777	39241	Drosophila melanogaster	JIL-1	CG6297 gene product from transcript CG6297-RA	2Ab17|CG6297|DmelCG6297|IL-1|JIL|JIL1|Jil-1|Jil1|MSK|Su(var)3-1|Su(var)3-103|Su-var(3)1|Su-var(3)103|anon-WO0118547.379|mAb2A	0	0
6778	39242	Drosophila melanogaster	CG6279	CG6279 gene product from transcript CG6279-RA	DmelCG6279	0	0
6779	39243	Drosophila melanogaster	CG6272	CG6272 gene product from transcript CG6272-RA	BcDNA:GH10915|CT19628|DmelCG6272	0	0
6780	39244	Drosophila melanogaster	APP-BP1	beta-Amyloid precursor protein binding protein 1	APPBP1|CG7828|DmelCG7828|anon-WO0140519.225|dAPP-BP1	0	0
6781	39245	Drosophila melanogaster	Elo68beta	Elongase 68beta	CG11801|DmelCG11801|elo68beta	0	0
6782	39246	Drosophila melanogaster	CG32071	CG32071 gene product from transcript CG32071-RA	CG6261|DmelCG32071	0	0
6783	39248	Drosophila melanogaster	CG12522	CG12522 gene product from transcript CG12522-RA	DmelCG12522	0	0
6784	39250	Drosophila melanogaster	CG7638	CG7638 gene product from transcript CG7638-RA	DmelCG7638	0	0
6785	39251	Drosophila melanogaster	Sod	Superoxide dismutase	CG11793|Cu|Cu-Zn SOD|Cu/Zn SOD|Cu/Zn sod|Cu/Zn superoxide dismutase|Cu/ZnSOD|CuSOD|CuZn SOD|CuZn-SOD|CuZn-SOD1|CuZnSOD|Cu[2+]/Zn[2+]SOD|DmelCG11793|G|SOD|SOD-1|SOD1|S[illegible]	0	0
6786	39252	Drosophila melanogaster	fd68A	forkhead domain 68A	CG11799|DmLD16137|DmMNF|DmelCG11799|FoxK|MNF|Mnf|fork head transcription factor long isoform|fork head transcription factor short isoform	0	0
6787	39253	Drosophila melanogaster	mRpL2	mitochondrial ribosomal protein L2	CG7636|DmelCG7636|L2	0	0
6788	39254	Drosophila melanogaster	Ufd1-like	Ubiquitin fusion-degradation 1-like	CG6233|DmelCG6233|UFD1|Udf1|anon-WO0118547.405|dUfd1|ufd1-like	0	0
6789	39255	Drosophila melanogaster	CG42575	CG42575 gene product from transcript CG42575-RA	CG7628|CR32078|DmelCG42575|Dmel_CG7628|Dmel_CR32078|Dromel_CG7628_FBtr0076213_mORF	0	0
6790	39256	Drosophila melanogaster	CG14143	CG14143 gene product from transcript CG14143-RA	DmelCG14143|chr3L_11128040_11128584.0	0	0
6791	39257	Drosophila melanogaster	CG6216	CG6216 gene product from transcript CG6216-RB	DmelCG6216	0	0
6792	39258	Drosophila melanogaster	CG32082	CG32082 gene product from transcript CG32082-RC	CG7624|DmelCG32082|anon-WO0118547.199	0	0
6793	39259	Drosophila melanogaster	wls	wntless	CG6210|DmelCG6210|Evi|Evi/Wls|Srt|Wls|Wls/Evi|evi|srt|wls/evi	0	0
6794	39260	Drosophila melanogaster	CG7616	CG7616 gene product from transcript CG7616-RA	DmelCG7616|FBgn 36142	0	0
6795	39261	Drosophila melanogaster	CG14142	CG14142 gene product from transcript CG14142-RC	DmelCG14142	0	0
6796	39262	Drosophila melanogaster	GlcAT-P	CG6207 gene product from transcript CG6207-RE	CG6207|DGlcAT-P|DmGlcAT-BSII|DmelCG6207	0	0
6797	39263	Drosophila melanogaster	CG7607	CG7607 gene product from transcript CG7607-RA	BcDNA:RH68451|CT23219|DmelCG7607	0	0
6798	39264	Drosophila melanogaster	CG14141	CG14141 gene product from transcript CG14141-RA	BcDNA:RH33338|CT33742|DmelCG14141	0	0
6799	39265	Drosophila melanogaster	Plod	procollagen lysyl hydroxylase	CG6199|DmelCG6199|LH3|anon-EST:ParkEST325|anon-WO0118547.416|dPlod	0	0
6800	39266	Drosophila melanogaster	Ube3a	Ubiquitin protein ligase E3A	AS|As|CG6190|DmelCG6190|Dube3a|UBE3A|dUBE3A|das|dube3A|dube3a	0	0
6801	39267	Drosophila melanogaster	CG7600	CG7600 gene product from transcript CG7600-RA	DmelCG7600	0	0
6802	39268	Drosophila melanogaster	CG42671	CG42671 gene product from transcript CG42671-RG	CG18490|CG34240|DmelCG42671|Dmel_CG18490|Dmel_CG34240	0	0
6803	39269	Drosophila melanogaster	Ir68a	Ionotropic receptor 68a	CG6185|CT19410|DmelCG6185|IR68a	0	0
6804	39270	Drosophila melanogaster	scyl	scylla	7590|CG7590|DmelCG7590|anon-WO0118547.215|scy	0	0
6805	39271	Drosophila melanogaster	CG6175	CG6175 gene product from transcript CG6175-RB	DmelCG6175|anon-WO0257455.23	0	0
6806	39272	Drosophila melanogaster	CG7573	CG7573 gene product from transcript CG7573-RB	DmelCG7573	0	0
6807	39273	Drosophila melanogaster	CG6168	CG6168 gene product from transcript CG6168-RB	DmelCG6168	0	0
6808	39274	Drosophila melanogaster	CG6163	CG6163 gene product from transcript CG6163-RA	DmelCG6163	0	0
6809	39275	Drosophila melanogaster	CG11726	CG11726 gene product from transcript CG11726-RA	DmelCG11726|cg11726	0	0
6810	39276	Drosophila melanogaster	CG7560	CG7560 gene product from transcript CG7560-RA	BEST:GH18008|DmelCG7560|gh18008	0	0
6811	39277	Drosophila melanogaster	CG6149	CG6149 gene product from transcript CG6149-RB	DmelCG6149	0	0
6812	39278	Drosophila melanogaster	CG7557	CG7557 gene product from transcript CG7557-RA	DmelCG7557|anon-WO0140519.44	0	0
6813	39279	Drosophila melanogaster	CG12289	CG12289 gene product from transcript CG12289-RA	BcDNA:AT04143|DmelCG12289	0	0
6814	39280	Drosophila melanogaster	CG7551	CG7551 gene product from transcript CG7551-RB	BcDNA:AT07524|DmelCG7551	0	0
6815	39281	Drosophila melanogaster	CG6140	CG6140 gene product from transcript CG6140-RA	DmelCG6140	0	0
6816	39282	Drosophila melanogaster	Vha16-2	Vacuolar H[+] ATPase subunit 16-2	CG32089|CG7547|DmelCG32089|V-ATPase|vha16-2	0	0
6817	39284	Drosophila melanogaster	chrb	charybde	BcDNA:GH09771|CG7533|DmelCG7533|EP(3)1035|anon-EST:Liang-2.51|char|clone 2.51	0	0
6818	39285	Drosophila melanogaster	Sgs8	Salivary gland secretion 8	68C GII|CG6132|DmelCG6132|Sgs|Sgs-8|sgs-8|sgs8	0	0
6819	39288	Drosophila melanogaster	Sgs3	Salivary gland secretion 3	68C GII|CG11720|DMSGS378|DmelCG11720|Muc68Cb|SGS-3|SGS378|Sgs|Sgs-3|sgs-3|sgs3	0	0
6820	39289	Drosophila melanogaster	CG7512	CG7512 gene product from transcript CG7512-RC	DmelCG7512	0	0
6821	39293	Drosophila melanogaster	Mob2	CG11711 gene product from transcript CG11711-RC	BEST:GH06341|BcDNA:GH13236|CG11711|CG11712|DMob2|DmelCG11711|Dmob1|Dmob2|Mob1|MobI|dmob2|gh06341	0	0
6822	39294	Drosophila melanogaster	CG7394	CG7394 gene product from transcript CG7394-RC	BcDNA:RE52392|D.M.UKP|DmelCG7394	0	0
6823	39297	Drosophila melanogaster	rt	rotated abdomen	CG6097|DPOMT1|DmelCG6097|POMT1|Rot_abd|dPOMT1|fs(3)neo1|pomt	0	0
6824	39300	Drosophila melanogaster	CG14137	CG14137 gene product from transcript CG14137-RA	DmelCG14137	0	0
6825	39301	Drosophila melanogaster	CG7368	CG7368 gene product from transcript CG7368-RB	DmelCG7368	0	0
6826	39302	Drosophila melanogaster	CG6091	CG6091 gene product from transcript CG6091-RF	DmelCG6091|ped	0	0
6827	39304	Drosophila melanogaster	CG6084	CG6084 gene product from transcript CG6084-RB	34F4T|DmelCG6084|ESTS:34F4T	0	0
6828	39305	Drosophila melanogaster	CG6083	CG6083 gene product from transcript CG6083-RA	DmelCG6083	0	0
6829	39306	Drosophila melanogaster	PCID2	PCI domain-containing protein 2	CG7351|DmelCG7351|dmPCID2	0	0
6830	39307	Drosophila melanogaster	CG32091	CG32091 gene product from transcript CG32091-RB	CG14136|CG7346|DmelCG32091	0	0
6831	39308	Drosophila melanogaster	CG6071	CG6071 gene product from transcript CG6071-RA	DmelCG6071	0	0
6832	39309	Drosophila melanogaster	CG11660	CG11660 gene product from transcript CG11660-RA	DmelCG11660	0	0
6833	39310	Drosophila melanogaster	CG7339	CG7339 gene product from transcript CG7339-RA	DmelCG7339	0	0
6834	39311	Drosophila melanogaster	CG32085	CG32085 gene product from transcript CG32085-RA	BcDNA:RH06780|CG14134|CG6060|CT33735|DmelCG32085	0	0
6835	39313	Drosophila melanogaster	TfIIEalpha	Transcription factor IIEalpha	CG10415|DmelCG10415|TFIIE|TFIIE-L|TFIIEalpha|TfIIE|TfIIEa|dTFIIE-L|dTFIIEL	0	0
6836	39314	Drosophila melanogaster	Sug	CG7334 gene product from transcript CG7334-RE	CG7334|DmelCG7334	0	0
6837	39315	Drosophila melanogaster	pallidin	CG14133 gene product from transcript CG14133-RB	CG14133|DmelCG14133	0	0
6838	39316	Drosophila melanogaster	CG14135	CG14135 gene product from transcript CG14135-RB	DmelCG14135	0	0
6839	39317	Drosophila melanogaster	CG11652	CG11652 gene product from transcript CG11652-RA	DmelCG11652	0	0
6840	39318	Drosophila melanogaster	CG6053	CG6053 gene product from transcript CG6053-RB	DmelCG6053	0	0
6841	39319	Drosophila melanogaster	CG11658	CG11658 gene product from transcript CG11658-RC	DmelCG11658|anon-WO0118547.413	0	0
6842	39320	Drosophila melanogaster	CG42630	CG42630 gene product from transcript CG42630-RA	CG7319|DmelCG42630|Dmel_CG7319	0	0
6843	39321	Drosophila melanogaster	crim	crimpled	CG6038|DmelCG6038	0	0
6844	39322	Drosophila melanogaster	Bmcp	CG7314 gene product from transcript CG7314-RB	CG7314|DmBMCP|DmUCP5|DmelCG7314|UCP5|dmUCP5|ucp5	0	0
6845	39323	Drosophila melanogaster	CG32087	CG32087 gene product from transcript CG32087-RA	CG7308|DmelCG32087	0	0
6846	39324	Drosophila melanogaster	Gr68a	Gustatory receptor 68a	68D.1|CG7303|DmelCG7303|GR68D.1|Gr68D1	0	0
6847	39325	Drosophila melanogaster	CG6024	CG6024 gene product from transcript CG6024-RD	CT18912|DmelCG6024	0	0
6848	39326	Drosophila melanogaster	Muc68D	Mucin 68D	CG6004|DmelCG6004|anon-WO0153538.47|anon-WO0153538.48	0	0
6849	39327	Drosophila melanogaster	Tim13	CG11611 gene product from transcript CG11611-RA	CG11611|DmelCG11611	0	0
6850	39328	Drosophila melanogaster	CG14131	CG14131 gene product from transcript CG14131-RA	DmelCG14131	0	0
6851	39329	Drosophila melanogaster	Pi3K68D	Phosphotidylinositol 3 kinase 68D	11621|BcDNA:LD15217|CG11621|Cpk|DmelCG11621|PI(3)K|PI3K|PI3K 68D|PI3K 68_D|PI3K-68D|PI3K-68D/E|PI3K68D|PI3K_68D|PI[[3]]K|Pi3K|cpk|dPI3K|dPIK	0	0
6852	39330	Drosophila melanogaster	CG5964	CG5964 gene product from transcript CG5964-RA	DmelCG5964	0	0
6853	39331	Drosophila melanogaster	CG10907	CG10907 gene product from transcript CG10907-RA	DmelCG10907	0	0
6854	39332	Drosophila melanogaster	Klp68D	Kinesin-like protein at 68D	CG7293|DmKlp68D|DmelCG7293|KIF3B|KLP-5|KLP5|KLP68D|KLP68Ddm|KLP[[64D/68D]]|Klp5|klp68d	0	0
6855	39333	Drosophila melanogaster	CG10361	CG10361 gene product from transcript CG10361-RA	DmelCG10361	0	0
6856	39334	Drosophila melanogaster	CG42255	CG42255 gene product from transcript CG42255-RA	CG32092|CG32094|CG5956|CT18701|DmelCG42255|Dmel_CG32092|Dmel_CG32094	0	0
6857	39335	Drosophila melanogaster	CG14130	CG14130 gene product from transcript CG14130-RA	DmelCG14130	0	0
6858	39336	Drosophila melanogaster	CG5946	CG5946 gene product from transcript CG5946-RB	DmelCG5946	0	0
6859	39337	Drosophila melanogaster	CG11597	CG11597 gene product from transcript CG11597-RC	DmelCG11597|PP4	0	0
6860	39338	Drosophila melanogaster	RpL10Ab	Ribosomal protein L10Ab	BcDNA:RH06366|CG7283|DmelCG7283|L10Ab|RpL10Ae|anon-EST:Posey188	0	0
6861	39339	Drosophila melanogaster	CG7264	CG7264 gene product from transcript CG7264-RA	DmelCG7264	0	0
6862	39340	Drosophila melanogaster	CycA	Cyclin A	0033/02|0040/24|0046/23|0104/07|0245/34|0521/06|0545/13|1329/16|1449/05|CG5940|CLD3|CYCA|Cyc A|DmcycA|DmelCG5940|S(rux)3B|anon-WO0140519.128|cycA|hari|l(3)03946|l(3)183|l(3)68Ea|l(3)j3[illegible]	0	0
6863	39343	Drosophila melanogaster	CG5906	CG5906 gene product from transcript CG5906-RA	BcDNA:AT16953|DmelCG5906	0	0
6864	39344	Drosophila melanogaster	CG14128	CG14128 gene product from transcript CG14128-RD	DmelCG14128	0	0
6865	39345	Drosophila melanogaster	CG14127	CG14127 gene product from transcript CG14127-RA	DmelCG14127	0	0
6866	39346	Drosophila melanogaster	CG5897	CG5897 gene product from transcript CG5897-RA	DmelCG5897	0	0
6867	39347	Drosophila melanogaster	CG11588	CG11588 gene product from transcript CG11588-RA	DmelCG11588	0	0
6868	39348	Drosophila melanogaster	CG5718	CG5718 gene product from transcript CG5718-RA	DmelCG5718	0	0
6869	39349	Drosophila melanogaster	byn	brachyenteron	Byn|CG7260|D-TRA|D-Trg|DTrg|Dm-BYN|DmelCG7260|Tra|Trg|apro|byn/apro|dm-Trg|trg	0	0
6870	39350	Drosophila melanogaster	CG14126	CG14126 gene product from transcript CG14126-RA	DmelCG14126	0	0
6871	39351	Drosophila melanogaster	Rpt4R	CG7257 gene product from transcript CG7257-RA	7257|CG7257|DmCG7257|Dm_Rpt4c|DmelCG7257	0	0
6872	39352	Drosophila melanogaster	CG5883	CG5883 gene product from transcript CG5883-RA	DmelCG5883	0	0
6873	39353	Drosophila melanogaster	CG7252	CG7252 gene product from transcript CG7252-RA	BcDNA:LP01340|DmelCG7252	0	0
6874	39354	Drosophila melanogaster	CG17826	CG17826 gene product from transcript CG17826-RA	DmelCG17826	0	0
6875	39355	Drosophila melanogaster	obst-G	obstructor-G	CG9781|DmelCG9781	0	0
6876	39356	Drosophila melanogaster	CG7248	CG7248 gene product from transcript CG7248-RA	DmelCG7248	0	0
6877	39357	Drosophila melanogaster	CG11570	CG11570 gene product from transcript CG11570-RB	DmelCG11570	0	0
6878	39359	Drosophila melanogaster	CG14125	CG14125 gene product from transcript CG14125-RA	DmelCG14125	0	0
6879	39360	Drosophila melanogaster	CG6947	CG6947 gene product from transcript CG6947-RA	DmelCG6947	0	0

6880	39361	Drosophila melanogaster	CG17824	CG17824 gene product from transcript CG17824-RA	DmelCG17824	0	0
6881	39362	Drosophila melanogaster	CG6938	CG6938 gene product from transcript CG6938-RB	DmelCG6938	0	0
6882	39363	Drosophila melanogaster	CG6931	CG6931 gene product from transcript CG6931-RA	DmelCG6931	0	0
6883	39364	Drosophila melanogaster	viaf	viral IAP-associated factor	CG18593|DmelCG18593|VIAF|anon-WO0134798.17|anon-WO0134798.18|viaf1	0	0
6884	39365	Drosophila melanogaster	Grip163	CG5688 gene product from transcript CG5688-RA	CG5688|DGrip163|Dgrip163|Dgrips163|DmelCG5688|GCP6|grip163	0	0
6885	39366	Drosophila melanogaster	Pop2	CG5684 gene product from transcript CG5684-RA	CAF1|CG5684|Caf1|DmelCG5684|POP2|pop2	0	0
6886	39367	Drosophila melanogaster	CG6928	CG6928 gene product from transcript CG6928-RB	DmelCG6928	0	0
6887	39368	Drosophila melanogaster	rols	rolling pebbles	ANTS|Ants|CG12277|CG17155|CG32096|CG5679|DmelCG32096|Rol/Ants|Rols|Rols7|anon-WO0257455.19|ants|ants/rols|l(3)08232|l(3)68Fd|l(3)j2A6|rolsants	0	0
6888	39369	Drosophila melanogaster	CG6793	CG6793 gene product from transcript CG6793-RA	DmelCG6793	0	0
6889	39373	Drosophila melanogaster	CG17154	CG17154 gene product from transcript CG17154-RA	DmelCG17154	0	0
6890	39374	Drosophila melanogaster	l(3)j2D3	lethal (3) j2D3	CG6801|DmelCG6801|l(3)68Fb|l(3)jD3	0	0
6891	39375	Drosophila melanogaster	ssp	sunspot	CG17153|DmelCG17153|Ssp	0	0
6892	39376	Drosophila melanogaster	CG11560	CG11560 gene product from transcript CG11560-RA	DmelCG11560	0	0
6893	39377	Drosophila melanogaster	yps	ypsilon schachtel	CG5654|DmelCG5654|YPS|Yps|p45|p57	0	0
6894	39378	Drosophila melanogaster	Ir68b	Ionotropic receptor 68b	CG17152|CT38092|DmelCG17152|IR68b	0	0
6895	39381	Drosophila melanogaster	CG11534	CG11534 gene product from transcript CG11534-RA	BEST:GH12489|DmelCG11534	0	0
6896	39382	Drosophila melanogaster	CG5645	CG5645 gene product from transcript CG5645-RA	DmelCG5645	0	0
6897	39383	Drosophila melanogaster	Atg12	Autophagy-specific gene 12	CG10861|DmelCG10861|atg12	0	0
6898	39385	Drosophila melanogaster	RhoGAP68F	CG6811 gene product from transcript CG6811-RA	CG 6811|CG6811|DmelCG6811	0	0
6899	39386	Drosophila melanogaster	CG5642	CG5642 gene product from transcript CG5642-RA	DmelCG5642	0	0
6900	39387	Drosophila melanogaster	Nrx-IV	Neurexin IV	CG6827|CT21123|DmelCG6827|Dnrx|NRX|NRX IV|Nrx|Nrx IV|NrxIV|Nx IV|l(3)68Ff|l(3)rsg23|nrx|nrx IV|nrx-IV|nrxIV|rsg23	0	0
6901	39388	Drosophila melanogaster	CG9760	CG9760 gene product from transcript CG9760-RC	DmelCG9760	0	0
6902	39389	Drosophila melanogaster	Rh7	Rhodopsin 7	CG5638|DMELRH7|DmelCG5638|Rh|anon-WO0170980.34|anon-WO0170980.35|rh7	0	0
6903	39390	Drosophila melanogaster	CAH2	Carbonic anhydrase 2	CG6906|CT21171|DmelCG6906	0	0
6904	39391	Drosophila melanogaster	CG6910	CG6910 gene product from transcript CG6910-RB	DmelCG6910	0	0
6905	39392	Drosophila melanogaster	Est-6	Esterase 6	CG6917|DmelCG6917|EST-5|EST-6|EST6|Est|Est 6|Est-5|Est-5B|Est-D|Est6|est-6|est6	0	0
6906	39393	Drosophila melanogaster	Est-P	Esterase P	CG17148|DmelCG17148|EST-5|ESTP|Est-5|Est-7|Est7|EstP|PsiEst-6|est-P|psiEst-6|yEst-6|yEst6	0	0
6907	39394	Drosophila melanogaster	thoc6	CG5632 gene product from transcript CG5632-RA	CG5632|DmelCG5632|THOC6	0	0
6908	39395	Drosophila melanogaster	CG11529	CG11529 gene product from transcript CG11529-RA	148640_at|DmelCG11529|SP84	0	0
6909	39396	Drosophila melanogaster	Adk1	Adenylate kinase-1	ADK-1|AK1|CG17146|DAK1|DmelCG17146|adk1|bs34e10.y1	0	0
6910	39397	Drosophila melanogaster	CG5626	CG5626 gene product from transcript CG5626-RA	DmelCG5626	0	0
6911	39399	Drosophila melanogaster	app	approximated	CG17144|CG42318|CG5620|CR32097|DmelCG42318|Dmel_CG17144|Dmel_CG5620	0	0
6912	39400	Drosophila melanogaster	CG32100	CG32100 gene product from transcript CG32100-RA	CG10429|CG18638|DmelCG32100	0	0
6913	39402	Drosophila melanogaster	Pbgs	Porphobilinogen synthase	CG10335|DmPBGS|DmelCG10335	0	0
6914	39403	Drosophila melanogaster	CG4300	CG4300 gene product from transcript CG4300-RB	DmelCG4300|anon-WO0118547.387	0	0
6915	39404	Drosophila melanogaster	CG10426	CG10426 gene product from transcript CG10426-RA	DmelCG10426|IPP	0	0
6916	39405	Drosophila melanogaster	CG4328	CG4328 gene product from transcript CG4328-RB	DmelCG4328	0	0
6917	39406	Drosophila melanogaster	CG32105	CG32105 gene product from transcript CG32105-RB	CG10432|CG17615|DmelCG32105	0	0
6918	39408	Drosophila melanogaster	CG10418	CG10418 gene product from transcript CG10418-RA	BcDNA:GM14851|DmelCG10418	0	0
6919	39409	Drosophila melanogaster	GRHRII	CG10698 gene product from transcript CG10698-RA	CG10698|Crz-R|DCR|DGRHRII|DmelCG10698|anon-WO0170980.196|anon-WO0170980.197	0	0
6920	39410	Drosophila melanogaster	Ncc69	sodium chloride cotransporter 69	BEST:CK01027|CG43567|CG4357|CK01027|DmelCG4357|NCC69	0	0
6921	39411	Drosophila melanogaster	Pbprp1	Pheromone-binding protein-related protein 1	69a|CG10436|DmelCG10436|Obp69a|PB-PRP1|PBP1|PBPRP-1|pbprp-1|pbprp1	0	0
6922	39413	Drosophila melanogaster	CG32104	CG32104 gene product from transcript CG32104-RB	CG14123|CG14124|DmelCG32104|anon-69Ag	0	0
6923	39414	Drosophila melanogaster	Smyd4	SET and MYND domain protein 4	CG14122|DmelCG14122|dSmyd4	0	0
6924	39415	Drosophila melanogaster	CG32103	CG32103 gene product from transcript CG32103-RB	CG18022|CG4392|DmelCG32103	0	0
6925	39417	Drosophila melanogaster	Gap69C	GTPase-activating protein 69C	ARF1-GAP|CG4237|DmelCG4237|GAP69C|dArf1-GAP|gap69|i51	0	0
6926	39418	Drosophila melanogaster	toe	twin of eyg	CG10704|DmelCG10704|Toe|anon-WO0172774.89	0	0
6927	39419	Drosophila melanogaster	eyg	eyegone	BK27|Bk27|CG10488|DmelCG10488|EYG|Eyg|lun	0	0
6928	39420	Drosophila melanogaster	CG10616	CG10616 gene product from transcript CG10616-RB	DmelCG10616	0	0
6929	39421	Drosophila melanogaster	CG10663	CG10663 gene product from transcript CG10663-RB	CT29868|DmelCG10663|SP30	0	0
6930	39422	Drosophila melanogaster	CG10660	CG10660 gene product from transcript CG10660-RA	DmelCG10660	0	0
6931	39423	Drosophila melanogaster	CG10657	CG10657 gene product from transcript CG10657-RA	BEST:LD32330|DmelCG10657	0	0
6932	39424	Drosophila melanogaster	CG10638	CG10638 gene product from transcript CG10638-RA	CG32101|DmelCG10638|anon-WO0172774.89	0	0
6933	39425	Drosophila melanogaster	CG10681	CG10681 gene product from transcript CG10681-RA	DmelCG10681	0	0
6934	39426	Drosophila melanogaster	CG10646	CG10646 gene product from transcript CG10646-RA	DmelCG10646|anon-WO0172774.52	0	0
6935	39428	Drosophila melanogaster	CG10654	CG10654 gene product from transcript CG10654-RA	DmelCG10654	0	0
6936	39429	Drosophila melanogaster	sti	sticky	CG10522|CIT-K|Citron K|DCK|DmelCG10522|STI|anon-WO0118547.388|cit|dck|l(3)7m-62|l(3)7m62|l(3)SG21	0	0
6937	39430	Drosophila melanogaster	tral	trailer hitch	BEST:GH08269|CG10686|DmLSm15|DmelCG10686|LSm15|Lsm15|Scd6p|TRAL|Tral|anon-EST:Posey139|anon-WO0172774.89|anon-WO02059370.24|p67|p70	0	0
6938	39431	Drosophila melanogaster	Pcaf	CG4107 gene product from transcript CG4107-RA	Ada4|CG4107|DmelCG4107|GCN5|Gcn5|Gcn5/PCAF|Gcn5/Pcaf|KAT|P/CAF|PCAF|anon-WO0172774.89|dGCN5|dGCN5 HAT|dGcn5|dKAT2|dPCAF|dmGCN5|dmHAG401|gcn5|p/CAF	0	0
6939	39432	Drosophila melanogaster	ver	verrocchio	1479/10|CG14121|DmelCG14121|anon-WO0172774.86|anon-WO0172774.87|anon-WO0172774.89|l(3)S147910	0	0
6940	39433	Drosophila melanogaster	eIF-2beta	Eukaryotic initiation factor 2beta	CG4153|D-eIF2beta|DmelCG4153|Eif2beta|IF2B|eIF-2|eIF2beta|elF2beta	0	0
6941	39434	Drosophila melanogaster	CG10627	CG10627 gene product from transcript CG10627-RA	DmelCG10627	0	0
6942	39435	Drosophila melanogaster	Tsf2	Transferrin 2	CG10620|DmelCG10620|MTf	0	0
6943	39436	Drosophila melanogaster	CG10688	CG10688 gene product from transcript CG10688-RA	DmelCG10688	0	0
6944	39437	Drosophila melanogaster	CG4069	CG4069 gene product from transcript CG4069-RA	DmelCG4069|anon-WO0172774.58	0	0
6945	39438	Drosophila melanogaster	sowah	sosondowah	BcDNA:GM09162|CG10632|DmelCG10632	0	0
6946	39439	Drosophila melanogaster	ara	araucan	Ara|CG10571|DmelCG10571|IRO-C|Iro|Iro-C|iro|iro-C	0	0
6947	39440	Drosophila melanogaster	caup	caupolican	CG10605|Caup|DmelCG10605|IRO-C|Iro|Iro-C|iro|iro-C	0	0
6948	39441	Drosophila melanogaster	mirr	mirror	CG10601|DH1|De1|De3|DmelCG10601|Group F|IRO-C|Iro|Iro-C|Mirr|Mrr|Sai|cre|crep|group F|iro|iro-C|l(3)69Ca|l(3)69Da|l(3)6D1|l(3)A5-3-42|mir|mrr	0	0
6949	39443	Drosophila melanogaster	Ptp69D	Protein tyrosine phosphatase 69D	CG10975|CT30751|DPTP|DPTP69D|DmelCG10975|Dptp69D|PTP69D|PTPase|Ptp|Ptp69d|R-PTP 69D|dptp69D|dptp69d|ptp69D|ptp69d	0	0
6950	39444	Drosophila melanogaster	CG32109	CG32109 gene product from transcript CG32109-RA	CG10982|DmelCG32109	0	0
6951	39445	Drosophila melanogaster	Klc	Kinesin light chain	CG5433|DKLC|DmelCG5433|KLC|kinesin|klc	0	0
6952	39446	Drosophila melanogaster	CG10984	CG10984 gene product from transcript CG10984-RC	DmelCG10984	0	0
6953	39447	Drosophila melanogaster	CG10973	CG10973 gene product from transcript CG10973-RA	DmelCG10973	0	0
6954	39448	Drosophila melanogaster	CG32113	CG32113 gene product from transcript CG32113-RB	CG11002|CG11003|DmelCG32113	0	0
6955	39450	Drosophila melanogaster	Hip1	Huntingtin interacting protein 1	CG10971|DmelCG10971|Hip1R	0	0
6956	39451	Drosophila melanogaster	CG10969	CG10969 gene product from transcript CG10969-RA	DmelCG10969	0	0
6957	39452	Drosophila melanogaster	CG17666	CG17666 gene product from transcript CG17666-RA	DmelCG17666|bs31g10.y1	0	0
6958	39453	Drosophila melanogaster	CG42709	CG42709 gene product from transcript CG42709-RC	CG17667|DmelCG42709|Dmel_CG17667|EP(3)3622|EP3622	0	0
6959	39454	Drosophila melanogaster	Atg1	Autophagy-specific gene 1	ATG1|CG10967|DK-4|DmATG1|DmelCG10967|ULK1|UNC 51-like|Unc-51|anon-WO0118547.287|atg1|dATG1|l(3)00305|unc-51|unc51	0	0
6960	39455	Drosophila melanogaster	Sap130	Sin3A-associated protein 130	BcDNA:LD21213|CG11006|DmelCG11006|SAP130|l(3)06924	0	0
6961	39456	Drosophila melanogaster	CG10754	CG10754 gene product from transcript CG10754-RA	DmelCG10754	0	0
6962	39457	Drosophila melanogaster	CG42588	CG42588 gene product from transcript CG42588-RA	BP1016|BcDNA:SD20456|CG11008|CG32108|CG34017|DmelCG42588|Dmel_CG11008|Dmel_CG32108|Dmel_CG34017	0	0
6963	39458	Drosophila melanogaster	CG10960	CG10960 gene product from transcript CG10960-RC	CT30701|DmelCG10960	0	0
6964	39459	Drosophila melanogaster	CG10948	CG10948 gene product from transcript CG10948-RB	CT30663|DmelCG10948|cg10948	0	0
6965	39460	Drosophila melanogaster	CG11009	CG11009 gene product from transcript CG11009-RB	DmelCG11009	0	0
6966	39461	Drosophila melanogaster	Ent3	Equilibrative nucleoside transporter 3	CG11010|DmENT3|DmelCG11010	0	0
6967	39462	Drosophila melanogaster	CG10943	CG10943 gene product from transcript CG10943-RA	DmelCG10943	0	0
6968	39463	Drosophila melanogaster	CG14120	CG14120 gene product from transcript CG14120-RA	DmelCG14120	0	0
6969	39464	Drosophila melanogaster	CG14119	CG14119 gene product from transcript CG14119-RA	DmelCG14119	0	0
6970	39465	Drosophila melanogaster	CG14118	CG14118 gene product from transcript CG14118-RA	DmelCG14118	0	0
6971	39466	Drosophila melanogaster	CG12520	CG12520 gene product from transcript CG12520-RB	DmelCG12520|FBgn0036324	0	0
6972	39467	Drosophila melanogaster	CG10752	CG10752 gene product from transcript CG10752-RA	DmelCG10752|anon-WO0140519.236	0	0
6973	39469	Drosophila melanogaster	CG10748	CG10748 gene product from transcript CG10748-RA	DmelCG10748	0	0
6974	39470	Drosophila melanogaster	CG10749	CG10749 gene product from transcript CG10749-RA	DmelCG10749	0	0
6975	39471	Drosophila melanogaster	CG11262	CG11262 gene product from transcript CG11262-RA	DmelCG11262	0	0
6976	39472	Drosophila melanogaster	CG11263	CG11263 gene product from transcript CG11263-RA	DmelCG11263	0	0
6977	39473	Drosophila melanogaster	CG14117	CG14117 gene product from transcript CG14117-RD	BcDNA:RE44251|DmelCG14117	0	0
6978	39474	Drosophila melanogaster	CG11261	CG11261 gene product from transcript CG11261-RA	CUL6|DmelCG11261	0	0
6979	39475	Drosophila melanogaster	MICAL-like	CG11259 gene product from transcript CG11259-RA	AAK93415|CG11259|DmelCG11259	0	0
6980	39476	Drosophila melanogaster	CG11267	CG11267 gene product from transcript CG11267-RA	DmelCG11267|Hsp10|anon-WO0118547.175	0	0
6981	39477	Drosophila melanogaster	mRpL20	mitochondrial ribosomal protein L20	CG11258|DmelCG11258|L20|anon-WO0118547.265	0	0
6982	39478	Drosophila melanogaster	ste14	isoprenylcysteine carboxylmethyltransferase	CG11268|DmelCG11268|DmelSte14|Ste14	0	0
6983	39479	Drosophila melanogaster	CG11255	CG11255 gene product from transcript CG11255-RA	DmelCG11255	0	0
6984	39480	Drosophila melanogaster	RpS12	Ribosomal protein S12	BcDNA:RH29995|CG11271|DmelCG11271|RS12|RpS12E|S12|l(3)s2783	0	0
6985	39481	Drosophila melanogaster	CG11253	CG11253 gene product from transcript CG11253-RA	DmelCG11253	0	0
6986	39483	Drosophila melanogaster	SRm160	CG11274 gene product from transcript CG11274-RA	CG11274|DmelCG11274|SRM160	0	0
6987	39484	Drosophila melanogaster	RpS4	Ribosomal protein S4	CG11276|DmelCG11276|M(3)69E|RPS4|SX4|anon-EST:Posey270|rpS4	0	0
6988	39485	Drosophila melanogaster	Syx13	Syntaxin 13	CG11278|Dm Syx13|DmSyx13|DmelCG11278|SYN13|Syx|ff16|ff16 (69D1-70A2)|l(3)01470	0	0
6989	39486	Drosophila melanogaster	CG11279	CG11279 gene product from transcript CG11279-RA	BcDNA:GM21080|DmelCG11279	0	0
6990	39487	Drosophila melanogaster	CG14115	CG14115 gene product from transcript CG14115-RA	DmelCG14115	0	0

6991	39489	Drosophila melanogaster	CG17300	CG17300 gene product from transcript CG17300-RA	DmelCG17300	0	0
6992	39490	Drosophila melanogaster	CG11251	CG11251 gene product from transcript CG11251-RA	DmelCG11251	0	0
6993	39491	Drosophila melanogaster	trn	tartan	0024/05|0641/17|CG11280|CT31487|DmelCG11280|anon-EST:fe2B1	0	0
6994	39492	Drosophila melanogaster	snky	sneaky	CG11281|DmelCG11281|ms(3)4|ms(3)ry4|ms(3)sneaky|ms(3)snky	0	0
6995	39493	Drosophila melanogaster	caps	capricious	BcDNA:LD07388|CAPS|CG11282|CT31495|Caps|DmelCG11282|l(3)02937|l(3)rM632	0	0
6996	39494	Drosophila melanogaster	Acp70A	Accessory gland peptide 70A	ACP|Acp-70A|Acp70|Acp70a|CG17673|DmelCG17673|DrmSP|PapB|SP|Spg70	0	0
6997	39495	Drosophila melanogaster	CG17687	CG17687 gene product from transcript CG17687-RA	DmelCG17687	0	0
6998	39496	Drosophila melanogaster	SNCF	SoxNeuro Co-Factor	BcDNA:RE54375|CG14112|DmelCG14112|HL6|SCNF	0	0
6999	39497	Drosophila melanogaster	CG14111	CG14111 gene product from transcript CG14111-RA	DmelCG14111	0	0
7000	39498	Drosophila melanogaster	CG14107	CG14107 gene product from transcript CG14107-RA	DmelCG14107	0	0
7001	39499	Drosophila melanogaster	ImpL1	Ecdysone-inducible gene L1	CG10717|DmelCG10717|IMP-L1|Imp-L1	0	0
7002	39500	Drosophila melanogaster	CG14110	CG14110 gene product from transcript CG14110-RA	DmelCG14110	0	0
7003	39501	Drosophila melanogaster	CG10171	CG10171 gene product from transcript CG10171-RB	DmelCG10171	0	0
7004	39502	Drosophila melanogaster	Poc1	Proteome of centrioles 1	CG10191|DmelCG10191|poc1	0	0
7005	39504	Drosophila melanogaster	CG10222	CG10222 gene product from transcript CG10222-RA	DmelCG10222	0	0
7006	39505	Drosophila melanogaster	flr	flare	Aip1|CG10724|DmelCG10724|aip1	0	0
7007	39506	Drosophila melanogaster	CG14106	CG14106 gene product from transcript CG14106-RA	DmelCG14106	0	0
7008	39507	Drosophila melanogaster	CG14105	CG14105 gene product from transcript CG14105-RA	DmelCG14105	0	0
7009	39508	Drosophila melanogaster	CG10713	CG10713 gene product from transcript CG10713-RA	CT28585|DmelCG10713	0	0
7010	39509	Drosophila melanogaster	CG10154	CG10154 gene product from transcript CG10154-RA	DmelCG10154	0	0
7011	39510	Drosophila melanogaster	CG10725	CG10725 gene product from transcript CG10725-RB	DmelCG10725	0	0
7012	39511	Drosophila melanogaster	CG10140	CG10140 gene product from transcript CG10140-RA	DmelCG10140	0	0
7013	39512	Drosophila melanogaster	CG14109	CG14109 gene product from transcript CG14109-RA	DmelCG14109	0	0
7014	39513	Drosophila melanogaster	CG10732	CG10732 gene product from transcript CG10732-RA	DmelCG10732|NEST:bs28e06	0	0
7015	39514	Drosophila melanogaster	CG10133	CG10133 gene product from transcript CG10133-RA	DmelCG10133	0	0
7016	39515	Drosophila melanogaster	CG10116	CG10116 gene product from transcript CG10116-RA	DmelCG10116	0	0
7017	39516	Drosophila melanogaster	CG10738	CG10738 gene product from transcript CG10738-RD	DmelCG10738|GYC	0	0
7018	39517	Drosophila melanogaster	CG10089	CG10089 gene product from transcript CG10089-RD	DmelCG10089	0	0
7019	39518	Drosophila melanogaster	stv	starvin	CG10745|CG14108|CG32130|DmelCG32130|GH04349|Stv|l(3)00543	0	0
7020	39520	Drosophila melanogaster	CG10083	CG10083 gene product from transcript CG10083-RA	DmelCG10083	0	0
7021	39521	Drosophila melanogaster	CG10741	CG10741 gene product from transcript CG10741-RB	DmelCG10741|NEST:bs23g10	0	0
7022	39522	Drosophila melanogaster	Spt20	CG17689 gene product from transcript CG17689-RC	CG17689|DmelCG17689	0	0
7023	39523	Drosophila melanogaster	Vps36	Vacuolar protein sorting 36	CG10711|DmelCG10711|l(3)L5212|vps36	0	0
7024	39524	Drosophila melanogaster	Liprin-beta	CG10743 gene product from transcript CG10743-RB	CG10743|DmelCG10743	0	0
7025	39525	Drosophila melanogaster	CG10710	CG10710 gene product from transcript CG10710-RA	DmelCG10710	0	0
7026	39527	Drosophila melanogaster	bru-3	bruno-3	Bru-3|CG10046|CG12478|Dm Bru-3|DmelCG12478|cg10046|cg12478|l(3)05871	0	0
7027	39528	Drosophila melanogaster	CG8757	CG8757 gene product from transcript CG8757-RB	DmelCG8757	0	0
7028	39529	Drosophila melanogaster	Hml	Hemolectin	CG7002|CT21553|DmelCG7002|d-hml|hml	0	0
7029	39530	Drosophila melanogaster	CG8745	CG8745 gene product from transcript CG8745-RA	DmelCG8745	0	0
7030	39531	Drosophila melanogaster	CG13737	CG13737 gene product from transcript CG13737-RA	DmelCG13737	0	0
7031	39533	Drosophila melanogaster	CG34400	CG34400 gene product from transcript CG34400-RD	CG17368|CG32131|CG8760|DmelCG34400|Dmel_CG17368|Dmel_CG32131|Dmel_CG8760|anon-AE003536.1	0	0
7032	39535	Drosophila melanogaster	CG8833	CG8833 gene product from transcript CG8833-RA	DmelCG8833|GPTC1	0	0
7033	39536	Drosophila melanogaster	Gl	Glued	CG9206|DYNA_DROME|DmelCG9206|Glu|P150|P150[Glued]|p150|p150/Glued|p150/glued|p150[Glued]|p150[glued]|t1	0	0
7034	39537	Drosophila melanogaster	CG32137	CG32137 gene product from transcript CG32137-RA	CG17365|CG17366|DmelCG32137	0	0
7035	39539	Drosophila melanogaster	Meics	Meiotic central spindle	CG8474|DmelCG8474	0	0
7036	39540	Drosophila melanogaster	ssp2	short spindle 2	CG9028|DmelCG9028|Ssp2	0	0
7037	39541	Drosophila melanogaster	CG13738	CG13738 gene product from transcript CG13738-RA	BcDNA:RE61257|DmelCG13738	0	0
7038	39542	Drosophila melanogaster	Hsc70-1	Heat shock protein cognate 1	143191_at|BcDNA:RH49358|CG8937|DmelCG8937|HSC-70|HSC1|HSC70|Hsc1|Hsc70|dhsc70|dhsc70-1|hsc70	0	0
7039	39543	Drosophila melanogaster	CG17364	CG17364 gene product from transcript CG17364-RA	DmelCG17364	0	0
7040	39545	Drosophila melanogaster	CG17362	CG17362 gene product from transcript CG17362-RA	DmelCG17362	0	0
7041	39546	Drosophila melanogaster	CG9040	CG9040 gene product from transcript CG9040-RA	BcDNA:LP10528|DmelCG9040	0	0
7042	39547	Drosophila melanogaster	26-29-p	26-29kD-proteinase	26/29kD-proteinase|CG8947|DmelCG8947|anon-SAGE:Wang-123|l(3)s3635	0	0
7043	39548	Drosophila melanogaster	CG17361	CG17361 gene product from transcript CG17361-RA	DmelCG17361	0	0
7044	39549	Drosophila melanogaster	CG17359	CG17359 gene product from transcript CG17359-RA	DmelCG17359	0	0
7045	39550	Drosophila melanogaster	CG8783	CG8783 gene product from transcript CG8783-RB	DmelCG8783	0	0
7046	39551	Drosophila melanogaster	CG9007	CG9007 gene product from transcript CG9007-RA	DmelCG9007	0	0
7047	39552	Drosophila melanogaster	ptip	CG32133 gene product from transcript CG32133-RA	CG32133|CG6532|CG8797|DmelCG32133	0	0
7048	39554	Drosophila melanogaster	endos	endosulfine	0670/06|CG6513|DmelCG6513|dendos|l(3)S067006	0	0
7049	39555	Drosophila melanogaster	CG6650	CG6650 gene product from transcript CG6650-RA	0670/06|DmelCG6650|anon-WO0172774.121|anon-WO0172774.122|anon-WO0172774.123|l(3)067006|l(3)S067006	0	0
7050	39556	Drosophila melanogaster	CG6661	CG6661 gene product from transcript CG6661-RA	CR33944|DmelCG6661|PUT2	0	0
7051	39557	Drosophila melanogaster	Hsc70Cb	CG6603 gene product from transcript CG6603-RG	0041/12|0318/20|1342/17|1348/02|1485/13|BEST:LD32772|CG6603|DmelCG6603|HSC-70|HSC70|HSP110-1|Hsc70|LD32772|anon-WO0118547.302|dHsp110|hsc70Cb|l(3)00082|l(3)07621|l(3)134217|l(3)1	0	0
7052	39558	Drosophila melanogaster	blue	bluestreak	CG6451|DmelCG6451|bls	0	0
7053	39559	Drosophila melanogaster	CG6833	CG6833 gene product from transcript CG6833-RA	DmelCG6833	0	0
7054	39560	Drosophila melanogaster	CG13484	CG13484 gene product from transcript CG13484-RA	DmelCG13484	0	0
7055	39561	Drosophila melanogaster	CG32138	CG32138 gene product from transcript CG32138-RA	CG13485|CG6807|DmelCG32138|anon-WO0172774.98	0	0
7056	39564	Drosophila melanogaster	btl	breathless	0844/01|BTL/FGFR2|Btl|CG32134|CG6714|CT20816|D-FGFR|DFGF-R1|DFR2|Dfr-2|DmHD-311|DmelCG32134|Dtk2|FGFR|FGFR1|Fgf-r|HD-311|Tk2|dFGFR|dev|dtk2|fgf-r|l(3)00208|lambdatop	0	0
7057	39565	Drosophila melanogaster	CG8100	CG8100 gene product from transcript CG8100-RA	DmelCG8100	0	0
7058	39566	Drosophila melanogaster	Fbp1	Fat body protein 1	CG17285|DmeFBP1|DmelCG17285|FBP1|Fbp-1|P1|fbp-1|fbp1	0	0
7059	39567	Drosophila melanogaster	Sox21a	CG7345 gene product from transcript CG7345-RA	CG7345|DmelCG7345|SOXB2.3|Sox B2-3|Sox21|SoxB2.3	0	0
7060	39569	Drosophila melanogaster	Sox21b	CG32139 gene product from transcript CG32139-RA	CG13483|CG32139|CG6419|DmelCG32139|SOXB2.2|Sox B2-2|SoxB2.2	0	0
7061	39570	Drosophila melanogaster	D	Dichaete	CG5893|DM10|DM23|DM33|DM36|DM63|DmelCG5893|SOX70|SOX70D|SOXB2.1|SOXDP|Sox B2-1|Sox70|Sox70D|Sry-rDM10|Sry-rDM23|Sry-rDM33|Sry-rDM36|Sry-rDM63|fish|l(3)LG9|loD	0	0
7062	39571	Drosophila melanogaster	nan	nanchung	CG5842|CT18317|DmelCG5842|Nan|OCR|TRPV	0	0
7063	39572	Drosophila melanogaster	nuf	nuclear fallout	CG32140|CG33991|CG7867|CIP-D2|Cy3-23|D2|DmelCG33991|EP3077|FIP3|NUF|Nuf|Nuf2p|anon-D2	0	0
7064	39573	Drosophila melanogaster	CG7768	CG7768 gene product from transcript CG7768-RA	DmelCG7768	0	0
7065	39574	Drosophila melanogaster	CG7924	CG7924 gene product from transcript CG7924-RA	DmelCG7924	0	0
7066	39575	Drosophila melanogaster	CG7906	CG7906 gene product from transcript CG7906-RA	DmelCG7906	0	0
7067	39577	Drosophila melanogaster	CG13482	CG13482 gene product from transcript CG13482-RA	DmelCG13482	0	0
7068	39579	Drosophila melanogaster	CG13481	CG13481 gene product from transcript CG13481-RB	DmelCG13481	0	0
7069	39580	Drosophila melanogaster	CG3868	CG3868 gene product from transcript CG3868-RA	DmelCG3868	0	0
7070	39581	Drosophila melanogaster	stwl	stonewall	CG3836|DmelCG3836|fs(3)02024|snw	0	0
7071	39582	Drosophila melanogaster	CG3919	CG3919 gene product from transcript CG3919-RB	DmelCG3919	0	0
7072	39583	Drosophila melanogaster	bbg	big bang	BBG|C96|CG42230|CG9587|CG9598|DmelCG42230|Dmel_CG9587|Dmel_CG9598	0	0
7073	39585	Drosophila melanogaster	CG9592	CG9592 gene product from transcript CG9592-RB	DmelCG9592	0	0
7074	39586	Drosophila melanogaster	CG4613	CG4613 gene product from transcript CG4613-RA	DmelCG4613|SP40	0	0
7075	39587	Drosophila melanogaster	Mpcp	Mitochondrial phosphate carrier protein	CG4994|DmelCG4994|MPCP|Mcpc|PpsM|l(3)00564	0	0
7076	39588	Drosophila melanogaster	CG9238	CG9238 gene product from transcript CG9238-RA	DmelCG9238|anon-WO0118547.400	0	0
7077	39592	Drosophila melanogaster	shd	shade	314a1|CG13478|CYP314A1|Cyp314a1|DmelCG13478|cyp314a1	0	0
7078	39593	Drosophila melanogaster	CG9628	CG9628 gene product from transcript CG9628-RA	DmelCG9628	0	0
7079	39594	Drosophila melanogaster	RecQ5	homolog of RecQ	CG4879|DQ1|Dm RECQ5/QE|DmRECQ5|DmelCG4879|Dq1|Q1|RECQ5|RECQ5/QE|RECQE|anon-EST:Posey293|dRecQ5|dmRecQ5b	0	0
7080	39596	Drosophila melanogaster	dlp	dally-like	CG17703|CG32146|CG5031|CT16138|D-gpcB|Dally-like|Dlp|Dly|DmelCG32146|dly	0	0
7081	39597	Drosophila melanogaster	CG4914	CG4914 gene product from transcript CG4914-RA	DmelCG4914|SP39	0	0
7082	39598	Drosophila melanogaster	CG5048	CG5048 gene product from transcript CG5048-RA	DmelCG5048|anon-WO0140519.238	0	0
7083	39599	Drosophila melanogaster	CG13477	CG13477 gene product from transcript CG13477-RA	DmelCG13477	0	0
7084	39600	Drosophila melanogaster	CG13474	CG13474 gene product from transcript CG13474-RA	BcDNA:AT29351|DmelCG13474	0	0
7085	39601	Drosophila melanogaster	CG17177	CG17177 gene product from transcript CG17177-RA	DmelCG17177	0	0
7086	39602	Drosophila melanogaster	CG13476	CG13476 gene product from transcript CG13476-RB	DmelCG13476	0	0
7087	39603	Drosophila melanogaster	CG13473	CG13473 gene product from transcript CG13473-RA	DmelCG13473	0	0
7088	39604	Drosophila melanogaster	CG13471	CG13471 gene product from transcript CG13471-RA	DmelCG13471	0	0
7089	39608	Drosophila melanogaster	CG9384	CG9384 gene product from transcript CG9384-RA	DmelCG9384	0	0
7090	39609	Drosophila melanogaster	CG17173	CG17173 gene product from transcript CG17173-RA	DmelCG17173	0	0
7091	39610	Drosophila melanogaster	mop	myopic	CG9311|DmelCG9311	0	0
7092	39611	Drosophila melanogaster	bmm	brummer	Atgl|Bmm|CG5295|DmelCG5295	0	0
7093	39612	Drosophila melanogaster	CG13472	CG13472 gene product from transcript CG13472-RA	DmelCG13472	0	0
7094	39613	Drosophila melanogaster	CG9425	CG9425 gene product from transcript CG9425-RB	DmelCG9425	0	0
7095	39615	Drosophila melanogaster	gnu	giant nuclei	CG5272|DmelCG5272|Gnu	0	0
7096	39616	Drosophila melanogaster	CG17839	CG17839 gene product from transcript CG17839-RB	CT39634|DmelCG17839	0	0
7097	39617	Drosophila melanogaster	DCX-EMAP	Doublecortin-domain-containing echinoderm-microtub	CG13466|CG13467|CG42247|DmelCG42247|Dmel_CG13466|Dmel_CG13467|anon-71Aa|dmEMAP	0	0
7098	39619	Drosophila melanogaster	Tom	Twin of m4	Bbu|CG5185|DmelCG5185|DmelCG5185|anon-EST:fe1F6|barbu|bbu/tom|tom	0	0
7099	39620	Drosophila melanogaster	Brd	Bearded	Brd-C|CG3096|DmelCG3096|DmelCG3096|brd	0	0
7100	39621	Drosophila melanogaster	Ocho	CG3396 gene product from transcript CG3396-RB	CG3396|CG5138|DmelCG3396|DmelCG3396|ocho	0	0
7101	39623	Drosophila melanogaster	CG3349	CG3349 gene product from transcript CG3349-RB	DmelCG3349	0	0

7102	39624	Drosophila melanogaster	CG5114	CG5114 gene product from transcript CG5114-RB	DmelCG5114	0	0
7103	39625	Drosophila melanogaster	mnd	minidiscs	CG3297|DmelCG3297|SG29|l(3)B5|l(3)SG29|l(3)ds-5|l(3)ds5|l(3)o52|md	0	0
7104	39626	Drosophila melanogaster	CG10006	CG10006 gene product from transcript CG10006-RB	DmelCG10006	0	0
7105	39627	Drosophila melanogaster	mRpL39	mitochondrial ribosomal protein L39	CG17166|DmelCG17166|MRP-L5|mRpL5	0	0
7106	39628	Drosophila melanogaster	Prosbeta2	Proteasome beta2 subunit	3329|CG3329|DTS-7|DTS7|DmelCG3329|Dts7|Prosbeta|Prosbeta 2|anon-EST:fe1E6|anon-EST:fe1G6|beta2|beta2_dm|l(3)DTS7	0	0
7107	39629	Drosophila melanogaster	Reck	Reversion-inducing-cysteine-rich protein with kazal mo	CG5392|DmelCG5392|RECK	0	0
7108	39630	Drosophila melanogaster	Sytbeta	Synaptotagmin beta	CG13462|CG3020|CG42333|DmelCG42333|Dmel_CG13462|Dmel_CG3020|Syt V|Syt beta|Syt13|SytV|dsytbeta|sytbeta	0	0
7109	39633	Drosophila melanogaster	CG18581	CG18581 gene product from transcript CG18581-RA	DmelCG18581	0	0
7110	39634	Drosophila melanogaster	CG12310	CG12310 gene product from transcript CG12310-RA	BcDNA:RE02926|DmelCG12310	0	0
7111	39635	Drosophila melanogaster	CG13461	CG13461 gene product from transcript CG13461-RA	DmelCG13461	0	0
7112	39636	Drosophila melanogaster	CG18649	CG18649 gene product from transcript CG18649-RA	BcDNA:RE73239|DmelCG18649	0	0
7113	39637	Drosophila melanogaster	CG13463	CG13463 gene product from transcript CG13463-RA	BcDNA:RE10167|DmelCG13463	0	0
7114	39638	Drosophila melanogaster	CG13460	CG13460 gene product from transcript CG13460-RB	DmelCG13460	0	0
7115	39641	Drosophila melanogaster	Or71a	Odorant receptor 71a	71B.1|71a|CG17871|DOR14|DmelCG17871|OR71a|Or71c|or71a	0	0
7116	39643	Drosophila melanogaster	CG6945	CG6945 gene product from transcript CG6945-RA	DmelCG6945	0	0
7117	39644	Drosophila melanogaster	Aats-gly	Glycyl-tRNA synthetase	Aat-gly|CG6778|DmelCG6778|GRS|GlyRS|gars|team	0	0
7118	39645	Drosophila melanogaster	CTPsyn	CTP synthase	CG6854|DmelCG6854|anon-WO0172774.90|anon-WO0172774.92	0	0
7119	39646	Drosophila melanogaster	CG13458	CG13458 gene product from transcript CG13458-RA	DmelCG13458	0	0
7120	39647	Drosophila melanogaster	Cep135	CG17081 gene product from transcript CG17081-RB	Bld10p|CG17081|DCep135|DCep135/Bld10|DmelCG17081|bld10|dbld10	0	0
7121	39648	Drosophila melanogaster	CG16959	CG16959 gene product from transcript CG16959-RB	CT21630|DmelCG16959	0	0
7122	39649	Drosophila melanogaster	CG13457	CG13457 gene product from transcript CG13457-RA	BcDNA:AT15860|BcDNA:AT16542|DmelCG13457	0	0
7123	39650	Drosophila melanogaster	CG12316	CG12316 gene product from transcript CG12316-RB	DmelCG12316	0	0
7124	39651	Drosophila melanogaster	Pdi	Protein disulfide isomerase	CG6988|DmelCG6988|Dpdi|EP(3)1022|PDI|dpdi|pdi	0	0
7125	39652	Drosophila melanogaster	CG6859	CG6859 gene product from transcript CG6859-RA	DmelCG6859	0	0
7126	39653	Drosophila melanogaster	FucTA	CG6869 gene product from transcript CG6869-RA	3-FucT|CG6869|Dm alpha1|DmelCG6869|anon-EST:Posey285	0	0
7127	39654	Drosophila melanogaster	Msh6	CG7003 gene product from transcript CG7003-RA	CG7003|DmelCG7003	0	0
7128	39655	Drosophila melanogaster	Prp31	CG6876 gene product from transcript CG6876-RA	CG6876|DmelCG6876|FBgn0036487|anon-EST:Liang-2.38|clone 2.38	0	0
7129	39656	Drosophila melanogaster	CG6878	CG6878 gene product from transcript CG6878-RA	DmelCG6878	0	0
7130	39657	Drosophila melanogaster	CG7011	CG7011 gene product from transcript CG7011-RA	DmelCG7011	0	0
7131	39658	Drosophila melanogaster	CG6888	CG6888 gene product from transcript CG6888-RA	6888|BcDNA:AT16346|DmelCG6888	0	0
7132	39659	Drosophila melanogaster	Tak1	TGF-beta activated kinase 1	CG1388|CG18492|D-TAK|D-tak|D10|DTAK1|DTak|DTak1|DmTak1|DmelCG18492|TAK|TAK-1|TAK1|Tak|Tak 1|dTAK|dTAK-1|dTAK1|dTak|dTak1|dtak1|tak|tak1	0	0
7133	39660	Drosophila melanogaster	Best4	Bestrophin 4	CG7259|CT22395|DmelCG7259|dbest4	0	0
7134	39661	Drosophila melanogaster	Best3	Bestrophin 3	CG12327|DmelCG12327|dbest3	0	0
7135	39662	Drosophila melanogaster	CG7255	CG7255 gene product from transcript CG7255-RF	DmelCG7255	0	0
7136	39663	Drosophila melanogaster	Toll-6	CG7250 gene product from transcript CG7250-RB	18w-like|CG7250|CT22359|DmelCG7250|TOLL 6|Tl-6|dTLR6|dToll6|toll|toll-6	0	0
7137	39665	Drosophila melanogaster	CG7804	CG7804 gene product from transcript CG7804-RA	DmelCG7804|cg7804	0	0
7138	39666	Drosophila melanogaster	ran-like	CG7815 gene product from transcript CG7815-RA	BcDNA:GH25818|CG7815|DmelCG7815|Ran-like|anon-WO0140519.150	0	0
7139	39667	Drosophila melanogaster	CG13455	CG13455 gene product from transcript CG13455-RA	DmelCG13455	0	0
7140	39668	Drosophila melanogaster	CG7276	CG7276 gene product from transcript CG7276-RB	DmelCG7276	0	0
7141	39669	Drosophila melanogaster	CG7275	CG7275 gene product from transcript CG7275-RA	DmelCG7275	0	0
7142	39670	Drosophila melanogaster	CG7272	CG7272 gene product from transcript CG7272-RA	DmelCG7272	0	0
7143	39671	Drosophila melanogaster	CG7857	CG7857 gene product from transcript CG7857-RA	DmelCG7857|anon-Becker1	0	0
7144	39672	Drosophila melanogaster	CG7841	CG7841 gene product from transcript CG7841-RA	DmelCG7841	0	0
7145	39673	Drosophila melanogaster	Z600	CG17962 gene product from transcript CG17962-RA	3.2|CG17962|DMZ60MEX|DmelCG17962|Frs|frs|z600	0	0
7146	39675	Drosophila melanogaster	Eip71CD	Ecdysone-induced protein 28/29kD	CG7266|DROZ60MEX|DmelCG7266|Eip28|Eip28 /29|Eip28/29|Eip71C|MsrA|eip28/29	0	0
7147	39676	Drosophila melanogaster	CG13454	CG13454 gene product from transcript CG13454-RA	DmelCG13454	0	0
7148	39677	Drosophila melanogaster	mex1	midgut expression 1	CG7936|DmelCG7936	0	0
7149	39678	Drosophila melanogaster	yellow-k	CG7463 gene product from transcript CG7463-RA	CG7463|DmelCG7463	0	0
7150	39679	Drosophila melanogaster	CG7945	CG7945 gene product from transcript CG7945-RB	CG17014|DmelCG7945	0	0
7151	39681	Drosophila melanogaster	obst-H	obstructor-H	CG17014|CG33983|CG7973|CG7973(short)|DmelCG33983|FBgn0036507	0	0
7152	39682	Drosophila melanogaster	CrebA	Cyclic-AMP response element binding protein A	BBF-2|BBF2_DROME|BOX B Binding Factor-2|Bbbf2|BcDNA:SD05937|CG7450|CRE-BP|CREB|CREB-A|CREB-a|CREBA|Creb|Creb A|Creb-A|DmelCG7450|creb|crebA|dCREB-A|dCREBA|dCreb-A|dCreb[illegible]	0	0
7153	39683	Drosophila melanogaster	AGO2	Argonaute 2	AG02|AGO 2|Ago-2|Ago2|CG13452|CG7439|Dm Ago2|DmelCG7439|ago|ago-2|ago2|dAGO2|dAgo2|eIF2C2	0	0
7154	39684	Drosophila melanogaster	CG7739	CG7739 gene product from transcript CG7739-RA	DmelCG7739	0	0
7155	39685	Drosophila melanogaster	CG7427	CG7427 gene product from transcript CG7427-RC	DmelCG7427	0	0
7156	39686	Drosophila melanogaster	CG6498	CG6498 gene product from transcript CG6498-RA	DmelCG6498|MAST 205|MAST205	0	0
7157	39687	Drosophila melanogaster	CG16979	CG16979 gene product from transcript CG16979-RA	DmelCG16979|UFSP2	0	0
7158	39688	Drosophila melanogaster	mrn	marionette	154538_at|CG7764|DmelCG7764|Dmp52|TFB2|Tfb2|anon-WO0118547.648|l(3)E35|ms(3)nc16|nc16|p52	0	0
7159	39689	Drosophila melanogaster	CG12301	CG12301 gene product from transcript CG12301-RA	DmelCG12301	0	0
7160	39690	Drosophila melanogaster	CG12304	CG12304 gene product from transcript CG12304-RA	DmelCG12304|p38	0	0
7161	39691	Drosophila melanogaster	CG7656	CG7656 gene product from transcript CG7656-RA	DmelCG7656	0	0
7162	39693	Drosophila melanogaster	RhoGAP71E	CG32149 gene product from transcript CG32149-RD	BcDNA:LD07615|CG16980|CG32149|CG7396|DmelCG32149|RhoGAP-71E1|l(3)j6B9	0	0
7163	39694	Drosophila melanogaster	CG7650	CG7650 gene product from transcript CG7650-RA	BcDNA:AT27057|DmelCG7650	0	0
7164	39695	Drosophila melanogaster	CG13449	CG13449 gene product from transcript CG13449-RA	DmelCG13449	0	0
7165	39696	Drosophila melanogaster	comm3	CG42334 gene product from transcript CG42334-RC	CG13446|CG13447|CG13448|CG33209|CG42334|DmelCG42334|Dmel_CG13446|Dmel_CG33209	0	0
7166	39697	Drosophila melanogaster	CG7372	CG7372 gene product from transcript CG7372-RA	DmelCG7372	0	0
7167	39700	Drosophila melanogaster	Eig71Ea	Ecdysone-induced gene 71Ea	CG16931|DmelCG16931|I|L71|L71-1|gene I|gene-I	0	0
7168	39701	Drosophila melanogaster	Eig71Eb	Ecdysone-induced gene 71Eb	CG7355|DmelCG7355|II|L71|L71-2|gene II|gene-II	0	0
7169	39703	Drosophila melanogaster	Eig71Ec	Ecdysone-induced gene 71Ec	CG7608|DmelCG7608|III|L71|L71-3|LP11175p|gene III|gene-III	0	0
7170	39704	Drosophila melanogaster	Eig71Ed	Ecdysone-induced gene 71Ed	BcDNA:LP04160|CG7350|DmelCG7350|IV|L71|L71-4|gene IV|gene-IV	0	0
7171	39706	Drosophila melanogaster	Eig71Ee	Ecdysone-induced gene 71Ee	CG7604|DmelCG7604|I71-7|L71|L71-7|VII|eig71Ee|gene VII|gene-VII|gp150	0	0
7172	39707	Drosophila melanogaster	Eig71Ef	Ecdysone-induced gene 71Ef	CG7599|DmelCG7599|EIG71-EF|L71|L71-5|V|gene V|gene-V	0	0
7173	39708	Drosophila melanogaster	Eig71Eg	Ecdysone-induced gene 71Eg	CG7336|DmelCG7336|L71|L71-6|VI|eig71Eg|gene VI|gene-VI	0	0
7174	39709	Drosophila melanogaster	Eig71Eh	CG7594 gene product from transcript CG7594-RB	CG7594|DmelCG7594|EIG71-H|L71|L71-8	0	0
7175	39710	Drosophila melanogaster	Eig71Ei	CG7327 gene product from transcript CG7327-RA	CG7327|DmelCG7327|EIG71-EI|L71|L71-9	0	0
7176	39711	Drosophila melanogaster	Eig71Ej	CG7588 gene product from transcript CG7588-RA	CG7588|DmelCG7588|L71|L71-10	0	0
7177	39712	Drosophila melanogaster	Eig71Ek	CG7325 gene product from transcript CG7325-RA	CG7325|DmelCG7325|EIG71-K|L71|L71-11	0	0
7178	39713	Drosophila melanogaster	CG7304	CG7304 gene product from transcript CG7304-RB	DmelCG7304	0	0
7179	39714	Drosophila melanogaster	CG7579	CG7579 gene product from transcript CG7579-RA	DmelCG7579	0	0
7180	39715	Drosophila melanogaster	pgant8	polypeptide GalNAc transferase 8	CG7297|DmelCG7297	0	0
7181	39716	Drosophila melanogaster	comm2	CG7554 gene product from transcript CG7554-RA	CG7554|DmelCG7554	0	0
7182	39717	Drosophila melanogaster	comm	commissureless	CG17943|Comm|DmelCG17943	0	0
7183	39719	Drosophila melanogaster	CG13445	CG13445 gene product from transcript CG13445-RA	DmelCG13445	0	0
7184	39720	Drosophila melanogaster	fwe	flower	CG6151|DmelCG6151|anon-WO0118547.242	0	0
7185	39721	Drosophila melanogaster	CkIIalpha-i1	CKII-alpha subunit interactor-1	CG6215|CKIIalpha-I1|CKIIalpha-il|DmA35|DmelCG6215|ZFP47|dCKIIalpha-i1	0	0
7186	39722	Drosophila melanogaster	Dcp2	Decapping protein 2	BEST:LD07107|CG6169|DCP2|DCp2p|DmelCG6169|LD07107|dDCP2|dDcp|dDcp2	0	0
7187	39724	Drosophila melanogaster	CG12713	CG12713 gene product from transcript CG12713-RA	DmelCG12713	0	0
7188	39725	Drosophila melanogaster	CG18081	CG18081 gene product from transcript CG18081-RB	BcDNA:RE63284|DmelCG18081	0	0
7189	39726	Drosophila melanogaster	CG15715	CG15715 gene product from transcript CG15715-RB	BcDNA:RE26825|DmelCG15715	0	0
7190	39727	Drosophila melanogaster	CG32150	CG32150 gene product from transcript CG32150-RA	CG12712|CG15714|DmelCG32150	0	0
7191	39729	Drosophila melanogaster	CG12486	CG12486 gene product from transcript CG12486-RA	DmelCG12486	0	0
7192	39730	Drosophila melanogaster	pHCl	CG33989 gene product from transcript CG33989-RI	BP1024|BcDNA:GH21757|CG13444|CG33989|CG6112|DmelCG33989|pHCL-A	0	0
7193	39732	Drosophila melanogaster	sff	sugar-free frosting	CG6114|DmelCG6114	0	0
7194	39733	Drosophila melanogaster	Pka-C3	cAMP-dependent protein kinase 3	5-23|CG6117|DC2|DmelCG6117|Dpck|PKA|PKA C3|PKA-C-3|PKA-C3|Pk72A|Pk?5	0	0
7195	39734	Drosophila melanogaster	GXIVsPLA2	CG17035 gene product from transcript CG17035-RC	CG17035|DmelCG17035	0	0
7196	39735	Drosophila melanogaster	elgi	early girl	CG17033|DmelCG17033	0	0
7197	39736	Drosophila melanogaster	CG17032	CG17032 gene product from transcript CG17032-RA	DmelCG17032	0	0
7198	39737	Drosophila melanogaster	l(3)72Ab	lethal (3) 72Ab	Brr2|CG5931|DmelCG5931|anon-EST:Liang-1.21|cg5931|clone 1.21	0	0
7199	39738	Drosophila melanogaster	CG10516	CG10516 gene product from transcript CG10516-RA	DmelCG10516	0	0
7200	39739	Drosophila melanogaster	CG17026	CG17026 gene product from transcript CG17026-RA	DmelCG17026|cg17026	0	0
7201	39740	Drosophila melanogaster	CG17029	CG17029 gene product from transcript CG17029-RA	DmelCG17029|IMP	0	0
7202	39741	Drosophila melanogaster	CG17028	CG17028 gene product from transcript CG17028-RA	DmelCG17028|IMP	0	0
7203	39742	Drosophila melanogaster	CG17027	CG17027 gene product from transcript CG17027-RA	152392_at|DmelCG17027|IMP	0	0
7204	39744	Drosophila melanogaster	brm	brahma	BRM|Brm|CG18438|CG5942|DmelCG5942|E(E2F)3A|dBRM|dSNF2|l(3)72Aa	0	0
7205	39745	Drosophila melanogaster	Arf72A	Arflike at 72A	72CDd|ARL1|ARL1_DROME|Arf|Arl1|BcDNA:GM20805|CG 6025|CG6025|DmelCG6025|arf72A|arf[72A]|arl|dARL1p|dArl1|l(3)72Ae|l(3)72CDd	0	0
7206	39746	Drosophila melanogaster	DNApol-delta	DNA-polymerase-delta	5949|CAA61369|CG5949|DNA Pol Delta|Dm Poldelta|DmelCG5949|l(3)72Ac	0	0
7207	39747	Drosophila melanogaster	Hip14	Huntingtin-interacting protein 14	3L1|CG 6017|CG6017|DmelCG6017|dHIP14|hip14|l(3)72Ad	0	0
7208	39748	Drosophila melanogaster	CG5830	CG5830 gene product from transcript CG5830-RA	DmelCG5830	0	0
7209	39749	Drosophila melanogaster	mRpS31	mitochondrial ribosomal protein S31	CG5904|DmelCG5904|MRP-S31|RpS31	0	0
7210	39750	Drosophila melanogaster	mib1	mind bomb 1	CG5841|D-mib|D-mib1|DmelCG5841|Dmib|MIB1|Mib|Mib1|dmib1|l(3)72CDa|l(3)72Db|mib|mind-bomb	0	0
7211	39751	Drosophila melanogaster	Notum	CG13076 gene product from transcript CG13076-RB	CG13076|DmelCG13076|Not|anon-WO0118547.482|anon-WO03062410.10|anon-WO03062410.8|l(3)72CDf|l(3)72Da|notum|wf	0	0
7212	39752	Drosophila melanogaster	CG5895	CG5895 gene product from transcript CG5895-RA	DmelCG5895	0	0

7213	39753	Drosophila melanogaster	th	thread	0736/01|1065/03|CG12284|D-IAP1|D-iap1|DIAP|DIAP I|DIAP-1|DIAP1|DIAPI|Diap|Diap-1|Diap-1/Th|Diap1|DmelCG12284|E(rpr)3-1|IAP|IAP1|Iap1|S048915|dIAP|dIAP-1|dIAP1|dIap1|diap|diap 1|diap-1|diap1	0	0
7214	39756	Drosophila melanogaster	CG13075	CG13075 gene product from transcript CG13075-RB	DmelCG13075	0	0
7215	39757	Drosophila melanogaster	Taspase1	Taspase 1	CG5241|DmelCG5241|anon-WO0118547.172|l(3)72Dl	0	0
7216	39758	Drosophila melanogaster	CG5235	CG5235 gene product from transcript CG5235-RA	DmelCG5235	0	0
7217	39759	Drosophila melanogaster	ClC-c	Chloride channel-c	CG5284|DmClC-c|DmClCc|DmelCG5284|anon-WO0138359.7	0	0
7218	39760	Drosophila melanogaster	CG13074	CG13074 gene product from transcript CG13074-RA	DmelCG13074	0	0
7219	39761	Drosophila melanogaster	CG5389	CG5389 gene product from transcript CG5389-RA	DmelCG5389	0	0
7220	39762	Drosophila melanogaster	CG5414	CG5414 gene product from transcript CG5414-RB	DmelCG5414	0	0
7221	39763	Drosophila melanogaster	IntS9	Integrator 9	CG5222|DmelCG5222|Int9|Ints9	0	0
7222	39764	Drosophila melanogaster	Zn72D	Zinc-finger protein at 72D	CG5215|DmelCG5215|l(3)72Dk	0	0
7223	39765	Drosophila melanogaster	Taf4	TBP-associated factor 4	CG5444|DmelCG5444|SR3-3|SY3-2 TAF110|TAF|TAF110|TAF4|TAFII110|TAF[II]110|TAF[[II]]|TAF[[II]]110|TFIID|TFIID 110|Taf110|Taf[[II]]110|d110|dTAF4|dTAFII110|dTAF[[II]]110|dmTAF4|l(3)72Dj|p110|taf110	0	0
7224	39766	Drosophila melanogaster	CG12272	CG12272 gene product from transcript CG12272-RB	DmelCG12272|p118	0	0
7225	39768	Drosophila melanogaster	SsRbeta	Signal sequence receptor beta	BcDNA.LD10457|BcDNA:LD10457|CG5474|DmelCG5474|anon-EST:Liang-1.1|clone 1.1|l(3)s1939	0	0
7226	39769	Drosophila melanogaster	l(3)72Dh	lethal (3) 72Dh	BcDNA:RE62842|CG5161|DmelCG5161|E(smoDN)B-left|Group B-left	0	0
7227	39770	Drosophila melanogaster	CG16838	CG16838 gene product from transcript CG16838-RC	DmelCG16838	0	0
7228	39771	Drosophila melanogaster	CG5157	CG5157 gene product from transcript CG5157-RA	DmelCG5157	0	0
7229	39772	Drosophila melanogaster	CG5151	CG5151 gene product from transcript CG5151-RE	DmelCG5151	0	0
7230	39773	Drosophila melanogaster	CG13073	CG13073 gene product from transcript CG13073-RB	DmelCG13073	0	0
7231	39774	Drosophila melanogaster	l(3)72Dn	lethal (3) 72Dn	CG5018|DmelCG5018|l(3)j5A4	0	0
7232	39775	Drosophila melanogaster	CG5027	CG5027 gene product from transcript CG5027-RA	DmelCG5027|LD24073p	0	0
7233	39776	Drosophila melanogaster	PDCD-5	CG13072 gene product from transcript CG13072-RA	BcDNA:RE17056|CG13072|DMPDCD-5|DmelCG13072	0	0
7234	39777	Drosophila melanogaster	MED10	Mediator complex subunit 10	BcDNA:SD24044|CG5057|DmelCG5057|Med10|Nut2|dMED10|dTRAP15|l(3)72Ds|med10	0	0
7235	39778	Drosophila melanogaster	l(3)72Dr	lethal (3) 72Dr	CG32154|CG5082|DmelCG32154	0	0
7236	39779	Drosophila melanogaster	CG13055	CG13055 gene product from transcript CG13055-RA	DmelCG13055	0	0
7237	39780	Drosophila melanogaster	CG13054	CG13054 gene product from transcript CG13054-RA	DmelCG13054	0	0
7238	39781	Drosophila melanogaster	CG13071	CG13071 gene product from transcript CG13071-RB	DmelCG13071	0	0
7239	39782	Drosophila melanogaster	CG13070	CG13070 gene product from transcript CG13070-RB	DmelCG13070	0	0
7240	39783	Drosophila melanogaster	CG4950	CG4950 gene product from transcript CG4950-RB	CT15890|DmelCG4950	0	0
7241	39784	Drosophila melanogaster	CG13068	CG13068 gene product from transcript CG13068-RA	DmelCG13068	0	0
7242	39785	Drosophila melanogaster	CG13067	CG13067 gene product from transcript CG13067-RA	DmelCG13067|anon-WO0153538.39|anon-WO0153538.46	0	0
7243	39786	Drosophila melanogaster	CG13065	CG13065 gene product from transcript CG13065-RA	BcDNA:RH56745|DmelCG13065	0	0
7244	39787	Drosophila melanogaster	CG13050	CG13050 gene product from transcript CG13050-RA	DmelCG13050	0	0
7245	39788	Drosophila melanogaster	CG13049	CG13049 gene product from transcript CG13049-RB	BcDNA:RE26528|DmelCG13049	0	0
7246	39789	Drosophila melanogaster	CG13048	CG13048 gene product from transcript CG13048-RA	DmelCG13048	0	0
7247	39790	Drosophila melanogaster	CG13047	CG13047 gene product from transcript CG13047-RB	DmelCG13047	0	0
7248	39791	Drosophila melanogaster	CG13046	CG13046 gene product from transcript CG13046-RB	DmelCG13046	0	0
7249	39792	Drosophila melanogaster	CG13045	CG13045 gene product from transcript CG13045-RB	DmelCG13045	0	0
7250	39793	Drosophila melanogaster	CG4962	CG4962 gene product from transcript CG4962-RA	CT15908|DmelCG4962	0	0
7251	39794	Drosophila melanogaster	CG4982	CG4982 gene product from transcript CG4982-RA	DmelCG4982	0	0
7252	39795	Drosophila melanogaster	CG13044	CG13044 gene product from transcript CG13044-RA	DmelCG13044	0	0
7253	39796	Drosophila melanogaster	CG13043	CG13043 gene product from transcript CG13043-RA	BcDNA:RH10371|CT32262|DmelCG13043	0	0
7254	39797	Drosophila melanogaster	CG13063	CG13063 gene product from transcript CG13063-RA	DmelCG13063	0	0
7255	39798	Drosophila melanogaster	CG13042	CG13042 gene product from transcript CG13042-RA	DmelCG13042	0	0
7256	39799	Drosophila melanogaster	CG13062	CG13062 gene product from transcript CG13062-RB	DmelCG13062	0	0
7257	39801	Drosophila melanogaster	CG13041	CG13041 gene product from transcript CG13041-RA	BcDNA:RE63063|DmelCG13041	0	0
7258	39802	Drosophila melanogaster	CG13060	CG13060 gene product from transcript CG13060-RA	BcDNA:RE40431|DmelCG13060	0	0
7259	39803	Drosophila melanogaster	CG13059	CG13059 gene product from transcript CG13059-RA	DmelCG13059	0	0
7260	39804	Drosophila melanogaster	CG13040	CG13040 gene product from transcript CG13040-RA	DmelCG13040	0	0
7261	39805	Drosophila melanogaster	CG13039	CG13039 gene product from transcript CG13039-RA	BcDNA:AT27312|DmelCG13039	0	0
7262	39806	Drosophila melanogaster	CG13058	CG13058 gene product from transcript CG13058-RA	DmelCG13058	0	0
7263	39807	Drosophila melanogaster	CG33060	CG33060 gene product from transcript CG33060-RB	CG13057|DmelCG33060	0	0
7264	39808	Drosophila melanogaster	CG4998	CG4998 gene product from transcript CG4998-RB	DmelCG4998|SPH66	0	0
7265	39809	Drosophila melanogaster	mRpS34	mitochondrial ribosomal protein S34	CG13037|CG42648|DmelCG42648|Dmel_CG13037|MRP-S34	0	0
7266	39810	Drosophila melanogaster	CG4925	CG4925 gene product from transcript CG4925-RA	DmelCG4925	0	0
7267	39811	Drosophila melanogaster	CG4933	CG4933 gene product from transcript CG4933-RA	DmelCG4933	0	0
7268	39812	Drosophila melanogaster	Pdh	Photoreceptor dehydrogenase	CG4899|DmelCG4899|pcdr|pdh	0	0
7269	39813	Drosophila melanogaster	CG4893	CG4893 gene product from transcript CG4893-RA	DmelCG4893	0	0
7270	39814	Drosophila melanogaster	Cpr72Ea	Cuticular protein 72Ea	CG4818|DmelCG4818	0	0
7271	39815	Drosophila melanogaster	Cpr72Eb	Cuticular protein 72Eb	CG12255|DmelCG12255	0	0
7272	39816	Drosophila melanogaster	Cpr72Ec	Cuticular protein 72Ec	CG4784|CT15377|DmelCG4784	0	0
7273	39817	Drosophila melanogaster	CG4842	CG4842 gene product from transcript CG4842-RA	DmelCG4842	0	0
7274	39818	Drosophila melanogaster	roq	roquin	CG16807|DmelCG16807|NEST:bs21b12	0	0
7275	39819	Drosophila melanogaster	CG4753	CG4753 gene product from transcript CG4753-RB	DmelCG4753	0	0
7276	39820	Drosophila melanogaster	CG4729	CG4729 gene product from transcript CG4729-RA	DmelCG4729	0	0
7277	39821	Drosophila melanogaster	CG4877	CG4877 gene product from transcript CG4877-RB	CG4689|DmelCG4877	0	0
7278	39823	Drosophila melanogaster	CG13036	CG13036 gene product from transcript CG13036-RA	DmelCG13036	0	0
7279	39824	Drosophila melanogaster	CG4680	CG4680 gene product from transcript CG4680-RA	DmelCG4680	0	0
7280	39825	Drosophila melanogaster	CG13035	CG13035 gene product from transcript CG13035-RA	DmelCG13035	0	0
7281	39826	Drosophila melanogaster	fax	failed axon connections	CG4609|DmelCG4609|Fax|anon-EST:Liang-1.34|anon-WO0172774.62|anon-WO0172774.63|anon-WO0172774.65|anon-WO0172774.66|anon-WO0172774.67|anon-WO0172774.68|anon-WO0172774.69	0	0
7282	39827	Drosophila melanogaster	TMS1	CG4672 gene product from transcript CG4672-RA	CG4672|DmelCG4672|TMS1d	0	0
7283	39828	Drosophila melanogaster	CG4573	CG4573 gene product from transcript CG4573-RA	DmelCG4573	0	0
7284	39829	Drosophila melanogaster	Aats-tyr	Tyrosyl-tRNA synthetase	CG4561|DmelCG4561|TyrRS|YRS|anon-EST:Posey261|dYARS	0	0
7285	39831	Drosophila melanogaster	dsx-c73A	doublesex cognate 73A	CG13034|CG32159|CG4541|DSC73|DmelCG32159|dsc73|dsx-c73|l(3)j10E8	0	0
7286	39833	Drosophila melanogaster	aos	argos	Aos|Argos|CG4531|DmAos|DmelCG4531|arg|gil|l(3)05845|l(3)05959|l(3)j5E11|l(3)rJ472|l(3)rJ812|l(3)rQ526|rlt|sty	0	0
7287	39834	Drosophila melanogaster	CG33158	CG33158 gene product from transcript CG33158-RB	CG10162|CG4638|DmelCG33158	0	0
7288	39835	Drosophila melanogaster	CG43373	CG43373 gene product from transcript CG43373-RB	AC|Ago2|CG12436|CG32158|CG42513|DmelCG43373|Dmel_CG12436|Dmel_CG32158|Dmel_CG42513	0	0
7289	39836	Drosophila melanogaster	st	scarlet	CG4314|DmelCG4314	0	0
7290	39839	Drosophila melanogaster	CG13033	CG13033 gene product from transcript CG13033-RA	BcDNA:RE50345|DmelCG13033	0	0
7291	39840	Drosophila melanogaster	CG4229	CG4229 gene product from transcript CG4229-RA	DmelCG4229	0	0
7292	39841	Drosophila melanogaster	Mipp1	Multiple inositol polyphosphate phosphatase 1	CG4123|CK2082|DmelCG4123	0	0
7293	39842	Drosophila melanogaster	mbf1	multiprotein bridging factor 1	BcDNA:GH10148|BcDNA:GM01267|CG4143|DmelCG4143|MBF-1|MBF1|MRE4|Mbf1	0	0
7294	39843	Drosophila melanogaster	nxf2	nuclear RNA export factor 2	CG4118|DmelCG4118|NXF2	0	0
7295	39844	Drosophila melanogaster	Smn	survival motor neuron	CG16725|DmSMN|DmelCG16725|SMN|dSMN|l(3)73Ao|smn	0	0
7296	39845	Drosophila melanogaster	Rpn12	CG4157 gene product from transcript CG4157-RA	CG4157|DmelCG4157|dRpn12|p30	0	0
7297	39846	Drosophila melanogaster	CG4169	CG4169 gene product from transcript CG4169-RA	BEST:GH10978|DmelCG4169|anon-EST:Posey245	0	0
7298	39847	Drosophila melanogaster	Syx8	Syntaxin 8	CG4109|Dm Syx8|DmSyx8|DmelCG4109|SYX8|Syx	0	0
7299	39849	Drosophila melanogaster	tra	transformer	CG16724|DmelCG16724|Dmtra|TRA|Tra	0	0
7300	39850	Drosophila melanogaster	spd-2	spindle defective 2	BcDNA:LD24702|CG17286|CG17286-PA|Cep192|D-SPD2|D-Spd2|DSPD-2|DSpd-2|DSpd2|DmelCG17286|Dspd-2|SPD-2|Spd-2|Spd2|anon-73Ba|dSpd2|spd2	0	0
7301	39854	Drosophila melanogaster	CG4098	CG4098 gene product from transcript CG4098-RA	CG4098-PA|DmelCG4098	0	0
7302	39855	Drosophila melanogaster	Pros26	Proteasome 26kD subunit	CG4097|DTS-5|DTS5|DmelCG4097|PSB1_DROME|Pros26-DTS|Prosbeta6|beta6|beta6_dm|l(3)73Ai|l(3)DTS-5|l(3)DTS5|pros26	0	0
7303	39856	Drosophila melanogaster	Su(P)	Suppressor of ref(2)P sterility	CG4086|DmelCG4086	0	0
7304	39857	Drosophila melanogaster	CG4101	CG4101 gene product from transcript CG4101-RA	DmelCG4101|anon-73B1|anon73B1	0	0
7305	39858	Drosophila melanogaster	Mo25	CG4083 gene product from transcript CG4083-RA	CG4083|DmelCG4083|Dmo25|dMo25|l(3)00274|mo25	0	0
7306	39860	Drosophila melanogaster	Baldspot	CG3971 gene product from transcript CG3971-RA	CG 3971|CG3971|DmelCG3971|NEST:bs18f10|NEST:bs18g10|blp|l(3)01895|l(3)02281|l(3)04106|l(3)neo21|l(3)neo22|noa	0	0
7307	39861	Drosophila melanogaster	Galpha73B	G protein alpha 73B	CG12232|DmelCG12232|Gf|Gfalpha	0	0
7308	39862	Drosophila melanogaster	zetaCOP	CG3948 gene product from transcript CG3948-RC	CG3948|CT13124|DmelCG3948|copz1|zeta-Cop|zetaCop	0	0
7309	39863	Drosophila melanogaster	CG13032	CG13032 gene product from transcript CG13032-RA	DmelCG13032	0	0
7310	39864	Drosophila melanogaster	Lasp	CG3849 gene product from transcript CG3849-RB	CG3849|DmelCG3849|anon-73Bd|cDNA B|lasp	0	0
7311	39865	Drosophila melanogaster	CG9692	CG9692 gene product from transcript CG9692-RB	DmelCG9692	0	0
7312	39866	Drosophila melanogaster	Dab	Disabled	CG9695|DmelCG9695|cDNA C|dab	0	0
7313	39867	Drosophila melanogaster	CG13031	CG13031 gene product from transcript CG13031-RA	DmelCG13031	0	0
7314	39868	Drosophila melanogaster	CG13026	CG13026 gene product from transcript CG13026-RB	DmelCG13026	0	0
7315	39869	Drosophila melanogaster	PGRP-SB2	CG9697 gene product from transcript CG9697-RA	CG9697|DmelCG9697	0	0
7316	39870	Drosophila melanogaster	PGRP-SB1	CG9681 gene product from transcript CG9681-RA	142657_at|CG9681|DmelCG9681|PGRP SB1	0	0
7317	39871	Drosophila melanogaster	Dbp73D	Dead box protein 73D	CG9680|DBP73D|DmRH7|DmelCG9680	0	0
7318	39872	Drosophila melanogaster	CG9701	CG9701 gene product from transcript CG9701-RA	DmelCG9701	0	0
7319	39873	Drosophila melanogaster	Nrt	Neurotactin	BP106|BcDNA:LD22004|CG9704|DAB|Dab|DmelCG9704|MabBP106|NRT|NT|Neur|Nrt BP106|dab|l(3)73Be|neu|nrt	0	0
7320	39874	Drosophila melanogaster	CG13025	CG13025 gene product from transcript CG13025-RA	DmelCG13025	0	0
7321	39875	Drosophila melanogaster	CG9705	CG9705 gene product from transcript CG9705-RA	DmelCG9705	0	0
7322	39876	Drosophila melanogaster	CG9706	CG9706 gene product from transcript CG9706-RA	DmelCG9706	0	0
7323	39877	Drosophila melanogaster	Int6	Int6 homologue	BcDNA.GM01233|BcDNA:GM01233|CG9677|DINT6|DmelCG9677|eIF-3p48/INT-6|eIF3-S6|eIF3-p48/INT6|l(3)10547|l(3)j9E8	0	0

7324	39878	Drosophila melanogaster	CG9674	CG9674 gene product from transcript CG9674-RD	DmelCG9674	0	0
7325	39879	Drosophila melanogaster	nudC	CG9710 gene product from transcript CG9710-RB	CG9710|DmelCG9710|DnudC|NUDC|NudC|Q9VVA6	0	0
7326	39880	Drosophila melanogaster	CG13024	CG13024 gene product from transcript CG13024-RC	BcDNA:RH61817|DmelCG13024	0	0
7327	39881	Drosophila melanogaster	TSG101	tumor suppressor protein 101	CG9712|DmTSG101|DmelCG9712|ESCRT-I|Tsg101|dTSg101|dTsg101|dVps23|ept|tsg101|vps23	0	0
7328	39882	Drosophila melanogaster	CG9669	CG9669 gene product from transcript CG9669-RB	DmelCG9669	0	0
7329	39883	Drosophila melanogaster	CG9715	CG9715 gene product from transcript CG9715-RA	DmelCG9715	0	0
7330	39884	Drosophila melanogaster	sina	seven in absentia	CG9949|DmelCG9949|SINA|Sina|anon-EST:fe2H7|sia	0	0
7331	39885	Drosophila melanogaster	sinah	sina homologue	CG13030|DmelCG13030|SinaH|anon-WO0140519.228|sina-h	0	0
7332	39886	Drosophila melanogaster	CG13029	CG13029 gene product from transcript CG13029-RC	BcDNA:AT31918|DmelCG13029|anon-WO0118547.38	0	0
7333	39887	Drosophila melanogaster	Rh4	Rhodopsin 4	CG9668|DMELRH4|Dm Rh4|DmelCG9668|FBgn0003250|RH4|Rh|rh4	0	0
7334	39888	Drosophila melanogaster	CG9951	CG9951 gene product from transcript CG9951-RA	DmelCG9951	0	0
7335	39889	Drosophila melanogaster	Lmpt	Limpet	BcDNA:RE37250|CG11914|CG11915|CG11916|CG32171|CG33256|CG42679|CG9959|DmelCG42679|Dmel_CG11915|Dmel_CG32171|Dmel_CG33256|lmpt	0	0
7336	39894	Drosophila melanogaster	CG13023	CG13023 gene product from transcript CG13023-RA	DmelCG13023	0	0
7337	39895	Drosophila melanogaster	CG11905	CG11905 gene product from transcript CG11905-RB	DmelCG11905	0	0
7338	39896	Drosophila melanogaster	CG13022	CG13022 gene product from transcript CG13022-RB	DmelCG13022	0	0
7339	39897	Drosophila melanogaster	Cpr73D	Cuticular protein 73D	CG9665|DmelCG9665	0	0
7340	39899	Drosophila melanogaster	Nc73EF	Neural conserved at 73EF	141810_at|CG11661|DmelCG11661|E1|FBgn0036682|anon-WO0118547.329	0	0
7341	39900	Drosophila melanogaster	Exn	Ephexin	BcDNA:GH03693|CG14060|CG3799|CG42665|DmelCG42665|Dmel_CG14060|Dmel_CG3799|exn	0	0
7342	39902	Drosophila melanogaster	CG3764	CG3764 gene product from transcript CG3764-RA	DmelCG3764	0	0
7343	39903	Drosophila melanogaster	CG6664	CG6664 gene product from transcript CG6664-RC	DmelCG6664	0	0
7344	39904	Drosophila melanogaster	CG7728	CG7728 gene product from transcript CG7728-RA	DmelCG7728	0	0
7345	39905	Drosophila melanogaster	CG6652	CG6652 gene product from transcript CG6652-RA	DmelCG6652	0	0
7346	39906	Drosophila melanogaster	a10	antennal protein 10	A10|CG6642|DmelCG6642|OS-D|OS-D/A10|Os-D|Phk-1	0	0
7347	39907	Drosophila melanogaster	Fit2	Fermitin 2	CG7729|DmelCG7729	0	0
7348	39908	Drosophila melanogaster	CG7730	CG7730 gene product from transcript CG7730-RC	DmelCG7730	0	0
7349	39909	Drosophila melanogaster	CG14059	CG14059 gene product from transcript CG14059-RB	DmelCG14059	0	0
7350	39910	Drosophila melanogaster	beg	bad egg	CG7842|DmelCG7842	0	0
7351	39914	Drosophila melanogaster	Papst2	PAPS transporter 2	CG7853|DmelCG7853|dPAPST2	0	0
7352	39915	Drosophila melanogaster	CG14057	CG14057 gene product from transcript CG14057-RA	DmelCG14057	0	0
7353	39916	Drosophila melanogaster	TpnC73F	Troponin C at 73F	CG7930|DmTnC3|DmTpnC73F|DmTpnCIa|DmelCG7930|TNC|TNC73F|TnC|TnC73F|TpnC Ia|TpnCIa	0	0
7354	39917	Drosophila melanogaster	rogdi	CG7725 gene product from transcript CG7725-RB	CG7725|DmelCG7725	0	0
7355	39918	Drosophila melanogaster	CG7724	CG7724 gene product from transcript CG7724-RA	DmelCG7724	0	0
7356	39919	Drosophila melanogaster	Rbp6	RNA-binding protein 6	BcDNA:RE25373|CG14055|CG14056|CG32169|CG6573|DmelCG32169|RRM6|rrm6	0	0
7357	39922	Drosophila melanogaster	CG6512	CG6512 gene product from transcript CG6512-RB	DmCG6512|DmelCG6512	0	0
7358	39923	Drosophila melanogaster	CG7707	CG7707 gene product from transcript CG7707-RA	DmelCG7707	0	0
7359	39924	Drosophila melanogaster	CG6497	CG6497 gene product from transcript CG6497-RA	DmelCG6497	0	0
7360	39925	Drosophila melanogaster	CG13723	CG13723 gene product from transcript CG13723-RA	DmelCG13723	0	0
7361	39926	Drosophila melanogaster	CG6485	CG6485 gene product from transcript CG6485-RA	DmelCG6485	0	0
7362	39927	Drosophila melanogaster	CG13724	CG13724 gene product from transcript CG13724-RA	DmelCG13724	0	0
7363	39928	Drosophila melanogaster	CG13725	CG13725 gene product from transcript CG13725-RA	DmelCG13725	0	0
7364	39929	Drosophila melanogaster	Or74a	Odorant receptor 74a	74A.1|74a|CG13726|DOR26|DmelCG13726	0	0
7365	39930	Drosophila melanogaster	CG6479	CG6479 gene product from transcript CG6479-RB	DmelCG6479	0	0
7366	39931	Drosophila melanogaster	CG13727	CG13727 gene product from transcript CG13727-RA	DmelCG13727	0	0
7367	39932	Drosophila melanogaster	brv2	brivido-2	CG16793|CT33194|DmelCG16793	0	0
7368	39933	Drosophila melanogaster	Mip	Myoinhibiting peptide precursor	ASB|AST-B|Ast-B|AstB|AstB-1|CG6456|DAP-B|DmMIP1|DmMIP2|DmMIP3|DmMIP4|DmMIP5|DmelCG6456|Drm-MIP|Drm-MIP-1|Drm-MIP-2|Drm-MIP-3|Drm-MIP-4|Drm-MIP-5|MIP|mip	0	0
7369	39934	Drosophila melanogaster	CG7692	CG7692 gene product from transcript CG7692-RA	DmelCG7692	0	0
7370	39935	Drosophila melanogaster	tap	target of Poxn	CG7659|DmelCG7659|Tap|bHLHc9|bps|l(3)01658	0	0
7371	39936	Drosophila melanogaster	Cad74A	CG6445 gene product from transcript CG6445-RA	CG6445|CT20086|Cad74B|DCad74B|DmelCG6445|cad74A	0	0
7372	39937	Drosophila melanogaster	CG13728	CG13728 gene product from transcript CG13728-RA	DmelCG13728	0	0
7373	39938	Drosophila melanogaster	CG13731	CG13731 gene product from transcript CG13731-RB	DmelCG13731	0	0
7374	39939	Drosophila melanogaster	blot	bloated tubules	CG3897|DmBLOT|DmelCG3897|l(3)1658	0	0
7375	39940	Drosophila melanogaster	sec3	CG3885 gene product from transcript CG3885-RA	CG3885|DmelCG3885|Sec3	0	0
7376	39941	Drosophila melanogaster	CG33051	CG33051 gene product from transcript CG33051-RA	CG3882|DmelCG33051	0	0
7377	39943	Drosophila melanogaster	frc	fringe connection	CG3874|DmelCG3874|FRC|UDP-GlcNAc/UDP-GlcUA|UST74C|anon-WO0172774.5|anon-WO0172774.7|dFRC|l(3)00073|l(3)02619|l(3)10098|l(3)L6750|l(3)S070006|l(3)j2E3|ust74C	0	0
7378	39944	Drosophila melanogaster	CG32176	CG32176 gene product from transcript CG32176-RA	CG13729|CG13730|DmelCG32176	0	0
7379	39945	Drosophila melanogaster	CG12229	CG12229 gene product from transcript CG12229-RA	DmelCG12229	0	0
7380	39947	Drosophila melanogaster	CG18265	CG18265 gene product from transcript CG18265-RA	DmelCG18265|ORE-10	0	0
7381	39948	Drosophila melanogaster	CG7603	CG7603 gene product from transcript CG7603-RA	DmelCG7603	0	0
7382	39949	Drosophila melanogaster	CG7589	CG7589 gene product from transcript CG7589-RA	DmelCG7589	0	0
7383	39950	Drosophila melanogaster	CG7580	CG7580 gene product from transcript CG7580-RA	BcDNA:AT13736|DmelCG7580	0	0
7384	39951	Drosophila melanogaster	CG13733	CG13733 gene product from transcript CG13733-RA	DmelCG13733	0	0
7385	39952	Drosophila melanogaster	qjt	quijote	CG13732|DmelCG13732|Quijote	0	0
7386	39953	Drosophila melanogaster	CG6333	CG6333 gene product from transcript CG6333-RB	DmelCG6333	0	0
7387	39954	Drosophila melanogaster	Oatp74D	Organic anion transporting polypeptide 74D	CG7571|DmelCG7571|oatp 74D	0	0
7388	39955	Drosophila melanogaster	U4-U6-60K	U4-U6 small nuclear riboprotein factor 60K	CG6322|DmelCG6322|Prp4|U4/U6-60K	0	0
7389	39956	Drosophila melanogaster	CG7564	CG7564 gene product from transcript CG7564-RA	DmelCG7564	0	0
7390	39957	Drosophila melanogaster	Edc3	Enhancer of decapping 3	CG6311|DmEDC3|DmelCG6311|EDC3|LSm16	0	0
7391	39958	Drosophila melanogaster	Nedd4	CG42279 gene product from transcript CG42279-RJ	CG32184|CG42279|CG7555|DNedd4|DmelCG42279|Dmel_CG32184|Dmel_CG7555|dNedd4|dNedd4-1|nedd4	0	0
7392	39959	Drosophila melanogaster	Jon74E	Jonah 74E	CG6298|CT19724|DmelCG6298|SP85	0	0
7393	39960	Drosophila melanogaster	CG7542	CG7542 gene product from transcript CG7542-RA	DmelCG7542|SP71	0	0
7394	39961	Drosophila melanogaster	CycT	Cyclin T	CG6292|Dmcyclin T|DmelCG6292|ORE-14|P-TEFb|anon-74EFc|dT|p124	0	0
7395	39962	Drosophila melanogaster	Eip74EF	Ecdysone-induced protein 74EF	3.3|CG32180|CG6273|CG6285|DmelCG32180|E-74|E74|E74-B|E74A|E74A/B|E74B|ECIP|Eip74 A|Eip74A|Eip74E|e74|eip74ef|l(3)24|l(3)neo24|mE74	0	0
7396	39964	Drosophila melanogaster	CG6259	CG6259 gene product from transcript CG6259-RA	DmelCG6259|anon-74EFb|bs36h07.y1	0	0
7397	39965	Drosophila melanogaster	CG7510	CG7510 gene product from transcript CG7510-RA	DmelCG7510|anon-74EFa	0	0
7398	39966	Drosophila melanogaster	CG7497	CG7497 gene product from transcript CG7497-RB	BcDNA:GH27361|CHED-related|DmelCG7497|anon-WO0170980.88|anon-WO0170980.89	0	0
7399	39969	Drosophila melanogaster	CG7484	CG7484 gene product from transcript CG7484-RB	BcDNA:SD16138|DmelCG7484|Sep15	0	0
7400	39970	Drosophila melanogaster	TORC	CG6064 gene product from transcript CG6064-RA	CG6064|CRTC|DmelCG6064|ORE-12|cg6064|dTORC	0	0
7401	39971	Drosophila melanogaster	CG6052	CG6052 gene product from transcript CG6052-RA	DmelCG6052	0	0
7402	39972	Drosophila melanogaster	Ccn	CG32183 gene product from transcript CG32183-RA	CG13735|CG13736|CG32183|DmelCG32183|dCCN	0	0
7403	39973	Drosophila melanogaster	CG7460	CG7460 gene product from transcript CG7460-RB	DmelCG7460	0	0
7404	39974	Drosophila melanogaster	CG6034	CG6034 gene product from transcript CG6034-RA	DmelCG6034	0	0
7405	39975	Drosophila melanogaster	Adgf-B	Adenosine deaminase-related growth factor B	ADGF-B|CG5998|DmelCG5998|adgfb	0	0
7406	39976	Drosophila melanogaster	Adgf-A	Adenosine deaminase-related growth factor A	ADGDF-A|ADGF-A|CG5992|DmelCG5992|adgf-a|adgfa	0	0
7407	39977	Drosophila melanogaster	CG42816	CG42816 gene product from transcript CG42816-RB	CG32186|CG5587|CG5944|DmelCG42816|Dmel_CG32186	0	0
7408	39978	Drosophila melanogaster	NUCB1	CG32190 gene product from transcript CG32190-RA	CG32190|CG5587|DmelCG32190|NucB1|nucb1	0	0
7409	39979	Drosophila melanogaster	CG5589	CG5589 gene product from transcript CG5589-RA	DmRH17|DmelCG5589|cg5589	0	0
7410	39980	Drosophila melanogaster	CG32187	CG32187 gene product from transcript CG32187-RA	CG5584|DPLG2|DmelCG32187	0	0
7411	39981	Drosophila melanogaster	cln3	CG5582 gene product from transcript CG5582-RA	CG5582|CLN3|DmelCG5582	0	0
7412	39982	Drosophila melanogaster	Ir75a	Ionotropic receptor 75a	CG14585|DmelCG14585|IR75a	0	0
7413	39983	Drosophila melanogaster	Ir75c	CG42642 gene product from transcript CG42642-RA	CG14586|CG14586-A|CG42642|DmelCG42642|Dmel_CG14586|IR75c	0	0
7414	39984	Drosophila melanogaster	Grd	Glycine receptor	CG7446|DmGABA(GRD)|DmelCG7446|GRD	0	0
7415	39985	Drosophila melanogaster	CG5577	CG5577 gene product from transcript CG5577-RA	DmelCG5577	0	0
7416	39986	Drosophila melanogaster	CG5567	CG5567 gene product from transcript CG5567-RA	DmelCG5567|Phos5567	0	0
7417	39987	Drosophila melanogaster	MED19	Mediator complex subunit 19	CG5546|DmelCG5546	0	0
7418	39988	Drosophila melanogaster	CG7430	CG7430 gene product from transcript CG7430-RA	DmelCG7430|E3	0	0
7419	39989	Drosophila melanogaster	CG7441	CG7441 gene product from transcript CG7441-RA	BEST:CK01295|DmelCG7441|GH31	0	0
7420	39990	Drosophila melanogaster	CG5535	CG5535 gene product from transcript CG5535-RC	DmelCG5535|anon-WO0118547.111	0	0
7421	39991	Drosophila melanogaster	CG7408	CG7408 gene product from transcript CG7408-RB	DmelCG7408	0	0
7422	39992	Drosophila melanogaster	CG5506	CG5506 gene product from transcript CG5506-RA	BcDNA:LP12049|DmelCG5506	0	0
7423	39993	Drosophila melanogaster	CG16775	CG16775 gene product from transcript CG16775-RB	DmelCG16775	0	0
7424	39994	Drosophila melanogaster	CG7402	CG7402 gene product from transcript CG7402-RA	DmelCG7402	0	0
7425	39995	Drosophila melanogaster	Tsp74F	Tetraspanin 74F	CG5492|Dm.Tsp74F|DmelCG5492	0	0
7426	39996	Drosophila melanogaster	Prestin	CG5485 gene product from transcript CG5485-RA	CG5485|DmelCG5485|dpres	0	0
7427	39997	Drosophila melanogaster	CG14353	CG14353 gene product from transcript CG14353-RA	DmelCG14353	0	0
7428	39998	Drosophila melanogaster	CG5290	CG5290 gene product from transcript CG5290-RA	DmelCG5290	0	0
7429	39999	Drosophila melanogaster	Eip75B	Ecdysone-induced protein 75B	57B|BcDNA:GM02640|CG8127|DmE75A|DmE75B|DmelCG8127|E75|E75-C|E75A|E75B|E75C|EP1121b|Eip75|Eip75 A|NR1D3|anon-WO0172774.31|anon-WO0172774.32|dE75|l(3)07041|l(3)j11A6|l(3)j12E8	0	0
7430	40000	Drosophila melanogaster	CG13698	CG13698 gene product from transcript CG13698-RB	DmelCG13698	0	0
7431	40001	Drosophila melanogaster	mRpS26	mitochondrial ribosomal protein S26	CG7354|DmelCG7354|MRP-S26	0	0
7432	40002	Drosophila melanogaster	CG5147	CG5147 gene product from transcript CG5147-RA	DmelCG5147	0	0
7433	40003	Drosophila melanogaster	mus304	mutagen-sensitive 304	ATRIP|CG7347|DmelCG7347|Mus304|anon-WO0140519.82|atrip|mus(3)304|mus-304	0	0
7434	40004	Drosophila melanogaster	CG7341	CG7341 gene product from transcript CG7341-RB	DmelCG7341	0	0

<table>
<thead><tr><th></th><th></th><th></th><th></th><th></th><th></th><th></th><th></th></tr></thead>
<tbody>
<tr><td>7435</td><td>40005</td><td>Drosophila melanogaster</td><td>Cyp312a1</td><td>CG5137 gene product from transcript CG5137-RA</td><td>312a1|CG5137|DmelCG5137|P450 IV</td><td>0</td><td>0</td></tr>
<tr><td>7436</td><td>40007</td><td>Drosophila melanogaster</td><td>CG7330</td><td>CG7330 gene product from transcript CG7330-RA</td><td>BcDNA:RE22290|DmelCG7330</td><td>0</td><td>0</td></tr>
<tr><td>7437</td><td>40008</td><td>Drosophila melanogaster</td><td>CG13699</td><td>CG13699 gene product from transcript CG13699-RA</td><td>DmelCG13699</td><td>0</td><td>0</td></tr>
<tr><td>7438</td><td>40009</td><td>Drosophila melanogaster</td><td>W</td><td>Wrinkled</td><td>CG5123|DmelCG5123|HID|Hid|Hid1|hid|hid1|his|l(3)05014</td><td>0</td><td>0</td></tr>
<tr><td>7439</td><td>40010</td><td>Drosophila melanogaster</td><td>CG7320</td><td>CG7320 gene product from transcript CG7320-RA</td><td>DmelCG7320</td><td>0</td><td>0</td></tr>
<tr><td>7440</td><td>40011</td><td>Drosophila melanogaster</td><td>CheA75a</td><td>Chemosensory protein A 75a</td><td>BcDNA:HL02449|CG7313|DmelCG7313</td><td>0</td><td>0</td></tr>
<tr><td>7441</td><td>40012</td><td>Drosophila melanogaster</td><td>CG5103</td><td>CG5103 gene product from transcript CG5103-RA</td><td>DmelCG5103</td><td>0</td><td>0</td></tr>
<tr><td>7442</td><td>40013</td><td>Drosophila melanogaster</td><td>CG13700</td><td>CG13700 gene product from transcript CG13700-RB</td><td>DmelCG13700</td><td>0</td><td>0</td></tr>
<tr><td>7443</td><td>40014</td><td>Drosophila melanogaster</td><td>grim</td><td>CG4345 gene product from transcript CG4345-RA</td><td>BcDNA:RE28551|CG4345|DmelCG4345|GRIM|Grim|gri</td><td>0</td><td>0</td></tr>
<tr><td>7444</td><td>40015</td><td>Drosophila melanogaster</td><td>rpr</td><td>reaper</td><td>CG4319|DmelCG4319|RPR|Rpr|anon-WO0162936.19|rp</td><td>0</td><td>0</td></tr>
<tr><td>7445</td><td>40016</td><td>Drosophila melanogaster</td><td>skl</td><td>sickle</td><td>BcDNA:RE14076|CG13701|DmelCG13701|Skl|meph|veto</td><td>0</td><td>0</td></tr>
<tr><td>7446</td><td>40017</td><td>Drosophila melanogaster</td><td>CG4306</td><td>CG4306 gene product from transcript CG4306-RA</td><td>DmelCG4306</td><td>0</td><td>0</td></tr>
<tr><td>7447</td><td>40019</td><td>Drosophila melanogaster</td><td>AICR2</td><td>allatostatin C receptor 2</td><td>ASTC-R2|CG13702|DmelCG13702|Drostar2|anon-WO0170980.154|anon-WO0170980.155</td><td>0</td><td>0</td></tr>
<tr><td>7448</td><td>40020</td><td>Drosophila melanogaster</td><td>star1</td><td>allatostatin C receptor 1</td><td>ASTC-R1|CG7285|DmelCG7285|Drostar1|anon-WO0131005.19|anon-WO0170980.79|anon-WO0170980.80</td><td>0</td><td>0</td></tr>
<tr><td>7449</td><td>40021</td><td>Drosophila melanogaster</td><td>CG7271</td><td>CG7271 gene product from transcript CG7271-RA</td><td>DmelCG7271</td><td>0</td><td>0</td></tr>
<tr><td>7450</td><td>40023</td><td>Drosophila melanogaster</td><td>CG4174</td><td>CG4174 gene product from transcript CG4174-RB</td><td>DmelCG4174</td><td>0</td><td>0</td></tr>
<tr><td>7451</td><td>40024</td><td>Drosophila melanogaster</td><td>CG18234</td><td>CG18234 gene product from transcript CG18234-RC</td><td>DmelCG18234</td><td>0</td><td>0</td></tr>
<tr><td>7452</td><td>40025</td><td>Drosophila melanogaster</td><td>CG18233</td><td>CG18233 gene product from transcript CG18233-RB</td><td>DmelCG18233</td><td>0</td><td>0</td></tr>
<tr><td>7453</td><td>40026</td><td>Drosophila melanogaster</td><td>CG18231</td><td>CG18231 gene product from transcript CG18231-RB</td><td>DmelCG18231</td><td>0</td><td>0</td></tr>
<tr><td>7454</td><td>40028</td><td>Drosophila melanogaster</td><td>CG32199</td><td>CG32199 gene product from transcript CG32199-RA</td><td>CG13382|DmelCG32199</td><td>0</td><td>0</td></tr>
<tr><td>7455</td><td>40029</td><td>Drosophila melanogaster</td><td>CG13380</td><td>CG13380 gene product from transcript CG13380-RB</td><td>DmelCG13380</td><td>0</td><td>0</td></tr>
<tr><td>7456</td><td>40030</td><td>Drosophila melanogaster</td><td>not</td><td>non-stop</td><td>CG4166|DmelCG4166|UBP8|USP22|anon-WO0257455.9|i155|l(3)02069|l(3)j9A6|nonstop</td><td>0</td><td>0</td></tr>
<tr><td>7457</td><td>40031</td><td>Drosophila melanogaster</td><td>bora</td><td>aurora borealis</td><td>CG6897|DmelCG6897</td><td>0</td><td>0</td></tr>
<tr><td>7458</td><td>40032</td><td>Drosophila melanogaster</td><td>MYPT-75D</td><td>CG6896 gene product from transcript CG6896-RA</td><td>CG6896|DmelCG6896</td><td>0</td><td>0</td></tr>
<tr><td>7459</td><td>40033</td><td>Drosophila melanogaster</td><td>GNBP2</td><td>Gram-negative bacteria binding protein 2</td><td>CG4144|DGNBP-2|DmelCG4144|GNBP|gnbp2</td><td>0</td><td>0</td></tr>
<tr><td>7460</td><td>40034</td><td>Drosophila melanogaster</td><td>GNBP1</td><td>Gram-negative bacteria binding protein 1</td><td>CG6895|DGNBP-1|DGNBP1|DmelCG6895|GNBP|GNBP 1|GNBP-1</td><td>0</td><td>0</td></tr>
<tr><td>7461</td><td>40035</td><td>Drosophila melanogaster</td><td>Sgf11</td><td>CG13379 gene product from transcript CG13379-RB</td><td>CG13379|DmelCG13379|dSgf11</td><td>0</td><td>0</td></tr>
<tr><td>7462</td><td>40036</td><td>Drosophila melanogaster</td><td>Chmp1</td><td>CG4108 gene product from transcript CG4108-RA</td><td>CG4108|CHMP1|DmelCG4108</td><td>0</td><td>0</td></tr>
<tr><td>7463</td><td>40037</td><td>Drosophila melanogaster</td><td>Cyp12c1</td><td>CG4120 gene product from transcript CG4120-RA</td><td>12c1|CG4120|DmelCG4120</td><td>0</td><td>0</td></tr>
<tr><td>7464</td><td>40038</td><td>Drosophila melanogaster</td><td>CG6893</td><td>CG6893 gene product from transcript CG6893-RB</td><td>DmelCG6893</td><td>0</td><td>0</td></tr>
<tr><td>7465</td><td>40039</td><td>Drosophila melanogaster</td><td>Dic4</td><td>Dicarboxylate carrier 4</td><td>CG18363|DmDic4p|DmelCG18363</td><td>0</td><td>0</td></tr>
<tr><td>7466</td><td>40040</td><td>Drosophila melanogaster</td><td>CG12477</td><td>CG12477 gene product from transcript CG12477-RA</td><td>DmelCG12477</td><td>0</td><td>0</td></tr>
<tr><td>7467</td><td>40041</td><td>Drosophila melanogaster</td><td>CG6885</td><td>CG6885 gene product from transcript CG6885-RA</td><td>DmelCG6885|anon-WO0118547.731</td><td>0</td><td>0</td></tr>
<tr><td>7468</td><td>40042</td><td>Drosophila melanogaster</td><td>MED11</td><td>Mediator complex subunit 11</td><td>BcDNA:GH01072|CG6884|DmelCG6884|Med11|Med21|anon-WO0153538.71|dMED21</td><td>0</td><td>0</td></tr>
<tr><td>7469</td><td>40043</td><td>Drosophila melanogaster</td><td>Nufip</td><td>CG4076 gene product from transcript CG4076-RA</td><td>CG4076|DmelCG4076|NUFIP</td><td>0</td><td>0</td></tr>
<tr><td>7470</td><td>40044</td><td>Drosophila melanogaster</td><td>Aut1</td><td>CG6877 gene product from transcript CG6877-RA</td><td>Atg3|CG6877|DmelCG6877|Draut1|atg3</td><td>0</td><td>0</td></tr>
<tr><td>7471</td><td>40045</td><td>Drosophila melanogaster</td><td>ftz-f1</td><td>ftz transcription factor 1</td><td>BFTZ-F1|CG13378|CG4059|DEP1|DmFTZ-F1|DmelCG4059|FIP1|FTZ-F1|FTZ-F1alpha|FTZF1|FTZf1alpha|Ftz-F1|Ftz-f1|NR5A3|alphaFTZ-F1|alphaFTZF1|alphaftz-f1|bFTZ-F1|beta FTZ-F1|beta-FTZ-F1|beta</td><td>0</td><td>0</td></tr>
<tr><td>7472</td><td>40046</td><td>Drosophila melanogaster</td><td>CG14073</td><td>CG14073 gene product from transcript CG14073-RB</td><td>DmelCG14073|Nirvana</td><td>0</td><td>0</td></tr>
<tr><td>7473</td><td>40047</td><td>Drosophila melanogaster</td><td>HipHop</td><td>HP1-HOAP-interacting protein</td><td>BcDNA:RE10558|CG6874|DmelCG6874|hiphop|l(3)neo26</td><td>0</td><td>0</td></tr>
<tr><td>7474</td><td>40048</td><td>Drosophila melanogaster</td><td>Cat</td><td>Catalase</td><td>CAT|CATA|CG6871|CT21282|CatA|DMCATHPO|DROCATHPO|DmelCG6871|U00145|bs36h11.y1|cat</td><td>0</td><td>0</td></tr>
<tr><td>7475</td><td>40049</td><td>Drosophila melanogaster</td><td>Indy</td><td>I'm not dead yet</td><td>BEST:LP01220|CG3979|DmelCG3979|INDY|anon-EST:fe3A7|anon-WO0172774.70|drIndy|indy</td><td>0</td><td>0</td></tr>
<tr><td>7476</td><td>40050</td><td>Drosophila melanogaster</td><td>CG6865</td><td>CG6865 gene product from transcript CG6865-RB</td><td>DmelCG6865|SP63</td><td>0</td><td>0</td></tr>
<tr><td>7477</td><td>40051</td><td>Drosophila melanogaster</td><td>CG14074</td><td>CG14074 gene product from transcript CG14074-RA</td><td>DmelCG14074</td><td>0</td><td>0</td></tr>
<tr><td>7478</td><td>40052</td><td>Drosophila melanogaster</td><td>dysb</td><td>dysbindin</td><td>CG6856|DmelCG6856</td><td>0</td><td>0</td></tr>
<tr><td>7479</td><td>40053</td><td>Drosophila melanogaster</td><td>CG6852</td><td>CG6852 gene product from transcript CG6852-RC</td><td>BcDNA:GH24739|DmelCG6852</td><td>0</td><td>0</td></tr>
<tr><td>7480</td><td>40054</td><td>Drosophila melanogaster</td><td>Rad9</td><td>CG3945 gene product from transcript CG3945-RA</td><td>CG3945|DmelCG3945|rad9</td><td>0</td><td>0</td></tr>
<tr><td>7481</td><td>40055</td><td>Drosophila melanogaster</td><td>Ugt</td><td>UDP-glucose-glycoprotein glucosyltransferase</td><td>CG6850|DUGT|DmelCG6850|GT|UGT|anon-WO0172774.73|anon-WO0172774.75</td><td>0</td><td>0</td></tr>
<tr><td>7482</td><td>40056</td><td>Drosophila melanogaster</td><td>CG3961</td><td>CG3961 gene product from transcript CG3961-RC</td><td>DmelCG3961</td><td>0</td><td>0</td></tr>
<tr><td>7483</td><td>40057</td><td>Drosophila melanogaster</td><td>CG11637</td><td>CG11637 gene product from transcript CG11637-RA</td><td>DmelCG11637|NijB</td><td>0</td><td>0</td></tr>
<tr><td>7484</td><td>40059</td><td>Drosophila melanogaster</td><td>CG3902</td><td>CG3902 gene product from transcript CG3902-RA</td><td>CG18168|CT12987|DmelCG3902</td><td>0</td><td>0</td></tr>
<tr><td>7485</td><td>40060</td><td>Drosophila melanogaster</td><td>RpL26</td><td>Ribosomal protein L26</td><td>CG6846|DmelCG6846|L26|RPL26</td><td>0</td><td>0</td></tr>
<tr><td>7486</td><td>40061</td><td>Drosophila melanogaster</td><td>CG3893</td><td>CG3893 gene product from transcript CG3893-RA</td><td>BcDNA:LD18018|DmelCG3893</td><td>0</td><td>0</td></tr>
<tr><td>7487</td><td>40062</td><td>Drosophila melanogaster</td><td>CG6843</td><td>CG6843 gene product from transcript CG6843-RA</td><td>DmelCG6843</td><td>0</td><td>0</td></tr>
<tr><td>7488</td><td>40063</td><td>Drosophila melanogaster</td><td>CSN1b</td><td>COP9 complex homolog subunit 1 b</td><td>CG3889|CH1-2|CSN1|CSN1B|Csn1B|DCH1-2|Dch1-2|DmelCG3889|csn1|dCSN1</td><td>0</td><td>0</td></tr>
<tr><td>7489</td><td>40064</td><td>Drosophila melanogaster</td><td>CG6841</td><td>CG6841 gene product from transcript CG6841-RA</td><td>BcDNA:LD43276|DmelCG6841|Prp6</td><td>0</td><td>0</td></tr>
<tr><td>7490</td><td>40065</td><td>Drosophila melanogaster</td><td>Ir75d</td><td>Ionotropic receptor 75d</td><td>CG14076|CT33663|DmelCG14076|IR75d</td><td>0</td><td>0</td></tr>
<tr><td>7491</td><td>40066</td><td>Drosophila melanogaster</td><td>CG14077</td><td>CG14077 gene product from transcript CG14077-RB</td><td>DmelCG14077|Vla</td><td>0</td><td>0</td></tr>
<tr><td>7492</td><td>40067</td><td>Drosophila melanogaster</td><td>CG6839</td><td>CG6839 gene product from transcript CG6839-RA</td><td>DmelCG6839</td><td>0</td><td>0</td></tr>
<tr><td>7493</td><td>40068</td><td>Drosophila melanogaster</td><td>CG18223</td><td>CG18223 gene product from transcript CG18223-RA</td><td>BcDNA:AT03311|DmelCG18223|SPH200</td><td>0</td><td>0</td></tr>
<tr><td>7494</td><td>40069</td><td>Drosophila melanogaster</td><td>CG3819</td><td>CG3819 gene product from transcript CG3819-RA</td><td>CT12789|DmelCG3819</td><td>0</td><td>0</td></tr>
<tr><td>7495</td><td>40070</td><td>Drosophila melanogaster</td><td>CG6836</td><td>CG6836 gene product from transcript CG6836-RA</td><td>DmelCG6836</td><td>0</td><td>0</td></tr>
<tr><td>7496</td><td>40071</td><td>Drosophila melanogaster</td><td>CG14075</td><td>CG14075 gene product from transcript CG14075-RA</td><td>DmelCG14075</td><td>0</td><td>0</td></tr>
<tr><td>7497</td><td>40072</td><td>Drosophila melanogaster</td><td>CG11619</td><td>CG11619 gene product from transcript CG11619-RA</td><td>DmelCG11619</td><td>0</td><td>0</td></tr>
<tr><td>7498</td><td>40073</td><td>Drosophila melanogaster</td><td>CG18135</td><td>CG18135 gene product from transcript CG18135-RC</td><td>DmelCG18135</td><td>0</td><td>0</td></tr>
<tr><td>7499</td><td>40074</td><td>Drosophila melanogaster</td><td>CG3808</td><td>CG3808 gene product from transcript CG3808-RA</td><td>DmelCG3808|cg3808</td><td>0</td><td>0</td></tr>
<tr><td>7500</td><td>40075</td><td>Drosophila melanogaster</td><td>CG18136</td><td>CG18136 gene product from transcript CG18136-RA</td><td>DmelCG18136</td><td>0</td><td>0</td></tr>
<tr><td>7501</td><td>40078</td><td>Drosophila melanogaster</td><td>Acp76A</td><td>Accessory gland-specific peptide 76A</td><td>Acp76|CG3801|DmelCG3801|Spn76A</td><td>0</td><td>0</td></tr>
<tr><td>7502</td><td>40079</td><td>Drosophila melanogaster</td><td>CG3797</td><td>CG3797 gene product from transcript CG3797-RA</td><td>DmelCG3797</td><td>0</td><td>0</td></tr>
<tr><td>7503</td><td>40080</td><td>Drosophila melanogaster</td><td>CG6812</td><td>CG6812 gene product from transcript CG6812-RA</td><td>DmelCG6812</td><td>0</td><td>0</td></tr>
<tr><td>7504</td><td>40081</td><td>Drosophila melanogaster</td><td>Mkp3</td><td>Mitogen-activated protein kinase phosphatase 3</td><td>CG14080|CG14081|DMKP-3|DmelCG14080|Dmkp3|MKP3|mkp-3|mkp3</td><td>0</td><td>0</td></tr>
<tr><td>7505</td><td>40083</td><td>Drosophila melanogaster</td><td>MESR6</td><td>Misexpression suppressor of ras 6</td><td>CG6818|DmelCG6818</td><td>0</td><td>0</td></tr>
<tr><td>7506</td><td>40084</td><td>Drosophila melanogaster</td><td>CG11577</td><td>CG11577 gene product from transcript CG11577-RA</td><td>DmelCG11577|anon-WO0118547.323</td><td>0</td><td>0</td></tr>
<tr><td>7507</td><td>40085</td><td>Drosophila melanogaster</td><td>CG10424</td><td>CG10424 gene product from transcript CG10424-RA</td><td>DmelCG10424</td><td>0</td><td>0</td></tr>
<tr><td>7508</td><td>40086</td><td>Drosophila melanogaster</td><td>CG14079</td><td>CG14079 gene product from transcript CG14079-RA</td><td>DmelCG14079</td><td>0</td><td>0</td></tr>
<tr><td>7509</td><td>40087</td><td>Drosophila melanogaster</td><td>Gem2</td><td>Gemin2</td><td>CG10419|DmelCG10419</td><td>0</td><td>0</td></tr>
<tr><td>7510</td><td>40088</td><td>Drosophila melanogaster</td><td>CG14082</td><td>CG14082 gene product from transcript CG14082-RB</td><td>DmelCG14082</td><td>0</td><td>0</td></tr>
<tr><td>7511</td><td>40090</td><td>Drosophila melanogaster</td><td>fz2</td><td>frizzled 2</td><td>CG9739|D-Fz2|D-fz2|DFZ2|DFz-2|DFz2|Dfrz2|Dfz2|Dfz[[2]]|Dm Fz2|DmelCG9739|FZ2|Fz2|dFz2|dfz(2)|dfz2|dfz2r|frz2|fz-2</td><td>0</td><td>0</td></tr>
<tr><td>7512</td><td>40091</td><td>Drosophila melanogaster</td><td>mRpL21</td><td>mitochondrial ribosomal protein L21</td><td>CG9730|DmelCG9730|MRP-L21</td><td>0</td><td>0</td></tr>
<tr><td>7513</td><td>40092</td><td>Drosophila melanogaster</td><td>rept</td><td>reptin</td><td>CG9750|DmelCG9750|REPT|Rept|Reptin|Tip48|Tip48/Reptin|anon-WO0142479.1|dRep|dReptin|drep|l(3)06945|rep</td><td>0</td><td>0</td></tr>
<tr><td>7514</td><td>40093</td><td>Drosophila melanogaster</td><td>nes</td><td>nessy</td><td>CG9655|DmelCG9655|Nes</td><td>0</td><td>0</td></tr>
<tr><td>7515</td><td>40094</td><td>Drosophila melanogaster</td><td>Bet1</td><td>CG14084 gene product from transcript CG14084-RB</td><td>BET1|BcDNA:RH40423|CG14084|Dm Bet1|DmelCG14084|G14084|bs16e01.y1</td><td>0</td><td>0</td></tr>
<tr><td>7516</td><td>40095</td><td>Drosophila melanogaster</td><td>Max</td><td>CG9648 gene product from transcript CG9648-RA</td><td>CG9648|DmelCG9648|MAX|bHLHd9|dMax|dmax|max</td><td>0</td><td>0</td></tr>
<tr><td>7517</td><td>40096</td><td>Drosophila melanogaster</td><td>CG9666</td><td>CG9666 gene product from transcript CG9666-RA</td><td>DmelCG9666|Dromel_CG9666_FBtr0074991_mORF</td><td>0</td><td>0</td></tr>
<tr><td>7518</td><td>40097</td><td>Drosophila melanogaster</td><td>CG9629</td><td>CG9629 gene product from transcript CG9629-RA</td><td>DmelCG9629</td><td>0</td><td>0</td></tr>
<tr><td>7519</td><td>40098</td><td>Drosophila melanogaster</td><td>CG14088</td><td>CG14088 gene product from transcript CG14088-RB</td><td>DmelCG14088|SPH199</td><td>0</td><td>0</td></tr>
<tr><td>7520</td><td>40099</td><td>Drosophila melanogaster</td><td>CG14085</td><td>CG14085 gene product from transcript CG14085-RA</td><td>DmelCG14085</td><td>0</td><td>0</td></tr>
<tr><td>7521</td><td>40100</td><td>Drosophila melanogaster</td><td>CG14086</td><td>CG14086 gene product from transcript CG14086-RB</td><td>DmelCG14086</td><td>0</td><td>0</td></tr>
<tr><td>7522</td><td>40101</td><td>Drosophila melanogaster</td><td>CG14089</td><td>CG14089 gene product from transcript CG14089-RA</td><td>DmelCG14089</td><td>0</td><td>0</td></tr>
<tr><td>7523</td><td>40102</td><td>Drosophila melanogaster</td><td>CG9619</td><td>CG9619 gene product from transcript CG9619-RA</td><td>149039_at|DmelCG9619</td><td>0</td><td>0</td></tr>
<tr><td>7524</td><td>40104</td><td>Drosophila melanogaster</td><td>pip</td><td>pipe</td><td>CG9614|DmelCG9614|Pipe|l(3)76BDp|pipe-ST2</td><td>0</td><td>0</td></tr>
<tr><td>7525</td><td>40105</td><td>Drosophila melanogaster</td><td>CG32206</td><td>CG32206 gene product from transcript CG32206-RB</td><td>CG14090|CG14091|CG14092|CG9600|DmelCG32206</td><td>0</td><td>0</td></tr>
<tr><td>7526</td><td>40109</td><td>Drosophila melanogaster</td><td>CG14087</td><td>CG14087 gene product from transcript CG14087-RB</td><td>DmelCG14087</td><td>0</td><td>0</td></tr>
<tr><td>7527</td><td>40111</td><td>Drosophila melanogaster</td><td>Chd3</td><td>CG9594 gene product from transcript CG9594-RA</td><td>CG9594|CHD-3|CHD3|DmCHD3|DmelCG9594|chd3|dCHD3</td><td>0</td><td>0</td></tr>
<tr><td>7528</td><td>40112</td><td>Drosophila melanogaster</td><td>CG14095</td><td>CG14095 gene product from transcript CG14095-RA</td><td>DmelCG14095</td><td>0</td><td>0</td></tr>
<tr><td>7529</td><td>40113</td><td>Drosophila melanogaster</td><td>CG14096</td><td>CG14096 gene product from transcript CG14096-RA</td><td>DmelCG14096</td><td>0</td><td>0</td></tr>
<tr><td>7530</td><td>40114</td><td>Drosophila melanogaster</td><td>CG12519</td><td>CG12519 gene product from transcript CG12519-RA</td><td>DmelCG12519</td><td>0</td><td>0</td></tr>
<tr><td>7531</td><td>40115</td><td>Drosophila melanogaster</td><td>CG18294</td><td>CG18294 gene product from transcript CG18294-RA</td><td>BcDNA:RE51966|DmelCG18294</td><td>0</td><td>0</td></tr>
<tr><td>7532</td><td>40116</td><td>Drosophila melanogaster</td><td>brv1</td><td>brivido-1</td><td>CG9472|CT26822|DmelCG9472</td><td>0</td><td>0</td></tr>
<tr><td>7533</td><td>40117</td><td>Drosophila melanogaster</td><td>CG9449</td><td>CG9449 gene product from transcript CG9449-RF</td><td>DmelCG9449</td><td>0</td><td>0</td></tr>
<tr><td>7534</td><td>40118</td><td>Drosophila melanogaster</td><td>CG9451</td><td>CG9451 gene product from transcript CG9451-RA</td><td>DmelCG9451</td><td>0</td><td>0</td></tr>
<tr><td>7535</td><td>40119</td><td>Drosophila melanogaster</td><td>CG9452</td><td>CG9452 gene product from transcript CG9452-RA</td><td>DmelCG9452</td><td>0</td><td>0</td></tr>
<tr><td>7536</td><td>40120</td><td>Drosophila melanogaster</td><td>Cpr76Ba</td><td>Cuticular protein 76Ba</td><td>CG9283|DmelCpr76Ba|DmelCG9283</td><td>0</td><td>0</td></tr>
<tr><td>7537</td><td>40121</td><td>Drosophila melanogaster</td><td>Cpr76Bb</td><td>Cuticular protein 76Bb</td><td>CG9290|DmelCpr76Bb|DmelCG9290</td><td>0</td><td>0</td></tr>
<tr><td>7538</td><td>40122</td><td>Drosophila melanogaster</td><td>Cpr76Bc</td><td>Cuticular protein 76Bc</td><td>CG9295|DmelCpr76Bc|DmelCG9295</td><td>0</td><td>0</td></tr>
<tr><td>7539</td><td>40123</td><td>Drosophila melanogaster</td><td>Cpr76Bd</td><td>Cuticular protein 76Bd</td><td>CG9299|DmelCpr76Bd|DmelCG9299</td><td>0</td><td>0</td></tr>
<tr><td>7540</td><td>40124</td><td>Drosophila melanogaster</td><td>CG9279</td><td>CG9279 gene product from transcript CG9279-RB</td><td>DmelCG9279</td><td>0</td><td>0</td></tr>
<tr><td>7541</td><td>40126</td><td>Drosophila melanogaster</td><td>Rcd7</td><td>Reduction in Cnn dots 7</td><td>CG14098|DmelCG14098</td><td>0</td><td>0</td></tr>
<tr><td>7542</td><td>40127</td><td>Drosophila melanogaster</td><td>l(3)76BDr</td><td>CG32210 gene product from transcript CG32210-RA</td><td>CG32210|CG9268|CG9274|DmelCG32210</td><td>0</td><td>0</td></tr>
<tr><td>7543</td><td>40128</td><td>Drosophila melanogaster</td><td>CG9300</td><td>CG9300 gene product from transcript CG9300-RA</td><td>DmelCG9300</td><td>0</td><td>0</td></tr>
<tr><td>7544</td><td>40129</td><td>Drosophila melanogaster</td><td>Shal</td><td>Shaker cognate l</td><td>CG9262|DmelCG9262|Sh-l|Shl|dShal|shal</td><td>0</td><td>0</td></tr>
<tr><td>7545</td><td>40130</td><td>Drosophila melanogaster</td><td>CG9231</td><td>CG9231 gene product from transcript CG9231-RA</td><td>DmelCG9231</td><td>0</td><td>0</td></tr>
</tbody>
</table>

<table>
<tr><td>7546</td><td>40131</td><td>Drosophila melanogaster</td><td>CG9330</td><td>CG9330 gene product from transcript CG9330-RA</td><td>DmelCG9330</td><td>0</td><td>0</td></tr>
<tr><td>7547</td><td>40132</td><td>Drosophila melanogaster</td><td>CG14100</td><td>CG14100 gene product from transcript CG14100-RA</td><td>DmelCG14100</td><td>0</td><td>0</td></tr>
<tr><td>7548</td><td>40133</td><td>Drosophila melanogaster</td><td>ash1</td><td>absent, small, or homeotic discs 1</td><td>ASH1|Ash|Ash 1|Ash1|CG8887|DmelCG8887|ash|ash-1|dash|l(3)76BDb|l(3)SG29a</td><td>0</td><td>0</td></tr>
<tr><td>7549</td><td>40134</td><td>Drosophila melanogaster</td><td>Taf6</td><td>TBP-associated factor 6</td><td>CG32211|CG9348|DmelCG32211|SR3-4B|TAF|TAF6|TAF60|TAF60/62|TAFII60|TAFII60(62)|TAFII62|TAF[[II]]|TAF[[II]]60|TAF[[II]]60/62|TAF[[II]]62|TFIID|TFIID 62|Taf-6|Taf60|Taf62|Taf[[II]]60|d60|dTAF6|dTAF62|dT</td><td>0</td><td>0</td></tr>
<tr><td>7550</td><td>40135</td><td>Drosophila melanogaster</td><td>CG9368</td><td>CG9368 gene product from transcript CG9368-RB</td><td>BcDNA:RH51268|DmelCG9368</td><td>0</td><td>0</td></tr>
<tr><td>7551</td><td>40136</td><td>Drosophila melanogaster</td><td>lush</td><td>CG8807 gene product from transcript CG8807-RA</td><td>76a|76c|76c(LUSH)|CG8807|DmelCG8807|LUSH|Lush|Obp76a|obp76a</td><td>0</td><td>0</td></tr>
<tr><td>7552</td><td>40137</td><td>Drosophila melanogaster</td><td>CG9372</td><td>CG9372 gene product from transcript CG9372-RA</td><td>DmelCG9372|SP34|c-SP34</td><td>0</td><td>0</td></tr>
<tr><td>7553</td><td>40138</td><td>Drosophila melanogaster</td><td>CG8798</td><td>CG8798 gene product from transcript CG8798-RC</td><td>DmelCG8798|anon-WO0118547.350</td><td>0</td><td>0</td></tr>
<tr><td>7554</td><td>40139</td><td>Drosophila melanogaster</td><td>CG9376</td><td>CG9376 gene product from transcript CG9376-RA</td><td>DmelCG9376</td><td>0</td><td>0</td></tr>
<tr><td>7555</td><td>40140</td><td>Drosophila melanogaster</td><td>l(3)76BDm</td><td>lethal (3) 76BDm</td><td>CG8793|DmelCG8793|l(3)L3809</td><td>0</td><td>0</td></tr>
<tr><td>7556</td><td>40141</td><td>Drosophila melanogaster</td><td>asf1</td><td>anti-silencing factor 1</td><td>ASF1|Asf1|CG9383|DmelCG9383|dASF/SF2|dASF1|dAsf1|l(3)76BDq</td><td>0</td><td>0</td></tr>
<tr><td>7557</td><td>40142</td><td>Drosophila melanogaster</td><td>CG9392</td><td>CG9392 gene product from transcript CG9392-RA</td><td>DmelCG9392</td><td>0</td><td>0</td></tr>
<tr><td>7558</td><td>40143</td><td>Drosophila melanogaster</td><td>wnd</td><td>wallenda</td><td>CG8789|DLK|DLK/ZPK|DmelCG8789|MAPKKK|Wnd</td><td>0</td><td>0</td></tr>
<tr><td>7559</td><td>40144</td><td>Drosophila melanogaster</td><td>CG8786</td><td>CG8786 gene product from transcript CG8786-RB</td><td>BcDNA:GM01527|DmelCG8786</td><td>0</td><td>0</td></tr>
<tr><td>7560</td><td>40145</td><td>Drosophila melanogaster</td><td>Oat</td><td>Ornithine aminotransferase precursor</td><td>151832_at|CG8782|DmelCG8782</td><td>0</td><td>0</td></tr>
<tr><td>7561</td><td>40146</td><td>Drosophila melanogaster</td><td>tey</td><td>teyrha-meyrha</td><td>CG8780|DmelCG8780</td><td>0</td><td>0</td></tr>
<tr><td>7562</td><td>40147</td><td>Drosophila melanogaster</td><td>CG8765</td><td>CG8765 gene product from transcript CG8765-RA</td><td>DmelCG8765</td><td>0</td><td>0</td></tr>
<tr><td>7563</td><td>40148</td><td>Drosophila melanogaster</td><td>Gbeta76C</td><td>G protein beta-subunit 76C</td><td>CG8770|DGqbeta|DmelCG8770|G[[beta]]|G[[beta]]e|G[q]|Galpha76C|Gbe|Gbeta|Gbeta[[e]]|Gbetae|Gbeta{e}|Gqbeta|eGbeta|eye Gbeta|gbe</td><td>0</td><td>0</td></tr>
<tr><td>7564</td><td>40149</td><td>Drosophila melanogaster</td><td>verm</td><td>vermiform</td><td>CG8756|DmCDA2|DmelCG8756|LCBP1|N63|Verm|hlm|l(3)76BDx</td><td>0</td><td>0</td></tr>
<tr><td>7565</td><td>40150</td><td>Drosophila melanogaster</td><td>serp</td><td>serpentine</td><td>CG32209|CG8747|CG8748|DmCDA1|DmelCG32209|Serp|l(3)76BDk</td><td>0</td><td>0</td></tr>
<tr><td>7566</td><td>40152</td><td>Drosophila melanogaster</td><td>trpml</td><td>transient receptor potential mucolipin</td><td>CG8743|CT25240|DmelCG8743|l(3)76BDi</td><td>0</td><td>0</td></tr>
<tr><td>7567</td><td>40153</td><td>Drosophila melanogaster</td><td>CG42637</td><td>CG42637 gene product from transcript CG42637-RB</td><td>DmelCG42637|Dmel_CG8742</td><td>0</td><td>0</td></tr>
<tr><td>7568</td><td>40154</td><td>Drosophila melanogaster</td><td>CG14101</td><td>CG14101 gene product from transcript CG14101-RA</td><td>DmelCG14101|anon-WO0140519.34</td><td>0</td><td>0</td></tr>
<tr><td>7569</td><td>40155</td><td>Drosophila melanogaster</td><td>HLH106</td><td>Helix loop helix protein 106</td><td>CG8522|DmelCG8522|HlH106|SREBP|Srebp|dSREBP|dSrebp|dsrebp|l(3)76BDw</td><td>0</td><td>0</td></tr>
<tr><td>7570</td><td>40156</td><td>Drosophila melanogaster</td><td>CG14102</td><td>CG14102 gene product from transcript CG14102-RA</td><td>DmelCG14102</td><td>0</td><td>0</td></tr>
<tr><td>7571</td><td>40157</td><td>Drosophila melanogaster</td><td>Ir76a</td><td>Ionotropic receptor 76a</td><td>CG34257|CG42584|CG8533|CT24909|DmelCG42584|Dmel_CG34257|Dmel_CG8533|IR76a</td><td>0</td><td>0</td></tr>
<tr><td>7572</td><td>40158</td><td>Drosophila melanogaster</td><td>CG14103</td><td>CG14103 gene product from transcript CG14103-RA</td><td>DmelCG14103</td><td>0</td><td>0</td></tr>
<tr><td>7573</td><td>40159</td><td>Drosophila melanogaster</td><td>CG15881</td><td>CG15881 gene product from transcript CG15881-RB</td><td>BcDNA:HL01254|DmelCG15881</td><td>0</td><td>0</td></tr>
<tr><td>7574</td><td>40160</td><td>Drosophila melanogaster</td><td>Kap-alpha1</td><td>karyopherin alpha1</td><td>CG8548|Dalpha1|DmelCG8548|IMPalpha1|Imp-alpha1|Kap alpha1|Kpna1|anon-WO0118547.276|imp alpha1|impalpha1</td><td>0</td><td>0</td></tr>
<tr><td>7575</td><td>40161</td><td>Drosophila melanogaster</td><td>Cyp305a1</td><td>CG8733 gene product from transcript CG8733-RA</td><td>305a1|AAF49108.1|CG8733|CYP305A1-Dm|DmelCG8733</td><td>0</td><td>0</td></tr>
<tr><td>7576</td><td>40162</td><td>Drosophila melanogaster</td><td>cyc</td><td>cycle</td><td>BMAL1|Bmal1|CG8727|CYC|CYCLE|Cyc|Cycle|DmelCG8727|MOP3|Mop3|bHLHe7|bMAL1|bmal1|dBMAL|dBMAL1|dBma1|dBmal|dCYC|dbmal|dbmal1</td><td>0</td><td>0</td></tr>
<tr><td>7577</td><td>40163</td><td>Drosophila melanogaster</td><td>Fibp</td><td>CG8660 gene product from transcript CG8660-RD</td><td>CG8660|DmelCG8660|DrFIBP|F1bp</td><td>0</td><td>0</td></tr>
<tr><td>7578</td><td>40164</td><td>Drosophila melanogaster</td><td>Deaf1</td><td>Deformed epidermal autoregulatory factor-1</td><td>CG8567|DEAF-1|DEAF1|Deaf-1|DmelCG8567|deaf1|l(3)76BDv</td><td>0</td><td>0</td></tr>
<tr><td>7579</td><td>40165</td><td>Drosophila melanogaster</td><td>trc</td><td>tricornered</td><td>0669/17|CG8637|DmelCG8637|NDR|Ndr|Trc|dmTRC|l(3)76BDa|l(3)S066917</td><td>0</td><td>0</td></tr>
<tr><td>7580</td><td>40167</td><td>Drosophila melanogaster</td><td>Papss</td><td>PAPS synthetase</td><td>CG8363|DmPAPSS|DmelCG8363|PAPS|PAPSS|Paps|l(3)76BDn|paps|papss</td><td>0</td><td>0</td></tr>
<tr><td>7581</td><td>40168</td><td>Drosophila melanogaster</td><td>Rab8</td><td>Rab-protein 8</td><td>AAF49101|CG8287|DRAB8|DRab8|Dm Rab8|DmRab8|DmelCG8287|Rab-r8|dm-Rab8|l(3)76BDu|rab8</td><td>0</td><td>0</td></tr>
<tr><td>7582</td><td>40169</td><td>Drosophila melanogaster</td><td>CG8334</td><td>CG8334 gene product from transcript CG8334-RA</td><td>DmelCG8334</td><td>0</td><td>0</td></tr>
<tr><td>7583</td><td>40170</td><td>Drosophila melanogaster</td><td>Mi-2</td><td>CG8103 gene product from transcript CG8103-RA</td><td>0006/06|0854/01|CG8103|Dm-Mi-2|DmelCG8103|MI-2|MI2|Mi 2|Mi2|Pha|dMI-2|dMi|dMi-2|dMi2|hip76|l(3)01058|l(3)76BDe|l(3)A154.3M3|l(3)L1243|l(3)S000606|l(3)S085401|l(3)j3D4|pha</td><td>0</td><td>0</td></tr>
<tr><td>7584</td><td>40171</td><td>Drosophila melanogaster</td><td>Su(Tpl)</td><td>CG32217 gene product from transcript CG32217-RB</td><td>0854/01|CG32217|CG8037|CR32216|DmelCG32217|ELL|Ell|SR3-4A|SS3-4|SY3-1|dELL|l (3)76BD|l(3)76BDs|l(3)S000606|l(3)S085401</td><td>0</td><td>0</td></tr>
<tr><td>7585</td><td>40172</td><td>Drosophila melanogaster</td><td>CG7757</td><td>CG7757 gene product from transcript CG7757-RA</td><td>DmelCG7757|PRPF3|Prp3</td><td>0</td><td>0</td></tr>
<tr><td>7586</td><td>40173</td><td>Drosophila melanogaster</td><td>Mtr3</td><td>CG8025 gene product from transcript CG8025-RA</td><td>CG8025|DmelCG8025|dMtr3|mtr3</td><td>0</td><td>0</td></tr>
<tr><td>7587</td><td>40174</td><td>Drosophila melanogaster</td><td>Rpn1</td><td>CG7762 gene product from transcript CG7762-RA</td><td>BcDNA:LD21723|CG7762|DmelCG7762|p97</td><td>0</td><td>0</td></tr>
<tr><td>7588</td><td>40176</td><td>Drosophila melanogaster</td><td>CG7770</td><td>CG7770 gene product from transcript CG7770-RA</td><td>BcDNA:GH28557|DmelCG7770</td><td>0</td><td>0</td></tr>
<tr><td>7589</td><td>40177</td><td>Drosophila melanogaster</td><td>Grasp65</td><td>CG7809 gene product from transcript CG7809-RA</td><td>CG7809|DmelCG7809|dGRASP|dGrasp65|dgrasp</td><td>0</td><td>0</td></tr>
<tr><td>7590</td><td>40178</td><td>Drosophila melanogaster</td><td>CG8004</td><td>CG8004 gene product from transcript CG8004-RA</td><td>DmelCG8004</td><td>0</td><td>0</td></tr>
<tr><td>7591</td><td>40179</td><td>Drosophila melanogaster</td><td>RhoGDI</td><td>CG7823 gene product from transcript CG7823-RA</td><td>CG7823|DmelCG7823</td><td>0</td><td>0</td></tr>
<tr><td>7592</td><td>40180</td><td>Drosophila melanogaster</td><td>Ac76E</td><td>Adenylyl cyclase 76E</td><td>AC 76E|CG7978|DAC2|DAC76E|DmelCG7978|ac76e</td><td>0</td><td>0</td></tr>
<tr><td>7593</td><td>40181</td><td>Drosophila melanogaster</td><td>CG14182</td><td>CG14182 gene product from transcript CG14182-RA</td><td>DmelCG14182</td><td>0</td><td>0</td></tr>
<tr><td>7594</td><td>40182</td><td>Drosophila melanogaster</td><td>CG17732</td><td>CG17732 gene product from transcript CG17732-RB</td><td>DmelCG17732</td><td>0</td><td>0</td></tr>
<tr><td>7595</td><td>40183</td><td>Drosophila melanogaster</td><td>hale</td><td>hale-bopp</td><td>CG7570|DmelCG7570</td><td>0</td><td>0</td></tr>
<tr><td>7596</td><td>40184</td><td>Drosophila melanogaster</td><td>schuy</td><td>schumacher-levy</td><td>CG17736|DmelCG17736</td><td>0</td><td>0</td></tr>
<tr><td>7597</td><td>40185</td><td>Drosophila melanogaster</td><td>fln</td><td>flightin</td><td>CG7445|DmelCG7445|Fln|Ftn|mp27</td><td>0</td><td>0</td></tr>
<tr><td>7598</td><td>40186</td><td>Drosophila melanogaster</td><td>Nca</td><td>Neurocalcin</td><td>CG7641|DmelCG7641|Ncalc|nca</td><td>0</td><td>0</td></tr>
<tr><td>7599</td><td>40187</td><td>Drosophila melanogaster</td><td>CG7646</td><td>CG7646 gene product from transcript CG7646-RB</td><td>BcDNA:RH69723|DmelCG7646|anon-EST:Posey160</td><td>0</td><td>0</td></tr>
<tr><td>7600</td><td>40188</td><td>Drosophila melanogaster</td><td>CG7433</td><td>CG7433 gene product from transcript CG7433-RB</td><td>149085_at|DmelCG7433</td><td>0</td><td>0</td></tr>
<tr><td>7601</td><td>40189</td><td>Drosophila melanogaster</td><td>Tom20</td><td>Translocase of outer membrane 20</td><td>CG7654|DmelCG7654|TOM20|anon-WO0118547.225|dtom20</td><td>0</td><td>0</td></tr>
<tr><td>7602</td><td>40190</td><td>Drosophila melanogaster</td><td>CG7668</td><td>CG7668 gene product from transcript CG7668-RA</td><td>BcDNA:GM07843|BcDNA:RE40293|DmelCG7668|FBpp0074673|FBpp0074674</td><td>0</td><td>0</td></tr>
<tr><td>7603</td><td>40191</td><td>Drosophila melanogaster</td><td>kug</td><td>kugelei</td><td>CG7749|CT23445|Cad76E|DCad76E|DmelCG7749|Ftl|fat2|ftl|kugel</td><td>0</td><td>0</td></tr>
<tr><td>7604</td><td>40192</td><td>Drosophila melanogaster</td><td>CG14183</td><td>CG14183 gene product from transcript CG14183-RA</td><td>DmelCG14183</td><td>0</td><td>0</td></tr>
<tr><td>7605</td><td>40193</td><td>Drosophila melanogaster</td><td>CG14184</td><td>CG14184 gene product from transcript CG14184-RA</td><td>DmelCG14184|anon-WO0118547.633|dp18|p18</td><td>0</td><td>0</td></tr>
<tr><td>7606</td><td>40195</td><td>Drosophila melanogaster</td><td>sNPF-R</td><td>short neuropeptide F receptor</td><td>CG18639|CG7395|CG7395/CG18639|CT22771|DmelCG7395|Drm-sNPF-R|GPCR60|NPFR76F|anon-WO0131005.1|sNPFR1</td><td>0</td><td>0</td></tr>
<tr><td>7607</td><td>40196</td><td>Drosophila melanogaster</td><td>CG14186</td><td>CG14186 gene product from transcript CG14186-RA</td><td>DmelCG14186</td><td>0</td><td>0</td></tr>
<tr><td>7608</td><td>40197</td><td>Drosophila melanogaster</td><td>CG14185</td><td>CG14185 gene product from transcript CG14185-RA</td><td>CT33794|DmelCG14185</td><td>0</td><td>0</td></tr>
<tr><td>7609</td><td>40198</td><td>Drosophila melanogaster</td><td>Ir76b</td><td>Ionotropic receptor 76b</td><td>CG7385|CT22733|DmelCG7385|IR76b</td><td>0</td><td>0</td></tr>
<tr><td>7610</td><td>40199</td><td>Drosophila melanogaster</td><td>CG14187</td><td>CG14187 gene product from transcript CG14187-RA</td><td>DmelCG14187</td><td>0</td><td>0</td></tr>
<tr><td>7611</td><td>40200</td><td>Drosophila melanogaster</td><td>CG7365</td><td>CG7365 gene product from transcript CG7365-RA</td><td>DmelCG7365</td><td>0</td><td>0</td></tr>
<tr><td>7612</td><td>40201</td><td>Drosophila melanogaster</td><td>gig</td><td>gigas</td><td>6975|C1|CG6975|DmelCG6975|ME 109|TSC2|Tsc2|Tsc2/gig|dTSC2|dTsc2|l(3)109|tsc2</td><td>0</td><td>0</td></tr>
<tr><td>7613</td><td>40202</td><td>Drosophila melanogaster</td><td>obst-J</td><td>obstructor-J</td><td>CG7348|DmelCG7348|anon-EST:ParkEST360</td><td>0</td><td>0</td></tr>
<tr><td>7614</td><td>40203</td><td>Drosophila melanogaster</td><td>CG7335</td><td>CG7335 gene product from transcript CG7335-RA</td><td>BcDNA:AT09463|DmelCG7335</td><td>0</td><td>0</td></tr>
<tr><td>7615</td><td>40204</td><td>Drosophila melanogaster</td><td>CG7328</td><td>CG7328 gene product from transcript CG7328-RA</td><td>DmelCG7328</td><td>0</td><td>0</td></tr>
<tr><td>7616</td><td>40205</td><td>Drosophila melanogaster</td><td>CG42674</td><td>CG42674 gene product from transcript CG42674-RE</td><td>CG13811|CG13812|CG32223|CG42348|CG7323|DmelCG42674|Dmel_CG13811|Dmel_CG32223|Dmel_CG42348|Dmel_CG7323</td><td>0</td><td>0</td></tr>
<tr><td>7617</td><td>40207</td><td>Drosophila melanogaster</td><td>CG6981</td><td>CG6981 gene product from transcript CG6981-RB</td><td>CT21613|DmelCG6981</td><td>0</td><td>0</td></tr>
<tr><td>7618</td><td>40209</td><td>Drosophila melanogaster</td><td>obst-F</td><td>obstructor-F</td><td>CG7306|DmelCG7306</td><td>0</td><td>0</td></tr>
<tr><td>7619</td><td>40210</td><td>Drosophila melanogaster</td><td>CG7298</td><td>CG7298 gene product from transcript CG7298-RA</td><td>DmelCG7298|anon-EST:ParkEST398</td><td>0</td><td>0</td></tr>
<tr><td>7620</td><td>40211</td><td>Drosophila melanogaster</td><td>CG7290</td><td>CG7290 gene product from transcript CG7290-RA</td><td>DmelCG7290</td><td>0</td><td>0</td></tr>
<tr><td>7621</td><td>40212</td><td>Drosophila melanogaster</td><td>CG6996</td><td>CG6996 gene product from transcript CG6996-RB</td><td>DmelCG6996</td><td>0</td><td>0</td></tr>
<tr><td>7622</td><td>40213</td><td>Drosophila melanogaster</td><td>CG7017</td><td>CG7017 gene product from transcript CG7017-RA</td><td>DmelCG7017</td><td>0</td><td>0</td></tr>
<tr><td>7623</td><td>40214</td><td>Drosophila melanogaster</td><td>CG6933</td><td>CG6933 gene product from transcript CG6933-RA</td><td>DmelCG6933</td><td>0</td><td>0</td></tr>
<tr><td>7624</td><td>40215</td><td>Drosophila melanogaster</td><td>CG17145</td><td>CG17145 gene product from transcript CG17145-RA</td><td>DmelCG17145</td><td>0</td><td>0</td></tr>
<tr><td>7625</td><td>40216</td><td>Drosophila melanogaster</td><td>CG17147</td><td>CG17147 gene product from transcript CG17147-RA</td><td>DmelCG17147</td><td>0</td><td>0</td></tr>
<tr><td>7626</td><td>40217</td><td>Drosophila melanogaster</td><td>Su(var)3-3</td><td>Suppressor of variegation 3-3</td><td>CG17149|Dmel/HDM|Dmel/LSD1|DmelCG17149|DmelLSD1|Hdm|Lsd1|SU(VAR)3-3|Su-var(3)3|Suvar(3)3|dLsd1|lsd1</td><td>0</td><td>0</td></tr>
<tr><td>7627</td><td>40218</td><td>Drosophila melanogaster</td><td>CG13813</td><td>CG13813 gene product from transcript CG13813-RA</td><td>DmelCG13813</td><td>0</td><td>0</td></tr>
<tr><td>7628</td><td>40220</td><td>Drosophila melanogaster</td><td>CG17233</td><td>CG17233 gene product from transcript CG17233-RG</td><td>CG6918|DmelCG17233</td><td>0</td><td>0</td></tr>
<tr><td>7629</td><td>40221</td><td>Drosophila melanogaster</td><td>Clc</td><td>Clathrin light chain</td><td>CG6948|CLC|DmelCG6948|clc</td><td>0</td><td>0</td></tr>
<tr><td>7630</td><td>40222</td><td>Drosophila melanogaster</td><td>CG6951</td><td>CG6951 gene product from transcript CG6951-RB</td><td>DmelCG6951|anon-WO0118547.579</td><td>0</td><td>0</td></tr>
<tr><td>7631</td><td>40223</td><td>Drosophila melanogaster</td><td>CG13814</td><td>CG13814 gene product from transcript CG13814-RA</td><td>DmelCG13814</td><td>0</td><td>0</td></tr>
<tr><td>7632</td><td>40224</td><td>Drosophila melanogaster</td><td>rdgC</td><td>retinal degeneration C</td><td>CG6571|DmelCG6571|DmrdgC-77B|RDGC|RDGC/PP5|RdgC|RdgC 77B</td><td>0</td><td>0</td></tr>
<tr><td>7633</td><td>40225</td><td>Drosophila melanogaster</td><td>gogo</td><td>golden goal</td><td>CG13815|CG13817|CG32227|CT33316|DmelCG32227|Gogo</td><td>0</td><td>0</td></tr>
<tr><td>7634</td><td>40226</td><td>Drosophila melanogaster</td><td>CG17122</td><td>CG17122 gene product from transcript CG17122-RA</td><td>DmelCG17122</td><td>0</td><td>0</td></tr>
<tr><td>7635</td><td>40228</td><td>Drosophila melanogaster</td><td>CG6480</td><td>CG6480 gene product from transcript CG6480-RA</td><td>DmelCG6480|FRG1</td><td>0</td><td>0</td></tr>
<tr><td>7636</td><td>40229</td><td>Drosophila melanogaster</td><td>CG32226</td><td>CG32226 gene product from transcript CG32226-RA</td><td>CG18565|CG6468|DmelCG32226</td><td>0</td><td>0</td></tr>
<tr><td>7637</td><td>40231</td><td>Drosophila melanogaster</td><td>CG6597</td><td>CG6597 gene product from transcript CG6597-RC</td><td>DmelCG6597</td><td>0</td><td>0</td></tr>
<tr><td>7638</td><td>40232</td><td>Drosophila melanogaster</td><td>polo</td><td>CG12306 gene product from transcript CG12306-RA</td><td>0256/04|1324/08|CG12306|DmelCG12306|POLO|POLO/PLK1|Polo|Polo kinase|anon-WO0172774.3|l(3)01673|l(3)77Aa|l(3)S025604|l(3)S132408</td><td>0</td><td>0</td></tr>
<tr><td>7639</td><td>40233</td><td>Drosophila melanogaster</td><td>Snap</td><td>Soluble NSF attachment protein</td><td>CG6625|DmelCG6625|SNAP|alpha-SNAP|alphaSnap|dSNAP|dalpha-SNAP|dalphaSNAP|dsnap|l(3)77ABa|l(3)77ABc|snap</td><td>0</td><td>0</td></tr>
<tr><td>7640</td><td>40234</td><td>Drosophila melanogaster</td><td>Spn77Ba</td><td>Serpin 77Ba</td><td>CG6680|DmelCG6680|l(3)77ABi|spn77Ba</td><td>0</td><td>0</td></tr>
<tr><td>7641</td><td>40235</td><td>Drosophila melanogaster</td><td>Spn77Bb</td><td>Serpin 77Bb</td><td>CG6266|CG6663|DmelCG6663</td><td>0</td><td>0</td></tr>
<tr><td>7642</td><td>40236</td><td>Drosophila melanogaster</td><td>Spn77Bc</td><td>Serpin 77Bc</td><td>CG6289|DmelCG6289</td><td>0</td><td>0</td></tr>
<tr><td>7643</td><td>40238</td><td>Drosophila melanogaster</td><td>CG6434</td><td>CG6434 gene product from transcript CG6434-RA</td><td>DmelCG6434</td><td>0</td><td>0</td></tr>
<tr><td>7644</td><td>40239</td><td>Drosophila melanogaster</td><td>CG5585</td><td>CG5585 gene product from transcript CG5585-RA</td><td>CG17952|DmelCG5585|RBBP5</td><td>0</td><td>0</td></tr>
<tr><td>7645</td><td>40240</td><td>Drosophila melanogaster</td><td>eRF1</td><td>eukaryotic release factor 1</td><td>CG5605|Dm0184|DmelCG5605|Group J|group J|l(3)00103|l(3)05637|l(3)77ABb|l(3)neo28</td><td>0</td><td>0</td></tr>
<tr><td>7646</td><td>40241</td><td>Drosophila melanogaster</td><td>CG5618</td><td>CG5618 gene product from transcript CG5618-RC</td><td>DmelCG5618</td><td>0</td><td>0</td></tr>
<tr><td>7647</td><td>40242</td><td>Drosophila melanogaster</td><td>kin17</td><td>CG5649 gene product from transcript CG5649-RA</td><td>CG5649|DmelCG5649</td><td>0</td><td>0</td></tr>
<tr><td>7648</td><td>40243</td><td>Drosophila melanogaster</td><td>CG5665</td><td>CG5665 gene product from transcript CG5665-RC</td><td>DmelCG5665|cg5665</td><td>0</td><td>0</td></tr>
<tr><td>7649</td><td>40245</td><td>Drosophila melanogaster</td><td>Toll-9</td><td>CG5528 gene product from transcript CG5528-RB</td><td>CG5528|CT17508|Dm Toll-9|DmelCG5528|TOLL 9|Tak/Toll-like|Tl-5|Tl-9|Toll 9|dTLR9|dToll9|toll</td><td>0</td><td>0</td></tr>
<tr><td>7650</td><td>40246</td><td>Drosophila melanogaster</td><td>in</td><td>inturned</td><td>B|CG16993|DmelCG16993</td><td>0</td><td>0</td></tr>
<tr><td>7651</td><td>40247</td><td>Drosophila melanogaster</td><td>CG13247</td><td>CG13247 gene product from transcript CG13247-RA</td><td>DmelCG13247</td><td>0</td><td>0</td></tr>
<tr><td>7652</td><td>40248</td><td>Drosophila melanogaster</td><td>Ide</td><td>Insulin degrading metalloproteinase</td><td>CG5517|DmelCG5517|IDE|dIDE</td><td>0</td><td>0</td></tr>
<tr><td>7653</td><td>40249</td><td>Drosophila melanogaster</td><td>RhoBTB</td><td>CG5701 gene product from transcript CG5701-RA</td><td>CG5701|DmelCG5701|RhoGTPase|RhoX</td><td>0</td><td>0</td></tr>
<tr><td>7654</td><td>40250</td><td>Drosophila melanogaster</td><td>CG5498</td><td>CG5498 gene product from transcript CG5498-RB</td><td>BcDNA:GH08385|DmelCG5498</td><td>0</td><td>0</td></tr>
<tr><td>7655</td><td>40252</td><td>Drosophila melanogaster</td><td>CG33969</td><td>CG33969 gene product from transcript CG33969-RC</td><td>CG32429|CG5571|DmelCG33969</td><td>0</td><td>0</td></tr>
<tr><td>7656</td><td>40254</td><td>Drosophila melanogaster</td><td>CG13248</td><td>CG13248 gene product from transcript CG13248-RA</td><td>DmelCG13248</td><td>0</td><td>0</td></tr>
</table>

7657	40255	Drosophila melanogaster	zye	CG5847 gene product from transcript CG5847-RA	CG5847|DmelCG5847	0	0
7658	40256	Drosophila melanogaster	CG5282	CG5282 gene product from transcript CG5282-RA	DmelCG5282	0	0
7659	40257	Drosophila melanogaster	CG5274	CG5274 gene product from transcript CG5274-RB	DmelCG5274	0	0
7660	40258	Drosophila melanogaster	CG5262	CG5262 gene product from transcript CG5262-RA	DmelCG5262|anon-WO0118547.347	0	0
7661	40259	Drosophila melanogaster	Las	Lipoic acid synthase	CG5231|DmelCG5231	0	0
7662	40260	Drosophila melanogaster	Psn	Presenilin	CG18803|CG5868|DPS|DPsn|DmPS|DmelCG18803|Dps|DrosPS|PS|PS1|dPsn|l(3)77CDb|pres|psn	0	0
7663	40261	Drosophila melanogaster	mRpL15	mitochondrial ribosomal protein L15	CG5219|DmelCG5219|L15|l(3)77CDc	0	0
7664	40262	Drosophila melanogaster	CG5872	CG5872 gene product from transcript CG5872-RA	DmelCG5872	0	0
7665	40263	Drosophila melanogaster	CG11796	CG11796 gene product from transcript CG11796-RA	DmelCG11796	0	0
7666	40264	Drosophila melanogaster	CG5910	CG5910 gene product from transcript CG5910-RA	CK02563|DmelCG5910	0	0
7667	40265	Drosophila melanogaster	CG5199	CG5199 gene product from transcript CG5199-RA	CUT8|DmelCG5199	0	0
7668	40266	Drosophila melanogaster	CG5195	CG5195 gene product from transcript CG5195-RA	CT16609|DmelCG5195|tartan/capricious-like	0	0
7669	40267	Drosophila melanogaster	CG5932	CG5932 gene product from transcript CG5932-RA	DmelCG5932	0	0
7670	40268	Drosophila melanogaster	CG5955	CG5955 gene product from transcript CG5955-RA	DmelCG5955	0	0
7671	40269	Drosophila melanogaster	CG5969	CG5969 gene product from transcript CG5969-RA	BcDNA:GM09417|BcDNA:RE53796|CG32426|DmelCG5969	0	0
7672	40270	Drosophila melanogaster	isoQC	iso Glutaminyl cyclase	CG32426|CG5976|DmelCG5976|isoDromeQC	0	0
7673	40271	Drosophila melanogaster	CG5130	CG5130 gene product from transcript CG5130-RA	DmelCG5130|anon-WO0118547.435	0	0
7674	40272	Drosophila melanogaster	CG6020	CG6020 gene product from transcript CG6020-RA	DmelCG6020	0	0
7675	40273	Drosophila melanogaster	CG32425	CG32425 gene product from transcript CG32425-RC	CG15869|CG5100|DmelCG32425	0	0
7676	40274	Drosophila melanogaster	CG18281	CG18281 gene product from transcript CG18281-RA	DmelCG18281	0	0
7677	40275	Drosophila melanogaster	CG17637	CG17637 gene product from transcript CG17637-RA	DmelCG17637	0	0
7678	40276	Drosophila melanogaster	CG5078	CG5078 gene product from transcript CG5078-RA	DmelCG5078	0	0
7679	40278	Drosophila melanogaster	CG5059	CG5059 gene product from transcript CG5059-RD	DmelCG5059	0	0
7680	40279	Drosophila melanogaster	mTerf3	CG5047 gene product from transcript CG5047-RA	CG5047|D-MTERF3|DmelCG5047	0	0
7681	40280	Drosophila melanogaster	CG5104	CG5104 gene product from transcript CG5104-RB	BcDNA:RH26504|DmelCG5104	0	0
7682	40281	Drosophila melanogaster	CG4825	CG4825 gene product from transcript CG4825-RA	DmelCG4825|ptdss1	0	0
7683	40282	Drosophila melanogaster	CG4858	CG4858 gene product from transcript CG4858-RA	DmelCG4858	0	0
7684	40283	Drosophila melanogaster	Rcd2	Reduction in Cnn dots 2	CG4786|CT15369|DmelCG4786	0	0
7685	40284	Drosophila melanogaster	CG13250	CG13250 gene product from transcript CG13250-RA	DmelCG13250	0	0
7686	40285	Drosophila melanogaster	knrl	knirps-like	CG4761|DmelCG4761|Knrl|NR0A2	0	0
7687	40286	Drosophila melanogaster	CG13251	CG13251 gene product from transcript CG13251-RA	DmelCG13251	0	0
7688	40287	Drosophila melanogaster	kni	knirps	CG4717|DmelCG4717|KNI|Kni|NR0A1|kn|ri	0	0
7689	40288	Drosophila melanogaster	cmpy	crimpy	CG13253|DmelCG13253	0	0
7690	40289	Drosophila melanogaster	CG13252	CG13252 gene product from transcript CG13252-RA	DmelCG13252	0	0
7691	40290	Drosophila melanogaster	CG4074	CG4074 gene product from transcript CG4074-RA	DmelCG4074	0	0
7692	40291	Drosophila melanogaster	CG4042	CG4042 gene product from transcript CG4042-RA	DmelCG4042	0	0
7693	40292	Drosophila melanogaster	Pitslre	CG4268 gene product from transcript CG4268-RA	CG4268|DmelCG4268|Gat|Gta|PITSLRE|cdk11|group 3|pitslre	0	0
7694	40293	Drosophila melanogaster	pex16	peroxin 16	CG3947|DmelCG3947	0	0
7695	40294	Drosophila melanogaster	CG4289	CG4289 gene product from transcript CG4289-RA	DmelCG4289	0	0
7696	40295	Drosophila melanogaster	CG11399	CG11399 gene product from transcript CG11399-RB	DmelCG11399	0	0
7697	40296	Drosophila melanogaster	CG11396	CG11396 gene product from transcript CG11396-RA	DmelCG11396	0	0
7698	40297	Drosophila melanogaster	Six4	CG3871 gene product from transcript CG3871-RA	CG3871|D-Six4|D-six4|DSix4|DmelCG3871|Dsix4|six4|six4/5	0	0
7699	40298	Drosophila melanogaster	CG3698	CG3698 gene product from transcript CG3698-RA	DmelCG3698	0	0
7700	40299	Drosophila melanogaster	CG4365	CG4365 gene product from transcript CG4365-RB	DmelCG4365	0	0
7701	40300	Drosophila melanogaster	Spc105R	Spc105-related	CG11451|DmSpc105|DmelCG11451|Spc105|dmKNL-1|dmSpc105R	0	0
7702	40301	Drosophila melanogaster	CG3634	CG3634 gene product from transcript CG3634-RA	DmelCG3634	0	0
7703	40302	Drosophila melanogaster	CG3680	CG3680 gene product from transcript CG3680-RA	DmelCG3680	0	0
7704	40303	Drosophila melanogaster	CG3618	CG3618 gene product from transcript CG3618-RB	DmelCG3618|GYCb100B	0	0
7705	40304	Drosophila melanogaster	Mst77F	Male-specific transcript 77F	18c|CG3354|DmelCG3354|R1-18c|ms(3)nc3|mst77F|nc3	0	0
7706	40305	Drosophila melanogaster	Pka-R1	cAMP-dependent protein kinase R1	18304|CG12452|CG18677|CG3263|CG42341|CdkR|DRI|DmelCG42341|Dmel_CG12452|Dmel_CG3263|PKA|PKA-R1|PKA-RI|PKAR|PKAi|PKAr|PKa-R1|Pka-RI|PkaR|RI|dPKA-RI|dr[[1]]|pKA|pkA|pka-R1	0	0
7707	40307	Drosophila melanogaster	CG3288	CG3288 gene product from transcript CG3288-RA	DmelCG3288	0	0
7708	40308	Drosophila melanogaster	CSN3	COP9 complex homolog subunit 3	CG18332|CH3|Csn3|DCH3|Dch3|DmelCG18332|FBgn0027055|dCSN3	0	0
7709	40309	Drosophila melanogaster	CG11456	CG11456 gene product from transcript CG11456-RB	DmelCG11456	0	0
7710	40310	Drosophila melanogaster	CG32432	CG32432 gene product from transcript CG32432-RA	CG13256|CG13257|CG3116|DmelCG32432|FBgn0037032	0	0
7711	40313	Drosophila melanogaster	CG10589	CG10589 gene product from transcript CG10589-RA	DmelCG10589|anon-WO0140519.139	0	0
7712	40314	Drosophila melanogaster	fng	fringe	CG10580|D-Fng|D-fng|Dfng|DmelCG10580|Fng|Frg|fg|frg|l(3)rG554	0	0
7713	40315	Drosophila melanogaster	CG10586	CG10586 gene product from transcript CG10586-RA	DmelCG10586|SP168	0	0
7714	40316	Drosophila melanogaster	CG10588	CG10588 gene product from transcript CG10588-RA	DmelCG10588	0	0
7715	40317	Drosophila melanogaster	CG11037	CG11037 gene product from transcript CG11037-RA	DmelCG11037|SP191	0	0
7716	40318	Drosophila melanogaster	CG10587	CG10587 gene product from transcript CG10587-RA	DmelCG10587|SP167	0	0
7717	40319	Drosophila melanogaster	CG12983	CG12983 gene product from transcript CG12983-RE	DmelCG12983|NEST:bs29a07|bs29a07.y1	0	0
7718	40321	Drosophila melanogaster	CG12984	CG12984 gene product from transcript CG12984-RA	DmelCG12984	0	0
7719	40323	Drosophila melanogaster	CG10585	CG10585 gene product from transcript CG10585-RA	DmelCG10585	0	0
7720	40324	Drosophila melanogaster	CG10584	CG10584 gene product from transcript CG10584-RA	DmelCG10584|anon-WO0118547.713	0	0
7721	40325	Drosophila melanogaster	Sin	Sex-lethal interactor	CG10582|DmelCG10582|sin	0	0
7722	40326	Drosophila melanogaster	CG10581	CG10581 gene product from transcript CG10581-RA	DmelCG10581	0	0
7723	40327	Drosophila melanogaster	siz	schizo	CG10577|CG12980|CG12981|CG32434|DmelCG32434|Loner|loner	0	0
7724	40330	Drosophila melanogaster	ko	knockout	CG10573|DmelCG10573	0	0
7725	40331	Drosophila melanogaster	CG10566	CG10566 gene product from transcript CG10566-RA	Dm ric-19|DmelCG10566	0	0
7726	40332	Drosophila melanogaster	CG10565	CG10565 gene product from transcript CG10565-RB	DmelCG10565	0	0
7727	40333	Drosophila melanogaster	Ac78C	Adenylyl cyclase 78C	AC 78C|AC78C|CG10554|CG10564|DAC78C|DAC78C-s|DmelCG10564	0	0
7728	40335	Drosophila melanogaster	CG42337	CG42337 gene product from transcript CG42337-RC	CG12976|CG12977|DmelCG42337|Dmel_CG12976|Dmel_CG12977	0	0
7729	40336	Drosophila melanogaster	park	parkin	CG10523|DmelCG10523|Dpark|SD01679|dpk	0	0
7730	40339	Drosophila melanogaster	CG10512	CG10512 gene product from transcript CG10512-RA	DmelCG10512	0	0
7731	40340	Drosophila melanogaster	CG33054	CG33054 gene product from transcript CG33054-RB	CG10167|CG10517|DmelCG33054	0	0
7732	40341	Drosophila melanogaster	CG10510	CG10510 gene product from transcript CG10510-RA	DmelCG10510	0	0
7733	40342	Drosophila melanogaster	CG10508	CG10508 gene product from transcript CG10508-RK	DmelCG10508	0	0
7734	40343	Drosophila melanogaster	CG12975	CG12975 gene product from transcript CG12975-RA	BcDNA:RE57810|DmelCG12975	0	0
7735	40345	Drosophila melanogaster	Eip78C	Ecdysone-induced protein 78C	CG18023|DR-78|DmelCG18023|E78|E78A|E78B|E78D|EIP78C|Eip78|Eip78D|NR1E1|anon-WO0172774.42|anon-WO0172774.44|dE78	0	0
7736	40346	Drosophila melanogaster	CG9391	CG9391 gene product from transcript CG9391-RC	142335_at|DmelCG9391|IMP	0	0
7737	40347	Drosophila melanogaster	CG9389	CG9389 gene product from transcript CG9389-RA	142336_at|DmelCG9389|IMP	0	0
7738	40348	Drosophila melanogaster	AcCoAS	Acetyl Coenzyme A synthase	ACS|AcCoAs|BEST:GH28401|CG9390|DmelCG9390|dACS	0	0
7739	40349	Drosophila melanogaster	ppl	pumpless	CG7758|DmelCG7758|l(3)00217|l(3)78Cb	0	0
7740	40350	Drosophila melanogaster	CG12974	CG12974 gene product from transcript CG12974-RB	DmelCG12974	0	0
7741	40351	Drosophila melanogaster	pzg	putzig	CG7752|DmelCG7752|Z4|p160	0	0
7742	40352	Drosophila melanogaster	Cpr78Ca	Cuticular protein 78Ca	CG11310|DmelCpr78Ca|DmelCG11310	0	0
7743	40353	Drosophila melanogaster	Cpr78Cb	Cuticular protein 78Cb	BcDNA:RE38359|CG7663|DmelCpr78Cb|DmelCG7663|anon-EST:ParkEST240	0	0
7744	40354	Drosophila melanogaster	Edg78E	Ecdysone-dependent gene 78E	CG7673|DMLCP78|DmelEdg78E|DmelCG7673|EDG-78|EDG78|EDG78E|Edg78|Edg78e	0	0
7745	40355	Drosophila melanogaster	Cpr78Cc	Cuticular protein 78Cc	BcDNA:RE32113|CG7658|DmelCpr78Cc|DmelCG7658|anon-EST:ParkEST167	0	0
7746	40356	Drosophila melanogaster	CG11309	CG11309 gene product from transcript CG11309-RA	DmelCG11309	0	0
7747	40357	Drosophila melanogaster	CG7632	CG7632 gene product from transcript CG7632-RA	DmelCG7632	0	0
7748	40358	Drosophila melanogaster	Pc	Polycomb	CG32443|CG7618|DmPc|DmelCG32443|PC|Pc-G|PcG|dPC	0	0
7749	40359	Drosophila melanogaster	Rab26	CG34410 gene product from transcript CG34410-RB	AAF51708|CG32439|CG34410|CG7605|DmRab26|DmelCG34410|Dmel_CG32439|Dmel_CG7605|rab26	0	0
7750	40360	Drosophila melanogaster	CG7338	CG7338 gene product from transcript CG7338-RA	DmelCG7338	0	0
7751	40361	Drosophila melanogaster	CG7324	CG7324 gene product from transcript CG7324-RA	DmelCG7324	0	0
7752	40362	Drosophila melanogaster	CG32436	CG32436 gene product from transcript CG32436-RA	CG12973|CG6968|DmelCG32436|HD35012	0	0
7753	40363	Drosophila melanogaster	ebd2	earthbound 2	CG12972|DmelCG12972	0	0
7754	40365	Drosophila melanogaster	CG12971	CG12971 gene product from transcript CG12971-RB	DmelCG12971	0	0
7755	40366	Drosophila melanogaster	CG32437	CG32437 gene product from transcript CG32437-RA	CG5051|DmelCG32437	0	0
7756	40367	Drosophila melanogaster	sa	spermatocyte arrest	CG11308|DmelCG11308|Sa|TAF-like|TAF8L|dTAF8L|gfb|ms(3)VO45|ms(3)VO5|ms(3)sa|tTAF	0	0
7757	40368	Drosophila melanogaster	rgn	regeneration	BcDNA:GH11973|CG6014|DmelCG6014|l(3)00534	0	0
7758	40369	Drosophila melanogaster	CG6049	CG6049 gene product from transcript CG6049-RC	DmelCG6049|Tat-SF1|cg6049|cus2	0	0
7759	40370	Drosophila melanogaster	Aef1	Adult enhancer factor 1	AEF-1|CG5683|DmelCG5683	0	0
7760	40371	Drosophila melanogaster	CG5664	CG5664 gene product from transcript CG5664-RA	DmelCG5664	0	0
7761	40372	Drosophila melanogaster	CG5656	CG5656 gene product from transcript CG5656-RA	DmelCG5656	0	0
7762	40373	Drosophila melanogaster	Syx6	Syntaxin 6	CG7736|CT23545|Dm Syx6|DmSyx6|DmelCG7736|SYN6|SYX6/10|Syx	0	0
7763	40374	Drosophila melanogaster	croc	crocodile	CG5069|DmelCG5069|Dmfd1|FD1|FoxC|fd1|fd78E	0	0
7764	40375	Drosophila melanogaster	Neu2	CG7204 gene product from transcript CG7204-RA	CG7204|DmelCG7204	0	0
7765	40376	Drosophila melanogaster	CG7202	CG7202 gene product from transcript CG7202-RA	DmelCG7202	0	0
7766	40377	Drosophila melanogaster	CG7519	CG7519 gene product from transcript CG7519-RA	DmelCG7519	0	0
7767	40378	Drosophila melanogaster	Hr78	Hormone-receptor-like in 78	CG7199|DHR78|Dhr78|DmelCG7199|HR78|Hr78D|NR2D1|XR78E/F	0	0

7768	40381	Drosophila melanogaster	Est-Q	Esterase Q	CG7529|DmelCG7529	0	0
7769	40382	Drosophila melanogaster	CG33214	CG33214 gene product from transcript CG33214-RA	CG7190|CG7193|CG7195|DmelCG33214|GLG1	0	0
7770	40383	Drosophila melanogaster	M6	CG7540 gene product from transcript CG7540-RA	CG7540|DmelCG7540|dM6a	0	0
7771	40384	Drosophila melanogaster	SAK	Sak kinase	CG7186|DmelCG7186|PLK4|Plk4|SAK/PLK|Sak|plk4|sak	0	0
7772	40385	Drosophila melanogaster	Cdk12	CG7597 gene product from transcript CG7597-RB	AC017581|CG7597|DmelCG7597|anon-WO0140519.165|cg7597|dCDK12|gi24668141	0	0
7773	40386	Drosophila melanogaster	CG7611	CG7611 gene product from transcript CG7611-RJ	DmelCG7611|WDR26	0	0
7774	40388	Drosophila melanogaster	Pros54	Proteasome 54kD subunit	7619|CG7619|DmS5a|DmelCG7619|Rpn10|Rpn10/p54|S5A|S5a|dRpn10|mu-54|p54	0	0
7775	40389	Drosophila melanogaster	VhaM9.7-b	Vacuolar H[+] ATPase subunit M9.7-b	CG7625|DmelCG7625|V-ATPase|Vha M9.7-2|VhaM9.7-2|anon-WO0118547.551|vhaM9.2-2|vhaM9.7-2	0	0
7776	40390	Drosophila melanogaster	CG7181	CG7181 gene product from transcript CG7181-RA	BcDNA:GH15688|DmelCG7181|VIII|anon-WO0118547.331	0	0
7777	40391	Drosophila melanogaster	Wnk	CG7177 gene product from transcript CG7177-RA	CG7177|DmelCG7177|cg7177|wnk	0	0
7778	40392	Drosophila melanogaster	CG7173	CG7173 gene product from transcript CG7173-RA	DmelCG7173	0	0
7779	40393	Drosophila melanogaster	capaR	capa receptor	CAP2B-R|CG14575|CapaR|Dm CG14575|DmelCG14575	0	0
7780	40394	Drosophila melanogaster	CG7634	CG7634 gene product from transcript CG7634-RA	DmelCG7634	0	0
7781	40395	Drosophila melanogaster	CG7172	CG7172 gene product from transcript CG7172-RA	DmelCG7172|anon-WO0118547.316	0	0
7782	40396	Drosophila melanogaster	CG32441	CG32441 gene product from transcript CG32441-RD	CG7783|DmelCG32441	0	0
7783	40398	Drosophila melanogaster	AP-2	CG7807 gene product from transcript CG7807-RB	AP-2alpha|CG7807|DAP-2|DmelCG7807|dAP-2|dAP-2alpha|l(3)1215	0	0
7784	40399	Drosophila melanogaster	S1P	CG7169 gene product from transcript CG7169-RA	CG7169|DmelCG7169|dS1P	0	0
7785	40400	Drosophila melanogaster	CG11307	CG11307 gene product from transcript CG11307-RA	DmelCG11307	0	0
7786	40401	Drosophila melanogaster	CG7166	CG7166 gene product from transcript CG7166-RB	CT22143|DmelCG7166	0	0
7787	40402	Drosophila melanogaster	CG11306	CG11306 gene product from transcript CG11306-RA	DmelCG11306	0	0
7788	40403	Drosophila melanogaster	MED1	Mediator complex subunit 1	CG7162|DmelCG7162|Med1|Med1/SOP3/TRAP220|Trap220|dTRAP220	0	0
7789	40404	Drosophila melanogaster	ORMDL	CG14577 gene product from transcript CG14577-RA	CG14577|DmelCG14577	0	0
7790	40406	Drosophila melanogaster	CG32444	CG32444 gene product from transcript CG32444-RA	BcDNA:GH08902|CG12562|CG14576|CT3430|DmelCG32444	0	0
7791	40408	Drosophila melanogaster	Cpr78E	Cuticular protein 78E	BcDNA:LP06027|CG7160|DmelCpr78E|DmelCG7160	0	0
7792	40409	Drosophila melanogaster	CG11249	CG11249 gene product from transcript CG11249-RA	DmelCG11249	0	0
7793	40410	Drosophila melanogaster	CG7158	CG7158 gene product from transcript CG7158-RA	DmelCG7158	0	0
7794	40411	Drosophila melanogaster	CG11248	CG11248 gene product from transcript CG11248-RB	DmelCG11248	0	0
7795	40412	Drosophila melanogaster	CG32447	CG32447 gene product from transcript CG32447-RB	CG14571|CG7155|DmelCG32447|anon-WO0170980.76|anon-WO0170980.77	0	0
7796	40414	Drosophila melanogaster	CG11247	CG11247 gene product from transcript CG11247-RC	DmelCG11247	0	0
7797	40415	Drosophila melanogaster	Rpb8	CG11246 gene product from transcript CG11246-RA	CG11246|DmelCG11246|RPB8	0	0
7798	40416	Drosophila melanogaster	CG14570	CG14570 gene product from transcript CG14570-RA	DmelCG14570	0	0
7799	40417	Drosophila melanogaster	CG14569	CG14569 gene product from transcript CG14569-RA	DmelCG14569	0	0
7800	40418	Drosophila melanogaster	CG14568	CG14568 gene product from transcript CG14568-RA	BcDNA:RE21944|DmelCG14568	0	0
7801	40419	Drosophila melanogaster	CG14573	CG14573 gene product from transcript CG14573-RA	DmelCG14573	0	0
7802	40420	Drosophila melanogaster	CG14567	CG14567 gene product from transcript CG14567-RA	DmelCG14567	0	0
7803	40421	Drosophila melanogaster	CG14566	CG14566 gene product from transcript CG14566-RA	DmelCG14566	0	0
7804	40422	Drosophila melanogaster	CG14572	CG14572 gene product from transcript CG14572-RA	DmelCG14572	0	0
7805	40423	Drosophila melanogaster	CG14565	CG14565 gene product from transcript CG14565-RA	DmelCG14565	0	0
7806	40424	Drosophila melanogaster	Syn1	Syntrophin-like 1	CG7151|CG7152|DmSYN-1|DmelCG7152	0	0
7807	40425	Drosophila melanogaster	CG14564	CG14564 gene product from transcript CG14564-RA	DmelCG14564	0	0
7808	40427	Drosophila melanogaster	CG7370	CG7370 gene product from transcript CG7370-RA	BcDNA:GM07065|DmelCG7370	0	0
7809	40428	Drosophila melanogaster	eg	eagle	CG7383|DmelCG7383|EGON|Eg|Egl|Egon|NR0A3|T1|eag|egl|egon|spy	0	0
7810	40429	Drosophila melanogaster	CycH	Cyclin H	142767_at|CG7405|DmelCG7405	0	0
7811	40430	Drosophila melanogaster	CG7407	CG7407 gene product from transcript CG7407-RA	BcDNA:LD29166|DmelCG7407	0	0
7812	40431	Drosophila melanogaster	CG7414	CG7414 gene product from transcript CG7414-RC	DmelCG7414|anon-WO0118547.243	0	0
7813	40432	Drosophila melanogaster	CG7148	CG7148 gene product from transcript CG7148-RA	BEST:GH10766|DmelCG7148	0	0
7814	40433	Drosophila melanogaster	Nopp140	CG7421 gene product from transcript CG7421-RB	CG7421|DmNopp140|DmelCG7421|nopp140	0	0
7815	40434	Drosophila melanogaster	P5CDh1	delta-1-Pyrroline-5-carboxylate dehydrogenase 1	CG7145|DmP5CDh1|DmelCG7145|P5CDh	0	0
7816	40435	Drosophila melanogaster	CG14563	CG14563 gene product from transcript CG14563-RA	DmelCG14563	0	0
7817	40436	Drosophila melanogaster	mub	mushroom-body expressed	CG7437|DmelCG7437|MRE17|Mub|anon-WO0172774.33|anon-WO0172774.35|anon-WO0172774.36|anon-WO0172774.37|l(3)04093|l(3)j6B10|l(3)j8B3	0	0
7818	40437	Drosophila melanogaster	CG7442	CG7442 gene product from transcript CG7442-RA	DmelCG7442	0	0
7819	40438	Drosophila melanogaster	DNApol-eta	CG7143 gene product from transcript CG7143-RA	AAF51794|CG7143|DmelCG7143|Dmpol eta|POLH|drad30A|pol h	0	0
7820	40439	Drosophila melanogaster	CG14562	CG14562 gene product from transcript CG14562-RA	DmelCG14562|anon-WO0140519.241	0	0
7821	40440	Drosophila melanogaster	CG7448	CG7448 gene product from transcript CG7448-RB	DmelCG7448	0	0
7822	40441	Drosophila melanogaster	CG7458	CG7458 gene product from transcript CG7458-RA	DmelCG7458	0	0
7823	40442	Drosophila melanogaster	CS-2	Chitin synthase 2	CG7464|ChSA|DmCS-2|DmeChSA|DmelCG7464	0	0
7824	40443	Drosophila melanogaster	CG7470	CG7470 gene product from transcript CG7470-RB	DmelCG7470	0	0
7825	40444	Drosophila melanogaster	Act79B	Actin 79B	143060_f_at|Actin|CG7478|D|DmelCG7478|act 79B|act79B|actin|mus79B	0	0
7826	40445	Drosophila melanogaster	CG7140	CG7140 gene product from transcript CG7140-RB	DmelCG7140	0	0
7827	40447	Drosophila melanogaster	CG14561	CG14561 gene product from transcript CG14561-RA	DmelCG14561	0	0
7828	40448	Drosophila melanogaster	CG7139	CG7139 gene product from transcript CG7139-RB	BcDNA:LD21293|DmelCG7139|anon-WO0118547.415	0	0
7829	40449	Drosophila melanogaster	CG7133	CG7133 gene product from transcript CG7133-RA	DmelCG7133	0	0
7830	40450	Drosophila melanogaster	CG7130	CG7130 gene product from transcript CG7130-RA	DmelCG7130	0	0
7831	40451	Drosophila melanogaster	RpLP0	Ribosomal protein LP0	AP3|Ap3|Ape|CG7490|DmelCG7490|LP0|P0|P35|PO|RLA0|RPL P0|RpP0|RpPO|Rplp0|l(3)0154|l(3)01544|p0	0	0
7832	40453	Drosophila melanogaster	olf413	CG12673 gene product from transcript CG12673-RB	CG12673|CG14461|CG7495|CR33185|DmelCG12673|Olf413	0	0
7833	40456	Drosophila melanogaster	Rich	RIC1 homolog	3L6|BcDNA:GH03694|BcDNAGH03694|CG9063|DmelCG9063|rich	0	0
7834	40457	Drosophila melanogaster	Ddx1	Dead-box-1	CG9054|DDX1|Dbp79E|DmDDX1|DmRH12|DmelCG9054|anon-79E	0	0
7835	40458	Drosophila melanogaster	CG11523	CG11523 gene product from transcript CG11523-RA	DmelCG11523	0	0
7836	40459	Drosophila melanogaster	Csp	Cysteine string protein	CG6395|CSP|CSP32|Cys|D-CSP|D.M.CSP|DCSP-1|Dcsp|DmelCG6395|MAB ab49|ab49|anon-WO02059370.50|csp|csp29|csp32|l(3)03988	0	0
7837	40461	Drosophila melanogaster	srpk79D	serine-arginine protein kinase at 79D	BEST:CK01209|CG11489|CG9085|CK01209|DmelCG11489|SRPK|SRPK1|Srpk79D|atc	0	0
7838	40462	Drosophila melanogaster	Hem	HEM-protein	CG5837|DmelCG5837|Kette|Kette/Nap1|Nap1|dhem|dhem-2|kette|kte|l(3)03335	0	0
7839	40464	Drosophila melanogaster	Ten-m	Tenascin major	2017|5301|BcDNA:AT25108|CG11452|CG5723|CT16449|DmelCG5723|Odz|Ten-mc|Ten79E|Ten[m]|l(3)00844|l(3)05301|l(3)05309|l(3)rJ307|l(3)rL201|l(3)rP126|odd(Oz)|odz|odz/ten-m|ten(m)|ten-m|ten-m/odz|	0	0
7840	40467	Drosophila melanogaster	CG11449	CG11449 gene product from transcript CG11449-RA	DmelCG11449	0	0
7841	40468	Drosophila melanogaster	laza	lazaro	CG11440|DmelCG11440|LPP-like|Laza|WUN-like	0	0
7842	40469	Drosophila melanogaster	CG11438	CG11438 gene product from transcript CG11438-RA	Css1alpha|DmelCG11438|LPP-like|WUN-like	0	0
7843	40470	Drosophila melanogaster	CG11437	CG11437 gene product from transcript CG11437-RA	DmelCG11437|LPP-like|WUN-like	0	0
7844	40471	Drosophila melanogaster	CG11426	CG11426 gene product from transcript CG11426-RA	Css1beta|DmelCG11426|LPP|LPP-like|WUN-like	0	0
7845	40472	Drosophila melanogaster	CG11425	CG11425 gene product from transcript CG11425-RA	DmelCG11425|LPP-like|WUN-like	0	0
7846	40474	Drosophila melanogaster	CG11404	CG11404 gene product from transcript CG11404-RA	BcDNA:GH11730|DmelCG11404	0	0
7847	40475	Drosophila melanogaster	Trxr-2	thioredoxin reductase 2	CG11401|Dm-TrxR|DmTrx|DmTrxR-2|DmelCG11401|Trxr2|trxr-2	0	0
7848	40476	Drosophila melanogaster	CG14459	CG14459 gene product from transcript CG14459-RB	DmelCG14459	0	0
7849	40477	Drosophila melanogaster	CG6914	CG6914 gene product from transcript CG6914-RA	BcDNA:AT13913|DmelCG6914	0	0
7850	40479	Drosophila melanogaster	CG14457	CG14457 gene product from transcript CG14457-RA	BcDNA:LP08817|CG18618|DmelCG14457	0	0
7851	40480	Drosophila melanogaster	CG14455	CG14455 gene product from transcript CG14455-RA	DmelCG14455	0	0
7852	40481	Drosophila melanogaster	CG14456	CG14456 gene product from transcript CG14456-RB	DmelCG14456	0	0
7853	40482	Drosophila melanogaster	CG14454	CG14454 gene product from transcript CG14454-RA	BcDNA:RE01153|CT34135|DmelCG14454	0	0
7854	40483	Drosophila melanogaster	CG12546	CG12546 gene product from transcript CG12546-RA	BcDNA:RE45259|DmelCG12546	0	0
7855	40484	Drosophila melanogaster	CG14453	CG14453 gene product from transcript CG14453-RA	BcDNA:RE13473|DmelCG14453	0	0
7856	40485	Drosophila melanogaster	CG14452	CG14452 gene product from transcript CG14452-RA	BcDNA:RE59202|DmelCG14452	0	0
7857	40486	Drosophila melanogaster	CG11370	CG11370 gene product from transcript CG11370-RA	DmelCG11370	0	0
7858	40487	Drosophila melanogaster	CG6838	CG6838 gene product from transcript CG6838-RC	DmelCG6838|arfgap1	0	0
7859	40488	Drosophila melanogaster	CG14451	CG14451 gene product from transcript CG14451-RA	DmelCG14451	0	0
7860	40489	Drosophila melanogaster	mael	maelstrom	CG11254|DmelCG11254|MAEL|mae1	0	0
7861	40490	Drosophila melanogaster	CG14450	CG14450 gene product from transcript CG14450-RA	DmelCG14450	0	0
7862	40491	Drosophila melanogaster	CG11367	CG11367 gene product from transcript CG11367-RA	DmelCG11367	0	0
7863	40492	Drosophila melanogaster	CG11241	CG11241 gene product from transcript CG11241-RB	DmelCG11241	0	0
7864	40495	Drosophila melanogaster	SPoCk	Secretory Pathway Calcium atpase	CG14449|CG32451|CG7651|DmelCG32451|SPoCK|anon-WO0118547.249	0	0
7865	40497	Drosophila melanogaster	CG14448	CG14448 gene product from transcript CG14448-RA	DmelCG14448	0	0
7866	40501	Drosophila melanogaster	CG11226	CG11226 gene product from transcript CG11226-RA	BcDNA:AT16729|DmelCG11226	0	0
7867	40502	Drosophila melanogaster	CG32459	CG32459 gene product from transcript CG32459-RA	CG8220|DmelCG32459|anon-WO0140519.31	0	0
7868	40503	Drosophila melanogaster	CG13239	CG13239 gene product from transcript CG13239-RA	DmelCG13239	0	0
7869	40504	Drosophila melanogaster	CG33169	CG33169 gene product from transcript CG33169-RA	BcDNA:RH56317|CG8244|DmelCG33169|EP3524	0	0
7870	40505	Drosophila melanogaster	CG11137	CG11137 gene product from transcript CG11137-RA	BcDNA:LD31024|CT31141|DmelCG11137	0	0
7871	40506	Drosophila melanogaster	Arf79F	ADP ribosylation factor 79F	ARF-1|ARF1|ARF1_DROME|Arf|Arf1|CG8385|Darf1|Dm Arf79F|DmelCG8385|dARF79F|dARFI|dArf1	0	0
7872	40507	Drosophila melanogaster	CG11109	CG11109 gene product from transcript CG11109-RB	CT31087|DmelCG11109	0	0
7873	40508	Drosophila melanogaster	Chro	Chromator	CG10712|Chiz|Chr|Chriz|DmelCG10712|anon-EST:fe2E3	0	0
7874	40509	Drosophila melanogaster	Ssl1	CG11115 gene product from transcript CG11115-RA	CG11115|DmelCG11115|SSL1|p44|ssl1	0	0
7875	40510	Drosophila melanogaster	slif	slimfast	CG11128|DmelCG11128|Slif	0	0
7876	40511	Drosophila melanogaster	CG11131	CG11131 gene product from transcript CG11131-RA	DmelCG11131	0	0
7877	40512	Drosophila melanogaster	CG11133	CG11133 gene product from transcript CG11133-RA	DmelCG11133	0	0
7878	40513	Drosophila melanogaster	CG12768	CG12768 gene product from transcript CG12768-RA	DmelCG12768	0	0

7879	40514	Drosophila melanogaster	Mes2	CG11100 gene product from transcript CG11100-RB	CG11100|DmelCG11100|adfR|mes2	0	0
7880	40515	Drosophila melanogaster	nrm	neuromusculin	CG32458|CG41452|CG43079|CG8779|CT16082|DmelCG43079|Dmel_CG32458|Dmel_CG41452|Dmel_CG8779	0	0
7881	40517	Drosophila melanogaster	alpha-Cat	alpha Catenin	CG17947|CT39986|Dalpha-Cat|Dalpha-cat|DalphaC|Dalphacat|Dmcatp|DmelCG17947|a-Cat|a-cat|alpha-CAT|alpha-cat|alpha-catenin|alpha-ctn|alphaCAT|alphaCat|alphacat	0	0
7882	40521	Drosophila melanogaster	nAcRalpha-80B	nicotinic Acetylcholine Receptor alpha 80B	CG12414|CG17552|Dalpha4|DmelCG12414|anon-WO0138359.1|nAChR|nAcRalpha-80|nAcRaplha-80B	0	0
7883	40522	Drosophila melanogaster	CG12581	CG12581 gene product from transcript CG12581-RA	DmelCG12581	0	0
7884	40524	Drosophila melanogaster	CG12582	CG12582 gene product from transcript CG12582-RD	CG12583|DmelCG12582	0	0
7885	40526	Drosophila melanogaster	CG14636	CG14636 gene product from transcript CG14636-RA	DmelCG14636	0	0
7886	40527	Drosophila melanogaster	aux	auxillin	CG1107|DmelCG1107|GAK|anon-WO0118547.393|auxilin|dAux	0	0
7887	40528	Drosophila melanogaster	DhpD	Dihydropterin deaminase	CG18143|DmelCG18143	0	0
7888	40529	Drosophila melanogaster	CG14641	CG14641 gene product from transcript CG14641-RA	DmelCG14641|cg14641	0	0
7889	40530	Drosophila melanogaster	abs	abstrakt	ABS|CG14637|DmRH23|DmelCG14637|anon-WO0118547.315|l(3)00620|l(3)04505|l(3)06862|l(3)06863	0	0
7890	40532	Drosophila melanogaster	CG14642	CG14642 gene product from transcript CG14642-RA	DmelCG14642|SP29	0	0
7891	40534	Drosophila melanogaster	TwdlF	TweedleF	CG14639|DmelCG14639	0	0
7892	40535	Drosophila melanogaster	TwdlG	TweedleG	CG14643|DmelCG14643	0	0
7893	40536	Drosophila melanogaster	CG14644	CG14644 gene product from transcript CG14644-RA	DmelCG14644|NEST:bs06h05	0	0
7894	40537	Drosophila melanogaster	TwdlV	TweedleV	CG14640|DmelCG14640	0	0
7895	40538	Drosophila melanogaster	CG1092	CG1092 gene product from transcript CG1092-RA	DmelCG1092	0	0
7896	40539	Drosophila melanogaster	CG9766	CG9766 gene product from transcript CG9766-RB	DmelCG9766	0	0
7897	40540	Drosophila melanogaster	CG9780	CG9780 gene product from transcript CG9780-RA	DmelCG9780	0	0
7898	40541	Drosophila melanogaster	MP1	Melanization Protease 1	BEST:GH02921|CG1102|DmelCG1102|SP25|proPO-AE	0	0
7899	40542	Drosophila melanogaster	vps24	Charged multivesicular body protein 3	CG9779|DmelCG9779|Vps24|dvps24	0	0
7900	40543	Drosophila melanogaster	CG9791	CG9791 gene product from transcript CG9791-RC	DmelCG9791	0	0
7901	40544	Drosophila melanogaster	Syt14	Synaptotagmin 14	CG9778|DmelCG9778|Syt 14|Syt16|dsyt14|syt14	0	0
7902	40545	Drosophila melanogaster	CG9795	CG9795 gene product from transcript CG9795-RD	DmelCG9795	0	0
7903	40546	Drosophila melanogaster	CG9776	CG9776 gene product from transcript CG9776-RA	BcDNA:LD19168|DmelCG9776	0	0
7904	40547	Drosophila melanogaster	CG1103	CG1103 gene product from transcript CG1103-RA	DmelCG1103|anon-WO0118547.152	0	0
7905	40548	Drosophila melanogaster	CG9772	CG9772 gene product from transcript CG9772-RA	153298_at|DmelCG9772|Skp2	0	0
7906	40549	Drosophila melanogaster	hkb	huckebein	5953|CG9768|DmelCG9768|HKB|Hkb|anon-82B3|gurt	0	0
7907	40551	Drosophila melanogaster	CG1090	CG1090 gene product from transcript CG1090-RB	DmelCG1090	0	0
7908	40552	Drosophila melanogaster	CG11739	CG11739 gene product from transcript CG11739-RC	DmelCG11739|anon-EST:Posey55	0	0
7909	40553	Drosophila melanogaster	Cont	Contactin	CG1084|CT1481|D-Axonin|DCONT|DmelCG1084|cont	0	0
7910	40554	Drosophila melanogaster	tub	tube	CG10520|DmelCG10520	0	0
7911	40555	Drosophila melanogaster	CG14646	CG14646 gene product from transcript CG14646-RA	DmelCG14646|anon-WO0118547.373	0	0
7912	40556	Drosophila melanogaster	CG9855	CG9855 gene product from transcript CG9855-RA	DmelCG9855	0	0
7913	40557	Drosophila melanogaster	CG9853	CG9853 gene product from transcript CG9853-RB	BEST:LD21829|CG41469|DmelCG9853|Dmel_CG41469	0	0
7914	40558	Drosophila melanogaster	CG14647	CG14647 gene product from transcript CG14647-RB	DmelCG14647	0	0
7915	40559	Drosophila melanogaster	lost	CG14648 gene product from transcript CG14648-RA	CG14648|DmelCG14648|Growl|anon-WO0118547.126|growl|nev	0	0
7916	40560	Drosophila melanogaster	CG32944	CG32944 gene product from transcript CG32944-RE	CG10532|CG9818|DmelCG32944	0	0
7917	40562	Drosophila melanogaster	Spargel	CG9809 gene product from transcript CG9809-RB	CG9809|DmelCG9809|Srl|cg9809|dPGC|dPGC-1|spargel	0	0
7918	40563	Drosophila melanogaster	eIF3-S10	CG9805 gene product from transcript CG9805-RA	CG9805|DmelCG9805|MMAP140|anon-WO0118547.261	0	0
7919	40564	Drosophila melanogaster	CG1074	CG1074 gene product from transcript CG1074-RA	DmelCG1074|anon-WO0118547.308	0	0
7920	40565	Drosophila melanogaster	CG9804	CG9804 gene product from transcript CG9804-RA	DmelCG9804	0	0
7921	40566	Drosophila melanogaster	CG14650	CG14650 gene product from transcript CG14650-RA	DmelCG14650	0	0
7922	40567	Drosophila melanogaster	CG31522	CG31522 gene product from transcript CG31522-RF	BcDNA:GH22993|BcDNA:LD10305|CG9798|DmelCG31522|anon-WO0118547.381	0	0
7923	40568	Drosophila melanogaster	CG14651	CG14651 gene product from transcript CG14651-RB	DmelCG14651	0	0
7924	40569	Drosophila melanogaster	Fip1	CG1078 gene product from transcript CG1078-RA	CG1078|DmelCG1078|FIP1|anon-EST:Liang-1.35|clone 1.35|dFip1	0	0
7925	40574	Drosophila melanogaster	Mur82C	Mucin related 82C	CG12586|DmelCG12586	0	0
7926	40575	Drosophila melanogaster	5-HT2	Serotonin receptor 2	5-HT2Dro|5-HT[2]Dro|5-HT[[2Dro]]|5-HT[[2]]|5-HT[[2]]Dro|5-ht2Dro|5HT-R82A|5HT-dro[[1]]|5HT2-Dro|5HT2Dro|5HT[[2Dro]]|CG1056|Dm5HT2|DmelCG1056|d5-HT2|l(3)82CDb	0	0
7927	40576	Drosophila melanogaster	CG9775	CG9775 gene product from transcript CG9775-RA	DmelCG9775	0	0
7928	40577	Drosophila melanogaster	MED31	Mediator complex subunit 31	BcDNA:GM28912|CG1057|DmelCG1057|Med31|Trap18|dSOH1|dTRAP18|p36	0	0
7929	40578	Drosophila melanogaster	slx1	nuclease slx1	CG18271|DmelCG18271|SLX1	0	0
7930	40579	Drosophila melanogaster	Dip2	Dorsal interacting protein 2	CG9771|DIP2|DmelCG9771|dip2|dmDIP2|sip4	0	0
7931	40580	Drosophila melanogaster	rpk	ripped pocket	CG1058|DmelCG1058|GNaC1|RPK|dGNaC1	0	0
7932	40581	Drosophila melanogaster	Karybeta3	Karyopherin beta 3	CG1059|DmelCG1059|Kary-beta3|l(3)82CDd|l(3)j3A4|l(3)j7E8	0	0
7933	40582	Drosophila melanogaster	CG12001	CG12001 gene product from transcript CG12001-RA	DmelCG12001	0	0
7934	40583	Drosophila melanogaster	CG43427	CG43427 gene product from transcript CG43427-RN	CG1054|CG1055|CG14653|CG18253|CG31531|CG31534|DmelCG43427|Dmel_CG31531|Dmel_CG31534|anon-WO0140519.95	0	0
7935	40587	Drosophila melanogaster	CG9769	CG9769 gene product from transcript CG9769-RA	DmelCG9769|eIF3-S5	0	0
7936	40588	Drosophila melanogaster	Nep2	Neprilysin 2	BcDNA:GH07643|CG9761|DmNEP2|DmelCG9761|NEP2|dNEP2|nep2	0	0
7937	40589	Drosophila melanogaster	tacc	transforming acidic coiled-coil protein	CG9765|D-TACC|D-tacc|DTACC|DmelCG9765|TACC|TACCdm|acc|d-tacc|dTACC|dtacc	0	0
7938	40590	Drosophila melanogaster	CG31530	CG31530 gene product from transcript CG31530-RA	CG12588|CG9767|DmelCG31530	0	0
7939	40592	Drosophila melanogaster	CG14654	CG14654 gene product from transcript CG14654-RA	DmelCG14654	0	0
7940	40593	Drosophila melanogaster	atms	antimeros	CG2503|DmelCG2503|PAF1|Paf1|anon-WO0172774.113|anon-WO0172774.114|anon-WO0172774.116|l(3)01456|l(3)02466|l(3)rK509	0	0
7941	40594	Drosophila melanogaster	CG14655	CG14655 gene product from transcript CG14655-RA	DmelCG14655	0	0
7942	40595	Drosophila melanogaster	CG17387	CG17387 gene product from transcript CG17387-RA	DmelCG17387	0	0
7943	40596	Drosophila melanogaster	CG42574	CG42574 gene product from transcript CG42574-RE	CG14656|CG17735|DmelCG42574|Dmel_CG14656|Dmel_CG17735	0	0
7944	40598	Drosophila melanogaster	Hus1-like	CG2525 gene product from transcript CG2525-RA	CG2525|DmelCG2525|Hus1|hus1	0	0
7945	40599	Drosophila melanogaster	CG1129	CG1129 gene product from transcript CG1129-RC	DmelCG1129	0	0
7946	40600	Drosophila melanogaster	CG1126	CG1126 gene product from transcript CG1126-RA	DmelCG1126	0	0
7947	40602	Drosophila melanogaster	CG14657	CG14657 gene product from transcript CG14657-RB	DmelCG14657	0	0
7948	40603	Drosophila melanogaster	CG14658	CG14658 gene product from transcript CG14658-RA	DmelCG14658	0	0
7949	40604	Drosophila melanogaster	CG14659	CG14659 gene product from transcript CG14659-RA	DmelCG14659	0	0
7950	40605	Drosophila melanogaster	opa	odd paired	CG1133|DmelCG1133|Opa	0	0
7951	40606	Drosophila melanogaster	laf	labial associated factor	C6|CG14660|DmelCG14660	0	0
7952	40607	Drosophila melanogaster	Gnf1	Germ line transcription factor 1	CG1119|DmRFC1|DmelCG1119|GTF-1|Gnf-1|Gtf1|ONF-CAACAA|RF-C 140|RFC|RFC1|RfC|RfC140|dRF-Cp140|dRFC140|gnf1|gtf1|rfc1	0	0
7953	40608	Drosophila melanogaster	Cdep	CG31536 gene product from transcript CG31536-RE	BcDNA:AT26639|BcDNA:RH50880|CDEP|CG1283|CG2008|CG31536|DmelCG31536|i23	0	0
7954	40610	Drosophila melanogaster	UbcD6	Ubiquitin conjugating enzyme	BcDNA:RE56673|CG2013|Dhr6|DmelCG2013|RAD6|Rad6	0	0
7955	40611	Drosophila melanogaster	CG14661	CG14661 gene product from transcript CG14661-RB	CT34439|DmelCG14661	0	0
7956	40612	Drosophila melanogaster	CG2016	CG2016 gene product from transcript CG2016-RB	DmelCG2016|anon-SAGE:Wang-083	0	0
7957	40613	Drosophila melanogaster	CG1124	CG1124 gene product from transcript CG1124-RA	CT1789|DmelCG1124	0	0
7958	40614	Drosophila melanogaster	CG14662	CG14662 gene product from transcript CG14662-RA	CT34440|DmelCG14662	0	0
7959	40615	Drosophila melanogaster	CG2022	CG2022 gene product from transcript CG2022-RB	DmelCG2022	0	0
7960	40616	Drosophila melanogaster	corto	CG2530 gene product from transcript CG2530-RC	7128|CG2530|CP-1|Ccf|Corto|DmelCG2530|anon-WO02059370.59|ccf|l(3)07128|l(3)neo31|l(3)neo32	0	0
7961	40617	Drosophila melanogaster	CG12007	CG12007 gene product from transcript CG12007-RA	DmelCG12007	0	0
7962	40618	Drosophila melanogaster	CG12590	CG12590 gene product from transcript CG12590-RA	DmelCG12590	0	0
7963	40619	Drosophila melanogaster	dpr16	CG12591 gene product from transcript CG12591-RA	CG12591|CT34444|DmelCG12591|Dpr-16	0	0
7964	40620	Drosophila melanogaster	cno	canoe	AF-6|CG2534|CG31537|CG42312|Canoe|Cno|DmelCG42312|Dmel_CG2534|Dmel_CG31537|anon-WO0172774.47|dlhA|lip|mis	0	0
7965	40621	Drosophila melanogaster	Prosbeta2R2	Proteasome beta2R2 subunit	CG12161|DmelCG12161	0	0
7966	40622	Drosophila melanogaster	CG1116	CG1116 gene product from transcript CG1116-RB	DmelCG1116|Rgp1|anon-WO0118547.337	0	0
7967	40623	Drosophila melanogaster	CG2604	CG2604 gene product from transcript CG2604-RC	CT40966|DmelCG2604	0	0
7968	40624	Drosophila melanogaster	CG1115	CG1115 gene product from transcript CG1115-RC	DmelCG1115|vps37B	0	0
7969	40626	Drosophila melanogaster	Mms19	CG12005 gene product from transcript CG12005-RB	CG12005|DmelCG12005|MMS19|NEST:bs07f08|dMMS19|mms19	0	0
7970	40627	Drosophila melanogaster	rtp	retinophilin	9C4|CG10233|DmelCG10233|anon-83ABa|retin|uta	0	0
7971	40628	Drosophila melanogaster	CG12163	CG12163 gene product from transcript CG12163-RB	DmelCG12163|anon-WO0118547.305	0	0
7972	40629	Drosophila melanogaster	CG1113	CG1113 gene product from transcript CG1113-RA	DmelCG1113	0	0
7973	40630	Drosophila melanogaster	CG12173	CG12173 gene product from transcript CG12173-RB	DmelCG12173	0	0
7974	40632	Drosophila melanogaster	Tim17a2	CG14666 gene product from transcript CG14666-RA	BcDNA:RH03914|CG14666|DmelCG14666	0	0
7975	40633	Drosophila melanogaster	Hph	HIF prolyl hydroxylase	0303/04|31543|CG1114|CG14665|CG31543|Dm.egl-9|DmelCG31543|anon-EST:Posey69|dHPH|dPHD2|dmHPH|fga|hph|l(3)02255|l(3)S030304	0	0
7976	40635	Drosophila melanogaster	Tim17b1	CG1158 gene product from transcript CG1158-RA	BcDNA:AT16284|CG1158|DmelCG1158	0	0
7977	40637	Drosophila melanogaster	CG11999	CG11999 gene product from transcript CG11999-RA	BEST:LD29996|CG-11999|DmelCG11999	0	0
7978	40638	Drosophila melanogaster	CG1161	CG1161 gene product from transcript CG1161-RB	DmelCG1161	0	0
7979	40639	Drosophila melanogaster	Prosbeta7	Proteasome beta7 subunit	12000|154275_at|CG12000|DmelCG12000|GC12000|Prosbeta4|anon-WO0118547.326|beta-4	0	0
7980	40640	Drosophila melanogaster	Cerk	Ceramide kinase	CG16708|DCERK|DmelCG16708|dcerk	0	0
7981	40642	Drosophila melanogaster	hd	humpty dumpty	CG2669|DmelCG2669|fs(3)272|fs(3)272-9|l(3)82Ff|l(3)82Fk	0	0
7982	40643	Drosophila melanogaster	CG14667	CG14667 gene product from transcript CG14667-RA	DmelCG14667	0	0
7983	40644	Drosophila melanogaster	7B2	CG1168 gene product from transcript CG1168-RB	CG1168|DmelCG1168|FBgn0040073|d7B2	0	0
7984	40646	Drosophila melanogaster	CG14668	CG14668 gene product from transcript CG14668-RA	DmelCG14668	0	0
7985	40647	Drosophila melanogaster	CG1172	CG1172 gene product from transcript CG1172-RB	DmelCG1172	0	0
7986	40648	Drosophila melanogaster	Or83a	Odorant receptor 83a	83A.1|83a|CG10612|DOR44|DmelCG10612	0	0
7987	40649	Drosophila melanogaster	CG2663	CG2663 gene product from transcript CG2663-RA	DmelCG2663	0	0
7988	40650	Drosophila melanogaster	Orco	Odorant receptor co-receptor	83A.2|83b|A45|CG10609|DOR45|DOR83b|DmelCG10609|OR49|OR83B|OR83b|Or83b|or83b|vainsA	0	0
7989	40651	Drosophila melanogaster	CG12147	CG12147 gene product from transcript CG12147-RA	DmelCG12147	0	0

7990	40652	Drosophila melanogaster	CG14669	CG14669 gene product from transcript CG14669-RA	DmelCG14669	0	0
7991	40653	Drosophila melanogaster	PEK	pancreatic eIF-2alpha kinase	CG2087|DPERK|DmPEK|DmelCG2087|DpERK|EIF2-like|PERK|Perk	0	0
7992	40654	Drosophila melanogaster	RpL35A	Ribosomal protein L35A	CG2099|DmelCG2099|L35a|anon-EST:Posey39	0	0
7993	40655	Drosophila melanogaster	CG12162	CG12162 gene product from transcript CG12162-RA	DmelCG12162	0	0
7994	40656	Drosophila melanogaster	mRpL44	mitochondrial ribosomal protein L44	CG2109|DmelCG2109|MRP-L44	0	0
7995	40657	Drosophila melanogaster	Snr1	Snf5-related 1	BAP45|CG1064|DmelCG1064|SNR1|Snr1/BAP45|l(3)01319|snr|snr1	0	0
7996	40659	Drosophila melanogaster	Hcs	Holocarboxylase synthetase	CG14670|DmelCG14670|HCS|HSC|NEST:bs27h05	0	0
7997	40660	Drosophila melanogaster	ksr	kinase suppressor of ras	CG2899|DmelCG2899|EK3-1|KSR|KSR-1|Ksr|Ksr-1|SR3-1|dKsr|l(3)j5E2	0	0
7998	40661	Drosophila melanogaster	CG31550	CG31550 gene product from transcript CG31550-RB	CG12167|CG12168|DmelCG31550	0	0
7999	40663	Drosophila melanogaster	CG31547	CG31547 gene product from transcript CG31547-RB	CG10544|CG2509|DmelCG31547	0	0
8000	40664	Drosophila melanogaster	Itp-r83A	Inositol 1,4,5,-tris-phosphate receptor	CG1063|DIP|DmInsP[[3R]]|DmInsP[[3]]R|DmelCG1063|IP|IP3R|IP[[3]] receptor|IP[[3]]R|ITP-r83A|InsP[[3]]|InsP[[3]]R|Insp3R|Itp-r|Itp-r42B|Itp-r[1]|anon-WO02059370.49|dip|itpr|itpr-83|l(3)05616|l(3)itpr|l(	0	0
8001	40665	Drosophila melanogaster	Nmdar1	NMDA receptor 1	CG2902|DMNMDAR1|DNMDAR-I|DNmdar|DmelCG2902|DrNR1|NMDA|NMDA R1|NMDAR1|NMdar1|NRI|Nmdar|dNR1|dnmdar-I|nmr	0	0
8002	40666	Drosophila melanogaster	CG2519	CG2519 gene product from transcript CG2519-RB	DmelCG2519	0	0
8003	40667	Drosophila melanogaster	mia	meiosis I arrest	CG10390|DmelCG10390|TAF|TAF60-2|TAF6L|TAF[[II]]|TAF[[II]]60-2|TFIID|Taf60-2|Taf6L|dTAF6L|dTAF[[II]]60|dTAF[[II]]60-2|dmTAF6b|ms(3)570	0	0
8004	40668	Drosophila melanogaster	Pcmt	Protein-L-isoaspartate (D-aspartate) O-methyltransfera	CG2152|DmelCG2152|PIMT|Pimt|dPIMT|pcmt	0	0
8005	40669	Drosophila melanogaster	Snm1	CG10018 gene product from transcript CG10018-RA	CG10018|DmelCG10018|SNM1|mus322	0	0
8006	40670	Drosophila melanogaster	Pi4KIIalpha	CG2929 gene product from transcript CG2929-RC	CG2929|DmelCG2929|PI4KII|PI4KIIalpha|PI4KIIbeta	0	0
8007	40671	Drosophila melanogaster	CG14671	CG14671 gene product from transcript CG14671-RA	DmelCG14671	0	0
8008	40672	Drosophila melanogaster	CG12746	CG12746 gene product from transcript CG12746-RD	DmelCG12746|WUN-like	0	0
8009	40673	Drosophila melanogaster	CG2931	CG2931 gene product from transcript CG2931-RA	DmelCG2931|cg2931	0	0
8010	40675	Drosophila melanogaster	CRMP	Collapsin Response Mediator Protein	BcDNA:HL02693|CG1411|DHP|DmelCG1411|EP(3)3238|EP3238|Pyd2|crmp|l(3)83Ba	0	0
8011	40676	Drosophila melanogaster	CG2926	CG2926 gene product from transcript CG2926-RA	DmelCG2926	0	0
8012	40677	Drosophila melanogaster	rev7	CG2948 gene product from transcript CG2948-RA	CG2948|DmREV7|DmelCG2948|mad2B	0	0
8013	40678	Drosophila melanogaster	noi	noisette	CG2925|DmelCG2925|Noi|SF3A3|SF3a[60]|U2|l(3)j3E7|ms3225	0	0
8014	40679	Drosophila melanogaster	Vha26	Vacuolar H[+]-ATPase 26kD E subunit	BcDNA.GH03683|BcDNA:GH03683|CG1088|DmelCG1088|V-ATPase|VhaE|anon-83Bb|vATPase|vha26	0	0
8015	40680	Drosophila melanogaster	exba	extra bases	CG2922|CT7240|Decp|DmelCG2922|Exba|Kra|anon-WO0172774.49|eIF|eIF-5C|eIF5|eIf-5c|ecp|eiF-5C|elF-5C|eIF5C|elf-5C|kra|l(3)03022|l(3)j9B6	0	0
8016	40681	Drosophila melanogaster	CG1427	CG1427 gene product from transcript CG1427-RC	DmelCG1427|SecS	0	0
8017	40682	Drosophila melanogaster	asl	asterless	Asl|CG2919|DmelCG2919|mecD|sdu	0	0
8018	40683	Drosophila melanogaster	Rga	Regena	CG2138|CG2161|DmelCG2161|NOT2|Not2|RGA|l(3)03834|not2	0	0
8019	40684	Drosophila melanogaster	Atu	Another transcription unit	CG1433|DmelCG1433|l(3)s1938	0	0
8020	40686	Drosophila melanogaster	CG2911	CG2911 gene product from transcript CG2911-RB	DmelCG2911	0	0
8021	40687	Drosophila melanogaster	RpL13A	Ribosomal protein L13A	CG1475|DmelCG1475|L13A|L13a2|M(3)83B|anon-EST:Posey125|anon-EST:fe1A5|bs25f04.y1|cg1475	0	0
8022	40689	Drosophila melanogaster	CG31549	CG31549 gene product from transcript CG31549-RA	CG11005|DmelCG31549	0	0
8023	40690	Drosophila melanogaster	CG12171	CG12171 gene product from transcript CG12171-RA	DmelCG12171|anon-WO0118547.83	0	0
8024	40691	Drosophila melanogaster	CG31546	CG31546 gene product from transcript CG31546-RA	CG2907|DmelCG31546|anon-WO0140519.189	0	0
8025	40692	Drosophila melanogaster	CG12170	CG12170 gene product from transcript CG12170-RA	DmelCG12170|anon-WO0118547.367	0	0
8026	40693	Drosophila melanogaster	MTA1-like	CG2244 gene product from transcript CG2244-RC	CG2244|DmelCG2244|MTA-like|dMTA|dMTA-1|dMTA1-like|p120	0	0
8027	40694	Drosophila melanogaster	sec23	CG1250 gene product from transcript CG1250-RA	CG1250|DmelCG1250|SEC23|Sec23|Sec23A|Sec23p|dSec23|dSec23p|dar2|dsec23|dsec23p|hau|l(3)j13C8	0	0
8028	40695	Drosophila melanogaster	elm	ethanol sensitive with low memory	CG2185|DmelCG2185	0	0
8029	40696	Drosophila melanogaster	MED27	Mediator complex subunit 27	CG1245|CRSP34|DmelCG1245|Med27|Trap37|dCRSP34|dCrsp34|dTRAP37|med27	0	0
8030	40697	Drosophila melanogaster	CG2182	CG2182 gene product from transcript CG2182-RA	DmelCG2182	0	0
8031	40698	Drosophila melanogaster	CG1109	CG1109 gene product from transcript CG1109-RA	BcDNA:LD24014|DmelCG1109	0	0
8032	40699	Drosophila melanogaster	Xe7	CG2179 gene product from transcript CG2179-RA	CG2179|DmelCG2179|l(3)02248	0	0
8033	40700	Drosophila melanogaster	CG1347	CG1347 gene product from transcript CG1347-RB	DmelCG1347	0	0
8034	40701	Drosophila melanogaster	Rab23	CG2108 gene product from transcript CG2108-RA	CG2108|DmRab23|DmelCG2108	0	0
8035	40702	Drosophila melanogaster	CG2104	CG2104 gene product from transcript CG2104-RA	DmelCG2104	0	0
8036	40704	Drosophila melanogaster	plx	pollux	CG1093|CG14674|CG34122|CG42612|DmelCG42612|Dmel_CG1093|Dmel_CG14674|Dmel_CG34122	0	0
8037	40706	Drosophila melanogaster	CG1239	CG1239 gene product from transcript CG1239-RA	DmelCG1239|cg1239	0	0
8038	40707	Drosophila melanogaster	CG2100	CG2100 gene product from transcript CG2100-RB	DmelCG2100	0	0
8039	40708	Drosophila melanogaster	CG1236	CG1236 gene product from transcript CG1236-RA	DmelCG1236	0	0
8040	40709	Drosophila melanogaster	Sym	Symplekin	CG2097|DmelCG2097|dSymp	0	0
8041	40710	Drosophila melanogaster	Madm	MLF1-adaptor molecule	BcDNA:LD28657|CG1098|DmelCG1098|MADM|anon-WO0118547.406|dMADM	0	0
8042	40711	Drosophila melanogaster	CG2091	CG2091 gene product from transcript CG2091-RA	DmelCG2091	0	0
8043	40712	Drosophila melanogaster	sec8	CG2095 gene product from transcript CG2095-RA	CG2095|DmelCG2095|Sec8|Sec8p|dsec8|fun	0	0
8044	40713	Drosophila melanogaster	CG2082	CG2082 gene product from transcript CG2082-RO	BcDNA:GH02439|DmelCG2082	0	0
8045	40714	Drosophila melanogaster	jagn	jagunal	CG10978|DmelCG10978	0	0
8046	40715	Drosophila melanogaster	kat-60L1	katanin p60-like 1	CG1193|DmCG1193|DmelCG1193	0	0
8047	40716	Drosophila melanogaster	CG2051	CG2051 gene product from transcript CG2051-RB	DmelCG2051|dmHAG409	0	0
8048	40717	Drosophila melanogaster	Rpn5	CG1100 gene product from transcript CG1100-RA	CG1100|DmelCG1100|p55	0	0
8049	40718	Drosophila melanogaster	CG1218	CG1218 gene product from transcript CG1218-RA	DmelCG1218	0	0
8050	40719	Drosophila melanogaster	CG2046	CG2046 gene product from transcript CG2046-RA	DmelCG2046	0	0
8051	40720	Drosophila melanogaster	CG10979	CG10979 gene product from transcript CG10979-RA	DmelCG10979	0	0
8052	40721	Drosophila melanogaster	CG11000	CG11000 gene product from transcript CG11000-RC	BcDNA:HL04389|CG31555|DmelCG11000	0	0
8053	40722	Drosophila melanogaster	CG42675	CG42675 gene product from transcript CG42675-RF	CG10999|CG31554|DmelCG42675|Dmel_CG10999|Dmel_CG31554|anon-WO0140519.247	0	0
8054	40723	Drosophila melanogaster	Hpr1	CG2031 gene product from transcript CG2031-RA	CG2031|DmelCG2031|HPR1	0	0
8055	40724	Drosophila melanogaster	CG2023	CG2023 gene product from transcript CG2023-RA	Cg2023|DmelCG2023	0	0
8056	40725	Drosophila melanogaster	dgrn	degringolade	CG10981|DmelCG10981	0	0
8057	40726	Drosophila melanogaster	glob3	globin 3	CG14675|DmeGb3|DmelCG14675	0	0
8058	40727	Drosophila melanogaster	CG1208	CG1208 gene product from transcript CG1208-RB	DmelCG1208	0	0
8059	40728	Drosophila melanogaster	CG1213	CG1213 gene product from transcript CG1213-RB	CT30783|DmelCG1213	0	0
8060	40729	Drosophila melanogaster	CG14676	CG14676 gene product from transcript CG14676-RA	DmelCG14676	0	0
8061	40732	Drosophila melanogaster	ECSIT	CG10610 gene product from transcript CG10610-RA	CG10610|DmECSIT|DmelCG10610|dECSIT	0	0
8062	40733	Drosophila melanogaster	CG2017	CG2017 gene product from transcript CG2017-RA	CG 2017|DmelCG2017	0	0
8063	40737	Drosophila melanogaster	Pbprp3	Pheromone-binding protein-related protein 3	83a|CG11421|DmelObp83a|DmelCG11421|OS-F|OS-F/PBP3|Obp33a|Obp83a|Os-F|PB-PRP3|PBP-H2|PBPRP-3|PBPRP3|obp83a|pbprp-3|pbprp3(OS-F)	0	0
8064	40738	Drosophila melanogaster	Os-E	Olfactory-specific E	83b|CG11422|DmelCG11422|OS-E|Obp83b|PBP-H1|obp83b	0	0
8065	40739	Drosophila melanogaster	Rm62	CG10279 gene product from transcript CG10279-RK	4136|CG10279|DmRH8|DmelCG10279|Dmp68|Lip|RM62|dmP68|l(3)01086|l(3)j3D2|l(3)j3D5|l(3)rG338|l(3)s5196|p68|rm62	0	0
8066	40740	Drosophila melanogaster	CG10280	CG10280 gene product from transcript CG10280-RA	DmelCG10280	0	0
8067	40741	Drosophila melanogaster	CG11459	CG11459 gene product from transcript CG11459-RA	DmelCG11459	0	0
8068	40743	Drosophila melanogaster	CG15580	CG15580 gene product from transcript CG15580-RC	DmelCG15580	0	0
8069	40744	Drosophila melanogaster	Or83c	Odorant receptor 83c	83D.1|83c|CG15581|DOR116|DmelCG15581	0	0
8070	40745	Drosophila melanogaster	Gasp	CG10287 gene product from transcript CG10287-RB	CG10287|DmelCG10287|anon-EST:ParkEST180|gammaBert27|gasp|obst-C	0	0
8071	40746	Drosophila melanogaster	Obp83cd	Odorant-binding protein 83cd	83c|83c-1|83c-2|83d|CG15582|DmelCG15582|Obp83c|Obp83d	0	0
8072	40747	Drosophila melanogaster	Obp83ef	Odorant-binding protein 83ef	83f|83f-1|83f-2|CG15583|CG31557|DmelCG31557|OBP83e|Obp83e|Obp83f	0	0
8073	40748	Drosophila melanogaster	Vha14-2	Vacuolar H[+] ATPase subunit 14-2	CG1076|DmelCG1076|vha14-2	0	0
8074	40749	Drosophila melanogaster	CG31559	CG31559 gene product from transcript CG31559-RA	CG11461|CG15584|DmelCG31559	0	0
8075	40751	Drosophila melanogaster	CG1077	CG1077 gene product from transcript CG1077-RA	DmelCG1077|anon-f	0	0
8076	40752	Drosophila melanogaster	Osi1	Osiris 1	CG15585|DmelCG15585|osi1	0	0
8077	40753	Drosophila melanogaster	CG31562	CG31562 gene product from transcript CG31562-RA	CG15587|CG15588|DmelCG31562	0	0
8078	40754	Drosophila melanogaster	NPFR1	neuropeptide F receptor	CG1147|DmelCG1147|NPFR|anon-WO0131005.3|anon-WO0131005.5|dNPFR1|npfr1|sNPFR1	0	0
8079	40755	Drosophila melanogaster	CG15589	CG15589 gene product from transcript CG15589-RA	DmelCG15589	0	0
8080	40756	Drosophila melanogaster	Osi2	Osiris 2	CG1148|DmelCG1148	0	0
8081	40757	Drosophila melanogaster	Osi3	Osiris 3	CG1150|DmelCG1150|osi3	0	0
8082	40758	Drosophila melanogaster	Osi4	Osiris 4	CG10303|DmelCG10303	0	0
8083	40759	Drosophila melanogaster	Osi5	Osiris 5	CG15590|DmelCG15590	0	0
8084	40760	Drosophila melanogaster	Osi6	Osiris 6	BcDNA:LD21503|CG1151|DmelCG1151	0	0
8085	40761	Drosophila melanogaster	Osi7	Osiris 7	CG1153|DmelCG1153|osi7	0	0
8086	40762	Drosophila melanogaster	Osi8	Osiris 8	CG15591|DmelCG15591	0	0
8087	40763	Drosophila melanogaster	Osi9	Osiris 9	CG15592|DmelCG15592	0	0
8088	40764	Drosophila melanogaster	Osi10	Osiris 10	CG15593|DmelCG15593	0	0
8089	40765	Drosophila melanogaster	Osi11	Osiris 11	CG15596|DmelCG15596|osi11	0	0
8090	40766	Drosophila melanogaster	Osi12	Osiris 12	CG1154|DmelCG1154|osi12	0	0
8091	40767	Drosophila melanogaster	CG15597	CG15597 gene product from transcript CG15597-RB	DmelCG15597	0	0
8092	40768	Drosophila melanogaster	CG15594	CG15594 gene product from transcript CG15594-RA	DmelCG15594	0	0
8093	40769	Drosophila melanogaster	Osi13	Osiris 13	CG15595|DmelCG15595	0	0
8094	40770	Drosophila melanogaster	Osi14	Osiris 14	BEST:LD24139|CG1155|DmelCG1155|SP558|Sp558|osi14	0	0
8095	40771	Drosophila melanogaster	Osi15	Osiris 15	CG1157|DmelCG1157	0	0
8096	40772	Drosophila melanogaster	CG31560	CG31560 gene product from transcript CG31560-RA	CG10314|DmelCG31560	0	0
8097	40774	Drosophila melanogaster	Osi17	Osiris 17	CG15598|DmelCG15598	0	0
8098	40775	Drosophila melanogaster	Osi18	Osiris 18	CG1169|DmelCG1169	0	0
8099	40776	Drosophila melanogaster	Osi19	Osiris 19	CG15189|DmelCG15189	0	0
8100	40777	Drosophila melanogaster	Osi20	Osiris 20	CG15188|DmelCG15188	0	0

<table>
<tr><td>8101</td><td>40778</td><td>Drosophila melanogaster</td><td>CG17917</td><td>CG17917 gene product from transcript CG17917-RA</td><td>DmelCG17917</td><td>0</td><td>0</td></tr>
<tr><td>8102</td><td>40779</td><td>Drosophila melanogaster</td><td>CG10298</td><td>CG10298 gene product from transcript CG10298-RA</td><td>BcDNA:GH28351|DmelCG10298</td><td>0</td><td>0</td></tr>
<tr><td>8103</td><td>40780</td><td>Drosophila melanogaster</td><td>CG17919</td><td>CG17919 gene product from transcript CG17919-RA</td><td>DmelCG17919</td><td>0</td><td>0</td></tr>
<tr><td>8104</td><td>40781</td><td>Drosophila melanogaster</td><td>SmD2</td><td>Small ribonucleoprotein particle protein SmD2</td><td>BcDNA:LD03002|CG1249|DmelCG1249|dD2|snRNP D2|snRNP2</td><td>0</td><td>0</td></tr>
<tr><td>8105</td><td>40782</td><td>Drosophila melanogaster</td><td>CG18048</td><td>CG18048 gene product from transcript CG18048-RA</td><td>DmelCG18048</td><td>0</td><td>0</td></tr>
<tr><td>8106</td><td>40783</td><td>Drosophila melanogaster</td><td>Hr83</td><td>Hormone receptor 83</td><td>CG10296|DHR83|DmelCG10296|FAX-1|NR2E5</td><td>0</td><td>0</td></tr>
<tr><td>8107</td><td>40785</td><td>Drosophila melanogaster</td><td>CG42724</td><td>CG42724 gene product from transcript CG42724-RJ</td><td>CG15187|CG31367|CG33097|DmelCG42724|Dmel_CG31367|Dmel_CG33097</td><td>0</td><td>0</td></tr>
<tr><td>8108</td><td>40786</td><td>Drosophila melanogaster</td><td>CG10286</td><td>CG10286 gene product from transcript CG10286-RA</td><td>DmelCG10286</td><td>0</td><td>0</td></tr>
<tr><td>8109</td><td>40787</td><td>Drosophila melanogaster</td><td>CG1041</td><td>CG1041 gene product from transcript CG1041-RA</td><td>DmelCG1041</td><td>0</td><td>0</td></tr>
<tr><td>8110</td><td>40788</td><td>Drosophila melanogaster</td><td>CG42564</td><td>CG42564 gene product from transcript CG42564-RA</td><td>CG10284|DmelCG42564|Dmel_CG10284</td><td>0</td><td>0</td></tr>
<tr><td>8111</td><td>40789</td><td>Drosophila melanogaster</td><td>CG1024</td><td>CG1024 gene product from transcript CG1024-RB</td><td>BcDNA:LD21969|DmelCG1024</td><td>0</td><td>0</td></tr>
<tr><td>8112</td><td>40790</td><td>Drosophila melanogaster</td><td>TfIIFalpha</td><td>Transcription factor IIFalpha</td><td>154355_at|CG10281|DmTFIIF|DmelCG10281|F5a|RAP74|TFIIF|TFIIF-L|TFIIFalpha|TfIIF</td><td>0</td><td>0</td></tr>
<tr><td>8113</td><td>40791</td><td>Drosophila melanogaster</td><td>CG10277</td><td>CG10277 gene product from transcript CG10277-RB</td><td>DmelCG10277</td><td>0</td><td>0</td></tr>
<tr><td>8114</td><td>40792</td><td>Drosophila melanogaster</td><td>CG1021</td><td>CG1021 gene product from transcript CG1021-RE</td><td>DmelCG1021|anon-WO0118547.375</td><td>0</td><td>0</td></tr>
<tr><td>8115</td><td>40793</td><td>Drosophila melanogaster</td><td>gpp</td><td>grappa</td><td>BcDNA:SD05230|CG10272|CG33324|CG42803|DmelCG42803|Dmel_CG10272|Dmel_CG33324|Gpp|anon-WO0118547.319|dDOT1L|dDot1|grp|l(3)03342|l(3)sA3484</td><td>0</td><td>0</td></tr>
<tr><td>8116</td><td>40794</td><td>Drosophila melanogaster</td><td>CG9727</td><td>CG9727 gene product from transcript CG9727-RA</td><td>DmelCG9727</td><td>0</td><td>0</td></tr>
<tr><td>8117</td><td>40795</td><td>Drosophila melanogaster</td><td>Zif</td><td>Zinc-finger protein</td><td>CG10267|DmelCG10267|anon-WO0118547.391</td><td>0</td><td>0</td></tr>
<tr><td>8118</td><td>40796</td><td>Drosophila melanogaster</td><td>Neurochondrin</td><td>CG2330 gene product from transcript CG2330-RA</td><td>CG2330|DmelCG2330</td><td>0</td><td>0</td></tr>
<tr><td>8119</td><td>40797</td><td>Drosophila melanogaster</td><td>CG15186</td><td>CG15186 gene product from transcript CG15186-RF</td><td>DmelCG15186</td><td>0</td><td>0</td></tr>
<tr><td>8120</td><td>40798</td><td>Drosophila melanogaster</td><td>CG15185</td><td>CG15185 gene product from transcript CG15185-RA</td><td>DmelCG15185</td><td>0</td><td>0</td></tr>
<tr><td>8121</td><td>40800</td><td>Drosophila melanogaster</td><td>dpr11</td><td>CG33202 gene product from transcript CG33202-RB</td><td>BcDNA:GH22307|CG15181|CG15182|CG15183|CG31309|CG33202|CT35098|DmelCG33202|Dpr-11</td><td>0</td><td>0</td></tr>
<tr><td>8122</td><td>40803</td><td>Drosophila melanogaster</td><td>CG1137</td><td>CG1137 gene product from transcript CG1137-RA</td><td>DmelCG1137|anon-WO0118547.86</td><td>0</td><td>0</td></tr>
<tr><td>8123</td><td>40804</td><td>Drosophila melanogaster</td><td>CG2336</td><td>CG2336 gene product from transcript CG2336-RA</td><td>DmelCG2336</td><td>0</td><td>0</td></tr>
<tr><td>8124</td><td>40805</td><td>Drosophila melanogaster</td><td>CG1138</td><td>CG1138 gene product from transcript CG1138-RA</td><td>DmelCG1138</td><td>0</td><td>0</td></tr>
<tr><td>8125</td><td>40806</td><td>Drosophila melanogaster</td><td>CG31482</td><td>CG31482 gene product from transcript CG31482-RA</td><td>CG2337|DmelCG31482</td><td>0</td><td>0</td></tr>
<tr><td>8126</td><td>40807</td><td>Drosophila melanogaster</td><td>glob2</td><td>globin 2</td><td>CG15180|DmeGb2|DmelCG15180|anon-SAGE:Wang-121</td><td>0</td><td>0</td></tr>
<tr><td>8127</td><td>40809</td><td>Drosophila melanogaster</td><td>sowi</td><td>solwind</td><td>CG15178|DmelCG15178</td><td>0</td><td>0</td></tr>
<tr><td>8128</td><td>40810</td><td>Drosophila melanogaster</td><td>CG15177</td><td>CG15177 gene product from transcript CG15177-RA</td><td>DmelCG15177</td><td>0</td><td>0</td></tr>
<tr><td>8129</td><td>40811</td><td>Drosophila melanogaster</td><td>sunz</td><td>sungrazer</td><td>BcDNA:AT31060|CG15179|DmelCG15179</td><td>0</td><td>0</td></tr>
<tr><td>8130</td><td>40812</td><td>Drosophila melanogaster</td><td>CG1315</td><td>CG1315 gene product from transcript CG1315-RA</td><td>BG:DS00004.14|DmelCG1315</td><td>0</td><td>0</td></tr>
<tr><td>8131</td><td>40813</td><td>Drosophila melanogaster</td><td>Taf1</td><td>TBP-associated factor 1</td><td>BG:DS00004.13|CG17603|DmelCG17603|EfW1|SR3-5|TAF|TAF1|TAF200|TAF230|TAF250|TAF250/230|TAFII-250|TAFII250|TAF[II]250|TAF[[II]]|TAF[[II]]230|TAF[[II]]250|TAF[[II]]250/230|TFIID|TFIID TAF250|Taf1</td><td>0</td><td>0</td></tr>
<tr><td>8132</td><td>40814</td><td>Drosophila melanogaster</td><td>CG1307</td><td>CG1307 gene product from transcript CG1307-RC</td><td>BG:DS00004.12|DmelCG1307</td><td>0</td><td>0</td></tr>
<tr><td>8133</td><td>40815</td><td>Drosophila melanogaster</td><td>twr</td><td>twisted bristles roughened eye</td><td>AF160889|BG:DS00004.11|BcDNA:GM04682|CG2358|DmelCG2358|EfW5|Ov15|Spase18-21|Spase18/21|l(3)05614|l(3)84Aa</td><td>0</td><td>0</td></tr>
<tr><td>8134</td><td>40816</td><td>Drosophila melanogaster</td><td>agt</td><td>O-6-alkylguanine-DNA alkyltransferase</td><td>Agt|BG:DS00004.10|CG1303|DmAGT|DmelCG1303</td><td>0</td><td>0</td></tr>
<tr><td>8135</td><td>40817</td><td>Drosophila melanogaster</td><td>lab</td><td>labial</td><td>BG:DS00004.9|CG1264|Dm lab|DmLab|DmelCG1264|Dmlab|EfR9|F121|F24|F90|F90-2|Lab|l(3)01241|l(3)84Ac|lb</td><td>0</td><td>0</td></tr>
<tr><td>8136</td><td>40818</td><td>Drosophila melanogaster</td><td>Edg84A</td><td>Ecdysone-dependent gene 84A</td><td>BG:DS00004.8|CG2345|DM-EDG84|DmelEDG84A|DmelCG2345|EDG-84|EDG-84A|EDG84|EDG84A|Edg84|cc1</td><td>0</td><td>0</td></tr>
<tr><td>8137</td><td>40819</td><td>Drosophila melanogaster</td><td>Ccp84Ag</td><td>CG2342 gene product from transcript CG2342-RA</td><td>BG:DS00004.7|BcDNA:RE04513|CG2342|Ccp|DmelCcp84Ag|DmelCG2342|cc2</td><td>0</td><td>0</td></tr>
<tr><td>8138</td><td>40820</td><td>Drosophila melanogaster</td><td>Ccp84Af</td><td>CG1331 gene product from transcript CG1331-RA</td><td>BG:DS00004.6|CG1331|Ccp|DmelCcp84Af|DmelCG1331|cc3</td><td>0</td><td>0</td></tr>
<tr><td>8139</td><td>40821</td><td>Drosophila melanogaster</td><td>Ccp84Ae</td><td>CG1330 gene product from transcript CG1330-RA</td><td>BG:DS00004.5|CG1330|Ccp|DmelCcp84Ae|DmelCG1330|cc4</td><td>0</td><td>0</td></tr>
<tr><td>8140</td><td>40822</td><td>Drosophila melanogaster</td><td>Ccp84Ad</td><td>CG2341 gene product from transcript CG2341-RA</td><td>BG:DS00004.4|CG2341|Ccp|DmelCcp84Ad|DmelCG2341|cc5</td><td>0</td><td>0</td></tr>
<tr><td>8141</td><td>40823</td><td>Drosophila melanogaster</td><td>Ccp84Ac</td><td>CG1327 gene product from transcript CG1327-RA</td><td>BG:DS00004.3|CG1327|Ccp|DmelCcp84Ac|DmelCG1327|cc6</td><td>0</td><td>0</td></tr>
<tr><td>8142</td><td>40824</td><td>Drosophila melanogaster</td><td>Ccp84Ab</td><td>CG1252 gene product from transcript CG1252-RA</td><td>BG:DS00004.2|CG1252|Ccp|DmelCcp84Ab|DmelCG1252|cc7</td><td>0</td><td>0</td></tr>
<tr><td>8143</td><td>40825</td><td>Drosophila melanogaster</td><td>Ccp84Aa</td><td>CG2360 gene product from transcript CG2360-RA</td><td>BG:DS00004.1|CG2360|Ccp|DmelCcp84Aa|DmelCG2360|cc8</td><td>0</td><td>0</td></tr>
<tr><td>8144</td><td>40826</td><td>Drosophila melanogaster</td><td>pb</td><td>proboscipedia</td><td>BG:DS01719.1|CG17880|CG31481|DmelCG31481|Dmpb|PB|Pb|l(3)04498|pd</td><td>0</td><td>0</td></tr>
<tr><td>8145</td><td>40827</td><td>Drosophila melanogaster</td><td>zen2</td><td>zerknullt-related</td><td>BG:DS00276.10|CG1048|DmelCG1048|z2|zpr</td><td>0</td><td>0</td></tr>
<tr><td>8146</td><td>40828</td><td>Drosophila melanogaster</td><td>zen</td><td>zerknullt</td><td>BG:DS00276.9|CG1046|DmelCG1046|ZEN VRE|Zen|l(3)84Ad|z1|zen1</td><td>0</td><td>0</td></tr>
<tr><td>8147</td><td>40830</td><td>Drosophila melanogaster</td><td>bcd</td><td>bicoid</td><td>BCD|BG:DS00276.7|Bcd|CG1034|DmelCG1034|bic|mum|prd4</td><td>0</td><td>0</td></tr>
<tr><td>8148</td><td>40831</td><td>Drosophila melanogaster</td><td>Ama</td><td>Amalgam</td><td>AMA|BG:DS00276.6|CG2198|CT7244|DmelCG2198|M109|ama</td><td>0</td><td>0</td></tr>
<tr><td>8149</td><td>40832</td><td>Drosophila melanogaster</td><td>Dfd</td><td>Deformed</td><td>BG:DS00276.5|CG2189|DmDfd|DmelCG2189|EbR11|dfd|l(3)84Ae</td><td>0</td><td>0</td></tr>
<tr><td>8150</td><td>40833</td><td>Drosophila melanogaster</td><td>Scr</td><td>Sex combs reduced</td><td>BG:DS07876.2|CG1030|DmScr|DmelCG1030|Msc|SCR|l(3)84Af|scr</td><td>0</td><td>0</td></tr>
<tr><td>8151</td><td>40834</td><td>Drosophila melanogaster</td><td>ftz</td><td>fushi tarazu</td><td>BG:DS07876.1|CG2047|Dm-Ftz|Dm-ftz|DmelCG2047|Dmftz|FTZ|Ftz|Ual|l(3)84Ag</td><td>0</td><td>0</td></tr>
<tr><td>8152</td><td>40835</td><td>Drosophila melanogaster</td><td>Antp</td><td>Antennapedia</td><td>3.4|ANT-C|ANT-P|ANTC|ANTP|Ant|AntP|AntP1|Antp P1|Antp P2|Antp1|Aus|BG:DS07700.1|CG1028|DMANTPE1|DRO15DC96Z|DmAntp|DmelCG1028|Hu|Ns|Scx|antp|l(3)84Ba</td><td>0</td><td>0</td></tr>
<tr><td>8153</td><td>40836</td><td>Drosophila melanogaster</td><td>Sodh-1</td><td>Sorbitol dehydrogenase 1</td><td>BG:DS00464.2|CG1982|DM_7298873|DmelCG1982|Sdh-1|Sodh</td><td>0</td><td>0</td></tr>
<tr><td>8154</td><td>40837</td><td>Drosophila melanogaster</td><td>CG1979</td><td>CG1979 gene product from transcript CG1979-RA</td><td>BG:DS00464.1|BcDNA:GH19145|DmelCG1979</td><td>0</td><td>0</td></tr>
<tr><td>8155</td><td>40838</td><td>Drosophila melanogaster</td><td>dj</td><td>don juan</td><td>CG1980|DJ|Dj|DmelCG1980|bs02h03.y1</td><td>0</td><td>0</td></tr>
<tr><td>8156</td><td>40839</td><td>Drosophila melanogaster</td><td>djl</td><td>don juan like</td><td>CG1984|DmelCG1984|Don Juan-like|dj like</td><td>0</td><td>0</td></tr>
<tr><td>8157</td><td>40840</td><td>Drosophila melanogaster</td><td>CG1988</td><td>CG1988 gene product from transcript CG1988-RB</td><td>DmelCG1988</td><td>0</td><td>0</td></tr>
<tr><td>8158</td><td>40841</td><td>Drosophila melanogaster</td><td>CG1105</td><td>CG1105 gene product from transcript CG1105-RA</td><td>DmelCG1105</td><td>0</td><td>0</td></tr>
<tr><td>8159</td><td>40842</td><td>Drosophila melanogaster</td><td>CG1965</td><td>CG1965 gene product from transcript CG1965-RA</td><td>DmelCG1965</td><td>0</td><td>0</td></tr>
<tr><td>8160</td><td>40843</td><td>Drosophila melanogaster</td><td>CG1104</td><td>CG1104 gene product from transcript CG1104-RC</td><td>DmelCG1104</td><td>0</td><td>0</td></tr>
<tr><td>8161</td><td>40844</td><td>Drosophila melanogaster</td><td>CG1943</td><td>CG1943 gene product from transcript CG1943-RA</td><td>DmelCG1943</td><td>0</td><td>0</td></tr>
<tr><td>8162</td><td>40846</td><td>Drosophila melanogaster</td><td>Dpck</td><td>Dephospho-CoA kinase</td><td>CG1939|DPCK|DmelCG1939|dDPCK</td><td>0</td><td>0</td></tr>
<tr><td>8163</td><td>40847</td><td>Drosophila melanogaster</td><td>CG1091</td><td>CG1091 gene product from transcript CG1091-RB</td><td>DmelCG1091|anon-WO0118547.222</td><td>0</td><td>0</td></tr>
<tr><td>8164</td><td>40848</td><td>Drosophila melanogaster</td><td>alphaTub84B</td><td>alpha-Tubulin at 84B</td><td>1t|ALPHA 84C|BEST:LD32507|CG1913|DM1alpha|DTA1|DmelCG1913|T|Talpha1|Tub|Tuba84B|Tubalpha1|a-tub84B|a1t|aTub84B|alpha-Tub|alpha-Tub84B|alpha-tub|alpha-tub84B|alpha-tubulin|alpha1|alph</td><td>0</td><td>0</td></tr>
<tr><td>8165</td><td>40849</td><td>Drosophila melanogaster</td><td>Mlp84B</td><td>Muscle LIM protein at 84B</td><td>CG1019|DLim-3|DmLIM-3|DmelCG1019|Mlmu84B|Mlp84b|dMlp84B|mlp|mlp84B</td><td>0</td><td>0</td></tr>
<tr><td>8166</td><td>40850</td><td>Drosophila melanogaster</td><td>Alh</td><td>Alhambra</td><td>AF10|CG1070|Dalf|DmelCG1070|anon-WO0118547.226|dAF10|l(3)84Be|l(3)g2|l(3)g5|l(3)j13A6|l(3)j8C8|l(3)j8c8|l(3)r13|l(3j8C8|stk</td><td>0</td><td>0</td></tr>
<tr><td>8167</td><td>40851</td><td>Drosophila melanogaster</td><td>CG10098</td><td>CG10098 gene product from transcript CG10098-RA</td><td>DmelCG10098</td><td>0</td><td>0</td></tr>
<tr><td>8168</td><td>40852</td><td>Drosophila melanogaster</td><td>CG10068</td><td>CG10068 gene product from transcript CG10068-RA</td><td>DmelCG10068</td><td>0</td><td>0</td></tr>
<tr><td>8169</td><td>40853</td><td>Drosophila melanogaster</td><td>CG31248</td><td>CG31248 gene product from transcript CG31248-RA</td><td>CG2640|DmelCG31248</td><td>0</td><td>0</td></tr>
<tr><td>8170</td><td>40856</td><td>Drosophila melanogaster</td><td>CG14610</td><td>CG14610 gene product from transcript CG14610-RB</td><td>DmelCG14610</td><td>0</td><td>0</td></tr>
<tr><td>8171</td><td>40857</td><td>Drosophila melanogaster</td><td>CG2656</td><td>CG2656 gene product from transcript CG2656-RA</td><td>DmelCG2656|NEST:bs33f03</td><td>0</td><td>0</td></tr>
<tr><td>8172</td><td>40858</td><td>Drosophila melanogaster</td><td>gfzf</td><td>GST-containing FLYWCH zinc-finger protein</td><td>CG10065|CG31329|CG31492|CG33546|DmelCG33546|GFZF|Su(Kpn)|cg10065|dGFZF|l(3)84Cc</td><td>0</td><td>0</td></tr>
<tr><td>8173</td><td>40859</td><td>Drosophila melanogaster</td><td>Sas-4</td><td>CG10061 gene product from transcript CG10061-RA</td><td>CG10061|D-Sas4|DSAS-4|DSas-4|DSas4|DmelCG10061|Dsas-4|SAS-4|SAS4|Sas4|d-SAS-4|dsas-4|l(3)S2214|l(3)s2214|sas-4</td><td>0</td><td>0</td></tr>
<tr><td>8174</td><td>40860</td><td>Drosophila melanogaster</td><td>MAGE</td><td>CG10059 gene product from transcript CG10059-RA</td><td>CG10059|DmelCG10059</td><td>0</td><td>0</td></tr>
<tr><td>8175</td><td>40861</td><td>Drosophila melanogaster</td><td>sas</td><td>stranded at second</td><td>CG2507|DmelCG2507|SAS|Sas|l(3)84Cd|rfd</td><td>0</td><td>0</td></tr>
<tr><td>8176</td><td>40862</td><td>Drosophila melanogaster</td><td>CG10055</td><td>CG10055 gene product from transcript CG10055-RA</td><td>DmelCG10055|RE52393</td><td>0</td><td>0</td></tr>
<tr><td>8177</td><td>40863</td><td>Drosophila melanogaster</td><td>lap</td><td>like-AP180</td><td>AP180|BcDNA:GM10787|CG2520|DmelCG2520|EP(3)3060|EP3060|LAP|Lap|dAP-3|l(3)neo34|lap/AP180|sig</td><td>0</td><td>0</td></tr>
<tr><td>8178</td><td>40864</td><td>Drosophila melanogaster</td><td>CG14609</td><td>CG14609 gene product from transcript CG14609-RA</td><td>DmelCG14609</td><td>0</td><td>0</td></tr>
<tr><td>8179</td><td>40865</td><td>Drosophila melanogaster</td><td>CG1288</td><td>CG1288 gene product from transcript CG1288-RA</td><td>BEST:LP10010|BcDNA:AT26093|DmelCG1288|anon-SAGE:Wang-119</td><td>0</td><td>0</td></tr>
<tr><td>8180</td><td>40866</td><td>Drosophila melanogaster</td><td>CG14606</td><td>CG14606 gene product from transcript CG14606-RA</td><td>DmelCG14606</td><td>0</td><td>0</td></tr>
<tr><td>8181</td><td>40867</td><td>Drosophila melanogaster</td><td>CG14605</td><td>CG14605 gene product from transcript CG14605-RC</td><td>BcDNA:AT16877|DmelCG14605</td><td>0</td><td>0</td></tr>
<tr><td>8182</td><td>40868</td><td>Drosophila melanogaster</td><td>CG14608</td><td>CG14608 gene product from transcript CG14608-RC</td><td>DmelCG14608</td><td>0</td><td>0</td></tr>
<tr><td>8183</td><td>40869</td><td>Drosophila melanogaster</td><td>CG14607</td><td>CG14607 gene product from transcript CG14607-RB</td><td>DmelCG14607</td><td>0</td><td>0</td></tr>
<tr><td>8184</td><td>40870</td><td>Drosophila melanogaster</td><td>CG1234</td><td>CG1234 gene product from transcript CG1234-RA</td><td>DmelCG1234</td><td>0</td><td>0</td></tr>
<tr><td>8185</td><td>40871</td><td>Drosophila melanogaster</td><td>CG10053</td><td>CG10053 gene product from transcript CG10053-RA</td><td>BEST:LD41878|BEST:LD45537|DmelCG10053</td><td>0</td><td>0</td></tr>
<tr><td>8186</td><td>40872</td><td>Drosophila melanogaster</td><td>CG1227</td><td>CG1227 gene product from transcript CG1227-RA</td><td>DmelCG1227|MPSK/PSK|Psk</td><td>0</td><td>0</td></tr>
<tr><td>8187</td><td>40873</td><td>Drosophila melanogaster</td><td>CG10050</td><td>CG10050 gene product from transcript CG10050-RA</td><td>DmelCG10050</td><td>0</td><td>0</td></tr>
<tr><td>8188</td><td>40874</td><td>Drosophila melanogaster</td><td>Pbprp4</td><td>Pheromone-binding protein-related protein 4</td><td>84a|CG1176|DmelCG1176|Obp84a|PBP4|PBPRP-4|PBPRP4|pbprp-4</td><td>0</td><td>0</td></tr>
<tr><td>8189</td><td>40875</td><td>Drosophila melanogaster</td><td>Gld</td><td>Glucose dehydrogenase</td><td>CG1152|DmelCG1152|GDH|GLD|Go|gld|glucose dehydrogenase|l(3)84Cg</td><td>0</td><td>0</td></tr>
<tr><td>8190</td><td>40876</td><td>Drosophila melanogaster</td><td>Syt4</td><td>Synaptotagmin 4</td><td>CG10047|D.Syt IV|DmelCG10047|SYT4|SytIV|dsyt4|syt 4|syt4|sytIV</td><td>0</td><td>0</td></tr>
<tr><td>8191</td><td>40877</td><td>Drosophila melanogaster</td><td>CG10032</td><td>CG10032 gene product from transcript CG10032-RA</td><td>DmelCG10032</td><td>0</td><td>0</td></tr>
<tr><td>8192</td><td>40879</td><td>Drosophila melanogaster</td><td>rn</td><td>rotund</td><td>5.3|CG10040|CG14600|CG14601|CG14603|CG32466|CG32467|CG42277|Dipr|DmelCG42277|Dmel_CG32466|Dmel_CG32467|P5.3|roe</td><td>0</td><td>0</td></tr>
<tr><td>8193</td><td>40883</td><td>Drosophila melanogaster</td><td>CG10029</td><td>CG10029 gene product from transcript CG10029-RA</td><td>DmelCG10029</td><td>0</td><td>0</td></tr>
<tr><td>8194</td><td>40884</td><td>Drosophila melanogaster</td><td>nxf4</td><td>nuclear export factor 4</td><td>BcDNA:AT07692|CG14604|CG31501|DmelCG31501</td><td>0</td><td>0</td></tr>
<tr><td>8195</td><td>40885</td><td>Drosophila melanogaster</td><td>CG17944</td><td>CG17944 gene product from transcript CG17944-RA</td><td>DmelCG17944</td><td>0</td><td>0</td></tr>
<tr><td>8196</td><td>40886</td><td>Drosophila melanogaster</td><td>Mst84Dd</td><td>Male-specific RNA 84Dd</td><td>CG17935|DmelCG17935|Mst|Mst ORF|Mst84|Mst87D|Mstprox|mst(3)gl-4</td><td>0</td><td>0</td></tr>
<tr><td>8197</td><td>40887</td><td>Drosophila melanogaster</td><td>Mst84Dc</td><td>Male-specific RNA 84Dc</td><td>CG17945|DmelCG17945|Mst|Mst ORF|Mst84|Mst84D|Mst87D|Mstprox|mst(3)gl-3</td><td>0</td><td>0</td></tr>
<tr><td>8198</td><td>40888</td><td>Drosophila melanogaster</td><td>Mst84Db</td><td>Male-specific RNA 84Db</td><td>BcDNA:AT08471|CG17934|DmelCG17934|Mst|Mst ORF|Mst84|Mst87D|Mstprox|NEST:bs22c06|bs22c06.y1|mst(3)gl-2</td><td>0</td><td>0</td></tr>
<tr><td>8199</td><td>40889</td><td>Drosophila melanogaster</td><td>Mst84Da</td><td>Male-specific RNA 84Da</td><td>CG17946|DmelCG17946|Mst|Mst ORF|Mst84|Mst87D|Mstprox|mst(3)gl-1</td><td>0</td><td>0</td></tr>
<tr><td>8200</td><td>40890</td><td>Drosophila melanogaster</td><td>MstProx</td><td>CG1149 gene product from transcript CG1149-RA</td><td>CG1149|DmMstProx|DmelCG1149|Tl-3|Toll-3|dTLR3|dToll3|mstprox|toll</td><td>0</td><td>0</td></tr>
<tr><td>8201</td><td>40891</td><td>Drosophila melanogaster</td><td>Ir84a</td><td>Ionotropic receptor 84a</td><td>CG10101|CT28433|DmelCG10101|IR84a</td><td>0</td><td>0</td></tr>
<tr><td>8202</td><td>40892</td><td>Drosophila melanogaster</td><td>wa-cup</td><td>walker cup</td><td>CG10113|DmelCG10113</td><td>0</td><td>0</td></tr>
<tr><td>8203</td><td>40893</td><td>Drosophila melanogaster</td><td>CG14598</td><td>CG14598 gene product from transcript CG14598-RA</td><td>DmelCG14598</td><td>0</td><td>0</td></tr>
<tr><td>8204</td><td>40894</td><td>Drosophila melanogaster</td><td>CG1142</td><td>CG1142 gene product from transcript CG1142-RA</td><td>DmelCG1142</td><td>0</td><td>0</td></tr>
<tr><td>8205</td><td>40896</td><td>Drosophila melanogaster</td><td>alpha-Est10</td><td>alpha-Esterase-10</td><td>CG1131|DmalphaE10|DmelCG1131|Esterase-10|K|aE10|alpha-Ests|alphaE10|anon-WO0172774.144</td><td>0</td><td>0</td></tr>
<tr><td>8206</td><td>40897</td><td>Drosophila melanogaster</td><td>alpha-Est9</td><td>alpha-Esterase-9</td><td>CG1128|DmalphaE9|DmelCG1128|Est9|J|aE9|alphaE9</td><td>0</td><td>0</td></tr>
<tr><td>8207</td><td>40898</td><td>Drosophila melanogaster</td><td>CG1287</td><td>CG1287 gene product from transcript CG1287-RA</td><td>BEST:GH03396|DmelCG1287</td><td>0</td><td>0</td></tr>
<tr><td>8208</td><td>40899</td><td>Drosophila melanogaster</td><td>alpha-Est8</td><td>alpha-Esterase-8</td><td>CG1121|DmalphaE8|DmelCG1121|Est8|I|aE8|alphaE8</td><td>0</td><td>0</td></tr>
<tr><td>8209</td><td>40900</td><td>Drosophila melanogaster</td><td>Ubc84D</td><td>Ubiquitin conjugating enzyme 84D</td><td>CG12799|DmelCG12799|ubc</td><td>0</td><td>0</td></tr>
<tr><td>8210</td><td>40901</td><td>Drosophila melanogaster</td><td>alpha-Est7</td><td>alpha-Esterase-7</td><td>Ali|CG1112|DmalphaE7|DmelCG1112|EST23|Est-23|Est23|H|Mce|aE7|ali|alphaE7|alphaest-7</td><td>0</td><td>0</td></tr>
<tr><td>8211</td><td>40902</td><td>Drosophila melanogaster</td><td>alpha-Est6</td><td>alpha-Esterase-6</td><td>CG1108|DmalphaE6|DmelCG1108|E|Esterase-6|F|G|aE6|alphaE6</td><td>0</td><td>0</td></tr>
</table>

<table>
<tr><td></td><td></td><td></td><td></td><td></td><td></td><td></td><td></td></tr>
<tr><td>8212</td><td>40903</td><td>Drosophila melanogaster</td><td>Rtnl2</td><td>CG1279 gene product from transcript CG1279-RC</td><td>CG1279|DmelCG1279|Tml84D|anon-WO0170980.134|rtnl2|trop</td><td>0</td><td>0</td></tr>
<tr><td>8213</td><td>40904</td><td>Drosophila melanogaster</td><td>alphaTub84D</td><td>alpha-Tubulin at 84D</td><td>3t|ALPHA 84D|CG2512|D.m.ALPHA-84D|DTA4|DmelCG2512|T|Tub|aTub84D|alpha-Tub|alpha-Tub84D|alpha-tub84D|alpha-tubulin|alpha3|alpha3t|alpha84D|alphaTUB|alphaTub|alphaTub3|alphaTub84</td><td>0</td><td>0</td></tr>
<tr><td>8214</td><td>40905</td><td>Drosophila melanogaster</td><td>alpha-Est5</td><td>alpha-Esterase-5</td><td>CG1089|D|DmalphaE5|DmelCG1089|EST-C|EST9|ESTC|Est-9|Est-C|Est-c|aE5|alphaE5</td><td>0</td><td>0</td></tr>
<tr><td>8215</td><td>40906</td><td>Drosophila melanogaster</td><td>alpha-Est4</td><td>alpha-Esterase-4</td><td>C|CG1082|DmalphaE4|DmelCG1082|FBgn0015572|aE4|alphaE4</td><td>0</td><td>0</td></tr>
<tr><td>8216</td><td>40907</td><td>Drosophila melanogaster</td><td>alpha-Est3</td><td>alpha-Esterase-3</td><td>B|CG1257|DmalphaE3|DmelCG1257|aE3|alpha-Ests|alphaE3</td><td>0</td><td>0</td></tr>
<tr><td>8217</td><td>40908</td><td>Drosophila melanogaster</td><td>alpha-Est2</td><td>alpha-Esterase-2</td><td>Alpha-Est2|B|CG2505|DmalphaE2|DmelCG2505|aE2|alpha-EST2|alphaE2</td><td>0</td><td>0</td></tr>
<tr><td>8218</td><td>40909</td><td>Drosophila melanogaster</td><td>alpha-Est1</td><td>alpha-Esterase-1</td><td>A|CG1031|DmalphaE1|DmelCG1031|Est1|aE1|alphaE1</td><td>0</td><td>0</td></tr>
<tr><td>8219</td><td>40912</td><td>Drosophila melanogaster</td><td>CG34127</td><td>CG34127 gene product from transcript CG34127-RB</td><td>CG1062|CG14597|CG18291|CG32465|DmelCG34127</td><td>0</td><td>0</td></tr>
<tr><td>8220</td><td>40913</td><td>Drosophila melanogaster</td><td>Nlg1</td><td>Neuroligin 1</td><td>CG12148|CG14596|CG31146|DmelCG31146|anon-WO0118547.256|dnlg1</td><td>0</td><td>0</td></tr>
<tr><td>8221</td><td>40915</td><td>Drosophila melanogaster</td><td>CG2616</td><td>CG2616 gene product from transcript CG2616-RA</td><td>DmelCG2616</td><td>0</td><td>0</td></tr>
<tr><td>8222</td><td>40916</td><td>Drosophila melanogaster</td><td>pyd3</td><td>CG3027 gene product from transcript CG3027-RA</td><td>CG3027|DmelCG3027|betaAS|betaASase</td><td>0</td><td>0</td></tr>
<tr><td>8223</td><td>40917</td><td>Drosophila melanogaster</td><td>CG10919</td><td>CG10919 gene product from transcript CG10919-RA</td><td>DmelCG10919</td><td>0</td><td>0</td></tr>
<tr><td>8224</td><td>40918</td><td>Drosophila melanogaster</td><td>Sp7</td><td>Serine protease 7</td><td>CG3066|DmelCG3066|MP2|PAE1|SP7|anon-Ryu|proPO-AE|sp7</td><td>0</td><td>0</td></tr>
<tr><td>8225</td><td>40919</td><td>Drosophila melanogaster</td><td>CG11286</td><td>CG11286 gene product from transcript CG11286-RA</td><td>DmelCG11286</td><td>0</td><td>0</td></tr>
<tr><td>8226</td><td>40920</td><td>Drosophila melanogaster</td><td>CG10086</td><td>CG10086 gene product from transcript CG10086-RA</td><td>DmelCG10086</td><td>0</td><td>0</td></tr>
<tr><td>8227</td><td>40921</td><td>Drosophila melanogaster</td><td>CG2641</td><td>CG2641 gene product from transcript CG2641-RA</td><td>DmelCG2641</td><td>0</td><td>0</td></tr>
<tr><td>8228</td><td>40922</td><td>Drosophila melanogaster</td><td>CG3014</td><td>CG3014 gene product from transcript CG3014-RB</td><td>DmelCG3014</td><td>0</td><td>0</td></tr>
<tr><td>8229</td><td>40923</td><td>Drosophila melanogaster</td><td>CG18268</td><td>CG18268 gene product from transcript CG18268-RA</td><td>DmelCG18268</td><td>0</td><td>0</td></tr>
<tr><td>8230</td><td>40924</td><td>Drosophila melanogaster</td><td>CG2993</td><td>CG2993 gene product from transcript CG2993-RA</td><td>DmelCG2993</td><td>0</td><td>0</td></tr>
<tr><td>8231</td><td>40925</td><td>Drosophila melanogaster</td><td>CG31472</td><td>CG31472 gene product from transcript CG31472-RA</td><td>CG2649|DmelCG31472</td><td>0</td><td>0</td></tr>
<tr><td>8232</td><td>40928</td><td>Drosophila melanogaster</td><td>CG17816</td><td>CG17816 gene product from transcript CG17816-RE</td><td>DmelCG17816</td><td>0</td><td>0</td></tr>
<tr><td>8233</td><td>40929</td><td>Drosophila melanogaster</td><td>CG10092</td><td>CG10092 gene product from transcript CG10092-RA</td><td>DmelCG10092|atl</td><td>0</td><td>0</td></tr>
<tr><td>8234</td><td>40931</td><td>Drosophila melanogaster</td><td>wtrw</td><td>water witch</td><td>153412_at|CG10409|CG17817|CG31284|CT29234|DmelCG31284</td><td>0</td><td>0</td></tr>
<tr><td>8235</td><td>40932</td><td>Drosophila melanogaster</td><td>mRpS9</td><td>mitochondrial ribosomal protein S9</td><td>CG2957|DmelCG2957|MRP-S9</td><td>0</td><td>0</td></tr>
<tr><td>8236</td><td>40933</td><td>Drosophila melanogaster</td><td>CG2943</td><td>CG2943 gene product from transcript CG2943-RA</td><td>DmelCG2943|NEST:bs09f06</td><td>0</td><td>0</td></tr>
<tr><td>8237</td><td>40934</td><td>Drosophila melanogaster</td><td>Taf7</td><td>TBP-associated factor 7</td><td>CG2670|DmelCG2670|E|TAF|TAF55|TAF7|TAF[[II]]|TAF[[II]]55|TFIID|Taf55|anon-84Ec|dTAF[[II]]55</td><td>0</td><td>0</td></tr>
<tr><td>8238</td><td>40935</td><td>Drosophila melanogaster</td><td>Prat</td><td>Phosphoribosylamidotransferase</td><td>CG2867|DmelCG2867</td><td>0</td><td>0</td></tr>
<tr><td>8239</td><td>40936</td><td>Drosophila melanogaster</td><td>CG2846</td><td>CG2846 gene product from transcript CG2846-RA</td><td>C|DmelCG2846|anon-84Ea</td><td>0</td><td>0</td></tr>
<tr><td>8240</td><td>40937</td><td>Drosophila melanogaster</td><td>CG2678</td><td>CG2678 gene product from transcript CG2678-RB</td><td>B|DmelCG2678|anon-84Eb</td><td>0</td><td>0</td></tr>
<tr><td>8241</td><td>40938</td><td>Drosophila melanogaster</td><td>CG10445</td><td>CG10445 gene product from transcript CG10445-RD</td><td>DmelCG10445</td><td>0</td><td>0</td></tr>
<tr><td>8242</td><td>40940</td><td>Drosophila melanogaster</td><td>dsx</td><td>doublesex</td><td>CG11094|DSX|Dmdsx|DmelCG11094|Dsx|Hr|ix-62c</td><td>0</td><td>0</td></tr>
<tr><td>8243</td><td>40941</td><td>Drosophila melanogaster</td><td>CD98hc</td><td>CD98 heavy chain</td><td>CG2791|DmelCG2791|cd98hc</td><td>0</td><td>0</td></tr>
<tr><td>8244</td><td>40942</td><td>Drosophila melanogaster</td><td>Os-C</td><td>CG3250 gene product from transcript CG3250-RC</td><td>CG3250|DmelCG3250|OS-C|Os-c</td><td>0</td><td>0</td></tr>
<tr><td>8245</td><td>40943</td><td>Drosophila melanogaster</td><td>CG2781</td><td>CG2781 gene product from transcript CG2781-RA</td><td>DmelCG2781</td><td>0</td><td>0</td></tr>
<tr><td>8246</td><td>40944</td><td>Drosophila melanogaster</td><td>CG14463</td><td>CG14463 gene product from transcript CG14463-RA</td><td>DmelCG14463</td><td>0</td><td>0</td></tr>
<tr><td>8247</td><td>40945</td><td>Drosophila melanogaster</td><td>CG2698</td><td>CG2698 gene product from transcript CG2698-RD</td><td>DmelCG2698</td><td>0</td><td>0</td></tr>
<tr><td>8248</td><td>40946</td><td>Drosophila melanogaster</td><td>CG2767</td><td>CG2767 gene product from transcript CG2767-RA</td><td>DmelCG2767</td><td>0</td><td>0</td></tr>
<tr><td>8249</td><td>40947</td><td>Drosophila melanogaster</td><td>CG3223</td><td>CG3223 gene product from transcript CG3223-RA</td><td>DmelCG3223</td><td>0</td><td>0</td></tr>
<tr><td>8250</td><td>40948</td><td>Drosophila melanogaster</td><td>CG10435</td><td>CG10435 gene product from transcript CG10435-RA</td><td>DmelCG10435</td><td>0</td><td>0</td></tr>
<tr><td>8251</td><td>40949</td><td>Drosophila melanogaster</td><td>Pbp95</td><td>PSEA-binding protein 95kD</td><td>BEST:LD28511|CG2702|DmPBP|DmPBP95|DmSNAP190|DmelCG2702|anon-WO0118547.359</td><td>0</td><td>0</td></tr>
<tr><td>8252</td><td>40950</td><td>Drosophila melanogaster</td><td>CG2747</td><td>CG2747 gene product from transcript CG2747-RD</td><td>DmelCG2747</td><td>0</td><td>0</td></tr>
<tr><td>8253</td><td>40952</td><td>Drosophila melanogaster</td><td>CG10903</td><td>CG10903 gene product from transcript CG10903-RA</td><td>DmelCG10903</td><td>0</td><td>0</td></tr>
<tr><td>8254</td><td>40953</td><td>Drosophila melanogaster</td><td>CG11035</td><td>CG11035 gene product from transcript CG11035-RA</td><td>DmelCG11035|anon-WO0118547.426</td><td>0</td><td>0</td></tr>
<tr><td>8255</td><td>40955</td><td>Drosophila melanogaster</td><td>CG7918</td><td>CG7918 gene product from transcript CG7918-RA</td><td>DmelCG7918|anon-WO0170980.91|anon-WO0170980.92</td><td>0</td><td>0</td></tr>
<tr><td>8256</td><td>40956</td><td>Drosophila melanogaster</td><td>CG7910</td><td>CG7910 gene product from transcript CG7910-RA</td><td>DmelCG7910</td><td>0</td><td>0</td></tr>
<tr><td>8257</td><td>40957</td><td>Drosophila melanogaster</td><td>CG7900</td><td>CG7900 gene product from transcript CG7900-RB</td><td>DmelCG7900</td><td>0</td><td>0</td></tr>
<tr><td>8258</td><td>40958</td><td>Drosophila melanogaster</td><td>puc</td><td>puckered</td><td>0238/03|1351/08|CG7850|DmelCG7850|JNK-DSP|PUC|PUC/MKP|Puc|hrt|l(3)84Eh|l(3)A251.1|l(3)j4E1|vco</td><td>0</td><td>0</td></tr>
<tr><td>8259</td><td>40959</td><td>Drosophila melanogaster</td><td>CG7878</td><td>CG7878 gene product from transcript CG7878-RA</td><td>DmRH26|DmelCG7878|cg7878</td><td>0</td><td>0</td></tr>
<tr><td>8260</td><td>40960</td><td>Drosophila melanogaster</td><td>CG9667</td><td>CG9667 gene product from transcript CG9667-RA</td><td>DmelCG9667</td><td>0</td><td>0</td></tr>
<tr><td>8261</td><td>40961</td><td>Drosophila melanogaster</td><td>Gie</td><td>GTPase indispensable for equal segregation of chrom</td><td>Arl8|BcDNA:LD29185|CG7891|DmelCG7891</td><td>0</td><td>0</td></tr>
<tr><td>8262</td><td>40962</td><td>Drosophila melanogaster</td><td>grn</td><td>grain</td><td>CG9656|CG9656-PA|DmelCG9656|GATAc|GRAIN|Gata-c|Gata2|Grn|dGATA-2|dGATAc|dGatac|gra|l(3)84Fa</td><td>0</td><td>0</td></tr>
<tr><td>8263</td><td>40963</td><td>Drosophila melanogaster</td><td>CG7800</td><td>CG7800 gene product from transcript CG7800-RA</td><td>CT23295|DmelCG7800</td><td>0</td><td>0</td></tr>
<tr><td>8264</td><td>40964</td><td>Drosophila melanogaster</td><td>CG18249</td><td>CG18249 gene product from transcript CG18249-RB</td><td>CT41336|DmelCG18249</td><td>0</td><td>0</td></tr>
<tr><td>8265</td><td>40965</td><td>Drosophila melanogaster</td><td>DNApol-iota</td><td>CG7602 gene product from transcript CG7602-RB</td><td>AAF54198|CG7602|DNA Pol II-iota|DNApol-i|DmelCG7602|drad30B</td><td>0</td><td>0</td></tr>
<tr><td>8266</td><td>40966</td><td>Drosophila melanogaster</td><td>Ada2b</td><td>CG9638 gene product from transcript CG9638-RA</td><td>ADA2b|Ada2B|Ada2S|BcDNA:LD24527|CG9638|DmelCG9638|ada2b|dADA2b|dAda2B|dAda2S|dAda2b</td><td>0</td><td>0</td></tr>
<tr><td>8267</td><td>40967</td><td>Drosophila melanogaster</td><td>CG9636</td><td>CG9636 gene product from transcript CG9636-RD</td><td>DmelCG9636</td><td>0</td><td>0</td></tr>
<tr><td>8268</td><td>40970</td><td>Drosophila melanogaster</td><td>mtg</td><td>mind the gap</td><td>0849/10|CG7549|DmelCG7549|l(3)S084910|l(3)S084910b</td><td>0</td><td>0</td></tr>
<tr><td>8269</td><td>40972</td><td>Drosophila melanogaster</td><td>RpA-70</td><td>Replication Protein A 70</td><td>CG9633|D-RPA70|D-SSB|DRP-A|DmRPA|DmelCG9633|RP-A|RPA|RPA 70|RPA1|RPA70|RpA70|Rpa-70|Ssb-70|dRP-A|dmrpa1|i164</td><td>0</td><td>0</td></tr>
<tr><td>8270</td><td>40973</td><td>Drosophila melanogaster</td><td>Mcm2</td><td>Minichromosome maintenance 2</td><td>CG7538|DmMCM2|DmMcm2|DmeMCM2|DmelCG7538|MCM2|MCM2-7|MCM2_DROME|McM2|PCR3|dMCM2|l(3)rL074</td><td>0</td><td>0</td></tr>
<tr><td>8271</td><td>40974</td><td>Drosophila melanogaster</td><td>CG9630</td><td>CG9630 gene product from transcript CG9630-RA</td><td>DmRH16|DmelCG9630|cg9630</td><td>0</td><td>0</td></tr>
<tr><td>8272</td><td>40975</td><td>Drosophila melanogaster</td><td>ato</td><td>atonal</td><td>ATO|At|Ato|CG7508|DmelCG7508|ato1|bHLHa10</td><td>0</td><td>0</td></tr>
<tr><td>8273</td><td>40976</td><td>Drosophila melanogaster</td><td>CG11671</td><td>CG11671 gene product from transcript CG11671-RA</td><td>BcDNA:GM23845|DmelCG11671</td><td>0</td><td>0</td></tr>
<tr><td>8274</td><td>40977</td><td>Drosophila melanogaster</td><td>CG11672</td><td>CG11672 gene product from transcript CG11672-RA</td><td>BcDNA:RE26525|DmelCG11672</td><td>0</td><td>0</td></tr>
<tr><td>8275</td><td>40978</td><td>Drosophila melanogaster</td><td>CG31463</td><td>CG31463 gene product from transcript CG31463-RA</td><td>CG11673|DmelCG31463</td><td>0</td><td>0</td></tr>
<tr><td>8276</td><td>40979</td><td>Drosophila melanogaster</td><td>CG9626</td><td>CG9626 gene product from transcript CG9626-RC</td><td>DmelCG9626</td><td>0</td><td>0</td></tr>
<tr><td>8277</td><td>40980</td><td>Drosophila melanogaster</td><td>mRpL1</td><td>mitochondrial ribosomal protein L1</td><td>CG7494|DmelCG7494|L1</td><td>0</td><td>0</td></tr>
<tr><td>8278</td><td>40981</td><td>Drosophila melanogaster</td><td>Gfr</td><td>CG9620 gene product from transcript CG9620-RA</td><td>CG9620|DmelCG9620|gfr</td><td>0</td><td>0</td></tr>
<tr><td>8279</td><td>40982</td><td>Drosophila melanogaster</td><td>Sgt1</td><td>CG9617 gene product from transcript CG9617-RA</td><td>CG9617|DmSgt1|DmelCG9617|Q9VHT3|sgt1</td><td>0</td><td>0</td></tr>
<tr><td>8280</td><td>40983</td><td>Drosophila melanogaster</td><td>tex</td><td>CG9615 gene product from transcript CG9615-RA</td><td>CG9615|DmelCG9615</td><td>0</td><td>0</td></tr>
<tr><td>8281</td><td>40984</td><td>Drosophila melanogaster</td><td>CG11693</td><td>CG11693 gene product from transcript CG11693-RB</td><td>DmelCG11693</td><td>0</td><td>0</td></tr>
<tr><td>8282</td><td>40985</td><td>Drosophila melanogaster</td><td>CG11694</td><td>CG11694 gene product from transcript CG11694-RA</td><td>DmelCG11694</td><td>0</td><td>0</td></tr>
<tr><td>8283</td><td>40986</td><td>Drosophila melanogaster</td><td>CG11698</td><td>CG11698 gene product from transcript CG11698-RA</td><td>DmelCG11698</td><td>0</td><td>0</td></tr>
<tr><td>8284</td><td>40987</td><td>Drosophila melanogaster</td><td>eIF4AIII</td><td>CG7483 gene product from transcript CG7483-RA</td><td>7483|CG7483|DmRH28|DmelCG7483|anon-WO0118547.364|eIF4A|eIF4A-III|l(3)84Fi</td><td>0</td><td>0</td></tr>
<tr><td>8285</td><td>40988</td><td>Drosophila melanogaster</td><td>Coq2</td><td>Coenzyme Q biosynthesis protein 2</td><td>CG9613|COQ2|DmelCG9613|coq2|sbo</td><td>0</td><td>0</td></tr>
<tr><td>8286</td><td>40989</td><td>Drosophila melanogaster</td><td>Ctr1B</td><td>Copper transporter 1B</td><td>CG7459|DmelCG7459|ctr1B</td><td>0</td><td>0</td></tr>
<tr><td>8287</td><td>40990</td><td>Drosophila melanogaster</td><td>Poxm</td><td>Pox meso</td><td>CG9610|DmelCG9610|P29|Pox-m|meso|poxm</td><td>0</td><td>0</td></tr>
<tr><td>8288</td><td>40991</td><td>Drosophila melanogaster</td><td>Or85a</td><td>Odorant receptor 85a</td><td>85A.4|85a|CG7454|DOR31|DmelCG7454|or85a</td><td>0</td><td>0</td></tr>
<tr><td>8289</td><td>40992</td><td>Drosophila melanogaster</td><td>CG7443</td><td>CG7443 gene product from transcript CG7443-RA</td><td>DmelCG7443</td><td>0</td><td>0</td></tr>
<tr><td>8290</td><td>40993</td><td>Drosophila melanogaster</td><td>Arf84F</td><td>ADP ribosylation factor 84F</td><td>ARL2|Arf|Arf84D|Arl2|Arl84F|CG 7435|CG7435|Dm Arf84F|DmelCG7435|Q06849|arl2</td><td>0</td><td>0</td></tr>
<tr><td>8291</td><td>40994</td><td>Drosophila melanogaster</td><td>CG9601</td><td>CG9601 gene product from transcript CG9601-RA</td><td>DmelCG9601</td><td>0</td><td>0</td></tr>
<tr><td>8292</td><td>40995</td><td>Drosophila melanogaster</td><td>CG18193</td><td>CG18193 gene product from transcript CG18193-RA</td><td>DmelCG18193|VIIa</td><td>0</td><td>0</td></tr>
<tr><td>8293</td><td>40996</td><td>Drosophila melanogaster</td><td>DppIII</td><td>Dipeptidyl aminopeptidase III</td><td>CG7415|DPP|DPP III|DmelCG7415</td><td>0</td><td>0</td></tr>
<tr><td>8294</td><td>40997</td><td>Drosophila melanogaster</td><td>CG7352</td><td>CG7352 gene product from transcript CG7352-RC</td><td>DmelCG7352</td><td>0</td><td>0</td></tr>
<tr><td>8295</td><td>40998</td><td>Drosophila melanogaster</td><td>Atg13</td><td>Autophagy-specific gene 13</td><td>BcDNA:HL01508|CG7331|DmelCG7331|atg13</td><td>0</td><td>0</td></tr>
<tr><td>8296</td><td>40999</td><td>Drosophila melanogaster</td><td>stck</td><td>steamer duck</td><td>CG7954|D-PINCH|DmelCG7954|PINCH|Pinch|dPINCH|dpin|pin85A|pinch|stk</td><td>0</td><td>0</td></tr>
<tr><td>8297</td><td>41000</td><td>Drosophila melanogaster</td><td>CG9684</td><td>CG9684 gene product from transcript CG9684-RA</td><td>DmelCG9684</td><td>0</td><td>0</td></tr>
<tr><td>8298</td><td>41001</td><td>Drosophila melanogaster</td><td>CG7963</td><td>CG7963 gene product from transcript CG7963-RB</td><td>DmelCG7963</td><td>0</td><td>0</td></tr>
<tr><td>8299</td><td>41006</td><td>Drosophila melanogaster</td><td>Obp85a</td><td>Odorant-binding protein 85a</td><td>CG11732|DmelCG11732</td><td>0</td><td>0</td></tr>
<tr><td>8300</td><td>41007</td><td>Drosophila melanogaster</td><td>Or85b</td><td>Odorant receptor 85b</td><td>85A.1|85b|CG11735|DOR115|DmelCG11735</td><td>0</td><td>0</td></tr>
<tr><td>8301</td><td>41008</td><td>Drosophila melanogaster</td><td>Or85c</td><td>Odorant receptor 85c</td><td>85A.3|85c|CG17911|DOR78|DmelCG17911</td><td>0</td><td>0</td></tr>
<tr><td>8302</td><td>41009</td><td>Drosophila melanogaster</td><td>CG11737</td><td>CG11737 gene product from transcript CG11737-RA</td><td>DmelCG11737</td><td>0</td><td>0</td></tr>
<tr><td>8303</td><td>41010</td><td>Drosophila melanogaster</td><td>CG31259</td><td>CG31259 gene product from transcript CG31259-RA</td><td>CG11740|DmelCG31259</td><td>0</td><td>0</td></tr>
<tr><td>8304</td><td>41011</td><td>Drosophila melanogaster</td><td>Or85d</td><td>Odorant receptor 85d</td><td>85.2|85A.2|85d|CG11742|DOR114|DmelCG11742</td><td>0</td><td>0</td></tr>
<tr><td>8305</td><td>41013</td><td>Drosophila melanogaster</td><td>pch2</td><td>CG31453 gene product from transcript CG31453-RA</td><td>CG11743|CG11744|CG31453|DmelCG31453</td><td>0</td><td>0</td></tr>
<tr><td>8306</td><td>41014</td><td>Drosophila melanogaster</td><td>Cenp-C</td><td>CG31258 gene product from transcript CG31258-RA</td><td>CENP-C|CG11745|CG11746|CG31258|CLD1|DmelCG31258|L1|l(3)85Aa</td><td>0</td><td>0</td></tr>
<tr><td>8307</td><td>41017</td><td>Drosophila melanogaster</td><td>CG42796</td><td>CG42796 gene product from transcript CG42796-RD</td><td>CG7994|CG8007|DmelCG42796|Dmel_CG7994|Dmel_CG8007|anon-WO0170980.94|anon-WO0170980.95|anon-WO0170980.97|anon-WO0170980.98</td><td>0</td><td>0</td></tr>
<tr><td>8308</td><td>41019</td><td>Drosophila melanogaster</td><td>Cyp313b1</td><td>CG9716 gene product from transcript CG9716-RA</td><td>313b1|CG9716|DmelCG9716</td><td>0</td><td>0</td></tr>
<tr><td>8309</td><td>41020</td><td>Drosophila melanogaster</td><td>Mkk4</td><td>MAP kinase kinase 4</td><td>142758_at|CG9738|CT27508|D-MKK4|DMKK4|DmelCG9738|JNKK2|MAPKK4|MKK4|Mpk4|SEK1/MKK4|dMKK4|mkk4</td><td>0</td><td>0</td></tr>
<tr><td>8310</td><td>41021</td><td>Drosophila melanogaster</td><td>CG8021</td><td>CG8021 gene product from transcript CG8021-RA</td><td>DmelCG8021|cg8021</td><td>0</td><td>0</td></tr>
<tr><td>8311</td><td>41022</td><td>Drosophila melanogaster</td><td>Dhod</td><td>Dihydroorotate dehydrogenase</td><td>CG9741|DHOD|DHODH|DmelCG9741|dhod|l(3)s3512</td><td>0</td><td>0</td></tr>
<tr><td>8312</td><td>41023</td><td>Drosophila melanogaster</td><td>CG11753</td><td>CG11753 gene product from transcript CG11753-RA</td><td>DmelCG11753</td><td>0</td><td>0</td></tr>
<tr><td>8313</td><td>41025</td><td>Drosophila melanogaster</td><td>p</td><td>pink</td><td>CG9770|DmelCG9770|dHPS5|snb</td><td>0</td><td>0</td></tr>
<tr><td>8314</td><td>41026</td><td>Drosophila melanogaster</td><td>CG8032</td><td>CG8032 gene product from transcript CG8032-RA</td><td>DmelCG8032</td><td>0</td><td>0</td></tr>
<tr><td>8315</td><td>41027</td><td>Drosophila melanogaster</td><td>CG8036</td><td>CG8036 gene product from transcript CG8036-RB</td><td>DmelCG8036|anon-WO0118547.344</td><td>0</td><td>0</td></tr>
<tr><td>8316</td><td>41028</td><td>Drosophila melanogaster</td><td>mRpL19</td><td>mitochondrial ribosomal protein L19</td><td>CG8039|DmelCG8039|L19</td><td>0</td><td>0</td></tr>
<tr><td>8317</td><td>41029</td><td>Drosophila melanogaster</td><td>CG9773</td><td>CG9773 gene product from transcript CG9773-RA</td><td>DmelCG9773</td><td>0</td><td>0</td></tr>
<tr><td>8318</td><td>41030</td><td>Drosophila melanogaster</td><td>CG8043</td><td>CG8043 gene product from transcript CG8043-RA</td><td>DmelCG8043</td><td>0</td><td>0</td></tr>
<tr><td>8319</td><td>41031</td><td>Drosophila melanogaster</td><td>CG11755</td><td>CG11755 gene product from transcript CG11755-RA</td><td>DmelCG11755</td><td>0</td><td>0</td></tr>
<tr><td>8320</td><td>41032</td><td>Drosophila melanogaster</td><td>hb</td><td>hunchback</td><td>CG9786|DmelCG9786|HB|Hb|Hunchback|R-pbx|Rg-bx|Rg-pbx|hbHLH|l(3)85Ah</td><td>0</td><td>0</td></tr>
<tr><td>8321</td><td>41033</td><td>Drosophila melanogaster</td><td>CG8112</td><td>CG8112 gene product from transcript CG8112-RA</td><td>DmelCG8112</td><td>0</td><td>0</td></tr>
<tr><td>8322</td><td>41034</td><td>Drosophila melanogaster</td><td>Cks85A</td><td>Cyclin-dependent kinase subunit 85A</td><td>BcDNA:GM01344|CG9790|Cks|DmelCG9790|cks85A</td><td>0</td><td>0</td></tr>
</table>

<table>
<tr><th></th><th></th><th></th><th></th><th></th><th></th><th></th><th></th></tr>
<tr><td>8323</td><td>41035</td><td>Drosophila melanogaster</td><td>CG8116</td><td>CG8116 gene product from transcript CG8116-RB</td><td>DmelCG8116|EP(3)3246|GH04517</td><td>0</td><td>0</td></tr>
<tr><td>8324</td><td>41036</td><td>Drosophila melanogaster</td><td>CG11760</td><td>CG11760 gene product from transcript CG11760-RB</td><td>BcDNA:LD16427|DmelCG11760</td><td>0</td><td>0</td></tr>
<tr><td>8325</td><td>41037</td><td>Drosophila melanogaster</td><td>CG8136</td><td>CG8136 gene product from transcript CG8136-RB</td><td>DmelCG8136|anon-WO0140519.254|anon-WO0140519.56</td><td>0</td><td>0</td></tr>
<tr><td>8326</td><td>41038</td><td>Drosophila melanogaster</td><td>CG8145</td><td>CG8145 gene product from transcript CG8145-RA</td><td>DmelCG8145</td><td>0</td><td>0</td></tr>
<tr><td>8327</td><td>41039</td><td>Drosophila melanogaster</td><td>CG11762</td><td>CG11762 gene product from transcript CG11762-RA</td><td>DmelCG11762|i36</td><td>0</td><td>0</td></tr>
<tr><td>8328</td><td>41040</td><td>Drosophila melanogaster</td><td>CG8159</td><td>CG8159 gene product from transcript CG8159-RB</td><td>DmelCG8159</td><td>0</td><td>0</td></tr>
<tr><td>8329</td><td>41041</td><td>Drosophila melanogaster</td><td>ranshi</td><td>CG9793 gene product from transcript CG9793-RA</td><td>CG9793|DmelCG9793</td><td>0</td><td>0</td></tr>
<tr><td>8330</td><td>41042</td><td>Drosophila melanogaster</td><td>CG9797</td><td>CG9797 gene product from transcript CG9797-RA</td><td>DmelCG9797</td><td>0</td><td>0</td></tr>
<tr><td>8331</td><td>41043</td><td>Drosophila melanogaster</td><td>CG8202</td><td>CG8202 gene product from transcript CG8202-RA</td><td>DmelCG8202</td><td>0</td><td>0</td></tr>
<tr><td>8332</td><td>41044</td><td>Drosophila melanogaster</td><td>CG9801</td><td>CG9801 gene product from transcript CG9801-RC</td><td>DmelCG9801</td><td>0</td><td>0</td></tr>
<tr><td>8333</td><td>41045</td><td>Drosophila melanogaster</td><td>CG8223</td><td>CG8223 gene product from transcript CG8223-RB</td><td>DmelCG8223</td><td>0</td><td>0</td></tr>
<tr><td>8334</td><td>41046</td><td>Drosophila melanogaster</td><td>CG11768</td><td>CG11768 gene product from transcript CG11768-RC</td><td>DmelCG11768</td><td>0</td><td>0</td></tr>
<tr><td>8335</td><td>41047</td><td>Drosophila melanogaster</td><td>CG8236</td><td>CG8236 gene product from transcript CG8236-RA</td><td>DmelCG8236</td><td>0</td><td>0</td></tr>
<tr><td>8336</td><td>41048</td><td>Drosophila melanogaster</td><td>CG13318</td><td>CG13318 gene product from transcript CG13318-RA</td><td>DmelCG13318|SPH101|c-SPH101</td><td>0</td><td>0</td></tr>
<tr><td>8337</td><td>41052</td><td>Drosophila melanogaster</td><td>Ir85a</td><td>Ionotropic receptor 85a</td><td>CG11775|CT32618|DmelCG11775|IR85a</td><td>0</td><td>0</td></tr>
<tr><td>8338</td><td>41054</td><td>Drosophila melanogaster</td><td>Tcp-1eta</td><td>CG8351 gene product from transcript CG8351-RA</td><td>CG8351|Cct7|DmelCG8351|Tcp-1-eta1</td><td>0</td><td>0</td></tr>
<tr><td>8339</td><td>41055</td><td>Drosophila melanogaster</td><td>CG9839</td><td>CG9839 gene product from transcript CG9839-RA</td><td>DmelCG9839</td><td>0</td><td>0</td></tr>
<tr><td>8340</td><td>41056</td><td>Drosophila melanogaster</td><td>CG8359</td><td>CG8359 gene product from transcript CG8359-RA</td><td>DmelCG8359</td><td>0</td><td>0</td></tr>
<tr><td>8341</td><td>41057</td><td>Drosophila melanogaster</td><td>CG9837</td><td>CG9837 gene product from transcript CG9837-RD</td><td>DmelCG9837</td><td>0</td><td>0</td></tr>
<tr><td>8342</td><td>41058</td><td>Drosophila melanogaster</td><td>CG9821</td><td>CG9821 gene product from transcript CG9821-RB</td><td>CG31442|DmelCG9821|Ov29|anon-WO0118547.399|anon-WO0118547.424</td><td>0</td><td>0</td></tr>
<tr><td>8343</td><td>41059</td><td>Drosophila melanogaster</td><td>CG9836</td><td>CG9836 gene product from transcript CG9836-RA</td><td>DmelCG9836</td><td>0</td><td>0</td></tr>
<tr><td>8344</td><td>41060</td><td>Drosophila melanogaster</td><td>CG8379</td><td>CG8379 gene product from transcript CG8379-RB</td><td>DmelCG8379|anon-WO0118547.250</td><td>0</td><td>0</td></tr>
<tr><td>8345</td><td>41062</td><td>Drosophila melanogaster</td><td>pyd</td><td>polychaetoid</td><td>CG11782|CG11962|CG12409|CG31349|CG43140|CG9729|CG9731|CG9763|CT36877|DZO-1|DmelCG43140|Dmel_CG31349|Dmel_CG9731|Pch|Pyd|Pyd/ZO-1|Tamou|ZO-1|ZO1|dzo-1|pyd(Z01)|tam|xvt</td><td>0</td><td>0</td></tr>
<tr><td>8346</td><td>41066</td><td>Drosophila melanogaster</td><td>osk</td><td>oskar</td><td>CG10901|Dm-osk|DmelCG10901|OSK|Osk|Oskar</td><td>0</td><td>0</td></tr>
<tr><td>8347</td><td>41067</td><td>Drosophila melanogaster</td><td>skap</td><td>skpA associated protein</td><td>CG11963|CGI 1963|DmelCG11963|SkAP|Skap</td><td>0</td><td>0</td></tr>
<tr><td>8348</td><td>41068</td><td>Drosophila melanogaster</td><td>CG11964</td><td>CG11964 gene product from transcript CG11964-RA</td><td>DmelCG11964</td><td>0</td><td>0</td></tr>
<tr><td>8349</td><td>41069</td><td>Drosophila melanogaster</td><td>CG11966</td><td>CG11966 gene product from transcript CG11966-RA</td><td>DmelCG11966</td><td>0</td><td>0</td></tr>
<tr><td>8350</td><td>41070</td><td>Drosophila melanogaster</td><td>CAHbeta</td><td>Carbonic anhydrase beta</td><td>CG11967|DmBCA|DmelCG11967|beta-CA</td><td>0</td><td>0</td></tr>
<tr><td>8351</td><td>41071</td><td>Drosophila melanogaster</td><td>RagA</td><td>CG11968 gene product from transcript CG11968-RA</td><td>CG11968|DmelCG11968|Gtr1|dRagA|dRagB</td><td>0</td><td>0</td></tr>
<tr><td>8352</td><td>41072</td><td>Drosophila melanogaster</td><td>CG11970</td><td>CG11970 gene product from transcript CG11970-RA</td><td>BcDNA:LD23876|DmelCG11970</td><td>0</td><td>0</td></tr>
<tr><td>8353</td><td>41073</td><td>Drosophila melanogaster</td><td>E(var)3-9</td><td>Enhancer of variegation 3-9</td><td>1E9|Anon1E9|CG11971|DmelCG11971|E-var(3)9|anon-EST:fe1E9|anon1E9</td><td>0</td><td>0</td></tr>
<tr><td>8354</td><td>41074</td><td>Drosophila melanogaster</td><td>CG11975</td><td>CG11975 gene product from transcript CG11975-RA</td><td>DmelCG11975</td><td>0</td><td>0</td></tr>
<tr><td>8355</td><td>41075</td><td>Drosophila melanogaster</td><td>CG31100</td><td>CG31100 gene product from transcript CG31100-RA</td><td>CG11976|CG11978|DmelCG31100</td><td>0</td><td>0</td></tr>
<tr><td>8356</td><td>41076</td><td>Drosophila melanogaster</td><td>CG11977</td><td>CG11977 gene product from transcript CG11977-RB</td><td>DmelCG11977</td><td>0</td><td>0</td></tr>
<tr><td>8357</td><td>41078</td><td>Drosophila melanogaster</td><td>CG11980</td><td>CG11980 gene product from transcript CG11980-RB</td><td>DmelCG11980|anon-WO0118547.327</td><td>0</td><td>0</td></tr>
<tr><td>8358</td><td>41079</td><td>Drosophila melanogaster</td><td>Prosbeta3</td><td>Proteasome beta3 subunit</td><td>11981|CG11981|DmelCG11981</td><td>0</td><td>0</td></tr>
<tr><td>8359</td><td>41080</td><td>Drosophila melanogaster</td><td>CG11982</td><td>CG11982 gene product from transcript CG11982-RA</td><td>DmelCG11982</td><td>0</td><td>0</td></tr>
<tr><td>8360</td><td>41081</td><td>Drosophila melanogaster</td><td>CG11983</td><td>CG11983 gene product from transcript CG11983-RA</td><td>DmelCG11983</td><td>0</td><td>0</td></tr>
<tr><td>8361</td><td>41082</td><td>Drosophila melanogaster</td><td>CG11984</td><td>CG11984 gene product from transcript CG11984-RD</td><td>DmelCG11984</td><td>0</td><td>0</td></tr>
<tr><td>8362</td><td>41083</td><td>Drosophila melanogaster</td><td>CG11986</td><td>CG11986 gene product from transcript CG11986-RA</td><td>DmelCG11986</td><td>0</td><td>0</td></tr>
<tr><td>8363</td><td>41084</td><td>Drosophila melanogaster</td><td>tgo</td><td>tango</td><td>ARNT|Arnt|CG11987|DARNT|DmelCG11987|HIF-1|HIF-1beta|HIF1b|Hif1|TGO|Tango|Tgo|arnt|bHLHe4|dARNT|dHIF-1beta|dTgo|darnt|prd7</td><td>0</td><td>0</td></tr>
<tr><td>8364</td><td>41085</td><td>Drosophila melanogaster</td><td>neur</td><td>neuralized</td><td>0328/12|0971/15|1293/09|1315/05|BEST:LD21494|CG11988|DmelCG11988|Neur|l(3)j6B12|l(3)j9B8|l(3)j9B9|l(3)j9C8|l(3)neo37|neu</td><td>0</td><td>0</td></tr>
<tr><td>8365</td><td>41086</td><td>Drosophila melanogaster</td><td>hyx</td><td>hyrax</td><td>CG11990|Cdc73|DmelCG11990|Hyx</td><td>0</td><td>0</td></tr>
<tr><td>8366</td><td>41087</td><td>Drosophila melanogaster</td><td>Rel</td><td>Relish</td><td>CG11992|DmelCG11992|NF-kappaB|NFkappaB|REL|RELI|Rel-p110|Rel/NF-kappaB|ird|ird4|l(3)neo36|rel|relish</td><td>0</td><td>0</td></tr>
<tr><td>8367</td><td>41089</td><td>Drosophila melanogaster</td><td>Mst85C</td><td>CG11993 gene product from transcript CG11993-RA</td><td>CG11993|DmelCG11993</td><td>0</td><td>0</td></tr>
<tr><td>8368</td><td>41090</td><td>Drosophila melanogaster</td><td>Kdm2</td><td>Lysine (K)-specific demethylase 2</td><td>CG11033|DmelCG11033|JHDM1|dKDM2</td><td>0</td><td>0</td></tr>
<tr><td>8369</td><td>41091</td><td>Drosophila melanogaster</td><td>CG18005</td><td>CG18005 gene product from transcript CG18005-RA</td><td>DmelCG18005</td><td>0</td><td>0</td></tr>
<tr><td>8370</td><td>41092</td><td>Drosophila melanogaster</td><td>Ada</td><td>Adenosine deaminase</td><td>ADA|CG11994|DmelCG11994|DrosADA|dADA</td><td>0</td><td>0</td></tr>
<tr><td>8371</td><td>41093</td><td>Drosophila melanogaster</td><td>CG11997</td><td>CG11997 gene product from transcript CG11997-RA</td><td>DmelCG11997</td><td>0</td><td>0</td></tr>
<tr><td>8372</td><td>41094</td><td>Drosophila melanogaster</td><td>pum</td><td>pumilio</td><td>CG9755|CG9763|DmelCG9755|EP(3)0883|PUM|Pum|anon-WO0172774.19|bem|fs(3)02003|l(3)01688|ova|ovt|pkl</td><td>0</td><td>0</td></tr>
<tr><td>8373</td><td>41095</td><td>Drosophila melanogaster</td><td>D1</td><td>D1 chromosomal protein</td><td>CG9745|DmelCG9745|EP(3)0473|RE69409|dD1</td><td>0</td><td>0</td></tr>
<tr><td>8374</td><td>41096</td><td>Drosophila melanogaster</td><td>ird1</td><td>immune response deficient 1</td><td>CG9746|DmelCG9746|Ird1|Vps15|dVps15|dVps15/p150|ird|vps15</td><td>0</td><td>0</td></tr>
<tr><td>8375</td><td>41097</td><td>Drosophila melanogaster</td><td>CG8420</td><td>CG8420 gene product from transcript CG8420-RA</td><td>DmelCG8420|anon-WO0140519.182</td><td>0</td><td>0</td></tr>
<tr><td>8376</td><td>41098</td><td>Drosophila melanogaster</td><td>CG16733</td><td>CG16733 gene product from transcript CG16733-RA</td><td>DmelCG16733</td><td>0</td><td>0</td></tr>
<tr><td>8377</td><td>41100</td><td>Drosophila melanogaster</td><td>CG16734</td><td>CG16734 gene product from transcript CG16734-RD</td><td>DmelCG16734</td><td>0</td><td>0</td></tr>
<tr><td>8378</td><td>41101</td><td>Drosophila melanogaster</td><td>CG16736</td><td>CG16736 gene product from transcript CG16736-RA</td><td>DmelCG16736</td><td>0</td><td>0</td></tr>
<tr><td>8379</td><td>41102</td><td>Drosophila melanogaster</td><td>CG9740</td><td>CG9740 gene product from transcript CG9740-RA</td><td>DmelCG9740</td><td>0</td><td>0</td></tr>
<tr><td>8380</td><td>41103</td><td>Drosophila melanogaster</td><td>CG8436</td><td>CG8436 gene product from transcript CG8436-RA</td><td>DmelCG8436</td><td>0</td><td>0</td></tr>
<tr><td>8381</td><td>41104</td><td>Drosophila melanogaster</td><td>VhaM8.9</td><td>CG8444 gene product from transcript CG8444-RA</td><td>ATP6AP2|CG8444|DmelCG8444|VhaM8-9|VhaPRR|dPRR|vhaM8.9</td><td>0</td><td>0</td></tr>
<tr><td>8382</td><td>41105</td><td>Drosophila melanogaster</td><td>sage</td><td>salivary gland-expressed bHLH</td><td>CG12952|DmelCG12952|Sage|bHLHc7</td><td>0</td><td>0</td></tr>
<tr><td>8383</td><td>41107</td><td>Drosophila melanogaster</td><td>Vps16A</td><td>Vacuolar protein sorting 16A</td><td>CG8454|DmVps16|DmelCG8454|Vps16|dVps16A|vps16A</td><td>0</td><td>0</td></tr>
<tr><td>8384</td><td>41108</td><td>Drosophila melanogaster</td><td>HP1e</td><td>CG8120 gene product from transcript CG8120-RA</td><td>CG8120|DmelCG8120|HP1E</td><td>0</td><td>0</td></tr>
<tr><td>8385</td><td>41109</td><td>Drosophila melanogaster</td><td>CG8861</td><td>CG8861 gene product from transcript CG8861-RB</td><td>DmelCG8861</td><td>0</td><td>0</td></tr>
<tr><td>8386</td><td>41110</td><td>Drosophila melanogaster</td><td>CG12951</td><td>CG12951 gene product from transcript CG12951-RA</td><td>DmelCG12951|SP87</td><td>0</td><td>0</td></tr>
<tr><td>8387</td><td>41111</td><td>Drosophila melanogaster</td><td>CG16749</td><td>CG16749 gene product from transcript CG16749-RA</td><td>DmelCG16749|SP91</td><td>0</td><td>0</td></tr>
<tr><td>8388</td><td>41112</td><td>Drosophila melanogaster</td><td>CG8866</td><td>CG8866 gene product from transcript CG8866-RA</td><td>DmelCG8866|UNC 51-like|dULK3</td><td>0</td><td>0</td></tr>
<tr><td>8389</td><td>41113</td><td>Drosophila melanogaster</td><td>CG8121</td><td>CG8121 gene product from transcript CG8121-RA</td><td>DmelCG8121|dmCG8121</td><td>0</td><td>0</td></tr>
<tr><td>8390</td><td>41114</td><td>Drosophila melanogaster</td><td>CG33936</td><td>CG33936 gene product from transcript CG33936-RB</td><td>BcDNA:RE70963|CG16750|CG16751|CG33187|CG33188|DmelCG33936|anon-85Da|drn</td><td>0</td><td>0</td></tr>
<tr><td>8391</td><td>41116</td><td>Drosophila melanogaster</td><td>CG18473</td><td>CG18473 gene product from transcript CG18473-RA</td><td>CG8125|DmelCG18473</td><td>0</td><td>0</td></tr>
<tr><td>8392</td><td>41117</td><td>Drosophila melanogaster</td><td>CG8129</td><td>CG8129 gene product from transcript CG8129-RB</td><td>CT24308|DmelCG8129</td><td>0</td><td>0</td></tr>
<tr><td>8393</td><td>41118</td><td>Drosophila melanogaster</td><td>Fps85D</td><td>Fps oncogene analog</td><td>CG8874|DFER|DFer|Dfer|Dfps|Dm FPS|DmHD-179|DmelCG8874|FER|FPS|Fer|HD-179|dFer|dfer|dfps|dfps 85D|dfps85D|fer|fps|fps85D</td><td>0</td><td>0</td></tr>
<tr><td>8394</td><td>41119</td><td>Drosophila melanogaster</td><td>Or85f</td><td>Odorant receptor 85f</td><td>85D.1|85f|CG16755|DOR32|DmelCG16755</td><td>0</td><td>0</td></tr>
<tr><td>8395</td><td>41120</td><td>Drosophila melanogaster</td><td>RpL34b</td><td>Ribosomal protein L34b</td><td>CG9354|DmelCG9354|L34b|RpL34|anon-EST:fe1C10|anon-EST:fe1E8</td><td>0</td><td>0</td></tr>
<tr><td>8396</td><td>41121</td><td>Drosophila melanogaster</td><td>CG8132</td><td>CG8132 gene product from transcript CG8132-RA</td><td>DmelCG8132</td><td>0</td><td>0</td></tr>
<tr><td>8397</td><td>41122</td><td>Drosophila melanogaster</td><td>CG9356</td><td>CG9356 gene product from transcript CG9356-RA</td><td>DmelCG9356</td><td>0</td><td>0</td></tr>
<tr><td>8398</td><td>41123</td><td>Drosophila melanogaster</td><td>CG8135</td><td>CG8135 gene product from transcript CG8135-RA</td><td>DmelCG8135</td><td>0</td><td>0</td></tr>
<tr><td>8399</td><td>41124</td><td>Drosophila melanogaster</td><td>betaTub85D</td><td>beta-Tubulin at 85D</td><td>2t|B2t|BETA 85D|BETA2|CG9359|D.m.BETA-85D|DTB4|Dmbeta2|DmelCG9359|Tub|beta(2)Tu|beta(2)Tub|beta-Tub85D|beta-tub|beta-tub85D|beta2|beta2t|beta2tub|beta85D|betaTub2|beta[[2]]-tubulin|ms(3)</td><td>0</td><td>0</td></tr>
<tr><td>8400</td><td>41125</td><td>Drosophila melanogaster</td><td>Task7</td><td>CG9361 gene product from transcript CG9361-RA</td><td>CG9361|DmelCG9361|K2P-TASK7|dTASK-7</td><td>0</td><td>0</td></tr>
<tr><td>8401</td><td>41126</td><td>Drosophila melanogaster</td><td>alpha-Man-II</td><td>alpha Mannosidase II</td><td>CG18474|CG18802|CG8139|DM-GII.1|DmelCG18802|GMII|GmII|MAN-2|Man|dGMII|dGMIIb</td><td>0</td><td>0</td></tr>
<tr><td>8402</td><td>41132</td><td>Drosophila melanogaster</td><td>CG9362</td><td>CG9362 gene product from transcript CG9362-RA</td><td>DmelCG9362</td><td>0</td><td>0</td></tr>
<tr><td>8403</td><td>41133</td><td>Drosophila melanogaster</td><td>CG9363</td><td>CG9363 gene product from transcript CG9363-RB</td><td>DmelCG9363|cg9363</td><td>0</td><td>0</td></tr>
<tr><td>8404</td><td>41134</td><td>Drosophila melanogaster</td><td>CG16779</td><td>CG16779 gene product from transcript CG16779-RA</td><td>DmelCG16779</td><td>0</td><td>0</td></tr>
<tr><td>8405</td><td>41135</td><td>Drosophila melanogaster</td><td>CG8147</td><td>CG8147 gene product from transcript CG8147-RA</td><td>154821_at|BEST:LD38109|DmelCG8147</td><td>0</td><td>0</td></tr>
<tr><td>8406</td><td>41136</td><td>Drosophila melanogaster</td><td>RhoL</td><td>rho-like</td><td>9366|CG9366|D-RhoL|DRhoL|DmRHO-L|DmelCG9366|Drac3|G|Mes1|Rac3|Rho-like|RhoL/Rac3|Rhol|anon-EST:CL1c2|rhoL|rhol|rol</td><td>0</td><td>0</td></tr>
<tr><td>8407</td><td>41137</td><td>Drosophila melanogaster</td><td>CG8149</td><td>CG8149 gene product from transcript CG8149-RA</td><td>DmelCG8149</td><td>0</td><td>0</td></tr>
<tr><td>8408</td><td>41138</td><td>Drosophila melanogaster</td><td>rump</td><td>rumpelstiltskin</td><td>CG9373|DmelCG9373|Hrp59|cg9373|hnRNP M|hrp59|p75</td><td>0</td><td>0</td></tr>
<tr><td>8409</td><td>41139</td><td>Drosophila melanogaster</td><td>Rlb1</td><td>CG8161 gene product from transcript CG8161-RA</td><td>CG8161|DmelCG8161|Ov30|Rlb</td><td>0</td><td>0</td></tr>
<tr><td>8410</td><td>41140</td><td>Drosophila melanogaster</td><td>Ras85D</td><td>Ras oncogene at 85D</td><td>C-ras1|CG9375|D-Ras|D-Ras1|D-ras-1|D-ras1|DRAS1|DRas|DRas85D/Ras|Dm Ras1|DmelCG9375|Dmras85D|Dras|Dras1|Dras85D|E(faf)|E(sev)3C|EK3-4|RAS|RAS1|RAS85D|RTK|Ras|Ras 85D|Ras-1|Ras1</td><td>0</td><td>0</td></tr>
<tr><td>8411</td><td>41141</td><td>Drosophila melanogaster</td><td>Rlc1</td><td>CG9378 gene product from transcript CG9378-RB</td><td>BcDNA:AT29239|CG9378|DmelCG9378|MRP-L47|mRpL47</td><td>0</td><td>0</td></tr>
<tr><td>8412</td><td>41143</td><td>Drosophila melanogaster</td><td>JHDM2</td><td>JmjC domain-containing histone demethylase 2</td><td>CG31123|CG8165|DmelCG8165</td><td>0</td><td>0</td></tr>
<tr><td>8413</td><td>41144</td><td>Drosophila melanogaster</td><td>by</td><td>blistery</td><td>3R-B|BcDNA:RE65113|CG9379|CT26639|DmelCG9379</td><td>0</td><td>0</td></tr>
<tr><td>8414</td><td>41145</td><td>Drosophila melanogaster</td><td>mura</td><td>murashka</td><td>CG9381|CG9382|DmelCG9381</td><td>0</td><td>0</td></tr>
<tr><td>8415</td><td>41147</td><td>Drosophila melanogaster</td><td>RnpS1</td><td>RNA-binding protein S1</td><td>CG16788|DEK|DmelCG16788|cg16788</td><td>0</td><td>0</td></tr>
<tr><td>8416</td><td>41148</td><td>Drosophila melanogaster</td><td>CG9386</td><td>CG9386 gene product from transcript CG9386-RA</td><td>DmelCG9386</td><td>0</td><td>0</td></tr>
<tr><td>8417</td><td>41149</td><td>Drosophila melanogaster</td><td>CG8199</td><td>CG8199 gene product from transcript CG8199-RA</td><td>DmelCG8199</td><td>0</td><td>0</td></tr>
<tr><td>8418</td><td>41150</td><td>Drosophila melanogaster</td><td>AP-47</td><td>CG9388 gene product from transcript CG9388-RA</td><td>AP-1mu1|AP47|CG9388|DmelCG9388|Dmu1|cg9388|mu1</td><td>0</td><td>0</td></tr>
<tr><td>8419</td><td>41151</td><td>Drosophila melanogaster</td><td>MBD-like</td><td>CG8208 gene product from transcript CG8208-RA</td><td>CG8208|Dm Mbd2/3|DmMbd2/3|DmelCG8208|MBD2-3|MBD2/3|dMBD-like|dMBD2/3|dMbD2/3</td><td>0</td><td>0</td></tr>
<tr><td>8420</td><td>41152</td><td>Drosophila melanogaster</td><td>CG9393</td><td>CG9393 gene product from transcript CG9393-RA</td><td>DmelCG9393</td><td>0</td><td>0</td></tr>
<tr><td>8421</td><td>41153</td><td>Drosophila melanogaster</td><td>Vps45</td><td>Vacuolar protein sorting 45</td><td>CG8228|DmVps45|DmelCG8228|MENE (3R)-C|MENE(3R)-C|VPS45|Vps45p|dVps45</td><td>0</td><td>0</td></tr>
<tr><td>8422</td><td>41154</td><td>Drosophila melanogaster</td><td>CG16789</td><td>CG16789 gene product from transcript CG16789-RA</td><td>DmelCG16789</td><td>0</td><td>0</td></tr>
<tr><td>8423</td><td>41155</td><td>Drosophila melanogaster</td><td>CG16790</td><td>CG16790 gene product from transcript CG16790-RA</td><td>DmelCG16790</td><td>0</td><td>0</td></tr>
<tr><td>8424</td><td>41156</td><td>Drosophila melanogaster</td><td>CG9396</td><td>CG9396 gene product from transcript CG9396-RA</td><td>DmelCG9396</td><td>0</td><td>0</td></tr>
<tr><td>8425</td><td>41157</td><td>Drosophila melanogaster</td><td>CG9399</td><td>CG9399 gene product from transcript CG9399-RC</td><td>DmelCG9399</td><td>0</td><td>0</td></tr>
<tr><td>8426</td><td>41158</td><td>Drosophila melanogaster</td><td>Kap-alpha3</td><td>karyopherin alpha3</td><td>0335/13|CG9423|Dalpha3|DmelCG9423|IMPalpha3|Imp alpha-3|KAP-ALPHA3|Kap alpha3|Kap-?3|Kpna3|dKap-alpha3|imp alpha3|imp-a3|imp-alpha3|l(3)S033513</td><td>0</td><td>0</td></tr>
<tr><td>8427</td><td>41159</td><td>Drosophila melanogaster</td><td>CG8273</td><td>CG8273 gene product from transcript CG8273-RA</td><td>DmelCG8273|cg8273</td><td>0</td><td>0</td></tr>
<tr><td>8428</td><td>41160</td><td>Drosophila melanogaster</td><td>CG8301</td><td>CG8301 gene product from transcript CG8301-RA</td><td>DmelCG8301</td><td>0</td><td>0</td></tr>
<tr><td>8429</td><td>41161</td><td>Drosophila melanogaster</td><td>P58IPK</td><td>CG8286 gene product from transcript CG8286-RA</td><td>BEST:SD05682|CG8286|DmelCG8286|SD05682.5prime|dIPK</td><td>0</td><td>0</td></tr>
<tr><td>8430</td><td>41162</td><td>Drosophila melanogaster</td><td>bocksbeutel</td><td>CG9424 gene product from transcript CG9424-RA</td><td>Bocks|Bocksbeutel|CG9424|DmelCG9424|emerin</td><td>0</td><td>0</td></tr>
<tr><td>8431</td><td>41163</td><td>Drosophila melanogaster</td><td>CG8312</td><td>CG8312 gene product from transcript CG8312-RA</td><td>DmelCG8312</td><td>0</td><td>0</td></tr>
<tr><td>8432</td><td>41164</td><td>Drosophila melanogaster</td><td>CG9427</td><td>CG9427 gene product from transcript CG9427-RA</td><td>DmelCG9427</td><td>0</td><td>0</td></tr>
<tr><td>8433</td><td>41165</td><td>Drosophila melanogaster</td><td>CG8319</td><td>CG8319 gene product from transcript CG8319-RA</td><td>DmelCG8319</td><td>0</td><td>0</td></tr>
</table>

8434	41166	Drosophila melanogaster	Crc	Calreticulin	CG9429|Clr|DmelCG9429|anon-EST:Liang-2.11|anon-EST:Liang-2.17|anon-EST:fe3D7|clone 2.11|clone 2.17|crc|eth|potp	0	0
8435	41167	Drosophila melanogaster	SpdS	Spermidine Synthase	CG8327|DmelCG8327	0	0
8436	41168	Drosophila melanogaster	Scm	Sex comb on midleg	CG9495|DmelCG9495|SCM|Su(z)302|l(3)85Ef|scm	0	0
8437	41169	Drosophila melanogaster	Fst	Frost	CG9434|DmelCG9434|frost	0	0
8438	41170	Drosophila melanogaster	Dh44	Diuretic hormone 44	CG8348|DH|DH 44|DH44|DH[[44]]|Dh|DmelCG8348|Drm-DH|Drome-DH[[44]]|drome-DH44	0	0
8439	41171	Drosophila melanogaster	CG9492	CG9492 gene product from transcript CG9492-RF	DmelCG9492	0	0
8440	41172	Drosophila melanogaster	CG8358	CG8358 gene product from transcript CG8358-RA	DmelCG8358	0	0
8441	41173	Drosophila melanogaster	CG16817	CG16817 gene product from transcript CG16817-RA	DmelCG16817|YC17	0	0
8442	41174	Drosophila melanogaster	nmdyn-D7	CG8362 gene product from transcript CG8362-RA	CG8362|DmelCG8362	0	0
8443	41175	Drosophila melanogaster	CG9444	CG9444 gene product from transcript CG9444-RA	DmelCG9444	0	0
8444	41176	Drosophila melanogaster	CG18542	CG18542 gene product from transcript CG18542-RA	DmelCG18542	0	0
8445	41177	Drosophila melanogaster	eca	eclair	CG33104|CG9443|DmelCG33104|p24	0	0
8446	41178	Drosophila melanogaster	Unc-115b	CG31332 gene product from transcript CG31332-RD	CG31332|CG9489|DmelCG31332|anon-WO0140519.9|unc-115	0	0
8447	41179	Drosophila melanogaster	trbd	trabid	CG9448|DmelCG9448	0	0
8448	41180	Drosophila melanogaster	dmt	dalmatian	0481/03|CG8374|DmelCG8374|Dmt|anon-WO0118547.317|s048103	0	0
8449	41181	Drosophila melanogaster	hyd	hyperplastic discs	CG5119|CG9484|DmelCG9484|HYD|Hyd|UBE 3A|c43|l(3)85Ea|l(3)C43|l(3)C43-3|l(3)SG33|l(3)c43|l[[3]]c43	0	0
8450	41182	Drosophila melanogaster	FoxP	Forkhead box P	CG16899|CG32937|CG43067|DmFoxP|Dmcg16899|DmelCG43067|Dmel_CG16899|Dmel_CG32937|fd85E	0	0
8451	41183	Drosophila melanogaster	alphaTub85E	alpha-Tubulin at 85E	2t|ALPHA 85E|CG9476|DTA3|DmelCG9476|T|Tub|a2t|aTub85E|alpha-Tub|alpha-Tub85E|alpha-tubulin|alpha2|alpha2t|alpha85E|alpha85E-tub|alphaTub|alphaTub2|alphaTub84E	0	0
8452	41184	Drosophila melanogaster	CG12950	CG12950 gene product from transcript CG12950-RA	CT32101|DmelCG12950	0	0
8453	41185	Drosophila melanogaster	Pnn	Pinin	CG8383|DmelCG8383	0	0
8454	41187	Drosophila melanogaster	CG12948	CG12948 gene product from transcript CG12948-RA	DmelCG12948	0	0
8455	41189	Drosophila melanogaster	CG16908	CG16908 gene product from transcript CG16908-RA	DmelCG16908	0	0
8456	41190	Drosophila melanogaster	Rpt3R	CG9475 gene product from transcript CG9475-RC	9475|CG9475|DmCG9475|Dm_Rpt3b|DmelCG9475	0	0
8457	41191	Drosophila melanogaster	CG8412	CG8412 gene product from transcript CG8412-RA	DmelCG8412	0	0
8458	41192	Drosophila melanogaster	CG8417	CG8417 gene product from transcript CG8417-RA	DmelCG8417	0	0
8459	41193	Drosophila melanogaster	Snap24	Synapse protein 24	CG9474|DmelCG9474|SNAP-24|SNAP24	0	0
8460	41194	Drosophila melanogaster	CG8478	CG8478 gene product from transcript CG8478-RD	DmelCG8478	0	0
8461	41195	Drosophila melanogaster	CG8481	CG8481 gene product from transcript CG8481-RB	DmelCG8481	0	0
8462	41197	Drosophila melanogaster	CG9471	CG9471 gene product from transcript CG9471-RB	BVR|DmelCG9471	0	0
8463	41198	Drosophila melanogaster	Whamy	CG12946 gene product from transcript CG12946-RB	CG12946|DmelCG12946	0	0
8464	41199	Drosophila melanogaster	topi	matotopetli	CG8484|DmelCG8484|Topi	0	0
8465	41200	Drosophila melanogaster	RpS29	Ribosomal protein S29	BcDNA:AT13329|BcDNA:AT28563|BcDNA:RH06643|CG8495|DmelCG8495|M(3)85E|M(3)LS5|anon-EST:fe3B4|anon-WO0118547.360	0	0
8466	41201	Drosophila melanogaster	CG12947	CG12947 gene product from transcript CG12947-RB	DmelCG12947	0	0
8467	41202	Drosophila melanogaster	MtnA	Metallothionein A	BcDNA:GH18460|CG9470|DmelCG9470|DrosMtn|MTN|Mtn|anon-WO0153538.42|anon-WO0153538.44|anon-WO0153538.49	0	0
8468	41203	Drosophila melanogaster	CG8500	CG8500 gene product from transcript CG8500-RA	DmelCG8500	0	0
8469	41204	Drosophila melanogaster	CG12945	CG12945 gene product from transcript CG12945-RB	DmelCG12945	0	0
8470	41205	Drosophila melanogaster	CG8507	CG8507 gene product from transcript CG8507-RA	DmelCG8507	0	0
8471	41206	Drosophila melanogaster	CG8516	CG8516 gene product from transcript CG8516-RA	DmelCG8516|FBgn0037757	0	0
8472	41207	Drosophila melanogaster	CG9467	CG9467 gene product from transcript CG9467-RA	DmelCG9467	0	0
8473	41208	Drosophila melanogaster	CG8526	CG8526 gene product from transcript CG8526-RA	DmelCG8526	0	0
8474	41209	Drosophila melanogaster	FBX011	FBX011 ortholog	CG9461|DmelCG9461|dFBX011	0	0
8475	41210	Drosophila melanogaster	CG8534	CG8534 gene product from transcript CG8534-RA	DmelCG8534	0	0
8476	41211	Drosophila melanogaster	eloF	elongase F	CG16905|DmelCG16905	0	0
8477	41212	Drosophila melanogaster	CG16904	CG16904 gene product from transcript CG16904-RA	DmelCG16904	0	0
8478	41213	Drosophila melanogaster	CG9459	CG9459 gene product from transcript CG9459-RA	CT26804|DmelCG9459	0	0
8479	41214	Drosophila melanogaster	CG9458	CG9458 gene product from transcript CG9458-RA	CT26802|DmelCG9458	0	0
8480	41215	Drosophila melanogaster	Teh1	tipE homolog 1	CG12806|DmelCG12806|TEH1	0	0
8481	41217	Drosophila melanogaster	Glut4EF	Glucose transporter 4 enhancer factor	BcDNA:GH25505|BcDNA:GM06804|BcDNA:RE66512|BcDNA:RH63124|CG11676|CG12802|CG12804|CG12805|CG32469|CG33975|CG34360|D-Glut4EF|DmelCG34360|Dmel_CG12802|Dmel_CG33975|s	0	0
8482	41218	Drosophila melanogaster	CG12419	CG12419 gene product from transcript CG12419-RA	DmelCG12419	0	0
8483	41219	Drosophila melanogaster	Art4	Arginine methyltransferase 4	CG5358|DART4|DmelCG5358	0	0
8484	41221	Drosophila melanogaster	Spn85F	Serpin 85F	CG12807|DmelCG12807	0	0
8485	41222	Drosophila melanogaster	CG5359	CG5359 gene product from transcript CG5359-RC	BcDNA:RE37471|DmelCG5359	0	0
8486	41225	Drosophila melanogaster	Mical	Molecule interacting with CasL	CG11685|CG11687|CG18484|CG18667|CG18668|CG33186|CG33190|CG33208|DmelCG33208|MICAL|mical|milch	0	0
8487	41226	Drosophila melanogaster	CG3909	CG3909 gene product from transcript CG3909-RA	BcDNA:LD21537|DmelCG3909	0	0
8488	41227	Drosophila melanogaster	CG11722	CG11722 gene product from transcript CG11722-RA	BcDNA:AT14909|DmelCG11722	0	0
8489	41228	Drosophila melanogaster	mtTFB2	mitochondrial transcription factor B2	CG3910|DmelCG3910|TFB2|d-TF2BM|d-mtTFB2	0	0
8490	41229	Drosophila melanogaster	CG12811	CG12811 gene product from transcript CG12811-RA	DmelCG12811	0	0
8491	41230	Drosophila melanogaster	CG3925	CG3925 gene product from transcript CG3925-RA	DmelCG3925	0	0
8492	41231	Drosophila melanogaster	Fancl	CG12812 gene product from transcript CG12812-RA	CG12812|DmelCG12812|FANCL	0	0
8493	41232	Drosophila melanogaster	Npc2d	Niemann-Pick type C-2d	CG12813|DmelCG12813|npc2d	0	0
8494	41233	Drosophila melanogaster	Npc2c	Niemann-Pick type C-2c	CG3934|DmML5A|DmelCG3934|npc2c	0	0
8495	41235	Drosophila melanogaster	nerfin-2	nervous fingers 2	CG12809|DmelCG12809|Nerfin	0	0
8496	41236	Drosophila melanogaster	CG5361	CG5361 gene product from transcript CG5361-RB	DmelCG5361	0	0
8497	41238	Drosophila melanogaster	CG3940	CG3940 gene product from transcript CG3940-RA	DmelCG3940	0	0
8498	41239	Drosophila melanogaster	CG6208	CG6208 gene product from transcript CG6208-RA	DmelCG6208|SNAP-31	0	0
8499	41242	Drosophila melanogaster	CG6241	CG6241 gene product from transcript CG6241-RA	DmelCG6241	0	0
8500	41244	Drosophila melanogaster	CG6254	CG6254 gene product from transcript CG6254-RA	DmelCG6254	0	0
8501	41246	Drosophila melanogaster	CG12814	CG12814 gene product from transcript CG12814-RC	CG12815|DmelCG12814	0	0
8502	41247	Drosophila melanogaster	Syn	Synapsin	CG3985|DmelCG3985|Dsyn-1|SYN|SYN1|SYN2|syn	0	0
8503	41248	Drosophila melanogaster	Timp	Tissue inhibitor of metalloproteases	CG6281|DTIMP|DmelCG6281|Dtimp|TIMP|dTIMP|timp	0	0
8504	41249	Drosophila melanogaster	CG12420	CG12420 gene product from transcript CG12420-RA	DmelCG12420	0	0
8505	41250	Drosophila melanogaster	CG12817	CG12817 gene product from transcript CG12817-RB	BcDNA:RE53107|DmelCG12817	0	0
8506	41252	Drosophila melanogaster	CG42795	CG42795 gene product from transcript CG42795-RA	CG12816|CG34108|CG3996|DmelCG42795|Dmel_CG34108|Dmel_CG3996	0	0
8507	41253	Drosophila melanogaster	CG3999	CG3999 gene product from transcript CG3999-RA	DmelCG3999	0	0
8508	41254	Drosophila melanogaster	Sirt6	CG6284 gene product from transcript CG6284-RB	CG6284|D.mel4|DmelCG6284|dSIRT6|dmSRT408	0	0
8509	41256	Drosophila melanogaster	CG43143	CG43143 gene product from transcript CG43143-RF	CG11870|CG11871|CG11872|DmelCG43143|Dmel_CG11870|Dmel_CG11872|EMK/KIAA0537|Omphk1	0	0
8510	41259	Drosophila melanogaster	CG6293	CG6293 gene product from transcript CG6293-RA	DmelCG6293	0	0
8511	41260	Drosophila melanogaster	Bruce	CG6303 gene product from transcript CG6303-RA	CG6303|DmelCG6303|bruce|dBRUCE|dBruce|dbruce	0	0
8512	41261	Drosophila melanogaster	CG12818	CG12818 gene product from transcript CG12818-RA	DmelCG12818	0	0
8513	41262	Drosophila melanogaster	sle	slender lobes	CG12819|CG12819-PA|DmelCG12819	0	0
8514	41263	Drosophila melanogaster	CG12592	CG12592 gene product from transcript CG12592-RA	CG12592-PA|DmelCG12592	0	0
8515	41264	Drosophila melanogaster	CG18545	CG18545 gene product from transcript CG18545-RA	CG18545-PA|DmelCG18545	0	0
8516	41265	Drosophila melanogaster	jumu	jumeau	5295|CG4029|DmelCG4029|Dmjumu|Dom|Dwhn|E(var)631|Jumu|Scim31|Whn|jumu/Dom|l(3)06142|l(3)06439	0	0
8517	41266	Drosophila melanogaster	Rfx	CG6312 gene product from transcript CG6312-RC	CG6312|DmelCG6312|RFX|dRFX|dRfx|rfx	0	0
8518	41268	Drosophila melanogaster	CG6325	CG6325 gene product from transcript CG6325-RA	DmelCG6325	0	0
8519	41269	Drosophila melanogaster	Irp-1B	Iron regulatory protein 1B	144037_at|C-Acon|CG6342|DmelCG6342|IRP-1|IRP-1B|IRP1|cAcon	0	0
8520	41270	Drosophila melanogaster	Rrp46	CG4043 gene product from transcript CG4043-RA	CG4043|DmelCG4043|dRrp46|rrp46	0	0
8521	41271	Drosophila melanogaster	CG6345	CG6345 gene product from transcript CG6345-RA	149631_at|DmelCG6345	0	0
8522	41272	Drosophila melanogaster	Cyp12e1	CG14680 gene product from transcript CG14680-RC	12e1|CG14680|DmelCG14680	0	0
8523	41273	Drosophila melanogaster	hth	homothorax	1323/07|1422/04|CG17117|Dm-HTH|DmelCG17117|Hth|Meis1|P53|anon-EST:Liang-2.13|clone 2.13|dtl|hth1|hth2|l(3)05745|l(3)86Ca	0	0
8524	41274	Drosophila melanogaster	CG6465	CG6465 gene product from transcript CG6465-RA	DmelCG6465	0	0
8525	41275	Drosophila melanogaster	CG14688	CG14688 gene product from transcript CG14688-RB	DmelCG14688	0	0
8526	41278	Drosophila melanogaster	CG14683	CG14683 gene product from transcript CG14683-RA	DmelCG14683	0	0
8527	41279	Drosophila melanogaster	pug	pugilist	CG4067|DmelCG4067|MTHFD|Pug|bs14h02.y1|pugD	0	0
8528	41280	Drosophila melanogaster	CG31391	CG31391 gene product from transcript CG31391-RA	BcDNA:AT04406|CG6471|DmelCG31391	0	0
8529	41281	Drosophila melanogaster	CG14684	CG14684 gene product from transcript CG14684-RA	DmelCG14684	0	0
8530	41282	Drosophila melanogaster	CG31278	CG31278 gene product from transcript CG31278-RA	CG6482|DmelCG31278|PDF	0	0
8531	41283	Drosophila melanogaster	CG14689	CG14689 gene product from transcript CG14689-RA	DmelCG14689	0	0
8532	41284	Drosophila melanogaster	CG4073	CG4073 gene product from transcript CG4073-RA	DmelCG4073	0	0
8533	41285	Drosophila melanogaster	tomboy20	CG14690 gene product from transcript CG14690-RA	CG14690|DmelCG14690	0	0
8534	41286	Drosophila melanogaster	Takr86C	Tachykinin-like receptor at 86C	CG6515|DmelCG6515|NKD|NKD12|NKr|Nkd|TAKR86|TAKR86C	0	0
8535	41287	Drosophila melanogaster	CG14691	CG14691 gene product from transcript CG14691-RA	DmelCG14691	0	0
8536	41288	Drosophila melanogaster	MED7	Mediator complex subunit 7	CG31390|CG6529|DmelCG31390|Med7|dMED7|dTRAP34	0	0
8537	41289	Drosophila melanogaster	Cap-H2	Chromosome associated protein H2	CG14685|CapH2|DmelCG14685|dCap-H2	0	0
8538	41290	Drosophila melanogaster	TfIIFbeta	Transcription factor TFIIFbeta	CG6538|DmelCG6538|F[[S]]|RAP30|T2FB_DROME|TFIIF|TFIIFbeta|TfIIF|dTFIIF30|l(3)j3C1	0	0
8539	41291	Drosophila melanogaster	fau	CG6544 gene product from transcript CG6544-RG	BcDNA:HL07933|CG6544|DmelCG6544|Fau	0	0
8540	41292	Drosophila melanogaster	Desi	Desiccate	CG14686|DmelCG14686	0	0
8541	41293	Drosophila melanogaster	mRpL37	mitochondrial ribosomal protein L37	CG42632|CG6547|DmelCG42632|Dmel_CG6547|MRP-L2|mRpL2a	0	0
8542	41294	Drosophila melanogaster	Rbp1	RNA-binding protein 1	CG17136|DmelCG17136|RBP1|RRM1|RRM11|Rbp-1|Rbp11|rbp1|rrm1|rrm11	0	0
8543	41295	Drosophila melanogaster	Art1	Arginine methyltransferase 1	CG6554|DART1|DmelCG6554	0	0
8544	41296	Drosophila melanogaster	Mcm5	Minichromosome maintenance 5	CG4082|DmCDC46|DmCDC465|DmMCM5|DmMcm5|DmeMCM5|DmelCG4082|MCM5|McM5|PCR4|mcm5	0	0

8545	41297	Drosophila melanogaster	CG14687	CG14687 gene product from transcript CG14687-RA	BcDNA:RE74890|DmelCG14687	0	0
8546	41298	Drosophila melanogaster	CG14692	CG14692 gene product from transcript CG14692-RC	DmelCG14692	0	0
8547	41299	Drosophila melanogaster	CG14693	CG14693 gene product from transcript CG14693-RC	DmelCG14693	0	0
8548	41300	Drosophila melanogaster	CG4089	CG4089 gene product from transcript CG4089-RA	DmelCG4089	0	0
8549	41302	Drosophila melanogaster	Cad86C	CG42601 gene product from transcript CG42601-RC	BcDNA:LP07955|CG31393|CG31425|CG33112|CG42601|CG4509|CG4655|CT14654|CT15041|DCad86C|DmelCG42601|Dmel_CG4509|Dmel_CG4655	0	0
8550	41303	Drosophila melanogaster	CG4565	CG4565 gene product from transcript CG4565-RB	DmelCG4565	0	0
8551	41304	Drosophila melanogaster	CG6567	CG6567 gene product from transcript CG6567-RB	DmelCG6567	0	0
8552	41305	Drosophila melanogaster	CG4511	CG4511 gene product from transcript CG4511-RA	DmelCG4511	0	0
8553	41306	Drosophila melanogaster	CG4570	CG4570 gene product from transcript CG4570-RA	DmelCG4570	0	0
8554	41307	Drosophila melanogaster	CG14694	CG14694 gene product from transcript CG14694-RA	DmelCG14694	0	0
8555	41308	Drosophila melanogaster	CG6574	CG6574 gene product from transcript CG6574-RA	DmelCG6574	0	0
8556	41309	Drosophila melanogaster	SelR	CG6584 gene product from transcript CG6584-RI	BcDNA:RE36040|BcDNA:RH61230|CG6584|DmelCG6584|MsrB|selR	0	0
8557	41310	Drosophila melanogaster	Tsp86D	Tetraspanin 86D	CG4591|Dm.Tsp86D|DmelCG4591	0	0
8558	41311	Drosophila melanogaster	Fdh	Formaldehyde dehydrogenase	ADHX|ADHX_DROME|CG6598|DmelCG6598|GSNOR|ODH|Odh|fdh|gfd|gfd/odh|odh	0	0
8559	41312	Drosophila melanogaster	CG4596	CG4596 gene product from transcript CG4596-RA	DmelCG4596	0	0
8560	41313	Drosophila melanogaster	Sodh-2	Sorbitol dehydrogenase-2	CG4649|DM_7299382|DmelCG4649|SODH-2|Sdh|Sdh-2	0	0
8561	41314	Drosophila melanogaster	CG14695	CG14695 gene product from transcript CG14695-RA	DmelCG14695	0	0
8562	41316	Drosophila melanogaster	Tpc1	Thiamine pyrophosphate carrier protein 1	CG6608|DmTpc1|DmelCG6608	0	0
8563	41317	Drosophila melanogaster	CG14696	CG14696 gene product from transcript CG14696-RA	DmelCG14696	0	0
8564	41318	Drosophila melanogaster	Adk3	Adenylate kinase-3	AK3|CG6612|DAK3|DmelCG6612|bs12h03.y1	0	0
8565	41319	Drosophila melanogaster	CG6621	CG6621 gene product from transcript CG6621-RB	DmelCG6621	0	0
8566	41320	Drosophila melanogaster	CG4674	CG4674 gene product from transcript CG4674-RA	CG32471|DmelCG4674	0	0
8567	41321	Drosophila melanogaster	CG4683	CG4683 gene product from transcript CG4683-RA	DmelCG4683	0	0
8568	41324	Drosophila melanogaster	CG6629	CG6629 gene product from transcript CG6629-RA	DmelCG6629	0	0
8569	41326	Drosophila melanogaster	CG4706	CG4706 gene product from transcript CG4706-RA	DmelCG4706	0	0
8570	41330	Drosophila melanogaster	CG4757	CG4757 gene product from transcript CG4757-RA	BcDNA:GH05741|DmelCG4757|GH05702|GH05741	0	0
8571	41333	Drosophila melanogaster	Ugt35b	UDP-glycosyltransferase 35b	AC 006491B|CG6649|DmeUgt35b|DmelCG6649|UDPGT2|UGT35b|Ugt35B|Ugt86Db|ugt35b	0	0
8572	41334	Drosophila melanogaster	Ugt35a	UDP-glycosyltransferase 35a	AC 006491F|CG6644|DmeUgt35a|DmelCG6644|UDPGT1|Ugt86Df	0	0
8573	41337	Drosophila melanogaster	CG18577	CG18577 gene product from transcript CG18577-RA	DmelCG18577	0	0
8574	41339	Drosophila melanogaster	cu	curled	CG31299|CG4782|CG4796|DmelCG31299|no|no(cu)|noc|noct|nocturnin	0	0
8575	41340	Drosophila melanogaster	SdhC	Succinate dehydrogenase C	BcDNA:RH04289|CG6666|DmelCG6666|sdhC	0	0
8576	41341	Drosophila melanogaster	Tctp	Translationally controlled tumor protein	CG4800|DmelCG4800|TCTP|dTCTP	0	0
8577	41342	Drosophila melanogaster	CG6672	CG6672 gene product from transcript CG6672-RA	DmelCG6672	0	0
8578	41343	Drosophila melanogaster	RpS25	Ribosomal protein S25	CG6684|D25|DmelCG6684|M(3)86D|M(3)S31|M(3)S32|M(3)s[32]|RpS31|S25|anon-EST:Posey260	0	0
8579	41344	Drosophila melanogaster	CG4820	CG4820 gene product from transcript CG4820-RB	DmelCG4820	0	0
8580	41345	Drosophila melanogaster	CG6689	CG6689 gene product from transcript CG6689-RA	DmelCG6689|dmCG6689	0	0
8581	41346	Drosophila melanogaster	CG6693	CG6693 gene product from transcript CG6693-RA	DmelCG6693	0	0
8582	41347	Drosophila melanogaster	RpL3	Ribosomal protein L3	CG4863|DmelCG4863|L3|Rpl3|anon-EST:GressD4|rpL3	0	0
8583	41348	Drosophila melanogaster	scpr-C	SCP-containing protein C	BEST:CK01680|CG5106|DmelCG5106|SCPR-C|Scpr-C|cg5106|scp-C	0	0
8584	41349	Drosophila melanogaster	CG17726	CG17726 gene product from transcript CG17726-RA	DmelCG17726	0	0
8585	41350	Drosophila melanogaster	CG10703	CG10703 gene product from transcript CG10703-RA	DmelCG10703|dGCC88	0	0
8586	41351	Drosophila melanogaster	CG17187	CG17187 gene product from transcript CG17187-RA	BcDNA:RE47242|DmelCG17187	0	0
8587	41352	Drosophila melanogaster	CG14701	CG14701 gene product from transcript CG14701-RA	DmelCG14701	0	0
8588	41353	Drosophila melanogaster	CG17184	CG17184 gene product from transcript CG17184-RB	DmelCG17184	0	0
8589	41354	Drosophila melanogaster	CG17721	CG17721 gene product from transcript CG17721-RA	BcDNA:LP09251|DmelCG17721	0	0
8590	41355	Drosophila melanogaster	CG31388	CG31388 gene product from transcript CG31388-RB	BcDNA:GH10523|CG17722|CG5135|DmelCG31388	0	0
8591	41357	Drosophila melanogaster	scpr-B	SCP-containing protein B	CG17210|DmelCG17210|SCPR-B|anon-WO0140519.115|cg17210|scp-B	0	0
8592	41358	Drosophila melanogaster	scpr-A	SCP-containing protein A	CG5207|DmelCG5207|SCPR-A|bs03f04.y1|cg5207|scp-A	0	0
8593	41359	Drosophila melanogaster	CG17734	CG17734 gene product from transcript CG17734-RA	BcDNA:RH54117|DmelCG17734	0	0
8594	41360	Drosophila melanogaster	CG5214	CG5214 gene product from transcript CG5214-RA	DmelCG5214	0	0
8595	41361	Drosophila melanogaster	KP78b	CG17216 gene product from transcript CG17216-RB	CG17216|DmelCG17216|KP78B|Kp78B|Kp78b	0	0
8596	41362	Drosophila melanogaster	KP78a	CG6715 gene product from transcript CG6715-RB	CG6715|DmelCG6715|KP78A|Kp78A	0	0
8597	41363	Drosophila melanogaster	pros	prospero	0244/09|0320/10|0441/16|0451/09|0563/18|0585/13|0664/07|0671/02|0763/13|0989/01|1135/07|1135/09|1167/13|1316/02|671/2|BcDNA:HL08040|CG17228|DMPROSPER|DROPROSA|DmelCG17228|PR	0	0
8598	41364	Drosophila melanogaster	mRpL40	mitochondrial ribosomal protein L40	CG5242|DmelCG5242|MRPL22(24)|mRpL22-24	0	0
8599	41365	Drosophila melanogaster	CG6719	CG6719 gene product from transcript CG6719-RA	BcDNA:LD34406|DmelCG6719|PAC10	0	0
8600	41366	Drosophila melanogaster	mus309	mutagen-sensitive 309	BLM|Blm|CG6920|DmBLM|DmBlm|DmKu70|Dmblm|DmelCG6920|Ku70|MUS309|RecQ|blm|mus 309|mus309/DmBLM	0	0
8601	41367	Drosophila melanogaster	Ranbp9	CG5252 gene product from transcript CG5252-RA	CG5252|DmelCG5252	0	0
8602	41368	Drosophila melanogaster	CG6723	CG6723 gene product from transcript CG6723-RA	DmelCG6723	0	0
8603	41369	Drosophila melanogaster	ninaG	CG6728 gene product from transcript CG6728-RB	CG6728|DmelCG6728|NinaG	0	0
8604	41370	Drosophila melanogaster	CG5270	CG5270 gene product from transcript CG5270-RB	DmelCG5270	0	0
8605	41371	Drosophila melanogaster	CG18643	CG18643 gene product from transcript CG18643-RB	DmelCG18643	0	0
8606	41372	Drosophila melanogaster	RpL24-like	Ribosomal protein L24-like	CG6764|DmelCG6764	0	0
8607	41373	Drosophila melanogaster	CG5276	CG5276 gene product from transcript CG5276-RA	DmelCG5276	0	0
8608	41374	Drosophila melanogaster	CG6744	CG6744 gene product from transcript CG6744-RA	DmelCG6744	0	0
8609	41375	Drosophila melanogaster	CG5281	CG5281 gene product from transcript CG5281-RA	DmelCG5281	0	0
8610	41376	Drosophila melanogaster	CG17230	CG17230 gene product from transcript CG17230-RA	BcDNA:GH24095|CG5286|DmelCG17230	0	0
8611	41377	Drosophila melanogaster	sals	sarcomere length short	CG14703|CG31374|CG6771|DmelCG31374	0	0
8612	41379	Drosophila melanogaster	PGRP-LB	Peptidoglycan recognition protein LB	CG14704|DmelCG14704	0	0
8613	41381	Drosophila melanogaster	dpr5	CG5308 gene product from transcript CG5308-RA	CG5308|CT16895|DmelCG5308|Dpr-5	0	0
8614	41383	Drosophila melanogaster	CG14708	CG14708 gene product from transcript CG14708-RA	DmelCG14708	0	0
8615	41384	Drosophila melanogaster	CG10898	CG10898 gene product from transcript CG10898-RA	DmelCG10898|anon-WO0118547.280	0	0
8616	41387	Drosophila melanogaster	Mrp4	Multidrug resistance protein 4 ortholog	BEST:CK01227|BEST:CK02472|CG14709|CK01227|Dme_CG14709|DmelCG14709|EP3221|dMRP4	0	0
8617	41388	Drosophila melanogaster	CG6790	CG6790 gene product from transcript CG6790-RA	DmelCG6790|NEST:bs22e02|anon-WO0140519.186	0	0
8618	41389	Drosophila melanogaster	CG5342	CG5342 gene product from transcript CG5342-RB	DmelCG5342	0	0
8619	41390	Drosophila melanogaster	wkd	whacked	CG5344|DmelCG5344|cg5344	0	0
8620	41391	Drosophila melanogaster	CG6791	CG6791 gene product from transcript CG6791-RA	DmelCG6791	0	0
8621	41392	Drosophila melanogaster	Jupiter	CG31363 gene product from transcript CG31363-RD	BEST:LP05168|BcDNA:GH06373|BcDNA:LD21358|BcDNA:LD43690|BcDNA:RH53211|CG17238|CG18762|CG18763|CG31363|DmelCG31363|anon-EST:Liang-1.76|anon-WO0140519.173|clone 1.76|jupit	0	0
8622	41393	Drosophila melanogaster	CG14710	CG14710 gene product from transcript CG14710-RB	DmelCG14710	0	0
8623	41394	Drosophila melanogaster	CG6808	CG6808 gene product from transcript CG6808-RA	CG31372|DmelCG6808	0	0
8624	41395	Drosophila melanogaster	CG14711	CG14711 gene product from transcript CG14711-RA	DmelCG14711	0	0
8625	41396	Drosophila melanogaster	CG6813	CG6813 gene product from transcript CG6813-RB	CG31372|DmelCG6813	0	0
8626	41397	Drosophila melanogaster	CG14712	CG14712 gene product from transcript CG14712-RA	DmelCG14712	0	0
8627	41398	Drosophila melanogaster	Csk	C-terminal Src kinase	CG17309|CG32852|CG42317|CSK|DCsk|DmelCG42317|Dmel_CG17309|Dmel_CG32852|csk|dCSK|dCsk|dcsk|flik|l(3)S017909|l(3)j1D8	0	0
8628	41399	Drosophila melanogaster	Elp1	Elongator complex protein 1	CG10535|D-elp1|DmelCG10535|ELP1|elp1	0	0
8629	41401	Drosophila melanogaster	CG42327	CG42327 gene product from transcript CG42327-RE	CG14713|CG14714|CG18479|DmelCG42327|Dmel_CG14713|Dmel_CG14714|anon-WO0118547.710	0	0
8630	41403	Drosophila melanogaster	CG14715	CG14715 gene product from transcript CG14715-RA	BcDNA:GM09283|BcDNA:RH50927|DmelCG14715	0	0
8631	41404	Drosophila melanogaster	CG18476	CG18476 gene product from transcript CG18476-RA	CG17822|DmelCG18476	0	0
8632	41406	Drosophila melanogaster	Taf12	TBP-associated factor 12	CG17358|DmelCG17358|T2DA_DROME|TAF|TAF12|TAF30a|TAF30alpha|TAF30alpha/28|TAF30alpha/28/22|TAFII30alpha/22|TAF[II]30alpha|TAF[[II]]|TAF[[II]]230|TAF[[II]]30alpha|TAF[[II]]30alpha/28 TAF[[II]]22	0	0
8633	41407	Drosophila melanogaster	Ho	Heme oxygenase	CG14716|CT34507|DmHO|DmelCG14716|HO|dHO	0	0
8634	41408	Drosophila melanogaster	CG6830	CG6830 gene product from transcript CG6830-RA	DmelCG6830	0	0
8635	41409	Drosophila melanogaster	CG6834	CG6834 gene product from transcript CG6834-RA	DmelCG6834	0	0
8636	41410	Drosophila melanogaster	CG6908	CG6908 gene product from transcript CG6908-RA	DmelCG6908	0	0
8637	41411	Drosophila melanogaster	Fer3	48 related 3	BcDNA:LD04689|CG6913|DmelCG6913|Dnato3|bHLHa32|nato3	0	0
8638	41412	Drosophila melanogaster	CG14717	CG14717 gene product from transcript CG14717-RA	DmelCG14717	0	0
8639	41413	Drosophila melanogaster	CG14718	CG14718 gene product from transcript CG14718-RA	DmelCG14718|cg14718	0	0
8640	41414	Drosophila melanogaster	I-t	inhibitor-t	CG14719|DmelCG14719|pp1d6	0	0
8641	41415	Drosophila melanogaster	CG14720	CG14720 gene product from transcript CG14720-RA	DmelCG14720	0	0
8642	41416	Drosophila melanogaster	CG12594	CG12594 gene product from transcript CG12594-RA	DmelCG12594	0	0
8643	41417	Drosophila melanogaster	CG14721	CG14721 gene product from transcript CG14721-RA	DmelCG14721	0	0
8644	41419	Drosophila melanogaster	CG14722	CG14722 gene product from transcript CG14722-RA	DmelCG14722	0	0
8645	41420	Drosophila melanogaster	CG6923	CG6923 gene product from transcript CG6923-RC	DmelCG6923	0	0
8646	41423	Drosophila melanogaster	l(3)neo38	lethal (3) neo38	BcDNA:SD16005|CG14725|CG31364|CG6930|DmelCG6930	0	0
8647	41425	Drosophila melanogaster	CG17360	CG17360 gene product from transcript CG17360-RA	BcDNA:RH29885|DmelCG17360	0	0
8648	41426	Drosophila melanogaster	HisCl1	Histamine-gated chloride channel subunit 1	CG14723|DMHisClalpha2|Dm-HCLB|DmHisCl1|DmelCG14723|hclB|hisCl1	0	0
8649	41427	Drosophila melanogaster	Sbf	SET domain binding factor	CG6939|DSbf1|DmelCG6939|SBF|Sbf1|dSbf1|sbf	0	0
8650	41428	Drosophila melanogaster	ClC-a	Chloride channel-a	CG14726|CG31116|CG6942|DmClC-2|DmClC-a|DmClCa|DmelCG31116|FBgn0037951	0	0
8651	41431	Drosophila melanogaster	glo	glorund	110/46|CG6946|DmelCG6946|Glo|anon-WO0118547.328|cg6946|l(3)S011046|l(3)S11046|p67|p67-K	0	0
8652	41432	Drosophila melanogaster	CoVa	Cytochrome c oxidase subunit Va	CG14724|COX|COXA_DROME|DmelCG14724|Va|tend	0	0
8653	41433	Drosophila melanogaster	CG6950	CG6950 gene product from transcript CG6950-RC	DmelCG6950	0	0
8654	41434	Drosophila melanogaster	CG6959	CG6959 gene product from transcript CG6959-RA	CT21563|DmelCG6959	0	0
8655	41436	Drosophila melanogaster	CG6962	CG6962 gene product from transcript CG6962-RA	DmelCG6962	0	0

8656	41437	Drosophila melanogaster	CG31368	CG31368 gene product from transcript CG31368-RD	CG14729|CG14730|DmelCG31368	0	0
8657	41438	Drosophila melanogaster	mthl5	methuselah-like 5	CG6965|DmelCG6965|Mth-5|anon-WO0170980.70|anon-WO0170980.71	0	0
8658	41440	Drosophila melanogaster	CG6971	CG6971 gene product from transcript CG6971-RA	DmelCG6971|anon-WO0140519.67	0	0
8659	41441	Drosophila melanogaster	Cad87A	CG6977 gene product from transcript CG6977-RA	CG6977|CT21611|DCad87A|DmelCG6977	0	0
8660	41442	Drosophila melanogaster	CG14731	CG14731 gene product from transcript CG14731-RA	DmelCG14731	0	0
8661	41443	Drosophila melanogaster	CG31211	CG31211 gene product from transcript CG31211-RB	CG14732|CG18546|DmelCG31211|anon-WO03070958.3|cDNA	0	0
8662	41445	Drosophila melanogaster	CG3281	CG3281 gene product from transcript CG3281-RA	87A7-9/1|DmelCG3281|anon-87Aa|anon-WO0140519.243	0	0
8663	41446	Drosophila melanogaster	aur	aurora	87A7-9/2|AurA|CG3068|DmelCG3068|aurA|ck10|ck[10]|l(3)87Ac|l(3)ck10	0	0
8664	41447	Drosophila melanogaster	CG12213	CG12213 gene product from transcript CG12213-RB	87A7-9/3|DmelCG12213|anon-87Ab	0	0
8665	41448	Drosophila melanogaster	CG18347	CG18347 gene product from transcript CG18347-RA	87A7-9/4|DmelCG18347|anon-87Ac	0	0
8666	41449	Drosophila melanogaster	CG12201	CG12201 gene product from transcript CG12201-RC	DmelCG12201	0	0
8667	41450	Drosophila melanogaster	Tango9	Transport and Golgi organization 9	87A7-9/5|CG10007|DmelCG10007|TANGO9|anon-87Ad	0	0
8668	41451	Drosophila melanogaster	CG10005	CG10005 gene product from transcript CG10005-RA	BcDNA:RE59626|DmelCG10005	0	0
8669	41452	Drosophila melanogaster	CG18547	CG18547 gene product from transcript CG18547-RA	DmelCG18547	0	0
8670	41453	Drosophila melanogaster	CG12224	CG12224 gene product from transcript CG12224-RB	DmelCG12224	0	0
8671	41454	Drosophila melanogaster	CG3397	CG3397 gene product from transcript CG3397-RA	DmelCG3397	0	0
8672	41455	Drosophila melanogaster	mfas	midline fasciclin	87A7-9/7|BEST:GH06752|CG3359|DmelCG3359|Mfas|anon-87Af|anon-EST:Liang-97|anon-WO0140519.174|clone 97|gh06752	0	0
8673	41456	Drosophila melanogaster	Tk	Tachykinin	CG14734|DTK|DTK-6|DTK1|DmelCG14734|Drm-TK|Drm-TK-1|Drm-TK-2|Drm-TK-3|Drm-TK-4|Drm-TK-5|Drm-TK-6|Dtk|TK|TK-1|dtk	0	0
8674	41457	Drosophila melanogaster	Ect3	CG3132 gene product from transcript CG3132-RA	87A7-9/8|CG3132|DmelCG3132|anon-87Ag	0	0
8675	41458	Drosophila melanogaster	KLHL18	CG3571 gene product from transcript CG3571-RA	CG3571|DmelCG3571|anon-WO0118547.179|dKLHL18	0	0
8676	41459	Drosophila melanogaster	CG3532	CG3532 gene product from transcript CG3532-RB	DmelCG3532|dGCC185	0	0
8677	41460	Drosophila melanogaster	CG3313	CG3313 gene product from transcript CG3313-RA	DmelCG3313	0	0
8678	41461	Drosophila melanogaster	Spt3	CG3169 gene product from transcript CG3169-RA	CG3169|DmelCG3169|SPT3|TFIID|dSpt3	0	0
8679	41462	Drosophila melanogaster	CG31358	CG31358 gene product from transcript CG31358-RA	CG17424|CG18141|CG18142|DmelCG31358	0	0
8680	41465	Drosophila melanogaster	ssp5	short spindle 5	CG14735|DmelCG14735|Ssp5|Tes59|anon-WO0140519.127	0	0
8681	41466	Drosophila melanogaster	CG14736	CG14736 gene product from transcript CG14736-RE	DmelCG14736	0	0
8682	41467	Drosophila melanogaster	CG14739	CG14739 gene product from transcript CG14739-RA	DmelCG14739	0	0
8683	41468	Drosophila melanogaster	CG14740	CG14740 gene product from transcript CG14740-RA	DmelCG14740|anon-WO0140519.178	0	0
8684	41469	Drosophila melanogaster	CG14741	CG14741 gene product from transcript CG14741-RC	DmelCG14741	0	0
8685	41470	Drosophila melanogaster	dpr17	CG31361 gene product from transcript CG31361-RA	CG14737|CG14738|CG31361|CT34531|DmelCG31361|Dpr-17	0	0
8686	41472	Drosophila melanogaster	CG4702	CG4702 gene product from transcript CG4702-RC	DmelCG4702	0	0
8687	41473	Drosophila melanogaster	dpr15	CG10095 gene product from transcript CG10095-RA	CG10095|CT28423|DmelCG10095|Dpr-15	0	0
8688	41474	Drosophila melanogaster	Cpn	Calphotin	77H5|Ag 72H5|Ag72H5|CG4795|DmelCG4795|Mab77H5|cap|cpn	0	0
8689	41475	Drosophila melanogaster	CG4810	CG4810 gene product from transcript CG4810-RA	149735_at|DmelCG4810|eIF3-S7	0	0
8690	41476	Drosophila melanogaster	CG3809	CG3809 gene product from transcript CG3809-RA	DmelCG3809|anon-WO0140519.193	0	0
8691	41477	Drosophila melanogaster	CG4830	CG4830 gene product from transcript CG4830-RA	DmelCG4830	0	0
8692	41479	Drosophila melanogaster	CG4848	CG4848 gene product from transcript CG4848-RA	DmelCG4848	0	0
8693	41480	Drosophila melanogaster	CG4860	CG4860 gene product from transcript CG4860-RA	DmelCG4860	0	0
8694	41481	Drosophila melanogaster	CG10014	CG10014 gene product from transcript CG10014-RA	DmelCG10014	0	0
8695	41482	Drosophila melanogaster	CG17404	CG17404 gene product from transcript CG17404-RB	DmelCG17404|SP149	0	0
8696	41483	Drosophila melanogaster	CG12256	CG12256 gene product from transcript CG12256-RA	DmelCG12256|SP133	0	0
8697	41484	Drosophila melanogaster	CG3916	CG3916 gene product from transcript CG3916-RA	DmelCG3916|SP154	0	0
8698	41486	Drosophila melanogaster	Cyp313a5	CG15807 gene product from transcript CG15807-RA	313a5|CG15807|DmelCG15807	0	0
8699	41487	Drosophila melanogaster	Cyp313a2	CG10094 gene product from transcript CG10094-RB	313a2|CG10094|DmelCG10094	0	0
8700	41488	Drosophila melanogaster	Cyp313a3	CG10093 gene product from transcript CG10093-RA	313a3|CG10093|DmelCG10093	0	0
8701	41489	Drosophila melanogaster	CG3942	CG3942 gene product from transcript CG3942-RA	DmelCG3942	0	0
8702	41490	Drosophila melanogaster	CG17738	CG17738 gene product from transcript CG17738-RA	DmelCG17738	0	0
8703	41491	Drosophila melanogaster	svp	seven up	0005/12|0042/01|0106/18|0114/20|0351/01|0598/01|0861/07|BcDNA:GH08189|CG11502|CG18158|DmelCG11502|NR2F3|SVP|SVP-1|SVP1|Svp|ck16|don|l(3)07842|l(3)87Bd|l(3)ck16|l(3)j2E2|l(3)rA028|l(3)r	0	0
8704	41493	Drosophila melanogaster	CG4066	CG4066 gene product from transcript CG4066-RA	DmelCG4066	0	0
8705	41494	Drosophila melanogaster	CG10013	CG10013 gene product from transcript CG10013-RA	DmelCG10013	0	0
8706	41495	Drosophila melanogaster	CG10038	CG10038 gene product from transcript CG10038-RB	DmelCG10038	0	0
8707	41496	Drosophila melanogaster	CG10041	CG10041 gene product from transcript CG10041-RA	DmelCG10041|SP178	0	0
8708	41498	Drosophila melanogaster	MBD-R2	CG10042 gene product from transcript CG10042-RB	CG10042|CG17393|Dm Tam3|DmelCG10042|MBD|MBDR2|TAM3|dMBD-R2	0	0
8709	41499	Drosophila melanogaster	CG4115	CG4115 gene product from transcript CG4115-RA	DmelCG4115	0	0
8710	41500	Drosophila melanogaster	Tim17a1	CG10090 gene product from transcript CG10090-RA	CG10090|DmelCG10090	0	0
8711	41502	Drosophila melanogaster	GstD9	Glutathione S transferase D9	CG10091|DmGSTD9|DmGstd9|DmelCG10091|GSTD9|gstD9	0	0
8712	41503	Drosophila melanogaster	GstD1	Glutathione S transferase D1	CG10045|D1|DmGST-1|DmGST1|DmGSTD1|DmGSTD1-1|DmGst1|DmelCG10045|G-S-T|GST|GST-1|GST1|GST1-1|GSTD1|GSTase|GSt1-1|Gst|Gst-D1|Gst1|Gst1-1|Gst1.1|GstD|T5|dmGSTD1|gst-D1-1|gs	0	0
8713	41511	Drosophila melanogaster	CG10035	CG10035 gene product from transcript CG10035-RA	DmelCG10035|anon-EST:Liang-20|clone 20	0	0
8714	41512	Drosophila melanogaster	CG17639	CG17639 gene product from transcript CG17639-RA	BcDNA:RE72392|DmelCG17639|cg17639	0	0
8715	41513	Drosophila melanogaster	CG34402	CG34402 gene product from transcript CG34402-RC	CG17756|CG17793|CG31218|CG4940|CT39356|DmelCG34402|Dmel_CG17793|Dmel_CG31218|Dmel_CG4940|SOL-1|sol-1	0	0
8716	41518	Drosophila melanogaster	lig3	DNA ligase III	CG17227|DmelCG17227|LIG3	0	0
8717	41520	Drosophila melanogaster	Cyp9f2	CG11466 gene product from transcript CG11466-RB	9f2|CG11466|Cyp 9f2|DmelCG11466|anon-EST:Posey150	0	0
8718	41521	Drosophila melanogaster	CG5167	CG5167 gene product from transcript CG5167-RA	DmelCG5167	0	0
8719	41522	Drosophila melanogaster	CG5196	CG5196 gene product from transcript CG5196-RA	DmelCG5196	0	0
8720	41524	Drosophila melanogaster	pps	protein partner of snf	CG6525|DmelCG6525|FBgn0082831|SPP|Spp|spp	0	0
8721	41525	Drosophila melanogaster	Scgbeta	Sarcoglycan beta	CG5657|DmSCG-beta|DmelCG5657|dscgbeta|scg-beta	0	0
8722	41526	Drosophila melanogaster	CG17202	CG17202 gene product from transcript CG17202-RA	BcDNA:RH21090|DmelCG17202	0	0
8723	41528	Drosophila melanogaster	Sas	Sialic acid phosphate synthase	CG5232|DmSAS|DmelCG5232|Neu5Ac|SAS|sas	0	0
8724	41529	Drosophila melanogaster	CG5641	CG5641 gene product from transcript CG5641-RA	DmelCG5641	0	0
8725	41530	Drosophila melanogaster	CG5245	CG5245 gene product from transcript CG5245-RA	DmelCG5245|anon-WO0118547.611	0	0
8726	41531	Drosophila melanogaster	Pros25	Proteasome 25kD subunit	153303_at|CG5266|DmelCG5266|PRC3|PROS Dm25|PROS-Dm25g|PSA2_DROME|Prosalpha2|Su(DTS)|Su(DTS)-1|alpha2_dm|dalpha2	0	0
8727	41532	Drosophila melanogaster	Aos1	CG12276 gene product from transcript CG12276-RA	Aos|CG12276|DmAos1|DmSAE1|DmSae1|Dmaos1|DmelCG12276|SAE1|Sae1	0	0
8728	41533	Drosophila melanogaster	CG5844	CG5844 gene product from transcript CG5844-RA	DmelCG5844	0	0
8729	41536	Drosophila melanogaster	desat2	CG5925 gene product from transcript CG5925-RA	7-11HD|CG5925|Desat2|DmelCG5925|Fad|desat|desat2/Fad|ds2	0	0
8730	41537	Drosophila melanogaster	CG5538	CG5538 gene product from transcript CG5538-RA	DmelCG5538|anon-WO0140519.84|bs11a11.y1	0	0
8731	41538	Drosophila melanogaster	CG18549	CG18549 gene product from transcript CG18549-RA	DmelCG18549	0	0
8732	41539	Drosophila melanogaster	CG5509	CG5509 gene product from transcript CG5509-RA	DmelCG5509	0	0
8733	41540	Drosophila melanogaster	trus	toys are us	CG5333|DmelCG5333	0	0
8734	41541	Drosophila melanogaster	CG5961	CG5961 gene product from transcript CG5961-RB	DmelCG5961	0	0
8735	41542	Drosophila melanogaster	CG12267	CG12267 gene product from transcript CG12267-RB	DmelCG12267	0	0
8736	41543	Drosophila melanogaster	CG5608	CG5608 gene product from transcript CG5608-RA	DmelCG5608	0	0
8737	41547	Drosophila melanogaster	hug	hugin	CG6371|DmelCG6371|Drm-MT2|Drm-PK-2|HUG|HUGgamma|Hug-gamma|hug gamma|hug-gamma|huggamma|pyrokinin/PBAN-like	0	0
8738	41549	Drosophila melanogaster	Octbeta2R	CG33976 gene product from transcript CG33976-RD	CG18553|CG33976|CG6989|DmCG6989|DmOctbeta2R|DmelCG33976|OctB2R|OctBeta2|anon-WO0170980.184|anon-WO0170980.185	0	0
8739	41550	Drosophila melanogaster	Vha55	Vacuolar H[+]-ATPase 55kD B subunit	1208/13|CG17369|DmelCG17369|SzA|V-ATPase|VAT-2|l(3)87Ca|l(3)SzA|l(3)j2E9|vha55	0	0
8740	41551	Drosophila melanogaster	CG6359	CG6359 gene product from transcript CG6359-RA	DmelCG6359	0	0
8741	41552	Drosophila melanogaster	CG18616	CG18616 gene product from transcript CG18616-RA	DmelCG18616|dNot10	0	0
8742	41554	Drosophila melanogaster	CG11598	CG11598 gene product from transcript CG11598-RB	DmelCG11598	0	0
8743	41555	Drosophila melanogaster	CG11600	CG11600 gene product from transcript CG11600-RB	DmelCG11600	0	0
8744	41556	Drosophila melanogaster	CG11608	CG11608 gene product from transcript CG11608-RB	DmelCG11608	0	0
8745	41557	Drosophila melanogaster	CG6753	CG6753 gene product from transcript CG6753-RB	DmelCG6753	0	0
8746	41558	Drosophila melanogaster	CG6234	CG6234 gene product from transcript CG6234-RA	DmelCG6234	0	0
8747	41559	Drosophila melanogaster	CG6225	CG6225 gene product from transcript CG6225-RB	DmelCG6225	0	0
8748	41560	Drosophila melanogaster	CG14395	CG14395 gene product from transcript CG14395-RB	DmelCG14395	0	0
8749	41561	Drosophila melanogaster	CG6188	CG6188 gene product from transcript CG6188-RA	DmelCG6188	0	0
8750	41562	Drosophila melanogaster	mbo	members only	1465/07|1465/7|CG6819|DNup88|DmelCG6819|Dnup 88|Dnup88|Mbo|Nup88|anon-WO0118547.263|dNup88|l(3)05043|l(3)S146507|nup88	0	0
8751	41563	Drosophila melanogaster	Cyp313a4	CG6802 gene product from transcript CG6802-RA	313a4|CG6802|DmelCG6802	0	0
8752	41565	Drosophila melanogaster	Su(fu)	Suppressor of fused	CG6054|DmelCG6054|SU(FU)|SUFU|Su(Fu)|SuFu|Sufu|su(fu)	0	0
8753	41566	Drosophila melanogaster	Arp87C	Actin-related protein 87C	ARP1|ARP87C|Actr87C|Arp|Arp-1|Arp1|Arp87c|CG6174|DmelCG6174|arp1|gdl|grid	0	0
8754	41567	Drosophila melanogaster	CG14391	CG14391 gene product from transcript CG14391-RA	BcDNA:AT22529|DmelCG14391	0	0
8755	41568	Drosophila melanogaster	CG14394	CG14394 gene product from transcript CG14394-RB	DmelCG14394|NijC	0	0
8756	41569	Drosophila melanogaster	Past1	Putative Achaete Scute Target 1	CG6148|DmelCG6148|Ehd|PAST|PAST-1|Past-1|dEHD	0	0
8757	41570	Drosophila melanogaster	CG12279	CG12279 gene product from transcript CG12279-RA	BcDNA:RH54517|DmelCG12279	0	0
8758	41571	Drosophila melanogaster	mus308	mutagen-sensitive 308	0443/09|AAF54858|CG6019|DmelCG6019|Mus308|Nuc3|POLQ|Pol theta|PolQ|mus-308	0	0
8759	41573	Drosophila melanogaster	CG5724	CG5724 gene product from transcript CG5724-RA	DmelCG5724	0	0
8760	41574	Drosophila melanogaster	CG5999	CG5999 gene product from transcript CG5999-RB	DmelCG5999	0	0
8761	41575	Drosophila melanogaster	beat-Vc	CG14390 gene product from transcript CG14390-RA	CG14390|DmelCG14390|beat Vc	0	0
8762	41576	Drosophila melanogaster	CG31345	CG31345 gene product from transcript CG31345-RA	CG10135|CG14388|DmelCG31345	0	0
8763	41578	Drosophila melanogaster	beat-Va	CG10134 gene product from transcript CG10134-RA	CG10134|DmelCG10134|beat Va	0	0
8764	41579	Drosophila melanogaster	CG10126	CG10126 gene product from transcript CG10126-RB	CT28495|DmelCG10126	0	0
8765	41580	Drosophila melanogaster	d-cup	davis-cup	CG14387|DmelCG14387	0	0
8766	41581	Drosophila melanogaster	CG10909	CG10909 gene product from transcript CG10909-RA	DmelCG10909	0	0

<table>
<tr><td>8767</td><td>41583</td><td>Drosophila melanogaster</td><td>beat-Vb</td><td>CG31298 gene product from transcript CG31298-RA</td><td>CG14385|CG14386|CG31298|DmelCG31298|beat Vb</td><td>0</td><td>0</td></tr>
<tr><td>8768</td><td>41585</td><td>Drosophila melanogaster</td><td>Spc25</td><td>CG7242 gene product from transcript CG7242-RA</td><td>BcDNA:LD37196|CG7242|Cal2|DmelCG7242|Mitch|S3|cal2|dmSpc25R|l(3)87Da|l(3)A34-1|l(3)A46-1|l(3)S3|l(3)c00064</td><td>0</td><td>0</td></tr>
<tr><td>8769</td><td>41586</td><td>Drosophila melanogaster</td><td>Cyp304a1</td><td>CG7241 gene product from transcript CG7241-RA</td><td>304a1|BcDNA:LP06355|CG7241|CYP304A1-Dm|DmelCG7241</td><td>0</td><td>0</td></tr>
<tr><td>8770</td><td>41587</td><td>Drosophila melanogaster</td><td>granny-smith</td><td>granny smith</td><td>BcDNA:LD41548|CG7340|DmelCG7340|grsm|l(3)c00064</td><td>0</td><td>0</td></tr>
<tr><td>8771</td><td>41588</td><td>Drosophila melanogaster</td><td>CG14384</td><td>CG14384 gene product from transcript CG14384-RA</td><td>DmelCG14384</td><td>0</td><td>0</td></tr>
<tr><td>8772</td><td>41589</td><td>Drosophila melanogaster</td><td>CG7381</td><td>CG7381 gene product from transcript CG7381-RD</td><td>CT22687|DmelCG7381</td><td>0</td><td>0</td></tr>
<tr><td>8773</td><td>41590</td><td>Drosophila melanogaster</td><td>CG7091</td><td>CG7091 gene product from transcript CG7091-RB</td><td>DmelCG7091</td><td>0</td><td>0</td></tr>
<tr><td>8774</td><td>41591</td><td>Drosophila melanogaster</td><td>Paip2</td><td>polyA-binding protein interacting protein 2</td><td>CG12358|DmelCG12358|dPaip2</td><td>0</td><td>0</td></tr>
<tr><td>8775</td><td>41592</td><td>Drosophila melanogaster</td><td>CG31342</td><td>CG31342 gene product from transcript CG31342-RC</td><td>CG18551|CG7472|DmelCG31342</td><td>0</td><td>0</td></tr>
<tr><td>8776</td><td>41593</td><td>Drosophila melanogaster</td><td>CG14383</td><td>CG14383 gene product from transcript CG14383-RA</td><td>DmelCG14383</td><td>0</td><td>0</td></tr>
<tr><td>8777</td><td>41595</td><td>Drosophila melanogaster</td><td>yellow-f</td><td>CG18550 gene product from transcript CG18550-RA</td><td>CG18550|DmelCG18550</td><td>0</td><td>0</td></tr>
<tr><td>8778</td><td>41596</td><td>Drosophila melanogaster</td><td>yellow-f2</td><td>CG8063 gene product from transcript CG8063-RA</td><td>CG8063|DmelCG8063</td><td>0</td><td>0</td></tr>
<tr><td>8779</td><td>41597</td><td>Drosophila melanogaster</td><td>CG7488</td><td>CG7488 gene product from transcript CG7488-RA</td><td>DmelCG7488</td><td>0</td><td>0</td></tr>
<tr><td>8780</td><td>41598</td><td>Drosophila melanogaster</td><td>CG17327</td><td>CG17327 gene product from transcript CG17327-RB</td><td>DmelCG17327</td><td>0</td><td>0</td></tr>
<tr><td>8781</td><td>41599</td><td>Drosophila melanogaster</td><td>CG7518</td><td>CG7518 gene product from transcript CG7518-RD</td><td>DmelCG7518</td><td>0</td><td>0</td></tr>
<tr><td>8782</td><td>41600</td><td>Drosophila melanogaster</td><td>CG11656</td><td>CG11656 gene product from transcript CG11656-RA</td><td>DmelCG11656|anon-WO0140519.199</td><td>0</td><td>0</td></tr>
<tr><td>8783</td><td>41601</td><td>Drosophila melanogaster</td><td>CG8031</td><td>CG8031 gene product from transcript CG8031-RA</td><td>DmelCG8031|dMEMO</td><td>0</td><td>0</td></tr>
<tr><td>8784</td><td>41602</td><td>Drosophila melanogaster</td><td>CtBP</td><td>C-terminal Binding Protein</td><td>0256/16|256/16|BcDNA:GH07572|CG7583|CTBP|Dm-CtBP|DmelCG7583|EP(3)3528|G9|RE01030|RE61183|ctbp|dCtBP|l(3)03463|l(3)03464|l(3)87DE|l(3)87De|l(3)G9</td><td>0</td><td>0</td></tr>
<tr><td>8785</td><td>41603</td><td>Drosophila melanogaster</td><td>CG12360</td><td>CG12360 gene product from transcript CG12360-RA</td><td>DmelCG12360</td><td>0</td><td>0</td></tr>
<tr><td>8786</td><td>41605</td><td>Drosophila melanogaster</td><td>ry</td><td>rosy</td><td>CG7642|DmelCG7642|XDH|XOR|Xdh|Xdh/ry</td><td>0</td><td>0</td></tr>
<tr><td>8787</td><td>41606</td><td>Drosophila melanogaster</td><td>CG11668</td><td>CG11668 gene product from transcript CG11668-RA</td><td>DmelCG11668|SP115</td><td>0</td><td>0</td></tr>
<tr><td>8788</td><td>41607</td><td>Drosophila melanogaster</td><td>snk</td><td>snake</td><td>CG7996|DmelCG7996|SNK|SP26|c-SP26|l(3)87Dg|me(3)4</td><td>0</td><td>0</td></tr>
<tr><td>8789</td><td>41608</td><td>Drosophila melanogaster</td><td>CG11670</td><td>CG11670 gene product from transcript CG11670-RE</td><td>DmelCG11670|SP27</td><td>0</td><td>0</td></tr>
<tr><td>8790</td><td>41609</td><td>Drosophila melanogaster</td><td>Hsc70-2</td><td>Heat shock protein cognate 2</td><td>CG7756|DmelCG7756|HSC-70|HSC2|HSC70|Hsc-2|Hsc2|Hsc70|Hsc70.2|dhsc70|hsc2|hsc70</td><td>0</td><td>0</td></tr>
<tr><td>8791</td><td>41610</td><td>Drosophila melanogaster</td><td>CG7966</td><td>CG7966 gene product from transcript CG7966-RA</td><td>DmelCG7966</td><td>0</td><td>0</td></tr>
<tr><td>8792</td><td>41611</td><td>Drosophila melanogaster</td><td>pic</td><td>piccolo</td><td>0263/16|1258/16|1366/03|153636_at|263/16|CG7769|D-DDB1|DDB-1|DDB1|Ddb1|DmelCG7769|d-ddb1|dDdb1|l(3)87Dh|l(3)S136603</td><td>0</td><td>0</td></tr>
<tr><td>8793</td><td>41612</td><td>Drosophila melanogaster</td><td>sim</td><td>single-minded</td><td>0483/09|0716/08|0899/14|0953/08|1002/10|1034/02|1050/13|1110/04|1111/10|1330/08|1479/04|CG7771|DmelCG7771|S8|SIM|Sim|bHLHe16|l(3)87Ea|l(3)E320|l(3)RD|l(3)S8|l(3)s8|schm|shm</td><td>0</td><td>0</td></tr>
<tr><td>8794</td><td>41615</td><td>Drosophila melanogaster</td><td>timeout</td><td>CG7855 gene product from transcript CG7855-RA</td><td>CG14381|CG14382|CG7855|CG8148|DmelCG7855|TIM2|dtim2|tim2</td><td>0</td><td>0</td></tr>
<tr><td>8795</td><td>41616</td><td>Drosophila melanogaster</td><td>2mit</td><td>CG42573 gene product from transcript CG42573-RA</td><td>CG10148|CG17319|CG42573|CT28541|CT38314|DmelCG42573|Dmel_CG10148|Dmel_CG17319</td><td>0</td><td>0</td></tr>
<tr><td>8796</td><td>41619</td><td>Drosophila melanogaster</td><td>CG8138</td><td>CG8138 gene product from transcript CG8138-RA</td><td>DmelCG8138</td><td>0</td><td>0</td></tr>
<tr><td>8797</td><td>41620</td><td>Drosophila melanogaster</td><td>CG8508</td><td>CG8508 gene product from transcript CG8508-RA</td><td>DmelCG8508|anon-WO0140519.216</td><td>0</td><td>0</td></tr>
<tr><td>8798</td><td>41621</td><td>Drosophila melanogaster</td><td>CG14380</td><td>CG14380 gene product from transcript CG14380-RB</td><td>DmelCG14380</td><td>0</td><td>0</td></tr>
<tr><td>8799</td><td>41622</td><td>Drosophila melanogaster</td><td>CG8141</td><td>CG8141 gene product from transcript CG8141-RA</td><td>BcDNA:AT26376|DmelCG8141</td><td>0</td><td>0</td></tr>
<tr><td>8800</td><td>41623</td><td>Drosophila melanogaster</td><td>CG8483</td><td>CG8483 gene product from transcript CG8483-RA</td><td>DmelCG8483</td><td>0</td><td>0</td></tr>
<tr><td>8801</td><td>41624</td><td>Drosophila melanogaster</td><td>CG8476</td><td>CG8476 gene product from transcript CG8476-RA</td><td>DmelCG8476</td><td>0</td><td>0</td></tr>
<tr><td>8802</td><td>41625</td><td>Drosophila melanogaster</td><td>Ace</td><td>Acetylcholine esterase</td><td>ACE|AChE|AcChE|AchE|CG17907|CHE|DmAChE|DmelCG17907|FBgn0000024|ace|ace-2|ache|dAChE|dmAChE|l(3)26|l(3)87Ed</td><td>0</td><td>0</td></tr>
<tr><td>8803</td><td>41626</td><td>Drosophila melanogaster</td><td>Ravus</td><td>CG15889 gene product from transcript CG15889-RA</td><td>CG15889|DmelCG15889</td><td>0</td><td>0</td></tr>
<tr><td>8804</td><td>41627</td><td>Drosophila melanogaster</td><td>Su(var)3-7</td><td>Suppressor of variegation 3-7</td><td>CG8599|DmSu(var)3-7|DmelCG8599|SU(VAR)3-7|Su(Var)3-7|Su(var)(3)3|Su(var)(3)7|Su-var(3)7|Suvar(3)7|l(3)87El</td><td>0</td><td>0</td></tr>
<tr><td>8805</td><td>41628</td><td>Drosophila melanogaster</td><td>CG8449</td><td>CG8449 gene product from transcript CG8449-RA</td><td>DmelCG8449</td><td>0</td><td>0</td></tr>
<tr><td>8806</td><td>41629</td><td>Drosophila melanogaster</td><td>CG8630</td><td>CG8630 gene product from transcript CG8630-RA</td><td>DmelCG8630</td><td>0</td><td>0</td></tr>
<tr><td>8807</td><td>41630</td><td>Drosophila melanogaster</td><td>CG15888</td><td>CG15888 gene product from transcript CG15888-RB</td><td>DmelCG15888</td><td>0</td><td>0</td></tr>
<tr><td>8808</td><td>41631</td><td>Drosophila melanogaster</td><td>CG15887</td><td>CG15887 gene product from transcript CG15887-RA</td><td>BcDNA:RE53127|DmelCG15887</td><td>0</td><td>0</td></tr>
<tr><td>8809</td><td>41632</td><td>Drosophila melanogaster</td><td>Osi22</td><td>Osiris 22</td><td>CG8644|DmelCG8644</td><td>0</td><td>0</td></tr>
<tr><td>8810</td><td>41633</td><td>Drosophila melanogaster</td><td>wntD</td><td>wnt inhibitor of Dorsal</td><td>CG8458|DWnt-8|DWnt8|Dm DWnt8|DmelCG8458|Wnt8|WntD|wnt8</td><td>0</td><td>0</td></tr>
<tr><td>8811</td><td>41634</td><td>Drosophila melanogaster</td><td>CG8773</td><td>CG8773 gene product from transcript CG8773-RB</td><td>DmelCG8773</td><td>0</td><td>0</td></tr>
<tr><td>8812</td><td>41635</td><td>Drosophila melanogaster</td><td>CG8774</td><td>CG8774 gene product from transcript CG8774-RB</td><td>DmelCG8774</td><td>0</td><td>0</td></tr>
<tr><td>8813</td><td>41636</td><td>Drosophila melanogaster</td><td>CG32473</td><td>CG32473 gene product from transcript CG32473-RA</td><td>CG11688|CG8775|DmelCG32473|GM08240p</td><td>0</td><td>0</td></tr>
<tr><td>8814</td><td>41638</td><td>Drosophila melanogaster</td><td>CG8795</td><td>CG8795 gene product from transcript CG8795-RC</td><td>Dm CG8795|DmelCG8795|anon-WO0170980.193|anon-WO0170980.194</td><td>0</td><td>0</td></tr>
<tr><td>8815</td><td>41639</td><td>Drosophila melanogaster</td><td>CG8784</td><td>CG8784 gene product from transcript CG8784-RA</td><td>Dm CG8784|DmelCG8784|anon-WO0170980.190|anon-WO0170980.191</td><td>0</td><td>0</td></tr>
<tr><td>8816</td><td>41640</td><td>Drosophila melanogaster</td><td>Dic1</td><td>Dicarboxylate carrier 1</td><td>BcDNA:GH02431|CG8790|DmDic1p|DmelCG8790</td><td>0</td><td>0</td></tr>
<tr><td>8817</td><td>41641</td><td>Drosophila melanogaster</td><td>poly</td><td>CG9829 gene product from transcript CG9829-RB</td><td>0081/31|CG9829|DmelCG9829|did|did/alt1|l(3)05137|l(3)S008131</td><td>0</td><td>0</td></tr>
<tr><td>8818</td><td>41642</td><td>Drosophila melanogaster</td><td>CheA87a</td><td>Chemosensory protein A 87a</td><td>CG14379|DmelCG14379</td><td>0</td><td>0</td></tr>
<tr><td>8819</td><td>41643</td><td>Drosophila melanogaster</td><td>Lip3</td><td>CG8823 gene product from transcript CG8823-RA</td><td>CG8823|DmelCG8823|Dmlip3|LIP3|LIP3Dm|dLip3|lip3</td><td>0</td><td>0</td></tr>
<tr><td>8820</td><td>41644</td><td>Drosophila melanogaster</td><td>CG9813</td><td>CG9813 gene product from transcript CG9813-RC</td><td>DmelCG9813|ORE-7</td><td>0</td><td>0</td></tr>
<tr><td>8821</td><td>41645</td><td>Drosophila melanogaster</td><td>CG8870</td><td>CG8870 gene product from transcript CG8870-RA</td><td>DmelCG8870|SP38</td><td>0</td><td>0</td></tr>
<tr><td>8822</td><td>41646</td><td>Drosophila melanogaster</td><td>Droj2</td><td>DnaJ-like-2</td><td>CG8863|DmelCG8863|anon-WO0118547.404</td><td>0</td><td>0</td></tr>
<tr><td>8823</td><td>41647</td><td>Drosophila melanogaster</td><td>CG9799</td><td>CG9799 gene product from transcript CG9799-RA</td><td>DmelCG9799</td><td>0</td><td>0</td></tr>
<tr><td>8824</td><td>41648</td><td>Drosophila melanogaster</td><td>CCHa2</td><td>CCHamide-2</td><td>BcDNA:RE10132|CG14375|Dm-CCHa2|DmelCG14375</td><td>0</td><td>0</td></tr>
<tr><td>8825</td><td>41649</td><td>Drosophila melanogaster</td><td>CG14377</td><td>CG14377 gene product from transcript CG14377-RA</td><td>DmelCG14377</td><td>0</td><td>0</td></tr>
<tr><td>8826</td><td>41650</td><td>Drosophila melanogaster</td><td>CG9796</td><td>CG9796 gene product from transcript CG9796-RA</td><td>DmelCG9796</td><td>0</td><td>0</td></tr>
<tr><td>8827</td><td>41651</td><td>Drosophila melanogaster</td><td>yellow-e3</td><td>CG17045 gene product from transcript CG17045-RA</td><td>CG17045|DmelCG17045</td><td>0</td><td>0</td></tr>
<tr><td>8828</td><td>41652</td><td>Drosophila melanogaster</td><td>yellow-e2</td><td>CG17044 gene product from transcript CG17044-RB</td><td>CG17044|DmelCG17044</td><td>0</td><td>0</td></tr>
<tr><td>8829</td><td>41653</td><td>Drosophila melanogaster</td><td>yellow-e</td><td>CG9792 gene product from transcript CG9792-RA</td><td>CG9792|DmelCG9792</td><td>0</td><td>0</td></tr>
<tr><td>8830</td><td>41654</td><td>Drosophila melanogaster</td><td>Ir87a</td><td>Ionotropic receptor 87a</td><td>CG14376|DmelCG14376|IR87a</td><td>0</td><td>0</td></tr>
<tr><td>8831</td><td>41656</td><td>Drosophila melanogaster</td><td>yrt</td><td>yurt</td><td>CG9764|DmelCG9764|Nbl4|Yrt|dlhB|l(3)87Ek|l(3)E19|l(3)m112|m112</td><td>0</td><td>0</td></tr>
<tr><td>8832</td><td>41657</td><td>Drosophila melanogaster</td><td>CG14372</td><td>CG14372 gene product from transcript CG14372-RD</td><td>BP1073|CT34011|DmelCG14372</td><td>0</td><td>0</td></tr>
<tr><td>8833</td><td>41658</td><td>Drosophila melanogaster</td><td>CG12538</td><td>CG12538 gene product from transcript CG12538-RA</td><td>DmelCG12538</td><td>0</td><td>0</td></tr>
<tr><td>8834</td><td>41659</td><td>Drosophila melanogaster</td><td>CG14370</td><td>CG14370 gene product from transcript CG14370-RA</td><td>DmelCG14370</td><td>0</td><td>0</td></tr>
<tr><td>8835</td><td>41660</td><td>Drosophila melanogaster</td><td>CG14369</td><td>CG14369 gene product from transcript CG14369-RA</td><td>DmelCG14369</td><td>0</td><td>0</td></tr>
<tr><td>8836</td><td>41661</td><td>Drosophila melanogaster</td><td>CG9759</td><td>CG9759 gene product from transcript CG9759-RA</td><td>BcDNA:GH02603|DmelCG9759</td><td>0</td><td>0</td></tr>
<tr><td>8837</td><td>41662</td><td>Drosophila melanogaster</td><td>CG9757</td><td>CG9757 gene product from transcript CG9757-RA</td><td>DmelCG9757|GCR 20|GCR-20|GCR20|Pen50DE|anon-Pen50DE|pen50DE</td><td>0</td><td>0</td></tr>
<tr><td>8838</td><td>41663</td><td>Drosophila melanogaster</td><td>CG9269</td><td>CG9269 gene product from transcript CG9269-RA</td><td>DmelCG9269</td><td>0</td><td>0</td></tr>
<tr><td>8839</td><td>41665</td><td>Drosophila melanogaster</td><td>CG10841</td><td>CG10841 gene product from transcript CG10841-RA</td><td>DmelCG10841|anon-WO0140519.42</td><td>0</td><td>0</td></tr>
<tr><td>8840</td><td>41666</td><td>Drosophila melanogaster</td><td>sqd</td><td>squid</td><td>BcDNA:GM03761|CG16901|CG17791|CR33328|DmelCG16901|Dmel_CR33328|HRP40|Hrb87|Hrb87Fb|Hrp40|RRM3|Rbp3|Sqd|Squid|fs(3)00048|hnRNP 1|hrp40|l(3)EP3631|l(3)j4B4|l(3)j6E3|l(3)j8E7|rrm3</td><td>0</td><td>0</td></tr>
<tr><td>8841</td><td>41668</td><td>Drosophila melanogaster</td><td>Rbp4</td><td>RNA-binding protein 4</td><td>CG9654|DmelCG9654|RRM4|TSR|Tsr|rrm4|tsr</td><td>0</td><td>0</td></tr>
<tr><td>8842</td><td>41670</td><td>Drosophila melanogaster</td><td>B52</td><td>CG10851 gene product from transcript CG10851-RM</td><td>B52/SRp55|B52/dSRp55|CG10851|DmelCG10851|E(Dfd)|E726|MabB52|RRM8|RSp55|Rbp8|SR55|SRp55|dSRp55|l(3)s2249|rrm8</td><td>0</td><td>0</td></tr>
<tr><td>8843</td><td>41671</td><td>Drosophila melanogaster</td><td>Task6</td><td>CG9637 gene product from transcript CG9637-RC</td><td>CG9637|DmelCG9637|K2P-TASK6|anon-WO0109301.11|anon-WO0109301.53|anon-WO0109301.54|anon-WO0109301.55|anon-WO0109301.56|anon-WO0109301.57|anon-WO0109301.58|anon-WO010</td><td>0</td><td>0</td></tr>
<tr><td>8844</td><td>41672</td><td>Drosophila melanogaster</td><td>CG9588</td><td>CG9588 gene product from transcript CG9588-RA</td><td>9588|DmelCG9588</td><td>0</td><td>0</td></tr>
<tr><td>8845</td><td>41673</td><td>Drosophila melanogaster</td><td>lkb1</td><td>CG9374 gene product from transcript CG9374-RJ</td><td>CG9374|DmLKB1|DmelCG9374|LKB1|LKB1/PEUTZ JEGHERS KINASE|Lkb1|PAR-4|PAR4|STK11|anon-EST:Posey135|dLKB1|dlkb1|par-4</td><td>0</td><td>0</td></tr>
<tr><td>8846</td><td>41674</td><td>Drosophila melanogaster</td><td>omd</td><td>oocyte maintenance defects</td><td>CG9591|DmelCG9591|IntS5|Omd</td><td>0</td><td>0</td></tr>
<tr><td>8847</td><td>41675</td><td>Drosophila melanogaster</td><td>flfl</td><td>falafel</td><td>CG9351|DmelCG9351|PP4R3|l(3)L4179</td><td>0</td><td>0</td></tr>
<tr><td>8848</td><td>41676</td><td>Drosophila melanogaster</td><td>CG14367</td><td>CG14367 gene product from transcript CG14367-RA</td><td>DmelCG14367</td><td>0</td><td>0</td></tr>
<tr><td>8849</td><td>41677</td><td>Drosophila melanogaster</td><td>f-cup</td><td>flyers-cup</td><td>BcDNA:GH09045|CG9611|DmelCG9611</td><td>0</td><td>0</td></tr>
<tr><td>8850</td><td>41678</td><td>Drosophila melanogaster</td><td>ninaB</td><td>neither inactivation nor afterpotential B</td><td>CG9347|DRPE65|DmelCG9347|RPE65|anon-WO0148162.1|anon-WO0148163.1|beta-diox</td><td>0</td><td>0</td></tr>
<tr><td>8851</td><td>41679</td><td>Drosophila melanogaster</td><td>Adgf-D</td><td>Adenosine deaminase-related growth factor D</td><td>ADGF-D|ADGFD|CG9621|DmelCG9621|adgfd</td><td>0</td><td>0</td></tr>
<tr><td>8852</td><td>41680</td><td>Drosophila melanogaster</td><td>Adgf-C</td><td>Adenosine deaminase-related growth factor C</td><td>ADGF-C|CG9345|DmelCG9345|adgfc</td><td>0</td><td>0</td></tr>
<tr><td>8853</td><td>41682</td><td>Drosophila melanogaster</td><td>CG9602</td><td>CG9602 gene product from transcript CG9602-RA</td><td>BcDNA:GH25305|DmelCG9602</td><td>0</td><td>0</td></tr>
<tr><td>8854</td><td>41686</td><td>Drosophila melanogaster</td><td>CG9312</td><td>CG9312 gene product from transcript CG9312-RA</td><td>CT26517|DmelCG9312</td><td>0</td><td>0</td></tr>
<tr><td>8855</td><td>41687</td><td>Drosophila melanogaster</td><td>Cht5</td><td>CG9307 gene product from transcript CG9307-RA</td><td>CG9307|DmCHT5|DmelCG9307</td><td>0</td><td>0</td></tr>
<tr><td>8856</td><td>41688</td><td>Drosophila melanogaster</td><td>CG9297</td><td>CG9297 gene product from transcript CG9297-RB</td><td>DmelCG9297</td><td>0</td><td>0</td></tr>
<tr><td>8857</td><td>41689</td><td>Drosophila melanogaster</td><td>CG9288</td><td>CG9288 gene product from transcript CG9288-RA</td><td>DmelCG9288|Dromel_CG9288_FBtr0082829_pyruvatekinasedomain_mORF</td><td>0</td><td>0</td></tr>
<tr><td>8858</td><td>41690</td><td>Drosophila melanogaster</td><td>CG9286</td><td>CG9286 gene product from transcript CG9286-RA</td><td>DmelCG9286</td><td>0</td><td>0</td></tr>
<tr><td>8859</td><td>41693</td><td>Drosophila melanogaster</td><td>Mst87F</td><td>Male-specific RNA 87F</td><td>BEST:GH23081|CG17956|DmelCG17956|Mst|bs35f12.y1|mst(3)87F|mst(3)gl-9|mst(3)gl9|mst-9|mst87F</td><td>0</td><td>0</td></tr>
<tr><td>8860</td><td>41694</td><td>Drosophila melanogaster</td><td>Nsf2</td><td>NEM-sensitive fusion protein 2</td><td>CG33101|CG9931|DmNSF2|DmelCG33101|NSF-2|NSF2|Nsf-2|dNSF|dNSF-2|dNSF2|dNsf2|dnsf2</td><td>0</td><td>0</td></tr>
<tr><td>8861</td><td>41695</td><td>Drosophila melanogaster</td><td>CG14362</td><td>CG14362 gene product from transcript CG14362-RA</td><td>DmelCG14362</td><td>0</td><td>0</td></tr>
<tr><td>8862</td><td>41697</td><td>Drosophila melanogaster</td><td>ems</td><td>empty spiracles</td><td>CG2988|DmelCG2988|E4|Ems|W13|ant</td><td>0</td><td>0</td></tr>
<tr><td>8863</td><td>41698</td><td>Drosophila melanogaster</td><td>Art9</td><td>Arginine methyltransferase 9</td><td>CG9929|DART9|DmelCG9929</td><td>0</td><td>0</td></tr>
<tr><td>8864</td><td>41699</td><td>Drosophila melanogaster</td><td>Art6</td><td>Arginine methyltransferase 6</td><td>CG9927|DART6|DmelCG9927</td><td>0</td><td>0</td></tr>
<tr><td>8865</td><td>41700</td><td>Drosophila melanogaster</td><td>CG9926</td><td>CG9926 gene product from transcript CG9926-RA</td><td>DmelCG9926</td><td>0</td><td>0</td></tr>
<tr><td>8866</td><td>41701</td><td>Drosophila melanogaster</td><td>Ipp</td><td>Inositol polyphosphate 1-phosphatase</td><td>CG3028|DmelCG3028|anon-WO02059370.48|ipp</td><td>0</td><td>0</td></tr>
<tr><td>8867</td><td>41702</td><td>Drosophila melanogaster</td><td>CG9925</td><td>CG9925 gene product from transcript CG9925-RA</td><td>DmelCG9925</td><td>0</td><td>0</td></tr>
<tr><td>8868</td><td>41703</td><td>Drosophila melanogaster</td><td>Orc2</td><td>Origin recognition complex subunit 2</td><td>CG3041|DmORC 2|DmORC2|DmOrc2p|DmelCG3041|ORC|ORC2|Orc-2|Orc2p|fs(3)293|fs(3)293-19|k43|l(3)88Ab|l(3)A5|l(3)K43|l(3)SG44|l(3)dsl5|l(3)k43|orc2|rDmORC</td><td>0</td><td>0</td></tr>
<tr><td>8869</td><td>41704</td><td>Drosophila melanogaster</td><td>rdx</td><td>roadkill</td><td>BEST:GM07940|CG10235|CG12537|CG9924|D-SPOP|DmelCG12537|Dmel_CG9924|Dromel_CG9924_FBtr0082871_rdx_mORF|HIB|Rdx|Spop|anon-WO0118547.577|hib|l(3)03477|l(3)2004|l(3)88Aa|l(3)A6|</td><td>0</td><td>0</td></tr>
<tr><td>8870</td><td>41706</td><td>Drosophila melanogaster</td><td>Cyp6d5</td><td>CG3050 gene product from transcript CG3050-RA</td><td>6d5|CG3050|DmelCG3050|cyp6d5</td><td>0</td><td>0</td></tr>
<tr><td>8871</td><td>41707</td><td>Drosophila melanogaster</td><td>CG3061</td><td>CG3061 gene product from transcript CG3061-RA</td><td>DmelCG3061</td><td>0</td><td>0</td></tr>
<tr><td>8872</td><td>41708</td><td>Drosophila melanogaster</td><td>CG9922</td><td>CG9922 gene product from transcript CG9922-RA</td><td>BcDNA:RE55111|DmelCG9922|NEST:bs28g09</td><td>0</td><td>0</td></tr>
<tr><td>8873</td><td>41709</td><td>Drosophila melanogaster</td><td>foxo</td><td>forkhead box, sub-group O</td><td>3143|Afx|CG3143|Dfoxo|DmQ95V5|DmelCG3143|FKHR|FOXO|FoxO|Foxo|Q95V55|anon-WO0118547.374|cg3143|dFOXO|dFoxO|dFoxo|dfoxo|fd88A</td><td>0</td><td>0</td></tr>
<tr><td>8874</td><td>41710</td><td>Drosophila melanogaster</td><td>Npc2b</td><td>Niemann-Pick type C-2b</td><td>CG3153|CT10556|DmML2|DmelCG3153|npc2b</td><td>0</td><td>0</td></tr>
<tr><td>8875</td><td>41711</td><td>Drosophila melanogaster</td><td>CCHa1</td><td>CCHamide-1</td><td>CG14358|Dm-CCHa1|DmelCG14358</td><td>0</td><td>0</td></tr>
<tr><td>8876</td><td>41712</td><td>Drosophila melanogaster</td><td>CG9920</td><td>CG9920 gene product from transcript CG9920-RA</td><td>BEST:GH20473|DmelCG9920|anon-WO0140519.48</td><td>0</td><td>0</td></tr>
<tr><td>8877</td><td>41713</td><td>Drosophila melanogaster</td><td>Pk1r</td><td>Pyrokinin 1 receptor</td><td>CG9918|DmelCG9918|anon-WO0170980.112|anon-WO0170980.113</td><td>0</td><td>0</td></tr>
</table>

8878	41714	Drosophila melanogaster	CG12402	CG12402 gene product from transcript CG12402-RA	DmelCG12402	0	0
8879	41715	Drosophila melanogaster	Or88a	Odorant receptor 88a	88A.1|88a|CG14360|DOR99|DmelCG14360	0	0
8880	41716	Drosophila melanogaster	CG14357	CG14357 gene product from transcript CG14357-RB	DmelCG14357	0	0
8881	41717	Drosophila melanogaster	Kif19A	CG9913 gene product from transcript CG9913-RB	CG9913|DmKIF19A|DmelCG9913|KIF19A	0	0
8882	41718	Drosophila melanogaster	Abi	Abelson interacting protein	Abi-1|Ablphilin|CG9749|DmelCG9749|abi|dAbi	0	0
8883	41719	Drosophila melanogaster	twf	twinfilin	A6|CG3172|DmelCG3172|Twf	0	0
8884	41720	Drosophila melanogaster	140up	upstream of RpII140	CG9852|DmRP140-upstream|DmRP140up|DmelCG9852|l(3)88Ba|l(3)Z6	0	0
8885	41721	Drosophila melanogaster	RpII140	RNA polymerase II 140kD subunit	CG3180|DmRP140|DmelCG3180|H5|II|Pol II|Pol IIc|PolII|PolIIc|RNA Pol II|RNA Pol IIc|RNA pol II|RNA polII|RNAP|RNAP II|RNApolII|RP140|RPB2_DROME|RPII140|RpII140[wimp]|Rpb2|RpII140|l(3)RpIII140|p	0	0
8886	41722	Drosophila melanogaster	CG14356	CG14356 gene product from transcript CG14356-RA	DmelCG14356	0	0
8887	41723	Drosophila melanogaster	CG14355	CG14355 gene product from transcript CG14355-RA	DmelCG14355	0	0
8888	41724	Drosophila melanogaster	CG9722	CG9722 gene product from transcript CG9722-RA	DmelCG9722	0	0
8889	41725	Drosophila melanogaster	CG3199	CG3199 gene product from transcript CG3199-RA	DmelCG3199	0	0
8890	41726	Drosophila melanogaster	DopR	Dopamine receptor	CG9652|D-Dop1|DA1|DmDOP1|DmDop1|Dmdop1|DmelCG9652|DopR1|DopR35F|dDA1|dDA1/DmDOP1|dumb	0	0
8891	41727	Drosophila melanogaster	CG9649	CG9649 gene product from transcript CG9649-RA	DmelCG9649|SP55	0	0
8892	41728	Drosophila melanogaster	CG31326	CG31326 gene product from transcript CG31326-RA	CG9645|DmelCG31326|SPH144|cg9645	0	0
8893	41729	Drosophila melanogaster	CG9631	CG9631 gene product from transcript CG9631-RA	BcDNA:GH08420|DmelCG9631|SP60	0	0
8894	41730	Drosophila melanogaster	CG9624	CG9624 gene product from transcript CG9624-RA	DmelCG9624	0	0
8895	41731	Drosophila melanogaster	CG9616	CG9616 gene product from transcript CG9616-RB	DmelCG9616	0	0
8896	41733	Drosophila melanogaster	Aats-met	Methionyl-tRNA synthetase	CG31322|CG8684|CG9612|DmelCG31322|MRS|MetRS	0	0
8897	41734	Drosophila melanogaster	CG14840	CG14840 gene product from transcript CG14840-RA	DmelCG14840	0	0
8898	41735	Drosophila melanogaster	CG14841	CG14841 gene product from transcript CG14841-RA	BcDNA:AT04690|DmelCG14841	0	0
8899	41736	Drosophila melanogaster	CG14839	CG14839 gene product from transcript CG14839-RA	DmelCG14839	0	0
8900	41737	Drosophila melanogaster	trx	trithorax	4720|4733|CG8651|DMTRXIII|DmelCG8651|NR0A5|R-bx|Rg-bx|TRX|Trx|Trx-G|l(3)bx[v]|l(3)j14A6|l(3)j3A2|l(3)s5452|trx-g|trxI and trxII	0	0
8901	41738	Drosophila melanogaster	CG12207	CG12207 gene product from transcript CG12207-RB	DmelCG12207	0	0
8902	41739	Drosophila melanogaster	CG3259	CG3259 gene product from transcript CG3259-RA	DmelCG3259	0	0
8903	41740	Drosophila melanogaster	su(Hw)	suppressor of Hairy wing	CG8573|DmelCG8573|SU(HW)|SUHW|Su(HW)|Su(Hw)|Su(hw)|Su[Hw]|su(HW)|su(hw)|suHw	0	0
8904	41741	Drosophila melanogaster	RpII15	RNA polymerase II 15kD subunit	CG3284|DmelCG3284|H5|Pol II|Pol-II|PolII|RNA Pol II|RNA pol II|RNA polII|RNAP|RNAP II|RNApolII|RPII15|RpII215|dRPB9|l(3)88Be|l(3)Z23|pol II|polII	0	0
8905	41742	Drosophila melanogaster	Crz	Corazonin	CG3302|COR|CRZ|Cora|Dm-Crz|DmelCG3302|Drm-COR|cor|crz	0	0
8906	41743	Drosophila melanogaster	pr-set7	CG3307 gene product from transcript CG3307-RC	CG3307|DmelCG3307|PR-SET7|PR-Set7|PR/SET07|Pr-set-7|Set7|Set8|dSET8|dSet8|l(3)neo41|pr-Set7	0	0
8907	41744	Drosophila melanogaster	CG8538	CG8538 gene product from transcript CG8538-RD	DmelCG8538	0	0
8908	41745	Drosophila melanogaster	CG3321	CG3321 gene product from transcript CG3321-RC	DmelCG3321|anon-88Bd|c550	0	0
8909	41746	Drosophila melanogaster	spn-B	spindle B	CG3325|DmelCG3325|Rad51|SpnB|XRCC3|rad51-like|spnB	0	0
8910	41747	Drosophila melanogaster	NK7.1	CG8524 gene product from transcript CG8524-RA	CG8524|DmelCG8524|NK-7.1	0	0
8911	41748	Drosophila melanogaster	soti	scotti	CG8489|DmelCG8489	0	0
8912	41749	Drosophila melanogaster	cv-c	crossveinless c	CG14845|CG14846|CG14847|CG14848|CG14849|CG31319|CG31330|CG34389|CG8480|DmelCG34389|Dmel_CG14846|Dmel_CG14847|Dmel_CG31319|Dmel_CG31330|RhoGAP88C|l(3)06951|rhoGAP8	0	0
8913	41756	Drosophila melanogaster	HtrA2	CG8464 gene product from transcript CG8464-RA	CG8464|DmHtrA2|DmelCG8464|HtrA2/Omi|Omi|dOmi|domi|htrA2|omi	0	0
8914	41757	Drosophila melanogaster	mRpL11	mitochondrial ribosomal protein L11	BcDNA:RE67638|CG3351|DmelCG3351|L11	0	0
8915	41758	Drosophila melanogaster	CG8461	CG8461 gene product from transcript CG8461-RA	DmelCG8461	0	0
8916	41759	Drosophila melanogaster	Cyp313a1	CG3360 gene product from transcript CG3360-RA	313a1|313a1p|CG3360|Cyp313a|Cyp313a1p|DmelCG3360	0	0
8917	41760	Drosophila melanogaster	Pde6	Phosphodiesterase 6	CG8279|DmPDE5/6|DmPDE6|DmelCG8279|PDE6	0	0
8918	41761	Drosophila melanogaster	CG14854	CG14854 gene product from transcript CG14854-RB	DmelCG14854	0	0
8919	41762	Drosophila melanogaster	CG14850	CG14850 gene product from transcript CG14850-RA	DmelCG14850	0	0
8920	41763	Drosophila melanogaster	CG14851	CG14851 gene product from transcript CG14851-RA	DmelCG14851	0	0
8921	41764	Drosophila melanogaster	CG8087	CG8087 gene product from transcript CG8087-RA	DmelCG8087	0	0
8922	41765	Drosophila melanogaster	CG14852	CG14852 gene product from transcript CG14852-RA	DmelCG14852	0	0
8923	41766	Drosophila melanogaster	CG8066	CG8066 gene product from transcript CG8066-RA	DmelCG8066	0	0
8924	41767	Drosophila melanogaster	Cys	Cystatin-like	BcDNA:RH72992|CG8050|DmelCG8050|anon-EST:Liang-2.34|clone 2.34	0	0
8925	41768	Drosophila melanogaster	CG7987	CG7987 gene product from transcript CG7987-RA	DmelCG7987|anon-WO0118547.245	0	0
8926	41770	Drosophila melanogaster	stumps	CG31317 gene product from transcript CG31317-RC	0837/10|C|CG18485|CG31317|CG3375|DOF|DmelCG31317|Dof|Dof/stumps/heartbroken|Hbr|Hbr/Dof|P1740|anon-EST:CL47|anon-estC|dof|dof/hbr|dof/hbr/sms|gene C|hbr|heartbroken/dof|i21|i28|l(3)099	0	0
8927	41771	Drosophila melanogaster	CG14853	CG14853 gene product from transcript CG14853-RB	DmelCG14853	0	0
8928	41772	Drosophila melanogaster	put	punt	Act-r|Atr|Atr-II|Atr88CD|AtrII|CG7904|DmelCG7904|PUNT|Punt|Put|STK-C|TGF-B|TGF-beta|Tgf-r|atrII|dlhC|l(3)10460|l(3)j5A5|pun	0	0
8929	41773	Drosophila melanogaster	His4r	Histone H4 replacement	BcDNA:RH52884|CG3379|DmelCG3379|H4r|His4-88CD|His4R	0	0
8930	41774	Drosophila melanogaster	Cad88C	CG3389 gene product from transcript CG3389-RA	CG3389|CT11389|DCad88C|Dm Cad88C|DmelCG3389|cad88C	0	0
8931	41775	Drosophila melanogaster	CG7886	CG7886 gene product from transcript CG7886-RA	DmelCG7886|anon-WO0140519.200	0	0
8932	41776	Drosophila melanogaster	l(3)L1231	lethal (3) L1231	BEST:LD14744|CG7832|DmelCG7832|LD14744	0	0
8933	41777	Drosophila melanogaster	CG3505	CG3505 gene product from transcript CG3505-RA	DmelCG3505|SPH9|proPO-AE	0	0
8934	41778	Drosophila melanogaster	CG3508	CG3508 gene product from transcript CG3508-RB	DmelCG3508	0	0
8935	41779	Drosophila melanogaster	Rad17	CG7825 gene product from transcript CG7825-RA	CG7825|DmRad17/24|DmelCG7825	0	0
8936	41780	Drosophila melanogaster	CG3509	CG3509 gene product from transcript CG3509-RA	DmelCG3509	0	0
8937	41783	Drosophila melanogaster	kibra	kibra ortholog	CG12600|CG33967|CG7552|DmelCG33967|Kibra|ORE-2|RE26350|l(3)02404|l(3)j5C3|l(3)neo42	0	0
8938	41784	Drosophila melanogaster	CG7530	CG7530 gene product from transcript CG7530-RB	DmelCG7530	0	0
8939	41785	Drosophila melanogaster	eff	effete	3527|6535|CG7425|DmelCG7425|E(Pc)88D|EFF|Eff|SS3-3|UBC4|UBCD-1|UBCD1|Ubc-D1|UbcD|UbcD1|anon-WO0118547.343|fs(3)01295|l(3)01462|l(3)j2C8|l(3)s1782|mei68|ms(3)01295|ms(3)1|ms(3)3|ms(	0	0
8940	41786	Drosophila melanogaster	smp-30	Senescence marker protein-30	CG7390|CT22723|Dca|DmelCG7390|Smp-30	0	0
8941	41787	Drosophila melanogaster	CG7362	CG7362 gene product from transcript CG7362-RA	DmelCG7362	0	0
8942	41788	Drosophila melanogaster	CG43162	CG43162 gene product from transcript CG43162-RA	CG33330|CG3563|DmelCG43162|Dmel_CG33330|Dmel_CG3563|anon-WO0153538.4|anon-WO0153538.5	0	0
8943	41789	Drosophila melanogaster	CG14855	CG14855 gene product from transcript CG14855-RA	DmelCG14855	0	0
8944	41790	Drosophila melanogaster	CG14856	CG14856 gene product from transcript CG14856-RA	DmelCG14856	0	0
8945	41791	Drosophila melanogaster	CG14857	CG14857 gene product from transcript CG14857-RA	DmelCG14857	0	0
8946	41795	Drosophila melanogaster	CG3610	CG3610 gene product from transcript CG3610-RA	DmelCG3610	0	0
8947	41796	Drosophila melanogaster	CG17304	CG17304 gene product from transcript CG17304-RA	DmelCG17304	0	0
8948	41797	Drosophila melanogaster	CG3631	CG3631 gene product from transcript CG3631-RD	DmelCG3631	0	0
8949	41798	Drosophila melanogaster	Rrp6	CG7292 gene product from transcript CG7292-RB	CG7292|DmRrp6|DmelCG7292|dRrp6|rrp6	0	0
8950	41800	Drosophila melanogaster	CG3731	CG3731 gene product from transcript CG3731-RA	DmelCG3731	0	0
8951	41801	Drosophila melanogaster	CycC	Cyclin C	CG1C_DROME|CG7281|CYCC|Cln3|Cyc-C|DmcycC|DmelCG7281|SRB11|cycC|cyclinC|dTRAP33	0	0
8952	41802	Drosophila melanogaster	CG7265	CG7265 gene product from transcript CG7265-RA	DmelCG7265	0	0
8953	41803	Drosophila melanogaster	CG14860	CG14860 gene product from transcript CG14860-RB	DmelCG14860	0	0
8954	41804	Drosophila melanogaster	CG7262	CG7262 gene product from transcript CG7262-RA	Cg7262|DmelCG7262	0	0
8955	41805	Drosophila melanogaster	CG3817	CG3817 gene product from transcript CG3817-RA	DmelCG3817	0	0
8956	41806	Drosophila melanogaster	VhaPPA1-2	Vacuolar H[+] ATPase subunit PPA1-2	CG7026|DmelCG7026|V-ATPase|vhaPPA1-2	0	0
8957	41807	Drosophila melanogaster	RpS5b	Ribosomal protein S5b	142157_at|CG7014|DmelCG7014	0	0
8958	41809	Drosophila melanogaster	CG3837	CG3837 gene product from transcript CG3837-RA	DmelCG3837	0	0
8959	41810	Drosophila melanogaster	CG14861	CG14861 gene product from transcript CG14861-RA	DmelCG14861	0	0
8960	41811	Drosophila melanogaster	RpL10Aa	Ribosomal protein L10Aa	BcDNA:AT11516|CG3843|DmelCG3843	0	0
8961	41815	Drosophila melanogaster	CG6974	CG6974 gene product from transcript CG6974-RA	DmelCG6974	0	0
8962	41816	Drosophila melanogaster	CG6966	CG6966 gene product from transcript CG6966-RB	BcDNA:AT05246|DmelCG6966	0	0
8963	41817	Drosophila melanogaster	CG42788	CG42788 gene product from transcript CG42788-RC	CG18442|CG31304|CG6934|CG6960|DmelCG42788|Dmel_CG31304|Dmel_CG6934|anon-WO0118547.79	0	0
8964	41820	Drosophila melanogaster	CG6912	CG6912 gene product from transcript CG6912-RA	DmelCG6912|anon-WO0118547.571|anon-WO0140519.208	0	0
8965	41821	Drosophila melanogaster	CG3984	CG3984 gene product from transcript CG3984-RA	DmelCG3984	0	0
8966	41822	Drosophila melanogaster	CG3987	CG3987 gene product from transcript CG3987-RA	DmelCG3987|anon-WO0140519.215	0	0
8967	41823	Drosophila melanogaster	CG6904	CG6904 gene product from transcript CG6904-RC	CG6940|DmelCG6904|anon-WO0118547.108	0	0
8968	41824	Drosophila melanogaster	Mf	Myofilin	BcDNA:GH23305|CG6803|DmMf|DmelCG6803|Zeelin1|z1	0	0
8969	41825	Drosophila melanogaster	Gyc88E	Guanylyl cyclase at 88E	CG4154|DmelCG4154|GYC|Gyc-88E	0	0
8970	41826	Drosophila melanogaster	CG6752	CG6752 gene product from transcript CG6752-RA	DmelCG6752	0	0
8971	41829	Drosophila melanogaster	Spn88Eb	Serpin 88Eb	CG6687|DmelCG6687	0	0
8972	41830	Drosophila melanogaster	CG4203	CG4203 gene product from transcript CG4203-RA	DmelCG4203|FBgn0038300	0	0
8973	41831	Drosophila melanogaster	CG6654	CG6654 gene product from transcript CG6654-RA	DmelCG6654	0	0
8974	41832	Drosophila melanogaster	CG4210	CG4210 gene product from transcript CG4210-RC	BcDNA:SD13446|BcDNA:SD26038|DmelCG4210|FBgn 38302	0	0
8975	41833	Drosophila melanogaster	SIDL	Shal Interactor of Di-Leucine Motif	CG6623|DmelCG6623	0	0
8976	41834	Drosophila melanogaster	CG12241	CG12241 gene product from transcript CG12241-RA	DmelCG12241	0	0
8977	41835	Drosophila melanogaster	CG31344	CG31344 gene product from transcript CG31344-RA	CG6572|DmelCG31344|anon-WO0118547.636	0	0
8978	41836	Drosophila melanogaster	Caf1	Chromatin assembly factor 1 subunit	154659_at|55|CAF-1|CAF1|CAF1-55|CAF1p55|CG4236|Caf-1|Caf1/p55|Caf1p55|DmelCG4236|MSI1/RbAp48/CAC3/LIN-53|NURF|NURF-55|NURF55|Nurf|Nurf 55|Nurf-55|Nurf55|P55|RbAp48|caf-1|caf1|ca	0	0
8979	41837	Drosophila melanogaster	Art3	Arginine methyltransferase 3	CG6563|DART3|DmelCG6563	0	0
8980	41838	Drosophila melanogaster	mRpS10	mitochondrial ribosomal protein S10	BcDNA:GM25447|CG4247|DmelCG4247|MRP-S10|MrpS10	0	0
8981	41839	Drosophila melanogaster	tefu	telomere fusion	ATM|CG6535|DmelCG6535|Tefu|atm|atm/tefu|dATM|tef	0	0
8982	41840	Drosophila melanogaster	Hsc70-4	Heat shock protein cognate 4	BAP74|CG4264|DmelCG4264|E(csp)1545|E(nd)195|HSC-70|HSC4|HSC70|HSC70-4|Hsc-4|Hsc4|Hsc4p|Hsc70|Hsp70|anon-WO0118547.237|bs17d06.y1|dhsc70|hsc4|hsc70|hsc70-4|hsp70|i190|l(3)03550|	0	0
8983	41841	Drosophila melanogaster	CG6499	CG6499 gene product from transcript CG6499-RB	DmelCG6499	0	0
8984	41842	Drosophila melanogaster	CG42404	CG42404 gene product from transcript CG42404-RE	BcDNA:SD12744|CG31154|CG34128|CG4285|DmelCG42404|Dmel_CG34128|Dmel_CG4285	0	0
8985	41843	Drosophila melanogaster	Su(var)3-9	Suppressor of variegation 3-9	CG6476|DmelCG6476|E(var)3-2|SU(VAR)3-9|SUV39|SUV39H1|Su(Var)3-9|Su(var)|Su(var) 3-9|Su(var)3-902|Su(var)3-9c|Su(var)306|Su(var)310|Su(var)328|Su-var(3)9|Su-var(3)902|SuVar3-9|Suv 3-9|Suvar(3)(	0	0
8986	41844	Drosophila melanogaster	Set	CG4299 gene product from transcript CG4299-RA	CG4299|DmelCG4299|SET	0	0
8987	41845	Drosophila melanogaster	Oscp	Oligomycin sensitivity-conferring protein	ATPO_DROME|CG4307|DmelCG4307|OSCP	0	0
8988	41846	Drosophila melanogaster	CG14864	CG14864 gene product from transcript CG14864-RB	BcDNA:AT13896|DmelCG14864	0	0

8989	41847	Drosophila melanogaster	CG4334	CG4334 gene product from transcript CG4334-RB	DmelCG4334	0	0
8990	41848	Drosophila melanogaster	Cp190	Centrosomal protein 190kD	AbS1-4|Bx-63|Bx63|CEN190|CG6384|CP-190|CP-60|CP190|Cen185|Cen190|DMAP-190|DMAP190|Dmap190|DmelCG6384|E(mod)4-1|Map190|Rb188|dmap190	0	0
8991	41849	Drosophila melanogaster	CG4338	CG4338 gene product from transcript CG4338-RA	DmelCG4338	0	0
8992	41850	Drosophila melanogaster	MRG15	CG6363 gene product from transcript CG6363-RA	CG6363|DmMRG15|DmelCG6363|Dmrg15|Mrg15|dMRG15|dMrg15|l(3)j6A3	0	0
8993	41851	Drosophila melanogaster	l(3)neo43	lethal (3) neo43	CG14865|DmelCG14865	0	0
8994	41852	Drosophila melanogaster	Tm1	Tropomyosin 1	1305/10|2299|BcDNA:GH09289|BcDNA:LD37158|BcDNA:SD21996|CG4898|Dm Tm1|Dm TmH33|Dm TmH34|DmTm1|DmelCG4898|PmI|TMII|Tm|TmH-33|TmH-34|TmH33|TmH34|TmII|Tmr33|Tmr34|TnH|T	0	0
8995	41853	Drosophila melanogaster	Tm2	Tropomyosin 2	CG4843|DROTROPI1|DmelCG4843|Ifm(3)3|Ifm(3)5|Ifm-TmI|TM2|Tm|Tm1|Tm127|TmI|Tn-H|dro Tm|l(3)nc99Eb|mTmI	0	0
8996	41854	Drosophila melanogaster	CG14866	CG14866 gene product from transcript CG14866-RA	DmelCG14866	0	0
8997	41855	Drosophila melanogaster	CG6276	CG6276 gene product from transcript CG6276-RA	DmelCG6276	0	0
8998	41857	Drosophila melanogaster	CG6236	CG6236 gene product from transcript CG6236-RB	DmelCG6236	0	0
8999	41858	Drosophila melanogaster	ea	easter	CG4920|DmelCG4920|EA|SP24|c-SP24	0	0
9000	41859	Drosophila melanogaster	mRpL9	mitochondrial ribosomal protein L9	CG31478|CG4923|DmelCG31478|L9	0	0
9001	41860	Drosophila melanogaster	FK506-bp1	FK506-binding protein 1	CG6226|DmelCG6226|FK506bp1|FKBP39|FKbp39|lFK506-bp1	0	0
9002	41861	Drosophila melanogaster	Sra-1	specifically Rac1-associated protein 1	CG4931|CYFIP|CYFIP1/2|DSra-1|DmelCG4931|PIR121|PIR121/Sra-1|Sra|Sra-1/CYFIP|Sra1|Sra1/PIR121|dSra|sra-1|sra1	0	0
9003	41862	Drosophila melanogaster	Aats-ser	Seryl-tRNA synthetase	Aat-Ser|CG4938|DmSRS2|DmelCG4938|SRS|SerRS|seryl-tRNA synthetase	0	0
9004	41863	Drosophila melanogaster	CG6218	CG6218 gene product from transcript CG6218-RA	DmelCG6218	0	0
9005	41864	Drosophila melanogaster	Surf4	Surfeit 4	CG6202|DmelCG6202|SURF-4|Surf-4	0	0
9006	41865	Drosophila melanogaster	CG31301	CG31301 gene product from transcript CG31301-RA	CG12258|DmelCG31301	0	0
9007	41866	Drosophila melanogaster	CG6196	CG6196 gene product from transcript CG6196-RA	BcDNA:RH37427|DmelCG6196	0	0
9008	41867	Drosophila melanogaster	CG5038	CG5038 gene product from transcript CG5038-RA	DmelCG5038	0	0
9009	41868	Drosophila melanogaster	CG6194	CG6194 gene product from transcript CG6194-RA	DmAtg4-like|DmelCG6194	0	0
9010	41869	Drosophila melanogaster	CG5044	CG5044 gene product from transcript CG5044-RB	CT16187|DmelCG5044	0	0
9011	41870	Drosophila melanogaster	ldlCp	ldlCp-related protein	CG6177|DmelCG6177|ldl-Cp	0	0
9012	41871	Drosophila melanogaster	Trax	Translin associated factor X	CG5063|DmelCG5063|trax	0	0
9013	41872	Drosophila melanogaster	CG6171	CG6171 gene product from transcript CG6171-RB	DmelCG6171|anon-Becker2	0	0
9014	41873	Drosophila melanogaster	CG34404	CG34404 gene product from transcript CG34404-RD	CG18496|CG6156|DmelCG34404|Dmel_CG18496|Dmel_CG6156|anon-FF	0	0
9015	41875	Drosophila melanogaster	CG14868	CG14868 gene product from transcript CG14868-RA	DmelCG14868	0	0
9016	41876	Drosophila melanogaster	CG5073	CG5073 gene product from transcript CG5073-RA	DmelCG5073	0	0
9017	41877	Drosophila melanogaster	CG6136	CG6136 gene product from transcript CG6136-RC	DmelCG6136	0	0
9018	41879	Drosophila melanogaster	h-cup	heineken-cup	CG6130|DmelCG6130|anon-WO0140519.172	0	0
9019	41880	Drosophila melanogaster	CG43073	CG43073 gene product from transcript CG43073-RF	CG14867|CG31302|CG5143|CG5152|DmelCG43073|Dmel_CG14867|Dmel_CG31302	0	0
9020	41882	Drosophila melanogaster	CG6125	CG6125 gene product from transcript CG6125-RB	DmelCG6125	0	0
9021	41883	Drosophila melanogaster	Atx2	Ataxin-2	ATX2|Atxain-2|BEST:GH27029|CG5166|CG5166a|Datx2|DmelCG5166|GH27029|SCA2|Sca2|anon-EST:fe2E1|anon-WO0118547.351|anon-WO0140519.1|atx2|dAtx2|dSCA2|l(3)06490	0	0
9022	41884	Drosophila melanogaster	CG6118	CG6118 gene product from transcript CG6118-RC	DmelCG6118	0	0
9023	41885	Drosophila melanogaster	Act88F	Actin 88F	Act(88F)|Act88-F|Act88f|Actin|CG5178|DmelCG5178|E|Ifm(3)7|act 88F|act88F|actin|rsd	0	0
9024	41887	Drosophila melanogaster	CG14869	CG14869 gene product from transcript CG14869-RB	CG6107|CT19033|DmelCG14869	0	0
9025	41888	Drosophila melanogaster	CG14870	CG14870 gene product from transcript CG14870-RA	DmelCG14870	0	0
9026	41889	Drosophila melanogaster	Rh6	Rhodopsin 6	CG5192|DMELRH6|Dm Rh6|DmelCG5192|R8|RH6|Rh|rh6	0	0
9027	41890	Drosophila melanogaster	CG14871	CG14871 gene product from transcript CG14871-RB	DmelCG14871	0	0
9028	41891	Drosophila melanogaster	CG5205	CG5205 gene product from transcript CG5205-RA	DmelCG5205|cg5205	0	0
9029	41892	Drosophila melanogaster	CG5213	CG5213 gene product from transcript CG5213-RA	DmelCG5213|cg5213	0	0
9030	41893	Drosophila melanogaster	CG14872	CG14872 gene product from transcript CG14872-RB	DmelCG14872	0	0
9031	41894	Drosophila melanogaster	CG18522	CG18522 gene product from transcript CG18522-RA	CT42272|DmAO1|DmelCG18522	0	0
9032	41895	Drosophila melanogaster	CG18519	CG18519 gene product from transcript CG18519-RA	DmAO2|DmelCG18519	0	0
9033	41896	Drosophila melanogaster	CG6045	CG6045 gene product from transcript CG6045-RA	DmAO3|DmelCG6045	0	0
9034	41897	Drosophila melanogaster	CG18516	CG18516 gene product from transcript CG18516-RA	DmAO4|DmelCG18516	0	0
9035	41899	Drosophila melanogaster	pxb	CG33207 gene product from transcript CG33207-RB	BcDNA:RE16319|CG14873|CG14874|CG33207|CG33207/Pxb|DmelCG33207|Pxb	0	0
9036	41900	Drosophila melanogaster	CG5399	CG5399 gene product from transcript CG5399-RA	DmelCG5399	0	0
9037	41901	Drosophila melanogaster	CG5404	CG5404 gene product from transcript CG5404-RA	DmelCG5404	0	0
9038	41902	Drosophila melanogaster	CG4520	CG4520 gene product from transcript CG4520-RA	DmelCG4520	0	0
9039	41903	Drosophila melanogaster	CG12784	CG12784 gene product from transcript CG12784-RA	DmelCG12784	0	0
9040	41904	Drosophila melanogaster	CG5623	CG5623 gene product from transcript CG5623-RA	BcDNA:AT10995|DmelCG5623	0	0
9041	41905	Drosophila melanogaster	CG4525	CG4525 gene product from transcript CG4525-RA	DmelCG4525	0	0
9042	41906	Drosophila melanogaster	CG5614	CG5614 gene product from transcript CG5614-RA	DmelCG5614	0	0
9043	41907	Drosophila melanogaster	CG9590	CG9590 gene product from transcript CG9590-RA	DmelCG9590	0	0
9044	41908	Drosophila melanogaster	CG9589	CG9589 gene product from transcript CG9589-RA	BcDNA:AT11468|DmelCG9589	0	0
9045	41910	Drosophila melanogaster	Acyp2	Acylphosphatase 2	AcPDro2|CG18505|DmelCG18505	0	0
9046	41911	Drosophila melanogaster	wah	waharan	CG4699|DmelCG4699|E(nos)|NSL1|l(3)06536|l(3)S009413	0	0
9047	41912	Drosophila melanogaster	CG9593	CG9593 gene product from transcript CG9593-RB	DmelCG9593|FBpp0082632	0	0
9048	41913	Drosophila melanogaster	alpha-Man-IIb	CG4606 gene product from transcript CG4606-RA	CG4606|DM-GII.2|DmelCG4606|GTD#24/1|Man'ase|alpha-M-II	0	0
9049	41914	Drosophila melanogaster	CG4576	CG4576 gene product from transcript CG4576-RA	DmelCG4576	0	0
9050	41916	Drosophila melanogaster	CG43317	CG43317 gene product from transcript CG43317-RA	CG14876|DmelCG43317|Dmel_CG14876	0	0
9051	41917	Drosophila melanogaster	Arpc3A	CG4560 gene product from transcript CG4560-RC	ARC-P21|ARPC|CG4560|DmelCG4560	0	0
9052	41919	Drosophila melanogaster	spn-E	spindle E	CG3158|DmelCG3158|SPN-E|Spn-E|fs(1)hls|fs(3)hls|hls|homeless|spn E|spn-E/hls|spnE	0	0
9053	41920	Drosophila melanogaster	Pbp45	PSEA-binding protein 45kD	CG9597|DmPBP|DmPBP45|DmSNAP43|DmelCG9597	0	0
9054	41921	Drosophila melanogaster	CG31150	CG31150 gene product from transcript CG31150-RA	CG9622|CG9625|CG9627|DmelCG31150	0	0
9055	41922	Drosophila melanogaster	CG4546	CG4546 gene product from transcript CG4546-RA	DmelCG4546	0	0
9056	41925	Drosophila melanogaster	Hmt-1	Heavy metal tolerance factor 1	CG4225|DmelCG4225|EP(3)3387|hmt-1	0	0
9057	41926	Drosophila melanogaster	CG9632	CG9632 gene product from transcript CG9632-RA	BcDNA:AT06908|DmelCG9632|NEST:bs19g07|bs19g07.y1	0	0
9058	41927	Drosophila melanogaster	CG31183	CG31183 gene product from transcript CG31183-RA	CG11846|CG4224|DmelCG31183|FBgn0038379|GYC	0	0
9059	41929	Drosophila melanogaster	CG14877	CG14877 gene product from transcript CG14877-RA	DmelCG14877	0	0
9060	41930	Drosophila melanogaster	glob1	globin 1	CG9734|DmHb|DmeGb1|DmeGlob1|DmelCG9734|Glob1|Hb|dmeglob1	0	0
9061	41931	Drosophila melanogaster	CG3303	CG3303 gene product from transcript CG3303-RA	DmelCG3303	0	0
9062	41932	Drosophila melanogaster	CG31291	CG31291 gene product from transcript CG31291-RB	CG14878|CG5552|DmelCG31291	0	0
9063	41934	Drosophila melanogaster	CG34276	CG34276 gene product from transcript CG34276-RA	CG5470|DmelCG34276	0	0
9064	41935	Drosophila melanogaster	CG4221	CG4221 gene product from transcript CG4221-RA	DmelCG4221	0	0
9065	41936	Drosophila melanogaster	CG5478	CG5478 gene product from transcript CG5478-RA	DmelCG5478	0	0
9066	41937	Drosophila melanogaster	blp	black pearl	BcDNA:RE11908|CG5268|DmelCG5268|l(3)01618	0	0
9067	41938	Drosophila melanogaster	Sap47	Synapse-associated protein 47kD	CG8884|DmelCG8884|SAP 47|SAP-47|SAP47|dsap-47|sap47	0	0
9068	41939	Drosophila melanogaster	CG4287	CG4287 gene product from transcript CG4287-RB	DmelCG4287	0	0
9069	41940	Drosophila melanogaster	CG5516	CG5516 gene product from transcript CG5516-RB	DmelCG5516	0	0
9070	41941	Drosophila melanogaster	Rbf2	Retinoblastoma-family protein 2	CG5083|DmelCG5083|RBF2|rbf2	0	0
9071	41942	Drosophila melanogaster	mor	moira	BAP155|CG18740|CG4275|DmelCG18740|E(E2F)3B|MOR|MOR/BAP155|Moira|Mor|Swi3D|dmMOIRA|l(3)89B1	0	0
9072	41943	Drosophila melanogaster	Hel89B	Helicase 89B	89B|BTAF1|CG4261|DmelCG4261|Helicase89B|Mot1	0	0
9073	41944	Drosophila melanogaster	srp	serpent	A7.1|ABF|Abf|CG3992|DmGATAb|DmelCG3992|GATA|GATAb|SRP|Srp|abf|dGATAb|l(3)01549|l(3)89B2|l(3)neo45|spt|srpD	0	0
9074	41945	Drosophila melanogaster	GATAe	CG10278 gene product from transcript CG10278-RB	CG10278|CG10278-PA|DmelCG10278|dGATA-E|dGATAe	0	0
9075	41948	Drosophila melanogaster	CG10264	CG10264 gene product from transcript CG10264-RA	DmelCG10264	0	0
9076	41949	Drosophila melanogaster	CG10407	CG10407 gene product from transcript CG10407-RA	DmelCG10407|cg10407	0	0
9077	41951	Drosophila melanogaster	CG5013	CG5013 gene product from transcript CG5013-RA	DmelCG5013	0	0
9078	41952	Drosophila melanogaster	msps	mini spindles	CG5000|Dis1|DmelCG5000|MSPS|Msps|TOG|XMAP215|anon-WO0140519.136	0	0
9079	41953	Drosophila melanogaster	CG10185	CG10185 gene product from transcript CG10185-RA	DmelCG10185	0	0
9080	41954	Drosophila melanogaster	sxe2	sex-specific enzyme 2	CG4979|DmelCG4979	0	0
9081	41955	Drosophila melanogaster	Mhcl	Myosin heavy chain-like	CG10218|CG10224|CG31045|DmPDZ|DmelCG31045|MHCL(PDZ)	0	0
9082	41957	Drosophila melanogaster	Akt1	CG4006 gene product from transcript CG4006-RA	AKT|AKT/PKB|AKT1|Akt|Akt/PKB|CG4006|D-Akt|DAKT1|DAKT1/PKB|DAkt|DAkt1|DPKB|DRAC-PK|DRAC-PK66|DRAC-PK85|Dakt|Dakt1|DmelCG4006|PKB|PKB/AKT|PKB/Akt|PKB/dAKT|RAC|RacPK|akt|a	0	0
9083	41958	Drosophila melanogaster	Sb	Stubble	CG4316|DmelCG4316|SP56|Sb-sbd|c-SP56|sbd|st-sb	0	0
9084	41959	Drosophila melanogaster	CG5903	CG5903 gene product from transcript CG5903-RA	DmelCG5903|anon-EST:Posey87|anon-WO0118547.99|bs05g07.y1	0	0
9085	41960	Drosophila melanogaster	CG5916	CG5916 gene product from transcript CG5916-RA	DmelCG5916	0	0
9086	41961	Drosophila melanogaster	Fer2	48 related 2	CG5952|DmelCG5952|bHLHa33	0	0
9087	41962	Drosophila melanogaster	CG6006	CG6006 gene product from transcript CG6006-RC	BcDNA:SD04753|DmelCG6006	0	0
9088	41963	Drosophila melanogaster	CG8925	CG8925 gene product from transcript CG8925-RA	DmelCG8925	0	0
9089	41964	Drosophila melanogaster	CG8927	CG8927 gene product from transcript CG8927-RA	DmelCG8927	0	0
9090	41965	Drosophila melanogaster	Bin1	Bicoid interacting protein 1	150001_at|BcDNA:RE02417|Bin 1|CG6046|DmelCG6046|SAP18|bin1|bin1/dSAP18|dSAP18|dSap18|dsap18|sap18	0	0
9091	41967	Drosophila melanogaster	CG6126	CG6126 gene product from transcript CG6126-RA	CT19169|DmelCG6126	0	0
9092	41968	Drosophila melanogaster	mtSSB	mitochondrial single stranded DNA-binding protein	0046/13|CG4337|DmelCG4337|DmmtSSB|SSB|SSB_DROME|c3a|lopo	0	0
9093	41971	Drosophila melanogaster	asun	asunder	CG6814|DmelCG6814|Mat89Bb	0	0
9094	41972	Drosophila melanogaster	Mst89B	CG6864 gene product from transcript CG6864-RA	CG6864|DmelCG6864	0	0
9095	41973	Drosophila melanogaster	Mat89Ba	Maternal transcript 89Ba	CG12785|DmelCG12785	0	0
9096	41975	Drosophila melanogaster	Zip3	Zinc/iron regulated transporter-related protein 3	CG6898|DmelCG6898|ZIP3	0	0
9097	41977	Drosophila melanogaster	CG6901	CG6901 gene product from transcript CG6901-RA	DmelCG6901	0	0
9098	41978	Drosophila melanogaster	CG17929	CG17929 gene product from transcript CG17929-RB	DmelCG17929	0	0
9099	41979	Drosophila melanogaster	CG17930	CG17930 gene product from transcript CG17930-RA	DmelCG17930	0	0

9100	41981	Drosophila melanogaster	pad	poils au dos	CG10309|DmelCG10309	0	0
9101	41982	Drosophila melanogaster	CG14879	CG14879 gene product from transcript CG14879-RB	BcDNA:RE20991|DmelCG14879	0	0
9102	41983	Drosophila melanogaster	Manf	Mesencephalic astrocyte-derived neurotrophic factor	ARP-like|CG7013|DmMANF|DmelCG7013|anon-EST:ParkEST188	0	0
9103	41984	Drosophila melanogaster	CG10311	CG10311 gene product from transcript CG10311-RA	BEST:GH22794|DmelCG10311|dmCG10311|gh22794	0	0
9104	41985	Drosophila melanogaster	CG42446	CG42446 gene product from transcript CG42446-RA	DmelCG42446	0	0
9105	41986	Drosophila melanogaster	CG14880	CG14880 gene product from transcript CG14880-RA	DmelCG14880	0	0
9106	41987	Drosophila melanogaster	CG10317	CG10317 gene product from transcript CG10317-RD	DmelCG10317	0	0
9107	41988	Drosophila melanogaster	ss	spineless	AHR|AhR|CG6993|DmelCG6993|SS|Ss|ssa	0	0
9108	41989	Drosophila melanogaster	CG17565	CG17565 gene product from transcript CG17565-RA	DmelCG17565	0	0
9109	41990	Drosophila melanogaster	CG14881	CG14881 gene product from transcript CG14881-RB	DmelCG14881	0	0
9110	41991	Drosophila melanogaster	mRpS33	mitochondrial ribosomal protein S33	CG10406|DmelCG10406|MRP-S33	0	0
9111	41992	Drosophila melanogaster	ema	endosomal maturation defective	CG12753|DmelCG12753	0	0
9112	41993	Drosophila melanogaster	CG14894	CG14894 gene product from transcript CG14894-RA	DmelCG14894	0	0
9113	41994	Drosophila melanogaster	CG14882	CG14882 gene product from transcript CG14882-RA	BcDNA:SD10961|DmelCG14882	0	0
9114	41995	Drosophila melanogaster	Pak3	CG14895 gene product from transcript CG14895-RC	CG14895|D-Pak3|DPAK3|DPak3|DmPAK3|DmelCG14895|PAK3|PaK3|anon-WO0118547.285|dPAK3|pak3	0	0
9115	41996	Drosophila melanogaster	CG10405	CG10405 gene product from transcript CG10405-RB	DmelCG10405|SP155	0	0
9116	41997	Drosophila melanogaster	CG14883	CG14883 gene product from transcript CG14883-RA	DmelCG14883	0	0
9117	42000	Drosophila melanogaster	CSN5	COP9 complex homolog subunit 5	BcDNA.LD14392|BcDNA:LD14392|CG14884|CH5|Csn5|DCH5|Dch5|DmelCG14884|JAB1/CSN5|Jab1|JadBp|csn5|dch5|jab1/csn5|l(3)89Cf|l(3)L4032|l(3)M203|quo	0	0
9118	42001	Drosophila melanogaster	Gyc-89Da	Guanylyl cyclase at 89Da	CG14885|DmelCG14885|GYCb	0	0
9119	42002	Drosophila melanogaster	Gyc-89Db	Guanylyl cyclase at 89Db	CG14886|DmelCG14886	0	0
9120	42003	Drosophila melanogaster	Dhfr	Dihydrofolate reductase	CG14887|DHFR|DmelCG14887|dhfr	0	0
9121	42004	Drosophila melanogaster	CG14898	CG14898 gene product from transcript CG14898-RA	BcDNA:RE23580|DmelCG14898	0	0
9122	42005	Drosophila melanogaster	Der-2	Derlin-2	CG14899|Cg14899|DER1|DmelCG14899	0	0
9123	42006	Drosophila melanogaster	Cad89D	CG14900 gene product from transcript CG14900-RB	CG14900|CT34724|DCad89D|DmelCG14900	0	0
9124	42007	Drosophila melanogaster	Gr89a	Gustatory receptor 89a	CG14901|DmelCG14901	0	0
9125	42008	Drosophila melanogaster	decay	death executioner caspase related to Apopain/Yama	CC3|CG14902|Cas3|Casp 3|Casp3|Caspase-3|DECAY|Decay|DmelCG14902|Drosophila executioner caspase related to Apopain/Yama|caspase 3|caspase-3	0	0
9126	42013	Drosophila melanogaster	CG14891	CG14891 gene product from transcript CG14891-RA	DmelCG14891	0	0
9127	42014	Drosophila melanogaster	CG14903	CG14903 gene product from transcript CG14903-RB	DmelCG14903	0	0
9128	42015	Drosophila melanogaster	Scp2	Sarcoplasmic calcium-binding protein 2	CE|CG14904|Cex|Dcabp-A.2|DmelCG14904|JHDK|SCP2|cex|dSCP2	0	0
9129	42016	Drosophila melanogaster	CG14892	CG14892 gene product from transcript CG14892-RA	DmelCG14892|SP175	0	0
9130	42017	Drosophila melanogaster	CG10345	CG10345 gene product from transcript CG10345-RA	BcDNA|BcDNA:GH08773|DmelCG10345	0	0
9131	42018	Drosophila melanogaster	npf	neuropeptide F	CG10342|Dm-NPF|DmelCG10342|Drm-NPF|NP-PP|NPF|NPF-A1|NPF-A2|NPF89D3|Npf|dNPF|dnpf	0	0
9132	42019	Drosophila melanogaster	CG12783	CG12783 gene product from transcript CG12783-RA	DmelCG12783	0	0
9133	42020	Drosophila melanogaster	CG10340	CG10340 gene product from transcript CG10340-RA	DmelCG10340|anon-EST:fe2A5|anon2A5	0	0
9134	42021	Drosophila melanogaster	CG17562	CG17562 gene product from transcript CG17562-RA	DmelCG17562	0	0
9135	42022	Drosophila melanogaster	CG17560	CG17560 gene product from transcript CG17560-RA	CT38785|DmelCG17560	0	0
9136	42023	Drosophila melanogaster	CG14893	CG14893 gene product from transcript CG14893-RB	CT34717|DmelCG14893	0	0
9137	42024	Drosophila melanogaster	CG14905	CG14905 gene product from transcript CG14905-RA	DmelCG14905	0	0
9138	42025	Drosophila melanogaster	Fas1	Fasciclin 1	CG6588|DROFAS|DROFAS1|DROFASI|DmelCG6588|Fas I|FasI|MRE24|fas I|fas-I|fasI|gasI	0	0
9139	42026	Drosophila melanogaster	nonA-l	nonA-like	BEST:LD09360|CG10328|DmelCG10328|LD09360|PSF|Z|nonA 1|nonA-1	0	0
9140	42027	Drosophila melanogaster	CG10326	CG10326 gene product from transcript CG10326-RA	DmelCG10326	0	0
9141	42028	Drosophila melanogaster	CG10324	CG10324 gene product from transcript CG10324-RA	DmelCG10324	0	0
9142	42029	Drosophila melanogaster	Cctgamma	CG8977 gene product from transcript CG8977-RC	CG8977|Cct-gamma|Cct3|Cctg|DmelCG8977|TCPG_DROME|Tcp-1-gamma|Tcp-1gamma|Y|geneY	0	0
9143	42030	Drosophila melanogaster	CG14906	CG14906 gene product from transcript CG14906-RB	DmelCG14906|ORF2|anon-89Eb	0	0
9144	42031	Drosophila melanogaster	CG14907	CG14907 gene product from transcript CG14907-RB	DmelCG14907	0	0
9145	42032	Drosophila melanogaster	modSP	modular serine protease	CG10100|CG17557|CG31217|CT28389|Dm-SP|DmelCG31217|LDL|Ldlr|ModSP|SP172|SP53|Ser89E|W|X	0	0
9146	42034	Drosophila melanogaster	Ubx	Ultrabithorax	BX-C|Bxl|CG10388|Cbx|DUbx|Dm Ubx|DmUbx|DmelCG10388|Hm|UBX|UBx|Uab|Ubx1b|abx|bx|bx[D]|bxd|bxd[D]|bxl|l(3)89Eb|pbx|ubx	0	0
9147	42036	Drosophila melanogaster	Glut3	Glucose transporter type 3	CG3853|DmelCG3853|GLU|glut-I	0	0
9148	42037	Drosophila melanogaster	abd-A	abdominal A	Abd-A|AbdA|Abda|BX-C|CG10325|Cbxd|DmabdA|Dmabda|DmelCG10325|Hab|abd A|abd-a|abdA|abd_a|abda|iab|iab-2|iab-3|iab-4|iab-5|iab2|iab3|iab4|l(3)89Ec	0	0
9149	42041	Drosophila melanogaster	VhaM9.7-d	Vacuolar H[+] ATPase subunit M9.7-d	CG14909|DmelCG14909|vhaM9.7-4	0	0
9150	42043	Drosophila melanogaster	Ahcy89E	Adenosylhomocysteinase 89E	AHCY|CG8956|DmelCG8956|S-AHCY|pH200	0	0
9151	42044	Drosophila melanogaster	CG18622	CG18622 gene product from transcript CG18622-RA	BcDNA:RH18149|DmelCG18622	0	0
9152	42045	Drosophila melanogaster	CG3678	CG3678 gene product from transcript CG3678-RA	DmelCG3678	0	0
9153	42046	Drosophila melanogaster	CG17556	CG17556 gene product from transcript CG17556-RA	DmelCG17556	0	0
9154	42047	Drosophila melanogaster	CG3534	CG3534 gene product from transcript CG3534-RA	DmelCG3534	0	0
9155	42048	Drosophila melanogaster	CG16941	CG16941 gene product from transcript CG16941-RA	DmelCG16941|SF3a1|SF3a120	0	0
9156	42049	Drosophila melanogaster	Irc	Immune-regulated catalase	CG8913|DmelCG8913|IRC	0	0
9157	42050	Drosophila melanogaster	CG8907	CG8907 gene product from transcript CG8907-RA	DmelCG8907|LP01469p	0	0
9158	42051	Drosophila melanogaster	CG3590	CG3590 gene product from transcript CG3590-RA	DmelCG3590	0	0
9159	42053	Drosophila melanogaster	CG5225	CG5225 gene product from transcript CG5225-RA	DmelCG5225	0	0
9160	42054	Drosophila melanogaster	CG4009	CG4009 gene product from transcript CG4009-RB	DmelCG4009	0	0
9161	42055	Drosophila melanogaster	CG18213	CG18213 gene product from transcript CG18213-RB	DmelCG18213	0	0
9162	42056	Drosophila melanogaster	CG5220	CG5220 gene product from transcript CG5220-RA	DmelCG5220	0	0
9163	42057	Drosophila melanogaster	CG3995	CG3995 gene product from transcript CG3995-RA	DmelCG3995	0	0
9164	42058	Drosophila melanogaster	Patr-1	Protein associated with topo II related - 1	BcDNA:LD27979|CG5208|DmelCG5208|HPat|LD27979|anon-WO0118547.282	0	0
9165	42059	Drosophila melanogaster	Dad	Daughters against dpp	1883|CG5201|DAD|Dad1|DmelCG5201|EP(3)3196|EP3196|anon-EST:fe1F5|dad|l(3)j1E4	0	0
9166	42060	Drosophila melanogaster	ns1	nucleostemin 1	CG3983|DmelCG3983|NS1|dmNS1	0	0
9167	42061	Drosophila melanogaster	mRpS11	mitochondrial ribosomal protein S11	BcDNA:RH70248|CG5184|DmelCG5184|GH1|MRP-S11	0	0
9168	42062	Drosophila melanogaster	Keap1	CG3962 gene product from transcript CG3962-RC	CG3962|DmelCG3962|KEAP1|dKEAP1|dKeap1|dkeap1|keap1	0	0
9169	42063	Drosophila melanogaster	kuk	kugelkern	CG5175|DmelCG5175|Kuk|char|kuk/char	0	0
9170	42064	Drosophila melanogaster	GckIII	Germinal centre kinase III	CG5169|DmelCG5169|GCKIII|STLK3|dGCKIII	0	0
9171	42065	Drosophila melanogaster	cal1	chromosome alignment defect 1	CAL1|CG5148|CLD2|Cal1|DmelCG5148	0	0
9172	42066	Drosophila melanogaster	cher	cheerio	CG3937|Cher|DmelCG3937|FLN|Fil|sko	0	0
9173	42067	Drosophila melanogaster	CG17477	CG17477 gene product from transcript CG17477-RA	DmelCG17477|SPH174	0	0
9174	42068	Drosophila melanogaster	CG31265	CG31265 gene product from transcript CG31265-RA	CG5233|DmelCG31265|SP126	0	0
9175	42069	Drosophila melanogaster	CG17475	CG17475 gene product from transcript CG17475-RA	DmelCG17475|SP131	0	0
9176	42070	Drosophila melanogaster	CG4053	CG4053 gene product from transcript CG4053-RB	DmelCG4053|SP185	0	0
9177	42071	Drosophila melanogaster	CG31266	CG31266 gene product from transcript CG31266-RB	CG5240|DmelCG31266|SPH146	0	0
9178	42072	Drosophila melanogaster	CG5246	CG5246 gene product from transcript CG5246-RA	BcDNA:LP06628|DmelCG5246|SP96	0	0
9179	42073	Drosophila melanogaster	CG5255	CG5255 gene product from transcript CG5255-RA	DmelCG5255|SP141	0	0
9180	42074	Drosophila melanogaster	CG5265	CG5265 gene product from transcript CG5265-RA	DmelCG5265	0	0
9181	42075	Drosophila melanogaster	TwdlW	TweedleW	CG4060|DmelCG4060	0	0
9182	42076	Drosophila melanogaster	m-cup	mann-cup	CG11896|DmelCG11896	0	0
9183	42077	Drosophila melanogaster	Det	Deterin	CG12265|DmelCG12265	0	0
9184	42078	Drosophila melanogaster	CG5285	CG5285 gene product from transcript CG5285-RA	DmelCG5285	0	0
9185	42079	Drosophila melanogaster	CG5292	CG5292 gene product from transcript CG5292-RA	BcDNA:RE41712|DmelCG5292	0	0
9186	42080	Drosophila melanogaster	Mur89F	Mucin related 89F	CG4090|CT13556|DmelCG4090	0	0
9187	42082	Drosophila melanogaster	beat-IIb	CG4135 gene product from transcript CG4135-RB	CG14337|CG4135|CT13620|DmelCG4135|beat IIb	0	0
9188	42084	Drosophila melanogaster	CG31418	CG31418 gene product from transcript CG31418-RA	CG5329|DmelCG31418	0	0
9189	42086	Drosophila melanogaster	beat-IIa	beaten path IIa	BcDNA:RE17794|CG14334|CG14335|CT33965|DmelCG14334|beat IIa	0	0
9190	42087	Drosophila melanogaster	Brf	CG31256 gene product from transcript CG31256-RA	BRF|BRF1|CG31256|CG4155|CG5419|DmelCG31256|TFIIIB	0	0
9191	42089	Drosophila melanogaster	lute	CG43226 gene product from transcript CG43226-RC	CG14333|CG43226|CG5319|DmelCG43226|Dmel_CG14333|Dmel_CG5319|anon-WO0118547.289	0	0
9192	42091	Drosophila melanogaster	sds22	CG5851 gene product from transcript CG5851-RA	CG5851|CT18355|DmelCG5851	0	0
9193	42092	Drosophila melanogaster	CREG	Cellular Repressor of E1A-stimulated Genes	BcDNA:LP11958|CG5413|DmelCG5413|creg|dCREG	0	0
9194	42093	Drosophila melanogaster	Sur-8	CG5407 gene product from transcript CG5407-RA	CG5407|CT17146|DmelCG5407|SUR-8|Sur8	0	0
9195	42094	Drosophila melanogaster	CG17283	CG17283 gene product from transcript CG17283-RA	DmelCG17283	0	0
9196	42095	Drosophila melanogaster	CG5860	CG5860 gene product from transcript CG5860-RA	DmelCG5860|FBgn0038506	0	0
9197	42096	Drosophila melanogaster	CG5863	CG5863 gene product from transcript CG5863-RA	DmelCG5863	0	0
9198	42097	Drosophila melanogaster	CG5866	CG5866 gene product from transcript CG5866-RB	DmelCG5866	0	0
9199	42098	Drosophila melanogaster	CG14332	CG14332 gene product from transcript CG14332-RA	DmelCG14332	0	0
9200	42099	Drosophila melanogaster	CG14331	CG14331 gene product from transcript CG14331-RB	DmelCG14331	0	0
9201	42100	Drosophila melanogaster	CG5873	CG5873 gene product from transcript CG5873-RA	DmelCG5873	0	0
9202	42101	Drosophila melanogaster	Eh	Eclosion hormone	CG5400|DMEHAB|DmelCG5400|Drm-EH|EH|EHAB|eh	0	0
9203	42102	Drosophila melanogaster	CG14330	CG14330 gene product from transcript CG14330-RA	DmelCG14330	0	0
9204	42103	Drosophila melanogaster	Dscam3	Down syndrome cell adhesion molecule 3	CG12536|CG31190|CG8106|CT33960|DmelCG31190|Dscam-like	0	0
9205	42105	Drosophila melanogaster	CG5823	CG5823 gene product from transcript CG5823-RA	DmelCG5823	0	0
9206	42106	Drosophila melanogaster	CG5840	CG5840 gene product from transcript CG5840-RB	CT18319|DmelCG5840	0	0
9207	42109	Drosophila melanogaster	Prx3	Peroxiredoxin 3	CG5826|DPx-5037|DPx5037|DmelCG5826|Prx5037|dPrx3|mTPx|mtTPx	0	0
9208	42110	Drosophila melanogaster	Hmx	H6-like-homeobox	CG34419|CG5829|CG5832|DHmx|DmelCG34419|Dmel_CG5829|Dmel_CG5832	0	0
9209	42112	Drosophila melanogaster	CG18139	CG18139 gene product from transcript CG18139-RA	DmelCG18139	0	0
9210	42113	Drosophila melanogaster	CG7587	CG7587 gene product from transcript CG7587-RA	DmelCG7587	0	0

9211	42114	Drosophila melanogaster	Sgs5	Salivary gland secretion 5	BcDNA:LP02306|CG7596|DmelCG7596|SGS-5|Sgs|Sgs-5|sgs5	0	0
9212	42115	Drosophila melanogaster	sll	slalom	1422/14|CG7623|DmelCG7623|anon-WO0118547.555|anon-WO0172774.85|l(3)S142214	0	0
9213	42116	Drosophila melanogaster	CG14329	CG14329 gene product from transcript CG14329-RA	DmelCG14329	0	0
9214	42117	Drosophila melanogaster	Edg91	Ecdysone-dependent gene 91	CG7539|DmelCG7539|EDG-91|EDG91	0	0
9215	42118	Drosophila melanogaster	CG14327	CG14327 gene product from transcript CG14327-RA	BcDNA:RE25364|DmelCG14327	0	0
9216	42119	Drosophila melanogaster	CG14324	CG14324 gene product from transcript CG14324-RA	BcDNA:RE34075|DmelCG14324	0	0
9217	42120	Drosophila melanogaster	CG14326	CG14326 gene product from transcript CG14326-RA	BcDNA:RE19820|DmelCG14326	0	0
9218	42121	Drosophila melanogaster	CG14323	CG14323 gene product from transcript CG14323-RA	DmelCG14323	0	0
9219	42122	Drosophila melanogaster	AttD	Attacin-D	Att|CG7629|DmelCG7629|att|att D|attD	0	0
9220	42123	Drosophila melanogaster	CG14325	CG14325 gene product from transcript CG14325-RB	DmelCG14325	0	0
9221	42124	Drosophila melanogaster	CG14322	CG14322 gene product from transcript CG14322-RA	DmelCG14322	0	0
9222	42125	Drosophila melanogaster	CG7523	CG7523 gene product from transcript CG7523-RA	DmelCG7523	0	0
9223	42126	Drosophila melanogaster	ald	altered disjunction	Ald/Mps1|CG7643|DmelCG7643|Mps1|TKK|dMps1|mps1	0	0
9224	42127	Drosophila melanogaster	alt	aluminum tubes	CG18212|DmelCG18212	0	0
9225	42128	Drosophila melanogaster	CG7655	CG7655 gene product from transcript CG7655-RA	DmelCG7655	0	0
9226	42129	Drosophila melanogaster	CG31249	CG31249 gene product from transcript CG31249-RA	CG7477|DmelCG31249|Q9VEG8	0	0
9227	42130	Drosophila melanogaster	osa	CG7467 gene product from transcript CG7467-RD	C819|CG7467|DmelCG7467|E(E2F)3C|OSA|Osa|anon-WO0118547.314|anon-WO0172774.126|eld|en(lz)4F/4H|l(3)00090|l(3)04539|l(3)j9C3|p300	0	0
9228	42131	Drosophila melanogaster	Pxt	Peroxinectin-like	CG7660|DmelCG7660|Dpxt|anon-WO0172774.128|pxd|pxt	0	0
9229	42132	Drosophila melanogaster	Atg8b	Autophagy-specific gene 8b	ATG8|ATG8b|Atg8|Atg8B|CG12334|DmelCG12334|atg8b	0	0
9230	42133	Drosophila melanogaster	Fsh	Fsh-Tsh-like receptor	CG7665|DLGR-1|DLGR1|DmelCG7665|FSH|FSH-TSH|Fsh/CG7665|LH/CG receptor|TSH	0	0
9231	42134	Drosophila melanogaster	CG14321	CG14321 gene product from transcript CG14321-RA	DmelCG14321	0	0
9232	42135	Drosophila melanogaster	TyrRII	Tyramine receptor II	CG16766|DmCG16766|DmelCG16766|anon-WO0170980.124|anon-WO0170980.125	0	0
9233	42136	Drosophila melanogaster	TyrR	Tyramine receptor	CG7431|DmCG7431|DmelCG7431|DrmTR|TR|anon-WO0170980.82|anon-WO0170980.83	0	0
9234	42137	Drosophila melanogaster	CG43102	CG43102 gene product from transcript CG43102-RA	CG14318|CG7397|DmelCG43102|Dmel_CG14318|Dmel_CG7397|FBgn0038543	0	0
9235	42139	Drosophila melanogaster	CG7713	CG7713 gene product from transcript CG7713-RA	DmelCG7713|dmCG7713	0	0
9236	42140	Drosophila melanogaster	CG7379	CG7379 gene product from transcript CG7379-RA	DmelCG7379|ING1	0	0
9237	42141	Drosophila melanogaster	CG17803	CG17803 gene product from transcript CG17803-RA	DmelCG17803	0	0
9238	42142	Drosophila melanogaster	CG17806	CG17806 gene product from transcript CG17806-RA	DmelCG17806	0	0
9239	42143	Drosophila melanogaster	CG17802	CG17802 gene product from transcript CG17802-RA	DmelCG17802	0	0
9240	42144	Drosophila melanogaster	CG17801	CG17801 gene product from transcript CG17801-RA	DmelCG17801	0	0
9241	42145	Drosophila melanogaster	CG7357	CG7357 gene product from transcript CG7357-RA	DmelCG7357	0	0
9242	42146	Drosophila melanogaster	CG18012	CG18012 gene product from transcript CG18012-RB	DmelCG18012	0	0
9243	42148	Drosophila melanogaster	tinc	tincar	CG14319|CG14320|CG31246|CG31247|CG7723|DmelCG31247|Dmel_CG31246	0	0
9244	42150	Drosophila melanogaster	Rim	CG33547 gene product from transcript CG33547-RG	CG33547|CG7301|CG7305|CG7321|DmelCG33547|RIM|UNC-10/RIM|dm-Rim	0	0
9245	42152	Drosophila melanogaster	CG12347	CG12347 gene product from transcript CG12347-RA	DmelCG12347	0	0
9246	42158	Drosophila melanogaster	CG7785	CG7785 gene product from transcript CG7785-RA	DmelCG7785	0	0
9247	42159	Drosophila melanogaster	CG7794	CG7794 gene product from transcript CG7794-RA	DmelCG7794|anon-WO0140519.191	0	0
9248	42160	Drosophila melanogaster	htl	heartless	CG7223|CT22273|CT39172|DFGF-R1|DFGF-R2|DFR-1|DFR1|DFR1/DFGF-R2|DPR3|DTRK(FR1)|Dfr-1|Dfr1|DmHD-38|DmelCG7223|Dtk1|EMS2|FGF-R2|FGFR|FGFR2|FR1|Fr1|HD-38|HTL/FGFR1|Htl|Tk1|dt	0	0
9249	42161	Drosophila melanogaster	CG14317	CG14317 gene product from transcript CG14317-RA	DmelCG14317|anon-WO0172774.95	0	0
9250	42162	Drosophila melanogaster	sr	stripe	CG7847|DmelCG7847|Sr|l(3)03999|l(3)06948|l(3)06948b	0	0
9251	42163	Drosophila melanogaster	CG14316	CG14316 gene product from transcript CG14316-RA	DmelCG14316	0	0
9252	42164	Drosophila melanogaster	CG14315	CG14315 gene product from transcript CG14315-RA	DmelCG14315	0	0
9253	42165	Drosophila melanogaster	CG7218	CG7218 gene product from transcript CG7218-RA	DmelCG7218	0	0
9254	42166	Drosophila melanogaster	Cbp20	cap binding protein 20	CBP20|CG12357|DmelCG12357	0	0
9255	42169	Drosophila melanogaster	PP2A-B'	CG7913 gene product from transcript CG7913-RK	B'|B'/PR61|B56-1|BcDNA:GM05554|CG 7913|CG7901|CG7913|DmelCG7913|PP2A|PP2A B'|PP2A-B|PP2A[B'-1]|anon-WO0118547.420|dB56-1|dPP2A|i234|wrd	0	0
9256	42171	Drosophila melanogaster	cdm	cadmus	3R23|CG 7212|CG7212|DmelCG7212|Imp13|imp13|sd-5	0	0
9257	42172	Drosophila melanogaster	CG7208	CG7208 gene product from transcript CG7208-RA	CG 7208|DmelCG7208	0	0
9258	42173	Drosophila melanogaster	Arp5	Actin-related protein 5	CG7940|DmelCG7940|dArp5	0	0
9259	42174	Drosophila melanogaster	CG12321	CG12321 gene product from transcript CG12321-RA	DmelCG12321	0	0
9260	42175	Drosophila melanogaster	MED17	Mediator complex subunit 17	CG7957|DmelCG7957|Med17|TRAP80|Trap80|dTRAP80|dTRAP80/SRB4|dTrap80|l(3)s2956|p70	0	0
9261	42176	Drosophila melanogaster	CG14313	CG14313 gene product from transcript CG14313-RA	DmelCG14313	0	0
9262	42177	Drosophila melanogaster	Ssdp	Sequence-specific single-stranded DNA-binding prote	CG7187|DmelCG7187|l(3)neo48|ssdp	0	0
9263	42178	Drosophila melanogaster	CG7985	CG7985 gene product from transcript CG7985-RA	BcDNA:GH04120|DmelCG7985	0	0
9264	42179	Drosophila melanogaster	CG14314	CG14314 gene product from transcript CG14314-RA	DmelCG14314	0	0
9265	42180	Drosophila melanogaster	CG7988	CG7988 gene product from transcript CG7988-RA	DmelCG7988	0	0
9266	42181	Drosophila melanogaster	CG7183	CG7183 gene product from transcript CG7183-RA	DmelCG7183	0	0
9267	42182	Drosophila melanogaster	CG7175	CG7175 gene product from transcript CG7175-RA	DmelCG7175	0	0
9268	42183	Drosophila melanogaster	CG7993	CG7993 gene product from transcript CG7993-RA	DmelCG7993	0	0
9269	42184	Drosophila melanogaster	CG7168	CG7168 gene product from transcript CG7168-RA	DmelCG7168	0	0
9270	42185	Drosophila melanogaster	Mdh2	Malate dehydrogenase 2	CG7998|DmelCG7998|MDH-2|Mdh|l(3)psg7|mMdh	0	0
9271	42186	Drosophila melanogaster	14-3-3epsilon	CG31196 gene product from transcript CG31196-RB	14-3-3|14-3-3 epsilon|14-3-3-e|14-3-30epsilon|14-3-3EPSILON|14-3-3e|14-3-3omicron|1433epsilon|143E_DROME|CG31196|CT24092|D14-3-3e|D14-3-3epsilon|DmelCG31196|EK3-5|PAR-5|PAR5|Par-5|	0	0
9272	42187	Drosophila melanogaster	CG7156	CG7156 gene product from transcript CG7156-RB	DmelCG7156	0	0
9273	42188	Drosophila melanogaster	CG18598	CG18598 gene product from transcript CG18598-RA	CT42539|DmelCG18598	0	0
9274	42189	Drosophila melanogaster	CG12320	CG12320 gene product from transcript CG12320-RA	DmelCG12320	0	0
9275	42191	Drosophila melanogaster	CG18599	CG18599 gene product from transcript CG18599-RA	DmelCG18599	0	0
9276	42192	Drosophila melanogaster	CG7146	CG7146 gene product from transcript CG7146-RA	DmelCG7146	0	0
9277	42194	Drosophila melanogaster	CG7142	CG7142 gene product from transcript CG7142-RA	DmelCG7142|SP132	0	0
9278	42195	Drosophila melanogaster	CG14312	CG14312 gene product from transcript CG14312-RA	DmelCG14312	0	0
9279	42196	Drosophila melanogaster	CG8064	CG8064 gene product from transcript CG8064-RA	DmelCG8064	0	0
9280	42197	Drosophila melanogaster	CG7131	CG7131 gene product from transcript CG7131-RA	DmelCG7131|anon-WO0140519.212	0	0
9281	42199	Drosophila melanogaster	Dlc90F	Dynein light chain 90F	CG12363|DmelCG12363|TcTex-1|Tctex|Tctex-1|bs35g12.y1|dlc90F|dtctex-1|l(3)04091|tctex|tctex-1	0	0
9282	42201	Drosophila melanogaster	CG18600	CG18600 gene product from transcript CG18600-RA	BEST:CK01205|CK01205|DmelCG18600	0	0
9283	42202	Drosophila melanogaster	CG7126	CG7126 gene product from transcript CG7126-RA	DmelCG7126	0	0
9284	42203	Drosophila melanogaster	PKD	Protein Kinase D	150131_at|CG7125|DmelCG7125|PKCm|dPKD	0	0
9285	42204	Drosophila melanogaster	CG31235	CG31235 gene product from transcript CG31235-RA	CG14311|CG7117|DmelCG31235	0	0
9286	42206	Drosophila melanogaster	CG15803	CG15803 gene product from transcript CG15803-RB	DmelCG15803	0	0
9287	42207	Drosophila melanogaster	CG7669	CG7669 gene product from transcript CG7669-RA	DmelCG7669|anon-WO0140519.198	0	0
9288	42208	Drosophila melanogaster	WRNexo	WRN exonuclease	CG7670|DmWRNexo|DmelCG7670	0	0
9289	42209	Drosophila melanogaster	Nup43	Nucleoporin 43	CG7671|DmelCG7671	0	0
9290	42210	Drosophila melanogaster	gl	glass	CG7672|DmelCG7672|E(sina)5|Glass|SS3-2|SY3-3|gla|none|rh	0	0
9291	42211	Drosophila melanogaster	CG7675	CG7675 gene product from transcript CG7675-RC	DmelCG7675	0	0
9292	42212	Drosophila melanogaster	CG14309	CG14309 gene product from transcript CG14309-RA	DmelCG14309	0	0
9293	42213	Drosophila melanogaster	cona	corona	CG7676|DmelCG7676	0	0
9294	42214	Drosophila melanogaster	Vha100-4	Vacuolar H[+] ATPase subunit 100-4	CG7678|DmelCG7678|vha100-4	0	0
9295	42215	Drosophila melanogaster	koko	kokopelli	CG31232|CG7682|CG7683|DmelCG31232|EP(3)1037	0	0
9296	42216	Drosophila melanogaster	Vha100-2	Vacuolar H[+] ATPase subunit 100-2	BcDNA:LD21735|CG18617|CG7679|DmelCG18617|V-ATPase|anon-WO0118547.296|vha100-2	0	0
9297	42218	Drosophila melanogaster	CG12333	CG12333 gene product from transcript CG12333-RA	DmelCG12333	0	0
9298	42220	Drosophila melanogaster	CG7685	CG7685 gene product from transcript CG7685-RA	DmelCG7685	0	0
9299	42221	Drosophila melanogaster	CG31122	CG31122 gene product from transcript CG31122-RA	CG14308|CG7687|DmelCG31122	0	0
9300	42222	Drosophila melanogaster	CG10864	CG10864 gene product from transcript CG10864-RA	DmelCG10864	0	0
9301	42226	Drosophila melanogaster	fru	fruitless	BTB-VI|BtbVI|CG14307|CG7688|CG7689|CG7690|CT22773|DmelCG14307|Dmfru|FRU|Fru|cg7688|cg7689|fty|ms(3)06411|sat	0	0
9302	42228	Drosophila melanogaster	CG7691	CG7691 gene product from transcript CG7691-RA	DmelCG7691	0	0
9303	42230	Drosophila melanogaster	CG7694	CG7694 gene product from transcript CG7694-RA	BcDNA:LP11469|DmelCG7694	0	0
9304	42231	Drosophila melanogaster	CG14306	CG14306 gene product from transcript CG14306-RB	DmelCG14306	0	0
9305	42232	Drosophila melanogaster	CG14304	CG14304 gene product from transcript CG14304-RA	DmelCG14304	0	0
9306	42233	Drosophila melanogaster	CG14305	CG14305 gene product from transcript CG14305-RA	DmelCG14305	0	0
9307	42234	Drosophila melanogaster	CG7695	CG7695 gene product from transcript CG7695-RA	DmelCG7695	0	0
9308	42235	Drosophila melanogaster	CG14301	CG14301 gene product from transcript CG14301-RB	DmelCG14301	0	0
9309	42236	Drosophila melanogaster	CG14303	CG14303 gene product from transcript CG14303-RA	DmelCG14303	0	0
9310	42237	Drosophila melanogaster	CG31224	CG31224 gene product from transcript CG31224-RA	CG17796|CG7696|DmelCG31224|l(3)c00083	0	0
9311	42239	Drosophila melanogaster	CstF-64	Cleavage stimulation factor 64 kilodalton subunit	CG7697|CStF 64|CstF 64|CstF64|DmelCG7697|dCstF64	0	0
9312	42240	Drosophila melanogaster	Cpsf73	Cleavage and polyadenylation specificity factor 73	CG7698|CPSF-73|CPSF73|DmelCG7698|cpsf-73|dCPSF-73	0	0
9313	42242	Drosophila melanogaster	CG7702	CG7702 gene product from transcript CG7702-RB	CT23351|DmelCG7702	0	0
9314	42243	Drosophila melanogaster	CG7705	CG7705 gene product from transcript CG7705-RA	DmelCG7705	0	0
9315	42244	Drosophila melanogaster	CG7706	CG7706 gene product from transcript CG7706-RA	DmelCG7706	0	0
9316	42245	Drosophila melanogaster	CG7708	CG7708 gene product from transcript CG7708-RA	DmelCG7708	0	0
9317	42246	Drosophila melanogaster	Muc91C	Mucin 91C	CG7709|DmelCG7709	0	0
9318	42248	Drosophila melanogaster	CG34282	CG34282 gene product from transcript CG34282-RA	CG7710|DmelCG34282	0	0
9319	42249	Drosophila melanogaster	Cha	Choline acetyltransferase	CG12345|CHAT|CLAT_DROME|CT23399|Cat|ChAT|ChAt|ChaT|DmelCG12345|cha|dChAT|l(3)91Cc	0	0
9320	42250	Drosophila melanogaster	CG7714	CG7714 gene product from transcript CG7714-RA	DmelCG7714|anon-EST:ParkEST066	0	0
9321	42251	Drosophila melanogaster	CG7715	CG7715 gene product from transcript CG7715-RA	DmelCG7715	0	0

9322	42252	Drosophila melanogaster	CG14302	CG14302 gene product from transcript CG14302-RA	BcDNA:LP02994|DmelCG14302	0	0
9323	42253	Drosophila melanogaster	Mekk1	CG7717 gene product from transcript CG7717-RB	CG7717|D-MEKK1|D-Mekk1|DmMEKK1|DmelCG7717|MEKK|MEKK1|MEKK4|Mekk1/4|dMEKK|dMEKK1|dMEKK4|dMekk1|mekk1|mekk1a|mekk1b	0	0
9324	42254	Drosophila melanogaster	CG7718	CG7718 gene product from transcript CG7718-RA	DmelCG7718	0	0
9325	42256	Drosophila melanogaster	CG14299	CG14299 gene product from transcript CG14299-RD	DmelCG14299	0	0
9326	42257	Drosophila melanogaster	CG7720	CG7720 gene product from transcript CG7720-RB	BcDNA:RH05973|DmelCG7720|ORE-8	0	0
9327	42258	Drosophila melanogaster	CG18208	CG18208 gene product from transcript CG18208-RA	DmelCG18208	0	0
9328	42259	Drosophila melanogaster	CG14298	CG14298 gene product from transcript CG14298-RA	DmelCG14298	0	0
9329	42260	Drosophila melanogaster	CG14297	CG14297 gene product from transcript CG14297-RA	DmelCG14297|LMW-PTP	0	0
9330	42261	Drosophila melanogaster	Rh2	Rhodopsin 2	CG16740|DMELRH2|Dm Rh2|DmelCG16740|RH2|Rh|rh2	0	0
9331	42262	Drosophila melanogaster	CG14294	CG14294 gene product from transcript CG14294-RA	BcDNA:AT18358|DmelCG14294	0	0
9332	42264	Drosophila melanogaster	CG14292	CG14292 gene product from transcript CG14292-RA	DmelCG14292	0	0
9333	42265	Drosophila melanogaster	endoA	endophilin A	CG14296|D-endo|D-endoA|Dendo-A|DmelCG14296|Endo|endo	0	0
9334	42266	Drosophila melanogaster	CG14291	CG14291 gene product from transcript CG14291-RA	DmelCG14291	0	0
9335	42267	Drosophila melanogaster	Xrp1	CG17836 gene product from transcript CG17836-RB	2515|CG17836|DmelCG17836|XRP1|anon-EST:Liang-2.44|clone 2.44|l(3)02515	0	0
9336	42268	Drosophila melanogaster	CG14290	CG14290 gene product from transcript CG14290-RB	DmelCG14290	0	0
9337	42269	Drosophila melanogaster	CG42613	CG42613 gene product from transcript CG42613-RE	CG14289|CG31149|CG31222|CG5449|CT17110|CT33918|DmelCG42613|Dmel_CG31149|Dmel_CG31222|tld-like	0	0
9338	42271	Drosophila melanogaster	CG12269	CG12269 gene product from transcript CG12269-RA	DmelCG12269|SCP-2	0	0
9339	42272	Drosophila melanogaster	CG5451	CG5451 gene product from transcript CG5451-RB	DmelCG5451	0	0
9340	42273	Drosophila melanogaster	dnk	deoxyribonucleoside kinase	CG5452|DM-dNK|DNK|Dm-dNK|DmdNK|DmelCG5452|dNK	0	0
9341	42274	Drosophila melanogaster	snRNP-U1-C	small ribonucleoprotein particle U1 subunit C	BcDNA:RH29440|C|CG5454|DmelCG5454|U1C|dU1-C|snRNP-U1|snRNPC	0	0
9342	42279	Drosophila melanogaster	CG6005	CG6005 gene product from transcript CG6005-RA	DmelCG6005	0	0
9343	42280	Drosophila melanogaster	CG14286	CG14286 gene product from transcript CG14286-RA	DmelCG14286	0	0
9344	42281	Drosophila melanogaster	CG14285	CG14285 gene product from transcript CG14285-RA	DmelCG14285	0	0
9345	42282	Drosophila melanogaster	NP15.6	CG6008 gene product from transcript CG6008-RA	CG6008|DmelCG6008|anon-WO0118547.281	0	0
9346	42283	Drosophila melanogaster	gatA	glutamyl-tRNA amidotransferase subunit A	0929/02|CG6007|DmelCG6007|bene|benedict|l(3)S092902	0	0
9347	42284	Drosophila melanogaster	P5cr	Pyrroline 5-carboyxlate reductase	CG6009|DmelCG6009|P5CR	0	0
9348	42285	Drosophila melanogaster	Prp18	CG6011 gene product from transcript CG6011-RA	CG6011|DmelCG6011|prp18	0	0
9349	42286	Drosophila melanogaster	CG6013	CG6013 gene product from transcript CG6013-RA	DmelCG6013	0	0
9350	42287	Drosophila melanogaster	CG6026	CG6026 gene product from transcript CG6026-RB	DmelCG6026	0	0
9351	42289	Drosophila melanogaster	cdi	center divider	91F|B9.3|CDI/TSKI|CG6027|Cdi|Cdi/TESK1|DmelCG6027|cdi/TESK1|l(3)07013	0	0
9352	42290	Drosophila melanogaster	mRpL55	mitochondrial ribosomal protein L55	BcDNA:RH10246|CG14283|DmelCG14283|MRP-L55|tmb	0	0
9353	42291	Drosophila melanogaster	ATPsyn-d	ATP synthase, subunit d	ATPsyn-D|ATPsyn-delta|CG6030|DmelCG6030	0	0
9354	42292	Drosophila melanogaster	CG6040	CG6040 gene product from transcript CG6040-RA	DmelCG6040	0	0
9355	42293	Drosophila melanogaster	Cyp12a5	CG11821 gene product from transcript CG11821-RA	12a5|CG11821|DmelCG11821	0	0
9356	42294	Drosophila melanogaster	Cyp12a4	CG6042 gene product from transcript CG6042-RA	12a4|CG6042|CYP12A4|DmelCG6042|P450|cyp12a4	0	0
9357	42295	Drosophila melanogaster	Ppcs	Phosphopantothenoylcysteine synthetase	BcDNA:GH03502|CG5629|DmelCG5629|GH03502|PPCS|anon-WO0118547.401|dPPCS	0	0
9358	42296	Drosophila melanogaster	CG5835	CG5835 gene product from transcript CG5835-RA	DmelCG5835	0	0
9359	42297	Drosophila melanogaster	nos	nanos	CG5637|DRONANOS|DmelCG5637|Dronanos|NANOS|NOS|Nos|l(3)07117|l(3)j3B6|l(3)j4B6|nNOS	0	0
9360	42298	Drosophila melanogaster	CG11779	CG11779 gene product from transcript CG11779-RB	BEST:GM03811|BcDNA:GM03811|CG117799|DmelCG11779|GH-18370	0	0
9361	42299	Drosophila melanogaster	CG42359	CG42359 gene product from transcript CG42359-RA	CG5558|DmelCG42359|Dmel_CG5558	0	0
9362	42300	Drosophila melanogaster	sqz	squeeze	CG5557|DmelCG5557|Sqz|anon-WO0118547.697|l(3)02102	0	0
9363	42301	Drosophila melanogaster	CG14282	CG14282 gene product from transcript CG14282-RA	DmelCG14282	0	0
9364	42302	Drosophila melanogaster	CG5555	CG5555 gene product from transcript CG5555-RA	DmelCG5555	0	0
9365	42303	Drosophila melanogaster	CG31475	CG31475 gene product from transcript CG31475-RA	CG14281|CG3768|DmelCG31475	0	0
9366	42305	Drosophila melanogaster	cry	cryptochrome	CG3772|CRY|Cry|DCry|Dm-CRY1|DmCRY|DmCRY1|Dmcry|DmelCG3772|anon-WO0140519.17|anon-WO0140519.19|anon-WO0140519.20|anon-WO0172774.15|cryb|dCRY|dCry|dcry	0	0
9367	42306	Drosophila melanogaster	vib	vibrator	0450/02|1104/16|CG5269|DmelCG5269|anon-WO0142479.3|anon-WO0172774.12|anon-WO0172774.16|anon-WO0172774.28|gio|l(3)S045002|l(3)S045002c|l(3)S110416|l(3)j4A3|l(3)j5A6|l(3)j7A3|l(3)j7C8	0	0
9368	42307	Drosophila melanogaster	CG11703	CG11703 gene product from transcript CG11703-RA	DmelCG11703|JYbeta2|anon-WO0140519.49	0	0
9369	42308	Drosophila melanogaster	CG5250	CG5250 gene product from transcript CG5250-RA	DmelCG5250	0	0
9370	42309	Drosophila melanogaster	CG3773	CG3773 gene product from transcript CG3773-RA	DmelCG3773|anon-EST:fe2D10|anon-EST:fe2D12	0	0
9371	42310	Drosophila melanogaster	unc79	CG5237 gene product from transcript CG5237-RB	CG5237|DUNC79|DmelCG5237|anon-WO0153538.74|anon-WO0172774.15|dunc79	0	0
9372	42311	Drosophila melanogaster	CG5217	CG5217 gene product from transcript CG5217-RA	DmelCG5217|anon-WO0140519.188	0	0
9373	42312	Drosophila melanogaster	CG14280	CG14280 gene product from transcript CG14280-RA	DmelCG14280	0	0
9374	42313	Drosophila melanogaster	Dl	Delta	0495/20|0926/11|1053/14|1119/09|1304/03|1423/11|1440/11|1485/04|C1|CG3619|CT12133|D|DL|DmelCG3619|E(ls)2|anon-WO0118547.269|delta|delta D1|dmDelta|l(3)05151|l(3)92Ab|l(3)j8C3	0	0
9375	42314	Drosophila melanogaster	Ino80	CG31212 gene product from transcript CG31212-RA	CG14279|CG18491|CG31212|CG3602|DmelCG31212|dINO80|dIno80	0	0
9376	42315	Drosophila melanogaster	CG3581	CG3581 gene product from transcript CG3581-RA	DmelCG3581	0	0
9377	42316	Drosophila melanogaster	CG31245	CG31245 gene product from transcript CG31245-RA	BcDNA:AT17586|CG3575|DmelCG31245	0	0
9378	42318	Drosophila melanogaster	CG3734	CG3734 gene product from transcript CG3734-RA	DmelCG3734	0	0
9379	42319	Drosophila melanogaster	CG18493	CG18493 gene product from transcript CG18493-RA	DmelCG18493	0	0
9380	42320	Drosophila melanogaster	CG3739	CG3739 gene product from transcript CG3739-RA	DmelCG3739	0	0
9381	42322	Drosophila melanogaster	CG5316	CG5316 gene product from transcript CG5316-RC	DmelCG5316	0	0
9382	42323	Drosophila melanogaster	CG11626	CG11626 gene product from transcript CG11626-RB	DmelCG11626	0	0
9383	42324	Drosophila melanogaster	CG3517	CG3517 gene product from transcript CG3517-RA	DmelCG3517	0	0
9384	42325	Drosophila melanogaster	CG31221	CG31221 gene product from transcript CG31221-RA	BcDNA:GH25289|CG11625|CG7237|DmelCG31221|anon-EST:Posey251	0	0
9385	42326	Drosophila melanogaster	CG6255	CG6255 gene product from transcript CG6255-RA	AAF55672|DmelCG6255|anon-WO0140519.197	0	0
9386	42327	Drosophila melanogaster	Dys	Dystrophin	CG17750|CG31175|CG34157|CG7240|CG7243|CG7344|DLP|DLP1|DLP186|DLP2|DLP3|DmDLP|DmDYS|DmelCG34157|Dp117|Dp186|Dp205|GI3046716|IDLP|det|dmDLP|dmDp186|dys	0	0
9387	42328	Drosophila melanogaster	CG15025	CG15025 gene product from transcript CG15025-RA	DmelCG15025	0	0
9388	42333	Drosophila melanogaster	Cpr92A	Cuticular protein 92A	CG6240|DmelCpr92A|DmelCG6240	0	0
9389	42334	Drosophila melanogaster	CG7333	CG7333 gene product from transcript CG7333-RB	DmelCG7333	0	0
9390	42335	Drosophila melanogaster	CG7342	CG7342 gene product from transcript CG7342-RB	DmelCG7342	0	0
9391	42336	Drosophila melanogaster	CG17751	CG17751 gene product from transcript CG17751-RB	DmelCG17751	0	0
9392	42337	Drosophila melanogaster	CG17752	CG17752 gene product from transcript CG17752-RA	DmelCG17752	0	0
9393	42338	Drosophila melanogaster	CG16727	CG16727 gene product from transcript CG16727-RA	DmelCG16727	0	0
9394	42339	Drosophila melanogaster	CG6231	CG6231 gene product from transcript CG6231-RC	DmelCG6231	0	0
9395	42340	Drosophila melanogaster	CG16718	CG16718 gene product from transcript CG16718-RA	DmelCG16718	0	0
9396	42341	Drosophila melanogaster	Vha13	Vacuolar H[+] ATPase G-subunit	BcDNA:AT14009|CG6213|DmelCG6213|V-ATPase|VhaG|l(3)05113|vha13	0	0
9397	42342	Drosophila melanogaster	Nup58	Nucleoporin 58	CG7360|Dm NUP58|DmelCG7360	0	0
9398	42343	Drosophila melanogaster	CG6195	CG6195 gene product from transcript CG6195-RA	BEST:GH15838|DmelCG6195	0	0
9399	42344	Drosophila melanogaster	CG31219	CG31219 gene product from transcript CG31219-RA	CG16731|DmelCG31219	0	0
9400	42345	Drosophila melanogaster	CG6184	CG6184 gene product from transcript CG6184-RD	DmelCG6184	0	0
9401	42347	Drosophila melanogaster	CG7432	CG7432 gene product from transcript CG7432-RB	DmelCG7432|SP19|c-SP19	0	0
9402	42348	Drosophila melanogaster	Naam	Nicotinamide amidase	AAM52663|CG31216|CG7444|CG7500|D-NAAM|DmelCG31216|anon-WO0172774.59|anon-WO0172774.60	0	0
9403	42350	Drosophila melanogaster	GluClalpha	CG7535 gene product from transcript CG7535-RG	BcDNA:HL07853|CG7535|Dm-GluCl|DmGlu|DmGluClalpha|DmelCG7535|DrosGlu-Cl-alpha|DrosGluCl|DrosGluCl-alpha|DrosGluCl-alpha1|GLUCL|GluCl|GluCla|dGluCl-alpha|glc	0	0
9404	42351	Drosophila melanogaster	CG6300	CG6300 gene product from transcript CG6300-RA	DmelCG6300	0	0
9405	42352	Drosophila melanogaster	CG11659	CG11659 gene product from transcript CG11659-RA	DmelCG11659	0	0
9406	42353	Drosophila melanogaster	CG11391	CG11391 gene product from transcript CG11391-RA	DmelCG11391	0	0
9407	42354	Drosophila melanogaster	CG11407	CG11407 gene product from transcript CG11407-RA	DmelCG11407	0	0
9408	42355	Drosophila melanogaster	CG11453	CG11453 gene product from transcript CG11453-RA	DmelCG11453	0	0
9409	42356	Drosophila melanogaster	bnl	branchless	Bnl|CG4608|DFGF|DmBnl|DmelCG4608|FGF|l(3)06916|l(3)neo52	0	0
9410	42357	Drosophila melanogaster	CG4662	CG4662 gene product from transcript CG4662-RB	DmelCG4662	0	0
9411	42358	Drosophila melanogaster	Ire1	Inositol-requiring enzyme-1	CG4583|DmelCG4583|IRE-like|IRE1|Ire|Ire-1|dIRE1|dire-1|ire-1	0	0
9412	42359	Drosophila melanogaster	CG11447	CG11447 gene product from transcript CG11447-RA	DmelCG11447	0	0
9413	42360	Drosophila melanogaster	CG4572	CG4572 gene product from transcript CG4572-RD	CT14806|DmelCG4572|anon-WO02059370.13	0	0
9414	42361	Drosophila melanogaster	CG4686	CG4686 gene product from transcript CG4686-RA	BcDNA:AT09463|DmelCG4686	0	0
9415	42362	Drosophila melanogaster	CG4562	CG4562 gene product from transcript CG4562-RB	CT14766|Dme_CG4562|DmelCG4562	0	0
9416	42363	Drosophila melanogaster	CG17186	CG17186 gene product from transcript CG17186-RA	DmelCG17186	0	0
9417	42364	Drosophila melanogaster	Arc42	CG4703 gene product from transcript CG4703-RA	CG4703|DmelCG4703|dARC42	0	0
9418	42366	Drosophila melanogaster	Pk92B	Protein kinase at 92B	ASK1|Ask1|CG4720|D-ASK|DASK|DASK1|DPk92B/ASK1|DmelCG4720|MEKK5/ASK1|dASK	0	0
9419	42367	Drosophila melanogaster	ninaE	neither inactivation nor afterpotential E	143283_at|1F9|BEST:GH11778|CG4550|DMELRH1|DRh1|Dm Rh1|DmRh1|DmelCG4550|FBgn0002940|Nina E|NinaE|R|RH1|Rh|Rh-1|Rh1|Rh1(ninaE)|Rh1/ninaE|opsin|ora|rh1|rh1/ninaE|rhodopsin	0	0
9420	42368	Drosophila melanogaster	CG4733	CG4733 gene product from transcript CG4733-RD	DmelCG4733|PP2A-B''|PR-72|PR72|dPR72	0	0
9421	42369	Drosophila melanogaster	CG4538	CG4538 gene product from transcript CG4538-RA	DmelCG4538	0	0
9422	42370	Drosophila melanogaster	Surf6	Surfeit 6	CG4510|DmelCG4510	0	0
9423	42371	Drosophila melanogaster	RhoGAP92B	CG4755 gene product from transcript CG4755-RA	CG4755|DmelCG4755	0	0
9424	42373	Drosophila melanogaster	CG4468	CG4468 gene product from transcript CG4468-RA	DmelCG4468	0	0
9425	42374	Drosophila melanogaster	CG4465	CG4465 gene product from transcript CG4465-RA	DmelCG4465	0	0
9426	42375	Drosophila melanogaster	CG4770	CG4770 gene product from transcript CG4770-RA	DmelCG4770	0	0
9427	42376	Drosophila melanogaster	CG4462	CG4462 gene product from transcript CG4462-RA	DmelCG4462	0	0
9428	42377	Drosophila melanogaster	CG4459	CG4459 gene product from transcript CG4459-RA	DmelCG4459	0	0
9429	42379	Drosophila melanogaster	mira	miranda	CG12249|DmelCG12249|MIR|MIRA|Mir|Mira|Miranda|mir	0	0
9430	42380	Drosophila melanogaster	Hs6st	Heparan sulfate 6-O-sulfotransferase	CG11683|CG17188|CG18533|CG4451|DmHs6st|DmelCG4451|dHS6ST|hs6st	0	0
9431	42381	Drosophila melanogaster	CG4783	CG4783 gene product from transcript CG4783-RA	DmelCG4783|GH26202	0	0
9432	42382	Drosophila melanogaster	CG31213	CG31213 gene product from transcript CG31213-RA	CG11460|CG4794|DmelCG31213	0	0

9433	42385	Drosophila melanogaster	MED25	Mediator complex subunit 25	Arc92|CG12254|DmelCG12254|LD07688|Med25|dARC92|dDrip97|med25	0	0
9434	42386	Drosophila melanogaster	CG17190	CG17190 gene product from transcript CG17190-RB	DmelCG17190	0	0
9435	42387	Drosophila melanogaster	CG4836	CG4836 gene product from transcript CG4836-RC	DM_7300579|DmelCG4836|anon-WO0140519.105|anon-WO0140519.33|bs15h04.y1|bs36e10.y1	0	0
9436	42388	Drosophila melanogaster	CG4433	CG4433 gene product from transcript CG4433-RC	DmelCG4433	0	0
9437	42389	Drosophila melanogaster	psidin	phagocyte signaling impaired	CG4845|DmelCG4845|ird16	0	0
9438	42390	Drosophila melanogaster	CG4424	CG4424 gene product from transcript CG4424-RA	DmelCG4424	0	0
9439	42391	Drosophila melanogaster	CG4854	CG4854 gene product from transcript CG4854-RA	DmelCG4854	0	0
9440	42392	Drosophila melanogaster	trem	trade embargo	CG4413|DmelCG4413	0	0
9441	42393	Drosophila melanogaster	CG4936	CG4936 gene product from transcript CG4936-RA	DmelCG4936	0	0
9442	42394	Drosophila melanogaster	CG10889	CG10889 gene product from transcript CG10889-RA	DmelCG10889	0	0
9443	42396	Drosophila melanogaster	CG4390	CG4390 gene product from transcript CG4390-RB	DmelCG4390	0	0
9444	42397	Drosophila melanogaster	CG4973	CG4973 gene product from transcript CG4973-RA	DmelCG4973	0	0
9445	42398	Drosophila melanogaster	Rh3	Rhodopsin 3	CG10888|DMELRH3|Dm Rh3|DmelCG10888|FBgn0003249|RH3|Rh|rh3	0	0
9446	42399	Drosophila melanogaster	CG10887	CG10887 gene product from transcript CG10887-RA	DmelCG10887	0	0
9447	42400	Drosophila melanogaster	CG5023	CG5023 gene product from transcript CG5023-RA	DmelCG5023	0	0
9448	42401	Drosophila melanogaster	CG17199	CG17199 gene product from transcript CG17199-RA	DmelCG17199	0	0
9449	42402	Drosophila melanogaster	CG34139	CG34139 gene product from transcript CG34139-RD	BcDNA:AT13703|CG15682|CG15683|CG31209|CG31499|CG5030|CG5035|DmelCG34139	0	0
9450	42409	Drosophila melanogaster	CG4367	CG4367 gene product from transcript CG4367-RA	DmelCG4367	0	0
9451	42410	Drosophila melanogaster	CG4362	CG4362 gene product from transcript CG4362-RA	DmelCG4362	0	0
9452	42412	Drosophila melanogaster	CG42668	CG42668 gene product from transcript CG42668-RK	CG15684|CG5077|DmelCG42668|Dmel_CG15684|Dmel_CG5077	0	0
9453	42413	Drosophila melanogaster	CG4360	CG4360 gene product from transcript CG4360-RA	DmelCG4360	0	0
9454	42414	Drosophila melanogaster	Sirt2	CG5085 gene product from transcript CG5085-RA	CG5085|D.mel2|DmelCG5085|dSIRT2	0	0
9455	42415	Drosophila melanogaster	Ir92a	Ionotropic receptor 92a	CG15685|DmelCG15685|IR92a	0	0
9456	42416	Drosophila melanogaster	MtnC	Metallothionein C	CG5097|DmelCG5097	0	0
9457	42421	Drosophila melanogaster	CG4335	CG4335 gene product from transcript CG4335-RA	DmelCG4335	0	0
9458	42422	Drosophila melanogaster	CG10881	CG10881 gene product from transcript CG10881-RA	DmelCG10881|cg10881|eIF3-S4	0	0
9459	42423	Drosophila melanogaster	Dic2	Dicarboxylate carrier 2	CG4323|DmDic2p|DmelCG4323	0	0
9460	42424	Drosophila melanogaster	MtnB	Metallothionein B	CG4312|DmelCG4312|DrosMto|MTN|MTO|Mto|mtnB	0	0
9461	42425	Drosophila melanogaster	Or92a	Odorant receptor 92a	92E.1|92a|CG17916|DOR111|DmelCG17916|OR 92a	0	0
9462	42426	Drosophila melanogaster	CG4288	CG4288 gene product from transcript CG4288-RB	DmelCG4288	0	0
9463	42428	Drosophila melanogaster	Stat92E	Signal-transducer and activator of transcription protein	CG4257|D-STAT|D-Stat|D-stat|D-stat/stat92E|DRODSRC|DSRC|DSTAT|DmSTAT|DmelCG4257|Dstat|Dstat 92E|Dstat92E|SD-stat|STAT|STAT 92E|STAT92E|Stat|Stat1alpha-like|d-STAT|dSTAT|dSTAT92E|dST	0	0
9464	42429	Drosophila melanogaster	att-ORFA	alternative testis transcripts open reading frame A	CG4241|DmelCG4241|att	0	0
9465	42431	Drosophila melanogaster	CG5191	CG5191 gene product from transcript CG5191-RE	DmelCG5191	0	0
9466	42432	Drosophila melanogaster	CG10877	CG10877 gene product from transcript CG10877-RA	DmelCG10877	0	0
9467	42433	Drosophila melanogaster	TFAM	mitochondrial transcription factor A	CG4217|D-mtTFA|DmelCG4217|Tfam|d-TFAM|d-mttfa|d-tfam|mtTFA|mttfa|tfam	0	0
9468	42434	Drosophila melanogaster	CG5412	CG5412 gene product from transcript CG5412-RA	DmelCG5412	0	0
9469	42435	Drosophila melanogaster	Elongin-B	Elongin B	CG4204|DmelCG4204	0	0
9470	42436	Drosophila melanogaster	Srp14	Signal recognition particle protein 14	BcDNA:RE48595|CG5417|DmelCG5417	0	0
9471	42437	Drosophila melanogaster	CG16953	CG16953 gene product from transcript CG16953-RB	DmelCG16953	0	0
9472	42438	Drosophila melanogaster	Sep2	Septin-2	CG4173|DmelCG4173|sep2	0	0
9473	42439	Drosophila melanogaster	Srp72	Signal recognition particle protein 72	CG5434|DmelCG5434	0	0
9474	42440	Drosophila melanogaster	CG4159	CG4159 gene product from transcript CG4159-RA	DmelCG4159	0	0
9475	42443	Drosophila melanogaster	CG15923	CG15923 gene product from transcript CG15923-RB	DmelCG15923	0	0
9476	42444	Drosophila melanogaster	CG5466	CG5466 gene product from transcript CG5466-RA	DmelCG5466	0	0
9477	42445	Drosophila melanogaster	H	Hairless	CG5460|DmelCG5460	0	0
9478	42446	Drosophila melanogaster	Pi3K92E	CG4141 gene product from transcript CG4141-RB	CG4141|DmelCG4141|Dmp110|Dp110|Dp110/PI3K|PI(3)K|PI-3 kinase|PI-3-K|PI3'K|PI3K|PI3K-92D|PI3K-92E/Dp110|PI3K-Dp110|PI3K-dp110|PI3K92E|PI3k|PI[[3]]K|Pi3K|Pi3K92D|Pi3Kp110|anon-92Ed|dP	0	0
9479	42447	Drosophila melanogaster	Lrrk	Leucine-rich repeat kinase	CG5483|CT17358|DmelCG5483|LRRK|LRRK2|dLRRK|lrrk	0	0
9480	42449	Drosophila melanogaster	Nep4	Neprilysin 4	CG4058|DmeNEP4|DmelCG4058|NEP4|dNEP4|nep4	0	0
9481	42450	Drosophila melanogaster	Cpr92F	Cuticular protein 92F	CG5494|DmelCG5494	0	0
9482	42451	Drosophila melanogaster	CG4000	CG4000 gene product from transcript CG4000-RA	DmelCG4000	0	0
9483	42452	Drosophila melanogaster	Oct-TyrR	Octopamine-Tyramine receptor	CG7485|DmTyrR|DmelCG7485|Dmoct/tyr|DrmOTR|OA-R|OAR_DROME|OTR|OcR|Ocr|OctoR1|OctyR99AB|TYR|TyR|Tyr|Tyr-dro|TyrR|hono|l(3)neo30	0	0
9484	42453	Drosophila melanogaster	cdc2c	CG10498 gene product from transcript CG10498-RC	CDC2C|CDC2c|CDK2|CDK2/CDC2c|CG10498|Cdc2c|Cdk2|Dcdc2c|DmCdc2|DmCdk2|Dmcdc2c|DmelCG10498|S(Sev-CycE)3A|cdk2|dCdk2	0	0
9485	42454	Drosophila melanogaster	CG17267	CG17267 gene product from transcript CG17267-RA	DmelCG17267	0	0
9486	42455	Drosophila melanogaster	CG42322	CG42322 gene product from transcript CG42322-RI	CG15688|CG15689|CG15690|CG31203|DmelCG42322|Dmel_CG15690|Dmel_CG31203	0	0
9487	42456	Drosophila melanogaster	CG31199	CG31199 gene product from transcript CG31199-RA	CG17837|DmelCG31199	0	0
9488	42457	Drosophila melanogaster	Pros28.1A	Proteasome 28kD subunit 1A	CG17268|DmelCG17268|Prosalpha4T1|a4-t1|alpha4t1|alpha4t1_dm|dalpha7|pros28.1A	0	0
9489	42460	Drosophila melanogaster	CG17838	CG17838 gene product from transcript CG17838-RA	AI945337|DmelCG17838|anon-WO0118547.613|cg17838|l(3)03806	0	0
9490	42462	Drosophila melanogaster	CG17270	CG17270 gene product from transcript CG17270-RA	DmelCG17270|anon-Liu	0	0
9491	42463	Drosophila melanogaster	CG17271	CG17271 gene product from transcript CG17271-RB	DmelCG17271	0	0
9492	42464	Drosophila melanogaster	RpS20	Ribosomal protein S20	CG15693|DmelCG15693|M(3)93A|Rps20|anon-EST:Posey111|anon-EST:Posey122|anon-EST:Posey147	0	0
9493	42465	Drosophila melanogaster	CG17272	CG17272 gene product from transcript CG17272-RA	BEST:GH27755|DmelCG17272	0	0
9494	42466	Drosophila melanogaster	CG17273	CG17273 gene product from transcript CG17273-RA	BcDNA:LD32788|DmelCG17273|PURA_DROME	0	0
9495	42467	Drosophila melanogaster	Synd	Syndapin	CG15694|CG33094|D-Synd|DmelCG33094|synd	0	0
9496	42468	Drosophila melanogaster	CG15695	CG15695 gene product from transcript CG15695-RA	DmelCG15695	0	0
9497	42469	Drosophila melanogaster	CG15696	CG15696 gene product from transcript CG15696-RA	DmelCG15696	0	0
9498	42470	Drosophila melanogaster	RpS30	Ribosomal protein S30	CG15697|DmelCG15697|M(3)93A|S30|anon-EST:Posey127	0	0
9499	42471	Drosophila melanogaster	Ir93a	Ionotropic receptor 93a	CG17274|CG17275|CG42315|CT35907|DmelCG42315|Dmel_CG17274|Dmel_CG17275|IR93a	0	0
9500	42473	Drosophila melanogaster	CG3822	CG3822 gene product from transcript CG3822-RA	CT12759|DmelCG3822	0	0
9501	42474	Drosophila melanogaster	TotB	Turandot B	BcDNA:LP06769|CG5609|DmelCG5609|tot	0	0
9502	42475	Drosophila melanogaster	CG10830	CG10830 gene product from transcript CG10830-RA	DmelCG10830	0	0
9503	42476	Drosophila melanogaster	CG5621	CG5621 gene product from transcript CG5621-RA	CT17756|DmelCG5621|anon-WO0138359.5	0	0
9504	42477	Drosophila melanogaster	CG31191	CG31191 gene product from transcript CG31191-RA	CG17276|CG17277|DmelCG31191	0	0
9505	42478	Drosophila melanogaster	CG5630	CG5630 gene product from transcript CG5630-RB	DmelCG5630	0	0
9506	42481	Drosophila melanogaster	Calx	Na/Ca-exchange protein	9C5|CALX|CALX1.1|CG5685|CalX|Dmel/Ncx|DmelCG5685|NCX|Ncx|anon-93ABa|calX|calx	0	0
9507	42482	Drosophila melanogaster	CG10827	CG10827 gene product from transcript CG10827-RA	DmelCG10827	0	0
9508	42483	Drosophila melanogaster	CG5697	CG5697 gene product from transcript CG5697-RA	BcDNA:RH32180|DmelCG5697	0	0
9509	42484	Drosophila melanogaster	Rlip	Ral interacting protein	CG11622|D-RLIP|DmelCG11622|RLIP|RhoGAP-93B3|dRLIP|dRalBP|dRlip	0	0
9510	42485	Drosophila melanogaster	Dhc93AB	Dynein heavy chain at 93AB	CG3723|DHC|Dhc|Dhc 93AB|DmelCG3723|dhc	0	0
9511	42486	Drosophila melanogaster	CG12278	CG12278 gene product from transcript CG12278-RA	DmelCG12278	0	0
9512	42487	Drosophila melanogaster	CG31189	CG31189 gene product from transcript CG31189-RB	CG7096|DmelCG31189	0	0
9513	42488	Drosophila melanogaster	CG7079	CG7079 gene product from transcript CG7079-RA	DmelCG7079|cg7079	0	0
9514	42489	Drosophila melanogaster	CG17279	CG17279 gene product from transcript CG17279-RB	DmelCG17279	0	0
9515	42490	Drosophila melanogaster	Mvl	Malvolio	CG3671|DmelCG3671|NRM2|dNRAMP|mvl	0	0
9516	42491	Drosophila melanogaster	Cortactin	CG3637 gene product from transcript CG3637-RD	CG3637|DmelCG3637|cortactin|cttn	0	0
9517	42492	Drosophila melanogaster	AnnIX	Annexin IX	Ann|Anx B9|AnxB9|CG5730|DmelCG5730|annexin	0	0
9518	42493	Drosophila melanogaster	r-l	rudimentary-like	CG3593|DmelCG3593|OMPDC|OPRT|UMPS|ral	0	0
9519	42494	Drosophila melanogaster	dmrt93B	doublesex-Mab related 93B	CG5737|DmelCG5737|dmrt93b	0	0
9520	42495	Drosophila melanogaster	CG7056	CG7056 gene product from transcript CG7056-RA	DmelCG7056	0	0
9521	42496	Drosophila melanogaster	RhoGAP93B	CG3421 gene product from transcript CG3421-RA	CG3421|CrGAP|DmelCG3421|Vilse|vilse	0	0
9522	42497	Drosophila melanogaster	CG7044	CG7044 gene product from transcript CG7044-RA	DmelCG7044	0	0
9523	42498	Drosophila melanogaster	CG5745	CG5745 gene product from transcript CG5745-RA	DmelCG5745	0	0
9524	42499	Drosophila melanogaster	sec15	CG7034 gene product from transcript CG7034-RA	3R41|CG7034|DmelCG7034|Sec15|Sec15p|dsec15	0	0
9525	42500	Drosophila melanogaster	rtet	tetracycline resistance	BcDNA:LD28419|CG5760|DmelCG5760|Drtet|anon-WO0119857.1|l(3)07086	0	0
9526	42501	Drosophila melanogaster	Rab11	Rab-protein 11	AAF55850|CG5771|DRAB11|DRab11|Dm Rab11|DmRab11|DmelCG5771|Drab11|RAB11|Rab-r11|l(3)93Bi|l(3)j2D1|rab-11|rab11|rlr7	0	0
9527	42502	Drosophila melanogaster	ppan	peter pan	CG5786|DmelCG5786|PPAN|Ppan|l(3)02231|l(3)j6B6	0	0
9528	42503	Drosophila melanogaster	CG17282	CG17282 gene product from transcript CG17282-RA	DmelCG17282	0	0
9529	42504	Drosophila melanogaster	slmb	supernumerary limbs	BcDNA:GM02031|CG3412|DmelCG3412|Slimb|Slimb/Beta-TRCP1|Slimb/beta-TRCP|Slmb|crd|ica|l(3)00295|shv|slimb	0	0
9530	42505	Drosophila melanogaster	CG5793	CG5793 gene product from transcript CG5793-RC	DmelCG5793|FBgn0038858	0	0
9531	42506	Drosophila melanogaster	Obp93a	Odorant-binding protein 93a	CG17284|DmelCG17284	0	0
9532	42507	Drosophila melanogaster	CG10825	CG10825 gene product from transcript CG10825-RC	DmelCG10825	0	0
9533	42508	Drosophila melanogaster	CG7009	CG7009 gene product from transcript CG7009-RA	DmelCG7009	0	0
9534	42509	Drosophila melanogaster	Ubpy	UBPY ortholog	CG5798|CT18196|DmelCG5798|dUBPY	0	0
9535	42510	Drosophila melanogaster	CG5802	CG5802 gene product from transcript CG5802-RB	DmelCG5802|NEST:bs03h03|anon-EST:fe1H6	0	0
9536	42512	Drosophila melanogaster	CG10824	CG10824 gene product from transcript CG10824-RA	CT11359|DmelCG10824	0	0
9537	42513	Drosophila melanogaster	CG5810	CG5810 gene product from transcript CG5810-RA	CT18214|DmelCG5810	0	0
9538	42514	Drosophila melanogaster	Snmp1	Sensory neuron membrane protein 1	CG7000|DmelCG7000|SNMP|SNMP1|Snmp|snmp1	0	0
9539	42515	Drosophila melanogaster	SNF4Agamma	SNF4/AMP-activated protein kinase gamma subunit	AMPgamma|CG17299|CG5806|DmelCG17299|EP3015b|FBgn0025803|SNF4|SNF4A|SNF4Ag|SNF4a|Snfg|Snfgamma|anon-WO0118547.338|anon-WO0118547.575|anon-WO0257455.21|loe	0	0
9540	42516	Drosophila melanogaster	CG5862	CG5862 gene product from transcript CG5862-RA	DmelCG5862	0	0
9541	42517	Drosophila melanogaster	CG3353	CG3353 gene product from transcript CG3353-RA	DmelCG3353	0	0
9542	42518	Drosophila melanogaster	Oga	O-GlcNAcase	CG5871|DmelCG5871	0	0
9543	42519	Drosophila melanogaster	CG3337	CG3337 gene product from transcript CG3337-RA	DmelCG3337	0	0

<table>
<tr><td>9544</td><td>42520</td><td>Drosophila melanogaster</td><td>Nelf-A</td><td>Negative elongation factor A</td><td>BcDNA:SD07139|CG5874|DmelCG5874|NELF|NELF-A</td><td>0</td><td>0</td></tr>
<tr><td>9545</td><td>42521</td><td>Drosophila melanogaster</td><td>e</td><td>ebony</td><td>CG3331|DmelCG3331</td><td>0</td><td>0</td></tr>
<tr><td>9546</td><td>42522</td><td>Drosophila melanogaster</td><td>CG5892</td><td>CG5892 gene product from transcript CG5892-RA</td><td>DmelCG5892</td><td>0</td><td>0</td></tr>
<tr><td>9547</td><td>42523</td><td>Drosophila melanogaster</td><td>ETHR</td><td>CG5911 gene product from transcript CG5911-RB</td><td>CG5911|CG5911a|CG5911b|Dm ETHRa|DmelCG5911|anon-WO0170980.37|anon-WO0170980.38</td><td>0</td><td>0</td></tr>
<tr><td>9548</td><td>42524</td><td>Drosophila melanogaster</td><td>Rab1</td><td>Rab-protein 1</td><td>AAF55873|CG3320|DRAB1|DRab1|Dm Rab1|DmRab1|DmelCG3320|RAB1a|Rab1a|dRab1|dar6|drab1|rab1</td><td>0</td><td>0</td></tr>
<tr><td>9549</td><td>42525</td><td>Drosophila melanogaster</td><td>AP-2sigma</td><td>CG6056 gene product from transcript CG6056-RA</td><td>AP2|CG6056|DmelCG6056|cg6056|sigma2</td><td>0</td><td>0</td></tr>
<tr><td>9550</td><td>42526</td><td>Drosophila melanogaster</td><td>CG5919</td><td>CG5919 gene product from transcript CG5919-RA</td><td>DmelCG5919</td><td>0</td><td>0</td></tr>
<tr><td>9551</td><td>42527</td><td>Drosophila melanogaster</td><td>CG3308</td><td>CG3308 gene product from transcript CG3308-RA</td><td>DmelCG3308</td><td>0</td><td>0</td></tr>
<tr><td>9552</td><td>42528</td><td>Drosophila melanogaster</td><td>CG3301</td><td>CG3301 gene product from transcript CG3301-RB</td><td>DmelCG3301</td><td>0</td><td>0</td></tr>
<tr><td>9553</td><td>42529</td><td>Drosophila melanogaster</td><td>CG17298</td><td>CG17298 gene product from transcript CG17298-RA</td><td>BcDNA:RH37844|CT35916|DmelCG17298</td><td>0</td><td>0</td></tr>
<tr><td>9554</td><td>42530</td><td>Drosophila melanogaster</td><td>SIFR</td><td>SIFamide receptor</td><td>CG10823|DmelCG10823|DrmSIFR|SIF-R|SIFa-R|anon-WO0170980.43|anon-WO0170980.44</td><td>0</td><td>0</td></tr>
<tr><td>9555</td><td>42531</td><td>Drosophila melanogaster</td><td>CG16791</td><td>CG16791 gene product from transcript CG16791-RA</td><td>DmelCG16791|anon-WO0172774.78</td><td>0</td><td>0</td></tr>
<tr><td>9556</td><td>42536</td><td>Drosophila melanogaster</td><td>tin</td><td>tinman</td><td>CG7895|DROHOXHK4|DROHOXNK4|DmNK-4|DmelCG7895|HOX|NK-4|NK-4/msh-2|NK4|NK4/msh-2|TIN|Tin|msh-2|msh2|tin/NK4</td><td>0</td><td>0</td></tr>
<tr><td>9557</td><td>42537</td><td>Drosophila melanogaster</td><td>bap</td><td>bagpipe</td><td>Bap|CG7902|DmelCG7902|NK-3|NK3|bgp|dNK-3|nk-3</td><td>0</td><td>0</td></tr>
<tr><td>9558</td><td>42538</td><td>Drosophila melanogaster</td><td>CG6475</td><td>CG6475 gene product from transcript CG6475-RC</td><td>DmelCG6475</td><td>0</td><td>0</td></tr>
<tr><td>9559</td><td>42539</td><td>Drosophila melanogaster</td><td>CG7907</td><td>CG7907 gene product from transcript CG7907-RB</td><td>DmelCG7907</td><td>0</td><td>0</td></tr>
<tr><td>9560</td><td>42540</td><td>Drosophila melanogaster</td><td>CheB93a</td><td>Chemosensory protein B 93a</td><td>CG15503|DmelCG15503</td><td>0</td><td>0</td></tr>
<tr><td>9561</td><td>42541</td><td>Drosophila melanogaster</td><td>lbl</td><td>ladybird late</td><td>CG6570|DmelCG6570|Lb|Lbl|NK?|NKch4|lb|nkch4</td><td>0</td><td>0</td></tr>
<tr><td>9562</td><td>42542</td><td>Drosophila melanogaster</td><td>lbe</td><td>ladybird early</td><td>CG6545|DmelCG6545|Hox11-D125|Lb|Lbe|NK?|lb</td><td>0</td><td>0</td></tr>
<tr><td>9563</td><td>42543</td><td>Drosophila melanogaster</td><td>CG7922</td><td>CG7922 gene product from transcript CG7922-RA</td><td>DmelCG7922|cg7922</td><td>0</td><td>0</td></tr>
<tr><td>9564</td><td>42544</td><td>Drosophila melanogaster</td><td>C15</td><td>CG7937 gene product from transcript CG7937-RA</td><td>311|93Bal|CG7937|Cll|DmelCG7937|Ect5|Hox11-311|c15|cll</td><td>0</td><td>0</td></tr>
<tr><td>9565</td><td>42545</td><td>Drosophila melanogaster</td><td>CG7956</td><td>CG7956 gene product from transcript CG7956-RA</td><td>DmelCG7956</td><td>0</td><td>0</td></tr>
<tr><td>9566</td><td>42547</td><td>Drosophila melanogaster</td><td>slou</td><td>slouch</td><td>CG6534|DmelCG6534|NK-1|NK1|NK1/S59|Nk1|S-59|S59|S59/NK-1|prd9|s59|slou/NK1</td><td>0</td><td>0</td></tr>
<tr><td>9567</td><td>42548</td><td>Drosophila melanogaster</td><td>CG15498</td><td>CG15498 gene product from transcript CG15498-RA</td><td>DmelCG15498</td><td>0</td><td>0</td></tr>
<tr><td>9568</td><td>42549</td><td>Drosophila melanogaster</td><td>InR</td><td>Insulin-like receptor</td><td>18402|CG18402|DIHR|DILR|DIR|DIRH|DIRbeta|DInR|DInr|Dir-a|Dir-b|DmelCG18402|INR|IR|Inr|Inr-alpha|Inr-beta|InsR|dINR|dIR|dIRH|dInR|dInr|dInsR|dinr|dir|er10|inr|insulin/insulin-like growth factor recepto</td><td>0</td><td>0</td></tr>
<tr><td>9569</td><td>42550</td><td>Drosophila melanogaster</td><td>E2f</td><td>E2F transcription factor</td><td>CG6376|DRTF1/E2F|DmE2F-1|DmelCG6376|Dp|E(Sev-CycE)3A|E(var)3-93E|E(var)3-95E|E(var)93E|E2F|E2F-1|E2F1|E2f1|Evar(3)164|dE2F|dE2F1|dE2f|dE2f1|de2f1|def21|drosE2F1|e2f|e2f1|l(3)07172|l(3)3B</td><td>0</td><td>0</td></tr>
<tr><td>9570</td><td>42551</td><td>Drosophila melanogaster</td><td>CG6353</td><td>CG6353 gene product from transcript CG6353-RA</td><td>BcDNA:SD28072|DmelCG6353</td><td>0</td><td>0</td></tr>
<tr><td>9571</td><td>42552</td><td>Drosophila melanogaster</td><td>CG15497</td><td>CG15497 gene product from transcript CG15497-RA</td><td>DmelCG15497</td><td>0</td><td>0</td></tr>
<tr><td>9572</td><td>42553</td><td>Drosophila melanogaster</td><td>DNApol-alpha180</td><td>DNA polymerase alpha 180kD</td><td>183 kDa pol alpha|AAB24152|CG6349|DNA Pol-alpha180|DNA polalpha|DNA-Polalpha|DNApol|DNApol-alpha|DNApola|DNApolalpha|DNApolalpha180|DmPolalpha|DmelCG6349|Dmpol alpha|E(mus304)|</td><td>0</td><td>0</td></tr>
<tr><td>9573</td><td>42554</td><td>Drosophila melanogaster</td><td>CG31176</td><td>CG31176 gene product from transcript CG31176-RA</td><td>CG15495|CG6328|DmelCG31176|EP(3)3662|EP3662|RE56061</td><td>0</td><td>0</td></tr>
<tr><td>9574</td><td>42556</td><td>Drosophila melanogaster</td><td>CG5849</td><td>CG5849 gene product from transcript CG5849-RB</td><td>DmelCG5849</td><td>0</td><td>0</td></tr>
<tr><td>9575</td><td>42557</td><td>Drosophila melanogaster</td><td>CG31233</td><td>CG31233 gene product from transcript CG31233-RA</td><td>CG5839|DmelCG31233|anon-WO0140519.125|anon-WO0140519.65</td><td>0</td><td>0</td></tr>
<tr><td>9576</td><td>42558</td><td>Drosophila melanogaster</td><td>CG42335</td><td>CG42335 gene product from transcript CG42335-RC</td><td>CG13420|CG31177|CG5845|DmelCG42335|Dmel_CG13420|Dmel_CG31177</td><td>0</td><td>0</td></tr>
<tr><td>9577</td><td>42560</td><td>Drosophila melanogaster</td><td>burs</td><td>bursicon</td><td>BURS|Bursalpha|CG13419|DmelCG13419|burs alpha|burs-alpha|bursalpha|frl</td><td>0</td><td>0</td></tr>
<tr><td>9578</td><td>42561</td><td>Drosophila melanogaster</td><td>GABA-B-R2</td><td>metabotropic GABA-B receptor subtype 2</td><td>BcDNA:GH07312|CG6706|D-GABA[[B]]R2|D-Gaba2|DmelCG6706|GABA[[B]]-R2|GABA[[B]]R2|GB2|GH07312|anon-WO0170980.58|anon-WO0170980.59</td><td>0</td><td>0</td></tr>
<tr><td>9579</td><td>42562</td><td>Drosophila melanogaster</td><td>CG6800</td><td>CG6800 gene product from transcript CG6800-RA</td><td>AC014407|DmelCG6800</td><td>0</td><td>0</td></tr>
<tr><td>9580</td><td>42563</td><td>Drosophila melanogaster</td><td>Rpl12</td><td>CG13418 gene product from transcript CG13418-RA</td><td>CG13418|DmelCG13418</td><td>0</td><td>0</td></tr>
<tr><td>9581</td><td>42564</td><td>Drosophila melanogaster</td><td>tsl</td><td>torso-like</td><td>CG6705|DmelCG6705|fs(3)00617</td><td>0</td><td>0</td></tr>
<tr><td>9582</td><td>42567</td><td>Drosophila melanogaster</td><td>CASK</td><td>CASK ortholog</td><td>CAKI|CAMGUK|CG13412|CG13413|CG6703|CMG|CMG/CASK|Caki|CamGUK|Camguk|Cask|Cmg|DLin-2|DmelCG6703|caki|camguk|cmg|dCASK</td><td>0</td><td>0</td></tr>
<tr><td>9583</td><td>42568</td><td>Drosophila melanogaster</td><td>Gr93a</td><td>Gustatory receptor 93a</td><td>93F.1|CG13417|DmelCG13417|GR93|GR93F.1|GR93a|Gr93F1</td><td>0</td><td>0</td></tr>
<tr><td>9584</td><td>42571</td><td>Drosophila melanogaster</td><td>glec</td><td>gliolectin</td><td>CG6575|DmelCG6575</td><td>0</td><td>0</td></tr>
<tr><td>9585</td><td>42572</td><td>Drosophila melanogaster</td><td>lsn</td><td>larsen</td><td>CG6637|DmelCG6637|Vps22|vps22</td><td>0</td><td>0</td></tr>
<tr><td>9586</td><td>42573</td><td>Drosophila melanogaster</td><td>CG6569</td><td>CG6569 gene product from transcript CG6569-RA</td><td>DmelCG6569</td><td>0</td><td>0</td></tr>
<tr><td>9587</td><td>42574</td><td>Drosophila melanogaster</td><td>Cby</td><td>Chibby</td><td>CG13415|DmelCG13415</td><td>0</td><td>0</td></tr>
<tr><td>9588</td><td>42575</td><td>Drosophila melanogaster</td><td>CG31178</td><td>CG31178 gene product from transcript CG31178-RB</td><td>BEST:LP09246|BcDNA:LP09246|CG13414|CT32770|DmelCG31178|Dromel_CG31178_FBtr0084138_mORF|Tes87</td><td>0</td><td>0</td></tr>
<tr><td>9589</td><td>42576</td><td>Drosophila melanogaster</td><td>CG6656</td><td>CG6656 gene product from transcript CG6656-RA</td><td>DmelCG6656</td><td>0</td><td>0</td></tr>
<tr><td>9590</td><td>42577</td><td>Drosophila melanogaster</td><td>CG31465</td><td>CG31465 gene product from transcript CG31465-RA</td><td>CG6670|DmelCG31465</td><td>0</td><td>0</td></tr>
<tr><td>9591</td><td>42578</td><td>Drosophila melanogaster</td><td>fit</td><td>female-specific independent of transformer</td><td>CG17820|DmelCG17820</td><td>0</td><td>0</td></tr>
<tr><td>9592</td><td>42579</td><td>Drosophila melanogaster</td><td>CG17819</td><td>CG17819 gene product from transcript CG17819-RA</td><td>BcDNA:LP07781|DmelCG17819</td><td>0</td><td>0</td></tr>
<tr><td>9593</td><td>42580</td><td>Drosophila melanogaster</td><td>dnd</td><td>dead end</td><td>Arl3|CG6560|DmelCG6560</td><td>0</td><td>0</td></tr>
<tr><td>9594</td><td>42581</td><td>Drosophila melanogaster</td><td>CG6678</td><td>CG6678 gene product from transcript CG6678-RA</td><td>DmelCG6678</td><td>0</td><td>0</td></tr>
<tr><td>9595</td><td>42582</td><td>Drosophila melanogaster</td><td>CG6690</td><td>CG6690 gene product from transcript CG6690-RA</td><td>DmelCG6690</td><td>0</td><td>0</td></tr>
<tr><td>9596</td><td>42583</td><td>Drosophila melanogaster</td><td>CG17843</td><td>CG17843 gene product from transcript CG17843-RA</td><td>DmQSOX2|DmelCG17843|anon-WO0140519.66</td><td>0</td><td>0</td></tr>
<tr><td>9597</td><td>42585</td><td>Drosophila melanogaster</td><td>CG6332</td><td>CG6332 gene product from transcript CG6332-RA</td><td>DmelCG6332|anon-WO0140519.231</td><td>0</td><td>0</td></tr>
<tr><td>9598</td><td>42586</td><td>Drosophila melanogaster</td><td>CG6439</td><td>CG6439 gene product from transcript CG6439-RA</td><td>DmelCG6439|FBgn0038922|Idh</td><td>0</td><td>0</td></tr>
<tr><td>9599</td><td>42587</td><td>Drosophila melanogaster</td><td>CG6455</td><td>CG6455 gene product from transcript CG6455-RC</td><td>DmelCG6455</td><td>0</td><td>0</td></tr>
<tr><td>9600</td><td>42588</td><td>Drosophila melanogaster</td><td>mRpL35</td><td>mitochondrial ribosomal protein L35</td><td>BcDNA:RE35766|CG13410|DmelCG13410|MRP-L35</td><td>0</td><td>0</td></tr>
<tr><td>9601</td><td>42589</td><td>Drosophila melanogaster</td><td>CG6028</td><td>CG6028 gene product from transcript CG6028-RA</td><td>DmelCG6028</td><td>0</td><td>0</td></tr>
<tr><td>9602</td><td>42590</td><td>Drosophila melanogaster</td><td>Cchl</td><td>Cytochrome c heme lyase</td><td>CG6022|DmelCG6022|cchl</td><td>0</td><td>0</td></tr>
<tr><td>9603</td><td>42591</td><td>Drosophila melanogaster</td><td>ND42</td><td>NADH:ubiquinone reductase 42kD subunit precursor</td><td>CG6343|DmelCG6343</td><td>0</td><td>0</td></tr>
<tr><td>9604</td><td>42592</td><td>Drosophila melanogaster</td><td>CG13409</td><td>CG13409 gene product from transcript CG13409-RA</td><td>DmelCG13409</td><td>0</td><td>0</td></tr>
<tr><td>9605</td><td>42593</td><td>Drosophila melanogaster</td><td>CG6015</td><td>CG6015 gene product from transcript CG6015-RA</td><td>DmelCG6015|prp17</td><td>0</td><td>0</td></tr>
<tr><td>9606</td><td>42594</td><td>Drosophila melanogaster</td><td>BG4</td><td>CG12297 gene product from transcript CG12297-RA</td><td>CG12297|DmelCG12297|FADD|Fadd|dFADD|dFadd|dfadd</td><td>0</td><td>0</td></tr>
<tr><td>9607</td><td>42595</td><td>Drosophila melanogaster</td><td>pit</td><td>pitchoune</td><td>CG6375|DmRH15|DmelCG6375|PIT</td><td>0</td><td>0</td></tr>
<tr><td>9608</td><td>42596</td><td>Drosophila melanogaster</td><td>how</td><td>held out wings</td><td>24B|CG10293|DmelCG10293|HOW|How|KH93F|P62|SZ1|Who/How|anon-EST:Liang-2.39|clone 2.39|l(3)j5B5|l(3)j5D5|l(3)s2612|qkr|qkr[93F]|sci|stru|who</td><td>0</td><td>0</td></tr>
<tr><td>9609</td><td>42597</td><td>Drosophila melanogaster</td><td>CG13408</td><td>CG13408 gene product from transcript CG13408-RA</td><td>DmelCG13408</td><td>0</td><td>0</td></tr>
<tr><td>9610</td><td>42598</td><td>Drosophila melanogaster</td><td>CG5778</td><td>CG5778 gene product from transcript CG5778-RB</td><td>BcDNA:GH13168|CT18134|DmelCG5778</td><td>0</td><td>0</td></tr>
<tr><td>9611</td><td>42599</td><td>Drosophila melanogaster</td><td>CG13407</td><td>CG13407 gene product from transcript CG13407-RA</td><td>DmelCG13407</td><td>0</td><td>0</td></tr>
<tr><td>9612</td><td>42600</td><td>Drosophila melanogaster</td><td>CG42390</td><td>CG42390 gene product from transcript CG42390-RB</td><td>CG33334|CG5740|DmelCG42390|Dmel_CG33334|Dmel_CG5740</td><td>0</td><td>0</td></tr>
<tr><td>9613</td><td>42601</td><td>Drosophila melanogaster</td><td>SKIP</td><td>Shal K[+] channel interacting protein</td><td>CG13863|CG13864|CG31163|CG5736|CG7086|CG7087|DmelCG31163</td><td>0</td><td>0</td></tr>
<tr><td>9614</td><td>42602</td><td>Drosophila melanogaster</td><td>Gld2</td><td>CG5732 gene product from transcript CG5732-RB</td><td>CG5732|Dm1|DmCG5732|DmGLD2|DmelCG5732</td><td>0</td><td>0</td></tr>
<tr><td>9615</td><td>42606</td><td>Drosophila melanogaster</td><td>CG7084</td><td>CG7084 gene product from transcript CG7084-RB</td><td>DmelCG7084</td><td>0</td><td>0</td></tr>
<tr><td>9616</td><td>42608</td><td>Drosophila melanogaster</td><td>CG34377</td><td>CG34377 gene product from transcript CG34377-RB</td><td>CG13858|CG13859|CG13860|CG13861|DmelCG34377|Dmel_CG13858|Dmel_CG13859|Dmel_CG13860|Dmel_CG13861</td><td>0</td><td>0</td></tr>
<tr><td>9617</td><td>42609</td><td>Drosophila melanogaster</td><td>CG7080</td><td>CG7080 gene product from transcript CG7080-RA</td><td>DmelCG7080</td><td>0</td><td>0</td></tr>
<tr><td>9618</td><td>42610</td><td>Drosophila melanogaster</td><td>CG13862</td><td>CG13862 gene product from transcript CG13862-RA</td><td>DmelCG13862</td><td>0</td><td>0</td></tr>
<tr><td>9619</td><td>42611</td><td>Drosophila melanogaster</td><td>CG5391</td><td>CG5391 gene product from transcript CG5391-RA</td><td>DmelCG5391</td><td>0</td><td>0</td></tr>
<tr><td>9620</td><td>42612</td><td>Drosophila melanogaster</td><td>CG5388</td><td>CG5388 gene product from transcript CG5388-RA</td><td>DmelCG5388</td><td>0</td><td>0</td></tr>
<tr><td>9621</td><td>42613</td><td>Drosophila melanogaster</td><td>CG5386</td><td>CG5386 gene product from transcript CG5386-RA</td><td>DmelCG5386</td><td>0</td><td>0</td></tr>
<tr><td>9622</td><td>42614</td><td>Drosophila melanogaster</td><td>CG7077</td><td>CG7077 gene product from transcript CG7077-RA</td><td>DmelCG7077</td><td>0</td><td>0</td></tr>
<tr><td>9623</td><td>42615</td><td>Drosophila melanogaster</td><td>sar1</td><td>CG7073 gene product from transcript CG7073-RB</td><td>5712|CG7073|Dm Sar1|DmelCG7073|NP_732719|SAR1|Sar1|anon-EST:Liang-1.48|bro5|clone 1.48|dar3|dsar1|l(3)05712</td><td>0</td><td>0</td></tr>
<tr><td>9624</td><td>42616</td><td>Drosophila melanogaster</td><td>PSR</td><td>phosphatidylserine receptor</td><td>BEST:LD22859|CG5383|DmelCG5383|L0022859|PS|dPSR|psr</td><td>0</td><td>0</td></tr>
<tr><td>9625</td><td>42617</td><td>Drosophila melanogaster</td><td>CG7071</td><td>CG7071 gene product from transcript CG7071-RA</td><td>BcDNA:RE40914|DmelCG7071|Dromel_CG7071_FBtr0084211_mORF</td><td>0</td><td>0</td></tr>
<tr><td>9626</td><td>42618</td><td>Drosophila melanogaster</td><td>CG5382</td><td>CG5382 gene product from transcript CG5382-RB</td><td>DmelCG5382</td><td>0</td><td>0</td></tr>
<tr><td>9627</td><td>42619</td><td>Drosophila melanogaster</td><td>CG5380</td><td>CG5380 gene product from transcript CG5380-RB</td><td>DmelCG5380</td><td>0</td><td>0</td></tr>
<tr><td>9628</td><td>42620</td><td>Drosophila melanogaster</td><td>PyK</td><td>Pyruvate kinase</td><td>CG7070|DmelCG7070|PYK|Pyk</td><td>0</td><td>0</td></tr>
<tr><td>9629</td><td>42621</td><td>Drosophila melanogaster</td><td>CG7069</td><td>CG7069 gene product from transcript CG7069-RA</td><td>DmelCG7069</td><td>0</td><td>0</td></tr>
<tr><td>9630</td><td>42622</td><td>Drosophila melanogaster</td><td>CG18596</td><td>CG18596 gene product from transcript CG18596-RA</td><td>DmelCG18596</td><td>0</td><td>0</td></tr>
<tr><td>9631</td><td>42624</td><td>Drosophila melanogaster</td><td>CG43343</td><td>CG43343 gene product from transcript CG43343-RB</td><td>CG18427|CG31169|DmelCG43343|Dmel_CG31169|NEST:bs15d11|NEST:bs16c09|gpdh-3</td><td>0</td><td>0</td></tr>
<tr><td>9632</td><td>42625</td><td>Drosophila melanogaster</td><td>CG5379</td><td>CG5379 gene product from transcript CG5379-RA</td><td>DmelCG5379</td><td>0</td><td>0</td></tr>
<tr><td>9633</td><td>42626</td><td>Drosophila melanogaster</td><td>CG7059</td><td>CG7059 gene product from transcript CG7059-RA</td><td>DmelCG7059</td><td>0</td><td>0</td></tr>
<tr><td>9634</td><td>42627</td><td>Drosophila melanogaster</td><td>CG13857</td><td>CG13857 gene product from transcript CG13857-RA</td><td>DmelCG13857</td><td>0</td><td>0</td></tr>
<tr><td>9635</td><td>42628</td><td>Drosophila melanogaster</td><td>CG13856</td><td>CG13856 gene product from transcript CG13856-RA</td><td>DmelCG13856</td><td>0</td><td>0</td></tr>
<tr><td>9636</td><td>42629</td><td>Drosophila melanogaster</td><td>CG13855</td><td>CG13855 gene product from transcript CG13855-RA</td><td>DmelCG13855</td><td>0</td><td>0</td></tr>
<tr><td>9637</td><td>42630</td><td>Drosophila melanogaster</td><td>CG13850</td><td>CG13850 gene product from transcript CG13850-RA</td><td>DmelCG13850|GH07286p</td><td>0</td><td>0</td></tr>
<tr><td>9638</td><td>42632</td><td>Drosophila melanogaster</td><td>lqfR</td><td>liquid facets-Related</td><td>CG13853|CG13854|CG31170|CG31285|CG42250|DmelCG42250|Dmel_CG31170|Dmel_CG31285|Epsin|LqfR|XII-10|epsin|epsin-like|epsin02|l(3)03685|l(3)A9|l(3)SG62|l(3)XII-10|l(3)dsl-9|l(3)dsl9</td><td>0</td><td>0</td></tr>
<tr><td>9639</td><td>42633</td><td>Drosophila melanogaster</td><td>Nop56</td><td>CG13849 gene product from transcript CG13849-RA</td><td>CG13849|DmNop56|DmelCG13849|Dnop56|FBgn0038964|nop56</td><td>0</td><td>0</td></tr>
<tr><td>9640</td><td>42634</td><td>Drosophila melanogaster</td><td>mats</td><td>mob as tumor suppressor</td><td>CG13852|DMob1|DmelCG13852|Dmob1|Mats|Mob1|anon-WO0118547.303|dmob1</td><td>0</td><td>0</td></tr>
<tr><td>9641</td><td>42635</td><td>Drosophila melanogaster</td><td>pinta</td><td>prolonged depolarization afterpotential (PDA) is not ap</td><td>CG13848|CT33362|DmelCG13848|Pinta</td><td>0</td><td>0</td></tr>
<tr><td>9642</td><td>42636</td><td>Drosophila melanogaster</td><td>CG13847</td><td>CG13847 gene product from transcript CG13847-RB</td><td>DmelCG13847</td><td>0</td><td>0</td></tr>
<tr><td>9643</td><td>42637</td><td>Drosophila melanogaster</td><td>CG12499</td><td>CG12499 gene product from transcript CG12499-RA</td><td>DmelCG12499</td><td>0</td><td>0</td></tr>
<tr><td>9644</td><td>42638</td><td>Drosophila melanogaster</td><td>CG34376</td><td>CG34376 gene product from transcript CG34376-RB</td><td>CG13845|CG13846|CG13851|CG31160|DmelCG34376|Dmel_CG13845|Dmel_CG31160</td><td>0</td><td>0</td></tr>
<tr><td>9645</td><td>42641</td><td>Drosophila melanogaster</td><td>Rpn7</td><td>CG5378 gene product from transcript CG5378-RA</td><td>141231_at|CG5378|DmelCG5378|anon-WO0172774.103|anon-WO0172774.106|p42A</td><td>0</td><td>0</td></tr>
<tr><td>9646</td><td>42642</td><td>Drosophila melanogaster</td><td>AP-50</td><td>CG7057 gene product from transcript CG7057-RB</td><td>AP-2mu|AP50|CG7057|DmelCG7057|Dmu2|MENE (3R)-D|MENE(3R)-D|anon-EST:Posey50|anon-WO0172774.104|cg7057|mu2</td><td>0</td><td>0</td></tr>
<tr><td>9647</td><td>42643</td><td>Drosophila melanogaster</td><td>CG7054</td><td>CG7054 gene product from transcript CG7054-RA</td><td>DmelCG7054</td><td>0</td><td>0</td></tr>
<tr><td>9648</td><td>42644</td><td>Drosophila melanogaster</td><td>Pebp1</td><td>Phosphatidylethanolamine-binding protein 1</td><td>BcDNA:LP12095|CG18594|DmelCG18594|PEBP1|T2</td><td>0</td><td>0</td></tr>
<tr><td>9649</td><td>42645</td><td>Drosophila melanogaster</td><td>CG5377</td><td>CG5377 gene product from transcript CG5377-RA</td><td>DmelCG5377</td><td>0</td><td>0</td></tr>
<tr><td>9650</td><td>42646</td><td>Drosophila melanogaster</td><td>Nrx-1</td><td>Neurexin 1</td><td>CG7050|CT21808|DmelCG7050|Nrx|Nrx1|dnrx|dnrx-1|nrx</td><td>0</td><td>0</td></tr>
<tr><td>9651</td><td>42647</td><td>Drosophila melanogaster</td><td>CG7048</td><td>CG7048 gene product from transcript CG7048-RB</td><td>DmelCG7048</td><td>0</td><td>0</td></tr>
<tr><td>9652</td><td>42648</td><td>Drosophila melanogaster</td><td>CG5376</td><td>CG5376 gene product from transcript CG5376-RA</td><td>DmelCG5376</td><td>0</td><td>0</td></tr>
<tr><td>9653</td><td>42649</td><td>Drosophila melanogaster</td><td>T-cp1</td><td>Tcp1-like</td><td>BEST:GH05123|CCT1|CG5374|DmelCG5374|T-cpl|TCP-1alpha|TCPA_DROME|Tcp-1|Tcp1|gh05123</td><td>0</td><td>0</td></tr>
<tr><td>9654</td><td>42650</td><td>Drosophila melanogaster</td><td>CG7045</td><td>CG7045 gene product from transcript CG7045-RA</td><td>DmelCG7045</td><td>0</td><td>0</td></tr>
</table>

9655	42651	Drosophila melanogaster	CG7046	CG7046 gene product from transcript CG7046-RB	BcDNA:AT28425|DmelCG7046	0	0
9656	42652	Drosophila melanogaster	oa2	octopamine receptor 2	CG6919|DmCG6919|DmOctBeta1R|DmelCG6919|Dmoa2|OA2|Oa2|OctBeta1|Octbeta1R|anon-WO0170980.67|anon-WO0170980.68	0	0
9657	42653	Drosophila melanogaster	CG5346	CG5346 gene product from transcript CG5346-RA	DmelCG5346	0	0
9658	42654	Drosophila melanogaster	CG33093	CG33093 gene product from transcript CG33093-RA	CG5340|DmelCG33093	0	0
9659	42655	Drosophila melanogaster	CG5326	CG5326 gene product from transcript CG5326-RA	DmelCG5326	0	0
9660	42656	Drosophila melanogaster	CG5315	CG5315 gene product from transcript CG5315-RA	DmelCG5315	0	0
9661	42657	Drosophila melanogaster	bond	james bond	CG6921|DmelCG6921	0	0
9662	42658	Drosophila melanogaster	CG5278	CG5278 gene product from transcript CG5278-RA	DmelCG5278	0	0
9663	42659	Drosophila melanogaster	CG33110	CG33110 gene product from transcript CG33110-RA	CG13844|CG6926|DmelCG33110	0	0
9664	42661	Drosophila melanogaster	CSN6	COP9 complex homolog subunit 6	CG6932|CH6|Dch6|DmelCG6932	0	0
9665	42662	Drosophila melanogaster	CG6937	CG6937 gene product from transcript CG6937-RA	DmelCG6937|cg6937	0	0
9666	42663	Drosophila melanogaster	Dph5	Diphthamide methyltransferase	BcDNA.LD12153|BcDNA:LD12153|CG31289|CG5275|DmelCG31289|l(3)L4910	0	0
9667	42664	Drosophila melanogaster	btn	buttonless	1261/13|CG5264|DmelCG5264|btn/MEOX	0	0
9668	42665	Drosophila melanogaster	CG31158	CG31158 gene product from transcript CG31158-RG	CG18185|CG6941|DmelCG31158|EFA6	0	0
9669	42668	Drosophila melanogaster	CG13843	CG13843 gene product from transcript CG13843-RA	DmelCG13843	0	0
9670	42669	Drosophila melanogaster	CG31156	CG31156 gene product from transcript CG31156-RA	CG5253|CG5257|DmelCG31156	0	0
9671	42670	Drosophila melanogaster	EF-G2	Elongation Factor G2	CG31159|CG6943|DmelCG31159|dEF-G2	0	0
9672	42671	Drosophila melanogaster	mRpL45	mitochondrial ribosomal protein L45	CG6949|DmelCG6949|MRP-L45	0	0
9673	42672	Drosophila melanogaster	loco	locomotion defects	CG17229|CG5248|DmelCG5248|Loco|dLoco	0	0
9674	42674	Drosophila melanogaster	CG42686	CG42686 gene product from transcript CG42686-RB	CG17622|CG17623|DmelCG42686|Dmel_CG17622|Dmel_CG17623	0	0
9675	42676	Drosophila melanogaster	CG6954	CG6954 gene product from transcript CG6954-RE	DmelCG6954	0	0
9676	42678	Drosophila melanogaster	CG17625	CG17625 gene product from transcript CG17625-RA	DmelCG17625	0	0
9677	42679	Drosophila melanogaster	wfs1	wolfram syndrome 1	CG4917|DmelCG4917	0	0
9678	42680	Drosophila melanogaster	Nup133	Nucleoporin 133	CG6958|DmelCG6958|Nup 133|nup133	0	0
9679	42681	Drosophila melanogaster	CG6969	CG6969 gene product from transcript CG6969-RA	DmelCG6969	0	0
9680	42682	Drosophila melanogaster	Cyp6d4	CG12800 gene product from transcript CG12800-RA	6d4|CG12800|DmelCG12800	0	0
9681	42683	Drosophila melanogaster	Ccap	Cardioacceleratory peptide	CCAP|CG4910|DmCCAP|DmelCG4910|ccap	0	0
9682	42684	Drosophila melanogaster	CG6972	CG6972 gene product from transcript CG6972-RA	DmelCG6972	0	0
9683	42685	Drosophila melanogaster	CG13842	CG13842 gene product from transcript CG13842-RA	DmelCG13842	0	0
9684	42686	Drosophila melanogaster	CG4907	CG4907 gene product from transcript CG4907-RA	DmelCG4907	0	0
9685	42687	Drosophila melanogaster	wge	winged eye	CG17621|CG31151|CG4902|DmelCG31151	0	0
9686	42689	Drosophila melanogaster	Irp-1A	Iron regulatory protein 1A	C-Acon|CG4900|DmelCG4900|IRP-1|IRP-1A|IRP1|cAcon	0	0
9687	42690	Drosophila melanogaster	CG4813	CG4813 gene product from transcript CG4813-RA	DmelCG4813	0	0
9688	42691	Drosophila melanogaster	CG6982	CG6982 gene product from transcript CG6982-RC	DmelCG6982	0	0
9689	42692	Drosophila melanogaster	Takl2	Tak1-like 2	CG4803|DmelCG4803|TAK	0	0
9690	42693	Drosophila melanogaster	Dcr-1	Dicer-1	CG4792|DCR1|DICER|Dcr|Dcr1|Dicer|Dicer1|DmelCG4792|cg4792|dcr 1|dcr-1|dcr1|dicer|dicer-1|dicer1	0	0
9691	42694	Drosophila melanogaster	CG6985	CG6985 gene product from transcript CG6985-RA	DmelCG6985	0	0
9692	42695	Drosophila melanogaster	vret	vreteno	CG4771|DmelCG4771	0	0
9693	42696	Drosophila melanogaster	HP1c	CG6990 gene product from transcript CG6990-RA	CG6990|DmelCG6990|HP1|HP1C|Hp1c|hp1c	0	0
9694	42697	Drosophila melanogaster	CG17141	CG17141 gene product from transcript CG17141-RA	DmelCG17141	0	0
9695	42698	Drosophila melanogaster	CG31139	CG31139 gene product from transcript CG31139-RA	BcDNA:AT07872|CG17138|DmelCG31139	0	0
9696	42699	Drosophila melanogaster	CG4725	CG4725 gene product from transcript CG4725-RA	DmelCG4725	0	0
9697	42700	Drosophila melanogaster	CG4723	CG4723 gene product from transcript CG4723-RA	DmelCG4723	0	0
9698	42701	Drosophila melanogaster	CG4721	CG4721 gene product from transcript CG4721-RA	DmelCG4721|anon-EST:Posey132	0	0
9699	42702	Drosophila melanogaster	CG7023	CG7023 gene product from transcript CG7023-RB	DmelCG7023	0	0
9700	42703	Drosophila melanogaster	CG7029	CG7029 gene product from transcript CG7029-RB	DmelCG7029	0	0
9701	42704	Drosophila melanogaster	CG7031	CG7031 gene product from transcript CG7031-RA	DmelCG7031	0	0
9702	42705	Drosophila melanogaster	CG13840	CG13840 gene product from transcript CG13840-RB	DmelCG13840	0	0
9703	42706	Drosophila melanogaster	CG4704	CG4704 gene product from transcript CG4704-RA	DmelCG4704	0	0
9704	42707	Drosophila melanogaster	klg	klingon	CG6669|CT20712|DmelCG6669|Klg|h214|l(3)rN712	0	0
9705	42708	Drosophila melanogaster	CG6660	CG6660 gene product from transcript CG6660-RA	DmelCG6660	0	0
9706	42709	Drosophila melanogaster	CG17244	CG17244 gene product from transcript CG17244-RA	BcDNA:RH17470|DmelCG17244	0	0
9707	42710	Drosophila melanogaster	Nha2	Na[+]/H[+] hydrogen antiporter 2	CG31052|CG43205|CG43442|CG4682|CG4693|DMG8-chr3R.34.001|DmNHA2|DmelCG43442|Dmel_CG31052|TANGO12|Tango12|tango12	0	0
9708	42711	Drosophila melanogaster	Or94a	Odorant receptor 94a	94D.1|94a|CG17241|DOR108|DmelCG17241	0	0
9709	42712	Drosophila melanogaster	Or94b	Odorant receptor 94b	94D.2|94b|CG6679|DOR109|DmelCG6679|anon-JP2002534971-A.31|anon-JP2002534971-A.32	0	0
9710	42715	Drosophila melanogaster	CG34375	CG34375 gene product from transcript CG34375-RA	CG13834|CG13835|DmelCG34375|Dmel_CG13834|Dmel_CG13835	0	0
9711	42716	Drosophila melanogaster	CG6688	CG6688 gene product from transcript CG6688-RA	DmelCG6688	0	0
9712	42717	Drosophila melanogaster	lmd	lame duck	CG4677|DmelCG4677|K|LMD|Lmd|anon-EST:CL2d4|gfl|minc	0	0
9713	42718	Drosophila melanogaster	CG13833	CG13833 gene product from transcript CG13833-RA	DmelCG13833	0	0
9714	42719	Drosophila melanogaster	CG13838	CG13838 gene product from transcript CG13838-RA	DmelCG13838	0	0
9715	42720	Drosophila melanogaster	CG13837	CG13837 gene product from transcript CG13837-RA	DmelCG13837	0	0
9716	42721	Drosophila melanogaster	CG17121	CG17121 gene product from transcript CG17121-RA	DmelCG17121	0	0
9717	42723	Drosophila melanogaster	CG17119	CG17119 gene product from transcript CG17119-RA	DmelCG17119	0	0
9718	42726	Drosophila melanogaster	CG6697	CG6697 gene product from transcript CG6697-RA	BcDNA:LD21504|DmelCG6697	0	0
9719	42727	Drosophila melanogaster	CG17111	CG17111 gene product from transcript CG17111-RB	DmelCG17111	0	0
9720	42728	Drosophila melanogaster	CG6726	CG6726 gene product from transcript CG6726-RB	DmelCG6726	0	0
9721	42729	Drosophila melanogaster	CG17110	CG17110 gene product from transcript CG17110-RA	DmelCG17110	0	0
9722	42730	Drosophila melanogaster	CG17109	CG17109 gene product from transcript CG17109-RA	DmelCG17109	0	0
9723	42731	Drosophila melanogaster	CG6733	CG6733 gene product from transcript CG6733-RA	DmelCG6733	0	0
9724	42732	Drosophila melanogaster	CG6738	CG6738 gene product from transcript CG6738-RA	DmelCG6738	0	0
9725	42733	Drosophila melanogaster	CG13830	CG13830 gene product from transcript CG13830-RD	DmelCG13830	0	0
9726	42734	Drosophila melanogaster	Rassf	Ras association family member	CG4656|DmelCG4656|RASSF|dRASSF	0	0
9727	42735	Drosophila melanogaster	cenB1A	centaurin beta 1A	CG6742|CenB1A|DmelCG6742	0	0
9728	42736	Drosophila melanogaster	CG31365	CG31365 gene product from transcript CG31365-RA	CG4639|DmelCG31365	0	0
9729	42737	Drosophila melanogaster	hh	hedgehog	CG4637|DmelCG4637|HH|Hh|Mir|Mrt|anon-WO0134654.19|anon-WO0182946.19|bar|bar-3|bar3|hg|l(3)hh|l(3)neo56|l(3)neo57	0	0
9730	42738	Drosophila melanogaster	unk	unkempt	CG4620|DmelCG4620	0	0
9731	42739	Drosophila melanogaster	VhaAC39-2	Vacuolar H[+] ATPase subunit AC39-2	CG4624|DmelCG4624|anon-WO0118547.227|vhaAC39-2	0	0
9732	42740	Drosophila melanogaster	CG13829	CG13829 gene product from transcript CG13829-RA	DmelCG13829	0	0
9733	42741	Drosophila melanogaster	CG13836	CG13836 gene product from transcript CG13836-RA	DmelCG13836	0	0
9734	42742	Drosophila melanogaster	Ir	Inwardly rectifying potassium channel	CG6747|DIR|Dir|DmelCG6747|Irk1|dKirI|ir|irk1	0	0
9735	42743	Drosophila melanogaster	cnc	cap-n-collar	5134|BcDNA:RE05559|CG13826|CG17894|CG43286|CG4566|CG4578|CNC|CNC_DROME|Cnc|DmelCG43286|Dmel_CG13826|Dmel_CG17894|anon-WO0153538.6|anon-WO0153538.7|l(3)03921|l(3)j5E7	0	0
9736	42745	Drosophila melanogaster	fzo	fuzzy onions	CG4568|DmelCG4568|anon-WO0125274.1|anon-WO0125274.25|anon-WO0125274.26	0	0
9737	42747	Drosophila melanogaster	CG4467	CG4467 gene product from transcript CG4467-RB	CG13825|DmelCG4467	0	0
9738	42748	Drosophila melanogaster	CG4449	CG4449 gene product from transcript CG4449-RB	DmelCG4449	0	0
9739	42749	Drosophila melanogaster	EloA	Elongin A	CG6755|DmelCG6755|Elo-A|dEloA	0	0
9740	42750	Drosophila melanogaster	wda	will decrease acetylation	CG4448|DmelCG4448|WFA	0	0
9741	42751	Drosophila melanogaster	CG13827	CG13827 gene product from transcript CG13827-RA	BcDNA:RE30473|DmelCG13827	0	0
9742	42752	Drosophila melanogaster	orb	oo18 RNA-binding protein	151666_s_at|CG10868|DmelCG10868|ORB|Orb|fs(3)00107|oo18	0	0
9743	42753	Drosophila melanogaster	cdc16	CG6759 gene product from transcript CG6759-RA	APC6|Apc6|CDC16|CG6759|Cdc16|Dmcdc16|DmelCG6759	0	0
9744	42754	Drosophila melanogaster	CG6763	CG6763 gene product from transcript CG6763-RA	DmelCG6763	0	0
9745	42755	Drosophila melanogaster	CG17083	CG17083 gene product from transcript CG17083-RA	DmelCG17083	0	0
9746	42756	Drosophila melanogaster	CG4434	CG4434 gene product from transcript CG4434-RA	DmelCG4434	0	0
9747	42757	Drosophila melanogaster	pnt	pointed	0123/09|0608/07|0998/12|3520|CG17077|D-Ets-2|D-ets-2|DMPOINT1A|DmelCG17077|E(E2F)3D|EK3-2|ETS2|EY3-1|Ets2|Ets58AB|Ets94F|PNT-P1|PNTP1|PNTP2|POINT|Pnt|Pnt-P1|PntP1|PntP2|Pointed-F	0	0
9748	42758	Drosophila melanogaster	DNApol-epsilon	DNA polymerase epsilon	6768|BAB17608|CG6768|DmelCG6768|pol-epsilon	0	0
9749	42759	Drosophila melanogaster	ATPsyn-Cf6	ATPase coupling factor 6	BcDNA:RH08870|CG4412|DmelCG4412	0	0
9750	42761	Drosophila melanogaster	RpS3	Ribosomal protein S3	6779|CG6779|DmRpS3|DmelCG6779|M(3)124|M(3)95A|M(3)B|M(3)B2|M(3)Fla|M(3)w|M(3R)w|M95A|M[[w]]|Mw|RS3|RpS|Rps3|S3|X72921|dS3|rpS3|rps3	0	0
9751	42762	Drosophila melanogaster	CG4408	CG4408 gene product from transcript CG4408-RA	BEST:GH09055|DmelCG4408	0	0
9752	42763	Drosophila melanogaster	CG42827	CG42827 gene product from transcript CG42827-RC	CG6784|DmelCG42827|Dmel_CG6784|cg6784	0	0
9753	42764	Drosophila melanogaster	CG4393	CG4393 gene product from transcript CG4393-RB	DmelCG4393	0	0
9754	42765	Drosophila melanogaster	Ir94e	Ionotropic receptor 94e	CG17379|CG42298|DmelCG42298|Dmel_CG17379|IR94e	0	0
9755	42766	Drosophila melanogaster	CG17380	CG17380 gene product from transcript CG17380-RB	BcDNA:AT18288|DmelCG17380	0	0
9756	42767	Drosophila melanogaster	CG4374	CG4374 gene product from transcript CG4374-RB	DmelCG4374	0	0
9757	42768	Drosophila melanogaster	Ir94g	Ionotropic receptor 94g	CG17381|DmelCG17381|IR94g	0	0
9758	42769	Drosophila melanogaster	Ir94h	Ionotropic receptor 94h	CG17382|DmelCG17382|IR94h	0	0
9759	42770	Drosophila melanogaster	Irk2	Inwardly rectifying potassium channel 2	CG10180|CG4370|DmelCG4370|dKirII|irk2	0	0
9760	42772	Drosophila melanogaster	CG10177	CG10177 gene product from transcript CG10177-RA	DmelCG10177	0	0
9761	42773	Drosophila melanogaster	CG10175	CG10175 gene product from transcript CG10175-RA	DmelCG10175	0	0
9762	42774	Drosophila melanogaster	CG10170	CG10170 gene product from transcript CG10170-RA	DmelCG10170	0	0
9763	42775	Drosophila melanogaster	CG16732	CG16732 gene product from transcript CG16732-RB	DmelCG16732	0	0
9764	42776	Drosophila melanogaster	CG10168	CG10168 gene product from transcript CG10168-RA	DmelCG10168	0	0
9765	42777	Drosophila melanogaster	CG10164	CG10164 gene product from transcript CG10164-RB	DmelCG10164|NEST:bs02f09	0	0

9766	42778	Drosophila melanogaster	beat-IV	CG10152 gene product from transcript CG10152-RA	CG10152|CG13823|DmelCG10152|beat IV	0	0
9767	42780	Drosophila melanogaster	CG10182	CG10182 gene product from transcript CG10182-RA	DmelCG10182	0	0
9768	42781	Drosophila melanogaster	CG16723	CG16723 gene product from transcript CG16723-RB	DmelCG16723	0	0
9769	42782	Drosophila melanogaster	CG10183	CG10183 gene product from transcript CG10183-RB	DmelCG10183	0	0
9770	42783	Drosophila melanogaster	CG10184	CG10184 gene product from transcript CG10184-RA	DmelCG10184	0	0
9771	42784	Drosophila melanogaster	CG31145	CG31145 gene product from transcript CG31145-RC	CG10187|CG13820|CG13821|DmelCG31145	0	0
9772	42787	Drosophila melanogaster	CG13822	CG13822 gene product from transcript CG13822-RA	DmelCG13822	0	0
9773	42788	Drosophila melanogaster	CG10157	CG10157 gene product from transcript CG10157-RA	DmelCG10157|anon-EST:Posey120	0	0
9774	42789	Drosophila melanogaster	eIF-3p66	Eukaryotic initiation factor 3 p66 subunit	153314_at|BEST:GM10514|CG10161|DmelCG10161|GM04979|GM10514|eIF3-S7|eiF3p66	0	0
9775	42790	Drosophila melanogaster	CG16710	CG16710 gene product from transcript CG16710-RB	DmelCG16710|SP16	0	0
9776	42791	Drosophila melanogaster	SPE	Spatzle-Processing Enzyme	CG16705|CT37173|DmelCG16705|SP4|Sp4|c-SP4|proPO-AE|spe	0	0
9777	42793	Drosophila melanogaster	CG10254	CG10254 gene product from transcript CG10254-RB	BcDNA:LD22087|DmelCG10254	0	0
9778	42794	Drosophila melanogaster	CG10252	CG10252 gene product from transcript CG10252-RA	BEST:GH13953|DmelCG10252|anon-WO0140519.133|bs30h03.y1	0	0
9779	42795	Drosophila melanogaster	VAChT	CG32848 gene product from transcript CG32848-RA	CG12345|CG32848|CT41182|DmelCG32848|VACHT|VACht|Vacht|vAChT|vacht	0	0
9780	42796	Drosophila melanogaster	CG31148	CG31148 gene product from transcript CG31148-RA	CG10299|CT28913|DmelCG31148|anon-WO0140519.234	0	0
9781	42797	Drosophila melanogaster	CG10301	CG10301 gene product from transcript CG10301-RB	DmelCG10301	0	0
9782	42798	Drosophila melanogaster	CG10300	CG10300 gene product from transcript CG10300-RA	DmelCG10300	0	0
9783	42799	Drosophila melanogaster	nau	nautilus	CG10250|DmelCG10250|Dmyd|MYOD|Mdrf|MyoD|NAU|Nau	0	0
9784	42800	Drosophila melanogaster	CG10232	CG10232 gene product from transcript CG10232-RB	DmelCG10232|SP14	0	0
9785	42801	Drosophila melanogaster	CG10365	CG10365 gene product from transcript CG10365-RC	DmelCG10365|anon-WO0118547.386	0	0
9786	42802	Drosophila melanogaster	Rpn9	CG10230 gene product from transcript CG10230-RB	0718/06|718/06|CG10230|CT28749|DmelCG10230|Rpn4|Rpn94|l(3)S071806|l(3)S071806b|p39A|rpn9	0	0
9787	42803	Drosophila melanogaster	Hmgcr	HMG Coenzyme A reductase	BcDNA:LD15354|CG10367|Clb|DmHMG-CoAr|DmelCG10367|HMGCR|HMGCR_Dm|HMGCoAR|HMGCoARr|HMGCoAr|HmG-CoAR|Hmg|bro1|bs32f05.y1|clb|hmgc|hmgcr|hmgrc|l(3)01152	0	0
9788	42804	Drosophila melanogaster	RanBP3	Ran binding protein 3	CG10225|DmelCG10225|GC10225	0	0
9789	42805	Drosophila melanogaster	Tbp-1	Tat-binding protein-1	BcDNA.GH12068|BcDNA:GH12068|CG10370|DmTBP-1|Dm_Rpt5a|Dm_Rpt5b|DmelCG10370|Pros26S|Pros26S-RS6A|Rpt5|TBP-1|Tbp1|anon-WO0153538.10|anon-WO0153538.8|anon-WO0153538.9|d	0	0
9790	42806	Drosophila melanogaster	Hrd3	CG10221 gene product from transcript CG10221-RA	BcDNA:LD23587|CG10221|DmelCG10221|HRD3	0	0
9791	42807	Drosophila melanogaster	Plip	PTEN-like phosphatase	150466_at|CG10371|DmelCG10371|PLIP|plip	0	0
9792	42808	Drosophila melanogaster	CG10219	CG10219 gene product from transcript CG10219-RA	BEST:GH27735|DmelCG10219	0	0
9793	42809	Drosophila melanogaster	CG10217	CG10217 gene product from transcript CG10217-RB	DmelCG10217	0	0
9794	42810	Drosophila melanogaster	Lsd-1	Lipid storage droplet-1	CG10374|DmelCG10374|LSD-1|LSDP1|Lsd1|Lsdp1|plin1	0	0
9795	42811	Drosophila melanogaster	CG10214	CG10214 gene product from transcript CG10214-RA	DmelCG10214	0	0
9796	42812	Drosophila melanogaster	CG10375	CG10375 gene product from transcript CG10375-RA	DmelCG10375	0	0
9797	42813	Drosophila melanogaster	tst	twister	CG10210|DmelCG10210|SKI2|Ski2|Ski2/tst|cg10210	0	0
9798	42814	Drosophila melanogaster	CG10208	CG10208 gene product from transcript CG10208-RA	CT28701|DmelCG10208	0	0
9799	42816	Drosophila melanogaster	Nup98	Nucleoporin 98	CG10198|CG10201|DmelCG10198|Nup 96|Nup 98|Nup96|Nup98-Nup96|Nup98/96|l(3)95BCd|nup145	0	0
9800	42817	Drosophila melanogaster	mbc	myoblast city	CG10379|DOCK180|Dm MBC|DmelCG10379|Dock180|MBC|MBC/DOCK180|Mbc|Su(rac)1|dock180	0	0
9801	42819	Drosophila melanogaster	eIF4G2	eukaryotic translation initiation factor 4G2	CG10192|DmelCG10192|ms(3)nc32|nc32|ofs	0	0
9802	42820	Drosophila melanogaster	CG34355	CG34355 gene product from transcript CG34355-RE	CG12492|CG13818|DmelCG34355|Dmel_CG12492	0	0
9803	42821	Drosophila melanogaster	Pli	Pellino	CG5212|DmelCG5212|PAP	0	0
9804	42822	Drosophila melanogaster	TfIIA-S	Transcription-factor-IIA-S	BcDNA:RE44302|CG5163|DmelCG5163|F121|TF-IIAS|TFIIA|TFIIA-S|dTFIIA|dTFIIA-S|tfiia-s	0	0
9805	42823	Drosophila melanogaster	CG13597	CG13597 gene product from transcript CG13597-RA	DmelCG13597	0	0
9806	42824	Drosophila melanogaster	sba	six-banded	AAF56201|CG13598|DMTF125|Dm Sba|DmelCG13598|F125|TF125|Tf125	0	0
9807	42825	Drosophila melanogaster	CG5857	CG5857 gene product from transcript CG5857-RA	DmelCG5857	0	0
9808	42826	Drosophila melanogaster	CG13601	CG13601 gene product from transcript CG13601-RA	DmelCG13601	0	0
9809	42827	Drosophila melanogaster	CG13602	CG13602 gene product from transcript CG13602-RB	BcDNA:AT03227|DmelCG13602	0	0
9810	42828	Drosophila melanogaster	Pros26.4	Proteasome 26S subunit subunit 4 ATPase	5289|CG5289|Dm-S4-1|DmP26S4|Dm_Rpt2|DmelCG5289|P26s4|Rpt2|S4|dRpt2|p54|p56|pros26.4	0	0
9811	42829	Drosophila melanogaster	CG13599	CG13599 gene product from transcript CG13599-RA	DmelCG13599	0	0
9812	42830	Drosophila melanogaster	RpS19b	Ribosomal protein S19b	BcDNA:SD22440|CG5338|DmelCG5338	0	0
9813	42831	Drosophila melanogaster	CG5854	CG5854 gene product from transcript CG5854-RA	DmelCG5854|anon-WO0118547.306	0	0
9814	42832	Drosophila melanogaster	Gdh	Glutamate dehydrogenase	CG5320|DHE3|DmelCG5320|GDH|GLU-D|GLUD|GLUD pre-mRNA|Glud|gdh	0	0
9815	42833	Drosophila melanogaster	CG12268	CG12268 gene product from transcript CG12268-RB	DmelCG12268	0	0
9816	42834	Drosophila melanogaster	Aats-glupro	Glutamyl-prolyl-tRNA synthetase	AATS|AATs-GluPro|Aa-tRNA-syn-glupro|Aats-glu|CG5394|DmelCG5394|EPRS|ERS|GluPro-RS|GluProRS|GluRS|aminoacyl-tRNA synthetase|glutamyl-prolyl-tRNA synthetase|p200	0	0
9817	42835	Drosophila melanogaster	AP-1sigma	CG5864 gene product from transcript CG5864-RA	AP-1[sigma]|CG5864|DmelCG5864|cg5864|sigma1	0	0
9818	42836	Drosophila melanogaster	CG31140	CG31140 gene product from transcript CG31140-RB	CG13600|CG31452|CG5875|DGKt|DmelCG31140	0	0
9819	42838	Drosophila melanogaster	CG13603	CG13603 gene product from transcript CG13603-RA	DmelCG13603	0	0
9820	42839	Drosophila melanogaster	CG5902	CG5902 gene product from transcript CG5902-RB	DmelCG5902|anon-WO0118547.372	0	0
9821	42840	Drosophila melanogaster	CG13604	CG13604 gene product from transcript CG13604-RD	DmelCG13604|UBASH3A	0	0
9822	42841	Drosophila melanogaster	Rab7	Rab-protein 7	AAF56218|CG5915|DRAB7|DRab7|Dm Rab7|DmRab7|DmelCG5915|Drab7|RAB7|Rab-7|Rab-r7|rab7	0	0
9823	42842	Drosophila melanogaster	LSm3	CG31184 gene product from transcript CG31184-RA	BcDNA:RE11655|CG31184|CG5926|DmelCG31184	0	0
9824	42843	Drosophila melanogaster	KrT95D	Krueppel target at 95D	CG5405|DmelCG5405	0	0
9825	42844	Drosophila melanogaster	Mta70	Mta70 homologue	CG5933|DmelCG5933|IME4|MTA70	0	0
9826	42845	Drosophila melanogaster	Miro	Mitochondrial Rho	B682|CG5410|DmMiro|DmelCG5410|dMiro|dmiro|miro	0	0
9827	42846	Drosophila melanogaster	spas	spastin	CG5977|D-spastin|DmelCG5977|Dspastin|Spas	0	0
9828	42848	Drosophila melanogaster	Rox8	CG5422 gene product from transcript CG5422-RD	CG5422|DmTIAR|DmelCG5422|ROX8|RRM12|Rbp12|Rox-8|TIAR|dTIAR|rox8|rrm12	0	0
9829	42849	Drosophila melanogaster	CG5991	CG5991 gene product from transcript CG5991-RA	BEST:LD21713|DmelCG5991|LD21713|Psd	0	0
9830	42850	Drosophila melanogaster	Atg6	Autophagy-specific gene 6	ATG6|Beclin-1|Beclin1|CG5429|DmAtg6|DmelCG5429|atg6|l(3)00096	0	0
9831	42851	Drosophila melanogaster	CG6000	CG6000 gene product from transcript CG6000-RB	BcDNA:RE30346|DmelCG6000	0	0
9832	42852	Drosophila melanogaster	Hsp68	Heat shock protein 68	68|CG5436|DmelCG5436|HSP68|Hsp-68|hsp68	0	0
9833	42853	Drosophila melanogaster	SMC1	CG6057 gene product from transcript CG6057-RA	CG6057|DmSMC1|DmelCG6057|Smc1|dSMC1|smc1	0	0
9834	42854	Drosophila melanogaster	Syx1A	Syntaxin 1A	CG10716|CG18615|CG31136|CG5448|CT30033|Dm Syx1|DmSyx1A|DmelCG31136|SYX1A|Syt|Syt1A|Syx|Syx-1A|Syx1|Syx1a|SyxA|anon-EST:Gibbs4|anon-WO02059370.54|dSyx1|l(3)06737|synt|syt-1A|s	0	0
9835	42855	Drosophila melanogaster	CG10694	CG10694 gene product from transcript CG10694-RA	DmelCG10694	0	0
9836	42857	Drosophila melanogaster	CG18428	CG18428 gene product from transcript CG18428-RB	DmelCG18428	0	0
9837	42858	Drosophila melanogaster	CG13605	CG13605 gene product from transcript CG13605-RB	DmelCG13605	0	0
9838	42859	Drosophila melanogaster	CG13607	CG13607 gene product from transcript CG13607-RC	150482_at|DmelCG13607	0	0
9839	42860	Drosophila melanogaster	CG6129	CG6129 gene product from transcript CG6129-RD	DmelCG6129	0	0
9840	42861	Drosophila melanogaster	CG5463	CG5463 gene product from transcript CG5463-RA	DmelCG5463	0	0
9841	42862	Drosophila melanogaster	Tsc1	CG6147 gene product from transcript CG6147-RA	CG6147|DmelCG6147|TSC|TSC1|dTSC|dTSC-1|dTSC1|dTsc1|rocky|tsc1	0	0
9842	42863	Drosophila melanogaster	sec10	CG6159 gene product from transcript CG6159-RA	CG6159|DmelCG6159|P71|Sec10|Sec10p|dSec10|dsec10	0	0
9843	42864	Drosophila melanogaster	Npc2f	Niemann-Pick type C-2f	BcDNA:LP08842|CG6164|DmelCG6164|npc2f	0	0
9844	42865	Drosophila melanogaster	kal-1	CG6173 gene product from transcript CG6173-RA	CG6173|CT19368|DmKal-1|DmelCG6173|Dmkal	0	0
9845	42866	Drosophila melanogaster	Mpk2	CG5475 gene product from transcript CG5475-RB	CG5475|D-MPK2|D-P38a|D-p38|D-p38 MAPK|D-p38a|DMPK2|DmMPK2|DmelCG5475|Dp38|Erk2|P38|dMPK2|dp38|dp38a|group 2|mpk2|p38|p38 alpha|p38A|p38a	0	0
9846	42867	Drosophila melanogaster	CG6178	CG6178 gene product from transcript CG6178-RA	DmelCG6178	0	0
9847	42869	Drosophila melanogaster	CG6182	CG6182 gene product from transcript CG6182-RA	BcDNA:LD17019|DmelCG6182	0	0
9848	42870	Drosophila melanogaster	mRpS24	mitochondrial ribosomal protein S24	CG13608|DmelCG13608|MRP-S24|MrpS24	0	0
9849	42871	Drosophila melanogaster	Apc2	Adenomatous polyposis coli tumor suppressor homol	APC|APC2|CG6193|D-APC2|Dm APC2|DmelCG6193|E-APC|E-APC dAPC2|apc2|d-APC2|dAPC2|dAPC2/E-APC|dApc2	0	0
9850	42872	Drosophila melanogaster	CG5510	CG5510 gene product from transcript CG5510-RA	DmelCG5510	0	0
9851	42873	Drosophila melanogaster	CG13606	CG13606 gene product from transcript CG13606-RB	DmelCG13606	0	0
9852	42874	Drosophila melanogaster	CHORD	CG6198 gene product from transcript CG6198-RA	CG6198|DmelCG6198|Q9U4A3|chp|mora	0	0
9853	42875	Drosophila melanogaster	CG5515	CG5515 gene product from transcript CG5515-RA	DmelCG5515	0	0
9854	42876	Drosophila melanogaster	CG5524	CG5524 gene product from transcript CG5524-RA	DmelCG5524	0	0
9855	42877	Drosophila melanogaster	CG6204	CG6204 gene product from transcript CG6204-RA	DmelCG6204|GH20028p	0	0
9856	42879	Drosophila melanogaster	CG17786	CG17786 gene product from transcript CG17786-RA	DmelCG17786	0	0
9857	42880	Drosophila melanogaster	twin	CG31137 gene product from transcript CG31137-RB	BEST:GH20274|CCR4|CG17741|CG31137|CG5534|CT39331|Ccr4|DmelCG31137|ccr4|twn	0	0
9858	42881	Drosophila melanogaster	cav	caravaggio	1G5|Anon1G5|CG6219|Caravaggio|DmelCG6219|HOAP|Hoap|anon fe 1G5|anon-EST:fe1G5|anon1G5|hoap|p55	0	0
9859	42882	Drosophila melanogaster	Spps	Sp1-like factor for pairing sensitive-silencing	CG5669|DmelCG5669	0	0
9860	42883	Drosophila melanogaster	CG13609	CG13609 gene product from transcript CG13609-RA	DmelCG13609	0	0
9861	42885	Drosophila melanogaster	Spase22-23	Spase 22/23-subunit	CG5677|DmelCG5677|Spase22/23	0	0
9862	42888	Drosophila melanogaster	CG5706	CG5706 gene product from transcript CG5706-RA	DmelCG5706	0	0
9863	42889	Drosophila melanogaster	jar	jaguar	95F|95F MHC|CG5695|Dm 95F|Dm95F|DmelCG5695|Dro95F|JAG|Jaguar|M6|MHC95F|MYOVI|Mhc95F|MyoVI|Myosin VI|jag|ms(3)06746|myosins VI	0	0
9864	42890	Drosophila melanogaster	Orct2	Organic cation transporter 2	CG13610|DmelCG13610|EP(3)1072|EP1072|cald|orct2	0	0
9865	42891	Drosophila melanogaster	Orct	Organic cation transporter	CG6331|DmelCG6331	0	0
9866	42892	Drosophila melanogaster	CG13611	CG13611 gene product from transcript CG13611-RA	DmelCG13611	0	0
9867	42893	Drosophila melanogaster	CG6356	CG6356 gene product from transcript CG6356-RA	DmelCG6356	0	0
9868	42894	Drosophila melanogaster	CG6364	CG6364 gene product from transcript CG6364-RD	CR42873|DmelCG6364|Dmel_CR42873	0	0
9869	42895	Drosophila melanogaster	CG5715	CG5715 gene product from transcript CG5715-RA	DmelCG5715	0	0
9870	42896	Drosophila melanogaster	crb	crumbs	0509/20|1384/04|CG6383|CRB|CT19912|Crb|Crbs|Crumbs|DmelCG6383|crumb|far|l(3)07207|l(3)S050920|l(3)S058104|l(3)j1B5	0	0
9871	42897	Drosophila melanogaster	CG5720	CG5720 gene product from transcript CG5720-RA	BEST:GH27517|BcDNA:LD27873|DmelCG5720|RE28534|anon-EST:Liang-1.43|clone 1.43	0	0
9872	42898	Drosophila melanogaster	BRWD3	CG31132 gene product from transcript CG31132-RA	CG18670|CG31132|CG6400|DmelCG31132|anon-WO0118547.520|anon-WO0118547.620|dBRWD3|l(3)05842|l(3)s5349|ram	0	0
9873	42899	Drosophila melanogaster	CG5728	CG5728 gene product from transcript CG5728-RA	DmelCG5728	0	0
9874	42900	Drosophila melanogaster	Dis3	CG6413 gene product from transcript CG6413-RA	CG6413|DmelCG6413|dDis3|dRrp44|dis3|taz	0	0
9875	42901	Drosophila melanogaster	CG6432	CG6432 gene product from transcript CG6432-RA	DmelCG6432	0	0
9876	42903	Drosophila melanogaster	CG5746	CG5746 gene product from transcript CG5746-RB	DmelCG5746	0	0

<table>
<tr><td>9877</td><td>42904</td><td>Drosophila melanogaster</td><td>CG6454</td><td>CG6454 gene product from transcript CG6454-RC</td><td>DmelCG6454</td><td>0</td><td>0</td></tr>
<tr><td>9878</td><td>42905</td><td>Drosophila melanogaster</td><td>Golgin84</td><td>CG17785 gene product from transcript CG17785-RA</td><td>CG17785|DmelCG17785|golgin</td><td>0</td><td>0</td></tr>
<tr><td>9879</td><td>42906</td><td>Drosophila melanogaster</td><td>CG18528</td><td>CG18528 gene product from transcript CG18528-RA</td><td>DmelCG18528</td><td>0</td><td>0</td></tr>
<tr><td>9880</td><td>42907</td><td>Drosophila melanogaster</td><td>CG5762</td><td>CG5762 gene product from transcript CG5762-RA</td><td>BEST:GH05452|DmelCG5762|anon-WO0140519.76</td><td>0</td><td>0</td></tr>
<tr><td>9881</td><td>42909</td><td>Drosophila melanogaster</td><td>CG17784</td><td>CG17784 gene product from transcript CG17784-RA</td><td>DmelCG17784</td><td>0</td><td>0</td></tr>
<tr><td>9882</td><td>42910</td><td>Drosophila melanogaster</td><td>CG13613</td><td>CG13613 gene product from transcript CG13613-RC</td><td>DmelCG13613</td><td>0</td><td>0</td></tr>
<tr><td>9883</td><td>42911</td><td>Drosophila melanogaster</td><td>CG13614</td><td>CG13614 gene product from transcript CG13614-RA</td><td>DmelCG13614</td><td>0</td><td>0</td></tr>
<tr><td>9884</td><td>42912</td><td>Drosophila melanogaster</td><td>CG17782</td><td>CG17782 gene product from transcript CG17782-RA</td><td>DmelCG17782</td><td>0</td><td>0</td></tr>
<tr><td>9885</td><td>42913</td><td>Drosophila melanogaster</td><td>CG17781</td><td>CG17781 gene product from transcript CG17781-RB</td><td>DmelCG17781</td><td>0</td><td>0</td></tr>
<tr><td>9886</td><td>42914</td><td>Drosophila melanogaster</td><td>CG17780</td><td>CG17780 gene product from transcript CG17780-RC</td><td>BcDNA:RE41041|DmelCG17780</td><td>0</td><td>0</td></tr>
<tr><td>9887</td><td>42915</td><td>Drosophila melanogaster</td><td>CG5768</td><td>CG5768 gene product from transcript CG5768-RB</td><td>DmelCG5768</td><td>0</td><td>0</td></tr>
<tr><td>9888</td><td>42916</td><td>Drosophila melanogaster</td><td>CG13615</td><td>CG13615 gene product from transcript CG13615-RA</td><td>DmelCG13615</td><td>0</td><td>0</td></tr>
<tr><td>9889</td><td>42917</td><td>Drosophila melanogaster</td><td>CG13616</td><td>CG13616 gene product from transcript CG13616-RA</td><td>DmelCG13616</td><td>0</td><td>0</td></tr>
<tr><td>9890</td><td>42918</td><td>Drosophila melanogaster</td><td>nAcRalpha-96Aa</td><td>nicotinic Acetylcholine Receptor alpha 96Aa</td><td>ALS|ALs|Acr96A|Acr96Aa|AcrB|CG5610|CG5610-PA|Dalpha1|DmelCG5610|alpha-like|alpha1|als|dALS|nAChR|nAcRs|nAcr96Aa</td><td>0</td><td>0</td></tr>
<tr><td>9891</td><td>42919</td><td>Drosophila melanogaster</td><td>nAcRalpha-96Ab</td><td>nicotinic Acetylcholine Receptor alpha 96Ab</td><td>Acr96Ab|AcrE|CG6844|CG6844-PA|D[a2]|Da2|Dalpha|Dalpha2|Dalpha2/SAD|DmelCG6844|SAD|alpha2|alpha[[2]]|da2|nAChR|nAChR Dalpha2|nAcRs|nAchRalpha-96Ab|nAcr96Ab|sad</td><td>0</td><td>0</td></tr>
<tr><td>9892</td><td>42920</td><td>Drosophila melanogaster</td><td>nAcRbeta-96A</td><td>nicotinic Acetylcholine Receptor beta 96A</td><td>Acr96Ac|AcrF|CG6798|Dbeta2|DmelCG6798|SBD|beta[[2]]|nAChR|nAcRbeta|nAcRs|nAcr96Ac|sbd</td><td>0</td><td>0</td></tr>
<tr><td>9893</td><td>42921</td><td>Drosophila melanogaster</td><td>CG13617</td><td>CG13617 gene product from transcript CG13617-RA</td><td>DmelCG13617</td><td>0</td><td>0</td></tr>
<tr><td>9894</td><td>42922</td><td>Drosophila melanogaster</td><td>Pp1alpha-96A</td><td>Protein phosphatase 1alpha at 96A</td><td>96A|CG6593|DmPp1-96A|DmelCG6593|PP1|PP1 96A|PP1 alpha96a|PP1alpha96A|PP1alpha[[1]]|PP1c|PP1c 96A|Pp1-96A|pp1alpha96A</td><td>0</td><td>0</td></tr>
<tr><td>9895</td><td>42923</td><td>Drosophila melanogaster</td><td>CG13622</td><td>CG13622 gene product from transcript CG13622-RB</td><td>DmelCG13622</td><td>0</td><td>0</td></tr>
<tr><td>9896</td><td>42924</td><td>Drosophila melanogaster</td><td>CG13618</td><td>CG13618 gene product from transcript CG13618-RA</td><td>DmelCG13618</td><td>0</td><td>0</td></tr>
<tr><td>9897</td><td>42925</td><td>Drosophila melanogaster</td><td>CG6607</td><td>CG6607 gene product from transcript CG6607-RA</td><td>DmelCG6607</td><td>0</td><td>0</td></tr>
<tr><td>9898</td><td>42926</td><td>Drosophila melanogaster</td><td>CG13623</td><td>CG13623 gene product from transcript CG13623-RA</td><td>BcDNA:GH27470|DmelCG13623</td><td>0</td><td>0</td></tr>
<tr><td>9899</td><td>42927</td><td>Drosophila melanogaster</td><td>ana1</td><td>anastral spindle 1</td><td>Ana1|CG6631|DmelCG6631|mecB</td><td>0</td><td>0</td></tr>
<tr><td>9900</td><td>42928</td><td>Drosophila melanogaster</td><td>CG5789</td><td>CG5789 gene product from transcript CG5789-RA</td><td>Dme_CG5789|DmelCG5789</td><td>0</td><td>0</td></tr>
<tr><td>9901</td><td>42929</td><td>Drosophila melanogaster</td><td>Esyt2</td><td>CG6643 gene product from transcript CG6643-RA</td><td>CG6643|DmelCG6643</td><td>0</td><td>0</td></tr>
<tr><td>9902</td><td>42930</td><td>Drosophila melanogaster</td><td>CG13624</td><td>CG13624 gene product from transcript CG13624-RD</td><td>DmelCG13624|anon-WO0153538.65</td><td>0</td><td>0</td></tr>
<tr><td>9903</td><td>42931</td><td>Drosophila melanogaster</td><td>CG13625</td><td>CG13625 gene product from transcript CG13625-RA</td><td>DmelCG13625|anon-WO0118547.376</td><td>0</td><td>0</td></tr>
<tr><td>9904</td><td>42933</td><td>Drosophila melanogaster</td><td>Syx18</td><td>Syntaxin 18</td><td>CG13626|Dm Ufe1|DmSyx18|DmelCG13626|Syx|Ufe1/Syntaxin 18|gtx</td><td>0</td><td>0</td></tr>
<tr><td>9905</td><td>42934</td><td>Drosophila melanogaster</td><td>atl</td><td>atlastin</td><td>ATL|CG6668|DmelCG6668</td><td>0</td><td>0</td></tr>
<tr><td>9906</td><td>42935</td><td>Drosophila melanogaster</td><td>CG5794</td><td>CG5794 gene product from transcript CG5794-RF</td><td>BcDNA:AT28817|DmelCG5794|NEST:bs19d10</td><td>0</td><td>0</td></tr>
<tr><td>9907</td><td>42936</td><td>Drosophila melanogaster</td><td>ash2</td><td>absent, small, or homeotic discs 2</td><td>1124/11|291.8|703|ASH2|Ash2|CG6677|DmelCG6677|ash-2|l(3)112411|l(3)S112411|l(3)SG65|mad</td><td>0</td><td>0</td></tr>
<tr><td>9908</td><td>42937</td><td>Drosophila melanogaster</td><td>CG6695</td><td>CG6695 gene product from transcript CG6695-RA</td><td>DmelCG6695</td><td>0</td><td>0</td></tr>
<tr><td>9909</td><td>42939</td><td>Drosophila melanogaster</td><td>Ppox</td><td>Protoporphyrinogen oxidase</td><td>BcDNA:LD41392|CG5796|DmelCG5796|ESTS:9F2T</td><td>0</td><td>0</td></tr>
<tr><td>9910</td><td>42940</td><td>Drosophila melanogaster</td><td>slo</td><td>slowpoke</td><td>BcDNA:GH10751|CG10693|DmelCG10693|Dslo|Slo|Slo1|dSlo|dslo|fSlo</td><td>0</td><td>0</td></tr>
<tr><td>9911</td><td>42941</td><td>Drosophila melanogaster</td><td>CG13627</td><td>CG13627 gene product from transcript CG13627-RA</td><td>DmelCG13627</td><td>0</td><td>0</td></tr>
<tr><td>9912</td><td>42942</td><td>Drosophila melanogaster</td><td>Rpb10</td><td>CG13628 gene product from transcript CG13628-RA</td><td>BcDNA:SD08670|CG13628|DmelCG13628|RPB10</td><td>0</td><td>0</td></tr>
<tr><td>9913</td><td>42943</td><td>Drosophila melanogaster</td><td>CG13630</td><td>CG13630 gene product from transcript CG13630-RA</td><td>DmelCG13630|MAP1A</td><td>0</td><td>0</td></tr>
<tr><td>9914</td><td>42944</td><td>Drosophila melanogaster</td><td>tok</td><td>tolkin</td><td>CG6863|CT21251|DmelCG6863|TOK|Tlr|Tlr-1|Tok|tldr-1|tldr1|tlr|tlr-1|tlr1</td><td>0</td><td>0</td></tr>
<tr><td>9915</td><td>42945</td><td>Drosophila melanogaster</td><td>tld</td><td>tolloid</td><td>CG6868|CT21278|DmelCG6868|TLD|Tld|Tolloid|dTld</td><td>0</td><td>0</td></tr>
<tr><td>9916</td><td>42946</td><td>Drosophila melanogaster</td><td>asp</td><td>abnormal spindle</td><td>ASP|Asp|CG6875|Dm Asp|DmelCG6875|anon-96Aa|anon-WO0118547.279</td><td>0</td><td>0</td></tr>
<tr><td>9917</td><td>42947</td><td>Drosophila melanogaster</td><td>Ast</td><td>Allatostatin</td><td>ASA|AST|AST-1|AST-A|Ast-A|AstA|AstA-1|BcDNA:RE16553|CG13633|DAP|DAP-A|DST-1A|DST-2A|DST-3A|DST-4A|DmelCG13633|Drm-AST-1|Drm-AST-2|Drm-AST-3|Drm-AST-4|Drm-AST-A|ast</td><td>0</td><td>0</td></tr>
<tr><td>9918</td><td>42948</td><td>Drosophila melanogaster</td><td>CG42331</td><td>CG42331 gene product from transcript CG42331-RB</td><td>CG13632|CG6879|DmelCG42331|Dmel_CG13632|Dmel_CG6879</td><td>0</td><td>0</td></tr>
<tr><td>9919</td><td>42950</td><td>Drosophila melanogaster</td><td>CG5805</td><td>CG5805 gene product from transcript CG5805-RA</td><td>DmelCG5805</td><td>0</td><td>0</td></tr>
<tr><td>9920</td><td>42951</td><td>Drosophila melanogaster</td><td>CG13634</td><td>CG13634 gene product from transcript CG13634-RA</td><td>DmelCG13634</td><td>0</td><td>0</td></tr>
<tr><td>9921</td><td>42952</td><td>Drosophila melanogaster</td><td>Ets96B</td><td>CG6892 gene product from transcript CG6892-RA</td><td>CG6892|DmelCG6892|Ets</td><td>0</td><td>0</td></tr>
<tr><td>9922</td><td>42953</td><td>Drosophila melanogaster</td><td>Ude</td><td>Uracil-DNA degrading factor</td><td>CG18410|DmelCG18410|UDE</td><td>0</td><td>0</td></tr>
<tr><td>9923</td><td>42954</td><td>Drosophila melanogaster</td><td>polybromo</td><td>CG11375 gene product from transcript CG11375-RA</td><td>BAF180|Bap180|CG11375|DmelCG11375|PB|anon-WO0140519.158|baf180|bap180</td><td>0</td><td>0</td></tr>
<tr><td>9924</td><td>42955</td><td>Drosophila melanogaster</td><td>CG6980</td><td>CG6980 gene product from transcript CG6980-RA</td><td>DmelCG6980|anon-WO0140519.171</td><td>0</td><td>0</td></tr>
<tr><td>9925</td><td>42956</td><td>Drosophila melanogaster</td><td>CG5807</td><td>CG5807 gene product from transcript CG5807-RA</td><td>BcDNA:GH12663|DmelCG5807|anon-WO0118547.355</td><td>0</td><td>0</td></tr>
<tr><td>9926</td><td>42957</td><td>Drosophila melanogaster</td><td>CG5808</td><td>CG5808 gene product from transcript CG5808-RA</td><td>BcDNA:GH01073|DmelCG5808|UKL|cg5808</td><td>0</td><td>0</td></tr>
<tr><td>9927</td><td>42958</td><td>Drosophila melanogaster</td><td>Saf-B</td><td>Scaffold attachment factor B</td><td>CG6995|DmelCG6995|LD22822|SAF-B|cg6995|saf-b</td><td>0</td><td>0</td></tr>
<tr><td>9928</td><td>42959</td><td>Drosophila melanogaster</td><td>niki</td><td>nimA-like kinase</td><td>CG10951|DmelCG10951|NEK1|Niki</td><td>0</td><td>0</td></tr>
<tr><td>9929</td><td>42960</td><td>Drosophila melanogaster</td><td>RabX4</td><td>CG31118 gene product from transcript CG31118-RA</td><td>AAF56345|CG13638|CG31118|DmRabX4|DmelCG31118|rabX4</td><td>0</td><td>0</td></tr>
<tr><td>9930</td><td>42961</td><td>Drosophila melanogaster</td><td>CG13636</td><td>CG13636 gene product from transcript CG13636-RB</td><td>BcDNA:RE05274|BcDNA:RE20019|DmelCG13636</td><td>0</td><td>0</td></tr>
<tr><td>9931</td><td>42962</td><td>Drosophila melanogaster</td><td>Esp</td><td>Epidermal stripes and patches</td><td>CG7005|DmelCG7005</td><td>0</td><td>0</td></tr>
<tr><td>9932</td><td>42963</td><td>Drosophila melanogaster</td><td>CG7006</td><td>CG7006 gene product from transcript CG7006-RA</td><td>BcDNA:GM12126|DmelCG7006|anon-WO0118547.410</td><td>0</td><td>0</td></tr>
<tr><td>9933</td><td>42964</td><td>Drosophila melanogaster</td><td>nct</td><td>nicastrin</td><td>CG7012|DmelCG7012|Nct|Nic|S5A|ST96B|agro|dNct|nic</td><td>0</td><td>0</td></tr>
<tr><td>9934</td><td>42965</td><td>Drosophila melanogaster</td><td>CG10899</td><td>CG10899 gene product from transcript CG10899-RA</td><td>BcDNA:AT02719|DmelCG10899</td><td>0</td><td>0</td></tr>
<tr><td>9935</td><td>42967</td><td>Drosophila melanogaster</td><td>CG13640</td><td>CG13640 gene product from transcript CG13640-RA</td><td>BcDNA:RE70528|DmelCG13640</td><td>0</td><td>0</td></tr>
<tr><td>9936</td><td>42968</td><td>Drosophila melanogaster</td><td>CG7016</td><td>CG7016 gene product from transcript CG7016-RA</td><td>DmelCG7016</td><td>0</td><td>0</td></tr>
<tr><td>9937</td><td>42969</td><td>Drosophila melanogaster</td><td>CG13641</td><td>CG13641 gene product from transcript CG13641-RA</td><td>DmelCG13641</td><td>0</td><td>0</td></tr>
<tr><td>9938</td><td>42970</td><td>Drosophila melanogaster</td><td>Ela</td><td>Elastin-like</td><td>CG7021|DmelCG7021</td><td>0</td><td>0</td></tr>
<tr><td>9939</td><td>42971</td><td>Drosophila melanogaster</td><td>CycB3</td><td>Cyclin B3</td><td>CG5814|Cyc B3|DmelCG5814|anon-WO0118547.414|cycB3|l(3)L6540</td><td>0</td><td>0</td></tr>
<tr><td>9940</td><td>42972</td><td>Drosophila melanogaster</td><td>CG3744</td><td>CG3744 gene product from transcript CG3744-RC</td><td>DmelCG3744</td><td>0</td><td>0</td></tr>
<tr><td>9941</td><td>42973</td><td>Drosophila melanogaster</td><td>CG11089</td><td>CG11089 gene product from transcript CG11089-RD</td><td>153761_at|BEST:GH11240|CT30991|DmelCG11089</td><td>0</td><td>0</td></tr>
<tr><td>9942</td><td>42975</td><td>Drosophila melanogaster</td><td>CG31121</td><td>CG31121 gene product from transcript CG31121-RA</td><td>BcDNA:GH19411|CG11764|CG18633|CG18655|DmelCG31121</td><td>0</td><td>0</td></tr>
<tr><td>9943</td><td>42976</td><td>Drosophila melanogaster</td><td>CG11069</td><td>CG11069 gene product from transcript CG11069-RA</td><td>DmelCG11069</td><td>0</td><td>0</td></tr>
<tr><td>9944</td><td>42978</td><td>Drosophila melanogaster</td><td>CG10845</td><td>CG10845 gene product from transcript CG10845-RA</td><td>DmelCG10845</td><td>0</td><td>0</td></tr>
<tr><td>9945</td><td>42979</td><td>Drosophila melanogaster</td><td>CG31120</td><td>CG31120 gene product from transcript CG31120-RB</td><td>CG13644|CG18761|CG6031|DmelCG31120</td><td>0</td><td>0</td></tr>
<tr><td>9946</td><td>42981</td><td>Drosophila melanogaster</td><td>CG11168</td><td>CG11168 gene product from transcript CG11168-RC</td><td>DmelCG11168</td><td>0</td><td>0</td></tr>
<tr><td>9947</td><td>42982</td><td>Drosophila melanogaster</td><td>CG11120</td><td>CG11120 gene product from transcript CG11120-RA</td><td>DmelCG11120</td><td>0</td><td>0</td></tr>
<tr><td>9948</td><td>42983</td><td>Drosophila melanogaster</td><td>Trf4-2</td><td>CG17462 gene product from transcript CG17462-RA</td><td>AAF56369|CG17462|DmTRF4-2|DmelCG17462</td><td>0</td><td>0</td></tr>
<tr><td>9949</td><td>42984</td><td>Drosophila melanogaster</td><td>CG11771</td><td>CG11771 gene product from transcript CG11771-RA</td><td>DmelCG11771|anon-EST:Posey100</td><td>0</td><td>0</td></tr>
<tr><td>9950</td><td>42985</td><td>Drosophila melanogaster</td><td>Osbp</td><td>Oxysterol binding protein</td><td>CG6708|DmelCG6708|OSBP-Dm|osbp</td><td>0</td><td>0</td></tr>
<tr><td>9951</td><td>42986</td><td>Drosophila melanogaster</td><td>ssh</td><td>slingshot</td><td>CG6238|DmelCG6238|MKP|Mkph|SSH|Ssh|l(3)01207</td><td>0</td><td>0</td></tr>
<tr><td>9952</td><td>42987</td><td>Drosophila melanogaster</td><td>Nmnat</td><td>Nicotinamide mononucleotide adenylyltransferase</td><td>3R4|AAL90149|CG13645|DmelCG13645|NMNAT|Nmnat1|NmnatB|dNmnat|nmnat</td><td>0</td><td>0</td></tr>
<tr><td>9953</td><td>42988</td><td>Drosophila melanogaster</td><td>CG13646</td><td>CG13646 gene product from transcript CG13646-RA</td><td>DmelCG13646</td><td>0</td><td>0</td></tr>
<tr><td>9954</td><td>42990</td><td>Drosophila melanogaster</td><td>tnc</td><td>tenectin</td><td>CG13648|DmelCG13648|Mur96B|Tnc</td><td>0</td><td>0</td></tr>
<tr><td>9955</td><td>42991</td><td>Drosophila melanogaster</td><td>beta4GalT7</td><td>beta-4-galactosyltransferase 7</td><td>CG11780|Dbeta4GalT7|DmelCG11780|GalTI|beta4Gal-T7|dbeta4GalT7|dbeta4GalTI</td><td>0</td><td>0</td></tr>
<tr><td>9956</td><td>42992</td><td>Drosophila melanogaster</td><td>CG11781</td><td>CG11781 gene product from transcript CG11781-RA</td><td>BcDNA:RE35789|DmelCG11781</td><td>0</td><td>0</td></tr>
<tr><td>9957</td><td>42993</td><td>Drosophila melanogaster</td><td>Hr96</td><td>Hormone receptor-like in 96</td><td>CG11783|DHR96|Dhr96|DmelCG11783|HR96|NR1J1</td><td>0</td><td>0</td></tr>
<tr><td>9958</td><td>42994</td><td>Drosophila melanogaster</td><td>Smg6</td><td>CG6369 gene product from transcript CG6369-RA</td><td>CG6369|DmelCG6369|SMG6|dSMG6</td><td>0</td><td>0</td></tr>
<tr><td>9959</td><td>42995</td><td>Drosophila melanogaster</td><td>CG6422</td><td>CG6422 gene product from transcript CG6422-RA</td><td>DmelCG6422</td><td>0</td><td>0</td></tr>
<tr><td>9960</td><td>42996</td><td>Drosophila melanogaster</td><td>bai</td><td>baiser</td><td>150547_at|BcDNA:RE52411|CG11785|DmelCG11785|p23|p24</td><td>0</td><td>0</td></tr>
<tr><td>9961</td><td>42998</td><td>Drosophila melanogaster</td><td>CG11786</td><td>CG11786 gene product from transcript CG11786-RB</td><td>DmelCG11786</td><td>0</td><td>0</td></tr>
<tr><td>9962</td><td>42999</td><td>Drosophila melanogaster</td><td>CG11790</td><td>CG11790 gene product from transcript CG11790-RB</td><td>DmelCG11790</td><td>0</td><td>0</td></tr>
<tr><td>9963</td><td>43000</td><td>Drosophila melanogaster</td><td>CG11791</td><td>CG11791 gene product from transcript CG11791-RB</td><td>DmelCG11791</td><td>0</td><td>0</td></tr>
<tr><td>9964</td><td>43001</td><td>Drosophila melanogaster</td><td>CG31109</td><td>CG31109 gene product from transcript CG31109-RA</td><td>BcDNA:RE17222|CG13649|DmelCG31109</td><td>0</td><td>0</td></tr>
<tr><td>9965</td><td>43002</td><td>Drosophila melanogaster</td><td>CG34349</td><td>CG34349 gene product from transcript CG34349-RC</td><td>BP1013|CG11819|CG34037|DmelCG34349|Dmel_CG11819|Dmel_CG34037</td><td>0</td><td>0</td></tr>
<tr><td>9966</td><td>43003</td><td>Drosophila melanogaster</td><td>veli</td><td>CG7662 gene product from transcript CG7662-RA</td><td>CG7662|D-veli|DLin-7|DmelCG7662|Dveli|Flin-7|Lin-7|Veli|d-Veli|dLin-7|dLin7|dVeli|lin-7|lin7</td><td>0</td><td>0</td></tr>
<tr><td>9967</td><td>43004</td><td>Drosophila melanogaster</td><td>PQBP1</td><td>Poly-glutamine tract binding protein 1</td><td>CG11820|DmelCG11820|PQBP-1|dPQBP-1|dPQBP1</td><td>0</td><td>0</td></tr>
<tr><td>9968</td><td>43005</td><td>Drosophila melanogaster</td><td>OstStt3</td><td>Oligosaccharyl transferase 3</td><td>BcDNA.GM01838|BcDNA:GM01838|CG7748|DmelCG7748|anon-WO03054008.7|l(3)j2D9</td><td>0</td><td>0</td></tr>
<tr><td>9969</td><td>43006</td><td>Drosophila melanogaster</td><td>CG11839</td><td>CG11839 gene product from transcript CG11839-RB</td><td>CT36969|DmelCG11839</td><td>0</td><td>0</td></tr>
<tr><td>9970</td><td>43007</td><td>Drosophila melanogaster</td><td>CG11836</td><td>CG11836 gene product from transcript CG11836-RB</td><td>DmelCG11836|SP23</td><td>0</td><td>0</td></tr>
<tr><td>9971</td><td>43008</td><td>Drosophila melanogaster</td><td>CG9996</td><td>CG9996 gene product from transcript CG9996-RB</td><td>DmelCG9996</td><td>0</td><td>0</td></tr>
<tr><td>9972</td><td>43009</td><td>Drosophila melanogaster</td><td>CG11920</td><td>CG11920 gene product from transcript CG11920-RA</td><td>DmelCG11920</td><td>0</td><td>0</td></tr>
<tr><td>9973</td><td>43010</td><td>Drosophila melanogaster</td><td>fd96Ca</td><td>forkhead domain 96Ca</td><td>CG11921|DmelCG11921|Dmfd4|FD4|fd4</td><td>0</td><td>0</td></tr>
<tr><td>9974</td><td>43011</td><td>Drosophila melanogaster</td><td>fd96Cb</td><td>forkhead domain 96Cb</td><td>CG11922|DmelCG11922|Dmfd5|FD5|fd5</td><td>0</td><td>0</td></tr>
<tr><td>9975</td><td>43012</td><td>Drosophila melanogaster</td><td>CG33095</td><td>CG33095 gene product from transcript CG33095-RB</td><td>CG11935|CG11938|DmelCG33095|FBgn0039275</td><td>0</td><td>0</td></tr>
<tr><td>9976</td><td>43014</td><td>Drosophila melanogaster</td><td>CG13650</td><td>CG13650 gene product from transcript CG13650-RB</td><td>DmelCG13650</td><td>0</td><td>0</td></tr>
<tr><td>9977</td><td>43015</td><td>Drosophila melanogaster</td><td>CG31108</td><td>CG31108 gene product from transcript CG31108-RA</td><td>CG10057|DmelCG31108</td><td>0</td><td>0</td></tr>
<tr><td>9978</td><td>43016</td><td>Drosophila melanogaster</td><td>vig2</td><td>CG11844 gene product from transcript CG11844-RB</td><td>BcDNA:SD06613|CG11844|DmelCG11844|anon-WO0118547.290</td><td>0</td><td>0</td></tr>
<tr><td>9979</td><td>43017</td><td>Drosophila melanogaster</td><td>Mocs2</td><td>Molybdopterin synthase 2</td><td>CG10238|DmelCG10238|Dromel_CG10238_FBtr0084782_mocs_mORF|MOCS2</td><td>0</td><td>0</td></tr>
<tr><td>9980</td><td>43018</td><td>Drosophila melanogaster</td><td>Clbn</td><td>Caliban</td><td>CG11847|DmelCG11847</td><td>0</td><td>0</td></tr>
<tr><td>9981</td><td>43019</td><td>Drosophila melanogaster</td><td>Bili</td><td>Band4.1 inhibitor LRP interactor</td><td>CG11848|DmelCG11848|dBili</td><td>0</td><td>0</td></tr>
<tr><td>9982</td><td>43020</td><td>Drosophila melanogaster</td><td>danr</td><td>distal antenna-related</td><td>CG13651|Danr|DmelCG13651|hern</td><td>0</td><td>0</td></tr>
<tr><td>9983</td><td>43023</td><td>Drosophila melanogaster</td><td>dan</td><td>distal antenna</td><td>CG11849|Dan|DmelCG11849|fer</td><td>0</td><td>0</td></tr>
<tr><td>9984</td><td>43025</td><td>Drosophila melanogaster</td><td>CG13653</td><td>CG13653 gene product from transcript CG13653-RA</td><td>DmelCG13653</td><td>0</td><td>0</td></tr>
<tr><td>9985</td><td>43026</td><td>Drosophila melanogaster</td><td>Cad96Ca</td><td>CG10244 gene product from transcript CG10244-RA</td><td>CG10239|CG10244|CT28779|DCad96Ca|Dm Cad96Ca|DmHD-14|DmelCG10244|FGFR1-like|HD-14|cad96C|stit</td><td>0</td><td>0</td></tr>
<tr><td>9986</td><td>43028</td><td>Drosophila melanogaster</td><td>CG13654</td><td>CG13654 gene product from transcript CG13654-RA</td><td>DmelCG13654</td><td>0</td><td>0</td></tr>
<tr><td>9987</td><td>43029</td><td>Drosophila melanogaster</td><td>CG13663</td><td>CG13663 gene product from transcript CG13663-RA</td><td>DmelCG13663</td><td>0</td><td>0</td></tr>
</table>

9988	43030	Drosophila melanogaster	CG31103	CG31103 gene product from transcript CG31103-RB	CG13655|DmelCG31103	0	0
9989	43031	Drosophila melanogaster	CG11851	CG11851 gene product from transcript CG11851-RA	DmelCG11851	0	0
9990	43033	Drosophila melanogaster	Cad96Cb	CG13664 gene product from transcript CG13664-RC	CG10421|CG13664|CT29262|DCad96Cb|DmelCG13664|cad96C|cad96Cb	0	0
9991	43034	Drosophila melanogaster	CG10420	CG10420 gene product from transcript CG10420-RA	DmelCG10420	0	0
9992	43035	Drosophila melanogaster	CG11852	CG11852 gene product from transcript CG11852-RA	CT33073|DmelCG11852	0	0
9993	43036	Drosophila melanogaster	to	takeout	CG11853|CT33074|DmelCG11853	0	0
9994	43037	Drosophila melanogaster	CG11854	CG11854 gene product from transcript CG11854-RA	DmelCG11854	0	0
9995	43038	Drosophila melanogaster	bam	bag of marbles	BAM|Bam|Bam-C|BamC|BamF|CG10422|DmelCG10422|alpha|fs(3)neo61|ham	0	0
9996	43039	Drosophila melanogaster	RpS27	Ribosomal protein S27	CG10423|DmelCG10423|M(3)96C|anon-EST:Posey295	0	0
9997	43040	Drosophila melanogaster	CG11875	CG11875 gene product from transcript CG11875-RA	DmelCG11875	0	0
9998	43041	Drosophila melanogaster	Nup358	Nucleoporin 358	CG11856|DmelCG11856|RanBP2|nup358	0	0
9999	43042	Drosophila melanogaster	CG11857	CG11857 gene product from transcript CG11857-RA	DmelCG11857	0	0
10000	43043	Drosophila melanogaster	CG10425	CG10425 gene product from transcript CG10425-RA	DmelCG10425	0	0
10001	43044	Drosophila melanogaster	CG11858	CG11858 gene product from transcript CG11858-RA	BcDNA:RH66629|DmelCG11858	0	0
10002	43046	Drosophila melanogaster	CG11859	CG11859 gene product from transcript CG11859-RA	DmelCG11859	0	0
10003	43050	Drosophila melanogaster	CG11878	CG11878 gene product from transcript CG11878-RA	CT33083|DmelCG11878	0	0
10004	43051	Drosophila melanogaster	CG10513	CG10513 gene product from transcript CG10513-RB	CT29500|DmelCG10513	0	0
10005	43052	Drosophila melanogaster	CG10514	CG10514 gene product from transcript CG10514-RA	CT29508|DmelCG10514	0	0
10006	43053	Drosophila melanogaster	CG11892	CG11892 gene product from transcript CG11892-RA	DmelCG11892	0	0
10007	43054	Drosophila melanogaster	CG31104	CG31104 gene product from transcript CG31104-RA	CG13657|DmelCG31104	0	0
10008	43055	Drosophila melanogaster	CG13658	CG13658 gene product from transcript CG13658-RB	DmelCG13658	0	0
10009	43056	Drosophila melanogaster	CG11893	CG11893 gene product from transcript CG11893-RA	DmelCG11893	0	0
10010	43057	Drosophila melanogaster	CG31098	CG31098 gene product from transcript CG31098-RA	BcDNA:GH27183|BcDNA:RE13429|CG10634|DmelCG31098	0	0
10011	43058	Drosophila melanogaster	CG31097	CG31097 gene product from transcript CG31097-RB	CG13665|DmelCG31097	0	0
10012	43059	Drosophila melanogaster	CG13659	CG13659 gene product from transcript CG13659-RA	DmelCG13659	0	0
10013	43060	Drosophila melanogaster	CG31370	CG31370 gene product from transcript CG31370-RB	CG13660|DmelCG31370	0	0
10014	43061	Drosophila melanogaster	CG10550	CG10550 gene product from transcript CG10550-RD	DmelCG10550	0	0
10015	43062	Drosophila melanogaster	CG31087	CG31087 gene product from transcript CG31087-RA	CG10551|DmelCG31087	0	0
10016	43063	Drosophila melanogaster	CG10559	CG10559 gene product from transcript CG10559-RB	DmelCG10559	0	0
10017	43064	Drosophila melanogaster	CG10553	CG10553 gene product from transcript CG10553-RA	CT29612|DmelCG10553	0	0
10018	43065	Drosophila melanogaster	CG10560	CG10560 gene product from transcript CG10560-RA	DmelCG10560	0	0
10019	43066	Drosophila melanogaster	CG10562	CG10562 gene product from transcript CG10562-RA	DmelCG10562	0	0
10020	43067	Drosophila melanogaster	CHKov1	CG10618 gene product from transcript CG10618-RB	BcDNA:GH03753|CG10618|DmelCG10618	0	0
10021	43068	Drosophila melanogaster	CHKov2	CG10675 gene product from transcript CG10675-RA	CG10675|DmelCG10675	0	0
10022	43069	Drosophila melanogaster	CG10669	CG10669 gene product from transcript CG10669-RB	DmelCG10669	0	0
10023	43070	Drosophila melanogaster	CG11902	CG11902 gene product from transcript CG11902-RB	DmelCG11902|anon-96CDa	0	0
10024	43071	Drosophila melanogaster	rha	CG11908 gene product from transcript CG11908-RA	CG11908|DmelCG11908|RADHA|anon-WO0140519.50	0	0
10025	43072	Drosophila melanogaster	tobi	target of brain insulin	CG11909|CT33098|DmelCG11909	0	0
10026	43073	Drosophila melanogaster	CG11913	CG11913 gene product from transcript CG11913-RB	CG111913|DmelCG11913	0	0
10027	43074	Drosophila melanogaster	alrm	astrocytic leucine-rich repeat molecule	CG11910|CT37074|DmelCG11910	0	0
10028	43077	Drosophila melanogaster	Vps33B	Vacuolar protein sorting 33B	CG5127|DmelCG5127|VPS33b|dVps33B	0	0
10029	43078	Drosophila melanogaster	CG4553	CG4553 gene product from transcript CG4553-RA	DmelCG4553	0	0
10030	43079	Drosophila melanogaster	MED28	Mediator complex subunit 28	BcDNA:GM31575|CG5121|DmelCG5121|Med23|Med28|dMED23	0	0
10031	43080	Drosophila melanogaster	XNP	CG4548 gene product from transcript CG4548-RA	ATR-X/XNP|CG4548|DATR-X|DATRX|DmelCG4548|EP(3)0635|EP635|dATRX|dXNP|xnp|xnp/atr-x	0	0
10032	43081	Drosophila melanogaster	CG5116	CG5116 gene product from transcript CG5116-RA	DmelCG5116|Dromel_CG5116_FBtr0084877_mORF	0	0
10033	43083	Drosophila melanogaster	CG5112	CG5112 gene product from transcript CG5112-RA	DmelCG5112	0	0
10034	43084	Drosophila melanogaster	CG5107	CG5107 gene product from transcript CG5107-RA	DmelCG5107|anon-WO0140519.57	0	0
10035	43085	Drosophila melanogaster	CG5111	CG5111 gene product from transcript CG5111-RA	DmelCG5111	0	0
10036	43086	Drosophila melanogaster	CG4582	CG4582 gene product from transcript CG4582-RA	DmelCG4582	0	0
10037	43087	Drosophila melanogaster	msi	musashi	CG5099|DMSIDNA|DmelCG5099|Msi|SIDNA|anon-EST:Liang-2.35|clone 2.35	0	0
10038	43088	Drosophila melanogaster	ymp	yellow-emperor	BcDNA:AT03213|CG12250|DmelCG12250	0	0
10039	43089	Drosophila melanogaster	CG5079	CG5079 gene product from transcript CG5079-RA	DmelCG5079	0	0
10040	43090	Drosophila melanogaster	CG5071	CG5071 gene product from transcript CG5071-RB	BcDNA:AT31683|DmelCG5071	0	0
10041	43091	Drosophila melanogaster	CG4673	CG4673 gene product from transcript CG4673-RB	DmelCG4673|NPL4|dNp14|npl4	0	0
10042	43092	Drosophila melanogaster	Ssadh	Succinic semialdehyde dehydrogenase	CG4685|DmSSADH|DmelCG4685|SSADH|anon-WO0118547.428	0	0
10043	43093	Drosophila melanogaster	jigr1	jing interacting gene regulatory 1	CG17383|DmelCG17383|JIGR-1|JIGR1|bs29f05.y1	0	0
10044	43095	Drosophila melanogaster	tankyrase	CG4719 gene product from transcript CG4719-RA	BcDNA:LD22548|CG17487|CG4719|DmelCG4719	0	0
10045	43096	Drosophila melanogaster	RASSF8	CG5053 gene product from transcript CG5053-RA	Boa|CG5053|DmelCG5053|dRASSF8	0	0
10046	43098	Drosophila melanogaster	Lgr3	CG31096 gene product from transcript CG31096-RA	CG31096|CG5042|CG5046|CT16193|DmelCG31096|anon-WO0170980.25|anon-WO0170980.26	0	0
10047	43099	Drosophila melanogaster	CG4730	CG4730 gene product from transcript CG4730-RA	DmelCG4730	0	0
10048	43100	Drosophila melanogaster	CG5039	CG5039 gene product from transcript CG5039-RA	BcDNA:LD35121|DmelCG5039	0	0
10049	43101	Drosophila melanogaster	CG4743	CG4743 gene product from transcript CG4743-RB	DmelCG4743	0	0
10050	43102	Drosophila melanogaster	CG5028	CG5028 gene product from transcript CG5028-RB	DmelCG5028	0	0
10051	43103	Drosophila melanogaster	RpL27	Ribosomal protein L27	CG4759|DmelCG4759|M(3)96CF|RpL27e	0	0
10052	43104	Drosophila melanogaster	CLS	cardiolipin synthase	CG4774|DmelCG4774	0	0
10053	43105	Drosophila melanogaster	LpR2	Lipophorin receptor 2	CG31092|CG4823|CG4834|DmelCG31092|LDLR|Lpr2|lpr2	0	0
10054	43110	Drosophila melanogaster	CG17198	CG17198 gene product from transcript CG17198-RA	DmelCG17198	0	0
10055	43111	Drosophila melanogaster	CG17197	CG17197 gene product from transcript CG17197-RB	CG17197-PA|DmelCG17197	0	0
10056	43112	Drosophila melanogaster	CG17196	CG17196 gene product from transcript CG17196-RB	CG17196-PA|DmelCG17196	0	0
10057	43113	Drosophila melanogaster	CG17195	CG17195 gene product from transcript CG17195-RA	CG17195-PA|DmelCG17195	0	0
10058	43114	Drosophila melanogaster	CG4956	CG4956 gene product from transcript CG4956-RA	DmelCG4956	0	0
10059	43115	Drosophila melanogaster	CG4960	CG4960 gene product from transcript CG4960-RA	BcDNA:AT31258|DmelCG4960	0	0
10060	43116	Drosophila melanogaster	CG31093	CG31093 gene product from transcript CG31093-RA	BcDNA:AT30660|CG4964|DmelCG31093	0	0
10061	43117	Drosophila melanogaster	CG5024	CG5024 gene product from transcript CG5024-RA	BcDNA:AT16150|DmelCG5024|gene1	0	0
10062	43118	Drosophila melanogaster	CG17770	CG17770 gene product from transcript CG17770-RA	DmelCG17770|gene2	0	0
10063	43120	Drosophila melanogaster	CG14354	CG14354 gene product from transcript CG14354-RA	DmelCG14354	0	0
10064	43121	Drosophila melanogaster	Mst57Dc	Male-specific RNA 57Dc	Acp57Dc|CG4986|DmelCG4986|Mst75Dc|mst(2)ag-1|mst-1	0	0
10065	43122	Drosophila melanogaster	Mst57Db	Male-specific RNA 57Db	CG5016|DmelCG5016|mst(2)ag-1|mst-1	0	0
10066	43124	Drosophila melanogaster	alpha4GT2	CG5878 gene product from transcript CG5878-RB	CG5878|DmelCG5878	0	0
10067	43125	Drosophila melanogaster	CG5886	CG5886 gene product from transcript CG5886-RA	DmelCG5886	0	0
10068	43126	Drosophila melanogaster	CG5890	CG5890 gene product from transcript CG5890-RB	DmelCG5890	0	0
10069	43127	Drosophila melanogaster	Tsp96F	Tetraspanin 96F	BcDNA:LD19727|CG6120|Dm.Tsp96F|DmelCG6120|LD19727	0	0
10070	43128	Drosophila melanogaster	CG17370	CG17370 gene product from transcript CG17370-RD	CG5901|DmelCG17370|SPPL3	0	0
10071	43130	Drosophila melanogaster	Lnk	CG17367 gene product from transcript CG17367-RC	CG 17367|CG17367|CG5908|DmelCG17367|dSH2B|lnk	0	0
10072	43132	Drosophila melanogaster	CG5913	CG5913 gene product from transcript CG5913-RA	DmelCG5913	0	0
10073	43133	Drosophila melanogaster	CG5948	CG5948 gene product from transcript CG5948-RA	DmelCG5948	0	0
10074	43134	Drosophila melanogaster	MCO3	multicopper oxidase 3	CG5959|DmelCG5959	0	0
10075	43135	Drosophila melanogaster	CG31089	CG31089 gene product from transcript CG31089-RA	CG5990|DmelCG31089	0	0
10076	43136	Drosophila melanogaster	CG42235	CG42235 gene product from transcript CG42235-RE	CG14539|CG31090|CG8932|CG8934|CG8951|CG8957|CG8966|DmelCG42235|Dmel_CG31090|Dmel_CG8932|Dmel_CG8934|Dmel_CG8957|Dmel_CG8966	0	0
10077	43142	Drosophila melanogaster	CG14546	CG14546 gene product from transcript CG14546-RA	DmelCG14546	0	0
10078	43145	Drosophila melanogaster	CG14540	CG14540 gene product from transcript CG14540-RA	DmelCG14540	0	0
10079	43146	Drosophila melanogaster	boss	bride of sevenless	BOSS|Bos|Boss|CG8285|DmelCG8285|F|X55887	0	0
10080	43148	Drosophila melanogaster	CG42261	CG42261 gene product from transcript CG42261-RA	CG14541|CG8968|DmelCG42261|Dmel_CG14541|Dmel_CG8968	0	0
10081	43149	Drosophila melanogaster	Nf1	Neurofibromin 1	CG8318|DmelCG8318|NF-1|NF1|Nf-1|dNF1|nf1	0	0
10082	43150	Drosophila melanogaster	HLHmdelta	E(spl) region transcript mdelta	CG8328|DmelCG8328|E(Spl) HLH-m delta|E(Spl)-HLH-mdelta|E(spl) mdelta|E(spl)-mdelta|E(spl)Mdelta|E(spl)delta|E(spl)mC|E(spl)mdelta|HLH-mdelta|HLHMD|HLHmC|bHLHc14|mC|mdelta|mdelta0.5	0	0
10083	43151	Drosophila melanogaster	HLHmgamma	E(spl) region transcript mgamma	CG8333|Dm-mB|DmelCG8333|E(Spl) HLH-m gamma|E(spl) m gamma|E(spl) mgamma|E(spl)-mgamma|E(spl)Mgamma|E(spl)gamma|E(spl)mB|E(spl)mgamma|HLH-mgamma|HLHmB|Mgamma|anon-EST:Li	0	0
10084	43152	Drosophila melanogaster	HLHmbeta	E(spl) region transcript mbeta	CG14548|Dm-mA|DmelCG14548|E(Spl) HLH-Mbeta|E(Spl)mbeta|E(spl)|E(spl) mbeta|E(spl)-Mbeta|E(spl)-mbeta|E(spl)Mbeta|E(spl)beta|E(spl)mA|E(spl)mbeta|HLH-mbeta|HLHmA|Mbeta|bHLHb34|m-beta|m	0	0
10085	43153	Drosophila melanogaster	malpha	E(spl) region transcript malpha	CG8337|DmelCG8337|DmelCG8337|E(spl)ma|E(spl)malpha|E(spl)mmalpha|HLHmalpha	0	0
10086	43154	Drosophila melanogaster	m1	E(spl) region transcript m1	BcDNA:RE44854|CG8342|DmelCG8342|E(spl)-m1|E(spl)m1|HLHm1	0	0
10087	43155	Drosophila melanogaster	m2	E(spl) region transcript m2	CG6104|DmelCG6104|DmelCG6104|E(Spl)m2|E(spl)m2|HLHm2	0	0
10088	43156	Drosophila melanogaster	HLHm3	E(spl) region transcript m3	CG8346|DmelCG8346|E(Spl)-HLH-m3|E(spl)-m3|E(spl)C|E(spl)C m3|E(spl)M3|E(spl)m3|E(spl)m3/m4|HLH-m3|HLHM3|bHLHc14|m3	0	0
10089	43157	Drosophila melanogaster	m4	E(spl) region transcript m4	CG6099|DmelCG6099|DmelCG6099|E(spl)m4|HLHm4	0	0
10090	43158	Drosophila melanogaster	HLHm5	E(spl) region transcript m5	CG6096|DmelCG6096|E(spl) transcription unit m5|E(spl)-m5|E(spl)M5|E(spl)m5|HLH-m5|bHLHb36|m5	0	0
10091	43159	Drosophila melanogaster	m6	E(spl) region transcript m6	CG8354|DmelCG8354|DmelCG8354|E(spl)M6|E(spl)m6|HLHm6|M6	0	0
10092	43160	Drosophila melanogaster	HLHm7	E(spl) region transcript m7	CG8361|DmelCG8361|E(SPL)m7|E(spl) m7|E(spl)-M7|E(spl)-m7|E(spl)M7|E(spl)m7|ESPL|En(spl)m7|HLH-m7|M7|bHLHb35|m7	0	0
10093	43161	Drosophila melanogaster	E(spl)	Enhancer of split	CG8365|DmelCG8365|E(Spl)|E(spl) m8|E(spl)-C|E(spl)-m8|E(spl)M8|E(spl)bHLH|E(spl)m8|En(spl)|En(spl)-C|Espl|HLHm8|Hes|M8|Su(H)[[m8]]|anon-EST:Liang-1.71|bHLHb36|clone 1.71|e(spl)|l(3)96Fd|m8	0	0
10094	43162	Drosophila melanogaster	gro	groucho	BEST:GM01575|BEST:LD15161|BcDNA.LD33829|BcDNA:LD33829|CG8384|DGro|DmelCG8384|E(spl)-WD|E(spl)[2]|E(spl)gro|E(spl)m9/m10|En-spl|GRO|Grg/TLE|Gro|Groucho|anon-WO0118547.385|dAES	0	0
10095	43163	Drosophila melanogaster	exo84	CG6095 gene product from transcript CG6095-RA	CG6095|DExo84|DmelCG6095|Exo84|Exo84p|anon-WO0153538.55|onr	0	0
10096	43164	Drosophila melanogaster	vps2	CG14542 gene product from transcript CG14542-RA	CG14542|Did4|DmelCG14542|NEST:bs02f06|Vps2|dvps2	0	0
10097	43165	Drosophila melanogaster	Dak1	CG6092 gene product from transcript CG6092-RA	CG6092|Dak1L|Dm.UMP-CMP kinase|DmelCG6092|anon-WO0118547.320|dak1	0	0
10098	43166	Drosophila melanogaster	Sld5	CG14549 gene product from transcript CG14549-RA	CG14549|DmelCG14549|anon-WO0172774.61	0	0

10099	43167	Drosophila melanogaster	CG14543	CG14543 gene product from transcript CG14543-RA	DmelCG14543	0	0
10100	43168	Drosophila melanogaster	CG14550	CG14550 gene product from transcript CG14550-RA	BcDNA:GM30016|DmelCG14550|Dromel_CG14550_FBtr0084974_mORF	0	0
10101	43169	Drosophila melanogaster	RpL34a	Ribosomal protein L34a	CG6090|DmelCG6090|L34|RpL34	0	0
10102	43170	Drosophila melanogaster	CG14544	CG14544 gene product from transcript CG14544-RA	DmelCG14544	0	0
10103	43171	Drosophila melanogaster	CG14551	CG14551 gene product from transcript CG14551-RA	DmelCG14551	0	0
10104	43174	Drosophila melanogaster	dys	dysfusion	CG12561|CG14552|CG14553|CG14554|CG32474|DmelCG32474|Dys|NXFL|dNXFL	0	0
10105	43176	Drosophila melanogaster	CG14556	CG14556 gene product from transcript CG14556-RB	DmelCG14556	0	0
10106	43177	Drosophila melanogaster	CG31324	CG31324 gene product from transcript CG31324-RA	BcDNA:RH68619|CG14555|CG14557|DmelCG31324	0	0
10107	43178	Drosophila melanogaster	CG6142	CG6142 gene product from transcript CG6142-RA	DmelCG6142	0	0
10108	43179	Drosophila melanogaster	CG31086	CG31086 gene product from transcript CG31086-RB	CG14558|DmelCG31086	0	0
10109	43180	Drosophila melanogaster	CG6073	CG6073 gene product from transcript CG6073-RA	DmelCG6073	0	0
10110	43181	Drosophila melanogaster	CG34129	CG34129 gene product from transcript CG34129-RA	CG6069|DmelCG34129|SPH192	0	0
10111	43182	Drosophila melanogaster	CG12290	CG12290 gene product from transcript CG12290-RA	DmelCG12290|anon-WO0170980.49|anon-WO0170980.50	0	0
10112	43183	Drosophila melanogaster	Ald	Aldolase	ALD|BcDNA:LP07735|CG6058|DmelCG6058|FBA|T12|T4|T6|T7|ald	0	0
10113	43184	Drosophila melanogaster	CG6154	CG6154 gene product from transcript CG6154-RD	DmelCG6154	0	0
10114	43185	Drosophila melanogaster	CG6036	CG6036 gene product from transcript CG6036-RA	DmelCG6036|PP2Cb	0	0
10115	43186	Drosophila melanogaster	CG33970	CG33970 gene product from transcript CG33970-RA	CG14559|CG31085|CG6162|CG6166|DmelCG33970	0	0
10116	43188	Drosophila melanogaster	CG14239	CG14239 gene product from transcript CG14239-RB	DmelCG14239	0	0
10117	43189	Drosophila melanogaster	CG5432	CG5432 gene product from transcript CG5432-RA	DmelCG5432	0	0
10118	43190	Drosophila melanogaster	tx	taxi	CG5441|Dei|Delilah|DmelCG5441|bHLHc10|dei|del|delilah	0	0
10119	43191	Drosophila melanogaster	Hex-t2	CG32849 gene product from transcript CG32849-RA	CG32849|CG5443|DmelCG32849|HK|Hex|anon-WO0140519.203	0	0
10120	43192	Drosophila melanogaster	CG5447	CG5447 gene product from transcript CG5447-RA	DmelCG5447	0	0
10121	43193	Drosophila melanogaster	Pdf	Pigment-dispersing factor	BcDNA:RH08487|CG6496|DmelCG6496|Drm-PDF|Drm-PDH|Drm-pdf|PAP|PDF|PDH|anon-WO0140519.261|anon-WO0140519.3|cPDH|pdf	0	0
10122	43194	Drosophila melanogaster	CG14237	CG14237 gene product from transcript CG14237-RA	DmelCG14237	0	0
10123	43195	Drosophila melanogaster	CG14238	CG14238 gene product from transcript CG14238-RA	DmelCG14238	0	0
10124	43196	Drosophila melanogaster	CG5455	CG5455 gene product from transcript CG5455-RA	CT17296|DmelCG5455	0	0
10125	43197	Drosophila melanogaster	CG6490	CG6490 gene product from transcript CG6490-RD	CT20213|DmelCG6490	0	0
10126	43200	Drosophila melanogaster	TwdlM	TweedleM	CG5468|DmelCG5468	0	0
10127	43201	Drosophila melanogaster	TwdlP	TweedleP	CG14240|DmelCG14240	0	0
10128	43202	Drosophila melanogaster	TwdlB	TweedleB	CG6478|DmelCG6478	0	0
10129	43203	Drosophila melanogaster	TwdlL	TweedleL	CG6447|DmelCG6447	0	0
10130	43204	Drosophila melanogaster	TwdlO	TweedleO	CG6452|DmelCG6452	0	0
10131	43205	Drosophila melanogaster	TwdlK	TweedleK	CG6460|DmelCG6460	0	0
10132	43206	Drosophila melanogaster	TwdlJ	TweedleJ	CG5471|DmelCG5471	0	0
10133	43207	Drosophila melanogaster	TwdlN	TweedleN	BcDNA:RE20092|BcDNA:RE59605|CG5476|DmelCG5476	0	0
10134	43208	Drosophila melanogaster	TwdlH	TweedleH	CG14241|CG31080|DmelCG31080	0	0
10135	43209	Drosophila melanogaster	TwdlS	TweedleS	CG14242|DmelCG14242	0	0
10136	43210	Drosophila melanogaster	TwdlD	TweedleD	CG14243|DmelCG14243	0	0
10137	43212	Drosophila melanogaster	Tb	Tubby	CG5480|DmelCG5480|Tu|TwdlA	0	0
10138	43213	Drosophila melanogaster	beat-VII	CG42242 gene product from transcript CG42242-RD	CG14248|CG14249|CG42242|DmelCG42242|Dmel_CG14248|Dmel_CG14249|beat VII	0	0
10139	43214	Drosophila melanogaster	TwdlQ	TweedleQ	CG14250|DmelCG14250	0	0
10140	43215	Drosophila melanogaster	amon	amontillado	Amon|CG6438|DMH#5|DmelCG6438|PC2|PPP|Ppp|dPC2	0	0
10141	43216	Drosophila melanogaster	CG6425	CG6425 gene product from transcript CG6425-RA	DmelCG6425	0	0
10142	43217	Drosophila melanogaster	CG5484	CG5484 gene product from transcript CG5484-RC	DmelCG5484|anon-WO0118547.366	0	0
10143	43218	Drosophila melanogaster	CG6420	CG6420 gene product from transcript CG6420-RA	DmelCG6420	0	0
10144	43220	Drosophila melanogaster	CG6403	CG6403 gene product from transcript CG6403-RA	DmelCG6403	0	0
10145	43221	Drosophila melanogaster	CG14247	CG14247 gene product from transcript CG14247-RA	DmelCG14247	0	0
10146	43222	Drosophila melanogaster	Tl	Toll	CG5490|CT17414|DmelCG5490|EP(3)1051|EP1051|Fs(1)Tl|Fs(3)Tl|T1|TL|Toll-1|Toll1|dToll|dToll1|mat(3)9|mel(3)10|mel(3)9|tl|toll|toll-1	0	0
10147	43223	Drosophila melanogaster	Lerp	lysosomal enzyme receptor protein	CG14255|CG14256|CG31072|CG6390|CG6396|DmelCG31072|LERP|anon-WO0118547.383	0	0
10148	43227	Drosophila melanogaster	IntS12	Integrator 12	CG5491|DmelCG5491|Int12	0	0
10149	43228	Drosophila melanogaster	ball	ballchen	BALL|Ball|BcDNA:LD09009|CG6386|DmelCG6386|NHK-1|NHK1|VRK|nhk-1|trip	0	0
10150	43229	Drosophila melanogaster	His2Av	Histone H2A variant	5499|CG5499|DmelCG5499|H2A.F/Z|H2A.X|H2A.Z|H2AV|H2AV_DROM|H2AX|H2AZ|H2Av|H2AvD|H2a.V|H2av|H2avD|HIS2AV|HIS2AVD|His2|His2AV|His2AvD|HisH2Av|Hist|Hist2av|gamma-H2Av|gamma-HI	0	0
10151	43230	Drosophila melanogaster	BM-40-SPARC	CG6378 gene product from transcript CG6378-RA	BM-40/SPARC|BM40|CG6378|CT19876|DmelCG6378|SPARC|anon-EST:Posey291|dSPARC|dsparc	0	0
10152	43231	Drosophila melanogaster	Rb97D	Ribonuclear protein at 97D	CG6354|DmelCG6354|Hrb97D|R16|Rb97D1|ms(3)03445|rbf	0	0
10153	43232	Drosophila melanogaster	ms(3)K81	male sterile (3) K81	CG14251|DmelCG14251|K81|ms(3)97Dg|ms(3)k81|ph	0	0
10154	43233	Drosophila melanogaster	CG5500	CG5500 gene product from transcript CG5500-RA	DmelCG5500	0	0
10155	43234	Drosophila melanogaster	ro	rough	CG6348|DROROUGH|DmelCG6348|Ro	0	0
10156	43235	Drosophila melanogaster	T48	Transcript 48	48|CG5507|DmelCG5507|Ubx-t48|anon-97D|t48	0	0
10157	43236	Drosophila melanogaster	Ets97D	Ets at 97D	CG6338|D-Elg|D-elg|DELG|Delg|DmelCG6338|Elg|Ets|d-elg|elg|tne|tny	0	0
10158	43238	Drosophila melanogaster	CG14252	CG14252 gene product from transcript CG14252-RA	DmelCG14252	0	0
10159	43239	Drosophila melanogaster	CG18472	CG18472 gene product from transcript CG18472-RA	DmelCG18472	0	0
10160	43240	Drosophila melanogaster	CG6330	CG6330 gene product from transcript CG6330-RB	DmelCG6330	0	0
10161	43241	Drosophila melanogaster	Tsp97E	Tetraspanin 97E	CG6323|Dm.Tsp97E|DmelCG6323	0	0
10162	43242	Drosophila melanogaster	CG5521	CG5521 gene product from transcript CG5521-RB	DmelCG5521|EP3527	0	0
10163	43243	Drosophila melanogaster	CG14253	CG14253 gene product from transcript CG14253-RB	DmelCG14253	0	0
10164	43244	Drosophila melanogaster	CG31075	CG31075 gene product from transcript CG31075-RA	150697_at|CG6309|CT19744|DmelCG31075	0	0
10165	43245	Drosophila melanogaster	TwdlC	TweedleC	CG14254|DmelCG14254|TwdlB	0	0
10166	43246	Drosophila melanogaster	CG6296	CG6296 gene product from transcript CG6296-RA	DmelL7|DmelCG6296	0	0
10167	43247	Drosophila melanogaster	CG6295	CG6295 gene product from transcript CG6295-RA	DmelL6|DmelCG6295	0	0
10168	43248	Drosophila melanogaster	CG17192	CG17192 gene product from transcript CG17192-RA	DmelL5|DmelCG17192	0	0
10169	43249	Drosophila melanogaster	CG17191	CG17191 gene product from transcript CG17191-RA	DmelL4|DmelCG17191	0	0
10170	43250	Drosophila melanogaster	CG6283	CG6283 gene product from transcript CG6283-RA	DmelL3|DmelCG6283	0	0
10171	43251	Drosophila melanogaster	CG6277	CG6277 gene product from transcript CG6277-RA	DmelL2|DmelCG6277	0	0
10172	43252	Drosophila melanogaster	CG6271	CG6271 gene product from transcript CG6271-RA	DmelL1|DmelCG6271	0	0
10173	43253	Drosophila melanogaster	NepYr	Neuropeptide Y receptor-like	CG5811|CG5811/107368|DmelCG5811|PR4|anon-WO0131005.13|anon-WO0131005.15	0	0
10174	43255	Drosophila melanogaster	Nep5	Neprilysin 5	CG6265|DmeNEP5|DmelCG6265|NEP5	0	0
10175	43256	Drosophila melanogaster	spz	spatzle	CG6134|CT19282|DmelCG6134|SPZ|Spz|mel(3)7	0	0
10176	43257	Drosophila melanogaster	CG14257	CG14257 gene product from transcript CG14257-RB	DmelCG14257	0	0
10177	43258	Drosophila melanogaster	Cpr97Ea	Cuticular protein 97Ea	CG6131|DmelCG6131	0	0
10178	43259	Drosophila melanogaster	Cpr97Eb	Cuticular protein 97Eb	CG15884|CT33880|DmelCG15884	0	0
10179	43260	Drosophila melanogaster	CG14258	CG14258 gene product from transcript CG14258-RA	DmelCG14258	0	0
10180	43261	Drosophila melanogaster	CG14259	CG14259 gene product from transcript CG14259-RA	DmelCG14259	0	0
10181	43262	Drosophila melanogaster	eater	CG6124 gene product from transcript CG6124-RB	C901-like|CG6124|CT19227|DmelCG6124|Eater	0	0
10182	43263	Drosophila melanogaster	CG17189	CG17189 gene product from transcript CG17189-RA	DmelCG17189|cg17189	0	0
10183	43264	Drosophila melanogaster	CG6074	CG6074 gene product from transcript CG6074-RA	CT19053|DmelCG6074	0	0
10184	43265	Drosophila melanogaster	gb	genderblind	CG6070|DmelCG6070|Gb|anon-WO0118547.295	0	0
10185	43266	Drosophila melanogaster	CG5815	CG5815 gene product from transcript CG5815-RB	BcDNA:GH07089|BcDNA:GH07346|DmelCG5815	0	0
10186	43267	Drosophila melanogaster	CG6066	CG6066 gene product from transcript CG6066-RA	DmelCG6066	0	0
10187	43268	Drosophila melanogaster	CG5880	CG5880 gene product from transcript CG5880-RA	DmelCG5880	0	0
10188	43269	Drosophila melanogaster	CG5882	CG5882 gene product from transcript CG5882-RA	DmelCG5882	0	0
10189	43270	Drosophila melanogaster	CG6059	CG6059 gene product from transcript CG6059-RA	DmelCG6059	0	0
10190	43271	Drosophila melanogaster	CG6051	CG6051 gene product from transcript CG6051-RC	DmelCG6051	0	0
10191	43273	Drosophila melanogaster	grass	Gram-positive Specific Serine protease	CG5896|DmelCG5896|Grass|SP1|SP2|c-SP1	0	0
10192	43274	Drosophila melanogaster	CG5909	CG5909 gene product from transcript CG5909-RA	DmelCG5909|SP8|c-SP8	0	0
10193	43275	Drosophila melanogaster	Ser	Serrate	Bd|CG6127|CT18858|DmelCG6127|Rpw|SER|mel(3)8|ser|std	0	0
10194	43276	Drosophila melanogaster	CG42813	CG42813 gene product from transcript CG42813-RA	CG31063|CG6001|DmelCG42813|Dmel_CG31063	0	0
10195	43277	Drosophila melanogaster	CG31064	CG31064 gene product from transcript CG31064-RB	CG14261|CG6001|DmelCG31064	0	0
10196	43278	Drosophila melanogaster	DNApol-alpha73	DNA polymerase alpha 73kD	CG5923|DNA pol-73|DNA polalpha|DNApol-alpha|DNApol-alpha72|DNApolalpha|DmelCG5923|Dmpol alpha|POLA|Pol|Polalpha|pol alpha|polalpha	0	0
10197	43279	Drosophila melanogaster	CG17991	CG17991 gene product from transcript CG17991-RA	DmelCG17991	0	0
10198	43281	Drosophila melanogaster	CG5984	CG5984 gene product from transcript CG5984-RA	DmelCG5984	0	0
10199	43282	Drosophila melanogaster	CG5987	CG5987 gene product from transcript CG5987-RA	DmelCG5987	0	0
10200	43283	Drosophila melanogaster	pll	pelle	CG5974|DmelCG5974|PELLE/IL-1|p11	0	0
10201	43284	Drosophila melanogaster	TfIIA-L	Transcription factor IIA L	CG5930|CT18631|DmelCG5930|TFIIA|TFIIA-L|dTFIIA|tfiia-l	0	0
10202	43286	Drosophila melanogaster	CG14262	CG14262 gene product from transcript CG14262-RA	DmelCG14262	0	0
10203	43287	Drosophila melanogaster	CG14260	CG14260 gene product from transcript CG14260-RA	DmelCG14260	0	0
10204	43288	Drosophila melanogaster	l(3)mbt	lethal (3) malignant brain tumor	CG5954|DmelCG5954|L(3)MBT|L(3)MBT/LIN-61|L(3)mbt|L3mbt|MBT|dl(3)MBT|l(3)malignant braintumor|mbt	0	0
10205	43289	Drosophila melanogaster	CG5934	CG5934 gene product from transcript CG5934-RA	DmelCG5934	0	0
10206	43290	Drosophila melanogaster	CG5938	CG5938 gene product from transcript CG5938-RD	BEST:LD40095|DmelCG5938	0	0
10207	43291	Drosophila melanogaster	mrt	martik	CG3361|DmelCG3361	0	0
10208	43292	Drosophila melanogaster	CG3368	CG3368 gene product from transcript CG3368-RA	DmelCG3368	0	0
10209	43293	Drosophila melanogaster	bigmax	CG3350 gene product from transcript CG3350-RA	BIGMAX|Bigmax|CG3350|DmMlx|DmelCG3350|bHLHd13	0	0

<table>
<tr><td>10210</td><td>43294</td><td>Drosophila melanogaster</td><td>Hmu</td><td>Hemomucin</td><td>CG3373|DmelCG3373|RRM5|Rbp5|SPH210|rrm5</td><td>0</td><td>0</td></tr>
<tr><td>10211</td><td>43295</td><td>Drosophila melanogaster</td><td>CG3339</td><td>CG3339 gene product from transcript CG3339-RD</td><td>DmelCG3339</td><td>0</td><td>0</td></tr>
<tr><td>10212</td><td>43296</td><td>Drosophila melanogaster</td><td>CG3330</td><td>CG3330 gene product from transcript CG3330-RA</td><td>DmelCG3330|anon-WO0140519.147</td><td>0</td><td>0</td></tr>
<tr><td>10213</td><td>43298</td><td>Drosophila melanogaster</td><td>CG42534</td><td>CG42534 gene product from transcript CG42534-RC</td><td>CG13980|CG14264|CG6599|DmelCG42534|Dmel_CG13980|Dmel_CG14264|Dmel_CG6599</td><td>0</td><td>0</td></tr>
<tr><td>10214</td><td>43300</td><td>Drosophila melanogaster</td><td>side</td><td>sidestep</td><td>CG12510|CG13973|CG13974|CG13975|CG13979|CG31062|CT33527|CT33528|DmelCG31062|Doe|Side|doe</td><td>0</td><td>0</td></tr>
<tr><td>10215</td><td>43303</td><td>Drosophila melanogaster</td><td>CG13978</td><td>CG13978 gene product from transcript CG13978-RA</td><td>DmelCG13978</td><td>0</td><td>0</td></tr>
<tr><td>10216</td><td>43304</td><td>Drosophila melanogaster</td><td>Cyp6a18</td><td>CG13977 gene product from transcript CG13977-RA</td><td>6a18|CG13977|DmelCG13977</td><td>0</td><td>0</td></tr>
<tr><td>10217</td><td>43305</td><td>Drosophila melanogaster</td><td>Gr98a</td><td>Gustatory receptor 98a</td><td>98B.4|CG13976|DmelCG13976|GR98B.4|Gr98B4</td><td>0</td><td>0</td></tr>
<tr><td>10218</td><td>43306</td><td>Drosophila melanogaster</td><td>CG5402</td><td>CG5402 gene product from transcript CG5402-RA</td><td>DmelCG5402|anon-WO0140519.87</td><td>0</td><td>0</td></tr>
<tr><td>10219</td><td>43307</td><td>Drosophila melanogaster</td><td>CG13972</td><td>CG13972 gene product from transcript CG13972-RA</td><td>DmelCG13972</td><td>0</td><td>0</td></tr>
<tr><td>10220</td><td>43308</td><td>Drosophila melanogaster</td><td>CG12885</td><td>CG12885 gene product from transcript CG12885-RA</td><td>DmelCG12885</td><td>0</td><td>0</td></tr>
<tr><td>10221</td><td>43309</td><td>Drosophila melanogaster</td><td>Gr98d</td><td>Gustatory receptor 98d</td><td>98B.1|CG12884|CG31061|DmelCG31061|GR98B.1|Gr98A1|Gr98B1</td><td>0</td><td>0</td></tr>
<tr><td>10222</td><td>43310</td><td>Drosophila melanogaster</td><td>Klp98A</td><td>Kinesin-like protein at 98A</td><td>CG5658|DmKlp98A|DmelCG5658|KIF16A|KLP6|KLP98A|Klp6</td><td>0</td><td>0</td></tr>
<tr><td>10223</td><td>43311</td><td>Drosophila melanogaster</td><td>CG5646</td><td>CG5646 gene product from transcript CG5646-RA</td><td>DmelCG5646</td><td>0</td><td>0</td></tr>
<tr><td>10224</td><td>43312</td><td>Drosophila melanogaster</td><td>wdb</td><td>widerborst</td><td>0318/07|B56-2|BEST:LD02456|BcDNA:LD34343|CG5643|DmelCG5643|EP3559|LD02456|PP2-AB|PP2A|PP2A[B'-2]|PP2a|Wdb|dB56-2|dPP2A|l(3)S031807</td><td>0</td><td>0</td></tr>
<tr><td>10225</td><td>43314</td><td>Drosophila melanogaster</td><td>CG5639</td><td>CG5639 gene product from transcript CG5639-RA</td><td>CT17816|DmelCG5639</td><td>0</td><td>0</td></tr>
<tr><td>10226</td><td>43315</td><td>Drosophila melanogaster</td><td>dsd</td><td>distracted</td><td>CG5634|CT17798|DmelCG5634</td><td>0</td><td>0</td></tr>
<tr><td>10227</td><td>43316</td><td>Drosophila melanogaster</td><td>CG5612</td><td>CG5612 gene product from transcript CG5612-RA</td><td>DmelCG5612</td><td>0</td><td>0</td></tr>
<tr><td>10228</td><td>43317</td><td>Drosophila melanogaster</td><td>Tusp</td><td>CG5586 gene product from transcript CG5586-RE</td><td>CG5586|DMG2-MB6.chr3R.24.043|DmelCG5586|TUSP</td><td>0</td><td>0</td></tr>
<tr><td>10229</td><td>43318</td><td>Drosophila melanogaster</td><td>CG5611</td><td>CG5611 gene product from transcript CG5611-RA</td><td>DmelCG5611</td><td>0</td><td>0</td></tr>
<tr><td>10230</td><td>43319</td><td>Drosophila melanogaster</td><td>Mtl</td><td>Mig-2-like</td><td>5588|CG5588|DmelCG5588|MTL|Mt1/Mig2-like|Rac|Rac GTPase|Rac-like|dMig-2|mtl</td><td>0</td><td>0</td></tr>
<tr><td>10231</td><td>43320</td><td>Drosophila melanogaster</td><td>CG31058</td><td>CG31058 gene product from transcript CG31058-RA</td><td>CG5606|DmelCG31058</td><td>0</td><td>0</td></tr>
<tr><td>10232</td><td>43321</td><td>Drosophila melanogaster</td><td>RpS10a</td><td>Ribosomal protein S10a</td><td>BcDNA:LD32148|CG12275|DmelCG12275|RpS10|S10</td><td>0</td><td>0</td></tr>
<tr><td>10233</td><td>43323</td><td>Drosophila melanogaster</td><td>Mlc1</td><td>Myosin alkali light chain 1</td><td>CG5596|DmMLC1|DmelCG5596|ELC|FBgn0002772|LC1|MLC-ALK|MLC1|Mlc-1|MlcI|Mlci|mlc|mlc-alk|mlc1|mlcl</td><td>0</td><td>0</td></tr>
<tr><td>10234</td><td>43324</td><td>Drosophila melanogaster</td><td>CG18437</td><td>CG18437 gene product from transcript CG18437-RB</td><td>DmelCG18437</td><td>0</td><td>0</td></tr>
<tr><td>10235</td><td>43325</td><td>Drosophila melanogaster</td><td>CG5590</td><td>CG5590 gene product from transcript CG5590-RA</td><td>DmelCG5590</td><td>0</td><td>0</td></tr>
<tr><td>10236</td><td>43326</td><td>Drosophila melanogaster</td><td>CG12883</td><td>CG12883 gene product from transcript CG12883-RA</td><td>DmelCG12883</td><td>0</td><td>0</td></tr>
<tr><td>10237</td><td>43327</td><td>Drosophila melanogaster</td><td>Sce</td><td>Sex combs extra</td><td>CG5595|DRING|Ding|DmelCG5595|Dring|RING|RING1|Ring|Ring/Sce|Rnf2|SCE|dRING|dRING1|dRing|dRing1|dring</td><td>0</td><td>0</td></tr>
<tr><td>10238</td><td>43328</td><td>Drosophila melanogaster</td><td>CG12880</td><td>CG12880 gene product from transcript CG12880-RA</td><td>BEST:LD29228|DmelCG12880</td><td>0</td><td>0</td></tr>
<tr><td>10239</td><td>43329</td><td>Drosophila melanogaster</td><td>CG43320</td><td>CG43320 gene product from transcript CG43320-RA</td><td>CG12879|DmelCG43320</td><td>0</td><td>0</td></tr>
<tr><td>10240</td><td>43330</td><td>Drosophila melanogaster</td><td>ALiX</td><td>ALG-2 interacting protein X</td><td>AIP1|ALIX|Aip1|CG12876|DAIP1|DmelCG12876</td><td>0</td><td>0</td></tr>
<tr><td>10241</td><td>43331</td><td>Drosophila melanogaster</td><td>btz</td><td>barentsz</td><td>Btz|CG12878|DmelCG12878|wkl</td><td>0</td><td>0</td></tr>
<tr><td>10242</td><td>43332</td><td>Drosophila melanogaster</td><td>CG12428</td><td>CG12428 gene product from transcript CG12428-RB</td><td>DmelCG12428</td><td>0</td><td>0</td></tr>
<tr><td>10243</td><td>43333</td><td>Drosophila melanogaster</td><td>CG12877</td><td>CG12877 gene product from transcript CG12877-RC</td><td>BcDNA:LD30051|DmelCG12877</td><td>0</td><td>0</td></tr>
<tr><td>10244</td><td>43334</td><td>Drosophila melanogaster</td><td>Ets98B</td><td>Ets at 98B</td><td>CG5583|D-Ets-4|D-ets-4|D-ets4|DmelCG5583|ETS4|Ets|Ets4|ets98B</td><td>0</td><td>0</td></tr>
<tr><td>10245</td><td>43341</td><td>Drosophila melanogaster</td><td>Or98a</td><td>Odorant receptor 98a</td><td>98B.1|98a|CG5540|DOR110|DmelCG5540</td><td>0</td><td>0</td></tr>
<tr><td>10246</td><td>43342</td><td>Drosophila melanogaster</td><td>CG12426</td><td>CG12426 gene product from transcript CG12426-RB</td><td>DmelCG12426</td><td>0</td><td>0</td></tr>
<tr><td>10247</td><td>43343</td><td>Drosophila melanogaster</td><td>CG5017</td><td>CG5017 gene product from transcript CG5017-RA</td><td>DmelCG5017</td><td>0</td><td>0</td></tr>
<tr><td>10248</td><td>43344</td><td>Drosophila melanogaster</td><td>CG5003</td><td>CG5003 gene product from transcript CG5003-RA</td><td>DmelCG5003</td><td>0</td><td>0</td></tr>
<tr><td>10249</td><td>43345</td><td>Drosophila melanogaster</td><td>mRpS22</td><td>mitochondrial ribosomal protein S22</td><td>AAF56757|CG12261|DmelCG12261|MRP-S22|MrpS22</td><td>0</td><td>0</td></tr>
<tr><td>10250</td><td>43347</td><td>Drosophila melanogaster</td><td>CG12259</td><td>CG12259 gene product from transcript CG12259-RA</td><td>DmelCG12259</td><td>0</td><td>0</td></tr>
<tr><td>10251</td><td>43348</td><td>Drosophila melanogaster</td><td>CG4980</td><td>CG4980 gene product from transcript CG4980-RA</td><td>DmelCG4980</td><td>0</td><td>0</td></tr>
<tr><td>10252</td><td>43349</td><td>Drosophila melanogaster</td><td>RpL4</td><td>Ribosomal protein L4</td><td>CG5502|DmelCG5502|M(3)98B|RpL1|anon-WO0172774.76|rpL1</td><td>0</td><td>0</td></tr>
<tr><td>10253</td><td>43350</td><td>Drosophila melanogaster</td><td>CG5508</td><td>CG5508 gene product from transcript CG5508-RA</td><td>152088_at|BcDNA:GH07066|DmelCG5508|GPAT1</td><td>0</td><td>0</td></tr>
<tr><td>10254</td><td>43351</td><td>Drosophila melanogaster</td><td>Mes-4</td><td>CG4976 gene product from transcript CG4976-RA</td><td>CG4976|DmelCG4976|dMES-4|dMes-4</td><td>0</td><td>0</td></tr>
<tr><td>10255</td><td>43352</td><td>Drosophila melanogaster</td><td>CG5514</td><td>CG5514 gene product from transcript CG5514-RC</td><td>DmelCG5514</td><td>0</td><td>0</td></tr>
<tr><td>10256</td><td>43353</td><td>Drosophila melanogaster</td><td>mfrn</td><td>mitoferrin</td><td>CG4963|DmelCG4963|HD35059|Mfrn|anon-EST:fe2H5|dmfrn</td><td>0</td><td>0</td></tr>
<tr><td>10257</td><td>43354</td><td>Drosophila melanogaster</td><td>Gp93</td><td>Glycoprotein 93</td><td>CG5520|CT17486|DmelCG5520|GP93|gp93|p93</td><td>0</td><td>0</td></tr>
<tr><td>10258</td><td>43355</td><td>Drosophila melanogaster</td><td>CG4951</td><td>CG4951 gene product from transcript CG4951-RB</td><td>DmelCG4951</td><td>0</td><td>0</td></tr>
<tr><td>10259</td><td>43356</td><td>Drosophila melanogaster</td><td>CG5527</td><td>CG5527 gene product from transcript CG5527-RA</td><td>DmelCG5527</td><td>0</td><td>0</td></tr>
<tr><td>10260</td><td>43357</td><td>Drosophila melanogaster</td><td>CG4884</td><td>CG4884 gene product from transcript CG4884-RA</td><td>DmelCG4884</td><td>0</td><td>0</td></tr>
<tr><td>10261</td><td>43358</td><td>Drosophila melanogaster</td><td>CG4849</td><td>CG4849 gene product from transcript CG4849-RA</td><td>DmelCG4849|eEF2</td><td>0</td><td>0</td></tr>
<tr><td>10262</td><td>43359</td><td>Drosophila melanogaster</td><td>betaTub97EF</td><td>beta-Tubulin at 97EF</td><td>4t|B4t|BETA 98B|CG4869|DTB1|Dmbeta4|DmelCG4869|T|Tub|beta-Tub|beta-Tub97EF|beta-tub|beta4t|beta97F|betaTub4|betaTub98BC|betaTub98C</td><td>0</td><td>0</td></tr>
<tr><td>10263</td><td>43360</td><td>Drosophila melanogaster</td><td>CG4815</td><td>CG4815 gene product from transcript CG4815-RA</td><td>DmelCG4815|SP189</td><td>0</td><td>0</td></tr>
<tr><td>10264</td><td>43362</td><td>Drosophila melanogaster</td><td>CG34362</td><td>CG34362 gene product from transcript CG34362-RD</td><td>BcDNA:RE10833|CG12870|CG12872|DmelCG34362|Dmel_CG12870|Dmel_CG12872|Tia1-like</td><td>0</td><td>0</td></tr>
<tr><td>10265</td><td>43367</td><td>Drosophila melanogaster</td><td>Mst98Cb</td><td>Male-specific RNA 98Cb</td><td>CG18396|DmelCG18396|MST98Cb|Mst|Mst98C|anon-WO0140519.46|mst(3)gl-13</td><td>0</td><td>0</td></tr>
<tr><td>10266</td><td>43369</td><td>Drosophila melanogaster</td><td>CG17856</td><td>CG17856 gene product from transcript CG17856-RA</td><td>DmelCG17856</td><td>0</td><td>0</td></tr>
<tr><td>10267</td><td>43370</td><td>Drosophila melanogaster</td><td>CG12516</td><td>CG12516 gene product from transcript CG12516-RB</td><td>DmelCG12516</td><td>0</td><td>0</td></tr>
<tr><td>10268</td><td>43373</td><td>Drosophila melanogaster</td><td>Gfat2</td><td>Glutamine:fructose-6-phosphate aminotransferase 2</td><td>CG1345|Dmel/GFAT2|DmelCG1345</td><td>0</td><td>0</td></tr>
<tr><td>10269</td><td>43374</td><td>Drosophila melanogaster</td><td>Moca-cyp</td><td>CG1866 gene product from transcript CG1866-RC</td><td>BEST:LD38313|CG1866|DmelCG1866|anon-WO0147981.13|anon-WO0147981.4|anon-WO0147981.5</td><td>0</td><td>0</td></tr>
<tr><td>10270</td><td>43375</td><td>Drosophila melanogaster</td><td>Or98b</td><td>Odorant receptor 98b</td><td>98C.1|98b|CG1867|DOR121|DmelCG1867|Or98bP</td><td>0</td><td>0</td></tr>
<tr><td>10271</td><td>43377</td><td>Drosophila melanogaster</td><td>beat-VI</td><td>CG14064 gene product from transcript CG14064-RA</td><td>CG12089|CG14064|DmelCG14064|beat VI</td><td>0</td><td>0</td></tr>
<tr><td>10272</td><td>43378</td><td>Drosophila melanogaster</td><td>CG1894</td><td>CG1894 gene product from transcript CG1894-RA</td><td>DmelCG1894|dmHAG407</td><td>0</td><td>0</td></tr>
<tr><td>10273</td><td>43379</td><td>Drosophila melanogaster</td><td>Dhc98D</td><td>Dynein heavy chain at 89D</td><td>CG1842|DHC|Dhc|DmelCG1842|dhc</td><td>0</td><td>0</td></tr>
<tr><td>10274</td><td>43382</td><td>Drosophila melanogaster</td><td>CG12413</td><td>CG12413 gene product from transcript CG12413-RA</td><td>DmelCG12413</td><td>0</td><td>0</td></tr>
<tr><td>10275</td><td>43383</td><td>Drosophila melanogaster</td><td>fkh</td><td>fork head</td><td>CG10002|DmelCG10002|Dmfkh|FKH|Fkh|Sebp2</td><td>0</td><td>0</td></tr>
<tr><td>10276</td><td>43384</td><td>Drosophila melanogaster</td><td>Noa36</td><td>CG10009 gene product from transcript CG10009-RA</td><td>CG10009|DmelCG10009|NOA 36|NOA36</td><td>0</td><td>0</td></tr>
<tr><td>10277</td><td>43385</td><td>Drosophila melanogaster</td><td>Hrb98DE</td><td>Heterogeneous nuclear ribonucleoprotein at 98DE</td><td>CG9983|DmelCG9983|E7Delta6|HDP(P9)|HRB98DE/hrp38|Hrb1|Pen9|anon-E7Delta6|hrb98DE|hrp38|p9</td><td>0</td><td>0</td></tr>
<tr><td>10278</td><td>43386</td><td>Drosophila melanogaster</td><td>CG9986</td><td>CG9986 gene product from transcript CG9986-RA</td><td>DmelCG9986</td><td>0</td><td>0</td></tr>
<tr><td>10279</td><td>43387</td><td>Drosophila melanogaster</td><td>CG10011</td><td>CG10011 gene product from transcript CG10011-RA</td><td>DmelCG10011</td><td>0</td><td>0</td></tr>
<tr><td>10280</td><td>43388</td><td>Drosophila melanogaster</td><td>CG9988</td><td>CG9988 gene product from transcript CG9988-RA</td><td>DmelCG9988</td><td>0</td><td>0</td></tr>
<tr><td>10281</td><td>43389</td><td>Drosophila melanogaster</td><td>CG14062</td><td>CG14062 gene product from transcript CG14062-RB</td><td>DmelCG14062|LP23408p</td><td>0</td><td>0</td></tr>
<tr><td>10282</td><td>43390</td><td>Drosophila melanogaster</td><td>CG9989</td><td>CG9989 gene product from transcript CG9989-RA</td><td>DmelCG9989</td><td>0</td><td>0</td></tr>
<tr><td>10283</td><td>43391</td><td>Drosophila melanogaster</td><td>CG9990</td><td>CG9990 gene product from transcript CG9990-RA</td><td>DmelCG9990</td><td>0</td><td>0</td></tr>
<tr><td>10284</td><td>43392</td><td>Drosophila melanogaster</td><td>htt</td><td>huntingtin</td><td>CG9995|DmelCG9995|HD|HTT|HsapHD|Htt|dHtt|dhtt</td><td>0</td><td>0</td></tr>
<tr><td>10285</td><td>43393</td><td>Drosophila melanogaster</td><td>AR-2</td><td>Allatostatin Receptor 2</td><td>CG10001|D.AlstR2|DAR|DAR-2|DmelCG10001|anon-WO0131005.7|anon-WO0170980.115|anon-WO0170980.116</td><td>0</td><td>0</td></tr>
<tr><td>10286</td><td>43394</td><td>Drosophila melanogaster</td><td>CG10000</td><td>CG10000 gene product from transcript CG10000-RA</td><td>DmelCG10000</td><td>0</td><td>0</td></tr>
<tr><td>10287</td><td>43395</td><td>Drosophila melanogaster</td><td>CG9997</td><td>CG9997 gene product from transcript CG9997-RA</td><td>DmelCG9997</td><td>0</td><td>0</td></tr>
<tr><td>10288</td><td>43396</td><td>Drosophila melanogaster</td><td>CG14061</td><td>CG14061 gene product from transcript CG14061-RA</td><td>DmelCG14061</td><td>0</td><td>0</td></tr>
<tr><td>10289</td><td>43397</td><td>Drosophila melanogaster</td><td>CG12558</td><td>CG12558 gene product from transcript CG12558-RA</td><td>DmelCG12558|SPH202</td><td>0</td><td>0</td></tr>
<tr><td>10290</td><td>43398</td><td>Drosophila melanogaster</td><td>wdn</td><td>wings down</td><td>CG1454|DmelCG1454|Sryc|pqp|sry h-l|sry-c|sry-h1</td><td>0</td><td>0</td></tr>
<tr><td>10291</td><td>43399</td><td>Drosophila melanogaster</td><td>CG1646</td><td>CG1646 gene product from transcript CG1646-RH</td><td>DmelCG1646|Prp39</td><td>0</td><td>0</td></tr>
<tr><td>10292</td><td>43400</td><td>Drosophila melanogaster</td><td>CG1523</td><td>CG1523 gene product from transcript CG1523-RA</td><td>DmelCG1523</td><td>0</td><td>0</td></tr>
<tr><td>10293</td><td>43401</td><td>Drosophila melanogaster</td><td>CG1647</td><td>CG1647 gene product from transcript CG1647-RA</td><td>DmelCG1647</td><td>0</td><td>0</td></tr>
<tr><td>10294</td><td>43402</td><td>Drosophila melanogaster</td><td>WASp</td><td>CG1520 gene product from transcript CG1520-RA</td><td>CG1520|D-WASP|Dm WASP|DmWASP|DmelCG1520|WASP|WSP|Wasp|Wsp|dWASP|wasp|wsp</td><td>0</td><td>0</td></tr>
<tr><td>10295</td><td>43404</td><td>Drosophila melanogaster</td><td>CG31048</td><td>CG31048 gene product from transcript CG31048-RB</td><td>CG11754|CG14530|Dm Dock4|DmelCG31048</td><td>0</td><td>0</td></tr>
<tr><td>10296</td><td>43408</td><td>Drosophila melanogaster</td><td>CG14529</td><td>CG14529 gene product from transcript CG14529-RA</td><td>DmelCG14529</td><td>0</td><td>0</td></tr>
<tr><td>10297</td><td>43410</td><td>Drosophila melanogaster</td><td>CG14528</td><td>CG14528 gene product from transcript CG14528-RA</td><td>DmelCG14528</td><td>0</td><td>0</td></tr>
<tr><td>10298</td><td>43411</td><td>Drosophila melanogaster</td><td>CG14523</td><td>CG14523 gene product from transcript CG14523-RA</td><td>DmelCG14523</td><td>0</td><td>0</td></tr>
<tr><td>10299</td><td>43412</td><td>Drosophila melanogaster</td><td>CG14527</td><td>CG14527 gene product from transcript CG14527-RA</td><td>DmelCG14527</td><td>0</td><td>0</td></tr>
<tr><td>10300</td><td>43413</td><td>Drosophila melanogaster</td><td>CG14526</td><td>CG14526 gene product from transcript CG14526-RB</td><td>BcDNA:GH07188|DmelCG14526</td><td>0</td><td>0</td></tr>
<tr><td>10301</td><td>43414</td><td>Drosophila melanogaster</td><td>CG33203</td><td>CG33203 gene product from transcript CG33203-RB</td><td>CG14522|CG14524|CG14525|CG31046|DmelCG33203</td><td>0</td><td>0</td></tr>
<tr><td>10302</td><td>43415</td><td>Drosophila melanogaster</td><td>Doa</td><td>Darkener of apricot</td><td>CG1658|CG31049|CG33204|CG33553|CG42320|DOA|DOA/CLK2|DmelCG42320|Dmel_CG33204|Dmel_CG33553|Msu|doa|l(3)01705</td><td>0</td><td>0</td></tr>
<tr><td>10303</td><td>43416</td><td>Drosophila melanogaster</td><td>CG11828</td><td>CG11828 gene product from transcript CG11828-RD</td><td>DmelCG11828</td><td>0</td><td>0</td></tr>
<tr><td>10304</td><td>43417</td><td>Drosophila melanogaster</td><td>CG14521</td><td>CG14521 gene product from transcript CG14521-RA</td><td>CT34248|DmelCG14521|anon-WO0140519.196</td><td>0</td><td>0</td></tr>
<tr><td>10305</td><td>43420</td><td>Drosophila melanogaster</td><td>CG1443</td><td>CG1443 gene product from transcript CG1443-RA</td><td>DmelCG1443</td><td>0</td><td>0</td></tr>
<tr><td>10306</td><td>43421</td><td>Drosophila melanogaster</td><td>CG14518</td><td>CG14518 gene product from transcript CG14518-RA</td><td>DmelCG14518</td><td>0</td><td>0</td></tr>
<tr><td>10307</td><td>43422</td><td>Drosophila melanogaster</td><td>eIF4E-6</td><td>CG1442 gene product from transcript CG1442-RA</td><td>BcDNA:RH55324|CG1442|DmelCG1442|eIF4E</td><td>0</td><td>0</td></tr>
<tr><td>10308</td><td>43423</td><td>Drosophila melanogaster</td><td>CG1951</td><td>CG1951 gene product from transcript CG1951-RA</td><td>DmelCG1951</td><td>0</td><td>0</td></tr>
<tr><td>10309</td><td>43424</td><td>Drosophila melanogaster</td><td>Ssl2</td><td>Strictosidine synthase-like 2</td><td>CG11833|DmSSl2|DmelCG11833|SSl2</td><td>0</td><td>0</td></tr>
<tr><td>10310</td><td>43425</td><td>Drosophila melanogaster</td><td>beta4GalNAcTB</td><td>CG14517 gene product from transcript CG14517-RA</td><td>CG14517|DmelCG14517|beta4GalTB|dbeta4GalTB</td><td>0</td><td>0</td></tr>
<tr><td>10311</td><td>43426</td><td>Drosophila melanogaster</td><td>Cpsf100</td><td>Cleavage and polyadenylation specificity factor 100</td><td>BcDNA:LD14168|CG1957|CPSF-100|CPSF100|DmelCG1957</td><td>0</td><td>0</td></tr>
<tr><td>10312</td><td>43427</td><td>Drosophila melanogaster</td><td>Slu7</td><td>CG1420 gene product from transcript CG1420-RA</td><td>CG1420|DmelCG1420|SLU7</td><td>0</td><td>0</td></tr>
<tr><td>10313</td><td>43428</td><td>Drosophila melanogaster</td><td>Pkc98E</td><td>Protein C kinase 98E</td><td>98F|CG1954|DmelCG1954|Dpkc3|Nc98F|PKC|PKC 98F|PKC d98F|PKC-98F|PKC98E|Pkc3|dPKC98F|nPKC</td><td>0</td><td>0</td></tr>
<tr><td>10314</td><td>43429</td><td>Drosophila melanogaster</td><td>CG11837</td><td>CG11837 gene product from transcript CG11837-RA</td><td>Dim1|DmelCG11837|FBgn0039627</td><td>0</td><td>0</td></tr>
<tr><td>10315</td><td>43430</td><td>Drosophila melanogaster</td><td>CG11841</td><td>CG11841 gene product from transcript CG11841-RA</td><td>BcDNA:GH26583|DmelCG11841|SP45|anon-WO0140519.18|anon-WO0140519.6</td><td>0</td><td>0</td></tr>
<tr><td>10316</td><td>43431</td><td>Drosophila melanogaster</td><td>CG11842</td><td>CG11842 gene product from transcript CG11842-RA</td><td>DmelCG11842|SP68</td><td>0</td><td>0</td></tr>
<tr><td>10317</td><td>43432</td><td>Drosophila melanogaster</td><td>CG11843</td><td>CG11843 gene product from transcript CG11843-RA</td><td>DmelCG11843|SP43</td><td>0</td><td>0</td></tr>
<tr><td>10318</td><td>43433</td><td>Drosophila melanogaster</td><td>Sirt7</td><td>CG11305 gene product from transcript CG11305-RA</td><td>CG11305|D.mel5|DmelCG11305|dSIRT7|dmSRT409</td><td>0</td><td>0</td></tr>
<tr><td>10319</td><td>43434</td><td>Drosophila melanogaster</td><td>Cul-5</td><td>Cullin-5</td><td>CG1401|CUL5|Cul5|DmelCG1401|cul-5|cul5</td><td>0</td><td>0</td></tr>
<tr><td>10320</td><td>43435</td><td>Drosophila melanogaster</td><td>CG11873</td><td>CG11873 gene product from transcript CG11873-RA</td><td>DmelCG11873|anon-WO0140519.162</td><td>0</td><td>0</td></tr>
</table>

<table>
<tr><th></th><th></th><th></th><th></th><th></th><th></th><th></th><th></th></tr>
<tr><td>10321</td><td>43436</td><td>Drosophila melanogaster</td><td>CG11874</td><td>CG11874 gene product from transcript CG11874-RA</td><td>DmelCG11874|ORE-11</td><td>0</td><td>0</td></tr>
<tr><td>10322</td><td>43437</td><td>Drosophila melanogaster</td><td>CG11876</td><td>CG11876 gene product from transcript CG11876-RA</td><td>DmelCG11876|E1beta|PDHB</td><td>0</td><td>0</td></tr>
<tr><td>10323</td><td>43438</td><td>Drosophila melanogaster</td><td>CG11877</td><td>CG11877 gene product from transcript CG11877-RA</td><td>DmelCG11877</td><td>0</td><td>0</td></tr>
<tr><td>10324</td><td>43439</td><td>Drosophila melanogaster</td><td>yemalpha</td><td>yemanuclein alpha</td><td>CG11879|CG14513|DmelCG14513|Yemalpha|yG4.5|yT4.5|yem|yem-alpha|yema</td><td>0</td><td>0</td></tr>
<tr><td>10325</td><td>43440</td><td>Drosophila melanogaster</td><td>CG11880</td><td>CG11880 gene product from transcript CG11880-RA</td><td>DmelCG11880</td><td>0</td><td>0</td></tr>
<tr><td>10326</td><td>43441</td><td>Drosophila melanogaster</td><td>dgt6</td><td>dim gamma-tubulin 6</td><td>CG11881|Dgt6|DmelCG11881|msd4</td><td>0</td><td>0</td></tr>
<tr><td>10327</td><td>43442</td><td>Drosophila melanogaster</td><td>Vha100-1</td><td>Vacuolar H[+] ATPase subunit 100-1</td><td>BcDNA:LD21248|CG1709|DmelCG1709|V-ATPase|V100|v100|vha|vha100-1</td><td>0</td><td>0</td></tr>
<tr><td>10328</td><td>43443</td><td>Drosophila melanogaster</td><td>CG14512</td><td>CG14512 gene product from transcript CG14512-RA</td><td>Alg13|DmelCG14512</td><td>0</td><td>0</td></tr>
<tr><td>10329</td><td>43444</td><td>Drosophila melanogaster</td><td>CG14516</td><td>CG14516 gene product from transcript CG14516-RB</td><td>DmelCG14516</td><td>0</td><td>0</td></tr>
<tr><td>10330</td><td>43445</td><td>Drosophila melanogaster</td><td>CG14511</td><td>CG14511 gene product from transcript CG14511-RA</td><td>DmelCG14511</td><td>0</td><td>0</td></tr>
<tr><td>10331</td><td>43446</td><td>Drosophila melanogaster</td><td>CG11882</td><td>CG11882 gene product from transcript CG11882-RA</td><td>DmelCG11882</td><td>0</td><td>0</td></tr>
<tr><td>10332</td><td>43447</td><td>Drosophila melanogaster</td><td>Pglym78</td><td>Phosphoglyceromutase</td><td>CG1721|DmelCG1721|PGAM|PGLYM|Pglym|pglym78</td><td>0</td><td>0</td></tr>
<tr><td>10333</td><td>43448</td><td>Drosophila melanogaster</td><td>Slbp</td><td>Stem-loop binding protein</td><td>CG11886|DmelCG11886|SLBP|dSLBP|dSLPB|slbp</td><td>0</td><td>0</td></tr>
<tr><td>10334</td><td>43449</td><td>Drosophila melanogaster</td><td>Rpn2</td><td>CG11888 gene product from transcript CG11888-RA</td><td>11888|CG11888|DmelCG11888|dRpn2|p110</td><td>0</td><td>0</td></tr>
<tr><td>10335</td><td>43450</td><td>Drosophila melanogaster</td><td>CG11897</td><td>CG11897 gene product from transcript CG11897-RA</td><td>DmelCG11897</td><td>0</td><td>0</td></tr>
<tr><td>10336</td><td>43451</td><td>Drosophila melanogaster</td><td>CG11898</td><td>CG11898 gene product from transcript CG11898-RB</td><td>Dme_CG11898|DmelCG11898</td><td>0</td><td>0</td></tr>
<tr><td>10337</td><td>43453</td><td>Drosophila melanogaster</td><td>CG14509</td><td>CG14509 gene product from transcript CG14509-RB</td><td>CG14510|DmelCG14509</td><td>0</td><td>0</td></tr>
<tr><td>10338</td><td>43454</td><td>Drosophila melanogaster</td><td>CG14515</td><td>CG14515 gene product from transcript CG14515-RA</td><td>DmelCG14515</td><td>0</td><td>0</td></tr>
<tr><td>10339</td><td>43456</td><td>Drosophila melanogaster</td><td>Mesh1</td><td>Metazoan SpoT homolog-1</td><td>CG11900|DmelCG11900|Q9VAM9</td><td>0</td><td>0</td></tr>
<tr><td>10340</td><td>43457</td><td>Drosophila melanogaster</td><td>CG14508</td><td>CG14508 gene product from transcript CG14508-RA</td><td>DmelCG14508</td><td>0</td><td>0</td></tr>
<tr><td>10341</td><td>43459</td><td>Drosophila melanogaster</td><td>CG1458</td><td>CG1458 gene product from transcript CG1458-RA</td><td>BcDNA:RE49709|DmelCG1458</td><td>0</td><td>0</td></tr>
<tr><td>10342</td><td>43460</td><td>Drosophila melanogaster</td><td>Brd8</td><td>CG14514 gene product from transcript CG14514-RA</td><td>CG14514|DmelCG14514|dBrd8</td><td>0</td><td>0</td></tr>
<tr><td>10343</td><td>43461</td><td>Drosophila melanogaster</td><td>CG14507</td><td>CG14507 gene product from transcript CG14507-RC</td><td>DmelCG14507</td><td>0</td><td>0</td></tr>
<tr><td>10344</td><td>43462</td><td>Drosophila melanogaster</td><td>CG15817</td><td>CG15817 gene product from transcript CG15817-RD</td><td>BcDNA:LD22910|DmelCG15817|LD22910</td><td>0</td><td>0</td></tr>
<tr><td>10345</td><td>43463</td><td>Drosophila melanogaster</td><td>CG11951</td><td>CG11951 gene product from transcript CG11951-RB</td><td>DmelCG11951</td><td>0</td><td>0</td></tr>
<tr><td>10346</td><td>43464</td><td>Drosophila melanogaster</td><td>CG31427</td><td>CG31427 gene product from transcript CG31427-RD</td><td>CG11955|DmelCG31427</td><td>0</td><td>0</td></tr>
<tr><td>10347</td><td>43466</td><td>Drosophila melanogaster</td><td>stg</td><td>string</td><td>0224/06|0245/03|0439/22|0730/13|0896/05|0967/05|0980/06|1083/13|1089/08|1143/02|5473|CDC25|CDC25[string]|CG1395|Cdc25|Cdc25[Stg]|Cdc25[String]|Cdc25[stg]|Cdc25[string]|DmelCG1395|EP12</td><td>0</td><td>0</td></tr>
<tr><td>10348</td><td>43467</td><td>Drosophila melanogaster</td><td>CG14506</td><td>CG14506 gene product from transcript CG14506-RA</td><td>DmelCG14506|cg14506</td><td>0</td><td>0</td></tr>
<tr><td>10349</td><td>43469</td><td>Drosophila melanogaster</td><td>Ptp99A</td><td>Protein tyrosine phosphatase 99A</td><td>CG11515|CG11516|CG11517|CG2005|CT6383|DPTP99A|DPTP[[99A]]|DmelCG11516|PTP99A|R-PTP 99A|dptp99A</td><td>0</td><td>0</td></tr>
<tr><td>10350</td><td>43471</td><td>Drosophila melanogaster</td><td>CG2321</td><td>CG2321 gene product from transcript CG2321-RA</td><td>DmelCG2321</td><td>0</td><td>0</td></tr>
<tr><td>10351</td><td>43472</td><td>Drosophila melanogaster</td><td>CG2006</td><td>CG2006 gene product from transcript CG2006-RA</td><td>DmelCG2006</td><td>0</td><td>0</td></tr>
<tr><td>10352</td><td>43473</td><td>Drosophila melanogaster</td><td>CG2310</td><td>CG2310 gene product from transcript CG2310-RA</td><td>DmelCG2310</td><td>0</td><td>0</td></tr>
<tr><td>10353</td><td>43474</td><td>Drosophila melanogaster</td><td>CG11501</td><td>CG11501 gene product from transcript CG11501-RA</td><td>BcDNA:RH38110|DmelCG11501</td><td>0</td><td>0</td></tr>
<tr><td>10354</td><td>43475</td><td>Drosophila melanogaster</td><td>CG2010</td><td>CG2010 gene product from transcript CG2010-RB</td><td>DmelCG2010</td><td>0</td><td>0</td></tr>
<tr><td>10355</td><td>43476</td><td>Drosophila melanogaster</td><td>Trc8</td><td>CG2304 gene product from transcript CG2304-RB</td><td>CG2304|DmelCG2304|Dtrc8|TRC8</td><td>0</td><td>0</td></tr>
<tr><td>10356</td><td>43477</td><td>Drosophila melanogaster</td><td>CG2014</td><td>CG2014 gene product from transcript CG2014-RA</td><td>DmelCG2014</td><td>0</td><td>0</td></tr>
<tr><td>10357</td><td>43479</td><td>Drosophila melanogaster</td><td>CG7567</td><td>CG7567 gene product from transcript CG7567-RA</td><td>BcDNA:RE47447|DmelCG7567</td><td>0</td><td>0</td></tr>
<tr><td>10358</td><td>43480</td><td>Drosophila melanogaster</td><td>CG11470</td><td>CG11470 gene product from transcript CG11470-RA</td><td>DmelCG11470</td><td>0</td><td>0</td></tr>
<tr><td>10359</td><td>43481</td><td>Drosophila melanogaster</td><td>alph</td><td>alphabet</td><td>CG1906|DmelCG1906|PP2Cb|SK3-1|pp2c99B</td><td>0</td><td>0</td></tr>
<tr><td>10360</td><td>43482</td><td>Drosophila melanogaster</td><td>CG7568</td><td>CG7568 gene product from transcript CG7568-RC</td><td>DmelCG7568</td><td>0</td><td>0</td></tr>
<tr><td>10361</td><td>43483</td><td>Drosophila melanogaster</td><td>CG1907</td><td>CG1907 gene product from transcript CG1907-RA</td><td>DmelCG1907</td><td>0</td><td>0</td></tr>
<tr><td>10362</td><td>43484</td><td>Drosophila melanogaster</td><td>DopR2</td><td>Dopamine receptor 2</td><td>CG18741|CG7569|DAMB|DOPR99B|Dar2|DmelCG18741|DopD1|DopR99B|DopRII</td><td>0</td><td>0</td></tr>
<tr><td>10363</td><td>43485</td><td>Drosophila melanogaster</td><td>ppk21</td><td>pickpocket 21</td><td>CG12048|DmelCG12048|PPK21</td><td>0</td><td>0</td></tr>
<tr><td>10364</td><td>43486</td><td>Drosophila melanogaster</td><td>ppk20</td><td>pickpocket 20</td><td>CG7577|DmelCG7577|PPK20</td><td>0</td><td>0</td></tr>
<tr><td>10365</td><td>43487</td><td>Drosophila melanogaster</td><td>CG18110</td><td>CG18110 gene product from transcript CG18110-RA</td><td>DmelCG18110</td><td>0</td><td>0</td></tr>
<tr><td>10366</td><td>43488</td><td>Drosophila melanogaster</td><td>Obp99a</td><td>Odorant-binding protein 99a</td><td>99a|BcDNA:RH70762|CG18111|DmelCG18111|OBP99a</td><td>0</td><td>0</td></tr>
<tr><td>10367</td><td>43489</td><td>Drosophila melanogaster</td><td>ppk19</td><td>pickpocket 19</td><td>CG18287|DmelCG18287|PPK19</td><td>0</td><td>0</td></tr>
<tr><td>10368</td><td>43490</td><td>Drosophila melanogaster</td><td>Bub3</td><td>CG7581 gene product from transcript CG7581-RA</td><td>CG7581|Dmbub3|DmelCG7581|bub3|dBUB3</td><td>0</td><td>0</td></tr>
<tr><td>10369</td><td>43491</td><td>Drosophila melanogaster</td><td>CAP-D2</td><td>CAP-D2 condensin subunit</td><td>CG1911|Cap-D2|CapD2|DmCAP-D2|DmelCG1911|dCap-D2</td><td>0</td><td>0</td></tr>
<tr><td>10370</td><td>43492</td><td>Drosophila melanogaster</td><td>CG7582</td><td>CG7582 gene product from transcript CG7582-RB</td><td>DmelCG7582</td><td>0</td><td>0</td></tr>
<tr><td>10371</td><td>43493</td><td>Drosophila melanogaster</td><td>Gycalpha99B</td><td>Guanylyl cyclase alpha-subunit at 99B</td><td>CG1912|Dgc1|Dgcalpha1|DmelCG1912|GCalpha1|GYCa 99B|Gcalpha1|Gycalpha-99B|Gycalpha63A|SGCalpha|dgc1|dgca1|dgcalpha1|gycalpha|sGC|sGCalpha1</td><td>0</td><td>0</td></tr>
<tr><td>10372</td><td>43494</td><td>Drosophila melanogaster</td><td>Obp99c</td><td>Odorant-binding protein 99c</td><td>99c|CG7584|DmelCG7584|Obp99b</td><td>0</td><td>0</td></tr>
<tr><td>10373</td><td>43495</td><td>Drosophila melanogaster</td><td>dmrt99B</td><td>doublesex-Mab related 99B</td><td>CG15504|DmelCG15504</td><td>0</td><td>0</td></tr>
<tr><td>10374</td><td>43496</td><td>Drosophila melanogaster</td><td>Obp99d</td><td>Odorant-binding protein 99d</td><td>99d|CG15505|CG155505|DmelCG15505|Obp99c</td><td>0</td><td>0</td></tr>
<tr><td>10375</td><td>43497</td><td>Drosophila melanogaster</td><td>Obp99b</td><td>Odorant-binding protein 99b</td><td>99b|BcDNA:LP06572|CG7592|DmelCG7592|OBP99b|Obp99d|tsx</td><td>0</td><td>0</td></tr>
<tr><td>10376</td><td>43498</td><td>Drosophila melanogaster</td><td>CG15506</td><td>CG15506 gene product from transcript CG15506-RA</td><td>DmelCG15506</td><td>0</td><td>0</td></tr>
<tr><td>10377</td><td>43499</td><td>Drosophila melanogaster</td><td>Pcd</td><td>pterin-4a-carbinolamine dehydratase</td><td>BcDNA:LP07248|CG1963|DmelCG1963|PCD</td><td>0</td><td>0</td></tr>
<tr><td>10378</td><td>43500</td><td>Drosophila melanogaster</td><td>CG7593</td><td>CG7593 gene product from transcript CG7593-RA</td><td>DmelCG7593</td><td>0</td><td>0</td></tr>
<tr><td>10379</td><td>43501</td><td>Drosophila melanogaster</td><td>Kul</td><td>Kuzbanian-like</td><td>ADAM10|CG1964|CT3146|DmelCG1964</td><td>0</td><td>0</td></tr>
<tr><td>10380</td><td>43502</td><td>Drosophila melanogaster</td><td>CG7601</td><td>CG7601 gene product from transcript CG7601-RA</td><td>BcDNA:GH06026|DmelCG7601</td><td>0</td><td>0</td></tr>
<tr><td>10381</td><td>43503</td><td>Drosophila melanogaster</td><td>CG7598</td><td>CG7598 gene product from transcript CG7598-RA</td><td>DmelCG7598</td><td>0</td><td>0</td></tr>
<tr><td>10382</td><td>43504</td><td>Drosophila melanogaster</td><td>CG1969</td><td>CG1969 gene product from transcript CG1969-RB</td><td>DmelCG1969</td><td>0</td><td>0</td></tr>
<tr><td>10383</td><td>43505</td><td>Drosophila melanogaster</td><td>CG7609</td><td>CG7609 gene product from transcript CG7609-RB</td><td>BcDNA:LD21720|DmelCG7609</td><td>0</td><td>0</td></tr>
<tr><td>10384</td><td>43506</td><td>Drosophila melanogaster</td><td>IntS11</td><td>Integrator 11</td><td>CG1972|DmelCG1972|Int11|dRC-68</td><td>0</td><td>0</td></tr>
<tr><td>10385</td><td>43507</td><td>Drosophila melanogaster</td><td>ATPsyn-gamma</td><td>ATP synthase-gamma chain</td><td>ATPG_DROME|ATPsyn-gam|CG7610|DmelCG7610</td><td>0</td><td>0</td></tr>
<tr><td>10386</td><td>43508</td><td>Drosophila melanogaster</td><td>yata</td><td>CG1973 gene product from transcript CG1973-RA</td><td>CG1973|DmelCG1973|Yata</td><td>0</td><td>0</td></tr>
<tr><td>10387</td><td>43511</td><td>Drosophila melanogaster</td><td>fig</td><td>fos intronic gene</td><td>CG7615|DmelCG7615</td><td>0</td><td>0</td></tr>
<tr><td>10388</td><td>43512</td><td>Drosophila melanogaster</td><td>sro</td><td>shroud</td><td>CG12068|DmelCG12068</td><td>0</td><td>0</td></tr>
<tr><td>10389</td><td>43513</td><td>Drosophila melanogaster</td><td>CG7837</td><td>CG7837 gene product from transcript CG7837-RA</td><td>DmelCG7837</td><td>0</td><td>0</td></tr>
<tr><td>10390</td><td>43514</td><td>Drosophila melanogaster</td><td>Ice</td><td>CG7788 gene product from transcript CG7788-RA</td><td>CG7788|DRICE|DmelCG7788|DrICE|DrIce|Drice|ICE|caspase 3|crice|drICE|drIce|drice|ice</td><td>0</td><td>0</td></tr>
<tr><td>10391</td><td>43515</td><td>Drosophila melanogaster</td><td>CG7834</td><td>CG7834 gene product from transcript CG7834-RA</td><td>DmelCG7834</td><td>0</td><td>0</td></tr>
<tr><td>10392</td><td>43516</td><td>Drosophila melanogaster</td><td>CG7789</td><td>CG7789 gene product from transcript CG7789-RA</td><td>DmelCG7789</td><td>0</td><td>0</td></tr>
<tr><td>10393</td><td>43517</td><td>Drosophila melanogaster</td><td>ncd</td><td>non-claret disjunctional</td><td>CG7831|DmNCD|DmNcd|DmelCG7831|KIFC1|Ncd|ca|ca[nd]|l(3)05884|ndc</td><td>0</td><td>0</td></tr>
<tr><td>10394</td><td>43518</td><td>Drosophila melanogaster</td><td>ca</td><td>claret</td><td>CG18133|CG18134|CG31037|CG7799|DmelCG31037|bw-p|bw[G]</td><td>0</td><td>0</td></tr>
<tr><td>10395</td><td>43521</td><td>Drosophila melanogaster</td><td>Vps16B</td><td>Vacuolar protein sorting 16B</td><td>CG18112|DmelCG18112|dVps16B|fob</td><td>0</td><td>0</td></tr>
<tr><td>10396</td><td>43522</td><td>Drosophila melanogaster</td><td>CG7829</td><td>CG7829 gene product from transcript CG7829-RA</td><td>DmelCG7829|SP136</td><td>0</td><td>0</td></tr>
<tr><td>10397</td><td>43523</td><td>Drosophila melanogaster</td><td>neo</td><td>neyo</td><td>CG7802|DmelCG7802</td><td>0</td><td>0</td></tr>
<tr><td>10398</td><td>43526</td><td>Drosophila melanogaster</td><td>CG15510</td><td>CG15510 gene product from transcript CG15510-RB</td><td>BcDNA:AT14346|DmelCG15510</td><td>0</td><td>0</td></tr>
<tr><td>10399</td><td>43528</td><td>Drosophila melanogaster</td><td>Cad99C</td><td>CG31009 gene product from transcript CG31009-RB</td><td>CA-10|CG15511|CG310034|CG31009|CG7805|CT35624|CT4930|DCad99C|Dm Cad99C|DmelCG31009|cad99C</td><td>0</td><td>0</td></tr>
<tr><td>10400</td><td>43529</td><td>Drosophila melanogaster</td><td>dgt1</td><td>dim gamma-tubulin 1</td><td>CG18041|Dgt1|DmelCG18041|NSL2</td><td>0</td><td>0</td></tr>
<tr><td>10401</td><td>43530</td><td>Drosophila melanogaster</td><td>CG7824</td><td>CG7824 gene product from transcript CG7824-RC</td><td>DmelCG7824</td><td>0</td><td>0</td></tr>
<tr><td>10402</td><td>43531</td><td>Drosophila melanogaster</td><td>CG15514</td><td>CG15514 gene product from transcript CG15514-RA</td><td>DmelCG15514</td><td>0</td><td>0</td></tr>
<tr><td>10403</td><td>43532</td><td>Drosophila melanogaster</td><td>RpS8</td><td>Ribosomal protein S8</td><td>BcDNA:RH06886|BcDNA:SD17528|CG7808|DmelCG7808|M(3)99B|Rps8|S8|anon-EST:Liang-1.56|anon-EST:fe1A2|clone 1.56</td><td>0</td><td>0</td></tr>
<tr><td>10404</td><td>43533</td><td>Drosophila melanogaster</td><td>CG7816</td><td>CG7816 gene product from transcript CG7816-RJ</td><td>DmelCG7816</td><td>0</td><td>0</td></tr>
<tr><td>10405</td><td>43535</td><td>Drosophila melanogaster</td><td>CG31038</td><td>CG31038 gene product from transcript CG31038-RA</td><td>CG15512|CG7854|DmelCG31038</td><td>0</td><td>0</td></tr>
<tr><td>10406</td><td>43537</td><td>Drosophila melanogaster</td><td>CG15517</td><td>CG15517 gene product from transcript CG15517-RA</td><td>DmelCG15517</td><td>0</td><td>0</td></tr>
<tr><td>10407</td><td>43538</td><td>Drosophila melanogaster</td><td>CG15515</td><td>CG15515 gene product from transcript CG15515-RA</td><td>BcDNA:RE21592|DmelCG15515</td><td>0</td><td>0</td></tr>
<tr><td>10408</td><td>43539</td><td>Drosophila melanogaster</td><td>CG31036</td><td>CG31036 gene product from transcript CG31036-RB</td><td>CG15516|CG15518|DmelCG31036</td><td>0</td><td>0</td></tr>
<tr><td>10409</td><td>43541</td><td>Drosophila melanogaster</td><td>capa</td><td>capability</td><td>CAP|CAP-2b|CAP2B-1|CAP2B-2|CAP2B-3|CAP2B/pyrokinin|CAP2b|CAP2b-1|CAP2b-2|CAP2b-3|CAPA|CAP[[2b]]|CG15520|Cap-1|Cap-2|Cap-2b|Cap-3|Capa|Capa-1|Capa-2|DmelCG15520|Drm-CAP-1|</td><td>0</td><td>0</td></tr>
<tr><td>10410</td><td>43542</td><td>Drosophila melanogaster</td><td>trp</td><td>transient receptor potential</td><td>559|CG7875|DmTRP|DmelCG7875|TRP|Trp|dTRP|dTrp|idl-B</td><td>0</td><td>0</td></tr>
<tr><td>10411</td><td>43543</td><td>Drosophila melanogaster</td><td>Jon99Ciii</td><td>Jonah 99Ciii</td><td>99Calpha|CG15519|CG31362|DmelCG31362|Jon99B|Jon99C|Jon99C1|Jon99C2|Jon99Calpha|Jon99Calphaii|Jon99Cbeta|Jon99Cbetaiii|JonC|SER2|SP143|Ser2|Ser5|Ser99Db</td><td>0</td><td>0</td></tr>
<tr><td>10412</td><td>43544</td><td>Drosophila melanogaster</td><td>Jon99Cii</td><td>Jonah 99Cii</td><td>99Calpha|CG31034|CG7877|DmelCG31034|Jon99B|Jon99C|Jon99C1|Jon99C2|Jon99Calpha|Jon99Calphai|Jon99Cbeta|Jon99Cbetaii|JonC|SER1|SP122|Ser1|Ser5|Ser99Da|SerI</td><td>0</td><td>0</td></tr>
<tr><td>10413</td><td>43545</td><td>Drosophila melanogaster</td><td>Jon99Ci</td><td>Jonah 99Ci</td><td>143341_at|99Calpha|CG17951|CG31039|DmelCG31039|Jon99B|Jon99C|Jon99C1|Jon99C2|Jon99Calpha|Jon99Calphai|Jon99Cbeta|Jon99Cbetai|JonC|SER3|SP150|Ser3|Ser99Dc|ser99Dc</td><td>0</td><td>0</td></tr>
<tr><td>10414</td><td>43546</td><td>Drosophila melanogaster</td><td>CG15522</td><td>CG15522 gene product from transcript CG15522-RA</td><td>DmelCG15522</td><td>0</td><td>0</td></tr>
<tr><td>10415</td><td>43547</td><td>Drosophila melanogaster</td><td>Cog7</td><td>CG31040 gene product from transcript CG31040-RA</td><td>CG31040|CG7880|DmelCG31040</td><td>0</td><td>0</td></tr>
<tr><td>10416</td><td>43548</td><td>Drosophila melanogaster</td><td>CG42557</td><td>CG42557 gene product from transcript CG42557-RA</td><td>CG7866|DmelCG42557|Dmel_CG7866</td><td>0</td><td>0</td></tr>
<tr><td>10417</td><td>43549</td><td>Drosophila melanogaster</td><td>eIF2B-alpha</td><td>CG7883 gene product from transcript CG7883-RA</td><td>CG7883|DeIF2Balpha|DmelCG7883</td><td>0</td><td>0</td></tr>
<tr><td>10418</td><td>43550</td><td>Drosophila melanogaster</td><td>CG15523</td><td>CG15523 gene product from transcript CG15523-RA</td><td>DmelCG15523</td><td>0</td><td>0</td></tr>
<tr><td>10419</td><td>43551</td><td>Drosophila melanogaster</td><td>Takr99D</td><td>Tachykinin-like receptor at 99D</td><td>CG7887|DTKR|DmelCG7887|TAKR99D|TKR99D|Tak-r|anon-WO0131005.11|anon-WO0131005.9|dtkr|l(3)s2222</td><td>0</td><td>0</td></tr>
<tr><td>10420</td><td>43552</td><td>Drosophila melanogaster</td><td>CG7896</td><td>CG7896 gene product from transcript CG7896-RA</td><td>CT23878|DmelCG7896|tartan/capricious-like</td><td>0</td><td>0</td></tr>
<tr><td>10421</td><td>43554</td><td>Drosophila melanogaster</td><td>CG7903</td><td>CG7903 gene product from transcript CG7903-RA</td><td>DmelCG7903|cg7903</td><td>0</td><td>0</td></tr>
<tr><td>10422</td><td>43555</td><td>Drosophila melanogaster</td><td>sas-6</td><td>spindle assembly abnormal 6</td><td>CG15524|DSAS-6|DSas-6|DSas6|DmelCG15524|Dsas6|SAS-6|SAS6|Sas-6|Sas6|dsas-6</td><td>0</td><td>0</td></tr>
<tr><td>10423</td><td>43556</td><td>Drosophila melanogaster</td><td>CG15525</td><td>CG15525 gene product from transcript CG15525-RA</td><td>BcDNA:SD23244|DmelCG15525</td><td>0</td><td>0</td></tr>
<tr><td>10424</td><td>43557</td><td>Drosophila melanogaster</td><td>CG11504</td><td>CG11504 gene product from transcript CG11504-RB</td><td>DmelCG11504</td><td>0</td><td>0</td></tr>
<tr><td>10425</td><td>43558</td><td>Drosophila melanogaster</td><td>Tace</td><td>CG7908 gene product from transcript CG7908-RB</td><td>CG7908|CT23908|DTACE|DmelCG7908|TACE</td><td>0</td><td>0</td></tr>
<tr><td>10426</td><td>43559</td><td>Drosophila melanogaster</td><td>CG7911</td><td>CG7911 gene product from transcript CG7911-RA</td><td>DmelCG7911</td><td>0</td><td>0</td></tr>
<tr><td>10427</td><td>43560</td><td>Drosophila melanogaster</td><td>Nlp</td><td>Nucleoplasmin</td><td>CG7917|CRP1|Crp1|DmelCG7917|dNLP|dNLP-S|p22</td><td>0</td><td>0</td></tr>
<tr><td>10428</td><td>43561</td><td>Drosophila melanogaster</td><td>CG7912</td><td>CG7912 gene product from transcript CG7912-RA</td><td>DmelCG7912</td><td>0</td><td>0</td></tr>
<tr><td>10429</td><td>43562</td><td>Drosophila melanogaster</td><td>CG7920</td><td>CG7920 gene product from transcript CG7920-RA</td><td>BEST:GH02390|DmelCG7920|GH02390.5prime|anon-WO0153538.36|anon-WO0153538.37|i130</td><td>0</td><td>0</td></tr>
<tr><td>10430</td><td>43563</td><td>Drosophila melanogaster</td><td>Mgat2</td><td>CG7921 gene product from transcript CG7921-RA</td><td>CG7921|DmelCG7921|GlcNAc-TII|GlcNAcT2|dMGAT2</td><td>0</td><td>0</td></tr>
<tr><td>10431</td><td>43564</td><td>Drosophila melanogaster</td><td>RpS28a</td><td>Ribosomal protein S28a</td><td>CG15527|DmelCG15527</td><td>0</td><td>0</td></tr>
</table>

10432	43565	Drosophila melanogaster	Axn	Axin	0442/30|CG7926|D-Axin|DAxin|Daxin|DmelCG7926|axin|axn|d-axin|dAXIN|dAxin|din|l(3)S044230	0	0
10433	43566	Drosophila melanogaster	CG7928	CG7928 gene product from transcript CG7928-RA	DmelCG7928	0	0
10434	43567	Drosophila melanogaster	ocn	ocnus	BcDNA:GH02250|CG7929|DmelCG7929|Ocn|bs29e10.y1	0	0
10435	43568	Drosophila melanogaster	janB	janus B	CG31024|CG7931|DmelCG7931|Jan|bs30f02.y1|jan|jan B|janus	0	0
10436	43569	Drosophila melanogaster	janA	janus A	BcDNA:AT12574|CG7933|DmelCG7933|Jan|Y|jan|jan A|janus	0	0
10437	43570	Drosophila melanogaster	Sry-beta	Serendipity beta	CG7938|DmelCG7938|Srybeta|ser|sry|sry beta|sry-b|sry-beta|sryb|srybeta	0	0
10438	43571	Drosophila melanogaster	Sry-alpha	Serendipity alpha	CG17957|DmelCG17957|Sry alpha|Sryalpha|ser|sry|sry alpha|sry-a|sry-alpha|srya|sryalpha	0	0
10439	43572	Drosophila melanogaster	Sry-delta	Serendipity delta	CG17958|DmelCG17958|EH8|Sry delta|Srydelta|anon-EH8|sry|sry delta|sry-delta|srydelta	0	0
10440	43573	Drosophila melanogaster	RpL32	Ribosomal protein L32	143250_at|BcDNA:RH03940|CG7939|DmelCG7939|L32|M(3)99D|RP-49|RP49|RPL32|Rp-49|Rp49|Rp49/RpL32|Rpl32|bs30a02.y1|rp49|rp49/RpL32	0	0
10441	43574	Drosophila melanogaster	CG7943	CG7943 gene product from transcript CG7943-RB	DmelCG7943	0	0
10442	43575	Drosophila melanogaster	CG15528	CG15528 gene product from transcript CG15528-RB	DmelCG15528	0	0
10443	43576	Drosophila melanogaster	CG7946	CG7946 gene product from transcript CG7946-RA	DmelCG7946	0	0
10444	43577	Drosophila melanogaster	spn-A	spindle A	CG7948|D37788|DMR|DMR/DroRAD51|DMR1|Dm Rad51|DmRAD51|DmRad51|DmelCG7948|Dmrad51|RA51|RA51_DROME|RAD51|Rad51|Rad51dm|Spn-A|dmRAD51|rad51|spnA	0	0
10445	43578	Drosophila melanogaster	CG15526	CG15526 gene product from transcript CG15526-RA	DmelCG15526	0	0
10446	43579	Drosophila melanogaster	CG7950	CG7950 gene product from transcript CG7950-RB	DmelCG7950|Dromel_CG7950_FBtr0085563_CHCHD4inmorf_mORF	0	0
10447	43580	Drosophila melanogaster	sima	similar	7951|CG7951|DMU43090|DmelCG7951|HIF-1|HIF-1alpha|HIF-alpha/sima|Hif-1A|SIMA|Sima|bHLHe56|dHIF-1alpha|dHIF-alpha|l(3)j11B7	0	0
10448	43581	Drosophila melanogaster	CG31025	CG31025 gene product from transcript CG31025-RA	CG2164|DmelCG31025|anon-WO0140519.94	0	0
10449	43582	Drosophila melanogaster	Tpi	Triose phosphate isomerase	CG2171|DmelCG2171|ND14|TPI|TPIS|sgk|tpi|wstd	0	0
10450	43583	Drosophila melanogaster	AdoR	Adenosine receptor	CG9753|DmelCG9753|adoR|anon-WO0170980.109|anon-WO0170980.110|dAdoR	0	0
10451	43584	Drosophila melanogaster	CG15529	CG15529 gene product from transcript CG15529-RA	DmelCG15529	0	0
10452	43585	Drosophila melanogaster	CG11498	CG11498 gene product from transcript CG11498-RB	DmelCG11498	0	0
10453	43586	Drosophila melanogaster	CG31028	CG31028 gene product from transcript CG31028-RC	CG2176|DmelCG31028	0	0
10454	43587	Drosophila melanogaster	Mlc2	Myosin light chain 2	CG2184|DmMLC2|DmelCG2184|Dmlc|Dmlc2|Ifm(3)99Eb|LC2|MLC|MLC 2|MLC-2|MLC2|MRLC|MYL2|RLC|RLC2|dro MLC-2|l(3)99Eb|mlc-2|mlc2	0	0
10455	43588	Drosophila melanogaster	CG1983	CG1983 gene product from transcript CG1983-RA	DmelCG1983	0	0
10456	43589	Drosophila melanogaster	CG15530	CG15530 gene product from transcript CG15530-RB	DmelCG15530	0	0
10457	43590	Drosophila melanogaster	Bet5	CG1359 gene product from transcript CG1359-RA	BET5|Bet5p|CG1359|DmelCG1359	0	0
10458	43591	Drosophila melanogaster	CG9747	CG9747 gene product from transcript CG9747-RA	DmelCG9747	0	0
10459	43592	Drosophila melanogaster	CG15531	CG15531 gene product from transcript CG15531-RA	DmelCG15531	0	0
10460	43593	Drosophila melanogaster	CG9743	CG9743 gene product from transcript CG9743-RA	DmelCG9743|anon-WO0118547.264	0	0
10461	43594	Drosophila melanogaster	RpS7	Ribosomal protein S7	CG1883|DmelCG1883|M(3)99E	0	0
10462	43595	Drosophila melanogaster	Anp	Andropin	And|CEC|CG1361|Cec|DmelCG1361|k-9	0	0
10463	43596	Drosophila melanogaster	CecA1	Cecropin A1	CEC|CG1365|CG4740|Cec|Cec A1|CecA|DmelCG1365|cec|cecA|cecA1|crpA1	0	0
10464	43597	Drosophila melanogaster	CecA2	Cecropin A2	CG1367|Cec|Cec A2|CecA|DmelCG1367|cec|cecA|cecA2|crpA2	0	0
10465	43598	Drosophila melanogaster	CecB	Cecropin B	CG1878|Cec|DmelCG1878|cec|cecB|cedcB|crpB	0	0
10466	43599	Drosophila melanogaster	CecC	Cecropin C	BcDNA:RH33561|CG1373|Cec|Cec-C|DmelCG1373|cec|cecC	0	0
10467	43600	Drosophila melanogaster	CG9737	CG9737 gene product from transcript CG9737-RA	DmelCG9737|SP5|c-SP5	0	0
10468	43601	Drosophila melanogaster	CG9733	CG9733 gene product from transcript CG9733-RA	DmelCG9733|SP10|c-SP10|proAE|proPO-AE	0	0
10469	43602	Drosophila melanogaster	CG9682	CG9682 gene product from transcript CG9682-RA	DmelCG9682|anon-WO0140519.240	0	0
10470	43603	Drosophila melanogaster	CG18404	CG18404 gene product from transcript CG18404-RA	DmelCG18404|anon-WO0140519.106	0	0
10471	43604	Drosophila melanogaster	hdc	headcase	CG15532|DmelCG15532|Hdc|l(3)B4-3-20	0	0
10472	43607	Drosophila melanogaster	CG2217	CG2217 gene product from transcript CG2217-RA	BcDNA:GH11688|DmelCG2217	0	0
10473	43608	Drosophila melanogaster	CG15535	CG15535 gene product from transcript CG15535-RA	DmelCG15535	0	0
10474	43609	Drosophila melanogaster	mRpS18C	mitochondrial ribosomal protein S18C	CG9688|DmelCG9688|MRP-S18-(1)|mRpS18a	0	0
10475	43610	Drosophila melanogaster	CG15536	CG15536 gene product from transcript CG15536-RA	DmelCG15536	0	0
10476	43611	Drosophila melanogaster	CG2218	CG2218 gene product from transcript CG2218-RA	DmelCG2218	0	0
10477	43612	Drosophila melanogaster	CG15533	CG15533 gene product from transcript CG15533-RA	DmelCG15533|aSMase	0	0
10478	43613	Drosophila melanogaster	CG15534	CG15534 gene product from transcript CG15534-RA	DmelCG15534|aSMase	0	0
10479	43614	Drosophila melanogaster	CG15537	CG15537 gene product from transcript CG15537-RB	DmelCG15537	0	0
10480	43615	Drosophila melanogaster	Osi23	Osiris 23	CG15538|DmelCG15538	0	0
10481	43616	Drosophila melanogaster	aralar1	CG2139 gene product from transcript CG2139-RC	BcDNA:GH07418|CG2139|DmAralar|DmelCG2139|aralar	0	0
10482	43617	Drosophila melanogaster	CG2224	CG2224 gene product from transcript CG2224-RA	Amsh|DmelCG2224	0	0
10483	43618	Drosophila melanogaster	CDase	Ceramidase	CG1471|DmelCG1471|anon-WO0118547.407|cdase|fcd|slab	0	0
10484	43620	Drosophila melanogaster	PH4alphaEFB	prolyl-4-hydroxylase-alpha EFB	BcDNA:SD05564|CG12088|CG1473|CG31022|DmelCG31022	0	0
10485	43621	Drosophila melanogaster	Jon99Fii	Jonah 99Fii	CG2229|DmelCG2229|Jon99F|SP135	0	0
10486	43622	Drosophila melanogaster	Jon99Fi	Jonah 99Fi	CG18030|DmelCG18030|Jon99F|SPH181	0	0
10487	43623	Drosophila melanogaster	PH4alphaSG2	prolyl-4-hydroxylase-alpha SG2	CG1546|DmelCG1546|SG2	0	0
10488	43624	Drosophila melanogaster	PH4alphaMP	prolyl-4-hydroxylase-alpha MP	59D7T|CG9726|DM59D7T|DmelCG9726|ESTS:59D7T	0	0
10489	43625	Drosophila melanogaster	PH4alphaNE1	prolyl-4-hydroxylase-alpha NE1	CG9728|DmelCG9728	0	0
10490	43626	Drosophila melanogaster	CG31371	CG31371 gene product from transcript CG31371-RC	CG15542|DmelCG31371	0	0
10491	43627	Drosophila melanogaster	CG15539	CG15539 gene product from transcript CG15539-RA	CG15539-NE4|DmelCG15539	0	0
10492	43628	Drosophila melanogaster	PH4alphaNE2	prolyl-4-hydroxylase-alpha NE2	CG9720|DmelCG9720	0	0
10493	43629	Drosophila melanogaster	CG9698	CG9698 gene product from transcript CG9698-RA	CG9698-E|DmelCG9698	0	0
10494	43630	Drosophila melanogaster	jdp	CG2239 gene product from transcript CG2239-RB	CG2239|DmelCG2239|JDP|dJDP|p25a	0	0
10495	43633	Drosophila melanogaster	tmod	tropomodulin	1309/10|CG11493|CG1539|CG15540|CT3919|CT41421|DmelCG1539|E42|Tmod|l(3)00848|l(3)S130910|spdo	0	0
10496	43634	Drosophila melanogaster	CG9702	CG9702 gene product from transcript CG9702-RA	DmelCG9702	0	0
10497	43635	Drosophila melanogaster	Rpt6R	CG2241 gene product from transcript CG2241-RA	CG2241|DmCG2241|Dm_Rpt6b|DmelCG2241	0	0
10498	43636	Drosophila melanogaster	CG9717	CG9717 gene product from transcript CG9717-RA	DmelCG9717	0	0
10499	43637	Drosophila melanogaster	CG2246	CG2246 gene product from transcript CG2246-RH	DmelCG2246	0	0
10500	43638	Drosophila melanogaster	CG31021	CG31021 gene product from transcript CG31021-RB	CG9708|DmelCG31021	0	0
10501	43639	Drosophila melanogaster	CG2267	CG2267 gene product from transcript CG2267-RA	DmelCG2267|anon-WO0140519.167	0	0
10502	43640	Drosophila melanogaster	PH4alphaPV	prolyl-4-hydroxylase-alpha PV	CG31015|CG9713|DmelCG31015	0	0
10503	43642	Drosophila melanogaster	Spn100A	Serpin 100A	CG1342|DmelCG1342	0	0
10504	43643	Drosophila melanogaster	CG12069	CG12069 gene product from transcript CG12069-RA	DmelCG12069	0	0
10505	43644	Drosophila melanogaster	Pka-C2	cAMP-dependent protein kinase 2	CG12066|CdkB|DC1|DC1a|DmelCG12066|PKA|PKA C2	0	0
10506	43645	Drosophila melanogaster	CG1340	CG1340 gene product from transcript CG1340-RA	DmelCG1340|cg1340|eIF4H	0	0
10507	43646	Drosophila melanogaster	CG11313	CG11313 gene product from transcript CG11313-RB	DmelCG11313|SP11|c-SP11	0	0
10508	43647	Drosophila melanogaster	CG15543	CG15543 gene product from transcript CG15543-RA	DmelCG15543	0	0
10509	43648	Drosophila melanogaster	Npc2g	Niemann-Pick type C-2g	BcDNA:RE56164|CG11314|DmelCG11314|npc2g	0	0
10510	43649	Drosophila melanogaster	Npc2h	Niemann-Pick type C-2h	BcDNA:GH26608|BcDNA:RH04252|BcDNA:RH68460|CG11315|DmML1A|DmelCG11315|npc2h	0	0
10511	43650	Drosophila melanogaster	zfh1	Zn finger homeodomain 1	CG1322|DmelCG1322|ZFH-1|ZFH1|Zfh-1|Zfh1|Zfh1a|l(3)00865|zfh-1|zfl-1	0	0
10512	43651	Drosophila melanogaster	wts	warts	CG12072|Dlats|DmelCG12072|LATS|Lats|Lats/Warts|MENE (3R)-G|WTS/LATS|Warts|Warts/Lats|Wts|Wts/Lats|dmLATS|l(3)100Aa|lats|lts|wart|warts/lats|wts/lats	0	0
10513	43652	Drosophila melanogaster	dj-1beta	CG1349 gene product from transcript CG1349-RA	CG1349|DJ-1|DJ-1B|DJ-1b|DJ-1beta|DmelCG1349	0	0
10514	43654	Drosophila melanogaster	cindr	CIN85 and CD2AP orthologue	BcDNA:GH03163|CD2AP|CG11316|CG1408|CG31012|Cindr|DmelCG31012	0	0
10515	43655	Drosophila melanogaster	CG15544	CG15544 gene product from transcript CG15544-RA	DmelCG15544	0	0
10516	43656	Drosophila melanogaster	tll	tailless	CG1378|DmelCG1378|NR2E2|TLL|Tll	0	0
10517	43657	Drosophila melanogaster	Cpr100A	Cuticular protein 100A	CG12045|DmelCpr100A|DmelCG12045	0	0
10518	43658	Drosophila melanogaster	CG15545	CG15545 gene product from transcript CG15545-RA	DmelCG15545	0	0
10519	43659	Drosophila melanogaster	CG15546	CG15546 gene product from transcript CG15546-RA	DmelCG15546	0	0
10520	43660	Drosophila melanogaster	CG12071	CG12071 gene product from transcript CG12071-RB	DmelCG12071	0	0
10521	43661	Drosophila melanogaster	CG15547	CG15547 gene product from transcript CG15547-RA	DmelCG15547	0	0
10522	43662	Drosophila melanogaster	Sap-r	Saposin-related	Apa|BcDNA:GH08312|CG12070|DAPA|DmelCG12070|Ov9|P110|Sap-R|Sapr|anon-EST:ParkEST270|sap-r	0	0
10523	43663	Drosophila melanogaster	Cyp4c3	Cytochrome P450-4c3	4C3|CG1438|DmelCG1438|P-450|P450|cyp4c3	0	0
10524	43664	Drosophila melanogaster	Ptx1	CG1447 gene product from transcript CG1447-RC	CG1447|D-Ptx1|DmelCG1447|Ptx|ptx1	0	0
10525	43665	Drosophila melanogaster	CG15549	CG15549 gene product from transcript CG15549-RA	BcDNA:AT07554|DmelCG15549	0	0
10526	43666	Drosophila melanogaster	CG15550	CG15550 gene product from transcript CG15550-RA	DmelCG15550	0	0
10527	43667	Drosophila melanogaster	CG15548	CG15548 gene product from transcript CG15548-RA	DmelCG15548	0	0
10528	43668	Drosophila melanogaster	Ctr1C	Copper transporter 1C	BcDNA:AT31946|CG15551|DmelCG15551|FBgn0039813	0	0
10529	43669	Drosophila melanogaster	5-HT7	Serotonin receptor 7	5-HT-dro|5-HT-dro1|5-HT7Dro|5-HT[7]Dro|5-HT[[7Dro]]|5-HT[[7]]|5-HT[[7]]Dro|5-HT[[dro1]]|5-HTdro1|5HT-R1|5HT-dro|5HT-dro1|5HT[[7Dro]]|5htdror|CG12073|DRO1|Dm5HTdro1|DmelCG12073	0	0
10530	43670	Drosophila melanogaster	CanA1	Calcineurin A1	CAN A1/PP2B|CG1455|CNA1|Can|Cana1|CnnA21EF|D14|DmCanA1-100B|DmelCG1455|PP2B|PP2B 21EF|PpD14|canA|pMY4	0	0
10531	43671	Drosophila melanogaster	Aph-4	Alkaline phosphatase 4	AP|Aph|CG1462|DmelCG1462|l(3)07028|pMY51	0	0
10532	43672	Drosophila melanogaster	l(3)03670	lethal (3) 03670	0205/14|0353/13|0812/02|0966/01|1274/08|CG1715|DmelCG1715|anon-l|l(3)3670|l(3)96601|l(3)S127408|pMY8|x94917	0	0
10533	43673	Drosophila melanogaster	dco	discs overgrown	0538/13|0915/10|1396/02|1447/01|1460/09|CG2048|CK1epsilon|CKIepsilon|DBT|DBT/CK1epsilon|DCO|DCO/CKIe|Dbt|Dco|Dco/CK1|DmelCG2048|ck1epsilon|dCKIepsilon|dbt|dco-1|ddbt|l(3)S053813|l(3)	0	0
10534	43676	Drosophila melanogaster	CG11317	CG11317 gene product from transcript CG11317-RA	DmelCG11317	0	0
10535	43677	Drosophila melanogaster	CG15553	CG15553 gene product from transcript CG15553-RC	DmelCG15553	0	0
10536	43678	Drosophila melanogaster	CG11318	CG11318 gene product from transcript CG11318-RA	DmelCG11318|anon-WO0170980.121|anon-WO0170980.122	0	0
10537	43679	Drosophila melanogaster	Prosalpha3T	Proteasome alpha3T subunit	CG1736|DmelCG1736|Pros20S-alpha3t|prosalpha3T	0	0
10538	43680	Drosophila melanogaster	CG15554	CG15554 gene product from transcript CG15554-RA	BcDNA:RE10288|DmelCG15554	0	0
10539	43681	Drosophila melanogaster	CG15556	CG15556 gene product from transcript CG15556-RA	DmelCG15556|anon-WO0170980.178|anon-WO0170980.179	0	0
10540	43682	Drosophila melanogaster	Gycbeta100B	Guanylyl cyclase beta-subunit at 100B	CG1470|Dgc beta 1|Dgcbeta1|DmelCG1470|Gycbeta-100B|dgcb1|dgcbeta1|gycbeta100B|sGCbeta1	0	0
10541	43683	Drosophila melanogaster	stops	slow termination of phototransduction	CG15557|CG15558|CG15559|CG31006|DmelCG31006|su(rdgB)69	0	0
10542	43687	Drosophila melanogaster	bnk	bottleneck	Bnk|CG1480|DmelCG1480	0	0

<table>
<tr><td>10543</td><td>43688</td><td>Drosophila melanogaster</td><td>CG31004</td><td>CG31004 gene product from transcript CG31004-RC</td><td>CG12074|CG15560|CT5150|DmelCG31004|anon-WO0140519.233</td><td>0</td><td>0</td></tr>
<tr><td>10544</td><td>43689</td><td>Drosophila melanogaster</td><td>CG1544</td><td>CG1544 gene product from transcript CG1544-RA</td><td>DmelCG1544</td><td>0</td><td>0</td></tr>
<tr><td>10545</td><td>43690</td><td>Drosophila melanogaster</td><td>chp</td><td>chaoptic</td><td>24B10|516|BSH|CG1744|CHP|CT5040|Chaoptin|Chp|DmelCG1744|MAb 24B10|MAb24B10|Mab24B10|Nsp|mAB24B10|mAb 24B10|mAb24B10</td><td>0</td><td>0</td></tr>
<tr><td>10546</td><td>43691</td><td>Drosophila melanogaster</td><td>CG1542</td><td>CG1542 gene product from transcript CG1542-RA</td><td>DmelCG1542</td><td>0</td><td>0</td></tr>
<tr><td>10547</td><td>43692</td><td>Drosophila melanogaster</td><td>CG15561</td><td>CG15561 gene product from transcript CG15561-RA</td><td>DmelCG15561</td><td>0</td><td>0</td></tr>
<tr><td>10548</td><td>43693</td><td>Drosophila melanogaster</td><td>CG1746</td><td>CG1746 gene product from transcript CG1746-RA</td><td>DmelCG1746|anon-EST:Posey224</td><td>0</td><td>0</td></tr>
<tr><td>10549</td><td>43694</td><td>Drosophila melanogaster</td><td>CG12054</td><td>CG12054 gene product from transcript CG12054-RA</td><td>DmelCG12054</td><td>0</td><td>0</td></tr>
<tr><td>10550</td><td>43695</td><td>Drosophila melanogaster</td><td>CG15563</td><td>CG15563 gene product from transcript CG15563-RA</td><td>DmelCG15563</td><td>0</td><td>0</td></tr>
<tr><td>10551</td><td>43696</td><td>Drosophila melanogaster</td><td>CG15564</td><td>CG15564 gene product from transcript CG15564-RA</td><td>DmelCG15564</td><td>0</td><td>0</td></tr>
<tr><td>10552</td><td>43697</td><td>Drosophila melanogaster</td><td>qless</td><td>CG31005 gene product from transcript CG31005-RA</td><td>CG15565|CG31005|CG3684|DmelCG31005|anon-WO0118547.422</td><td>0</td><td>0</td></tr>
<tr><td>10553</td><td>43698</td><td>Drosophila melanogaster</td><td>mRpL32</td><td>mitochondrial ribosomal protein L32</td><td>BcDNA:LD41522|CG12220|DmelCG12220|L32</td><td>0</td><td>0</td></tr>
<tr><td>10554</td><td>43699</td><td>Drosophila melanogaster</td><td>CG1750</td><td>CG1750 gene product from transcript CG1750-RA</td><td>DmelCG1750</td><td>0</td><td>0</td></tr>
<tr><td>10555</td><td>43700</td><td>Drosophila melanogaster</td><td>spn-F</td><td>CG12114 gene product from transcript CG12114-RA</td><td>1130/03|CG12114|DmelCG12114|Spn-F|l(3)S113003b</td><td>0</td><td>0</td></tr>
<tr><td>10556</td><td>43701</td><td>Drosophila melanogaster</td><td>CG3669</td><td>CG3669 gene product from transcript CG3669-RB</td><td>DmelCG3669</td><td>0</td><td>0</td></tr>
<tr><td>10557</td><td>43702</td><td>Drosophila melanogaster</td><td>CG15555</td><td>CG15555 gene product from transcript CG15555-RA</td><td>DmelCG15555</td><td>0</td><td>0</td></tr>
<tr><td>10558</td><td>43703</td><td>Drosophila melanogaster</td><td>CG11340</td><td>CG11340 gene product from transcript CG11340-RA</td><td>DmelCG11340</td><td>0</td><td>0</td></tr>
<tr><td>10559</td><td>43707</td><td>Drosophila melanogaster</td><td>CG1607</td><td>CG1607 gene product from transcript CG1607-RA</td><td>DmelCG1607</td><td>0</td><td>0</td></tr>
<tr><td>10560</td><td>43708</td><td>Drosophila melanogaster</td><td>CG31002</td><td>CG31002 gene product from transcript CG31002-RA</td><td>CG15569|CG31002-PA|DmelCG31002</td><td>0</td><td>0</td></tr>
<tr><td>10561</td><td>43709</td><td>Drosophila melanogaster</td><td>Gcn2</td><td>CG1609 gene product from transcript CG1609-RA</td><td>CG1609|DGCN2|DmelCG1609|EIF2|GCN2|dGCN2</td><td>0</td><td>0</td></tr>
<tr><td>10562</td><td>43710</td><td>Drosophila melanogaster</td><td>CG11337</td><td>CG11337 gene product from transcript CG11337-RD</td><td>DmelCG11337</td><td>0</td><td>0</td></tr>
<tr><td>10563</td><td>43712</td><td>Drosophila melanogaster</td><td>lox</td><td>lysyl oxidase-like</td><td>CG11335|DmLOXL-1|DmelCG11335|Dmloxl-1|Lox|dmlox-1</td><td>0</td><td>0</td></tr>
<tr><td>10564</td><td>43713</td><td>Drosophila melanogaster</td><td>CG11334</td><td>CG11334 gene product from transcript CG11334-RA</td><td>DmelCG11334</td><td>0</td><td>0</td></tr>
<tr><td>10565</td><td>43714</td><td>Drosophila melanogaster</td><td>CG11333</td><td>CG11333 gene product from transcript CG11333-RA</td><td>BcDNA:AT02774|DmelCG11333</td><td>0</td><td>0</td></tr>
<tr><td>10566</td><td>43715</td><td>Drosophila melanogaster</td><td>mey</td><td>morpheyus</td><td>CG12063|DmelCG12063</td><td>0</td><td>0</td></tr>
<tr><td>10567</td><td>43716</td><td>Drosophila melanogaster</td><td>nyo</td><td>nyobe</td><td>CG1499|DmelCG1499|anon-EST:Liang-2.47|clone 2.47</td><td>0</td><td>0</td></tr>
<tr><td>10568</td><td>43717</td><td>Drosophila melanogaster</td><td>gammaCop</td><td>gamma-coatomer protein</td><td>0573/02|573.02|CG1528|COPIgamma|DmelCG1528|anon-WO0118547.334|copg|gamma-COP|gammaCOP|l(3)S057302|l(3)S057302b</td><td>0</td><td>0</td></tr>
<tr><td>10569</td><td>43718</td><td>Drosophila melanogaster</td><td>pygo</td><td>pygopus</td><td>BEST:LD21971|CG11518|Dm Pygo|DmelCG11518|Pygo|anon-WO02077023.1|dPygo|gam|pNB5.2|pyg|pypo</td><td>0</td><td>0</td></tr>
<tr><td>10570</td><td>43719</td><td>Drosophila melanogaster</td><td>rod</td><td>rough deal</td><td>CG1569|DmelCG1569|EP3408|ROD|Rod|pNB20A</td><td>0</td><td>0</td></tr>
<tr><td>10571</td><td>43720</td><td>Drosophila melanogaster</td><td>CG1635</td><td>CG1635 gene product from transcript CG1635-RA</td><td>DmelCG1635</td><td>0</td><td>0</td></tr>
<tr><td>10572</td><td>43721</td><td>Drosophila melanogaster</td><td>CG1638</td><td>CG1638 gene product from transcript CG1638-RB</td><td>DmelCG1638|bs31f06.y1</td><td>0</td><td>0</td></tr>
<tr><td>10573</td><td>43722</td><td>Drosophila melanogaster</td><td>CG1774</td><td>CG1774 gene product from transcript CG1774-RA</td><td>DmelCG1774</td><td>0</td><td>0</td></tr>
<tr><td>10574</td><td>43723</td><td>Drosophila melanogaster</td><td>RpL6</td><td>Ribosomal protein L6</td><td>CG11522|DmelCG11522|M(3)100CF</td><td>0</td><td>0</td></tr>
<tr><td>10575</td><td>43724</td><td>Drosophila melanogaster</td><td>CycG</td><td>Cyclin G</td><td>CG 11525|CG11525|DmelCG11525|anon-EST:Liang-1.25|anon-EST:Liang-2.24|clone 1.25|clone 2.24|dCycG|i205|i261</td><td>0</td><td>0</td></tr>
<tr><td>10576</td><td>43725</td><td>Drosophila melanogaster</td><td>Med</td><td>Medea</td><td>CG1775|DmelCG1775|E(zen)3|MED|SMAD4|Smad4|anon-EST:Posey121|dSmad4|l(3)11m-254|l(3)12m-137|l(3)SG36|l(3)SG70|l(3)XIIm137|med|medea</td><td>0</td><td>0</td></tr>
<tr><td>10577</td><td>43726</td><td>Drosophila melanogaster</td><td>CG11539</td><td>CG11539 gene product from transcript CG11539-RA</td><td>DmelCG11539</td><td>0</td><td>0</td></tr>
<tr><td>10578</td><td>43727</td><td>Drosophila melanogaster</td><td>CG1792</td><td>CG1792 gene product from transcript CG1792-RA</td><td>DmelCG1792</td><td>0</td><td>0</td></tr>
<tr><td>10579</td><td>43728</td><td>Drosophila melanogaster</td><td>pasha</td><td>partner of drosha</td><td>CG1800|DGCR8|DmelCG1800|Pasha|cg1800|dmDGCR8</td><td>0</td><td>0</td></tr>
<tr><td>10580</td><td>43729</td><td>Drosophila melanogaster</td><td>kek6</td><td>CG1804 gene product from transcript CG1804-RA</td><td>CG1804|DmelCG1804</td><td>0</td><td>0</td></tr>
<tr><td>10581</td><td>43730</td><td>Drosophila melanogaster</td><td>CG1815</td><td>CG1815 gene product from transcript CG1815-RA</td><td>DmelCG1815</td><td>0</td><td>0</td></tr>
<tr><td>10582</td><td>43731</td><td>Drosophila melanogaster</td><td>CG11550</td><td>CG11550 gene product from transcript CG11550-RA</td><td>DmelCG11550</td><td>0</td><td>0</td></tr>
<tr><td>10583</td><td>43734</td><td>Drosophila melanogaster</td><td>CstF-50</td><td>CG2261 gene product from transcript CG2261-RA</td><td>CG2261|CstF50|DmelCG2261</td><td>0</td><td>0</td></tr>
<tr><td>10584</td><td>43735</td><td>Drosophila melanogaster</td><td>CG11563</td><td>CG11563 gene product from transcript CG11563-RA</td><td>BcDNA:RE07994|DmelCG11563</td><td>0</td><td>0</td></tr>
<tr><td>10585</td><td>43736</td><td>Drosophila melanogaster</td><td>Ef1alpha100E</td><td>Elongation factor 1alpha100E</td><td>CG1873|DmelCG1873|EF-1|EF-1alpha|EF-1alpha F2|EF1-alpha|EF1alpha|EF1alpha100|Ef1 alpha100E|Ef1a48|Ef1alpha2|Efla2|F2|eEF1alpha|ef-1alpha</td><td>0</td><td>0</td></tr>
<tr><td>10586</td><td>43737</td><td>Drosophila melanogaster</td><td>CG1890</td><td>CG1890 gene product from transcript CG1890-RA</td><td>DmelCG1890</td><td>0</td><td>0</td></tr>
<tr><td>10587</td><td>43738</td><td>Drosophila melanogaster</td><td>CG1896</td><td>CG1896 gene product from transcript CG1896-RA</td><td>DmelCG1896</td><td>0</td><td>0</td></tr>
<tr><td>10588</td><td>43739</td><td>Drosophila melanogaster</td><td>awd</td><td>abnormal wing discs</td><td>1084/08|Awd|BcDNA:GM19775|BcDNA:RH27794|CG2210|DmelCG2210|K-pn|Kpn|NDKB|NDPK|Nm23/awd|anon-EST:Liang-2.27|anon-EST:Liang-2.28|anon-WO0172774.80|anon-WO0172774.82|clone 2</td><td>0</td><td>0</td></tr>
<tr><td>10589</td><td>43740</td><td>Drosophila melanogaster</td><td>nero</td><td>CG2245 gene product from transcript CG2245-RA</td><td>CG2245|DOHH|DmelCG2245|l(3)s1921</td><td>0</td><td>0</td></tr>
<tr><td>10590</td><td>43741</td><td>Drosophila melanogaster</td><td>CG1910</td><td>CG1910 gene product from transcript CG1910-RB</td><td>1A3|Anon1A3|DmelCG1910|anon-EST:fe1A3|anon-WO0118547.398|anon-WO0118547.745|anon1A3</td><td>0</td><td>0</td></tr>
<tr><td>10591</td><td>43742</td><td>Drosophila melanogaster</td><td>salt</td><td>salty dog</td><td>CG2196|DmelCG2196</td><td>0</td><td>0</td></tr>
<tr><td>10592</td><td>43743</td><td>Drosophila melanogaster</td><td>CG2187</td><td>CG2187 gene product from transcript CG2187-RA</td><td>D3-100EF|DmelCG2187|anon-100EF-D3</td><td>0</td><td>0</td></tr>
<tr><td>10593</td><td>43744</td><td>Drosophila melanogaster</td><td>Smvt</td><td>Sodium-dependent multivitamin transporter</td><td>CG2191|DmelCG2191|SMVT|smvt</td><td>0</td><td>0</td></tr>
<tr><td>10594</td><td>43745</td><td>Drosophila melanogaster</td><td>CG2150</td><td>CG2150 gene product from transcript CG2150-RA</td><td>DmelCG2150|GCR 1|GCR1|Pen100DE|anon-Pen100DE|pen100DE</td><td>0</td><td>0</td></tr>
<tr><td>10595</td><td>43746</td><td>Drosophila melanogaster</td><td>CG2135</td><td>CG2135 gene product from transcript CG2135-RA</td><td>DmelCG2135</td><td>0</td><td>0</td></tr>
<tr><td>10596</td><td>43747</td><td>Drosophila melanogaster</td><td>sip3</td><td>septin interacting protein 3</td><td>CG1937|DmelCG1937|HRD1|Hrd1|Sip3|dSyno</td><td>0</td><td>0</td></tr>
<tr><td>10597</td><td>43748</td><td>Drosophila melanogaster</td><td>CG2126</td><td>CG2126 gene product from transcript CG2126-RA</td><td>DmelCG2126|anon-WO0118547.412</td><td>0</td><td>0</td></tr>
<tr><td>10598</td><td>43749</td><td>Drosophila melanogaster</td><td>faf</td><td>fat facets</td><td>BcDNA.LD22582|BcDNA:LD22582|CG1945|DmelCG1945|Faf|Fat</td><td>0</td><td>0</td></tr>
<tr><td>10599</td><td>43750</td><td>Drosophila melanogaster</td><td>CG2118</td><td>CG2118 gene product from transcript CG2118-RC</td><td>DmelCG2118|MCC|mcc</td><td>0</td><td>0</td></tr>
<tr><td>10600</td><td>43751</td><td>Drosophila melanogaster</td><td>Acf1</td><td>ATP-dependent chromatin assembly factor large subu</td><td>ACF|ACF/CHRAC|ACF1|Acf|Acf-1|CAF|CG1966|CHRAC|CHRAC-175|DmelCG1966|acf1|dACF|dCHRAC|p170/p185</td><td>0</td><td>0</td></tr>
<tr><td>10601</td><td>43753</td><td>Drosophila melanogaster</td><td>Ir100a</td><td>Ionotropic receptor 100a</td><td>CG11575|DmelCG11575|IR100a</td><td>0</td><td>0</td></tr>
<tr><td>10602</td><td>43754</td><td>Drosophila melanogaster</td><td>CG42233</td><td>CG42233 gene product from transcript CG42233-RB</td><td>CG11574|CG2112|DmelCG42233|Dmel_CG11574|Dmel_CG2112</td><td>0</td><td>0</td></tr>
<tr><td>10603</td><td>43755</td><td>Drosophila melanogaster</td><td>CG1971</td><td>CG1971 gene product from transcript CG1971-RA</td><td>DmelCG1971</td><td>0</td><td>0</td></tr>
<tr><td>10604</td><td>43756</td><td>Drosophila melanogaster</td><td>CG11576</td><td>CG11576 gene product from transcript CG11576-RA</td><td>DmelCG11576</td><td>0</td><td>0</td></tr>
<tr><td>10605</td><td>43757</td><td>Drosophila melanogaster</td><td>ferrochelatase</td><td>CG2098 gene product from transcript CG2098-RA</td><td>CG2098|DmelCG2098</td><td>0</td><td>0</td></tr>
<tr><td>10606</td><td>43758</td><td>Drosophila melanogaster</td><td>RhoGAP100F</td><td>CG1976 gene product from transcript CG1976-RD</td><td>1976|CG1976|CG1976-PA|DSyd-1|DSyd1|DmelCG1976</td><td>0</td><td>0</td></tr>
<tr><td>10607</td><td>43761</td><td>Drosophila melanogaster</td><td>CG2003</td><td>CG2003 gene product from transcript CG2003-RA</td><td>DmelCG2003</td><td>0</td><td>0</td></tr>
<tr><td>10608</td><td>43762</td><td>Drosophila melanogaster</td><td>CG2053</td><td>CG2053 gene product from transcript CG2053-RA</td><td>DmelCG2053</td><td>0</td><td>0</td></tr>
<tr><td>10609</td><td>43764</td><td>Drosophila melanogaster</td><td>mod</td><td>modulo</td><td>0952/14|CG2050|DmelCG2050|Mod|ms(3)100EF</td><td>0</td><td>0</td></tr>
<tr><td>10610</td><td>43765</td><td>Drosophila melanogaster</td><td>Map205</td><td>Microtubule-associated protein 205</td><td>205-kDa MAP|205K MAP|205kD MAP|205kDa MAP|CG1483|DmelCG1483|MAP205|MAP4|map205</td><td>0</td><td>0</td></tr>
<tr><td>10611</td><td>43766</td><td>Drosophila melanogaster</td><td>plexB</td><td>plexin B</td><td>CG17245|DmelCG17245|PLEXB|PlexB|plex|plex B</td><td>0</td><td>0</td></tr>
<tr><td>10612</td><td>43767</td><td>Drosophila melanogaster</td><td>ci</td><td>cubitus interruptus</td><td>CG2125|CI|CID|Ce|Ci|Ci-155|Ci/GLI|Ci/Gli|Ci155|CiD|Ci[D]|DmelCG2125|Gli|Siah|ci-D|ci155|ciD|ci[D]|l(4)102ABc|l(4)13|l(4)17</td><td>0</td><td>0</td></tr>
<tr><td>10613</td><td>43768</td><td>Drosophila melanogaster</td><td>RpS3A</td><td>Ribosomal protein S3A</td><td>C3|CG2168|DmelCG2168|M(4)101|M(4)57g|M(4)[57g]|M57g|M[57g]|RPS3A|RPS3a|RpS3a|anon-EST:Liang-2.29|clone 2.29|l(4)102ABa</td><td>0</td><td>0</td></tr>
<tr><td>10614</td><td>43769</td><td>Drosophila melanogaster</td><td>pan</td><td>pangolin</td><td>CG17964|CG32005|CG34403|DTCF|DTcf|Dm Pan|DmelCG34403|Dmel_CG17964|Dmel_CG32005|IA5|LEF-1|LEF/TCF|LEF/TCF-1|LEF1|LEF1/TCF|Lef|Lef1|PAN|Pan|TCF|TCF/LEF|TCF/LEF1|Tcf|Tcf-1|Tcf/LE</td><td>0</td><td>0</td></tr>
<tr><td>10615</td><td>43770</td><td>Drosophila melanogaster</td><td>Ank</td><td>Ankyrin</td><td>ANK|CG1651|CG32008|DAnk1|Dank1|DmelCG1651|ank|dank1</td><td>0</td><td>0</td></tr>
<tr><td>10616</td><td>43771</td><td>Drosophila melanogaster</td><td>CG2219</td><td>CG2219 gene product from transcript CG2219-RA</td><td>ARL4B|BcDNA:AT26185|CG 2219|DmelCG2219</td><td>0</td><td>0</td></tr>
<tr><td>10617</td><td>43772</td><td>Drosophila melanogaster</td><td>CG2316</td><td>CG2316 gene product from transcript CG2316-RD</td><td>DmelCG2316</td><td>0</td><td>0</td></tr>
<tr><td>10618</td><td>43774</td><td>Drosophila melanogaster</td><td>CG31998</td><td>CG31998 gene product from transcript CG31998-RB</td><td>CG11572|CG11578|DmelCG31998</td><td>0</td><td>0</td></tr>
<tr><td>10619</td><td>43775</td><td>Drosophila melanogaster</td><td>Crk</td><td>CG1587 gene product from transcript CG1587-RD</td><td>CG1587|CRK|D-CRK|D-Crk|DCrk|Dcrk|DmelCG1587|crk|dCRK|dCrk</td><td>0</td><td>0</td></tr>
<tr><td>10620</td><td>43777</td><td>Drosophila melanogaster</td><td>CG31999</td><td>CG31999 gene product from transcript CG31999-RA</td><td>CG10323|CG11288|CT31491|CT7856|DmelCG31999</td><td>0</td><td>0</td></tr>
<tr><td>10621</td><td>43779</td><td>Drosophila melanogaster</td><td>yellow-h</td><td>CG1629 gene product from transcript CG1629-RA</td><td>CG1629|DmelCG1629</td><td>0</td><td>0</td></tr>
<tr><td>10622</td><td>43780</td><td>Drosophila melanogaster</td><td>CG1674</td><td>CG1674 gene product from transcript CG1674-RB</td><td>DmelCG1674</td><td>0</td><td>0</td></tr>
<tr><td>10623</td><td>43781</td><td>Drosophila melanogaster</td><td>RhoGAP102A</td><td>CG42316 gene product from transcript CG42316-RD</td><td>BP1050|CG1748|CG34013|CG42316|DmelCG42316|Dmel_CG1748|Dmel_CG34013</td><td>0</td><td>0</td></tr>
<tr><td>10624</td><td>43782</td><td>Drosophila melanogaster</td><td>NfI</td><td>Nuclear factor I</td><td>CG2380|DmelCG2380|NFI</td><td>0</td><td>0</td></tr>
<tr><td>10625</td><td>43783</td><td>Drosophila melanogaster</td><td>Syt7</td><td>CG2381 gene product from transcript CG2381-RB</td><td>CG10322|CG2381|DmelCG2381|SYT7|Syt VII|dsyt7|syt7</td><td>0</td><td>0</td></tr>
<tr><td>10626</td><td>43785</td><td>Drosophila melanogaster</td><td>Rad23</td><td>CG1836 gene product from transcript CG1836-RB</td><td>CG1836|DHR23|DmelCG1836|RAD23|rad23</td><td>0</td><td>0</td></tr>
<tr><td>10627</td><td>43786</td><td>Drosophila melanogaster</td><td>CG2177</td><td>CG2177 gene product from transcript CG2177-RA</td><td>DmelCG2177</td><td>0</td><td>0</td></tr>
<tr><td>10628</td><td>43787</td><td>Drosophila melanogaster</td><td>PMCA</td><td>plasma membrane calcium ATPase</td><td>BEST:CK|BEST:CK01140|BEST:CK02288|BP1021|CG2165|CG34036|CG42314|CK02288|DmelCG42314|Dmel_CG2165|Dmel_CG34036</td><td>0</td><td>0</td></tr>
<tr><td>10629</td><td>43788</td><td>Drosophila melanogaster</td><td>Hcf</td><td>Host cell factor</td><td>CG1710|DmelCG1710|HCF|anon-WO0172774.48|dHCF</td><td>0</td><td>0</td></tr>
<tr><td>10630</td><td>43789</td><td>Drosophila melanogaster</td><td>Lin29</td><td>CG2052 gene product from transcript CG2052-RC</td><td>CG10204|CG2052|DmLin29|DmelCG2052|dati</td><td>0</td><td>0</td></tr>
<tr><td>10631</td><td>43791</td><td>Drosophila melanogaster</td><td>lgs</td><td>legless</td><td>BCL9|CG2041|Dm Lgs|DmelCG2041|Lgl|Lgs</td><td>0</td><td>0</td></tr>
<tr><td>10632</td><td>43792</td><td>Drosophila melanogaster</td><td>CaMKI</td><td>Calcium/calmodulin-dependent protein kinase I</td><td>CG1495|Calcium/calmodulin-dependent protein kinase|CamKI|DmelCG1495|caMKI|camKI|dCKI</td><td>0</td><td>0</td></tr>
<tr><td>10633</td><td>43793</td><td>Drosophila melanogaster</td><td>bip2</td><td>CG2009 gene product from transcript CG2009-RA</td><td>BIP2|Bip2|CG2009|DmTAF3|DmelCG2009|TAF155|TAF3|TAFII155|TAF[[II]]155|TFIID|bip-II|dBIP2|dTAFII3|dTAF[[II]]155|i163|i31</td><td>0</td><td>0</td></tr>
<tr><td>10634</td><td>43794</td><td>Drosophila melanogaster</td><td>Asator</td><td>CG11533 gene product from transcript CG11533-RF</td><td>CG11533|CK I|CKI-like|DmelCG11533</td><td>0</td><td>0</td></tr>
<tr><td>10635</td><td>43795</td><td>Drosophila melanogaster</td><td>zfh2</td><td>Zn finger homeodomain 2</td><td>CG1449|DmelCG1449|ZFH-2|ZFH2|Zfh-2|Zfh2|zfh-2</td><td>0</td><td>0</td></tr>
<tr><td>10636</td><td>43796</td><td>Drosophila melanogaster</td><td>Thd1</td><td>CG1981 gene product from transcript CG1981-RA</td><td>1981|CG1981|DmelCG1981|G/T-mismatch-specific-thymine-DNA-glycosylase</td><td>0</td><td>0</td></tr>
<tr><td>10637</td><td>43797</td><td>Drosophila melanogaster</td><td>Pur-alpha</td><td>Purine-rich binding protein-alpha</td><td>CG1507|DmelCG1507|PURalpha|Pur-a|pur-alpha</td><td>0</td><td>0</td></tr>
<tr><td>10638</td><td>43798</td><td>Drosophila melanogaster</td><td>CG1970</td><td>CG1970 gene product from transcript CG1970-RA</td><td>DmelCG1970</td><td>0</td><td>0</td></tr>
<tr><td>10639</td><td>43799</td><td>Drosophila melanogaster</td><td>Ephrin</td><td>CG1862 gene product from transcript CG1862-RA</td><td>CG1862|DmelCG1862</td><td>0</td><td>0</td></tr>
<tr><td>10640</td><td>43800</td><td>Drosophila melanogaster</td><td>CG1909</td><td>CG1909 gene product from transcript CG1909-RA</td><td>DmelCG1909</td><td>0</td><td>0</td></tr>
<tr><td>10641</td><td>43803</td><td>Drosophila melanogaster</td><td>Eph</td><td>Eph receptor tyrosine kinase</td><td>CG1511|CT3831|DEK|Dek|Dek7|Deph|DmelCG1511|Ek7|RPTK Dek7|dek|eph</td><td>0</td><td>0</td></tr>
<tr><td>10642</td><td>43804</td><td>Drosophila melanogaster</td><td>mav</td><td>maverick</td><td>CG1901|DmelCG1901|MAV|Mav|TGF-b</td><td>0</td><td>0</td></tr>
<tr><td>10643</td><td>43805</td><td>Drosophila melanogaster</td><td>CG1732</td><td>CG1732 gene product from transcript CG1732-RA</td><td>CG1732a|CG6218|DmGAT|DmelCG1732|anon-WO0149848.11|dGAT</td><td>0</td><td>0</td></tr>
<tr><td>10644</td><td>43806</td><td>Drosophila melanogaster</td><td>CG9935</td><td>CG9935 gene product from transcript CG9935-RC</td><td>CT36399|DmelCG9935</td><td>0</td><td>0</td></tr>
<tr><td>10645</td><td>43808</td><td>Drosophila melanogaster</td><td>gw</td><td>gawky</td><td>CG11484|CG31992|CG9905|DmelCG31992|GW182|Gw|anon-EST:Posey205|dGW182</td><td>0</td><td>0</td></tr>
<tr><td>10646</td><td>43809</td><td>Drosophila melanogaster</td><td>Slip1</td><td>CG1783 gene product from transcript CG1783-RA</td><td>CG1783|DmelCG1783|dSLIP1</td><td>0</td><td>0</td></tr>
<tr><td>10647</td><td>43810</td><td>Drosophila melanogaster</td><td>CG11360</td><td>CG11360 gene product from transcript CG11360-RA</td><td>DmelCG11360|cg11360</td><td>0</td><td>0</td></tr>
<tr><td>10648</td><td>43811</td><td>Drosophila melanogaster</td><td>myoglianin</td><td>CG1838 gene product from transcript CG1838-RD</td><td>BcDNA:LD02307|CG1838|DmelCG1838|MYO|Myo|Myoglianin|myg</td><td>0</td><td>0</td></tr>
<tr><td>10649</td><td>43812</td><td>Drosophila melanogaster</td><td>ey</td><td>eyeless</td><td>CG1464|DPax-6|DmelCG1464|EY|EYEL|Ey|Ey/Pax6|Pax-6|Pax6|eye|l(4)102CDh|l(4)102CDr|l(4)33|pax6</td><td>0</td><td>0</td></tr>
<tr><td>10650</td><td>43814</td><td>Drosophila melanogaster</td><td>bt</td><td>bent</td><td>39c-18|Bt|CG10285|CG1479|CG32019|CT3598|CT8086|DmelCG32019|Prj|l(2)2|l(2)23|l(4)102CDa|l(4)2|l(4)21|l(4)23|l(4)37|l(4)38|l(4)PT-2|titin</td><td>0</td><td>0</td></tr>
<tr><td>10651</td><td>43816</td><td>Drosophila melanogaster</td><td>MED26</td><td>Mediator complex subunit 26</td><td>ARC 70|Arc70|CG1793|CG1823|DmelCG1793|Med26|dARC70</td><td>0</td><td>0</td></tr>
<tr><td>10652</td><td>43819</td><td>Drosophila melanogaster</td><td>pho</td><td>pleiohomeotic</td><td>CG17743|DmelCG17743|PHO|Pho|YY1|dYY1|l(4)102EFc|l(4)29|l(4)BU-2|l(4)OC-1|l(4)pho</td><td>0</td><td>0</td></tr>
<tr><td>10653</td><td>43820</td><td>Drosophila melanogaster</td><td>Kif3C</td><td>CG17461 gene product from transcript CG17461-RA</td><td>CG17459|CG17461|CP38609|DmKIF3C|DmelCG17461|KIF24|KIF3C|KIF3Cdm|Kif3c</td><td>0</td><td>0</td></tr>
</table>

<table>
<tr><td>10654</td><td>43822</td><td>Drosophila melanogaster</td><td>CG11155</td><td>CG11155 gene product from transcript CG11155-RA</td><td>CT30863|DmelCG11155</td><td>0</td><td>0</td></tr>
<tr><td>10655</td><td>43823</td><td>Drosophila melanogaster</td><td>Arf102F</td><td>ADP ribosylation factor 102F</td><td>ARF2|ARFII|Arf102E|CG11027|DmelCG11027|dARFII</td><td>0</td><td>0</td></tr>
<tr><td>10656</td><td>43824</td><td>Drosophila melanogaster</td><td>cals</td><td>calsyntenin-1</td><td>CALS|CG11059|CT30877|Cad102F|Cals|DCad102F|DmelCG11059</td><td>0</td><td>0</td></tr>
<tr><td>10657</td><td>43825</td><td>Drosophila melanogaster</td><td>sv</td><td>shaven</td><td>CG11049|Cat|D-Pax2|D-pax-2|D-pax2|DPax-2|DmelCG11049|Dpax-2|Dpax2|PAX2|Pax2|Pax2/5/8|Pax258|Sv|d-Pax2|dPax2|dPax258|en(lz)4G/l|l(4)40|pax2/sparkling|pol|shaven/sparkling|spa|spa-sv</td><td>0</td><td>0</td></tr>
<tr><td>10658</td><td>43826</td><td>Drosophila melanogaster</td><td>Actbeta</td><td>Activin-beta</td><td>Act|Activin|CG11062|DmelCG11062|actbeta|activin-beta|anon-WO02059370.79|beta-Activin|dACT|dAct|dact</td><td>0</td><td>0</td></tr>
<tr><td>10659</td><td>43827</td><td>Drosophila melanogaster</td><td>Rfabg</td><td>Retinoid- and fatty acid-binding glycoprotein</td><td>ApoL1|ApoL2|ApoLI|ApoLII|CG11064|DRBP|DmelCG11064|Lipophorin|Lpp|RFABG|RFABP|RFBP|RfaBP|RfaBp|T3|lipophorin|rfabg</td><td>0</td><td>0</td></tr>
<tr><td>10660</td><td>43828</td><td>Drosophila melanogaster</td><td>CaMKII</td><td>Calcium/calmodulin-dependent protein kinase II</td><td>CAMKII|CAMKIIalpha|CAMKIId|CDPK1|CG18069|Ca2+/calmodulin-dependent protein kinase II|CaM|CaM II|CaM KII|CaM kinase|CaMK II|CaMK-II|Calcium/calmodulin-dependent protein kinase|Cam|Cam</td><td>0</td><td>0</td></tr>
<tr><td>10661</td><td>43829</td><td>Drosophila melanogaster</td><td>ATPsyn-beta</td><td>ATP synthase-beta</td><td>ATP|ATP-syn-B|ATP-synbeta|ATPB|ATPase beta|ATPsyn b|ATPsyn-&bgr|ATPsyn-b|CG11154|DmelCG11154|beta-ATPase</td><td>0</td><td>0</td></tr>
<tr><td>10662</td><td>43830</td><td>Drosophila melanogaster</td><td>CG11076</td><td>CG11076 gene product from transcript CG11076-RC</td><td>DmelCG11076</td><td>0</td><td>0</td></tr>
<tr><td>10663</td><td>43831</td><td>Drosophila melanogaster</td><td>CG11077</td><td>CG11077 gene product from transcript CG11077-RA</td><td>DmelCG11077</td><td>0</td><td>0</td></tr>
<tr><td>10664</td><td>43832</td><td>Drosophila melanogaster</td><td>plexA</td><td>plexin A</td><td>BcDNA:GM05237|CG11081|D-Plex A|DPlexA|DmelCG11081|Plex1|PlexA|PlexA1|plex|plex A</td><td>0</td><td>0</td></tr>
<tr><td>10665</td><td>43833</td><td>Drosophila melanogaster</td><td>toy</td><td>twin of eyeless</td><td>CG11186|DmelCG11186|Pax-6|Toy|l(4)102CDg|l(4)8</td><td>0</td><td>0</td></tr>
<tr><td>10666</td><td>43835</td><td>Drosophila melanogaster</td><td>CG11093</td><td>CG11093 gene product from transcript CG11093-RB</td><td>DmelCG11093</td><td>0</td><td>0</td></tr>
<tr><td>10667</td><td>43836</td><td>Drosophila melanogaster</td><td>CG32016</td><td>CG32016 gene product from transcript CG32016-RB</td><td>CG11097|CG11232|DmelCG32016</td><td>0</td><td>0</td></tr>
<tr><td>10668</td><td>43838</td><td>Drosophila melanogaster</td><td>mGluRA</td><td>metabotropic glutamate receptor</td><td>CAA67993.1_Dr|CG11144|DmGlu-A|DmGlu-RA|DmGluA|DmGluAR|DmGluR|DmGluRA|DmGluR[[A]]|DmGlu[[A]]R|DmelCG11144|Glu-RA|GluR|dmGluRA|mGluR</td><td>0</td><td>0</td></tr>
<tr><td>10669</td><td>43839</td><td>Drosophila melanogaster</td><td>eIF4G</td><td>eukaryotic translation initiation factor 4G</td><td>CG10811|DmelCG10811|EIF4G|Eif4G|deIF4G|eIF-4G|eIF4g|p200</td><td>0</td><td>0</td></tr>
<tr><td>10670</td><td>43841</td><td>Drosophila melanogaster</td><td>unc-13</td><td>CG2999 gene product from transcript CG2999-RD</td><td>CG2999|DUNC-13|DmelCG2999|Dunc-13|Dunc13|Munc-13|Munc-13/UNC-13|UNC-13|UNC-13-B|Unc-13|dUNC-13|dUnc-13|drunc13|dunc-13|l(4)ry16</td><td>0</td><td>0</td></tr>
<tr><td>10671</td><td>43842</td><td>Drosophila melanogaster</td><td>CG11148</td><td>CG11148 gene product from transcript CG11148-RD</td><td>DmelCG11148</td><td>0</td><td>0</td></tr>
<tr><td>10672</td><td>43843</td><td>Drosophila melanogaster</td><td>fd102C</td><td>forkhead domain 102C</td><td>CG11152|Dmcg11152|DmelCG11152</td><td>0</td><td>0</td></tr>
<tr><td>10673</td><td>43844</td><td>Drosophila melanogaster</td><td>Sox102F</td><td>CG11153 gene product from transcript CG11153-RB</td><td>CG11153|DmelCG11153|SOX102D|SOXD|Sox D|Sox102D|SoxD</td><td>0</td><td>0</td></tr>
<tr><td>10674</td><td>43845</td><td>Drosophila melanogaster</td><td>CG11231</td><td>CG11231 gene product from transcript CG11231-RA</td><td>DmelCG11231</td><td>0</td><td>0</td></tr>
<tr><td>10675</td><td>43851</td><td>Drosophila melanogaster</td><td>A</td><td>Abnormal abdomen</td><td>-</td><td>0</td><td>0</td></tr>
<tr><td>10676</td><td>43852</td><td>Drosophila melanogaster</td><td>a</td><td>arc</td><td>Arc|CG13505|CG6741|DmelCG6741|bran</td><td>0</td><td>0</td></tr>
<tr><td>10677</td><td>43856</td><td>Drosophila melanogaster</td><td>nej</td><td>nejire</td><td>CBP|CBP/p300|CBP_|CG15319|CG15321|Cbp|Crbp|DmelCG15319|Dmel_CG15321|Nej|Nejire|anon-WO0147981.11|anon-WO03040301.89|cbp|dCBP|dKAT3|dmCBP|l(1)G0112|l(1)G0350|l(1)G0470|nej CE</td><td>0</td><td>0</td></tr>
<tr><td>10678</td><td>43860</td><td>Drosophila melanogaster</td><td>eppy</td><td>epilated periorbitally</td><td>-</td><td>0</td><td>0</td></tr>
<tr><td>10679</td><td>43862</td><td>Drosophila melanogaster</td><td>bl</td><td>bancal</td><td>CG13425|DmelCG13425|Hrb57A|Q18|dhnRNP K|l(2)k08305</td><td>0</td><td>0</td></tr>
<tr><td>10680</td><td>43864</td><td>Drosophila melanogaster</td><td>RpL10</td><td>Ribosomal protein L10</td><td>BcDNA:LD24589|CG17521|DQM|DmelCG17521|L10|M(3)80|Qm|anon-EST:Posey179</td><td>0</td><td>0</td></tr>
<tr><td>10681</td><td>43867</td><td>Drosophila melanogaster</td><td>Atf3</td><td>Activating transcription factor 3</td><td>A3-3|ATF3|CG11405|DmelCG11405|EG:33C11.1|atf3|dATF3</td><td>0</td><td>0</td></tr>
<tr><td>10682</td><td>43869</td><td>Drosophila melanogaster</td><td>amx</td><td>almondex</td><td>BcDNA:GH02974|CG12127|DmelCG12127|fs(1)amx</td><td>0</td><td>0</td></tr>
<tr><td>10683</td><td>43870</td><td>Drosophila melanogaster</td><td>bis</td><td>bistre</td><td>-</td><td>0</td><td>0</td></tr>
<tr><td>10684</td><td>43872</td><td>Drosophila melanogaster</td><td>Ppn</td><td>Papilin</td><td>CG1540|CG18436|CG33103|CT3186|CT41988|DmelCG33103|ppl|ppn</td><td>0</td><td>0</td></tr>
<tr><td>10685</td><td>43873</td><td>Drosophila melanogaster</td><td>ras</td><td>raspberry</td><td>CG11485|CG1799|DmelCG1799|EP(X)1093|IMPDH|IMPdH|LD02673|LD06825|l(1)9Eb|l(1)G0002|l(1)G0056|l(1)G0098|l(1)G0127|l(1)G0238|l(1)G0351|l(1)G0380|l(1)G0388|l(1)G0391|l(1)G0436|l(1)G0482|ras-l|r</td><td>0</td><td>0</td></tr>
<tr><td>10686</td><td>43875</td><td>Drosophila melanogaster</td><td>Ret</td><td>Ret oncogene</td><td>CG1061|CG14396|D-ret|DRET|DRet|DmHD-59|DmelCG14396|Dret|HD-59|MEN2|RET|Reto|dRET|dRet|ret</td><td>0</td><td>0</td></tr>
<tr><td>10687</td><td>43877</td><td>Drosophila melanogaster</td><td>muc</td><td>midline uncoordinated</td><td>-</td><td>0</td><td>0</td></tr>
<tr><td>10688</td><td>43880</td><td>Drosophila melanogaster</td><td>deg</td><td>degarni</td><td>-</td><td>0</td><td>0</td></tr>
<tr><td>10689</td><td>43881</td><td>Drosophila melanogaster</td><td>dkn</td><td>deathknell</td><td>-</td><td>0</td><td>0</td></tr>
<tr><td>10690</td><td>43882</td><td>Drosophila melanogaster</td><td>fez</td><td>freeze</td><td>-</td><td>0</td><td>0</td></tr>
<tr><td>10691</td><td>43883</td><td>Drosophila melanogaster</td><td>hrp</td><td>hyperpolarizing receptor potential</td><td>-</td><td>0</td><td>0</td></tr>
<tr><td>10692</td><td>43884</td><td>Drosophila melanogaster</td><td>oho51</td><td>overgrown hematopoietic organs 51</td><td>l(2)211/15|l(2)211/5</td><td>0</td><td>0</td></tr>
<tr><td>10693</td><td>43885</td><td>Drosophila melanogaster</td><td>tru</td><td>troupa</td><td>-</td><td>0</td><td>0</td></tr>
<tr><td>10694</td><td>43886</td><td>Drosophila melanogaster</td><td>ttp</td><td>tip-tap</td><td>-</td><td>0</td><td>0</td></tr>
<tr><td>10695</td><td>43888</td><td>Drosophila melanogaster</td><td>atx</td><td>abortex</td><td>-</td><td>0</td><td>0</td></tr>
<tr><td>10696</td><td>43889</td><td>Drosophila melanogaster</td><td>elg</td><td>elongatus</td><td>-</td><td>0</td><td>0</td></tr>
<tr><td>10697</td><td>43890</td><td>Drosophila melanogaster</td><td>obx</td><td>obelix</td><td>-</td><td>0</td><td>0</td></tr>
<tr><td>10698</td><td>43891</td><td>Drosophila melanogaster</td><td>rtg</td><td>rotated genitalia</td><td>-</td><td>0</td><td>0</td></tr>
<tr><td>10699</td><td>43892</td><td>Drosophila melanogaster</td><td>sif</td><td>still life</td><td>CG32414|CG34418|CG5256|CG5406|DmelCG34418|Dmel_CG32414|Dmel_CG5406|RhoGEF|SIF|Sif|slf</td><td>0</td><td>0</td></tr>
<tr><td>10700</td><td>43895</td><td>Drosophila melanogaster</td><td>E23</td><td>Early gene at 23</td><td>CG15410|CG3327|DmelCG3327</td><td>0</td><td>0</td></tr>
<tr><td>10701</td><td>43896</td><td>Drosophila melanogaster</td><td>cro</td><td>croaker</td><td>-</td><td>0</td><td>0</td></tr>
<tr><td>10702</td><td>43897</td><td>Drosophila melanogaster</td><td>vid</td><td>visual system disorganizer</td><td>-</td><td>0</td><td>0</td></tr>
<tr><td>10703</td><td>43898</td><td>Drosophila melanogaster</td><td>pla</td><td>palla</td><td>-</td><td>0</td><td>0</td></tr>
<tr><td>10704</td><td>43900</td><td>Drosophila melanogaster</td><td>Crg-1</td><td>Circadianly Regulated Gene</td><td>CG32788|Crg1|DmelCG32788|crg1</td><td>0</td><td>0</td></tr>
<tr><td>10705</td><td>43901</td><td>Drosophila melanogaster</td><td>chb</td><td>chromosome bows</td><td>0686/07|BcDNA:LD31673|CG32435|CLASP|Chb|DMAP160|DmelCG32435|MAST|MAST/Orbit|MESR7|Mast|NEST:bs13a06|NEST:bs17d05|NEST:bs28g05|Orbit|Orbit/MAST|Orbit/Mast|anon-WO0104295|c</td><td>0</td><td>0</td></tr>
<tr><td>10706</td><td>43904</td><td>Drosophila melanogaster</td><td>SNF1A</td><td>SNF1A/AMP-activated protein kinase</td><td>AMPK|AMPKalpha|CG3051|DmAMPK alpha|DmelCG3051|EG:132E8.2|FBgn0023169|Gprk-4|Gprk4|ampkalpha|dAMPKa|dAMPKalpha|snf1a</td><td>0</td><td>0</td></tr>
<tr><td>10707</td><td>43905</td><td>Drosophila melanogaster</td><td>syd</td><td>sunday driver</td><td>CG8110|DmelCG8110|JIP-3</td><td>0</td><td>0</td></tr>
<tr><td>10708</td><td>43906</td><td>Drosophila melanogaster</td><td>skd</td><td>skuld</td><td>CG9936|DmelCG9936|LD28662|MED13|Med13|Pap|Pap/Trap|SPH203|Scad78|Skd|Skd/Med13|Skd/Pap/Bli|Srb9/AMIB/PAP/TRAP240|TRAP240|Trap240|bli|bls|dMED13|dTRAP240|dTrap240|dmMED13|l(</td><td>0</td><td>0</td></tr>
<tr><td>10709</td><td>43907</td><td>Drosophila melanogaster</td><td>eel</td><td>eel</td><td>-</td><td>0</td><td>0</td></tr>
<tr><td>10710</td><td>43908</td><td>Drosophila melanogaster</td><td>ami</td><td>anosmic</td><td>-</td><td>0</td><td>0</td></tr>
<tr><td>10711</td><td>43911</td><td>Drosophila melanogaster</td><td>oop</td><td>out of place</td><td>-</td><td>0</td><td>0</td></tr>
<tr><td>10712</td><td>43912</td><td>Drosophila melanogaster</td><td>SF1</td><td>Splicing factor 1</td><td>CG5836|DmelCG5836|cg5836|dSF1|p70</td><td>0</td><td>0</td></tr>
<tr><td>10713</td><td>43913</td><td>Drosophila melanogaster</td><td>Gyk</td><td>Glycerol kinase</td><td>CG18374|DmelCG18374</td><td>0</td><td>0</td></tr>
<tr><td>10714</td><td>43914</td><td>Drosophila melanogaster</td><td>maz</td><td>matzerath</td><td>-</td><td>0</td><td>0</td></tr>
<tr><td>10715</td><td>43916</td><td>Drosophila melanogaster</td><td>rok</td><td>Rho-kinase</td><td>CG9774|DROK|DRhk|DRok|DmelCG9774|Drok|ROCK|ROCK-1|ROCK1|ROK|ROKalpha/beta|Rhk|RhoK|Rock|Rok|dROK|dRok|drok</td><td>0</td><td>0</td></tr>
<tr><td>10716</td><td>43918</td><td>Drosophila melanogaster</td><td>noe</td><td>CG32172 gene product from transcript CG32172-RC</td><td>CG32172|DmelCG32172|Noe|anon-EST:Posey101|anon-EST:Posey151|anon-EST:Posey243|anon-EST:Posey292|anon-EST:Posey35|anon-WO02059370.51</td><td>0</td><td>0</td></tr>
<tr><td>10717</td><td>43919</td><td>Drosophila melanogaster</td><td>mog</td><td>mogura</td><td>mgr</td><td>0</td><td>0</td></tr>
<tr><td>10718</td><td>43923</td><td>Drosophila melanogaster</td><td>axo</td><td>axotactin</td><td>CG13715|CG13717|CG18296|CG32234|CG42664|CG43225|CT1277|DmelCG43225|Dmel_CG13715|Dmel_CG13717|Dmel_CG18296|Dmel_CG32234|Dmel_CG42664</td><td>0</td><td>0</td></tr>
<tr><td>10719</td><td>43924</td><td>Drosophila melanogaster</td><td>jim</td><td>CG11352 gene product from transcript CG11352-RB</td><td>BcDNA:SD14927|CG11352|DmelCG11352|Jim|OVK|ovfc.K|ovk</td><td>0</td><td>0</td></tr>
<tr><td>10720</td><td>43926</td><td>Drosophila melanogaster</td><td>fan</td><td>farinelli</td><td>CG7919|DmelCG7919</td><td>0</td><td>0</td></tr>
<tr><td>10721</td><td>43927</td><td>Drosophila melanogaster</td><td>fal</td><td>falten</td><td>CG9670|DmelCG9670</td><td>0</td><td>0</td></tr>
<tr><td>10722</td><td>43928</td><td>Drosophila melanogaster</td><td>chm</td><td>chameau</td><td>CG5229|Chm|DmelCG5229|HAT|HAT1|dmHAG404</td><td>0</td><td>0</td></tr>
<tr><td>10723</td><td>43930</td><td>Drosophila melanogaster</td><td>prc</td><td>pericardin</td><td>BEST:CK01593|CG5700|CK01593|DmelCG5700|EC11|Pc|Prc|pc</td><td>0</td><td>0</td></tr>
<tr><td>10724</td><td>43932</td><td>Drosophila melanogaster</td><td>yew</td><td>yew</td><td>-</td><td>0</td><td>0</td></tr>
<tr><td>10725</td><td>43934</td><td>Drosophila melanogaster</td><td>gom</td><td>gomdanji</td><td>CG6727|DmelCG6727</td><td>0</td><td>0</td></tr>
<tr><td>10726</td><td>43935</td><td>Drosophila melanogaster</td><td>NtR</td><td>CG6698 gene product from transcript CG6698-RA</td><td>CG6698|DNtR|DmelCG6698|anon-WO0138359.3</td><td>0</td><td>0</td></tr>
<tr><td>10727</td><td>43936</td><td>Drosophila melanogaster</td><td>Men-b</td><td>Malic enzyme b</td><td>CG5889|DmelCG5889|MDH|Mdh|anon-EST:Posey79</td><td>0</td><td>0</td></tr>
<tr><td>10728</td><td>43938</td><td>Drosophila melanogaster</td><td>cl</td><td>clot</td><td>BcDNA:RE06767|CG11024|DmelCG11024</td><td>0</td><td>0</td></tr>
<tr><td>10729</td><td>43939</td><td>Drosophila melanogaster</td><td>Cy</td><td>Curly</td><td>l(2)23Bb</td><td>0</td><td>0</td></tr>
<tr><td>10730</td><td>43940</td><td>Drosophila melanogaster</td><td>fd</td><td>furled</td><td>-</td><td>0</td><td>0</td></tr>
<tr><td>10731</td><td>43941</td><td>Drosophila melanogaster</td><td>mh</td><td>maternal haploid</td><td>fs(1)1182|fs(1)A1182</td><td>0</td><td>0</td></tr>
<tr><td>10732</td><td>43942</td><td>Drosophila melanogaster</td><td>nw</td><td>narrow</td><td>-</td><td>0</td><td>0</td></tr>
<tr><td>10733</td><td>43943</td><td>Drosophila melanogaster</td><td>or</td><td>orange</td><td>AP-3sigma|CG3029|DmelCG3029|Or|cg3029|sigma3</td><td>0</td><td>0</td></tr>
<tr><td>10734</td><td>43944</td><td>Drosophila melanogaster</td><td>sbr</td><td>small bristles</td><td>CG1664|CG17335|CT4634|DNXF-1|Dm nxf1|DmNXF1|DmelCG1664|NXF1|NXF1-p15|PABPr|dTap|l(1)9Fd|l(1)G1|l(1)ts|l(1)ts403|nxf1|sbt</td><td>0</td><td>0</td></tr>
<tr><td>10735</td><td>43946</td><td>Drosophila melanogaster</td><td>wb</td><td>wing blister</td><td>2]]|A1|BEST:CK02229|BG:DS03792.1|BG:DS03792.2|CG15288|CG32970|CG42677|CK02229|CT35236|DLAM|DmelCG42677|Dmel_CG15288|Dmel_CG32970|LM-A/alpha2|alpha[[1|fus5|l(2)09437|l(2)34Fb|</td><td>0</td><td>0</td></tr>
<tr><td>10736</td><td>43947</td><td>Drosophila melanogaster</td><td>Bt</td><td>Branchlet</td><td>-</td><td>0</td><td>0</td></tr>
<tr><td>10737</td><td>43949</td><td>Drosophila melanogaster</td><td>Hi</td><td>Hirsute</td><td>-</td><td>0</td><td>0</td></tr>
<tr><td>10738</td><td>43950</td><td>Drosophila melanogaster</td><td>CdsA</td><td>CDP diglyceride synthetase</td><td>CDS|CG7962|DmelCG7962|cds|cdsA|eye-CDS|eye-cds</td><td>0</td><td>0</td></tr>
<tr><td>10739</td><td>43951</td><td>Drosophila melanogaster</td><td>e(r)</td><td>enhancer of rudimentary</td><td>CG1871|DmelCG1871|ER|ER_DROME</td><td>0</td><td>0</td></tr>
<tr><td>10740</td><td>43952</td><td>Drosophila melanogaster</td><td>MED20</td><td>Mediator complex subunit 20</td><td>CG18267|CG18780|DmelCG18780|Med20|Srb2/TRFP|Tmr|TrfP|Trfp|dTrap26|dTrfp|l(2)28DE|med20|p29</td><td>0</td><td>0</td></tr>
<tr><td>10741</td><td>43953</td><td>Drosophila melanogaster</td><td>stld</td><td>stalled</td><td>-</td><td>0</td><td>0</td></tr>
<tr><td>10742</td><td>43955</td><td>Drosophila melanogaster</td><td>osho</td><td>one shot</td><td>-</td><td>0</td><td>0</td></tr>
<tr><td>10743</td><td>43956</td><td>Drosophila melanogaster</td><td>CU61</td><td>Cible d'Ubx 61</td><td>61|Cu61</td><td>0</td><td>0</td></tr>
<tr><td>10744</td><td>43958</td><td>Drosophila melanogaster</td><td>spag</td><td>spaghetti</td><td>CG13570|DmelCG13570|l(2)k12101</td><td>0</td><td>0</td></tr>
<tr><td>10745</td><td>43960</td><td>Drosophila melanogaster</td><td>toto</td><td>toto</td><td>-</td><td>0</td><td>0</td></tr>
<tr><td>10746</td><td>43972</td><td>Drosophila melanogaster</td><td>fldA</td><td>flight defective A</td><td>unnamed</td><td>0</td><td>0</td></tr>
<tr><td>10747</td><td>43973</td><td>Drosophila melanogaster</td><td>Lip1</td><td>Lipase 1</td><td>CG7279|DmelCG7279|Dmlip1|LIP1Dm|Lip|dLip1|lip1</td><td>0</td><td>0</td></tr>
<tr><td>10748</td><td>43974</td><td>Drosophila melanogaster</td><td>trio</td><td>CG18214 gene product from transcript CG18214-RA</td><td>0368/10|0959/14|1372/03|1386/06|BEST:LD36950|CG18214|CG9208|DTrio|DmelCG18214|M89|TRIO|Trio|UNC-73/Trio|l(3)036810|l(3)6D104|l(3)S036810|l(3)S095914|l(3)S137203|l(3)S138606|l(3)S[1372/3]|l</td><td>0</td><td>0</td></tr>
<tr><td>10749</td><td>43978</td><td>Drosophila melanogaster</td><td>Plap</td><td>Phospholipase A2 activator protein</td><td>CG5105|DmelCG5105|UFD3|dPLAP|dmPLAP|plap</td><td>0</td><td>0</td></tr>
<tr><td>10750</td><td>43980</td><td>Drosophila melanogaster</td><td>Lip2</td><td>Lipase 2</td><td>CG17116|DmelCG17116|Dmlip2|lip2</td><td>0</td><td>0</td></tr>
<tr><td>10751</td><td>43981</td><td>Drosophila melanogaster</td><td>DIP1</td><td>DISCO Interacting Protein 1</td><td>CG17686|DmelCG17686|TO34|TO67|UbxBP1|clot 10495|clot 2020|dip1|dip1a|klt</td><td>0</td><td>0</td></tr>
<tr><td>10752</td><td>43982</td><td>Drosophila melanogaster</td><td>Oamb</td><td>Octopamine receptor in mushroom bodies</td><td>CG15698|CG3856|Dm-OAMB|DmOAMB|DmOCT1B|DmOctalpha1Rb|DmelCG3856|Dmoa1|GPCR|OAMB|OAMB-1a|OAMB-AS|OAMB-K3|Oamb a|Oamb b|oamb</td><td>0</td><td>0</td></tr>
<tr><td>10753</td><td>43984</td><td>Drosophila melanogaster</td><td>Sdic1</td><td>Sperm-specific dynein intermediate chain 1</td><td>CG9580|DmelCG9580|Sdic:CG9580</td><td>0</td><td>0</td></tr>
<tr><td>10754</td><td>43986</td><td>Drosophila melanogaster</td><td>pana</td><td>pollyanna</td><td>fret</td><td>0</td><td>0</td></tr>
<tr><td>10755</td><td>43988</td><td>Drosophila melanogaster</td><td>fret</td><td>free thinker</td><td>thag</td><td>0</td><td>0</td></tr>
<tr><td>10756</td><td>43989</td><td>Drosophila melanogaster</td><td>fldB</td><td>flight defective B</td><td>AD170</td><td>0</td><td>0</td></tr>
<tr><td>10757</td><td>43990</td><td>Drosophila melanogaster</td><td>Sep5</td><td>CG2916 gene product from transcript CG2916-RB</td><td>CG2916|DmelCG2916|Sep|sep5</td><td>0</td><td>0</td></tr>
<tr><td>10758</td><td>43991</td><td>Drosophila melanogaster</td><td>Pten</td><td>CG5671 gene product from transcript CG5671-RB</td><td>CG5671|DPTEN|DmelCG5671|PTEN|PTEN3|dPTEN|dPten|dpten|pten</td><td>0</td><td>0</td></tr>
<tr><td>10759</td><td>43997</td><td>Drosophila melanogaster</td><td>jbug</td><td>jitterbug</td><td>AAF46896|CG11605|CG13525|CG30092|CG3550|DmelCG30092|Jbug</td><td>0</td><td>0</td></tr>
<tr><td>10760</td><td>43999</td><td>Drosophila melanogaster</td><td>trbl</td><td>tribbles</td><td>CG5408|DmelCG5408|trb</td><td>0</td><td>0</td></tr>
<tr><td>10761</td><td>44000</td><td>Drosophila melanogaster</td><td>Spt6</td><td>CG12225 gene product from transcript CG12225-RA</td><td>CG12225|DmelCG12225|Dspt6|SPT6|dSpt6|dspt6|l(1)G0063|spt 6|spt6</td><td>0</td><td>0</td></tr>
<tr><td>10762</td><td>44001</td><td>Drosophila melanogaster</td><td>Sucb</td><td>CG10622 gene product from transcript CG10622-RA</td><td>142911_at|CG10622|DmelCG10622|SucB|sucB|sucb</td><td>0</td><td>0</td></tr>
<tr><td>10763</td><td>44002</td><td>Drosophila melanogaster</td><td>Sras</td><td>severas</td><td>CG4852|DmelCG4852|FAC2_DROME</td><td>0</td><td>0</td></tr>
<tr><td>10764</td><td>44005</td><td>Drosophila melanogaster</td><td>NHP2</td><td>CG5258 gene product from transcript CG5258-RA</td><td>CG5258|DmelCG5258|FBgn0029148</td><td>0</td><td>0</td></tr>
</table>

<table>
<tr><th></th><th></th><th></th><th></th><th></th><th></th><th></th><th></th></tr>
<tr><td>10765</td><td>44006</td><td>Drosophila melanogaster</td><td>Fad2</td><td>CG7923 gene product from transcript CG7923-RA</td><td>CG7923|DmelCG7923|desatF|fad|fad2</td><td>0</td><td>0</td></tr>
<tr><td>10766</td><td>44007</td><td>Drosophila melanogaster</td><td>COQ7</td><td>CG14437 gene product from transcript CG14437-RA</td><td>CG14437|Dclk1|DmelCG14437</td><td>0</td><td>0</td></tr>
<tr><td>10767</td><td>44008</td><td>Drosophila melanogaster</td><td>Hex-C</td><td>Hexokinase C</td><td>CG8094|CT22069|DM2|DmelCG8094|Fk|HEX|HEX-3|HEX-C|HK|Hex|Hex-3|Hex-c|Hk</td><td>0</td><td>0</td></tr>
<tr><td>10768</td><td>44009</td><td>Drosophila melanogaster</td><td>pcv</td><td>posterior crossvein</td><td>-</td><td>0</td><td>0</td></tr>
<tr><td>10769</td><td>44010</td><td>Drosophila melanogaster</td><td>Pgm</td><td>Phosphogluconate mutase</td><td>CG5165|DmelCG5165|PGM|PGM-1|Pgm-1|pgm</td><td>0</td><td>0</td></tr>
<tr><td>10770</td><td>44011</td><td>Drosophila melanogaster</td><td>pwn</td><td>pawn</td><td>CG11101|CT31067|DmelCG11101|l(2)pwn</td><td>0</td><td>0</td></tr>
<tr><td>10771</td><td>44012</td><td>Drosophila melanogaster</td><td>seg</td><td>short egg</td><td>-</td><td>0</td><td>0</td></tr>
<tr><td>10772</td><td>44013</td><td>Drosophila melanogaster</td><td>sls</td><td>sallimus</td><td>0020/01|CG18242|CG18245|CG18857|CG1915|CT41299|D-Titin|D-titin|DmelCG1915|KZ|Ket|MCP|SLS|Sls|Titin|anon-CREST|d-titin|ket|kettin|l(3)62Ca|l(3)Ca|l(3)S002001|l(3)dre8|l(3)j1D7|l(3)rL182|sal|sam|ti</td><td>0</td><td>0</td></tr>
<tr><td>10773</td><td>44014</td><td>Drosophila melanogaster</td><td>sol</td><td>small optic lobes</td><td>CG1391|CalpD|DmSol|DmelCG1391|PC79|SOL|Sol|nofE|w5000</td><td>0</td><td>0</td></tr>
<tr><td>10774</td><td>44015</td><td>Drosophila melanogaster</td><td>U</td><td>Upturned</td><td>-</td><td>0</td><td>0</td></tr>
<tr><td>10775</td><td>44017</td><td>Drosophila melanogaster</td><td>ads</td><td>all discs small</td><td>-</td><td>0</td><td>0</td></tr>
<tr><td>10776</td><td>44018</td><td>Drosophila melanogaster</td><td>cas</td><td>castor</td><td>CG2102|Cas|DmelCG2102|l(3)j1C2|l(3)neo33|ming</td><td>0</td><td>0</td></tr>
<tr><td>10777</td><td>44019</td><td>Drosophila melanogaster</td><td>dcm</td><td>dark carmine</td><td>d-cm</td><td>0</td><td>0</td></tr>
<tr><td>10778</td><td>44020</td><td>Drosophila melanogaster</td><td>Hup</td><td>Humeral patch</td><td>-</td><td>0</td><td>0</td></tr>
<tr><td>10779</td><td>44021</td><td>Drosophila melanogaster</td><td>nmd</td><td>no mitochondrial derivative</td><td>CG5395|DmCG5395|DmelCG5395|Msp|Msp1|anon-EST:Liang-1.79|clone 1.79|l(2)08774|ms(2)4|ms(2)ry4</td><td>0</td><td>0</td></tr>
<tr><td>10780</td><td>44025</td><td>Drosophila melanogaster</td><td>Nc94B</td><td>Neural conserved at 94B</td><td>-</td><td>0</td><td>0</td></tr>
<tr><td>10781</td><td>44027</td><td>Drosophila melanogaster</td><td>sbb</td><td>scribbler</td><td>Bks|CG5580|DmelCG5580|GM04742|Group IIa|Sbb|bks|disc6|l(2)04440|mtv|sbb/mtv</td><td>0</td><td>0</td></tr>
<tr><td>10782</td><td>44029</td><td>Drosophila melanogaster</td><td>Aly</td><td>CG1101 gene product from transcript CG1101-RA</td><td>BcDNA:LD24793|CG1101|DmREF1|DmelCG1101|REF1|cg1101|dmREF1|l(3)02267|ref1</td><td>0</td><td>0</td></tr>
<tr><td>10783</td><td>44030</td><td>Drosophila melanogaster</td><td>msn</td><td>misshapen</td><td>6286|CG16973|DMSN|DmelCG16973|HGK|JNKKKK|MESR5|MSN|Msn|NIK|Nik|l(3)03349|l(3)06946|l(3)6286|l(3)j1E2|msm</td><td>0</td><td>0</td></tr>
<tr><td>10784</td><td>44033</td><td>Drosophila melanogaster</td><td>Inr-A</td><td>Inverse-regulator-Adh</td><td>-</td><td>0</td><td>0</td></tr>
<tr><td>10785</td><td>44034</td><td>Drosophila melanogaster</td><td>l(1)x</td><td>lethal (1) x</td><td>l(x)</td><td>0</td><td>0</td></tr>
<tr><td>10786</td><td>44035</td><td>Drosophila melanogaster</td><td>spb</td><td>split brain</td><td>-</td><td>0</td><td>0</td></tr>
<tr><td>10787</td><td>44038</td><td>Drosophila melanogaster</td><td>Hf</td><td>Helical Factor</td><td>CG10658|DHF|DmelCG10658|GH21|Os9|dhf</td><td>0</td><td>0</td></tr>
<tr><td>10788</td><td>44039</td><td>Drosophila melanogaster</td><td>Pak</td><td>PAK-kinase</td><td>CG10295|D-Pak|DPAK|DPak|DmPAK1|DmelCG10295|Dpak|Dpak1|PAK|PAK1|PAK2|PaK|Pak1|dPAK|dPAK1|dPak|dpak|dpak1|p65[PAK]|pak|pak1</td><td>0</td><td>0</td></tr>
<tr><td>10789</td><td>44041</td><td>Drosophila melanogaster</td><td>hty</td><td>hypersprouty</td><td>-</td><td>0</td><td>0</td></tr>
<tr><td>10790</td><td>44043</td><td>Drosophila melanogaster</td><td>ost</td><td>odd-striped</td><td>-</td><td>0</td><td>0</td></tr>
<tr><td>10791</td><td>44044</td><td>Drosophila melanogaster</td><td>raz</td><td>razorback</td><td>-</td><td>0</td><td>0</td></tr>
<tr><td>10792</td><td>44045</td><td>Drosophila melanogaster</td><td>rip</td><td>ripcord</td><td>VIII|unnamed</td><td>0</td><td>0</td></tr>
<tr><td>10793</td><td>44046</td><td>Drosophila melanogaster</td><td>sun</td><td>stunted</td><td>BcDNA:RE19513|BcDNA:RH48911|CG9032|DmelCG9032|Sun|l(1)13Fe|l(1)XV</td><td>0</td><td>0</td></tr>
<tr><td>10794</td><td>44050</td><td>Drosophila melanogaster</td><td>Lcch2</td><td>Ligand-gated chloride channel homolog 2</td><td>LCCH2</td><td>0</td><td>0</td></tr>
<tr><td>10795</td><td>44051</td><td>Drosophila melanogaster</td><td>jal</td><td>jalapeno</td><td>-</td><td>0</td><td>0</td></tr>
<tr><td>10796</td><td>44052</td><td>Drosophila melanogaster</td><td>Shc</td><td>SHC-adaptor protein</td><td>CG3715|D-Shc|DSHC|DmelCG3715|dShc|dshc|shc</td><td>0</td><td>0</td></tr>
<tr><td>10797</td><td>44054</td><td>Drosophila melanogaster</td><td>crl</td><td>courtless</td><td>CG4443|DmelCG4443|Ubc7|col</td><td>0</td><td>0</td></tr>
<tr><td>10798</td><td>44055</td><td>Drosophila melanogaster</td><td>srg</td><td>semirough</td><td>-</td><td>0</td><td>0</td></tr>
<tr><td>10799</td><td>44056</td><td>Drosophila melanogaster</td><td>teg</td><td>tegamino</td><td>-</td><td>0</td><td>0</td></tr>
<tr><td>10800</td><td>44057</td><td>Drosophila melanogaster</td><td>veg</td><td>vegetable</td><td>CG6657|DmelCG6657</td><td>0</td><td>0</td></tr>
<tr><td>10801</td><td>44058</td><td>Drosophila melanogaster</td><td>Dot</td><td>Dorothy</td><td>CG2788|DmelCG2788</td><td>0</td><td>0</td></tr>
<tr><td>10802</td><td>44059</td><td>Drosophila melanogaster</td><td>RpL30</td><td>Ribosomal protein L30</td><td>BcDNA:RE25263|BcDNA:RH09938|CG10652|DmelCG10652|L30|RPL30|Rpl30|anon-EST:fe1D4|l(2)01265|l(2)SH0229|l(2)SH2 0229|l(2)k09918|plume</td><td>0</td><td>0</td></tr>
<tr><td>10803</td><td>44063</td><td>Drosophila melanogaster</td><td>ynd</td><td>yande</td><td>-</td><td>0</td><td>0</td></tr>
<tr><td>10804</td><td>44064</td><td>Drosophila melanogaster</td><td>Grt</td><td>Glucose repressible transcripts</td><td>-</td><td>0</td><td>0</td></tr>
<tr><td>10805</td><td>44066</td><td>Drosophila melanogaster</td><td>mng</td><td>mango</td><td>-</td><td>0</td><td>0</td></tr>
<tr><td>10806</td><td>44067</td><td>Drosophila melanogaster</td><td>ifx</td><td>iron felix</td><td>-</td><td>0</td><td>0</td></tr>
<tr><td>10807</td><td>44071</td><td>Drosophila melanogaster</td><td>Mcr</td><td>Macroglobulin complement-related</td><td>CG7586|DmelCG7586|alpha-2-Mrp|anon-28DE</td><td>0</td><td>0</td></tr>
<tr><td>10808</td><td>44072</td><td>Drosophila melanogaster</td><td>ran</td><td>CG1404 gene product from transcript CG1404-RA</td><td>AAF30287|CG1404|DmelCG1404|Ran|dran|l(1)G0075|ran10A</td><td>0</td><td>0</td></tr>
<tr><td>10809</td><td>44073</td><td>Drosophila melanogaster</td><td>peo</td><td>pendolino</td><td>pen</td><td>0</td><td>0</td></tr>
<tr><td>10810</td><td>44074</td><td>Drosophila melanogaster</td><td>oap</td><td>ombre-a-paupieres</td><td>-</td><td>0</td><td>0</td></tr>
<tr><td>10811</td><td>44079</td><td>Drosophila melanogaster</td><td>Ag5r2</td><td>Antigen 5-related 2</td><td>Agr2|CG9540|DmelCG9540</td><td>0</td><td>0</td></tr>
<tr><td>10812</td><td>44082</td><td>Drosophila melanogaster</td><td>tondo</td><td>tondo</td><td>ton</td><td>0</td><td>0</td></tr>
<tr><td>10813</td><td>44083</td><td>Drosophila melanogaster</td><td>tak</td><td>takahe</td><td>bee</td><td>0</td><td>0</td></tr>
<tr><td>10814</td><td>44084</td><td>Drosophila melanogaster</td><td>chi</td><td>chianti</td><td>-</td><td>0</td><td>0</td></tr>
<tr><td>10815</td><td>44086</td><td>Drosophila melanogaster</td><td>Dmn</td><td>Dynamitin</td><td>CG8269|DmelCG8269|P50|dDyn|dmn|dyna|l(2)k16109|l(2)k16218|p50|p50/Dmn|p50/dynamitin</td><td>0</td><td>0</td></tr>
<tr><td>10816</td><td>44087</td><td>Drosophila melanogaster</td><td>Rs1</td><td>CG2173 gene product from transcript CG2173-RA</td><td>153844_at|BcDNA:GM05306|CG2173|DmRH21|DmelCG2173|Drs1|cg2173|l(2)k09514</td><td>0</td><td>0</td></tr>
<tr><td>10817</td><td>44088</td><td>Drosophila melanogaster</td><td>dc</td><td>dosach</td><td>dos</td><td>0</td><td>0</td></tr>
<tr><td>10818</td><td>44090</td><td>Drosophila melanogaster</td><td>Scf</td><td>Scruffy</td><td>-</td><td>0</td><td>0</td></tr>
<tr><td>10819</td><td>44094</td><td>Drosophila melanogaster</td><td>Ocu</td><td>Oculess</td><td>-</td><td>0</td><td>0</td></tr>
<tr><td>10820</td><td>44095</td><td>Drosophila melanogaster</td><td>fus</td><td>fusilli</td><td>CG8205|DmelCG8205|E(cact[E10])7|cg8205</td><td>0</td><td>0</td></tr>
<tr><td>10821</td><td>44096</td><td>Drosophila melanogaster</td><td>wit</td><td>wishful thinking</td><td>1262/15|ALK3/BMPRII|CG10776|DmelCG10776|SE20|STK-D|Stk-D|WIT|Wit|l(3)64Aa|l(3)S126215|l(3)SH12</td><td>0</td><td>0</td></tr>
<tr><td>10822</td><td>44097</td><td>Drosophila melanogaster</td><td>sns</td><td>sticks and stones</td><td>43-49|CG12495|CG13752|CG13753|CG13754|CG13755|CG18464|CG2385|CG33141|CG8278|CT33230|CT33232|DmelCG33141|SNS|Sens|Sns|rost|sns20</td><td>0</td><td>0</td></tr>
<tr><td>10823</td><td>44098</td><td>Drosophila melanogaster</td><td>rasp</td><td>CG11495 gene product from transcript CG11495-RA</td><td>7F21|CG11495|DmelCG11495|RASP|Rasp|cmn|cnm|l(3)63Bg|l(3)M47|l(3)SG3|l(3)m47|rsp|sig/ski|sit|ski|ski/sit</td><td>0</td><td>0</td></tr>
<tr><td>10824</td><td>44099</td><td>Drosophila melanogaster</td><td>grr</td><td>grater</td><td>-</td><td>0</td><td>0</td></tr>
<tr><td>10825</td><td>44100</td><td>Drosophila melanogaster</td><td>Patj</td><td>CG12021 gene product from transcript CG12021-RC</td><td>BcDNA:LD22238|CG12021|D-Patj|DLT|DPATJ|DPatj|Dlt|DmelCG12021|Dpatj|PATJ|PaTJ|PatJ|Patj/Dlt|dPATJ|dPatj|dlt|dlt:discs lost|patj</td><td>0</td><td>0</td></tr>
<tr><td>10826</td><td>44104</td><td>Drosophila melanogaster</td><td>nate</td><td>not enough anterior extension</td><td>-</td><td>0</td><td>0</td></tr>
<tr><td>10827</td><td>44105</td><td>Drosophila melanogaster</td><td>jok</td><td>just odd knobs</td><td>-</td><td>0</td><td>0</td></tr>
<tr><td>10828</td><td>44108</td><td>Drosophila melanogaster</td><td>Optix</td><td>CG18455 gene product from transcript CG18455-RA</td><td>CG18455|D-Six3|DmelCG18455|Dsix3|OPTIX|Six3|anon-WO0153538.79|opt|optix|opx</td><td>0</td><td>0</td></tr>
<tr><td>10829</td><td>44109</td><td>Drosophila melanogaster</td><td>tbi</td><td>tibi</td><td>-</td><td>0</td><td>0</td></tr>
<tr><td>10830</td><td>44110</td><td>Drosophila melanogaster</td><td>Mgstl</td><td>Microsomal glutathione S-transferase-like</td><td>BcDNA:RE10696|BcDNA:RH59553|CG1742|DmelCG1742|Mgst1</td><td>0</td><td>0</td></tr>
<tr><td>10831</td><td>44111</td><td>Drosophila melanogaster</td><td>slpr</td><td>slipper</td><td>CG2272|D-MLK|DmelCG2272|JNKKK|MLK|MLK2|Mlk2|Slpr|dMLK|dMLK2</td><td>0</td><td>0</td></tr>
<tr><td>10832</td><td>44115</td><td>Drosophila melanogaster</td><td>Rgl</td><td>Ral guanine nucleotide exchange factor</td><td>CG8865|DRgl|DmelCG8865|RalGDS|dRGL|rgl</td><td>0</td><td>0</td></tr>
<tr><td>10833</td><td>44117</td><td>Drosophila melanogaster</td><td>bgm</td><td>bubblegum</td><td>2a9hom|BG:DS01514.2|BG:DS0514.2|CG4501|DmelCG4501</td><td>0</td><td>0</td></tr>
<tr><td>10834</td><td>44118</td><td>Drosophila melanogaster</td><td>vih</td><td>vihar</td><td>CG10682|DmelCG10682|anon-WO0172774.54|anon-WO0172774.55|anon-WO0172774.56</td><td>0</td><td>0</td></tr>
<tr><td>10835</td><td>44120</td><td>Drosophila melanogaster</td><td>cof</td><td>condensed figures</td><td>-</td><td>0</td><td>0</td></tr>
<tr><td>10836</td><td>44121</td><td>Drosophila melanogaster</td><td>TotA</td><td>Turandot A</td><td>CG31509|DmelCG31509|Tot|Tot-A|Turt|tot|totA</td><td>0</td><td>0</td></tr>
<tr><td>10837</td><td>44122</td><td>Drosophila melanogaster</td><td>ren</td><td>renegade</td><td>-</td><td>0</td><td>0</td></tr>
<tr><td>10838</td><td>44123</td><td>Drosophila melanogaster</td><td>bls</td><td>bractless</td><td>-</td><td>0</td><td>0</td></tr>
<tr><td>10839</td><td>44125</td><td>Drosophila melanogaster</td><td>ETH</td><td>Ecdysis triggering hormone</td><td>CG18105|DmETH|DmelCG18105|Drm-ETH-1|Drm-ETH-2|ETH-1|ETH1|ETH2|eth</td><td>0</td><td>0</td></tr>
<tr><td>10840</td><td>44126</td><td>Drosophila melanogaster</td><td>AlstR</td><td>Allatostatin Receptor</td><td>CG2872|D.AlstR1|DAR-1|DGR|DmelCG2872|EG:121E7.2|GR</td><td>0</td><td>0</td></tr>
<tr><td>10841</td><td>44127</td><td>Drosophila melanogaster</td><td>mab-21</td><td>CG4746 gene product from transcript CG4746-RA</td><td>CG4746|DmelCG4746|mab-2</td><td>0</td><td>0</td></tr>
<tr><td>10842</td><td>44128</td><td>Drosophila melanogaster</td><td>CG34441</td><td>CG34441 gene product from transcript CG34441-RA</td><td>CG18042|DmelCG34441|Dmel_CG18042|Dromel_CG18042_FBtr0079695_lmg_mORF</td><td>0</td><td>0</td></tr>
<tr><td>10843</td><td>44129</td><td>Drosophila melanogaster</td><td>hbs</td><td>hibris</td><td>CG7449|CT22841|DmelCG7449|Hbs|icon</td><td>0</td><td>0</td></tr>
<tr><td>10844</td><td>44131</td><td>Drosophila melanogaster</td><td>Mkrn1</td><td>Makorin 1</td><td>CG7184|DmelCG7184|MKRN1</td><td>0</td><td>0</td></tr>
<tr><td>10845</td><td>44132</td><td>Drosophila melanogaster</td><td>slmo</td><td>slowmo</td><td>CG9131|CT26166|DmelCG9131|kisir</td><td>0</td><td>0</td></tr>
<tr><td>10846</td><td>44133</td><td>Drosophila melanogaster</td><td>At</td><td>Attenuated</td><td>-</td><td>0</td><td>0</td></tr>
<tr><td>10847</td><td>44134</td><td>Drosophila melanogaster</td><td>bv</td><td>brevis</td><td>bs</td><td>0</td><td>0</td></tr>
<tr><td>10848</td><td>44135</td><td>Drosophila melanogaster</td><td>ect</td><td>ectodermal</td><td>CG11965|CG6611|DROECT|Difl|DmelCG6611|lfl</td><td>0</td><td>0</td></tr>
<tr><td>10849</td><td>44136</td><td>Drosophila melanogaster</td><td>ecl</td><td>echinus-like</td><td>-</td><td>0</td><td>0</td></tr>
<tr><td>10850</td><td>44137</td><td>Drosophila melanogaster</td><td>fltB</td><td>flight defective B</td><td>-</td><td>0</td><td>0</td></tr>
<tr><td>10851</td><td>44141</td><td>Drosophila melanogaster</td><td>msf</td><td>misformed</td><td>-</td><td>0</td><td>0</td></tr>
<tr><td>10852</td><td>44142</td><td>Drosophila melanogaster</td><td>Rf</td><td>Roof</td><td>-</td><td>0</td><td>0</td></tr>
<tr><td>10853</td><td>44143</td><td>Drosophila melanogaster</td><td>slrp</td><td>slow receptor potential</td><td>-</td><td>0</td><td>0</td></tr>
<tr><td>10854</td><td>44144</td><td>Drosophila melanogaster</td><td>stw</td><td>straw</td><td>str</td><td>0</td><td>0</td></tr>
<tr><td>10855</td><td>44146</td><td>Drosophila melanogaster</td><td>tnk</td><td>tonock</td><td>-</td><td>0</td><td>0</td></tr>
<tr><td>10856</td><td>44147</td><td>Drosophila melanogaster</td><td>ef</td><td>elfin</td><td>-</td><td>0</td><td>0</td></tr>
<tr><td>10857</td><td>44148</td><td>Drosophila melanogaster</td><td>PpD5</td><td>Protein phosphatase D5</td><td>CG10138|D5|DmelCG10138|anon-WO0140519.107</td><td>0</td><td>0</td></tr>
<tr><td>10858</td><td>44149</td><td>Drosophila melanogaster</td><td>Acon</td><td>Aconitase</td><td>ACON-1|AconM|BEST:GH10550|CG9244|DmelCG9244|FBgn0010100|M-Acon|i204|i59|l(2)07054|m-acon|mAc|mAcon</td><td>0</td><td>0</td></tr>
<tr><td>10859</td><td>44150</td><td>Drosophila melanogaster</td><td>RpS15Aa</td><td>Ribosomal protein S15Aa</td><td>CG2033|DmelCG2033|M(1)11F|RpS15A|RpS15a|S15|S15Aa|anon-EST:Posey44</td><td>0</td><td>0</td></tr>
<tr><td>10860</td><td>44154</td><td>Drosophila melanogaster</td><td>Hmgs</td><td>HMG Coenzyme A synthase</td><td>CG16796|CG4311|DmelCG4311|Ov25|l(2)06214</td><td>0</td><td>0</td></tr>
<tr><td>10861</td><td>44155</td><td>Drosophila melanogaster</td><td>Sip1</td><td>SRY interacting protein 1</td><td>CG10939|DmelCG10939|EBP50/NHERF1|l(2)06373</td><td>0</td><td>0</td></tr>
<tr><td>10862</td><td>44158</td><td>Drosophila melanogaster</td><td>fos84E</td><td>fos-related gene at 84E</td><td>unnamed</td><td>0</td><td>0</td></tr>
<tr><td>10863</td><td>44160</td><td>Drosophila melanogaster</td><td>sw</td><td>short wing</td><td>17-234|CG18000|Cdic|DIC|Dic|Dic19B|Dic19C|DmelCG18000|IC|IC74|cDIC|dic|l(1)19Bb</td><td>0</td><td>0</td></tr>
<tr><td>10864</td><td>44164</td><td>Drosophila melanogaster</td><td>grb</td><td>gorbun</td><td>gor</td><td>0</td><td>0</td></tr>
<tr><td>10865</td><td>44166</td><td>Drosophila melanogaster</td><td>l(2)talc</td><td>lethal (2) tumorous antennal, labial, clypeo-labral imagi</td><td>l(2)106/22|l(2)k10622</td><td>0</td><td>0</td></tr>
<tr><td>10866</td><td>44167</td><td>Drosophila melanogaster</td><td>nok</td><td>next-of-kin</td><td>-</td><td>0</td><td>0</td></tr>
<tr><td>10867</td><td>44170</td><td>Drosophila melanogaster</td><td>fod</td><td>friend of diaphanous</td><td>-</td><td>0</td><td>0</td></tr>
<tr><td>10868</td><td>44171</td><td>Drosophila melanogaster</td><td>ptls</td><td>pathless</td><td>ptl</td><td>0</td><td>0</td></tr>
<tr><td>10869</td><td>44172</td><td>Drosophila melanogaster</td><td>chrw</td><td>chrowded</td><td>AAF47018|CG3870|Chrw|DmRabX1|DmelCG3870|RAB5c|RabX1|chr|l(2)k06908</td><td>0</td><td>0</td></tr>
<tr><td>10870</td><td>44173</td><td>Drosophila melanogaster</td><td>hoip</td><td>hoi-polloi</td><td>CG3949|DmelCG3949</td><td>0</td><td>0</td></tr>
<tr><td>10871</td><td>44175</td><td>Drosophila melanogaster</td><td>ros</td><td>rosario</td><td>fs(3)ros</td><td>0</td><td>0</td></tr>
<tr><td>10872</td><td>44180</td><td>Drosophila melanogaster</td><td>Etr1</td><td>ETR-1-like</td><td>etr-1</td><td>0</td><td>0</td></tr>
<tr><td>10873</td><td>44181</td><td>Drosophila melanogaster</td><td>I-55</td><td>screen I - clone 55</td><td>-</td><td>0</td><td>0</td></tr>
<tr><td>10874</td><td>44183</td><td>Drosophila melanogaster</td><td>ScpX</td><td>Sterol carrier protein X-related thiolase</td><td>CG17320|DmelCG17320|SCP-x</td><td>0</td><td>0</td></tr>
<tr><td>10875</td><td>44187</td><td>Drosophila melanogaster</td><td>l(1)5ABa</td><td>lethal (1) 5ABa</td><td>unnamed</td><td>0</td><td>0</td></tr>
</table>

10876	44189	Drosophila melanogaster	smi26D	smell impaired 26D	-	0	0
10877	44190	Drosophila melanogaster	smi27E	smell impaired 27E	-	0	0
10878	44191	Drosophila melanogaster	smi28E	smell impaired 28E	-	0	0
10879	44192	Drosophila melanogaster	smi45E	smell impaired 45E	-	0	0
10880	44193	Drosophila melanogaster	smi51A	smell impaired 51A	-	0	0
10881	44195	Drosophila melanogaster	smi61A	smell impaired 61A	-	0	0
10882	44196	Drosophila melanogaster	smi65A	smell impaired 65A	-	0	0
10883	44197	Drosophila melanogaster	smi79E	smell impaired 79E	-	0	0
10884	44199	Drosophila melanogaster	smi98B	smell impaired 98B	-	0	0
10885	44200	Drosophila melanogaster	tilA	touch insensitive larva A	-	0	0
10886	44201	Drosophila melanogaster	tilC	touch insensitive larva C	-	0	0
10887	44202	Drosophila melanogaster	tilD	touch insensitive larva D	-	0	0
10888	44205	Drosophila melanogaster	spen	split ends	136/24|BcDNA:GM01870|CG18497|DmelCG18497|E(E2F)2A|E(Raf)2A|E(Sev-CycE)2A|EK2-9|EY2-7|En(yan[ACT])2-7|Splitends|l(2)03350|l(2)k06805|l(2)k07612|l(2)k08102|poc|rno|spe|yip1	0	0
10889	44206	Drosophila melanogaster	oho48E	overgrown hematopoietic organs 48E	l(2)131/17	0	0
10890	44207	Drosophila melanogaster	ND23	NADH:ubiquinone reductase 23kD subunit precursor	CG3944|DmelCG3944	0	0
10891	44218	Drosophila melanogaster	Clch	Chloride channel	unnamed	0	0
10892	44219	Drosophila melanogaster	Sr-CII	Scavenger receptor class C, type II	CG8856|CT25430|DmelCG8856|SR-CII|dSR-CII	0	0
10893	44224	Drosophila melanogaster	cv-3	crossveinless 3	-	0	0
10894	44226	Drosophila melanogaster	Xbp1	X box binding protein-1	138/3|CG 9415|CG9415|DmelCG9415|Dxbp-1|XBP-1|XBP1|Xbp-1|anon-EST:Liang-1.41|clone 1.41|dXbp-1|l(2)k13803|xbp-1|xbp1	0	0
10895	44227	Drosophila melanogaster	Fatp	Fatty acid (long chain) transport protein	CG7400|DmelCG7400|Dromel_CG7400_FBtr0080133_fatp_mORF|anon-WO0121795.74|fatp|l(2)k10307	0	0
10896	44228	Drosophila melanogaster	Pdsw	CG8844 gene product from transcript CG8844-RA	BcDNA:RE65754|CG8844|DmelCG8844|anon-EST:Posey91|l(2)k10101|pdsw	0	0
10897	44231	Drosophila melanogaster	l(1)B6.1	lethal (1) B6.1	B6.1	0	0
10898	44233	Drosophila melanogaster	fray	frayed	4624|CG7693|DmelCG7693|STE20-like/SPAK|dSTRAD|l(3)07551|lag	0	0
10899	44235	Drosophila melanogaster	bon	bonus	0241/08|0249/12|0434/20|0487/06|150268_at|150269_at|Bon|Bonus|CG15687|CG5206|DmelCG5206|anon-WO0118547.418|l(3)S043420	0	0
10900	44238	Drosophila melanogaster	sip1	septin interacting protein 1	BcDNA:GH11690|CG7238|DmelCG7238|GH11690	0	0
10901	44239	Drosophila melanogaster	quag	quagmire	-	0	0
10902	44241	Drosophila melanogaster	SM-20d	Similar to rat Smooth Muscle-20	SM-20	0	0
10903	44246	Drosophila melanogaster	roam	roaming	-	0	0
10904	44247	Drosophila melanogaster	knax	knobby axons	-	0	0
10905	44248	Drosophila melanogaster	fsf	fruitless stimulation factor	-	0	0
10906	44249	Drosophila melanogaster	dalm2	dalmatian-2	dalm	0	0
10907	44250	Drosophila melanogaster	Cpsf160	Cleavage and polyadenylation specificity factor 160	CG10110|CPSF|CPSF160|Cpsf|DmelCG10110|anon-WO0118547.217|cpsf|dCPSF-160	0	0
10908	44251	Drosophila melanogaster	RpL8	Ribosomal protein L8	CG1263|DmelCG1263|L8|M(3)62F|M(3)LS2|anon-EST:Posey5|rpL8	0	0
10909	44252	Drosophila melanogaster	qkr58C	quaking related 58C	-	0	0
10910	44253	Drosophila melanogaster	fliH	flightless H	-	0	0
10911	44254	Drosophila melanogaster	bab2	bric a brac 2	BAB2|BTB-II|Bab2|BtbII|CG13911|CG9102|DmelCG9102|bab|bab-II	0	0
10912	44257	Drosophila melanogaster	sur	schmalspur	-	0	0
10913	44258	Drosophila melanogaster	ps	pasilla	BcDNA:RE47781|CG16765|CG16776|CG16777|CG18509|CG42670|CG8144|DmelCG42670|Dmel_CG16765|Dmel_CG16777|EP(3)3406|PS|cg8144|l(3)10615	0	0
10914	44259	Drosophila melanogaster	nbs	CG6754 gene product from transcript CG6754-RD	6754|CG6754|DmelCG6754|Dnbs1|NBS|NBS1|Nbs|Nbs1|l(3)67BDp|l(3)67BDr|l(3)e77A1	0	0
10915	44260	Drosophila melanogaster	eIF-1A	Eukaryotic initiation factor 1A	CG8053|D-eIF1A|D-eIF4c|DmelCG8053|EP(3)0935|EP935|Group I|anon-EST:Liang-2.3|anon-EST:Posey4|clone 2.3|eIF-4c|eIF1A|group I	0	0
10916	44261	Drosophila melanogaster	eIF5B	CG10840 gene product from transcript CG10840-RE	A1|CG10840|CG14970|DmelCG10840|IF2|anon-WO0118547.362|cIF2|dIF2|yIF2	0	0
10917	44263	Drosophila melanogaster	Sara	Smad anchor for receptor activation	CG15667|DmelCG15667|SARA|dSARA|sara	0	0
10918	44265	Drosophila melanogaster	reggie-1	reggie-1	reggie1/flo2	0	0
10919	44266	Drosophila melanogaster	inx3	innexin 3	CG1448|Dm-inx3|DmelCG1448|INX-3|Inx3|Ix3|inx-3	0	0
10920	44268	Drosophila melanogaster	sut3	sugar transporter 3	CG17976|DmelCG17976	0	0
10921	44269	Drosophila melanogaster	sut2	sugar transporter 2	CG17975|DmelCG17975	0	0
10922	44270	Drosophila melanogaster	slug	slug	-	0	0
10923	44271	Drosophila melanogaster	xmas-2	CG32562 gene product from transcript CG32562-RA	CG32562|CG8919|DmelCG32562|Xmas-2	0	0
10924	44272	Drosophila melanogaster	tio	tiptop	CG12630|DmelCG12630|Tio	0	0
10925	44273	Drosophila melanogaster	limber	limber	-	0	0
10926	44274	Drosophila melanogaster	disp	dispatched	CG2019|Disp|DmelCG2019|anon-WO0146227.1	0	0
10927	44275	Drosophila melanogaster	SoxN	SoxNeuro	CG18024|DM64|DmelCG18024|SOX29F|SOXB1|Sox B1|Sry-rDM64|soxN	0	0
10928	44276	Drosophila melanogaster	Crtp	Caldesmon-related protein	CG13580|DmelCG13580	0	0
10929	44277	Drosophila melanogaster	abr	abero	-	0	0
10930	44279	Drosophila melanogaster	arr	arrow	Arr|BEST:CK00539|CG5912|CK00539|CT15575|Dm Arr|DmelCG5912|lrp6|LRP|LRP/Arr|LRP5/6|LRP6|l(2)k08131	0	0
10931	44281	Drosophila melanogaster	eql	equatorial-less	-	0	0
10932	44282	Drosophila melanogaster	err	erratic	l(2)31El	0	0
10933	44283	Drosophila melanogaster	Ext	Extras	-	0	0
10934	44284	Drosophila melanogaster	fltC	flight defective C	-	0	0
10935	44285	Drosophila melanogaster	fltD	flight defective D	-	0	0
10936	44286	Drosophila melanogaster	fltE	flight defective E	-	0	0
10937	44287	Drosophila melanogaster	fltF	flight defective F	-	0	0
10938	44288	Drosophila melanogaster	fltG	flight defective G	-	0	0
10939	44289	Drosophila melanogaster	flw	flapwing	CG15305|CG2096|DmPp1-9C|DmelCG2096|FLW|FLW/PP1B|PP-1|PP1|PP1 9C|PP1 b9C|PP1 beta9c|PP19C|PP1B_DROME|PP1beta|PP1beta-9C|PP1beta9C|PP1c|Pp1beta-9C|anon-WO03040301.120|l(	0	0
10940	44290	Drosophila melanogaster	hdp-b	heldup b	hdp	0	0
10941	44291	Drosophila melanogaster	Idh	Isocitrate dehydrogenase	CG7176|CT22171|DmelCG7176|IDH|IDH-NADP|Idh-NADP|idh|isocitrate dehydrogenase|l(3)L3852	0	0
10942	44292	Drosophila melanogaster	irreD	irregular optic chiasma D	-	0	0
10943	44293	Drosophila melanogaster	J	Jammed	-	0	0
10944	44294	Drosophila melanogaster	M(2)31A	Minute (2) 31A	M(2)30A|M(2)LS3|M(2)e|M(2)fs|M[[e]]|RpL13|RpL7	0	0
10945	44295	Drosophila melanogaster	min	mini	mi	0	0
10946	44296	Drosophila melanogaster	mk	murky	fs(1)M7	0	0
10947	44297	Drosophila melanogaster	ome	omega	BEST:LD21715|CG17705|CG32143|CG32145|CG42280|CG9370|DmelCG42280|Dmel_CG32143|Dmel_CG32145|LD21715	0	0
10948	44299	Drosophila melanogaster	slgB	sluggish B	-	0	0
10949	44301	Drosophila melanogaster	tbd	tiny bristloid	-	0	0
10950	44302	Drosophila melanogaster	tur	turnip	-	0	0
10951	44304	Drosophila melanogaster	ven	venation	-	0	0
10952	44305	Drosophila melanogaster	vtw	vertical wings	-	0	0
10953	44307	Drosophila melanogaster	Vha16-1	Vacuolar H[+] ATPase subunit 16-1	BcDNA:SD02875|CG3161|DmelCG3161|DucC|EP2372|MRE15|V-ATPase|Vha1|Vha16|Vhac|vha16|vha16-1	0	0
10954	44309	Drosophila melanogaster	vt	verticals	-	0	0
10955	44310	Drosophila melanogaster	crs	cru sterile	-	0	0
10956	44311	Drosophila melanogaster	gusC	gustatory C	-	0	0
10957	44312	Drosophila melanogaster	ggy	groggy	-	0	0
10958	44313	Drosophila melanogaster	Gtd	Glucose tasting-defective	GT-1	0	0
10959	44315	Drosophila melanogaster	pie	pineapple eye	CG5354|DmelCG5354|l(2)31Ek|l(2)54|mat(2)QM47|mat(2)ea-E|mat(2)earlyQM47	0	0
10960	44317	Drosophila melanogaster	CanB	Calcineurin B	BcDNA:RH11383|CG4209|CNB|Can|DmelCG4209|canB-4F|dCnB1	0	0
10961	44319	Drosophila melanogaster	ret	reticulated	Dret2|dRet	0	0
10962	44323	Drosophila melanogaster	Csr	Chaser	-	0	0
10963	44324	Drosophila melanogaster	Dms	Dromyosuppressin	CG6440|DMS|DmelCG6440|Drm-MS|Drome-MS|FLRFa|MS|Nems|cg6440|dms	0	0
10964	44325	Drosophila melanogaster	Low	Lightener of white	-	0	0
10965	44326	Drosophila melanogaster	mRpL12	mitochondrial ribosomal protein L12	BcDNA:RE53508|CG5012|DmelCG5012|L7|L7/12A|L7/L12|Mrp17|l(3)10534|l(3)j4B2|l(3)rL424|mRpL7-L12	0	0
10966	44327	Drosophila melanogaster	m11	E(spl) region transcript m11	-	0	0
10967	44331	Drosophila melanogaster	Spf	Spadefoot	-	0	0
10968	44333	Drosophila melanogaster	l(1)1P2	lethal (1) 1P2	-	0	0
10969	44334	Drosophila melanogaster	l(1)3P5	lethal (1) 3P5	-	0	0
10970	44335	Drosophila melanogaster	l(1)HA6	lethal (1) HA6	HA6	0	0
10971	44336	Drosophila melanogaster	l(1)LB5	lethal (1) LB5	LB5	0	0
10972	44337	Drosophila melanogaster	l(1)U10	lethal (1) U10	U-10	0	0
10973	44338	Drosophila melanogaster	l(3)2	lethal (3) 2	-	0	0
10974	44339	Drosophila melanogaster	imd	immune deficiency	BG5|CG5576|DmelCG5576|IMD|Imd|anon-WO0172774.166|shadok	0	0
10975	44340	Drosophila melanogaster	Nc98F	Neural conserved at 98F	NC98F	0	0
10976	44341	Drosophila melanogaster	dfk	distal of forkhead	dfh	0	0
10977	44343	Drosophila melanogaster	l(2)X	lethal (2) X	-	0	0
10978	44345	Drosophila melanogaster	tete	tete	c47|te	0	0
10979	44346	Drosophila melanogaster	trimmed	trimmed	-	0	0
10980	44349	Drosophila melanogaster	tlr-2	tld-related-2	tlr	0	0
10981	44350	Drosophila melanogaster	Chn	Cha's neighbour	chn	0	0
10982	44351	Drosophila melanogaster	Eig4F	Ecdysone-induced gene 4F	4F	0	0
10983	44352	Drosophila melanogaster	Mst23Ea	Male-specific transcript 23Ea	-	0	0
10984	44353	Drosophila melanogaster	shark	SH2 ankyrin repeat kinase	CG18247|DmelCG18247|Dtk7|SHARK|SYK/SHARK|Shark|Syk|Tk7|dtk7|l(2)W4|l(2R)W4	0	0
10985	44354	Drosophila melanogaster	biz	bizarre	SB8	0	0
10986	44355	Drosophila melanogaster	drl	derailed	CG10758|CG17348|DRL|DmelCG17348|Drl|lin|lio|lio/drl	0	0

10987	44356	Drosophila melanogaster	l(1)1Fg	lethal (1) 1Fg	4-134|D42		0	0
10988	44357	Drosophila melanogaster	l(1)1Fh	lethal (1) 1Fh	13-80		0	0
10989	44358	Drosophila melanogaster	pdg	pigment cell degeneration	-		0	0
10990	44359	Drosophila melanogaster	sda	slamdance	BcDNA:GH07466|CG5518|DmelCG5518|Sda		0	0
10991	44360	Drosophila melanogaster	Rab-RP4	Rab-related protein 4	AAF46057|CG3129|DRABR4|DRabRP4|DmRab18|DmelCG3129|Rab-r4|Rab18|Rab18/RP4		0	0
10992	44363	Drosophila melanogaster	l(1)6Ef	lethal (1) 6Ef	fs/l		0	0
10993	44364	Drosophila melanogaster	l(1)6Fd	lethal (1) 6Fd	l		0	0
10994	44367	Drosophila melanogaster	SR3-6	Suppressor of Ras85D 3-6	-		0	0
10995	44369	Drosophila melanogaster	limbo	limbo	lio		0	0
10996	44377	Drosophila melanogaster	Indf	Indifferent	-		0	0
10997	44378	Drosophila melanogaster	oro	oroshigane	E(B)2A		0	0
10998	44379	Drosophila melanogaster	moa	moa	l(2)59ABd|sac		0	0
10999	44380	Drosophila melanogaster	fok	fledgling of Klp38B	38B.11|CG10746|DmelCG10746|Fok|l(2)k07614		0	0
11000	44381	Drosophila melanogaster	Tim17b2	Translocase inner membrane 17	BG:DS01845.2|BcDNA:AT05822|BcDNA:AT13511|CG15257|DmelCG15257|Tim17|dmTim17		0	0
11001	44382	Drosophila melanogaster	Sr-CIII	Scavenger receptor class C, type III	CG31962|DmelCG31962|SR-CIII|dSR-CIII		0	0
11002	44384	Drosophila melanogaster	atg	adjacent-to-grapes	-		0	0
11003	44385	Drosophila melanogaster	Ufo	Ultra female overexpression	-		0	0
11004	44386	Drosophila melanogaster	xen	xenicid	-		0	0
11005	44387	Drosophila melanogaster	l(1)3Db	lethal (1) 3Db	X1		0	0
11006	44388	Drosophila melanogaster	auk	auk	gon		0	0
11007	44389	Drosophila melanogaster	bub	bubblewing	-		0	0
11008	44390	Drosophila melanogaster	RFeSP	Rieske iron-sulfur protein	CG7361|DmelCG7361|H|Noble|RISP|RfeSP|anon-EST:Posey219|l(2)k11704		0	0
11009	44391	Drosophila melanogaster	Nnp-1	CG12396 gene product from transcript CG12396-RA	CG12396|DmelCG12396|NNP-1|Nnp1|l(2)k07826|nnp-1		0	0
11010	44392	Drosophila melanogaster	Rpp30	RNaseP protein p30	#19/1|CG11606|DmelCG11606|RNase P|l(2)k01901		0	0
11011	44393	Drosophila melanogaster	l(2)254	lethal (2) 254		254	0	0
11012	44394	Drosophila melanogaster	l(1)L35.1	lethal (1) L35.1	L35.1		0	0
11013	44395	Drosophila melanogaster	l(1)L19.1	lethal (1) L19.1	L19.1		0	0
11014	44396	Drosophila melanogaster	l(1)J67.3	lethal (1) J67.3	J67.3		0	0
11015	44397	Drosophila melanogaster	l(1)H22.1	lethal (1) H22.1	H22.1		0	0
11016	44398	Drosophila melanogaster	l(1)D47.6	lethal (1) D47.6	D47.6		0	0
11017	44399	Drosophila melanogaster	l(1)B22.5	lethal (1) B22.5	B22.5		0	0
11018	44400	Drosophila melanogaster	l(1)B10.2	lethal (1) B10.2	-		0	0
11019	44401	Drosophila melanogaster	l(1)A36.3	lethal (1) A36.3	A36.3		0	0
11020	44402	Drosophila melanogaster	l(1)3Ai	lethal (1) 3Ai	l[4]		0	0
11021	44409	Drosophila melanogaster	Cbp80	cap binding protein 80	CBP80|CG7035|DmelCG7035|EG:84H4.3		0	0
11022	44410	Drosophila melanogaster	ned	no endurance	-		0	0
11023	44411	Drosophila melanogaster	l(1)3Bg	lethal (1) 3Bg	B2/50.8		0	0
11024	44412	Drosophila melanogaster	l(1)3Aq	lethal (1) 3Aq	1200D14		0	0
11025	44413	Drosophila melanogaster	l(1)3Ap	lethal (1) 3Ap	1200D2b		0	0
11026	44414	Drosophila melanogaster	l(1)3Ao	lethal (1) 3Ao	A2/4		0	0
11027	44415	Drosophila melanogaster	l(1)3An	lethal (1) 3An	C3/47.16		0	0
11028	44416	Drosophila melanogaster	l(1)3Am	lethal (1) 3Am	B2/45.10		0	0
11029	44417	Drosophila melanogaster	l(1)3Al	lethal (1) 3Al	C2/5.6		0	0
11030	44418	Drosophila melanogaster	l(1)3Ak	lethal (1) 3Ak	1200D15a|DCE-4|DCE-7		0	0
11031	44419	Drosophila melanogaster	l(1)3Aj	lethal (1) 3Aj	A2/3		0	0
11032	44420	Drosophila melanogaster	l(1)2Fg	lethal (1) 2Fg	AS2/11|B2/27.14		0	0
11033	44421	Drosophila melanogaster	l(1)2Ff	lethal (1) 2Ff	1200E17a		0	0
11034	44422	Drosophila melanogaster	l(1)2Ed	lethal (1) 2Ed	E3/34.12		0	0
11035	44423	Drosophila melanogaster	l(1)2Ec	lethal (1) 2Ec	D3/36.6		0	0
11036	44424	Drosophila melanogaster	l(1)2Dm	lethal (1) 2Dm	B2/35.2|C3/50.71		0	0
11037	44425	Drosophila melanogaster	l(1)2Dl	lethal (1) 2Dl	B2/34.27|C2/5.11|E3/36.11		0	0
11038	44426	Drosophila melanogaster	l(1)2Dk	lethal (1) 2Dk	1200B2		0	0
11039	44427	Drosophila melanogaster	l(1)2Dj	lethal (1) 2Dj	800D22|800F24|B3/52.19		0	0
11040	44428	Drosophila melanogaster	l(1)1Eg	lethal (1) 1Eg	DCE-17|DCE-21		0	0
11041	44431	Drosophila melanogaster	obk	obake	-		0	0
11042	44432	Drosophila melanogaster	ird5	immune response deficient 5	CG4201|DIK|DLAK|DmIKK|DmIKKBeta|DmIKKb|DmIKKbeta|DmIkkbeta|DmelCG4201|Dmikkb|Dmikkbeta|IK|IKK|IKK-beta|IKKB|IKKb|IKKbeta|IRD-5|Ik|Ikkbeta|Ird-5|Ird5|LRK|Lak|anon-89Bd|dIKK|dIKK-beta		0	0
11043	44433	Drosophila melanogaster	Hs2st	heparan sulfate 2-O-sulfotransferase	CG10234|DmHs2st|DmelCG10234|HS2ST|Hs2st-2|dHS2ST|dmHS-2OST|dmHs2st		0	0
11044	44437	Drosophila melanogaster	sec13	CG6773 gene product from transcript CG6773-RA	BEST:CK00043|BcDNA:LD03471|CG6773|CK00043|DmelCG6773|Dsec13|SEC13|Sec13|l(3)01031		0	0
11045	44438	Drosophila melanogaster	EfTuM	Elongation factor Tu mitochondrial	CG6050|DmelCG6050|efTuM|eftu-M|l(3)L4569|mtEF-Tu		0	0
11046	44441	Drosophila melanogaster	Bem46	CG18642 gene product from transcript CG18642-RA	CG18642|DmelCG18642|c1.6		0	0
11047	44446	Drosophila melanogaster	Hdac3	Histone deacetylase 3	CG2128|DHDAC3|DmHDAC3|DmelCG2128|Dromel_CG2128_FBtr0078767_hdac3_mORF|HDAC|HDAC3|dHDAC3|dmHDA402|hdac3		0	0
11048	44448	Drosophila melanogaster	scrib	scribbled	0424/05|CG31082|CG42614|CG43398|CG5462|CG5467|CT17324|DmelCG43398|Dmel_CG31082|Dmel_CG42614|Dmel_CG5462|Dmel_CG5467|MENE (3R)-F|SCR|Scb|Scrb|Scrib|ird|ird15|l(3)673|l(3)S042		0	0
11049	44451	Drosophila melanogaster	ear	ENL/AF9-related	CG4913|DmelCG4913|ENL/AF-9|ENL/AF9|dENL/AF9		0	0
11050	44474	Drosophila melanogaster	l(1)2Cg	lethal (1) 2Cg	unnamed		0	0
11051	44481	Drosophila melanogaster	Met75Cb	CG18064 gene product from transcript CG18064-RA	CG18064|DmelCG18064|bs14h01.y1		0	0
11052	44482	Drosophila melanogaster	Met75Ca	CG32197 gene product from transcript CG32197-RA	CG32197|DmelCG32197		0	0
11053	44483	Drosophila melanogaster	KaiRIA	CG18039 gene product from transcript CG18039-RA	CG18039|CT13538|DGluRIID|DKaiR-IA|DmelCG18039|Dro GluRIA|GluR-IID|GluRIA|GluRIID|Kai-RIA|brec		0	0
11054	44484	Drosophila melanogaster	Glu-RIB	Glutamate receptor IB	CG4481|DGluR-IB|DmelCG4481|Glu-R1B|GluRIb|dglur-IB		0	0
11055	44496	Drosophila melanogaster	Uba2	Smt3 activating enzyme 2	CG7528|DmSAE2|DmSae2|DmUba2|DmelCG7528|Dmuba2|L36|SAE2|Sae2|UBA2|Ubc2|dUba2|sip2|uba2		0	0
11056	44497	Drosophila melanogaster	Tollo	CG6890 gene product from transcript CG6890-RA	CG6890|CT21344|DmelCG6890|TOLL 8|Tl-8|Toll-8|dTLR8|dToll8|toll|tollo|tollo/toll-8		0	0
11057	44498	Drosophila melanogaster	Surf1	Surfeit 1	BcDNA:GH23574|CG9943|DmelCG9943		0	0
11058	44501	Drosophila melanogaster	ag	agametic	-		0	0
11059	44502	Drosophila melanogaster	amb	amber	-		0	0
11060	44503	Drosophila melanogaster	ar	abdomen rotatum	-		0	0
11061	44504	Drosophila melanogaster	bhe	broad head	-		0	0
11062	44505	Drosophila melanogaster	br	broad	2B5|2Bc|A18|BR|BR-C|BR-C Z1|BR-c|BRC|BRC-Z1|BRC-Z2|BRC-Z3|BRC-Z4|BR_C|Br|Br-C|Br-C Z2|Br-C-Z3|Br-Z4|BrC-Z1|CG11491|CG11511|CG11514|DmelCG11491|EG:123F11.1|EG:17A9.1|EG:25D2		0	0
11063	44506	Drosophila melanogaster	brc	brachymacrochaetae	C93|DCE-12		0	0
11064	44508	Drosophila melanogaster	cho	chocolate	-		0	0
11065	44509	Drosophila melanogaster	clw	clubwing	-		0	0
11066	44510	Drosophila melanogaster	cv	crossveinless	CG12410|DmelCG12410|Tsg2|cv-1|tsg2		0	0
11067	44511	Drosophila melanogaster	dd	displaced	-		0	0
11068	44512	Drosophila melanogaster	dn	doughnut	-		0	0
11069	44513	Drosophila melanogaster	CG42640	CG42640 gene product from transcript CG42640-RA	CG2952|DmePPO3|DmelCG42640|Dmel_CG2952|Dox-A3|PPO3|Pro-PO3|proPO59		0	0
11070	44514	Drosophila melanogaster	fap	female abdomen pattern	-		0	0
11071	44518	Drosophila melanogaster	lis	lisis	-		0	0
11072	44519	Drosophila melanogaster	Ki	Kinked	-		0	0
11073	44520	Drosophila melanogaster	l(1)C	lethal (1) C	-		0	0
11074	44521	Drosophila melanogaster	lac	lacquered	-		0	0
11075	44522	Drosophila melanogaster	lea	leak	CG14347|CG14348|CG5481|CG5574|CT17326|D-Robo2|D-robo2|DmelCG5481|ROBO2|Robo 2|Robo-2|Robo2|anon-EST:Liang-1.75|clone 1.75|dRobo-2|fus4|robo-2|robo2		0	0
11076	44524	Drosophila melanogaster	mdl	male diplolethal	-		0	0
11077	44525	Drosophila melanogaster	MR	Male recombination factor	MRF		0	0
11078	44527	Drosophila melanogaster	nob	no bridge	-		0	0
11079	44528	Drosophila melanogaster	pt	platinum	fs(1)M47|l(1)VA70|pa		0	0
11080	44529	Drosophila melanogaster	ptr	proximal to raf	CG2841|DmelCG2841|EG:BACH48C10.5		0	0
11081	44530	Drosophila melanogaster	gua1	guanosine 1	gua-1		0	0
11082	44531	Drosophila melanogaster	rg	rugose	AKAP|Akap550|CG15466|CG6775|DAKAP550|DmelCG6775|fas		0	0
11083	44534	Drosophila melanogaster	sam	sperm amotile	-		0	0
11084	44535	Drosophila melanogaster	slc	slim chaetae	-		0	0
11085	44536	Drosophila melanogaster	tn	thin	-		0	0
11086	44537	Drosophila melanogaster	Tu	Turned-up wing	-		0	0
11087	44538	Drosophila melanogaster	ung	uniungula	-		0	0
11088	44539	Drosophila melanogaster	vs	vesiculated	bw		0	0
11089	44540	Drosophila melanogaster	ct	cut	BcDNA:GH10590|CG11387|CT|Ct|Cut|DmelCG11387|kf|l(1)7Ba|l(1)7Bb|l(1)VE614		0	0
11090	44542	Drosophila melanogaster	Wk	Walker	-		0	0
11091	44543	Drosophila melanogaster	enc	encore	BB129|CG10847|DmelCG10847|Enc|fs(3)63F-64A		0	0
11092	44544	Drosophila melanogaster	Hnf4	Hepatocyte nuclear factor 4	CG9310|DmHNF4|DmelCG9310|HNF-4|HNF-4(D)|HNF4|HNF4alpha|Hnf-4|Hnf-4h|NR2A4|dHNF-4|dHNF4		0	0
11093	44545	Drosophila melanogaster	pch	parched	EC1|EC2|l(1)1Aa|l(1)[d]|lEC1|l[EC1]		0	0
11094	44546	Drosophila melanogaster	l(1)v3	lethal (1) v3	l(1)3		0	0
11095	44547	Drosophila melanogaster	flam	flamenco	-		0	0
11096	44548	Drosophila melanogaster	lola	longitudinals lacking	BEST:LD03274|BTB-IV|BcDNA:LD17006|BcDNA:RH31485|BtbIV|CG12052|CG18376|CG18378|CG18379|CG18380|CG18381|CG30012|CG30013|CG30014|DmelCG12052|LD03274|Lola|NEST:bs06a08|bs		0	0
11097	44549	Drosophila melanogaster	l(1)2Db	lethal (1) 2Db	l(1)107		0	0

11098	44550	Drosophila melanogaster	l(1)2Dc	lethal (1) 2Dc	l(1)405	0	0
11099	44551	Drosophila melanogaster	uni	unicorn	-	0	0
11100	44552	Drosophila melanogaster	ptl	pointless	-	0	0
11101	44554	Drosophila melanogaster	Bzd	Buzidau	CG13761|DmelCG13761|EG:BACR7C10.4	0	0
11102	44566	Drosophila melanogaster	l(1)3PP4	lethal (1) 3PP4	-	0	0
11103	44567	Drosophila melanogaster	l(1)8PP9	lethal (1) 8PP9	-	0	0
11104	44573	Drosophila melanogaster	MAPk-Ak2	MAP kinase activated protein-kinase-2	CG3086|DmMAPKAPK-2|DmelCG3086|MAPKAP|MAPKAPK-2|MK2|dMK2	0	0
11105	44585	Drosophila melanogaster	l(2)31Aa	lethal (2) 31Aa	-	0	0
11106	44586	Drosophila melanogaster	l(2)31Ab	lethal (2) 31Ab	-	0	0
11107	44587	Drosophila melanogaster	l(2)31Bb	lethal (2) 31Bb	-	0	0
11108	44588	Drosophila melanogaster	l(2)31Bc	lethal (2) 31Bc	-	0	0
11109	44589	Drosophila melanogaster	l(2)31Ca	lethal (2) 31Ca	-	0	0
11110	44590	Drosophila melanogaster	l(2)31Cb	lethal (2) 31Cb	-	0	0
11111	44591	Drosophila melanogaster	l(2)31Da	lethal (2) 31Da	-	0	0
11112	44592	Drosophila melanogaster	l(2)31Db	lethal (2) 31Db	-	0	0
11113	44593	Drosophila melanogaster	l(2)31Eb	lethal (2) 31Eb	-	0	0
11114	44594	Drosophila melanogaster	l(2)31Ec	lethal (2) 31Ec	-	0	0
11115	44595	Drosophila melanogaster	l(2)31Ed	lethal (2) 31Ed	-	0	0
11116	44596	Drosophila melanogaster	l(2)31Ee	lethal (2) 31Ee	-	0	0
11117	44597	Drosophila melanogaster	l(2)31Ef	lethal (2) 31Ef	31Ef	0	0
11118	44598	Drosophila melanogaster	l(2)31Eg	lethal (2) 31Eg	-	0	0
11119	44599	Drosophila melanogaster	l(2)31Ej	lethal (2) 31Ej	-	0	0
11120	44600	Drosophila melanogaster	l(2)31Em	lethal (2) 31Em	-	0	0
11121	44601	Drosophila melanogaster	l(2)31En	lethal (2) 31En	-	0	0
11122	44602	Drosophila melanogaster	l(2)31Eo	lethal (2) 31Eo	-	0	0
11123	44603	Drosophila melanogaster	l(2)32Aa	lethal (2) 32Aa	-	0	0
11124	44604	Drosophila melanogaster	l(3)94Fa	lethal (3) 94Fa	unnamed	0	0
11125	44605	Drosophila melanogaster	l(3)ry15	lethal (3) ry15	-	0	0
11126	44606	Drosophila melanogaster	l(3)ry50	lethal (3) ry50	-	0	0
11127	44607	Drosophila melanogaster	l(3)ry60	lethal (3) ry60	-	0	0
11128	44608	Drosophila melanogaster	l(3)ry73	lethal (3) ry73	-	0	0
11129	44609	Drosophila melanogaster	l(3)ry74	lethal (3) ry74	-	0	0
11130	44610	Drosophila melanogaster	l(3)ry84	lethal (3) ry84	-	0	0
11131	44611	Drosophila melanogaster	l(3)ry87	lethal (3) ry87	-	0	0
11132	44612	Drosophila melanogaster	l(3)ry98	lethal (3) ry98	-	0	0
11133	44613	Drosophila melanogaster	l(3)ry99	lethal (3) ry99	-	0	0
11134	44615	Drosophila melanogaster	rdgE	retinal degeneration E	-	0	0
11135	44620	Drosophila melanogaster	CG14630	CG14630 gene product from transcript CG14630-RA	B|DmelCG14630|EG:BACR7A4.9|anon-1Eb	0	0
11136	44631	Drosophila melanogaster	Ag5r	Antigen 5-related	Agr|Agr5|CG9538|DmelCG9538|agr|anon-WO0140519.74	0	0
11137	44632	Drosophila melanogaster	Cyp4e1	Cytochrome P450-4e1	4e1|CG2062|CYP4E1|DmelCG2062|P-450|P450	0	0
11138	44633	Drosophila melanogaster	God	Godzilla	-	0	0
11139	44637	Drosophila melanogaster	cutlet	CG33122 gene product from transcript CG33122-RA	CG10030|CG33122|DmelCG33122|ctl	0	0
11140	44638	Drosophila melanogaster	l(1)7Do	lethal (1) 7Do	-	0	0
11141	44639	Drosophila melanogaster	l(1)7Fg	lethal (1) 7Fg	-	0	0
11142	44640	Drosophila melanogaster	l(1)9Fi	lethal (1) 9Fi	-	0	0
11143	44641	Drosophila melanogaster	l(1)9Fj	lethal (1) 9Fj	-	0	0
11144	44642	Drosophila melanogaster	Apc	APC-like	APC|APC1|Apc1|CG1451|D-APC|D-APC1|DAPC|Dm APC1|DmelCG1451|apc|apc1|d-APC|dAPC|dAPC1|dApc	0	0
11145	44643	Drosophila melanogaster	Cnx99A	Calnexin 99A	CG11958|Cnx|DmelCG11958|cnx	0	0
11146	44645	Drosophila melanogaster	Eig82F	Ecdysone induced gene at 82F	-	0	0
11147	44646	Drosophila melanogaster	Mei66F	Meiotic 66F-67A	-	0	0
11148	44652	Drosophila melanogaster	dfc	defaced	-	0	0
11149	44653	Drosophila melanogaster	wech	CG42396 gene product from transcript CG42396-RC	CG1624|CG42396|DmelCG42396|dpld|lin-41	0	0
11150	44654	Drosophila melanogaster	furrow	furrow	-	0	0
11151	44655	Drosophila melanogaster	l(2)55Da	lethal (2) 55Da	-	0	0
11152	44656	Drosophila melanogaster	l(2)55Db	lethal (2) 55Db	-	0	0
11153	44657	Drosophila melanogaster	l(3)61Da	lethal (3) 61Da	-	0	0
11154	44658	Drosophila melanogaster	l(3)87Em	lethal (3) 87Em	-	0	0
11155	44660	Drosophila melanogaster	xmas-1	CG32561 gene product from transcript CG32561-RA	CG32561|CG8919|DmelCG32561|l(1)15Ea|m(1)3|ms(1)3|ms3|xmas	0	0
11156	44663	Drosophila melanogaster	l(1)1Bl	lethal (1) 1Bl	unnamed	0	0
11157	44668	Drosophila melanogaster	SmD1	Small ribonucleoprotein particle protein SmD1	BcDNA:RE39488|CG10753|DmelCG10753|dD1|l(2)SH3 003|l(3)SH003|smd1|snRNP|snRNP-69D|snRNP69D	0	0
11158	44669	Drosophila melanogaster	cwo	clockwork orange	AAF24476.1|CG17100|CWO|DmelCG17100|GM05287|bHLHe49	0	0
11159	44670	Drosophila melanogaster	len	leopard	-	0	0
11160	44672	Drosophila melanogaster	Lk6	CG17342 gene product from transcript CG17342-RA	CG17342|CG6929|DmelCG17342|LK6|MESR8|MNK/LK6|anon-WO0172774.84|lk6	0	0
11161	44674	Drosophila melanogaster	l(3)92Eb	lethal (3) 92Eb	HSH11	0	0
11162	44675	Drosophila melanogaster	l(1)47	lethal (1) 47	-	0	0
11163	44676	Drosophila melanogaster	l(1)38	lethal (1) 38	-	0	0
11164	44679	Drosophila melanogaster	ergic53	CG6822 gene product from transcript CG6822-RB	CG6822|DmelCG6822|dERGIC53|dergic53|l(3)S1760|l(3)s1760|rhea	0	0
11165	44688	Drosophila melanogaster	l(3)98Dm	lethal (3) 98Dm	M217	0	0
11166	44689	Drosophila melanogaster	l(3)98Dl	lethal (3) 98Dl	-	0	0
11167	44690	Drosophila melanogaster	l(3)98Dk	lethal (3) 98Dk	M31	0	0
11168	44691	Drosophila melanogaster	l(3)98Dj	lethal (3) 98Dj	M316	0	0
11169	44692	Drosophila melanogaster	l(3)98Di	lethal (3) 98Di	M311	0	0
11170	44693	Drosophila melanogaster	l(3)98Dh	lethal (3) 98Dh	M315	0	0
11171	44694	Drosophila melanogaster	l(3)98Dd	lethal (3) 98Dd	M33|l(3)98D-Ed|l(3)M3-3	0	0
11172	44695	Drosophila melanogaster	l(3)98Dc	lethal (3) 98Dc	M318|l(3)98D-Ec|l(3)M3-18	0	0
11173	44696	Drosophila melanogaster	l(3)98Da	lethal (3) 98Da	M313|l(3)98D-Ea|l(3)M3-13	0	0
11174	44697	Drosophila melanogaster	l(2)PW27	lethal (2) PW27	l(2)PW#27	0	0
11175	44698	Drosophila melanogaster	cass	cassowary	bad|cas	0	0
11176	44699	Drosophila melanogaster	blis	blisterwing	2L-F	0	0
11177	44701	Drosophila melanogaster	Coprox	Coproporphyrinogen oxidase	BcDNA:GM14838|CG3433|DmelCG3433|l(2)k10617|l(2)k11018	0	0
11178	44702	Drosophila melanogaster	Vha36-1	Vacuolar H[+] ATPase subunit 36-1	CG8186|DmelCG8186|V-ATPase|Vha36|VhaD|anon-WO0153538.20|anon-WO0153538.21|anon-WO0153538.28|anon-WO02059370.27|l(2)k07207|vha36|vha36-1	0	0
11179	44703	Drosophila melanogaster	lolal	lola like	25/12|Ban|CG5738|DmelCG5738|EP0647|EP647|LOLAL|Lolal|anon-EST:fe3D3|anon-WO0172774.186|ban|bat|l(2)k02512|l(2)kO2512	0	0
11180	44704	Drosophila melanogaster	l(1)M120.1	lethal (1) M120.1	M120.1	0	0
11181	44705	Drosophila melanogaster	l(1)L9.8	lethal (1) L9.8	L9.8	0	0
11182	44709	Drosophila melanogaster	l(3)82Ca	lethal (3) 82Ca	unnamed	0	0
11183	44711	Drosophila melanogaster	l(2)46Ej	lethal (2) 46Ej	H	0	0
11184	44712	Drosophila melanogaster	l(2)46Ei	lethal (2) 46Ei	S	0	0
11185	44714	Drosophila melanogaster	l(2)46Eg	lethal (2) 46Eg	W	0	0
11186	44717	Drosophila melanogaster	l(1)3ABb	lethal (1) 3ABb	1200C6	0	0
11187	44718	Drosophila melanogaster	l(1)3ABa	lethal (1) 3ABa	A2/7|D3/22.61	0	0
11188	44719	Drosophila melanogaster	l(1)1Aj	lethal (1) 1Aj	1200C30b	0	0
11189	44721	Drosophila melanogaster	l(3)98Fa	lethal (3) 98Fa	unnamed	0	0
11190	44722	Drosophila melanogaster	l(2)55Ca	lethal (2) 55Ca	unnamed	0	0
11191	44723	Drosophila melanogaster	l(2)21Bf	lethal (2) 21Bf	unnamed	0	0
11192	44724	Drosophila melanogaster	san	separation anxiety	CG12352|DmAAF34715|DmelCG12352|atado|span	0	0
11193	44726	Drosophila melanogaster	hopR-90E	hop-responsive-90E	unnamed	0	0
11194	44728	Drosophila melanogaster	MED6	Mediator complex subunit 6	BcDNA:LD15729|CG9473|DmelCG9473|Med6|Trap32|anon-EST:Posey279|dMED6|dMed6|dTRAP32|p35	0	0
11195	44730	Drosophila melanogaster	rambl	rambling	-	0	0
11196	44736	Drosophila melanogaster	CG3719	CG3719 gene product from transcript CG3719-RA	BcDNA:LD03613|DmelCG3719|EG:132E8.3	0	0
11197	44737	Drosophila melanogaster	qkr58E-7	quaking related 58E-7	-	0	0
11198	44738	Drosophila melanogaster	qkr58E-6	quaking related 58E-6	-	0	0
11199	44739	Drosophila melanogaster	qkr58E-5	quaking related 58E-5	-	0	0
11200	44740	Drosophila melanogaster	qkr58E-4	quaking related 58E-4	-	0	0
11201	44742	Drosophila melanogaster	l(3)tb	lethal (3) tumorous brain	-	0	0
11202	44747	Drosophila melanogaster	msk	moleskin	61|CG7935|CIP-61|DIM-7|Dim-7|Dim7|DmRanBP7|DmelCG7935|Imp7|Imp7/8|Msk|S(ls)2|dim-7|dim7	0	0
11203	44748	Drosophila melanogaster	NiPp1	Nuclear inhibitor of Protein phosphatase 1	CG8980|DmelCG8980|NIPP-1Dm|NIPP1|NIPP1Dm|NiPp-1|nipp1	0	0
11204	44750	Drosophila melanogaster	Ady43A	CG1851 gene product from transcript CG1851-RA	Adk43A|Ady|CG1851|DmelCG1851|ady43A	0	0
11205	44752	Drosophila melanogaster	l(2)18E4	lethal (2) 18E4	-	0	0
11206	44753	Drosophila melanogaster	l(3)99Ba	lethal (3) 99Ba	unnamed	0	0
11207	44754	Drosophila melanogaster	l(3)93Ea	lethal (3) 93Ea	unnamed	0	0
11208	44755	Drosophila melanogaster	l(3)88Ae	lethal (3) 88Ae	unnamed	0	0

11209	44756	Drosophila melanogaster	l(3)87Di	lethal (3) 87Di	unnamed	0	0
11210	44757	Drosophila melanogaster	l(3)77Ea	lethal (3) 77Ea	unnamed	0	0
11211	44758	Drosophila melanogaster	l(3)64Da	lethal (3) 64Da	unnamed	0	0
11212	44759	Drosophila melanogaster	l(3)61Db	lethal (3) 61Db	unnamed	0	0
11213	44760	Drosophila melanogaster	l(2)51Fb	lethal (2) 51Fb	unnamed	0	0
11214	44761	Drosophila melanogaster	l(2)30Ca	lethal (2) 30Ca	unnamed	0	0
11215	44762	Drosophila melanogaster	l(2)23Ea	lethal (2) 23Ea	unnamed	0	0
11216	44763	Drosophila melanogaster	l(3)94Ba	lethal (3) 94Ba	unnamed	0	0
11217	44764	Drosophila melanogaster	l(3)93Bj	lethal (3) 93Bj	unnamed	0	0
11218	44765	Drosophila melanogaster	l(3)90Ea	lethal (3) 90Ea	unnamed	0	0
11219	44766	Drosophila melanogaster	l(3)87Ce	lethal (3) 87Ce	unnamed	0	0
11220	44767	Drosophila melanogaster	l(3)84Ch	lethal (3) 84Ch	unnamed	0	0
11221	44768	Drosophila melanogaster	l(3)82Cb	lethal (3) 82Cb	unnamed	0	0
11222	44769	Drosophila melanogaster	l(3)79Ba	lethal (3) 79Ba	unnamed	0	0
11223	44771	Drosophila melanogaster	l(3)66Ea	lethal (3) 66Ea	unnamed	0	0
11224	44772	Drosophila melanogaster	l(3)64Ao	lethal (3) 64Ao	unnamed	0	0
11225	44773	Drosophila melanogaster	l(2)56Da	lethal (2) 56Da	unnamed	0	0
11226	44775	Drosophila melanogaster	l(2)W9	lethal (2) W9	l(2R)W9	0	0
11227	44776	Drosophila melanogaster	l(2)W7	lethal (2) W7	l(2R)W7	0	0
11228	44777	Drosophila melanogaster	l(2)W6	lethal (2) W6	l(2R)W6	0	0
11229	44780	Drosophila melanogaster	l(2)W3	lethal (2) W3	l(2R)W3	0	0
11230	44781	Drosophila melanogaster	l(2)W2	lethal (2) W2	l(2R)W2	0	0
11231	44782	Drosophila melanogaster	l(2)W1	lethal (2) W1	l(2R)W1	0	0
11232	44783	Drosophila melanogaster	RpL37A	Ribosomal protein L37A	BcDNA:RE23595|BcDNA:RH41593|CG5827|DmL37a|DmelCG5827|L37A|M(2)25C|M(2)S1|RpL37a|l(2)25Cb	0	0
11233	44785	Drosophila melanogaster	tan	tantalus	CG6586|DmelCG6586	0	0
11234	44786	Drosophila melanogaster	nerfin-1	nervous fingers 1	CG13906|DmelCG13906|Nerfin|Nerfin-1	0	0
11235	44787	Drosophila melanogaster	miple2	CG18321 gene product from transcript CG18321-RA	CG18321|DmelCG18321	0	0
11236	44790	Drosophila melanogaster	TwdlT	TweedleT	CG5812|DmelCG5812|GCR(ich)	0	0
11237	44791	Drosophila melanogaster	Ef1gamma	CG11901 gene product from transcript CG11901-RC	CG11901|DmelCG11901|EF1gamma|Ef1Bggr|eEF1gamma	0	0
11238	44792	Drosophila melanogaster	aa	anarista	-	0	0
11239	44793	Drosophila melanogaster	abo	abnormal oocyte	CG6093|DmelCG6093	0	0
11240	44795	Drosophila melanogaster	Alp	Abnormal leg pattern	-	0	0
11241	44797	Drosophila melanogaster	arth	arthritic	-	0	0
11242	44798	Drosophila melanogaster	B	Bar	Bar|BarH1|bar	0	0
11243	44799	Drosophila melanogaster	bas	bang-sensitive	-	0	0
11244	44800	Drosophila melanogaster	bly	bellyache	-	0	0
11245	44801	Drosophila melanogaster	bsk	basket	Bsk|CG5680|D-JNK|D-junk|DBSK/JNK|DJNK|DJNK/bsk|DmelCG5680|JNK|JNK/SAPK|Jnk|Junk|SAPKa|c-Jun|dJNK|jnk	0	0
11246	44802	Drosophila melanogaster	c	curved	C|MLCK|Strn-mlck|mlck	0	0
11247	44803	Drosophila melanogaster	cbd	central body defect	-	0	0
11248	44804	Drosophila melanogaster	con	condensed	-	0	0
11249	44805	Drosophila melanogaster	ctl	coatless	-	0	0
11250	44806	Drosophila melanogaster	cx	curlex	-	0	0
11251	44807	Drosophila melanogaster	dal	daughterless-like	-	0	0
11252	44808	Drosophila melanogaster	den	denervated	-	0	0
11253	44809	Drosophila melanogaster	dvr	divers	-	0	0
11254	44811	Drosophila melanogaster	E(bx)	Enhancer of bithorax	CG10894|CG17135|CG32346|CG32478|CG7022|DmelCG32346|E(BX)|E(Bx)|En-bx|NURF|NURF-215|NURF-301|NURF215|NURF301|NuRF301|Nurf 301|Nurf-215|Nurf301|dNURF|dNURF301|l(3)122|l(3)ry1	0	0
11255	44812	Drosophila melanogaster	E(da)	Enhancer of daughterless	-	0	0
11256	44813	Drosophila melanogaster	E(S)	Enhancer of Star	-	0	0
11257	44814	Drosophila melanogaster	E(SD)	Enhancer of Segregation Distorter	E(Sd)	0	0
11258	44815	Drosophila melanogaster	Flu	Flutter	-	0	0
11259	44816	Drosophila melanogaster	fmf	factor for male fertility	-	0	0
11260	44817	Drosophila melanogaster	for	foraging	142251_at|BcDNA:GM08338|CG10033|DG2|Dg2|DmelCG10033|FOR/PKG|For|PKG|Pkg2|Pkg24A|anon-WO0140519.260|anon-WO02059370.47|dg2|l(2)06860	0	0
11261	44818	Drosophila melanogaster	fs(1)M8	female sterile (1) M8	-	0	0
11262	44819	Drosophila melanogaster	g	garnet	CG10986|CG11197|DmelCG10986|cg11197|delta|l(1)Mc19	0	0
11263	44820	Drosophila melanogaster	grt	grotle	gr	0	0
11264	44821	Drosophila melanogaster	Hiv	Haploinviable	unnamed	0	0
11265	44825	Drosophila melanogaster	irreB	irregular optic chiasma B	-	0	0
11266	44826	Drosophila melanogaster	j	jaunty	-	0	0
11267	44827	Drosophila melanogaster	kdu	kudu	-	0	0
11268	44830	Drosophila melanogaster	kto	kohtalo	CG8491|DmelCG8491|Kto|Kto/Med12|MED12|Med12|SRB8|Srb8/KTO/TRAP230|Trap230|dTRAP230|dTrap230|l(3)76BDc|med12/kto|siren1	0	0
11269	44831	Drosophila melanogaster	L	Lobe	CG10109|DmelCG10109|fee	0	0
11270	44832	Drosophila melanogaster	soz	sozzled	A72|EA41|l(1)20Bb	0	0
11271	44835	Drosophila melanogaster	m	miniature	CG9369|DmelCG9369|Min|min	0	0
11272	44836	Drosophila melanogaster	mit	mitotic loss inducer	-	0	0
11273	44837	Drosophila melanogaster	M(Sd)	Modifier of Sd	M(SD)	0	0
11274	44839	Drosophila melanogaster	mud	mushroom body defect	CG12047|DmelCG12047|KS63|Mud|NuMA	0	0
11275	44840	Drosophila melanogaster	mul	multiple	l(1)ESHS2	0	0
11276	44841	Drosophila melanogaster	mw	mottler of white	-	0	0
11277	44843	Drosophila melanogaster	nkd	naked cuticle	CG11614|CG18224|DmelCG11614|Nkd|naked	0	0
11278	44846	Drosophila melanogaster	Oce	Ocellarless	-	0	0
11279	44848	Drosophila melanogaster	plu	plutonium	CG9183|DmelCG9183|PLU|Plu	0	0
11280	44849	Drosophila melanogaster	pnr	pannier	CG3978|CG3978-PA|DmelCG3978|GATA|GATAa|GATAa/Pannier|PNR|Pnr|dGATAa|dGATAa/pannier|l(3)89B3|prn	0	0
11281	44850	Drosophila melanogaster	ptg	pentagon	3D18|fs(1)M71	0	0
11282	44851	Drosophila melanogaster	raw	CG12437 gene product from transcript CG12437-RB	CG12437|CG9321|DmelCG12437|cyr|ensh|l(2)06825|l(2)k03514	0	0
11283	44854	Drosophila melanogaster	rex	rapid exhaustion	-	0	0
11284	44855	Drosophila melanogaster	rib	ribbon	CG7230|DmelCG7230|fus1|gene 5	0	0
11285	44856	Drosophila melanogaster	ru	roughoid	CG1214|DmelCG1214|Rho-3|Rho-related [62A1]|rho-3|rho3	0	0
11286	44857	Drosophila melanogaster	s	sable	eb	0	0
11287	44858	Drosophila melanogaster	sad	shadow	315a1|CG14728|CYP315A1|Cyp315a1|DmelCG14728|Sad	0	0
11288	44859	Drosophila melanogaster	scg	scrambled egg	-	0	0
11289	44860	Drosophila melanogaster	scp	scooped	-	0	0
11290	44861	Drosophila melanogaster	sdt	stardust	BP1063|CG12657|CG12658|CG15339|CG15340|CG15341|CG15342|CG1617|CG32717|DmelCG32717|PALS1|Pals1|Sdt|Std|anon-EST:fe2E6|l(1)7Ef|pal1|std	0	0
11291	44865	Drosophila melanogaster	sie	sieve	-	0	0
11292	44866	Drosophila melanogaster	spg	sponge	mat(3)6	0	0
11293	44867	Drosophila melanogaster	sph	sparse hairs	18|26|27|30|4P1|R-9-5|X-4|l(1)R-9-5|l(1)X-4|l(1)sph|l(1)x-4|l4P1|lX4|x-4	0	0
11294	44869	Drosophila melanogaster	St-SD	Stabilizer of Segregation Distorter	St(SD)	0	0
11295	44870	Drosophila melanogaster	sub	subito	CG12298|DmelCG12298|Dub|KIF20A|SUB|mei-1794	0	0
11296	44873	Drosophila melanogaster	Gr5a	Gustatory receptor 5a	CG15779|Dmel5a|DmelCG15779|GR5A|GRLU.7|Gr5|Gr5A1|GrLU7|LU.7|Tre|han|tre	0	0
11297	44874	Drosophila melanogaster	Tul	Turneduplike	-	0	0
11298	44875	Drosophila melanogaster	tx	taxi	-	0	0
11299	44876	Drosophila melanogaster	uex	unextended	41Ad|EMS45-73|GroupIII|l(2)41Ad|l(2)EMS34-7|l(2R)A'|l(2R)EMS45-73	0	0
11300	44877	Drosophila melanogaster	wdl	wavoid-like	-	0	0
11301	44878	Drosophila melanogaster	olfF	olfactory F	-	0	0
11302	44879	Drosophila melanogaster	rhi	rhino	CG10683|DmelCG10683|HP1D|rno	0	0
11303	44880	Drosophila melanogaster	gustC	gustatory-defective C	-	0	0
11304	44881	Drosophila melanogaster	gustD	gustatory-defective D	-	0	0
11305	44882	Drosophila melanogaster	prd2	paired-like 2	-	0	0
11306	44883	Drosophila melanogaster	exb	extreme bar	Kra	0	0
11307	44884	Drosophila melanogaster	fur	fused rhabdomeres	-	0	0
11308	44885	Drosophila melanogaster	mys	myospheroid	BetaPS|CG1560|CT40473|DmelCG1560|EM28|MAb6G11|Mys|PS|PS[[beta]]|PSbeta|bPS|b[[PS]]|beta-Int|beta-PS|beta-int|beta1|betaPS|betaPS Int|betaPS-integrin|betaPS1|beta[[PS]]|beta[[PS]]-integrin|be	0	0
11309	44886	Drosophila melanogaster	Esm	Enhancer of split mimic	-	0	0
11310	44887	Drosophila melanogaster	mdy	midway	CG13273|CG17937|CG17938|CG31991|CT39962|DGAT|Dgat1|DmelCG31991	0	0
11311	44888	Drosophila melanogaster	Zhr	Zygotic hybrid rescue	Zhr[ts]|zhr	0	0
11312	44889	Drosophila melanogaster	ph-d	polyhomeotic distal	CG3895|DROZFP|DmelCG3895|EG:BACN25G24.3|PH|PH-d|PHD|Ph|PhD|ph|ph-D|ph[D]|ph[d]|phd|phm	0	0
11313	44890	Drosophila melanogaster	rls	restless	-	0	0
11314	44892	Drosophila melanogaster	crip	cripple	-	0	0
11315	44893	Drosophila melanogaster	dipp-7	defective in phototaxis plasticity 7	-	0	0
11316	44895	Drosophila melanogaster	l(1)184	lethal (1) 184	-	0	0
11317	44897	Drosophila melanogaster	dsl	diskless	l(1)C154|l(1)discless|l(1)discless-1	0	0
11318	44898	Drosophila melanogaster	slf	schlaff	-	0	0
11319	44900	Drosophila melanogaster	CG42569	CG42569 gene product from transcript CG42569-RA	CG12058|DmelCG42569|Dmel_CG12058	0	0

11320	44901	Drosophila melanogaster	l(1)TK105	lethal (1) TK105	l105	0	0
11321	44902	Drosophila melanogaster	S63	S63	S 63	0	0
11322	44903	Drosophila melanogaster	Hdl	Haplo-, diplo-lethal	-	0	0
11323	44904	Drosophila melanogaster	cex	central complex	-	0	0
11324	44907	Drosophila melanogaster	sib	sibless	-	0	0
11325	44910	Drosophila melanogaster	unc-45	CG2708 gene product from transcript CG2708-RA	CG2708|DmUNC-45|DmUnc-45|DmelCG2708|Tom34|dUNC-45|dunc-45|l(3)03692	0	0
11326	44911	Drosophila melanogaster	gru	gruyere	-	0	0
11327	44912	Drosophila melanogaster	mlt	mulet	ms(2)4210	0	0
11328	44913	Drosophila melanogaster	And	Androcam	ACaM|Acam|CG17769|Camr97A|DmelCG17769	0	0
11329	44915	Drosophila melanogaster	DNApol-alpha60	DNA polymerase alpha 60kD	BcDNA:GM13640|CG5553|DNA polalpha|DNAprim|DNAprimase|DmelCG5553|Dmpol alpha|GM13640|Pol|S(faf)240|l(3)77ABe|l(3)j10B2|pol alpha	0	0
11330	44920	Drosophila melanogaster	Hsp70Ab	Heat-shock-protein-70Ab	CG18743|DMHSP7A2|Dm-hsp70|DmelCG18743|GRP78|HSP70|Hsp 70|Hsp70|Hsp70-Ab|Hsp70A|Hsp70aB|Hsp70ab|dHsp70|dhsp70|hs70|hsp 70|hsp-70|hsp70|hsp70 87A|hsp70(87A)|hsp70A|hsp70Ab|	0	0
11331	44921	Drosophila melanogaster	Hsp70Ba	Heat-shock-protein-70Ba	CG31449|Dm-hsp70|DmelCG31449|HSP70|Hsp 70|Hsp70|Hsp70(87C)|Hsp70B|dHsp70|dhsp70|hsp-70|hsp70|hsp70 87C|hsp70 Ba|hsp70B|hsp70Ba|hsp70b	0	0
11332	44924	Drosophila melanogaster	l(1)1PP18	lethal (1) 1PP18	-	0	0
11333	44925	Drosophila melanogaster	l(1)4Fb	lethal (1) 4Fb	-	0	0
11334	44927	Drosophila melanogaster	l(1)6PP19	lethal (1) 6PP19	-	0	0
11335	44932	Drosophila melanogaster	pbr	postscutellar bristles	psb	0	0
11336	44936	Drosophila melanogaster	Eip93F	CG18389 gene product from transcript CG18389-RB	CG18389|DmelCG18389|E93|anon-WO02059370.58	0	0
11337	44937	Drosophila melanogaster	Inr-b	Inverse regulator b	-	0	0
11338	44938	Drosophila melanogaster	four-play	four-play	-	0	0
11339	44942	Drosophila melanogaster	l(2)DA2	lethal (2) DA2	DA2	0	0
11340	44943	Drosophila melanogaster	l(2)DB1	lethal (2) DB1	DB1	0	0
11341	44944	Drosophila melanogaster	l(2)DB2	lethal (2) DB2	DB2	0	0
11342	44945	Drosophila melanogaster	l(2)DB4	lethal (2) DB4	DB4	0	0
11343	44946	Drosophila melanogaster	l(2)FE3	lethal (2) FE3	FE3	0	0
11344	44947	Drosophila melanogaster	oho55DE	overgrown hematopoietic organs 55DE	l(2)30/7|l(2)k03007|l(2)k13104|oho55D,E	0	0
11345	44948	Drosophila melanogaster	l(3)neo14	lethal (3) neo14	-	0	0
11346	44949	Drosophila melanogaster	l(3)ry102	lethal (3) ry102	-	0	0
11347	44950	Drosophila melanogaster	l(3)ry107	lethal (3) ry107	-	0	0
11348	44951	Drosophila melanogaster	l(3)ry111	lethal (3) ry111	-	0	0
11349	44952	Drosophila melanogaster	l(3)ry114	lethal (3) ry114	-	0	0
11350	44953	Drosophila melanogaster	l(3)ry116	lethal (3) ry116	-	0	0
11351	44954	Drosophila melanogaster	l(3)ry146	lethal (3) ry146	-	0	0
11352	44955	Drosophila melanogaster	l(3)ry151	lethal (3) ry151	-	0	0
11353	44956	Drosophila melanogaster	l(3)ry3	lethal (3) ry3	-	0	0
11354	44957	Drosophila melanogaster	l(3)ry6	lethal (3) ry6	unnamed	0	0
11355	44963	Drosophila melanogaster	l(3)90DEa	lethal (3) 90DEa	l(3)5D4	0	0
11356	44964	Drosophila melanogaster	Actn3	alpha actinin 3	CG8953|DmelCG8953|ORF1|acta-3|dabp	0	0
11357	44965	Drosophila melanogaster	Fer2LCH	Ferritin 2 light chain homologue	2LCH|CG1469|DmelCG1469|Fer-H|Fer2LC|FerLCH|LCH|anon-EST:ParkEST264|fer2lch|l(3)00035|l(3)07016|l(3)j2A3|l(3)neo60|l(3)neo63|l(3)s2083	0	0
11358	44967	Drosophila melanogaster	l(1)10Bp	lethal (1) 10Bp	-	0	0
11359	44968	Drosophila melanogaster	l(1)4ACa	lethal (1) 4ACa	-	0	0
11360	44969	Drosophila melanogaster	l(1)9BDa	lethal (1) 9BDa	-	0	0
11361	44970	Drosophila melanogaster	l(1)9Fk	lethal (1) 9Fk	-	0	0
11362	44971	Drosophila melanogaster	l(1)9Fl	lethal (1) 9Fl	-	0	0
11363	44974	Drosophila melanogaster	cln	clown	-	0	0
11364	44975	Drosophila melanogaster	l(1)m-C	lethal (1) m-Caggese	-	0	0
11365	44976	Drosophila melanogaster	l(2)55BCa	lethal (2) 55BCa	-	0	0
11366	44977	Drosophila melanogaster	l(3)843	lethal (3) 843	-	0	0
11367	44978	Drosophila melanogaster	Tom40	Translocase of outer membrane 40	CG12157|DmelCG12157|dtom40|l(1)G0216|mit|ms(1)15|ms15|tom40	0	0
11368	44980	Drosophila melanogaster	l(3)X1	lethal (3) X1	X1	0	0
11369	44981	Drosophila melanogaster	l(3)X4	lethal (3) X4	X4	0	0
11370	44982	Drosophila melanogaster	l(1)1Ai	lethal (1) 1Ai	HM17	0	0
11371	44985	Drosophila melanogaster	smu	small mushroom bodies	-	0	0
11372	45012	Drosophila melanogaster	Vha68-2	V-ATPase 69 kDa subunit 2	CG3762|DmelCG3762|MENE-R6|V-ATPase|l(2)01510|l(2)s4214|vha67-3|vha68-2	0	0
11373	45013	Drosophila melanogaster	l(3)9C3	lethal (3) 9C3	-	0	0
11374	45016	Drosophila melanogaster	Mst57Ca	Male-specific transcript 57C	unnamed	0	0
11375	45017	Drosophila melanogaster	Lcp65Af	CG10533 gene product from transcript CG10533-RA	CG10533|DCP7|DMLCP65f|DmelLcp65Af|DmelCG10533|Lcp-f	0	0
11376	45018	Drosophila melanogaster	Lcp65Ae	CG10529 gene product from transcript CG10529-RA	CG10529|DCP6|DMLCP65e|DmelLcp65Ae|DmelCG10529|Lcp-e	0	0
11377	45019	Drosophila melanogaster	fs(2)al	female sterile (2) al	fs(2L)al	0	0
11378	45021	Drosophila melanogaster	Zfrp8	Zinc finger protein RP-8	137/5|CG3260|DmelCG3260|PDCD2|l(2)k13705|zfrp8	0	0
11379	45022	Drosophila melanogaster	l(1)M37.2	lethal (1) M37.2	M37.2	0	0
11380	45023	Drosophila melanogaster	l(1)L62.4	lethal (1) L62.4	L62.4	0	0
11381	45024	Drosophila melanogaster	l(1)L48.3	lethal (1) L48.3	L48.3	0	0
11382	45025	Drosophila melanogaster	l(1)H30.1	lethal (1) H30.1	H30.1	0	0
11383	45026	Drosophila melanogaster	l(1)B23.2	lethal (1) B23.2	B23.2	0	0
11384	45027	Drosophila melanogaster	l(1)A27.2	lethal (1) A27.2	A27.2	0	0
11385	45028	Drosophila melanogaster	l(1)A13.3	lethal (1) A13.3	A13.3	0	0
11386	45031	Drosophila melanogaster	Pp4-19C	Protein phosphatase 19C	CG1459|CG1596|CG18339|CG32505|DmPpp4-19C|DmelCG32505|PP1|PP4|PP4 19C|PP4C|PPP4c|PPX|PpX|pp4	0	0
11387	45032	Drosophila melanogaster	l(3)69ABa	lethal (3) 69ABa	unnamed	0	0
11388	45039	Drosophila melanogaster	Sox100B	CG15552 gene product from transcript CG15552-RA	CG12098|CG15552|CG18674|CT6009|DmelCG15552|SOX100B|SOXE|Sox100|Sox9|SoxB	0	0
11389	45040	Drosophila melanogaster	Spn43Ab	Serine protease inhibitor 43Ab	CG1865|DmelCG1865|SER2|Ser2|anon-WO0118057.2|spn43Ab	0	0
11390	45041	Drosophila melanogaster	Spn43Aa	Serine protease inhibitor 43Aa	CG12172|DmelCG12172|SER1|Ser1|Spn43 Aa|Spn43A|anon-WO0118057.1	0	0
11391	45042	Drosophila melanogaster	SS3-6	Suppressor of GMR-sina 3-6	-	0	0
11392	45043	Drosophila melanogaster	SS3-5	Suppressor of GMR-sina 3-5	-	0	0
11393	45044	Drosophila melanogaster	SS3-1	Suppressor of GMR-sina 3-1	-	0	0
11394	45045	Drosophila melanogaster	ES3-1	Enhancer of GMR-sina 3-1	-	0	0
11395	45046	Drosophila melanogaster	ES2-1	Enhancer of GMR-sina 2-1	-	0	0
11396	45047	Drosophila melanogaster	ES1-1	Enhancer of GMR-sina 1-1	-	0	0
11397	45064	Drosophila melanogaster	CG6133	CG6133 gene product from transcript CG6133-RA	DmelCG6133|EG:EG0007.9	0	0
11398	45067	Drosophila melanogaster	bae	batone	-	0	0
11399	45068	Drosophila melanogaster	l(2)3A2	lethal (2) 3A2	-	0	0
11400	45069	Drosophila melanogaster	l(2)11B9	lethal (2) 11B9	-	0	0
11401	45104	Drosophila melanogaster	l(3)89ABa	lethal (3) 89ABa	unnamed	0	0
11402	45105	Drosophila melanogaster	l(3)88EFa	lethal (3) 88EFa	unnamed	0	0
11403	45106	Drosophila melanogaster	l(3)87ABa	lethal (3) 87ABa	unnamed	0	0
11404	45107	Drosophila melanogaster	l(3)76BCa	lethal (3) 76BCa	unnamed	0	0
11405	45108	Drosophila melanogaster	l(3)71DEd	lethal (3) 71DEd	-	0	0
11406	45109	Drosophila melanogaster	l(3)71DEc	lethal (3) 71DEc	1(3)71DEc	0	0
11407	45110	Drosophila melanogaster	l(3)71DEb	lethal (3) 71DEb	-	0	0
11408	45111	Drosophila melanogaster	l(3)71DEa	lethal (3) 71DEa	-	0	0
11409	45112	Drosophila melanogaster	l(2)56CDa	lethal (2) 56CDa	unnamed	0	0
11410	45113	Drosophila melanogaster	l(2)39CDa	lethal (2) 39CDa	unnamed	0	0
11411	45174	Drosophila melanogaster	l(2)32BCa	lethal (2) 32BCa	-	0	0
11412	45175	Drosophila melanogaster	l(3)S081003	lethal (3) S081003	0073/29|0528/04|0693/07|0770/08|0810/03|1199/06|1225/01|1459/11|1472/07|1473/10|l(3)S145911	0	0
11413	45176	Drosophila melanogaster	l(3)S003405	lethal (3) S003405	-	0	0
11414	45233	Drosophila melanogaster	capt	capulet	Acap|BcDNA:LD24380|CAP|CG33979|CG5061|CG5074|Cap|Capt|Dcap|DmelCG33979|acu|cap|capping protein|cyclase-associated protein|l(2)06955|punc	0	0
11415	45234	Drosophila melanogaster	Ggamma30A	G protein gamma30A	CG18511|CG3694|DmelCG3694|G-gamma30A|G[[gamma]]|Galpha30A|Ggamma|Ggamma[[e]]|Ggammae	0	0
11416	45236	Drosophila melanogaster	l(2)W19	lethal (2) W19	l(2R)W19	0	0
11417	45237	Drosophila melanogaster	l(2)W18	lethal (2) W18	l(2R)W18	0	0
11418	45238	Drosophila melanogaster	l(2)W17	lethal (2) W17	l(2R)W17	0	0
11419	45239	Drosophila melanogaster	l(2)W16	lethal (2) W16	l(2R)W16	0	0
11420	45240	Drosophila melanogaster	l(2)W15	lethal (2) W15	l(2R)W15	0	0
11421	45241	Drosophila melanogaster	l(2)W14	lethal (2) W14	l(2R)W14	0	0
11422	45242	Drosophila melanogaster	l(2)W13	lethal (2) W13	l(2R)W13	0	0
11423	45244	Drosophila melanogaster	l(2)W11	lethal (2) W11	l(2R)W11	0	0
11424	45245	Drosophila melanogaster	l(2)W10	lethal (2) W10	l(2R)W10	0	0
11425	45247	Drosophila melanogaster	VhaPPA1-1	Vacuolar H[+] ATPase subunit PPA1-1	CG7007|DmelCG7007|V-ATPase|vhaPPA1-1	0	0
11426	45248	Drosophila melanogaster	Nckx30C	CG18660 gene product from transcript CG18660-RD	CG18660|CG4106|DmelCG18660|potassium-dependent sodium/calcium	0	0
11427	45249	Drosophila melanogaster	Ef1beta	Elongation factor 1 beta	154264_at|BcDNA.LD24492|BcDNA:LD24492|CG6341|DmelCG6341|EG:EG0003.7|Elf 1 beta	0	0
11428	45250	Drosophila melanogaster	deltaCOP	delta-coatomer protein	63B12S|ARCN1|Arch|CG14813|DM63B12S|Delta COP|DeltaCop|DmelCG14813|EG:63B12.10|ESTS:63B12S|anon-WO03040301.246|delta-Cop|deltaCop|l(1)G0051|l(1)G0301|l(1)G0450	0	0
11429	45268	Drosophila melanogaster	cathD	CG1548 gene product from transcript CG1548-RA	CATH|CG1548|CT3996|CathD|DmelCG1548|T17	0	0
11430	45269	Drosophila melanogaster	Prosbeta5	Proteasome beta5 subunit	CG12323|DmelCG12323|LD08717|Pros b5|anon-WO0118547.116|beta-5|dbeta5	0	0

11431	45270	Drosophila melanogaster	abd	abdominal	-	0	0
11432	45272	Drosophila melanogaster	aperB	abnormal proboscis extension reflex B	-	0	0
11433	45273	Drosophila melanogaster	aperC	abnormal proboscis extension reflex C	-	0	0
11434	45274	Drosophila melanogaster	Bl	Bristle	Sy	0	0
11435	45275	Drosophila melanogaster	cd	cardinal	-	0	0
11436	45276	Drosophila melanogaster	cgd	congested	-	0	0
11437	45277	Drosophila melanogaster	cp	clipped	E(nd)88	0	0
11438	45278	Drosophila melanogaster	csw	corkscrew	19-106|CG3954|CSW|Csw|Csw/Shp2|DmelCG3954|E(sev)1A|EG:BACN25G24.2|SHP-2|Shp-2|Shp2/csw|anon-WO03040301.207|anon-WO03040301.209|anon-WO03040301.219|l(1)2Db|l(1)2Dd|l(1)G017	0	0
11439	45279	Drosophila melanogaster	cu	curled	-	0	0
11440	45280	Drosophila melanogaster	cv-2	crossveinless 2	BMPER|CG15671|CT35855|Cv 2|Cv2|DmelCG15671|cv 2	0	0
11441	45281	Drosophila melanogaster	db	dark body	-	0	0
11442	45282	Drosophila melanogaster	dib	disembodied	302a1|CG12028|CYP302A1|Cyp|Cyp302a1|DmelCG12028|l(3)64Ak|l(3)SH14|l(3)dib	0	0
11443	45283	Drosophila melanogaster	Dk	Daughter killer	-	0	0
11444	45285	Drosophila melanogaster	Dr	Drop	153515_at|99B|CG1897|DmelCG1897|Msh|Msx|l(3)FA30|ltt|msh|msh-1|msh1	0	0
11445	45286	Drosophila melanogaster	dv	divergent	aea	0	0
11446	45290	Drosophila melanogaster	ee	extra eye	-	0	0
11447	45292	Drosophila melanogaster	eo	extra organs	12|17|A7|E54|N-30|l(1)E-54|lA7|lN-30	0	0
11448	45293	Drosophila melanogaster	ff	fluff	-	0	0
11449	45294	Drosophila melanogaster	fliC	flightless C	-	0	0
11450	45295	Drosophila melanogaster	fliD	flightless D	-	0	0
11451	45296	Drosophila melanogaster	flz	filzig	-	0	0
11452	45297	Drosophila melanogaster	fs(1)C4	female sterile (1) C4	-	0	0
11453	45298	Drosophila melanogaster	fs(1)C5	female sterile (1) C5	-	0	0
11454	45299	Drosophila melanogaster	fs(1)C6	female sterile (1) C6	-	0	0
11455	45300	Drosophila melanogaster	fs(1)de1	female sterile (1) defective eggs 1	de1	0	0
11456	45301	Drosophila melanogaster	fs(1)M11	female sterile (1) M11	-	0	0
11457	45302	Drosophila melanogaster	fs(1)M40	female sterile (1) M40	-	0	0
11458	45303	Drosophila melanogaster	fs(1)M41	female sterile (1) M41	unnamed	0	0
11459	45304	Drosophila melanogaster	fs(1)ne1	female sterile (1) no eggs 1	ne1	0	0
11460	45307	Drosophila melanogaster	fz	frizzled	CG17697|CG3646|DFZ1|DFz|DFz1|Dfz1|Dm Fz1|DmelCG17697|FZ|Frz|Fz|Fz1|frz|fz1|fz[[1]]	0	0
11461	45308	Drosophila melanogaster	gg	goggle	-	0	0
11462	45309	Drosophila melanogaster	fliF	flightless F	gmp	0	0
11463	45310	Drosophila melanogaster	gs(2)M	grandchildless M	-	0	0
11464	45311	Drosophila melanogaster	GustR	Gustatory R	-	0	0
11465	45312	Drosophila melanogaster	gv	grooved	gs	0	0
11466	45316	Drosophila melanogaster	hy	humpy	-	0	0
11467	45317	Drosophila melanogaster	jv	javelin	-	0	0
11468	45318	Drosophila melanogaster	kn	knot	CG10197|Col|Col/Kn|DmelCG10197|Kn|col|collier	0	0
11469	45320	Drosophila melanogaster	trol	terribly reduced optic lobes	BcDNA:GM02481|CG12497|CG33675|CG33950|CG7981|CT23996|DmelCG33950|EG:BACR25B3.1|EG:BACR25B3.10|EG:BACR25B3.11|EG:BACR25B3.2|GC7891|Pcan|Trol|ZW-1|anon-WO0153538.72|l	0	0
11470	45322	Drosophila melanogaster	l(3)87Ae	lethal (3) 87Ae	ck12|l(3)ck12	0	0
11471	45324	Drosophila melanogaster	lf	little fly	12|9|l152|lN5|lT2-14a	0	0
11472	45325	Drosophila melanogaster	lin	lines	CG11770|DmelCG11770|Lin	0	0
11473	45326	Drosophila melanogaster	Lsp2	Larval serum protein 2	C23|CG11538|CG6806|DmeLSP2|DmelCG6806|LHP|LSP 2|LSP-2|LSP2|Lsp-2|Pt-1	0	0
11474	45328	Drosophila melanogaster	sens	senseless	1228/04|CG10714|CG32120|DmelCG32120|Ly|Ly-1|SENS|Sen|Sens|l(3)70Ad|sen|sense	0	0
11475	45329	Drosophila melanogaster	RpL12	Ribosomal protein L12	CG3195|DmelCG3195|L12|M(2)60B|Rpl12|anon-EST:Posey236	0	0
11476	45330	Drosophila melanogaster	ma	maroon	-	0	0
11477	45331	Drosophila melanogaster	mapA	midgut amylase pattern AMG	-	0	0
11478	45332	Drosophila melanogaster	mapP	midgut amylase pattern PMG	map|map-PMG	0	0
11479	45334	Drosophila melanogaster	mex	Malic enzyme modifier	-	0	0
11480	45336	Drosophila melanogaster	mu1	mutator 1	mu	0	0
11481	45337	Drosophila melanogaster	mus112	mutagen-sensitive 112	-	0	0
11482	45338	Drosophila melanogaster	na	narrow abdomen	CG1517|Dma1U|Dmalpha1U|DmelCG1517|Na|alpha1U|har|har 38|har 85|har38|har85|harA	0	0
11483	45339	Drosophila melanogaster	net	CG11450 gene product from transcript CG11450-RB	CG11450|DmelCG11450|Group IId|Shout|bHLHc42|shout	0	0
11484	45340	Drosophila melanogaster	nrd	neural disrupted	l(2)23Aa	0	0
11485	45341	Drosophila melanogaster	ny	notchy	-	0	0
11486	45342	Drosophila melanogaster	ot	outheld	-	0	0
11487	45343	Drosophila melanogaster	pk	prickle	CG11084|CG12830|DmelCG11084|Pk|Pk-Sple|Sple|pk-sple|sple	0	0
11488	45344	Drosophila melanogaster	pl	pleated	-	0	0
11489	45346	Drosophila melanogaster	prat	pratfall	-	0	0
11490	45348	Drosophila melanogaster	pre	presto	-	0	0
11491	45349	Drosophila melanogaster	pu	pupal	pup	0	0
11492	45350	Drosophila melanogaster	qs	quicksilver	ftd|su(b)|su(b)[18]	0	0
11493	45351	Drosophila melanogaster	qui	quit	-	0	0
11494	45352	Drosophila melanogaster	rd	reduced	-	0	0
11495	45353	Drosophila melanogaster	Rex	Ribosomal exchange	Su(Rex)	0	0
11496	45354	Drosophila melanogaster	rol	reduced optic lobes	-	0	0
11497	45355	Drosophila melanogaster	rs	rose	-	0	0
11498	45358	Drosophila melanogaster	sf	safranin	dki	0	0
11499	45359	Drosophila melanogaster	shr	shrunken	-	0	0
11500	45360	Drosophila melanogaster	shu	shutdown	CG4735|DmelCG4735	0	0
11501	45363	Drosophila melanogaster	su(b)	suppressor of black	-	0	0
11502	45365	Drosophila melanogaster	tc	tiny chaetae	-	0	0
11503	45366	Drosophila melanogaster	tft	tufts	-	0	0
11504	45367	Drosophila melanogaster	tk	thick	-	0	0
11505	45368	Drosophila melanogaster	Treh	Trehalase	87D3T|CG9364|DmelCG9364|ESTS:87D3T|TRE|TREH|Tre|anon-EST:Posey159|anon-sts38	0	0
11506	45369	Drosophila melanogaster	ty	tiny	-	0	0
11507	45370	Drosophila melanogaster	un	uneven	-	0	0
11508	45371	Drosophila melanogaster	wo	white ocelli	-	0	0
11509	45374	Drosophila melanogaster	fwd	four wheel drive	CG7004|DmelCG7004|PI4K|anon-WO0118547.259	0	0
11510	45375	Drosophila melanogaster	prd1	paired-like 1	-	0	0
11511	45376	Drosophila melanogaster	prd5	paired-like 5	-	0	0
11512	45377	Drosophila melanogaster	prd8	paired-like 8	-	0	0
11513	45378	Drosophila melanogaster	l(1)air1	lethal aberrant immune response 1	air1|l(1)air[1]	0	0
11514	45379	Drosophila melanogaster	blnd	bland	-	0	0
11515	45380	Drosophila melanogaster	spin	spinster	25/11|CG8428|DmelCG8428|Spin|bnch|l(2)10403|l(2)W5|l(2)k02511|l(2)k09905|l(2R)W5	0	0
11516	45381	Drosophila melanogaster	mX	E(spl) region transcript mX	-	0	0
11517	45382	Drosophila melanogaster	ogre	optic ganglion reduced	CB8|CG3039|Dm-inx1|DmelCG3039|Inx1|cb-8|inx-1|inx1|jnL3|l(1)6Ec|l(1)jnL3|l(1)ogre	0	0
11518	45383	Drosophila melanogaster	Fs(2)M	Female sterile of Meyer	Fs	0	0
11519	45384	Drosophila melanogaster	sat	satin	-	0	0
11520	45385	Drosophila melanogaster	l(2)S3	lethal of Seto 3	-	0	0
11521	45386	Drosophila melanogaster	l(3)SG83	lethal (3) SG83	-	0	0
11522	45387	Drosophila melanogaster	l(1)adl3	lethal (1) adl3	l(1)adl-3	0	0
11523	45388	Drosophila melanogaster	fliK	flightless K	EM15	0	0
11524	45389	Drosophila melanogaster	l(1)v47	lethal (1) v47	l(1)47	0	0
11525	45390	Drosophila melanogaster	l(1)v306	lethal (1) v306	l(1)306	0	0
11526	45391	Drosophila melanogaster	l(1)v459	lethal (1) v459	l(1)459	0	0
11527	45392	Drosophila melanogaster	belt	belt	-	0	0
11528	45395	Drosophila melanogaster	lawc	leg arista wing complex	lawC	0	0
11529	45396	Drosophila melanogaster	E5	CG9930 gene product from transcript CG9930-RA	CG9930|DmelCG9930	0	0
11530	45398	Drosophila melanogaster	Aldh-III	Aldehyde dehydrogenase type III	ALDH-III|CG11140|CT41571|Dhap|DmelCG11140|FALDH|l(2)03610	0	0
11531	45399	Drosophila melanogaster	Aats-trp	Tryptophanyl-tRNA synthetase	85D-WRS|CG9735|DmelCG9735|TrpRS|WRS|WRS-85D|l(3)03559|l(3)03560|l(3)3559|wars	0	0
11532	45400	Drosophila melanogaster	disd	dispersed	-	0	0
11533	45401	Drosophila melanogaster	ox	oxen	BcDNA:RH44662|CG8764|DmelCG8764|QCR9|Qcr10|Qcr9|ms(2)00815	0	0
11534	45403	Drosophila melanogaster	sbn	scatterbrained	-	0	0
11535	45407	Drosophila melanogaster	l(3)j2B1	lethal (3) j2B1	-	0	0
11536	45409	Drosophila melanogaster	l(3)j2C3	lethal (3) j2C3	-	0	0
11537	45414	Drosophila melanogaster	l(3)j4B9	lethal (3) j4B9	-	0	0
11538	45417	Drosophila melanogaster	l(3)j5B2	lethal (3) j5B2	-	0	0
11539	45420	Drosophila melanogaster	l(3)j6B4	lethal (3) j6B4	l(3)J6B4	0	0
11540	45421	Drosophila melanogaster	l(3)j7C3	lethal (3) j7C3	-	0	0
11541	45426	Drosophila melanogaster	l(3)neo1	lethal (3) neo1	-	0	0

11542	45427	Drosophila melanogaster	l(3)neo2	lethal (3) neo2	-	0	0
11543	45428	Drosophila melanogaster	l(3)neo3	lethal (3) neo3	-	0	0
11544	45429	Drosophila melanogaster	l(3)neo8	lethal (3) neo8	-	0	0
11545	45430	Drosophila melanogaster	l(3)neo9	lethal (3) neo9	-	0	0
11546	45431	Drosophila melanogaster	boas	beads on a string	-	0	0
11547	45432	Drosophila melanogaster	can	cannonball	CG6577|DmelCG6577|TAF5L|dTAF5L|dTAF[[II]]80|dmTAF5b	0	0
11548	45433	Drosophila melanogaster	grap	grape	-	0	0
11549	45435	Drosophila melanogaster	mrr	myosin rod-related	-	0	0
11550	45436	Drosophila melanogaster	prp	prickly pear	-	0	0
11551	45438	Drosophila melanogaster	l(3)j1B8	lethal (3) j1B8	-	0	0
11552	45441	Drosophila melanogaster	l(3)j6C3	lethal (3) j6C3	-	0	0
11553	45445	Drosophila melanogaster	l(1)12P3	lethal (1) 12P3	-	0	0
11554	45448	Drosophila melanogaster	l(1)14Ae	lethal (1) 14Ae	I|l(1)I	0	0
11555	45449	Drosophila melanogaster	l(1)14Ag	lethal (1) 14Ag	II|l(1)II	0	0
11556	45451	Drosophila melanogaster	l(1)14Ah	lethal (1) 14Ah	X|l(1)X	0	0
11557	45453	Drosophila melanogaster	l(1)14Ai	lethal (1) 14Ai	XII|l(1)XII|mn4	0	0
11558	45454	Drosophila melanogaster	l(1)14Af	lethal (1) 14Af	XIII|l(1)XIII	0	0
11559	45455	Drosophila melanogaster	l(1)14Ad	lethal (1) 14Ad	XIV|l(1)XIV	0	0
11560	45456	Drosophila melanogaster	l(2)ry50	lethal (2) ry50	l(2)50|unnamed	0	0
11561	45457	Drosophila melanogaster	l(2)ry51	lethal (2) ry51	l(2)51|unnamed	0	0
11562	45458	Drosophila melanogaster	l(3)ry59	lethal (3) ry59	l(3)59	0	0
11563	45459	Drosophila melanogaster	l(3)ry69	lethal (3) ry69	l(3)69	0	0
11564	45460	Drosophila melanogaster	pex1	peroxin 1	0848/07|CG6760|DmCAB51031.1|DmelCG6760|anon-WO0172774.100|anon-WO0172774.101|anon-WO0172774.102|anon-WO0172774.96|anon-WO0172774.98|anon-WO0172774.99|l(3)02402|l(3)0848(	0	0
11565	45461	Drosophila melanogaster	l(3)ry78	lethal (3) ry78	l(3)78	0	0
11566	45463	Drosophila melanogaster	l(3)78Da	lethal (3) 78Da	-	0	0
11567	45464	Drosophila melanogaster	l(3)82Fa	lethal (3) 82Fa	-	0	0
11568	45465	Drosophila melanogaster	l(3)82Fb	lethal (3) 82Fb	-	0	0
11569	45466	Drosophila melanogaster	l(3)82Fc	lethal (3) 82Fc	-	0	0
11570	45467	Drosophila melanogaster	l(3)82Fd	CG32464 gene product from transcript CG32464-RI	CG10196|CG10199|CG32464|DmelCG32464|L(3)82Fd|L82|anon-EST:Posey263|l(3)82FD	0	0
11571	45468	Drosophila melanogaster	l(3)82Fe	lethal (3) 82Fe	-	0	0
11572	45469	Drosophila melanogaster	l(3)ry85	lethal (3) ry85	l(3)85	0	0
11573	45470	Drosophila melanogaster	l(3)92Ea	lethal (3) 92Ea	AS416	0	0
11574	45471	Drosophila melanogaster	l(3)97De	lethal (3) 97De	-	0	0
11575	45472	Drosophila melanogaster	l(3)97Df	lethal (3) 97Df	-	0	0
11576	45473	Drosophila melanogaster	l(3)W33B	lethal (3) W33B	-	0	0
11577	45477	Drosophila melanogaster	l(1)16Ff	lethal (1) 16Ff	-	0	0
11578	45478	Drosophila melanogaster	l(1)16Fh	lethal (1) 16Fh	HL-XI	0	0
11579	45479	Drosophila melanogaster	l(1)16Fi	lethal (1) 16Fi	-	0	0
11580	45480	Drosophila melanogaster	l(1)16Fk	lethal (1) 16Fk	HL-XIII	0	0
11581	45481	Drosophila melanogaster	l(1)16Fl	lethal (1) 16Fl	-	0	0
11582	45482	Drosophila melanogaster	l(1)17Aa	lethal (1) 17Aa	-	0	0
11583	45483	Drosophila melanogaster	l(1)17Ab	lethal (1) 17Ab	-	0	0
11584	45484	Drosophila melanogaster	l(2)31Ep	lethal (2) 31Ep	-	0	0
11585	45485	Drosophila melanogaster	l(2)31Eq	lethal (2) 31Eq	-	0	0
11586	45486	Drosophila melanogaster	l(2)31Er	lethal (2) 31Er	-	0	0
11587	45487	Drosophila melanogaster	l(2)31Es	lethal (2) 31Es	-	0	0
11588	45488	Drosophila melanogaster	l(2)31Et	lethal (2) 31Et	-	0	0
11589	45489	Drosophila melanogaster	l(2)31Eu	lethal (2) 31Eu	-	0	0
11590	45490	Drosophila melanogaster	l(2)31Fa	lethal (2) 31Fa	-	0	0
11591	45491	Drosophila melanogaster	l(2)32Ab	lethal (2) 32Ab	-	0	0
11592	45492	Drosophila melanogaster	l(2)32Ac	lethal (2) 32Ac	-	0	0
11593	45493	Drosophila melanogaster	l(2)32Ad	lethal (2) 32Ad	-	0	0
11594	45494	Drosophila melanogaster	l(2)32Ae	lethal (2) 32Ae	-	0	0
11595	45495	Drosophila melanogaster	l(2)32Af	lethal (2) 32Af	-	0	0
11596	45497	Drosophila melanogaster	l(2)N7-6	lethal (2) N7-6	N7-6	0	0
11597	45498	Drosophila melanogaster	l(2)N7-8	lethal (2) N7-8	N7-8	0	0
11598	45499	Drosophila melanogaster	l(2)k08225	lethal (2) k08225	l(2)82/25	0	0
11599	45500	Drosophila melanogaster	l(2)k08634	lethal (2) k08634	l(2)86/34	0	0
11600	45501	Drosophila melanogaster	l(2)k11512	lethal (2) k11512	l(2)115/12	0	0
11601	45503	Drosophila melanogaster	l(3)64Ab	lethal (3) 64Ab	l(3)SH15|l(3)SH19	0	0
11602	45504	Drosophila melanogaster	l(3)64Ac	lethal (3) 64Ac	-	0	0
11603	45505	Drosophila melanogaster	l(3)64Ad	lethal (3) 64Ad	-	0	0
11604	45506	Drosophila melanogaster	l(3)64Af	lethal (3) 64Af	-	0	0
11605	45507	Drosophila melanogaster	l(3)7-8	lethal (3) 7-8	-	0	0
11606	45508	Drosophila melanogaster	l(3)neo53a	lethal (3) neo53a	-	0	0
11607	45509	Drosophila melanogaster	l(3)neo53b	lethal (3) neo53b	-	0	0
11608	45510	Drosophila melanogaster	l(3)neo58a	lethal (3) neo58a	-	0	0
11609	45511	Drosophila melanogaster	l(3)ry125a	lethal (3) ry125a	-	0	0
11610	45512	Drosophila melanogaster	l(3)ry125b	lethal (3) ry125b	-	0	0
11611	45513	Drosophila melanogaster	sov	small ovary	-	0	0
11612	45524	Drosophila melanogaster	Cyp4p1	Cytochrome P450-4p1	4p1|CG10842|DmelCG10842|P-450|P450|anon-WO0140519.35	0	0
11613	45525	Drosophila melanogaster	eIF-4E	Eukaryotic initiation factor 4E	0260/09|0587/11|0589/11|0919/12|1004/13|2|7238|CBP|CG4035|CT13384|CT39424|CT39426|D-eIF4E|DmelCG4035|Eif4E|Eif4e|IF4E|dEif4e|deIF-4E|deIF4E|eIF 4E|eIF-4E1|eIF-4E2|eIF-4EII|eIF-4e|eIF4E|eII	0	0
11614	45531	Drosophila melanogaster	l(1)14Db	lethal (1) 14Db	-	0	0
11615	45532	Drosophila melanogaster	l(2)37Dk	lethal (2) 37Dk	l(2)E144,M	0	0
11616	45533	Drosophila melanogaster	l(2)37Dl	lethal (2) 37Dl	l(2)E34	0	0
11617	45534	Drosophila melanogaster	l(2)37Dm	lethal (2) 37Dm	l(2)E12	0	0
11618	45535	Drosophila melanogaster	l(2)44Fa	lethal (2) 44Fa	-	0	0
11619	45536	Drosophila melanogaster	l(2)44Fb	lethal (2) 44Fb	-	0	0
11620	45537	Drosophila melanogaster	l(2)44Fc	lethal (2) 44Fc	-	0	0
11621	45538	Drosophila melanogaster	l(2)45Aa	lethal (2) 45Aa	-	0	0
11622	45541	Drosophila melanogaster	l(2)45Bb	lethal (2) 45Bb	-	0	0
11623	45544	Drosophila melanogaster	l(2)45Cb	lethal (2) 45Cb	-	0	0
11624	45550	Drosophila melanogaster	l(2)46Cl	lethal (2) 46Cl	T	0	0
11625	45551	Drosophila melanogaster	l(2)46Cm	lethal (2) 46Cm	U	0	0
11626	45552	Drosophila melanogaster	l(2)46Cn	lethal (2) 46Cn	X	0	0
11627	45553	Drosophila melanogaster	l(3)95Aa	lethal (3) 95Aa	TnM2|unnamed	0	0
11628	45556	Drosophila melanogaster	Cyp6a17	CG10241 gene product from transcript CG10241-RA	61D6S|6a17|CG10241|DmelCG10241|ESTS:61D6S|cyp6a17	0	0
11629	45568	Drosophila melanogaster	l(2)40Aa	lethal (2) 40Aa	unnamed	0	0
11630	45570	Drosophila melanogaster	l(3)63Ec	lethal (3) 63Ec	l(3)SH2|63Ec	0	0
11631	45571	Drosophila melanogaster	l(3)63Ed	lethal (3) 63Ed	l(3)SH5	0	0
11632	45572	Drosophila melanogaster	l(3)63Ee	lethal (3) 63Ee	l(3)SH1	0	0
11633	45573	Drosophila melanogaster	l(3)63Fa	lethal (3) 63Fa	l(3)SH8	0	0
11634	45574	Drosophila melanogaster	ida	imaginal discs arrested	APC5|Apc5|CG10850|DmelCG10850|IDA|l(3)63Fb|l(3)SH7	0	0
11635	45576	Drosophila melanogaster	l(3)63Fd	lethal (3) 63Fd	l(3)SH6	0	0
11636	45577	Drosophila melanogaster	Faa	Fumarylacetoacetase	CG14993|DmelCG14993|dfaa|l(3)64Al|l(3)SH11	0	0
11637	45578	Drosophila melanogaster	l(3)64Am	lethal (3) 64Am	l(3)SH13	0	0
11638	45580	Drosophila melanogaster	l(3)85Ak	lethal (3) 85Ak	-	0	0
11639	45581	Drosophila melanogaster	l(3)89Bm	lethal (3) 89Bm	-	0	0
11640	45582	Drosophila melanogaster	l(3)89Bn	lethal (3) 89Bn	-	0	0
11641	45583	Drosophila melanogaster	l(3)89Ce	lethal (3) 89Ce	-	0	0
11642	45585	Drosophila melanogaster	l(3)89Ek	lethal (3) 89Ek	-	0	0
11643	45586	Drosophila melanogaster	l(3)89El	lethal (3) 89El	l(3)M171	0	0
11644	45587	Drosophila melanogaster	nompA	no mechanoreceptor potential A	CG13207|DmelCG13207|NOMPA|Nomp-A|NompA|nomp	0	0
11645	45588	Drosophila melanogaster	Pdp1	PAR-domain protein 1	CG17888|DmelCG17888|PDP1|PDP1epsilon|Pdp-1|Pdp1epsilon|anon-WO0140519.206|pdp|pdp1|pdp1epsilon	0	0
11646	45589	Drosophila melanogaster	Rst(2)Lb	Resistance to Leptopilina boulardi	Rlb|rlb	0	0
11647	45591	Drosophila melanogaster	l(2)39Aa	lethal (2) 39Aa	-	0	0
11648	45592	Drosophila melanogaster	l(2)39Ad	lethal (2) 39Ad	-	0	0
11649	45593	Drosophila melanogaster	l(2)39Ah	lethal (2) 39Ah	l243	0	0
11650	45594	Drosophila melanogaster	l(3)X12	lethal (3) X12	X12	0	0
11651	45595	Drosophila melanogaster	l(3)X2	lethal (3) X2	X2	0	0
11652	45596	Drosophila melanogaster	l(3)89Bq	lethal (3) 89Bq	l(3)89Bo	0	0

<table>
<tr><td>11653</td><td>45597</td><td>Drosophila melanogaster</td><td>l(3)89Br</td><td>lethal (3) 89Br</td><td>l(3)89Bp</td><td>0</td><td>0</td></tr>
<tr><td>11654</td><td>45598</td><td>Drosophila melanogaster</td><td>l(3)89Bp</td><td>lethal (3) 89Bp</td><td>l(3)89Bn</td><td>0</td><td>0</td></tr>
<tr><td>11655</td><td>45599</td><td>Drosophila melanogaster</td><td>l(3)89Bo</td><td>lethal (3) 89Bo</td><td>l(3)89Bm</td><td>0</td><td>0</td></tr>
<tr><td>11656</td><td>45600</td><td>Drosophila melanogaster</td><td>l(1)EA65</td><td>lethal (1) EA65</td><td>EA65</td><td>0</td><td>0</td></tr>
<tr><td>11657</td><td>45610</td><td>Drosophila melanogaster</td><td>l(3)69Ai</td><td>lethal (3) 69Ai</td><td>l(3)69Ab</td><td>0</td><td>0</td></tr>
<tr><td>11658</td><td>45611</td><td>Drosophila melanogaster</td><td>l(3)69Ah</td><td>lethal (3) 69Ah</td><td>l(3)69Aa</td><td>0</td><td>0</td></tr>
<tr><td>11659</td><td>45612</td><td>Drosophila melanogaster</td><td>l(3)68Fi</td><td>lethal (3) 68Fi</td><td>l(3)68Fe</td><td>0</td><td>0</td></tr>
<tr><td>11660</td><td>45613</td><td>Drosophila melanogaster</td><td>l(3)68Fh</td><td>lethal (3) 68Fh</td><td>l(3)68Fc</td><td>0</td><td>0</td></tr>
<tr><td>11661</td><td>45614</td><td>Drosophila melanogaster</td><td>l(3)68Fg</td><td>lethal (3) 68Fg</td><td>l(3)68Fa</td><td>0</td><td>0</td></tr>
<tr><td>11662</td><td>45615</td><td>Drosophila melanogaster</td><td>l(2)42Bb</td><td>lethal (2) 42Bb</td><td>6G1|l(2)6G1</td><td>0</td><td>0</td></tr>
<tr><td>11663</td><td>45616</td><td>Drosophila melanogaster</td><td>l(1)83B</td><td>lethal (1) 83B</td><td>-</td><td>0</td><td>0</td></tr>
<tr><td>11664</td><td>45617</td><td>Drosophila melanogaster</td><td>l(1)83A</td><td>lethal (1) 83A</td><td>-</td><td>0</td><td>0</td></tr>
<tr><td>11665</td><td>45618</td><td>Drosophila melanogaster</td><td>l(1)36</td><td>lethal (1) 36</td><td>-</td><td>0</td><td>0</td></tr>
<tr><td>11666</td><td>45619</td><td>Drosophila melanogaster</td><td>l(1)29</td><td>lethal (1) 29</td><td>-</td><td>0</td><td>0</td></tr>
<tr><td>11667</td><td>45620</td><td>Drosophila melanogaster</td><td>l(1)24</td><td>lethal (1) 24</td><td>-</td><td>0</td><td>0</td></tr>
<tr><td>11668</td><td>45621</td><td>Drosophila melanogaster</td><td>l(1)22</td><td>lethal (1) 22</td><td>-</td><td>0</td><td>0</td></tr>
<tr><td>11669</td><td>45622</td><td>Drosophila melanogaster</td><td>l(1)16</td><td>lethal (1) 16</td><td>-</td><td>0</td><td>0</td></tr>
<tr><td>11670</td><td>45623</td><td>Drosophila melanogaster</td><td>l(1)14</td><td>lethal (1) 14</td><td>-</td><td>0</td><td>0</td></tr>
<tr><td>11671</td><td>45624</td><td>Drosophila melanogaster</td><td>l(1)04</td><td>lethal (1) 04</td><td>-</td><td>0</td><td>0</td></tr>
<tr><td>11672</td><td>45655</td><td>Drosophila melanogaster</td><td>dom</td><td>domino</td><td>27/4|CG9696|DmelCG9696|Dom|DomA|anon-WO0172774.190|l(2)81/8|l(2)k02704|l(2)k08108|p400</td><td>0</td><td>0</td></tr>
<tr><td>11673</td><td>45668</td><td>Drosophila melanogaster</td><td>Vha68-1</td><td>Vacuolar H[+] ATPase subunit 68-1</td><td>CG12403|DmelCG12403|V-ATPase|Vha|Vha-68-1|Vha68|vha67-2|vha68-1</td><td>0</td><td>0</td></tr>
<tr><td>11674</td><td>45670</td><td>Drosophila melanogaster</td><td>l(3)CB24</td><td>lethal (3) CB24</td><td>-</td><td>0</td><td>0</td></tr>
<tr><td>11675</td><td>45673</td><td>Drosophila melanogaster</td><td>l(2)CA61</td><td>lethal (2) CA61</td><td>-</td><td>0</td><td>0</td></tr>
<tr><td>11676</td><td>45674</td><td>Drosophila melanogaster</td><td>l(2)21Be</td><td>lethal (2) 21Be</td><td>21Be</td><td>0</td><td>0</td></tr>
<tr><td>11677</td><td>45680</td><td>Drosophila melanogaster</td><td>Tapdelta</td><td>Translocon-associated protein delta</td><td>BcDNA:GM12291|CG9035|DmelCG9035|TRAPdelta|l(2)k17005</td><td>0</td><td>0</td></tr>
<tr><td>11678</td><td>45682</td><td>Drosophila melanogaster</td><td>eIF-3p40</td><td>Eukaryotic initiation factor 3 p40 subunit</td><td>BcDNA:GM14618|CG9124|DmelCG9124|eIF-3|eIF3-S3|eIf3-p44|l(2)k09003</td><td>0</td><td>0</td></tr>
<tr><td>11679</td><td>45683</td><td>Drosophila melanogaster</td><td>milt</td><td>milton</td><td>CG13777|CG31630|CG43227|DmelCG43227|Dmel_CG13777|Dmel_CG31630|anon-WO0153538.19|l(2)k04704</td><td>0</td><td>0</td></tr>
<tr><td>11680</td><td>45684</td><td>Drosophila melanogaster</td><td>l(2)IR12</td><td>lethal (2) IR12</td><td>-</td><td>0</td><td>0</td></tr>
<tr><td>11681</td><td>45686</td><td>Drosophila melanogaster</td><td>l(1)M123.2</td><td>lethal (1) M123.2</td><td>M123.2</td><td>0</td><td>0</td></tr>
<tr><td>11682</td><td>45687</td><td>Drosophila melanogaster</td><td>l(1)D39.5</td><td>lethal (1) D39.5</td><td>D39.5</td><td>0</td><td>0</td></tr>
<tr><td>11683</td><td>45688</td><td>Drosophila melanogaster</td><td>l(1)B8.5</td><td>lethal (1) B8.5</td><td>B8.5</td><td>0</td><td>0</td></tr>
<tr><td>11684</td><td>45695</td><td>Drosophila melanogaster</td><td>l(2)46Ec</td><td>lethal (2) 46Ec</td><td>5.2|G|unnamed</td><td>0</td><td>0</td></tr>
<tr><td>11685</td><td>45698</td><td>Drosophila melanogaster</td><td>l(1)20Ag</td><td>lethal (1) 20Ag</td><td>B2/45.8</td><td>0</td><td>0</td></tr>
<tr><td>11686</td><td>45699</td><td>Drosophila melanogaster</td><td>l(1)19Fh</td><td>lethal (1) 19Fh</td><td>B2/45.4</td><td>0</td><td>0</td></tr>
<tr><td>11687</td><td>45700</td><td>Drosophila melanogaster</td><td>l(1)19Eg</td><td>lethal (1) 19Eg</td><td>B2/32.16</td><td>0</td><td>0</td></tr>
<tr><td>11688</td><td>45701</td><td>Drosophila melanogaster</td><td>l(1)19Cd</td><td>lethal (1) 19Cd</td><td>800D9</td><td>0</td><td>0</td></tr>
<tr><td>11689</td><td>45702</td><td>Drosophila melanogaster</td><td>eggroll</td><td>eggroll</td><td>-</td><td>0</td><td>0</td></tr>
<tr><td>11690</td><td>45706</td><td>Drosophila melanogaster</td><td>Cyp4d14</td><td>CG3540 gene product from transcript CG3540-RA</td><td>4d14|CG3540|CYP4D14|DmelCG3540|EG:152A3.2</td><td>0</td><td>0</td></tr>
<tr><td>11691</td><td>45707</td><td>Drosophila melanogaster</td><td>CG18031</td><td>CG18031 gene product from transcript CG18031-RA</td><td>DmelCG18031|EG:103B4.2</td><td>0</td><td>0</td></tr>
<tr><td>11692</td><td>45708</td><td>Drosophila melanogaster</td><td>l(2)39Ea</td><td>lethal (2) 39Ea</td><td>-</td><td>0</td><td>0</td></tr>
<tr><td>11693</td><td>45715</td><td>Drosophila melanogaster</td><td>l(2)k04102</td><td>lethal (2) k04102</td><td>41/2</td><td>0</td><td>0</td></tr>
<tr><td>11694</td><td>45717</td><td>Drosophila melanogaster</td><td>l(3)63Dc</td><td>lethal (3) 63Dc</td><td>unnamed</td><td>0</td><td>0</td></tr>
<tr><td>11695</td><td>45718</td><td>Drosophila melanogaster</td><td>l(3)63Db</td><td>lethal (3) 63Db</td><td>c.g.15</td><td>0</td><td>0</td></tr>
<tr><td>11696</td><td>45719</td><td>Drosophila melanogaster</td><td>l(2)k11560</td><td>lethal (2) k11560</td><td>115/60</td><td>0</td><td>0</td></tr>
<tr><td>11697</td><td>45720</td><td>Drosophila melanogaster</td><td>l(2)k09931</td><td>lethal (2) k09931</td><td>99/31</td><td>0</td><td>0</td></tr>
<tr><td>11698</td><td>45721</td><td>Drosophila melanogaster</td><td>l(2)k07822</td><td>lethal (2) k07822</td><td>78/22</td><td>0</td><td>0</td></tr>
<tr><td>11699</td><td>45722</td><td>Drosophila melanogaster</td><td>l(2)k07813</td><td>lethal (2) k07813</td><td>78/13</td><td>0</td><td>0</td></tr>
<tr><td>11700</td><td>45723</td><td>Drosophila melanogaster</td><td>l(2)k07620</td><td>lethal (2) k07620</td><td>76/20</td><td>0</td><td>0</td></tr>
<tr><td>11701</td><td>45724</td><td>Drosophila melanogaster</td><td>l(2)k07403</td><td>lethal (2) k07403</td><td>74/3</td><td>0</td><td>0</td></tr>
<tr><td>11702</td><td>45725</td><td>Drosophila melanogaster</td><td>l(1)LefEM7</td><td>lethal (1) LefEM7</td><td>EM7</td><td>0</td><td>0</td></tr>
<tr><td>11703</td><td>45726</td><td>Drosophila melanogaster</td><td>l(1)LefEM3</td><td>lethal (1) LefEM3</td><td>EM3</td><td>0</td><td>0</td></tr>
<tr><td>11704</td><td>45727</td><td>Drosophila melanogaster</td><td>l(1)LefEM2</td><td>lethal (1) LefEM2</td><td>EM2</td><td>0</td><td>0</td></tr>
<tr><td>11705</td><td>45728</td><td>Drosophila melanogaster</td><td>l(1)LefEM1</td><td>lethal (1) LefEM1</td><td>EM1</td><td>0</td><td>0</td></tr>
<tr><td>11706</td><td>45731</td><td>Drosophila melanogaster</td><td>l(2)52Fe</td><td>lethal (2) 52Fe</td><td>E6.17</td><td>0</td><td>0</td></tr>
<tr><td>11707</td><td>45732</td><td>Drosophila melanogaster</td><td>l(2)52Fd</td><td>lethal (2) 52Fd</td><td>E3.27</td><td>0</td><td>0</td></tr>
<tr><td>11708</td><td>45733</td><td>Drosophila melanogaster</td><td>l(2)52Fc</td><td>lethal (2) 52Fc</td><td>D8.13</td><td>0</td><td>0</td></tr>
<tr><td>11709</td><td>45734</td><td>Drosophila melanogaster</td><td>l(2)52Fb</td><td>lethal (2) 52Fb</td><td>D2.33</td><td>0</td><td>0</td></tr>
<tr><td>11710</td><td>45739</td><td>Drosophila melanogaster</td><td>SuUR</td><td>Suppressor of Under-Replication</td><td>CG7869|DmelCG7869|Dromel_CG7869_FBtr0076203_suur_mORF|SU(UR)|SUUR|Su(UR)|Su(UR)ES|su(UR)ES</td><td>0</td><td>0</td></tr>
<tr><td>11711</td><td>45740</td><td>Drosophila melanogaster</td><td>l(3)88Ad</td><td>lethal (3) 88Ad</td><td>-</td><td>0</td><td>0</td></tr>
<tr><td>11712</td><td>45741</td><td>Drosophila melanogaster</td><td>l(3)82Eb</td><td>lethal (3) 82Eb</td><td>-</td><td>0</td><td>0</td></tr>
<tr><td>11713</td><td>45742</td><td>Drosophila melanogaster</td><td>l(3)82Ea</td><td>lethal (3) 82Ea</td><td>-</td><td>0</td><td>0</td></tr>
<tr><td>11714</td><td>45743</td><td>Drosophila melanogaster</td><td>l(3)82Db</td><td>lethal (3) 82Db</td><td>-</td><td>0</td><td>0</td></tr>
<tr><td>11715</td><td>45744</td><td>Drosophila melanogaster</td><td>l(3)82Da</td><td>lethal (3) 82Da</td><td>-</td><td>0</td><td>0</td></tr>
<tr><td>11716</td><td>45745</td><td>Drosophila melanogaster</td><td>l(3)73Bh</td><td>lethal (3) 73Bh</td><td>-</td><td>0</td><td>0</td></tr>
<tr><td>11717</td><td>45746</td><td>Drosophila melanogaster</td><td>l(3)73Bg</td><td>lethal (3) 73Bg</td><td>-</td><td>0</td><td>0</td></tr>
<tr><td>11718</td><td>45747</td><td>Drosophila melanogaster</td><td>l(3)73Bf</td><td>lethal (3) 73Bf</td><td>-</td><td>0</td><td>0</td></tr>
<tr><td>11719</td><td>45749</td><td>Drosophila melanogaster</td><td>l(3)73An</td><td>lethal (3) 73An</td><td>-</td><td>0</td><td>0</td></tr>
<tr><td>11720</td><td>45750</td><td>Drosophila melanogaster</td><td>l(3)73Am</td><td>lethal (3) 73Am</td><td>-</td><td>0</td><td>0</td></tr>
<tr><td>11721</td><td>45751</td><td>Drosophila melanogaster</td><td>l(3)73Al</td><td>lethal (3) 73Al</td><td>-</td><td>0</td><td>0</td></tr>
<tr><td>11722</td><td>45752</td><td>Drosophila melanogaster</td><td>l(3)73Ak</td><td>lethal (3) 73Ak</td><td>-</td><td>0</td><td>0</td></tr>
<tr><td>11723</td><td>45753</td><td>Drosophila melanogaster</td><td>l(3)72Fc</td><td>lethal (3) 72Fc</td><td>-</td><td>0</td><td>0</td></tr>
<tr><td>11724</td><td>45754</td><td>Drosophila melanogaster</td><td>l(3)72Fb</td><td>lethal (3) 72Fb</td><td>-</td><td>0</td><td>0</td></tr>
<tr><td>11725</td><td>45755</td><td>Drosophila melanogaster</td><td>l(3)72Fa</td><td>lethal (3) 72Fa</td><td>-</td><td>0</td><td>0</td></tr>
<tr><td>11726</td><td>45757</td><td>Drosophila melanogaster</td><td>beta'Cop</td><td>beta'-coatomer protein</td><td>CG6699|COPIbeta'|DmelCG6699|beta'-COP|beta'COP|dbeta'COP</td><td>0</td><td>0</td></tr>
<tr><td>11727</td><td>45758</td><td>Drosophila melanogaster</td><td>l(3)93Dm</td><td>lethal (3) 93Dm</td><td>unnamed</td><td>0</td><td>0</td></tr>
<tr><td>11728</td><td>45759</td><td>Drosophila melanogaster</td><td>l(3)93Dl</td><td>lethal (3) 93Dl</td><td>E23|unnamed</td><td>0</td><td>0</td></tr>
<tr><td>11729</td><td>45760</td><td>Drosophila melanogaster</td><td>l(3)93Dk</td><td>lethal (3) 93Dk</td><td>unnamed</td><td>0</td><td>0</td></tr>
<tr><td>11730</td><td>45761</td><td>Drosophila melanogaster</td><td>l(3)89En</td><td>lethal (3) 89En</td><td>l(3)M2511</td><td>0</td><td>0</td></tr>
<tr><td>11731</td><td>45762</td><td>Drosophila melanogaster</td><td>l(3)89Em</td><td>lethal (3) 89Em</td><td>l(3)M96</td><td>0</td><td>0</td></tr>
<tr><td>11732</td><td>45763</td><td>Drosophila melanogaster</td><td>l(3)89Df</td><td>lethal (3) 89Df</td><td>l(3)M2922</td><td>0</td><td>0</td></tr>
<tr><td>11733</td><td>45764</td><td>Drosophila melanogaster</td><td>l(3)89De</td><td>lethal (3) 89De</td><td>l(3)M188</td><td>0</td><td>0</td></tr>
<tr><td>11734</td><td>45765</td><td>Drosophila melanogaster</td><td>l(3)89Dd</td><td>lethal (3) 89Dd</td><td>l(3)M175</td><td>0</td><td>0</td></tr>
<tr><td>11735</td><td>45766</td><td>Drosophila melanogaster</td><td>l(3)89Ci</td><td>lethal (3) 89Ci</td><td>l(3)M238</td><td>0</td><td>0</td></tr>
<tr><td>11736</td><td>45767</td><td>Drosophila melanogaster</td><td>l(3)89Ch</td><td>lethal (3) 89Ch</td><td>l(3)M2912</td><td>0</td><td>0</td></tr>
<tr><td>11737</td><td>45768</td><td>Drosophila melanogaster</td><td>l(3)89Cg</td><td>lethal (3) 89Cg</td><td>l(3)M191</td><td>0</td><td>0</td></tr>
<tr><td>11738</td><td>45769</td><td>Drosophila melanogaster</td><td>l(3)89Ad</td><td>lethal (3) 89Ad</td><td>-</td><td>0</td><td>0</td></tr>
<tr><td>11739</td><td>45770</td><td>Drosophila melanogaster</td><td>l(3)89Ac</td><td>lethal (3) 89Ac</td><td>-</td><td>0</td><td>0</td></tr>
<tr><td>11740</td><td>45771</td><td>Drosophila melanogaster</td><td>l(3)89Ab</td><td>lethal (3) 89Ab</td><td>-</td><td>0</td><td>0</td></tr>
<tr><td>11741</td><td>45772</td><td>Drosophila melanogaster</td><td>l(2)37Ad</td><td>lethal (2) 37Ad</td><td>l(2)02660|l(2)02660r</td><td>0</td><td>0</td></tr>
<tr><td>11742</td><td>45775</td><td>Drosophila melanogaster</td><td>mei-P26</td><td>CG12218 gene product from transcript CG12218-RA</td><td>AF145661|BcDNA:GH10646|CG12218|DmelCG12218|MEI-P26|Mei-P26</td><td>0</td><td>0</td></tr>
<tr><td>11743</td><td>45779</td><td>Drosophila melanogaster</td><td>l(2)17E1</td><td>lethal (2) 17E1</td><td>-</td><td>0</td><td>0</td></tr>
<tr><td>11744</td><td>45780</td><td>Drosophila melanogaster</td><td>Prosalpha1</td><td>Proteasome alpha1 subunit</td><td>18495|CG18495|DmelCG18495|PSA6_DROME|Prosalpha6|l(2)SH2 2342|l(2)SH2342|prosalpha1</td><td>0</td><td>0</td></tr>
<tr><td>11745</td><td>45783</td><td>Drosophila melanogaster</td><td>Aats-val</td><td>Valyl-tRNA synthetase</td><td>BEST:LD45671|CG4062|DmelCG4062|VRS|ValRS|l(2)03531|l(2)rI255|ld45671</td><td>0</td><td>0</td></tr>
<tr><td>11746</td><td>45784</td><td>Drosophila melanogaster</td><td>Aats-thr</td><td>Threonyl-tRNA synthetase</td><td>42/3|CG5353|DmelCG5353|P539|TRS|ThrRS|l(2)k04203|threonyl-tRNA synthetase</td><td>0</td><td>0</td></tr>
<tr><td>11747</td><td>45785</td><td>Drosophila melanogaster</td><td>Aats-ile</td><td>Isoleucyl-tRNA synthetase</td><td>CG11471|DmelCG11471|IRS|IleRS|l(3)00827</td><td>0</td><td>0</td></tr>
<tr><td>11748</td><td>45786</td><td>Drosophila melanogaster</td><td>Aats-gln</td><td>Glutaminyl-tRNA synthetase</td><td>Aast-g1n|BcDNA:GH11673|CG10506|DmelCG10506|GlnRS|QRS|l(3)05461|qars</td><td>0</td><td>0</td></tr>
<tr><td>11749</td><td>45787</td><td>Drosophila melanogaster</td><td>l(3)70Ae</td><td>lethal (3) 70Ae</td><td>Ly-2</td><td>0</td><td>0</td></tr>
<tr><td>11750</td><td>45789</td><td>Drosophila melanogaster</td><td>l(2)PN-6</td><td>lethal (2) PN-6</td><td>-</td><td>0</td><td>0</td></tr>
<tr><td>11751</td><td>45790</td><td>Drosophila melanogaster</td><td>l(2)KC-E</td><td>l(2)Karp and Cherbas E</td><td>E</td><td>0</td><td>0</td></tr>
<tr><td>11752</td><td>45791</td><td>Drosophila melanogaster</td><td>l(2)KC-D</td><td>l(2)Karp and Cherbas D</td><td>D</td><td>0</td><td>0</td></tr>
<tr><td>11753</td><td>45792</td><td>Drosophila melanogaster</td><td>l(2)KC-C</td><td>l(2)Karp and Cherbas C</td><td>C</td><td>0</td><td>0</td></tr>
<tr><td>11754</td><td>45793</td><td>Drosophila melanogaster</td><td>l(2)KC-B</td><td>l(2)Karp and Cherbas B</td><td>B</td><td>0</td><td>0</td></tr>
<tr><td>11755</td><td>45794</td><td>Drosophila melanogaster</td><td>l(2)KC-A</td><td>l(2)Karp and Cherbas A</td><td>A</td><td>0</td><td>0</td></tr>
<tr><td>11756</td><td>45795</td><td>Drosophila melanogaster</td><td>l(2)60Ai</td><td>lethal (2) 60Ai</td><td>-</td><td>0</td><td>0</td></tr>
<tr><td>11757</td><td>45796</td><td>Drosophila melanogaster</td><td>l(2)60Ah</td><td>lethal (2) 60Ah</td><td>ET/141|unnamed</td><td>0</td><td>0</td></tr>
<tr><td>11758</td><td>45797</td><td>Drosophila melanogaster</td><td>l(2)60Ag</td><td>lethal (2) 60Ag</td><td>unnamed</td><td>0</td><td>0</td></tr>
<tr><td>11759</td><td>45798</td><td>Drosophila melanogaster</td><td>l(2)60Af</td><td>lethal (2) 60Af</td><td>unnamed</td><td>0</td><td>0</td></tr>
<tr><td>11760</td><td>45802</td><td>Drosophila melanogaster</td><td>l(3)72Dq</td><td>lethal (3) 72Dq</td><td>unnamed</td><td>0</td><td>0</td></tr>
<tr><td>11761</td><td>45803</td><td>Drosophila melanogaster</td><td>l(2)46Ck</td><td>lethal (2) 46Ck</td><td>Q</td><td>0</td><td>0</td></tr>
<tr><td>11762</td><td>45810</td><td>Drosophila melanogaster</td><td>l(1)10Ak</td><td>lethal (1) 10Ak</td><td>unnamed</td><td>0</td><td>0</td></tr>
<tr><td>11763</td><td>45815</td><td>Drosophila melanogaster</td><td>Spn27A</td><td>Serpin 27A</td><td>CG11331|Dm-Spn27A|DmelCG11331|EST1|Spn27|sp27A|spn27A</td><td>0</td><td>0</td></tr>
</table>

11764	45816	Drosophila melanogaster	onecut	CG1922 gene product from transcript CG1922-RA	A1|CG1922|DmelCG1922	0	0
11765	45821	Drosophila melanogaster	Abl	Abl tyrosine kinase	4674|ABL|Abl1|AblK|Ableson|Am ABL|C-abl|CG4032|D-abl|D-ash|DAbl|DROTKABL3|Dabl|Dash|Ddash/abl|DmelCG4032|Dsrc7|abl|abl1|c-abl|cAbl|dAbl|l(3)04674|l(3)73Ba|l(3)c-abl	0	0
11766	45823	Drosophila melanogaster	aor	abdominal one reduced	-	0	0
11767	45825	Drosophila melanogaster	as	ascute	-	0	0
11768	45826	Drosophila melanogaster	bel	belle	BEL|Bel|CG9748|DmRH6|DmelCG9748|L3|anon-85Ab|cap|cg9748|l(3)85Ac|l(3)L3|l(3)L4740|l(3)S047110|l(3)S097074|ms(3)Pneo85A|ms(3)neo30	0	0
11769	45827	Drosophila melanogaster	bic	bicaudal	107/12|5A|BcDNA.GM05329|BcDNA:GM05329|Bic|Btf|CG3644|DmelCG3644|E(2)Bic|E(Bic)|l(2)49Da|l(2)k10712|l(2)vr22|vr22	0	0
11770	45828	Drosophila melanogaster	blo	bloated	ba|kit	0	0
11771	45829	Drosophila melanogaster	bul	bulge	-	0	0
11772	45830	Drosophila melanogaster	bur	burgundy	CG9242|DmelCG9242|ES2-2|GMPS|Gmps|P174|anon-WO0118547.193|anon-WO0118547.204|gua2|l(2)10523	0	0
11773	45831	Drosophila melanogaster	cab	cabbage	-	0	0
11774	45832	Drosophila melanogaster	cau	cauliflower	-	0	0
11775	45833	Drosophila melanogaster	cel	celibate	-	0	0
11776	45836	Drosophila melanogaster	Cma	Comma	-	0	0
11777	45837	Drosophila melanogaster	cop	copper	-	0	0
11778	45840	Drosophila melanogaster	cpo	couch potato	1824|3603|5339|6015|BcDNA:AT31405|CG12349|CG18434|CG18435|CG31243|CPO|Cpo|DmelCG31243|E(sda)N|Line N|ORE-17|cg18435|l(3)01432|l(3)6015|l(3)j4A1|l(3)j4D4|l(3)j7E2|l(3)j9B3|l(3)rJ553|l(3)s23	0	0
11779	45841	Drosophila melanogaster	crt	crumpled tips	-	0	0
11780	45845	Drosophila melanogaster	Dsk	Drosulfakinin	BcDNA:RH46857|CCK|CG18090|DSK|DSK-I|DmelCG18090|Drm-SK|Drm-SK-0|Drm-SK-1|Drm-SK-2|DrmSK-I|DrmSK-II|SK-0|dsk	0	0
11781	45846	Drosophila melanogaster	dsp	dispersed	-	0	0
11782	45848	Drosophila melanogaster	e(y)2	enhancer of yellow 2	CG15191|DmelCG15191|E(y)2|ENY2|d|e(y)[2]	0	0
11783	45850	Drosophila melanogaster	e(y)5	enhancer of yellow 5	-	0	0
11784	45851	Drosophila melanogaster	e(y)6	enhancer of yellow 6	-	0	0
11785	45852	Drosophila melanogaster	febA	feeding behavior A	-	0	0
11786	45853	Drosophila melanogaster	fl	fluted	-	0	0
11787	45854	Drosophila melanogaster	fl(1)302	female lethal 302	-	0	0
11788	45855	Drosophila melanogaster	fs(1)C3	female sterile (1) C3	-	0	0
11789	45856	Drosophila melanogaster	fs(1)M15	female sterile (1) M15	-	0	0
11790	45857	Drosophila melanogaster	fs(1)M39	female sterile (1) M39	-	0	0
11791	45858	Drosophila melanogaster	fs(1)M48	female sterile (1) M48	-	0	0
11792	45859	Drosophila melanogaster	fs(1)M68	female sterile (1) M68	unnamed	0	0
11793	45860	Drosophila melanogaster	fs(1)M70	female sterile (1) M70	-	0	0
11794	45861	Drosophila melanogaster	fs(1)M74	female sterile (1) M74	-	0	0
11795	45862	Drosophila melanogaster	fs(1)M76	female sterile (1) M76	-	0	0
11796	45863	Drosophila melanogaster	fs(1)M77	female sterile (1) M77	-	0	0
11797	45864	Drosophila melanogaster	fs(1)ne8	female sterile (1) no eggs 8	ne8	0	0
11798	45865	Drosophila melanogaster	fs(1)se	female sterile (1) small eggs	-	0	0
11799	45871	Drosophila melanogaster	grau	grauzone	CG3282|CG33133|DmelCG33133	0	0
11800	45872	Drosophila melanogaster	gy	gouty legs	-	0	0
11801	45873	Drosophila melanogaster	hal	halted	-	0	0
11802	45874	Drosophila melanogaster	hap	hapless	-	0	0
11803	45875	Drosophila melanogaster	Hex-A	Hexokinase A	CG3001|DM1|DmelCG3001|HEX|HEX-A|HK|Hex|Hex-A:B	0	0
11804	45879	Drosophila melanogaster	ImpE1	Ecdysone-inducible gene E1	BcDNA:RE39082|CG13668|CG32356|CG7116|CT22003|CT33103|DmelCG32356|IMP-E1|Imp-E1	0	0
11805	45880	Drosophila melanogaster	ImpL3	Ecdysone-inducible gene L3	CG10160|DmelCG10160|IMP-L3|IMPL3|Imp-L3|Impl3|LDH|Ld|Ldh|dLdha|dmLDH	0	0
11806	45881	Drosophila melanogaster	ix	intersex	CG13201|DmelCG13201|MED29	0	0
11807	45882	Drosophila melanogaster	jvl	javelinlike	-	0	0
11808	45883	Drosophila melanogaster	kar	karmoisin	CG12286|DmelCG12286	0	0
11809	45884	Drosophila melanogaster	kkv	krotzkopf verkehrt	CG2666|CS-1|CS-1/kkv|ChSB|DmCS-1|DmeChSB|DmelCG2666|Kkv|blimp|l(3)82Fh	0	0
11810	45885	Drosophila melanogaster	stout	strung out	JNXL1|jnL1	0	0
11811	45886	Drosophila melanogaster	l(1)6Fb	lethal (1) 6Fb	-	0	0
11812	45887	Drosophila melanogaster	l(1)6Fc	lethal (1) 6Fc	-	0	0
11813	45888	Drosophila melanogaster	l(1)7Be	lethal (1) 7Be	-	0	0
11814	45889	Drosophila melanogaster	l(1)7Bf	lethal (1) 7Bf	-	0	0
11815	45890	Drosophila melanogaster	l(1)7Cf	lethal (1) 7Cf	-	0	0
11816	45891	Drosophila melanogaster	l(1)7Cg	lethal (1) 7Cg	-	0	0
11817	45893	Drosophila melanogaster	ld	loboid	-	0	0
11818	45894	Drosophila melanogaster	lds	lodestar	CG2684|DmF2|DmelCG2684|Fs(3)Hor|Fs(3)Horka|Fs(3)Sz11|Horka|Lds|NTef2|early|lod	0	0
11819	45895	Drosophila melanogaster	lfl	little fly-like	10|11|13|B-56|B-96|J22|l(1)B56	0	0
11820	45896	Drosophila melanogaster	lon	longevity	-	0	0
11821	45899	Drosophila melanogaster	M(1)3E	Minute (1) 3E	-	0	0
11822	45901	Drosophila melanogaster	Me	Moire	Mo	0	0
11823	45902	Drosophila melanogaster	mel	melanized	-	0	0
11824	45903	Drosophila melanogaster	mfd	myofibrillar defect	-	0	0
11825	45904	Drosophila melanogaster	mi	minus	-	0	0
11826	45905	Drosophila melanogaster	Mpe	Monoplane	Mpl	0	0
11827	45906	Drosophila melanogaster	mr	morula	APC2|APC2/mr|Apc2|CG3060|DmelCG3060|dAPC2	0	0
11828	45908	Drosophila melanogaster	nec	necrotic	CG1857|DmelCG1857|Nec|SER3|Ser3|Spn43Ac|anon-WO0118057.3	0	0
11829	45909	Drosophila melanogaster	olfB	olfactory B	-	0	0
11830	45910	Drosophila melanogaster	ort	ora transientless	CG7411|DMHisClalpha1|Dm-hclA|DmHisCl2|DmelCG7411|GluCl|HisCl2|HisCl|HisCl2|Hist1|Ort|hclA|hisCl2|ora	0	0
11831	45911	Drosophila melanogaster	pa	patulous	-	0	0
11832	45912	Drosophila melanogaster	pal	paternal loss inducer	-	0	0
11833	45913	Drosophila melanogaster	pd	purpleoid	-	0	0
11834	45914	Drosophila melanogaster	pdf	pod foot	-	0	0
11835	45915	Drosophila melanogaster	plk	plucked	-	0	0
11836	45916	Drosophila melanogaster	plo	pillow	pil	0	0
11837	45917	Drosophila melanogaster	pph	polyphenic	-	0	0
11838	45918	Drosophila melanogaster	Px	Plexate	-	0	0
11839	45919	Drosophila melanogaster	qrt	quartet	l(3)C21|l(3)c21R|l(3)c21r|l(3)ds-1	0	0
11840	45921	Drosophila melanogaster	r(ADH)	regulator of ADH	-	0	0
11841	45922	Drosophila melanogaster	rap	retina aberrant in pattern	APC/C[Fzr/Cdh1]|CDH1|CG3000|Cdh1|Cdh1/Fzr|Cdh1[Fzr]|Cdh[Fzr]|DmelCG3000|Fxr|Fzr|Fzr/rap|Hec/Cdh[Fzr]|Rap/Fzr|anon-WO03040301.193|cdh1|dFZR|fzr|l(1)G0326|l(1)G0418|rap/Fzr|rap/fizzy-related	0	0
11842	45923	Drosophila melanogaster	red	red Malpighian tubules	-	0	0
11843	45924	Drosophila melanogaster	Rev	Revolute	-	0	0
11844	45927	Drosophila melanogaster	sesE	stress-sensitive E	-	0	0
11845	45928	Drosophila melanogaster	shi	shibire	CG18102|DYN|Ddyn|Ddyn3|Ddyn4|DmelCG18102|Dyn|Shi|anon-WO0153538.12|anon-WO0153538.13|anon-WO0153538.14|dDyn|dyn|l(1)VII|l(1)shi	0	0
11846	45929	Drosophila melanogaster	slm	slim	-	0	0
11847	45930	Drosophila melanogaster	sp	speck	-	0	0
11848	45931	Drosophila melanogaster	spir	spire	38C.34|38C.37|CG10076|CG18621|DmelCG10076|Spir|Spire|l(2)08327|p150-Spir|p150Spir	0	0
11849	45932	Drosophila melanogaster	Spl	Splayed	-	0	0
11850	45936	Drosophila melanogaster	Su(stn)	Suppressor of stoned	-	0	0
11851	45937	Drosophila melanogaster	Su(var)	Suppressor of variegation	Su-V	0	0
11852	45939	Drosophila melanogaster	ta	tapered	-	0	0
11853	45940	Drosophila melanogaster	Tch	Thin chaetae	-	0	0
11854	45942	Drosophila melanogaster	Tpl	Triplolethal	-	0	0
11855	45943	Drosophila melanogaster	twg	twisted genitals	-	0	0
11856	45944	Drosophila melanogaster	tyl	tinylike	ty-l	0	0
11857	45945	Drosophila melanogaster	urd	urdur	-	0	0
11858	45946	Drosophila melanogaster	vac	vacuolated	-	0	0
11859	45947	Drosophila melanogaster	vao	varied outspread	-	0	0
11860	45948	Drosophila melanogaster	vin	vin	-	0	0
11861	45950	Drosophila melanogaster	Vn	Vein	-	0	0
11862	45951	Drosophila melanogaster	wap	wings apart	15|16|19|20|21|22|Q217|Q464|l(1)A-200|l(1)Q217|l(1)Q464	0	0
11863	45952	Drosophila melanogaster	wrl	whirligig	nc4	0	0
11864	45953	Drosophila melanogaster	wt	welt	-	0	0
11865	45954	Drosophila melanogaster	wy	wavy	-	0	0
11866	45956	Drosophila melanogaster	cwi	crumpled wings	-	0	0
11867	45957	Drosophila melanogaster	ovt	ovarian tumor	-	0	0
11868	45958	Drosophila melanogaster	hum	humilis	-	0	0
11869	45959	Drosophila melanogaster	mts	microtubule star	5559|CG7109|DmPp2A-28D|DmelCG7109|ER2-6|MTS/PP2A|Mts|PP2|PP2A|PP2A 28D|PP2A C|PP2A-C|PP2A[C]|PP2A[[C]]|PP2Ac|PP2a|PP2a 28D|Pp2A|Pp2A-28D|dPP2A|l(2)02496|l(2)s5286	0	0
11870	45960	Drosophila melanogaster	aly	always early	143450_at|Aly|CG2075|DmelCG2075|ms(3)2|ms(3)ry2	0	0
11871	45961	Drosophila melanogaster	Pep	Protein on ecdysone puffs	CG6143|DmelCG6143|PEP|PEP/X4|X4/PEP|p100|pep	0	0
11872	45962	Drosophila melanogaster	msopa	male-specific opa containing gene	CG14560|DmelCG14560	0	0
11873	45963	Drosophila melanogaster	gustA	gustatory-defective A	gust-A	0	0
11874	45964	Drosophila melanogaster	prd10	paired-like 10	-	0	0

<table>
<tr><td>11875</td><td>45965</td><td>Drosophila melanogaster</td><td>prd11</td><td>paired-like 11</td><td>-</td><td>0</td><td>0</td></tr>
<tr><td>11876</td><td>45966</td><td>Drosophila melanogaster</td><td>str</td><td>stripes</td><td>-</td><td>0</td><td>0</td></tr>
<tr><td>11877</td><td>45969</td><td>Drosophila melanogaster</td><td>gwl</td><td>greatwall</td><td>1J|CG7719|CG7719-related|CT23435|DmelCG7719|EP0515|Pk91C|Pk?2|anon-WO2004063362.73|scant</td><td>0</td><td>0</td></tr>
<tr><td>11878</td><td>45970</td><td>Drosophila melanogaster</td><td>Pk17E</td><td>Protein kinase-like 17E</td><td>3-10|BIN4|Bin4|CG7001|DmelCG7001|Pk?4|bin4|pk17e</td><td>0</td><td>0</td></tr>
<tr><td>11879</td><td>45973</td><td>Drosophila melanogaster</td><td>fs(1)N</td><td>female sterile (1) Nasrat</td><td>371|8D8.1|CG11411|DmelCG11411|EG:8D8.1|Fs(1)N|Nas|fs(1)A371|fs(1)K1540|fs(1)M6|fs(1)Nas|fs(1)Nasrat|fs(1)[nas]</td><td>0</td><td>0</td></tr>
<tr><td>11880</td><td>45974</td><td>Drosophila melanogaster</td><td>mbd</td><td>mushroom body deranged</td><td>-</td><td>0</td><td>0</td></tr>
<tr><td>11881</td><td>45975</td><td>Drosophila melanogaster</td><td>mei-I1</td><td>meiotic I1</td><td>-</td><td>0</td><td>0</td></tr>
<tr><td>11882</td><td>45976</td><td>Drosophila melanogaster</td><td>retn</td><td>retained</td><td>2535|BcDNA:LD35748|CG5403|DRI|Dead Ringer/Retain|DmelCG5403|Dri|EY2-10|dri|l(2)02535|retn/dri</td><td>0</td><td>0</td></tr>
<tr><td>11883</td><td>45977</td><td>Drosophila melanogaster</td><td>fir</td><td>fireworks</td><td>-</td><td>0</td><td>0</td></tr>
<tr><td>11884</td><td>45980</td><td>Drosophila melanogaster</td><td>elA</td><td>elbow A</td><td>el|el[A]|elb</td><td>0</td><td>0</td></tr>
<tr><td>11885</td><td>45983</td><td>Drosophila melanogaster</td><td>abg</td><td>ambiguous</td><td>-</td><td>0</td><td>0</td></tr>
<tr><td>11886</td><td>45984</td><td>Drosophila melanogaster</td><td>coq</td><td>coquilled</td><td>-</td><td>0</td><td>0</td></tr>
<tr><td>11887</td><td>45985</td><td>Drosophila melanogaster</td><td>lar</td><td>larvaphile</td><td>-</td><td>0</td><td>0</td></tr>
<tr><td>11888</td><td>45986</td><td>Drosophila melanogaster</td><td>plp</td><td>polyphase</td><td>-</td><td>0</td><td>0</td></tr>
<tr><td>11889</td><td>45987</td><td>Drosophila melanogaster</td><td>juv</td><td>juvenile</td><td>-</td><td>0</td><td>0</td></tr>
<tr><td>11890</td><td>45988</td><td>Drosophila melanogaster</td><td>ado</td><td>adolescent</td><td>l(1)4P1</td><td>0</td><td>0</td></tr>
<tr><td>11891</td><td>45989</td><td>Drosophila melanogaster</td><td>l(2)CA6</td><td>lethal (2) CA6</td><td>l(2)br6</td><td>0</td><td>0</td></tr>
<tr><td>11892</td><td>45990</td><td>Drosophila melanogaster</td><td>l(2)S3a</td><td>lethal of Seto 3a</td><td>-</td><td>0</td><td>0</td></tr>
<tr><td>11893</td><td>45991</td><td>Drosophila melanogaster</td><td>l(2)S32</td><td>lethal of Seto 32</td><td>-</td><td>0</td><td>0</td></tr>
<tr><td>11894</td><td>45992</td><td>Drosophila melanogaster</td><td>l(2)S55</td><td>lethal of Seto 55</td><td>-</td><td>0</td><td>0</td></tr>
<tr><td>11895</td><td>45993</td><td>Drosophila melanogaster</td><td>M(1)7BC</td><td>Minute (1) 7BC</td><td>-</td><td>0</td><td>0</td></tr>
<tr><td>11896</td><td>45994</td><td>Drosophila melanogaster</td><td>fs(1)M3</td><td>female sterile (1) M3</td><td>CG4790|DmelCG4790|Fs(1)K646|fs(1)K646|fs(1)ph|fs(1)phl|fs(1)pole hole|fs(1)polehole</td><td>0</td><td>0</td></tr>
<tr><td>11897</td><td>45995</td><td>Drosophila melanogaster</td><td>gat</td><td>gate</td><td>psi 2|psi-2|psi2</td><td>0</td><td>0</td></tr>
<tr><td>11898</td><td>45996</td><td>Drosophila melanogaster</td><td>l(1)adl4</td><td>lethal (1) adl4</td><td>l(1)adl-4</td><td>0</td><td>0</td></tr>
<tr><td>11899</td><td>45997</td><td>Drosophila melanogaster</td><td>l(1)adl5</td><td>lethal (1) adl5</td><td>l(1)adl-5</td><td>0</td><td>0</td></tr>
<tr><td>11900</td><td>45998</td><td>Drosophila melanogaster</td><td>l(1)adl6</td><td>lethal (1) adl6</td><td>l(1)adl-6</td><td>0</td><td>0</td></tr>
<tr><td>11901</td><td>45999</td><td>Drosophila melanogaster</td><td>l(1)v455</td><td>lethal (1) v455</td><td>l(1)455</td><td>0</td><td>0</td></tr>
<tr><td>11902</td><td>46000</td><td>Drosophila melanogaster</td><td>scw</td><td>screw</td><td>BcDNA:LD30945|CG31695|DmelCG31695|SCW|Scw|l(2)37Ff|l(2)IG76|mor2</td><td>0</td><td>0</td></tr>
<tr><td>11903</td><td>46001</td><td>Drosophila melanogaster</td><td>Nc18A</td><td>Neural conserved at 18A</td><td>NC18A</td><td>0</td><td>0</td></tr>
<tr><td>11904</td><td>46002</td><td>Drosophila melanogaster</td><td>l(1)TK128</td><td>lethal (1) TK128</td><td>l128</td><td>0</td><td>0</td></tr>
<tr><td>11905</td><td>46003</td><td>Drosophila melanogaster</td><td>L12</td><td>L12</td><td>-</td><td>0</td><td>0</td></tr>
<tr><td>11906</td><td>46004</td><td>Drosophila melanogaster</td><td>66Dem</td><td>66Dem</td><td>-</td><td>0</td><td>0</td></tr>
<tr><td>11907</td><td>46005</td><td>Drosophila melanogaster</td><td>94Che</td><td>94Che</td><td>-</td><td>0</td><td>0</td></tr>
<tr><td>11908</td><td>46006</td><td>Drosophila melanogaster</td><td>east</td><td>enhanced adult sensory threshold</td><td>CG4399|DmelCG4399|EAST|EG:133E12.4|East|anon-WO03040301.224|l(1)G0014|l(1)G0500|su(w[sp])</td><td>0</td><td>0</td></tr>
<tr><td>11909</td><td>46007</td><td>Drosophila melanogaster</td><td>pur1</td><td>purine requiring</td><td>pur-1</td><td>0</td><td>0</td></tr>
<tr><td>11910</td><td>46008</td><td>Drosophila melanogaster</td><td>Gel</td><td>Gelsolin</td><td>CG1106|DGS|DmelCG1106|gel</td><td>0</td><td>0</td></tr>
<tr><td>11911</td><td>46010</td><td>Drosophila melanogaster</td><td>l(2)00064</td><td>lethal (2) 00064</td><td>-</td><td>0</td><td>0</td></tr>
<tr><td>11912</td><td>46012</td><td>Drosophila melanogaster</td><td>l(2)00572</td><td>lethal (2) 00572</td><td>-</td><td>0</td><td>0</td></tr>
<tr><td>11913</td><td>46015</td><td>Drosophila melanogaster</td><td>tutl</td><td>turtle</td><td>CG15426|CG15427|CG15428|CT35489|CT35490|DmelCG15427|jf4|l(2)01085|l(2)24Df|l(2)jf4</td><td>0</td><td>0</td></tr>
<tr><td>11914</td><td>46017</td><td>Drosophila melanogaster</td><td>l(2)01289</td><td>lethal (2) 01289</td><td>CG30444|CG9432|CG9435|DmelCG9432</td><td>0</td><td>0</td></tr>
<tr><td>11915</td><td>46020</td><td>Drosophila melanogaster</td><td>NAT1</td><td>CG3845 gene product from transcript CG3845-RA</td><td>CG3845|DmelCG3845|dNAT1|l(2)01424</td><td>0</td><td>0</td></tr>
<tr><td>11916</td><td>46023</td><td>Drosophila melanogaster</td><td>l(2)01482</td><td>lethal (2) 01482</td><td>-</td><td>0</td><td>0</td></tr>
<tr><td>11917</td><td>46027</td><td>Drosophila melanogaster</td><td>kermit</td><td>CG11546 gene product from transcript CG11546-RB</td><td>BEST:LD04728|CG11546|DmelCG11546|LD04728|dGIPC|dKermit|l(2)02045</td><td>0</td><td>0</td></tr>
<tr><td>11918</td><td>46029</td><td>Drosophila melanogaster</td><td>l(2)02321</td><td>lethal (2) 02321</td><td>-</td><td>0</td><td>0</td></tr>
<tr><td>11919</td><td>46032</td><td>Drosophila melanogaster</td><td>Bka</td><td>Bekka</td><td>BcDNA:RE14949|CG4539|DmelCG4539|l(2)02695|mip</td><td>0</td><td>0</td></tr>
<tr><td>11920</td><td>46035</td><td>Drosophila melanogaster</td><td>CCS</td><td>CG17753 gene product from transcript CG17753-RB</td><td>CCD|CG-17753|CG17753|Ccs|DmCCS|DmelCG17753|anon-EST:Posey231|l(2)03221</td><td>0</td><td>0</td></tr>
<tr><td>11921</td><td>46038</td><td>Drosophila melanogaster</td><td>l(2)03709</td><td>lethal (2) 03709</td><td>CG15081|DmelCG15081|anon-WO0118547.157|anon-WO02079478.4</td><td>0</td><td>0</td></tr>
<tr><td>11922</td><td>46039</td><td>Drosophila melanogaster</td><td>l(2)03728</td><td>lethal (2) 03728</td><td>-</td><td>0</td><td>0</td></tr>
<tr><td>11923</td><td>46040</td><td>Drosophila melanogaster</td><td>cype</td><td>cyclope</td><td>BcDNA:GH04604|CG14028|CG18323|COXVIc|Cyc|DmelCG14028|cyc|l(2)03771</td><td>0</td><td>0</td></tr>
<tr><td>11924</td><td>46041</td><td>Drosophila melanogaster</td><td>l(2)03788</td><td>lethal (2) 03788</td><td>-</td><td>0</td><td>0</td></tr>
<tr><td>11925</td><td>46043</td><td>Drosophila melanogaster</td><td>l(2)03845</td><td>lethal (2) 03845</td><td>-</td><td>0</td><td>0</td></tr>
<tr><td>11926</td><td>46055</td><td>Drosophila melanogaster</td><td>l(2)04884</td><td>lethal (2) 04884</td><td>-</td><td>0</td><td>0</td></tr>
<tr><td>11927</td><td>46056</td><td>Drosophila melanogaster</td><td>l(2)05006</td><td>lethal (2) 05006</td><td>-</td><td>0</td><td>0</td></tr>
<tr><td>11928</td><td>46058</td><td>Drosophila melanogaster</td><td>Prosbeta1</td><td>Proteasome beta1 subunit</td><td>8392|CG8392|DmelCG8392|l(2)05070</td><td>0</td><td>0</td></tr>
<tr><td>11929</td><td>46059</td><td>Drosophila melanogaster</td><td>Sply</td><td>Sphingosine-1-phosphate lyase</td><td>CG8946|DmelCG8946|SPL|Spl|anon-WO0142479.5|l(2)05091|spl|sply</td><td>0</td><td>0</td></tr>
<tr><td>11930</td><td>46061</td><td>Drosophila melanogaster</td><td>l(2)05315</td><td>lethal (2) 05315</td><td>-</td><td>0</td><td>0</td></tr>
<tr><td>11931</td><td>46063</td><td>Drosophila melanogaster</td><td>l(2)05337</td><td>lethal (2) 05337</td><td>unnamed</td><td>0</td><td>0</td></tr>
<tr><td>11932</td><td>46066</td><td>Drosophila melanogaster</td><td>l(2)05714</td><td>lethal (2) 05714</td><td>CG8886|DmelCG8886|l(2)05715</td><td>0</td><td>0</td></tr>
<tr><td>11933</td><td>46068</td><td>Drosophila melanogaster</td><td>Acsl</td><td>Acyl-CoA synthetase long-chain</td><td>7447|CG8732|DmelCG8732|FACS|FCS|anon-WO0172774.173|dAcsl|dFAC|l(2)05847|l(2)44DEa|l(2)7447|l(2)SH0465|l(2)SH2 0465|l(2)k05304</td><td>0</td><td>0</td></tr>
<tr><td>11934</td><td>46069</td><td>Drosophila melanogaster</td><td>l(2)06225</td><td>lethal (2) 06225</td><td>BcDNA:RE20862|BcDNA:RH28372|CG6105|DmelCG6105|l(2)06225|anon-EST:Posey10</td><td>0</td><td>0</td></tr>
<tr><td>11935</td><td>46070</td><td>Drosophila melanogaster</td><td>l(2)06250</td><td>lethal (2) 06250</td><td>-</td><td>0</td><td>0</td></tr>
<tr><td>11936</td><td>46071</td><td>Drosophila melanogaster</td><td>l(2)06253</td><td>lethal (2) 06253</td><td>unnamed</td><td>0</td><td>0</td></tr>
<tr><td>11937</td><td>46073</td><td>Drosophila melanogaster</td><td>l(2)06320</td><td>lethal (2) 06320</td><td>-</td><td>0</td><td>0</td></tr>
<tr><td>11938</td><td>46074</td><td>Drosophila melanogaster</td><td>l(2)06496</td><td>lethal (2) 06496</td><td>CG9893|DmelCG9893|p24</td><td>0</td><td>0</td></tr>
<tr><td>11939</td><td>46077</td><td>Drosophila melanogaster</td><td>l(2)06935</td><td>lethal (2) 06935</td><td>-</td><td>0</td><td>0</td></tr>
<tr><td>11940</td><td>46078</td><td>Drosophila melanogaster</td><td>vlc</td><td>vulcan</td><td>CG8390|DmelCG8390|X|anon-41Fa|anon-WO0118547.98|l(2)07022</td><td>0</td><td>0</td></tr>
<tr><td>11941</td><td>46080</td><td>Drosophila melanogaster</td><td>Sec61beta</td><td>CG10130 gene product from transcript CG10130-RA</td><td>CG10130|DSec61beta|DmelCG10130|Sec1|Sec61|Secbeta61|l(2)07214|sec61b|sec61beta</td><td>0</td><td>0</td></tr>
<tr><td>11942</td><td>46085</td><td>Drosophila melanogaster</td><td>l(2)08717</td><td>lethal (2) 08717</td><td>8717|CG15095|DmelCG15095|lethal(2)08717</td><td>0</td><td>0</td></tr>
<tr><td>11943</td><td>46087</td><td>Drosophila melanogaster</td><td>l(2)09639</td><td>lethal (2) 09639</td><td>-</td><td>0</td><td>0</td></tr>
<tr><td>11944</td><td>46090</td><td>Drosophila melanogaster</td><td>l(2)10425</td><td>lethal (2) 10425</td><td>-</td><td>0</td><td>0</td></tr>
<tr><td>11945</td><td>46091</td><td>Drosophila melanogaster</td><td>Nup214</td><td>Nucleoporin 214</td><td>CAN|CAN/Nup214|CG3820|DCAN|DNup214|DmelCG3820|Nup 214|dCAN|l(2)10444|nup214</td><td>0</td><td>0</td></tr>
<tr><td>11946</td><td>46094</td><td>Drosophila melanogaster</td><td>l(2)10521</td><td>lethal (2) 10521</td><td>-</td><td>0</td><td>0</td></tr>
<tr><td>11947</td><td>46099</td><td>Drosophila melanogaster</td><td>l(2)s1976</td><td>lethal (2) s1976</td><td>-</td><td>0</td><td>0</td></tr>
<tr><td>11948</td><td>46102</td><td>Drosophila melanogaster</td><td>l(2)s4199</td><td>lethal (2) s4199</td><td>-</td><td>0</td><td>0</td></tr>
<tr><td>11949</td><td>46105</td><td>Drosophila melanogaster</td><td>l(2)s4989</td><td>lethal (2) s4989</td><td>-</td><td>0</td><td>0</td></tr>
<tr><td>11950</td><td>46115</td><td>Drosophila melanogaster</td><td>l(3)00714</td><td>lethal (3) 00714</td><td>-</td><td>0</td><td>0</td></tr>
<tr><td>11951</td><td>46116</td><td>Drosophila melanogaster</td><td>l(3)00716</td><td>lethal (3) 00716</td><td>-</td><td>0</td><td>0</td></tr>
<tr><td>11952</td><td>46120</td><td>Drosophila melanogaster</td><td>l(3)01107</td><td>lethal (3) 01107</td><td>-</td><td>0</td><td>0</td></tr>
<tr><td>11953</td><td>46121</td><td>Drosophila melanogaster</td><td>l(3)01239</td><td>lethal (3) 01239</td><td>CG6302|DmelCG6302|P01239|l(3)j9B4</td><td>0</td><td>0</td></tr>
<tr><td>11954</td><td>46123</td><td>Drosophila melanogaster</td><td>l(3)01374</td><td>lethal (3) 01374</td><td>-</td><td>0</td><td>0</td></tr>
<tr><td>11955</td><td>46129</td><td>Drosophila melanogaster</td><td>l(3)01728</td><td>lethal (3) 01728</td><td>-</td><td>0</td><td>0</td></tr>
<tr><td>11956</td><td>46134</td><td>Drosophila melanogaster</td><td>l(3)02104</td><td>lethal (3) 02104</td><td>-</td><td>0</td><td>0</td></tr>
<tr><td>11957</td><td>46139</td><td>Drosophila melanogaster</td><td>l(3)02634</td><td>lethal (3) 02634</td><td>-</td><td>0</td><td>0</td></tr>
<tr><td>11958</td><td>46140</td><td>Drosophila melanogaster</td><td>l(3)02640</td><td>lethal (3) 02640</td><td>2640|3544|CG9165|DmelCG9165|PBGD|anon-WO0153538.70|l(3)3544|l(3)7E130|l(3)j4E3</td><td>0</td><td>0</td></tr>
<tr><td>11959</td><td>46144</td><td>Drosophila melanogaster</td><td>l(3)03076</td><td>lethal (3) 03076</td><td>-</td><td>0</td><td>0</td></tr>
<tr><td>11960</td><td>46145</td><td>Drosophila melanogaster</td><td>l(3)03077</td><td>lethal (3) 03077</td><td>-</td><td>0</td><td>0</td></tr>
<tr><td>11961</td><td>46148</td><td>Drosophila melanogaster</td><td>l(3)03471</td><td>lethal (3) 03471</td><td>-</td><td>0</td><td>0</td></tr>
<tr><td>11962</td><td>46151</td><td>Drosophila melanogaster</td><td>l(3)03691</td><td>lethal (3) 03691</td><td>-</td><td>0</td><td>0</td></tr>
<tr><td>11963</td><td>46155</td><td>Drosophila melanogaster</td><td>CG31195</td><td>CG31195 gene product from transcript CG31195-RB</td><td>DmelCG31195|l(3)03806</td><td>0</td><td>0</td></tr>
<tr><td>11964</td><td>46156</td><td>Drosophila melanogaster</td><td>Gug</td><td>Grunge</td><td>Atro|CG6964|DmelCG6964|GUG|atro|gug|l(3)01323|l(3)03928|l(3)j5A3|l(3)rO116</td><td>0</td><td>0</td></tr>
<tr><td>11965</td><td>46158</td><td>Drosophila melanogaster</td><td>l(3)04053</td><td>lethal (3) 04053</td><td>CG11238|DmelCG11238|l(3)j8B2</td><td>0</td><td>0</td></tr>
<tr><td>11966</td><td>46167</td><td>Drosophila melanogaster</td><td>l(3)04713</td><td>lethal (3) 04713</td><td>unnamed</td><td>0</td><td>0</td></tr>
<tr><td>11967</td><td>46168</td><td>Drosophila melanogaster</td><td>l(3)04837</td><td>lethal (3) 04837</td><td>-</td><td>0</td><td>0</td></tr>
<tr><td>11968</td><td>46173</td><td>Drosophila melanogaster</td><td>l(3)05279</td><td>lethal (3) 05279</td><td>-</td><td>0</td><td>0</td></tr>
<tr><td>11969</td><td>46174</td><td>Drosophila melanogaster</td><td>l(3)05430</td><td>lethal (3) 05430</td><td>-</td><td>0</td><td>0</td></tr>
<tr><td>11970</td><td>46184</td><td>Drosophila melanogaster</td><td>l(3)06465</td><td>lethal (3) 06465</td><td>-</td><td>0</td><td>0</td></tr>
<tr><td>11971</td><td>46187</td><td>Drosophila melanogaster</td><td>sinu</td><td>sinuous</td><td>6524|CG10624|DmelCG10624|Sinu|i8|l(3)06524</td><td>0</td><td>0</td></tr>
<tr><td>11972</td><td>46189</td><td>Drosophila melanogaster</td><td>l(3)06664</td><td>lethal (3) 06664</td><td>-</td><td>0</td><td>0</td></tr>
<tr><td>11973</td><td>46194</td><td>Drosophila melanogaster</td><td>Spn</td><td>Spinophilin</td><td>BcDNA:GM16129|CG16757|DmelCG16757|E62|EL62|c62E-5|l(3)06911|spn</td><td>0</td><td>0</td></tr>
<tr><td>11974</td><td>46201</td><td>Drosophila melanogaster</td><td>l(3)07882</td><td>lethal (3) 07882</td><td>CG5824|DmelCG5824</td><td>0</td><td>0</td></tr>
<tr><td>11975</td><td>46204</td><td>Drosophila melanogaster</td><td>l(3)08724</td><td>lethal (3) 08724</td><td>-</td><td>0</td><td>0</td></tr>
<tr><td>11976</td><td>46208</td><td>Drosophila melanogaster</td><td>l(3)09904c</td><td>lethal (3) 09904c</td><td>unnamed</td><td>0</td><td>0</td></tr>
<tr><td>11977</td><td>46211</td><td>Drosophila melanogaster</td><td>stretch</td><td>stretched out</td><td>l(3)10477</td><td>0</td><td>0</td></tr>
<tr><td>11978</td><td>46215</td><td>Drosophila melanogaster</td><td>l(3)s1696</td><td>lethal (3) s1696</td><td>-</td><td>0</td><td>0</td></tr>
<tr><td>11979</td><td>46218</td><td>Drosophila melanogaster</td><td>l(3)s1922</td><td>lethal (3) s1922</td><td>-</td><td>0</td><td>0</td></tr>
<tr><td>11980</td><td>46221</td><td>Drosophila melanogaster</td><td>l(3)s2253</td><td>lethal (3) s2253</td><td>-</td><td>0</td><td>0</td></tr>
<tr><td>11981</td><td>46222</td><td>Drosophila melanogaster</td><td>l(3)s2383</td><td>lethal (3) s2383</td><td>-</td><td>0</td><td>0</td></tr>
<tr><td>11982</td><td>46224</td><td>Drosophila melanogaster</td><td>l(3)s2564</td><td>lethal (3) s2564</td><td>-</td><td>0</td><td>0</td></tr>
<tr><td>11983</td><td>46225</td><td>Drosophila melanogaster</td><td>l(3)s2681</td><td>lethal (3) s2681</td><td>unnamed</td><td>0</td><td>0</td></tr>
<tr><td>11984</td><td>46228</td><td>Drosophila melanogaster</td><td>l(1)DM-1</td><td>lethal (1) DM-1</td><td>-</td><td>0</td><td>0</td></tr>
<tr><td>11985</td><td>46229</td><td>Drosophila melanogaster</td><td>l(1)DM-3</td><td>lethal (1) DM-3</td><td>-</td><td>0</td><td>0</td></tr>
</table>

11986	46230	Drosophila melanogaster	l(1)DM-4	lethal (1) DM-4	-	0	0
11987	46231	Drosophila melanogaster	l(1)291-3	lethal (1) 291-3	291-3	0	0
11988	46232	Drosophila melanogaster	l(1)291-9	lethal (1) 291-9	291-9	0	0
11989	46234	Drosophila melanogaster	fbl	fumble	CG5725|DmelCG5725|PANK|dPANK	0	0
11990	46236	Drosophila melanogaster	bet	betel	-	0	0
11991	46240	Drosophila melanogaster	gor	gorp	-	0	0
11992	46242	Drosophila melanogaster	pto	pistachio	-	0	0
11993	46243	Drosophila melanogaster	poe	purity of essence	3420|CG14472|Calo|DmelCG14472|POE|calo|ms(2)01659|ms(2)27C|ms(2)3420|ms(2)5970|push	0	0
11994	46245	Drosophila melanogaster	srh	short rhabdomeres	-	0	0
11995	46246	Drosophila melanogaster	Pglym87	CG17645 gene product from transcript CG17645-RA	CG17645|DmelCG17645|Pglym|pglym87	0	0
11996	46248	Drosophila melanogaster	l(3)j2C5	lethal (3) j2C5	-	0	0
11997	46256	Drosophila melanogaster	l(3)neo4	lethal (3) neo4	-	0	0
11998	46258	Drosophila melanogaster	l(3)neo6	lethal (3) neo6	-	0	0
11999	46259	Drosophila melanogaster	l(3)neo10	lethal (3) neo10	-	0	0
12000	46260	Drosophila melanogaster	l(3)neo18	lethal (3) neo18	CG9762|DmelCG9762	0	0
12001	46268	Drosophila melanogaster	l(3)neo46	lethal (3) neo46	-	0	0
12002	46269	Drosophila melanogaster	l(3)neo47	lethal (3) neo47	-	0	0
12003	46271	Drosophila melanogaster	l(3)neo49	lethal (3) neo49	-	0	0
12004	46273	Drosophila melanogaster	l(3)neo55	lethal (3) neo55	-	0	0
12005	46274	Drosophila melanogaster	l(3)neo59	lethal (3) neo59	-	0	0
12006	46277	Drosophila melanogaster	l(3)rK145	lethal (3) rK145	-	0	0
12007	46279	Drosophila melanogaster	l(2)06424	lethal (2) 06424	-	0	0
12008	46280	Drosophila melanogaster	l(3)00945	lethal (3) 00945	-	0	0
12009	46281	Drosophila melanogaster	ams	adult male-specific transcript	-	0	0
12010	46285	Drosophila melanogaster	P13	P13	Dmcks	0	0
12011	46290	Drosophila melanogaster	l(3)05050	lethal (3) 05050	-	0	0
12012	46293	Drosophila melanogaster	l(3)rI809	lethal (3) rI809	-	0	0
12013	46297	Drosophila melanogaster	l(3)rQ303	lethal (3) rQ303	-	0	0
12014	46299	Drosophila melanogaster	l(3)s2714	lethal (3) s2714	-	0	0
12015	46301	Drosophila melanogaster	l(3)s3246	lethal (3) s3246	-	0	0
12016	46318	Drosophila melanogaster	l(1)1P3	lethal (1) 1P3	-	0	0
12017	46319	Drosophila melanogaster	l(1)5PP10	lethal (1) 5PP10	-	0	0
12018	46320	Drosophila melanogaster	l(2)23AB2	lethal (2) 23AB2	l(2)23Ae	0	0
12019	46322	Drosophila melanogaster	l(2)23AB4	lethal (2) 23AB4	l(2)23Af	0	0
12020	46325	Drosophila melanogaster	l(2)23AB7	lethal (2) 23AB7	-	0	0
12021	46326	Drosophila melanogaster	l(2)dtl	lethal-(2)-denticleless	CG11295|Cdt2|DTL83|DmelCG11295|L2DTL	0	0
12022	46327	Drosophila melanogaster	l(3)ry119	lethal (3) ry119	l(3)119	0	0
12023	46329	Drosophila melanogaster	l(3)ry133	lethal (3) ry133	l(3)133	0	0
12024	46330	Drosophila melanogaster	l(3)ry141	lethal (3) ry141	l(3)141	0	0
12025	46331	Drosophila melanogaster	l(3)ry160	lethal (3) ry160	l(3)160	0	0
12026	46333	Drosophila melanogaster	l(3)82Fg	lethal (3) 82Fg	-	0	0
12027	46335	Drosophila melanogaster	l(3)82Fj	lethal (3) 82Fj	-	0	0
12028	46337	Drosophila melanogaster	l(3)L1042	lethal (3) L1042	-	0	0
12029	46339	Drosophila melanogaster	l(3)L2290	lethal (3) L2290	-	0	0
12030	46341	Drosophila melanogaster	l(3)L5150	lethal (3) L5150	-	0	0
12031	46342	Drosophila melanogaster	l(3)neo11	lethal (3) neo11	-	0	0
12032	46343	Drosophila melanogaster	l(3)neo12	lethal (3) neo12	-	0	0
12033	46345	Drosophila melanogaster	l(3)neo15	lethal (3) neo15	-	0	0
12034	46346	Drosophila melanogaster	l(3)neo16	lethal (3) neo16	-	0	0
12035	46347	Drosophila melanogaster	l(3)neo23	lethal (3) neo23	-	0	0
12036	46348	Drosophila melanogaster	l(3)neo29	lethal (3) neo29	-	0	0
12037	46349	Drosophila melanogaster	l(3)neo51	lethal (3) neo51	-	0	0
12038	46353	Drosophila melanogaster	l(3)neo44	lethal (3) neo44	neo44	0	0
12039	46362	Drosophila melanogaster	l(1)14Cd	lethal (1) 14Cd	l(1)14Ce	0	0
12040	46363	Drosophila melanogaster	l(1)14Ce	lethal (1) 14Ce	l(1)14Cf	0	0
12041	46364	Drosophila melanogaster	l(2)31Ba	lethal (2) 31Ba	-	0	0
12042	46365	Drosophila melanogaster	l(2)vsp	lethal (2) very small puparia	l(2)60A-G	0	0
12043	46366	Drosophila melanogaster	l(3)64Ae	lethal (3) 64Ae	l(3)SH20	0	0
12044	46368	Drosophila melanogaster	l(3)64Ba	lethal (3) 64Ba	-	0	0
12045	46369	Drosophila melanogaster	l(3)64Bb	lethal (3) 64Bb	-	0	0
12046	46370	Drosophila melanogaster	l(3)64Bc	lethal (3) 64Bc	-	0	0
12047	46371	Drosophila melanogaster	l(3)64Bd	lethal (3) 64Bd	-	0	0
12048	46372	Drosophila melanogaster	l(3)64Be	lethal (3) 64Be	-	0	0
12049	46373	Drosophila melanogaster	l(3)64Bf	lethal (3) 64Bf	-	0	0
12050	46374	Drosophila melanogaster	l(3)64Bg	lethal (3) 64Bg	-	0	0
12051	46375	Drosophila melanogaster	l(3)64Bh	lethal (3) 64Bh	-	0	0
12052	46376	Drosophila melanogaster	l(3)64Bi	lethal (3) 64Bi	-	0	0
12053	46377	Drosophila melanogaster	l(3)64Bj	lethal (3) 64Bj	-	0	0
12054	46378	Drosophila melanogaster	l(3)7-19	lethal (3) 7-19	-	0	0
12055	46379	Drosophila melanogaster	l(3)ry127	lethal (3) ry127	-	0	0
12056	46386	Drosophila melanogaster	CG13397	CG13397 gene product from transcript CG13397-RA	172F5T|DmelCG13397|ESTS:172F5T|anon-sts19	0	0
12057	46391	Drosophila melanogaster	CG11899	CG11899 gene product from transcript CG11899-RA	39C10S|DmelCG11899|ESTS:39C10S|Ov8|anon-sts29	0	0
12058	46411	Drosophila melanogaster	l(1)13Fa	lethal (1) 13Fa	V|l(1)V	0	0
12059	46412	Drosophila melanogaster	l(1)13Fc	lethal (1) 13Fc	VIII|l(1)VIII	0	0
12060	46413	Drosophila melanogaster	l(1)13Fd	lethal (1) 13Fd	IX|l(1)IX	0	0
12061	46414	Drosophila melanogaster	E(var)53E	Enhancer of variegation 53E	E(var)2-53E	0	0
12062	46415	Drosophila melanogaster	Fer1HCH	Ferritin 1 heavy chain homologue	1HCH|BEST:LD36673|CG2216|DmelCG2216|FER1|FER1HCH|Fer-L|Fer1|Fer1HC|FerHCH|HCH|T21|T9|fer1hch|l(3)00451|l(3)j10B4	0	0
12063	46416	Drosophila melanogaster	M(1)15DEF	Minute (1) 15DEF	-	0	0
12064	46426	Drosophila melanogaster	cub	currant bun	Bg|l(2)37Bg	0	0
12065	46427	Drosophila melanogaster	l(1)14ABa	lethal (1) 14ABa	-	0	0
12066	46428	Drosophila melanogaster	l(1)15ACa	lethal (1) 15ACa	-	0	0
12067	46429	Drosophila melanogaster	l(1)15DFa	lethal (1) 15DFa	-	0	0
12068	46430	Drosophila melanogaster	l(1)15DFb	lethal (1) 15DFb	-	0	0
12069	46431	Drosophila melanogaster	l(1)16ACa	lethal (1) 16ACa	-	0	0
12070	46432	Drosophila melanogaster	l(1)16ACb	lethal (1) 16ACb	-	0	0
12071	46433	Drosophila melanogaster	l(1)HM32	lethal (1) HM32	-	0	0
12072	46434	Drosophila melanogaster	l(2)24CDa	lethal (2) 24CDa	l(2)d	0	0
12073	46435	Drosophila melanogaster	l(2)24CDb	lethal (2) 24CDb	l(2)g	0	0
12074	46436	Drosophila melanogaster	l(2)37CDa	lethal (2) 37CDa	l(2)E142,M	0	0
12075	46437	Drosophila melanogaster	l(2)37CDb	lethal (2) 37CDb	l(2)E137	0	0
12076	46438	Drosophila melanogaster	l(2)37CDd	lethal (2) 37CDd	l(2)E2	0	0
12077	46439	Drosophila melanogaster	l(2)37CDe	lethal (2) 37CDe	l(2)E107	0	0
12078	46440	Drosophila melanogaster	l(2)37CDf	lethal (2) 37CDf	l(2)E27	0	0
12079	46441	Drosophila melanogaster	l(2)45ABa	lethal (2) 45ABa	-	0	0
12080	46442	Drosophila melanogaster	l(2)45ABb	lethal (2) 45ABb	-	0	0
12081	46443	Drosophila melanogaster	l(2)45Ab	lethal (2) 45Ab	-	0	0
12082	46444	Drosophila melanogaster	l(2)45Ac	lethal (2) 45Ac	-	0	0
12083	46445	Drosophila melanogaster	l(3)65ACa	lethal (3) 65ACa	I	0	0
12084	46446	Drosophila melanogaster	l(3)65ACb	lethal (3) 65ACb	II	0	0
12085	46447	Drosophila melanogaster	l(3)65ACc	lethal (3) 65ACc	III	0	0
12086	46448	Drosophila melanogaster	l(3)65ACd	lethal (3) 65ACd	IV	0	0
12087	46449	Drosophila melanogaster	l(3)65ACe	lethal (3) 65ACe	V	0	0
12088	46450	Drosophila melanogaster	l(3)65ACf	lethal (3) 65ACf	VI|l(3)k65ACf	0	0
12089	46451	Drosophila melanogaster	l(3)65CEa	lethal (3) 65CEa	X	0	0
12090	46452	Drosophila melanogaster	l(3)65CEb	lethal (3) 65CEb	XI	0	0
12091	46453	Drosophila melanogaster	l(3)65CEc	lethal (3) 65CEc	XII	0	0
12092	46455	Drosophila melanogaster	l(3)65CEf	lethal (3) 65CEf	XV	0	0
12093	46456	Drosophila melanogaster	CanB2	Calcineurin B2	CG11217|CS2-1|DmelCG11217|canB2|dCNB2|dCnB2	0	0
12094	46472	Drosophila melanogaster	l(2)35Fg	lethal (2) 35Fg	-	0	0
12095	46473	Drosophila melanogaster	l(2)46Co	lethal (2) 46Co	group 3	0	0
12096	46474	Drosophila melanogaster	l(2)46Cp	lethal (2) 46Cp	group 4	0	0

12097	46475	Drosophila melanogaster	l(2)46DEa	lethal (2) 46DEa	group 5		0	0
12098	46476	Drosophila melanogaster	l(2)46DEb	lethal (2) 46DEb	group 6		0	0
12099	46477	Drosophila melanogaster	l(2)46DEc	lethal (2) 46DEc	group 7		0	0
12100	46478	Drosophila melanogaster	l(2)46DEd	lethal (2) 46DEd	group 8		0	0
12101	46479	Drosophila melanogaster	l(2)PC4-C	lethal (2) PC4-C	-		0	0
12102	46480	Drosophila melanogaster	l(2)PC4-N	lethal (2) PC4-N	fs(2)PC4-N		0	0
12103	46481	Drosophila melanogaster	l(2)PC4-R	lethal (2) PC4-R	-		0	0
12104	46482	Drosophila melanogaster	l(2)PC4-S	lethal (2) PC4-S	-		0	0
12105	46484	Drosophila melanogaster	l(3)73CFa	lethal (3) 73CFa	-		0	0
12106	46498	Drosophila melanogaster	l(2)39ADa	lethal (2) 39ADa	B819		0	0
12107	46499	Drosophila melanogaster	l(2)39ADb	lethal (2) 39ADb	J50		0	0
12108	46500	Drosophila melanogaster	l(2)39ADc	lethal (2) 39ADc	K490		0	0
12109	46501	Drosophila melanogaster	l(2)39ADd	lethal (2) 39ADd	H221		0	0
12110	46502	Drosophila melanogaster	l(2)39ADe	lethal (2) 39ADe	N6		0	0
12111	46503	Drosophila melanogaster	l(2)39DEa	lethal (2) 39DEa	A447		0	0
12112	46504	Drosophila melanogaster	l(2)39DEb	lethal (2) 39DEb	C87		0	0
12113	46505	Drosophila melanogaster	l(2)39DEc	lethal (2) 39DEc	D490		0	0
12114	46506	Drosophila melanogaster	l(2)39DEd	lethal (2) 39DEd	H173		0	0
12115	46507	Drosophila melanogaster	l(2)39DEe	lethal (2) 39DEe	C704		0	0
12116	46508	Drosophila melanogaster	l(2)39DEf	lethal (2) 39DEf	I4		0	0
12117	46509	Drosophila melanogaster	l(2)39DEg	lethal (2) 39DEg	K213		0	0
12118	46510	Drosophila melanogaster	l(2)39DEh	lethal (2) 39DEh	D95		0	0
12119	46511	Drosophila melanogaster	l(2)39DEi	lethal (2) 39DEi	D241		0	0
12120	46512	Drosophila melanogaster	l(2)39DEj	lethal (2) 39DEj	D244		0	0
12121	46513	Drosophila melanogaster	l(2)39DEk	lethal (2) 39DEk	D282		0	0
12122	46514	Drosophila melanogaster	l(2)39DEl	lethal (2) 39DEl	K234		0	0
12123	46515	Drosophila melanogaster	l(2)39DEm	lethal (2) 39DEm	K236		0	0
12124	46516	Drosophila melanogaster	l(2)39DEn	lethal (2) 39DEn	L110		0	0
12125	46517	Drosophila melanogaster	l(2)39DEo	lethal (2) 39DEo	L138		0	0
12126	46519	Drosophila melanogaster	l(3)72CDb	lethal (3) 72CDb	-		0	0
12127	46520	Drosophila melanogaster	l(3)72CDc	lethal (3) 72CDc	-		0	0
12128	46524	Drosophila melanogaster	l(3)X27	lethal (3) X27	X27		0	0
12129	46525	Drosophila melanogaster	l(3)X39	lethal (3) X39	X39		0	0
12130	46526	Drosophila melanogaster	l(1)HM442	lethal (1) HM442	HM-442		0	0
12131	46527	Drosophila melanogaster	l(1)HM409	lethal (1) HM409	-		0	0
12132	46546	Drosophila melanogaster	mwr	more wright	-		0	0
12133	46547	Drosophila melanogaster	l(2)42Bc	lethal (2) 42Bc	5CC1|l(2)5CC1		0	0
12134	46548	Drosophila melanogaster	l(2)42Bd	lethal (2) 42Bd	4LL1|l(2)4LL1		0	0
12135	46558	Drosophila melanogaster	l(2)46CFn	lethal (2) 46CFn	19.22|unnamed		0	0
12136	46570	Drosophila melanogaster	l(2)46CFb	lethal (2) 46CFb	1.11|unnamed		0	0
12137	46572	Drosophila melanogaster	l(1)A17	lethal (1) A17	-		0	0
12138	46573	Drosophila melanogaster	l(1)A13	lethal (1) A13	-		0	0
12139	46574	Drosophila melanogaster	l(1)A10	lethal (1) A10	-		0	0
12140	46575	Drosophila melanogaster	l(1)815-9	lethal (1) 815-9	-		0	0
12141	46576	Drosophila melanogaster	l(1)815-3	lethal (1) 815-3	-		0	0
12142	46577	Drosophila melanogaster	l(1)75B5	lethal (1) 75B5	-		0	0
12143	46578	Drosophila melanogaster	l(1)54B2	lethal (1) 54B2	-		0	0
12144	46579	Drosophila melanogaster	l(1)54B1	lethal (1) 54B1	-		0	0
12145	46580	Drosophila melanogaster	l(1)32	lethal (1) 32	-		0	0
12146	46581	Drosophila melanogaster	l(1)318	lethal (1) 318	-		0	0
12147	46582	Drosophila melanogaster	l(1)30	lethal (1) 30	-		0	0
12148	46583	Drosophila melanogaster	l(1)218	lethal (1) 218	-		0	0
12149	46584	Drosophila melanogaster	l(1)17	lethal (1) 17	-		0	0
12150	46585	Drosophila melanogaster	l(1)08	lethal (1) 08	-		0	0
12151	46586	Drosophila melanogaster	l(1)02	lethal (1) 02	-		0	0
12152	46609	Drosophila melanogaster	l(3)06266	lethal (3) 06266	unnamed		0	0
12153	46610	Drosophila melanogaster	l(2)39DEp	lethal (2) 39DEp	I259		0	0
12154	46614	Drosophila melanogaster	l(3)1A1	lethal (3) 1A1	1A1		0	0
12155	46651	Drosophila melanogaster	l(3)03647	lethal (3) 03647	-		0	0
12156	46652	Drosophila melanogaster	l(2)21Ba1	lethal (2) 21Ba1	21Ba1		0	0
12157	46656	Drosophila melanogaster	l(3)95Ca	lethal (3) 95Ca	-		0	0
12158	46657	Drosophila melanogaster	l(3)95Ba	lethal (3) 95Ba	-		0	0
12159	46660	Drosophila melanogaster	l(2)k03002a	lethal (2) k03002a	-		0	0
12160	46662	Drosophila melanogaster	l(2)09851	lethal (2) 09851	CG12792|DmelCG12792|GRWD1|l(2)09851|l(2)k08138		0	0
12161	46664	Drosophila melanogaster	l(1)L60.6	lethal (1) L60.6	L60.6		0	0
12162	46665	Drosophila melanogaster	l(1)L55.6	lethal (1) L55.6	L55.6		0	0
12163	46666	Drosophila melanogaster	l(1)J30.4	lethal (1) J30.4	J30.4		0	0
12164	46667	Drosophila melanogaster	l(1)D10.2	lethal (1) D10.2	D10.2		0	0
12165	46668	Drosophila melanogaster	l(1)B74.4	lethal (1) B74.4	B74.4		0	0
12166	46669	Drosophila melanogaster	l(1)B64.2	lethal (1) B64.2	-		0	0
12167	46670	Drosophila melanogaster	l(1)A33.3	lethal (1) A33.3	A33.3		0	0
12168	46677	Drosophila melanogaster	E(faf)bE365	Enhancer of faf bE365	-		0	0
12169	46678	Drosophila melanogaster	E(faf)bE160	Enhancer of faf bE160	-		0	0
12170	46679	Drosophila melanogaster	l(3)s9999	lethal (3) s9999	-		0	0
12171	46680	Drosophila melanogaster	l(3)S113105	lethal (3) S113105	1131/05|1131/5|unnamed		0	0
12172	46683	Drosophila melanogaster	l(1)20Cc	lethal (1) 20Cc	C2/5.5		0	0
12173	46684	Drosophila melanogaster	l(1)20Bd	lethal (1) 20Bd	C2/5.3		0	0
12174	46685	Drosophila melanogaster	l(1)20Aj	lethal (1) 20Aj	1200E7b		0	0
12175	46686	Drosophila melanogaster	l(1)20Ai	lethal (1) 20Ai	C2/44.1		0	0
12176	46687	Drosophila melanogaster	l(1)20Ah	lethal (1) 20Ah	A2/25		0	0
12177	46717	Drosophila melanogaster	arg	arginase	CG18104|DmelCG18104|EG:171D11.4|EG:65F1.3		0	0
12178	46719	Drosophila melanogaster	dlt	discs lost	CG1977|CG32315|Dlt|DmelCG32315|Van|dre1|l(3)04276|l(3)62Ba|l(3)dre1		0	0
12179	46724	Drosophila melanogaster	l(3)L0499	lethal (3) L0499	-		0	0
12180	46734	Drosophila melanogaster	l(3)L3051	lethal (3) L3051	-		0	0
12181	46737	Drosophila melanogaster	l(3)L3659	lethal (3) L3659	1(3)L3659		0	0
12182	46738	Drosophila melanogaster	l(3)L3930	lethal (3) L3930	-		0	0
12183	46747	Drosophila melanogaster	l(3)L6332	lethal (3) L6332	-		0	0
12184	46761	Drosophila melanogaster	l(1)LefEM27	lethal (1) LefEM27	EM27		0	0
12185	46762	Drosophila melanogaster	l(1)LefEM26	lethal (1) LefEM26	EM26		0	0
12186	46763	Drosophila melanogaster	l(1)HF326	lethal (1) HF326	unnamed		0	0
12187	46764	Drosophila melanogaster	l(1)DC711	lethal (1) DC711	unnamed		0	0
12188	46775	Drosophila melanogaster	l(3)96Bg	lethal (3) 96Bg	-		0	0
12189	46776	Drosophila melanogaster	l(3)96Bf	lethal (3) 96Bf	-		0	0
12190	46777	Drosophila melanogaster	l(3)96Be	lethal (3) 96Be	-		0	0
12191	46778	Drosophila melanogaster	l(3)96Bd	lethal (3) 96Bd	-		0	0
12192	46779	Drosophila melanogaster	l(3)96Bc	lethal (3) 96Bc	-		0	0
12193	46780	Drosophila melanogaster	l(3)96Bb	lethal (3) 96Bb	-		0	0
12194	46781	Drosophila melanogaster	l(3)96Ba	lethal (3) 96Ba	-		0	0
12195	46782	Drosophila melanogaster	l(2)03055	lethal (2) 03055		3055	0	0
12196	46789	Drosophila melanogaster	l(3)82EFc	lethal (3) 82EFc	-		0	0
12197	46790	Drosophila melanogaster	l(3)82EFb	lethal (3) 82EFb	-		0	0
12198	46791	Drosophila melanogaster	l(3)82EFa	lethal (3) 82EFa	-		0	0
12199	46793	Drosophila melanogaster	l(3)82CDc	lethal (3) 82CDc	-		0	0
12200	46795	Drosophila melanogaster	l(2)59ABc	lethal (2) 59ABc	-		0	0
12201	46796	Drosophila melanogaster	l(2)59ABb	lethal (2) 59ABb	-		0	0
12202	46805	Drosophila melanogaster	l(3)99Fa	lethal (3) 99Fa	-		0	0
12203	46806	Drosophila melanogaster	l(3)95Az	lethal (3) 95Az	-		0	0
12204	46807	Drosophila melanogaster	l(3)95Ay	lethal (3) 95Ay	-		0	0
12205	46808	Drosophila melanogaster	l(3)95Ax	lethal (3) 95Ax	-		0	0
12206	46809	Drosophila melanogaster	l(3)89Bu	lethal (3) 89Bu	l(3)M1511		0	0
12207	46813	Drosophila melanogaster	CG6421	CG6421 gene product from transcript CG6421-RA	DmelCG6421|EG:EG0003.8		0	0

12208	46827	Drosophila melanogaster	l(3)71EFa	lethal (3) 71EFa	-	0	0
12209	46828	Drosophila melanogaster	l(2)PN-11	lethal (2) PN-11	-	0	0
12210	46829	Drosophila melanogaster	l(2)60A-O	lethal (2) 60A-O	-	0	0
12211	46830	Drosophila melanogaster	l(2)60A-N	lethal (2) 60A-N	unnamed	0	0
12212	46831	Drosophila melanogaster	l(2)60A-M	lethal (2) 60A-M	-	0	0
12213	46832	Drosophila melanogaster	l(2)60A-L	lethal (2) 60A-L	-	0	0
12214	46833	Drosophila melanogaster	l(2)60A-K	lethal (2) 60A-K	-	0	0
12215	46834	Drosophila melanogaster	l(2)60A-I	lethal (2) 60A-I	unnamed	0	0
12216	46835	Drosophila melanogaster	l(2)60A-H	lethal (2) 60A-H	unnamed	0	0
12217	46836	Drosophila melanogaster	l(2)60A-F	lethal (2) 60A-F	-	0	0
12218	46837	Drosophila melanogaster	l(2)60A-E	lethal (2) 60A-E	-	0	0
12219	46838	Drosophila melanogaster	l(2)60A-D	lethal (2) 60A-D	-	0	0
12220	46839	Drosophila melanogaster	l(2)60A-C	lethal (2) 60A-C	unnamed	0	0
12221	46840	Drosophila melanogaster	l(2)60A-B	lethal (2) 60A-B	-	0	0
12222	46841	Drosophila melanogaster	l(2)60A-A	lethal (2) 60A-A	-	0	0
12223	46876	Drosophila melanogaster	l(1)G0090	lethal (1) G0090	-	0	0
12224	46883	Drosophila melanogaster	l(1)G0013	lethal (1) G0013	-	0	0
12225	46884	Drosophila melanogaster	l(3)S147506	lethal (3) S147506	-	0	0
12226	46885	Drosophila melanogaster	l(3)S147006	lethal (3) S147006	1470/06	0	0
12227	46886	Drosophila melanogaster	l(3)S146004	lethal (3) S146004	1460/04	0	0
12228	46887	Drosophila melanogaster	l(3)S145710a	lethal (3) S145710a	0479/06|1067/10	0	0
12229	46888	Drosophila melanogaster	l(3)S145709	lethal (3) S145709	1457/09	0	0
12230	46889	Drosophila melanogaster	l(3)S144607b	lethal (3) S144607b	1446/07	0	0
12231	46891	Drosophila melanogaster	l(3)S142909	lethal (3) S142909	1429/09	0	0
12232	46892	Drosophila melanogaster	l(3)S141110	lethal (3) S141110	1411/10	0	0
12233	46894	Drosophila melanogaster	l(3)S137908	lethal (3) S137908	1379/08	0	0
12234	46895	Drosophila melanogaster	l(3)S135710	lethal (3) S135710	1357/10	0	0
12235	46896	Drosophila melanogaster	l(3)S132703	lethal (3) S132703	1327/03	0	0
12236	46897	Drosophila melanogaster	l(3)S126801	lethal (3) S126801	1268/01	0	0
12237	46898	Drosophila melanogaster	l(3)S126014	lethal (3) S126014	-	0	0
12238	46899	Drosophila melanogaster	l(3)S123208	lethal (3) S123208	0542/11|1232/08|1282/02	0	0
12239	46900	Drosophila melanogaster	l(3)S122404	lethal (3) S122404	1224/04	0	0
12240	46901	Drosophila melanogaster	l(3)S119715	lethal (3) S119715	1197/15	0	0
12241	46902	Drosophila melanogaster	l(3)S119608	lethal (3) S119608	1196/08	0	0
12242	46903	Drosophila melanogaster	l(3)S119307b	lethal (3) S119307b	1337/05	0	0
12243	46904	Drosophila melanogaster	l(3)S119005	lethal (3) S119005	1190/05	0	0
12244	46905	Drosophila melanogaster	l(3)S116415	lethal (3) S116415	1164/15	0	0
12245	46906	Drosophila melanogaster	l(3)S115415	lethal (3) S115415	l(3)s036809	0	0
12246	46907	Drosophila melanogaster	l(3)S114709	lethal (3) S114709	-	0	0
12247	46908	Drosophila melanogaster	l(3)S110416	lethal (3) S110416	1104/16	0	0
12248	46909	Drosophila melanogaster	l(3)S108310a	lethal (3) S108310a	1083/10	0	0
12249	46911	Drosophila melanogaster	l(3)S106605	lethal (3) S106605	1066/05	0	0
12250	46912	Drosophila melanogaster	l(3)S104103	lethal (3) S104103	1041/03	0	0
12251	46914	Drosophila melanogaster	l(3)S103909	lethal (3) S103909	1039/09	0	0
12252	46915	Drosophila melanogaster	l(3)S103710	lethal (3) S103710	1037/10	0	0
12253	46916	Drosophila melanogaster	l(3)S103616	lethal (3) S103616	1036/16	0	0
12254	46917	Drosophila melanogaster	l(3)S103603	lethal (3) S103603	1036/03	0	0
12255	46918	Drosophila melanogaster	l(3)S103408	lethal (3) S103408	1034/08	0	0
12256	46919	Drosophila melanogaster	l(3)S103114	lethal (3) S103114	1031/14	0	0
12257	46922	Drosophila melanogaster	l(3)S101902	lethal (3) S101902	1019/02	0	0
12258	46923	Drosophila melanogaster	l(3)S101712	lethal (3) S101712	1017/12	0	0
12259	46924	Drosophila melanogaster	l(3)S100604	lethal (3) S100604	1006/04	0	0
12260	46925	Drosophila melanogaster	l(3)S100209	lethal (3) S100209	1002/09	0	0
12261	46926	Drosophila melanogaster	l(3)S099312	lethal (3) S099312	-	0	0
12262	46927	Drosophila melanogaster	l(3)S098105	lethal (3) S098105	0981/05	0	0
12263	46928	Drosophila melanogaster	l(3)S097001	lethal (3) S097001	0970/01|1087/09|1450/06	0	0
12264	46929	Drosophila melanogaster	l(3)S096611	lethal (3) S096611	0966/11	0	0
12265	46931	Drosophila melanogaster	l(3)S096108	lethal (3) S096108	0961/08	0	0
12266	46933	Drosophila melanogaster	l(3)S095109	lethal (3) S095109	0951/09	0	0
12267	46934	Drosophila melanogaster	l(3)S094003	lethal (3) S094003	0940/03	0	0
12268	46935	Drosophila melanogaster	l(3)S093909	lethal (3) S093909	0939/09	0	0
12269	46936	Drosophila melanogaster	l(3)S093411	lethal (3) S093411	0934/11	0	0
12270	46937	Drosophila melanogaster	l(3)S092416	lethal (3) S092416	0924/16	0	0
12271	46938	Drosophila melanogaster	l(3)S092405	lethal (3) S092405	0924/05	0	0
12272	46939	Drosophila melanogaster	l(3)S091306	lethal (3) S091306	0913/06	0	0
12273	46940	Drosophila melanogaster	l(3)S090302	lethal (3) S090302	0903/02	0	0
12274	46941	Drosophila melanogaster	l(3)S090001	lethal (3) S090001	0900/01	0	0
12275	46943	Drosophila melanogaster	l(3)S088205	lethal (3) S088205	0882/05	0	0
12276	46944	Drosophila melanogaster	l(3)S086802	lethal (3) S086802	0868/02	0	0
12277	46945	Drosophila melanogaster	l(3)S086405	lethal (3) S086405	0864/05	0	0
12278	46947	Drosophila melanogaster	l(3)S085301	lethal (3) S085301	0853/01	0	0
12279	46948	Drosophila melanogaster	l(3)S083705	lethal (3) S083705	0837/05	0	0
12280	46949	Drosophila melanogaster	l(3)S082106	lethal (3) S082106	0821/06	0	0
12281	46950	Drosophila melanogaster	l(3)S079311b	lethal (3) S079311b	0793/11	0	0
12282	46951	Drosophila melanogaster	l(3)S074103	lethal (3) S074103	0741/03	0	0
12283	46952	Drosophila melanogaster	l(3)S073615	lethal (3) S073615	0736/15	0	0
12284	46953	Drosophila melanogaster	l(3)S073002a	lethal (3) S073002a	1152/14|1323/04|1382/10	0	0
12285	46954	Drosophila melanogaster	l(3)S072611	lethal (3) S072611	0726/11	0	0
12286	46956	Drosophila melanogaster	l(3)S071710	lethal (3) S071710	0717/10	0	0
12287	46957	Drosophila melanogaster	l(3)S070103	lethal (3) S070103	0701/03	0	0
12288	46960	Drosophila melanogaster	l(3)S068911	lethal (3) S068911	0689/11	0	0
12289	46961	Drosophila melanogaster	l(3)S068609	lethal (3) S068609	-	0	0
12290	46962	Drosophila melanogaster	l(3)S065103	lethal (3) S065103	0651/03	0	0
12291	46963	Drosophila melanogaster	l(3)S064316b	lethal (3) S064316b	0643/16	0	0
12292	46964	Drosophila melanogaster	l(3)S064301	lethal (3) S064301	-	0	0
12293	46966	Drosophila melanogaster	l(3)S061207	lethal (3) S061207	0612/07|0671/10|1226/12	0	0
12294	46967	Drosophila melanogaster	l(3)S059706	lethal (3) S059706	-	0	0
12295	46970	Drosophila melanogaster	l(3)S057809a	lethal (3) S057809a	0578/09|l(3)s057809	0	0
12296	46972	Drosophila melanogaster	l(3)S056113	lethal (3) S056113	0561/13	0	0
12297	46973	Drosophila melanogaster	l(3)S051311	lethal (3) S051311	0513/11	0	0
12298	46974	Drosophila melanogaster	l(3)S051309	lethal (3) S051309	0513/09	0	0
12299	46975	Drosophila melanogaster	l(3)S049405a	lethal (3) S049405a	0494/05	0	0
12300	46977	Drosophila melanogaster	l(3)S048318	lethal (3) S048318	0483/18	0	0
12301	46978	Drosophila melanogaster	l(3)S048301a	lethal (3) S048301a	0483/01	0	0
12302	46979	Drosophila melanogaster	l(3)S047916	lethal (3) S047916	0479/16	0	0
12303	46980	Drosophila melanogaster	l(3)S047110	lethal (3) S047110	-	0	0
12304	46981	Drosophila melanogaster	l(3)S045608	lethal (3) S045608	0456/08	0	0
12305	46982	Drosophila melanogaster	l(3)S045230	lethal (3) S045230	0452/30	0	0
12306	46983	Drosophila melanogaster	l(3)S045002c	lethal (3) S045002c	0450/02	0	0
12307	46984	Drosophila melanogaster	l(3)S044823	lethal (3) S044823	0448/23	0	0
12308	46985	Drosophila melanogaster	l(3)S044601	lethal (3) S044601	0446/01	0	0
12309	46986	Drosophila melanogaster	l(3)S042420	lethal (3) S042420	0424/20	0	0
12310	46988	Drosophila melanogaster	l(3)S042228	lethal (3) S042228	0422/28	0	0
12311	46989	Drosophila melanogaster	l(3)S041415	lethal (3) S041415	0414/15	0	0
12312	46990	Drosophila melanogaster	l(3)S036912	lethal (3) S036912	0369/12	0	0
12313	46992	Drosophila melanogaster	l(3)S034305	lethal (3) S034305	0343/05	0	0
12314	46993	Drosophila melanogaster	l(3)S030305	lethal (3) S030305	0303/05	0	0
12315	46994	Drosophila melanogaster	l(3)S027707	lethal (3) S027707	0277/07	0	0
12316	46998	Drosophila melanogaster	l(3)S024639b	lethal (3) S024639b	0246/39	0	0
12317	46999	Drosophila melanogaster	l(3)S024535b	lethal (3) S024535b	0245/35|88A	0	0
12318	47000	Drosophila melanogaster	l(3)S024506	lethal (3) S024506	0245/06	0	0

12319	47001	Drosophila melanogaster	l(3)S024135	lethal (3) S024135	0241/35	0	0
12320	47002	Drosophila melanogaster	l(3)S024020a	lethal (3) S024020a	0240/20	0	0
12321	47003	Drosophila melanogaster	l(3)S023941c	lethal (3) S023941c	0239/41	0	0
12322	47004	Drosophila melanogaster	l(3)S023825	lethal (3) S023825	0238/16|0238/20|0238/25|0238/26|0291/20|0300/04|0428/24|0610/02|0621/12|0685/02|0690/07|0743/09|0816/07|0826/09|1165/12	0	0
12323	47005	Drosophila melanogaster	l(3)S023549	lethal (3) S023549	0235/49|0901/01|l(3)S090101	0	0
12324	47006	Drosophila melanogaster	l(3)S023426	lethal (3) S023426	0234/26	0	0
12325	47007	Drosophila melanogaster	l(3)S022930	lethal (3) S022930	0229/30	0	0
12326	47009	Drosophila melanogaster	l(3)S022564	lethal (3) S022564	-	0	0
12327	47010	Drosophila melanogaster	l(3)S022378	lethal (3) S022378	0223/78	0	0
12328	47011	Drosophila melanogaster	l(3)S022348	lethal (3) S022348	0223/48	0	0
12329	47012	Drosophila melanogaster	l(3)S022231b	lethal (3) S022231b	0222/31|l(3)S022231	0	0
12330	47013	Drosophila melanogaster	l(3)S020514a	lethal (3) S020514a	0205/14	0	0
12331	47014	Drosophila melanogaster	l(3)S019203	lethal (3) S019203	0019/06|0192/03|0229/11|0281/08|0955/15|1003/03|1026/09|1145/13|1232/13|1367/03|1417/15|1426/01|l(3)S141715	0	0
12332	47015	Drosophila melanogaster	l(3)S018801	lethal (3) S018801	0188/01	0	0
12333	47016	Drosophila melanogaster	l(3)S018405	lethal (3) S018405	0184/05	0	0
12334	47017	Drosophila melanogaster	l(3)S015601b	lethal (3) S015601b	0156/01	0	0
12335	47018	Drosophila melanogaster	l(3)S012719	lethal (3) S012719	0127/19	0	0
12336	47020	Drosophila melanogaster	l(3)S011028	lethal (3) S011028	0110/28	0	0
12337	47023	Drosophila melanogaster	l(3)S011001c	lethal (3) S011001c	0110/01	0	0
12338	47025	Drosophila melanogaster	l(3)S010102a	lethal (3) S010102a	0101/02	0	0
12339	47026	Drosophila melanogaster	l(3)S009720	lethal (3) S009720	0097/20	0	0
12340	47027	Drosophila melanogaster	l(3)S008418	lethal (3) S008418	0084/18|0092/23|0256/37|1193/13|1398/10|1483/18	0	0
12341	47028	Drosophila melanogaster	l(3)S007934	lethal (3) S007934	0079/34|0363/02|1175/05|l(3)s007934	0	0
12342	47029	Drosophila melanogaster	l(3)S007412	lethal (3) S007412	0074/12|l(3)61Ea	0	0
12343	47030	Drosophila melanogaster	l(3)S007403a	lethal (3) S007403a	0074/03|l(3)s007403	0	0
12344	47032	Drosophila melanogaster	l(3)S005042	lethal (3) S005042	0050/42	0	0
12345	47033	Drosophila melanogaster	l(3)S005028	lethal (3) S005028	l(3)S127405	0	0
12346	47034	Drosophila melanogaster	l(3)S004624	lethal (3) S004624	0245/33|0475/22	0	0
12347	47035	Drosophila melanogaster	l(3)S004021	lethal (3) S004021	0040/21	0	0
12348	47036	Drosophila melanogaster	l(3)S003220	lethal (3) S003220	0016/04|0032/20|0248/36|0797/09	0	0
12349	47038	Drosophila melanogaster	l(3)S001925	lethal (3) S001925	0019/25	0	0
12350	47039	Drosophila melanogaster	l(3)S001717	lethal (3) S001717	0017/17	0	0
12351	47040	Drosophila melanogaster	l(3)S001620a	lethal (3) S001620a	0016/20	0	0
12352	47041	Drosophila melanogaster	l(3)S001405	lethal (3) S001405	0014/05	0	0
12353	47043	Drosophila melanogaster	l(3)S000107	lethal (3) S000107	0001/07|0082/23|0122/24|0143/27|0194/18|0232/04|0251/32|0263/09|0661/21|1118/02|1118/16	0	0
12354	47044	Drosophila melanogaster	l(3)76BDg	lethal (3) 76BDg	-	0	0
12355	47063	Drosophila melanogaster	l(2)s9998	lethal (2) s9998	-	0	0
12356	47065	Drosophila melanogaster	qsm	quasimodo	CG13432|CG13433|DmelCG13432|EP2356|EP2587|l(2)05510|l(2)k009920|lethal (2) 05510	0	0
12357	47066	Drosophila melanogaster	l(2)05095a	lethal (2) 05095a	-	0	0
12358	47068	Drosophila melanogaster	l(2)01034	lethal (2) 01034	-	0	0
12359	47069	Drosophila melanogaster	ifm(2)RU5	indirect flight muscle (2) RU5	-	0	0
12360	47070	Drosophila melanogaster	ifm(2)RU4	indirect flight muscle (2) RU4	-	0	0
12361	47071	Drosophila melanogaster	ifm(2)RU3	indirect flight muscle (2) RU3	-	0	0
12362	47072	Drosophila melanogaster	ifm(2)RU2	indirect flight muscle (2) RU2	-	0	0
12363	47079	Drosophila melanogaster	abb	abbreviated	-	0	0
12364	47080	Drosophila melanogaster	acj6	abnormal chemosensory jump 6	ACJ6|Acj6|CG9151|DmelCG9151|I-POU|Ipou|tI-POU	0	0
12365	47082	Drosophila melanogaster	Adf1	Adh transcription factor 1	Adf 1|Adf-1|CG15845|DmelCG15845|adf-1|adf1|l(2)01349|l(2)04065|nal	0	0
12366	47084	Drosophila melanogaster	Bpt	Blackpatch	-	0	0
12367	47085	Drosophila melanogaster	btdl	buttonhead like	btd-l	0	0
12368	47091	Drosophila melanogaster	comt	comatose	CG 1618|CG1618|DmNSF|DmelCG1618|NSF|NSF-1|NSF1|Nsf|com|dNSF|dNSF-1|dNSF1|dNsf1|dnsf1	0	0
12369	47093	Drosophila melanogaster	dw-24E	dwarf 24F	dw-24F	0	0
12370	47094	Drosophila melanogaster	eay	early	-	0	0
12371	47096	Drosophila melanogaster	E(f)	Enhancer of forked	If	0	0
12372	47097	Drosophila melanogaster	fliG	flightless G	-	0	0
12373	47100	Drosophila melanogaster	fs(1)A59	female sterile (1) A59	-	0	0
12374	47101	Drosophila melanogaster	fs(1)A1242	female sterile (1) A1242	fs(1)1242	0	0
12375	47102	Drosophila melanogaster	fs(1)de2	female sterile (1) defective eggs 2	de2	0	0
12376	47103	Drosophila melanogaster	fs(1)de5	female sterile (1) defective eggs 5	de5	0	0
12377	47104	Drosophila melanogaster	fs(1)de6	female sterile (1) defective eggs 6	de6	0	0
12378	47105	Drosophila melanogaster	fs(1)de8	female sterile (1) defective eggs 8	de8	0	0
12379	47106	Drosophila melanogaster	fs(1)de11	female sterile (1) defective eggs 11	de11	0	0
12380	47107	Drosophila melanogaster	fs(1)M13	female sterile (1) M13	-	0	0
12381	47108	Drosophila melanogaster	fs(1)M21	female sterile (1) M21	-	0	0
12382	47109	Drosophila melanogaster	fs(1)M24	female sterile (1) M24	-	0	0
12383	47110	Drosophila melanogaster	fs(1)M27	female sterile (1) M27	-	0	0
12384	47111	Drosophila melanogaster	fs(1)M28	female sterile (1) M28	-	0	0
12385	47112	Drosophila melanogaster	fs(1)M49	female sterile (1) M49	-	0	0
12386	47113	Drosophila melanogaster	fs(1)M52	female sterile (1) M52	-	0	0
12387	47114	Drosophila melanogaster	fs(1)M63	female sterile (1) M63	-	0	0
12388	47115	Drosophila melanogaster	fs(1)M95	female sterile (1) M95	-	0	0
12389	47116	Drosophila melanogaster	fs(1)ne4	female sterile (1) no eggs 4	ne4	0	0
12390	47117	Drosophila melanogaster	fs(1)ne7	female sterile (1) no eggs 7	ne7	0	0
12391	47118	Drosophila melanogaster	fs(1)ne10	female sterile (1) no eggs 10	ne10	0	0
12392	47119	Drosophila melanogaster	Fs(2)Kom	Female sterile (2) Kompolt	Kom	0	0
12393	47120	Drosophila melanogaster	mama	maternal metaphase arrest	mat(2)Ito-DF6	0	0
12394	47121	Drosophila melanogaster	dup	double parked	19k|CDT1|CDT1/DUP|CG8171|Cdt1|DUP|DUP/CDT1|DmelCG8171|Dup|anon-WO0257455.31|cdt1|cdt1/dup|fs(2)51Fa|fs(2)PA77|fs(2)ItoPA77|l(2)51Ec|l(2)k03308	0	0
12395	47122	Drosophila melanogaster	Fs(3)Jut	Female sterile (3) Jutas	Fs(3)Jutas|Jut	0	0
12396	47123	Drosophila melanogaster	Fs(3)Kar	Female sterile (3) Kartal	Fs(3)Kartal|Kar	0	0
12397	47124	Drosophila melanogaster	Fs(3)Kel	Female sterile (3) Keled	Fs(3)Keled	0	0
12398	47125	Drosophila melanogaster	Fs(3)Pil	Female sterile (3) Pilis	Fs(3)Pilis	0	0
12399	47126	Drosophila melanogaster	Fs(3)Ros	Female sterile (3) Rosd	Fs(3)Rosd	0	0
12400	47127	Drosophila melanogaster	Fs(3)Zol	Female sterile (3) Zolta	Fs(3)Zolta	0	0
12401	47129	Drosophila melanogaster	gustE	gustatory-defective E	-	0	0
12402	47130	Drosophila melanogaster	gvl	grooveless	-	0	0
12403	47132	Drosophila melanogaster	hv	heavy vein	-	0	0
12404	47133	Drosophila melanogaster	irreA	irregular optic chiasma A	-	0	0
12405	47137	Drosophila melanogaster	knk	knickkopf	CG6217|DmelCG6217|Gnarled|Knk|gnar|l(3)85Eg	0	0
12406	47138	Drosophila melanogaster	l(1)1Ac	lethal (1) 1Ac	J1|l(1)Ac|l(1)J1|l(1)Jl|lJ1	0	0
12407	47139	Drosophila melanogaster	l(1)1Ad	lethal (1) 1Ad	EC2|l(1)2804|lEC2|l[EC2]	0	0
12408	47140	Drosophila melanogaster	l(1)1Af	lethal (1) 1Af	-	0	0
12409	47141	Drosophila melanogaster	Exp6	Exportin 6	CG3923|DmelCG3923|EC4|EG:165H7.3|Exp1|l(1)1Bb|l(1)Bb|l(1)C84|l(1)EC4|l(1)G4	0	0
12410	47142	Drosophila melanogaster	l(1)1Bc	lethal (1) 1Bc	EC5|l(1)EC5	0	0
12411	47143	Drosophila melanogaster	l(1)1Bg	lethal (1) 1Bg	l(1)Bg	0	0
12412	47144	Drosophila melanogaster	l(1)1Ef	lethal (1) 1Ef	C24	0	0
12413	47145	Drosophila melanogaster	l(1)1Fc	lethal (1) 1Fc	JA29	0	0
12414	47146	Drosophila melanogaster	l(1)1Ff	lethal (1) 1Ff	-	0	0
12415	47147	Drosophila melanogaster	l(1)2Fb	lethal (1) 2Fb	JC155|N4|N9|VA172|l68	0	0
12416	47148	Drosophila melanogaster	l(1)2Fc	lethal (1) 2Fc	HF330|N12|N7|l(1)N9|l46	0	0
12417	47149	Drosophila melanogaster	l(1)2Fd	lethal (1) 2Fd	D82|EC226|N11|N6|l(1)EC226|l26	0	0
12418	47151	Drosophila melanogaster	l(1)3Ag	lethal (1) 3Ag	ZW-13|l(1)zw13|zw-13|zw13	0	0
12419	47152	Drosophila melanogaster	l(1)3Ah	lethal (1) 3Ah	ZW-2|l(1)zw2|zw-2|zw2	0	0
12420	47153	Drosophila melanogaster	l(1)3Bc	lethal (1) 3Bc	ZW-12|l(1)zw12|zw-12|zw12	0	0
12421	47154	Drosophila melanogaster	l(1)3Bf	lethal (1) 3Bf	ZW-11|l(1)zw11|l[a5]|zw-11|zw11	0	0
12422	47155	Drosophila melanogaster	l(1)3Da	lethal (1) 3Da	RA55	0	0
12423	47156	Drosophila melanogaster	l(1)3Eb	lethal (1) 3Eb	GF332	0	0
12424	47157	Drosophila melanogaster	l(1)3Ec	lethal (1) 3Ec	-	0	0
12425	47158	Drosophila melanogaster	l(1)3Ed	lethal (1) 3Ed	JC43	0	0
12426	47159	Drosophila melanogaster	l(1)3Ee	lethal (1) 3Ee	-	0	0
12427	47160	Drosophila melanogaster	l(1)carot3	lethal (1) carot3	34|l(1)34|l34	0	0
12428	47161	Drosophila melanogaster	l(1)CP9	lethal (1) CP9	-	0	0
12429	47162	Drosophila melanogaster	l(2)cg	lethal (2) with comb gap	-	0	0

12430	47163	Drosophila melanogaster	soba	suppressor of bulge and argos	-	0	0
12431	47164	Drosophila melanogaster	l(3)S4	lethal (3) S4	-	0	0
12432	47165	Drosophila melanogaster	ll	lanceolate	-	0	0
12433	47166	Drosophila melanogaster	M(1)8F	Minute (1) 8F	M(1)8E/F|RpS28b	0	0
12434	47167	Drosophila melanogaster	M(2)53	Minute (2) 53	M(2)53-1|M(2)53C|M(2)S-7|M(2)S7|M(2R)S7|RpLP2|RpS15	0	0
12435	47168	Drosophila melanogaster	M(3)62A	Minute (3) 62A	M(3)LS1|RpL23A	0	0
12436	47169	Drosophila melanogaster	mar	metaphase arrest	-	0	0
12437	47172	Drosophila melanogaster	mell	melanized-like	-	0	0
12438	47173	Drosophila melanogaster	Men	Malic enzyme	CG10120|DmelCG10120|MDH|ME|Mdh-NADP|Me|anon-WO0118547.278|mem|men	0	0
12439	47174	Drosophila melanogaster	mesA	messy A	l(3)mes-1A|mes-A|messy	0	0
12440	47175	Drosophila melanogaster	mgr	merry-go-round	-	0	0
12441	47176	Drosophila melanogaster	mit(1)21	mitotic 21	-	0	0
12442	47177	Drosophila melanogaster	msd(gl)	modifier of sexual dimorphism of glass	-	0	0
12443	47178	Drosophila melanogaster	Mst25F	Male-specific RNA 25F	-	0	0
12444	47179	Drosophila melanogaster	Mst47A	Male-specific RNA 47A	mst-325	0	0
12445	47180	Drosophila melanogaster	Mst51F	Male-specific RNA 51F	mst-35	0	0
12446	47181	Drosophila melanogaster	Mst66D	Male-specific RNA 66D	-	0	0
12447	47182	Drosophila melanogaster	mus102	mutagen-sensitive 102	mus 102|mus(1)102|mus-102	0	0
12448	47183	Drosophila melanogaster	mus105	mutagen-sensitive 105	l(1)d deg-1|mus(1)105|mus-105	0	0
12449	47184	Drosophila melanogaster	mus109	mutagen-sensitive 109	mus(1)107|mus(1)109|mus-109|mus107	0	0
12450	47185	Drosophila melanogaster	mus111	mutagen-sensitive 111	mus(1)111	0	0
12451	47186	Drosophila melanogaster	mus205	mutagen-sensitive 205	154926_at|AAF59191|CG1925|DhRev3|DmREV3|DmelCG1925|Dmpolzeta|Mus205|mus 205|mus(2)205|mus-205	0	0
12452	47187	Drosophila melanogaster	mus306	mutagen-sensitive 306	-	0	0
12453	47188	Drosophila melanogaster	mus307	mutagen-sensitive 307	-	0	0
12454	47190	Drosophila melanogaster	Pin	Pin	-	0	0
12455	47191	Drosophila melanogaster	Pr	Prickly	-	0	0
12456	47192	Drosophila melanogaster	Raf2	Raf oncogene 2	Draf-2	0	0
12457	47193	Drosophila melanogaster	rvn	ravenoid	-	0	0
12458	47194	Drosophila melanogaster	Scn	Scutenick	-	0	0
12459	47195	Drosophila melanogaster	sesD	stress-sensitive D	-	0	0
12460	47196	Drosophila melanogaster	sesF	stress-sensitive F	-	0	0
12461	47198	Drosophila melanogaster	Sgs7	Salivary gland secretion 7	68C GIII|CG18087|DmelCG18087|Sgs-7|sgs-7|sgs7	0	0
12462	47199	Drosophila melanogaster	sic	sichel	-	0	0
12463	47202	Drosophila melanogaster	stf	stormfront	-	0	0
12464	47203	Drosophila melanogaster	Su(S)	Suppressor of Star	-	0	0
12465	47204	Drosophila melanogaster	su(sc)	suppressor of scute	-	0	0
12466	47205	Drosophila melanogaster	Su(ss)	Suppressor of spineless	-	0	0
12467	47206	Drosophila melanogaster	Ta	Thickened arista	-	0	0
12468	47207	Drosophila melanogaster	Tcr	Third chromosome alpha methyl dopa-resistant	-	0	0
12469	47208	Drosophila melanogaster	term	terminus	Bsg75C|CG4216|DmelCG4216|Term|ter	0	0
12470	47209	Drosophila melanogaster	tlc	tender little chaetae	-	0	0
12471	47210	Drosophila melanogaster	tuh1	tumorous head on 1	tuh|tuh-1	0	0
12472	47211	Drosophila melanogaster	tyr1	tyrosine 1	tyr-1	0	0
12473	47212	Drosophila melanogaster	uncl	uncoordinated-like	17|24|25|Q456|R-10-10|l(1)Q-456|l(1)R-10-10|l(1)R10-10	0	0
12474	47213	Drosophila melanogaster	Bsg95C	Blastoderm-specific transcript 95C	-	0	0
12475	47214	Drosophila melanogaster	Gsg71A	Gastrula-specific transcript 71A	-	0	0
12476	47216	Drosophila melanogaster	Klp54D	Kinesin-like protein at 54D	CG15844|CG43323|DmelCG43323|Dmel_CG15844|KLP1|Klp1	0	0
12477	47218	Drosophila melanogaster	RpS14a	Ribosomal protein S14a	CG1524|DmelCG1524|RPS14A|RpS14|RpS14A|S14|S14a|anon-EST:Posey115|anon-EST:Posey131|anon-EST:Posey154|anon-EST:Posey77|anon-WO03040301.136|anon-WO03040301.138|rpS14A	0	0
12478	47219	Drosophila melanogaster	RpS14b	Ribosomal protein S14b	CG1527|DmelCG1527|RPS14B|RpS14|RpS14B|S14|S14b|anon-EST:Posey115|anon-EST:Posey131|anon-EST:Posey154|anon-EST:Posey77|rpS14B	0	0
12479	47220	Drosophila melanogaster	Fur1	Furin 1	CG10772|Dfur1|Dfurin1|DmelCG10772|Furin|Furl|Klip1|dFur1|dFurin 1|dKLIP-1|fur-1|fur1|fur[1]|furin|l(3)rL205	0	0
12480	47223	Drosophila melanogaster	wapm	wings apart mimic	-	0	0
12481	47224	Drosophila melanogaster	Indnc	In dunce	indnc	0	0
12482	47228	Drosophila melanogaster	l(1)3C3	lethal (1) 3C3	-	0	0
12483	47229	Drosophila melanogaster	l(1)J413	lethal (1) J413	-	0	0
12484	47230	Drosophila melanogaster	l(2)DTS6	lethal (2) DTS6	-	0	0
12485	47231	Drosophila melanogaster	ms(2)73d	male sterile (2) 73d	-	0	0
12486	47232	Drosophila melanogaster	l(1)adl7	lethal (1) adl7	l(1)adl-7	0	0
12487	47233	Drosophila melanogaster	l(1)adl8	lethal (1) adl8	l(1)adl-8	0	0
12488	47234	Drosophila melanogaster	l(1)adl9	lethal (1) adl9	l(1)adl-9	0	0
12489	47235	Drosophila melanogaster	l(1)v11	lethal (1) v11	l(1)11	0	0
12490	47236	Drosophila melanogaster	l(1)v25	lethal (1) v25	l(1)25	0	0
12491	47237	Drosophila melanogaster	l(1)v75	lethal (1) v75	l(1)75	0	0
12492	47238	Drosophila melanogaster	l(1)v135	lethal (1) v135	l(1)135	0	0
12493	47239	Drosophila melanogaster	l(1)v150	lethal (1) v150	l(1)150	0	0
12494	47240	Drosophila melanogaster	l(1)v163	lethal (1) v163	l(1)163	0	0
12495	47241	Drosophila melanogaster	l(1)v219	lethal (1) v219	l(1)219	0	0
12496	47242	Drosophila melanogaster	l(1)v223	lethal (1) v223	l(1)223	0	0
12497	47243	Drosophila melanogaster	l(1)v453	lethal (1) v453	l(1)453	0	0
12498	47244	Drosophila melanogaster	mcd2	muscle cell death 2	mcd-2	0	0
12499	47245	Drosophila melanogaster	Nc34CD	Neural conserved at 34CD	NC34CD	0	0
12500	47246	Drosophila melanogaster	l(1)TK134	lethal (1) TK134	l134	0	0
12501	47247	Drosophila melanogaster	l(1)TK235	lethal (1) TK235	l235	0	0
12502	47248	Drosophila melanogaster	spno	supernova	-	0	0
12503	47249	Drosophila melanogaster	woc	without children	CG5965|DmelCG5965|Woc	0	0
12504	47250	Drosophila melanogaster	esp	extra sensory photoreceptor cells	-	0	0
12505	47251	Drosophila melanogaster	Gtp-bp	GTP-binding protein	BEST:CK00309|CG2522|CK00309|DmelCG2522|GTP-bp|SrpRalpha|mAb 13D5	0	0
12506	47253	Drosophila melanogaster	l(2)01810	lethal (2) 01810	CG5304|DmelCG5304|anon-WO0118547.88|lethal(2)01810	0	0
12507	47260	Drosophila melanogaster	l(3)05822	lethal (3) 05822	5822|CG7129|DmelCG7129|L(3)05822|LD12970|clot 3142|l(3)5822	0	0
12508	47263	Drosophila melanogaster	l(1)291-95	lethal (1) 291-95	291-95	0	0
12509	47266	Drosophila melanogaster	cash	cashews	cas	0	0
12510	47267	Drosophila melanogaster	goul	goulash	-	0	0
12511	47268	Drosophila melanogaster	hall	halley	hal	0	0
12512	47272	Drosophila melanogaster	cbx	crossbronx	CG10536|Cbx|DmelCG10536|Ft1|ms(2)05704|ms(2)46C	0	0
12513	47273	Drosophila melanogaster	l(3)j1A1	lethal (3) j1A1	-	0	0
12514	47278	Drosophila melanogaster	l(3)j9E7	lethal (3) j9E7	-	0	0
12515	47280	Drosophila melanogaster	l(3)neo39	lethal (3) neo39	-	0	0
12516	47283	Drosophila melanogaster	SrpRbeta	Signal recognition particle receptor beta	BcDNA:GM04779|CG33162|CG5950|Dm SRbeta|DmelCG33162|SRbeta|l(3)rK561|l(3)rL537|srpRbeta	0	0
12517	47284	Drosophila melanogaster	ded	depleted	-	0	0
12518	47285	Drosophila melanogaster	repo	reversed polarity	3702|AbRK2|CG31240|CG8045(CT24072)|CT24072|DmelCG31240|REPO|RK2|Repo|l(3)03702|rk2	0	0
12519	47297	Drosophila melanogaster	l(1)12P2	lethal (1) 12P2	-	0	0
12520	47299	Drosophila melanogaster	l(1)6PP7	lethal (1) 6PP7	-	0	0
12521	47300	Drosophila melanogaster	l(2)ry28	lethal (2) ry28	l(2)28|unnamed	0	0
12522	47301	Drosophila melanogaster	l(2)35Ab	lethal (2) 35Ab	-	0	0
12523	47302	Drosophila melanogaster	l(2)ry36	lethal (2) ry36	l(2)36|unnamed	0	0
12524	47303	Drosophila melanogaster	l(2)ry53	lethal (2) ry53	l(2)53|unnamed	0	0
12525	47304	Drosophila melanogaster	l(3)ry16	lethal (3) ry16	l(3)16	0	0
12526	47305	Drosophila melanogaster	l(3)ry82	lethal (3) ry82	l(3)82	0	0
12527	47306	Drosophila melanogaster	l(3)82Fi	lethal (3) 82Fi	-	0	0
12528	47307	Drosophila melanogaster	l(3)ry93	lethal (3) ry93	l(3)93|unnamed	0	0
12529	47308	Drosophila melanogaster	l(3)97Da	lethal (3) 97Da	-	0	0
12530	47309	Drosophila melanogaster	l(3)97Db	lethal (3) 97Db	l(3)155	0	0
12531	47310	Drosophila melanogaster	l(3)97Dc	lethal (3) 97Dc	-	0	0
12532	47314	Drosophila melanogaster	E(sina)46C	Enhancer of seven in absentia at 46C	-	0	0
12533	47320	Drosophila melanogaster	hni	he's not interested	-	0	0
12534	47334	Drosophila melanogaster	l(2)23Ab	lethal (2) 23Ab	-	0	0
12535	47335	Drosophila melanogaster	l(2)46Ca	lethal (2) 46Ca	I	0	0
12536	47336	Drosophila melanogaster	l(2)46Cb	lethal (2) 46Cb	II	0	0
12537	47337	Drosophila melanogaster	l(2)46Cc	lethal (2) 46Cc	III	0	0
12538	47338	Drosophila melanogaster	l(2)46Da	lethal (2) 46Da	VII	0	0
12539	47339	Drosophila melanogaster	l(2)46Db	lethal (2) 46Db	VIII	0	0
12540	47340	Drosophila melanogaster	l(2)46Dc	lethal (2) 46Dc	IX	0	0

12541	47341	Drosophila melanogaster	l(2)46Dd	lethal (2) 46Dd	X	0	0
12542	47342	Drosophila melanogaster	l(2)46De	lethal (2) 46De	XI	0	0
12543	47343	Drosophila melanogaster	l(2)46Df	lethal (2) 46Df	XII	0	0
12544	47344	Drosophila melanogaster	l(2)46Dg	lethal (2) 46Dg	XIII	0	0
12545	47345	Drosophila melanogaster	l(1)14BCa	lethal (1) 14BCa	l(1)14-12|l(1)14Ce	0	0
12546	47346	Drosophila melanogaster	l(2)44Fe	lethal (2) 44Fe	-	0	0
12547	47347	Drosophila melanogaster	l(3)EGX4	lethal (3) EGX4	EGX4|PGX4	0	0
12548	47352	Drosophila melanogaster	Su(Kr|If])	Suppressor of Kruppel-Irregular facets	-	0	0
12549	47356	Drosophila melanogaster	l(2)PC4-51	lethal (2) PC4-51	l(2)PC4-G	0	0
12550	47359	Drosophila melanogaster	l(3)89Bt	lethal (3) 89Bt	l(3)89Br	0	0
12551	47360	Drosophila melanogaster	l(1)15Fa	lethal (1) 15Fa	-	0	0
12552	47361	Drosophila melanogaster	l(1)15Eb	lethal (1) 15Eb	-	0	0
12553	47362	Drosophila melanogaster	l(1)15De	lethal (1) 15De	-	0	0
12554	47363	Drosophila melanogaster	l(1)15Dd	lethal (1) 15Dd	-	0	0
12555	47364	Drosophila melanogaster	l(1)15Dc	lethal (1) 15Dc	-	0	0
12556	47365	Drosophila melanogaster	l(1)15Db	lethal (1) 15Db	-	0	0
12557	47366	Drosophila melanogaster	l(1)15Da	lethal (1) 15Da	-	0	0
12558	47367	Drosophila melanogaster	l(1)15Bc	lethal (1) 15Bc	-	0	0
12559	47368	Drosophila melanogaster	l(1)15Bb	lethal (1) 15Bb	-	0	0
12560	47370	Drosophila melanogaster	l(3)LG12	lethal (3) LG12	-	0	0
12561	47375	Drosophila melanogaster	l(1)89B	lethal (1) 89B	-	0	0
12562	47376	Drosophila melanogaster	l(1)827	lethal (1) 827	-	0	0
12563	47377	Drosophila melanogaster	l(1)48B	lethal (1) 48B	-	0	0
12564	47378	Drosophila melanogaster	l(1)100	lethal (1) 100	-	0	0
12565	47384	Drosophila melanogaster	sra	sarah	89B|89B11|CG6072|DmelCG6072|Sra|anon-WO0118547.92|anon-WO0172774.10|l(3)05203|nebula|nla	0	0
12566	47385	Drosophila melanogaster	l(2)k06407	lethal (2) k06407	lp	0	0
12567	47390	Drosophila melanogaster	l(3)95BCc	lethal (3) 95BCc	-	0	0
12568	47391	Drosophila melanogaster	l(3)95BCb	lethal (3) 95BCb	-	0	0
12569	47392	Drosophila melanogaster	l(3)95BCa	lethal (3) 95BCa	-	0	0
12570	47394	Drosophila melanogaster	l(2)k17031	lethal (2) k17031	l(2)k17035	0	0
12571	47396	Drosophila melanogaster	Tor	Target of rapamycin	5092|CG5092|CT16317|CT24745|CT24817|DmTOR|DmelCG5092|FRAP/TOR|TOR|dTOR|dTor|dtor|l(2)k17004|mTOR|tor	0	0
12572	47399	Drosophila melanogaster	l(2)k16918	lethal (2) k16918	CG3920|DmelCG3920	0	0
12573	47401	Drosophila melanogaster	l(2)k16801	lethal (2) k16801	-	0	0
12574	47406	Drosophila melanogaster	l(2)k16525	lethal (2) k16525	-	0	0
12575	47408	Drosophila melanogaster	Vps28	Vacuolar protein sorting 28	CG12770|DmVps28|DmelCG12770|Dvps28|ESCRT-I|ESCRT-II|dVps28|dvps28|l(2)k16503|vps28	0	0
12576	47412	Drosophila melanogaster	l(2)k16210	lethal (2) k16210	-	0	0
12577	47413	Drosophila melanogaster	l(2)k16207	lethal (2) k16207	-	0	0
12578	47415	Drosophila melanogaster	l(2)k16105	lethal (2) k16105	unnamed	0	0
12579	47417	Drosophila melanogaster	l(2)k16005	lethal (2) k16005	-	0	0
12580	47423	Drosophila melanogaster	l(2)k15206	lethal (2) k15206	-	0	0
12581	47425	Drosophila melanogaster	l(2)k14817	lethal (2) k14817	-	0	0
12582	47426	Drosophila melanogaster	l(2)k14816	lethal (2) k14816	-	0	0
12583	47430	Drosophila melanogaster	l(2)k14614	lethal (2) k14614	-	0	0
12584	47431	Drosophila melanogaster	l(2)k14602	lethal (2) k14602	-	0	0
12585	47432	Drosophila melanogaster	l(2)k14601	lethal (2) k14601	-	0	0
12586	47433	Drosophila melanogaster	l(2)k14517	lethal (2) k14517	-	0	0
12587	47442	Drosophila melanogaster	l(2)k13805	lethal (2) k13805	-	0	0
12588	47456	Drosophila melanogaster	l(2)k13116	lethal (2) k13116	-	0	0
12589	47457	Drosophila melanogaster	ytr	yantar	CG18426|DmelCG18426|b(2)gcn|bgcn|l(2)60A-F|l(2)k13108|l(2)k13214	0	0
12590	47459	Drosophila melanogaster	l(2)k12408	lethal (2) k12408	-	0	0
12591	47461	Drosophila melanogaster	l(2)k12004	lethal (2) k12004	-	0	0
12592	47462	Drosophila melanogaster	l(2)k11702	lethal (2) k11702	unnamed	0	0
12593	47463	Drosophila melanogaster	l(2)k11537	lethal (2) k11537	-	0	0
12594	47466	Drosophila melanogaster	l(2)k11502	lethal (2) k11502	-	0	0
12595	47469	Drosophila melanogaster	l(2)k11325	lethal (2) k11325	-	0	0
12596	47472	Drosophila melanogaster	l(2)k11303	lethal (2) k11303	-	0	0
12597	47479	Drosophila melanogaster	l(2)k11038	lethal (2) k11038	-	0	0
12598	47480	Drosophila melanogaster	l(2)k11021	lethal (2) k11021	-	0	0
12599	47481	Drosophila melanogaster	l(2)k10914	lethal (2) k10914	-	0	0
12600	47482	Drosophila melanogaster	l(2)k10816	lethal (2) k10816	unnamed	0	0
12601	47487	Drosophila melanogaster	l(2)k10411	lethal (2) k10411	-	0	0
12602	47491	Drosophila melanogaster	l(2)k10237	lethal (2) k10237	-	0	0
12603	47495	Drosophila melanogaster	l(2)k10209	lethal (2) k10209	unnamed	0	0
12604	47501	Drosophila melanogaster	l(2)k10012	lethal (2) k10012	-	0	0
12605	47510	Drosophila melanogaster	l(2)k09903	lethal (2) k09903	-	0	0
12606	47514	Drosophila melanogaster	l(2)k09624	lethal (2) k09624	unnamed	0	0
12607	47517	Drosophila melanogaster	l(2)k09601	lethal (2) k09601	-	0	0
12608	47521	Drosophila melanogaster	l(2)k09310	lethal (2) k09310	-	0	0
12609	47522	Drosophila melanogaster	l(2)k09303	lethal (2) k09303	unnamed	0	0
12610	47523	Drosophila melanogaster	l(2)k09238	lethal (2) k09238	-	0	0
12611	47527	Drosophila melanogaster	l(2)k09107	lethal (2) k09107	unnamed	0	0
12612	47531	Drosophila melanogaster	l(2)k09021	lethal (2) k09021	-	0	0
12613	47533	Drosophila melanogaster	l(2)k09005	lethal (2) k09005	-	0	0
12614	47535	Drosophila melanogaster	l(2)k08914	lethal (2) k08914	-	0	0
12615	47541	Drosophila melanogaster	l(2)k08611	lethal (2) k08611	-	0	0
12616	47544	Drosophila melanogaster	l(2)k08320	lethal (2) k08320	-	0	0
12617	47545	Drosophila melanogaster	l(2)k08317	lethal (2) k08317	-	0	0
12618	47547	Drosophila melanogaster	l(2)k08306	lethal (2) k08306	-	0	0
12619	47548	Drosophila melanogaster	l(2)k08222	lethal (2) k08222	-	0	0
12620	47552	Drosophila melanogaster	l(2)k08046	lethal (2) k08046	-	0	0
12621	47560	Drosophila melanogaster	l(2)k07713	lethal (2) k07713	-	0	0
12622	47562	Drosophila melanogaster	l(2)k07526	lethal (2) k07526	-	0	0
12623	47563	Drosophila melanogaster	l(2)k07525	lethal (2) k07525	-	0	0
12624	47568	Drosophila melanogaster	l(2)k07332	lethal (2) k07332	unnamed	0	0
12625	47571	Drosophila melanogaster	l(2)k07245	lethal (2) k07245	unnamed	0	0
12626	47573	Drosophila melanogaster	l(2)k07219	lethal (2) k07219	-	0	0
12627	47575	Drosophila melanogaster	l(2)k07204	lethal (2) k07204	-	0	0
12628	47581	Drosophila melanogaster	l(2)k07109a	lethal (2) k07109a	-	0	0
12629	47585	Drosophila melanogaster	l(2)k06918	lethal (2) k06918	-	0	0
12630	47586	Drosophila melanogaster	l(2)k06917	lethal (2) k06917	-	0	0
12631	47593	Drosophila melanogaster	l(2)k06408	lethal (2) k06408	unnamed	0	0
12632	47594	Drosophila melanogaster	aPKC	atypical protein kinase C	2R-141-24|2R-265-2|2R-417-13|2R-69-30|CG10261|CG30475|CG42783|DaPKC|DmelCG42783|Dmel_CG10261|Dmel_CG30475|PKCl|PKCzeta|a-PKC|aPKC-zeta|aPKCzeta|alphaPKCzeta|apkc|daPKC|da	0	0
12633	47601	Drosophila melanogaster	l(2)k06107	lethal (2) k06107	-	0	0
12634	47603	Drosophila melanogaster	l(2)k06019	lethal (2) k06019	-	0	0
12635	47604	Drosophila melanogaster	l(2)k06006	lethal (2) k06006	-	0	0
12636	47607	Drosophila melanogaster	l(2)k05816	lethal (2) k05816	-	0	0
12637	47611	Drosophila melanogaster	Gpo-1	Glycerophosphate oxidase-1	CG18786|CG8256|DmelCG8256|GPO|GPO-1|Gpo|alpha-GPO|alphaGPO|anon-WO0118547.114|l(2)k05713	0	0
12638	47613	Drosophila melanogaster	l(2)k05614	lethal (2) k05614	-	0	0
12639	47617	Drosophila melanogaster	l(2)k05416	lethal (2) k05416	-	0	0
12640	47618	Drosophila melanogaster	l(2)k05404	lethal (2) k05404	-	0	0
12641	47623	Drosophila melanogaster	l(2)k05104	lethal (2) k05104	-	0	0
12642	47624	Drosophila melanogaster	l(2)k05103	lethal (2) k05103	-	0	0
12643	47626	Drosophila melanogaster	l(2)k04810	lethal (2) k04810	unnamed	0	0
12644	47627	Drosophila melanogaster	l(2)k04512	lethal (2) k04512	unnamed	0	0
12645	47636	Drosophila melanogaster	l(2)k03303	lethal (2) k03303	-	0	0
12646	47643	Drosophila melanogaster	l(2)k03105	lethal (2) k03105	-	0	0
12647	47647	Drosophila melanogaster	l(2)k02505	lethal (2) k02505	-	0	0
12648	47648	Drosophila melanogaster	l(2)k02501	lethal (2) k02501	-	0	0
12649	47653	Drosophila melanogaster	l(2)k01206	lethal (2) k01206	-	0	0
12650	47666	Drosophila melanogaster	l(2)k00224	lethal (2) k00224	-	0	0
12651	47667	Drosophila melanogaster	l(2)k00213	lethal (2) k00213	-	0	0

12652	47671	Drosophila melanogaster	l(2)05559b	lethal (2) 05559b	-	0	0
12653	47672	Drosophila melanogaster	l(2)04518b	lethal (2) 04518b	-	0	0
12654	47673	Drosophila melanogaster	l(1)M52.3	lethal (1) M52.3	M52.3	0	0
12655	47674	Drosophila melanogaster	l(1)D36.5	lethal (1) D36.5	D36.5	0	0
12656	47676	Drosophila melanogaster	hypnos-3	hypoxia, anoxia, sensitive 3	-	0	0
12657	47677	Drosophila melanogaster	E(faf)E337	Enhancer of faf E337	-	0	0
12658	47678	Drosophila melanogaster	l(1)20BFa	lethal (1) 20BFa	400C20a	0	0
12659	47687	Drosophila melanogaster	dsx-c98D	doublesex cognate 98D	dsx-c98	0	0
12660	47698	Drosophila melanogaster	l(2)k09033	lethal (2) k09033	unnamed	0	0
12661	47699	Drosophila melanogaster	l(2)k05627	lethal (2) k05627	-	0	0
12662	47703	Drosophila melanogaster	l(2)38Eb	lethal (2) 38Eb	-	0	0
12663	47705	Drosophila melanogaster	l(2)38De	lethal (2) 38De	-	0	0
12664	47706	Drosophila melanogaster	l(2)38Dd	lethal (2) 38Dd	-	0	0
12665	47707	Drosophila melanogaster	l(2)38Dc	lethal (2) 38Dc	-	0	0
12666	47708	Drosophila melanogaster	l(2)38Db	lethal (2) 38Db	-	0	0
12667	47709	Drosophila melanogaster	l(2)38Da	lethal (2) 38Da	-	0	0
12668	47710	Drosophila melanogaster	ms(3)85E	male sterile (3) 85E	ms(3)KM27	0	0
12669	47718	Drosophila melanogaster	CG17829	CG17829 gene product from transcript CG17829-RA	DmelCG17829|EG:115C2.6	0	0
12670	47719	Drosophila melanogaster	l(3)93DFi	lethal (3) 93DFi	unnamed	0	0
12671	47720	Drosophila melanogaster	l(3)93DFh	lethal (3) 93DFh	unnamed	0	0
12672	47721	Drosophila melanogaster	l(3)93DFg	lethal (3) 93DFg	unnamed	0	0
12673	47722	Drosophila melanogaster	l(3)93DFf	lethal (3) 93DFf	unnamed	0	0
12674	47723	Drosophila melanogaster	l(3)93DFe	lethal (3) 93DFe	unnamed	0	0
12675	47724	Drosophila melanogaster	l(3)93DFd	lethal (3) 93DFd	unnamed	0	0
12676	47725	Drosophila melanogaster	l(3)93DFc	lethal (3) 93DFc	unnamed	0	0
12677	47726	Drosophila melanogaster	l(3)93DFb	lethal (3) 93DFb	unnamed	0	0
12678	47727	Drosophila melanogaster	l(3)93DFa	lethal (3) 93DFa	unnamed	0	0
12679	47728	Drosophila melanogaster	l(2)k04206	lethal (2) k04206	-	0	0
12680	47729	Drosophila melanogaster	CG16833	CG16833 gene product from transcript CG16833-RG	BEST:LD13681|DmelCG16833|LD13681	0	0
12681	47730	Drosophila melanogaster	CG12253	CG12253 gene product from transcript CG12253-RA	BEST:LD08487|DPLG1|DmelCG12253|LD08487	0	0
12682	47732	Drosophila melanogaster	slow-phase	slow phase	sph	0	0
12683	47733	Drosophila melanogaster	l(3)06734	lethal (3) 06734	-	0	0
12684	47735	Drosophila melanogaster	l(1)G0329	lethal (1) G0329	-	0	0
12685	47746	Drosophila melanogaster	Lk	Leucokinin	CG13480|DLK|DmelCG13480|Drm-KIN|LK|Leuk|leucokinin|leukokinin|lk|pp	0	0
12686	47748	Drosophila melanogaster	l(3)L7160a	lethal (3) L7160a	l(3)L7160	0	0
12687	47749	Drosophila melanogaster	l(3)L4222a	lethal (3) L4222a	l(3)L4222	0	0
12688	47750	Drosophila melanogaster	l(3)05218	lethal (3) 05218	-	0	0
12689	47752	Drosophila melanogaster	l(2)k07521	lethal (2) k07521	-	0	0
12690	47753	Drosophila melanogaster	l(2)10565a	lethal (2) 10565a	l(2)10565	0	0
12691	47754	Drosophila melanogaster	l(2)04207a	lethal (2) 04207a	l(2)04207	0	0
12692	47755	Drosophila melanogaster	l(2)00434a	lethal (2) 00434a	l(2)00434	0	0
12693	47757	Drosophila melanogaster	betaggt-II	beta subunit of type II geranylgeranyl transferase	CG18627|DmelCG18627|GGT-II beta|betaggt|betaggt II	0	0
12694	47762	Drosophila melanogaster	FKBP59	FK506-binding protein FKBP59	CG4535|DmelCG4535|FKBP59-bp1|anon-WO0118547.161|dFKBP59|l(2)k00424|l(2)k09010	0	0
12695	47763	Drosophila melanogaster	Abd-B	Abdominal B	9|ABD-B|ABDB|Abd B|Abd-b|Abd0B|AbdB|AbdB(CA)[[26]]|AbdB-I|AbdB-II|AdbB|BX-C|CG10291|CG11648|DROABDB|DmAbdB|DmelCG11648|Droabdb|FAB|Fab-7|Fab-8|Fab7|IAB 5|IAB5|IAB8|Iab-7|MCP	0	0
12696	47764	Drosophila melanogaster	Amy-p	Amylase proximal	AMY|Amy|Amy(p)|Amy-proximal|Amy1|Amy2|Amy4|Amy5|AmyA|CG18640|CG18730|DmelCG18730|alpha-AMY|alpha-Amy|amy|amy-p	0	0
12697	47765	Drosophila melanogaster	c(3)G	crossover suppressor on 3 of Gowen	C(3)G|C3G|CG17604|DmelCG17604|c(3)g|c3G|cx	0	0
12698	47766	Drosophila melanogaster	clv-2	cloven thorax 2	Clv	0	0
12699	47767	Drosophila melanogaster	crc	cryptocephal	929|ATF-4|ATF4/crc|CG8669|DmelCG8669|dATF-4|l(2)crc	0	0
12700	47768	Drosophila melanogaster	crib	cribble	-	0	0
12701	47769	Drosophila melanogaster	Dip-C	Dipeptidase C	CG5663|DmelCG5663|dipC	0	0
12702	47770	Drosophila melanogaster	E(ade2)	Enhancer of ade2	-	0	0
12703	47771	Drosophila melanogaster	Edg64CD	Ecdysone-dependent gene 64CD	Edg64	0	0
12704	47773	Drosophila melanogaster	febB	feeding behavior B	-	0	0
12705	47774	Drosophila melanogaster	fs(1)A473	female sterile (1) A473	fs(1)473	0	0
12706	47775	Drosophila melanogaster	fs(1)A508	female sterile (1) A508	fs(1)508	0	0
12707	47776	Drosophila melanogaster	fs(1)de10	female sterile (1) defective eggs 10	de10	0	0
12708	47777	Drosophila melanogaster	fs(1)de13	female sterile (1) defective eggs 13	de13	0	0
12709	47778	Drosophila melanogaster	fs(1)de14	female sterile (1) defective eggs 14	de14	0	0
12710	47779	Drosophila melanogaster	fs(1)de16	female sterile (1) defective eggs 16	de16	0	0
12711	47780	Drosophila melanogaster	fs(1)de17	female sterile (1) defective eggs 17	de17	0	0
12712	47781	Drosophila melanogaster	fs(1)K10	female sterile (1) K10	CG3218|DmelCG3218|EG:30B8.5|K-10|K10|fs(1)M9|fs(1)k10|sqd	0	0
12713	47782	Drosophila melanogaster	fs(1)K79	female sterile (1) K79	-	0	0
12714	47783	Drosophila melanogaster	fs(1)K254	female sterile (1) K254	-	0	0
12715	47784	Drosophila melanogaster	fs(1)K741	female sterile (1) K741	-	0	0
12716	47785	Drosophila melanogaster	fs(1)M17	female sterile (1) M17	-	0	0
12717	47786	Drosophila melanogaster	fs(1)M111	female sterile (1) M111	-	0	0
12718	47787	Drosophila melanogaster	Fs(3)Bak	Female sterile (3) Baksa	Bak|Fs(3)Baksa	0	0
12719	47788	Drosophila melanogaster	Fs(3)Ber	Female sterile (3) Bercel	Fs(3)Bercel	0	0
12720	47789	Drosophila melanogaster	Fs(3)Boj	Female sterile (3) Bojla	Boj|Fs(3)Bojla	0	0
12721	47790	Drosophila melanogaster	Fs(3)Dam	Female sterile (3) Damasa	Dam|Fs(3)Damasa	0	0
12722	47791	Drosophila melanogaster	Fs(3)Ger	Female sterile (3) Gerec	Fs(3)Gerec|Ger	0	0
12723	47792	Drosophila melanogaster	Fs(3)Hod	Female sterile (3) Hodos	Fs(3)Hodos|Hod	0	0
12724	47794	Drosophila melanogaster	Fs(3)Kev	Female sterile (3) Keve	Fs(3)Keve	0	0
12725	47795	Drosophila melanogaster	Fs(3)Kun	Female sterile (3) Kun	Kan	0	0
12726	47797	Drosophila melanogaster	Fs(3)Pal	Female sterile (3) Palat	Fs(3)Palat	0	0
12727	47798	Drosophila melanogaster	Fs(3)Tev	Female sterile (3) Tevel	Fs(3)Tevel	0	0
12728	47799	Drosophila melanogaster	Fs(3)Var	Female sterile (3) Varas	Fs(3)Varas|Var	0	0
12729	47800	Drosophila melanogaster	Fs(3)Zer	Female sterile (3) Zerind	Fs(3)Zerind|Zer	0	0
12730	47801	Drosophila melanogaster	Fs(3)Zom	Female sterile (3) Zombor	Fs(3)Zombor|Zom	0	0
12731	47802	Drosophila melanogaster	gustB	gustatory-defective B	-	0	0
12732	47803	Drosophila melanogaster	gustM	gustatory-defective M	-	0	0
12733	47804	Drosophila melanogaster	hat	half out	-	0	0
12734	47805	Drosophila melanogaster	intro	introverted	23|Q56|R9-13|YT1|l(1)Q56|unnamed	0	0
12735	47807	Drosophila melanogaster	l(1)3Bb	lethal (1) 3Bb	ZW-6|l(1)zw6|zw-6|zw6	0	0
12736	47808	Drosophila melanogaster	l(1)3Bd	lethal (1) 3Bd	ZW-7|l(1)zw7|zw-7|zw7	0	0
12737	47809	Drosophila melanogaster	l(1)6Da	lethal (1) 6Da	-	0	0
12738	47810	Drosophila melanogaster	l(1)6Db	lethal (1) 6Db	EM24	0	0
12739	47811	Drosophila melanogaster	l(1)6Dc	lethal (1) 6Dc	EM25	0	0
12740	47812	Drosophila melanogaster	l(1)6Dd	lethal (1) 6Dd	-	0	0
12741	47813	Drosophila melanogaster	l(1)6De	lethal (1) 6De	-	0	0
12742	47814	Drosophila melanogaster	l(1)6Df	lethal (1) 6Df	-	0	0
12743	47815	Drosophila melanogaster	l(1)6Dg	lethal (1) 6Dg	-	0	0
12744	47816	Drosophila melanogaster	l(1)6Dh	lethal (1) 6Dh	-	0	0
12745	47818	Drosophila melanogaster	l(1)6Eb	lethal (1) 6Eb	JNLX|jnLX	0	0
12746	47819	Drosophila melanogaster	l(1)6Ed	lethal (1) 6Ed	JNL2|jnL2|l(1)jnL2	0	0
12747	47820	Drosophila melanogaster	l(1)7Aa	lethal (1) 7Aa	RA62|jnR1|l(1)jnR1	0	0
12748	47821	Drosophila melanogaster	l(1)7Ab	lethal (1) 7Ab	jnR2|l(1)jnR2	0	0
12749	47822	Drosophila melanogaster	l(1)7Ad	lethal (1) 7Ad	jnR4|l(1)jnR4	0	0
12750	47823	Drosophila melanogaster	l(1)7Bc	lethal (1) 7Bc	-	0	0
12751	47824	Drosophila melanogaster	l(1)7Bd	lethal (1) 7Bd	-	0	0
12752	47825	Drosophila melanogaster	l(1)7Ca	lethal (1) 7Ca	-	0	0
12753	47826	Drosophila melanogaster	l(1)7Cb	lethal (1) 7Cb	-	0	0
12754	47827	Drosophila melanogaster	l(1)7Cc	lethal (1) 7Cc	EC271	0	0
12755	47828	Drosophila melanogaster	l(1)7Cd	lethal (1) 7Cd	-	0	0
12756	47829	Drosophila melanogaster	l(1)7Ce	lethal (1) 7Ce	-	0	0
12757	47830	Drosophila melanogaster	l(1)7Ch	lethal (1) 7Ch	-	0	0
12758	47831	Drosophila melanogaster	l(1)7Dh	lethal (1) 7Dh	-	0	0
12759	47832	Drosophila melanogaster	l(1)7Eb	lethal (1) 7Eb	JA4	0	0
12760	47833	Drosophila melanogaster	l(1)7Ec	lethal (1) 7Ec	-	0	0
12761	47834	Drosophila melanogaster	l(1)7Ed	lethal (1) 7Ed	-	0	0
12762	47835	Drosophila melanogaster	l(1)7Ee	lethal (1) 7Ee	-	0	0

<table>
<tr><td>12763</td><td>47836</td><td>Drosophila melanogaster</td><td>l(1)7Fd</td><td>lethal (1) 7Fd</td><td>l(1)VA67</td><td>0</td><td>0</td></tr>
<tr><td>12764</td><td>47837</td><td>Drosophila melanogaster</td><td>l(1)8Aa</td><td>lethal (1) 8Aa</td><td>HC179|l(1)VE709</td><td>0</td><td>0</td></tr>
<tr><td>12765</td><td>47838</td><td>Drosophila melanogaster</td><td>l(1)8Ad</td><td>lethal (1) 8Ad</td><td>l(1)A86</td><td>0</td><td>0</td></tr>
<tr><td>12766</td><td>47839</td><td>Drosophila melanogaster</td><td>l(1)9Ea</td><td>lethal (1) 9Ea</td><td>EM31</td><td>0</td><td>0</td></tr>
<tr><td>12767</td><td>47840</td><td>Drosophila melanogaster</td><td>l(1)9Ec</td><td>lethal (1) 9Ec</td><td>-</td><td>0</td><td>0</td></tr>
<tr><td>12768</td><td>47841</td><td>Drosophila melanogaster</td><td>l(1)9Ee</td><td>lethal (1) 9Ee</td><td>-</td><td>0</td><td>0</td></tr>
<tr><td>12769</td><td>47842</td><td>Drosophila melanogaster</td><td>l(1)9Fb</td><td>lethal (1) 9Fb</td><td>-</td><td>0</td><td>0</td></tr>
<tr><td>12770</td><td>47843</td><td>Drosophila melanogaster</td><td>l(2)Su(H)</td><td>lethal (2) Su(H)</td><td>l(2)su(H)</td><td>0</td><td>0</td></tr>
<tr><td>12771</td><td>47844</td><td>Drosophila melanogaster</td><td>l(3)F34</td><td>lethal (3) F34</td><td>l(3)34</td><td>0</td><td>0</td></tr>
<tr><td>12772</td><td>47846</td><td>Drosophila melanogaster</td><td>M(1)5A</td><td>Minute (1) 5A</td><td>RpL35</td><td>0</td><td>0</td></tr>
<tr><td>12773</td><td>47847</td><td>Drosophila melanogaster</td><td>M(2)44C</td><td>Minute (2) 44C</td><td>M(2)38b</td><td>0</td><td>0</td></tr>
<tr><td>12774</td><td>47848</td><td>Drosophila melanogaster</td><td>mab</td><td>malformed abdomen</td><td>Ebmab</td><td>0</td><td>0</td></tr>
<tr><td>12775</td><td>47849</td><td>Drosophila melanogaster</td><td>Mab4A11</td><td>Monoclonal antibody 4A11</td><td>4A11</td><td>0</td><td>0</td></tr>
<tr><td>12776</td><td>47850</td><td>Drosophila melanogaster</td><td>ms(1)401</td><td>male sterile (1) 401</td><td>-</td><td>0</td><td>0</td></tr>
<tr><td>12777</td><td>47851</td><td>Drosophila melanogaster</td><td>ms(1)V7</td><td>male sterile (1) V7</td><td>-</td><td>0</td><td>0</td></tr>
<tr><td>12778</td><td>47852</td><td>Drosophila melanogaster</td><td>Acp95EF</td><td>Accessory gland-specific peptide 95EF</td><td>Acp|CG17924|DTA-D|DTA-E|DmelCG17924|Mst95E|Mst95F|mst 316|mst(3)ag-3|mst-3|mst316|mst316F</td><td>0</td><td>0</td></tr>
<tr><td>12779</td><td>47853</td><td>Drosophila melanogaster</td><td>Mst95EF</td><td>Male-specific RNA 95EF</td><td>-</td><td>0</td><td>0</td></tr>
<tr><td>12780</td><td>47854</td><td>Drosophila melanogaster</td><td>Mst98Ca</td><td>Male-specific RNA 98Ca</td><td>CG11719|DmelCG11719|MST98Ca|Mst|Mst98C|Mst98CE|mst(3)gl-12|mst336</td><td>0</td><td>0</td></tr>
<tr><td>12781</td><td>47858</td><td>Drosophila melanogaster</td><td>ota2</td><td>olfactory trap abnormal 2</td><td>-</td><td>0</td><td>0</td></tr>
<tr><td>12782</td><td>47859</td><td>Drosophila melanogaster</td><td>ota7</td><td>olfactory trap abnormal 7</td><td>-</td><td>0</td><td>0</td></tr>
<tr><td>12783</td><td>47862</td><td>Drosophila melanogaster</td><td>rue</td><td>ruffled eye</td><td>-</td><td>0</td><td>0</td></tr>
<tr><td>12784</td><td>47865</td><td>Drosophila melanogaster</td><td>su(Cbx)</td><td>suppressor of Contrabithorax</td><td>-</td><td>0</td><td>0</td></tr>
<tr><td>12785</td><td>47866</td><td>Drosophila melanogaster</td><td>Su(Gl)77</td><td>Suppressor of Glued 77</td><td>-</td><td>0</td><td>0</td></tr>
<tr><td>12786</td><td>47868</td><td>Drosophila melanogaster</td><td>tta</td><td>tetanic</td><td>-</td><td>0</td><td>0</td></tr>
<tr><td>12787</td><td>47869</td><td>Drosophila melanogaster</td><td>vir</td><td>virilizer</td><td>CG3496|DmelCG3496|Vir</td><td>0</td><td>0</td></tr>
<tr><td>12788</td><td>47870</td><td>Drosophila melanogaster</td><td>Inr-a</td><td>Inverse regulator a</td><td>-</td><td>0</td><td>0</td></tr>
<tr><td>12789</td><td>47871</td><td>Drosophila melanogaster</td><td>M(1)7C</td><td>Minute</td><td>-</td><td>0</td><td>0</td></tr>
<tr><td>12790</td><td>47872</td><td>Drosophila melanogaster</td><td>DebC</td><td>Developmental embryonic C</td><td>Deb|Deb-C</td><td>0</td><td>0</td></tr>
<tr><td>12791</td><td>47873</td><td>Drosophila melanogaster</td><td>bgcn</td><td>benign gonial cell neoplasm</td><td>BGCN|Bgcn|CG10330|CG10331|CG30170|DmelCG30170|PNBP|b(2)cgn|b(2)gcn|bcgn|ben(2)gcn|cg10331|fs(2)eo-C|fs(2)eo6|fs(2)eoQS2|gcn|pep</td><td>0</td><td>0</td></tr>
<tr><td>12792</td><td>47874</td><td>Drosophila melanogaster</td><td>mit(1)15</td><td>mitotic 15</td><td>CG9900|DmelCG9900|EG:BACR7C10.3|Mit(1)15|ZW-10|ZW10|Zw10|abe|dZW10|l(1)3Af|l(1)6-99|l(1)zw10|mit(l)15|zw-10|zw10</td><td>0</td><td>0</td></tr>
<tr><td>12793</td><td>47875</td><td>Drosophila melanogaster</td><td>E(lz)</td><td>Enhancer of lozenge</td><td>-</td><td>0</td><td>0</td></tr>
<tr><td>12794</td><td>47877</td><td>Drosophila melanogaster</td><td>tws</td><td>twins</td><td>1466/06|2414|B/PR55|CG6235|DPR55|DmelCG6235|PP2A|PP2A[B]|PP2a|PR55|Pp2A-85F|Tws|aar|dPP2A|dPR55|l(3)01436|l(3)02414|l(3)S022205b|l(3)S022361|l(3)S022440|l(3)S023013|l(3)S023141a|l(3)S0</td><td>0</td><td>0</td></tr>
<tr><td>12795</td><td>47878</td><td>Drosophila melanogaster</td><td>Eip63F-1</td><td>Ecdysone-induced protein 63F 1</td><td>63F-1|CG15855|Ca[2+] binding protein|DmelCG15855|E63-1|E63F|Eip63F1|Eip63Fa|eip63F-1</td><td>0</td><td>0</td></tr>
<tr><td>12796</td><td>47880</td><td>Drosophila melanogaster</td><td>l(1)4Ea</td><td>lethal (1) 4Ea</td><td>-</td><td>0</td><td>0</td></tr>
<tr><td>12797</td><td>47881</td><td>Drosophila melanogaster</td><td>l(3)R12</td><td>lethal (3) R12</td><td>-</td><td>0</td><td>0</td></tr>
<tr><td>12798</td><td>47882</td><td>Drosophila melanogaster</td><td>fs(1)A457</td><td>female sterile (1) A457</td><td>fs(1)457|fs(1)M22</td><td>0</td><td>0</td></tr>
<tr><td>12799</td><td>47883</td><td>Drosophila melanogaster</td><td>l(1)adl10</td><td>lethal (1) adl10</td><td>l(1)adl-10</td><td>0</td><td>0</td></tr>
<tr><td>12800</td><td>47884</td><td>Drosophila melanogaster</td><td>l(1)adl12</td><td>lethal (1) adl12</td><td>l(1)adl-12</td><td>0</td><td>0</td></tr>
<tr><td>12801</td><td>47885</td><td>Drosophila melanogaster</td><td>l(1)v129</td><td>lethal (1) v129</td><td>l(1)129</td><td>0</td><td>0</td></tr>
<tr><td>12802</td><td>47886</td><td>Drosophila melanogaster</td><td>l(1)v361</td><td>lethal (1) v361</td><td>l(1)361</td><td>0</td><td>0</td></tr>
<tr><td>12803</td><td>47887</td><td>Drosophila melanogaster</td><td>l(1)v454</td><td>lethal (1) v454</td><td>l(1)454</td><td>0</td><td>0</td></tr>
<tr><td>12804</td><td>47888</td><td>Drosophila melanogaster</td><td>l(1)v463</td><td>lethal (1) v463</td><td>l(1)463</td><td>0</td><td>0</td></tr>
<tr><td>12805</td><td>47889</td><td>Drosophila melanogaster</td><td>yemG4</td><td>yema gene 4</td><td>unnamed|yG4|yT4</td><td>0</td><td>0</td></tr>
<tr><td>12806</td><td>47890</td><td>Drosophila melanogaster</td><td>neuro</td><td>neuromuscular</td><td>-</td><td>0</td><td>0</td></tr>
<tr><td>12807</td><td>47891</td><td>Drosophila melanogaster</td><td>mcd1</td><td>muscle cell death 1</td><td>mcd-1</td><td>0</td><td>0</td></tr>
<tr><td>12808</td><td>47892</td><td>Drosophila melanogaster</td><td>l(1)TK136</td><td>lethal (1) TK136</td><td>l136</td><td>0</td><td>0</td></tr>
<tr><td>12809</td><td>47893</td><td>Drosophila melanogaster</td><td>l(1)TK207</td><td>lethal (1) TK207</td><td>l207</td><td>0</td><td>0</td></tr>
<tr><td>12810</td><td>47894</td><td>Drosophila melanogaster</td><td>hbn</td><td>homeobrain</td><td>57San|CG10614|CG15647|CG15648|CG33152|DmelCG33152</td><td>0</td><td>0</td></tr>
<tr><td>12811</td><td>47895</td><td>Drosophila melanogaster</td><td>Nmdmc</td><td>NAD-dependent methylenetetrahydrofolate dehydroge</td><td>151767_at|CG18466|DNMDMC|DmelCG18466|Dnmdmc|dNMDMC|nmdmc</td><td>0</td><td>0</td></tr>
<tr><td>12812</td><td>47898</td><td>Drosophila melanogaster</td><td>Mst75Ca</td><td>Male-specific RNA 75Ca</td><td>Mst75C|mst-2</td><td>0</td><td>0</td></tr>
<tr><td>12813</td><td>47899</td><td>Drosophila melanogaster</td><td>Mst75Cb</td><td>Male-specific RNA 75Cb</td><td>Mst75C|mst-2</td><td>0</td><td>0</td></tr>
<tr><td>12814</td><td>47900</td><td>Drosophila melanogaster</td><td>Taf5</td><td>TBP-associated factor 5</td><td>CG7704|DmelCG7704|TAF|TAF5|TAF80|TAF80/85|TAFII80|TAFII85|TAF[II]80|TAF[[II]]|TAF[[II]]80|TAF[[II]]80/85|TAF[[II]]85|TFIID|TFIID 85|Taf80|Taf85|Taf[[II]]80|d80|dTAF5|dTAFII85|dTAF[[II]]80|dTAFii80|dmT</td><td>0</td><td>0</td></tr>
<tr><td>12815</td><td>47901</td><td>Drosophila melanogaster</td><td>betaTry</td><td>betaTrypsin</td><td>CG18211|DmBetaTry|DmelCG18211|SP119|Try|beta</td><td>0</td><td>0</td></tr>
<tr><td>12816</td><td>47902</td><td>Drosophila melanogaster</td><td>meso17A</td><td>mesoderm specific 17A</td><td>Dmeso17A</td><td>0</td><td>0</td></tr>
<tr><td>12817</td><td>47903</td><td>Drosophila melanogaster</td><td>l(2)00297</td><td>lethal (2) 00297</td><td>-</td><td>0</td><td>0</td></tr>
<tr><td>12818</td><td>47905</td><td>Drosophila melanogaster</td><td>l(2)03659</td><td>lethal (2) 03659</td><td>A|CG11803|CG8799|Dme_CG8799|DmelCG8799|hspr</td><td>0</td><td>0</td></tr>
<tr><td>12819</td><td>47906</td><td>Drosophila melanogaster</td><td>Dek</td><td>CG5935 gene product from transcript CG5935-RA</td><td>CG593|CG5935|DEK|DmelCG5935|EG:EG0003.6|anon-WO0172774.191|anon-WO0172774.192|anon-WO0172774.194|dDEK|dek|l(2)04154|l(2)k09907</td><td>0</td><td>0</td></tr>
<tr><td>12820</td><td>47909</td><td>Drosophila melanogaster</td><td>l(2)07206</td><td>lethal (2) 07206</td><td>-</td><td>0</td><td>0</td></tr>
<tr><td>12821</td><td>47914</td><td>Drosophila melanogaster</td><td>l(3)01949</td><td>lethal (3) 01949</td><td>unnamed</td><td>0</td><td>0</td></tr>
<tr><td>12822</td><td>47917</td><td>Drosophila melanogaster</td><td>l(3)02732</td><td>lethal (3) 02732</td><td>-</td><td>0</td><td>0</td></tr>
<tr><td>12823</td><td>47918</td><td>Drosophila melanogaster</td><td>l(3)02946</td><td>lethal (3) 02946</td><td>-</td><td>0</td><td>0</td></tr>
<tr><td>12824</td><td>47920</td><td>Drosophila melanogaster</td><td>l(3)03847</td><td>lethal (3) 03847</td><td>unnamed</td><td>0</td><td>0</td></tr>
<tr><td>12825</td><td>47926</td><td>Drosophila melanogaster</td><td>l(3)06404</td><td>lethal (3) 06404</td><td>-</td><td>0</td><td>0</td></tr>
<tr><td>12826</td><td>47929</td><td>Drosophila melanogaster</td><td>l(3)06658</td><td>lethal (3) 06658</td><td>-</td><td>0</td><td>0</td></tr>
<tr><td>12827</td><td>47934</td><td>Drosophila melanogaster</td><td>l(3)s2017</td><td>lethal (3) s2017</td><td>-</td><td>0</td><td>0</td></tr>
<tr><td>12828</td><td>47936</td><td>Drosophila melanogaster</td><td>l(1)291-5</td><td>lethal (1) 291-5</td><td>291-5</td><td>0</td><td>0</td></tr>
<tr><td>12829</td><td>47937</td><td>Drosophila melanogaster</td><td>l(1)291-7</td><td>lethal (1) 291-7</td><td>291-7</td><td>0</td><td>0</td></tr>
<tr><td>12830</td><td>47938</td><td>Drosophila melanogaster</td><td>l(1)291-68</td><td>lethal (1) 291-68</td><td>291-68</td><td>0</td><td>0</td></tr>
<tr><td>12831</td><td>47939</td><td>Drosophila melanogaster</td><td>l(1)291-100</td><td>lethal (1) 291-100</td><td>291-100</td><td>0</td><td>0</td></tr>
<tr><td>12832</td><td>47941</td><td>Drosophila melanogaster</td><td>shk</td><td>shank</td><td>ms(3)07735</td><td>0</td><td>0</td></tr>
<tr><td>12833</td><td>47942</td><td>Drosophila melanogaster</td><td>scat</td><td>scattered</td><td>CG3766|DmelCG3766|ms(2)05289|ms(2)30B</td><td>0</td><td>0</td></tr>
<tr><td>12834</td><td>47944</td><td>Drosophila melanogaster</td><td>l(3)j13E7</td><td>lethal (3) j13E7</td><td>-</td><td>0</td><td>0</td></tr>
<tr><td>12835</td><td>47945</td><td>Drosophila melanogaster</td><td>l(3)j15E7</td><td>lethal (3) j15E7</td><td>-</td><td>0</td><td>0</td></tr>
<tr><td>12836</td><td>47947</td><td>Drosophila melanogaster</td><td>l(3)neo40</td><td>lethal (3) neo40</td><td>-</td><td>0</td><td>0</td></tr>
<tr><td>12837</td><td>47948</td><td>Drosophila melanogaster</td><td>l(3)neo58b</td><td>lethal (3) neo58b</td><td>-</td><td>0</td><td>0</td></tr>
<tr><td>12838</td><td>47951</td><td>Drosophila melanogaster</td><td>l(3)00707</td><td>lethal (3) 00707</td><td>-</td><td>0</td><td>0</td></tr>
<tr><td>12839</td><td>47952</td><td>Drosophila melanogaster</td><td>Dlw</td><td>Dorsal wing</td><td>-</td><td>0</td><td>0</td></tr>
<tr><td>12840</td><td>47954</td><td>Drosophila melanogaster</td><td>Mst57Da</td><td>Male-specific RNA 57Da</td><td>BcDNA:GH19893|CG9074|DmelCG9074|Mst57D|bs30d07.y1|mst(2)ag-1|mst-1|mst57D</td><td>0</td><td>0</td></tr>
<tr><td>12841</td><td>47955</td><td>Drosophila melanogaster</td><td>ST2</td><td>ST2</td><td>-</td><td>0</td><td>0</td></tr>
<tr><td>12842</td><td>47966</td><td>Drosophila melanogaster</td><td>l(3)100Ab</td><td>lethal (3) 100Ab</td><td>-</td><td>0</td><td>0</td></tr>
<tr><td>12843</td><td>47967</td><td>Drosophila melanogaster</td><td>l(3)ry103</td><td>lethal (3) ry103</td><td>l(3)103</td><td>0</td><td>0</td></tr>
<tr><td>12844</td><td>47968</td><td>Drosophila melanogaster</td><td>l(3)B3-3-21</td><td>lethal (3) B3-3-21</td><td>-</td><td>0</td><td>0</td></tr>
<tr><td>12845</td><td>47969</td><td>Drosophila melanogaster</td><td>l(3)B7-3-32</td><td>lethal (3) B7-3-32</td><td>-</td><td>0</td><td>0</td></tr>
<tr><td>12846</td><td>47970</td><td>Drosophila melanogaster</td><td>l(3)E7-3-58</td><td>lethal (3) E7-3-58</td><td>-</td><td>0</td><td>0</td></tr>
<tr><td>12847</td><td>47975</td><td>Drosophila melanogaster</td><td>l(1)9-38-1</td><td>lethal (1) 9-38-1</td><td>l(1)14Cc</td><td>0</td><td>0</td></tr>
<tr><td>12848</td><td>47976</td><td>Drosophila melanogaster</td><td>l(2)60Aa</td><td>lethal (2) 60Aa</td><td>-</td><td>0</td><td>0</td></tr>
<tr><td>12849</td><td>47977</td><td>Drosophila melanogaster</td><td>l(2)60Ab</td><td>lethal (2) 60Ab</td><td>-</td><td>0</td><td>0</td></tr>
<tr><td>12850</td><td>47978</td><td>Drosophila melanogaster</td><td>l(2)60Ac</td><td>lethal (2) 60Ac</td><td>-</td><td>0</td><td>0</td></tr>
<tr><td>12851</td><td>47979</td><td>Drosophila melanogaster</td><td>l(2)60Ad</td><td>lethal (2) 60Ad</td><td>-</td><td>0</td><td>0</td></tr>
<tr><td>12852</td><td>47980</td><td>Drosophila melanogaster</td><td>l(2)60Ae</td><td>lethal (2) 60Ae</td><td>-</td><td>0</td><td>0</td></tr>
<tr><td>12853</td><td>47981</td><td>Drosophila melanogaster</td><td>l(2)k04301a</td><td>lethal (2) k04301a</td><td>l(2)43/1|l(2)k00202</td><td>0</td><td>0</td></tr>
<tr><td>12854</td><td>47990</td><td>Drosophila melanogaster</td><td>l(2)46Cd</td><td>lethal (2) 46Cd</td><td>433|IV</td><td>0</td><td>0</td></tr>
<tr><td>12855</td><td>47996</td><td>Drosophila melanogaster</td><td>l(3)EGX13</td><td>lethal (3) EGX13</td><td>-</td><td>0</td><td>0</td></tr>
<tr><td>12856</td><td>47998</td><td>Drosophila melanogaster</td><td>rin</td><td>rasputin</td><td>CG9412|DmelCG9412|Nts|Rin|cg9412|rasp</td><td>0</td><td>0</td></tr>
<tr><td>12857</td><td>48025</td><td>Drosophila melanogaster</td><td>l(2)PC4-427</td><td>lethal (2) PC4-427</td><td>l(2)PC4-M</td><td>0</td><td>0</td></tr>
<tr><td>12858</td><td>48026</td><td>Drosophila melanogaster</td><td>l(2)PC4-D</td><td>lethal (2) PC4-D</td><td>-</td><td>0</td><td>0</td></tr>
<tr><td>12859</td><td>48027</td><td>Drosophila melanogaster</td><td>l(2)PC4-E</td><td>lethal (2) PC4-E</td><td>l(2)PC4-H</td><td>0</td><td>0</td></tr>
<tr><td>12860</td><td>48028</td><td>Drosophila melanogaster</td><td>l(2)PC4-F</td><td>lethal (2) PC4-F</td><td>-</td><td>0</td><td>0</td></tr>
<tr><td>12861</td><td>48029</td><td>Drosophila melanogaster</td><td>l(2)PC4-G</td><td>lethal (2) PC4-G</td><td>l(2)PC4-J</td><td>0</td><td>0</td></tr>
<tr><td>12862</td><td>48030</td><td>Drosophila melanogaster</td><td>l(2)PC4-H</td><td>lethal (2) PC4-H</td><td>l(2)PC4-K</td><td>0</td><td>0</td></tr>
<tr><td>12863</td><td>48031</td><td>Drosophila melanogaster</td><td>l(2)PC4-K</td><td>lethal (2) PC4-K</td><td>l(2)PC4-L</td><td>0</td><td>0</td></tr>
<tr><td>12864</td><td>48032</td><td>Drosophila melanogaster</td><td>l(2)PC4-M</td><td>lethal (2) PC4-M</td><td>l(2)PC4-E</td><td>0</td><td>0</td></tr>
<tr><td>12865</td><td>48033</td><td>Drosophila melanogaster</td><td>l(2)PC4-P</td><td>lethal (2) PC4-P</td><td>-</td><td>0</td><td>0</td></tr>
<tr><td>12866</td><td>48034</td><td>Drosophila melanogaster</td><td>l(2)PC4-Q</td><td>lethal (2) PC4-Q</td><td>-</td><td>0</td><td>0</td></tr>
<tr><td>12867</td><td>48035</td><td>Drosophila melanogaster</td><td>l(3)03884</td><td>lethal (3) 03884</td><td>l(3)3884|unnamed</td><td>0</td><td>0</td></tr>
<tr><td>12868</td><td>48040</td><td>Drosophila melanogaster</td><td>l(3)64Cb</td><td>lethal (3) 64Cb</td><td>-</td><td>0</td><td>0</td></tr>
<tr><td>12869</td><td>48042</td><td>Drosophila melanogaster</td><td>l(3)64Cd</td><td>lethal (3) 64Cd</td><td>-</td><td>0</td><td>0</td></tr>
<tr><td>12870</td><td>48043</td><td>Drosophila melanogaster</td><td>l(3)64Ce</td><td>lethal (3) 64Ce</td><td>-</td><td>0</td><td>0</td></tr>
<tr><td>12871</td><td>48044</td><td>Drosophila melanogaster</td><td>l(3)64Cf</td><td>lethal (3) 64Cf</td><td>-</td><td>0</td><td>0</td></tr>
<tr><td>12872</td><td>48045</td><td>Drosophila melanogaster</td><td>l(3)64Cg</td><td>lethal (3) 64Cg</td><td>-</td><td>0</td><td>0</td></tr>
<tr><td>12873</td><td>48046</td><td>Drosophila melanogaster</td><td>l(3)64Ch</td><td>lethal (3) 64Ch</td><td>-</td><td>0</td><td>0</td></tr>
</table>

12874	48047	Drosophila melanogaster	misn	missensed	-	0	0
12875	48048	Drosophila melanogaster	l(1)10Bt	lethal (1) 10Bt	HM416	0	0
12876	48049	Drosophila melanogaster	l(1)10Bs	lethal (1) 10Bs	HM22	0	0
12877	48050	Drosophila melanogaster	l(1)10Br	lethal (1) 10Br	HM12	0	0
12878	48051	Drosophila melanogaster	l(1)10Bq	lethal (1) 10Bq	HM10	0	0
12879	48060	Drosophila melanogaster	l(1)XR49	lethal (1) XR49	-	0	0
12880	48061	Drosophila melanogaster	l(1)75B2	lethal (1) 75B2	-	0	0
12881	48062	Drosophila melanogaster	l(1)5g11	lethal (1) 5g11	-	0	0
12882	48065	Drosophila melanogaster	l(3)96Ac	lethal (3) 96Ac	291.6	0	0
12883	48066	Drosophila melanogaster	l(3)96Ab	lethal (3) 96Ab	299.1	0	0
12884	48069	Drosophila melanogaster	Cyp6a9-rAF4-2	Cytochrome P450-6a9 related AF4-2	AF4-2|P-450|P450	0	0
12885	48071	Drosophila melanogaster	Su(z)12	CG8013 gene product from transcript CG8013-RA	CG8013|DmelCG8013|SU(Z)12|Su(Z)12|Su(z)[12]|l(3)76BDo|su(z)12	0	0
12886	48074	Drosophila melanogaster	l(2)k17019b	lethal (2) k17019b	-	0	0
12887	48075	Drosophila melanogaster	l(2)k14603b	lethal (2) k14603b	-	0	0
12888	48076	Drosophila melanogaster	l(2)k12502b	lethal (2) k12502b	-	0	0
12889	48080	Drosophila melanogaster	l(2)k08931	lethal (2) k08931	-	0	0
12890	48083	Drosophila melanogaster	l(2)k07312b	lethal (2) k07312b	-	0	0
12891	48084	Drosophila melanogaster	l(2)k06608b	lethal (2) k06608b	-	0	0
12892	48085	Drosophila melanogaster	l(2)45Bc	lethal (2) 45Bc	l(2)k05611b	0	0
12893	48088	Drosophila melanogaster	l(2)k05125a	lethal (2) k05125a	-	0	0
12894	48089	Drosophila melanogaster	l(2)k04909b	lethal (2) k04909b	-	0	0
12895	48091	Drosophila melanogaster	l(2)k03906a	lethal (2) k03906a	l(2)k03906	0	0
12896	48092	Drosophila melanogaster	l(2)k03501	lethal (2) k03501	-	0	0
12897	48093	Drosophila melanogaster	l(2)k02107a	lethal (2) k02107a	l(2)k02107	0	0
12898	48095	Drosophila melanogaster	l(3)GPMG-30	lethal (3) GPMG-30	GPMG-30	0	0
12899	48096	Drosophila melanogaster	l(2)GPMG-18	lethal (2) GPMG-18	GPMG-18	0	0
12900	48098	Drosophila melanogaster	E(faf)E263	Enhancer of faf E263	-	0	0
12901	48099	Drosophila melanogaster	spongecake	spongecake	-	0	0
12902	48112	Drosophila melanogaster	l(4)102Ca	lethal (4) 102Ca	-	0	0
12903	48113	Drosophila melanogaster	l(1)LefEM20	lethal (1) LefEM20	EM20	0	0
12904	48114	Drosophila melanogaster	Dipr	Distal into proximal	-	0	0
12905	48115	Drosophila melanogaster	l(2)k14209	lethal (2) k14209	-	0	0
12906	48116	Drosophila melanogaster	l(2)k03906b	lethal (2) k03906b	l(2)k03906	0	0
12907	48117	Drosophila melanogaster	l(2)38EFf	lethal (2) 38EFf	-	0	0
12908	48118	Drosophila melanogaster	l(2)38EFe	lethal (2) 38EFe	-	0	0
12909	48120	Drosophila melanogaster	l(2)38EFc	lethal (2) 38EFc	-	0	0
12910	48121	Drosophila melanogaster	l(2)38EFb	lethal (2) 38EFb	-	0	0
12911	48127	Drosophila melanogaster	l(2)k09017a	lethal (2) k09017a	-	0	0
12912	48128	Drosophila melanogaster	l(3)S148213	lethal (3) S148213	1482/13	0	0
12913	48129	Drosophila melanogaster	l(3)S147913	lethal (3) S147913	1479/13	0	0
12914	48130	Drosophila melanogaster	l(3)S145110	lethal (3) S145110	1451/10|l(3)s145110	0	0
12915	48131	Drosophila melanogaster	l(3)S142916	lethal (3) S142916	0510/01|0925/01|1400/09|1429/16	0	0
12916	48132	Drosophila melanogaster	l(3)S140410	lethal (3) S140410	l(3)s140410	0	0
12917	48133	Drosophila melanogaster	l(3)S139308b	lethal (3) S139308b	1393/08	0	0
12918	48134	Drosophila melanogaster	l(3)S139101	lethal (3) S139101	1391/01	0	0
12919	48135	Drosophila melanogaster	l(3)S138815	lethal (3) S138815	1388/15	0	0
12920	48136	Drosophila melanogaster	l(3)S137212	lethal (3) S137212	1372/12	0	0
12921	48138	Drosophila melanogaster	l(3)S134303	lethal (3) S134303	1343/03	0	0
12922	48141	Drosophila melanogaster	l(3)S128711	lethal (3) S128711	1287/11	0	0
12923	48142	Drosophila melanogaster	l(3)S127416	lethal (3) S127416	1274/16	0	0
12924	48145	Drosophila melanogaster	l(3)S121910b	lethal (3) S121910b	1219/10	0	0
12925	48147	Drosophila melanogaster	l(3)S115909	lethal (3) S115909	1159/09	0	0
12926	48148	Drosophila melanogaster	l(3)S112110	lethal (3) S112110	1121/10	0	0
12927	48149	Drosophila melanogaster	l(3)S104913b	lethal (3) S104913b	1049/13	0	0
12928	48150	Drosophila melanogaster	l(3)S104115	lethal (3) S104115	-	0	0
12929	48151	Drosophila melanogaster	l(3)S103505b	lethal (3) S103505b	1035/05	0	0
12930	48152	Drosophila melanogaster	l(3)S102315	lethal (3) S102315	1023/15	0	0
12931	48153	Drosophila melanogaster	l(3)S102211	lethal (3) S102211	1022/11	0	0
12932	48154	Drosophila melanogaster	l(3)S101310	lethal (3) S101310	1013/10	0	0
12933	48155	Drosophila melanogaster	l(3)S101206b	lethal (3) S101206b	1012/06	0	0
12934	48156	Drosophila melanogaster	l(3)S100201	lethal (3) S100201	1002/01	0	0
12935	48159	Drosophila melanogaster	l(3)S096901b	lethal (3) S096901b	0969/01	0	0
12936	48162	Drosophila melanogaster	l(3)S094603	lethal (3) S094603	0946/03	0	0
12937	48163	Drosophila melanogaster	l(3)S094310	lethal (3) S094310	0943/10|943/10	0	0
12938	48164	Drosophila melanogaster	l(3)S094204	lethal (3) S094204	0942/04	0	0
12939	48166	Drosophila melanogaster	l(3)S092708	lethal (3) S092708	0927/08	0	0
12940	48167	Drosophila melanogaster	l(3)S091014	lethal (3) S091014	0910/14	0	0
12941	48168	Drosophila melanogaster	l(3)S090609	lethal (3) S090609	0906/09	0	0
12942	48169	Drosophila melanogaster	l(3)S090314	lethal (3) S090314	0903/14	0	0
12943	48172	Drosophila melanogaster	l(3)S086909	lethal (3) S086909	0869/09	0	0
12944	48173	Drosophila melanogaster	l(3)S086112	lethal (3) S086112	0861/12	0	0
12945	48174	Drosophila melanogaster	l(3)S085212	lethal (3) S085212	0852/12	0	0
12946	48175	Drosophila melanogaster	l(3)S085009	lethal (3) S085009	0850/09	0	0
12947	48176	Drosophila melanogaster	l(3)S084805	lethal (3) S084805	0848/05	0	0
12948	48177	Drosophila melanogaster	l(3)S084402	lethal (3) S084402	0844/02	0	0
12949	48178	Drosophila melanogaster	l(3)S078514	lethal (3) S078514	0785/14	0	0
12950	48179	Drosophila melanogaster	l(3)S075101	lethal (3) S075101	0751/01	0	0
12951	48180	Drosophila melanogaster	l(3)S074706	lethal (3) S074706	0747/06	0	0
12952	48181	Drosophila melanogaster	l(3)S074606b	lethal (3) S074606b	0746/06	0	0
12953	48182	Drosophila melanogaster	l(3)S072703b	lethal (3) S072703b	0727/03	0	0
12954	48183	Drosophila melanogaster	l(3)S072613a	lethal (3) S072613a	0726/13|l(3)S072613	0	0
12955	48184	Drosophila melanogaster	l(3)S071501	lethal (3) S071501	0715/01	0	0
12956	48186	Drosophila melanogaster	l(3)S066317	lethal (3) S066317	0663/17	0	0
12957	48187	Drosophila melanogaster	l(3)S063813	lethal (3) S063813	0638/13	0	0
12958	48188	Drosophila melanogaster	l(3)S060313	lethal (3) S060313	l(3)s001813	0	0
12959	48189	Drosophila melanogaster	l(3)S058209b	lethal (3) S058209b	0582/09	0	0
12960	48191	Drosophila melanogaster	l(3)S051007a	lethal (3) S051007a	0510/07	0	0
12961	48192	Drosophila melanogaster	l(3)S047431b	lethal (3) S047431b	0474/31	0	0
12962	48193	Drosophila melanogaster	l(3)S047418	lethal (3) S047418	0474/18	0	0
12963	48194	Drosophila melanogaster	l(3)S045902	lethal (3) S045902	0459/02	0	0
12964	48195	Drosophila melanogaster	l(3)S045507b	lethal (3) S045507b	0455/07	0	0
12965	48196	Drosophila melanogaster	l(3)S044422	lethal (3) S044422	0444/22	0	0
12966	48197	Drosophila melanogaster	l(3)S041919	lethal (3) S041919	0419/19	0	0
12967	48198	Drosophila melanogaster	l(3)S041303	lethal (3) S041303	0413/03|l(3)041303	0	0
12968	48200	Drosophila melanogaster	l(3)S032412	lethal (3) S032412	0324/12	0	0
12969	48202	Drosophila melanogaster	l(3)S027302b	lethal (3) S027302b	0273/02	0	0
12970	48203	Drosophila melanogaster	l(3)S024151	lethal (3) S024151	0241/51	0	0
12971	48204	Drosophila melanogaster	l(3)S024120	lethal (3) S024120	0241/20	0	0
12972	48205	Drosophila melanogaster	l(3)S023635	lethal (3) S023635	0236/35|0553/10|0726/03	0	0
12973	48206	Drosophila melanogaster	l(3)S014308	lethal (3) S014308	0143/08	0	0
12974	48207	Drosophila melanogaster	l(3)S008224	lethal (3) S008224	0082/24	0	0
12975	48208	Drosophila melanogaster	l(3)S007902	lethal (3) S007902	0079/02	0	0
12976	48209	Drosophila melanogaster	l(3)S007608	lethal (3) S007608	0076/08	0	0
12977	48210	Drosophila melanogaster	l(3)S007302	lethal (3) S007302	0073/02	0	0
12978	48211	Drosophila melanogaster	l(3)S005818	lethal (3) S005818	0058/18|58/18	0	0
12979	48212	Drosophila melanogaster	l(3)S003606	lethal (3) S003606	0036/06|l(3)s003606	0	0
12980	48213	Drosophila melanogaster	l(3)S003006	lethal (3) S003006	0030/06|30/6	0	0
12981	48214	Drosophila melanogaster	l(3)S000721	lethal (3) S000721	0007/21	0	0
12982	48216	Drosophila melanogaster	l(2)k05113a	lethal (2) k05113a	l(2)k05113	0	0
12983	48217	Drosophila melanogaster	l(2)k04301b	lethal (2) k04301b	-	0	0
12984	48219	Drosophila melanogaster	ms(3)mntr	male sterile minotaurus	-	0	0

12985	48224	Drosophila melanogaster	Aprt	Adenine phosphoribosyltransferase	BcDNA:LP07986|CG18315|DmelCG18315|aprt	0	0
12986	48228	Drosophila melanogaster	DNaseII	Deoxyribonuclease II	CG7780|DNase|DNase 1|DNase-1|DNase1|DmelCG7780|dDNase II	0	0
12987	48231	Drosophila melanogaster	Edg43A	Ecdysone-dependent gene at 43A	EDg43	0	0
12988	48232	Drosophila melanogaster	e(sei)	enhancer of seizure	-	0	0
12989	48233	Drosophila melanogaster	fs(1)A273	female sterile (1) A273	fs(1)K1075|fs(1)M114	0	0
12990	48234	Drosophila melanogaster	fs(1)A1561	female sterile (1) A1561	fs(1)1561	0	0
12991	48235	Drosophila melanogaster	fs(1)M14	female sterile (1) M14	-	0	0
12992	48237	Drosophila melanogaster	fs(1)M29	female sterile (1) M29	fs(1)151	0	0
12993	48238	Drosophila melanogaster	fs(1)M79	female sterile (1) M79	-	0	0
12994	48239	Drosophila melanogaster	ImpE3	Ecdysone-inducible gene E3	CG11046|CG2723|DmelCG2723|IMP-E3|Imp-E3	0	0
12995	48241	Drosophila melanogaster	l(1)1Bh	lethal (1) 1Bh	-	0	0
12996	48242	Drosophila melanogaster	l(1)1Bk	lethal (1) 1Bk	HM441	0	0
12997	48243	Drosophila melanogaster	l(1)1Da	lethal (1) 1Da	HM-454|HM454	0	0
12998	48244	Drosophila melanogaster	l(1)1Dc	lethal (1) 1Dc	JC45	0	0
12999	48245	Drosophila melanogaster	l(1)1Ea	lethal (1) 1Ea	-	0	0
13000	48246	Drosophila melanogaster	l(1)1Eb	lethal (1) 1Eb	HM-14	0	0
13001	48247	Drosophila melanogaster	l(1)1Ec	lethal (1) 1Ec	28/6A|EA1	0	0
13002	48248	Drosophila melanogaster	l(1)1Ed	lethal (1) 1Ed	-	0	0
13003	48249	Drosophila melanogaster	l(1)1Ee	lethal (1) 1Ee	-	0	0
13004	48250	Drosophila melanogaster	l(1)1Fa	lethal (1) 1Fa	-	0	0
13005	48251	Drosophila melanogaster	l(1)1Fd	lethal (1) 1Fd	-	0	0
13006	48252	Drosophila melanogaster	l(1)1Fe	lethal (1) 1Fe	-	0	0
13007	48253	Drosophila melanogaster	l(1)2Ac	lethal (1) 2Ac	l(1)2Aa	0	0
13008	48254	Drosophila melanogaster	l(1)2Ad	lethal (1) 2Ad	A60|l(1)BA11	0	0
13009	48255	Drosophila melanogaster	l(1)2Ae	lethal (1) 2Ae	l(1)BA12	0	0
13010	48256	Drosophila melanogaster	l(1)2Ca	lethal (1) 2Ca	-	0	0
13011	48257	Drosophila melanogaster	l(1)2Cc	lethal (1) 2Cc	l(1)VE651	0	0
13012	48258	Drosophila melanogaster	l(1)2Cd	lethal (1) 2Cd	-	0	0
13013	48259	Drosophila melanogaster	l(1)2Ce	lethal (1) 2Ce	l(1)GF316	0	0
13014	48260	Drosophila melanogaster	l(1)2Da	lethal (1) 2Da	l(1)DF967	0	0
13015	48261	Drosophila melanogaster	l(1)2Dg	lethal (1) 2Dg	N3|l(1)90	0	0
13016	48262	Drosophila melanogaster	l(1)2Ea	lethal (1) 2Ea	JA127|N3|N7|l(1)JA127|l70	0	0
13017	48263	Drosophila melanogaster	l(1)air7	lethal aberrant immune response 7	RC61|air7|jnR3|l(1)air[7]|l(1)jnR3	0	0
13018	48264	Drosophila melanogaster	l(1)7Dc	lethal (1) 7Dc	-	0	0
13019	48265	Drosophila melanogaster	l(1)7Dd	lethal (1) 7Dd	-	0	0
13020	48266	Drosophila melanogaster	l(1)7De	lethal (1) 7De	-	0	0
13021	48267	Drosophila melanogaster	l(1)7Df	lethal (1) 7Df	-	0	0
13022	48268	Drosophila melanogaster	l(1)7Dg	lethal (1) 7Dg	EM29	0	0
13023	48269	Drosophila melanogaster	l(1)7Di	lethal (1) 7Di	-	0	0
13024	48270	Drosophila melanogaster	l(1)7Dj	lethal (1) 7Dj	-	0	0
13025	48271	Drosophila melanogaster	l(1)7Dl	lethal (1) 7Dl	-	0	0
13026	48272	Drosophila melanogaster	l(1)7Fc	lethal (1) 7Fc	JC18|l(1)GE208	0	0
13027	48274	Drosophila melanogaster	l(1)14Ea	lethal (1) 14Ea	k22a|l(1)k22a	0	0
13028	48275	Drosophila melanogaster	l(1)15Ba	lethal (1) 15Ba	l(1)g27a	0	0
13029	48276	Drosophila melanogaster	l(1)18Fa	lethal (1) 18Fa	-	0	0
13030	48277	Drosophila melanogaster	l(1)19Ac	lethal (1) 19Ac	EM19	0	0
13031	48278	Drosophila melanogaster	l(1)19Ad	lethal (1) 19Ad	-	0	0
13032	48279	Drosophila melanogaster	l(1)20Ca	lethal (1) 20Ca	13E3|33|P-19|P19|l(1)13E3|l(1)D-19|l(1)P-19|l(1)P19|l13E3|unnamed	0	0
13033	48280	Drosophila melanogaster	l(1)20Cb	lethal (1) 20Cb	20|Q463|l(1)20|l(1)Q463|l20|lQ463|unnamed	0	0
13034	48281	Drosophila melanogaster	l(1)air4	lethal aberrant immune response 4	air4	0	0
13035	48282	Drosophila melanogaster	l(1)carot2	lethal (1) carot2	l(1)5	0	0
13036	48283	Drosophila melanogaster	l(1)CP7	lethal (1) CP7	-	0	0
13037	48284	Drosophila melanogaster	l(1)CP8	lethal (1) CP8	-	0	0
13038	48285	Drosophila melanogaster	l(1)EA61	lethal (1) EA61	-	0	0
13039	48286	Drosophila melanogaster	l(1)VE807	lethal (1) VE807	-	0	0
13040	48287	Drosophila melanogaster	l(2)41Aa	lethal (2) 41Aa	41Aa|GroupI|l(2)EMS31|l(2R)B|l(2R)EMS-31	0	0
13041	48288	Drosophila melanogaster	l(2)41Ab	lethal (2) 41Ab	41Ab|GroupI|l(2)EMS45-10|l(2R)B	0	0
13042	48290	Drosophila melanogaster	l(3)5G83	lethal (3) 5G83	-	0	0
13043	48291	Drosophila melanogaster	l(3)67Ac	lethal (3) 67Ac	-	0	0
13044	48292	Drosophila melanogaster	l(3)67Ad	lethal (3) 67Ad	-	0	0
13045	48293	Drosophila melanogaster	l(3)80Fa	lethal (3) 80Fa	80Fa|l(3)Lh8|l8	0	0
13046	48294	Drosophila melanogaster	l(3)80Fb	lethal (3) 80Fb	80Fb|l(3)Lh7|l7	0	0
13047	48295	Drosophila melanogaster	l(3)80Fc	lethal (3) 80Fc	80Fc|l(3)Lh6|l6	0	0
13048	48296	Drosophila melanogaster	l(3)80Fd	lethal (3) 80Fd	80Fd|l(3)Lh5|l5B	0	0
13049	48297	Drosophila melanogaster	l(3)80Ff	lethal (3) 80Ff	80Ff|l(3)Lh4B|l4B	0	0
13050	48298	Drosophila melanogaster	l(3)80Fg	lethal (3) 80Fg	80Fg|l(3)Lh4A|l4A	0	0
13051	48301	Drosophila melanogaster	l(3)80Fj	lethal (3) 80Fj	80Fj|l(3)Lh1|l1|lethal 1	0	0
13052	48302	Drosophila melanogaster	l(3)S2	lethal (3) S2	-	0	0
13053	48303	Drosophila melanogaster	M(2)56CD	Minute (2) 56CD	M(2)b|RpL11	0	0
13054	48304	Drosophila melanogaster	M(3)63B	Minute (3) 63B	M(3)LS3|RpL28|l(3)63Bf|l(3)63bf	0	0
13055	48305	Drosophila melanogaster	M(3)96CF	Minute (3) 96CF	M(3)j|M[[j]]|RpL27	0	0
13056	48306	Drosophila melanogaster	mesB	messy B	0303/02|l(3)mes-4B|mes-B|messy	0	0
13057	48307	Drosophila melanogaster	ms(1)7	male sterile (1) 7	-	0	0
13058	48309	Drosophila melanogaster	mus101	mutagen-sensitive 101	CG11156|DmMus101|DmelCG11156|Mus101|TOPBP1|anon-WO0148202.1|fs(1)K451|mus 101|mus(1)101|mus-101	0	0
13059	48311	Drosophila melanogaster	Pkc53E	Protein C kinase 53E	CG6622|DPKC|DmelCG6622|Dpkc1|PKC|PKC 53B|PKC 53E|PKC-53B|PKC-53E|PKC1|PKC53E|Pkc1|dPKC|dPKC53E(br)|pkc53e	0	0
13060	48312	Drosophila melanogaster	RpII18	RNA polymerase II 18kD subunit	BcDNA:RH21608|CG1163|Dm6|DmelCG1163|H5|Pol II|PolII|RNA Pol II|RNA pol II|RNA polII|RNAP|RNAP II|RNApolII|RPB6_DROME|RPII18|RpABC14|RpII18|pol II|polII	0	0
13061	48313	Drosophila melanogaster	Rst(3)amd	alpha methyl dopa-resistant on 3	-	0	0
13062	48314	Drosophila melanogaster	Su(SD)X	Suppressor of SD on the X	su(SD)|supp-X(SD)	0	0
13063	48315	Drosophila melanogaster	Su(z)3	Suppressor of zeste 3	Su(z)2D	0	0
13064	48316	Drosophila melanogaster	alphaTry	alphaTrypsin	CG18444|DmAlphaTry|DmelCG18444|SP102|Try|alpha	0	0
13065	48317	Drosophila melanogaster	ttk	tramtrack	0037/17|0250/25|0438/31|0702/07|1049/07|1119/04|1184/16|1209/05|1209/10|1260/10|1281/04|1325/15|1372/08|1396/14|1418/06|3540|5125|5311|CG11558|CG1856|DmelCG1856|E(yan)100D|FTZ-F2|T	0	0
13066	48319	Drosophila melanogaster	l(2)Ta	lethal (2) with Thickened arista	-	0	0
13067	48320	Drosophila melanogaster	pmst	pmst	PMST	0	0
13068	48321	Drosophila melanogaster	l(1)air5	lethal aberrant immune response 5	-	0	0
13069	48322	Drosophila melanogaster	l(1)air6	lethal aberrant immune response 6	air6|l(1)air[6]	0	0
13070	48323	Drosophila melanogaster	l(1)air9	lethal aberrant immune response 9	air9|l(1)air[9]	0	0
13071	48324	Drosophila melanogaster	fs(1)B4	female sterile (1) B4	-	0	0
13072	48326	Drosophila melanogaster	E(A[53g])	Enhancer of abnormal abdomen-53g	E(A)	0	0
13073	48327	Drosophila melanogaster	l(1)2Dh	lethal (1) 2Dh	N2|N5	0	0
13074	48328	Drosophila melanogaster	l(1)2Di	lethal (1) 2Di	N6	0	0
13075	48329	Drosophila melanogaster	yemG3a	yema gene 3a	unnamed|yG3a|yT3a	0	0
13076	48330	Drosophila melanogaster	yemG3b	yema gene 3b	unnamed|yG3b|yT3b	0	0
13077	48331	Drosophila melanogaster	yemG3c	yema gene 3c	unnamed|yG0.7|yG3c|yG3c/0.7|yT0.7|yT3c	0	0
13078	48332	Drosophila melanogaster	l(1)TK205	lethal (1) TK205	l205	0	0
13079	48333	Drosophila melanogaster	l(1)TK218	lethal (1) TK218	l218	0	0
13080	48334	Drosophila melanogaster	E(Dl)9P39.3	Enhancer of Delta 9P39.3	-	0	0
13081	48335	Drosophila melanogaster	GstD2	Glutathione S transferase D2	CG4181|DmGST21|DmGSTD2|DmelCG4181|GST|GST D2|GSTD2|Gst|GstD21|gstD21	0	0
13082	48336	Drosophila melanogaster	GstD3	Glutathione S transferase D3	BcDNA:LP11313|CG4381|D3|DmGST22|DmGSTD3|DmelCG4381|GST-D3|GSTD3|Gst|GstD22|gstD22	0	0
13083	48337	Drosophila melanogaster	GstD4	Glutathione S transferase D4	CG11512|DmGST23|DmGSTD4|DmGstd4|DmelCG11512|GST|GSTD4|Gst|GstD23|gstD23	0	0
13084	48338	Drosophila melanogaster	GstD5	Glutathione S transferase D5	CG12242|D5|DmGST24|DmGSTD5|DmelCG12242|GST|GSTD5|Gst|GstD24|gstD24	0	0
13085	48339	Drosophila melanogaster	GstD6	Glutathione S transferase D6	CG4423|DmGST25|DmGSTD6|DmGstd6|DmelCG4423|GST|GSTD6|Gst|GstD25|gstD25	0	0
13086	48340	Drosophila melanogaster	GstD7	Glutathione S transferase D7	CG4371|DmGST26|DmGSTD7|DmGstd7|DmelCG4371|GSTD7|Gst|GstD26|gstD26	0	0
13087	48341	Drosophila melanogaster	GstD8	Glutathione S transferase D8	CG4421|DmGST27|DmGSTD8|DmelCG4421|GST|GST D27|GSTD8|Gst|GstD27|gstD27	0	0
13088	48343	Drosophila melanogaster	deltaTry	deltaTrypsin	CG12351|DmDeltaTry|DmelCG12351|delta	0	0
13089	48345	Drosophila melanogaster	meso60A	mesoderm specific 60A	-	0	0
13090	48347	Drosophila melanogaster	l(3)j00015	lethal (3) j00015	-	0	0
13091	48348	Drosophila melanogaster	l(3)AFA7	lethal (3) AFA7	-	0	0
13092	48349	Drosophila melanogaster	l(1)291-11	lethal (1) 291-11	291-11	0	0
13093	48351	Drosophila melanogaster	ms(2)43C	male sterile (2)43C	-	0	0
13094	48360	Drosophila melanogaster	l(1)rp1	lethal (1) rp1	-	0	0
13095	48362	Drosophila melanogaster	l(3)B12-3-31	lethal (3) B12-3-31	-	0	0

13096	48363	Drosophila melanogaster	E(nd)195	Enhancer(notchoid)195	-	0	0
13097	48370	Drosophila melanogaster	l(2)PC4-A	lethal (2) PC4-A	-	0	0
13098	48371	Drosophila melanogaster	l(2)PC4-B	lethal (2) PC4-B	-	0	0
13099	48372	Drosophila melanogaster	l(3)100CDa	lethal (3) 100CDa	-	0	0
13100	48373	Drosophila melanogaster	l(2)k10201	lethal (2) k10201	B|CG13951|CG8803|DmelCG13951	0	0
13101	48375	Drosophila melanogaster	l(1)HM452	lethal (1) HM452	-	0	0
13102	48376	Drosophila melanogaster	l(3)96Aa	lethal (3) 96Aa	291.5	0	0
13103	48377	Drosophila melanogaster	l(2)50CDa	lethal (2) 50CDa	-	0	0
13104	48378	Drosophila melanogaster	l(1)815-5	lethal (1) 815-5	-	0	0
13105	48379	Drosophila melanogaster	l(1)6c-21	lethal (1) 6c-21	-	0	0
13106	48380	Drosophila melanogaster	l(2)k01015	lethal (2) k01015	l(2)01015	0	0
13107	48381	Drosophila melanogaster	Lcp65Ab2	CG18773 gene product from transcript CG18773-RA	BcDNA:RE39879|CG18773|CP5|DCP-beta|DmelCG18773|LCP-5|LCP5|L[[3]]CP5|Lcp-b|Lcp-b2|Lcp5|Lcp65A-b|Lcp65Ab	0	0
13108	48382	Drosophila melanogaster	Lcp65Ab1	CG32400 gene product from transcript CG32400-RA	CG18776|CG32400|CP5|DCP-alpha|DmelCG32400|LCP-5|LCP10|LCP5|L[[3]]CP5|Lcp-b|Lcp-b1|Lcp10|Lcp5|Lcp65A-b|Lcp65Ab|Lcp65Abl|Rho	0	0
13109	48384	Drosophila melanogaster	l(2)k17003	lethal (2) k17003	unnamed	0	0
13110	48385	Drosophila melanogaster	l(2)k15819	lethal (2) k15819	unnamed	0	0
13111	48387	Drosophila melanogaster	l(2)k14710	lethal (2) k14710	BcDNA:LD21719|CG8325|DmelCG8325|l(2)k15603	0	0
13112	48390	Drosophila melanogaster	l(2)k12701	lethal (2) k12701	-	0	0
13113	48395	Drosophila melanogaster	l(2)k11009	lethal (2) k11009	-	0	0
13114	48396	Drosophila melanogaster	l(2)k10825	lethal (2) k10825	-	0	0
13115	48400	Drosophila melanogaster	l(2)k09318	lethal (2) k09318	-	0	0
13116	48401	Drosophila melanogaster	l(2)k09217	lethal (2) k09217	unnamed	0	0
13117	48402	Drosophila melanogaster	l(2)k09216	lethal (2) k09216	-	0	0
13118	48404	Drosophila melanogaster	l(2)k08612	lethal (2) k08612	-	0	0
13119	48410	Drosophila melanogaster	l(2)k05419a	lethal (2) k05419a	l(2)k05419	0	0
13120	48416	Drosophila melanogaster	l(3)S101804	lethal (3) S101804	1018/04|unnamed	0	0
13121	48417	Drosophila melanogaster	l(3)S001407b	lethal (3) S001407b	0014/07|14/7	0	0
13122	48418	Drosophila melanogaster	l(2)k00301	lethal (2) k00301	-	0	0
13123	48421	Drosophila melanogaster	CG3558	CG3558 gene product from transcript CG3558-RC	BEST:LD23852|DmelCG3558|FBgn0025681|LD23852|c4.3	0	0
13124	48422	Drosophila melanogaster	CG5707	CG5707 gene product from transcript CG5707-RA	BEST:HL03644|DmelCG5707|HL03644	0	0
13125	48423	Drosophila melanogaster	l(3)S148011a	lethal (3) S148011a	1480/11	0	0
13126	48424	Drosophila melanogaster	l(3)S145511	lethal (3) S145511	1455/11	0	0
13127	48425	Drosophila melanogaster	l(3)S144912	lethal (3) S144912	1449/12	0	0
13128	48426	Drosophila melanogaster	l(3)S113208a	lethal (3) S113208a	1132/08|1132/8	0	0
13129	48427	Drosophila melanogaster	l(3)S096404	lethal (3) S096404	0964/04	0	0
13130	48428	Drosophila melanogaster	l(3)S090417	lethal (3) S090417	0904/17	0	0
13131	48429	Drosophila melanogaster	l(3)S059011	lethal (3) S059011	0590/11	0	0
13132	48430	Drosophila melanogaster	l(3)S042622	lethal (3) S042622	0426/22	0	0
13133	48431	Drosophila melanogaster	l(3)S041706	lethal (3) S041706	0417/06	0	0
13134	48432	Drosophila melanogaster	l(3)S027607	lethal (3) S027607	0276/07	0	0
13135	48433	Drosophila melanogaster	l(3)S027344	lethal (3) S027344	0273/44	0	0
13136	48434	Drosophila melanogaster	l(3)S026420	lethal (3) S026420	0264/20	0	0
13137	48435	Drosophila melanogaster	l(3)S026115b	lethal (3) S026115b	0261/15	0	0
13138	48437	Drosophila melanogaster	l(3)S020114	lethal (3) S020114	0201/14	0	0
13139	48438	Drosophila melanogaster	l(3)S005343	lethal (3) S005343	0053/43	0	0
13140	48440	Drosophila melanogaster	CG31672	CG31672 gene product from transcript CG31672-RA	BEST:LD15963|CG4248|DmelCG31672|n(2)k09932	0	0
13141	48441	Drosophila melanogaster	CG3287	CG3287 gene product from transcript CG3287-RB	BEST:LD11166|DmelCG3287|n(2)09967	0	0
13142	48445	Drosophila melanogaster	Acph-1	Acid phosphatase 1	ACP|ACPH|APH|Acph|CG7899|DmelCG7899	0	0
13143	48448	Drosophila melanogaster	CkIIalpha	Casein kinase II alpha subunit	CG17520|CK II|CK-2|CK-II|CK-II alpha|CK2|CK2 alpha|CK2-alpha|CK2a|CK2alpha|CKII|CKII alpha|CKII-alpha|CKIIa|CKIIalfa|CKIIalpha|Cask-II-a|Ck2alpha|CkII|DmCK2alpha|DmCKIIalpha|DmelCG17520|Ti	0	0
13144	48450	Drosophila melanogaster	Dip-B	Dipeptidase B	CG9285|DipB|DmelCG9285	0	0
13145	48453	Drosophila melanogaster	fs(1)A120	female sterile (1) A120	fs(1)120|fs(1)571	0	0
13146	48454	Drosophila melanogaster	fs(1)BP	female sterile (1) BP	fs(1)BP2	0	0
13147	48455	Drosophila melanogaster	fs(1)M46	female sterile (1) M46	-	0	0
13148	48456	Drosophila melanogaster	ibx	icebox	fs(1)M72	0	0
13149	48457	Drosophila melanogaster	fs(1)M73	female sterile (1) M73	-	0	0
13150	48458	Drosophila melanogaster	fs(1)M78	female sterile (1) M78	-	0	0
13151	48459	Drosophila melanogaster	fs(1)M93	female sterile (1) M93	-	0	0
13152	48460	Drosophila melanogaster	fs(1)M94	female sterile (1) M94	-	0	0
13153	48461	Drosophila melanogaster	fs(1)M122	female sterile (1) M122	-	0	0
13154	48462	Drosophila melanogaster	fs(1)MY18	female sterile (1) MY18	fs(1)MY-18	0	0
13155	48463	Drosophila melanogaster	l(1)1Ca	lethal (1) 1Ca	-	0	0
13156	48464	Drosophila melanogaster	l(1)1EFa	lethal (1) 1EFa	l(1)BA1	0	0
13157	48465	Drosophila melanogaster	l(1)1EFb	lethal (1) 1EFb	l(1)BA2	0	0
13158	48466	Drosophila melanogaster	l(1)1EFc	lethal (1) 1EFc	l(1)BA3	0	0
13159	48467	Drosophila melanogaster	l(1)1EFd	lethal (1) 1EFd	l(1)BA4	0	0
13160	48468	Drosophila melanogaster	l(1)1EFe	lethal (1) 1EFe	l(1)BA5	0	0
13161	48469	Drosophila melanogaster	l(1)1EFf	lethal (1) 1EFf	l(1)BA6	0	0
13162	48470	Drosophila melanogaster	l(1)1EFg	lethal (1) 1EFg	l(1)BA7	0	0
13163	48471	Drosophila melanogaster	l(1)1EFh	lethal (1) 1EFh	l(1)BA8	0	0
13164	48472	Drosophila melanogaster	l(1)1EFi	lethal (1) 1EFi	l(1)BA9	0	0
13165	48473	Drosophila melanogaster	l(1)1EFj	lethal (1) 1EFj	l(1)BA10	0	0
13166	48474	Drosophila melanogaster	l(1)2Bu	lethal (1) 2Bu	10-153-2|l(1)10-153-2	0	0
13167	48475	Drosophila melanogaster	l(1)7Dk	lethal (1) 7Dk	-	0	0
13168	48476	Drosophila melanogaster	l(1)7Dm	lethal (1) 7Dm	RA5	0	0
13169	48478	Drosophila melanogaster	l(1)9Ff	lethal (1) 9Ff	-	0	0
13170	48479	Drosophila melanogaster	l(1)9Fg	lethal (1) 9Fg	l(1)BP3	0	0
13171	48480	Drosophila melanogaster	l(1)9Fh	lethal (1) 9Fh	-	0	0
13172	48481	Drosophila melanogaster	l(1)dd4	lethal (1) discs degenerate 4	CG10988|DGrip91|Dd4|Dgrip9|Dgrip91|Dgrips91|DmelCG10988|GCP3|GRIP91|Grip91|anon-WO03040301.197|dGRIP91|dd4|l(1)G0122|l(1)d deg-4|l(1)d.deg.-4|l(1)d.deg.4	0	0
13173	48482	Drosophila melanogaster	l(1)ESHS14	lethal (1) ESHS14	2/16B/1	0	0
13174	48483	Drosophila melanogaster	l(1)ESHS15	lethal (1) ESHS15	-	0	0
13175	48484	Drosophila melanogaster	l(1)ESHS16	lethal (1) ESHS16	-	0	0
13176	48485	Drosophila melanogaster	l(1)ESHS17	lethal (1) ESHS17	-	0	0
13177	48486	Drosophila melanogaster	l(1)ESHS20	lethal (1) ESHS20	-	0	0
13178	48487	Drosophila melanogaster	l(1)ESHS21	lethal (1) ESHS21	-	0	0
13179	48488	Drosophila melanogaster	l(1)ESHS22	lethal (1) ESHS22	-	0	0
13180	48489	Drosophila melanogaster	l(1)ESHS23	lethal (1) ESHS23	-	0	0
13181	48490	Drosophila melanogaster	l(1)10Ci	lethal (1) 10Ci	-	0	0
13182	48491	Drosophila melanogaster	l(2)21Ea	lethal (2) 21Ea	l(2)neh-8	0	0
13183	48492	Drosophila melanogaster	l(2)21Fa	lethal (2) 21Fa	l(2)neh-9	0	0
13184	48493	Drosophila melanogaster	l(2)21Fb	lethal (2) 21Fb	l(2)neh-10	0	0
13185	48494	Drosophila melanogaster	l(2)21Fc	lethal (2) 21Fc	l(2)neh-11	0	0
13186	48495	Drosophila melanogaster	l(2)21Fd	lethal (2) 21Fd	l(2)neh-12	0	0
13187	48496	Drosophila melanogaster	l(2)21Fe	lethal (2) 21Fe	l(2)neh-13	0	0
13188	48497	Drosophila melanogaster	l(2)22Aa	lethal (2) 22Aa	l(2)neh-14	0	0
13189	48498	Drosophila melanogaster	l(2)22Ab	lethal (2) 22Ab	l(2)neh-15	0	0
13190	48499	Drosophila melanogaster	l(2)22Ac	lethal (2) 22Ac	l(2)neh-16	0	0
13191	48500	Drosophila melanogaster	l(2)22Ad	lethal (2) 22Ad	l(2)neh-17	0	0
13192	48501	Drosophila melanogaster	l(2)22Ae	lethal (2) 22Ae	l(2)neh-18	0	0
13193	48502	Drosophila melanogaster	l(2)37Fd	lethal (2) 37Fd	-	0	0
13194	48504	Drosophila melanogaster	l(3)62Be	lethal (3) 62Be	l(3)neo7|neo7	0	0
13195	48505	Drosophila melanogaster	l(3)63Aa	lethal (3) 63Aa	-	0	0
13196	48506	Drosophila melanogaster	l(3)63Ab	lethal (3) 63Ab	-	0	0
13197	48507	Drosophila melanogaster	l(3)63Ba	lethal (3) 63Ba	-	0	0
13198	48508	Drosophila melanogaster	l(3)63Bb	lethal (3) 63Bb	-	0	0
13199	48509	Drosophila melanogaster	l(3)63Bc	lethal (3) 63Bc	-	0	0
13200	48510	Drosophila melanogaster	l(3)63Bd	lethal (3) 63Bd	-	0	0
13201	48511	Drosophila melanogaster	l(3)63Be	lethal (3) 63Be	-	0	0
13202	48513	Drosophila melanogaster	l(3)85Ee	lethal (3) 85Ee	-	0	0
13203	48515	Drosophila melanogaster	l(3)F31	lethal (3) F31	l(3)31	0	0
13204	48516	Drosophila melanogaster	l(3)KX305	lethal (3) KX305	-	0	0
13205	48517	Drosophila melanogaster	M(1)11F	Minute (1) 11F	RpS15Aa	0	0
13206	48518	Drosophila melanogaster	M(1)13A	Minute (1) 13A	RpL37a	0	0

13207	48519	Drosophila melanogaster	M(1)18C	Minute (1) 18C	M(1)n|M[[n]]	0	0
13208	48520	Drosophila melanogaster	M(2)24D	Minute (2) 24D	M(2)LS2|RpL40	0	0
13209	48521	Drosophila melanogaster	M(2)24F	Minute (2) 24F	M|M(2)24F-1|M(2)Z|M(2)z|M(2L)z|M[[z]]|Mz	0	0
13210	48522	Drosophila melanogaster	M(2)39F	Minute (2) 39F	M(2)H|M(2L)H|RpL21	0	0
13211	48523	Drosophila melanogaster	M(3)69E	Minute (3) 69E	M(3)h|M(3)h[y]|M(3L)h|M[33d30]|Mh|RpS4	0	0
13212	48524	Drosophila melanogaster	M(3)76A	Minute (3) 76A	M(3)S34	0	0
13213	48525	Drosophila melanogaster	M(3)80	Minute (3) 80	M(3)LS4|M(3)LS4[Q-III]|Q-III|QIII|Qm|RpL10	0	0
13214	48527	Drosophila melanogaster	M(3)96C	Minute (3) 96C	M(3)be|M(3R)be|RpS27	0	0
13215	48528	Drosophila melanogaster	Mab77H5	Monoclonal antibody 77H5	77H5	0	0
13216	48529	Drosophila melanogaster	mat(2)syn-E	maternal effect syncytial blastoderm arrest E	PJ60|mat(2)synPJ50	0	0
13217	48531	Drosophila melanogaster	Pp1-13C	Protein phosphatase 1 at 13C	13C|CG9156|DmPp1-13C|DmelCG9156|PP1|PP1 13C|PP1 beta13c|PP1-13C|PP13C|PP1alpha-13C|PP1alpha13C|PP1c|Pp1|pp1-13C	0	0
13218	48532	Drosophila melanogaster	PpY-55A	Protein phosphatase Y at 55A	CG10930|D19|DmPpY-55A|DmelCG10930|PPY|PPY 55A|PpD19	0	0
13219	48533	Drosophila melanogaster	Su(crc)	Suppressor of cryptocephal	Su(crc)1	0	0
13220	48534	Drosophila melanogaster	tu(1)Sz	tumor (1) Sz	Tu-Sz|tu-Sz	0	0
13221	48535	Drosophila melanogaster	Hrb87F	Heterogeneous nuclear ribonucleoprotein at 87F	CG12749|DmelCG12749|HRB87F|HRB87F/hrp36|HRP36|Hrb2|Hrb85CD|Hrb87Fa|Hrb87f|Hrp36|P11|P11/Hrb87F|Q14|Q16|hnRNP36|hrp36|hrp40|p38	0	0
13222	48537	Drosophila melanogaster	l(1)air2	lethal aberrant immune response 2	air2	0	0
13223	48538	Drosophila melanogaster	l(1)air3	lethal aberrant immune response 3	-	0	0
13224	48539	Drosophila melanogaster	l(1)air10	lethal aberrant immune response 10	air10	0	0
13225	48540	Drosophila melanogaster	l(1)air11	lethal aberrant immune response 11	air11|l(1)air[11]	0	0
13226	48541	Drosophila melanogaster	l(1)air12	lethal aberrant immune response 12	-	0	0
13227	48542	Drosophila melanogaster	fs(1)A5	female sterile (1) A5	-	0	0
13228	48544	Drosophila melanogaster	l(3)63Bi	lethal (3) 63Bi	-	0	0
13229	48545	Drosophila melanogaster	l(3)63Bh	lethal (3) 63Bh	-	0	0
13230	48546	Drosophila melanogaster	l(3)63Da	lethal (3) 63Da	-	0	0
13231	48547	Drosophila melanogaster	l(2)hs	lethal heat-sensitive	-	0	0
13232	48548	Drosophila melanogaster	Bsg99D	Blastoderm-specific transcript 99D	-	0	0
13233	48551	Drosophila melanogaster	l(1)4Fa	lethal (1) 4Fa	-	0	0
13234	48552	Drosophila melanogaster	Sam-S	S-adenosylmethionine Synthetase	CG2674|DmelCG2674|M(2)21A-B|M(2)21AB|METK|SamS|Su(z)5|m(2)21ab	0	0
13235	48553	Drosophila melanogaster	M(3)96A	Minute (3) 96A	-	0	0
13236	48554	Drosophila melanogaster	M(3)S35	Minute (3) S35	-	0	0
13237	48555	Drosophila melanogaster	fs(2)lto4	female sterile (2) lto4	-	0	0
13238	48556	Drosophila melanogaster	M(2)25C	Minute (2) 25C	M(2)S1	0	0
13239	48557	Drosophila melanogaster	M(2)36F	Minute (2) 36F	M(2)m|M[[s]]	0	0
13240	48561	Drosophila melanogaster	yemG9.5	yema gene 9.5	unnamed|yG9.5|yT9.5	0	0
13241	48562	Drosophila melanogaster	yemG2.8	yema gene 2.8	unnamed|yG2.6|yG2.8|yG2.8/2.6|yT2.6|yT2.8	0	0
13242	48563	Drosophila melanogaster	yemG3.4	yema gene 3.4	unnamed|yG3.4|yT3.4	0	0
13243	48564	Drosophila melanogaster	fs(2)lto1	female sterile (2) lto1	PN48	0	0
13244	48565	Drosophila melanogaster	l(1)TK308	lethal (1) TK308	l308	0	0
13245	48566	Drosophila melanogaster	l(1)TK313	lethal (1) TK313	l313	0	0
13246	48568	Drosophila melanogaster	mat(2)N	maternal lethal (2) Notchlike	l(2)31Ei	0	0
13247	48569	Drosophila melanogaster	Prl	Proxless	-	0	0
13248	48571	Drosophila melanogaster	heph	hephaestus	CG2094|CG2290|CG31000|DmelCG31000|PTB|Ptb|dPTB|dmPTB|ema|l(3)03429|l(3)j11B9|ptb	0	0
13249	48572	Drosophila melanogaster	Hsp60B	Heat shock protein 60 related	152031_at|CG2830|DmelCG2830|Hsp60b|anon-WO0140519.61|hsp60B|ms(2)06619|ms(2)21D	0	0
13250	48575	Drosophila melanogaster	Mse9.4	Modifier of split & Enhancer of split 9.4	MSE9.4	0	0
13251	48581	Drosophila melanogaster	Hsp70Aa	Heat-shock-protein-70Aa	87A|CG31366|Dm-hsp70|DmelCG31366|HSP70|Hsp 70|Hsp-70Aa|Hsp70|Hsp70A|dHsp70|dhsp70|dhsp70Aa|hs70|hsp 70|hsp-70|hsp70|hsp70 87A|hsp70 Aa|hsp70(87A)|hsp70A|hsp70Aa|hsp70a	0	0
13252	48582	Drosophila melanogaster	Hsp70Bb	Heat-shock-protein-70Bb	CG31359|DMHSP7D1|Dm-hsp70|DmelCG31359|HSP70|Hsp 70|Hsp70|Hsp70(87C)|Hsp70A7d|Hsp70B|Hsp70Bbc|Hsp70Bc|dHsp70|dhsp70|hsp-70|hsp70|hsp70 87C|hsp70B|hsp70Bb	0	0
[truncated: 1,658,686 more chars]
